# Early History of Mammals Is Elucidated with the ENCODE Multiple Species Sequencing Data

**DOI:** 10.1371/journal.pgen.0030002

**Published:** 2007-01-05

**Authors:** Sergey Nikolaev, Juan I Montoya-Burgos, Elliott H Margulies, NISC Comparative Sequencing Program, Jacques Rougemont, Bruno Nyffeler, Stylianos E Antonarakis

**Affiliations:** 1 Department of Genetic Medicine and Development, University of Geneva, Geneva, Switzerland; 2 Department of Zoology and Animal Biology, University of Geneva, Geneva, Switzerland; 3 Genome Technology Branch, National Human Genome Research Institute, National Institutes of Health, Bethesda, Maryland, United States of America; 4 Intramural Sequencing Center, National Human Genome Research Institute, National Institutes of Health, Bethesda, Maryland, United States of America; 5 Swiss Institute of Bioinformatics, Lausanne, Switzerland; Cornell University, United States of America

## Abstract

Understanding the early evolution of placental mammals is one of the most challenging issues in mammalian phylogeny. Here, we addressed this question by using the sequence data of the ENCODE consortium, which include 1% of mammalian genomes in 18 species belonging to all main mammalian lineages. Phylogenetic reconstructions based on an unprecedented amount of coding sequences taken from 218 genes resulted in a highly supported tree placing the root of Placentalia between Afrotheria and Exafroplacentalia (Afrotheria hypothesis). This topology was validated by the phylogenetic analysis of a new class of genomic phylogenetic markers, the conserved noncoding sequences. Applying the tests of alternative topologies on the coding sequence dataset resulted in the rejection of the Atlantogenata hypothesis (Xenarthra grouping with Afrotheria), while this test rejected the second alternative scenario, the Epitheria hypothesis (Xenarthra at the base), when using the noncoding sequence dataset. Thus, the two datasets support the Afrotheria hypothesis; however, none can reject both of the remaining topological alternatives.

## Author Summary

Application of molecular phylogenetic methods drastically changed the conception of relationships within mammals. Recent molecular phylogenetic studies have shown that living placental mammals belong to one of the three subgroups: Boreoeutheria, Afrotheria, or Xenarthra, but the relations between these are still unknown. In a previous analysis using 16 genes, Boreoeutheria and Xenarthra grouped together. However, a study based on LINE insertions supported the grouping of Boreoeutheria and Afrotheria. To resolve this discrepancy, we applied sequence data from 1% of a genome in a subset of 18 mammalian species. We used concatenated coding sequence data from 218 genes encompassing 205 kilobases of DNA sequence. Phylogenetic analyses have shown Afrotheria as a basal group of Placentalia with high statistical support. To further validate these results, we analyzed a new phylogenetic marker: conserved noncoding sequence alignments (430 kilobases), which resulted in the same position of the placental root. Topological tests rejected the possibility of Afrotheria-Xenarthra grouping with the coding sequence dataset and Boreoeutheria-Afrotheria grouping with the noncoding sequence dataset. Ascertaining the relationships between mammals is of great importance for the investigation of evolutionary behavior of the different functional genomic elements.

## Introduction

The relationships among mammalian lineages have recently been revised using multiple gene phylogenetic analyses leading to the recognition of four main placental clades: Euarchontoglires, Laurasiatheria, Xenarthra, and Afrotheria. Humans and other Primates and Dermoptera, Scandentia, and Glires (Rodentia and Lagomorpha) form the Euarchontoglires [1,2]. The Laurasiatheria comprise the Cetartiodactyla, Perissodactyla, Carnivora, Chiroptera, Eulipotyphla, and Pholidota [3]. The Euarchontoglires and Laurasiatheria are sister groups, and together they form the Boreoeutheria [2]. The American lineage Xenarthra groups anteaters, armadillos, and sloths [4]. The remaining placental lineage is called Afrotheria due to the African origin of its stem members and includes elephants, Sirenia, Hyracoidea, Tubulidentata, Macroscelida, Tenrecidae, and Chrysochloridae [2]. It is still unclear, however, how Afrotheria, Xenarthra, and Boreoeutheria are interrelated, though most molecular studies favor Afrotheria as the basal placental lineage (e.g., [2,5,6])**.**

The three possible scenarios for the position of the root of placental mammals are: (1) Afrotheria at the base of Placentalia (Xenarthra grouping with Boreoeutheria in the Exafroplacentalia); this hypothesis emerged from molecular phylogenetic studies [2,7]. Due to a limited amount of available comparative information this topology was not significantly supported, and Shimodaira-Hasegawa tests did not reject alternative topologies [8]. (2) Epitherian hypothesis, where Xenarthra are at the base of placental mammals and Afrotheria are grouped with other placental mammals. This hypothesis is based on morphological features, uniting Afrotheria and Boreoeutheria, such as developed penis and absence of vaginal longitudinal divisions [9]. Recently, molecular characters consisting of two LINE insertions were found to be shared by Boreoeutheria and Afrotheria and absent in Xenarthra [10]. (3) Atlantogenata hypothesis, based on the analysis of vertebrate mtDNA protein sequences favors the grouping of Afrotheria and Xenarthra [11].

Understanding the early evolution of placental mammals remains a major challenge not only for evolutionary biology but also for genomic, developmental, and biomedical research [12]. Resolving the placental root is an important question that has not been unambiguously answered even by the analysis of numerous genes and a wide taxonomic sampling [2,13]. The reason may lie in the limited amount of phylogenetic information tracing back to early placental divergences that may have been compressed in time [14] as suggested by the fossil record [15]. Adding a substantial amount of sequence data may help in resolving the order of these closely spaced cladogenetic events.

To investigate the early evolution of placental mammals, we analyzed the ENCODE [16] dataset of coding sequences (CDSs) comprising 218 orthologous genes taken from 18 mammalian species representing all main lineages (Table S1). In addition, we have investigated the phylogenetic properties of evolutionarily constrained DNA sequences that do not overlap known CDSs. Such conserved noncoding sequences (CNCs) have been established by genomic scale comparisons between human and mouse [17–19]. CNCs are mostly not repetitive and cover approximately 3% of mammalian genomes, which is roughly two times more than CDSs [19–23]. CNCs seem to be under an equal or even stronger purifying selection than CDSs [21,23]. These qualities could make CNCs a potentially powerful class of phylogenetic markers to solve ancient cladogenetic events.

## Results/Discussion

To settle the debate about the position of the root of placental mammals, we used the ENCODE consortium sequencing data, covering 1% of human genome and orthologous genomic regions in other mammals [23,24]. We created two independent alignments: the first from CDSs, the second from CNCs. Both alignments were prepared in a way in which in every column there are positions for at least one representative of each major mammalian group (Primates, Glires, Laurasiatheria, Xenarthra, Afrotheria, Marsupialia, and Monotremata). The final alignment of concatenated CDSs contained 204,786 base pairs (bp) belonging to 218 genes while the concatenated CNC alignment included 429,675 bp coming from all individual CNCs longer than 50 bp (the CNC alignment is more than twice as large as the CDS alignment). With this unprecedented amount of sequence information (at least 39 times larger than previous studies) we assessed mammalian phylogeny using *Monodelphis* (Metatheria) and *Platypus* (Monotremata) as an out-group.

**Figure 1:**
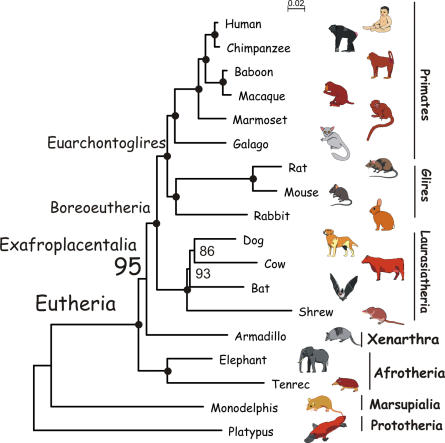
ML Phylogenetic Tree of Mammals The topology and branch lengths shown here are based on concatenated alignments of coding exons (68,262 codons). Branch lengths are scaled to the number of substitutions per site. Bootstrap support values are indicated at each node, with solid-color dots indicating 100% support.

The two independent datasets, CDS and CNC, were analyzed using maximum likelihood (ML) methods as implemented in PHYML and a general time-reversible (GTR) + gamma (G) + proportion of invariable sites (I) model of sequence evolution as defined by ModelTest [25,26]. Both datasets converged to the same highly supported topology (Figure 1). All major mammalian lineages known so far are reconstructed: Primates, Glires, Eurarchontoglires, Laurasiatheria, Boreoeutheria (B), Xenarthra (X), and Afrotheria (A). The bootstrap support for those nodes is 100% on trees from both phylogenetic markers. Moreover, our phylogenetic analyses provide a clear topological solution to the long-standing question of the position of mammalian root: placental mammals are split onto Afrotheria on one side and Exafroplacentalia on the other side with a high statistical support (CDS amino acids [aa]: 95% bootstrap proportion [BP]; CDS nucleotides: 88% BP; CNC: 73% BP). The phylogeny based on CNCs fully corroborates the CDS-based phylogeny. The consistent results of the two non-intersecting datasets provide additional support for the settlement of the debate regarding the position of the placental root.

The two remaining alternative topologies of the position of the root of placentals: (1) the Epitheria hypothesis (X [A,B]), and (2) the Atlantogenata hypothesis ([A,X] B), were confronted to the ML topology (Figure 1) using approximately unbiased, Kishino-Hasegawa, and Shimodaira-Hasegawa topological tests as implemented in the CONSEL package [27]. The results of ML analysis with GTR + G + I model performed with baseml program implemented in PAML package [28] (Table 1) using the CDS_DNA dataset show that none of the alternative topologies can be significantly rejected. Using the CDS_AA dataset the Atlantogenata theory can be rejected (*p* < 0.00001) while the Epitheria theory cannot (Table 1). Only using the CNC dataset, we can reject the Epitheria theory (*p* < 0.01) while the two other topologies are almost equally possible. The combined dataset from CNC and CDS_DNA 1 and 2 (first and second positions from CDS_DNA) alignments places Afrotheria at the base and allows us to reject only the Epitheria but not the Atlantogenata hypotheses.

**Table 1:**
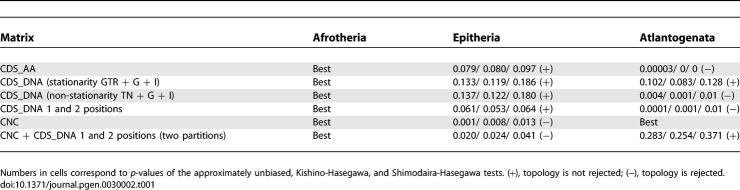
Topological Tests of Alternative Hypotheses of Placental Root

Taken together, these results suggest that the root of the placental lineage lies between the Afrotheria and the group formed by all other Placentalia. Thus, it is only by the combined use of the CNC and CDS datasets (taken from 1% of mammalian genome) that a reasonable amount of evidence supporting the root of the tree is provided. We conclude that CNCs are powerful phylogenetic markers that can be complementary to CDS markers in phylogenetic reconstructions.

By concatenating the CNC and CDS_DNA 1 and 2 datasets, we obtain a DNA matrix that is approximately four times larger than when using CDS_DNA 1 and 2 alignments alone (592,027 bp, comprised of one fourth of CDS data and three fourths of CNC data). The bootstrap support for the placental root between Afrotheria and Exafroplacentalia using the combined data provides a confidence of 98% of BP (the individual BP supports were 95% and 73% using CDS_DNA 1 and 2 and CNC data alone, respectively). Thus, the inclusion of the CNC data has a significant impact in determining the most likely topology of Mammalia.

In order to test if a partitioning of our concatenated dataset might affect our results, we calculated the ML scores for the three alternative topologies using PAML and partitioning CNC versus CDS_DNA 1 and 2 (Table 1). We found that the topology with Afrotheria at the base is the best; and using CONSEL, the Epitheria hypothesis is rejected while the Atlantogenata hypothesis is not significantly rejected.

Third codon positions of CDSs are known to saturate over evolutionary time, possibly at the placental evolutionary scale. To test this, we first applied a codon model (GTR + G) that assigns different values to all parameters for first, second, and third codon positions, as implemented in baseml. We also tested the exclusion of the third position from the analysis (CDS_DNA 1 and 2) (Table 1). Both analyses increased the robustness of our results. In all analyses, the Afrotheria clade remained at the base of Placentalia (with 95% BP support if excluding third position); the Atlantogenata hypothesis was rejected with *p* < 0.01 in both cases (codon model or excluding third position). In both analyses, topological tests did not reject the Epitheria hypothesis, yet with the CDS_DNA 1 and 2 data, the *p-*value is close to the significance threshold of 5% (see Table 1).

Problems with base compositional differences are important to address in studies where taxonomic sampling is sparse rather than dense. In the concatenated dataset CNC + CDS_DNA 1 and 2, the homogeneity chi-square test for base composition rejected base stationarity. Therefore, we assessed the impact of base composition by using baseml to perform likelihood estimates of the three competing topologies under a non-stationarity model (nhomo = 3 option) with TN93 + G + I. The best ML score was obtained for the topology with Afrotheria at the base (Table 1), suggesting that our results are not sensitive to non-stationarity of base composition.

Distal out-groups may influence the branching order of the basal in-group lineages. One way of exploring the potential impact of the out-group sampling on the rooting of Placentalia is to delete either *Monodelphys* or *Platypus.* Deleting *Monodelphys* favors the topology with the Afrotheria at the base for all datasets, while deleting *Platypus* favors the Epitheria hypothesis (for CDS-derived datasets) or the Atlantogenata hypothesis (CNC dataset). We further tested for the potential impact of the long branch of *Tenrec* on the rooting of the Placentalia by its deletion. All three alternative possibilities were found depending on the three datasets. There is, therefore, no clear evidence that a long-branch attraction artifact favors the topology with Afrotheria at the base.

To test if our phylogenetic searches were sensitive to the starting tree, we repeated the analysis for CDS_AA, CDS_DNA, and CNC using different starting trees (Afrotheria, Epitheria, and Atlantogenata). In all cases the tree with Afrotheria at the base was retrieved. To further test if PHYML NNI could result in the best topology, we performed PHYML SPR analysis for three datasets, CDS_DNA, CDS_AA, and CNC. All three resulted in the trees with Afrotheria at the base with support of 92%, 95%, and 65%, respectively.

**Figure 2:**
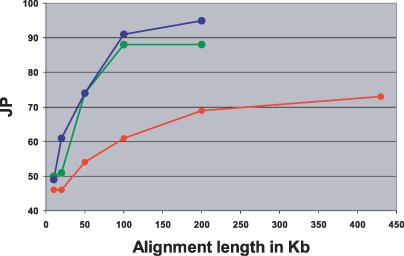
Jacknife Support for Exafroplacentalia Depending on the Alignment Length Red, CNC; green, CDS_DNA; blue, CDS_AA.

Our analysis demonstrated that mammalian CNCs contain abundant signal for phylogenetic studies. In order to assess the phylogenetic signal as compared to CDS, we used two approaches: (1) jacknife analysis, (Figure 2); and (2) likelihood mapping (Figure S1). With jacknife analysis, the relative amount of phylogenetic signal is measured in CNC and CDS datasets (both DNA and AA) by systematically reducing the length of the initial alignment and measuring jacknife supports. We generated a CNC alignment of equivalent length to that of CDS_DNA alignment (205 kilobases [kb]) and for both sets we generated four gradually reduced datasets, comprising 100 kb, 50 kb, 20 kb, and 10 kb (for CDS_AA the length was calculated for the corresponding DNA alignment). We observed that for almost all nodes of the tree, even 5% of initial alignment comprising 10 kb (3,300 aa) is sufficient to assess highly supported phylogenetic relationship with jacknife proportion (JP) of near 100%. Among the less stable nodes of the tree is that of Exafroplacentalia. In the CNC and CDS datasets, the JP support of the Exafroplacentalia declines drastically with the reduction of the length of the alignments (Figure 2). Only with around a 100-kb sequence alignment (for both coding and noncoding sequence) is it possible to reconstruct the basal Exafroplacentalia group with support between 60% and 90% and 90% JP. This result explains why the majority of previous studies addressing the question of placental root were unable to give a conclusive answer, since these studies were conducted using a maximum alignment length of 16.4 kb [2], that is, approximately six times less than needed according to our estimates.

Likelihood mapping [29] was used as a second test of assessing the quality of the phylogenetic signal contained in CNCs as compared to CDSs. In the 18 species datasets used in this study, the 3,060 possible quartets were phylogenetically analyzed. The proportion of resolved quartets indicates the amount of information in the dataset. For this test, the length of the CDS and CNC alignments were 205 kb and 430 kb, respectively.

The results showed a similar performance of the CNC dataset which gave 99.97% of resolution of 3,060 quartets (one quartet remained unresolved, 0.03%); the CDS_DNA dataset (for which six quartets were unresolved, 0.2%); and CDS_AA (one quartet unresolved, 0.03%).

Overall, the results suggest that CNCs are equally powerful phylogenetic markers as CDSs, and hence they could be used in parallel with CDSs, to maximize the statistical support of phylogenetic trees.

The recent study of retroposed LINE elements in mammals revealed a number of insertions supporting all major mammalian clades that are also supported by our analyses [10]. Two insertions (L1MB5) common for Boreoeutheria and Afrotheria that are absent in Xenarthra were found supporting the Epitheria hypothesis (X [A,B]). The analysis of rare genomic changes, such as the insertion of retroposed elements, are thought to be exceptionally useful markers due to their ambiguity-free phylogenetic information, because the coincidence of orthologous insertions of retroposed elements belonging to the same type is unlikely [30]. However, little is known about the frequency of retroposon loss by small-scale deletions. Because extant Xenarthra radiated quite recently (during the Tertiary) from a 35-million-y-long standing stem lineage [31], the probability of deletion of one or more retroposons in this 35-million-y period of time is not negligible. This explanation may reconcile the findings by Kriegs et al. [10] and the phylogenomic results presented here. Another possible explanation comes from the fact that the splitting among Afrotheria, Xenarthra, and Boreoeutheria occurred in a relatively short period of time (estimated 5–10 million y [2]), and therefore incomplete lineage sorting [32,33] may also explain the observation of Kriegs et al.

Although our study provides strong evidence for the rooting of Placentalia between Afrotheria and Exafroplacentalia, the phylogenetic signal supporting this hypothesis in 1% of mammalian genome is not sufficiently conclusive. The final resolution of the placental root might come with the addition in the genomic datasets of Xenarthra and Afrotheria species with short branch lengths. Previous phylogenetic studies suggest that short branches are expected for some xenarthrans: Choloepus spp. (two-toed sloth), Cyclopes didactylus (silky anteater), Cabassous spp. (naked-tailed armadillo); and afrotherians: Dugong dugong (Dugong), Chrysochloris spp. (golden mole), Talpa spp. (mole).

## Materials and Methods

The phylogenetic analyses were based on the ENCODE TBA alignments [23]. The CDS_DNA alignment (Dataset S1) was created by concatenating the longest transcript per gene for all ENCODE targets and keeping a single reading frame. The CDS_DNA dataset was translated into amino acids. The CNC alignment (Dataset S2) was prepared by the concatenation of all CNCs >50 bp, as identified by the ENCODE Multi-Species Sequence Analysis group [23,24]. For the CNC dataset, we have screened visually for clearly misaligned parts of sequences and have deleted them. For the CDSs, we translated the DNA alignment, and the stretches with stop codons we deleted from both DNA and AA alignments. Due to the large amount of missing data, we kept only sites for which at least one representative of each main lineage was present. In this way we preserved 100% of data for Xenarthra, Marsupialia, and Monotremata, and we have full coverage across taxa for Primates, Glires, Laurasiatheria, and Afrotheria. The individual maximal amount of missing data is 32%. The amount of data included in the alignments for each species is shown in Table S1 [23].

To gain the maximum amount of positions, we selected only mammalian species with substantial amount of data. A total of 18 species representing the major mammalian groups (Table S2) were included; *Platypus* was used as an out-group. For the CDS, the length of DNA alignment is 204,786 bp, and the length of amino acid alignment is 68,262 aa positions; CNC alignments comprise 429,675 bp.

All phylogenies were performed using ML method [34] with PHYML software using NNI and SPR branch swapping methods, or imposing different starting topologies [25,35]. The GTR + G + I model of sequence evolution [36–38] was selected as the best fitting model to the data using ModelTest 3.7 program [26]. Statistical support was assessed with bootstrap analysis [39].

Baseml and Codeml programs implemented in the PAML package [28] were used to calculate ML scores for three competing topologies (Afrotheria, Epitheria, and Atlantogenata) using different models of sequence evolution. Approximately unbiased, Kishino-Hasegawa, and Shimodaira-Hasegawa topological tests (as implemented in the CONSEL package [27]) were used to test alternative rooting of placental animals.

To assess the amount of phylogenetic signal in CDS and CNC datasets we performed a jacknife analysis. For that, with the seqboot.exe program implemented in PHYLIP package [40], we created 100 jacknife replicates of the following lengths: 205 kb (68 kilo–amino acids [Kaa]), 100 kb (33 Kaa), 50 kb (17 Kaa), 20 kb (7 Kaa), and 10 kb (3.3 Kaa) from CDS_DNA, CDS_AA, and CNC matrices. With PHYML program we reconstructed trees from those replicates, and with consense.exe (PHYLIP package) we calculated JPs (Figure 2).

Using the likelihood mapping method [29], we performed a direct comparison of the amount of signal between CNC, CDS_DNA, and CDS_AA matrices by the analysis of ML for the fully resolved tree topologies that could be computed for four sequences.

## Supporting Information

Dataset S1 The CDS_DNA Alignment with the Length 204,786 bp in a Subset of 18 Species This alignment was generated by concatenating the longest transcript per gene for all ENCODE targets and by keeping a single reading frame. Clearly misaligned DNA parts were manually deleted.
(4.9 MB DOC).

Dataset S2 The CNC Alignment with the Length 429,675 bp in a Subset of 18 Species This alignment was prepared by the concatenation of all CNCs >50 bp, as identified by the ENCODE Multi-Species Sequence Analysis group [23,24]. Clearly misaligned DNA parts were manually deleted.
(10.5 MB DOC).

Figure S1 Direct Comparison of Amount of Phylogenetic Signal between CDS_DNA, CDS_AA, and CNC Datasets Numbers in the corners of triangles indicate the percentage of resolved quartets of species in the alignment. Numbers in the middle of the triangle and on the sides indicate the percentage of unresolved quartets.
(133 KB JPG).

Table S1 Amount of Data Included in CNC and CDS Datasets per Species (51 KB DOC).

Table S2 Latin Names of the Species (34 KB DOC).

## Abbreviations

aa: amino acid
bp: base pair
BP: bootstrap proportion
CDS: coding sequence
CNC: conserved noncoding sequence
G: gamma
GTR: general time-reversible
I: proportion of invariable sites
JP: jackknife proportion
Kaa: kilo–amino acids
kb: kilobase
ML: maximum likelihood
