# Supplementary material for: Early History of Mammals Is Elucidated with the ENCODE Multiple Species Sequencing Data
Source: PLoS Genet. 2007 Jan 5;3(1):e2. doi: 10.1371/journal.pgen.0030002 (PMC1761045; doi:10.1371/journal.pgen.0030002)
Supplement: Supplementary file 3 [file pgen.0030002.st001.doc]

Table S1. Amount of data included into CNC and CDS datasets per species.

| Name | CNC | | CDS | |
| --- | --- | --- | --- | --- |
|  | length (bp) | % of missing data | length (bp) | % of missing data |
| Human | 429675 | 0 | 204774 | 0 |
| Chimpanzee | 417452 | 3 | 196170 | 4 |
| Baboon | 419938 | 2 | 196622 | 4 |
| Macaque | 398582 | 7 | 183198 | 11 |
| Marmoset | 402705 | 6 | 192309 | 6 |
| Galago | 407795 | 5 | 183216 | 11 |
| Rat | 404335 | 6 | 194335 | 5 |
| Mouse | 420016 | 2 | 201871 | 1 |
| Rabbit | 352870 | 18 | 159635 | 21 |
| Dog | 426561 | 1 | 202943 | 1 |
| Cow | 291914 | 32 | 157588 | 23 |
| Bat | 393379 | 8 | 183342 | 10 |
| Shrew | 339413 | 21 | 157144 | 23 |
| Armadillo | 429675 | 0 | 204526 | 0 |
| Elephant | 349849 | 19 | 163425 | 20 |
| Tenrec | 316012 | 26 | 157551 | 23 |
| Monodelphis | 429675 | 0 | 204180 | 0 |
| Platypus | 429675 | 0 | 204023 | 0 |
