# Supplementary material for: Early History of Mammals Is Elucidated with the ENCODE Multiple Species Sequencing Data
Source: PLoS Genet. 2007 Jan 5;3(1):e2. doi: 10.1371/journal.pgen.0030002 (PMC1761045; doi:10.1371/journal.pgen.0030002)
Supplement: Supplementary file 4 [file pgen.0030002.st002.doc]

Table S2. Latin names of the species.

| Species common name | Species Latin name |
| --- | --- |
| Human | *Homo sapiens* |
| Chimpanzee | *Pan troglodytes* |
| Baboon (olive) | *Papio cynocephalus anubis* |
| Macaque | *Macaca mulatta* |
| Marmoset (white-tufted ear) | *Callithrix jacchus* |
| Galago (small-eared) | *Otolemur garnetti* |
| Rat | *Rattus norvegicus* |
| Mouse | *Mus musculus* |
| Rabbit | *Oryctolagus cuniculus* |
| Dog | *Canis familiaris* |
| Bovine (Cow) | *Bos taurus* |
| Bat (greater horseshoe) | *Rhinolophus ferrumequinum* |
| Shrew (European common) | *Sorex araneus* |
| Armadillo (nine-banded) | *Dasypus novemcinctus* |
| Elephant (African) | *Loxodonta africana* |
| Tenrec (lesser hedgehog) | *Echinops telfairi* |
| Opossum | *Monodelphis domestica* |
| Platypus (duck-billed) | *Ornithorhynchus anatinus* |
