# Supplementary material for: Early History of Mammals Is Elucidated with the ENCODE Multiple Species Sequencing Data
Source: PLoS Genet. 2007 Jan 5;3(1):e2. doi: 10.1371/journal.pgen.0030002 (PMC1761045; doi:10.1371/journal.pgen.0030002)
Supplement: Supplementary file 1 [file pgen.0030002.sd001.doc]

18 204786

human	CGAGGCCTGC CAGTGGCTGG CGGAGGCGAG AGTAGCGAGA GCGAGGATGA

chimp	CGAGGCCTGC CAGTGGCTGG CGGAGGCGAG AGTAGCGAGA GCGAGGATGA

baboon	CGCGGCCTGC CAGTGGCCGG CGGAGGGGAG AGTAGCGAGA GCGAGGATGA

macaque	CGCGGCCTGC CAGTGGCCGG CGGAGGGGAG AGTAGCGAGA GCGAGGATGA

marmoset	CGGGGCCTGG CAGTAGCCGG TGGAGGCGAG AGTAGCGAGA GTGAGGATGA

galago	CGCGGCCTGG CAGTGGCTGG CGGAGGCGAG AGTAGCGAGA GCGAGGATGA

rat	AGGGGCTTGG CAGTGGCTGG TGGAGGCGAG AGCAGCGATA GTGAGGATGA

mouse	AGGGGCTTGG CAGTGGCTGG TGGAGGCGAG AGCAGCGATA GTGAGGATGA

rabbit	CGTGGGCTGG CAGTGGCTGG TGGAGGAGAG AGTAGCGACA GCGAGGATGA

cow	CGCGGTCTAG CAGTGGCCGG TGGAGGCGAG AGTAGTGACA GTGAGGATGA

dog	CGCAGCCAGG CGGTGGCCGG TGGAGGCGAG AGTAGCGAGA GCGAGGATGA

rfbat	CGCGGCCTGG CAGTGGCCGG TGGAGGCGAG ACCAGCGACA GCGAGGATGA

shrew	CGTGGCCTGG CTGTGGCCGG AGGAGGCGAG AGCAGCGAGA GCGAGGATGA

armadillo	CGAGGCTTGG CAGTAGCTGG CGGAGGCGAG AGTACTGATA GCGAGGATGA

elephant	CGCGGATTGC CGGTGGCCGG AGGAGGCGAG AGTAGCGAGA GCGAGGATGA

tenrec	TGGGGCCCGG CGGTGGCCGG CGGAGGGGAG AGTAGCGAGA GTGAGGATGA

monodelphi	GGTTCCCTGG TCGTGGCAGG TGGAGGCGAG AGCAGCGACA GCGAAGAAGA

platypus	-gcgggccgg ccgtgccggg gggcggcgAG AGCAGCGACA GCGACGAGGA

TGGCTGGGAG ATTGGGTATC TCGACCGGAC GTCTCAGAAA TTGAAAAGGC

CGGCTGGGAG ATTGGGTATC TCGACCGGAC GTCTCAGAAA TTGAAAGGGC

CGGCTGGGAG ATTGGGTATC TCGACCGGAC GTCTCAGAAA TTGAAAGGGC

CGGCTGGGAG ATTGGGTATC TCGACCGGAC GTCTCAGAAA TTGAAAGGGC

CGGCTGGGAG ATTGGGTATC TCGACCGGAC GTCTCAGAAA TTGAAAGGGC

CGGCTGGGAG ATAGGCTATC TCGACCGGGC ATCTCAGAAA TTAAAAGGTC

CGGCTGGGAT ATTGGGTATC TCGACCGGTC CTCTCAGAAA TTAAAAAGGT

CGGCTGGGAT ATTGGGTATC TCGACCGGTC CTCTCAGAAA TTAAAAAGGT

TGGCTGGGAG ATCGGGTATC TCGACCGACC GCCTCAGAAA TTAAAAGGAC

CGGCTGGGAG ATTGGGTATC TCGACCGGAA ATCTCAGAAA TTAAAAGGAC

CGGCTGGGAG ATTGGGTATC TCGACCGGGC GGTTCAGAAA TTAAAAGGGC

CGGCTGGGAG ATTGGGTATC TCGATCGTGC AGCTCAGAAA TTGAAAGGAC

CGGCTGGGAG ATCGGGTATC TGGATCGCGC AGCACAGAAA TTAAAAGGCC

CGGCTGGGAG ATTGGGTATC TAGACCGGGC ATCTCAGAAA TTAACAGGGC

CGGCTGGGAG ATTGGCTATC TCGACCGGGC ATCTCAGAAA TTAACGAGTC

TGGCTGGGAA ATTGGGTATC TCGACCGGGC ACCTCAGAAA TTTACAGCGC

CTACTGGGAT ATAGGGTACA TGGATCGGGC GCCTCAGACA ATGAAAGGGT

CGGCTGGGAC ATCGGGTACA CGGAGCGGCC CGACAAGCAG TTGAAAGACT

TATTACCCAT TGAAGAAAAG AAAGAAAAAT TTAAGAAAGC AATGACCATC

TGTTACCCAT TGAAGAAAAG AAAGAAAAAT TTAAGAAAGC AATGACCATC

TGTTACCCAT TGAGGAAAAG AAAGAAAAAT TTAAGAAAGC AATGACCATC

TGTTACCCAT TGAGGAAAAG AAAGAAAAAT TTAAGAAAGC AATGACCATC

TGTTACCCAT TGAAGAAAAG AAAGAAAAAT TTAAGAAAGC ATTGACCACC

CGTTACCTAT TGAAGAAAAG AATGAAACTT TTAAGAAAGC ATTGACCACA

CTTTACCCGT TGAAGAGAAG AACGAAACAT TCAAGAAAGC ACTGACCACC

CTTTACCCGT TGAAGAGAAG AACGAGACAT TTAAGAAAGC ACTGACCACC

CATTACCCAT AGAAGAGAAG AATGAAACAT TTAAGAAAGC ATTGACCACA

CGTTACCAGT TGAAGAAAGG CAGGAAACAT TTAAGAAGGC TTTGACCACA

CGTTACTCCC CGAAGAAAAG AATGAGACGT TTAAAAAAGC ACTGACCTCA

CGTTACCCAC TGAGGAAAAG AATGAAACAT TTAAGAAAGC ATTGACCACA

CATTACCCAC CGAAGACAAG AATGAAACAT TTAAGAAAGC ATTGACCACG

TGTTACCCAC TGAAGAAAAG AATGAAACGT TTAAGAAAGC ATTGACCACT

CGTTACCCAC TGAAGAAAAG AATGAAATAT TTAAGAAAGC ATTGACCACA

CGTTACCTGC TGAAGAAAAG AATGAAATGT TTAAGAAAGC ATTGACCACA

CATCAGTGAC TGAAGAAAAA GATGAAACCT TTAAAAAAGC TTTGACTTCA

CATTACTGAG CGAAGAGAAG GATGAGGTAT TGAAGAGAGC TTTGACTACG

GGAGATGTTT CATTGGTCCA GGAGCTCCTA GATTCTGGCA TTAGTGTAGA

GGAGATGTTT CATTGGTCCA GGAGCTCCTA GATTCTGGCA TTAGTGTAGA

GGAGATGTTT CATTGGTCCA GGAGCTCCTA GATTCTGGCA TTAGTGTAGA

GGAGATGTTT CATTGGTCCA GGAGCTCCTA GATTCTGGCA TTAGTGTAGA

GGAGATGTTT CATTGGTCCT GGAGCTCCTA GATTCTGGCA TTAGTGTAGA

GGAGATATTT CGTTGGTCCA GGAACTCCTA GATTCTGGCA TCAGTGTCGA

GGAGATATTT CCTTAGTGAA GGAACTCCTG GATTCTGGCA TTAATGTAGA

GGAGATATTT CTTTAGTTAA AGAACTCCTG GATTCTGGCA TTAATGTGGA

GGAGATACTT CTCTGGTGAA GGAGCTCCTA GATTCTGGTA TTAGTGTGGA

GGCAATATTT CATTGGTCGA GGAGCTCCTA GATTCTGGCA TCAGTGTAGA

GGAGATACTT CATTGGTCAA GGAGCTCCTG GATTCCGGCA TTAGTGTCGA

GGAGATATTT TACTGGTCAA GGAGCTCCTA AATTCTGGCA TTAGTGTAGA

GGAGATATTT CGTTGGTCCA GGAGCTCCTC GATTCTG--- ----------

GGAGATATTT CATTGGTGGA GGAACTCCTA GATTCTGGCA TTAGTATAGA

GGAGATATTT CGTTGGTCGA GGAGCTCCTA GATTCTGGCA TCAGTGTAGA

GGAGATACTT CGTTGGTTGA AGAGCTCCTA AACGCTGGCA TCAGTGTGGA

GGAGATATTT CACTGGTTGA AGAACTTCTA AATTCTGGAA TGAATGTAGA

GGTGATGGTT CACTACTTGA AGAGCTCCTA AATTCTGGCA TGCAAGTCGA

TTCCAACTTT CAGTATGGAT GGACTCCCCT TATGTATGCT GCTAGTGTTG

TTCCACCTTT CAGTATGGAT GGACTCCCCT TATGTATGCT GCTAGTGTTG

TTCCACCTTT CAGTATGGAT GGACTCCCCT TATGTATGCT GCTAGTGTTG

TTCCACCTTT CAGTATGGAT GGACTCCCCT TATGTATGCT GCTAGTGTTG

CGCTACCTTT CGATATGGAT GGACTCCCCT TATGTATGCT GCTAGCGTTG

CTCCAGTTTT CGGTACGGAT GGACTCCCCT TATGTACGCT GCTAGTGTTG

TTCCAGCTTC CGCTATGGAT GGACCCCTCT TATGTATGCA GCTAGTGTGG

CTCCAGCTTC CGCTATGGAT GGACCCCTCT TATGTATGCC GCTAGTGTGG

TTCCAGTTTT CGATATGGAT GGACTCCCCT TATGTATGCT GCTAGTGTTG

TACCAGCTTT CAGTATGGTT GGACTTCCCT TATGTATGCT GCTAGTGTTT

TTCCAGCTTT CGGTATGGAT GGACTCCACT TATGTATGCT GCTAGTGTTT

CTCCAGCTTT CGATATGGAT GGACTCCCCT CATGTATGCT GCTAGCGTTT

---------- ---------- ---------- ---------- ----------

TTCCAGTTTT CGGTATGGAT GGACTCCTCT TATGTATGCT GCTAGCGTGG

GTCCAGCTTT CGATATGGAT GGACTCCCCT TATGTATGCT GCTAGCGTTG

CTCCAGCTTT CGGTATGGGT GGACACCCCT TATGTTTGCT GCAAGCATTG

ATCCAACTTT CATTTTGGAT GGACTCCTCT TATGTATGCT GCTAATATTG

CTCAAGTTTT CGGTTCGGAT GGACTCCCCT TATGTATGCT GCTAGCATCG

CCAATGCAGA GCTGGTTCGG GTCCTTTTGG ACAGAGGTGC TAATGCAAGC

CCAATGCAGA GCTGGTTCGG GTCCTTTTGG ACAGAGGTGC TAATGCAAGC

CCAATGCAGA ACTGGTTCGG GTCCTTCTGG ACAGAGGTGC TAATGCAAGC

CCAATGCAGA ACTGGTTCGA GTCCTTCTGG ACAGAGGTGC TAATGCAAGC

CCAATGCAGA GCTGGTTCGG GTCCTTTTGG ACAGAGGTGC TAATGCAAGC

CCAACGTAGA GCTCGTTCGG GTCCTTTTGG ACAGAGGTGC TAATGCAAGC

CCAATGTAGA GCTGGTTCGG TTCCTTTTGG ACAGGGGTGC TAATGCAAGC

CCAATGCAGA GCTGGTTCGC TTCCTTTTGG ACAGAGGTGC TAATGCCAGC

CTAATGTGGA GCTGGTGCGG GTTCTTTTGG ATAGAGGTGC TAATGCAAGC

CCAATGTAGA ACTGGTTCGG GTCCTTCTGG ACAGAGGTGC TAATGCAAGC

CCAATGTAGA ACTGGTTCGG GTCCTTTTGG ACAAAGGTGC TAATGCAAGC

CCAATGTCGA GTTGGTTCGG GTCCTTTTGG ACAGAGGTGC TAATGCAAGC

---------- ---------- ---------- ---------- ----------

CCAATGTAGA GCTGGTTCGG GTCCTTTTGG ACAGAGGTGC CAATGCAAGC

CCAATGTAGA GCTGGTTCGG GTCCTTTTGG ACAGAGGTGC TAATGCAAGC

CCAACGTCAA CCTGGTCCGG GTCCTTTTGA ACAGAGGTGC TAATGCAAGC

CTAATGTGGA GTTGGTGCGT ATCTTGTTGG ACAGAGGGGC TAATGCAAGC

CTAATGTTGA TCTGGTGCGT ATCCTGTTGG ACAGAGGTGC AAATGCAAGC

TTTGAGAAGG ATAAGCAAAG TATTTTGATA ACTGCATGTT CTGCTCATGG

TTTGAGAAGG ATAAGCAAAG TATTTTGATA ACTGCATGTT CTGCTCATGG

TTTGAGAAGG ATAAGCAAAC TATTTTGATA ACTGCATGTT CTGCTCATGG

TTTGAGAAGG ATAAGCAAAC TATTTTGATA ACTGCATGTT CTGCTTATGG

TTTGAGAAGG ATAAGCAAAC TATTTTGATA ACTGCATGTT CTGCTCATGG

TTTGATAAGG ATAAGCAGAC TATTTTGATA ACTGCGTGTT CTGCTCGTGG

TTTGATAAGG ACAAGTTAAC CGTTTTGATA ACTGCTTGCT CTGCTCGTGG

TTTGATAAGG ACAAGTTAAC CATTTTGATA AGTGCTTGTT CTGCTCGTGG

TTTGATAAGG ATAAACAAAC TATTTTGATA ACTGCATGTT CTGCTCGTGG

TTTGATAAGG ATAAGCAAAC TGTTTTGATT ACTGCATGTT CTGCTCGTGG

TTTGATAAGG ATAAGCAAAC TATTTTGATA ACTGCGTGTT CTGCATGTGG

TTTGATAAAG ATAAGCAAAC GATTCTGATA ACCGCATGTT CTGCTCGTGG

---------- ATAAGCAGAC AATTTTGATA ACTGCATGTT CTGCTCGTGG

TTCGATAAGG ATAAGCAAAC TATTTTGATA ACTGCATGTT CAGCTCGTGG

TTTGAGAAGG ATAAGCACAC TGTTTTGATA ACTGCGTGTT CTGCTCGAGG

TTTGAAAAGG ATAAGCAAAC TGTTTTGATG ACTGCGTGTT CTGCTCGTGG

TTTGATAAGG ATCAGTATAC TATTTTGATG GCTGCATGTT CTGCACGTGC

TTTTCAAAAG ATCAACACAC TGTTCTGATG GCTGCATGTT CAGCTCGAGT

CTCAGAGGAA CAGATCTTGA AGTGTGTAGA ACTACTACTT TCAAGAAATG

CTCAGAGGAA CAGATCTTGA AGTGTGTAGA ACTACTACTT TCAAGAAATG

CTCAGAGGAA CAGATCTTGA AGTGTGTAGA GCTACTACTT TCAAGAAATG

CTCAGAGGAA CAGATCTTGA AGTGTGTAGA GCTACTACTT TCAAGAAATG

CTCAGAGGAA CAGATCTTGA AATGTGTAGA GCTACTACTT TCAAGAAATG

CTCAGAGGAA CAGATCTTAA AGTGTGTAGA GCTGTTACTT TCAAGAAATG

CTCAGAGGAA CAAGTCTTGA AGTGTGTAGA GTTACTACTT TCACGAAATG

CTCAGAGGAG CAAGTCTTGA AATGTGTAGA GTTACTACTT TCAAGAAATG

CTCAGAGGAG CAGATCTTGA AGTGTGTAGA GCTATTACTT TCAAGAAATG

CTCAGAGGAA AAGATCTTGA AGTGTATAGA GCTACTACTT TCAAGAAATG

CTCAGAGGAA CAGATCTTAA AGTGTGTAGA GCTACTACTT TCAAGAAATG

CTCAGAAGAA CAGATCTTGA AGTGTGTGGA GCTGTTACTT TCAAGAAATG

CTCAGAAGAA CAGATCTTGA AGTGTGTAGA ACTACTACTT TCCAGAAATG

CTCAGAGGAA CAGATCTTGA AGTGTGTAGA ACTACTACTT TCAAGAAATG

CTCACAGGAA CAGATCTTGA AGTGTGTGGA GCTACTGCTT TCAAGAAATG

CTCACAGGAA CAGATCATAA AGTGTGTAGA GCTACTACTT TCCCGAAATG

TTCAGAGGGT CAGATTTTAA AGAGTGTAGA ATTGCTACTT TCGAGAAATG

TCCAGAAGAA CGGATTTTGA AGACTGCAGA GCTACTTCTT TCAAGAAATG

CTGATCCAAA TGTTGCTTGT AGGAGACTTA TGACCCCAAT CATGTATGCT

CTGATCCAAA TGTTGCTTGT AGGAGACTTA TGACCCCAAT CATGTATGCT

CTGATCCAAA TGTTGCTTGT AGGAGACTTA TGACCCCAAT CATGTATGCT

CTGATCCAAA TGTTGCTTGT AGGAGACTTA TGACCCCAAT CATGTATGCT

CTGATCCAAA TGTTGCTTGT AGGAGACTTA TGACCCCAAT CATGTATGCT

CTGATCCAAA TGTTGCTTGT AGGAGACTTA TGACTCCAAT CATGTATGCT

CGGATCCTAA TGTTGCTTGT AGGAGACTTA TGACTCCAAT CATGTATGCT

CGGACCCAAA TACTGCTTGT AGGAGACTTA TGACGCCAAT CATGTATGCT

CTGACCCAAA TGTTGCTTGT AGGAGACTTA TGACTCCCAT CATGTATGCT

CTGATCCAAA TGTCGCTTGT AGGAGACTTA TGACACCAAT CATGTACGCT

CTGATCCAAA TGTTCCTTGT AGGAGACTTA TGACTCCAAT CATGTATGCT

CTGATCCAAA TGTTCCTTGT AGGAGACTCA TGACTCCAAT CATGTACGCT

CTGATCCAAA TGTTGCTTGT AGGAGATTTA TGACTCCAAT CATGTATGCT

CTGATCCAAA TGTTGCTTGT AGGAGACTTA TGACTCCAGT CATGTATGCT

CTGATCCAAA TGTTGCTTGT AG-------- ---------- ----------

CTGACCCAAA TGTTGCTTGT AGGAGACAGA TGACTCCAAT CATGTATGCT

CTAACCCAAA TGTTGCTTGC AGAAAATGTA TGACTCCAAT CATGTATGCA

CCAGCCCTAA TGCTACTTGC AGGAAACGCA TGTCTCCACT GATGTATGCA

GCTCGAGATG GTCACACCCA GGTTGTTGCT CTCCTTGTTG CTCATGGAGC

GCTCGAGATG GTCACACCCA GGTTGTTGCT CTCCTTGTTG CTCATGGAGC

GCTCGAGATG GTCACACCCA GGTTGTTGCT CTCCTTGTTG CTCATGGAGC

GCTCGAGATG GTCACACCCA GGTTGTTGCT CTCCTTGTTG CTCATGGAGC

GCTCGAGATG GTCACACCCA GGTTGTTGCT CTCCTTGTTG CTAGTGGAGC

GCCCGGGATG GTCACCCTCA GGTCGTTGCT CTCCTCGTTG CTCATGGAGC

GCCCGAGATG GTCATACTCA GGTCGTTGCT CTGCTTGTTG CTCATGGAGC

GCCCGAGACG GTCATACTCA GGTTGTGGCT CTGCTTGTGG CTCACGGAGC

GCTCGGGATG GTCACCCTCA AGTTGTTGCT CTGCTTGTTG CTCATGGAGC

GCACGTGACG GCCACCCTCA GGTCGTTGCT CTCCTTGTTG CTCATGGAGC

GCACGAGATG GTCACCCTCA GGTTGTTGCT CTCCTTGTTG CTCATGGAGC

GCCCGCGACG GTCACACCCA GGTTGTCGCT CTCCTTGTTG CTCACGGAGC

GCACGGGACG GTCACCCTCA GGTGGTTGCG CTCCTTGTTG CTCATGGAGC

GCACGAGCTG GTCACCCTCA GGTTGTTGCT GTCCTTGTTG CTTATGGAGC

---------- ---------- ---------- ---------- ----------

GCCCGAGGTG GTCACCCTCA GGTTGTTGCT CTTCTTGTTG CGCATGGAGC

GCACGAGAAG GCCATCCTCA GGTTGTTGCT CTCCTTGTTG CTCATGGAGC

GCTCGAGAAG GTCATTCTCA GTTGGTGGCT CTTCTCGTTG GACACGGAGC

AGAAGTTAAT ACCCAGGATG AGAATGGTTA CACTGCTTTA ACGTGGGCAG

AGAAGTTAAT ACCCAGGATG AGAATGGTTA CACTGCTTTA ACGTGGGCAG

AGAAGTTAAT ACCCAGGATG AGAATGGTTA CACTGCTTTA ACATGGGCAG

AGAAGTTAAT ACCCAGGATG AGAATGGTTA CACTGCTTTA ACATGGGCAG

AGAAGTTAAT ACCCAGGATG AGAATGGTTA CACTGCTTTA ACGTGGGCAG

AGAAGTTAAT ACCCAGGATG AGAATGGCTA CACTGCTTTA ACATGGGCAG

AGAAGTCAAC GCCCAGGATG AGAATGGTTA TACTGCCTTA ACATGGGCAG

CGAAGTTAAC GCCCAGGATG AGAATGGTTA TACTGCCTTA ACATGGGCAG

AGAAGTTAAT ATCCAGGATG AGAATGGTTA TACTGCTTTA ACATGGGCAG

AGAAGTGAAT ACCCAGGACG AGAACGGCTA TACTGCTTTA ACATGGGCAG

AGAAGTTAAT ACCCAGGATG AGAATGGTTA TACTGCTTTA ACATGGGCAG

AGAAGTCAAT ACACAGGATG AGAACGGTTA CACTGCTTTA ACATGGGCAG

AGAAGTCAAT ATTCAAGATG AGAATGGTTA TACTGCTCTA ACATGGGCTG

AGAGGTTAAT ACCCAGGATG AGAACGGTTA TACTGCTTTA ACATGGGCAG

---------- ---------- ---------- ---------- ----------

AGAAGTTAAT GCCCAGGATG AGAATGGCTA CACTGCTTTA ACGTGGGCAG

AGAAATAAAT GCCCAAGATG AAAATGGTTA TACAGCCTTA ATGTGGGCAG

AGAGATAAAT GCCCAAGACG ATAACGGGTA TACAGCTCTT GCATGGGCAG

CACGTCAGGG TCATAAAAAT ATAGTTTTGA AGTTGCTTGA ACTTGGAGCT

CACGTCAGGG TCATAAAAAT ATAGTTTTGA AGTTGCTTGA ACTTGGAGCT

CACGTCAGGG TCATAAAAAT ATAGTTTTGA AGTTGCTTGA ACTTGGAGCT

CACGTCAGGG TCATAAAAAT ATAGTTTTGA AGTTGCTTGA ACTTGGAGCT

CACGTCAGGG TCATAAAAGT ATAGTTTTGA AGTTGCTTGA ACTTGGAGCT

CGCGTCAGGG TCATAAAAAT GTAGTTTTGA AGTTGCTTGA ACTTGGAGCT

CACGGCAGGG TCATAAAAAT GTAATTCTGA AGTTGCTTGA ACTCGGAGCT

CACGGCAGGG TCATAAAAAT GTAATTCTGA AGTTGCTTGA ACTGGGAGCT

CACGTCAGGG ACATAAAAGT GTAGTTTTGA AGTTGCTTGA ACTTGGAGCT

CACGTCAGGG TCATAAAAAT GTAGTTTTGA AGTTGCTTGA ACTTGGAGCT

CACGGCAGGG TCATAAAAAT GTAGTTTTGA AGTTGCTTGA ACTTGGAGCT

CACGTCAGGG TCATAAAAAT GTAGTTTTGA AGTTGCTTGA ACTTGGAGCT

CACGTCAGGG TCATAAAAAC GTCGTTTTAA AGTTGCTTGA ACTTGGAGCT

CACGTCAGGG TCATAAAAAT GTAATTTTGA AGTTACTTGA ACTTGGAGCT

---------- ---------- ---------- ---------- ----------

CATATCAGGG TCATAAAAAT GTTATTTTGA AGTTGCTTGA ACTTGGAGCT

CACGTCAGGG TCATAAAAGT GTAGTTTTGA AGTTGCTTGA ACTGGGAGCT

CTCGTCATGG TCATAAAACT ACGGTTTTAA AGTTGCTTGA ACTTGGAGCT

AATAAAATGC TACAAACCAA AGATGGAAAG ATGCCAAGTG AGATTGCAAA

AATAAAATGC TACAAACCAA AGATGGAAAG ATGCCAAGTG AGATTGCAAA

AATAAAATGC TACAAACCAA AGATGGAAAG ATGCCAAGTG AGATTGCAAA

AATAAAATGC TACAAACCAA AGATGGAAAG ATGCCAAGTG AGATTGCAAA

AATAAAATGT TACAAACCAA AGATGGGAAG CTGCCCAGTG AGATTGCAAA

AATAAAATGC TACAAACCAA AGATGGAAAG ATACCAAGCG AAATTGCAAA

AATAAAATGC TACAGACCAA AGATGGCAGG ACACCAAGTG AGATTGCAAA

AATAAAATGC TACAGACCAA AGATGGCAGA ACACCAAGCG AGATTGCAAA

AACAAAACTC TGCAAACCAA AGATGGAAAG ACGCCAAGTG AGATTGCAAA

AATAAAATGA TACAAACCAA AGATGGAAAG ACACCAAGTG AAATTGCAAA

AATAAAATGC TACAGACCAA AGATGGAAAG ACACCAAGTG AGATTGCAAA

AATAAAATGC TACAAACCAA AGATGGAAAG ACACCAAGCG AGATTGCAAA

AATAAAACAC TGCAAACTAA ---------- ---------- ----------

GATAAAATGC TACAAACCAA AGATGGAAAG ACACCCAGTG AAATTGCAAA

---------- ---------- ---------- ---------- ----------

AATAAAATGC TACAAACCAA AGATGGAAAG ACACCAAGTG AGATTGCAAA

AATAAAACAA TACAAACCAA AGATGGAAAG ACAGCGGGAG AGATTGCCAA

GATAAAACTC TACAAACCCA AGATGGAAAG ACACCAGCAG AGATTGCTAA

AAGAAACAAA CATCATGAGA GTTCATATAC AGCATTTGGA GATCTGGAAG

AAGAAACAAA CATCATGAGA GTTCATATAC AGCATTTGGA GATCTGGAAG

AAGAAACAAA CATCATGAGA GTTCATATAC AGCATTTGGA GATCTGGAAG

AAGAAACAAA CATCATGAGA GTTCATATAC AGCATTTGGA GATCTGGAAG

AAGAAACAAA CATCATGAGA GTTCATATGC AGCATTTGGA GATCTGGAAG

AAGAAATAAA CATCTTGAGA GTTCATATAC AGCTTTTGGA GATCTGGAAA

AAGAAATAAA CATCTTGAGA GTCCATATAC AGCGTTTGGA GATCTGGAGA

AAGAAATAAA CATCTTGAGA GCCCGTATAC AGCGTTTGGA GATCTGGAAA

AAGAAATAAA CATCTTGAGA GTTCTTATAC AGCATTTGGA GATCTGGAAA

AAGAAATAAA CATCTAGAGA GTTCATATAC AGCATTTGGA GATCTGGAAA

AAGAAATAAA CATCTAGAGA GTTCATATAC AGCATTTGGA GATCTGGAAA

AAGAAATAAA CATGTAGAGA GTTCATATAC AGCACTTGGA GATCTGGAAA

---------- ---------A G--------- ---------- ----------

AAGAAATAAA CATCTAGAGA GTTCATATGC AGCATTTGAA GATCTGGAAA

---------- ---------- ---------- ---------- ----------

TAGAAATAAA CATCCAGAG- -TTCATACAC AGCATTTGGA GAGTTGGACT

AAGAAATAAA CATCCAGAGA GTTCATATAC AGCTTTTGGT GATCTTGAAC

AAGAAATAAA CACCCAGAGA GTTCATATTC CGCTTTTGGC GATCTGGAGA

TATTTTTACA TGGTCTTGGA CTTGAACATA TGACAGATTT ACTAAAGATG

TATTTTTACA TGGTCTTGGA CTTGAACATA TGACAGATAT ACTAAAGATG

TATTTTTACA TGGTATTGGA CTTGAACATA TGACAGATTT ACTAAAGATG

TATTTTTACA TGGTATTGGA CTTGAACATA TGACAGATTT ACTAAAGATG

TATTTTTACA TGGTCTTGGA CTTGAACATA TGACAGATTT ATTAAAGATG

TATTTTTACA TGGTCTTGGA CTTGAACACA TGACAGATTT ATTAAAGATG

TATTTTTACA TGGCCTTGGA CTGGAACATA TGACAGACTT ATTAAAGATG

TATTTTTACA TGGCCTTGGA CTTGAACATA TGACAGACTC ATTAAAGATG

TATTTTTACA TGGTCTTGGA CTTGAACACA TGACAGATTT ATTAAAGATG

TATTTTTACA TGGTCTTGGA CTTGAACATA TGACAGATTT ATTGAAGATG

TATTTTTACA TGGTCTTGGA CTTGAACATA TGATGGATTT ATTAAAGATG

TATTTTTGCA CGGTCTTGGA CTTGAACATA TGACAGATTT ATTAAAGATG

---------- ---------- ---------- ---------- ----------

TATTTTTACA TGGTCTTGGA CTTGAACATA TGACAGATTT ATTAAAGATG

---------- ---------- ---------- ---------- -------ATG

TATTTTTACA TGGTCTTGGA CTTGAACATA TGACAGATTT ATTAAAGATG

TATTTTTACA TGGACTTGGA CTTGAACATA TGACAGAATT ACTAAAGATG

TATTTTTGCA CGGTCTTCAG CTTGAACACT TGACAGAGTT ACTAAAGATG

TCTGGGGGCA AATACGTAGA CTCGGAGGGA CATCTCTACA CCGTTCCCAT

TCTGGGGGCA AATACGTAGA CTCGGAGGGA CATCTCTACA CCGTTCCCAT

TCTGGGGGCA AATACGTAGA CTCGGAGGGA CATCTCTACA CCGTTCCCAT

TCTGGGGGCA AATACGTAGA CTCGGAGGGA CATCTCTACA CCGTTCCCAT

TCTGGGGGCA AATACGTAGA CTCGGAGGGA CATCTCTACA CCGTTCCCAT

TCTGGGGGCA AATACGTAGA CTCCGAGGGA CATCTCTATA CCGTTCCCAT

TCTGGGGGTA AATACGTAGA CTCCGAGGGA CATCTCTACA CTGTTCCCAT

TCTGGGGGCA AATACGTAGA CTCCGAGGGA CATCTCTACA CTGTTCCCAT

TCTGGGGGCA AATACGTAGA CTCCGAGGGA CATCTCTACA CTGTTCCCAT

TCGGGGGGCA AATACGTAGA CTCAGAGGGA CATCTCTACA CTGTTCCCAT

TCGTGGGGCA AATACGTAGA CTCCGAGGGG CACCTCTACA CCGTTCCCAT

TCTGGGGGCA AATACGTAGA CTCCGAGGGA CATCTCTACA CTGTTCCCAT

---------- ---------- ---------- ---------- ----------

TCTGGGGGCA AATACGTAGA CTCCGAGGGA CATCTCTACA CCGCTCCCAT

TCTGGGGGCA AATACGTAGA TTCCGAGGGA CATCTCTATA CCGCTCCCAT

TCTGGGGGCA AATACGTAGA CTCCGAGGGA CATCTCTACA CCCTTCCCAT

TCTGGTGGCA AATACATAGA CTCAGAGGGG CTTCTCTACT CGGCGCCCAT

TCTGGGGGCA AGTACGTAGA CGCGGAGGGG CTGCTCTACT CCGTGCCCAG

CCGGGAACAG GGCAACATCT ACAAGCCCAA CAACAAGGCC ATGGCAGACG

CCGGGAACAG GGCAACATCT ACAAGCCCAA CAACAAGGCC ATGGCAGACG

CCGGGAACAG GGCAACATCT ACAAGCCCAA CAACAAGGCC ATGGCAGACG

CCGGGAACAG GGCAACATCT ACAAGCCCAA CAACAAGGCC ATGGCAGACG

CCGGGAGCAG GGCAACATCT ACAAGCCCAA CAACAAGGCC ATGGCAGACG

CCGGGAACAG GGCAACATCT ACAAGCCCAA CAACAAGGCC ATGGCAGAGG

CCGGGAACAG GGCAACATCT ACAAGCCCAA CAACAAGGCC ATGGCAGACG

CCGGGAACAG GGCAACATCT ACAAGCCCAA CAACAAGGCC ATGGCAGACG

CCGGGAACAG GGCAACATCT ACAAGCCCAA CAACAAGGCC ATGGCCGACG

CCGGGAACAG GGCAACATCT ACAAGCCCAA CAACAAGGCT ATGGCAGAGG

CCGGGAGCAG GGCAACATCT ACAAGCCCAA CAACAAGGCC ATGGCGGAGG

CCGGGAACAG GGCAACATCT ACAAGCCCAA CAACAAGACC ATGGCAGACG

---------- ---------- ---------- ---------- ----------

CAGGGAACAG GGCAACATCT ACAAGCCCAA CAACAAGGCC ATGGCAGAGG

CCGGGAACAG GGCAACATCT ACAAGCCCAA CAACAGGGAC ATGGCAGAGG

CCGGGAGCAG GGCAACATCT ACAAGCCCAA CAACAAGGCC ATGGCCGAAG

CCGAGAACAG GGAAATATCT ACAAGCCCAA CAATAAGACC ATGGCCGACG

CCGGGACCAG GGCAACATCT ACAAGCCGCA CAACAAGACC ATGGCAGACG

AGCTGAGCGA GAAGCAAGTG TACGACGCGC ACACCAAGGA GATCGACCTG

AGCTGAGCGA GAAGCAAGTG TACGACGCGC ACACCAAGGA GATCGACCTG

AGCTGAGCGA GAAGCAAGTG TACGACGCGC ACACCAAGGA GATCGACCTG

AGCTGAGCGA GAAGCAAGTG TACGACGCGC ACACCAAGGA GATCGACCTG

AACTGAGTGA GAAGCAAGTT TACGACGCGC ACACCAAGGA GATCGACCTG

AGGTGAACGA GAAGCAAGTC TACGACGCGC ACACCAAAGA GATCGACCTG

AGGTGAATGA GAAGCAAGTG TACGACGCGC ACACCAAGGA GATTGATCTG

AGGTGACTGA GAAGCAAGTG TATGACGCGC ACACCAAGGA GATTGACCTG

AGGTGAACGA GAAGCAAGTG TACGACGCGC ACACCAAGGA AATCGACCTG

AAATGAACGA GAAGCAAGTG TACGACGCGC ACACCAAGGA GATAGATCTG

AGATGAGCGA GAAGCAGGTG TACGACGCGC ACACCAAGGA AATCGACCTG

AAATGAACGA AAAGCAAGTG TACGACGCGC ACACCAAGGA GATCGACCTG

---------- ---------- ---------- ---------- ----------

AGATGAATGA AAAGCAAGTG TACGACGCGC ACACCAAGGA GATCGACCTG

AGATGCACGA GAAGCAAGTG TACGACGCAC ACACCAAGGA GATCGACCTG

ACATGAACGA GAAGCAAGTG TACGACGCGC ACACCAAGGA GATCGACCTG

AAATGAACGA GAAGCAAATG TACGATGCAC ACACCAAGGA GATCGACCTG

ACCTGCCCGA GAAGCAGCTG TATGACGCGC ACACCAAGGA GATCGATCTG

GTCAACCGCG ACCCTAAACA CCTCAACGAT GACGTGGTCA AGATTGACTT

GTCAACCGCG ACCCTAAACA CCTCAACGAT GACGTGGTCA AGATTGACTT

GTCAACCGCG ACCCTAAACA CCTCAACGAC GACGTGGTCA AGATTGACTT

GTCAACCGCG ACCCTAAACA CCTCAACGAC GACGTGGTCA AG--------

GTCAACCGCG ACCCCAAACA CCTCAACGAC GATGTGGTCA AGATTGACTT

GTCAACCGCG ACCCCAAGCA TCTCAACGAC GACGTGGTCA AGATTGACTT

GTCAACCGCG ACCCCAAGCA TCTCAACGAC GACGTGGTCA AGATTGACTT

GTCAACCGCG ACCCCAAGCA TCTCAACGAC GACGTGGTCA AGATTGACTT

GTCAACCGCG ACCCCAAGCA TCTCAACGAC GACGTGGTCA AGATCGACTT

GTCAACCGCG ACCCCAAGCA TCTCAACGAC GACGTGGTCA AGATTGATTT

GTCAACCGCG ACCCCAAGCA TCTCAACGAC GACGTGGTCA AGATTGATTT

GTCAACCGCG ACCCCAAGCA TCTCAACGAC GACGTGGTCA AGATTGATTT

---------- ---------- ---------- ---------- --ATTGATTT

GTCAACCGCG ACCCCAAGCA TCTCAACGAC GACGTGGTCA AGATTGATTT

GTCAATCGCG ACCCCAAGCA TCTCAACGAC GACGTGGTCA AGATTGATTT

GTCAACCGCG ACCCCAAGCA TCTCAACGAC GACGTGGTCA AGATTGACTT

GTCAACAGAG ACCCCAAGCA TCTCAATGAC GATATGGTCA AGATTGATTT

GTCAACCGGG ACCCCAAGCA CCTCAACGAC GATGTGGTCA AGATCGACTT

TGAAGATGTG ATTGCAGAAC CAGAAGGGAC ACACAGTTTT GACGGCATTT

TGAAGATGTG ATTGCAGAAC CAGAAGGGAC ACACAGTTTT GACGGCATTT

TGAAGATGTG ATTGCAGAAC CAGAAGGGAC ACACAGTTTC GATGGCATTT

---------- ---------- ---------- ---------- ----------

TGAAGATGTG ATTGCAGAAC CAGAAGGGAC ACACAGTTTC GACGGCATTT

TGAAGATGTG ATTGCAGAAC CAGAAGGGAC ACATAGTTTT GATGGCATTT

TGAAGATGTG ATTGCGGAAC CAGAAGGGAC ACACAGTTTC GACGGCATCT

TGAAGATGTG ATTGCAGAAC CAGAAGGGAC ACACAGTTTC GACGGCATCT

TGAAGATGTG ATTGCAGAAC CAGAAGGGAC ACACAGTTTC GACGGCATCT

TGAAGATGTG ATTGCAGAAC CAGAAGGAAC ACACAGTTTC GATGGCATCT

TGAAGATGTG ATTGCAGAAC CAGAAGGAAC ACACAGTTTT GATGGCATCT

TGAAGATGTG ATTGCAGAGC CAGAAGGGAC ACACAGTTTT GACGGCATCT

TGAAGATGTG ATCGCTGAAC CAGAAGGAAC ACACAGTTTT GATGGCATCT

TGAAGATGTG ATTGCAGAAC CAGAAGGGAC ACACAGTTTT GATGGCATCT

TGAAGATGTG ATTGCAGAAC CAGAAGGGAC ACACAGTTTC GATGGCATCT

CGAAGATGTA ATTGCAGAGC CAGAAGGGAC TCACAGTTTC GACGGCATCT

TGAAGATGTG ATAGCAGAAC CAGAAGGAAC ACACAGCTTT GATGGAATCT

CGAAGACGTG ATTGCCGAGC CCGAGGGCAC ACACAGCTTT GACGGGATCT

GGAAGGCCAG CTTCACCACC TTCACTGTGA CGAAATACTG GTTTTACCGC

GGAAGGCCAG CTTCACCACC TTCACTGTGA CGAAATACTG GTTTTACCGC

GGAAGGCCAG CTTCACCACC TTCACTGTGA CGAAATACTG GTTTTACCGC

---------- ---------- ---------- ---------- ----------

GGAAGGCCAG CTTCACCACC TTCACTGTGA CGAAATACTG GTTTTACCGC

GGAAGGCCAG CTTCACCACC TTCACTGTGA CAAAATACTG GTTTTACCGC

GGAAGGCCAG CTTCACCACC TTCACTGTGA CAAAATATTG GTTTTACCGC

GGAAGGCCAG CTTCACCACC TTCACTGTGA CAAAATATTG GTTTTACCGC

GGAAGGCCAG CTTCACCACC TTCACTGTGA CGAAATACTG GTTTTACCGC

GGAAGGCCAG CTTCACCACC TTCACTGTGA CAAAGTACTG GTTTTACCGT

GGAAGGCCAG CTTCACCACC TTCACTGTGA CAAAATACTG GTTTTACCGC

GGAAGGCCAG CTTCACCACC TTCACTGTGA CAAAGTACTG GTTTTACCGC

GGAAGGCCAG CTTCACCACC TTCACTGTGA CGAAATATTG GTTTTACCGC

GGAAGGCCAG CTTCACCACC TTCACCGTGA CGAAATACTG GTTTTACCGC

GGAAGGCCAG CTTCACCACC TTCACTGTGA CAAAATACTG GTTTTATCGC

GGAAGGCCAG CTTCACCACC TTCACTGTGA CGAAATACTG GTTTTACCGT

GGAAGGCAAG CTTTACCACC TTCACTGTGA CGAAATACTG GTTTTACCGC

GGAAGGCCAG CTTCACCACC TTCACCGTGA CAAAATACTG GTTTTACCGC

TTGCTGTCTG CCCTCTTTGG CATCCCGATG GCACTCATCT GGGGCATTTA

TTGCTGTCTG CCCTCTTTGG CATCCCGATG GCACTCATCT GGGGCATTTA

TTGCTGTCTG CCCTCTTTGG CATCCCAATG GCGCTCGTCT GGGGCATTTA

---------- ---------- ---------- ---------- ----------

CTGCTGTCTG CCCTCTTTGG CATTCCGATG GCGCTCATCT GGGGTATTTA

TTGCTGTCTG CGCTGTTCGG CATCCCAATG GCACTGATCT GGGGCATTTA

TTGCTGTCTA CCATCTTCGG CATCCCTATG GCGCTCATCT GGGGCATCTA

TTGTTGTCTA CGATCTTCGG CATCCCAATG GCACTCATCT GGGGCATTTA

CTACTGTCTA CCATCTTTGG CATCCCAATG GCACTCATCT GGGGCATTTA

TTGCTGTCTG CCCTCTTTGG CATCCCAATG GCACTCATCT GGGGCATTTA

TTGCTGTCTG CCCTCTTTGG CATCCCAATG GCACTCATAT GGGGCATTTA

CTGCTGTCTG CCGTCTTTGG CATCCCGATG GCCCTCATCT GGGGCATTTA

TTGCTGTCTA CCCTCTTTGG CATCCCAATG GCACTCATCT GGGGCCTTTA

TTGCTGTCTA CCCTCTTTGG CATCCCAATG GCACTCATAT GGGGCATTTA

TTGCTGTCTG CCCTCTTCGG CATCCCGATG GCGCTCATCT GGGGCATTTA

CTGCTCTCGG GCATCTTTGG CATCCCGATG GCACTCATCT GGGGCGTCTA

TTGCTCTCTG CACTCTTCGG CATACCAATG GCACTCATCT GGGGCATTTA

TTGCTCTCGG CCCTCTTGGG CATCCCCTTG GCCCTCCTCT GGGGCATCTA

CTTCGCCATT CTCTCTTTCC TGCACATCTG GGCAGTTGTA CCATGCATTA

CTTCGCCATT CTCTCTTTCC TGCACATCTG GGCAGTTGTA CCATGCATTA

CTTCGCCATT CTCTCTTTCC TGCACATCTG GGCAGTTGTA CCATGCATTA

---------- ---------- ---------- ---------- ----------

CTTTGCCATT CTCTCTTTCC TGCACATCTG GGCAGTTGTC CCATGCATTA

CTTCGCCATT CTCTCTTTCC TGCACATCTG GGCAGTTGTA CCGTGCATTA

CTTTGCCATC CTCTCTTTCC TGCACATCTG GGCAGTTGTA CCGTGCATCA

CTTTGCCATT CTCTCCTTCC TGCACATCTG GGCGGTTGTA CCGTGCATCA

CTTTGCCATT CTCTCTTTCC TGCACATCTG GGCAGTTGTG CCTTGCATTA

CTTTGCCATT CTCTCTTTCC TGCACATCTG GGCAGTTGTA CCATGCATTA

CTTTGCCATT CTTTCTTTCC TGCACATCTG GGCAGTTGTG CCGTGCATTA

CTTTGCCATC GTGTCCTTCC TGCACATCTG GGTGGTCGTG CCCTACATTA

CTTTGCCGTT CTCTCTTTCC TGCACATCTG GGCAGTCGTA CCATGTATTA

CTTTGCCATT CTCTCTTTCC TGCACATCTG GGCAGTTGTA CCGTGCATTA

CTTCGCCATT CTCTCTTTCC TGCACATCTG GGCAGTCGTA CCATGCATTA

CTTTGCCATC CTCTCTTTCC TGCACATCTG GGCGGTTGTG CCATGCATCA

CTTTGCCATC CTTTCCTTCT TGCACATCTG GGCAGTTGTG CCATGCATCA

CTTTGCCATC CTCTCCTTCC TGCACATCTG GGCCGTGGTG CCCTGCATCC

AGAGCTTCCT GATTGAGATT CAGTGCATCA GCCGTGTCTA TTCCATCTAC

AGAGCTTCCT GATTGAGATT CAGTGCATCA GCCGTGTCTA TTCCATCTAC

AGAGCTTCCT GATTGAGATT CAGTGCATCA GCCGTGTCTA TTCCATCTAC

---------- ---------- ---------- ---------- ----------

AGAGCTTCCT GATTGAGATT CAGTGCATCA GCCGTGTCTA TTCCATCTAC

AGAGCTTCCT GATCGAGATT CAGTGCATCA GCCGCGTCTA TTCCATCTAC

AGAGCTTCCT GATTGAGATT CAGTGCATCA GCCGTGTCTA TTCCATCTAC

AGAGCTTCCT GATTGAGATT CAGTGCATCA GCCGCGTCTA CTCCATCTAC

AGAGTTTCCT GATTGAGATT CAGTGCATCA GCCGAGTCTA TTCCATCTAC

AGAGTTTCCT GATTGAGATT CAGTGCATCA GCCGTGTCTA TTCCATCTAC

AGAGTTTCCT GATTGAGATT CAGTGCATCA GCCGTGTCTA TTCCATCTAC

AGAGCTTCCT GATTGAGATT CAGTGCATCA GCCGTGTCTA TTCCATCTAC

AGAGTTTCCT GATTGAGATT CAGTGCATCA GCCGTGTCTA TTCCATCTAC

AGAGTTTCCT GATTGAGATT CAGTGCATCG GCCGTGTCTA TTCCATCTAC

AGAGTTTCCT GATTGAGATC CAGTGCATCA GCCGTGTCTA TTCCATCTAC

AGAGCTTCCT GATCGAGATC CAGTGCATCA GCCGTGTCTA TTCCATCTAC

GGAGCTACCT GATTGAAATC CAATGTATCA GTCGGATCTA TTCAATCTGC

GGAGCTACCT GATAGAGATC CAGTGCATCA GCCGCGTCTA CTCCATCTGC

GTCCACACCG TCTGTGACCC ACTCTTTGAA GCTGTTGGGA AAATATTCAG

GTCCACACCG TCTGTGACCC ACTCTTTGAA GCTGTTGGGA AAATATTCAG

GTCCACACCG TCTGTGACCC ACTCTTTGAA GCTGTTGGGA AAATATTCAG

---------- ---------- ---------- ---------- ----------

ATCCACACCG TCTGTGACCC ACTCTTCGAA GCTATTGGGA AAATATTCAG

GTACACACCT TCTGCGACCC ACTCTTTGAA GCTATTGGCA AAATATTCAG

GTCCACACCT TCTGTGATCC ACTCTTTGAA GCGATTGGCA AGATATTCAG

GTCCATACCT TCTGCGATCC ACTCTTTGAA GCTATTGGCA AGATATTCAG

GTCCACACCT TCTGTGATCC ACTCTTTGAA GCTATTGGCA AAATATTCAG

GTCCACACCT TCTGTGACCC GCTGTTTGAG GCTATTGGCA AAATATTCAG

GTCCACACCT TCTGTGACCC GTTCTTTGAG GCTGTTGGCA AAATATTCAG

ATCCACACCT TCTGTGACCC GCTCTTTGAG GCTTTTGGCA AAGTATTCAG

ATCCACACCT TCTGTGACCC ACTCTTTGAG GCTATTGGCA AAATATTCAG

ATCCACACCT TCTGTGACCC ACTCTTCGAG GCTGTTGGCA AACTATTCAG

GTCCACACCT TCTGTGACCC GCTCTTTGAG GCCATTGGCA AAATATTCAG

GTCCACACCT TCTGCGACCC GCTCTTTGAG GCCATTGGCA AAATATTCAG

ATCCATACCT TCTGTGACCC ACTCTTTGAG GCTATTGGCA AAATATTCAG

ATCCACACCT TCTGCGACCC ACTCTTCGAG GCCATCGGCA AGGTGTTCAG

CAATGTCCGC ATCAACTTGC AGAAAGAAAT AAAGCTGGGC TTCGAGGATG

CAATGTCCGC ATCAACTTGC AGAAAGAAAT A--------- ----------

CAATGTCCGC ATCAACTTGC AGAAAGAAAT AAAGCTGGGC TTCGAGGATG

---------- ---------- ---------- ----CTGGGC TTCGAGGATG

CAATGTCCGC ATCAGCTTGC AGAAAGAAAT AAAGCTGGGC TTCGAGGATG

CAATATCCGC ATCAACATGC AGAAAGAAAT AAAGGTGGGC TTCGAGGATG

CAATATCCGC ATCAGCACGC AGAAAGAGAT AAAGCTAGGC TTCGAGGATC

CAACATCCGC ATCAGCACGC AGAAAGAGAT AAAGCTAGGC TTCGAGGATC

CAATGTCCGC ATCAGTATGC AGAAAGAAAT AAAGCTAGGC TTTGAGGATG

CAATATCCGC ATCAACACGC AGAAAGAAAT AAAGGTGGGA TTCGAGGATG

CAATATCCGC ATCAACATGC AGAAAGAAAC ACAGGTGGGC TTCGAGGACG

CAATATCCGC ATCAACACGC AGAAAGAAAT AAAGGTGGGC TTCGAGGATG

CAATATCCAC ATCAACATGC AGAAAGAAAT A--------- ----------

CAATATCCGT ATCAACATGC AGAAGGAAAT ACAGCTGGGT TTCGAGGACG

CAGTATCCGC ATCAACATGC AGAAAGAAAT A---ATTGAT TTTGAAGATG

CAACATCCGC ATCAGCACGC AGAAAGAAAT ACAGATTGAC TTCGAAGATG

TCATGTCCGG ATCGCCTTAC AGAAAGAAAT AaggATGGGT TTTGAGGATG

CAACATCCGA GCCACGGTCC AGAAAGAAAT AcgaATCGAC TTCGAAGACG

TGATCGCAGA GCCGGTGACT ACGCACTCCT TTGACAAAGT GTGGATCTGC

---------- ---------- ---------- ---------- ----------

TGATCGCAGA GCCGGTGACT ACGCACTCCT TTGACAAAGT GTGGATCTGC

TGATCGCAGA GCCGGTGACT ACGCACTCCT TTGACAAAGT GTGGATCTGC

TGATCGCAGA GCCGGTGACT ACGCACTCCT TTGACAAAGT GTGGATCTGC

TGATCGCAGA GCCGGTGACT ACGCACTCCT TTGACAAAGT GTGGATCTGC

TGATCGCAGA ACCTCCGACT ACACACTCCT TTGACAAAGT GTGGATCTGC

TGATTGCAGA GCCTGAGACT ACACACTCCT TTGACAAAGT GTGGATCTGC

TGATCGCCGA GCCGGTGACT ACGCACTCCT TTGACAAAGT GTGGATCTGC

TGATTGCAGA GCCAGTGTCT ACGCACTCCT TTGACAAAGT GTGGATCTGC

TGATCGCGGA GCCCGTGTCT ACGCACTCCT TTGACAAAGT GTGGATTTGC

TGATCGCAGA GCCCGAGTCT ACTCACTCCT TAGACAAAGT ATGGATCTGC

---------- ---------- ---------- ---------- ----------

TGATCGCCGA GCCCGTGTCT ACGCACTCCT TAGACAAAGT GTGGATCTGC

TGATTGCAGA ACCAGAAGGG ACACACAGTT TCGATGGCAT CTGGAAGGCC

TAATTGCAGA GCCAGAAGGG ACTCACAGTT TCGACGGCAT CTGGAAGGCC

TGATTGGAGA GCCCGAGTCC ACCCACAGTT TTGATAAAGT TTGGATCTGC

TGATTGCCGA GCCCGAGGGC ACACACAGCT TTGACGGGAT CTGGAAGGCC

AGCCATGCCC TCTTTGAAAT CAGCAAATAC GTAATGTACA AGTTCCTGAC

---------- ---------- ---------- ---------- ----------

AGCCATGCCC TCTTTGAAAT CAGCAAATAC GTAATGTACA AGTTCCTGAC

AGCCATGCCC TCTTTGAAAT CAGCAAATAC GTAATGTACA AGTTCCTGAC

AGCCATGCCC TCTTTGAAAT CAGCAAATAC GTAATGTACA AGTTCCTGAC

AGCCATGCCC TCTTTGAAAT CAGCAAATAC GTGCTGTACA AGTTCCTAAC

AGCCACGCGC TCTTTGAAAT CAGCAAATAC GTGATCTACA AGTTCCTGAC

AGCCATGCTC TCTTTGAAAT CAGCAAATAT GTGATGTACA AGTTCCTGAC

AGCCATGCCC TCTTTGAAAT CAGCAAGTAC GTGCTGTACA AGTTCCTGAC

AGCCACGCTC TATTTGAAAT GAGCAAATAT GTGATCTACA AGTTCCTGAC

AGCCATGCCC TGTTTGAGGT CAGCAAGTAC GTGATCTACA AGTTCCTGAC

AGCCACGCCC TCTTTGAAGT CAGCAAATAT GTGATTTACA AGTTCCTGAC

---------- ---------- ---------- ---------- ----------

AGCCATGCCC TCTTTGAAAT CAGCAAGTAC ATAATCTACA AGTTCCTGAC

AGCTTCACCA CCTTCACTGT GACAAAATAC TGGTTTTATC GCTTGCTGTC

AGCTTCACCA CCTTCACTGT GACGAAATAC TGGTTTTACC GTCTGCTCTC

AGCCATGCCC TCTTTGAGGT CAGCAAGTAC CTGATCTACA AGGTCCTAAC

AGCTTCACCA CCTTCACCGT GACAAAATAC TGGTTTTACC GCTTGCTCTC

GGTGTTCCTG GCCATTCCCC TGGCCTTCAT TGCGGGAATT CTCTTTGCCA

---------- ---------- ---------- ---GGGAATT CTCTGTGCCA

GGTGTTCCTG GCCATTCCCC TGGCCTTCAT TGCGGGAATT CTCTTTGCCA

GGTGTTCCTG GCCATTCCCC TGGCCTTCAT TGCGGGAATT CTCTTTGCCA

GGTGTTCCTG GCCATCCCTC TGGCCTTCCT TGCGGGAATT CTCTTTGCCA

CGTGTTCCTG GCCATCCCCC TGGCCTTCGT CGCGGGAATT CTCTTTGCCA

GGTGTTTCTA GCCATCCCCT TGGCCTTCAT TGCGGGTATC CTGTTTGCTA

CGTATTTCTG GCCATCCCCT TGGCCTTCAT TGCGGGTATC CTGTTTGCTA

GGTATTCCTG GCCATCCCCC TGGCCTTCGC CGCGGGAGTT CTCTTTGCCG

GGTGTTCCTG GCTATTCCCT TGGCTTTCGC TGCGGGAATT CTCTTTGCCA

GTTGCTCCTG GCGATGCCCA TGGCCTTCGC GGCAGGGGTT CTCTTCGCCA

GTTGTTCCTG GCTATCCCCC TGGCCTTTGC TGCGGGAATT CTCTTTGCCA

---------- ---------- ---------- ---------- ----------

GGTGTTCCTG GCGATTCCCC TGGCCTTCGC TGCTGGAATT CTCTTTGCCA

TGCCCTCTTC GGCATCCCGA TGGCGCTCAT CTGGGGCATT TACTTCGCCA

GGGCATCTTT GGCATCCCGA TGGCACTCAT CTGGGGCGTC TACTTTGCCA

TGTGCTCCTG GCTATCCCAT TGGCCTTTGT GGCAGGAATT CTCTTTGCAA

GGCCCTCTTG GGCATCCCCT TGGCCCTCCT CTGGGGCATC TACTTTGCCA

CCCTCAGCTG TCTGCACATC TGGATTTTAA TGCCTTTTGT AAAGACCTGC

CGCTCAGGTG TCTGCACATC TGGATTTTAA TGCCTTTTGT AAAGACCTGC

CCCTCAGCTG TCTGCACATC TGGATTTTAA TGCCTTTTGT AAAGACCTGC

CCCTCAGCTG TCTGCACATC TGGATTTTAA TGCCTTTTGT AAAGACCTGC

CCCTCAGCTG CCTGCACATC TGGATTATAA TGCCTTTTGT AAAGACCTGC

CCCTCAGCTG CCTGCACATC TGGATTATAA TGCCCTTTGT AAAGACCTGC

CCCTCAGCTG TCTGCACATC TGGATCCTAA TGCCTTTTGT GAAGACCTGC

CCCTCAGCTG TCTGCACATC TGGATCCTGA TGCCTTTTGT GAAGACCTGC

TCCTCAGCTG TCTACACATC TGGATTTTGA TGCCTTTCGT AAAGACCTGC

CCCTCAGCTG TCTGCATATC TGGATTATAA TGCCTTTTGT AAAGACCTGC

CCCTCAGCTG CCTGCACATC TGGATTATAA TGCCTTTCGT GAAGACCTGC

CCCTCAGCTG TCTGCACATT TGGATTGTCA TGCCATTTGT AAAGACGTGC

---------- ---------- --GATTTTAA TGCCTTTTGT AAAGACCTGC

CCCTCAGCTG TCTGCACATC TGGATTACAA TGCCTTTTGT AAAGACCTGC

TTCTCTCTTT CCTGCACATC TG-------- ---------- ----------

TCCTCTCTTT CCTGCACATC TGGATTATAA TGCCTTTTGT AAAGACCTGC

CTCTCAGCTG CCTGCACATC TGGATTGTGG TGCCTTTCGT AAAGACCTGT

TCCTCTCCTT CCTGCACATC TGGATCGTGA TGCCTTTCGT AAAGACCTGC

CTAATGGTTC TGCCTTCAGT GCAGACAATA TGGAAGAGTG TGACAGATGT

CTAATGGTTC TGCCTTCAGT GCAGACAATA TGGAAGAGTG TGACAGATGT

CTAATGGTTC TGCCTTCAGT GCAGACAATA TGGAAGAGTG TGACAGATGT

CTAATGGTTC TGCCTTCAGT GCAGACAATA TGGAAGAGTG TGACAGATGT

CTAATGGTTC TGCCTTCGGT GCAGACAATA TGGAAGAGTG TGACAGATGC

CTAATGGTTC TGCCTTCGGT GCAGACAATA TGGAAGAGTG TGACCGACGT

CTCATGGTCC TGCCTTCCGT GCAGACGATA TGGAAGAGTG TGACAGATGT

CTAATGGTCT TGCCTTCCGT GCAGACAATA TGGAAGAGTG TGACAGACGT

TTAATGGTCC TGCCTTCAGT GCAGACGATA TGGAGGAGTG TGACAGATGT

CTAATGGTCC TGCCTTCAGT GCAGACAATA TGGAAGAGTG TGACAGATGT

CTCATGGTCC TGCCTTCGGT GCAGACCATA TGGAAGAGTG TAACAGATGC

CTCATGGTCC TGCCTTCCGT ACAGACGATA TGGAAGAGCG TGACAGATGT

CTAATGGTCC TACCTTCTGT GCAGACAATA TGGAGGAGTG TGACAGATGT

CTAATGGTCC TGCCTTCGGT GCAGACAATA TGGAAAAGTG TGACAGATGT

---------- ---------- ---------- ---------- ----------

CTAATGGTCT TGCCTTCAGT GCAGACAATA TGGAAGAGCG TGACAGATGT

TTAATGGTCT TGCCATCAGT ACAGACGGTA TGGCACAGCA TTACAGATGG

CTCATGGTCC TGCCTTCCGT GCAGGCGGTG TGGAAGAGCG GGACCAGTGT

TATCATTGCT CCATTGTGTA CGAGCGTAGG ACGATGCTTC TCTTCTGTCA

TATCATTGCT CCATTGTGTA CGAGCGTAGG ACGATGCTTC TCTTCTGTCA

TTTCATTGCT CCATTGTGTA CAAGCATAGG ACGAAGCTTC TCTTCTGTCA

TTTCATTGCT CCATTGTGTA CAAGCGTAGG ACGAAGCTTC TCTTCTGTCA

TATCATTGCT CCATTGTGTA CGAGCATAGG ACGAAGCTTC TCTTCTGTCA

TATCATCGCC CCATTGTGTA CAAGCGTGGG ACGAAGCTTC TCTTCTATCA

TGTCATTGGC CCATTGTGTA CAAGTGTAGG GCGCATCTTC TCCTCTGTCA

TGTCATTGGC CCATTGTGTA CAAGTGTGGG CCGCAGCTTC TCATCGGTCA

TGTCATTGCG CCACTGTGTG CAAGCATAGG ACGAAGCTTC TCTTCCGTCG

TGTCATTGCC CCATTGTGTA CGAGCATAGG ACGCAGCTTC TCTTCTGTCA

TGTCATTGCC CCGTTGTGTT CAAGTGTAGG ACGCAGCTTC TCTTCTGTCA

GGTTATTGCC CCACTGTGTG CGAGCGTGGG ACGCAGCTTC TCTTCTGTCA

TGTCATTGCC CCTTTGTGTG CAAGCATAGG ACGCAGCTTC TCTTCTGTCA

TGCCATTGCC CCATTGTGTA CGAGCGTAGG ACGCAGCTTC TCTTCTGTCA

---------- ---------- ---------- ---------- ----------

TGTCATTGCA CCATTGTGCA CAAGCGTGGG ACGCAGCTTC TCTTCTGTCA

CTTCATTGCT CCATTGTATA AAAGCATGGG ACTCATTTTC TCTTCAATAA

CCTGGTGGCC CCGCTGTGTC AGAGCTTCGG CAAGATCTTC TCCTCGGTCC

GCCTGCAACT GAGCCAGGAT ATGCAGAGGT CGCCTCTGGA AAAGGCCAGC

GCCTGCAACT GAGCCAGGAT ATGCAGAGGT CGCCTCTGGA AAAGGCCAGC

GCCTGCAACT GAGCCAGGAC ATGCAGAGGT CGCCTCTGGA AAAGGCCAGC

GCCTGCAACT GAGCCAGGAC ATGCAGAGGT CGCCTCTGGA AAAGGCCAGC

GCCTGCAACT GAGCCAGGAC ATGCAGAGGT CGCCTCTGGA AAAGGCCAGC

GCCTGCGACT GAGCCAAGAC ATGCAGAGGT CGCCTCTGGA AAAGGCCAGC

GCATGCAACT GAGCCATGAC ATGCAGAAGT CGCCTCTGGA GAAAGCCAGC

GCATGCAACT GAGCCACGAC ATGCAGAAGT CGCCTTTGGA GAAAGCCAGC

GCTTGCAGCT GAGTCACGAC ATGCAGAAGT CGCCTCTGGA GAAGGCCGGC

GCCTGCAACT GAGCCACGAT ATGCAGAGGT CGCCTCTGGA AAAGGCCAGC

GCTTGCAAGT GAGTCACGAC ATGCAGAGGT CGCCCCTGGA AAAGGCCAGC

GCATGCAGCT GAGCCGCGAC ATGCAGAGGT CGCCTCTGGA AAAGGCCAGC

ATATGCAGCT GAGCCATGAC ATGCAGAGGT CGCCCCTGGA AAAGGCCGGC

GCCTGCAACT GAGCCACGAC ATGCAGAGGT CGCCTCTGGA AAAAGCCAGC

---------- ---------- ATGCAGAAGT CGCCTTTGGA AAGGGCCAGC

GCCTGCAGCT GAGCCAGGAC ---------- ---------- ----------

GCCTGAGACT AGGTCCTGAG ATGCAGAGAT CACCCCTGGA AAAGGCAAAT

GGCTGAGACT GGGCCCCAGT ATGCAGAGAT CACCGCTGGA GCGAGCCAAC

GTTGTCTCCA AACTTTTTTT CAGCTGGACC AGACCAATTT TGAGGAAAGG

GTTGTCTCCA AACTTTTTTT CAGCTGGACC AGACCAATTT TGAGGAAAGG

GTTGTCTCCA AACTTTTTTT CAGCTGGACC AGACCAATTT TGAGGAAAGG

GTTGTCTCCA AACTTTTTTT CAGCTGGACC AGACCAATTT TGAGGAAAGG

GTTGTCTCCA AACTTTTTTT CAGCTGGACC AGACCAATTT TGAAGAAAGG

GTCTTCTCCA AACTTTTTTT CAGCTGGACC AGACCAATTT TGAGGAAAGG

TTTATCTCCA AACTCTTCTT CAGCTGGACC ACACCAATTT TGAGAAAAGG

TTTATCTCCA AACTCTTCTT CAGCTGGACC ACACCAATTT TGAGGAAAGG

GTCCTCTCCA AACTTTTTTT CAGCTGGACT AGACCGATTT TGAGAAAAGG

GTCGTCTCCA AACTTTTTTT CAGCTGGACC AGACCAATTT TGAAGAAAGG

GTCCTCTCCA AACTTTTCTT CAGCTGGACC AGACCAATTT TGATAAAAGG

GTCATCTCCA AACTTTTTTT CAGCTGGACC ATACCAATTT TGAAGAAAGG

GTCATCTCCA AACTTTTTTT CAG------- ---------- ----------

GTCATCTCCA AACTTTTCTT CAGCTGGACC AGACCAATTT TGAGGAAAGG

GTCATCTCCA AACTTTTCTT CAGCTGGCCC GGACCAATTT TGAGGAAAGG

---------- ---------- ---CTGGACC AGACCAATTT TGCGCAAAGG

CTCCTCTCGA AACTTTTCTT CAGTTGGACT AGACCAATTT TGAGTAAAGG

CTCTTCTCCA AACTTTTCTT CAGCTGGACG AAACCGATTT TGAAGAAAGG

ATACAGACAG CGCCTGGAAT TGTCAGACAT ATACCAAATC CCTTCTGTTG

ATACAGACAG CGCCTGGAAT TGTCAGACAT ATACCAAATC CCTTCTGTTG

ATACAGACAG CGCCTGGAAT TGTCAGATAT ATACCAAATC CCTTCTGCTG

ATACAGACAG CGCCTGGAAT TGTCAGATAT ATACCAAATC CCTTCTGCTG

ATACAGACAG CGCCTGGAAT TGTCAGACAT ATACCAAATC CCTTCTGCTG

GTACAGACAG CGCCTGGAAT TGTCAGACAT TTACCAGATT CCTTCTGCTG

GTACAGACAC CACTTGGAGC TGTCAGACAT ATACCAAGCC CCTTCTTCTG

GTACAGACAC CACTTGGAGT TGTCAGACAT ATACCAAGCC CCTTCTGCTG

ATACAGACAA CGCCTGGAAT TGTCAGACAT TTACCAAATC CCTTCTGCTG

ATACAGACAG CGCTTGGAAT TGTCAGACAT ATACCATATC TCTTCTTCTG

CTATAGACAA CGCCTGGAAT TATCAGACAT ATACCAAGTC CCTTCTACTG

GTACAGGCAG CGCCTGGAGT TGTCGGACAT ATACCAAATC TCTTCCGCTG

---------- ---------- ---------- ---------- ----------

GTACAGACAG CGCCTGGAAT TGTCAGACAT ATACCAAATA CCTTCTGCTG

TTACAGACAG CACCTGAAAT TATCAGACAT ATACCAAATC CCATCTGTTG

GTACAGGCAG CGCCTGGAAC TATCAGACAT ATACCAAATT CCATCTGTGG

TTTCAGAAAA CGCTTGGAAT TGTCAGACAT ATATCAAATT CCCTCATCTA

ATATAGACAA CATCTGGAAT TGTCCGACAT ATATCAAATT CCCACCGCTG

ATTCTGCTGA CAATCTATCT GAAAAATTGG AAAGAGAATG GGATAGAGAG

ATTCTGCTGA CAATCTATCT GAAAAATTGG AAAGAGAATG GGATAGAGAG

ATTCTGCTGA CAATCTATCT GAAAAATTGG AAAGAGAATG GGATAGAGAG

ATTCTGCTGA CAATCTATCT GAAAAATTGG AAAGAGAATG GGATAGAGAG

ATTCTGCTGA CAATCTATCT GAAAAATTGG AAAGAGAATG GGATAGAGAG

ATTCTGCTGA CAATCTATCT GAAAAATTGG AAAGAGAATG GGATAGAGAG

ATTCAGCTGA CCACCTGTCT GAAAAGCTAG AAAGAGAATG GGACAGGGAA

ATTCAGCTGA CCACTTGTCT GAAAAACTAG AAAGAGAATG GGACAGAGAA

ATTCTGCAGA CAACCTGTCT GAGAAGCTGG AGAGAGAATG GGATAGAGAG

ATTCTGCTGA CAATCTATCT GAAAAATTGG AAAGAGAATG GGACAGAGAA

ATTCTGCTGA TCATCTGTCT GAAAAATTGG AAAGAGAATG GGACAGAGAG

ATTCTGCTGA CAATCTCTCA GAAAAATTGG AAAGAGAATG GGACAGAGAG

---------- ---------- ---------- ----AGAATG GGACAGAGAG

ATTCTGCTGA CAATCTATCT GAAAAATTGG AAAGAGAATG GGACAGAGAG

ATTCTGCTGA CAATCTCTCT GAAAAACTGG AAAGAGAATG GGACAGAGAG

ATTCTGCTGA CAATCTCTCT GAAAAACTGG AAAGAGAATG GGACAGAGAG

ACTCTGCAGA CAATCTATCT GAAAAGTTAG AAAGGGAATG GGACAGAGAG

ATTCAGCTGA CAACCTATCT GAAAAACTAG AAAGGGAATG GGACAGAGAG

CTGGCTTCAA AGAAAAATCC TAAACTCATT AATGCCCTTC GGCGATGTTT

CTGGCTTCAA AGAAAAATCC TAAACTCATT AACGCCCTTC GGCGATGTTT

CTGGCTTCAA AGAAAAATCC CAAACTCATT AATGCCCTTC GGCGATGCTT

CTGGCTTCAA AGAAAAATCC CAAACTCATT AATGCCCTTC GGCGATGCTT

CTGGCTTCAA AGAAAAACCC CAAACTCATT AATGCCCTTC GGCGATGCTT

CTGGCTTCAA AGAAAAATCC CAAACTCATT AATGCCCTTC GGCGATGCTT

CAAGCTTCCA AAAAGAAGCC CCAGCTTATC CACGCCCTCC GGCGATGCTT

CAAGCTTCAA AAAAGAATCC CCAGCTTATC CACGCCCTTC GGCGATGCTT

CTGGCTTCAA AGAAAAAACC CAAGCTCATT AATGCCCTTC GGAGGTGCTT

CTGGCTTCAA AGAAAAATCC CAAACTCATA AATGCCCTTC GACGATGCTT

CTGGCATCAA AGAAGAACCC CAAACTTATT AATGCCCTTC GGCGATGCTT

CTGGCATCCA AGAAAAATCC GAAACTCATT AATGCCCTTC AGCGATGCTT

CTGGCTTCCA AAAAAAATCC CAAACTAATT AATGCCCTTC GACGATGCTT

CTGGCTTCAA AGAAAAATCC CAAACTCATT AATGCTCTTC GACGATGCTT

CTGGCTTCCA AGAAAAATCC CAAACTCATT AATGCCCTTC GGCGATGCTT

CTGGCTTCAA AGAAAAATCC CACACTCATT AATGCCCTTC GACGATGTTT

CTGGCATCAA AGAAAAAACC CAAACTCATT AATGCCATTA GACGATGTTT

CTGGCTTCCA AGAAGAACCC CAAACTCATT AATGCCCTTC GACGATGCTT

TTTCTGGAGA TTTATGTTCT ATGGAATCTT TTTATATTTA GGGGAAGTCA

TTTCTGGAGA TTTATGTTCT ATGGAATCTT TTTATATTTA GGGGAAGTCA

TTTCTGGAGA TTTATGTTCT ATGGAATCTT GTTATATTTA GGGGAAGTCA

TTTCTGGAGA TTTATGTTCT ATGGAATCTT GTTATATTTA GGGGAAGTCA

TTTCTGGAGA TTTACATTCT ATGGAATCTT GTTATATTTA GGGGAAGTCA

TTTCTGGAGA TTTATGTTCT ATGGAATCTT ATTGTATTTA GGGGAGGTCA

TGTCTGGAGA TTCGTCTTCT ACGGAGTCTT GTTATACCTC GGGGAAGTCA

TTTCTGGAGA TTCCTCTTCT ATGGAATTTT GCTATACCTA GGGGAAGTCA

TTTCTGGAGA TTTATGTTCT ATGGAATCTT ATTATATTTA GGGGAAGTCA

TTTCTGGAGA TTTATGTTCT ATGGAATCAT ATTATATTTA GGGGAAGTCA

TTTCTGGAGA TTTGCGTTCT ATGGAATCTT ATTATATTTA GGGGAAGTCA

TTTCTGGAGA TTTACGTTCT ATGGAATCTT ACTATATTTA GGGGAAGTCA

TTTCTGGAAA TTTATGTTCT ATGGAATCTT ATTATATTTA GGGGAAGTGA

TTTCTGGAGA TTTACGTTCT ATGGGATTAT ATTGTATTTA GGGGAAGTCA

TTTCTGGAGA TTTGTGTTCT ATGGAATCTT GTTATATTTA GGGGAAGTCA

TTTCGGGAGA TTTATGTTCT ATGGAATCTT GCTCTATTTA GGGGAGGTGA

TTTCTGGAGA TTTGTGTTTC ATGGAATCAT ATTATACTTA GGGGAAGTAA

CTTCTGGAGA TTCATGTTCT ATGGACTCTT GCTGTATTTA GGGGAGGTGA

CCAAAGCAGT ACAGCCTCTC TTACTGGGAA GAATCATAGC TTCCTATGAC

CCAAAGCAGT ACAGCCTCTC TTACTGGGAA GAATCATAGC TTCCTATGAC

CCAAAGCAGT ACAGCCTCTC TTACTGGGAA GAATCATAGC TTCCTATGAC

CCAAAGCAGT ACAGCCTCTC TTACTGGGAA GAATCATAGC TTCCTATGAC

CCAAAGCAGT ACAACCTCTC TTACTAGGAA GAATCATAGC TTCCTATGAC

CCAAAGCAGT ACAGCCTCTC TTACTGGGGA GAATCATAGC TTCCTACGAT

CCAAGGCTGT CCAGCCTGTC TTGCTAGGAA GAATCATAGC ATCCTATGAT

CCAAGGCTGT CCAGCCTGTC TTGCTAGGAA GAATCATAGC ATCCTATGAT

CCAAAGCCGT ACAGCCTCTC CTATTGGGGC GAATCATAGC TTCCTATGAT

CCAAAGCAGT CCAGCCTCTC TTACTGGGAA GAATCATAGC TTCCTATGAC

CCAAAGCAGT CCAGCCTCTC TTACTTGGAA GAATCATAGC TTCCTATGAT

CCAAAGCAAT CCAGCCTCTC CTACTGGGAA GAATCATAGC TTCCTACGAT

CCAAAGCAGT CCAGCCACTC TTACTCGGAA GAATCATAGC TTCCTACGAT

CCAAAGCTGT GCAGCCTCTC TTACTGGGAA GAATTATAGC TTCCTATGAT

CCAAGGCAGT CCAGCCACTC TTACTGGGAA GAATCATAGC TTCCTATGAT

CCAAAGCAGT ACAGCCACTC CTACTGGGAA GAATTATAGC TTCCTATGAT

CCAAAGCAGT ACAGCCTCTA TTACTGGGAA GAATCATAGC CTCCTATGAT

CCAAGGCAGT GCAGCCTCTC CTCCTGGGGC GAATCATTGC CTCCTATGAC

CCGGATAACA AGGAGGAACG CTCTATCGCG ATTTATCTAG GCATAGGCTT

CCGGATAACA AGGAGGAACG CTCTATCGCG ATTTATCTAG GCATAGGCTT

CCGGATAACA AGGAGGAACG CTCTATTGCG ATTTATCTAG GCATAGGCTT

CCGGATAACA AGGAGGAACG CTCTATTGCG ATTTATCTAG GCATAGGCTT

CCAGATAACA AGACAGAACG CTCTATTGCG ATTTACCTGG GCATAGGCTT

CCAGACAACA AGGTGGAGCG TTCCATAGCG ATCTATCTAG GCATAGGCTT

CCAGACAACA CGGAGGAGCG TTCCATTGCC ATTTACCTAG GCATAGGCTT

CCAGAAAACA AGGTGGAACG TTCCATTGCC ATTTACCTTG GCATAGGCTT

CCAGACAACA AGGTGGAGCG CTCCATTGCC ATTTACCTGG GCATAGGTCT

CCAGATAACA AGGTGGAACG CTCCATTGCC ATTTACTTAG GCATAGGCCT

CCAGATAACA AGCAGGAACG CTCCATTGCA ATCTACCTAG CCATTGGCTT

CCAGATAACA AGATGGAACG GTCCATTGCG ATTTACCTAG GCATAGGCTT

CCAGATAACA CTGCGGAACG CTCTATTGCC ATTTATCTAG GCATAGGCTT

CCAGATAACA AGGTGGAACG CTCCATTGCA ATTTACCTTG CCGTTGGCTT

CCAGATAACA AGGTGGAACG CTCCATTGCG ATTTACTTGG CCATAGGCCT

CCAGATAACC AGGCAGAACG CTCCATTGCC TTGTACCTGG CCGCAGGCTT

CCTGACAACG AAGTAGAACG TGCCATTGCA ATATATCTAG GCATAGGCCT

CCAGACAACG CTCACGAACG CTCTATCGCG TACTACCTGG GCATCGGCCT

ATGCCTTCTC TTTATTGTGA GGACACTGCT CCTACACCCA GCCATTTTTG

ATGCCTTCTC TTTA------ ---------- ---------- ----------

ATGCCTTCTC TTTATCGTGA GGACACTGCT CTTACACCCA GCCATTTTTG

ATGCCTTCTC TTTATCGTGA GGACACTGCT CTTACACCCA GCCATTTTTG

ATGCCTTCTC TTTATTGTGA GGACACTGCT TCTACACCCA GCCATTTTTG

ATGCCTTCTC TTTATCGTGA GGACACTGCT TCTACACCCA GCCATTTTTG

ATGCCTCCTC TTCATTGTCA GGACACTGCT TCTGCACCCA GCTATTTTTG

ATGCCTTCTC TTCATTGTCA GGACACTGCT TCTTCACCCA GCTATTTTTG

GTGCTTACTC TTTGTTGTGA GGACCCTACT CCTACACCCA GCAATTTTTG

ATGCCTTCTC TTTATCGTGA GGACACTGCT CCTGCACCCA GCCATTTTTG

ATGCCTTCTC TTTATCATGA GGCCGCTGCT CCTGCACCCA GCCATTTTTG

ATGCCTGCTC TTTATCATGA GGACACTGCT CTTGCACCCC GCCATTTTTG

ATGCCTTCTC TTTATCGTGA GAACACTGCT CCTGCATCCA GCCATTTTTG

GTGCCTTCTG TTCGTTGTGA GGACGCTGCT CCTGCACCCA GCCATTTTTG

GTGCCTTCTC TTCATCGTGA GGACGCTCCT CCTACACCCA GCCATTTTTG

GTGCCTTCTC TTCATTGTGA GGACACTGCT CCTGCACCCA GCCATTTTTG

GTGCCTTCTC TTTATAGTGA GGACACTTCT TCTACACCCT GCCATCTTTG

GTGTCTTCTC TTTATTGTGC GCACCCTGTT GCTGCACCCT GCCGTTTTTG

GCCTTCATCA CATTGGAATG CAGATGAGAA TAGCTATGTT TAGTTTGATT

---------- ---------- ---------- ---------- ----------

GCCTTCATCA CATTGGAATG CAGATGAGAA TAGCTATGTT TAGTTTGATT

GCCTTCATCA CATTGGAATG CAGATGAGAA TAGCTATGTT TAGTTTGATT

GCCTTCATCA CATTGGAATG CAGATGAGAA TAGCTATGTT TAGTTTGATT

GCCTTCATCA CATTGGAATG CAGATGAGAA TAGCTATGTT TAGTTTGATT

GCCTTCATCA CATTGGAATG CAGATGAGAA TAGCCATGTT TAGCTTGATT

GCCTTCATCG CATTGGAATG CAGATGAGAA CAGCTATGTT TAGCTTGATT

GTCTTCATCA TATTGGAATG CAGATGAGAA TAGCCATGTT CAGTTTGATT

GTCTCCATCA CATTGGAATG CAGATGAGAA TAGCTATGTT TAGTTTGATT

GCCTTCATCA CATTGGAATG CAGATCAGAA TAGCTATGTT TAGTTTGATT

GCCTTCATCA CATTGGGATG CAAATGAGGA TAGCTTTGTT TAGTTTGATT

GCCTTCATCA CATTGGAATG AAAATGAGAA TAGCTATGTT CAGTTTAATT

GACTTCATCA CATTGGAATG CAGATGAGAA TAGCGATGTT TAGCTTGATT

GCCTCCATCA CATGGGAATG CAGATGAGAA TAGCTATGTT TAGTTTGATT

GCCTCCATCA TATTGGAATG CAGATGAGAA TAGCTATGTT TAGTTTGATT

GCCTTCATCA CATGGGAATG CAGATGAGAA TAGCCCTGTT TAGTCTGATT

GCCTCCATCA CATCGGAATG CAGATGAGGA TCGCCCTGTT CAGTCTGATT

TATAAGAAGA CTTTAAAGCT GTCAAGCCGT GTTCTAGATA AAATAAGTAT

---------A CTTTAAAGCT GTCAAGCCGT GTTCTAGATA AAATAAGTAT

TATAAGAAGA CTTTAAAGCT GTCAAGCCGT GTTCTAGATA AAATAAGTAT

TATAAGAAGA CTTTAAAGCT GTCAAGCCGT GTTCTAGATA AAATAAGTAT

TATAAGAAGA CTTTAAAGCT GTCGAGCCGT GTTCTAGATA AAATAAGTAT

TATAAGAAGA TTTTAAAGCT GTCAAGCCGT GTTCTAGATA AAATAAGTAT

TATAAGAAGA CTTTAAAGTT GTCAAGCCGT GTTCTTGATA AAATAAGTAT

TATAAGAAGA CTTTAAAGTT GTCAAGCCGC GTTCTTGATA AAATAAGTAT

TATAAGAAGA CTTTAAAGCT GTCAAGCCGT GTTCTAGATA AAATAAGTAT

TATAAGAAGA CTTTGAAGCT ATCAAGCCGT GTTCTGGATA AAATAAGTAT

TATAAGAAGA CTTTAAAGCT GTCAAGCCGT GTTCTGGATA AAATAAGTAT

TATAAGAAGA CTTTAAAGCT GTCAAGCCGT GTTCTAGATA AAATAAGTAT

TATAAGAAG- ---------- ---------- ---------- ----------

TATAAGAAGA CCTTAAAACT GTCAAGCCGT GTTTTAGATA AAATAAGTAT

TATAAGAAGA CTTTAAAACT GTCCAGCCGT GTTCTTGATA AAATAAGTAT

TATAAGAAGA CTTTAAAACT TTCGAGCCGG GTTCTAGATA AAATAAGTAT

TACAAAAAGA CCTTAAAACT GTCAAGCCGT GTTCTAGATA AAATAAGCAC

TACAAAAAGA CTTTAAAATT GTCAAGCCGA GTCCTGGATA AAATAAGCAC

TGGACAACTT GTTAGTCTCC TTTCCAACAA CCTGAACAAA TTTGATGAAG

TGGACAACTT G-TAGTCTCC TTTCCAACAA CC-GGACAGA ATTGATG-AG

TGGACAACTT GTTAGTCTCC TTTCCAACAA CCTGAACAAA TTTGATGAAG

TGGACAACTT GTTAGTCTCC TTTCCAACAA CCTGAACAAA TTTGATGAAG

TGGACAACTT GTTAGTCTCC TTTCCAACAA CCTGAACAAA TTTGATGAAG

TGGACAGCTT GTAAGTCTCC TTTCCAACAA CCTGAACAAA TTTGATGAAG

TGGACAACTT ATTAGTCTTC TTTCCAACAA CCTGAACAAA TTTGATGAAG

TGGACAACTT GTTAGTCTTC TTTCCAACAA CCTGAACAAA TTTGATGAAG

TGGGCAACTT ATTAGTCTCC TTTCCAACAA CCTGAACAAA TTTGATGAAG

TGGACAACTT GTTAGTCTCC TTTCCAACAA CCTGAACAAA TTTGATGAAG

TGGACAACTT GTTAGTCTCC TTTCCAACAA CCTGAACAAA TTCGATGAAG

TGGACAACTT GTTAGTCTCC TTTCCAACAA TCTGAACAAA TTTGATGAAG

---------- ---------- ---------- ---------- ---------G

TGGACAACTT GTTAGTCTCC TTTCCAACAA CCTGAACAAA TTTGATGAAG

TGGACAACTT GTCAGTCTCC TTTCCAACAA CCTGAACAAA TTTGATGAAG

TGGACAACTT GTTAGTCTCC TTTCCAACAA CCTGAACAAA TTTGATGAA-

TGGACAGCTT ATCAGTCTCC TTTCCAACAA TCTGAACAAG TTTGATGAAG

CGGACAGCTC GTTAGCCTTC TTTCAAACAA CTTGAACAAA TTTGATGAAG

GACTTGCATT GGCACATTTC GTGTGGATCG CTCCTTTGCA AGTGGCACTC

GACTTGCATT GGCACATTTC GTGTGGATCG CTCCTTTGCA AGTGGCACTC

GACTTGCATT GGCACATTTC GTGTGGATCG TTCCTCTGCA AGTGGCACTC

GACTTGCATT GGCACATTTC GTGTGGATCG TTCCTCTGCA AGTGGCACTC

GACTTGCATT GGCACATTTC GTGTGGATCG CTCCTTTGCA AGTGGCACTC

GACTTGCTTT GGCTCATTTC GTGTGGATCG CCCCTTTGCA AGTAACGCTT

GACTTGCCTT GGCACATTTT ATATGGATTG CTCCTTTACA AGTGGTTCTT

GACTTGCCTT GGCACATTTT ATATGGATTG CTCCTTTACA AGTGACTCTT

GGCTTGCGCT GGCCCATTTC GTGTGGATTT CACCTCTGCA AGTGACTCTG

GACTCGCACT GGCACATTTT GTGTGGATCG CTCCTCTGCA AGTGACGCTG

GACTTGCACT GGCGCATTTC GTGTGGATTG CTCCTCTACA AGTGACGCTC

GACTTGCGTT GGCCCATTTC GTGTGGATTG CTCCTTTACA AGTGATGCTG

GACTTGCCTT GGCACATTTT GTGTGGATTG CTCCTTTACA AGTGACACTT

GCCTAGCATT AGCACATTTC GTGTGGATCG CTCCTTTACA AGTGACGCTG

GACTTGCACT GGCACATTTT GTGTGGATTG CTCCTTTACA AGTGACACTT

---------- ---------- ---------- ---------- -----CGCTC

GGCTGGCCCT GGCACATTTT GTCTGGATTG TACCTTTACA AGTGGTGCTC

GTCTGGCGCT GGCCCATTTT GTGTGGATCG CGCCTCTCCA GGTGATGCTG

CTCATGGGGC TAATCTGGGA GTTGTTACAG GCGTCTGCCT TCTGTGGACT

CTCATGGGGC TAATCTGGGA GTTGTTACAG GCATCTGCCT TCTGTGGACT

CTCATGGGGC TAATCTGGGA GTTGTTACAG GCCTCTGCCT TCTGTGGACT

CTCATGGGGC TAATCTGGGA GTTGTTACAG GCCTCTGCCT TCTGTGGACT

CTCATGGGGC TAATCTGGGA GTTGTTACAG GCCTCTGCCT TCTGTGGACT

CTGATGGGGC TGTTGTGGGA ATTGCTACAG GCCTCTGCCT TCTGCGGACT

CTGATGGGGC TTCTCTGGGA CTTGTTACAG TTCTCAGCCT TCTGTGGCCT

CTGATGGGGC TTCTCTGGGA CTTGTTACAG TTCTCAGCCT TCTGTGGCCT

CTGATGGGGC TGCTCTGGGA ACTACTACAG GCCTCTGCCT TCTGCGGCCT

CTCATGGGGC TGCTCTGGGA GTTGTTGCAG GCCTTCACCT TCTGTGGGCT

CTGATGGGGC TGCTCTGGGA TTTGTTACAA GCCTCTGCCT TCTGTGGTCT

CTGATGGGGC TGCTTTGGGA GCTGTTGCAG GCCTCCGCCT TCTGCGGGCT

CTAATGGGGC TACTCTGGGA GTTGTTACAG GCCTCTGCCT TCTGTGGACT

CTGATGGGAC TGCTGTGGGA TTTGCTCCAG GCCTCTGCCT TCAGTGGACT

CTGATGGGGC TGATCTGGGA CCTGCTCCAG GCCTCTGCCT TCTGTGGACT

TTGATGGGGC TGCTCTGGGA GCTGCTCCAG GCCTCGGCCT TCTGCGGACT

CTGATGGGAC TTCTTTGGGA TTTGCTACAA GCCTCAGCAT TTTGTGGACT

CTGATGGGGC TGCTGTGGGA TCTGTTGCAG GCCTCCGCCT TCTGCGGCTT

TGGTTTCCTG ATAGTCCTTG CCCTTTTTCA GGCTGGGCTA GGGAGAATGA

TGGTTTCCTG ATAGTCCTTG CCCTTTTTCA GGCTGGGCTA GGGAGAATGA

TGGTTTCCTG ATAGTCCTTG CCCTTTTTCA GGCTGGGCTA GGGAGAATGA

TGGTTTCCTG ATAGTCCTTG CCCTTTTTCA GGCTGGGCTA GGGAGAATGA

TGGTTTCCTG ATAGTTCTTG CCCTTTTTCA GGCTGGGCTG GGGAGAATGA

TGGGTTCCTC ATAGTTCTTG CCCTTGTTCA AGCTGGATTA GGGAGAATGA

TGGTTTACTG ATAGTCTTGG TTATTTTTCA AGCTATCTTA GGGAAGATGA

TGGTTTACTG ATAATCCTGG TTATTTTTCA AGCTATCCTA GGGAAGATGA

TGCTTTCCTA ATAGTCCTTG CCCTTGTTCA AGCCGGGCTA GGGAGAATGA

TGCTTTCCTC ATAGTCCTCG CCCTTCTTCA AGCCGGATTA GGGAAAATGA

TGCTTTCCTC ATAGTCCTCG CCCTTGTCCA AGCTGGTTTG GGAAGAATGA

CGCTTTCCTC ATAGTCCTCG CCCTGCTTCA AGCCGGGCTA GGGAGAATGA

TGCTGTTCTG ATAGTTCTAG CCCTTTTCCA ATCTGGCTTA GGAAGAATGA

TGGTGTCCTG ATCATCCTCG CCTGTTTTCA AGCTGGGTTT GGGAGAATGA

TGCTTTCCTG ATAATTGTCG CCCTTGGTCA AGCTGGGTTA GGAAGAATGA

TGCCTTCCTG GTGGTCCTGG CCCTTTTCCA AGCCGGCCTC GGGAGGATGA

TGCTTTCCTA ATAGTACTTG CTCTGTTCCA AGCCTGGTTA GGACAAATGA

GGCAGTCCTC GTCGTCCTTG TCCTCTTCCA AGCCTGGCTG GGGCACAGGA

TGATGAAGTA CAGAGATCAG AGAGCTGGGA AGATCAGTGA AAGACTTGTG

TGATGAAGTA CAGAGATCAG AGAGCTGGGA AGATCAGTGA AAGACTTGTG

TGATGAAGTA CAGAGATCAG AGAGCTGGGA AGATCAATGA AAGACTTGTG

TGATGAAGTA CAGAGATCAG AGAGCTGGGA AGATCAATGA AAGACTTGTG

TGATGAAGTA CAGAGATCAA AGAGCTGGAA AGATCAATGA AAGACTTGTG

TGATGAAGTA CAGAGATCAG AGAGCAGGAA AGATCAATGA AAGACTTGTG

TGGTGAAGTA CAGAGATAAA AGAGCTGCAA AGATCAATGA AAGACTTGTG

TGGTGAAGTA CAGAGATCAG AGAGCTGCAA AGATCAATGA AAGACTCGTG

TGATGAAGTA CAGGGATCAG AGAGCTGGAA AGATCAATGA GAGACTAGTG

TGATGAAGTA CAGAGATCAG AGAGCTGGAA AGATCAATGA AAGACTGGTG

TAATGAAGTA CAGAGATCAG AGAGCTGGAA AGATCAATGA AAGACTCGTG

TGATGAAGTA CAGAGATCAG AGAGCTGGAA AGATCAATGA AAGACTTGTG

TGATGAAATA CAGAGATAAG AGAGCTGGAA AGATCAATGA AAGGCTTGTA

TGATGAAATA CAGAGATCAG AGAGCTGGAA AGATCAATGA AAGACTTGTG

TGATGAAGTA CAGAGATAAG AGAGCTGGAA AGATCAATGA AAGACTTGTG

TGATGAAGTA CAG------- ---------- ---------- ----------

TGATGAAATA CAGAGAAAGG AGAGCAGGAA AGATAAATGA AAGACTTGTG

TGATGAAGTA CAGAGACAGA AGAGCCGGAA AGATAAATGA GAGACTTGTG

ATTACCTCAG AAATGATTGA AAATATCCAA TCTGTTAAGG CATACTGCTG

ATTACCTCAG AAATGATTGA AAATATCCAA TCTGTTAAGG CATACTGCTG

ATTACCTCAG AAATGATTGA AAATATCCAA TCTGTGAAGG CATACTGCTG

ATTACCTCAG AAATGATTGA AAATATCCAA TCTGTGAAGG CATACTGCTG

ATTACATCAG AAATGATTGA AAATATCCAA TCTGTGAAGG CATACTGCTG

ATCACCTCAG AAATGATTGA AAATATCCAG TCTGTGAAGG CATACTGCTG

ATCACATCAG AAGTTATTGA CAATATCTAT TCTGTTAAGG CATATTGTTG

ATCACATCAG AAATTATTGA TAATATCTAT TCTGTTAAGG CATATTGTTG

ATCACTTCAG AAATGATTGA AAACATCCAA TCTGTTAAAG CATACTGCTG

ATAACGTCAG AAATGATTGA AAATATCCAA TCAGTTAAGG CATACTGCTG

ATCACCTCAG AAGTGATTGA AAATATCCAA TCTGTTAAGG CATACTGCTG

ATCACCTCAG AAATGATTGA AAATATCCAA TCTGTTAAGG CATACTGCTG

ATCACCTCGG AAATGATTGA AAATATACAG TCTGTTAAGG CATACTGTTG

ATCACCTCAG AAATGATTGA AAATATTCAG TCTGTCAAGG CATACTGCTG

ATCACCTCTG AAATGATTGA AAATATCCAA TCTGTGAAGG CATACTGCTG

---------- ---------- ---------- ---------- ----------

ATAACTTCAG AAATGATTGA CAATATACAG TCTGTTAAGG CATATTGCTG

ATCACCGCAG AAATTATTGA GAATATCCAA TCCGTTAAAG CCTACTGTTG

GGAAGAAGCA ATGGAAAAAA TGATTGAAAA CTTAAGACAA ACAGAACTGA

GGAAGAAGCA ATGGAAAAAA TGATTGAAAA CCTAAGACAA ACAGAACTGA

GGAAGAAGCA ATGGAAAAAA TGATTGAAAA CCTAAGACAA ACAGAACTGA

GGAAGAAGCA ATGGAAAAAA TGATTGAAAA CCTAAGACAA ACAGAACTGA

GGAAGAAGCA ATGGAAAAAA TGATTGAAAA CCTAAGACAA ACAGAACTGA

GGAGGAAGCA ATGGAAAAAA TGATTGAAAA CCTAAGACAA ACAGAACTGA

GGAATCAGCG ATGGAGAAGA TAATTGAAAG CTTGAGAGAG GAGGAACTGA

GGAATCAGCG ATGGAGAAAA TGATTGAAAA CTTGAGAGAG GTGGAGCTGA

GGAGGAAGCG ATGGAAAAAA TGATTGAAAA CCTAAGACAA ACAGAACTGA

GGAAGAAGCA ATGGAAAAAA TCATTGAAAA CCTAAGACAA ACAGAACTGA

GGAGGAAGCA ATGGAAAAAA TAATTGAAAA CATAAGACAA ACAGAGCTGA

GGAGGAAGCA ATGGAAAAAA TGATTGAAAA CCTAAGACAA ACAGAACTGA

GGAGGAAGCA ATGGAAAAAA TAATTGAAAA CCTCAGACAA ACAGAACTGA

GGAGGAGGCA CTGGAAAAAA TGATTGAAAA TTTCAGACAG TCGGAACTGA

GGAGGAAGCA ATGGAAAAAA TGATCGAAAA CCTAAGACAA ATAGAGCTGA

---------- ---------- ---------- ---------- ----------

GGAGGAGGCA ATGGAAAATA TGATTGAAAA TATAAGAGAA ACTGAACTGA

GGAGGAAGCA ATGGAAAACA TGATTGAAAG CCTCAGAGAA ACGGAACTGA

AACTGACTCG GAAGGCAGCC TATGTGAGAT ACTTCAATAG CTCAGCCTTC

AACTGACTCG GAAGGCAGCC TATGTGAGAT ACTTCAATAG CTCAGCCTTC

AACTAACTCG GAAGGCAGCC TATGTGAGAT ACTTCAATAG CTCAGCCTTC

AACTAACTCG GAAGGCAGCC TATGTGAGAT ACTTCAATAG CTCAGCCTTC

AACTGACTCG GAAGGCAGCC TATGTGAGAT ACTTCAATAG CTCAGCCTTC

AGCTGACCCG GAAGGCAGCC TATGTGAGAT ACTTCAATAG CTCAGCCTTC

AAATGACCCG GAGGTCAGCC TATATGAGGT TCTTCACTAG CTCTGCCTTC

AAATGACCCG GAAGGCGGCC TATATGAGGT TCTTCACTAG CTCTGCCTTC

AACTGACCCG GAAAGCAGCC TACGTGAGAT ACTTCAACAG CTCAGCCTTC

AACTCACCCG GAAGGCAGCC TATGTGAGAT ACCTCAATAG CTCAGCCTTC

AACTGACCCG GAAGGCAGCC CATGTGAGAT ACTTCAATAG CTCAGCCTTC

GACTGACCCG GAAGGCTGCC TATGTGAGAT ACGTCAACAG CTCAGCATTC

AACTGACCCG GAAGGCAGCC TATGTGAGAT ACTTCAATAG CTCAGCTTTC

GACTGACTCG GAAGGCAGCC TATGTGAGAT ACTTCAACAG CTCAGCTTTC

GACTGACCCG GAAGGCAGCC TATGTGAGAT ACTTCAACAG CGCAGCCTTC

---TGACCCG GAAGGCAGCC TATGTGAGAT ACTTCAACAG CTCAGCCTTC

AATTGACCCG AAAAGCCGCA TATGTGAGAT ACTTCAATAG CTCAGCCTTC

AGCTGACCCG AAAGGCTGCT TATATGAGGT ACTTCAATAG CTCAGCCTTC

TTCTTCTCAG GGTTCTTTGT GGTGTTTTTA TCTGTGCTTC CCTATGCACT

TTCTTCTCAG GGTTCTTTGT GGTGTTTTTA TCTGTGCTTC CCTATGCACT

TTCTTCTCAG GGTTCTTTGT GGTGTTTTTA TCTGTGCTTC CCTATGCACT

TTCTTCTCAG GGTTCTTTGT GGTGTTTTTA TCTGTGCTTC CCTATGCACT

TTCTTCTCAG GGTTCTTTGT GGTGTTTTTA TCTGTGCTTC CATATGCACT

TTCTTCTCAG GGTTCTTTGT GGTGTTTTTA TCGGTCCTTC CTTATGCACT

TTCTTTTCAG GGTTTTTTGT AGTCTTTTTA TCTGTGCTTC CCTACACAGT

TTCTTTTCAG GGTTCTTTGT AGTCTTTCTA TCTGTGCTTC CCTACACAGT

TTCTTCTCAG GGTTCTTTGT GGTGTTTTTA TCTGTGCTTC CTTACGCACT

TTCTTCTCAG GGTTCTTTGT GGTATTTTTA TCTGTGCTTC CTTATGCACT

TTCTTCTCAG GGTTCTTTGT GGTGTCTTTA TCTGTGCTTC CCTATGCACT

TTCTTCTCGG GGTTCTTTGT GGTGTTTTTA TCTGTGCTTC CCTATGCACT

TTCTTCTCAG GCTTTTTCGT AGTGTTTTTG TCCGTGCTTC CCTACGCGTT

TTCTTCTCAG GGTTTTTTGT AGTGTTTTTA TCTGTGCTTC CCTATGCACT

TTCTTCTCAG GGTTCTTCGT GGTGTTTTTA TCTGTGCTGC CCTACGCAAT

TTCTTCTCAG GGTTCTTCGT GGTGTTTCTG TCTGTGCTAC CTTACGCACT

TTCTTCTCAG GTTTCTTTGT TGTATTTCTA GCTGTGCTTC CTTATGCCTT

TTCTTCTCGG GGTTCTTCGT GGTGTTCCTG TCGGTGCTAC CCTCCATGCT

AATCAAAGGA ATCATCCTCC GGAAAATATT CACCACCATC TCATTCTGCA

AATCAAAGGA ATCATCCTCC GGAAAATATT CACCACCATC TCATTCTGCA

AATCAAAGGA ATCGTCCTCC GAAAAATATT CACAACCATA TCATTCTGCA

AATCAAAGGA ATCGTCCTCC GAAAAATATT CACAACCATA TCATTCTGCA

AATCAAAGGA ATCATCCTCC GAAAAATCTT TACAACCATC TCATTCTGCA

GATCAAGGGA ATAATCCTTC GGAAAATATT CACAACCATC TCATTCTGCA

CATCAATGGA ATCGTCCTCC GAAAAATATT CACAACCATT TCATTCTGCA

CATCAACGGA ATCGTCCTAC GAAAAATATT CACAACCATT TCATTCTGCA

TACCAAAGGA ATCATCCTGC GCAAAATATT CACCACCATC TCATTCTGCA

GCTCAAAGGA ATCATCCTTC GAAAAATATT CACAACCATC TCATTCTGCA

GATCAAAACA ATCATCCTTC GCAAAATATT CACAACCATC TCATTCTGCA

GATCAAAGGA ATCATCCTCC GAAAAATATT CACAACCATC TCGTTCTGCA

GCTCAAAGGC ATTATCCTTC GAAAAATATT CACCACCATC TCATTCTGCA

GATCAAAGGA ATCATCCTTC GCAAAATATT CACAACCATC TCATTCTGCA

GCTCAAAGGA ATCATCCTTC GAAAAATATT CACTACCATC TCATTCTGCA

GCTCAAGGGA ATCATCCTTC GAAAAATATT CACCACTATT TCATTCTGCA

GATGAAAGGA ATTATCCTGC GAAAAATATT TACAACCATC TCATTCTGTA

GACCAAGGGA ATTGTCCTCC GCAAGATATT CACCACCATC TCGTTCTGCA

TTGTTCTGCG CATGGCGGTC ACTCGGCAAT TTCCCTGGGC TGTACAAACA

TTGTTCTGCG CATGGCGGTC ACTCGGCAAT TTCCCTGGGC TGTACAAACA

TTGTTCTGCG CATGGCGGTC ACTCGGCAAT TTCCCTGGGC TGTACAAACA

TTGTTCTGCG CATGGCGGTC ACTCGGCAAT TTCCCTGGGC TGTACAAACA

TTGTTCTGCG CATGGCAGTC ACTCGACAAT TTCCCTGGGC TGTACAAACA

TCGTTCTGCG CATGGCTGTC ACTCGGCAGT TCCCCTGGGC TGTGCAAACA

TTGTCCTGCG TATGTCAGTC ACACGGCAAT TCCCCACTGC TGTACAGATA

TTGTCCTACG TATGTCAGTC ACACGGCAGT TCCCCACTGC CGTACAGATA

TCGTTCTGCG CATGGCCGTT ACTCGGCAGT TCCCCTGGGC TGTGCAAACA

TCGTTCTGCG CATGGCAGTT ACTCGGCAGT TCCCCTGGGC TGTACAAACT

TCGTTTTGCG CATGGCAGTC ACTCGGCAAT TTCCCTGGGC TGTACAAACA

TCGTCCTGCG CATGGCAGTC ACTCGGCAAT TCCCCTGGGC TGTGCAAACG

TTGTTCTGCG CATGACAGTC ACACGGCAGT TTCCTTGGGC TGTGCAAACA

TTGTTCTGCG CATGGCAGTC ACACGGCAAT TCCCCTGGGC TGTTCAAACA

TCGTTCTGCG CATGGCTGTC ACTCGGCAGT TCCCCTGGGC TGTGCAAACG

TCGTTCTGCG CATGTCTGTC ACTCGGCAGT TCCCCTGGGC TGTGCAAACC

TTGTTCTTCG AATGGCAGTA ACTCGGCAGT TTCCCTGGGC TGTACAAACC

TTGTACTGCG GATGGCGGTC ACCCGGCAGT TCCCCTGGGC CGTGCAGATG

TGGTATGACT CTCTTGGAGC AATAAACAAA ATACAGGATT TCTTACAAAA

TGGTATGACT CTCTTGGAGC AATAAACAAA ATACAGGATT TCTTACAAAA

TGGTATGACT CTCTTGGAGC AATAAACAAA ATACAGGATT TCTTACAAAA

TGGTATGACT CTCTTGGAGC AATAAACAAA ATACAGGATT TCTTACAAAA

TGGTATGACT CTCTTGGAGC AATAAACAAA ATACAGGATT TCTTACAAAA

TGGTATGACT CTCTTGGAGC AATAAACAAA ATACAGGACT TTTTACAAAA

TGGTATGATT CTTTGGGAAT GATAAGAAAA ATACAGGATT TCCTACAGAC

TGGTATGATT CTTTTGGAAT GATAAGAAAA ATACAGGATT TCCTGCAGAA

TGGTATGACT CGCTTGGAGC AATAAACAAA ATCCAGGATT TCTTACAAAA

TGGTATGATT CTCTGGGAGC AATAAACAAA ATACAGGATT TCTTACAAAA

TGGTATGACT CTCTTGGAGC AATAAACAAA ATACAGGATT TCTTACAAAA

TGGTATGACT CTCTTGGAGC AATAAATAAA ATACAGGAAT TCTTACAAAA

TGGTACGACT CTCTTGGAGC CATCAACAAA ATACAGGATT TCTTGCAAAA

TGGTATGACT CACTTGGAGC AATAAACAAA ATACAGGACT TCTTACAAAA

TGGTATGACT CGCTCGGAGC AATAAACAAA ATACAGGATT TCTTACAAAA

TGGTATGACT CATTTGCAGC AATAAATAAA ATACAGGATT TTTTACAAAA

TGGTATGATT CTCTTGGAGC AATAAACAAA ATACAGGATT TCTTACAAAA

TGGTACGACT CCATCGGAGC GATTTACAAG ATACAGGATT TCCTTCAAAA

GCAAGAATAT AAGACATTGG AATATAACTT AACGACTACA GAAGTAGTGA

GCAAGAATAT AAGACATTGG AATATAACTT AACGACTACA GAAGTAGTGA

GCAAGAATAT AAGACATTGG AATATAACTT AACAACTACA GAAGTAGTGA

GCAAGAATAT AAGACATTGG AATATAACTT AACAACTACA GAAGTAGTGA

GCAGGAATAT AAGACATTGG AATATAATTT AACAACTACA GAAGTAGTGA

GCAAGAGTAT AAGACCTTGG AATATAACTT AACAACTACA GAGGTAGTGA

TCAAGAATAT AAAGTGCTGG AGTATAACTT AATGTTCACA GGCCTAGTCA

ACAAGAGTAT AAAGTACTGG AGTATAACTT AATGACCACA GGCATAATCA

GCAAGAATAT AAAACACTGG AATACAACTT AACAACTACA GAGGTCGTGA

GCAAGAATAT AAGACATTGG AATATAACTT AACAACTACA GATGTAGTGA

GCAAGAGTAT AAGACGTTGG AATATAACTT AACAACTACG GAAGTAATGA

GCAAGAGCAT AAGACGTTGG AATATAACTT AACAACCACA GAGGTAGTGA

GCAAGAGTAT AAGACATTGG AGTATAACTT AACAACTACA GAAGTAGAGA

ACAAGAGTAT AAGTCATTGG AGTATAACTT AACAACTACA GATGTAGTGA

GCAAGAGTAT AAGACGTTGG AATATAACTT AACAACTACA GATGTGGTGA

GCAAGAGTAT CAGACCTTGG AATACAACTT GACTACAACA GATGTTGTGA

AGAAGAATAC AAAACATTGG AATATAACTT AACAACCACA GAAGTTATGA

AGAAGAGTAC AAGACATTGG AATACAACTT AACAACTACA GATGTTGTGA

TGGAGAATGT AACAGCCTTC TGGGAGGAGG GATTTGGGGA ATTATTTGAG

TGGAGAATGT AACAGCCTTC TGGGAGGAGG GATTTGGGGA ATTATTTGAG

TGGAGAATGT AACAGCCTTC TGGGAGGAGG GATTTGGGGA ATTATTTGAG

TGGAGAATGT AACAGCCTTC TGGGAGGAGG GATTTGGGGA ATTATTTGAG

TGGAGAATGT AACAGCCTTC TGGGAGGAGG GATTTGGGGA ATTATTTGAG

TGGAGAACGT AACAGCCTTC TGGGAGGAGG GAATTGGAGA ATTATTTGAG

TGGAGAATGT AACAGCATTT TGGGAGGAGG GATTTCAGGA ATTACTGGAG

TGGAAAATGT AACAGCATTT TGGGAGGAGG GATTTGGGGA ATTACTGGAG

TGGATAATGT AACAGCCTTC TGGGAAGAGG GATTTGGGGA ATTATTTGAG

TGGAGAATGT AACAGCCTTC TGGGAGGAGG GATTTAGTAA ATTATTTGAG

TGGAGAATGT AACAGCCTTC TGGGAGGAGG GATTTGGGGA ATTACTTGAG

TGGAGAATGT AACAGCCTTC TGGGAGGAGG GGGTTGGGGA GTTATTTGAG

TGGAGAATGC AACAGCTTAC TGGGAAGAGG GACTTGGGGG ATTATTTGAA

TGGAGACTGT AACAGCCTTC TGGGAGGAGC GATTTGGAGA ATTATTTGAG

TGGAAAATGT GACAGCCTTC TGGGAGGAGG GATTTGGGGA ATTATTTGAG

TGGAAAACGT AACAGCCTTC TGGGAAGAGG GATTTGGGGA ATTACTTGAG

TGGAAAATAT AACAGCATTC TGGGATGAGG GATTTGGAGA ATTATTTGTA

TGGAAAACGT AACAGCTTTC TGGGACGAGG GATTCGGGGA ATTGCTCGTC

AAAGCAAAAC AAAACAATAA CAATAGAAAA ACTTCTAATG GTGATGACAG

AAAGCAAAAC AAAACAATAA CAATAGAAAA ACTTCTAATG GTGATGACAG

AAAGCAAAAC AAAACAATAG CAATAGGAAA ACTTCTAATG ATAATGACAG

AAAGCAAAAC AAAACAATAG CAATAGAAAA ACTTCTAATG ATGATGACAG

AAAGCAAAAC AAAACAATAA CAATAGAAAA ACTTCTAATG ACGATAACAA

AAAGCAAAAC AAAACAATGA CAATAGAAAA ATTTCCAATG GTGATAACAG

AAAGTACAAC TAAACAATGA TGACAGAAAA ACTTCCAATG GTGAGAACCA

AAAGTACAAC AAAGCAATGG TGACAGAAAA CATTCCAGTG ATGAGAACAA

AAAGCAAAGC AAAACAATAG TGATAGAAAA ATCTCCAATG GTGATAACAA

AAAGCAAAAG AAAATAATAA CAGTAGAAAA ATTTCCAATG GTGATAACAG

AAAGCAAAAG AAAACAGTAA TGACAGAAAA ATTTCCAATG GGGACAACAG

AATGCAAAGC AGAACAATAA CAGTGGAAAC ATTTCCAGTG GTGATAACCG

AAAGCAAAAG AAAATAATAA TAATAGTGGC ATTTCCAATG ATGATAACAG

AAAGCAAAAC AAAATAATAA CAACAGAAAA ATTTCCAATG TTGATAATAG

AAAGCAAAAC AAAATAATAA CAATAGAGAA ATTTCCAATG GTGATAACAG

AAAGCAAAAC AAAATAATAA CAACAGCGAC ATCCCCAATG GTGACAGCAG

AAGGCAAAAC AGAATAATAG TGATGTGAAA ATTTCCAGTG GTGATAATGG

AAAGCAAAGC AAAATGACAG TAGCCGAAAA GTCTCCAGTG ATGACAAAAA

CCTCTTCTTC AGTAATTTCT CACTTCTTGG TACTCCTGTC CTGAAAGATA

CCTCTTCTTC AGTAATTTCT CACTTCTTGG TACTCCTGTC CTGAAAGATA

CCTCTTCTTC AGTAATTTCT CACTTCTTGG TACTCCTGTC CTGAAAGATA

CCTCTTCTTC AGTAATTTCT CACTTCTTGG TACTCCTGTC CTGAAAGATA

CCTCTTCTTC AGTAATTTCT CACTTCTTGG TACTCCTGTC CTGAAAGATA

CCTCTTCTTC AGTAATTTAG CACTTCTTGG TGCTCCTGTC CTAAAAGACA

CCTCAGTTTT AGTCATCTCT GTCTAGTGGG AAATCCTGTG TTGAAAAACA

TGTCAGTTTC AGTCATCTCT GCCTTGTGGG AAATCCTGTG CTGAAAAACA

CCTCTTCTTC AGTAATTTCT CACTTCTTGG TGCTCCTGTC TTGAAAGATA

CCTCTTCTTC AGTAACTT-- -ACTTCTTGG TACTCCTGTC CTGAAAGATA

CCTCTTCTTC AGCAACTTCT CACTTCTTGG TACTCCTGTC CTAAAAGACA

CCTCTTCTTC AGTAATTTCG CAATTCTTAG TACTCCTGTG CTGAGAGGTA

CCTCTTCTTC AGCAACTTTT CACTTCTTGG TACTCCTGTC CTGAAAGATA

CCTCTTCTTC AGTAACTTCT CACTTCTTGG CACTCCTGTC CTTAAAGATA

CCTCTTCTTC AGTAACTTCT CACTTCTTGG TGCTCCTGTC CTGAAGGATA

CCTCTTCTTC AGTAACTTCG CACTACTCGG CACTCCTGTG CTCAGGGACA

CCTCTTCTTT AGCAACTTCT CACTTCTTGG TACTCCTGTC CTTAAAAATA

CCTCATCTTT AGTAACATCT CGCTCCTCGG TCCCCCTGTC CTCAAAGACA

TTAATTTCAA GATAGAAAGA GGACAGTTGT TGGCGGTTGC TGGATCCACT

TTAATTTCAA GATAGAAAGA GGACAGTTGT TGGCGGTTGC TGGATCCACT

TTAATTTCAA GATAGAAAGA GGACAGTTGT TGGCGGTTGC TGGATCCACT

TTAATTTCAA GATAGAAAGA GGACAGTTGT TGGCGGTTGC TGGATCCACT

TTAATTTCAA GATAGAAAGA GGACAGTTGT TGGCGGTTGC TGGATCTACT

TCAATTTCAA GATAGAAAGG GGACAGTTGT TGGCGGTGGC TGGATCTACT

TCAATTTGAA TATAAAGAAA GGAGAGATGT TGGCTATTAC TGGATCTACT

TCAATTTGAA TATAGAGAAA GGAGAGATGT TGGCTATTAC TGGATCTACT

TCAGCTTCAA GATAGAAAGA GGGCAGCTGT TGGCGGTGGC TGGATCTACT

TCAGTTTCAA GATAGAAAGA GGACAGTTGT TGGCAGTTGC TGGATCTACG

TCAATTTCAA GATAGAAAGA GGACAGTTGT TGGCGGTTGC TGGATCTACT

TCAATTTCAA GGTAGAAAGA GGACAGTTGT TGGCTGTTGC GGGATCTACT

TCAATTTTAA AATAGAAAGA GGACAATTGT TGGCAGTTGC AGGATCTACT

TTAATTTCAA GATAGAAAGA GGACAATTAT TGGCAGTTGC TGGATCTACT

TCAATTTCAA GATAGAAAGA GGACAGTTAT TGGCGGTTGC TGGATCTACT

TCAATTTCAA GATCGAGAAA GGACAGCTGT TGGCAGTTGC TGGGTCTACT

TCAGTTTCAA GATAGAAAAG GGACAATTGT TGGCTGTTGC TGGATCTACT

TCAGTTTCAA GATAGAAAAA GGACAGTTGT TGGCTGTAGC TGGATCTACT

GGAGCAGGCA AGACTTCACT TCTAATGGTG ATTATGGGAG AACTGGAGCC

GGAGCAGGCA AGACTTCACT TCTAATGATG ATTATGGGAG AACTGGAGCC

GGAGCAGGCA AGACTTCACT TCTAATGATG ATTATGGGAG AACTGGAGCC

GGAGCAGGCA AGACTTCACT TCTAATGATG ATTATGGGAG AACTGGAGCC

GGAGCAGGCA AGACTTCACT TCTAATGATG ATTATGGGAG AACTGGAGCC

GGAGCAGGCA AGACTTCACT TCTAATGATG ATTATGGGAG AACTGGAGCC

GGAGCAGGAA AGACATCACT CCTGATGCTG ATACTGGGAG AACTGGAAGC

GGATCAGGAA AGACATCACT CCTGATGTTG ATTTTGGGAG AACTGGAAGC

GGTGCTGGAA AGACTTCGCT TCTGATGATG ATTATGGGAG AGTTGGAGCC

GGAGCAGGCA AGACTTCACT TCTCATGATG ATTATGGGAG AATTGGAACC

GGAGCAGGCA AGACTTCACT CCTAATGATG ATTATGGGAG AACTGGAGCC

GGAGCCGGCA AGACTTCACT TCTGATGATG ATTATGGGAG AACTGGAACC

GGAGCGGGCA AGACCTCTCT TCTAATGATG ATTATGGGAG AACTGGAGCC

GGAGCAGGCA AGACTTCCCT TTTAATGATG ATTATGGGAG AACTAGAGCC

GGAGCAGGCA AGACTTCACT TCTAATGATG ATTATGGGAG AACTGGAGCC

GGAGCAGGCA AGACCTCACT TCTGATGATG ATTATGGGAG AACTGGAGCC

GGGGCAGGCA AGACTTCACT TTTAATGATG ATCATGGGAG AATTGGAACC

GGGGCAGGCA AGACCTCACT GCTGATGATG ATCATGGGTG AGCTGGAGCC

TTCAGAGGGT AAAATTAAGC ACAGTGGAAG AATTTCATTC TGTTCTCAGT

TTCAGAGGGT AAAATTAAGC ACAGTGGAAG AATTTCATTC TGTTCTCAGT

TTCAGAGGGT AAAATTAAGC ACAGTGGAAG AATTTCATTC TGTTCTCAGT

TTCAGAGGGT AAAATTAAGC ACAGTGGAAG AATTTCATTC TGTTCTCAGT

TTCAGAGGGT AAAATTAAGC ACAGTGGAAG AATTTCATTC TGCTCTCAGT

TTCAGAGGGT AAAATTAAGC ACAGTGGAAG GATTTCATTC TGCTCTCAGT

GTCAGAGGGA ATTATTAAGC ACAGTGGAAG AGTTTCATTC TCCTCTCAAA

TTCAGAGGGA ATTATTAAGC ACAGTGGAAG AGTTTCATTC TGCTCTCAAT

TTCAGAGGGT AAAATTAAGC ACAGTGGAAG AATATCATTC TGCTCTCAGT

TTCAGAGGGT AAAATTAAGC ACAGTGGAAG AATTTCATTC TGCTCTCAGT

TTCAGAGGGT AAAATTAAGC ACAGTGGAAG AATTTCATTC TGCTCTCAGT

TTCAGAGGGC AAAATTAAGC ACAGTGGAAG AATTTCCTTC TGCTCTCAGT

TTCAGAAGGT AAAATTAAGC ACAGTGGAAG AATTTCATTC TGCCCTCAGT

CTCAGAGGGT AAAATTAAGC ACAGTGGGAG AATTTCGTTC TGCTCTCAGT

CTCAGAGGGT AAAATTAAGC ACAGTGGAAG AATTTCATTC TGTTCTCAGT

CTCGGAAGGC ACAATCAGGC ACAGTGGGAG AATTTCCTTC TGCTCCCAGT

TTCAGAGGGC AAAATTAAGC ACAGCGGAAG GATTTCCTTT TGTTCTCAGT

TTTGGAAGGC AAAATCAAGC ACAGCGGAAG GATCTCCTTC TCTCCCCAAT

TTTCCTGGAT TATGCCTGGC ACCATTAAAG AAAATATCAT CTTTGGTGTT

TTTCCTGGAT TATGCCTGGC ACCATTAAAG AAAATATCAT CTTTGGTGTT

TTTCCTGGAT TATGCCTGGC ACCATTAAAG AAAATATCAT CTTTGGTGTT

TTTCCTGGAT TATGCCTGGC ACCATTAAAG AAAATATCAT CTTTGGTGTT

TCTCCTGGAT TATGCCTGGA ACCATTAAAG AAAATATCAT CTTTGGTGTT

TTTCCTGGAT CATGCCTGGC ACCATTAAAG AAAACATCAT CTTTGGTGTT

TTTCTTGGAT TATGCCGGGT ACTATCAAAG AAAATATCAT CTTTGGTGTT

TTTCTTGGAT TATGCCGGGT ACTATCAAAG AAAATATCAT CTTTGGTGTT

TTTCCTGGAT CATGCCTGGC ACCATTAAAG AGAACATCAT CTTCGGTGTT

ATTCCTGGAT CATGCCTGGA ACCATTAAAG ATAACATCAT CTTTGGTGTT

TTTCTTGGAT TATGCCTGGC ACCATTAAAG AAAACATCAT CTTTGGTGTT

TTTCATGGAT TATGCCTGGC ACCATTAAAG AAAACATCAT CTTTGGTGTT

TTTCCTGGAT CATGCCTGGA ACTATAAAAG AAAATATCAT CTTTGGTGTT

TTTCCTGGAT CATGCCTGGT ACCATTAAAG AAAACATCAT CTTTGGTGTT

TCTCCTGGAT CATGCCTGGC ACCATTAAAG AAAACATCAT CTTTGGTGTT

TCTCCTGGAT CATGCCCGGC ACCATTAAAG AGAACATCGT CTTCGGGGTT

TCTCTTGGAT CATGCCTGGT ACTATTAAAG AAAACATTAT TTTTGGTGTT

TCTCGTGGAT CATGCCAGGA ACCATCAAGG AGAACATCGT CTTCGGGGTT

TCCTATGATG AATATAGATA CAGAAGCGTC ATCAAAGCAT GCCAACTAGA

TCCTATGATG AATATAGATA CAGAAGCGTC ATCAAAGCAT GCCAACTAGA

TCCTATGATG AATACAGATA CAGAAGTGTC ATCAAAGCAT GCCAACTAGA

TCCTATGATG AATACAGATA CAGAAGTGTC ATCAAAGCAT GCCAACTAGA

TCCTATGATG AATACAGATA TAGGAGTGTC ATCAAAGCAT GCCAACTAGA

TCCTATGATG AGTATAGATA TAGGAGTGTT ATCAAAGCAT GCCAACTAGA

TCCTATGATG AGTACAGATA TAAGAGTGTT GTCAAAGCTT GCCAACTACA

TCCTATGATG AGTACAGATA TAAGAGTGTT GTCAAAGCTT GCCAACTACA

TCCTACGATG AGTATAGATA TAAGAGTGTC ATCAAAGCTT GCCAACTAGA

TCCTATGATG AATATAGATA TAGAAGTGTC ATCAAAGCAT GCCAACTAGA

TCCTATGATG AGTATAGATA TAGGAGTGTC ATCAAAGCAT GCCAACTAGA

TCCTATGATG AGTATAGATA TAGGAGTGTC ATCAAAGCAT GCCAACTAGA

TCCTATGATG AATACAGATA TAAGAGTGTC ATCAAAGCAT GTCAATTAGA

TGTTATGATG AGTATAGATA CAGGAGTGTC ATCAAAGCCT GCCAACTAGT

TCCTATGATG AGTATAGATA TAGGAGTGTC ATCAAAGCCT GCCAGCTAGA

TCCTATGACG AGTACAGATA CAGGAGTGTC GTCAAAGCCT GCCAACTCGA

TCTTATGATG AATACCGATA TAGAAGTGTC ATCAAAGCCT GCCAATTAGA

TCTTTCGATG AGTACCGATA CAGGAGTGTC ATCAAGGCCT GCCAATTGGA

AGAGGACATC TCCAAGTTTG CAGAGAAAGA CAATATAGTT CTTGGAGAAG

AGAGGACATC TCCAAGTTTG CAGAGAAAGA CAATATAGTT CTTGGAGAAG

AGAGGACATC TCCAAGTTTG CAGAGAAAGA CAATATAGTT CTTGGAGAAG

AGAGGACATC TCCAAGTTTG CAGAGAAAGA CAATATAGTT CTTGGAGAAG

AGAGGACATC TCCAAGTTTG CAGAGAAAGA CAATATAGTT CTTGGAGAAG

AGAGGATATC TCCAAGTTTG CCGAGAAAGA CAACATAGTT CTTGGAGAAG

GGAGGACATC ACCAAGTTTG CAGAACAAGA CAACACAGTT CTTGGAGAAG

GCAGGACATC ACCAAGTTTG CAGAACAAGA CAACACAGTT CTTGGAGAAG

AGAGGACATC TCCAAGTTTA CAGAGAAAGA CAATACAGTT CTTGGAGAAG

AGAGGACATC TCCAAGTTTG CAGAGAAAGA CAACGTAGTT CTCGGAGAAG

GGAGGACATT TCCAAGTTTG CAGAGAAGGA CAACATCGTT CTTGGAGAAG

AGAGGACATC TCCAAGTTTG CAGAGAAAGA CAATATAGTT CTTGGAGAAG

AGAGGACATC TCCAAATTTG CTGAGAAAGA CAACATAGTT CTTGGAGAAG

GGAGGACATC TCCAAGTTTG CAGAGAAAGA CAATACAGTT CTTGGAGAAG

AGAGGACATT TCCAAGTTTG CAGAGAAAGA CAATATAGTT CTTGGAGAGG

AGAG------ ---------- ---------- ---------- ----------

AGAGGACATA TCAAAGTTTG CGGAAAAAGA CAACACAGTT CTTGGAGAAG

GGAGGAAATC TCCAAGTTTG CAGAAAAAGA TAATACAGTC CTGGGGGAAG

GTGGAATCAC ACTGAGTGGA GGTCAACGAG CAAGAATTTC TTTAGCAAGA

GTGGAATCAC ACTGAGTGGA GGTCAACGAG CAAGAATTTC TTTAGCAAGA

GTGGAATCAC ACTGAGTGGA GGTCAACGAG CAAGAATTTC TTTAGCAAGA

GTGGAATCAC ACTGAGTGGA GGTCAACGAG CAAGAATTTC TTTAGCAAGA

GTGGAATCAC ATTGAGTGGA GGTCAACGAG CAAGAATTTC TTTAGCAAGA

GCGGAATCAC ACTGAGTGGA GGTCAGCGAG CAAGAATCTC TTTAGCAAGA

GTGGAGTCAC ACTCAGTGGA GGTCAACGTG CAAGAATTTC TTTAGCAAGA

GTGGAGTCAC ACTGAGTGGA GGTCAGCGTG CAAGGATTTC TTTAGCAAGA

GTGGAATTAC TCTGAGTGGA GGTCAACGAG CAAGAATTTC TTTAGCAAGA

GTGGAATCAC ACTGAGTGGA GGTCAGCGAG CGAGAATTTC TTTAGCAAGA

GTGGCGTCAC GCTGAGCGGA GGGCAAAGAG CAAGAATTTC CCTAGCAAGA

GTGGGATCAC ACTGAGTGGA GGGCAGCGAG CAAGAATTTC TTTAGCAAGA

GTGGAATCAC CCTGAGTGGA GGTCAGCGAG CCAGAATTTC TTTAGCAAGA

GTGGGATCAC ATTGAGTGGA GGTCAACGAG CAAGAATTTC TTTAGCAAGA

GTGGAATCAC ACTGAGTGGA GGGCAACGAG CCAGAATCTC GTTAGCAAGG

---------- ---------- ---------- ---------- ----------

GTGGAATCAC TCTGAGTGGA GGTCAACGAG CAAGAATTTC TTTAGCAAGA

GTGGCATCAC CTTGAGTGGA GGTCAGCGGG CCAGAATTTC ATTAGCCAGA

GCAGTATACA AAGATGCTGA TTTGTATTTA TTAGACTCTC CTTTTGGATA

GCAGTATACA AAGATGCTGA TTTGTATTTA TTAGACTCTC CTTTTGGATA

GCAGTATACA AAGATGCTGA TTTGTATTTA TTAGACTCTC CTTTTGGATA

GCAGTATACA AAGATGCTGA TTTGTATTTA TTAGACTCTC CTTTTGGATA

GCTGTATACA AAGATGCTGA TTTGTATTTA TTAGACTCTC CTTTTGGATA

GCAGTATATA AAGATGCTGA CTTGTACCTT TTAGACTCTC CTTTTGGATA

GCAGTATATA AAGATGCTGA TTTGTACCTG TTAGATTCCC CCTTCGGATA

GCAGTATATA AAGATGCTGA TTTGTACCTA TTAGATTCCC CTTTTGGATA

GCAGTATACA AAGATGCTGA TTTGTACCTA TTGGACTCTC CTTTTGGATA

GCAGTATACA AAGATGCTGA TTTGTACCTA TTAGACTCTC CTTTTGGATA

GCAGTATATA AAGATGCTGA TTTATATCTC TTAGACTCTC CTTTTGGATA

GCAGTATATA AAGATGCTGA TTTGTACCTA TTAGACTCTC CTTTTGGATA

GCAGTATACA AAGATGCTGA TCTGTACCTA TTGGACTCTC CTTTTGGTTA

GCAGTATACA AAGACGCTGA TTTGTACCTC TTAGACTCTC CTTTTGGATA

GCAGTGTACA AAGATGCTGA TTTGTACCTA TTAGACTCTC CTTTTGGATA

---------- ---------- ---------- ---------- ----------

GCAATATACA AAGATGCTGA TTTATACCTC TTAGATTCCC CATTTGGCTA

GCAGTGTACA AAGATGCTGA CTTATATCTC CTAGACTCTC CTTTTGGCTA

CCTAGATGTT TTAACAGAAA AAGAAATATT TGAAAGCTGT GTCTGTAAAC

CCTAGATGTT TTAACAGAAA AAGAAATATT TGAAAGCTGT GTCTGTAAAC

CCTAGATGTT TTAACAGAAA AAGAAATATT TGAAAGCTGT GTCTGTAAAC

CCTAGATGTT TTAACAGAAA AAGAAATATT TGAAAG---- ----------

CCTAGATGTT TTAACAGAAA AAGAAATATT TGAAAGCTGT GTCTGTAAAC

CTTAGATGTT TTAACAGAAA AAGAAATATT TGAAAGCTGT GTCTGTAAAC

TCTAGATGTT TTAACTGAAG AACAAATATT TGAAAGTTGT GTCTGTAAAT

TCTAGATGTT TTTACTGAAG AACAAGTATT TGAAAGCTGT GTTTGTAAAT

TCTAGATGTC TTAACAGAAA AAGAAATATT TGAGAGCTGT GTCTGTAAAT

TCTAGATGTT TTAACAGAGA AAGAAATATT TGAAAGCTGT ATCTGTAAAT

CCTGGATGTT TTAACAGAAA AAGAAATATT TGAAAGCTGT GTCTGTAAAT

CCTAGATGTT TTAACAGAAA AAGAAATATT TGAAAGGTGT GTCTGTAAAC

CCTAGATGTT TTAACAGAAA AAGAAATATT TGAAAGCTGT GTCTGTAAAT

CCTAGATGTT TTAACAGAAA AAGAAATATT TGAAAGCTGT GTCTGTAAAT

CCTAGATGTT TTAACAGAAA AAGAAATTTT TGAAAGCTGT GTCTGTAAAT

---------- ---------- ---------- ------CTGT GTTTGTAAAT

TTTAGATATG TTAACTGAAA AGGAGATATT TGAAAGCTGT GTTTGCAAGC

CTTGGATGTG CTTACGGAAA AGGAAATATT TGAAAGCTGT GTCTGCAAGC

TGATGGCTAA CAAAACTAGG ATTTTGGTCA CTTCTAAAAT GGAACATTTA

TGATGGCTAA CAAAACTAGG ATTTTGGTCA CTTCTAAAAT GGAACATTTA

TGATGGCTAA CAAAACTAGG ATTCTGGTCA CTTCTAAAAT GGAACATTTA

---------- ---------- ---------- ---------- ----------

TGATGGCTAA CAAAACTAGG ATTCTGGTCA CTTCCAAAAT GGAACATTTA

TGATGGCTAA CAAAACTAGG ATTTTGGTAA CTTCTAAAAT GGAACATTTA

TGATGGCCAG CAAAACTAGG ATTCTGGTCA CATCTAAAAT GGAGCAGTTA

TGATGGCCAA CAAAACTAGG ATTTTGGTTA CATCTAAAAT GGAACACTTA

TGATGGCCAA CAAAACTAGA ATTTTGGTCA CTTCTAAAAT GGAACATTTA

TGATGGCTAA CAAAACTAGG ATTTTGGTCA CTTCTAAAAT GGAGCACTTA

TGATGGCTAA CAAAACTAGG ATCTTGGTCA CTTCTAAAAT GGAACATTTA

TGATGGCTAA CAAAACTAGG ATTTTAGTCA CTTCTAAAAT GGAACATTTA

TGATGGCTAA CAAAACTAGG ATTTTGGTCA CTTCTAAAAT GGAGCATTTA

TGATGGCTAA CAAAACTAGG ATTTTGGTTA CTTCCAAAAT GGAACATTTA

TGATGGCGAA CAAAACTAGA ATTTTGGTCA CTTCTAAAAT GGAACATTTA

TGATGGCGAA CAAAACTAGG ATTTTGGTCA CTTCTAAAAT GGAACATTTA

TGATGGCAAA CAAGACCAGG ATATTGATCA CTTCTAAAAT GGAACATTTA

TTATGGCCAA TAAAACGAGG ATCCTCGTCA CTTCCAAAAT GGAACACTTG

AAGAAAGCTG ACAAAATATT AATTTTGCAT GAAGGTAGCA GCTATTTTTA

AAGAAAGCTG ACAAAATATT AATTTTGCAT GAAGGTAGCA GCTATTTTTA

AAGAAAGCTG ACAAAATATT AATTTTACAT GAAGGTAGCA GCTATTTTTA

---------- ---------- ---------- ---------- ----------

AAGAAAGCTG ACAAAATACT AATTTTACAT GAAGGTAGCA GCTATTTTTA

AAGAAAGCTG ACAAAATATT AATTTTACAT GAAGGTAGCA GCTATTTTTA

AAGAAAGCTG ACAAGATACT CATTTTGCAT GAGGGCAGCA GCTATTTTTA

AGGAAAGCTG ACAAAATACT AATTTTGCAT CAGGGCAGTA GCTATTTTTA

AAGAAAGCTG ACAAAATATT AATTTTACAT GAAGGCAGCA GCTATTTTTA

AAGAAAGCTG ACAAAATATT AATTTTACAT GAAGGTAGCA TCTATTTTTA

AAGAAAGCTG ACAAAGTCTT AATTTTACAT GAAGGTAGCT GCTATTTTTA

AAGAAAGCTG ACAAAATATT AATTTTACAT GAAGGTAGCA GCTATTTTTA

AAGAAAGCCG ACAAAATATT AATTTTACAT GAAGGGAGCA GCTATTTTTA

AAGAAAGCTG ACAAAATACT AATTTTACAT GAAGGTAGCA GCTATTTTTA

AAGAAAGCTG ACAAAATATT AATTTTACAT GAAGGTAGCA GCTATTTTTA

AGGAAAGCCG ACAAAATACT AATTTTGCAT GAAGGTAGCA GTTATTTTTA

AAGAAAGCTG ACAAGATATT AATATTACAT GAAGGCAGCT GCTATTTCTA

AAGAAAGCCG ACAAAATCTT AATTCTGCAC GAAGGCAGTT GCTATTTCTA

TGGGACATTT TCAGAACTCC AAAATCTACA GCCAGACTTT AGCTCAAAAC

TGGGACATTT TCAGAACTCC AAAATCTACG GCCAGACTTT AGCTCAAAAC

TGGAACATTT TCAGAACTCC AAAATCTACG GCCAGACTTT AGCTCAAAAC

---------- ---------- ---------- ---------- ----------

TGGGACATTT TCAGAACTAC AAAATCTACG GCCAGACTTT AGCTCAAAAC

TGGCACATTT TCTGAACTAC AAAATCTACG GCCGGACTTT AGCTCAAAGC

TGGGACGTTT TCTGAACTAC AAAGTCTACG TCCAGACTTC AGTTCCAAAC

TGGGACATTT TCTGAGCTAC AAAGTCTACG TCCAGACTTC AGTTCGAAAC

TGGTACTTTT TCTGAACTCC AAAGTCTACG GCCGGATTTT AGCTCAAAGC

TGGGACATTT TCTGAATTAC AAAATCAGCG GCCCGACTTC AGCTCGAAGC

TGGGACATTT TCTGAATTAC AAAGTCTCCG GCCCGACTTC AGCTCGAAGC

TGGGACGTTT TCTGAATTAC TAAATCTACG GCCTGACTTT AGCTCAAAGC

TGGAACGTTT TCTGAACTGC AAAACCTGCG GCCAGACTTC AGCTCAAAGC

TGGAACATTT TCTGAACTAC AAAATCTACG GCCAGACTTC AGCTCAAAGC

TGGGACATTT TCTGAACTAC AAACTCTACG GCCAGACTTC AGCTCGGAGC

TGGGACATTT TCTGAACTAC AAAACCTGCG GCCAGACTTC AGCTCAAAGC

TGGAGCCTTT TCTGATCTTC AAAATATGCG TCCTGACTTT AGCTCAAAAC

CGGCGCGTTT TCAGAACTTC AGAACCTGCG GCCAGACTTC AGCTCCAAGC

TCATGGGATG TGATTCTTTC GACCAATTTA GTGCAGAAAG AAGAAATTCA

TCATGGGATG TGATTCTTTC GACCAATTTA GTGCAGAAAG AAGAAATTCA

TCATGGGATA TGATTCTTTC GACCAATTTA GTGCAGAAAG AAGAAATTCA

---------- ---------- ---------- ---------- ----------

TCATGGGATA TGATTCTTTT GACCAATTTA GTTCAGAAAG AAGAAATTCA

TCATGGGATT TGATTCTTTC GACCAGTTTA GTGCAGAAAG AAGAAATTCC

TCATGGGGTA TGATACTTTT GACCAGTTTA CTGAGGAAAG AAGGAGTTCA

TCATGGGGTA TGATACTTTT GACCAGTTTA CTGAGGAAAG AAGAAGTTCA

TCATGGGATA TGATTCTTTT GACCAGTTTA GTGCAGAAAG AAGAAATTCA

TCATGGGATG TGACACTTTC GACCAGTTTA CTGCAGAACG AAGAAATTCA

TCATGGGCTA TGATTCTTTT GACCAGTTCA GTCCAGAAAG AAGAAACTCC

TCATGGGATA TGATTCTTTC GACCAGTTTA GTGCAGAAAG AAGAAATTCA

TCATGGGATA TGATTCTTTT GATCAGTTTA GTGCAGAAAG AAGAAATTCC

TCATGGGATA TGATTCTTTT GACCAGTTTA GTGCAGAAAG AAGAAATTCA

TCATGGGATA TGATTCTTTC GATCAGTTTA GTGCAGAAAG AAGAAATTCA

TCATGGGCTA TGATTCTTTC GACCACTTCA GCGCAGACAG AAGAAACTCC

TCATGGGATA TGATTCTTTT GACCAATTCA GTGCAGAAAG AAGAAGTTCA

TCATGGGCTA CGACTCCTTC GACCAGTTCA GCGCCGAAAG GAGAAACTCC

ATCCTAACTG AGACCTTACA CCGTTTCTCA TTAGAAGGAG ATGCTCCTGT

ATCCTAACTG AGACCTTACG CCGTTTCTCA TTAGAAGGAG ATGCTCCTGT

ATCCTAACTG AGACCTTGCG CCGTTTCTCA TTAGAAGGAG ATGCTCCTGT

---------- ---------- ---------- ---------- ----------

ATCCTCACTG AGACCTTACG CCGTTTCTCT TTAGAAGGAG ATGCTCCTGT

ATCCTCACTG AGACTCTGCG CCGTTTCTCA TTAGAAGGAG ACGCCGCTGT

ATTCTAACTG AGACCTTACG CAGGTTCTCA GTGGA---CG ATGCCTCTAC

ATTCTAACTG AGACCTTACG CAGGTTCTCA GTAGA---CG ATTCCTCTGC

ATCCTAACTG AGACCTTACG GCGTTTCTCA TTAGAAGGAG ATGCTAGCAT

ATCATAACTG AGACTTTACG GCGTTTCTCA TTAGAAGGAG ATACTTCTGT

ATCATCACTG AGACTTTACG GCGTTTCTCC TTAGAAGGAG ATGCGGCCGT

ATCTTAACTG AGACTTTACG GCGTTTCTCA TTTGAAGGAG ATGCTGCTGG

ATCCTAACGG AGACTTTGCG GCGTTTCTCA CTGGAAGGGG ATGCTGCCGT

ATTATAACTG AAACCCTGCG GCGTTTTTCA TTAGAAGGAG ATGCTGCTCA

ATCCTAACGG AGACCTTACG GCGTTTCTCA TTAGAAGGAG A---TACTGT

ATCATCACGG AGACCTTGCG GCGCTTCTCT CTAGAAGGAG AGGCTGCTGT

ATCCTAACAG AAACCCTGAG ACGTTTCTCA ATAGAAGGTG ATACTGCTGT

ATCCTGACGG AAACCCTGCG GCGTTTCTCC CTGGACGGCG ATGCCGCCGG

CTCCTGGACA GAAACAAAAA AACAATCTTT TAAACAGACT GGAGAGTTTG

CTCCTGGACA GAAACAAAAA AACAATCTTT TAAACAGACT GGAGAGTTTG

CTCCTGGACC GAAACAAAAA AACAATCTTT TAAACAGACT GGAGAGTTTG

---------- ---------- ---------- ---------- ----------

CTCCTGGACA GAAACAAAAA AACAATCTTT TAAACAGACT GGAGAGTTTG

CTCCAGGAAT GAGACGAAAA AACAATCTTT TAAACAGACT GGAGAGATTG

CACCTGGAAC AAAGCC---A AACAGTCATT TAGACAGACT GGAGAGTTTG

CCCGTGGAGC AAACCC---A AACAGTCGTT TAGACAGACT GGAGAGGTGG

CTCCTGGAAT GATACCAGAA AACAATCTTT TAAACAGAAT GGAGAGTTGG

GTCCTGGAAT GAAACGAAAA AGCCTTCTTT TAAACAGACT GGAGAGTTTG

GCCCTGGAAT GAAACAAAAA AGCAATCTTT TAAACAGACT GGAGAGTTTG

CCCCTGGAAT GAACCGAAAA AACAGTCTTT TAAACAGACT GGAGAGTTCG

CTCCTGGAAT GAAGCCAGAA AGCAATCTTT TAAACAGACG GGGGAGTTTG

TTCCTGGAAT GAAACAAAAA AACAATCTTT TAAACAGACT GGAGAGTTTG

CTCCTGGAAT GAAACAAAAA AGCAGTCTTT TAAACAAGCT GGAGAGTTTG

CTCCTGGACT GACAACAGAA AGCAGTCTTT TAAGCAGACC GCAGAGATCA

CTCCTGGAAT GAAGGAAAAA AGCAATCTTT TAAACAAACT GGAGATTTTG

CTCCTGGAAT GAAACTAAGA AGCAGTCGTT CAAGCAGACG GGAGAGTTTG

GGGAAAAAAG GAAGAATTCT ATTCTCAATC CAATCAACTC TATACGAAAA

GGGAAAAAAG GAAGAATTCT ATTCTCAATC CAATCAACTC TATACGAAAA

GGGAAAAAAG GAAGAATTCT ATTCTCAATC CAATCAACTC TATACGAAAA

---------- ---------- ---------- ---------- ----------

GGGAGAAAAG AAAGAATTCT ATTCTCAATT CCATCAACTC TATACGAAAA

GAGAAAAAAG AAAGAATTCT ATTCTCAATC CAATCAACTC TATAAGGAAA

GAGAAAAAAG GAAGAACTCT ATTCTAAGTT CATTCAGCTC TGTGAAGAAA

GAGAAAAAAG GAAGAACTCT ATTCTAAATT CATTCAGCTC TGTAAGGAAA

GGGAAAAACG GAAGAACTCT ATTCTCAATC CAGTCAACTC TATGAGAAAA

GTGAAAAAAG GAAGAACTCC ATTCTCAGTT CAATCAACTC TATAAGGAAA

GTGAAAAAAG GAAGAACTCT ATTCTTAATC CAATCAACTC CATAAGAAAA

GTGAAAAAAG GAAGAGCTCT GTTCTCAATC CAATCAATGC TATAAAAAGA

GTGAAAAAAG GAAGAACTCT GTTCTCAATC CAATCAACTC TCTGCGCAAG

GTGAAAAAAG AAAGAACTCT ATTCTCAATC CAATCAACTC CCTAAGAAGA

GTGAAAAAAG GAAGAACTCT ATTCTCAATT CAATCAACTC CATAAGAAAA

GTGAAAAAAG GAAGAACTCT ATTCTCAATT CCATCAACTC CATGAGAAAG

GGGAAAGAAG AAAGAATTCT ATTCTTAGTT CACTGAACTC CATAAGGAAA

GCGAAAGAAG AAAGAACTCC ATCCTCAACC CGCTCAACTC CATCCGGAAG

TTTTCCATTG TGCAAAAGAC TCCCTTACAA ATGAATGGCA TCGAAGAGGA

TTTTCCATTG TGCAAAAGAC TCCCTTACAA ATGAATGGCA TCGAAGAGGA

TTTTCCATTG TCCAAAAGAC TCCCTTACAA ATGAATGGCA TCGAAGAGGA

---------- ---------- ---------- ---------- ----------

TTTTCCATTG TACAAAAGAC TCCCTTACAA ATGAATGGCA TCGAAGAGGA

TTTTCTGTTG TACAAAAGAC TCCACTCCCA ATGAATGGCA TTGAGGAGGA

ATTTCCATTG TGCAAAAGAC TCCATTGT-- -------CCA TAGAAGGAGA

ATTTCCATTG TGCAAAAGAC TCCATTAT-- -------GTA TCGATGGAGA

TTTTCTATTG TACCAAAAAC TCCTTTACAA ATGAATGGCA TTGAAGAAGA

TTTTCAGTTG TACAAAAGAC TTCATTACAA ATGAATGGTA TCGAAGGAGC

TTTTCAATTG TACAAAAGAC CCCGTTACCC ATGAATGGCA TCGAAGGAGA

TTTTCAATTG TACAAAAGAC TCCATTACAA ATGAATGGAA TTGAAGAAGA

TTTTCGATTG CTCAAAAGGC GCCGTTGCAA ATGAACGGTA TTGAAGAAGA

ATTTCAATAA CACAAAAGAC TCCATTGCCA ATGAATGGCA TTGATGAGGA

TTTTCAGTAG TACAAAAGAC TCCACTACAA ATGAATGGTA TTGATGAAGA

TTTTCCATTG TCCAGAAGGC TCCGCTGCCA ATGAACGGCA TCGACAAGGA

TTTTCAGTAG TGCAAAAGAG TCAACCGCAA ATGAATGGCA TTGAGGAAAA

TTCTCCCTGG TCCAGAAGGC TCCGCTCCAG ATGAACTGCA TCGACGAGAC

TTCTGATGAG CCTTTAGAGA GAAGGCTGTC CTTAGTACCA GATTCTGAGC

TTCTGATGAG CCTTTAGAGA GAAGGCTGTC CTTAGTACCA GATTCTGAGC

TTCTGATGAG CCTTTAGAGA GAAGGCTGTC CTTAGTTCCA GATTCTGAGC

---------- ---------- ---------- ---------- ----------

TTCTGATGAG CCTTTAGAGA GAAGGCTCTC CTTAGTTCCA GATTCTGAGC

CTCGGAGGAG CCCGTGGAGA GAAGGCTGTC CTTAGTTCCA GACTCCGAGC

ATCTGATGAC CTCCAAGAAA GGAGACTGTC CCTGGTTCCA GACTCTGAAC

GTCTGATGAT CTCCAAGAAA AGAGACTGTC CCTAGTTCCG GATTCTGAAC

TTCGGACGCA TCCATAGAGC GGAGGCTGTC CTTGGTTCCA GATTCAGAAC

TGCTGATGCG CCTTTAGAGA GAAGACTATC CTTAGTTCCC CATTCTGAAC

GTCTGAGGTG CCAGTAGAGC GAAGGCTGTC CTTATTTCCC GACTGTGAGC

CTCTGAAGAG TCTTCAGAGA GAAGGCTGTC CTTAGTTCCG GATTCTGAGC

CTCGGAGGGG TCTTCAGAAA GAAGACTGTC CCTGATTCCC GATTCTGAGC

TTCTGGTGAA ACTTTAGAGA GAAGACTGTC CTTAGTTCCA GATTGTGAGC

TTCTGACGAG CCTTTGGAGA GAAGGCTGTC CCTAGTTCCG GATTCTGAGC

CTCTGATGAG CCCCTGGAGA GAAGGCTGTC CTTAGTGCCC GACTCTGAGC

TGATGATGAG CCCTTAGAAA GGAGGTTGTC TTTGGTGCCA GATTCTGAGC

CCTGGACGAG CCGCCGGAGC GGAAGCTTTC CCTGGTACCC GACAGCGAGC

AGGGAGAGGC GATACTGCCT CGCATCAGCG TGATCAGCAC TGGCCCCACG

AGGGAGAGGC GATACTGCCT CGCATCAGCG TGATCAGCAC TGGCCCCACG

AGGGAGAGGT GATACTCCCT CGCATCAGCG TGATCAGCAC TGGCCCCACG

---------- ---------- ---------- ---------- ----------

AGGGAGAAGC AATACTACCT CGCATCAGCG TGATCAACAC TGGCCCCGCA

AGGGGGAGGC CATCCTGCCT CGAAGCAACG TGTTCAACAC TGGCCCCACG

ATGGAGAGGC CGCTCTGCCT CGCAGCAACA TGATCACCGC CGGGCCCACG

AGGGGGAGGC TGCTCTGCCG CGCAGCAACA TGATCGCCAC CGGCCCCACA

AGGGAGAGGC GATTCTACCT CGAAGCAACA TGATCAACAC TGGCCCCATG

CTGGAGAGGG GATCCTGCCT CGGAGCAATG CAGTCAACAG TGGCCCCACA

AAGGAGAGGC CATCCTGCCT CGGAGCAACA TGATCAACAC TGGCCCCACG

AGGGAGAGAC CATCCTGCCT CGAAGTAACC TAATAAACAC TGGTCCCACA

AGGGGGAGGC CATCCTTCCT CGGAGCAATG TCTTCAACAC TGGTCCCATG

AGGGAGAAGG TATCCTTCCT CGGAGTAACT TGATTAACAC TGGCCCCACG

AAGGAGAGGC CATCCTACCT CGAAGTAACG TGATCAACAC GGGTCCCACA

AGGGGGAGGT CATCCTGCCC AGAAGTAATG TGATCCACAC GGGCCCCACG

AGGGAGAGGC AATTCTGCCC CGGAGTAATA TCATGAATAC AGGTCCAACA

CAGGAGAGGC CATCCTCCCC CGCAGCAACA TGCTAAACCC TGGGCCGGTG

CTTCAGGCAC GAAGGAGGCA GTCTGTCCTG AACCTGATGA CACACTCAGT

CTTCAGGCAC GAAGGAGGCA GTCTGTTCTG AACCTGATGA CACACTCAGT

CTTCAGGCAC GGAGGAGGCA GTCTGTTCTG AACCTGATGA CACACTCAGT

---------- ---------- ---------- ---------- ----------

CTTCAGGTAC GAAGGAGACA GTCTGTTCTG AACATGATGA CACACTCAGT

TTTCAGGGAC GAAGGAGGCA GTCTGTTTTG AACCTGATGA CTCATTCCGT

TTTCCAGGCA GAAGAAGACA GTCTGTTTTG GATCTTATGA CATTCACACC

TTTCCAGGCA GAAGAAGACA GTCTGTTTTG GATCTGATGA CGTTCACACC

CTGCAGGGCT GCAGGAGGCA GTCGGTTCTG AACCTGATGA CACACTCGGT

TTTCTGGGAG GGAGGAGGCA GTCCGTTCTG AACCTTATGA CCGGCTCAGT

CTCCGGAGAC AACGAAGACA GTCTGTTCTG AACCTGATGA CGCGCTCCGT

TTTCAGGGAC GAAGGAGGCA GTCTGTTCTG AACCTGATGA CACACTCAGT

CTCCAGGGCC GCAGGAGGCA GTCTGTTCTG AACCTGATGA CGCACTCGGG

CTTCAGAGAG GAAGAAGGCA ATCTGTTCTG AACTTGATGA CACACTCTGG

TTTCAGAGAC GAAGAAGACA GTCTGTTCTC AACCTGATGA CACGCCCAAT

TTTCAGAGGC GGAGGAGACA GTCTGTGCTG AACCTGATGA CCCGCTCCAT

TTCCAAGGTC GAAGGAGACA GTCAGTACTG AACTTGATGA CCCGCCCAGT

CTCCGAGCCC GGAGGAGGCA GTCGGTCCTG AATCTGATGA CCCGGCCCAT

TAACCAAGGT CAGAACATTC ACCGAAAGAC AACAGCATCC ACACGAAAAG

TAACCAAGGT CAGAACATTC ACCGAAAGAC AACAGCATCC ACACGAAAAG

TAACCAAGGT CAGAGCATTC ACAGAAAGAC AGCAGCATCC ACACGAAAAG

---------- ---------- ---------- ---------- ----------

TAACCAAGGT CAGAGCATTC ACCGAAAGAC AACAGCATCC ACACGAAAAG

TAACCAAGGT CAAAACATTC ACCGAAAGAC TGCAGCATCT ACACGTAAGA

CAGCTCAGTC TCCAGCAGTC TCCAGAGGAC CAGAGCTTCT ATTCGAAAAA

CAACTCAGGC TCCAGCAATC TTCAGAGGAC CAGAACTTCT ATTCGAAAAA

CAGCCAAGGT CCCAGCATTT ACAGAAGGAC AACCACATCC GCACGAAAAA

GAACCAAGGT CAAAGCATTC ATCGAAAGAC AGCGACATCC ACACGAAAAA

GAACCAAGGT CAGAGCATTC ACCGAAGGAC AACAGCATCC ACGCGAAAAA

GAACCAAGGT CAAATCGTTC ACAGAAGCAC AGCAGCATCC ACAAGAAAAA

GAACCAAGGT TCAAATATTC ACCGAAGGGC CACAGCAGCT GCTCGAAAGA

CAACCAAGGG CAAAACATTC ACAGGAGGAC AACAGCATTC ACACGAAAAA

TAACCAAGGT CAAAACATTC ACCGAAGGAC AGCAGCATCC GCACGAAAAA

CCACCAAGGC CAAAACAGTC ACCGCAAGAC AGCAGCATCC TCACGAAAGA

TTGCCAAGGT CAAAACATGT ATAAAACGGG GAATGCTGCC A---GGAAGA

TCACCAAGGC TCGAGCCTTT ACAAGAAGGG CAGCGCCTCG GCCCGCAAGA

TGTCACTGGC CCCTCAGGCA AACTTGACTG AACTGGATAT ATATTCAAGA

TGTCACTGGC CCCTCAGGCA AACTTGACTG AACTGGATAT ATATTCAAGA

TGTCACTGGC CCCTCAGGCA AACTTGACTG AACTGGATAT ATATTCAAGA

---------- ---------- ---------- ---------- ----------

TGTCACTGGC CCCTCAGGCA AACCTGACTG AGCTGGATAT ATATTCAAGA

TGTCACTGGC CCCGCAGGCG AATCTGACGG AAATGGATAT ATATTCAAGA

TATCCTTAGC CCCTCGGATA AGCTTAAAGG AAGAGGATAT ATATTCAAGG

TCTCCTTAGT CCCTCAGATA AGCTTAAATG AAGTGGATGT ATATTCAAGG

TGTCTCTGGC CCCTCAGACA AACTTGACTG AAATGGACAT ATATTCAAGA

TGTCACTGGC TCCTCAGGCA AGCTTAGCCG AAATAGATAT ATATTCAAGA

TGTCACTGGC CCCTCAGGCA AATCTAACTG AAATGGATAT ATATTCCAGA

TGTCACTGGC CCCTCAGGAG AACTTCACTG AAATGGATAT ATATTCAAGA

TGTCCCTGGC CCCCCAGGCG AACTTCACCG AGATGGATAT ATACTCAAGA

TGTCACTGGC CCCTCAGGCT AACTTAGTTG AAATGGACAT ATATTCAAGA

TGTCACTGGC CCCTCAGGCT AACTTAACTG AAATGGATAT ATATTCAAGA

TGTCTATGGC CCCGCAAGCC AGCCTGACAG AGATGGACAT ATATTCAAGA

TGTCCATGGC CCCCCAGTCT AAGTTATCTG AAATTGATAT TTACTCCCGA

TGTCCGTGGC CCCTCAGTCC AATATGTCGG AGATGGATAT CTATTCCCGG

AGGTTATCTC AAGAAACTGG CTTGGAAATA AGTGAAGAAA TTAACGAAGA

AGGTTATCTC AAGAAACTGG CTTGGAAATA AGTGAAGAAA TTAACGAAGA

AGGTTATCTC AAGAAACTGG CTTGGAAATA AGTGAAGAAA TTAATGAAGA

---------- ---------- ---------- ---------- ----------

AGGTTATCTC AAGAAACTGG CTTGGAAATA AGTGAAGAAA TTAATGAAGA

CGGTTATCTC AGGAAAGTAG CTTGGAAATA AGTGAAGAAA TTAATGAAGA

CGATTATCGC AAGATAGCAC ACTGAACATC ACCGAAGAAA TTAATGAAGA

AGATTATCGC AAGATAGCAC ACTGAACATC ACTGAAGAAA TTAACGAAGA

CGGTTATCTC AAGAAAGTGG CTTGGAAATT AGTGAAGAGA TTAATGAAGA

CGGTTATCTC AAGATACTGG CTTGGAAATA AGTGAAGAAA TTAATGAAGA

CGGTTATCTC AAGACAGTGG CTTGGAAATA AGTGAAGAAA TTAATGAAGA

CGGTTATCTC AAGACAATGG CTTGGAAATA AGTGAAGAAA TAAATGAAGA

AGGTTATCTC AAGACAGCAT CTTGGAAATT AGCGAAGAAA TCAATGAGGA

CGATTATCTC AAGACAGTGG TTTGGAAATA AGTGAAGAAA TTAATGAAGA

AGATTATCTC AAGACAGTGG CTTGGAAATA AGTGAAGAAA TTAATGAAGA

AGACTCTCGC AAGACAGCGG CTTAGAAATA AGCGAAGAAA TTAATGAAGA

CGTTTATCTC AAGATAGTGG CATGGATATA AGTGATGAAA TTAATGAAGA

CGCCTCTCAA AAGACAGCGG ATTGGAAATC AGCGAGGAGA TTAACGAGGA

AGACTTAAAG GAGTGCTTTT TTGATGATAT GGAGAGCATA CCAGCAGTGA

AGACTTAAAG GAGTGCTTTT TTGATGATAT GGAGAGCATA CCAGCAGTGA

AGACTTAAAG GAGTGCTTTT TTGATGATAT GGAGAGCATA CCAGCGGTGA

---------- ---------- ---------- ---------- ----------

AGACTTAAAG GAGTGCTTTT TCGATGATAT GGAGAGCATA CCAGCAGTGA

ATATTTAAAG GAGTGCTTTT GTGATGATGT GGAGAACATT CCAGCAGTGA

AGATTTAAAG GAGTGTTTTT TTGATGATAT GGTAAAGATC CCCACGGTGA

AGATTTAAAG GAGTGTTTTC TTGATGATGT GATCAAGATA CCCCCGGTGA

AGACTTAAAG GAATGCTTTA TTGATGATGT GGACAGCATA CCTACAGTGA

AGATTTAAGG GATTGCTTTT TTGATGATGT GGAGAACATA CCAGCAGTGA

GGATTTAAAG GAGTGCTTTT TTGATGATGT CGAGAGCATA CCACCAGTGA

AGATTTAAAG GAGTGCCTTT TTGATGATTT GGAGAGCATA CCAGCAGTGA

AGACCTAAAG GACTGTTTTT TTGATGATGT GGACAACATG CCAACAGTGA

AGATTTAAAG GAGTGCTTTT TGGATGATGT GGAGAGCATT CCAGCAGTGA

AGATTTAAAG GAGTGCTTTT TTGACGACGT GGAGAACATG CCACCAGTGA

GGATCTAAAG GAATGCTTTT TGGACGACGT GGAGAGCATA CCGGCCGTGA

AGATTTAAAG GAGTGGTTTT TTGATGATGT AGAAAACATC CCAGCTGTTA

AGATTTAAAG GAGTGCTTTA TTGATGACAT AGAGAGCACG ACTTCTGTGA

CTACATGGAA CACATACCTT CGATATATTA CTGTCCACAA GAGCTTAATT

CTACATGGAA CACATACCTT CGATATATTA CTGTCCACAA GAGCTTAATT

CTACATGGAA CACATACCTT CGATATATTA CTGTCCACAA GAGCTTAATT

---------- ---------- ---------- ---------- ----------

CTACATGGAA CACATACCTT CGATACATTA CTCTCCACAA GAGCTTAATT

CTACCTGGAA CACATACCTT AGATACCTAG CTGTCAACAA GAGCTTAAGT

CAACATGGAA CACATACCTT CGATATTTCA CGCTCCACAG AGGCTTATTT

CAACATGGAA CACATACCTA CGATATTTTA CTCTCCATAA AGGCTTACTG

CCACATGGAA TACATACCTT CGGTATATTA CTGTCCACAG GAGCTTGATT

CTACATGGAA CACATACCTT CGATATATTA CTGTCCACAA GAGCTTAATG

CTACATGGAA CACATACCTT CGATATATTA CTGTCCACAA GAACTTAATT

CTACGTGGAA CACATACCTT AGATATATTA CTGTCCACAA GAACTTAATT

CCACATGGAA CACATACCTT CGATATATTA CTGTTCATAA GAATTTAATT

CCACATGGAA CACATATCTT CGATACATTA CTGTCCATAA GAGATTAATT

CCACATGGAA CACATACCTT CGATATGTTA CTGTCCACAA GAGCTTAATT

CCACGTGGAA CACGTACCTT CGCTACATTA CCGTCCACAA GAGCTTAATT

CCACATGGAA CACCTACCTC AGATATATCA CTATTCATAA GAACTTAGTT

CCACATGGAA TACATATCTC AGATACATGA CCATTCACAA GAAGTTGATT

TTTGTGCTAA TTTGGTGCTT AGTAATTTTT CTGGCAGAGG TGGCTGCTTC

TTTGTGCTAA TTTGGTGCTT AGTGATTTTT CTGGCAGAGG TGGCTGCTTC

TTTGTGCTAA TTTGGTGCTT GGTAATTTTT CTGGCTGAGG TGGCTGCTTC

---------- ---------- ---------- ---------G TGGCTGCTTC

TTTGTGCTAA TTTGGTGCTT AGTAATTTTT CTGGCTGAGG TGGCTGCTTC

TTGGTACTAA TTTGGTGCTT AGTTATTTTT CTGGCTGAGG TGGCAATTTC

GCAGTGCTGA TTTGGTGCGT GCTGGTTTTT CTGGTTGAGG TGGCTGCTTC

CTAGTGCTGA TTTGGTGCGT ACTGGTTTTT CTGGTTGAGG TGGCTGCTTC

TTTGTGCTAA TTTGGTGCAT AGTTATTTTT CTAGCTGAGG TGGCTGCCTC

TTTGTGCTAA TTTGGTGCTT AGTCGTTTTT CTGGTTGAGG TGGCCGCTTC

TTTGTGCTAA TTTGGTGCTT AGTTATTTTT CTGGCTGAGG TGGCTGTTTC

TTTGTGCTCA TTTGGTGCTT AGTTATTTTT CTGGCTGAGG TGGCTGCTTC

TTGGTGCTAA TTTGGTGTTT AGTTATTTAT CTCGCTGAGG TGGCTCTGTC

TTTGTGCTAA TTTGGTGCTT TGTTGTTTTT CTGATTGAGG TGGCTGCTTC

TTTGTGCTAA TTTGGTGTTT AGTTGTTTTT CTGGCTGAGG TGGCTGTTTC

GTTGTGCTCA TTTGGTGTGT CGCCATCTTT CTGGCTGAGG TGGCTCTTTC

TTTGTCTTAA TTTGGTGCCT CGTTATTTTT CTGGTTGAGG TGGCTGCTTC

TTTGTGCTAA TGATGTGCCT AGTCATCTTT TTGATTGAGG TGGCTGCGTC

TTTGGTTGTG CTGTGGCTCC TTGGAAACAC TCCTGACAAA GGGAATAGTA

TTTGGTTGTG CTGTGGCTCC TTGGAAACAC TCCTGACAAA GGGAATAGTA

TTTGGTTGTG CTGTGGTTCC TTGGAAACAC TCCTGACAAA GGGAATAGTA

TTTGGTTGTG CTGTGGTTCC TTGGAAACAC TCCTGACAAA GGGAATAGTA

TTTGGTTGTG TTGTGGCTCC TTAGAAACAC TCCTGACAAA GGGAATAGTA

TTTGGCGGTG TTGTTGCTAC TTGACAAGAG CCCTAGCAAA GGGAACGGCA

TTTATTTGTG TTGTGGTTGC TTAAAAACAA CCCCGGTGGA AACAATGGCA

TTTATTTGTG TTATGGTTGC TTAAAAACAA CCCTAGTGGA AACAATGGTA

CTTGGTTGTG TTGTGGCTAT TTGGAAACAC TGCTGACAAA GAGAATAGTA

TTTGGTTGTG TTGTGCCTAT TTCCGAAAAT ATTTGACAAA GGAAACAGTA

ATTGGTTGTA TTGTGGATAC TTAGAAACTT GTCTGACAAA GGGAATAGCA

GCTGGTTGCG TTTTGGCTAA TTGAAAAAAC ACGTGACAAA GGGAATAGTA

TCTGGTGGTG CTCTGGCTAT TTGAACCGAA TCCTGACAAA GGGAATAGTA

TTTGGTTCTG TTGTGTCTAC TTAGTAAAGT ATCTGACAAA GGGAATACTA

TTTGGTTGTG TTGTATCTAC TTAGGACATC ATCTGACAAA GGGAATAATA

TTTGGTCGTG TTGTGGCTGC TTCGAAGTTC CTCTGACAAA GGAAATAGCA

TTTGGTTGGG CTTTGGCTCC TTAAGGACAC CTCTGATAAA ACAAATGGTA

TTTGGTTGGG CTGTGTTTAT TCAAAGAGGC CTC---AAGA ATGAATTCCA

CTCATAGTAG AAATAACAGC TATGCAGTGA TTATCACCAG CACCAGTTCG

CTCATAGTAG AAATAACAGC TATGCAGTGA TTATCACCAG CACCAGTTCG

CTTATAGTAG AAATAACAGC TATGCAGTGA TTATCACCAG AACCAGTTCG

CTTATAGTAG AAATAACAGC TATGCAGTGA TTATCACCAG AACCAGTTCG

CTTATAGTAG AAATAACAGC TATGCAGTGA TTATCACCAA CACCAGTTCA

CTGCCAGTGG GAATGGCAGC TCCGCCGTGA TCATCACCAG CACCAGCTCC

CTAAAATTGC CAATACCTCC TATGTTGTGG TCATCACCAG TTCCAGCTTC

CTAAAATTTC CAATAGCTCC TATGTTGTGA TCATCACCAG TACCAGTTTC

CAAAAAGTGG GAACAGCAGC TATGCTGTGA TTATCACCAA CACCAGCTCT

CTAAGAGTGC AAATAACAGT TATGCAGTGA TCATCACCAG CACCAGCTCG

CTCAGAGCGT AAACAGCAGT TATGCAGTGA TCTTCACCAG TACCAGCGCA

CCAGGAGCAC AAATAACACC TCCCCTGTCA TCATCACCAG CACCAGCGCC

CTGAGATTTC AAATAGCACC TATGCCGTGA TCATCACTGA CACCAGCAAA

CTAAGAGTGC CAATGACAGC TCTGCAGTGA TCATCACCAG CACCAGCTCG

CTACTAACGC AAATAGCAGC TACGGAGTGA TCGTCACTAA CACCAGCTCA

CTACTAATGC CACTAACAGC TATGGCATCA TCGTCACTGG CACCAGCTCG

CAAATGGGAC CAATAATACC ATTGGAGTCA TTATCACTGA CACCAGCAAA

CCAGTAATCT AAACCACAGG TTCGCCGTGA TCGTCACCAA CACGAGCACC

TATTATGTGT TTTACATTTA CGTGGGAGTA GCCGACACTT TGCTTGCTAT

TATTATGTGT TTTACATTTA CGTGGGAGTA GCCGACACTT TGCTTGCTAT

TATTATGTGT TTTACATTTA CGTGGGAGTA GCCGACACTT TGCTTGCTAT

TATTATGTGT TTTACATTTA CGTGGGAGTA GCCGACACTT TGCTTGCTAT

TATTATGTGT TTTACATTTA CGTGGGAGTT GCTGACACTT TGCTTGCTCT

TACTACTTGT TCTACATTTA CGTGGGAGTA GCTGACACTT TGCTTGCTCT

TATTACATTT TTTACATTTA CGTGGGAGTG GCTGACACTT TGCTTGCTCT

TATTATATTT TTTACATTTA CGTGGGAGTG GCTGACACTT TGCTTGCCCT

TACTACTTTT TTTACATTTA TGTGGGAGTA GCCGACACCT TGCTTGCATT

TATTATATTT TTTACATTTA TGTGGGAGTG GCTGACACTT TGCTTGCTCT

TATTATATTT TCTACATTTA TGTGGGAGTA GCTGATACTT TGCTTGCTCT

TTCTATATGT TTTACATTTA CGTGGGAGTA GCTGACAGTT TGCTCGCTCT

TTTTATATTA TTTACATTTA TGTGGGAGTC GCTGACACTT TGCTTGCTCT

TTTTACTTTT TGTACATTTA CGTGGGAGTA GCTGACACTT TCCTCGCTCT

TATTATCTTT TGTACATTTA TGTGGGAATA GCTGACAGTT TGTTTGCCCT

TTTTATGTTT TCTACATCTA TGTGGGAGTA GCCGACACTC TGCTGGCCCT

TACTACTTGT TTTATATATA TGTGGGAGTA GCTGACACAT TCTTTGCCCT

TATTACATGT TTTATATTTA TGTGGGGGTA GCAGACACCT TGCTTGCCCT

GGGATTCTTC AGAGGTCTAC CACTGGTGCA TACTCTAATC ACAGTGTCGA

GGGATTCTTC AGAGGTCTAC CACTGGTGCA TACTCTAATC ACAGTGTCGA

GGGATTCTTC AGAGGTCTAC CACTGGTGCA TACTCTAATC ACAGTGTCGA

GGGATTCTTC AGAGGTCTAC CACTGGTGCA TACTCTAATC ACAGTGTCGA

GGGATTCTTC AGAGGTCTAC CACTGGTGCA TACCCTAATC ACAGTGTCGA

GGGCTTCTTC AGAGGTCTGC CCCTGGTGCA TACTCTCATC ACAGTGTCAA

GAGCCTCTTC AGAGGTTTGC CCCTGGTGCA TACGTTAATC ACAGCATCAA

GAGCCTCTTC AGAGGTTTGC CGCTGGTGCA TACGTTAATC ACAGCATCAA

GGGATTATTC AGAGGTTTGC CACTGGTGCA TACTCTAATC ACAGTGTCGA

AGGACTCTTC AGAGGTTTAC CACTGGTGCA TACTCTAATC ACAGTGTCAA

GGGACTCTTC AGAGGTTTAC CACTGGTGCA TACTCTAATC ACAGTGTCGA

GGGATTCCTC CGAGGCTTAC CACTGGTGCA TACGCTAATC ACAGTATCGA

GGGGATTTTC AGAGGTCTGC CACTGGTACA CACTCTGATC ACAGTGTCAA

GGGACTCTTC AGGGGTTTAC CACTGGTGCA CACTCTAATT ACAGTGTCGA

GGCAATCTTC AGAGGTTTAC CACTGGTGCA TACCCTAATC AAAGTGTCAA

GGGATTCGCT AGGGGTTTGC CACTGGTACA TGCTCTCATC AAAGTGTCGA

AGGAATGCTT AGAGGCTTAC CACTGGTGCA TACACTAATA TCGGTCTCCA

GGGGTTCCTC CGTGGACTCC CCCTGGTGCA CAGCCTGATA TCCGTTTCCA

AAATTTTACA CCACAAAATG TTACATTCTG TTCTTCAAGC ACCTATGTCA

AAATTTTACA CCACAAAATG TTACATTCTG TTCTTCAAGC ACCTATGTCA

AAATTTTACA CCACAAAATG TTACATTCTG TTCTTCAAGC ACCTATGTCA

AAATTTTACA CCACAAAATG TTACATTCTG TTCTTCAAGC ACCTATGTCA

AAATTTTACA CCACAAAATG TTACATTCTG TTCTTCAAGC ACCTATGTCA

AAATTTTACA CCACAGAATG TTGCATTCTG TTCTTCGAGC GCCTATGTCA

AAATTTTGCA CAGGAAAATG TTACATTCTA TTCTTCATGC CCCTATGTCA

AAATTTTGCA CAGGAAAATG TTACACTCCA TTCTTCACGC CCCTATGTCG

AAATTTTACA CCACAAAATG TTACATTCTG TTCTTCAGGC GCCCATGTCA

AAACTTTACA CCACAAAATG CTACAGTCTG TTCTTCAAGC CCCTATGTCA

AAATTTTACA CCACAAAATG TTACATTCTG TTCTTCAAGC ACCTATGTCA

AAATCTTACA CCAGAAAATG TTACATTCTG TTCTTCATGC ACCTATGTCG

AAATATTACA CCACAAAATG CTGCATTCTG TTCTTCATGC ACCTATGTCA

AAATTTTACA CCACAAAATG TTACATTCTG TTCTTCAAGC ACCTATGTCA

AAACTTTACA CCACAAAATG TTACGTTCTA TTCTTCAAGC ACCTATGTCA

ACATTTTACA CCGCAAAATG TTGCATTCTG TTCTTCAAGC ACCTATGTCG

AAATTCTACA TCACAAAATG TTATGCTCTG TTTTGAAAGC ACCTATGTCA

AAATTCTCCA CCAGAAGATG CTGCATTCGG TCCTGCAGGC GCCCATGTCG

ACCCTCAACA CGTTGAAAGC AGGTGGGATT CTTAATAGAT TCTCCAAAGA

ACCCTCAACA CGTTGAAAGC AGGTGGGATT CTTAATAGAT TCTCCAAAGA

ACCCTCAACA CGTTGAAAGC AGGTGGGATT CTTAATAGAT TCTCCAAAGA

ACCCTCAACA CGTTGAAAGC AGGTGGGATT CTTAATAGAT TCTCCAAAGA

ACCCTCAACA CATTGAAAGC AGGTGGGATT CTTAATAGAT TCTCCAAAGA

ACCCTCAACA TGTTAAAAGC AGGTGGGATT CTGAATAGAT TCTCCAAAGA

ACCTTCAACA AGCTGAAAGC AGGTGGGATT CTTAACAGAT TCTCCAAAGA

ACCATCAGCA AGCTGAAAGC AGGTGGGATT CTTAACAGAT TCTCCAAAGA

ACACTCAACA CATTGAAAGC AGGTGGGATT CTTAATAGAT TCTCCAAGGA

ACCCTCAACA CGTTGAAAAC AGGTGGGATT CTTAATAGAT TCTCCAAAGA

ACCCTCAACA CACTGAAAGC AGGTGGGATT CTTAATAGAT TCTCCAAAGA

ACCCTGAACA CCTTGAAAGC AGGTGCGATT CTTAATAGAT TTTCCAAAGA

ACCCTC-ACG CACTGAAAGC AGGTGGGATT CTTAATAGAT TCTCCAAAGA

ACCTTAAACA CATTAAAAGC AGGTGGGATA CTTAATAGAT TCTCCAAAGA

ACGTTCAACA CATTGAAAGC AGGTAGGATT CTTAATAGAT TCTCCAAAGA

ACCTTCAACA CGTTGAAAGC AG-------- ---------- ----------

ACCTTCAACA CTTTGAAAGC AGGGGGAATT CTGAACCGTT TCTCCAAAGA

ACTTTCAACA CTTTGAAAAC AGGTAGCATT CTCAACAGAT TCTCCAAAGA

TATAGCAATT TTGGATGACC TTCTGCCTCT TACCATATTT GACTTCATCC

TATAGCAATT TTGGATGACC TTCTGCCTCT TACCATATTT GACTTCATCC

TATAGCAATT TTGGATGATC TTCTGCCTCT TACCATATTT GACTTCATCC

TATAGCAATT TTGGATGATC TTCTGCCTCT TACCATATTT GACTTCATCC

TATAGCAATT TTGGATGATC TTCTGCCTCT TACCATATTT GACTTCATCC

CATAGCGATC TTGGATGACC TCCTGCCTCT TACCATATTT GACTTCGTCC

TATAGCAATT TTGGATGACT TTCTGCCTCT TACCATATTT GACTTCATCC

TATAGCAATT TTGGATGACT TTCTGCCTCT TACCATTTTT GACTTCATTC

TATAGCCATT CTGGATGATC TGCTGCCTCT TACCATATTT GACTTCATCC

TATAGCAGTT TTGGATGATC TTCTGCCTCT TACCATATTT GATTTTGTTC

TATAGCGATT TTGGATGATC TTCTGCCCCT TACCATATTT GACTTCATCC

TATAGCAATT TTGGATGATC TTCTGCCTCT TACCATATTT GACTTCATCC

TATAGCAATT TTGGATGATC TTCTGCCTCT TACCATATTT GACTTCATTC

TATAGCAATT TTGGATGATC TTCTGCCTCT TACCATATTT GACTTCATCC

TATAGCAATT TTGGATGATC TTCTGCCTCT TACCATGTTT GACTTCATCC

---------- ---------- ---------- ---------- ----------

TATAGCAATT CTGGATGACC TCCTGCCACT TACAATATTT GACTTCATCC

TATGGCCATT TTGGATGACC TTCTGCCACT TACAATATTT GACTTCATTC

AGTTGTTATT AATTGTGATT GGAGCTATAG CAGTTGTCGC AGTTTTACAA

AGTTGTTATT AATTGTGATT GGAGCTATAG CAGTTGTCGC AGTTTTACAA

AGTTGTTATT AATTGTGATT GGAGCTATAG CAGTTGTCGC GGTTTTACAA

AGTTGTTATT AATTGTGATT GGAGCTATAG CAGTTGTCGC GGTTTTACAA

AGTTGTTATT AATTGTGATT GGAGCTATAG CAGTTGTCTC GGTTTTACAG

AGTTGTTACT AATCGTGATT GGAGCTGTGG CAGTTGTCTC GGTTTTGCAA

AGTTGCTGTT CATTGTGGTC GGAGCAATAA TAGTTGTCTC GGCTTTACAA

AGTTGGTGTT CATTGTGATT GGAGCTATAA TAGTCGTCTC GGCATTACAA

AGTTATTGTT AATTGTGGTT GGAGCCATAG CAGTGGTTTC GGTTTTACAA

AGTTGTTATT AATTGTGATC GGAGCTGTGG TGGTCGTCTC AGTTTTACAG

AGTTGTTATT AATTGTGATC GGAGCTGTGG CGGTGGTGTC GGTTTTACAG

AGTTGGTGTT AATTGTGATT GGAGCTGTAG TGGTGGTCTC GATTTTAAAG

AGTTGGTTTT AATTGTGATC GGAGCTGTGA CAGTGGTCTC GGTTTTACAA

AGCTGCTGTT AATTGTGATT GGTGCTGTAG CCGTAGTCTC GATTTTAAAA

AGTTGTTATT AATTGTGATT GGCGCTGTAG TAGTCGTCTC TGTTTTACAA

---------- ---------- ---------- ---------- ----------

AGTTAATACT GATTGTATTT GGAGCTCTCA TTGTAGTGTC AGCCATACGA

AGCTACTGCT GATTGTGATT GGAGCCGTGA CAGTCGTGTC AGCTTTACAA

CCCTACATCT TTGTTGCAAC AGTGCCAGTG ATAGTGGCTT TTATTATGTT

CCTTACATCT TTGTTGCAAC AGTGCCAGTG ATAGTGGCTT TTATTATGTT

CCCTACATCT TTGTTGCAAC AGTGCCAGTG ATAGTGGCTT TTATTATGTT

CCCTACATCT TTGTTGCAAC AGTGCCAGTG ATAGTGGCTT TTATTATGTT

CCCTACATCT TTCTTGCAAC AGTGCCAGTG ATAGCAGCTT TTGTTTTATT

CCTTACATCT TCCTTGCCAC GGTGCCGGTG ATAGCGGCTT TTGTTATCTT

CCCTACATCT TCCTAGCAAC GGTGCCAGGG CTAGCAGTCT TCATTTTACT

CCCTACATCT TCCTAGCAAC GGTGCCAGGG CTAGTAGTCT TTATTTTACT

CCTTACATCT TCCTAGCAAC AGTTCCAGTG ATAGCAGCCT TCATTTTATT

CCCTACATCT TCCTAGCTAC AGTGCCAGTG ATAGCGGCTT TTATTCTCTT

CCCTACATCT TCCTAGCGAC AGTGCCAGTG ATAGCGGCCT TTATTATCCT

CCCTACATCT TCCTAGCAGC AGTGCCAGTG ATCATTGCTT TTGTGATACT

CCCTACATCT TCCTAGCAAC AGTGCCAGTG ATAGCTGCTT TTATTTTATT

CCCTACATCT TCCTAGCAAC AGTGCCTGTG ATAGTTGCGT TTGTTCTATT

CCCTACATCT TCCTGGCAAC AGTGCCCGTG ATAGCTGCGT TTATTATACT

---------- ---------- ---------- ---------- ----------

CCATATATCT TTCTGGCAAC AATGCCCGTG ATAGCAGCCT TTATTATGTT

CCCTACATCT TCCTCGCCTC TGTGCCAGTG GTAATAGCCT TTGTATTGTT

GAGAGCATAT TTCCTCCAAA CCTCACAGCA ACTCAAACAA CTGGAATCTG

GAGAGCATAT TTCCTCCAAA CCTCACAGCA ACTCAAACAA CTGGAATCTG

GAGAGCATAT TTTCTCCAAA CCTCACAGCA ACTCAAACAA CTGGAATCTG

GAGAGCATAT TTTCTCCAAA CCTCACAGCA ACTCAAACAA CTGGAATCTG

GAGAGCATAT TTCCTCCAAA CCTCACAGCA ACTCAAACAA CTAGAATCTG

GAGAGGATAC TTCCTACACA CCTCACAGCA ACTCAAACAA CTGGAATCTG

GAGGGCATAC TTCCTTCATA CCTCCCAACA GCTCAAACAA CTGGAATCTG

GAGGGCCTAC TTCCTTCATA CAGCACAGCA GCTCAAACAA CTGGAATCTG

GAGAGCGTAC TTCCTCCATA CCTCACAGCA ACTCAAGCAA CTGGAATCTG

GAGAGCCTAC TTCCTCCACA CCTCCCAGCA ACTCAAGCAG CTGGAATCTG

GCGGGCCTAC TTCCTCCACA CCTCACAGCA ACTCAAGCAA CTGGAATCTG

GAGAGCCTAC TTCCTGCAGA CCTCCCAGCA ACTCAAACAA CTGGAATCAG

GAGAGCCTAC TTCCTCCACA CCTCACAGCA GCTCAAACAA CTAGAGTCTG

GAGAGCATAC TTCCTTCACA CCTCACAGCA ACTCAAACAA CTGGAATCTG

GAGAGCATAC TTCCTCCACA CCTCACAGCA GCTCAAACAA TTGGAATCTG

---------- ---------- ---------- ---------- ----------

AAGGGCATAT TTCCTCCAAA CCTCCCAGCA GCTAAAACAA CTGGAATCTG

GAGAGCATAC TTTCTCCGCA CATCCCAGCA ACTTAAACAA CTGGAATCTG

AAGGCAGGAG TCCAATTTTC ACTCATCTTG TTACAAGCTT AAAAGGACTA

AAGGCAGGAG TCCAATTTTC ACTCATCTTG TTACAAGCTT AAAAGGACTA

AAGGCAGGAG TCCAATTTTC ACTCATCTTG TTACAAGCTT AAAAGGACTA

AAGGCAGGAG TCCAATTTTC ACTCATCTTG TTACAAGCTT AAAAGGACTA

CAGGCAGGAG TCCAATTTTC ACTCATCTTG TTACAAGTTT AAAAGGACTA

AAGGCAGGAG TCCAATTTTC ACTCATCTGG TTACAAGCTT AAAAGGACTA

AAGGCAGGAG TCCAATTTTC ACGCACCTTG TTACAAGCTT AAAAGGACTG

AAGGCAGGAG TCCAATTTTC ACCCACCTTG TGACAAGCTT AAAAGGACTC

AAGGTAGGAG TCCAATTTTC ACTCATCTTG TTACAAGCTT GAAAGGATTA

AAGGCAGGAG TCCAATTTTC ACTCATCTTG TTACAAGTTT AAAAGGACTA

AAGGCAGGAG TCCAATTTTC ACTCATCTTC TTACAAGTTT AAAAGGACTG

AAGGCAGGAG TCCAATTTTC ACTCATCTGG TTACAAGCTT AAAAGGCCTA

AAGGCAGAAC TCCAATTTTC ACTCATCTTG TTACAAGCTT AAAAGGACTG

AAGCCAGAAG CCCAATTTTC ACTCATCTTG TTACAAGTTT AAAAGGACTG

AAGCCAGGAG TCCCATTTTC ACTCATCTTG TTACAAGCGT AAAAGGACTA

---------- ---------- ---------- ---------- ----------

AAGCCAGGAC TCCAATTTTT ACTCATCTGG TTACAAGTTT AAAGGGACTG

AAGCCCGGAG TCCAATATTT ACTCATCTTA TTACAAGCTT AAAGGGGCTA

TGGACACTTC GTGCCTTCGG ACGGCAGCCT TACTTTGAAA CTCTGTTCCA

TGGACACTTC GTGCCTTCGG ACGGCAGCCT TACTTTGAAA CTCTGTTCCA

TGGACACTTC GTGCCTTCGG ACGGCAGCCT TACTTTGAAA CTCTGTTCCA

TGGACACTTC GTGCCTTCGG ACGGCAGCCT TACTTTGAAA CTCTGTTCCA

TGGACACTTC GTGCCTTCGG ACGGCAGCCT TACTTTGAAA CTCTGTTCCA

TGGACACTTC GTGCCTTCGG TCGTCAGCCT TACTTTGAAA CTCTTTTCCA

TGGACACTTC GAGCCTTCCG ACGCCAGACT TACTTTGAAA CCCTATTCCA

TGGACACTTC GAGCCTTCCG ACGCCAGACT TACTTTGAAA CTCTGTTCCA

TGGACACTTC GTGCCTTTGG ACGGCAGCCT TACTTTGAAA CTCTGTTCCA

TGGACACTTC GTGCCTTTGG ACGGCAGCCT TACTTTGAAA CTTTATTCCA

TGGACACTTC GGGCCTTTGG ACGCCAGCCT TACTTTGAGA CTTTGTTTCA

TGGACACTGC GTGCCTTCGG ACGTCAGCCT TACTTTGAAA CTCTGTTCCA

TGGACACTTC GTGCTTTTGG GCGACAGCCT TACTTTGAAA CTCTGTTCCA

TGGACACTTC GTGCCTTTGG ACGTCAGCCT TACTTTGAAG CTCTGTTCCA

TGGACGCTCC GTGCCTTTGG ACGCCAGCCT TACTTTGAAA CTCTGTTCCA

---------- ---------- ---------- ---------- ----------

TGGACTCTAC GAGCTTTCGG ACGCCAACCG TACTTTGAGA CTTTGTTCCA

TGGACATTGC GTGCATTTGG ACGCCAAACG TATTTTGAAG CGCTGTTCCA

CAAAGCTCTG AATTTACATA CTGCCAACTG GTTCTTGTAC CTGTCAACAC

CAAAGCTCTG AATTTACATA CTGCCAACTG GTTCTTGTAC CTGTCAACAC

CAAAGCTCTG AATTTACATA CTGCCAACTG GTTCTTGTAC CTATCAACAC

CAAAGCTCTG AATTTACATA CTGCCAACTG GTTCTTGTAC CTATCAACAC

CAAAGCTCTG AATTTACATA CTGCCAACTG GTTCTTGTAC CTATCAACAC

CAAAGCTCTG AATTTACATA CTGCCAACTG GTTCTTGTAC CTGTCAACCC

CAAAGCTCTG AATTTGCACA CTGCCAACTG GTTTATGTAT CTGGCAACCT

CAAAGCTCTG AATTTGCACA CTGCCAACTG GTTTATGTAT CTGGCAACCT

CAAAGCTCTG AATTTACACA CTGCCAACTG GTTCTTGTAT CTGTCAACAC

CAAAGCTCTG AATTTACATA CTGCCAACTG GTTCTTGTAT CTGTCAACAC

CAAAGCTCTG AACTTACATA CTGCCAACTG GTTCTTGTAC CTGTCAACGC

CAAAGCTTTG AATTTACATA CTGCCACCTG GTTCTTATAC CTGTCAACAC

TAAAGCGCTA AACTTACATA CAGCCAACTG GTTTTTGTAC CTATCAACTT

CAAAGCTCTG AATTTGCATA CTGCCAACTG GTTCCTGTAC CTATCAACAC

CAAAGCTCTG AATTTACACA CTGCCAACTG GTTCTTGTAC CTGTCGACAC

---------- ---------- ---------- ---------- ----------

TAAAGCTCTG AATTTACACA CAGCAAATTG GTTCCTATAC CTGTCCACAC

CAAGGCTCTG AATTTACACA CCGCCAACTG GTTCCTGTAT CTGTCCACTC

TGCGCTGGTT CCAAATGAGA ATAGAAATGA TTTTTGTCAT CTTCTTCATT

TGCGCTGGTT CCAAATGAGA ATAGAAATGA TTTTTGTCAT CTTCTTCATT

TGCGCTGGTT CCAAATGAGA ATAGAAATGA TTTTTGTCAT CTTCTTCATT

TGCGCTGGTT CCAAATGAGA ATAGAAATGA TTTTTGTCAT CTTCTTCATT

TGCGCTGGTT CCAAATGAGA ATAGAAATGA TTTTTGTCAT CTTCTTCATT

TGCGCTGGTT CCAAATGCGA ATAGAAATGA TATTTGTCGT CTTCTTCATT

TGCGCTGGTT CCAGATGAGA ATAGACATGA TTTTCGTCCT CTTCTTCATT

TGCGCTGGTT CCAAATGAGA ATAGACATGA TATTTGTCCT CTTCTTCATT

TACGCTGGTT CCAAATGAGG ATAGAAATGA TTTTTGTCCT CTTCTTCATT

TGCGCTGGTT CCAAATGAGA ATAGAAATGA TTTTTGTCAT CTTCTTCATT

TGCGCTGGTT CCAAATGAGA ATGGAAATTA TTTTTGTCAT ATTCTTCATT

TGCGCTGGTT CCAAATGAGA ATAGAGATGA TTTTCGTCGT CTTCTTCGTT

TGCGCTGGTT CCAAATGAGG ATAGAAATTA TTTTTGTCCT CTTCTTCATT

TACGCTGGTT CCAAATGAGG ATAGAAATGA TTTTTGTCAT CTTCTTCATT

TTCGCTGGTT CCAAATGAGA ATAGAAATTA TTTTTGTCAT CTTCTTCATT

---------- ---------- ---------- ---------- ----------

TCCGCTGGTT CCAAATGAGA ATAGAAATTG TTTTTGTCAT CTTCTTCATT

TCCGCTGGTT CCAAATGAGG ATAGAAATGG TATTTGTCAT CTTCTTCATT

GCTGTTACCT TCATTTCCAT TTTAACAACA GGAGAAGGAG AAGGAAGAGT

GCTGTTACCT TCATTTCCAT TTTAACAACA GGAGAAGGAG AAGGAAGAGT

GCTGTTACCT TCATTTCCAT TTTAACAACA GGAGAAGGAG AAGGAACAGT

GCTGTTACCT TCATTTCCAT TTTAACAACA GGAGAAGGAG AAGGAACAGT

GCTGTTACCT TCATTTCCAT TTTAACTACA GGAGAAGGAG AAGGAACAGT

GCTGTTACCT TCATTTCCAT TTTAACAACA GGTGAAGGAG AAGGAACCGT

GTTGTTACCT TCATCTCCAT TTTAACAACA GGTGAAGGAG AAGGAACGAC

GTTGTTACCT TCATCTCCAT TTTAACAACA GGTGAAGGAG AAGGAACAGC

GCCGTAGCCT TCATTTCCAT TTTAACAACA GGTGAAGGAG AAGGAAGAGT

GCTGTTACCT TCATTTCCAT TTTAACAACA GGTGAAGGAG AAGGAAGAGT

GCTGTTACCT TCATCTCCAT TTTAACAACA GGTGAAGGAG AAGGAACAGT

GCTGTTACCT TCATTTCTAT TTTAACAACA GGTGAAGGAG AAGGAACGGT

GCCGTTACCT TCATTTCCAT TTTAACAACA GGTGAAGGAC AAGGACAAAT

GCTGTTACCT TCATTTCCAT TTTAACAACA GGTGAAGGAG AAGGAACAGT

GCTGTTACCT TCGTTTCCAT TTTAACAACA GGTGAAGGAG AAGGAACAAT

---------- ---------- ---------- ---------- ----------

GCTGTGACCT TCATCTCCAT TTTAACTACA GGAAATGGAG AAGGAGAAAT

CTGGTGACCT TCATCTCCAT TTTAACCACA GGTGATGGAG AAGGGAAAGT

TGGTATTATC CTGACTTTAG CCATGAATAT CATGAGTACA TTGCAGTGGG

TGGTATTATC CTGACTTTAG CCATGAATAT CATGAGTACA TTGCAGTGGG

TGGTATTATC CTGACTTTAG CCATGAATAT CATGAGTACA TTGCAGTGGG

TGGTATTATC CTGACTTTAG CCATGAATAT CATGAGTACA TTGCAGTGGG

TGGTATTATA CTGACTTTAG CCATGAATAT CATGAGTACA TTGCAGTGGG

TGGAATTATT CTGACTTTAG CCATGAATAT CATGGGGACA TTGCAGTGGG

TGGTATTATT CTAACTTTAG CTATGAATAT TATGAGTACT TTGCAATGGG

TGGTATTATT CTAACTTTAG CTATGAATAT CATGAGTACT TTGCAGTGGG

TGGTATTATT CTAACTTTAG CCATGAATAT CATGAGTACG TTGCAGTGGG

TGGGATTATT CTAACTTTAG CCATGAATAT CATGGGTACA TTGCAGTGGG

TGGTATTATT CTAACATTAG CCATGAATAT CATGGGTACG TTGCAGTGGG

TGGCATTATT CTAACATTAG CCATGAATAT CATGAGTACA TTGCAGTGGG

TGGTATTGTA CTAACTTTAG CCATGAATAT TATGAATACA TTGCAGTGGG

TGGTATTATT CTAACTTTAG CCATGAATAT CATGAGTACA TTGCAGTGGG

TGGCATTATT CTAACATTAG CCATGAATAT CATGAACACA TTGCAGTGGG

---------- ---------- ---------- ---------- ----------

TGGAATTCTT CTAACTTTAG CCATGAATAT CATGAGTACC TTGCAGTGGG

TGGAATTGTT CTAACATTAG CCATGAACAT CATGGGTACA TTGCAGTGGG

CTGTAAACTC CAGCATAGAT GTGGATAGCT TGATGCGATC TGTGAGCCGA

CTGTAAACTC CAGCATAGAT GTGGATAGCT TGATGCGATC TGTGAGCCGA

CTGTAAACTC CAGCATAGAT GTGGATAGCT TGATGCGATC TGTGAGCCGA

CTGTAAACTC CAGCATAGAT GTGGATAGCT TGATGCGATC TGTGAGCCGA

CTGTAAACTC CAGCATAGAT GTGGATAGCT TGATGCGATC TGTGAGCCGA

CTGTGAACTC CAGCATAGAC GTGGATAGCT TGATGCGATC TGTGAGCCGA

CTGTGAACTC AAGCATTGAT ACAGATAGCT TGATGCGATC TGTGAGCAGA

CTGTGAACTC AAGCATTGAT ACAGATAGCT TGATGCGATC TGTGAGCAGA

CTGTGAACTC CAGCATAGAT GTAGATAGCT TGATGCGATC TGTGAGTCGA

CTGTAAACTC TAGCATAGAT GTGGATAGCT TGATGCGATC TGTGAGCCGA

CTGTAAACTC CAGCATAGAG GTGGACAGCT TGATGCGATC GGTGAGCCGA

CTGTGAACTC CAGCATAGAT GTGGATAGCT TGATGCGATC TGTGAGCCGA

CTGTCAATTC CAGCATAGAT GTGGATAGCT TGATGCGATC TGTGAGCCGA

CTGTGAACTC CAGCATAGAT GTGGATAGTT TGATGCGATC TGTGAGCCGA

CTGTAAACTC CAGCATAGAT GTGGACAGTT TGATGAGATC TGTGAGCCGA

---------- ---------- ---------- ---------- ----------

CTGTGAACTC CAGCATAGAT GTGGACAGTT TGATGCGGTC TGTGAGCCGA

CAGTAAACGC GAGCATTGAT GTGGACAGTT TGATGCGGTC TGTGAGCAGA

GTCTTTAAGT TCATTGACAT GCCAACAGAA GGTAAACCTA CCAAGAAACC

GTCTTTAAGT TCATTGACAT GCCAACAGAA GGTAAACCTA CCAAGAAACC

GTCTTTAAGT TCATTGACAT GCCAACAGAA GGTAAACCTA CCAGGAAACC

GTCTTTAAGT TCATTGACAT GCCAACAGAA GGTAAACCTA CCAGGAAACC

GTCTTTAAGT TCATTGACAT GCCAACAGAA GGTAAACCTA CCAAGAAAGC

GTCTTTAAGT TTATTGATAT GCCGACAGAA GGTAGATCTA CCAAGAAACC

GTCTTTAAGT TTATTGATAT ACAAACAGAA AGTATATGTA CCAAGAAACT

GTGTTTAAGT TTATTGATAT ACAAACAGAA AGTATGTACA CACAGAAACT

GTCTTTAAGT TTATTGACAT GCCAACAGAA ACTAAGTCTA CCAAAAAACC

GTCTTTAAGT TTATTGATAT GCCAACAGAT GGTAAACCTA ACAATAGACC

GTCTTCAAGT TTATTGACAT GCCTACAGAA AGTAAACCTA CCAAGAAACC

GTCTTTAAAT TTATTGACAT GCCAGCAGAA AGTAAATATA CCAAGAAACC

GTCTTCAAAT TTATTGATAT GCCAACAGAA AGTAAACCTA CCAAGAAGCC

GTCTTTAAGT TTGTTGACAT ACCAACAGAA AATAAGCCTA CCAAAAAACT

ATCTTTAAGT TTATCGACAT GCCAACAGAA AGTAAACCTG CCAAAAAACC

---------- ---------- ---------- ---------- ----------

GTATTTAAAT TCATTGATAT GCCAGCAGAA CCTCTACCTA CCAAAAAATC

GTATTTAAAT TCATCGACAT GCCAACGGAG CACAGGCCCA CCC---ACCC

ATACAAGAAT GGCCAACTCT CGAAAGTTAT GATTATTGAG AATTCACACG

ATACAAGAAT GGCCAACTCT CGAAAGTTAT GATTATTGAG AATTCACACG

ATACAAGAAT GGCCAACTCT CGAAAGTTAT GGTTATTGAG AATTCACACG

ATACAAGAAT GGCCAACTCT CGAAAGTTAT GGTTATTGAG AATTCACACG

ATACAAGAAT GGCCAGCTCT CAAAAGTTAT GATTATTGAG AATTCACACG

ATCCAAGGAT TGCCAGCTCT CAAAAGTTAT GATTTTTGAG AATCTACATG

ACATAGTGAA GAC---TCAC CTAATGCTTT AGTCATTAAG AATGAACATG

ACCTAGAGAA GGA---TCAT CTGACGTTTT AGTCATTAAG AATGAGCATG

ATCCAGTAAC TGCCAGCTAT CAAAAGTTAT GATTATTGAG AATCAACATG

ATCCAAGGAT AGTCAGCCCT CAAAAGTTAT GATCATTGAG AATCAACATG

ATCCAAGGAT GCCCAACTCT CAAAAGTTAC GATTACTGAG AATCGGCATG

ATACAAGGAT GGCCAGCTCT CGAAAGTTAT GATTCTCGAG AATCAACATG

ATCCAAGGGT GGTCCACTCT CAGAAGTTAT GATTATTGAG AATCAACAT-

GCCCAAGGAT GGCCAGCTTT CCAAAGTCAT GATTATTGAG AATCAGCACG

ACCCAAGGAT GGCCAGCTGT CCAAAGTTAT GATTATTGAG AACCAGCACG

---------- ---------- ---------- ---------- ----------

ATTGAAGAAA AACCAGCTTT CACAAGTTCT GATCATTGAG AATGAGCACG

AGCAAAGAAC AAAGACATCT CTGGAGTCCT GATCATTGAG AACCAGCATG

TGAAGAAAGA TGACATCTGG CCCTCAGGGG GCCAAATGAC TGTCAAAGAT

TGAAGAAAGA TTACATCTGG CCCTCAGGGG GCCAAATGAC TGTCAAAGAT

TGAAGAAAGA TGACATCTGG CCCTCAGGGG GCCAAATGAC CGTCAAAGAT

TGAAGAAAGA TGACATCTGG CCCTCGGGGG GCCAAATGAC GGTCAAAGAT

TGAAGAAAGA TGACATCTGG CCCTCAGGGG GTCAAATGAC CATCAAAGAC

TGAAGAAAGA TGACATCTGG CCTTCAGGAG GCCAAATGAC CGTCAAAGAC

TGAAGAAATG TGATACCTGG CCCTCTGGAG GTGAAATGGT TGTCAAAGAC

TGAAGAAAAG TGATATCTGG CCCTCTGGAG GCGAAATGGT TGTCAAAGAC

TGAAGAAAGA TGACGTCTGG CCCTCAGGAG GCCAAATGAC TGTCAAAGGT

TGAAGAAAGA TGACATCTGG CCCTCAGGGG GCCAAATGAC TGTCAAAGAC

TGAGGGAAGA TGATATCTGG CCCTCAGGGG GCCAAATGAC TGTCAAAGAC

TGAAGAAAGA TGACATCTGG CCCTCAGGGG GCCAAATTAC CGTCAAAGAC

CAAAAAAAGA GGATATCTGG CCCTCAGGTG GCCAAATGAC CATCAAAGAC

TGAAGAAAGA TGACATCTGG CCCTCTGGGG GCCAAATGAC TGTCAAAGAC

TGAAGAAAGA TTACAACTGG CCCTCAGGGG GTCACATGAC TGTCAAAGAC

---------- ---------- ---------- ---------- ----------

TAAATAAAGA AAA---CTGG CCCTCTGGAG GTCAGATGAT TGTCAAAGAC

TGAAGAGAGA GAAGAACTGG CCTTCAGGAG GCCAAATGAC CGTCCAGGAC

CTCACAGCAA AATACACAGA AGGTGGAAAT GCCATATTAG AGAACATTTC

CTCACAGCAA AATACACAGA AGGTGGAAAT GCCATATTAG AGAACATTTC

CTCACAGCAA AATACACAGA AGGTGGAAAT CCCATATTAG AGAACATTTC

CTCACAGCAA AATACACAGA AGGTGGAAAT CCCATATTAG AGAACATTTC

CTCACAGCAA AATACATAGA AGGTGGAAAT GCCATATTAG AGAACATTTC

CTCACAGCAA GATATATAGA CAGTGGGAAT GCCATATTGG AGAACATTTC

CTTACTGTGA AATATGTGGA TGATGGGAAT GCCATATTAG AGAACATTTC

CTTACTGTGA AATACATGGA TGATGGAAAT GCCGTATTAG AGAACATTTC

CTCACAGCAA AATATATAGA CAGCGGAAAT GCCATCCTGG AGAACATTTC

CTCACAGCAA AGTATACAGA TGGAGGGAAT GCCATACTAG AGAACATATC

CTCACAGCAA AGTATATAGA TGGTGGGAAT GCCATATTAG AGAACATTTC

CTCACAGCAA AATATATAGA TGGTGGGAAT GCCATATTAG AGAACATTTC

CTCACTGCAA AATATATAGA TGGAGGGAAT GCCATACTAG AGAACATCTC

CTTACAGCAA AATATATAGA TGGTGGGAAT GCCATATTAG AGAACATTTC

CTCACAGCAA AATATATAGA TGGTGGCAAT GCCATATTAG AGAACATTTC

---------- ---------- ---------- ---------- ----------

CTCACAGCCA AATATATAGA TGGTGGGAAT GCTGTATTAG AGAACATTTC

CTCACAGCCA AGTACCTAGA TGGAGGTCCG GCCATCTTGG ACAATATCTC

CTTCTCAATA AGTCCTGGCC AGAGGGTGGG CCTCTTGGGA AGAACTGGAT

CTTCTCAATA AGTCCTGGCC AGAGGGTGGG CCTCTTGGGA AGAACTGGAT

CTTCTCAATA AGTCCTGGCC AGAGGGTGGG CCTCTTGGGA AGAACTGGAT

CTTCTCAATA AGTCCTGGCC AGAGGGTGGG CCTCTTGGGA AGAACTGGAT

CTTCTCAATA AGTCCTGGCC AGAGGGTGGG CCTCTTGGGA AGAACTGGAT

CTTCTCAATA AGTCCCGGAC AGAGGGTGGG CCTCCTGGGA AGAACTGGAG

TTTTTCAATA AGTCCTGGCC AGAGGGTGGG GCTCTTAGGA AGAACTGGCT

TTTTTCAATA AGTCCTGGAC AGAGGGTGGG GCTCTTAGGA AGAACTGGAT

CTTCTCAATA AGTCCTGGCC AGAGGGTGGG CCTCTTAGGA AGAACTGGCT

CTTCTCAATA AGTCCCGGCC AGAGGGTGGG CCTCTTGGGA AGAACTGGAT

CTTCTCAATA AGTCCTGGCC AGAGGGTGGG CCTCTTGGGA AGAACTGGAT

CTTCTCAATA AGTCCTGGCC AAAGGGTGGG TCTCTTGGGA AGAACTGGAT

CTTCTCTATA AGTCCTGGCC AGAGGGTGGG TCTTTTGGGA AGAACTGGGT

ATTCTCAATA AGTCCTGGCC AGAGGGTGGG CCTCTTGGGA AGAACTGGAT

CTTCTCAATA AGTCCTGGCC AGAGGGTGGG CCTGTTGGGA AGAACTGGAT

---------- ---------- -----GTGGG CCTGTTGGGA AGAACTGGAT

CTTCTCAATA AGTCCTGGGC AGAGGGTGGG CCTCTTGGGC AGAACTGGTT

CTTTTCGATA AGTTCCGGTC AGAGGGTGGG CCTGCTGGGA AGAACGGGCT

CAGGGAAGAG TACTTTGTTA TCAGCTTTTT TGAGACTACT GAACACTGAA

CAGGGAAGAG TACTTTGTTA TCAGCTTTTT TGAGACTACT GAACACTGAA

CGGGGAAGAG TACTTTGTTA TCAGCTTTTT TGAGACTACT GAACACTGAA

CAGGGAAGAG TACTTTGTTA TCAGCTTTTT TGAGACTACT GAACACTGAA

CAGGGAAGAG TACTTTGTTA TCAGCTTTTT TGAGACTACT GAACACTGAA

CGGGAAAAAG TACTTTGTTA TCTGCTTTTT TGAGACTCCT GAACACTGAA

CAGGAAAAAG TACTTTGCTG TCAGCCTTTT TACGAATGTT GAACATTAAA

CAGGAAAAAG TACTTTGCTT TCAGCATTTT TACGAATGTT GAACATTAAA

CAGGGAAGAG TACTTTGTTA TCAGCATTTT TGAGACTACT GAACACTGAA

CAGGGAAGAG CACTTTGTTA CTGGCTTTTT TGAGACTGCT GAATACCAAA

CAGGGAAAAG TACTTTGTTA TCTGCTCTTT TGAGACTGGT GAACACTGAG

CAGGGAAGAG CACTCTGTTA TCGGCTCTTC TGAGACTGCT GAACACCGAG

CAGGGAAGAG TACTTTGTTA TCGGCTCTTT TGAGACTGCT GAATACGGAA

CAGGGAAAAG CACTTTGTTG TCAGCTTTTT TGAGACTGCT AAACACTAAG

CAGGGAAAAG CACTTTGTTA TCAGCTTTTT TGAGGCTGCT GAACACTGAA

CAGGGAAAAG CACACTGTTA TTGGCTTTCC TGAGGCTGCT GAACACCGCC

CTGGAAAAAG CACCTTGTTA GCTGCATTTT TAAGACTACT GAACACAGAA

CAGGGAAGAG CACTTTATTG TTCGCCTTTT TGAGGCTGCT GAACACTGAA

GGAGAAATCC AGATCGATGG TGTGTCTTGG GATTCAATAA CTTTGCAACA

GGAGAAATCC AGATCGATGG TGTGTCTTGG GATTCAATAA CTTTGCAACA

GGAGAAATCC AGATTGATGG TGTGTCTTGG GATTCAATAA CTTTGCAACA

GGAGAAATCC AGATCGATGG TGTGTCTTGG GATTCAATAA CTTTGCAACA

GGAGAAATCC AGATTGATGG TGTGTCTTGG GATTCAATAA CTTTGCAACA

GGAGATATCC AGATCGATGG CGTGTCTTGG GATTCAATAA CTTTGCAACA

GGCGAAATAC AGATCGATGG TGTCTCCTGG AACTCAATGA CCTTACAAGA

GGTGATATAG AGATTGATGG TGTCTCATGG AATTCAGTGA CCTTACAAGA

GGAGAAATCC AGATTGATGG TGTGTCTTGG GATTCGATCA CTTTGCAGCA

GGAGAAATCC AAATAGATGG TGTGTCTTGG GATTCAATTA CTTTGCAACA

GGAGAAATCC AAATAGATGG CGTGTCTTGG GACTCAATCC CTTTGCAAGA

GGAGAGATCC AAGTAGACGG AGTGTCTTGG GAGTCGGTGA CGGTGCAGCA

GGAGAAATCC AAATTGATGG TGTGTCTTGG GATTCAATAA CTGTACAACA

GGAGAAATTC AGATTGATGG CGTGTCTTGG GATTCAATAA CTTTGCAAGA

GGAGAAATTC AGATTGATGG TGTGTCCTGG GATTCAATAA CTTTGCAACA

GGAGAAATCC AGATTGATGG GGTGTCCTGG GACTCAATCA CTTTGCAGCA

GGAGAAATGC AGATTGATGG TATATCTTGG AATTCAATAC CCTTGCAGCA

GGAGATATCC GGATCGATGG CATATCCTGG GACGCAGTAC CTGTGCAGCA

GTGGAGGAAA GCCTTTGGAG TGATACCACA GAAAGTATTT ATTTTTTCTG

GTGGAGGAAA GCCTTTGGAG TGATACCGCA GAAAGTATTT ATCTTTTCTG

GTGGAGGAAA GCCTTTGGAG TGATACCACA GAAAGTATTT ATCTTTTCTG

GTGGAGGAAA GCCTTTGGAG TGATACCACA GAAAGTATTT ATCTTTTCTG

GTGGAGGAAA GCCTTTGGAG TGATACCACA GAAAGTATTT ATCTTTACTG

GTGGAGGAAA GCCTTTGGAG TGATACCACA GAAAGTGTTT ATCTTTTCTG

ATGGAGAAAA GCTTTTGGAG TGATAACACA GAAAGTATTT ATCTTTTCTG

ATGGAGGAAA GCTTTCGGAG TGATAACACA GAAAGTATTT ATCTTTTCTG

GTGGAGAAAA GCCTTTGGAG TGATCCCACA GAAAGTATTT ATCTTTTCTG

ATGGAGGAAG GCCTTTGGAG TCATACCACA GAAAGTATTC ATCTTTTCTG

ATGGAGGAGA GCCTTTGGGG TGATCCCACA GAAAGTATTT ATATTCTCTG

GTGGAGAAGA GCCTTTGGAG TGATCCCGCA GAAAGTATTT ATCTTTTCTG

GTGGAGGAAA GCCTTTGGAG TCATACCACA GAAAGTATTT ATATTTAATG

GTGGAGGAAA GCCTTTGGAG TGATACCACA GAAAGTATTT ATCTTTTCTG

GTGGAGGAAA GCCTTTGGAG TGATCCCGCA G--------- ----------

GTGGAGGAAA GCCTTTGGGG TGATCCCACA GAAAGTATTC ATCTTTTCTG

GTGGAGAAAA GCATTTGGAG TAATACCACA GAAAGTATTT GTATTTTCTG

GTGGAGGAAG GCGTTTGGAG TAATACCTCA GAAAGTATTC ATATTTTCCG

GAACATTTAG AAAAAACTTG GATCCCTATG AACAGTGGAG TGATCAAGAA

GAACATTTAG AAAAAACTTG GATCCCTATG AACAGTGGAG TGATCAAGAA

GAACATTTAG AAAAAACTTG GATCCCTATG AACAGTGGAG TGATCAAGAA

GAACATTTAG AAAAAACTTG GATCCCTATG AACAGTGGAG TGATCAAGAA

GAACATTTAG AAAAAACTTG GATCCCTATG AACAGTGGAG TGATCAAGAA

GAACATTTAG AAAAAACTTG GATCCCTATG AACAGTGGAG TGATCAAGAA

GAACATTCAG ACAAAACTTG GATCCCAATG GAAAATGGAG AGATGAAGAA

GAACATTCAG ACAAAACCTG GATCCCAATG GAAAATGGAA AGATGAAGAA

GAACATTTAG AAAAAACTTG GATCCCTATG AACAATGGAG TGATCAAGAA

GAACATTTAG AAAAAACTTG GATCCCTATG GACAGTGGAG TGATCAAGAA

GAACATTTAG AAAAAACTTG GATCCCTATG GACAGTGGAA TGATCAAGAA

GAACATTTAG AAAAAACTTG GATCCCTATG GACAATGGAA TGATCAAGAA

GAACGTTTAG AAAAAACCTG GATCCCTATG ATCAGTGGAA TGATCAAGAA

GAACATTTAG AAAAAACTTG GATCCCTATG GACAATGGAG TGATCAAGAA

---------- ---------- ---------- ---------- ----------

GAACATTTAG AAAAAACTTG GATCCCTATG AACAGTGGAG TGATGAAGAA

GGACATTTAG AAAAAATCTG GATCCTTATG GGCAATGGAG CGATCATGAA

GAAGTTTTCG AAAGAACCTG GATCCCTATG GGCAGTGGAC AGACCAGGAA

ATATGGAAAG TTGCAGATGA GGTAACATAC CAAATAATTA GAAGAACTCT

ATATGGAAAG TTGCAGATGA GGTAGCATAC CAAATAATTA GAAGAACTCT

ATATGGAAAG TTGCAGATGA GGTAACATAC CAGATAATTA GAAGAACTCT

ATATGGAAAG TTGCAGATGA GGTAACATAC CAGATAATTA GAAGAACTCT

ATATGGAAAG TTGCAGATGA GGTAACATAC CAAATAATTA GAAGAGCTCT

ATATGGAAAG TTGCAGATGA GATAACATAC CAGATAATTA GAAGAACTCT

ATATGGAAAG TTGCAGATCA GATAACATAC CAAGTGATTC GACGAGTTCT

ATATGGAAAG TTGCAGATGA GATAACATAC CAAGTCATTC GACGAGTTCT

ATATGGAAAG TTGCAGATGA GATAACATAC CAAATAATTC GGAGAACTCT

ATATGGAAAG TTGCAGATGA GATAACATAC CAGATCATTC GAAGAACCCT

ATATGGAAAG TTGCGGATGA GATAACATAC CAAATCATTC GAAGAACCCT

ATATGGAAAG TTGCAGACGA GATAACATAC CAAATCATTC GCAGAGCTCT

ATCTGGAAAG TTACAGAAGA GATAACATAT CAAATCATTC GAAGAACTCT

ATATGGAAAG TTGCTGATGA GATAACTTAC CAAATCATTC GAAGAACGCT

---------- ---------- ---------- ---------- ----------

ATATGGAAAG TTGCAGATCA G--AACATAC CAAATCATCC GAAGAACTCT

TTATGGAAAG TTGCAGATGA GATAACATAT CAAGTCATTC GGAGAACACT

CTATGGAAAG TGGCAGAGGA GATAACATAC CAGGTCATTC GCAAAACTCT

AAAACAAGCA TTTGCTGATT GCACAGTAAT TCTCTGTGAA CACAGGATAG

AAAACAAGCA TTTGCTGATT GCACAGTAAT TCTCTGTGAA CACAGGATAG

AAAACAAGCA TTTGCTGACT GCACAGTAAT CCTTTGTGAA CACAGGATAG

AAAACAAGCA TTTGCTGACT GCACAGTAAT CCTCTGTGAA CACAGGATAG

AAAACAAGCG TTTGCTGATT GCACAGTAAT CCTCTGTGAA CACAGGATAG

AAAACAAGCA TTTGCTGACT GCACAGTAAT CCTTTGTGAA CACAGGATAG

AAGACAAGCC TTCGCTGGTT GCACAGTAGT CCTCTGTGAA CACAGGATAG

AAAACAAGCC TTCGCTGGTT GCACAGTCAT CCTCTGTGAA CACAGGATAG

GAAACAAGCA TTTGCTGATT GCACAGTAAT CCTCTGTGAA CACAGGATAG

AAAACAAGCA TTTGCTAATT GCACAGTAAT CCTCTCTGAA CACAGGATAG

AAAGCAAGCG TTTGCAGATT GCACAGTGAT CCTTTCTGAA CACAGGATAG

AAAACAAGCG TTTGCTGATT GCACCGTGAT CCTCTGTGAA CACAGGATAG

AAAGCAAGCA TTTGCTGATT GCACAGTAAT CCTCTGTGAG CACAGGATAG

AAAACAAGCA TTTGCTGATT GCACAGTAAT CCTCTGTGAG CACAGGATAG

---------- ---------- ---------- ---------- ----------

AAAACAAGCA TTTGCTGATT GCACAGTCAT CCTCTGTGAG CACAGGATAG

GAAGAATGCA TTTGCCAACT GCACTGTAAT CCTCTCTGAA CACAGGATAG

AAAGCAAGCA TTTACCAACT GCACAGTCAT CCTCTCAGAA CACAGGATAG

AAGCAATGCT GGAATGCCAA CAATTTTTGG TCATAGAAGA GAACAAAGTG

AAGCAATGCT GGAATGCCAA CAATTTTTGG TCATAGAAGA GAACAAAGTG

AAGCAATGCT GGAATGCCAA CAATTTTTGG TCATAGAAGA GAACAAAGTG

AAGCAATGCT GGAATGCCAA CAATTTTTGG TCATAGAAGA GAACAAAGTG

AAGCAATGCT GGAATGCCAG CAATTTTTGG TCATAGAAGA GAACAAAGTG

AAGCAATGTT GGAATGTCAA CGATTTTTAG TCATAGAAGA GAACAAAGTG

AAGCAATGTT GGACTGTCAG CGATTTTTGG TCATAGAGCA GGGCAATGTC

AAGCGATGTT GGATTGCCAG CGATTTTTGG TCATAGAAGA GAGCAATGTC

AGGCAATGTT GGAATGCCAA CGATTTCTGG TCATAGAAGA GAACACAGTG

AAGCAATGTT GGAATGTCAA CGATTTTTGG TCATAGAGGA GAACAAGGTG

AAGCGATGTT GGAATGTCAA CGATTCTTGG TCATCGAGGA CAGCAGGCTG

AAGCAATGTT GGAATGCCAG CGATTTTTGG TCATAGAGGA GAACAAAGTA

AGGCAATGTT GGAATGCCAA CGATTTTTAG TCATAGAGGA GAACAAAGTG

AAGCAATGTT GGAATGTCAA CGATTTTTGG TCATAGAGGA GAACAAAGTG

---------- ---------- ---------- ---------- ----------

AAGCGATGTT GGAATGTCAG CGCTTTTTGG TCATAGAGGA GAACAAAGTG

AAGCAATGCT GGAGTGTCAG CGATTTTTGG TCATAGAAGA GAACAAAGTG

AAGCAATGCT CGAGTGTCAA CGCTTTCTGG TGATAGAGGA GAACAAGGTC

CGGCAGTACG ATTCCATCCA GAAACTGCTG AACGAGAGGA GCCTCTTCCG

CGGCAGTACG ATTCCATCCA GAAACTGCTG AACGAGAGGA GCCTCTTCCG

CGGCAGTATG ATTCCATCCA GAAACTGCTA AATGAGAGGA GCCTCTTCCG

CGGCAGTATG ATTCCATCCA GAAACTGCTG AATGAGAGGA GCCTCTTCCG

CGGCAGTATG ATTCCATCCA GAAACTGCTG AACGAGAAGA GCCTCTTCCG

CGGCAGTACG ACTCCATCCA GAAACTGCTG AGCGAGAAGA GCCTCTTCCG

TGGCAGTATG AATCCCTCCA GGCACTTCTG AGCGAGAAGA GTGTCTTCCA

TGGCAGTACG ACTCCCTTCA GGCACTTCTG AGTGAGAAGA GTATCTTCCA

CGGCAGTACG AATCCATCCA GAAATTGCTG AGTGAGAAGA GCCTCTTCCG

CGGCAGTATG ATTCCATCCA GAGGATGCTG AGCGAGAAGA GCCTCTTCCG

CGGCAGTTCG AGTCCATCCA GAGGCTGCTG AGCGAGAGGA GCGCCTTCCG

CGGCAGTACG ATTCCATGCA GAAGCTTCTG AGCGAAAAGA GCCTCTTCCA

CGGCAGTATG ACTCCATCCA GAAGCTGCTG AGTGAGAAGA GCCTCTTCCG

CGGCAGTACG ATTCCATCCA GAGGCTGCTG AGCGAGAAGA GCCTCTTCCG

---------- ---------- ---------- ---------- ----------

CGGCAGTACG ATTCCATCCA GAAGCTGCTG AGCGAGAAGA GCCTCTTCCG

CGTCAGTACG AGTCAATCCA GAAGCTGGTG AATGAGAAGA GTCTCTACAG

CGGCAGTACG AGTCTATCCA GAAGCTGCTG AATGAGAAGA GCGTCTTCAA

GCAAGCCATC AGCCCCTCCG ACAGGGTGAA GCTCTTTCCC CACCGGAACT

GCAAGCCATC AGCCCCTCCG ACAGGGTGAA GCTCTTTCCC CACCGGAACT

GCAAGCCATT AGCCCCTCCG ACAGGGTGAA GCTCTTTCCC CACCGGAACT

GCAAGCCATC AGCCCCTCCG ACAGGGTGAA GCTCTTTCCC CACCGGAACT

GCAAGCCATC AGCCACTCAG ACAGGGTGAA GCTCTTTCCC CACCGGAACT

ACAGGCCATC AGCCCCTCAG ACAGGATGAA GCTCTTCCCC CGCCGGAACT

GCGAGCCCTT AGCTCCTCAG AAAAGATGAA GCTCTTCCAC GGCCGGCACT

GCAGGCCATT AGCTCCTCGG AAAAGATGAG GTTCTTCCAG GGCCGCCACT

GCAGGCCATC AGCTCCTCGG ACAGGGCAAA GCTCTTCCCC CACAGGAACT

GCAGGCCATC AGCCCCGCAG ACCGCTTGAA GCTCTTGCCC CACCGGAACT

GCAGGCCATC GGGCCCCCCG AGCGGCCCGG CCTCCTCCCG CACCGGCTCT

GCAGGCCATC AGCCCCTCGG ACCGGCTGAA GCTCTTCCCC CAGCGGAGCT

CCAGGCCATC AGCTCCTCTG ACCGGTTGAA GCTCTTCCCA CACCGAAATT

GCAGGCTATC AGCCCCTCAG ACCGTGTGAA GCTCTTCCCC CACCAGAACT

---------- ---------- ---------- ---------- ----------

GCAGGCCATC AGCCCTTCGG ATCGTCTGAA GCTCTTCCCA CACAGAAACT

GCAAGCCATC AGTCACTCAG ACAGGATGAA GCTCTTCCCC CACCGGAACT

GCAGGCCATC AGCCACTCGG ACAAAATGAA GCTGTTCCCC CGCCGGAACT

CAAGCAAGTG CAAGTCTAAG CCCCAGATTG CTGCTCTGAA AGAGGAGACA

CAAGCAAGTG CAAGTCTAAG CCCCAAATTG CTGCTCTGAA AGAGGAGACA

CAAGCAAGTG CAAGTCTCAG CCCCAAATTG CTGCTCTGAA AGAGGAGACA

CAAGCAAGTG CAAGACTCAG CCCCAAATTG CTGCTCTGAA AGAGGAGACA

CAAGCAAGTA CAAGTCTCGG CCCCAAATTG CTTCTCTCAA AGAGGAGACA

CCAGCAAGCA CAAGTCGCGA TCCCCAATCA CTGCCCTGAA AGAGGAGACA

CCAGCAAGCA GAAGCCTCGG ACACAGATCA CTGCTGTGAA AGAGGAGACG

CCAGCAAGCA CAAGCCTCGG ACGCAAATTA CTGCTCTGAA AGAGGAGACA

CAAGCAAGCA CAAGTCTCGG CCCCAGATCA CTGCTCTGAA GGAGGAGGCA

CGAGCAGGCA GAGGTCTCGG TCCAACATCG CGGCTCTGAA GGAGGAGACA

CCAGCCGGCA GAGGTCCCCT TCCAGGATCG CGGCCCTGAA GGAGGAGACG

CCAGCAAGCA CAGGTCTCGA GCCCAGATCA CTGCCCTGAA GGAGGAGGCA

CCAGCAAGTA CAAATCCCGA TCCAAAATCA CTGCCCTCAA GGAGGAGACG

CTGGCAAGCA CAAGTCTCGA TCCAAAATCA CAGCTCTAAA GGAGGAGACA

---------- ---------- ---------- ---------- ----------

CCAGCAAATA CAAGTCTCGG TCCAAAATCG CCGCTCTGAC GGAGGAGACG

CGAGCAGGCA CAAGTCCCGA GCCAAAATCA CTGCCTTGAA GGAAGAAACA

CGAGCAAGCG CTTGTCCAGG CCCAAAATCA CGGCCCTCCA GGAGGAGACG

GAAGAAGAGG TGCAAGATAC AAGGCTTAAA AAAGAGTTTG ATGTGGATAC

GAAGAAGAGG TGCAAGATAC AAGGCTTAAA AAAGAGTTTG ATGTGGATAC

GAAGAAGAGG TGCAAGATAC AAGGCTTAAA AAAGAGTTTG ATGTGGATAC

GAAGAAGAGG TGCAAGATAC AAGGCTTAAA AAAGAGTTTG ATGTGGATAC

GAAGAAGAGG TGCAAGAAAC AAGGCTTAAA AAAGAGTTTG ATGTGGATAC

GAAGAAGAGG TGCAAGAGAC CAGGCTGAAA AAAGAGTTTG ATGTGGACAC

GAGGAAGAAG TGCAAGAGAC CCGGCTCAAA AAGGAGTTTG ATGTGGACAC

GAAGAAGAAG TTCAAGAAAC CCGTCTCAAA AAGGAATTTG ATGTGGACAC

GAAGAAGAAG TGCAAGAAAC AAGGCTCAAA AAGGAGTTTG ACGTGGACAC

GAAGAAGAGG TGCAAGAAAC AAAGCTT--- ---------- ----------

GAAGACGAGG TGCAGGACAC AAGGCTTAAA AAAGAGTTTG ATGTGGACAC

GAAGAAGAAG TGCAAGACAC CAGGCTGAAA CAAGATTTTG ATGTGGATGC

GAAGAAGAGG TGCAAGAAAC ACGGCTTAAA AAAGAATTTG ATGTGGACAC

GAAGAAGAGG TGCAAGACAC AAGACTTACG AAAGAGTTTG ATGTGGACAC

---------- ---------- ---------- ---------- ----------

GAAGAAGAGG TGCAAGAAAC GCGGCTCAGA AAAGAGTTTG ATGTGGACAC

GAAGAAGAGG TACAGGAAAC CCGACTTAAA AAAGAATTTG ATGTGGACAC

GAAGAGGAAG TGCAGGACAC CAGACTCAAA AAGGAGTTCG ACGTGGACAC

TCTCAGTAAA TCCGAGCTGC GGATGCTCCT CAGCGTGATG GAAGGGGAGC

TCTCAGTAAA TCCGAGCTGC GGATGCTCCT CAGCGTGATG GAAGGGGAGC

TCTCAGTAAA TCCGAGCTGC GGATGCTCCT CAGCGTGATG GAAGGGGAGC

TCTCAGTAAA TCCGAGCTGC GGATGCTCCT CAGCGTGATG GAAGGGGAGC

TCTCAGTAAA TCGGAGCTGC GGATGCTCCT CAGCGTGATG GAAGGGGAGC

CCTCAGTAAG ACAGAGCTGC GGATGCTTCT GAGCGTGATG GAAGGGGAGC

CCTCAGCAAA TCAGAGCTGC GGATGCTCCT CAGTGTGATG GAAGGTGAAC

GCTCAGCAAA TCAGAGCTGC GGATGCTCCT TAGCGTGATG GAAGGTGAAC

CCTGAGTAAG TCAGAGCTGC GGATGCTTCT CAGCGTGATG GAAGGGGAGC

---------- ---------- ---------- ---------- ----------

CCTCAGTAAA TCAGAGCTCC GGATGCTCCT CAGCGTGATG GAAGGGGAGC

GCTCAGTAAG TCAGAGCTCC GCATGCTCCT CAGTGTGATG GAAGGGGAGC

TCTCAGCAAA CCAGAGCTTC GGATGCTCCT GAGCGTGATG GAGGGAGAGC

ACTCAGTAAA TCAGAGCTGA GGATGCTCCT AAGCGTGATG GAAGGGGAAC

---------- ---------- ---------- ---------- ----------

GCTCAGTAAG TCTGAGCTGC GGATGCTCCT CAGCGTGATG GAAGGAGAAC

ACTCAGTAAA CCAGAGCTGC GAATGCTCCT CAGTGTAATG GAAGGAGAAC

CCTCAGCAAA GCTGAACTGC GGATGCTCCT GAGCGTCATG GAAGGGGAGC

TGGAGGCCAG AGACCTTGTC ATCGAGGCCC TGCGGGCTCG CAGGAAGGAG

TGGAGGCCAG AGACCTTGTC ATCGAGGCCC TGCGGGCTCG CAGGAAGGAG

TGGAGGCCAG AGACCTCGTC ATCGAGGCCC TGCGGGCTCG CAGGAAGGAG

TGGAGGCCAG AGACCTCGTC ATCGAGGCCC TGCGGGCTCG CAGGAAGGAG

TGGAGGCCAG AGACCTCGTC ATCGAGGCCC TGCGGGCTCG CAGGAAGGAG

TGGAAGCCAG AGACCTGGTC ATCGAGGCCC TCCGAGCTCG CAGGAAGGAA

TAGAAGCCAG AGACCTCGTG ATTGAGGCCC TGCGGGCTCG CAGGAAGGAG

TAGAAGCCAG AGACCTGGTG ATTGAGGCCC TGAGGGCTCG CAGGAAGGAG

TGGAGGCCAG AGACCTTGTC ATCGAGGCCC TGCGGGCTCG CAGGAAGGAG

---------- ---------- ---------- -----GCTCG CAGGAAGGAG

TGGAGGCCAG AGACCTGGTC ATCGAGGCCC TGCGGGCTCG CAGGAAGGAG

TGGAGGCCAG AGACCTGGTC ATCGAGGCCC TACGGGCTCG CAGAAAGGAG

TGGAGGCCAG AGACCTGGTC ATCGAGGCCC TGAGGGCACG CCGGAAGGAG

TGGAGGCCAG AGACCTCGTT ATTGAGGCCC TGCGGGCACG CAGGAAAGAG

---------- ---------- ---------- -----GCTCG CCGGAAGGAG

TGGAGGCCAG AGATCTCGTC ATCGAGGCCC TGCGGGCTCG GCGGAAGGAG

TGGAAGCGAG AGACCTTGTC ATAGAAGCAT TGAGGGCACG AAGGAAGGAG

TGGAAGCCAG GGATGTCGTC ATTGAGGCCC TGAGGGCTCG CAGAAAAGAG

GTATTTATCC AGGAACGGTA TGGGAGATTT AATCTAAATG ACCCGTTCCT

GTATTTATCC AGGAACGGTA TGGGAGATTT AATCTAAATG ACCCGTTCCT

GTATTTATCC AGGAACGGTA TGGGAGATTT AATCTAAATG ACCCGTTCCT

GTATTTATCC AGGAACGGTA TGGGAGATTT AATCTAAATG ACCCATTCCT

GTATTTATCC AGGAACGGTA TGGGAGATTT AATCTAAATG ACCCGTTCTT

GTATTTATCC AGGAACGGTA TGGGAGATTT AATCTAAACG ACCCATTTTT

GTGTTCATCC AGGAACGTTA TGGGAGATTT AATCTAAACG ACCCTTTCCT

GTGTTCATCC AGGAACGGTA TGGGAGGTTT AATCTAAATG ACCCCTTCCT

GTGTTTATCC AGGAACGGTA TGGGAGATTT AATCTAAACG ACCCGTTCTT

GTATTTATCC AGGAACGGTA TGGGAGATTT AATTTAAATG ACCCATTCCT

GTATTTATCC AGGAACGGTA TGGGAGATTT AATCTGAATG ACCCATTCTT

GTATTTATCC AGGAACGGTA TGGAAGATTT AATTTAAATG ACCCATTCTT

GTGTTTATCC AGGAACGGTA TGGAAGATTT AATTTAAACG ACCCATTCTT

GTATTTATCC AAGAGCGGTA TGGAAGATTT AATCTAAATG ACCCATTCCT

GTATTTATCC AGGAACGGTA TGGGAGATTT AACTTAAATG ACCCATTCCT

GTATTTATCC AGGAACGATA TGGGAGATTT AACTTAAATG ACCCATTCCT

GTCTTTGTTC AAGAACGATA TGGAAGGTTT AACCTAAATG ACCCATTCCT

GTGTTTATCC AGGAACGATA TGGAAGGTTT AACCTCAATG ACCCATTCCT

GGCACTCCAG AGAGACTATG AAGCAGGTGC TGGTGACAAA GAGAAGCCAG

GGCACTCCAG AGAGACTATG AAGCAGGTGC TGGTGACAAA GAGAAGCCAG

GGCACTCCAG AGAGACTATG AAGCAGGTGC TGGTGACAAA GAGAAGCCAG

GGCACTCCAG AGAGACTATG AAGCAGGTGC TGGTGACAAA GAGAAGCCAG

GGCACTCCAG AGAGACTATG AAGCAGGTGC TGGTGACAAA GAGAAGCCAG

GGCGCTCCAG CGAGACTATG AAGCAGGTGC TGGGGACAAA GAGAAGCCGG

GGCACTCCAG AGAGACTATG AAGCGGGTGC TGGTGACAAA GAGAAGCCAG

GGCACTTCAG AGAGACTATG AAGCAGGTCC TGGTGACAAA GAGAAGCCAG

GGCACTCCAA AGAGACTATG AAGCAGGTGC CAGTGACAAA GAGAAGCCTG

GGCACTACAG AGAGACTATG AAGCAGGTGC CAGCGATAAA GAGAAGCCAG

GGCACTGCAG AGAGACTATG AGGCAGGTGC CGGCGAGAAA GAGAAGCCAG

GGCACTGCAG AGAGACTATG AAGCAGGGGC CGGTGATAAA GAGAAGCCAG

GGCACTCCAG AGAGACTATG AAGCAGGTGC CAGTGACAAA GAGAAGCCCG

GGCACTGCAG AGAGACTATG AAGCAGGTGC TGGTGAGAAA GAGAAGCCAG

GGCACTGCAG AGGGACTATG AAGCAGGTGC CAGGGAGAAA GAGAAACCGG

GGCGCTCCAG AGAGACTATG AAGCAGGTGC TGGGGATAAA GA-AAGCCAG

GGCTCTGCAG AGGGACTATG AAGCAGGTGC TGGTGATGCA GAGAAGCCAG

GGCTCTGCAG AGAGACTACG AAGCAGGTGC TGGTGAAGCA GAGAAGCCCG

TTTGTACCAA CCCCCTCTCC ATCCTTGAAG CAGTCATGGC CCACTGCAAG

TTTGTACCAA CCCCCTCTCC ATCCTTGAAG CAGTCATGGC CCACTGCAAG

TGTGTACCAA CCCCCTCTCC ATCCTTGAAG CAGTCATGGC CCACTGCAAG

TGTGTACCAA CCCCCTCTCC ATCCTTGAAG CAGTCATGGC CCACTGCAAG

TTTGTACCAA CCCCCTCTCC ATCCTTGAAG CAGTTATGGC CCACTGCAGG

TTTGTACCAA CCCCCTCTCC ATCCTTGAAG CAGTCATGGC CCACTGCAGA

TCTGCACCAA CCCACTCTCC ATTCTTGAAG CCGTCATGGC CCACTGCAGA

TCTGCACCAA TCCACTCTCC ATCCTTGAAG CTGTCATGGC CCACTGCAGA

TTTGCACCAA CCCCCTGTCC ATCCTCGAAG CAGTCATGGC CCACTGCAGG

TTTGTACCAA CCCCCTCTCC ATCCTCGAAG CAGTCATGGC ACACTGCAGA

TTTGTACCAA CCCCCTCTCC ATCCTTGAAG CGGTCATGGC CCACTGCAGA

TTTGCGCCAA CCCCCTCTCC ATCCTTGAAG CAGTCATGGC ACACTGCAGA

TTTGCACCAA CCCACTCTCG ATCCTTGAAG CCGTCATGGC CCACTGCAGG

TTTGTACCAA CCCCCTTTCC ATCCTTGAAG CTGTCATGGC CCACTGCAGA

TTTGTACCAA CCCCCTCTCC ATCCTTGAAG CTGTCATGGC CCACTGCAGA

TTTGTACCAA CCCGCTCTCC ATCCTGGAAG CCGTCATGGC CCACTGCAGG

TTTGTACCAA CCCCCTCTCC ATTTTGGATG CTGTCATGGC TCACTGCAGG

TCTGTACCAA CCCCCTCTCC ATTTTGGAGG CCGTCATGGC TCACTGCCGG

AAAATGCAAG AAAGGATGTC CGCACAGCTG GCTGCTGCTG AGAGCAGACA

AAAATGCAAG AAAGGATGTC CGCACAGCTG GCTGCTGCTG AGAGCAGACA

AAAATGCAAG AAAGGATGTC CGCACAGCTG GCTGCTGCTG AGAGCAGACA

AAAATGCAAG AAAGGATGTC CGCACAGCTG GCTGCTGCTG AGAGCAGACA

AAAATGCAAG AAAGGATGTC CGCACAGCTG GCTGCTGCTG AGAGCAGACA

AAAATGCAGG AAAGGATGGC CACACAGCTG GCTGCTGCTG AGAGCAGACA

AAAATGCAAG AAAGAATGTC CGCACAGCTG GCTGCCGCTG AGAGTAGGCA

AAAATGCAAG AAAGGATGTC CGCACAGCTG GTGGCCGCTG AGAGCAGACA

AAAATGCAGG AACGGATGTC CACACAGCTG GCTGCCGCCG AGAGCAGACA

AAAATGCAAG AAAGGATGTC CACACAGCTG GCTGCTGCTG AGAGCAGACA

AAGATGCAGG AGAGGATGTC CACGCAGCTG GCTGCTGCTG AAAGCCGACA

AAAATGCAAG AAAGGATGTC CACACAGCTG GCTGCTGCTG AGAGCAGACA

AAAATGCAGG AGAGAATGGC CGCCCAGCTT GCTGCTGCCG AGAGCAGACA

AAAATGCAGG AGAGGATGTC CACACAGCTG GCTGCTGCTG AGAGCAGACA

AAAATGCAGG AGCGGATGTC CACACAGCTG GCTGCTGCTG AGAGCAGGCA

AAGATGCAGG AGCGGATGTC CACACAGCTG GCTGCCGCTG AGAGCAGGCA

AAAATGCAGG CGAGAATGTC CACACAGCTG GCTGCTGCTG AAAACAGACA

AAAATGCAAG AGAGGATGTC CGCCCAGCTG GCCGCCGCAG AGAGCAGGCA

AAAGAAGCTG GAAATGGAGA AGCTTCAGCT ACAAGCCCTT GAGCAAGAGC

AAAGAAGCTG GAAATGGAGA AGCTTCAGCT ACAAGCCCTT GAGCAAGAGC

AAAGAAGCTG GAAATGGAGA AGCTTCAGCT ACAAGCCCTT GAGCAAGAGC

AAAGAAGCTG GAAATGGAGA AGCTTCAGCT ACAAGCCCTT GAGCAAGAGC

AAAGAAGCTG GAAATGGAAA AGCTTCAGCT ACAAGCCCTT GAGCAAGAGC

AAAAAAGCTG GAAATGGAAA AGCTTCAGCT ACAAGCCCTG GAGCAAGAGC

GAAAAAGTTG GAGATGGAAA AGCTCCAGCT TCAGGCCCTT GAACAGGAGC

AAAAAAGTTG GAGATGGAAA AGCTCCAGCT CCAGGCCCTT GAACAGGAGC

GAAGAAGCTG GAAATGGAAA AGCTTCAGCT GCAAGCCCTG GACCAGGAGC

AAAGAAGCTG GAGATGGAGA AGCTTCAGCT CCAAGCACTT GAGCAGGAGC

AAAGAAGCTG GAGATGGAGA AGCTGCAGCT GCAGGCGCTG GAGCAGGAGC

AAAAAAGCTG GAGATGGAAA AGCTGCAGCT GCAAGCCCTT GAGTTAGAGC

AAAGAAGCTG GAGACGGAGA AGCTGCAGTT ACAGGCACTG GAGCAGGAGC

AAAGAAGCTG GAAATGGAAA AGCTTCAGCT ACAAGCCCTT GAGCAGGAGC

AAAGAAGCTG GAAATGGAAA AGCTTCAGCT CCAGGCCCTT GAGCAGGAGC

GAAGAAGCTG GAAATGGAGA AGCTGCAGCT CCAGACCCTG GAGCAGGAGC

AAAGAAGCTG GAAATGGAAA AGCTGCAATT ACAGACATTG GAGCAGGAGC

GAAGAAGTTG GAAATGGAGA AGTCGCAGTT GCACGTCCTC CAGCAGGAGC

ACAAGAAGCT GGCTGCCCGC CTTGAGGAAG AGCGTGGCAA GAACAAGCAG

ACAAGAAGCT GGCTGCCCGC CTTGAGGAAG AGCGTGGCAA GAACAAGCAG

ACAAGAAGCT GGCCGCCCGC CTCGAGGAAG AGCGCGGCAA GAACAAGCAG

ACAAGAAGCT GGCCGCCCGC CTCGAGGAAG AGCGCGGCAA GAATAAGCAG

ACAAGAAGCT GGCCGCCCGC CTTGAGGAAG AGCGCGGCAA GAACAAGCAG

ACAAGAAGCT GGCGGCCCGC CTGGAGGAGG AGCGGGGCAA AAACAAGCAG

ACAAGAAGCT GGCTGCCCAC CTGGAGGAAG AGAGGGGCAA GAACAAGCAC

ACAAGAAGCT GGCTGCCCAC CTGGAGGAAG AGCGGGGCAA GAACAAGCAC

ACCAGAAGCT GGCAGCTCGC CTGGAGGAGG AGCGCGGCAA GAACCGGCAC

ACAAGAAGTT GGCTGCCCGC CTGGAGGAGG AGCGAGGCAA GAACAAGCAC

ACAAGAAGCT GGCGGCCCGC CTGGAGGAGG AGCGCGGCAA GAACAAGCAC

ACAAGAAGCT GGCTGCCCGC CTGGAGGAGG AGCGAGGCAA GAACAAGCAC

ACAAGAAGCT GGCGGCGCGC CTGGAGGAGG AGCGCACCAA GAACAAGCAC

ACAAGAAGCT GGCCACCCGC CTCGAGGAGG AACGTGGCAA GAACAAGCAC

ACAAGAAGCT GGCCGCCCGC CTCGAGGAGG AACGTGGCAA GAACAAGCAC

ACAAGACGCT GGCTGCGCGG CTGGAGGAGG AGCGGGTCAA GAACAAGCAC

ACAAGAAGTT ATCTGCTCGC CTGGAGGATG AACGTGGTAA AAATAAGCAT

ACAGGAAGCT CTCTGCCCGC CTCGAAGACG AACGGGGCAA GAACAAACAG

GTGGTCCTGA TGCTGGTCAA AGAGTGCAAG CAGCTCTCAG GCAAAGTCAT

GTGGTCCTGA TGCTGGTCAA AGAGTGCAAG CAGCTCTCAG GCAAAGTCAT

GTGGTCTTGA TGCTGGTCAA AGAGTGCAAG CAGCTCTCCG GCAAGGTCAT

GTGGTCCTGA TGCTGGTCAA AGAGTGCAAG CAGCTCTCCG GCAAGGTCAT

GTGGTCCTGA TGCTCGTCAA AGAGTGCAAG CAGCTCTCAG GCAGAGTCAT

GTGGTCCTGC TGCTGGTCAA GGAGTGTAAA CAGCTCTCGG GCAGAGTCAT

GTGGTCCTGA TGCTGGTGAA GGAGTGCAAG CAGCTCTCAG GCAAAGTCGT

GTGGTCCTGA TGCTGGTGAA GGAGTGTAAG CAGCTCTCGG GCAAAGTCGT

GTGGTGCTGA TGCTGGTGAA GGAGTGTAAG CAGCTCTCGG GCAAGGTCAT

GTGGTCCTGA TGCTGGTCAA GGAGTGCAAG CAGCTCTCCG GGAAAGTCCT

GTGGTGCTGA TGCTGGTCAA GGAGTGCAAG CAGCTGTCGG GCAAGGTCAT

GTGGTACTGA TGCTCGTCAA GGAGTGCAAA CAGCTCTCAG GCAGGGTCAT

GTGGTGCTCA TGCTGGTCAA GGAGTGTAAG CAGCTGTCGG GCAGAGTGCT

GTGGTCCTGA TGCTTGTAAA GGAGTGTAAA CAGCTCTCTG GGAAAGTCAT

GTTGTCCTGA TGCTGGTGAA GGAGTGCAAG CAGCTCTCAG GCAAAGTCAT

GTGGTGCTGA TGCTGGTCAA GGAGTGCAAG CAGCTCTCGG GCAAGGTCAT

GTTGTCTTAA TGCTGGTCAA GGAGTGCAAA CAGTTGTCTG GTAAAATCAT

GTGGTGCTCA TGCTGGTCAA AGAGTGCAAG CAGCTTTCCA GCAAGGTGGG

AGAGGAGGCC CAGAAGCTCG AAGACGTAAT GGCCAAACTG GAAGAGGAAA

AGAGGAGGCC CAGAAGCTCG AAGACATAAT GGCCAAACTG GAAGAGGAAA

AGAGGAGGCC CAGAAGCTCG AAGACATAAT GGCCAAACTG GAAGAGGAAA

AGAGGAGGCC CAGAAGCTCG AAGACATAAT GGCCAAACTG GAAGAGGAAA

AGAGGAGGCC CAGAAGCTGG AAGACATAAT GGCCAAACTG GAAGAGGAAA

CGAGGAGGCC CAGAGGCTAG AGGAGGTCAT GGCCAAGCTG GAAGAGGAGA

AGAGGAAGCT CAGAAGCTGG AGGAAGTCAT GGTGAAATTG GAGGAGGAAA

GGAGGAGGCC CAGAAGCTGG AGGAAGTCAT GGCGCAACTG GAGGAGGAAA

CGAGGAGGCC CAGAAGCTGG AGGAGGTCAT GGCCAGCCTG GAAGAGGAGA

AGAGGAGGCC CAGAAGCTGG AAGAGGTGAT GGCCAAACTG GAAGAGGAGA

CGAGGAGGCG CGCAAGCTGG ACGCCGCCGT GTCCCAGCTG GAGGAGGAGC

CGAGGAGGCC CAGAAGCTGG AAGACGTCAT GGCCAAACTG GATGAGGAGA

CGAGGAGGCG CAGAAGCTCG ACGAGGTCAT GGGCGAGCTG GAAGAGGAGA

AGAGGAAGCC CAGAAGTTAG AAGAAGTAAT GACCAAACTG GAGGAGGAAA

TGAAGAGGCC CAGAAGGTAG AAGAAGTCAT GGCCCAACTG GAAGAGGAAA

CGAGGAGGCC CAGAAGGTGG AAGAAGCCCT GGCCCAGCTG GAGGAGGAGA

AGAGGAAGCC CAGAAGCTGG AAGAAGTCAT GGCCAGTCTT GAAGAGGAAA

CGAGGAAGGT GAGAAGCTGG AGGAAGCGGC CTCCAAGCTG GATGCCGAAA

AGAAAAAGAC GAATGAATTA GAAGAGGAAC TCTCCGCTGA GAAACGAAGA

AGAAAAAGAC GAATGAATTA GAAGAGGAAC TCTCCGCTGA GAAACGAAGA

AGAAAAAGAC AAATGAATTA GAAGAAGAAC TCTCCGCTGA GAAACGAAGA

AGAAAAAGAC AAATGAATTA GAAGAAGAAC TCTCTGCTGA GAAACGAAGA

AGAAAAAGAC GAATGAATTG GAAGAGGAAC TCTCTGCTGA GAAACGAAGA

AGAAAAGGAC GAGTGAATTA GAAGAGGAGC TCTCTGCCGA AAAACGAAGA

AGAAAAAGAC AAGTGAGTTA GAAGACCAGC TCTCCGCTGA GAAACAGAGA

AGAAAAAGAC AAGTGAGTTA GAAGAGCAGC TCTCAGCTGA GAAACAGAGA

AGAAGAAGAC GAACGACTTG GAAGAGCAGC TGTGCACTGA GAAACGCCGA

AGAAAAAGAC CAGTGCGTTA GAGGAGGAAC TCGCCACGGA GAAACGAAGA

GGGCGCGCGG CCGGGCCCTG CAGGAGGAGC TGGGCGCCGA GCGGCGGAGG

AGAAGAAGAC GAGTGCGTTA GAAGAGGAGC TTTCCACCGA GAAACGAAGA

AGAAGAAGAC CAGCGCGCTG GAGGAGGAGC TCTCGGCCGA GAAGCGCCGG

AGAAAAAGAC AAATGAACTG GAAGAGGAAC TCTCTGCGGA GAAACGAAGA

AGAAAAGGAC AAATGAATTA GAAGAGGAAC TCTCCACTGA GAAACGAAGA

AGAAAAGGGC CAGTGGCCTG GAGGAGGAGC TCTCCGGAGA GAAGCGAAGG

AGAAAAAAAC AAATGAATTA GAGGAAGCAC TTACTGCAGA GAAACAAAGA

AGAGAAAGAC GGGCGAGCTA GAAGGGGCCC TGTCGGCCGA GAGGCAGAAG

AGCACAGAAA TGGAAGCTCA GATGGAAAAA CAACTCTCTG AGTTTGACAC

AGCACAGAAA TGGAAGCTCA GATGGAAAAA CAACTCTCTG AGTTTGACAC

AGCACAGAAA TGGAAGCTCA GATGGAAAAA CAGCTCTCTG AGTTTGACAC

AGCACAGAAA TGGAAGCTCA GATGGAAAAA CAGCTCTCTG AGTTTGACAC

AGCTCAGAAA TGGAAGCTCA GATGGAAAAA CAACTCTCTG AGTTTGATAC

AGCACAGAAA TGGAAGCTCA GATGGAAAAA CAGCTCTCTG AGTTCGATAC

AGCGCTGGAA TGGAAGCTCA GCTGGAGAAA CAGCTCTTTG AGTTTGATAC

AGCTCTGGAA TGGAAGCTCA GTTGGAGAAA CAGCTCTCCG AGTTTGACAC

AGCGCCGAGA TGGAAGCGCA GATGGAAAAG CAGCTCTCCG AGTTCGACAC

AGCACAGAAA TGGAAGCTCA GATGGAAAAG CAGCTCTCGG AGTTTGACAC

GGCGCAGACC TGGAGGCGCA CCTGGAGAAG CAGCTGTCGG ACTCGGACAC

AGCACCGACA TGGAAGCTCA GATGGAAAAA CAGCTCTCTG AGTTTGACAC

GGCGCGGAGA TGGAGGCGCA GCTGGAGAAG CAGCTCTCCG AGTTCGACAC

AGCACAGAAA TGGAAGCTCA GCTGGAGAAA CAGCTCTCTG AGTTCGATAT

AGCACAGAAA TGGAGGCTCA GATGGAAAAA CAGCTCTCTG AGTTTGATAC

AGTGCGGAGA TGGAAGCTCA GATGGAAAAG CAGCTGTCCG AGTTTGATAC

AGTGCAGAAA TGGAAGCACA GATGGAGAAA CAACTCTCTG AATTTGATAC

AGCTCCCAGA TGGAAGCCCG CATGGAGAAG CAGCTCTCCG AATTCGACAC

TGAGCGGGAA CAGCTTCGTG CCAAGCTGAA CCGGGAAGAA GCACACACCA

CGAGCGGGAA CAGCTTCGTG CCAAGCTGAA CCGGGAAGAA GCACACACCA

CGAGCGGGAA CAGCTTCGTG CCAAGCTGAA CCGGGAAGAA GCACACACCA

CGAGCGGGAA CAGCTTCGTG CCAAGCTGAA CCGGGAAGAA GCACACACCA

CGAGCGGGAA CAGCTTCGTG CCAAGCTGAA CCGAGAAGAA GCACACACCA

GGAACGGGAG CAGCTCCGTG CCAAGCTGAG CCGGGAAGAA GCGCACACCA

GGAACGGGAG CAGCTGCGGG CCAAGCTGAC CCGAGAAGAA GCACACACCA

GGAACGGGAG CAGCTGCGCG CCAAGCTGAG CCGAGAGGAA GCACACACCA

GGAACGGGAG CAGCTGCGTG CCAAGCTGAA CCGAGAGGAA GCCCACACCA

GGAGCGGGAA CAGCTTCGTG CCAAGCTGCA CCGAGAAGAA GCACACACCA

GGAGCGCGAG CAGCTGCGCG CCCGGCTGCA CCGCGAGGAG GCGCTGGCCG

CGAACGGGAG CAGCTTCGTG CCAAGCTGCA CCGAGAAGAA GCACACACCG

GGAGCGCGAG CAGCTGCGGG CCAAGCTGCA CCGGGAAGAG GCGCACACGG

CGAAAGGGAG CAGCTTCGTG CTAAGCTGAA CCGAGAGGAA GCACACACCA

CGAAAGGGAG CAGCTTCGTG CCAAGTTGAA CCGCGAGGAA GCACACACGA

CGAGCGGGAG CAGCTCCGTG CCAAGCTGAG CCGCGAGGAA GCACACACGA

AGAGAGGGAG CAACTCCGAG CCAAGCTTAA CAGAGAGGAA GCACACACAA

GGAGCGGGAG CAGCTGCGGG CCAAGCTGAG CAGGGAGGAA GCGCTCACGG

CTGACCTCAA AGAGGAGATA GACAAGATGA GGAAAATGAT TGAGCAACTG

CTGACCTCAA AGAGGAGATA GACAAGATGA GGAAAATGAT TGAGCAACTG

CTGACCTCAA AGAGGAGATA GACAAGATGA AGAAAATGAT TGAGCAACTG

CTGACCTCAA AGAGGAGATA GACAAGATGA AGAAAATGAT TGAGCAACTG

CTGACCTCAA AGAGGAGATA GACAAGATGA AGAAAATGAT TGAGCAACTG

CTGACCTCAA GGAGGAGGTC GACAAGATGA AGAAAATGAT CGAGCAGCTG

CCGACCTCAA AGAGGAGATA GACAAGATGA AGAAAATGAT GGAGCAAATG

CCGACCTCAA AGAGGAGATA GACAAGATGA AGAAAATGAT GGAGCAAATG

CCGACCTCAA AGAGGAGATA GACAAGATGA AGAAGATGAT CGAGCAACTG

CTGACCTCAA AGAGGAGATA GACAAGATGA AGAAGATGAT CGAGCAGCTG

CGG------- ---------- ---------- ---------- ----------

CAGACCTCAA AGAGGAGATA GACAAGATGA AGAAAATGAT TGAGCAGCTG

CCGACCTCAA GGAGGAGATC GACAAGCTGA AGAAGATGAT CGAGCAGCTG

CTGACCTCAA AGAGGAGATA GACAAGATGA AGAAAATGAT TGAGCAGCTG

CTGACCTCAA AGAGGAGATA GACAAGATGA AGAAGATGAT CGAGCAACTG

CTGACCTCAA AGGGGAGATC GACAAGATGA AGAAGATGAC TGAGCAGCTG

CTGACCTCAA AGAAGAGATA GACAAGATGA AAAAAATGAT CGAGCAACTG

CCGACCTCAG GGAGGAGATC GACAAGATGA GAAAGACGAT CGAGCAGCTG

AAAAGGGGAA GTGACAGCAA ACCAAGCCTC TCTCTTCCAC GGAAGACAAA

AAAAGGGGAA GTGACAGCAA ACCAAGCCTC TCTCTTCCAC GGAAGACAAA

AAAAGGGGAA GTGACAGTAA ACCAAGCCTC TCTCTTCCAC GGAAGACAAA

AAAAGGGGAA GTGACAGTAA ACCAAGC--- ---------- ----------

AAAAGGGGAA GTGACAGCAA ACCAAGCCTC TCTCTTCCCC GGAAGACGAA

AAAAGGGGAG GCGACGGCAA ACCCAGCCTC TCTCTCCCAC GGAAGACCAA

AAGAAAGGCA ACGATGGGAA GCCCGGACTC TCCCTCCCCC GGAAGACAAA

AAGAAGGGAA GTGATGGCAA GCCCGGCCTC TCCCTCCCAC GGAAGACAAA

AAAAGGGGGA GCGACAGCAA ACCGAGTCTG TCTCTCCCGC GCAAGACAAA

AAAAAGGGAA ATGACAGCAA ACCCAGCCTC TCCCTCCCCC GGAAGACAAA

---------- ---------- ---------- ---------- ----------

AAAAGGGGAA GTGACAGTAA ACCAAGCCTC TCTCTCCCCC GGAAGACAAA

AAAAAGGGCC CGGACAGCAA GCCCAGCCTC CCCCTCGCCC GGAAGGCAAA

AAGCGGGGAA ATGACAGCAA ACCAAGCCTC TCTCTCCCCC GGAAGACAAA

AAAAGGGGAA ATGACAGCAA ACCAAGCCTC TCTCTTCCCC GGAAGGTGAA

AAAAGGGGAA ACGACAGCAA GCCCAGTCTC TCTCTTCCTC GGAAGGCGAA

AAGAAGGGAA ACGACAGCAA ACCGAGCCTT TCTCTCCCCC GCAAGACAAA

AGGAAGGGCA ACGACCACAA ACCGAGCCTC TCCCTCCCCC GGAAAGCGAA

AGATAGGCGT TTGGTTTCCA TATCTGTGGG AACAGAAGGA ACTGTGACAA

AGATAGGCGT TTGGTTTCCA TATCTGTGGG AACAGAAGGA ACTGTGACAA

AGATAGGCGT TTGGTTTCCA TATCTGTGGG AACAGAAGGA ACTGTGACAA

---------- ---------- ---------- ---------- ----------

AGATAGGCGT TTGGTTTCCA TATCTGTGGG AACAGAAGGA ACTGTGATGA

AGACAGGCGC TTGGTTTCCG CGTCTGTGGG GACAGAAGGG CCGCTGACGA

AGATAAGCGA CTGGCTTCCA TATCTGTGGC AACAGAAGGG CCTGTGACAA

AGATAAGCGC CTGGCTTCCA TATCTGTGGC AACAGAAGGG CCTGTGACAA

AGACAGGCGT CTGGCTTCTG TGTCTGTGGC CACCGAAGGA CCGGTGACGA

AGATAGACGT TTGGTGTCCA TATCTGTGGC AACAGAAGGC CCAATGACAA

---------- ---------- ---------- ---------- ----------

AGATAGGCGT TTGGTCTCCA TATCTGTAGG AACAGAAGGA CCTATGACGA

GGACCGGCGG GCCGTCTCCA TCTCGGTGGC CACGGAAGGA CCGGCGACCC

AGATAGGCGT TTGGTGTCCA TATCTGTGGG AACAGAAGGA CCTGTGACAA

AGATAGGCGT TTGGTTTCCG TATCAGTGGG AACAGAAGGA CCTGTGACAA

AGATAGGCGT TGGGTTTCCG TATCCGTGGG CACAGAAGGC CCTGGGACAG

AGACAAACGT TTGGTTTCTA TATCTGTGGG AACAGAAGGA CCTACATCAA

AGACAGGCGC TCCGTGTCCG TGTCCGTGGG GACCGAGGGG CCGGCCTCGC

GGTCTGTTGC ATGCCAGACA GACCTAGTGA CAGAAAATGC TGACCACATG

GGTCTGTTGC ATGCCAGACA GACCTAGTGA CAGAAAGTGC GGACCACATG

GGTCTGTTGC ATGCCAGACA GACCTAGTGA CAGAAAGTGC TGACCATGTG

---------- ---------- ---------- ---------- ----------

GGTCTGTTGC TTGCCAGACA GACCTAGTGA CAGAAAGTGC TGACCACGTG

GGTCTGTGGC CTGCCAGACA GACCTGGCTG TGGAAAGCGC CGAGCCCGTG

GGTCTGTTGC TTGCCAGACA GATGTAGTGA CAGAAAGCAC TGACCCTGTG

GGTCCGTTGC TTGCCAGACA GATGTAGTGA CAGAAAGCAC TGACCCTGTG

GGGCTGTTGC TTGTCAGACA GACCCAGTGA CCGAAAGCAC TGACCACGTG

GATCTGTTGC GTGCCAGACA GACCTAGTGG TGGAGACTGC TGAGCCCCTG

---------- ---------- ---------- ---------- ----------

GGTCTGTTGC TTGCCAGACA GACCCAGTGA TAGAAAGCAC TGATCATGTG

GCTCTGTCGC TTGCCAGACA GATCCCGTGA CAGAAAGCGC CGACCCCGAC

GGTCTGTTGC TTGCCAGACA GACCTGGTGA TAGAAAGCAC TGACCACGTG

GGTCTGTTGC TTGCCAAACT GACCTAGTGG TCGAAAGCAC TGACCACGTG

GGTCTGTGGC ATGTCAGACC GACCTGGGGG TGGAAAGCAC CGACCACGTG

GGTCGGTTGC TTGCCAGACC GACACCATGG TGGATAGTAC TGACCCTGTT

GCGCAGCGGC CTGCCAGACC GACCCAGTGG TGGAGGGCGC AGACCCGGTG

AAAAAGTTGC CTTTAACCAT GCCTGTAAAA CCTTCCACAG GGAGTCCCCT

AAAAAGTTGC CTTTAACTAT GCCTGTAAAA CCTTCCACAG GGAGTCCCCT

AAAAAGTTGC CTTTAACCAT GCCTGTAAAA CCTTCCACAG GGAGCCCCCT

---------- ---------- ---------- --------AG GGAGCCCCCT

AAGAAGTTGC CTTTAACCAT GCCTGTAAAA CCTTCCACAG GGAGCCCCCT

AAGAAGTCGC CTTTGACTGT CCATGTCAAG CCCTCCCCCG GCAGCCCCCA

AAGAAATTGC CTTTGTCTGT ACCCATAAAA CCTTCCACAG GGAGCCCCCT

AAGAAGTTGC CTTTGACTGT ACCCATAAAG CCTTCCACAG GGAGCCCCCT

AGGAAGTTGC CTTTGACGGT ACCTGCCAAG CCCTCGGCCG GGAGCCCCCT

AAGAAGTTGC CTTTGAGCGT GCCCGTAAAG CCTGCCACGG GGAGCCCCCT

---------- ---------- ---------- ---------- ----------

AAGAAGTTGC CTTTGACTGT GCCTGTAAAG CCTTCCACAG GGAGCCCCCT

GAGAAGTTGC CCTTGACCGT ACCCGTCAAG CCCCCCGCGA GAGCCCCTCT

AAGAAGTTGC CTTTGACTGT ACCTGTAAAG CCTTCGACAG GAAGCCCCCT

AAGAAGTTGC CTTTGACTGT ACCTGTCAAG CCTTCTGCAG GGAGCCCCCT

AAGAAGTTGC CTCTGACGGT GCCTGCCAAG CCTGCTGCCG GGAGCCCCCT

AAGAAGTTGC CTTTGACTGT ACCCATAAAG CCTTCAACAG CAAGCCCCCT

AAGAAGCCCC CCGTGGCGGT CCCCGCGAAG CCCTCCTCAG CCGGCCCCCT

AGTTTCTGCA AATGCAAAAG GGAGCGTGTG CAGACCAGGT ATTGACAGGC

AGTTTCTGCA AATGCAAAAG GGAGCGTGTG CAGACCAGGT ATTGACAGGC

AGTTTCTGCA AATGCAAAAG GGAGCGTGTG CAGACCAGGT ATTGATAGGC

AGTTTCTGCA AATGCAAAAG GGAGCGTGTG CAGACCAGGT ATGGACAGGC

AGCTTCTGCA AATGCAAAAG GAAGCGTGTG CAGACCAGGT ATTGACAGGC

CGTCTCG--- ---GTGAAAG GGAGCGGGGG CAAACCGGGT ATGGACAGGC

AGTTTCCACA AACACAAAAG GGAATGTGGG CAGACCAGGT ATTGATAGAC

AGTTCCCACA AACACAAAAG GGAATGTGGG CAGACCAGGT ATTGATAGGC

CGTGTCTGCC AACACAAAAG GGAACGTGTG CAGACCAGGT ATGGACAGGC

AGTCTCCGCC GGTGCCAAGG GGAATGCCTG CAGACCGGGT ATCGAGAGGC

---------- ---------- ---------- ---------- ----------

AGTTTCTGCA AATGCAAAAG GGAATGCATG CAGGCCAGGT ATTGACAGGC

CGTTCCTGCG AATGCAAAAG GGGGGTTGTG C--------- ----------

CGTTTCCGCA CCTGCAAAAG GGAATGTGTG CAGACCAGGT ATTGACAGGC

TGTTTCTGCC AGTGCAAAAG GGAATG---- -AGGCCCAGT GTTGACAGGC

CGTAGCTGCA AGTGCAAAGG GGAGTGGGGG CAGGCCTGGG GTTGACAGAC

CGTTTCTGGC AATGCAAAAG GCAGTGTTTG CAAACCAGTT ATTGACAGGC

GGCTTCTGGG ACCACCAAAG GGGGCGTTGG GAAGCCCAGC GTGGGTCGGC

AGGCTTCCTA TGGTGACTTG CCAAGTGCAA ACAAAATTGA GGAAAATGGA

AGGCTTCCTA TGGTGACTTG CCAAGTGCAA ACAAAATTGA GGAAAATGGA

AGGCTTCCCA CGGTGACTTG CCAAGTGCAA ACAGAATTGA GGAAAATGGA

AGGCTTCCCA CGGTGACTTG CCAAGTGCAA ACAGAATTGA GGAAAATGGA

AGGCTTCCCA TGGTGACTTG TCAAGTGCAA ACAGAATTGA GGAAAATGGA

AGGCTTCCCA CGGTGAGCTG CCAAGTGCAA GCAGAATTGA GGAAAATGGA

AAGCTTCCCA CAGTGACTT- TCAAGTGCAA GTAGAATTGA GGAAAACGGA

AGTCTTCCCA CAGTGACTT- TCAAGTGCAA ACAGAATTGA GGAGAATGGA

AGGCCTCCCA CGGGGACTTA GCAAGTGCAA GCCGAATGGA GGCGAATGGA

AGGTTTCCCA CGGTGACTTG CCAAGTGCAA ACAGAATTGA GGAAAATGGA

---------- ---------- ---------- ---------- ----------

AGGCTTCCCA TGGTGACTTG CCAAGTGCAA CCAGAGTTGA GGAAAATGGA

-GGCTTCCCA CGGGGACTCG CCAGGTGCAA ACAGACTAGA GGAAAATGGG

AGGCTTCCCA TGGTGACTTG CCAAATGCAA ACAGAATCGA GGAAAATGGA

AGGCTTCCCA TGGTGACTTG CCAAGTGTGA ACAAAACTGA GGAGAATGGA

AGGCTTCCCA TGCTGACTTG CCGAGTACAA ACAAAATTGA GGAAAATGGA

AGTCTTCCTA CAGTGATTTG CCAAGTCCAA ACAGAATTGA GGAAAATGGA

AGTTTTCCCA CGGCGACTT- -CCGGTCCAC CGCGGATCCA GGAAAATGGA

CCAAGCACTG GCTCAACACC AGATCCAACC AGTAGCACAC CCCCACTTCC

CCAAGCACTG GCTCAACACC AGATCCAACC AGTAGCACAC CCCCACTTCC

CCAAGCACTG ACTCAACACC AGATCCAACC AGTAGCACAC CCCCACTTCC

CCAAGCACTG ACTCAACACC AGATCCAACC AGTAGCACAC CCCCACTTCC

CCAAGCACTG GCTCAACACC AGATCCAACC AGTAGCATAC CCCCACTTCC

CCGAGCCCGG gctccacgcc ggacgcgccg ggcagcgccg ccccgc----

CCAAGTGCAG GCAATGCCCC TGACCTATCA AACAGCACAC CCTCAACTCC

CCAAGCACAG GCAATGCCCC TGATCTATCA AATAGCACCC CCTCAACTCC

CCAAGCCCTG GCTCCACACC GGATCTCACA AGTAGCACGC CCCCTATTCC

CCGAGCACCG GCTCAACACC AGATTTAACC AGTAGCCCAA GCCCACTACC

---------- ---------- ---------- ---------- ----------

CCGAGCACTG ACTCAGCACC AGATTTAACC AATAGCACAC CCCCCGTTCC

CCCAGCCCCG GC-------- ---------- ---------- ----------

CCTAGTATTG GCTCAACACC AGATTTAGCA AGTAGCACAC CCCCTCTTCC

CCGAGTACTG GGTCAACACC AGATTTACCA AGCAGCACAC CCCCTCTTCC

CCGAGTACTA GCTCAGGGCC AGATACACCA AGCAGCACAG CCCCTCTTCC

CCAAGTACTG GCTCTACACC AGTTTTACCA AGTAGCACAT CCCCCCTTCC

CCGAGCTCCG GTTCGACCCC CGAGCCGACG GGTAGTGCCC TCCCCCTTCT

CAGTAACGCT GCCCCTCCCA CCGCTCCTCA GAACTCGCAA GCTCCACCTA

CAGTAACGCT GCCCCTCCCA CCGCTCCTCA GAACTCGCAA GCTCCACCTA

CAGTAATGCT GCCCCTCCCA CCGCTCCTCA GAACTCCCAG GCTCCACCTA

CAGTAATGCT GCCCCTCCCA CCGCTCCTCA GAACTCCCAG GCTCCACCTA

CAGTAACGCT GCCCCTTCCA CTACTCCTCA GAACTCACAA GCTCCACCTA

---------- ---------- ---------- --------ca gcgccgccc-

TAGTGGAACT GCCCCTGCTG CTGCACCCCA GAACTCACAA GCTCCTCCTG

TAGTAGCACT GCCCCTGCCG CTGCACCCCA GAACTCACAA GCTCCTACTG

CAGTGGCACC ACTCCTGCCC CGGCCACCCA GAGCGTGCCC GCAGCGCCTG

CAGTGCCGTT TCCCCGGCCT CC-------- ---------- ----------

---------- ---------- ---------- ---------- ----------

CAGTAGCACT GCCCCTCCCG CCGCTCCCCA GAGCTCACAA GCTCCAC---

---------- ---------G CCGCTCCCCA GGGCCAGCCC GCCGCACCCG

CAGTAATGCT GCCCCTCCCA CCGCTTCCCA GAACTCACAA GCTTCCTCTA

CAATAACACT GCACCTCCCG CCGCATCCCA GAACTCGCAG GCTTCTTCTT

CAGTAACGCT GCCCCTCCCG CCGCCGCCCA GAACCCGCAG GCTCCTTCTA

CAGTAATGCT GCCCCTTCCA CTGCTTCTCA GAACTCACTA GCTTCATCTA

AAACAACGCC CCTCCTCCCG CTGCTTCTCA GAACTCGCTC ACCTCTTCCA

TGCACAGTTT ACATTCACCA TGTGCCAACA CCTCTTTGCA TCCAGGTCTA

TGCACAGTTT ACATTCACCA TGTGCCAACA CCTCTTTGCA TCCAGGTCTA

TGCACAGTTT ACATTCACCA TGTGCCAACG CCTCTTTGCA TCCAGGTCTA

TGCACAGTTT ACATTCACCA TGTGCCAACG CCTCTTTGCA TCCAGGTCTA

TGCACAGTTT ACATTCACCA TGTGCCAACG CCTCTTTGCA TCCAGGTCTA

cgcacAGTTT CCACTCCCCG TGTGCCAGCG CGCCCCCGCA CCCGGGCCTG

TGCACAGTTT ACATTCACCG TGTGCCAACA CT------CA TCCAGGTCTC

TGCACAGTTT ACATTCACCA TGTGCCAACA CT------CA TCCAGGCCTC

GGCACGGCCT ACACTCACCG TGTGCCACCA CGGCTTTGCA TCCAGGCCTC

CGCACAGTTT ACATTCACCG AGTGCCAGCG CGCCTCTGCA TCCGGGTCTG

---------- ---------- ---------- ---------- ----------

TGCACAGTTT ACATTCACCG AGTGCCAATG CGTCTCTGCA TCCAGGTTTA

CGCACAGTTT ACATTCACCG TGTGCCAATG CGTCTCTGCA CCCGGGGCTG

TGCACAGTTT ACATTCACCA TGTGCCAATG CATCTTTGCA TCCAGGTCCA

TGCACAGTTT ACATTCACCA TGTGCCAACG CGTCTTTGCA TCCAGGTGTA

CGCACACTGT CCATTCACCA TGTGCCAATG CGGCTTTGCA TCCGCCTCTA

TGCACAGTTC ACATTCACCA TGTGCCAATG CATCTTTGCA TCCAGGTCCA

CGCCCAACCT CCATTCACCG TGTGCCAACG CGGCTTCGTA CCCCGCGCTC

AACCCACGAA TCCAAGCAGC TAGATTTAGA TTTCAGGGCA ATGCTAACGA

AACCCACGAA TCCAAGCAGC TAGATTTAGA TTTCAGGGCA ATGCTAACGA

AACCCACGAA TCCAAGCAGC TAGATTTAGA TTTCAGGGCA ATGCTAACGA

AACCCACGAA TCCAAGCAGC TAGATTTAGA TTTCAGGGCA ATGCTAACGA

AACCCACGAA TCCAAGCAGC TAGATTTAGA TTTCAGGGCA ATGCTAATGA

AACCCGCGAA TCCAGGCCGC TAGATTCCGA TTTCAGGGCA ATGCTAATGA

AACCCACGCA TCCAAGCAGC TAGATTTAGA TTTCAGGGCA ATGCAAATGA

AACCCACGCA TCCAAGCAGC TAGATTCAGA TTTCAGGGCA ATGCAAATGA

AACCCACGCA TCCAAGCAGC CAGGTTTAGA TTTCAAGGCA ACGCAAACGA

AACCCACGCA TCCAAGCGGC TCGATTTCGA TTTCAGGGCA ATGCTAATGA

---------- ---------- ---------- ---------- --------GA

AACCCACGAA TCCAAGCAGC TAGGTTTAGA TTCCAGGGCA ATGCTAATGA

AACCCGCGCG TCCAGGCCGC GAGGTTCCGA TTCCAGGGCA ATGCCAATGA

AACCCACGAA TCCAAGCAGC TAGATTTAGA TTTCAGGGCA ATGCTAATGA

AACCCACGAA TCCAAGCAGT TAGATTTAGA TTTCAGGGCA ATGCTAATGA

ACCCCACGGG TCCAAGCAGT GAGGTTTAGA TTTCAGGGCA ATGCTAATGA

AACCCACGTG TCCAAGCAGC TAGATTTAGA TTTCAGGGCA ATGCCAATGA

AATCCGCGGG TCCAAGCGGC CCGGTTCAGG TTCCAGGGCA ACGCCAATGA

CCCAGACCAA AATGGAAATA CTACCCAAAG TCCTCCGTCA AGAGATGTCT

CCCAGACCAA AATGGAAATA CTACCCAAAG TCCTCCGTCA AGAGATGTCT

CCCAGACCAA AATGGAAATA CTACCCAAAG TCCTCCGTCA AGAGATATGT

CCCAGACCAA AATGGAAATA CTACCCAAAG TCCTCCGTCA AGAGATATGT

CCCAGACCAA AATGGAAATA CTACCCAAAG TCCTCCGTCA AGAGATGTCT

CCCAGACCAA AACGGAAATA CTACCCCAAG TCCTCCATCT AGAGACGTCT

CCCAGACCAG AATGGAAATA ACACTCAAAG TCCTCCCTCC AGAGATGTTT

CCCAGACCAG AATGGAAATA ACACTCAAAG CCCTCCCTCG AGAGATGTCT

CCCCGACCAA AATGGAAACA CCACCCAGAG TCCTCCATCA AGAGACGTCT

CCCAGACCAA AATGGAAACA CCACCCAAAG CCCTCCATCC AGAGAAGTCT

CCCGGACCAG AACGGGAACA CCACGCAGAG CCCCCCGTCC AGGGACGTGT

CCCAGACCAA AATGGAAATA CTACCCAAAG CCCTCCATCC AGAGATGTCT

CCCCGACCAA AACGGAAACA CCACGCAGAG TCCTCCTTCC AGGGATGTGT

CCAAGACCAA AATGGAAACA CTACTCAAAG TCCTCCATCA AGGGATGTTT

CCAAGACCAA AATGGAAATA CGACGCAGAG TCCTCCATCA AGAGATGTGT

CCAGGACCAA AATGGAAACA ACACTCAAAG TCCCCCTTCC AGAGATGTCT

CCAAGACCAA AATGGAAACA CTACCCAAAG CCCTCCATCA AGAGATGTCT

CCAAGACCAG AATGGGAACA CCACCCAGAG CCCTCCATCC AGGGAAGTCT

CGCCTACAAG TCGTGACAAC CTAGTGGCCA AACAACTAGC TCGGAATACT

CGCCTACAAG TCGTGACAAC CTAGTGGCCA AACAACTAGC TCGGAATACT

CACCTACAAG TCGTGACAAC CTAGTGGCCA AACAACTAGC TCGGAATACT

CACCTACAAG TCGTGACAAC CTAGTGGCCA AACAACTAGC TCGGAATACT

CGCCTACAAG TCGTGACAAC CTAGTGGCCA AACAACTGGC TCGGAATACT

CTCCTACAAG TCGTGACAAC CTAGTGGCCA AACAACTAGC TCGGAATACT

CTCCTACGAG TCGTGACAAC CTAGTAGCCA AGCAGCTAGC TCGGAATACT

CTCCTACGAG TCGTGACAAC CTAGTAGCCA AGCAGCTAGC TCGGAATACT

CTCCTACAAG TCGCGACAAC CTAGTGGCCA AACAGCTAGC TCGGAACACT

CTCCTACAAG TCGTGACAAC CTAGTGGCCA AACAGCTTGC TCGGAATACT

CTCCCACGAG TCGTGACAAC CTCGTGGCCA AACAGCTGGC CCGGAACACG

CTCCTACAAG TCGTGACAAC CTAGTGGCCA AACAGCTAGC TCGGAATACT

CCCCGACGAG TCGCGACAAC CTGGTGGCCA AACAGCTGGC TCGCAATACC

CCCCTACAAG CCGTGACAAC CTAGTGGCCA AACAGCTGGC TCGGAATACT

CTCCTACAAG TCGTGACAAC CTAGTGGCCA AACAGCTCGC TCGGAATACT

CTCCCACGAG TCGTGACAAC CTAGTGGCCA AACAGCTCGC TCGGAACACC

CTCCCACGAG TCGTGACAAC TTAGTAGCCA AACAGCTAGC TCGGAATACT

CCCCCACCAG CCGGGACAAC TTGGTCGTCA GGCAGCTGGC CCGCAACACG

GTGACCCAAG CACTGTCAAG ATTTACAAGC CCTCAAGCAG GTGCTCCCTC

GTGACCCAAG CACTGTCAAG ATTTACAAGC CCTCAAGCAG GTGCTCCCTC

GTGACCCAAG CACTGTCAAG ATTTACAAGC CCTCAAGCAG GTGCTCCCTC

GTGACCCAAG CACTGTCAAG ATTTACAAGC CCTCAAGCAG GTGCTCCCTC

GTGACCCAAG CACTGTCAAG ATTTACAGGA CCTCAAGCAG GTGCTCCCCC

GTGACCCAAG CACTGTCGAG ATTTACAAGC CCTCCAGCGG GTGCTGCCCC

GTGACCCAAG CGCTGTCCAG ATTTACCAGC CCTCAAGCAG GTGCTTCTTC

GTGACCCAAG CGCTGTCCAG ATTTACCAGC CCTCAAGCGG GTGCTTCTTC

GTGACCCAAG CTCTGTCGAG GTTCACAAGT CCTCAAGTGG GTACTCCCCC

GTGACCCAAG CACTGTCGAG ATTTACAAGC CCCCCGGCGG GAGCACCCCC

GTGACCCAGG CGCTGTCGAG GTTCACGGGC CCCCAGGCAg ggccgccccc

GTGACCCAAG CACTGTCAAG ATTTACAGGC CCTCCAGCAG GAGCGCCCCC

GTGACCCAGG CTCTGTCAAG ATTCACCAGC CCTCAAACGG GAACGCCCCC

GTGACCCAAG CGCTGTCAAG ATTTACGAGC CCTCAAGCGA GTGCTTCCCC

GTGACCCAAG CGCTGTCAAG ATTTACAGGC CCTCAAGTGG GTGCTTCTGC

GTGACCCAAG CGCTGTCAAG ATTTACAAGC CCTCAAGCAG GTGCTCCCCC

GTCACCCAAG CACTGTCAAG ATTTACAAGC CCTCAAGTGG GGGCTGCAGT

GTCACCCAAG CGCTCTCCAG GTTCACCGGC CCCCAGGCCG GGGCCCCCGC

AAGGCCTGGA GTGCCCCCAA CAGGGGATGT TGGCACCCAC CCTCCAGTTG

AAGGCCTGGA GCGCCCCCAA CAGGGGATGT TGGCACCCAC CCTCCAGTTG

AAGGCCTGGA GCGCCCCCAA CAGGGGATGT TGGCACTCAC CCTCCAGTCG

AAGGCCTGGA GCGCCCCCAA CAGGGGATGT TGGCACTCAC CCTCCAGTCG

AAGGCCTGGA GCGCCCCCAA CAGGGGATGT CAGCACCCAC CCATCAGTCG

AAGGCCAGGA GCGTCCCCCA CAGGGGACGG GGGCGCCTAC CCTCCAGTGG

AAGGCTGGGA GCATCTCCAG GAGGCGATGC TGGCACCTGC CCTCCAGTTG

AAGACTTGGA GTATCTCCAG GAGGGGACGC TGGCACCTGC CCTCCAGTTG

CAGGCCGGGA GTGCCCCCGA CAGGGGACGT GGCCACCCAC CCTCCAGTCA

GAGACCTGGA GCGTCCCCGA CGGGGGACGT TGGCACCTAC CCCCCGGTTG

caggcccggg ggggccccgg cggcgGGCAC CGGCACCTGC CCTCCCGTCG

AAGGCACGGA GTGCCCCCCT CGGGGGACGT TGGCACCTAC CCTCCCGTTG

CAGGCCAGGA GGCCCCCCGA CGGGAGACGC GGGCACCCAC CTTCCAGTCG

GAGGCCTGGA GCTCCCCCCA CAGGGGACAT TGGCACCCAC CCTGCAGTCA

CAGGCCTGGA GCACCCACCA CGGGGGACAT CAGCACCCAC CCCCCAGTTG

CAGGCCGGGG GCACCCCCCA CGGGGGAGGG CAGCACCCAC CCTCCAGTTG

GAGACCTGGA GCACCCCATC CAGTAGAAGC TGGCACCTGT CCCCCTGTTG

CAGCCCCAGA GCCCCCCATC CGGGGGAAGG AGGGACCGGC CCGCCCTCCG

GTCGGACCAG TTTAAAGACT CATGGTGTAG CACGAGTTGA CAGAGGAAAT

GTCGGACCAG TTTAAAGACT CATGGTGTAG CACGAGTTGA CAGAGGAAAT

GTCGGACCAG TTTAAAGACT CACGGTGTTG CACGAGTTGA CAGAGGAAAT

GTCGGACCAG TTTAAAGACT CACAGTGTTG CACGAGTTGA CAGAGGAAAT

GTCGGACCAG TGTAAAGACA CATGGCATAG CACGAGTTGA CAGAGGAAAC

GGCGGACGGG TTTAAAGACT CCCGGTGTAG CAAGAGTCGA CAGAGGAAAT

GTCGAACCGG TTTAAAGACT CCAGGGGCAG CACGAGTTGA CAGAGGAAAT

GTCGAACCGG TTTAAAGACT CCCGGGGCAG CAAGAGTTGA CAGAGGAAAT

GTCGGAGTGG TTTGAAGACT CCCGGCGGAG CGCGAGTCGA TAGAGGAAAT

GTCGAACCAG TTTAAAGACT CCTGGTGGGG CCCGAGTTGA CAGAGGAAAC

GTCGGACCAG CCTAAAGACT CCCGGGGTAG CCCGAGTCGA CAGAGGAAAC

GTCGGACCAA TGTCAAGACT CCCGGTGTGG CACGAGTTGA CCGAGGAAAC

GTCGGACCGG TTTAAAGACT CCCAGCGCAG CTCGAGTTGA CCGAGGAAAC

GTCGGACCAG TTTAAAGACA CCTGGTTTAG CACGAGTTGA CCGAGGAAAT

GTCGGACCAG TTTAAAGACT TCTGGTGTAG CACGAGTTGA CAGAGGAAAT

GTCGGACCAG TTTAAAGACT CCCGGTGCAG CACGTGTTGA CAGAGGAAAC

GTAGAATGAG TTTAAAGACT CCCAGTGTAC CAAGAGTTGA CCGAGGAAAT

GCGGGACGAG CGCCAAGACT CCCAACGCCC CACGTGTCGA CCGGGGGAAC

CCTCCTCCTA TCCCTCCAAA AAAGCCAGGG CTCTCCCAAA CTCCTTCTCC

CCTCCTCCTA TCCCTCCAAA AAAGCCAGGG CTCTCCCAAA CTCCTTCTCC

CCTCCTCCTA TCCCTCCAAA AAAGCCAGGG CTCTCCCAAA CTCCTTCTCC

CCTCCTCCTA TCCCTCCAAA AAAGCCAGGG CTCTCCCAAA CTCCTTCTCC

CCTCCTCCTA TCCCTCCAAA AAAGCCAGGG CTCTCCCAAA CGCCTTCTCC

CCTCCTCCTA TCCCTCCAAA AAAGCCAGGC CTCTCCCAAA CTCCTTCTCC

CCTCCTCCTA TCCCTCCCAA AAAGCCAGGG CTCTCCCAAA CTCCTTCTCC

CCTCCTCCTA TCCCTCCCAA AAAGCCAGGG CTCTCCCAAA CTCCTTCTCC

CCTCCTCCCA TCCCCCCGAA AAAACCAGGG CTGTCCCAGA CGCCTTCTCC

CCTCCTCCCA TCCCTCCAAA AAAGCCAGGG CTCTCCCAAA CTCCTTCTCC

CCTCCTCCCA TCCCTCCAAA AAAGCCAGGG CTCTCCCAGA CTCCTTCTCC

CCTCCTCCTA TCCCGCCAAA AAAGCCAGGG CTGTCCCAAG CTCCTTCTCC

CCGCCTCCCA TCCCTCCCAA AAAGCCAGGG CTCTCCCAAG CCCCTTCTCC

CCTCCTCCTA TCCCTCCAAA AAAGCCAGGG CTCTCCCAAG CTCCTTCTCC

CCTCCTCCCA TCCCTCCAAA AAAGCCAGGG CTCTCCCAAA CTCCTTCTCC

CCGCCCCCCA TCCCTCCAAA AAAGCCAGGG CTCTCCCAGA CTCCTTCTCC

CCTCCTCCCA TCCCACCAAA AAAGCCAGGG CTCTCCCAAA CTCCTTCACC

CCTCCGCCCA TCCCTCCGAA AAAGCCAGGG CTGTCCCAGA CCCCTTCGCC

ACCACACCCC CAACTCAAGG TTATTATAGA CAGCAGCAGG GCCTCGAACA

ACCACACCCC CAACTCAAGG TTATTATAGA CAGCAGCAGG GCCTCGAACA

ACCACACCCC CAACTCAAGG TTATTATAGA CAGCAGCAGG GCCTCGAACA

ACCACACCCC CAACTCAAGG TTATTATAGA CAGCAGCAGG GCCTCGAACA

ACCACACCCC CAACTCAAGG TTATTATAGA TAGCAGCAGG GCCTCGAACA

ACCACACCCC CAACTCAAGG TTATTATGGA TAGCAGCAGG GCCTCCAATG

ACCACACCCT CAACTGA--- ---------- --------GG GCCTCCAATG

GCCACACCCC CAACTGA--- ---------- --------GG GCCTCCAATG

ACCCCACCCC CAACTCAAGA TTCCGGTGGA CAGCAGCAGG GCCTCCAGTG

ACCGCACCCC CAACTCAAGG TTATCATGGA TAGCAGCAGG GCCTCCAGCA

GCCACACCCC CAGCTCAAGG TCCTCCTGGA TAGCAG---G GCCCCCAGCG

ACCCCACCCC CAACTCAAGG TCGTTATGGA TAGCAGCAGG GCCCCCAGTG

CCCACACCCC CAACTCAAGG CTATCATGGA T---AGCAGG GCCTCCAGTG

ACCACACCCC CAGCTCAAGG CTATTATGGA TAGCAGCAGG GCCTCCAATG

ACCGCACCCC CAACTCAAGG TTATTATGGA TAGCAGCAGG GCCTCAAATG

ACCACACCCC CAACTCAAAG TTCTCCTGGA TAGCAGCCGG GCCTCGAACG

ACCACACCCT CAAATCAAAG TTATTATGGA TAGCAGCAGA TCCTCTAATG

TCCCCACCCC CAGCTCAAGG TCCTCAAGGA CAGCGGCCGC CCAGCCAACG

CAGGGGCCAA AGTTGATAAC AAAACTGTGG CTTCGACTCC TTCCAGTTTG

CAGGGGCCAA AGTTGATAAC AAAACTGTGG CTTCGACTCC TTCCAGTTTG

CAGGGGCCAA AGTTGATAAC AAAACTGTGG CTTCGCCTCC TTCCAGTTTG

CAGGGGCCAA AGTTGATAAC AAAACTGTGG CTTCGCCTCC TTCCAGTTTG

CAGGGGCCAA AGGTGATAAC AAAACTGTGG CTTCGCCTCC TTCCAGTTTG

CAGGGGCCAA AGTTGATAAC AAAACTGTGG CTTCACCTCC TTCCAGCTTG

CAGGGGCCAA AGTTGATAAC AAAATTGTGG CTTCACCTCC CTCTACATTG

CAGGGGCCAA AGTTGATAAC AAAATTGTGG CTTCACCTCC CTCTACTTTG

CGGGGGCCAA AGTTGAAAAC AAAACTGTGG CTTCGCCTCC TTCCAGCTTG

CAGGGATCAA AGCTGATAAC AAAACTGTGG CTTCGCCTCC TTCCAGTTTG

CAGGGGCCAA AGTTGATAAC AAAACTCTGG CTTCGCCTCC TTCCAGTGGG

CAGGGGCCAA AGTGGATAAC AAAACTGTGG CTTCGCCTCC TTCCAGTTTG

CGGGGGCCAA AGTCGATAAC AAAACTGTGG CATCACCTCC TTCCACCCTG

CAGGGGCCAA AGTTGATAAC AAAACTGTAG CTTCACCTCC TGCCAGTTTG

CAGGGGCCAA AGTTG---AT AAAACTGTGG CTTCGCCTCC TACCAGTTTG

CAGGGCCCAA AGTTGATCAT AAAACGGTGG CTTTGCCTCC CACCAGTGTG

CAGGGGCCAA AGTTGATAAC AAAACCACGA CTTCATCTCC TTCCAGTTCA

CGGGGGCCAA AGTCGATAGC AAAACCGGGG TGCCGCCTCC TTCCGCCCCG

CCACAAGGGA ACAGGGTGAT CAATGAGGAG AACCTTCCTA AGTCATCCTC

CCACAAGGGA ACAGGGTGAT CAATGAGGAG AACCTTCCTA AGTCATCCTC

CCACAAGGGA ACAGGGTGAC CAATGAGGAC AACCTTCCTA AGTCATCCTC

CCACAAGGGA ACAGGGTGAC CAATGAGGAC AACCTTCCTA AGTCATCCTC

CCACAAGGGA ACAGGGTGAT CAATGAGGAG AACCTTCCTA AGTCATCCTC

CCACAAGGGA GCAGGGTAAT AAATGAGGAG AACCTCCCTA AATCGTCCTC

CCACAAGGGA CCAAGGTAGT AAATGAGGAG AACGTCCCTA AGTCATCCTC

CCACAAGGGA CCAAGGTAGT AAATGAGGAG AACGTCCCTA AGTCGTCCTC

CCCCCCGGGA GCAGGGTCAT TACGGAAGAG AACCCCCCTA AGTCTTCCTC

CCACAAGGGA ACAGGGTAAT AAATGAGGAG AATCTCTCTA AATCTTCCTC

CCACAAGGGA ACAGGGTAAT AAGTGAGGAG ACTCTCCCTA AGTCATCTTC

CCCCCAGGGA ACAGGGTCAT AAATGAGGAG AACCTCCCCA AACCAACCAC

CCCCAAGGGA GCAGGGTCGT AACAGAGGAG AATCTCACTA AGTCATCTTC

CCACAAGGGA ACAGGGTAAT AAATGAGGAG AACCTTCCTA AGTCTTCCTC

CCACAAGGGA ACAGGGTAAT AAATGAGGAG AACCTCCCTA AGTCATCCTC

CCACAAGGGA ACAGGGTCAT AAATGAGGAG AGCCTCCCTA AGTCATCCTC

CCACAAGGAA TTAGGGTAAT AAATGAGGAA AACCTCTCTA AGTCACCCTC

CCCCACGGAA TCAGGGTAAT GAACGAGGAA AACCTCGCTA AGTCTTCCTC

CCCTCAGCTG CCACCAAAAC CATCCATAGA TTTAACTGTG GCACCTGCAG

CCCTCAGCTG CCACCAAAAC CATCCATAGA TTTAACTGTG GCACCTGCAG

CCCTCAGCTG CCACCAAAAC CATCCATAGA TTTAACTGTG GCACCTGCAG

CCCTCAGCTG CCACCAAAAC CATCCATAGA TTTAACTGTG GCACCTGCAG

CCCTCAGCTG CCACCAAAAC CATCCATAGA TTTAACTGTG GCACCTGCAG

CCCTCAGCTG CCACCAAAAC CATCCATAGA TTTAACTGTG GCACCTGCAG

CCCTCAGCTG CCACCAAAAC CATCCATAGA TTTAACTGTG GCATCTGCAG

CCCTCAGCTG CCACCAAAAC CGTCCATAGA TTTAACTGTG GCACCTGCAG

CCCTCAGCTG CCACCAAAGC CGTCCATAGA GCTAACTGTG GCACCTGCAG

CCCTCAGCTG CCACCAAAAC CATCCATAGA TTTAACTGTG GCACCCGCAG

CCCTCAGCTG CCACCAAAAC CGTCCATAGA TTTAACTGTG GCACCGGCAG

CCCTCAGCTG CCACCAAAAC CATCCATAGA TTTAACTGTG GCACCGGCAG

CCCTCAGCTG CCACCAAAAC CGTCCATAGA TTTAACTGTG GCACCTGCCG

CCCTCAGCTG CCACCAAAAC CATCCATAGA TTTAACTGTG GCACCTGCAG

CCCTCAGCTG CCACCAAAAC CATCCATAGA TTTAACTGTG GCACCTGCAG

CCCCCAGCTG CCACCAAAAC CCTCCATAGA TTTAACTGTG GCACCCGCAG

CCCTCAGCTG CCACCAAAAC CAACCATAGA TTTAACTGTG GCACCTGCAG

CCCCCAGCCG CCCCCCAAAC CGGCGGGAGA CTTAGCTGCG GTGCCCGCGG

GCTGTGCCGT TTCAGCCCTG GCCACGTCTC AGGTGGGTGC CTGGCCTGCT

GCTGTGCCGT TTCAGCCCTG GCCACGTCTC AGGTGGGTGC CTGGCCTGCT

GCTGTACCGT TTCAGCCCTG GCCACATCTC AGGTGGGTGC CTGGCCTGCT

GCTGTACCGT TTCAGCCCTG GCCACATCTC AGGTGGGTGC CTGGCCTGCT

GCTGTGCCGT TTCAGCCCTG GCCACGTCTC AGGTGGGTGC CTGGCCTGCT

GCTGTGCCGT TTCAGCCCTG GCCACGTCTC AGGTGGGTGC CTGGCCTGCT

GCTGTCCCGT TTCGGCCCTG GCCACGTCTC AGGTGGGTGC CTGGCCTGCT

GCTGTCCCGT TTCGGCCCTG GCCACGTCTC AGGTGGGTGC CTGGCCTGCT

GCTGTGCCGT TTCCGCCCTG GCTGCGTCTC AGGTGGGTGC CTGGCCTGCT

GCTGTGCCGT TTCAGCCCTG GCCACATCTC AGGTGGGTGC CTGGCCTGCT

GCTGTGCCGT TGCGGCCCTG GCCACGTCTC AGGTGGGTGC CCGGCCTGCT

GCTGTGCTGT GTCAGCCCTG GCCACGTCTC AGGTGGGTGC CTGGCCTGCT

GCTGTGCCGT TTCAGCCCTG GCCACGTCTC AGGTGGGTGC CCGGCCTGCT

GCTGTGCCGT TTCAGCCCTG GCCACGTCTC AGGTGGGTGC CCGGCCTGCT

GCTGTGCCGT TTCGGCCCTG GCCACGTCTC AGGTGGGTGC CTGGCGTGCA

GCTGTGCCGT TTCAGCCCTG GCCACGTCTC AGGTGGGTGC CTGGCGTGCT

GCTGTGCCAT TTCAGCCATG GCCACTTCTC AGGTGGGTGC CTGGCCTTCC

GCTGCGCCCT GCCCGCCGTG GCCGCGTCTC AGGTGGGTGC CTGCCTGGCT

GCAACCCCCG GACTGAACCA ACCTGCATGT TCAGACAGTT CCCTTGTCAT

GCAACCCCCG GACTGAACCA ACCTGCATGT TCAGACAGTT CCCTTGTCAT

GCAACCCCCG GACTGAACCA ACCTGCATGT TCAGACAGTT CCCTTGTCAT

GCAACCCCCG GACTGAACCA ACCTGCATGT TCAGACAGTT CCCTTGTCAT

GCAACCCCCG GACTGAACCA ACCTGCATGT TCAGACAGTT CCCTTATCAT

GAAACCCCTG GACTGAACCA CCCTGCATGT TCAGACAGTT CCCTTGTCAT

GAAACCCCTG GACTGAACCA ACCCGCATGT ACAGACAGCT CCCTTGTCAT

GGAACCCCTG GACTGAACCA ACCCGCATGT TCAGACAGCT CCCTTGTCAT

GAAACCCTTG GACTGAAGCC ACCTGCATGT TCAGACAGTT CCCTTGTCAT

GAAACCCCTG GACTGAACCA ACCCGCATGT TCAGAAAGTT CCCTTGTCAT

GAAACCCCTG GACCGAACCA ACCTGCATGT TCAGAGCGTT CCCTTGTCAT

GAAACCCCGG GACTGAACCA ACCTGCATGT TCAGAACGTT CCCTTGTCAT

GAAACCCCTG GACTGAACCA GCCTGCATGT TCAGAACGTT CCCCTGTCCT

GAAACCCCTG GACCGACCCA ACCTGCATGT TCAGACAGCT CCCTTGTCAT

GAAACCCCTG GACTGAACCA ACCTGCATGT TCAGACAGTT CCCTTGTCAT

GAAACCCCTG GACTGAAACC ACCTGCATGT TCAGACAGAC CCCTTGTCAT

GAAACCCCTG GACTGAGCCA ACCTGCATGT TCCGAAAATC CCCTTGTCAT

GCAACCC-GG GACCGAGCCA GCCTGCATGT TCAGAAAGTA GCCATGCCAT

TCCTACCACC ATTGCCTTTT GCTCTTCCAT AAACCCCGTT AGTGCCTCAT

TCCTACCACC ATTGCCTTTT GCTCTTCCAT AAACCCCGTT AGTGTCTCAT

TCCTACCACC ATTGCCTTTT GCTCTTCCAT AAACCCCGTT AGTGCCTCAT

TCCTACCACC ATTGCCTTTT GCTCTTCCAT AAACCCCGTT AGTGCCTCAT

TCCCACCACC ATTGCCTTTT GCTCTTCCAT AAACCCCGTT AGTGCCTCAT

TCCCACCACC ACTGCCTCTC GCTCTGCCAT AAACCCCGTT AGTGCCTCAT

TCCCACCACC GTTGCCTTTC GCTCTTCCAT AAACCCTGTT AGTGCCTCAT

TCCCGCCACC GTTGCCTTTT GCTCTTCCAT AAACCCTGTT AGTGCCTCAT

TCCCAACACC ATTGCCTTTC GCTCTTCCAT AAACCCTGTT AGTGCCTCAA

TCCCACCACC ACTGCCTTTC GCTCTTCCAT AAACCCTGTT AGTGCCTCAT

TCCCACCACC ATTGCCTTTT GCTCTTCCAT AAACCCTGTT AGTGCCTCAT

TCCCACCACC ATTGCCTTTT GCTCTTCCAT AAACCCTGTT AGTGCCTCAT

CCCCAGCACC ATTGCCTTTC GCTCTCCCAT AAACCCTGTT AGTGCCGCAT

TCCCACCACC ATTGCCTTTC GCTCTTCCAT AAACCCTGTT AGTGCTTCAG

TCCCACCACC ATTGCCTTTT GCTCTTCCAT AAACCCTGTT AGTGCCTCAT

TCCCACCACC ATTGCCTTCC GCTGTCCCAT AAACCCTGTT AGTGCCTCAT

TCCCACCACC ATTGCCTGTA GCTCCTCCAT AAACCCCGTT AGTGCCTCAT

CCCCACCGCC ATTGCCTGTA GCTCCTCCAT AAACCCCGTT AGTGCCTCAT

CCTGTAGACC AGGTGCCTCA GACAGCCTCC TGGTAACAGC ATCAGGCTGG

CCTGTAGACC AGGTGCCTCA GACAGCCTCC TGGTAACAGC ATCAGGCTGG

CCTGTAGACC AGGTGCCTCA GACAGCCTCC TGGTAACAGC ATCAGGCTGG

CCTGTAGACC AGGTGCCTCA GACAGCCTCC TGGTAACAGC ATCAGGCTGG

CCTGTAGACC AGGTGCCTCA GACAGCCTCC TGGTAACAGC ATCAGGCTGG

CCTGTAGCCC AGGTGCCTCA GACAGCCTCC TGGTAACAGC ATCAGGCTGG

CCCGTAGCCC CGGTGCCTCA GACAGCCTCT TGGTAACAGC ATCAGGCTGG

CCCGTAGCCC CGGTGCCTCA GACAGCCTCT TGGTAGCAGC ATCAGGCTGG

CCAGTAGGCC AGGTGCCTCA GACAGCCTCC TGGTAACAGC ATCAGGCTGG

CCCGTAGAGC AGGTGCCTCT GACAGCCTCC TGGTAACAGC ATCAGGCTGG

CCTGTAGAGC AGGTGCCTCA GACAGCCTCC TGGTAACAGC ATCAGGCTGG

CCTGTAGAGC AGGTGCCTCA GACAGCCTCC TGGTAACAGC ATCAGGCTGG

CCTGTAGAGC AGGTGCCTCA GACAGCCTCC TGGTAACAGC ATCAGGCTGG

CCTCTAGACC AGGCGCCTCA GACAGCCTCC TGGTAACAGC ATCAGGCTGG

CCTGTAGACC AGGTGCCTCA GACAGCCTCC TGGTAACAGC ATCAGGCTGG

CCTGTAGACC AGGTGCCTCA GACAGCCTCC TGGTAACAGC ATCAGGCTGG

CCTGTAGACC ATGTGCCTCA GACAGCCTCC TGGTAACAGC ATCAGGCTGG

CCTGTGCACC CTGGGCCTCC CACAGCCTCC TGGTAGCAGC CTCAGGGTGG

TCACCCTCCC TAACCCCTTT GCTAATGAGT GGTGGTCCTG CCCCCCTGGC

TCACCCTCCC TAACCCCTTT GCTAATGAGT GGTGGTCCTG CCCCCCTGGC

TCACCCTCCC TAACCCCTTT GCTAATGAGT GGTGGTCCTG CCCCCCTGGC

TCACCCTCCC TAACCCCTTT GCTAATGAGT GGTGGTCCTG CCCCCCTGGC

TCACCCTCCC TAACCCCTTT GCTAATGAGT GGTGGTCCTG CCCCCCTGGC

TCACCCTCCC TAACCCCTTT GCTAATGAGT GGTGGTCCTG CCCCCCTGGC

TCACCCTCCC TAACCCCTTT GCTAATGAGT GGTGGTCCTG CCCCCCTGGC

TCACCCTCCC TAACCCCTTT GCTAATGAGT GGTGGTCCTG CCCCCCTGGC

TCACCCTCCC TAACCCCTTT GCTAATGAGT GGTGGTCCTG CCCCCCTGGC

TCACCCTCCC TAACCCCTTT GCTAATGAGT GGTGGTCCTG CCCCCCTGGC

TCACCCTCCC TAACCCCTTT GCTAATGAGT GGTGGTCCTG CCCCCCTGGC

TCACCCTCCC TAACCTTTTT GCTAATGAGT GGTGGTCCTG CCCCCCTGGC

TCACCCTCCC TAACCCCTTT GCTAATGAGT GGTGGTCCTG CCCCCCTGGC

TCACCCTCCC TAACCCCTTT GCTAATGAGT GGTGGTCCTG CCCCCCTGGC

TCACCCTCCC TAACCCCTTT GCTAATGAGT GGTGGTCCTG CCCCCCTGGC

TCACCCTCCC TAACCCCTTT GCTAATGAGT GGTGGTCCTG CCCCCCTGGC

TCACCCTCCC TAACCCCTTT GCTAATGAGT GGTGGTCCTG CCTCCCTGGC

CCCCCCTCCC TAACCCCTTT GCTAACAAGT TGTGGTCCTG TGTCCCTGGG

TGGCAGGCCC ACCCTTCTTC AGCAAGCTGC TGCCCAGGGA AATGTCACTT

TGGCAGGCCC ACCCTTCTTC AGCAAGCTGC TGCCCAGGGA AATGTCACTT

TGGCAGGCCC ACCCTTCTTC AGCAAGCTGC TGCCCAGGGA AATGTCACTT

TGGCAGGCCC ACCCTTCTTC AGCAAGCTGC TGCCCAGGGA AATGTCACTT

TGGCAGGCCC ACCCTTCTTC AGCAAGCTGC TGCCCAGGGA AATGTCACTT

TGGCAGGCCC ACCCTTCTTC AGCAAGCTGC TGCCCAGGGA AATGTCACTT

TGGCAGGCCC ACCCTTCTTC AGCAAGCTGC TGCCCAGGGA AATGTCACTC

TGGCAGGCCC ACCCTTCTTC AGCAAGCTGC TGCCCAGGGA AATGTCACTT

TGGCAGGCCC ACCCTTCTTC AGCAAGCTGC TGCCCAGGGA AATGTCACTT

TGGCAGGCCC ACCCTTCTTC AGCAAGCTGC TGCCCAGGGA AATGTCACTT

TGGCAGGCCC ACCCTTCTTC AGCAAGCTGC TGCCCAGGGA AATGTCACTT

TGGCAGGCCA ACCCTTCTTC AGCAAGCTGC TGCCCAGGGA AATGTCACTT

TGGCAGGCCC ACCCTTCTTC AGCAAGCTGC TGCCCAGGGA AATGTCACTT

TGGCAGGCCC ACCCTTCTTC AGCAAGCTGC TGCCCAGGGA AATGTCACTT

TGGCAGGCCC ACCCTTCTTC AGCAAGCTGC TGCCCAGGGA AATGTCACTT

TGGCAGGCCC ACCCTTCTTC AGCAAGCTGC TGCCCAGGGA AATGTCACTT

TGGCAGGTCC ACCCTTCTTC AGCAAGCTGC TGCCCAGGGA AATGTCACTT

TGGCAGGCCC ACCCGTCTTC ACCAAGCTGC TGCCCAGGGA AATGTCACTT

TATTATCAAT GCTGCTTAAT GAAGAAGGAC TGGACATTAA TTACTCCTGT

TATTATCAAT GCTGCTTAAT GAAGAAGGAC TGGACATTAA TTACTCCTGT

TATTATCAAT GCTGCTTAAT GAAGAAGGAC TGGACATTAA TTACTCCTGT

TATTATCAAT GCTGCTTAAT GAAGAAGGAC TGGACATTAA TTACTCCTGT

TATTATCAAT GCTGCTTAAT GAAGAAGGAC TGGACATTAA TTACTCCTGT

TATTATCAAT GCTGCTTAAT GAAGAAGGAC TGGACATTAA TTACTCCTGT

TATTATCAAT GCTGCTTAAT GAAGAAGGAC TGGACATAAA TTACTGCTGT

TATTATCAAT GCTGCTTAAT GAAGAAGGAC TGGACATAAA TTACTCCTGC

TATTATCAAT GCTGCTTAAT GAAGAAGGAC TGGACACTAA TTACTCCTGT

TATTATCAAT GCTGCTTAAT GAAGAAGGAC TGGACATTAA TTACTCCTGT

TATTATCAAT GCTGCTTAAT GAAGAAGGAC TGGACATTAA TTACTCCTGT

TATTGTCAAT GCTGCTTAAT GAAGAAGGAC TGGACATTAA TTACTCCTGT

TATTATCAAT GCTGCTTAAT GAAGAAGGAC TGGACATTAA TTACTCCTGT

TATTGTCAAT GCTGCTTAAT GAAGAAGGAC TGGACATTAA TTACTCCTGT

TATTATCAAT GCTGCTTAAT GAAGAAGGAC TGGACATTAA TTACTCCTGT

TACTGTCAAT GCTGCTTAAT GAAGAAGGAC TCGACATCAA TCACTCTTGC

TATTATCAAT GCTGCTTAAT GAAGAAGGAC TGGACATTAA TTACTCCTGT

TATTGTCTGT GCTGCTTAAT GAAGAAGGAC TGGACATTAA TCACGCCTGT

GAAGATGGCC ATTCTGCCTT GTATTCTGCT GCTAAGAATG GACATACAGA

GAAGATGGCC ATTCTGCCTT GTATTCTGCT GCTAAGAATG GACATACAGA

GAAGATGGCC ATTCTGCCTT GTATTCTGCT GCTAAGAATG GACATACAGA

GAAGATGGCC ATTCTGCCTT GTATTCTGCT GCTAAGAATG GACATACAGA

GAAGATGGCC ATTCTGCCTT GTATTCTGCT GCTAAGAATG GACATACAGA

GAAGATGGCC ATTCTGCCTT GTATTCTGCT GCTAAGAATG GACATACAGA

GAAGATAGCC ATTCTGCCTT GTATTCTGCT GCTAAGAATG GACATACAGA

GAAGATGGCC ATTCTGCCTT GTATTCTGCT GCTAAGAATG GACATACAGA

GAAGATGGCC ATTCTGCCTT GTATTCTGCT GCTACGAATG GACATGCAGA

GAAGATGGCC ATTCTGCCTT GTATTCTGCT GCTAAGAATG GACATACAGA

GAAGATGGCC ATTCTGCCTT GTATTCTGCT GCTAAGAATG GACATACAGA

GAAGATGGCC ATTCTGCCTT GTATTCTGCT GCTAAGAATG GACATACAGA

GAAGATGGCC ATTCTGCCTT GTATTCTGCT GCTAAGAATG GACATACAGA

GAAGATGGCC ATTCTGCCTT GTATTCTGCT GCTAAGAATG GACATACAGA

GAAGATGGCC ATTCTGCCTT GTATTCTGCT GCTAAGAATG GACATACAGA

GAAGATGGCC ATTCTGCCTT GTATTCTGCT GCTAAGAATG GACATACAGA

GAAGATGGCT CTTCTGCCTT GTATTCTGCT GCTAAGAATG GACATACAGA

GAAGATGGCT CTTCTGCCTT GTATTCTGCT GCTAAGAATG GACATACAGA

CTGTGTGAGA TTGCTGCTGA GTGCAGAAGC CCAAGTCAAT GCTGCTGATA

CTGTGTGAGA TTGCTGCTGA GTGCAGAAGC CCAAGTCAAT GCTGCTGATA

CTGTGTGAGA TTGCTGCTGA GTGCAGAAGC CCAAATCAAT GCTGCTGATA

CTGTGTGAGA TTGCTGCTGA GTGCAGAAGC CCAAATCAAT GCTGCTGATA

CTGTGTGAGA TTGCTGCTGA GTGCAGAAGC CCAAGTCAAT GCTGCTGATA

CTGTGTGAGA TTGCTGCTGA ATGCAGAAGC CCAAGTCAAT GCTGCTGATA

CTGTGTGCGA TTGCTGCTGA ATGCAGAAGC CCGCGTCGAC GCTGCTGATA

CTGTGTGCGA TTGCTGCTGA ATGCAGAAGC CCGAGTCGAC GCTGCTGATA

CTGTGTGAGA TTGCTGCTGA ATGCAGAAGC CCAAGTCAAT GCTGCTGAGA

CTGTGTGAGA TTGCTGCTGA ATGCAGAAGC CCAAGTCAAT GCTGCTGATA

CTGTGTGAGA TTGCTGCTGA ATGCAGAAGC CCAAGTCAAT GCTGCTGATA

TTGTGTGAGA TTGCTGCTGA ATGCAGAAGC CCAAGTCGAT GCTGCTGATA

CTGTGTGAGA TTGCTGCTGA ATGCAGAAGC CCAAGTCAGC GCTGCTGATA

CTGTGTGAGA TTGCTGCTGA ATGCAAAAGC CCAAATTGAT GCTGCTGATA

CTGTGTGAGA TTGCTGCTGA ATGCAGAAGC CCAAGTCAAC GCTGCTGATA

CTGTGTGAGA TTGCTGCTGA ATGCAGAAGC CCAAGTCAAT GCTGCTGATA

CTGTGTGAGA TTGCTGCTGA ATGCAGAAGC ACAAGTTAAT ACTGCTGATA

CTGTGTGAGA TTGCTGCTGA ATGCCAATGC GCAAGTTGAT GATGCCGATA

AAAATGGCTT CACACCCTTG TGTGCTGCAG CTGCTCAGGG ACATTTCGAG

AAAATGGCTT CACACCCTTG TGTGCTGCAG CTGCTCAGGG ACATTTCGAG

AAAATGGCTT CACACCCTTG TGTGCTGCAG CTGCTCAGGG ACATTTCGAG

AAAATGGCTT CACACCCTTG TGTGCTGCAG CTGCTCAGGG ACATTTCGAG

AAAATGGCTT CACACCCTTG TGTGCTGCAG CTGCTCAGGG ACATTTCGAG

AAAATGGCTT CACACCCTTG TGTGCTGCAG CTGCTCAGGG ACATTTCGAG

AAAATGGCTT CACACCCTTG TGCGTTGCAG CCGCTCAGGG ACATTTTGAA

AAAATGGCTT CACACCCTTG TGCGTCGCAG CTGCTCAGGG ACATTTTGAA

AGAACGGCTT CACACCCTTG TGTGCCGCAG CTGCTCAGGG ACATTTCGAG

AAAATGGCTT CACACCCTTG TGTGCTGCAG CTGCTCAGGG ACATTTCAAG

AAAATGGCTT CACACCCTTG TGTGCTGCAG CTGCTCAGGG ACATTTCAAG

AAAATGGCTT CACACCCTTG TGTGCTGCAG CTGCTCAGGG ACATTTCAAG

AAAACGGCTT TACACCCTTG TGTGCTGCAG CTGCACAGGG ACACTTCAAG

AAAATGGCTT CACACCCTTG TGTGCTGCAG CTGCTCAGGG ACATTTCGAG

AAAACGGCTT CACACCCTTG TGTGCCGCAG CTGCTCAGGG ACATGACAAG

ACGATGGCTT CACACCCGTG TGTGCTGCAG CTGCTCAGGG ACACGTCAAA

AAAATGGCTT CACACCCTTG TGTTCAGCAG CTGCTCAGGG ACATTTCAAG

AAAATGGCTT CACCCCCTTG TGTTCAGCAG CCGCTCAGGG ACATGTTAAG

TGTGTAGAAT TATTAATTTC ATATGATGCT AACATTAATC ATGCTGCTGA

TGTGTAGAAT TATTAATTTC ATATGATGCT AACATTAATC ATGCTGCTGA

TGTGTAGAAT TATTAATTGC ATATGATGCT AACATTAATC ACGCTGCTGA

TGTGTAGAAT TATTAATTGC ATATGATGCT AACATTAATC ATGCTGCTGA

TGTGTAGAAT TATTAATAGC ATATGATGCT AATATTAATC ATGCTGCTGA

TGTGTAGAAT TATTGATTGC ATATGATGCT CACATTAATC ACGCTGCTGA

TGTATAGAAT TACTAACTGC ATACAATGCC AACATTAATC ACTCTGCTGC

TGTATAGAAT TACTAACTGC ATACAATGCC AACATTAATC ACTCTGCTGC

TGTTTAGAAT TACTACTTGC ATCTGATGCT GATGTCAATC ATGCTGCTGA

TGTGTAGAAT TATTAATTGC ATATGATGCT AATATTAATC ATGCTGCTGA

TGTGTAGAAT TATTAATTGC ATATGATGCC AACATTAATC ATGCTGCTGA

TGTGTAGAAT TATTAACTGC ATATGATGCT GACATTAATC ATACTGCTGA

TGTGTAGAAT TACTAATTGC ATACGATGCT AACATTAATC ATGCTGCTGA

TGTGTAGAAT TATTAATTGC ATATCATGCT AACATTGATC ATGCTGCTGA

TGTGTAGAAT TACTAATTGC ATATCGTGCT AACATTAACC ATGCTGCTGA

TGTGTAGAAT TATTAGTTGC ATACCATGCT AACATTAATC ATGCTGCTGC

TGTGCAGAAT TACTAATTGC ATATCATGCT GACATTAACC ATGCTGCTGA

TGTGCAGAAT TACTAATTGC ATATCACGCT GACATTAACC ATGCTGCTGA

TGGAGGACAG ACACCTCTAT ACCTGGCCTG TAAAAATGGA AATAAAGAAT

TGGAGGACAG ACACCTCTAT ACCTGGCCTG TAAAAATGGA AATAAAGAAT

TGGAGGACAG ACACCTCTAT ACCTGGCCTG TAAAAATGAA AATAAAGAAT

TGGAGGACAG ACACCTCTAT ACCTGGCCTG TAAAAATGAA AATAAAGAAT

TGGAGGACAG ACACCTCTAT ACCTGGCCTG TAAAAATGGA AATAAAGAAT

TGAAGGACAG ACACCTCTAT ACCTGGCCTG TAAAAACGGA AATAAAGAAT

TGGAGGACAG ACACCTCTGT ACCTGGCCTG TAAAACTGGA AATAAGGAAT

TGGAGGACAG ACACCTTTGT ACTTGGCCTG TAAAAATGGA AATAAGGAAT

TGGAGGACAG ACACCTCTAT ACCTGGCCTG TAAAAATGGA AATACAGACT

TGGAGGACAG ACACCTCTAT ACCTGGCCTG TAAAAATGGA AATAAAGAAT

TGAAGGACAG ACACCTCTAT ACCTGGCCTG TAAAAATGGA AATAAGGAGT

TGGAGGACAG ACACCTCTAT ACCTGGCCTG TAAAAATGGA AATAAAGAAT

TGGAGGACAG ACACCTCTAT ACCTGGCCTG TAAAAATGGA AACAAAGAAT

TGGAGGACAG ACACCTCTGT ACCTGGCCTG TAAAAATGGA AATAAAGAAT

TGGAGGACAG ACACCTCTAT ACCTGGCCTG TAAAAATGGA AATAAAGAAT

TGAAGGACAG ACACCTCTCT ACCTGGCCTG TAAAAATGGA AATAAAGAAT

AGGAGGACAA ACACCTCTAT ACCTGGCCTG TAAAAATGGA AATAATGAAT

AGGAGGTCAG ACACCTCTGT ACCTGGCCTG TAAAAACGGA AATAACGAAT

GTATTAAACT CTTGTTGGAA GCTGGAACCA ATCGAAGTGT AAAAACCACA

GTATTAAACT CTTGTTGGAA GCTGGAACCA ATCGAAGTGT AAAAACCACA

GTATTAAACT CTTGTTGGAA GCCGGAACCA ACCGAAGTGT AAAAACCACA

GTATTAAACT CTTGTTGGAA GCCGGAACCA ACCGAAGTGT AAAAACCACA

GTATTAGACT CTTGTTGGAA GCTGGAACCG ATCGAAGTGT AAAAACCACA

GTATTAAACT CTTGTTGGAA GCTGGAACTA ACCGAAATGT AAAAACCAGA

GTATCAAACT CCTGCTGGAA GCTGGCACTG ACCGGAGTAT AAAAACCAGA

GTATCAAACT CCTGCTGGAA GCTGGCACTG ACCGGAGCAT AAAAACCAGA

GTATTAAACT CTTGTTGGAA GCTGGAACTG ACCGAAGTAT AAAAACCAGA

GTATTAAACT CTTGTTGGAA GCTGGAACTG ACCGAAGTGT AAAAACCAGA

GTATTAAATA CCTACTGGAA GCCGGAACTG ACCGAAGTGT GGAAACCAGA

GTATTAAACT CTTGTTGGAA GCTGGAAGTG ACCGAAGCGT AAAAACTAGT

GTATCAAACT CTTGTTAGAA GCTGGAGCTG ACCGGAGTGT AAAAACCAGA

GTATTAAAGT CCTACTGGAA GCTGGAACAG ACCGAAGTGT AAAAACCAGA

GTATTAAACT CTTGTTGGAA GCTGGAACTG ACCGAAGTGT AAAAACCAGA

GTATTAAACT CTTATTGGAA GCTGGAACAG ACCGAAGTGT AAAAACCAGA

GTATTAAACT ATTGTTGGAA GCTGGAACAG ACCGAAATGT AAAAACCAGT

GTATCAAACT CCTGCTGGAA GCTGGAACAG ACCGAAGTAT AACAACCAGT

GATGGCTGGA CACCAGTTCA CGCAGCTGTG GACACTGGTA ATGTGGACAG

GATGGCTGGA CACCAGTTCA CGCAGCTGTG GACACTGGTA ATGTGGACAG

GATGGCTGGA CACCAGTTCA TGCAGCTGTG GACACTGGTA ATGTGGACAG

GATGGCTGGA CACCAGTTCA TGCAGCTGTG GACACTGGTA ATGTGGACAG

GATGGCTGGA CACCCGTTCA CGCAGCTGTG GACACTGGTA ATGTGGACAG

GATGGCTGGA CACCAGTTCA TGCAGTTGTG GACACTGGTG ATGTGGACAG

GATGGCTGGA CACCAATTCA TGCAGCCGTG GACACTGGTA ATGTGGACAG

GATGGCTGGA CGCCAATTCA CGCAGCTGTG GACACTGGTA ATGTGGACAG

GATGGCTGGA CACCAGTGCA TGCAGCCGTG GACACTGGTA ATGTGGACAG

GATGGCTGGA CACCAATTCA TGCAGCCGTG GACACTGGTA ATGTGGACAG

GATGGCTGGA CACCGGTTCA TGCAGCTGTG GACACTGGTA ATGTGGACAG

GATGGCTGGA CACCACTGCA CGCAGCTGTG GACACTGGTA ATGTGGACAG

GATGGCTGGA CACCAGTTCA TGCAGCTGTG GACACTGGCA ATGTGGACAG

GATGGCTGGA CACCAGTTCA TGCAGCTGTG GACACTGGAA ATGTGGACAG

GATGGCTGGA CACCAGTTCA CGCAGCTGTG GACACTGGTA ATGTGGATAG

GATGGCTGGA CACCAGTTCA CGCTGCGGTG GACACTGGTA GTGTGGATGG

GATGGTTGGA CCCCAATTCA TGCTGCTGTG GATTCTGGCA ATGTGGACAG

GATGGCTGGA CCCCAGTCCA CGCCGCTGTG GATTCTGGAA ACGTGGACAG

CCTCAAGCTT CTTATGTACC ATAGAATACC AGCTCATGGA AATTCTGACT

CCTCAAGCTT CTTATGTACC ATAGAATACC AGCTCGTGGA AATTCTGACT

CCTCAAGCTT CTTATGTACC ATAGAATACC AGCTTGTGGA AATTCTGACT

CCTCAAGCTT CTTATGTACC ATAGAATACC AGCTTGTGGA AATTCTGACT

CCTCAAGCTT CTTATGTACC ACAGAGTACC AGCTCATGGA AACTCTGACT

CCTCAAACTT CTGATGTATC ATAGAGCACC AGCTCGTGGA AACTCTGACT

CCTCAAGCTA TTGATGTACC ATAGAGTGCC AGCCCCTGGA AACTCTAGCT

CCTCAAGCTC CTGATGTACC ATAGAGTGCG AGCCCATGGA AACTCTAGCT

CCTGAAGCTC CTCATGTACT ATCAAGCACC AGCTCGGGGG AACTCTGCCC

CCTCAAGCTT CTTATGTACC ACAGAGCCCC AGCCCATGGA AACAAGGACT

CCTCAAGCTT CTCATGTACC ATAGAACACC AGCTAGCGGG AACTCTGGCT

CGTCAAGCTT CTCATGTACC ATAGCGCACC AGCTCGTGGC CACTTTGGCT

TCTCAAGCTT CTTATGTACC ATCGAGC--- ---TGGTGGA AACTCTGACT

CCTCAAGCTT CTTATGTACC ACAGAGCACC AACTCTTGGA CACTCTGACT

CCTCAAGCTT CTTATGTACC ACAGAGCACC AGCTTGTGGA GACAGGGACT

CCTCAAGCTC CTCCTGTACC ACAGAGCACC GGCTTGTGGG GATGGGGATT

TCTGAAGCTC CTTATGTACT ATGGGGTAAC TGATAATGGA AACCTTGACT

CCTCACGCTC CTCATGTACT ACGGAGGGCC GGAGAGCGAG AATTCTGGCT

TGGATGGAGG AGAAGAGAGT TCCAAGCCTG TTGTTCCTGC AGACCTCATT

TGGATGGAGG AGAAGAGAGT TGCAAGCCTG TTGTTCCTGC AGACCTCATT

TGGATGGAGG AGAAGAGAGT TTCAAGCCTG TTGTTCCTGC AGACCTCATT

TGGATGGAGG AGAAGAGAGT TTCAAGCCTG TTGTTCCTGC AGACCTCATT

TGGATGAAGG AGAAGAGAGT TCCAAGCCTG TTGTTACTGC AGACTTCATT

TGGATGAAGG AGAAGAGAGT TCCAAGCCTG TTATTCCTGC AGACCTCATT

TGAATGGAGG ---TGAGAGC TCTAAGCCTG TGGTTCCTGC GGACCTCATT

TGAATGGAGG ---AGAGAGC TCTAAGCCTG TGGTTCCTGC AGACCTAATT

GGGATGGTGG GGAAGAGAGT TCCGAACCCG TTGTTTCTGC AGATCTCATT

TGGATCAAGA AGAAGAGCAT TCCAAGCCTG TTGTTCCTGC AGACCTCATC

TGGATGAAGG AGAAGAGAGT CCCAAGCCTG TGGTTCCTGC AGACCTCGTT

TGGATGGAGG AGAAGGGAGT GCCAAGCCTG TGGTTCCTGC AGACCTCATT

TG---CAAGG AGAAGGCAGC TCCAAGCCCA TTGTTCCCGA GGAGCTCATT

TGGATCAAGG ACAAGAGGGT GCCAAGCCTG TTGTTCCCAC AGACCTCATT

TGGATGGAGG AGGAGAACGT GTCAAGCCTG TTGTTCCTGC AGACCTCATT

TGGATGAAGG AGAAGCAAAC CCCACGCCCG TTATCCCCGC CGACCTCATT

TGGATTGCAG AGAGAAGAAT ACCAAGCCTA TTGTTCCTAC AGATCTTATC

TGGGGAGCCG AGAGGAGAG- -CCATGCCTG TCATTTCGGC CGATCTCATC

AACCACGCCA ACAGAGAAGG CTGGACTGCT GCCCACATTG CTGCTTCCAA

AACCACGCCA ACAGAGAAGG CTGGACTGCT GCCCACATTG CTGCTTCCAA

AACCACGCCA ACAGAGAAGG CTGGACTGCT GCCCACATTG CTGCTTCCAA

AACCACGCCA ACAGAGAAGG CTGGACTGCT GCCCACATCG CTGCTTCCAA

AACCACGCCA ACAGAGAAGG CTGGACTGCT GCCCACATTG CTGCTTCCAA

AACTATGCCA ACAGAGAAGG CTGGACTGCT GCCCATATTG CTGCTTCCAA

AACCACGCTG ACAAAGAAGG CTGGACTGCT GCCCATATTG CTGCTTCCAA

AACCACGCTG ACAAAGAAGG CTGGACTGCT GCCCACATTG CTGCCTCCAA

AACCACGCCG ATAGAGAGGG CTGGACTGCT GCCCACATCG CTGCTTCGAA

AACCACGCTG ACAGTGAAGG CTGGACTGCT GCCCATATTG CTGCTTCAAA

AACCATGCTG ACAGAGAAGG CTGGACCGCT GCCCATATTG CTGCTTCGAA

AACCAGGCCG ACAGAGAAGG CTGGACCGCT GCCCATATTG CTGCTTCAAA

AACCACGCTG ACAGAGAGGG CTGGACCGCT GCCCACATCG CTGCTTCTAA

AACCACGCCA ACAGAGAAGG CTGGACTGCT GCTCATATTG CTGCCTCAAA

AACCATGCTG ACAGAGAAGG CTGGACTGCT GCCCACATTG CTGCCTCAAA

AACCACGCCA ACAGGGAGGG CTGGACGGCT GCCCACATTG CTGCTTCGAA

AACCATGCTG ACAAGGAAGG CTGGACTGCT GCCCACATAG CCGCATCAAA

AACCAGGCTG ACAAGGAGGG TTGGACGGCT GCCCACATTG CGGCTTCGAA

AGGTTTTAAG AACTGCCTAG AAATCTTGTG TAGGCACGGA GGGCTTGAGC

AGGTTTTAAG AACTGCCTAG AAATCTTGTG TAGGCACGGA GGGCTTGAGC

AGGTTTTAAG AACTGCCTAG AAATCTTGTG TAGACACGGA GGGCTTGAGC

AGGTTTTAAG AACTGCCTAG AAATCTTGTG TAGACACGGA GGGCTTGAGC

AGGCTTTAAG AACTGCCTAG AAATCTTGTG TAGGCACGGA GGGCTTGAGA

AGGTTTTAAG AACTGCCTAG AAATCTTGTG TAGACACAGA GGGCTTGAAC

AGGTTTCAAG ---------- ---------- ---------- ----------

AGGTTTCAAG AACTGCCTAG AAGTCTTGTG TAGGCATGGA GGACTAGAGC

AGGCTTTAAG AACTGCCTAG AAATCTTATG TAGGCACGGA GGGCTGGAGC

GGGTTTTAAG AACTGCCTAG AAGTCTTGTG TAGGCACGGA GGGCTGGAGC

AGGTTTTAAG AACTGCCTGG AAATCTTGTG TAAGCACGGA GGGCTTGAGC

GGGTTTTAAG AACTGCCTAG AAATCTTGTG TAGGCATGCA GGGCTGGAGC

AGGGTTCAAG AACTGCCTTG AAATATTATG TAGGCACGGA GGGCTGGAGC

AGGCTTTAAG AACTGCCTAG AAATCTTATG TAGGCACGGA GGGCTCGAGC

AGGTTTTAAG AACTGCTTAG AAATCTTGTG TCAGCATGGA GGGCTGGAGC

AGGTTTTAAG ---------- ---------- ---------- ----------

AGGTCTTAAG AACTGCCTAG AAATCTTGTG TAGCCATGGT GGACTTGAGG

AGGTTTGAAG AATTGCCTAG AAATCCTCTG CGGTCACGGC AGGCTGGAGG

CAGAAAGGAG AGACAAGTGC AATCGGACTG TGCATGATGT TGCCACTGAT

CAGAAAGGAG AGACAAGTGC AATCGGACTG TGCATGATGT TGCCACTGAT

CAGAAAGGAG AGACAAGTGC AATCGGACTG TGCATGATGT TGCCACTGAT

CAGAAAGGAG AGACAAGTGC AATCGGACTG TGCATGATGT TGCCACTGAT

CGGAGAGGAG AGACAAGTGC AATCGGACTG TGCATGATGT TGCCACTGAT

CAGAAAAGAG AGACAAGTGC AATCGGACTG TACATGATGT TGCCACCGAT

---------- ---------- ---------- ---------- ----------

CGGAAAGGAG AGACAAGTGC AATCGGACTG TGCACGATGT TGCCACTGAT

CAGAAAAGAG AGACAAGTGC AATCGGACTG TGCATGATGT TGCTACTGAT

CGGAAAGAAG AGATAAGTGT AATCGGACCG CACACGATGT TGCTACTGAT

CAGAAAGGAG AGATAAGTGC AATAGGACCG TGCATGATGT CGCCACCGAT

CGGACAGGAG AGAGAAGTGC AATCGGACTG TGCACGATGT GGCCACCGAC

CAGAAAGGAG AGACAAATGC AATCGGACTG TGCATGATGT GGCCACCGAT

CTGAAAGGAA AGATAAGTGC AATCGGACTG TGCATGATGT TGCCACTGAT

CTGAAAGGAA CGATAAGTGC AATCGGACTG TTCACGACGT TGCCACTGAT

---------- ---------- AATCGGACTG TACATGACGT CGCCACAGAT

CGGAAAAGAG AGATAAGTGC AATCGGACTG TGCATGATGT TGCTACTGAT

CTGAAAGGAA AGATAAGTGC GATCGAACTG CGCATGACGT GGCAACAGAT

GACTGCAAGC ATTTGCTGGA GAATCTGAAT GCTCTTAAAA TACCCTTAAG

GACTGCAAGC ATTTGCTGGA GAATCTGAAT GCTCTTAAAA TACCCTTAAG

GACTGCAAGC ATTTGCTGGA GAATCTGAAT GCTCTTAAAA TACCCTTAAG

GACTGCAAGC ATTTGCTGGA GAATCTGAAT GCTCTTAAAA TACCCTTAAG

GACTGCAAGC ATTTGCTGGA GAATCTGAAT GCTCTTAAAA TACCCTTAAG

GACTGCAAAC ATTTGCTGGA GAATCTGAAT GCTCTTAAAA TACCCGTAAG

---------- ---------- --------AT GCTCTTAAAA TACCCTTAAG

GACTGCAAGC ATTTGCTGGA GAATCTGAAT GCTCTTAAAA TACCCTTAAG

GACTGCAAGC ACTTGCTGGA GAATCTGAAT GCTCTTAAAA TACCCTTAAG

GACTGCAAGC ACCTGCTGGA GAATCTGAAT GCTCTTAAAA TACCCTTAAG

GACTGCAAGC ATCTGCTGGA GAACCTGAAC GCTCTTAAAA TACCTTTAAG

GACTGCAAAC ACCTGCTGGA GAATCTGCAT GCTTTTAAAA TACCCTTAAG

GACTGCAAAC ATTTGCTGGA GAATCTGCAT GCTCTGAAAA TACCCCTTAG

GACTGCAAGC ACCTATTGGA GAATCTGAAT GCTCTTAAAA TACCCTTAAG

GACTGCAAGC ATCTGCTGGA GAATCTGAAT GCTCTTAAAA TACCCTTAAG

GACTGCAAAC ACCTGCTGGA GAATTGGAAT GCTCTTAAAA TACCCTTAAG

GACTGCAAAC ATCTTCTAGA AAACTTGAAT GCTCTTAAAA TACCCTTAAG

GACTGCAAGC ATCTTCTGGA GAACCTCAAT GCTTTGAAAA TATCCGTAAG

GATTTCAGTG GGTGAGATTG AACCAAGCAA CTATGGTTCT GATGACTTGG

GATTTCAGTG GGTGAGATTG AACCAAGCAA CTATGGTTCT GATGACTTGG

AATTTCAGTG GGTGAGATTG AACCAAGCAA CTATGGTTCT GATGACTTGG

GATTTCAGTG GGTGAGATTG AACCAAGCAA CTATGGTTCT GATGACTTGG

GATTTCAGTG GGTGAGATTG AACCAAGCAA CTATGGTTCT GATGACTTTG

GATTTCAGTG GGTGAGATTC AACCAGGCAA CTATGGTTCC AATGACTTTG

GATTTCCGTG GGTGAAATTC AACCAAGCAA CGACGGTTCT GACGACTTTG

GATTTCCGTG GGTGAGATTC AACCAAGCAA CGATGTTTCT GACGACTTTG

GATTTCCGTA GGTGAGATTC AACCAGACAA CTGTGGTTCC GATGATTTTG

GATTTCAGTG GGTGAGACTG AACCAGGCAA CTGCGGTGCT GATGACTTTG

GATTTCAGTG GGTGAGATAC CCCCAGGCAC CTATGGTTCA GATGACTTTG

GATTTCAGTG GGTGAAGTTC AACCAGACAT CTATTGTTCT GATGACTTTG

GATTTCAGTG GGTGAAATCC AACCAAGCAC CTATGGTTCA AATGACTTCG

GATATCAGTG GGTGAGACTC AACCAGGCAG CTATGGTTGC GATGATTTTG

GATTTCAATG AGTGAGACCC AACGAGACAG CTTTGGTTCT GATGACTTTG

GATCTCAGTG GGCGAGACTC AAGGAGACAA CTCCGGTCCC GATGAGTTTG

GATTTCAATT AGTGAACAAC ATCCAGCCAA CTATAGTCCT GACGATTTTG

AATTTCTGTT GGTGAGAAAC AGCCAGCCGT TTGTGGTTCC GATGATTTTG

AATGTGAAAA CACAATATGT GCTTTAAATA TCCGCAAACA GACATCATGG

AATGTGAAAA CACAATATGT GCTTTAAATA TCCGCAAACA GACATCATGG

AATGCGAAAA CACAATATGT GCTTTAAATA TCCGCAAACA GACATCATGG

AATGCGAAAA CACAATATGT GCTTTAAATA TCCGCAAACA GACATCATGG

AATGCGAAAA CACAATATGT GCTTTAAATA TCCGCAAACA GACATCATGG

AATGCGAAAA CACAATATGT GCTTTACATA TCCGCAAACA GACTTCATGG

AATGTGAACA CACAATATGC ACTTTAAATA TCCGCAAACA GACATCATGG

AATGTGAACA TACGATATGC ACTTTAAATA TCCGCAAACA GACATCATGG

AATGCGAAAA CATAATATGT ACTTTAAGTA TCCGCAAACA GACATCGTGG

AATGTGAAAG CACAATATGT GCTTTAAATA TTCGCAAACA GACATCATGG

AATGTGAAAA CACAATATGC GCTTTAAATA TCCGCAAACA GACATCATGG

AATGTGAAAA CACAATATGT ATTTTAAATA TCCGCAAACA GACATCATGG

AGTGCGAGAA CACAATATGT GTTTTAAATA TCCGCAAACA GACATCATGG

AATGTGAAAA CACAATATGT GCTTTAAATA TCCACAAGCA GACATCATGG

AATGCGAAAA CACAATATGG GCTTTAACTA TCCGCAGACA GACATCCTGG

AAAGTGAACA CACAATATGC GCGTTAAATA TCCGCAAACC AACATCATGG

AAACTGAAAA CACGATATGC GCTTTAATTA TCCGCAAACA GACATCTTGG

AAGCAGAAAA CACAATATGT GCTTTAAATA TCCGTAAACA GACATCGTGG

GATGATTTTT CAAAAGCAGT GAGTCAAGCT CTGACAAATC ATTTCCAGGC

GATGATTTTT CAAAAGCAGT GAGTCAAGCT CTGACAAATC ATTTCCAGGC

GATGATTTTT CAAAAGCAGT GAGTCAAGCT CTGACAAATC ATTTCCAAGC

GATGATTTTT CAAAAGCAGT GAGTCAAGCT CTGACAAATC ATTTCCAAGC

GATGATTTTT CAAAAGCAGT GAGTCAAGCT CTGACAAATC ATTTCCAGGC

GATGATTTTT CAAAAGCAGT GAGTCAAGCT CTGACAAATC ATTTCCAAGC

GAAGATTTTT CAAAAGCAGT GAGTCAGGCT CTGACAAATC ACTTCCAAGC

GAAGATTTTT CCAAAGCAGT GAGTCAGGCT CTGACAAATC ATTTCCAAGC

GATGATTTTT CAAAAGCAGT GAGTCAAGCT CTGACAAATC ATTTTCAAGC

GATGATTTTT CAAAAGCAGT GAGTCAAGCT CTGACAAACC ATTTCCAAGC

GATGATTTTT CAAAAGCAGT GAGTCAAGCT CTGACAAATC ATTTCCAAGC

GATGATTTTT CAAAAGCAGT GAGTCAAGCT CTGACAAATC ATTTCCAAGC

GATGATTTTT CAAAAGCAGT GAGTCAAGCT CTGACAAATC ATTTCCAGGC

GATGATTTTT CAAAAGCAGT GAGTCAAGCT CTGACAAATC ATTTCCAAGC

GATGATTTTT CAAAAGGAGT GATTCAAGCT CTGACAAATC ATTTCCAAGC

GACGATTTTT CGAAAGCAGT GAGTCAAGCT CTGACCAGTC ATTTCCAAGC

GATGATCTTT CAAAAGCAAT GAGTCAAGCT TTGACAAACC ATTTCCAGGC

GATGATTTTT CCAAAGCTGT GAGTCAGGCT GTGACGAACC ATTTCCAAGC

AATCTCTTCT GATGGATGGT GGAGTCTGGA AGACGTGACT TGCAATAACA

AATCTCTTCT GATGGATGGT GGAGTCTGGA AGACGTGACT TGCAATAACA

AATCTCTTCT GATGGATGGT GGAGTCTGGA AGACGTGACT TGCAATAACA

AATCTCTTCT GATGGATGGT GGAGTCTGGA AGACGTGACT TGCAATAACA

AATCTCTTCT GATGGATGGT GGAGTCTGGA AGACATGACT TGTAATAACA

CATCTCTTCT GATGGATGGT GGAGTTTGGA AGACACGGCC CTTAATGACA

CATCTCTTCT GATGGATGTT GGGGTCTGGA AGATGGGACA CTTAATAACA

CATCTCTTCT GATGGATGGT GGAGTCTGGA AGATGGGACA TTTAATAATG

AATCTCTTCT GATGGGTGGT GGAGTCTGGA AGACGTGGCA TTTAATAACA

AATCTCTTCT GATGGATGGT GGAGCCTGGA AGACATGACG TTTAGTAGCA

AATCTCATCT GATGGATGGC GGAGTCTGGA GGATGTGACG TTTAATAACA

CATCTCTTCT GATGGATGGT GGAGTCTGGA AGATGTGACG TGTAATAACA

AATCTCTTCT GATGGATGGC GGAGCCTGGA AGACATGACA TTTAATGATA

AATCTCTTCT GATGGATGGT GGAGTCTGGA AGATATGACA TTTAATAACA

AATCTCTTCT GATGGATGGT GGAGCCTGGA AGACGTGACG TTTAATAACA

GATCTCTTCT GATGGATGGT GGAGTCTGGA GGACGTGACG CTCAACAGCC

AGTCTCTTCT GATGGATGGA GAAATCCAGA AGACTTGACA CTTAATAACA

AATCTCTTCT GATGGATGGA AGCGACTAGA AGACTTGACG TTTAATAACG

CCACTGATTC CAACATCGGC CTCAGTGCAA GAAGCATACG ATCCATCACG

CCACTGATTC CAACATTGGC CTCAGTGCAA GAAGCATACG ATCCATCACG

CCACCAATTC CAACATCGGC CTCAGTGCAA CAAGCATACG ATCCATCACA

CCACCAATTC CAACATCGGC CTCAGTGCAA CAAGCATACG ATCCATCACG

CTACTGATTC CAACATTGGC CTCAGTGCAA GAAGCATACG ATCCATCACG

CCGCTGACTC CAACATCGGC CTCAGCACAA GCAGCGTGCG GGCCATCATG

CGACTGACTC CTGCATCGGT CTTGGCACCA GCAGCATACA ATCCATCATG

CTACTGACTC CTGCATCGGT CTTGGCACCA GCAGTATACG ATCCATCATG

CCACCGACTC TAGCATCGGC CTCAGCGCAA GCAGCGTACG ATCCATCACG

CCACCGACTC CAGCATTGGG CTCAGTGCAA GCAGTGTGCG ATCCATCACC

CGACCGACTC CAGCATTGGC CTCAGTACAA GCAGCGTGCG ATCCGTCATG

CTGCTGACTC CAGCATTGGC CTCGGTGCGA GCAGCGTGCG GTCTGTCACA

CTACCGACTC CAGCATCGGC CTCGGTGCAA GCAGCGTGAG ATCCATCATG

CCCCTGAACC GAGCATCGGC CTCAGTGCAA GAAGTATACT ATCCATCACG

CCACTGAATC CAGCATCGGC CTCGGTGCAA GTAGCGTACT ATCCATCATG

CCGCTGAAGC CAGCATCGGC CTCAGTGCCA CCAGCGTCCT GTCCATCACA

CTACTGAATC CAACATCGGC CTAAATCTGA GCAGCATCCG ATCCATCAAA

CTACTGAATC CAGCGTTGGC CTCAGTGTGA GCAGTATCCT ATCAGTCAAA

CTAGGAAATG TGCCGTGGTC AGTGGGTCAG AGCTTCGCGC AGTCCCCGTG

CTAGGAAATG TGCCGTGGTC AGTGGGTCAG AGCTTCGCGC AGTCCCCGTG

CTAGGAAATG TGCCATGGTC AGTGGGTCAG AGCTTCACGC AGTCCCCGTG

CTAGGAAATG TGCCATGGTC AGTGGGTCAG AGCTTCACGC AGTCCCCGTG

CTAGGAAATG TGCCATGGTC AGTGGGTCAG AGCTTTGTGC AGTCCCCGTG

CTAGGACATG TGCCATGGTC TTCTGGTCAG AGCTTTACCC AGTCCCCGTG

CTAGGAAGTA TTCCGTGGTC AACTGGTCAG AGCTTTTCCC AGTCCCCGTG

TTAGGAAGTA TGCCGTGGTC AACTGGTCAG AGCTTCTCCC AGTCCCCGTG

CTAGGAAATA TGCCGTGGCC AGCAGGTCGG AGCTTCGCCC CGTCCCCGTG

TTAGGAAATG TGCCATGGTC AGCAGGTCAG AGCTTCACCC AGTCCCCATG

CTAGGAAATG TGCCATGGTC AGCAGGTCAG AGCTTTTCCC AGTCCCCGTG

CTAGGAAATG TGCCGTGGTC AGTGGGTCAG AGCTTCGCCC AGTCCCCGTG

CTAGGAAATG TGCCATGGTC AGCAGGTCAG AGCTTCCCCC TGTCCCCGTG

CTAGGAAATG TGCCATGGTC AGCAGGTCAG TGCTTCACCC AGGCCCCATG

CTAGGAAGTG TATCGTGGTC ACCTGGTCAG AGCTTCGCCC AGTCCCCATG

CTAGGAAACG TGCCATGGTC AGTTGGGCAG AGCTTCACCC AGTCCCCGTG

CTAGGAAATA TATCATGGTC GATAGGCCAG ATTTTGCCCC AATCACCATG

CTAGGAAGTG TGACGTGGTC AACAGGTCAG AGTTTTTCCC AGCCTCCGTG

GGACTTTATG AGGAAGAATA AGGCAGAGCA CATCACTGTG CTTTTGTCAG

GGACTTTATG AGGAAGAATA AGGCAGAGCA CATCACTGTG CTTTTGTCAG

GGACTTTATG AGGAAGAATA AGGCAGAGCA CATCACTGTG CTTTTGTCAG

GGACTTTATG AGGAAGAATA AGGCAGAGCA CATCACTGTG CTTTTGTCAG

GGACTTTATG ATAAAGAATA AGGCAGAGCA CATCACTGTG CTTTTGTCAG

GGACTTTATG AAGAGGAACA AGGTGGAGCA GGTCACCGTG CTTCTGTCAG

GGACTTCATG AAGAAGAAGA AGGTGGAGCA GGTCACAGTG CTCTTGTCAG

GGACTTCTTG AAAAAGAAGA AGGTGGAGCA GGTCCTGGCG CTCTTGTCAG

GGACTTCGTG AGGAAGAACA AGACAGAGCA AGTCACGGCG CTTCTGTCAG

GGACTTCATG AGAACGAATA AAGCAGAGCA GGTCACTGTG CTTTTGTCAG

GGACTTTATG AAGAAGAATA AAGCCGAGCA AGTCACTGTG CTTTTATCAG

GGACTTTCTG AGGAAGAACA AAGCAGAGCA GGTCTCTGTG CTTTTGTCAG

GGACTTGATG AAGAAGAATG AAGCAGAGCA GGTCACTGTG CTTTTGTCAG

GGACTTCATG AGGAAGAATA AGGCAGAGCA GGTCACTGTG CTTTTGTCAG

GGACTTTCTG AAGAAGAATA AAGCTGAGCA GGTTACTGTG CTTTTGTCAG

GGACTTCCTG AGGAAGCATC AGGCCGAGCA GGTCACTGTG CTCTTGTCAG

GGATTTTATG AGGAAGAATA AAACTGACCA TATAACTGTG TTTTTTTCAG

GGACTTCCTG CAAAAGAACA AGGTGGAACA TGTAACCGTA TTTTTGTCAG

GTCCTCAAGA AGGCTGTCTC AGTAGTGTGA CTTATGCCTC CATGATCCCT

GTCCTCAAGA AGGCTGCCTC AGTAGTGTGA CTTATGCCTC CATGATCCCT

GTCCTCAAGA AGGCTGCCTC AGTAGTGTGA CTTATGCCTC CATGATCCCT

GTCCTCAAGA AGGCTGCCTC AGTAGTGTGA CTTATGCCTC CATGATCCCT

GTCCTCAAGA AGGCTGCCTC AGTAGTGTGA CTTATGCCTC CATGATCCCT

GTCCTCAGGA AGGCTGCCTC AGTAGTGTGA CTTACGCTTC CATGATCCCT

GTCCCCAAGA AGGCTGCCTC AGTAGTGTGA CTTATACATC CATGATCCCA

GTCCCCAAGA AGGCTGCCTC AGTAGTGTGA CTTACGCATC CATGATCCCA

GCCCTCAAGA AGGCTGCCTT AGTAGTGTGA CGTACGCATC CATGATTCCT

GGCCTCAGGA GGGCTGCCTC AGTAGTGTGA CCTACGCGTC TATGATCCCT

GTCCTCAAGA AGGCTGCCTC AGTAGTGTGA CTTACGCGTC AATGATCCCT

GTCCTCAAGA AGGCTGCCTC AGCAGTGTGA CCTACGCATC TCTGATCCCT

GTCCTCGAGA AGGCTGCCTC TATAGTGTGA CGTACACATC CATGATCCCT

GTCCTCAAGA AGGCTGCCTC AATAGTGTGA CTTATGCATC CATGATTCCT

GTCCTCAAGA AGGCTGCCTC AGTAGTGTGA CTTACGCATC TATGATCCCT

GTCCCCAAGA AGGCTGCCTC AGTAGTGTGA CGTACGCATC TATGATCCCT

GTCCTCAAGA AGGCTGCCTC AGCAGTGTGA CTTATGCATC CATGATCCCT

GACCTCAAGA AGGCTGCCTC AGTAGTGTGA CTTACGCATC CATGATCCCT

CTCCAGATGA TGCAGAACTA CCTCAGGCTG GTTGAGCAAT ATCATAATGT

CTCCAGATGA TGCAGAACTA CCTCAGGCTG GTTGAGCAAT ATCATAATGT

CTCCAGATGA TGCAGAACTA CCTCAGGCTG GTTGAGCAAT ATCATAATGT

CTCCAGATGA TGCAGAACTA CCTCAGGCTG GTTGAGCAAT ATCATAATGT

CTTAAGATGA TGCAGAACTA CCTCAGGCTG GTTGAGCAAT ATCATAATGT

CTTCACATGC TGCAGAACTA CCTCAGGCTG ATTGAGCAGT ATCGTAATGT

CTTCAGATGC TGCAGAACTA CCTCAGGCTG GTTGAGCAAT ATCATAATGT

CTTCAGATGC TGCAGAACTA CCTCAGGCTG GTTGAGCAAT ATCATAATGT

CTGAAGACGC TGCAGAACTA CCTCCGGCTG GTCGAGCAAT ATCATAATGT

CTTCAGATGC TGCAGAACTA CCTCAGACTG GTCGAGCAAT ATCATAATGT

CTTCAGATGC TACAGAACTA CCTCAGGCTG GTCGAGCAAT ACCATAATGT

CTGCAGGTGC TGCAGAACTA CCTCCGGCTG GTTGAGCAAT ATCATAACGT

CTGCAGATGC TGCAGGACTA CCTCAGGCTG GTTGAACAAT ATCATAATGT

CTTCAGATGC TGCAGAACTA CCTCAGGCTG GTCGAGCAAT ATCATAATGT

CTTCCAATGC TGCAGAACTA CCTCAGGCTG GTCGAGCAAT ATCATAACGT

CTTCCTGTGC TGCAGAACTA CCTAAGGCTG GTCGAGCAAT ATCACACTGT

CTCCAGATGC TACAGAACTA CCTCCGATTG GTTGAGCAAT ATCATAATGT

CTTCAGATGC TACAGAACTA CCTCCGGCTG GTGGAGCAAT ATCATAACGT

CATTTTCCAC GGCCCAGAAG GAAGCTTGCA AGACTACATA GTACATCAGC

CATTTTCCAC GGCCCAGAAG GAAGCTTGCA AGACTACATA GTACATCAGC

CATTTTCCAC GGCCCAGAAG GAAGCTTGCA AGACTACATA GTACATCAGC

CATTTTCCAC GGCCCAGAAG GAAGCTTGCA AGACTACATA GTACATCAGC

CATTTTCCAC GGCCCAGAAG GAAGTTTGCA AGACTACATA GTACATCAGC

CATTTTCCAC GGCCCAGAAG GAAGCTTGCA AGACTACATA GTACATCAGC

CATTTTCCAT GGCCCAGAAG GAAGCTTGCA AGACTACATA GCAAATCAGC

CATTTTCCAT GGACCAGAAG GAAGCTTGCA AGACTACATA GCAAATCAGC

CATATTTCAT GGCCCTGAAG GAAGCTTGCA AGACTTCATA GCACATCAGC

CATTTTCCAT GGCCCAGAAG GGAGCTTGCA AGACTACATC GCCCATCAGC

CATTTTCCAT GGTCCAGAAG GAAGCTTGCA AGACTACATA GCACATCAGC

CATCTTCCAT GGCCCAGAAG GAAGCTTGCA AAACTACATA GCACATCAGC

CATTTTTCAC GGCCCTGAAG GAAGCCTACA AGACTACATA GCGCATCAAC

CATTTTCCAT GGCCCAGAAG GAAGCTTGCA AGACTACATA GCCCATCAGC

CATTTTCCAT GGCCCAGAAG GAAGCTTGCA AGACTACATA GCACATCAGC

CATTTTCCAT GGTCCAGAAG GAAGCTTGCA AGACTACATA GCGCTCCAGC

TATCTTTCAT GGCCCAGAAG GAAGCTTGCA AGACTACATA GCACATCAGC

GGTCTTCCAT GGCCCTGAAG GAAGCTTGCA AGACTATATA GCCCATCAGA

TTGCACTCTG CCTGAAGCAC AGACAAATGG CTGCAGGATT CTCCTGTGAA

TTGCACTCTG CCTGAAGCAC AGACAAATGG CTGCAGGATT CTCCTGTGAA

TTGCACTCTG CCTGAAGCAC AGACAAATGG CTGCAGGATT CTCCTGTGAA

TTGCCCTCTG CCTGAAGCAC AGACAAATGG CTGCGGGATT CTCCTGTGAA

TTGCACTCTG CCTGAAGCAC AGACAAATGG CTGCGGGATT CTCCTGTGAA

TTGCACTCTG CCTGAAGCAT AGACAAATGG CTGCAGGATT CTCCTGTGAA

TTGCCCTCTG CATGAAGCAC AGACAGATGG CTGCAGGATT CCCCTGTGAA

TTGCCCTCTG CATGAAGTAC AGACAGATGG CTGCAGGATT CCCCTGTGAA

TTGCACTCTG CATGAAGCAC AGACAAATGG CTGCAGGGTT TTCCTGTGAA

TTGCACTCTG CCTGAAGCAC AGACAAATGG CTGCAGGATT CCCCTGTGAA

TTGCACTCTG CATGAAGCAC AGACAAATGG CTGCGGGATT CACCTGTGAA

TTGCACTCTG TATGAAGCAC AGACAGATTG CTGCAGGATT CTCCTGTGAA

TTGCCCTCTG CATGAAGCAC AGACAAATGG CTGCAGGATT CCCCTGTGAA

TTGCTCTCTG CATGAAGCAT AGGCAAATGG CTGCAGGATT CTCCTGTGAG

TTGCACTCTG CATGAAGCAC AGACAAATGG CTGTGGGTTT CTCCTGTGAA

TTGCACTCTG CATGAAGCAC AGACAGGCGG CTGTGGGGTT CTCATGTGAT

TCGCACTCTG CATGAAGCAA AAACAAAGAG CTGCAGGATT CACCTGTGAA

TAGCACTCTG CCTGAAGCAC AAGCAGACAG CTGCAGGATT CCCTTGTGAA

ATAGTGAGAG CTGAAGTAGA TGCTGGTTTT TCCAAGGAAC AGCTACTAGA

ATAGTGAGAG CTGAAGTAGA TGCTGGTTTT TCCAAGGAAC AGCTACTAGA

ATAGTGAGAG CTGAAGTAGA TGCTGGCTTT TCCAAGGAAC AGCTGCTAGA

ATAGTGAGAG CTGAAGTCGA TGCTGGCTTT TCCAAGGAAC AGCTGCTAGA

ATAGTGAAAG CTGAAGTAGA TGCTGGTTTT TCCAAGAAAC AGCTACTAGA

ATAGTGACAG CTGAAGTAGA TGCTGGTTTC TCCAAGGAAC AGCTGGTAGA

ATAGTGAGAG CTGAGGTGGA TTCAGGTTTC TCCAAGGAAC AGCTAGTAGA

ATAGTGAGAG CCGAGGTGGA TTCGGGTTTC TCCAAAGAGC AGCTAGTAGA

ATAGTAAGAG CTGAAGTGGA TGCAGGTTTC TCCAAGGAGC AGCTATTAGA

ATAGTGAGAG CTGAAGTGGA TGCTGATTTC TCCAAGGAAC AGCTAGTAGA

ATAGTGAGAG CCGAAGTGGA TGCTGGTTTC TCCAAGGAAC AGCTCGTAGA

ATAGTGAGAG CCCAAGTGGA TGCGAGTTTC TCCAAGGAAC AGCTTGCGGA

ATAGTGAGAG TTGAAGTGGA TGCTGGTTTC TCCAAGGAAC AGCTGGTAGA

ATAGTGAGAG CTGAAGTGGA TGCTGGTTTT TCCAAGGAAC AGCTAATAGA

ATAGTGAGAG CTGAAGTGGA TGCTGCTTTC TGCAAGGAAC AGCTAGTCGA

ATAGTTAGAG CTGATGTTGA TGCTGGTTTC TCCAAAGAAC AGCTGGTCGA

ATAGTGAGAG CTGAAGTGGA TGCTGAGTTC TCTAAGCATC AGCTAGTCGA

ATAGTGAAAG CCGAAGTGGA CACCAACTTC TCCAAGGAGC AGCTAGTGGA

CCTGTTCATT AGTAGCGCTT GTCTGATCCC AGTGAAACAA TCTCCCAGTA

CCTGTTCATT AGTAGCGCTT GTCTGATCCC AGTGAAACAA TCTCCCAGTA

CCTGTTCATC AGTAGCGCTT GTCTGATTCC AGTGAAACAG TCTCCCAGTA

CCTATTCATC AGTAGCGCTT GTCTGATTCC AGTGAAACAA TCTCCCAGTA

CCTGTTCATC AGTAGCGCTT GTCTGATCCC AGTGAAACAA TCTCCTGTTA

CCTGTTCATC AGTAGCGCTT GTCTGATCCC AGTGAAACAG TCTCCTGTTA

CGTGTTCATC AGTAATGCCT GTCTGATCCC AGTAAAGCAG TTCCCTGTTA

CGTGTTCATC AGGAATGCTT GTCTCATCCC AGTGAAGCAG TTCCCTGTTA

CCTGTTCATC AGTAGTGCTT GTCTGATCCC AGTGAAACAA TCTCCTGTAA

TCTGTTCATC AGTAGTGCGT GTCTGATCCC AGTGAAACAA TCTCCTGCTA

CCTGTTCATC AGTAGTGCTT GTCTGATCCC AGTGAAACAG TCCCCTGTTA

CCTATTCATC AGTAGTGCAT GTCTGATCCC AGTGAAGCAA TCTCCTGTGA

CCTGTTTATC AGTAGCGCAT GTTTGATCCC AGTGAAACAA GCTCCTGTCA

CCTCTTCATC AATAGCGCTT GTCTGATCCC TGTGAAACAG TCTCCTGTTA

CCTGTTTATC AGGAACGCTT GTCTGATCCC AGTGAAACAA TCTCCTGGTA

CCTGTTCATC AAGAGCGCCT GTCTGATCCC AGCGAAGCAA TCGCCGGCTA

ATTATTCATC AGTAGTGCTT GTCTCATCCC AGTGAAACAG CCTCCTGTAA

GTTATTCATC AATAGCGCTT GTCTGATCCC TGTGAAACAG CCTCCTGTGA

AGAAGAAAAT CATCATCATT TTAGAAAATT TAGAAAAATC TTCACTGTCG

AGAAGAAAAT CATCATCATT TTAGAAAATT TAGAAAAATC TTCACTGTCG

AGAAGAAAAT CATCATCATT TTAGAGAATT TGGAAAAATC TTCACTGTCT

AGAAGAAAAT CATCATCATT TTAGAGAATT TGGAAAAATC TTCACTGTCT

AGAAGAAAAT CATCATCATT TTAGAGAATT TGGAAAAATC TTCATTGTTT

AGAAGAAAAT CATCATCATC TTAGAGAATC TGGAAAAATC TTCATTGTCT

AGAAGAAAAT AATCGTCATC TTGGAAAATT TGGAAAAATC ATCATTGTCT

AGAAGAAAAT AATCGTCATC TTGGAAAATT TGGAAAAATC TTCATTGTCT

AGAAGAAAAT CATCATCATC TTAGAAAATT TGGAGAATTC TTCCTTGTCT

ACAAGAAAAT CATCGTCATC TTAGAGAACT TGGAAAAATC TTCATTGTCT

ACAAGAAAGT CATCGTCATC TTAGAGAATT TGGAAAAATC TTCGTTGTCG

ACAAGAAAAT CATCATCATC TTAGAGAATT TGGAAAAATC TTCCATGTCT

ACAAGAAAAT TATCATTATC TTAGAGAATC TGGAAAAATG TTCATTGTCA

CCAAGAAAAT CATCATCATC TTAGAGAATT TGGAAAAATC CTCTTTGTCT

ACAAGAAAGT CATCGTCATC TTGGAGAACT TGGAGAAATC GTCATTGTCT

GCAAGAAAAC CATCATCATC TTGGAGAATT TGGAGAGAGC GTCGTTGTCT

GTAAGAAAAT CATCATCATC CTGGAGAAGT TGGAAAATGC TTCCTTATCT

GCAAAAAAGT CATCGTCATC CTAGAGAATT TGGAAAAAGC TTCATTATCA

GAGTTATTGA GGGACTTTTT GGCACCTCTT GAAAATCGCA GCACTGAAAG

GAGTTATTGA GGGACTTTTT GGCACCTCTT GAAAATCGCA GCACTGAAAG

GAGTTATTGA GGGACTTTTT GGCACCTCTT GAAAATCGCA GCACTGAAAG

GAGTTATTGA GGGACTTTTT GGCACCTCTT GAAAATCGCA GCACTGAAAG

GAGTTATTGA GGGACTTTTT GGCACCTCTT GAAAATCGCA GCACTGAAAG

GAGTTACTGG GGGATTTTCT GGCACCTCTG GAAAATCGCA GCACTGAGAG

GAGTTACTGG GGGACTTTTT GGCACCTTTG GAGAATCGCA GCACTGAAAG

GAGTTACTGG GGGACTTTCT GGCACCTTTG GAAAATCGCA GCACTGAAAG

GAATTATTGG GGGACTTTTT GGCACCTCTT GAAAATCGCA GCACAGAAAG

GAGTTACTGG GGGACTTTTT GGGACCTCTG GAAAATCACA GCACTGAAAG

GAGTTACTGG GAGACTTCCT GGCACCTCTG GAAAATCGCA GCACTGAAAG

GAGTTATTGG GGGACTTTTT GGCACCTCTG GAGAATCGCA GCCCTGAAAG

GAGTTATTGG GGGACTTTTT GGCACCTCTG GAAAATCGCA GCACTGAAAG

GAGTTATTGG GGGACTTTTT GGCACCTCTC GAAAATCGCA GCACTGAAAG

GAGTTATTGG GGGACTTTCT GGCGCCTCTT GAAAATCGCA GCACTGAAAG

GAACTCTTGG GGGACTTTCT GGCACCTCTG GAAAATCGCA GCCCTGAAAG

GAGCTATTAG GAGACTTTCT GGCACCTCTA GAAAATCGTA GCACTGAAAA

GAGTTGTTGG GAGACTTTCT AGCTCCCCTT GAGAACCGTA GCAGTGAAAA

CCCCTGCACT TTCCAAAAAG GAAATGGACT GTCCGAATGT TATTACTTTC

CCCCTGCACT TTCCAAAAAG GAAATGGACT GTCCGAATGT TATTACTTTC

CCCCTGCACT TTCCAAAAAG GAAATGGAAT GTCCGAATGT TATTACTTTC

CCCCTGCACT TTCCAAAAAG GAAATGGAAT GTCCGAATGT TACTACTTTC

TCCCTGCACT TTCCACAAAG GAAATGGAAT GTCTGAGTGT TATTACTTTC

CCCCTGCACT GTCCAGAAAG GAAACGGAAT GTCTGCATGT TACTACTTTC

CCCATGCACT TTCCAGAAAG GAAATGGAAC ATCTGAATGC TATTATTTCC

CCCCTGCACT TTCCAAAAAG GAAATGGAAC ATCCGAATGC TATTATTTCC

CCCATGCACT TTCCAAAAAG GAAATGGAGC ATCCGAGTGC TATTATTTTC

CCCCTGTACC TTCCAAAAAG GAAACGGAAT GTCTGAATGC TACTACTTTC

CCCCTGGACC TTTCAAAAAG GAAATGGAAC GTCTGAATGC TACTACTTTC

CCCCTGTACT TTTCATAAAG GAAACGGAAC GTCGGGATGT TACTACTTTC

CCCATGTACT TTTCAAAGAG GAAATGGGAC AGCCGAATGT TACTATTTTC

CCCCTGTACT TTTCAAAAAG GAAATGGAAC ATCTGAATGT TATTACTTTC

CCCCTGTACT TTTCAAAAAG GAAATGGAAC ATCTGAATGT TATTACTTCC

CCCCTATACG TTTCACAAAG GAAATGGAAC ATCCGAATGT TACTACTTCC

CCCCTGTACT TTTCAAAAAG GAAATGGAAC ATCTGAGTCC TATTACTTTC

TCCCTATACT TTTCAAAAAG GAAATGGAGT GGCCAATTCT TATTACTTCC

ATGAGAACTG CTTTCTGATG GGAACCATCG CCAAGGCCTG TCTCCAGGGC

ATGAGAACTG CTTTCTGATG GGAACCATCG CCAAGGCCTG TCTCCAGGGC

ATGAGAACTG CTTTCTGATG GGAACCATCG CCAAGGCCTG TCTCCAGGGC

ATGAGAACTG CTTTCTGATG GGAACCATCG CCAAGGCCTG TCTCCAGGGC

ATGAGAACTG CTTTCTGATG GGAACCATCG CCAAGGCCTG TCTCCAGGGC

ACGAGAACTG CTTTCTGATG GGAACCATTG CCAAGGCCTG TCTCCAGGGC

ATGAGAACTG CTTTCTGCTG GGAACCCTCG CCAAGGCTTG TCTCCAGGGC

ATGAGAACTG CTTTCTGGTG GGAACCATTG CCAAAGCCTG TCTCCAGGGC

ATGAGAACTG CTTCCTGATG GGGACCATTG CCAAGGCCTG TCTGCAGGGC

ATGAGAACTG CTTTCTGATG GGAACAATCG CCAAGGCCTG TCTCCAGGGC

ATGAAAACTG CTTTCTCATG GGAACCATTG CCAAGGCCTG TCTCCAGGGC

ACGAGCACTG CTTCCTGATG GGCACCGTTG CCAAGGCCTG CCTGCAGGGC

ATGAGAACTG CTTTCTGATG GGAACCATCG CCAAGGCCTG CCTCCAGGGC

ATGAGAACTG CTTTTTGATG GGAACCATTG CCAAGACCTG TCTCCAGGGC

ACGAGAACTG TTTTCTGATG GGAACCATTG CAAAGGCATG TCTCCAGGGC

ATGAGAACTG CTTTCTGGTG GGAACCATCG CCAAGGCCTG TCTCCAGGGC

ATGAGAACTG CTTTCTGATG GGGACAATTG CCAAATCCCG TCTCCAGGGC

ATGAAAACTG CTTTCTGGTG GGAACCATTG CAAAGTCGTG TCTCCAGGGA

TCCGACTTGC TGGTGCAGCA GCATTTCCGC TGGGTGCAGC TGCGGTGGGA

TCCGACTTGC TGGTGCAGCA GCATTTCCGC TGGGTGCAGC TGCGGTGGGA

TCTGACTTGC TAGTGCAGCA GCATTTCCGC TGGGTGCAGC TGCGGTGGGA

TCCGACTTGC TGGTGCAGCA GCATTTCCGC TGGGTGCAGC TGCGGTGGGA

TCTGACTTGC TGGTACAGCA GCATTTCCGC TGGGTGCAGC TGCGGTGGGA

TCCGAGCTGC TGGTGCAGCA GCATTTCCGC TGGGTGCAGC TGCGGTGGGA

TCTGACCTGC TGGTGCAGCA ACACTTCCGC TGGGTTCAGC TGCGCTGGGA

TCTGACCTGC TGGTACAGCA ACACTTCCGC TGGGTTCAGC TGCGCTGGGA

TCTGACCTGC TGGTGCAGCA GCACTTCCGC TGGGTACAGC TGCGGTGGGA

TCCGACTTGC TGGTGCAGCA GCATTTCCGC TGGGTGCAGC TGCGCTGGGA

TCTGACTTGC CGGTGCAGCA GCATTTCCGC TGGGTTCAGC TGCGGTGGGA

TCTGACCTGC TGGTGCAGCA GCATTTCCGC TGGGTGCAGC TGCGGTGGGA

TCGGACCTGC TGGTGCAGCA GCATTTCCGC TGGGTTCAGC TGCGGTGGGA

TCTGATTTGC TGGTGCAGCA GCATTTCCGC TGGGTTCAGC TACGGTGGGA

TCCGACCTAC TGGTGCAGCA GCATTTCCGC TGGGTCCAGC TGCGGTGGGA

CCCGACCTGC TGGTGCAGCA ACACTTCCGC TGGGTCCAGT TGCGGTGGGA

TCTGATTTGG TGGTCCAGCA GCATTTCCGC TGGGTTCAGC TAAGATGGGA

TCTGATCTCT TAGTGCAGCA ACATTTCCGC TGGGTTCAGT TAAGATGGGA

TGGCGAGCCC ATGCAAGGAC TGCTGCAGAG GTTCTTACGA AGGAAAGTTG

TGGCGAGCCC ATGCAAGGAC TGCTGCAGAG GTTCTTACGA AGGAAAGTTG

TGGCGAGCCC ATGCAAGGAC TGCTGCAGAG GTTCTTACGA AGGAAAGTTG

TGGCGAGCCC ATGCAAGGAC TGCTGCAGAG GTTCTTACGA AGGAAAGTTG

TGGTGAGCCC ATGCAAGGAC TGCTGCAGAG GTTCTTACGG AGGAAAGTTG

TGGAGAGCCC ATGCAAGGGC TGCTGCAGCG GTTCTTAAGA AGGAAAGTTG

TTGCGAGCCC AGTCAAGGAC TGCTTCAGAG GTTCCTCCGA AGAAAAGCAG

TTGCGAGCCC ATTCAAGGAT TGCTTCAGAG GTTCTTACGA AGGAAAGTCG

TGGCGAGCCC ATGCAAGGAC TGCTGCCCAG GTTCCTGAGA AGGAAAGTTG

CGGCGAGCCC ATGCAGGGGC TGCTGCAGAG GTTCCTCAGG AGGAAGGTTG

CGGCGAGCCC ATGCAGGGAC TGCTACAGAG GTTCCTACGA AGGAAAGTAG

CGGCGAGCCC ATGCAGAGTC TGCTGCCCAG GTTCCTAAGA AGGAGAGCTG

TGGAGAGCCC ATGCAGGGGC TGCTGCAGAG GTTCTTAAGG AGGAAAGTGG

TGGCGAGCCC ATGCACGGTC TGCTGCAGAG GTTCTTAAGA AGGAAAGTTG

TGGCGAGCCC ATGCAGGGTC TGCTGCAGAG GTTCTTAAGA AGGAAAGCTG

TGGCGAGCCC ATGCAGGGTC TGCTGCAGAG GTTCTTAAGA AGGAAAGTTG

TGGAGAACCA ATGCATGGCT TGCTACAGAG GTTTCTAAGA AGGAAAGTTT

TGGAGAGCCC ATCCACGGGC TCCTGCAAAG GTTTTTAAGA AGGAAAGTAA

TGAATAAGTT CAAAGGTCAG GCGCCCTCCC CCTGCGATCC TGTGTGCAAG

TGAATAAGTT CAAAGGTCAG GCGCCCTCCC CCTGCGATCC TGTGTGCAAG

TGAATAAGTT CAAAGGTCAG GCGCCCTCAC CCTGCGATCC TGTGTGTAAG

TGAATAAGTT CAAAGGTCAG GCGCCCTCAC CCTGCGATCC TGTGTGCAAG

TGAATAAGTT CAGAGGTCAG GTGCCTCCAC CCTGCGATCC TGTGTGCAAG

TAAATAAGTT CAGGGGTCAG GTGCCCTCAC CCTGTGATCT TGTGTGCAAG

TCAGCAAGTT CAGAGGTCAG CTGCCCGCAC CCTGTGATCC TGTGTGTAAA

TCAGCAAGTT CAGAGGTCAG CTGCCTGCAC CCTGTGATCC TGTATGTAAG

TGAATAAGTT CAGAGGTCAG GTGCCTGCAC CCTGCGACCC CGTGCACAAG

TGAATAAGTT CAGAGGTCAG GTGCCCTCTC CCTGCGACCC CGTGTGCAAG

TGAATAAGTT CAGGGGTCAG GTGCCCTCGG CCTGTGATCC TGTGTGCAAG

TGAATAAGTT CAGAGGTCAG GTACCCTCTC CCTGTGATCC CGGGTGCAAA

TGAATAAGTT CAGAGGTCAG CTGCCGTCAC CCTGTGACCC TGTGTGCAAG

TCAATAAGTT CAGAGGTCAG GCACCCTCAC CCTGTGATCC CGTGTGCAAG

TGAATAAGTT CCGAGGAAAG CTGTCCTCTC CCCGAGATCC TGTGTGCAAG

TGAATAAGTT CCGAGGAAAG CTCCCCTCGC CTGGCGAACC CGTGTGCAAG

TGAATAAGTT CAGAGGTAAA GTACCTTCAC CCTGTGACCC CGTGTGCAAG

TGAATAAGTT CCGAGGGAAA GTGCCTTCTC CCTGTGATCC CGTGTGCAAG

ATTGTCGACT GGGCTCTGTC CGTCTGGCGT CAGCTTAACT CCTGCCTGGC

ATTGTCGACT GGGCTCTGTC CGTCTGGCGT CAGCTTAACT CCTGCCTGGC

ATTGTCGACT GGGCTCTGTC CGTCTGGCGT CAGCTTAACT CCTGCCTGGC

ATTGTCGACT GGGCTCTGTC CGTCTGGCGT CAGCTTAACT CCTGCCTGGC

ATTGTCGACT GGGCTCTATC TGTCTGGCGT CAGCTTAACT CCTGCCTGTC

ACCGTCGCCT GGGCCCTGTC TGTCTGGCGT CAGCTCAACT CCTGCCTGGC

ATTGTGGACT GGGTTATCTC TGTCTGGAGG CAGCTCAACT CCTGCCTGGC

ATTGTGGACT GGGCTCTGTC TGTCTGGAGG CAGCTCAACT CCTGTCTGGC

ACCGTAGCCT GGGCACTGTC CGTCTGGCGA CAGCTCAACT CCTGCCTGGC

ACGGTCGACT GGGCCCTGGC CGTGTGGCGC CAGCTCAACT CGTGCCTGGC

ACCGTCGACT GGGCCCTGGC CGTCTGGCGC CAGCTGAACT CCTGCCTGGC

GCAGTCGACT GGGCCGCAGC CGTCTGGCGC CAGCTCAACT CCTGCCTGAC

ATTGTCGACT GGGCTCTGGC TGTCTGGCGC CAGCTCAACT CCTGCCTGGC

ATGGTCGACT GGGCCCTGGC TGTCTGGCAC CAACTCAACT CCTGCCTGGC

ACAGTCGACT GGGCCCTGTC GGTCTGGCGC CAGCTCAACT CCTGCCTGGC

ATGGTCGACT GGGCCCTGTC CGTCTGGCGC CAGCTCAACT CCTGCCTCTC

ACTGTAGACT GGATTCTTGC TGTCTGGCAC CAGTTGAACT CCTGCTTGTC

ATTATCGATT GGATCCTTAC CGTCTGGCAC CAGCTGAACT CCTGTTTGTC

CCGCTTGGGC ACACCTGAAG CACTTCTTGG ACCAAAATAT TTCCTGTCTT

CCGCTTGGGC ACACCTGAAG CACTTCTTGG ACCAAAATAT TTCCTGTCTT

CCGCTTGGGC ACACCTGAAG CACTTCTTGG ACCAAAATAT TTCCTGTCTT

CCGCTTGGGC ACACCTGAAG CACTTCTTGG ACCAGAATAT TTCCTGTCTT

CCGCTTGGGC ACCCCTGAAG CACTTCTTGG ACCAAAGTAT TTCCTGTCTT

CCGCTTGGGT ACACCTGAAG CTCTTCTTGG GCCGAAGTAT TTCCTGTCTT

CCGCTTGGGT ACACCGGAAG CACTTCTTGG ACCCAAGTAT TTCCTGTCTT

CCGCTTGGGT ACACCAGAAG CACTTCTTGG ACCAAAGTAT TTCCTGTCGT

CCACCTGGGC ACACCTGAAG CACTTCTCGG CCCAAAGTAT TTCCTGTCTT

CCGCCTGGGC ACGCCGGAAG CACTTCTCGG GCCACAGCAT TTCCTGTCTT

CCGCCTGGGC ACACCTGAAG CACTTCTTGG ACCAAAGTAT TTCCTGTCTT

CCGCTTGGGC ACACCTGAAG CACTTCTGGG GCCAAAGTAC TTCCTGTCAT

CCGCTTGGGC ACCCCTGAAG CGCTCCTGGG GCCAAAGTAT TTCCTGTCTT

CCGCTTGGGC ACCCCAGATG CGCTTCTTGG GCCAAAGTAT TTCCTGTCTT

CCGCTTGGGC ACACCTGAAG CGCTTCTTGG GCCAAAGTAT TTCCTGTCTT

GCACCTGGGC ACACCTGAAG CACTGCTGGG ACCGAAGTGT TTCCTGTCTT

CCGGTTAGGC ACACCTGAAG CACTGATTGG GCCAAAATAT TTCCTCTCCT

CCGCCTGGGC ACTCCAGAGG CCCTTATAGG GCCCAGGTAT TTCCTGTCAT

GTCCTGTAGT TCCTGGGCAT GCCCAAGTGA CAGTGAAGTG GATGTCTAAG

GTCCTGTAGT TCCTGGGCAT GCCCAAGTGA CAGTGAAGTG GATGTCTAAG

GTCCTGTAGT TCCTGGACAT GCCCAAGTGA CAGTGAAGTG GATGTCTAAG

GTCCTGTAGT TCCTGGACAT GCCCAAGTGA CAGTGAAGTG GATGTCTAAG

GTCCTGTAGT CCCTGGACAT GCCCAAGTGA CAGTGAAATG GATGTCTAAG

GTCCTGTAAT CCCAGGACAT GCCCAAGCAA CAGTGAAGTG GATGTCTAAG

GTCCAGTAGT CCCGGGACAT GCCCAAGCAA CAGTGAAGTG GATGTCTAAG

GTCCAGTAGT CCCGGGACAT GCCCAGGCAA CAGTGAAGTG GATGTCTAAG

GTCCTGTGGT CCCTGGACGT GCCCAAGCGA CAGTGAAGTG GATGTCCAAG

GTCCTGTAGT CCCGGGGCAC GCCCAAGCAA CAGTGAAGTG GATGTCTAAG

GTCCTGTAGT CCCAGGACAT GCCCAAGCAA CAGTGAAGTG GATGGCTAAG

GTCCTGTCAT CCCAGGGCAT GCCCAAGTGA CGGTGAAGTG GATGTGTAAG

GTCCTGTAGT CCCAGGACAT GCCCAAGCAA CAGTGAAGTG GATGTCTCAA

GCCCTGTAGT CCCAGGACAC GCCCAAGTGA CAGTAAAGTG GATGTCTAAG

GTCCAGTCGT CCCAGGACAT GCCCAAGCAA CAGTGAAGTG GATGTCCAAG

GTCCGGTAGT CCCCGGACAT GCACAAGCAA CAGTGAAGTG GATGTCCAAG

GTCCGGTAGT TCCAGGACAT GCCCAGGTGA CAGTGAAGTG GATGTCCAAA

GCCCAGTGGT TCCAGGACAT GCCCATGTGA CTGTGAAGTG GATGTCCAAA

CTGTGGAATG GCGTCATCGC ACCCAGAGTT CAAGAAGCAA TATTGTCAAG

CTGTGGAATG GCGTCATCGC ACCCAGAGTT CAAGAAGCAA TATTGTCAAG

CTGTGGAACG GCGTCATCGC GCCCAGAGTT CAAGAAGCAA TATTGTCAAG

CTGTGGAACG GCGTCATCGC GCCCAGAGTT CAAGAAGCAA TATTGTCAAG

CTGTGGAATG GCGTCATCAC ACCCAGAGTT CAAGAAGCAA TATTGTCAAG

CTGTGGAATG CAGTCATCGC ACCCAGAGTT CAAGAAGCAA TATTGTCAAG

CTGTGGAACG CCATCATTGC TCCCCGAGTT CAAGAAGCAA TACTGTCAAG

CTGTGGAATG CTGTCATTGC TCCCAGAGTT CAAGAAGCAA TATTGTCAAG

CTGTGGAACG CTGTCATCGC GCCCAGAGTT CAAGCAGCAA TCCTGTCGCG

CTATGGAATG CTGTCATTGC ACCCAGAGTT CAAGAAGCAA TATTGTCAAG

CTGTGGAACG CCATCATCGC GCCCAGGGTT CAAGAAGCAA TATTGTCAAG

CTGTGGAACG CTGTCATTGC ACCCAGAGTT CAAGAAGCAA TTTTGTCAAG

CTGTGGAATG CCGTGATCAC TCCCAGAGTG CAAGAAGCAA TATTATCAAG

CTGTGGAACG CCATCATTGC ACCCAAAGTT CAAGAAGCAA TATTGTCAAG

CTGTGGAATG CCGTTATTGC ACCCAGAGTT CAAGAAGCAA TATTGTCAAA

CTGTGGAACG CCGTCATTGC CCCCAGAGTG CAAGATGCCA TACTGTCAAG

TTGTGGAATG CTGTCATTGC TCCCAGAGTT CAAGAAGCGA TACTGTCGAG

TTATGGAATG CCGTGATCGC TCCCAAAGTT CAAGAAGCCA TACTTTCGAG

AGCCTCTGTG AAAAGACAAA CAACTGCTAA AAGACACCCT AGCCAAGGAC

AGCCTCTGTG AAAAGACAAA CAACTGCTAA AAGACACCCT AGCCAAGGAC

AGCCTCTGTG AAAAGACAAA CAACTGCTAA AAGACACCCT AGCCAAGGAC

AGCCTCTGTG AAAAGACAAA CAACTGCTAA AAGACACCCG AGCCAAGGAC

AGCTTCTGTG AAAAGACAAA CAATTGCTAA AAGACACCCT AGCCAAGGAC

AGCCTCTATG AAGAGACAAA CAACTGCTAA GAAACACCCT AGCCAAGGGC

AGCCGCCATG AACAAACAAA CAGCTTCGAA GAAGCACCCT AGCCAAGGGC

AGCCTCCATG AACAAACAAA CAGCTTCTAA GAAGTACCCT AGCCAAGGAC

AGCCTCAGTG AAGAGACAGA CGGCGGCTAA GAAACACCCC AGCCACGGGC

AGCCTCTGTG AAAAGACAGA CAACT---AA AA---ACCCC AGCCAAGGGC

AGCCTCTGTG AAAAGACCTA CAGCTGCTAA GAGCCACCCC AGCCAAGGAC

AGCCTCTGTG GAAAGACACA CAACTGCGAA GAAAACCCCT AGCCAAGGAC

AGCCTCTGTG AAAAGACAGA TAGCTGCCAA AAAACACCCC AATCAAGGAC

AGCCTCTGTG AAAAGACAAA CAACTGCTAA AAAACCCCCT AGCCAAGGAC

AGCCTCTGTG AAAAGACAAA CAAATGCTAA AAAACACCCC AGCCAAGGAC

AGCCTCTGTG AACAGACAAA CAGCTGCTAA GAAACACCCC AGCCCCGGGC

AGCATCTGTG AAAAGGCAGA CAACTGCTAG GAAGCATCCC AGCCAAGGAC

AGCCTCTGTG AAGAGAC--- CAGCAGTCAG ACCGAGTCCC AGCCAAGGGC

AGCAGGCTGT GGTCAAAGCT GCTCTCAGCA TCTTGCTAAA TAAAGCTGTA

AGCAGGCTGT GGTCAAAGCT GCTCTCAGCA TCTTGCTAAA TAAAGCTGTA

AGCAGGCTGT GGTCAAAGCT GCTCTCAGCA TCTTGCTAAA TAAAGCTGTA

AGCAGGCTGT GGTCAAAGCT GCTCTCAGCA TCTTGCTAAA TAAAGCTGTA

AGCAGGCTGT GGTCAAAGCT GCTCTCAGCA TCTTGCTAAA CAAAGCTGTA

AGCAGGCGGT GGTGAAAGCT GCTCTCAGCA TCTTGCTTAA TAAAGCTGTG

AGCAGGCTGT GGTGAGGGCT GCTCTCAGCA TTCTGCTTAA TAAAGCCATT

AGCAGGCTGT GGTGAGGGCC GCCCTCAGCA TTCTGCTTAA TAAAGCGGTT

AGCAGGCTGT GGTGAAAGCT GCTCTCAGCA TCTTACTCAA TAAAGCTGTT

AGCAGGCTGT AGTTAGAGCT GCTCTCAGCA TTTTGCTTAA TAAAGCTGTT

AGCAGGCCGT GGTCAAAGCT GCGCTCAGCA TCTTGCTCAA TAAAGCCGTG

AGCAGGCTGT GGTTAAGGCG GCTCTCAGCA TATTGCTCAA TAAAGCTGTC

AGCAGGCTGT GGTTAAAGCC GCCCTCAGCA TCCTGCTCAA TAAAGCTGTT

AGCAGGCTGT GGTTAAAGCT GCTCTCAGCA TCTTGCTCAA TAAAGCTGTT

AGCAAGCTGT CGTTAAAGCT GCTCTCAGCA TCTTGCTTAA TAAAGCTGTT

AGCAGGCTGT GGTGAAAGCT GCTCTCAGCA TCTTGCTTCA CAAAGCTGTG

AGCAAGCAGT GGTGAAGGCT GCTCTCAGTA TTTTGCTAAA TAAGGCAGTC

AGCAAGCCGT GGTCAAAGCA GCCCTCAGCA TCTTGCTGAA CAAGGCCATT

CTGCATGGCT GTCCCCTCCC CAGAGCAGAG CTAGACCAGC ATACAGCTGA

CTGCATGGCT GTCCCCTCCC CAGAGCAGAG CTAGACCAGC ATACAGCTGA

CTGCATGGCT GTCCCCTCCC CAGAGCAGAA CTAGACCAGC ATACAGCTGA

CTGCATGGCT GTCCCCTCCC CAGAGCAGAA CTAGACCAGC ATACAGCTGA

CTGCATGGCT GTCCCCTCCC TAGAGCAGAG CTAGAACAGC ATAGAGCTGA

CTACACGGCT GTCCCCTCCC CAGAGCAGAG CTTGACCAGC ACACAGCTGA

CTGCATGGCT GTCCCCTTCC CAGGACAGAG CTGGACCAGC AGATAGCAGA

CTGCATGGCT GTCCCCTTCC CAGGGCAGAG CTGGATCAGC AGATCGCAGA

CTGCACGGTT GTCCCCTCCC GAGAGCAGAG CTAGACCAGC ACACAGCGGA

CTGCATGGCT GTCCCCTCCA GAGAGCAGAG CTGGACCAGC ATACAGCTGA

CTGCACGGCT GTCCCCTGCA GACCCCAGAG CTGGACCAGC ATACAGCTGA

CTGCACGGCT GTCCCCTCCC GAGAGCAGAG CTAGACCAGC ATACAGCTGA

CTGCATGGCT GTCCCCTTCA GAGGGCAG-- ---------- ----------

CTGCATGGCT GTCCCCTCCT GAGAGCAGAG CTCGACCAGT ATATAACTGA

CTGCACGGCT GTCCCCTCCC AAGAGCAGAG CTAGACCAGT ATATTGCTGA

CTGCACGGCT GCCCCCTCCC GCAAGCAGAG CTCGACCAGT CTATGGCTGA

CTGCATGGCT GTCCTCTCCC CAGAGCAGAG CTCGATCAGT ATATAGCTGA

CTACATGGCT GTCCTCTCCC CAGAGCAGAG TTGGACCAGT ATATGGCTGA

TTTCAAAGGA GGAAGTTTCC CTTTATCCAT AGTTTCCAGT TATAACACTT

TTTCAAAGGA GGAAGTTTCC CTTTATCCAT AGTTTCCAGT TATAACACTT

TTTCAAAGGA GGAAGTTTCC CTTTATCCAT AGTTTCCAGT TATAACAGTT

TTTCAAAGGA GGAAGTTTCC CTTTATCCAT AGTTTCCAGT TATAACAGTT

TTTCAAAGGA GGAAGTTTCC CTTTATCCAT AGTTTCCAGT TATAACAGTT

TTTCAAGGGA GGAAGTTTTC CTTTATCTTT GGTTTCCAGT TATAATAGCT

CTTCAAAGGG GGAAGTTTCC CTCTGTCAAT AGTTTCAAGT TATA------

CTTCAAAGGG GGCAGTTTTC CTCTGTCAAT AGTGTCAAGT TATA------

TTTCAGAGGG GGAAGTTTTC CTCTATCCAT TGTTTCAAGT TACAACAGCT

TTTCAAAGGA GGAAGCTTCC CTCTGTCCAT AGTTTCAAGT TACAGCAGTT

TTTCAAAGGA GGAGGTTTCC CTTTATCTAT AGTCTCAAGT TACAACAGTT

TTTCAAAGGA GGAAGTTTCC CTTTATCCAT AGTTTCAAGT TACAACTGTT

---------- ---------- ---------- ---------- ----------

TTTCAAGGGA GGAAGTTTCC CTTTATCCAT AGTTTCAAGT TACAACAGTT

GTTCAAAGGA GGAAGTTTCC CTTTATCCAT AGTTTCAAGT TATAGCAGTT

CTTCAAAGGA GGAAGTTTCC CTTTGTCCCT CGTTTCAAGT TACAGCAGTT

TTTCAAAGGA GGAAGCTTCC CCCTCTCCAT GGTTTCAAGT TACAACAATT

GTTCCGGAGT GGGTGCTTCC CATTATCCAT GGTTTCCAGC TACAGCGGTA

GTAACAAGAA GAAAGGAGAG AGTGGTGCCT GGAGAAAGGT GAACACCAGT

GTAACAAGAA GAAAGGAGAG AGTGGTGCCT GGAGAAAGGT GAACACCAGT

GTAACAAAAA GAAAGGAGAA AGTGGTGCCT GGAGAAAGGT GAACACCAGT

GTAACAAAAA GAAAGGAGAG AGTGGTGCCT GGAGAAAGGT GAACACCAGT

GCAGCAAGAA GAAAGGAGAG AGTGGTGCCT GGAGAAAGGT GAACACCAGT

GTAGCAAGAA GAAAG---AA AGTGGTGCCT GGAGAAAGGT GAACACCAGT

---GCAAGAA GAAAGGAGAG AGTGCAGCCT GGAGAAAGGT GAACACCAGT

---GCAAAAA GAAAGTGGAG AGCGGCGCCT GGAGAAAGGT GAACACCAGT

GCAGCAAGAA GAAAGGAGAG AGCGGGGCCT GGAGAAAGGT GAGCACCAGT

GCAGCAAGAA GAAAG---AG AGTGGTGCCT GGAGAAAGGT GAGCACCAGT

GTAGCAGAAA GAAAG---AG AGTGGTGCCT GGAGAAGAGT GAGCACCAGT

GTAGCAAGAA GAAAGGAGAG AATGGTACCT GGCGAAAGGT GAGCACGAGT

---------- ---------- ---------- ---------- ----------

GTAGTAAGAA AAAAGGAGAG AACGGTGCCT GGAGAAAGGT GAGCACCAAT

GTGGCAAGAA GAAAGGGGAG AATGGTGCCT GGAGAAAGGT GAGCACCAGT

GTGGCAAAAA GAAAGGAGAG AATGGCGCGT GGAGAAAAGT GAGCACCAGT

GTGGCAAGAA GAAAGGGGAT AATAATTCCT GGAGAAAGGT GAACACTAGT

GTCACAGGAA GAAAGGAGAG AGTGGGTCCT GGAGGAAAGT CAACACCAGC

CCTCGCAGGA AGTCTGGCCG CTTCTCTTTA CCCACCTGGA ATAAGCCAGA

CCTCGCAGGA AGTCTGGCCG CTTCTCTTTA CCCACCTGGA ATAAGCCAGA

CCTCGCAGGA AGTCTGGCCG CTTCTCTTTA CCCACCTGGA ATAAGCCAGA

CCTCGCAGGA AGTCTGGCCG CTTCTCTTTA CCCACCTGGA ATAAGCCAGA

CCTCGAAGGA AGTCTGGCCG CTTCTCTTTA CCCACCTGGA ATAAGCCAGA

CCTCGCAGGA AATCTGGCCG TTTCTCTTCT CCCACCTGGA ATAAGGCAGA

CCTCGCAAGA AACCTGGGCA CTTCTCCTCA CCCATGTGGA ACAAGCCAGA

CCTCGAAAGA AGCCTGGGCA CTTCTCCTCA CCCACGTGGA ACAAGCCAGA

CCCCGCAAGA AGTCTGGCCG CTTCTCCTCA CCCATTTGGA ATGAGCCCGA

CCTCGCAAGA AGTCTGGCCG GTTCTCTTCA CCCACCTGGA ACAAGCCAGA

CCTCGCAAGA AGTCCGGCCG CTACTCTCCA CCCTCCTGGA ATAGGCCGGG

CCTCGCAAAA AGTCTGGCCA TTTCTCTTCA CCCACCTGGA ATAAGCCGGA

---------- ---------- ---------- ---------- ----------

CCTCGCAAGA AGTCAGGCCG CTTCTCTTCA CCGACCTGGA GTAAGCCAGA

CCTCGCAAGA AGTCTGGCCG CTTCCCTTCA CCCACCTGGA GTAAGCCAGA

CCTCGCAAGA AGTCTGGTTG GTTCTTTTCA CCCACCTGGA GTAAGCCGGA

CCCCGAAAGA AGTCTGGTCA TTCCACTTCA CAATCCTGGA GCAAACAAGA

CCTCGAAAGA AATCCGGCCT CTCCTCTTCC CAGACCTGGA CTAAGCAAGA

CCTAAGCACT GAAGGTATGA AAAATAAGAC TATATCACAG CTGAATTGTA

CCTAAGCACT GAAGGTATGA AAAATAAGAC TATATCACAG CTGAATTGTA

CCTAAGCACT GAAGGTATAA AAAATAAGAC TATATCACAG CTGAATTATA

CCTAAGCACC GAAGGTATAA AAAATAAGAC TATATCACAG CTGAATTATA

CCTAAGCACC GAAGGAATAA AAAATAAGAC CCTATCACAG CTGAATTGTA

CCCGAGCAGC GAAGGTCTAA AAAATAAGAC GATATCACAG CTGAATTGTA

CCTGAAGCAT GAAGGTATGA GAAACAAGAG TGTACCACAT CTGAATATTA

CCCGAAGCGT GAAGGTATGA GGAACAAGAC TATACCACAC CTGAATACTA

CCTCAGCCCG GGAGGTATAA AAAATAAGGC TATATCACAG CTGAACTGTG

CTTGAGTGAA GAAGGTATCA AAAGTAACAC TATATTACAG CTGAATTGTA

CCTGAGCGAG GGAGGTATCA AAATTAAAGC TCTGTCTCAG CTGAATTATA

CCTGAATGAG GAAGGTATCA GAAATACGAC TACATCACAG CTGAATTGTA

---------- ----GCATCA AGAATAAGAC CATCTCACAG CTGAATTGTA

CCTGGGTGAG GAAGGTACAA AAAATAAGAC TATGTCGCAG CCAAATTGTA

CCTGAGTGAT GAAGGTATAA AAAATAAGAC CGTATCACAG CTGAATTGTA

CCTGAGTGAT GAAGGTATAA AAAGTAAGAC GATATCACAA CCGAACTGTA

GATAAGTCCA GAAGGTACAA AAAGTAAGAA CACACTGCAG CCAAATTGCA

GGCAACGAAG GATGGTGTAA GAAATGACAC ---AGGACAT CAGAATGGCA

ACAGGAATGC TTCTCTGTCA AAACAAAAGT CTTTAGAGAA TGATCTATCA

ACAGGAATGC TTCTCTGTCA AAACAAAAGT CTTTAGAGAA TGATCTATCA

ACAGGAATGC TTCTCTGTCA AAACAAAAGT CTTTAGAGAA TGATCTGTCA

ACAGGAAT-- ----CTGTCG AAACAAAAGT CTTTAGAGCA TGATCTGTCA

ACAGGAATGC TTCTCTGTCA AAACAAATGT CTTTAGAGAA TGATGTATCA

ACAAGAACGC TTCTCTGTCA AAACAAAAGT CTTTAGAGAA CGATCTGTCC

ACAGGAGCTC CTCTCTGTCA AAGCAACAGT CCTTAGAGAA TGATCTATCC

ACAGGAACTC CTCTCTGTCG AAGCAACAGT CCTTAGAGAA TGATCTATCT

GAAGGAACAC TTCCCTATCA AAACAAAAGT CTTTAGAGAA CGAGCTGTCG

ACAGGAATGC TTCGCTGTCA AACCAAAAGT CTTTAGAGAA TGATCTGTCA

ACAGGAATGC TTCTCTGTCG AAACAAAAGT CTCTCGAGAA TGATCTGTCA

ACAGGAATGC TTCTCTGTCA AAACAAAAGT CTTTAGAGAA TGATCTATCA

ACAGGAATAT TTCACTGTCA AAACAAAAGT CTATAGAAAA TGACTTATCA

ACAGGATTGC TTCTCTGTCA AAACAAAAGT CCTCAGAGAA TGATCCATCA

ACAGGAATGC TTCTCTGTCA AGACAAAAGT GTTTAGAGAA TGACCTGTCG

ACCGAAATGC CTCTCTATCA AGACAAAAGT GTTTGGAGAA TGATCTGTCG

ACCGGATTGC TTCTCTAATG AAACAAAACT CTTTAGAGAA TGACCAACCA

ACAGCATCGC TTCTCTAGTA AAACAAAAAT CTTTAGAGAA CGGCCATCCA

CTGACGTTGA ATTTGGATCA GAGACTCTCT CTGGGTTCAG ATGACGAAGC

CTGACGTTGA ATTTGGATCA GAGACTCTCT CTGGGTTCAG ATGACGAAGC

CTGACGTTGA ATTTGGATCA GAGACTCTCT CTGGGTTCGG ATGACGAAGC

CTGACGTTGA ATTTGGATCA GAGACTCTCT CTGGGTTCGG ATGACGAAGC

CTGACGTTGA ATTTGGATCA GAGACTCTCT CTGGGTTCAG ATGATGAAGC

CTGATGTTGA ATTTGGATCA GAGACTCTCT CTGGGCTCAG ATGATGAAGT

ATGACATTGA CTTTGGACCA CAGACTCTCT TTGGGTTCAG ATGATGAGGC

GTGACATTGA CTTTGGACCA CAGACTCTCT TTGGGTTCAG ATGATGAAGC

TTGACGTTGA ATTTGGATCA GAGGTTCTCT CTGGGCTCAG ACGATGCAGC

TTGACCTTGA ATCTGGATCA AAGACTCTCT CTGGGTTCGG ATGATGAAGC

TTGACCTTGA ATTTGGAACA AAGGCTCTCT CTGGGTTCGG ACGACGAAGC

TCGACATTGA CCTTGGATCA AAAACTCTAT CTGGGTTCAG ACGATGAAGC

TTGACATTG- -----GATCA AAGACTCTCT CTGTGTTCCG ATGATGAAAT

TCGATGTTGA ATCTGGACCA AAGACTCTCT CTAGGTTCTG ATGATGAAGC

TTGACGTTGA ATCTGGATCA AAGACTCTCT CTGGGTTCAG ATGATGAAGC

TTGGCGTTGA ATCTGGACCA AAGGCTCTCT CTGGGCTCCG ATGACGAAGC

CAAGGATTGA ATCTGGAGCA AAGATTGTCT CTCGGTTCAG ACGATGAGGT

CAAGTGTTAC ATCTAGATCA AAGGCTGTCT CTAGGTTCCG ATGACGAGGT

AGATCTTGTC AAGGAACTTC AGAGCATGTG CTCCAGCAAG TCTGAGTCTG

AGATCTTGTC AAGGAACTTC AGAGCATGTG CTCCAGCAAG TCTGAGTCTG

AGATCTTGTC AAGGAACTTC AGAGCATGTG CTCCAGCAAG TCTGAGTCTG

AGATCTTGTC AAGGAACTTC AGAGCATGTG CTCCAGCAAG TCTGAGTCTG

AGATCTTGTC AAGGAACTTC AGAGCATGTG CTCCAGCAAG TCTGAGTCTG

GGATCTGGTG AAGGAACTTC AGAGTATGTG CTCCAGCAAG TCTGAATCTG

AGATCTGGTC AAAGAACTCC AGAGTATGTG TTCCAGCAAA TCTGAGTCTG

AGATCTGGTC AAAGAACTCC AGAGTATGTG TTCCAGCAAA TCTGAGTCTG

AGATCTCGTC AAGGAGCTTC AGAGCATGTG CTCCAGCAAG TCGGAATCTG

AGATCTTGTC AAGGAACTTC AGAGCATGTG CTCTAGCAAA TCTGAATCTG

CGACCTCGTC CAGGAGCTTC AGAGTATGTG CTCCAGCAAA TCTGAATCTG

TGACCTTATC AAGGAACTTC AGAGTATGTG CTCCAGCAAA TCCGAATCTG

GGACCTTGTT AAAGAACTTC AGAGTATGTG CTCCAGCAAA TCAGAATCTG

AGACCTTGTT AAGGAACTTC AGAGTATGTG TTCCAGCAAA TCGGAATCTG

CGACCTTGTT AAGGAACTTC AGAGCATGTG TTCCAGCAAA TCTGAATCTG

AGACCTCGTT AAGGAACTGC AGAGCATGTG CTCTAGCAAA TCCGAGTCTG

AGACCTTGTG AAAGAACTTC AGAGTATGTG TTCCAGCAAA TCTGAATCTG

AGACCTTGTG AGGGAACTTC AGAGCATGTG TTCCAGTAAA TCTGAATCTG

ATATCAGCAA GATTGCTGAT TCCAGGGATG ATTTAAGGAT GTTTGATAGT

ATATCAGCAA GATTGCTGAT TCCAGGGATG ATTTAAGGAT GTTTGATAGT

ATATCAGCAA GATTGCTGAT TCCAGGGATG ATTTAAGGAT GTTTGATAGT

ATATCAGCAA GATTGCTGAT TCCAGGGATG ATTTAAGGAT GTTTGATAGT

ATATCAGCAA GATTGCTGAT TCCAGGGATG ATTTAAGGAT GTTTGATAGT

ATATAAGCAA GATTGCTGAT TCCAGGGATG ATTTAAGGAC GTTTGATAGT

ATATAAGCAA GATTGCTGAT TCCAGGGAAG ACTTAAGGAC ATTTGATAGT

ATATAAGTAA GATTGCTGAT TCCAGGGATG ACTTGAGGAA GTTTGATAGT

ACCTAAGCAA GATTGCTGAT TCCAGGGAGG AGTTAAGGAC GTTTCATAGT

ATATAAGCAA GATTGCTGAT TCCAGGGATG ATTTAAGGAG GTTTGATAGT

ACATAAGCAA GATCGCTGAC TCCAGGGATG ATTTAAGGAG GTTTGATAGT

ATATAAGCAA GATTGCTGAT TCCAGGGATG ATTTAAGGAG GTTTGATAGT

ACATAAGCAA GATTGCTGAC TCCAGGGATG ATTTGAGGAG ATTTGATAGT

ATATAAGCAA GATTGCTGAT TCCAGGGATG ATTTAAGGAG GTTTGATAGT

ATATAAGCAA GATTGCTGAT TCCAGGGATG ATTTAAGGAG ATTTGATAGC

ATATAAGCAA GATTGCTGAC ACCAGGGATG ATTTAGGGAG GTTTGATAGT

ATATCAGCAA GATTGTGGAT TCCAGGGATG ACTTGAGGAT ATTTGGTTGC

ACATAAGCAA GATTGCAGAT TCCAAGGATG ATTTCAGAAT GTTTGGCAGC

TCTGGAAACA ACCCTGTACT TTCAGCAACT ATTAATAATC TGAGAATGCC

TCTGGAAACA ACCCTGTACT TTCAGCAACT ATTAATAATC TGAGAATGCC

TCTGGAAACA ACCCTGTACT TTCAGCAACT ATTAATAATC TGAGAATGCC

TCTGGAAACA ACCCTGTACT TTCAGCAACT ATTAATAATC TGAGAATGCC

TCTGGAAACA ACCCTGTATT TTCAGCAACT ATTAATAATC TGAGAATGCC

TCAGGAAACA ACCCTGCATT TTCAGCAACT GTTAATAATC CAAGAATGCC

TCAAGAACCA ACCCTGTGAC TTCAGCACCT GT---TAACC TGAGGATGCC

TCAAGAACCA ACCCTGGGAC TTCAGCACCT CT---TAACC TGAGGACACC

TCAGGAAGCA ATCCTGCCTT TTCAGCACCT GTCAATAATC CAAGAATGCC

TCAGGAAACA ACCCTGTGTT TTCAGCAACT GTTAATAATC CAAGAATGCC

TCAGGAAACA GCCCTGCCTT TTCAGCAACT GT---TAACC CAAGAATGCC

TCAAGAAACA ACCCTACATT TTCAGCAACT GTTAATAATC TAAGAATGCC

TCAGGAACTA GCCCTGGATT TTCAGCAACA GCCAATAACC CAAGGATGCC

TCAGAAAACA GCCCTGCATT TTCAGCAGCT ATTAATAATC TTAGAATGCC

TCCAGAAACA GCCCTGCATT TTCAGCAACT GTTAATAATC CACGAATGCC

TCCAGAAACC GCCCTGCACC CTCAGCAACT GTTACGAATC CAAGGATGCC

TCACAAAATA GCCCTGGGTT TTCAGCAGCC ATTACTAATC CAAGTTTGCC

TCCCGGACTG ATCCTGAGTT TTCACCGACC ATGTCTGATC GAAGTTTGCC

AGTGTCACAA AAGGAGGTCA GTCCTCTCAG CAGCCATCAA ACTACTGAAT

AGTGTCACAA AAGGAGGTCA GTCCTCTCAG CAGCCATCAA ACTACTGAAT

AGTGTCACAA AAGGAGGTCA GTCCTCTCAG CAGCCATCAA ACTACTGAAT

AGTGTCACAA AAGGAGGTCA GTCCTCTCAG CAGCCATCAA ACTACTGAAT

AGTGTCACAA AAGGAGGTCT GTCCTCTCAG CAGCCATCAC ACTACTGAAT

AGTGTCACAA AAGGAGGTTA GTCCTCTCAG CAGCCACCAA ACTACCGAAT

AGTGCCACAG AAGGAAGCCA GTCCTCTCAG CAGCCATCAA ACTACCGAAT

AGTGCCACAG AAGGAAGCCA GTCCTCCCAG CAGCCGTCAA ACTGCCGAAT

AGTGGCACCA AAGGAGGTCA GTCCTCTCAG CAGCCATCAA GCTACCGAAT

AGTGTCACAA AAGGAGGTCA GTCCTCTCAG CAGCCATCAA ACAACCGAAT

AGTGTCACCA AAGGAGCCCA GTCCTCTCAG CAGCCACCAA GCGGCCGAAT

AGTGTCAGAG AAGGAGGTCA GTCCTCTCAG CAGCCATCAA ACTACCGAAT

AGTGTCGCCA AAGGAGGTCA GTCCTCTCAG CAGCCATCAA ACTACCGAGT

AGTGTCACAA AAGGAGGTCA GTCCTGTCAG CAGCCATCAA ACTACAAAAC

AGTGTCACAA AAGGAGGTCA GTCCTCTCAG CAGCCATCAA ACTACAGAAT

AGTGTCACAA AAGGAGGTCA GTCCTCTCAG CAGCCATCAA ACTATGGAAC

AGTGTCACAA AAGGATGTCT GTCCTCTCAG CAGCAGTCCA ACTGTAGAAT

AAGCTCCGAA AAGGAGGTCT GTCCTCTTAG CAGCAATCCA ACTCTTGAAT

GCAGCAACAG TAAATCAAAG ACTGAGTTGG GTGTTTCAAG AGTTAAATCT

GCAGCAACAG TAAATCAAAG ACTGAGTTGG GTGTTTCAAG AGTTAAATCT

GCAGCAACAG TAAATCAAAG ACTGAGTTGG GTGTTTCAAG AGTTAAATCT

GCAGCAACAG TAAATCAAAG ACTGAGTTGG GTGTTTCAAG AGTTAAATCT

GCAGCAACAG TAAATCAAAG ACTGAGTTGG GTGTTTCAAG AGTTAAATCT

GCAGCAACAA TAAATCCAAG CCTGAGTCGG GTGTTTCAAG AGCTAAATCT

GCAGCAACAG TAAATCCAAG ACTGAGTTGG GTGTCTCAAG AGTTAAATCT

GCAGCAACAG TAAATCAAAG ACTGAGATGG GTGTCTCAAG CGTTAAATCT

GCAGCACCAG TAAATCAAAG ACTGAGTTGG GTGTTTCAAG AGTTAAATCT

GCAGCAACAG TAAATCAAAG ACTGAGTTGG GTGTTTCAAG AGTTAAATCT

GCAGCAACAG GCAATCCAAG ACGGAGTTGG GTGTTTCGAG AGTTAAATCT

GCAACGACAG TAAATCCAAG ACTGAGTCGG GTGTTTCAAG AGTTAAATCT

GCAGCAACAA TAAATCAAAG ACTGAGTTGG GTGTATCAAG AGTTAAATCT

GCAGCACCAG TACGTCAAAG ACTGAGTTGA GTGTTTCAAG AGTTAAATCT

GCAGCAATAG TAAATTAAAG ACCGAGTTGG GTGTTTCAAG AGTGAAATCT

GCAGCAACAG GACGCTAAAG ACCGAGTTGG GCGTTTCAAG AGTTAAATCT

GCAGTATCAG CAAACCAAAA ATTGAGTTGG GCGTTTCAAG AGTAAAATCT

GCAGCAACAA CACACCGAAA CCCGAGTCGG GGGTTTCGAG AGTGAAATCT

TTTCTTCCTG TTCCTAGAAG TAAAGTCACC CAGTGTTCCC AGAACACCAA

TTTCTTCCTG TTCCTAGAAG TAAAGTCACC CAGTGTTCCC AGAACACCAA

TTTCTTCCTG TTCCTAGAAG TAAAGTCACC CTGTGTTCCC AGAACACCAA

TTTCTTCCTG TTCCTAGAAG TAAAGTCACC CTGTGTTCCC AGAACACCAA

TTTCTTCCTG TTCCTAGAAG TAAAGTCACC CAGTGTTCCC AGAACACCAA

TTTCTTCCTG TTCCTAGAAG TAAAGCCACC CAGTGTTCCC AGAACACCAA

TTTCTTCCTG TTCCTAGAAG TAAAGTCGCC CAGTGTTCCC AGAACACCAA

TTTCTTCCTG TTCCTAGAAG CAAAGTCGCT CAGTGTTCCC AGAACACCAA

TTTCTTCCTG TTCCTAGAAG TAAAATCGCC CAGTGTTCCC AGAACACCAA

TTTCTTCCTG TTCCTAGAAG TAAAGTCACC CAGTGTTCCC AGAACACCAA

TTTCTTCCTG TTCCTAGAAG TAAAGCCACC CAGTGTTCCC CGAACACCAA

TTTCTTCCTG TTCCTCAAAG TAAAGCCACC CTGTGTTCCC AGAACACCAA

TTTCTTCCTG TTCCTAGAAG TAAAGTCACC CAGTGTTCCC AGAACACCAA

TTTCTTCCTG TTCCTAGAAG TAAAGTCTCC CAGTGTTCCC AGAACACCAA

TTCCTTCCTG TTCCTAGAAG TAAAGTCGCC CAGTGTTCCC AGAACACCAG

TTTCTCCCTG TTCCTAGAAG TAAAATCACC CAGTGCTCCC AGAACACCAA

TTTCTCCCTG TACCTAGAAA TAAAGTCACC CAGTGTTCTC CGAACACCAA

TTCCTCCCTG TGCCCAGGAA TAAAGTCGCA CAGTGTTCTC CGAACCCCAA

AAGAAGCAGC AGCAGCAGTA ATACAAGGCA AATAGAAAAC AACAACTCAA

AAGAAGCAGC AGCAGCAGTA ATACAAGGCA AATAGAAAAC AACAACTCAA

AAGAAGCAGC AGCAGCAGTA ATACAAGGCA AATAGAAAAC AACAACTCAA

AAGAAGCAGC AGCAGCAGTA ATACAAGGCA AATAGAAAAC AACAACTCAA

AAGAAGCAGC AGCAGCAGTA ATACAAGGCA AATAGAAAAC AACAACTCAA

AAGAAGCAGC AGCAGCAGTA ATACAAGGCA AATAGAAAAC AACAACTCAA

AAGAAGCAGC AGCAGCAGCA ATACAAGGCA ACTAGAAAAT AACAACTCCA

AAGAAACAGC AGCAGCAGCA ATACAAGGCA ACTAGAAAAC AACAACTCCA

AAGAAGCAGC AGCAGCAGTA ATACAAGGCA ACCAGAAAAC AACAACTCCA

AAGAAGCAGC AGCAGCAGTA ATACAAGGCA AATAGAAAAC AACAACTC--

AAGAAGCAGC AGCAGCAGTA ATACAAGGCA AACCGAAAAC AACAACTCAA

AAGAAGCAGC AGCAGCAGTA ATACAAGGCA AATAGAAAAC AACAACTCAA

AAGAAGCAGC AGCAGCAGTA ATACAAGGCA AATAGAAAAC AACAACACAA

AAGAAGCAGC AGCAGCAGTA ATACAAGGCA AACAGAAAAT AACAACTCAA

AAGAAGCAGC AGCAGCAGTA ATACAAGGCA AATAGAAAAT AACAACTCAA

ACGAAGCAGC AGCAGCAGTA ATACAAGGCA AATAGAAAAT AACAACTCAA

AAGAAGCAGC AGCAGCAGTA ATACAAGGCA AATAGAAGGA AATAACTCAA

AAGAAGCAGC AGCAGCAGTA ATACAAGGCA AAGAGAAAAC AACAACTTAA

AAGAAGTGAA TTGGAACTTA CACAAAAATG AACACCTAGA AAAACCTAAC

AAGAAGAGAA TTGGAACTTA CACAAAAATG AACACCTAGA AAAACCTAAC

AAGAAGAGAA TTGGAACTTA CACAAAAATG AACACCTAGA CAAACATAAC

AAGAAGAGAA TTGGAACTTA CACAAAAATG AACACCTAGA CAAACCTAAC

AAGAAGAGAA TTGGAACTTA CACAAAAATG AACACCTAGA AAAACCTAAC

AAGAAGAGAA TTGGAACTTA CACAAAAACG AACAAATAGA AAAACCTAAC

AAGAAGAGAA TTGGAACGTA GACAAACACG AACACGTAGA AAAACGTAAC

AAGAAGAGAA TTGGACCTTA GACAAACACG AACAAGTAGA AAAACCAAAC

AAGAAGAGAA TTGGAACCTA CACAAACATG AACAAGTAGA AAAACCTAAC

---AAGAGAT TTGGAACCTA CGCAAAAATG AACAAGTAGA AAAATCTAGC

AAGAAGAGAT T---AACTTC CACAAAAACG AACAAGTCGC AAAACCAAAC

AAGAAGAGAT TTGGAACTTA CACAAAAATG AACAAGTAGA AAAACCTAAC

AAGAAGATAT TTGGAATTTA CGCAAAAACG AA---GTAGA AAAACCTAAC

AAGAAGAGAT TTGGAACTTA TGCAAAAATG AACAAGTACA AAAACCTAAC

AAGACGAGAT TTGGAACTTA CGCAAAAATG AACAAGTAGA AAAACCTAAC

AAGACGAGAT TTGGAACTTA CGCAACAATG AACAAATAGA AAAACCTAAC

AAGAAGAGAT TTGGAACTCA CGCCAAAATG TGCAAATAGA AAAAACACAC

AAGAAGAGTT TTGGGTCTTA CGCAAAAACA TACAA-TAGG TATACCTAAC

AAAATGAATG TGAATATGAA GTATCAGCTT CCCAACTTCA CCGCGGAAAC

AAAATGAATG TGAATATGAA GTATCAGCTT CCCAACTTCA CCGCGGAAAC

AAAATGAATG TGAATATGAA GTATCAGCTT CCCAACTTCA CCGCGGAAAC

AAAATGAATG TGAATATGAA GTATCAGCTT CCCAACTTCA CCGCGGAAAC

AAAATGAATG TGAATATGAA ATATCAGCTT CCCAACTTCA CTGCAGAAAC

AAAATGAACG TGGATATGAA GTATCAGCTT CCCAACTTCA CCGCGGAAAC

AAAATGAACG TGAACATGAA GTACCAGCTT CCCAACTTCA CCGCAGAAAC

AAAATGAACG TGAACATGAA GTATCAGCTC CCCAACTTCA CGGCAGAAAC

ACAATGAATG TGAACATGAA GTATCAGCTT CCCAACTTCA CCGCTGAAAC

AAAATGAATG TGAACATGCA ATATCAGCTT CCTAACTTCA CTGCAGAAAC

CAAATGAATG TGAACATGAA GTATCAGCTT CCCAACTTCA CTGCCGAAAC

AAAATGAACG TGGACATGAA GTACCAGCTG CCCAACTTCA CCGCGGAAAC

AAAATGAATG TGAACATGAA GTATCAGCTT CCCAACTTCA CTGCCGAGAC

AAAATGAATG TGAATATGAA GTACCAGCTT CCCAACTTCA CTGCAGAAAC

AAAATGAATG TGAACATGAA GTATCAGCTT CCCAACTTCA CTGCAGACAC

CAA------- ---------- ---------- ---------- ----------

AAAATGGACG TTAATAGGAA GTATCCACTT CCCAACTTTA TTGCAGAAAC

CAAACAGATG TCAACGTGAG CTACTCGCTG CCCCACTTCA TCGCGAATAC

ACCCATCCAG AATGTCATTC TACATGAGCA TCACATTTTC CTTGGTGCCA

ACCCATCCAG AATGTCATTC TACATGAGCA TCACATTTTC CTCGGTGCCA

AGCCATCCAG AATGTCATTC TACATGAGCA TCACATTTTC CTCGGTGCCA

AGCCATCCAG AATGTCATTC TACATGAGCA TCACATTTTC CTCGGTGCCA

ACCCATCCAG AATGTCATTC TACATGAGCA TCACATTTTC CTTGGTGCCA

ACCCATTCAG AATATCGTTC TCCATGAGCA TCACATTTAC CTCGGTGCCA

CCCCATCCAC AATGTCGTCC TCCATGGGCA CCATATTTAT CTCGGAGCCA

CCCCATCCAG AATGTCGTCC TACACGGCCA TCATATTTAT CTCGGAGCCA

GCCCATCCAG AACGTCCTTC TGCACCAGCA TCACGTCTAC CTCGGTGCCA

ATCCATTCAG AATGTTGTTT TGCACAAACA TCACATTTAC CTCGGTGCCA

ACCCATCCAG AATGTTGTTT TACACAAGCA TCATATTTAC CTTGGTGCAG

GCCCATCCAG AATGTCGTCG TGCACCAGCA CTACATTTAC CTCGGTGCCG

GCCCATCCAG CATGTGGTTC TGCATAAGCA TCATATTTAC CTCGGGGCTG

ACCCATCCAG AATGTCGTTT TACACAAGCA TCACATTTAC CTCGGTGCCG

ACCCATCCAG AACGTCGTTT TGCATGAGCA TCACATTTTC CTTGGTGCCA

---------- ---------- ---------- ---------- ----------

TGAGATCCAA AACATTATTT TACACAATAA CCACATCTTC CTGGGTGCAG

CACCCTCCTA AATATCGTCG TCCACCGGGA TGACCTCTAT CTGGGCGCCG

CTAACTACAT TTATGTTTTA GACCTTCAGA AGGTTGCTGA GTACAAGACT

CTAACTACAT TTATGTTTTA GACCTTCAAA AGGTTGCTGA GTACAAGACT

CCAACTACAT TTATGTTTTA GACCTTCAGA AGGTTGCTGA GTACAAGACC

CCAACTACAT TTATGTTTTA GACCTTCAGA AGGTTGCTGA GTACAAGACC

CTAACTACAT TTATGTTTTA GACCTTCAGA AGGTTGCTGA GTACAGGACG

TTAACTACAT TTACGTGTTA GACCTGCAGA AGGTTGCAGA ATACAAGACT

CAAACTACAT TTATGTTTTA GACCTTCAGA AGGTATCTGA GTTCAAGACC

CAAACTACAT TTATGTTTTA GACCTTCAGA AGGTATCCGA ATTCAAGACC

TCAACCACAT CTACGTTTTA GACCTTCAGA AGGTTGCTGA ATACACGACC

TTAACTACAT TTACGTTTTA GACCTTCAGA AGGTTGCTGA GTACAAGACT

TTAACTATAT TTACGTTTTA GACCTTCAGA AGGTTGCTGA GTACAAGACT

TTAACTACAT CTACGTCTTG GACCTTCAGA AGGTCGCTGA GTACAGGACC

TGAACTCCCT TTATGTTTTA GACCTGCAGA AGGTTGCCGA ATACAAAACA

TTAACTACAT TTACGTCTTA GACCTTCAAA AGGTTGCTGA GTATAAGACC

TTAACAACAT TTATGTTCTA GACCTCCAGA AGGTTGCTGA GTACAAGACC

---------- ---------- ---------- ---------- ----------

TCAACAAGAT TTATGTCTTA -ATCTCCATA ATGTTTCTGA GTACAAGACA

TCAACAAGAT CTACGTCTTA -ACCTCCAGG ACTTCGTAGA GTACCCGACT

GGGCCTGTGC TGGAACACCC AGATTGTTTC CCATGTCAGG ACTGCAGCAG

GGGCCTGTGC TGGAACACCC AGATTGTTTC CCATGTCAGG ACTGCAGCAG

GGGCCTGTGC TGGAACACCC AGATTGTTTC CCGTGTCAGG ACTGCAGCAG

GGGCCTGTGC TGGAACACCC AGATTGTTTC CCGTGTCAGG ACTGCAGCAG

GGGCCTGTGC TAGAACACCC AGATTGTTTC CCATGTCAGG ACTGCAGCAG

GGGCCTGTGC TGGAACACCC AGATTGTTTC CCGTGTCAGG ACTGCAGCGA

GGGCCCGTGG TGGAACACCC AGATTGTTTT CCTTGTCAGG ACTGCAGCAG

GGGCCCGTGT TGGAACACCC AGATTGTTTA CCTTGTCGGG ACTGCAGCAG

GGGCCCGTGC TGGAACACCC GGACTGTTTG CCGTGTCAGG ACTGCAGCAG

GGGCCTGTGC TGGAACACCC AGATTGTTTC CCATGTCAGG ACTGCAGCCA

GGGCCCGTGC TGGAACACCC AGATTGTTCC CCATGTCAGG ACTGCAGCCA

GGGCCGGTGC TGGAACACCC GGAGTGCTTG CCGTGCCAGG ACTGCAGCCA

GGCCCGGTGC TGGAACACCC AGACTGCTTG CCCTGCCAGG ACTGCCACCA

GGGCCTGTGT TGGAACACCC AGATTGTGTC CCGTGTCAGA ACTGCAGCAG

GGGCCCGTGT TGGAGCACCC AGATTGTTTG CCGTGTCAGG ACTGCAGTAG

---------- ---------- ---------- ---------- ----------

GGACCTGTGC TAGAACACCC GGACTGTGCT CCATGTCAAG ACTGTAGTCA

GGGCCAGTGT TGGAACATCC TGACTGCGCT CCATGTGAGA ACTGCTCCGC

CAAAGCCAAT TTATCAGGAG GTGTTTGGAA AGATAACATC AACATGGCTC

CAAAGCCAAT TTATCAGGAG GTGTTTGGAA AGATAACATC AACATGGCTC

CAAAGCCAAT TTATCAGGAG GCGTTTGGAA AGATAACATC AACATGGCTC

CAAAGCCAAT TTATCAGGAG GCGTTTGGAA AGATAACATC AACATGGCTC

CAAAGCCAAT TTATCAGGAG GAGTTTGGAA AGATAACATC AACATGGCTC

CAAGGCCAAT TTATCAGGTG GCATTTGGAA AGATAACGTC AACATGGCTC

CAAAGCCAAT GTGTCAGGAG GTGTTTGGAA AGACAACGTC AACATGGCGC

CAAAGCCAAT TCATCAGGAG GGGTTTGGAA AGACAACATC AACATGGCTC

CAAAGCCAAT TCCTCAGGTG CTGTTTGGAA AGATAACATC AACCTGGCTC

TAAAGCCAAT TTATCAGGTG GTGTTTGGAA AGATAACATC AACATGGCTC

CAAAGCCAAT TTATCAGGTG GTGTTTGGGA AGATAACATC AACATGGCTC

CAAGGCCAAT GCGTCAGGGG GCGTCTGGGC AGATAACGTC AACATGGCCC

CAAAGCCAAT TCATCAGGTG GCATTTGGAA AGATAACGTC AACATGGCAC

CAAAGCCAAT TTGTCAGGCA GTGCTTGGAG AGATAACATC AACATGGCTC

CAAAGCCAAT TTATCAGGTA GTGTTTGGAA AGATAACATC AACATGGCCC

---------- --CTCGCGGG GCGCCTGGAG AGAGAACGTG AACATGGCCC

CAAAGCTAAC TTATCTGGTA GCATTTGGAA GGATAATGTC AACAAAGCCT

CATAGCCCAT TCGTCGAAAG CAGACTTGAG GGATAACGTC AACATGGCCC

TAGTTGTCGA CACCTACTAT GATGATCAAC TCATTAGCTG TGGCAGCGTC

TAGTTGTCGA CACCTACTAT GATGATCAAC TCATTAGCTG TGGCAGCGTC

TGGTTGTCGA CACCTACTAT GATGATCAAC TCATTAGCTG TGGCAGCGTC

TGGTTGTCGA CACCTACTAT GATGATCAAC TCATTAGCTG TGGCAGCGTC

TGGTTGTCGA CACCTACTAT GATGACCAAC TCATTAGCTG TGGCAGCGTC

TGCTTGTCGA CACCTACTAC GATGACCAAC TCATCAGCTG TGGCAGCGTC

TGCTTGTTGA CACTTACTAT GACGACCAGC TCATCAGCTG TGGCAGCGTC

TGCTTGTTGA CACATACTAT GATGATCAAC TCATTAGCTG TGGCAGTGTC

TGCTTGTTGA CAAGTACTAT GATGACCAAC TCATTAGCTG TGGCAGCGTC

TGCTTGTTGA CACGTACTAT GATGATCAAC TCATTAGCTG TGGCAGTGTC

TGCTTGTTGA CACATACTAC GATGACCAAC TCATTAGCTG TGGCAGTGTC

TGCTGGTGGA CACGTACTAC GACGACCAGC TCATCAGCTG CGGCAGCGTC

TGCTTGTTGA CACATACTAC GATGACCAGC TCATTAGCTG TGGCAGCGTC

TGCTTGTTGA CACATACTAC GACGACCAGC TCATTAGCTG TGGCAGCGTC

TGCTTGTTGA CACATACTAT GATGACCAAC TCATTACCTG TGGCAGCGTC

TGCTGGTGCA AACCTACTCT GACGACCGAC TCATCAGCTG CGGCAGCGTC

TGCTCGTTGA TACTTACTAT GACGATCAGC TTATTAGTTG TGGTAGTGTT

TGCTGATCGA GACCTACTAC TATGAGCAAC TGATCATCTG CGGGAGTGTG

AACAGAGGGA CCTGCCAGCG ACATGTCTTT CCCCACAATC ATACTGCTGA

AACAGAGGGA CCTGCCAGCG ACATGTCTTT CCCCACAATC ATACTGCTGA

AACAGAGGGA CCTGCCAGCG ACATGTCTTT CCCCATAATC ATACTGCTGA

AACAGAGGGA CCTGCCAGCG ACATGTCTTT CCCCATAATC ATACTGCTGA

AACAGAGGAA CCTGCCAGCG ACACGTCTTT CCCCACAATC ATACTGCTGA

AACAGAGGGG CCTGCCAGCG CCATGTCCTT CCACGCAATA ACGCAGCTGA

AACAGAGGGA CCTGCCAAAG GCATGTCCTT CCTCCTGACA ATGCTGCCGA

AACAGAGGGA CTTGCCAGCG GCATGTCCTT CCTCCTGACA ATTCTGCTGA

AACAGAGGGA CCTGCCAGCG ACACGTCCTC CCACCTGATA ATCCTGCTGA

CACAGAGGGA CCTGCCAGCG ACACGTCCTT CCACCCAACA ATACTGCAGA

CACAGAGGGA CCTGCCAGCG ACATATCCTT CCACCCAGCA ATATTGCTGA

CGCAGAGGCA CGTGCCGGCG GCACGTGCTG CGCTCCGAC- --GCGGCGGA

CACAGAGGGA CCTGCCAGCG ACACGTGCTG CCCCCGGACA ATACTGCTGA

AACAGAGGGA CCTGCCAGCG GCACGTCTTT CCACCCCATA ATACTGCTGA

AACAGGGGGA CCTGCCAGCG GCACGTCCTA CCACCTGATA ATCCTGCTGA

CACCGGGGCA CCTGCCAGCG GCACGTGCTG CCCCCCGATA ACCCCGCGGA

AATCGAGGGA CCTGCCAGCG GCATGTGTTT CAACCTAATA ATACTGCTGA

GATGGAGGGG CCTGCTGGAG GTTCCCCCTG CCTCTTGGGG AGCCGACTGA

CATACAGTCG GAGGTTCACT GCATATTCTC CCCACAGATA GAAGAGCCCA

CATACAGTCG GAGGTTCACT GCATATTCTC CCCACAGATA GAAGAGCCCA

CATACAGTCG GAGGTTCACT GCATATTCTC CCCACAGATA GAAGAGCCCA

CATACAGTCG GAGGTTCACT GCATATTCTC CCCACAGATA GAAGAGCCCA

CATTCAGTCG GAGGTTCACT GCATATTCTC CGCGCAGACA GAAGAGCCCA

CATACAGTCG AAGGTTCATT GCATGTTCTC CCCGCAGGCA GAGGAGCCAG

CATTCAGTCC GAGGTTCACT GCATGTTCTC CCCACTTGCG GAAGAGTCAG

CATCCAGTCT GAGGTCCACT GCATGTTCTC CCCA------ GAGGAGTCAG

CATACAGTCG GGGGTTTACT GTATGTTCTC CCCACAGGCA GAAGAGCCTG

CATAGAGTCG GAGGTTCACT GCATGTACTC TCCTCAGGCA GAAGAGACCA

CATACAGTCG GAAGTGCATT GCATGTACTC CTCACAGGCA GAAGAGCCCA

CATCCGGTCC GAGGTGCACT GCATGTTCTC GCCGCAGGCG GAGGAGCCCG

CATCCACTCG GAGGTTCACT GCATGTACTC CTCACAGGCG GAAGAGCCGA

CATACAGTCA GAGGTTCGTT GTATGTACTC CCCACAGCCA GAAGAGCCCA

CATACACTCG AAGGTGCATT GCATGTACTC CCCACAGGCA GAGGAGCCCA

CATCCAGGCG GAGGTCCATT GCATGCACTC CCCGCGCGCG GAGGAGGCCA

CATACAGTCA AATGTTTATT GTATATACTC CCCACAGATG GAGGAGTCCC

CTTCCACTCC AAAAAGCACT GCATGTTCTC CCACCAGGCC GAAGAGTCCA

GCCAGTGTCC TGACTGTGTG GTGAGCGCCC TGGGAGCCAA AGTCCTTTCA

GCCAGTGTCC TGACTGTGTG GTGAGCGCCC TGGGAGCCAA AGTCCTTTCA

GCCAGTGCCC TGACTGTGTG GTGAGCGCCC TGGGAGCCAA AGTCCTTTCA

ACCAGTGCCC TGACTGTGTG GTGAGCGCCC TGGGAGCCAA AGTCCTTTCA

GCCAGTGTCC TGACTGCATG GTGAGCGCCC TGGGAACCAA AGTCCTTTTA

GCCAGTGTCC TGACTGTGTG GTGAGTGCCC TGGGAGCCAA AGTCCTCCTG

GCCAGTGTCC CGACTGTGTA GTGAGTGCCC TGGGAGCCAA AGTCCTCCTG

GGCAGTGTCC TGACTGTGTA GTGAGTGCCC TCGGAGCCAA AGTCCTCCTG

GCCAGTGTCC TGACTGCGTG GTGAGCCCCC TGGGAGCCAA AGTTCTCCTA

ACCAGTGTCC TGACTGTGTG GTGAGTGCCC TGGGAACCAA AGTCCTACTG

GCCAGTGCCC TGACTGTGTG GTGAGTGCTC TAGGAACCAA AGTCCTGATA

GCCGCTGTCC CGACTGTGTG GTGAGCGCGC TGGGGACCAA GGTGCTGCTT

ACCAGTGTCC CGACTGCGTG GTGAGCGCCC TGGGAACCAA AGTCCTGCTG

GCCAGTGTCC CGACTGTGTG GTGAGTGCCC TGGGAACCAA AGTCCTTCTG

GCAAGTGTCC TGACTGCGTG GTGAGCGCTC TGGGAACCAA AGTCCTACTT

GCCAGTGTCC CGACTGCGTG GTCAGTGCCC TGGGCACCAA GGTGCTCCTG

TCCAATGTCC TGACTGTGTG GTAAGCACTT TAGGTACTAA AGTTCTGCTG

AAGACTGCCC CGACTGCATC GTGAGCACGC TGGGAACCAA AGTCCTGCTG

TCTGTAAAGG ACCGGTTCAT CAACTTCTTT GTAGGCAATA CCATAAATTC

TCTGTAAAGG ACCGGTTCAT CAACTTCTTT GTAGGCAATA CCATAAATTC

TCTGTAAAGG ACCGGTTCAT CAACTTCTTT GTAGGCAATA CCATAAATTC

TCTGTAAAGG ACCGGTTCAT CAACTTCTTT GTAGGCAATA CCATAAATTC

TCTGTAAAGG ACCGGTTCCT CAACTTCTTT GTAGGCAATA CCATAAATTC

TCTGAAAAGG ACCGCTTCAT CAACTTCTTT GTAGGCAATA CCATAAATTC

TCTGAAAAGG ACCGGTTCAT CAATTTCTTC GTGGGGAATA CGATAAACTC

TCGGAAAAGG ACCGGTTCAT CAATTTCTTT GTGGGGAATA CGATCAATTC

TCCAAAAAGC AGCGCTTCAT CTACTTCTTT GTGGGCAATA CCATAAATTC

TCTGAAAAGG ATCGATTCAT CAACTTCTTC GTGGGCAATA CCATAAATTC

TCAGAAAAGG ACCGGTTCAT CAACTTCTTC GTAGGCAATA CCATAAATTC

TCCGAGAAGG ACCGCTTCAT CAACTTCTTC GTGGGCAACA CCATCAGCTC

TCGGAGAAGG AGCGTTTCAT CAACTTCTTT GTGGGCAACA CTATCAACTC

ACCGTAAAGG ACCGTTTCAT CAACTTCTTT GTGGGCAATA CCATAAATTC

ACCGAGAAGG ACAGGTTCAT CAACTTCTTT GTAGGTAATA CTGTCAATTC

GCCGAGAAGC AGCGCTTTGT CAACTTCTTT GTGGGCAACA CCCTCAATGG

TCAGAAAAAG AACGATTTAT AAACTTCTTT GTGGGCAATA CCATTAACTC

GTCGAGAAAG ACCGATTTGT CAACTTCTTT GTGGGCAATA CCATCAACTC

TTCTTATTTC CCAGATCATC CATTGCATTC GATATCAGTG AGAAGGCTAA

TTCTTATTTC CCAGATCATC CGTTGCATTC AATATCAGTG AGAAGGCTAA

TTCTTATTTC CCACATCATC CGTTGCATTC GATATCAGTG AGAAGGCTAA

TTCTTATTTC CCACATCATC CGTTGCATTC GATATCAGTG AGAAGGCTAA

TTCTTACTTC CCAGATCATT CATTGCATTC GATATCAGTG AGAAGGCTAA

TTCTTATTTT CCAAATCATC CATTGCATTC AATATCAGTT AGAAGGCTAA

TTCCTACCCT CCCGATTATT CATTGCATTC AATATCGGTG AGGCGGCTGA

CTCCTATCCT CCTGGTTATT CACTGCATTC GATATCGGTG AGACGGCTGA

TTCTTACCTT CCAGATCACT CATTGCATTC GATATCAGTG AGAAGGCTGA

TTCTTACCTT CCAGATTATA TATTGCACTC GATATCAGTG AGAAGGCTAA

CTCGGACCAT CCAGATCATT CATTGCATTC GATATCGGTG AGAAGGCTAA

GGCCCACCTG CCGGGCCGCG CGTTACATTC GATATCGGTG AGGAGGCTGA

TTCTTATCCT CCCAATCATT CCTTGCATTC TATCTCCGTG AGACGGCTGA

TTCTTACCTT CCCGATCATT CCTTGCATTC AATATCAGTG AGAAGGCTCA

TTCCTACCTT CCAGATCATT CATTGCATTC GATATCGGTG AGAAGGCTAA

CTCCTCGCTC CCAGGCCACG CACTGCATTC CATATCAGTG AGAAGGATCA

TTCTGA---T CCCGACAATT CTTTACATTC AATATCTGTC AGGAGGTTAA

GTCTTCTCAC CCTTTCTCGT CTCTGCATTC CATCTCCGTG AGGCGGCTGA

AGGAAACGAA AGATGGTTTT ATGTTTTTGA CGGACCAGTC CTACATTGAT

AGGAAACGAA AGATGGTTTT ATGTTTTTGA CAGACCAGTC CTACATTGAT

AGGAAACGAA AGATGGTTTT ATGTTTTTGA CGGACCAGTC CTACATTGAT

AGGAAACGAA AGATGGTTTT ATGTTTTTGA CGGACCAGTC CTACATTGAT

AGGAAACAAA AGATGGTTTT ATGTTTTTGA CGGACCAGTC CTACGTTGAT

AGGAAACACA GGATGGTTTT AAGTTTTTGA CGGACCAGTC CTATATTGAT

AGGAAACCCA GGACGGTTTT AAGTTTTTGA CAGACCAGTC CTACATTGAT

AGGAAACCCA AGATGGTTTT AAGTTTTTGA CAGACCAGTC CTATATTGAT

AGGAAACACA GGATGGTTTT AAGTTTCTGA CGGATCAGTC CTATATTGAT

AGGAAACACA AGATGGTTTT AAGTTTTTGA CAGACCAATC CTATATTGAT

AGGAAACGCA AGATGGGTTC AAGTTTTTGA CAGACCAGTC TTACATTGAT

AGGAGACGCA AGACGGCTTT AAGTTTCTGA CAGACCAGTC CTACATTGAT

AGGAAACGCA AGATGGTTTC AAGTTCTTGA CAGACCAGTC CTATATTGAT

AGGAAACACA AGATGGTTTT AAGTTTTTGA CAGACCAGTC CTATATTGAT

AGGAAACGCA AGATGGATTT AAGTTTTTGA CGGACCAGTC CTATATTGAT

AGGAGACCCA GGATGGCTTT AAGTTTCTGA CAGACAAGTC CTACATCGAC

AGGAAACACA AGACGGTTTT AAATTTTTGA CAGACCAGTC CTATATTGAT

AGGAAACTCT GGATGGCTTT AAGTTCTTGA CGGATCAGTC TTACATCGAC

GTTTTACCTG AGTTCAGAGA TTCTTACCCC ATTAAGTATG TCCATGCCTT

GTTTTACCTG AGTTCAGAGA TTCTTACCCC ATTAAGTATG TCCATGCCTT

GTTTTACCTG AGTTCAGAGA TTCTTACCCC ATTAAGTACA TCCATGCTTT

GTTTTACCTG AGTTCAGAGA TTCTTACCCC ATTAAGTACA TCCATGCTTT

GTTTTACCTG AGTTCAGAGA TTCTTACCCC ATTAAGTACG TCCATGCCTT

GTTTTACCTG AGTTCCGAGA CTCTTACCCC ATTAAGTATG TCCATGCCTT

GTCCTGCCAG AATTCCGAGA TTCCTACCCC ATCAAGTACA TACATGCCTT

GTCTTACCAG AATTCCAAGA TTCCTACCCC ATAAAGTACA TACATGCCTT

GTCCTACCCG AGTTCCGAGA TTCCTACCCC ATTAAGTATG TCCACGCTTT

GTTCTACCTG AACTCCGAGA TTCCTACCCC ATTAAGTATG TCCACGCCTT

GTTCTACCGG AGTTCAGAGA CTCCTACCCC ATTAAATATG TCCACGCCTT

GTTTTACCCG AGTTCCGAGA TTCTTACCCC ATTAAGTATG TCCACGCCTT

GTCCTGCCTG AGTTCCGAGA CTCTTACCCC GTTAAATACA TCCATGCCTT

GTTCTACCTG AGTTCCGAGA TTCTTACCCC ATTAAGTATG TCCATGCCTT

GTTCTACCTG AGTTCCGAGA TTCGTACCCC ATTAAGTATG TCCATGCCTT

GTGCTCCCCG AGTTCCAAGC CTCCTACCCC ATTAAGTACG TCCACGCCTT

GTTCTACCAG AATTCCAAGA TTCTTACCCC ATTAAATACA TACATGCCTT

GTTCTGCCGC AGTTCCGAGA TTCGTACCCC ATCAAGTACA TCCACGCTTT

TGAAAGCAAC AATTTTATTT ACTTCTTGAC GGTCCAAAGG GAAACTCTAG

TGAAAGCAAC AATTTTATTT ACTTCTTGAC GGTCCAAAGG GAAACTCTAG

TGAAAGCAAC AATTTTATTT ACTTCTTGAC GGTCCAAAGG GAAACTCTAA

TGAAAGCAAC AATTTTATTT ACTTCTTGAC GGTCCAAAGG GAAACTCTAA

TGAAAGCAAC AATTTTATTT ACTTCTTGAC GGTCCAAAGG GAAACTCTAA

TGAAAGCAAC CATTTTATTT ACTTTCTGAC CGTCCAAAGG GAAACTCTGG

CGAAAGCAAC CATTTTATCT ACTTTCTGAC TGTCCAGAAG GAAACCCTAG

CGAAAGCAAC CATTTTATTT ACTTTCTGAC TGTCCAAAAG GAAACTCTAG

TGAAAGCAAC CATTTTATTT ACTTCCTGAC GGTCCAAAAG GAAACTCTAG

TGAAAGCAAC CATTTTATTT ACTTTTTGAC GGTCCAGCGG GAAACTCTAG

TGAAAGCAAC CACTTTATTT ACTTTTTGAC AGTCCAGCGA GAAACTCTAG

TGAAAGCAAC CATTTTATTT ACTTCTTGAC GGTCCAACGG GAAACTCTCG

CGAGAGCAAC CATTTCATTT ACTTCCTGAC AGTCCAGCGG GAAACGCTGG

TGAAAGCAAC CATTTTATTT ACTTTTTGAC AGTCCAACGG GAAACCCTAG

CAAACACAAC CAGTTTATTT ACTTTTTGAC TGTCCAACGG GAAACTCTAG

CGAAAGCAAC CGCTTTATTT ACTTTCTCAC GGTCCAGCGA GAAACGCTGG

TGAAAGCAGT GACTTCATTT ACTTTTTAAC AGTCCAAAGA GAAACTCTGG

TGAGAGTAAT CAGTTCATCT ATTTCCTGAC CGTACAGAAA GAAACTGTGG

ATGCTCAGAC TTTTCACACA AGAATAATCA GGTTCTGTTC CATAAACTCT

ATGCTCAGAC TTTTCACACA AGAATAATCA GGTTCTGTTC CATAAACTCT

ATGCTCAGAC TTTTCACACA AGAATAATCA GGTTCTGTTC CTTAAACTCT

ATGCTCAGAC TTTTCACACA AGAATAATCA GGTTCTGTTC CTTAAACTCT

ATGCTCAGAC TTTTCACACA AGAATAATCA GGTTCTGTTC CATAAACTCT

ACTCTCAGAC TTTTCATACT CGAATAATCA GGTTCTGTTC CGTAGACTCT

ATGCTCAGAC TTTCCATACA AGAATAATCA GGTTCTGTTC TGTAGACTCT

ATGCTCAGAC TTTTCATACA AGAATAATCA GGTTCTGTTC CGTAGACTCT

ATGCTCAGAC TTTCCACACA AGAATCATCA GGTTCTGTTC CAAGGACTCC

ATGCTCAGAC TTTTCACACA AGAATAATCA GGTTCTGTTC CGCAGACTCT

ATGCTCAGAC TTTTCACACG AGAATAATCA GGTTCTGTTC TGTAGACTCT

ATGCTCAGAC TTTTCACACG AGAATCGTCA GGTTCTGCTC CGTAGACTCG

ATGCTCAGAC TTTTCACACG AGAATAATCA GGTTCTGTTC CACAGACTCC

ATTCTCAGAC TTTTCACACA AGAATAATCA GATTCTGTTC CGTAGACTCT

AATCTCAGAC CTTTCACACA AGAATCATCA GGTTCTGTTC TGTAGACTCT

ACTCGCCGTC CTTCCACACG CGTATCATCA GGTTCTGCTC GGCGGACTCG

AATCTCAGAC TTATCACACA AGAATAATCA GGTTCTGTTC AGTTGACTCT

AATCCCAAAC TTTCCACACA AGAATTATCC GGTTCTGTTC AATGGATTCG

GGATTGCATT CCTACATGGA AATGCCTCTG GAGTGTATTC TCACAGAAAA

GGATTGCATT CCTACATGGA AATGCCTCTG GAGTGTATTC TCACAGAAAA

GGATTGCACT CCTACATGGA AATGCCTCTA GAGTGTATTC TCACAGAAAA

GGATTGCACT CCTACATGGA AATGCCTCTA GAGTGTATTC TCACAGAAAA

GCATTGCATT CCTACATGGA AATGCCTCTG GAGTGTATTC TCACAGAAAA

GGGCTGCATT CGTACATGGA AATGCCCCTG GAGTGTATTC TCACCGAAAA

GGGTTGCACT CCTACATGGA AATGCCTCTG GAGTGCATTC TGACGGAAAA

GGGTTGCACT CCTACATGGA AATGCCCCTG GAATGCATCC TGACAGAAAA

GGCTTGCATT CCTACATGGA AATGCCTCTG GAGTGTATTC TCACGGAGAA

GGATTGCATT CGTACATGGA AATGCCTCTG GAGTgtattc tgacggaaaa

GGATTGCATT CCTACATGGA AATGCCTCTG gagtgtattc tcacggaaaa

GGCTTGCATT CCTACGTGGA AATGCCCCTG GAGTGTATTC TCACAGAAAA

GGGTTGCATT CTTACATGGA GATGCCTCTG GAGTGTATTC TCACAGAGAA

GGATTGCATT CCTACATGGA AATGCCTCTT GAGTGTATTC TCACTGAAAA

GGATTGCATT CCTACATGGA AATGCCTCTG GAGTGTATTC TCACTGAGAA

GGCTTGCACT CCTACATGGA AATGCCCCTG GAGTGCATCC TCACCGAGAA

GGATTGCATT CATATATGGA AATGCCTCTT GAATGTATTC TTACCGAAAA

GAATTACATT CCTACATGGA AATGCCTCTC GAATGCATCC TCACAGAGAA

GAGAAAAAAG AGATCCACAA AGAAGGAAGT GTTTAATATA CTTCAGGCTG

GAGAAAAAAG AGATCCACAA AGAAGGAAGT GTTTAATATC CTTCAGGCTG

GAGAAAAAAG AGATCCACAA AGAAGGAAGT GTTTAATATC CTTCAGGCTG

GAGAAAAAAG AGATCCACAA AGAAGGAAGT GTTTAATATC CTTCAGGCTG

GCGAAAAAAG AGATCCACAA AAAAGGAAGT GTTTAATATC CTTCAGGCTG

GAGAAGAAAG AGATCCACAA GGAAGGAAGT ATTCAACATC CTCCAAGCTG

AAGAAGAAAG AGATCCACAA GGGAAGAAGT GTTTAATATC CTCCAAGCCG

AAGAAGGAAG AGATCCACAA GGGAAGAAGT GTTTAATATC CTCCAAGCCG

GAGGAGGAAG AGAGCCACCA GGGAGGAAGT GTTTAATATC CTTCAGGCTG

gagaagaaag agatccacaa agcaggaagt gtttaatatt ctccaagctg

gagaagaaag agatccacaa gggaggaagt gtttaatatt ctccaagctg

GAGGAGGAGG AGATCCGCAA GGTCGGAAGT GTTCAATATC CTCCAAGCTG

GAGAAGAAAG AGGGCCACAA GGGAGGAAGT GTTCAATATT CTCCAAGCTG

GAGAAGAAAG AGATCCACAA GACAGGAGGT GTTTAATATT CTTCAGGCTG

GAGAAGGAAG AGGTCAGCCA GGGAGGAGGT GTTTAATATC CTTCAGGCTG

GCGCCGCAAG CGGGCCCTGC GCAGCGAGGT GTTCAACGTG CTGCAGGCCG

GAGAAGAAAA AGGTCAACAA GGGAAGAAGT ATTTAACATT CTACAAGCAG

GAGGAGGAGG AGGTCCACCA ACAAGGAAGT GTTCAATGTC CTCCAGGCTG

CGTATGTCAG CAAGCCTGGG GCCCAGCTTG CTAGACAAAT AGGAGCCAGC

CGTATGTCAG CAAGCCTGGG GCCCAGCTTG CTAGACAAAT AGGAGCCAGC

CATATGTCAG CAAGCCCGGG GCCCAGCTTG CTAGACAAAT AGGAGCCAGC

CATATGTCAG CAAGCCCGGG GCCCAGCTTG CTAGACAAAT AGGAGCCAGC

CATATGTCAG CAAGCCTGGG GCCCAACTTG CGAGGCAAAT AGGAGCCAGC

CATACGTCAG TAAGCCTGGG GCCCACCTTG CCAAGCAGAT AGGAGTCAGT

CATATGTCAG TAAACCGGGG GCCAATCTTG CTAAGCAAAT AGGGGCCAGC

CGTATGTCAG TAAACCAGGG GCCAATCTTG CTAAGCAAAT AGGAGCTAGC

CATATGTCAG TAAGCCTGGG GCCCATCTTG CTAGGCAGAT TGGAGCCACC

catatgtcag taagcctggg gctcagCTCG CTAGGCAAAT AGGTGCCAGC

catatgtcag taagCCTGGG GCCCATCTCG CTAAACAAAT AGGTGCCAAC

CCTATGTCGG CAAGCCTGGG ACCCATCTCG CCAACCAAAT AGGGGTGAAC

CGTATGTCAG TAAACCCGGG GCCCATCTCG CTAAGCAAAT AGGTGCCAAC

CATATGTCAG TAAGCCTGGG GCCCATCTCG CTAAGCAAAT AGGTGCCAGC

CCTACGTCAG TAAGCCTGGG GCCTATCTCG CTAAGCAAAT AGGTGCCCTC

CGTACGTGGG CAAGCCCGGG GCGCAACTGG CCAAGCAGAT CGGCGCCAGC

CATATGTAAG TAAACCTGGT GCACATCTGG CTAAGCAAAT AGGTGTTGGG

CCTATGTCAG TAAGCCAGGG GCCCATCTGG CTCGGCAAAT AGGAACCGGA

CTGAATGATG ACATTCTTTT CGGGGTGTTC GCACAAAGCA AGCCAGATTC

CTGAATGATG ACATTCTTTT CGGGGTGTTC GCACAAAGCA AGCCAGATTC

CTGAATGATG ACATTCTCTT TGGGGTGTTC GCACAAAGCA AGCCAGATTC

CTGAATGATG ACATTCTCTT TGGGGTGTTC GCACAAAGCA AGCCAGATTC

CTGAATGATG ACATTCTCTT CGGGGTGTTT GCACAAAGCA AGCCAGATTC

GTGAATGATG ACATTCTTTT TGGGGTGTTC GCACAAAGCA AGCCAGATTC

CCGTATGATG ACATTCTCTA CGGGGTGTTT GCACAAAGCA AGCCAGATTC

CCTTCTGATG ACATTCTCTT CGGGGTGTTT GCACAAAGCA AGCCAGATTC

CCGAATGATG ACATTCTCTA TGGGGTGTTC GCCCAGAGCA AACCAGATTC

CTGAATGATG ACATTCTGTA CGGGGTGTTT GCACAAAGCA AGCCAGATTC

CTGAATGATG ACATTCTCTA TGGAGTGTTC GCACAAAGCA AGCCAGATTC

CTGGACGATG ACATTCTCTA CGCGGTGTTC GCACAAAGCA AGCCGGATTC

CCGAATGATG ACATTCTCTA CGGGGTGTTT GCCCAGAGCA AGCCGGATTC

CCGAATGATG ACATTCTCTA CGGGGTGTTT GCACAGAGCA AGCCAGATTC

CCAGATGATG ACATTCTCTA TGGGGTGTTC GCACAAAGCA AGCTAGATTC

GCCCACGACG ACATTCTCTA CGGCGTGTTC TCGCAGAGCC GGCCCGACTC

CCCAATGATG ACATTATCTT TGGAGTTTTT GCACAAAGTA AACCAGACTC

TTAAATGATG ACATTCTCTA TGGGGTGTTT GCGCAGAGTA AGCCAGACTC

TGCCGAACCA ATGGATCGAT CTGCCATGTG TGCATTCCCT ATCAAATATG

TGCCGAACCA ATGGATCGAT CTGCCATGTG TGCATTCCCT ATCAAATATG

TGCCGAACCA ATGGATCGAT CTGCAATGTG TGCATTCCCT ATCAAATATG

TGCCGAACCA ATGGATCGAT CTGCAATGTG TGCATTCCCT ATCAAATATG

TGCCGAACCA ATGGATCGAT CGGCCGTGTG TGCATTCCCT ATCAAATATG

TGCTGAACCG ATGAATCGAT CAGCCGTCTG TGCATTCCCT ATCAAATACG

TGCTGAGCCC ATGAACCGAT CAGCGGTCTG TGCATTCCCC ATCAAATATG

TGCTGAACCT GTGAATCGAT CAGCAGTCTG TGCATTCCCC ATCAAATATG

CGCTGAACCC ATGAATCGAT CTGCCGTCTG TGCATTCCCT ATCAAATATA

CTCAGAACCA ATGAATCGTT CTGCTGTCTG TGCATTCCCT GTCAAATATG

TGCTGAACCA ATGAATCGCT CTGCCGTCTG TGCGTTCCCT ATCAAATATG

CGCCGAACCG ATGAATCGCT CCGCCGTCTG TGCGTTCCCG ATCAAGTTGG

TGCCGAGCCG ATGAACCGCT CTGCAGTCTG TGCATTCCCT ATCAAGTATG

TGCTGAACCA ATGAATCGAT CCGCTGTCTG TGCATTCCCG ATCAAATATG

AGCGGAACCA ATGAATCGAT CTGCCGTCTG TGCATTCCCA ATCAAATATG

GGCCGAGCCC ACCGACCGCT CCGCCCTCTG TGCCTTTCCT GTCAAATATG

TTCAGAACCA ACCAAGAGAT CAGCCGTCTG TGCATTTCCA ATCAAGTTTG

CGCAGAACCC ATGAACCGAT CTGCTATCTG CGCATTCCCC ATCAAATACA

TCAACGACTT CTTCAACAAG ATCGTCAACA AAAACAATGT GAGATGTCTC

TCAACGACTT CTTCAACAAG ATCGTCAACA AAAACAATGT GAGATGTCTC

TCAACGACTT CTTCAACAAG ATCGTCAACA AAAACAATGT GAGATGTCTC

TCAACGACTT CTTCAACAAG ATCGTCAACA AAAACAATGT GAGATGTCTC

TCAATGACTT TTTCAATAAG ATCGTCAACA AAAACAATGT GAGATGTCTC

TCAATGAATT CTTCAACAAG ATTGTCAACA AAAACAATGT GAGATGCCTC

TCAATGACTT CTTCAACAAG ATTGTCAACA AAAACAACGT ACGGTGTCTC

TCAATGACTT CTTCAACAAG ATTGTCAACA AAAACAACGT GAGATGTCTC

TCAATGAATT CTTCAACAAG ATCGTCAACA AAAACAACGT GAAATGTCTC

TCAATGAGTT CTTCAACAAA ATCGTCAACA AAAACAATGT GAGATGTCTC

TCAATGAATT CTTCAACAAG ATCGTCAACA AAAACAATGT GAGATGTCTT

TCAATGAATT CTTCAACAAG ATCGTCAACA AAAACAACGT GAGATGTCTC

TCAATGAATT CTTCAACAAG ATTGTCAACA AAAACAATGT GAGGTGTCTC

TCAACGAATT CTTCAACAAG ATCGTCAACA AAAATAACGT GAGATGTCTC

TCAATGACTT CTTCAACAAG ATTGTCAACA AAAACAATGT GAGATGTCTC

TCGATGAGTT CTTCCATAGG ATCGTCAACA AGAACAACGT CAGGTGTCTC

TCAACGATTT CTTTAACAAA ATTGTGAACA AGAATAACGT GAAATGTCTT

TCAATGAGTT CTTCAGCAAG ATTGTCAACA AGAACAACGT GAGGTGTCTG

CAGCATTTTT ACGGACCCAA TCATGAGCAC TGCTTTAATA GGACACTTCT

CAGCATTTTT ACGGACCCAA TCATGAGCAC TGTTTTAATA GGACACTTCT

CAGCATTTTT ATGGACCCAA TCATGAGCAC TGTTTTAATA GGACACTTCT

CAGCATTTTT ATGGACCCAA TCATGAGCAC TGTTTTAATA GGACACTTCT

CAGCATTTTT ACGGACCCAA TCATGAGCAC TGTTTCAATA GGACATTTCA

CAACATTTTT ATGGACCAAA TCATGAGCAC TGCTTTAACA GGACACTTCT

CAGCATTTTT ATGGACCCAA CCACGAGCAC TGTTTCAATA GGACCCTGCT

CAGCATTTTT ACGGACCCAA CCATGAGCAC TGTTTCAATA GGACCCTGCT

CAGCATTTTT ATGGACCCAA TCATGAGCAC TGCTTCAATA GGACACTTTT

CAGCACTTTT ATGGCCCCAA CCATGAACAC TGCTTCAATA GGACACTTTT

CAGCACTTTT ATGGACCCAA CCACGAACAC TGCTTTAATA GGACACTTTT

CAGCATTTTT ATGGACCGAA CCACGAGCCC TGCTTCAATA GGACGCTTCT

CAGCACTTCT ATGGACCCAA CCACGAGCAC TGCTTTAACA GA--------

CAGCATTTTT ATGGGCCCAA CCATGAACAC TGCTTTAACA GGACACTTTT

CAGCATTTTT ATGGACCCAA CCATGAACAC TGCTTTAATA GGACGCTGTT

CAGCACTTCT ACGGGCCCAA CCACCTGCAC TGCTTTAATC GGACACTTTT

CAGCATTTTT ATGGGAACAA TCACAAGCAC TGTTTTAATA GGACATTCTT

CAACACTTTT ATGGGAACCA CCACAGACAC TGTGCGAATA GGACTTTCCA

GAGAAATTCA TCAGGCTGTG AAGCGCGCCG TGATGAATAT CGAACAGAGT

GAGAAATTCA TCAAGCTGTG AAGCGCGCCG TGATGAATAT CGAACAGAGT

GAGAAATTCA TCAGGCTGTG AAGCGCGCCG TGATGAATAT CGAGCAGAGT

GAGAAATTCA TCAGGCTGTG AAGCGCGCCG TGATGAATAT CGAGCAGAGT

GAGAAATTTA CTAGGCTGTG AAGCACGCCA CGATGAATAT CGAACAGAGT

AAGAAATTCA TCAGGCTGTG AAGTACGCAG TGATGAATAT CGGACAGAGT

GAGAAATTCA TCGGGCTGCG AAGTGCGCAG TGACGAGTAC CGGACGGAGT

GAGAAACTCT TCCGGCTGTG AAGCGCGCAG TGACGAGTAT CGGACAGAGT

GAGGAATTCG TCAGACTGTG AGGCACGCAG CGATGAATAC CGTACAGAGC

GAGAAATTCA TCAGGATGTG AAGTGCGCAA TGATGAATAT CGAACGGAGT

GAGAAATTCA TCGGGCTGTG AAGCGCGCAA TGATGAATAT CGAACGGAGT

GAGAATTCCA TCAGGCTGTG AAGTGCGCAG TGATGAGTAT CGGACGGAGT

---------- ---------- ---------- ---------- ----------

GAGAAATTCG TCAGGCTGTG AAGTACGCAG TGATGAATAT CGAACGGAGT

GAGAAATTCA TCGGGCTGTG AAGTGCGCAG GGATGAATAT CGAACAGAGT

GAGAAACTCA TCCGGCTGTG AAGTGCGCAG CGATGAATAT CGGACAGAGT

AAGAAACTCA TCAGGTTGTG AAGCAAGAGA TGATGAATAT CGAACAGAAT

GTGGAATTCT TCGGGCTGCG AGGTGCGTGA TGACGAATAC CGCATGGAGT

TTACCACAGC TTTGCAGCGC GTTGACTTAT TCATGGGTCA ATTCAGCGAA

TTACCACAGC TTTGCAGCGC GTTGACTTAT TCATGGGTCA ATTCAGCGAA

TTACCACAGC TTTGCAACGT GTTGACTTAT TCATGGGTCA ATTCAGCGAA

TTACCACAGC TTTGCAGCGC GTTGACTTAT TCATGGGTCA ATTCAGCGAA

TTACCACAGC TTTGCAGCGC ATTGACTTAT TCGCAGGTCA ATTCAACAAA

TTACCACAGC TCTGCAACGT GTCGACTTAT TCATGGGCCA ATTCAACCAA

TTACCACGGC GCTGCAGCGT GTGGATTTAT TCATGGGCCG GCTCAACCAT

TTACCACGGC TTTGCAGCGC GTCGACTTAT TCATGGGCCG GCTTAACCAA

TGACCACCGC GCTGCAGCGT GTGGACTTAT TCATGGGCCA GTTCAACCAA

TTACCACAGC TTTGCCACGC GTTGACTTAT TCACGGGCCA GTTCAACCAA

TCACTACAGC TTTGCAGCGC GTTGACTTAT TCATGGGCCA GTTCAACCAA

TCACCACCGC TTTGCAGCGC GTCGACTTAT TCATGGGCCA GTTCAACCGA

---------- ---------- ---------- ---------- ----------

TTACCACGGC TTTGCAGCGT GTTGACTTAT TTATGGGCCA GTTCAACCAA

TTACCACAGC TTTGCAACGC GTTGACTTAT TCACGGGCCA GTTCAACCAG

TTACCACCGC TCTGCAGCGC ATTGACTTAT CCGCGGGCCA CTTCAGTCAA

TTACTACAGG CTTGCAGCGT ATTGACTTAT TCACAGGACA ATTCAAGCAA

TCACCACCCC TTTACAGCGC GTCGACCTGT CCATGGGGAA GTTCAGTCAT

GTCCTCTTAA CATCTATATC CACCTTCATT AAAGGAGACC TCACCATAGC

GTCCTCTTAA CATCTATATC CACCTTCATT AAAGGAGACC TCACCATAGC

GTCCTCTTAA CATCTATATC CACCTTCGTG AAAGGAGACC TCACCATCGC

GTCCTCTTAA CATCTATATC CACCTTCGTG AAAGGAGACC TCACCATCGC

GTCCTCTTAA CGTCTATATC CACCTTCATT AAAGGAGACC TCACCATTGC

GTCCTCTTAA CATCTATATC TACTTTCATT AAAGGCGACC TCACCATTGC

GTACTCTTGA CGTCTATCTC TACCTTCATC AAAGGTGACC TCACCATTGC

GTGCTCCTGA CATCCATCTC CACCTTCATC AAAGGTGACC TCACCATTGC

GTCCTCTTAA CATCCATCTC CACCTTCATT AAAGGCGACC TCACCATTGC

GTCCTCTTAA CATCTATATC CACCTTTATC AAAGGAGATC TCACCATCGC

GTCCTCTTAA CGTCTATATC CACCTTCATC AAAGGAGACC TCACCATTGC

GTCCTCCTGA CATCTGTGTC GACGTTCATT GCAGGCGACC TCACCATCGC

---------- ---------- ---------- ---------- ----------

GTCCTTCTAA CATCTATATC CACCTTCATT AAAGGTGACC TCACCATTGC

GTCCTCTTAA CATCCATATC CACCTTCATT AAAGGAAACC TCACGATTGC

GTCCTCTTAA CTTCCATATC CACCTTCATT AAGGGAGACC TCACGATTGC

GTCCTTTTAA CATCGATATC CACCTTTATC AAAGGAAACC TAACTATTGC

GTCCTCCTGA CGTCGATATC CACCTTCATC AAAGGGAACC TCACCGTGGC

TAATCTTGGG ACATCAGAGG GTCGCTTCAT GCAGGTTGTG GTTTCTCGAG

TAATCTTGGG ACATCAGAGG GTCGCTTCAT GCAGGTTGTG GTTTCTCGAG

TAATCTTGGG ACATCAGAGG GTCGCTTCAT GCAGGTTGTG GTTTCTCGAG

TAATCTTGGG ACATCAGAGG GTCGCTTCAT GCAGGTTGTG GTTTCTCGAG

TAATCTTGGG ACATCAGAGG GCCGCTTCAT CCAGATTGTG GTTTCTCGAG

TAATCTTGGG ACATCAGAGG GTCGCTTCAT GCAGATTATG GTTTCTCGAG

TAATCTAGGG ACATCAGAAG GTCGCTTCAT GCAGGTGGTG CTCTCTCGCG

TAATCTAGGG ACGTCAGAAG GTCGCTTCAT GCAGGTGGTG CTCTCTCGAG

TAACCTCGGG ACCTCAGAGG GTCGTTTCAT GCAGGTTGTG ATTTCTCGAA

TAATCTCGGG ACATCAGAAG GTCGATTCAT GCAGGTTGTG GTTTCTCGAG

TAATCTTGGG ACGTCCGAGG GTCGCTTCAT GCAGGTCGTG GTTTCTCGAG

CAACCTCGGG ACGTCGGAGG GCCGCTTCAT GCAGGTTGTG GTTTTTCGAG

---------- ---------- ---------- ----GTTGTG GTTTCCCGAG

TAATCTTGGG ACATCAGAGG GCCGTTTCAT GCAGGTTGTG GTCTCTCGAG

TAACCTTGGG ACCTCAGAGG GTCGTTTCAT GCAGGTTGTG GTTTCTCGAG

CAATCTCGGA ACGTCCGAGG GTCGCTTCAT GCAGGTTGTG GTTTCTCGAG

AAACCTTGGA ACCTCAGATG GCCGATTTAT GCAGGTTGTG GTTTCTCGAG

CAATCTTGGC ACCGCCGAGG GGCGATTCAT GCAGGTTGTG ATTTCTCGCG

GACCATCAAC CCCTCATGTG AATTTTCTCC TGGACTCCCA TCCAGTGTCT

GACCATCAAC CCCTCATGTG AATTTTCTCC TGGACTCCCA TCCAGTGTCT

GACCATCAAC CCCTCATGTG AATTTTCTCC TGGACTCTCA CCCGGTGTCT

GACCATCAAC CCCTCATGTG AATTTTCTCC TGGACTCTCA CCCGGTGTCT

TACCATCAAA CCCTCATGTG AATTTTCTCC TGGACTCCCA CCCCGTGTCT

GATCCTCAAC CCCTCATGTG AACTTTCACC TGGATTCCCA CCCTGTGTCT

CACATTTCAC CCCCCATGTG AATTTCCTCC TGGATTCCTA TCCTGTGTCT

CACACCTCAC TCCTCATGTG AACTTCCTCC TGGACTCCCA TCCTGTATCT

TACTGAACGT CCCTCATGTG AATTTTCGCC TGGATTCCCA CCCTGTGTCT

GATCATTAAC TCCTCATGTG AATTTTCACC TGGATTCCCA CCCTGTGTCT

GATTGTCGAC CCCTCACGTG AACTTCCGCC TGGACTCCCA CCCCGTGTCT

GATCATCAAC CCCTCATGTG AATTTTCGCC TGGATTCCCA CCCCGTGTCT

GATCATCAAC TCCTCATGTG AATTTCCTCC TGGATTCCCA CCCTGTGTCT

TATTGCCAGC CCCTCATGTG AATTTCCTCT TGGATTCCAA TCCTGTGTCT

GATTGACGAC ACCGCATGTG AACTTTCGCC TGGATTCCCA TGCTGTGTCC

GATCGTGGAC TCCTCACGTG GACTTCCGCC TGGACTCCCA TGCTGTGTCT

GACAATCAAC CCCTCATGTG AATTTTCAAC TGGATTCCCA TCCTGTTTCT

ATCCACCTTC CCCTCATGTG GATTTTCTTC TGGACAACCA CCCGGTCTCC

CCAGAAGTGA TTGTGGAGCA TACATTAAAC CAAAATGGCT ACACACTGGT

CCAGAAGTGA TTGTGGAGCA TACATTAAAC CAGAATGGCT ACACACTGGT

CCAGAAGTGA TTGTGGAGCA TCCATTAAAC CAGAATGGCT ACACACTGGT

CCAGAAGTGA TTGTGGAGCA TCCATTAAAC CAGAATGGCT ACACACTGGT

CCAGAAGTGA TCGTGGAGCA TCCGTTAAAC CAGAATGGCT ATACACTGGT

CCAGAAGTGA TCGTGGAGCA CACAGTGAAC CAGAATGGCT ACACGCTGGT

CCGGAAGTTA TTGTCGAACA TCCATCAAAT CAAAATGGCT ATACCTTGGT

CCAGAAGTTA TTGTTGAGCA TCCATCAAAT CAAAATGGCT ATACATTGGT

CCAGAAGTAG TTGTGGAGCA CCCGTTAAAC CAGAATGGCT ACACACTGGT

CCAGAAGTGA TTGTGGAGCA CCCGCTAAAC CAGAATGGCT ATACACTGGT

CCAGAAGCAA TTGTGGAGCA CCCACTAAAC CAAAACGGCT ACACACTCGT

CCAGAAATGA TTGTGCAGCA CCCATTAAAC CAAAATGGCT ACACACTGGT

CCAGAAGTGA TTGTAGAACA CTCCTCGGAA CAAGATGGTT ACACATTAGT

CTAGAAGCAC TTGTGGAGGA CCCAGTAAAC CAAAACGGCT ACACACTGGT

CCTGAGGTTA TTTTGGAACA CCCATTAAAC CAAAATGGCT ACACGCTGGT

CCG------- ---------- ---------- ---------- ----------

CCAGAAGTGA TTGTTCAACA TCCTCTAAAT ACAGATGGCT TCACATTGGT

CCCGAAGTCA TCGTGGTCAA TTCTTCAATG CCAGATGGCT ACACGCTGGT

TATCACTGGG AAGAAGATCA CGAAGATCCC ATTGAATGGC TTGGGCTGCA

TGTCACTGGG AAGAAGATCA CGAAGATCCC ATTGAATGGC TTGGGCTGCA

TGTCACGGGG AAGAAGATCA CCAAGATCCC ATTGAATGGC TTGGGCTGCA

TGTCACGGGG AAGAAGATCA CCAAGATCCC ATTGAATGGC TTGGGCTGCA

TGTCACTGGG AAGAAGATCA CCAAGATCCC ATTGAATGGT TTGGGCTGCA

TGTCACCGGG AAGAAGATCT CCAAGATCCC ACTGAATGGC TTGGGCTGCG

GGTCACAGGG AAGAAGATCA CCAAGATTCC ACTGAATGGC CTAGGCTGTG

TGTCACAGGA AAGAAGATCA CCAAGATTCC ATTGAATGGC CTGGGCTGTG

GGTCACAGGA AACAAGATTA CCAAAATCCC ATTGAATGGG TTGGGCTGTG

TGTCACTGGG AAGAAGATCA CCAAGATCCC GCTGAATGGC CTGGGCTGTG

TGTCACTGGG AAGAAGATCA CCAGGATCCC ACTGAATGGC TTAGGCTGTG

CGTCACTGGG AAGACGATCA CCAAGATCCC GCTGAATGGG CTAGGCTGTG

TGTCACGGGG AAGAAGATCA CCAAGATCCC GCTGAACGGC CTGGGCTGTG

TGTCACTGGG AAGAAAATAA CCAGGATCCC ATTGGATGGC TTGGGCTGTG

TATCACTGGG AAGAAGATCA CCAAGATCCC GTTGGATGGC TTGGGCTGCG

---------- ------ATAA CCAAGATCCC GCTGGACGGC TTGGGCTGCG

TATCACTGGG AAGAAGATAA CCAAGATCCC ACTGAATGGC CTAGGCTGTG

CATCACGGGC AAGAAGATAA CGAAGATCCC ACTGAATGGC CTTGGCTGTG

GACATTTCCA GTCCTGCAGT CAATGCCTCT CTGCCCCACC CTTTGTTCAG

GACATTTCCA GTCCTGCAGT CAGTGCCTCT CTGCCCCACC CTTTGTTCAG

GACATTTCCA GTCCTGCAGT CAGTGCCTCT CTGCCCCACC CTTTGTGCAG

GACATTTCCA GTCCTGCAGT CAGTGCCTCT CTGCCCCACC CTTTGTGCAG

GACATTTCCA GTCCTGCAGT CAGTGCCTCT CTGCTCCTTC CTTTGTGCAG

AACATTTCCA GTCCTGCAGT CAGTGTCTGT CCGCTCCTCC CTTTGTCCAG

GGCATTTCCA GTCCTGCAGT CAGTGTCTCT CTGCCCCCTA CTTTATACAG

GACATTTCCA ATCCTGCAGT CAGTGCCTCT CTGCCCCTTA CTTTATACAG

GACATTTCCA GTCCTGCAGT CAGTGTCTCT CCGCCCCTCC CTTTGTGCAA

AACATTTCCA GTCCTGCAGT CAGTGCCTCT CAGCCCCTTC CTTTGTGCAA

AGCATTTTCA GTCCTGCAGT CAGTGTCTCT CCGCCCCTCC CTTTGTGCAG

AACACTTCCA GTCCTGCAGC CAGTGTCTCT CCGCCCCTCC CTTTGTGCAG

AGCATTTTCA GTCCTGCAGT CAGTGTCTCT CTGCCCCTGC CTTTGTGCAG

GTCACTTCCA GTCCTGCAGT CAGTGTCTCT CCGCCCCTCC CTTCGTGCAG

ACCATTTCCA GTCCTGCAGT CAGTGTCTCT CCGCCCCTTC CTTTGTGCAG

AGCACTTCCA GTCCTGCAGC CAGTGTCTCT CTGCCCCGCC CTTTGTGCAG

ATCATTTCAA ATCCTGCAGT CAGTGCCTCT CAGCCCCTTC TTTTGTGCAG

ATCACTTCAA GTCCTGCAGG CAGTGTCTGT CGGCTCCCTC TTTCGTGCAG

TGTGGCTGGT GCCACGACAA ATGTGTGCGA TCGGAGGAAT GCCTGAGCGG

TGTGGCTGGT GCCACGACAA ATGTGTGCGA TCGGAGGAAT GCCTGAGCGG

TGTGGCTGGT GCCACGACAA ATGTGTGCGA TCGGAGGAAT GCCCCAGTGG

TGTGGCTGGT GCCACGACAA ATGTGTGCGA TCGGAGGAGT GCCCCAGTGG

TGTGGCTGGT GCCACGATAA ATGTGTGCGA TCAGAGGAAT GCTCCAGCGG

TGTGGCTGGT GCCACGATAA ATGTGTGCGA TCAGAGGAAT GCCCCAGCGG

TGTGGCTGGT GCCACAATCG GTGTGTGCAT TCCAATGAAT GCCCCAGCGG

TGTGGCTGGT GCCACAATCA ATGTGTGCGT TTTGATGAAT GCCCCAGCGG

TGTGGCTGGT GCCATGACAA GTGTGTGCCG TCAGAGGAAT GCCCCAGCGG

TGTGGCTGGT GCCATGATAA ATGTGTGCGA TTGGAGGAAT GCTCCAGTGG

TGTGGCTGGT GCCACGATAG ATGTGTGCAC CTGGAGGAAT GTCCCACTGG

TGTGGCTGGT GCCACAACAA ATGTGTGCGA TCGGAGGAAT GCCCCAGTGG

TGTGGCTGGT GCCAGGATAA ATGTGTGCAA GTGGAGGGAT GCCCCAGTGG

TGTGGCTGGT GCCAGGATAA ATGTGTGCAA GTGGAGGAAT GTCCCAATGG

TGTGGCTGGT GCCACAATAA GTGTGCGCGA GCAGAGGAAT GCCCCAACGG

TGTGGCTGGT GCCACGATAA GTGTGCCCGA GCCGAGGACT GCCCCAACGG

TGCACTTGGT GCCATAATCA ATGTGTGAAA TCTGAGGAAT GTGTCACCGA

TGCGGTTGGT GTGACGACCG GTGCACTACT GCAGAGAAGT GCGATTATGG

GACATGGACT CAACAGATCT GTCTGCCTGC AATCTACAAG GTTTTCCCAA

GACATGGACT CAACAGATCT GTCTGCCTGC AATCTACAAG GTTTTCCCAA

GACATGGACT CAACAGATCT GTCTGCCTGC AATCTACAAG GTTTTCCCAA

GACATGGACT CAACAGATCT GTCTGCCTGC AATCTACAAG GTTTTCCCAA

GACATGGACT CAAGAGACCT GTCTGCCTAC AATCTACAAG GTTTTCCCAA

GTCGTGGACG CAAGAGACCT GCCGGCCTGC GGTCCACAAG GTTTTCCCAA

TACATGGACT CAAGAGATCT GTCTGCCAGC AGTTTATAAG GTTTTCCCCA

TACATGGACT CAAGAGATCT GTCTGCCGGC GGTTTATAAG GTGTTCCCCA

GACGTGGACT CAAGAGATCT GTCTGCCTGC AATCTACAAG GTTTTCCCAG

AACGTGGACT CAAGAGACCT GTCTGCCAAC AATCTACAAG GTTTTTCCAA

AGCGTGGACT CAGGAGGTCT GTCTGCCTGC AATCTATGAG GTTTTCCCAA

AGTATGGACT CAAGACGTCT GTCATCCTAC AATCTACGAG GTTTTCCCGA

AACGTGGACT CAAGAGACCT GTCTGCCGAC AATCTACAAG GTTTTCCCAA

GACATGGACT CAAGAGATCT GTCTGCCTAC AGTTTATGAG GTTTTCCCAA

GATGTGGACT CAAGAGATCT GTCTGCCGAC GATTTATGAG GTTTTCCCAA

GACATGGACT CAAGAGATCT GCCTGCCGAC GATTTATGAG ----------

GCTAGGGAAT CAGGATATTT GTCTGCCTAC CATTTATGAG ATTCTCCCAA

GCTGTGGACC CAAGACACCT GCCCACCCAC AATTTTTGAG ATAATCCCCA

ATAGTGCACC CCTTGAAGGA GGGACAAGGC TGACCATATG TGGCTGGGAC

ATAGTGCACC CCTTGAAGGA GGGACAAGGC TGACCATATG TGGCTGGGAC

CTAGTGCACC CCTTGAAGGA GGGACAAGGC TGACCATATG TGGCTGGGAC

CTAGTGCACC CCTTGAAGGA GGGACAAGAC TGACCATATG TGGCTGGGAC

CTAGTGCACC CCTTGAAGGA GGGACAAGGC TGACCATATG TGGCTGGGAC

CTAGCGCACC CCTGGAAGGG GGAACAAGGC TGACTATATG TGGCTGGGAC

CTAGTGCACC CCTCGAAGGA GGAACAATGC TGACCATATG TGGCTGGGAC

CCAGCGCGCC CCTTGAAGGA GGAACAGTGT TGACCATATG TGGCTGGGAC

CCAGTGCACC CCTTGAGGGA GGGACCACAC TGACCATCTG TGGCTGGGAC

CTAGTGCCCC CCTTGAAGGA GGGACAACAC TGACTGTTTG TGGCTGGGAC

CTAGTGCACC CCTGGAAGGA GGGACAGTGC TGACTGTATG TGGCTGGGAC

CTACTGCCCC CCTGGAAGGA GGGACAACGC TGACCATATG TGGCTGGGAC

CCAGTGCACC TCTTGAAGGA GGAACAACAT TGACAGTATG TGGCTGGGAC

CCAGTGCACC CCTTGAAGGA GGGACAATGC TGACCATCTG TGGTTGGGAC

CTAGTGCACC CCTTGAAGGA GGGACAACGC TGACAGTATG TGGCTGGGAC

---------- ---------- ---------- ---------- ----------

CTAGTGCACC CCTGGAGGGT GGTACAACTT TGACGGTGTG TGGCTGGGAT

GCAGTGCACC CCTGGAAGGA GGAACCATGC TGACTGTGTG TGGCTGGGAC

TTTGGATTTC GGAGGAATAA TAAATTTGAT TTAAAGAAAA CTAGAGTTCT

TTTGGATTTC GGAGGAATAA TAAATTTGAT TTAAAGAAAA CTAGAGTTCT

TTTGGATTTC GGAGGAATAA TAAATTTGAT TTAAAGAAAA CTAGAGTTCT

TTTGGATTTC GGAGGAATAA TAAATTTGAT TTAAAGAAAA CTAGAGTTCT

TTTGGATTTC GGAGGAATAA TAAATTCGAT TTAAAGAAAA CTAGAGTTCT

TTTGGATTCC GGAGGAATAA TAAATTTGAC TTAAAGAAAA CCAGAGTTCT

TTTGGATTCA AGAAGAATAA TAAATTTGAT TTAAGGAAAA CCAAAGTTCT

TTTGGATTCA GGAAGAATAA TAAATTTGAT TTAAGGAAAA CCAAAGTTCT

TTTGGGTTCA GGAAGAATAA TAAATTTGAT TTAAAGAAAA CCAAAGTCCT

TTTGGATTCA AGAGGAATAA TAAATTTGAT TTGAAGAAAA CCAGAGTTCT

TTCGGATTCA GGAGGAATAA TAAATTTGAT TTAAAGAAAA CCAAAGTTTT

TTTGGATTCA GGAGGAATAA TAAATTTGAT TTAAAGAAAA CCAGAATTCT

TTTGGATTCC GGAGGAATAA TAAATTTGAT CTAAAGAAAA CCAAGGTTCT

TTTGGATTCA GGAGGAATAA TAAATTTGAT TTAAAGAAAA CTCGAGTTCT

TTTGGATTCA GGAGGAATAA TAAATTTGAT TTAAAGAAAA CCAGAGTTCT

---------- ---------- ---------- ---------- ----------

TTTGGATTAA GAAAAAATAA TAAATTTGAT TTTAGAAAAA CCAAAGTTCT

TTCGGATTCC AAAAGAATAA CAAGTTTGAG CTGAGGAAAA CCAAGGTTCT

CCTTGGAAAT GAGAGCTGCA CCTTGACTTT AAGTGAGAGC ACGATGAATA

CCTTGGAAAT GAGAGCTGCA CCTTGACTTT AAGTGAGAGC ACGATGAATA

CCTTGGAAAT GAGAGCTGCA CCTTGACTTT AAGTGAGAGC ACGATGAATA

CCTTGGAAAT GAGAGCTGCA CCTTGACTTT AAGTGAGAGC ACGATGAATA

CCTTGGAAAT GAGAGCTGCA CCTTGACCTT AAGTGAGAGC ACAATGAATA

CCTTGGAAAT GAGAGCTGTA CCTTGACCTT GAGTGAGAGC ACCACAAACA

GCTTGGCAAC GAGAGCTGTA CCTTGACCTT AAGCGAGAGC ACGACAAATA

GCTTGGCAAC GAGAGCTGTA CCTTGACCTT AAGCGAGAGC ACGACAAATA

CCTTGGAAAT GAAAGCTGTG CCTTGAACTT AAGTGAGAGC ACGACAAATA

CCTTGGCAAT GAGAGCTGCA CCTTAACCTT AACTGAGAGC ACAACAAACA

CCTTGGAAAT GAGAGCTGCA CCTTGACCTT AAGTGAGAGC ACAACAAATA

GCTTGGAAAT GAGAGCTGCA CCTTGACCTT AAGTGAGAGT ACAGCAAATA

CCTTGGAAAT GAGAGCTGCA CGTTAACCTT GAGTGAGAGC ACAACAAATA

CGTTGGAAAT GAAAGTTGCA CCTTGACCTT AAGCGAGAGC ACAACAAATA

CATTGGAAAT GATAGCTGCA CCTTGACCTT GAGCGAGAGC ACAACAAATA

---------- ---------- ---------- ---------- ----------

CATTGGAAAT GAGAGCTGCA TATTGAATAC AAGTGAGAGC ACTACCAATA

CGTCGGCAAC CAGAGCTGCA CTCTGTTCTT CAACGAGAGC TCCACAAACA

CATTGAAATG CACAGTTGGT CCTGCCATGA ATAAGCATTT CAATATGTCC

CATTGAAATG CACAGTTGGT CCTGCCATGA ATAAGCATTT CAATATGTCC

TATTGAAATG CACAGTTGGT CCTGCCATGA ATAAACATTT CAATATGTCC

CATTGAAATG CACAGTTGGT CCTGCCATGA ATAAACATTT CAATATGTCC

CATTGAAATG CACAGTTGGT CCTGCCATGA ATGAGCGTTT CAATATGTCC

CATTGAAATG CACAGTTGGT CCTGCAATGA ATGAGCACTT CAATATGTCC

CGTTGAAATG CACAGTTGGC CCCGCGATGA GTGAGCACTT CAATGTGTCT

CGTTGAAATG CACAGTTGGT CCCGCGATGA GTGAGCACTT CAATGTGTCT

CGTTGAAATG CACAGTTGGT GCTACAACGC ATGAGCATTT CAATATGTCC

TGTTGAAATG CACAGTTGGT CCTGCAATGA ATGAACATTT CAATATGTCC

TGCTGAAATG CACAGTTGGC CCTGCAGTGA ACGAGCATTT CAATATATCC

CGTTGAAATG CACAGTTGGT CCTGCAATGA GCGAGCACCT CAATCTGTCC

TGTTAAAATG CACAGTTGGT CCTTCAATCA ATGAACATTT TAATATGTCA

CGTTGAAATG CACGGTTGGT CATGCAATGA ATGAGAATTT TAATATGTCC

CGTTGAAATG CACAGTAGGT CCTGCAATGA ATAAGCATTT CAATCTGTCC

---------- ---------- ---------- ---------- ----------

AGTTGAAATG TACTGTTGGT CCTGCTAAGA ATCTGCATTT CAATGTATCC

AGCTGAAATG CACTGCCGGG CCTTCCACCC AGCCCCGCCT CAACATCTCC

ATAATTATTT CAAATGGCCA CGGGACAACA CAATACAGTA CATTCTCCTA

ATAATTATTT CAAATGGCCA CGGGACAACA CAATACAGTA CATTCTCCTA

ATAATTATTT CAAATGGCCA CGGGACAACA CAATACAGTA CATTCTCCTA

ATAATTATTT CAAATGGCCA TGGGACAACA CAATACAGTA CATTTTCCTA

ATAATTATTT CAAACGCCCA CGGGACAACA CAATACAGTA CATTTTCCTA

ATAGTTATTT CAAATGGTCG GGAGACCACA CAATATAGTA CATTTTCCTA

GTGATCGTCT CAAACAGTCG AGAGACAACA CAGTACAGTG CGTTTTCCTA

GTAATTATCT CAAACAGTCG AGAGACGACG CAATACAGTG CATTCTCCTA

ATAACTGTTT CAAACAGTCG AGGGAGAACA CAGTATAGTA CATTTTCTTA

ATAGTTATTT CAAACAGCCG TGGGTCAGTA GAGTATAGTG CGTTTTCCTA

ATAATTATTT CAAATGGTCG AGGGACAGCA CAATATAGTA CATTTTCGTA

ATAATTATTT CAAGCGGTCG AGGCACAGCA CAATATACTG CATTTTCTTA

CTAACTGTTT CAAATGGTCG AGAGGCTGCA CAATATAGTA CATTTTCTTA

ATAATCATTT CAAATGGTCG AAGGACAGTG CATTATAATG CATTTTCCTA

ATCATTATTT CAAATGGTCG AGGGACAGCA CGATATAGGA CATTTTCCTA

---------- ---------- ---------- ---------- ----------

ATCTCTGTTT CAAATGGTCG AGAAGCAACA CAATTTAATA CATTCTCTTA

ATCAGTGTTT CCAATGCCCA AGGCGGAAGA CGATCCAAAA CGTTCTCGTA

TGTGGATCCT GTAATAACAA GTATTTCGCC GAAATACGGT CCTATGGCTG

TGTGGATCCT GTAATAACAA GTATTTCGCC GAAATACGGT CCTATGGCTG

TGTGGATCCT ATAATAACAA GTATTTCGCC AAAATATGGC CCTATGGCTG

TGTGGATCCT ATAATAACAA GTATTTCGCC AAAATATGGC CCTATGGCTG

TGTGGACCCT ATAATAACAA GTATTTCCCC GAGATACGGT CCTATGTCTG

TGTGGATCCC GTAATCACAA GTATTTCTCC GAGTTATGGT CCTAAGGCCG

TGTGGATCCT GTAATAACAA GTATTTCTCC AAGGTATGGT CCTCATGCCG

TGTAGATCCT GTAATAACAA GCATTTCTCC GAGGTACGGC CCTCAGGCTG

TGTGGCTCCT GTAATAACAA GTATTTCTCC AAGTTATGGC CCCAAGGCTG

TGTGGATCCT ATAATAACAA GTATTTCTCC AAATTATGGT CCAAAAACTG

TGTGGATCCT ATTATAACAA GTATTTCTCC AAGTTATGGT CCCAAGAATG

TGTGGATCCT ATCATAACAA GTGTTTCTCC AAGTTACGGT CCTAAGACTG

TGTTGATCCC ATAATAACAA GTATTTCTCC AAGTTATGGC CCTCAGACTG

TGTGGATCCT ATAATAACAA GTATTTTTCC AAAGTATGGT CCTAAGGCTG

TGTGGAACCT GTAATTACGA GTATTTCTCC AAGTTATGGT CCTAAGGCTG

---------- ---------- ---------- ---------- ----------

TGTGGATCCT ATAATAACAA ATATTTCTCC AAGCTATGGT CCCAAGGCTG

CGTGGACCCT AAGATAACCA GTATTTCTCC CCGCTATGGT CCCATAGCTG

GTGGCACTTT ACTTACTTTA ACTGGAAATT ACCTAAACAG TGGGAATTCT

GTGGCACTTT ACTTACTTTA ACTGGAAATT ACCTAAACAG TGGGAATTCT

GTGGCACTTT ACTTACTTTA ACTGGAAATT ACCTAAACAG TGGGAATTCT

GTGGCACTTT ACTTACTTTA ACTGGAAATT ACCTAAACAG TGGGAATTCT

GTGGCACTTT ACTTACTTTA ACCGGAAATT ACCTAAACAG TGGGAATTCT

GTGGTACTTT ACTTACGTTA ATTGGAAAGT ACCTAAACAG TGGGAATTCG

GAGGCACCTT ACTCACTTTG ACTGGAAAAT ACCTCAACAG CGGCAATTCT

GAGGCACCTT ACTCACTCTT ACTGGGAAAT ACCTCAACAG TGGCAATTCT

GTGGCACTTT ACTCACTTTA ACTGGAAAAT ACCTGGACAG TGGAAATTCC

GTGGCACTTT ACTTACATTA ACTGGAAAGC ACCTAAACAG TGGAAATTCA

GTGGCACCTT GCTCACTTTA ACTGGAAAAT ACCTCAACAG TGGGAATTCT

GTGGCACTTT ACTTACTTTA ACTGGAAAGT ACCTAAACAG TGGGAATTCT

GCAGTACTTT ACTTACTTTA ACTGGAAAAT ACCTCAACAG TGGAAATTCT

GTGGCACTTT ACTTACCTTA ACTGGAAAGT ACCTAAACAG TGGCAATTCT

GAGGCACTTT GGTTACTTTA ACTGGAAAGT ACCTAAACAG TGGGAACTCT

---------- ---------- ---------- ---------- ----------

GTGGCACATT ACTTACCTTA ACTGGGAAAT ATCTAAACAG TGGAAATTCT

GAGGAACTTT ACTCacctta actgggaagt acctgaactg tggaagtTCC

AGACACATTT CAATTGGTGG AAAAACATGT ACTTTAAAAA GTGTGTCAAA

AGACACATTT CAATTGGTGG AAAAACATGT ACTTTAAAAA GTGTGTCAAA

AGACACATTT CAATTGGTGG AAAAACGTGT ACTTTAAAAA GTGTGTCAAA

AGACACATTT CAATTGGTGG AAAAACATGT ACTTTAAAAA GTGTGTCAAA

AGACACATTT CAATTGGTGG AAAAACATGT ACTTTAAAAA GTGTGTCAAA

AGACACATTT CAATTGGTGG AAAAACATGT ACTTTAAAAA GTGTGTCAAA

AGACACATTT CAATCGGAGG GAAAACATGT ACTTTAAAAA GTGTATCAGA

AGACACATTT CAATTGGAGG GAAAACATGT ACTTTAAAAA GTGTATCAGA

AGACACATTT CAATTGGTGG AAAAACATGT ACTTTAAAAA GCATGTCAAA

AGACACATTT CAATTGGTGG AAAAACATGT ACTTTAAAAA GTGTTTCACA

AGACACATTT CAATGGGTGG AAAAACATGT ACTTTAAAAA GTGTGTCAGA

AGACACATTT CAATTGGTGG AAAAACATGT TCTTTAAAAA GTGTGTCCGA

AGACACATTT CAATTGGTGG AAAGACGTGT ACCTTAAAAA GTGTGTCAGA

AGACATATTT CAATTGGTGG AAAAACATGT ACTTTAAAAA GCGTGTCAAA

AGACACATTT CAATTGGTGG AAAAACGTGT ACTTTAAAAA GTGTGTCAGA

---------- ---------- ---------- ---------- ----------

AGATGTATTT CAATTGGTGG AAAATCGTGT ATTTTAAAAA GTGTGTCTCA

AGACACATTT CCATCGGCGG AAAAACATGC ACCTTGAGGA GTGTGTCGGA

CAGTATTCTT GAATGTTATA CCCCAGCCCA AACCATTTCA ACTGAGTTTG

CAGTATTCTT GAATGTTATA CCCCAGCCCA AACCATTTCA ACTGAGTTTG

CAGTATTCTC GAATGTTATA CCCCAGCCCA AACCATTTCA ACTGAGTTTG

CAGTATTCTC GAATGTTATA CCCCAGCCCA AACCATTTCA ACTGAGTTTG

CAGTATTCTT GAATGTTATA CCCCAGCCCA AAGCATTTCA ACTGAGTTTC

TAGTATTCTT GAATGTTATA CCCCTGCCCA AATCATTTCA ACTGAGTTTC

TAGCATTCTC GAATGCTACA CCCCAGGCCA CACCGTCTCT GCCGAGTTTC

TAGTATTCTT GAATGCTACA CCCCAGCCCA AACTACCTCT GATGAGTTTC

CAGTATTCTT GAGTGTTATA CTCCAGCCCA AACCATTTCA ACTGAATTTC

TAGTATTCTC GAATGTTATA CCCCAGCCCA AAGTGCTCCA ACTGAGTTTT

TAGTATTCTC GAATGTTATA CCCCAGCTCA AGCCACTGCA ACTGAGTTTC

GAGTGTTCTT GAATGTTATA CCCCAGCCCA AACCACTACA ACTGAGTTTC

TAGCACTCTC GAGTGTTATA CCCCAGCCCA AACCACTTCA ACTGAGTTTC

TAGTATTCTC GAATGTTATA CCCCAGCCCA AATCATTTCA ACTGAGTTTC

TAGTGTTCTC GAATGTTATA CCCCAGCCCA AAGCATTTCA ACTGACTTTC

---------- ---------- ---------- -----TTTCA GCTGACTTTC

TAGCAGTCTA GAATGCTACA CTCCTCCTCA AACCATTCCA AATGAGTACC

ACACGTTCTA GAATGCTACA CGCCTTCCCA GACCGTACCC ACCCTCTATG

CTGTTAAATT GAAAATTGAC TTAGCCAACC GAGAGACAAG CATCTTCAGT

CTGTTAAATT GAAAATTGAC TTAGCCAACC GAGAGACAAG CATCTTCAGT

CTGTTAAATT GAAAATTGAC TTAGCCAACC GAGAGACAAG CATCTTCAGT

CTGTTAAATT GAAAATTGAC TTAGCCAACC GAGAGACAAG CATCTTCAGT

CTGTTAAATT GAAAATTGAC TTAGCCAACC GAGAGACAAG CATCTTCAGT

CTGTTAAATT GAAAATTGAC TTAGCCATCC GAGAGGCGAG CAGCTTCAGT

CCGTGAAATT GAAAATCGAC CTGGCTGACC GAGTGACAAG CAGCTTCAGT

CTGTGAAATT GAAGATTGAC TTGGCTAACC GAGAGACCAG CAGCTTCAGT

CTGTTAAATT GGAAATCGAC TTAGCCAGCC GAGAAACCAG CAGCTTCAGT

CTGTTAAGTT GAAAATTGAC TTAGCCAACC GAGAGGTGAA CAGCTTCATT

CTATTAAATT GAAAATTGAC CTAGCCAACC GAGAGATGAA CAGCTTCAGT

CTATTAAATT AAAAATTGAC TTAGCCAACC GAGAGACGAA CAGCTTCAAT

CAGTTAAACT GAAAATTGAC TTAGCCAACC GAGAGACGGA CAGCTTCAGT

CTATTAAATT GAAAATTGAC TTAGCCAACC GAGAGGCCTC TAGCTTCAGT

CCGTCAAACT GAAAATTGAC TTAGCCAACC GAGAGGCCTA CAGCTTCAGT

CTGTTAAGTT GAAGATTGAC TTAGCCAACC GAGAGGCCTA CAGCTTCAGT

CTATTAAAAT GAAAATTGAC TTAGCCAATC GAGAAGCCTA CAGTTTTACA

CGGTTAAGAT GAAGATCGAC TTGGCCAATC GCGAAGCCGG CACTTTCATC

TACCGTGAAG ATCCCATTGT CTATGAAATT CATCCAACCA AATCTTTTAT

TACCGGGAAG ATCCCATTGT CTATGAAATT CATCCAACCA AATCTTTTAT

TACCGGGAAG ACCCCATTGT CTATGAAATT CATCCAACCA AATCTTTTAT

TACCGGGAAG ACCCCATTGT CTATGAAATT CATCCAACCA AATCTTTTAT

TACCGGGAAG ACCCTATTGT CTATGAAATT TATCCAACCA AATCTTTTGT

TACCAGGAAG ATCCCATCGT CTATGAAATT CATCCAATGA AATCTTTTGT

TACCGGGAAG ACCCGGTTGT CTCTGAAATC CACCCGACCA AATCTTTTAT

TACCGGGAAG ACCCCGTTGT CTATGAAATC CACCCGACCA AATCTTTTAT

TACCGGGAAG ACCCCATTGT GGATGAATTT TATCCAACCA AATCTTTTAT

TACCGGGAAG ACCCCATTGT CTATGAAATA CATCCCACCA AATCTTTTAT

TACCAGGAAG ACCCCATTGT CTATGCAATT CATCCAACGA AATCTTTTAT

TACCGGGAAG ACCCCATTGT CGATGAAATT CACCCAACCA AATCTTTTAT

TACCGGGATG ACCCCATTGT CTATGAAATT CACCCAACCA AATCTTTTAT

TACCGAGAAG ACCCCATTGT CTATGAAATT CATCCAACCA AATCTTTTCT

TACCAGGAAG ACCCTACTGT CTATGAAATC CATCCAAACA AATCTTTTAT

TACCAGGAAA ATCCCCTTGT CGTTGAAATC CATCCAACCA AATCTTTTGT

TACAAAGAGG ACCCCGTTGT TCATGAAATC CATCCAACCA AATCTTTTCT

TACAAGGAGG ACCCCGTCAT CCATGAGATT CACCCAACCA AATCATTCCT

TAGTCCTCTC AACATTGTCA GTTTTCTATT TTGCTTTGCC AGTGGTGGGA

TAGTCCTCTC AACATTGTCA GTTTTCTATT TTGCTTTGCC AGTGGTGGGA

TAGTCTTCTC AATATTGTCA ATTTTCTATT TTTCTTTGCC AGTGGTGGGA

TAGTCCTCTC AATATTGTCA ATTTTCTATT TTGCTTTGCC AGTGGTGGGA

TAGTCCTCTC AATATTGTCA ATTTTCTATT TCGATTTGCC AGTGGTGGGA

TAGACCTCTT ATTATTATAA TTTTTCTGTT TTGTTTTGCC AGTGGTGGGA

CAGT-CTCTG AATATT-TCC GTTTTCCGTT TTATTTGACC AGTGGTGGAA

TAGC-CTCTG AATATT-CCT GTTTTCTATT TTGTTTTACC AGTGGTGGAA

TAGT-ACTTG TATTTT-TCT ATTTTTTTTT TTTTTTTGCC AGTGGTGGAA

TAGTCCTCTC A---TAGCTA TTTTTCTATT TTATTTTGCC AGTGGTGGGA

TAGTCCTCTC AATATTGCTA TTTTTCTGTT TTATTTTGCC AGTGGTGGGA

TAGTCCTCTT AATGTTGTTG TTTTTCTGTT TTATTTTGCT AGTGGTGGGA

CAGGCCTCTT CGTCATACTG CTTTTCTATT TTCCCTTACC AGTGGTGGGA

CAGTCCTCTC AATATAACCA TTTTTCAATT TTGTTTTACC AGCGGTGGCA

TAGTCGTCTC AATATTGCCA TTTTTCTATT TTATTTTGCC AGTGGTGGAA

CAGTCCTCTC ACCATCACCA TTTTCCTGTT TTATTTTGCC AGCGGTGGAA

TAG-TTTCTC AATATTATCA CTTTTCTATT TTATTTTATC AGTGGTGGAA

TAGTCTTCTT GATACTGCCA TGTTTCCATT TTGTTTTGCC AGTGGCGGAA

GCACAATAAC AGGTGTTGGG AAAAACCTGA ATTCAGTTAG TGTCCCGAGA

GCACAATAAC AGGTGTTGGG AAAAACCTGA ATTCAGTTAG TGTCCCGAGG

GCACAATAAC AGGTGTTGGG AAAAACCTGC ATTCAGTTAG TGTCCCGAGG

GCACAATAAC AGGTGTTGGG AAAAACCTGC ATTCAGTTAG TGTCCCGAGG

GCACAATAAC AGGTATTGGG AAAAACCTGA ATTCAGTTAG TGTTCCGAGG

GCACAATAAC AGGTGTTGGA AAAAACCTGA ATTCTGTTAG TGTGCCCAGG

GCACAATAAC GGGGATTGGA AAGAACCTGA ATTCAGTTAG CACCCCAAAG

GCACAATAAC GGGTATTGGG AAGACCCTGA ACTCGGTTAG CCTCCCAAAG

GCACCATAAC AGGTGTTGGA AAAAACCTGG ATTCAGTTAG TGTCCCAAGG

GTACAATAAC AGGTGTTGGA AAAAACCTGA ATTCAGTTAG TGTCCTAAGG

GCACAATAAC AGCTGTTGGA AAAAACCTGA ATTCAGTGAG TGTCCTGAGG

GCACAATAAC AGGTGTTGGA AAAAACCTGA ATTCAGTTAG TGGCCTGAGG

GCACAATAAC AGGTGTTGGA AAAAATCTGG ATTCGGTGAG TGTCCTGAGG

GCACAATAAC AGGTGTTGGG AAAAACCTGA ATTCAGTTAG TGTCACAAGG

GCACAATAAC AGGTATTGGA AAAAACCTGA ATTCAGTTAG TGTCCCAAGG

GCACAATAAC AGTTGTTGGA AAAAATCTGA ATTCAGTTAG TGTCCCAAGG

GCACAATAAC AGGTTTTGGG AAAAACCTAA ATTCTGCTAG TGTTCCCAAG

GCACAATCAC CGGATTTGGG AAAAACCTCA ACGGATCCAG TGCTCCTAAG

ATGGTCATAA ATGTGCATGA AGCAGGAAGG AACTTTACAG TGGCATGTCA

ATGGTCATAA ATGTGCATGA AGCAGGAAGG AACTTTACAG TGGCATGTCA

ATGGTCATAA ATGTGCATGA AGCAGGAAGG AACTTTACAG TGGCATGTCA

ATGGTCATAA ATGTGCATGA AGCAGGAAGG AACTTTACAG TGGCATGTCA

ATGGTCATAA ATCTGCATGA AGCAAAAAGG AACTTTACTG TGGCATGTCA

ATGGTGATAC ATGTGCGTGA AGCAGGAAGG GACTTTACAG TGGCGTGTCA

CTGGTAATAG AAGTGCATGA CGTGGGCGTG AACTACACCG TGGCGTGCCA

CTGGTAATAG ATGTGCATGA AGTGGGTGTG AACTACACAG TGGCATGTCA

ATGGTAATAA GTGTGCACGA AGCAGGAAGT AACTTCACAG TGGCATGTCA

ATGGTAATAA ATGTGCATGA AGCCGGAAGG AACTTTACAG TGGCATGTCA

ATGGTAATAG ATGTCCATGA AACAAGAAGG AACTTTACAG TGGCATGTCA

ATGGTAATAA GTGTGCGTGA AGCAGGAAGG AACTTTACTG TGGCATGTCA

ATGGTGATAA CCGTACACGA AGCAGGAAGG AACTTTACAG TGGCATGTCA

ATGGTCATAA ATGTCCATGA AGCAAGAAAG AATTTTACAG TGGCCTGTCA

ATGGTAATAA ATGTCCAGGA AGCAGGAAGG AACTTTACAG TG--------

ATGATCATAA ACGTCCATGA AGCGGAAATG AACTTCACAG TGGCATGTCA

ATGGTAATAA ATGTTCCTCA AGTAGAAAGA AACTTCACAG TGGCATGTAA

ATGATCATCC AAGTCGCAGA AGTGGGCAGG AACTTCACAG TAGCATGTCA

ACATCGCTCT AATTCAGAGA TAATCTGTTG TACCACTCCT TCCCTGCAAC

ACATCGCTCT AATTCAGAGA TAATCTGTTG TACCACTCCT TCCCTGCAAC

GCATCGCTCT AATTCAGAGA TAATCTGCTG TACCACTCCT TCCCTGCAAC

GCATCGCTCT AATTCAGAGA TAATCTGCTG TACCACTCCT TCCCTGCAAC

GCATCGCTCT AATTCAGAGA TAATCTGCTG TACCACTCCT TCCCTGCAAC

GCATCGCTCT AATGCAGAGA TCCTCTGCTG CACCACTCCT TCACTGCAAC

ACATCGCTCG AGTTCAGAGA TCATCTGCTG CACCACTCCT TCCCTGCAAC

GCATCGCTCA AATTCAGAGA TCATCTGCTG CACTACTCCT TCACTGAAAC

GCATCGGTCC AACTCAGAGA TCATCTGCTG CACCACTCCT TCACTGCAAC

ACATCGCTCT AATTCAGAGA TAATCTGCTG TACAACTCCT TCAATAGAAC

ACATCGCTCT AATTCAGAGA TAATCTGTTG TACGACTCCT TCACTGCAAC

ACACCGCTCT AATTCAGAGA TAATCTGCTG TACAACTCCT TCACTCCAAC

ACATCGCTCT AATTCAGAGA TAATCTGCTG TACCACTCCT TCATTGCAAC

ACATCGCTCT AATTCAGAGA TAATGTGCTG TACAACTCCT TCGCTGCAAC

---------- ---------- ---------- ---------- ----------

ACAGCGCTCT AATTCAGAGC TAATCTGCTG TACGACTCCT TCACTGCAAC

ACAGCGCTCT AATTCAGAGA TAATATGTTG TTCAACTCCT TCCTTACAAC

AAGTCGTTCT AATGCAGAAA TAATCTGTTG TACGACTCCT TCACTGCAGC

AGCTGAATCT GCAACTCCCC CTGAAAACCA AAGCCTTTTT CATGTTAGAT

AGCTGAATCT GCAACTCCCC CTGAAAACCA AAGCCTTTTT CATGTTAGAT

AGCTGAATCT GCAACTCCCC CTGAAAACCA AAGCCTTTTT CATGTTAGAT

AGCTGAATCT GCAACTCCCT CTGAAAACCA AAGCCTTTTT CATGTTAGAT

AGCTGAATCT GCAACTCCCC CTGAAAACCA AAGCCTTTTT CATGTTAGAT

AGCTGAATCT GCAACTCCCC CTCAAAACCA AAGCCTTTTT CATGTTAGAC

AGCTGGACCT GCAACTCCCC CTGAAGACCA AAGCCTTCTT CCTGCTGGAC

AGCTGGGCCT GCAACTCCCC CTGAAGACCA AAGCCTTCTT CCTGTTAGAC

AGCTGAATCT GCACCTCCCC CTGAAAACCA AAGCCTTTTT CATGTTGGAT

AGCTGAACCT ACAACTCCCC CTGAAAACCA AAGCCTTTTT CATGTTAGAT

AGCTGAATCT GCAACTCCCT CTGAAAACCA AAGCCTTTTT CATGTTAGAT

AGCTGAATCT GCAGCTCCCC CTGACAACCA AGGCCTTTTT CATGTTAGAC

AGCTGAATCT GCAGCTCCCC CTGACAACCA AGGCCTTTTT CATGTTAGAT

AGCTGAATCT GCAACTTCCC CTGAAAACCA GAGCCTTTTT CGTGTTAGAT

---------- ---------- ---------- ---------- ----------

AGCTGGATCT GCAACTCCCC CTGAAAGCCA CGGCCTTCTT CATGCTAGAT

AGCTAAATCT GCAACCCCCC TTGGAAACAA AAGCTCTTTT CATATTTGAT

AACTAAATCT GCCACCCCCC TTCAAAACGA AAGCATTTTT TGTATTTGAT

GGGATCCTTT CCAAATACTT TGATCTCATT TATGTACATA ATCCTGTGTT

GGGATCCTTT CCAAATACTT TGATCTCATT TATGTACATA ATCCTGTGTT

GGGATCCTTT CCAAATACTT TGATCTCATT TATGTACATA ATCCTGTGTT

GGGATCCTTT CCAAATACTT TGATCTCATT TATGTACATA ATCCTGTGTT

GGGATCCTTT CCAAATACTT TGATCTCATT TATGTACATA ATCCTGTGTT

GGGGTTCTTT CCAAATACTT TGATCTCATT TATGTACATA ATCCTGTGTT

GGGATCCTTT CCAAACACTT TGATCTCACT TATGTACATG ATCCTATGTT

GGGATTCTTT CCAAACACTT TGATCTCACT TATGTGCATA ATCCTGTGTT

GGGATCCTTT CCAAACACTT TGATCTCACT TATGTACATA ATCCTGTGTT

GGGATCCATT CCAAATACTT TGATCTCATT TATGTACATA ATCCTGTGTT

GGGATCCATT CCAAATACTT TGATCTCATT TATGTACATA ATCCTGTGTT

GGGATCCATT CCAAATACTT TGATCTCATT TATGTACACG ATCCTGTGTT

GGGATCCATT CCAAATACTT TGATCTCATT TATGTACATA ATCCCGTGTT

GGGATCTATT CCAATTACTT TGATCTCATT TATGTACATA ATCCTGTGTT

---------- ---------- ---------- ---------- ----------

GGGATCCATT CCAGGGACTT TGATCTCATT TATGTACCTA ATCCTGTGTT

GGTATCCATT CCACTTACTT TGATCTCATT TATGTCCATA ATCCTGTATT

GGGGTCCAGT CTGCTAACTT TGACCTCATT TATGTGCACA ATCCAGTGTT

TAAGCCTTTT GAAAAGCCAG TGATGATCTC AATGGGCAAT GAAAATGTAC

TAAGCCTTTT GAAAAGCCAG TGATGATCTC AATGGGCAAT GAAAATGTAC

TAAGCCTTTT GAAAAGCCAG TGATGATCTC AATGGGCAAT GAAAATGTAC

TAAGCCTTTT GAAAAGCCAG TAATGATCTC AATGGGCAAT GAAAATGTAC

TAAGCCTTTT GAAAAGCCAG TGATGATCTC AATAGGCAAT GAAAATGTAC

TAAGCCTTTT GAAAAGCCAG TGATGATCTC AATGGGCAAT GAAAATATCC

TAAGCCTTTT GAAAAGCCAG TAATGATCTC CATGGGCAAT GAGAATGTAG

TGAGCCTTTT GAAAAGCCAG TAATGATCTC AATAGGCAAT GAAAATGTAG

TAAGCCTTTT GAAAAGCCAG TGATGATCTC AATGGGCAAT GAAAATGTAC

TAAGCCTTTT GAAAAGCCAG TGATGATCTC AGTAGGCAAT GAAAATGTAC

TAAGCCTTTT GAAAAGCCAG TGATGATCTC AATAGGCAAT GAAAATGTAC

TAAGCTTTTT GAAAAGCCAG TGATGATCTC AATAGGCAAT GAAAATGTCC

TAAGCCTTTT GAAAAGCCAG TGAAGATCTC AATAGGCAAT GAAAATGTAC

TAAGCCTTTT GAAAAGCCAG TGATGATCTC AATGGGCCGA GAAAATGTGC

---------- ---------- ---------- ---------- ----------

TAAGCTTTTT GAAAAGCCAG TGATGATCTC AATGGGCAAT GAGAATGTAC

TAAGCCTTTT GAAAAGCCAG TGATGATCTC AATGGGCAAT GAAAATGTAC

TAAGCTGTTT GAAAAGCCAG TGATGATCTC AATGGGCAAC AAACATGTAC

TGGAAATTAA GTGGAAGCAA GCAATTTCTT CAACCGTCCT TGGAAAAGTA

TGGAAATTAA GTGGAAGCAA GCAATTTCTT CAACCGTCCT TGGAAAAGTA

TGGAAATTAA GTGGAAGCAA GCAATTTCTT CAACCGTCCT TGGAAAAGTA

TGGAAATTAA GTGGAAGCAA GCAATTTCTT CAACCGTCCT TGGAAAAGTA

TGGAAATTAA GTGGAAGCAA GCAATTTCTT CAACCGTTCT TGGAAAAGTA

TGGAAATTAA GTGGAAGCAA GCAGTTTCTT CAACCGTCCT TGGAAAAGTA

TGGAAATTAA GTGGAAGCAA GCAGTCTCTT CAACTGTCCT TGGAAAAGTG

TGGAAATTAA GTGGAAGCAA GCAGTCTCTT CAACTGTTCT TGGAAAAGTG

TGGAAATTAA GTGGAAGCAA GCAGTTTCTT CAACTGTCCT TGGAAAAGTA

TGGAAATTAA GTGGAAGCAA GCAATTTCTT CAACTGTTCT TGGAAAAGTG

TGGAAATTAA GTGGAAGCAA GCAGTTTCTT CAACCGTCCT TGGAAAAGTA

TGGAAATTAA GTGGAAGCAA GCAGTTTCTT CAACTATCCT TGGAAAAGTA

TGGAAATTAA GTGGAAGCAA GCAGTTTCTT CAACTATTCT TGGAAAAGTT

TGGAAATTAA GTGGAAGCAA GCAGTTTCTT CATATATCCT TGGAAAAGTA

---------- ---------- ---------- ---------- ----------

TGGAAATTAA GTGGAAGCAA GCAGTTTCTT CAACTGTCCT TGGAAAAGTA

TGGAAATTAA GTGGAAGCAA GCAATTTCTT CAACTGTCCT TGGAAAAGTG

TGGAAATTAA GTGGCAGCAG GCAGTCTCCT CGACCATCCT TGGAAAGGTG

ATAGTTCAAC CAGATCAGAA TTTCACAGGA TTGATTGCTG GTGTTGTCTC

ATAGTTCAAC CAGATCAGAA TTTCACAGGA TTGATTGCTG GTGTTGTCTC

ATAGTTCAAC CAGATCAGAA TTTCACAGGA TTGATTGCTG GTGTTGTCTC

ATAGTTCAAC CAGATCAGAA TTTCACCGGA TTGATTGCTG GTGTTGTCTC

ATAGTTCAAC CAGATCAGAA TTTCACAGGA TTGGTTGCTG GTGTTGTCTC

ATAGTTCAAC CAGATCAGAA TTTCACAGGG TTGATTGTTG GTGTTGTCTC

ATCGTTCAAC CGGATCAGAA TTTTGCAGGA TTGATCATTG GTGCGGTCTC

ATCGTTCAAC CGGATCAGAA TTTTGCAGGA TTGATCATTG GTGCGGTCTC

ATAGTTCAAC CAGATCAGAA TTTTATGGGA TTGATTGTTG GTGGTGTCTC

ATAGTTCAAC CAGATCAGAA TTTCACAGGA TTGATTGTTG GTGTTGTCTC

ATAGTTCAAC CAGATCAGAA TTTCACAGGA TTGATTGCTG GTGTTATCTC

ATAGTTCAAC CAGATCAGAA TTTCACAGGA TTGATTGTTG GTGTTATCTC

GTAGTTCAAC CAGATCAGAA TCTCACGGGA ATGATTGTTG GTATTGTTTT

ATAGTTCAAT CAGATCAGAG TTTCACAGGA GTGATTGTTG GTGTTGTTGC

---------- ---------- ---------- ---------- ----------

ATAGTTCAGC CGGATCAGAA TTTCACAGGA TTGATTGTTG GTGTTGTCTC

ATAGTACAAC CAGACCAGAA TTTCACAGGA CTCATTGTTG GAGTCGTCTT

ATTGTTCTCC CTGACCAGAA TTTCACAGGA CTAATCGCCG GAGTGGCCTC

AATATCAACA GCACTGTTAT TACTACTTGG GTTTTTCCTG TGGCTGAAAA

AATATCAATA GCACTGTTAT TACTACTTGG GTTTTTCCTG TGGCTGAAAA

AATATCAATA GCACTCTTAC TACTACTTGG GCTTTTCCTG TGGCTGAAAA

AATATCAATA GCACTCTTAC TACTACTTGG GCTTTTCCTG TGGCTGAAAA

AATCTCAATA GCACTCTTAT TACTACTTGG GCTTTTCCTG TGGCTGAAAA

AATATCAATA ATACTCTTAT TACTACTGGG ACTTTTCCTG TGGCTGAAAA

AATATCAGTG GTAGTTTTGT TAGTATCCGG GCTCTTCCTG TGGCTGAGAA

AATATCAGTA GTAGTTTTGT TATTATCCGG GCTCTTCCTG TGGATGAGAA

CATATCAATA ATACTTTTAT TATTACTTGG ACTGTTTTTG TGGCTAAAAA

AATATCAATA ATACTCTTAC TGTTACTCGG GCTTTTCCTG TGGCTGAAAA

AATATCAACA ATAGTCTTAT TATTACTCGG ACTTTTCCTG TGGCTGAAAA

AATATCAGTA CTACTTTCGT TATTGTTCGG ACTTTTCCTG TGGCTGAAAA

AATATCAGTA ATGCTCTTAC TACTACTTGG CCTTTTCCTG TGGTGGAAAA

AATATCAATA ATACTCTTAT TATTACTCGG ACTTTTCCTG TGGCTAAAAA

---------- ---------- ---------- ---------- ----------

AATATCAGTC ATACTCTTAT CATCCCTGGG GCTTTTCCTG TGGCTGAAAA

GATATCATTA TTGCTCTTGT TACTACTAGG GCTTTTCCTG TGGCTGAAAA

CATCTCGGTC TTACTCTTGT TATTCCTGGG CCTTTTTCTG TGGATGAAAA

AGAGAAAGCA AATTAAAGAT CTGGGCAGTG AATTAGTTCG CTACGATGCA

AGAGAAAGCA AATTAAAGAT CTGGGCAGTG AATTAGTTCG CTACGATGCA

AGAGAAAGCA AATTAAAGAT CTGGGCAGTG AATTAGTTCG CTATGATGCA

AGAGAAAGCA AATTAAAGAT CTGGGCAGTG AATTAGTTCG CTATGATGCA

AGAAAAAGCA AATTAAAGAT CTGGGCAGTG AATTAGTTCG CTATGATGCA

AAAGAAAGCA AAACAAAGAT CCAGGCAGTG AATTAGTTCG CTATGATGCA

AGAGAAAGCA ---TAAAGAT CTGGGCAGTG AATTAGTTCG CTATGACGCA

AGAGAAAGCA ---TAAAGAT CTGGGCAGTG AATTAGTTCG CTATGACGCA

AGAAAAAGCG CATTAAAGAT CTGGGCAGTG AATTAGTTCG CTATGATGCA

AAAGGAAGCA AATTAAAGAT CTGGGCAGTG AATTAGTTCG CTATGATGCA

GGAAAAAGCA AATTAAAGAT CTGGGCAGTG AATTAGTTCG CTATGATGCA

GGAGAAAGCA AATTAAAGAT CTGGGCAGTG AATTAGTTCG CTATGATGCA

AGAGAAAGCA AATTAAAGAT CTGGGCAGTG AATTAGTTCG CTATGATGCA

AGAAAAAGCA AATTAAAGAT CTGGGCAGTG AATTAGTTCG TTATGATGCA

---------- ---------- ---------- ---------- ----------

AGAGAAAGCA AATTAAAGAT CTGGGCAGTG AATTAGTTTG CTATGACGCA

AGAGGAAGCA GATTAAAGAT CTGGGCAGTG ATTTAGTTCG CTATGATGCA

AGAAGAAACA AATTAAAGAT CTGGGCAGTG AATTAGTTCG CTATGACCCA

AGAGTACACA CTCCTCATTT GGATAGGCTT GTAAGTGCCC GAAGTGTAAG

AGAGTACACA CTCCTCATTT GGATAGGCTC GTAAGTGCCC GAAGTGTAAG

AGAGTACACA CTCCTCATTT GGATAGGCTT GTAAGTGCCC GAAGTGTAAG

AGAGTACACA CTCCTCATTT GGATAGGCTT GTAAGTGCCC GAAGTGTAAG

AGAGTACACA CTCCTCATTT GGATAGGCTT GTAAGTGCCC GAAGTGTAAG

AGAGTACACA CTCCTCATCT GGATAGGCTT GTAAGTGCCC GAAGTGTAAG

AGAGTACACA CTCCTCATTT GGATAGGCTT GTAAGTGCCC GAAGTGTAAG

AGAGTACACA CTCCTCATTT GGATAGGCTT GTAAGTGCCC GAAGTGTAAG

AGAGTTCACA CTCCTCATTT GGATAGGCTT GTAAGTGCCC GAAGTGTGAG

AGAGTACACA CTCCACATTT GGATAGGCTT GTCAGTGCCC GAAGTGTAAG

AGAGTACACA CTCCTCATTT GGATAGGCTT GTAAGTGCCC GAAGTGTAAG

CGAATACACA CTCCTCACTT GGATAGGCTC GTAAGTGCCC GAAGTATAAG

AGAGTACACA CTCCTCATTT GGATAGGCTT GTAAGTGCCC GAAGTGTAAG

AGAGTACACA CTCCTCATTT GGATAGGCTT GTAAGTGCCC GAAGTGTAAG

---------- ---------- ---------- ---------- ----------

AGAGTCCACA CTCCTCACTT GGATCGGCTT GTAAGTGCCC GAAGTGTAAG

AGAGTACACA CTCCTCACTT GGATAGGCTT GTAAGCGCCC GAAGTGTAAG

AGAGTTCACA CGCCTCACCT GGACCGCCTT GTAAGCGCCA GGAGTATCAG

CCCAACTACA GAAATGGTTT CAAATGAATC TGTAGACTAC CGAGCTACTT

CCCAACTACA GAAATGGTTT CAAATGAATC TGTAGACTAC CGAGCTACTT

CCCAACAACA GAAATGGTTT CAAATGAATC TGTAGACTAC CGAGCTACTT

CCCAACAACA GAAATGGTTT CAAATGAATC TGTAGACTAC CGAGCTACTT

CCCAACTACA GAAATGGTTT CAAATGAATC TGTAGACTAC CGAGCTACTT

CCCAACTACA GAAATGGTGT CAAATGAATC TGTAGACTAC CGAGCTACTT

CCCAACTACA GAGATGGTCT CAAATGAGTC TGTAGACTAC AGAGCTACTT

TCCAACTACA GAGATGGTTT CAAATGAGTC TGTAGACTAC AGAGCTACTT

CCCAACTACA GAAATGGTTT CAAATGAATC TGTAGACTAC AGAGCTACTT

CCCAACTACA GAAATGGTTT CCAATGAATC TGTAGACTAC CGAGCTACTT

CCCAACTACA GAAATGGTTT CAAATGAATC TGTAGACTAC CGAGCTACTT

CCCAACCACA GAAATGGTCT CAAATGAATC TGTAGACTAC CGCGCTACTT

CCCAACTACA GAAATGGTTT CAAATGAATC TGTAGACTAC AGAGCTACTT

CCCAACTACA GAAATGGTTT CAAATGAATC TGTAGACTAC CGAGCTACTT

---------- ---------- ---------- ---------- ----------

CCCAACTACG GAAATGGTTT CAAATGAGTC CGTGGACTAC CGGGCCACAT

CCCTACCACG GAAATGGTTT CCAATGAATC TGTAGACTAC AGAGCAACAT

CCCCACGGCG GAAATGGTTT CCAATGAATC TGTGGACTAC CGAGCGACGT

TTCCAGAAGA TCAGTTTCCT AATTCATCTC AGAACGGTTC ATGCCGACAA

TTCCAGAAGA TCAGTTTCCT AATTCATCTC AGAACGGTTC ATGCCGACAA

TTCCAGAAGA TCAGTTTCCT AACTCATCTC AGAACGGTTC ATGCCGACAA

TTCCAGAAGA TCAGTTTCCT AATTCATCTC AGAACGGTTC ATGCCGACAA

TTCCAGAAGA TCAGTTTCCT AACTCATCTC AGAATGGATC ATGCCGACAA

TTCCAGAAGA CCAGTTTCCT AACTCATCTC AGAATGGATC ATGCAGACAA

TTCCAGAAGA CCAGTTTCCC AACTCCTCTC AGAATGGAGC CTGCAGACAA

TTCCAGAAGA CCAGTTTCCC AACTCCTCTC AGAATGGAGC ATGCAGACAA

TTCCAGAAGA CCAGTTTCCT AACTCATCTC AGAATGGATC ATGCAGACAA

TTCCAGAAGA CCAGTTTCCT AATGCATCTC AGAATGGGTC ATGCAGACAA

TTCCAGAAGA CCAGTTTCCT AATTCATCTC AGAATGGATC ATGCAGACAA

TTCCAGAAGA CCAGTTTGCT AACTCATCCC AGAATGGATC CTGCAGACAA

TTCCAGAAGA CCAGTTTCCT AACTCATCTC AGAACGGATC TTGCAGACAA

TTCCAGAAGA CCAATTTCCT AACTCATCTC AGAATGGATC ATGCAGGCAA

---------- ---------- ---------- ---------- ----------

TTCCAGAAGA CCAGTTTCCT AACTCATCTC AGAACGGATC ATGCCGACAA

TTCCAGAAGA TCAGTTTCCA AATTCTTCCC AGAATGGATC CTGCAGACAA

TCCCAGAAGA TCAGTTTCCC AATTCCTCTC AGAACGGATC ATGCAGACAA

GTGCAGTATC CTCTGACAGA CATGTCCCCC ATCCTAACTA GTGGGGACTC

GTGCAGTATC CTCTGACAGA CATGTCCCCC ATCCTAACTA GTGGGGACTC

GTGCAGTATC CTCTGACAGA CATGTCCCCC ATCCTGACTA GTGGGGACTC

GTGCAGTATC CTCTGACAGA CATGTCCCCC ATCCTGACTA GTGGGGACTC

GTGCAGTATC CTCTGACAGA CATGTCCCCT ATCCTGACTA GTGGGGACTC

GTGCAGTATC CTCTCACAGA CTTGTCCCCC ATCCTAACTA GCGGGGACTC

GTGCAGTATC CTCTGACAGA TCTGTCCCCC ATCCTGACGA GTGGAGACTC

GTGCAATACC CTCTGACAGA CCTGTCCCCT ATCCTGACAA GTGGAGACTC

GTGCAGTATC CTCTGACGGA CCTGTCTCCC ATCCTAACTA GTGGAGACTC

GTGCAGTATC CTCTGACAGA TCTATCCCCC ATCTTAACTA GTGGGGACTC

GTACAATATC CTCTGACGGA CCTGTCCCCC ATGCTTACTA GTGGGGACTC

GTGCAGTGCC CTCTGATGGA CCTCTCCCCC ATCCTGCCTG GTGGAGACTC

GTTCAGTATC CTCTGACGGA CCTATCCCCC ATCCTCACTA GTGGGGATTC

GTGCAGTATC CACTGATGGA CCTCTCCCCC ATTCTAACTA GTGGGGACTC

---------- ---------- ---------- ---------- ----------

GTGCAGTACC CCCTGCCGGA CCTGTCCCCC ATTCTAACTA GTGGGGACTC

GCTCATTATC CTCTGACAGA TTTGTCCCCC ATCCTAACCA GTGGAGACTC

GCCCATTATC CCCTGGCCGA CCTGTCGCCC ATTCTCTCCA GTGGGGACTC

TGATATATCC AGTCCATTAC TGCAAAATAC TGTCCACATT GACCTCAGTG

TGATATATCC AGTCCATTAC TGCAAAATAC TGTCCACATT GACCTCAGTG

TGATATATCC AGTCCATTAC TGCAAAACAC TGTCCACATT GACCTCAGCG

TGATATATCC AGTCCATTAC TGCAAAACAC TGTCCACATT GACCTCAGCG

TGATATATCC AGTCCATTAC TGCAAAATAC TGTCCACATT GACCTCAGTG

TGATATATCC AGTCCATTAC TGCAAAATAC TGTCCACATT GACCTCAGTG

TGATATATCC AGCCCATTAC TACAAAACAC TGTTCACATT GACCTCAGCG

TGATATATCC AGCCCATTAC TACAAAATAC TGTTCACATT GACCTCAGTG

TGATATATCC AGTCCATTAC TGCAAAATAC TGTCCACATT GACCTCAGTG

TGATATATCC AGTCCATTAC TGCAAAATAC CGTCCACATC GACCTCAGTG

TGATATATCC AGTCCATTAT TGCAAAATAC TGTCCACATT GACCTCAGTG

TGACATATCC AGTCCGTTGC TGCAAAATAC CGTTCGCATT GACCTCAGTG

TGATATATCC AGTCCATTAC TGCAAAATAG TGTCCACATT GACCTCAGTG

TGATATATCC AGTCCATTAC TGCAAAATGT TGTCCACATT GATCTCAGTG

---------- ---------- ---------- ---------- ----------

TGACATCTCC AGTCCATTAC TGCAGAACAC TGTCCATATT GACCTCAGCG

AGATCTCTCC AGTCCGCTGC TTCAGAATCA CGTCCACATT GATATCAGCG

CAACCCATCT AGCCCACTTC TACAGAGCAA CGTTCACATT GACATCAGCG

CTCTAAATCC AGAGCTGGTC CAGGCAGTGC AGCATGTAGT GATTGGGCCC

CTCTAAATCC AGAGCTGGTC CAGGCAGTGC AGCATGTAGT GATTGGGCCC

CTCTAAATCC AGAGCTGGTC CAGGCAGTGC AGCATGTAGT GATTGGGCCC

CTCTAAATCC AGAGCTGGTC CAGGCAGTGC AGCATGTAGT GATTGGGCCC

CTCTAAATCC AGAGCTGGTC CAGGCAGTGC AGCATGTAGT GATTGGGCCC

CTCTAAATCC AGAGCTAGTC CAGGCAGTCC AGCACGTCGT GATTGGGCCC

CTCTAAATCC AGAGCTGGTC CAAGCGGTGC AGCACGTAGT GATTGGACCC

CTCTAAATCC AGAGCTGGTC CAAGCAGTTC AGCACGTAGT GATTGGACCC

CTTTAAATCC AGAGCTGGTC CAAGCAGTCC AGCACGTAGT GATTGGGCCA

CTCTAAATCC AGAGCTGGTC CAGGCAGTTC AGCACGTAGT AATTGGGCCG

CTCTAAATCC AGAGCTGGTG CAGGCAGTCC AGCATGTAGT GATTGGGCCC

CACTGAATCC GGAGCTGGTT CAGGCGGTCC AGCACGTGGT GATTGGGCCT

CTCTAAATCC AGAGCTGGTC CAGGCAGTGC AGCACGTAGT AATTGGGCCC

CTCTAAATCC AGAGCTGGTC CAAGAAGTCC AACACGTAGT GATTGGGCCC

---------- ---------- ---------- ---------- ----------

CTCTAAACCC TGAGTTGGTC CAAGCCGTCC AGCACGTCGT GATCGGGCCC

CTTTAAATCC TGAGCTGGTC CAAGCAGTTC AGCATGTGGT AATTGGGCCA

CCCTGAACCC AGATCTAGTC CAAGCCGTTC AGCACGTGGT GATCGGACCA

AGTAGCCTGA TTGTGCATTT CAATGAAGTC ATAGGAAGAG GGCATTTTGG

AGTAGCCTGA TTGTGCATTT CAATGAAGTC ATAGGAAGAG GGCATTTTGG

AGTAGCCTGA TTGTGCATTT CAATGAAGTC ATAGGAAGAG GGCATTTTGG

AGTAGCCTGA TTGTGCATTT CAATGAAGTC ATAGGAAGAG ----------

AGTAGCCTGA TTGTGCATTT CAATGAAGTC ATAGGAAGAG GGCATTTTGG

AGTAGCCTGA TTGTGCATTT CAATGAAGTC ATAGGAAGAG GCCATTTTGG

AGTAGCCTGA TTGTGCATTT CAATGAAGTC ATAGGAAGAG GGCATTTTGG

AGCAGCCTGA TTGTGCATTT CAATGAAGTC ATAGGAAGAG GGCATTTTGG

AGTAGCCTGA TTGTGCATTT CAGTGAAGTC ATAGGAAGAG GGCATTTTGG

AGCAGTCTGA TTGTACATTT CAATGAAGTC ATAGGAAGAG GACATTTTGG

AGTAGCCTGA TTGTGCATTT CAATGAAGTC ATAGGAAGAG GACATTTTGG

AGCCGCCTGA TTGTGCATTT CAATGAGGTC ATAGGAAGAG GACATTTTGG

AGCAGCTTGA TTGTGCATTT CAATGAAGTC ATAGGAAGAG GACATTTTGG

AGTAGCCTGA TTGTGCATTT CAATGAAGTC ATAGGAAGAG GGCATTTTGG

---------- ---------- ---------- ---------- ----------

AGCAGCCTGA TTGTGCATTT CAATGAAGTC ATAGGAAGAG GCCATTTCGG

AACAGCCTGA TTGTACATTT CAATGAAATC ATAGGAAGAG GGCATTTTGG

GACAGCCTGA TGGTGCATTT CACTGAAATC ATAGGAAGAG GTCATTTTGG

TTGTGTATAT CATGGGACTT TGTTGGACAA TGATGGCAAG AAAATTCACT

TTGTGTATAT CATGGGACTT TGTTGGACAA TGATGGCAAG AAAATTCACT

TTGTGTATAT CATGGGACTT TGTTGGACAA TGATGGCAAG AAAATTCACT

---------- ---------- ---------- ---------- ----------

TTGTGTGTAT CATGGGACTT TGCTGGACAA TGATGGCAAG AAAATTCACT

TTGTGTGTAT CACGGGACTT TGTTGGACAA TGATGGCAAG AAAATTCACT

CTGTGTCTAT CATGGGACTT TGTTGGACAG TGACGGAAAG AAAATTCACT

CTGTGTCTAT CATGGGACTT TGCTGGACAA TGACGGAAAG AAAATTCACT

TTGTGTATAT CATGGGACTT TGTTGGACAA TGATGGCAAG AAAATTCACT

GTGTGTATAT CATGGAACTT TGTTGGACAA CGATGATAAG AAAATTCACT

GTGTGTATAC CATGGGACTT TGTTGGACAA TGACGACAAA AAAATTCACT

TTGTGTGTAT CATGGGACTT TGTTGGATAA TGATGACAAG AAAATTCACT

GTGTGTGTAT CATGGAACCT TGCTGGACAA TGATGACAAG AAAATCCATT

GTGTGTATAT CATGGGACTT TGTTGGACAG TGACGACAAG AAAATTCACT

---------- ---------- ---------- ---------- ----------

GTGTGTGTAT CATGGGACTC TGTTGGACAA TGATGACAGG AAAATCCACT

GTGTGTATAT CATGGGACTT TATTGGATAA TGATGACAAG AAGATTCATT

GTGTGTGTAT CACGGAACTC TGTTGGATAA TGATGACAAA AAAATCCACT

GTGCTGTGAA ATCCTTGAAC AGAAATCCAA CTGTAAAAGA TCTTATTGGC

GTGCTGTGAA ATCCTTGAAC AGAAATCCAA CTGTAAAAGA TCTTATTGGC

GTGCTGTGAA ATCCTTGAAC AGAAATCCAA CTGTAAAAGA TCTTATTGGC

---------- ---------- -GAAATCCAA CTGTAAAAGA TCTTATTGGC

GTGCTGTGAA ATCCTTGAAC AGAAATCCAA CTGTAAAAGA TCTTATTGGC

GTGCTGTGAA ATCCTTGAAT AGAAACCCAA CTGTAAAAGA TCTTATTGGC

GTGCTGTGAA ATCCTTGAAC AGAAACCCAA CTGTGAAAGA TCTTATAGGA

GTGCTGTGAA ATCCTTAAAT AGAAATCCAA CTGTGAAAGA TCTTATAGGA

GTGCTGTGAA ATCTTTGAAT AGAAACCCGA CTGTAAAAGA TCTTATTGGC

GTGCTGTGAA ATCCTTGAAT AGAAACCCAA CTGTAAAAGA TCTTATTGGC

GTGCTGTGAA ATCCCTGAAT AGAAACCCAA CTGTAAAAGA TCTTATTGGC

GTGCTGTGAA ATCCTTGAAT AGAAACCCAA CTGTAAAAGA TCTTATTGGC

GTGCTGTGAA GTCCTTGAAT AGAAGTCCAA CTGTAAAAGA TCTCATTGGC

GTGCTGTGAA ATCTTTGAAT AGAAACCCAA CTGTAAAAGA TCTTATTGGC

---------- ---------- ---------- ---------- ----------

GTGCTGTGAA GTCCTTGAAT C--AGCCCCA CTGTGAAAGA TCTCATTGGC

GTGCTGTGAA ATCCTTGAAT AGAAATCCAA CAGTGAAGGA CCTCATTGGT

GTGCTGTGAA GTCCTTGAAT AGAAATCCCA CCGTGAAAGA CCTCATCGGA

TTTGGTCTTC AAGTAGCCAA AGGCATGAAA TATCTTGCAA GCAAAAAGTT

TTTGGTCTTC AAGTAGCCAA AGGCATGAAA TATCTTGCAA GCAAAAAGTT

TTTGGTCTTC AAGTAGCCAA AGGCATGAAA TATCTTGCAA GCAAAAAGTT

TTTGGTCTTC AAGTAGCCAA AGGCATGAAA TATCTTGCAA GCAAAAAGTT

TTTGGTCTTC AAGTAGCCAA AGGCATGAAA TATCTTGCAA GCAAAAAGTT

TTTGGTCTTC AAGTAGCCAA AGGCATGAAA TATCTTGCAA GCAAAAAGTT

TTCGGTCTTC AAGTAGCCAA GGGCATGAAA TATCTTGCCA GCAAAAAGTT

TTTGGCCTTC AAGTAGCCAA AGGCATGAAA TATCTTGCCA GCAAAAAGTT

TTTGGTCTTC AAGTAGCAAA AGGCATGAAA TACCTTGCAA GCAAAAAGTT

TTTGGTCTTC AAGTAGCCAA AGGCATGGAA TATCTTGCAA GCAAAAAGTT

TTTGGTCTTC AAGTAGCCAA AGGCATGAAA TATCTTGCAA GCAAAAAGTT

TTTGGCCTTC AAGTAGCCAA AGGCATGAAA TATCTTGCAA GCAAAAAGTT

TTTGGTCTTC AAGTAGCCAA AGGCATGAAA TATCTTGCAA GCAAAAAGTT

TTTGGTCTGC AAGTAGCCAA AGGCATGAAA TATCTTGCAA GCAAAAAGTT

---------- ---------- ---------- ---------- ----------

TTTGGTCTTC AAGTCGCCAA GGGCATGAAA TATCTTGCCA GCAAAAAGTT

TTTGGCCTTC AAGTAGCAAA AGGCATGAAA TACCTTGCAA GCAAAAAGTT

TTTGGCCTCC AAGTGGCCAA AGGTATGAAA TACCTTGCCA GCAAAAAGTT

TGTCCACAGA GACTTGGCTG CAAGAAACTG TATGCTGGAT GAAAAATTCA

TGTCCACAGA GACTTGGCTG CAAGAAACTG TATGCTGGAT GAAAAATTCA

TGTCCACAGA GACTTGGCTG CAAGAAACTG TATGCTGGAT GAAAAATTCA

TGTCCACAGA GACTTGGCTG CAAGAAACTG TATGCTGGAT GAAAAATTCA

TGTCCACAGA GACTTGGCTG CAAGAAACTG TATGCTGGAT GAAAAATTCA

TGTGCACAGA GACTTGGCTG CAAGAAACTG CATGCTGGAT GAGAATTTCA

TGTCCACAGA GACTTAGCTG CAAGAAACTG CATGTTGGAT GAAAAATTCA

TGTCCACAGA GACTTAGCTG CAAGAAACTG CATGTTGGAT GAAAAATTCA

TGTCCACAGA GACTTGGCTG CAAGAAACTG TATGTTGGAT GAAAAATTCA

TGTCCACAGA GACTTGGCTG CAAGAAACTG TATGCTGGAT GAAAAATTCA

TGTCCACAGA GACTTGGCTG CAAGAAACTG TATGCTGGAT GAAAAATTCA

TGTCCACAGA GACTTGGCCG CAAGAAACTG TATGCTGGAT GAAAACTTCA

TGTCCACAGA GACTTGGCTG CAAGAAACTG TAT------- ---------A

TGTCCACAGA GACTTGGCTG CAAGAAACTG TATGCTGGAT GAAAAATTCA

---------- ---------- ---------- ---------- ----------

TGTCCACAGA GACCTGGCCG CCAGGAACTG TATGCTGGAT GGAAAATTCA

CGTACACAGA GACTTGGCTG CAAGAAATTG TATGCTGGAT GAAAAATTTA

TGTCCACCGA GACTTGGCTG CCCGAAATTG TATGCTGGAT GAAAAATTCA

CAGTCAAGGT TGCTGATTTT GGTCTTGCCA GAGACATGTA TGATAAAGAA

CAGTCAAGGT TGCTGATTTT GGTCTTGCCA GAGACATGTA TGATAAAGAA

CAGTCAAGGT TGCTGATTTT GGTCTTGCCA GAGACATGTA TGATAAAGAA

CAGTCAAGGT TGCTGATTTT GGTCTTGCCA GAGACATGTA TGATAAAGAA

CTGTCAAGGT TGCTGATTTT GGCCTTGCCA GAGATATGTA TGATAAAGAA

CTGTCAAAGT TGCTGACTTT GGCCTTGCCA GAGACATGTA TGATAAAGAA

CTGTCAAGGT TGCTGATTTC GGTCTTGCCA GAGACATGTA CGACAAAGAG

CTGTCAAGGT TGCTGATTTC GGTCTTGCCA GAGACATGTA CGATAAAGAG

CTGTCAAAGT TGCTGATTTT GGTCTTGCCC GAGATATGTA TGATAAAGAA

CCGTCAAGGT TGCTGATTTT GGTCTTGCCA GAGACGTGTA TGATAAGGAA

CTGTCAAGGT TGCTGATTTT GGTCTTGCCA GAGACATGTA TGATAAAGAA

CTGTCAAGGT TGCTGATTTC GGTCTTGCCA GAGACGTGTA TGATAAAGAA

CTGTTAAGGT TGCTGATTTT GGTCTTGCCA GAGATATATA TGATAAGGAA

CTGTTAAGGT GGCTGACTTT GGTCTTGCCA GAGATATGTA TGATAAAGAG

---------- ---------- ---------- ---------- ----------

CCGTCAAAGT TGCCGATTTT GGTCTTGCCC GGGATATGTA TGATAAGGAA

CTGTCAAGGT TGCTGATTTT GGGCTTGCCA GAGATATGTA TGATAAAGAA

CTGTCAAGGT GGCTGATTTT GGCCTGGCCC GAGATATGTA CGATAAAGAA

TACTATAGTG TACACAACAA AACAGGTGCA AAGCTGCCAG TGAAGTGGAT

TACTATAGTG TACACAACAA AACAGGTGCA AAGCTGCCAG TGAAGTGGAT

TACTATAGTG TGCACAACAA AACAGGTGCA AAGCTGCCAG TGAAGTGGAT

TACTATAGTG TGCACAACAA AACAGGTGCA AAGCTGCCAG TGAAGTGGAT

TACTATAGTG TACACAACAA AACAGGTGCA AAGCTGCCGG TGAAGTGGAT

TACTACAGTG TACATAACAA GACAGGTGCC AAGCTGCCCG TGAAGTGGAT

TATTATAGCG TCCACAACAA AACGGGTGCG AAACTACCGG TGAAGTGGAT

TACTATAGTG TCCACAACAA GACGGGTGCC AAGCTACCAG TAAAGTGGAT

TACTATAGTG TACACAACAA AACAGGTGCA AAACTGCCAG TGAAGTGGAT

TACTATAGCG TACACAACAA AACAGGTGCA AAACTGCCAG TGAAATGGAT

TACTACAGCG TACACAACAA AACAGGCGCC AAACTACCAG TGAAGTGGAT

TACTACAGTG TCCACAACAA AACAGGCGCC AAGCTGCCTG TGAAGTGGAT

TACTATAGTG TCCACAACAA AACAGGAGCG AAACTGCCAG TGAAGTGGAT

TACTATAGTG TGCATAATAA AACAGGTGCA AAACTGCCAG TGAAGTGGAT

---------- ---------- ---------- ---------- ----------

TACTACAGTG TGCACAACAA AACCGGGGCG AAGCTGCCAG TGAAGTGGAT

TATTATAGTG TACACAATAA AACAGGAGCA AAACTGCCAG TGAAATGGAT

TATTACAGCG TGCACAATAA AACGGGAGCC AAGCTGCCAG TGAAATGGAT

GGCTTTGGAA AGTCTGCAAA CTCAAAAGTT TACCACCAAG TCAGATGTGT

GGCTTTGGAA AGTCTGCAAA CTCAAAAGTT TACCACCAAG TCAGATGTGT

GGCTTTGGAA AGTCTGCAAA CTCAAAAGTT TACCACCAAG TCAGATGTGT

GGCTTTGGAA AGTCTGCAAA CTCAAAAGTT TACCACCAAG TCAGATGTGT

GGCTCTGGAA AGTCTACAAA CTCAAAAGTT TACCACCAAG TCAGACGTGT

GGCTCTGGAA AGTCTGCAGA CGCAGAAGTT CACCACCAAG TCAGACGTGT

GGCTTTAGAG AGTCTGCAGA CGCAAAAGTT CACCACCAAG TCAGACGTGT

GGCTTTAGAG AGTCTGCAAA CGCAGAAGTT CACCACCAAG TCAGATGTGT

GGCTTTAGAA AGTCTGCAAA CTCAGAAGTT TACAACCAAA TCTGATGTGT

GGCTTTAGAA AGCCTGCAGA CTCAGAAATT TACCACCAAG TCAGATGTGT

GGCTTTAGAA AGTCTGCAAA CTCAGAAGTT TACCACCAAG TCAGATGTGT

GGCTTTGGAA AGTCTGCAGA CTCAGAAGTT CACCACCAAG TCAGATGTGT

GGCTTTAGAA AGTCTACAAA CTCAGAAGTT CACCACCAAG TCAGATGTGT

GGCTTTGGAA AGTCTGCAAA CTCAGAAGTT TACCACCAAG TCAGATGTGT

---------- ---------- ---------- ---------- ---------T

GGCTCTGGAG AGCCTGCAGA CGCAGAAGTT CACCACCAAG TCAGACGTGT

GGCTTTAGAA AGTCTACAAA CTCAGAAGTT TACTACAAAG TCAGATGTGT

GGCTCTCGAA AGTCTGCAGA CACAGAAGTT CACCTCCAAG TCAGATGTGT

GGTCCTTTGG CGTGCTCCTC TGGGAGCTGA TGACAAGAGG AGCCCCACCT

GGTCCTTTGG CGTGCTCCTC TGGGAGCTGA TGACAAGAGG AGCCCCACCT

GGTCCTTTGG TGTGCTCCTC TGGGAGCTCA TGACAAGAGG AGCCCCACCC

GGTCCTTTGG TGTGCTCCTC TGGGAGCTCA TGACAAGAGG AGCCCCACCC

GGTCCTTTGG CGTACTCCTC TGGGAGCTGA TGACAAGAGG AGCCCCACCT

GGTCCTTTGG TGTGCTCCTC TGGGAGCTGA TGACGAGAGG AGCCCCACCT

GGTCCTTCGG TGTGCTTCTC TGGGAGCTCA TGACGAGAGG AGCCCCTCCT

GGTCCTTTGG TGTGCTCCTC TGGGAGCTCA TGACGAGAGG AGCCCCTCCT

GGTCCTTCGG CGTGCTCCTG TGGGAGCTGA TGACAAGAGG AGCCCCACCT

GGTCCTTTGG CGTGCTTCTC TGGGAACTGA TGACAAGAGG AGCACCACCT

GGTCCTTTGG CGTGCTCCTC TGGGAACTGA TGACAAGAGG AGCACCACCT

GGTCCTTTGG CGTGCTCCTC TGGGAACTGA TGACAAGAGG TGCCCCGCCT

GGTCCTTTGG TGTGCTCCTC TGGGAGCTGA TGACAAGAGG GGCACCGCCT

GGTCCTTTGG TGTGCTGCTC TGGGAACTGA TGACAAGAGG CGCACCACCT

GGTCCTTTGG TGTGCTTCTC TGGGAACTGA TGACAAGGGG AGCTCCACCT

GGTCCTTTGG TGTGCTCCTC TGGGAACTGA TGACAAGAGG AGCGCCCCCG

GGTCGTTTGG AGTGCTTCTC TGGGAATTAA TGACCAGAGG AGCACCTCCT

GGTCCTTCGG CGTCCTTCTC TGGGAGCTGA TGACACGAGG CGCTCCCCCG

TATCCTGACG TAAACACCTT TGATATAACT GTTTACTTGT TGCAAGGGAG

TATCCTGATG TGAACACCTT TGATATAACT GTTTACTTGT TGCAAGGGAG

TATCCTGACG TGAACACCTT TGATATAACT GTTTACTTGT TGCAAGGGAG

TATCCTGACG TGAACACCTT TGATATAACT GTTTACTTGT TGCAAGGGAG

TATCCTGATG TGAACACTTT CGATATAACT GTTTACCTGT TGCAAGGGAG

TACCCTGACG TCAACACCTT CGATATAACT GTTTACTTGT TACAAGGCAG

TATCCTGACG TGAACACATT TGATATCACT ATATACCTGT TGCAAGGCAG

TATCCCGACG TGAACACATT TGATATCACT ATCTACCTGT TGCAAGGCAG

TACCCTGATG TGAACACCTT TGACATAACC GTGTACCTGC TGCAAGGCAG

TATCCTGACG TCAACACCTT TGACATAACA GTTTACTTGT TGCAAGGAAG

TATCCTGACG TCAACACCTT TGATATAACA GTTTACTTGT TGCAAGGCAG

TATCCTGATG TCAACACCTT TGATATAACA GTTTACTTGT TGCAAGGCAG

TATCCAGACG TCAATACATT TGACATCACA ATCTACTTGT TGCAAGGCAG

TATCCTGATG TGAACACATT TGACATCACA GTTTACTTAT TGCAAGGCAG

TATCCTGATG TAAACACCTT TGATATAACA GTTTACTTAT TGCAAGGAAG

TATCCCGACG TAAACACCTT CGATATAACG GTTTACTTAT TGCAAGGAAG

TATCCTGATG TGAATACCTT TGATATAACA GTTTACTTAT TGCAAGGTCG

TATCCCGACG TGAACACTTT CGATATAACG GTCTACCTGC TGCAAGGAAG

AAGACTCCTA CAACCCGAAT ACTGCCCAGA CCCCTTATAT GAAGTAATGC

AAGGCTCCTA CAACCCGAAT ACTGCCCAGA CCCCTTATAC GAAGTAATGC

AAGGCTCCTA CAACCTGAAT ACTGCCCAGA CCCCTTATAT GAAGTAATGC

AAGGCTCCTA CAACCCGAAT ACTGCCCAGA CCCCTTATAT GAAGTAATGC

AAGGCTCCTG CAACCCGAAT ACTGCCCAGA CCCCTTATAT GAAGTCATGC

AAGGCTCTTA CAACCCGAAT ACTGCCCAGA CCCCTTGTAT GAAGTGATGC

AAGACTCTTG CAACCAGAGT ACTGTCCAGA CGCCTTGTAT GAAGTGATGC

AAGACTCTTG CAACCAGAAT ACTGTCCAGA CGCCTTGTAC GAAGTGATGC

GAGACTCCTG CAACCCGAAT ACTGCCCAGA TGCCTTGTAC GAAGTGATGC

AAGGCTCCTA CAACCTGAAT ACTGCCCAGA TCCCTTGTAT GAAGTGATGC

AAGGCTCCTG CAACCCGAAT ACTGCCCAGA TCCCTTATAC GAAGTGATGC

AAGGCTCTTG CAACCCGAGT ACTGCCCAGA TCACCTGTAT GAAGTGATGC

GAGGCTCTTA CAACCTGAAT ACTGCCCAGA TCCCTTGTAT GAAGTGATGT

AAGGCTCTTA CAACCTGAAT ACTGCCCAGA TCGCTTGTAT GAAGTGATGC

GAGGCTTTTA CAACCTGAAT ACTGCCCCGA TCCCTTGTAT GAAGTGATGC

ACGGCTCTTA CAACCTGAAT ACTGCCCAGA TCCCTTGTAT GAAGTGATGC

AAGGCTCTTG CAGCCTGAAT ACTGCCCAGA TCCACTGTAT GAAGTCATGC

GAGGCTGCTA CAGCCAGAAT ACTGTCCGGA CCCACTATTT GAAGTCATGC

TAAAATGCTG GCACCCTAAA GCCGAAATGC GCCCATCCTT TTCTGAACTG

TAAAATGCTG GCACCCTAAA GCCGAAATGC GCCCATCCTT TTCTGAACTG

TAAAATGCTG GCACCCTAAA GCAGAAATGC GCCCATCCTT TTCTGAACTG

TAAAATGCTG GCACCCTAAA GCAGAAATGC GCCCATCCTT TTCTGAACTG

TAAAATGCTG GCACCCTAAA GCTGAAATGC GCCCATCCTT TTCCGAACTC

TAAAATGCTG GCACCCTAAA GCCGAAATGC GCCCATCCTT TTCTGAACTG

TAAAATGCTG GCACCCCAAA GCAGAAATGC GCCCATCGTT TTCTGAACTG

TAAAATGCTG GCACCCCAAA GCGGAAATGC GCCCGTCCTT TTCCGAACTG

TAAAGTGCTG GCACCCGAAA GCCGAAATGC GCCCGTCCTT CTCAGAACTG

TGAAATGCTG GCACCCTAAA GCTGAACTGC GCCCATCCTT TTCTGAACTG

TAAAATGCTG GCACCCTAGA GCTGAACTGC GCCCATCTTT TTCTGAACTG

TAAAATGCTG GCACCCGAAA GCTGAGCAGC GCCCTTCTTT TGCTGAGCTG

TAAAATGCTG GCACCCTAAA GCTGAACTGC GCCCATCCTT CTCTGAACTG

TAAAATGCTG GCACCCTAAA GCCGAAATGC GCCCATCCTT TTCTGAACTC

TAAAATGCTG GCATCCTAAA GCTGAAATGC GTCCATCCTT TTCTGAACTG

TTAAATGCTG GCACCCCAAG GCCGAAATGC GCCCGTCCTT TACTGAGCTG

TAAAGTGCTG GCACCCCAAA GCTGAAATGC GTCCCTCCTT CTCTGAATTG

TAAAATGCTG GCATCCCAAC GCTGAAATGC GCCCGTCCTT CTCGGAACTG

GTGTCCCGGA TATCAGCGAT CTTCTCTACT TTCATTGGGG AGCACTATGT

GTGTCCCGGA TATCAGCGAT CTTCTCTACT TTCATTGGGG AGCACTATGT

GTGTCCCGGA TATCAGCGAT CTTCTCTACT TTCATTGGGG AGCACTACGT

GTGTCCCGGA TATCAGCGAT CTTCTCTACT TTCATTGGGG AGCACTACGT

GTGTCCCGGA TATCAGCGAT CTTCTCTACT TTCATCGGGG AGCACTATGT

GTCTCTAGGA TATCGGCAAT CTTCTCTACT TTCATCGGAG AACACTACGT

GTCTCCAGGA TATCCTCAAT CTTCTCCACT TTCATTGGCG AGCACTATGT

GTCTCCAGGA TATCCTCAAT CTTCTCCACG TTCATTGGGG AACACTACGT

GTCTCCAGGA TATCCACGAT CTTCTCTACT TTCATTGGGG AGCACTATGT

GTCTCCAGGA TATCCGTGAT ATTCTCTACT TTCATTGGGG AGCACTATGT

GTCTCCAGGA TATCAGCAAT ATTCTCTACT TTCATTGGGG AGCACTATGT

GTCTCCAGGA TATCAGCGAT ATTCTCTGCT TTCATCGGGG AGCATTATGT

GTCTCCAGGA TATCGGGGAT ATTCTCCACT TTCATCGGGG AACATTATGT

GTCTCCAGGA TATCAGCGAT ATTCTCTACT TTCATCGGGG AACACTACGT

GTGTCGCGGA TATCAGCGAT ATTCTCTACT TTCATCGGGG AGCACTATGT

GTCTCCCGGA TCTCAGCCAT ATTCTCCACA TTCATCGGGG AGCACTATGT

GTGTCAAAGA TATCAGTCAT ATTCTCTACA TTCATTGGAG AGCATTATGT

GTTTCAAAGA TAGCGGTCAT CTTCTCCACT TTCATCGGCG AGCATTACGT

CCATGTGAAC GCTACTTATG TGAACGTAAA ATGTGTCGCT CCGTATCCTT

CCATGTGAAC GCTACTTATG TGAACGTAAA ATGTGTCGCT CCATATCCTT

CCATGTAAAC GCTACTTATG TGAACGTAAA ATGTGTCGCT CCATATCCTT

CCATGTAAAC GCTACTTATG TGAACGTAAA ATGTGTCGCT CCATATCCTT

CCATGTGAAC GCTACTTATG TGAACGTAAA ATGTGTCGCT CCATATCCTT

CCATGTGAAT GCTACTTATG TGAATGTGAA ATGTGTTGCT CCATATCCCT

CCATGTGAAC GCTACTTATG TGAATGTAAA ATGTGTTGCT CCATATCCTT

CCACGTGAAC GCTACTTATG TGAATGTAAA ATGTGTTGCT CCATATCCTT

CCACGTGAAT GCGACTTACG TGAACGTGAA ATGTGTGGCT CCCTATCCTT

CCATGTGAAT GCTACTTACG TGAACGTCAA ATGTGTCGCT CCATATCCTT

CCATGTGAAC GCCACTTATG TGAATGTCAA ATGTGTTGCT CCATACCCTT

CCACGTGAAT GCCACCTATG TGAACGTCAA ATGTGTCGCT CCATACCCTT

CCATGTGAAC ACTACTTATG TGAATGTCAA GTGTGTCGCT CCCTATCCTT

CCACGTGAAT GCTACTTATG TGAATGTAAA ATGCATCGCT CCGTATCCTT

CCACGTGAAC ACTACTTACG TGAATGTGAA ATGTGTCGCT CCATATCCTT

CCACGTGAAC GCTACATACG TGAATGTCAA GTGTGTCGCT CCATACCCTT

TCACGTGAAT GCAACCTACG TCAATGTGAA GTGTGTCGCT CCCTACCCTT

CCACGTGAAC GCCACCTACG TCAACGTCAA GTGCGTGGCG CCCTACCCAT

CTCTGTTGTC AGAAGATAAC GCTGATGATG AGGTGGACAC ACCAGCCTCC

CTCTGTTGTC AGAAGATAAC GCTGATGATG AGGTGGACAC ACCAGCCTCC

CTCTGTTGTC AGAAGATAAC GCTGATGACG AGGTGGACAC ACCAGCCTCC

CTCTGTTGTC AGAAGATAAC GCTGATGACG AGGTGGACAC ACCAGCCTCC

CTCTGTTGTC ACAAGATAAC ACTGATGGCG AGGTGGACAC ACCGGCCTCT

CTCTGCTGTC ACAAGATAAC ATCGATGGTG AGGTGGACAC A---GCCCCC

CTCTGTTGCC ACAAGACAAC ATTGACGGCG AAGCGAACAC ACCAGCCCCC

CTCTGTTGCC ACAAGACAAC ATTGATGGCG AGGGGAACAC ACCAGCCCAC

CTCTGCTGTC ACAGGATAAC GTGGATGGCA CAGTGGACAC GACAGTCC-T

CTCTGTTGTC ACAAGATAAT GTCAGTGGCG AGGATGACAC AGTGCCCCCC

CTCTCTTGTC ACAAGATAAC ATTGATGGCG AGGGGGACAC ATCACCTCCC

GTCTGTTGTC ACAAGATCAC GTTGATGGTG AGGGAGACAC ATCAAGCCCC

CTCTTTTGTC CCAAGGTAAT GTTGAAGGCC AGGGGGACAC ACAAGACACT

CTCTCTTGTC ACAAGATGAC CTCGACGGTG AGGTGGACAC ACCAGCCTCC

CTCTCTTGTC GCAAGATAGC GTGGATGATG AGGTGGACAC ACACCACCCC

CGCTCTTGTC ACACGACACG GTGGATGGCG AGGTGGACAC A-CCCCTCCC

CCCTTTTGTC ACAAGACAAC ATCGACAGGG AGGTGGACAC CACAGGGTGT

CCCTCCTGTC CCAGGACGCC CCAGACAGGG TTGTGGACAC ACTCGCCTTG

TTCGAGACAT GGTACATGAG AGCTACAGGC TCCTCCAGGG TGATGTGCGA

TTCGAGACAT GGTACATGAG AGCTACAGGC TCCTCCAGGG TGATGTGCGA

TTCGAGACAT GGTACATGAG AGCTACAGGC TCCTCCAGGG TGATGTGCGA

TTCGAGACAT GGTACATGAA AGCTACAGGC TCCTCCAGGG TGATGTGCGA

TTCGAGACAT GGTACATGGG AGCTACAGGC TCCTCCAGGG TGATGTGTGA

TCCGGGACAT GGTACATGAG AGCTACAGGC TCCTCCAGGG TGATGTGTGA

TTCGAAAC-T GGTACATGAG AGCGACAGGC TCCTCCAGGG TGATGTGTGA

TTCGAAAC-T GGTACATGAG AGCTACAGGC TCCTCCAGGG TGATGTGTGA

ATCGCAAC-T GGTACATGAG AGCTACAGAC TCCTCCAGGG TGATGTGTGA

TTCGAAACAT GGTACATGAG AGCTACGGGC TCCTCCAGGG TGATGTGTGA

TTCGAAACGT GGTACATGAG AGCTACAGGC TCCTCCAGGG TGATGTGTGA

TTCGAAACAT GGTACATGAA AGCTACAGGC TCTTCCAGGG TGATGTGTGA

CTCGAAACAT G--------- ---------- ---------- ----------

CTCGAAACAT GGTACATGAG GGCTACAGGC TCCTCCAGGG TGATGTGTGA

TTCAGAACA- ---------- ---------- ---------- ----------

CTCGGAACA- -GTACATGAG AGCTACAGGC ACCTCCAGGG TGATGTGTGA

ACCGAAAGAT GGTACATGAG AGCTGTGGGC TCCTCAAGGG TCATGTGTGA

TTCGAGACAT GGTACATGAG AGCGGCGGGC TCCTCGAGGG TGATGTGTGA

TAATGTGCCA GGCCTGGTGA GCAGCCAGCG GCAGCTGTGT CACCGACATC

TAATGTGCCA GGCCTGGTGA GCAGCCAGCG GCAGCTGTGT CACCGACATC

TAATGTGCCA GGCCTGGTGA GCAGCCAGCG GCAGCTGTGT CACAGACATC

TAATGTGCCA GGCCTGGTGA GCAGCCAGCG GCAGCTGTGT CACCGACATC

TAATGTGCCA GGCCTGGTGA GCAGCCAGCG GCAGCTGTGT CACCGACACC

CAATGTGCCC GGCCTGGTAA GCCGCCAGCG GCAGCTGTGC CACCGGCACC

CAATGTGCCA GGTCTGGTGA GCCGCCAGCG TCAGCTGTGC CACCGACACC

CAATGTGCCA GGCCTGGTGA GCCGGCAGCG TCAGCTGTGC CACCGACACC

CAATGTGCCA GGCCTGGTGA GCCGCCAGCG GCAGCTGTGC CACCGACACC

CAACGTGCCA GGCCTGGTGA GCCACCAACG GCAGCTGTGC CACCGACACC

CAATGTGCCA GGCCTGGTGA GCCGCCAGCG CCAGCTGTGC CACCGACACC

CAATGTGCCA GGCCTGGTGA GCCGCCAGCG GCAGCTGTGC CACCGACACC

---------- ---------- ---------- ---------- ----------

CAATGTGCCA GGCCTGGTGA GCCGCCAGCG TCAGCTGTGC CACCGCCACC

---------- ---------- ---------- ---------- ----------

CAATGTGCCA GGCTTGGTGA GCCGCCAGCG CCAACTGTGC CACCGACACC

CAACGTGCCA GGCTTGGTCA GCCGCCAGCG TCAGCTGTGC CACCGGCACC

CAACGTGCCA GGCCTGGTCA GCCGCCAGCG GCAGCTGTGC CACCGGCACC

CAGATGTGAT GCGTGCCATT AGCCAGGGCG TGGCCGAGTG GACAGCAGAA

CAGATGTGAT GCGTGCCATT AGCCAGGGCG TGGCCGAGTG GACAGCAGAA

CAGATGTGAT GCGTGCCATT AGCCAGGGCG TGGCTGAGTG GACAGCAGAA

CAGATGTGAT GCGTGCCATT AGCCAGGGCG TGGCCGAGTG GACAGCGGAA

CAGATGTGAT GCGTGCCATT GGCCTGGGCG TGACCGAGTG GACTTCGGAA

CGGACGTGAT GCGCGCCATT GGCCTGGGTG TCGCAGAGTG GACAGCAGAG

CAGACGTGAT GCGTGCCATT GGCCTGGGCG TGGCTGAGTG GACAGCAGAG

CAGATGTGAT GCGTGCCATT GGCCTGGGTG TGGCTGAGTG GACTGCAGAG

CGGACGTGAT GCGTGCCATA GGCCTGGGTG TGGCCGAGTG GACAGCAGAG

CGGATGTGAT GCGTGCCATC GGCCTGGGCG TTACTGAGTG GACGATGGAG

CGGACGTGAT GCGTGCCATT GGGCTGGGCG TGGCCGAGTG GACAGCAGAG

CCGACGTGAT GCGTGCCATT GGCCTGGGTG TGGCCGAGTG GACAGCTGAG

---------- ---------- ---------- ---------- ----------

CAGATGTGAT GCGAGCCATT GGCCTGGGTG TGGCTGAATG GACAGCAGAG

---------- ---------- ---------- ---------- ----------

CGGATGTAAT GCGTGCCATT GGCCTGGGGG TGGCCGAGTG GACGGCAGAA

CAGAGGTGAT GCGCTCCATC GGCCTGGGTG TGGCGGAGTG GACCGCGGAG

CGGAGGTCAT GCGCTCCATC GGCCTGGGCA TCGCCGAGTG GACAGCGGAG

TGCCAGCACC AGTTCCGCCA GCACCGCTGG AATTGCAACA CCCTGGACAG

TGCCAGCACC AGTTCCGCCA GCACCGCTGG AATTGCAACA CCCTGGACAG

TGCCAGCACC AGTTCCGCCA GCACCGCTGG AATTGCAACA CCCTGGACAG

TGCCAGCACC AGTTCCGCCA GCACCGCTGG AATTGCAACA CCCTGGACAG

TGCCAGTACC AGTTCCGCCA GCACCGCTGG AATTGCAACA CTCTGGACAG

TGCCAGCACC AGTTCCGCCA GCACCGCTGG AACTGCAACA CCCTAGACAG

TGCCAGCATC AGTTCCGCCA GCATCGCTGG AACTGCAACA CCCTGGACAG

TGCCAACACC AGTTCCGCCA GCATCGCTGG AACTGCAACA CCCTGGACAG

TGCCAGTATC AGTTCCGACA GCACCGCTGG AACTGCAACA CCCTGGACAG

TGCCAGCACC AGTTCCGCCA GCACCGCTGG AACTGCAACA CCCTGGACAG

TGCCAGCACC AGTTCCGCCA GCACCGCTGG AACTGCAACA CGCTGGACAG

TGCCAGCACC AGTTCCGCCA GCACCGCTGG AACTGCAACA CCCTCGACCG

---------- ---------- ---------- ---------- ----------

TGCCAGCACC AGTTCCGCCA GCACCGTTGG AACTGCAACA CCCTAGACAG

---------- ---------- ---------- ---------- ----------

TGCCAGCACC AGTTCCGACA GCACCGCTGG AACTGTGACA CCCTGGACAG

TGTCAGCACC AGTTTCGCCA GCACCGCTGG AACTGCCACA CCCTGGACAG

TGTCAGCACC AGTTCCGCCA ACACCGATGG AACTGCAACA CCTTGGATCG

GGATCACAGC CTTTTTGGCA GGGTCCTACT CCGAAGTAGT CGGGAATCTG

GGATCACAGC CTCTTTGGCA GGGTCCTACT CCGAAGTAGT CGGGAATCTG

GGATCACAGC CTCTTTGGCA GGGTCCTACT CCGAAGTAGT CGGGAATCTG

GGATCACAGC CTCTTTGGCA GGGTCCTACT CCGAAGTAGT CGGGAATCTG

GGATCACAGC CTCTTTGGCA GGGTCCTACT CCGAAGTAGT CGGGAATCTG

GGATCACAGC CTCTTCGGCA GGGTCCTGCT CCGAAGTAGT CGTGAATCTG

AGACCACAGC CTCTTTGGCC GGGTCCTCCT CCGAAGTAGC CGGGAATCGG

AGATCACAGC CTCTTTGGCC GGGTCCTCCT CCGAAGTAGT CGGGAATCGG

GGATCACGGC CTCTTTGGCA GGGTTCTGCT CCGAAGTAGT CGGGAATCTG

GGATCACAGC CTCTTCGGCA GGGTCCTGCT CCGAAGTAGC CGGGAATCTG

GGATCACAGC CTCTTTGGCA GGGTCCTGCT CCGAAGTAGT CGGGAATCTG

CGACCACAGC CTCTTCGGCA GGGTCCTGCT CCGAAGTAGT CGGGAATCTG

---------- ---------- ---------- -----GTAGT CGGGAATCTG

GGACCACAGC CTCTTTGGCA GGGTCCTGCT CCGAAGTAGT CGGGAATCTG

---------- ---------- ---------- ---------- ----------

AGACCACAGC CTCTTTGGCC GGTTGCTGCT CCGAAGTAGT CGGGAATCTG

GGACCACAAT CTCTTCGGCA AAGTTCTGCT CCGAAGTAGT CGAGAAGCCG

GGACCACAGC CTGTTCGGCC GAGTCTTGCT GCGCAGTAGC CGGGAGGCTG

CCTTTGTTTA TGCCATCTCC TCAGCTGGAG TTGTATTTGC CATCACCAGG

CCTTTGTTTA TGCCATCTCC TCAGCTGGAG TTGTATTTGC CATCACCAGG

CCTTTGTTTA TGCCATCTCC TCAGCTGGAG TTGTATTTGC CATCACCAGG

CCTTTGTTTA TGCCATCTCC TCAGCTGGAG TTGTATTTGC CATCACCAGG

CCTTTGTTTA TGCCATCTCC TCAGCTGGAG TCGTATTTGC CGTCACCAGG

CCTTTGTTTA TGCCATCTCC TCAGCTGGAG TTGTATTTGC CATCACCAGG

CCTTTGTTTA CGCCATCTCC TCAGCTGGCG TTGTATTTGC CATCACCAGG

CCTTTGTTTA CGCCATCTCT TCAGCTGGCG TTGTATTTGC CATCACCAGG

CCTTTGTATA CGCCATCTCC TCAGCTGGAG TTGTATTTGC CATCACCAGG

CGTTTGTTTA CGCCATCTCT TCAGCTGGAG TTGTATTTGC CATCACCAGG

CCTTTGTTTA CGCCGTCTCC TCAGCTGGAG TTGTGTTTGC CATCACCAGG

CCTTTGTTTA CGCCATCTCC TCAGCTGGAG TTGTATTTGC CGTCACCAGG

CCTTTGTTTA CGCCATCTCC TCAGCTGGAG TTGTATTTGC CATCACCAGG

CCTTTGTTTA CGCCATCTCT TCAGCTGGAG TTGTATTTGC CATCACCAGG

---------- ---------- ---------- ---------- ----------

CCTTTGTTTA CGCCATCTCC TCAGCCGGAG TTGTATTTGC TATCACCAGG

CCTTCGTGTA CGCCATCTCC TCCGCCGGCG TCGTGTTTGC CATCACCAGA

CTTTCGTCTA TGCCATCTCC TCTGCCGGAG TTGTCTTTGC CATCACCCGG

GCCTGTAGCC AAGGAGAAGT AAAATCCTGT TCCTGTGATC CAAAGAAGAT

GCCTGTAGCC AAGGAGAAGT AAAATCCTGT TCCTGTGATC CAAAGAAGAT

GCCTGTAGCC AAGGAGAAGT AAAATCCTGT TCCTGTGATC CAAAGAAGAT

GCCTGTAGCC AAGGAGAAGT AAAATCCTGT TCCTGTGATC CAAAGAAGAT

GCCTGTAGCC AAGGAGAAGT AAAATCCTGT TCCTGTGATC CAAAGAAGAT

GCCTGTAGCC AAGGAGAATT AAAATCCTGT TCCTGTGATC CAAAGAAGAA

GCCTGTAGCC AAGGAGAATT AAAGTCCTGT TCCTGCGATC CAAAGAAGAA

GCCTGTAGCC AAGGAGAATT AAAGTCCTGC TCCTGTGATC CAAAGAAGAA

GCCTGTAGCC AAGGAGAATT AAAATCCTGT TCCTGTGATC CAAAGAAGAA

GCCTGTAGCC AAGGAGAATT AAAATCCTGT TCCTGTGACC CAAAGAAGAA

GCCTGTAGCC AAGGAGAGTT AAAATCCTGT TCCTGTGATC CAAAGAAGAA

GCCTGTAGCC AAGGAGAATT AAAATCCTGT TCCTGTGATC CCAAGAAGAA

GCCTGTAGCC AAGAGAA--T AAAATCCTGT TC-TGTGATC CGAAGAAGA-

GCCTGTAGCC AAGGAGAGTT AAAATCCTGT TCCTGTGATC CAAAGAAGAA

---------- ---------- ---------- ---------- ----------

GCCTGTAGCC AAGGAGAGTT AAAATCCTGT TCCTGTGATC CAAAGAAGAA

GCCTGTAGCC AGGGAGAACT AAAATCTTGC TCCTGCGACc caaagaagaa

GCCTGCAGCC AAGGGGAGCT CAAGTCCTGT TCCTGTGACC CCAAGAAGAA

GGGAAGCGCC AAGGACAGCA AAGGCATTTT TGATTGGGGT GGCTGCAGTG

GGGAAGCGCC AAGGACAGCA AAGGCATTTT TGATTGGGGT GGCTGCAGTG

GGGAAGCGCC AAGGACAGCA AGGGCATTTT TGATTGGGGT GGCTGCAGTG

GGGAAGCGCC AAGGACAGCA AGGGCATTTT TGATTGGGGT GGCTGCAGTG

GGGAAGTGGC AAGGACAGCA AGGGCGTTTT TGATTGGGGT GGCTGCAGTG

GGGAACCGCC AAGGACAGCA AGGGCACTTT CGACTGGGGT GGCTGCAGTG

AGGGAGTGGC AAGGACAGCA AAGGTACCTT CGACTGGGGC GGCTGTAGTG

AGGAAGTGCC AAGGACAGCA AAGGCACCTT CGACTGGGGT GGCTGCAGTG

AGGAACTGCC AAGGACAGCA AGGGCACCTT CGACTGGGGT GGCTGCAGTG

GGGAACCGCC AAGGATAACA AGGGCACTTT TGACTGGGGT GGCTGCAGTG

GGGAACCGCC AAGGACAGCA AGGGCAGTTT CGACTGGGGT GGCTGCAGCG

GGGGACCGCC AAGGACAGCA AGGGCACTTT CGACTGGGGC GGCTGCAGTG

GGGAACAGCC AAGGACAGCA AAGGCACTTT CGACTGGGGG GGCTGCAGTG

GGGAACCGCC AAGGACAGCA AGGGCACTTT CGATTGGGGT GGCTGCAGTG

---------- ---------- ---------- ---------- ----------

GGGTACTGCC AAGGACAGCA GGGGCACTTT CGATTGGGGT GGCTGCAGCG

aggaacttcc aaagacagca aaggcacttT TGATTGGGGC GGATGCAGCG

AGGCTCTGCC AAGGACAGCA AGGGCACTTT CGACTGGGGC GGATGCAGCG

ATAACATTGA CTATGGGATC AAATTTGCCC GCGCATTTGT GGATGCaaag

ATAACATTGA CTATGGGATC AAATTTGCCC GCGCATTTGT GGATGCaaag

ATAACATTGA CTATGGGATC AAATTTGCCC GCGCATTCGT GGATGCCaag

ATAACATTGA CTATGGGATC AAATTTGCCC GCGCATTCGT GGATGCCaag

ATAACATTGA CTATGGGATC AAGTTTGCCC GCGCATTTGT GGATGCCAAG

ACAACATTGA CTATGGGATC AAATTTGCCC GAGCATTTGT GGATGCCAAG

ACAACATTGA CTACGGGATC AAATTCGCCC GTGCATTTGT AGATGCCaag

ACAATATTGA CTACGGGATC AAGTTTGCCC GTGCCTTTGT AGATGCCAAG

ATAACATTGA CTATGGGATC AAGTTTGCCC GAGCCTTTGT GGATGCCaag

ATAACATTGA CTATGGGATC AAGTTTGCCA GAGCGTTTGT GGATGCCAAG

ATAACATTGA CTATGGGATC AAATTTGCCC GAGCCTTCGT GGATGCCAAG

ACAACATTGA CTATGGGATC AAGTTTGCGC GGGCGTTCGT GGACGCCAAG

ATAACAT-GA CTATGGGATC AAGTTTGCCC GAGCGTTTGT GGATGCCAAG

ATAATATTGA CCATGGAATC AAATTTGCCC GTGCATTTGT GGATGCCaag

---------- ---------- ---------- ---------- ----------

ACAACATCGA TTATGGGGTC AAGTTTGCCC GCGCCTTCGT GGATGCCAAG

ATAACATTGA CTATGGCATC AAATTTGCCC GAGCGTTTGT GGATGCCAAG

ATAACATTGA CTATGGCATC AAGTTCGCCC GTGCGTTTGT GGACGCCAAG

gaaaggaaag gaaaggaTGC CAGAGCCCTG ATGAATCTTC ACAACAACAG

gaaaggaaag gaaaggaTGC CAGAGCCCTG ATGAATCTTC ACAACAACAG

gaaaggaaag gaaaggaTGC CAGAGCCCTG ATGAATCTTC ATAACAACAG

gaaaggaaag gaaaggaTGC CAGAGCCCTG ATGAATCTTC ATAACAACAG

GAGAGGAAAG GAAAGGATGC CAGAGCCCTG ATGAATCTTC ATAACAACAG

GAAAGAAAAG GAAAGGACGC CAGAGCCCTG ATGAATCTTC ACAACAACAG

gaaaggaaag gaaaggaTGC CAGAGCCCTG ATGAATCTTC ACAACAACAG

GAGAGGAAAG GCAAGGATGC CAGAGCCCTG ATGAACCTTC ACAACAACAG

gaaaggaaag gaaaggaTGC CAGAGCGCTG ATGAATCTTC ACAACAACAG

GAAAGGAAAG GCAAGGATGC CAGAGCCCTG ATGAATCTTC ACAACAACCG

GAAAGGAAGG GAAAGGATGC CAGAGCCCTG ATGAACCTTC ACAACAACAG

GAGCGGAAGG GCAAGGACGC CAGAGCGCTG ATGAACGTGC ACAACAACAG

GAAAGGAAAG GAAAGGATGC CAGGGCCCTG ATGAATCTCC ACAACAACAG

gaaaggaaag gaaaggaCGC CAGAGCCCTG ATGAACCTTC ACAACAACAG

---------- ---------- ---------- ---------- ----------

GAGAAGAAAG GGAAGGACGC CAGGGCGCTG ATGAACCTTC ACAACAACAG

GAAAGGAAAG GAAAAGATGC CCGAGCACTG ATGAACCTTC ACAACAACAG

GAGAGGAAAG GAAAAGATGC CAGAGCCCTA ATGAATCTTC ACAACAACCG

AGCTGGCAGG AAGGCTGTAA AGCGGTTCTT GAAACAAGAG TGCAAGTGCC

AGCTGGCAGG AAGGCTGTAA AGCGGTTCTT GAAACAAGAG TGCAAGTGCC

AGCTGGCAGG AAGGCTGTAA AGCGGTTCTT GAAACAAGAG TGCAAATGCC

AGCTGGCAGG AAGGCTGTAA AGCGGTTCTT GAAACAAGAG TGCAAATGCC

AGCTGGCAGG AAGTCTGTAA AGCGGTTCTT GAAACAAGAG TGCAAGTGCC

AGCTGGCAGG AAGGCTGTAA AGCGGTTCTT GAAACAAGAA TGCAAGTGTC

AGCTGGAAGG AAGGCTGTAA AGCGCTTCCT GAAACAAGAA TGCAAGTGTC

AGCTGGAAGG AAGGCTGTAA AGCGCTTCTT GAAACAAGAA TGCAAGTGTC

AGCGGGCAGG AAGGCTGTAA AGCGGTTCTT GAAACAAGAG TGCAAGTGCC

AGCCGGCAGG AAGGCTGTAA AGAGGTTCTT GAAACAGGAG TGCAAGTGTC

AGCCGGCCGG AAGGCGGTGA AGCGGTTCTT GAAGCAGGAA TGCAAGTGTC

AGCCGGCCGG AAGGCTGTGA AGCGCTTCCT GAAACAAGAG TGCAAGTGTC

AGCCGGCAGG AAGGCTGTCA AGCGCTTTTT GAAGCAGGAG TGCAAGTGCC

AGCCGGGAGG AAGGCTGTAA AGCGGTTCTT GAAACAAGAG TGCAAGTGTC

---------- ---------- ---------- ---------- ----------

AGCCGGCAGG AAGGCGGTCA AGCGCTTCTT GAAACAAGAG TGCAAATGTC

AGCTGGGAGG AAGGCTGTAA AGCGGTTCTT GAAACAAGAG TGCAAATGCC

TGCAGGAAGA AAGGCCGTCA AGCGGTTCCT GAAGCAAGAG TGTAAATGCC

ACGGGGTGAG CGGCTCATGT ACTCTCAGGA CATGCTGGCT GGCCATGGCC

ACGGGGTGAG CGGCTCATGT ACTCTCAGGA CATGCTGGCT GGCCATGGCC

ATGGGGTGAG CGGCTCGTGT ACTCTCAGGA CATGCTGGCT GGCCATGGCC

ATGGGGTGAG CGGCTCGTGT ACTCTCAGGA CATGCTGGCT GGCCATGGCC

ACGGGGTGAG CGGCTCGTGT TCTCTCAGGA CATGCTGGCT GGCCATGGCC

ACGGTGTGAG TGGCTCCTGC ACCCTGAGGA CATGCTGGCT GGCCATGGCC

ATGGCGTGAG CGGTTCCTGT ACTCTGAGGA CATGCTGGCT GGCCATGGCT

ATGGTGTGAG TGGCTCCTGT ACTCTGAGGA CATGCTGGCT GGCCATGGCT

ACGGTGTGAG CGGCTCGTGT ACTCTGCGGA CGTGCTGGCT GGCCATGGCC

ATGGCGTGAG CGGTTCCTGT ACCCTGAGGA CCTGCTGGCT GGCCATGGCT

ACGGCGTGAG TGGCTCGTGT ACGCTGAGGA CATGCTGGCT GGCCATGGCG

ATGGCGTGAG TGGCTCATGT ACGCTGAGGA CCTGCTGGCT GGCCATGGCT

ACGGTGTGAG TGGCTCCTGC ACTTTGAGGA CGTGCTGGCT GGCCATGGCT

ACGGCGTGAG TGGTTCCTGT ACTCTGAGGA CATGCTGGCT GGCCATGGCT

---------- ---------- ---------- ---------- ----------

ACGGGGTGAG CG-CTCCTGT ACTCTGAGGA CGTGCTGGCT GGCCATGGCC

ATGGGGTGAG TGGATCCTGC ACCCTGAGGA CCTGTTGGCT GGCCATGGCT

ATGGGGTGAG TGGATCCTGT ACTCTGAGGA CCTGTTGGCT CGCCATGGCG

GACTTCAGGA AAACGGGCGA TTATCTCTGG AGGAAGTACA ATGGGGCCAT

GACTTCAGGA AAACGGGCGA TTATCTCTGG AGGAAGTACA ATGGGGCCAT

GACTTCAGGA AAACGGGCGA TTATCTCTGG AGGAAGTACA ATGGGGCCAT

GACTTCAGGA AAACGGGCGA TTATCTCTGG AGGAAGTACA ATGGGGCCAT

GACTTCAGGA AAACGGGAGA TTATCTCTGG AGGAAGTACA ATGGGGCCAT

GACTTCAGAA AGACGGGCTC CTATCTCTGG AGGAAGTACA ACGGAGCCAT

GACTTCAGGA AAACAGGCGA CTATCTCTGG AGGAAGTACA ACGGGGCCAT

GACTTCAGGA AAACAGGCGA CTATCTCTGG AGGAAGTACA ATGGGGCCAT

GACTTCAGGA AAACGGGCGA TTACCTCTGG AGGAAGTACA ATGGAGCCAT

GACTTCAGGA AAACGGGCAA TTATCTCTGG AGGAAGTACA ATGGGGCCAT

GACTTCAGAA AAACGGGCGA TTATCTCTGG AGGAAGTACA ACGGGGCCAT

GACTTCAGGA AAACGGGCGA TTACCTGTGG AGGAGGTACA ACGGCGCCAT

GACTTCAGAA AAACAGGCGA TTATCTCTGG AGGAAGTACA ACGGGGCCAT

GACTTCAGGA AAACGGGTGA CTATCTCTGG AGGAAGTACA ATGGAGCCAT

---------- ---------- ---------- ---------- ----------

GACTTCAGAA AGACGGGCGA TTATCTCTGG AGGAAGTACA ACGGGGCCAT

GACTTTAGAA AGACGGGAGA TTATCTCTGG AAGAAGTACA ATGGGGCCAT

GACTTTAGGA AAACAGGAGA TTATCTGTGG AGAAAATACA ACGGGGCCAT

CCAGGTGGTC ATGAACCAGG ATGGCACAGG TTTCACTGTG GCTAACGAGA

CCAGGTGGTC ATGAACCAGG ATGGCACAGG TTTCACTGTG GCTAACGAGA

CCAGGTAGTC ATGAACCAGG ATGGCACAGG TTTCACTGTG GCTAACGAGA

CCAGGTGGTC ATGAACCAGG ATGGTACAGG TTTCACTGTG GCTAACGAGA

CCAGGTGGTC ATGAACCAGG ATGGCACAGG TTTCACTGTG GCTAACGAGA

CCAGGTGGTC ATGAACCAGG ATGGCACTGG CTTCACAGTA GCTAACAAGA

CCAGGTGGTC ATGAACCAGG ACGGCACTGG CTTCACTGTA GCCAATAAGA

CCAGGTAGTC ATGAACCAGG ATGGCACTGG CTTCACTGTA GCCAATAAGA

CCAGGTCGTC ATGAACCAGG ATGGCACTGG TTTCACTGTG GCTAACAAGA

CCAGGTTGTC ATGAACCAGG ATGGCACTGG TTTCACTGTG GCTAACAAGA

CCAGGTCGTC ATGAACCAGG ATGGCACGGG GTTCACGGTG GCTAACAAGA

CCAGGTCGTC ATGAATCAGG ATGGCACTGG TTTCACTGTG GCTAACAAGA

CCAGGTCGTC ATGGACCAGG ATGGCACCGG CTTCACTGTG GCTAACAAGC

CCAGGTCGTC ATGAACCAAG ATGGCACTGG TTTCACTGTG GCTAACAAGA

---------- ---------- ---------- ---------- ----------

CCAGGTCGTC ATGAACCAGG ACGGCACCGG TTTCACTGTG GCCAACAAGA

CCAAGTGGTC ATGAATCAAG ATGGCACTGG TTTCACTGTG GCTAACAAGA

TCAGGTGGTC ATGAATCAAG ATGGGACCGG CTTCACCGTG GCAAACAAGA

GGTTTAAGAA GCCAACGAAA AATGACCTCG TGTATTTTGA GAATTCTCCA

GGTTTAAGAA GCCAACAAAA AATGACCTCG TGTATTTTGA GAATTCTCCA

GGTTTAAGAA GCCAACCAAA AATGACCTCG TGTATTTTGA GAATTCTCCA

GGTTTAAGAA GCCAACAAAA AATGACCTCG TGTATTTTGA GAATTCTCCA

GGTTTAAGAA GCCAACAAAA AATGACCTCG TGTATTTTGA GAATTCTCCA

GGTTTAAGAA ACCAACAAAA AATGACCTCG TGTATTTTGA GAATTCTCCA

GGTTTAAGAA GCCAACGAAA AATGACCTTG TGTATTTTGA GAATTCTCCA

GGTTTAAGAA GCCAACGAAA AATGACCTCG TGTATTTTGA GAATTCTCCA

GGTTTAAGAA GCCAACGAAA AATGACCTTG TGTACTTTGA GAATTCTCCA

GGTTTAAAAA GCCAACGAAA AATGACCTCG TGTATTTTGA GAATTCTCCA

GGTTCAAGAA GCCAACCAAA AATGACCTCG TGTATTTTGA GAATTCTCCA

GGTTTAAGAA ACCAACAAAA AACGACCTCG TATATTTTGA GAATTCTCCA

GCTTTAAGAA GCCCACGAAA AATGACCTTG TGTACTTTGA GAATTCTCCT

GGTTTAAGAA ACCAACGAAA AATGACCTTG TATATTTTGA GAATTCTCCA

---------- ---------- ---------- ---------- ------TCCA

GGTTCAAGAA GCCAACGAAA AATGACCTTG TGTACTTTGA GAATTC-CCA

GATTTAAGAA GCCAACCAAA AATGACCTGG TCTATTTTGA GAATTCTCCA

GGTTCAAGAA ACCAACCAAA AATGACTTAG TGTATTTTGA GAACTCTCCA

GACTACTGTA TCAGGGACCG AGAGGCAGGC TCCCTGGGTA CAGCAGGCCG

GACTACTGTA TCAGGGACCG AGAAGCAGGC TCCCTGGGTA CAGCAGGCCG

GACTACTGTA TCAGGGACCG AGAGGCAGGC TCCCTGGGTA CAGCAGGCCG

GACTACTGTA TCAGGGACCG AGAGGCAGGC TCCCTGGGTA CAGCAGGCCG

GACTACTGTA TCAGGGACCG AGAGACAGGC TCCCTGGGTA CAGCAGGCCG

GACTACTGTA TCAGGGACCG AGAGGCAGGC TCCCTGGGTA CAGCAGGCCG

GACTACTGTA TCAGGGACCG AGAGGCAGGC TCCCTGGGTA CAGCAGGCCG

GACTACTGTA TCAGGGACCG AGAGGCAGGC TCCCTGGGTA CAGCGGGCCG

GACTACTGTA TCAGGGACCG AGAGGCAGGC TCCCCGGGTA CAGCAGGTCG

GACTACTGTA TCAGGGACCG AGATGCAGGC TCCCTGGGTA CAGCGGGCCG

GACTATTGTA TCAGGGACCG AGACGCAGGC TCCCCGGGGA CGGCGGGCCG

GACTACTGTA TCAGGGACCG AGATGCAGGC TCCCTGGGTA CAGCAGGACG

GACTACTGTA TCCGGGACCG AGATGCAGGC TCCTTGGGTA CAGCAGGCCG

GACTACTGTA TCAGGGACCG AGATGCAGGC TCTCTGGGTA CAGCAGGCCG

GACTACTGTA TCAGGGACCG AGACGCAGGC TCCCCGGGTA CAGCAGGTCG

GACTACTGTA TCAGGGACCG GGATGCAGGC TCCCCAGGTA CAGCAGGCCG

GACTATTGTA TCAGAGACCG AGATGCAGGT TCCCTGGGTA CTGCAGGTCG

GACTACTGTA TCAAGGACCG GGATGCAGGC TCCCTAGGGA CGGCGGGCCG

TGTGTGCAAC CTGACTTCCC GGGGCATGGA CAGCTGTGAA GTCATGTGCT

TGTGTGCAAC CTGACTTCCC GGGGCATGGA CAGCTGTGAA GTCATGTGCT

TGTGTGCAAC CTGACTTCCC GGGGCATGGA CAGCTGCGAA GTCATGTGCT

TGTGTGCAAC CTGACTTCCC GGGGCATGGA CAGCTGCGAA GTCATGTGCT

TGTGTGCAAC CTGACTTCCC GGGGCATGGA CAGCTGTGAA GTCATGTGCT

TGTGTGCAAC CTGACGTCCC GGGGCATGGA CAGCTGTGAA GTCATGTGCT

TGTGTGCAAC TTGACTTCCC GAGGCATGGA CAGCTGCGAA GTTATGTGTT

TGTGTGCAAC TTGACTTCCC GAGGCATGGA CAGCTGCGAA GTTATGTGTT

TGTGTGCAAC CTGACGTCCA GAGGCATGGA CAGCTGTGAA GTCATGTGCT

TGTGTGCAAC CTGACTTCCC GGGGCATGGA CAGCTGCGAA GTCATGTGCT

CGTGTGCAAC CTGACTTCCC GCGGCATGGA CAGCTGCGAG GTCATGTGCT

TGTGTGCAAC CTGACCTCCC GAGGCATGGA CAGCTGTGAA GTCATGTGCT

TGTGTGCAAC CTGACTTCCC GAGGCATGGA CAGCTGTGAA GTCATGTGCT

TGTGTGCAAC CTGACTTCCC GAGGCATGGA CAGCTGCGAA GTCATGTGCT

AGTGTGCAAC CTGACTTCCC GAGGCATGGA CAGCTGCGAA GTCATGTGCT

AGTCTGCAAC CTGACCTCCC GAGGCATGGA CAGCTGCGAA GTCATGTGCT

GGTGTGCAAC CTGACCTCCC GTGGCATGGA CAGCTGTGAA GTCATGTGCT

GGTGTGCAAC CTGACGTCCC GCGGCATGGA CAGCTGCGAG GTCATGTGCT

GTGGGAGAGG CTACGACACC TCCCATGTCA CCCGGATGAC CAAGTGTGGG

GTGGGAGAGG CTACGACACC TCCCATGTCA CCCGGATGAC CAAGTGTGGG

GTGGGAGAGG CTACGACACC TCCCATGTCA CCCGGATGAC CAAGTGTGGG

GTGGGAGAGG CTACGACACC TCCCATGTCA CCCGGATGAC CAAGTGTGGG

GTGGAAGAGG CTACGACACC TCCCATGTCA CCCGGATGAT CAAGTGTGGG

GCGGGAGAGG CTATGACACC TCCCGTGTCA CCCGGATGAC CAAGTGCGAG

GTGGGAGAGG CTATGACACA TCCCATGTCA CCCGGATGAC CAAGTGTGAG

GTGGGAGAGG CTATGACACA TCCCACGTCA CCCGGATGAC CAAGTGTGAG

GCGGGAGAGG CTACGACACC TCCCGCGTCA CCCGGATGAC CAAGTGCGAG

GTGGCAGAGG CTACGACACC TCCCACATTA CCCGGAAGAC CAAGTGTGAG

GCGGGAGAGG CTACGACACG TCCCGCGTCA CGCGGATGAC CAAGTGCGAG

GCGGGAGAGG CTACGACACG TCCCGCGTCA CGAGGATGAC CAAGTGTGAG

GCGGGAGAGG CTATGACACC TCCCGAGTCA CCCGGATGAC CAAATGCGAG

GCGGGAGAGG CTACGACACC TCCCATGTCA CCCGGATGAC CAAATGTGAG

GCGGGAGAGG CTACGACACC TCTCGCATCA CCCGGATGAC CAAGTGCGAG

GCGGGAGAGG CTACGACACC TCCCGCGTCA CCCGCATGAC CAAGTGTGAG

GCGGAAGGGG CTATGACACT TCCCGGGTCA CCCGAATGAC CAAATGCGAG

GCGGGAGGGG CTACGACACG GCCCGCGTCA CCAGGATGAC CAAGTGCGAG

TGTAAGTTCC ACTGGTGCTG CGCCGTGCGC TGTCAGGACT GCCTGGAAGC

TGTAAGTTCC ACTGGTGCTG CGCCGTGCGC TGTCAGGACT GCCTGGAAGC

TGTAAGTTCC ACTGGTGCTG CGCCGTGCGC TGTCAGGACT GCCTGGAGGC

TGTAAGTTCC ACTGGTGCTG CGCCGTGCGC TGTCAGGACT GCCTGGAGGC

TGTAAGTTCC ACTGGTGCTG TGCCGTGCGC TGTCAGGACT GCCTGGAAGC

TGTAAGTTCC ACTGGTGCTG CGCCGTCCGC TGTCAGGACT GCCTGGAGGC

TGTAAATTCC ACTGGTGCTG TGCCGTGCGC TGTCAGGACT GCCTGGAAGC

TGTAAATTCC ACTGGTGCTG TGCCGTGCGC TGTCAGGACT GCCTGGAGGC

TGTAAGTTCC ACTGGTGCTG TGCCGTGCGT TGCCAGGACT GCCTGGAGGC

TGTAAGTTCC ACTGGTGCTG TGCCGTGCGC TGCCAGGACT GCGTGGAGGC

TGTAAGTTCC ACTGGTGCTG CGCCGTGCGC TGTCAGGACT GCCTGGAGGC

TGTAAGTTCC ACTGGTGCTG TGCTGTGCGT TGTCAGGACT GCCTGGAGGC

TGTAAGTTCC ACTGGTGCTG CGCCGTGCGC TGTCAGGACT GCCTGGAGGC

TGTAAATTCC ACTGGTGCTG TGCTGTGCGC TGTCAGGACT GCCTGGAGGC

TGTAAGTTCC ACTGGTGCTG CGCCGTGCGC TGTCAGGACT GCCTGGAGGC

TGTAAGTTCC ACTGGTGCTG TGCCGTGCGC TGTCAGGACT GCCTGGAGGC

TGTAAGTTCC ATTGGTGCTG TGCTGTGCGC TGTCAGGACT GTTTGGAAGT

TGCAAGTTCC ACTGGTGCTG CGCCGTCCGC TGCCAGGACT GCCTGGAAGC

TCTGGATGTG CACACATGCA AGGCCCCCAA GAACGCTGAC TGGACAACCG

TCTGGATGTG CACACATGCA AGGCCCCCAA GAACGCTGAC TGGACAACCC

CCTGGATGTG CACACATGCA AGGCCCCCAA GAACGCTGAC TGGACAACCC

CCTGGATGTG CACACATGCA AGGCCCCCAA GAACGCTGAC TGGACAACCC

CCTGGACGTG CACACATGCA AGGCACCCAA GAACGCTGAC TGGAAAAGCC

CTTGGATGTG CACACATGCA AGGCCCCCAA GAACGCCGAC TGGACAACCC

CCTGGACGTG CACACATGCA AGGCCCCCAA GAGTGCCGAC TGGACAACTC

CCTGGACGTG CACACATGCA AGGCCCCCAA GAGTGCCGAC TGGGCGACGC

CCTGGACGTG CACACATGCA AGGCTCCCAA GAGTGCTGAC TGGACAACCC

CCTGGACGTG CACACGTGCA AGGCCCCGAA GAGCCCCGAC TGGGCTGCTC

CCTAGACGTG CACACGTGCA AGGCCCCCAC GAGCGCCGAC TGGGCGGCTC

CCTGGACGTA CACACATGCA AGGCTCCGAA GAGTGCCGAC TGGGCAGCTC

CCTCGATGTG CACACGTGCA AGGCCCCCAA GAGCGCTGAT TGGGCAGCTC

TCTGGACGTG CACACATGCA AGGCCCCCAA GAGTGCTGAC TGGGCAGTTC

TCTGGATGTG CACACGTGCA AGGCCCCCAA GAGCGCCGAC TGGGCAGCTC

TCTGGACGTG CACACCTGCA AAGCCCCCAA GAGCGCCGAC TGGGCGGTGC

AGTGGACGTG CACACGTGCA AAGCCCCCAA GAGTCCTGAT TGGGTGGCAC

CCTCGACGTG CACACGTGCA AAGCCCCCAG CAGCGCCAGC GAGGGCGCCC

CTACAATGGG CCGCGAGTTT GGGAATCTGA CGCGGATGCG GCATGTGATC

CTACAATGGG CCGCGAGTTT GGGAATCTGA CGCGGATGCG GCATGTGATC

CCACAATGGG CCGCGAGTTT GGGAATCTGA CGCGGATTCG GCATGTGATC

CCACAATGGG CCGCGAGTTT GGGAATCTGA CGCGGATTCG GCATGTGATC

CCACAATGGG TCGCGAATTT GGGAATCTGA CGCGGATGCG GCATGTGATC

CCACAATGGG CCGCGAGTTT GGGAACCTGA CGCGGATGCG GCATGTGATC

CTACAATGGG CCGCGAGTTT GGGAACCTGA CGCGGATACG GCATGTGATC

CTACAATGGG CCGCGAGTTT GGGAACCTGG CGCGGATACG GCACGTGATC

CAACAATGGG CCGCGAGTTT GGGAACCTGA CGCGGGTGCG GCATGTGATC

CGACAATGGG CCGCCAGTTT GGGCATCTGA CACGGGTGCG GCATGTGATC

CCACAATGGG CCGCGAGTTT GGGAACCTGA CCCGGATGCG GCATGTGATC

CCACA----- ---------- ---------- ---------- ----------

CCACTATGGG CCGCGAGTTC GGGCACCTGG CGCGGATGCG ACATGTGATC

CCACAATGGG TCGCGAATTT GGGAACCTGA CGCGGATGCG GCATGTGATC

CTACAATGGG CCGCGAGTTT GGGAACCTGG CGCGGGTGCG GCACGTGATC

CCACA----- ---------- ---------- ---------- ----------

CCACAATGGG CCGCGAGTTT GGGAACCTAA TCCGGATAAG GCATATCATT

CCACAATGGG TCGTGAGTTC GGAAACCTAA TAAAAGTGAG GCATATCATT

AGCTACAGCT TGTCACCGTT CGAGCAGCGC GCCTATCCGC ACGTCTTCAC

AGCTACAGCT TGTCACCTTT CGAGCAGCGC GCCTATCCGC ACGTCTTCAC

ACCTACAGCT TGTCACCTTT CGAGCAGCGC GCCTTGCCGC ATGTTTTCAG

ACCTACAGCT TGTCACCTTT CGAGCAGCGC GCCTTGCCGC ACGTTTTCAG

TCCTACAGCT TGTCACCTTT CGAGCAGCGC GCCTTCCCGC ACGTCTTCTC

ACCTACAGCT TGTCACCCTT CGAGCAGCGC GCTTTCCCGC ACTACTTCAC

TCCTACAGCT TGTCGCCCTT TGAGCAGCGC GCCTTCCCGC ACTATTTCAG

TCCTACAGCT TGTCGCCCTT TGAGCAGCGC GCCTTCCCAA GCTATTTCAG

AGCTACAGCT TGTCGCCCTT CGAGCAGCGC GCCTTCCCGC ACTTCTTCAG

ACCTACAGCT TGTCGCCCTT CGAGCAGCGC GCCTTCCCGC ACTACTTCAG

ACCTACAGTT TGTCGCCCTT CGAGCAGCGC GCCTTCCCGC ACTACTTCAG

---------- ---------- ---------- ---------- ----------

TCCTACAGCC TGTCTCCCTT CGAACAGCGC GCCTTCCCGC ACTACTTCAG

ACCTACAGCT TGTCACCCTT TGAGCAGCGC GCCTTTCCGC ACTACTTCAG

ACCTACAGCT TGTCGCCCTT CGAACAGCGC GCTTTCCCTC ACTACTTCAC

---------- ---------- ---------- ---------- ----------

TCCTACAGCC TGTCCCCGTT CGAGCAGAGG GCCTTCCCCA ACTACTTCTC

TCCTATAGCC TGTCGCCATT TGAGCAGAGG GCATTCCCCA ACTTCTTCAT

TAAAGGAATC CCCAATGTTC TGCGCCGCAT TCGGGAGTCT TTCTTTCGCG

TAAAGGAATC CCCAACGTTC TGCGCCGCAT TCGGGAGTCT TTCTTTCGCG

TAAAGGAGTC CCCAACATGC TGCGCCGCGT TCGGGAGTCT TTCTTTCGCA

TAAAGGAGTC CCCAACATGC TGCGCCGCGT TCGGGAGTCT TTCTTTCGCA

TAAAGGAGTC CCCAACGTGG TGCGCCGCAC TCGGGAGTCT TTCCTTCGCG

TAAGGGAATC CCCAACATGA TTCGCCGCAT TCAGGCATCT TTCCTTCGGG

CAAAGGCATC CCCAACGTGC TGCGCCGCAC TCGCGAGCGC ATCCTGCGCG

CAAAGGCATC CCCAACGTGC TGCGCCGCAC TCGCGAGCGC ATCCTGCGCG

CAAGGGCATC CCCAACCTGC TGCGCCGCAC CCGGGAGTCC GTGCTTCGCG

CAAGGGCATC CCCAACGTTC TGCGCCGAAC TCGGGCGTGC ATCCTTCGCG

CAAAGGCATC CCCAACGTGC TGCGCCGTAC GCGGGCGTGC GCCCTTCGCG

---------- ---------- ---------- ---------- ----------

CAAGGGCATC CCCAACGTGC TGCGCCGCAC GCAGGCGTCC ATCCTTCGCG

CAAGGGTATC CCGAACGTGC TGCGCCGCAC TCGGGCGTCC ATCCTTCGCG

CAAGGGCATC CCCAACGCGC TGCGCCGTAT TCGGGCGTCC ATCCTGCGGG

---------- ---------- ---------- ---------- ----------

AAAGGGCATC CCCAACATGA TCCGCCGGGT GCAGGACTCC ATCTTGCGGG

CAAAGGCATT CCGAATGTCT TACGGCGAAC GCGAGAGTCC ATCCTGAAGG

TGGTGCCGCA GTTTGTAGTG TTTTATCTTA TCTACACATG GGGGACTGAA

TGGTGCCGCA GTTTGTAGTG TTTTATCTTA TCTACACATG GGGGACTGAA

TGGTGCCGCA GTTTGTAGTG TTTTATCTTC TCTACAAATG GGGGACTGAA

TGGTGCCGCA GTTTGTAGTG TTTTATCTTA TCTACAAATG GGGGAGTGAA

TGGTGCCGCC GTTTATAGCG TTTTATCTTC TCTACACATG GGGGACTGAA

TGGCGCCGC- ---------- ---------- ---------- ----------

TGGCGCCGCC ATTTGTACTG TTCTATCTGA TCTACACATG GGGCAACCAG

TGGCGCCGCC GTTTGTAGTG GTCTACCTGA TCTACACATG GGGCAACCAG

TGGTGCCCCC ATTTGTCGTG TTTTACCTTG TCTACACCTG GGGGAACGAG

TCGCGCCGCC GTTCGTAGCG TTTTATCTTG TCTACACATG GGGAACGCAG

TCGTGCCCCC GTTCATAGCG TTTTACCTTG TCTACACATG GGGAACCCAG

---------- ---------- ---------- ---------- ----------

TGGTCCCGCC ATTTATAGGG TTTTACCTTG TCTACACGTG GGGAACCCAG

TCATGCCGCC ATTTGTAGCA TTTTATCTTA TCTACACATG GGGAAACGAG

TCGCGCCCCC ATTTGTAGTG TTCTATTTTG TCTACACATG GGGGACCCAG

---------- ---------- ---------- ---------- ----------

TCGCCCCCCC ATTTATAGGC TTTTACCTCC TCTACACCTG GGGAAACCAG

TAGCCCCTCC ATTTGTAGCC TTTTATATGC TGTACACCTG GGGCAATGAA

GAGTTCGAGA GATCCAAGAG GAAGAATCCA GCTGCCTATG AAAATGACAA

GAGTTCGAGA GATCCAAGAG GAAGAATCCA GCTGCCTATG AAAATGACAA

GAGGTCGAGA AATCCAAGAG GAAGAATCCA GCTGCCTATG AAAATGACAA

GAGGTCGAGA AATCCAAGAA GAAGAATCCA GCTGCCTATG AAAATGACAA

GAGTTTGAGC GATCCAAGAG GAAGAATCCA GCTGACTATG AAAATGACAA

---------- ---------- ---------- ---------- ----------

GAGTTTGCGC AGTCCAAACG GAAGAATCCA GCTAAGTACG AGAATGACAA

GAGTTTGAGC AGTCGAAAAG GAAGAATCCA GCCATGTATG AAAATGACAA

GAGTTTGAGA AATCCAAGAG GAAGAATCCC GCCGCCTACG AACATGACAA

GAGTTTGAGA AATCGAAGAG GAAGAATCCA GCTGCCTATG AAAATGACAG

GAATTTGAGA AATCCAAGAG GAAGAATCCA GCTGCCTATG AAAATGACAA

---------- ---------- ---------- ---------- ----------

GAATTTGAGA AATCCAAGAG GAAGAATCCA GCCGACTATG AAAATGACTC

GAGTTTGAGA AATCCAAGAG GAAGAATCCA GCTGCCTATG AAAATGACAA

GAGTTTGAAA AATCCAAGAG GAAAAATCCA GCCGACTATG AAAATGACAA

---------- ---------- ---------- ---------- ----------

GAGTTTGAGA ATTCAAAGAA GAAGAAGCCA GATGCCTTTG AGGAAGACAA

GAATTTGAAA AGTCCAAGAG GAAGAACCCC GCTGACTATG AAAACGACAA

AATGTGGCAC CTCAAACTTT GTGCAGTCCT CATGATCTTC CTGTTGCTGT

AATGTGGCAC CTCAAACTTT GTGCAGTCCT CATGATCTTC CTGTTGCTGT

AATGTGGCAC CTCAAACTTT GTGCAGTCCT CATGATCTTC CTCTTGCTGT

AATGTGGCAC CTCAAACTTT GTGCAGTCCT CATGATCTTC CTCTTGCTGT

AATGTGGCAC CTCAAACTTT GTGCACTGCT CATGATCTTC CTGTTGCTGT

-ATGTGGCAC CTCAAACTCT GTGCAGTGCT CATGATCTGC CTGCTGCTCT

GATGCTACAG CTAAAACTCT TTGCAGTGCT TCTGACTTGC CTGCTGCTGC

GATGCTACAG CTAAAACTCT TTGCAGTGCT CCTGACTTGC CTGCTGCTGC

GATGTGGCAC CTCAAACTCT TTGCAGTGCT CATGATCTGC CTATTGCTGT

AATGTGGCAC CTCAAACTCT TTGCAGTACT CATGATCTGC CTGTTGCTGT

AATGTGGCAC CTCAAACTCT TTGCAGTGCT CATGATCTGC TTGCTGCTGT

-ATGTGGCAC CTCAAACTCG TTGCACTGCT TGTGATCTGT CTGTTGCTGT

AATGTGGTTC CTCAAACTCT TTGCAGTGCT CATGCTCTGC CTACTGCTCG

AATGTGGCAC CTCAAACTCT TGGCAGTGCT TATGACCTGC CTGCTGCTGT

GATGTGGCCC CTCAGGCTCT TTGCAGTGCT CATGACCTGC CTCCTGCTGT

-ATGTTGCAC CTCAAACTCT TTGCAGTGCT TTTGACCTGC CTGTTGCTGT

GATGGGGCAT TTTAAGCTCT TAGCCCTGTT CATCATGTGC CTGCTGCTCC

GAGGAGGCGG CTGAATCTCT GGGCAGCTCT CCTGCTGTGC TTGGGGCTCC

TGGGCCAGAT AGATGGCTCC CCAATACCAG AAGTGAGTTC GGCAAAGAGA

TGGGCCAGAT AGATGGCTCC CCAATACCAG AAGTGAGTTC AGCAAAGAGA

TGGGCCAGAT AGATGGCTCC CCAATAACAG AAGTGAGTTC AGCAAAGAGA

TGGGCCAGAC AGATGGCTCC CCAATACCAG AAGTGAGTTC AGCAAAGAGA

TGGGCCAGAT AAATGGCTCC CCAATACCAG AAGTGAGTTC AGCAAAGAGA

TGGGCCAGGT AGAAGGTTCC CCAATACCAC AACTGAGTTC AGCAAAGAGA

TGGGCCAGGC CCAAAGCTCC CCAGTACCAG AACTGAGTTC AGCAAAGAGA

TGGGTCAGGT CAATAGTTCC CCAGTACCAG AAGTGAGTTC AGCAAAGAGA

TGGGCCAGGT AGATGGCTCC CCAGTACCAG AGCTGAGCTC AGCAAAGAGG

TAGCCCAGGT AGATGGCTCT CCAATACCAC AACAGAGTTC AGCAAAGAGA

TGAACCAGGT AGATGGCTCC CCCATAATGG AACTGAGTTC AACCAAGAGA

TGGGCCAGGT AGATGGCTCT CCAATACCAG AGCTGAGTTC GGCAAAGAGA

TGGGCCAGGT AAATGGCTCC CCAGTACCAG AACTGACTGC TTCAAAAATA

TGGGCCAGGT AGATGGCTCA CCAGTATCAG AACTGAGCTC AGTAAAAAGA

TAGGCCAGGT AGATGGTTCC CCAGTACCAG AACTGAGCTC AGCAAAGAGA

TAGGCCAGGT AGACGGCTCC CCAGTTCCGG CACTGGGTCC AGCAAAGAGA

TGAGCCAGGT GGTTTCATCT CCCTTGCCTC AACAGAGATC AGTCCAGAGA

TAACCCAGGT GCACTGTTCG CCACTGCCCG AGACACATTC TCCGCACCTG

AGGCCACGGA GAATGACCCC ATTTTGGAGA GGGGTTTCCC TCAGGCCTAT

AGGCCACGGA GAATGACCCC ATTTTGGAGA GGGGTTTCCC TCAGGCCTAT

AGGCCACGGA GAATGACCCC ATTTTGGAGA GGGGTTTCCC TCAGGCCCAT

AGGCCACGGA GAATGACCCC ATTTTGGAGA GGGGTTTCCC TCAGGCCCAT

AGGCCACAGA GAATGACCCC ATTTTGGAGA GGGGTTTCCC TCAGGCCCAT

AGGCTACGAA GAATGACCCC ATTTTGGAGA GGGGTTTCCC TCAGGCCCAT

A---CCCGGA GAATGACCCC ATTTTGGAGA GGGGTTTCCC TCAGACCCAT

T---CCCGGA GAATGACCCC ATTTTGGAGA GGGGTTTCCC TCAGGCCCAT

CGGCCACGGA GAATGACCCC GTTTTGGAGA GGGGTCTCCC TCAGGCCCAT

AGGCCGAGGA GAATGACCCC ATTTTGGAGA GCGGTTTCCC TCAGGCCCAT

AGGCCACGGA GAATGACCCC ATTTTGGAGA GGGGTTTCCC TAAGGCCCAT

AGGCTGCGGA GAATGACCCC CTTTTGGAGA GGGGTTTCCC TCAGGCCCAT

AGGCATCGGA GAATGACACC GTTTTGGAGA GGGGTTTCCC TCAGGCCCAT

AGGCTGAGGA GAATGACCCC ATTTTGGAGA GAGGTCTCCC TCAGGCGTAT

AGGCTGAGGC GAATGACCCC ATTTTGGAGA GGAGTGTCCC TTAGGCCTAT

AGATTGCAGA GAATGACCCC GTTTTGGAGA GGAGTGTCCC TTAGGCCTAT

AGACTAAGAA GGATGACTCC CTTCTGGAGA GGTGTCTCCC TTAGGCCCAT

CGGATGAAGA GGATGACCCC GTTTTGGCGA GGGGTCTCCC TCCGGCCCAT

TGGAGCCTCC TGCCGGGATG ATTCTGAGTG TATCACAAGG CTATGCAGAA

TGGAGCCTCC TGCCGGGATG ATTCTGAGTG TATCACAAGG CTATGCAGAA

TGGAGCCTCC TGCCGGGATG ATTCTGAGTG TATCACAAGG CTATGCAGAA

TGGAGCCTCC TGCCGGGATG ATTCTGAGTG TATCACAAGG CTATGCAGAA

TGGAGCCTCC TGCCGGGATG ATTCTGAGTG TATCACAAGG CTATGCAGAA

TGGTGCCTCC TGTCGGGACA ATTCTGAGTG TATCACAAGG CTCTGCAGAA

TGGTGCCTCA TGTCGGGATG ATTCGGAGTG TATCACAAGA CTATGCAAAA

TGGTGCCTCG TGCCGGGATG ACTCTGAGTG TATCACAAGA CTATGCAGAA

TGGAGCCTCG TGTCGGGACA ATGCTGAGTG TGTCACAAGG CTATGCAGAA

TGGAGCCTCC TGTCGGGATG ATTCTGAATG TATCACAAGG CTATGCAGAA

TGGATCCTCC TGCCGGGACG ATTCTGAGTG TATCACGAGG CTGTGCAGAA

TGGAGCCTCT TGCCGGGACG ATTCTGAGTG TATCACCAGG CTATGCAGAA

TGGAGCTTCC TGTCGGGACG ATTCTGAGTG CATCACGAAA CTATGCAGAA

TGGAGCTTCC TGCATGGACA ATTCTGAATG TACCACCAGG CTATGCAGAA

TGGAGCCTCC TGTCGGGATG AATCTGAATG TATCACAAGG CTATGCAGAA

TGGAGCCTCC TGCCGAGATG ATTCTGAATG TACCACGAGA TTGTGCAG--

AGGGGCATCC TGTCGTGATG ACTCTGAGTG CATCACAAGA CTATGCAGGA

TGGGGCCTCC TGCCGAGACA ACTCGGAATG CATCACGCTG CTCTGCAGAA

AAAGACGCTG TTCCTTAAGT GTGGCCCAGG AAGAGTCTCA GTCCAATTTT

AAAGACGCTG TTCCTTAAGT GTGGCCCAGG AAGAGTCTCA GTCCAATTTT

AAAGACGCTG TTCCTTAAGT GTGGCCCAGG AAGAATCTCA GTCCAATTTT

AAAGACGCTG TTCCTTAAGT GTGGCCCAGG AAGAATCTCA GTCCAATTTT

AACGACGTTG TTCCTTAAGT GTGGCCCAGG AAGATTCTCA GTCCAATTTT

AAAGACGCTG TTCCCTAACT GTGGCCCAGG AA-------- ----------

GACGACGCTG TTCCCTGAGT GTGGCCCAGG AGGAGTCTCA GTCTAACTTT

AAAGACGCTG TTCCCTAAGT GTGGCCCAGG AGGAGTCGCA ATCTAACTTT

AAAGACGCTG CTCCCTGAGT GTGGCCCAGG AAGAGCCTCA GTCCAACTTT

AAAGACGCTG CTCCCTAAGT GTGGCCCAGG AAGAGTCTCA GTCCAGTTTT

AAAGACGCTG TTCCCTAAGT GTGGCCCAGG AAGAATCTCA GTCCAGTTTT

AAAGACGCTG TTCCCTAAGT GTGGCCCAGG AGGAATCTCA ATCCAACTTT

AAAGACGCTG TTCTCTAAGT GTGGCCCAGG AAGAAACGCA GTCCAATTTC

AAAGACGCTG TTCCTTGAGT GTGGCCCAGG AAGAGTCTCA GTCCAGTTTT

AAAGACGCTG TTCCCTAAGC GTGGCCCAGG AA-------- ----------

---------- ---------- ---------- --GAGTCTCA ATCTAATTTT

AAAGACGCTG TTTGCTCAAT GTGACCCAAG AAGAATCTCA ATCAAATTTT

AGAGTCGTTG CTCTCTGAAT ATTTCCCCAG AGGAGTCTCA GTCCAATTTT

GGCACTGGAG AACAAAAAAA AACTTCAAAT GGGCACCAGT CTAAATCTAT

GGCACTGGAG AACAAAAAAA AACTTCAAAT GGGCACCAGT CTAAATCTAT

GGCCCTGGAG AACAAAAAAA AACTTCAAAT GGGCACCAGT CTAAATCTAT

GGCCCTGGAG AACAAAAAAA AACTTCAAAT GGGCACCAGT CTAAATCTAT

GGCCCTGGAG AACAAAAAAA AACTTCAAAT GGGCACCAGT CTAAATCTAT

---------- ---------- ---------- ---------- ----------

GGCTCTGGAG AACAAAAAAA AACATCAAAT GGACACCAGT CAAAATCTAT

GGCCCTGGAG AACAAAAAAA AACATCAAAT GGACACCAGT CAAAATCTAT

GGTCCTGGAG AACAAA---- ---------- ---------- -------TAT

GGCCCTGGAG AACAAAAAAA AACTTCAAAT GCACACCAGT CTAAATCTAT

GGCCCTGGAG AACAAAAAAA AACTTCAAAT GGACACCAGT CTAAATCTAT

GGTCCTGGAG AACAAAAAAA AACTTCAAAT GGACATCAGT CTAAATCTAT

GGCCCTGGAG AACAAAAAAA AACTTCAAAT GGGCACCAGT CTAAATCTAT

GGCCCTGGAG AGCAAAAAAA AACTTCAAAT GGACACCAGT CTAAATCTAT

---------- ---------- ---------- ---------- -------TAT

GGCCCTGGAG AGCAAA---A GACTTCAAAT GGACACCAGT CTAAATCTAT

GGCCCTGGAG AACAAAAAAA AACTTCAAAT GGGCATCAGT CCAAATCTAT

GGTACTGGAG AACAAAAAAA AACATCAAAT GGTCACCAGG CCAAATCTAT

GTTAAAAGAT GACTTAAAAC TAAGCAGCAG TGAAGACAGT GATGGGGAAC

GTTAAAAGAT GACTTAAAAC TAAGCAGCAG TGAAGACAGT GATGGGGAAC

GTTAAAAGAT GACTTAAAAC TAAGCAGCAG TGAAGACAGT GATGGGGAAC

GTTAAAAGAT GACTTAAAAC TAAGCAGCAG TGAAGACAGT GATGGGGAAC

GTTAAAAGAT GACTTAAAAC TAAGCAGCAG TGAAGACAGT GATGGGGAAC

---------- ---------- ---------- ---------- ----------

GTTAAAAGAT GACTTAAAAC TAAGCAGCAG TGAAGACAGT GATGGGGAAC

GTTAAAAGAT GACTTAAAAC TAAGCAGCAG TGAAGACAGT GATGGGGAAC

GTTAAAAGAT GACTTAAAAC TCAGCAGCAG TGAAGACAGT GATGGGGAAC

GTTAAAAGAT GACTTAAAGC TAAGCAGCAG TGAAGACAGT GATGGAGAAC

GTTAAAAGAT GACTTAAAAC TAAGCAGCAG TGAAGACAGC GATGGGGAGC

GTTAAAAGAT GACTTAAAGC TAAGCAGCAG TGAAGACAGT GATGGGGAAC

GTTAAAAGAT GACTTAAAAA TAAGCAGCAG TGAAGACAGT GATGGGGAAC

GTTAAAAGAT GACTTAAAAC TAAGCAGCAG TGAAGACAGT GATGGGGAAC

GTTAAAAGAT GACTTAAAAC TAAGCAGCAG TGAAGACAGT GATGGGGAAC

GTTAAAAGAT GACTTAAAAC TAAGCAGCAG TGAAGACAGT GATGGGGAAC

GTTAAAGGAT GATTTAAAAC TTAGCAGCAG TGAAGATAGT GATGGAGAGC

GTTAAAAGAT GACTTAAAAC TCAGCAGCAG TGAAGACAGT GATGGGGAAC

AGGATTGTGA TAAGACAATG CCGAGGAGTA CACCAGGAAG TAACTCTGAA

AGGATTGTGA TAAGACAATG CCGAGGAGTA CACCAGGAAG TAACTCTGAA

AGGATTGTGA TAAGACAATG CCGAGGAGTA CACCAGGAAG TAATTCTGAA

AGGATTGTGA TAAGACAATG CCGAGGAGTA CACCAGGAAG TAATTCTGAA

AGGATTGTGA TAAGACAATG CCAAGGAGCA CACCTGGAAG TAACTCTGAA

---------- ---------- ---------- ---------- ----------

AGGATTGTGA TAAGACAATG CCAAGGAGTA CACCAGGAAG TAACTCTGAG

AGGATTGTGA TAAGACAATG CCAAGGAGTA CACCAGGAAG TAACTCTGAA

AGGATTGTGA TAAAACAATG CCAAGAAGTA CACCAGGAAG TAACTCTGAA

AGGATTGTGA TAAGACAATG CCAAGGAGTA CACCAGGAAG TAACTCTGAA

AGGATTGTGA TAAGACAATG CCAAGGAGTA CACCAGGAAG TAACTCTGAA

AGGATTGTGA TAAGACAATG CCAAGGAGTA CACCAGGAAG TAACTCTGAA

AGGATTGTGA TAAGACAATG CCAAGGAGTA CACCAGGGAG ----------

AGGATTGTGA TAAGACAATG CCAAGGAGTA CACCAGGAAG TAATTCTGAA

AGGATTGTGA TAAGACGATG CCACGGAGTA CACCAGGAAG TAACTCTGAA

AGGATTGTGA TAAGACCATG CCAAGGAGCA CACCAGGAAG TAATTCTGAG

AGGATTGTGA TAAGACTGTG CCAAGGAGTA CACCAGGAAG TAATTCTGAA

AGGATTGTGA TAAAACAGTG CCAAGGAGTA CCCCTGGAAG TAATTCTGAA

CCTTCACACC ATAATAGTGA AGGAGCAGAT AACTCCAGGG ATGATTCTAG

CCTTCACACC ATAATAGTGA AGGAGCAGAT AACTCCAGGG ATGATTCTAG

CCTTCACATC ATAATAGTGA AGGAGCAGAT AACTCCAGGG ATGATTCTAG

CCTTCACATC ATAATAGTGA AGGAGCAGAT AACTCCAGGG ATGATTCTAG

CCTTCACACC ATAATAGTGA AGGAGCAGAT AACTCCAGGG ATGATTCTAG

---------- ---------- ---------- ---------- ----------

CCTTCACACC ATAATAGTGA GGGAGCAGAT AACTCCAGGG ATGACTCAAG

CCTTCACACC ATAACAGTGA GGGAGCAGAC AACTCCAGGG ATGACTCGAG

CCTTCACATC ATAATAGTGA AGGGGCAGAT AACTCCAGAG ATGATTCTAG

CCTTCACACC ATAATAGTGA AGGGGCAGAT AACTCCAGGG ATGATTCTAG

CCTTCACACC ATAATAGTGA AGGAGCTGAT AACTCCAGGG ATGATTCTAG

CCTTCACACC ACAACAGTGA AGGGGCAGAA AACTCACGGG ATGACTCCAG

---------- ---------- ---------- ---------- ----------

CCTTCACACC ATAATAGTGA AGGAGCAGAT AACTCTAGAG ATGATTCTAG

CCTTCACACC ATAATAGTGA AGGAGCAGAT AACTCCAGGG ATGATTCTAG

CCTTCACACC ATAACAGTGA AGGAGCAGAT AACTCCAGAG ATGATTCTAG

CCTTCACACC ACAATAGCGA AGGAGCGGAT AATTCCAGGG ATGACTCAAG

CCGTCACACC ATAATAGTGA AGGAGCAGAT AATTCCAGGG ATGACTCGAG

TAGCCACAGT GGATCTGAAA GCAGCTCTGG ATCTGACTCA GAGAGTGAAA

TAGCCACAGT GGATCTGAAA GCAGCTCTGG ATCTGACTCA GAGAGTGAAA

TAGCCACAGT GGATCTGAAA GCAGCTCTGG ATCTGACTCA GAGAGTGAAA

TAGCCACAGT GGATCTGAAA GCAGCTCTGG ATCTGACTCA GAGAGTGAAA

TAGCCACAGT GGATCTGAAA GCAGCTCTGG ATCTGACTCA GAGAGTGAAA

---------- ---------- ---------- ---------- ----------

CAGCCACAGT GGGTCTGAAA GCAGCTCTGG GTCCGACTCC GAGAGTGAAA

CAGCCACAGT GGATCTGAGA GCAGCTCTGG GTCTGACTCG GAGAGTGAGA

CAGCCACAGT GGGTCTGAAA GCAGCTCTGG ATCTGACTCC GAGAGTGAAA

CAGCCACAGT GGATCTGAAA GCAGCTCTGG ATCTGACTCA GAGAGTGAAA

CAGCCACAGT GGATCTGAAA GCAGCTCTGG ATCTGACTCA GAGAGTGAAA

CAGCCACAGT GGGTCTGAAA GCAGCTCTGG ATCTGACTCA GAGAGTGAAA

---------- ---------- ---------- ---------- ----------

CAGCCATAGT GGATCTGAAA GCAGCTCTGG ATCTGACTCA GAGAGTGAAA

CAGCCACAGT GGATCTGAAA GCAGCTCTGG ATCTGACTCC GAGAGTGAAA

CAGCCATAGT GGATCTGAAA GCAGCTCTGG ATCTGACTCA GAGAGCGAAA

TAGCCATAGT GGATCTGAAA GCAGTTCTGG ATCTGACTCG GAGAGTGAAA

CAGCCATAGT GGATCCGAAA GCAGTTCCGG ATCTGACTCG GAAAGCGAGA

GTAGTTCCAG TGACAGTGAG GCAAATGAGC CATCCCAGAG TGCATCTCCC

GTAGTTCCAG TGACAGTGAG GCAAATGAGC CATCCCAGAG TGCATCTCCC

GTAGTTCCAG TGACAGTGAG GCAAATGAGC CATCCCAGAG TGCATCTCCT

GTAGTTCCAG TGACAGTGAG GCAAATGAGC CATCCCAGAG TGCATCTCCT

GTAGTTCCAG TGACAGTGAG GCAAATGAGC CATCCCAGAG TGCATCTCCT

---------- ---------- ---------- ---------- ----------

GCAGCTCCAG TGACAGTGAG GCAAATGAGC CATCCCAGAG CGCATCCCCT

GCAGCTCCAG TGACAGTGAG GCTAATGAGC CGTCGCAGAG CGCATCTCCT

GTAGTTCCAG TGATAGTGAG GCAAATGAGC CATCCCAGAG TGCATCTCCT

GTAGTTCCAG TGACAGTGAG GCAAATGAGC CATCCCAGAG TGCATCTCCT

GTAGCTCCAG TGACAGTGAG GCAAATGAAC CATCCCAGAG TGCATCTCCT

GTAGTTCCAG TGACAGTGAG GCAAACGAGC CATCCCAGAG TGCATCTCCT

---------- ---------- ---------- ---------- ----------

GTAGTTCCAG TGACAGTGAG GCAAATGAGC CATCCCAGAG TGCATCTCCT

GTAGTTCCAG TGACAGTGAG GCAAATGAGC CATCCCAGAG TGCATCTCCT

GTAGTTCCAG TGACAGTGAG GCCAATGAGC CATCCCAGAG TGCATCTCCT

GTAGTTCCAG TGACAGTGAG GCCAATGAAC CATCACAGAG TGCATCTCCT

GTAGTTCCAG TGACAGCGAG GCTAACGAGC CATCCCAGAG TGCGTCTCCC

GAGCCTGAAC CCCCGCCAAC AAACAAATGG CAACTTGATA ATTGGCTGAA

GAG------- ---------- ---------- ---------- ----------

GAGCCTGAAC CCCCGCCAAC AAACAAATGG CAACTTGATA ATTGGCTGAA

GAGCCTGAAC CCCCGCCAAC AAACAAATGG CAACTTGATA ATTGGCTGAA

GAGCCTGAAC CCCCGCCAAC AAACAAATGG CAACTTGATA ATTGGTTGAA

---------- ---------- ---------- ---------- ----------

GAGCCTGAAC CACCACCAAC CAACAAGTGG CAACTTGATA ATTGGTTGAA

GAGCCTGAAC CACCACCAAC AAACAAGTGG CAACTTGATA ATTGGTTGAA

GAGCCTGAAC CACCACCAAC AAACAAATGG CAACTAGATA ATTGGTTGAA

GAGCCTGAAC CACCTCCCAC AAATAAATGG CAACTTGATA ACTGGCTGAA

GAGCCTGAAC CACCACCAAC AAACAAATGG CAACTTGATA ATTGGTTAAA

GAGCCCGAAC CACCACCTAC AAACAAATGG CAACTTGATA ATTGGTTGAA

---------- ---------- ---------- ---------- ----------

GAGCCTGAAC CTCCACCAAC AAACAAATGG CAACTTGATA ATTGGTTGAA

GAGCCTGAAC CACCACCAAC AAACAAATGG CAACTGGATA ATTGGTTGAA

GAGCCTGAAC CACCACCAAC AAACAAATGG CAGCTTGATA ACTGGTTGAA

GAGCCTGAAC CACCACCAAC AAATAAATGG CAACTTGACA ATTGGCTGAA

GAGCCCGAGC CACCCCCAAC CAACAAATGG CAACTGGACA ATTGGTTGAA

TAAAGTGAAC CCACATAAAG TGTCACCCGC CTCTTCAGTG GACAGTAACA

---------- ---------- ---------- ---------- ----------

TAAAGTGAAC CCACATAAAG TGTCACCCGC TTCTTCAGTG GACAGTAACA

TAAAGTGAAC CCACATAAAG TGTCACCCGC TTCTTCAGTG GACAGTAACA

TAAGGTGAAC CCACATAAAG TGTCACCCGC TTCTTCAGTG GACAGTAATA

---------- ---------- ---------- ---------- ----------

CAAAGTGAAC CCACATAAAG TGTCCCCAGC TTCCTCTGTA GATAGCAACA

TAAAGTGAAC CCACATAAAG TATCCCCAGC TTCCTCTGTA GACAGCAACA

TAAAGTGAAC CCGCATAAGG TGTCTCCAGC TTCTTCTGTG GATAGTAACA

TAAAGTAAAT CCACATAAAG TATCACCAGC TTCTTCTGTG GACAGTAATA

TAAAGTGAAC CCACATAAAG TGTCACCAGC TTCTTCTGTG GACAGTAACA

TAAAGTGAAC CCACATAAAG TGTCACCAGC TTCTTCTGTG GACAGTAACA

---------- ---------- ---------- ---------- ----------

TAAAGTGAAC CCACATAAAG TATCACCAGC TTCTTCCGTG GACAGTAACA

TAAAGTGAAC CCACACAAAG TATCACCAGC TTCATCTGTG GACAGTAACA

TAAAGTGAAC CCCCACAAAG TGTCTCCAGC GTCTTCTGTG GATAGCAACA

TAAAGTGAAT CCACATAAAG TGTCACCAGC TTCTTCTGTA GATAGCAATA

TAAGGTGAAT CCACACAAAG TGTCACCTGC TTCCTCTGTG GACAGCAACA

TCCCATCATC TCAAGGCTAC AAAAAGGAAG GCCGAGAGCA GGGCACTGGG

---------- ---------- ---------- ---------- ----------

TCCCATCATC TCAAGGCTAC AAAAAGGAAG GCCGAGAGCA GGGCACTGGG

TCCCATCATC TCAAGGCTAC AAAAAGGAAG GCCGAGAGCA GGGCACTGGG

TCCCATCATC TCAAGGTTAC AAAAAGGAAG GCAGAGAGCA GGGCACTGGG

---------- ---------- ---------- ---------- ----------

TCCCATCGTC TCAAGCCTAC AAGAAAGAAG GCCGAGAGCA GGGCACTGCC

TCCCATCTTC TCAAGCCTAC AAGAAAGAAG GCCGGGAGCA GGGCACTGCC

TCCCATCATC TCAAGGCTAC AAAAAGGAGG GCCGAGAACA GAGCACTGGG

TCCCATCATC TCAAGGCTAT AAAAAGGAAG GCCGAGAACA GGGCACAGGG

TCCCGTCATC TCAAGGCTAT AAAAAGGAAG GCCGAGAACA GGGCACTGGG

TCCCATCATC TCAAGGCTAT AAAAAGGAAG GCCGAGAGCA GGGCACTGGG

---------- ---------- ---------- ---------- ----------

TCCCATCATC TCAAGGATAT AAAAAGGAAG GCCGAGAGCA GGGCACGGGG

TCCCATCATC TCAAGGCTAT AAAAAGGAAG GCCGAGAACA GGGCACTGGG

TCCCGTCATC TCAAGGCTAT AAAAAGGAAG GCCGGGAACA GAGTGCTGGG

TCCCATCTTC TCAAGGTTAT AAAAAGGAAG GTCGGGAGCA AGGACCAGGA

TCCCCTCCTC ACAAGGTTAT AAGAAGGAGG GTCGGGAGCA ATGCCCTGGG

AATAGCTACA CTGATACAAG TGGACCTAAA GAAACGAGTT CCGCTACTCC

---------- ---------- ---------- ---------- ----------

AATAGCTATA CTGATACAAG TGGACCTAAA GAAACGAGTT CCGCTACTCC

AATAGCTACA CTGATACAAG TGGACCTAAA GAAACGAGTT CCGCTACTCC

AATAGCTACA CTGATACGAC TGGACCCAAA GAAACGAGTT CCGCTACTCC

---------- ---------- ---------- ---------- ----------

AGTAACTACA CAGATCCAGG TGGGGCCAAA GAAGCGAGCT CTGCCACTCC

AGTAACTACA CAGATCCAGG GGGGACAAAA GAAACGAGCT CTGCCACCCC

AATAGCTACA CGGAGACGAG TGCACCTAAA GAAACAAGTT CTGCTACTCC

AATAGCTACA CAGATCCAGG TGGTCCTAAA GAAACAAGTT CTGCTACTCC

AATAGCTATA CTGATCCAGG CGGACCTAAA GAAACAAGTT CTGCTACTCC

AATAGCTACA CTGATCCAAG TGGAGCTAAA GAAACTAGTT CCACTACTCC

---------- ---------- ---------- ---------- ----------

AATAGCTACA CTGATCCAGG TGGACCTAAA GAAACAAGTT CCACTACTCC

AATAGCTACA CTGATCCAGG TGGACCTAAA GAAACGAGTT CCGCTACTCC

AACAGCTACC CTGACACAGG GGGACCGAAA GAAACGAGTT CTGCCACTCC

AGTGGCTACA TTGAGCAAAA TGGACCTAAG GAGTCCAATT C---TACTCC

AGTGGCTACG TCGATCAGAC AGGGCCTAAG GATTCCAACT CCTCCACTCC

GGGACGAGAC TCCAAAACCA TCCAAAAGGG ATCAGAAAGT GGGCGTGGGA

---------- ---------- ---------- ---------- ----------

TGGACGAGAC TCCAAAACCA TCCAAAAGGG ATCAGAAAGT GGGCGTGGGA

TGGACGAGAC TCCAAAACCA TCCAAAAGGG ATCAGAAAGT GGGCGTGGGA

TGGACGAGAC TCCAAAACCA TCCAAAAGGG ATCAGAAAGT GGGCGTGGGA

---------- ---------- ---------- ---------- ----------

GGGACGAGAC TCTAAAACTA TCCAAAAAGG ATCAGAAAGT GGCCGTGGGA

AGGACGAGAC TCTAAAACCA TCCAAAAGGG ATCAGAAAGT GGCCGTGGGA

TGGGCGAGAC TCTAAAACCA TCCAAAAGGG ATCAGAAAGT GGGCGCGGAA

TGGACGAGAC TCCAAAACTG TCCAAAAGGG ATCAGAAAGT GGGCGTGGGA

TGGACGAGAC TCCAAAACTG TACAAAAGGG ATCAGAAAGT GGGCGCGGGA

TGGACGAGAT TCCAAAACCG TCCAAAAGGG ATCAGAAAGT GGGCGTGGGA

---------- ---------- ---------- ---------- ----------

TGGACGAGAC TCCAAAACCA TCCAAAAGGG ATCAGAAAGT GGGCGAGGGA

TGGGCGAGAC TCCAAACCCA TCCAAAAGGG ATCAGAAGGT GGACGAGGGA

AGGACGAGAC TCCAAAACGA CCCAAAAGGG ATCAGAAAGT GGACGAGGGA

TGGACGAGAT TCCAAAACTG TTCAAAAAGG ATCAGAGAGT GGTCGTGGGA

CGGCCGAGAT TCCAAAACCG TCCAAAAAGG ATCCGAGGGT GGTCGCGGCA

GGCAGAAATC TCCTGCACAG AGTGACAGCA CAACACAGAG AAGAACTGTA

---------- ---------- ---------- -------GGA AAAAATTAAG

GGCAGAAATC TCCTGCACAG AGTGACAGCA CAACACAGAG AAGAACTGTA

GGCAGAAATC TCCTGCACAG AGTGACAGCA CAACACAGAG AAGAACTGTA

GACAGAAATC TCCTGCTCAG AGTGACAGCA CAACACAGAG AAGAACTGTA

---------- ---------- ---------- ---------- ----------

GGCAGAAGTC TCCTGCACAG AGTGACAGTA CCACACAGAG GAGAACTGTT

GGCAGAAGTC TCCTGCACAG AGCGACAGTA CCACTCAGAG GAGAACTGTC

GGCAGAAGTC TCCTGCACAG AGTGACAGCA CAACACAGAG GAGAACTGTA

GGCAAAAGTC TCCTGCCCAG AGTGACAGCA CAGCACAGAG GAGAACTGTA

GGCAGAAGTC CCCTGCACAG AGTGACAGCA CAACACAGAG GAGAACTGTA

GGCAGAAGTC TCCTGCACAG AGTGACAGCA CAACACAGAG GAGAACTGTA

---------- ---------- ---------- ---------- ----------

GGCAGAAGTC TCCTGCACAG AGTGATAGTG CAACACAGAG GAGAACTGTA

GACAGAAGTC TCCTGCACAG AGTGACAGTA CAACACAGAG GAGAACTGTA

GACAGAAGTC TCCTGCCCAG AGTGACAGCA CAACCCAGAG GCGAACTGTA

GACAAAAGTC CCCAGCACAG AGTGATAACA CTACACAGAG AAGAACTGTA

GACAAAAGTC TCCCGCCCAG AGTGATAACA CGACCCAAAG AAGAACTGTG

GGCAAAAAAC AACCCAAAAA GGCTGAGAAG GCAGCTGCTG AAGAGCCTCG

GGCAAAAACA AACCCAAAAA GGGGGAGAAG GCAGTTGTTA AAGAGCCTTT

GGCAAAAAAC AACCCAAAAA GGCTGAGAAG ACAGCTGCTG AAGAGCCTCG

GGCAAAAAAC AACCCAAAAA GGCTGAGAAG ACAGCTGCTG AAGAGCCTCG

GGCAAAAAAC AACCCAAAAA GGCTGAGAAG GCAGCTGCTG AAGAGCCTCG

---------- ---------- ---------- ---------- ----------

GGCAAAAAAC AACCCAAAAA AACTGAGAAG TCAGCTGCTG AAGAGCCCCG

GGCAAAAAAC AACCCAAAAA ACCTGAGAAG TCAGCTGCTG AAGAGCCTCG

GGCAAAAAAC AACCCAAAAA GGCCGAGAAG GCAGCTGTTG AAGAACCCCG

GGGAAAAAAC AACCCAAAAA GACTGAGAAG GCAGCTGCTG AAGAGCCCCG

GGCAAAAAAC AACCCAAAAA GGCTGAGAAG GCAGCCACTG AAGAGCCCCG

GGCAAAAAAC AACCCAAAAA GGCTGAGAAG GCAGCCGCTG AAGAGCCCCG

---------- ---------- ---------- ---------- ----------

GGCAAAAAAC AACCCAAAAA AGCTGAGAAA GCAGTTGCTG AAGAGCCTCG

GGCAAAAAAC AACCCAAAAA GGCTGAGAAA GCAGCTGCCG AAGAGCCCCG

GGCAAAAAAC AACTCAAAAA GGCGGAGAAG GCAGCTGCAG AAGAGCCCCG

GGAAAAAAAC AACCCAAAAA AACGGAGAAG GCAGGTATAG AGGAGCCTCG

GGGAAGAAGC AGCCCAAAAA GGCAGAGAAA GCAGCTGTTG AGGAACCTAG

TGGAGGCCTG AAGATAGAAA GTGAAACCCC TGTAGACTTG GCTAGCAGCA

GGGGGGCCTG AAGAAAGAAA GTGAAACCCC CGTGGACTTG GCTAGCAGCA

TGGAGGCCTG AAGATAGAAA GTGAAACTCC TGTAGACTTG GCTAGCAGCA

TGGAGGCCTG AAGATAGAAA GTGAAACTCC TGTAGACTTG GCTAGCAGCA

TGGAGGCCTG AAGATAGAAA GTGAAACCCC TGTAGACTTG GCTAGCAGCA

---------- ---------- ---------- ---------- ----------

AGGAGGCCTG AAGATAGAAA GTGAGACCCC TGTGGACATG GCTACCAGCA

TGGAGGCCTG AAGATAGAAA GTGAGACCCC TGTGGACATG GCTGCCAGCA

TGGAGGCCTG AAGATAGAAA GTGAGACGCC TGTCGACATG GCTGCCAGCA

TGGAGGCCTG AAGATAGAGA GTGAAACTCC TGTAGACATG GCTACCAGCA

GGGAGGCCTG AAGATAGAAA GCGAAACCCC CGTAGACATG GCTACCAGCA

CGGAGGCTTG AAGATAGAAA GTGAAACCCC CGTAGACATG GCTACCAGCA

---------- ---------- ---------- ---------- ----------

TGGAGGCCTG AAGATAGAAA GTGAAACTCC AGTAGACATG GCTACCAACA

TGGAGGCCTG AAGATAGAAA GTGAAACCCC AGTCGACATG GCCACCAGCA

AGGAGGCCTG AAGATCGAAA GTGAAACTCC AGTAGACATG GCCACCAGTA

AGGTGGCCTA AAAATAGAAA GTGAAACCCC TGTGGACATG ACAACAAGTA

GGGAGGACTA AAAATAGAAA GTGAAACCCC CCTGGATATG GCCACAAGCA

TGCCCTCCAG CAGACACAAA GCAGCCACCA AAGGCTCAAG GAAACCCAAT

TGCCCTCCAG CAGACACAAG GCAGCCACCA AGGGGTCAAG GAAACCCAAT

TGCCCTCCAG CAGACACAAA GCAGCCACCA AAGGCTCAAG GAAACCCAAT

TGCCCTCCAG CAGACACAAA GCAGCCACCA AAGGCTCAAG GAAACCCAAT

TGCCCTCCAG CAGACACAAA GCAGCCACCA AAGGCTCAAG GAAACCCAAT

---------- ---------- ---------- ---------- ----------

TGCCCTCCAG CAGGCATAAG GCAGCCACCA AGGGCTCAAG GAAACCTAAC

TGCCCTCCAG CAGGCACAAA GCAGCCACCA AGGGCTCGAG GAAACCCAAC

TGCCCTCCAG CAGACACAAA GCAGCCACCA AAGGCTCAAG GAAACCCAAT

TGCCCTCCAG CAGACACAAA GCGGCCACCA AAGGCTCAAG AAAACCCAAT

TGCCTTCCAG CAGACACAAA GCAGCCACCA AAGGCTCAAG GAAACCCAAT

TGCCTTCCAG CAGACACAAA GCAGCCACCA AAGGCTCAAG GAAACCCAAC

---------- ---------- ---------- ---------- ----------

TGCCCTCCAG CAGACACAAA GCAGCCACCA AAGGCTCAAG GAAACCCAAT

TGCCCTCCAG CAGACACAAG GCAGCCACCA AAGGCTCTAG GAAACCCAAT

TGCGCTCCAG CAGACACAAG GCAGCCACCA AAGGCTCGAG GAAGCCCAAC

TGCCCTCCAA TAGGCACAAG GCAGCCACAA AAGGCTCAAG AAAACCCAAT

TGCCCTCAAA CAGGCACAAG GCAGCCACAA AAGGCTCCAG GAAGCCCAAC

ATAAAGAAGG AGTCTAAGTC TTCCCCTCGA CCTACAGCAG AGAAAAAGAA

AAAAGAAGGG AGTGTAAGTC TTTCCCT--- CCTACAGCAG AGAAAAAGAA

ATAAAGAAGG AGTCTAAGTC TTCCCCTCGA CCTACAGCAG AGAAAAAGAA

ATAAAGAAGG AGTCTAAGTC TTCCCCTCGA CCTACAGCAG AGAAAAAGAA

ATAAAGAAGG AGTCTAAGTC TTCCCCTCGA CCTACAGCAG AGAAAAAGAA

---------- ---------- ---------- ---------- ----------

ATCAAAAAGG AGTCGAAATC TTCCCCTCGA CCTACAGCAG AGAAAAAGAA

ATCAAAAAGG AATCAAAATC TTCTCCTCGA CCTACGGCAG AGAAGAAGAA

ATAAAGAAGG AGTCTAAATC TTCCCCTCGG CCTCCAGCAG AGAAAAAGAA

ATAAAAAAGG AGTCCAAATC TTCCCCTCGA CCTTCAGCAG AGAAAAAGAA

ATCAAAAAGG AGTCCAAATC TTCCCCTCGA CCATCTGCAG AGAAGAAGAA

ATAAAAAAAG AGTCCAAATC TTCCCCTCGA CCTTCAGCAG AGAAAAAGAA

---------- ---------- ---------- ---------- ----------

ATAAAGAAGG AGTCCAAATC TTCCCCTCGA CCTACAGCAG AGAAAAAGAA

ATAAAGAAGG AGTCCAAATC TTCCCCTCGA CCTACAGCAG AGAAAAAGAA

ATAAAGAA-G AGTCCAAATC TTCCCCTCGA CCGACAGCAG AGAAGAAGAA

ATAAAAAAGG AACCCAAATC TTCCCCAAGA TCAACAACAG AAAAAAAGAA

ATAAAGAAGG AACCCAAGTC ATCCCCTAGG CCCACGACGG AGAAAAAGAA

ATATAAGTCA ACAAGTAAAT CTTCCCAGAA ATCAAGGGAA ATCATAGAAA

ATATAAGTCA ACAAGTAAAT CTTCCCAGAA ATCAAGGGAA ATCATAGAAA

ATATAAATCA ACAAGTAAAT CTTCCCAGAA ATCAAGGGAA ATCATAGAAA

ATATAAATCA ACAAGTAAAT CTTCCCAGAA ATCAAGGGAA ATCATAGAAA

ATATAAGTCG ACAAGTAAAT CTTCCCAGAA ATCAAGGGAA ATCATAGAAA

---------- ---------- ---------- ---------- ----------

ATACAAGTCA GCAAGTAAAT CCTCCCAGAA ATCTCGGGAA ATCATAGAAA

GTACAAGTCA GCCAGTAAAC CGTCCCAGAA GTCTCGGGAG ATCATAGAGA

ATACAAGTCA ACGAGTAAGT CTTCCCAGAA ATCAAGGGAA ATCATAGAAA

ATACAAGTCA ACAAGTAAAT CTTCCCAGAA ATCAAGGGAA ATTATAGAAA

ATACAAGTCA ACGAGTAAAT CTTCCCAGAA ATCAAGGGAA ATCATAGAAA

ATACAAGTCA ACGAGTAAAT CTTCCCAGAA ATCAAGGGAA ATCATAGAAA

---------- ---------- ---------- ---------- ----------

ATACAAGTCA ACGAGCAAAT CTTCCCAGAA ATCAAGGGAA ATCATAGAAA

ATACAAGTCA ACGAGTAAAT CTTGTCAGAA ATCAAGGGAA ATCATTGAAA

ATACAAGTCA ACAAGCAAAT CTTCCCAGAA ATCGA----- ----------

GTATAAGTCA ACAAGTAAAT CTTCCCAAAA ATCTAGGGAA ATCATAGAAA

ATACAAGTCA ACAAGCAAAT CTTCCCAGAA ATCTCGGGAA ATCATAGAAA

CAGATACCTC ATCCTCAGAT TCAGATGAAA GTGAGAGCCT TCCTCCTTCC

CAGATACCTC ATCCTCAGAT TCAGATGAAA GTGAGAGCCT TCCTCCTTCC

CAGATACCTC ATCCTCAGAT TCAGATGAAA GCGAGAGCCT TCCTCCTTCC

CAGATACCTC ATCCTCAGAT TCAGATGAAA GCGAGAGCCT TCCTCCTTCC

CAGATACCTC ATCCTCAGAT TCAGATGAAA GTGAGAGCCT TCCTCCTTCC

---------- ---------- ---------- ---------- ----------

CAGATAGCTC CTCCTCAGAT TCAGATGAGA GGGAGAGCCT CCCTCCCTCC

CTGACACCTC GTCCTCAGAC TCGGACGGCA GCGAGAGCCT GCCTCCCTCC

CAGATACCTC ATCCTCAGAT TCAGATGAAA GTGAGAGCCT TCCTCCTTCC

CAGATACCTC ATCCTCAGAT TCGGATGAAA GCGAGAGCCT TCCTCCTTCC

CAGATACCTC ATCCTCAGAT TCGGATGAAA GCGAGAGCCT TCCTCCTTCC

CAGATACCTC ATCCTCAGAT TCGGATGAAA GCGAGAGCCT TCCTCCTTCC

---------- ---------- ---------- ---------- ----------

CAGATACCTC ATCCTCAGAT TCAGATGAAA GTGAGAGCCT TCCTCCTTCC

CAGATACCTC ATCCTCAGAT TCGGATGAAA GTGAGAGCCT TCCTCCCTCC

---------- ---------- ---------- ---------- ----------

CAGATACTTC ATCCTCTGAT TCTGATGAAA GTGAGGGCCT TTTTCCTTCT

CAGATACCTC ATCCTCTGAT TCTGATGAAA GCGAGAGCCT CCCGCCTTCA

TCACAAACTC CTAAGTACCC CGAGAGCAAT AGGACTCCTG TTAAACCCTC

TCACAAACTC CTAAGTACCC CGAGAGCAAT AGGACTCCTG TTAAACCCTC

TCACAAACTC CTAAGTACCC CGAGAGCAAT AGGACTCCTG TTAAGCCCTC

TCACAAACTC CTAAGTACCC CGAGAGCAAT AGGACTCCTG TTAAGCCCTC

TCACAAACTC CTAAGTACCC CGAGAGTAAT AGGACTCCTG TTAAACCCTC

---------- ---------- ---------- ---------- ----------

TCCCAGACTC CTAAGTACCC TGAGAGCAAT AGGACTCCTG TCAAACCCTC

TCACAGACTC CTAAGTACCC TGAAAGCAAC AGGACTCCTG TCAAACCCTC

TCTCAAACTC CTAAGTACCC TGACAGCAAT AGGACTCCTG TTAAACCCTC

TCACAAACTC CTAAGTACCC TGAGAGTAAT AGGACTCCCG TTAAACCCTC

TCACAAACTC CTAAGTACCC TGAGAGTAAC AGGACTCCCG TGAAACCCTC

TCACAAACTC CTAAGTACCC TGAGAGTAAT AGGACTCCCG TTAAGCCCTC

---------- ---------- ---------- ---------- ----------

TCACAAACAC CTAAGTACCC TGAGAGCAAT AGGACTCCTG TTAAACCCTC

TCACAAACTC CTAAGTATCC TGAGAGCAGT AGGACTCCTG TTAAGCCTTC

---------- ---------- ---------- ---------- ----------

TCACAAACTC CTAAATATCC TGAGAGCAAC AGAACTCCTG TTAAACCCTC

TCACAAACTC CTAAATATCC CGAGAGCAAT AGGACTCCTG TTAAACCCTC

CTCAGTGGAG GAAGAAGATA GCTTTTTTCG GCAACGAATG TTCTCTCCTA

CTCAGTGGAG GAAGAAGATA GCTTTTTTCG GCAACGAATG TTCTCTCCTA

CTCAGTGGAG GAAGAAGATA GCTTTTTTCG GCAACGAATG TTCTCTCCTA

CTCAGTGGAG GAAGAAGATA GCTTTTTTCG GCAACGAATG TTCTCTCCTA

CTCAATGGAG GAAGAAGATA GCTTTTTTCG GCAACGAATG TTCTCTCCTA

---------- ---------- ---------- ---------- ----------

CTCGGCGGAG GAAGACGATA GCTTTTTCCG GCAGCGGATG TTCTCTCCTA

CTCAGTGGAG GAAGAAGATA GCTTTTTTCG GCAACGAATG TTCTCTCCTA

CTCAGTGGAG GAAGAAGATA GCTTTTTTCG GCAGCGAATG TTCTCTCCTA

CTCAGTGGAG GAAGAAGATA GCTTTTTTCG GCAACGAATG TTCTCTCCTA

CTCAGTGGAG GAAGAAGATA GCTTTTTTCG GCAACGAATG TTCTCTCCTA

CTCAGTGGAG GAAGAAGATA GCTTTTTTCG GCAACGTATG TTCTCTCCTA

---------- ---------- ---------- ---------- ----------

CTCAGTGGAG GAAGAAGACA GCTTTTTTCG GCAACGAATG TTCTCTCCTA

CTCAGTAGAG GAAGAAGACA GCTTTTTCCG GCAACGAATG TTCTCTCCTA

---------- ---------- ---------- ---------- ----------

CTCAGTAGAG GAGGAAGATA GCTTTTTTCG GCAAAGAATG TTCTCTCCTA

CTCCATGGAG GAGGAAGACA GCTTTTTTCG GCAGAGAATG TTCTCTCCTA

TGGAAGAGAA GGAACTTCTT TCACCCCTCA GTGAGCCTGA TGACAGGTAC

TGGAAGAGAA GGAACTTCTT TCACCCCTCA GTGAGCCTGA TGACAGGTAC

TGGAAGAGAA GGAACTTCTT TCACCCCTCA GTGAGCCTGA TGACAGGTAT

TGGAAGAGAA GGAACTTCTT TCACCCCTCA GTGAGCCTGA TGACAGGTAT

TGGAAGAGAA GGAACTTCTT TCACCCCTCA GTGAGCCTGA TGACAGGTAT

---------- ---------- ---------- ---------- ----------

TGGAAGAGAA GGAGCTCCTT TCCCCACTCA GTGACCCTGA TGACAGGTAC

TGGAAGAGAA GGAGCTCCTT TCCCCACTCA GTGAGCCTGA TGACAGGTAT

TGGAAGAGAA GGAACTTCTT TCACCCCTTA GTGAGCCTGA TGACAGGTAT

TGGAAGAGAA GGAACTTCTT TCACCCCTCA GTGAGCCTGA TGACAGGTAT

TGGAAGAGAA GGAACTTCTT TCACCCCTCA GTGAGCCTGA TGACAGGTAT

TGGAAGAGAA GGAACTTCTT TCACCCCTTA GTGAGCCTGA TGACAGGTAT

---------- ---------- ---------- ---------- ----------

TGGAAGAGAA GGAACTTCTT TCACCCCTCA GTGAGCCTGA TGACAGGTAT

TGGAAGAGAA GGAACTTCTT TCACCCCTCA GTGAGCCTGA TGACAGGTAT

---------- ---------- ---------- ---------- ----------

TGGAAGAGAA GGAACTCCTT TCTCCACTTA GTGATCCTGA TGACAGGTAT

TGGAAGAGAA GGAACTCCTT TCACCACTCA GTGAGCCTGA CGACAGGTAT

CCACTTATTG TGAAGATTGA CCTGAATCTT TTGACTAGAA TACCAGGAAA

CCACTTATTG TGAAGATTGA CCTGAATCTT TTGACTAGAA TACCAGGAAA

CCACTTATTG TGAAGATTGA CCTGAATCTT TTGACTAGAA TACCAGGAAA

CCACTTATTG TGAAGATTGA CCTGAATCTT TTGACTAGAA TACCAGGAAA

CCACTTATTG TGAAGATTGA CCTGAATCTT TTGACTAGAA TACCAGGAAA

---------- ---------- ---------- ---------- ----------

CCACTCATTG TGAAGATAGA CCTGAATCTT TTGACTAGAA TACCCGGGAA

CCACTCATTG TGAAGATTGA CCTCAATCTT CTGACTCGAA TACCCGGGAA

CCACTTATTG TGAAGATTGA CCTGAATCTT TTGACTAGAA TACCAGGAAA

CCACTTATTG TGAAGATTGA CCTGAATCTC TTGACTAGAA TACCAGGAAA

CCACTTATTG TGAAGATTGA CCTGAATCTT TTGACCAGAA TACCAGGAAA

CCACTTATTG TGAAGATTGA CCTGAATCTC TTGACTAGAA TACCAGGAAA

---------- ---------- ---------- ---------- ----------

CCACTTATTG TGAAGATTGA CCTGAATCTC TTAACTAGAA TACCAGGAAA

CCGCTTATTG TGAAGATTGA CCTGAATCTC TTGACTAGAA TACCAGGAAA

---------- ---------- ---------- ---------- ----------

CCACTTATTG TGAAGATTGA CCTGAGTCTC TTGTCAAGAA TACCAGGGAA

CCTCTCATTG TGAAGATTGA CCTGAATCTC TTGTCCAGAA TACCAGGGAA

GCCTTACAAA GAAACAGAGC CGCCCAAGGG GGAAAAGAAA AATGTGCCAG

GCCTTACAAA GAAACAGAGC CGCCCAAGGG GGAAAAGAAA AATGTGCCAG

GCCTTACAAA GAAACAGAGC CGCCCAAGGG GGAAAAGAAA AATGTGCCAG

GCCTTACAAA GAAACAGAGC CGCCCAAGGG GGAAAAGAAA AATGTGCCAG

GCCTTACAAA GAAACAGAGC CACCCAAGGG GGAAAAGAAA AATGTGCCAG

---------- ---------- ---------- ---------- ----------

GCCTTACAAA GAAACAGAGC CACCCAAGGG GGAAAAGAAA AATGTTCCAG

GCCTTACAAA GAAACAGAGC CACCCAAGGG GGAAAAGAAA AATGTTCCAG

GCCTTACAAA GAGACAGAGC CACCTAAGGG GGAAAAGAAA AATGTGCCAG

GCCTTACAAA GAAACAGAGC CACCCAAGGG GGAAAAGAAA AATGTGCCAG

GCCTTACAAA GAAACAGAGC CACCCAAGGG GGAAAAGAAA AATGTGCCAG

GCCTTACAAA GAAACAGAGC CACCCAAGGG GGAAAAGAAA AATGTGCCAG

---------- ---------- ---------- ---------- ----------

GCCTTACAAA GAAACAGAAC CACCCAAGGG AGAAAAGAAA AATATTCCAG

GCCTTACAAA GAAACAGAGC CGCCCAAGGG GGAAAAGAAA AATGTGCCAG

---------- ---------- ---------- ---------- ----------

GCCTTACAAA GAAATGGAGC CACCCAAGAT AGAAAAGAAA AATGTGCCTG

GCCTTACAAA GAAACTGAGC CACCTAAGGT GGAAAAGAAA ACTGTGCCAG

AAAAGCACAC GAGAGAGGCT CAGAAACAAG CCTCAGAAAA AGTTTCCAAC

AAAAGCACAC GAGAGAGGCT CAGAAACAAG CCTCAGAAAA AGTTTCCAAC

AAAAGCACAC GAGAGAGGCT CAGAAACAAG CCTCAGAAAA AGTTTCCAAC

AAAAGCACAC GAGAGAGGCT CAGAAACAAG CCTCAGAAAA AGTTTCCAAC

AAAAGCACAC GAGAGAGGCT CAGAAACAAG CCTCAGAAAA AGTTTCCAAC

---------- ---------- ---------- ---------- ----------

AAAAACACTC AAGAGAGGTG CAGAAGCAAG CCTCAGAAAA GGCTTCCAAC

AGAAACACTC GAGAGAGGTG CAGAAGCAAG CCTCAGAAAA GGCTTCCAAC

AAAAGCACAC AAGAGAGGCC CAGAAACAAG CCTCAGAGAA AGTGTCCAAC

AAAAGCACAC AAGAGAGGCT CAGAAACAAG CCTCAGAGAA AGTTTCCAAC

AAAAGCACAC AAGAGAGGCT CAGAAACAAG CCTCAGAAAA AGTTTCCAAC

AAAAGCACAC AAGAGAGGCT CAGAAACAAG CCTCAGAAAA AGTTTCCAAC

---------- ---------- ---------- ---------- ----------

AAAAACACAC GAGAGAGGCT CAGAAACAAG CCTCAGAAAA AGTTTCCAAC

AAAAGCACAC GAGAGAGGCT CAGAAACAAG CCTCAGAAAA GGTTTCCAAC

---------- ---------- ---------- ---------- ----------

AGAAGCACAC AAGAGAAACT CAGAAACAAG TCTCAGATAA AGGTTCCACC

AAAAGCACCC GAGAGACCCA CAGAAACAAG CCTCAGACAA AGTCTCTAAC

AAAGGCAAGA GGAAGCATAA GGAGTCATCT AAGCAGAGTG CTGCAAAAGA

AAAGGCAAGA GGAAGCATAA GGAGTCATCT AAGCAGAGTG CTGCAAAAGA

AAAGGCAAGA GGAAGCATAA GGAGTCATCT AAGCAGAGTG CTGCAAAAGA

AAAGGCAAGA GGAAGCATAA GGAGTCATCT AAGCAGAGTG CTGCAAAAGA

AAAGGCAAGA GAAAGCATAA GGAGTCATCT AAGCAGAGTG CTGCAAAAGA

---------- ---------- ---------- ---------- ----------

AAAGGCAAGA GGAAACACAA GGAGTCATCG AAGCAGAGTA GTACAAAAGA

AAAGGCAAGA GGAAACACAA GGAGTCATCT AAGCAGAGTA GTACAAAAGA

AAAGGCAAGA GGAAACATAA GGAGTCATCT AAGCAGAGTA CTGCAAAAGA

AAAGGCAAGA GGAAACATAA GGAGTCATCT AAACAGAGTG CTTCTAAAGA

AAAGGCAAGA GGAAACATAA GGAGTCATCT AAGCAAAGTG CTGCAAAAGA

AAAGGCAAGA GGAAACATAA GGAGTCGTCT AAGCAGAGTG CAGCAAAAGA

---------- ---------- ---GTCATCG AAGCAGAGTG TTGCAAAAGA

AAAGGCAAGA GGAAACATAA GGAGTCCTCT AAGCAAAGTG CTGCAAAAGA

AAAGGCAAGA GGAAACATAA GGAGTCATCT AAGCAGAGCA CTGCAAAAGA

---------- ---------- ---GTCATCC AAACAGAGTG CTGCAAAAGA

AAGGGAAAGA GGAAGCATAA GGAGTCATCC AAACAGAGTA CTGTGAAAGA

AAGGGCAAGA GGAAACATAA G--GTCATCC AAACCAAGTG CTATAAAAGA

AAAGGATTTG TTGCCTTCTC CCGCTGGGCC TGTTCCTTCA AAAGATCCAA

AAAGGATTTG TTGCCTTCTC CCGCTGGGCC TGTTCCTTCA AAAGATCCAA

AAAGGATTTG TTGCCTTCTC CTGCTGGGCC TGTTCCTTCA AAAGATCCAA

AAAGGATTTG TTGCCTTCTC CTGCTGGGCC TGTTCCTTCA AAAGATCCAA

AAAGGATTTG TTGCCTTCTC CCGCTGGGCC TGTTCCTTCA AAAGATCCAA

---------- ---------- ---------- ---------- ----------

AAAGGACTTG CTACCTTCAC CTGCTGGGCC TGTTCCTTCA AAAGATTCAA

AAAGGACTTG CTACCTTCAC CTGCTGGGCC CATTCTTTCA AAAGATTCAA

AAAAGATTTG TTGCCTTCTC CTGCTGGGCC TGTTCCTTCA AAAGATCCAA

AAAAGATTTA TTGCCTTCTC CTGCTGGGCC TGTTCCTTCA AAAGATCCAA

AAAGGATTTG TTGCCGTCTC CTGCTGGGCC CGTTCCTTCA AAAGACCCCA

AAAGGATTTG TTGCCTTCTC CTGCTGGGCC TGTTCCTTCA AAAGATCAAA

AAAGGATTTG TTGCCTTCTC CTGCTGCTCC TGTTCCTTCA AAAGATCCAA

AAAGGATTTG TTGCCTTCTC CTGCTGGGCC TGTTCCTTCA AAAGATCCAA

AAAGGATTTG TTGCCTTCTC CTGCTGCGCC TGTTCCTTCA AAAGATCCAA

AAAGGATTTG TTGCCTTCTC CTGCCGGGCC TGTTCCTTCA AAAGATCCAA

GAAGGATCCA TTACCTTCTC CTGCTGGACC TATTCCCATA AAAGACTCAA

GAAGGATCTT TTGCCTTCCC CAGCTGCACC TATTCCCCCA AAAGATCCAA

AAACAGAGCA TGGCTCTCGG AAGAGGACTA TTAGTCAGTC TTCTTCCTTA

AAACAGAGCA TGGCTCTCGG AAGAGGACTA TTAGTCAGTC TTCTTCCTTA

AAACAGAGCA TGGCTCTCGG AAGAGGACTA TTAGTCAGTC TTCTTCCTTA

AAACAGAGCA TGGCTCTCGG AAGAGGACTA TTAGTCAGTC TTCTTCCTTA

AAACAGAGCA TGGCTCTAGG AAGAGGACTA TTAGTCAGTC TTCTTCCTTA

---------- ---------- ---------- ---------- ----------

AAACAGAGCA TGGCTCTCGG AAGAGGACTG TCAGTCAGTC ATCATCCTTA

AAACAGAGCA TGGTTCTCGG AAGAGGACTG TCAGTCAGTC GTCGTCCTTA

AATCAGAGCA TAGTTCTCGA AAGAGGACCA TTAGTCAGTC ATCTTCCTTG

AAACAGAGCA TGGCTCTCGG AAGAGGACTG TTAGTCAGTC TTCTTCCTTA

AACCAGAGCA TGGCTCTCGG AAGAGGACTA TTAGTCAGTC ATCATCATTA

AAACAGAGCA TGGCTCTCGG AAGAGGACTA TTAGTCAGTC TTCTTCCTTA

AAACTGAGCA TAGCTCTCGG AAGAGGACTA TTAGTCAGTC TTCTTCTTTG

AAGTAGAACA TGGCTCTAGG AAGCGGACTA TTAGTCAGTC TTCTTCCTTA

AAACAGAGCA TAGCTCTCGG AAGAGGACTA TTAGTCAGTC TTCCTCCCTA

AAACAGAGCA TGGCTCTCGG AAGAGGACTA TTAGTCAGTC TTCCTCCCTA

AAACTGAGCA TGGATCCAGG AAGAGAACAA TCAGCCAGTC GTCGTCTTTA

AACCAGAGCA CGGGTCCCGG AAGAGGACAG TCAGCCAATC GTCTTCCTTA

AAGTCAAGCA GTAACAGCAA CAAGGAGACG AGTGGCAGCA AAAACAGTTC

AAGTCAAGCA GTAACAGCAC CAAGGAGACG AGTGGCAGCA AAAACAGTTC

AAGTCAAGCA GTAACAGCA- --AGGAGACT AGTGGCAGCA GAAACAGTTC

AAGTCAAGCA GTAACAGCA- --AGGAGACT AGTGGCAGCA GAAACAGTTC

AAGTCAAGCA GTAACAGCAA CAAGGAGACA AGTGGCAGCA AAAACAGTTC

---------- ---------- ---------- ---------- ----------

AAGTCCAGCA GTACCAGCAA CAAAGAGAAC AGTGGCAGCA AGAGCACTTC

AAGTCCAGTG GGACCAGCAG CAAAGAGAAC AGTGGCAGCA AGAGCACCTC

AAGTCAAGCA GTAACAGCAA TAAGGAGAAC AGTGGCAGCA AAAGCAGTTC

AAGTCAAGTA ATAACAGCAA CAAGGAAAAT AGTGCCAGCA AAAACAGTTC

AAGTCCGGTG CTAACAGCAA CAAAGAGAAC AGTGCGAGCA GGAACAGCTC

AAGTCAAGTA ATAACAGCAA CAAGGAGAAC AGTGGGAGCA AAAACAGTTC

AAGTCAAGTA ATAACAGCAA CAAGGAGAAT AGTAGCAGCA AAAGCAGTTC

AAGTCAAGCA GTAACAGCAA CAAGGAGAAT AGTGGCAGCA AAAACAGTTC

AAGTCAAGCA ATAATAACAA CAAGGAGAAT AGTGGCAGCA AAAATAGTTC

AAGTCTAGCA GTAATAGCAA CAAGGAGAAC AGCAGCAGCA AAAGTGGCTC

AAATCTACCA GTAGCAGCAA CAAGGAGAGT AGTG---GTA AAAATAGTTC

AAATCTAGCA GCAACAGTAT CAAGGAGAGC A---GCAGTA AAAACAGTTC

CTCCACATCA AAGCAGAAGA AGACCGAAGG GAAGACTTCC AGTAGCTCCA

CTCCACATCA AAGCAGAAGA AGACCGAAGG GAAGACTTCC AGTAGCTCCA

CTCTACATCC AAGCAGAAGA AGACCGAAGG GAAGACTTCC AGTAGTTCCA

CTCTACATCC AAGCAGAAGA AGACCGAAGG GAAGACTTCC AGTAGTTCCA

TTCCACATCC AAGCAGAAGA AGACCGAAGG GAAGACTTCT AGTAACTCCA

---------- ---------- ---------- ---------- ----------

CTCCACAGCA AAACAGAAGA AGACTGAAGG GAAGGTTTCC AGCAGCTCCA

CTCCACAGCG AAGCAGAAGA AGACTGAAGG CAAGGGTCCC AGCAGCTCCA

CTCCACATCA AAGCTGAAAA AGACTGAAGG GAAGACCTCA AGTAGCTCCA

CTCCGCATCA AAGCAGAAGA GGACTGAAGG GAAGACTTCC AGTAGCTCTA

CTCCACATCC AAGCAGAAGA AGACTGAGGG GAAGACCTCC AGTAGCTCCA

CTCCACGTCA AAGCAGAAGA AAACTGAAGG GAAGACTTCC AGTAGCTCTA

CTCTGCATCA AAGCAGAAGA AGACTGAAGG GAAGACGTCC GGTAGCTCGA

CTCTACATCA AAGCAGAAGA AGACTGAAGG GAAGACTTCC AGTAACTCCA

CTCCACATCA AAGCAGAAGA AGACTGAAGG GAAGACTTCC AGTAGCTCCA

CTCCACATCC AAGCAGAAGA AGACTGACGG GAAGACCTCC AGTAGCTCCA

TTCTACTTCC AAACAGAAAA AGACAGAAGG GAAAGTTTTT AGTGGTACCA

TTCCTCTTCA AAGCAAAAAA AGACAGAAGG AAAGGTTTCT ACCAGTTCCA

AGGAGGTTAA GGAAAAGGCT CCAAGTAGCT CCTCTAACTG TCCTCCATCT

AGGAGGTTAA GGAAAAGGCT CCAAGTAGCT CCTCTAACTG TCCTCCATCT

AGGAGGTTAA GGAAAAGGCT CCAAGTAGCT CCTCTAATTG TCCTCCATCT

AGGAGGTTAA GGAAAAGGCT CCAAGTAGCT CCTCTAATTG TCCTCCATCT

AGGAGGTTAA GGAAAAGGCT CCAAGTAGCT CCTCTCACTG TCCTCCATCT

---------- ---------- ---------- ---------- ----------

AAGAGGCCAA GGAGAAGGCT CCAAATAGCT CCTCTAATTG TCCTCCGTCT

AGGAGGCCAA GGAGAAGGCT CCAAATAGCT CCTCTAATTG TCCTCCGTCT

AGGAGGTTAA G--------- ---------- ---------- ----------

AGGAGGTCAA GGAAAAAGCT CCAAATAGTT CTTCTAACTG TCCTCCATCT

AGGAGGTCAA GGAAAAGGCT CCAAATAGCT CCTCTAACTG TCCTTCATCT

AGGAGGTCAA GGAAAAGGCT TCAAATAGTT CCTCTAACTG TCCTCCATCT

AGGAAACTAA GGAAAAGACT TCAAATAGCT CCTCAAACTG TCCTTCATCT

AGGAGATCAA GGAAAAGGCT CCAAATAGCT CCTCTAACTG TCCTCCATCT

AGGAGGTCAA GGATAAGGCT CCAAATAGCT CCTCTAACTG CCCTCCATCT

AGGAGACCAA G--------- ---------- ---------- ----------

AGGAAGCCAA GGAAAAGGCT CCAAGTAGTT CTTCGAACTA TCCTCCAGCT

AGGAAACCAA GGAAAAAAAT CCAAACAACT CTTCAAATTG TCCTCCATCT

GCACCAACTC TTGATTCTTC TAAGCCTCGG AGAACAAAGC TTGTCTTTGA

GCACCAACTC TTGATTCTTC TAAGCCTCGG AGAACAAAGC TTGTCTTTGA

GCACCAACTC TTGATTCTTC TAAGCCTCGG AGAACAAAGC TTGTCTTTGA

GCACCAACTC TTGATTCTTC TAAGCCTCGG AGAACAAAGC TTGTCTTTGA

GCACCAACTC TTGATTCTTC TAAGCCTCGG AGAACAAAGC TTGTCTTTGA

---------- ---------- ---------- ---------- ----------

ACACCAACTC CTGATTCTTC GAAGCCTCGA AGAACGAAGC TCGCCTTTGA

ACACCAACTT CTGAGTCTTC GAAGCCTCGG AGAACAAAGC TTGCCTTTGA

---------- ---------- ---------- --------GC TCGCCTTTGA

GCACCAACTC CTGATGCTTC TAAGCCTCGG AGAACGAAGC TTGCCTTTGA

ACACCAACTC CTGATGCTTC TAAGCCTCGG AGAACAAAGC TTGCCTTTGA

ACACCAACTC CTGATGCTTC TAAGCCTCGG AGAACAAAGC TTGTCTTTGA

ACACCAACTT CTGAAGCTTC TAAACCTCGG AGAACAAAGC TTGCCTTTGA

ACACCAACTC CTGATGCGTC TAAGTCTCGG AGAACAAAGC TTGCATTTGA

ACACCAACTC CTGATGCTTC TAAGCCTCGG AGAACAAAGC TTGCCTTTGA

---------- ---------- ---------- ---------- ----------

ACACAAATTC CTGATGGCTC CAAAACACGA AGAGCAAAGC TTGCTTTTGA

ACAACAACTC CTGACAGTTC CAAGTCCCGA AGAGCAAAGC TTGCTTTTGA

TGACAGAAAT TATTCAGCAG ACCATTATTT ACAAGAAGCA AAAAAGCTAA

TGACAGAAAT TATTCAGCAG ACCATTATTT ACAAGAAGCA AAAAAGCTAA

TGACAGAAAT TATTCAGCAG ACCATTATTT ACAAGAAGCA AAAAAGCTAA

TGACAG---- ---------- ---------- ---------- ----------

TGACAGAAAT TATTCAGCAG ACCATTATCT ACAAGAAGCA AAAAAGCTAA

---------- ---------- ---------- ---------- ----------

TGACAGAAAT TATTCAGCAG ACCATTATTT ACAAGAAGCA AAAAAACTCA

TGACAGAAAT TATTCAGCAG ACCATTATTT ACAAGAAGCA AAAAAACTCA

TGACAGAAGT TACTCCGCAG ACCATTATTT ACAAGAAGCA AAAAAGTTAA

TGACAGAAAT TATTCAGCAG ACCATTATTT ACAAGAAGCA AAAAAGCTAA

TGACAGAAAT TATTCAGCAG ACCATTATTT ACAAGAAGCA AAAAAGCTAA

CGACAGAAAT TATTCAGCAG ACCATTATTT ACAAGAAGCA AAAAAGCTAA

TGACAGAAAT TATTCAGCAG ATCATTATTT GCAAGAAGCA AAAAAGCTAA

CGACAGAAAT TATTCAGCAG ACCATTATTT ACAAGAAGCA AAAAAGCTAA

CGACAGGAAT TATTCAGCAG ACCATTATTT ACAGGAAGCA AAAAAGCTAA

---------- ---------- ---------- ---------- ----------

TGACAGGATT TACTCAGCTG ACCATTATTT ACAAGAAGCT AAAAAGCTAA

TGACAGGAGC TATTCAGCAG ATCATTACTT ACAAGAAGCA AAAAAACTAA

AGCACAATGC AGATGCATTG TCTGATAGGT TTGAGAAAGC TGTATACTAT

AGCACAATGC AGATGCATTG TCTGATAGGT TTGAGAAAGC TGTATACTAT

AGCACAATGC AGATGCATTG TCTGATAGGT TTGAGAAAGC TGTATACTAT

---------- ---------- ---------- ---------- ----------

AGCACAATGC AGATGCATTG TCTGATAGGT TTGAGAAAGC TGTATACTAT

---------- ---------- ---------- ---------- ----------

AACACAATGC AGATGCTTTG TCTGATAGGT TTGAGAAAGC TGTGTACTAC

AACACAATGC AGATGCTTTG TCTGACAGGT TTGAGAAAGC CGTGTACTAC

AGCACAATGC AGATGCATTG TCTGATAGGT TTGAGAAAGC TGTGTACTAT

AGCACAATGC AGATGCATTG TCTGATAGGT TTGAGAAAGC TGTATACTAT

AGCACAATGC AGATGCATTG TCTGATAGGT TTGAGAAAGC TGTATACTAT

AGCACAATGC AGATGCTTTG TCTGACAGGT TTGAGAAGGC TGTGTACTAT

AGCACAATGC CGATGCATTG TCTGACAGGT TTGAGAAAGC TGTGTACTAT

AGCATAATGC AGATGCATTG TCTGATAGGT TTGAGAAAGC AGTATACTAT

AGCACAATGC AGATGCATTG TCTGATAGGT TTGAGAAAGC AGTGTACTAT

---------- ---------- ---------- ---------- ----------

AGCACAATGC GGATGCATTG TCTGATAGAT TTGAGAAAGC TGTCTATTAT

AGCACAATGC AGATGCGTTG TCCGATAGGT TTGAGAAAGC GGTCTTCTAC

CTTGATGCTG TGGTATCTTT CATTGAATGT GGGAATGCAT TAGAGAAGAA

CTTGATGCTG TGGTATCTTT CATTGAATGT GGGAATGCAT TAGAGAAGAA

CTTGATGCTG TGGTATCTTT CATTGAATGT GGGAATGCAT TAGAGAAAAA

---------- ---------- ---------- ---------- ------AAAA

CTTGATGCTG TGGTATCTTT CATTGAATGT GGGAATGCAT TAGAGAAAAA

---------- ---------- ---------- ---------- ----------

CTTGATGCCG TAGTGTCTTT CATTGAATGT GGGAATGCAT TAGAGAAGAA

CTTGATGCTG TAGTGTCTTT CATTGAGTGT GGGAATGCAT TAGAGAAGAA

CTTGATGCTG TGGTATCTTT CATTGAATGT GGGAACGCTT TGGAGAAAAA

CTTGATGCTG TGGTATCTTT CATTGAATGT GGGAATGCAT TAGAGAAAAA

CTTGATGCTG TGGTATCTTT CATTGAATGT GGGAATGCAT TAGAGAAAAA

CTTGATGCCG TGGTGTCTTT CATTGAATGT GGGAATGCAT TAGAGAAAAA

CTTGATGCTG TGGTATCTTT CATAGAATGT GGGAATGCGT TAGAGAAAAA

CTTGATGCTG TGGTATCTTT CATTGAATGT GGGAATGCCT TGGAGAAAAA

CTTGATGCTG TGGTATCTTT CATTGAATGT GGGAATGCAT TAGAGAAAAA

---------- ---------- ---------- ---------- ----------

CTTGATGCTG TGGTATCTTT CATTGAATGT GGAAATGCTT TGGAGAAAAA

CTTGATGCTG TTGTGTCTTT CATTGAATGT GGGAATGCAT TGGAGAAAAA

TGCTCAGGAA TCCAAATCCC CATTCCCTAT GTATTCAGAG ACGGTGGATC

TGCTCAGGAA TCCAAATCCC CATTCCCTAT GTATTCAGAG ACGGTGGATC

TGCTCAGGAA TCCAAATCCC CATTCCCTAT GTATTCAGAG ACAGTGGATC

TGCTCAGGAA TCCAAATCCC CATTCCCTAT GTATTCAGAG ACAGTGGATC

TGCTCAGGAA TCCAAATCCC CATTCCCTAT GTATTCAGAG ACGGTGGATC

---------- ---------- ---------- ---------- ----------

CGCTCAGGAA TCCAAGTCTC CATTCCCTAT GTACTCAGAC ACAGTGGAGC

CGCTCAGGAA TCCAAGTCCC CATTCCCTAT GTACTCAGAC ACGGTGGAGC

TGCTCAGGAA TCTAAATCCC CATTCCCCAT GTATTCAGAG ACAGTGGAGC

TGCTCAGGAA TCCAAATCCC CATTCCTTAT GTATTCAGAA ACGGTGGAGC

TGCTCAGGAA TCCAAATCCC CATTCCCTAT GTATTCAGAG ACAGTGGAGC

TGCTCAGGAA TCCAAATCCC CATTTCTTAT GTATTCAGAG ACGGTGGATC

TGCTCAGGAA TCCAAGTCCC CATTCCCTAT GTATTCAGAG ACAGCAGATC

TGCTCAGGAA TCCAAGTCCC CATTCCCTAT GTATTCAGAG ACGGTGGAAC

TGCTCAGGAA TCCAAGTCCC CATTCCTTAT GTATTCAGAG ACGGTGGATC

---------- ---------- ---------- ---------- ----------

TGCTCAGGAA TCCAAATCTC CATTCCTTAT GTATTCAGAA ACTGTGGAGT

CGCTCAGGAA TCCAAATCCC CGTTCCCTAT GTATTCAGAA ACTGTGGAAC

TCATCAAATA CACTATGAAG CTAAAGAATT ACTTGGCACC AGATGCTACA

TCATCAAATA CACTATGAAG CTAAAGAATT ACTTGGCACC AGATGCTACA

TCATCAAATA CACTATGAAG CTAAAGAATT ACTTGGCACC AGATGCTACA

TCATCAAATA CACTATGAAG CTAAAGAATT ACTTGGCACC AGATGCTACA

TCATCAAATA CACTATGAAG CTAAAGAACT ACTTGGCACC AGATGCTACA

---------- ---------- ---------- ---------- ----------

TTATTAAATA CACTATGAAA CTAAAGAATT ATTTGGCGCC AGATGCTACA

TCATTAAGTA CACTATGAAA CTAAAGAATT ATTTGGCACC AGATGCTACA

TCATCAAGTA CACTATGAAG CTGAAGAGTT ACTTAGCACC AGACGCCACA

TCATCAAATA CACTATGAAG CTAAAGAATT ACTTGGCACC AGATGCTACA

TCATCAAATA CACTATGAAG CTAAAGAATT ACTTGGCACC TGATGCTACA

TCATCAAATA CACTATGAAG CTAAAGAATT ACTTAGCACC AGATGCTACA

TCATCAAATA CACTATGAAA CTAAAGAGTT ACTTGGCACC AGACGCTACC

TTATCAAATA TACTATGAAG CTAAAGAATT ACTTGGCTCC AGATGCTACA

TCATCAAATA CACTATGAAG TTAAAGAATT ACTTGGCGCC AGATGCTACA

---------- ---------- ---------- ---------- ----------

TAATTAAATA TACTATGAAG CTGAAGAATT ACCTGGCACC AGATGCTACA

TAATTAAATA CACTATGAAG CTGAAGAATT ACTTGGCACC AGATGCCACA

GCTGCAGATA AACGACTCAC AGTACTTTGC CTGCGATGCG AGTCTTTGCT

GCTGCAGATA AACGACTCAC AGTACTTTGC CTGCGATGCG AGTCTTTGCT

GCTGCAGATA AACGACTCAC AGTACTTTGC CTGCGATGCG AGTCTTTGCT

GCTGCAGATA AACGACTCAC AGTACTTTGC CTGCGATGCG AGTCTTTGCT

GCTGCAGATA AACGACTCAC AGTGCTTTGC CTGCGATGCG AGTCTTTGCT

---------- ---------- ---------- ---------- ----------

GCTGCAGACA AGAGACTCAC AGTACTTTGC CTGAGATGCC AGTCATTGTT

GCTGCAGACA AGAGACTCAC AGTACTTTGC CTGAGATGCC AGTCATTATT

GCTGCAGATA AGAGACTCAC GGTGCTCTGC CTGCGATGCC AGTCTTTGCT

GCTGCAGATA AAAGACTTAC AGTGCTTTGC CTGCGATGCC AGTCTTTGCT

GCTGCAGATA AAAGGCTCAC AGTACTTTGC CTGCGATGCC AGTCTTTGCT

GCAGCAGATA AAAGACTCAC AGTACTTTGC CTACGGTGCG AGTCTTTGCT

GCTGCAGATA AAAGACTTAC AGTACTTTGC CTACGGTGTC AGTCTTTGCT

GTTGCAGATA AAAGACTCAC AGTACTTTGC CTACGATGCC AGTCTTTGCT

GCTGCAGATA AAAGACTCAC AATACTTTGC CTACGATGCC AGTCTTTGCT

---------- ---------- ---------- ---------- ----------

TCTGCAGATA AAAGGCTGGC AGTTCTTTGC CTTCGATGCC AGTCCTTATT

GCTGCAGATA AAAGACTAGC CGTGCTATGT CTGCGATGCC AGTCCCTGTT

GTACCTGAGG CTGTTCAAAC TGAAGAAGGA AAATGCTCTG AAGTACTCAA

GTACCTGAGG CTGTTCAAAC TGAAGAAGGA AAATGCTCTG AAGTACTCAA

GTACCTGAGG CTTTTCAAAC TGAAGAAAGA AAATGCTTTG AAGTACTCAA

GTACCTGAGG CTTTTCAAAC TGAAGAAAGA AAATGCTTTG AAGTACTCAA

GTACCTGAGG CTATTCAAAC TGAAGAAGGA AAATGCTCTG AAGTACTCAA

---------- ---------- ---------- ---------- ----------

GTACCTGAGG CTGTTCAAAC TGAAGAAGGA GAACGCTCTG AAGTACTCCA

GTACCTGAGG CTGTTCAAAC TGAAGAAGGA GAACGCTCTC AAGTACTCCA

GTACCTGAGG CTATTCAAGC TGAAGAAGGA GAATGCTCTG AAGTACTCAA

GTACCTGAGG CTCTTCAAAC TGAAGAAAGA AACTGCTCTG AAGTATTCAA

GTATCTGAGG CTATTCAAAC TGAAGAAGGA GAATGCTCTG AAGTACTCAA

GTACCTGAGG CTATTCAAAC TGAAGAAGGA AAATGCTCTG AAGTACTCCA

ATACCTGAGG CTATTCAAAC TGAAGAAGGA AAATGCTTTG AGGTACTCCA

TTACCTGAGG TTATTCAAAC TGAAGAAGGA GAATGCTCTG AAGTACTCCA

GTACCTGAGG CTATTCAAAC TGAAGAAGGA AAATGCTCTG AAGTACTCAA

---------G CTATTCAAAC TGAAGAAGGA GAATGCTCTC AAGTACTCCA

GTACCTGAGG TTATTTAAGC TGAAGAAAGA AAATGCTTTG AAATACTCAA

GTACCTGAGG TTGTTCAAGC TGAAGAAAGA GAGTGCGTTG AAATACTCAA

AGACACTGAC AGAGCACCTG AAGAATTCTT ATAATAATTC TCAAGCACCA

AGACACTGAC AGAGCACCTG AAGAATTCTT ATAATAATTC TCAAGCACCA

AGACACTGAC AGAGCACCTG AAGAATTCTT ATAATAATTC TCAAGCACCA

AGACACTGAC AGAGCACCTG AAGAATTCTT ATAATAATTC TCAAGCACCA

AGACACTGAC AGAGCACCTG AAGAATTCTT ACAATAATTC TCAAGCACCA

---------- ---------- ---------- ---------- ----------

AGACGCTGAC TGAGCACCTG AAGAATTCTT ACAGTAATTC TCAAGCACCT

AGACGCTAAC TGAGCACCTG AAGAATTCTT ACAGTAATTC TCAAGCACCT

AGACACTGAC AGAGCACCTC AAGAATTCTT ATAATAATTC TCAAGCACCA

AGACACTGAC AGAGCATCTG AAGAATTCTT ACAATAACTC TCAAGCACCA

AGACACTGAC AGAGCACCTG AAGAATTCTT ACAATAATTC TCAAGCACCA

AGACACTGAC GGAGCACCTG AAGAATTCTT ATAATAATTC TCAAGCACCA

AGACACTGAC AGAGCACCTG AAG------- ---------- ----------

AGACACTGAC AGAACACCTG AAGAATTCTT ATAATAATTC TCAGGCTCCA

AGACACTGAC GGAGCATCTG AAGAATTCTT ACAATAATTC TCAAGCTCCA

AGACCCTGAC AGAGCATCTG AAGAATTCTT ACAATAATTC TCAAGCTCCG

AAACATTAAC TGAACATTTA AAGAATTCTT ATAATAATTC TCAGGCTCCA

AAACACTGAC GGAACATCTA AAGAACTCCT ATAATAATTC GCAAGCTCCC

TCGCCTGGCT TGGGAAGCAA AGCTGTGGGG ATGCCTTCCC CTGTTTCTCC

TCGCCTGGCT TGGGAAGCAA AGCTGTGGGG ATGCCTTCCC CTGTTTCTCC

TCGCCTGGCT TGGGAAGCAA AGCTGTGGGG ATGCCTTCCC CTGTTTCTCC

TCACCTGGCT TGGGAAGCAA AGCTGTGGGG ATGCCTTCCC CTGTTTCTCC

TCGCCTGGTT TGGGAAGCAA AGCTGTGGGG ATGCCTTCCC CTGTTTCTCC

---------- ---------- ---------- ---------- ----------

TCACCTGGCT TGGGGAGCAA AGCTGTTGGG ATGCCCTCCC CTGTTTCTCC

TCACCCGGCT TGGGGAGCAA AGCTGTTGGG ATGCCCTCCC CTGTTTCTCC

TCCCCTGGCT TGGGAAGCAA AACTGTTGGC ATGCCTTCCC CAATTTCTCC

TCACCAGGCT TGGGAAGCAA AGCTGTTGGG ATGCCTTCCC CTGTTTCTCC

TCACCTGGCT TGGGAACCAA AGCCGTTGGG ATGCCTTCCC CTGTTTCTCC

TCACCTGGCT TGGGAAGCAA AGCTGTTGGG ATGCCTTCCC CTGTTTCTCC

---------- -------CAA AGCTGTTGGG ATGCCATCCC CTGTTTCTCC

TCACCTGGCT TGGGAAGCAA AGCTGTTGGG ATGCCTTCCC CTGTTTCTCC

TCACCTGGCT TGGGAAGCAA AGCTGTTGGG ATGCCTTCCC CTGTTTCTCC

TCACCTGGCT TGGGAAGCAA AGCCGCTGGG ATGCCTTCCC CTGTGTCACC

TCACCTGGCA TGGGAAGCAA AGCTGTTGGG ATGCCTTCCC CTGTTTCTCC

TCACCTGGCA TAGGAAGCAA AGCCGCCGGG ATGCCCTCGC CTGTCTCCCC

AAAGCTGTCA CCAGGCAATT CAGGAAATTA TTCATCTGGG GCCAGTAGTG

AAAGCTGTCA CCAGGCAATT CAGGAAATTA TTCATCTGGG GCCAGTAGTG

AAAGCTGTCA CCAGGTAATT CAGGAAATTA TTCATCTGGG GCCAGTAGTG

AAAGCTGTCA CCAGGCAATT CAGGAAATTA TTCATCTGGG GCCAGTAGTG

AAAGCTGTCA CCAGGCAATT CAGGAAATTA CTCATCTGGG GCCAGTAGTG

---------- ---------- ---------- ---------- ----------

AAAGCTGTCA CCAGGCAATT CTGGAAGTTA CTCTTCTGGG GGAAGTAGTG

AAAACTGTCA CCAGGCAATT CTGGAAGTTA CTCTTCTGGG GGAAGTAGTG

AAAGACGTCA CCAGCCAATT CAAAAAAAGA ATTCTCCGGG GCCAGTAGTG

AAAGCTGTCA CCAGGCAATT CAGGAAATTA TTCTTCTGGA GCCAGTAGTG

AAAGTTGTCG CCAGGCAATT CAGGAAATTA TTCTTCAGGG GCCAGTAGTG

AAAGCTGTCT CCAGGCAATT CAGGAAGTTA CTCTTCTGGG GCCAGTAGTG

AAAGCTGTCA CCAGGTAATT CAGGAAATTA TTCTTCTGGA GCCAGTAGTG

AAAGCTATCG CCAGGCAATT CAGGAAATTA TTCTTCTGGT GCCAGTAGTG

AAAACTGTCA CCAGGCAATT CAGGAAATTA CTCTTCTGGA GCCAGTAGTG

AAAACTGTCA CCAGGCAATT CCGGAAACTA CTCTTCTGGA GCCAGCGGTG

AAAGCTCTCT CCTGGCAATT CAGGAAATTA TACTTCAGGG GCAAGCAGCA

CAAGCTTTCT CCGGGCAATT CAGGAAACTA CTCCTCAGGG GCCAGCAACC

CTTCTGCAAG TGGTTCTTCA GTGACCATTC CACAGAAGAT CCACCAGATG

CTTCTGCAAG TGGTTCTTCA GTGACCATTC CACAGAAGAT CCACCAGATG

CTTCTGCAAG TGGCTCTTCA GTGACCATTC CACAGAAGAT CCACCAGATG

CTTCTGCAAG TGGCTCTTCA GTGACCATTC CACAGAAGAT CCACCAGATG

CTTCTGCAAG TGGTTCTTCA GTGACCATTC CACAGAAGAT CCACCAGATG

---------- ---------- ---------- ---------- ----------

CTTCAGCAAG TGGTTCTTCG GTGACCATTC CACAGAAAAT CCACCAGATG

CTTCAGCAAG TGGTTCTTCA GTGACCATTC CACAGAAAAT CCACCAGATG

CTTCAGCAAC CACCTCATCC ATTACCATCC CACAGAAGAT CCACCAGA--

CTTCAGCAAG TGGTTCTTCA GTGACCATTC CACAGAGGAT TCACCACATG

CTTCAGCAAG TGGTTCTTCA GTGACCATTC CACAGAGGAT CCACCAGATG

CTTCAGCAAG TGGTTCTTCA GTGACCATTC CACACAGGAT CCACCACATG

CTTCAGCAAG TGGTTCTTCG GTGACCATTC CACAGAGGAT CCACCATATG

CTTCAGCAAG TGGTTCTTCA GTGACCATTC CACAGAAGAT CCACCAGATG

CTTCAGCTAG TGGCTCTTCA GTGACCATCC CACAGAGGAT CCACCAGATG

CTTCAGCAAG TGGATCTTCC GTGACCATTC CACAGAAGAT CCACCAGATG

CTTCAGGGAG CAGTTCTTCA GTAACTATTC CACAGAAGAT CCACCAGATG

CTTCGGGGAG CGGTTCTTCG GTGACAATTC CGCAGAGGAT CCACCAGATG

GCAGCCAGCT ATGTTCAGGT CACATCCAAC TTCCTCTATG CCACCGAAAT

GCAGCCAGCT ATGTTCAGGT CACATCCAAC TTCCTCTATG CCACCGAAAT

GCAGCCAGCT ATGTTCAGGT CACGTCCAAC TTCCTCTATG CCACCGAAAT

GCAGCCAGCT ATGTTCAGGT CACGTCCAAC TTCCTCTATG CCACTGAAAT

GCAGCCAGCT ATGTTCAGGT CACATCCAAC TTCCTCTATG CCACCGAAAT

---------- ---------- ---------- ---------- ----------

GCAGCAAGCT ATGTCCAGGT TACATCTAAC TTCCTCTATG CCACAGAAAT

GCAGCAAGCT ATGTCCAGGT TACATCTAAC TTCCTCTATG CCACAGAAAT

---------- ---------- -----CCAAC TTCCTCTATG CCACCGAAAT

GCAGCCAGCT ATGTTCAGGT TACATCCAAC TTCCTCTATG CCACTGAAAT

GCAGCCAGCT ATGTTCAAGT TACATCTAAC TTTCTCTATG CCACGGAAAT

GCAGCCAGCT ATGTCCAGGT CACATCCAAC TTCCTCTATG CCACAGAAAT

GCAGCCAGCT ATGTTCAGGT TACATCCAAC TTCCTCTATG CCATTGAAAT

GCAGCCAGCT ATGTTCAGGT TACATCCAAC TTCCTCTATG CCACTGAAAT

GCAGCCAGCT ATGTTCAGGT TACATCCAAC TTCCTCTATG CCACTGAAAT

GCAGCCAGCT ATGTTCAGGT TACATCTAAC TTCCTCTATG CCACTGAAAT

GCAGCCAACT ATGTTCAGGT CACATCCAAC TTCCTTTATG CCACTGAAAT

GCGGCCAGCT ACGTCCAGGT CACCTCCAAC TTCCTCTACG CGACTGAAAT

TTGGGACCAA GCTGAACAGC TTTCCAAAGA GCAAAAAGAA TTCTTTGCTG

TTGGGACCAA GCTGAACAGC TTTCCAAAGA GCAAAAAGAA TTCTTTGCTG

TTGGGACCAA GCTGAACAGC TTTCCAAAGA GCAAAAAGAA TTCTTTGCTG

TTGGGACCAA GCTGAACAGC TTTCCAAAGA GCAAAAAGAA TTCTTTGCTG

TTGGGACCAA GCTGAACAAC TTTCCAAAGA GCAAAAAGAA TTCTTTGCTG

---------- ---------- ---------- ---------- ---TTTGCTG

TTGGGACCAA GCTGAACAGC TTTCCAAAGA ACAAAAAGAA TTCTTTGCTG

TTGGGACCAA GCTGAACAGC TTTCCAAAGA ACAAAAAGAA TTCTTTGCTG

CTGGGACCAA GCTGAACAGC TTTCCAAAGA GCAAAAAGAA TTCTTTGCTG

TTGGGACCAA GCTGAACAGC TTTCCAAAGA GCAAAAAGAA TTCTTTGCTG

TTGGGACCAA GCTGAACAGC TTTCCAAAGA GCAGAAAGAA TTCTTTGCTG

TTGGGACCAA GCTGAACAGC TTTCCAAAGA GCAAAAAGAA TTCTTTGCTG

TTGGGACCAA GCTGAACAGC TTTCCAAAGA GCAAAAAGAA TTCTTTGCTG

TTGGGACCAA GCAGAACAGC TTTCCAAAGA ACAAAAAGAA TTCTTTGCTG

TTGGGACCAA GCGGAACAGC TTTCCAAAGA GCAAAAAGAA TTCTTTGCTG

TTGGGACCAA GCTGAACAGC TTTCCAAAGA GCAAAAAGAA TTCTTTGCTG

CTGGGACCAA GCTGAACAGC TTTTGAAGGA GCAAAAAGAG TTCTTTGCTG

TTGGGACCAA GCCGAACAGC TTTCCAAAGA GCAAAAAGAA TTCTTTGCTG

AACTGGATAA AGTAATGGGC CCTCTCATCT TTAATGCAAG CATCATGACA

AACTGGATAA AGTAATGGGC CCTCTCATCT TTAATGCAAG CATCATGACA

AACTGGATAA AGTAATGGGC CCTCTCATCT TTAATGCAAG CATCATGACA

AACTGGATAA AGTAATGGGC CCTCTCATCT TTAATGCAAG CATCATGACA

AACTGGATAA AGTAATGGGT CCTCTCATCT TTAATGCAAG CATCATGACA

AACTGGATAA AGTAATGGGT CCTCTCATCT TTAATGCAAG CATCATGACA

AACTGGACAA AGTGATGGGC CCTCTCATCT TTAATGCAAG CATCATGACA

AACTGGACAA AGTGATGGGC CCTCTCATCT TTAATGCAAG CATCATGACA

AACTGGATAA AGTAATGGGC CCGCTCATCT TTAATGCAAG CATCATGACA

AACTGGATAA AGTAATGGGC CCTCTCATCT TTAATGCAAG CATCATGACA

AACTGGATAA AGTGATGGGC CCTCTCATCT TTAATGCGAG CATCATGACA

AACTGGATAA AGTAATGGGC CCTCTCATCT TTAACGCAAG CATCATGACA

AACTGGATAA AGTAATGGGC CCTCTCATCT TTAATGCAAG CATCATGACA

AACTGGATAA AGTAATGGGA CCTCTCATCT TTAATGCAAG CATCATGACA

AACTGGATAA AGTAATGGGC CCTCTCATCT TTAATGCAAG CATCATGACA

AACTGGATAA AGTAATGGGC CCTCTCATCT TTAATGCAAG CATCATGACA

AACTGGATAA AGTTATGGGC CCTCTCATCT TTAATTCAAG CATCATGACA

AACTGGATAA AGTTATGGGC CCGCTCATCT TTAATTCAAG CATCATGACA

GATCTAGTTC GTTATACCCG GCAGGGACTG CACTGGCTTC GCCAGGATGC

GATCTAGTTC GTTATACCCG GCAGGGACTG CACTGGCTTC GCCAGGATGC

GATCTAGTTC GTTATACCCG GCAGGGACTG CACTGGCTTC GCCAGGATGC

GATCTAGTTC GTTATACCCG GCAGGGACTG CACTGGCTTC GCCAGGATGC

GATCTAGTTC GTTACACCCG GCAGGGACTG CACTGGCTTC GCCAGGATGC

GATCTAGTTC GTTATACCCG GCAGGGACTG CACTGGCTTC GCCAGGATGC

GATCTGGCTC GTTACACCCG GCAGGGACTG CATTGGCTTC GGCAGGATGC

GATCTGGCTC GTTACACCCG GCAGGGACTG CATTGGCTTC GGCAGGATGC

GATCTGGTTC GTTATACCCG GCAGGGACTG CACTGGCTTC GCCAGGATAC

GACCTAGTTC GTTACACCCG GCAGGGACTG CACTGGCTTC GCCAGGATGC

GACCTAGTTC GATACACCCG GCAGGGACTG CACTGGCTTC GCCAGGATGC

GACCTAGTTC GTTACACCCG GCAGGGACTG CACTGGCTTC GCCAGGATGC

GACCTAATTC GCTATACCCG GCAGGGACTA CACTGGCTTC GCCAGGATGC

GACCTAGTCC GTTACACCCG GCAGGGATTA CACTGGCTTC GCCAGGACGC

GACCTAGTTC GTTACACCCG GCAGGGACTG CACTGGCTTC GCCAGGATGC

GACCTAGTTC GTTACACCCG GCAGGGACTG CACTGGCTTC GCCAGGATGC

GACCTAGTTC GTTACACCCG GCAGGGACTT CACTGGCTTC GTCTGGATGC

GACCTAGTTC GTTACACCCG GCAGGGACTC CACTGGTTGC GTCAGGATGC

CAAGTTGATA TCTGACCTCT ATATTGGGAC GCCGCTGGAC TTCTTGACTA

CAAGTTGATA TCTGACCTCT ATATTGGGAC GCCGCTGGAC TTCTTGACTA

CAAGTTGGTA TCTGACCTCT ATATTGGGAC GCCGCTGGAC TTCTTGACTA

CAAGTTGGTA TCTGACCTCT ATATTGGGAC GCCGCTGGAC TTCTTGACTA

CAAGTTGATA TCTGACCTCT ATATCGGGAC GCCGCTGGAC TTCTTGACTG

CAAGTTGATA TCTGACCTCT ATATTGGGAC GCCGCTGGAT TTCTTGATTA

CAAGCTGATA TCTAACCTCC ACGTGGGAAC GTCGCTGGAT TTCTTGATTA

CAAGCTGATA TCTGACCTCC ACGCGGGAAT GTCGCTGGGT TTCTTGATTA

CAAGTTCAAG TCTGACCTGT ATCTCGCCAC GCCACTGGAT TTCTTGACTC

CAAGTTGATA TCC------- ---------- ---------- ----------

CAAGTTGATA TCTGACCTCT ATATTGGGAC GCCGCTGGAC TTCTTGACCA

CAAGTTGATA TCGGACCTCT ATATTGGGAC GCCGCTGGAC TTCTTGACCA

CAAATTGATA TCTGACCTC- --ACTGGGAC GCCGATGGAA TTCTTGGTCA

CAAATTGATA TCTGACCTCT ATATTGGGAC GCCGCTGGAC TTCTTGACTA

CAAGTTAATA TCTGACCTCT ATATCGGGAT ACCGCTGGAC TTCTTGACTA

CAAGTTGATC TCTGACCTCT GTGTCGGGAT GCCGCTGGAC TGCAGGACTA

CAAGTTAATA TCTGATCCTT TCACAGCCAC AATGAGGGAC TTCTTGTTTG

CAAGTCGATA CCTGACCTTT ACACAGCAAC ACCGCTTGAT TTCTTGAACA

TATTCCATGC CCTGGTGGTC CTGAGCTGGC CCCATGTGTT GGAGCTGCTG

TATTCCATGC CCTGGTGGTC CTGAACTGGC CCCATGTGTT GGAGCTGCCG

TATTCCATGC CCTGGTGGTC CTGAGCTGGC CCCATGTGTT GGAGCTGCTG

TATTCCATGC CCTGGTGGTC CTGAGCTGGC CCCATGTGTT GGAGCTGCTG

TATTTCATGC CCTGGTGGTC CTGAGCTGGC CCCATGTGTT GGAGCTGCTG

TATTCCATAC CCTGGTGCTC CTGAGCTGGC CCCGTGTGAA GGAGCTGATA

TATTCCACGC CCTGATGGTC CGGAGCCGGC CCCGTGTGGA GGAACTGTTG

TATTCCACGC CCTGATGGTC CGGAGCTGGC CCCGTGTGGT GGAACTGTGG

TGTTCCACGC CCTAAGGATT CTGAGC---C CCCAAGTGAT GGAGCTGCTG

---------- ---------- ---------- ---------- ----------

TATTCCACGC CCTGCTGGTC CTGGGCTGGC CCCACGTGGT GGAGCTGCTG

TATTCCATGC CCTGGTGGTC GTGGGCTGGC CCCACATGAT GGAGCTGCTG

TGTTCCACAC CCTGGTGGCT CTGGACCGGC CCCGAGTGAA AGAGCTGCTG

TATTCCACGC CCTGGTGGTC CTGGGCTGGC CCCACGTGTT GGACCTGCTG

TATTCCACAC CCTGGTGGTA CTGGGCTGGC CCCACGTGGT GGAGCTGCTG

GATTCTACAG CCTGGTGGTA CTGGGC-GGC CCCGCGTGAT GGAGCTGCTG

T-TTCCAGAT TATGGTGCTA TCCAACTGTT CCCATCTACT AAATGAGCTG

CCTTCCACGC CATGCTGATG TCCAACTGGC CCCGTCTACT GAAGGCGTCG

CCTCAGAGGA ATCCTTCCCT CCACGTCGCA TCCCTGACCA GGCAGCTGCA

CCTCAGAAGA ATCCTTCCCT CCACCTCGCA TCCCTGACCA GGCAGCTGCA

CCTCAGAGGA ATCCTTCCCT CCACGTCGCA TCCCTGACCA GGCAGCTGCA

CCTCAGAGGA ATCCTTCCCT CCACGTCGCA TCCCTGACCA GGCAGCTGCA

CCTCAGAGGA ATCCTTCCCT CCACGTCGCA TCCCTGACCA GGCAGCTGCA

CCTCAGAGGA ATCCTTCCCG CCACGTTGCA TCCCTGACCA GACAGTTGCA

CCTCAGAGGA ATCCTTCCCT CCACGTCGCG TCCCTGACCA GGCAGCTACA

CCTCAGAGGA ATCCTTCCCT CCACGTCGCG TCCCTGACCA GGCAGCTGCA

CCTCAGAGGA ATCCTTCCCT CCACGTCGCG TCCCTGACCA GGCAGCTGCA

---------- ---------- ---------- ---------- ----------

CCTCAGAGGA ATCCTTCCCT CCACGTCGCG TCCCTGACCA GGCAGCTGCA

CCTCAGAGGA ATCCTTCCCT CCACGTCGCG TCCCTGACCA GGCAGCTGCA

CCTCAGAGGA AGCCTTCCCT TCACGTCGCG TCCATGAACA GGCACTTGCA

CCTCAGAGGA ATCCTTCCCT CCACGTCGCG TCCCTGACCA GGCAGCTGCA

CCTCAGAGGA ATCCTTCCCG CCACGTCGCG TCCCTGACCA GGCAGCTGCA

CCTCAGAGGA ATCCTTCCCG CCACGTCGTG TCCCTGACCA GGCAGCTGCA

CCTCAGATGA ATCGTTCCTG CCCCGCCGGA TTCTTGTTCA GGCAGTTGCA

CCTCAGATGA ATCCTTCCCG CCACGTTGCG TTCTTGACCA GGCAGCTGCA

GCACTGTATG GCGGGCCACC AGCTGCTGCA GTTCAAGGGC TCCACACTGG

GCACTGTATG GGGGGCCCAC CACTGCTTCA GTTCAAGGGC TCCACACTGG

GCACTGTATG GCGGGCCACC AGCTGCTGCA GTTCAAGGGC TCCACACTGG

GCACTGTATG GCGGGCCACC AGCTGCTGCA GTTCAAGGGC TCCACACTGG

GCACTGTATG GCGGGCCACC AGCTGCTGCA GTTCAAGGGC TCCACACTGG

GCACTGTATG GCAGGCCACC AGCTGCTGCA GTTCAAGGGC TCCACACTGG

GCACTGGATG GCAGGCCACC AGCTGCTGCA GTTCAAGGGT TCCACATTTG

GCACTGGATG GCAGGCCACC AGCTGCTGCA GTTCAAGGGC TCCACATTTG

GCACTGTATG GCCGGCCACC AGCTGCTGCA GTTCAAGGGC TCCACACTGG

---------- ---------- ---------- ---------- ----------

GCACTGTATG GCGGGCCACC AGCTGCTGCA GTTCAAGGGC TCCACACTGG

GCACTGTATG GCGGGCCACC AACTGCTGCA GTTCAAGGGC TCCACACTGG

GCACTGGCTG GCGGGCCACC GAGTGCTGCA ATTCAAGGGT TCCACAC---

GCACTGTATG GCGGGCCACC AGCTGCTGCA GTTCAAGGGC TCCACACTGG

GCACTGTATG GCGGGCCACC AGCTGCTGCA GTTCAAGGGC TCCACACTGG

GCACTGGATG GCGGGCCACC AGCTGCTGCA GTTCAAGGGC TCCACACTGG

GCACTGTATG GCTTGCCACC AGCTACTGTA CTTTAAGGGC TCCACGCTGG

GCACTGTATG GCATGCCACC AGCTATTGCA GTTCAAGGGC TCCACGCTGG

CCTTGGTCAT CATCACCTTA GAGCTGGAGA GGATGGCGCT GGCCGCCGCC

CCTTGGTCAT CATCCCCTT- ---------- --ATGGCGCT GGCCGCCGCC

CCTTGGTCAT CATCACCTTA GAGCTGGAGA GGATGGCCCT Ggccgccgcc

CCTTGGTCAT CATCACCTTA GAGCTGGAGA GGATGGCCCT Ggccgccgcc

CCTTGGTCAT CATCACCTTA GAGTTGGAGA GGATGGCGCT Ggccgccgcc

CCTTTGTCAT CATCACCTTA GAGTTGGAGA GGATGGCGCT AGCCGCCGCC

CTTTGGTCAT CATCACCTTA GAGCTGGAGA GGATGGCGCT GGCCGCTGCT

CTTTAGTCAT CATCACCTTA GAGTTGGAGA GGATGGCGCT Ggccgccgct

CCTTGGTCAT CATCACCTTA GAGTTGGAGA GG-------- ----------

---------- ---------- ---------- --ATGGCACT GGCCGCGGCC

CCTTGGTCAT CATCACCTTA GAGCTGGAGA GGATGGCGCT Ggccgccgcc

CCTTGGTCAT CATCACCTTA GAGTTGGAGA GGATGGCGCT CGCCGCGGCC

---------- ---------- ---------- ---------- ----------

CCTTGGTCAT CATCACCTTA GAGCTGGAGA GGATGGCCCT Agccgccgcc

CCTTGGTCAT CATCACCTTA GAGTTGGAGA GGATGGCGCT AGCCGCGGCC

CCTTGGTCAT CATCACCTTA GAGTTGGATA GG-------- ----------

ACCTTGTGAT CATCCCCTTA GCGCTGGAAA GGatggcgct ggcagcggcg

CTTTGGTGAT CATCACCTTA GAGCTGGAGA GGGAGGCCCA GGGGCCCGTC

GCCGCCGCTG CGGCTGCCGG GGTGAGCCAG GCGGCGGTGC TGGGCTTCCT

GCCGCCGCTG CGGCTGCCGG GGTGAGCCAG GCGGCGGTGC TGGGCTTCCT

gccgccgctg cggccgccgG GGTGAGCCAG GCAGCGGTGC TGGGCTTCCT

gccgccgctg cggccgccgG GGTGAGCCAG GCAGCGGTGC TGGGCTTCCT

gccgccgctg cggctgccgG GGTGAGCCAG GCGGCAGTGC TGGGCTTCCT

GCAGCGGCTG CGGCCGCCGG GGTGAGCCAG GCTGCAGTGC TGGGCTTCCT

GCGGCCGCCG CCGCGGCAGG AGTGAGCCAG GCGGCGGTGC TGGGCTTCCT

gccgccgctg ctgccgcAGG GGTGAGCCAG GCGGCGGTGT TGGGCTTCCT

---------- ---------- ---------- ---------- ----------

GCGGCTGCTG CGGCCGCCGG AGTGAGCCAG GCGGCGGTGC TGGGTTTCCT

gccgccgccg ccgccgccgG GGTGAGCCAG GCGGCCGTGC TGGGCTTCCT

GCCGCTGC-- ---------- GGCGAGCCAG GCGGCGGTGC TGGGCTTCCT

---------- ---------- ---------- ---------- ----------

gcggccgccg cggccgccgG AGTGAGCCAG GCGGCAGTGC TGGGCTTCCT

GCCGCTGCCG CGGCTGCCGG GGTGAGCCAG GCGGCAGTGC TGGGGTTCCT

---------- ---------- ---------- ---------- ----------

gcagcggccg cggccgcagg ggtgAGTCAG GCAGCGGTGC TGGGCTTCCT

CGGGCCGGTG AGCCCGCTGT GGTGGGTCTT GCGGAGGCTG AGGGGTCCAC

GCAGGAGCAC GGCGGGAAGG TGCGCAACTC CGAGCTGCTG AGCCGCTTCA

GCAGGAGCAC G--------- ---------- ---------- ----------

GCAGGAGCAC GGCGGGAAGG TGCGCAACTC CGAGCTGCTG AGCCGCTTCA

GCAGGAGCAC GGCGGGAAGG TGCGCAACTC TGAGCTGTTG AGCCGCTTCA

GCAGGAGCGC GGCGGGAAGG TGCGCAACTC GGAGCTGCTG AGTCGCTTCA

GCAAGAGCGC GGCGGCAAGG TGCCCAACGC GGAACTGCTG AGTCGCTTCA

GCGGGAGCAC GGCGGGCAGG TGCGCAACTC CGAGCTGCTG AGCCGCTTCA

GCGGGAGCAC GGCGGGCAGG TGCGCAACTC CGAGCTGCTG AGCCGCTTCA

---------- ---------- ---------- ---------- ----------

GCAGGAGAAC GGCGGGAAGG TGCGCAACTC TGAGCTGCTG AGCCGCTTCA

GCTGGAGCAC GGCGGCCAGG TGCGCAGCTG CGACCTGCTG AGCCGCTTCA

GCAGGAGCAC GGCGGAAAGG TGCGCAGCTC GGAGCTGGTG AGCCGCTTCA

---------- ---------- ---------- ---------- ----------

GCAGGAGCGC GGCGGCCAGG TGCGCAACTC GGAGCTGCTG AGCCGCTTCA

GCAGGAGCGC GGCGGGAAGG TGCGTAACTC CGAGCTGCTG AGTCGCTTCA

---------- ---------- ---------- ---------- ----------

GCAGGAGCGT GGGGGCAAGG TACGCAATTC GGAGCTCCTG AGCCGCTTCA

GGAGGTCCAG AGGGGGAGGC CGAGGAGCTC GAagaggccg aggggcccac

AGGTGAAGGA GCTCGACGGC GTCAAGTTCG TGAAGAAGCC CCGGGGGGCA

--GTGAAGGA GGTCGACGGC CTCAAGTTCG TGAAGAAGCC CCGG------

AGGTGAAGGA GCTCGACGGC GTCAAGTTCG TGAAGAAGCC GCGGGGGGCA

AGGTGAAGGA GCTCGACGGC GTCAAGTTCG TGAAGAAGCC GCGGGGGGCA

AGGTAAAGGA GCTCGACGGC GTCAAGTTCG TGAAGAAGCT GCAGAGGACA

AGGTGAAGGA GCTCGACGGC GTGAAGTTCG TTAAGAAGCC ACGG------

AGGTGAAGGA GCTGGACGGG GTCAAGTTCG TGAAGAAGCC CCGGCCCACG

AGGTGAAGGA GCTGGATGGG GTCAAGTTCG TGAAGAAGCC CCGGCCCACG

---------- ---------- ---------- ---------- ----gccccg

AAGTGAAGGA GCTCGACGGC GTCAAATTCG TGAAGAAGTC GCGG------

AGGTGAAGGA GTTGGACGGC GTCAAGTTCG TGAAGAGACT gcgg---GCG

AGGTGAAGGA GCTCGACGGC GTCAAGTTCG TGAAGAAGCC GCGGGGGGCG

---------- ---------- ---------- ---------- ----------

AGGTGAAGGA GCTTGACGGC GTCAAGTTCG TGAAAAAGCC GCGGAGGATG

AGGTGAAGGA GTTCGACGGC GTCAAGTTCG TGAAAAAGCC GCGGGGGACT

-GGTGAAGGA CCTGGACGGC GTCAAGTTCG TGGAAAAGCC GCCGAGGCC-

AGGTGAAAGA ACAGGACGGG GTGAAATTCG TGAAGAAGCT GCGGGCGGTG

g-gccgagga gcccgaagtt gaggggtccg t--agaggcc gca---AGAG

GCGAAAGGGC CGCCGCAGCA GAAGCCCTGT ATGCTGCCGG TGCGCTGCGT

---------- ---------- --------GT ATGCTGCCCG TGCGATTCGT

GCGAAAGGGC CGTCGCAGCA GAAACCGTGT ATGCTGCCCG TGCGCTGCGT

GCGAAAGGGC CGTCGCAGCA GAAACCCTGT ATGCTGCCCG TGCGCTGCGT

GCGAAAGGGC CGCCACCGCA GAAGCCCTGC ATGCTGCCGG TGCGCTGCGT

-----ACGCG CGCCGCCGCA AAAGCCCTGC ATGCTGCCGG TGCGCTGCGT

GCGCAAG--- TGCCACCGAA GAAGCCTTGC ATGCTGCCGG TGCGCTGCGT

GCGCAAG--- TGCCACCGCA GAAGCCTTGC ATGCTGCCGG TGCGCTGCGT

gggc------ cgccgccgcc AAAGTCCTGC ATGCTGCCGG TGCGCTGCGT

-CTCGGGGGC CACCGCCTCA GAAGCCTTGC ATGCTGCCTG TGCGCTGCGT

GCCCGACGGC CGCCGCCCCC CAAGCCCTGC ATGCTGCCCG TGCGCTGCGT

ACGACAGGGC CGCCTCCGCA GAAGCCCTGC ATGCTGCCCG TGCGCTGCGT

---------- ---------- ---------- ---------- ----------

AAGGAAGGGC CGCCGCCGCC GAAGCCCTGC ATGCTGCCCG TGCGCTGCGT

GCCAATGGGC CGCCGCCGCC GAAGCCGTGC ATGCTGCCGG TGCGCTGCTT

-CAAAAGGAc cgccgccgcc cAAGCCCTGC ATGCTGCCGG TGCGCTACGT

CCAGAGGAGC TTCCCCCGCT CAAGCCCCGC ATGCTGCCGG TGCGCTGTGT

GGCGAGGAGC CAGCGGCCCC GAAGCCCTGC ATGCTGCCCA TCCGCTGTCT

CCCGGCCGCC ACGCTCCGCC TCCGGGCGGA GGAGCCCGGC CTGCGCCGGC

CCCGGCCGCC ACGCTCCGCC TCCGGGCGGA GGAGCCGGGC CTGCGCCGGC

CCCGGCCGCC ACGCTCCGCA TCCGGGCGGA GGAGCAGGGC CTGCGCCGGC

CCCGGCCGCC ACGCTCCGCA TCCGGGCGGA GGAGCAGGGC CTGCGCCGGC

CCCGGGCGCC ACGCTCCGCA TCCGGGCGGA GGAGCAGGGC CTGCGCCGGC

CCCGGGCGCT GCGCTCCGGC TCCGAGCCGA GGACCAGGGC CTGCGCCGGC

CCCTGGCGCG GCGCTGCGGA TCCGCGCGGA GGAGCAGGGC CTGCGCCGGC

TCCTGGCGCG GCGCTGCGGA TCCGCGCGGA GGAGCAGGGC CTGCGCCGGC

GCCGGGCGCC GCGCTCCGCG TCCGGGCCGA GGAGCAGGGC CTGCGCCGGC

CCCCGGCTCG GCGCTGCGGA TCCGGGCTGA GGAGCAGGGC CTGCGCAGGC

CCCGGGCGCC GCGCTCCGGA TCCGGGCCGA GGAGCAGGGC CTGCGCGTGC

CCCTGGCCCG GCGTTTCGGA TCCGGGCCGA GGAGCAGGGC CTGCGCCGGC

---------- ---------- ---------- ---------- ----------

CCCGGCGGCC GCGCTCCGGG TCCGGGCGGA GGAGCAGGGC CTGCGCCGGC

CCCGGGCGCC GCGCTCCGGG TCCGGGGCGA GGAGCACGGC CTGCGCCGAC

GCCGGGAGCC GCGCTCCGGG TGCGCGCCGA GGATCAGGGC CTGCGCCGGC

CCCAT---CG GTGGTCCGGA TCCGGGCGGA GGAGCAGGGC CTGCGGCGAC

CCCGC---CG ACGATCCGCG TCCGGGCAGC GGAGCGGGGC CTGAGGCGGC

AGCTGTCGGA GGAGCCGAGC CCGCGGAGGC GGCTCTCGGT GGAAGAGTCC

AGCTGTCGGA GGAGCCGAGC CCGCGGAGGC GGCTCTCGGT GGAAGAGTCC

AGCTGTCGGA GGAGCCGAGC CCGCGGAGGC GGCTGTCGGT GGAAGAGTCC

AGCTGTCGGA GGAGCCGAGC CCGCGGAGGC GGCTGTCGGT GGAAGAGTCC

AGCTGTCAGA GGAGCCGAGC CCGCGGAGGC GGCTCTCGGT GGAGGAGTCC

AGCTGTCGGA GGAACCCAGC CCGCGGAGGC GGCTGTCGGT AGAGGAGTCC

AGCGGTCGGA GGAGCCGAGC CCGCGGCGGA GGCTCTCGGT AGAGGAGTCC

AGCGCTCGGA GGAGCCGAGC CCACGGCGGA GGCTTTCGGT AGAGGAGTCG

AGCTGTCGGA GGAGCCGAGC CCGCGCAGGA GGCTGTCGGT GGAGGAGACC

AGCTGTCGGA GGAGCCCAGT CCGCGGCGGC GGCTCTCGGT TGAGGAGTCC

AGCTGTCGGA GGAGCCGAGC CCGCGCCGGC GGCTGTCGGT GGAGGAGGCC

AGCTGTCAGA GGAGCCGAGC CCGCGGCGGC TGGTCTCGGT AGAGGAGTCT

---------- -----CAAAC CCGCGCCGAC GGCTGTCAGT GGAGGAGCCG

AGCTGTCGGA GGAGCCGAGC CCCCGGCGGC GGCTCTCGGT GGAGGAGGCC

AGCTGTCGGA GGAGCCGAGC CCGAGGCGGC GGCTCTCAGT GGAGGAGTCC

AGCAGTCGGA GGAGCCCAGC CCGCGGCGCC GCCTCTCCGT GGAGGAGTCC

AGCTGTCGGA AGAGCCATCC CTGCGGCGGC GACTCTCGGT GGAGGAGAC-

AGAGCTCCGA GGAGCCGGGC CCTC--CGGC GCCTGTCGGC GGACGAGCC-

GGCCGCTCCC CGCACCTGAG GCGCCTGTCG CGCGCCGGCC CGCGTCTGCT

GGCCGCTCCC CGCACCTGAG GCGCCTGTCG CGCGCCGGCC CGCGTCTGCT

GGCCGCTCCC CGCACCTGAG GCGCCTGTCG CGCGCCGGCC CGCGTCTGCT

GGCCGCTCCC CGCACCTGAG GCGCCTGTCG CGCGCCGGCC CGCGTCTGCT

GGCCGCTCCC CGCACCTGCG GCGTCTGTCG CGCGCCGGCC CGCGTCTGCT

GGCCGCTCTC CGCACCTGAG GCGCCTGTCG CGTGCTGGCC CGCGTTTGCT

GGCCGCTCCC CGCATCTCAG GCGCCTGTCG CGCGCCGGCC CGCGCCTGCT

GGCCGTTCCC CGCATCTCAG GCGCCTGTCC CGCGCCGGTC CGCGCCTGCT

GGCCGCTCCC CGCACCTGCG ACGCCTGTCG CGCGCCGGCC CCCGCCTGCT

GGCCGCTCCC CTCACCTGAG GCGCCTGTCG CGCGCCGGTC CGCGCCTGCT

GGCCGCTCCC CGCACCTGAG GCGCCTGCCG CGCGCAGGCC CGCGCCTGCT

-----CTCCC CGCATCTGAG ACGCCTGTCG CGCGCCGGCC CGCGCCTGCT

AGCCACTCCC CGTATTTGAG GCGCCCGTCG CGCGCCGGCC CGCGGCTGCT

GGCCGATCCC CGCACCTGAG GCGCCTATCG CGCGCCGGGC CGCGCCTGCT

GGACGCTCCC CGCACCTGAG GCGCCTGTCG CGCGCAGGCC CGCGTCTCCT

ACTCGCTCCC CGCACCTGAG GCGCCTGTCA CGGCCAAACG CGCGGCTGCT

GGTCGGTCCC CTCACCTCCG GCGCCTGTCC CGGGCCGGCC CGCGCCTGCT

--GCGCTCCC CGGGCTTGAG GCGGCCCCTG CGGGCAGCTC CGCGCCTGCT

GAGCCCTGAC GCCGAGGAGT TGCCCGCCGC GCCGCCGCCG TCCGCGGTGC

GAGCCCTGAC GCCGAGGAGG TGCCCGCCGC GCCGCCGCCG TCCGCGGTGC

GAGCCCCGAC GCCGAGGAGG TGCCCGCCGC GCCGCCGCCG TCCGCCGTGC

GAGCCCCGAC GCCGAGGAGG TGCCCGCCGC GCCGCCGCCG TCCGCCGTGC

GAGCCCCGAC GCCGAGGAGG TGCCCGCCGC GCCACCGCCG TCCGCCGTGC

CAGCCCGGAC ACCGAGGAGG TGCCCGCCGC GCCGCCGCCG TCCGCAGTAC

AAGTCCGGAC ATGGAGGAGG TGCCCATCGC GCCGCCACCG CCCGCGGTGC

GAGTCCGGAC ACGGAGGAGA TGCCCGTCGC GCCGCTACCG TCCGCGGTGC

GAGCCCCGAC ACCGAGGAgg cgcccgccgc gccgccgccg ccCGCCGTGC

GAGCCCCGAC ACTGAGGAGG CTCCGGCTGC CCCGCCGCCG TCCGCCGTGC

AAGCCCGGAC GCGGAGGAAG Tgcccgccgc gccgccgccg cccgccgTGC

GAGCCCCGAT ACCGAGGAGG TGCCCCCCGC GCCGCCGCCG TCCCCCGTGC

GAGTCCCGAC GCGGACGAcg cgcccgccgc gccgccgccg gccgccgTGC

GAGCCCCGAC AATGAGGAAG CAcccgccgc ggcgccgccg ccCGCCGTGC

GAGCCCCGAC GCCGAAGAGG TGCCAGCCGC GCCGCCGCCG TCTGCCCTGC

GAGCCCCGAC GCCGAGGAGG TGCCCGCCGC GCCGCCCCCG TCCGCCCTGC

GAGCCCCGAG GCCGAGGAGC CGCCCATCCC CCAGCCGCCA CCCACCGTGC

GAGCCCCGAc gccgacgacg ctgccgccgc accgccgccA TCCGTCGTAC

CCCTGGAGCC GTCCGAGCAC GAGTGGCTCG TGCGGACTGC CGGGGGCCGC

CCCTGGAGCC GTCCGAGCAC GAGTGGCTCG TGCGGACTGC CGGGGGCCGC

CCCTGGAGCC GTCAGAGCAC GAGTGGCTCG TGCGGACGGC CGGGGGCCGC

CCCTGGAGCC GTCAGAGCAC GAGTGGCTCG TGCGGACGGC CGGGGGCCGC

CACTGGAGCC GTCCGAGCAC GAGTGGCTCG TCCGGACGGC CGGGGGCCGC

CCCTGGACCC GTCTGAGCAC GAGTGGCTCG TGAGGGCGGC CAGTGGTCAC

CCCTGGAGCC CACGGAGCAC GAATGGCTCG TGCGCACGGC CAGCGGTCGT

CCCTGGAGCC CACGGAGCAC GAGTGGCTGG TGCGCACGGC CAGCGGTCGT

CCCTGGAGCC GGCCGAGCAC GAGTGGCTGG TGCGGGCGGC CGGCGGCCGC

CCCTGGAGCC GGCCGAACAC GAGTGGCTAG TGCGGGCGGC TGGGGGCCGC

CCCTGGAGCC CACGGAGCAC GAGTGGCTGG TGCGGGCGGC GGGGGGCTGC

CCCTGGAGCC GACCGAGCAC GAGTGGCTAG TGCGGGCAGC CTGTGGCCGC

CCCTGGAGCC GGCCGAGCAC GAGTGGCTGG TGCGCGTGGC CGGCGGCCAC

CCCTGGAGCA GGCCGAGCAC GAGTGGCTGG TACGAGCGGC AGCTGGCCGG

CCCTGGAGCC GGCCGAGCAC GAGTGGCTAG TGCGGGCGGC CGGTGGCCGC

CCCTGGAGCC GGCCGAGCAC GAGTGGCTGG TGCGCGCCGC CGGCGGCCGC

CTTTGGAGCC AGCCGAGCAC GAGTGGCTGG TGAGGGCGGC TGGAGGCCGC

CCCTGGAGCC GGCGGAGCAC GAGTGGCTGG TGCGGAGCGC GGCGGGCCGC

TGGACCCACC AGCTGCACGG GCTGCTGCTG CGCGACCGCG GCTTGGCGGC

TGGACCCACC AGCTGCACGG GCTGCTGCTG CGCGACCGCG GCCTGGCGGC

TGGACCCACC AGCTGCACGG GCTGCTGCTG CGCGACCGTG GCCTGGCGGC

TGGACCCACC AGCTGCACGG GCTGCTGCTG CGCGACCGTG GCCTGGCGGC

TGGACCCACC AGCTGCACGG GCTGCTGCTG CGCGACCGCG GCCTGGCGGC

TGGACCCACC AACTCCACGG GATGCTGCTG CGCGACGGCG GCCTGGCGGC

TGGAGCCACC AGCTACACGG GCTGCTCCTG CGGGACTGTG GCCTGGCTGC

TGGAGTCACC AGCTGCACGG GTTGCTCTTG CGGGACCGCG GCCTGGCTGC

TGGACCCACC AGCTGCACGG GCTGCTGCTG CGCGACCGCG GCCTGGCGGC

TGGACCCACC AGCTGCACGG GCTGCTGCTG CGCGACCTCG GCCTGGCAGC

TGGACCCACC AGCTGCACGG GCTGCTGCTG CGCGACAGCG GCCTGGCGGC

TGGACCCACC AGCTGCACGG GCTGCTGCTG CGCGACTGCG GCCTGGCGGC

TGGTCCCACC AGCTGCACGG GCTGCTCCTG CGCGACAGCG GCCTGGCGAC

TGGACCCACC AGCTGCATGG GCTGCTGCTG CGCGACCGCG GCCTGGCGGC

TGGACTCACC AGTTGCACGG GCTGCTGCTG CGCGACTGCG GCCTGGCAGC

TGGACTCACC AGTTGCACGG GCTGCTGCTG CGCGACTGCA GCCTGGCAGC

TGGACTCACC AGTTGCATGG GTTACTGCTG CGGGATCGGA GTCTGGCAAC

TGGACCCACC AGCTCCACGG GCTGCTGCTG CGGGACAGCG GGCTGGCGGC

CAAGCGCGAC TTCATGTCTG GCTTCACGGC CCTGCATTGG GCCGCCAAGA

CAAGCGCGAC TTCATGTCTG GCTTCACGGC CCTGCATTGG GCCGCCAAGA

CAAGCGCGAC TTCATGTCTG GCTTCACCGC CCTGCATTGG GCCGCCAAGA

CAAGCGCGAC TTCATGTCTG GCTTCACGGC CCTGCATTGG GCCGCCAAGA

CAAGCGCGAC TTCATGTCTG GCTTCACCGC CCTGCATTGG GCCGCCAAGA

CAAGCGCGAT TTCATCTCTG GCTTCACGGC TCTACATTGG GCCGCCAAGA

CAAGCGCGAC TTCATGTCGG GCTTCACCGC CCTACACTGG GCAGCCAAGA

CAAGCGCGAC TTCATGTCCG GCTTCACCGC GCTACACTGG GCGGCCAAGA

CAAGCGCGAC TTCATGTCCG GCTTCACGGC CCTGCACTGG GCCGCCAAGA

CAAACGCGAC TTCATGTCTG GTTTCACAGC CCTGCACTGG GCCGCTAAGA

CAAGCGCGAC TTCATGTCGG GCTTCACGGC CCTGCACTGG GCCGCCAAGA

CAAGTGCGAC TTCATGTCTG GTTTCACGGC CCTGCATTGG GCCGCCAAGA

CAAGCGCGAC TTCATCTCCG GCTTCACCGC CCTGCACTGG GCCGCCAAGA

CAAGCGCGAC TTCATGTCGG GCTTCACGGC CCTGCATTGG GCCGCCAAGA

CAAGCGCGAC TTCATATCGG GCTTCACTGC CCTGCACTGG GCCGCCAAGA

CAAGCGCGAC TTCATCTCGG GCTTCACTGC CCTGCACTGG GCCGCCAAGA

CAAACGCGAC TTCATGTCGG GATTCACGGC CCTGCACTGG GCCGCCAAGA

CAAGCGCGAC TTCACATCGG GCTTCACCGC CCTGCACTGG GCCGCCAAGA

GCGGCGACGG CGAGATGGCG CTGCAGCTGG TGGAGGTCGC GCGGCGCAGT

GCGGCGACGG CGAGATGGCG CTGCAGCTGG TGGAGGTCGC GCGGCGCAGT

GCGGCAACGG CGAGATGGCG CTGCAGCTGG TGGAGGTCGC GCGGCGCAGT

GCGGCGACGG CGAGATGGCG CTGCAGCTGG TGGAAGTAGC GCGGCGCAGT

GCGGCGACGG CGAGATGGCA CTGAAGCTGG TGGAGGTCGC GCAGCTCGGT

GCGGCGACCG GGAGATGGCG CTGCAGGTGG TGGAGGTGGC CCGGCGTGGG

GCGGCGACCG GGAGATGGCT CTACAGCTGG TGGAGGTGGC GCGGCGTGGG

ACGGCGACCG GGAGATGGCT TTACAGCTGG TGGAGGTGGC GCGGCGTGGA

GCGGCGACCG CGAGATGGCG CTGCAGCTGG TGGAggcggc gcggcgcggg

GCGGCGACCT GGAGATGGTG CAGCAGCTGG TGGAGGTCGC GCGGCGTGGG

GCGGCGACCG CGAGATGGCG CTGCAGCTGG TGGAGGTGGC GCGCCGAGGG

GCGGCGACCG CGAGATGGTG CTGCAGCTGG TGGAAGTTGC GCGGCGTGGG

GCGGCGACCG CGAGATGGCG CTGCAGCTGG TGGAGGTggc gcggcgcggc

GTGGCGACCG CGAGATGGCA CTGCAGCTGG TGGAGGTCGC GCGGCGTACG

GCGGCGACAG TGAGATGGTG CAGCAGCTGG TGGAAGTTGC GCTTCGCGGC

GCGGCGACGG GGAGATGGCT CTGCAGCTGG TGGAAGTcgc gcggcgcggg

GCGGTGACCG CGAGATGGCG CTGCAGCTGG TGGAAGTAGC GCGCCGCGGG

GCGGCGATCG CGAGATGGCA CTGCGCCTGG TGGAGGTGGC GCGGCGCGGT

GGCGCACCAG TCGACGTGAA CGCACGCTCC CACGGCGGCT ACACGCCGCT

GGCGCACCAG TCGACGTGAA CGCACGCTCC CACGGCGGCT ACACGCCGCT

GGCGCGCCAG TCGACGTGAA CGCGCGCTCC CACGGCGGTT ACACGCCGCT

GGCGCGCCAG TCGACGTGAA CGCGCGCTCC CACGGCGGTT ACACGCCGCT

GGCGCGCCAG TCGACGTGAA CGCGCGCTCC CACGGCGGCT ACACGCCTCT

GGCGCGCCGG TCGATGTAAA TGCGCGCTCG CACGGCGGCT ACACACCCCT

GGCGCGCCCG TGGACGTGAA CGCGCGCTCG CACGGTGGCT ACACGCCGCT

GGCGCGCCCG TGGACGTGAA CGCGCGCTCT CACGGTGGCT ACACGCCGCT

ggcgcgcccg TCGACGTGAA CGCGCGCTCG CACGGCGGCT ACACGCCGCT

GGCGCACGGG TCGACGTCAA CGCGCGCTCG CACGGCGGCT ATACGCCGCT

GGCGCGCGGG TGGACGTGAA CGCGCGCTCG CACGGCGGCT ACACGCCGCT

GGCGCGCCAG TCGACGTGAA CGCGCGCTCA CATGGCGGCT ATACGCCCCT

ggcgcgcgcg TGGACGTGAA CGCGCGCTCC CACGGCGGCT ACACGCCCCT

GGCGCCCCGG TCGACGTGAA TGCGCGCTCG CACGGCGGGT ACACTCCGCT

GGCACGCCGG TGGACGTGAA CGCACGTTCG CATGGCGGCT ACACTCCACT

ggcgccccgg TCGACGTGAA TGCGCGCTCC CACGGCGGCT ACACGCCGCT

GGGGCGCCTG TCGACGTGAA CGCCCGTTCC CACGGAGGTT ACACTCCCTT

GGCGCGCCCG TCGACGTCAA CGCCCGCTCC CACGGGGGCT ACACCCCGCT

GCACCTGGCT GCACTGCACG GCCACGAGGA CGCTGCCGTG CTGCTGGTGG

GCACCTGGCT GCGCTGCACG GCCACGAGGA CGCTGCCGTG CTGCTGGTGG

GCACCTGGCT GCGCTGCACG GCCACGAGGA CGCCGCTGTG CTGCTGGTGG

GCACCTGGCT GCGCTGCACG GCCACGAGGA CGCCGCTGTG CTGCTGGTGG

GCACCTGGCT GTGCTGCACG GCCACGAGGA CGCCGCTGTG CTGCTGGTGG

GCACCTGGCC GCTCTGCACG GCCACGAGGA CGCTGCCGTG CTGCTAGTGG

GCACCTGGCG GCTCTGCACG GCCACGAGGA TGCTGCTGTG CTGCTAGTGG

GCACCTGGCG GCTCTGCACG GCCACGAGGA TGCTGCTGTG CTGCTAGTGG

GCACCTTGCC GCGCTGCACG GCCACGAGGA CGCCGCCGTG CTGCTCGTCG

GCATTTGGCG GCGCTGCACG GCCACGAGGA TGCGGCCGTG CTCTTGGTAG

GCACCTGGCC GCCCTGCACG GCCGCGAGGA CGCCGCCGTG CTGCTGGTGG

GCACCTGGCC TCTCTGCACC GCCACGAGGA TGTCGCCATG CTGCTGGTAG

GCACCTGGCG GCGCAGCACG GCCACGAGGA GGCGGCCGTG CTGCTGGTGA

GCACCTGGCT GCGCTGCATG GCCACGAGGA TGCCGCCGTG CTGCTGGTGG

GCATCTGGCT GCGCTGCACG GCCGCGAGGA TGCCGCGGTG CTGCTGGTGG

CCACCTGGCG GTACTGCACG GCCACGAGGA CGCGGCGGTG CTGCTGGTGG

GCACCTGGCT GCCCTTCACG GCCACGAAGA TGCTGCCATC CTGTTGGTGG

GCACCTGGCC GCGCCGGCGG AGCACGAGTG -GCTGGTGCG GAGCGCGGCG

TGCGTCTGGG TGCCCAGGTG CACGTGCGTG ATCACAGCGG GCGTCGCGCC

TGCGTCTGGG TGCCCAGGTG CACGTGCGTG ATCACAGCGG GCGTCGCGCC

TGCGCTTGGG TGCCCAGGTG AACGTGCGTG ACCACAGCGG GCGTCGCGCC

TGCGCTTGGG TGCCCAGGTG AACGTGCGTG ACCACAGCGG GCGTCGCGCC

TGCGTCTGGG TGCCCATGTG CACGTGCGTG ACCACAGCGG ACGTCGCGCC

TCCGTCTGGG TGCACAGGTG CACGTGCGTG ACCACAGCGG CCGTCGAGCT

TGCGCCTGGG CGCCCAAGTG CACGTCCGCG ACTACAGCGG CCGGCGAGCC

TGCGCTTGGG TGCCCAAGTG CACGTCCGCG ACTACAGCGG CCGGCGTGCC

TGCGCCTCGG CGCGCAGGTG CACGTGCGCG ACCACAGCGG GCGGCGCGCC

TCCGCTTGGG TGCGCAGGTG CACGTGCGCG ACCACAGCGG ACGGCGCGCT

TGCGCCTGGG CGCGCAGGTG CACGTGCGCG ACCACAGCGG GCGGCGCGCC

---GCCTGGG TGCACACGTA CACGTGCGCG ACCACAGTGG GCGGCGCGCC

AGGACCTGGG CGCGCAGGTG CGCGTGCGCG ACCACAGCGG CCGCCGCGCC

CGCAACTGGG CGCGCAGGTG CATGTACGCG ACCACAGCGG ACGGCGCGCC

ATCGCCTGGG TGCGCAGGTG CACGTGCGCG ACCACAGCGG GCGGCGGGCC

CTCACCTGGG GGCGCAGGTG CACGTGCGCG ACCACAGCGG GCGGCGAGCC

AGCGCCTCGG AGCGCAGGTG CGAGTCCGCG ACCACAGCGG GCGTCGAGCC

GGCCGCTGGA CCCACCAGTG CACGTACGTG ACCACAGCGG TCGCCGCGCC

TACCAGTACC TGCGGCCCGG CTCCTCGTAC GCGCTGCGCC GCCTTCTTGG

TACCAGTACC TGCGGCCCGG CTCCTCGTAC GCGCTGCGCC GCCTTCTTGG

TACCAGTACC TGCGGCCTGG CTCCTCGTAC GCGTTGCGCC GCCTTCTTGG

TACCATTACC TGCGGCCTGG CTCCTCGTAC GCGTTGCGCC GCCTTCTTGG

TACCAGTACC TGAGGCCCGG CTCTTCGTAT GCGCTGCGCC GCCTACTTGG

TACCAGTACT TGCGGCCCGG CTCCTCGTAT GCGCTGCGAC GCCTACTCGG

TACCAGTACT TGCGGCCTGG CTCCTCCTAC GCGCTGAGGC GTTTACTAGG

TACCAGTACC TACGGCCTGG CTCCTCCTAC GCGCTGAGGC GTTTACTGGG

TACCAGTACC TGCGGCCCGG CTCCTCCTAC GCGCTG---C GCCCGCCTGG

TACCAGTACC TGCAGTCGGG CGCCTCGTAT GCTCTGCGTC GCCTACTTGG

TACCAGTACC TGCCGCCCGG CGCCTCGTAC GCGCTCCGCC GCCTCCTCGG

TACCAGTACC TGTCGCCCGG CGCCTCGTAT GCAGTGCGCC GTGTACTCGG

TACCAGTACC TGCCGCCCGG CGCCTCGTAC GCGCTGCGCC TGCTGCTCGG

TACCAGTACC TGCGGCCCGG CACCTCGTAC GCGCTGCGCC GCCTACTCGG

TACCAGTACC TGCGGCCTGG CTCCTCGTAC GCGCTGCGCC GCTTACTCGG

TACCAGTACC TGCGGCCGGG GGCCTCCTAT TCCCTGCGCC GCCTACTCGG

TACCAGTACC TGCGGCCTGG CGCCTCATAC GCCCTTCGTT TGTTGCTAGG

TACCAGTATC TGCGCCCGGG GGCGTCGGAA GGCGTGCGTC GCCTGCTGGG

CGATCCAGGC CTGCGAGGCA CCACGGAGCC AGATGCTGCC AGGCGCCCCG

CGATCCAGGC CTGCGAGGCA CCACGGAGCC AGATGCTGCC AGGCGCCCCG

CGATCCAGGC CTGCGAAGCA CCACGGAGCC AGACGCTGCC AGGCGCCCGG

CGATCCAGGC CTGCGAAGCA CCACGGAGCC AGACGCTGCC AGGCGCCCGG

CGATCCAAGC CTGCCAGGCA CCACAGAGCT AGATGCTGCC AGGCGCCCGG

CGACCCGGGC CTGCGAGCCA CGCCAGAGCC AGATGCGACC CGGCGCCCAG

TGACCCTGGC CTGCGATCTA CGACGGAGCC TGATGTGTCT CGGCACCCCG

TGACCCTGGC CTGCGATCTA TGATGGAGCC TGATGTGTCT CGGCACCCCG

CGGCCGGGCC CTGCG--CCG GCGCCGAGCT CGAC------ ----------

CGACCCTGGC CTGCGAGGTT CGACCGAACC CGATGCGGCC CGGCGCCCCG

CGACCCCTGC CTTCCAGGCT CTGCCGACGC CGACGCGGCC CGGCGCCCGG

CGACCCCCGC CTGCAAAGCT CTATGGAACT GTATGCGGCC CGGAGCCCAG

CGACCCCGAC CTGCGCAGCC CT---GAGGC CGAGGCGGCC CGGCGCCCGG

AGACCCCGGC CTGCGCAGCG CGACCGAGCC AGATGCGGCC CGGCGCCCCG

CGACCCGAGT CTGCGCGGGG CGACCGAGCC GGATGCGGCC CGGCCCCCAG

TGACCCTAGC CCGCGCGGCG CGACCGAGCC CGA-GCGGCC CGGCGCCCCG

TGACCCTGCC CTTCGAGGCC CCACTGAGCA TGG--CAGCC AAAAGGCCTG

CGACCCCCGC CTGCGAGGCC CGCCCGACCC CCAC-CCGCC AGGCGGTCGG

TACAGGTGGC CGCCACCATC CTCAGTTCCA CCACCAGTGC ATTTCTGGGC

TACAGGTGGC CGCCACCATC CTCAGTTCCA CCACCAGTGC ATTTCTGGGC

TGCAGGTGGC CGCCACCATC CTCAGTTCCA CCACCAGTGC ATTTTTGGGC

TGCAGGTGGC CGCCACCATC CTCAGTTCCA CCACCAGTGC ATTTTTGGGC

TGCAGGTGGC CGCCACCATC CTCAGTTCCA CCACCAGTGC ATTTCTAGGC

TGCAGGTTGC TGCCACCATC CTCGGTTCCA CCACCAGTGC GTTTCTGGGT

TGCAGGTGGC CGCCACCATC CTCGGCTCCA CCACTAGCGC GTTTCTGGGC

TGCAGGTAGC CGCCACCATC CTCAGCTCCA CCACTAGTGC GTTTCTGGGC

---------- ---------- ---------- ---------- ----------

TGCAGGTGGC GGCCACCATC CTCAGTTCCA CCACCAGCGC GTTTCTGGGC

TGCAGGTGGC CGCCACCATC CTCAGTTCCA CCACCAGCGC GTTCCTGGGC

TGCAGGTGGC CGCCACCATC CTCAGTTCCA CCACCAGCGC GTTCCTGGGT

TCCAGGTGGC CGCCACCATC CTCAGTTCCA CCACCAGCGC GTTCCTGGGC

TGCAGGTGGC TGCCACCATC CTCGGTTCCA CCACCAGCGC GTTTCTGGGC

TGCAGGTGGC CGCCACCATC CTCAGCTCCA CTACCAGCAC GTTTCTGGGC

TGCAAGTGGC CGCCACCATC CTCAGCAGCA CCACCAGCGC GTTTCTGGGC

TGCAGGTGGC CGCCTCCATT CTTAGCTCCA CTACTAGCGC CTTCCTGGGT

TCCAGGTGGC CGCCTCCATC CTTGGCTCCA CCACCAGCGC CTTCCTGGGT

GTCCTGGCTG ACGACCTCAT GCTCCAGGAC CTGGCCCGAG GCTTGAAGAA

GTCCTGGCTG ACGACCTCAT GCTCCAGGAC CTGGCCCGAG GCTTGAAGAA

GTCCTGGCCG ACGACCTCAT GCTCCAGGAC CTGGCCCGAG GCTTGAAGAA

GTCCTGGCCG ACGACCTCAT GCTCCAGGAC CTGGCCCGAG GCTTGAAGAA

GTCCTGGCCG ACGACCTCAT GCTCCAGGAC CTGGCCCGAG GCTTGAAGAA

GTCCTGGCGG ACGACATCAT GCTCCAGGAC CTGGCCCGAG GCTTGAGGAA

GTCCTGGCCG ATGACTTGAT GCTCCAGGAC CTGGCCCGTG GCTTGAAGAA

GTCCTGGCCG ATGACTTGAT GCTCCAGGAC CTGGCTCGTG GCTTGAAGAA

---------- ---------- ---------- ---------- ----------

GTCCTGGCTG ATGACCTTAT GCTCCAGGAT CTGGCTCGAG GCATGAGAAA

GTCCTGGCCG ACGACCTGAT GCTCCAAGAC CTGGCTCGAG GCATGAGGAA

GTTCTGGCCG ACGACCTGCT GCTCCAGGAC CTGGCACGAG GCTTCAAGAA

GTCCTCACCG AGGACCCCAT GCTCCAGGAA CTGACCCGCG GCTTGAAGAA

GTCCTGGCAG ACGACCTGAT GCTCCAGGAC CTAGCGCGGG GCTTGAGAAA

GTCTTGGCTG ACGACCTGAT GCTCCAGGAC CTGGCTCGCG GCCTGAGGAA

GTCCTGGCCG ATGACTTGAT GTTCCAGGAC CTGGCTCGAG GTCTCAAGAA

GTGCTGGCTG ATGACCTGAT GCTGCAGGAC CTTGCCCGAG GCATCAGGAA

GTCCTGGCCG ACGACTTGGC CTTCCAGGAC CTGGCCCGGG GCCTGCGGAA

GTCGAGCTCC TTCAGCAAGT TCTTGAGCGC CTCGCCCATG GCTCCACGTA

GTCGAGCTCC TTCAGCAAGT TCTTGAGCGC CTCGCCCATG GCTCCACGTA

GTCGAGCTCC TTCAGTAAGT TCTTGAGCGC GTCGCCCATG GCTCCACGTA

GTCGAGCTCC TTCAGTAAGT TCTTGAGCGC GTCGCCCATG GCTCCACGTA

GTCAAGCTCC TTCAGCAAGT TCTTGAGCGC CTCGCCCATG GCTCCACGTA

GTCCAGCTCC TTCAGCAAAT TTTTGAGCGC CTCGCCCATG GCTCCACGTA

ATCAAGCTCC TTCAGCAAGT TCTTGGGTGC CTCGCCCATG GCTCCCCGAA

GTCAAGTTCC TTCAGCAAGT TCTTGGGTGC CTCGCCCATG GCTCCCCGAA

---------- ---------- ---------- ---------- ----------

GTCAAGCTCC TTAAACAAAT TCTTGGGAGC CTCGCCCATG GCTCCTCGTA

GTCGGGCTCC CTTAGCAAGT TCTTGGGTGC CTCGCCCACA GCTCCACGGA

GTCGGGCTCC TTCAGCAGGT TCTTGGGTGC GTCGCCCATT GTTCCTCGTA

GTCCAGTTCC TTCAGCAAGT TACTGGGTGC CTCCCCGCTG GCGCCTCGTA

GTCTGGCTCC TTAAGCAAGT TCTTGGGCGC CTCGCCCATG GCCCCGCGCA

GTCCGGCTCC TTCAGCAAGT TCCTGGGCGC CTCACCCATG GCTCCACGCA

GTCCAGTTCC TTCAGCAAGT TCTTG---GC GTCGCCCATG GTTTCACGCA

ATCTGGCTCC TTTAGTAAGT TCCTGGGACC CTCTCCCCTG GTGCCCCGAA

GTCCGGCTCC TTCAGCAAGG GAAAGGGCAC CTCCCCCTTG CCCTCCCGCA

AAAAGACAAA GATCCGCGGT GGTCTGCCAG CCTTCTCACC TACTCCGGGG

AAAAGACAAA GATCCGCGGT GGTCTGCCAG CCTTCTCACC TACTCCGGGG

AAAAGACAAA GATCCGCGGT GGTCTGCCAG CCTTCTCACC TACTCCGGGG

AAAAGACAAA GATCCGCGGT GGTCTGCCAG CCTTCTCACC TACTCCGGGG

AAAAGATGAA GATCCGTGGT GGTCTGCCAG CCTTCTCACC TACTCCGGGG

AAAAGACAAA GATCCGCGGT GGCCTTCCAG CCTTCTCTCC TACTCCAGGG

AAAAGACCAA GATTCGTGGT GGCCTGCCAT CCTTCACTCC CACTCCAGGA

AAAAGACCAA GATTCGCGGT GGCCTGCCGT CCTTCACCTC CACTCCAGGA

---------- ---------- ---------- ---------- ----------

AAAAGACAAA GATCCGCGGC AGTCTGCCAG CCTTTTCACC CACTCCAGGA

AAAAGACGAA GACCCGCAGC GGCCTGCCGG CTTTCCCACC TACTCCCGGG

AAAAGACGAA GATTCGAAGT GGCCTGCCAG CCTTTTCATC CACTTTGGGG

AAAAGACCAA GACCCGCGGG GTCCTGCCGG CCTTTGGCAC CACTGCCGGG

AAAAGACCAA AAACCGCGGT GCCCTGCCAA CCTTCTCACC CAGTCCAGGG

AAAAGACCAA GACCCGCAGT GGCCTTCCGT CCTTCCCAGC CACTTCAGGG

AAAAGACCAA GATCCGAGGG GCCCTGCCCT CTTTCTCAAC CACCCCGAGG

AGAAGGCCAA GGCCAGGCCG GGCCTCCCCA CCTTCCCTTC CACTACAGGG

AGAAGCCCAA GGCCTGGGGG GGCTTCCCCA CCTTCTC-CC GGGCCCGAGG

CCTTTAGCTG GTCTAGTGCC CAGTTTTCCT CCAACAACCG AGTTTGTCCT

CCTTTAGCTG GTCTAGTGCC CAGTTTGCCT CCAACAACCG AGTTTGTCCT

CCTTTAGCTG GTCTAGTGCC CAGTTTGCCT CCAACAACCG AGTTTGTCCT

CCTTTAGCTG GTCTAGTGCC CAGTTTGCCT CCAACAACCG AGTTTGTCCT

CCTTTAGCTG GTCTAGTGCC CACTTTGCCC CCAACCACCG AGTTTGTCCT

CCTTTTGCTG GTCTAGTGCC CAGTCTGCCT CCCACAACCG AGTTAGTCCT

CCTTTGGCTG GTTTAGTGCC AAGTCTGCCC CCTCCAACCG AGATTGCCCT

CCTTTAGCTG GTTTAGTGCC CAGTCTGCCC CCTCCAACCG AGATTGCTCT

---------- ---------- ---------- ---------G ----------

GCCTTGGCTG GTCTAGTGCC CAGCCTACCC CCAGCAACCG AGTTCTTCCT

CCCCTGGCTG GGCTCGTGCC CAGTCTCCCG CCCCCAACCG AGTTCATCCT

CCTTTCGCTG GTCTAATGCC CAGTTTGCC- CTTACAAACG AGTTTGTTTT

CCTTTCGCCG GTCTGGTGCC CACTCTGCCA CCCACAACC- AGTTAATCCT

CCATTAGCTG GGTTAGTGCC TAGCCTACCC CCCACAACAG AGTTCGTCCT

CCCCTAGCCG GGCTAATGCC CAGCTTGCCC CCCACAACCG AGTTTGTCCT

CCATTCGCTG GCCTAGTGCC CAGCCTGCCC CCCACAACCG ----------

TCAAAGGCTG GCCAGGCACT GACCTCTTCC AAGGCCATTG AGTTTGCCCT

GCACCGGGAA GCGCAGGCCC GTCTATGCCT CTTCCAGGC- AGTTTGCCCT

CTCACTTCTG GAGAAGATGC AGACACAGGA GATCCTGAGG ATACTGCGAC

CTCACTTCTG GAGAAGATGC AGACACAGGA GATCCTGAGG ATACTGCGAC

CTCACTTCTG GAGAAGATGC AGACACAGGA GATCCTGAGG ATACTGCGAC

CTCACTTCTG GAGAAGATGC AGACACAGGA GATCCTGAGG ATACTGCGAC

CTCACTTCTG GAGAAGATGC AGACACAGGA GATCCTGAGG ATACTGCGGC

CTCACTTCTG GAGAAGATGC AGACCCAGGA GATCCTGAGG ACACTGCGGC

GTCACTTCTG GAGAAGATGC AGACCCAGGA GATCCTGAGG ATCCTGCGGC

CTCACTTCTG GAGAAGATGC AGACCCAGGA GATCCTGAGG ATCCTGCGGC

---------- ---------- ---------- ---------- ----------

CTCGCTTCTG GAGAAGATGC AGACTCAGGA GATCCTGAGG ATGCTGCGGC

CTCACTTCTG GAGAAAATGC AGACACAGGA GATCTTGAGG TTGCTGCGGC

CTCGCTTCTG GAGAAGATGC AGACACAGGA GATCCTGCGG ATGCTGCGGC

CTCACTTCTG GAGAAGATGC AGACACAGGA GATCCTAAAG ATACTGCGGC

CTCACTGCTG GAGAAGATGC AGACACAGGA GATCCTGAGG ATGTTGCGGT

TTCGCTTCTG GAGAAGATGC AGACACAGGA GATCCTCAGG ATGCTGCAGC

---------- ---------- ---------- ---------- ----------

TTCAATCCTG GAGAAGATGC AAGCTCAGGA GATCCTGAGG AGTCTGCGAC

TTCGATCCTG GAGAAGATGC AAGCGCAGGA GATCTTGCGG AGCCTGCGGC

TGCCTGAGCT AGGTGACTTG GGACAGTTTT TCCGCAGCCT CTCGGCCACC

TGCCTGAGCT AGGTGACTTG GGACAGTTTT TCCGCAGCCT CTCGGCCACC

TGCCTGAGCT AGGCGACTTA GGCCAGTTTT TCCGCAGCCT CTCGGCCACC

TGCCTGAGCT AGGCGACTTA GGCCAGTTTT TCCGCAGCCT CTCGGCCACC

TGCCTGAGCT AGGCGACTTG GGCCAGTTTT TCCGCAGCCT CTCGGCCACC

TGCCCGAGCT GGGTGACTTG GGCCAGTTTT TCCGCAGCCT CTCAGCTACC

TGCCCGAGCT ATCGGACTTG GGCCAGTTTT TCCGCAGCCT CTCAGCCACC

TGCCCGAGCT ATCGGACTTG GGCCAGTTTT TCCGCAGCCT CTCAGCTACC

---------- ---------- ---------- ---------- ----------

TGCCCGAGCT GGCTGACTTG GGCCAGTTTT TCCGCAGCCT CTCAGCCACC

TGCCCGAGCT GGGTGACTTG GGCCAATTTT TCCGCAGCCT CTCAGCCACT

TGCCCGAGCT GGGTGACTTG GGTCAGTTTT TCCGCAGTCT TTCAGCCACC

TGCCCGAGCT GGCTGACCTG GGCCAGTTTT TCCGCAGTTT CTCAGCCACC

TGCCCGAGCT GGGTGACTTG GGCCAGTTTT TCCGCAGCCT CTCAGCCACC

TGCCTGAGCT GGGTGACTTG GGCCAGTTTT TCTGCAGCCT CTCAGCCACC

---------- ---------- ---------- ---------- ----------

TGCCAGAGTT GGATGATTTC AGCCAGTTTT TGCGTAACTT GCCGGCCACC

TGCCCGAGTT CGATGACTTC AGCCAGTTCT TCCGCAGCCT GCCGGCCACC

ACCCTCGTGA GTATGGGTGC CCTGGCTGCC ATCCTTGCCT ACTGGTTCAC

ACCCTCGTGA GTATGGGTGC CCTGGCTGCC ATCCTTGCCT ACTGGTTCAC

ACCCTCGTGA GTATGGGTGC CCTGGCCGCC ATCCTCGCCT ACTGGTTCAC

ACCCTCGTGA GTATGGGTGC CCTGGCCGCC ATCCTCGCCT ACTGGTTCAC

ACCCTTGTGA GTATGGGTGC CCTGGCCGCC ATCCTCGCTT ACTGGTTCAC

ACCCTCGTGA GTATGGGTGC ACTGGCCGCC ATCCTCGCCT ACTGGTTCAC

ACCCTTGTGA GTATGGGTGC ACTGGCTGCC ATCCTTGCAT ACTGGCTCAC

ACCCTCGTGA GTGTGGGTGC ACTGGCTGCC GTCCTTGCCT ACTGGCTCAC

---------- ---------- ---------- ---------- ----------

ACCCTCGTGA GTATGGGTGC ACTAGCCGCC ATCCTCGCCT ACTGGTTCAC

ACCCTCGTGA GTGTGGGTGC GCTGGCTGCC ATCCTCGCCT ACTGGTTCAC

ACCCTCGTGA GTATGGGTGC GTTGGCTGCC ATCCTCGCCT ACTGGTTCAC

ACCCTCGTGA GTATGGGCGC ACTGGCAGCC CTCCTCGCCT ACTGGTTCAC

ACCCTCGTGA GTATGGGTGC CCTGGCCGCC ATCCTTGCCT ACTGGTTCAC

ACCCTCGTGA GTATGGGTGC CCTGGCTGCC ATCCTCGCCT ACTGGTTCAC

---------- ---------- ---------- ---------- ----------

ACATTAGTGG GTATCGGGGC CTTTGCTGCT CTGCTGGCCT ATTGGTTTGC

ACGCTGGTGG GCATCGGGGC CTTCGCTGCT CTCCTGGCCT ACTGGTTCGC

TCACCGGCCA AAGGCCTTGC AGCCGCCATG CAACCTCCTG ATGCAGTCAG

TCACCGGCCA AAGGCCTTGC AGCCGCCATG CAACCTCCTG ATGCAGTCAG

TCACCGGCCA AAGGCCTTGC AGCCACCATG CAACCTCCTG ATGCAGTCAG

TCACCGGCCA AAGGCCTTGC AGCCACCATG CAACCTCCTG ATGCAGTCAG

TCACCGGCCA AAGGCTTTGC AGCCACCATG CAACCTCCTG ATGCAGTCAG

TCACCGGCCA AAGGCCTTGC AGCCTCCCTG CAACCTCCTG ATGCAGTCGG

TCACCGGCCA AAGGCCTTGC AACCACCATG CAACCTCCTG ATGCAGTCGG

TCACCGGCCA AAGGCCTTGC AACCACCATG CAACCTCCTG AAGCAGTCGG

---------- ---------- ---------- ---------- ----------

TCACCGACCG AAGGCCTTAA AGCCACCATG CAATCTCCTG ATGCAGTCAG

TCACCGGCCA AAGGCCTTAC AACCACCATG CAACCTCCTG ATGCAGTCAG

TCACCGGCCA AAGGCGTTGC AACCACCGTG CAACCTCCTG ATGCAGTCAG

TCACCGGCCC AAGGCCTTGC AGCCGCCATG TGACCTCCTG ATGCAGTCAG

TCATCGGCCA AAGGCCTTAC AACCACCGTG TGACCTCCTG AGGCAGTCGG

TCATCGGCCA AAGGCCTTGC AACCACCATG TGACCTCCTG AGGCAGTCGG

---------- ---------- ---------- ---------- ----------

TAATCGGCCA AAAGCTGTTA AGCCACCATG TGACCTCCTG ATGCAGTCTG

TAGCCGGCCC AAAGCCCTGA AGCCACCCTG TGACCTCCTG ATGCAGTCTA

AAGAAGTAGA GGACAGTGGC GGGGCACGGC GATCTGTGAT TGGGTCTGGC

AAGAAGTAGA GGACAGTGGC GGGGCACGGC GATCTGTGAT TGGGTCTGGC

AAGAAGTAGA GGACAGTGGC GGGGCACGGC GATCTGTGAT TGGGTCTGGC

AAGAAGTAGA GGACAGTGGC GGGGCACGGC GATCTGTGAT TGGGTCTGGC

AAGAAGTAGA GGACAGTGGC GGGGCAAAGC GATCTGTGAT TGGGTCTGGC

AAGAAGTAGA GGAGAGTGGC AGGGCCCGGA GATCTGTGAT TGGGGGTAGC

AAGAAGTAGA GGACAGTGGT GGAGCCCGGC GATCTGTGAT TGGGGATTGC

AAGAAGTGGA GGACGGTGGT GGAGCCCGGC GATCTGTGAT TGGGGGTTGC

---------- -GACAGTGGT GGGGCTCGGC GATCTGTGAT CGGTGGTGGC

AAGAGGTGGA GGACAGTGGT GGGGCCCGGC GATCCGTAAT AGGGGACAGC

AGGAAGTGGA GGACAGTGGT GGGGCCCGGC GATCTGTGAT CGGGGATGGC

AAGAAGTGGA GGACAGCGGT GGGGCCCGGC GATCCGTGAT TGGGGATGGC

AGGAAGTGGA GGACAGTGGT GGGGCACGAC GATCTGTGAT TGGGGATGGC

AGGAAGTGGA GGACAGTGGT GGAGCCAGGC GGTCTGTGAT CGGAAGTGGC

AGGAAGTGGA GGGCAGTGAT GGAGCCCGGA GATCTGTAAT CGGGGGTAGC

---------- ---------- ---------- ---------- ----------

AAGAAGCAGA GGGCTTTGAT GGAGCTCGGC GGTCTGTGAT CGGAGGCAGC

AAGAGGTGGA GGGCTGTGAT GGAGCGAGAA GATCCGTGAT AGGAGGCAGC

CCTCAGCTAC TTACCCACTA CTATGATGAT GCCCGGACCA TGTACCAGGT

CCTCAGCTAC TTACCCACTA CTATGATGAC GCCCGGACCA TGTACCAGGT

CCTCAGCTAC TTACCCACTA CTATGATGAT GCCCGGACCA TGTACCAGGT

CCTCAGCTAC TTACCCACTA CTATGATGAT GCCCGGACCA TGTACCAGGT

TCTCAGCTAC TTACCCACTA CTACGACGAT GCCCGGACCA TGTACCACGT

CCTCACCTGC TTACCCACTA CTATGATGAT GCTCGGACCA TGTACCAGGT

ACTCAGTTGC TTACCCACTA CTATGACGAT GCCCGGACGA TGTACCAGGT

ACTCAATTGC TTACCCATTA CTATGACGAT GCCCGGACCA TGTACCAGGT

CCTCAGCTGC TCACCCACTA CTACGAGGAC GCCCGGACCA TGTACCACGT

CCGGAGCTGT TCACCCACTA CTATGACGAT GCCAGGACCA TGTACGAGGT

CCTCAATTGC TCACCCACTA CTATGATGAT GCCAGAACCA TGTACCAGGT

CCTCAGCTGC TCACCCACTA CTACGATGAC GCCAGGACTA TGTACCAGGT

CCGCAGCTTC TGACCCACTA CTATGACGAC GCCCGAACCA TGTACCAGGT

CCTCAACTGC TTACCCACTA CTATGAAGAT GCCCAGACCA TGTACCAGGT

CCTCAGCTCC TCACCCACTA CTATGATGAC GCCCGAACCA TGTACCAGGT

---------- ---------- ---------- ---------- ----------

CCTAAACTGC TGAACCATTA CTACGATGAT GCTCGGACCA TGTATGAAGT

CCCCAGCTGC TCACTCACTT CTATGATGAT GCTCGCACCA TGTACGAAGT

GTTCCGCCGT GGGCTTAGCA TCTCAGGGAA TGGGCCCTGT CTTGGTTTCA

GTTCCGCCGT GGGCTTAGCA TCTCAGGGAA TGGGCCCTGT CTTGGTTTCA

GTTCCGCCGT GGGCTTAGCA TCTCAG---- ---------- ----------

GTTCCGCCGT GGGCTTAGCA TCTCAGGGAA TGGGCCCTGT CTTGGTTTCA

GTTCCGCCGT GGGCTTAGCA TCTCAGGGAA TGGGCCCTGT CTTGGTTTCA

GTTCCGCCGT GGGCTTAGCA TCTCAGGGAA TGGACCCTGT CTTGGCTTTA

GTTCCGACGT GGGCTTAGCA TCTCAGGGAA TGGACCCTGT CTTGGCTTCC

GTTCCGCCGT GGGCTTAGCA TCTCAGGGAA TGGACCCTGT CTTGGCTTCC

GTTCCGCCGA GGGCTCAGCA TCTCACGGAA CGGGCCCTGT CTTGGCTTCA

GTTCCACCGT GGGCTTAGGA TCTCAGGGAA TGGACCCTGT CTTGGCTTCA

GTTCCGCCGT GGGCTTAGCA TCTCAGGGAA TGGACCCTGT CTTGGCTTCA

GTTCCGTCGT GGGCTTAGCA TCTCAGGGAA TGGACCCTGC CTTGGCTTCA

GTTCTGTCGT GGGCTTCGCA TCTCAGGAAA TGGCCCATGT CTCGGCTTCA

GTTCCGCCGT GGGCTTAGCA TCTCAGGGAA TGGGCCTTGC CTTGGCTCCA

GTTCCACCGT GGGCTTAGAA TCTCAGGGAA TGGGCCCTGC CTTGGCTCCA

---------- ---------- ---------- ---------- ----------

CTTCCAGAGG GGGCTGAGCA TCTCTGGAGA TGGTCCCTGC CTTGGATCCA

TTTCAGACGG GGATTGAACA TTTCTGAGAA TGGCCCTTGT CTAGGATCCA

GGAAGCCTAA GCAGCCTTAC CAGTGGCTGT CCTACCAGGA GGTGGCCGAC

GGAAGCCTAA GCAGCCTTAC CAGTGGTTGT CCTACCAGGA GGTGGCCGAC

---------- ---------- -----GCTGT CCTACCAGGA GGTGGCCGAC

GGAAGCCCAA CCAGCCTTAC CAGTGGCTGT CCTACCAGGA GGTGGCCGAC

GGAAGCCCAA GCAGCCTTAC CAGTGGCTGT CCTACCAGGA GGTGGCCGAC

GGAAGCCCAA GCAGCCTTAC CAGTGGCTGT CCTACCAGGA AGTGGCCGAC

GGAAGCCAGA GCAACCTTAC CAGTGGCTGT CCTATCAAGA GGTGGCCAAA

GGAAGCCAGA GCAACCTTAC CAGTGGCTGT CCTACCAAGA GGTGGCCAAA

GAAAGCCCAA GCAGCCATAC CAGTGGCTGT CCTACCAGGA GGTGGTGGAC

GGAATCCCAA GCAGCCTTAT CAGTGGCTGT CCTACCAGGA GGTGGCTGAC

GGAAGCCCAA ACAACCTTAT CAGTGGCTGT CCTATCAGGA GGTGGCCGAC

GGAAGCCCAA GCAGCCTTAT CAGTGGCTGT CCTACCAGGA GGTGGCCGAC

GGAAGCCCAA GCAGCCTTAC CAGTGGCTGT CCTACCAGGA GGTGGCTGAC

GGAAGCCCAA GCAGCCTTAT CAGTGGCTGT CCTACCAGGA GGTGGCGGAC

GGAAGCCCAA GCAGCCTTAC CAGTGGCTGT CCTACCAGGA GGTGGCTGAC

---------- ---------- ---------- ---------- ----------

GGAAGCCCAA GCAGCCATAT CAGTGGTTGT CCTACAGAGA GGTGGCAGAC

GAAGACCCAA GCAGCCTTAT CAGTGGTTAT CCTACAAAGA GGTGGCTAAA

AGGGCTGAAT TTCTGGGGTC CGGACTTCTC CAGCACAATT GTAAAGCATG

AGGGCTGAAT TTCTGGGGTC CGGACTTCTC CAGCACAATT GTAAAGCATG

CGGGCTGAAT TTCTGGGGTC TGGACTTCTC CAGCACAATT GTAAAGCATG

CGGGCTGAAT TTCTGGGGTC TGGACTTCTC CAGCATAATT GTAAAGCATG

AGGGCTGAAT TTCTGGGGTC CGGACTTCTC CAGCACAATT GTAAAGCATG

AGGGCTGAAT TTCTGGGGTC CGGACTGCTC CAGCACAACT GTAAAGCATG

AGGGCTGAAT TCCTGGGGTC AGGACTTCTC CAGCATGACT GTAAAGTGGG

AGGGCTGAAT TTCTGGGGTC GGGGCTTCTC CAGCACGACT GTAAAGTGGG

AGAGCTGAGT TTCTGGGGTC TGGACTGCTG CGGCACAATT GTAAAGCATC

AGGGCTGAAT TTCTAGGGTC TGGACTTCTC CAGCACAATT GTAAACCATG

AGGGCTGAAT TTCTAGGGTC TGGACTTCTC CAACACAATT GTAAACCGTG

AGGGCTGAAT TTCTAGGGTC CGGACTTCTC CAGCACAATT GCAAAGCAAG

AGGGCTGAAT TTCTGGGGTC TGGACTTCTC CAGCACAATT GTAAAGCAAG

AGGGCTGAGT TTCTGGGGTC TGGACTTCTC CAGCACAACT GTAAATCATC

AGGGCTGAAT TCCTAGGGTC CGGACTTCTC CAGCACAATT GTAAAGCATC

---------- ---------- ---------- ---------- ----------

AGAGCAGAGT TTCTGGGATC AGGACTCCTC CAACATGACT GTAAACCATC

AGGGCAGAAT TCTTGGGGTC AGGACTGCTC CACCATGGCT GTAAACCATC

CACTGATCAG TTTATTGGTG TTTTTGCACA AAATCGGCCA GAGTGGATCA

CACCGATCAG TTTATTGGAG TTTTTGCACA AAATCGGCCA GAGTGGATCA

CACCGATCAG TTTATTGGTG TTTTTGCACA AAATCGGCCA GAGTGGATCA

CACCGATCAG TTTATTGGTG TTTTTGCACA AAATCGGCCA GAGTGGATCA

CACAGATCAG TTTATTGGTG TTTTTGCACA AAATCGGCCA GAGTGGATCA

TACGGATCAG TTTATTGGTG TTTTCGCACA AAATCGGCCA GAGTGGATCA

CACAGAGCAG TTCATTGGCG TTTTTGCACA AAATCGGCCA GAGTGGATCA

CACAGAGCAG TTCGTTGGTG TTTTTGCACA AAATCGGCCG GAGTGGATCA

TACAGAGCAG TTTATTGGTG TTTTTGCACA AAACCGGCCA GAGTGGATCA

TACAGATCAG TTTATCGGCG TTTTCGCACA AAATAGACCA GAGTGGATCA

TACGGATCAG TTTATTGGCG TTTTTGCACA AAATCGGCCA GAGTGGATCA

TACAGATCAG TTCATCGGCG TTTTTGCACA AAATCGGCCA GAGTGGATCA

CACAGATCAG TTTATCGGCG TTTTTGCACA AAATCGGCCA GAGTGGATCA

TACAGATCAG TTTATTGGAG TTTTTGCACA AAATCGGCCA GAGTGGATCA

TTCAGATCAG TTTATTGGTG TATTTGCACA AAATCGACCA GAGTGGATCA

---------- ---------- ---------- ---------- ----------

AACAGAACAG TTTATTGGTG TCTTTGCTCA AAACCGGCCA GAGTGGATCA

GTCCGATCAG TTTATCGGCG TCTTCGCACA GAACCGGCCA GAGTGGATCA

TTGTGGAGCT GGCCTGCTAC ACATATTCCA TGGTGGTGGT CCCGCTCTAT

TTGCGGAGCT GGCCTGCTAC ACATATTCCA TGGTGGTGGT CCCACTCTAT

TTGCGGAGCT GGCCTGCTAC ACATATTCCA TGGTGGTGGT CCCACTCTAT

TTGTGGAGCT GGCCTGCTAC ACATATTCCA TGGTGGTGGT CCCGCTCTAT

TTGCGGAGCT GGCCTGCTAC ACATATTCCA TGGTGGTGGT CCCTCTCTAT

TTGCAGAGCT GGCCTGTTAC ACGTATTCCA TGGTGGTGGT CCCGCTGTAT

TTGCTGAGCT GGCCTGCTAC ACATATTCCA TGGTGGTGGT ACCGCTCTAT

TTGCCGAGCT GGCCTGCTAC ACGTATTCCA TGGTGGTGGT ACCGCTCTAC

TTGCGGAGCT GGCCTGCTAC ACGTATTCTA TGGTGGTGGT CCCACTCTAT

TCGCAGAGCT TGCCTGTTAC ACATACTCTA TGGTGGTGGT CCCTCTATAT

TTGCAGAGCT TGCCTGCTAC ACGTATTCTA TGGTGGTGGT CCCACTCTAT

TTGCTGAGCT TGCCTGCTAC ACCTACTCCA TGGTGGTGGT CCCGCTGTAT

TCTCGGAGCT GGCCTGCTAC ACATATTCCA TGGTGGTGGT CCCGCTCTAT

TCGCAGAGCT GGCCTGCTAC ACGTACTCCA TGGTGGTCGT CCCGCTCTAC

TCGCAGAGCT AGCCTGCTAC ACATATTCCA TGGTGGTGGT CCCACTGTAT

---------- ---------- ---------- ---------- ----------

TCTCGGAGCT GGCCTGCTAC ACCTACTCCA TGGTGGTGGT TCCACTATAT

TTTCTGAGTT GGCTTGTTAT ACCTACTCCA TGGTGGTGGT GCCTCTGTAC

GACACCCTGG GCCCTGGGGC TATCCGCTAC ATCATCAATA CAGCGGACAT

GACACCCTGG GCCCTGGGGC TATCCGCTAC ATCATCAATA CAGCGGACAT

GACACCCTGG GCCCTGGAGC TATCCGCTAC ATCATCAATA CAGCGGACAT

GACACCCTGG GCCCTGGAGC TATCCGCTAC ATCATCAATA CAGCAGACAT

GACACCCTGG GCCCTGGGGC TATCTGCTAC ATCATCAATA CAGCGGACAT

GACACCCTGG GCCCTGGGGC TATCCGCTAT ATCATCAATA CAGCGGACAT

GACACACTGG GCCCAGGGTC TATCCGCTAC ATCATCAATA CAGCGGACAT

GACACCCTGG GCCCAGGGTC TATCAGCTAC ATCATCAATA CTGCGGACAT

GACACCCTGG GCCCTGGGGC CATCCGCTAC ATCATCAATA CAGCGGACAT

GACACCCTGG GCCCTGGGGC AGTCCGCTAC ATCATCAACA CAGCTGACAT

GACACCCTGG GCCCTGGTGC TATCCGCTAC ATCATCAGCA CAGCTGACAT

GACACGCTGG GCCCCGGGGC TATACGCTAC ATCATCAACA CAGCTGACAT

GACACCCTGG GCCCTGGGGC CATCCGCTAC ATCATCAATA CAGCGGACAT

GACACCCTGG GCCCGGGGGC CATCCGCTAC ATCATCAACA CAGCGGACAT

GACACCCTGG GCCCCGGGGC CATCCACTAC ATCATCAACA CAGCGGACAT

---------- ---------- ---------- ---------- ----------

GACACCTTAG GCCCCGGTGC CATTCGCTAC ATTATCAACA CAGCTGATAT

GACACACTGG GGCCTGGAGC CATTCGCTAC ATTATTAACA CAGCTGATAT

CAGCACCGTG ATTGTGGACA AACCTCAGAA GGCTGTGCTT CTGCTAGAGC

CAGCACCGTG ATTGTGGACA AACCTCAGAA GGCTGTGCTT CTGCTAGAGC

CAGCACCGTG ATTGTGGACA AACCTCAGAA GGCTGTGCTT CTGCTAGAGC

CAGCACCGTG ATTGTGGACA AACCTCAGAA GGCTGTGCTT CTGCTAGAGC

CAGCACTGTG ATTGTGGACA AACCTCAGAA GGCTGTGCTT CTGCTACAGC

CAGCACTGTG ATTGTGGATA AACCTCAGAA GGCTATGCTT CTGCTGGAGC

CTGCACTGTA ATCGTGGATA AGCCGCATAA GGCTATACTC CTGCTAGAAC

TTGCACGGTA ATCGTCGATA AACCCCACAA GGCAACACTT CTGCTGGAAC

CAGCACCGTG ATCGTGGACA AAGCTCAGAA GGCTGGCCTA CTGCTGGAGC

CAGCACCGTG ATCGTGGATA AACCTCAGAA AGCTGTGGTT CTCCTGGAGC

CAGCACCGTG GTGGTGGATA AACCTCAGAA GGCCGTGCTT CTGCTAGAAC

CAGCACCGTG ATTGTGGATA AACCTCAGAA GGCCCTGCTT CTGCTGGAGC

CAGCACCGTG ATTGTGGACA AGCCTCAGAA -GCCGTGACT CTGCTGGAGC

CAGCACCGTG ATCGTCGACA AACCCCAGAA GGCTGTACTT CTGCTAGAAC

TAGCACCGTG ATTGTGGATA AACCTCAGAA GGCTGTGCTT CTGCTAGAGC

---------- ---------- ---------- ---------- ----------

TGCCACAGTG ATTTGTGATA AACCCGAAAA AGCCATCCTT CTCCTGGAAC

CGCCACCGTC ATCTGTGACA AGCCAGAGAA GGCTACCCTT CTCCTAGAAC

ATGTGGAGAG GAAGGAGACT CCAGGCCTCA AGCTGATCAT CCTCATGGAC

ATGTGGAGAG GAAGGAGACT CCAGGCCTCA AGCTGATCAT CCTCATGGAC

ATGTGGAGAG GAAGGAGACT CCAGGCCTCA AGCTGATTAT CCTCATGGAC

ATGTGGAGAG GAAGGAGACT CCAGGCCTCA AGCTGATTAT CCTCATGGAC

ATGTGGAGAG GAAGGAGACT CCAGGCCTCA AGCTGATCAT CCTCATGGAC

ATGTGGAGAG GAAGGAAACT CCAGGCCTCA AGCTAATTAT CCTCATGGAC

ACGTGGAGAG GAAGGAGACT CCGGGCCTCA AGCTGGTCAT CCTCATGGAG

ACGTGGAGAG GAAGGAGACT CCGGGCCTCA AGCTGGTCAT CCTCATGGAG

ACGTGGAGAA GAAGGAGACT CCAGGGCTCA AGCTGATCAT CCTCATGGAG

ACGTGGAGAG GAAGGAGACG CCAGGGCTCA AGCTGATCAT CCTCATGGAG

ATGTGGAGAG GAAGGAGACT CCGGGGCTCA AGCTCATCAT CATCATGGAA

ACGTGGAGAG GAAGGAGACT CCAGGGCTCA AGCTTATCAT CCTCATGGAA

ATGTGGAGAG GAAGGAGACT CC-TGGCTCA AGCTCATCAT CCTCATGGAA

ATGTGCGGAA GAACGAGACT CCAGGGCTCA AGCTGATCAT CCTCATGGAA

ATGTGGAGAA GAAGGAGACT CCAGGGCTCA AGCTGATCAT CATCATGGAG

---------- ---------- ---------- ---------- ----------

ATGTGGAGAG AAAGGAAATT CCTGGCCTGA AATTCATTAT TCTCATCGAA

ACGTGGAAAG AAAGGACACT CCGGGCCTGA AATTCATTAT CCTCATCAAT

CCATTCGAAG AAGCCCTGAA AGAGAGAGGG CAGAAGTGCG GGGTGGTCAT

CCATTTGAAG AAGCCCTGAA AGAGAGAGGG CAGAAGTGTG GGGTGGTCAT

CCATTTGAAG AAGCCCTGAA AGAGAGAGGG CAGAAGTGCG GGGTGGTCAT

CCATTTGAAG AAGCCCTGAA AGAGAGAGGG CAGAAGTGCG GGGTGGTCAT

CCATTCGAAG AAACCCTGAA AGAGAGAGGG CAGAAGTGCG GGGTGGTCAT

CCATTTGAGG AAGACCTGAA AGAAAGAGGG CAGAAGTGTG GGGTGGTCGT

CCATTTGACG ATGCCCTGAG AGAGAGGGGA AAGAAGTGCG GGGTGGACAT

CCGTTTGAGG ACGCCCTGAG AGAGAGAGGA AAGAAGTGCG GGGTGGACAT

CCGTTCGAGG AAGCCCTGAG AGAGCGGGGG AAGAAGTGTG GGGTGCTCCT

CCATTCGAGG AGGCCCTGAA AGACAGAGGG CAAGAGTGCG GGGTGGTCAT

CCGTTCGAGG AGGCTCTGAA AGAAAGAGGG CAGGAGTGCG GGGTGATCAT

CCGTTCGAGG AAGCTCTGAA AGACAGAGGG CAGGAGTGCG GGGTGGTCAT

CCGTTCGAGG ACGCTCTGA- ---------- ---------- ----------

CCGTTCGAGG AAGACCTGAA AGAGAAAGGG CAGGAGTGCG GGGTGGTCGT

CCGTTCGAGG AAGCCCTGAA AGAGAGAGGG CAGGAGTGCG GGGTGGTCAT

---------- ---------- ---------- ---------- ----------

TCACTTGAAA AGGACTTGAA AGAAAGAGGG AAGAAATGTG GCGTGGAGAT

GCATTTGGGA ATGACTTGAA GGAGCGAGGG AAAAAATGTG GCGTGCTCAT

TAAGTCCATG CAGGCCGTGG AGGACTGTGG CCAAGAGAAT CACCAGGCTC

TAAGTCCATG CAGGCCGTGG AGGACTGTGG CCAAGAGAAT CACCAGGCTC

TAAGTCCATG CAGGCCGTGG AGGACTGTGG CCAAGAGAAT CACCATGCTC

TAAGTCCATG CAGGCCGTGG AGGACTGTGG CCAAGAGAAT CACCATGCTC

TAAGTCCATG CAGGCTGTGG AGGACTGTGG CCAAGAGAAT CACCATGTTC

CAAGTCCATG CAGGCTATGG AGGACTGTGG CCAAGAGAAT CACCATGTTC

CAAGTCCATG CAGGCTATAG AGGACTCCGG CCAAGAGAAT CATCGTGTCC

CAAGTCCATG CAGGCTATAG AGGACTGTGG CCGAGAGAAT CATCATGCCC

CAAGTCCATG CGGGCTGTGG AGGACTGTGG CCAGGAGAAC CACTGTGCTC

TAAGTCCATG CAGGATGTGG AGGACTGTGG CCAGCAGAAT CACTGTGATC

TAAGTCCATG CAAGCTGTGG AGGAATGTGG CCAACAGAAT CATCATGCTC

CAAGTCCATG CAGGTTGTGG AGGACTGTGG CCAACAGAAT CATCACGTCC

---------- ---------- ---------- ---------- ----------

TAAGTCCATG CAGGCCGTGG AGGACTGTGG CCAGGAGAAT CTTCACACTC

TAAGTCCATG CAGGCTGTGG AGGACTGTGG CCAAGGGAAT TATCATGCTC

---------- ---------- ---------- ---------- ----------

TCAGTCCATG AAAGAAGTTG AGGATTGTGG ACGAGAAAAT CATCACCCTC

CCAGTCTATG CAGGAAGTCG AGGATTATGG AAGAGAGAAC CACCAGGCTC

CTGTGCCCCC GCAGCCTGAT GACCTCTCCA TTGTGTGTTT CACAAGCGGC

CTGTGCCCCC GCAGCCTGAT GACCTCTCCA TTGTGTGTTT CACAAGCGGC

CTGTGCCCCC ACAGCCTGAT GACCTCTCCA TTGTGTGTTT CACAAGCGGC

CTGTGCCCCC ACAGCCTGAT GACCTCTCCA TTGTGTGTTT CACAAGCGGC

CTGTGCCCCC GCAGCCTGAT GACCTCTCCA TTGTGTGTTT CACAAGCGGC

CTGTGCCCCC GCAGCCTGAT GACCTCTCCA TTGTGTGTTT CACAAGCGGC

CCGTGCCCCC ACGGCCCGAT GACCTCTCCA TTGTGTGTTT CACGAGCGGT

CCGTGCCCCC ACGGCCTGAT GACCTCTCCA TCGTGTGTTT CACAAGTGGT

CTGTG----- ---------- ---------- ---------- ----------

---------- ---------- ---------- ---------- ----------

CTGTGCCCCC GAAGCCCAGC GACCTCTCCA TTGTGTGTTT TACAAGCGGC

CTGTGCCCCC AAAGCCCAGC GACCTCTCCA TTGTGTGTTT CACAAGCGGC

-----CCCCC GGAGCCCTGC GACCTCTCCA TTGTGTGTTT CACCAGCGGC

CTGTGCCGCC ACGGCCAGAT GACCTCTCAA TTGTGTGTTT CACAAGTGGC

CTGTGCCCCC CCAGCCTGAT GACCTCTCGA TTGTATGTTT CACAAGCGGT

---------- ---------- ---------- ---------- ----------

CTGTGCCTCC TCGACCAGAA GACCTTTCAA TCGTGTGTTT CACTAGTGGT

CTGTGCCTCC CCGGCCGGAA GACCTGTCAA TTGTGTGTTT CACCAGTGGC

ACGACAGGGA ACCCAAAAGG TGCGATGCTC ACCCATGGGA ACGTGGTGGC

ACGACAGGGA ACCCAAAAGG TGCGATGCTC ACCCATGGGA ACGTGGTGGC

ACAACAGGGA ACCCAAAAGG TGCGATGCTC ACCCATGGGA ACGTGGTGGC

ACAACAGGGA ACCCAAAAGG TGCGATGCTC ACCCATGGGA ACGTGGTGGC

ACAACAGGGA ACCCAAAAGG TGCGATGCTC ACCCATGGGA ACGTGGTGGC

ACAACAGGGA ACCCAAAAGG TGCGATGCTC ACCCATGGGA ACGTGGTGGC

ACAACAGGGA ACCCCAAAGG TGCGATGCTC ACCCACGGGA ACGTGGTGGC

ACAACAGGGA ACCCCAAAGG TGCAATGCTC ACCCACGGGA ACGTGGTGGC

-------GGA ACCCAAAAGG TGCGATGCTC ACCCATGGGA ACGTGGTGGC

---------- ---------- ---------- ---------- ----------

ACAACAG--- ---------- ---------- ---------- ----------

ACGACAGGGG ACCCAAAAGG TGCGATGCTC ACGCATGGGA ACGTGGTGGC

ACCACAGGGA ACCCAAAAGG TGCGATGCTC ACCCATGGGA ACGTGGTGGC

ACGACAGGTA ACCCAAAAGG GGCGATGCTC ACCCATGGGA ACGTGGTGGC

ACAACAGGGA ACCCAAAAGG TGCGATGCTC ACCCATGGAA ACGTGGTGGC

-------GGA ACCCGAAAGG CGCGATGCTC ACGCATGGAA ACGTGGTGGC

ACCACAGGGA ACCCGAAAGG TGCGATGCTC ACTCATGGGA ATGTGGTTGC

ACTACAGGAA ACCCCAAAGG TGCGATGCTA ACCCATGGAA ATGTCGTTGC

TGATTTCTCA GGCTTTCTGA AAGTGACAGA GAAAGTGATC TTTCCGAGAC

TGATTTCTCA GGCTTTCTGA AAGTGACAGA GAAAGTGATC TTTCCGAGAC

TGATTTCTCA GGCTTTCTGA AAGTGACAGA GAAAGTGATC TTTCCGAGAC

TGATTTCTCA GGCTTTCTGA AAGTGACAGA GAAAGTGATC TTTCCGAGAC

CGATTTCTCA GGCTTTCTGA AAGTGACAGA GAAAGTGATC TTTCCGAGAC

TGATTTCTCA GGCTTTCTGA AAGTGACAGA GAAAGTGATC TTTCCGAGAC

CGATTTCTCG GGCTTTCTGA AAGTCACAGA GAAAGTGATC TTTCCGAGAC

CGATTTCTCG GGCTTTCTGA AAGTGACAGA GAAAGTGATC TTTCCGAGAC

TGATTTCTCT GGCTTTCTGA AAGTGACAGA GAAAGTGATC TTTCCGAGAC

---------- ---------- ---------- ---------- ----------

---------- ---------- ---------- -AAAGTGATC TTTCCGAGAC

TGATTTCTCA GGCTTTCTGA AAGTGACAGA GAAAGTGATC TTTCCGAGAC

TGATTTCTCA GGCTTTCTAA AAGTGACAGA GAAAGTGATC TTTCCGAGAC

TGATTTCTCA GGCTTTCTGA AAGTGACAGA GAAAGTGATC TTTCCGAGAC

TGATTTCTCA GGCTTTCTGA AAGTGACAGA GAAAGTGATC TTTCCGAGAC

TGATTTCTCA GGCTTTCTGA AAGTGACCGA G--------- ----------

TGATTTTTCA GGCTTTCTGA AAGTCACAGA GAAAGTGATC TTTCCGAGAC

TGATTTTTCA GGCTTTTTGA AAGTGACAGA GAAAGTGATC TTTCCGAGAC

AGGACGATGT GCTCATCTCC TTCCTGCCTC TGGCTCACAT GTTTGAGAGA

AGGACGATGT GCTCATCTCC TTCCTGCCTC TGGCTCACAT GTTTGAGAGA

AGGACGATGT GCTCATCTCC TTCCTGCCTC TGGCTCACAT GTTTGAGAGA

AGGACGATGT GCTCATCTCC TTCCTGCCTC TGGCTCACAT GTTTGAGAGA

AGGACGATGT GCTCATCTCC TTCCTGCCTC TGGCTCACAT GTTTGAGAGA

AGGACGATGT GCTCATCTCC TTCCTGCCTC TGGCTCACAT GTTTGAGAGA

AGGACGATGT GCTCATCTCC TTCCTGCCTC TGGCTCACAT GTTTGAGAGA

AGGACGATGT GCTCATCTCC TTCCTGCCTC TGGCTCACAT GTTTGAGAGA

AGGACGATGT GCTCATCTCC TTCCTGCCTC TGGCTCACAT GTTTGAGAGA

---------- ---------- ---------- ---------- ----------

AGGACGATGT GCTCATCTCC TTCCTGCCTC TGGCTCACAT GTTTGAGAGA

AGGACGATGT GCTCGTCTCC TTCCTGCCTC TGGCTCACAT GTTTGAGAGA

AGGACGATGT GCTCATCTCC TTCCTGCCTC TGGCTCACAT GTTTGAGAGA

AGGACGATGT GCTCATCTCC TTCCTGCCTC TGGCTCACAT GTTTGAGAGA

AGGACGATGT GCTCATCTCC TTCCTGCCTT TGGCTCACAT GTTTGAGAGA

---------- ---------- ---------- ---------- ----------

AGGACGATGT GCTCATCTCC TTCCTGCCTC TGGCTCACAT GTTTGAAAGA

AGGACGATGT GCTCATCTCC TTCCTGCCTC TGGCTCACAT GTTTGAAAGA

GTAATCCAGT CTGTCGTCTA TTGCCACGGA GGGCGTGTTG GCTTCTTCCA

GTAATCCAGT CTGTCGTCTA TTGCCACGGA GGGCGTGTTG GCTTCTTCCA

GTAATCCAGT CTGTCGTCTA TTGCCATGGA GGGCGCGTTG GCTTCTTCCA

GTAATCCAGT CTGTCGTCTA TTGCCACGGA GGGCGCGTTG GCTTCTTCCA

GTAATCCAGT CTGTTGTCTA TTGCCATGGA GGGCGTGTTG GCTTCTTCCA

GTAATCCAGT CTGTCGTCTA CTGCCACGGA GGACGTGTTG GCTTCTTCCA

GTGATCCAGT CTGTTGTCTA CTGCCATGGA GGGCGTGTGG GCTTCTTCCA

GTGATCCAGT CTGTTGTCTA CTGCCACGGA GGGCGCGTGG GCTTCTTCCA

GTGATCCAGT CTGTTGTCTA CTGCCATGGA GGGCGTGTCG GCTTCTTCCA

---------- ---------- ---------- ---------- ----------

GTAATCCAGT CTGTTGTCTA CTGCCATGGG GGACGTGTCG GCTTCTTCCA

GTGATCCAGT CTGTTGTCTA CTGCCATGGA GGGCGTGTTG GCTTCTTCCA

GTGATCCAGT CTGTCGTCTA CTGCCATGGA GGACGCGTTG GCTTCTTCCA

GTAATCCAGT CTGTTGTCTA CTGCCACGGA GGGCGCGTCG GCTTCTTCCA

GTAATCCAGT CTGTTGTTTA CTGCCATGGA GGACGCGTCG GCTTCTTCCA

---------T CTGTTGTGTA CTGCCACGGA GGGCGCGTTG GCTTCTTCCA

GTTATACAGT CAGTAGTGTA CTGCCATGGG GGAAGAGTTG GATTCTTCCA

GTTATACAGT CCGTGGTCTA TTGCCATGGG GGACGAGTTG GATTCTTTCA

GGGAGATATC CGCCTTCTCT CAGATGACAT GAAGGCTCTA TGCCCCACCA

GGGAGACATC CGCCTTCTCT CAGATGACAT GAAGGCTCTA TGCCCCACCA

GGGAGACATC CGCCTCCTCT CAGATGACAT GAAGGCTCTA TGCCCCACCA

GGGAGACATC CGCCTCCTCT CAGATGACAT GAAGGCTCTA TGCCCCACCA

GGGAGACATC CGCCTCCTCT CAGATGACAT GAAGGCTCTA TGCCCCACCA

AGGGGACATC CGCCTCCTCT CAGATGACAT GCAGGCTCTA CACCCCACCA

GGGAGACATC CGCCTCCTCT CAGACGACAT GAAAGCTCTC CGCCCCACCA

GGGAGACATC CGCCTCCTCT CAGATGACAT GAAGGCTCTC CGCCCTACCA

GGGAGATATC CGCCTCCTCT CGGATGACAT GAAGGCTCTC TGCCCCACCA

---------- ---------- ---------- ---------- ----------

GGGAGACATC CGTCTCCTCT CAGATGACAT GAAGGCTCTG TGCCCCACTA

GGGAGACATC CGTCTCCTCT CAGATGACAT GAAAGCTCTA TGCCCCACCA

GGGAGATATC CGTCTCCTCT CAGATGACAT GAAGGCTCTG TGCCCCACTA

GGGAGACATC CGCCTCCTCT CAGATGACAT GAAAGCTCTG TGCCCCACCA

GGGAGATATC CGCCTCCTCT CTGATGACAT GAAGGCTCTG TGCCCCACCA

GGGAGACATC CGCCTCCTGT CTGACGACAT GAAGGCTCTG TGCCCCACCA

AGGAGACATT CGCCTTTTGT CAGATGACAT GAAAGCTCTA CATCCCACTA

AGGAGATATT CGTCTTTTAT CTGATGATAT GAAAGCCCTG CGTCCCACCA

TCTTCCCTGT GGTCCCACGA CTGCTGAACC GGATGTACGA CAAGATCTTC

TCTTCCCTGT GGTCCCACGA CTGCTGAACC GGATGTACGA CAAGATCTTC

TCTTCCCTGT GGTCCCACGA CTGCTGAACC GGATGTACGA CAAGATCTTC

TCTTCCCTGT GGTCCCACGA CTGCTGAACC GGATGTATGA CAAGATCTTC

TCTTCCCTGT GGTACCACGA CTGCTAAACC GGATGTACGA CAAGATCTTC

TCTTCCCTGT GGTCCCACGA CTGCTGAACC GGATGTACGA CAAGATCTTC

TCTTCCCTGT GGTCCCACGG CTGCTGAATC GGATGTATGA CAAGATCTTC

TCTTCCCTGT GGTCCCACGG CTGCTGAATC GGATGTATGA CAAGATCTTC

TCTTCCCAGT AGTGCCACGA CTGCTGAACC GGATGTACGA CAAGATCTTC

---------- ---------- ---------- ---------- ----------

TCTTCCCTGT GGTCCCTCGA TTGCTGAACC GGATGTATGA CAAGATCTTC

TCTTCCCTGT GGTCCCACGA CTGCTGAACC GGATGTATGA CAAGATCTTC

TCTTCCCTGT GGTCCCACGC CTGCTGAACC GGATGTATGA CAAGATCTTC

TCTTTCCTGT GGTCCCACGA CTGCTGAACC GGATGTATGA CAAGATCTTC

TTTTCCCTGT GGTCCCACGA CTGCTGAACC GGATGTATGA CAAGATCTTC

TCTTCCCAGT GGTCCCACGG CTGCTGAACC GGATGTATGA CAAGATCTTC

TCTTCCCAGT GGTCCCCAGA CTGCTGAACA GAATGTATGA CAAGATCTTC

TTTTTCCAGT TGTCCCAAGG CTGTTGAACC GAATGTATGA TAAGATCTTC

AGCCAGGCAA ACACACCATT AAAGCGCTGG CTCCTGGAGT TTGCAGCAAA

AGCCAGGCAA ACACACCATT AAAGCGCTGG CTCCTGGAGT TTGCAGCAAA

AGCCAGGCAA ACACACCATT AAAGCGCTGG CTCCTGGAGT TTGCAGCAAA

AGCCAGGCAA ACACACCATT AAAGCGCTGG CTCCTGGAGT TTGCAGCAAA

AGCCAGGCAA ACACACCATT AAAGCGCTGG CTCCTGGAGT TTGCAGCAAA

AGCCAGGCAA ACACACCGTT AAAGCGCTGG CTCCTGGAAT TTGCAGCAAA

CACCAGGCAG ACACCTCACT GAAGCGCTGG CTCCTGGAGT TTGCAGCAAA

CACCAGGCAG ACACCTCACT AAAGCGCTGG CTCCTGGAGT TTGCAGCAAA

AGCCAGGCGG ACGGCCCGAT AAAGCGCTGG CTCCTGGAGT TTGCAGCCAA

---------- ---------- ---------- ---------- ----------

AGCCAGGCAG ACACACGACT AAAGCGCTGG CTCCTGGAGT TTGCAGCAAA

AGCCAGGCAG ACACACCACT AAAGCGTTGG CTCCTGGAGT TTGCGGCGAA

AGTCAGGCAG ACACACCATT AAAGCGCTGG CTTCTGGAGT TTGCTGCGAA

AGCCAGGCAG ACACACCATT AAAGCGCTGG CTCCTGGAGT TTGCAGCAAA

AGCCAGGCAA ATACACCACT GAAGCGCTGG CTCCTGGAGT TTGCGGCAAA

AGCCAGGCAA ACACGCCACT AAAGCGCTGG GTCCTGGAGT TTGTGGCGAA

AGCCAAGCAA ATACTCCATT GAAACGGTGG CTCCTGGAAT TTGCAGCAAA

AGCCAGGCTG ATACGCCTCT AAAACGCTGG CTTCTAGAAT TTGCAGCAAA

GCGTAAGCAA GCCGAGGTCC GGAGTGGAAT CATCAGGAAT GATAGTATCT

GCGTAAGCAA GCCGAGGTCC GGAGTGGAAT CATCAGGAAT GATAGTATCT

GCGTAAGCAG GCCGAGGTGC GGAGTGGAAT CATCAGGAAT GATAGTATCT

GCGTAAGCAG GCCGAGGTGC GGAGTGGAAT CATCAGGAAT GATAGTATCT

GCGTAAACAA GCCGAGGTCC GGAGTAGAAT CATCAGGAAT GATAGTATCT

GCGAAAGCAA GCTGAGATCC GGAGTGGAAT CATCAGGAAT GATAGTATCT

GCGCAAGCAG GCTGAGGTCC GGAGCGGAAT CATCAGAAAC AATAGTATCT

GCGCAAGCAG GCAGAGGTCC GGAGCGGAAT CATCAGAAAC AATAGTATCT

GCGCAAGCAG GCTGAGGTCC GGAGTGGGAT CGTCAGGAAT GACAGTATCT

---------- ---------- ---------- ---------- ----------

ACGTAAGCAG GCTGAGGTCC GGAGTGGAAT CATCAGGAAT GATAGTATCT

GCGTAAACAG GCTGAGGTCC GGAGTGGAAT CATCAGGAAT GATAGCATCT

GCGTAAACAG GCTGAAATTC GCTGTGGAAT CATCAGGAAC AACAGTATCT

GCGTAAACAG GCCGAGGTTC GGAGTGGAAT CATCAGAAAT GATAGTATCT

GCGTAAGCAG GCCGAGGTCC GGAGTGGAAT CATCAGAAAT GATAGTATCT

GCGTAAACAG GCCGAGGTCC GCAGCGGAAT CATCAGAAAT GATAGTATCT

GCGTAAGAAG GCTGAAGTTC GAAGTGGAAT CATTAGAAAT GATAGTATAT

ACGTAAAAAG GCCGAGGTTC GGAGTGGAAT CATTAGAAAT GACAGTATAT

GGGATGAACT CTTCTTTAAT AAGATTCAGG CCAGTCTTGG TGGGTGTGTG

GGGATGAACT CTTCTTTAAT AAGATTCAGG CCAGTCTTGG TGGGTGTGTG

GGGATGAACT CTTCTTTAAT AAGATTCAGG CCAGTCTTGG TGGGTGTGTG

GGGATGAACT CTTCTTTAAT AAGATTCAGG CCAGTCTTGG TGGGTGTGTG

GGGATGAACT CTTCTTTAAT AAGATTCAGG CCAGTCTTGG TGGGTGCGTG

GGGATGAACT CTTCTTTAAT AAGATTCAGG CCAGTCTTGG TGGATGTGTG

GGGATGAACT CTTCTTTAAT AAGATTCAGG CCAGTCTTGG AGGGCACGTG

GGGATGAACT CTTCTTTAAT AAGATTCAGG CCAGTCTTGG TGGGCATGTG

GGGATGAGCT CTTCTTTAAA AAGATCCAGG CCAGCCTTGG TGGGTGTGTG

---------- ---------- ---------- ---------- ----------

GGGATGAACT CTTCTTTAAT AAGATTCAGG CCAGTCTTGG AGGATGCGTG

GGGACGAACT CTTCTTTAAT AAGATTCAGG CCAGTCTTGG CGGGTGTGTA

GGGATGAACT CTTCTTTAAT AAGATTCAGG CCAGTCTTGG GGGAAGCGTA

GGGATGAACT CTTCTTTAAC AAAATTCAGG CCAGCCTTGG TGGGTGCGTA

GGGATGAACT CTTCTTTAAT AAAATTCAGG CCAGCCTTGG CGGGCGCGTG

GGGATGAACT CTTCTTCAAT AAGATTCAGG CCAGTCTTGG TGGGTGCGTG

GGGATGAGCT TTTTTTTAAT AAGATTCAGG CTAGTCTTGG TGGGTGTGTT

GGGATGAACT TTTCTTTAAT AAGATTCAGG CAAGCCTCGG GGGCTGTGTA

CGGATGATTG TTACTGGAGC AGCCCCAGCA TCACCAACAG TTCTGGGATT

CGGATGATTG TTACTGGAGC AGCCCCAGCA TCACCAACAG TTCTGGGATT

CGGATGATCG TTACTGGAGC AGCCCCCGCA TCACCAACAG TTCTGGGATT

CGGATGATCG TTACTGGAGC AGCCCCCGCA TCACCAACAG TTCTGGGATT

CGGATGATTG TTACTGGAGC AGCCCCAGCA TCACCAACAG TTCTGGGATT

CGGATGATTG TTACTGGAGC AGCCCCTGCA TCACCAGCAG TCCTAGGATT

AGAATGATTG TTACCGGAGC AGCCCCCGCA TCACCAACAG TCCTGGGATT

AGGATGATTG TCACTGGAGC AGCCCCCGCG TCACCAACGG TTCTGGGATT

CGGATGATTG TTACTGGAGC AGCCCCCGCG TCGCCAACAG TCCTGGGATT

---------- ---------- ---------- ---------- ----------

AGGATGATTG TCACCGGAGC AGCCCCTGCA TCACCGACAG TCCTGGGATT

CGCATGATAG TGACTGGAGC AGCCCCCGCA TCGCCGGCCG TCCTGGGATT

AGGATGATTG TTACTGGAGC AGCCCCCGCC TCTCCAGCAG TCCTGGGATT

CGGATGATTG TTACTGGAGC AGCCCCCGCA TCACCAACAG TCCTGGGATT

CGGATGATTG TCACTGGAGC AGCGCCTGCA TCGCCAGCAG TCCTGGGATT

CGAATGATTG TCACTGGAGC GGCGCCCGCA TCACCAGCAG TCTTGGGATT

AGAATGATTG TCACTGGAGC AGCTCCTGCA TCTCCCACAG TTTTGGGTTT

AGGATGATTG TCACCGGTGC GGCTCCCGCA TCTCCTACTG TCCTCGGTTT

TCTCCGGGCA GCTCTAGGGT GCCAGGTTTA TGAAGGTTAT GGCCAAACTG

TCTCCGGGCA GCTCTAGGGT GCCAGGTTTA TGAAGGTTAT GGCCAAACTG

TCTCCGGGCA GCTCTAGGGT GCCAGGTTTA TGAAGGTTAT GGCCAAACTG

TCTCCGGGCA GCTCTAGGGT GCCAGGTTTA TGAAGGTTAT GGCCAAACTG

TCTCCGGGCA GCTCTAGGGT GCCAGGTTTA TGAAGGTTAT GGCCAAACTG

CCTCCGGGCA GCTCTAGGGT GCCAGGTTTA TGAAGGTTAT GGCCAAACTG

CCTACGAGCA GCTCTGGGGT GCCAGGTCTA TGAAGGTTAT GGGCAAACTG

CCTACGAGCA GCTCTGGGGT GCCAGGTCTA TGAAGGTTAT GGGCAAACTG

CCTCCGGGCC GCTCTGGGCT GCCAG----- ---------- ----------

---------- ---------- ---------- ---------- ----------

CCTCCGGGCT GCTCTAGGAT GCCAGGTTTA TGAAGGTTAT GGCCAAACTG

CCTCCGTGCA GCTCTGGGCT GCCAGGTTTA TGAAGGTTAC GGCCAAACCG

CCTCCGAGCA GCTCTGGGAT GCCAGGTTTA TGAAGGTTAT GGCCAAACAG

CCTCCGGGCA GCTCTGGGGT GCCAGGTTTA TGAAGGTTAC GGCCAAACTG

CCTCCGGGCA GCTCTGGGGT GCCAGGTTTA TGAAGGTTAC GGCCAAACTG

CCTCAGAGCA GCTCTGGGGT GCCAG----- ---------- ----------

TCTTCGTGCA GCCCTGGGGT GTCAGGTATA TGAAGGTTAT GGCCAAACTG

CCTTCGGGCA GCTTTGGGGT GCCAAGTTTA TGAAGGTTAT GGCCAAACTG

AGTGCACAGC TGGATGTACC TTCACCACTC CTGGCGACTG GACCTCAGGG

AGTGCACAGC TGGATGTACC TTCACCACGC CTGGCGACTG GACCTCAGGG

AGTGCACAGC TGGATGTACC TTCACCACAC CTGGCGACTG GACCTCAGGG

AGTGCACAGC TGGATGTACC TTCACCACAC CTGGAGACTG GACCTCAGGG

AGTGCACAGC TGGATGTACC TTCACCACGC CTGGGGACTG GACCTCAGGG

AGTGCACAGC TGGATGTACC TTCACCACGC CAGGAGACTG GACCTCAGGG

AATGCACAGC TGGGTGTACC TTCACAACGC CAGGGGACTG GACATCAGGG

AATGCACAGC TGGGTGTACC TTCACAACGC CAGGGGACTG GACATCAGGG

---------- ---------- ---------- ---------- --------GG

---------- ---------- ---------- ---------- ----------

AGTGCACCGC TGGATGTACC TTCACCACAC CTGGGGATTG GACCTCAGGG

AGTGCACAGC TGGATGTACC TTTACCACAC CTGGGGACTG GACGTCAGGG

AGTGCACAGC TGGATGTACC TTTACCACAC CCGGGGACTG GACCTCAGGG

AGTGCACAGC TGGATGCACC TTCACCACAC CTGGGGACTG GACTTCAGGG

AGTGCACAGC TGGATGTACC TTCACCACGC CCGGAGACTG GACCTCAGGG

---------- ---------- ---------- ---------- ----------

AGTGTACAGC TGGGTGTACC TTCACAACTC CAGGGGACTG GACCTCAGGT

AGTGTACTGC TGGATGCACT TTTACTACTC CAGGTGACTG GACTTCAGGT

CACGTAGGGG CGCCACTTCC CTGCAATCAT ATCAAGCTCG TTGATGTTGA

CACGTAGGGG CGCCACTTCC CTGCAATCAT ATCAAGCTCG TTGATGTTGA

CACGTAGGGG CACCGCTTCC CTGCAATCAT ATCAAGCTCG TTGATGTTGA

CACGTAGGGG CACCGCTTCC CTGCAATCAT ATCAAGCTCG TTGATGTTGA

CACGTAGGGG CGCCACTTCC CTGCAATCAT ATCAAGCTCG TTGATGTTGA

CATGTAGGGG CACCACTGCC TTGCAATCAC ATCAAGCTTG TTGATGTCGA

CACGTGGGAG CCCCTCTGCC CTGCAACCAC ATCAAGCTGG TCGACGCAGA

CATGTAGGGG CACCTCTGCC CTGCAACCAC ATCAAGCTGG TCGATGCAGA

CACGTAGGGG CGCCTCTGCC CTGCAACCAC ATCAAACTTG TCGACGTTGA

---------- ---------- ---------- ---------- ----------

CATGTAGGGG CACCTCTGCC CTGCAATCAT ATCAAGCTCA TCGATGTTGA

CACGTAGGCG CGCCTTTGCC CTGCAATCAC ATCAAGCTCA TCGATGTTGA

CATGTTGGGG CTCCCCTGCC CTGCAATCAT ATCAAGCTGA TTGATGTGGA

CATGTAGGGG CTCCTCTGCC CTGCAATCAT ATCAAGCTCA TCGATGTCGA

CATGTCGGCG CACCTCTGCC TTGCAATCAT ATCAAGCTCA TGGATGTTGA

---------- ---------- ---------- ---------- ----------

CATGTGGGTG CACCATTACC CTGCAATTAC ATCAAACTGA TAGATGTGGA

CACGTTGGGG CTCCTTTGCC CTGTAATTTC ATCAAGTTGA CAGATGTGGA

GGAACTGAAC TACTGGGCCT GCAAAGGAGA GGGAGAGATA TGTGTGAGAG

GGAACTGAAC TACTGGGCCT GCAAAGGAGA GGGAGAGATA TGTGTGAGAG

GGAACTGAAC TATTGGGCCT GCAAAGGAGA GGGAGAGATA TGTGTGAGAG

GGAACTGAAC TATTGGGCCT GCAAAGGAGA GGGAGAGATA TGTGTGAGAG

GGAACTGAAC TATTGGGCCT GCAAAGGAGA GGGAGAGATA TGTGTGAGAG

AGAACTGAAC TACTGGGCCT GCAAAGGAGA GGGAGAGATA TGTGTGAGAG

GGAACTCAAC TACTGGACCA GCAAAGGAGA AGGAGAGATC TGTGTGAAAG

GGAACTCAAC TACTGGACCT GCAAAGGAGA AGGAGAGATA TGTGTGAAAG

GGAACTGAAC TACTGGACCT GCAAAGGAGA GGGCGAGATA TGTGTGAGAG

---------- ---------- ---------- ---------- ----------

GGAACTGAAC TATTGGACCT GCAAAGGAGA GGGAGAGATA TGTGTGAGAG

GGAACTGAAC TATTGGACCT ACAAAGGAGA GGGAGAGATA TGCGTGAGAG

GGAACTGAAC TATTGGACCA GCAAAGGCGA AGGCGAGATA TGTGTGAGAG

GGAACTGAAC TATTGGACCA GCAAAGGGGA GGGAGAGATA TGTGTGAGAG

GGAGCTGAAC TATTGGACCT ACAAAGGAGA GGGAGAGATA TGTGTGAGGG

---------- ---------- ---------- -------ATA TGCGTGAGAG

AGAACTGAAC TATTTTGCCT CCAAAGGAGA GGGAGAGATC TGTGTGAAAG

AGAACTGAAC TATTTTGCTT CCAAAGGAGA GGGAGAGATA TGCGTCAAGG

GACCAAATGT GTTCAAAGGC TACTTGAAAG ATCCAGACAG GACGAAGGAG

GACCAAATGT GTTCAAAGGC TATTTGAAAG ATCCAGACAG GACGAAGGAG

GACCAAATGT GTTCAAAGGC TACTTGAAAG ATCCAGACAG GACGAAGGAG

GACCAAATGT GTTCAAAGGC TACTTGAAAG ATCCAGACAG GACGAAGGAG

GACCAAACGT GTTCAAAGGC TACTTGAAAG ATTCAGACAG GACAAAGGAG

GACCAAATGT GTTCAAGGGC TACTTGAAAG ATCAGGACAG GACAAATGAG

GACCAAATGT GTTCAAAGGC TACTTAAAAG ACGAGGACAG GACAAAGGAG

GACCAAATGT GTTCAAAGGC TACTTAAAAG ACGAGGACAG GACAAAGGAG

GACCAAATGT ATTCAAAGGC TACTTGAAAG ATCAGAACAG GACGAAGGAG

---------- ---------- ---------- ---------- ----------

GACCAAATGT GTTCAAAGGT TACTTGAAAG ATCCAGACAG GACAAAGGAA

GACCAAATGT GTTCAAAGGC TACTTGAAAG ATCCAGACAA GACAAAGGAG

GACCAAATGT ATTCAAGGGC TACTTGAAAG ATCCTGAGAG GACAAAAGAG

GACCAAATGT GTTCAAAGGT TACTTGAAAG ATGAAGAGAA GACAAAGGAG

GACCAAATGT GTTTAAAGGT TACTTGAAAG ATCGGGAGAA GACGAAAGAG

GACCAAATGT GTTTAAAGGT TACTTGAAAG ATACAGAGAA GACAAAAGAG

GCCCAAATGT TTTCAAAGGT TATCTCAAAG ACAAGGAGAA GACTGATGAA

GACCAAATGT TTTCAAGGGT TATTTGAAAG ATGATGAGAA GACTAATGAG

GCCCTGGACA GCGATGGCTG GCTTCACACT GGAGACATCG GAAAATGGCT

GCCCTGGACA GCGATGGCTG GCTTCACACT GGAGACATCG GAAAATGGCT

GCCCTGGACA GCGATGGCTG GCTTCACACT GGAGACATCG GAAAATGGCT

GCCCTGGACA GCGATGGCTG GCTTCACACT GGAGACATCG GAAAATGGCT

GCCCTGGACA GTGATGGCTG GCTTCACACT GGAGATATTG GGAAATGGCT

GCCTTGGACA GTGATGGCTG GCTTCACACT GGAGACATCG GGAAGTGGCT

GCCCTGGACA GTGACGGCTG GCTTCACACG GGAGACATTG GAAAATGGCT

GCCCTGGACA GCGACGGCTG GCTTCACACT GGAGACATTG GGAAATGGCT

GCCCTGGATA GTGACGGCTG GCTTCACACT GGAGACATCG GCAAGTGGCT

---------- ---------- ---------- ---------- ----------

GCCCTGGACA GCGACGGCTG GCTTCACACT GGGGACATTG GCAAGTGGCT

GCTCTGGACA GTGACGGCTG GCTTCACACT GGGGACATTG GAAAGTGGCT

GCCTTAGACA GCGACGGCTG GCTTCACACT GGAGACATTG GCAAGTGGCT

GCCCTGGACA GTGATGGCTG GCTTCACACT GGAGACATTG GGAAATGGCT

GCCCTGGATA GCGATGGCTG GCTTCACACT GGAGACATTG GAAAATGGCT

GCCCTGGACC GTGATGGTTG GCTTCACACT GGAGACATTG GACGATGGCT

GCACTGGACA AGGATGGCTG GCTTCATACT GGAGATATTG GGAAGTGGTT

GCTCTGGACC AGGAAGGCTG GCTTCACACA GGAGACATCG GAAAGTGGTT

GCCGGCAGGA ACTCTTAAAA TTATTGATCG GAAAAAGCAT ATATTTAAAC

GCCGGCAGGA ACTCTTAAAA TTATTGATCG GAAAAAGCAT ATATTTAAAC

GCCGGCAGGA ACTCTTAAAG TTATTGATCG GAAGAAGCAC ATATTTAAAC

GCCG------ ---------- ---------- ---------- ----------

GCCGGCAGGA ACTCTTAAAA TTATTGATCG GAAAAAGCAC ATATTTAAAC

GCCGGCAGGA ACTCTTAAAA TTATTGATCG GAAAAAGCAC ATATTTAAAC

GCCGGAGGGA ACGCTTAAAA TCATCGATCG GAAAAAGCAC ATATTTAAAC

GCCGGAGGGA ACACTCAAAA TCATTGATCG GAAAAAGCAC ATATTTAAAC

TCCGGAAGGA ACTCTTAAAA TTATTGACCG GAAAAAGCAC ATATTTAAAC

---------- ---------- ---------- ---------- ----------

GCCGGCAGGA ACTCTTAAAA TTATCGATCG GAAAAAGCAC ATATTTAAAC

GCCGGCGGGA ACTCTTAAAA TTATTGATCG GAAGAAGCAC ATATTTAAAC

GCCGGCAGGA ACTCTTAAAA TTATTGATCG GAAAAAGCAC ATATTTAAAC

GCCGGCAGGA ACACTTAAAG TTATTGATCG GAAAAAGCAC ATATTTAAAC

GCCGGCAGGA ACACTTAAAA TTATTGATCG GAAAAAGCAC ATATTTAAAC

GCCG------ ---------- ---------- ---------- ----------

ACCTACAGGA ACTCTTAAAA TTATTGATCG GAAAAAGCAC ATATTTAAAC

ACCGACTGGA ACTCTTAAAA TTATTGATCG AAAAAAGCAC ATATTTAAAC

TTGCTCAGGG AGAATATGTT GCACCCGAGA AGATTGAGAA CATCTACATC

TTGCTCAGGG AGAATATGTT GCACCCGAGA AGATTGAGAA CATCTACATC

TTGCTCAGGG AGAATATGTT GCACCCGAGA AGATTGAGAA CATCTACATC

---------- ---------- ---------- ---------- ----------

TTGCTCAGGG AGAATATGTT GCACCCGAGA AGATTGAGAA CATCTACGTC

TTGCTCAGGG GGAATATGTT GCACCTGAGA AGATTGAGAA CATCTACATC

TTGCTCAGGG GGAATACGTT GCGCCCGAGA AGATTGAGAA CATCTACATC

TTGCTCAGGG GGAATATGTT GCGCCAGAGA AGATCGAGAA CATCTACATC

TTGCTCAGGG AGAATATGTT GCACCCGAGA AGATTGAGAA CATCTACATC

---------- ---------- ---------- ---------- ----------

TCGCTCAGGG AGAATATATT GCACCCGAGA AGATTGAAAA CATCTACATA

TGGCTCAGGG AGAGTATGTT GCGCCCGAGA AGATTGAGAA CATCTACATC

TCGCTCAGGG AGAGTATGTG GCACCTGAGA AGATCGAGAA TATCTACATC

TTGCTCAGGG AGAATATGTT GCACCGGAAA AGATTGAGAA CATCTACATC

TTGCTCAGGG AGAATATGTC GCACCCGAGA AGATTGAGAA CATCTACATC

---------- ---------- ---------- ---------- ----------

TTGCTCAGGG AGAATACATT GCTCCAGAAA AGATTGAAAA TATCTACATT

TTGCCCAGGG GGAATATATT GCCCCAGAAA AAATTGAGAA TATCTACATC

CGGAGCCAAC CTGTGGCGCA AATCTATGTC CATGGGGACA GCTTAAAGGC

CGGAGCCAAC CTGTGGCGCA AATCTATGTC CATGGGGACA GCTTAAAGGC

CGGAGCCAAC CTGTGGCACA AATCTATGTA CATGGGGACA GCTTAAAGGC

---------- ---------- ---------- ---------- --------GC

CGGAGCCAAC CTGTAGCACA AATCTATGTC CATGGGGACA GCTTAAAGGC

CGGAGCCAGC CTGTGGCACA AGTCTACGTC CATGGGGACA GTTTAAAGGC

CGAAGTGAGC CTGTGGCGCA AATCTACGTC CATGGGGACA GCTTAAAG--

CGGAGTGAGC CTGTGGCACA AATCTACGTC CACGGGGACA GCTTAAAGGC

CGAAGTGAGC CTGTGGCACA GGTCTATGTC CATGGGGACA GCTTAAAGGC

---------- ---------- ---------- ---------- ----------

CGGAGTGAGC CCGTGGCACA AGTCTATGTC CATGGGAACA GCCTAAAGGC

CGGAGTGAGC CTGTGGCACA AATCTATGTC CATGGGGACA GCCTGAAGGC

CGTTGTGAAC CTGTGGCACA AATCTATGTC CACGGGGACA GCTTAAAG--

CGGAGTGAGC CTGTGGCACA AATCTATGTC CACGGGGACA GCTTAAAGGC

CGGAGTGAAC CTGTGGCACA AGTATATGTC CATGGAGACA GTTTAAAGGC

---------- ---------- ---------- ---------- --------GC

CGAAGTGAAC CCGTAGCTCA GACGTACGTG CATGGAGACA GCTTACAGGC

CGAAGTGAAC CAGTTGCTCA GATTTACGTC CACGGGGACA GCTTACAGGC

CTTTTTGGTA GGCATTGTTG TGCCTGACCC TGAAGTTATG CCCTCCTGGG

CTTTTTGGTA GGCATTGTTG TGCCTGACCC TGAAGTTATG CCCTCCTGGG

CTTTTTGGTA GGCATTGTTG TGCCTGACCC TGAAGTTATG CCCTCCTGGG

CTTTTTGGTA GGCATTGTTG TGCCTGACCC TGAAGTTATG CCCTCCTGGG

CTTTTTGGTA GGCATTGTCG TACCTGACCC TGAAGTTATG CCCTCCTGGG

CTTTTTGGTG GGTATTGTTG TGCCTGACCC TGAAGTCATG CCCTCATGGG

---------- ---------- ---------- ---------- ----------

CTTTTTGGTT GGCATTGTCG TGCCTGACCC TGAAGTCATG CCTTCCTGGG

CTTTTTGGTG GGCGTTGTTG TGCCTGACCC TGAGGTGATG CCCACTTGGG

---------- ---------- ---------- ---------- ----------

CTATTTGGTG GGTATTGTTG TGCCTGACCC TGAGGTCATG CCCACATGGG

CTTTCTGGTG GGCATTGTCG TGCCTGACCC TGAAATCATG CCAGCTTGGG

---------- ---------- ---------- ---------- ----------

CTTTTTGGTG GGCATTGTTG TGCCTGACCC TGAAGTCATG CCCTATTGGG

CTTTTTGGTG GGCATCGTGG TACCCGACCC TGAAGTCATG CCCTCCTGGG

CTTCCTGGTG GGCATTGTCG TTCCCGACCC GGAAATCATG CCTGCCTGGG

CTTCTTGGTG GGTATTGTTG TGCCTGATTC TGAAGTCATG CCTAGTTGGG

TTTCTTAGTG GGCATTGTTG TACCTGACCC TGAAGTCTTG CCAGGTTGGG

CCCAGAAGAG AGGAATTGAA GGAACATATG CAGATCTCTG CACAAATAAG

CCCAGAAGAG AGGAATTGAA GGAACATATG CAGATCTCTG CACAAATAAG

CCCAGAAGAG AGGAATTGAA GGAACATATG CAGATCTCTG CACAAGTAAG

CCCAGAAGAG AGGAATTGAA GGAACATATG CAGATCTCTG CACAAGTAAG

CCCAGAAGAG AGGAATTGAA GGAACATATG CAGATCTCTG CACAAGTAAG

CCCAGAAGAG AGGAATTGGA GGGACATATG CAGAGCTCTG CACAAGTAAG

---------- ---------- ---------- ---------- ----------

CTCAGAAGAA AGGAATCGAA GGGACCTATC AGGAACTCTG CATGAAAAAG

CCCAGAAGAG GGGGATTGAA GGGACCTATA CACAGCTCTG CATAAATAAG

---------- ---------- ---------- ---------- ----------

CCCAGAAGAG AGGAATTGAA GGCTCATACA CAGAGCTCTG TGCGAATAAG

CCCAGAAGAG AGGCATTGAA GGGACGTACG TGGAGCTCTG CGCCAATAAG

---------- ---------- ---------- ---------- ----------

CTCAGAAGAG AGGATTTGAG GGAACATATG CAGAGCTCTG CACAAATAAG

CCCAGAAGAA AGGATTTGAA GGGACATATG AAGAGCTCTG TACAAATAAG

CCCAGAAGAA AGGATTTGAA GGGACGTATG AAGAGCTCTG TGCAAATAAG

CGAAGAAGAG AGGATTTGAT GGGACGTATG CAGAGCTCTG TCAAGACAAG

CAAAGAAGAG AGGCCTTGAA GGAGCATATG CAGAACTCTG TGAAGATAAG

GATCTGAAGA AAGCCATTTT GGAAGATATG GTGAGGTTAG GAAAAGAAAG

GATCTGAAGA AAGCCATTTT GGAAGATATG GTGAGGTTAG GAAAAGAAAG

GATCTGAAGA AAGCCATTTT GGAAGATATG GTGAGGTTAG GAAAAGAAAG

GATCTGAAGA AAGCCATTTT GGAAGATATG GTGAGGTTAG GAAAAGAAAG

GATCTGAAGA AAGCCATTTT GGAAGATATG GTGAGGTTAG GAAAAGAAAG

GATCTGAAGA AAGCCATATT GGAAGATATG GTGAGGTTAG GAAAAGAAAG

GAATTGAAGA AAGCCATTCT GGATGACATG GTGATGTTGG GGAAAGAAAG

GAATTGAAGA AAGCCATTCT GGATGACATG GTGATGCTGG GGAAAGAAAG

GAACTGAAGA AAGCCATTTT GGAAGATTTG GTGAGGTTAG GAAAAGAAAG

---------- ---------- ---------- ---------- ----------

GAGCTGAAAA AAGCCATTTT GGAAGATATG GTGAGGTTAG GAAAAGAGAG

GAGCTGAAGA AAGCCATTTT GGAAGATATG ATGAGGTTAG GAAAAGAAAG

GAGCTGAAGA AGGACATCTT GGATGATTTG GTGAGGTTAG GAAAAGAAGG

GAGCTGAAGA AAGCCATTTT GGAAGATCTG GTGAGGTTAG GAAAAGAAAG

GAGGTGAAGA AAGCCATTTT GGACGATTTG GTGAGGCTAG GAAAGGCAGG

---------- ---------- ---------- ---------- ----------

GAGCTGAAGC AAGCTATAAT GGATGATATG GTAAGACTAG GAAAAGAAAG

GAACTGAAGC AAGCTATAAT GGATGACTTG GTGAGGTTAG GAAAAGAAAG

TGGACTCCAT TCTTTTGAGC AGGTTAAAGC CATTCACATC CATTCTGACA

TGGACTCCAT TCTTTTGAGC AGGTTAAAGC CATTCACATC CATTCTGACA

TGGACTCCAT TCTTTTGAGC AGGTTAAAGC CATTCACATC CATTCTGACA

TGGACTCCAT TCTTTTGAGC AGGTTAAAGC CATTCACATC CATTCTGACA

TGGACTCCAT TCTTTTGAGC AGGTTAAAGC CATTCACATC CATTCTGACA

TGGACTCCAT TCTTTTGAAC AGGTTAAAGC CATTCACATC CATTCTGACA

TGGACTGCAT TCTTTTGAAC AGGTTAAAGC CATTTACATC CATTGTGACA

CGGACTGCAT TCTTTTGAAC AGGTTAAAGC CATTTACATC CATTGTGACA

TGGACTCCAT TCTTTTGAAC AGGTTAAAGC CATTCACATC CATTCTGACA

---------- ---------- ---------- ---------- ----------

CGGACTCCAT TCATTTGAAC AGGTTAAAGC CATTCACATC CACTCTGACA

CGGACTCCAT TCTTTTGAAC AGGTTAAAGC CATTCACATC CATTCTGACA

TGGACTCCAT TCTTTTGAAC AGGTTAAAGC CATTCACATC CACCGTGACA

TGGACTCCAT TCTTTTGAAC AGGTTAAAGC CATTTACATC CACTCTGATA

TGGACTCTAT TCTTTTGAAC AGGTTAAAGC CATTCACATC CACACTGACA

---------- ---------- --GTTAAAGC CATTCACATC CATTCTGACA

TGGACTTCAT TCCTTTGAAC AGGTTAAAGC TATTTACATA CACTCTGACA

TGGACTTCAT TCTTTTGAAC AGGTTAAAGC TATTTACATC CACTCTGACA

TGTTCTCAGT TCAAAATGGC TTGCTGACAC CAACACTAAA AGCTAAGAGA

TGTTCTCAGT TCAAAATGGC TTGTTGACAC CAACACTAAA AGCTAAGAGA

TGTTCTCAGT TCAAAATGGC TTGCTGACAC CAACACTAAA AGCTAAGAGA

TGTTCTCAGT TCAAAATGGC TTGCTGACAC CAACACTAAA AGCTAAGAGA

TGTTCTCTGT TCAAAATGGC TTGCTGACAC CAACACTAAA GGCTAAAAGA

TGTTCTCAGT TCAAAATGGC TTGCTGACAC CAACACTAAA GGCTAAGAGA

TGTTCTCAGT TCAAAATGGC TTGCTGACAC CAACACTAAA GGCTAAGAGG

TGTTCTCTGT TCAAAATGGT CTGCTGACAC CAACACTAAA GGCTAAGAGA

TGTTCTCAGT TCAAAATGGC CTGCTGACAC CAACACTAAA GGCTAAGAGA

---------- ---------- ---------- ---------- ----------

TGTTCTCAGT TCAGAATGGC TTGCTGACAC CAACACTAAA GGCTAAGAGA

TGTTCTCAGT TCAAAACGGC TTGCTGACAC CGACACTAAA GGCTAAGAGA

TGTTCTCAGT TCAAAATGGC TTGCTGACAC CAACATTAAA AGCTAAGAGA

TGTTCTCAGT TCAAAATGGC TTGCTGACAC CAACACTAAA GGCGAAGAGA

TGTTCTCAGT TCAAAATGGC TTGCTGACAC CAACGTTAAA GGCTAAGAGA

TGTTCTCAGT TCAAAACGGC TTGTTGACAC CAACACTAAA GGCTAAGAGA

TGTTCTCAGT ACAAAATGGC TTGCTGACAC CAACACTAAA GGCTAAGAGA

TGTTTTCAGT GCAAAACGGC TTGCTGACAC CAACATTAAA GGCTAAGAGA

CCTGAGCTGA GAGAGTACTT CAAAAAACAA ATAGAAGAGC TTTACTCAAT

CCTGAGCTGA GAGAGTACTT CAAAAAACAG ATAGAAGAGC TTTACTCAAT

CCTGAGCTGA GAGAGTACTT CAAAAAACAA ATAGAAGAGC TTTACTCAAT

CCTGAGCTGA GAGAGTACTT CAAAAAACAA ATAGAAGAGC TTTACTCAAT

CCTGAGCTGA GAGAGTACTT CAAAAAACAA ATAGAAGAGC TTTACTCAAT

CCTGAGCTGA GAGAGTACTT CAAAAAACAA ATAGAAGAGC TTTACTCAAT

CCCGAGCTGA GAGAGTACTT CAAAAAGCAA ATAGAAGAGC TGTACTCAAT

CCGGAGCTGA GAGAGTACTT CAAAAAGCAA ATAGAAGAGC TTTACTTAGT

CCCGAGCTGA GGGAGTACTT CAAAAAGCAA ATAGAAGAGC TTTACTCAAT

---------- ---------- ---------- ---------- ----------

CCTGAGCTGA GAGAGTACTT CAAAAAACAA ATAGAAGAGC TTTACTCAAT

CCTGAACTGA GAGAGTACTT CAAAAAACAA ATAGAAGAGC TTTACTCAAC

CCTGAGCTGA GAGAATACTT CAAAAGACAA ATAGAAGAGC TTTACTCAAT

CCCGAGTTGA GAGAGTACTT CAAAAAACAA ATAGAAGAGC TTTACTCAAT

CCCCAGCTGA GAGAGTACTT CAGAACACAA ATAGAAGACC TTTATTCAGT

CCCCAACTGA GAGAGTACTT CAGAACGCAA ATAGAAGAGC TTTACTCAGG

CCAGAACTGA GAGATTACTT CAAAAAACAA ATAGAAGAGC TTTATTCAAC

CCAGAACTGA GAGATTACTT CAAAAAACAA ATAGAAGAGC TTTATTCAAC

CTCCATGATG GCACGTCCCA ACAAATTCCT CCTTTGGTTT TGCTGCTTTG

CTCCATGATG GCACTTCCCA ACAAATTCCT CCTTTGGTTT TGCTGCTTTG

CTCCATGATG GCGCTTCCCA ACAAATTTCT CCTTTGGTTT TACTGCTTTG

CTCCATGATG GCGCTTCCCA ACAAATTTCT CCTGTGGTTT TACTGCTTTG

CTCCATGATG GCGCTTCCCA GCAAATTACT CCTTTGGTTT TGCTGCTTTG

CTCCATGATG GCACTTCCTG GTGAATTCCT CCTTCGGTTT TGCTGTTTTG

CTCCATGATG GCATTTCCCA GCAGATTCCT GTTGGGGGTT TGCTGCTTTG

CTCCGTGATG GCACTTCCCA GCAACTTCCT GTTGGGGGTT TGCTGCTTTG

CTCCGTGATG GCGCTTCCCA GCAAATTCCT TCTGTGGTTT TGCTGTCTTG

-------ATG GCGCTTCCCA ACAAATTCTT CCTTTGGTTT TGCTGCTTTG

CTCCATGATG GCACTTCTCA GCAACTTCTT CCTTTGGTTT TTCTGCTTAA

CTCCATGATG GGGCTTCCCA GCAAATGCTT TTTTGGGTTT TGTTGCTTTT

CTCCATAATG GCGCTTCCAC ACAGAT---T CCTTTGGTTT GGCTGCGTGG

CTCCATGATG GCACTTCCCA GCAAATTCCT TCCTTGGTTT TGCTGCTTTG

CTTCGTAATG GCTCTTGCTA GTGAATTCCT CTTTTGGTCT TGTTGCTTTG

CTCCATAATG GCCCTTCCCG GCACATTGCT CCTTTGGTGG TGTTGCTTTG

CTCAATGATG GAAATGATCA GCACATTTAT CCTCTACTTT TGCTACTCTG

CTCCATGATG GCTGTTCCAT GTGTTCTTCT TCTTTG---T TGCTGTTATT

CCTGGCTGTG TTTTCCTATT AGCCTTGGTT CTCAGGCTTC TGGGGGAGAA

CCTGGCTGTG TTTTCCTATT AGCCTTGGTT CTCAGGCTTC TGGGGGAGAA

CCTGGCTGTG TTTTCCTGTT AGCCTTGGTT CTCAGGCTTC TGGGGGAGAA

CCTGGCTGTG TTTTCCTGTT AGCCTTGGTT CTCAGGCTTC TGGGGGAGAA

CCTGGCTATG TTTTCCTATT AGTGTTGGTT CTCAGGCTTC TAGGGGAGCA

CCTGGCTATG TTTTTCTAGT AGCCTTGGTT CTCAGGCTTC TAGGAGAGAA

CCTGGCTGTG TCTTCTTATT AGCCTTAGCT CTCAGGCTTC CACGGGAGAG

CCTGGCTGTG TTTTCTTAGT AGCCTTAGCT CTCAGGCTTC TACTGAAGAG

CCTGCATGTG TTTTTCAGCT AGCTTTGGTT CCCAGCCTCC CAGGGCAGAG

CCTGGCTCTG TTTTCCTATT AGCCTTGATT CTCAGCCTTC TAGGGGAGAA

CGTTGCTGTG TTTTCCTATT AGTCTTGGTT CTCAAGTTTC GAGAGGAGAA

CCTGGCTATG TTTTCTCTCT AACGTTGGTT CTCAGGCTTC TAGGAGAGAA

CTTGGCTGTG TTTTCCTATT ATTCTTGGTT CTCAGGCCTC CAGGGTTGAA

TCTGCCTGTG TTTTTCTACT AGCCTTGACT GTCAGGCTTC TAGGGGAGAA

CCTGGCTGTG TTTTTCTATT AGCTTTGGTT CTCAGGCTTC TAACGGAGAA

CCTGGCTGTG TT---CTATT AGCTACGGTT CTCAGGGTTC TCAGGGGGAA

TCTGGATATT TTCCTCCCTT AGCCTTGGGT CTCCAACCCT TGAGGATTCC

TGTGGTTCTT ACCTCTTGTG AGTGTGGGTT CCCCAATATC AAGGGG---A

TCTGGGGCTA TGCCTTGGTC CTTGCTGCAG CATATAGATG AGAGAGACAG

TCTGGGGCTA TGCCTTGGTC CTTGCTACAG CATATAGATG AGAGAGACAG

TCTGGGGCTA CACCTTGGTC CTTGCTGCAG CCTATAGATG AGAGAGACAG

TCTGGGGCTA CACCTTGGTC CTTGCTGCAG CCTATAGATG AGAGAGACAG

TCTGAGGCTA AGCCTTGGTC CTTGCTGCAG CCTATAGAGG AGAGAGACAG

TCTGAGGCTG AGCCTTGGTC CTTGCTGCAG CCTGCAGACA GGAGGGAGAG

TCTGAGGCAG ACCCCTGGTC CTTGCTGTTG CCTGTAGATG GGACTGACAG

TCTGAGGCAG ACCCCTGGTC CTTGCTGCTG CCTGTAGATG GGACTGACAG

TCTGAGGTTG AGCCTGGGTC CTCGTTGTGG CCTGCAGATG GGAGAGACCG

TCTGAGGCTG AGACTTGGTC CTTGCTGAAG CATCTAGATG GGAGACACAG

TCTGTGGCTG AGCCTTGGTC ATTGCTGCGG CCTCCAGATG GAAGAGACAG

TCTGAGGCGG AACCTTGGTC CTTGCTGCAG CCTCTAGATG GGAAAGACAG

TCTGGGGCTG CGCCCTGGTC TTTGCTGCAG CCGTTAGATG GGAGAGAGAG

CCTGAGGCTG AGCCTTGGTC CTTGCTCCAT CCTCTAGATG GGAGAGACCG

CCTGAGGCTG AACCTTGGTC CTTGCTGCAG TCTCAAGATG GAAGAGACAG

CCTGAGGCTG AGCCTTGGTC CTTGCTTCAG CCTTTCGATA GCAGAGACCC

GTTGAGGCTG AACCTCTGCC CTGGGAACAG CCTCTAGATA GAGAAAATAG

TCCAAGGTTG AGCCTCTGTT TTTTCTGCTA CCCTTGGAAG GTGGAAATAG

AGCTGGCCTC CTTCCCGCGC TTTTCAAAGT TCTATCTGTT GGGCGAGGTG

AGCTGGCCTC CTTCCTGCGC TTTTCAAAGT TCTATCTGTT GGGCGAGGTG

AGCTGGCCTC CTTCCCCCGC TTTTCAAAGT TCTATCTGTT GGGCGAGGTG

AGCTGGCCTC CTTCCCCCGC TTTTCAAAGT TCTATCTGTT GGGCGAGGTG

AGCTGGCCTC CTTCCCCCGC TTTTCAAAGT TCTCTCTGGT GGGCGAGGTG

CTCTGGCCTC CTTCCCCCAC TCTTCAAAGT CCTATCTGAT GGGCAAGGTG

GTCTGGCCTC TTGCCCCCCC TCTTCAAGGT TCTCTCTGAT AGGCGAGGTG

GTCTGGCCTC TTGCCCCCCC TCTTTAAGGT TCTATCTGAT AGGCGAGGTG

ACCTGGCCTC CTTCCCCCTC TCTTCAAAGT TCTGTCTGAT GGGCGAGGTG

ACCTGGTCTC CTTTCCCCTC TCTTAAACGT TCTGTATGAT GGGCAAGGGA

ATCTGGCTTC CTTTCTCCTC TTTTCAAGGT TCTATATGAT GGGCAAGGTG

AGCTGGCCTC TTTCCCCCTC TCTTTAAGGT TCTGTACGAT GGGCAAGGTG

ACCTGGCCTC CTCCCGCCTC TCTTCAAGGT TCTCTATGAC GAGCAAGGTG

ATCTGGCCTT CTTCCCCCTC TCTTCAAAAT TCTGTCTGAT GAACGAGAAG

ATCTGGCCTC CTTCCCTCTC CCTTCAATGT TCCATCTGAT GAGCAAGGTG

CTCTGGCCTC CTTCCCCCTC TCTTCAAGGT TCTGTCTGAC GAGCGAGATG

CCCTGGTCTC CCGCCACCCC TCCTCAAGGT CTTAACTAAC CGCCATCCTG

AACGGACCTC TTGTCACCCC TCCTCCAGGT TCTATCTGAT AGCCAAAGGG

GGTCACCTAG GCTGCAGCCA GACTCCAGAG CTTTGCACTA CATGAAGAAG

GGTCACCTAG GCTGCAGCCA GACTCCAGAG CTTTGCACTA CATGAAGAAG

GGGCACCTAG GCTGCAACCA GACTCCAGAG CTTTGCACTA CATGAAGAAT

GGGCACCTAG GCTGCAACCA GACTCCAGAG CTTTGCACTA CATGAAGAAT

GGGCACCTAG GCTGCAGCCA GACTCCAGAG CTTTGCACTA CATGAAGAAG

GGGCACCTAG ACTACAGCCA GACTCCAGAG CCTTGCAATA CATGAAGAAG

AGACCCCTAA GCTGCAGCCT GACTCCAGAG CTCTCTACTA CATGAAAAAG

AGACCCCTAA GCTGCAGCCT GACTCCAGAG CACTCTACTA CATGAAAAAG

AGGCCCCTAG GCTGCAGCCA GACTCGAGAG CGTTGCAGTA CATGAAGAAG

ACCCCCC-AG GCTTCAGCCA GATGACAGAG CTTTGAGCTA CATGAAGAGG

GCACGCC--- ---------- -----TAGAG CTTTGCGCTA CATGAAGAGG

GGGCCCCGAG GCTGCAGCCA GACTCCAGAG ATTTGCGCTA CATGAAGAGG

GGACCCCTAG GCTGCAGCCT GATTCCCGGG CTTTGCGCTA CATGAAGAGG

GGATCCCTAG GCTGCAGCCA GACACCAGGG CTTTACGCTA CATGAAGAGG

GGACACGAAG GCTGCAGCCA GACTCCAGAG CTTTGCGCTA CATGAAGAGG

GGCCACCGAG GCTGCAGCCA GACTCGAGAG CTCTGCGCTA CATGAAGAGG

GGGCACCGAG GCTACAACCT GACTCAAGAT CCCTGCGTTA CATGAAGAGG

GGATGCGATC ACTGCAGCCT GATTCCGCGG CCCTGGGCTT CATGAAGAGA

CTCTATAAGA CATATGCTAC CAAGGAAGGG ATTCCTAAAT CCAATAGAAG

CTCTATAAGA CATATGCTAC CAAGGAAGGG ATTCCTAAAT CCAATAGAAG

CTCTATAAGA CATACGCTAC CAAGGAAGGG ATTCCTAAAT CCAATAGAAG

CTCTATAAGA CATACGCTAC CAAGGAAGGG ATTCCTAAAT CCAATAGAAG

CTCTATAACA CATATGCTAC GAAGGAAGGG ATTCCTAAAT CCAATAGAAG

CTCTATAAGG AATATGCTAC TAAGGAAGGG ATTCCCAAAT CCAACAGAAG

CTCTATAAGA CGTATGCTAC CAAAGAGGGG GTTCCTAAAC CCAGCAGAAG

CTCTATAAGA CGTATGCTAC CAAAGAGGGG GTTCCCAAAC CCAGCAGAAG

CTTTATAAAG CTTACGCTAC CAAGGAGGGG ATTCCTAAAG CCAACAGAAA

CTCTATAAAG CATATGCTAC CAAGGAGGGG ACCCCTAAAT CCAACAGAAG

CTCTATAAGG CCTGTGCTAC CAAGGAGGGG ATCCCTAAAT CCAACAGAAG

CTCTATAAGG CATACGCTAC CAAGGAGGGG ATCCCTAAGT CCAACAGAAG

CTCTATAAGG CATACGCAAC CAAAG----- ---------- ----------

CTCTATAAGG CATATGCTAC CAAGGAGGGA ATCCCTAAAT CCAACAGAAG

CTCTATAAGG CATATGCTAC CAAGGAGGGG ATCCCTAAAT CCGACAGACA

CTCTATAAGG CATATGCTAC CAAGGAGGGG ATCCCTAAAT CCACCAGAAG

CTTTATAAAG CATATGCCAC CAAAGAAGGA ATCCCTAAGT CTGACAGAAG

CTCTACCGGA TGTATGCTTC CAAAGAAGGC ATCCCAAAGT CCAATAGGAG

TCACCTCTAC AACACTGTTC GGCTCTTCAC CCCCTGTACC CGGCACAAGC

TCACCTCTAC AACACTGTTC GGCTCTTCAC CCCCTGTACC CGGCACAAGC

TCACCTCTAC AACACTGTTC GGCTCTTCAC CCCCTGTACC CAGCACAAGC

TCACCTCTAC AATACTGTTC GGCTCTTCAC CCCCTGTACC CAGCACAAGC

TCACCTCTAC AACACTATTC GGCTCTTCAC CCCCTGTACC CAGCACAAGC

TCACCTCTAC AATACTATTC GGCTCTTCAC CCCCTGTGCC CAGCACAAGC

TCACCTCTAC AATACCGTCC GGCTCTTCAG CCCCTGTGCC CAGCAGGAGC

TCACCTCTAC AATACCGTCC GGCTCTTCAG TCCCTGTGCC CAGCAAGAGC

TCACCTCTAC AACACAGTTC GACTCTTCAC ACCCTGGGTC CAGCAGAAGC

CCACCTCTAC AACACTGTTC GGCTCTTCAC CCCCTGTGCT CAGCACAAGC

CCCTCTTTAC AACACTGTTC GACTCTTCAC GTCCTGTGCC CAGCACACGC

TCACGTCTCC AACACCATTC GGCTATTCAC CCCCTATATC CAGCGCAAGT

---------- ---------- ---------- ---------- ----------

TCACCTCTAC AACACTGTTC GGCTCTTCAC CTCCTGTGTC CAGCACAAGC

TCACCTCTAC AACACTATTC GGCTCCTCAC ACCCTGTGCC CAGCACAGGC

TCACCTCTAC AACACTGTTC GGCTCTTCAC GCCCGGTGCC CAGCACAAGC

TCACCTCTAC AACACCGTCC GTCTCTTCAC GCCATGTGCC CAGCACCAAC

TCACTTCTAC AATACAGTTC GGCTCTTTAC TCCGGATGCC AAGCCCAAGC

AGGCTCCTGG AGACCAGGTA ACAGGAATCC TTCCATCAGT GGAACTGCTA

AGGCTCCTGG AGACCAGGTA ACAGGAATCC TTCCATCAGT GGAACTGCTA

AGGTTCCTGG AGACCAGGTA ACAGGAATCC TTCCATCAGT GGACCTGTTA

AGGTTCCTGG AGACCAGGTA ACAGGAATCC TTCCATCAGT GGACCTGTTA

AGGCTCCTGG AGACCAGGTA ACAGGAATCC TTCCATCAGT GGACCTGCTA

AGGCTCCTGG AGACCAGGTG ACAGGAATCC TTCCATCAGG GGATCTGCTG

AGGCTCCCAG CAACCAGATG ACAGGACCCC TGCCAATGGT GGACCTGCTA

AGGCACCCAG CAACCAGGTG ACAGGACCGC TGCCGATGGT GGACCTGCTG

AGGCTCCAGG ACGCCCGGAA ACAGGAGTCC TGCCGCCCGC GGACCTGCTG

AGGCTCCTGG GGACCAGGCA GCAGGAACCC TTCCATCAGT GGATCTGCTG

AGGCTCCTGG GGACCAGGCG ACAGGCTCTG TTCCACCTGT GGACCTGCTC

GGGTTCCTGC AGACCAGGTG ACAGGAACCG CTCCGTCCGT GGACCTGCTG

---------- ---------- ----GAACCC TTTCATCCGT GGACCTGCTG

AGGTTCCTGT GGACCAAGTG CCAGGAATCT TTCCATCACT GGACCTGCTG

AAACTCTTGG CAACCAGATG ACAGGAATCC TTCCATCACT GGACCTGCTG

AAACGCCTGA GAACCAGGTG ACAGGCATCC T---GTCTGT GGAGCTGCTG

AGCCCTCTTG GGGTCAAGTC ACAGGAGTTC TTCAGTCAAT GGATCTGCTA

AGGACGCTGA AGACCAAAGA ATGGGACTTC CGGGCTCCGC AGATCTAACA

TTTAACCTGG ATCGCATTAC TACCGTTGAA CACTTACTCA AGTCAGTCTT

TTTAACCTGG ATCGCATTAC TACCGTTGAA CGCTTACTCA AGTCAGTCTT

TTTAACCTGG ATCGCATTAC TACCGTTGAA CACTTACTCA AGTCAGTCTT

TTTAACCTGG ATCGCATTAC TACCGTTGAA CACTTACTCA AGTCAGTCTT

TTTAACCTGG ATCGCATTAC TACCGTTGAA CACTTACTCA AGTCAGTCTT

TTTAACCTGG ATCGTGTTAC CGCTGTTGAA CACTTACTCA AGTCAGTCTT

TTTAACCTGG ACCGGGTGAC TGCCATGGAA CACTTACTCA AATCGGTCTT

TTTAACCTGG ACCGGGTGAC TGCCATGGAA CACTTGCTCA AATCGGTCTT

-TTAACCTGG ATCGCGTTAC TGCCGTGGAA CACCTGATCA AGTCGGTCCT

TTTAACCTGG ATCGTGTTAC TGTTGTGGAA CATTTATTCA AGTCAGTCTT

TTTAACCTGG ATCGTGTTCC TCCCGTTGAA CACTTACTGA AGTCGGGTTT

TTTAACCTGG ACCGTGTTAC TGCTGTGGAA CACTTACTGA AGTCCGTCTT

TTTAACCTGG ATCGTGTGAG TGCCGTTGAA CACTTACTGA AATCGGTCTT

TTTAACCTGG ATCGTGTTTC TGTTCTTGAA CACTTACTCA AGTCCATCTT

TTTAACCTGG ATAGTGTTCC TGCTCTCGAA CACTTACTCA AGTCAGTCTT

TTTCACCTGG ACAGTGTCAC CGCGCCCGAG CACTTACTCA AGTCGGTCTT

TTTAACCTGG ACCGTATTAC TGACCATGAA TATTTGCTCA AGTCTGTTTT

TTCAACCTGG ATCGCGTTAC AGTCGTGGAA CACTTACTCA AGTCTGTCTT

GCTGTACAAT ATCAACAACT CAGTTTCTTT TTCCTCTGCT GTCAAATGTG

GCTGTACAAT ATCAACAACT CAGTTTCTTT TTCCTCTGCT GTCAAATGTG

GTTGTACACT ATCAACAACT CAGTTTCTTT TTCCTCTGCT GTCAAATGTG

GTTGTACACT ATCAACAACT CAGTTTCTTT TTCCTCTGCC GTCAAATGTG

GCTGTACGCT ATCAGTGACT CAGTTTCTTT TTCCTCTGCT ATCAAATGTA

GCTGTACACT ATCAACAACT CAGTTTCCTT TTCCTCTCCT ATTAAATGTG

GCTATACACT CTGAACAACT CCGCTGCTTC CTCCTCCACT GTGACCTGTG

GCTATACACT CTGAACAACT CTGCCTCTTC CTCCTCCACT GTGACCTGTA

GCTCTACACT CTCGACAGCT CCGTCTCTGT CTCCAGTGCT GTGCAGTGTG

GCTATATACT TTCAACAACT CCATTTCTTT TCCCTTTCCT GTTAAATGTA

GCTGTACTCT TTCAGCAATT CCATTTCTTT CCCCTCTGCC GTTAAATGTC

GCTGTACACC TTCAACAACT CTGTTTCTTT TCCCTCTGCT GTCAAATGCG

GCTCTACACG TTCAATAACT CCCTGTCTTT TCCCTCTACT GCTAAATGTG

ACTATACACT TTCAAGGACT CCATCTCTTT TCCCTCTGCT GTTAAATGTG

TCTATACACT TTCAACAACT CAGTTTCTTT TCCCTCTGCT GTTAAATGTA

GCGGTACACT TTCAACGACT GGGCCTCTCT GCCGGCCGCC GCTCACTGCA

GCTATATTCT TTGAAGAATC CAGTTTCTTT TTCTTCCACA CCCAAGTGTA

GCTCTACTCT TTCAACAGTT CTGTTCCTTT TCCCTCCGCC ACCGTGTGCC

TGTGCAATCT AATGATAAAG GAGCCAAAGT CTTCTAGCAG GACTCTCGGC

TGTGCAATCT AATGATAAAG GAGTCAAAGT CTTCTAGCAG GACTCTTGGC

TGTGCAACCT AATGATAAAG GAGCCAAAGT TTTCTAGCAA GACTCTCCAC

TGTGCAACCT AATGATAAAG GAGCCAAAGT TTTC------ ----------

TGTGCAACCT GATGATGAAG GAACCAAAGT TTTCTAGCAA GACACTCCCC

TATGCAACCT GGTGATAAAA GACCCAAAAT CTTCTGGCAA GACTCTCTCT

TGTGTGACCT TGTGGTAAAG GAGCCCATGT CTTCCAGCAA GGCTACCCCA

TGTGTGACCT TGTGGTAAAG GAGGCCATGT CTTCTGGCAG GGCACCCCCA

TGTGCAGCC- ---------- ---------- ---------- ----------

TATGCAACCT GGTGATAAAA GAGCCAGAGT TTTCTAGCAA GACTCTCCCT

TGTGCCACCT GGTGATCAAA GAGCCAGAGT TTTCTAGCTG GACCCCCCAG

GGTACAGCCT GGTGATGAAC GAGCCAGAGT CGTCTCCTAA GGCTCGCCCT

TGTGCAACCT GGTGATAAAA GAGCCAGAAT TTCCCCTCAA GTCTCTTCCA

AGTGTGACCT TGTGATAAAG GAGCCAAAGT CTTCTAGTGA GGCTTTCCCT

TGTGCAACAT GATGATAATG GAACCAAAGT CTTCCAGCAA GACTGTCCCT

CGGGCAGCCT GGAGGTCGGA GAAGCGGGGT CTTCCGGCAA GACGACCACC

TGTGCCACCT CGTTGTAAAG GAACCTGAAT GTTCTAGCCC ATTTTGTTTT

CGTGCCAGCT AGTTGTAAAG GAGCAGGAAT CTTCTAGCCA AAAGTGTTCT

AGAGCTCCAT ACTCATTTAC CGGAAAGAAA CACAAATGGA TTCAGATTGA

AGAGCTCCAT ACTCATTTAC CGGAAAGAAA CACAAATGGA TTCAGATTGA

AGAGCTCTGT ACTCATTTAC CGGAAAGAAA CACAAATGGA TTGAGATTGA

---------- ---------- ---------- ---------- ----------

AGAGGTCCAT ACTCATTTAC CGGAAAGAAA CACAAGTGGA TTGAGATTGA

AGAGCTCCAC ATTCCTTTAC TGAAAAGAAA CATAAATGGA TTGAGATTGA

AGAGCGCCAT ACTCATTTAC C--GAGGAAA CACAGGTGGA TTGAGATGGA

AGAGCACCGT ACTCATTCAC C--GAAGAAA CACAGATGGA TTGAGATTGA

---------- ---------- ---------- ---------- ----------

AGAGCTCCAT ACTCATTTAC CAGAAAGAAA TACAAATGGA TTGAGATTGA

AGAGCTCCAT CCTTATTGAC CAAAAAGAAA TACAAATGGT TTGAGGTTGA

CCAGCTCCAC GCTCCTTTAC CAGAAAGAAG TACACGTGGG TGGAGGTTGA

AGAG------ ------TTAC TCAGAAGAAG CACAAATGGC TCGAGATGGA

AGAGCTCCAA ACTCATTTAC -AGAAAGAAA CACAAATGGA TTGAGCTTGA

AGAGCTCCAT ACTCATTTAC CAGAAAGAAA CACAAGTGGA TTGAGATTGA

GGGGCCCCGG ACGCCTTTCC CAGAAAGAAG CGCCAATGGA TCGAGATGGA

GGGTCTCCCC GGTCCTTCAC TAGAAAAAAT CACAAGTGGA TTGAAATAGA

AGGGCTCCTC ATTCACTGAA TAGAAAAAAG AGAAGGTGGG TTGAAATCGA

TGTGACCAGC CTCCTTCAAC CTTTAGTGGC CTCCAACAAG AGAAGTATTC

TGTGACCAGC CTCCTTCAAC CTTTAGTGGC CTCCAACAAG AGAAGTATTC

TGTGACCAGC CTCCTTCAAC CTTTAGTGGC CTCCAACAAG AGAAGTATTC

---------- ---------- ---------- ---------- ----------

TGTGACTAGC CTCCTTCAAC CTTTAGTGGC CTCCAACAAG AGAAGTGTTC

CATAACCACC CTCCTTCAGC CTCTAGTGGC CGCCAACAAG AAGA---TTC

TGTGACCTCC CTCCTTCAGC CCCTGGTGGC CTCAAGCGAG AGGAGCATTC

TGTGACCTCC CTCCTTCAGC CCCTAGTGAC CTCCAGCGAG AGGAGCATTC

---------- ---------- ---------- ---------- ----------

TGTGACAGCT CCTCTTGAGC CTCTGGTGGC CTCCCACAAG AGGAATATTC

TGTGACCACT GCTCTCCGGC CTCTAGTGGC CTTGCACAAG AGAAGTCTTC

TGTGACCACT CTCCTTCAGC CTCTAGTGGC TTCTGACAAG AGAAGTATTC

TGTGACCAGT CTCCTTCAAC CTTTCGTGGC CTCCAACAAG AGAAGCATTC

TGTGACTACT CTCCTTCAGC CCCTGGTGGC CTCTAACGAG AGAAGTATTC

TGTGACCACT CTCCTTCAGC CTCTGGTTGC TTCCAAGAAA CAAAGTATTC

TGTCACTCCG CTCCTGCAGC CTCTAGTGGC CTCCAACAAG CGAAACATCC

TGTGACGACT TTGCTTCAGC CTCTAGTTGC CTCCAACAAG AGAAATATCC

TGTGACTACT CTACTTTGGC CTCTGATTGC TGCGAACAAG AGGAGTGTTA

ACATGTCTAT AAATTTTACT TGCATGAAAG ACCAGAATGG TTTGTTTAAC

ACATGTCTAT AAATTTTACT TGCATGAAAG ACCAGAATGG TTTGTTTAAC

ATATGTCTAT AAATTTTACA TGCATGAAAG ACCAGAATGG TTTGTTTAAC

---------- ---------- ---------- ---------- ----------

ACATGTCTGT AAATTTTACA TGCATGAAAG ACCAGAATGG TTTGTTTAAC

ACATGTCTGT CAATTTTACC TGCCCGAGAG ACCAGGATGG CCCGTTTAAC

ACCTATCTGT CAATTTTACA TGCACAAGAG ACGAGAATGG AACGTTTAAT

ACCTGTCTGT CAATTTTACA TGCACAAAAG ACGAGGACGG AGTGTTTAGC

---------- ---------- ---------- --CAGGAGGG CCCCTTGAAC

ACATGTCTGT AAATTTTACA TGTGTGAAAG ACCGGGACAG CCTGTTTAAC

ACATGTCTGT AAATTTTACC TGTGTGAGAA ACCAGGATGG GCCGTGGAAT

ACATGTCTGT AAATGTTACC TGCGTGAAAG ACCCGGACAG TCCATTGAAG

TCATGGCCGT GAATTTGACT TGCATGAAAG AGCAGGACGG CACATGGAAC

ACATGTCTGT AAACTTTACC TGCGTGAATG ACCAGGAGAG TCCGTTTAAC

ACATGTCTGT AAATTTTACA TGTGAGAAAG ACAAGGACGG TCCATTTAAC

GCATGTCTGT CACCGTTGTG TGTGAGGAAG GC-GGGCTGG GCCGTTCAAC

ACATAGCTCT GAATTTCACC TGTCTCAAAA ACCAGGATGA GTTATTTGAT

AAATGGCTGT GAACTTCACT TGCCTGGAAG --CAGAACGA GACCCTAAAC

ATGACTCTGA ACACAACTGA GAAGGAAACC TTCTGCAGGG CTGCGACTGT

ATGACTCTGA ACACAACTGA GAAGGAAACC TTCTGCAGGG CTGCGACTGT

ATGACTCTGA ACACAACTGA GAAGGAAACC TTCTGCAGGG CTGCGACTGT

---------A ACACAACTGA GAAGGAAACC TTCTGCAGGG CTGCGACTGT

ATGACTGTGA ACACAACTGA GAAGGAAACC TTCTGCAGGG CTGCGACTGT

ATGACTCTGA ACACAACTGA GAAGGAAATA TTCTGCAGGG CTGCGACTGT

ATGCCCTCAA ACACCACGGA GAACGAGCTC ATCTGCAGGG CTTCCAGGGT

ATGCCCTCAA ACACCACAGA GAGTGAGCTC GTCTGTAGGG CTTCCAAGGT

TTAACGCTAA ACACAACCGA GAGAGAGGCC GTCTGCAGGG CCGCGACTGC

ATGACTCTC- ---------- ---------- ---------- ----------

AGGACGCTGA ACACAAGCGA TAAGGAAATC TTCTGCAGAG CTGCTACTGT

CTGCCTCTGA ACACAACTGA GAAGGAAGCC TTCTGCAGGG CCGCAATGGA

TTGACTCTGA ATACAACCAG GACGGAACTC TTCTGCAGGG CCGCTACGGT

ATGTCTCTGA GCACAACTGA GAATGAAACC TTCTGCAGAG TTGCGTCTGT

ATGCCTCTGA ACACAACCGA GAAAGAAACC TTCTGCAGAG CTACTACTGT

ACGTCTCTGA ACACAACGGA GAACGGAACC TTATGCAGGG CCACGACTGT

ATGGCGTTAA CAAGGAATGA CTTTGAAACA TTATGCAGAG CTGCCACGGT

ACGGCGCTGA GCCCCAAAGA TTTAGCAACG CTCTGCCAGG CCACCAGCGT

GCTCCGGCAG TGTCCTGTGA AGGAAGCCAA CCAGAGTACG TTGGAAAACT

GCTCCGGCAG TGTCCTGTGA AGGAAGCCAA CCAGAGTACG TTGGAAGACT

GCTCCGGCAG TGTCCTGTGA AGGAAGCCAG CCAGAGTACG TTGGAAGACT

GCTCCGGCAG TGTCCTGTGA AGGAAGCCAG CCAGAGTACG TTGGAAGACT

GCTCCGGCAG TGTCCTGTGA AGGAAGCCGA CCAGACTATG TTGAAAGACT

GCTTCGGCAA TGTCCTGTGA ATGACGCCAT GCAGAGCACA CTGAAAGACT

GCTTCGCAAA TGCACCGTGA ATGAGTCCAC GCTCACAACA CTGAAAGACT

GCTTCGCATA TGCACCATGA ATGAGTCCAA GTCCACATCA CTGAAAGACT

ACTCCGGCAG TGTCCTGGGA AGGAAGCCAG ACAGACTACA CTGGAAGACT

---------- ---------- ---------- ---------- ----------

ACTGCGGCAG TGTTCTATGA ATGAAATCAA GAAGAGTACA CTGAAAGACT

GCTTCGGAAG TGCCCTGTGA ATGAAACCAA GAAGATCACC TTGAAAGAAC

GCTCCGGCAG ---------- ---------- ---------- ----------

GCTTCAGAAG TGTCCTATGA ATGAAGCTAA GACGTGTACG CTGAAAGACC

GCTTTGGCAG TGTCCTGTGA ATGAAGACAG GACGACTACA TTGAAAGACT

GCTGTGGCAG TGTCCTATGA ATGAAAACAG GACCAGCACG TTGAAAGAGT

GCTCCAAGAG TGTCCTGTGA ATGAAACCAA GATTATTAAA TTGCAAGATT

GCTCCAACAG TGTCCGGTGA CCAGAACCAA CAGCATCCAA CTGAAGAAAT

TCTTGGAAAG GCTAAAGACG ATCATGAGAG AGAAATATTC AAAGTGTAGC

TCTTGGAAAG GCTAAAGACG ATCATGAGAG AGAAATATTC AAAGTGTAGC

TCTTGGAAAG GCTAAAGACG ATCATGAAAG AGAAATATTC AAAGTGTAGC

TCTTGGAAAG GCTAAAGACG ATCATGAAAG AGAAATATTC AAAGTGTAGC

TCTTGGAAAG GCTAAAGATG ATCATGAAAG AGATATATTC AAAGTGTAGC

TCTTGGAAAG GCTAAAGACG ACCATGAAAG AGAAATATTC AAAGTATAGC

TCCTGGAAAG CCTAAAAAGC ATCCTACGAG GGAAATACTT GCAGTCCACT

TCCTGGAAAG CCTAAAGAGC ATCATGCAAA TGGATTACTC G---TACGCC

TCTTAGACAG GCTAAAGACC GCCATGCAAG AGAAATACTC AAAGCGTAGC

---------- ---------- ---------- ---------- ----------

TCTTGGAAAG GCTAAAAGTG ATCATGCAGA AGAAATACTA CAGGCATAGC

TCTTGGAAAG GCTAAAGCAG ACAATGAAAA AGAAATACGC CAAGGGTAGC

---------- ---------- ---------- ---------- ----------

TCTTGGAAAG GCTAAAGATG ATCATGAAGG AAAAATACTC AAAGTGTA--

TCTTGGAAAG GCTAAAGACG ATCATGAAGG AGAAATATTC AAAGTGTAGA

TCTTGGAAAG CCTAAAGGAC ATCATGAAGA AGAAATATTC AAAGTGTGGA

TCCTGAGCAG ACTAAAAACC ATCCATCAGA AGATATTTTC AGAGTTTA--

TCCTGGAAAA GCTGAAGAAC ATCTCCCAGA AGATCTATTC CAGCACTGGC

ATGCCCATCA CTCGGATGCG CATGAGACCC TGGCTAGAGA TGCAGATTAA

ATGCCCATCA CTCGGATGCG CATGAGACCC TGGCTAGAGA TGCAGATTAA

ATGCCCATCA CTCGGATGCG CATGAGACCC TGGCTAGAGA TGCAGATTAA

ATGCCCATCA CTCGGATGCG CATGAGACCC TGGCTAGAGA TGCAGATTAA

ATGCCCATCA CTCGGATGCG CATGAGACCC TGGCTAGAGA TGCAGATTAA

ATGCCCATCA CTCGGATGCG CATGAGACCC TGGCTAGAGA TGCAGATTAA

ATGCCTATCA CTCGGATGCG AATGAGACCC TGGCTAGAGA TGCAGATTAA

ATGCCAATCA CTCGAATGCG GATGAGACCC TGGCTAGAGA TGCAGATTAA

ATGCCCATCA CTCGGATGCG CATGAGACCC TGGCTAGAGA TGCAGATTAA

ATGCCCATCA CTCGGATGCG CATGAGACCT TGGCTAGAGA TGCAGATTAA

ATGCCCATCA CTCGGATGCG CATGAGACCC TGGCTAGAGA TGCAGATTAA

ATGCCCATCA CTCGCATGCG CATGAGACCC TGGCTAGAGA TGCAGATTAA

---------- ---------- ---------- ---------- ----------

ATGCCCATCA CTCGGATGCG CATGAGACCC TGGCTAGAGA TGCAGATTAA

---------- ---------- ---------- ---------- ----------

ATGCCCATCA CTCGGATGCG CATGAGACCC TGGCTAGAGA TGCAGATTAA

ATGCCCATTG CCAGAATGCG CATGAGACCC TGGCTAGAGA TGCAGATTAA

ATGCCGACAA CACGAATGCG CATGAGACCC TGGCTGGAGT TGCAGATCAA

TTCCAACCAA ATCCCGGGGC TCATCTGGAT TAATAAAGGC CGATACAAAG

TTCCAACCAA ATCCCGGGGC TCATCTGGAT TAATAAAGGC CGATACAAAG

TTCCAACCAA ATCCCGGGGC TCATCTGGAT TAATAAAGGC CGATACAAAG

TTCCAACCAA ATCCCGGGGC TCATCTGGAT TAATAAAGGC CGATACAAAG

TTCCAACCAA ATCCCGGGGC TCATCTGGAT TAATAAAGGC CGATACAAAG

TTCCAACCAA ATCCCAGGGC TGACCTGGAT TAATAAAGGC CGATACAAAG

TTCCAACCAA ATTCCAGGGC TGAGCTGGAT CAATAAAGGC CGATACAAAG

TTCCAACCAA ATCCCAGGGC TGATCTGGAT CAATAAAGGC CGATACAAAG

TTCCAACCAG ATCCCCGGGC TGATCTGGAT TAATAAAGGC CGATACAAAG

TTCCAACCAA ATCCCAGGGC TGATCTGGAT TAATAAAGGC CGATACAAAG

TTCCAACCAA ATCCCAGGGC TGATCTGGAT TAATAAAGGT CGATACAAAG

TTCCAACCAG ATCCCAGGGC TGATCTGGAT TAATAAAGGC CGATACAAAG

---------- ---------- ---------- -------G-- ----------

TTCCAACCAA ATTCCGGGGC TGATCTGGAT TAATAAAGGT CGATACAAAG

---------- ---------- ---------- ---------- ----------

TTCCAACCAA ATCCCAGGGC TGACCTGGAT TAATAAA-GC CGATACAAAG

TTCCAACCAA ATCCCAGGCC TGACCTGGAT TAACAAGGGT CGCTACAAAG

TTCCAATCAG ATCCCAGGCC TTACGTGGAT CAATAAGGGG AGATACAAGG

CAGGGGAAAA GGAGCCAGAT CCCAAGACGT GGAAGGCCAA CTTTCGCTGT

CAGGGGAAAA GGAGCCAGAT CCCAAGACGT GGAAGGCCAA CTTTCGCTGT

CAGGGGAAAA GGAACCAGAT CCCAAGACGT GGAAGGCCAA CTTTCGCTGT

CAGGGGAAAA GGAACCAGAT CCCAAGACGT GGAN------ ------CTGT

CAGGGGAAAA GGAGCCAGAC CCCAAAACAT GGAAGGCCAA CTTTCGCTGT

CAGGGGAAAA GGAGCCAGAT CCCAAGACAT GGAAGGCCAA CTTTCGCTGT

CAGGGGAAAA AGAGCCAGAT CCCAAGACTT GGAAGGCAAA CTTCCGGTGT

CAGGAGAAAA AGAGCCAGAT CCCAAGACAT GGAAGGCAAA CTTCCGTTGT

CTGGGGAAAA GGAGCCAGAT CCCAAGACGT GGAAGGCCAA CTTCCGCTGT

CAGGGGAAAA GGAGCCGGAT CCCAAGACCT GGAAGGCCAA CTTTCGCTGC

CCGGGGAAAA GGAGCCAGAT CCTAAGACAT GGAAGGCCAA CTTTCGCTGT

CAGGGGAAAA GGAGCCAGAT CCCAAGACAT GGAAGGCCAA CTTCCGCTGT

---------- ---------- ---------- ---------- ----------

CAGGAGAAAA GGAGCCGGAT CCCAAGACGT GGAAGGCCAA TTTTCGCTGC

---------- ---------- ---------- ---------- ----------

CAGGGGAAAA GGAGACAGAC CCCAAGACGT GGAAAGCCAA CTTCCGCTGC

TTGGGGAGAG GGAACCAGAC CCTAAGACAT GGAAGGCCAA TTTCCGCTGT

TCGGAGAAAA GGAGCCAGAC CCCAAGACCT GGAAGGCCAA CTTCCGCTGC

GCCATGAACT CCCTGCCAGA TATCGAGGAG GTGAAAGACC AGAGCAGGAA

GCCATGAACT CCCTGCCAGA TATCGAGGAG GTGAAAGACC AGAGCAGGAA

GCCATGAACT CCCTGCCAGA TATTGAGGAG GTGAAGGACC AGAGCAGGAA

GCCATGAACT CCCTGCCAGA TATTGAGGAG GTGAAGGACC AGAGCAGGAA

GCCATGAACT CCCTGCCAGA TATTGAGGAG GTGAAGGACC AGAGCAGGAA

GCCATGAACT CCCTGCCAGA TATTGAGGAG GTGAAGGACC AGAGCAGGAA

GCCATGAACT CCCTACCAGA CATCGAGGAA GTGAAGGACC AGAGCAGGAA

GCCATGAACT CCCTGCCAGA CATCGAGGAA GTGAAGGATC AGAGTAGGAA

GCCATGAACT CCCTGCCAGA CATCGAGGAG GTGAAGGACC AGAGCAGGAA

GCCATGAACT CGCTGCCAGA TATTGAGGAG GTGAAGGACC AGAGCAGGAA

GCCATGAACT CCCTGCCAGA TATTGAGGAA GTGAAGGACC AGAGCAGGAA

GCCATGAACT CCTTGCCGGA CATCGAGGAA GTTAAGGACC AAAGCAGAAA

---------- ---------- ---------- ---------- ----------

GCCATGAACT CCCTGCCAGA CATTGAGGAG GTGAAGGACC AGAGCAGGAA

---------- ---------- ---------- ---------- ----------

GCCATGAACT CGCTGCCGGA CATCGAGGAG GTGAAGGACC AGAGCAGGAA

GCCATGAATT CCCTCCCTGA CATAGAGGAA GTGAAGGACC AAAGCAGGAA

GCCATGAACT CCCTGCCCGA CATCGAGGAG GTGAAGGACC AGAGCAGGAA

CAAGGGCAGC TCAGCTGTGC GAGTGTACCG GATGCTTCCA CCTCTCACCA

CAAGGGCAGC TCAGCTGTGC GAGTGTACAG GATGCTTCCA CCTCTCACCA

CAAGGGCAGC TCAGCTGTGC GAGTGTACCG GATGCTTCCA CCTCTCACCA

CAAGGGCAGC TCAGCTGTGC GAGTGTACAG GATGCTTCCA CCTCTCACCA

CAAGGGTAGT TCAGCTGTGC GGGTGTACCG GATGCTTCCA CCTCTCAACA

CAAGGGCAGC TCTGCCGTGC GGGTGTACCG GATGCTCCCA CCTCTCACCA

CAAGGGCAGC TCTGCTGTAC GCGTGTACCG GATGCTGCCA CCCCTCACCA

CAAGGGCAGC TCTGCTGTGC GGGTGTACCG GATGCTGCCA CCCCTCACCA

CAAGGGCAGC TCGGCCGTGC GGGTGTACCG GATGCTCCCG CCCCTCACCA

CAAGGGCAGT TCAGCTGTGC GGGTGTACCG GATGCTCCCA CCCCTCACCA

CAAAGGCAGC TCAGCTGTGC GGGTGTACCG GATGCTCCCA CCCCTCACCA

CAAAGGCAGC TCAGCTGTGC GGGTGTATCG GATGCTCCCA CCCCTCACCA

---------- ---------- ---------- ---------- ----------

CAAGGGCAGT TCAGCTGTGC GGGTGTACCG GATGCTTCCA CCCCTCACCA

---------- ---------- ---------- ---------- ----------

CAAGGGCAGC TCAGCTGTGC GGGTGTACCG CATGCTCCCC CCGCTGACCA

CAAGGGAAGC TCAGCTGTGA GAGTATACCG AATGCTCCCG CCTCTCACCA

CAAAGGCTCC ACTGCCGTCC GCGTCTATCG GATGCTTCCG CCTTTGTCCA

AGAACCAGAG AAAAGAAAGA AAGTCGAAGT CCAGCCGAGA TGCTAAGAGC

AGAACCAGAG AAAAGAAAGA AAGTCGAAGT CCAGCCGAGA TGCTAAGAGC

AGAACCAGAG AAAAGAAAGA AAGTCGAAGT CCAGCCGAGA TGCTAAGAGC

AGAACCAGAG AAAAGAAAGA AAGTCGAAGT CCAGCCGAGA TGCTAAGAGC

AGAACCAGAG AAAAGAAAGA AAGTCGAAGT CCAGCCGAGA T---AAGAGC

AGAACCAGAG AAAAGAGAGA AAGTCCAAGT CCAGCCGAGA TGCTAAGAGC

AG-------- -----AGAGA AAGTCCAAGT CCAGCCGTGA CACTAAGAGC

GGAACCAGAG GAAAGAGAGA AAGTCCAAGT CCAGCCGAGA CACTAAGAGC

GGAACCAGAG GAAAGAGAGA AAGTCCAAGT CCACCCGAGA TGCTAAGGGC

AGAGCCAGAG GAAAGAGAGA AAGTCCAAGT CCAGCCGAGA CGCTAGGAGC

AGAACCAGAG AAAAGAGAGA AAGTCCAAGT CCAGCCGAGA TGCTAAGAGC

AAAACCAGAG GAAAGAGAGA AAGTCCAAGT CCAGCCGAGA CACTAAGAGC

---------- ---------- ---------- ---------- ----------

AGAACCAGAG GAAAGAGAGG AAATCCAAGT CCAGCCGAGA TGCTAAGAGC

---------- ---------- ---------- ---------- ----------

AGAACCAGAG GAAAGAAAGA AAGTCCAAGT CTAGCCGAGA GACCAAGAGC

AGAACCAGAG GAAAGAGAGA AAGTCCAAGT CATCCAAAGA TTCTAGAAGC

AGAGCCAGAG AAAAGAGAGA AAGACCAAGA CCTCAAGAGA CTCCAGAAAC

AAGGCCAAGA GGAAGTCATG TGGGGATTCC AGCCCTGATA CCTTCTCTGA

AAGGCCAAGA GGAAGTCATG TGGGGATTCC AGCCCTGATA CCTTCTCTGA

AAGGCCAAGA GGAAGTCATG TGGGGATTCC AGCCCTGATA CCTTCTCTGA

AAGGCCAAGA GGAAGTCATG TGGGGATTCC AGCCCTGATA CCTTCTCTGA

AAGGTCAAGA GGAAGTTATG TGGGGATTCC AGCCCTGATA CCTTCTCTGA

AAGGCCAAAA GGAAGTCATA TGGGGATTCT AGCCCTGATA CCTTCTCTGA

AAAACCAAGA GGAAGCTGTG CGGAGATTCT AGCCCTGACA CCTTATCTGA

AAAACCAAGA GGAAGCTGTG TGGAGATGTT AGCCCGGACA CTTTCTCTGA

AAGGCCAAGA GGAAGTCCTG TGGGGATTCC AGCCCAGACA CCTTCTCTGA

AAGGCCAAGA AGAAGCCATA TGGGGAATAC AGCCCCGATA CCTTCTCTGA

AAGGCCAAGA GGAAGTCATG TGGTGACTCC AGTCCTGACA CCTTCTCTGA

AAGGCCAAGA GGAAGTCATG TGGGGACTCC AGCCCCGATA CCTTCTCCGA

---------- -----TCCTG TCGCGACTCT AGCCCCGATG CCTTGTCTGA

AAGACCAAGA GGAAGTCGTG TGGGGATTCC AGCCCCGATA CCTTCTCTGA

---------- ---------- ---------- ---------- ----------

AAGGCCAAGA GGAAGTCATG TGGGGACTCC AGCCCTGATA CCTTCTCCGA

AAGAGCAAGA GAAAGTCATA TGCAGAATCC AGTCCAGACA CCCAATCAGA

AAGAATAAGA GAAAGGTGTA TGAAGAGTTC AGCCCGGAGG CCAAGTCAGA

TGGACTCAGC AGCTCCACTC TGCCTGATGA CCACAGCAGC TACACAGTTC

TGGACTCAGC AGCTCCACTC TGCCTGATGA CCACAGCAGC TACACAGTTC

TGGACTCAGC AGCTCCACTC TGCCTGATGA CCACAGCAGC TACACAGCTC

TGGACTCAGC AGCTCCACTC TGCCTGATGA CCACAGCAGC TACACAGCTC

TGGACTCAGC AGCTCCACTC TGCCTGATGA CCACAGCAGC TATACAACTC

TGGCCTCAGC AGCTCCACCC TGCCTGATGA CCACAGCAGC TACACAGCTC

CGGACTGAGC AGCTCTACTC TGCCTGATGA CCACAGCAGT TACACAGCTC

TGGACTCAGC AGCTCTACCC TACCTGATGA CCACAGCAGT TACACCACTC

TGGACTCAGC AGCTCCACCC TGCCTGATGA CCACAGTGGC TACACTGCCC

TGGACTCAGT AGCTCCACCC TGCCTGATGA CCACAGCAAC TACACAGTTC

TGGACTCAGC ACCTCCACCC TGCCCGATGA CCACAGCAGC TACACAGCTC

TGGCCTCAGC AGCTCCACCC TGCCTGACGA CCACAGCAGC TACACAGCTC

TGGGCTCAGC AGCTCCACCC TGCCTGACGA TCACAGCAGC TACACGGCTC

TGGACTCAGC AGTTCCACCC TGCCTGATGA CCACAGCAGC TACACAGCTC

---------- ---------- ---------- ---------- ----------

CGGGCTCAGC AGCTCCACTC TGCCCGATGA CCACAGCGGC TACACAGCTC

TGGGCTTAGC AGTTCAACTC TGCCTGATGA CCACAGTGGA TACACAGCTC

AGCATTAAAC AGCTCCACAC TACCAGATGA CCACAACGGT TACACAGCTC

CAGGCTACAT GCAGGACTTG GAGGTGGAGC AGGCCCTGAC TCCAGCACTG

CAGGCTACAT GCAGGACTTG GAGGTGGAGC GGGCCCTGAC TCCAGCACTG

CGGGCTACAT GCAGGACTTG GAGGTGGAGC GGGCCCTGAC TCCAGCACTG

CGGGCTACAT GCAGGACTTG GAGGTGGAGC GGGCCCTGAC TCCAGCACTG

CGGGCTACAT GCAGGACTTG GAGGTGGAGC GGGCCCTGAC TCCAGCACTG

AGGGCTATCT GCAGGACTTG GAGGTGGAGC GGGCCCTGAC TCCAGCACTT

AGGGATACCT GCAGGACTTG GACATGGACA GGGACATTAC CCCAGCTCTG

AGGGCTACCT GCAGGACTTG GATATGGAAA GGGACATAAC TCCAGCACTG

AAGGCTACAT TCAGGACTCG GAAGTGGAAC AGGCCCTCAC TCCAGCACTG

GCAGCTACAT GCAGGACCTG GACATCGAAC GGACTCTCAC TCCAGCGCTG

ACGGCTACAT GCAGGATTTG GAGGTTGAGC GGGACCTTAC TCCAGCATTG

AGGGCTACAT GCAGGACATG GAGGTTGAAC GGGCCCTTAC TCCAGCACTG

AGGGCTACCT GCAGGACTTA GACATGGACA GGGCCCTCAC TCCAGAGCTC

AGGGATACCT GCAGGACTTG GACGTGGAAC AGGCCCTCAC TCCAGCACTG

---------- ---------- ---------- ---------- ----------

ACGTCTGCTT CGAGGACCTG GATATGGAAC AGGCACTCAC TCCAGCACTG

AGGTTTATCC TCAGGACATG GAAGTGGAAA GCTCCTTCAC TTTGGAACTC

ACAACTATGC CCAGGAAGCC GAGGTCGAGA GATCCATCAC TTTAGCCAGG

TCGCCATGTC CGGACAGCAC CAGTGATCTG TACAACTTCC AGGTGTCACC

TCGCCGTGTC CGGACAGCAC CAGTGATCTG TACAACTTCC AGGTGTCACC

TCGCCGTGTC CGGACAGCAC CAGTGATCTG TACAACTTCC AGGTGTCACC

TCGCCGTGTC CGGACAGCAC CAGTGATCTG TACAACTTCC AGGTGTCACC

TCGTCGTGTC CGGACAGCAC CAGTGATCTG TACAACTTCC AGGTGTCACC

TCCCCATGTC CAGATAGCAC CAGTGACCTG TACAGCTTCC AGGTGTCACC

TCACCGTGCC CAGACAGCAC CACTGATCTG TACAACTTGC AGGTGTCGCC

TCACCGTGTC CAGATAGCAC CACTGATCTG TATAACCTAC AGGTGTCACC

TCACCACATC CGGACAGCAC CAGTGACCTG TACAGCTTCC AGGTGTCTCC

TCACCGTGTC CAGACAGCAC CAGCGACCTG TACAACTTTC AGGTGTCGCC

TCACCGTGTC CGGACAGCAC CAGTGACCTC TACAGCTTCC AGGTGTCACC

TCACCATGTC CGGATAGCAC CAGCGACCTG TACAATTTCC AGGTGTCGCC

TCATCCTGTC CAGACAGTAC CAGTGACCTG TACAACTTCC AGGTGTCGCC

TCACCGTGTC CTGACAGCAC CAGTGAGCTG TATAACTTCC AGGTGTCGCC

---------- ---------- ---------- ---------- ----------

TCACCGTGTC CCGACAGCAC CAGTGACCTG TACAACTTAC AGGTGTCGCC

TCCTCCTGTC CTGATAGCAC CAATGACATG TACACCTTCC AGGTGTCGCC

AGGTTCAA-G CGGACAGCAA CAGCGAGCTC TCC-CCTTCC CCTTGCCACC

CATGCCCTCC ACCTCTGAAG CTATCAATAA ATCAGAGAAG CCAGAAAGCT

CATGCCCTCC ACCTCTGAAG CTATCAATAA ATCAGAGAAG CCAGAAAGCT

CATGCCCTCC ACCTCTGAAG CTATCAATAA ATCAGAGAAG CCAGAAAGCT

CATGCCCTCC ACCTCTGAAG CTATCAATAA ATCAGAGAAG CCAGAAAGCT

TATGCCCTCC ACTTCTGAAG CTATCAATAA ATCAGAGAAG CCAGAAAGCT

CATGCCCTCT GCCTCTGAAG CTATCAATAA ATCAGAGAAG CCAGAAAGCT

CATGCCCTCC ACCTCTGAAG CTATCAATAA ATCAGAGAAG CCAGAAAGCT

CATGCCTTCC ACCTCCGAAG CCATCAATAA GTCGGAGAAG CCGGAAAGCT

CATGCCCTCC GCCTCTGAAG CT-------- ---------- ----------

CATGCCCTCC ACCTCTGAAG CT-------- ---------- ----------

TATGCCCTCC ACTTCTGAAG CCATCAATAA ATCTGAGAAG CCTGAGAGCT

CATGCCCTCC ACCTCTGAAG CCATCAATAA ATCAGAGAAG CCGGAGAGCT

TATGCCTTCC TCTTCTGAAG CAATCAATAA ATCAGAGAAG CCAGAAAGCT

GATGCCCTCC ACCTCTGAAG CTATCAACAA ATCAGAGAAG CCGGAAAGTT

---------- ---------- --ATCAACAA GTCAGAGAAA CCGGAAAGCT

CATGCCTTCC ACTTCTGAAG ---------- ---------- ----------

ATTGCCCTCC AGCTCTGATG CAATCAACAA ATCAGAGAAG CCTGAAAGTT

AGAGCCCGGC TCCTCTGGAG CGATCGAAAA ACCAGAAAAA CCTGAAAGCT

GCGATAATGT GAAGGTTGTT GTTAGGTGCC GGCCCCTCAA TGAGAGAGAG

GCGATAATGT GAAGGTTGTT GTTCGGTGCC GGCCCCTCAA TGAGAGAGAG

GCGATAATGT GAAGGTTGTT GTTAGATGCC GGCCCCTCAA TGAGAGAGAG

GCGATAATGT GAAGGTTGTT GTTAGGTGCC GGCCCCTCAA TGAGAGAGAG

GCGATAATGT GAAGGTTGTT GTTAGGTGCC GGCCCCTCAA TGAGAGAGAG

GTGATAATGT GAAGGTCGTT GTTAGGTGCC GGCCCCTCAA TGAGAGAGAG

GCGACAACGT GAAGGTAGTT GTCAGGTGCC GACCTCTCAA CGAGAGAGAG

GCGATAATGT GAAGGTGGTG GTTAGGTGCC GGCCTCTCAA TGAGAGAGAG

---------- ---------- ---------- ---------- ----------

---------- ---------- ---------- ---------- ----------

GTGATAATGT GAAGGTCGTT GTTAGGTGCC GGCCCCTCAA CGAGAGAGAG

GTGACAACGT GAAGGTGGTG GTTAGGTGTC GGCCCCTCAA TGAGAGGGAG

GTGATAATGT GAAGGTGGTT GTTAGATGCC GGCCCCTCAA TGAGAGAGAG

GCGATAATGT GAAGGTCGTT GTTAGGTGCC GGCCCCTCAA TGAGAGAGAG

GCGACAACGT GAAGGTTGTT GTTAGGTGCC GGCCCCTCAA TGAGAGAGAG

---------- ---------- ---------- ---------- ----------

GCGACAATGT CAAAGTTGTT GTTCGATGTC GACCCTTCAA CGAGAGAGAA

GTGACAACGT GAAAGTTGTG GTTCGATGCC GTCCGTTTAA TGAGCGGGAG

AAATCAATGT GCTACAAACA GGCTGTCAGT GTGGATGAGA TGAGGGGAAC

AAATCAATGT GCTACAAACA TGCAGGCAGT GTGGATGAGA TGAGGGGAAC

AAATCAATGT GCTACAAACA GGCTGTCAGT GTGGATGAGA TGAGGGGAAC

AAATCAATGT GCTACAAACA GGCTGTCAGT GTGGATGAGA TGAGGGGAAC

AAATCAATGT GCTATAAACA AGCTGTCAGT GTGGATGAGA TGAGGGGAAC

AAATCAATGT GCTACAAACA GGCTGTCAGT GTGGATGAGA TGAGGGGAAC

AAATCAATGT GCTACAGACA GGCCGTCAGC GTGGATGAAA TGCGGGGGAC

AAGTCAATGT GCTATAGACA GGCCGTCAGC GTGGATGAAA TGAGGGGAAC

---------- ---------- ---------- ---------- ----------

---------- ---------- ---------- ---------- ----------

AAATCAATGT GCTATAAACA GGCTGTCAGT GTCGATGAGA TGAGGGGAAC

AAATCAATGT GCTACAAACA GGCTGTCAGT GTGGATGAGA TGAGGGGAAC

AAATCAATGT GCTACAGACA GGCTGTCAGT GTGGATGAAA TGAGGGGAAC

AAATCCATGT GCTACAAACA GGCCGTCAGT GTGGATGAGA TGAGGGGAAC

AAATCAATGT GCTATAAGCA GGCTGTCAGT GTGGATGAGA TGAGGGGAAC

---------- ---------- ---------- ---------- ----------

AAAGCAATGT GCTACAAATT GTCTGTTAAT GTGGATGAGA TGAGAGGAAC

AAGGCTATGT GTTACAAACT CTCCGTTAGC GTGGATGAGA TGAGGGGAAC

TATCACTGTA CATAAGACTG ATTCTTCCAA TGAACCTCCA AAGACATTTA

TATCACTGTA CATAAGACTG ATTCTTCCAA TGAACCTCCA AAGACA-TTA

TATCACTGTA CATAAGACTG ATTCTTCCAA TGAACCTCCG AAGACATTTA

TATCACTGTA CATAAGACTG ATTCTTCCAA TGAACCTCCG AAGACATTTA

TATCACTGTA CATAAAACTG ACTCTTCCAA TGAACCTCCG AAGACATTTA

TATCACTGTA CACAAGACTG ATTCTTCCAA TGAACCTCCA AAGACATTTA

TATCACCGTC CATAAGACCG ACTCTTCCAA CGAACCTCCG AAGACATTTA

TATCACCGTC CATAAGACCG ATTCCTCCAA CGAACCTCCA AAGACATTTA

---------- ---------- ---------- ---------- ----------

---------- ---------- ---------- ---------- ----------

TATTACTGTA CATAAGACTG ATTCTTCCAA TGAACCTCCA AAGACATTTA

TATCACTGTG CATAAGACTG ATTCTTCCAA TGAACCTCCA AAGACATTTA

TATCACTGTT CATAAGACTG ACTCTTCCAA TGAACCTCCA AAGACATTTA

TATCACTGTA CATAAGACTG ATTCTTCCAA TGAACCTCCG AAGACATTTA

TATCACTGTA CATAAGACTG ATTCTTTCAA TGAACCTCCA AAGACATTTA

---------- ---------- ---------- ---------- ----------

TATTACTGTG CACAAAACAG ATTCTTCCAA CGAACCTCCA AAGACATTTA

AATTACAGTG AATAAAACAG ATTCTGCCAA TGAACCTCCA AAGACATTTA

CTTTTGATAC TGTTTTTGGA CCAGAGAGTA AACAACTTGA TGTTTATAAC

CTTTTGATAC TGTTTTTGGA C-AGAGAGTA AACAACTTGA TGTTTATAAC

CTTTTGATAC TGTTTTTGGA CCAGAGAGTA AACAACTTGA TGTTTATAAC

CTTTTGATAC TGTTTTTGGA CCAGAGAGTA AACAACTTGA TGTTTATAAC

CTTTTGATAC CGTTTTTGGA CCAGAGAGTA AACAACTTGA TGTTTATAAC

CTTTTGATAC TGTTTTTGGA CCAGAGAGTA AACAACTTGA TGTTTATAAC

CTTTCGATAC TGTTTTTGGA CCAGAGAGTA AACAACTTGA TGTATATAAC

CTTTCGATAC TGTTTTTGGA CCAGAAAGTA AACAACTTGA TGTGTATAAC

---------- ---------- ---------- ---------- ----------

---------- ---------- ---------- ---------- ----------

CTTTTGATAC TGTTTTTGGA CCAGAGAGTA AACAACTTGA TGTTTATAAC

CTTTTGATAC TGTTTTTGGA CCAGAGAGTA AACAGCTTGA TGTGTATAAC

CTTTTGATAC TGTTTTTGGA CCGGAGAGTA AACAACTTGA TGTTTATAAC

CTTTTGATAC TGTTTTTGGA CCAGAGAGTA AACAACTTGA TGTCTATAAC

CTTTTGATAC GGTTTTTGGA CCAGAGAGTA AACAACTTGA TGTTTATAAC

---------- ---------- ---------- ---------- ----------

CATTTGACAC AGTTTTTGGA CCAGATAGTA AACAACTTGA TGTTTACAAC

CGTTTGACAC TGTTTTTGGC CCAGAGAGTA AACAGCTCGA TGTCTATAAC

TTAACTGCAA GACCTATTAT TGATTCTGTA CTTGAAGGCT ACAATGGGAC

TTAACTGCAA GACCTATTAT TGATTCTGTA CTTGAAGGCT ACAATGGGAC

TTAACTGCAA GACCTATTAT TGATTCTGTA CTTGAAGGCT ACAATGGGAC

TTAACTGCAA GACCTATTAT TGATTCTGTA CTTGAAGGCT ACAATGGGAC

TTAACTGCAA GACCTATTAT TGATTCTGTA CTTGAAGGCT ACAATGGGAC

TTAACTGCAA GACCTATTAT TGATTCTGTT CTTGAAGGCT ACAATGGAAC

TTGACTGCAA GACCAATTAT TGATTCTGTC CTGGAAGGCT ACAATGGGAC

TTAACTGCAA GACCAATTAT TGATTCTGTT CTGGAAGGCT ACAATGGGAC

---------- ---------- ---------- ---------- ------GGAC

---------- ---------- ---------- ---------- ----------

TTAACTGCAA GACCTATTAT TGATTCTGTT CTTGAAGGCT ACAATGGGAC

TTAACGGCAA GACCTATTAT TGATTCTGTG CTTGAAGGCT ACAATGGGAC

TTAACTGCAA GACCCATTAT TGATTCTGTT CTTGAAGGCT ACAATG----

TTAACTGCAA GGCCTATTAT TGATTCTGTT CTTGAAGGTT ACAATGGGAC

TTAACCGCAA GGCCTATTAT TGATTCAGTT CTTGAAGGCT ACAATGGAAC

---------- ---------- -------gtt ---------- ----TGGAAC

CTAACAGCAA GACCCATTAT TGATTCTGTG CTAGAAGGCT ACAATGGTAC

TTGACTGCAA GGCCTATTAT TGACTCTGTT CTGGAAGGCT ATAATGGAAC

TATTTTTGCA TATGGACAAA CCGGAACAGG CAAAACTTTT ACCATGGAAG

TATTTTTGCA TATGGACAAA CCGGAACAGG CAAAACTTTT ACCATGGAAG

TATTTTTGCA TATGGACAAA CTGGAACAGG CAAAACTTTT ACCATGGAAG

TATTTTTGCA TATGGACAAA CTGGAACAGG CAAAACTTTT ACCATGGAAG

TATTTTTGCA TATGGACAAA CTGGAACAGG CAAAACTTTT ACCATGGAAG

TATTTTTGCA TATGGACAAA CTGGAACAGG CAAAACTTTT ACCATGGAAG

CATTTTTGCA TATGGACAGA CTGGGACAGG CAAGACTTTC ACCATGGAAG

CATCTTTGCA TATGGACAGA CTGGGACAGG CAAGACTTTC ACCATGGAAG

TATTTTTGCA TATGGTCAAA CTGGAACAGG CAAAACTTTC ACCATGGAAG

---------- ---------- ---------- ---------- ----------

TATTTTTGCA TATGGACAAA CTGGAACAGG CAAAACTTTT ACCATGGAAG

TATTTTTGCA TATGGACAAA CTGGAACAGG CAAAACGTTT ACCATGGAAG

---------- ---------- ---------- ---------- ----------

TATTTTTGCT TATGGACAAA CTGGAACAGG CAAAACTTTT ACCATGGAAG

TATTTTTGCA TATGGACAAA CTGGAACAGG AAAAACTTTT ACCATGGAAG

GATTTTTGCA TATGGACAAA CGGGAACAGG CAAAACCTTC ACCATGGAAG

TATATTTGCA TATGGACAGA CTGGAACTGG CAAAACATTT ACCATGGAAG

TATTTTTGCA TATGGACAGA CTGGAACAGG CAAAACCTTT ACAATGGAAG

GTGTTCGAGC TATTCCTGAA CTTAGAGGAA TAATTCCCAA TTCATTTGCT

GTGTTCGAGC TATTCCTGAA CTTAGAGGAA TAATTCCCAA TTCATTTGCT

GTGTTCGAGC TGTTCCTGAA CTTAGAGGAA TAATTCCCAA TTCATTTGCT

GTGTTCGAGC TGTTCCTGAA CTTAGAGGAA TAATTCCCAA TTCATTTGCT

GTGTTCGAGC TGTTCCTGAA CTTAGAGGAA TAATTCCCAA TTCATTTGCT

GTGTTCGAGC TGTTCCTGAA CTTAGAGGAA TAATTCCCAA TTCATTTGCT

GTGTTCGAGC AGTGCCGGGG CTCAGAGGGG TCATCCCCAA CTCATTTGCT

GCGTTCGAGC AGTACCGGGG CTCAGAGGGG TCATCCCCAA CTCATTTGCT

GTGTTCGAGC TGTTCCTGAA CTTAGAGGAA TCATTCCCAA TTCATTTGCT

---------- ---------- ---------- ---------- ----------

GTGTTCGAGC AGTTCCTGAA CTTAGAGGAA TTATTCCAAA TTCATTTGCT

GTGTTCGAGC TGTTCCTGAA CTTAGAGGAA TCATTCCAAA TTCATTTGCT

---------- ---------- ---------- ---------- ----------

GTGTTCGAGC TGTTCCTGAA CTTAGAGGAA TCATTCCAAA TTCATTTGCT

GTGTTCGTGC TGTTCCCGAA CTTAGAGGAA TCATTCCAAA TTCATTTGCT

GTGTTCGAGC TGTTCCTGAA CTCAGAGGAA TCATTCCAAA CTCATTCGCT

GTGTACGAGC TGTACCTGAA CTTAGAGGAA TTATCCCAAA TTCATTTGCT

GTGTGCGAGC TGTGCCTGAA CTTAGAGGAA TCATCCCAAA TTCATTTGCC

CACATATTTG GTCATATTGC AAAAGCGGAG GGTGATACAA GATTTTTGGT

CACATATTTG GTCATATTGC AAAAGCGGAG GGTGATACAA GATTTTTGGT

CACATATTTG GTCATATTGC AAAAGCAGAG GGTGATACAA GATTTTTGGT

CACATATTTG GTCATATTGC AAAAGCAGAG GGTGATACAA GATTTTTGGT

CACATATTTG GTCATATTGC AAAAGCAGAG GGTGATACAA GATTTTTGGT

CACATATTTG GTCATATTGC AAAAGCAGAG GGTGACACAA GATTTTTGGT

CACATATTTG GTCATATCGC AAAAGCAGAG GGCGACACAA GGTTTCTGGT

CACATATTTG GTCACATCGC AAAAGCAGAG GGCGACACAA GGTTTTTGGT

CATATATTTG GTCATATTGC AAAGGCAGAG GGTGACACAA GGTTTTTGGT

---------- ---------- ---------- ---------- ----------

CACATATTTG GTCACATTGC TAAAGCAGAG GGTGATACAA GATTTTTGGT

CACATATTTG GTCATATTGC AAAAGCAGAG GGCGACACAA GATTTTTGGT

---------- ---------- ---------- ---------- -ATTTTTGGT

CACATATTTG GTCATATTGC AAAAGCAGAG GGTGATACAA GATTTTTGGT

CACATATTTG GTCATATTGC AAAGGCAGAG GGTGATACAA GATTTTTGGT

CACATATTTG GTCATATTGC AAAAGCAGAA GGCGATACAA GATTTCTGGT

CATATATTTG GTCATATTGC AAAAGCAGAG GGGGATACAA GGTTTTTGGT

CATATTTTTG GGCATATTGC AAAAGCAGAG GGGGACACAA GATTTCTGGT

TCGAGTGTCT TATTTGGAAA TATATAATGA AGAAGTTCGT GACCTTTTGG

TCGAGTGTCT TATTTGGAAA TATATAATGA AGAAGTTCGT GACCTTTTGG

TCGAGTGTCT TATTTGGAAA TATATAATGA AGAAGTTCGT GACCTTTTGG

TCGAGTGTCT TATTTGGAAA TATATAATGA AGAAGTTCGT GACCTTTTGG

TCGAGTGTCT TATTTGGAAA TATATAATGA AGAAGTTCGT GACCTTTTGG

TCGTGTGTCT TATTTGGAAA TATATAATGA AGAAGTTCGT GACCTTTTGG

TCGAGTGTCA TACTTAGAGA TATATAATGA AGAAGTCCGA GACCTTCTGG

TCGAGTGTCA TACTTGGAGA TATATAATGA AGAAGTTCGA GACCTTTTGG

TCGAGTTTCT TATTTGGAAA TATATAATGA AGAAGTTCGT GACCTTTTGG

---------- ---------- ---------- ---------- ----------

TCGAGTGTCA TATTTGGAAA TATATAATGA GGAAGTCCGT GACCTTTTGG

TCGAGTGTCC TATTTGGAAA TATATAATGA AGAAGTTCGT GACCTTTTGG

TCGAGTGTCT TATTTGGAAA TATATAATGA AGAAGTTCGT GATCTTTTGG

TCGAGTATCT TATTTGGAAA TATATAATGA AGAAGTTCGT GACCTTTTGG

TCGAGTGTCT TATTTGGAAA TATATAATGA AGAGGTTCGT GACCTTTTGG

TCGAGTATCT TATTTGGAAA TATATAATGA AGAAGTTCGT GACCTTTTGG

TCGAGTATCT TATTTGGAAA TTTATAATGA AGAAGTGCGT GACCTTCTAG

GCGAGTATCC TATTTGGAAA TTTATAACGA AGAAGTGCGT GACCTGCTAG

GCAAGGATCA GACACAAAGG TTAGAGGTTA AAGAAAGACC TGATGTGGGA

GCAAGGATCA GACACAAAGG TTAGAGGTTA AAGAAAGACC TGATGTGGGA

GCAAGGATCA GACACAAAGG TTAGAGGTTA AAGAAAGACC TGATGTGGGA

GCAAGGATCA GACACAAAGG TTAGAGGTTA AAGAAAGACC TGATGTGGGA

GAAAGGATCA GACACAAAGG TTAGAGGTTA AAGAAAGACC TGATGTGGGA

GCAAGGATCA GACACAAAGG TTAGAGGTTA AAGAAAGACC TGATGTGGGA

GTAAAGATCA GACACAGAGG TTGGAGGTTA AAGAAAGGCC TGACGTGGGA

GTAAAGATCA GACCCAGAGG TTAGAGGTTA AAGAAAGGCC TGATGTGGGA

GCAAGGATCA GACACAGAGG TTAGAGGTTA AAGAAAGACC TGATGTGGGA

---------- ---------- ---------- ---------- ----------

GCAAGGATCA GACACAGAGG TTAGAGGTTA AAGAAAGACC TGATGTGGGA

GCAAGGATCA GACACAAAGG TTAGAGGTTA AAGAAAGACC TGATGTGGGA

GCAAGGATCA GACACAGAGA TTAGAGGTTA AAGAAAGACC TGATGTAGGA

GCAAGGATCA GACACAGAGG TTAGAGGTTA AAGAAAGACC TGATGTGGGA

GCAAGGACCA GACACAGAGG TTAGAGGTTA AAGAAAGACC TGATGTGGGA

GCAAGGATCA AACACAGAGG TTAGAGGTTA AAGAAAGACC TGATGTGGGA

GAAAGGATCA GACACAAAGA TTAGAGGTTA AAGAAAGACC TGATGTAGGA

GAAAAGACCA GACCCAGAGA TTAGAGGTTA AAGAAAGACC TGATGTGGGA

GTTTATATCA AAGATTTATC AGCTTATGTG GTAAATAATG CTGATGATAT

GTTTATATCA AAGATTTATC AGCTTATGTG GTAAATAATG CTGATGATAT

GTTTATATCA AAGATTTATC AGCTTATGTG GTAAATAATG CTGATGATAT

GTTTATATCA AAGATTTATC AGCTTATGTG GTAAATAATG CTGATGATAT

GTTTATATCA AAGATTTATC AGCTTATGTG GTAAATAATG CTGATGATAT

GTTTATATCA AAGATTTATC AGCTTATGTG GTAAATAATG CTGATGATAT

GTATATATCA AAGATTTATC AGCATATGTA GTCAACAATG CTGACGACAT

GTATATATCA AAGACTTATC AGCATATGTA GTCAACAACG CTGACGACAT

GTTTATATCA AAGATTTATC AGCTTATGTT GTAAACAATG CTGATGATAT

---------- ---------- ---------- ---------- ----------

GTTTATATCA AAGATTTATC GGCTTACGTG GTAAATAATG CTGATGATAT

GTTTATATCA AAGATTTATC AGCTTACGTG GTAAATAATG CCGATGATAT

GTTTATATCA AAGATTTATC AGCTTACGTG GTAAATAATG CTGATGATAT

GTTTATATCA AAGATTTATC AGCTTATGTA GTAAATAATG CTGATGATAT

GTTTATATCA AAGATTTATC AGCTTACGTG GTAAATAATG CTGATGATAT

GTTTATATTA AAGATTTGTC AGCTTACGTG GTAAATAATG CCGATGATAT

GTTTACATCA AAGATCTGTC AGCTTATGTG GTAAATAATG CAGATGATAT

GTTTACATCA AAGATCTGTC AGCTTATGTT GTGAACAATG CAGATGATAT

GGATAGAATT ATGACGCTAG GCCACAAAAA TCGTTCTGTT GGTACTAATA

GGATAGAATT ATGACGCTAG GCCACAAAAA TCGTTCTGTT GGTACTAATA

GGATAGAATT ATGACACTAG GCCACAAAAA TCGTTCTGTT GGTACTAATA

GGATAGAATT ATGACACTAG GCCACAAAAA TC-------- ----------

GGATAGAATT ATGACACTAG GCCATAAAAA TCGTTCTGTT GGTACTAATA

GGATAGAATT ATGACACTAG GCCACAAAAA CCGTTCTGTT GGTACTAATA

GGATAGAATC ATGACTCTGG GCCACAAAAA CCGTTCCGTT GGTACCAATA

GGATAGAATC ATGACTCTAG GCCACAAAAA CCGTTCTGTC GGTACTAATA

GGATAGGATT ATGACTCTAG GCCACAAAAA TC-------- ----------

---------- ---------- ---------- ---------- ----------

GGATAGAATT ATGACACTAG GCCACAAAAA TCGTTCTGTT GGTACTAATA

GGATAGAATT ATGACACTAG GCCACAAAAA TCGTTCTGTT GGCACTAATA

GGATAGAATT ATGACACTAG GCCACAAAAA TCGTTCTGTT GGTACTAATA

GGATAGAATT ATGACACTAG GGCACAAAAA TCGTTCTGTT GGTACTAATA

GGATAGAATT ATGACACTAG GCCACAAAAA TCGTTCTGTT GGTACTAATA

GGACAGGATT ATGACATTAG GCCACAAGAA TCGTTCTGTT GGTACTAATA

GGATAGAATT ATGACATTAG GCCACAAAAA TCAAACCGTT GATACTAACA

GGATAGAATT ATGACGTTGG GCCACAAAAA TCGCTCCGTC GGCACCAATA

TGAACGAAGA TCTTGCTGGT TCAGAAAGAC AGGCAAAAAC TGGAGCTACT

TGAACGAAGA TCTTGCTGGT TCAGAAAGAC AGGCAAAAAC TGGAGCTACT

TGAATGAAGA TCTTGCTGGT TCAGAAAGGC AGGCAAAAAC TGGAGCTACT

---------- -------GGT TCAGAAAGGC AGGCAAAAAC TGGAGCTACT

TGAACGAAGA TCTTGCT--- ---------- ---------- -----CTACT

TGAATGAAGA TCTTGCTGGT TCAGAAAGAC AAGCAAAAAC TGGAGCTACT

TGAACGAGGA TCTTGCTGGG TCAGAAAGAC AAGCAAAAAC TGGAGCCACA

TGAACGAGGA TCTGGCTggg tcagaaagac aagcaaaAAC TGGAGCCACA

---------- -------GGT TCAGAAAGAC AAGCAAAAAC TGGAGCTACT

---------- ---------- ---------- ---------- ----------

TGAATGAAGA TCTTGCTGGT TCAGAAAGAC AAGCAAAAAC TGGAGCAACT

TGAACGAAGA TCTTGCTGGT TCAGAAAGAC AAGCAAAAAC TGGAGCTACT

TGAACGAAGA TCTTGCTGGT TCAGAAAGAC AAGCAAAAAC TGGAGCTACT

TGAATGAAGA TCTTGCTGGT TCAGAAAGAC AAGCAAAAAC TGGAGCTACT

TGAATGAAGA TCTTGCTGGT TCAGAAAGGC AAGGAAAAAC TGGAGCTACT

TGAATGAGGA TCTTGCGGGT TCAGAAAGAC AAGCAAAAAC TGGAGCTACT

TGAA--A--- TTTTAATGGT TCAGAAAGAC AGGCAAAAAC TGGAGCTACT

TGAATGAAGA TCTGGCGGGA TCAGAAAGGC AGGCGAAGAC TGGAGCTACA

GGACAGCGCC TAAAGGAAGC TACAAAAATC AATCTTTCAC TTTCCACCCT

GGACAGCGCC TAAAGGAAGC TACAAAAATC AATCTTTCAC TTTCCACCCT

GGACAGCGCC TAAAGGAAGC TACAAAAATC AATCTTTCAC TTTCCACCCT

GGACAGCGCC TAAAGGAAGC TACAAAAATC AATCTTTCAC TTTCCACCCT

GGACAGCGCC TAAAGGAAGC TACAAAAATC AATCTTTCAT TTTCCACCCT

GGACAACGCC TAAAGGAAGC TACAAAAATC AATCTTTCAC TTTCCACCCT

GGGCAGCGCC TCAAAGAAGC TACCAAAATC AACCTTTCAT TGTCTACCTT

GGACAGCGCC TCAAGGAAGC TACCAAAATC AACCTTTCAT TGTCTACCCT

GGACAGCGCT TAAAGGAAGC TACAAAAATT AATCTTTCTC TTTCTACGCT

---------- ---------- ---------- ---------- ----------

GGACAGCGCC TAAAAGAAGC CACAAAAATC AATCTTTCGC TTTCCACCCT

GGACAGCGCC TGAAGGAAGC TACAAAAATC AACCTTTCGC TTTCCACCCT

GGACAGCGCC TAAAGGAAGC TACAAAAATC AATCTTTCAC TTTCTACCCT

GGACAGCGCC TAAAGGAAGC TACAAAAATC AATCTTTCAC TTTCCACCCT

GGGCAACGCC TAAAGGAAGC TACAAAAATC AATCTTTCAC TTTCCACCCT

GGGCAGCGAC TAAAGGAAGC CACAAAAATC AATCTTTCCC TGTCCACCCT

GGACAACGAC TGAAAGAAGC AACGAAAATT AACCTTTCAC TTTCCACTCT

GGGCAGCGGC TAAAGGAAGC AACCAAAATT AACCTTTCCC TTTCCACCCT

TGGTAATGTA ATTTCTGCCT TGGTTGATGG AAAAAGCACT CATGTGCCTT

TGGTAATGTA ATTTCTGCCT TGGTTGATGG AAAAAGCACT CATGTGCCTT

TGGTAATGTA ATTTCTGCCT TGGTTGATGG AAAAAGCACT CATGTGCCTT

TGGTAATGTA ATTTCTGCCT TGGTTGATGG AAAAAGCACT CATGTGCCTT

TGGTAATGTA ATTTCTGCCT TGGTTGATGG AAAAAGCACT CATGTGCCTT

TGGTAATGTA ATTTCTGCCT TGGTTGATGG AAAAAGCACT CATGTGCCTT

GGGCAATGTC ATTTCTGCCT TGGTGGATGG AAAAAGCACT CATGTGCCGT

GGGCAACGTT ATTTCTGCCT TGGTGGATGG AAAAAGCACT CACGTGCCGT

TGGCAATGTA ATTTCTGCTT TGGTTGATGG GAAAAGTACA CATGTGCCTT

---------- ---------- ---------- ---------- ----------

TGGTAATGTA ATTTCTGCCT TGGTTGACGG AAAAAGCACT CATGTTCCAT

TGGCAATGTG ATTTCTGCCT TGGTTGATGG AAAAAGCACT CATGTGCCTT

TGGTAATGTA ATTTCTGCCT TGGTTGATGG AAAAAGCACT CATGTGCCTT

TGGTAATGTA ATTTCTGCCT TGGTTGATGG AAAAAGCACT CATGTGCCTT

TGGTAATGTA ATTTCTGCCT TGGTTGATGG AAAAAGCACT CATGTGCCTT

CGGGAACGTC ATCTCTGCGC TAGTCGATGG AAAGAGCACC CACGTGCCGT

GGGTAATGTA ATTTCTGCCT TGGTTGATGG GAAAAGCACT CATGTACCAT

TGGCAATGTA ATCTCCGCTT TGGTGGATGG GAAAAGTACC CACGTGCCCT

ATCGTAACTC TAAACTGACT CGTCTTCTTC AGGATTCCTT AGGAGGAAAT

ATCGTAACTC TAAACTGACT CGTCTTCTTC AGGATTCCTT AGGAGGAAAT

ATCGTAACTC TAAATTGACT CGTCTTCTTC AGGATTCCTT AGGAGGAAAT

ATCGTAACTC TAAATTGACT CGTCTTCTTC AGGATTCCTT AGGAGGAAAT

ATCGTAACTC GAAATTGACT CGTCTTCTTC AGGATTCCTT AGGAGGAAAT

ATCGTAACTC TAAATTGACA CGTCTTCTTC AGGATTCTTT AGGAGGAAAT

ATCGGAACTC TAAACTGACA CGTCTCCTGC AGGATTCCTT AGGAGGGAAC

ATCGGAACTC CAAACTGACC CGTCTCCTGC AGGACTCCTT AGGAGGGAAC

ACCGTAACTC TAAATTGACT CGACTTCTTC AGGATTCCTT AGGAGGAAAT

---------- ---------- ---------- ---------- ----------

ATCGCAACTC AAAACTGACT CGTCTTCTTC AGGATTCCTT AGGAGGAAAT

ATCGTAACTC TAAATTGACT CGTCTTCTTC AGGACTCCTT AGGAGGAAAT

ATCGTAACTC TAAATTGACC CGTCTTCTTC AGGATTCCTT AGGAGGAAAT

ATCGTAACTC TAAATTGACT CGTCTTCTTC AGGATTCCTT AGGAGGAAAT

ATCGTAACTC TAAGTTGACT CGTCTTCTTC AGGATTCCTT AGGAGGAAAT

ATCGTAACTC TAAGTTGACT CGCCTCCTTC AAGACTCTTT AGGAGGAAAC

ATCGAAATTC AAAATTGACT CGCCTTCTTC AGGACTCCTT AGGAGGAAAC

ATCGTAATTC AAAATTGACC CGTCTTCTTC AGGATTCCCT AGGAGGCAAC

TCAAAAACCA TGATGTGTGC AAATATTGGG CCAGCAGATT ACAATTATGA

TCAAAAACCA TGATGTGTGC AAATATTGGG CCAGCAGATT ACAATTATGA

TCAAAAACCA TGATGTGTGC AAATATTGGG CCAGCAGATT ACAATTATGA

TCAAAAACCA TGATGTGTGC AAATATTGGG CCAGCAGATT ACAATTATGA

TCAAAAACTA TGATGTGTGC AAATATTGGG CCAGCAGATT ACAATTATGA

TCAAAAACCA TGATGTGTGC AAATATTGGG CCAGCAGATT ACAATTATGA

TCAAAAACCA TGATGTGTGC AAATATTGGG CCAGCAGATT ATAATTATGA

TCAAAAACCA TGATGTGTGC AAATATTGGG CCAGCAGATT ATAATTATGA

TCAAAAACCA TGATGTGTGC AAATATTGGG CCAGCAGATT ATAATTATGA

---------- ---------- ---------- ---------- ----------

TCAAAAACCA TGATGTGTGC AAATATTGGG CCAGCAGATT ACAATTATGA

TCAAAAACCA TGATGTGTGC AAATATTGGG CCAGCAGATT ACAATTATGA

TCAAAAACCA TGATGTGTGC AAATATTGGG CCAGCCGACT ACAATTACGA

TCAAAAACCA TGATGTGTGC AAATATTGGG CCAGCAGATT ACAATTATGA

TCAAAAACCA TGATGTGTGC AAATATTGGG CCAGCAGATT ACAATTATGA

TCAAAAACCA TGATGTGTGC AAATATTGGG CCAGCAGATT ACAATTATGA

TCAAAAACCA TGATGTGTGC AAATATTGGG CCTGCAGACT ACAATTATGA

TCAAAAACCA TGATGTGTGC AAATATCGGT CCAGCAGACT ACAATTACGA

TGAAACTATC AGTACATTAC GGTATGCCAA TCGTGCTAAG AATATTAAAA

TGAAACTATC AGTACATTAC GGTATGCCAA TCGTGCTAAG AATATTAAAA

TGAAACGATC AGTACATTAC GGTATGCCAA TCGTGCTAAG AATATTAAAA

TGAAACGATC AGTACATTAC GGTATGCCAA TCGTGCTAAG AATATTAAAA

TGAAACTATC AGTACATTAC GGTATGCCAA TCGTGCTAAG AATATTAAAA

TGAAACTATC AGTACATTAC GGTATGCTAA CCGTGCTAAG AATATTAAAA

TGAAACCATC AGTACCTTGC GGTATGCCAA CCGCGCCAAG AACATTAAAA

TGAAACTATC AGCACCTTGC GGTATGCCAA CCGCGCCAAG AACATTAAAA

TGAAACTATT AGTACATTAC GGTATGCCAA CCGTGCTAAG AATATTAAAA

---------- ---------- ---------- ---------- ----------

TGAAACTATC AGTACATTGC GGTATGCTAA CCGTGCTAAG AATATTAAAA

TGAAACTATC AGTACATTAC GGTATGCTAA CCGTGCTAAG AATATTAAAA

CGAAACTATC AGTACGTTAC GGTACGCCAA CCGTGCTAAG AATATTAAAA

TGAAACTATC AGTACATTAC GGTATGCCAA CCGTGCTAAA AATATTAAAA

TGAAACTATC AGTACTTTAC GCTATGCCAA CCGTGCTAAA AATATTAAAA

TGAAACTATC AGTACGTTAC GGTATGCCAA CCGTGCTAAA AATATCAAAA

TGAGACTATC AGTACATTGC GATATGCTAA CCGTGCTAAA AATATTAAGA

CGAGACCATC AGTACATTAC GGTACGCTAA CCGTGCTAAA AATATCAAGA

ATAAAGCTAG AATTAATGAA GATCCAAAGG ATGCTTTGCT GCGTCAGTTC

ATAAAGCTAG AATTAATGAA GATCCAAAGG ATGCTTTGCT GCGTCAGTTC

ATAAAGCTAG AATTAATGAA GATCCAAAGG ATGCTTTGCT GCGTCAGTTC

ATAAAGCTAG AATTAATGAA GATCCAAAGG ATGCTTTGCT GCGTCAGTTC

ATAAAGCTAG AATTAATGAA GATCCAAAGG ATGCTTTGCT GCGTCAGTTC

ATAAAGCTAG AATTAATGAA GATCCAAAGG ATGCTCTGCT TCGTCAGTTT

ACAAAGCTAG AATTAACGAA GACCCAAAGG ACGCTCTGCT TCGCCAGTTT

ACAAAGCGAG AATCAATGAG GACCCAAAGG ATGCTCTGCT CCGCCAGTTT

ATAAAGCTAG AATTAATGAA GATCCAAAGG ATGCTTTGCT CCGTCAGTTT

---------- ---------- ---------- ---------- ----------

ATAAAGCTAG AATTAATGAA GATCCAAAAG ATGCTTTGCT TCGCCAGTTT

ATAAAGCTAG AATTAATGAA GATCCAAAAG ATGCTTTGCT TCGTCAATTT

ATAAAGCTAG AATTAATGAA GATCCAAAGG ATGCTTTGCT TCGTCAATTT

ATAAAGCTAG AATTAATGAA GATCCAAAGG ATGCTTTACT TCGTCAGTTT

ATAAAGCTAG AATTAATGAA GATCCAAAGG ATGCTTTGCT TCGTCAGTTT

ATAAAGCTAG AATTAATGAA GATCCAAAGG ATGCTTTACT TCGTCAATTT

ATAAAGCTAG AATTAATGAA GATCCAAAGG ATGCTTTGCT TCGGCAGTTT

ACAAGGCTAG AATTAATGAG GATCCAAAGG ACGCTTTGCT TCGCCAGTTT

CAGAAAGAAA TAGAAGAACT GAAAAAGAAG CTTGAAGAAG GAGAAGAAAT

CAGAAAGAAA TAGAAGAACT GAAAAAGAAG CTTGAAGAAG GAGAAGAAAT

CAGAAAGAAA TAGAAGAACT GAAAAAGAAA CTTGAAGAAG GAGAAGAAAT

CAGAAAGAAA TAGAAGAACT GAAAAAGAAA CTTGAAGAAG GAGAAGAAAT

CAGAAAGAAA TAGAAGAACT GAAAAAGAAG CTTGAAGAAG GAGAAGAAAT

CAGAAAGAAA TAGAAGAACT GAAAAAAAAG CTCGAAGAAG GGGAAGAAAT

CAGAAAGAAA TAGAAGAGCT GAAGAAAAAG CTTGAGGAAG GGGAAGAAGT

CAGAAAGAAA TCGAAGAGCT GAAGAAAAAG CTTGAGGAAG GGGAAGAAGT

CAGAAAGAAA TAGAAGAATT GAAGAAAAAG CTTGAAGAAG GGGAAGAAAT

---------- ---------- ---------- ---------- ----------

CAGAAAGAAA TAGAAGAACT GAAAAAGAAG CTTGAAGAAG GGGAAGAAAT

CAGAAAGAAA TAGAAGAATT GAAAAAAAAG CTTGAAGAAG GGGAAGAAAT

CAGAAAGAAA TAGAAGAACT GAAAAAAAAA CTTGAGGAAG GGGAAGAAAT

CAGAAAGAAA TAGAAGAACT GAAAAAAAAG CTTGAAGAAG GAGAAGAAAT

CAGAAAGAAA TAGAAGAACT TAAAAAAAAG CTTGAAGAAG GGGAAGAAAT

CAGAAAGAAA TAGAAGAACT GAAAAAAAAA CTTGAAGAAG GGGAAGAAAT

CAGAAAGAAA TAGAAGAACT CAAAAAAAAG CTTGAAGAAG GGGAAGAGAT

CAGAAAGAAA TAGAAGAACT CAAAAAAAAG CTTGAAGAAG GTGAAGAAAT

ATCAGGCTCT GATATCAGTG GGTCAGAGGA AGATGATGAT GAGGGTGAGG

ATCAGGCTCT GATATCAGTG GGTCAGAGGA AGATGATGAT GAGGGTGAGG

ATCAGGCTCT GATATCAGTG GGTCagagga agatgatgat gaaggtgagg

ATCAGGCTCT GATATCAGTG GGTCagagga agatgatgat gaaggtgagg

ATCAGGCTCT GATATAAGTG GATCAGAGGA AGATGATGAT GAGGGTGAGG

ATCAGGCTCT GATATCAGTG GGTCAGAGGA AGATGATGAT GATGGTGAGA

GTCAGGCTCT GACATCAGTG GGTCAGAGGA GGATGACAAC GAGGGTGAAC

GTCAGGCTCT GACATCAGTG GGTCAGAGGA GGATGAC--- GAGGGTGAAC

ATCGGGCTCT GATATCAGTG GTTCAGGGGA GGAGGATGAT GAGGGTGAGG

---------- ---------- ---------- ---------- ----------

ATCAGGTTCT GATATCAGTG GGTCGGAGGA AGAGGATGAT GAGGGTGAGG

ATCAGGCTCT GATATCAGTG GGTCAGAGGA GGATGATGAT GAGGGTGAAG

ATCAGGCTCT GATATCAGTG GTTCagagga agatgatgaa gaaggtgagg

ATCAGGCTCC GATATCAGTG GATCAGAGGA GGAAGACGAT GAGGGTGAGA

ATCTGGATCT GATATCAGTG AGTCagagga agaagatgat gaggatgaag

ATCAGGATCT GATATCAGTG GGTCAgagga ggaggaggat gagggtgaag

TTCAGGCTCT GATGTCAGTG GATCAGAAGA TGATCAAGAT GAGGCTGAGG

CTCAGGCTCC GATATCAGTG GATCAGATGA GGAAGATGAC GAGGGGGAAG

TTGGAGAAGA TGGAGAGAAA AGGAAAAAAA GAAGGGATCA AGCAGGAAAA

TTGGAGAAGA TGGAGAGAAA AGGAAAAAAA GAAGGGATCA AGCAGGAAAA

ttggagaaga tggagagaaa aggaaaaaaa gaagGGATCA AGCAGGAAAA

ttggagaaga tggagagaaa aggaaaaaaa gaagGGATCA AGCAGGAAAA

TTGGAGAAGA TGGAGAGAAA AGGAAAAAA- GAAAGG---- -----GTAAA

TTGGAGAAGA TGGAGAGAAA AGGAAAAAAA GAAGAGATCA AGCAGGTAAA

TTGGAGAAGA TGGAGAAAAA AGGAAAAAGA GAAGGGACCA AGCAGGTAAA

TTGGAGAAGA TGGAGAAAAA AAGAAAAAGA GAAGGGACCA AGCAGGTAAA

TTGGAGAAGA TGGAGAGAAA AGGAAAAAAA GAAGGGATCA AGCAG-----

---------- ---------- ---------- ---------- ----------

TTGGAGAAGA TGGAGAGAAA AGGAAAAAAA GAAGGGATCA AGCAGGTAAA

TTGGAGAAGA TGGAGAGAAA AGGAAAAAAA GGAGGGATCA AGCAGGTAAA

ttggagaaga tggagagaaa aggaaaaaaa gaCGGGATCA AGCAG-----

TTGGAGAAGA TGGAGAAAAA AGGAAAAAAA GAAGGGATCA AGCAGGTAAA

ttggagaaga cggagagaag aggaaaaaga gaagGGATCA AGCAGGTAAA

tcggagagga tggagagaag aggaaaaaaa gaagggATCA AGCAGGTAAA

CTGGAGAACA TGGAGAAAAA AGGAAAAAGA AAAAGGATCA AGCAGGTAAA

TTGGGGAAGA TGGAGAAAAG CGGAAGAAAC GAAGGGATCA AACAGGTAAA

AAGAAAGTCT CCCCAGACAA GATGATTGAA ATGCAAGCAA AAATTGATGA

AAGAAAGTCT CCCCAGACAA GATGATTGAA ATGCAAGCAA AAATTGATGA

AAGAAAGTAT CCCCAGACAA GATGATTGAA ATGCAAGCAA AAATTGATGA

AAGAAAGTAT CCCCAGACAA GATGATTGAA ATGCAAGCAA AAATTGATGA

AAGAAAGTCT CCCCAGATAA GATGATTGAA ATGCAAGCAA AAATTGATGA

AAGAAAGTTT CTCCAGATAA AATGGTTGAA ATGCAAGCAA AAATTGATGA

AAGAAAGTTT CCCCAGATAA GATGATGGAA ATGCAAGCGA AAATTGATGA

AAGAAAGTTT CCCCAGATAA GATGGTGGAA ATGCAAGCGA AAATTGACGA

---------- ---------- ---------- ---------- ----------

---------- ---------- ---------- ---------- ----------

AAGAAAGTGT CCCCAGATAA GATGGTTGAA ATGCAAGCAA AAATTGATGA

AAGAAAGTTT CCCCTGATAA GATGGTTGAA ATGCAAGCAA AAATTGATGA

---------- ---------- ---------- ---------- ----------

AAGAAAGTTT CCCCAGATAA GATGGTTGAA ATGCAAGCAA AAATTGATGA

AAGAAAGTTT CTCCAGATAA GATGGTTGAA ATGCAAGCAA AAATTGATGA

AAGAAGGTTT CTCCAGATAA GATGGTTGAA ATGCAAGCAA AAATTGAAGA

AAGAAGGTTT CTCCAGATAA GATGGTTGAA ATGCAAGCAA AAATTGATGA

AAGAAGGTTT CCCCAGACAA GATGGTGGAG ATGCAAGCTA AGATCGACGA

GGAGAGAAAA GCACTTGAAA CAAAGCTCGA CATGGAAGAA GAAGAAAGAA

GGAGAGAAAA GCACTTGAAA CAAAGCTCGA CATGGAAGAA GAAGAAAGAA

GGAGAGAAAA GCACTTGAAA CAAAGCTTGA CATGGAAGAA GAAGAAAGAA

GGAGAGAAAA GCACTTGAAA CAAAGCTCGA CATGGAAGAA GAAGAAAGAA

GGAGAGAAAA GCACTTGAAA CAAAGCTTGA CATGGAAGAA GAAGAAAGAA

AGAGAGAAAA GCACTTGAAA CAAAGCTTGA CATGGAAGAA GAAGAAAGAA

GGAGAGAAAA GCACTGGAAA CCAAACTCGA CATGGAGGAA GAAGAAAGAA

GGAGAGAAAA GCACTGGAAA CCAAACTTGA CATGGAAGAA GAAGAAAGAA

---------- ---------- ---------- ---------- ----------

---------- ---------- ---------- ---------- ----------

GGAGAGAAAA GCACTTGAAA CAAAGCTTGA TATGGAAGAA GAAGAAAGAA

GGAGAGAAAA GCACTTGAAA CAAAGCTCGA TATGGAAGAA GAAGAAAGAA

---------- -----TGAAA CAAAGCTTGA CATGGAAGAA GAAGAAAGGA

GGAGAGGAAA GCACTTGAAA CAAAGCTTGA CATGGAAGAA GAAGAAAGAA

GGAGAGGAAA GCACTTGAAA CAAAGCTTGA CATGGAGGAA GAAGAAAGAA

GGAGAGGAAA GCACTTGAAA CAAAGCTCGA CATGGAGGAA GAAGAACGGA

GGAAAGGAAA GCACTTGAAA CCAAACTTGA TATGGAAGAA GAAGAAAGAA

GGAGAGGAAG GCCCTTGAGA CCAAACTCGA CATGGAAGAA GAAGAAAGAA

ACAAGGCTAG AGCTGAATTA GAGAAACGGG AAAAAGATCT TCTTAAAGCC

ACAAGGCTAG AGCTGAATTA GAGAAACGGG AAAAAGATCT TCTTAAAGCC

ACAAGGCTAG AGCTGAATTA GAGAAACGAG AAAAAGATCT TCTTAAAGCC

ACAAGGCTAG AGCTGAATTA GAGAAACGAG AAAAAGATCT TCTTAAAGCC

ACAAGGCTAG AGCTGAATTA GAGAAACGGG AAAAAGATCT TCTTAAAGCC

ACAAGGCTAG AGCTGAACTA GAGAAACGGG AAAAAGATCT ACTTAAAGCC

ACAAGGCTAG AGCTGAGCTG GAGCGACGGG AGAAGGACCT TCTGAAAGCC

ACAAGGCTAG AGCTGAACTA GAGAGACGGG AGAAGGACCT TCTGAAAGCC

---------- ---------- ---------- ---------- ----------

---------- ---------- ---------- ---------- ----------

ACAAGGCTAG AGCTGAATTA GAGAAACGGG AGAAAGATCT TCTTAAGGCC

ACAAGGCTAG AGCTGAATTA GAGAAACGGG AAAAGGATCT TCTTAAGGCC

ATAAGGCTAG AGCTGAATTA GAGAAACGGG AAAAAGATCT TCTTAAGGCC

ACAAGGCTAG AGCTGAATTA GAGAAACGGG AAAAAGATCT TCTTAAGGCT

ACAAAGCTAG AGCTGAGTTA GAGAAACGAG AAAAAGATCT TCTTAAGGCT

ACAAGGCTAG AGCGGAGCTG GAGAAACGAG AAAAGGATCT TCTTAAGGCG

ATAAAGCCAG AGCTGAACTG GAAAAAAGGG AGAAGGACCT TCTTAAAGCC

ACAAAGCTAG AGCTGAGCTG GAGAGACGGG AGAAAGATCT GCTTAAAGCG

CAACAAGAGC ATCAGTCTTT GCTGGAAAAA TTATCTGCCC TGGAAAAGAA

CAACAAGAGC ATCAGTCTTT GCTGGAAAAA TTATCTGCCC TGGAAAAGAA

CAACAAGAGC ATCAGTCTTT GCTGGAAAAA TTATCTGCCC TGGAAAAGAA

CA-------- ---------- ---------- ---------- ----------

CAACAAGAAC ATCAGTCTTT GCTGGAAAAA TTGTCTGCCC TGGAAAAGAA

CAACAAGAGC ATCAGTCTTT GCTAGAAAAA TTATCTGCGC TGGAGAAGAA

CAACAAGAGC ATCAGTCTTT GCTGGAAAAA CTGTCTGCGC TGGAGAAGAA

CAACAAGAGC ATCAGTCTTT GCTGGAAAAG CTGTCTGCTT TGGAAAAGAA

--ACAAGAGC ATCAGTCTTT GCTGGAAAAA TTATCTGCAC TGGAGAAGAA

---------- ---------- ---------- ---------- ----------

CAACAAGAAC ATCAGTCTTT GCTAGAGAAA TTATCTGCCC TGGAGAAGAA

CAACAGGAGC ATCAGTCTTT GTTAGAGAAA TTATCTGCAC TGGAGAAGAA

CAACAAGAGC ATCAGACTTT GCTAGAGAAG TTATCTGCAT TGGAGAAGAA

CAACAAGAGC ATCAGTCTTT GTTGGAAAAG TTGTCTGCTC TTGAAAAGAA

CAACAAGAGC ATCAGTCTTT GCTGGAAAAG TTATCTGCAC TGGAGAAGAA

CAGCAAGAGC ATCAGTCTTT GCTGGAAA-G CTATCTGCAC TGGAGAAGAA

CAACAAGAGC ATCAGTCACT TTTGGAAAAA CTATCTGCAC TGGAAAAGAA

CAACAGGAAC ATCAGTCATT GCTGGAAAAA TTGTCTGCCC TGGAGAAGAA

GGTAATTGTT GGTGGGGTTG ACTTGTTGGC CAAAGCTGAG GAACAAGAGA

GGTAATTGTT GGTGGGGTTG ACTTGTTGGC CAAAGCTGAG GAACAAGAGA

GGTAATTGTT GGTGGGGTTG ATTTGTTGGC CAAAGCTGAG GAACAAGAGA

---------- ---------- ---------- ---------- ----------

GGTAATTGTT GGTGGGGTTG ATTTGTTGGC CAAAGCTGAG GAACAAGAGA

GGTAATTGTT GGTGGTGTTG ATTTGTTGGC CAAAGCAGAG GAACAAGAGA

GGTGATTGTC GGTGGTGTGG ACTTGTTGGC CAAAGCTGAG GAGCAAGAGA

GGTCATTGTC GGCGGCGTGG ACCTGCTGGC CAAGGCTGAG GAGCAAGAGA

GGTCATTGTT GGTGGTGTTG ATTTGTTGGC CAAAGCTGAA GAACAAGAAA

---------- ---------- ---------- ---------- ----------

GGTAATTGTT GGTGGTGTTG ATTTGTTGGC AAAAGCTGAG GAACAAGAGA

GGTGATTGTT GGGGGTGTTG ATTTGTTGGC GAAAGCTGAG GAACAAGAGA

GGTGATTGTT GGTGGTGTAG ATTTATTAGC AAAAGCCGAG GAACAAGAAA

GGTCATTGTT GGTGGTGTTG ATTTGTTAGC AAAAGCTGAG GAACAAGAGA

GGTCATTGTT GGTGGTGTTG ATTTGTTGGC AAAAGCTGAG GAACAAGAGA

GGTGATTGTT GGTGGTGTTG ATTTGCTGGC GAAAGCCGAG GAACAAGAGA

GGTAATTGTT GGTGGTGTTG ACTTGTTGGC TAAAGCTGAG GAGCAAGAGA

GGTGATTGTT GGTGGTGTGG ATTTGTTGGC CAAGGCTGAG GAGCAAGAGA

AACTTCTTGA AGAATCTAAC ATGGAACTGG AAGAAAGGAG GAAAAGAGCA

AACTTCTTGA AGAATCTAAC ATGGAACTGG AAGAAAGGAG GAAAAGAGCA

AACTTCTTGA AGAATCTAAC ATGGAACTGG AAGAAAGGAG GAAAAGAGCA

---------- ---------- ---------- ---------- ----------

AACTTCTTGA AGAATCTAAC ATGGAACTGG AAGAAAGGAG GAAAAGAGCA

AACTTCTTGA AGAATCTAAT ATGGAACTGG AAGAAAGAAG GAAAAGAGCA

AGCTTCTCGA GGAGTCCAAT ATGGAGCTGG AGGAGCGGAG GAAAAGAGCT

AGCTTCTGGA GGAGTCCAAC ATGGAGCTGG AGGAGAGGAG GAGACGAGCT

AACTTCTTGA AGAATCTAAT ATGGAGCTGG AAGAAAGGAG GAAAAGAGCA

---------- ---------- ---------- ---------- ----------

AACTTCTTGA AGAATCTAAT ATGGAACTGG AAGAACGGAG GAAAAGAGCA

AACTGCTTGA AGAATCTAAT ATGGAACTGG AAGAAAGGAG GAAGAGAGCC

AACTTCTTGA AGAATCCAAT ATGGAACTGG AAGAAAGGAG GAAAAGAGCA

AACTTCTTGA AGAATCTAAT ATGGAACTGG AAGAAAGGAG GAAAAGAGCC

AACTTCTTGA AGAATCTAAT ATGGAATTGG AAGAAAGGCG GAAAAGAGCA

AACTTCTTGA AGAATCTAAC ATGGAGCTAG AAGAAAGGCG GAAAAGAGCA

AACTTCTAGA GGAATCTAAC ATGGAACTAG AAGAAAGAAG GAAAAGAGCA

AGCTTCTGGA AGAATCCAAC ATGGAGCTGG AGGAAAGGCG GAAAAGAGCA

GAGCAACTTC GCAGAGAACT TGAGGAAAAA GAGCAAGAAG ATATTGAAGA

GAGCAACTTC GCAGAGAACT TGAGGAAAAA GAGCAAGAAG ATATTGAAGA

GAGCAACTTC GCAGAGAACT TGAGGAAAAA GAGCAAGAAG ATATTGAAGA

---------- ---------- ---------- ---------- ----------

GAGCAACTTC GCAGAGAACT TGAGGAAAAA GAGCAAGAAG ATATTGAAGA

GAGCAACTTC GCAGAGAACT TGAAGAAAAA GAGCAAGAAG ATATTGAAGA

GAGCAACTTC GGAAAGAACT AGAGGAAAAA GAGCAAGAAG ACATCGAGGA

GAGCAGCTTC GGAAAGAGCT GGAGGAAAAG GAGCAAGAAG ACATCGAGGA

GAGCAACTTC GCAAAGAACT CGAGGAGAAA GAGCAAGAAG ATATTGAAGA

---------- ---------- ---------- ---------- ----------

GAGCAACTTC GCAAAGAACT TGAGGAAAAA GAGCAAGAAG ATATTGAAGA

GAGCAACTTC GCAGAGAACT TGAGGAAAAA GAGCAAGAAG ATATTGAAGA

GAGCAGCTTC GCAGAGAACT TGAAGAAAAA GAGCCAAAAA ATAATATAAT

GAACAGCTTC GCAGAGAACT TGAGGAAAAA GAGCAAGAAG ATATTGAAGA

GAGCAGCTTC GCAGAGAACT TGAGGAAAAA GAGATAGAAT TTAATGAAGA

GAACAGCTCC GCAGAGAGCT TGAGGAAAAA GAGATAAAAT TTAGTGAAGA

GAACAACTTC GAAGAGAGCT TGAGGAAAAA GAGCAAGAAG ATATTGAAGA

GAGCAACTTC GCAGAGAGCT GGAGGAAAAA GAGCAAGAAG ATATCGAAGA

AAAATATACC AGTTTGCAAG AGGAAGCACA GGGAAAGACC AAGAAGTTAA

AAAATATACC AGTTTGCAAG AGGAAGCACA GGGAAAGACC AAGAAGTTAA

AAAATATACC AGCTTGCAAG AGGAAGCACA GGGAAAGACC AAGAAGTTAA

---------- ---------- ---------- ---------- ----------

AAAATATACC AGTTTGCAAG AGGAAGCACA GGGGAAGACC AAGAAATTAA

AAAATATACC AGTTTGCAAG AGGAAGCACA GGGAAAGACT AAGAAGTTAA

AAAATATACC AGTCTGCAAG AGGAGGCACA AGGAAAGACC AAGAAATTAA

AAAATATACC AGTCTGCAAG AGGAGGCACA AGGAAAGACC AAGAAATTAA

AAAATATACC AGTTTGCAAG AAGAAGCACA GGGAAAGACC AAGAAATTAA

---------- ---------- ---------- ---------- ----------

AAAATATACC AGCTTGCAAG AGGAAGCACA GGGAAAAACC AAGAAATTAA

AAAATATACC AGTTTGCAAG AAGAAGCACA GGGAAAGACC AAGAAATTAA

AAAACAAACA AGT------- ---------- ---------- ----------

AAAATATACC AGTTTACAAG AGGAGGCTCA GGGAAAGACG AAGAAATTGA

AAAGTTTTCC AATTTACAAG AGGAAGCTCA GGGAAAGACG AAGAAATTAA

AAAGCGTGCC CGTTTGCAAG AGGAAGCTCA GGGAAAGACC AAGAAGTTAA

GAAATATACT AGTTTACAGG AAGAAGCTCA GGGAAAGACC AAGAAGTTGA

GAAATACACC AGTCTGCAGG AGGAAGCTCA GGGCAAGACC AAGAAATTAA

AGAAAGTTTG GACTATGCTG ATGGCTGCAA AGTCAGAGAT GGCTGATCTC

AGAAAGTTTG GACTATGCTG ATGGCTGCAA AGTCAGAGAT GGCTGATCTC

AGAAAGTTTG GACTATGCTG ATGGCTGCAA AATCAGAGAT GGCTGATCTT

---------- ---------- ---------- ---------- ----------

AGAAAGTGTG GACTATGCTG ATGGCTGCAA AGTCGGAGAT GGCTGATCTC

AGAAAGTTTG GACAATGCTG ATGGCTGCAA AGTCAGAGAT GGCTGATCTT

AGAAAGTCTG GACCATGCTG ATGGCTGCAA AGTCAGAGAT GGCTGATCTC

AGAAAGTCTG GACCATGCTG ATGGCTGCAA AGTCAGAGAT GGCTGATCTC

AGAAAGTGTG GACAATGCTG ATGGCTGCAA AGTCGGAG-- ----------

---------- ---------- ---------- ---------- ----------

AGAAAGTTTG GACTATGCTA ATGGCTGCAA AATCAGAGAT GGCTGATCTC

AGAAAGTTTG GACTATGCTT ATGGCTGCAA AGTCAGAGAT GGCTGATCTC

---------- ---------- ---------- --------AT GGCTGATCTT

AGAAAGTTTG GACTATGCTG ATGGCTGCAA AGTCAGAGAT GGCCGATCTC

AGAAAGTTTG GACTATGTTG ATGGCTGCAA AGTCAGAGAT GGCCGATCTC

AGAAAGTCTG GACTATGCTG ATGGCCGCAA AATCTGAGAT GGCGGATCTC

AGAAAGTTTG GACCATGCTA ATGGCTGCAA AGTCAGAGAT GGCAGATCTT

AGAAAATAAA GACGATGCTA ATGGCTGCAA AGTCAGAGAT GGCAGATCTC

CAACAAGAAC ATCAGAGGGA AATTGAAGGC CTACTGGAGA ACATTCGGCA

CAACAAGAAC ATCAGAGGGA AATTGAAGGC CTACTGGAGA ACATTCGGCA

CAACAGGAAC ATCAGAGGGA AATTGAAGGC CTACTGGAGA ATATTCGGCA

---------- ---------- ---------- ---------- ----------

CAACAAGAAC ATCAGAGGGA AATTGAGGGC CTACTGGAGA ATATTCGGCA

CAACAGGAAC ATCAGAGGGA AATTGAAGGC CTTCTGGAGA ACATTCGGCA

CAGCAAGAGC ATCAGAGGGA AATTGAAGGC CTCCTGGAGA ACATTCGGCA

CAGCAGGAGC ATCAGAGGGA GATCGAAGGC CTCCTGGAGA ACATCCGGCA

---------- ---------- ---------- ---------- ----------

---------- ---------- ---------- ---------- ----------

CAACAGGAGC ATCAGAGGGA AATTGAAGGC CTACTGGAGA ATATTCGACA

CAACAGGAAC ACCAGCGGGA AATTGAAGGC CTGCTGGAGA ACATTCGACA

CAACAAGAAC ATCAGAGAGA AATTGAAGGA CTATTGGAAA ATATCCGACA

CAGCAAGAAC ATCAGCGAGA AATTGAAGGC CTACTGGAGA ACATTCGACA

CAACAAGAAC ATCAGAGAGA AATTGAAGGC CTACTGGAGA ACATTCGACA

CAGCAAGAAC ATCAGAGGGA AATAGAAGGT TTACTGGAGA ACATCCGACA

CAACAAGAAC ATCAGAGGGA AATTGAAGGC CTTCTGGAAA ACATTAGACA

CAGCAAGAAC ATCAGCGAGA AATTGAAGGC CTCCTGGAGA ACATCCGACA

ACTTAGCCGG GAGCTTCGAC TTCAGATGCT TATTATTGAT AACTTTATAC

ACTTAGCCGG GAGCTTCGAC TTCAGATGCT TATTATTGAT AACTTTATAC

ACTTAGCCGG GAGCTTCGAC TTCAGATGCT TATTATTGAT AATTTTATAC

---------- ---------- ---------- ---------- ----------

ACTTAGCCGG GAGCTTCGAC TTCAGATGCT TATTATTGAT AACTTTATAC

GCTTAGCCGG GAGCTTCGAC TTCAGATGCT TATTATTGAT AACTTTATAC

GCTCAGCCGT GAGCTCCGAC TTCAGATGCT CATCATTGAT AACTTCATAC

GCTCAGCCGG GAGCTCCGTC TTCAGATGCT CATCATTGAT AACTTCATAC

---------- ---------- ---------- ---------- ----------

---------- ---------- ---------- ---------- ----------

ACTTAGCCGG GAGCTCCGAC TTCAGATGCT TATCATTGAT AACTTTATAC

ACTTAGCAGG GAGCTTCGAC TTCAGATGCT CATTATCGAT AACTTTATAC

ACTTAGTCGG GAACTTCGAC TTCAGATGCT CATTATTGAT AACTTTATAC

ACTTAGCCGG GAGCTTCGAC TTCAGATGCT TATTATTGAT AACTTTATAC

ACTTAGCCGG GAGCTTCGAC TTCAGATGCT TATTATTGAT AACTTTATAC

GCTTAGTCGG GAGCTTCGAC TTCAGATGCT TATCATCGAT AACTTTATAC

GCTCAGCAGA GAACTTCGCC TTCAGATGCT TATTATTGAC AACTTTATTC

GCTCAGCAGG GAACTGAGAC TCCAAATGCT TATCATAGAC AACTTCATTC

CTCGGGATTA TCAGGAAATG ATTGAAAACT ATGTCAATGA AGACATAGGA

CTCGGGATTA TCAGGAAATG ATTGAAAACT ATGTCAATGA AGACATAGGA

CTCGGGATTA TCAGGAAATG ATTGAAAACT ATGTCAATGA AGACATAGGA

---------- ---------- ---------- ---------- ----------

CTCGGGATTA TCAGGAAATG ATTGAAAACT ACGTCAATGA AGACATAGGA

CTCAGGATTA TCAGGAAATG ATCGAAAACT ATGTCAATGA AGACATAGGA

CTCAGGACTA CCAGGAAATG ATTGAAAACT ACGTCAATGA AGACATAGGA

CTCAGGATTA CCAGGAAATG ATTGAAAACT ACGTCAATGA AGACATAGGA

---------- ----GAAATG ATTGAAAATT ATGTCAATGA AGACATTGGA

---------- ---------- ---------- ---------- ----------

CTCAAGATTA TCAGGAAATG ATTGAAAACT ATGTCAATGA AGACATAGGA

CTCAGGATTA TCAGGAAATG ATTGAAAACT ATGTCAATGA AGACATAGGA

CCCAGGATTA TCAGGAAATG ATTGGAAACT ATGTCAATGA AGACATAGGA

CTCAGGATTA TCAGGAAATG ATTGAAAACT ATGTCAATGA AGACATAGGA

CTCAGGATTA TCAGGAAATG ATTGAAAACT ATGTCAATGA AGACATAGGA

CTCAGGATTA CCAGGAAATG ATTGAAAATT ATGTCAATGA AGACATAGGA

CTCAGGATTA TCAGCAAATT AT-GAAAAAA ATGT---TGA AAATAGAGTA

CTCACGACTA CCAGGAAATG ATTGAAAATT ATGTTAATGA AGACATCGGA

GAACAGCTAA AATGTGTTGC TTATACAGGA AATAACATGA GGAAGCAAAC

GAACAGCTAA AATGTGTTGC TTATACAGGA AATAACATGA GGAAGCAAAC

GAACAGCTAA AATGTGTTGC CTATACAGGA AATAACATGA GGAAGCAAAC

---------- ---------- ---------- ---------- ----------

GAACAACTAA AATGTGTTGC CTACACGGGA AATAACATGA GGAAGCAAAC

GAACAGCTAA AATGTGTTGC ATACACAGGA AATAACATGA GGAAGCAAAC

GAACAGCTGA AATGTGTGGC CTACACAGGA AATAACATGA GGAAACAAAC

GAACAGCTAA AATGTGTGGC CTACACAGGA AATAACATGA GGAAACAAAC

GAACAGTTGA AATGTGTTGC CTACACAGGG AATAACATGA GGAAGCAAAC

---------- ---------- ---------- ---------- ----------

GAACAGCTAA AATGTGTTGC TTACACAGGA AATAACATGA GGAAGCAGAC

GAACAGCTAA AATGTGTTGC CTACACAGGA AATAACATGA GGAAGCAGAC

GAACAGCTAA AATGTGTTGC CTATACAGGA AATAATATGC GGAAACAGAC

GAACAGTTAA AATGTGTTGC CTACACAGGA AATAACATGA GGAAGCAAAC

GAACAGCTAA AATGTGTTGC CTACACAGGA AATAACATGA GGAAGCAGAC

GAACAGCTAA AATGTGTTGC TTATACAGGA AATAACATGA GGAAACAGAC

TTTTGGCTGA AATGTGTTGC CTACACAGGA AATAATATGA GAAAGCAGAC

GAACAGTTGA AATGCGTTGC ATACACGGGA AATAACATGA GAAAGCAGAC

CCCAGTACCT GATAAAAAGG AGAAAGATCC CTTTGAGGTG GACCTTTCTC

CCCAGTACCT GATAAAAAGG AGAAAGATCC CTTTGAGGTG GACCTTTCTC

CCCAGTACCT GATAAAAAGG AGAAAGATCC CTTTGAAGTG GACCTTTCTC

---------- ---------- ---------- ---------- ----------

CCCAGTACCT GATAAAAAGG AAAAAGATCC CTTTGAAGTA GACCTTTCTC

CCCAGTACCT GATAAAAAGG AGAAAGATCC CTTTGAAGTG GACCTTTCCC

TCCAGTCCCT GACAAGAAGG AGAGAGACCC ATTTGAAGTA GACCTTTCCC

TCCAGTCCCT GACAAGAAGG AGAGAGACCC ATTTGAAGTC GACCTTTCCC

TCCAGTACCT GATAAAAAGG AGAAAGAT-- ---------- ----------

---------- ---------- ---------- ---------- ----------

TCCAGCTCCT GATAAAAAAG AGAAAGATCC CTTTGAAGTG GACCTTTCCC

TCCAGTTCCT GATAAAAAAG AGAAAGATCC CTTTGAAGTG GACCTTTCCC

TCCAGTTTCT GATAAAAAAG AGAAAGATCC CTTTGAAGTG GACCTTTCTC

TCCAGTACCT GATAAAAAGG AAAAAGATCC CTTTGAAGTA GACCTTTCCC

CCCAGTACCT GATAAAAAGG AGAAAGATCC CTTTGAAGTG GACCTTTCTC

CCCTGTACCT GACAAAAAGG AGAAAGATCC CTTCGAGGTG GACCTTTCCC

TCCAGTACCT GATAAAAAGG AAAAAGATCC TTTTGAAGTA GACCTTTCCC

TCCAATACCT GACAAAAAGG AGAAGGATCC CTTTGAAGTG GACCTTTCTC

ACGTGTATCT TGCCTATACT GAGGAGAGTC TGCGTCAGTC TTTGATGAAA

ACGTGTATCT TGCCTATACT GAGGAGAGTC TGCGTCAGTC TTTGATGAAA

ATGTGTATCT TGCCTATACT GAGGAGAGTC TGCGTCAGTC TTTGATGAAA

---------- ---------- ---------- ---------- ----------

ACGTGTATCT CGCCTATACT GAGGAGAGTC TCCGTCAGTC TTTGATGAAA

ACGTGTATCT TGCCTATACT GAGGAGAGTC TACGTCAGTC TCTGATGAAA

ACGTGTACCT CGCCTATACA GAGGAGAGTT TGCGTCAGTC TCTGATGAAG

ACGTGTACCT CGCCTATACA GAGGAGAGTC TGCGTCAGTC TCTGATGAAG

---------- ---------- ---------- ---------- ----------

---------- ---------- ---------- ---------- ----------

ACGTGTATCT TGCCTACACT GAGGAGAGTC TCCGGCAGTC CTTGATGAAA

ACGTGTATCT TGCCTATACT GAGGAGAGCC TGCGGCAGTC CTTGATGAAA

ATGTGTATCT CGCCTATACT GAAGAGAGTC TGCGACAGTC TTTGATGAAA

ATGTGTATCT TGCTTATACC GAGGAGAGCT TACGGCAGTC CTTGATGAAA

ATGTGTATCT CGCCTACACA GAGGAAAGCC TGCGGCAGTC CTTGATGAAA

ATGTGTATCT CGCCTACACA GAGGAGAGTC TGCGGCAGTC CCTGATGAAG

ATGTGTATTT AGCTTATACT GAGGAAAGCC TCAGACAATC TTTGATGAAA

ATGTGTACTT GGCCTACACA GAAGAAAGTC TGAGGCAGTC TCTGATGAAA

CTAGAAAGAC CACGAACTTC AAAGGGGAAA GCAAGGCCAA AGACAGGGAG

CTAGAAAGAC CACGAACTTC AAAGGGGAAA GCAAGGCCAA AGACAGGGAG

CTAGAAAGAC CACGAACTTC AAAGGGGAAA GCCAGGCCAA AGACAGGGAG

---------- ---------- ---------- ---------- ----------

CTAGAAAGAC CACGAACTTC AAAGGGGAAA GCAAGGCCAA AGACAGGGAG

TTAGAGAGGC CACGGACTTC TAAGGGGAAG GCACGGCCCA AGACAGGGAG

TTAGAAAGGC CACGGACCTC CAAGGGAAAG GCCAGGCCAA AGACCGGTAG

TTAGAAAGGC CACGGACCTC CAAGGGGAAA GCAAGGCCAA AGATGGGGCG

---------- ---------- ---------- ---------- ----------

---------- ---------- ---------- ---------- ----------

TTAGAGAGAC CACGGACTTC TAAGGGGAAA GCAAGGCCAA AGACAGGGCG

TTGGAGAGGC CACGGACCTC AAAGGGAAAA GCAAGGCCAA AGACAGGGAG

TTAGAACGGC CACGAACTTC AAAGGGAAAG GCAAGGCCCA AGACAGGGAG

TTAGAAAGGC CACGGACTTC AAAGGGGAAA GCAAGGCCAA AGACTGGGAG

TTAGAAAGGC CACGGACTTC AAAGGGAAAA GGAAGGCCAA AGACAGGGAG

TTAGAAAGGC CACGGACTTC AAAGGGGAAA GGAAGGCCCA AGACCGGGAG

TTAGAGAGGC CACGCACTTC AAAGGGAAAG TCAAGGCCAA AAACTGGAAG

CTGGAGAGGC CAAGGACTTC GAAAGGAAAG TCAAGGCCCA AAACggggag

AAGAATGAAA CTCTGGGTGT CTGCATTGCT GATGGCCTGG TTTGGTGTCC

AAGAATGAAA CTCTGGGTGT CTGCATTGCT GATGGCCTGG TTTGGTGTCC

AAGAATGAAA CTCTGGGTGT CTGCATTGCT GATGGCCTGG TTTGGTGTCC

----ATGAAA CTCTGGGTGT CTGCATTGCT GATGGCCTGG TTTGGTGTCC

AAGAATGAAA CTCTGGGTGT CTACATTGTT GATGGCCTGG TCTGGTGTCC

AAGAATGAAA CTCCAGGAGT CCATGTTGCT GATGGCCTGG TTTGGTGTTC

AAGAATGAAG CTCCAGGTGT TGGTGTTGCT GATGTCCTGG TTTGGTGTCC

AAGAATGAAG CTCCAGGTGT TGGTGTTGCT GATGTCCTGG TTCGGTGTCC

----ATGAAA CCCTGGGTGT CCACGTTGCT GATGGCCTGG TTTGCTGCCC

----ATGAAA CCCTGGGAGT CCACGTTGCT GGTGGCCTGG TTTGGTGTCC

AAGAATGAAA CGCTGGGTGT CCACATTGCT GGTGGCCTGG TTTGGTGTCC

AAGAATGAAA CTCCGGGTGT CTGCATTGCT GGTGGCCTGG TTTGGTGTCC

AAGAATGAAT CCTTGGGTGT CTGCCTTGCT GATGGCCTGG TTTAGTGTCC

AAGAATGAAA CTCCGGGCAT CCATGCTGCT GCTGGCCTGG TTTGGTGTCG

AAGA------ ---------- ---------- ---------- ----------

ACG-ATGAGA CCCCAGGTGT CCGTGCTGCT GCTGATCTGG CTTGGTGTCC

AAGAATGAAG ACCTGCATCT CCCCTTTGCT CCTGGCCTGG ATTAGTTTTC

aag-ATGCAT CTCTGGGTCC CGTCTCTGCT GTGGGCCTGC CTCAGTACCT

TGAGCTGTGT GCAGGCCGAA TTCTTCACCT CTATTGGGCA CATGACTGAC

TGAGCTGTGT GCAGGCCGAA TTCTTCACCT CTATTGGGCA CATGACTGAC

TGAGCTGTGT GCAGGCCGAA TTCTTCACCT CTATTGGGCA CATGACTGAC

TGAGCTGTGT GCAGGCCGAA TTCTTCACCT CTATTGGGCA CATGACTGAC

TGAGCTGTGT GCAGGCTGAA TTCTTCACCT CTATTGGGCA CATGACTGAC

TGAGTAGTGT GCAGGCTGAA TTCTTCACCT CTATTGGCCA CATGACTGAC

TGAGCTGGGT GCAGGCGGAA TTCTTCACCT CCATTGGGCA CATGACCGAC

TGAGCTGGGT GCAGGCAGAA TTCTTCACCT CCATTGGGCA CATGACCGAT

TGAGCTGTGC ATGGGCTGAA TTCTTCACCT CTATTGGGCA CATGACCGAC

TGAGCTGCGT GCAGGCTGAA TTCTTCACTT CTATTGGACA CATGACAGAC

TGAGCTGTGT GCAGGCTGAA TTCTTCACTT CTATTGGGCA CATGACTGAC

TGGGCTGTGT GCAGGCCGAG TTCTTTACCT CTATCGGGCA CATGACGGAC

TGTGCTCCGT GCAGGCTGAA TTCTTCACCT CGATCGGGCA CATGACAGAC

TGGGATGCGT GCGGGCTGAG TTCTTCACCT CCATCGGACA CATGACAGAC

---------- ---------- ---------- ---------- ----------

TGGGCTGTGT GGGGGCCGAG TTCTTCACGT CCATTGGGCA CATGACGGAC

TACACTGCAC ATGGGCTGAG TTCTTCACCT CAATTGGGCA GATGACAGAC

GCGTTCATGT CCAGGCCGAG TTCTTCACCT CCATAGGGCA AATGACAGAC

CTGATTTATG CAGAGAAAGA GCTGGTGCAG TCTCTGAAAG AGTACATCCT

CTGATTTATG CAGAGAAAGA GCTGGTGCAG TCTCTGAAAG AGTACATCCT

CTGATTTATG CAGAAAAAGA GCTGGTGCAG TCTCTGAAGG AGTACATCCT

CTGATTTATG CAGAAAAAGA GCTGGTGCAG TCTCTGAAGG AGTACATCCT

CTGATTTATG CAGAGAAAGA GCTGGTGCAA TCTCTGAAGG AGTACATCCT

CTGATTTATG CAGAGAAGGA TCTGGTGCAG TCTCTGAAAG AGTACATCCT

CTGATTTATG CAGAGAAGGA CCTCGTCCAG TCTCTGAAGG AGTACATCCT

CTGATTTACG CAGAGAAGGA CCTGGTACAG TCTCTGAAGG AGTACATCCT

CTGATCTATG CTGAGAAAGA CCTGGTGCAC GCACTGAAGG AGTACATCCT

CTGATTTATG CAGAGAAGGA CCTGGTGCAG TCCCTGAAGG AGTACATCCT

CTAATTTATG CAGAGAAGGA CCTGGTGCAG TCTCTGAAGG GGTACATCCT

CTGATTTATG CAGAGAAGGA CCTGGTGCAG TCGCTGAAGG AGTACATTCT

CTGATTTATG CGGAGCAGGA CCTGGTACAG TCTCTGAAGG AGTACATCCT

CTGATTTATG CAGAGAAAGA CCTGGTACAG TCTCTGAAGG AGTACATCCT

---------- ---------- ---------- ---------- ----------

CTGATCTATG CAGAGAAGGA TCTGGTGAAG TCCCTGAAGG AGTACATCCT

TTGATTTATG CAGAGAAAGA CCTGGTTCAA TCTCTGAAAG AATATATAAG

CTGATTTATG CAGAGAAAGA CCTGGTGCAG TCTTTGAAGG AATACATCCA

TGTGGAGGAA GCCAAGCTTT CCAAGATTAA GAGCTGGGCC AACAAAATGG

TGTGGAGGAA GCCAAGCTTT CCAAGATTAA GAGCTGGGCC AACAAAATGG

TGTGGAGGAA GCCAAGCTTT CCAAGATTAA GAGCTGGGCC AACAAAATGG

TGTGGAGGAA GCCAAGCTTT CCAAGATTAA GAGCTGGGCC AACAAAATGG

TGTAGAGGAA GCCAAGCTTT CCAAGATTAA GAGCTGGGCC AACAAAATGG

TGTGGAGGAA GCCAAGCTGT CCAAGATTAA GAGCTGGGCC AACAAGATGG

GGCAGAGGAA GCCAAGCTCT CCAAGATTAA GAGCTGGGCC AGTAAGATGG

TGTGGAGGAA GCCAAGCTCG CCAAGATTAA GAGCTGGGCC AGCAAGATGG

TGCGGAGGAG GCCAAACTCT CTAAGATCAA GAGATGGGCA AGCAAGATGG

GGTGGAGGAA GCCAAGCTCT CCAAGATTAA GAGCTGGGCT GACAAAATGG

TGTGGAGGAA GCCAAACTCT CTAAGATTAA GAGCTGGGCC AACAAGATGG

TGAGGAGGAA GCCAAGCTCT CCAAGATTAA GAGCTGGGCC AACAAAATGG

GGTGGAGGAA GCTAAACTCT CCAAGATTAA GAGTTGGGCC AACAAAATGG

TGTGGAAGAA GCCAAGCTTT CCAAGATCAA GAGTTGGGCC AACAAATTGG

---------- ---------- ---------- ---------- ----------

AGCAGAGGAG GCCAAGCTCG CCCAGATTAA GAGTTGGGCC ACCAAAATGG

AGCAGAAGAA GCCAGACTCT TCAAGATTAA AAGCTGGGCT GAAAAAATGG

GGTGGAAGAG GCCAAGCTCT CGAAAATTAA AAGCTGGGCT GAAAAAATGG

AAGCCTTGAC TAGCAAGTCA GCTGCTGATG CTGAGGGCTA CCTGGCTCAC

AAGCCTTGAC TAGCAAGTCA GCTGCTGATG CTGAGGGCTA CCTGGCTCAC

AAGCCTTGAC TAGCAAGTCA GCTGCTGACG CTGAGGGCTA CCTGGCCCAC

AAGCCTTGAC TAGCAAGTCA GCTGCTGACG CTGAGGGCTA CCTGGCCCAC

AAGCCTTGAC TAGCAAGTCA GCTGCTGATG CTGAGGGCTA CCTGGCCCAC

ACGCCCTGAC CAGCAAGTCG GCTGCTGACC CTGAGGGCTA CCTGGCTCAC

AGGCCCTGAC CAGCAAGTCA GCTGCTGACC CCGAGGGCTA CCTGGCCCAT

AAGCCCTGAC CAGCAGATCA GCTGCCGACC CCGAGGGCTA CCTGGCTCAT

AAGCCTATAC CAGCAAGTCA GCTGCTGACC CCGAGGGCTA CCTTGCCCAT

AAGCCCTGAC CAGCAAGTCG GCTGCTGACC CTGAGGGCTA CCTGGCCCAC

AAGCCTTGAC CAGCAAGTCA GCTGCTGACC CTGAAGGTTA CCTGGCTCAC

AAGCCCTGAC CAGCAAGTCG GCTGCTGACC CCGAGGGCTA CCTGGCCCAC

AGGCCCTGAC CAGCAAGTCA GCAGCTGACC CTGAGGGCTA CCTCGCCCAC

AATCTCTCAC CAGCAAGTCA GCTGCTGACC CTGAGGGCTA TCTGGCCCAC

---------- ---------- ---------- ---------- ----------

AAGCGCTGAC CAGCAAGTCG ACGGCTGACC CTGAGGGCTA CCTGGCACAC

ATATTCTGAC AAGCAAATCA GCTTCTGATC CTGAAGGCTA CTTGTCTCAT

ACTTGCTGAC AAGCAAGTCC ACGTCAGATC CTGAGGGCTA CCTGGCACAC

CCTGTGAATG CCTACAAACT GGTGAAGCGG CTAAACACAG ACTGGCCTGC

CCTGTGAATG CCTACAAACT GGTGAAGCGG CTAAACACAG ACTGGCCTGC

CCTGTGAATG CCTACAAACT GGTGAAGCGG CTAAACACAG ACTGGCCTGC

CCTGTGAATG CCTACAAACT GGTGAAGCGG CTAAACACAG ACTGGCCTGC

CCTGTGAATG CCTACAAACT GGTGAAGCGG CTAAACACAG ACTGGCCTGC

CCTGTGAATG CCTACAAACT GGTGAAGCGG CTCAATACGG ACTGGCCTGC

CCTGTGAACG CCTATAAGCT GGTGAAGCGA TTGAACACAG ACTGGCCTGC

CCTGTGAATG CCTACAAGCT GGTGAAGCGG TTGAACACAG ACTGGCCTGC

CCTGTGAACG CCTACAAACT GGTTAAGCGA CTGAACTCAG ACTGGCCTGC

CCTGTGAATG CCTATAAACT GGTGAAGCGG CTAAACACGG ACTGGCCTGC

CCCGTGAATG CCTATAAACT TGTGAAGCGG CTCAACACAG ACTGGCCTGC

CCTGTGAATG CCTATAAACT GGTGAAGCGG CTGAACACAG ACTGGCCCGC

CCCGTGAATG CCTACAAACT GGTGAAGCGG CTGAACACAG ACTGGCTGGC

CCTGTGAATG CCTACAAACT GGTGAAGCGG CTGAACACAG ACTGGCCGGC

---------- ---------- ---------- ---------- ----------

CCGGTGAACG CCTACAAACT GGTGAAGCGG CTGAACACAG ACTGGCCTGC

CCAGTAAATG CCTACAAGCT GGTGAAGCGT CTAAATACAG ATTGGCCAGC

CCGGTGAATG CCTACAAGCT GGTGAAGCGA CTAAACACGG ATTGGCTGGC

GCTGGAGGAC CTTGTCCTGC AGGACTCAGC TGCAGGTTTT ATCGCCAACC

GCTGGAGGAC CTTGTCCTGC AGGACTCAGC TGCAGGTTTT ATCGCCAACC

GCTGGAGGAC CTTGTCCTGC AGGACTCAGC TGCAGGTTTT ATCGCCAACC

ACTAGAGGAC CTTGTCCTGC AGGACTCAGC TGCAGG-TTT ATCGCC-ACC

GCTGGAGGAC CTTGTCCTGC AGGACTCTGC TGCAGGTTTT ATCGCCAACC

ACTGGAAGAC CTTGTCCTGC AGGACTCAGC TGCAGGTTTC ATCGCCAACC

CCTGGGGGAC CTTGTCCTCC AGGACGCGGC TGCAGGTTTT GTGGCTAACC

ACTGGGGGAC CTTGTCCTTC AGGATGCTTC GGCAGGTTTT GTCGCTAACC

GCTAGAAGAC CTTGTGCTGC AAGACCCAGC CACAGGTTTC ATTGCCAACC

ACTGGAGGAC CTTGTCCTGC AGAACTCGGC CGCAGATTTT ATTGCAAACC

ACTGGAGGAT CTGGTCCTGC AGAACTCGGC TGCAGGTTTT ATTGCCAACC

CCTGGAGGAC CTGGTCCTGC AGGACTCTGC CGCAGGTTTT ATTGCCAACC

GCTGGAGGAC CTTGTTCTGC AGGATTCTGC CGCAG----- ----------

GCTGCACGAC CTCGTCCTGC AGGACTCAGC CACAGGCTTT CTTGCCAACC

---------- ---------- ---------- ---------- ----------

CCTGGAGGAC CTTGTCTTGC AAGACTCGGC AGCAGGCTTT ATTGGCAACC

TCTGGAGAAC ATGGTCTTGC AGGATTCAAC CTCTGATTTT ATTGCCAATT

CCTGGAGCAC TTAGTCCTCG AGGATGTGAC GAATGGTTTC ATCGCCAACC

TCTCTGTGCA GCGGCAGTTC TTCCCCACTG ATGAGGACGA GATAGGAGCT

TCTCTGTGCA GCGGCAGTTC TTCCCCACTG ATGAGGACGA GATAGGAGCT

TCTCTGTGCA GCGGCAGTTC TTCCCCACTG ACGAGGATGA GATAGGAGCT

TCTC-GGGCA -CGGCAG-TC TCCCCCGATG A--------- ----------

TCTCTGTGCA GCGGCAGTTC TTCCCCACTG ATGAGGACGA GATAGGAGCT

TCTCAGTGCA GCGGCAGTTC TTTCCTACTG ATGAGGACGA GATGGGGGCT

TCTCAGTTCA GCGGCAGTTC TTCCCCACTG ACGAGGACGA GTCTGGAGCT

TCTCAGTTCA GCGGCAATTC TTCCCCACTG ATGAGGACGA GTCTGGAGCT

TCTCAATGCA GCGGCACTTC TTTCCCACTG ATGAGGATGA GGCTGGAGCC

TGTCAGTGCA GCGACAGTTC TTCCCCACCG ATGAAGATGA AACAGGAGCC

TCTCTGTGCA GCGGCAGTTC TTCCCCACGG ACGAGGACGA AATGGGAGCC

TCTCTGTACA GCGACAGTTC TTCCCCACTG ACGAGGACGA AACAGGAGCT

---------- ---------- ---------- ---------- ----------

TCACAGTGCA GCGGCAATTC TTCCCCACTG ACGAGGATGA AACAGGAGCT

---------- ---------- ---------- ---------- ----------

TCTCCGTGCA GCGGCAGTTC TTCCCCACAG ACGAGGATGA AACGGGAGCT

TCACCATGCA GCGCCAGCTA TTCCCCACAG AAGAGGATGA AACAGGAGCA

TCACAGTGCA GCGACAGTTC TTCCCCACGG AGGAGGATGA GACCGGAGCA

GCCAAAGCCC TGATGAGACT TCAGGACACA TACAGGCTGG ACCCAGGCAC

GCCAAAGCCC TGATGAGACT TCAGGACACA TACAGGCTGG ACCCAGGCAC

GCCAAAGCCC TGATGAGACT TCAGGACACA TACAGGCTGG ACCCAGACAC

---------- ---------- ---------- ---------- ----------

GCCAAAGCCC TGATGAGACT TCAGGACACA TATAGGCTGG ACCCAGACAC

GCCAAAGCCC TGATGAGACT TCAGGACACA TACAGGCTGG ACCCAGACAC

GCCAGAGCCC TGATGCGACT TCAGGACACC TACAAACTGG ATCCGGACAT

GCCAGAGCCC TGATGAGACT TCAGGACACG TACAAACTGG ATCCGGACAC

GCCAAGGCCC TGATGAGACT TCAGGACACC TACAAGCTGG ACCCAGCCAC

GCCAAGGCCT TGATGAGACT TCAGGATACA TACAAACTGG ACCCAGATAC

GCCAAGGCCC TGATGAGACT TCAGGACACA TACAAGCTGG ATCCAGACAC

GCCAAGGCCC TGATGAGGCT TCAGGACACA TACAAGCTGG ACCCAGACAC

---------- ---------- ---------- ---------- ----------

GCCAAGGCCC TGATGAGGCT TCAGGACACG TACCGGCTGG ACACAGACAC

---------- ---------- ---------- ---------- ----------

GCCAAGGCTC TCATGAGGCT TCAGGACACG TACAAACTGG ATCCGGACAC

GCAATAGCAT TAATGCGCCT TCAGGACACA TACAAACTGG ATCCTGACAC

GCCAAGGCCT TAATGCGTCT GCAAGACACT TACAAACTGG ACCCGGACAC

AATTTCCAGA GGGGAACTTC CAGGAACCAA GTACCAGGCA ATGCTGAGTG

AATTTCCAGA GGGGAACTTC CAGGAACCAA GTACCAGGCA ATGCTGAGTG

AATTTCCAGA GGAGAACTTC CAGGAACCAA GTACCAGGCA GTGCTGAGTG

---------- ---------- ---GAACCAA GTACCAGGCA GTGCTGAGTG

AATTTCCAGA GGAGAACTTC CAGGAACCAA GTACCAGGCA ATGCTGAGTG

AATTTCCAGA GGGGAGCTTC CAGGCACTAA GTACCAGGCA GTGCTGAATG

GATTTCTAGA GGGGAGCTTC CAGGAACAAA GTACCAGGCC ATGCTGAGTG

GATTTCCAGA GGGGAACTTC CAGGCACAAA GTACCAGGCC ATGCTGAGTG

GATTTCCAGA GGGGAGCTTC CAGGAACTAA GTACCAGGCA ACGCTGAGTG

AATTTCCAAA GGGGAACTTC CAGGAACCAA ATACCAGGCC ATGCTGAGTG

AATTTCCAAA GGGCAACTTC CAGGAACCAA GTACCAGGCC GTGCTGAGTG

GATTTCCAAA GGGGAACTTC CAGGAACCAA GTACCAGGCA GTGATGAGTG

---------- ---------- ---------- ---------- ----------

GATTTCCAAA GGGGAGCTTC CAGGAACCAA ATACCAGGCA ACACTGAGTG

---------- ---------- ---------- ---------C ACCCTGAGTA

CATTTCCAAA GGGGCACTTC CTGGAACCAA GTACCAGGCC ACCCTGAGTG

TATTTCCAAG GGGGATCTTC CAGGTACCAA ATACCGTACC ACACTGAGTG

CATTGCTAAA GGGGACCTTC CAGGGACCAA GTACAGATCG ACGCTGACGG

TGGATGACTG CTTTGGGATG GGCCGCTCGG CCTACAATGA AGGGGACTAT

TGGATGACTG CTTTGGGATG GGCCGCTCGG CCTACAATGA AGGGGACTAT

TGGATGACTG CTTTGGGATG GGCCGCTCAG CCTACAATGA AGGGGATTAT

TGGATGACTG CTTTGGGATG GGCCGCTCAG CCTACAATGA AGGGGACTAT

TAGATGACTG CTTTGGGATG GGCCGCTCAG CCTATAATGA AGGGGACTAT

CAGATGACTG CTTTGGGATG GGCCGCTCGG CCTACAACGA AGGGGACTAT

TGGATGACTG CTTTGGGATG GGCCGCTCAG CTTACAACGA AGGAGACTAT

TGGACGACTG CTTTGGGCTG GGCCGCTCAG CTTACAATGA AGGAGACTAT

TGGACGACTG CTTCGGGATG GGCCGCTCGG CCTACAATGA AGGGGACTAC

TGGATGACTG CTTTGGGATG GGCCGCTCGG CCTACAACGA AGGCGACTAT

TGGATGACTG CTTTGGGATG GGCCGCTCAG CCTATAATGA AGGTGACTAT

CGGATGACTG CTTTGGGATG GGCCGCTCAG CTTACAATGA GGGGGACTAT

---------- ---------- ---------- ---------- ----------

TGGACGACTG CTTTGGGATG GGCCGCTTGG CCTACAATGA AGGAGACTAT

TTGATGACTG CTTCATACTG GGGCAGACAG CCCACCATAT CAAGGACTTT

TGGACCACTG CTTTGGTATG GGCCGCTTGG TCCACAATGA GGGGGACTAC

CAGATGACAC CTTTGGCATG GGTAAGATAG CCTACAATAA TGGGGACTAT

CAGACGATTG CTTCGGCATG GGCAAGACGG CCTACAATGA CGGGGATTAC

TATCATACGG TGTTGTGGAT GGAGCAGGTG CTAAAGCAGC TTGATGCCGG

TATCATACGG TGTTGTGGAT GGAGCAGGTG CTAAAGCAGC TTGATGCCGG

TATCATACGG TGTTGTGGAT GGAGCAGGTG CTAAAGCAGC TTGATGCTGG

TATCATACGG TGTTGTGGAT GGAGCAGGTG CTAAAGCAGC TTGATGCTGG

TATCATACGG TGATGTGGAT GGAGCAGGTG CTAAAGCAGC TTGATGCTGG

TATCACACGG TGCTATGGAT GGAGCAGGTG CTCAAGCAGC TTGATGCTGG

TACCACACCG TGTTGTGGAT GGAGCAGGTG CTGAAGCAGC TGGATGCTGG

TACCATACTG TGCTGTGGAT GGAGCAGGTA CTGAAGCAGC TCGATGCTGG

TATCACACGG TGCTGTGGAT GGAGCAGGTG CTGAAGCAGC TCGATGCTGG

TACCACACGG TGTTGTGGAT GGAACAGGTG CTAAAGCAGC TCGATGCTGG

TACCACACAG TCTTGTGGAT GGAGCAGGTA CTGAAGCAGC TCGATGCTGG

TACCACACGG TGCTGTGGAT GGAGCAGGTG CTGAAGCAGC TTGATGCTGG

---------- ---------- ---------- ---------- ----------

TATCATACAG TCTTGTGGAT GGAGCAAGTG CTGAAGCAGC TTGATGCAGG

TATCACTCAA TGCTTTGGAT A------GCA GTGAAGAAGC TGAATGCAAG

TACCACACTG TGCTGTGGAT GGAGCAGGTG CTGAAGCAGC TCGACGCGGA

TACCACACAG TCCTGTGGAT GCAGCAGGCA CTGAAGCAAC ACGATGCTGG

TATCACACGG TGCTTTGGAT GCAGCAGGCC CTGAAGCAGC ACGACCAGGG

GGAGGAGGCC ACCACAACCA AGTCACAGGT GCTGGACTAC CTCAGCTATG

GGAGGAGGCC ACCACAACCA AGTCCCAGGT GCTGGACTAC CTCAGCTATG

GGAGGAGGCC ACCACAACCA AGTCCCAGGT GCTGGACTAC CTCAGCTATG

GGAGGAGGCC ACCACAACCA AGTCCCAGGT GCTGGACTAC CTCAGCTATG

GGAGGAGGCC ACCACAACCA AGTCCCAGGT GCTGGACTAC CTCAGCTATG

GGAGGAGGCC ACCACGACCA AGTCCCAGGT GCTGGACTAC CTCAGCTATG

GGAGGAGGCC ACTGTTACCA AATCCCTGGT GCTGGACTAC CTGAGCTATG

GGAGGAGGCC ACTGTTACCA AGTCCCTGGT GCTGGACTAC CTGAGCTATG

CGAGGAGGGC ACCACGACCA AATCTCAGGT GCTGGACTAC CTCAGCTATG

GGAGGAGGCC ACCACATCCA AGGCCCAGGT GCTGGACTAT CTGAGCTACG

AGAGGAGGCC ACCACATCCA AGGCCCAGGT GCTAGACTAC CTGAGCTATG

GGAGGAGGCC ACCGCAACCA AGGCCCAGGT GCTGGACTAC CTGAGCTATG

---------- ---------- ---------- ---------- ----------

GGAGGAGGCC AGCACAACCA AGGCCCAGGT GCTTGACTAC CTCAGCTATG

AGAGCACAGC ACTATCTCTA AGGCC---GT CCTGGATTAT CTCAGTAATG

GGAGGAGGCC ACCGTGGCCA AGGCCCAGGT GCTGGACTAC CTCAGCTACG

TGAGGATGCT ACCACATCTA AAGTCGAAAT CTTGGATTAC CTCAGCTATG

GGAGGATGCA GTCACCTCCA AGGTGGAGGT CTTGGATTAC CTCAGCTATG

CTGTCTTCCA GTTGGGTGAT CTGCACCGTG CCCTGGAGCT CACCCGCCGC

CTGTCTTCCA GTTGGGTGAT CTGCACCGTG CCCTGGAGCT CACCCGCCGC

CTGTCTTTCA GTTGGGTGAT CTGCACCGTG CCCTGGAGCT CACCCGCCGC

CTGTCTTTCA GTTGGGTGAT CTGCACCGTG CCCTGGAGCT CACCCGCCGC

CTGTCTTCCA GTTGGGTGAC CTGCATCGTG CCCTGGAACT CACTCGTCGC

CTGTCTTCCA GTTGGGTGAT CTGCACCGTG CCCTGGAGCT CACCCGCCGC

CCGTCTTCCA GCTGGGTGAC CTGCACCGTG CGGTGGAACT CACCCGTCGC

CTGTCTTCCA ACTGGGTGAC CTGCACCGTG CTGTGGAACT CACCCGCCGC

CCGTCTTCCA GTTGGGTGAC CTGCATCGTG CCCTGGAGCT CACCCGCCGC

CTGTCTTCCA GTTGGGTGAC CTGCACCGTG CCGTGGAGCT CACCCGCCGC

CTGTCTTCCA GTTGGGTGAC CTGCACCGTG CCCTGGAGCT TACCCGCCGC

CTGTCTTCCA GCTGGGCGAC CTGCACCGTG CCCTGGAGCT CACCCGCCGC

---------- ---------- ---------- ---------- ----------

CTGTCTTCCA GCTGGGAGAC ATGCACCGGG CCCTGGAGCT CACCCGCCGC

GCAGCTTCTG CTTGGGC-AC CTGCACAGGA TTGTGGACTT CACCCTTCAA

CCGTCTTCCA GCTGGGCGAC CTGCCCCGGG CCCTGGAGCT CACACGCCGT

CTGTCTTTCA GCTTGGAGAC ATTCACAGGG CTCTAGAATT CACCCGACGC

CCGTTTTTCA GCTCGGAGAT CTACACAGAG CGGTGGAGCT CACCAGGCGC

CTGCTCTCCC TTGACCCAAG CCACGAACGA GCTGGAGGGA ATCTGCGGTA

CTGCTCTCCC TTGACCCAAG CCATGAACGA GCTGGAGGGA ATCTGCGGTA

CTGCTCTCCC TTGACCCAAG CCATGAACGA GCTGGAGGGA ATCTGCGGTA

CTGCTCTCCC TTGACCCAAG CCATGAACGA GCTGGAGGGA ATCTGCGGTA

CTCGTCTCCC TTGACCCAAG CCATGAACGA GCTGGAGGGA ATCTGCGGTA

CTTCTCTCCC TCGATCCGAG CCATGAACGA GCTGGAGGAA ATCTGCGGTA

CTGCTCTCTC TTGACCCGAG CCATGAACGA GCCGGAGGGA ACCTGCGGTA

CTGCTCTCTC TTGACCCAAG CCACGAACGA GCTGGAGGGA ATCTGCGGTA

CTGCTCTCAC TTGATCCAAG CCATGAGCGA GCTGGAGGGA ATCTGCGCTA

CTGCTCTCCC TTGACCCGAG CCATGAACGA GCTGGAGGGA ATCTGCACTA

CTGCTCTCTC TTGACCCAAG CCATGAACGA GCTGGAGGGA ATCTGCGGTA

CTGCTGTCCC TTGACCCAAG CCATGAACGA GCTGGAGGGA ATCTGCGGTA

---------- ---AGCCGAG CCACGAACGA GCTGGAGGCA ATCTGCGGTA

CTGCTCTCCC TTGACCCAAG CCATGAACGG GCTGGAGGGA ATCTGCGGTA

CTGCTTTCTC TTG------- ---------- ---------- ----------

CTGCTCTCCC TTGACCCGAG CCACGAACGG GCTGGAGGGA ACCTGCGGTA

TTGATTTCTC TTGACCCCAA TCATGAAAGA GCCGGGGGGA ATCTGCGGTA

TTGATTTCGC TGGACTCCAG TCACGAACGA GCTGGAGGAA ACTTACGATA

CTTTGAGCAG TTATTGGAGG AAGAGAGAGA AAAAACGTTA ACAAATCAGA

CTTTGAGCAG TTATTGGAGG AAGAGAGAGA AAAAACGTTA TCAAATCAGA

CTTTGAGCAG TTATTGGAGG AAGAGAGAGA AAAAATGTTA TCAAATCAGA

CTTTGAGCAG TTATTGGAGG AAGAAAGAGA AAAAATGTTA TCAAATCAGA

CTTTGAGCAG TTATTGGAGG AAGAAAGAGA AAAAATGTTA TCAAATCAGA

CTTTGAGCAC TTGTTGGAGG AAGAAAGAGA AAAAATGTTA TCAAATAAGA

CTTTGAGCGG CTGTTAGAGG AAGAAAGAGG AAAATCATTG TCGAATCAGA

CTTTGAACGG TTGTTAGAGG AAGAAAGAGG GAAATCACTG TCAAATCAGA

CTTTGAGCGG TTGTTGGAGG AACAAAGAGG AAAGTCGTTA TTGAATCAGA

CTTTGAACGG TTGTTGGAAG AAGAAAGAGA AAAAATGTTA TCGAATCACA

CTTTGAACGG TTGCTGGAGG AAGAAAGAGA AAAAATGTTA TTAAATCAGA

CTTTGAACGG TTGTTGGAGG AAGAAAGGGA AAAAATAGTG TCAAATCAGA

CTTCGAACGA CTGTTGGAGG AAGAGAGAGA GAAAACAGTA TTGAATCAGA

CTTTGAACGG TTGTTAGAGG AAGAAAGAGA AAAACTGTTA TCCAATCAGA

---------- ---------- ---------- ---------- ----------

CTTTGAACAC TTGTTGGAAG AAGAAAGGCA GCAAACGTTA CCGAACCAGA

CTTTGAGAAG CTAATAGAGG AAGAAAGACT AGGAAAGACA TTAAATAAGA

TTTTGAGAAA TTGCTCGAAG AAGAAAGGAT GGAGAAGCCT CTAAACAGGA

CAGAAGCTGA GCTAGCAACC CCAGAAGGCA TCTATGAGAG GCCTGTGGAC

CAGAAGCTGA GCTAGCAACC CCAGAAGGCA TCTATGAGAG GCCTGTGGAC

CAGAAGCTGA GCTAGCAACC CCAGAAGGCA TCTATGAGAG GCCTGTGGAC

CAGAAGCTGA ACTAGCAACC CCAGAAGGCA TCTATGAGAG GCCTGTGGAC

CAGAAGCTGA GCTAGCAACC CCAGAAGGCA TCTATGAGAG GCCTGTGGAC

CAGAAGCTGA GCTGGCAACC CAGGAAGGCA TCTATGAGAG GCCGGTGGAC

CGGACGCTGG ACTAGCATCG CAGGAAAACT TGTACGAGAG GCCTGTAGAC

CAGACGCCGG ACTGGCCACC CAGGAAAACT TGTACGAGAG GCCCACGGAC

CAGAAGCTGT GGCAGTGACC CAGGAAGGCA TCTACGAGAG GCCTGTGGAC

CAGAAGCTGA GCTTGCATCC CAGCAAGGCA TATACGAGAG GCCTGTGGAC

CAGAAGCTGG ACTGGCCACG CAGGAAAGCA TCTATGAGAG GCCTGTGGAC

CAGAAGCTGA GCCAGCAAGC CAGGAAGGCA TCTATGAGAG GCCTGTGGAC

CGGGAGCTGA GCCTGCCACC CAGGAAGGCT TCTATGAGAG GCCCTCGGAC

CAGAAGCTGA GCCAACCACC CAGGAAGGCA TCTATGAGAG GCCTGCGGAC

---------- ---------- ---------- ---------- ----------

CGGAAGCCGA GCCAGCTGCC CAGGATGGCA TTTACGAGAG GCCCGCGGAC

CAGAGACTGA ACCGGCAACA CAGGGAGCTT TCTATCAGAG GCCTTCAGAC

CATCAGCTAA ACCAGCGACA CATGGAGGGA TCTATGAAAG ACCTCCGGAT

TACCTGCCTG AGAGGGATGT TTACGAGAGC CTCTGTCGTG GGGAGGGTGT

TACCTGCCTG AGAGGGATAT TTACGAGAGC CTCTGTCGTG GGGAGGGTGT

TACCTGCCTG AGAGGGATGT TTACGAGAGC CTCTGTCGTG GGGAGGGTGT

TACCTGCCTG AGAGGGATGT TTACGAGAGC CTCTGTCGTG GGGAGGGTGT

TACCTGCCTG AGAGGGATGT TTACGAGAGC CTCTGTCGTG GGGAGGGTGT

TACCTGCCTG AGAGGGAGGT GTATGAGAGC CTCTGCCGTG GGGAGGGTGT

TACCTGCCCG AGAGGGACGT GTATGAGAGC CTGTGTCGAG GGGAGGGCAT

TACCTGCCTG AGAGGGATGT GTACGAGAGC CTGTGTCGAG GGGAGGGCGT

TACCTGCCCG AGAGGGATGT GTATGAGAGC CTGTGTCGTG GGGAGGGCGT

TACCTGCCGG AGAGGGATGT CTACGAGAGC CTCTGTCGTG GGGAGGGTGT

TACCTGCCTG AGAGGGATGT TTATGAGAGC CTCTGTCGTG GGGAGGGTGT

TACCTGCCCG AGAGAGATGT CTACGAGAGC CTCTGTCGTG GGGAGGGTGT

TACCTGCCCG AGAGGGACGT GTACGAGAGC CTCTGTCGAG GGGAGGGCGT

TACCTGCCCG AGAGGGATGT CTATGAGAGC CTCTGTCGTG GGGAGGGTGT

---------- ---------- ---------- ---------- ----------

TACCTGCCCG AGAGGGACAT CTACGAGGGC CTCTGTCGTG GGGAGGGCGT

TACCTGCCTG AGAGAGAAGT CTATGAGGCC CTCTGTAGAG GGGAAGGCAT

TACCTGCCCG AAAGGGATGT GTATGAAGGG TTATGCAGAG GAGAGGGTGT

CAAACTGACA CCCCGTAGAC AGAAGAGGCT TTTCTGTAGG TACCACCATG

CAAACTGACA CCCCGTAGAC AGAAGAGGCT TTTCTGTAGG TACCACCATG

CAAACTGACA CCCCGGAGAC AGAAGAGGCT TTTCTGTAGG TACCACCATG

CAAACTGACA CCCCGGAGAC AGAAGAGGCT TTTCTGTAGG TACCACCATG

CAAACTGACA CCCCGGAGGC AGAAGAGGCT TTTCTGTAGG TACCACCATG

CAAATTGACA CCCCGGAGGC AAAAGAGGCT CTTCTGTAGG TACCACCATG

CAAAATGACA CCCCGGAGGC AGAAGAGGCT TTTCTGTAGA TACCACCATG

GAAACTGACA CCCCGGAGGC AGAAGAAGCT TTTCTGTAGG TACCATCATG

CAAACTGACC CCCCGGAGAC AGAAGAGGCT TTTCTGCAGG TACCACGATG

CAAACTGACC CCCCGAAGGC AGAAGAGGCT CTTCTGTAGG TATCACCATG

CAAACTGACT CCCCGGAGGC AGAAGAGGCT TTTCTGTAGG TACCACCATG

CAAACTGACG CCCCGGAGGC AGAAGAGGCT TTTCTGTAGG TACCACCATG

CAAACTGACG CCCCGGAGGC AGAAGAGGCT TTTCTGTAGG TACCACCATG

CAAACTGACA CCCCGGAGGC AGAAGAGGCT TTTCTGTAGA TATCACCATG

---------- ---------- ---------- ---------- ----------

CAAACTGACG CCCCGGAGGC AGAAGAGGCT TTTCTGTAGA TACCATCATG

TAAACTGACA CCTCAGAGAA GGAAGAGACT TTTCTGTAGA TACCACGATA

CAAGCTGACA CCCCGGAGGC AGAAGAGACT TTTCTGCAGA TATCATGATG

GCAACAGGGC CCCACAGCTG CTCATTGCCC CCTTCAAAGA GGAGGACGAG

GCAACAGGGC CCCACAGCTG CTCATTGCCC CCTTCAAAGA GGAGGACGAG

GCAACAGGGC CCCACAGCTG CTCATCGCCC CCTTCAAAGA GGAGGATGAG

GCAACAGGGC CCCACAGCTG CTCATCGCCC CCTTCAAAGA GGAGGATGAG

GCAACAGAGC TTCACAGCTG CTCATCGCCC CCTTCAAAGA GGAGGACGAG

GCAACAGGGC TCCACAGCTG CTCATCGCCC CCTTCAAAGA GGAGGACGAG

GAAACAGAGT GCCACAGCTC CTCATTGCCC CCTTCAAAGA GGAAGACGAG

GAAACAGAGT GCCACAGCTC CTCATCGCCC CCTTCAAAGA GGAAGACGAG

GCAATGGGGC GCCGCAGCTG CTCATCGCGC CCTTCAAGGA GGAGGACGAG

GCAACAGGGT GCCGCAGCTG CTCATCGCCC CCTTCAAAGA GGAGGATGAG

GCAACAGGAC ACCACAGCTG CTCATCGCCC CCTTCAAAGA GGAGGATGAG

GCAACAGGAC GCCACAGCTG CTCATTGCCC CCTTCAAAGA GGAGGACGAG

GCCACGGGGC GCCACAGCTG CTCATCGCCC CCTTCAAAGA GGAGGATGAG

GCAACAGGAC TCCCCAGCTG CTCATCGCCC CCTTTAAAGA GGAAGATGAG

---------- ---------- ---------- ---------- ----------

GCCACAGGAC CCCACAGCTG CTCATCGCCC CTTTCAAAGA GGAGGACGAG

GCAATAAGAC ACCCCAGCTG CTGATTGCAC CCTTTAAGGA GGAAGACGAG

GCAACAGAAC CCCCCAGCTG CTCATCGCCC CCTTTAAGGA AGAGGATGAG

TGGGACAGCC CGCACATCGT CAGGTACTAC GATGTCATGT CTGATGAGGA

TGGGACAGCC CGCACATCGT CAGGTACTAC GATGTCATGT CTGATGAGGA

TGGGACAGCC CACACATCGT CAGGTACTAC GATGTCATGT CTGATGAGGA

TGGGACAGCC CACACATCGT CAGGTACTAC GATGTCATGT CTGATGAGGA

TGGGACAGCC CGCACATCGT GAGGTACTAT GATGTCATGT CTGATGAGGA

TGGGACAGCC CACACATCGT CAGGTACTAC GATGTCATGT CAGACGAGGA

TGGGACAGCC CACACATCGT CAGGTACTAT GATGTGATGT CGGACGAAGA

TGGGACAGCC CACACATCGT CAGGTACTAT GATGTGATGT CCGACGAAGA

TGGGACAGCC CGCACATCGT CAGGTACTAC GACGTCATGT CTGATGAGGA

TGGGACAGCC CGCACATCGT CAGGTACTAC GACGTCATGT CTGACGAGGA

TGGGACAGCC CGCATATCGT CAGGTATTAT GATGTCATGT CTGATGAGGA

TGGGACAGTC CGCACATCGT CAGGTACTAC GACGTCATGT CTGATGAGGA

TGGGACAGCC CGCACATCGT CAGGTACTAC GATGTCATGT CTGATGAGGA

TGGGATAGCC CACACATCGT GCGGTACTAT GACATCATGT CTGACGAGGA

---------- ---------- ---------- ---------- ----------

TGGGACAGCC CACACATCGT CAGGTACTAC GATGTCATGT CTGATGAGGA

TGGGACAGCC CACACATCGT GAGATACTAC GATGTCTTAT CAGATGAGGA

TGGGATAGCC CTCATATCGT CCGCTATTAC GACGTCCTGT CCGATGAAGA

AATCGAGAGG ATCAAGGAGA TCGCAAAACC TAAAATGAGT GATGTAGAAG

AATCGAGAGG ATCAAGGAGA TCGCAAAACC TAAAATGAGT GATGTAGAAG

AATCGAGAGG ATCAAGGAGA TCGCAAAACC TAAAATGAGT GATGTAGAAG

AATCGAGAGG ATCAAGGAGA TCGCAAAACC TAAAATGAGT GATGTAGAAG

AATCGAGAGG ATCAAGGAGA TCGCAAAACC TAAAATGAGT GATGTAGAAG

AATCGAGAGG ATCAAGGAGA TTGCAAAACC CAAAATGAGT GATGTAGAAG

GATCGAGAGG ATCAAGGAGA TTGCTAAGCC CAAAATGAGT GATGTTGAAG

AATCGAGAGG ATCAAGGAGA TTGCTAAGCC CAAAATGAGC GATGTCGAAG

GATCGAGAGG ATCAAGGAGA TTGCGAAACC CAAGATGAGT GATGTAGAAG

AATCGAGAGG ATCAAGGAGA TTGCGAAACC CAAAATGAGT GATGTAGAAG

AATCGAGAGG ATCAAGGAGA TCGCGAAACC CAAAATGAGT GATGTAGAAG

AATCGAGAAG ATCAAGGAGA TCGCGAAACC CAAAATGAGT GATGTAGAAG

AATCGAGAGG ATCAAGGAGA TTGCAAAACC TAAGATGAGT GATGTAGAAG

AATCGAGAGG ATCAAGGAGA TTGCGAAACC CAAAATGAGT GATGTAGAAG

---------- ---------- ---------- ---------- ----------

AATCGAGAGG ATCAAGGAGC TTGCGAAGCC CAAAATGAGC GATGTGGAAG

GATTGAGAAG ATCAAGGAAA TTTCCAAGCC TAAAATGAGT GATGTTGAAG

GATTGAGAAG ATTAAAGAGC TGGCTAAACC CAAGATGAGT GACGTCGAAG

CTGGTGGTGC CACCGGTACA GCTGTGTTCT GGTACAACCT CTTGCGGAGC

CTGGTGGTGC CACCGGTACA GCTGTGTTCT GGTACAACCT CTTGCGGAGC

CTGGTGGTGC CACCGGTACA GCTGTGTTCT GGTACAACCT CTTGCGGAGC

CTGGTGGTGC CACCGGTACA GCTGTATTCT GGTACAACCT CTTGCGGAGC

CTGGTGGTGC CACTGGTACA GCTGTATTCT GGTACAACCT CTTGCGGAGT

CTGGTGGTGC CACTGGTACA GCTGTATTCT GGTACAACCT CTTGCGGAGT

CTGGTGGCGC CACTGGCACG GCTGTATTTT GGTATAACCT TCTTCGGAGC

CTGGTGGTGC CACCGGCACA GCTGTATTCT GGTACAACCT TCTTCGCAGT

CTGGTGGTGC CACTGGCACA GCTGTATTCT GGTACAACCT TCTGCGGAGT

CTGGTGGTGC CACCGGCACA GCTGTATTCT GGTACAACCT CCTACGGAGT

CTGGTGGTGC CACTGGTACA GCTGTATTCT GGTACAACCT TCTGCGGAGT

CTGGGGGTGC CACCGGCACA GCAGTATTCT GGTACAATCT CCTCAGGAGT

CAGGGGGTGC CACCGGCACC GCTGTGTTCT GGTACAACCT CCTGCGGAGT

CTGGTGGTGC CACTGGCACA GCCGTGTTCT GGTACAACCT CCTGCGGAGT

---------- ----GGCACA GGCTTGTTCT GGTATAA--- CCTGTGGAGT

CTGGTGGTGC CAC-GGCACA GCTGTGTTCT GGTACAATCT CCTGCGGAGT

CTGGTGGAGC CACAGGCACA TCAGTATTCT GGTACAATCT CTTCCGCAGT

CCGGTGGTGC CACAGGGACA GCGGTGTTTT GGTATAATCT GTTCAGAAGC

GGGGAAGGTG ACTACCGAAC AAGACATGCT GCCTGCCCTG TGCTTGTGGG

GGGGAAGGTG ACTACCGAAC AAGACATGCT GCCTGCCCTG TGCTTGTGGG

GGGGAAGGTG ACTACCGAAC AAGACACGCT GCCTGCCCTG TGCTTGTGGG

GGGGAAGGTG ACTACCGAAC AAGACACGCT GCCTGCCCTG TGCTTGTGGG

GGGGAAGGTG ACTACCGAAC AAGACATGCT GCCTGCCCTG TGCTTGTGGG

GGAGAAGGTG ACTACCGAAC AAGACATGCT GCCTGCCCTG TGCTTGTGGG

GGGGAAGGCG ATTATCGAAC GAGGCACGCG GCCTGCCCTG TGCTTGTGGG

GGGGAAGGTG ATTATCGGAC GAGACATGCA GCCTGCCCTG TGCTTGTGGG

GGGGAAGGTG ACTACCGAAC AAGACACGCC GCCTGCCCTG TGCTTGTGGG

GGGGAAGGTG ACTATCGAAC AAGACATGCT GCCTGCCCTG TGCTTGTGGG

GGGGAAGGTG ACTACCGTAC AAGACACGCT GCCTGTCCTG TGCTTGTGGG

GGGGAAGGTG ACTACCGAAC GAGACACGCT GCCTGCCCCG TACTTGTGGG

GGGGAAGGGG ACTATCGGAC GAGGCATGCG GCCTGCCCCG TGCTTGTGGG

GGGGAAGGCG ACTACCGGAC AAGACATGCT GCCTGCCCCG TGCTTGTGGG

GAGAAACGCA GCCATCTGAT GAGACATGCT GCTTGCCTTG TGCTTGTGGG

GGCGAAGGCG ACTACCGGAC GAGACACGCT GCCTGCCCCG TGCTCGTGGG

GGGGAGTGTG ACTACCGGAC AAGACATGCA GCCTGTCCAG TGCTTGTTGG

GGAGAGGGAG ATTACAGGAC ACGACATGCG GCTTGTCCGG TACTAGTAGG

CTGCAAGTGG GTCTCCAATA AGTGGTTCCA TGAACGAGGA CAGGAGTTCT

CTGCAAGTGG GTCTCCAATA AGTGGTTCCA TGAACGAGGA CAGGAGTTCT

CTGCAAGTGG GTCTCCAATA AGTGGTTCCA TGAACGAGGA CAGGAGTTCT

CTGCAAGTGG GTCTCCAATA AGTGGTTCCA TGAACGAGGA CAGGAGTTCT

CTGCAAGTGG GTCTCCAATA AGTGGTTCCA TGAACGAGGA CAGGAGTTCT

CTGCAAGTGG GTCTCCAATA AGTGGTTCCA TGAACGAGGA CAAGAGTTCT

CTGCAAGTGG GTCTCCAATA AGTGGTTCCA TGAGCGAGGA CAGGAGTTCT

CTGCAAGTGG GTCTCCAACA AGTGGTTCCA TGAGCGAGGA CAGGAGTTCT

TTGCAAGTGG GTTTCCAACA AGTGGTTTCA TGAACGAGGA CAGGAGTTCC

CTGCAAGTGG GTCTCCAATA AGTGGTTCCA TGAACGAGGA CAGGAATTCT

CTGCAAGTGG GTCTCCAATA AGTGGTTCCA TGAACGAGGA CAGGAATTCT

CTGCAAGTGG GTCTCCAATA AGTGGTTCCA TGAACGAGGC CAGGAGTTCT

CTGCAAGTGG GTCTCCAATA AATGGTTCCA TGAGCGAGGA CAGGAGTTCT

CTGCAAGTGG GTCTCCAATA AATGGTTCCA TGAACGAGGA CAGGAATTCT

CTACGAG--- GTTTCCAATA GGTGGTTCTG TGAG---GGA CAAGAGTTCT

CTGCAAGTGG GTTTCCAATA AGTGGTTCCA CGAGCGAGGA CAGGAGTTCT

CAGTAAATGG GTTTCCAATA AATGGTTCCA TGAGCGGGGG CAGGAGTTCT

GTGCAAGTGG GTTTCAAATA AATGGTTCCA TGAACGAGGA CAGGAATTCT

TGAGACCTTG TGGATCAACA GAAGTTGACA GAGGCCTCCC GCGGAGCCGG

TGAGACCTTG TGGATCAACA GAAGTTGACA GAGGCCTCCC GCGGAGCCGG

TGAGACCTTG TGGATCAACA GAAGTTGACA GAGGCCTCCC GCGGAGCCGG

TGAGACCTTG TGGATCAACA GAAGTTGACA GAGGCCTCCC ACGGAGCCGG

TGAGGCCTTG TGGATCAACA GAAGTTGACA GGGGCCTCCC GCGGAGCCGG

TGAGGCCTTG TGGATCAACA GAAGTTGACA GAGGCCTCCC GTGGACCCGG

TGAGGCCTTG TGGGACAACG GAAGTCGAT- ---------- ----------

TAAGACCTTG TGGAACAACG GAAGTTGATA GGGTTCTCCC TCGGAACCGG

TGAGACCTTG TGGATCAACA GAAGTTGAC- ---------- ----------

TGAGGCCGTG TGGATCGACA GAAGTTGACA GAGGCCTTCC GAGAAGTCGC

TGAGGCCATG TGGATCAACA GAAGTTGACA GAGGCCTCCC GCGAAGCCGT

TGAGGCCGTG TGGATCAACA GAAGTCGACA GAATCCTCTC TCGGAACCGC

TGAGGCCATG TGGATCAACA GAAGTTGACA GGGGCCTCTC ACGGAGCCGA

TGCGGCCTTG TGGATCCACA GAAGTTGACA GAAGCCTCCC ACGGATCCGC

TAAGAC---- TGGATCAACA GAAGTTGAC- ---------- ----------

TGAGGCCGTG TGGATCAACA GAGGTCGACA GAAGCCTCTC TCGGAGTCGG

TGAGACGATG TGGATCAACA GAAGTCGACA GATCCCTTCC AAGGAGCAGG

TGCGGCCTTG CGGATCCACA GAAGTTGAT- GAGGTCCCTC CCGGAACAGG

GACTGCGTCG ACCTGGGCTC CGAGGTGTAC AGGATGCTGC GGGAGCCAGC

GACTGCGTCG ACCTGGGCTC CGAGGTGTAC AGGATGCTGC GGGAGCCGGC

GACTGCGTCG ACCTGGGCTC CGAGGTGTAC AGGATGCTGC GGGAGCCGGC

GACTGCGTCG ACCTGGGCTC CGAGGTGTAC AGGATGCTGC GGGAGCCGGC

GACTGCGTCG ACCTGGGCTC CGAGGTGTAC AGGATGCTAC GGGAGCCAGC

GACTGCGTGG ACCTGGGCTC GGAGGTGTAC AGGATGCTGC GGGAGCCCGC

---------- ---------- ---------- ---------- ----------

GACTGCGTAG ACCTGGGTTC AGAGGTATAC AGGATGTTAA GGGAGCCAGC

---------- ---------- ---------- ---------- ----------

GACTGCGTCG ACCTGGGCTC AGAGGTGTAC AGGATGCTTC GGGAGCCAGC

GACTGCGTGG ACCTGGGCTC AGAGGTGTAC AGGATGCTGC GAGAGCCGGC

GACTGTGTCG ACCTGGGCTC GGAAGTGTAC AGGATGCTGA GGGAGCCCGC

GACTGCGTGG ACATGGGCTC TGAGGTGTAC CGGATGCTCC AGGAGCCAGC

GACGGCGTCG ACCTGGGATC CAGTGTGTAC AGGATGCTGC AGAAACCCGC

---------- ---------- ---------- ---------- ----------

GACAGCATTG ACCTGGGCTC TGACGTGTAC AGGATGCTGC GGGAGCCGGC

AATGGCATTG ATGTGGAATC TGATGTATAT AAAATGCTCC AGGAATATGG

AACGGCATCG ATGTGGAGTC AGAGGTCTAC AAGATGCTCC AGGATCCCAG

CGAGCCCGCC GCGGAGCCCA AGCAGTCAGG CTCCTTCCGC TACTTGCAGG

CGAGCCCGCC GCGGAGCCCA AGCAGTCAGG CTCCTTCCGC TACTTGCAGG

TGAGTCCGCT GCGGAGCCCA AGCAGTC--- ---------- ----------

GGAGTCCGCT GTGGAGCCCA AGCAGTCAGG CTCCTTCCGC TACTTGCAGG

AGAACCCGCT GCGGAGCCCA AGCAGTCAGG CTCCTTCCGC TACTTGCAGG

CGAGCCCGCC ACCGAGCCCA AGCAGTCAGG CTCCTTCCGC TACTTGCAGG

---------- ---------- ---------- ---------- ----------

AGAGCCCGCC TCGGAGCCGA AACAGTCAGG ATCCTTCCGC TACCTGCAGG

---------- ---------- ---------- ---------- ----------

CGAGCCTGCT GCGGAGCCCA AGCAGTCAGG CTCCTTCCGC TATTTGCAGG

TGAACCCGCT TCGGAGCCCA AGCAGTCAGG CTCCTTCCGC TACTTGCAGG

CGAGCCCGCT GCAGAGCCCA AGCAGTCAGG CTCCTTCCGC TACTTGCAGG

CGAGCCCTCA GCAGAGCCCA AGCAGTCAGG CTCCTTCCGC TACTTGCAGG

TGAGCCCGCC GTGGAGCCCA AGCAGTCAGG CTCCTTCCGC TACCTGCAGG

---------- ---------- ---------- ---------- ----------

TGAGCCCACT TCTGAACCCA AACAGTCCGG CTCCTTCCGA TACCTGCAGG

AGAACCTGCC TCAGAGCCCA AGCAGTCTGG CTCCTTCAGA TACCTACAGG

CGAGCCCGCT CCGGCGCCCA AACAGTGTGG CTCCTTCAGA TATCTGCAGG

GCATGCTAGA GGCCGGCGAG GGCGGGAATG CAGAGCCACG GAGCCTCTCC

GCATGCTAGA GGCCGGCGAG GGCGGGAATG CAGAGCCACG GAGCCTCTCC

---------- ---------- ------AATG CAGAGCCACG GAGCCTCTCC

GCATGCTAGA GGCCGGCGAG GGTGGGAATG CAGAGCCACG GAGCCTCTCC

GCATGCTGGA GGCCGGCGAG AGCGGAAATG CAGAGGCACG GAGCCTCTCC

GCATGCTAGA GGCCGGTGAG GGCGGGAGTG CAGAGCCACG GAGCCTCTCC

---------- ---------- ------AACG CAGAGCCTCG GAGCCTGTCC

GCATGCTAGA GGCTGGCGAG GGCGGGAACG CAGAGCCTCG GAGCCTGTCC

---------- ---------- ---------- ---------- ----------

GCATGCTGGA GGCGGGCGAG GGCGGG---- ---------- ----------

GCATGCTAGA GGCCGGGGAG GGTGGGAATG CAGAGCCACG GAACCTCTCC

GCATGCTGGA GGCCAGCGAG GGCGGAAATG CAGAGCCACG GAGCCTCTCC

GCATGCTGGA GGCCGGCGAG GGGGGGAACG CGGAGCCGCG GCCCCTCTCC

GCATGCTAGA GGCTGGGGAA GGCGGGAATG CAGAGCCGCG GAACCTCTCC

---------- ---------- ------AATG CAGAGCCGCG GAGCCTCTCC

GCATGCTAGA GGCTGGCCAG GGTG--AATG CAGAACTGCG AAATCTCTCC

GCATGTTAGA AGCTGGAGAG AATGGAAAAG AAGAGATGCG CATCCTCAAT

GCAAGATGGA GGCCGGGAGA GACCGTAGCG AACAGAAGCG GAGTCTTTCG

CTGGGCGGCC ATGTGGGTTT CGACAGCCTC CCCGACCAGC TGGTCAGCAA

CTGGGCGGCC ATGTGGGTTT CGACAGCCTC CCCGACCAGC TGGTCAGCAA

CTGGGCGGCC ATGTGGGTTT TGACAGCCTC CCCGACCAGC TGGTCAGCAA

CTGGGCGGCC ATGTGGGTTT TGACAGCCTC CCCGACCAGC TGGTCAGCAA

CTGGGCGGCC ATGTGGGCTT TGACAGCCTC CCTGACCAGC TGGTCAGCAA

CTGGGCGGCC ATGTGGGCTT TGACAGCCTC CCCGACCAGC TGGTCAGCAA

CTGGGTGGCC ATGTGGGCTT CGACAGCCTC CCTGACCAGC TGGTCAGCAA

CTGGGCGGCC ATGTTGGGTT TGACAGCCTC CCTGACCAGC TGGTCAGCAA

---------- ---------- ---------- ---------- ----------

---------- ---------- ---------- ---------- ----------

CTTGGTGGTC ATGTGGGCTT CGACAGCCTC CCGGATCAGC TTGTCAGCAA

CTGGGCGGCC ACGTGGGCTT TGACAGCCTC CCTGACCAGC TGGTCAGCAA

CTGGGCGGCC ATGTGGGCTT CGACAGCCTC CCCGACCAGC TGGTCAGCAA

ATGGGGGGCC ACGTGGGCTT CGACAGCCTC CCGGACCAGC TGGTCAGCAA

CTCGGGGGCC ATGTGGGCTT CGACAGCCTT CCCGACCAGC TGGTCAGCAA

CTGGGGGGCC ATGTGGGCTT CGACAGCCTC CCCGACCAGC TGGTCAGCAA

CTGGGAGGCC ATGTGGGTTT TGACAGCCTC CCAGACCAGC TGGTCAGTAA

CTCGGAGGCC ACGTTGGCTT TGACAGCCTC CCGGACCAGC TGGTCAGCAA

GTCGGTCACT CAGGGCTTCA GCTTCAACAT CCTCTGTGTG GGGGAGACCG

GTCGGTCACT CAGGGCTTCA GCTTCAACAT CCTCTGTGTG GGGGAGACCG

GTCGGTCACT CAGGGCTTCA GCTTCAACAT CCTCTGTGTG GGGGAGACTG

GTCGGTCACT CAGGGCTTCA GCTTCAACAT CCTCTGTGTG GGGGAGACTG

GTCGGTCACT CAGGGCTTCA GCTTCAACAT ACTCTGTGTG GGGGAGACCG

GTCGGTCACT CAGGGCTTCA GCTTCAACAT CCTCTGTGTG GGGGAGACTG

GTCAGTCACT CAGGGCTTCA GCTTCAACAT TCTCTGTGTG GGGGAAACTG

GTCAGTCACT CAGGGCTTCA GCTTCAACAT ACTCTGTGTG GGCGAAACTG

---------- ---------- ---------- ---------- ----------

---------- ---------- ---------- ---------- ----------

GTCAGTCACA CAGGGCTTCA GCTTTAACAT CCTCTGTGTG GGGGAGACTG

GTCGGTCACT CAGGGCTTCA GCTTCAATAT CCTTTGTGTG GGGGAGACTG

GGCGGTCACT CAGGGCTTCA GCTTCAACAT CCTCTGTGTG G--------G

GTCGGTCACT CAGGGCTTCA GCTTCAACAT CCTCTGTGTG GGGGAGACTG

GTCGGTCACT CAGGGCTTCA GCTTCAACAT CCTCTGTGTG GGGGAGACTG

GTCGGTCACT CAGGGCTTCA GCTTCAACAT CCTCTGTGTG GGGGAGACTG

GTCCGTATCT CAGGGCTTCA GCTTTCACAT CCTTTGTGTG GGGGAAACAG

ATCGGTCAAC CAGGGCTTCA GCTTCAACAT CCTCTGTGTC GGGGAGACGG

GCATTGGCAA ATCCACACTG ATGAACACAC TCTTCAACAC GACCTTCGAG

GCATTGGCAA ATCCACACTG ATGAACACAC TCTTCAACAC GACCTTCGAG

GCATTGGCAA ATCTACACTG ATGAACACGC TCTTCAACAC GACCTTCGAG

GCATTGGCAA ATCCACACTG ATGAACACGC TCTTCAACAC GACCTTCGAG

GCATTGGCAA ATCCACACTG ATGAACACGC TCTTCAACAC AACCTTCGAG

GCATTGGCAA ATCCACATTG ATGAACACAC TCTTCAACAC GACCTTCGAG

GGATTGGCAA GTCCACACTA ATGAACACGC TCTTCAACAC GACCTTTGAG

GGATTGGCAA GTCCACGCTA ATGAACACAC TCTTCAACAC GACCTTTGAG

---------- ---------- ---------- ---------- ----------

---------- ---------- ---------- ---------- ----------

GCATTGGCAA GTCCACACTG ATGAACACAC TCTTCAACAC AACCTTTGAG

GCATTGGCAA GTCCACACTG ATGAACACAC TCTTCAACAC GACCTTCGAG

GCATCGGCAA GTCCACCCTG ATGAACACGC TCTTCAACAC CACCTTCGAG

GCATTGGCAA GTCCACACTG ATGAACACGC TCTTTAACAC GACCTTCGAG

GCATTGGCAA ATCCACGCTG ATGAACACGC TCTTCAACAC AACCTTTGAG

GCATTGGCAA GTCCACGCTG ATGAACACGC TCTTCAACAC CACATTCGAG

GCATTGGAAA GTCTACACTG ATGAACACAC TCTTCAACAC TATATTTGAG

GCATTGGGAA GTCCACCCTG ATGAACACGC TGTTCAACAC TACGTTTGAG

ACTGAGGAAG CCAGTCACCA TGAGGCATGC GTGCGCCTGC GGCCCCAGAC

ACTGAGGAAG CCAGTCACCA TGAGGCATGC GTACACCTGC GGCCCCAGAC

ACTGAGGAAG CCAGTCACCA TGAGGCATGC GTGCGCCTGC GGCCCCAGAC

ACTGAGGAAG CCAGTCACCA TGAGGCATGC GTGTGCCTGC GGCCCCAGAC

ACTGAGGAAG CCAGTCACCA TGAGGCGTGT GTGCGCCTGC GGCCCCAGAC

ACTGAGGAAG CCAGTCACCA CGAGGCATGT GTGCGCCTGC GGCCCCAGAC

ACTGAAGAAG CCAGTCACCA TGAAGAGTGT GTACGCCTGC GGCCTCAGAC

ACTGAAGAAG CCAGTCACCA TGAAGAGTGT GTGCGCCTGC GGCCTCAGAC

---------- ---------- ---------- ---------- ----------

---------- ---------- ---------- ---------- ----------

ACAGAAGAAG CCAGTCATCA TGAGGAGTGT GTGCGCCTAC GGCCCCAGAC

ACCGAGGAAG CCAGTCACCA CGAGGAGTGT GTGCGCCTGC GGCCCCAGAC

ACCGAGGAGG CCAGTCACCA CGAGGAGTGC GTGCGTCTGC GGCCCCAGAC

ACTGAGGAAG CCAGCCACCA TGAGGAGTGC GTGCGCCTGC GGCCCCAGAC

ACCGAGGAAG CCAGTCACCA TGAGGAGTGC GTGCGCCTGC GGCCCCAGAC

ACGGAGGAGG CCAGCCACCA TGAGGAGTGT GTGCGCCTGC GGCCCCAGAC

ACAGAAGAGT CTGGACACTA TGAGACCTGT GTACGCCTCC GGCCACGCAC

ACGGAGGAGG CCAGCCACTA TGAGAACTGC GTCCGGCTGC AGCCGCGCAC

CTATGACCTC CAGGAGAGCA ACGTGCAGCT CAAGCTGACC ATTGTGGATG

CTACGACCTC CAGGAGAGCA ACGTGCAGCT CAAGCTGACC ATTGTGGATG

CTACGACCTC CAGGAGAGCA ACGTGCAGCT CAAGCTGACC ATTGTGGATG

CTACGACCTC CAGGAGAGCA ACGTGCAGCT CAAGCTGACC ATTGTGGATG

CTATGACCTC CAGGAGAGCA ATGTGCAGCT CAAGCTGACC ATTGTGGACG

CTACGACCTC CAAGAGAGCA ACGTGCACCT CAAGCTGACC ATTGTGGATG

CTATGACCTC CAAGAGAGTA ACGTTCATCT CAAACTGACC ATCGTGGATG

CTATGACCTC CAAGAGAGTA ACGTTCATCT CAAGCTGACC ATCGTGGATG

---------- ---------- ---------- ---------- ----------

---------- ---------- ---------- ---------- ----------

CTATGACCTC CAAGAGAGCA ATGTGCACCT CAAGCTGACC ATTGTGGACG

CTATGACCTC CAAGAGAGCA ACGTGCAGCT CAAGCTGACC ATCGTGGACG

CTACGACCTC CAGGAGAGCA ACGTGCACCT CAAGCTGACC ATCGTGGATG

CTACACCCTG CAAGAGAGCA ACGTGCAGCT GAAGCTGACC ATCGTGGACG

CTATGACCTC CAGGAGAGCA ACGTGCAGCT CAAGCTGACC ATTGTGGACG

ATACAACCTC CAGGAGAGCA ACGTGCAGCT CAAGCTGACC ATCGTGGACG

TTATGATCTC CAGGAAAGCA ATGTGCACCT TAAATTGACC ATTGTGGATG

CTACGACCTG CAGGAAAGCA GCGTGCAGCT CAAGCTGACC ATCGTGGATG

CCGTGGGCTT TGGGGATCAG ATCAATAAGG ATGAGAGTTA CAGGCCCATA

CCGTGGGCTT TGGGGATCAG ATCAATAAGG ATGAGAGTTA CAGGCCCATA

CCGTGGGCTT TGGGGATCAG ATCAATAAGG ATGAGAGTTA CAGGCCCATA

CCGTGGGCTT TGGGGATCAG ATCAATAAGG ATGAGAGTTA CAGGCCCATA

CCGTGGGCTT CGGGGATCAG ATCAATAAGG ATGAGAGTTA CAGGCCTATT

CTGTGGGCTT CGGGGATCAG ATCAACAAAG ATGAGAGTTA CAGGCCCATA

CTGTGGGCTT TGGGGATCAG ATCAATAAGG ATGACAGTTA CAGGCCCATA

CTGTGGGCTT TGGCGATCAG ATCAATAAGG ATGACAGTTA CAGGCCCATA

---------- ---------- ---------- ---------- ----------

---------- ---------- ---------- ---------- ----------

CTGTGGGCTT CGGGGATCAG ATCAATAAGG ATGAGAGTTA CAGGCCTATC

CTGTGGGCTT CGGGGACCAG ATCAATAAGG ATGAGAGTTA CAGGCCCATA

CCGTGGGCTT CGGGGATCAG ATCAACAAGG ATGAGAGTTA CAGGCCCATC

CCGTGGGCTT CGGGGACCAG ATCAACAAGG ATGACAGTTA CAGGCCCATC

CTGTGGGCTT CGGAGATCAA ATCAATAAGG ATGAGAGTTA CAGGCCCATT

CTGTGGGCTT CGGGGATCAG ATCAATAAGG ACGAGAGTTA CAGGCCCATC

CAGTCGGTTT TGGAGACCAG ATCAATAAGG ATGAGAGTTA CAGGCCTATA

CCGTTGGCTT CGGAGACCAG ATCAACAAGG ACGAGAGTTA CAGGCCAATA

GTTGACTACA TCGATGCGCA GTTTGAAAAT TATCTGCAGG AGGAGCTGAA

GTTGACTACA TCGATGCGCA GTTTGAAAAT TATCTGCAGG AGGAGCTGAA

GTTGACTACA TCGATGCGCA GTTTGAAAAT TATCTGCAGG AGGAGCTGAA

GTTGACTACA TCGATGCGCA GTTTGAAAAT TATCTGCAGG AGGAGCTGAA

GTTGACTACA TCGATGCGCA GTTTGAAAAC TATCTCCAGG AGGAGCTGAA

GTTGACTACA TCGATGCGCA GTTTGAAAAC TATCTCCAGG AGGAGCTGAA

GTTGATTACA TCGACACGCA GTTTGAAAAC TATCTACAGG AGGAGTTGAA

GTTGACTACA TCGACGCGCA GTTTGAAAAC TATCTGCAGG AGGAGTTGAA

---------- ---------- ---------- ---------- ----------

---------- ---------- ---------- ---------- ----------

GTCGACTACA TCGATGCGCA GTTTGAAAAC TACCTACAGG AGGAGCTGAA

GTCGACTACA TTGATGCGCA GTTTGAAAGC TATCTGCAGG AAGAGCTGAA

GTCGACTACA TTGATGCGCA GTTTGAAAAC TACCTGCAGG AGGAGCTGAA

GTTGACTACA TCGACGCGCA GTTTGAAAAC TATCTACAGG AGGAGCTGAA

GTTGATTACA TCGACGCACA GTTTGAAAAC TATCTGCAAG AGGAGCTGAA

GTCGACTACA TTGATGCGCA GTTTGAAAAC TATCTGCAAG AGGAGCTGAA

GTTGACTACA TTGATACGCA GTTTGAAAAC TACCTTCAGG AAGAGCTGAA

GTTGATTACA TTGATACGCA GTTTGAAAAC TACTTGCAAG AAGAACTGAA

GATCCGCCGC TCGCTCTTCG ACTACCATGA CACAAGGATC CACGTTTGCC

GATCCGCCGC TCGCTCTTCG ACTACCATGA CACAAGGATC CACGTTTGCC

GATCCGCCGC TCGCTCTTCG ACTACCATGA CACAAGGATC CACGTTTGCC

GATCCGCCGC TCGCTCTTCG ACTACCATGA CACAAGGATC CACGTTTGCC

GATCCGCCGC TCGCTTTTCG ACTACCATGA CACGAGGATC CACGTTTGCC

GATCCGCCGC TCGCTCTTCG ACTACCATGA CACTAGGATC CATGTTTGCC

GATTCGCCGT TCCCTCTTTG ACTACCATGA CACGAGGATC CACGTTTGCC

GATCCGCCGT TCCCTCTTTG ACTATCACGA CACGAGGATC CATGTTTGCC

---------- ---------- ---------- ---------- ----------

---------- ---------- ---------- ---------- ----------

GATCCGCCGC TCACTCTTTG ACTACCATGA CACAAGGATC CACGTCTGCC

GATTCGCCGC TCGCTCTTCG ACTACCATGA CACCAGGGTC CACGTGTGCC

GATCCGCCGC TCACTCTTCG ACTACCACGA CACGAGGATC CACGTGTGCC

GATCCGCCGC TCGCTCTTTG ACTACCATGA CACGAGGATC CACGTTTGCC

GATCCGCCGT TCCCTCTTTG ATTATCATGA CACGAGGATC CACGTTTGCC

GATCCGGCGC TCCCTCTTCG ACTACCATGA CACCAGGATC CACGTCTGCC

GATTCAGCGC TCTCTATTTG ACTATCATGA CACGCGGATC CATGTCTGTC

GATCCGGCGG GCCCTGTTCA ACTACCACGA CACCAGGATC CACGTCTGCC

TCTACTTCAT CACGCCCACA GGGCACTCCC TGAAGTCTCT AGATCTAGTG

TCTACTTCAT CACGCCCACA GGGCACTCCC TGAAGTCTCT AGATCTAGTG

TCTACTTCAT CACGCCCACG GGGCACTCCC TGAAGTCCCT GGATCTGGTG

TCTACTTCAT CACGCCCACG GGGCACTCCC TGAAGTCCCT GGATCTGGTG

TCTACTTTAT CACGCCCACG GGGCACTCCC TGAAGTCCCT GGATCTGGTG

TCTACTTCAT CACACCCACA GGGCACTCCC TGAAGTCCCT TGATCTGGTG

TCTACTTTAT CACACCCACC GGGCACTCCC TGAAGTCCCT GGATCTGGTG

TCTACTTCAT CACGCCCACC GGGCACTCCC TGAAGTCCCT GGATCTGGTG

---------- ---------- ---------- ---------- ----------

---------- ---------- ---------- ---------- ----------

TCTACTTTAT CACACCCACA GGGCACTCCC TCAAGTCCCT GGATCTGGTG

TCTACTTCAT CACGCCCACC GGGCACTCCC TCAAGTCCCT GGACCTGGTG

TCTACTTCAT CACCCCCACC GGCCACTCCC TCAAGTCCCT GGATCTGGTC

TCTACTTCAT CACACCCACG GGGCACTCCC TCAAGTCCCT GGACCTGGTG

TCTACTTCAT CACGCCCACG GGGCATTCTC TCAAGTCCCT GGACCTGGTG

TCTACTTCAT CACCCCCACG GGCCACTCCC TCAAGTCCCT GGACCTGGTG

TCTATTTCAT CACACCCACT GGACATTCCC TCAAGTCGCT GGACCTGGTG

TTTACTTCAT CACCCCCACC GGACATTCAC TCAAGTCCCT GGACTTGGTG

ACCATGAAGA AACTAGACAG CAAGGTGAAC ATTATTCCCA TCATCGCCAA

ACCATGAAGA AACTAGACAG CAAGGTGAAC ATTATTCCCA TCATCGCCAA

ACCATGAAGA AACTAGATAG CAAGGTGAAC ATTATTCCCA TCATCGCCAA

ACCATGAAGA AACTAGATAG CAAGGTGAAC ATTATTCCCA TCATCGCCAA

ACCATGAAGA AACTAGACAG CAAGGTGAAC ATTATTCCCA TCATCGCCAA

ACTATGAAGA AACTCGACAG CAAGGTGAAC ATTATTCCCA TCATTGCCAA

ACCATGAAGA AACTAGATAG CAAGGTGAAC ATAATCCCCA TCATTGCCAA

ACCATGAAGA AACTAGATAG CAAGGTGAAT ATAATCCCCA TCATTGCCAA

---------- ---------- ---------- ---------- ----------

---------- ---------- ---------- ---------- ----------

ACCATGAAGA AACTAGACAG CAAGGTGAAC ATTATTCCCA TCATTGCCAA

ACCATGAAGA AACTGGACAG CAAGGTGAAC ATTATTCCCA TCATTGCCAA

ACCATGAAGA AGCTTGACAG CAAGGTGAAC ATCATCCCCA TCATTGCCAA

ACTATGAAGA AACTGGACAG CAAGGTGAAC ATTATTCCCA TCATCGCCAA

ACCATGAAGA AACTGGACAG CAAGGTGAAC ATTATCCCCA TCATCGCCAA

ACCATGAAGA AGCTGGACAG CAAGGTGAAC ATCATCCCCA TCATTGCCAA

ACCATGAAGA AGCTGGATAG CAAGGTGAAC ATTATCCCTA TCATTGCCAA

ACCATGAAAA AACTGGATAG TAAGGTGAAC ATCATCCCCA TAATCGCCAA

GGCTGACACC ATCTCCAAGA GCGAGCTCCA CAAGTTCAAG ATCAAGATCA

GGCTGACACC ATCTCCAAGA GCGAGCTCCA CAAGTTCAAG ATCAAGATCA

GGCTGACACC ATCTCCAAGA GCGAGCTCCA CAAGTTCAAG ATCAAGATCA

GGCTGATACC ATCTCCAAGA GCGAGCTCCA CAAGTTCAAG ATCAAGATCA

GGCTGACACC ATCTCCAAGA GTGAACTCCA CAAGTTCAAG ATCAAGATCA

GGCCGACACC ATCTCCAAGA GCGAGCTCCA CAAGTTCAAG ATCAAGATCA

GGCTGACACC ATCTCCAAGA GCGAGCTCCA CAAGTTCAAG ATCAAGATCA

GGCTGACACC ATCTCCAAGA GCGAGCTCCA CAAGTTCAAA ATCAAGATCA

---------- ---------- ---------- ---------- ----------

---------- ---------- ---------- ---------- ----------

GGCTGACACC ATCTCCAAGA GTGAGCTCCA CAAGTTCAAG ATCAAGATCA

GGCTGACACC ATCTCCAAGA GTGAGCTCCA CAAGTTCAAG ATCAAGATCA

GGCCGACACC ATCTCCAAGA GTGAGCTCCA CAAGTTCAAG ATCAAGATCA

GGCTGATACC ATCTCCAAGA GCGAGCTCCA CAAGTTCAAG ATCAAGATCA

GGCTGACACC ATCTCCAAGA GTGAGCTCCA CAAGTTCAAG ATCAAGATCA

GGCGGACACC ATCTCC---A GCGAGCTTCA CAAGTTC--- ATCGAGATCA

AGCTGACACC ATCTCCAAGA GTGAACTGCA TAAGTTCAAA AGCAAGATCA

GGCCGACACC ATTTCCAAGA GCGAGTTGCA CAAGTTCAAG ATCAAGATCA

TGGGCGAGTT GGTCAGCAAT GGGGTCCAGA TCTACCAGTT CCCCACGGAT

TGGGCGAGTT GGTCAGCAAC GGGGTCCAGA TCTACCAGTT TCCCACGGAT

TGGGCGAGCT GGTCAGCAAC GGGGTCCAGA TCTACCAGTT TCCCACGGAT

TGGGCGAGCT GGTCAGCAAC GGGGTCCAGA TCTACCAGTT TCCCACGGAT

TGGGCGAGCT GGTCAGCAAC GGGGTCCAGA TCTACCAGTT TCCCACGGAT

TGGGTGAGCT GGTCAGCAAC GGGGTCCAGA TCTACCAGTT TCCCACAGAT

TGGGTGAGCT GGTCAGCAAT GGAGTCCAGA TCTACCAGTT TCCCACCGAC

TGGGTGAGCT GGTCAGCAAC GGAGTCCAGA TCTACCAGTT TCCCACCGAT

---------- ---------- ---------- ---------- ----------

---------- ---------- ---------- ---------- ----------

TGGGCGAGCT GGTCAGCAAT GGGGTTCAGA TCTACCAGTT TCCCACTGAT

TGGGCGAGCT GGTCAGCAAT GGGGTCCAGA TCTACCAGTT CCCCACTGAT

TGGGCGAGCT GGTTAGCAAC GGGGTCCAGA TCTACCAGTT CCCCACCGAT

TGGGCGAGCT GGTCAGCAAT GGGGTCCAGA TCTACCAATT CCCCACTGAT

TGGGCGAGCT GGTCAGCAAT GGGGTCCAGA TCTACCAGTT TCCCACTGAT

TGGGCGAGCT CGTCTGCAAC GCGGTCCAGA TCTA------ ----------

TGAGTGAACT GGATCAAAAT GGAGTCCAGA TCTATCAGTT CCCTACAGAT

TGGGTGAGCT GGTCAGCAAC GGTGTCCAGA TCTACCAGTT CCCCACGGAC

GATGAGGCTG TTGCAGAGAT TAACGCAGTC ATGAATGCAC ATCTGCCCTT

GACGAGGCTG TTGCAGAGAT TAACGCAGTC ATGAATGCAC ATCTGCCCTT

GACGAGGCTG TTGCAGAGAT TAACGCAGTC ATGAATGCAC ATCTGCCCTT

GACGAGGCTG TTGCAGAGAT TAACGCAGTC ATGAATGCAC ATCTGCCCTT

GACGAGGCTG TTGCAGAGAT TAACGCAGTC ATGAATGCAC ACCTGCCCTT

GATGAGGCAG TTGCAGAGAT TAATGCAGTT ATGAACGCAC ACCTGCCCTT

GATGAGGCTG TCGCCGAGAT TAATGCGGTC ATGAATGCAC ACTTGCCTTT

GACGAGGCTG TCGCCGAGAT TAATGCAGTC ATGAACGCAC ACCTGCCTTT

---------- ---------- ---------- ---------- ----------

---------- ---------- ---------- ---------- ----------

GATGAGGCTG TTGCTGAGAT TAACGCAGTC ATGAATGCAC ACTTGCCCTT

GACGAGGCTG TTGCAGAGAT TAACGCAGTC ATGAATGCAC ACCTGCCCTT

GACGAGGCTG TGGCAGAGAT CAATGCAGTC ATGAAT---- ----------

GACGAGGCTG TTGCAGAGAT TAACGCAGTC ATGAATGCAC ACCTGCCTTT

GATGAGGCTG TTGCAGAGAT AAACGCAGTC ATGAATGCAC ACCTGCCCTT

---------- ---------- ---------- ---------- ----------

GATGAAGCTG TGGCAGAGAT CAATGCTGTA ATGAATGCCC ATCTTCCCTT

GATGAGACGG TAGCAGAGAT CAATGCCGTC ATGAATGCTC ATCTACCTTT

TGCCGTGGTG GGCAGCACCG AGGAGGTGAA GGTGGGGAAC AAGCTGGTCC

TGCCGTGGTG GGCAGCACCG AGGAGGTGAA GGTGGGGAAC AAGCTGGTTC

CGCCGTAGTG GGCAGCACCG AGGAGGTGAA GGTGGGGAAC AAGCTGGTCC

CGCCGTAGTG GGCAGCACCG AGGAGGTGAA GGTGGGGAAC AAGCTGGTCC

TGCCGTGGTG GGCAGCACCG AGGAGGTGAA GGTGGGGAAC AAACTGGTCC

TGCTGTGGTG GGCAGCACCG AGGAGGTGAA GGTGGGAAAC AAGCTGGTCC

TGCCGTGGTG GGCAGCACAG AGGAGGTGAA GGTGGGGAAC AAGCTGGTTC

TGCCGTGGTG GGCAGCACAG AGGAGGTGAA GGTGGGGAAT AAGCTGGTTC

---------- ---------- ---------- ---------- ----------

---------- ---------- ---------- ---------- ----------

CGCTGTGGTG GGCAGCACTG AGGAGGTGAA GGTGGGGAAC AAGCTGGTCC

CGCCGTGGTG GGCAGCACCG AGGAGGTGAA GGTGGGGAAC AAGCTGGTGC

---------- ---------- ---------- ---------- ----------

TGCGGTGGTT GGCAGCACCG AGGAGGTGAA GGTGGGGAAC AAGCTGGTCC

TGCTGTAGTG GGCAGCACCG AGGAGGTGAA GGTGGGGAAC AAGCTGGTCC

---------- ---------- ---------- ---------- ----------

TGCTGTGGTT GGCAGTACTG ATGAAGTAAA AGTTGGGAAC AAACTGGTTC

CGCCGTGGTC GGCAGCACAG AAGAGGTAAA AGTCGGGAAC AAACTGGTGC

GAGCACGGCA GTACCCCTGG GGAGTGGTGC AGGTGGAGAA TGAGAATCAC

GAGCACGGCA GTACCCCTGG GGAGTGGTGC AGGTGGAGAA TGAGAATCAC

GAGCACGGCA GTACCCCTGG GGAGTGGTGC AGGTGGAGAA TGAGAATCAC

GAGCACGGCA GTACCCCTGG GGAGTGGTGC AGGTGGAGAA TGAGAATCAC

GAGCACGGCA GTACCCTTGG GGAGTGGTGC AGGTGGAGAA TGAGAATCAC

GAGCACGGCA GTACCCTTGG GGAGTGGTGC AGGTGGAGAA TGAGAATCAC

GAGCACGACA GTACCCCTGG GGCGTGGTGC AGGTGGAGAA CGAGAACCAC

GAGCACGACA GTACCCTTGG GGTGTGGTGC AGGTGGAGAA TGAGAATCAC

---------- ---------- ---------- ---------- ----------

---------- ---------- ---------- ---------- ----------

GAGCACGGCA GTACCCTTGG GGAGTGGTGC AGGTGGAGAA CGAGAATCAC

GAGCGCGGCA GTACCCGTGG GGAGTGGTGC AGGTGGAGAA TGAGAATCAC

---------- ---------- ---------- ---------- ----------

GAGCACGGCA GTACCCTTGG GGAGTGGTGC AGGTGGAGAA CGAGAACCAC

GAGCACGGCA GTACCCTTGG GGAGTAGTCC AGGTGGAGAA CGAGAATCAC

---------- ---------- ---------- ---------- ----------

GAGCTCGGCA GTACCCCTGG GGTGTTGTGC AAGTGGAGAA TGAGAGTCAC

GGGCTCGACA GTACCCGTGG GGGGTCGTAC AAGTGGAGAA CGAGAGTCAC

TGCGACTTCG TGAAGCTGCG GGAGATGTTG ATCCGGGTGA ACATGGAAGA

TGCGACTTCG TGAAGCTGCG GGAGATGTTG ATCCGGGTGA ACATGGAAGA

TGCGACTTCG TGAAGCTGCG GGAAATGTTG ATCCGGGTGA ACATGGAAGA

TGCGACTTCG TGAAGCTGCG GGAAATGTTG ATCCGGGTGA ACATGGAAGA

TGCGACTTCG TGAAGCTGCG GGAGATGTTG ATCCGGGTGA ACATGGAGGA

TGCGACTTTG TGAAGCTCCG GGAGATGTTG ATCCGGGTGA ACATGGAGGA

TGTGACTTCG TGAAGTTGCG GGAGATGTTG ATCCGGGTGA ACATGGAGGA

TGTGACTTCG TGAAGCTGCG GGAGATGCTG ATCCGGGTGA ACATGGAGGA

---------- ---------- ---------- ---------- ----------

---------- ---------- ---------- ---------- ----------

TGCGACTTTG TGAAGCTGCG GGAGATGCTG ATCCGGGTGA ACATGGAGGA

TGCGATTTCG TGAAGCTCCG GGAGATGCTG ATCCGCGTGA ACATGGAGGA

---------- ---------- ---------- ---------- ----------

TGTGACTTCG TGAAGCTCCG GGAGATGCTG ATCAGGGTGA ACATGGAGGA

TGTGACTTTG TGAAGCTTCG GGAGATGCTG ATTCGGGTGA ACATGGAAGA

---------- ---------- ---------- ---------- ----------

TGTGACTTTG TGAAACTCCG GGAGATGCTG ATCCGTGTGA ACATGGAGGA

TGTGACTTTG TGAAGCTGCG GGAGATGCTG ATCCGGGTGA ACATGGAAGA

CCTCCGCGAG CAGACCCACA GCCGGCACTA CGAGCTCTAC CGGCGCTGCA

CCTCCGCGAG CAGACCCACA GCCGGCACTA CGAGCTCTAC CGGCGCTGCA

CCTCCGCGAG CAGACCCACA GCCGGCACTA CGAGCTCTAC CGGCGCTGCA

CCTCCGCGAG CAGACCCACA GCCGGCACTA CGAGCTCTAC CGGCGCTGCA

CCTCCGTGAG CAGACCCACA GCCGGCACTA TGAGCTGTAC CGGCGCTGCA

CCTCCGGGAG CAGACCCACA GTCGGCACTA TGAGCTCTAC CGGCGCTGCA

CCTGCGTGAG CAGACCCACA GTCGGCACTA CGAGCTCTAC CGGCGCTGCA

CCTGCGCGAG CAGACCCACA GCCGGCACTA CGAGCTCTAC CGCCGCTGCA

---------- ---------- ---------- ---------- ----------

---------- ---------- ---------- ---------- ----------

CCTCCGAGAG CAGACCCACA GTCGGCACTA CGAGCTCTAC CGGCGCTGCA

CCTCCGCGAG CAGACGCACA GTCGGCACTA TGAGCTCTAC AGGCGCTGCA

---------- ---------- ---------- ---------- ----------

CCTCCGCGAG CAGACCCACA GCCGGCACTA CGAGCTCTAT CGGCGCTGCA

CCTCCGTGAG CAGACCCACA GTCGGCACTA TGAGCTCTAC CGACGCTGCA

---------- ---------- ---------- ---------- ----------

TCTCCGAGAA CAGACACACA GCAAACACTA TGAATTGTAC CGGCGCCATA

TCTGCGTGAA CAGACCCACA CCCGCCACTA CGAGCTCTAC CGGCGTTGCA

AGTTGGAGGA GATGGGCTTT CAGGACAGCG ATGGTGACAG CCAGCCCTTC

AGTTGGAGGA GATGGGCTTT CAGGACAGCG ATGGTGACAG CCAGCCCTTC

AGTTGGAGGA GATGGGCTTC CAGGACAGCG ATGGTGACAG CCAGCCCTTC

AGTTGGAGGA GATGGGCTTC CAGGACAGTG ATGGTGACAG CCAGCCCTTC

AGTTGGAGGA GATGGGCTTT CAGGACAGCG ATGGTGACAG TCAGCCCTTC

AGTTGGAGGA GATGGGCTTC CAGGACAGCG ATGGTGACAG TCAGCCCTTC

AGTTGGAGGA GATGGGCTTC CAGGACAGTG ATGGTGACAG CCAGCCCTTC

AGTTGGAGGA GATGGGCTTC CAGGACAGTG ACGGTGACAG CCAGCCCTTC

---------- ---------- ---------- ---------- ----------

---------- ---------- ---------- ---------- ----------

AGCTGGAGCA GATGGGCTTC CAGGACAGCG ACGGTGACAG CCAGCCCTTC

AGTTGGAGGA GATGGGCTTC CAGGACAGCG ACGGGGACAG CCAGCCCTTC

---------- ---------- ---------- ---------- ----------

AGTTGGAGGA GATGGGCTTC CAGGACAGCG ACGGTGACAG CCAGCCCTTC

AATTGGAAGA GATGGGCTTC CAAGACAGCG ATGGTGACAG CCAGCCCTTC

---------- ---------- ---------- ---------- ----------

AGCTTGAAGA GATGGGCTTC CAGGACACAG CTGCAGAGAG CAAGCCTGTT

AGCTGGAGGA GATGGGCTTC AAGGACACCG ACCCGGACAA CCAGCCGTTC

AGCCTACAAG AGACATACGA GGCCAAGAGG AAGGAGTTCC TAAGTGAGCT

AGCCTACAAG AGACATACGA GGCCAAGAGG AAGGAGTTCC TAAGTGAGCT

AGCCTACAAG AGACATATGA GGCCAAGAGG AAGGAATTCC TGAGTGAGCT

AGCCTACAAG AGACATATGA GGCCAAGAGG AAGGAATTCC TGAGTGAGCT

AGCCTACAAG AGACATATGA GGCCAAGAGG AAGGAGTTCC TGAGTGAGCT

AGTCTACAAG AGACATATGA AGCCAAGAGG AAGGAGTTTC TGAGTGAGCT

AGCCTCCAAG AGACGTACGA GGCAAAGAGG AAGGAGTTCC TGAGCGAGCT

AGCCTCCAAG AGACATATGA GGCAAAGAGG AAGGAGTTCC TAAGCGAGCT

---------- ---------- ---------- ---------- ----------

---------- ---------- ---------- ---------- ----------

AGCCTGCAGG AGACCTACGA GGCCAAGCGC AAGGAGTTCC TGAGTGAGCT

AGCCTACAGG AGACCTACGA GGCCAAGAGG AAGGAGTTCC TGAGTGAGCT

---------- ---------- ---------- ---------- ----------

AGCCTACAGG AGACATACGA GGCCAAGAGG AAGGAGTTCC TGAGTGAGCT

AGCCTACAGG AGACATATGA GGCCAAGAGG AAGGAGTTCT TGAGTGAGCT

---------- ---------- ---------- ---------- ----------

AGCCTCCAGG AGACCTATGA AACCAGGAGA AAGGAGTTCC TAGGTGAGCT

AGCCTCCAAG AGACATATGA GACGAAGAGG AAAGAGTTTC TCGGGGAGCT

GCAGAGGAAG GAGGAAGAGA TGAGGCAGAT GTTTGTCAAC AAAGTGAAGG

GCAGAGGAAG GAGGAAGAGA TGAGGCAGAT GTTTGTCAAC AAAGTGAAGG

GCAGAGGAAG GAGGAAGAGA TGAGGCAGAT GTTTGTCAAC AAAGTGAAGG

GCAGAGGAAG GAGGAAGAGA TGAGGCAGAT GTTTGTCAAC AAAGTGAAGG

GCAGAGGAAG GAGGAAGAGA TGAGGCAGAT GTTTGTCAAC AAAGTGAAGG

GCAGAGGAAG GAGGAAGAGA TGAGGCAGAT GTTCGTCAAC AAAGTGAAGG

ACAGAGGAAG GAGGAAGAGA TGAGGCAGAT GTTTGTCAAC AAAGTGAAGG

GCAGAGGAAG GAGGAAGAGA TGCGACAGAT GTTTGTCAAC AAAGTCAAGG

---------- ---------- ---------- ---------- ----------

---------- ---------- ---------- ---------- ----------

GCAGAGGAAG GAGGAGGAGA TGAGGCAGAT GTTTGTCAAC AAAGTGAAGG

GCAGAGGAAG GAGGAAGAGA TGAGGCAGAT GTTTGTCAAC AAAGTGAAGG

---------- ---------- ---------- ---------- ----------

GCAGAGGAAG GAGGAAGAGA TGAGGCAGAT GTTTGTTAAC AAAGTGAAGG

GCAGAGGAAG GAGGAAGAGA TGAGGCAGAT GTTCGTCAAC AAAGTGAAGG

---------- ---------- ---------- ---------- ----------

GCAGAAGAAG GAAGAAGAGA TGAGACAGAT GTTTGTGAAC AAGGTGAAGG

GCAGCGGAAG GAGGAGGAAA TGAGGCAAAT GTTTGTCAAC AAAGTGAAAG

AGACAGAGCT GGAGCTGAAG GAGAAGGAAA GGGAGCTCCA TGAGAAGTTT

AGACAGAGCT GGAGCTGAAG GAGAAGGAAA GGGAGCTCCA TGAGAAGTTT

AGACAGAGCT GGAGCTGAAG GAGAAGGAAA GGGAGCTCCA TGAGAAGTTT

AGACAGAGCT GGAGCTGAAG GAGAAGGAAA GGGAGCTCCA TGAGAAGTTT

AGACAGAGCT GGAGCTGAAG GAGAAGGAAA GGGAGCTTCA TGAGAAGTTT

AGACAGAGCT GGAATTGAAG GAGAAGGAAA GGGAGCTCCA TGAGAAGTTC

AGACAGAGCT TGAATTGAAG GAGAAGGAAC GGGAGCTCCA TGAGAAGTTT

AGACAGAGCT TGAGCTGAAG GAGAAGGAAC GGGAGCTCCA TGAGAAGTTT

---------- ---------- ---------- ---------- ----------

---------- ---------- ---------- ---------- ----------

AGACAGAACT GGAGCTGAAG GAGAAAGAGA GGGAGCTCCA TGAGAAGTTT

AGACAGAACT GGAGCTGAAG GAGAAGGAAA GGGAGCTCCA CGAGAAGTTT

---------- ---------- ---------- ---------- ----------

AGACAGAGCT GGAGTTGAAA GAGAAGGAAA GGGAGCTCCA TGAGAAGTTT

AGACGGAGCT GGAGTTGAAG GAGAAGGAAA GGGAGCTTCA TGAGAAGTTT

---------- ---------- ---------- ---------- ----------

AGACAGAGTT GGAACTGAAA GAGAAAGAGA AGGAGCTTCA TGAGAAATTT

AGACTGAGTC GGAGCTGAAG GAGAAGGAGA GGGAGCTCCA CGAGAAGTTT

GAGCACCTGA AGCGGGTCCA CCAGGAGGAG AAGCGCAAGG TGGAGGAAAA

GAGCACCTGA AGCGGGTCCA CCAGGAGGAG AAGCGCAAGG TGGAGGAAAA

GAGCACCTGA AGCGGGTCCA CCAGGAGGAG AAGCGCAAGG TGGAGGAAAA

GAGCACCTGA AGCGGGTCCA CCAGGAGGAG AAGCGCAAGG TGGAGGAAAA

GAGCACCTGA AGCGGGTCCA CCAGGAGGAG AAGCGCAAGG TGGAGGAAAA

GAGCACCTGA AGCGGGTCCA CCAGGAGGAG AAGCGCAAGG TGGAGGAGAA

GAGCACCTGA AGCGGATCCA CCAGGAGGAG AAGCGCAAGG TAGAGGAGAA

GAGCACCTGA AGCGAATCCA CCAGGAGGAG AAGCGCAAGG TAGAGGAGAA

---------- ---------- ---GGAGGAG AAGCGCAAGG TGGAGGAGAA

---------- ---------- ---------- ---------- ----------

GAGCACCTTA AGCGGATCCA CCAGGAGGAG AAGCGCAAGG TGGAGGAGAA

GAGCACCTCA AGCGGGTCCA CCAGGAGGAA AAGCGGAAGG TGGAGGAGAA

---------- ---------- ---------- ---------- ----------

GAGCACCTCA AGCGGGTCCA TCAGGAGGAG AAGCGCAAAG TGGAGGAGAA

GAGCACCTGA AGCGGGTCCA CC-GGAGGAG AAGCGCAAGG TGGAGGAGAA

---------A AGCGGGTGCA CCCGGAGGAG AAGCGCAGGG TGGAGGAGAA

GAGCTCTTAA AGAGGACTCA CCAGGAAGAA AAGCGGAAGG TGGAAGAAAA

GAGCACCTAA AGAGGATACA CCaggaggag aagaggaagg tggaggagaa

GCGCCGGGAA CTGGAGGAGG AGACCAACGC CTTCAATCGC CGGAAGGCTG

GCGCCGGGAA CTGGAGGAGG AGACCAACGC CTTCAATCGC CGGAAGGCTG

ACGCCGGGAA CTGGAGGAGG AGACCAACGC CTTCAATCGC CGGAAGGCTG

ACGCCGGGAA CTGGAGGAGG AGACCAACGC CTTCAATCGC CGGAAGGCTG

GCGCCGCGAG CTGGAGGAGG AGACCAATGC CTTCAACCGC CGGAAGGCCG

GCGCCGGGAA CTGGAGGAGG AGACCAACGC CTTCAACCGC AGGAAGGCTG

GCGCAGGGAG CTGGAAGAGG AGACCAACGC CTTCAACTGC CGGAAGGCAG

GCGCAGGGAG CTGGAAGAGG AGACCAACGC CTTCAACTGC CGGAAGGCGG

GCGCCGGGAG CTGCTGCATA AGACCCGCGC CTTCAGCCGC AGGAAGG---

---------- ---------- ---------- ---------- ----------

GCGGCGGGAG CTGGAGGAGG AGACGAACGC CTTCAACCGC AGGAAGGCCG

GCGCCGGGAG CTGGAGGAGG AGACCAACGC CTTCAACCGC AGGAAGGCCG

---------- ---------- ---------- ---------- ----------

GCGGCGGGAA CTGGAAGAGG AGACCAACGC CTTCAACCGC AGGAAGGCCG

GCGGCGGGAG CTGGAGGAGG AGACCAATGC CTTCAATCGC AGAAAGGCAG

GCGGCAGGAG CTGGAGGAGG AGACCAGCGC CTTTACCCGC AGAGAAGCAG

ACGACGGCAA CTTGAGGAGG AGAAAATTGC CTTCAATCGG AGGAAGGCAG

gagaagggag ctggaggagg agaTGAACGC TTTCAACCGG CGGAAAGTGG

CGGTGGAGGC CCTGCAGTCG CAGGCCTTGC ACGCCACCTC GCAGCAGCCC

CGGTGGAGGC CCTGCAGTCG CAGGCCTTGC ACGCCACCTC GCAGCAGCCC

CGGTGGAGGC CCTGCAGTCG CAGGCCTTGC ACGCCACCTC GCAGCAGCCC

CGGTGGAGGC CCTGCAGTCG CAGGCCTTGC ACGCCACCTC GCAGCAGCCC

CGGTGGAGGC CCTGCAGTCG CAGGCCTTGC ACGCCACCTC GCAGCAGCCC

CGGTGGAGGC CCTGCAGTCA CAGGCCTTGC ACGCCACCTC ACAGCAGCCC

CCATGGAGGC CCTGCAGTCA CAGGCCTTAC ATGCCACTTC ACAGCAGCCT

CCATGGAGGC CCTGCAGTCA CAGGCCTTGC ATGCCACTTC ACAGCAGCCT

---TGGAGGC CCTTCAGTCG CAGGCCCTGC AGGCCACCTC GCAGCAGCCG

---------- ---------- ---------- ---------- ----------

CTGTGGAGGC CCTGCAGTCC CAGGCTTTGC ATGCCACCTC CCAGCAGCCT

CGGTGGAGGC CCTGCAGTCT CAGGCCTTGC ACGCCACCTT GCAGCAGCCT

---------- ---------- ---------- ---------- ----------

CAGTGGAGGC CCTGCAGTCG CAGGCCTTGC AAGCCACCTC GCAGCAGCCC

CTGTGGAGGC TCTGCAGTCG CAGGCCTTTC ATGCCACCTC ACAGCAGCCT

CCGTGGAGGC CCTGCAGTCG CAGGCCTTCC ACGCCACCTC GCAGCAGCCT

CTGTTGAGGC CATGCAGGTC CAAGCCTTAC AGGCCACCTC ACAGCAACCA

CCGTGGAAAC CCTGCAATCT CAGTCCCTGC AAGCCACCTC CCAGCAGCCC

CTGAGGAAGG ACAAGGACAA GAAGAACGGA AGCCTCTCCA GCTCTAAGGT

CTGAGGAAGG ACAAGGACAA GAAGAA---- ---------- -----AAGGG

CTGAGGAAGG ACAAGGACAA GAAGAACGGA AGCCTCTCCA GCTCTAAGGT

CTGAGGAAGG ACAAGGACAA GAAGAACGGA AGCCTCTCCA GCTCTAAGGT

CTGAGGAAGG ACAAGGACAA GAAGAACGGA AGCCTCTCCA ACTCTAAGGT

CTGAGGAAGG ACAAGGACAA GAAGAACGGA AGCCTCTCCA ACTCTAAGGT

CTGAGGAAAG ACAAGGACAA GAAGAACGGA AGCCTCTCCA GCTCTAAGGT

CTGAGGAAAG ACAAGGACAA GAAGAACGCA AGCCTCTCCA ACTCTAAGGT

CTGCGCAAGG ACAAGGACAA GAAGAACGGA AGCCTCTCCA ACTCTAAGGT

---------- ---------- ---------- ---------- ----------

CTGAGGAAGG ACAAGGACAA GAAGAACGGA AGCCTCTCCA GCTCTAAGGT

CTGAGGAAGG ACAAGGACAA GAAGAACGGA AGCCTCTCCA ACTCTAAGGT

---------- ---------- ---------- ---------- ----------

CTGAGGAAAG ACAAGGACAA GAAGAACGGA AGCCTCTCCA GCTCTAAGGT

CTGAGGAAGG ACAAAGACAA GAAGAACGGA AGCCTCTCCA ACTCTAAGGT

CTGCGAAAAG ACAAAGACAA GAAGAACGGA AGCCTATCCA ACTCTAAGGT

TTGAGAAGAG ACAAGGACAA GAAAAA---A --CCTTTCCA GCGCTAAGGG

CTGAAAAAGG ACAAAGACAA GAAAAACACA AGCCTCTCCA GCTCTAAGGT

GATGATGACC AAGGCCAGTG TGGAGCCCTT GAACTGCAGC AGCTGGTGGC

GATGATGACC AAGGCCAGTG TGGAGCCCTT GAAC---AGC AGTGGTGGGC

GATGATGACC AAGGCCAGTG TGGAGCCCTT GAACTGCAGC AGCTGGTGGC

GATGATGACC AAGGCCAGTG TGGAGCCCTT GAACTGCAGC AGCTGGTGGC

GATGATGACC AAGGCCAGTG TGGAGCCCTT GAACTGCAGC AGCTGGTGGC

GATGATGACA AAGGCCAGTG TGGAGCCCTT GAACTGCAGC AGCTGGTGGC

GATGATGACC AAAGCAAATG TGGAACCCTT GAACTGCAGC AGCTGGTGGC

GATGATGACT AAAGCAAATG TGGAGCCCTT GAACTGCAGC AGCTGGTGGC

GATGATGACC AAGGCCAGTG TGGAGCCCTT GAACTGCAGC AGCTGGTGGC

---------- ---------- ---------- ---------- ----------

GATGATGACC AAGGCCAGTG TGGAGCCCTT GAACTGCAGC AGCTGGTGGC

GATGATGACC AAGGCCAGTG TGGAGCCCTT GAACTGCAGC AGTTGGTGGC

---------- -------GTG TGGAACCCTT GAATTGCAGC ACTCGGTGGC

GATGATGACC AAGGCCAGTG TGGAGCCCTT GAACTGCAGC AGCTGGTGGC

GATGATGACC AAGGCCAGTG TGGAGCCCTT GAACTGCAGC AGCTGGTGGC

GATGATGACC AAGGCCAGTG TGGAGCCCTT GAACTGCAGC AGCTGGTGGC

GATGATCACA AGGTCCAGCG TGGAGCCCTT AAAGTACAGT AGCTGGTGGC

GATGATCACC AAAGCCAGCG TGGAGCCCTT GAACTGCAAT AGCTGGTGGC

CCGCCATACA GTGCTGCAGC TGCCTGGTCA GGGATGCGAC GTGGAGGGAA

CCCCCATACA GTGCTGCAGC TGCCTGGTCA GGGATGCGAG GTGGAGGGAA

CCGCCATACA GTGCTGCAGC TGCCTGGTCA GGGATGCGAC GTGGAGGGAA

CCGCCATACA GTGCTGCAGC TGCCTGGTCA GGGATGCGAC GTGGAGGGAA

CCGCCATACA GTGCTGCAGC TGCCTGGTCA GGGATGCGAC GTGGAGGGAA

CTGCCATACA GTGCTGCAAC TGTCTGGTCA GGGATGCAAC GTGGCGGGAA

CTGCCATCCA GTGCTGTAGC TGCCTGGTCA GGGACGCGAC GTGGAGGGAA

CTGCCATCCA GTGCTGCAGC TGCCTGGTCA GGGACGCGAC GTGGAGGGAA

CGGCCATACA GTGCTGCAGC TGCCTGGTCA GGGACGCGAC GTGGAGGGAA

---------- ---------- ---------- ---------- ----------

CCGCCATACA GTGCTGCAGC TGCCTGGTCA GGGACGCGAC GTGGAGGGAA

CCGCCATACA GTGCTGCAGC TGCCTGGTCA GGGACGCGAC GTGGAGGGAA

CCGCCAGCCA GTGCTGCAAG TGCCTGTTCA TGGACGCGAC G---AGGGAA

CCGCCATACA GTGCTGCAGC TGCCTGGTCA GGGACGCGAC GTGGAGGGAA

CCGCCATACA GTGCTGCAGC TGCCTGGTCA GGGACGCGAC GTGGCGGGAA

CCGCCATCCA GTGCTGCAGC TGCCTGGTCA GGGACACGAC GTGGCGGGAA

AAGCCATACA GTGCTGCAAC TGCCTGAACA AGAATCCGGC GGGGCAGGAA

ATGCCATACA GTGCTGCAGC TGCCTGGTCA AGAACGCAAC GTGGCGGGAA

GGATTCCTCA TGCGGGACTA CGACGAGGTG ATCGCCTTCC TGGGCGAGTG

GGATTCTTCA TGCGGGACTA CGACGAGGTG ATCGCCTTCC TGGGCGAGTG

GGATTCCTCA TGCGGGACTA CGACGAGGCG ATCGCCTTCC TGGGCGAGTG

GGATTCCTCA TGCGGGACTA CGACGAGGCG ATCGCCTTCC TGGGCGAGTG

GGATTCCTCA TGCGCGACTA CGACGAGGCG ACCGCCTTCC TGGGGGAGTG

GGATTCCTCA TGCGGGACTA CGACGAGGTG ATCGCCTTCC TTGGCGAGTG

GGATTCCTCA TGCGGGACTA CGACGAGGTG ACCGCCTTCC TGGGCGAGTG

GGATTCCTCA TGCGGGACTA CGACGAGGTG ACCGCCTTCC TAGGCGAGTG

GGATTCCTCA TGCGGGACTA CGACGAGGTG ACCGCGTTTC TGGGCGAGTG

---------A TGCAGGACTA CGACGAAGTG ACCGCCTTCC TAGGCGAGTG

GGATTCCTCA TGCGGGACTA CGACGAGGTG ACCACCTTCC TGGGCGAGTG

GGATTCCTCA TGCGGGACTA CGACGAGGTG ACCGCCTTCC TGGGCGAGTG

GGCTTCCTCA TGCGGGACTA CGACGAGGTG ACCGCCTTCC TGGGCCAGTG

GGATTCCTCA TGCGTGACTA CGACGAGGCG ATCGCCTTCC TGGGCGAGTG

GGATTCCTC- ---------- ---------- ---------- ----------

GGATTCCTCA TGCGTGACTA CGACGAGGTG ACCTCCTTCC TGGGCGAGTG

CGATTCATCA TGCGGGACTA CGACGAGGTG ACAGCCTTCC TGGGCGAGTG

GGATTCATCA TGAGGGACTA CGACGAGGCG ACGGCCTTCC TGGGCGAGTG

GGGGCCCTTC CAGCGCCTCA TCTTCTTCCT GCTCAGCGCC AGCATCATCC

GGGGCCCTTC CAGCGCCTCA TCTTCTTCCT GCTCAGCGCC AGCATCATCC

GGGACCCTTC CAGCGGCTCA TCTTCTTCCT GCTCAGCGCC AGCATCATCC

GGGACCCTTC CAGCGGCTCA TCTTCTTCCT GCTCAGCGCC AGCATCATCC

GGGGCCCTTC CAGCGCCTCA TCTTCTTCCT GCTCAGCGCC AGCATCATCC

GGGGCCCTTC CAGCGCCTCA TCTTCTTCCT GCTCAGCGCC AGCATCATCC

GGGGCCCTTC CAGCGCCTCA TTTTCTTCCT GCTCAGCGCC AGCATCATCC

GGGGCCCTTC CAGCGCCTCA TCTTCTTCCT GCTCAGCGCC AGCATCATCC

GGGGCCCTTC CAGCGCCTCA TCTTCTTCCT GCTCAGCGCC AGCATCATCC

GGGGCCCTTT CAGCGCCTCA TCTTCTTCCT GCTCAGCGCC AGCATCATCC

GGGGCCCTTC CAGCGCCTCA TCTTCTTCCT GCTCAGCGCC AGCATCATCC

GGGGCCGTTC CAGCGCCTCA TCTTCTTCTT GCTCAGCGCC AGCATCATCC

GGGGCCCTTC CAGCGCCTCA TCTTCTTCCT GCTCAGCGCC AGCATCATCC

GGGGCCCTTC CAGCGCCTCA TCTTCTTCCT GCTCAGCGCC AGCATCATCC

---------- ---------- ---------- ---------- ----------

GGGGCCCTTC CAGCGCCTCG TCTTCTTCCT GCTCAGCGCC AGCATCGTCC

GGGACCCTTC CAGCGCCTCA TCTTCTTCCT GCTCAGCTCC AGCATCATCC

GGGCCCCTTC CAGCGGCTGG TCTTCTTCCT GCTGAGCGCC AGCATCATCC

CCAATGGCTT CAATGGTATG TCAGTCGTGT TCCTGGCGGG GACCCCGGAG

CCAATGGCTT CAATGGTATG TCAGTCGTGT TCCTGGCGGG GACCCCGGAG

CCAATGGCTT CAATGGTATG TCAGTCGTGT TCCTGGCGGG GACCCCAGAG

CCAATGGCTT CAATGGTATG TCAGTCGTGT TCCTGGCGGG GACCCCAGAG

CCAACGGCTT CAATGGTATG TCAGTCGTGT TCCTGGCGGG GACCCCAGAG

CCAACGGCTT CAATGGTATG TCAGTCGTGT TCCTAGCTGG GACCCCGGAG

CCAATGGCTT CAATGGTATG TCTATCGTGT TCCTGGCGGG GACCCCGGAG

CCAATGGCTT CAATGGTATG TCCATCGTGT TCCTGGCGGG GACCCCGGAG

CAAACGGCTT CAATGGGATG TCAGTGGTGT TCCTGGCGGG GACCCCCGAG

CCAACGGCTT CAATGGCCTG TCGTCCGTGT TCTTTACGGC CACCCCGGAA

CCAACGGCTT CAATGGAATG TCAGCCGTGT TCCTGGCCGC CACCCCCGAG

CCAACGGCTT CAATGGTATG TCAGCCGTGT TCCTGACGGG GACCCCGGAG

CCAACGGCTT CAACGGGATG TCAGTCGTGT TCCTGACGGG GACCCCGGAG

CCAATGGCTT CAACGGTATG TCAGCCGTGT TCCTGGCGGG GACCCCCGAG

---------- ---------- ---------- ---------- ----------

CCAACGGGTT CAACGGCCTG TCAGCCGTGT TCCTGACGGC GACACCGCAG

CCAACGGTTT CAATGGCATA TCCGTAGTGT TCCTGGCGGG GATCCCCGAA

CCAACGGCTT CAACGGCATG TCGGCCGTCT TCCTGGCGGG GACCCCCGAG

CACCGCTGTC GAGTGCCGGA CGCCGCGAAC CTGAGCAGCG CCTGGCGCAA

CACCGCTGTC GAGTGCCGGA CGCCGCGAAC CTGAGCAGCG CCTGGCGCAA

CACCGCTGCC GGGTGCCTGA CGCCGCGAAC CTGAGCAGCG CCTGGCGCAA

CACCGCTGCC GGGTGCCTGA CGCCGCGAAC CTGAGCAGCG CCTGGCGCAA

CACCACTGCC GGGTGCCGGA CGCTGCGAAC CTGAGCAGCG CCTGGCGCAA

CACCACTGCC GGGTGCCGGA CACCGCGAAC CTGAGCAGCG CGTGGCGTAA

CACCGTTGCC TTGTGCCTCA CACCGTGAAC TTGAGCAGCG CGTGGCGCAA

CACCGTTGCC TTGTGCCTCA CACCGTGAAC CTGAGCAGCG CGTGGCGCAA

CACCGCTGCC GGGTGCCGGA GTCGGCGAAC CTGAGCAGCG CGTGGCGCAA

CACCACTGCC GGGTGCCGGA CACCGCGAAC CTGAGCAGCG CGTGGCGCAA

CACCGCTGCC GGGTGCCCGA CGCCGCGAAC CTGAGCCGCG CGTGGCGCAA

CACCGCTGCC GGGTGCCAGA CACCGCGAAC CTGAGCAGCG CGTGGCGCAA

CACCGCTGCC GAGTGCCGGC CACCGCGAAC CTGAGCGACG CGTGGCGCAA

CACCGCTGCC GGGTGCCCGA CGCGGCGAAC CTGAGCAGCG CCTGGCGCAA

---------- ---------- ---------- ---------- ----------

CACCGGTGCC GGGTGCCGGA GTCGGCCAAC CTGAGCGGCG CGTGGCGCAA

CACCACTGCC GGGTGCCCGC CGGGGCAAAC CTGAGCAGCG AGTGGCGTAA

CACCGGTGCT CGGTGCCGGC GGCGGCCAAC CTGAGCGCCG AGTGGCGGAA

CAACAGTGTC CCGCTGCGGC TGCGGGACGG CCGCGAGGTG CCCCACAGCT

CAACAGTGTC CCGCTGCGGC TGCGGGACGG CCGCGAGGTG CCCCACAGCT

CAACAGTGTC CCGCTGCGGC TGCGGGACGG CCGCGAGGTG CTCCACAGCT

CAACAGTGTC CCGCTGCGGC TGCGGGACGG CCGCGAGGTG CTCCACAGCT

CCACAGTGTC CCGCTGCGGC TGCAGGACGG CCGCGAGGTG CCCCACAGCT

CCACAGTATC CCGGTGCGGC TGCAGGACGG TCGCGAGGTG ATCCATAGCT

CCACAGTATC CCGTTGGAGA CGAAGGACGG ACGACAGGTG CCTCAGAGCT

CCACAGTATC CCGTTGGAGA CGAAGGACGG ACGACAGGTG CCTCAGAAAT

CCACAGTGTC CCGCTGCGGC TTCAGGACGG CCGCGAGGTG CCTCACAGCT

CCACAGTGTC CCCATGCGGC TGCAGGACGG CCGCGAGGTG CCTCAGAGCT

CCACAGTATC CCGCTGCGGC TGCAGGACGG CCGCGAGGTG CCGCACAGCT

CCACAGTGTC CCGCTGCGGC TGCAGAACGG TCATGAGGTG CCTCACAGCT

CCACAGTATC CCGCTGCAGG TGCGGGACGG CCGCGAGGTG CCCCACAACT

CCACAGTATC CCGCTGCGGC TGCAGGACGG CCGCGAGGTG CCCCACAGCT

---------- ---------- ---------- ---------- ----------

CCACAGTGTC CCGCTGCGGC TGCAGGACGG CCGCGAGGTG CCGCACAGCT

CCATAGTATC CCGCTGCAGC TGCAGGACGG CCGGGAAGTG CCCAGACAGT

CCACAGCATC CCGCTGGAgg cgcggggcgc ggggcggggg cccagccgct

GCAGCCGCTA CCGGCTCGCC ACCATCGCCA ACTTCTCGGC GCTCGGGCTG

GCAGCCGCTA CAGGCTCGCC ACCATCGCCA ACTTCTCGGC GCTCGGGCTG

GCCGCCGCTA CCGGCTCGCC ACCATCGCCA ACTTCTCGGC GCTCGGGCTA

GCCGCCGCTA CCGGCTCGCC ACCATCGCCA ACTTCTCGGC GCTCGGGCTA

GCCGCCGCTA CCGGCTCGCC ACCATCGCCA ACTTCTCTGC GCTCGGGTTG

GCCGCCGCTA CCGGCTCGCC ACCATCGCCA ACTTCTCAGC GCTCGGGCTG

GCCGCCGCTA CCGACTGGCC ACCATCGCCA ACTTCTCTGC GCTGGGGCTG

GCCGCCGCTA CCGACTGGCC ACCATCGCCA ACTTCTCTGA GCTAGGGCTG

GCCGGCGCTA CCGGCTCGCC GCCATCGCCA ACTTCTCGGC GCTGGGGCTG

GCCGCCGCTA CCGACTCGCT ATGATGGTCA ACTTCTCGGA GCTCGGGCTG

GCCGGCGCTA CCGGCTCGCG GCGATCGCCA ACTTCTCGGC GCTCGGGCTG

GCAGGCGCTA CCGGCTCGAG GCGATCTCCA ACTTCTCGGC GCTCGGGCTG

GCCGCCGCTA TCAGCTCGCG GCCATCGCCA ACTTCTCGGC GCTCGGGCTG

GCCGCCGCTA CCGGCTCGCA GAGCTCGCCA ACTTCTCGGC GCTCGGGTTG

---------- ---------- ---------- ---------- ----------

GCCGCCGCTA CAAGCTGGCC GCGCTCGCCA ACTTCTCGGC GCTCGGGCTG

GCCAGCGCTA CCGGCTCGCC GCGTTGGCCA ACTTCTCCGC CATGGGGCTA

gccgccgcTA CCGGCTGCAG GCCCTGCGCA ACTTCTCCGC CCTGGGGCTG

GAGCCGGGGC GCGACGTGGA CCTGGGGCAG CTGGAGCAGG AGAGCTGCCT

GAGCCGGGGC GCGACGTGGA CCTGGGGCAG CTGGAGCAGG AGAGCTGCCT

GAGCCGGGGC GCGACGTGGA CCTGGGACAG CTGGAGCAGG AGAGCTGCCT

GAGCCGGGGC GCGACGTGGA CCTGGGACAG CTGGAGCAGG AGAGCTGCCT

GAGCCGGGGC GCGACGTGGA CCTGGGGCAG CTGGAGCAGG AGAGCTGCCT

GAGCCGGGGC GCGACGTGAA TCTGGAGCAG CTGGAGCAGG AGAACTGCCT

GAGCCGGGAC GGGACGTGGA CCTGGAGCAG CTGGAGCAAG AGAACTGCCT

GAGCCGGGGC GGGACGTGGA CCTGGAGCAG CTGGAGCAGG AGAGCTGCCT

GAGCCCGGGC GCGACGTGGA CCTGGAGCAG CTGGAGCAGG AGAGCTGCTT

GAGCCGGGGC GCGATGTGGA CCTAGAGCAG CTGGAGCAGG AGGGCTGCCT

GAGCCGGAGC GCGATGTGGA CCTGGAGCAG CTGGAGCAGG AGAGCTGCCT

GAGCCGGGGC GCGATGTGGA CCTGGAGCAA TTGGAGCAGG AGAGCTGCCT

GAGCCGGGGC GCGATGTGGA CCTGGAGCGG CTGGAGCAGG AGGGCTGCCT

GAGCCCGGGC GCGACGTGGA TCTGGCGCAG CTGGAGCAGG AGAGCTGCCT

---------- ---------- ---------- ---------- ----------

CTGCCGGGGC GCGACGTGAA CCTGACGGAG CTGGAGCAGG AGAGCTGCCT

GTGCCCGGTA GGGACGTGAA CCTGAGTCAG GTGGAGCTGG AGCCCTGTCT

GCGCCCGGCA CCGACGTCGA CCTGGCCCGC CTGGAGCAGG AGAAGTGTCT

GGATGGCTGG GAGTTCAGCC AGGACGTCTA CCTGTCCACC GTCGTGACCG

GGATGGCTGG GAGTTCAGCC AGGACGTCTA CCTGTTCACC GTCGTGACCG

GGATGGCTGG GAATTCAGCC AAGACGTCTA CTGGTCCACC GTCGTGACTG

GGATGGCTGG GAGTTCAGCC AAGACGTCTA CTGGTCCACC GTCGTGACTG

GGATGGCTGG GAGTTCAGCC AGGACGTCTA CTCGTCCACC GTCGTGACCG

AGATGGCTGG GAGTTCAGCC AGGACGTCTA TCTGTCCACC ATCGTGACCG

GGATGGCTGG GAGTACAACA AGGACGTCTT CCTGTCCACC ATCGTGACAG

GGATGGCTGG GAGTACGACA AGGACGTCTT CCTGTCCACC ATCGTGACAG

GGATGGCTGG GAGTACAGCC AGGACGTGTA CCTGTCCACC ATCGTGACCG

GGATGGCTGG GAGTTCAGCC AGGACATCTA CCTGTCCACC ATCGTCACCG

GGATGGCTGG GAGTTCAGCC AGGACGTCTA CCAGTCCACC ATCGTGACCG

GGATGGCTGG GAGTTCAGCC AGGACGTCTA CCTGTCCACC ATCGTGACCG

GGATGGCTGG GAGTACAGCC AGGACGTCTT CCTGTCCACC ATCGTCACCG

GGACGGCTGG GAGTTCAGCC AGGACGTTTA CCAGTCCACC ATCGTGACCG

---------- ---------- ---------- ---------- ----------

GGACGGCTGG GAGTTCAGCC AGGACGTCTA CCTGTCCACC ATCGTCACCG

GGACGGCTGG GAATACAGCC AGGACACATA CCTGTCCACC ATCGTCACTG

GGACGGCTGG GACTACGGCC GCGACGTCTA CCTGTCCACC GTCGTCACCG

AGTGGAATCT GGTGTGTGAG GACAACTGGA AGGTGCCCCT CACCACCTCC

AGTGGAATCT GGTGTGTGAG GACAACTGGA AGGTGCCCCT CACCACCTCC

AGTGGAATCT GGTGTGTGAG GACAACTGGA AGGTGCCCCT CACCACCTCC

AGTGGAATCT GGTGTGTGAG GACAACTGGA AGGTGCCCCT CACCACCTCC

AGTGGAATCT GGTGTGTGGG GACGACTGGA AGGTGCCTCT CACCACCTCC

AGTGGAACCT GGTATGTGAG GAAGACTGGA GGATACCTCT CACCACCTCC

AGTGGGACCT GGTGTGTAAG GATGACTGGA AAGCCCCACT CACCACCTCC

AGTGGGACCT GGTGTGTAAG GATGACTGGA AAGCCCCACT CACCACCTCC

AGTGGAACCT GGTGTGCGAG GATGACTGGA AGACTCCGCT CACCACCTCC

AGTGGAGTCT GGTGTGTGAG GAGGATTGGA AGACGCCCCT CACCACTTCC

AGTGGAGTCT AGTGTGTGAG GATGATTGGA AGACGCCCCT CACCTCCTCC

AGTGGAATCT GGTGTGTGAG GAAGACTGGA AGACGCCCCT CACCTCCTCC

AGTGGAATCT GGTATGTGAG GAGAACTGGA AGACGCCCCT GACCACGTCC

AGTGGAACCT GGTGTGCGAC GATGACTGGA AGACGCCCCT CACCACCTCC

---------- ---------- ---------- ---------- ----------

AGTGGAGCCT GGTGTGCGAG CACGACTGGA AGACGCCCCT CACCACCTCC

AGTGGAATAT AGTGTGTGAT GAAGACTGGA AACCACCACT AACGACCTCA

AGTGGGATCT GGTGTGTGAC AACGACTGGA AGACTCCTCT CACGACGTCC

CTGTTCTTCG TAGGCGTGCT CCTCGGCTCC TTCGTGTCCG GGCAGCTGTC

CTGTTCTTCG TAGGCGTGCT CCTCGGCTCC TTCGTGTCCG GGCAGCTGTC

CTGTTCTTCG TAGGAGTGCT CCTCGGCTCC TTCGTTTCCG GGCAACTGTC

CTGTTCTTCG TAGGAGTGCT CCTCGGCTCC TTCCTTTCCG GGCAACTGTC

CTGTTCTTCG TAGGCGTGCT CCTCGGCTCC TTTGTGTCTG GGCAGCTGTC

CTGTTCTTCG TAGGCGTGCT CCTTGGCTCC TTTGTGTCTG GGCAGCTGTC

TTGTTTTTCG TGGGTGTGCT GATGGGCTCC TTCATTTCGG GACAGCTCTC

TTGTTTTTCG TGGGTGTGCT GATGGGCTCC TTCATTTCAG GACAGCTCTC

CTGTTCTTCG TGGGCGTGCT TATCGGCTCC TTCGTGTCCG GCCAGCTGTC

CTGTTCTTTG TAGGCGTGCT TCTCGGCTCC TTTGTGTCTG GGCAGCTGTC

CTGTTCTTTG TGGGTGTGCT CCTTGGCTCC TTTGTGTCTG GGCAGCTATC

CTGTTCTTTG TAGGCGTTCT CCTTGGTTCC TTTGTGTCCG GGCAGCTATC

CTATTCTTCG TGGGTGTGCT CCTCGGCTCC TTCGTGTCCG GGCAGCTGTC

CTGTTCTTCG TAGGCGTGCT CCTTGGCTCC TTCGTGTCCG GGCAGCTCTC

---------- ---------- ---------- ---------- ----------

CTGTTTTTCC TGGGCGTGCT CTTCGGCTCC TTCGTGTCCG GGCAGCTGTC

TTATTCTTCC TGGGTGTGTT GATGGGATCC TTCATATCAG GACAACTCTC

CTGTTCTTTG TGGGCGTGCT CATCGGATCC TTCATATCGG GACAACTCTC

AGACAGGTTT GGCAGGAAGA ACGTTCTCTT CGCAACCATG GCTGTACAGA

AGACAGGTTT GGCAGGAAGA ACGTTCTCTT CGCAACCATG GCTGTACAGA

AGACAGGTTT GGCAGGAAGA ACGTTCTCTT CGCAACCATG GCTGTACAGA

AGACAGGTTT GGCAGGAAGA ACGTTCTCTT CGCAACCATG GCTGTACAGA

AGACAGGTTC GGCAGGAAAA ATGTTCTCTT CGCAACTATG GCTATACAGA

AGACAGGTTT GGCAGGAAGA ACATTCTCTT CACGACCATG GCTGTGCAAA

AGACAGGTTT GGTCGCAAGA ATGTGCTGTT TTTGACCATG GGCATGCAGA

AGACAGGTTT GGTCGCAAGA ATGTGCTGTT TTTGACCATG GGCATGCAGA

CGACAGGTTT GGTCGGAAGA ACGTGCTCTT TGTCACCATG GGCATGCAGA

AGACAGATTT GGCAGAAAGA CCATCCTCTT CGCAACCATG GCTGTGCAGA

TGACAGGTTT GGCAGGAAGA ACATCCTCTT TGCAACCATG GCTGTGCAGA

AGACAGGTTT GGCAGGAAGA ATGTCCTCTT TGCAACCATG GCTGTACAGA

AGACAGGTTC GGCCGGAAGA ATGTGCTGTT CGTGACCATG GGCATGCAGA

AGACAGGTTT GGCAGGAAGC GCATCCTCTT TGCAACCATG GCTGTGCAGA

---------- ---------- ---------- ---------- ----------

AGACAGGTTT GGCAGGAAGA GCATCCTCTT CAGCACCATG GCCGTGCAGA

AGACAGGTTC GGCAGGAAGA ACATCCTCTT TGCGACAATG GCTGTGCAGA

AGACAGGTTT GGCCGGAAAA ATGTCCTGTT TGTGACCATG GGCATCCAGA

CTGGCTTCAG CTTCCTGCAG ATTTTCTCCA TCAGCTGGGA GATGTTCACT

CTGGCTTCAG CTTCCTGCAG ATTTTCTCCA TCAGCTGGGA GATGTTCACT

CTGGCTTCAG CTTCCTGCAG ATTTTCTCCA TCAGCTGGGA GATGTTCACT

CTGGCTTCAG CTTCCTGCAG ATTTTCTCCA TCAGCTGGGA GATGTTCACT

CTGGCTTCAG CTTCCTGCAG ATTTTCTCCA TCAGCTGGGA GATGTTCACT

CGGGCTTCAG CTTCCTGCAG ATTTTCTCCA TCAACTGGGA GATGTTCACC

CTGGCTTCAG CTTCCTGCAG CTCTTCTCTG TGAACTTCGA GATGTTTACA

CTGGCTTCAG CTTCCTGCAG GTCTTCTCTG TGAACTTCGA GATGTTTACA

CGGGCTTCAG CTTTGTGCAG GTCTTCTCAA AGAACTTCGA GATGTTTGCC

CTGGCTTCAG CTTTCTGCAG GTTTTTTCCA TCAACTGGGA GATGTTCACT

CTGGCTTCAG CTTCCTGCAG ATTTTCTCCA TCAACTGGGA GATGTTTACT

CTGGCTTCAG CTTCCTGCAG ATTTTCTCCA TCAACTGGGA GATGTTCACT

CAGGCTTCAG CTTCCTGCAG GTCTTCTCGA AGAACTTTGA GATGTTTTCC

CTGGCTTCAG CTTCCTGCAG ATTTTCTCCA TCAACTGGGA GATGTTCACT

---------- ---------- ---------- ---------- ----------

CTGGCTTCAG CTTCCTGCAA ATTTTCTCCG TCAACTGGGA GATGTTCACT

CGGGCTTCAG TTTCCTACAG ATCTTCTCCA TCAACTGGGA GATGTTCACT

CGGGCTTCAG CTTCGTGCAG GTTTTCTCGA CGAGCTATGA GATGTTCTCG

GTGTTATTTG TCATCGTGGG CATGGGCCAG ATCTCCAACT ATGTGGTAGC

GTGTTATTTG TCATCGTGGG CATGGGCCAG ATCTCCAACT ATGTGGTAGC

GTGTTATTTC TCATCGTGGG CATGGGCCAG ATCTCCAACT ATGTGGTAGC

GTGTTATTTC TCATCGTGGG CATGGGCCAG ATCTCCAACT ATGTGGTAGC

GTGCTATTTG TCATTGTGGG CATGGGCCAG ATCTCCAATT ATGTGGTGGC

GCGCTATTTG TCATCGTGGG CGTGGGCCAG ATCTCCAACT ACGTGGTAGC

GTGCTTTTTG TCCTTGTTGG CATGGGTCAG ATCTCCAACT ACGTGGCAGC

GTGCTTTTTG TCCTTGTTGG CATGGGTCAG ATCTCCAACT ACGTGGCAGC

GTGCTGTTTG TCCTCGTGGG CATGGGCCAG ATCTCCAACT ACGTGGCAGC

GTGTTATTTG TCATCGTCGG CATGGGCCAG ATCTCCAACT ACGTGGTGGC

GTGTTATTTG TCATCGTTGG CATGGGCCAG ATCTCCAACT ATGTGGTGGC

GTGTTATTTG TCATCGTCGG CATGGGCCAG ATCTCCAACT ATGTGGTGGC

GTGCTGTTTT TCCTTGTAGG CATGGGCCAG ATCTCCAACT ATGTGGCAGC

ATATTGTTCG TCATCGTGGG CATGGGCCAG ATCTCCAACT ACGTGGTGGC

---------- ---------- ---------- ---------- ----------

GTGCTGTTTG TCATCGTGGG CATGGGCCAG ATCTCCAACT ACGTGGTGGC

GTGCTGTTTG TCATCGTGGG CATGGGCCAG ATCTCCAACT ATGTAGTGGC

GTCCTTTTTC TCATCGTGGG CATGGGCCAG ATCTCCAATT ACGTGGCGGC

CTTCATACTA GGAACAGAAA TTCTTGGCAA GTCAGTTCGT ATTATATTCT

CTTCATACTA GGAACAGAAA TTCTTGGCAA GTCAGTTCGT ATTATATTCT

CTTCATACTA GGAACAGAAA TTCTTGGCAA GTCAGTTCGT ATTATATTCT

CTTCATACTA GGAACAGAAA TTCTTGGCAA GTCAGTTCGT ATAATATTCT

TTTCATACTA GGAACAGAAA TTCTTGGCAA GTCAGTTCGT ATTATATTCT

CTTTATACTA GGAACAGAAA TTCTTGGCAA GTCAGTTCGT ATTATATTCT

ATTTGTCCTG GGGACTGAAA TCCTGAGCAA GTCGGTTCGC ATCCTCTTCT

ATTTGTCCTG GGAACTGAAA TCCTGAGCAA GTCGGTTCGC ATCATCTTCT

GTTTGTCCTG GGAACAGAAA TTCTTGGCAA GTCAGTTCGT ATTCTATTCT

CTTCATACTA GGAACAGAAA TTCTTGGCAA GTCAGTTCGT ATTATATTCT

CTTCATTCTA GGAACTGAAA TTCTGGGCAA GTCAGTTCGT ATTATATTCT

CTTCATACTC GGAACAGAAA TCCTTGGCAA GTCAGTGCGT ATTATATTCT

ATTTGTCCTG GGAACAGAAA TTCTTGGCAA GTCAGTTCGT ATTATATTCT

CTTCATACTA GGAACAGAAA TTCTTGGCAA GTCAGTTCGT ATTATATTCT

---------- ---------- ---------- ---------- ----------

CTTCATACTA GGAACAGAAA TTCTTGGCAA GTCAGTTCGT ATTATATTCT

CTTCATACTA GGAACAGAGA TACTTGGCAA ATCAGTTCGT ATTATATTCT

TTTTGTCCTC GGAACAGAAA TTCTTGGCAA ATCAGTTCGC ATTATATTCG

CTACATTAGG AGTGTGCACA TTTTTTGCAG TTGGCTATAT GCTGCTGCCA

CTACATTAGG AGTGTGCACA TTTTTTGCAG TTGGCTATAT GCTGCTGCCA

CTACATTAGG AGTATGCACA TTTTTTGCAG TTGGCTATAT GCTGCTGCCA

CTACGTTAGG AGTATGCATA TTTTATGCAT TTGGCTACAT GGTGCTCCCA

CTACGTTAGG AGTATGCACC TTTTTTGCAG TTGGCTATAT GCTGCTGCCA

CTACGTTAGG AGTGTGCACA TTTTTTGCAG TCGGCTACAT GCTGCTGCCA

CCACATTAGG AGTCTGTACA TTTTTTGCCA TCGGCTACAT GGTGCTGCCG

CCACATTAGG AGTCTGTACA TTTTTTGCAA TCGGCTACAT GGTCCTGCCA

CTACGTTAGG AGTGTGCACA TTTTTTGCAG TGGGCTACAT GCTGCTGCCA

CTACGTTAGG AGTGTGCACA TTTTTTGCAA TTGGCTACAT GCTGCTGCCA

CTACATTAGG AGTGTGCACA TTTTTTGCAG TTGGCTACAT GCTGCTGCCA

CTACGTTAGG AGTGTGCATA TTTTTTGCAG TTGGCTACAT GCTGCTGCCA

CCACGTTAGG AGTGTGCACA TTTTTTGCAG TTGGCTACAT GCTGCTGCCC

CCACGTTAGG CGTGTGCACA TTTTTTGCAG TTGGCTACAT GCTGCTGCCG

---------- ---------- ---------- ---------- ----------

CCACGTTAGG AGTGTGCACA TTCTTTGCAG TTGGCTACAT GCTGCTGCCA

CTACATTAGG AGTTTGCATA TTTTTTGCAA TTGGCTACAT GTTGCTGCCA

CTACGTTAGG AATTTGCATA TTTTTTGCAA TTGGCTACAT GTTGCTGCCC

CTGTTTGCTT ACTTCATCAG AGACTGGCGG ATGCTGCTGC TGGCGCTGAC

CTGTTTGCTT ACTTCATCAG AGACTGGCGG ATGCTGCTGC TGGCGCTGAC

CTGTTTGCTT ACTTCATCAG AGACTGGCGG ATGCTGCTGC TGGCGCTGAC

CTGTTTGCTT ACTTCATCCG AGACTGGCGA ATACTGCTGG TGGCACTGAC

CTGTTTGCTT ACCTCATCAG AGACTGGCGG ATGCTGCTGC TGGCGCTGAC

CTGTTTGCTT ACTTCATCAG AGACTGGCGG ATGCTGCTGC TGGCGCTGAC

CTGTTTGCAT ACTTCATCCG AGACTGGAGG ATGCTGCTGC TGGCACTGAC

CTGTTTGCAT ACTTCATCAG AGACTGGAGG ATGCTGCTGC TGGCCCTGAC

CTGTTTGCTT ACTTCATCAG AGATTGGCGG ATGCTGCTGC TGGCGCTGAC

CTGTTTGCTT ACTTCATCAG AGACTGGCGG ATGCTGCTGC TGGCGCTGAC

CTGTTTGCTT ACTTCATCAG AGACTGGCGG ATGCTGCTGC TGGCGCTGAC

CTGTTTGCTT ACTTCATCAG AGACTGGCGG AAGCTGCTTC TGGCGCTGAC

CTGTTTGCTT ACTTCATCAG AGACTGGCGG ATGCTGCTGC TGGCGCTGAC

CTGTTTGCTT ACTTCATCAG AGACTGGCGG ATGCTGCTGC TGGCGCTGAC

---------- ---------- ---------- ---------- ----------

CTGTTTGCTT ACTTCATCCG AGACTGGCGG ATGCTGCTGC TGGCGCTGAC

CTGTTTGCTT ACTTCATCAG AGACTGGCGG ATGTTGCTAC TGGCTCTGAC

CTATTTGCTT ACTTCATCAG AGACTGGCGG ATGTTGCTAC TGGCTCTTAC

GGTGCCGGGA GTGCTGTGTG TCCCGCTGTG GTGGATGCGG GACTACGACG

GGTGCCGGGA GTGCTGTGTG TCCCGCTGTG GTGGATGCGG GACTACGACG

GGTGCCGGGA GTGCTGTGCG TCCCGCTGTG GTGGATGCGG GACTACGACG

GATGCCAGGG GTGCTGTGCG TGGCACTCTG GTGGATGCGG GACTACGACG

GGTGCCGGGG GTGCTGTGTG TCCCGCTGTG GTGGATGCGG GACTACGACG

GGTGCCGGGG GTGCTGTGCG TCCCGCTGTG GTGGATGCGG GACTACGACG

ACTGCCTGGG CTGTTCTGTG TCCCCCTGTG GTGGATGCGG GACTACGACG

ACTGCCTGGG CTATTTTGTG TCCCCCTGTG GTGGATGCGG GACTACGACG

GGTGCCGGGG GTGCTGTGTG TCCCGCTGTG GTGGATGCGG GACTACGACG

GGTGCCGGGG GTGCTGTGTG TCCCGCTGTG GTGGATGCAG GACTACGACG

GCTGCCGGGG GTGCTGTGCA TCCCTCTGTG GTGGATGCGG GACTACGACG

GGTGCCCGGG GTGCTGTGCG TCCCGCTGTG GTGGATGCGG GACTACGACG

GGTGCCGGGG GTGCTGTGCG TCCCGCTGTG GTGGATGCGG GACTACGACG

GGTGCCCGGG GTGCTGTGCG TCCCGCTGTG GTG-ATGCGT GACTACGACG

---------- ---------- ---------- ---------- ----------

GGTGCCGGGG GTGCTGTGCG TCCCGCTGTG GTGGATGCGT GACTACGACG

GGTGCCGGGG GTGCTGTGTG TCCCGCTGTG GTGGATGCGG GACTACGACG

AATACCGGGG GTGTTCTGCG TGCCGCTGTG GTGGATGAGG GACTACGACG

AGGTGACCGC CTTCCTGGGC GAGTGGGGGC CCTTCCAGCG CCTCATCTTC

AGGTGACCGC CTTCCTGGGC GAGTGGGGGC CCTTCCAGCG CCTCATCTTC

AAGTGACTGC CTTCCTGGGC GAGTGGGGGC CTTTCCAGCG CCTCATCTTC

AGGTGACCGC CTTCCTGGGC GAGTGGGGGC CTTTCCAGCG CCTCATCTTG

AGGTGACCGC CTTCCTGGGC GAGTGGGGGC CCTTCCAGCG CCTCATCTTC

AGGTGACCGC CTTCCTTGGC GAGTGGGGGC CCTTCCAACG CCTCATCTTC

AGGTGACCGC CTTCCTGGGC GAGTGGGGGC CCTTCCAGCG CCTCATTTTC

AGGTGACCGC CTTCCTAGGC GAGTGGGGGC CCTTCCAGCG CCTCATCTTC

AGGTGACCGC GTTTCTGGGC GAGTGGGGGC CCTTCCAGCG CCTCATCTTC

AAGTGACCGC CTTCCTAGGC GAGTGGGGGC CCTTTCAGCG CCTCATCTTC

AGGTGACCAC CTTCCTGGGC GAGTGGGGGC CCTTCCAGCG CCTCATCTTC

AGGTGACCGC CTTCCTGGGC GAGTGGGGGC CGTTCCAGCG CCTCATCTTC

AGGTGACCGC CTTCCTGGGC CAGTGGGGGC CCTTCCAGCG CCTCATCTTC

AGGCGATCGC CTTCCTGGGC GAGTGGGGGC CCTTCCAGCG CCTCATCTTC

---------- ---------- ---------- ---------- ----------

AGGTGACCTC CTTCCTGGGC GAGTGGGGGC CCTTCCAGCG CCTCGTCTTC

AGGTGACAGC CTTCCTGGGC GAGTGGGGAC CCTTCCAGCG CCTCATCTTC

AGGCGACGGC CTTCCTGGGC GAGTGGGGCC CCTTCCAGCG GCTGGTCTTC

TTCCTGCTCA GCGCCAGCAT CATCCCCAAT GGCTTCACCG GCCTGTCCTC

TTCCTGCTCA GCGCCAGCAT CATCCCCAAT GGCTTCACCG GCCTGTCCTC

TTCCTGCTCA GCGCCAGCAT CATCCCTAAT GGCTTCACCG GCCTGTCCTC

TTCCTGCTCA GCGCCAGCAT CATCCCTAAT GGCTTCACCG GCCTGTCCTC

TTCCTGCTCA GCGCCAGCAT CATCCCCAAT GGCTTCACCG GCCTGTCCTC

TTCCTGCTCA GCGCCAGCAT CATCCCCAAC GGCTTCAGCG GCCTGTCGTC

TTCCTGCTCA GCGCCAGCAT CATCCCCAAT GGCTTCAATG GTATGTCTAT

TTCCTGCTCA GCGCCAGCAT CATCCCCAAT GGCTTCAATG GTATGTCCAT

TTCCTGCTCA GCGCCAGCAT CATCCCAAAC GGCTTCAATG GGATGTCAGT

TTCCTGCTCA GCGCCAGCAT CATCCCCAAC GGCTTCAATG GCCTGTCGTC

TTCCTGCTCA GCGCCAGCAT CATCCCCAAC GGCTTCAATG GAATGTCAGC

TTCTTGCTCA GCGCCAGCAT CATCCCCAAC GGCTTCAATG GTATGTCAGC

TTCCTGCTCA GCGCCAGCAT CATCCCCAAC GGCTTCAACG GGATGTCAGT

TTCCTGCTCA GCGCCAGCAT CATCCCCAAT GGCTTCAACG GTATGTCAGC

---------- ---------- ---------- ---------- ----------

TTCCTGCTCA GCGCCAGCAT CGTCCCCAAC GGGTTCAACG GCCTGTCAGC

TTCCTGCTCA GCTCCAGCAT CATCCCCAAC GGTTTCAATG GCATATCCGT

TTCCTGCTGA GCGCCAGCAT CATCCCCAAC GGCTTCAACG GCATGTCGGC

CGTGTTCCTG ATAGCGACCC CGGAGCACCG CTGCCGGGTG CCGGACGCCG

CGTGTTCCTG ATAGCGACCC CGGAGCACCG CTGCCGGGTG CCGGACGCCG

CGTGTTCCTG ATAGCGACCC CGGAGCACCG CTGCCGGGTG CCGGACGCCG

CGTGTTCCTG ATAGCGACCC CGGAGCACCG CTGCCGGGTG CCGGACGCCG

CGTGTTCCTG ATAGGGACCC CGGCGCACCG CTGCCGGGTG CCGGACGCCG

CGTGTTCCTA GCTGGGACCC CGGAGCACCA CTGCCGGGTG CCGGACACCG

CGTGTTCCTG GCGGGGACCC CGGAGCACCG TTGCCTTGTG CCTCACACCG

CGTGTTCCTG GCGGGGACCC CGGAGCACCG TTGCCTTGTG CCTCACACCG

GGTGTTCCTG GCGGGGACCC CCGAGCACCG CTGCCGGGTG CCGGAGTCGG

CGTGTTCTTT ACGGCCACCC CGGAACACCA CTGCCGGGTG CCGGACACCG

CGTGTTCCTG GCCGCCACCC CCGAGCACCG CTGCCGGGTG CCCGACGCCG

CGTGTTCCTG ACGGGGACCC CGGAGCACCG CTGCCGGGTG CCAGACACCG

CGTGTTCCTG ACGGGGACCC CGGAGCACCG CTGCCGAGTG CCGGCCACCG

CGTGTTCCTG GCGGGGACCC CCGAGCACCG CTGCCGGGTG CCCGACGCGG

---------- ---------- ---------- ---------- ----------

CGTGTTCCTG ACGGCGACAC CGCAGCACCG GTGCCGGGTG CCGGAGTCGG

AGTGTTCCTG GCGGGGATCC CCGAACACCA CTGCCGGGTG CCCGCCGGGG

CGTCTTCCTG GCGGGGACCC CCGAGCACCG GTGCTCGGTG CCGGCGGCGG

CGAACCTGAG CAGCGCCTGG CGCAACCACA CTGTCCCACT GCGGCTGCGG

CGAACCTGAG CAGCGCCTGG CGCAACCACA CTGTCCCGCT GCGGCTGCGG

CGAACCTAAG CAGCGCCTGG CGCAACAACA GTGTCCCACT GCGGCTGCGG

CGAACCTAAG CAGCGCCTGG CGCAACCACA GTGTCCCACT GCGGCTGCGG

CTAACCTGAG CAGCGCCTGG CGCAACCACA GTGTCCCGCT GCGGCTGCAG

CGAACCTGAG CAGCTCGTGG CGCAACCACA GTATCCCGGT GCGGCTGCAG

TGAACTTGAG CAGCGCGTGG CGCAACCACA GTATCCCGTT GGAGACGAAG

TGAACCTGAG CAGCGCGTGG CGCAACCACA GTATCCCGTT GGAGACGAAG

CGAACCTGAG CAGCGCGTGG CGCAACCACA GTGTCCCGCT GCGGCTTCAG

CGAACCTGAG CAGCGCGTGG CGCAACCACA GTGTCCCCAT GCGGCTGCAG

CGAACCTGAG CCGCGCGTGG CGCAACCACA GTATCCCGCT GCGGCTGCAG

CGAACCTGAG CAGCGCGTGG CGCAACCACA GTGTCCCGCT GCGGCTGCAG

CGAACCTGAG CGACGCGTGG CGCAACCACA GTATCCCGCT GCAGGTGCGG

CGAACCTGAG CAGCGCCTGG CGCAACCACA GTATCCCGCT GCGGCTGCAG

---------- ---------- ---------- ---------- ----------

CCAACCTGAG CGGCGCGTGG CGCAACCACA GTGTCCCGCT GCGGCTGCAG

CAAACCTGAG CAGCGAGTGG CGTAACCATA GTATCCCGCT GCAGCTGCAG

CCAACCTGAG CGCCGAGTGG CGGAACCACA GCATCCCGCT GGAggcgcgg

GACGGCCGCG AGGTGCCCCA CAGCTGCCGC CGCTACCGGC TCGCCACCAT

GACGGCCGCG AGGTGCCCCA CAGCTGCCGC CGCTACCGGC TCGCCACCAT

GACGGCCGCG AGGTGCCCCA CAGCTGCCGC CGCTACCGGC TCGCCACCAT

GACGGCCGCG AGGTGCCCCA CAGCTGCCGC CGCTACCGGC TCGCCACCAT

GACGGCCGCG AGGTGCCCCA CAGCTGCCGC CGCTACCGGC TCGCCACCAT

GACGGTCGCG AGTTGCTCCA TAGCTGCCGC CGCTACCGGC TCGCCACCAT

GACGGACGAC AGGTGCCTCA GAGCTGCCGC CGCTACCGAC TGGCCACCAT

GACGGACGAC AGGTGCCTCA GAAATGCCGC CGCTACCGAC TGGCCACCAT

GACGGCCGCG AGGTGCCTCA CAGCTGCCGG CGCTACCGGC TCGCCGCCAT

GACGGCCGCG AGGTGCCTCA GAGCTGCCGC CGCTACCGAC TCGCTATGAT

GACGGCCGCG AGGTGCCGCA CAGCTGCCGG CGCTACCGGC TCGCGGCGAT

AACGGTCATG AGGTGCCTCA CAGCTGCAGG CGCTACCGGC TCGAGGCGAT

GACGGCCGCG AGGTGCCCCA CAACTGCCGC CGCTATCAGC TCGCGGCCAT

GACGGCCGCG AGGTGCCCCA CAGCTGCCGC CGCTACCGGC TCGCAGAGCT

---------- ---------- ---------- ---------- ----------

GACGGCCGCG AGGTGCCGCA CAGCTGCCGC CGCTACAAGC TGGCCGCGCT

GACGGCCGGG AAGTGCCCAG ACAGTGCCAG CGCTACCGGC TCGCCGCGTT

ggcgcggggc gggggcccag ccgctgccgc cgcTACCGGC TGCAGGCCCT

CGCCAACTTC TCGGCGCTTG GGCTGGAGCC GGGGCGCGAC GTGGACCTGG

CGCCAACTTC TCGGCGCTCG GGCTGGAGCC GGGGCGCGAC GTGGACCTGG

CGCCAACTTC TCGGCGCTCG GGCTGGAGCC GGGGCGTGAC GTGGACCTGG

CGCCAACTTC TCGGCGCTCG GGCTGGAGCC GGGGCGCGAC GTGGACCTGG

CGCCAACTTC TCGGCGCTCG GGCTGGAGCC GGGGCGCGAC GTGGACCTGG

CGCCAACTTC TCAGCACTCG GGCTGGAACC GGGGCGTGAC GTGGACCTGG

CGCCAACTTC TCTGCGCTGG GGCTGGAGCC GGGACGGGAC GTGGACCTGG

CGCCAACTTC TCTGAGCTAG GGCTGGAGCC GGGGCGGGAC GTGGACCTGG

CGCCAACTTC TCGGCGCTGG GGCTGGAGCC CGGGCGCGAC GTGGACCTGG

GGTCAACTTC TCGGAGCTCG GGCTGGAGCC GGGGCGCGAT GTGGACCTAG

CGCCAACTTC TCGGCGCTCG GGCTGGAGCC GGAGCGCGAT GTGGACCTGG

CTCCAACTTC TCGGCGCTCG GGCTGGAGCC GGGGCGCGAT GTGGACCTGG

CGCCAACTTC TCGGCGCTCG GGCTGGAGCC GGGGCGCGAT GTGGACCTGG

CGCCAACTTC TCGGCGCTCG GGTTGGAGCC CGGGCGCGAC GTGGATCTGG

---------- ---------- ---------- ---------- ----------

CGCCAACTTC TCGGCGCTCG GGCTGCTGCC GGGGCGCGAC GTGAACCTGA

GGCCAACTTC TCCGCCATGG GGCTAGTGCC CGGTAGGGAC GTGAACCTGA

GCGCAACTTC TCCGCCCTGG GGCTGGCGCC CGGCACCGAC GTCGACCTGG

GGCAGCTGGA GCAGGAGAGC TGTCTGGATG GCTGGGAGTT CAGTCAGGAC

GGCAGCTGGA GCAGGAGAGC TGTCTGGATG GCTGGGAGTT CAGTCAGGAC

GGCAACTGGA GCTGGAGAGC TGCCTGGATG GCTGGGAGTT CAGCCGGGAC

GGCAACTGGA GCTGGAGAGC TGCCTGGATG GCTGGGAGTT CAGCCGGGAC

GGCAGCTGGA GCTGGAGAGC TGCCTGGATG GCTGGGAGTT CAGCCAGGAC

AGCAGCTGGA GCAGGAGAAC TGCCTAGATG GCTGGGAGTT CAGCCAGGAC

AGCAGCTGGA GCAAGAGAAC TGCCTGGATG GCTGGGAGTA CAACAAGGAC

AGCAGCTGGA GCAGGAGAGC TGCCTGGATG GCTGGGAGTA CGACAAGGAC

AGCAGCTGGA GCAGGAGAGC TGCTTGGATG GCTGGGAGTA CAGCCAGGAC

AGCAGCTGGA GCAGGAGGGC TGCCTGGATG GCTGGGAGTT CAGCCAGGAC

AGCAGCTGGA GCAGGAGAGC TGCCTGGATG GCTGGGAGTT CAGCCAGGAC

AGCAATTGGA GCAGGAGAGC TGCCTGGATG GCTGGGAGTT CAGCCAGGAC

AGCGGCTGGA GCAGGAGGGC TGCCTGGATG GCTGGGAGTA CAGCCAGGAC

CGCAGCTGGA GCAGGAGAGC TGCCTGGACG GCTGGGAGTT CAGCCAGGAC

---------- ---------- ---------- ---------- ----------

CGGAGCTGGA GCAGGAGAGC TGCCTGGACG GCTGGGAGTT CAGCCAGGAC

GTCAGGTGGA GCTGGAGCCC TGTCTGGACG GCTGGGAATA CAGCCAGGAC

CCCGCCTGGA GCAGGAGAAG TGTCTGGACG GCTGGGACTA CGGCCGCGAC

GTCTACCTGT CCACCATTGT GACCGAGTGG AACCTGGTGT GTGAGGACGA

GTCTACCTGT CCACCATTGT GACTGAGTGG AACCTGGTGT GTGAGGACGA

GTCTATCTGT CCACCATTGT GACCGAGTGG AACCTGGTGT GTGAGGACGA

GTCTATCTGT CCACCATTGT GACCGAGTGG AACCTGGTGT GTGAGGACGA

GTCTACCTGT CCACCATCGT GACCGAGTGG AACCTGGTGT GTGAGGACGA

GTCTACCTGT CCACCATCGT GACCGAGTGG AACCTGGTAT GTGAGGACGA

GTCTTCCTGT CCACCATCGT GACAGAGTGG AACCTGGTGT GTAACGATGC

GTCTTCCTGT CCACCATCGT GACAGAGTGG GACCTGGTGT GTAAGGATGA

GTGTACCTGT CCACCATCGT GACCGAGTGG AACCTGGTGT GCGAGGATGA

ATCTACCTGT CCACCATCGT CACCGAGTGG AACCTGGTGT GTGAGGACGA

GTCTACCAGT CCACCATCGT GACCGAGTGG AACCTGGTGT GTGAGGACGA

GTCTACCTGT CCACCATCGT GACCGAGTGG AATCTGGTGT GTGAGGAAGA

GTCTTCCTGT CCACCATCGT CACCGAGTGG AACCTGGTAT GCGATGACGA

GTTTACCAGT CCACCATCGT GACCGAGTGG AACCTGGTGT GCGACGATGA

---------- ---------- ---------- ---------- ----------

GTCTACCTGT CCACCATCGT CACCGAGTGG AGCCTGGTGT GCGAGCACGA

ACATACCTGT CCACCATCGT CACTGAGTGG GACCTGGTCT GTGAGGATGA

GTCTACCTGT CCACCGTCGT CACCGAGTGG AACCTGGTGT GTGATGATGA

CTGGAAGGCC CCACTCACAA TCTCCTTGTT CTTCGTGGGT GTGCTGTTGG

CTGGAAGGCC CCACTCACAA TCTCCTTGTT CTTCGTGGGT GTGCTGTTGG

CTGGAAGGCC CCACTCACGA TCTCCTTGTT CTTCGTGGGT GTGCTGTTGG

CTGGAAGGCC CCACTCACGA TCTCCTTGTT CTTCGTGGGT GTGCTGTTGG

CTGGAAGGCC CCACTCACCA TCTCCTTGTT CTTCGTGGGT GTGCTGTTGG

CTGGAAGGCC CCGCTCACGA TCTCCATGTT TTTCGTGGGC GTGCTATTGG

CTGGAAAGCC CCACTCACCA CCTCCATGTT TTACGTGGGT GTGCTCTTAG

CTGGAAAGCC CCACTCACCA CCTCCTTCTT TTACGTGGGT GTGCTCTTAG

CTGGAAGACT CCGCTCACCA CCTCCCTGTT CTTCGTGGGC GTGCTTATCG

CTGGAAGGCC CCGCTCACAA TCTCCTTGTT TTTCGTGGGT GTGCTGATGG

CTGGAAGGCC CCACTCACGG TCTCCTTGTT TTTCGTGGGT GTGCTGGTGA

CTGGAAGACG CCCCTCACCT CCTCCCTGTT CTTTGTAGGC GTTCTCCTTG

CTGGAAGGCC CCGCTCACCA TCTCCTTATT TTTCGGGGGC GTGCTCGTGG

CTGGAAGACG CCCCTCACCA CCTCCCTGTT CTTCGTAGGC GTGCTCCTTG

---------- ---------- ---------- ---------- ----------

CTGGAAGACG CCCCTCACCA CCTCCCTGTT TTTCCTGGGC GTGCTCTTCG

CTGGAAAGCT CCACTAACTA CCTCCTTATT TTTTGTGGGT GTGCTTATCG

CTGGAAAGCT CCACTAACTA CCTCCTTATT CTTTGTGGGT GTGCTTATCG

GCTCCTTCAT TTCAGGGCAG CTGTCAGACA GGTTTGGCCG GAAGAATGTG

GCTCCTTCAT TTCAGGGCAG CTGTCAGACA GGTTTGGCCG GAAGAATGTG

GCTCCTTTGT TTCAGGGCAG CTGTCAGACA GGTTTGGCCG GAAGAATGTG

GCTCCTTCGT TTCAGGGCAG CTATCAGACA GGTTTGGCCG GAAGAATGTG

GCTCCTTCAT TTCGGGGCAG CTGTCAGACA GGTTTGGCCG GAAGAATGTG

GCTCCTTCAT TTCAGGGCAG CTCTCAGACA GGTTCGGTCG GAAGAAAGTG

GCTCCTTCAT TTCGGGACAG CTCTCAGACA GGTTTGGTCG CAAGAATGTG

GCTCCTTCAT TTCGGGACAG CTCTCAGACA GGTTTGGTCG CAAGAATGTG

GCTCCTTCGT GTCCGGCCAG CTGTCCGACA GGTTTGGTCG GAAGAACGTG

GCTCCTTCAT TTCGGGGCAG CTCTCAGACA GGTTTGGTCG GAAGAACGTG

GCTCCTTCAT TTCGGGGCAG CTCTCAGACA GGTTTGGCCG GAAGAATGTG

GTTCCTTTGT GTCCGGGCAG CTATCAGACA GGTTTGGTCG GAAGAACGTG

GCTCCTTCAT CTCAGGGCAG CTCTCAGACA GGTTCGGCCG GAAGAATGTG

GCTCCTTCGT GTCCGGGCAG CTCTCAGACA GGTTTGGCAG GAAGCGCATC

---------- ---------- ---------- ---------- ----------

GCTCCTTCGT GTCCGGGCAG CTGTCAGACA GATTTGGACG GAAGAATGTG

GATCCTTTAT TTCAGGACAG CTCTCAGACA AGTTTGGACG GAAGAATGTA

GATCCTTTGT TTCGGGACAG CTCTCAGACA GGTTTGGCCG GAAAAATGTC

CTGTTCGTGA CCATGGGCAT GCAGACAGGC TTCAGCTTCC TGCAGATCTT

CTGTTCGTGA CCATGGGCAT GCAGACAGGC TTCAGCTTCC TGCAGATCTT

CTGTTCATGA CCATGGGCAT GCAGACAGGC TTCAGCTTCC TGCAGATCTT

CTGTTCGTGA CCATGGGCAT GCAGACAGGC TTCAGCTTCC TGCAGATCTT

CTGTTTGTGA CCATGGGCAT GCAGACAGGC TTCAGCTTCC TGCAGCTCTT

CTGTTTGTAA CCATGGCCAT GCAGACAGGC TTCAGCTTCC TGCAGGTCTT

CTGTTTTTGA CCATGGGCAT GCAGACTGGC TTCAGCTTCC TGCAGCTCTT

CTGTTTTTGA CCATGGGCAT GCAGACTGGC TTCAGCTTCC TGCAGGTCTT

CTCTTTGTCA CCATGGGCAT GCAGACGGGC TTCAGCTTTG TGCAGGTCTT

CTGTTTGTGA CGATGGGCAT GCAGACAGGC TTCAGCTTCC TGCAGATCTT

CTCTTTGTGA CCATGGGCAT GCAGACGGGC TTCAGCTTCC TGCAGATTTT

CTGTTTGTGA CCATGGGCAT GCAGACGGGC TTCAGCTTCC TGCAGGTCTT

CTGTTCGTGA CCATGGGCAT GCAGACAGGC TTCAGCTTCC TGCAGGTCTT

CTCTTTGCAA CCATGGCTGT GCAGACTGGC TTCAGCTTCC TGCAGATTTT

---------- ---------- ---------- ---------- ----------

CTGTTAGTGA CCATGGGCAT ACAGACGGGC TTCAGCTTCC TGCAGGTCTT

CTATTTGTGA CAATGGGGAT GCAGACAGGA TTTAGTTTTC TACAAATCTT

CTGTTTGTGA CCATGGGCAT CCAGACGGGC TTCAGCTTCG TGCAGGTTTT

CTCGAAGAAT TTTGAGATGT TTGTCGTGCT GTTTGTCCTT GTAGGCATGG

CTCGAAGAAT TTTGAGATGT TTGTCGTGCT GTTTGTCCTT GTAGGCATGG

CTCAAAAAAC TTTGAGATGT TTGTCGTGCT GTTTGTCCTT GTAGGCATGG

CTCAAAAAAC TTTGAGATGT TTGTCATGCT GTTTGTCCTT GTAGGCATGG

CTCGAAGAAC TTTGAGATGT TTGTCGTGCT GTTTGTCTTT GTAGGCATGG

CTCGAAGAAC TTCGAGATGT TTATCGTGCT GTTTACCTTT GTAGGCATGG

CTCTGTGAAC TTCGAGATGT TTACAGTGCT TTTTGTCCTT GTTGGCATGG

CTCTGTGAAC TTCGAGATGT TTACAGTGCT TTTTGTCCTT GTTGGCATGG

CTCAAAGAAC TTCGAGATGT TTGCCGTGCT GTTTGTCCTC GTGGGCATGG

CTCGAAGAAC TTTGAGATGT TTACCGTGCT GTTTTTCCTT GTAGGCATGG

CTCAAAGAAC TTTGAGATGT TTACTGTGCT TTTTGTCCTT GTGGGCATGG

CTCCAAGAAC TTCGAGATGT TCACCGTGCT GTTTATCCTT GTAGGCATGG

CTCGAAGAAC TTTGAGATGT TTTCCGTGCT GTTTTTCCTT GTAGGCATGG

CTCCATCAAC TGGGAGATGT TCACTATATT GTTCGTCATC GTGGGCATGG

---------- ---------- ---------- ---------- ----------

CTCCACCAAC TTTGAGATGT ATGCTGTGCT GTACTGCGAC GTGGGCATGG

CTCAACAAGC TTTGAGATGT TCTCAGTGCT ATTTGTCCTC GTGGGCATGG

CTCGACGAGC TATGAGATGT TCTCGGTCCT TTTTCTCATC GTGGGCATGG

GCCAGATCTC CAACTATGTG GCAGCATTTG TCCTGGGGAC AGAAATTCTT

GCCAGATCTC CAACTATGTG GCAGCATTTG TCCTGGGGAC AGAAATTCTT

GCCAGATCTC CAACTATGTG GCAGCATTTG TCCTGGGGAC AGAAATTCTT

GCCAGATCTC CAACTATGTG GCAGCATTTG TCCTGGGAAC AGAAATTCTT

GCCAGATCTC CAACTACGTG GCAGCATTTG TCCTGGGGAC AGAAATTCTT

GCCAGATTTC CAACTATGTG GCAGCATTTG TCCTGGGAAC AGAAATTCTT

GTCAGATCTC CAACTACGTG GCAGCATTTG TCCTGGGAAC AGAAATTCTT

GTCAGATCTC CAACTACGTG GCAGCATTTG TCCTGGGAAC AGAAATTCTT

GCCAGATCTC CAACTACGTG GCAGCGTTTG TCCTGGGAAC CGAAATTCTC

GCCAGATCTC CAACTATGTG GCAGCATTTG TCCTGGGAAC AGAAATTCTT

GCCAGATCTC CAACTATGTG GCAGCATTTG TCCTGGGAAC AGAAATTCTT

GCCAGATCTC CAACTATGTG GCAGCATTTG TCTTGGGAAC AGAAATCCTT

GCCAGATCTC CAACTATGTG GCAGCATTTG TCCTGGGAAC AGAAATTCTT

GCCAGATCTC CAACTACGTG GTGGCCTTCA TACTAGGAAC AGAAATTCTT

---------- ---------- ---------- ---------- ----------

GCCAGATCTC CAACTACGTG GCAGCGTTTG TCCTGGGAAC AGAAATTCTT

GACAGATCTC TAACTATGTG GCTGCTTTTG TTCTTGGAAC AGAAATTCTG

GCCAGATCTC CAATTACGTG GCGGCTTTTG TCCTCGGAAC AGAAATTCTG

GGCAAGTCAG TTCGTATAAT ATTCTCTACG TTAGGAGTGT GCATATTTTA

GGCAAGTCAG TTCGTATAAT ATTCTCTACG TTAGGAGTGT GCATATTTTA

GGCAAGTCAG TTCGTATAAT ATTCTCTACG TTAGGAGTAT GCATATTTTA

GGCAAGTCAG TTCGTATAAT ATTCTCTACG TTAGGAGTAT GCATATTTTA

GGCAAGTCAG TTCGTATAAT ATTCTCTACG TTAGGAGTGT GCATATTTTA

GGCAAGTCAA TTCGTATTAT ATTCTCCACG TTAGGAGTGT GCATATTTTA

TCCAAGTCAA TTCGAATTAT ATTCGCCACC TTAGGAGTTT GCATATTTTA

TCCAAGTCAA TTCGAATTAT ATTCGCCACC TTAGGAGTTT GCATATTTTA

AGCAAGTCGA TCCGGATTGT GTTCTCCACA CTGGGCGTGT GCATATTCTA

GGCAAGTCAG TTCGTATTAT ATTCTCTACG TTAGGCGTGT GCATATTTTA

GGCAAATCGG TTCGTATTAT ATTCTCTACG TTAGGAGTGT GCATATTTTA

GGCAAGTCAG TGCGTATTAT ATTCTCTACG TTAGGAGTGT GCATATTTTA

GGCAAGTCAG TTCGTATTAT ATTCTCCACG TTAGGAGTGT GCACATTTTT

GGCAAGTCAG TTCGTATTAT ATTCTCCACG TTAGGCGTGT GCACATTTTT

---------- ---------- ---------- ---------- ----------

GGCAAGTCAG CCCGCATTAT ATTCTCCACG TTAGGAGTGT GCATATTTTA

GGCAAATCAA TTAGAATAAT ATTCTCCACA TTGGGAGTTT GCATATTTTA

GGCAAAGAGG TTCGCATCAT ATACTCCACA ATAGGTGTGT GCATATTTTA

TGCATTTGGC TACATGGTGC TGCCACTGTT TGCTTACTTC ATCCGAGACT

TGCATTTGGC TACATGGTGC TGCCACTGTT TGCTTACTTC ATCCGAGACT

TGCATTTGGC TACATGGTGC TGCCACTGTT TGCTTACTTC ATCCGAGACT

TGCATTTGGC TACATGGTGC TCCCACTGTT TGCTTACTTC ATCCGAGACT

TGCATTTGGC TACATGGTGC TGCCACTGTT TGCTTACTTC ATCCGAGACT

TGCCTTCGGC TACATGGTGC TGCCACTGTT TGCTTACTTC ATCAGAGACT

TGCATTTGGC TTCATGGTGC TGCCTCTGTT TGCATACTTC ATCAGAGACT

TGCGTTTGGC TTCATGGTGC TGCCACTGTT TGCATACTTC ATCAGAGACT

CGCCGTGGGC TACATGGTGC TGCCGCTGTT CGCCTACTTC ATCAGAGACT

TGCGTTTGGC TACATGCTGC TGCCACTGTT TGCTTACTTC ATCAGAGACT

TGCATTTGGC TACATGATGC TGCCACTATT TGCCTACTTC ATCAGAGACT

TGCAGTTGGC TACATGCTGC TGCCACTGTT TGCTTACTTC ATCAGAGACT

TGCAGTTGGC TACATGCTGC TGCCCCTGTT TGCTTACTTC ATCAGAGACT

TGCAGTTGGC TACATGCTGC TGCCGCTGTT TGCTTACTTC ATCAGAGACT

---------- ---------- ---------- ---------- ----------

TTCCATTGGC TACATGCTGC TGCCACTGTT TGCCTACTTC ATCCGAGACT

TGCAATTGGC TACATGCTCC TGCCTCTGTT TGCTTATTTC ATTCGAGAAT

TGCCGTGGGC TACATGTTGC TGCCCCTATT CGCTTACTTC ATCAGACACT

GGCGGATGCT GCTGGTGGCG CTGACGATGC CGGGGGTGCT ATGCGTGGCA

GGCGGATGCT GCTGGTGGCG CTGACGATGC CGGGGGTGCT GTGCGTGGCA

GGCGAATACT GCTGGTGGCA CTGACGATGC CGGGGGTGCT GTGCGTGGCA

GGCGAATACT GCTGGTGGCA CTGACGATGC CAGGGGTGCT GTGCGTGGCA

GGCGGATGCT GCTGCTGGCG CTGACGGTGC CAGGGGTGCT GTGTGTGGCA

GGCGGATGCT GCTGCTGGCA CTGACGGTGC CAGGGGTACT ATGCGTGGCA

GGAGGATGCT GCTGCTTGCG CTCACTGTAC CAGGGGTGCT GTGTGGGGCT

GGAGGATGCT GCTGCTGGCG CTCACTGTGC CAGGGGTGCT ATGTGGGGCT

GGCGGATGCT GCTGCTGGCG CTGACGGTGC CCGGGGTGCT GTGTGCGGCC

GGCGGATGCT GCTGCTGGCG CTGACGGTGC CGGGGGTGCT CTGTGCAGCG

GGCGGATGCT GCTGCTGGCA CTGACGCTAC CAGGGGTGCT GTGTGCAGCT

GGCGGAAACT GCTTCTGGCT CTGACGGTGC CCGGGGTGCT GTGCGCGGCG

GGCGGATGCT GCTGCTGGCG CTGACGGTGC CGGGGGTGCT GTGCGTCCCG

GGCGGATGCT GCTGCTGGCG CTGACGGTGC CCGGGGTGCT GTGCGTCCCG

---------- ---------- ---------- ---------- ----------

GGCGGATGCT GCTGCTGGCG CTGACGGTGC CGGGGGCACT GTGCTTAGCG

GGAGGATGTT ATTACTGGCC CTAACTCTTC CAGGCTTGCT TTGTTTCCCA

GGCGGATGTT ACTACTGGCT CTTACAGTGC CTGGACTGTT CTATATTCCA

CTCTGGTGGA TGTGTACAAA GACAATCCCA GTCCTCTGGG GATGTTTCCT

CTCTGGTG-A TGTGTACAAA GACAATCCCA GTCCTCTGGG GATGTTTCCT

CTCTGGTGGA TGTGTACAAA GACAATCCCA GTCCTCTGGG GATGTTTCCT

CTCTGGTGGA TGTGTACAAA GACAATCCCA GTCCTCTGGG GATGTTTCCT

CTCTGGTGGA TGTGTACAAA GATAATCCCA GTCCTCTGGG GATGTTTCCT

CTCTGGTGGA TGCGTACAAA GGCAATCCCA GTCCTCTGGG GGTGCTTCCT

CTCTGGTGGA TGCGAACAAA GCAGGTCCCA GTCCTCGGGG CATGCTTCCT

CTCTGGTGGA TGCGGACAAA GCAGGTCCCA GTCCTCTGGG CATGCTTTCT

CTGTGGTGG- ---------- ---------- ---------- ----------

CTGTGGTGGA TGTGTACAAA GATAGTCCAA GTCCTCTGGG GATGTTTCCT

CTCTGGTGGA TGCGTACAAA GGCAGTTCAA GTCCTCTGGG GATGTTTCTT

CTCTGGTGG- ---------- ---------- ---------- ----------

CTGTGGTGGA TGCATGCCAA GGCGGTCCCG GTGCTCTGGG GATGTTTCCT

CTGTGGTG-A TGCCTACAAA GCCGGTCCAA GTGCTCTGGG GCTGTTTCCT

---------- ---------- ---------- ---------- ----------

CTTTGGTGGA TGTATGCAAG GATGGTCCAG TTGCTGTGGG GCTGTTTCTT

CTATGGTGGa tggaagcaat gaagatcctg atccttTGGG GTTATTTCCT

CTGTGGTGGA TGGAAACAAG GCAGATTCTG ACTGTTTGGG GCTTTTTTCT

CCTGTGGAAT CTCTATGTCT CATCCTCTCA GACCATTTAC CCTGGAATCA

CCTGTGGAAT CTCTATGTCT CATCCTCTCA GACCATTTAC CCTGGAATCA

CCTGTGGCAT CTCTGCCTCT CATCCTCTCA GACCATTTAC CCTGGAATCA

CCTGTGGCAT CTCTGCCTCT CATCCTCTCA GACCATTTAC CCTGGAATCA

CCTGTGGAAT CTCTACATAT CATCTTCTTA CACCATTTAC CCTGGAATCA

CCTCTGGAAT CTCTGCACCT CATCCTCTCA GACCATTTAC CCTGGAATCA

CCTGTGGAGT CTCTACATCG CCTCCTCCCA GACTGTTTAC CCTGGAATCA

CCTATGGAGT CTCTACATTG CCTCCTCCCA GACTGTCTAC CCTGGAATCA

---------- ---------- ---------- ---------- ----------

CCTGTGGAAT CTCTACATCT CATCTGCTCA GGCCATTTAC CCTGGAATCA

CCTGTGGAAT CTCTGCAGCT CATCCACGCA GACCATTTAC CCTGGAATCA

---------- ---------- ---------- ---------- ----------

CCTGTGGAAT GTCTACGCCT CCTCCTCTGA GGCTGCTTAC CCTGGAATCA

CCTGTGGAAT CTCTACATCT TATCTTCTCA GACCACATAC CCAGGAATCA

---------- ---------- ---------- ---------- ----------

CCTGTGGAGC CTCAGCATTG CATCTTCTCA GACCATTTAC CCTGGAATCA

CTTGTGGAAC CCACTTACCC AATCAACTCA GC---TTTAC CCTGGAATCA

ACTGTGGAAC CTCTTAACTG CATCTTCTTC GCTCAACCAG CCAGGGATCA

AGGCAAGGAT TACTCAGAGG GCACTTGACT ATGGTGTTCA AGCTGGAATG

AGGCAAGGAT TACTCAGAGG GCACTTGACT ATGGTGTTCA AGCTGGAATG

AGGCAAGGAT TACTCAGAGG GCACTTGACT ATGGTGTTCA AGCTGGAATG

AGGCAAGGAT TACTCAGAGG GCACTTGACT ATGGTGTTCA AGCTGGAATG

AGGCAAGGAT TACTCAGAGG GCACTTGACT ATGGTGTTCA AGCTGGAATG

AGGTGAGAGC TACTCAGCGC GCACTGGACT ATGGGGTTCA GGCTGGCATG

AGGCGAGGAT CACCCAGCGT GCACTCGACT ATGGTCTTCA AGTTGGGATG

CGGCGAGGAT CACCCAGCGT GCACTAGACT ATGGTCTTCA AGTTGGGATG

---------- ---------- ---------- ---------- ----------

AGGCGAGGGC TACTCAGCGG GCCCTGGACT ATGCTGTTCA AGCTGGAATG

AGGCCAGGGT TACTCAGCGG GCTCTGGACT ATGGCATTCA AGCTGGAATG

---------- ---------- ---------- ---------- ----------

AGGCCAGGAT TACACAGCGG GCTCTGGACT ATGGTGTCCA AGCTGGGCTA

AGGCGAGGGC TACTCAGAGG TTGCTGGACT ATGGCGTGCA AGTTGGAATG

---------- ---------- ---------- ---GCATTCA AGCTGGGATG

AGGCAAGGAT CACCCAGCGG GCACTGGACT ATGGCATTCA AGCTGGGCTG

AAGTGAGGAT TACTCAGAAG GGCCTGGACT ATGGTATACA GGCTGCAATG

AAGTGAGGAT CACTCAGAAG GCCCTTGACT ATGGTGTACA GGCTGGAATG

AAGATGATTG AGCAAATGCT AAAAGAAAAG AAACTCCCAG ATTTAAGCGG

AAGATGATTG AGCAAATGCT AAAAGAAAAG AAACTCCCAG ATTTAAGTGG

GAGATAATTG AGCGAATGCT GAAAGAAAAC AACCTCCCAA ATTTAAGCGG

GAGATAATTG AGCGCATGCT AAAAGAAAAG AACCTCCCAG ATTTAAGGGG

GAGATGATTG AGCAGATGCT AAAGCAAAAG AACCTCCCAG ATTTAAGCGG

GAGATGATTG AACACATGGT CAAGGAAAAG AACATCCCAG ATATAAATGG

GAGATACTGG AGCAGCTGGC AAAGGAAATC GTCATCCCAG ATTTGAACGG

AAGGTACTGG AGCAATTGGC AAAGGAAATC GTCATCCCAG ATTTGAACGG

---------- ---------- ---------- ---------- ----------

GAGATGATTG AACAAATGGC CAAGGAAAGA AAGCTCCCAG ATTTAAAAGG

GAGATGATTG AGCAAATGAT TAAGGAAAAA AACATCCCAG ACCTAAAAGG

---------- ---------- ---------- ---------- ----------

GAGATGATGG AGCAAATGCT CAAGGAGAGA AACATCCCGG ACTTAAAAGG

GAGACAATTG AGCAAATGTT AAAGGAAAGG AAGATGCCAG ATTTAAATGG

GAGACAATTG AGCAAATGAT AATGGAAAAG AGCATCCCAG ATTTAAAAGG

GAGACAGTTG AGCAAATGAT CATGGAGAGA GACATCCCCA GTTTAAACGG

GAAGCAATTG AGGTGATAAT GAAGACAAAT GGGATTCCGG ATTTCAAAGG

GGGATCCTGG AGAAGTTAAT AAAGAAAAAG AGAATACCAG ATTTTAAAGG

TTCTGAGTCT CTTGAATTTC TAAAAGTTGA TTATGTAAAC TACAATTTTT

TTCTGAGTCT CTTGAATTTC TAAAAGTTGA TTATGTAAAC TACAATTTTT

TTCTGAGTCT CTTGAATTTC TAAAGGTTGA TTACGTAAAC TACAATTTTT

TTCTGAGTCT CTTGAATTTC TAAAGGTTGA TTACGTAAAC TACAATTTTT

TTCTGAGTCT CTTGAATTTC TAAAGGTTGA TTATGTGAAC TATAATTTTT

TTCCGAGTCT CTTGACTTCC TAAAGGTTGA TTATGTGAAC TACAACTTTT

TTCTGAGTCT CTTAAGTTTC TGAAGATCGA CTATGTAAAG TACAACTTCT

TTCTGAGTCT CTTAAATTTC TGAAGATTGA CTATGTAAAG TACAACTTCT

---------- ---------- ---------- ---------- ----------

TTCTGAGTCT CTTGAATTTG TGAAAATCGA TTATGTGAAC TACAATTTTA

TTCTGAATCT CTAGAATTTC TGAAGGTTGA TTATGTGAAC TATAATTTTT

---------- ---------- ---------- ---------- ----------

CTCGGAAACC CTGGAATTTC TGAAGGTTGA TTATGTGGAC TACAACTTTT

TTCTGAGTCT CTTGAATTTC TGAAGGTGGA TTATGTGGAC TACAATTTTT

TTCTGAATCT CTTGAATTTC TGAAGGTTGA TTATGTGGGC TACAATTTTT

TTCTGAGTCT CTTGAGTTTC TGCAGCTGGA TTATGTGAAC TACAACTTTT

CTCTGAATCT CTTGACTTTT TAAAGGTTGA CTATGTTGAC TATAATTTTT

CTCTGAAACC CTCGATTTTC TGAAAGTTGA ATACATCGAC TACAATTTTT

CAAATATAAA AATCAGTGCC TTTTCATTTC CAAATACCTC ATTGGCTTTT

CAAATATAAA AATCAGTGCC TTTTCATTTC CAAATACCTC ATTGGCTTTT

CAAATATAAA AATCAGGGCC TTTTCATTTC CAAATACCTC ATTGGCTTTT

CAAATATAAA AATCAGGGCC TTTTCATTTC CAAATACCTC ATTGGCTTTT

CAAATATAAA AATCAATGCC TTTTCATTTC CAAATACCTC ACTGGCTTTT

CAAACATCAA AATCAATGCC TTTTCATTTC CAAATACGTC ATTAGCCTTT

CAAACATAAA AATCAATGCC TTTTCATTCC CAAACACCTC ACTGGCCTTT

CAAACATAAA AATCAATGCC TTCTCATTCC CAAATACCTC ACTGGCCTTC

----CATAAA AATCAATGCC TTTTCATTTC CAAATACTTC CTTAGCCTTC

CAAACATAAA AATCAATGCC TTTTCATTTC CAAATACTTC ACTCTCCTTT

CAAACATAAA AATCAATGTC TTTTCATTTC CAAATACTTC ATTAGCCTTT

---------- ---------- ---------- ---------- ----------

CAAACATATC GATCAATGCC TTTTCATTTC CAAACACGTC ATTGACCTTC

CAAACATAAA AATCAATGCC TTTTCATTTC CAAATTCTTC ATTAGCCTTT

CAACCATAAA AATCAAGGCC TTTTCATTTC CAAATACTTC ATTAGCCTTT

CAAACATAAA AATCAAGGCT TTTTCATTTC CAAACACTTC CCTGGCCTTC

CAAACATAAA AATCAACATG TTCTCATTTC CAAATATTTC ATTAACCCCT

CAAACATTCG ATTCAGTGCC TTTTCATTTC CAAATACTTC ACTGATTCTT

GTGCCTGGAG TGGGAATCAA AGCGCTAACC AACCATGGCA CTGCCAACAT

GTGCCTGGAG TGGGAATCAA AGCGCTAACC AACCATGGCA CTGCCAACAT

GTGCCTGGGG TGGGAATCAA AGCGCTAACC AACCACGGCA CTGCCAACAT

GTGCCTGGGG TGGGAATCAA AGCGCTAACC AACCACGGCA CTGCCAACAT

GTGCCAGGAG TGGGAATCAA AGCACTAACC AACCATGGCA CTGCCAACAT

GTGCCTGGAG TGGGGATCAA AGTCCTGACC AACCACGGCT CTGCCAATAT

GTACCTGGGG TAGGAATCAG AGCTCTGAGC AATCACGGCA CTGCCAACAT

GTACCTGGGG TAGGCATCAG AGCACTGAGC AACCACGGCA CTGCCAACAT

GTGCCTGGGG TGGGAATCAA AGCACTGACC ---------- ----------

GTTCCTGGTG TCGGAATCAA GGCTCTGACC AACCATGGAA CTACCAACAT

GTGCCTGGAG TGGGAATCAA GGCACTGACC AACCATGGCA CAGCCAACAT

---------- ---------- ---------- ---------- ----------

GTGCCTGGGG TGGGAATCAG AGCCCTGACC AACCACGGCA CCGCCAACAT

GTACCCGGAG TGGGAATCAA AGTGCTGACC AACCACGGCA CTGCCAACAT

GTGCCTGGAG TGGGAATCAA AGTGCTCACC AATCACGGCA CAGCCAACAT

GAGCCTGGCA TGGGGATCAA AGTACTCACC AATCATGGCA TGGCCAACAT

GTGCCTGAAA CTGGAATTAA AATATTAACC AATCATGGTT CAACAAACAT

GTACCTGGAA CTGGGATTAA AGCGTTAACC AATCACGGTT CTGCAAACAT

CAGCACAGAC TGGGGGTTCG AGTCTCCACT TTTCCAAGAC ACAGGAGGGG

CAGCACAGAC TGGGGGTTCG AGTCTCCACT TTTCCAAGAC ACAGGAGGGG

CAGCACAGAC TGGGAGTTCG AGTCTCCACT TTTCCAAGAC ACAGGAGGAG

CAGCACAGAC TGGGAGTTCG AGTCTCCACT TTTCCAAGAC ACAGGAGGAG

CAGCACAGAC TGGGAATTCA AGTCTCCAAT TTTCCAAGAC ACAGGAGGAG

CAGCACAGAC TGGGAAATCA AGTCTCCACT TTTCCAAGAT GCAGGAGGGG

CAGCACCAAC TGGGACGTCA AGGCTCCACT TTTCCAAGAC TCGGGAGCTG

CAGCACCAAC TGGAGCGTCA AGGCTCCACT TTTCCGAGAC TCGGGAGCTG

---------- ---------- ---------- ---CAAAGAC GCAGGAGGAG

CAGCACAGAC TGGGAAGTCA AGTCTCCACT TTTCCAAGAC ACAGGAGCAG

CAGTACCAAC TGGGATGTCA AATCTCCACT CTTTCAAGAC ACAGGAGAAG

---------- ---------- ---------- ---------- ----------

CAGCACCGAC TGGGAATTCA AGTCTCCACT CTTCCAAGAT TTTGGAGGGG

CAGCACCGAC TGGGCGGTCA GCTCTCCACT TTTCCAAGAT GCAGGAGCGG

CAGCACACAC TGGGAAGTCA AGTCTCCACT TTTCCAAGAT GCAGGAGGAG

CAGCACCCAC TGGGACGTCA AGTCTCCGCT CTT------- ----------

CAGCATGGGA TGGGAAATAA TGTCTCCACT TTTAAAAGAT GGAGGAGGAG

CAGCACAGAC TGGGAAATAA AGTCCCCACT TTTCAAAGAT GAAGGAGGAG

CTGATCTGTT TCTCTCCGGA GTCTACTTTA CCGGTATCAT TATCCTAACC

CTGATCTGTT TCTCTCCGGA GTCTACTTTA CCGGTATCAT TATCCTAACC

CTGATTTGTT TCTCTCCGGA GTCTACTTTA CTGGTATCAT CATCCTAACC

CTGATTTGTT TCTCTCCGGA GTCTACTTTA CTGGTATCAT CATCCTAACC

CTGATCTGTT TCTGTCCGGA GTCTACTTTA CTGGTATCAT TATTCTAACC

CTGATCTCTT TCTCTCTGGA GTCTACTTTA TCGGTATTGT TATCCTGACC

CCGATCTGTT TCTCTCTGGA ATCTACTTTA CCGGCATCAT CGTCTTCACC

CCAATCTGTT CCTCTCTGGA ATCTATTTCA CTGGCATCGT TGCCTTTACC

CAAATCTGTT CCTCTCCGGG GTCTACTTTA CTGGTAACAT TATCCTAAAC

CTGACCTGTT TCTCTCTGGG GTCTATTTCA CTGGTATAGT TGTCCTGTCC

CTGATTTGTT TCTGTCTGGG GTCTACTTCA CTGGTATCAT TGTCCTCTCC

---------- ---------- ---------- ---------- ----------

CTGATCTGTT CCTCTCTGGG GTCTTCTTCT CGGGCACTGT TGTCCTGACC

CTGATCTGTT CCTCTCTGGG GGCTTCTTCA CCGGTATCGT TATTCTGTCC

CCGATCTGTT TCTCTCTGGG GCCTTCTTCA CTGGTATCCT CATCCTGTCC

---------- ---------- ---------- ---------- ----------

CTACTCTGTT TCTGGGTGAG ATCTTTTTCT CTGGTCTTGG TAACCTGTCC

CTTCTCTGTT CCTCTCTGAG GTGTTTTTCA CGGGCATCAT CAACTTGTCC

CGAAATGACT TTGGTCATCC TACCCTGAAG CTCCAAGATT GCTACGCCCA

CGAAATGACT TTGGTCATCC TACCCTGAAG CTCCAAGATT GCTACGCCCA

CGAAATGACT TTGGTCATCC TACACTGAAG CTCCAAGATT GCTACGCCCA

CGAAATGACT TTGGTCATCC TACACTGAAG CTCCAAGATT GCTACGCCCA

CGAAACGACT TTGGTCATCC TATCCTGAAG CTCCAAGATT GCTATGCCCA

CGAAATGACT CTGGGCATCC AATCCTGAGG CTCCAAGACT GCTACGCCCA

CGGAATGACT TTGGTCACCC AGCTCTGGAA CTGCAAGACT GCCATGTACA

CGTAATGACT TTGGTTACCC AGCTCTGGAA CTCCAAGACT GCCATGTTCA

CGAAATGACT TTGGTCAGCC TCTCCTGAAG CTTGAAGACT GCTATGCCCA

CGAAATGACT TTGGTCACCC GATCCTGAAG CTCCAAGACT GCTATGCTCA

CAAAATGACT TTGGTCACCC AATCCTGAAG CTCCAAGACT GTTATGCCCA

---------- ---------- ---------- ---------- ----------

CGGAATGACT TTGGCCACCC AATCCTGAAA CTCCAGGACT GCCACGCCCA

CGAAATAACT TCGGTAAACC AACCCTGAAG CTCCAAGACT GCTACGCTCA

CGAAATGACT TTGGTCACCC AACCCTGAAG CTCCAAGACT GCCATGCTCA

---------- ---------- ---------- ---------- ----------

CGGAGTGATA CAGGCCACCC AAGCATGCAT TTAGTAGATT GCCATGTTCA

CGGAGCGCCA TGGGCCACCC GACCATGAAG CTCGAAGACT GTTACGTTCA

ACTGAGCCAT GCCCACGTCT CATTTTCCGG AGAACTCAGT GTTCTGTATA

ACTGAGCCAT GCCCACGTCT CATTTTCCGG AGAACTCAGT GTTCTGTATA

AGTGAGCCAT GCCCATGTCT CATTTTCCGG AGAACTCAGT GTTCTGTATA

AGTGAGCCAT GCCCATGTCT CATTTTCCGG AGAACTCAGT GTTCTGTATA

AGTGAGCCAC GCCCACGTTT CATTTTCCGG AGAACTCAGT GTCCTGTATA

AGTGAGCCAC GCCCATGTCT CATTTTCTGG AGAGCTCAGT GTCCTATATA

AGTGAGCCAC GCCCGAGTCT CATTTTCTGG AGGCCTCAGT GTCCTGTATA

AGTGAGCCAT GCCCGAGTCT CATTTTTCGG AAGTCTCAGT GCCCTGTATA

AGTGAGCCAT GCCCACGTCT CATTTTTTGG ACAGTTCAGT GTCCTGTATA

AGTGAGCCAT GCTGACGTCT CGTTTTCTGG AGAGCTCAGT GTCCTATATA

AGTGAGCCAT GCCCACGTGT CATTTTCTGG AGAACTCAGT GTCCTGTACA

---------- ---------- ---------- ---------- ----------

AGTGAGCCAC GCCCACGTCA CATTCTCCGG AGAGCTCAGT GTCCTGTATA

AGTGAGCCAT GCCCACGTCT CCTTTTCCGG AGATCTCAGT GTTCTGTATA

AGTGAGCCAC GCCCATGTCT CGTTCTCTGG AGAACTCAGT GTCCTGTATA

---------- ---------- ---------- ---------- ----------

AGTTGGTCAT GCCCACATAT CATTTTCAGG AGAACTCAGT GCCCTATATA

CATCAGTCAT GCCCACATTG CGTTTTCAGG AGAACTCAGT CCCCTCTATA

ACTCCTTTGC TGAGCCCATG GAGAAACCCA TTTTAAAGAA CTTAAATGAA

ACTCCTTTGC TGAGCCCATG GAGAAACCCA TTTTAAAGAA CTTAAATGAA

ACTCCTTTGC TGAGCCCATG GAGAAACCCA TTTTAAAGAA CTTAAATGAA

ACTCCTTTGC TGAGCCCATG GAGAAACCCA TTTTAAAGAA CTTAAATGAA

ACTCATTTGC TGAACCCATG GAGAAACCCA TTTTAAAGAA CCTAAATGAA

ACTCCTTTGC TGACCCCATG GAAAAACCCA TTTTAAAGAA CTTAAATGAA

ACTCCTTTGC TGAGCCCATG GAGAAACCCA TTCTGAAGAA TTTAAATGAA

ACTCCTTTGC TGAGCCCATG GAGAAACCCA TTCTAAAGAA TTTAAATGAA

ACTCCTTTGC TGAGCCCATG GAGAAACCCA TTTTAAAGAA CTTAAATGAA

ACTCCTTCGC TGAGCCCATG GAGAAACCCA TTTTAAAGAA ATTAAATGAA

ACTCCTTTGC TGAGCCCATG GAAAAACCCA TTTTAAAGAA CTTAAATGAA

---------- ---------- ---------- ---------- ----------

ACTCCTTCGC TGAACCCATG GAGAAACCCA TTTTAAAGAA TTTAAATGAA

ACTCCTTTGC TGGGGCCATG GAGAAACCCA TTTTAAGGCA TTTAAATGAA

ACCCCTTTGC TGAGCCCATG GAGAAACCCA TTTTAAAGAA CTTAAATGAA

---------- ---------- ---------- ---------- ----------

ACTCCTTTGC ACATCCAATG GAGAAACCTA TTCTGAAGAC TTTGAATAAA

ACTCCTTCTC TGAGCCAATG GAGAAACCCA TCCTGAAGAA CTTGAATGAA

ATGCTCTGTC CCATTATTGC AAGTGAAGTC AAAGCGCTAA ATGCCAACCT

ATGCTCTGTC CCATTATTGC AAGTGAAGTC AAAGCGCTAA ATGCCAACCT

ATGCTCTGTC CCATTATTGC AAGTGAAGTC AAAGCGCTAA ATGCCAACCT

ATGCTCTGTC CCATTATTGC AAGTGAAGTC AAAGCGCTAA ATGCCAACCT

ATGCTCTGTC CTATTATTGC AAATGAAGTC GAAGCACTAA ATGCCAACCT

ATGCTGTGTC CTATTATCAC AAGAGAGGTT GAAGCGCTAA ATGCCAATCT

ATGCTTTGTC CCATTGCCAT CAGCCAAGTG GAAGAGTTCA ACGTCAACAT

ATGCTTTGTC CCATTGCCAT CAGCCAAGTG GAACAGTTCA ATGTCAACAT

ATGCTCTGCC CCATTATCAC AGGCGAGGTT GAAGCACTGA ACGTCAATCT

ATGCTCTGTC CCATTATCAC AGGTGAGGTG GAGGCACTGA ATGCCAATCT

ATGCTCTGTC CCATTATCAC GAGTGAGGTG GAAGCCCTGA ATGCCAATCT

---------- ---------- ---------- ---------- ----------

ATGCTGTGCC CCATCGTCAC CAGCGAGGTG GAAGCCCTGA ACGCCAACCT

ATGCTCTGTC CCATTATTGT GCATGAAGTT GAAGCACTAA ATGCCAATCT

ATGCTCTGTC CCATGATCAC CAATGAGGTC GAAGCACTAA GTGCTAATCT

---CTCTGTC CCATCATCAT TGGTGAGGTT GAAGGGCTCA ATGCTAATCT

AGGTTATGTA CAATTATCAC GGATCAGTTT GAAGAAATAA ATGCAAATAT

AAATTATGTC CAATTATCAA AGACGAAATG GAAAGGATAA ATGCTGAGCT

CAGCACACTG GAGGTTTTAA CCAAGATTGA CAACTACACT CTGCTGGATT

CAGCACACTG GAGGTTTTAA CCAAGATTGA CAACTACACT CTGCTGGATT

CAGCACACTG GAGGTTTTAA CCAAGATTGA CAACTACACT CTGTTGGATT

CAGCACACTG GAGGTTTTAA CCAAGATTGA CAACTACACT CTGTTGGATT

CAGCACACTG GAGGTTTTAA CCAGGATTGA CAACTATACT CTGCTGGATT

CAGCACACTG GAAGTTTTAA CCAAGATCGA CAACTATACT GTGCTGGATT

CAGCGCGCTG GAGGTTTTAA CCAAGATTGA CAACTACACT GTGCTGGATT

CAGCGCACTG GAGGTTTTAA CCAAGATTGA CAACTACACA GTGCTGGATT

CAGCACACTG GAGGTCCTAA CCAAGATTGA CAACTACACT CTTCTGGATT

CAGCATGCTG GAGGTTTTAA GAAAGATTGA CAACTATACT CTGCTGGATT

CAGCATGCTA GAGGTTTTAA CCAAGATTGA CAACTATACT CTGCTGGATT

---------- ---------- ---------- ---------- ----------

CAGCATGCTG GAGGTGTTAA CCAAGATCGA CAACTATACT ATCCTGGATT

CAGCATGCTG GAGGTTTTAA CCAAGATTGA CAACTATACT CTGCTGGATT

CAGCATGCTG GAGGTTCTAA CCAAGATCAA CAACCATACT CTGCTGGATT

CAGCATGCTA GAAGTTCTAA CCAGGATCAA CAACCACACT CTGCTGGATT

CAGCTCTCTG GAGGTTGTAA CCAAGTTTGG TGAGGATGTT GTGCTAGATT

CCGAACACTG GATGTCCTAA CCAGGATTGA TGAAAATACT CTGTTAGATT

ACTCCCTAAT CAGTTCTCCA GAAATTACTG AGAACTACCT TGACCTGAAC

ACTCCCTAAT CAGTTCTCCA GAAATTACTG AGAACTACCT TGACCTGAAC

ACTCCCTAAT CAGTTCTCCA GAAATTACTG AGAACTACCT TGACCTGAAC

ACTCCCTAAT CAGTTCTCCA GAAATTACTG AGAACTACCT TGACCTGAAC

ACTCCCTAAT CAGTTCTCCA GAAATTACTG AGAACTACCT TGACCTGAAC

ATTCCCTAAT CGATTCTCCA GAAATTACTG AAAACTACCT TGACCTGAAC

GTTCTCTGAT CAGTCCTCCG GAAGTCACCG AGAACCACCT TGACTTTCAT

GTTCCCTGAT CAGTCCTCCA GAAATCACCG AGAACCACCT TGACTTTAAT

ATTCCATAAT CGATTCTCCA GAAATTACTG AGAACTCCCT TGACGTGAAT

ATTCCCTAAT CAGTTCCCCA GAAATTACTG AGAATTACTT TGACCTCAAT

ATTCCCTCAT CAGTTCTCCG GAAATTACTG AGAATTATAT CGAGCTGAAT

---------- ---------- ---------- ---------- ----------

ATTCCCTAAT CAGTTTTCCA GAAGTTGCTG AGAATTACAT GGATCTGAAT

TTTCCCTAAT CGATTCTCCA GCTGTTCATG AGAACTCCAT TGACCTGAAT

ATTCCCTGAT CAATTCTCCA GAAATTACTG AGAATTACAT GGACCTGAAT

ATTCCCTACT CGATGCTCCA GAGATTACTG AGAACTACAT GGACCTGAAT

ATTCCCTTTT GGAGCCTCCA GAAATCACCC AGTCATCCAT TGACCTGCAA

ATTCTCTCAT CAACTCCCCG GAAATTCAAG AGTCAGCCAT CGACATGGAT

TTGAAGGGTG TATTCTACCC ACTGGAAAAC CTCACCGACC CCCCCTTCTC

TTGAAGGGTG TATTCTACCC ACTGGAAAAC CTCACCGACC CCCCCTTCTC

TTGAAGGGCG TATTCTACCC ACTGGAAAAC CTCGCCGACC CACCCTTCTC

TTGAAGGGCG TATTCTACCC ACTGGAAAAC CTCGCCGACC CGCCCTTCTC

TTGAAGGGTG TATTCTACCC GCTGGAAAAC CTTGTTGACC CCTACTTCTC

TTGAAGGGCG TGTTCTACCC ACTGGAAGAC CTCACCGACC CCCCATTCTC

TTGAAGGGTG CGTTCTACCC ACTGGAAAGC CTTGTTGACC CTCCCTTCAC

TTGAAGGGTG CGTTCTACCC TCTGGAAAGC CTCGTGGACC CTCCCTTCAC

TTTAAG---- ---------- ---------- ---------- ----------

TTGAAGGGCG TTTTCTACCC GTTGAAAAGT CTCCGATATC CCCCCTTCCC

TTGAAGGGTG TATTCTACCC ACTGGACAAC CTCACTGACC CCCCCTTCTT

---------- ---------- ---------- ---------- ----------

TTGAAG---- ---------- ---------- ---------- ----------

TTGAAGGGCA CAGTCTACCC ACTGGACAAT GTCACCGACC CCCCCTTCTC

TTGAAGGGCA CATTCTACCC ACTGGAAAAC CTTACTGACC CTCCCTTCTC

CTGAAGGGCA CATTCTACCC TCTGGACAAC CTGGCTGACC CGCCCTTCGC

TTGAAGGGCA CATTCTACCC AGTGGAAAAT TTTACTGACC CTCCATTCCA

TTGAAGGGCA CATTCTATCT AATGGAAAAT TTCACTGACC CTCCTTTCAG

ACCAGTTCCT TTTGTGCTCC CAGAACGCAG CAACTCCATG CTCTACATTG

ACCAGTTCCT TTTGTGCTCC CAGAACGCAG CAACTCCATG CTCTACATTG

ACCAGTTCCT TTTGTGCTCC CAGAACGCAG CAACTCCATG CTCTACATTG

ACCAGTTCCT TTTGTGCTCC CAGAACGCAG CAACTCCATG CTCTACATTG

CCCAGTTCCT TTTGTGCTCC CAGAACGCAG CAACTCCATG CTCTACATAG

ACCAGTTCCT TTCGTGCTCC CGGAACGTAC TGACTCCATG CTCTACATTG

ACCAGCTCCC TTCGAGCTCA CAGAGAGCAG AGATTCCATG CTCTACATTG

ACCAGCTCCC TTCCATCTCC CAGAGAGCAG AGATTCCATG CTCTACATTG

---------- ---------- ---------- ---------- ----------

ATCAGTTCCT TTTGTGCTCC CGGAACGCCG TGACTCTATG CTCTACATTG

GCCAGTTCCT TTTGCACTGC CAGAACGCAG TGACTCTATG CTCTATGTTG

---------- ---------- ---------- ---------- ----------

---------- ---------- ---------- ---------- ----------

ACCAGTTCCT TTCATGCTCC CAGAAATCAC TGACTCAATG CTCTACATCG

ACCAGTTCCA TTTACACTTC CTGAACGCAG TGACTCTATG CTCTACGTTG

CCCAGTTCCC TTCACACTCC CAGAGCGCAC TGACGCTATG CTCTACCTGG

ACCAGATCCA TTCACACTCC CAGAGCGCAG TGATTCGATG CTTTACCTTG

GCCAGCTCCT TTCACTCTGC CAGACCTCAA TGACTTCATG GTGTACATCG

GAATCGCCGA GTATTTCTTT AAATCTGCGT CCTTTGCTCA TTTCACAGCT

GAATCGCCGA GTATTTCTTT AAATCTGCGT CCTTTGCTCA TTTCACAGCT

GAATCGCCGA GTATTTCTTT AAATCTGCGT CCTTTGCTTA TTTCACAGCT

GAATCGCCGA GTATTTCTTT AAATCTGCGT CCTTTGCTTA TTTCACAGCT

GAATCGCCGA GTATTTCTTT AAATCTGCAT CATTTGCTTA TTTCACAGCT

GAATCTCCGA GTATTTCTTT AAGTCGGCTT CCTTTGCTTA TTTCATTGCT

GCGTCTCTGA GTAC------ ---------- ---------- ----------

GCATCTCTGA GTACTTCTTT AAATCTGCGT CATTTGCTCA CTATGTATCT

---------- ---------- ---------- ---------- ----------

GAATCTCCGA GTATTTCTTT AAATCTGCAT CCTATGCTTA TTTTACAGCT

GAATCTCCGA GTTTTTCTTT AAATCTGCAT CCTTTGCTTA CTTTATAGCT

---------- ---------- ---------- ---------- ----------

---------- ---------- ---------- ---------- ----------

GAATCTCTGA ATATTTCTTT CGATCTGCAT CCCTTGCTTA TTTCACAGCC

GGATCTCCGA CTATTTCTTT AAATCTGCAT CCTTTGCTCA CTTTACAGCT

GCATCTCCGA GTATTTTTTT AAATCCGCAT CCTTCGCCCA CTTCATGGCT

GAATCTCAGA GTATTTCTTT AAGTCTGCAT CATTCGCATA TTTCACCACT

GAATCTCAGA ATATTTCTTT AGGTCTGCAT CATTTGCCTA TTTCACAGCA

GGGGTTTTCA ATGTCACTCT CTCCACCGAA GAGATTTCCA ACCATTTTGT

GGGGCTTTCA ATGTCACTCT CTCCACCGAA GAGATTTCCA ACCATTTTGT

GGGGCTTTCA ATGTCACTCT CTCCACCAAA GAGATTTCCA ACCATTTTGT

GGGGCTTTCA ATGTCACTCT CTCCACCAAA GAGATTTCCA ACCATTTTGT

GGGGCTTTCA ATGTCACTCT CTCCACCAAA GAGATTTCCA ACCACTTTGT

GGGGCTTTCG ACGTCATGCT CTCCACCAGA GAGATTTCCA ACCACTTTGT

---------- ---------- ---------- ---ATTTCCA ACTATTTCAA

GGGGCTCTTG GTACCACGCT GTCCACTAGG GAGATTTCCA ACTATTTCAG

---------- ---------- ---------- ---------- ----------

GGGGCTTTCA GCTTCACTCT CACCACAAAA GAGATTTCCA ACCATTTGCT

GGGGCCTTCA ACGTCACTCT CTCCATGAAA GAGATTTCCA ACCATTTGGT

---------- ---------- ---------- ---------- ----------

---------- ----CACCCT GTCCACGGAA GAGATTTCCA AGCACTTTGC

GGGGCTTTCA ATGTCACGCT CTCCACAAAA GAGATTTCCA GTCATTTGTT

GGGGCTTTCA ATGTCACACT CTCCACAAAG GAGATTTCCA ATCATTTAGT

GGGGCTTTCA ATGTCACGCT CTCCACCAAG GAG------- ----------

GGGGCCTTCG ACATCACTCT GACCACAAAA GAGCTATACA AGCACCTAAT

GGGGTCTTCA ACATCACCTT GACGACGAAA GAGATTTCCA AGCATTTTCC

TCAAAACTCT CAAGGCCTTG GCAACGTGCT CTCCCGGATT GCAGAGATCT

TCAAAACTCT CAAGGCCTTG GCAACGTGCT CTCCCGGATT GCAGAGATCT

TCAAAACTCT CAAGGCCTTG GCAATGTGCT CTCCCGGATT GCAGAGATCT

TCAAAACTCT CAAGGCCTTG GCAATGTGCT CTCCCGGATT GCAGAGATCT

TCAAAACTCT CAAGGCCTTG GCAATGTGCT CTCCCGGATT GCAGAGATCT

TCAAAACTCT CAAGGCCTTG GCAACGTGCT CTCCCGGATC GCTGAGATAT

TCAAAACGCT CAAGGAATTG GCAGCGTGCT CTCCAAGATT GCGGAGATCT

TCAAAATGTT CAAGGATTTG GCAGCGTACT CTCCAAGATT GCAGAGATCT

---------- ---------- ---------- ---------- ----------

TCACAACTCT CAAGCCCATG GCAACATGCT CTCCCGGATT GCAGAACTCT

TCAAAACTCT CAAGGTCTTG GCAACATGCT CTCCCGGATT GCAGAAATCT

---------- ---------- ---------- ---------- ----------

TCAGAATTCT CCAGGCATTG GCAACATGCT CTCCTGGATC GCTGAGAGCT

TCAAAACTCT CCAGGCCTAG GCATTGTGAT TTCCCAGATT GCTGAGCTCT

TCAAAACTCC CAAGGTTTTG GCAACATGCT GTCCCGGATT GCAGAGATCT

---------- ---------- ---------- -------ATT GCAGAGGCCT

TCAAAACCCC CAGGGCATTG GCAGTTTGTT TTCTCAGATT GCAGAACTAT

TCAAAACTCC AAAAACTTTG GCAGGGTGAT TTCTCAGATT GCAGAAATAT

ACATCTTGTC CCAGCCCTTC ATGGTGAGGA TCATGGCCAC AGAGCCTCCC

ACATCTTGTC CCAGCCCTTC ATGGTGAGGA TCATGGCCAC AGAGCCTCCC

ACATCTTGTC CCAGCCCTTC ATGGTGAGGA TAATGGCCAC AGAGCCTCCC

ACATCTTGTC CCAGCCCTTC ATGGTGAGGA TAATGGCCAC AGAGCCTCCC

ACATCTTGTC CCAGCCCTTC ATGGTGAGGA TCATGGCCAC AGAGCCTCCC

ACATCTTGTC CCAGCCCTTC ATGGTGCGGA TCATGGCCAC AAAGCCCCCT

ACATCTTGTC CCAGCCATTC ATGCTGCAGA TCATGGCCAC AGGGCCTCCT

ATGTCTTGTC CCAGCCGTTC ATACTGCAGA TGATGGCCAC AGGGCCTCCT

---------- ---------- ---------- ---------- ----------

ACATCTTGTC CAAGCCCTTC ATGGTACAGG TCATGGCAAC AGAGCCTCCT

ACATCTTGTC CCAGCCCTTC ATGGTACGGG TCATGGCAAC GGAGCCTCCC

---------- ---------- ---------- ---------- ----------

ACATCTTGTC ACAGCCCTTC ATGATGCGGA TCATGGCCAC GGAGCCTCCC

ACATCTTTTC CAAGACCTTC AAGGTACAAC TCATGGCAAC GGAGCCTCCT

ACATCTTGTC CCAGCCCTTC ATGGTCCAGA TCATGGCAAC AGAGCCTCCT

ACATATTGTC CCAGCCCTTC ATGGTCCGCA TCATGGCGAC AGAACCTCCT

ATATCATGTC TCGGCCACTG ATGGTGAGAA TTCTGACCAC TGAACCCCCA

ATTTAAATAC TGAGCCGTTT ATAGCAATGG TCGTGGTTAC TTCTCCGCCA

ATAATCAATC TACAACCAGG CAATTTCACC CTGGACATCC CTGCCTCCAT

ATGATCAATC TACAACCAGG CAATTTCACC CTGGACATCC CTGCCTCCAT

GTGATCAATC TACAACCAGG CAACTTCACC CTGGACATCC CTGCCTCCAT

ATAATCAATC TACAACCAGG CAACTTCACC CTGGACATCC CTGCCTCCAT

GTGATCAATC TACAACCAGG CAACTTCACC CTGGACATCC CTGCCTCCAT

GTGGTCCATT TACTACCAGG TAACTTCACC CTGGACATCC CTGCCTCCAT

GTGGTCAACT TACACCCAAA CAATTTCAGC CTGGAGCTCC CTGCCGCTGT

ATGGTCAACT TACAACGAAA CAATTTCAGC CTGGAGTTCC CTGCTGCTGT

---------- ---------- ---------- ---------- ----------

GTGATCAGTT TACTACCAGG TAACTTCACC CTGGACATCC CCGCCTCCAT

GTGATCAGTT TGCTACCTGG TAACTTCACC CTGGACATCC CTGCCTCCAT

---------- ---------- ---------- ---------- ----------

ATTGTCCATT TGCTACCTGG CAACTTCACC CTGGACATCC CGGCCTCCAT

GTGATCAATT TACTGAAGGA TAACTTCACC CTGGACATCC CTGCCTCCAT

GTGATCAATT TACAACCGGG TAATTTCACC TTGGATATCC CTGCCTCCAT

GCGGTCAGCT TACAGCCGGG GAACTTCACC CTGGACATCC CCGCCTCCAT

GTGATCAATT TAGAACCAAA CAATTTCACC CTCAACATCC CTGCTTCTAT

GCAGTCAATT TACAACCAGG CAACTTTACC GTAGAAATCC CAGGCTCCAT

CATGATGCTC ACCCAACCCA AGAACTCCAC AGTTGAAACC ATCGTTTCCA

CATGATGCTC ACCCAACCCA AGAACTCCAC AGTTGAAACC ATCGTTTCCA

CATGATGCTC ACCCAACCCA AGAACTCCAC AGTTGAAACC ATCGTTTCCA

CATGATGCTC ACCCAACCCA AGAACTCCAC CGTTGAAACC ATTGTTTCCA

CATGATGCTC ACCCAAACCA AGAACTCCAC AGTTGAAACC ATCGTTTCCA

CATCATGCTT ACGCAACCCA AGAACTCTAC GGTGGAAACC ATCGTTTCCA

CATCATGCTC ACTCAACCGG AGAACTCTAC CATCCAACCC ATCGTCTCCA

CATCATGCTC ACTCAACTGG ATAACTCTAC CATCCAGCCC ATCGTCTCCA

---------- ---------- ---------- ---------- ----------

CATGATACTC ACCCAGCCCG AGAACTCGAC TGCAGAAACC ATCGTTTCCA

CGTGATCCTC ACCCAGCCTG AGAACTCAAC TGCTGAAGCC ATTGTGTCCA

---------- ---------- ---------- ---------- ----------

CATGATTCTC ACCAAGACTG AGAACGCGAC CACAGAGAAC ATTGTCTCCA

CGTGATATTC ACCCAGTCCG AGAACTCAAC AGCGAACGCC ATTGTTTCCA

CATGGTGCTC ACCCAACCCA AGAACTCAAC TGTTGAAACC ATCGTTTCCA

CATGATGCTC ACCCAGTCGG AGAATTCCAC CACTGAAACC ATCGTTTCCA

TGTGATGATG ACCAAACCTA AAAACACCAC CGAGGAAGTG ATCGTCTCTA

CAAGATGTTT ACCCAACCAG AAAATTCAAC TGCGGAAACC ATTCTTTCAA

TGGACTTCGT TGCTAGTACC AGTGTTGGCC TGGTTATTTT GGGACAAAGA

TGGACTTCGT TGCTAGTACC AGTGTTGGCC TGGTTATTTT GGGACAAAGA

TGGACTTCGT TGCTAGTACC AGTGTTGGCC TGGTTATTTT GGGACAAAGA

TGGACTTCGT TGCTAGTACC AGTGTTGGCC TGGTTATTTT GGGACAAAGA

TGGACTTCGT TGCTAGTACG AGTGTAGGCC TGGTTATTTT GGGACAAAGA

TGGACTTTGT TGCTAGTACC AGTGTTGGCC TGGCTATTTT GGGACAAAGA

TGGACTTTGT TGCTAGTACC AGTGTTGGCC TGGCTATTTT GGGACAAAAA

TGGACTTCGT TGCTAGTACC AGTGTTGGCC TGGCTATTTT GGGACAAAAA

---------- ---------- ---------- ---------- ----------

TGGACTTTGT TGCTAGTACC AATGTTGCCC TGGCTATTTT GGGACAAAGA

TGGATTTTGT TGCTAGTACT AGTGTTGGCC TGGTTATCTT GGGACAAAGA

---------- ---------- ---------- ---------- ----------

TGGACTTCGT TGCCAGCACC AGTGTGGGCC TCGTCATCTT GGGACAAAAA

TGGACTTTGT TGCTAGTACC AGTGTTGGTC TGGTTATACT GGGACAAAGA

TGGACTTTGT TGCTAGTACC AGTGTCGGCC TGGTGATTTT GGGACAAAGA

TGGACTTTGT TGCTAGTACC AGCGTCGGCC TGGTTATCCT GGGACAAAGG

TGGATTTTAT GGCAAGTACC AGTGTAGACC TGGCTATTTT TGGACAAAGG

TGAATTTTGT AGCCAGTACC AGCGTTGATC TGGTTATTTT AGGACAGAAG

CTGGTCTGCT CCTTGTCTCT GAACAGATTC CGCCTTGCTT TGCCAGAGTC

CTGGTCTGCT CCTTGTCTCT GAACAG-TTC CGCCTTGCTC TGCCAGAGTC

CTGGTCTGCT CCTTGTCTCT GAACAGATTC CGCCTTGCTT TGCCAGAGTC

CTGGTCTGCT CCTTGTCTCT GAACAGATTC CGCCTTGCTT TGCCAGAGTC

CTGGTGTGCT CCTTGTCTct gaacagATTC CGCCTCGCTT TGCCAGAGTC

CTGATCTGCT CCTTGTCTCT GAACAG---- ---------- -----gagct

CTGATCTGCT CCTTATCTTT GAACAGATTC CGCCTCTCTT TGCCAGAGAA

CTGATCTGCT CCTTATCTTT GAACAGATTC CGCCTGTCTT TGCCAGAGAA

---------- ---------- ---------- ---------- ----------

CTGATCTGCT CTTTATCTCT GAACAGATTC CGCCTTTCTT TGCCAGAGTC

CTGGTCTGCT CGTTGTCTCT GAACAGATTC CGCCTCTCTT TGCCAGAGTC

---------- ---------- ---------- ---------- ----------

CTGATCTGCT CCTTGTCTCT GAACAGATTC CGCCTTTCTC TGCCGGAGAT

CTGATCTGCT CCTTGTCTCT GAACAGATTC CGCCTCTCTT TGCCAGAGTC

CTGGTCTGCT CCTTATCTCT GAACAGATTC CGCCTCTCTT TGCCAGAATC

CTGGTCTGCT CCTTATCGCT GAACAG---- ---------- ----------

TTGATCTGCT CCCTATCTCT GAACAGGTTC CAGCTCTCTG GACCAGAATC

CTGATCTGCT CCTTATCTCT GAACAGATTC CGCCTCTCTG TGCCCCAGTC

CAATCGCAGC AACATTGAGG TCTTGAGGTT TGAAAATATT CTATCGTCCA

CAATCGCAGC AACATTGAGG TCTTGAGGTT CGAAAATATT CTATCCTCCA

CAATCGCAGC AATATTGAGG TCTTGAGGTT TGAAAATATT CTGTCCTCTA

CAATCGCAGC AATATTGAGG TCTTGAGGTT TGAAAATATT CTGTCCTCTA

CAATCGCAGC AATATTGAGG TCTTGAGGTT TGAAAATATT CTGTCCTCTA

caggc----- ---------G TCTTGAGGTT TGAAAACATT CTGTCCTCCA

CAGCCCACGG GATATTAAGG TTGTGAGATT TGAGAATATC CTGTCTTCCA

CAGCCAACGA GATGCTAAGG TTGTGAGATT TGAGAATATC CTGTCTTCCA

---------- ---------- ---------- ---------- ----------

CAATCGCGGC AATATTGAGG TCTTGAGGTT TGAGAACATT CTGTCCTCCA

CAGTCGCAGC AATATTGAGG TCTTGAGGTT TGAGAATATT CTGTCCTCCA

---------- ---------- ---------- ---------- ----------

CGATCGCAGC AATATTGAG- ---------- ---------- ----------

CAATCACAGC AACATTGAGG TGTTGAGATT TGAGAATATT CTGTCCACCA

CAATCGCAGC AGTATTGAGG TCTTGAGGTT TGAGAATATT CTGTCTTCAA

---------- ---------G TCCTGAGGTT TGAGAACATC CTGTCTTCCA

TAATCGTAGC TCTATTGAGG TCCTGAGATT TGAGAATATT CTCTCCTCTA

CAATCGAAGC TCTATAGAGA TGCTGAGATT TGAGAATATT CTGTCCTCCG

TTCTTCACTT TGGAGTCCTC CCACTGGCCA ATGCAAAATT GCAGCAAGGA

TTCTTCACTT TGGAGTCCTC CCACTGGCCA ATGCAAAATT GCAGCAAGGA

TTCTTCACTT TGGAGTCCTC CCACTGGCCA ATGCAAAATT GCAGCAAGGA

TTCTTCACTT TGGAGTGCTC CCACTGGCCA ATGCAAAATT GCAGCAAGGA

TTCTTCACTT TGGAGTCCTC CCACTGGCAA ATGCAAAATT GCAGCAAGGA

TTCTTCACTT CGGAGTCCTC CCACTGGCCA ATGCAAAATT GCAGCAAGGA

TTCTTCACTT CGGAGTCCTC CCACTGGCCA ACACAAAATT GCAGCAAGGG

TTCTTCACTT CGGCGTCCTC CCACTGGCCA ACACAAAACT GCAGCAAGGA

---------- ---------- ---------- ---------- ----------

TTCTCCACTT TGGAGTCCTC CCACTAGCCA ATGCAAAATT ACAGCAAGGA

TTCTCCACTT TGGAATCCTC CCACTGGCCA ACGCAAAATT GCAGCAAGGA

---------- ---------- ---------- ---------- ----------

---------- ---------- ---------- ---CCAAATT GCAGCAAGGA

TTCTTCACTT TGGCGTCCTC CCACTGGCCA ATGCAAAGCT GCAGCAAGGA

TTCTTCACTT TGGAGTGCTT CCACTGGTCA ACGCAAAATT GCAGCGAGGG

TCCTTCACTT CGGAGTGCTG CCGCTGGCCA ATGCCAAATT GCAACAGGGA

TCCTTCACTT TGGAGTCATC CCATTGGCAA ATGCAAGATT ACAGCAAGGA

TCCTGCATTT CGGGCTCATC CCGCTGGCCA ACGCAAAATT ACAGAAAGGA

TTTCCTCTGT CCAATCCACA CAAATTCTTA TTCGTCAATT CAGATATTGA

TTTCCTCTGC CCAATCCACA CAAAATCTCA TTCATCAATT CAGATATTGA

TTTCCTCTGC CCAATCCGCA CAAAATCTCA TTCGTCAATT CAGATATTGA

TTTCCTCTGC CCAATCCGCA CAAAATCTCA TTCGTCAATT CAGATATTGA

TTTCCTCTGC CCAATCCACA CAAAATTTCA TTCGTCAATT CAGATATTGA

TTTCCTCTGC CCAACCCACA CAAAATCTCA TTTGTCAATT CAGATATTGA

TTTCCTCTTC CCAACCCCTA CAATATCTCA TTCGTCAACT CGGATATTGA

TTTCCTCTTC CCAACCCCTA CAATATCTCA TTCATCAATT CGGATATTGA

---------- ---------- ---------- ---------- ----------

TTTCCTCTAC CCAACCCACA CAAAATCTCA CTCGTCAATT CAGATATTGA

ATTCCTCTGC CCAACCCACA CAAAATCTCA TTTGTCAATT CAGATGTTGA

---------- ---------- ---------- ---------- ----------

ATTCCCCTGC CCAACCCATA CAATATCTCC CTGACCAAAT CGGAGATTGA

TTTCCTCTGC CCAACCCACA CAAAATCTCA TTCGTCAATT CAAATATTGA

TTTCCTCTGC CCAACCCGTA CAAAATCTCA TTCGTCAATT CAGATATCGA

TTTCCTCTGC CCAATCCGTA CAAGATCTCC TTGGTCAATT CAAATATTGA

TTCCCCCTGC CTAACCCACA TCAGATCTCT CTTGTCAATT CTGAGATTGA

TTCCCTTTGC CTAACCTGGA CCAGATTACC CTTGTTAATT CCGACATTGA

AGTTCTTGAG GGTTTCCTTT TGATTTCCAC CGACCTGAAG TATGAAACAA

AGTCCTTGAG GGTTTCCTTT TGATTTCCAC CGACCTGAAG TATGAAACAA

AGTTCTTGAG GGTTTCCTTT TGATTTCCAC CGACCTGAAG TATGAAACAA

AGTTCTTGAG GGTTTCCTTT TGATTTCCAC CGACCTGAAG TATGAAACAA

GGTTCTTGAG GGTTTCCTTT TGATTTCCAC CGACCTGAAG TATGAAACA-

GGTTCTTGAG GGTTTCCTTT TGATTTCCAC TGACCTGAAG TATGAAACAA

AGTTCTTGAG GGTTTCCTTC TGATTTCTAC TGACGTGGCA CATGAAACAA

AGTTCTTGAG GGTTACCTTC TGGTTTCTAG TGACCTGGCA TATGACACAA

---------- ---------- ---------- ---------- ----------

AGTTCTTGAG GGTTTCCTTT TGATCTCCAC CGACCTGAAG TATAAAACAA

AGTTTTTGAG GGTTTCCTTT TGATTTCCAC TGACCTGAAG TATGAAACAA

---------- ---------- ---------- ---------- ---------A

AGTCCTTGAG GGCTTCCTTT TGATTTCCAC GGACCTGAAG CATGAAACGA

AGTTCTTGAG GGTTTCCTTT TGATTTCTAC CGACCTGAAG TATGAAGAAA

GGTTCTTGAG GGTTTCCTTT TGATTTCCTC CGACCTGAAG TATGAACCAA

AGTGTTTGAG GGTTTCCTGC TGATTTCCAC CGACCTGAAG TATGAACAG-

AGTTCACAAG GGTTTCCTTC TGATTTCCAC TGACCTCAGA TATGCTC--A

AGTTCATCAG GGATTCCTCT TGGTTTCCAC TGATGTTCAC TACAATCCTA

TGAGAACAAC AAAGGTCTAC AAACTCGTCA TCCACAAGAA GGGCTTTGGG

TGAGAACAAC AAAGGTCTAC AAACTCGTCA TCCACAAGAA GGGCTTTGGG

TGAGAACAAC AAAGGTCTAC AAACTCGTCA TCCACAAGAA GGGCTTTGGG

TGAGAACAAC AAAGGTCTAC AAACTCGTCA TCCACAAGAA GGGCTTTGGG

---------- ---------- ---------- ---------- ----------

TGAGAACAAC TAAGGTCTAC AAACTCGTCA TCCACAAGAA GGGTTTTGGG

TGAGAACCAC AAAGGTCTAC AAACTTGTCA TCCACAAGAA GGGCTTTGGG

TGAGAACCAC AAAGGTCTAC AAACTTGTCA TCCACAAGAA GGGCTTTGGG

---------- ---------- ---------- ---------- ----------

TGAGAACAAC AAAGGTCTAC AAACTCGTCA TCCACAAGAA GGGCTTCGGG

TGAGAACAAC AAAAGTCTAC AAACTTGTCA TCCACAAGAA GGGCTTTGGG

TGAGAACAAC AAAAGTCTAC AAACTGGTCA TCCACAAGAA GGGCTTTGGG

TGAGAACAAC AAAGGTCTAC AAACTTGTCA TCCACAAGAA GGGCTTCGGG

TGAGAACAAC AAAGGTCTAC AAACTTGTCA TCCACAAGAA GGGCTTTGGG

TGAGAACAAC GAAAGTCTAC AAACTCGTCA TCCACAAGAA GGGCTTTGGG

---------- ---------- ---------- ---------- ----------

TGAGGACCAA TAAGGTCTAC AAGCTCGTCA TACACAAGAA GGGATTTGGG

TGAGAACCAC GAAGGTCTAC AAACTCGTTA TACACAAGAA GGGCTTTGGG

GGCAGTGATG ATGAGCTAGT TGTGAACCCC AAAGTGTTCC CTCACATCAA

GGCAGTGATG ATGAGCTAGT TGTGAACCCC AAAGTGTTCC CTCACATCAA

GGCAGTGATG ATGAGCTAGT TGTGAACCCC AAAGTGTTTC CTCACATCAA

GGCAGTGATG ATGAGCTAGT TGTGAACCCC AAAGTGTTTC CTCACATCAA

---------- ---------- ---------- ---------- ----------

GGCAGTGATG ATGAGCTAGT TGTGAACCCA AAAGTGTTTC CTCACATCAA

GGCAGTGATG ATGAGCTGGT CGTGAACCCT AAAGTCTTTC CTCACATCAA

GGCAGTGATG ATGAGCTAGT CGTGAACCCT AAAGTATTTC CTCACATCAA

-------ACG ATGAGCTGGT TGTGAACCCC AAAGTGTTTC CTCACATCAA

GGCAGTGACG ATGAGCTAGT TGTGAACCCC AAAGTATTTC CACACATCAA

GGCAGTGATG ATGAGCTAGT CATGAACCCT AAAGTGTTTC CTCACATCAA

GGCAGTGATG ATGAGCTGGT AATGAACCCC AAAGTCTTTC CTCACATCAA

GGCAGTGATG ATGAGTTGGT TATGAACCCC AAAGTGTTTC CTCACATCAA

GGCAGTGATG ATGAGTTAGT CGTGAACCCC AGAGTGTTTC CTCATATCAA

GGCAGTGACG ATGAGCTGGT TGTGAACCCC AAAGTGTTTC CCCACATTAA

-------ATG ATGAGCTGGT TGTAAACCCC AAAGTGTTTC CTCACATCAA

GGCAGCGATG ATGAGCTCAT GGTGAACCCC AAAGTGTTCC CCCAAATTAA

GGTAGTGATG ATGAGCTGAT GGTGAACCCC AAAGTCTTTC CACAGATTAA

GCTTGGAGAC ATTGTAGAGA TTGCACACCC CAACGATGAA TACAGCAGAG

GCTTGGAGAC ATTGTAGAGA TTGCACACCC CAACGATGAA TACAGCAGAG

GCTTGGAGAC ATTGTAGAGA TTGCACACCC CAACGATGAA TACAGCAGAG

GCTTGGAGAC ATTGTAGAGA TTGCACACCC CAACGATGAA TACAGCAGAG

---------- ---------- ---------- ---------- ------AGAG

GCTTGGAGAC ATTGTAGAGA TTGCTCACCC CAACGATGAA TTCAGCAGGG

ACTTGGAGAC ATTGTGGAGA TCGCTCACCC CAATGATGAA TACAGTAGGG

ACTTGGAGAC ATTGTGGAGA TTGCTCACCC CAATGATGAG TACAGTAGGG

GCTTGGAGAC TTATTGGAGA TTGCTCACCC CAATGACGAA TACAGC----

GCTTGGAGAC ATCGTGGAGA TTGCCCACCC CAACGATGAG TACAGCAGGG

GCTTGGAGAC ATTGTGGAGA TTGCTCACCC CAATGATGAA TACAGCAGGG

GCTTGGAGAC ATTGTGGAGA TTGCTCACCC CAACGATGAG TACAGCAGGG

GCTTGGGGAC ATCGTGGAGA TTGCTCACCC CAACGATGAA TACAG-AG--

GCTTGGAGAT ATTGTAGAGA TTGCTCACCC CAACGATGAA TACAGCAGGG

GCTCGGAGAC ATTGTGGAGA TTGCTCACCC CAACGATGAA TACAG---GG

GCTGGGAGAC ATCGTGGAGA TTGCTCACCC CAACGATGAG TACAG-----

GCTGGGGGAC ATTGTGGAGA TCGCACACCC CAATGACGAG TACAGCCGGG

ACTGGGAGAC ATTGTGGAAA TTGCACACCC TAATGATGAG TACAGCCGGG

CACAGGCTGG TGAACTGTGG GTTAAGAATG AGAAGGTCAT GTGTGGCTAC

CACAGGCTGG TGAACTGTGG GTTAAGAATG AGAAGGTCAT GTGTGGTTAC

CACAGGCTGG TGAGCTGTGG GTTAAGAATG AGAAGGTCAT GTGTGGTTAC

CACAGGCTGG TGAGCTGTGG GTTAAGAATG AGAAGGTCAT GTGTGGTTAC

CACAGGCTGG TGAGCTGTGG GTCAAGAATG AAAAGGTCAT GTGTGGTTAC

CACAAGCTGG TGAGCTATGG GTCAAGAATG AAAAAGTCAT GTGTGGTTAT

CACAGGCTGG TGAGCTGTGG GTCAAGAATG AGAAGGTCAT GTGTGGTTAC

CACAGGCTGG TGAGCTGTGG GTCAAGAATG AGAAGGTCAT GTGTGGTTAC

---------- ---------- ---------- ---------- ----------

CACAGGCTGG TGAGCTGTGG GTCAAGAATG AGAAGGTCAT GTGTGGTTAT

CACAGGCTGG TGAGCTGTGG GTCAAGAATG AGAAAGTCAT GTGTGGTTAT

CACAGGCTGG TGAGCTGTGG GTCAAGAATG AGAAGGTTAT GTGTGGTTAT

---------- ---------- ---------- ---------- ----------

CACAGGCTGG TGAACTATGG GTCAAGAATG AGAAGGTCAT GTGTGGTTAT

CACAGGCTGG TGAGCTGTGG GTCAAGAATG AGAAGGTCAT GTGTGGTTAT

---------- ---------- ---------- ---------- ----------

CCCAGGCAGG TGAGCTGTGG GTGAAGAGTG AAAAAGTCAT GTGTGGGTAC

CACAGGCTGG TGAATTTGGG GTTAAAAGTG AAAAGGTCAT GTGTGGGTAC

ATCAGTGAAG ATACCAGGGT GGTGTTTCGT TCTACGTCGG CTATGGTTTA

ATCAGTGAAG ATACCAGGGT GGTGTTTCGT TCTACGTCGG CTATGGTTTA

ATCAGTGAAG ATACCAGGGT GGTGTTTCGT TCTACGTCGG CTATGGTTTA

ATCAGTGAAG ATACCAGGGT GGTGTTTCGT TCTACGTCGG CTATGGTTTA

ATCAGTGAAG ATACCAGGGT GGTGTTTCGT TCTACATCAG CTATGGTTTA

ATTAGCGAAG ATACCAGGGT GGTGTTCCGT TCTACGTCGG CTATGGTTTA

ATTAGTGAAG AGACCAGGGT GGTGTTCCGT TCTACGTCGG CTATGGTTTA

ATTAGTGAAG AGACCAGGGT GGTGTTCCGT TCTACGTCGG CTATGGTTTA

---------- --------GT GGTGTTCCGT TCTACGTCAG CTATGGTTTA

ATCAGCGAAG ATACCAGGGT GGTGTTCCGT TCTACGTCGG CTATGGTTTA

ATCAGTGAAG ATACCAGGGT GGTGTTCCGT TCTACGTCGG CTATGGTTTA

ATCAGTGAAG ATACCAGGGT GGTGTTCCGT TCTACGTCGG CTATGGTTTA

---------- --------GT GGTGTTTCGC TCTACGTCAG CTATGGTTTA

ATCAGTGAAG ATACCAGGGT GGTATTCCGT TCTACGTCGG CTATGGTTTA

ATCAGTGAAG ATACAAGGGT GGTGTTTCGT TCTACATCGG CTATGGTTTA

---------- ---------- ---------- ---------- ----------

ATCAGTGAAG ACACACGGGT GGTGTTCCGC TCGACTTCAG CGATGGTTTA

ATCAGTGAGG ACACAAGGGT GGTGTTCCGC TCAACTTCAG CTATGGTCTA

CATATTTATT CAGATGAGCT GTGAAATGTG GGATTTTGAT ATTTATGGGG

CATATTTATT CAGATGAGCT GTGAAATGTG GGATTTTGAT ATTTATGGGG

CATATTTATT CAGATGAGCT GTGAAATGTG GGATTTTGAT ATTTATGGGG

CATATTTATT CAGATGAGCT GTGAAATGTG GGATTTTGAT ATTTATGGGG

CATATTTATT CAGATGAGCT GTGAAATGTG GGATTTTGAT ATTTATGGGG

CATATTTATT CAGATGAGCT GTGAAATGTG GGATTTTGAT ATTTATGGGG

CATATTTATT CAGATGAGCT GTGAAATGTG GGATTTTGAT ATTTATGGGG

CATATTTATT CAGATGAGCT GTGAAATGTG GGATTTTGAT ATTTATGGGG

CATATTTATT CAGATGAGCT GTGAAATGTG GGATTTTGAT ATTTATGTGG

CATATTTATT CAGATGAGCT GTGAAATGTG GGATTTTGAT ATTTATGGGG

CATATTTATT CAGATGAGCT GTGAAATGTG GGATTTCGAT ATTTATGGGG

CATATTTATT CAGATGAGCT GTGAAATGTG GGATTTTGAT ATTTATGGAG

CATATTTATT CAGATGAGCT GTGAAATGTG GGACTTCGAT ATTTATGGGG

CATATTTATT CAGATGAGCT GTGAAATGTG GGATTTTGAT ATTTATGGGG

CATATTTATT CAGATGAGCT GTGAAATGTG GGATTTTGAT ATTTATGGAG

---------- ---------- ---------- ---------- ----------

TATATTTATT CAGATGAGCT GTGAAATGTG GGATTTCGAT ATTTATGGAG

TATATTTATC CAGATGAGCT GTGAAATGTG GGATTTTGAT ATTTATGGAG

ATTTGTATTT TGAGAAAGCT GTGAATGGTT TCCTTGCTGA TCTATTTACC

ATTTGTATTT TGAGAAAGCT GTGAATGGTT TCCTTGCTGA TCTATTTACC

ATTTGTATTT TGAGAAAGCT GTGAATGGTT TCCTTGCTGA TCTATTTACC

ATTTGTATTT TGAGAAAGCT GTGAATGGTT TCCTTGCTGA TCTATTTACC

ATTTGTATTT TGAGAAAGCT GTGAATGGTT TCCTTGCTGA TCTATTTACC

ATCTGTATTT CGAGAAAGCT GTGAATGGTT TCCTTGCTGA TCTATTTACC

ATCTGTACTT TGAGAAAGCT GTGAATGGTT TCCTTGCCGA CCTGTTTACT

ATCTGTACTT TGAGAAAGCT GTGAACGGTT TCCTCGCCGA CCTGTTTACT

AGGGGTATTT TGAGAAAGCT GTGAATGGTT TCCTTGCTGA TCTGTTTACC

ATCTGTATTT TGAGAAAGCT GTGAATGGTT TCCTTGCTGA TCTCTTTACC

ATCTATATTT TGAGAAAGCT GTGAATGGTT TCCTTGCTGA TCTCTTTACC

ATCTATATTT TGAGAAAGCT GTGAATGGTT TCCTTGCTGA TCTCTTTACC

ATTTGTATTT TGAGAAAGCT GTGAATGGTT TCCTTGCTGA CCTTTTTACC

ATCTGTATTT TGAGAAAGCT GTGAATGGTT TCCTTGCTGA TCTCTTTACC

ATTTGTATTT TGAGAAAGCT GTGAATGGTT TCCTTGCTGA TCTCTTTATC

---------- ---------- ---------- ---------- ----------

ATTTGTATTT TGAAAAAGCT GTGAATGGTT TCCTTGCTGA CCTCTTTACC

ACCTGTATTT TGAGAAAGCT GTGAATGGTT TTCTTGCTGA TCTCTTTACT

AAGTGGAAGG AGAAGAACTG TAGTCATGAA GTGACAGTGG TCCTGTTTTC

AAGTGGAAG- ---------- ---------- ---------- ----------

AAGTGGAAG- ---------- ---------- ---------- ----------

AAGTGGAAGG AGAAGAACTG TAGTCATGAG GTGACAGTGG TCCTGTTTTC

AAGTGGAAGG AGAAGAACTG TAGTCATGAA GTGACAGTGG TCCTGTTTTC

AAATGGAAGG AAAAGAATTG TAGTCATGAA GTAACAGTGG TCCTATTTTC

AAGTGGAAGG AGAAGAACTG TAGCCATGAA GTGACTGTGG TTCTGTTTTC

AAATGGAAGG AGAAGAACTG TAGTCATGAA GTGACTGTGG TCCTGTTTTC

AAGTGGAAGG AGAAGAATTG TAGTCATGAA GTGACGGTGG TCCTGTTTTC

AAGTGGAAGG AGAAGAACTG CAGTCATGAA GTGACAGTGG TCCTGTTTTC

AAGTGGAAGG AGAAGAACTG TAGTCATGAA GTGACAGTGG TCCTATTTTC

AAGTGGAAGG AGAAGAACTG TAGTCATGAA GTGACAGTGG TCCTATTTTC

AAGTGGAAGG AGAAGAACTG TAGTCATGAA GTGACAGTGG TCCTGTTTTC

AAGTGGAAGG AGAAGAACTG TAGTCATGAA GTGACAGTGG TCCTGTTTTC

AAGTGGAAGG ACAAGAACTG TAGTCATGAA GTGACAGTGG TACTATTTTC

---------G AGAAGAACTG TAGCCATGAA GTGACAGTGG TCCTGTTTTC

AAGTGGAAGG AAAAGAACTG TAGCCACGAA GTGACCGTGG TCCTGTTTTC

AAGTGGAAGG AAAAGAATTG TAGTCACGAA GTCACAGTGG TGCTATTCTC

TAGAACTTTC TATGATGCAA AATCTGTTGA TGAATTTCCT GAAATAAACC

---------- ---------- ---------- ---------- ----------

---------- ---------- ---------- ---------- ----------

TAGAACTTTC TATGATGCAA AATCTGTTGA TGAATTTCCT GAGATAAACC

TAGAACTTTC TATGATGCAA AATCTGTTGA TGAATTTCCT GAAATAAACC

CAGAACTTTC TATGATGCAA AATCTATTGA TGAATTTCCT GAAATAAACC

TAGAACTTTT TATGATGCAA AATCTATTGA TGAATTTCCT GAAGTAAACC

CAGAACTTTC TATGATGCAA AATCTATTGA TGAATTTCCT GAAATAAACC

TAGAACTTTC TATGATGCAA AATCTACTGA TGAATTTCCT GAAGTAAACC

TAGGACTTTT TATGATGCGA AATCTCTTGA TGAATTTCCT GAAATAAACC

TAGAACTTTT TATGATGCAA AATCTCTTGA TGAATTTCCT GAAATAAACC

TAGAACTTTC TATGATGCAA AATCTGTTGA TGAATTTCCT GAAGTAAACC

TAGAACTTTC TATGATGCAA AATCTCTTGA TGAATTTCCT GAAATAAACC

TAGAACTTTC TATGATGCAA AATCTATTGA TGAATTTCCT GAAATAAACC

TAGAACATTC TATGATGCAA AATCTATTGA TGAGTTTCCC GAAGTAAGCC

TAGAACTTTC TATGATGCGA AATCTATTGA TGAGTTTCCT GAAGTAAACC

CAGGACATTC TATGATGCCA AGTCCATTGA TGAGTTTCCC GAGACACACC

TAGGACCTTC TATGATGCAA AATCAATAGA TGAGTTTCCA GAAATACATC

GAGCCTCAAT TCGACAGGAT CACAAGGGGA GATTCTATGA AGACTTTTAC

---------- ---------- ---------- ---------- ----------

---------- ---------- ---------- ---------- ----------

GAGCCTCAAT TCGACAGGAT CATAAGGGGA GATTCTATGA AGACTTTTAC

GAGCCTCAAT TCGACAGGAT CACAAAGGGA GATTCTATGA AGACTTTTAC

GAGCCTCAAT TCGACAAGAT CACAAGGGAC GGTTCTATGA AGACTTTTAC

GAGCTTCAAT TCAACAGGAT CATAAAGGGA GATTCTACGA GGACTTTTAC

GAGCTTCAAT TCAAGAGGAT CACAAAGGGA GATTCTATGA GGACTTTTAC

GAGCCTCAAT TAGACAGGAC CACAAGGGGC GATTCTATGA AGACTTTTAC

GAGCCTCAAT TAGGCAGGAT CACAAGGGGC GATTCTATGA AGACTTTTAC

GAGCCTCAAT TCGACAGGAT CACAAGGGAC GGTACTATGA AGACTTTTAT

GACCCTCAAT TCGACAGGAT CACAAGGGAC GATTCTATGA AGACTTTTAT

GAGCCTCAAT TCGACAGGAT CACAAGGGTC GATTCTATGA AGACTTTTAT

GAACCTCAAT TCGACAGGAT CACAAGGGTC GATTCTATGA AGACTTCTAC

GAGCCTCAAT TCGACAGGAT CACAAAGGAC GATTCTATGA AGACTTTTAC

GAGCCTCAAT TAGACAGGAT CACAAGGGGC GATTCTATGA AGATTTTTAC

GAGCCTCAAT TCGCCAGGAT CATGAAGGCC GATTCTATGA AGACTTTTAC

GAGCTTCAAT TCGACAGGAT CATGAAGGCA GATTCTATGA AGACTTTTAC

AAAGTGGTGG TGCAGAATGA GAGAAGAGAA GAATGGACTT CACTTCTCGT

--AGTGGTGG TGCAGAATGA GAGAAGAGAA GAATGGACTT CACTTCTCGT

---------- ---------- ---------- ---------- ----------

AAAGTGGTGG TGCAGAATGA GAGAAGAGAA GAATGGACTT CACTTCTCGT

AAAGTAGTGG TACAGAATGA GAGAAGAGAA GAATGGACTT CACTTCTTGT

AAAGTGGTGG TGCAGAATGA GAGAAGAGAA GAATGGACTT CGCTTCTTGT

AAAGTGGTGG TGCAGAACGA GAGAAGGGAA GAGTGGACGT CACTCCTTGT

AAAGTGGTGG TGCAGAATGA GAGAAGGGAA GAGTGGACTT CACTCCTCGT

AAAGTAGTGG TGCAGAATGA GAGAAGAGAA GAGTGGACCT CCCTCCTTGT

AAGGTGGTGG TGCAGAACGA GAGGAGGGAA GAGTGGACCT CACTCCTCGT

AAAGTGGTGG TGCAGAATGA GAGGAGAGAA GAGTGGACGT CACTCCTTGT

AAAGTGGTGG TACAGAATGA GAGGAGAGAA GAGTGGACTT CACTCCTTGT

AAAGTGGTGG TGCAGAACGA GAGGAGAGAA GAGTGGACGT CACTGCTTGT

AAAGTGGTGG TGCAAAATGA GAGAAGAGAA GAGTGGACTT CACTCCTTGT

AAAGTGGTGG TGCAGAATGA GAGAAGAGAA GAATGGACTT CACTTCTTGT

AAAGTGGTGG TGCAGAATGA GAGACGGGAA GAGTGG---- ----------

AAAGTAGTGG TTCAGAACGA GAGACGAGAA GAGTGGACGT CGCTCTTAGT

AAGGTGGTGG TTCAGAATGA GAGGCGAGAA GAGTGGACGT CACTGCTCGT

AACCATTAAA AAACTCTTCA TCCAGTATCC AGTGTTGGTG CGACTGGAAC

AACCATTAAA AAACTCTTCA TCCAGTATCC AGTGTTGGTG CGACTGGAAC

---------- ---------- ---------- ---------- ----------

GACCATTAAA AAACTCTTCA TCCAGTATCC AGTGTTGGTG CGACTGGAAC

GACCATTAAA AAACTCTTCA TCCAGTATCC AGTGTTGGTG CGGCTGGAAC

GACCATTAAA AAACTCTTCA TCCAGTATCC AGTGTTGGTG CGCCTGGAGC

GACCATTAAG AAACTCTTCA TCCAGTATCC AGTGTTGGTT CGACTGGAAC

GACCATTAAA AAACTCTTCA TCCAGTATCC AGTGTTGGTG CGACTGGAAC

GACCATTAAA AAGCTCTTCA TCCAGTATCC AGTGTTGGTG CGACTGGAAC

GACCATCAAA AAGCTCTTCA TCCAGTACCC AGTGCTGGTG CGACTGGAAC

GACCATTAAA AAACTCTTCA TCCAGTATCC AGTGTTGGTA CGACTGGAAC

GACCATTAAA AAACTCTTCA TCCAGTATCC AGTGTTGGTG CGGCTGGAAC

GACCATTAAA AAACTCTTCA TCCAGTATCC AGTGTTGGTG CGACTGGAAC

GACCATTAAA AAACTCTTCA TCCAGTATCC AGTGTTGGTG CGACTGGAAC

GACCATTAAA AAACTCTTCA TCCAGTATCC AGTGTTGGTG CGACTGGAAC

---------- ---------- ---------- ---------- ----------

GACCATTAAG AAGCTCTTTG TCCAGTACCC AGTGCTGGTG CGGCTGGACC

GACTATTAAA AAACTCTTCA TCCAGTACCC GGTGCTGGTA CGATTGGAAC

AGGCAGAGGG CTTTCCTCAA GGAGATAATT CTACCTCAGC ACAAGGAAAC

AGGCAGAGGG CTTTCCTCAA GGAGATAATT CTACCTCAGC ACAAGGAAAC

---------- ---------- ---------- ---------- ----------

AGGCAGAGGG CTTTCCTCAA GGAGACAATT CTACCTCAGC ACAAGGAAAC

AGGCAGAGGG CTTTCCTCAA GGAGACAATT CTACCTCAGC ACAAGGAAAC

AGGCAGAGGG CTTTCCTCAT GGAGATAATT CTACCTCAGC ACAAGGAAAC

AGGCAGGGGG CTTTCCTCAA GGAGACAATT CTACCTCAGC ACAAGGAAAC

AGGCAGGGGG CTTTCCTCAA GGAGACAATT CTACCTCAGC ACAAGGAAAC

AGGCAGAGGG CTTTCCTCAA GGAGACAATT CTACCTCAGC CCAAGGAAAC

AGGCAGAGGG CTTTCCCCAG GGAGACAATG CTACCTCAGC ACAAGGAAAC

AGGCAGAGGG CTTTCCTCAA GGCGACAATT CTACCTCAGC ACAAGGAAAC

AGGCAGAGGG CTTTCCTCAA GGAGACAATT CTACCTCAGC ACAAGGAAAC

AGGCAG---- ---------- ---------- ---------- ----------

AGGCAGAGGG CTTTCCTCAA GGAGACAATT CTACCTCAGC ACAAGGGAAC

AGGCAGAGGA CTTTCCTCAA GGAGACAATT CTACCTCAGC ACAAGGAAAC

--------GG CTTTCCTCAA GGAGACAATT CTACCTCAGC ACAAGGAAAC

GGGCAGAGGG CTTTCCTCAG GGTGACAATT CTACCTCGGC CCAAGGGAAC

AGGCAGAGGG CTTTCCTCGA GGTGACAATT CTACCTCAGC TCAAGGGAAC

TACCTGGAGG CCATCAATCT GTCATTCAAT GTGTTTGATA AGCACTACAT

TACCTGGAGG CCATCAATCT GTCATTCAAT GTGTTTGATA AGCACTACAT

---------- ---------- ---------- -TGTTTGATA AGCATTACAT

TACCTGGAGG CCATCAATCT GTCATTCAAT GTGTTTGATA AGCATTACAT

TACCTGGAGG CCATCAATCT GTCATTCAAT GTGTTTGATA AGCACTACAT

TACCTAGAGG CCATCAATCT GTCATTCAAT GTGTTTGACA AGCACTACAT

TACCTAGAGG CCATCAACTT GTCATTCAAT GTGTTTGACA AGCACTACAT

TACCTAGAGG CCATCAACTT GTCATTCAAT GTGTTTGACA AGCACTACAT

TACCTAGAGG CCATCAACCT GTCATTCAAT G--------- ----------

TACCTGGAGG CCATCAACCT GTCCTTCAAC GTGTTTGACA AGCACTACAT

TACCTAGAGG CCATCAATTT GTCATTCAAT GTGTTTGACA AGCACTACAT

TACCTAGAGG CCATCAATCT GTCATTCAAT GTGTTTGACA AGCACTACAT

---------- ---------- ---------- -TGTTTGACA AGCACTACAT

TACCTAGAGG CCATCAATCT GTCATTCAAT GTGTTCGACA AGCACTATAT

TACCTAGAGG CCATCAATCT GTCATTCAAT GTGTTTGACA AGCATTACAT

TACCTAGAGG CCATCAATCT ATCATTCAAT GTGTTTGACA AGCATTACAT

TACTTGGAGG CCATCAACCT GTCATTCAAT GTGTTTGACA AGCACTATAT

TACTTAGAGG CCATAAATCT GTCGTTCAAT GTGTTTGACA AGCATTACAT

CAACCGCAAC TTTGACCGAA CTGGGCAGAT GTCAGTGGTG ATCACGCCCG

CAACCGCAAC TTTGACCGAA CTGGACAGAT GTCAGTGGTG ATCACGCCCG

CAACCGCAAC TTTGACCGAA CGGGGCAGAT GTCAGTGGTG ATCACGCCCG

CAACCGCAAC TTTGACCGAA CGGGTCAGAT GTCAGTGGTG ATCACGCCCG

CAACCGCAAT TTTGACCGAA CGGGGCAGAT GTCAGTGGTA ATCACGCCTG

CAACCGCAAC TTTGACCGAA CTGGACAGAT GTCTGTGGTG ATCACGCCTG

CAACCGAAAC TTTGACCGAA CTGGGCAGAT GTCCGTGGTG ATCACGCCGG

CAACCGAAAC TTTGACCGAA CTGGGCAGAT GTCTGTGGTG ATCACGCCGG

---------- ---------- ---------- ---------- ----------

CAACCGCAAC TTTGACCGAA CCGGGCAGAT GTCTGTGGTG ATCACACCCG

CAACCGCAAC TTTGACCGAA CTGGGCAGAT GTCTGTGGTG ATCACGCCTG

CAACCGCAAC TTTGACCGAA CTGGGCAGAT GTCCGTGGTG ATCACACCTG

CAACCGCAAC TTTGACCGGA CGGGCCAGAT GTCTGTGGTG ATCACCCCTG

CAACCGCAAC TTTGACCGGA CCGGACAGAT GTCTGTGGTG ATCACGCCCG

CAACCGAAAC TTTGACCGGA CGGGGCAGAT GTCTGTGGTG ATCACACCTG

CAACCGCAAC TTTGACCGGA CGGGGCAGAT GTCTGTGGTG ATTACACCCG

CAACCGTAAC TTTGACCGGA CGGGGCAGAT GTCGGTGGTC ATCACCCCCG

CAACCGGAAC TTTGACCGGA CTGGACAGAT GTCTGTTGTT ATCACGCCCG

GGGTGGGTGT CTTTGAAGTG GACCGCCTAC TCATGATCCT GACCAAGCAG

GGGTGGGTGT CTTTGAAGTG GACCGCCTAC TCATGATCCT GACCAAGCAG

GGGTGGGTGT CTTTGAGGTG GACCGCCTAC TCATGATCTT GACCAAGCAG

GGGTGGGTGT CTTTGAGGTG GACCGCCTAC TCATGATCTT GACCAAGCAG

GGGTGGGTGT CTTTGAAGTG GACCGCCTAC TCATGATCTT GACCAAGCAG

GGGTGGGTGT CTTTGAAGTG GACCGCCTAC TCATGATCCT GACCAAGCAG

GGGTGGGTGT CTTTGAAGTG GACCGCCTCC TCATGATCTT GACCAAGCAG

GGGTGGGTGT CTTTGAAGTG GACCGCCTAC TCATGATCTT GACCAAGCAG

---------- ---------- ---------- ---------- -----AACAG

GGGTGGGCGT GTTCGAGGTG GACCGGCTGC TCATGATCCT GACCAAGCAG

GGGTGGGTGT TTTTGAAGTG GACCGTCTAC TTATGATCTT GACCAAGCAG

GGGTGGGTGT GTTCGAAGTA GACCGTCTCC TCATGATCTT GACCAAGCAG

GGGTGGGTGT GTTTGAAGTG GACCGCCTGC TCATGATCCT GACCAAGCAG

GAGTGGGTGT GTTTGAAGTG GACCGATTAC TCATGATCTT GACCAAGCAG

GGGTGGGTGT GTTTGAAGTG GACCGCTTAC TCATGATCTT GACCAAGCAG

GAGTGGGTGT GTTTGAAGTA GATCGCTTAC TCATGATCTT GACCAAGCAG

GGGTCGGCGT GTTTGAGGTC GACCGGCTGC TCATGATCCT GACCAAGCAG

GGGTAGGAGT GTTTGAAGTT GACCGGTTAC TCATGATTCT GACCAAACAG

CGGATGATAG ATAATGGAAT TGGTGTGGAT TTGGTGTGCA TGGGAGAGCA

CGGATGATAG ATAATGGAAT TGGTGTGGAC TTGGTGTGCA TGGGAGAGCA

CGGATGATAG ATAATGGAAT TGGTGTGGAC TTGGTGTGTA TGGGAGAGCA

CGGATGATAG ATAATGGAAT TGGTGTGGAC TTGGTGTGTA TGGGAGAGCA

CGGATGATAG ATAATGGAAT TGGCGTCGAC TTGGTGTGCA TGGGAGAGCA

AGGATGATAG ATAATGGAAT TGGTGTGGAC TTGGTATGCA TGGGAGAGCA

CGGATGATCG ATAATGGAAT TGGTGTGGAC TTAGTGTGCA TGGGAGAGCA

CGGATGATTG ATAATGGAAT TGGTGTGGAC TTAGTGTGCA TGGGAGAGCA

CGGATGATAG ATAACGGAAT TGGCGTGGAC TTGGTGTGCA TGGGAGAGCA

CGGATGATAG ATAACGGAAT CGGTGTGGAC CTGGTGTGCA TGGGGGAGCA

CGGATGATAG ATAATGGAAT TGGTGTGGAC TTGGTGTGCA TGGGGGAGCA

CGGATGATAG ATAACGGAAT TGGTGTCGAC TTGGTGTGCA TGGGGGAGCA

CGGATGATAG ACAATG---- ---------- ---------- ----------

CGGATGATAG ATAATGGAAT TGGAGTAGAC TTGGTGTGCA TGGGGGAGCA

CGGATGATAG ATAATGGAAT TGGTGTGGAC TTGGTGTGCA TGGGGGAGCA

CGGATGATAG ATAACGGAAT TGGTGTGGAC TTAGTATGTA TGGGAGAGCA

CGGATGATCG ACAACGGGAT CGGCGTGGAC TTGGTGTGCA TGGGGGAGCA

CGCATGATAG ACAACGGAAT TGGTGTGGAT TTGGTGTGCA TGGGGGAGCA

ACCGTTACAT GCTGTCCCAT TGTTCAAGCT CCATAATCGG AGTGCTCCCC

ACCATTACAT GCTGTCCCAT TGTTCAAGCT CCATAATCGG AGTGCTCCCC

ACCGTTACAT GCTGTCCCAT TGTTCAAGCT CCATAATCGG AGTGCTCCCC

ACCGTTACAT GCTGTCCCAT TGTTCAAGCT CCATAATCGG AGTGCTCCCC

ACCATTACAT GCTGTCCCAT TGTTCAAGCT CCATAATCGG AGTGCTCCCC

ACCATTACAT GCTGTCCCAT TGTTCAAGTT GCATAATCGG AGCGCTCCTC

GCCATTACAC GCTGTGCCAT TATTCAAGCT GCACAACCGG AGTGTGCCAC

GCCACTACAT GCTGTGCCAT TATTCAAGCT ACATAACCGG AGTGTTCCGA

ACCATTGCAC GCTGTCCCAT TGTTCAAGCT GCATAATCGG AGCACTCCTC

GCCGCTGCAC GCTGTCCCGT TGTTCAAGCT GCACAATCGG AGTGCCCCTC

ACCATTACAT GCTGTCCCGC TGTTTAAGCT GCATAATCGA AGTGCACCTC

GCCATTACAT GCTGTCCCAT TGTTCAAGCT GCATAATAGG AGCGTTCCTC

---------- ---------- ---------- ---------- ----------

ACCGTTGCAT GCTGTCCCAT TGTTCAAGCT GCATAATCGG AGCACTCCTC

ACCATTACAT GCTGTCCCAT TGTTCAAGCT ACATAATCGG AGTGCTCCTC

ACCGTTACAC GCTGTTCCAT TGTTCAAGCT CCATAATCGG AGTGTTCCTC

GCCGCTCCAT GCGGTGCCGC TGTTTAAGCT GCATAATCGA AGCGGCCCCC

ACCACTGCAT GCTGTGCCAT TATTTAAGCT TCATAATCGC TGTGGTCCCC

GTGATTCTCG TCTGGGCGAT GACTATAATA TCCCTCACTG GATAAACCAC

GGGATTCTCG TCTGGGCGAT GACTATAATA TCCCTCACTG GATAAACCAC

GGGATTCTCG TCTGGGCGAT GACTATAATA TCCCTCACTG GATAAACCAC

GGGATTCTCG TCTGGGCGAT GACTATAATA TCCCTCACTG GATAAACCAC

GGGATTCTCG TCTGGGCGAT GACTATAATA TCCCTCACTG GATAAACCAC

GGGATTCTCG TCTGGGTGAT GACTATAATA TTCCTCACTG GATAAACCAC

GGGATTCTCG GCTGGGCGAT GACTATAATA TTCCTCACTG GATAAACCAC

GGGATTCTCG ACTGGGTGAT GATTATAATA TTCCTCACTG GATAAACCAT

GCGATTCTCG TCTGGGCGAT GACTATAATA TTCCTCACTG GATAAACCAC

ATGACTCCCG TCTGGGGGAT GACTACAACA TCCCTCACTG GATAAACCAC

GTGATTCTCG TCTGGGCGAT GACTATAATA TTCCTCACTG GATAAACCAC

GCGATTCTTG TCTGGGTGAC GACTATAATA TTCCTCACTG GATAAACCAC

---------- ---------- ---------- ---------- ----------

GGGACTCTCG TCCAGGTGAT GACTACAATA TTCCTCACTG GATAAACCAC

GAGATTCTCG TCTGGGTGAT GACTATAATA TTCCTCACTG GATAAACCAC

GGGACTCTCG TCTGGGTGAT GACTATAATA TTCCTCACTG GATAAACCAC

GGGATTCCCG CCTCGGGGAC GACTACAACA TCCCTCACTG GATAAACCAC

GGGATTCCCG CTTAGGAGAT GACTATAATA TTCCTCACTG GATAAATCAT

AGTTTCGGAA AGAAGCCCGC CTCTGAGAAA GCAAAAAATG GCCGTGATAC

AGTTTCGGAA AGAAGCCCGC CTCTGAGAAA GCAAAAAATG GCCGTGATAC

AGTTTCGGAA AGAAGCCTGC CTCTGAGAAA GCAAAAAATG GCCGTGATAC

AGTTTCGGAA AGAAGCCTGC CTCTGAGAAA GCAAAAAATG GCCGTGATAC

AGTTTCGGAA AGAAGCCTGC CTCTGAGAGA GCGAAAAATG GCCGTGATAC

AGTTTCGGAA AGAAGCCTGC CTCTGAGAAA GCAAAAAATG GCCGTGATAC

AGTTTCGGAA AGAAGTCTGC CACTGAGAAA ACCAAAAATG GCCGTGATAC

AGTTTCGGAA AGAAGTCTGC CTCTGAGAAA ACCAAAAATG GTCGTGATAC

AGTTTCGGAA AGAAG----- ---------- ---------- ----------

AGtttc---- -----CCCAC CTGTGAGAAA GCAAAAAATG GCCGTGATAC

AGTTTCGGAA AGAAGCCTGC CTCTGAGAAA GCAAAAAATG GCCGTGATAC

AGTTTCGGAA AGAAGCCTGC CTCTGAGAAA GCAAAAAATG GCCGTGATAC

--TTTCGGAA AGAAGCCTGC CTCTGAGAAA GCAAAAAATG GCCGGGATAC

AGTTTC--AA AAAAGCCTGC CTCTGAGAAA GCAAAAAATG GCCGTGATAC

AGTTTTGGAA AGAAGCCTAC CTCTGAGAAA ACAAAAAATG GCCGTGATAC

AGTTTTGGAA AGAAGTGTGC TTCTGAGAAA GCAAAGAATG GCCGTGATAC

AGCTTCGGAA GGAAGCCTGC AACCGAAAAG GCAAAAAACG GCCGGGATAC

AGTTTCGGAA GGAAGCCAGC TGCAGAGAAA GCAAAAAACG GCCGGGATAC

ATCTCTCGGG AGTCCAAAAG AATCTGAGAA CGCCCTTCCC ATCCAAGTAG

GTctctcGGG AGTCCAAAAG AATCTGAGAA CGCCCTTCCC ATCCAAGTAG

ATCTCTCGGG AGTCCAAAAG AATCTGAGAA CGCCCTTCCC ATCCAAGTAG

ATCTCTCGGG AGTCCAAAAG AATCTGAGAA CGCCCTTCCC ATCCAAGTAG

ATCTCTCGGA AGTCCAAAAG AATCTGAGAA CGCCCTTCCC ATCCAAGTAG

ATCTCTCGGG TCTCCAAAAG AATCTGAGAA CGCCCTTCCC ATCCAAGTAG

ATCTCTTGGG GCTCCAAAGG AATCTGAAAA CACCCTTCCC ATCCAAGTAG

ATCTCTCGGG ACTCCAAAGG AATCTGAAAA CACCCTTCCC ATCCAAGTAG

--CTCTCGGA ACTCCAAAAG AATCTGAGAA CGCCCTTCCC ATCCAAGTGG

ATCTCTCGGG ACCCCCAAAG AATCTGAGAA TGCCCTTCCC ATCCAGGTGG

ATCTCTCGGG ACTCCAAAAG AATCTGAGAA CGCCCTTCCC ATCCAAGTAG

GTCTCTTGGG ACGCCGAAAG AATCTGAGAA CGCCCTTCCC ATCCAAGTAG

ATCTCTCGGA ACTCCGAAAG AGTCTGAGAA TGCCCTTCCC ATCCAAGTGG

ATCCCTCGGG ACTCCCAAAG ATTCTGAGAA TGCCCTTCCC ATCCAAGTAG

GTCTCTGGGG ACTCCCAAAG AAGCTGAGAA CACCCTTCCC ATCCAAGTAG

TTCTCTGGGG ACTCCCAAAG AGGCTGAGAA CGCGCTTCCC ATCCAAGTAG

CTCCATCGGG ACCCCAAAGG AATCAGAGAA TGCCCTGCCC ATCCAGGTGG

CTCTCTTGGG ACTCCAAAGG AATCTGAAAA TGCTCTTCCC ATCCAAGTGG

ATTATGACGC CTATGACGCT CAAGTGTTCA GGCTGCCCGG CCCATCCCGG

ATTATGACGC CTATGACGCT CAAGTGTTCA GGCTGCCCGG CCCATCCCGG

ATTATGATGC CTATGACGCT CAAGTGTTCA GGCTGCCCGG CCCATCCCGG

ATTATGATGC CTATGACGCT CAAGTGTTCA GGCTGCCCGG CCCATCCCGG

ATTATGACGC CTATGACGCT CAAGTGTTCA GGCTGCCCGG CCCATCCCGG

ATTATGATGC CTATGATGCT CAAGTGTTCA GGCTGCCCGG CCCATCCCGG

ATTATGATGC CTACGATGCC CAAGTATTCC GGCTGCCTGG TCCATCCCGG

ATTATGATGC CTACGATGCT CAAGTGTTCA GGCTGCCTGG CCCATCCCGG

ATTACGATGC CTACGATGCG CAAGTGTTCA GGCTGCCTGG CCCGTCCCGG

ATTACGATGC TTACGATGCA CAAGTCTTCA GGCTCCCTGG CCCCTCCCGG

ATTATGATGC CTATGATGCC CAAGTGTTCA GGCTGCCAGG CCCATCCCGG

ATTATGATGC CTACGACGCT CAAGTGTTCA GGCTGCCAGG CCCATCCCGG

ACTACGATGC CTATGATGCA CAAGTGTTCA GGCTGCCTGG TCCATCTCGG

ATTATGATGC CTACGACGCT CAAGTGTTCA GGCTTCCAGG CCCATCCCGA

ATTATGATGC CTACGATGCT CAAGTGTTTA GGCTGCCAGG CCCATCCCGA

ATTATGATGC CTACGACGCC CGAGTGTTCA GGCTGCCAGG CCCAGCCCGG

ACTATGATGC ATACGATGCC CAAGTGTTCA GGCTGCCTGG CCCGGCCCGT

ATTATGACGG CTACGATGCC CAAGTGTTCA GACTGCCTGG CCCATCACGA

GCCCAGTGCC TCACCACCTG CAGATCTGTG CGAGAGCGAG AGAGTCACAG

GCCCAGTGCC TCGCCACCTG CAGATCTGTG CGAGAGCGAG AGAGTCACAG

GCCCAGTGCC TCGCCACCTG CAGGTCTGTG CGAGAGCGAG AGAATCACAG

GCCCAGTGCC TCGCCACCTG CAG------- ---------- ----------

GCTCAGTGCC TCGCCACCTG CAGGTCTGTG CGAGAGCGAG AGAGTCACAG

GCTCAGCGCC TCGCCACCTG CAGGTCCGTG CGAGAGCGGG AAAGTCATAG

GCCCAGCGAC TCGCCACCTG CAGGTCTGTG AGAGAGCAGG AAAATCACAA

GCTCAGCGCC TCGCCACCTG CAGGTCTGTG AGAGAACAGG AGAATCACAG

GCTCAGCGCC TCGCCACCTG CAGGGCCGTG CGGGAGCGGG AGCATCACAG

GCTCAGCGCC TCAGCACCTG CAGGTCTGTG CGAGAGCGGG AGAGTCACAG

GCTCAGCGCC TCGCCACCTG CAGGTCTGTG CGAGAACGGG ACAGTCACAA

GCTCAGCGCC TCGCCACCTG CAGGTCTGTG CGAGAAAGGG AAAGTCACAG

GCTCAGCGCC TCGCCACCTG CAGGTCTGTT CGAGAGCGAG AGAATCACAG

GCTCAGCGCC TTGCCACCTG CAGGTCTGTT AGAGAGCGGG AGAGTCAGAG

GCACAGCGCC TCGCCACCTG CAGGTCTGTG AGAGAGCGGG AGAGTCACAG

GCACAGCGCC TCGCCACTTG CAGGTCTGTG AGAGAGCGGG AGAGCCACAG

GCCCAGCGCC TCACCACCTT CAGGTCTGTG AGGGAACGTG AGAGCCATGG

GCCCAGCGCC TCGTCACCTT CAGATCAGTG AGGGAGCGGG AATGCCACAC

TCGAAAGAGT GCCAGCTCCT GTGATGTTTC ATCCAGCCCT TCCCTACCAA

TCGAAAGAGT GCCAGCTCCT GTGATGTTTC ATCCAGCCCT TCCCTACCAA

TCGAAAGAGT GCCAGCTCCT GTGATGTTTC ATCCAGCCCT TCCCTACCAA

---------- ---------- ---------- ---------- ----------

TCGAAAGAGT GCCAGCTCCT GTGATGTTTC ATCCAGCCCT TCCCTACCAA

TCGAAAGAGC GCCAGCTCCT GCGATGTCTC ATCCAGCCCT TCCCTGCCCA

TCGCAAAAGT GCCAGCTCCT GTGACGTCTC CTCCAGCCCT TCCTTGCCAA

TCGCAAAAGT GCAAGCTCCT GTGATGTCTC ATCCAGCCCT TCCCTGCCAA

TGGAAAGAGT GCCAGCTCCT GTGACGTCTC ATGCAGCCCT TCCCTGCCTA

CCGGAAGAGC GCCAGCTCCT GCGACGTTTC ATCCAGCCCT TCGCTGCCCG

TCGAAAGAGT GCCAGCTCCT GTGATGTCTC ATCCAGCCCT TCCCTGCCCA

TCGAAAGAGT GCCAGCTCCT GTGACGTCTC ATCCAGCCCT TCCATGCCCA

TCGAAAGAGC AGCAGCTTTT GTGACGTCTC CTCCAGCCCT TCCCTGCCCA

CCGAAAGAGC GCCAGCTCCT GCGATGTCTC ATCCAGCCCT TCGCTGCCAA

TCGAAAGAGC GCCAGCTCCT GTGATGTCTC CTCCAGCCCT TCCCTGCCAA

TCGAAAGAGT GGCAGCTTCT GCGATGTCTC CTCCAGCCCC TCCATGCTGA

CCGGAAGAGC TCCAGCAGCT TCGACGTGTC ATCCAGCCCC TCGGTGCAGA

CCGGAAGAGC TCCAGCTCCT ACGACGTCTC GTCCAGCCCT TCGCTGCAGA

GCCGCACACT GCCCACTGAG GAAGTGAGGA GCCAGGCTTC TGACGACAGC

GCCGCACACT GCCCACTGAG GAAGTGAGGA GCCAGGCTTC TGACGACAGC

GCCGCACACT ACCCACTGAG GAAGTGAGGA GCCAGGCTTC TGATGACAGC

---------- ---------- ---------- ---------- ----------

GCCGCACACT GCCCACTGAG GAAGTGAGGA GCCAGGCTTC TGACGACAGC

GCCGTGCACT GCCCTCTGAG GAAGTGAGAA GCCAGGCTTC TGATGACAGC

GCCGTGCACT GCCCACTGAG GAAGTGAGGA GCCAGGCTTC AGACGACAGC

GCCGTGCACT GCCCACTGAG GAAGTGAGGA GCCAGGCTTC TGATGACAGC

GCCGCGCACT GCCCACTGAG GAAGTGAGGA GCCAGGCTTC CGACGACAGC

GCCGGACGCT GCCCACGGAG GAGGTGAGGA GCCAGGCGTC TGACGACAGC

GCCGTGCACT GCCCACTGAA GAAGTGAGGA GCCAGGCTTC TGACGACAGC

ACCGTGCGCT GCCCAGTGAG GAAGTGAGGA GCCAGGCTTC TGATGACAGC

GCCGTGTGCT GCCCACCGAG GAAGTGAGGA GCCAGGCTTC TGACGACAGT

ACCGGGCACT GCCCACGGAG GAAGTGAGGA GCCAGGCTTC TGATGACAGC

GCCGTGCGCT GCCTGCGGAG GAAGTGAGGA GCCAGGCTTC TGATGATAGC

GCCGGGCATT GCCTGCCGAG GAAGTGCGCA GCCAGACCTC TGACGACAGC

GCCGTACCCT GCTGCCCGAG GAGGGGAGGA GCCAGGCCTC AGACGACAGC

GCCGCGCCCT GCCAGCCGAG GAGGTGAGGA GCCAGGCCTC CGACGACAGC

TCCCTAGGCA AGAGTGCCAA CATCCTGATG ATCCCACACC CCCACCTGCA

TCCCTAGGCA AGAGTGCCAA CATCCTGATG ATCCCACACC CCCACCTGCA

TCCCTGGGCA AGAGCGCCAA CATCCTGATG ATCCCACACC CCCACCTGCA

---------- ---------- ---------- ---------- ----------

TCCTTGGGCA AGAGTGCCAA CATCCTGATG ATCCCACACC CCCACCTGCA

TCCCTGGGCA AGAGTACCAA CATCCTGATG ATCCCACACC CCCACTTGCA

TCCCTGGGCA AGAGCACCAA CATCCTGATG ATCCCTAACC CCCATCTGCA

TCCCTGGGCA AGAGCACCAA CATCCTGATG ATCCCTAACC CCCACCTGCA

TCCCTGGGCA AGAGTGCCAA CATCCTGATG ATCCCACACC CCCACCTGCA

TCGCTGGGCA GGAGCGCCAA CATCCTGGTG ATCCCTCACC CCCACCTGCA

TCCCTGGGCA GGAGTGCCAA TATCCTGATG ATCCCACACC CCCACCTGCA

TCCCTGGGCA GGAGTGCCAA CATCCTGATG ATCCCACACC CCCACCTGCA

TCCCTGGGCA GGAGCGCCAA CATCCTGATG ATCCCACACC CGCACCTGCA

TCCCTGGGCA GGAGCGCCAA CATCCTGATG ATCCCACACC CCCACCTGCA

TCCCTGGGCA GGAGTGCCAA CATCCTGATG ATCCCACACC CCCACCTGCA

TCTCTGGGCA GGAGCGCCAA TATCCTGATG ATCCCACACT CCCATCTGCA

TCCCTGGGCA AGGGCTCCCA CATCCTGATG ATCCCCCACT CCCACCTGCA

TCCTTGGGAA AGAGCTCCAA CATTCTGATG ATTCCCCACT CTCACCTCCA

CCAGTATGAA GTCAGCAGCT CCTTGGGATA CACCAGCACT CGAGATGTCC

CCAGTATGAA GTCAGCAGCT CCTTGGGATA CACCAGCACT CGAGATGTCC

CCAGTATGAA GTCAGCAGCT CCTTGGGATA CACCAGCACT CGAGATGTCC

---------- ---------- ---------- ---------- ----ATGTCC

CCAGTACGAA GTCAGCAGCT CCTTGGGCTA CACCAGCACT CGAGATATCC

CCAGTATGAA GTCAGCAGCT CCCTGGGCTA CACCAGCACT CGAGATGTCC

CCAGTATGAA GTCAGCAGCT CACTGGGCTA CACCAGCACC CGAGATGTCC

CCAGTATGAA GTCAGCAGCT CACTGGGCTA CACCAGCACC CGAGATGTCC

CCAGTATGAA GTCAGCAGCT CCTTGGGCTA CACCAGCACT CGAGACGCCC

CCAGTACGAA GTCAGCAGCT CCCTGGGCTA CACCAGCACT CGAGACGTCC

CCAGTATGAA GTCAGCAGTT CCTTGGGCTA TACCAGCACT CGAGATGTCC

CCAGTATGAA GTCAGCAGCT CCTTGGGCTA CACCAGCACT CGAGACATCC

CCAGTATGAA GTCAGCAGCT CCTTGGGTTA CACCAGCACT CGAGACGTCC

CCAGTATGAA GTTAGCAGCA GCTTGGGCTA TACCAGCACT CGAGATGTCC

CCCGTATGAA GTCAGCAGCT CCTTAGGCTA CACCAGCACT CGAGATGTCC

CCAGTATGAA GTCAGCAGTT CTTTGGGCTA CACCAGCACT CGAGATGTCC

CCAGTATGAC GTCAGCAGCT CTCTCGGCTA CCCCAGCACC CGAGATGTCC

CCAGTACGAA GTCAGCAGCT CCTTGGGCTA CACCAGCACC CGAGATGTCC

TGGAGAACAT GATGGAGCCA CCACAGCGAG ACTCCAGTGC ACCAGGGAGG

TGGAGAACAT GATGGAGCCA CCGCAGCGAG ACTCCAGTGC ACCAGGGAGG

TGGAGAACAT GATGGAGCCA CCACAGCGAG ACTCCAGTGC ACCAGGGAGG

TGGAGAACAT GATGGAGCCA CCACAGCGAG ACTCCAGTGC ACCAGGGAGG

TGGAGAACAT GATGGAGCCA CCACAGCGAG ACTCCAGTGC ACCAGGGAGG

TGGAGAACAT GATGGAGCCA CCACAGAGAG ATTCCAGCGC ACCAGGGAGG

TGGAGAACAT GATAGAACCA CCACAGCGGG ACTCTAGTGC ACCTGGAAGG

TGGAGAACAT GATAGAACCC CCACAGAGGG ACTCCAGTGC ACCAGGAAGG

TGGAGAACAT GATGGAGCCG CCGCAGCGAG ACTCCAGCGC ACCAGGGAGG

TGGAGAACAT GATGGAGCCA CCACAGCGGG ATTCCAGCGC ACCGGGCAGG

TGGAGAACAT GATGGAGCCA CCACAGCGAG ACTCTAGCGC ACCAGGGAGG

TGGAGAACAT GATGGAGCCA TCGCAGCGAG ACTCCAGCGC GCCGGGGAGG

TGGAGAACAT GGTGGAGCCA CCACAGCGAG ACTCCAGCGC GCCAGGGAGG

TCGAGAACAT GATGGAGCCA CCACAGCGAG ATTCCAGTGC ACCAGGGAGG

TGGAGAACAT GATGGAGACT TCACAGCGAG ACTCCAGCGC TCCAGGGCGG

TGGAGAACCT GATGGAGGCC TCGCAGCGAG ATTCCAGTGC TCCGGGCCGG

TGGAGAACCT GATGGAGTCC CAGCAGCGAG ACTCAAGTGC GCCTGGGAGG

TGGAGAACAT GATGGAGACG CAACAGCGGG ACTCCAGTGC ACCAGGGAGG

TTTCACGTTG GCAGTGCAGA ATCCATGCTG CATGTTCGAC CTGGTGGATA

TTTCACGTTG GCAGTGCAGA ATCCATGCTG CATGTTCGAC CTGGTGGATA

TTCCACGTTG GCAGTGCAGA ATCCATGCTG CATGTTCGAC CTGGTGGATA

TTCCACGTTG GCAGTGCAGA ATCCATGCTG CATGTTCGAC CTGGTGGATA

TTTCACGTTG GCAGCGCAGA ATCCATGCTG CATGTACGAC CTGGTGGATA

TTCCATGTGG GCAGCGCAGA ATCCATGCTC CATGTCCGGC CTGGTGGATA

TTCCACGTGG GCAGTGCAGA GTCCATGCTA CATGTTCGAC CTGGAGGCTA

TTCCATGTGG GTAGTGCAGA GTCCATGCTC CATGTCCGAC CTGGAGGATA

TTCCACGTAG GCAGTGCAGA GTCCATGCTA CACGTTCGAC CTGGCGGATA

TTCCACGTGG GCAGCGCAGA GTCCATGCTG CATGTCCGCC CCGGGGGGTA

TTCCATGTAG GCAGTGCGGA ATCCATGCTG CATGTTCGAC CCGGGGGATA

TTCCACGTGG GCAGCGCAGA GTCCATGCTG CATGTTCGAC CTGGGGGATA

TTCCACGTAG GCAGCGCGGA ATCCATGCTG CACGTTCGAC CTGGGGGATA

TTCCATGTAG GCAGCGCTGA ATCCATGCTA CATGTGCGGC CTGGTGGCTA

TTCCATGTAG GCAGTGCGGA GTCCATGCTA CACGTTCGGC AGGGTGGATA

TTCCATGTAG GTAGTGCGGA GTCCATGCTG CACGTCCGGC CTGGTGGATA

TTCCACGTGG GCAGTGCCGA GTCCATGCTG CACGTGCGTC CTGGCGGCTA

TTCCACGTGG GCAGTGCAGA ATCCATGCTG CATGTCCGTC CTGGCGGATT

CACGCCCCAG AGAGCACTGA TTAACCCCTT CGCTCCCTCT CGGATGCCCA

CACGCCCCAG AGAGCACTGA TTAACCCCTT CGCTCCCTCT CGGATGCCCA

CACGCCCCAG AGAGCACTGA TTAACCCCTT CGCTCCCTCT CGAATGCCCA

CACGCCCCAG AGAGCACTGA TTAACCCCTT CGCTCCCTCT CGAATGCCCA

CACGCCTCAG AGAGCACTGA TTAACCCCTT CGCCCCCTCT CGGATGCCCA

CACCCCCCAG CGAGCGCTGA TTAACCCCTT TGCCCCCTCT CGAATGCCCA

CACCCCTCAG AGAGCACTGA TTAACCCCTT CGCCCCCTCA AGAATGCCCA

CACGCCTCAG AGAGCGCTGA TTAACCCCTT CGCCCCCTCG AGGATGCCCA

CACGCCGCAG CGAGCGCTGA TTAACCCCTT CGCCCCCTCA CGGATGCCCA

CACGCCCCAG CGGGCACTGA TTAACCCCTT TGCGCCCTCA CGGATGCCCA

CACGCCCCAG CGGGCGCTCA TTAACCCCTT TGCCCCCTCA CGAATGCCCA

CACACCCCAG CGGGCGCTCA TCAACCCCTT TGCCCCCTCT CGAATGCCCA

CACGCCGCAG CGGGCGCTGA TTAACCCTTT TGCCCCCTCA CGCATGCCGA

CACACCCCAA CGAGCACTGA TTAACCCCTT TGCCCCCTCA CGAATGCCCA

TACACCCCAG CGAGCACTGA TTAACCCCTT CGCCCCCTCT CGAATGCCCA

CACACCCCAG CGGGCGCTGA TTAACCCCTT CGCCCCCTCG AGAATGCCGA

CACGCCCCAG CGAGCGCTGA TCAACCCCTT TGCTCCGTCC CGCATGCCCA

CACCCCTCAG CGGGCTCTGA TCAATCCCTT TGCGCCGTCT CGCATGCCAA

TGAAGCTTAC GTCCAACAGA AGGCGCTGGA TGCACACTTT TCCTGTGGGG

TGAAGCTTAC ATCCAACAGA AGGCGCTGGA TGCACACTTT TCCTGTGGGA

TGAAGCTTAC GTCCAACAGA AGGCGCTGGA TGCACACTTT TCCTGTGGGA

TGAAGCTTAC GTCCAACAGA AGGCGCTGGA TGCACACTTT TCCTGTGGGA

TGAAGCTCAC GTCCAACAGA AGGCGGTGGA TGCACACATT TCCTGTGGGA

TGAAGCTCAC ATCCAACAGA AGGCGCTGGA TGCACACTTT TCCTGTGGGA

TGAAGCTCAC CTCCAACAGA AGGCGCTGGA TGCATACTTT TCCTGTAGGA

TGAAGCTCAC CTCCAACAGA AGGCGCTGGA TGCACACTTT TCCTGTAGGC

TGAAGCTGAC GTCCAACAGA AGGCGCTGGA TGCACACCTT TCCTGTGGGA

TGAAGCTCAC GTCCAACAGG AGGCGCTGGA TGCACACTTT CCCTGTGGGA

TGAAGCTCAC ATCCAACAGA AGACGCTGGA TGCACACTTT TCCTGTGgGA

TGAAGCTCAC ATCCAACAGA AGACGCTGGA TGCACACTTT TCCTGTGGGA

TGAAGCTCAC GTCCAACCGA AGACGCTGGA TGCACACGTT TCCTGTGGGA

TGAAGCTCAC TTCCAACAGA AGACGCTGGA TGCACACTTT TCCTGTGGGA

TGAAGCTGAC TTCCAACAGA AGGCGCTGGA TGCATACCTT CCCTGTGGGA

TGAAACTGAC TTCCAACAGA AGGCGCTGGA TGCACACCTT CCCTGTGGGA

TGAAGCTGAC CTCCAACCGG AGGAGGTGGA TGCACACCTT CCCCGTGGGT

TGAAGCTTAC TTCTAACAGA AGACGCTGGA TGCACACCTT CCCTGTTGGT

CCATCCGGAG AAGCCATCCA GATCCACCAC CAGACCCGAC AGAATATGGC

CCATCCGGAG AAGCCATCCA GATCCACCAC CAGACCCGAC AGAATATGGC

CCATCCGGAG AAGCCATCCA GATCCACCAC CAGACCCGAC AGAATATGGC

CCATCCGGAG AAGCCATCCA GATCCACCAC CAGACCCGAC AGAATATGGC

CCATCCGGAG AAGCCATCCA GATCCACCAC CAGACCCGAC AGAATATGGC

CCATCCGGAG AGGCCATCCA GATCCATCAC CAGACCCGAC AGAATATGGC

CCATCTGGAG AGGCCATCCA GATCCATCAT CAAACCCGGC AAAACATGGC

CCATCTGGAG AGGCCATCCA GATCCATCAT CAGACCCGGC AAAACATGGC

CCATCCGGGG AGGCCATCCA GATCCATCAC CAGACCCGGC AGAATATGGC

CCGTCGGGAG AGGCCATTCA GATCCATCAC CAGACCCGAC AGAATATGGC

CCATCCGGAG AGGCCATCCA GATCCATCAC CAGACCCGAC AGAATATGGC

CCGTCCGGAG AGGCCATCCA GATCCATCAC CAGACCCGAC AGAATATGGC

CCATCGGGAG AGGCCATCCA GATCCACCAC CAGACCCGGC AGAATATGGC

CCTTCCGGAG AAGCCATCCA GATCCATCAC CAGACCCGAC AGAACATGGT

CCATCCGGAG AGGCCATCCA GATCCATCAT CAGACCCGAC AGAACATGGC

CCCTCCGGAG AGGCCATCCA GATCCATCAC CAGACCCGGC AGAACATGGT

CATCTGGGAG AAGCCATCCA GATCCATCAC CAGACTCGCC AGACATGGGT

CCATCCGGAG AAGCCATTCA GATTCATCAC CAGACTCGCC AGAACATGGC

GGAGCTACAA GGCAGCGGGC AGAGGGATCC AACTCACTCC TCTGCAGAGC

GGAGCTACAA GGCAGCGGGC AGAGGGATCC AACTCACTCC TCTGCAGAGC

GGAGCTGCAA GGCAGTGGGC AGAGGGACCC AACTCATTCC TCTGCAGAGC

GGAGCTGCAA GGCAGTGGGC AGAGGGACCC AACTCATTCC TCTGCAGAGC

CGAGCTGCAG GGCAGCGGGC AGAGGGACCC AACTCACTCC TCTGCAGAGC

GGAGCTGCAG GGCAGCGGGC AGAGGGACCC AACTCACTCC TCTGCAGAGC

AGAGCTGCAG GGCAGCAGGC AGCGGGACCC CACCCACTCC TCTGCAGAAC

AGAGCTGCAG GGCAGCAGGC AGAGGGACCC TACCCACTCC TCTGCAGAGC

GGAGCTGCAG GACAGCGGGC AGAGGGACCC AGCCCACTCC TCGGCAGAGC

AGAGCTGCAG GGCAGCGGGC AGAGGGACCT GACCCATTCC TCCGCGGAGC

GGAGCTACAG GGCAGCGGGC AGAGGGACCC AGCTCACTCC TCTGCAGAGC

GGAGCTGCAG GGCAGCGGGC AGAGGGATCC CACTCACTCC TCTGCAGAGC

AGAGCTGCAG GGCAGCGGGC AGCGGGACCT AACTCATTCC TCTGCAGAGC

GGAGCTGCAG GGCAGCAGGC AGAGGGACCC AACTCACTCC TCTGCAGAGC

GGAGCTGCAG GGCAGCGGGC AGAGGGACCC AACCCACTCC TCTGCCGAGC

GGAGCTGCAG GGCAGCGGGC AGAGAGACCC AATCCACTCC TCTGCAGAGC

GGAGCTCCAG GGCAGGGGGC AGCGGGATCC CACTCACTCA TCTGCAGAGC

AGAACTTCAA GGCAGTGGGC AGAGGGACCT GACACACTCT TCTGCGGAGC

TGCTGGAGTT AGCATATCAT GAAGCTGCTG GAAGGCACAG CAATTCCCGC

TGCTGGAGTT AGCATATCAT GAAGCTGCTG GAAGGCACAG CAATTCCCGC

TGCTGGAGTT AGCATATCAT GAAGCTGCTG GAAGGCACAG CAATTCCCGC

TGCTGGAGTT AGCATATCAT GAAGCTGCTG GAAGGCACAG CAATTCCCGC

TGCTGGAGTT AGCATATCAC GAAGCTGCTG GAAGGCACAG CAATTCCTGC

TGCTGGAGTT AGCATATCAT GAAGCTGCTG GAAGGCACAG CACTTCCCAC

TGTTGGAGCT GGCATATCAT GAAGCCGCAG GAAGGCACAG CACTTCCCGA

TGTTGGAGTT GGCATACCAT GAAGCTGCCG GGAGGCACAG CACTTCCCGC

TGCTGGAGTT AGCGTATCAC GAAGCTGCTG GAAGGCACAG CACATCCCGC

TGCTGGAGCT GGCATACCAC GAGGCCGCCG GCAGGCACAG CACTTCCCGC

TGCTGGAGTT AGCATATCAG GAAGCTGCTG GAAGGCACAG CACTTCCCGC

TGCTGGAGTT AGCATATCAT GAAGCTGCTG GAAGGCACAG CACCTCCCGC

TGCTGGAGTT AGCGTATCAT GAAGCTGCTG GAAGGCACAG CACTTCCCGC

TGCTGGAGTT AGCCTACCAT GAAGCTGCTG GAAGGCACAG CACGTCCCGC

TGCTGGAGTT AGCGTATCAT GAAGCTGCTG GAAGGCACAG CTCTTCCCGC

TGCTGGAGCT AGCATATCAC GAAGCTGCCG GAAGGCACAG CACTTCCCGC

TGCTGGAGCT GGCATACCAC GAAGCGGCAG GAAGGCATCT CCCTTCCCGC

TGCTGGAGTT AGCGTATAAT GAAGCGGCTG GCAGGCACAT CGCTTCTCGG

CAGCCTGGTG ACGGCATGTC CTTCTTGAAC TTCAGTGGAA CAGAGGAGCT

CAGCCTGGTG ACGGCATGTC CTTCTTGAAC TTCAGTGGAA CAGAGGAGCT

CAGCCTGGTG ACGGCATGTC CTTCTTGAAC TTCAGTGGAA CAGAAGAACT

CAGCCTGGTG ACGGCATGTC CTTCTTGAAC TTCAGTGGAA CAGAAGAACT

CAGCCTGGTG ACGGCATGCC TTTCTTGAAC TTCAGTGGAA CAGAAGAGCT

CAGCCTGGTG ACGGCATGTC CTTCTTGAAC TTCAGTGGAA CAGAAGAGCT

CAGCCTGGTG ACAGCATGTC ---CTTGAAC TTCAGTGGAA CGGAAGAGCT

CAGCCTGGTG ACAGCATGTC ---CTTGAAC TTCAGTGGAA CGGAAGAGCT

CAGCCTGGCG ACGGCCTGTC TTTCCTGAGC TTCAGCGGAA CAGA------

CAGCCCGGCG ACGGCACGTC CTTCTTGAGC TTCGCTGGGA CGGAGGAGCT

CAGCCTGGTG ACAGCATGTC CTTCTTGAGC TTCAGTGGAA CAGAAGAGCT

CAGCCTGGTG ACGACATGTC CTTCTTGAAC TTCAGCGGAA CTGAAGAACT

CAGCCTGGTG ACGGCATGTC CTTCTTGAAC TTCAGTGGAA CGGAAGAGCT

CAGCCAGGTG ACCGCATGTC CTTCTTGAAC TTCAATGGAA CAGAAGAGCT

CAGCCTGGTG ACAGCATGTC CTTCTTGAAC TTCAGTGGAA CAGAAGAGCT

CAGTCTGGTG ACAACATGTC CTTCTTGAAC TTCAGTGGAA CAGAAGCGCT

CAGCCTGGGG ACAGCGCATC CTTCCTGAAT TTCAGTGGAT CTAACGAGTT

CAGCCTGGCG ACAGCTTGTC CTTCCTGAGC TTCAGTGGAA CAGAAGAATT

TTCTGTCGGC CTGCTTAGCA ACAGTGGTGC AGGTAATCCT AGGACCCAGA

TTCTGTCGGC CTGCTTAGCA ACAGTGGTGC AGGTAATCCT AGGACCCAGA

TTCTGTCGGT CTGCTTAGCA ACAGTGGTGC AGGTAATCCT AGGACCCAGA

TTCTGTCGGT CTGCTTAGCA ACAGTGGTGC AGGTAATCCT AGGACCCAGA

TTCTGTCAGC CTGCTTAGCA ACAGTGGTGC AGGTAATCCT AGGACCCAGA

TTCTGTCAGC CTGCTTAGCA ACAGTGGTGC AGGTAATCCT AGGACCCAGA

TTCTGTCAGC CTGCTTAGCA ACAGCGGCAC AGGTAATCCT AGGAACCAGA

TTCTGTCAGC CTGCTTAGCA ACAGCAGTAC AGGTAATCCT AGGACCCAGA

---------- ---------- -CAGCGGCAC AGCTAATCCT AGGACCCAGA

CTCGGTTAGC CTGCTCCGTG GCAGTGGTGC AGATAGTCCT CGGACGCAGA

TTCTGTCAGC CTGCTTAGCA ACAGTGGTGC AGGTAATCCT AGAACTCAGA

TTCTGTCAAC CTGCTTAGCA ACAGTGGTTC AGGTAATCCT AGGACCCAGA

CTCTGTCGGC CTGCTTAGCA ACAATGGTGC AGGTAATCCT AGGACCCAAA

TTCTGTCAGC CTGATTAGCA ACAGTGGTGC AGGTAATCCT AGGACTCAGA

TTCTGTCAGC CTGCTTAGCA ACAGTGGTGC AGGTAATCCT AGGACCCAGA

CTCTGTCAGC CTGCTTAGCA ACAGCTGTGC AG-------- ----------

TTCTGCCCGC CTCCTCAGCA ATAGTGCGAC AGG---ACCC AGGAGCCAGA

TTCTGTCAGC C---TTAGCA ACCACAGTGC AGGTAATCCT AGGACCCAGA

ATAAGGATTC TCTAGAGGAC AGTGTTTCTA CCGACCCAAT GCCAGGCTTC

ATAAGGATTC TCTAGAGGAC AGTGTTTCTA CCGACCCAAT GCCAGGCTTC

ATAAGGATTC TCTAGAGGAC AGTGTTTCTA CCGACCCAAT GCCAGGCTTC

ATAAGGATTC TCTAGAGGAC AGTGTTTCTA CCGACCCAAT GCCAGGCTTC

ATAAGGATTC TCTAGAGGAC AGTGTTTCTA CCGACCCAAT GCCAGGCTTC

ATAAGGATTC TCTAGAGGAC AATGTTTCTA CCGAACCAAT GCCAGGCTTC

ATAAGGACTC TCTAGAGGAT GGTGTTTCTA CCGACCCAAT GCCAGGCTTC

ACAAGGACTC CCTAGAGGAC AGTGTTTCTA CCGACCCAAT GCCAGGCTTC

ATAAGGATTC GTTAGAGGAC AATGTTTCTA CCGACCCAA- ----------

ATAAGGATTC TCCAGAGGAC AGTGTGTCTA CCGACCCAAT GCCAGGCTTC

ATAAGGATTC TCTAGAGGAC AGTGTTTCTA CCGACCCAAT GCCAGGCTTC

ATAAGGATTC TCTAGAGGAC AATGTTTCTA CCGACCCAAT GCCAGGCTTC

ATAAGGATTC GCTAGAAGAC AGTGTTTCTA CCGACCCAAT GCCAGGCTTC

ATAAGGATTC TCTGGAGGAC AATGTTTCTA CCGACCCAAT GCCAGGCTTC

ATAAGGATTC TCTAGAGGAC AGTGTCTCTA CCGACCCAAT GCCAGGCTTC

---------- ---------- ---------- ---------- ----------

ACAAGGATTC TCTAGAAGAC AGTGTGTCCG CCGATCCTAT GCCGGGCTTC

ACAAGGACTC TGTAGAAGAC AATGTATCGG CCGATCCAAT GCCAGGCTTC

TGTTGCACAG TTGGAGTGGA CTGGAAGTCT CTCACTACTC CGGCGTGCCT

TGTTGCACAG TTGGAGTGGA CTGGAAGTCT CTCACTACTC CGGCGTGCCT

TGTTGCACAG TTGGAGTGGA CTGGAAGTCT CTCACTACTC CGGCGTGCCT

TGTTGCACAG TTGGAGTGGA CTGGAAGTCT CTCACTACTC CGGCGTGCCT

TGTTGCACAG TTGGAGTGGA CTGGAAGTCT CTCACTACTC CGGCGTGCCT

TGTTGCACAG TTGGAGTGGA CTGGAAGTCT CTCACTACTC CGGCGTGCCT

TGTTGCACAG TTGGAGTGGA TTGGAAGTCT CTTACTACTC CTGCATGCCT

TGTTGCACAG TTGGAGTGGA TTGGAAGTCT CTTACTACTC CTGCATGCCT

---------- ---------- ---------- ---------- ---------T

TGTTGCACAG TTGGTGTGGA CTGGAAGTCA CTCACGACTC CCGCGTGCCT

TGTTGCACAG TTGGAGTGGA CTGGAAGTCT CTCACTACTC CGGCGTGCCT

TGTTGCACAG TGGGAGTGGA CTGGAAGTCT CTCACTACTC CGGCGTGCCT

TGTTGCACAG TTGGAGTGGA CTGGAAGTCT CTCACTACTC CGGCTTGCCT

TGTTGCACAG TTGGAGTGGA CTGGAAGTCT CTCACTACTC CGGCGTGCCT

TGTTGCACAG TTGGAGTGGA CTGGAAATCT CTCACTACTC CGGCATGCCT

---------- ---------- ---------- ---------- ----------

TGTTGCACAG TGGGAGTGGA CTGGAAGTCT CTCACGACTC CGGCGTGCCT

TGTTGCACAG TTGGAGTGGA CTGGAAGTCT CTCACTACCC CGGCCTGCCT

CCCCCTTACC ACCGACTACT TCCCTGACCG CCAGGGCCTG CAGAATGACT

CCCCCTTACC ACCGACTACT TCCCTGACCG CCAGGGCCTG CAGAATGACT

CCCCCTCACC ACCGACTACT TCCCTGACCG CCAGGGCCTG CAGAATGACT

CCCCCTCACC ACCGACTACT TCCCTGACCG CCAGGGCCTG CAGAATGACT

CCCCCTCACC ACCGACTACT TCCCTGACCG CCAGGGCCTG CAGAATGACT

CCCCCTCACC ACCGACTACT TCCCTGACCG CCAGGGCCTC CAGAACGACT

TCCCCTCACC ACTGACTACT TCCCGGACCG TCAGGGCCTG CAGAATGACT

TCCCCTCACC ACTGACTACT TCCCGGACCG TCAGGGCCTG CAGAATGACT

CCCCCTCACC ACCGACTACT TCCCTGACCG CCAGGCCCTG CAGAACGACT

CCCCCTCACC ACGGACTACT TCCCCGACCG CCAGGGCCTA CAGAATGACT

CCCCCTCACC ACCGACTACT TCCCCGACCG CCATCGCCTG CAGAACGACT

CCCGCTCACC ACCGACTACT TCCCCGACCG CCAGGGCTTG CAGAACGACT

CCCCCTCACC ACCGACTACT TCCCTGACCG CCAGGGCCTA CAGAACGACT

CCCCCTTACC ACCGACTACT TCCCTGACCG CCAGGGCCTG CAGAATGACT

GCCCCTCACC ACCGACTACT TCCCCGACCG CCAGAGCCTG CAGAACGACT

---------- ---------- ---------- ---------- ----------

GCCCCTCACC ACCGACTACT TCCCAGACCG CCAGGGCCTT CAGAACGACT

CCCCCTCACT ACGGACTACT TCCCGGATCG CCAGGGCCTG CAAAATGATT

ACACAGAGGG CTGTTATGAT CTCCTTCCAG AAGCAGACAT CGACAGGAGG

ACACAGAGGG CTGTTATGAT CTCCTTCCAG AAGCAGACAT CGACAGGAGG

ACACAGAGGG CTGTTATGAT CTCCTTCCAG AAGCAGACAT CGACAGGAGG

ACACAGAGGG CTGTTATGAT CTCCTTCCAG AAGCAGACAT CGACAGGAGG

ACACAGAGGG CTGTTATGAT CTCCTCCCAG AAGCAGACAT CGACAGGAGG

ACACAGAGGG CTGTTATGAT CTCCTCCCGG AAGCAGATAT GGACAGGAGG

ACACAGAGGG CTGCTATGAT CTCCTCCCAG AAGCAGACAT GGACAGGAGG

ACACAGAGGG CTGCTATGAT CTCCTCCCAG AAGCAGACAT GGACAGGAGG

ACACCGAGGG CTGCTACGAC CTGCTCCCGG AGGCGGACAT CGACAGGAGG

ACACGGAGGG CTGCTACGAT CTCCTCCCGG AGGCTGACAT CGACAGGAGG

ACACAGAGGG CTGTTACGAT CTCCTTCCAG AAGCAGATAT TGACAGGAGG

ATACAGAGGG CTGTTATGAC CTCCTCCCGG AAGCGGACAT GGACAGGAGA

ACACAGAGGG CTGTTATGAT CTTCTCCCGG AAGCAGACAT GGACAGGAGG

ACACTGAAGG CTGTTATGAT CTCCTCCCAG AAACGGACAC AGACAGGAGG

ACACGGAAGG CTGCTACGAT CTCCTCCCAG AAACAGACAT TGACAGGCGG

---------- ---------- ---------- ---------- ------GAGG

ACACCGAGGG CTGTTACGAC CTCCTCCCGG AGGCCGACAT GGACAGGAGG

ACACCGAAGG CTGTTACGAC CTGCTCCCGG AGGCGGATAT TGACAGGAGA

GACGAAGATG GTGTGCAGAT GACAGCCCAG CAGGTATTTG AAGAGTTTAT

GACGAAGATG GTGTGCAGAT GACAGCCCAG CAGGTGTTTG AAGAGTTTAT

GACGAAGACG GTGTGCAGAT GACAGCCCAG CAGGTGTTTG AAGAGTTTAT

GACGAAGACG GTGTGCAGAT GACAGCCCAG CAGGTGTTTG AAGAGTTTAT

GATGAAGAGG GTGTGCAGAT GACAGCCCAG CAGGTGTTTG AAGAGTTTAT

GACGAGGAGG GTGTACAGAT GACGGCCCAG CAGGTGTTTG AAGAATTCAT

GACGAGGAGG GCGTTCAAAT GACAGCCCAG CAAGTGTTTG AAGAGTTCAT

GATGAGGAGG GTGTTCAAAT GACAGCCCAG CAAGTGTTTG AAGAGTTCAT

GACGAGGAGG GCGTGCAGAT GACGGCCCAG CAGGTGTTTG AAGAGTTCAT

GATGAGGAGG GCGTGCAGAT GACGGCCCAG CAGGTATTTG AAGAGTTCAT

GATGAGGAGG GTGTGCAGAT GACGGCCCAG CAGGTGTTTG AAGAGTTCAT

GACGAGGAGG GTGTGCAGAT GACGGCTCAG CAGGTGTTTG AAGAGTTCAT

GATGAGGAGG GTGTACAGAT GACAGCCCAG CAAGTGTTTG AGGAGTTCAT

GACGAGGAAG GTGTGCAGAT GACAGCTCAG CAGGTGTTTG AGGAGTTTAT

GATGAGGAGG GCGTGCAGAT GACAGCCCAG CAGGTGTTTG AAGAGTTCAT

GATGAGGAGG GTGTGCAGAT GACAGCCCAG CAGGTGTTTG AAGAGTTCAT

GATGAAGAGG GCATGCAGAT GACTGCCCAG CAGGTGTTCG AGGAGTTTAT

GATGAAGAGG GTGTGCAGAT GACAGCTCAG CAAGTGTTTG AAGAATTTAT

TTGCCAACGT CTCATGCAGG GCTACCAAAT CATAGTGCAG CCCAAGACAC

TTGCCAACGT CTCATGCAGG GCTACCAAAT CATAGTGCAG CCCAAGACAC

TTGCCAGCGT CTCATGCAGG GCTACCAAAT CATAGTGCAG CCCAAGGCAC

TTGCCAGCGT CTCATGCAGG GCTACCAAAT CATAGTGCAG CCCAAGGCAC

TTGCCAACGT CTCATGCAGG GCTACCAAAT CATAGTGCAG CCCAAGGCAC

TTGCCAGCGT CTCATGCAGG GGTATCAAAT CATAGTGCAG CCCAAGGCAC

CTGCCAGCGT CTCATGCAGG GTTACCAAAT CATAGTACAG CCAAAGACCC

CTGCCAGCGT CTCATGCAGG GTTACCAAAT CATAGTACAG CCAAAGACCC

ATGTCAGCGC CTCATGCAGG GCTACCAGAT CATAGTGCAG CCCAAGGCCC

CTGCCAGCGG CTCATGCAGG GTTACCAGAT CATCGTTCAG CCCCGCGCAC

CTGCCAGCGT CTCATGCAGG GCTACCAAAT CATAGTGCAG CCCAAGGCAC

TTGCCAGCGT CTCATGCAGG GCTATCAAAT CATAGTGCAG CCCAAGGCCC

CTGCCAGCGC CTCATGCAGG GCTACCAGAT CATAGTGCAG CCCAAAGCCC

TTGCCAGCGT CTCATGCAGG GCTACCAGAT CATAGTGCAG CCCAGGGCGC

TTGCCAGCGG CTCATGCAGG GCTACCAAAT CATAGTGCAG CCCAAGGCAC

TTGCCAGCGG CTCATGCAGG GCTACCAAAT CATAGTGCAG CCCAAGGCCC

TTGCCAACGT CTCATGCAGG GCTACCAGAT CATCGTACAG CCAAAGCCCC

TTGCCAGCGT CTTATGCAGG GCTACCAGAT TATAGTTCAG CCTAAACCAC

AGAAACCCAA TCCTGCTGTC CCGCCCCCGC TGAGCAGTAG CCCACTCTAT

AGAAACCCAA TCCCGCCGTC CCGCCCCCGC TGAGCAGTAG CCCACTCTAT

AAAAACCCAA CCCTGCCGTC CCACCCCCGC TGAGCAGTAG CCCACTCTAT

AAAAACCCAA CCCTGCCGTC CCACCCCCGC TGAGCAGTAG CCCACTCTAT

AGAAACCTAA CCCCGCCGTC CCTCCACCGC TGAGCAGTAG CCCACTCTAT

AGAAACCCAA CTCCACTGTC CCACCTCCGC TGAGCAGTAG CCCACTCTAT

AGAAGCCCAG CACTACAGTC CCACCCCCGC TGAGCAGTAG CCCACTCTAT

AGAAACCCAA CACCACAGTC CCACCCCCAT TGAGCAGTAG CCCACTCTAT

AGAAGCCCAA CCCCGCGGTC CCACCCCCGC TGAGCAGCAG CCCCCTCTAC

AGAAGCCCAG CCCTGCTGGC CCACCGCCAC TCAGCAGCAG CCCGCTCTAC

AAAAATCCAA CCCCGTCGTC CCACCCCCAC TGAGCAGCAG CCCGCTCTAT

AGAAAGCCAA CCCGGCCGGC CCGCCCCCAC TGAGCAGCAG TCCCCTCTAT

CCAAACCCAG CCCTGCCGTC CCGCCGCCCC TCAGCAGCAG CCCGCTCTAT

CGAGACCTGC CCCTACTCTC CCACCCCCGT TGAGCAGCAG CCCACTCTAT

AGAAATCCAA CTCTGTCGTC CCTCCCCCGC TGAGCAGCAG CCCGCTCTAT

AGAAATCCAC CCCCATCGTG CCACCCCCGC TAAGCAGCAG CCCACTCTAT

AGAAACCCAA CCCGGCTGTG GCCCCTCCCC TGAGTAGCAG CCCCCTCTAC

AGAAACCAAA TCCAACCGTT CCACCGCCAC TTAGTAGCAG CCCCCTCTAT

AGCCGAGGCC TTGTGTCCCG AAACCGCCCT GAGGAGGAGG ACCAGTATTG

AGCCGAGGCC TTGTGTCCCG AAACCGCCCC GAGGAGGAGG ACCAGTATTG

AGCCGAGGCC TTGTGTCCCG AAACCGCCCC GAGGAGGAGG ACCAGTATTG

AGCCGAGGCC TTGTGTCCCG AAACCGCCCC GAGGAGGAGG ACCAGTATTG

AGCCGAGGGC TTGTGTCCCG AAACCGCCCT GAGGAGGAGG ACCAGTATTG

AGCCGAGGTC TTGTTTCCCG AAACCGCCCT GAGGAAGAGG ACCAGTATTG

AGCAGAGGCC TTGTGTCCCG AAACCGCCCT GAAGAGGAGG GCCAGTATTG

AGCAGAGGCC TTGTGTCCCG AAATCGCCCT GAGGAGGAAG GCCAGTATTG

AGCCGAG--- ---------- ---------- ---------- ----------

AGCCGGGGCC TTGTGTCCCG AAACCGCCCG GAAGAGGAAG ACCAGTACTG

AGTCGAGGCC TTGTGTCCCG AAACCGCCCT GAGGAGGAGG ACCAATACTG

GGCCGAGGCC TTGTGTCCCG AAACCGCCCT GAGGAAGAGG ACCAGTACTG

AGCCGAGGTC TCGTGTCCCG TAACCGCCCC GAGGAAGAAG ACCAGTATTG

AGCCGAGGCC TTGTGTCCCG AAACCGCCCT GAAGAGGAAG ATCAGTACTG

AGCCGAGGCC TTGTGTCCCG AAACCGCCCC GAGGAGGAGG ACCAGTACTG

AGCCGAG--- ---------- ---------- ---------- ----------

AGCCGGGGCC TCGTGTCCCG AAACCGTCCT GAGGAGGAAG ACCAGTATTG

AGTAGAGGGC TTGTGTCTCG TAACCGCCCC GAGGAGGAAG ACCAATACTG

GCTGAGTATG GGCAGAACGT TCCACAAAGT GACGCTGAAG GATAAGATGA

GCTGAGTATG GGCAGAACGT TCCACAAAGT GACGCTGAAG GATAAGATGA

GCTGAGTATG GGCAGAACGT TCCACAAAGT GACGCTGAAG GATAAGATGA

GCTGAGTATG GGCAGAACGT TCCACAAAGT GACGCTGAAG GATAAGATGA

GCTGAGTATG GGCAGAACGT TCCACAAAGT GACACTGAAG GATAAGATGA

GCTGAGTATG GGCAGAACTT TCCACAAAGT GACACTGAAG GATAAGATGA

GCTGAGTATG GGCAGAACCT TCCATAAGGT GACACTTAAG GACAAGATGA

GTTAAGTATG GGCAGAACTT TCCATAAGGT GACACTCAAG GACAAGATGA

---------- ---------- ---------- ---------- ----------

GCTGAGCATG GGCAGAACCT TCCACAAAGT CACACTGAAA GACAAGATGA

GCTGAGTATG GGCAGAACGT TCCACAAAGT GACGCTGAAA GATAAGATGA

GTTGAGTATG GGCAGAACTT TCCACAAAGT GACACTGAAG GATAAGATGA

GCTGAGTATG GGCAGAACTT TCCACAAGGT GACACTGAAG GATAAGATGA

GCTGAGTATG GGCAGAACTT TCCACAAAGT GACGCTGAAG GATAAGATCA

GCTGAGTATG GGCAGAACTT TCCACAAAGT GACCCTGAAG GATAAGATCA

---------- ---------- ---------- ---------- ----------

GCTGAGTATG GGTCGCACTT TCCACAAAGT GACGTTGAAG GACAAAATCA

GCTGAGCATG GGTCGTACTT TTCACAAAGT AACGCTGAAG GATAAAATCA

TCACAGTGAC GCGATACCTT CCCAAGTATC CTTATGAATC TGCCCAGATC

TCACAGTGAC GCGATACCTT CCCAAGTATC CTTATGAATC TGCCCAGATC

TCACAGTGAC GCGATACCTT CCCAAGTATC CTTATGAATC TGCCCAGATC

TCACAGTGAC ACGATACCTT CCCAAGTATC CTTATGAATC TGCCCAGATC

TTACAGTGAC ACGATACCTT CCCAAGTATC CTTATGAATC TGCCCAGATC

TCACAGTGAC ACGGTACCTT CCCAAGTACC CTTATGAATC TGCCCAGATC

TCACAGTGAC TCGTTACCTT CCCAAGTACC CTTATGAATC TGCCCAGATC

TCACAGTAAC TCGTTACCTT CCCAAGTACC CTTATGAATC TGCCCAGATC

---------- ---------- -----GTACC CTTATGAATC CGCCCAGATC

TCACAGTGAC CCGCTACCTT CCCAAGTACC CGTACGAGTC CGCCCAGATC

TCACAGTGAC ACGTTACCTT CCCAAGTACC CTTATGAATC TGCCCAGATC

TCACAGTGAC ACGGTACCTT CCCAAGTACC CTTATGAGTC TGCCCAGATC

TCACAGTCAC ACGCTATCTT CCCAAGTACC CTTATGAATC TGCCCAGATC

TCACAGTGAC GCGATACCTC CCCAAGTATC CTTATGAATC TGCTCAGATC

TCACAGTGAC ACGATACCTT CCCAAGTACC CTTATGAATC TGCCCAGATC

---------- ---------- ---------- ---------- ----------

TCACAGTCAC CCGCTACCTG CCCAAGTATC CCTACGAGTC TGCCCAGATC

TCACCGTCAC CCGGTACCTA CCCAAGTACC CCTATGAATC TGCCCAGATC

CACTACACCT ACAGCCTCTG TCCTTCCCAC TCAGACTCAG AGTTCGTCTC

CACTACACCT ACAGCCTCTG TCCTTCCCAC TCAGACTCAG AGTTCGTCTC

CACTATACCT ACAGCCTCTG TCCTTCCCAC TCAGACTCAG AGTTCGTCTC

CACTATACCT ACAGCCTCTG TCCTTCCCAC TCAGACTCAG AGTTCGTCTC

CACTACACCT ACAGCCTCTG TCCTTCACAC TCAGACTCAG AGTTCGTCTC

CACTACACCT ATAGCCTCTG CCCTTCCCAC TCAGACTCAG AGTTTGTATC

CATTACACCT ACAGCCTCTG CCCTTCCCAC TCTGACTCAG AGTTTGTCTC

CATTACACCT ACAGCCTCTG CCCTTCCCAC TCTGACTCGG AGTTTGTCTC

CACTACGCCT ACAGCCTCTG CCCTTCCCAT TCGGACTCGG AGTTCGTCCC

CACTACACCT ACAGCCTCTG CCCCTCCCAC TCCGATTCAG AGTTCGTCTC

CACTACACCT ACAGTCTCTG CCCTTCCCAC TCAGACTCGG AGTTCGTCTC

CACTACACCT ACAGCCTCTG CCCTTCCCAC TCAGACTCGG AGTTTGTCTC

CACTACACCT ACAGCCTCTG CCCCTCCCAC TCTGACTCAG AGTTTGTCTC

CACTATACCT ATAGCCTCTG CCCTTCCCAC TCAGACTCAG AATTCGTCTC

CACTACACAT ATAGCCTCTG CCCCTCCCAC TCAGACTCAG AGTTCGTCTC

---------- ---------- ---------- ---------- ----------

AACTATACCT ACAGCCTGTG CCCCTCGCAC TCCGACTCAG AGTTTGTGTC

AACTACACCT ACAGCTTGTG TCCCTCCCAT TCAGACTCCG AATTTGTCTC

CTGCTGGGTG GAATTCTCCC ACGAACGGCT GGAGGAGTAC AAGTGGAATT

CTGCTGGGTA GAATTCTCCC ACGAACGGCT GGAGGAGTAC AAGTGGAATT

CTGCTGGGTG GAATTCTCCC ATGAACGGCT GGAGGAGTAC AAGTGGAATT

CTGCTGGGTG GAATTCTCCC ATGAACGGCT GGAGGAGTAC AAGTGGAATT

CTGCTGGGTG GAATTCTCCC ATGAACGGCT GGAGGAGTAC AAGTGGAATT

CTGTTGGGTG GAATTCTCCC -CGAACGGCT GGAGGAGTAC AAATGGAATT

CTGTTGGGTG GAATTCTGCC ATGAACGGCT AGAGGAATAC AAGTGGAATT

CTGTTGGGTG GATTTCTGCC ACGAACGGCT CGAGGAATAC AAGTGGAATT

CTGCTGGGTG GAGTTCTCCC ACGAGCGGCT GGAGGAGTAC AAGTGGAATT

CTGTTGGGTG GAATTCTCGC ATGAGCGACT GGAGGAGTAC AAGTGGAATT

TTGTTGGGTG GAATTCTCCC ATGAGCGGCT GGAGGAGTAC AAGTGGAATT

CTGTTGGGTG GAATTCTCCC ACGAGCGGCT GGAGGAGTAC AAGTGGAATT

TTGCTGGGTG GAATTCTCTC ACGAACGGCT GGAGGAGTAC AAGTGGAATT

TTGCTGGGTG GAATTCTCCC ATGAACGGCT GGAGGAGTAC AAGTGGAATT

CTGTTGGGTA GAATTCTCCC ACGAACGGCT GGAGGAGTAC AAGTGGAATT

---------- ---------- ---------- ---------- ----------

CTGCTGGGTG GAGTTCTCCC ACGAACGCCT GGAAGAATAC AAGTGGAATT

TTGCTGGGTG GAGTTCTCCC ATGAACGACT GGAGGAGTAT AAGTGGAATT

ACTTAGATCA GTATATCTGT TCTGCCGGCT CTGAAGACTT CAGCTTAATT

ACTTAGATCA GTATATCTGT TCTGCCGGCT CTGAAGACTT CAGCTTAATT

ACTTAGATCA GTATATCTGT TCTGCCGGCT CTGAAGATTT CAGCTTAATT

ACTTAGATCA GTATATCTGT TCTGCCGGCT CTGAAGATTT CAGCTTAATT

ACTTAGATCA GTATATCTGT TCTGCAGGCT CTGAAGACTT CAGCTTAATT

ACTTAGATCA GTATATCTGT TCTGCAGGCT CCGAGGACTT CAGCTTAATT

ACTTAGATCA GTACATTTGT TCTGCCGGCT CCGAAGATTT CAGCTTAATC

ACTTAGATCA GTATATTTGT TCTGCTGGCT CTGAAGACTT CAGCTTAATC

ACTTGGACCA GTACATCTGC TCTGCCGGCT CCGAGGACTT CAGCTTAATT

ACTTAGATCA GTATATCTGC TCTGCCGGCT CTGAGGACTT CAGCTTAATC

ACTTAGATCA GTATATCTGT TCTGCTGGCT CTGAGGACTT CAGCTTAATT

ACTTAGATCA GTATATCTGT TCCACCGGCT CTGAGGATTT CAGCTTAATT

ACTTAGATCA GTATATTTGC TCTGCTGGCT CCGAGGACTT CAG-------

ACCTCGATCA GTATATTTGC TCCGCTGGCT CTGAGGACTT CAGCTTAATT

ACTTGGATCA GTATATCTGT TCCGCTGGCT CCGAGGACTT TAGCTTAATT

---------- ---------- ---------- ---------- ----------

ACCTGGATCA GTACATCTGC TCGGCGGGCT CCGAGGACTT cagTTTAATT

ATTTAGATCA ATATATCTGC TCTGCTGGCT CCGAGGACTT CAGCTTAATT

GAGTCCCTGA AGTTCTGGAG GACCCGCTTC CTGCTGCTGC CAGCCTGTGT

GAGTCCCTGA AGTTCTGGAG GACCCGCTTC CTGCTGCTGC CAGCCTGTGT

GAGTCTCTGA AGTTCTGGAG GACCCGCTTC CTGCTGCTGC CAGCCTGTGT

GAGTCTCTGA AGTTCTGGAG GACCCGCTTC CTGCTGCTGC CAGCCTGTGT

GAGTCCCTAA AGTTCTGGAG GACCCGATTC CTGCTGCTGC CAGCCTGTGT

GAGTCCCTGA AGTTCTGGAG GACCCGCTTT CTGCTGCTGC CAGCCTGTGT

GAGTCTCTGA AGTTCTGGAG AACCCGATTT CTACTACTGC CAGCCTGTGT

GAGTCTCTGA AGTTCTGGAG AACCCGATTT TTACTACTGC CAGCCTGTGT

GAGTCTCTGA AGTTCTGGAG AACCCGCTTC CTGCTGCTGC CAGCCTGCGT

GAGTCCCTGA AGTTCTGGAG GACACGCTTC CTGCTGCTGC CGGCCTGCGT

GAGTCTCTGA AGTTCTGGAG GACCCGCTTC CTGCTGCTGC CAGCCTGTGT

GAGTCTCTGA AGTTCTGGAG GACCCGATTC CTGCTGCTGC CAGCCTGTGT

---------- ---------- ---------- ---------- ----------

GAGTCCCTGA AGTTCTGGAG GACCCGCTTC CTGCTGCTGC CAGCCTGTGT

GAGTCGCTGA AGTTCTGGAG GACCCGCTTC CTGCTGCTGC CAGCCTGTGT

---------- ---------- ---------- ---------- ----------

GAGTCGCTGA AATTCTGGAG AACGCGCTTC CTGCTGCTCC CAGCATGCGT

GAGTCTCTGA AGTTCTGGAG GACCCGCTTC CTGCTGCTTC CTGCCTGCAT

CACCGCCACC AAGCGCATCA CGGAGGGGGA GGCCCACTGC GACATCTATG

CACCGCCACC AAGCGCATCA CGGAGGGGGA GGCCCACTGC GACATCTATG

CACCGCCACC AAGCGCATCA CAGAGGGGGA GGCCCACTGT GACATCTATG

CACCGCCACC AAGCGCATCA CAGAGGGGGA GGCCCACTGC GACATCTATG

CACCGCCACC AAGCGCATCA CGGAGGGAGA GGCCCACTGC GACATCTATG

CACTGCCACC AAGCGCATCA CAGAAGGGGA GGTACACTGC GACATCTATG

CACTGCCACC AAGCGCATCA CAGAAGGGGA AGTGCACTGT GACATCTACG

TACTGCCACC AAGCGCATCA CAGAAGGGGA GGTGCACTGT GACATCTATG

CACTGCCACC AAGCGTATCA CAGAGGGCGA GGCCCACTGC GACATCTATG

CACTGCCACC AAGCGCATCA CGGAGGGAGA GGCGCACTGC GACATCTACG

CACTGCCACT AAGCGCATCA CAGAAGGGGA GGCCCACTGC GACATCTATG

CACTGCCACC AAGCGCATCA CAGAGGGGGA GGCCCACTGC GACATCTACG

---------- ---------- ---------- ---------- ----------

CACCGCCACC AAGCGCATCA CAGAGGGGGA AGCCCACTGC GACATCTACG

CACTGCCACC AAGCGCATCA CAGAGGGGGA AGCCCACTGC GACATCTATG

---------- ---------- ---------- ---------- ----------

CACTGCTACC AAACGCATCA CAGAAGGGGA GGCGCACTGT GACATCTACG

CACCGCCACC AAGCGCATCA CTGAGGGCGA GTCCCACTGT GACATCTACG

GGGACAGGCC CCGTGCAGAC GAGGACGAGT GGCAACTCCT GGATGGTTTT

GGGACAGGCC CCGTGCAGAC GAGGACGAGT GGCAACTCCT GGATGGTTTT

GGGACAGGCC CCGTGCAGAC GAGGACGAAT GGCAACTCCT GGATGGCTTT

GGGACAGGCC CCGTGCAGAC GAGGACGAAT GGCAACTCCT GGATGGCTTT

GGGACAGGCC CCGTGCAGAC GAGGATGAGT GGCAACTCCT GGATGGCTTC

GGGACAGACC TCGTGCTGAT GAGGACGAGT GGCAACTTCT GGATGGTTTT

GGGACAAACC CCGTGCAGAT GAAGATGAGT GGCAGCTTCT AGATGGCTTT

GGGACAAACC CCGTGCAGAT GAAGATGAGT GGCAGCTTCT AGATGGCTTT

GGGACAGGCC CCGAGCTGAC GAGGACGAGT GGCAGCTTCT GGACGGCTTT

GGGACCGGCC GCGGGCCGAC GAGGATGAGT GGCAGCTCCT GGACGGCTTC

GGGACAGGCC TCGGGCAGAC GAGGAGGAAT GGCAGCTCTT GGATGGTTTT

GGGACAGGCC TCGGGCAGAT GAGGAGGAGT GGCAGCTCCT GGATGGCTTT

---------- ---------- ---------- ---------- ----------

GGGACAGGCC CCGGGCAGAT GAGGAGGAGT GGCAGCTCCT AGACGGTTTC

GGGACAAGCC CCGGGCAGAT GAGGAAGAGT GGCAGCTCCT CGACGGCTTT

---------- ---------- ---------- ---------- ----------

GGGAGAAGCC CCGCTCGGAT GAGGAAGAGT GGCAGCTCTT GGATGGCTTC

GGGACAAACC CCGCTCTGAT GAGGAAGAGT GGCAGCTCTT AGATGGCTTT

GTCCGCTTTG TGGAGGGCTT GAATCGCATT CGCAGGCGGC ATCGCTCGGA

GTCCGCTTTG TGGAGGGCTT GAATCGCATT CGCAGGCGGC ATCGCTCGGA

ATCCGCTTTG TGGAGGGCTT GAATCGCATT CGCAGGCGGC ATCGCTCGGA

ATCCGCTTTG TGGAGGGCTT GAATCGCATT CGCAGGCGGC ATCGCTCGGA

ATCCGCTTTG TGGAGGGCTT GAACCGCATT CGCAGGCGGC ATCGCTCAGA

ATCCGCTTTG TGGAAGGCTT GAATCGGATC CGCAGGCGGC ATCGGTCAGA

ATTCGCTTTG TAGAGGGCTT GAACCGCATC CGCAGGCGCC ACCGGTCAGA

ATTCGCTTTG TAGAGGGCTT AAATCGCATC CGCAGGCGCC ACCGCTCAGA

ATCCGCTTTG TGGAGGGCTT GAACCGGATC CGCAGGCGGC ACCGCTCAGA

ATCCGCTTCG TGGAGGGCCT GAACCGCATC CGCCGGCGGC ACCGCTCGGA

ATCCGCTTCG TGGAGGGTTT GAACCGGATC CGCAGGCGAC ATCGCTCAGA

ATCCGCTTTG TGGAGGGCTT GAACCGGATC CGCAGGCGCC ATCGCTCCGA

---------- ---------- ---------- ---------- ----------

ATCCGCTTTG TGGAGGGTTT GAACCGGATC CGCAGGCGGC ATCGCTCAGA

ATCCGCTTTG TGGAGGGCCT AAACCGGATC CGCAGGCGGC ACCGCTCTGA

---------- ---------- ---------- ---------- ----------

ATCCGCTTCA TGGAAGGCTT GAATCGCATC CGGCGGCGCC ACCGGTCAGA

ATCCGTTTCA TGGAAGGTTT GAACCGGATT CGAAGGCGTC ATCGATCAGA

TCGCATGATG CGGAAAGGGA CCGCCATGAA AGGCTTGCAG ATGACTGGGC

TCGCATGATG CGGAAAGGGA CCGCCATGAA AGGCTTGCAG ATGACTGGGC

TCGCATGATG CGGAAAGGGA CCGCCATGAA AGGCTTGCAG ATGACCGGGC

TCGCATGATG CGGAAAGGGA CCGCCATGAA AGGCTTGCAG ATGACCGGGC

TCGCATGATG CGGAAAGGGA CCACCATGAA AGGCTTACAG ATGACCGGGC

TCGCATGATG CGGAAAGGGG CCACAATGAA AGGCTTGCAG ATGACTGGGC

CCGCATGATT CGGAAAGGGA CTGCCATGAA AGGCTTGCAG ATGACCGGGC

CCGCATGATT CGGAAGGGGA CTGCCATGAA AGGCTTGCAG ATGACCGGGC

CCGCATGATG CGGAAAGGGG CCACCATGAA GGGCTTACAG ATGACCGGGC

CCGCATGATG CGGAAAGGGG CCACCATGAA AGGCTTGCAG ATGGCCGGGC

TCGCATGATG CGGAAAGGGA CCGCCATGAA AGGTTTGCAG ATGACTGGGC

TCGCATGATG CGGAAAGGGA CCTCCATGAA AGGCTTGCAG GTGACCGGAC

---------- ---AAGGGGA CAACCATGAA AGGTCTGCAG ATGCCCGGGC

TCGCATGATA CGGAAAGGGA CCACCATGAA AGGCTTGCAG ATGACTGGGC

TCGCATGATG CGGAAAGGGA CCACCATGAA AGGCTTGCAG ATGACTGGGC

---------- ---AAAGGAG CTGCCATGAA AGGCTTGCAG ATGACCGGGC

CCGCATGATA CGGAAAGGGG CTGCTATGAA GGGCTTGCAG ATGGCTGGCC

TCGCATGATT CGGAAAGGGA CCACTATGAA AGGCCTGCAG ATGGCTGGCC

CCATTTCCAC GCATTCTCTG GAGTCAACTG CACCCCCAGT GGGGAAGAAG

CCATTTCCAC GCATTCTCTG GAGTCAACTG CACCCCCAGT GGGGAAGAAG

CCATTTCCAC ACATTCTCTG GAGTCAGCCG CACCCCCAGT GGGGAAGAAG

CCATTTCCAC ACATTCTCTG GAGTCAGCCG CACCCCCAGT GGGGAAGAAG

CCATTTCCAC ACACTCTCTG GAGTCAACTG CACCCCCAGT GGGGAAGAAG

CTCTTCCCAC ACACACTCTT GAGTCAGCTG GCCCTCCGGT TGGGAAGAAG

CCATCTCCGC ACACTCTCTC GAGTCAACGG GCCCCCCAGT CGGGAAGAAA

CCATCTCTGC ACACTCTCTC GAGGCAGCAG GCCCTCCAGT CGGCAAGAAA

CCATTCCCAC ACACTCCCTG GAGTCGGCGG GGCCCCCTGT CGGGAAGAAG

CCATCCCCAC GCACTCTCTC GAGTCGGCTG GCCCCCCGGT TGGGAAGAAG

CCATTGCCAC ACACTCCCTT GAGTCGACCG GCCCCCCAGT TGGGAAGAAG

CCATTTCCAC GCACTCCCTC GAGTCAACCG GCCCCCCGGT TGGGAAGAAG

CCATTGCTAC CCATCCCCTC GAGTCAGCTG GCCCTCCTGT TGGGAAGAAG

CCATTTCCAC TCACTCGCTC GAGTCGGCCG GGCCCCCGGT TGGAAAGAAG

CCATTTCCAC GCACTCCCTC GAGTCTGCTG GCCCCCCAGT TGGGAAGAAG

CCATTTCTAC ACACTCCCTT GAGTCTGCCG TCCCCCCGGT TGGGAAGAAG

CACTCTCTGC CCACTCTCTG GAGCCGCCCG GACCACCGGT TGGAAAGAAG

CCATCTCCAC CCACTCCCTC GAACCCCCGG GACCACCAGT CGGGAAGAAG

GGAACCTCAG CTCTCTCTGC CCTGTTGGAG ATGGAGGCCA GTCAGAAGTG

GGAACCTCAG CTCTCTCTGC CCTGTTGGAG ATGGAGGCCA GTCAGAAGTG

GGAACCTCGG CTCTCTCTGC CCTGTTGGAG ATGGAGGCCA GTCAGAAGTG

GGAACCTCGG CTCTCTCTGC CCTGTTGGAG ATGGAGGCCA GTCAGAAGTG

GGAACCTCAG CTCTCTCTGC CCTGTTGGAG ATGGAGGCCA GTCAGAAGTG

GGAACCTCAG CTCTGTCTGC CCTGCTGGAG ATGGAGGCCA CTCAGAAGTG

GGAACCTCAG CTCTGTCTGC CCTGCTGGAG ATGGAGGCTA GTCAGAAGAG

GGAACCTCAG CTCTGTCTGC CCTGCTGGAG ATGGAAGCTA GTCAGAAGAG

GGAGCCTCAG CTCTGTCCGC CCTGCTGGAG ATGGAGGCCA GCCAGAAGTG

GGAACCTCAG CTCTGTCTGC CCTGCTGGAG ATGGAGGCCA GTCAGAAGTG

GGAACCTCAG CTCTGTCCGC CCTGTTGGAG ATGGAGGCCA GTCAGAAGTG

GGAACCTCAG CTCTGTCTGC CCTGTTGGAG ATGGAGGCCA GTCAGAAGTG

GGGACCTCAG CGCTGTCTGC CCTGTTGGAG ATGGAGGCCA GTCAGAAGTG

GGGACATCCG CTCTGTCCGC CTTGCTGGAA ATGGAGGCCA GTCAGAAGTG

GGAACCTCAG CTCTGTCTGC CTTGTTGGAG ATGGAGGCCA GTCAGAAGTG

GGAACGTCCG CTCTGTCTGC TCTGTTGGAG ATGGAGGCCA GTCAGAAGTG

GGGACGTCTG TCCTCTCGGC TTTGCTGGAA ATGGAAGCCA GTCAGAAGTG

GGAACCTCAG CGCTCTCTGC CTTGCTGGAA ATGGAAGCCA GTCAGAAATC

CCTGGGAGAA CAGCAGGCAG CTGTGCATGG TGGGAAGAGC TCCGCCCAGT

CCTGGGAGAA CAGCAGGCAG CTGTGCATGG TGGGAAGAGC TCCGCCCAGT

CCTGGGAGAA CAGCAGGCAG CTGTGCATGG TGGGAAGAGC TCCGCCCAGT

CCTGGGAGAA CAGCAGGCAG CTGTGCATGG TGGGAAGAGC TCCGCCCAGT

CCTAGGAGAA CAGCAGGCAG TTGTGCATGG TGGGAAGAGC TCCGCCCAAC

CCTGGGAGAA CAGCAGGCAG CTGCACACGG TGGGAAGAGC TCCACTCAGT

CCTAGGAGAA CAGCAGACAA GTGTGCATGG G---AAAAGC TCTACTCAGC

CCTAGGAGAA CAGCAGACGA CTGTGCACGG A---AAAAGC TCCACTCAGC

CCTGGGAGAC CAGCAGGCGG CCGTGCTCGG GGGGAAGAGC GCCGCCCAGC

CCTGGGAGAG CAGCAGGCAG CGGCACACGG GGTCAAGAGC TCCGCCCCGC

CCTAGGAGAA CAGCAGGCAG CTGTGCACGG TGGCAAGAGC GCCTCCCAGC

CCTGGGAGAA CAGCAGGCAG CTGTGCATGG TGGCAAGAGC TCCGCCCAGC

CCTGGGAGAA CAGCAGGCAG CTGTGCACGG CGGCAAGAGC TCTACCCAGC

CCTGGGAGAA CAGCAGGCAG CTGGGCATGG TGGGAAGAGC TCCATCCAGC

CCTTGGAGAA CAGCAGGCAG CAGTGCATGG TGGGAAGAGC TCCACCCAGC

CCTCGGTGAG CAGCAGGCTG CGGTGCATGC TGGTAAGAGC TCCGCTCAGT

TTTGGGAGAG CAGCAGGCCA GCCTGCATGC CTGCAAGGGC CCTGCCCATC

CCTGGGAGAT CAGCAAGGAG CACTGCATGT TGGAAAGAAT TCTGTTCAGT

CAGCCGAGAG CAGCAGCGTT GCCATGACTC CCACCTACAT GGACAGCCCA

CAGCCGAGAG CGGCAGCGTT GCCATGACTC CCACCTACAT GGACAGCCCA

CAGCGGAGAG CAGCAGCGTC GCCATGACTC CCACCTACAT GGACAGCCCA

CAGCCGAGAG CAGCAGCGTC GCCATGACTC CCACCTACAT GGACAGCCCA

CAGCCGAGAG CAGCAGCGTT GCCATGACTC CCACCTACAT GGACAGCCCA

CAGCGGAGAG CAGCAGTGTT GCCATGACTC CCACCCATGT GGACAGCCCA

CAGCTGAGAA CAGCAGTGTT GCCATGACTC CCACCTATGT GGACAGCCCA

CAGCTGAGAA CAGCAGTGTT GCCATGACTC CCACCTATGT GGACAGCCCA

CAGCCGAGAG CGGCAGCGTT GCCATGACTC CCACCTACGT GGACAGCCCA

CAGCCGAGAG TGGCGGTGTA GCCATGACTC CCACCTATGT GGACAGCCCG

CAGCTGAGAG CAGCAGCGTT GCCATGACTC CCACCTACGT GGACAGCCCA

CCACCGAGAG CAGCAGCGTT GCCATGACTC CCACCTACGT GGACAGCCCA

CAGCCGAGAG CAGTAGCGTT GCTATGACTC CCACCTACGT GGATAGCCCA

CCACCGAAAG CGGCAGTGTT GCCATGACTC CCACCTACGT GGACAGCCCA

CTGCAGAGAG CAGCAGTGTT GCCATGACAC CCACATACGT GGACAGCCCA

CCACAGAGAG CAACAGTGTT GCCATGACAC TCACCTACGT -GACAGCCCA

CAGCAGAGAA TAGTGGTGTG GCCCTCACAC CGACTTACCT GGACAGCCCC

CAATGGAGAG TAGCAGTATT TCTATCACAC CCACTTACCT GGACAGTCCT

CGAAAGGACG GGGCCTTCTT TATGGAGTTT GTCCGCAGCC CACGCACAGC

CGAAAGGACG GGGCCTTCTT TATGGAGTTT GTCCGCAGCC CACGCACAGC

CGAAAGGACG GGGCCTTCTT TATGGAGTTT GTCCGCAGCC CACGCACAGC

CGAAAGGACG GGGCCTTCTT TATGGAGTTT GTCCGCAGCC CACGCACAGC

CGAAAGGACG GGGCCTTCTT TATGGAGTTT GTCCGCAGCC CACGCACAGC

CGGAAGGACA GGGCCTTCTT TATGGAGTTT GTCCGCAGCC CACGCACAGC

CGCAAGGACG GGGCCTTCTT TATGGAGTTT GTCCGCAGCC CACGCACAGC

CGCAAGGACG GGGCCTTCTT TATGGAGTTT GTCCGCAGCC CACGCACAGC

CGAAAGGACG GGGCCTTCTT TATGGAGTTT GTCCGCAGCC CACGCACGGC

CGAAAGGACG GGGCCTTCTT TATGGAGTTT GTCCGCAGCC CGCGCACAGC

CGAAAGGACG GGGCCTTCTT TATGGAGTTT GTCCGCAGCC CTCGCACAGC

CGAAAGGATG GGGCCTTCTT TATGGAGTTT GTCCGCAGCC CGCGCACAGC

CGAAAG---- ------TCTT TATGGAGTTT GTCCGCAGCC CTCGAACAGC

CGAAAGGACG GGGCCTTCTT TATGGAGTTT GTCCGCAGCC CACGCACAGC

CGAAAGGATG GGGCCTTCTT TATGGAGTTT GTCCGCAGCC CACGCACAGC

CGAAAGGATG GGGCCTTCTT TACGGAGTTT GTCCGCAGCC CACGCACAGT

CGAAAGGAGG GGGCCTTCTT TACGGAGTCT GTCCGCAGCC CACGTACAGC

CGCAAGGATG GGGCCTTCTT TATGGAGTTT GTTCGCAGCC CACGTGCCGC

ATCGTCCGCC TTCTACCCTC AGGTATCTGT GGACCAAACA GCCACTCCTA

ATCGTCCGCC TTCTACCCTC AGGTATCTGT GGACCAAACA GCCACTCCTA

ATCGTCCGCC TTCTACCCTC AGGTATCTGT GGACCAAACA GCCACTCCTA

ATCGTCCGCC TTCTACCCTC AGGTATCTGT GGACCAAACA GCCACTCCTG

ATCGTCCACC TTCTACCCTC AGGTATCTGT GGACCAAACG GTCACTCCTG

ATCGTCCACC TTCTACCCTC AGATGTCTAT GGAACAAACA GTCCCTCCGT

ATCATCTGCC TTCTACCCTC AGGCATCTGT GGACCAAACA GCTCCTCTGG

ATCATCTGCC TTCTACCCTC AGGCATCTGT GGACCAAACA GCTCCTCTGG

ATCGTCCGCC TTCTACCCTC AGGTATCTTT GGACCAAACA GCCCCTCCCG

ATCATCCGCC TTCTACCCGC AGGTGTCTCT GGACCAGACG GCCGCCCCGG

GTCATCCGCC TTCTACCCAC AGGTGTCTTT GGACCAGACG GCCACCCCGG

ATCGTCCGCC TCCTACTCTC AGGTGTCTGT GGACCAGACG GCCGTCCCGA

ATCGTCTACC TTCCACTCAC AGGTGTCTGT GGACCAGACG GCTGCGCCAC

ATCGTCTGCC TTCTACCCAC AGGTGTCTGT GGACCAAACA GTTGCTCCAG

ATCGTCTGCC TTCTACCCAC AGGTGTCTGT AGACCAGACG GCTGCTCCAG

ATCGGCCGCC TTCTACCTGC AGGTATCTGT GGACCAGACG GCTGTTCCAG

GCCATCTGCC TTCTACCCCC AGGTCTCGGT GGATCAGAGT GCCACTCCAG

CTCTTCCACC TTCTACCCAC AGGTTTCAGT GGACCAGACC GTTCCTCCCA

TGTTGGACGG CACCAGTTTG GGCATATGCA CAGGCCAATC CATGGACAGA

TGTTGGACGG CACCAGTTTG GGCGTATGCA CAGGCCAATC CATGGACAGA

TGTTGGACGG CACCAGTTTG GGCATAAGCA CAGGCCAATC CGTGGACAGA

TGTTGGACGG CACCAGTTTG GGCATAAGCA CAGGCCAATC CGTGGACAGA

TGTTAGACAG CACCAGTTTG GGCATAAGCA CAGGCCAATC TGTGGACAGA

CATTGGATGG CACCAGTTTG GGGGTAAGCA CAGGCCAATC CGTGGACAGA

TGTTGGACAG CACCAGTTTA GGGGTCAGCA CGTGCCAGTC CGTGGATAGA

TGTTAGACAG CACCAGTTTA GGGGTCAGCA CAGGCCAGCC CATGGATAGA

TGTTGGATGG CACCAGTTTG GGGATAACCA TGGGCCAGTC TGTGGACAGA

TGCTGGACAG CAGCAGCTTG GGGGTGAGCA CAGGCCAGTC CGTGGACAGG

TGTTAGACAG CACCAGTTTA GGGGTAAGCA CAGGCCAGTC TGTGGACAGA

TGTTGGACGG TACCAGTTTG GGGGTAAGCG CAGGTCAATC CGTGGACAGA

TGTTGGATGG CTCCAGTCTG GGGATAAGCA CAGGCCAGTC CATGGACAGA

TCTTGGACGG CACCAGTTTG GGGGTAAGCA CTGGCCAATC CATGGACAGA

TCTTGGATGG CACTAGCCTG GGGGTAAGCA CAGGCCAATC CATGGACAGA

TCTTGGATGG CACGAGCTTG GGGCTAAGCA CAGGCCAGTC CGTGGACAGA

CCTTAGATGG CACCAGCCTG GGCATCAGCG TGGGCCAGCC CATGGACAGA

GCTTAGATGG CTCCAACCTG GGGATAAATG CAGGCCAGTC AACTGATAGA

GGCAACAGCC AGACCTTTGG GAACTCCCAG AACATAGGAG AACAGGGCTA

GGCAACAGCC AGACCTTTGG GAACTCCCAG AACATAGGAG AACAGGGCTA

GGCAACAGCC AGACCTTTGG GAACTCCCAG AACATAGGAG AACAGGGCTA

GGCAACAGCC AGACCTTTGG GAACTCCCAG AACATAGGAG AACAGGGCTA

GGCAACAGCC AGACCTTTGG GAACTCCCAG AACATAGGAG AACAGGGCTA

GGCAACAGTC AGGCCTTTGG GAACTCCCAG AACATAGGAG AACAGGGATT

GGCAATAACC AGACCTTTGG GAACTCCCAG AACATAG--- AACAGGCCTT

GGCAACAACC AGACCTTTGG GAACTCCCAG AACATAG--- AACAGGCCTT

GGCAACAGCC AGACCCTGGG GAACGCACAG AATGTGGGGG AACAGGGCTT

GGCAACAGCC AGACCTTCGG GAACTTGCAG AGCTTGGGGG AACAGGGCTT

GGCAACAGCC AGGCCTTTGG GAACTCCCAG AACTCAGGGG AACAGAGCTT

GGCAACAGCC AGACCTTTGG GAACTCTCAG AACTCAGGGG AACAGGGCTT

GTCAACAGCC AGACCGTTGG GAACACACAG AGCTCAGGGG AACAGGGCTT

GGCAACAGCC AGACCTTTGG GAACTCCCAG AATGCTGGGG AACAGGGCCT

GGCAACAGCC AGACCTTTGG GAACTCCCAG AATGCAGGGG AACAGGGCTT

GGCACCAGCC AGACCTTTGG GAACTCCCAG AACGCAGGGG AACAGGCCTT

GGCAGCACGC AAGCCTTTGG GAATGCCCAG GCTGCCGGAG AGCAGGGGTA

AGCAGCACCC AGCCCTTTGG GAGCTCCCAG AATTTAGGGG AACAGGGCTA

CTCCTCCACA AACTCCAGTG ACAGCAGCAA AATGCTGGAA GAGCAGCTCA

CTCCTCCACA AACTCCAGTG ACAGCAGCAA AATGCTGGAA GAGCAGCTCA

CTCCTCCACA AACTCCAGTG ACAGCAGCAA AATGCTGGAA GAGCAGCTCA

CTCCTCCACA AACTCCAGTG ATAGCAGCAA AATGCTGGAA GAGCAGCTCA

CTCCTCCACA AACTCCAGTG ACAGCAGCAA AATGCTGGAA GAGCAGCTCA

CACCTCTGCA AACTCCAGTG ACAACAGCAA AATGCTGGAA GAGCAGTTCA

TCCCTCTACA GGCTCTGGAG ACGGCAGCAA AATGCTGGAA GAGCAGCTCA

TCCCTCTGCA AACTCTGGTG ACTACAGCAA AATGCTGGAA GAGCAGCTCA

CCCCTCCACC AACTCCAGCG ACAGCAG-AA AATGCTGGAA GAGCAGCTCA

CCCCTCCGCA AACTCCAGCG ATAGCGGTAA AATGCTGGAA GAGCAGCTCA

CCCCTCTGCA AACTCCAGTG ATAGCAGCAA AATGCTGGAA GAGCAACTCA

CACCTCCGCA AACCCCAGTG ACAGCAGCAA AATGCTGGAA GAGCAGCTCA

CCCCTCCGCA AATCCCAGCG ACAGCAGCAA AATGCTGGAA GAGCAGCTCA

TCCTTCTGCA AACCCCAGTG ACAGCAGCAA AATGCTAGAA GAGCAGCTGA

CCCCTCCGCA AACCCTAGTG ACAACAGCAA AATGCTAGAA GAGCAACTCA

CCCCTCCGCA AACCCCAG-- -CGACAGCAA AATGCTGGAA GAACAACTCA

CCCCCTGGCA AACCCTAGCG ATGGCAGCAA AATGCTGGAG GAGCAGCTCA

TGTCCCAGCA AGTTCCATTG ACAGCAG-AA AATGTTGGAG GAGCAGCTGA

TCACACATGC ATCTGGCGAA GCCTGGCGGA CCTTCATCTA CGGCTTCTAT

TCACACATGC ATCTGGCGAA GCCTGGCGGA CCTTCATCTA CGGCTTCTAT

TCACACACGC ATCTGGCGAA GCCTGGCGGA CCTTCATCTA CGGCTTCTAT

TCACACACGC ATCTGGCGAA GCCTGGCGGA CCTTCATCTA CGGCTTCTAT

TCACACACGC ATCTGGCGAA GCCTGGCGGA CCTTCATCTA CGGCTTCTAT

TCATGCATGC ATCTGGGGAA GCCTGGCGGA CCTTCATCTA TGGCTTCTAT

TAGCGCATGC ATCTGGAGAA GCATGGCGAA CCTTCGTCTA CGGCTTCTAT

TAACGCATGC ATCTGGTGAA GCATGGCGGA CCTTCATCTA TGGCTTCTAT

TCACACACGC ATCTGGGGAA GCCTGGCGGA CCTTCATCTA CGGCTTCTAC

TTACACACGC CTCTGGTGAA GCCTGGCGGA CCTTCATCTA CGGCTTCTAT

TCACACACGC ATCTGGTGAA GTCTGGCGGA CCTTCATCTA TGGCTTCTAT

TCACGCACGC ATCTGGGGAA GTCTGGCGGA CCTTCATCTA CGGCTTCTAT

TCACGCACGC GTCTGGTGAA GTCTGGCGGA CCTTTATCTA TGGCTTCTAT

TCACGCACGC ATCTGGTGAA GTCTTGCGGA CCTTCATCTA CGGCTTCTAC

TCACACATGC CTCCGGTGAA GTTTTGCGGA CCTTCATCTA CGGTTTCTAT

TCATACATGC ATCTGGCGAA GCTTTGCGGA CCTTCATCTA TGGCTTCTAT

TTACCCACGC CTCGGGGGAG GCCCTGCGCA CCTTCATCTA CGGCTTCTAC

TCACACATGC CTCTGGGGAA GCCCTGCGAA CCTTCATTTA TGGATTCTAT

TTCTACAAGA TAGTAACGGA CAAAGAGCCC GACCGAGTGG CCATGCAGCA

TTCTACAAGA TAGTAACGGA CAAAGAGCCT GACCGAGTGG CCATGCAGCA

TTCTACAAGA TAGTAACGGA GAAAGAGCCC GACCGAGTGG CCATGCAGCA

TTCTACAAGA TAGTAACGGA GAAAGAGCCC GACCGAGTGG CCATGCAGCA

TTCTACAAGA TAGTAACGGA CAAAGAGCCC GACCGAGTGG CCATGCAGCA

TTCTACAAGA TCGTAGTGGA CAAAGAGCCA GACCGAGTGG CCATGCAGCA

TTCTACAAGA TAGTCACGGA CAGAGAGCCC GAACGAGTGG CTATGCAGCA

TTCTACAAGA TAGTAATGGA CAAAGAACCG GAAAGAGTGG CTATGCAGCA

TTCTACAAGA TCGTAATGGA CAAAGAACCC GACCGAGTGA CCATGCAGCA

TTCTACAAGA TCGTGATGGA CAGAGATCCT GAGCGAGTGG CTGTGCAGCA

TTCTACAAGA TAGTCATGGA CAAAGAACCT GACCGAGTGG CCATGCAGCA

TTCTACAAGA TAGTCATGGA CAAAGAGCCC GACCGAGCGG CCATGCAGCA

TTCTACAAGA TAGTGATGGA CAAAGAGCCC GACAGAGTGG CCCTGCAGCA

TTCTACAAGA TAGTAATGGA CAAAGAGCCT GACCGAGTGG CCATGCAGCA

TTCTACAAGA TAGTAACGGA CAAAGAGCCC GACCGAGTGG CCATGCAGCA

TTCTACAAGA TAGCGATGGA CAGAGAGCCC GATCGAGTGA CCATGCAGCA

TTCTATAAGA TCGTGGCCGA ACGCGAGCCC GAGCGAGGGG GCCTGCAGCA

TTCTACAAGA TAGTCATGGA CAAGGAGCCA GACCGAGTGG GCATGCAGCA

GCCCGCCACC ACCTGGCACA CAGCAGGAGT GGACGACTTC GCCAGCTTCC

GCCTGCCACC ACCTGGCACA CAGCAGGAGT GGACGACTTC GCCAGCTTCC

GCCCGCCACC ACCTGGCACA CAGCAGGAGT GGACGACTTC GCCAGCTTCC

GCCCGCCACC ACCTGGCACA CAGCAGGAGT GGACGACTTC GCCAGCTTCC

GCCGGCCACC ACCTGGCACA CAGCAGGAGT GGATGACTTC GCCAGCTTCC

GCCTGCCGCC ACCTGGCACA CAGCAGGGGG AGACGACTTC GCCAGCTTCC

GCCCTCTGCC CCATGGCACA CAGCAGGAGT GGATGACTTT GCCAGCTTCC

GCCCTCTGCC CCCTGGTACA CAGCAGGAGC GGATGACTTT GCCAGCTTCC

GCCCACCACC ACCTGGCACA CGGCGGGGGT GGACGACTTT GCCAGCTTCC

AC---CCACC ACCTGGCACA CGACGGCAGT GGACGACTTC GCCAGCTTCC

GC---CCACC ACCTGGCACA CGACGGCGGG GGAGGACTTC GCCAGCTTCC

GC---CTACC ACCTGGCACA CCGCAGCGGT GGGCGACTTC GCCAGCTTCC

GC------CC GCCTGGCACA CGACAGCGGT GGACGACTTC GCCAGCTTCC

GC---CCGCC ACCTGGCACA CGACAGCGGT GGACGACTTC ACCAGCTTCC

GC---CTACC ACGTGGCACA CGACAGCAGT AGACGACTTC ACCAGCTTCC

GC---CGGTC ACCTGGCACA CGACAAGCAA GGATGACTTC GCCAGCTTCC

GC---CCGCG GCCTGGCACT CGGCGGCCAT GGAGGACTTC ACGGCCTTCC

GC---CTGCC GCTTGGCACA CAGCAGCAGT GGATGATTTT ACCACCTTCC

AGCGCAAGTG GTTTGAGGTG GCCTTTGTGG CAGAAGAGCT CGTGCACTCT

AGCGCAAGTG GTTTGAGGTG GCCTTTGTGG CAGAAGAGCT CGTGCACTCT

AGCGCAAGTG GTTTGAGGTG GCCTTTGTGG CAGAAGAGCT CGTGCACTCT

AGCGCAAGTG GTTTGAGGTG GCCTTTGTGG CAGAAGAGCT CGTGCACTCT

AGCGCAAGTG GTTTGAGGTG GCCTTTGTGG CAGAAGAGCT CGTGCACTCT

AGCGTAAGTG GTTTGAGGTG GCCTTTGTGG CAGAAGAACT CCTGCACTCC

AACGCAAGTG GTTTGAGGTG GCCTTTGTGG CAGAAGAGCT TGTGCACTCC

AACGCAAGTG GTTTGAGGTG GCCTTTGTGG CAGAAGAGCT TGTGCACTCT

AGCGCAAGTG GTTTGAGGTG GCCTTCGTGG CCGAAGAGCT GCTGCACTCC

AGCGCAAGTG GTTCGAGGTG GCCTTCGTGG CAGAGGAGCT CCTGCACTCG

AGCGCAAGTG GTTTGAGGTG GCCTTTGTGG CCGAAGAGCT GTTGCACTCC

AGCGCAAGTG GTTCGAGGTG GCCTTTGTGG CTGAAGAGCT CTTGCACTCT

AGCGCAAGTG GTTTGAGGTG GCCTTTGTGG CCGAAGAGCT CTTGCACTCG

AGCGAAAGTG GTTTGAGGTA GCCTTTGTGG CCGAAGAGCT CCTTCACTCT

AGCGGAAGTG GTTCGAGGTG GCCTTTGTGG CAGAAGAGCG TTTGCACTCG

AGCGGAAGTG GTTCGAGGTA GCCTTTGTGG CAGAAGAGCG TTTGCACTCT

AGCGGAAGTG GTTCGAGGTG GCCTTCGTGG CCGAGGAGCT GCTGCACTCG

AGAGGAAGTG GTTTGAGGTA GCGTTTGTAG CAGAAGAGCT CTTGCACTCT

GAGATTCCTG CCTTTCTCCT GCCCTGGCTG CCTAGCCGGC CAGCCTCCTA

GAGATTCCTG CCTTTCTCCT GCCCTGGCTG CCTAGCCGGC CAGCCTCCTA

GAGATTCCTG CCTTTCTCCT GCCCTGGCTA CCCAGCCGGC CAGCCTCCTA

GAGATTCCTG CCTTTCTCCT GCCCTGGCTA CCCAGCCGGC CAGCCTCCTA

GAGATTCCTG CCTTTCTCCT GCCCTGGCTG CCCAGCCGGC CAGCCTCTTA

GAGATTCCTG CCTTCCTCCT GCCCTGGCTG CCGAGCCGGC CAGCCTCATA

GAGATTCCTG CCTTCCTCCT GCCCTGGCTA CCTAGCCGGC CAGCCTCTTA

GAGATTCCTG CCTTCCTCCT GCCCTGGCTA CCTAGCCGGC CAGCCTCTTA

GAGATCCCCG CCTTCCTCCT GCCCTGGCTG CCCAGCCGGC CAGCCTCCTA

GAGATCCCCG CCTTCCTGCT GCCCTGGCTG CCCAGCCGGC CAGCCTCCTA

GAGATCCCTG CTTTCCTCTT GCCCTGGCTG CCCAGCCGAC CAGCCTCCTA

GAGATTCCCG CCTTCCTCCT GCCCTGGTTG CCCAGCCGGC CAGCCTCCTA

GAGATTCCGG CTTTCCTGCT GCCCTGGCTG CCCAGCCGAC CTGCCTCCTA

GAGATCCCCG CCTTCCTCCT GCCCTGGCTG CCCAGCCGGC CAGCCTCCTA

GAGATCCCTG CCTTCCTCCT GCCCTGGCTG CCCAGTCGAC CAGCCTCCTA

GAGATTCCCG CCTTCCTCTT GCCCTGGCTG CCCAGCCGGC CGGCCTCCTA

GAGATCCCGG CCTTCCTGCT GCCCTGGCTG CCCAGCCGCC CCGCGTCCTA

GAAATCCCAG GATTCCTCCT GCCTTGGCTG CCCAGCCGCC CAGCCTCTTA

TGCAAGTAGG CACAGCTCCT TTAGCCGAAG TTTTGGAGGA CGGAGCCAGG

TGCAAGTAGG CACAGCTCCT TTAGCCGAAG TTTTGGAGGA CGGAGCCAGG

TGCAAGTAGG CACAGCTCCT TTAGCCGAAG TTTTGGAGGA CGGAGCCAGG

TGCAAGTAGG CACAGCTCCT TTAGCCGAAG TTTTGGAGGA CGGAGCCAGG

TGCAAGTAGG CACAGCTCCT TTAGCCGAAG TTTTGGAGGA CGGAGCCAGG

TGCAAGCAGG CACAGCTCCT TCAGCCGCAG TTTTGGAGGA CGGAGCCAGG

CGCAAGCAGG CACAGCTCCT TTAGCCGAAG TTTTGGAGGA CGGAGCCAGG

TGCAAGCAGA CACAGCTCCT TTAGCCGAAG TTTTGGAGGA CGGAGCCAGG

CGCAAGCAGG CACAGCTCCT TTAGCCGCAG CTTTGGAGGA CGGAGCCAGG

CGCCAGCAGG CACAGCTCCT TTAGCCGCAG CTTTGGGGGC CGCAGCCAGG

TGCCAGCAGG CACAGCTCCT TCAGCCGCAG CTTTGGAGGG CGGAGCCAGG

TGCAAGCAGG CACAGCTCCT TCAGCCGCAG CTTTGGAGGA CGGAGCCAGG

TGCAAGCAGG CACAGCTCCT TCAGCCGCAG CTTCGGCGGC CGGAGCCAGG

TGCAAGCAGG CACAGCTCCT TTAGCCGCAG TTTTGGAGGA CGGAGCCAGG

CGCAAGCAGG CACAGCTCCT TCAGCCGCAG CTTTGGAGGA CGGAGCCAGG

CGCAAGCAGG CACAGCTCCT TCAGCCGCAG CTTCGGGGGA CGGAGCCAGG

CGCCAGCCGC CACAGCTCCT TCAGCCGCAG CTTCGGCGGG AGGAGCCAGG

CGCAAGTAGG CACAGCTCCT TCAGTCGCAG TTTTGGAGGA CGGAGCCAAG

CGGCAGCACT TTTAGCTGCC ACTGTCCCAG AGCAGAGGAC TGTGACCCTG

CGGCAGCACT TTTAGCTGCC ACTGTCCCAG AGCAGAGGAC TGTGACCCTG

CAGCAGCACT TTTAGCTGCC ACTGTCCCAG AGCAGAGGAC TGTGACCCTG

CAGCAGCACT TTTAG----- ---------- ---------- ----------

CAGCTGCACT TTTAGCTGCC ACTGTCCCAG AGCAGAGGAC TGTGACCCTG

CAGCCGCGCT CTTAGCTGCC ACTGTCCCAG AGCAGAGGAC TGTGACCCTG

CAGCTGCACT GTTAGCTGCC ACTGTCCCAG AGCAGAGGAC TGTGACCCTG

CAGCTGCGCT GTTAGCTGCT ACTGTCCCAG AGCAGAGGAC TGTGACCCTG

CAGCTGCGCT TCTAGCCGCC ACGGTCCCAG AGCAGAGGAC CGTCACCCTG

CCGCCGCGCT CTTGGCTGCC ACTGTCCCGG AGCAGAGGAC CGTGACCCTG

CAGCTGCACT TTTAGCTGCC ACTGTCCCAG AGCAGAGGAC TGTGACCCTG

CAGCTGCCCT TTTAGCGGCT ACAGTCCCGG AACAGAGGAC TGTGACCCTG

CTGCTGCGCT CTTAGCTGCC ACCGTCCCTG AGCAAAGGAC AGTCACCCTG

CAGCCGCACT TTTAGCTGCC ACTGTCCCAG AGCAGAGGAC TGTGACCCTG

CAGCCGCACT TTTAGCTGCC ACTGTCCCAG AGCAGAGGAC GGTGACCCTG

CAGCCGCACT TTTAGCTGCC ACTGTCCCAG AGCAGAGGAC CGTGACCCTA

CGGCCGCACT CCTAGCAGCC ACTGTGCCCG AGCAGAGAAC GGTGACCCTG

CAGCTGCACT ATTAGCTGCC ACAGTACCAG AGCAGCGGAC AGTGACCCTG

GATGTTGACG TGAACAACCG CACAGACCGG CTGGAGTGGT GCAGCTGTTA

GATGTTGACG TGAACAACCG CACAGACCGG CTGGAGTGGT GCAGCTGTTA

GATGTTGATG TGAACAACCG CACAGACCGG CTGGAGTGGT GCAGCTGTTA

---------- ---------- ---------- ---------- ----------

GATGTTGATG TGAACAACCG CACAGACCGG CTGGAGTGGT GCAGCTGTTA

GATGTTGATG TGAACAACCG TACAGACCGG CTGGAGTGGT GCAGCTGTTA

GATGTCGATG TGAACAACCG AACGGACCGG CTGGAGTGGT GCAGCTGTTA

GATGTTGATG TGAACAACCG AACGGACCGG CTGGAATGGT GCAGCTGTTA

GACGTCGACG TGAACAACCG CACGGACCGG CTGGAGTGGT GCAGCTGCTA

GACGTGGACG TGAACAACCG CACGGACCGG CTGGAGTGGT GCAGCTGTTA

GATGTGGACG TGAACAACCG CACAGACCGG CTGGAGTGGT GCAGCTGTTA

GACGTGGACG TGAACAACCG CACAGACCGG CTGGAGTGGT GCAGCTGTTA

GATGTCGATG TGAACAACCG CACAGACAGG CTGGAATGGT GTAGCTGCTA

GATGTCGATG TGAACAACCG CACAGACCGG CTGGAGTGGT GCAGCTGTTA

GACGTCGATG TGAACAACCG CACAGACCGG CTCGAGTGGT GCAGCTGCTA

GATGTTGATG TGAACAATCG CACAGATCGG CTGGAGTGGT GCAGCTGCTA

GACGTGGACG TGAACAACCG CACCGACCGG CTGGAGTGGT GCAGCTGCTA

GATGTCGATG TGAACAACCG CACAGACCGG CTGGAGTGGT GTAGTTGTTA

TTACCATGGC AACTTTTCTC TGAATGCAGC CTTTGAGATC AAGCTGCACT

TTACCATGGC AACTTTTCTC TGAACGCAGC CTTTGAGATC AAGCTGCACT

TTACCATGGC AACTTTTCTC TGAACGCAGC CTTTGAGATC AAGCTGCACT

---------- ---------- ---------- ---------- ----------

TTACCATGGC AACTTTTCTC TGAACGCAGC CTTTGAGATC AAGCTGCACT

TTACCATGGC AACTTCTCCC TGAATGCAGC CTTCGAGATC AAGCTGCACT

CTACCACGGA AACTTCTCTC TCAACGCAGC CTTTGAAATC AAGCTACACT

CTACCATGGA AACTTCTCTC TCAATGCCGC CTTTGAGATC AAGCTACACT

CTACCATGGC AACTTCTCCC TGAACGCAGC CTTCGAGATC AAGCTGCACT

CTACCACGGC AACTTCTCTC TGAACGCGGC CTTCGAGATC AAGCTGCACT

TTATCATGGC AATTTCTCCC TGAATGCAGC TTTTGAGATC AAGCTCCACT

TTACCATGGC AACTTCTCCT TGAATGCAGC CTTCGAGATC AAGCTCCACT

TTACCATGGA AACTTCTCCC TGAACGCAGC CTTTGAGATC AAGCTCCACT

TTACCATGGC AACTTTTCCC TGAACGCAGC TTTCGAGATC AAGCTACACT

TTACCATGGC AACTTCTCCC TGAATGCGGC TTTCGAGATC AAGCTACACT

TTACCACGGC AACTTTTCCT TGAATGCAGC TTTTGAGATC AAGCTCCACT

CTACCACGGG AACTTCTCCC TCAACGCCGC CTTCGAGATC AAGCTGCACT

TTATCACGGC AACTTCTCCC TGAATGCTGC CTTTGAGATC AAACTTCACT

GGATGGCGGT GACCGCAGCA GTACTCTTCG AGATGGTCCA AGGTTGGCAT

GGATGGCGGT GACCGCAGCA GTACTCTTCG AGATGGTCCA AGGTTGGCAT

GGATGGCGGT GACCGCAGCA GTACTCTTCG AGATGGTCCA AGGTTGGCAT

---------- ---------- ---------- ---------- ----------

GGATGGCAGT GACCGCAGCA GTACTCTTCG AGATGGTCCA AGGTTGGCAT

GGATGGCAGT GACCGCTGCG GTACTCTTTG AGATGGTCCA AGGTTGGCAT

GGATGGCAGT GACTGCCACA GTGCTGTTTG AGATGGTCCA AGGTTGGCAT

GGATGGCAGT GACTGCCACA GTGCTGTTTG AGATGGTCCA AGGTTGGCAT

GGATGGCAGT GACTGCGGCA GTGCTCTTCG AGATGGTCCA GGGCTGGCAC

GGATGGCTGT GACCGCCGCC GTGCTCTTTG AGATGGTTCA AGGTTGGCAC

GGATGGCTGT GACTGCTGCG GTCCTCTTCG AGATGGTCCA GGGTTGGCAT

GGATGGCGGT GACTGCTGCG GTCCTCTTCG AGATGGTCCA AGGTTGGCAT

GGATGGCGGT GACCGCCGCA GTCCTCTTTG AGATGGTCCA AGGCTGGCAT

GGATGGCAGT GACAGCGGCA GTGCTCTTCG AGATGGTCCA AGGCTGGCAT

GGATGGCAGT GACCTCTGCA GTGCTTTTTG AGATGGTCCA AGGTTGGCAT

GGATGGCGGT GACAGCTGCC GTGCTCTTTG AGATGGTCCA AGGTTGGCAT

GGATGGCCGT GACGGCCGCC GTGCTCTCCG AGATGGTTCA GGGTTGGCAC

GGATGGCGGT GACTGCTGCT GTACTCTTTG AGATGGTTCA AGGTTGGCAT

CGGAAAGCCA CCTCCTGTGG CTTCTTGTTA GTCCCAGTTT TGGAGGGGCC

CGGAAAGCCA CCTCCTGTGG CTTCTTGTTA GTCCCAGTTT TGGAGGGGCC

CGGAAAGCCA CCTCCTGTGG CTTTTTGTTG GTCCCAGTTT TGGAGGGGCC

---------- ---------- ---------- ---------- ----------

CGGAAAGCCA CCTCCTGTGG TTTTTTGTTG GTCCCGGTTT TGGAAGGGCC

CGGAAAGCTA CCTCCTGTGG CTTTTTGTTG GTCCCGGTTT TAGAGGGGCC

CGAAAAGCTA CATCCTGCGG CTTCTTGTTA GTCCCGGTTT TGGAGGGTCC

CGAAAAGCTA CCTCCTGTGG CTTCTTGCTG GTCCCAGTTT TGGAGGGTCC

CGGAAGGCCA CCTCCTGTGG CTTTTTGTTG GTCCCGGTGT TGGAGGGTCC

CGCAAGGCCA CCTCCTGTGG CTTTTTGTTG GTGCCGGTTT TGGAGGGGCC

CGCAAAGCCA CTTCCTGCGG CTTTTTATTG GTCCCTGTTC TGGAGGGTCC

CGAAAAGCCA CCTCTTGTGG CTTTTTATTG GTCCCGGTTT TGGAAGGTCC

CGCAAGGCCA CCTCTTGTGG CTTTTTGCTA GTCCCGGTGT TGGAGGGCCC

CGAAAGGCCA CCTCCTGTGG CTTCCTGCTG GTCCCCGTTT TGGAAGGTCC

CGAAAAGCTA CCTCCTGTGG TTTTCTGCTG GTCCCAGTTT TGGAGGGACC

CGAAAAGCCA CCTCCTGTGG TTTTCTGCTG GTCCCGGTCT TGGAAGGTCC

CGGAAGGCCA CGTCCTGTGG CTTTTTGTTA GTGCCAGTTC TGGAAGGCCC

CGGAAAGCCA CCTCCTGCGG CTTCTTATTG GTTCCAGTTT TGGAAGGCCC

TTTTGCACTG CCCAGTTACC TGTATGGCGA CCCCCTTCGT GCCCAGCTCT

TTTTGCACTG CCCAGTTACC TGTATGGCGA CCCCCTTCGT GCCCAGCTCT

TTTTGCACTG CCCAGTTACC TGTATGGCGA CCCCCTTCGT GCCCAGCTCT

---------- ---------- ---------- ---------- ----------

TTTTGCACTG CCCAGTTACC TGTATGGTGA CCCACTTCGT GCCCAGCTCT

TTTCGCACTG CCCAGCTACC TGTATGGAGA CCCCCTACGA GCTCAGCTCT

TTTTGCACTG CCCAGTTACC TGTACGGTGA CCCCTTGAGG GCCCAGCTCT

TTTTGCTCTG CCCAGTTACC TGTATGGTGA TCCCCTGAGG GCCCAGCTCT

TTTTGCGCTG CCCAGTTACC TGTACGGTGA CCCGCTGCGA GCCCAGCTCT

CTTTGCTCTG CCCAGTTACC TGTACGGCGA CCCGCTCCGG GCCCAGCTGT

CTTTGCGCTG CCCAGTTACC TGTATGGCGA TCCACTTCGA GCCCAGCTCT

CTTTGCTCTG CCCAGTTACC TGTATGGCGA TCCGCTCCGA GCCCAGCTCT

CTTTGCGCTG CCCAGTTACC TGTACGGCGA CCCGCTGCGA GCCCAGCTCT

TTTTGCACTG CCCAGTTACC TTTATGGCGA TCCCCTTCGT GCCCAGCTCT

CTTTGCACTG CCCAGTTACC TGTATGGTGA TCCCCTTCGT GCCCAGCTCT

CTTCGCACTG CCCAGCTACC TGTACGGCGA CCCCCTTCGG GCCCAGCTCT

GTTTGCCCTG CCCAGCTACC TGTATGGAGA CCCGCTGCGA GCCCAGCTGT

ATTTGCACTA CCCAGTTACC TCTATGGCGA CCCACTTCGA GCCCAGTTGT

TCATCCCACT CAACATCAGC TGCTTGCTCA AGGAGGGCAG CGAGCACCTG

TCATCCCACT CAACATCAGC TGCTTGCTCA AGGAGGGCAG CGAGCACCTG

TCATCCCACT CAACATCAGC TGCTTGCTCA AGGAGGGCAG CGAGCACCTG

---------- ---------- ---------- ---------- ----------

TCATCCCACT CAACATCAGC TGCTTGCTAA AGGAGGGCAG CGAGCACCTG

TCATCCCGCT CAACGTCAGC TGCTTGCTCA AGGAGGGCAG TGAGCACCTG

TCATCCCGCT CAACCTCGGC TGCTTGCTCA AGGAGGGCAG CGAGCACCTG

TCATCCCGCT CAACCTCAGC TGCTTGCTCA AGGAGGGCAG CGAGCACCTG

TCATCCCGCT CAACATCAGC TGTCTGCTCA AGGAGGGCAG CGAGCACCTG

TCATCCCACT CAACATCAGC TGCCTGCTCA AGGAGGGCAG CGAGCACCTG

TCATCCCACT CAACACCAGC TGCCTGCTCA AGGAGGGCAG TGAGCACCTG

TCATCCCACT CAACGTCAGC TGTCTGCTCA AGGAGGGCAG TGAGCACCTG

TCATCCCGCT CAACATCAGC TGCCTGCTCA AGGAAGGCAG CGAGCACCTG

TCATCCCACT CAACATCAGC TGCCTGCTCA AGGAGGGCAG TGAGCACCTG

TCATCCCACT CAACATCAGC TGTCTGCTCA AGGAGGGCAG TGAGCACCTG

TCATCCCACT CAGCGTCAGC TGCCTGCTCA AGGAGGGCAG TGAGCACCTG

TTATCCCACT GAACATCAGC TGCCTGCTGA AGGAGGGCAG CGAGCACCTG

TCATTCCGCT CAACATCAGC TGCTTACTGA AGGAAGGCAG TGAACACCTC

TTTGATAGCT TTGAACCCGA AACGTACTGG GATCGAATGC ACCTCTTCCA

TTTGATA--- ---------- ---------- ---------- ----------

TTTGATAGCT TTGAACCGGA AACGTACTGG GATCGAATGC ACCTCTTCCA

-------GCT TTGAACCGGA AACGTACTGG GATCGAATGC ACCTCTTCCA

TTTGATAGCT TTGAACCAGA AACATACTGG GATCGAATGC ATCTCTTTCA

TTCGATAGCT TTGAACCAGA AACATACTGG GATCGAATGC ACCTTTTCCA

TTCGATAGCT TTGAACCAGA GACATATTGG GATCGAATGC ACCTTTTCCA

TTCGATAGCT TTGAACCAGA GACATATTGG GATCGAATGC ATCTTTTCCA

TTTGACA--- ---------- ---------- ---------- ----------

TTTGACGGCT TTGAACCAGA AACATACTGG GACCGAATGC ACCTTTTCCA

TTTGATAGCT TCGAGCCAGA AACATACTGG GATCGAATGC ACCTTTTCCA

TTTGACAGCT TTGAACCGGA GACATACTGG GATCGAATGC ACCTTTTCCA

TTTGACAGTT TTGAGCCAGA AACATACTGG GATCGAATGC ACCTTTTCCA

TTTGACAGCT TTGAACCCGA AACATACTGG GATCGAATGC ACCTTTTCCA

TTTGATAGCT TTGAACCAGA GACATACTGG GATCGAATGC ACCTTTTCCA

TTTGATAGCT TTGAACCAGA GACATACTGG GATCGGATGC ATCTTTTCCA

TTTGACAGCT TTGAACCAGA GACTTACTGG GATCGGATGC ACCTCTTCCA

TTCGATAGTT TTGAACCCGA AACTTACTGG GATCGAATGC ATCTCTTTCA

GGAAGCCATT GCACACAGGT TTGGGTTTGT ACAAGATAAA TATTCTGCCT

---------- --------GT TTGGGTTTGT ACAAGATAAA TATTCTGCCT

GGAAGCCATT GCACACAGGT TTGGGTTTGT ACAAGATAAA TATTCTGCCT

GGAAGCCATT GCACACAG-- ---------- ---------- ----------

GGAAGCCATT GCACACAGGT TTGGATTTGT ACAAGATAAA TATTCTGCTT

GGAAGCCATT GCACACAGGT TTGGGTTTGT ACAGGATAAA TACTCTGCCT

GGAAGCCATT GCACACAGGT TTGGATTTGT GCAAGATAAA TATTCTGCCT

GGAAGCCATT GCACACAGGT TTGGATTTGT GCAAGATAAA TATTCTGTCT

---------- --------GT TTGGCTTCGT ACAAGATAAA TACTCTGCCT

GGAAGCCATC GCGCACAGGT TTGGGTTTGT TCAAGATAAG TATTCTGCGT

GGAAGCCATT GCACACAGGT TTGGATTTGT GCAAGATAAA TATTCCGCCT

GGAAGCCATC GCACACAGGT TTGGGTTTGT ACAAGATAAA TATTCTGCGT

GGAAGCCATA GCACACAG-- --------GT GCATGACAAA TATTCTGCCT

GGAGGCCATT GCACACAGGT TTGGGTTTGT ACAAGATAAA TATTCTGCCT

GGAAGCCATT GCACACAGGT TTGGGTTTGT ACAAGATAAA TATTCTGCCT

GGAAGCCATT GCACACAG-- ---------- ---------- ----------

AGAAGCTGTG GCTCACAGAT TCGGATTTGT ACAAGACAAA TATTCTGCCT

GGAGGCCATT GTTCACAGGT TTGGATTCGT GCAGGATAAA TACTCTGCTT

CTGCTTTTAA CTTCCCTGCT GAGAACAAGC CTCAGTATAT CCACGTTACA

CTGCTTTTAA CTTCCCTGCT GAGAACAAGC CTCAGTATAT CCACGTTACA

CTGCTTTTAA CTTCCCTGCT GAGAACAAGC CTCAGTACAT CCACGTTACA

---------- ---------- ---------- ---------- ----------

CTGCCTTTAA CTTCCCTGCT GAGAACAAGC CTCAGTACAT CCACGTTACA

CTGCCTTTAA CTTTCCTGCT GAGAACAAGC CTCAGTACAT CCATGTTACA

CCGCGTTTAA CTTCCCTGCT GAGAATAAAC CACAGTATAT CCACGTCACA

CCGCTTTTAA CTTCCCTGCT GAGAATAAGC CACAGTATAT CCATGTCACA

CCGCCTTTAA CTTCCCAGCT ---------- ---------- ----------

CCGCTTTCAA CTTCCCAGCC GAGAACAAGC CCCAGTACAT CCATGTCACA

CTGCTTTTAA CTTCCCTGCT GAGAACAAGC CTCAGTATAT CCATGTTACA

CTGCTTTTAA CTTTCCAGCT GAGAACAAGC CTCAGTACAT CCACGTTACA

CTGCTTTTAA CTTTCCTGCC GAGAACAAGC CTCAGTACAT TCACGTTACA

CTGCTTTTAA CTTCCCTGCT GAGAACAAGC CTCAGTACAT CCATGTTACA

CTGCTTTTAA CTTCCCTGCT GAGAACAAGC CTCAGTACAT CCACGTTACA

---------- ---------- ---------- ---------- ----------

CCGCCTTCAA CTTCCCTTCT GAGAACAAGC CCCAGTACAT CCACGTCACA

CTGCTTTCAA CTTTCCATCA GAGAATAAAC CACAGTATAT CCATGTTACA

GGAACAGTGT TTCTGCAGCT GCCCTACTCC AAGCGCAAGT TCTCAGGGCA

GGAACAGTGT TTCTGCAGCT GCCCTACTCC AAGCGCAAGT TCTCAAGGCA

GGAACAGTGT TTCTGCAGCT GCCCTATTCC AAGCGCAAGT TCTCAGGGCA

---------- ---------- ---------- ---------- ----------

GGAACAGTGT TTCTGCAGCT GCCCTACTCC AAGCGCAAGT TCTCGGGGCA

GGAACGGTAT TTCTGCAGCT GCCGTACTCC AAGCGCAAGT TCTCAGGGCA

GGAACAGTGT TTCTTCAGCT GCCGTACTCC AAACGCAAGT TTTCTGGGCA

GGAACGGTGT TCCTTCAGCT GCCGTACTCC AAACGCAAGT TTTCAGGGCA

---------- ---------- ---------- ---------- ----------

GGGACAGTGT TCCTGCAGCT GCCCTACTCC AAGCGCAAGT TCTCAGGGCA

GGAACAGTGT TCCTGCAGCT GCCTTACTCC AGGCGCAAGT TCTCCGGGCA

GGAACAGTGT TCCTGCAGCT GCCCTACTCC AAGCGCAAGT TCTCCGGGCA

GGAACAGTCT TCCTGCAACT GCCCTACTCC AAGCGCAAGT TCGCGGGCCA

GGGACAGTGT TTCTGCAGCT GCCCTACTCC AAGCGCAAGT TCTCGGGACA

GGAACAGTGT TTCTGCAGCT GCCCTACTCC AAGCGGAAGT TCTCAGGGCA

-GAACAGTGT TTCTGCAGCT GCCCTACTCG AAGCGGAAGT TCTCAGGACA

GGGACTGTCT TCCTGCAGCT GCCCTACTCC AAGCGCAAGT TCTCGGGCCA

GGGACCGTCT TCCTGCAACT ACCCTACTCC AAGAGGAAGT TCTCTGGACA

GCAGCGGCGG CGGCGGAACT CCACCAGCTC CACCAACCAG AACATGTTCT

GCAGCGGCGG CGGCGGAACT CCACCAGCTC CACCAACCAG AACATGTTCT

GCAGCGGCGG CGGCGGAACT CCACCAGCTC CACCAACCAG AACATGTTCT

---------- ---------- ---------- ---------- ----------

GCAGCGGCGG CGGCGGAACT CCACCAGCTC CACCAACCAG AACATGTTCT

GCAGCGGCGG CGGCGGAACT CCACCAGCTC CACCAACCAG AACATGTTCT

GCAGCGGCGG CGACGGAACT CTACCAGCTC CACCAACCAA AACATGTTCT

GCAGCGGCGG CGACGGAACT CTACCAGCTC CACCAACCAA AACATGTTCT

---------- ---------- ---------- ---------- ----------

GCCGCGGCGG CGCCGCAACT CCACCAGCTC CGCCAGCCAG ACCCTGTTCT

GCAGCGGCGG CGGCGGAACT CCACCAGTTC CACCAACCAG AACTTGTTCT

GCAGCGGCGA CGGCGGAACT CCACCAGCTC CACCAACCAG AACATGTTCT

GCCGCGGCGG CGACGGAATT CCACCAGCTC CACCAGCCAG AACATGTTCT

ACAGCGGCGG CGGCGGAACT CCACCAGTTC CACCAACCAG AACATGTTCT

GCAGCGTCGG CGGCGGAACT CCACCAGCTC TACCGGCCAG AACATGTTCT

GCAGCGGCGG AGGCGGAACT CCACCAGCTC CACCAGCCAG AACATGTTCT

GCAGCGGCGC AGGCGCAACT CGACCAGCTC CACCGGCCAG AACATGTTCT

GCAACGGCGC CGACGGAATT CCACCAGCTC CACCAACCAG AACATGTTCT

GCGAGGAGCG GGTCGGCTAC AACTGGGCCT ACAACACCAT GCTCACCAAA

GCGAGGAGCG GGTCGGCTAC AACTGGGCCT ACAACACCAT GCTCACCAAA

GCGAGGAGCG GGTCGGCTAC AACTGGGCCT ACAACACCAT GCTAACCAAG

---------- ---------- ---------- ---------- ----------

GCGAGGAGCG GGTCGGCTAC AACTGGGCCT ACAACACCAT GCTCACCAAG

GCGAGGAGCG GGTTGGCTAC AACTGGGCCT ACAACACGAT GCTGACCAAG

GTGAGGAGCG GGTTGGCTAC AACTGGGCCT ATAACACCAT GCTGACCAAG

GTGAGGAGCG GGTTGGCTAC AACTGGGCCT ACAACACCAT GCTGACCAAG

---------- ---------- ---------- ---------- ----------

GCGAGGAGCG CGTGGGCTAC AACTGGGCCT ACAACACCAT GCTCACCAAG

GTGAGGAGCG TGTTGGCTAC AACTGGGCCT ACAACACCAT GCTGACCAAG

GCGAGGAGCG GGTCGGCTAT AACTGGGCCT ACAACACCAT GCTGACCAAG

GCGAGGAGCG GGTGGGCTAC AACTGGGCCT ACAACACCAT GCTGACCAAG

GCGAGGAGCG GGTTGGTTAC AACTGGGCCT ACAACACCAT GCTGACCAAG

GCGAGGAGCG GGTTGGCTAC AACTGGGCCT ATAACACCAT GCTGACCAAG

GCGAGGAGCG GGTGGGCTAC AACTGGGCCT ACAACACCAT GCTGACCAAG

GCGAGGAGCG CGTGGGCTAC AACTGGGCCT ACAACACCAT GCTGACCAAG

GTGAGGAGCG GGTGGGTTAC AACTGGGCCT ACAACACTAT GCTGACTAAA

ACATGGCGCT CCAGCGCCAC AGGGGATGAA AAGTTTGCTG ATCGGCTGCT

ACATGGCGCT CCAGCGCCAC AGGGGATGAA AAGTTTGCTG ATCGGCTGCT

ACATGGCGCT CCAGTGCCAC AGGGGATGAA AAGTTTGCTG ATCGGCTGCT

---------- ---------- ---------- ---------- ----------

ACATGGCGCT CCAGTGCCAC AGGGGATGAA AAATTTGCTG ATCGGCTGCT

ACATGGCGCT CCAGTGCCAC GGGGGATGAG AAATTTGCCG ACCGGCTGCT

ACATGGCGCT CTAGTGCCAC GGGGGATGAG AAGTTTGCCG ATCGGCTGCT

ACATGGCGCT CTAGTGCCAC AGGGGACGAG AAGTTTGCTG ATCGGCTGCT

---------- ---------- ---------- ---------- ----------

ACCTGGCGCT CCAGCGCCAC CGGCGACGAG AGGTTCGCCG ACCGGCTGCT

ACATGGCGTT CTAGTGCCAC GGGGGATGAG AAGTTTGCTG ATCGGCTGCT

ACGTGGCGCT CCAGCGCCAC AGGGGACGAG AAGTTTGCTG ATCGGCTGCT

ACGTGGCGCT CCAGCGCCAC GGGGGACGAG AAGTTTGCCG ACCGGCTGCT

ACGTGGCGCT CCAGCGCCAC GGGAGACGAG AAGTTTGCTG ACCGGCTGCT

ACATGGCGCT CCAGTGCCAC AGGGGACGAG AAGTTTGCCG ACCGGTTGCT

ACCTGGCGCT CCAGTGCCAC CGGGGACGAG AAGTTTGCCG ACCGGCTGCT

ACGTGGCGCT CCAGCGCCAC CGGGGACGAG AAGTTTGCCG ACCGGCTGCT

ACCTGGCGCT CCAGCGCCAC GGGCGACGAG AAGTTTGCCG ATAGGCTGCT

GAAGGACTTC ACGGACTTCT GCATCAACCG TGACAACCGG CTGGTCACGT

GAAGGACTTC ACGGACTTCT GCATCAACCG TGACAACCGG CTGGTCACGT

GAAGGACTTC ACAGACTTCT GCATCAACCG TGACAACCGT CTGGTCACGT

---------- ---------- ---------- ---------- ----------

GAAGGACTTC ACAGACTTCT GCATCAACCG TGACAACCGG CTGGTTACAT

GAAGGACTTC ACAGACTTCT GCATTAACCG TGACAACCGG CTGGTCACGT

GAAGGACTTC ACGGATTTCT GTATCAACCG TGACAACCGC CTGGTCACAT

GAAGGACTTC ACGGATTTCT GCATCAACCG TGACAACCGA CTGGTCACGT

---------- ---------- ---------- ---------- ----------

GAAGGACTTC ACAGACTTCT GTGGCAACCG CGACAGCCGG CTGGTGGCCT

CAGGGATTTC ACGGACTTCT GCATCAACCG CGACAACCGG CTGGTCACAT

GAAGGACTTC ACGGATTTCT GTATCAACCG CGACAACCGA CTCGTCACGT

GAAGGACTTC ACGGACTTTT GCGTCAATCG TGACAACCGC CTGGTGACGT

GAAGGACTTC ACAGACTTCT GCATCAACCG AGACAACCGG CTGGTCACAT

GAAGGACTTC ACGGACTTCT GTGTCAACCA AGACAACCGG CTGGTCACGT

GAAGGACTTC ACCGACTTCT GTGTCAACCG AGACAACCGG CTGGTCACAT

CAAGGACTTC ACCGACTTCT GCGTCAACCG CGACAACCGC CTCGTGGCGT

GAAGGACTTT ACGGAGTTCT GTGTTAACCG AGACAACAGG CTGGTCGCCT

TCTGGACAAG TTGCCTGGAG AAGATGCATG CCAGTGCCCC GGTGGCCCGA

TCTGGACAAG TTGCCTGGAG AAGATGCATG CCAGTGCCCC GG--------

TCTGGACAAG TTGCCTGGAG AAGATGCATG CCAGTGCCCC GGTGGCCCGA

---------- ---------- ---------- ---------- -GTGGCCCGA

TCTGGACAAG TTGCCTAGAG AAGATGCATG CCAGTGCCCC GGTGGCCCGA

TCTGGACAAG TTGCCTGGAG AAGATGCACG CCAGTGCCCC T---------

TCTGGACAAA CTGCCTGGAG AAGATGCACG CCAGCGCTCC CGTGGCCCGA

TCTGGACAAA CTGCCTGGAG AAGATGCATG CCAGTGCTCC AGTGGCCCGA

---------- ---------- ---------- ---------- -G--------

TCTGGGCCAG CTGCCTGGAG AAGATGCACG CCAGCGCCCC GGTGGCCAGA

TCTGGATGAG CTGCCTGGAG AAGATGCATG CCAGTGCCCC GGTGGCCCGA

TCTGGACGAG CTGCCTGGAG AAGATGCATG CCAGCGCCCC G---------

TCTGGACGAG CTGCCTGGAG AAGATGCATG CCAGCGCCCC GGTGGCCCGG

TCTGGGCAAG TTGCCTGGAG AAGATGCATG CCAGTGCCCC A-TGGCCCGA

TCTGGACAAG TTGCCTGGAG AAGATGCATG CCAGTGCCCC AGTGGCTCGA

TTTGGACAAG CTGCCTGGAG AAGATGCACG CCAGTGCCCC C---------

TCTGGGCCAG CTGTGTGGAC AAGATGCACG CCAGCGCGCC CGTGGCACGG

TCTGGACCGG CTGTCTGGAG AAAATGCATG CCAGTGCCCC CGTGGCTCGA

ACCTGTAACT TGATCTTGAT TGTTCTGGAT GTCCTGAAAC CTTTGGGACA

---------- ---------- ---------- ---------- ----------

ACCTGTAACT TGATCTTGAT TGTTCTGGAT GTCCTGAAAC CTTTGGGACA

ACCTGTAACT TGATCTTGAT TGTTCTGGAT GTCCTGAAAC CTTTGGGACA

ACCTGTAACT TGATCCTGAT TGTTTTGGAT GTCCTGAAAC CCTTGGGACA

---------- ---------- ---------- ---------- ----------

ACGTGTAATT TGATCTTGAT TGTTCTGGAT GTCCTAAAAC CCTTGGGGCA

ACGTGTAATT TGATTTTGAT TGTTCTGGAT GTCCTAAAAC CCTTGGGACA

---------- ---------- ---------- ---------- ----------

ACGTGTAACT TGATCTTGAT TGTTCTGGAT GTCCTGAAAC CCTTGGGACA

ACATGTAACT TGATCCTTAT TGTTCTGGAT GTCCTGAAAC CCTTGGGACA

---------- ---------- ---------- ---------- ----------

ACCTGTAACT TGATTCTGAT TGTTCTGGAT GTTCTGAAAC CCTTGGGACA

ACCTGTAACT TGATCCTGAT TGTTCTGGAT GTCCTTAAAC CCTTGGGGCA

ACCTGTAACT TGATCCTGAT TGTTCTGGAT GTCCTGAAAC CCTTGGGACA

---------- ---------- ---------- ---------- ----------

ACCTGTAATT TAATTCTGAT TGTTCTGGAT GTGCTGAAGC CGCTGGGACA

ACCTGTAACT TGATCCTGAT TGTCCTGGAT GTACTGAAGC CCCTCGGACA

TAAGAAGATA ATTGAAAATG AGCTGGAAGG CTTTGGCATT CGCTTGAACA

---------- ---------- ---------- ---------- ----------

TAAGAAGATA ATTGAAAATG AGCTGGAAGG CTTTGGCATT CGCTTGAACA

TAAGAAGATA ATTGAAAATG AGCTGGAAGG CTTTGGCATT CGCTTGAACA

TAAGAAGATA ATTGAAAATG AACTGGAAGG CTTTGGCATT CGCTTGAACA

---------- ---------- ---------- ---------- ----------

CAAGAAGATA ATTGAAAATG AGTTGGAAGG CTTTGGGATT CGTTTGAACA

TAAGAAGATA ATTGAAAATG AGTTGGAAGG CTTTGGGATT CGCTTGAACA

---------- ---------- ---------- ---------- ----------

TAAGAAGATA ATTGAAAATG AGCTGGAAGG CTTTGGCATT CGCTTGAACA

TAAAAAGATA ATTGAAAATG AGCTGGAAGG CTTTGGCATT CGTTTGAACA

---------- ---------- ---------- ---------- ----------

TAAGAAGATA ATTGAAAATG AGCTGGAAGG CTTTGGTATT CGCTTAAACA

TAAGAAGATA ATTGAAAATG AGCTGGAGGG TTTTGGCATT CGCTTGAACA

TAAGAAGATA ATTGAAAACG AGCTGGAAGG CTTTGGTATT CGCTTGAACA

---------- ---------- ---------- ---------- ----------

CAAGAAGATC ATCGAGAATG AGCTCGAGGG CTTTGGGATC CGCTTAAACA

CAAGAAAATC ATTGAGAATG AACTGGAAGG CTTTGGAATC CGTTTGAACA

GCAAACCCCC CAACATTGGC TTTAAGAAGA AGGACAAGGG AGGCATTAAT

---------- ---------- ---------- ---------- ----------

GCAAACCCCC CAACATTGGC TTTAAGAAGA AAGACAAGGG AGGCATTAAT

GCAAACCCCC CAACATTGGC TTTAAGAAGA AAGACAAGGG AGGCATTAAT

GCAAACCCCC CAACATTGGC TTTAAGAAGA AGGACAAGGG AGGCATTAAT

---------- ---------- ---------- ---------- ----------

GCAAACCCCC CAACATTGGC TTTAAGAAGA AAGACAAGGG AGGCATTAAT

GCAAACCCCC CAACATTGGC TTTAAGAAGA AAGACAAGGG AGGCATTAAC

---------- ---------- ---------- ---------- ----------

GCAAACCCCC CAACATTGGC TTTAAGAAGA AGGATAAAGG AGGCATTAAT

GCAAACCCCC CAACATTGGC TTTAAGAAGA AGGATAAAGG AGGCATTAAT

---------- ---------- ---------- ---------- ----------

GCAAACCCCC TAACATTGGC TTTAAGAAAA AGGATAAAGG AGGCATTAAT

GCAAGCCTCC CAACATCGGC TTTAAGAAGA AGGATAAAGG AGGCATTAAT

GCAAAGCCCC CAACATTGGC TTTAAGAAGA AGGACAAGGG AGGCATTAAT

---------- ---------- ---------- ---------- ----------

GCAAGCCCCC CAACATAGGC TTTAAGAAGA AGGACAAAGG AGGCATCAAC

GCAAACCTCC CAACATTGGC TTTAAGAAGA AGGACAAAGG AGGCATCAAT

CTCACAGCCA CTTGCCCCCA GAGTGAGCTG GATGCTGAAA CTGTGAAGAG

---------- --TGCCCCCA GAGTGAGCTG GATGCTGAGA CTGTGAAGAG

CTCACAGCCA CTTGCCCCCA GAGTGAGCTA GATGCTGAAA CTGTGAAGAG

CTCACAGCCA CTTGCCCCCA GAGTGAGCTA GATGCTGAAA CTGTGAAGAG

CTCACAGCTA CTTGCCCCCA GAGTGAGCTG GATGCTGAAA CTGTGAAAAG

---------- ---------- ---------- ---------- ----------

CTCACAGCCA CTTGTCCTCA GAGTGAACTA GATGCTGAAA CGGTGAAGAG

CTCACAGCCa ctTGTCCTCA GAGTGAACTG GATGCTGAAA CAGTGAAGAG

---------- ---------- ---------- ---------- ----------

CTCACAGCCA CTTGCCCTCA GAGTGAGCTG GATGCTGAAA CTGTGAAGAG

CTCACGGCCA CTTGCCCTCA GAGTGAGCTG GATGCTGAGA CTGTGAAGAG

---------- ---------- ---------- ---------- ----------

CTCACAGCCA CTTGCCCTCA GAGTGAGCTG GATGCTGAAA CTGTGAAGAG

CTCACAGCCA CTTGCCCACA AAGTGAGCTG GATGCTGACA CTGTGAAGAG

CTCACAGCCA CCTGCCCTCA GAGTGAGCTG GATGCTGAAA CTGTGAAGAG

---------- ---------- ---------- ---------- ----------

CTCACGGCCA CGTGCCCTCA GAGTGAGCTC GATGCTGACA CCGTGAAGAG

CTCACAGCTA CTTGCCCTCA GAGTGAACTG GATGCAGAGA CAGTGAAGAG

CATTCTGGCT GAATACAAGA TTCATAATGC CGATGTGACT CTACGTAGTG

CATTCTGGCT GAATACAAGA TTCATAATGC CGATGTGACT CTACGTAGTG

CATTCTGGCC GAATACAAGA TTCATAATGC TGATGTGACT CTACGTAGTG

CATTCTGGCC GAATACAAGA TTCATAATGC TGATGTGACT CTACGTAGTG

CATTCTGGCC GAATACAAGA TTCATAATGC TGATGTGACT CTACGTAGTG

---------- ---------- ---------- ---------- ----------

CATTCTGGCT GAATATAAAA TTCATAATGC TGATGTAACT TTGCGTAGTG

CATTCTGGCT GAATATAAGA TTCATAATGC TGATGTGACT TTGCGTAGTG

---------- ---------- ---------- ---------- ----------

CATTCTGGCT GAGTACAAAA TCCATAATGC TGATGTGACT CTGCGTAGTG

CATTCTGGCT GAATACAAAA TTCACAATGC TGATGTGACG CTGCGTAGTG

---------- ---------- ---------- ---------- ----------

CATTCTGGCT GAATACAAAA TTCATAATGC TGATGTGACT CTGCGTAGCG

CATCTTGGCG GAATACAAAA TTCATAATGC TGATGTGACT CTGCGTAGTG

CATTCTGGCT GAATACAAAA TTCATAATGC CGATGTGACT CTGCGAAGCG

---------- ---------- ---------- ---------- ----------

TATCCTGGCA GAGTACAAAA TCCACAACGC TGATGTGACC CTGCGCAGCG

CATTTTAGCA GAATATAAAA TCCACAATGC TGATGTGACC CTACGCAGCG

ATGCTACAGC TGATGACCTC ATTGATGTGG TGGAAGGAAA CAGAGTTTAT

ATGCTACAGC TGATGACCTC ATTGATGTGG TGGAAGGAAA CAGAGTTTAT

ATGCTACAGC TGATGACCTC ATTGATGTGG TGGAAGGAAA CAGAGTTTAT

ATGCTACAGC TGATGACCTC ATTGATGTGG TGGAAGGAAA CAGAGTTTAT

ATGCTACAGC TGATGACCTC ATTGATGTGG TGGAAGGAAA CAGAGTTTAT

---------- ---------- ---------- ---------- ----------

ATGCCACAGC TGATGACCTC ATCGACGTGG TTGAAGGAAA CAGAGTTTAT

ATGCCACAGC TGACGACCTC ATTGACGTGG TCGAAGGGAA CAGAGTTTAT

---------- ---------- ---------- ---------- ----------

ACGCCACAGC AGATGACCTC ATTGATGTGG TGGAAGGAAA CAGAGTCTAT

ATGCCACAGC AGACGACCTC ATCGATGTGG TGGAAGGAAA CAGAGTTTAT

---------- ---------- ---------- ---------- ----------

ATGCCACAGC AGATGACCTC ATTGATGTAG TGGAAGGAAA CAG-------

ATGCCACAGC TGATGACCTC ATTGATGTGG TGGAAGGAAA CAGAGTTTAT

ATGCCACAGC TGATGACCTC ATTGATGTGG TGGAAGGAAA TAGAGTTTAT

---------- ---------- ---------- ---------- ----------

ACGCCACGGC CGACGACCTC ATTGATGTGG TGGAAGGCAA CAGAGTTTAC

ATGCCACTGC CGATGACCTC ATTGATGTGG TGGAAGGCAA CAGAGTTTAC

ATCCCCTGTA TCTATGTGTT AAATAAGATT GACCAAATCT CCATTGAGGA

ATCCCCTGTA TCTATGTGTT AAATAAGATT GACCAAATCT CCATTGAGGA

ATCCCCTGTA TCTATGTGTT AAATAAGATT GACCAAATCT CCATTGAGGA

ATCCCCTGTA TCTATGTGTT AAATAAGATT GACCAAATCT CCATTGAGGA

ATCCCCTGTA TCTATGTGTT AAATAAGATT GACCAAATCT CCATTGAGGA

---------- ---------- ---------- ---------- ----------

ATTCCCTGTA TCTATGTATT AAATAAGATT GATCAGATCT CCATTGAGGA

ATTCCCTGTA TCTATGTATT AAATAAGATT GATCAGATCT CCATTGAGGA

---------- ---------- ---------- ---------- ----------

ATCCCCTGTA TCTATGTGTT AAATAAGATT GATCAGATTT CCATTGAGGA

ATCCCTTGTA TCTATGTGTT GAATAAGATT GATCAGATCT CCATTGAGGA

---------- ---------- ---------- ---------- ----------

---------- ---------- ---------- ---------- ----------

ATCCCCTGTA TCTATGTGTT AAATAAGATT GACCAGATCT CCATTGAGGA

ATCCCCTGTA TCTATGTGTT AAATAAGATT GATCAGATCT CCATTGAGGA

---------- ---------- ---------- ---------- ----------

ATCCCTTGTA TCTACGTGCT GAACAAGATC GACCAGATCT CCATCGAGGA

ATCCCTTGCA TTTATGTGCT GAATAAGATT GATCAGATCT CCATCGAGGA

ATTGGATATC ATCTATAAGG TGCCTCACTG TGTACCCATC TCTGCCCATC

ATTGGATATC ATCTATAAGG TGCCTCACTG TGTACCCATC TCTGCCCATC

ATTGGATATC ATCTATAAGG TGCCTCACTG TGTACCCATC TCTGCCCATC

ATTGGATATC ATCTATAAGG TGCCTCACTG TGTACCCATC TCTGCCCATC

ATTGGATATC ATCTATAAGG TGCCTCACTG TGTACCCATC TCTGCCCATC

---------- ---------- ---------- ---------- ----------

GTTGGATATT ATATATAAGG TGCCTCACTG TGTACCCATC TCTGCTCATC

GTTGGATATT ATCTATAAGG TGCCTCACTG TGTACCCATC TCTGCTCATC

---------- ---------- ---------- ---------- ----------

GTTGGATATT ATCTATAAGG TGCCACACTG TGTACCCATC TCTGCCCATC

ATTGGATATC ATCTATAAGG TGCCTCACTG TGTACCCATT TCTGCCCATC

---------- ---------- ---------- ---------- ----------

---------- ---------- ---------- ---------- ----------

ATTGGATATC ATCTATAAGG TACCTCACTG CGTACCCATC TCTGCCCACC

ATTGGATATC ATCTATAAGG TACCTCACTG TGTACCCATC TCTGCCCACC

---------- ---------- ---------- ---------- ----------

GCTGGACATC ATCTATAAGG TGCCGCACTG TGTGCCCATC TCTGCTCACC

GCTCGACATT ATCTACAAGG TGCCTCATTG CGTGCCGATC TCCGCCCATC

ACCGCTGGAA TTTTGATGAC CTATTGGAAA AGATCTGGGA CTATCTGAAA

ACCGCTGGAA TTTTGATGAC CTATTGGAAA AGATCTGGGA CTATCTGAAA

ACCGCTGGAA TTTTGATGAC CTATTGGAAA AGATCTGGGA CTATCTGAAA

ACCGCTGGAA TTTTGATGAC CTATTGGAAA AGATCTGGGA CTATCTGAAA

ACCGTTGGAA TTTTGATGAC CTATTGGAAA AGATCTGGGA CTATCTGAAA

---------- ---------- ---------- ---------- ----------

ACCGCTGGAA TTTTGATGAC CTTTTGGAGA AGATCTGGGA CTATCTGAAA

ACCGCTGGAA TTTTGATGAC CTTTTGGAGA AAATCTGGGA CTATCTGAAA

---------- ---------- ---------- ---------- ----------

ACCGCTGGAA TTTTGATGAC CTGTTGGAAA AGATCTGGGA CTATCTGAAA

ACCGCTGGAA TTTTGATGAC CTATTGGAAA AGATCTGGGA CTATCTGAAA

---------- ---------- ---------- ---------- ----------

---------- ---------- ---------- ---------- ----------

ACCGTTGGAA TTTTGATGAC CTGTTGGAAA AGATCTGGGA CTATCTGAAA

ACCGCTGGAA TTTTGATGAC CTGTTGGAAA AGATCTGGGA CTATCTGAAA

---------- ---------- ---------- ---------- ----------

ACCGCTGGAA TTTTGATGAC CTGCTCGAGA GAATCTGGGA CTACCTGCGC

ACCGTTGGAA CTTTGATGAC CTGCTGGAGA GGATTTGGGA TTACTTGAGA

CTAGTGAGAA TTTACACCAA ACCCAAAGGC CAGTTACCAG ATTACACATC

CTAGTGAGAA TTTACACCAA ACCCAAAGGC CAGTTACCAG ATTACACATC

CTAGTGAGAA TTTACACCAA ACCCAAAGGC CAATTACCAG ATTACACATC

CTAGTGAGAA TTTACACCAA ACCCAAAGGC CAATTACCAG ATTACACATC

CTAGTGAGAA TTTACACCAA ACCCAAAGGC CAGTTACCAG ATTACACATC

---------- ---------- ---------- ---------- ----------

CTAGTGAGAA TTTACACTAA ACCCAAAGGC CAGTTACCAG ATTACACGTC

CTAGTGAGAA TTTACACCAA ACCGAAAGGC CAGTTACCAG ATTACACATC

---------- ---------- ---------- ---------- ----------

CTAGTGAGGA TTTACACCAA ACCCAAGGGC CAGTTGCCCG ATTACACGTC

CTAGTGAGGA TTTACACCAA ACCCAAAGGC CAGTTGCCTG ATTATACATC

---------- ---------- ---------- ---------- ----------

---------- -TTATACAAA ACCCAAAGGG CAGTTGCCAG ATTACACATC

CTAGTAAGGA TTTACACCAA GCCCAAAGGC CAGTTACCAG ACTACACATC

CTAGTGAGGA TTTACACAAA ACCCAAAGGC CAGTTACCAG ACTACACATC

---------- ---------- ---------- ---------- ----------

CTAGTGAGGA TCTACACCAA GCCCAAGGGG CAGCTGCCAG ACTACACCTC

CTAGTGCGAA TCTACACCAA ACCCAAAGGC CAGTTACCAG ACTATACATC

CCCAGTGGTG CTTCCTTACT CCAGGACCAC AGTGGAGGAT TTCTGCATGA

CCCAGTGGTG CTTCCTTACT CCAGGACCAC AGTGGAGGAT TTCTGCATGA

CCCAGTGGTA CTTCCTTACT CCAGGACCAC AGTGGAGGAT TTCTGCATGA

CCCAGTGGTA CTTCCTTACT CCAGGACCAC AGTGGAGGAT TTCTGCATGA

CCCAGTGGTG CTTCCTTACT CCAGGACCAC AGTGGAGGAT TTCTGCATGA

---------- ---------- ---------- ---------- ----------

TCCAGTAGTC CTGCCTTATT CTAGGACCAC GGTGGAAGAT TTCTGCATGA

TCCAGTAGTG CTGCCTTATT CTAGGACCAC TGTGGAAGAT TTCTGCATGA

---------- ---------- ---------- ---------- ----------

TCCAGTGGTG CTGCCTTACT CCAGGACCAC GGTGGAGGAT TTCTGCATGA

TCCAGTGGTG CTGCCTTATT CCAGGACTAC AGTGGAGGAT TTCTGCATGA

---------- ---------- ---------- ---------- ----------

TCCAGTGGTG CTGCCTTATT CCAGGACCAC AGTAGAGGAT TTCTGCATGA

CCCAGTGGTG CTGCCTGACT CCAGGACCAC AGTGGAGGAT TTCTGCATGA

CCCGGTTGTG CTGCCTGACT CCAGGACCAC GGTGGAGGAT TTCTGCATGA

---------- ---------- ---------- ---------- ----------

CCCAGTGGTG CTGCCTGACT CCCGGACCAC AGTGGAGGAT TTCTGTATGA

CCCAGTGGTG CTGCCCGACT CCCGGACTAC AGTAGAGGAT TTTTGCATGA

AGATTCACAA AAATCTTATC AAAGAATTTA AATATGCTCT GGTCTGGGGT

AGATTCACAA AAATCTTATC AAAGAATTTA AATATGCTTT GGTCTGGGGT

AGATTCACAA AAATCTTATC AAAGAATTTA AATATGCTCT GGTCTGGGGT

AGATTCACAA AAATCTTATC AAAGAATTTA AATATGCTCT GGTCTGGGGT

AGATTCACAA AAATCTTATC AAAGAATTTA AATATGCTCT GGTCTGGGGT

---------- ---------- ---------- ---------- ----------

AGATTCACAA AAATCTGATC AAAGAATTTA AATATGCTTT GGTCTGGGGT

AGATTCACAA AAATCTCATC AAAGAATTTA AATATGCTTT GGTCTGGGGT

---------- ---------- ---------- ----CGCTCT GGTCTGGGGT

AGATTCACAA AAATCTTATC AAAGAATTTA AATATGCTCT GGTGTGGGGT

AGATTCATAA AAATCTTATC AAAGAATTTA AATACGCTTT GGTTTGGGGT

---------- ---------- ---------- ---------- ----------

AGATTCATAA AAATCTTATT AAAGAATTCA AATATGCTCT GGTCTGGGGT

AGATTCACAA AAATCTTATC AAAGAATTTA AATATGCTCT GGTCTGGGGT

AGATTCACAA AAATCTTATC AAAGAATTTA AATACGCTCT GGTCTGGGGT

---------- ---------- ---------- ---------- ----------

AGATCCATAA AAACCTCATC AAAGAATTCA AGTATGCGCT GGTCTGGGGT

AAATTCACAA GAATCTCATC AAAGAATTCA AGTATGCTCT GGTATGGGGT

CTCTCTGTGA AACACAATCC TCAGAAAGTG GGTAAAGACC ATACGTTGGA

CTCTCTGTGA AACACAATCC TCAGAAAGTG GGTAAAGACC ATACGTTGGA

CTGTCTGTGA AACACAATCC TCAGAAAGTG GGTAAAGACC ATACGTTGGA

CTGTCTGTGA AACACAATCC TCAGAAAGTG GGTAAAGACC ATACGTTGGA

CTCTCTGTGA AACACAATCC TCAGAAAGTG GGTAAAGACC ATACGTTGGA

---------- ---------- ---------- ---------- ----------

CTCTCTGTGA AACATAATCC TCAGAAAGTG GGTAAAGACC ATACGTTGGA

CTGTCTGTGA AACATAATCC TCAGAAAGTG GGTAAAGACC ACACATTGGA

CTCTCTGTGA AGCACAACCC TCAGAAAGTG GGTAAAGACC ACACGCTGGA

CTGTCTGTGA AACACAATCC TCAAAAAGTG GGTAAAGACC ATACGTTGGA

CTGTCTGTGA AACATAATCC TCAAAAAGTG GGTAAAGACC ATACGTTGGA

---------- ---------- ---------- ---------- ----------

CTCTCTGTGA AACATAATCC TCAAAAAGTG GGTAAAGACC ATACGTTGGA

CTCTCTGTGA AGCACAATCC TCAGAAAGTG GGTAAAGACC ATACATTGGA

CTGTCTGTGA AACACAATCC TCAGAAAGTG GGTAAAGATC ATACATTGGA

---------- ---------- ---------- ---------- ----------

CTGTCTGTGA AGCACAACCC TCAGAAGGTG GGGAAAGACC ACACATTGGA

CTGTCTGTGA AGCATAATCC CCAGAAAGTA GGCAAAGATC ACACAATGGA

GGATGAGGAT GTCATTCAAA TTGTGAAGAA GATGGATAGG AGAAGTATGG

GGACGAGGAT GTCATTCAAA TTGTGAAGAA GATGGATAGG AGAAGTATGG

GGACGAGGAT GTCATTCAAA TTGTGAAGAA GATGGATAGG AGAAGTGTGG

GGACGAGGAT GTCATTCAAA TTGTGAAGAA GATGGATAGG AGAAGTGTGG

GGATGAGGAT GTCATTCAAA TTGTGAAGAA GATGGATAGG AGAAGTGTGG

---------- ---------- ---------- ---------- ----------

AGATGAAGAT GTCATTCAGA TCGTGAAGAA GATGGAGAAG AGAAGTGTGG

AGATGAAGAC GTCATTCAGA TTGTGAAGAA GATGGAGAA- --AAGTGTGG

GGATGAGGAC GTCATTCAGA TTGTGAAGAA GATGGATAGG AGAAGTGTGG

GGACGAGGAT GTCATTCAGA TTGTGAAGAA GATGGAT--- AGAAGTGTGG

GGACGAGGAT GTCATTCAGA TTGTGAAGAA GATGGATAGG AGAAGTGTTG

---------- ---------- ---------- -ATGGATAGG AGAAGTGTGG

GGATGAGGAT GTCATTCAGA TTGTGAAGAA GATGGATAGG AAAAGTTTGG

GGACGAGGAT GTCATTCAGA TTGTGAAGAA GATGGATAGG AGAAGTGTGG

AGACGAGGAT GTCATTCAGA TTGTGAAGAA GATGGATAGG AGAAGTGTGG

---------- ---------- ---------- ---------- ----------

AGACGAGGAC GTCATTCAGA TCGTCAAGAA GATGGACAGA AGAGGGGTGG

GGACGAAGAT GTCATTCAGA TTGTGAAGAA AATGGAGAGG AAAGGTGGGG

GTGAAACAGA AAGTGGAGAT GCTTTCCTTG ACCTGAAGAA GCCTCCTGCC

GTGAAACAGA AAGTGGAGAT GCTTTCCTTG ACCTGAAGAA GCCTCCTGCC

GTGAAACAGA AAATGGAGAT GCTTTCCTTG ACCTGAAGAA GCCACCTGCC

GTGAAACAGA AAATGGAGAT ACTTTCCTTG ACCTGAAGAA GCCACCTGCC

GTGAAACAGA AAATGGAGAT GCTTTCCTTG ACCTGAAGAA GCTGCCTGCC

---------- ---------- ---------- ---------- ----------

ATGAAACAGA AAATGGAGAT GCTTTCCTTG ACCTGAAGAA GCTGCCCACC

CTGAAACAGA AAATGGAGAT GCTTTCCTTG AGCTGAAGAA ACTGCCCACC

GGGAAACAGA AAATGGAGAT GCTTTCGTTG ACCTGAAGAA GCTGCGGGCC

GTGAAACAGA AAATGGAGAT GCTTTTCTCG ACCTGAAGAA GCAACCCGCT

GTGAAACAGA AAACGGAGAT GCTTTCCTTG ACCTGAAGAA GCAACCAGCC

GCGAAACAGA AAATGGAGAT GCTTTCCTTG ACTTGAAGAA GCAGCCGGCC

GTGAAACGGA AAATGGAGAT GCTTGCCTTG ACCTGAAGAA GCAGCCGGCC

GTGAAACAGA AAATGGAGAT GCCTTCCTTG ACCTGAAGAA GCTGCCGGCC

GTGAAACAGA AAATGGAGAT GCTTTCCTTG ACCCGAAGAA GCTGCCGCCC

---------- ---------- ---------- ---------- ----------

GAGACACGGA AAATGGCGAA GCCTTCCTGG ACCTGAAGAA GCCACCCGCA

GAGAAACAGA AAATGGTGAT GCCTTCCTTG ACCTGAAGAG GACTCCAGCA

TCCAAATGCC CCCATCGCTA TACAAAAGAA GAACTCTTGG ATATAAAAGA

TCCAAATGCC CCCATCGCTA TACAAAAGAA GAACTCTTGG ATATAAAAGA

TCCAAATGCC CCCATCGCTA TACAAAAGAA GAACTCTTGG ATATAAAAGA

TCCAAATGCC CCCATCGCTA TACAAAA--- ---------- ----------

TCCAAATGCC CCCATCGCTA TACAAAAGAA GAACTCTTGG ATATAAAAGA

---------- ---------- ---------- ---------- ----------

TCCAAATCCC CCCACCGCTA TACGAAAGAG GAGCTCTTGG ATATAAAAGA

TCCAAATCCC CCCATCGCTA TACGAAAGAG GAGCTCTTGG ATATAAAAGA

TCCAAATCAC CGCATCGTTA CACGAAA--- ---------- ----------

TCCAGATCCC CCCATCGCTA TACAAAAGAG GAGCTCTTGG ATATTAAAGA

TCCAAATCCC CCCATCGCTA TACAAAAGAG GAGCTCTTGG ATATAAAAGA

TCGAAATCCC CCCATCGCTA TACAAAAGAG GAGCTTTTGG ATATAAAAGA

TCCAAATCCC CCCATCGCTA TACAAAA--- ---------- ----------

TCCAAATCCC CCCATCGCTA TACAAAAGAG GAACTCTTGG ATATAAAAGA

TCCAAATCCC CCCATCGCTA TACAAAAGAG GAGCTCTTGG ATATAAAAGA

---------- ---------- ---------- ---------- ----------

TCCAAGTCCC CCCATCGTTA CACAAAAGAG GAACTTTTGG ACATTAAAGA

GCCAAATCCC CCCATCGTTA CACGAAAGAA GAACTCTTGG ATATTAAAGA

ACTCCCCCAT TCCAAACAGA GGCCTTCATG CCTTTCTGAA AAATATGACA

ACTCCCCCAT TCCAAACAGA GGCCTTCGTG CCTTTCTGAA AAATATGACA

ACTCCCCCAT TCCAAACAGA GGCCTTCGTG CCTTTCTGAA AAATATGACA

---------- ---------- ---------- ---------- ----------

ACTCCCCCAT TCCAAACAGA GGCCTTCATG CCTTTCTGAA AAATATGACA

---------- ---------- ---------- ---------- ----------

ACGCCCATAC TCCAAACAGA GGCCATCATG CCTCTCTGAA AAATATGACA

ACGTCCATAC TCCAAGCAGA GGCCATCATG CCTCTCTGAA AAATATGACA

---------- ---------- ---------- ---------- ----------

ACGCCCTCAT TCCAAACAGA GGCCTTCGTG CCTCTCTGAG AAATATGACA

ACGCCCTCAT TCCAAACAAA GACCATCATG CCTCTCTGAA AAATATGACA

ACGCCCTCAT TCCAAACAAA GGCCTTCATG CCTCTCTGAA AAATACGACA

---------- ---------- ---------- ---------- ----------

ACGCCCCCAT TCCAAACAAA GGCCATCATG CCTCTCTGAA AAATATGACA

ACATCCCCAT TCGAAACAGA GGCCTTCATG CCTCTCTGAA AAATACGACA

---------- ---------- ---------- ---------- ----------

ACGTCCTCAC TCTAAACAGA GGCCTTCGTG CCTTTCTGAG AAATATGACA

ACGCCCCCAC TCTAAGCAGA GACCTCCATG TCTTTCTGAA AAATATGACA

GTGATGGTGT CTGGGACCCT GAGAAGTGGC ATGCCTCTCT CTACCCAGCT

GTGATGGTGT CTGGGACCCT GAGAAGTGGC ATGCCTCTCT CTACCCAGCT

GTGATGGTGT CTGGGACCCT GAGAAGTGGC ATGCCTCTCT CTACCCAGCT

---------- ---------- ---------- ---------- ----------

GTGATGGTGT CTGGGACCCT GAGAAGTGGC ATGCCTCTCT CTACCCGGCT

---------- ---------- ---------- ---------- ----------

GTGATGGTGT CTGGGACCCT GAGAAGTGGC ATGCCTCTCT TTACCCAGCG

GTGATGGTGT CTGGGACCCT GAGAAGTGGC ATGCCTCTCT CTACCCAGCC

-CGATGGTGT CTGGGACCCT GAGAAGTGGC ACGCTTCTCT CTACCCAGCT

GTGATGGTGT CTGGGACCCT GAGAAGTGGC ACGCCTCCCT CTACCCAGCT

GTGACGGTGT CTGGGACCCT GAAAAGTGGC ATGCCTCTCT CTACCCAGCT

GTGATGGCGT CTGGGACCCC GAGAAGTGGC ATGCCTCTCT TTACCCAGCT

-TGATGGTGT CTGGGACCCT GAGAAGTGGC ATGCCTCTCT CTATCCAGCT

GTGATGGCGT CTGGGACCCT GAGAAGTGGC ATGCCTCTCT CTACCCAGCT

GCGATGGCGT CTGGGACCCT GAGAAGTGGC ATGCCTCTCT CTACCCAGCT

---------- ---------- ---------- ---------- ----------

GCGATGGCGT CTGGGATCCT GAGAAGTGGC ATGCATCTCT TTACCCAAGC

GTGATGGTGT CTGGGACCCT GAGAAATGGC ATGCCTCTCT CTACCCAAGT

TCAGGGCGGA GCTCACCAGT GGAAAGTCTG AAGAAAGAGT TGGATACAGA

TCAGGGCGGA GCTCACCAGT GGAAAGTCTG AAGAAAGAGT TGGATACAGA

TCAGGGCGAA GCTCGCCAGT GGAAAGTCTG AAGAAAGAGT TGGATACAGA

---------- ---------- ---------- ---------- ----------

TCAGGGCGGA GCTCACCAGT GGAAAGTCTG AAGAAAGAGT TGGATACAGA

---------- ---------- ---------- ---------- ----------

TCAGGGCGCA GCTCACCAGT GGAAAGTCTG AAGAAAGAGC CGGAATCAGA

TCGGGGCGGA GCTCACCAGT GGAAAGTCTG AAGAAAGAGT CAGAATCCGA

TCAGGGCGAA GCTCACCGGT GGAAAGTCTG AAGAAAGAGT TGGAAACAGA

TCAGGGCGGA GCTCACCGGT GGAAAGCCTG AAGAAAGAGC TGGACACGGA

TCAGGGCGGA ACTCGCCAGT GGAAAGTCTG AAGAAAGAGT TGGATGCAGA

TCAGGACGGA GCTCACCAGT GGAAAGTGTG AAGAAAGAGT TGGATACAGA

TCAGGGCGGA GTTCACCAGT GGAAAGTCTG AAGAAAGATT TGGATACAGA

TCAGAACGGA GCTCACCAGT GGAAAGTCTA AAGAAAGAGG TGGATATAGA

TCAGGGCGGA GCTCGCCAGT GGAAAGTCTG AAGAAAGAGC TCGATACAGA

---------- ---------- ---------- ---------- ----------

TCAGGGAGAA GTTCTCCAGT AGAAAGCTTC AAGAAAGAGC TGGACTTGGA

TCGGGGCGAA GTTCTCCAGT GGAAAGCTTC AAAAAAGATT TGGATTCAGA

CCGGCCTTCC CTGGTGCGCA GGATAGTAGA TCCACGAGAG CGTGTGAAAG

CCGGCCTTCC CTGGTGCGCA GGATAGTAGA TCCACGAGAG GGTGTGAAAG

CCGGCCTTCC CTGGTGCGCA GGATAGTAGA TCCACGAGAG CGTGTGAAAG

---------- ---------- ---------- ---------- ----------

CCGGCCTTCC CTGGTGCGCA GGATAGTAGA TCCACGAGAG CGTGTAAAAG

---------- ---------- ---------- ---------- ----------

TCGGCCTTCT CTGGTGCGCA GAATAGCAGA TCCACGAGAG CGAGTGAAAG

TCGACCTTCT CTGGTGCGCA GAATAGCAGA TCCACGAGAG CGAGTGAAAG

CCGACCTTCC CTGGTGCGCA GGATAGTAGA CCCACGGGAG CGCGTGAAAG

CCGGCCTTCC CTGGTGCGCA GGATAGTAGA TCCACGAGAG CGTGTGAAGG

GCGGCCTTCT CTTGTGCGCA GAATAGCAGA TCCACGAGAG CGCGTGAAAG

CCGGCCTTCC CTGGTGCGCA GGATAGTAGA TCCACGAGAG CGCGTAAAAG

CCGGCCTTCC CTGGTGCGCA AGATAGTAGA TCCACGGGAA CGTGTGAAAG

CCGGCCTTCC CTGGTGCGCA GGATAGTAGA TCCACGAGAG CGCGTGAAAG

CCGGCCTTCT CTGGCACGTA GGATAGTAGA TCCACGAGAG CGTGTGAAAG

---------- ---------- ---------- ---------- ----------

GCGACCTCCT CTGATGCGAA GGACAGCAGA TCCAAGAGAG CGGGTTAAAG

CCGGACTCCC CTTATGCGTC GCCTAGTGGA TCCCAGAGAG CGAGTAAAAG

AAGATGACTT AGATGTTGTT CTCAGCCCTC AGAGACGGAG CTTTGGAGGG

AAGATGACTT AGATGTTGTT CTCAGCCCTC AGAGACGGAG CTTTGGAGGG

AAGATGACTT AGATGTTGTT CTCAGCCCTC AGAGACGGAG CTTTGGAGGG

---------- ---------- ---------- ---------- ----------

AAGATGACTT AGATGTTGTT CTCAGCCCTC AGAGGCGGAG CTTTGGAGGG

---------- ---------- ---------- ---------- ----------

AAGATGACTT AGATGTTGTT CTCAGCCCAC AGAGACGAAG TTTTGGCGGT

AAGATGACTT AGATGTTGTT CTCAGCCCAC AGAGACGAAG CTTTGGAGGT

AAGACGACTT AGATGTTGTT CTCAGCCCAC AGAGACGCAG CTTTGGCGGC

AAGATGACTT AGATGTTGTT CTCAGCCCAC AGAGACGCAG CTTTGGAGGG

AAGATGACTT AGATGTTGTT CTCAGCCCAC AAAGACGAAG CTTTGGAGGG

AAGATGACTT AGATGTTGTT CTCAGCCCAC AGAGACGAAG CTTTGGAGGG

AAGATGACTT AGATGTTGTT CTCAGCCCAC AGAGACGAAG CTTTGGTGGG

AAGATGACTT AGATGTTGTT CTCAGCCCAC AGAGACGAAG CTTTGGGGGG

AAGACGACTT AGATGTTGTT CTCAGTCCAC AGAGACGAAG CTTTGGAGGA

---------- ---------- ---------- ---------- ----------

AAGATGATTT AGACGTTGTT CTAAGTCCCC AGAGGCGCAG TTTTGGAGGG

ATGATGATTT GGATGTAGTC TTAAGCCCCC AGAGGCGCAG TTTTGGGGGA

GGCTGCCACG TGACAGCCGC TGTTAGCTCC CGGCGCTCAG GAAGTCCATT

GGCTGCCACG TGACAGCTGC TGTTAGCTCC CGGCGCTCAG GAAGTCCATT

GGCTGCCACG TGACAGCTGC TGTTAGCTCC CGGCGCTCAG GAAGTCCATT

---------- ---------- ---------- ---------- ----------

GGCTGCCACG TGACAGCTGC TGTTAGTTCT CGGCGCTCAG GAAGTCCATT

---------- ---------- ---------- ---------- ----------

GGCTGCCATG TAACAGCTGC TGTTAGCTCC CGGCGCTCTG GCAGTCCATT

GGCTGCCATG TGACAGCTGC TGTTAGCTCC CGACGATCTG GCAGTCCATT

GGCTGCCACG TAACAGCTGC CGTTAGCTCC CGGCGCTCAG GCAGTCCCTT

GGCTGCCACG TGACGGCGGC CGTCAGCTCC CGGCGCTCAG GAAGCCCGCT

GGCTGCCACG TGACGGCTGC TGTTAGTTCC CGGCACTCAG GAAGCCCGTT

GGCTGCCACG TGACGGCTGC TGTTAGCTCC CGGCGCTCAG GAAGCCCGTT

GGCTGCCACG TGACAGCAGC TGTTAGCTCC CGGCGCTCAG GAAGCCCTCT

GGCTGCCATG TGACGGCTGC TGTTAGCTCC CGACGCTCAG GAAGCCCATT

GGCTGCCACG TGACAGCTGC CGTTAGCTCC CGGCACTCAG GAAGCCCATT

---------- ---------- ---------- ---------- ----------

GGCTGTCATG TCACAACCTC TGTCAGCTCC CGCAGATCAG GGAGCCCATT

GGTTGTCATG TAACAACAAC CGTTAGCTCA CGACGATCAG GGAGTCCTTT

AGAGAAAGAT AGTGATGGGC TTCGTCTGCT TGGTGGACGT AGGATTGGCA

AGAGAAAGAT AGTGATGGGC TTCGTCTGCT TGGTGGACGT AGGATTGGCA

AGAGAAAGAT AATGATGGGC TTCGTCTGCT TGGTGGACGT AGGATTGGCA

---------- ---------- ---------- ---------- ----------

AGAAAAAGAC AGTGATGGGC TTCGTCTGCT TGGTGGACGT AGGATTGGCA

---------- ---------- ---------- ---------- ----------

GGAGAAAGAC AGTGATGGGC TTCGCCTGCT TGGTGGACGA AGAATTGGCA

GGAGAAAGAC AGTGATGGAC TTCGCCTGCT TGGTGGACGA AGAATTGGCA

AGAGAAAGAC AGCGATGGAC TTCGCCTGCT TGGCGGGCGT CGCATCGGCA

GGAGAAAGAC GGCGACGGGC TCCGCCTGCT TGGCGGACGC AGGATCGGCA

GGAAAAAGAA AATGATGGGC TTCGCCTGCT TGGCGGACGA AGGATTGGCA

GGAGAAAGAC AGTGATGGGC TCCGCCTTCT TGGGGGACGA AGGATTGGCA

GGAAAAAGAT AGTGATGGAC TTCGCCTGCT TGGTGGAAGG AGGATTGGGA

GGAGAAAGAT AATGATGGAC TTCGTCTGCT TGGCGGACGC AGGATTGGCA

GGAGAAAGAC AGTGATGGGC TTCGCCTGCT TGGTGGACGC AGGATTGGCA

---------- ---------- ---------- ---------- ----------

GGAAAAGGAC AATGATGGTG TTCGAATGAT TGGAGGTCGT AGGATCGGCA

GGAAAAAGAA AATGATGGTG TGCGAATTAT TGGAGGGCGT AGAATTGGCA

GTGGGAGGAT AATCTCTGCC GAGAAGGATC ACCGTCTTAG CGATAAGGAC

GTGGGAGGAT AATCTCTGCC GAGAAGGATC ACCGTCTTAG CGATAAGGAC

GTGGGAGGAT AATCTCTGCC GAGAAGGATC ACCGTCTTAG CGATAAGGAC

---------- ---------- ---------- ---------- ----------

GTGGGAGGAT AATCTCTGCC GAGAAGGATC ACCGTCTTAG CGATAAGGAC

---------- ---------- ---------- ---------- ----------

GTGGAAGAAT AATTTCTGCC GAGAAGGATC ACCGTCTTAG CGATAAGGAC

GTGGAAGGAT AATTTCTGCC GAAAAGGATC ACCGTCTCAG TGATAAGGAC

GTGGAAGGAT AGTCTCTGGC GAGAAGGACC ACCGTCTCAG CGAGAAGGAC

GCGGGAGGAT CATCTCTGCT GAGAAGGATC ACCGTCTTGG CGAGAAGGAC

GTGGGAGGAT AATCTCTGCT GAGAAGGATC ACCGTCTTAG TGATAAGGAG

GTGGGAGGAT AATCTCTGCT GAAAAGGATC ACCGCCTTAA CGATAAGGAC

GCGGAAGGAT TATCTCTGCT GAGAAGGATC ACCGTCTTAG TGATAAGGAC

GTGGGAGAAT AATCTCTGCC GAGAAGGATC ACCGTCTTAG TGATAAGGAC

GTGGGAGAAT AATCTCTGCC GAGAAGGATC ACCGTCTTAG TGATAAGGAC

---------- ---------- ---------- ---------- ----------

GTGGGAGGAT CATCTCTT-- -ACAAAGACC ACCGAAGTGG GGACAAAGAC

GTGGAAGAAT AATATCTGCC GATAAAGATC ACCG---TGG AGAAAAGGAC

CTGCGGGACT TGAGAGACAG AGACCGAGAG AGGGACTTCA AGGACAAGCG

CTGCGGGACT TGAGAGACAG AGACCGAGAG AGGGACTTCA AGGACAAGCG

CTGCGAGACT TGAGAGACAG AGACCGAGAG AGGGACTTCA AGGACAAGCG

---------- ---------- ---------- ---------- ----------

CTGCGGGACT TGAGAGACAG AGACCGAGAG AGGGACTTCA AGGACAAGCG

---------- ---------- ---------- ---------- ----------

CTACGAGACT TGAGAGACCG AGACAGGGAG AGGGACTACA AGGACAAGCG

CTGCGAGACT TGAGAGACCG AGACAGAGAG AGGGACTACA AGGACAAACG

CTGCGGGACC TGAGAGACAG AGACCGCGAG AGGGACCACA AGGACAAGCG

CTGCGAGACT TGAGAGAGAG AGACCGCGAG AGGGACTACA AGGACAAGCG

CTGCGGGACT TGAGAGACAG AGACCGAGAG AGAGACTATA AGGACAAGCG

CTGCGGGACT TGAGAGACAG AGACCGAGAG AGGGACTACA AGGACAAGCG

CTTCGGGACT TAAGAGACAG AGATCGAGAG AGAGACTACA AGGACAAGCG

CTGCGGGACT TGAGAGACAG AGACCGCGAG AGGGACTACA AGGACAAGCG

CTTCGGGACA TGAGAGACAG AGACCGTGAG AGGGACTACA AGGACAAGCG

---------- ---------- ---------- ---------- ----------

TTAAGGGACT GTAGAGACAG AGATCGGGAG AGAGATTATA AGGACAAGCG

TTGAGAGATT CCAGAGACAG AGATCGCGAG AGGGACTACA AGGACAAACG

TTTCAGGAGA GAGTTTGGAG ATAGTAAGCG TGTCTTTGGT GAGCGTAGAA

TTTCAGGAGA GAGTTTGGAG ATAGTAAGCG TGTCTTTGGT GAGCGTAGAA

TTTCAGGAGA GAGTTTGGAG ATAGTAAGCG TGTCTTTGGC GAGCGTAGAA

---------- ---------- ---------- ---------- ----------

TTTCAGGAGA GAGTTTGGGG ATAGTAAGCG TGTCTTTGGT GAACGCAGAA

---------- ---------- ---------- ---------- ----------

TTTCAGGAGG GAGTTTGGAG ATAGTAAACG TGTCTTTGGT GAGCGTAGAA

TTTCAGGAGG GAGTTTGGAG ATAGTAAACG TGTCTTTGGT GAGCGTAGAA

CTTCCGGAGG GAGTTTGGGG ATAGTAAGCG TGTCTTTGGC GAGCGTAGAA

CTTCAGGAGG GAGTTTGTGG ATAGTAAGCG TGTCTTTGGT GAGCGTAGAA

ATTCAGGAGG GAGTTTGGGG ATAGTAAGCG TGTCTTTGGT GAGCGAAGAA

TTTCAGGAGG GAGTTTGGCG ATAGTAAGCG TGTCTTTGGT GAGCGTAGAA

GTTTCGGAGG GAGTTTGGGG ATAGTAAGCG TGTCTTTGGT GAACGTCGAA

TTTCAGGAGG GAGTTTGGGG ATAGTAAGCG TGTCTTTGGT GAACGTAGAA

TTTTAGGAGA GAGTTTGGGG ATAGTAAGCG AGTCTTTGGC GAGCGTAGAA

---------- ---------- ---------- ---------- ----------

TTTCCGGAGA GAGTTTGGTG ACAGCAAACG TATATTTGGA GAGCGCAGAA

TTTTAGGAGA GAGTTTGGGG AAAGTAAGCG TGTCTTTGGA GAACGTAGAA

GAAATGATTC TTACACAGAA GAAGAACCAG AGTGGTTCTC TGCTGGACCC

GAAATGATTC TTACACAGAA GAAGAACCAG AGTGGTTCTC TGCTGGACCC

GAAATGATTC TTATACAGAA GAAGAACCAG AGTGGTTCTC TGCTGGACCC

---------- ---------- ---------- ---------- ----------

GAAATGATTC TTACACAGAA GAAGAACCAG AGTGGTTCTC AGCTGGACCC

---------- ---------- ---------- ---------- ----------

GAAACGATTC ATACACTGAG GAAGAACCAG AATGGTTTTC AGCTGGACCC

GAAACGATTC ATACACTGAG GAAGAACCAG AATGGTTTTC AGCTGGACCC

GGAATGATTC TTACACGGAA GAAGAACCAG AGTGGTTCTC AGCTGGACCC

GAAACGATTC CTACACAGAA GAAGAACCAG AATGGTTCTC AGCTGGACCC

GAAATGATTC CTACACAGAA GAAGAACCAG AATGGTTCTC AGCTGGACCC

GAAATGATTC CTACACAGAA GAAGAACCCG AATGGTTCTC AGCCGGACCC

GAAATGATTC CTACACAGAG GAAGAACCAG AATGGTTCTC AGCTGGACCC

GAAATGATTC CTACACAGAA GAAGAACCAG AATGGTTCTC AGCTGGACCC

GAAACGATTC TTACACGGAA GAAGAACCAG AATGGTTCTC AGCTGGACCT

---------- ---------- ---------- ---------- ----------

GAAATGATTC CTATACAGAA GAAGAGCCAG AGTGGTTTTC AGCTGGACCC

GAAATGATTC CTATACTGAA GAGGAACCTG AATGGTTCTC TGCTGGACCG

ACAAGTCAGT CTGAAACCAT CGAACTGACT GGCTTTGATG ATAAGATACT

ACAAGTCAGT CTGAAACCAT CGAACTGACT GGCTTTGATG ATAAGATACT

ACAAGTCAGT CTGAAACCAT CGAACTGACT GGCTTCGATG ATAAGATACT

---------- ---------- ---------- ---------- ----------

ACAAGTCAGT CTGAAACCAT CGAACTGACT GGCTTTGATG ATAAGATACT

---------- ---------- ---------- ---------- ----------

ACCAGTCAGT CTGAAACCAT AGAGCTGACT GGCTTTGATG ATAAGATTCT

ACCAGTCAGT CTGAAACCAT AGAGCTGACT GGCTTTGATG ATAAGATTCT

ACGAGTCAGT CTGAAACCAT TGAACTTACT GGCTTTGATG ATAAGATACT

ACGAGTCAGT CCGAAACCAT TGAGCTGACT GGCTTTGATG ATAAGATACT

ACAAGTCAAT CTGAAACCAT TGAGCTGACT GGCTTTGATG ATAAGATACT

ACAAGTCAGT CTGAAACCAT TGAGCTGACT GGCTTTGATG ATAAGATACT

ACAAGTCAGT CTGAAACCAT TGAACTGACT GGTTTCGATG ATAAGATACT

ACAAGTCAGT CAGAAACCAT TGAGCTGACT GGCTTTGATG ATAAGATATT

ACAAGTCAGT CTGAAACCAT TGAGCTTACT GGCTTTGATG ATAAGATACT

---------- ---------- ---------- ---------- ----------

ACAAGTCAGT CTGAAACCAT TGAGCTCACT GGCTTTGATG ACAAAATTCT

ACAAGTCAGT CTGAAACTAT TGAGCTTACA GGATTTGATG ATAAAATACT

AGAAGAAGAT CACAAAGGGA GAAAAAGAAC AAGGCGACGG ACAGCCTCTG

AGAAGAAGAT CACAAAGGGA GAAAAAGAAC AAGGCGACGG ACAGCCTCTG

AGAAGAAGAT CACAAAGGGA GAAAAAGAAC AAGGCGACGG ACAGCCTCTG

---------- ---------- ---------- ---------- ----------

AGAAGAAGAT CACAAAGGGA GAAAAAGAAC AAGGCGACGG ACAGCCTCTG

---------- ---------- ---------- ---------- ----------

AGAGGAGGAT CACAAAGGGA GAAAACGAAC AAGGCGACGG ACTGCCTCTG

AGAGGAGGAT CACAAAGGCA GAAAAAGAAC AAGGCGACGG ACTGCCTCTG

AGAAGAAGAT CACAAGGGGA GAAAAAGGAC GAGACGGAGG ACGGCCTCGG

GGAAGAAGAC CATAAGGGGA GAAAAAGAAC GCGGCGACGA ACAGCCTCTG

GGAAGAAGAT CACAAGGGGA GAAAGAGAAC AAGGCGAAGG ACGGCCTCCG

GGAAGAAGAT CACAAAGGGA GAAAAAGAAC AAGGCGACGG ACAGCCTCTG

GGAAGAAGAT CACAAGGGGA GAAAAAGAAC GAGGCGACGG ACAGCATCTG

AGAAGAAGAT CACAAAGGGA GAAAACGAAC AAGGCGACGG ACAGCCTCTA

AGAAGAAGAT CACAAAGGGA GAAAAAGAAC AAGGCGACGG ACAGCCTCTG

---------- ---------- ---------- ---------- ----------

GGAAGAAGAT CACAAAGGGA GAAAAAGAAC GCGGCGACGT GCCATGTCTG

AGAAGAAGAT CATAAAGGAC GAAAACGAAC AAGACGGCGC ACAGCATCCG

TGAAGGAAGG TATAGTAGAG TGCAATGGAG GAGTGGCCGA AGAGGATGAA

TGAAGGAAGG TATAGTAGAG TGCAATGGAG GAGTGGCCGA AGAGGATGAA

TGAAGGAAGG TATAGTAGAG TGCAATGGAG GAGTGGCCGA AGAAGATGAA

---------- ---------- ---------- ---------- ----------

TGAAGGAAGG TATAATAGAG TGCAATGGAG GAGTGGCCGA AGAGGATGAG

---------- ---------- ---------- ---------- ----------

TGAGGGAAGG CATAGTGGAG TGCAATGGAG GGGTGGCTGA AGAGGACGAG

TGAAGGAAGG CATAGTGGAG TGCAATGGAG GGGTGGCTGA AGAGGACGAG

TGAAGGAAG- ---------- ---------- ---------- ----------

TGAAGGAAGG CATAGTCGAG TGCAATGGAG GAGTGGCTGA AGAGGATGAG

TGAAGGAAGG CATAGTCGAG TGCAATGGAG GAGTGGCCGA AGAGGATGAG

TGAAGGAAGG CATAGTCGAG TGCAATGGAG GAGTGGCCGA AGAGCACGAG

TGAAAGAAG- ---------- ---------- ---------- ----------

TGAAGGAAGG CATAATCGAG TGCAATGGAG GAGTGGCCGA AGAGGATGAG

TGAAGGAAGG CATAGTCGAG TGCAATGGAG GAGTGGCCGA AGAGGATGAG

---------- ---------- ---------- ---------- ----------

TGAAGGAAGG AATAGTAGAA TACAATGGAG GCGTTGCAGA AGAGGATGAA

TGAAGGAAGG AATAGTAGAA TGCAATGGTG GAGTGGCAGA AGAGGATGAA

GTGGAGGTCA TCCTTGCACA GGAGCCTGCG GCTGATCAGG AAGTGCCAAG

GTGGAGGTCA TCCTTGCACA GGAGCCTGCG GCTGATCAGG AAGTGCCAAG

GTGGAGGTCA TCCTTGCACA GGAGCCTGCA GCTGATCAGG AAGTGCCAAG

---------- ---------- ---------- ---------- ----------

GTAGAGGTCA TCCTTGCACA GGAGCCTGCG GCTGATCAAG AAGTGCCAAG

---------- ---------- ---------- ---------- ----------

GTGGAAGTCA TTCTGGCCCA GGAGCCCTCT GCTGATCAGG AAGTTCCAAG

GTGGAAGTCA TTCTGGCCCA GGAGCCCTCT GCTGATCAGG AAGTTCCAAG

---------- ---------- ---------- ---------- ----------

GTGGAGGTCA TCCTCGAACA GGAGCCTGCT GCTGATCAGG AAGTGCCAAG

GTGGAGGTCA TCCTCGCACA GGAGCCTGCT GCTGATCAGG AAGTGCCAAG

GTGGAGGTCA TCCTCGCACA CGAGCCTGCT GCTGATCAGG AAGTGCCGAG

---------- ---------- ---------- ---------- ----------

GTGGAGGTCA TCCTGGCACA GGAGCCTGCT GCTGATCAGG AAGTGCCAAG

GTGGAGGTCA TTCTTGCACA GGAGCCTGCT GCTGATCAGG AGGTGCCAAG

---------- ---------- ---------- ---------- ----------

GTGGAGGTCA TTCTTGCCCA GGAGCCCGCT GCAGATCAGG AAGTGCCCAG

GTGGAGGTCA TTCTTGCTCA GGAGACAGCA GCCGATCAGG AGGTGCCCAG

GGATGCTGTC TTGCCTGAGC AGTCCCCAGG AGACTTTGAC TTTAATGAGT

GGATGCCGTC TTGCCTGAGC AGTCCCCAGG AGACTTTGAC TTTAATGAGT

GGATGCCATC TTGCCTGAGC AGTCCCCAGG AGAATTTGAC TTTAATGAGT

---------- ---------- ---------- ---------- ----------

GGATGCTGTC TTGCCTGAGC AGTCCCCAGG AGAATTTGAC TTTAACGAGT

---------- ---------- ---------- ---------- ----------

GGATGTCATC CTACCTGAGC AGTCCCCAGG AGAATTTGAC TTTAATGAAT

GGATGTCATC CTACCTGAGC AGTCCCCAGG AGAATTTGAC TTTAATGAAT

---------- ---------- ---------- ---------- ----------

GGAGGCCGTC TTGCCCGAGC AGTCCCCGGG AGAATTTGAC TTTAATGAAT

GGACGCCGTC TTGCCTGAGC AGTCCCCAGG AGAATTTGAC TTTAATGAGT

GGACGCCGTC TTGCCTGAGC AATCCCCAGG AGAATTTGAC TTTAATGAGT

---------- ---------- ---------- ---------- ----------

GGATGCCGTC TTGCCCGAGC AGTCCCCAGG AGAATTTGAC TTTAATGAGT

GGACGCCGTC TTGCCTGAGC AGTCCCCAGG AGAATTTGAC TTTAATGAGT

---------- ---------- ---------- ---------- ----------

GGAAGCAGTG CTGCCTGAAC AGTCGCCAGG AGAATTTGAT TTTAATGAGT

AGAAGCAGTG CTGCCAGAAC AAGCACCTGG AGAATTCGAT TTCAATGAGT

TCTTTAACCT TGATAAGGTG CCATGCTTGG CTTCGATGAT AGAAGATGTT

TCTTTAACCT TGATAAGGTG CCATGCTTGG CTTCGATGAT AGAAGATGTT

TCTTTAACCT TGATAAGGTG CCATGCTTGG CTTCGATGAT AGAAGATGTT

----TAACCT TGATAAGGTG CCATGCTTGG CTTCGATGAT AGAAGATGTT

TCTTTAACCT TGATAAGGTG CCAGGCTTGG CTTCGATGAT AGAAGATGTT

---------- ---------- ---------- ---------- ----------

TCTTTAACCT TGATAAGGTG CCATGCTTGG CTTCGATGAT AGAAGATGTT

TCTTTAACCT TGATAAGGTG CCATGCTTGG CTTCGATGAT AGAAGATGTT

---------- ---------- ---------- -----ATGAT AGAAGACGTC

TCTTTAACCT TGATAAGGTG CCATGCTTGG CTTCGATGAT AGAAGATGTT

TCTTTAACCT TGATAAAGTG CCAGGCTTGG CTTCGATGAT AGAAGATGTT

TCTTTAACCT TGATAAGGTG CCGTGCTTGG CTTCGATGAT AGAAGATGTT

---------- ---------- ---------- -----ATGAT CGAAGACGTT

TCTTTAACCT TGAAAAGGTG CCATGCTTGG CTTCGATGAT AGAAGATGTT

TCTTTAACCT TGATAAGGTG CCGTGCTTGG CTTCGATAAT AGAAGATGTT

---------- ---------- ---------- -----ATGAT AGAAGATGTT

TCTTTAACCT TGATAAGGTG CCAGGCCTGG CCTCGATGAT AGAAGATGTT

TCTTTAACCT CGACAAAGTC CCTGGTTTGG CTTCGATGAT TGAAGACGTT

TTGGGAGAAG GGTCAGTCTC TGCCAGTCGG TTCAGTAGGT GGTTCTCTAA

TTGGGAGAAG GGTCAGTCTC TGCCAGTCGG TTCAGTAGGT GGTTCTCTAA

TTGGGAGAAG GGTCAGTCTC TGCCAGTCGG TTCAGTAGGT GGTTCTCTAA

TTGGGAGAAG GGTCAGTCTC TGCCAGTCGG TTCAGTAGGT GGTTCTCTAA

TTGGGAGAAG GGTCAGTCTC TGCCAGTCGG TTCAGTAGGT GGTTCTCTAA

---------- ---------- ---------- ---------- ----------

CTGGGAGAAG GATCAGTCTC TGCCAGCCGC TTCAGTAGGT GGTTCTCTAA

CTGGGGGAAG GATCGGTCTC TGCCAGCCGC TTCAGTAGGT GGTTCTCTAA

TTGGGAGAAG GCTCCGTCTC TGCCAGTCGG TTCAGTAGGT GGTTCTCCAA

TTGGGAGAAG GTTCAGTCTC TGCCAGTCGG TTCAGTAGGT GGTTCTCTAA

TTGGGAGAAG GATCAGTCTC TGCAAGTCGG TTCAGTAGAT GGTTCTCTAA

TTGGGAGAAG GGTCCGTCTC TGCCAGTCGG TTCAGTAGGT GGTTCTCTAA

TTGGGAGAAG GGTCTGTCTC TGCCAGTCGG TTCAGTAGGT GGTTCTCTAA

TTGGGAGAAG GGTCAGTCTC TGCCAGTCGG TTTAGTAGGT GGTTCTCTAA

TTGGGGGAAG GATCAGTCTC TGCCAGTCGG TTCAGTAGGT GGTTCTCTAA

CTAGGGGAAG GGTCCGTCTC GGCCAGCCGC TTTAGTAGGT GGTTTTCTAA

CTCGGAGAAG GTTCCGTCTC TGCCAGCAGA TTTAGTAGGT GGTTTTCCAA

CTGGGAGAAG GTTCAGTCTC TGCCAGCAGG TTCAGTAGGT GGTTCTCCAA

CCCGAGCAGA TCAGGAAGCC GATCCAGCAG TCTTGGGTCA ACACCACATG

CCCGAGCAGA TCAGGAAGCC GATCCAGCAG TCTTGGGTCA ACACCACATG

CCCGAGCAGA TCAGGAAGCC GATCCAGCAG TCTTGGGTCA ACACCACATG

CCCGAGCAGA TCAGGAAGCC GATCCAGCAG TCTTGGGTCA ACACCACATG

CCCGAGCAGA TCAGGAAGCC GATCCAGCAG TCTTGGGTCA ACACCACACG

---------- ---------- ---------- ---------- ----------

CCCAAGCCGG TCAGGGAGCA GATCCAGCAG CCTCGGATCC ACACCCCATG

CCCAAGCCGG TCAGGGAGCA GATCCAGCAG CCTTGGATCC ACACCCCATG

CCCGAGCCAG TCAGGGAGCC GATCCAGCAG CCTTGGGTCG ACGCCACATG

CCCGAGCCGA TCAGGAAGCC GATCCAGCAG CCTTGGGTCA ACACCTCATG

TCCGAGCCGA TCAGGAAGCC GATCCAGCAG TCTTGGGTCA ACACCACATG

CCCGAGCCGA TCAGGAAGCC GATCCAGCAG TCTTGGGTCA ACACCACATG

CCCAAGTCGA TCAGGAAGCC GA-------- ---------- ----------

CCCAAGCCGA TCAGGAAGCC GGTCCAGCAG TCTTGGGTCA ACGCCACATG

CCCGAGCCGA TCAGGAAGCC GGTCCAGCAG TCTTGGGTCA ACACCACATG

CCCCAGCCGA TCAGGAAGCC GGTCCAGCAG TCTTGGGTCA ACACCACATG

TCCTAGTCGA TCAGGAAGTC GATCCAGCAG CCTTGGGTCA ACACCTCATG

TCCTAGTCGA TCCGGAAGCC GGTCCAGTAG TTTAGGATCA ACACCGCATG

AAGAGCTAGA GAGACTTGCA GGTCTGGAGC AAGCCATCCT CTCTCCTGGA

AAGAGCTAGA GAGACTTGCA GGTCTGGAGC AAGCCATCCT CTCTCCTGGA

AAGAGCTAGA GAGACTTGCA GGTCTGGAGC AAGCCATCCT CTCTCCTGGA

AAGAGCTAGA GAGACTTGCA GGTCTGGAGC AAGCCATCCT CTCTCCTGGA

AAGAGCTAGA GAGACTTGCA GGTCTGGAGC AAGCCATCAT CTCTCCTGGA

---------- ---------- ---------- ---------- ----------

AAGAACTTGA GAGACTGGCA GGTTTGGAGC AAGCCGTCCT CTCTCCTGGA

AAGAACTTGA GAGACTGGCA GGTTTGGAGC AAGCCGTCCT CTCTCCTGGA

AAGAGCTGGA GAGACTTGCA GGTCTGGAGC AAGCCATCCT CTCTCCTGGA

AAGAACTGGA GAGACTTGCA GGTCTGGAAC AAGCCATACT CTCTCCTGGA

AAGAACTGGA GAGACTAGCA GGTCTGGAAC AAGCAATCCT CTCTCCTGGA

AAGAACTGGA GAGACTTGCA GGTCTGGAGC AAGCCATCCT CTCTCCTGGG

---------- ---------- -GTCTAGAGC AAGCCATACT CTCTCCTGGA

AAGAACTGGA GAGACTTGCA GGTCTAGAGC AAGCCATCCT CTCTCCTGGA

AAGAACTGGA GAGGCTTGCA GGTCTGGAGC AAGCCATCCT CTCTCCTGGA

AAGAACTGGA GAGACTTGCA GGTCTGGAGC AGGTCATCCT CTCTCCTGCA

AGGAATTGGA GAGACTTGCA GGTTTGGAAC AAGCAATTCT ATCCCCAGGC

AGGAATTAGA AAGACTAGCA GGTCTGGAGC AAGCTATTCT CTCCCCAGGC

CAGAACTCGG GGAATTACTT TGCTCCTATA CCATTGGAAG ACCATGCTGA

CAGAACTCGG GGAATTACTT TGCTCCTATA CCATTGGAAG ACCATGCTGA

CAGAACTCGG GGAATTACTT TGCTCCTATA CCATTGGAAG ACCATACTGA

CAGAACTCGG GGAATTACTT TGCTCCTATA CCATTGGAAG ACCATACTGA

CAGAACTCGG GGAATTACTT TGCTCCTATA CCATTGGAAG ACCATACTGA

---------- ---------- ---------- ---------- ----------

CAGAACTCGG GGAATTATTT TGCTCCTATA CCATCAGAAG ACCATGCTGA

CAGAACTCGG GGAATTATTT TGCTCCTATA CCATCGGAAG ACCATGCTGA

CAGAACTCGG GGAATTATTT TGCTCCTATA CCATTGGAAG ACCATGCTGA

CAGAACTCGG GGAATTATTT TGCTCCTATA CCATTAGAAG ACCATGCTGA

CAGAACTCGG GGAATTATTT TGCTCCTATC CCACTGGAAG ACCACACTGA

CAGAATTCGG GGAATTATTT TGCTCCTATA CCATTGGAAG ACCATGCTGA

CAGAATTCGG GGAATTATTT TGCTCCTATA CAATTGGAAG ATCATGCTGA

CAGAACTCAG GAAATTATTT TGCTCCTATA CCATTGGAAG ACCATGCTGA

CAGAACTCGG GGAATTACTT TGCTCCTATA CCATTGGAAG ATAATGCTGA

CAGAACTCGG GGAATTACTT TGCTCCTATA CCATCAGAAG ATCATGCTGA

CAGAACTCAG GAAATTACTT TGCTCCTATC CCATTAGAAG ACCATGCAGA

CAGAACTCGG GAAACTACTT TGCTCCTATA CCATTGGAAG ATCACTCTGA

AAATAAAGTG GATATTTTAG AAATGCTACA GAAAGCCAAA GTGGATTTGA

AAATAAAGTG GATATTTTAG AAATGCTACA GAAAGCCAAA GTGGATTTGA

AAATAAAGTG GATATTTTAG AAATGCTACA GAAAGCCAAA GTGGATTTGA

AAATAAAGTG GATATTTTAG AAATGCTACA GAAAGCCAAA GTGGATTTGA

AAATAAAGTG GATATTTTAG AAATGCTACA GAAAGCCAAA GTGGATTTGA

---------- ---------- ---------- ---------- ----------

AAATAAAGTG GACATTTTAG AAATGCTACA GAAAGCCAAA GTGGATTTAA

AAATAAAGTG GACATTTTAG AAATGCTACA GAAAGCCAAA GTAGATTTAA

AAATAAAGTA GACATTTTAG AAATGCTACA GAAAGCCAAA GTGGATTTAA

AACTAAAGTG GATATTCTAG AAATGCTACA GAAAGCCAAA GTGGATTTAA

AAATAAAGTG GATATTTTAG AAATGCTGCA GAAGGCCAAA GTGGATTTAA

AAATAAAGTG GATATTTTAG AAATGCTACA GAAAGCCAAA GTGGATTTAA

AAATAAAGTA GATATTTTAG AAATGCTACA GAAAGCCAAA GTGGATTTAA

AAATAAAGTG GATATTTTAG AAATGCTACA GAAAGCCAAA GTGGACTTAA

AAATAAGGTG GATATTTTAG AAATGCTACA GAAAGCCAAA GTGGACTTAA

AAATAAAGTG GATATTTTAG AAATGCTACA GAAAGCCAAA GTGGACTTAA

AAACAAGGTG GACATTCTAG AAATGCTTCA GAAGGCCAAA GTGGACCTAA

AAACAAAGTG GATATTCTAG AAATGCTACA GAAAGCAAAA GTAGACTTAA

AACCTCTTCT TTCCAGCCTT TCTGCAAATA AAGAAAAACT TAAAGAAAGC

AACCTCTTCT TTCCAGCCTT TCTGCAAATA AAGAAAAACT TAAAGAAAGC

AACCTCTTCT TTCTAGCCTT TCTGCAAATA AAGAAAAACT TAAAGAAAGC

AACCTCTTCT TTCTAGCCTT TCTGCAAATA AAGAAAAACT TAAAGAAAGC

AACCGCTTCT TTCCAGCCTT TCTGCAAATA AAGAAAAACT TAAAGAAAGC

---------- ---------- ---------- ---------- ----------

AACCCCTTCT TTCTAGCCTT TCTGCAAATA AAGAAAAACT TAAAGAAAGC

AACCTCTTCT TTCTAGCCTT TCTGCAAATA AAGAAAAACT TAAGGAGAGC

AACCTCTTCT TTCCAGCCTT TCTGCAAATA AAGAAAAACT TAAGGAGAGC

AGCCTCTTCT TTCCAGCCTT TCTGCAAATA AAGAAAAGCT TAAAGAGAGC

AACCTCTTCT TTCCAGTCTT TCTGCAAATA AAGAAAAGCT TAAGGAAAGC

AACCTCTTCT TTCCAGCCTT TCTGCAAATA AAGAAAAACT TAAAGAGAGC

AACCTCTTCT TTCCAGCCTT TCTGCAAATA AAGAAAAACT TAAAGAGAGC

AACCTCTTCT CTCCAGCCTT TCTGCAAATA AAGAAAAACT TAAAGAAAGT

AACCTCTTCT TTCCAGCCTT TCTGCAAATA AAGAAAAACT TAAAGAAAGC

AACCTCTTCT CTCCAGCCTT TCTGCAAATA AAGAAAAGCT TAAAGAAAGC

AGCCCCTCCT CTCCAACCTT TCTGCCAACA AGGAAAAGCT GAAAGAAAGC

AACCTCTCCT CTCAAGTCTC TCCGCCAACA AGGAAAAGCT TAAAGAGAGC

TCACATTCAG GGGTTGTGCT TTCAGTGGAG GAGGTAGAAG CAGGTCTGAA

TCACATTCAG GGGTTGTGCT TTCAGTGGAG GAGGTAGAAG CAGGTCTGAA

TCACATTCAG GGGTTGTGCT TTCCGTGGAG GAGGTAGAAG CAGGTCTGAA

TCACATTCAG GGGTTGTGCT TTCCGTGGAG GAGGTAGAAG CAGGTCTGAA

TCACATTCAG GGGTCGTGCT TTCAGTGGAA GAGGTAGAAG CAGGGCTGAA

---------- ---------- ---------- ---------- ----------

TCACATTCAG GGGTTGTACT TTCAGTGGAA GAGGTAGAAG CCGGGCTGAA

TCACATTCAG GGGTTGTACT TTCAGTGGAA GAGGTAGAAG CAGGGCTGAA

TCACATTCGG GGGTCGTGCT TTCCGTGGAA GAGGTGGAAG CAGGGCTCAC

TCACATTCAG GGGTTGTGCT TTCCGTGGAG GAGGTGGAAG CAGGGCTCAA

TCACATTCAG GAGTTGTGCT GTCAGTGGAA GAGGTAGAAG CAGGGCTCAA

TCACATTCAG GGGTTGTGCT TTCAGTGGAA GAGGTAGAAG CAGGGCTCAA

TCACACTCAG GAGTTGTACT TTCGGTGGAA GAGGTAGAAG CTGGGCTCAA

TCACATTCAG GAGTTGTGCT TTCTGTGGAA GAAGTCGAAG CAGGGCTTAA

TCGCATTCAG GGGTCGTGCT TTCAGTGGAA GAGGTAGAAG CAGGGCTCAA

TCACATTCAG GGGTTGTGCT CTCAGTGGAA GAGGTAGAAG CAGGGCTCAA

TCGCATACAG GGGTTGTGCT TTCGGTGGAA GAGGTGGAAG CTGGGCTCAA

TCGCATTCCG GAGTTGTGCT GTCAGTGGAA GAAGTAGAAG CTGGGCTGAA

GGGCTTGAAG GTTGACCAGC AAGTGAAGAA TTCAACTCCC TTCATGGCAG

GGGCTTGAAG GTTGACCAGC AAGTGAAGAA TTCAACTCCC TTCATGGCAG

GGGCTTGAAG GTGGACCAGC AAGTGAAGAA TTCAACTCCC TTCATGGCAG

GGGCTTGAAG GTGGACCAGC AAGTGAAGAA TTCAACTCCC TTCATGGCAG

GGGCTTGAAG GTGGACCAGC AAGTGAAGAA TTCAACTCCC TTCATGGCAG

---------- ---------- ---------- ---------- ----------

GGGCTTGAAG GTGGACCAGC AAATAAAGAA TTCAACTCCC TTCATGGCAG

GGGCTTGAAA GTGGACCAGC AAATGAAGAA TTCAACTCCC TTCATGGCAG

GGGCCTGAAG GTGGACCAGC AAGTGAAGAA CTCGACTCCC TTCATGGCAG

GGGCTTGAAG GTTGACCAGC AAGTGAAGAA TTCGACCCCC TTTATGGCAG

GGGCTTGAAG GTGGACCAGC AAGTGAAGAA TTCGACTCCC TTCATGGCAG

GGGCTTGAAG ATGGACCAGC AAGTGAAAAA TTCAACTCCC TTCATGGCAG

GGGCTTGAAA GTGGATCAAC AAATGAAGAA TTCAACTCCC TTCATGGCAG

GGGCTTGAAG GTGGACCAGC AAGTGAAGAA TTCAACTCCC TTCATGGCAG

GGGCTTGAAG GTGGACCAGC AAGTGAAGAA TTCAACTCCC TTCATGGCAG

GGGCTTGAAG GTGGACCAGC ACATGAAGAA TTCAACTCCT TTCATGGCAG

GGGCCTGAGA GTAGACCAGC AAGGGAAGAG TGGCCCACCC TTCCTGGCTG

AGGCCTGAAA GTGGATCAGC AGGGGAAAAT CTCTACACCT TTCATGGCAG

AACACCTAGA AGAGACCTTG AGTGCCGTAA CCAACAATCG ACAACTGAAG

AACACCTAGA AGAGACCTTG AGTGCTGTAA CCAACAATCG ACAACTGAAG

AACACCTAGA AGAGACCTTG AGTGCTGTAA CCAACAATCG ACAACTCAAG

AACACCTAGA AGAGACCTTG AGTGCTGTAA CCAACAATCG ACAACTCAAG

AACACCTAGA AGAGACCTTG AATGCTGTAA CCAACAATCG ACAACTCAAG

---------- ---------- ---------- ---------- ----------

AACATTTAGA GGAGACCCTG AGTGCTGTGA CTAGCAATCG GCAGCTCAAG

AACATTTAGA GGAGACCCTG AGTGCTGCGA GTAGCAATAG ACAGCTCAAG

AGCACTTAGA GGAGCCCCTG AGTGCCGGAA CCAGCAGTCG GCCACTCAAG

AACATTTAGA GGAGACCCTG AGTACTGTGG CCAGCAATCG ACAGCTCAAA

AACACTTAGA GGAGACCTTG AGTGCTGTAA CCAGCAATCG ACAGCTTAAG

AACACTTAGA GGAGACCCTG AGTGCTGTAA CCAGCAATCG ACAGCTCAAA

AACAGTTAGA GGAGACTCTG AGTGCTGTAA CCAGCAATCG GCAGCTCAAG

AACACTTAGA GGAGACCCTG AGTGCTGTAG CCAGCAATCG GCAGCTCAAA

AACATTTAGA GGAGACTCTA AATGCTGTAA CAAGCAGTCG GCAGCTCAAA

AACATTTAGA GGAGACCCTG AGTGCTGTAA CAAGCAACCG GCAGCTCAGA

AGCGTGCAGA GGAGGCCCTG AGTGCCGTGG CCAGCTCCCG GCCGCTCACA

AGCATTTGGA GGAGGCGCTA ACTGCAGTGG CTAGCTCTAG ACAACTCAAA

AAAGACGGAG ACATGACTGC GTTCAACAAG CTAGTGAGCA CAATGAAGGC

AAAGACGGAG ACATGACTGC GTTCAACAAG CTAGTGAGCA CAATGAAGGC

AAAGACGGAG ACATGACTGC GTTCAACAAG CTAGTGAGCA CCATGAAGGC

AAAGACGGAG ACATGACTGC GTTCAACAAG CTAGTGAGCA CAATGAAGGC

AAAGATGGAG ACATGACTGC ATTCAACAAG CTAGTGAGCA CAATGAAGGC

---------- ---------- ---------- ---------- ----------

AAAGATGGAG ATATGACTGC ATTCAACAAA CTAGTGAACA CCATGAAGGC

AAAGATGGAG ATATGACTGC ATTCAACAAG CTAGTGAACA CCATGAAGGC

AAAGACGGCG ATATGACTGC TTTCAACAAG CTAGTGAGCA CCATGAAGGC

AAAGACGGAG ACATGACTGC GTTCAACAAG CTAGTGAGCA CCATGAAGGC

AAAGATGGAG ATATGACTGC ATTCAACAAA CTAGTGAGCA CCATGAAGGC

AAAGATGGAG ATATGACTGC ATTCAACAAG TTAGTGAGCA CCATGAAGGC

AAAGATGGAG ATATGACTGC TTTCAACAAA CTTGTGAGCA CCATGAAGGC

AAGGATGGAG ATATGACTGC TTTCAACAAG CTGGTGAGCA CCATGAAGGC

AAGGATGGAG ATATGACTGC GTTCAACAAA CTAGTGAGCA CCATGAAGGC

AAGGACGGCG ATATGACTGC ATTCAACAAG CTAGTGAGCA GCATGAAAGC

AAAGACGGTG ACATGTCTGC CTTTAACAAG CTCGTGAGCA CCATGAAGGC

AAAGATGGTG ATATGTCTGC ATTTAACAAG CTAGTGAGCA GCATGAAGGC

AAGTGGGACT TTGCCTTCTC AGCCCAAAGT CAGCCGAAAC CTTGAAAGCC

AAGTGGGACT TTGCCTTCTC AGCCCAAAGT CAGCCGAAAC CTTGAAAGCC

AAGTGGGACT TTGCCTTCTC AGCCCAAAGT CAGCCGAAAC CTTGAAAGCC

AAGTGGGACT TTGCCTTCTC AGCCCAAAGT CAGCCGAAAC CTTGAAAGCC

AAGTGGGACT TTGCCTTCTC AGCCCAAAGT CAGCCGAAAC CTTGAAAGCC

---------- ---------- ---------- ---------- ----------

AAGTGGGACT TTGCCTACCC AGCCCAAAGT CAGTCGAAAT CTTGAAAGTC

AAGTGGAACT TTGCCTACCC AGCCCAAAGT CAGTCGAAAC GTTGAAAGTC

GAGCGGGACT CTGCCTTCTC AGCCCAAAGT CAGC------ ----------

AAGTGGGACT TTGCCTTCTC AGCCTAAAGT CAGTCGAACT CTTGAAAGCC

AAGTGGAACT TTGCCTTCTC AGCCCAAAGT CAGCCGAAAC CTTGAAAGCC

AAGTGGGACT TTGCCTTCTC AGCCCAAAGT CAGCCGAAAC CTTGAAAGCC

AAGTGGGACT TTGCCTTCTC AGCCCAAAAT CAGCCGAAAC CTTGAAAGCC

AAGTGGGACT TTGCCTTCAC AGCCCAAAGT CAGCCGAAAC CTTGAAAGCC

AAGTGGGACT TTGCCTTCAC AGCCCAAAGC CAATCGAAAC CTTGAAAGCC

AAGCGGGACT CTGCCCTCGC AGCCCAAAGC CAGT------ ----------

CAGTGGCACT CTGCCATCCC AGCCCAAAGT CAATCGAAAC CTTGAAAGCC

AAGTGGGACT TTACCTTCAC AGCCCAAAGT CAATCGAAAT CTCGAAAGTC

ATTTGATGTC CCCTGCTGAG ATTCCAGGCC AGCCTGTCCC TAAGAACATC

ATTTGATGTC CCCTGCTGAG ATTCCAGGCC AGCCTGTCCC TAAGAACATC

ATTTGATGTC CCCTGCTGAG ATTCCAGGCC AGCCTGTCCC TAAGAACATC

ATTTGATGTC CCCTGCTGAG ATTCCAGGCC AGCCTGTCCC TAAGAACATC

ATTTGATGTC CCCTGCTGAG ATTCCAGGCC AGCCTGTCCC TAAGAACATT

---------- ---------- ---------- ---------- ----------

ACTTGTTGGC TCCTGCTGAG ATTCCAGGCC AGCCTGTCCC TAAAAACATC

ACTTATTGGC CCCTGCTGAG ATTCCAGGCC AGCCTGTTTC TAAAAACATC

---------- ---------- ---------- ---------- ----------

ATTTGATGTC CCCTGCTGAG ATTCCAGGCC AGCCTGTCCC TAAGAACATC

ATTTGATGTC CCCTGCTGAG ATTCCAGGCC AGCCTGTCCC TAAGAACATC

ATTTGCTGTC CGCTGCCGAG AGTCCGGGCC GGCCTGTCCC TAAGAACATC

ACTTGATGTC CCCTGCTGAG ATTCCAGGCC AGCCTGTCCC TAAGAACATC

ATTTGATGTC CCCTGCTGAG ATCCCAGGCC AGCCTGTTCC GAAGAACATC

ATTTGATGTC CCCTGCGGAG ATACCAGGCC AGCCTGTCCC TAAGAACATC

---------- ---------- ---------- ---------- ----------

ATTTGATGTC CCATGCTGAG ATTCCCGGGC AACCTCTCCC TAAGAACATC

ATTTGATGTC CCATCCAGAG ATCCCAAGTC AGCCTCTTCC AAAGAACATC

CTGCAGGAAC TTCTGGGTCA ACCAGTTCAG AGACCTGCTT CTTCCAATCT

CTGCAGGAAC TTCTGGGTCA ACCAGTTCAG AGACCTGCTT CTTCCAATCT

CTGCAGGAAC TTCTGGGTCA ACCAGTTCAG AGACCTGCTT CTTCCAATCT

CTGCAG---- ---------- ---------- ---------- ----------

CTGCAGGAAC TTCTGGGTCA ACCAGTTCAG AGACCTGCTT CTTCCAATCT

---------- ---------- ---------- ---------- ----------

CTACAGGAAC TTCTGGGTCA ACCGGTTCAG AGACCTGCTT CTTCCAATCT

CTACAGGAAC TTCTGGGTCA ACCGGTTCAG AGACCCGCTT CTTCCAATCT

------GAAC TCCTGGGTCA GCCCGTTCAG AGACCTGCGT CTTCCAATCT

TTGCAGGAAC TTCTGGGCCC ACCTGTTCAG AGACCTGCTT CTTCCAATCT

CTGCAGGAAC TTCTGGGTCC ACCAGTTCAG AGACCTGCTT CTTCCAATCT

CTGCAGGAAT TACTGGGGCC ACCAGTTCAG AGACCTGCTT CTTCCAATCT

TTGCAGGAAC TTCTGGGTCC ACCAGTTCCG AGACCTGCGT CTTCCAATCT

CTGCAGGAAC TTCTGGGTCC ACCAGTCCAG AGACCTGCTT CTTCCAATCT

CTCCAGGAAC TTCTGGGTCC ACCAGTTCAG AGACCTGCGT CTTCCAATCT

------GAAC TTCTGGGTCC ACCAGTTCAG AGACCGGCTT CTTCTAATCT

TTACAGGAGC TTCTGGGTCC ACCAGTTACA AGACCTGCTT CTTCTCATCT

TTGCAGGAAC TTCTAGGTCC ACCAGTCACC AGACCTGCTT CATCTAATCT

TCTGAGTGGC CTTATGGGGA GCTTGGAGCC TACAACATCT TTACTGGGCC

TCTGAGTGGC CTTATGGGGA GCTTGGAGCC TACAACATCT TTACTGGGCC

TCTGAGTGGC CTTATGGGGA GCTTGGAGCC TACAACATCT TTACTGGGCC

---------- ---------- ---------- ---------- ----------

TCTGAGTGGC CTTATGGGGA GCTTGGAGCC TACAACATCT TTACTGGGCC

---------- ---------- ---------- ---------- ----------

TCTCAGTGGC CTTATGGGGA GCTTGGAGCC TACTGCATCT TTATTGAGCC

TCTCAGTGGC CTTATGGGGA GCTTGGAGGC TACTGCATCT TTATTGAGCC

TCTGAGTGGC CTCATGGGAA GCTTGGAGCC TACGACATCG ATCCTGGGCC

CCTGAGTGGC CTTATGGGGA GCCTGGAGCC TACGACATCA TTACTGGGCC

TCTGAGTGGC CTTATGGGGA GTTTGGAACC TACAACACCT TTACTGGGCC

TCTGAGTGGC CTTATGGGGA GCTTGGAGCC CACAACATCT TTGCTTGGCC

TCTGAGTGGC CTTATGGGGA GCTTGGAGCA TACCACATCT TTACTGGGCC

TCTGAGCGGC CTTATGGGGA GCCTGGAGCC TACAGCACCT TTACTGGGCC

TCTGAGTGGC CTTATGGGGA GCTTGGAGCC TACAACATCT TTACTAGGCC

TCTGAGTGGC CTGATGGGGA GCTTGGAGCC TACAGCATCT TTACTGGGCC

CCTGAGTGGC CTTATGGGGA GTCTGGAACC AACAGCATCC TTGCTGGGCC

TCTTAGTGGC CTAATGGGGA GTTTGGAACC TACTGCAAAT TTACTAGGCC

AAAGAGCACC CTCTCCTCCC TTGTCACAGG TGTTTCAAAC TCGAGCAGCC

AAAGAGCACC CTCTCCTCCC TTGTCACAGG TGTTTCAAAC TCGAGCAGCC

AAAGAGCACC CTCTCCTCCC TTGTCACAGG TGTTTCAAAC TCGAGCAGCC

---------- ---------- ---------- ---------- ----------

AAAGAGCACC CTCTCCTCCC TTGTCACAGG TGTTTCAAAC TCGAGCAGCC

---------- ---------- ---------- ---------- ----------

AAAGAGCACC CTCTCCTCCA ATGTCACAGG TGTTTCGAAC TCAAGCCGCC

AAAGAGCGCC CTCTCCTCCA ATGTCACAGG TATTTCGAAC TCAAGCAGCC

AGAGGGCACC GTCTCCCCCC TTGTCACAGG TGTTTCAAAC TCGAGCAGCC

AGAGAGCACC CTCTCCTCCA TTGTCACAGG TGTTTCAGAC GAGAGCAGCC

AAAGAGCACC CTCTCCTCCC TTGTCACAGG TGTTTCAAAC ACGAGCAGCT

AAAGGGCACC CTCTCCTCCC CTGTCACAGG TGTTTCAGAC ACGAGCAGCC

AAAGAGCACC TTCTCCTCCC TTGTCACAGG TGTTTCAGAC ACGAGCAGCC

AAAGAGCACC TTCTCCTCCC TTGTCACAGG TGTTTCAGAC ACGAGCAGCT

AAAGAGCACC CACTCCTCCC TTATCACAGG TGTTTCAATC GCGAGCAGCC

AAAGGGCACC CTCTCCTCCC TTGTCACAGG CATTTCAAAC TCGAGCAGCC

AGAGAGCCCC CTCTCCTCCC ATTTCACAGG TGTTTCCAAC TCGAGCAGCC

AAAGAGCATC CTCTCCCCCC ATCTCACAGG TGTTTCCAAC TCGTGCTTCT

TCAGCTGACT ACCTTCGCCC AAGAATACCA TCACCAATTG GTTTCACACC

TCAGCTGACT ACCTTCGCCC AAGAATACCA TCACCAATTG GTTTCACACC

TCAGCTGACT ACCTTCGCCC AAGAATACCA TCACCAATTG GTTTCACACC

---------- ---------- ---------- ---------- ----------

TCAGCAGACT ACCTTCGCCC AAGAATACCA TCACCAATTG GTTTCACAAC

---------- ---------- ---------- ---------- ----------

TCAGCAGACT ACCTTCATCC AAGGATACCA TCACCCATTG GTTTCCCATC

TCAGCAGACT ACCTTCATCC AAGGATACCA TCACCAATAG GTTTCCCTTC

TCAGCAGACT ACCTTCGCCC AAGAATACCG TCACCGATTG CTTTCACGCC

TCAGCAGACT ATCTTCGTCC CAGAATACCA TCACCAATTG GTTTCACGTC

TCAGCAGACT ACCTTCGTCC TAGAATACCA TCACCAGTTG GTTTCACACC

TCAGCAGACT ACCTTCGTCC TCGAATACCA TCACCAATTG ----------

TCGGCAGATT ACCTGCGTCC TAGAATACCA TCACCTATTG GTTTCACAGC

TCAGCAGACT ACCTTCGCCC CAGAATACCA TCACCAGTTG GTTTCACCCC

TCGGCAGACT ACCTTCGCCC TAGAATATCT TCACCAGTTG GTTTTACAGC

TCGGCAGACT ACCTTCGCCC TAGAATATCT TCACCAGTTG GTTTCCCACC

TCTGCAGACT ACTTGCGCCC TAGGATACCC TCACCCATTG GTTTTGGACC

TCAGCAGACT ATCTTCGACC CCGAATTCCT TCACCACTTG GCTTTGGACC

AGGACCACAG CAGCTACTCG GAGATCCATT CCAAGGCATG CGCAAACCCA

AGGACCACAG CAGCTACTCG GAGATCCATT CCAAGGCATG CGCAAACCCA

AGGACCACAG CAGCTACTCG GAGATCCATT CCAAGGCATG CGCAAACCCA

---------- ---------- ---------- ---------- ----------

AGGACCACAG CAGCTACTCG GAGATCCATT CCAAGGCATG CGCAAACCCA

---------- ---------- ---------- ---------- ----------

AGGACCCCAG CAACTACTTG GAGATCCATT CCAAGGCATG CGCAAGCCCA

AGGACCCCAG CAACTACTTG GAGATCCATT CCAAGGCATG CGCAAGCCCA

GGGACCACAG CCGCTACTTG GAGACCCATT CCAAGGCACA CGCAAGCCCA

AGGACCACAG CAGCTTCTCG GAGATCCATT CCAAGGCATG CGCAAGCCCA

AGGACCACAG CAGCTACTTG GAGATCCATT CCAAGGCATG CGCAAGCCCA

---------- ---------- ---------- ---------- ----------

AGGACCACAG CAACTACTTG GAGATCCATT TCAAGGCATG CGCAAGTCCA

AGGGCCACAG CAGCTGCTCG GAGATCCATT CCAAGGCATG CGCAAACCTA

AGGGCCACAG CAGCTGCTCG GAGACCCGTT CCAAGGCACG CGCAAGCCCG

AGGACCACCG CAGCTTCTTG GGGACCCGTT CCAAGGCATG CGCAAGCCCA

CAGTTCTCAG CAGTTGCTTG GAGATCCATT TCATGGAATG CGCAAATCGA

AAGCTCTCAA CAGTTGCTTG GGGATCCATT TCAAGGATTG CGGAAACCAA

TGAGCCCCAT CACAGCCCAG ATGAGCCAGC TGGAGTTGCA ACAGGCAGCT

TGAGCCCCAT CACGGCCCAG ATGAGCCAGC TGGAGTTGCA ACAGGCAGCT

TGAGCCCCAT CACGGCCCAG ATGAGCCAGC TGGAGTTGCA ACAGGCAGCT

---------- ---------- ---------- ---------- ----------

TGAGCCCCAT CACGGCCCAG ATGAGCCAGC TGGAGATACA ACAGGCAGCT

---------- ---------- ---------- ---------- ----------

TGAGTCCTGT CACAGCCCAG ATGAGCCAGC TAGAACTGCA GCAAGCTGCT

TGAGTCCTGT CTCAGCCCAG ATGAGCCAGC TAGAATTGCA GCAAGCTGCT

CGAGCCCGGT CTCGGCCCAG ATGAGCCAGC TGGAGTTGCA GCAGTCTGCC

TGAGCCCAGT CACGGCCCAG ATGAGCCAGC TGGAATTGCA ACAGGCAGCT

TGAGCCCGGT CACGGCCCAG ATGAGCCAGC TAGAATTGCA ACAGGCAGCT

---------- ---------- ---------- ---------- ----------

TGAGCCCTGT TACAGCCCAG ATGAGCCAGC TGGAACTGCA GCAGGCAGCT

TGAGCCCTGT TGCAGCCCAG ATGAGCCAGC TGGAGTTGCA ACAGGCCGCT

TGAGTCCTGT CACAGCCCAG ATGAGCCAGC TGGAGTTGCA ACAGGCCACT

TGAGCCCCAT CACAGTCCAG ATGAGCCAGT TGGAGTTGCA ACAGGCCGCT

TGAGTCCTGT TGCTGCCCAG ATGAGCCAGC TGGAGCTGCA GCACCCTGCC

TGAGCCCTGT TGCTGCACAG ATGAGTCAGT TAGAGTTGCA GCAGGCTGCA

TTAGAAGGGC TGGCCTTGCC ACATGACCTT GCTGTACAGG CAGCAAACTT

TTAGAAGGGC TGGCCTTGCC ACATGACCTT GCTGTACAGG CAGCAAACTT

TTAGAAGGGC TGGCCTTGCC ACATGACCTT GCTGTACAGG CAGCAAACTT

---------- ---------- ---------- ---------- ----------

TTAGAGGGGT TGGCCTTGCC ACATGATCTG GCTGTACAGG CAGCAAACTT

---------- ---------- ---------- ---------- ----------

TTAGAAGGGC TGACCCTGCC ACATGACCTT GCAGTACAGA CCACACCCTT

TTGGAGGGGC TGGCCCTACC ACATGACCTT GCAGTCCAGA CAGCACCCTT

CTGGAGGGGC TGGCCCTGCC TCACGACCTT GCAGTGCAGG CTGCAAGCTT

TTGGAGGGGC TGGCCGTGCC ACATGACCTC GCGCTGCAGG CAGCAAACTT

TTGGAGGGGC TGGCCCTGCC ACACGACCTT GCAATGCAGG CAGCAAACTT

---------- ---------- ---------- ---------- ----------

TTGGAGGGAC TGGCCTTACC ACATGACCTT ACAGTGCCAG CAGCAAATTT

TTGGAGGGGT TGGCCTTGCC TCATGACCTT GCAATGCAGG CAGCAAACTT

TTGGAGGGGC TGGCTTTGCC ACATGACCTT GCAATGCAGG CAGCAAACTT

CTGGAGGGGC TGGCCTTGCC ACATGACCTT GCAATGCAGA CAGCAAACTT

TTGGAGGGAC TCAACCTGCC TCATGAGGTA GCCATGCAGG CAGCAAGCTT

CTAGAAGGGC TCGCGTTGCC TCATGAACTA GCAATGCAGA CGACTAATTT

CTACCAGCCT GGTTTTGGCA AACCACAGGT GGACAGAACC AGAGATGGAT

CTACCAGCCT GGTTTTGGCA AACCACAGGT GGACAGAACC AGAGATGGAT

CTACCAGCCT GGTTTTGGCA AACCACAGGT GGACAGAACC AGAGATGGAT

---------- ---------- ---------- ---------- ----------

CTATCAACCT GGTTTTGGCA AACCACAGGT GGACAGAACC AGAGATGGAT

---------- ---------- ---------- ---------- ----------

CTACCAGCCT GGTTTTAGCA AACCTCAAGT GGACAGAACC AGAGATGGGC

CTACCAGCCT GGTTTTAGCA AACCACAAGT GGACAGAACC AGAGATGGGC

CTACCAGCCT GGTCTTGGCA AGCCACAGGT GGACAGAACC AGAGATGGAT

CTACCAGCCT GGTTTTGGCA AACCGCAGGT GGACAGAACC AGAGATGGAT

TTACCAACCT GGTTTTGGCA AGCCACAAGT GGACAGAACC AGAGATGGAT

---------- ---------- ---------- ---------- ----------

CTATCAGCCT GGTTTTGGCA AACCACAGAT GGACAGAACC CGAGATGGGT

CTACCAGCCT GGTTTTGGCA AACCACAGGT GGACAGAACC AGAGATGGAT

CTACCAGCCT GGTTTTGGCA AACCACAGGT GGACAGAACC AGAGATGGAT

CTACCAGCCT GGTTTTGGCA AACCACAAGT GGACAGAACC AGAGATGGAT

TTACCAACCT GGCTTCGGCA AACCCCAAAT GGACAAAAGC AGAGATGGCT

CTACCAGCCT GGCTTTGGCA AATCCCAGAT TGACAAAAAC AGAGATGGCT

TCAGAAACAG GCAACAGCGA GTGACCAAGT CACCAGCACC CGTGCATCGA

TCAGAAACAG GCAACAGCGA GTGACCAAGT CACCAGCACC CGTGCATCGA

TCAGAAACAG GCAACAGCGA GTGACCAAGT CACCAGCACC AGTGCATCGA

---------- ---------- ---------- ---------- ----------

TCAGAAACAG GCAACAGCGA GTGACCAAGT CACCAGCACC AGTGCATCGA

---------- ---------- ---------- ---------- ----------

TCAGAAACAG GCAACAGCGA ATGACAAAGT CACCAGCACC CATGCACCGA

TCAGAAACAG GCAACAGCGA ATGTCAAAGT CACCAGCACC CATGCATGGT

TCAGAAACAG GCAGCAGCGA GTGACCAAGT CACCAGCACC CGTGCACCGA

TCAGAAACAG GCAACAGCGA GTGACCAAGT CACCAGCACC CGTGCACCGA

TCAGAAACAG GCAACAGCGA GTGACCAAGT CACCAGCACC CGTGCACCGA

---------- ---------- ---------- ---------- ----------

TCAGAAATAG GCAACAGCGA GTGACCAAGT CACCAGCACC TGTGCACCGA

TCAGAAACAG GCAGCAGCGG GTGACCAAAT CGCCAGCACC TGTGCACCGA

TCAGAAACAG GCAGCAGCGA GTGACCAAGT CGCCAGCACC CGTACATCAA

ACAGAAACAG GCAACAGCGA GTGACCAAGT CGCCAGCAAA CATGCATCAA

TTAGGAACAG GCAGCAGCGT GTGGCCAAGT CGCCAGCCCC TGTGCATCGT

TTAAGAACAG ACAACAGAGA GTGATTAAAT CTCCTGCACC TGGACATCGA

GGGAATTCCT CTTCCCCTGC CCCTGCTGCC TCCATCACAA GCATGCTTTC

GGGAATTCCT CTTCCCCTGC CCCTGCTGCC TCCATCACAA GCATGCTTTC

GGGAATTCCT CTTCCCCTGC CCCTGCTGCC TCCATCACAA GCATGCTTTC

---------- ---------- ---------- ---------- ----------

GGGAATTCCT CTTCCCCCGC CCCTGCTGCC TCCATCACAA GCATGCTTTC

---------- ---------- ---------- ---------- ----------

GGAAATTCCT CTTCCCCAGC TCCCGCGGCC TCCATCACGA GCATGCTTTC

GGAAATTCCT CTTCCCCAGC TCCTGCTGCC TCCATCACAA GCATGCTTTC

GGGAATTCCT CTTCCCCCGC GCCTTCTGCC TCCATCACAA GCATGCTCTC

GGGAATTCCT CTTCCCCTGC CCCAGCCGCC TCCATCACAA GCATGCTTTC

GGGAATTCCT CTTCCCCTGC CCCTGCTGCC TCCATCACAA GCATGCTTTC

---------- ---------- ---------- ---------- ----------

GGGAGTTCTT CTTCCCCTGC CCCTGCCGCC TCCATTACAA GCATGCTTTC

GGGAATGCCT CTTCTCCAGC CCCTGCTGCC TCCATCACAA GCATGCTTTC

GGGAATTCCT CTTCCCCTGC CCCCTCTGCC TCCATCACAA GCATGCTTTC

GGGAATTCCT CTTCCCCTGC TCCCTCGGCC TCCGTCACAA GCATGCTTTC

GGGAATTCAG CATCTCCTGC TCCCACTGCT GCTGTCACCA GCATGCTATC

GGAAATTCAG CTTCTCCTGC TCCCAGTGCA TCCATCACTA GCATGCTGTC

TCCTTCCTTT ACCCCTACCT CAGTGATTCG TAAGATGTAC GAGAGCAAAG

TCCTTCCTTT ACCCCTACCT CAGTGATTCG TAAGATGTAC GAGAGCAAAG

TCCTTCCTTT ACCCCTACCT CAGTGATTCG TAAGATGTAC GAGAGCAAAG

---------- ---------- ---------- ---------- ----------

TCCTTCCTTT ACCCCTACCT CAGTGATTCG TAAGATGTAC GAGAGCAAAG

---------- ---------- ---------- ---------- ----------

TCCTTCCTTC ACCCCTACCT CAGTGATCCG CAAGATGTAT GAAAGCAGAG

TCCTTCCTTC ACCCCTACCT CAGTGATCCG GAAGATGTAT GAAAGCAGAG

TCCTTCCTTC ACCCCTACCT CAGTGATCCG TAAGATGTAC GAGAGCAAAG

TCCTTCCTTC ACTCCTACCT CAGTGATTCG TAAGATGTAT GAGAGCAAAG

TCCTTCCTTC ACCCCTACCT CAGTGATTCG TAAGATGTAT GAGAGCAAAG

---------- ---------- ---------- ---------- ----------

TCCTTCCTTC ACCCCTACCT CAGTGATTCG TAAGATGTAT GAGAGCAAAG

TCCTTCCTTC ACCCCTACCT CAGTGATTCG TAAGATGTAC GAGAGCAAAG

TCCTTCCTTC ACCCCTACCT CAGTGATTCG TAAGATGTAC GAGAGCAAAG

TCCCTCCTTC ACCCCTACCT CGGTGATTCG AAAGATGTTC GAGAGCAAAG

TCCTTCCTTC ACTCCTACCT CAGTGATCCG CAGGATGTAT GAAAGCAAAG

TCCTTCCTTT ACCCCTACCT CAGTGATACG AAAGATGTAT GAAAGCAAAG

AGAAAAGCAA GGAGGAGCCA GCATCTGGAA AAGCAGCTCT TGGTGACAGT

AGAAAAGCAA GGAGGAGCCA GCATCTGGAA AAGCAGCTCT TGGTGACAGT

AGAAAAGCAA GGAGGAGCCA ATATCTGGAA AAGCAGCTCT TGGTGACAGT

---------- ---------- ---------- ---------- ----------

AGAAGAGCAA GGAGGAGCCA GCATCTGGAA AAGCAGCTCT TGGTGACAGT

---------- ---------- ---------- ---------- ----------

AGAAAAGCAA GGAGGAAATA GCACCCGGAA CAGTGGTTCC TGGTGATGGT

AGAAAACCAA GGAGGAAATG GCACCTGGAA TGGTGGTTCC TGGTGATGGT

AGAAAACCAA GGAGGAGCCA GCAGCCGGGA AAGCCGCTGG GAGTGAGAGT

AGAAAAGCAA GGAGGAACCA ACCTCTGGAA AAGCAGCTCT TGGTGACGGT

AAAAAAGCAA GGAGGAGCCG GCATCTGGAA AAGCAGCTCT TGGTGACAGT

---------- ---------- ---------- ---------- ----------

AGAAAAGCAA AGAGGAGCCA GCATCTGGAA AAACAGCTCT TGGCGATTGC

AGAAAAGCAA GGAGGAGCCA ACATCTGGAA AAGCGACTCT TGGTGACAGC

AGAAAAGCAA GGAGGAGCCA GCATCTGGAA AAGCAGCTCT CAGTGATAGT

AGAAAAGCAA GGAGGAGCCT GCCTCTGGGA AAGCCGCTCT TGGTGACCAT

AGAAGAACAA AGAAGACCCT ATTTCTGGAA AACCAGC--- CAGTGATGGG

AGAAAAGTAA AGAGGAACCA ACTCCTGGAA AACCAAC--- AGGTGATGGG

AAAGAGGATA CTCAGAAGGC CAGTGAAGAA AACCTCCTGT CAAGCTCTGT

AAAGAGGATA CTCAGAAGGC CAGTGAAGAA AACCTCCTGT CAAGCTCTGT

AAAGAGGATA CTCAGAAGGC CAGTGAAGAG AACCTCCTGT CCAGCTCTGT

---------- ---------- ---------- ---------- ----------

AAAGAGGATA CTCAGAAGTC TAGTGAAGAG AACCTCCTGT CAAGCTCTCT

---------- ---------- ---------- ---------- ----------

AAAGAGGATG CTCAGAAGGC CAGCGAAGAA AACCTGCTGT CAAATTCCAT

AAAGAGGATA CTCAGAAGAC CAGCGAAGAG AACCTGCTGT CAAATCCCAT

AAAGAGGATG CGCAGAAGGC CAGTGAAGAA AACCTCCTGT CGAACTCTCT

AAAGAGGACA CCCAGAAGCC CAGTGAAGAG AACCTCCTGT CAAGCTCTGT

AAAGAAGACA CTCAGAAGGC TAGTGAAGAG AATCTGCTGT CAAACTCTGT

---------- ---------- ---------- ---------- ----------

AAAGAAGATA CACAGAAGAC CAATGAAGAG AATCTCCTGT CAAACTCTCT

AAAGAGGACA CTCAGAAGGC CAGTGAAGAG AACATCCTGT CAAACTCTGT

AAAGAGGACA CTCACAAGGC CAGTGAAGAG AACCGCCTGT CAACCTCTGT

AAAGAGGGCA CTCAGGAGGC CAGTGAAGAC AACCTGCTGT CAAACCCCAT

AAAGAAGAAG GCCAGAGGGC AAATGAAGAG AACCTTCTGT TGAGTTCCAT

AAAGATGAAA ATTCAAGGAC AAATGAAGAT CACCTCCTTT CGAGTTCCGT

ACCCAGTGCC GATCGAGACT CTTCTCCCAC TACAAATTCC AAACTGTCAG

ACCCAGTGCC GATCGAGACT CTTCTCCCAC TACAAATTCC AAACTGTCAG

ACCCAGTGCC GATCGAGACT CTTCTCCCAC TACAAATCCA AAACTGTCAG

---------- ---------- ---------- ---------- ----------

ACCCAGTGCT GATCGAGGCT CTTCTCCCAC TACAAATCCG AAACTATCAG

---------- ---------- ---------- ---------- ----------

ATCCAATTCT GATCAAGACT CTTCTCCTAC GGCAAATCCT AAACTTTCAA

ACCCAATACT GATCAAGACT CTTCT---AC GACAAATCCT AAGCTTTCAA

ACCCAGTGCT GATCGGGACT CCTCGCCCAC TACAAATCCC AAACTGTCAG

GCCCACTGCT GATCGGGACT CTTCTCCCAC CACAAATCCC AAATTGTCAA

GCCCAGCGCT GATCGTGACT CTTCTCCTAC TACAAATCCC AAACTTTCAA

---------- ---------- ---------- ---------- ----------

GTCCAGTGCT GATCGAGACT CTTCTCCTAC TACAAATCCC AAACTTTCAA

GCCCAATGCT GATAGAGACT CTTCTCCCAC TACAAATCCC AAACTGTCAA

GCCCACTGCT GATCGAGACT CTTCTCCCAC TACAAACTCC AAGCTGTCAA

GTCCAGTGCT GATCGAGACT CTTCTCCCAC GACAAATCCC AAGATGTCAA

GGCGAATTCT GACCGAGACA CTTCCCCTAC CATGAACCCG AAACTCTCCA

AGTGAACTCG GATCAAGATG CTATCCCCCC CCTAAGTACC AAACTGTCAG

CATTACAGAG GTCTTCGTGT TCCACCCCAC TGTCCCAGGC CAACCGTTAC

CATTACAGAG GTCTTCGTGT TCCACCCCAC TGTCCCAGAC CAACCGTTAC

CGTTACAGAG GTCTTCGTGT TCCACCCCAC TGTCCCAGAC CAGCCGTTAC

---------- ---------- ---------- ---------- ----------

CGTTACAGAG GTCTTCGTGT TCCACCCCAC TGTCCCAGAC CAACCGTTAC

---------- ---------- ---------- ---------- ----------

CATTACAGCG GTCTTCATGC TCCACCCCAC TGTCCCAGAC CAACCGTTAT

CATTACAGCG GTCTTCATGT TCCACCCCAC TCTCCCAGAC CAGCCGTTAT

CGTTACAGCG GTCTTCGTGT TCCACACCAC TGTCCCAGAC CACCAGTTAC

CCCTGCAGCG GTTTTCCTGT TCCACCCCAC TGTCCCAGAC CAACCGTTAC

CGTTGCAGCG GTCTTCGTGT TCTACCCCAC TGTCTCAAAC CAACCGTTAC

---------- ---------- ---------- ---------- ----------

CATTGCGAC- --CTTCATGT TCCACTCCAC TGTCCCAGAA CAACCGCTAC

CGTTGCAGCG GTCTTCATGT TCCATCCCAC TATCCCAGAC CAACCGTTAC

CGTTGCAGCG GTCTTCGTGT TCTACCCCAC TGTCTCAAAC CAACCGTTAC

CGTTGCAGCG GTCTTCCTGC TCTACCCCTC TGTCCCAAAC CAACCGTTAC

CGCTGCAGCG ATCTGCATCT TCCACCCCCC TGTCCCAGGC CAGCCGCTAC

CACTTCAGCG CTCGGCATGT TCCACCCCAC TGTCTCAAAC CAACCGTTAC

ACCAAAGAAC AAGATTATCG ACCTAAAGCA ACTGGGAGAA AAACACCCAC

ACCAAAGAAC AAGATTATCG ACCTAAAGCA ACTGGGAGAA AAACACCCAC

ACCAAAGAAC AAGATTATCG ACCTAAAGCA ACTGGGAGAA AAACACCCAC

---------- ---------- ---------- ---------- ----------

ACCAAAGAAC AAGATTATCG ACCTAAAGCA GCTGGGAGAA AAACACCCAC

---------- ---------- ---------- ---------- ----------

ACCAAAGAAC AAGATTATCG CCCTAAAGCA GCTGGGAGGA AAACACCTAC

ACCAAAGAAC AAGATTATCG ACCTAAAACA GCAGGAAGGA AAACACCTAC

ACCAAAGAAC AAGATTACCG CCCTAAAGCA ACTGGGAGGA AAACGCCCAC

ACCAAAGAAC AAGATTATCG ACCTAAAGCA ACGGGGAGGA AGACACCCAC

ACCAAAGAAC AAGATTATCG ACCTAAAGCA ACTGGGAGAA AAACCCCCAC

---------- ---------- ---------- ---------- ----------

ACCAAAGAAC AAGATTACCG ACCTAAACCA ACTGGGAGAA AAACTCCTAC

ACCAAAGAAC AAGATTATCG ACCTAAAACA ACTGGGAGAA AAACCCCCAC

ACCAAAGAAC AAGATTATCG ACCTAAGGGA ACTGGGAGAA AAACACCCAC

ACCAAAGAAC AAGATTATCG ACCTAAAGCA GCCGGGAGGA AAACACCCAC

ACCAAAGAAC AGGACTATCG GCCTAAGGCA GCAGGCAGAA AGACTCCCAC

ACCAAAGAGC AAGACTACAG GCCAAAATCA ACTGGTCGGA AGACTCCTAC

CTTGGCATCC CCAGTTCCTA CAACACCTTT TCTCCGCCCT GTCCACCAAG

CTTGGCATCC CCAGTTCCTA CAACACCTTT TCTCCGCCCT GTCCACCAAG

CTTGGCATCC CCAGTTCCTA CAACACCTTT TCTCCGCCCT GTCCACCAAG

---------- ---------- ---------- ---------- ----------

CTTGGCATCC CCAGTTCCTG CAACACCCTT TCTCCGCCCT GTCCACCAAG

---------- ---------- ---------- ---------- ----------

CTTGGCATCC CCAGTCCCAG GAACACCTTT TCTCCGCCCT ACCCACCAAG

CTTGGCATCC CCAGTCCCAG GAACACCTTT TCTCCGCCCT ACCCACCAAG

CTTGGCGTCC CCGGTTCCAG GAACACCTTT CCTCCGCCCC GTCCACCAAG

CCTGGCATCC CCCGTTCCAG GAACACCTTT TCTCCGCCCT GTCCACCAAG

CTTGGCATCC CCAGTTCCAG GAACACCTTT TCTCCGACCT GTCCACCAAG

---------- ---------- ---------- ---------- ----------

TTTGGCATCA CCAGTTCCAG GAACACCTTT TCTCCGCCCT GTCCATCAAG

CTTGGCTTCC CCAGTTCCAG GAACACCTTT TCTCCGCCCT GTCCACCAAG

CTTGGCTTCC CCAGTTCCAG GAGCACCTTT TCTCCGCCCT GTCCACCAAG

CTTGGCCTCC CCAGTTCCAG GAGCACCTTT TCTCCGCCCT GTCCACCAAG

CCTGGCCTCC CCAGTGCCAG GGGCACCTTT CCTACGTCCT GTCCACCAGG

GATGGCATCC CCAATACCAG GGACTCCTTT TCTCCGGCCG GTTCACCAGG

TTCCCCTTGT CCCCCATGTC CCTATGGTTA GGCCTGCTCA CCAGCTTCAC

TTCCCCTTGT CCCCCATGTC CCTATGGTTA GGCCTGCTCA CCAGCTTCAC

TTCCCCTTGT CCCCCATGTC CCTATGGTTA GGCCTGCTCA CCAGCTTCAC

---------- ---------- ---------- ---------- ----------

TTCCCCTTGT CCCCCACGTC CCTATGGTTC GGCCTGCTCA CCAGCTTCAC

---------- ---------- ---------- ---------- ----------

TTCCCCTCGT CCCTCATGTT CCTATTGTTC GACCTGCACA TCAGCTTCAC

TTCCCCTTGT CCCTCATGTT CCTATTGTTC GACCTGCACA CCAGCTTCAC

TTCCCCTCGT TCCCCACGTC CCTATTGTCC GGCCTGCTCA CCAGCTTCAC

TCCCCCTGGT CCCCCACGTC CCCATCGTTC GGCCCGCTCA CCAGCTTCAC

TTCCCCTTGT CCCCCATGTC CCAATCGTTC GGCCTACTCA CCAGCTTCAC

---------- ---------- ---------- ---------- ----------

TTCCCCTTGT CCCCCATGTT CCAATTGTTC GGCCTGCTCA CCAGCTCCAC

TTCCCCTTGT CCCCCATGTA CCAATCGTTC GGCCTGCGCA TCAGCTTCAC

TTCCCCTTGT CCCCCATGTA CCGATCGTTC GGCCTGCTCA CCAGCTTCAC

TCCCCCTGGT CCCCCATGTA CCAATCGTTC GACCTGCTCA CCAGCTTCAC

TCCCGCTTGT CTCCCATGTT CCAATTGTTC GGCCTGCTCA TCAGTTTCAC

TGCCGCTTGT GTCCCACGTT CCAGTCGTTC GCCCTTCCCA CCAATTGCAC

CCAGGGTTGG TACAGAGGAT GCTGGCCCAG GGAGTACATC CACAGCATCT

CCAGGGTTGG TACAGAGGAT GCTGGCCCAG GGAGTACATC CACAGCATCT

CCAGGGTTGG TACAGAGGAT GCTGGCCCAG GGAGTACATC CACAGCATCT

---------- ---------- ---------- ---------- ----------

CCAGGATTGG TACAGAGAAT GCTGGCACAG GGAGTACATC CACAGCATCT

---------- ---------- ---------- ---------- ----------

CCAGGATTAG TCCAGAGGTT GATAGCCCAA GGGGTGCATC CACAGCATCT

CCAGGATTAG TCCAAAGGTT GATAGCCCAA GGGGTACATC CACAGCATCT

CCGGGGCTGG TTCAGAGGAT GCTGGCCCAA GGAGTCCATC CACAGCATCT

CCAGGGTTGG TCCAAAGGAT GCTGGCCCAG GGAGTACATC CACAGCATCT

CCAGGGTTGG TCCAGAGGAT GCTGGCCCAG GGAGTACATC CACAGCATCT

---------- ---------- ---------- ---------- ----------

CCAGGATTGG TCCAAAGGAT GCTGGCCCAG GGAGTACATC CACAGCATCT

CCAGGCTTGG TCCAAAGGAT GCTGGCCCAG GGAGTACATC CACAGCATCT

CCAGGGCTGG TCCAAAGGAT GCTTGCCCAG GGAGTACACC CACAGCATCT

CCAGGCCTGG TCCAAAGGAT GCATATTCA- ---------- -ACAGCATCT

CCGGGACTGG TCCAAAGGAT GCTGGCCCAG GGGGTCCATC CTCAGCATCT

CCAGGGCTGG TCCAAAGAAT GTTGGCACAG GGAGTGCATC CACAACATCT

TCCAAGTTTG CTCCAAACTG GTGTGCTTCC TCCTGGGATG GACTTGAGTC

TCCAAGTTTG CTCCAAACTG GTGTGCTTCC TCCTGGGATG GACTTGAGTC

TCCAAGTTTG CTCCAAACTG GTGTGCTTCC TCCTGGGATG GACTTGACTC

---------- ---------- GTGTGCTTCC TCCTGGGATG GACTTGACTC

TCCGAATTTG CTCCAAGCTG GCGTGCTTCC TCCTGGGATG GACTTGACTC

---------- ---------- ---------- ---------- ----------

TCCAGGTTTG CTCCAAGCTG GTGTGCTTCC TCCTGGGATA GACATAGCTC

TCCAAGTTTG CTCCAAGCTG GTGTGCTTCC TCCTGGGATA GACATGGCTC

CCCAAACCTG CTGCAAGCTG GTGTGCTTCC TCCTGGGATG GACCTGACTC

TCCAAGTTTG CTCCAAGCTG GTGTGCTTCC CCCTGGGATG GACCTGACCC

TCCAAGTTTG CTCCAGGCTG GTGTGCTTCC TCCTGGAATC GACCTGACTC

---------- ---------- ---------- ---------- ----------

TACAAATTTG CTCCAAGCTG GTGTGCTTCC TCCTGGGATG GATCTGACTC

TCCAAGTTTG CTCCAAGCTG GTGTGCTTCC TCCAGGGATG GACCTGACTC

TCCAAGTTTG CTCCAAGCTG GTGTGCTTCC TCCTGGGATG GACCTGACTC

TCCAAGTTTG CTCCAAGCTG GTATGCTTCC ACCAGGGATG GACCTGACGC

CCCAACGCTG CTTCAAGCCG GTATGCTTCC TCCTGGGATG GACTTGTCTC

TCCATCTTTA CTGCAGGCTG GCATGATTCC TCCTGGGGTG GACTTGTCTC

ATTTACAGGG AATATCTGGC CCCATCCTGG GTCAGCCCTT TTACCCTTTA

ATTTACAGGG AATATCTGGC CCCATCCTGG GTCAGCCCTT TTACCCTTTA

ATTTACAGGG AATATCTGGC CCTATCCTGG GTCAGCCCTT TTACCCTTTA

ATTTACAGGG AATATCTGGC CCTATCCTGG GTCAGCCCTT TTACCCTTTA

ATTTACAGGG AATATCTGGC CCTATCCTGG GTCAGCCCTT TTACCCTTTA

---------- ---------- ---------- ---------- ----------

CTTTACAGGG ACTATCTGGC CCACTCCTAG GTCAACCCTT GTACCCTTTG

CTTTACAGGG GCTATCTGGC CCACTCCTGG GTCAACCCTT GTACCCTTTG

ATTTACAGGG ATTATCTGGC CCAATTCTGG GTCAGCCCTT TTACCCTCTG

ATTTACAGGG ACTGTCTGGC CCAATCCTGA GTCAACCCTT TTACCCTCTA

CTTTACAGGG AATATCTGGC CCAATCCTGA GTCAACCCTT TTACCCTTTA

---------- ---------- ---------- ---------- ----------

ATTTACAAGG ACTATCTGGC CCAATTCTGG GTCAACCATT TTACCCTATA

ATTTACAGGG AATATCTGGT CCAATCCTGG GTCAGCCCTT TTACCCTTTA

ATTTACAAGG AATATCTGCC CCTATCCTGG GTCAGCCTTT TTACCCTTTA

ATTTACAGGG TATATCTGGC CCAATCCTGG GTCAGCCTTT TTACCCTTTA

ATTTGCAGGG AATCTCTGGC CCTCTCCTGG GGCAACCCTT TTACCCCCTC

ACTTGCAAGG AATATCTGGT CCCATTCTGG GGCAACCATT TTACCCATTA

CCTGCTGCTA GTCACCCTCT CTTAAACCCT CGTCCTGGAA CACCTCTGCA

CCTGCTGCTA GTCACCCTCT CTTAAACCCT CGTCCTGGAA CACCTCTGCA

CCTGCTGCTA GTCACCCTCT CTTAAACCCT CGTCCTGGAA CACCCCTGCA

CCTGCTGCTA GTCACCCTCT CTTAAACCCT CGTCCTGGAA CACCTCTGCA

CCTGCTGCTA GTCACCCTCT CTTAAACCCT CGTCCTGGAA CACCTCTGCA

---------- ---------- ---------- ---------- ----------

TCTGCTGCTA GTCACCCTCT CCTAAACCCT CGTCCTGGGA CCCCTCTGCA

TCTGCTGCTA GTCACCCTCT CCTAAACCCT CGTCCTGGGA CCCCTCTGCA

CCTGCTGCTA GTCACCCTCT CCTAAACCCT CGTCCTGGGA CACCTTTGCA

CCTGCCACTA GTCACCCTCT CCTAAACCCT CGTCCTGGGA CACCTCTGCA

CCTACGGCTA GTCACCCTCT CCTAAACCCT CGCCCTGGGA CACCTCTGCA

---------- ---------- ---------- ---------- ----------

CCTGCCCCTA GCCACCCTCT CCTAAACCCT CGTCCTGGGA CACCTTTGCA

CCCACCACTA GTCACCCTCT CCTAAACCCT CGTCCAGGGA CACCACTGCA

CCCGCTGTTA GTCACCCTCT CCTAAACCCT CGTCCTGGGA CACCTTTGCA

CCCGCTGCTA GTCACCCTCT ACTAAACCCT CGTCCCGGGA CACCTCTGCA

CCTGCAGCCA GTCACCCTCT CTTGACCCCT CGCTCGGGGA CCCCCCTGCA

CCCACTGCTA GCCACCCCCT CTTGAACCCA CGCTCTGGTA CACCCCTGCA

TCTGGCAATG GTGCAACAGC AGCTACAGCG CTCAGTTCTG CATCCTCCAG

TCTGGCAATG GTGCAACAGC AGCTACAGCG CTCAGTTCTG CATCCTCCAG

TCTGGCAATG GTGCAACAGC AGCTACAGCG CTCAGTTCTG CATCCTCCAG

TCTGGCAATG GTGCAACAGC AGCTACAGCG CTCAGTTCTG CATCCTCCAG

TCTGGCAATG ATGCAACAGC AGCTACAGCG CTCAGTTTTG CATCCTCCAG

---------- ---------- ---------- ---------- ----------

TTTGGCAGTG ATGCAGCAGC AGCTACAGCG CTCAGTTCTG CATCCTCCAG

TTTGGCGGTG ATGCAGCAGC AGCTACAGCG CTCAGTTCTG CATCCTCCAG

TCTGGCAATG ATGCAGCAGC AGCTACAGCG CTCAG----- ----------

TCTGGCAATG ATGCAGCAGC AGCTACAGCG CTCAGTTCTT CATCCTCCAG

TCTGGCAATG ATGCAGCAGC AACTACAGCG CTCAGTTCTT CATCCTCCAG

---------- ---------- ---------- ---------- ----------

TCTGGCAGTG ATGCAACAGC AGCTACAGCG CTCAGTTCTT CATCCTCCAG

TCTGGCAATG ATGCAACAGC AGCTACAGCG CTCAGTTCTG CATCCTCCAG

TCTGGCAATG ATGCAGCAGC AGTTACAGCG CTCAGTTCTG CATCCTCCAG

TCTGGCAATG ATGCAGCAGC AGTTACAGCG CTCAG----- ----------

GCTCGCGGTG ATGCAGCAGC AGCTGCAGCG GTCAGTCCTC CATCCCCCGG

GCTGGCAATG ATGCAACAAC AACTCCAGCG GTCAGTCTTA CATTCTCCAA

GCTCTGGTTC CCATGCAGCA GCTGTCAGCG TTCAGACAAC CCCTCAGAAC

GCTCTGGTTC CCATGCAGCA GCTGTCAGCG TTCAGACAAC CCCTCAGAAC

GCTCTGGTTC CCAGGCAGCA GCTGTCAGCG TTCAGACAAC TCCTCAGAAC

GCTCTGGTTC CCAGGCAGCA GCTGTCAGCG TTCAGACAAC TCCTCAGAAC

GCTCTGGTTC CCAGGCAGCA GCTGTCAGTG TTCAAACAGC CCCTCAGAAT

---------- ---------- ---------- ---------- ----------

GCTCTAGTTC CCAGGCAGCA GCTATCAACG TTCAGA---C TCCTCCGAAT

GCTCTAGTTC CCAGGCAGCA GCTATCAGCG TTCAGA---C TCCTCAGAAT

---------- ---------- ---------- ---------- ----------

GCTCTGGTTC CCAGGCAGCT GCTGTCAGCG TTCAGACGAC TCCTCAGAAT

GCTCTGGTTC CCCGGCAGCA GCTGTCAGCG TTCAGACAAC CCCTCAGAAT

---------- ---------- ---------- ---------- ----------

GCTCTGGTTC CCAGGCAGCA GCTGTCAGCA TTCAGACAAC CCCTCAGAAT

GTTCTGGTTC CCAGGCAGCA GCCGTTGGCG TTCAGACAAC CCCCCAGAAT

GCTCCGGTTC CCAGGCAGCA GCTGTCAGCA TTCCGACAAC TCCCCAAAAT

---------- ---------- ---------- ---------- ----------

GCTCTGGCTC ACAGGGAACA GCCATTGGTG TCCAGACGCC CCCCCAGAAC

GCTCCAGCTC CCAGGGAATG ACTGGTGGTG GACAAACCAC CTCCCAGAAC

GTGCCCAGCC GGTCAGGCCT GCCCCACATG CACTCCCAGC TGGAGCATCG

GTGCCCAGCC GGTCAGGCCT GCCCCACATG CACTCCCAGC TGGAGCATCG

ATGCCCAGCC GGTCGGGCCT GCCCCACATG CACTCCCAGC TGGAGCATCG

ATGCCCAGCC GGTCGGGCCT GCCCCACATG CACTCCCAGC TGGAGCATCG

GTGCCCAGCC GGTCAGGCCT GCCCCACATG CACTCCCAGC TGGAGCATCG

---------- ---------- ---------- ---------- ----------

GTACCCAGCC GGTCAGGCCT GCCCCATATG CACTCCCAGC TGGAGCATCG

GTACCCAGCC GGTCGGGCAT GCCCCACATG CACTCCCAGC TGGAGCATCG

---------- ---------- ---------- ---------- ----------

GTGCCCAGCC GGTCAGGCCT GCCGCATGTG CACTCCCAGC TCGAGCACCG

GTGCCCACCA GGTCAGGCCT GCCCCACGTG CACTCCCAGC TGGAACATCG

---------- ---------- ---------- ---------- ----------

GTGCCCAGTC GGTCAGGCCT GCCCCACATG CACTCCCAGC TGGAGCACCG

GTGCCCAGCC GGTCAGGCCT GCCCCACATG CACTCTCAGC TGGAGCACCG

GTGCCCAGCC GGTCAGGCCT GCCCCACGTG CACTCCCAGC TGGAGCACCG

---------- ---------- ---------- ---------- ----------

CTGTCGGGCC GGCCGGGCCT GCCCCACATT CCCCCACAGC TGGAGCACCG

ACGCCTGGTC GTTCTGGACT GCCTCATGTA CACTCGCAGC TCGACCATCG

CCCCAGCCAG AGGAGCAGCT CCCCTGTGGG CCTTGCCAAA TGGTTTGGCT

CCCCAGCCAG AGGAGCAGCT CCCCTGTGGG CCTTGCCAAA TGGTTTGGCT

CCCCAGCCAG AGGAGCAGCT CCCCTGTGGG CCTTGCCAAA TGGTTTGGCT

CCCCAGCCAG AGGAGCAGCT CCCCTGTGGG CCTTGCCAAA TGGTTTGGCT

CCCCAGCCAG AGGAGCAGCT CCCCCGTGGG ACTCGCCAAA TGGTTTGGCT

---------- ---------- ---------- ---------- ----------

CACCAGCCAA AGGAGCAGCT CCCCCGTGGG TCTTGCCAAG TGGTTTGGCT

TACCAGCCAA AGGAGCAGCT CCCCTGTGGG CCTTGCCAAA TGGTTTGGCT

---------- ---------- ---------- ---------- ----------

CCCCAGCCAG AGGAGCAGCT CCCCTGTGGG CCTCGCCAAA TGGTTTGGCT

CCCCAGCCAG AGGAGCAGCT CCCCAGTAGG CCTCGCTAAG TGGTTTGGCT

---------- ---------- ---------- ---------- ----------

CCCCAGCCAG AGGAGCAGCT CCCCAGTGGG CCTCGCCAAA TGGTTTGGCT

CACCAGCCAG AGGAGCAGCT CCCCTGTGGG CCTCGCCAAG TGGTTTGGTT

CTCCAACCAG AGGAGCACCT CCCCGGTGGG CCTTGCCAAA TGGTTTGGCT

---------- ---------- ---------- ---------- --GTTTAGCT

CCCCAGCCAG AGGAGCACCT CCCCTGTGGG CTTGGCCAAG TGGTTCGGCT

CTCCAGCCAG CGAAGTGGTT CTCCTGTTGG CCTTGCAAAA TGGTTTGGCT

CAGATGTGCT ACAGCAACCC CTGCCCTCCA TGCCCGCCAA AGTTATCAGT

CAGATGTGCT ACAGCAACCC CTGCCCTCCA TGCCCGCCAA AGTTATCAGT

CAGATGTGCT ACAGCAACCC CTGCCTTCGA TGCCTGCCAA AGTTATCAGT

CAGATGTGCT ACAGCAACCC CTGCCTTCGA TGCCTGCCAA AGTTATCAGT

CAGATGTGCT ACAGCAACCC CTGCCCTCCA TGCCCACCAA AGTTATCAGT

---------- ---------- ---------- ---------- ----------

CAGATGTGCT ACAGCAGCCT CTGCCCTCCA TGCCCACCAA AGTCATCAGT

CAGATGTGCT ACAGCAGCCT CTGCCCTCCA TGCCCACCAA AGTCATCAGT

---------- ---------- ---------- ---------- ----------

CAGATGTGCT ACAGCAGCCC CTGCCCTCCA TGCCCACCAA AGTCATCAGT

CAGATGTGCT ACAGCAACCC CTGCCCTCCA TGCCTGCCAA AGTCATCAGT

---------- ---------- ---------- ---------- ----------

CGGATGTGTT ACAGCAGCCT CTGCCCTCCA TGCCCGCCAA AGTCATCAGT

CAGACGTGCT GCAGCAGCCC CTGCCCTCTA TGCCCGCCAA AGTCATCAGT

CAGATGTGCT GCAGCAGCCC CTCCCCTCTA TGCCCGCCAA AGTCATCAGC

CAGATGTGCT GCAGCA-CCC TTGCCTTCTA TGCCTGCCAA AGTCATCAGT

CAGACGTGCT GCAGCAGCCC CTGCCCTCGA TGCCCTCCAA AGTCATCAGC

CGGATGTCTT GCAACAACCT CTCCCTTCTA TGCCCTCCAA AGTTATCAGC

GTAGATGAAT TGGAATACCG ACAGCGTTCT AATACCCGAT TTACAATTAC

GTAGATGAAT TGGAATACCG ACAGCGTTCT AATACCCGAT TTACAATTAC

GTAGATGAAT TGGAATACCG ACAGGGTTCT GATACCCGAT TTACAATTAC

GTAGATGAAT TGGAATACCG ACAGGGTTCT GATACCCGAT TTACAATTAC

GTAGATGAAT TGGAATACCG ACA-TGTTCT GATACCCGAT TTGCAATTAC

---------- ---------- ----GGTTCT GATACCCGAT TTGCAATTAC

GTAGATGAAC TGGAATATAG ACA-TGTTCT GATACCCGAT TTGCAATTAC

GTAGATGAAC TGGAATATAG ACA-CGTTCT GATACCCGAT TTGCAATTAC

---------- ---------- ---------- ---------- ----------

GTAGATGAAC TGGAGTACCG ACAG------ ---------- ----------

GTAGATGAAT TGGAGTACCG TCAAGGTTCT GATACCCGGT TTGCAATCAC

---------- ---------- ---------- ---------- ----------

GTAGATGAAT TGGAATACCG ACAG--TTCC GATGCCCGGT TTGCAATTAC

GTAGATGAAC TGGAATACCG ACAGGGTTCT GATACCCGGT TTGCAATTAC

GTAGATGAGT TGGAATACCG ACAGGGTTCT GATACCCGGT TTGCAATTAC

GTAGACGAGC TGGAGTACCG ACCG--TTCT GATACCCGGT TTGCAATTAC

GTGGATGAGC TGGAGTATAG GCAG--TTCT GACATCCAGT TTGCAGTAAC

GTAGATGAAC TGGAATATCG TCAG--CCCT GATACCCGTT TTTCAATAAC

ATTGAACTAC AAGGATCCCC TCACTGGAGA TGAAGAGACC TTGGCTTCAT

ATTGAACTAC AAGGATCCCC TCACTGGAGA TGAAGAGACC TTGGCTTCAT

ATTGAACTAC AAGGATGCCC TCACTGGAGA TGAAGAGACC TTGGCTTCCT

ATTGAACTAC AAGGATGCCC TCACTGGAGA TGAAGAGACC TTGGCTTCCT

ATTGAACAAC AAGGATGCCC TCACTGGAGA TGAAGAGACC TTGGCTTCAT

ATTGAACAAC AAGGATGCCC TTACTGGAGA TGAAGAGACC TTGGCTTCAT

ATTGAACAAC AAGGATGCCC TCACTGGAGA TGAAGAGACC TTGGCTTCAT

ATTGAACAAC AAGGATGCCC TCACTGGAGA TGAAGAGACC TTGGCTTCAT

---------- ---------- ---------- ---------- ----------

---------- ---------- ---------- ---------- ----------

ATTGAACAAC AAGGATGCCC TCACTGGAGA TGAAGAGACC TTGGCTTCAT

---------- ---------- ---------- ---------- ----------

ATTGAACAAC AAGGATGCCC TCACTGGAGA TGAAGAGACC CTGGCTTCAT

GTTGAACAAG AAGGATGCCC TCACTGGCGA TGAAGAGACT TTGGCTTCAT

ATTGAACAAC AAGGATGCCC TCACTGAAGA TGAAGAGACC TTGGCTTCAT

GTTGAACAAC AAGGATGTCC TCACTGGAGA TGAAGAGACC TTGGCTTCCT

ATTGAACAGC AAGGATGCCC TCACTGAAGA TGAAAAGACC TTGGCGGAAT

ACTGAA-AAG AAAGATGCCC TCACTGAAGA TAACGAGACC TTGGCTTCAT

ATGGGATTGT TTCTGGGGAC TTGATATGTT TGATTCTTCA AGATGACATT

ATGGGATTGT TTCTGGGGAC TTGATATGTT TGATTCTTCA AGATGACATT

ATGGGATTGT TTCTGGGGAC TTGATATGTT TGATTCTTCA AGATGACATT

ATGGGATTGT TTCTGGGGAC TTGATATGTT TGATTCTTCA AGATGACATT

ATGGGATTGT TTCTGGGGAC TTGATATGTT TGGTTCTTGA AGATGCCATT

ATGGGATTGT TTCTGGGGAC TTGATATGTT TGATTCTTGA AGATGCCATT

ATGGGATTGT TTCTGGGGAC TTGATATGTT TGGTTCTTGA AGATGAAATG

ATGGGATTGT TTCTGGGGAC TTGATATGTT TGGTTCTTGA AGATGACATG

---------- ---------- ---------- ---------- ----------

---------- ---------- ---------- ---------- ----------

ATGGGATTGT TTCTGGGGAC TTGATATGTT TGATTCTTGA AGATGCCATT

---------- ---------- ---------- ---------- ----------

ATGGGATTGT TTCTGGGGAC TTGATATGTT TGATTCTTGA AGATGCCATT

ATGGGATTGT TTCTGGGGAC TTGATATGTT TGATTCTTGA AGATGCCATT

ATGGGATTGT TTCTGGGGAC TTGATATGTT TGATTCTTGA AGATGCCATT

TTGGGATTGT TTCTGGGGAC TTGCTATGTT TGGTTCTTGA AGATGCCATT

ATGGGATTGT TTGTGGGGAC TTGATATGTT TAGTTCTTGA AGAGGCCACT

ACGGAATTGT CTGCGGGGAC CTGATATGTG TAGTCCTCGA AGATGCCA--

AATATACCTT CATCCACAGA TTCAGAGCAT TCTTCACTCC AGAATAATGA

AATATACCTT CATCCACAGA TTCAGAGCAT TCTTCACTCC AGAATAATGA

AATTTACCTT CATCCACAGA TTCAGAGCAT TCTTCACTCC AGAATAATGA

AATTTACCTT CATCCACAGA TTCAGAGCAT TCTTCACTCC AGAATAATGA

AATTTACCTT CATCCACAGA TTCAGAGCAT TCTTCACTCC AGAATAATGA

AATTTACCTT CATCCACAGA TTCAGAACAT TCCTCACTCC AGAATAATGA

AACTTACCTT CATCTACAGA TTCAGAGCAT TCCTCTCTCC AGAATAATGA

AACTTACCTT CATCTACAGA TACAGAGCAT TCCTCACTCC AGGATAATGA

---------- ---------- ---------- ---------- ----------

---------- ---------- ---------- ---------- ----------

AACTTACCTT CATCCACAGA TTCAGAGCAT TCCTCACTCC AGAATAATGA

---------- ---------- ---------- ---------- ----------

AATTTACCTT CATCCACAGA TTCAGAGCAT TCCTCACTCC AGAATAATGA

AACTTACCTT CATCCACAGA TTCAGAGCAT CCCTCACTTC AGAGTAGTGA

AACTTACCTT CATCCACAGA TTCAGAGCAT TCCTCACTCC AGAATAACGA

AACTTGCCTT CATCCACAGA TTCAGAGCAT TCCTCACTCC AGAATAACGA

-ACTTGCCTT CAT---TAGA TTTAGCACAT TCCTCATTCG CGAGTAATGA

-GCCTCCCTT CACCCACAGA TTCAGCTCTT TCCGGACTTC AGGATAGCGA

GCAACCCTCT TTGGCCACCA GCCAGGATGA ACAAGATTCA TTCCAGTCTG

GCAACCCTCT TTGGCCACCA GCCAGGATGA ACAAGATTCA TTCCAGTCTG

CCAACCCTCT TTGGCCACCA GCCAGGATGA ACAAGATTCA TTCCAGTCTG

CCAACCCTCT TTGGCCACCA GCCAGGATGA ACAAGATTCA TTCCAGTCTG

CCAACCCTCT TTGGCCACTA GCCAGGGTGA ACAAGATTCA TTCCAGTCTG

CCAACCCTCT TTGGCCGCCA GCCAGGATGA ACAAGATTCA TTTCAATCTG

CCAACCTCCT TTGGCTGCCA CCCCAGATGA ACAAGATTCA TCCCAATATG

CCAACCTTCT TTGGCTGCCA CCCCAGATGA ACAAGATTCA TCCCCATTTG

---------- ---------- ---------- ---------- ----------

---------- ---------- ---------- ---------- ----------

CCCACCCTCT TTGGCCGCCA GCCAGAATGA ACAGAATTCA TTCCAAACTG

---------- ---------- ---------- ---------- ----------

CCAACCCTCT CTGGCCGCCA GCCAGAACGA AGAGGGTTCA GTGCAGGCCG

CCCATCTTCT TTGGCTGCCA GCCAAGGTGA ACAAGATTCA TTCCAATCTG

CCAACCCTCT TTGGCCGCCA GCCAAGATGA ACAAGATTCG TTCCAGTCTG

CCAAGCCCCT TTGGCCGCCA GCCCTGATGA ACAAGATTCG TCCCAGTCTG

TCAGCCCTCT TTGGCCACCA GCCAAGGTGA AAAGGACCCA T---ATTCTG

GTCTCCCTCC TTGACCGCCA G-CAGGCTCG AGA--ATCCG TCTCAAGATG

GTGTTTGGAA TGACGACAGT ATGGGGGCAC TGAACCTACC AGATGTATTT

GTGTTTGGAA TGACGACAGT ATGGGGGCAC TGAACCTACC AGATGTATTT

ATGTTTGGAA TGACGACAGT ACGGGGGCAC TGAGCCTACC AGATGTATTT

ATGTTTGGAA TGACGACAGT ACGGGGGCAC TGAGCCTACC AGATGTATTT

ATGTCTGGAA TGACGACAAT ATGGGGGCAC TGAACCTACC AGATGTATTT

ATGTCTGGAA TGATGATAGT ATGGGGGCAC TGAACTTGCC AGACGCATTT

ATGCCTGGAC CGATGACAGT ATGGGGGTGC TGAACCTCCC AGATGTGTTT

ATGCCTGGAC TGATGACAGT ATGGGGGTAC TGAACCTTCC AGATGTGTTT

---------- ---------- ---A------ ---------- ----------

---------- ---------- ---GGGGCAC TGAACCTACC AGATGTATTC

ATGTCTGGAA TGAGGACAAT ATGGGGGCCC TGAACCTGCC AGACGTATTT

---------- ---------- ---------- ---------- ----------

ATGTCAGGAG TGATGACAGC ATGGGGGCAC TGAGCCTGCC GGATGTATTT

ATGTCTGGAA TGATGATAGT ATG-TGGCAC TAAACTTACC AGATGTATTT

ACGCCGGGGA TGATGACAGT ATG------- ---------- ----------

GTGTCGGCAA TGGCGACAGT GTGG---CCC TGAACCTCCC AGATGCGTTT

CTGTCCAGAG TGCAGAGAGC ATGGAGGCTT TAAACCTGCC TGATGTGTTT

ATGTCCAGAA TGACGACTTT ACGGAGGCCC TGAACCTCCC TGATGTTTTT

GGGTTGGTCG TCCTCCCATT GGAACTGAAA CTACGGATCT TCCGACTTCT

GGGTTGGTCG TCCTCCCATT GGAACTGAAA CTACGGATCT TCCGACTTCT

GGGTTGGTCG TCCTCCCCTT GGAACTGAAA CTACGGATCT TCCGACTTCT

GGGTTGGTCG TCCTCCCGTT GGAACTGAAA CTACGGATCT TCCGACTTCT

GGTTTGGTCG TCCTCCCATT GGAACTGAAA CTACGGATCT TCCGACTCTT

GGGCTGGTTG TCCTTCCATT GGAGTTAAAA CTACGGATCT TCCGACTTTT

GGGCTGGTTG TCCTTCCACT GGAGCTGAAA CTACGCATCT TCCGACTTCT

GGGCTGGTTG TCCTTCCACT GGAGCTGAAA CTACGCATCT TCCGACTTTT

---------- ---------- ---------- ---------- ----------

GGGTTGGTAG TCCTCCCGTT GGAGCTGAAA CTGCGGATCT TCCGACTTTT

GGGTTGGTGG TCCTCCCATT GGAGCTGAAG CTGCGGATCT TCCGACTTTT

---------- ---------- ---------- ---------- ----------

GGGCTGCTGG TCCTTCCACT GGAGCTGAAA CTACGGATAT TTCGACTTCT

GGGTTAGTAG TGCTCCCATT GGAGCTGAAA CTGCGGATCT TTCGACTTTT

---------- ---------- ---------- ---------- ----------

GGGCTGCTAG TCCTCCCACT GGAGCTGAAG CTGCGCATCT TCCGACTTTT

GGTTTAGTCG TCCTTCCATT GGAATTGAAA CTCCGGATTT TCCGGCTCTT

GGCTTGGTGG TACTCCCTCT GGAACTGAAA CTCCGGATTT TCCGCCTGCT

GGATGTTCGT TCCGTCTTGT CTTTGTCTGC GGTTTGTCGT GACCTCTTTA

GGATGTTCGT TCCGTCTTGT CTTTGTCTGC GGTTTGTCGT GACCTCTTTA

GGATGTTCGT TCCGTCTTGT CTTTGTCTGC AGTTTGTCGT GACCTCTTTA

GGATGTTCGT TCCGTCTTGT CTTTGTCTGC AGTTTGTCGT GACCTCTTTA

GGATGTTCGT TCCATCCTGT CTTTGTCTGC AGTTTGTCGT GACCTCTTTA

GGATGTTCGT TCTGTCCTGT CTTTGTCTGC GGTTTGTCGT GACCTCTTCA

GGATGTTCAT TCCGTCCTGG CTCTGTCTGC AGTCTGCCAT GACCTCCTCA

GGATGTTCAT TCTGTCCTGG CCCTGTCTGC AGTCTGTCAT GACCTCCTCA

---------- ---------- ---------- ---------- ----------

GGACGTTCGT TCTGTCCTGT CTCTGTCTGC AGTTTGTCGT GACCTCTGTA

GGATGTTCGT TCTGTTCTCT CTTTGTCTGC GGTTTGTCGT GACCTCCTTA

---------- ---------- ---------- ---------- ----------

GGATGTTCGC TCGGTTCTGT CCTTGTCTGC AGTTTGCCAC GATCTCTACA

GGATATTCGT TCCGTCCTCT CTTTGTCTGC AGTTTGTCGT GATCTCTTTA

---------- ---------- ---------- ---------- ----------

GGATGTCCGC TCTGTCCTGT CCCTGTCGGC CGTGTGTCGT GACCTCTTCA

AGATGTCCGT TCAGTCCTGG CTCTGTCTGC TGTTTGTCGT GACCTTTTCA

GGACGTCCGT TCTGTCCTTT CTCTGTCCGC TGTCTGCCGA GACCTCTTAA

CTGCTTCAAA TGACCCACTC CTGTGGAGGT TTTTATATCT GCGTGATTTT

CTGCTTCAAA TGACCCACTC CTGTGGAGGT TTTTATATCT GCGTGATTTT

CTGCTTCAAA TGACCCGCTT CTGTGGAGGT TTTTATATCT GCGTGATTTT

CTGCTTCAAA TGACCCACTT TTGTGGAGGT TTTTGTATCT GCGTGATTTT

CTGCTTCAAA TGACCCACTC CTGTGGAGGT TTTTATATCT TCGTGATTTT

TTGCTTCCAA TGACCCACTC CTGTGGAGGT GTTTGTATCT GCGGGATTTT

TTGCATCAAA TGACCCACTG CTGTGGAGGT GCTTGTATCT GCGGGATTTT

TTGCGTCAAA TGACCCACTG CTGTGGAGGT GTTTGTATCT GCGGGATTTT

---------- ---------- ---------- ---------- ----------

TTACTTCAAA TGATCAACTT CTGTGGAGGT GTTTGTATCT GCGGGATTTT

TTGCTTCAAA TGACCAGCTG CTCTGGAGGT GTTTATATCT GAGGGATTTT

---------- ---------- ---------- ---------- ----------

TTACATCCAA CGACCAGCTC CTTTGGAGGT GCTTGTATCT GCGGGATTTC

TTGCTTCAAA TGACCAACTC CTGTGGAGGT GTTTGTATCT GCGTGATTTT

---------- ---------- ---------- ---------- ----------

TTGCGTCCAA TGACCAGCTC CTGTGGAGGT ATTTGCTTCT GCGGGATTTT

ATGCTTCAAA TGACCAGCTC CTGTGGAGGT TTATGTACTT AAGAGATTTT

CGGCTTCCAA TGACCAGCTC TTGTGGAGGT TTATGTATCT TCGGGACTTT

CGAGACAGCG ATGCCAGAAA AATCCACACT GCCCCTGCCC GAACCATGTT

CGAGAC--CG ATGCCAGAAA AATCCACACT GCCCCTGCCC GAACCATGTT

CGAGACAGCG ATGCCAGAAA AGTCCACACT GCTCCTGCCC GAACCACGTT

CGAGACAG-- ---------- ---------- ---------- ----------

CGAGACAGCG ATGCCAGAAA AATCCACACT GCCCCTACCC GAAGCATGTT

CGAGAT---- ---------- ---------- ---------- ----------

CGAGACAGCG GGGTCCGAAA AGTCCATACC GCTCCTCTGC GAACCCTGTT

CGAGATAGCG GTGTCAGAAA TTTCCATACT GCTCCTGTCC GATCCTTGTT

---------G ATGCCAGAAA TGTTCACACT GCCCCTGCCC GAGCCTTGTT

CGAGATAGGA ATGCCAGGAA AGTCCACACG GCCCCTGCCC GCACCTCGTC

CGAGATAGCA ATGCCAGAAG AGTCCACACT GCCTCTGCCC GCACCTTGTT

------AGCA ATACCAGGAA AGTCCACACT GCCCCTGCTC GAACCTTGTT

CGAGATAGCA ATGCCAGAAA AGTTCACACT GCCCCAGCTC GGTCCTTGTC

CGAGATAGCG ATGCCAGAAA AGTCCACACT GCCCCTGTCC GAACCTTGTT

------AG-- ---------- ---------- ---------- ----------

CCAG--AG-- ATGCCAGAAG AGTGCACACG CTTTCTGCTC GTGCCTTGAA

CGAGAT--CG ATGCAAGGAA GTTCCACACG GCCGCTACCC GGACACTGTT

AGAGAT--CG ATGCAAGAAA TTTCCACACT GCCCCTGCCC GGGCATTTCT

CCTGCTGCGT CCCCTGCCCA TTCTGTTGGT GACAGGCGGC GGGTATGCAG

CCTGCTGCGT CCCCTGCCCA TTCTGTTGGC GACAGGCGGC GGGTATGCAG

CCTGCTGCGT CCCCTGCCCA TTCTGTTGGC GACAGGTGGC GGGTATGCAG

---------- ---------- ---------- ---------- ----------

CCTGCTGCGC CCCCTGCCCA TTCTGTTGGC AACAGGCGGC GGATATGCAG

---------- ---------- ---------- ---------- ----------

CCTGCTCCGC CCCGTGCCCA TCCTGTTGGC AGCAGGGGGC GGGTATGCTG

CCTGCTGCGC CCTGTGCCCA TCCTGTTGGC AACAGGTGGA GGGTATGCCG

CCGGCTGCGC CCCCTGCCCA TTCTGTTGGC GACAGGCGGA GGGTATGCAG

CCTGCTGCGC CCCCTGCCCA TTCTGGTCGC CACGGCTGGC GGGTATGCAG

CCTGCTACGG CCCCTGCCCA TATTGTTGGT GACAGGCAGT GGCTATGCAG

CCTGCTGCGC CCCCTTCCGT TTTTGTTTGC GACAGGCGGA GGGTATGCAG

TTTGCTGCGC CCCCTGCGCA TTCTTTTTGT GACGGGCGGT GGATATGCAG

CCGCCTGCGC CCGCTGCCTA TTCTCTTGGT GACAGGTGGA GGATATGCAG

---------- ---------- ---------- ---------- ----------

CCTGCTGCGG CCCGGGCCCG TTCTGTTGGC AACAGGCGGT GGCTATGCAG

CCGCCTGCGC CCATTCTACT TCTTGGTGGC TACAGGTGGC GGCTATGTAG

CCGTCTGCGC CCATTCCACT TTCTAGTTGC CACAGGAGGT GGGTATGCAG

GGTACCGGCA GTATGAGAAG TACAGGGAGC GAGAGCTGGA GAAGCTGGGA

GGTACCGGCA GTATGAGAAA TACAGGGAGC GAGAGCTGGA GAAGCTGGGA

GGTACCGGCA GTATGAGAAG TACAGGGAGC GAGAGCTGGA GAAGCTGGGA

---------- ---------- ---------- ---------- ----------

GGTACCGGCA GTATGAGAAG TACAGGGAGC GAGAGCTGGA GAAGCTGGGA

---------- ---------- ---------- ---------- ----------

GGTACCGGCA GTACGAGAAG TACAGGGAGC GGCAGCTCGA GAAGCTGGGG

GGTACCGGCA GTATGAGAAG TACAGGGAAC GGAAGCTTGA GAAGCTGGGC

GGTACCGGCA GTATGAGAAG TACAGGGAGC GAGAGCTGGA GAGGCTGGGA

GGTACCGGCA GTACGAGAAG TACCGGGAGC GAGAGCTGGA GAAGCTGGGC

GTTACCGGCA GTATGAGAAG TACAGGGAGC GAGAGCTGGA GAAGCTGGGG

GTTATCGGCA GTATGAGAAG TACAGGGAGC GAGAGCTGGA GAAGCTGGGA

GCTACCGGCA GTATGAGAAG TACAGGGAGC AAGAGCTGGA GAAGATGGGA

GGTACCGGCA GTATGAGAAG TACAGGGAGC AAGAGCTGGA GAAGCTGGGA

---------- ---------- ---------- ---------- ----------

GGTACCGGCA GTATGTGAAG TACAGGGAGC GGGAGCTGGA GAAGCTGGGA

GGTACCGCCA GTACGAGAAG TACAGAGATC GAGAGCTGGA GAGGCTGGGA

GATACCGTCA GTATGTGAAA TACCGAGATC ATCAGTTGGA GAAACTGGGA

TTGGAGATTC CACCCAAACT TGCTGGTCAC TGGGAGGTTG AAGGTGTTTT

TTGGAGATTC CACCCAAACT TGCTGGTCAC TGGGAGGTTG AAGGTGTTTT

TTGGAGATTC CACCCAAACT TGCTGGTCAC TGGGAGGTTG AGGGCGTTTT

---------- ---------- ---------- ------GTTG AGGGCGTTTT

TTGGAGATTC CACCCAAACT CGCTGGTCAC TGGGAGGTTG AAGGCGTTTT

---------- ---------- ---------- ------GTTG AAGGAGTCTT

TTGGAGATCC CGCCCAAGCT TGCTAGTCAC TGGGAGGTTG AAGGGGTGTT

TTGGAGATCC CGCCCAAACT TGCTAGTCAC TGGGAGGTTG AAGGAGTGTT

CTGGAGATTC CACCCAAACT TGCTGGTCAC TGGGAG---- ----------

TTGGACGTCC CGCCCAAGCT TGCTGGTCAC TGGGAGGTTG AAGGAGTTTT

TTGGAGATTC CACCCAAACT TGCTGGTCAC TGGGAGGTGG AAGGCGTTTT

TTGGAGATTC CACCCAAACT CGCTGGTCAC TGGGAG---- ----------

GTGGAGATTC CACCCAGGAT CGCTGGCCAC TGGGAGGTTG AAGGCGTTTT

TTGGAGATTC CACCCAAACT TGCTGGTCAC TGGGAGGTTG AAGGAGTTTT

---------- ---------- ---------- ------GTGG AAGGAGTTTT

CTGGAAATTC CACTCAGACT TGCTGGTGAA TGGGAG---- ----------

CTGGAGGTGC CACCCAGGCT TGCGGGAGAA TGGGAGGTGG AAGGAGTGTT

ATTGAAATAC CACCCCAAAT TGCAGGCGAA TGGGAGGTGG AAGGAGTATT

CTATGTGAAT GATGCTCTGG AGAAATTGAT GTTTGAGGAA TTAAGGAATG

CTATGTGAAT GATGCTCTGG AGAAATTGAT GTTTGAGGAA TTAAGGAATG

CTACGTGAAT GATGCTCTGG AGAAATTGAT GTTTGAGGAA TTAAGGAATG

CTACGTGAAT GATGCTCTGG AGAAATTGAT GTTTGAGGAA TTAAGGAATG

CTATGTGAAT GATGCTCTGG AGAAACTGAT GTTTGAGGAA TTAAGGAATG

CTATGTGAAT GATGCTCTGG AGAAATTGAT GTTTGAGGAA TTAAGGAATG

TTATGTGAAT GACGCTCTGG AAAAGCTCAT GTTTGAGGAG TTACGGAATG

TTATGTGAAT GATGCTCTGG AAAAACTAAT GTTTGAGGAA TTAAGGAACG

---------- ---------- ---------- ---------- ----------

CTATGTGAAT GATTCTCTGG AAAAATTAAT GTTTGAAGAA TTAAGGAATG

CTATGTGAAT GATGCTCTGG AAAAATTAAT GTTTGAGGAA TTAAGAAATG

---------- ---------- ---------- ---------- ----------

CTATGTGAAC GATTCTCTGG AAAAATTAAT GTTCGAAGAA TTGCGGAATG

CTATGTGAAT GATGCTCTGG AAAAGTTAAT GTTTGAGGAG TTAAGGAACG

CTATGTGAAT GATGCCTTGG AAAAATTAAT GTTTGAGGAG TTGCGGAATG

---------- ---------- ---------- ---------- ----------

CTATGTAAAT GATCCACTTG AGAAATTAAT GTTTGAGGAG TTAAGAAATG

CTATGTGAAC GATCCTCTAG AGAAGCTCAT GTTTGAGGAG TTAAGAAATG

CCTGTCGAGG TGGTGGTGTT GGTGGCTTCC TGCCAGCCAT GAAACAGATT

CCTGTCGAGG TGGTGGTGTT GGTGGCTTCC TGCCAGCCAT GAAACAGATT

CCTGTCGAGG TGGTGGTGTT GGTGGCTTCT TGCCAGCCAT GAAACAGATT

CCTGTCGAGG TGGTGGTGTT GGTGGCTTCT TGCCAGCCAT GAAACAGATT

CCTGTCGAGG TGGTGGTGTT GGTGGCTTCC TGCCAGCCAT GAAACAGATT

CCTGTCGTGG TGGTGGTGTT GGTGGCTTCT TGCCAGCCAT GAAACAGATT

CCTGTCGAGG TGGTGGTGTT GGTGGCTTCC TGCCAGCCAT GAAGCAGATT

CCTGTCGAGG TGGTGGTGTT GGTGGCTTTC TGCCAGCCAT GAAGCAGATT

---------- ---------- ---------- ---------- ----------

CCTGTCGAGG TGGTGGTGTT GGTGGCTTCC TGCCAGCCAT GAAACAAATT

CCTGTCGAGG TGGTGGTGTT GGTGGCTTCC TACCAGCCAT GAAACAAATT

---------- ---------- ---------- ---------- ----------

CCTGCCGAGG TGGTGGGGTT GGAGGCTTCC TGCCTGCTAT GAAGCAGATC

CCTGTCGAGG TGGTGGTGTT GGTGGCTTCC TGCCAGCCAT GAAACAGATT

CCTGCCGAGG TGGTGGTGTT GGTGGCTTCC TGCCAGCCAT GAAACAGATT

---------- ---------- ---------- ---------- ----------

CCTGCCGAGG TGGGGGTGTC GGTGGCTTCC TGCCAGCAAT GAAACAGATT

CCTGCCGAGG TGGTGGTGCT GGTGGCTTCC TGCCGGCCAT GAAGCAGATC

GGCAATGTGG CAGCCCTGCC TGGAATTGTT CATCGATCTA TTGGGCTTCC

GGCAATGTGG CAGCCCTGCC TGGAATTGTT CATCGATCTA TTGGGCTTCC

GGCAACGTGG CAGCCCTGCC TGGAATTGTT CATCGATCTA TTGGGCTTCC

GGCAACGTGG CAGCCCTGCC TGGAATTGTT CATCGATCTA TTGGGCTTCC

GGCAATGTGG CAGCCCTCCC TGGAATTGTT CATCGATCTA TTGGGCTTCC

GGCAACGTGG CAGCCCTGCC TGGGATAGTT CATCGATCAA TTGGGCTTCC

GGCAATGTGG CAGCCCTGCC TGGAATAGTT CATCGGTCTA TTGGGCTTCC

GGCAATGTGG CAGCCCTGCC TGGAATAGTT CATCGGTCTA TCGGGCTTCC

---------- ---------- ---------- ---------- ----------

GGCAATGTGG CCGCCCTGCC TGGGATTGTT CATCGATCCA TCGGTCTTCC

GGCAACGTGG CAGCCCTGCC TGGGATTGTT CATAGATCTA TTGGGCTTCC

---------- ---------- ---------- ---------- ----------

GGCAACGTGG CAGCCCTGCC TGGGATTGTT CATAGATCTA TCGGGCTTCC

GGCAATGTGG CCGCCCTGCC TGGGATTGTT CATCGATCTA TTGGACTTCC

GGCAATGTGG CAGCCCTGCC TGGGATTGTT CATCGTTCCA TTGGTCTTCC

---------- ---------- ---------- ---------- ----------

GGCAATGTGG CAGCCCTACC TGGGATTGTT CATAGATCTA TTGGGCTTCC

GGCAACGTGG CCGCCCTGCC CGGGATCGTT CACCGATCTA TTGGGCTTCC

TGATGTCCAT TCAGGATATG GGTTTGCTAT TGGGAACATG GCAGCCTTTG

TGATGTCCAT TCAGGATATG GGTTTGCTAT TGGGAACATG GCAGCCTTTG

TGATGTCCAT TCAGGATATG GATTTGCTAT TGGGAACATG GCAGCCTTTG

TGATGTCCAT TCAGGATATG GATTTGCTAT TGGGAACATG GCAGCCTTTG

TGATGTCCAT TCAGGATATG GGTTTGCCAT TGGAAATATG GCAGCCTTTG

TGATGTCCAT TCAGGCTATG GGTTTGCCAT TGGGAATATG GCAGCCTTTG

TGATGTCCAC TCAGGCTACG GGTTTGCCAT AGGGAACATG GCTGCCTTTG

TGATGTCCAT TCAGGCTATG GGTTTGCCAT AGGGAACATG GCTGCCTTTG

---------- ---------- ---------- ---------- ----------

TGATGTCCAT TCAGGTTATG GGTTTGCTAT TGGAAATATG GCAGCCTTTG

TGATGTCCAT TCAGGTTATG GGTTTGCTAT TGGGAATATG GCAGCCTTCG

---------- ---------- ---------- ---------- ----------

AGATGTCCAT TCAGGGTACG GGTTCGCTAT TGGCAACATG GCGGCCTTTG

TGATGTCCAT TCCGGTTATG GGTTTGCTAT TGGGAATATG GCAGCTTTTG

AGACGTCCAC TCAGGCTATG GGTTTGCTAT TGGGAATATG GCAGCCTTTG

---------- ---------- ---------- ---------- ----------

TGATGTCCAT TCTGGTTATG GATTCGCAAT TGGGAACATG GCTGCTTTTG

CGACGTCCAC TCTGGTTATG GATTTGCCAT TGGGAATATG GCCGCCTTTG

ATATGAATGA CCCTGAAGCA GTAGTATCCC CAGGTGGTGT CGGGTTTGAC

ATATGAATGA CCCTGAAGCA GTAGTATCCC CAGGTGGTGT CGGGTTTGAC

ATATGAATGA CTCTGAAGCA GTGGTATCCC CAGGTGGTGT CGGGTTTGAC

ATATGAATGA CTCTGAAGCA GTGGTATCCC CAGGTGGTGT CGGGTTTGAC

ATATGAATGA CCCTGAAGCA GTGGTATCCC CAGGTGGTGT CGGGTTTGAC

ATATGAATGA CCCTGAAGCA GTGGTATCCC CAGGTGGTGT CGGATTTGAT

ATATGAATGA CCCTGAGGCA GTTGTATCCC CAGGTGGTGT CGGATTTGAT

ATATGAATGA CCCTGAGGCC GTTGTATCCC CAGGTGGTGT CGGATTTGAT

---------- ---------- ---------- ---------- ----------

ATATGAACGA CCCTGAAGCA GTGGTATCCC CAGGTGGTGT TGGGTTTGAC

ATATGAATGA CCCCGAAGCA GTGGTATCCC CAGGTGGTGT CGGATTTGAC

---------- ---------- ---------- ---------- ----------

ACATGAGCGA CCCCGAAGCA GTGGTGTCTC CAGGTGGTGT CGGCTTCGAT

ATATGAGTGA CCCTGAAGCA GTGGTATCCC CAGGGGGTGT CGGATTTGAC

ACATGAATGA CCCTGACGCA GTGGTTTCAC CAGGTGGCGT CGGATTTGAT

---------- ---------- ---------- ---------- ----------

ATATGAGTGA CCCAGAAGCT GTGGTGTCAC CAGGTGGTGT CGGATTTGAT

ACATGAGTGA CCCAGAAGCG GTGGTGTCCC CAGGTGGCGT CGGATTTGAT

ATCAACTGTG GTGTCCGCTT GCTAAGAACC AATTTAGATG AAAGTGATGT

ATCAACTGTG GTGTCCGCTT GCTAAGAACC AATTTAGATG AAAGTGATGT

ATCAACTGTG GTGTCCGCTT GCTAAGAACC AATTTAGATG AAAGTGATGT

ATCAACTGTG GTGTCCGCTT GCTAAGAACC AATTTAGATG AAAGTGATGT

ATCAACTGTG GTGTCCGCTT GCTCAGAACC AATTTAGACG AAAGTGATGT

ATCAACTGTG GTGTCCGCTT GCTAAGAACC AATTTAGATG AAAGTGATGT

ATTAACTGTG GTGTCCGCTT GCTAAGAACC AATTTAGATG AGAGCGATGT

ATTAACTGTG GTGTCCGCTT GCTAAGAACC AATTTAGATG AGAGCGATGT

---------- ---------- ---------- ---------- ----------

ATTAACTGTG GTGTCCGCTT GCTGAGAACC AATTTAGATG AAAGTGATGT

ATCAACTGTG GTGTCCGCTT GCTAAGAACC AATTTAGATG AAAGCGATGT

---------- ---------- ---------- ---------- ----------

ATCAACTGCG GGGTGCGCCT GCTGAGAACC AACCTGGACG AGAGCGACGT

ATAAACTGTG GTGTCCGCTT GCTAAGAACC AATTTAGATG AAAGTGATGT

ATCAACTGTG GTGTCCGTTT GCTGAGAACC AATTTAGATG AAAGTGATGT

---------- ---------- ---------- ---------- ----------

ATCAACTGTG GTGTCCGATT GCTGAGAACC AACCTTGATG AAAGTGATGT

ATCAACTGTG GCGTCCGCTT GCTGAGAACC AACTTGGATG AAAGTGACGT

CCAGCCTGTG AAGGAGCAAC TTGCCCAAGC TATGTTTGAC CACATTCCTG

CCAGCCTGTG AAGGAGCAAC TTGCCCAAGC TATGTTTGAC CACATTCCTG

CCAGCCTGTG AAGGAGCAAC TTGCCCAAGC TATGTTTGAC CACATTCCTG

CCAGCCTGTG AAGGAGCAAC TTGCCCAAGC TATGTTTGAC CACATTCCTG

CCAGCCTGTG AAGGAGCAAC TTGCCCAAGC TATGTTTGAC CACATTCCTG

CCAGCCTGTG AAGGAGCAAC TTGCCCAAGC TATGTTTGAC CACATTCCTG

ACAGCCTGTG AAGGAACAAC TTGCCCAAGC TATGTTTGAC CACATCCCTG

ACAGCCTGTG AAGGAACAAC TTGCCCAAGC TATGTTTGAC CACATCCCTG

---------- ---------- ---------- ---------- ----------

TCAGCCTGTG AAAGAGCAAC TTGCCCAAGC TATGTTTGAC CACATTCCTG

CCAGCCTGTG AAGGAGCAGC TTGCCCAAGC TATGTTTGAC CATATTCCTG

---------- ---------- ---------- ---------- ----------

GCAGCCCGTC AAGGAGCAGC TGGCCCAGGC CATGTTTGAC CACATCCCTG

CCAGCCTGTG AAGGAGCAGC TTGCCCAAGC TATGTTTGAC CACATTCCTG

TCAGCCTGTG AAGGAGCAAC TTGCCCAAGC TATGTTTGAC CACATTCCTG

---------- ---------- ---------- ---------- ----------

CCAGCCTGTG AAGGAACAAC TAGCCCAGGC CATGTTTGAC CACATTCCTG

CCAGCCGGTG AAGGAGCAGC TCGCCCAGGC CATGTTTGAC CACATCCCCG

TTGGGGTGGG GTCAAAAGGT GTCATCCCAA TGAATGCCAA AGACTTGGAG

TTGGGGTGGG GTCAAAAGGT GTCATCCCAA TGAATGCCAA AGACTTGGAG

TTGGGGTGGG ATCAAAAGGT GTCATCCCAA TGAATGCCAA AGACTTGGAG

TTGGGGTGGG ATCAAAAGGT GTCATCCCAA TGAATGCCAA AGACTTGGAG

TTGGGGTAGG ATCAAAAGGT GTCATCCCAA TGAATGCCAA ----------

TTGGGGTGGG ATCAAAAGGC GTCATCCCAA TGAATGCCAA AGACTTGGAG

TCGGGGTGGG ATCGAAAGGT GTCATTCCAA TGAATGCCAA AGACTTGGAG

TTGGGGTGGG ATCAAAAGGT GTCATTCCAA TGAATGCCAA AGACTTGGAG

---------- ---------- ---------- ---------- ----------

TGGGAGTGGG GTCAAAAGGT GTCATCCCAA TGAATGCCAA AGACTTGGAG

TTGGAGTGGG ATCAAAAGGT GTCATCCCCA TGAGTGCCAA AGACTTGGAG

---------- ---------- ---------- ---------- ----------

TCGGTGTCGG CTCCAAAGGC GTCATCCCGA TGAATGCCAA AGACCTGGAG

TTGGAGTAGG ATCAAAAGGT GTCATCCCAA TGAATGCCAA AGACTTGGAG

TTGGGGTGGG ATCAAAAGGT GTCATCCCAA TGAATGCCAA AGACTTGGAG

---------- ---------- ---------- ---------- ----------

TTGGGGTGGG ATCAAAAGGC GTTATCCCAA TGAATGCCAA GGACTTGGAG

TCGGGGTGGG ATCTAAGGGG GTGATCCCCA TGAATGCCAA GGACCTAGAA

GAGGCCTTGG AGATGGGGGT GGACTGGTCC TTAAGAGAAG GGTATGCCTG

GAGGCCTTGG AGATGGGGGT GGACTGGTCC TTAAGAGAAG GGTATGCCTG

GAGGCCTTGG AGATGGGGGT GGACTGGTCC TTAAGAGAAG GGTATGCCTG

GAGGCCTTGG AGATGGGGGT GGACTGGTCC TTAAGAGAAG GGTATGCCTG

---------- ---------- ---------- ---------- ----------

GAGGCCTTGG AGATGGGTGT GGACTGGTCC CTGAGAGAAG GCTATGCCTG

GAGGCATTGG AGATGGGTGT GGACTGGTCC CTGAGAGAAG GCTATGCCTG

GAGGCATTGG AGATGGGGGT GGACTGGTCC CTGAGGGAAG GCTATGCCTG

---------- ---------- ---------- ---------- ----------

GAGGCCTTGG AGATGGGTGT GGACTGGTCC CTGAGAGAAG GCTATGCCTG

GAGGCCTTAG AGATGGGTGT GGATTGGTCC CTGAGGGAAG GCTATGCCTG

---------- ---------- ---------- ---------- ----------

GAGGCCTTGG AGATGGGCGT GGACTGGTCC CTGCGGGAAG GCTACGCCTG

GAGGCCTTGG AGATGGGTGT GGACTGGTCC TTGAGAGAAG GGTATGCCTG

GAGGCCTTGG AGATGGGTGT GGACTGGTCC TTGAGAGAAG GCTATGCCTG

---------- ---------- ---------- ---------- ----------

GAGGCTCTGG AGATGGGGGT GGACTGGTCT CTACGAGAAG GGTATGCCTG

GAGGCCCTGG AGATGGGTGT GGACTGGTCC TTACGAGAGG GCTATGCCTG

GGCTGAAGAC AAGGAGCACT GCGAGGAGTA CGGAAGGATG CTGCAGGCTG

GGCTGAAGAC AAGGAGCACT GCGAGGAGTA CGGAAGGATG CTGCAAGCTG

GGCTGAAGAC AAGGAGCACT GCGAGGAGTA TGGAAGGATG CTGCAAGCTG

GGCTGAAGAC AAGGAGCACT GCGAGGAGTA TGGAAGGATG CTGCAAGCTG

---------- ---------- ---------- ---------- ----------

GGCTGAAGAC AAGGAGCACT GTGAGGAGTA CGGAAGGATG CTGCAAGCTG

GGCTGAGGAC AAGGAGCACT GTGAGGAGTA TGGAAGGATG CTCCAAGCCG

GGCTGAAGAC AAGGAGCACT GTGAGGAGTA TGGAAGGATG CTGCAAGCCG

---------- ---------- ---------- ---------- ----------

GGCAGAGGAC AAGGAGCACT GTGAGGAGTA TGGAAGGATG CTGCAAGCTG

GGCTGAGGAC AAGGAGCACT GTGAGGAGTA CGGCAGGATG TTGCAGGCCG

---------- ---------- ---------- ---------- ----------

GGCGGAGGAC AAGGAGCACT GTGAGGAGTA CGGGAGGATG CTGCAGGCCG

GGCTGAAGAC AAGGAGCACT GTGAGGAGTA TGGAAGGATG CTGCAAGCTG

GGCCGAAGAC AAGGAGCACT GTGAGGAGTA TGGAAGGATG CTGCAGGCTG

---------- ---------- ---------- ---------- ----------

GGCTGAGGAT AAGGAGCATT GTGAGGAATA TGGGCGTATG CTGCAAGCTG

GGCCGAGGAC AAGGAGCACT GTGAGGAGTA CGGACGGATG CTGCAAGCTG

ACCCCAATAA AGTTTCTGCA AGGGCGAAGA AAAGAGGCCT TCCTCAGTTG

ACCCCAATAA GGTTTCTGCA AGGGCTAAGA AAAGAGGCCT TCCTCAGTTG

ACCCCAATAA GGTTTCTGCA AGGGCTAAGA AAAGAGGCCT TCCTCAGTTG

ACCCCAATAA GGTTTCTGCA AGGGCTAAGA AAAGAGGCCT TCCTCAGTTG

---------- ---------- ---------- ---------- -------TTG

ACCCCACCAA GGTCTCTGCA AGGGCTAAGA AAAGAGGCCT TCCTCAGTTG

ACCCCAATAA GGTCTCACCC AGGGCAAAGA AAAGGGGCCT TCCTCAGTTG

ACCCCAATAA GGTCTCACCC AGGGCAAAGA AAAGGGGCCT TCCTCAGTTG

---------- ---------- ---------- ---------- ----------

ATCCCAATAA AGTCTCAGCC AGGGCTAAAA AAAGAGGCCT TCCCCAGTTG

ACCCCAATAA AGTCTCTGCA AGGGCTAAGA AAAGAGGCCT TCCTCAGTTG

---------- ---------- ---------- ---------- ----------

ACCCCAACAA GGTGTCTGCC AGGGCCAAGA AAAGAGGCCT GCCTCAGTTG

ACCCCAATAA AGTTTCTGCA AGGGCCAAAA AAAGAGGCCT TCCTCAGCTG

ACCCCAACAA GGTTTCTGCA AGGGCCAAAA AAAGGGGCCT CCCTCAGTTG

---------- ---------- ---------- ---------- ----------

ACCCCAATAA GGTCTCAGCC AGGGCTAAAA AGAGAGGCCT TCCTCAGCTT

ACCCCAACAA GGTGTCCGCA CGGGCTAAGA AGAGAGGCCT GCCCCAGCTA

GGGACCCTGG GAGCAGGCAA CCATTATGCA GAAATCCAGG TTGTGGATGA

GGGACCCTGG GAGCAGGCAA CCATTATGCA GAAATCCAGG TTGTGGATGA

GGGACCCTGG GAGCAGGCAA CCATTATGCA GAAATCCAGG TTGTGGATGA

GGGACCCTGG GAGCAGGCAA CCATTATGCA GAAATCCAGG TTGTGGATGA

GGGACCCTGG GAGCAGGCAA CCATTATGCA GAAATCCAGG TTGTGGATGA

GGGACGCTGG GAGCAGGCAA TCACTATGCA GAAATCCAGG TTGTGGATGA

GGGACCCTGG GAGCAGGCAA CCATTATGCA GAGATCCAGG TTGTAGATGA

GGGACCCTGG GAGCAGGCAA CCATTATGCA GAAATCCAGG TTGTAGATGA

---------- ---------- ---------- ---------- ----------

GGGACTCTGG GAGCAGGCAA CCACTATGCA GAAATCCAGG TTGTGGATGA

GGAACCCTGG GAGCAGGCAA CCACTATGCA GAAATCCAGG TTGTGGATGA

---------- ---------- ---------- ---------- ----------

GGGACCCTGG GCGCAGGCAA CCACTATGCA GAAATCCAGG TTGTGGATGA

GGGACCTTAG GAGCAGGCAA CCATTACGCA GAAATCCAGG TTGTGGATGA

GGGACCCTGG GAGCTGGCAA CCATTATGCA GAAATCCAGG TTGTGGATGA

---------- ---------- ---------- ---------- ----------

GGGACCCTGG GAGCAGGAAA TCATTATGCT GAAATCCAAG TTGTGGATGA

GGGACCCTGG GAGCGGGAAA CCACTATGCC GAAATCCAGG TGGTGGATGA

GATTTTCAAT GAGTATGCTG CTAAAAAAAT GGGCATCGAC CATAAGGGAC

GATTTTCAAT GAGTATGCTG CTAAAAAAAT GGGCATCGAC CATAAGGGAC

GATTTTCAAT GAGTACGCTG CTAAAAAAAT GGGCATCGAC CATAAGGGAC

GATTTTCAAT GAGTACGCTG CTAAAAAAAT GGGCATCGAC CATAAGGGAC

GATTTTCAAT GAGTACGCTG CTAAGAAAAT GGGCATCGAC CGTAAGGGAC

GATTTTCAAT GAGTATGCTG CTAAGAAAAT GGGCATCGAC CATAAGGGAC

GATTTTCAAC GAGTATGCTG CCAAGAAGAT GGGCATCGAC CATAAGGGAC

GATTTTCAAT GAGTATGCCG CCAAGAAGAT GGGCATCGAC CATAAGGGAC

---------- ---------- ---------- ---------- ----------

GATTTTCAAC GAGTATGCTG CTAAGAAAAT GGGCATTGAC CATAAGGGAC

GATTTTCAAT GAGTATGCTG CTAAGAAAAT GGGCATCGAC CATAAGGGAC

---------- ---------- ---------- ---------- ----------

AATCTTCAAT GAGTACGCCG CCAAGAAGAT GGGCATTGAC CACAAGGGGC

GATTTTCAAT GAGTATGCTG CTAAGAAAAT GGGCATCGAC CACAAGGGAC

GATTTTCAAC GAGTATGCTG CTAAGAAAAT GGGCATCGAC CACAAGGGAC

---------- ---------- ---------- ---------- ----------

TATTTATAAT GAGTACGCTG CCAAGAAGAT GGGCATTGAC CACAAGGGAC

GATTTTTAAC GAGTATGCCG CCAAAAAAAT GGGCATCGAC CACAAAGGGC

AGGTGTGTGT GATGATCCAC AGTGGAAGCA GAGGCTTGGG CCACCAAGTA

AGGTGTGTGT GATGATCCAC AGTGGAAGCA GAGGCTTGGG CCACCAAGTA

AGGTGTGTGT GATGATCCAC AGTGGAAGCA GAGGCTTGGG CCACCAAGTA

AGGTGTGTGT GATGATCCAC AGTGGAAGCA GAGGCTTGGG CCACCAAGTA

AGGTGTGTGT GATGATCCAC AGTGGAAGCA GAGGCCTGGG CCACCAAGTA

AGGTGTGTGT GATGATCCAC AGTGGAAGCA GAGGCTTGGG CCACCAAGTA

AGGTGTGCGT GATGATCCAC AGCGGGAGCA GAGGCTTGGG CCATCAAGTA

AGGTGTGTGT GATGATCCAC AGTGGAAGCA GAGGCTTGGG CCACCAAGTA

---------- ---------- ---------- ---------- ----------

AGGTGTGTGT GATGATCCAC AGTGGAAGCA GAGGCTTGGG CCACCAAGTT

AAGTGTGTGT GATGATCCAC AGTGGAAGCA GAGGCTTGGG CCACCAAGTA

---------- ---------- ---------- ---------- ----------

AGGTGTGTGT GATGATCCAC AGCGGAAGCC GGGGCTTGGG CCACCAGGTC

AGGTGTGTGT GATGATCCAC AGTGGCAGCA GAGGCTTGGG CCACCAAGTA

AGGTGTGTGT GATGATCCAC AGCGGAAGCA GAGGCTTGGG CCACCAAGTA

---------- ---------- ---------- ---------- ----------

AGGTGTGTGT GATGATCCAC AGTGGCAGCA GAGGCCTGGG CCATCAAGTA

AAGTGTGCGT GATGATCCAC AGCGGCAGCA GAGGTCTGGG CCATCAGGTC

GCCACAGATG CGCTGGTAGC TATGGAGAAG GCCATGAAGA GAGACAAGAT

GCCACAGATG CGCTGGTAGC TATGGAGAAG GCCATGAAGA GAGACAAGAT

GCCACAGATG CACTGGTAGC TATGGAGAAG GCCATGAAGA GAGACAAGAT

GCCACAGATG CACTGGTAGC TATGGAGAAG GCCATGAAGA GAGACAAGAT

GCCACAGATG CTCTGGTAGC TATGGAGAAG GCCATGAAGA GAGACAAGAT

GCCACAGATG CGCTGGTAGC TATGGAAAAG GCCATGAAGA GAGACAAGAT

GCTACAGACG CACTGGTAGC TATGGAGAAA GCCATGAAGA GAGACAAGAT

GCTACAGATG CACTGGTAGC TATGGAAAAG GCCATGAAGA GAGACAAGAT

-------ATG CACTGGTGGC TATGGAAAAA GCCATGAAGA GAGACAAGAT

GCCACAGATG CACTTGTAGC TATGGAAAAA GCCATGAAGA GAGACAAGAT

GCCACAGATG CACTGGTAGC TATGGAAAAA GCCATGAAAA GAGACAAGAT

---------- ---------- ---------- ---------- ----------

GCCACAG--- ---------- ---------- ---------- ----------

GCCACAGATG CACTGGTAGC TATGGAAAAA GCCATGAAGA GAGACAAAAT

GCCACAGATG CACTGGTAGC CATGGAAAAA GCCATGAAGA GAGACAAGAT

---------- ---------- ---------- ---------- ----------

GCTACAGATG CACTGGTAGC GATGGAAAAA GCTATGAAGA GAGACAAGAT

GCTACAGATG CCCTGGTGGC TATGGAAAAG GCTATGAAAC GGGACAAGAT

TATAGTCAAT GATCGGCAGT TGGCTTGTGC TCGAATCGCT TCCCCAGAGG

TATAGTCAAT GATCGGCAGT TGGCTTGTGC TCGAATCGCT TCCCCAGAGG

TATAGTCAAT GATCGGCAGT TGGCTTGTGC TCGGATCGCT TCACCAGAGG

TATAGTCAAT GATCGGCAGT TGGCTTGTGC TCGGATCGCT TCACCAGAGG

TATAGTCAAT GATCGGCAGT TGGCCTGTGC TCGAATCGCT TCCCCAGAGG

TATAGTCAAC GACCGGCAGT TGGCTTGTGC ACGAATCGCT TCCCCAGAGG

TATAGTCAAT GACCGGCAGC TGGCGTGTGC TCGGATTGCA TCCCCAGAGG

TATAGTCAAT GACCGGCAGT TGGCTTGTGC TCGGATTGCA TCCCCAGAGG

CATCGTAAAT GACCGTCAGC TGGCTTGTGC ACGAATCGCT TCCGTGGAAG

TATAGTCAAT GACCGTCAGT TGGCTTGTGC TCGAATTGCT TCCCCAGAGG

TATAGTCAAC GACCGGCAGT TGGCTTGTGC TCGGATTGCT TCCCCAGAGG

---------- ---------- ---------- ---------- ----------

---------- ---------- ---------- ---------- ----------

CATAGTCAAT GACCGCCAGC TGGCTTGTGC CCGAATCGCT TCTGCTGAGG

TATAGTCAAT GACCGTCAGC TGGCATGTGC TCGAATCGCT TCCACGGAGG

---------- ---------- ---------- ---------- ----------

TATTGTCAAT GACCGTCAGT TAGCATGCGC GAGGATCAGC TCCCCAGAGG

CATAGTGAAT GACCGCCAGC TGGCCTGCGC CCGAATCTCC TCTCCCGAGG

GTCAAGACTA TCTGAAGGGA ATGGCTTTCG CCAAGGTCTT CAACACAACC

GTCAAGACTA TCTGAAGGGA ATGGCTTTTG CCAAGGTCTT CAACACAACC

GTCAGGACTA CCTGAAGGGA ATGGCTTTTG CCAAGGTCTT CAACACAACC

GTCAGGACTA CCTGAAGGGA ATGGCTTTTG CCAAGGTCTT CAACACAACC

GTCAAGACTA CCTAAAGGGA ATGGCTTTTG CCAAGGTCTT CAACACAACC

GTCAGGACTA CCTGAAGGGA ATGGCTTTTG CCAAGGTCTT CAACACAACC

GACAAGACTA TCTAAAGGGA ATGGCTTTTG CCAAAGTCTT CAACACAACC

GACAAGACTA TCTAAAGGGA ATGGCTTTTG CCAAAGTCTT CAACACAACC

GTCAGGACTA CCTGAAGGGA ATGGCTTTTG CCAAGGTCTT CAACACAACC

GTCAGGACTA CCTGAAGGGA ATGGCTTTTG CCAAGGTCTT CAACACAACC

GTCAGGACTA CCTGAAGGGG ATGGCTTTTG CCAAGGTCTT CAACACAACC

---------- ---------- ---------- ---------- ----------

---------- ---------- ---------- ---------- ----------

GTCAGGACTA CCTGAAGGGA AT-GCTTTTG CCAAAGTCTT CAACACAACC

GTCAGGACTA CCTGAAGGGA ATGGCCTTTG CCAAGGTCTT CAACACAACC

---------- ---------- ---------- ---------- ----------

GGCAGGATTA CCTAAAGGGG ATGGCTTTTG CCAAGGTTTT CAACACAACT

GACAGGACTA CCTGAAGGGA ATGGCCTTTG CCAAAGTCTT CAACACAACC

CCTGATGACT TGGACCTACA TGTGATCTAT GATGTTTCTC ACAACATTGC

CCTGATGACT TGGACCTACA TGTGATCTAT GATGTTTCTC ACAACATTGC

CCTGATGACT TGGACCTGCA CGTGATCTAT GATGTTTCTC ACAACATTGC

CCTGATGACT TGGACCTGCA CGTGATCTAT GATGTTTCTC ACAACATTGC

CCTGATGACT TGGACCTCCA TGTGATCTAT GATGTTTCTC ACAACATTGC

CCTGATGACT TGGACCTGCA CGTGATCTAT GATGTTTCTC ACAACATTGC

CCTGACGACC TGGACCTGCA TGTGATTTAT GATGTTTCTC ACAACATCGC

CCTGATGACC TGGACCTGCA TGTGATCTAT GATGTGTCGC ACAATATCGC

CCTGATGACT TGGACCTGCA CGTGATCTAT GATGTCTCTC ACAACATCGC

CCTGATGACT TGGACCTGCA TGTGATCTAT GATGTTTCTC ACAATATTGC

CCTGATGACT TGGATCTACA TGTGATCTAT GATGTTTCTC ACAATATTGC

---------- ---------- ---------- ---------- ----------

---------- ---------- ---------- ---------- ----------

CCTGATGACT TGGATCTACA CGTGATCTAC GACGTTTCTC ACAATATTGC

CCTGATGACT TGGACCTACA TGTGATCTAT GATGTTTCTC ACAATATTGC

---------- ---------- ---------- ---------- ----------

CCTGATGACC TGGACCTCCA CGTGATCTAT GACGTCTCCC ACAACATCGC

CCTGATGACC TGGACCTACA TGTGATTTAT GATGTATCTC ACAATATTGC

CAAAGTGGAG CAGCATGTGG TGGACGGAAA GGAACGGACA CTGTTAGTAC

CAAAGTGGAG CAGCATGTGG TGGACGGAAA GGAACGGACA CTGTTAGTAC

CAAAGTGGAG CAGCATGTAG TGGACGGGAA GGAGCGGACA CTGTTAGTAC

CAAAGTGGAG CAGCATGTAG TGGACGGGAA GGAGCGGACA CTGTTAGTAC

CAAAGTGGAG CAGCATGTGG TGGACGGAAA GGAACGGACA CTGTTAGTAC

CAAAGTGGAG CAGCATGTGG TGGACGGGAA GGAGCGGACG CTGTTAGTGC

CAAAGTGGAG CAGCACGTGG TAGACGGAAA GGAGCGGACG CTGTTGGTGC

CAAAGTGGAG CAGCACGTGG TGGATGGGAA GGAACGGACG CTGCTGGTGC

CAAGGTCGAG CAGCACGTGG TGGACGGGAA GGAGCGGACG CTGCTGGTGC

CAAAGTAGAA CAGCATGTGG TGGACGGGAA GGAGCGGACT CTGTTAGTAC

CAAAGTAGAA CAGCACGTGG TGGATGGAAA GGAACGGACA CTGTTGGTAC

---------- ---------- ---------- ---------- ----------

---------- ---------- ---------- ---------- ----------

CAAAGTAGAG CAGCATGTGG TGGATGGAAA GGAACGGACG CTGCTGGTGC

CAAGATAGAG CAGCACGTTG TGGACGGAAA GGAGCGGACG CTGTTGGTGC

---------- ---------- ---------- ---------- ----------

CAAGGTGGAA CAGCATGTGG TGGATGGGAA AGAGCGGACA TTGTTGGTGC

CAAGGTAGAA CAGCATGTGG TGGATGGAAA AGAGCGAACT CTTCTGGTAC

ACAGGAAGGG ATCCACCCGC GCTTTCCCTC CTCACCATCC CCTCATTGCT

ACAGGAAGGG ATCCACCCGC GCTTTCCCTC CTCACCATCC CCTCATTGCT

ACAGGAAGGG ATCCACCCGC GCTTTCCCTC CTCACCATCC CCTCATAGCT

ACAGGAAGGG ATCCACCCGC GCCTTCCCTC CTCACCATCC CCTCATAGCT

ACAGGAAGGG ATCCACCCGC GCGTTCCCTC CTCACCATCC ACTCATTGCT

ACCGGAAGGG GTCCACCCGC GCCTTCCCTC CTCACCATCC CCTCATTGCT

ACAGGAAAGG GTCCACCCGA GCTTTCCCTC CTCACCATCC CCTCATTGCT

ACAGGAAGGG ATCCACCCGT GCTTTCCCGC CTCACCACCC CCTCATTGCT

ACAGGAAGGG ATCCACCCGG GCTTTCCCTC CTCACCATCC CCTCATCGCG

ACAGGAAGGG GTCCACCCGA GCCTTCCCTC CTCACCATCC CCTCATTGCG

ACAGGAAGGG ATCCACTCGG GCTTTCCCTC CTCACCATCC CCTCATTGCT

---------- ---------- ---------- ---------- ----------

---------- ---------- ---------- ---------- ----------

ACAGGAAGGG GTCCACCCGT GCATTCCCTC CTCATCACCC CCTCATTGCT

ACAGGAAGGG ATCCACCCGG GCTTTCCCGC CTCACCACCC CCTCATTGCC

---------- ---------- ---------- ---------- ----------

ACAGGAAGGG CTCCACCCGG GCCTTCCCCC CACACCATCC CCTCATTGCT

ACAGGAAAGG GTCCACCAGG GCTTTCCCTC CTCACCATCC CCTCATTGCT

GTTGATTACC AACTCACTGG ACAGCCAGTG CTCATTGGTG GCACCATGGG

GTTGATTACC AACTCACTGG ACAGCCAGTG CTCATTGGTG GCACCATGGG

GTTGATTACC AACTCACTGG ACAACCAGTG CTCATTGGTG GCACCATGGG

GTTGATTACC AACTCACTGG ACAACCAGTG CTCATTGGTG GCACCATGGG

GTTGATTACC AACTCACTGG ACAACCAGTA CTCATTGGTG GCACCATGGG

GTAGATTACC AACTCACTGG ACAACCAGTA CTCATTGGTG GCACCATGGG

GTTGATTACC AGCTCACTGG ACAACCAGTG CTTATCGGTG GCACCATGGG

GTGGATTATC AACTCACAGG ACAACCAGTG CTTATTGGTG GCACCATGGG

GTTGATTACC AGCTCACTGG ACAACCAGTG CTCATTGGTG GCACCATGGG

GTTGATTACC AACTTACCGG ACAACCAGTG CTCATTGGTG GCACCATGGG

GTGGATTACC AACTCACTGG ACAGCCGGTG CTCATTGGTG GCACCATGGG

---------- ---------- ---------- ---------- ----------

---------- --CTCACGGG ACAGCCAGTG CTCATTGGTG GCACCATGGG

GTTGATTACC AGCTCACGGG ACAGCCTGTA CTCATTGGTG GCACCATGGG

GTGGATTACC AGCTCACTGG ACAGCCGGTG CTCATCGGTG GCACCATGGG

---------- ---------- ---------- ---------- ----------

GTGGATTACC AGCTCACTGG ACAGCCAGTC CTCATTGGGG GAACCATGGG

GTTGATTATC AGCTGACCGG ACAGCCCGTG CTGATCGGTG GGACCATGGG

AACCTGTAGT TATGTTCTTA CTGGCACTGA ACAGGGCATG ACTGAGACCT

AACCTGTAGT TACGTTCTTA CTGGCACTGA ACAGGGCATG ACTGAGACCT

AACCTGTAGT TATGTTCTTA CTGGCACTGA ACAGGGCATG ACTGAGACCT

AACCTGTAGT TATGTTCTTA CTGGCACTGA ACAGGGCATG ACTGAGACCT

AACCTGTAGT TATGTTCTTA CTGGCACTGA ACAGGGCATG ACTGAGACCT

AACCTGTAGT TACGTTCTTA CAGGCACTGA GCAGGGCATG ACTGAGACCT

GACCTGTAGT TATGTTCTGA CTGGCACTGA ACAAGGCATG ACTGAGACCT

GACCTGTAGT TACGTTCTGA CTGGCACTGA ACAAGGCATG ACTGAGACCT

AACCTGCAGT TACGT----- ---------- ---------- ----------

AACCTGTAGC TATGTTCTTA CTGGTACTGA GCAGGGCATG ACTGAAACCT

CACCTGCAGT TACGTTCTTA CGGGCACCGA GCAGGGCATG ACGGAGACCT

---------- ---------- ---------- ---------- ----------

GACCTGTAGT TATGTCCTGA CTGGCACCGA ACAGGGCATG ACTGAGACCT

AACCTGTAGT TACGTTCTTA CTGGCACGGA ACAGGGCATG ACAGAAACCT

AACCTGCAGT TATGTACTTA CTGGCACTGA ACAGGGCATG ACTGAAACCT

---------- ---------- ---------- ---------- ----------

AACCTGTAGC TATGTCCTTA CTGGGACTGA GCAGGGCATG ACTGAGACCT

GACCTGCAGC TATGTACTCA CCGGCACCGA GCAGGGCATG ACCGAGACCT

TTGGAACAAC CTGTCATGGA GCGGGCCGTG CATTGTCCCG AGCAAAATCT

TTGGAACAAC CTGTCATGGA GCGGGCCGTG CATTGTCCCG AGCAAAATCT

TTGGAACAAC CTGTCATGGA GCGGGCCGTG CATTGTCCCG AGCAAAATCT

TTGGAACAAC CTGTCATGGA GCGGGCCGTG CATTGTCCCG AGCAAAATCT

TTGGAACAAC CTGTCATGGA GCGGGCCGTG CATTGTCCCG AGCAAAATCT

TTGGAACAAC CTGTCATGGA GCGGGCCGTG CATTGTCCCG GGCAAAATCT

TTGGAACAAC CTGTCATGGA GCGGGCCGTG CTTTGTCCAG AGCAAAATCA

TTGGAACAAC CTGTCATGGA GCGGGCCGTG CTTTGTCCAG AGCAAAATCA

---------- ---------- ---GGCCGTG CATTGTCCCG AGCAAAATCT

TTGGAACAAC TTGTCATGGA GCGGGCCGTG CACTGTCCCG AGCAAAGTCA

TTGGGACCAC TTGCCATGGA GCGGGCCGTG CATTGTCCCG AGCAAAATCT

---------- ---------- ---------- ---------- ----------

TTGGAACAAC CTGCCACGGA GCG------- ---------- ----------

TTGGAACAAC ATGTCATGGA GCGGGCCGTG CATTGTCCCG AGCTAAATCA

TTGGAACCAC CTGTCACGGA GCGGGCCGTG CATTGTCTCG AGCGAAATCT

---------- ---------- ---------- ---------- ----------

TTGGAACAAC CTGCCATGGA GCAGGTCGTG CATTGTCCCG AGCAAAATCT

TCGGAACAAC CTGCCACGGA GCTGGCCGTG CACTGTCCCG AGCAAAATCT

CGACGTAATT TAGATTTCCA GGATGTCTTA GACAAATTGG CAGATATGGG

CGACGTAATT TAGATTTCCA GGATGTCTTA GACAAATTGG CAGATATGGG

CGACGTAATT TAGATTTCCA GGATGTTTTA GACAAATTGG CAGATATGGG

CGACGTAATT TAGATTTCCA GGATGTTTTA GACAAATTGG CAGATATGGG

CGACGTAATT TAGATTTCCA GGATGTCTTA GACAAATTGG CAGATATGGG

CGACGTAATT TAGATTTCCA GGATGTCTTA GACAAATTGG CAGATATGGG

CGTCGTAATT TAGATTTCCA AGATGTCTTA GACAAGCTGG CAGACATGGG

CGTCGTAACT TAGATTTCCA AGATGTCTTA GACAAACTGG CAGACATGGG

CGGCGTAATC TAGATTTCCA GGATGTCTTA GACAAACTGG CAGATATGGG

AGACGTAATT TAGATTTCCA GGATGTCCTC GACAAATTGG CAGACATGGG

CGTCGTAATT TAGATTTCCA GGATGTCTTG GACAAACTGG CAGATATGGG

---------- ---------- ---------- ---------- ----------

---------- ---------- ---------- ---------- ----------

CGACGTAATT TAGATTTCCA GGATGTCTTG GACAAATTGG CAGATATGGG

CGGCGTAATT TAGACTTCCA GGATGTCTTG GATAAATTGG CAGATATGGG

---------- ---------- ---------- ---------- ----------

CGACGTAACT TAGACTTCCA GGATGTTCTA GATAAATTGG CTGACATGGG

CGACGAAATT TGGACTTCCA GGACGTTTTA GATAAACTGG CAGACATGGG

AATTGCGATC CGTGTTGCCT CACCCAAACT GGTTATGGAA GAGGCTCCTG

AATTGCGATC CGTGTTGCCT CACCCAAACT GGTTATGGAA GAGGCTCCTG

AATTGCAATC CGTGTTGCCT CACCCAAACT GGTTATGGAA GAGGCTCCTG

AATTGCAATC CGTGTTGCCT CACCCAAACT GGTGATGGAA GAGGCTCCTG

AATTGCAATC CGTGTTGCTT CACCCAAACT GGTTATGGAA GAGGCTCCTG

AATCTCAATC CGTGTTGCCT CACCCAAACT GGTTATGGAA GAAGCTCCTG

AATCGCCATC CGGGTTGCGT CCCCCAAGCT GGTTATGGAA GAGGCTCCAG

AATTGCAATC CGGGTTGCTT CCCCCAAGCT GGTTATGGAA GAGGCACCAG

GATCGCAATC CGTGTTGCCT CGCCCAAACT GGTTATGGAA GAG-------

AATTGCAATC CGTGTCGCCT CACCCAAGCT GGTAATGGAA GAGGCCCCTG

AATCGCAATC CGAGTTGCCT CACCCAAACT GGTTATGGAA GAGGCTCCTG

---------- ---------- ---------- ---------- ----------

---------- ---------- ---------- ---------- ---GCTCCAG

GATTGCAATC CGTGTTGCCT CACCCAAACT GGTTATGGAA GAGGCTCCTG

AATTGCAATC CGTGTGGCCT CACCCAAACT AGTAATGGAA GAGGCTCCTG

---------- ---------- ---------- ---------- ----------

CATCGCCATC AGGGTTGCTT CCCCTAAATT GGTGATGGAG GAGGCTCCAG

AATCGCGATC CGCGTTGCTT CACCCAAACT GGTGATGGAA GAGGCTCCGG

AGTCCTATAA GAATGTGACA GATGTGGTAA ATACCTGCCA TGATGCTGGA

AGTCCTATAA GAATGTGACA GATGTGGTAA ATACCTGCCA TGATGCTGGA

AGTCCTATAA GAATGTGACA GATGTGGTGA ATACCTGCCA TGATGCTGGA

AGTCCTATAA GAATGTGACA GATGTGGTGA ATACCTGCCA TGATGCTGGA

AGTCCTATAA GAATGTGACA GATGTGGTGA ATACCTGCCA TGATGCTGGA

AGTCCTATAA GAACGTGACA GATGTGGTGA ACACCTGCCA TGACGCTGGG

AATCATATAA GAATGTGACA GACGTCGTGA ACACTTGCCA TGATGCTGGG

AGTCCTATAA GAATGTGACA GACGTCGTGA ACACCTGCCA TGATGCTGGG

---------- ---------- ---------- ---------- ----------

AGTCCTATAA GAACGTGACG GATGTGGTGA ACACCTGCCA TGATGCCGGA

AGTCCTATAA GAATGTGACG GATGTGGTGA ACACCTGCCA TGATGCTGGA

---------- ---------- ---------- ---------- ----------

AGTCCTATAA GAATGTGACC GACGTGGTGA ACACCTGCCA TGATGCCGGC

AATCCTACAA GAACGTGACC GACGTGGTCA ACACCTGCCA TGACGCTGGA

AATCCTATAA GAATGTAACA GATGTGGTGA ACACCTGCCA TGATGCTGGA

---------- ---------- ---------- ---------- ----------

AATCCTATAA AAACGTGACA GATGTGGTGA ATACTTGCCA TGATGCTGGA

AATCCTATAA AAACGTCACA GATGTGGTGA ATACCTGTCA CGATGCCGGC

ATCAGCAAGA AAGCCATTAA ACTGAGACCA ATTGCTGTGA TCAAAGGAAT

ATCAGCAAGA AAGCCATTAA ACTAAGACCA ATTGCTGTGA TCAAAGGAAT

ATCAGCAAGA AAGCCATTAA ACTGAGACCA ATTGCTGTGA TCAAAGGAAT

ATCAGCAAGA AAGCCATTAA ACTGAGACCA ATTGCTGTGA TCAAAGGA--

ATCAGCAAGA AAGCCATTAA ACTGAGACCA ATTGCTGTGA TCAAAGGAAT

ATCAGCAAGA AAGCCATTAA ACTGAGACCA GTCGCGGTTA TCAAAGGGAT

ATCAGCAAGA AGGCCATTAA ACTGAGACCA ATTGCTGTTA TTAAAGGAAT

ATCAGCAAGA AGGCCATTAA ACTGAGACCA ATTGCTGTTA TTAAAGGGAT

---------- ---------- ---------- ---------- --------AT

ATCAGCAAGA AGGCCATTAA ACTGAGGCCA ATTGCTGTTA TCAAAGGAAT

ATCAGCAAGA AGGCCATTAA ACTGAGACCG ATTGCTGTGA TCAAAGGAAT

---------- ---------- ---------- ---------- --------AT

ATCAGCAAGA AGGCCATTAA ACTCAGACCG ATCGCTGTGA TCAAAGGGAT

ATCAGCAAGA AGGCCATTAA ACTGCGACCG ATTGCTGTTA TCAAAGGCAT

ATCAGTAAGA AGACCATCAA ACTGAGGCCA ATTGCTGTTA TCAAAGGCAT

---------- ---------- ---------- ---------- --------AT

ATCAGCAAGA AGGCCATCAA ACTCAGACCA ATTGCAGTTA TCAAAGGCAT

ATTAGTAAGA AAGCCATTAA ACTGAGACCT ATCGCTGTTA TCAAAGGCAT

GCTGTCATCT GGAGTAGAGA CTCAGCCAGT TCCACTTGAT TCCTCCATGT

GCTGTCATCT GGAGTAGAGA CTCAGCCAGT TCCACTTGAT TCCTCCATGT

GCTGTCATCT GGAGTAGAGA CTCAGCCAGT TCCACTTGAT TCCTCCATGT

---------- ---------- ---------- ---------- ----------

GCTGTCATCG GGAGTGGAGA CTCAGCCAGT TCCACTTGAT TCCTCCATGT

GCTGTCATCC GGAATAGAGA CTCAGCCAGT TCCACTTGAT TCCTCCATGT

GCTGTCATCT GGAGTAGAGA CTCAGCCAGT TCCACTTGGT TCCTCCATGT

GCTGTCATCT GGAGTAGAGA CCCAGCCAGT TCCACTTGAT TCCTCCATGT

GCTGTCATCC GGAATAGAGA CTCAGCCAGT TCCGCTTGAT TCCTCCATGT

GCTGTCATCC GGCGTGGAGA CCCAGCCCGT TCCACTTGAT TCCTCCATGT

GCTGTCATCC GGAGTAGAGA CTCAGCCAGT TCCACTTGAT TCCTCCATGT

GCTGTCATCC GGAGTAGAGA CTCAGCCAGT TCCACTTGAT TCCTCCATGT

GCTGTCATCT GGAGTGGAGA CTCAGCCAGT TCCACTTGAT TCCTCCATGT

GCTGTCATCG GGAGTAGAGA CTCAGCCAGC TCCTCTTGAT TCCTCCATGT

GCTGTCATCC GGAGTAGAGA CTCAGCCAGT TCCACTTGAT TCCTCCATGT

GCTGTCATCC GGAGTAGAGA TTCAGCCAGT TCCACTTGAT TCCTCCATGT

GCTGTCATCT GGCATAGAGA CGCAGCCAGT CCCTCTTGAT TCCCCCATGT

GCTGACATCC GGAGCCGGGA CCCAGCCAGC CCCACTTGAG TCCTCCATGT

CTGCCGTGGT ACAGGAATTA TACTCTGAAC TCCCAGTAAG TGTCTCCAGA

CTGCCGTGGT ACAGGAATTA TACTCTGAAC TCCCAGTAAG TGTCTCCAGA

CTGCCGTGGT ACAGGAATTA TACTCTGAAC TCCCAGTAAG TGTCTCCAGA

---------- ---------- ---------- ---------- ----------

CTGCCGTGGT ACAGGAATTA TACTCTGAAC TCCCAGTAAG CGTCTCCAAA

CTGCCGTGGT ACAGGAATTA TACTCTGAAC TCCCAGTAAG TGTCTCCAAA

CTGCTGTGGT ACAGGAGTTG TACTCTGGAC TCTCAGTAAG TGTCTCCAAA

CTGCTGTGGT ACAGGAATTG TACTCGGAAC TCTCAGTAAG TGTCTCCAAA

CTGCCGTGGT ACAGGAATTA TACTCTGAAC TCCCAGTAAG TGTCTCCAAA

CTGCTGAGGT GCAGGAATTA TACTCTGAAC TCCCAGGGAG TGCCTCCAAA

CTGCCGTGGT ACAGGAATTA TACTCTGAAC TCCCTGTGAG TGTCTCCAAA

CTGCCGTGGT ACAGGAATTA TACTCTGAAC TCCCAGTAAG TGTCTCCAAA

CTGCCGTGGT ACAGGAATTA TACGCTGACC TCCCAGTAAG TGTCTCCAAA

CTGCCGTGGT ACAGGAATTA TTCTCTGAAC TCCCAGTAAG TGTCTCCAAA

CTGCAGTGGT AGAGGAAGTA TACTCTGAAC TCCCAGTGAG TGTCTCCAAA

CTGCTGTGGT ACAGGAATTA GACTCTGAAC TCCCAGTAAG TGTCTCCAAA

CTGCAGGGGC CCAGGAGTTG TACCCGGAAC TTCCAGTGAG CGTCTCCCCG

CTGGCGTGGC ACAAGAATTG TATAATGAAC TTCCTGTCAG CATCTCCCCT

GAGCTTCATG CTGACCCTGA GCCAAGTGTG ATTCCAGATG TAAAACCTGG

GAGCTTCATG CTGACCCTGA GCCAAGTGTG ATTCCAGATG TAAAACCTGG

GAGCTTCATG CTGATCCTGA GCCAAGTGTG ATTCCAAATG TAAAACCTGG

---------- ---------- ---------- ---------- ----------

GAGCTTCATG CTGACCCTGA GCCAAGTGTG ATTCCAGATG TAAAACCTGG

GAGCTTCATG CTGATTCTGA GCCAAGTGTG ATTCCGGATG TAAAACCTGG

GAGCTTCATG CTGAGCAGGA GCCGAGTATG ACTCCAGATC TAAAGCCTGG

GAGCTTCATG CTGAGCAGGA GCCAAGTAAG ATTGCAGATG TAAAGCCTGG

GAGCCTCATG CTGACTCTGA GCCGAGTGTG ATTCCAGATA GGAAACCTGG

GAGCTTCATG CTGACCCCGA GCCGAGTGCA ACTCCAGATG TAAAACCCGG

GAGCTTCATG CTGACCCTGA GCCAAGTGTG ATTCCAGATG TAAAACCTGG

GAGCTTCATG CTGACCCTGT GCCAAGTGTG ATTCCAGATG TAAAACCTGG

GAGCTTCATG CTGACCATGA GCCAAGTGTG ATTCCAGATG TAAAACCTGC

GAGCTTCATG CTGACTCTGA ACCAAGTGTG ATACCAGATG TAAAACCCGG

GAGCTTCATG CTGACTCTGA GCCAAGTGGG ATTCCAGATG TAAATCCCGG

GAGCTTCATG CTGATTCTGA GCCAAATGGG ATTCCACATG TAAAACCCAG

GAGCTTTCTG CCGAGTTTGA GCCAAGTATT GTTCTAGATG CAAAACCTGA

GAGCTGCCTG CTGAGCGTGA GCCAAGTGTT AATCCAGATG CAAAACCTGA

AGCCTCAAGC TCTCTTTTAA ATAGGGCATT GCCCTTGGAG CTGCAGAGGA

AGCCTCAAGC TCTCTTTTAA ATAGGGCATT GCCCTTGGAG CTGCAGAGGA

AGCCTCAAGC CCTCTTTTAA GTAGGGCATT GCCCTTGGAG CTGCAGAGGA

---------- ---------- ---------- ---------- ----------

AGCGTCAAGC TCTCTTTTAA GTAGCGCATT GCCCTTGGAG CTGCAGAGGA

AGCCTCAAGC TCTCTTGTAA GTAGGGCATT GCCCTTGGAG CTGCAGAGGA

AGCCTCAAGC TCTCCCATAG GCAGGGCATC ACCCCAGGGC TTGCAGAGGA

AGCCTCACGC TCTCCTCTAG GCAGGGCCTC ACCCCTGGGG TTGCAGAGGA

AGCCCCAAGC TCTCTTGTGA GTAGGGCATT GCCCTTGGAG CTGCAGAGGA

AGCCTCAAGC TCTCGTGTAA GTAGGGCTGT GCCCTCGGAG CTGCAGAGGA

AGCCTCAAGC TCTCTTATAA GTAGGGCAGT GCCCTTGGAG CTGCAGAGGA

AGCCTCAAGC TCTCTTGTAA GTAGGGCAGT GCCCTTGGAG CTGCAGAGGA

AGCCTCGTGC TCTCCTGTCA GTAGGGCGGT GCCCTTGGAG CTGCAGAGGA

AGTCTCAAGG GCTTTTTTAA GTAGGACATT GCCCTTGGAG CTACAGAGGA

AGCCTCAGGG GCGCTTATAA GTAGGACACT GCCCTTGGAG CAGCAGAGGA

AGCCTCGAGG GCTCGTTTGA GTAGGACATT ACCTCTGGAA CAGCAGAGGA

AGCCTCGAGC TCCTTGGT-- -CCGGACTTT GCCCGTAGAG CCCCAGAGAA

AGCCCCAAGC TCTCCGTTAG GCAGTAAATT GCCAGCAAAG CAGCCCGAGA

CTCATGTGGA GAGTTGTTGT GAAGAAACCG AGACCTTGGA TCATGGGAGT

CTCATGTGGA GAGTTGTTGT GAAGAAACCG AGACGTTGGA TCATGGGAGT

CTCGTGTGGA GAGTTGTTGT GAAGAAACCG AAACCTTGGA TCCTGGGAGT

---------- ---------- ---------- ---------- ----------

TTCATGCGGA GAGTTGTTGT GAAGAAAACG AGACGTTGGA TCATGGGAGT

CTCATGCAAA ATGTTGTGGT GAAGAAACCG AGACCTTGAA TCATGGGGGT

CTCATGCAGA AGGTTGTTGT GAAGAAAGCG AAACCGTGGA TTATGGGGGC

CTCATGCAGA AGGTTGTCGA GAAGAAAGCG AGACCTTGGA TTGTCGGGGC

CTCACGCGGA ACGCTGTGGT GAAGAAACCG AGAGCCTGGA TCGTGGGGGT

CTCCTGCGGA CAGTTGTCGT GAAGAAGCCA AGACCTCGGA CCATGGGGGT

CTCGTGCGGA AAGTTGTTGT GAAGAAACTG GCACCTTGGA TCATGGGGGT

CTCATGCGGA AAGTTGTTGC GAAGAAACCG AGAACTCGGA TCATGGGGGT

CCCGTTCGGA AAGTTG---C GAAGAAACCG CAACCTTGGA CCATGGGGCT

CTCATGTGGA AAGTTG---T GAAGAAACCG AGACCTTGGA TGATGGGGGT

CTCATGTGGA AAGTTGTTGT GAAGAAAACG AGACCTTGGA TGATGGGGGT

CTCGTGTGAA AA---GTTGT GAAGAAAACG AGACCTTGGA TGATGGGGGT

CTCATGTGGA AAATTGCTCT CCAGAAA--G GGACCCTGGA TGTTAGGGGG

CTCATGGTGA AAAATGGCGC TCAAAAACCG AGACTCTGGA CCACGGGGAC

GAGCCTGGGC GATGTGGGCT AGTGGACTCC ACAGCAGGAG GTTCTGTGGC

GAGCCTGGGC GATGTGGGCT AGTGGACTCC ACAGCAGGAG GTTCTGTGGC

GAGCCTGGGC AATGTGGGTT AGTGGACTCC ACAGCAGGAG GTTCTGTGGC

---------- ---------- ---------- ---------- ----------

GAGCCTGGGC GATATGGGCT AGTGGACTCC ACAGCAGGAG GTTCTGTGGC

GAGCCTGGGC AGTGTGGGCT AGTGGACTCC ACAGCAGGAG GCTCTGTGGC

AAGACTGGGT GGTGTGGACT AGTGGACCCT A---CAAGAG GTCCTATGGC

AAGAATGGGT GGTGCGGACT AGTGGATCCT A---CAAGAG ATCCTATGGC

GCGCCTGGGA GGTGTGGGCT AGTGGACCCC ACAGCAGGAG GTTCTCTGGC

GAGCCTGGGC AGTGTGGGCT GGTGGACCCC TCAACAGAAG GTCCTGTGGC

GAGCCTGGGC GGTGTGGGCT GGTGGACTCC ACAGCAGGAG GTTCTGTGGC

GAGCCTGGGC GGTGTGGGCT GGTGGACTCC ACAGGAGGCA GTTCTGTGGC

GAGCCTAGGC GGTGTGGGCT GGTGGACTCC ACAGCACGAG GTTCTTTAGC

GTGCCTGGGC GGTGTGGGCT GGTGGACTGC ACATCAGGAG GCTCTGTGGC

GAGCCTGGGT GGTGTGGGCT GGTGGACTCC ACAGCAGGAG GCTCTGTGGC

GAGCCTGGGA GGTGTGGGAT GGTGGACTCT ACAGCAGGAG GTGCTATGGC

AAACCAAGAT GTTGTGGACT GGCAGGCTCC CCAGCCAGAG ATGCTGTGGC

AAAGCTGGGT GTCCTAAGCT GGCAGAATCC ACAGCCAGAG AACCGGTGGC

ATCTGGGATC TTGGATAGGG CAAAGAGAAG CGAGAGCATG GAGCCAAAGG

ATCTGGGATC TTGGATAGGG CAAAGAGAAG CAAGAGCATG GAGCCAAAGG

ATCTGGGATC TTGGATAGGG CAAAGAGAAG CAAGAGCATG GAGCCAAAGG

---------- ---------- ---------- ---------- ----------

ATCTGGGATC TTGGATAGGG CAAACAGAAG CAAGAGCATG GAGCCAAAGG

TTCTGGGATC TTGGATAGGG AAGAGAACAC CAAGAGCATG GAGCCAAAGG

TTATGAGGTC TTGGATAGGG AAGAGAGACC CAAAAGCATG GAGCCAAAGG

TTATGAGATC TTGGATAGGG AAGAGAGACC CAAGAGCATG GAGCCAAAGG

TTCTGGGATC TTGGATAGGA AAGAGAAAGC CAAGAGCATG GAGCTAACGG

TCCTGGGAGC CTGGATAGGG AAGCGAAAGC CAAGAGCCTG GAGCAAAAGG

TTCTGGGATC TTGGATAGGA AAGAGAAAAC CAAGAGCGTG GAGCTAAAGG

TTCTGGGATC TTGGCTAGGG AAGAGAAAAC CAAGAGCATG GAGCTAAAGG

TTCTGGCATC TTGGATAGGG AAGAGAAAAC CAAGAGCTCA GAGCTAAAGG

TTCTGGGATT TTGGATAGGG CGGAGAAAAC CAAGAGCATG GAGCTAAAGG

TTCTGGCATC TTGGATAGGG AAGAGAAAAC CAAAAACATG GAGCTAAAGG

GTCTGTGATC TTGGATAGGG AAGCGAAAAC CCAAGA---- ----------

TTCAGGAGTC TTTGAGAAAG AGAGTCAAGC AGAGAGTGTG GAGCAGAAGT

TTCTGGAGAC TTTGACAGTA AAGAAAAACG CGGG--CATG GAGAAGCCAG

TCTTCAGAGA TCCAGGGGGC CAGGCAGGAA TTATAAGAGA ACCCTCTGAG

TCTTCAGAGA TCCAGGGGGC CAGGCAGGAA TTATAAGAGA ACCCTCTGAG

TCTTCAGAGA TCCAGGGGGC CAGGCGGAAA TTGTAAGAGA ACCTTCTGAG

---------- ---------- ---------- ---------- ----------

TCTTCAGAGA TCAAGGGGGG CAGATGGAAA TTATAAAAGA ACCCTCAGAG

TCTTCAGATA TCAAGGGGAC CAGGCGGAAA TTATGAGAGA TCCTTGTGAG

TCTTCAGAGA TCAAGGAGAC CAGGCGCAAG TTTTCAGAGG CCCATGTGAG

TCTTCAGAGA TCAAGGAGAC CAGGCACAAG TTTTCAGAGA CCCATGTGAG

TCTTCAGAGC TGAAGGGGAC CAGGCGGAAG GTAGAAGAGA CCCCTGTGAG

TCTTCAGAGA TGAAGGGGAC CAGGCAGAGG TGGTGAGAGA CCCCTGTGAG

TCGTCAGAGA TCAAGGGGAC CAGGCGGAAA TTGTAAGAGA CCCCTGTGAG

TCTTCAGAGA TCGAGGGAAC CAGGTGGAAA TTGTAAGAGA CCTCTGTGAG

TCTTCAGAGA TGAAGGGGAC CAGGAGGAAG TTGTAAGAGA GCCCTGTGAG

CCTTCAGAGA TCAAGGGAAG CAGGAAGAAA TTTTAAGAGA CCCAAGTGA-

TCTTCAGAGA TCGAGGGAAC CAGGAAGAAA TTGTAAGAGA CCTCTGTGAG

---------- ---------- ---------- ---------- ----------

CCTCGAACAA CCATGGGGAT CAGGAGGAAA CTGTGAGAGC CTCTGACAAG

ATTTGCCGAA TAGTGGGGAG TGGGATGAAA TTGTAAAAG- --TCAGGAAA

CAACATTCCA CAGCTGCTGA AGAAAAGACT AGCCCGAGTC AGGAAGGAAA

CAACATTCCA CAGCTGCTGA AGAAAAGACT AGCCCGAGTC AGG-------

CAACATTCCA CAGCTGCTGA AGAAAAGATT AGCCCAAGTC AGGAAGGAAA

---------- ---------- ---------- ---------- ---AAGGAAA

CAACATTCCA CAGCTGCTGA AGAAAAGATT AGCGCAAGTC AGGAAGGAAA

CAACATTCCA CAGCTGCTGA AGAAAAGATC AGCCCAAATC AGGAAGGAAA

CAACATTCCA CAACTACTAA GGAAAAGATC TGCCCCAGTC AGGAAGGAAA

CAACATTCCA CAGCTACTAA AGAAAAGATC TGCCCCTGTC AGGAAGGAAA

CAGCATTGCG CAGCTGCTGA AGACAGGATC AGCCCGAGCC AGGAGCAAAG

CAGTATCCCA CAGCTGCTGG AGAACAGCCC CGCCCCGGCC AGGAAGGAAA

CAACATTCCA CAGCTGCTGA AGAAAAGATC AGCCTAAGTC AGGAAGAAAA

CAACATTCCA CAGCAGCTGA AGCAAAGATC AGCCCAAGTC AGGAAGGAAA

CAGCATTCCA CAGCCGCTGA AGAAAAGATC AGCCCAAGTC AG--------

--ACATTCCA CAGCTGCTGA AGAAAAGATC AGCCCAAGTC AGGAAGGGAA

CAACATTCCA CAGCTGCTGA AGGAAAGATC AGCCTAAGGC AGGTAGGAAA

---------- --------GA AGAAAAGATC A---CAAGTC AGG-------

CAGCCTTCCC CAGCTGTGGA AGGAGCGTGG GGCTCATGCC AG-AAGGAAA

GAGCATTCCG TGGATGCCGA GGAAAAGTGT TGTTCAAATG AGGGAGGAAG

TGTACAGATT ACAGCTGAAA CTCTGCTAAA ATCCGCCGAA GTACAAGGTA

---------- ---------- -----CTAAA ATCCACCGAA GTA-AAGGTA

TGTACAGATT ACAGCTGAAA CTCTGCTAAA ATCCACTGAA GTACAAGGTA

TGTACAGATT ACAGCTGAAA CTCTGCTAAA ATCCACTGAA GTACAAGGTA

TGTACAGATT ACAGCTGAAA CTCTGCTAAA ATCTACTGAA GTACAAGGTA

TGTACAAATT ATAACCAAAA CTCTGCCAAA ATCCATTGAA GTACAAGGTA

TGTACACAAT ACAGCTGGAA CTCTGCCAGA AGCCACTGAA GGACGAGGTA

TGTACACAAT ACAACTGCAG CTCTGCCAGA ACTCACAGAA GGACAAGGTA

TGTGCAGATT ATAAATGAAA GCGTGGTAGA ACCCCCTGCA GTACAAGGTA

TGTACAAATC ACAGCTGAAA CTCTTCTGAC ATCTACTGAG GTCCAAGTTA

TGTACAACTT ACAACTGGAA CTCTGCTAAA ATCTACTGAA GTACAAGGTA

TGTACAAATT ACAACTGAAA CTCTCCTAAA ATCTACTGAA GTACAAGGTA

---------- ---------- ---------- ---------- ----------

TGTACAGATT ACAACTGAAA CTCTGCTAAA ATCCCCAGAA GTACAAGGTA

TGTACAGATT ACAACTGAAA CTCTACTAAA ATCCATTGAA ATACAAGGTA

---------- ---------- ---------- ---------- ----------

CGTACAGGTT ACAACAGAAA CTCTGCCAAA ACCTACTGAA GAACAAGGTA

CGAGCAGGTT ACAACAGAAA CTCCATTAAA ACCTATTGAA GTACAAGGTA

TGAAGGTCAA TGGGACTAAG ACGGATAATA AAAATGGCAA TGTGAGTAAA

TGAAGGTCAA TGGGACTAAG ACGGATAATA AAAATGGCAA TGTGAGTAAA

TGAAGGTCAA TGGGACTAAG ACGGATAATA AAAATGGCAA TGTGAGTAAA

TGAAGGTCAA TGGGACTAAG ACAGATAATA AAAACGGCAA TGTGAGTAAA

TGAAGACCAG TGGGACTAAG ATGGATAATA AAAATGGCAA TGtgatcgat

TGAAGGTCAG TGGCACTAAG ATGGATAATA AGAATGGCAA TGTGAGTAAA

TGAAAGCCAG AGTAACTA-- GGGGACTGTT GG-GTGGCAC TGTGAGTCAG

TGAAAGCCAG AGGAACCAAG GGGGACGGT- ---GTGGCAC TGTGAGACAA

TGAAGGTCAA TGGGACTGAG ACGGACAGTG AGAACGGCAA TGTGAGTAAA

TGAAGGTCCA TGGAACTAAG GTGGACGATG AGAATGGCCG TGTGAGTCAG

TGAAGGTCAA TGGGACTAAG ACGGATTATA AGAATGGCAA TGTGAGTAAA

TGAAGGTCAA TGGGACTAAG ACGGATAATG AGAATGGCAA TGTGAGTAAA

---------- ---------- ---------- ---------- ----------

TGAAGGTCAA TGGGACTAAG ACGGATAATA GGAATGGCAA TGTGAGTAAA

TGAAGGTCAA TGAGGCTAAG ATGGATAATA GGCATGGCAA CTTGAGTAAA

---------- ---------- ---------- ---------- ----------

TGAAGGTCAA TGGGACTGAG ACCATTAG-- -GAATGACAA AGTGAGTAAA

CCAAGGTCAA TGGGACTAGG ACGATTAATA GGAGTGACAA AGTGAGTAGA

GATCTCTCAG CTGGATGCGG TGAATTCCAA GAAGTAGACA AAATCATGAC

GATCTCTCAG CTGGATGCGG CAAATTCCCA GAAGTAGACA AAATCATGAC

GATCTCTCAG CTGGATGCAG CGAATTCCCA GAAGTAGACA AAATCATGAA

GATCTCTCAG CTGGATGCAG CGAATTCCCA GAAGTAGACA AAATCATGAA

gatctctcag ctggatgCAG CGAATTCCCA GAGGTAGACA AAATCGTGAC

GGTCTTTCAG CTGGGTGCAG CGAATTCCCA GAAGTAAACA AAATCATGAC

GATCTGTCAG CCAGGGGTAG TAAGCACCCA GGAGTAGACA GGAGCATGAC

CATCTGACAG CTGGGAGCAG TGCACATCCA GGAGTAGATA GGAGCATGAC

GGTGTCTCAG CTGGGCACAG CAAAGACCCA GACGTAGACA GAACCATGGC

GGTCTCTTGG CTGGGTGTCG TGGACACTCA GAGACAGACG AAATCATGAC

GATCTCTCAT CTGGGTTCAG CGAATACCCA GAGATAGACA AAATCATGAC

GGTCTCTCGG CTGGGTGCAG CGAATACCCA GAAGTAGACA AAATCATGAC

---------- ---------- ---------- ---------- ----------

GGTCTTTCAG CTGGGTGCAG CAAATACCCA GAAATAGACA AAATTTTGAC

GGTCTCTCCA CTGGGTGCAG TGAATACCCA GAAGTAGACA AAATCATGAC

---------- ---------- ---------- ---------- ----------

AGTCTCCCAA CTGGGTTCAT CGACTGCCCA GATAGAGACA AAATCATGAC

GAATGCCCAG CTGAGCCCGG TGAATGCCCA GATGTAGACA AAAGCATGGC

CAGTGATGAG GTTTCAGAAA CCAGCACATT AGTTACCCCA GAACCTTTAA

CAGTGATGAG GTTTCAGAAA CCAGCACATT AGTTACCCCA GAACCTTTAA

CAGTGATGAG GTTTCAGAAA CCAGCACATT AGTTACCCCA GAACCTTTAA

CAGTGATGAG GTTTCAGAAA CCAGCACATT AGTTACCCCA GAACCTTTAA

CAGTGATCAG GTTTCAGAAA CCAGCACGTT AGTTACCCTA GAACCATTAA

CAGTGGTGAG GTTTCAGAAA CCAACAGATT AGTTACCCTA GAGCCTTTAA

CAGTAGTGAG CTTTGGGAAA CCAGAGTTTT AGGTAC-ACA GAGCCTGTAA

CAGCAATGAG TTTTGGGAAA ACATAGCTTT CATTACCACA GAACCTGTAA

CAGTGGTGAG GTTTCAGAAA CCAGCACGTT AATTTCCCTA AAGCCTTTAA

CAGTGCTGAG GTTTCAGAGT CCAGCACCTT AGTTTCTCTA GAGCCTTTAA

CAGTGGTGAG GTTTCAGAAA CCAGCACATT AGTTTCCCTA GAGCCTTTAA

CAGTAGTGAG GTTTCAGAAA CCAGCACATT AGTTTCCCTA GAGCCTTTAA

---------- ---------- ---------- ---------- ----------

CAGTGGTGAG GTTTCAGAAA AGAGCACATT AGTTTCCCTA GAACCTTTAA

CAGTGGTGAA GTTTTAGAAA CCAGCACGTT AGTTTCCCTA GAGCCTTTAA

---------- ---------- ---------- ---------- ----------

CAGTGGTGAG GGTTCAGAAA CCAGCACCCT AGTTTCCCTA GAGCCTTTAA

CACTGGTGAG GTTTCAGAAA CCAGCACCTT AGTTTCCCCA GAGCCTTTAA

CCTTTGTGGA CCCTGTATTA ACAGAAGCAA CTCCTAAAGA AGAGGAACGG

CCTTTGTGGA CCCTGTATTA ACAGAAGCAA CTCCTAAAGA AGAGGAACGG

TCTTTGTGGA CCCTGTATTA ACAGAAGCAA CTCCTAAAGA AGAAGAACAG

CCTTTGTGGA CCCTGTATTA ACAGAAGCAA CTCCTAAAGA AGAAGAACAG

CCTTTGTGGA CCTTGGATTA ACAGAAGCAA CTCCTAAAGA AAAAGAACAG

CCTTCGTGGA CCCTGGATTA ACAGAAGCAA CTTCTAACGA GGAAGAACTC

CCTTTGTGGA CCCTGAATTA ACAAAAGCAA CTCTTAAAGA A---------

CCTTTGTGGA C--------- --AAAAGCAA CTCTTAAACA A---------

ATTTTATGGA CCCTGGATTA ACAGAAGCAA CTCCTAAGGA AGGAGCACAG

ACATTGTGGA CCCTGGATTA ACAGAAGCAA ATCCAAAAGA A-ACAAACAG

CCTTTGTGGA CCCTGGAATA ACAGAAGCAA CTTCTAAAGA AGATGAGCAG

ACTTTGTGGA CCCTGGATTA ACAGAAGCAA CTCCTAAAGA AGATGAACAG

---------- ---------- ---------- ---------- ----------

TCCTTGTGGA CCCTGGATTA AGAGAAGCAA CTTCTAAAGA AGAATTATAC

CCTTTGTGGA CTCTGGATTA ACAGAAGCAA CTTCTAAAGA AGAAAAACAG

---------- ---------- ---------- ---------- ----------

CCTCTGTGGA GCCTGAATTG ACAAAAGCGC CTTCAAAAGA AGAAGATCAA

CCTTTGCAGA AGCTGGGTTA ACAGAAGCAC CTTCCGAAGA AGAAGAACAG

CTTTTGATTC CTAGGAGTGA AAGAGGTGGA CCTTTTCTTT TTAATGCCAG

CTTTTGATTC CTAGGAGTGA AAGAGGTGGA CCTTTTCTTT TTAATGCCAG

CTTTTGATTC CTAGGAGTGA GAGAGGTGGA CCTTTTCTTA TTAATGCCAG

CTTTTGATTC CTAGGAGTGA GAGAGGTGGA CCTTTTCTTA TTAATGCCAG

CTTTTGATTC CCAGGAGTGA AAGAGGTGGA CCTTTTCTTA TTAATGCCAG

CTTTTGAATC CCAGGAGTGA AAGAAGGGAA CTCTTTCCTG TTATTGCCAC

---------- ---------- ---------- ---------- ----------

---------- ---------- ---------- ---------- ----------

CTTTTGAGTC CTGTTAGTGA ACGAGGAGAA CTCTCCGTTA TTAACGGCAG

CCTTTGAACC CTAGAAGTGA AAGAG---AA CTCTTCCTTG TTAACACCAG

TTTTTGAACC GCAGGAGTGA AAGAGGAGAA CTCTTTCTTG GTAATGCCAG

TTTTTGAACC CCAGGAGTGA AAGAGAGGAA CTCTTTCTTG TTAATGCCAG

---------- ---------- ---------- ---------- ----------

CTTTTGAACC CCAGGAGTGA AAGGGGTGAG GTCTTCCTTG TTAATGACAG

CGTTTGAGCC CTGGGAGTGA AAGGGGAGAA CTCTCTCCTT TTAATCCTGC

---------- ---------- ---------- ---------- ----------

GCTCTGAGTG TCAGGAGCCA AAGTAGAGAA GTACTTCCTG TTGATGCCA-

CTCCTGCATC CCACATGTGA AGATGGAAAT CTGCTTAATG CTGTTGCTGA

GGAACCAGAA AAGGAGATTA GTTGTTCTGG TGAAAAAGAG CCTGTTGTTT

GGAACCAGAA AAGGAGTTTA GTTGTTCTGG TGAAAAAGAG CCTGTTGTTT

GGAACCAGAA AAGGAGATTA GTGGTTCTGG TGAAAAAGAG CCTGTTGTTT

GGAACCGGAA AAGGAGATTA GTGGTTCTGG TGAAAAAGAG CCTGTTGTTT

GCAACCAGAA AAGGAGATTA GT---TCTGG TGAAAAAGAG CCTATTGTTT

ACAGCCAGAA AAGGAGATTG GTTGTTGTGG TGGGAAAAAA ACTGTTGATT

---------- ---------- ----TTGTAG AGAAGAAAGG ACTGTGGAAT

---------- ---------- ----TTGTGG AGAAGAAAGG GCTGTGGAAT

GCAGCCAGTC AGGGATGCCA GTTG------ --------AA ACTGTTGATT

GCAACCAGGA GAGAATTCTA GTTGTTCTGG AAAAAAAGAG ACTACTGTCT

GCAACCAAGA GAGGATGCTA GTCGTTCTGG TGAAAAAGAG ACTGTTGCCT

AAAACCAGAA GAGGATGCCA GTCGTTCTGG AGAAAAAGAG ACTGTTGCCT

---------- ---------- ---------- ---------- ----------

ACAACCAGAA GAGAATGCTA GTAATTCTAG TGAAAAAGAG TCTGTTGACT

TCAGCCAGAA GAGGATGCTA GTTGTTCTGG TGAAAAAGAG ATTGTTGACT

---------- ---------- ---------- ---------- ----------

--AGCCTGGA GGAGAAGAAA ATGGTTCTAG TGATGAACAC CATCCTCATG

GCAACAAGAA GAGCAGATTA G---CTCTGG CAACGGGGAA ACCACTGACA

CTCCAAAGGA AAATATCAAT AACTGCATTC AAGACAGTCT CCATACAGGG

CTCCAAAGGA AAATATCAAT AACTGCATTC AAGACAGTCT CCATACAGGG

CCCCAAAGGA AAATATCAAT GACTGCATTC AAGACAGTCT CCATACAGGG

CCCCAAAGGA AAATATCAAT GACTGCATTC AAGACAGTCT CCATACAGGG

CCTCAAAAGA AAATATCAAT AACTACATTC GAGACAGTCT TCATACAGGG

CTCCAAAAGA AAATATCAAT AACTGCATTC AGGGCGGTCT CCATACAGAG

CC-------- -AGTAACAGC AACTGCACTC TAGGCTGTCC CCGTATAGAC

CC-------- -AGTAACAGC AACTGCACTG AAGGTTGTCC CTGTACAGAC

CTCCAAAAGA AAATATCAGT AACCACGTGC AAGGCAGTCT CCATGCAGAG

TCCCCAAAGA AAATGCCGAT CACTACCCTC GAGGCAGTCC CCATATAGGC

CCTCCAAAGA GAATATCAGT AACTGCATTC AAGGCAGTAT CCATACAGAC

CCCCAAAAGA AAATATCGAT AACTGCATTC AAGGCAGTAT CCATACAGAC

---------- ---------- ---------- ---------- ----------

CCCTAAAAAA AAATGCCAAT AACTGCATTC AAGGCAATAT TTATACAGAG

CTCCAAAGGA AAATATCAAT AACTACATTC AAGGCCGTAT CCATACGGAA

---------- ---------- -----CATTC AAGGTAGTGT CCATAAAGAA

ATCAAAGAGA ATGTGCCGAT ATCTGGGCTC GAGGAAGTAT CCAGCAAGAG

CCTCAGAAGT AATCAGCAGG AACCTAAGAC AAGAAGGCAC CCACTTGAAG

AACTCTAGTT CTTTTACTGA AGCCAAAAAT GATAAAATTA CTGTACACGT

AGCTCTAGTT CTTTTACTGA AGCCAAAAAT GATAAAATTA CTGTACACGT

AGCTCTAGTT CTTTTACTGA AGCCAAAAGT GATAAAATTA CTTTACACAT

AGCTCTAGTT CTTTTACTGA AGCCAAAAGT GATAAAATTA CTTTACACAT

AGCTCTAGTT CTTTTACTGA AGCCAAGAAT GATAAAATTA CTTTACATGT

AGCTGTAGTT CTTTTACTGA AGCTAAGAAT GGTAAAATAA CTTTAGACAT

AGCTGTAGTT CTCCTACTGA AGCCAAGAAT GATAAAATTA CTTTAAACCT

AGCTGTACTT CTTCTACTGA AGCCAAAACC GATAAAATTA CTTTAAACCT

AGCCCTGCAT CTTCTCCTGA AGCCAACAAT GATAAAGTCA CTTCAGATAC

TGCTCCAATT CTTTTACTAA AGCCAAAAAT GATAAAATCA CTTTAGATGT

AGCTCTAGTT TGTTCACTGA AGCCAAAAAT GATAAAAACA CGTTAGACAT

AGCTCTAGTT CTTTTACTGA AGCCAAAAAA GATAGAATCA CTTTAGACAT

---------- ---------- ---------- ---------- ----------

AGCTCTAGCT CTTTTGCTGA AGCCGAAAAT GATATAATCA CTTTAGACAT

AGCCTTTATT CTTCTACTGA AGCCGAAAAT AATAAAATGA CTTCAGACAT

AGCGCTAGTT CTGTCACTGA AGCCGAAAGT GATAGTATCA CTACAGACAT

AACTCTGTT- -TCCTTCTGA AG---GAGAT CCTAAAAGCC CTTCAGAAAT

AACTCATGTC CTGTTGCCGA ATCCGAAGAT GACAGAatca ttttagaaaa

TCAAGGTAAC TTGACAAACC CTGAGGACCA TAAAGAAACT TTTACTAATA

TCAAGGTAAC TTGACAAACC CTGAGGACCA TAAAGAAACT TTTACTAATA

TCAAGGTAAC TTGACAAACC TTGAGGACCA TAAAGAAACT TCTACTAATA

TCAAGGGAAT TTGACAAACC TTGAGGACCA TAAAGAAACT TCTACTAATA

TCAAGGTAAC TTAACATACC CTGAGGACCA TAAAGAAACT TTTACTAATA

TCAAGAAAAC TTGACAAACT CTGAGGACCA TAGAGAAACT TTTATTAATA

TGATGGTAAT TTGATAAATT CTGAGGACCA TAGGAAAACT GTTGTGGATG

TCATGGTAAT TTGATAAACT CTGAGGACCA TAGAAAAACT GTTG---ATG

TCAAGGTAAT ---------- ---------- -----AAAC- ----------

TCACGGTAGC TTGGCAAACA ATGAGGACCA CAGAGGAACT TTTGCTGAAA

TCAGGGTGGT TTGACAAACC ATGAGGACCA TAGAGAAACT TCTACTGGTA

TCAAGTTAGC TTAACAAATC ATGAGGACCA TAGAGAAACT CTTACTAATA

---------- ---------- ---------- ---------- ----------

TCAAAGTAGC TTGACAAACC ATGAGGACCA TAAAGAAACT TGTACTAATA

TCAAGGAAGC TTCATAAACT GTGAGGACCA TACAGAAACA TTTGCTAATA

TCAAGGAAGC TTGACAAACC ATGAGAACCA TAGAGAAACT TTTGCTAATA

TCAAGAAAAT CTCATAAAAC ATGAAAGCAA GAGGGTAACC TGCCCTTGTG

gcaagatcat ttgaaaaagc aagaGTGCCA GAGGAAAATC TGTACCAGTG

TGAGCCATCC TAACTCCTTA GTCAGCATCC AGAGAAATCT GGAAGGCAAC

TGAGCCATCC TAACTCCTTA GTCAGCATCC AGAGAAATCT GGAAAGCAAC

TGAGCCATCC AAACTCCTTA GTCAGCATCC AGAGAAATCT GGAAAGCAGC

TGAGCCATCC AAACTCCTTA GTCAACATCC AGAGAAATCT GGAAAGCAAC

TGAGCCATCC AAACTCCTTA GTTACCATCC AGAGAAATCT GGAAAGCAAC

TGAGCCATCC AAACTCCTTA GTCAGCATTC AGAAAAATCT GCAAGGTGAT

TGACCCATCC TCAGTCTGTA GTCAGCCTCC AGAGGAAGCT G---GGCGAC

TGACCCATCC TAAGTCTGTA GTCAGCATCC AGAGGAGTCT G---GGTGAC

---------- -GACTCCTCA GTTAGCATCC AGAGAAATCT GGACGGTGAC

TGAGCCAACC AGACTCCTTA GTCCGTACCC AGAGAAATCT AGAAGGCAGA

TGAGCCATTC AAATTCATT- --CTGCTCCA AGAGAAATCT AGAAGATGAC

GGAGCCATCC A----CCTTA GTCATCACCC AGAGAAATCT GGAAGGCAAC

---------- -CATACTTTA GTTAGAACTC AGATGGGTCT GGACAGCAGC

AAAGCCATCC AAATTCCTTA GTCAACCTCC AGAAAAATAT GGAAGCCTAC

TTAATCATTC AGACTCCTTA GTCAATATCC AGAGGAGTCT GGAAAACAAC

TTAGCCATCC GAACTCTTTA GTCAGTATCC AGCAGAATCT GGACAGTAGC

ATAGTCACCA AAGTTCTTTG GTCAGCAGCC AAAGAGGCCT AGGGAGCAGT

GTACTCATC- -AATTTTTCA GCCAGCCACC AGAAGTGTTT GGATAATGGC

ACCCAGGAAA GAAAATCCAT TGTGAGTTTA ATGCCAGAGG ATCAAATAAG

ACCCAGGAAA GAAAATCCAT TGTGAGTTTA ATGCCAGAGG ATCAAATAAG

ACCCAGGAAA GAAAATCCAT TGTGAGTTTA ATGCCAGAGG ATCAGATAAG

ACCCAGGAAA GAAAATCCAT TGTGAGTTTA ATGCCAGAGG ATCAGATAAG

TCCCAGGAAA GAAAATCCAT TGTGAGTTTA ATGCCAGAGG ATCAGATAAG

ACCCATGAAA GAAAATCTCT GGTGAGTTTA ATGCCAGAAG ATCAGATAAG

TGTCCGGAAA GAAAGCCCCT TCAGAGCTCG ATGCCAGAAG ATCCAGTAAG

TCTCTGGAAA GAAAGCCTCC TCAGAGTTTG ATGTCAGAAG ACCCAGGAAG

ACTCAGGAAA GAAAATCCCT TCTGAGTTTA AGGCCAGAAG ATCAGATAAG

CCCCAGGAAG GGAAATCCCT TGTGAGTTTA ACGCCAGAGG ACCAGATAAA

ACCCAGGAAA GAAAATCCCT TGTGAGTTTA ATGCATGAGG ACCAGATAAG

ACCCAGGAAA AAAAATCCCT TTCGAATTTA ATGCCGGAGG ACCAGATAAG

ACCCAGGAAA GAAAATCCCT TGTGAATTTA AAGCCTGAGG ACCCAATAAG

ACCAAGAAAA GAAAATCCCT TTTGTGTTTA AAGCCAGAGA ACCAGTTAAG

ACCCAGAAAA GAAAATCCCT TGTGAATACA ATGCCAGAGG ACCAGATAAT

GCGCATAAAA GAAAAACCCT TGTGAA---- ---------- -CCAGTTACG

ACCAAGAAAA GAAACCCCAC TGCTTGTTCT GTGCCAGATA ACCCTCCTAA

TCTAAGCGAA AAGAGCCCCC TGCCAGTATA GGGGCAGAAG AGCCACTCAT

TCCTGTAAGT GAGGTATTAA AACCCAAGCA AGGTACTGCT CTGCCTGAGT

TCCTATAAGT GAGGTATTAA AACCCAAGCA AGGTACTGCT CTGCCTGAGT

TCCTGTAAGT GAGGTATTAA AACCCAAGAA AGGTACTGCG CTGCCTGAAT

TCCTGTAAGT GAGGTATTAA AACCCAAGAA AGGTACTGCA CTGCCTGAAT

TCCTGTAAGT GAGGTATTAA AACCCAAGAA GGGTACTGCT CTGCCTGAGT

TCCTATAAAT GAGGTTTTAA AGCCCTGCAA AGATACTGTT CAGCCTGGGT

TTCTGAAAAT GAAATATCAA AACCCAAGGA AGGTACTGCA AAA-------

TTCTGAAAAT GAAATATCAA AACCCAAGAA AGGTACTGCA AAA-------

TTCTATGAAT GATGGATCCA AACCCCAGGA AGGTACTACT CAGGCTGAGT

TTCTATAAAT GAGATGTCTA AGCCCAAGAA AGACATTACT CCACCTGAGT

TCTTATGAAT GAGGTATCAA AACCCAAGAA AGATGTTGCT CAGCCTGAGT

TTCTGTAAAT GAGGGATCAA AACCCAAGAA AGATACTGCT CAGCCTGAGT

GCCAATGAAT GAGTTAGCAA AACCAGAGAA TGATACTGCT CAGCCTGAGT

CCCAACAAAT GAGATATCAG AACCCAAGAA TGATATTGTT CAGTCTGAGT

TCCTATAAAT GAAGTGTCAA AACCCAAATA TGGTACTGTT CAGCCTGAGT

TCCTCTAAAT GAGCTATCAA AACTCAAGAA TGATACTATT CAGCCTGAGT

GCCTATAAGG AAAGTCTCAG AGGTCAGCAG TGTTCTTGCT AG---TGAAG

TACATTAATT AAAGTAGTGG AAAACAGTCG AGCCACTTCG CAGCCTAAAT

CAGAAAAAGT TGATGATATT CCACTTTTAG ATGAACAGAG CATAGCCTGT

CAGAAAAAGT TGATGATATT CCACTTTTAG ATGAACAGAG CATAGCCTGT

CAGAAAAAGT TGATGATATT CCACTTTTAG ATGAACAGAG CATAGCCTGT

CGGAAAAAGT TGATGATATT CCACTTTTAG ATGAACAGAG CATAGCCTGT

CAGAAAAAGT TGATGATATT CCAATTTTTG ATGAACAGAG CATAGCCTGT

CGGAAAAAGC TGACAGTATT CCTCGTTTAG ATGAACAGAG CATTGACTGT

CAGAAAAACC TGACAGCATT TCCCATTTTG ATGGACCCAT CACCGTTTGT

CAGGAAAACC TGACAGCATC TCACATTTTG ATGAACCCAT CACCACCTGT

CAGAAAAAGG TGGTGATATT CCATGTTCAG ATGAACAGAG CATTACCTGT

CAGAAATAGC CGACCATGGT CCACATTTAG GTAAACAGAG CGCTGCCTGG

TAGAACAAGC TGATGATAGT TCACATTTAG GTGAACGGAA CATTGCCTAT

CAGAAAAAGC GGACAATATT CCACATTTAG AAGAACAAAG CATTGCCTAT

CTGGAAAAAA TGACAATATT CCACA----- -GAGCCAGAT CATTGCCTGT

CAGAAAAAAC TTATGATAAT CCACATTTAG ATGAACAGAT CATTGCCTGT

CAGAAAAAAC TGATGATATT CCACGTTTAG ATGAACAGAG CATTGCTTGT

CAGAAAAAAC TGAGGGTAGT CCACATTCAG ATGAGCAGAG CCGTGCTTGC

CAAAAGACAC TGATGTTATT CCATTTGTAG ATGGACAGAT AATGGCTTAT

TAAATGAAA- --CTGGTATT CCAGAAGTAG ACAGACAAAA CCTTACTAAT

GAGATGAATG AACTTTCTTG TACCAATGAA CTGGTTGTAA ACAAAGTAGA

GAGATGAATG AACTTTCTTG TACCAATGAA CTGGTTGTAA ACAAAGTAGA

GAGATGAATG AATTTTCTTG TACCAATGAA CTGGTTGTAA ACAAAGTAGA

GAGATGAATG AATTTTCTTG TACCAATGAA CTGGTTGTAA ACAAAGTAGA

GAGATGAATG AACTTTCTTG TACCAATGAA CTGGTTGTAA ACAAAGTAGA

GAGATGAATA AACTTTCTTG TACTAGCAAA CTGATTATAA ATAATGTAGA

GAGATGAGTG AATGTTCTCC TAACAGAGAA CTGGTTGCAC ACAAAATAGA

GAGATGAGTA AACGTTCTCC TAACCGAGCC CTGGTTGCAC ACAAAATAGA

GAGATGAATA AACTTTCTTA TACCAATGAA CTCGTTGTAA ACAAACTCGA

GAGGTGAATA AATTTCCTCA TGACAACAAA CTGGTTGTAA ACAAAACAGG

GAGATGAATG AACTTCCTTG CACCAATGAA CTGGTTGTAA ACAAAATAGA

GAGATGGATG AAGCTCCTTG TACCGAAGAA CTAGCTGTAA ACAAAATAGA

GAGATAAATG AACTTCCTTC TACTAAGGAA CTAGCTGTTA ACAAAGTAGA

GAGATGAATG AACTTTGTTG TACTGATGAA CTGGCTGTAA ACAAAGTAGA

GAAGTAAATG AACTTTCTTG TACTGATGAA CTAGTTGTAA ACAAAGTAGA

GCAGTGAATG GACTTTCCTG TACTGATGAA CTAGCTGTAA GCA---CAGA

GGACAGAATG AGCTTGCTTA TCCTGCTGAG TCCAGAGAAG ACAAGGGGGA

GAAATGAGTG AGCTTTCTTG CCCTGTTGAT TCAGTCTCAA GGGGGATGAA

AAGTGAATGT GTTTTAAATC AAGTGTCCCT TAATTCTCCA ACTGACAACA

AAGTGAATGT GTTTTAAATC AAGTGTCCCT TAATTCTCCA ACTGACAACA

AAGTGAATGT GTTTTAAATC AAGTGTCCCT TAATTCTCCA ACTGACAACA

AAGTGAATGT GTTTTAAATC AAGTGTCCCT TAATTTTCCA ACTGACAACA

AAGTGAATGT GTTTTAAGTC AAGTGTTCCT TAATTCTCCA ACTGACAACA

AAGTGAATGT GTTTTAAATC AAGTGTCCCT TAATTCTCCA ACTGACAACA

ACATGAATGT GTTTCACATC AAGTGTCCCT TAGTTCTACA GCTGACAACA

GCATGAATGT GTTTCACATC AAGTGTCCCT TAGTTCTACA ACTGGCAACA

AAACGAATGT GTTTT---TC AAGTGCCCCT TAATTCTCCA ACAGATAACA

AAGTGAATGT GTTTTAAATC AAGTGTCCCT TAATTCTCCA AGCG---ACA

AAGTGAATGT GTTTTAAATC AAGTGCCCCT TAATTCTCCA GCTA---ATA

AAGTGAATGT CTTTTAAATC AAGTGTCCCT TAATTCTCCA ACTG---AGG

AAATGAATGT GTTATAAATC AAGTGTCCCT TAATTCTCCA GCTA---ATG

AAATGAATGT GTTTTAAATC AAGTGTCCCT TAATTCTCCC ACTGACAACA

AAGTGAATGT GTTTTAAATC AAGTGTCCCT TAATTCTCCA TCTGACAACA

ACGTGAATGT GTATTAAATC AAGTGTCCCT TAATTCTCCA ACTGACAACA

GCTTTTCTGT GTCTCATCTA AGATGTCCTT TGATGCTACA GCTCCCACTG

GAGCACAGAT GCTTCAACTG AAAAGTCTCC CAACTCT--G GAGGACAGTG

AAGAGATGCC TTTAGCAACA GGCAGAGATG CCCATCAGAG CCATCACCCT

AAGAGATGCC TTTAGCAACA GGCAGAGATG CCCATCAGAG CCATCACCCT

AAGAGATGCC TTTAGCAACA GGCAGAGATG CCCATCAGAG CCATCATCCT

AAGAGATGCC TTTAGCAACA GGCAGAGATG CCCATCAGAG CCATCATCCT

AAGAGATGCC TTTAAAAACA GGCAAAGATG CCCAACAGAG CCATCATCCT

AAGAGATGCC TTTAGAAACA AGCAAAGATG CCCAGCCAAG CCATCACCCT

GAGAAATGCC TTCAGCAGCA TGCAAAGATG TTCAAGAGAG CCGTCG---T

GAGAAATGCC TTCAGCAACA TGCAGAGATG TTCAAGAGAG CCACCA---T

AAGAGATGCC TTTCCCAGCA AGTGAGGGTG TCCAACAGAG CCATCAGCCT

AAGAGATGCC TGTGGCAGTG AGCGGGGGTT CCCGCCAGAG CCAGCGCCCT

AAGAGATGCC TTTAGCAACA AGCGAGGATT CCCAACAGAG CCATCACCCT

AAGAGTTGCC TTTAGCAACA AGCAAGGATT CCCAACAGAG CCATCACCCT

AGGAAATGCC TTTAGCAGCA AGCAAGAACT CTCTACAGAG CCATCGCCCT

AAGAGATGCC TTTAGCAACA AGTGAGGATT CCCAACAGAG CCCTCACCCT

AAGAGATGCT TAGAGCAAGA CGTGAGGATT TCCAACAGAG TCATCACTAT

AAGAGATTCC TAGAGCAATG GGTGAGGATT TCTATCAGAC CCATCTTCGT

AAGACCTCCA TAAGAGAACA AGTGAGGCCT CTCAGCAACA GGCTGACTCT

AAGAGTGGTC TGTAGAAAAA AGTATGGATT GTAGCCAAAA CAGTTGCCGT

CCATTAGAGG GTAGAGCAGA TGTCATTGCT GATATACAAA CCATTCCCAT

CCATTAGAGG GTAGAGCAGA TGTCATTGCT GATATACGAA CCATTCCCAT

CCATTAGAGG GTGGAGCAGA TGTCATTGCT GATATACAAA CCATTCCCGT

CCATTAGAGG GTGGAGCAGA TGTCATTGCT GATATACAAA CCATTCCCGT

CCATTAGAGG GTGGAGCAGA TGTCACTGCT GTTACACAAA CCGTTCCCAT

TCATTAGAGG ATGGAGCAGA TGTTTTTACT AATGCCCAAA CCATTCCCAT

TTTCTGGAGA ATGGAGTAAA TGTTACCCCT GACACTCAAA CCATTCCTGC

TCTCTGGAGA ATGGAGTAAA TGTTACCCCT GATACTCAAA CCATTCCTGC

CCTTCAGAGG ACGGAGCAGA TGTCATTGCT GGTACTCAGA CCATTCCCAT

CCATCAGAGG ATGGCACAGC TGTCACTGCC GGAACCCACC CCATTCCTGT

CCATTAGAGG ATGGAGTAGA TGTCATTGCT GATACCCAAA CCATTTCCAT

CCGTTAGAGG ACAAAGCAGA TGTCATTGCT GGTACCCAAA CCATTCCGAT

CCATTAAAGG ATGGAGTGGA CACCATTGCT GATACCCCGA TCATGTCCAT

CCTTTAGAGG ATGGAGCAGA CACTATCTCT GATACCCAAA CTGTTCCCAT

CCATTTGAGG TTGCAGCAGA CATCATTGCT AATACCCAGA CCGTTCCCAT

CCATTAGAGG ACAGAGCACA TGCCATCACT GATACCCAAA CCATTCCCAT

TTGTTGGAGG ATGGAGACAA GGTCATTGCC AACCAGCAAA CCATTCTGAT

GCTTTAGAAG ATAGTAACAA AGTAATCGGT GACCAGCAAG CCACCCTCAT

TCAGACAAAA ATAAAAGACA TCTCTCCACC AGGTAACCAA ACCTGTGGTG

TCAGACAAAA ATAAAAGACA TCTCTCCACC GGGTAACCAA ACCTGTGGTG

TGAGACAAAA GTGAAAGACA TCTCTCCACC AGGTAACCAA ACCTGTGGTG

TGAGACAAAA GTGAAAGACA TCTCTCCACC AGGTAACCAA ACCTGTGGTG

TCAGACAAAA ATGAAAGACG TCTCTTCACC AGGTAATAAA ACCTGTGGTG

TAAGACAAAA TTGAAAGACA TCTCTCCACC ATGTGACAAA ATCTGTGGTG

TGAGACAAAA ACGGaagcca tgtctccaca aggtgacaaa gccTATGGTG

TGAGACAAAA ATGGAAGCCA TGTCTCCACA AGGTGACAGA GCCTGTGGTG

TGAGACAAAA GCAAAAGACC ACTCTCCACC AGGTCACAAG ACCTGTGGTG

GAAAACAAAG ACGGAAGACA TCTCTCCACC AAGTGACAGA ACCTGTGGTG

GAAGACAAAA ATGAAAGACA TCTCTCCATC AGGTGACAAA ACCTGTGGTG

GAAGACAATA ATGAAAGACA TCTCTCCACC AGATGGCAAA AGCTGTGGTG

GAACACAGAA GTGAAAGAGA TCTCTCCAGC AGGTGACAAA ACATGTGGTG

TGAGACAAAT AGGAAAAACA TCTCTCTACC AGGTGACAAA GCCTGTGATG

TAAGACTAGA ATGAAAGACA TCTCTCCACC AGGTGACAAA ACCTGTGATG

TAAGACTAGA ATGAAAGACA TCTCTCTACT AGGTGACAAA ACCTGTGGTG

CCAAACAAGA ATGAAAGATG CCTTCTGCCC AGGTTCCACT GCCTGCCGTG

CAAAGCAAAT ATAAAAGACC AGTGTTCACC AGTTGTTAAA ACTTGTTGCT

CCTCTTCAAA CTGTCCCACC AACATCAAAC CAGTAAGCCT AGAGAGAAAA

CCTCTTCAAA CTGTCCCACC AACATCAAAC CAGTAAGCCT AGAGAGAAAA

CCTCTTCAAA CAGTCCCACC AACATCAAAC CAGGAAGCCT AGAGAGAAAA

CCTCTTCAAA CAGTCCCACC AACATCAAAC CAGGAAGCCT AGAGAGAAAA

CCTCTTCAAA CAATCCCACC AACATCAAAC CAGTAAGCCT AGAGAGAAAA

CCTCTTCAAG CAGTCCTACC AACATCAAAC CAGAAAACCT AGAGAGAAAA

CCTCCTCATT CATACAAACC AACATCAAAA CAAGCAGCTC AGAAGGGCAA

CCTCTTCATT CATACAAACC AACAACAAAA CAAGAAGCTC AGAAGGGCAA

CCACCTCAAA CAGTCCAACC AACATCAAAC CAGGAAGCCT TGAAGGAGAG

CCTCTTCAA- ---------- -ACAGCAGAT CAGAACGCCT AGAAAGAAAG

CCTCTTCAAG CAACCCTACC AACATCAAAA CAGGAAGCCT AGAAACAAAG

CCTCTTCAAA CACTCCCTCC AACATTAAAC CAGGAAGCCC AGAAAGTAAA

CCTCTTCAAA CAGTCTCACC AATATCGAAC TAGGAAGCCA AGAAAAGAAG

CCTCTTCAAA CAGTTCCACC AACATCA--- TAGGAAGCCT AGAAAAAAAA

CTTCTTCAAA CAGTTCAAAG AGCATCAAAC TAGGAAGCCT GGAAAGAAAC

CCTCTTCAAA CAATTCCAAC AACAGGAAGC CAGG------ ----------

CCTCTTCCCA GAATTACGTC AGTGCTAAAG CAGTGAACTC CCAAAGGATG

TTCCTTCAAG TAGTCCCAAT AATGTCATAC CAGTGGGGCT CGCCAGTATA

AAGGAAATGG CTGATTCAGG AACAAAATCA AATAAGAGAG AAGCAGCTGG

AAAGAAATGG CTGATTCAGG AACAAAATCA AATAAGAGAG AAGCAGCTGG

AAAGAAATGG CTGATTCAGG AACAAAATCA AATAAGAGAG AAGCAGCTGG

AAAGAAATGG CTGATTCAGG AACAAAATCA AATAAGAGAG AAGCAGCTGG

AAAGAAATGG CTGATTCAGG AACAAAATCA AATAAGAGAG ACGCAGCTGG

AATAAAATGG CTGATTTAAG AACAAAGTCA AGTAAGAAAG AAGTAGCTGG

AAAGAAATGA CTGGTG---- -------TCA AGTGTGAGAG GAGCGGCTGG

AAAGAAATGA CTGCTG---- -------TCA AGTGTGAGAG GAGCAGCTGC

AGAGAAACGG CTGATTCGGG AACTGAATCa gataagaaag aagcagctgg

ACCGAAGCAG CTAACTCGGG AGTAGGTTCA GATAAGAGCA AAGTGGCAGC

AAAGAAATGG CTGATTCTGG AATAGTATCA AGTAAGAAAG AAGCAGCAGG

AACGAAATGG CTGATTCAGG AACAAAATCA AGTAAGGAAG AAGCAGCAGG

AGGGGAAGGG TGGGTTCAGG AACAGAATCA GGTAGAGAAG CAGCAGCTGG

GAAGAAATTA CTGATTCAGG AGCAGAATCA AGTAAGAAAG GAGTTTC---

AAACAAGTGG CTGATTCAGG AATAGAATCA AGTAAGAATG AAGCACCTGG

---------- ---------- ---------- ---------- ----------

CA--AAATAA CAGAGTCAGG AAAACTG-CA CTTGAAAAAG AAGGAGCAGA

GAGACAGTTA TCGATTCAGA GAGTGAA-TA AATAACAGGG AGACGTTTAA

CTTTCCTCAA GTCTCTGTCA TAGAATGTCA CAGCGTTCAA TCTCAGGATA

C-TTCCTC-- ---------- ---------- ---------- ----------

CTTTCCTCAA GCCTCTGTCA TAGAATGTCA CAGTGTTCAA TCTCAGGATA

CTTTCCTCAA GCCTCTGTCA TAGAATGTCA CAGTGTTCAA TCTCAGGATA

CTTTCCTCAA GTCTCTGTCA TAGAATGTCA CAGTGTTCAA TCTCAGGATA

CTTTCCTCAA GTCTCTGTTG AAGAATGTCA AAATGTTCGA TCTCAGGATA

CTCCTCCC-- ----TTGTCA GAGAATGTCA AAATGTTCAG TCTCGGGATC

CTCTTCCT-- --CTTTGTCA CAGAGTGTCA AAATGTTCAG TCTCAGGATA

ttcttcac-- ----ctGTCA TAGAATGTAC GAATGTTCCA TCTCAGCGTA

CTTGCCTGA- GTCTCAGTCC GGGAACGTCA GAGCGTTCCG TCTCAGGATC

CTTGCTTC-A GTCTCTGCTA TGGAATGTCA AAGTGCTCAA TCTCAGGTTC

CTTGCTTC-A ATCCCTGTCA TGCAATGTCA AAGTGTTCAA TCTCAGGATC

CTTTCCTC-A ACCTTTATTG TGGAATGTCA GAGTGTTCAA TCTCAGGATC

CTTTCCTCAA ACATCTGTCA TGGAATGTCA AAGTGTTCAA TTTCAGAATC

CTTTCCTCAA GTGTCTTTCA TGGAATGTCA AAATGTTCAT TCTCAGGATA

---------- ---------- ---------- ---------- ----------

ATTATCTGAA GCTCCTGTCT TAGATCATGA AAGTGCTCAA TTTCATAGTA

CCATTTCAAA CTTTCTTTCA AGGAATGTGA AGCTTCTCCA TCTGAAGTTA

TCTCTTGTCA TCGTGTAAGA AAAAATGTAT CCAACATGTG TGCTGCTTTC

---------- ---------- ---------- ---------- ----------

TCTCTTGTCA TTGTGTAAGA AAAAATGCAT CCAACATGTG TGCTGCTTTC

TCTCTTGTCA TTGTGTAAGA AAAAATGCAT CCAACATGTG TGCTGCTTTC

TCTCTTGTCA TTGTATAAGA AAACATGTAT CTAACATGTG TGCTGCTTTC

CCTCTCGTCA TTGCGTAAGA CATAATGTAC CAAACATCTT TGCTACTTTT

TCTTCTGTCA TTGCATAACA CAAGATGCAT CTAACATGTG CGCTGCCTAT

TCTTCTGTCA CTGCATAACA CGAGATGCAT CTAACATGTG TGCTGCCTGT

CCTCCTGTCA TTGTGTGAGA AAAAGTGTAC CAAACACGTG TGCTGCCTTT

TCTCTTGCCC TTGTGTGAAG AAAAACGTCC CAAGCAGGTG TGCTGCCCTG

TCTCTTGTCA TTATGTAAGT AAAAATGCAC AAAGCATGTG TGCTACTTTT

TCTCTTGCCA TTGTGTAAGA AAAAATGCAC CAAGCGTGTG TGCTACTTTT

TCTTGTGCCA TTGTGTGGGG AAAAATGCAC CAATCACGGG TGCTGCCTCT

TCT---GTCA TTGTGCGAGT AAAAATGCAC CCAGCATGTG TTCTGCTTTT

AAACCAGCCA TTGTGTAAGT AAAAATGCAT CAGGCATCTG TACTAATTTT

---------- ---------- ---------- ---------- ----------

GTCATTGCCA CCCTAGAGTT GAGAATGCCC TAGACATGTG CGTTACCTAC

ATTTTTACTC TGGTGGGGTT CAGCATGTAC C-AACGTGTG TTCCGTTTCT

AAGTCCAGCA AAATCAGCCT GCAAGTTGAT AACTCTTTGA TAACAAAATA

---------- ---------- GCAAGTTGAT AACTCTTTGA TAACAAAATA

AAGTCCAGCA AAATCAGCCT GCAAGTTGAT AACTCTTTGA TAACAAAATA

AAGTCCAGCA AAATCAGCCT GCAAGTTGAT AACTCTTTGA TAACAAAATA

AAGTCCAGCA AAATCAACCT GCAAGTTGAT AACTCTTTGA TAACAAAATA

GAGTCCAGCA AAGTCAGCCT GAAAGTTAAC AACTCTTTGA TAACAAAATA

GAACCTAGCA AAGATATTCT GAAAGTTAGC AACT--GTAA AA-----ATG

GAACTGAGAA AAGATTCTCT GAAAGTTAGC AACT--GTAA AATCAA-ATG

GTGTCTGGCA GAACCACTGT GGACATCAAT AACTTGGCAG CCTCAAGACC

GAGCCCAGCA GAACGACCCT GGGCGTTGAG AACTGTCTGT TAACCAAGTA

GAGTCCAGCA GAATCATCCC AAACGTTGAG AACTCTTTGA TGACTACGTG

GAGTCCAGCA GAATCATCCT GGAAGTTGAA AACTCTCTGA TAGCCAAATA

GAGTCCAGCC AAATCACTCC CAGAGTGGAA AATTCTTTGG CAAACAAGTA

GAATCCAGCA AAATCATTGT AAAAGTTAAA AAAAATTGTA TAGCAAAATG

GAGTCTAGCA AAATCATCCT GGAAGTTGCA AAGCCTTTGA TAACAAAATG

---------- ---------- ---------- ---------- ----------

AACTCTACTA CAATCTCCCC AGAAGCTAAG AATAAGTCTG TATTGTCTTC

GAGACCAGCA GAATCTTATC AGAAGCTGAG ATTCCTTCCA AATGGACACC

TGAAAATGCA TTTCAGCACC GTGATCACTG CTGCCAAGGA AGCAGCTGTA

TGAAAATGCA TTTCAGCACC GTGATCACTG CTGCCAAGGA AGCAGCTGTA

TGAAAATGCA TTTCAGCACC GTGCTTACCG ATGCCAAGGA AGCAGCTGTA

TGAAAATGCA TTTCAGCACC GTGCTCACCG ATGCCAAGGA AGCAGCTGTA

TAAAAATGCA TTTCAGCACA GTCATCACCA CTGCCAAGGA AGCAGCTGTA

TGAAAGTGCA TTTCAACACA GCACCAGCCA CACTCAAGGG AACAGCTATG

TGGAAATGCA TCTCA----- ---------- ------AAGA AGCACC----

TGGAAATACA TCTCAGGATG GTAATCACTG TTCCCAAAGA AGCACC----

CGAAAATGAG CACCG----- -TGTTCTTTG CTCCCAGAGA AGTGCCTGTA

TGAAGATACC TTTCAGCATA TCGATCACCA CTCCCAAGGG TGCAGCTGTA

TGAAGATGCA TTTCAGCACA GCAATCGCCA TTCCCAAGGA AGCACCCATA

TGAGAATGCA TTTCAGCACA GTGATCTCCA GTCCCCAGGA AGCACCCATA

TGAAAATGCT TGTCGGCACG GTGATCAC-- -TCCCCAGGA AGCATGCACA

TGAAAATGCA TTTTGGCATG GTGATCACGA CTCCCAAGGA AGCACACATA

TGAGAATGCA TGTCAGCACA GCAATCACCA CTCCCAAGGA AGCGCCTGCA

---------- ---------- ---------- ---------- ----------

TGAAGAAATA TTGGAGCCCA GTGATCCTCA GTGTTCGGAA AGCTTCCTTG

TAAGCATGCC ACAACATCCA GTGAACACTT CTGTGAAAGA AGGAACCATA

AAGTGAGTTA CACATCACAG GAAAGGGAAG ATGGGAAAGA AACGAATGGC

AAGTGAGTTA CACATCACAG GAAAGGGAAG ATGGGAAAGA AACGAATGGC

AAGTGAGTTA TACATCAGGG GAAAGGGAAG ATGGGAGAGA AACGAATGGC

AAGTGAGTTA TACATCAGGG GAAAGGGAAG ATGGGAGAGA AACGAATGGC

AAGTGAGTTA TACATCAGAG GAAAGGGAAG ATGGGAAAGA AACTAATGGC

AAGTTAGTGG TACAGCAGAG GGAAGTGATG ATGGGAAAGA AACTAAAGGC

---------- ----CTCTTG GAGAGTGAAG CTGGGAGAGA GATGCAAAGC

---------- ----CTCTTG GAGAGTGAAG CTGGGAGAGA AGTGCAAAGC

AAGTGATGTG TACATCAGAG GAAAGTGAAG ATGAGGCGGA AACTAAAGAC

GAAAGAATTG TGCATCGGAG GAAAGTGAGG GTGAGGGAGA AACTGAAGGC

AAGTGAGTTA CACATTGGAG GACAGTGAAG CTAGGGGAGA AACTAGAGGC

AAGTGAGTTA TACACGGGAG GAAAGTGAAG ATGGGAGGGA AACTAAAGGC

GAGTGGGTGA TACACCAGAA GAAAGTGATA ATGGTAAAGA AGTTAAAGAC

AAGTTAGTTA TACACCAGAA GAAAGTAAAG GTAGCAGAGA AACTAAAGGC

AAGTGAATTA TACATCAGAG GAAGGTGAAG ATGGGAGAGA AATTAAAGAT

---------- ---------- ---------- ---------- ----------

ATGCGAGACA TCAAGCAGGG GAAAGTAAAA CTGTGAAAGA AATGGAAGAT

AAGTAACCAA TGCATTAGAG AAAGATGACG AGAGAATCAA AGTTGAAGAT

AGCCTTCCAG GAGATAAGAT CAGAAACAAA ATGGTAGCAG GCTTGTTAAA

AGCCTTCCAG GAGATAAGAT CAGAAACAAA ATGGTAGCAG GCTTGTTAAA

AGCCTTCCAG GAGATAAGAT CAGAAACAAA ATGACAGCAT GCTTGTTAAA

AGCCTTCCAG GAGATAAGAT CAGAAACAAA ATGACAGCAT GCTTGTTAAA

AGCCTTCCAG GAAATAAGAT CAGAAACAAA ATGACAGCAG ACTTGTTAAA

AGCCTTTCCA GAGATGAGAT AAAAAATGAA ATGATAGCAG TCCTGTTAAA

AGTATCCTAG GAGGCACAGT CAGAGGTTCA GCGACAGCAG CTGTGTCACA

AGCTTTCTAG AAGGCAAGGT CAGAAGTGCA GTGGCAGCAG CTGTGACACA

AGCCTTCTAG GAGATAAGTT CAGAAACAGA ACGGCAGCAG GGATGTTAAA

AGCCTTCCTG GAGGTGAGGC CAGAGATGAA ATGATGGCAG GCACGTTAAA

AGCCTCGCAG AAAATAAGAT GAGAAACGAA ATGGCAGCAG ACGTGTTAAA

AGCCTTCCAG GAGATAAGAT CGGAAATGAA AGGACAG--- ----------

AGCTTTCTAG GAGATAAGGT CAGAAACAAG CCCGAAGTAG GCGTGTTTAA

AGCCTTCAAG AAGATAACCA CAGAGACAAA ATGGCAGCAG GCATATTCAG

AGCCTTCAAG AAGATCAGAT CAGAAAGGAA ATGACAGTAG GCACATTAAG

---------- ---------- ---------- ---------- ----------

AGCTTTCAAG AAGTCAAGAT TAGAAATGAA GTGGTTTCCT GTTCATTACT

GATGTTCATA AAGATCCCAC CAGCAATGCA ATGGGTGCAC ATTTAGTGGT

TAGTGGAATT TCAAACAAAA CCATTTCCGT ATCCAAAGAA ACTGTATTTT

TAGTGGAATT TCAAACAAAA CCATTTCCGT ATCCAAAGAA ACTGTATTTT

TAGTGGAATT TCAAACAAAA TCATTTCCGT ATCCAAAGAA ACTGTATTTT

TAGTGGAATT TCAAACAAAA TCATTTCCGT ATCCAAAGAA ACTGTATTTT

TAGTGGAATT TCAAACAAAA CCATTTCGAT ATCCAAAGAA ACTGTATTTT

TACTGGCATT TCAAACAAAG CTGTTAAGAT ACCCAAAGAG ACTGTACTTT

TAGTG---CT CCTCTGAACG CAG---CTCA ACACAGAGAC ACTGAGATTT

TACTG---CT TCTCTGAACA CAG---CCCA ACGCATAGAC ACTGAGATTT

CAACGGATCT TCAGCCAAAG CCA---CCCT ACCCGGAGAG ACTGCGTTTT

CAGTGAAGCT CCAGACCAAA CCA-----AC ACCCAAAGAG ACTGTGTTCT

TGGTGAAGCT TCTGACAAGA CCATTACCGT ACCTAAAGAG ACTGTGTTTT

-----AAGCT TCAAATGAAA CCATTACCAT ACCCAAAGAG ACTGTGTTTT

TAGTGAAGCT TCAAACAAGA CCATTACCAT ATCCAAAAAG AGTGTGTTTT

TAATGGAGCT CCAAACAAAA CCATTACCAT ACCCAAAGAG ACTGCATTTT

TAATGGAGCC CCAATCAAAA CCATTCCCTT ACCCAAAGAG ACTGTATTTT

---------- ---------- --------CC ACCCGATTCG GCTGTCTTTT

CAGCAGAGCC TTAAACAAAA ATACATTGAC ATGCAAGGAG ATTATGTTTT

CAGCCAAGAG GTAAACAGAG CCATCCCCAC AGTGAATGGG AAGAAGACTT

GTAAGTATAA CATCTCTGAT CATGCTATAC AAGAACTAAA CCAGACTGTA

GTAAGTATAA CATCTCTGAT CATGCTATAC AAGAACTAAA CCAGACTGTA

GTAAGTATAA CATCTCTGAT CATGCTATAC AAGAACTAAA CCAGACTGTA

GTAAGTATAA CATCTCTGAT CATGCTATAC AAGAACTAAA CCAGACTGTA

GTAAGTATAA CATCTCTGAT CATGCTATAC AAGAACTAAA CCAGACTGTA

GTAAGTATAA CATCTCTGAT TATGCTGCAC AAGAACTAAC TCAATCTGCA

ATAAACACAG CATCTCTGCT TGTGACACAC AAGAACTAAG CCAATCTGTA

ATAAACACAG CACCTCTGCC TGTGACACAC AAGAACTAAA GCAATCTGCA

GTAAATATAA CATCTCTGAT TGTGCTGCAC AGGGACTAAA CCAATCTGCA

GTCAACGTGA TGTTTCTGCT CGTGTCGCAC AAGAATTAAA CCAACCCACA

GCAAGTATAA TGTCTCTGAC TGTGCCACAC CAGAACTAAA CTTATTTGCA

GTAAATATAA CATCTCTGAT TGTACCACAC AGGAACTAAA CCAATCTGGA

GTAAGTGTGA CATATCTGGC TGTGCCGT-- ---------- ----------

GTAAATATAA CATCTCTGAT TGTGCTGCAC AAGATCTAAA CCAATCTGCA

GTAAGTATGA CATCTCTGAT TATGTTTCAC AAGGACTAAA CCAATCTGCA

GTAAGCATAG CATTTCTGAT TGTGTTATAC AAGGACTCAA CCAATCTGTA

GTGAGTCCCA TCCTTCTGAA AATATTTT-- -AGAAAGCAA GCTATCCTCA

GTGAATTTAA CCTCTCTAAG GGTGATATTT CAGAGAACAG CGAATCCTCT

AACATTCCAG GTCCTGAAAA AGTGTTGGAC CAGTCTCCTA CTGTTATGTT

AACATTCCAG GTCCTGAAAA AGTGTTGGAC CAGTCTCCTA CTGTTATGTT

AACATTCCAA GTCCTGAAAA AGTGTTGGAC CAGTCTCCTA CTGTTATGTT

AACATTCCAA GTCCTGAAAA AGTGTTGGAC CAGTCTCCTA CTGTTATGTT

AACATTCCAA GTCCGGAAAA AATGTTGGAC CAGTCTCCTC CTGTTATGTT

AACATTCCAA GTCCTGAAGT ATTGTTGGAC CAGACTCCTA CTGATATGTT

AACATTCCAA GTCCTGAAAC AGTAGTGGAC CAGTCTCTGA GTGTTAGTTC

AACATTCCAA GTCCTGAAAC ATTAGTGGAC CAGTCTCTGA GTATTCTTTC

AACATTCCAA GTCCTGAGAA ATTGTTGGAG CAGGCTCCTA CTGTTACGTT

AACATTCCAA GTCCTGAAAA ACTGTTTGGC CAGTTTCCTC CCATAATGTT

AACATTCCAA GCCCTGAAAA ATTGTTGGAC CAGTCTCCTA GTATTATGTT

AACATTCCAA ATCCCGAGAA ACTGTTGGAC CAGTCTCCTA CTGTTATGTT

---------- ---------- ---------- ---------- ----------

AACATTCCAA GTACTGAAAA ATTGTTGGTC CAGTCTCCTA ATATCATGTT

AACATTCCAA GTCCTGAAAA ATTGTTGGAC CAGTCTCCTA ATGTTATGTC

GACATTCCAA ATCCTGAAAA ACTGTTGGAC CAGTCCCCTA ATTTATTGCC

AACATTCCAA ATCCTAAGGA GCGGATGGTC CCATGTCTTA ACACAGAGCA

GGCATTCCAA GTCCTGAAAT GTTGTTGGAC CCATCTCTTC AGTTGGAATT

CTCCAGTTTT AAAAATGTAA AATCAGTTGA AACACTCGAT CAGAAGGCAG

CTCCAATTTT AAAAATGTAA AATCAGTTGA AACACTCGAT CAGAAGGCAG

TTCCAGTTTT AAAAACATAA AAGCAGTTGA AACACTCGAT CAGAAGGCAG

TTCCAGTTTT AAAAACGTAA AAGCAGTTGA AACACTCGAT CAGAAGGCAG

CTCCAGTTTT AAAAACGTAA AAGCAGTTGA AACACTCAAT CAGAAGGCAG

TACCAATTTT AAAAACACAA AAGCAGCTGA AACTTTTGAT CACAAGGCAG

ATCTCATTTC AAAGCCATGC CAGCAGCTGA AAGTCTTCAC CAGAAAGCAG

ATCTCATTTT AAAACCATG- --GCAGCTGA AAATCTTCAC CAGAAAGCAG

CTC-CAGTTT GAAAACATGA AAGCAGTTGA AACTGTCCAT CGTAAGGCAG

CTCCGGTGTT AAGAACGTGA AAGCAGCTGA AACTCCCAAG CAGAAGGCAG

TCCCAGTTTC AAAAACTTGA GAGCATTTGA AACTCTTGAT CAGAAAGCAG

CTCCAGTTTT AAAAACATGA AAACAGTTGA AACTCCTGAT CAGAAGACAG

---------- ---------- ---------- ---------- ----------

CTCCAATTTT AAAAACATGG AAGCAAATAA AACTCTTGAT CAGACGTCAG

CTCCAGTTTT AAAAGCAAAA TGGCAGAAGA ACCTC----- ----AGACAG

C---AGTTTT AAGAGCATGA CACCAGAAGG ACCTCCAGGC CAGAAGGAAG

CTCAGGTGTT AGAATTATAA AAGCAGATGG GACTGTTGGT CAGGTGACAA

CTCAGGCTTT AACAATGTGA AAGCAGATGA ATCTATTATT CAGGTGGCAA

ATGAAGTCCT TGACTGTCAG AGTAACCAAA ACAGACCAGA TGAATGCAAA

ATGAAGTCCT TGACTGTCAG AGTAACCAAA ACAGACCAGA TGAATGCAAA

ATGAAGTTCT TGATTGTCAG AGTAACCAAA ACAGACCAGA TGAATGCAAA

ATGAAGTTCT TGATTGTCAT AGTAACCAAA ACAGACCAGA TGAATGCAAA

ATGAAGTTCT TGACTCTCAG AGTAACCAAA ACAGACCAGA TGAATGCAGA

ATGAAGTTCT TAACTGCCAG AGTAACCAAA ACAAACCTAA TAACGGCAGA

GTGAAGTCTC TGACTGTCAG AGTAGCCAAA ATAGTCTAGA CAAATGCAGA

ATGAAGTCTC TGACTGTCAG AGTAACCAAA ATATTCTAGA CAAGTGCAGA

AGGACGTCCT TGACTGGCAG AGTAAACAAA GCAGACCAGG TGGATGCAGA

ATGACGCCCT CGGCTCCCAG GGTAACCCAG GCGGACCCAA TGAATGCAGA

ATGAAGTCCT TGACTGCCAG GTTAACCAAA ACAGACCAGA TGAATGCAGA

ATGAAGTCCT TGACTGCCAG CGTAATCCAT ACAGACCAGC TGAATGCAGA

---------- ---------- ---------- ---------- ----------

AAGAAGTCCT TGACTGGCAG AGTAACCAAA ACAGACCAGA TGAATGCAAA

ATGAAGTCCT TGACTGCCAG AGTAACCTAA ACAGACCAGA TGAATGGAGA

GTGAAGTCCT TGACTGCCAG AGGAACCAAA GCAGACCAGA TGCATGGAGA

ATCAAGGCCT TAACTGCCAG AGTGACCTTA ACAGTCCAAT CGAATGCTCG

ATTGTGGCCT TGATGACCGA AGTGACCTTA ACCTTTCAGC TGCATACAGA

AGTGAAGGTC AGTCAGCCAA GGAGATGCTA AGTAGTGACC AGAGAGAGAC

AGTGAAGGTC AGTCAGCCAA GGAGATGCTA AGTAGTGACC AGAGAGAGAC

AGTGAAGGTC AGTCAGCCAA GGAGATGCTA GGTAGTGACC ACAGAGAGAC

AGTGAAGGTC AGTCAGCCAA GGAGATGCTA GGTAGTGACC ACGGAGAGAC

AGTGAAGGTC AGTCAGCTAA GGAGACACTA GGTAGTGACC AGAGAGAGAC

AATGAAGACA AACCAACTAA GGCGATGCTA GGTAGTAACC AGAAAGACAC

TGTGAAGGCA AGCCAGCCAG GGAAGGACTA AATGGTGACT ACCGAGAGAC

TGTGAAGGTA AGCCAGCCGG GGAAGGACTA AATGGTGACT TTGGAGAGAC

TGTGAAGATA GGCCAGCTGA GGAGGCACTG GAGAGGGAAG GGATAGAGAC

GGTGAAGGTA ACCCAGCTGA GGAGACGCTC GGCCGTGACC AGTGCAAGTC

AGTGAAGATA AACCAGCTAA GGAGATACTA GGTGGTGACA AAAGAGAGAC

TGTGAAGATA AACCAGCTAA GGAGACACTA GGTAGTGACC AGAGAGAGAC

---------- ---------- ---------- ---------- ----------

AGTGGCATTA AACCAGCTAA GGAGACCCTA CATAGTGACC AACGAGAGAC

AGTGAAGATA AACCAGCTAA GGAGACAAAA GATAGTGACG AGAGAAAGAG

AAGGAAGAGA AACCAGCTAA GAATACCCCG GATGGTGAGC AGAAAGAGAC

AATGAAAATA AATCCATTAA GGAATCTTTG AATAGTCGTA AAAATGAGGC

AATGAAAATG AATTAGTTAA GCAGAAATTA AGTGATATTA AAAGAGAGCA

TGTCACCGAG CCTCACGGGG AGGTAAACCA CAACCAAAAG GATCTGCTGG

TGTCACCGAG CCTCACAGGG AGGTAAACCA CAACCAAAAG GATCTGCTGG

TGTCACCGAG CCTCACGGGG AGGTAAACCA CAACCAAAAG GATCTACTGG

TGTCACCGAG CCTCACGGGG AGGTAAACCA CAACCAAAAG GATCTGCTGG

TGTCACCAAG CTTCAGGGAG AGGTAAGCCA CAATCAAAAA GATCTGTTGG

CATCACAGAT CCTAATGGGA CAGTACGCCA TCACAGAAAG GATCTGCCGG

CATC------ CCT------G AGGTAAGCCA TAAGCAAAAG GATCGGATAG

GGAC------ CCT------G AGGTCAGCCA CAGGCAGAAG GATCTGACAG

CCTCACAGGG CCTCACAGAG AAGGAAGTCA CAACCCACAG GATCAGCTAA

GCCCACAGAG CCTGACAGAG AGGCGAGCCA CAGCCTGAAG GCTCCACCCG

TGTCACAGAA CTTAACAGAG AGATAAGCCA CAACCAAAAT GAACTGCTAG

CGTCACAGAG CCTGTCAGAG AGGTAAGCCA CAACCAAAAG GATCTGCCAA

---------- ---------- ---------- ---------- ----------

TGTCACAGAG ACTAATGGAG GGGTAAGCCA CAGCCAAAAG GATCTGCTAG

TGTCATAGAG CCCAACAGAG AACTAAGCCA CAACCAAAAG GATCTGATTA

TGCCACAGAG CCTAGCAGCG AGGGAAGCCA CAATCAAAAG GCTCTGATGA

TCTTGCAAAA CCTATCAAAC GGTTGAACTA CCACCAGAAA GAACTGCTAG

AGACACAGAA TCTACAGAAG GGATAAGCCC TGTGCAGCAA GAAGTGCAAG

TCAGCTCAGG CAGTAATAAC TCACTACCTT GTGGTAGTCC AAAGAAATGC

TCAGCTCAGG CAGTAATAAC TCACTACCTT GTGGTAGTCC AAAGAAATGC

TCAGCTCAGG CAGTAATAAC TCACTACCTT GTGGTAGTCC AAAGAAAGGC

TCAGCTCAGG CAGTAATAAC TCACTACCTT GTGGTAGTCC AAAGAAAGGC

TCAGCTCAGG CAGTAATAAC TCACTACCTT GTGGTAGTCC AAAAAAAGAC

TCATTTCAAT CAGTAATATC CCACAGCTTT GTGGTAGACC AAAGAAAGGT

TCTGTTCAGG CAGTAAAGTC CCTCTGTCTT GTGGCAGTTT AAAGCAAAAA

TCAGCTCAGG CAGTAAAGTC CCTCTGTCTT GTGGTGGTTT AAAGATAAAA

TCACTTCAGG CAGTAGTAAC CCTCTGTCTA GTG---GTCC AAAGAAAGGC

TTGGTTCAGG CAGTAACAGT TCAGCATCTG GCAGGAGCCC CAAGAAAGG-

TTGGTTCAGA CAGGAACAAC CCATTGTCCA GTGGTAGTTC AAAGAAAGGC

TTGGTTCAGG CAACAACAAC TC---GTCTC GTGGTAGTCC AAAGGAAGGC

---------- ---------- ---------- ---------- ----------

CTGGCTCAGG CAATAATAAC CGACTCTGTT GTAGAAGTCC AAAGAAAGGC

CTTGTTCAGG CAATAATAAC CCACCCTCTC GTGGTAGTCC AAAGAAAGAT

CGGGTTCAGG CAAAAATAGC TCGCCCTCTT TTAGTACTCC AAAGAAAGTT

TTAATGACTT GAGTGACATC CAACTATCTT GCAGTCTTCC AAAGAAAACC

TTGCTAGAGA CAGTAGCATG CAGCTGCCTT GTGCACTTTC GAAGGCAGAT

AATTTGAAAG GAGCCTTTGT CAAGATGTCT GGTTGTGATG AGTCCACAGA

AATTTGAAAG GAGCCTTTGT CAAGATGTCT GGTTGTGATG AGTCCACAGA

AATTTGAAAG GAGCCTTTGT CAAGATGTCT GGTTGTGATG AGTCCACAGA

AATTTGAAAG GAGCCTTTGT CAAGATGTCT GGTTGTGATG AGTCCACAGA

AATTTGAAAG GAGACATTGT CAAGATTTCT GGTTGTGATG AGTCCACAGA

GATTTGAAAG GAGACTTTGA AATTATTTCT GGTTGTGAAG AGTCCACAGA

GACCCCAAAA GAGCCTTTAA AAATATTCCT GGTTCTGAAG AGTTCACTCA

GATCCCAAAA GAGCCTTTAT AGATATTCCT GGTTCTGAAG AGTTCACACA

AACGCAGAAG CAGCCATTGG AAGTGTTTCT GGTTGTGAAG AGTCCACACA

---------- -GGGCTGTGG GAGGACTTCT GATTGTGCGG AGCCCACAGA

TATTTGAAAG GAGACTCTGA ATGTACTTCT GGTTGTGAGG CATCCACAGA

AGTTTGAAAG GAGCCTTTGA AAGGATTTCT GGTTGTGAGG AGTCCACAGA

---------- ---------- ---------- ---------- ----------

AATTTTCAAG GAGACTTTGG AAGTATTCTT TGTTCTGAAG AGTCCTCAGA

GATTTTAAAG ---ACTTTGA GAATATTCGT GCCTGTGAAG ACTCCTCAGA

GATTCTAAAG GTGACTTTGA AAATAATCCT GCTTGTAAAG ACTTCACAGA

ATTTTAAAAA CTTCCCTTAA AAGTACCCTT GATAATGAAG TATCTGCCGT

ATTC---AAG GAAACCTTAA AAGTGGGGTG TCGTGTGAAA AATCAACATT

AGGTATGGTA GACATCGTCT ACTGTAGTAA TAAGCTTGAA GGTGTGCTGG

AGGTATGGTA GACATCGTCT ACTGTAGTAA TAAGCTTGAA GGTGTGCTGG

AAGTGTGGTA GACATCGTCT ACTGTAGTAA TAAGCTTGAA GGTGTGCTGG

AAGTGTGGTA GACATCGTCT ACTGTAGTAA TAAGCTTGAA GGTGTGCTGG

AGGTATGGCA AATATCGTCT ACTGTAATAA TAAGCCTGAA GGTGTGCTGG

CGGTATGATG GATGCCATAT ACTGTGGTAA TAAACCTGGA GGCATGTTGG

CAGTATGCTA GATGTGGTAT GTTGCAGAGG TGAGCCTGAG GGAGCGCAGG

CAGTATGCTA GATGTCATAT GTTGCACAGA TGAGCCTGAG GGAGTGCTGG

CGGAATAGTA GATGCTGTCT GTTGTAGTAG TGAACCTGAA GGCATGCTGG

TGGGACAGCA CATGTGGTCT CCTGTAGTGA TGAGCCCGAA GGCATTCTGG

TGGCATGGTA GACATCATCT ACTGTAGTAA TATGTCTGAA GGTGTGTTGG

CGGTACTAAA GACATCGTCT ACTGTAGTGA TAAGCCTGAA GGCATGCTGG

---------- ---------- ---------- ---------- ----------

CAGTATGGTA GCCCTCGTCT TTCATAATAA TAAGCCTGAA GAAATACTAG

CAACGTGGTA GACACTGTCT GCTGTAGCAG TAAGCCTGAA GGAGTGCTGG

CAGCATGGTA GACCCTATCT ACTTGATTAA TAAGCCTGAA GGCATGCTGG

GTGCAGAGTA GACGTCATCT ----TAGTAA TAAACTTGAA GGGACGTTGG

TGGCAAGGTA GATATTAATC ACTGTAGGGA TAACTCTGAA GAAACATCAG

ACGTGAAGGC ATCTAATCTA CTGGATTGTG GTGCAAGGCA AGAGAAACTG

ACGTGAAGGC ATCTAATCTA CTGGATTGTG GTGCAAGGCA AGAGAAACTG

ATGTGAAGGC ATCTAATCCA CTGGATTGTG GTGCAAGGCA AGAGAAACTG

ATGTGAAGGC ATCTAATCCA CTGGATTGTG GTGCAAGGCA AGAGAAACTG

ACGTTAAAGC ATCTAATCCA CTGGATTGTG ---CAAGGCA AGAGAGACTG

CCGTGACAGC ATCTAGTGTA CTTGACAGTG TAGCAAGGCC AGATAGATCG

GTCTAGAAGC ATCTAGTCTA TCTGGTCATT GTACAAGGCA CGAGTCACTG

GTGTGGAAGC ATCTAGTCTG TCTGGTCATT GTACAAGGCA AGATACACTG

GTGTGGAAGT ATCTGGCACG CTTGGTAGTG CCATAAGGCA AGGTAGAGTG

ATGGAAGGGT TTCTGGTACT CTCACTGGGG GTGCAAGGCA CGCCAGGGCA

ACTTAAGAGC ATCTAGTATT CTTGACAGTG GTACAAGGCA AGACAGACTG

ACATAAGAGC ATCTAACACA CGTGATGGTG GTGCATGGCA AGATGGACTA

---------- ---------- ---------- ---------- ----------

ATGCAAACTC ATCTGGTACA TTTGGTAGTG GTGCAAGGCA AGAAAAGCTG

ACAGAAAGGC ATGTAGCGCA CTTGATAGCA GTGCAGGGCA AGATAAACCA

GTGGAAAGGC AGCTGTTACA CTTGACAACA GTGCAAGGCA AGATAAACCA

AAATGAAAGA ATCTGAATCA ACTGGAAGTC ACAGAAGACA AAAAGAGCCC

AAGTAAAACA GCCCGATTCA GTTTGTAGTA GGGCAAAGCA AGATGAATCT

GCATTTCAAG AGGACTCCAG GAGTACCTTG TCTCGGAGAG AACTGGATGC

GCATTCCAAG AGGACTCCAG GAGTACCTTG TCTCGGAGAG AACTGGATGC

GCATTCCAAG AGGACTCAAG GAGTACCTTG TCTCAGAGAG AACTGGATGC

GCATTCCAAG AGGACTCAAG GAGTACCTTG GCTCAGAGAG AACTGGATGC

GCATTCCAAG AGGACTCAAG GAGTACCTTG TATCAAAGAG AACTGGATGC

CGATTTGTGG AAACCTCGAG CAGTACCTTG TCTCAGAGAG AACCGACTGC

GCACACCATG AGACTTTAAG AAGTACCTTA TCTCGAGGAG AACTGAATGT

GCATACCACG AGACTATAAG AAGTACCTCA TTTCAAGGAG AACTGAATGT

ACATTCCAAG AAACCTTAAG GAGTACCTTG TCTCAGAGAG ------ATAT

ACGTCGCTGG AAGCCTCAGC GAGTACGCTG TCCCAGAGGG CGCCTGTCGC

ACATTACAGG AAACCTCAGT GAGTACTCAA TCTCAAAGAG AACTAAATGC

GTATTACATG AAACCTCGAG GAATACCCTG TCTCAGAGAG CACGGAATGC

---------- ---------- ---------- ---------- ----------

GCATTATACA AAACCTCAAG GAGTACTTTG CCTCAGAGAG AACTGAATGC

GCATTACCTG AAAGCTCAAG GAGTGCCCCA TCTCAGAGAG AGCTGAATGC

GCATTCAGTA AAACCTCAAG ATGTGCTTTG TCTCCGCAAG CGCTCCATGT

CCCTTGCATG GACCGTTGAG GAGTGACCAG CCTCGGGGAG AAGTCCATGT

GCATTAGATG AAACATCAAG GCTCAACTGG CCTCAAAGAA AATTTAGTAT

TGCACATACA GGAACAACTG GTCAGGATTC AGATTTCCCA GTTACTGCTG

TGCACATACA GGAACAACTG GTCAGGATTC AGATTTCCCA GTTACTGCTG

TGCACATACA GGAACAACTG GCCAGGACTC AGATTTCCCA GTTACTGCTG

TGCACATACA GGAACAACTG GCCAGGACTC AGATTTCCCA GTTACTGCTG

TGCCCATATA GCAACAATTG GCCACGACTC CAATTTCTCA GATGCTGCTG

TGCATTTATG GGAAGGACTG ACCAGGGCTC AGATTTCCCA GATGCTGCTG

TGTATTTATA GGTACACCTG GCTGGCACTC AGAACTCCCA AATGCTGCTG

TGAATGTCAA GGTACA---- ---------- -GAACTCCCA AATGCTGCTG

TGCATTTACA GCAACAACTG ACCAGGACTC AGCCTTCCCA AATGCTACTG

TGCATTTATA GGAGTGATTG ACCAGGAGTC GGATTTTCAA GATGCTGCTT

TGCATTTATC AGAATGATTG GCCAGGACTC AGATTTTCCA GATGCTGCTT

TGCATTTAT- -----GGTTG ACCAGGATTC AGATCTCCCA AATGCTACTT

---------- ---------- ---------- ---------- ----------

TGAATTTACA AAAATGTTTG AGCGGGATTC AAATTTCCCA CATGCTACTT

TGGATTTACA AGAATGATTG ACCAGAACTC AGATTTCCCT GATGCTACTA

TGCACATACA GGAAAGCATG ACCAGACTTC AGATACCCAC TGTGCTACTT

TGCATTTGAA AGACTGATTG TCTCAGACTC TGTTTTACCA TGTGCTTCTT

TGTAGTTGAA AGTATGGTTG AGCGAGATTC AGATTTAACG GGTACTGTTT

CTTCTACAGT GGACTTTCTC AAAATAAAAA AATCATGTGA AGAAAACGTA

CTTTTACAGT GGACTTTCTC AAAATAAAAA AATCATGTGA AGAAAACATA

CTTCTACAGT GGACTTTCTC AAAATAAAA- --TCATGTGA AGAAAACGTG

CTTCTACAGT GGACTTTCTC AAAATAAAA- --TCATGTGA AGAAAACGTG

CTTCAATAGT GGGCTTTCTT GAACTAAAAA AATCATATGA AGAAAACATA

GTTCTACAGT GGAGTCTCTT GAAATAAAAA AATCATGTGA AGAGAGACCA

CTTCTACAGT AGACTCTCCT GAGATGGAAG AATCACATGA AGAAAAAGTG

CTTCTTCAGT AGACTCTTCT GAAATACAAA AATCACATGA AGAAAAAGTA

CTTCTGTAGT AGAATCTCTT GAAATAAAAA AATCATGTGA AGAGAAAGTT

CTTCTACATC AGACTCTCTC AATATTAAAA AATCATGTGA AGAGAAAGTA

CCTCTAAAGG GGAGCCTCTG GAAATTAAAA AATCATGTGA AGAGAAAGTA

CCTCTACAGT GGAATCTCTT GAAATAAAAA AATCATGTGA AGAGAAAGTC

---------- ---------- ---------- ---------- ----------

CCTCTGCAGT GGAATCTCTT GAAATAAAAA AATCATGTAA AGAGAAAGTA

CCTGTACAGT GGAATCTCTT GAAATAAAAA AACCATGTGA AGAGAAAGTA

CGTCTCCAGT GGACTCTCTT GCAATAAAAA AATCACGTGA AGAGAAAGTA

CCTCTAATAT GAAATCTCCA AAAATCATAC CATCATGTAA AGGGAAGAGA

CTTCCAAGAC TGAATCTCTT GAAGTAATAC AATCATGGGA AGTAAAAAAG

TGCAGATGTG AAATGGAAAA GTGTCCAGAC TCTTGTGCCC ATGAGATGGA

TGCAGATGTG AAATGGAAAA GTGTCCAGAC TCTTGTGCCC ATGAGATGGA

TGCAAATGTG AAATGGAAAA GTGTTCAGAC TCTTGTGCCC ATGAGATGGA

TGCAAATGTG AAATGGAAAA GTGTTCAGAC TCTTGTGCCC ATGAGATGGA

TGCAGATGTG AAATGGAAAA ATGTCCAGAT TCTTGTGCCC ATGAGATGGA

TGTAGATCCG AAATGGAAAA GTGTTCAGA- ---------- ----GATAGA

TGCAAATATG AAGTAGAAGA ATGTCCAGAC TC---TGCGC ATGAAGTAAA

TGCAACTATG AAATGGAAGA CTGTCCAGAC TC---TGCTC ATGAAGTAAA

TGCCAATGTG AAATAGAAAA ATGTCCAGAC TCTTGTGCGC ATAAGAAGGA

TGGAGATGTG AAATGGAAGT GTGTCCAGGC CCTTGTGTGC CTGACACGGG

TACAGGTGTG AAATGGAAGT GTGTCCAGAC TCTTGTGCGC ATGACATAGA

TGCAGATGTG AAATGAAAGT GTGTCCAGAC TCTAGTGCCA ATGAGATAGA

---------- ---------- ---------- ---------- ----------

TGCAGATATG AAATGGAAGG GTGTACAGAT TTTTGTGCCC ATGCAATGGC

TGCAGATGTG AAATGGACTT GTGTACAGAT TCTTGTGCCC ATGAGGCAGA

GGCCTATGTG AAATGAAATT ATGTATCGAC TCTTGTGCTC ATGAGCCAA-

TGCTGG-GTG AATCAGGAGA GTGCCCAGGC ACATGTGCCG ATGGGGTGGA

TGCAAATGTG AAATAGAAGA ATGCCCAGAC TCTCGTGTGT CTAATATAGA

GTCTGTTGCA GATCATGAAC CAAATAAAAG AATATTGGGC AGAGTAAATT

GTCTGTTGCA GATCATGAAC CAAATAAAAG AATATTGGGC AGAGTAAATT

GTCTGTTGCA GATCATGAAC CAAATAAAAG AATATTGGGC AGAGTAAATG

GTCTGTTGCA GATCATGAAC CAAATAAAAG AATATTGGGC AGAGTAAATA

GTCTGTTGCA GATCATGAAT CAAATAATAG AATATTGGAC AGAGTAAAGA

GCCTATTTCA GATCATGAAC CAAATATAAG AATATTGGAT GGAGTAAATG

ATCTATTGAA GATCATGAGC CACATAGAAG AGCGCCGGAT AGAGAAGGCG

ATCTGTTGTA GATCATGAAC CAAATATAAG AGCACTGGAT AGAGCAGGTG

GTCTTTTGCA GATCATGAAC CAAATATAAG AATATTGGAC AGAGTAAAGC

GTCTGTTGCA GATCATGAAC CA-ATGTAAG AGTATTGGGT AAAATAAATA

GTCTATTGCA GATCATGAAC TA-ATTTAAG AACATTGGAT AGAGTAAATG

GTCTGTTGCA GATCATGAAC CATATATAAG ACTATTGGAT AGAGTCAATG

---------- ---------- ---ATGTAAG AATATTGGAT AGAGTTAATG

ATGTGTTGCA GATCATGAGC CAAATATAAG AGTATTAGAT AGAGTGAATA

GTCTGCTGCA GATCATGAAC CGGATGTAAG AGTGTTAGGT AGAGCAAATG

---------- ---------- ---------G GGTATTGGAT AGAGTGAATG

AACTATTACC AATCATGAGC CAAATACAGA GTTGTCTGAT ACTGTGAATG

ATCTTTTCCC AATCATGAGC CAAGAAGAAA AATTGGGGGT GCGGTTAATA

TGTCTTTAAA TGATAGCCAT TATGGACAGC AAGATAAAGG AACATCTCTC

TGTCTTTAAA TGATAGCCAT TATGGACAGC AACATAAAGG AACATCTCTC

TGTCTTTAAG CGATACCCAT TATGGACAGC AACATAAAGG AACGTCTCTC

TGTCTTTAAG CGATACCCAT TATGGACAGC AACATAAAGG AACGTCTCTC

TGTCTTTAAA TCATATCCAT TATGGACAGC AAGATAAAGA AACGTCTCTC

TGTCTTTAAA TTATAATCAT CATAAACAGC TAAGTAAAGG AGCATCTCTG

TGTCTTTAAA TGACATTCAT TGTGAGCATC AAGGTAAAGG AGGATCCATG

TATCTTTAAA TTCCATTCAT TGTGAGCATC AAGGTAAAGG AGGATCCATG

TGTCTTTAAA TGATGTTTGT CCTGAACAGC AAAGTAAAGG AGCATTTCTG

TGTCTTTAAA TTACATTCAT CATGAAGAGC AAGTTAAAGA AGCATCCCTG

TATCCTTAGA TTATATTCAT CATAAAAAGC AACTTAAAGA AGCATCCCGT

TGTCTTTAAA TTATATTCAT CATGAACAGC AAGTTAAAGA AGCATCTCTG

TGTCTTTGAA TCATATTCAT CATGGACTGC AAGTTACAGG CTCGTCTCGG

TGTCTTTAAA TTATATTCCT CATGAGCAGC AAGTTAAAGG AACATCTCTG

TGTCTTTAAA TTGTATTCAT CATGAACAGC AAGTTAAAGG AGCATCTCTG

TGTCTTTAAA TTGTGTTCAT CCTGAAGAGA ACGTTAAAGA AGCATCTCTG

TGTCATTGGA GAGGATTCGT CATGGGCAGA CTGTTAAAGG AATGCTTCCA

TTTTATTAAA CGGCCCTCAT CATGGAGAAA TGCTTAAGAG CACACCTTCA

AGAGAAACAC AAGAAATGAC TGAAGGATCA AGACTAGAAC CAAACTCTGA

AGAGAAACAC AAGAAGTGAC TGAAGGATCA AGACTAGAAC TAAACTCTGA

AGAGAAACAC AAGAAATGAC TGAAGGATCA AGACTAGAAC TAAACTCTGA

AGAGAAACAC AAGAAATGAC TGAAGGATCA AGACTAGAAC TAAACTCTGA

AGAGAAACAC AAGAAATGAC TGAAGGATCA AGACTAGAAA TAAGCTCTGA

AGAGAAACAG AAGAAGTGAC TGAAGGATCA AGATTAGAAG TAAATTCTGG

ACAGAGGGAC TGAGACT--- ---------- -----CGAAA CAAAGGCTGA

ATAGAAGGGC TGAGACT--- ---------- -----TGAAG CAAATTCTGA

AAAGAAACAC AAAGAATGAC CGAGGGCGCA AAAGCAGAAA TAAATGTTCT

AGAGAAACAC AAGGAACAAT TGAAGGATCA AGACTAGAAA TAAATCCTGA

AGAGAAACAC AAGGAACGAT TGAAGGATCA AGACCAGAAA TAAATTCTGA

AGAGAAACAC AAAGAATAAT TGAAAGATCA AGACTAGAAA TAAATTCTGA

AGAGAAAAGC AAGGAATGAC TGAAGGATCG AGTTGTGAAG TAAATTCTGA

AGAAAACTGC AAGTAATGGG TGAAGAATCA AGATTAGAAA TAAATTCTGA

AGAGAACCAC AAGCAGTGGA TAAAGGATCA AGACTAGAAA TAAATTCTGA

GAAGAAATGC ACGTCACGGA CAAAGGATCA AGACTAGAAA TTAATTCTGT

GGGGAGGCCA GAGGGACCAC TGGTGGGTTA AGTATGCAAA TGATTTCTGC

GAAGAAATGC CAGAAACACC CGACACATCA GGTTTTCATA ATCATTCCGG

GTTTGGCAAA GAAAGTATTT CCTCAAAAGA GTCGATGTCT CTGAAATCCA

GTTTGGCAAA GAAAGTATTT CCTCAAAAGA GTTGATGTTT CTGAAATCCA

GTTTGGCAAA GAAAGTATTT CCTCAAAAGA GTTGATGTCT CTGAAGTCAG

GTTTGGCAAA GAAAGTATTT CCTCAAAAGA GTTGATGTCT CTGAAGTCAA

GTTTGGCAAA GAAAGTATTT CCTCAAAAGA GTTAATGTCT CTGAAATCCA

GTTTGACAAA GAAAATGTTT CCCTGCAAGA CTTGATGTTT CTAGAATCCG

GTTTGGCAAG AAGAAATTAT CCTTAAAAGA CTCAGTGTCT ------TCTA

GTTTGGCAAG AGGAAATTAT CCTTAAAAGA CTCAGTGTCT ------TCTG

CCTTGGCAAG GAAAATATTT CCTCAAAAGA GT---TGTCT CTGGAATCCA

GCAAGACAAA GAAAATATTT CTTCAGTAGA GTTGACATCT CCGAAATCCA

GTTTGACAAG GAAAATATTT CCTTGAAACA GTTACTGCCT CTGCAAACCA

GTTTGACAAG GAAAATATTC CCTCAAAAGA GTTGATGTCT CCGAAATCCA

GTTTGACAAG GAAAACGTTT CCTCTAGAGA ATTGACAGCT TTGGGATCCA

GTTTGACAAG GAAAA---TT TTTCAAAAGA GTCATTGTTT CTAGGATCTA

GTTTGGGAAG GAAAATATTT CTCCAAAAGA GTTGATATGT CTGCGCTCCA

GTTTGACAAA GACAATATTT CTTCGAAGGA TTTGATGTAT CTATGCTCCA

ACCTGATGAT GAAGATGTTT ATTCTAAAGA GCCAATGT-- -TGGAATCTA

TTGTGATAAA GAAGATATCT CTTCAAAAGA GTCTAGATTT TCACAACTAG

TTGAAATAAT GCCTTCTAAT TCAGAAACTA ATGTTAACAG TGAAGAAACT

TTGAAATAAT GCCTTCTAAT TCAGAAACTA ATGTTAACAG TGAAGAAACT

TTAAAATAAT GCCTTCTAAT TCAGAAACTA ATTTTAACAG TGAAGAAACT

TTGAAATAAT GCCTTCTAAT TCAGAAACTA ATATTAACAG TGAAGAAACT

TTGAAATAAT GCCTTCTAAT TCAGAAACTA ATAATAACAG TGAAGAAACT

TTGAGATAAT GCCTGCTAAT TCAGAAACTA ATATTAATAG TGAAGGAACT

TCAAGGTGGT ACCTTCTCAT TCACCAGCTG ATGTTCCTGG TGAAATGCCT

TCAAGATGGT GCCTTCTCAT TCACCAGCTG GTATTTTTGA TGAAATGCCT

CTGAGATAAT GCCTGCTAAA TCAGGAAAAA ATATTAAAAG TGAAGAAACT

CTGAGATAAT GCTGTCTAAA TCAGAAACAA TTATTAATAG TGAAGAAACT

CTGAGATAAT GCCTTCTAAT TCAGAAAGGA ATGTTAATAG TGAGGAAACT

TTGAGAGAAT GCCTTCTAGT TCAGAAGCAA ATATTAAGAG TGAAGAAACT

TTGGGA---- --CTTCTAAC TCAGAAGCTG ATTTTAATAG TGAAGAAACT

CTGAAATAAT GCCTTCTAAT GCAGAAACCA ATATTACTGG TGAAGAAACT

TTGAGAGACT ACCTTTTAAT TCAGAAACTA ATGTTAATAA TGGAGAAACT

TTCATAGAAT GCCTTCCGTT TCTGAAAGTA ATGTAAATAA TGGGAAAAC-

CTGAGTCAAT GCCTTCCAAT TCAGCCAGTG GTTCAGATGG TGAAAAACCA

TTGAAAAAGT ACCCTCC--T TCGGAAACCA GTTTAAATAG AGATAAATTT

GACCTGAAAA ATCTTTGTAA ACCAAAAGAT GGTGAAATGC TTTGTCATTT

GACCTGAAAA ATCTTTGTAA ACCAAAAGAT GGTGAAATGC TTTGTCATTT

GACCTGAAAA ATCTTTGTAA ACCAAATGAT GGTGAAATGC TTTGTCATTT

GACCTGAAAA ATCTTTGTAA ACCAAATGAT GGTGAAATGC TTTGTCATTT

GACCTGAAAA ATCTTTTTAA GCCAAAAGAT GGTGAAATGC TTTGTTATTT

GACCTGAAAA CTCCATTTAA ACCAAAAGTT GATGAAATGC TTCATCATTT

AACTTGAAAC ATCACTTTAA ACCCAAAGAT GGTAAAGTGC TTTGTCATTT

AACTCAAAAC ATCACTTTAA ACCCAAAGAT GATGAAATGC TTTGTCATTT

GCCCTGAAAA ACATTCTCAA GCCAAAAGAT GATGAAACGC TTTGTCATTT

GACCCCCAAA ACCTTTTTAA GCCAATAGAC GGTGAAATGC CTTGT--CTC

GACCTGGAAA ATATTTATAA ACCA------ GAAGAAATGC TTTGT--TTT

GACCTTAAAA TTCTTTGTAA GCCAAAAGAC GGTGAAATGC TTTGT--TTT

AGCCTGGAAA GTTATGTTAA GCCAAAAGAG GGCAAACAGC TTTGT--TCT

GATCTGAAAG ATCTTCTTAA ACCAAATGAT GGTAAAATGC TTTGCCATTT

CACCCAAAAA ATGTTTTTAA ACCAAAAGAT GGTGAAATAC TTTGTCACTT

---------- ---------- ---------- ---------- -----CCCTT

GAAACAAAAC ATCTTTCAGA ATCCGTAGAT GTCAAAGTAC TCACTACTTC

GGACAGACAG GGTGTTCTGT ACAAGCAGGT GACCGAATGC TTT--CATTT

GCCTTTGACA GTGAAAACAG ATATTAAAGT GAAAGGAGAA GAAACCGAAG

GCCTTTGACA GTGAAAACAG ATATTAAAGT GAAAGGAGAA GAAACCGAAG

GCCTTTGACA GTGAAAACAG AAATTAAAGT GAAAG---AA GAAACCGAAG

GCCTTTGACA GTGAAAACAG AAATTAAAGT GAAAG---AA GAAACCGAAG

GCCTTTGACA GTGAAAACAG AAACTAAAGT GAAAGGAGAA GAAACTCAGG

GCCTTTGACA ATGAAAACAG AATCTAAAGT GATAGGAAAA GAAACTGAAG

GCCTTTGACA ATGGAAACAG CAATTAAAAT GAATAAAGAA GAAACTGAAG

GCCTATGACA ATGGAAACAG CAATTAGAAT GAATAAAGAA GAAACTGAAA

GCCTTTGGCA GTGGAAACAG AAGCTACCA- --------GA GAACCTGAAG

GCCTTTGATG CTAGATGCAG AAACTAATGT GAAAGGAGAA GACACTGCAG

GCCTCTGACA GTGGAAACAG AAACTAAGTT GAAAGGCGAA AAAACTGAAG

GCCGTTGACG ATGGAAACCG AAACTAAAGT GACAG---AG GAAACCGAAG

GCTTTCAACA GTGGAAACAG AAGCTGCAGG GCACAGACAA GAAACTAAAG

GCCTTTGACT TTGGAATCAG AAGCTAAGGT GAAAGGAGAA GAAACCAAAG

GCCTTTGACA TTGGGAACAG AAGCTAAAGT GAAAAGAGAA GAAATCAAAG

GCCGTTAACA TTGGAAACAG AAGCTAACGT GAAAAAGGAA GAAA------

CTCTTTTCAA TTGAATTCAA AAAATAAAAT GACCAGAGGA GAGAATAAAG

ACCTTCTGAG ATTAATTCGG GAAGCAAAGC AAATAGGAAA GAGATTGAAG

AGCATCAGAG GGGACGACTG GGTTACTTAA CTGTTGGGGA GCAATCTGAG

AGCATCAGAG GGGACGACTG GGTTACTTAA CTGTTGGGGA GCAATCTAAG

AGCATCAGAG GGGACTACTG GGTGACTTAA CTGTTGGGGA GCAATCTGAG

AGCATCAGAG GGGACTACTG GGTGACTTAA CTGTTGGGGA GCAATCTGAG

AGCATCAAAG GGGACCACCG GGTGACTTAA CTGTTGGGGA GCAATCTGAG

AATATCAGAG GGAACCACTG GGTTGTTTAC CTGTTGGGAA GAAATCTGAA

AACATCAGAA GGGACTACTG GGTCACATAG CTGTTGGGGA AGCAGCTGAG

AACATCAGAC TGGACTACTG GGTCACATAC CTGTTGGAAA AGCATCTGAG

AGTCTCAGAG TGGACGAGTG GGGCCCGGAA CCATTGGGGA GGCATCTGAT

AGCAGCAGAG CGGGCCGTGG GGTCAGTTTC CTGCCGGGCA GGAGTCGCAA

AACATCAGAG GGGACCCCTG GGTCATTTAA CTGTCATGGA AGAGTCTGAG

AACATCAGAG GAGATCACCG CGTCACTTCA CTGTTGGGGA GAAATCTGAG

A---CCAGAG GGACTCACTG GATCACCAGA CTGCTGAGGA GGAAGCT---

ACCATCTGAA GGGTCCTCTG GGTCACTTAG CTGTTGAGGA GGAATCTGAA

AACACTGGAG GGGACCACTG GGTCACTTCA TTATTGGGGA GAAATCAAAA

---------- ---------- ---------- ---------- ----------

AACACCACCG AGGAACACTA AATGATTTCG AAGTCAGCGA GGAATCTGAA

AACA-CAACT GGGAACAGTG AATCATTCAT CAATTGAAGA GGTAGCTAGA

GAGTTGGTTA CCAGAGAAAC TGGCGATGGC GATCCCGTGA GCAACATCTC

GAGTTGGTTA CCAGAGAAAC TGGCGATGGC GATCCCGTGA GCAACATCTC

GAGATGGTTA CCAGAGAAGC TGGCAATGGC GATCATGTGA GCAACATCTC

GAGATGGTTA CCAGAGAAGC TGGCAATGGC GATCATGGGA GCAACATCTC

GAGATGGGTA CCAGAGAAAC TGGCGGTGGC TATCATGTGA ACAACATCTC

GAGATGGTTA TCAGAGA--- TAATGATACT GCTTGTATGA GCAAGATTTC

GAGACCATCA CCAGAGCAGG TCATGATGGT AA-------- -------TTC

GAGACCA--- ---GAGAAGG TCATGGTGGT AA-------- -------TTC

GTGAGTA--- CCAGAGAGGG CGGTGGTGGT GATGACACGA GTGAGATTTC

GAGATGGCAA TCAGGGAAGG CGGTCTCGCT GATGTCCCAA GCCAGGCATC

GAGATGATTA CCAGAGAAGG TGGTCATGGT AACAAAGGAA GAGAGATTTC

GAGGTGATTA CCAGAGAAGA TGGTTGTGGT GGTAATATGA GTGAGATTTC

GGGATGATTA CCCGAGAAGG TAATCGTGGT GATATTACAA ATGAGATTCC

GAGATGATTA CCAGAGAAGA CAGTGATAAT GATAATATGA CCGAGACTAC

GAGATGATGA CTGGAGAAGA TGGTGATATT GATAATAAGA GCAAGATTTC

---------- ---------- ---------- ---------- ----------

GGGGAAACTT TGAGTAAAGA TGGTGGTGAT AAATTTTCAA GTG---TCTG

GAAAGAATCA CTAGAGATGA CGAATGTGAT AAATATGCAA ACGAGGCCTG

TCAGACCCAT TTTAATGAAA TCACCCTAGC AAAGCTGGCC AAGGACAGCA

TCAGACCCAT TTTAATGAAA TCACCCTAGC AAAGCTGGCC AAGGACAGCA

TCAGACCCAT GTTAATGAAA TCACCCTAGC AAAGCTGGCC AAGGACAATA

TCAGACCCAT GTTAATGAAA TCACCCTAGC AAAGCTGGCC AAGGGCAACA

TCAGACCCAC ATTAATGAAA TCACCCTAGC ACAGTTGGCC AAGAACAGCA

TCAGACTCAC TCCAAAGAAA TCACCTTAGC AAAGCTGGCC CAGGTGGACA

TCAGACCCAC GTGGAAGAAG TCACCCTAAG AAAGCCAACC AAGGACGAAA

TCAGACCCAC TTGGGAGCAA TCACCCTAAG AAAGCCATCC AAGGACAGAA

CCAGGC---C TTTAAAGAAA TCACCGTAGC AAAGCCAGCC AAGGAGGA--

TCAGACCCAC CTTACGGAAG TCACCTCAGC AAAGCCGGCC ACGGGCGCCT

TCAGACCCAC TTGAAGGAAA TCCCCCTAGC AAAGCTGGCC AAGGGTGGTA

TCAGGTCCAC TGTAAAGAAA GCACCCCGGC AAAACTTGCC AGGGGTGACT

TCAGGCCCAT ATTAAAGAAA TTGCCACCGA AAAACTGGAC CAGGGTGCCA

TCAGACCCAC TTTAAAGAAA TCACCCTAGC AAAGATGGCC AAGGATGACA

TCAGACCCAC TTTAAGGAAA TCCCCCTAGC AAAGCTGCCC GGGGGTGACA

---------- ---------- ---------- ---------- ----------

TGGACCTCAT TTTGAGGATA TCATAGGAAC AAAGAAGACC AAGAGTAAGC

TCCGAATTGC TT-AAGGATA TTAAAAGGGC CAAGATTTTC AAGGATGATG

TTGTGGCACA GACTCAGAAG TTAGAAGACC AGAAGGAGGA AAGGTTACAT

TTGTGGCACA GACTCAGAAG TTAGAAGACC AGAAGGAGGA AAGCTTACAT

CTGTGGCACA GACTCAGAAG TTAGAAGACC AGAAGAAGGA AAGTTTACAT

TTGTGGCACA GACTCAGAAG TTAGAAGACC AGAAGGAGGA AAGCTTACAT

TTGTGGCACA GACTCAGAAG TTGGAAGACC AGAAGGAGGT AAGCTTAAAT

TTGCTGCCCT TACTCAGAAG GT-------- -------GGA AAAGTTAGGC

TTGCTAGACA GCTTCAGAGG TTGAAATCCC CAAAAGAAGA AAGCTTATGT

TTGCTAGACA GCTTCAGTGG TTGAAATACC CAAAAGAAGA AAGCTTATGT

---------- ---------- --------TC CAAAGGAGGA AAGCTTCCGC

CTGCCGTGGG GCCTCAGAAG CCCATTGACG TGGAGGAGGA AAGCTCGTGT

CTACTGCACA GTTTCAGAAG TTGGAAGATC CAGAGGAGGG AAGCTTGTGT

TTGCTGCACA GTTTCAGAAG TTGAAAGACC CAGAGGAGGA AACCT---GT

CTGCTGTACA GCAGCCGGCG TTGAAAGGAC CCAGGGAAGA AAGTGTACAT

TTGCTGCACA GTCTCAGAAA TTGAAAGACC CAAAGATGGA AAGCTTATAT

TCGCAGCATG TTCTCAGAAG TTAAAAGACC CAGAGATGGA AAGCTTGTAT

---------- ---------- ---------- ---------- ----------

CTTTTCTTCA GTCTCTAAAG GTGGATGATT CAGAGGCAGG ACAACAAGAT

CTGCGTCATG GCCTTTGAGG ATCAGAAGTT CAGAAATGGC CTCTCTGGAC

CATCCATTAA GGAAGGACAC TGAGTCATGC ACAAGTCCTT GCCTTCTTGG

CATCCATTAA GGAAGGACAC TGAGTCATGC ACAAGTCCTT GCCTTCTTGG

CATCCATTAA AGAAGGACAT TGAGTCATGC ACAAGTCCTT GCCTTCTTGG

CATCCATTAA AGAAGGACAT TGAGTCATGC ACAAGTCCTT GCCTTCTTGG

CATCCATTTA AGAAGTACAT TGAGTCATGC ACAAGTTCTT GCCTTCTTGG

CATCCTCTAA AGAAGGACAT TGAGTTGTGC ATAGGTCCTT GCCTTCCTGG

CCTTCATTAA AGAAAGACAC TCAATTATAC ACAGGTACTT GCCTTCCTGG

CATTCATTAA AGAAAGACAC TGAGTTATGC ACAGGTACTT GCCTTCCTGG

CATCCGTTAA AG---GACGC TGCCTTGTAC ACGGGCCCTT GCCTCCCTGG

CACCCGCTGA GAAAGGACAC AGAGTCATGC ACATGCCCCG GCCCCCGCAC

CGTCCATTTA AAAAGGACAT GGAGTCGTGC ACAGGTCCTT ACCTTCATGG

CACCCACTAA AAAAGGACAT TGAGTTGTGT ATAGGTCCTT GCCTTCGTGG

CAGCCAGTGA AAAAGAGCAC TGAATTGTGC ACAGCTCCT- -----CGTGC

CATCCATTAA AGAAAGACAC TGAGTTGTCC GCAGGTCCTT GTCTTCCTGG

CATCCATTGA AGAAGGACTT CAAGTTGCCC GCAGGCCATT GCCTTCCTGG

---------- ---------- ---------- ---------- ----------

CTTCATTTAT GTTCCACCAT TGAGCTATCT TTAGGTCCTC ATCTCCCCAT

CGTCGACTAG GTAATTGCAT TGATTTACCC GCAGATCCTG GCCTCCCGGC

TGCCCCTCGG AAAGCACAAG ACCCCTCCTC TGCTGGGTGT GATCAAATAC

TGCCCCTCGG AAAGCACAAG ACCCCTCCTC TGCTGGGTGT GATCAAATAC

TGCCCCTCGG AAAGCAGAGG ACCCCTCCTC TGCTGGGTGT GATCAAATAC

TGCCCCTTGG AAAGCAGAGG ACCCCTCCTC TGCTGGGTGT GATCAAATAC

TGCCCCTCAG AAAGCACAAG ACCCCTCCTC TGCTGGGTGT GATCAAATAC

TGCCTCCAGG AAAGCACAAG ACCCTAACAC TGCTAGGTGT GATCAGATAC

TACCTCCTGG AAAGAAGGTG ACCCCACTTT TGCTGGAGGT GATCGAATAC

TGCCTCCTGG AAAGAACATG ACCCCAGCTT TGCTGGAGGT GACCGAATAC

TGACCCCCAG AAAGTGCAGG ATCCCTCCTC TGCTGGGTGT GATCCGATCT

TGCCCCTCGG CGTGCTCAAG ACCCCAGCTC CACGGGGCGG GAGGCCTTCC

TGCCCCTCAG AAAGCACAAG ACCCCAACTG TGCTGGGTGT GATGAAATAC

TGCCCCCCAG AAAGCACAAG ACCCCGGCCC TGCTAGGTGT GATGAAACAC

CGACCCCCAG AAAGCGCAGG ACCCTGTCGC TGCTGGGCGT GATGAAGTAC

CACCCTTCAG AAAGCACAAG ATCCTAACTC TGTTGGGTGT GATGAAATAC

TGTCCCCCAG AAAGCACAAA ACCCCCGCTG TGCTGGGTGT GATGAAATAC

---------- ---------- ---------- ---------- ----------

CTCCAACATG ATACCCCAAG ATACCAGCTC TGCTGGATGT GAAGAAATGC

TTCCCAGATG ATACCTCAAA GCGACAACTC CGCGAGGGGT GACGAAAATC

ATGGTGCCTT TGCGAAGCTT CCCTTAAAGA AGCAGCCCCA TCGAACTTGT

ATGGTGCCTT TGCGAAGCTT CCCTTAAAGA AGCAGCCCCA TCGAACTTGT

ATGGTGCCTT TGCGAAGCTT CCCTTAAAGA AGCAGCCCCA TCGAACTTGT

ATGGTGCCTT TGCGAAGCTT CCCTTAAAGA AGCAGCCCCA TCGAACTTGT

GTGGTGCCTT TGCAAAGCTT CCTTTAAAGA AGCAGCCCCA TCGAACTTGT

ATGGTGCCTT TGGGAAGCTT CCCTTAAAGA AGCAGCCCCA TCGAACATGT

ACGGTGCCTT CGTGACGCTT CCTGTAAAGA AGCAGCCTCA TCGAACGTGT

ACGGTGCCTT TGTGAAGCTT TCTGTAAAGA AGCAGCCGCA TCGGACATGT

GTGGTGCCTT TGGGAAGCTT CCTGTAAAGA AGCAGCCCCA CCGAAGGTGT

ACGGTGCCTT TGGGAGGCTG CCCTTAAAGA AGCAGCCCCC CCGAACCTGC

ATGGTGCCTT TGGAAAGCTT CCCTTAAAGA AGCAGCCCCA TCGAACATGT

ACAGTGCCTC TGGGAAGCTT CCGTTAAAGA AGCAGCCCCA TCGAACATGT

ACGGTGCCTT TGGGAGGCTT CCCATAAAGA AGCAGCCCCA TCGAACGTGT

ACGGTGCCTT TGGAAAGCTT CCTTTAAAGA AACAGCCCCA TCGAGCATGT

ACGGTGCCTT TGAGAAGCTT CCCTTAAAGA AGCAGCCCCA TCGAACATGT

---------- ---------- ---------- ---------- ----------

GTGGTACCTT TGCGAAACTC CCATTAAAGA AAAAACTAGG TTGGACATGT

CAGGTGCCTT TGCGA---TT CCAGTAAAGA AACAGCCCAG TCGAAAATGT

AAGAAAGTTT CCTATCAGGA GCAAATCATT GTTGGGAGAA AAATAGGTAA

AAGAAAGTTT CCTATCAGGA GCAAATCATT GTTGGGAGAA AAATAGGTAA

AAGAAGGTTT CCTATCAGGA GCAAACCATT GTGGGGAGAA AAATAGGTAA

AAGAAAGTTT CCTATCAGGA GCAAACCATT GTGGGGAGAA AAATAGGTAA

AAGAAAGTTT CCAGTCAGGA GCAAAGCAGT GTGGGGAGAA AAATAGGTAA

AAGAAAGTTT CCCGTCAGGA GCAAGTCAGT GTGGGGAGAA AAATAAGCAA

AAGAGAATTT CTTGTCAGGA GCCGGTCACT GTGCGGAGAA AAATAAGTAA

AAGAGAATTT CCTGTCAGGA GCCGGTCACT GTGCGGAGAA AAATAAGTAA

AAGAGAGTTT CCTGTCAGGA GCAAGTCAGT GTGGGGAGAA GAATGAGTAG

AAGAAAGTCT CCTGCCAGGA GCTGGTCTCC GTAGGGAGGA AAAGGAGTAG

AAGAAAGTTT CCTGTCAGGA GCAAGTCAAC CTGGGGAAAA AAATAAGTAA

AAGAAAGTTT CCTGTCAGGA GCAAGTCAAC ATGGGGAGAA AATTAAGTAA

AAGAAAGCTT TCAGCCAGGA GCAAGTCAAC ATGGGGAGAA AAATAAGCAG

AAAAAAGTTT CCTGTCAGGA GCAAGTCAAG ATGAGAAGGA AAATAAGTAA

AAAAGAGCTT CCTGTCAGGA TCAAGTCATG GTGGGAAGAA AAGTAAGTAA

---------- ---------- ---------- ---------- ----------

AAAAATGTCA TTCATCAAGA GCACATCAAG ACTGGAAGAA AGATGAATAG

AAAAAAGTTC CATCTCAAGA GCAAATCAAG ACCATAAGAA GAAAGAG---

AATCAGAAGT TCTGCCTTTT TAAAGAGTTC TTCCAATCCC ATCCCCACAA

AATCAGAAGT TCTGCCTTTT TAAAGAGTTC CTCCAATCCC ATCCCCACAA

AATCAGAAGT TCTGCCTTTT TAAAGAGTTC CTCCAATGCC GTCCCCACAA

AATCAGAAGT TCTGCCTTTT TAAAGAGTTC CTCCAATGCT GTCCCCACAA

AATCAGAAGT TCTGCCTTTC TGAAGAGTTC CTCCAATCCC AACCCAACAA

AATTAGAAGT TCTGCCTTTG TAAAGAGTTC CTCTGATCCC ATCTCCACAA

AGTGAGAAAT TCTGATTGTG GAAAGAGTTT CTCTAACCCA GTTCCCACAA

AGTGAGAAAT TCTGATTGTG GAAAGAGTTT CTCTAACCCA ATCCCCACAA

AGCGAGCTGC TCTGCCTTTG TAAAGGGTCC CTCTGCGCCC ATCCCCACAA

AATTGCACAT TCTGCTTTCC TAAAGAGTTC CTCTGAGACC ATCCCCACAA

AATCAGGAGT TCGGCCATTT TAAAGAGTTC CTCTGAAACC ATCCCCACAA

AGTCAGAAAT TCTGCCTTTT TAAAGCATTC CTCAGAAACC GTCCCCACAA

AATCAGAAGT GCTCCCTTCT TAAAGAGTTC CTCTGAAACC ATCCCCACAA

AATCAGAAGT CCTGCCTTTA TAATGAGTTT CTCCGAAACC ATCCCCACAA

AATTGGCAGC TCCACGTTTT TAAAGAGCTC CTCAGAAACC ATCCCCACAA

---------- ---------- ---------- ---------- ----------

TTTCAAAAAG TGGATTCTTT TAAAGAGTGC TTCAGAAACC GTCTCAGTCA

AATTCAAAGT TCAGGTTTTT TAAGAAATTC TTCTGAAACG ATTCTTAAAA

AAGCGCACAG ACTTCTCAGT TTGTGTACTC TGTCTGCACC TACACGATTA

AAGCGCACAG ACTTCTCAGT TTGTGTACTC TGTCTGCACC TACACGATTA

AAGCGCACAG ACTTCTCAGT ATGTGCACTC TGTCTGCACC TACACGATTA

AAGCGCACAG ACTTCTCAGT ATGTGCACTC TGTCTGCACC TACACGATTA

AAGCACACAG ACTTCTCAGT TTGTGTACTG TGTCTGCACC TACACGATTA

AAGTACATAG ACTTCTTAGT TCAAGCGCTG TGTCTCCACC TACACAATTA

AAGCACACAG ACTTCTCAGC CCATGTACCG TGCCTG---C CAAGCCATTG

AAGCACACAG ACTTCTCAGC CCATGTACTG TGCCTG---C CAAGCCATTG

AAGCCTACAG GCTTCTCAGT TCACGGGTTG TCTCTGTGCC CACACGGTCA

AAGCACACAG GTTTCTCACT TCCCGTGCCA TGTCTGTACC TGCGTGCCCA

AAGCACACAG GTTTCTCAGT TCACGTGCTG TGCCTGCACC CGCACAGTTG

AAGCACACAG ACTTTTCAGT TCACATACCG CATCTGCACC CTCAAGACTA

AAGCACACCG ATTTCTTAGT TCACAGTTTG CGTCTGCACC CACGCAATTG

TAGAGCACAA GCTTCTCAGT TCTTGTGCTG CATCTGCACC TGCACAGCTG

AAGAACCCAG ACTTCTCAGT TCATGTGCAG TGTCTGTATC TGTACAATTG

---------- ---------- ---------- ---------- ----------

AAGAACACAA AGTCCTCAGC TCCTTTACTC CTCCTCCATC ATCATCTTCA

GAGAACACGA ACTCTTTAGT T---GCTCTC TTGGTGCATC TAAGCTACCA

GAATTGGTTA GTCACATACC AAAGCAGATG TCTACACCGT GCCATCCCTT

GAATTGGTTA GTCATATACC AAAGCAGATG TCTACACCGT GCCATCCCTT

GAATTGATTA GTCACATACC AAAGCAGACA GCTACACCGT GCCATCCCTT

GAATTGATTA GTCACATACC AAAGCAGACA GCTACACCGT GCCATCCCTT

GAATCAATTA GCCACATACC AAAGCAGACA ACTATACCAT GCCATCCCTT

GAATTGATTA GCCACATACC AAAGCAGAAG GCTACACCAT GCCATCCTTT

GAATTGCGTG GCCACACACC AAAGCCGAAG GCTACTCTGT GCCACTCCCT

GAATTGCGCA GCCACATACC AAAGCCGAAG GCTACTCTGT GCCATTCCTT

GAGTCCATCA GCCACATACC AAAGCAGAGG GCTACTCCGT GCCATCTCCT

GAGCTTGTGA GCCACGTACC AAAGCTGAAG GCTGCCCCGG GCCCACCCTC

GAATTATTGA GCCACATACC AAAGCAGAAG GCGTCTCTGT GCCATCCCTT

GAATTAGTTA GCCACATACC AAAGCAGAAG GCTACGCCGT GCCATCTCCT

GAAAAAGTCC GCCACACACC AAAGCAGAAG GTATCTCCGT GCCACTCTTT

GAATTAATTA GCCACATACC AAAGCAGAAG GCTACTCCGT GCCATCCCTT

GAATCAACTA GCCACATACC AAAGCAGAGG GGTGCTCCGT GCCAGGTTTT

---------- ---------- ---------- ---------- ----------

G---TGGCCA ACCATATGCC CAAGCCGAAA GCCACAAGAC GCCTTCTGCT

GAATCGATCG ACCGTATGCC AAAGCAGAGG GCCACTGCCT GTAGCCTGTG

GAGGAGCCTG AATTTTAGGA AGACTACCAA AGAATCAGCC TTACTAAACA

GAGGAGCCTG AATTTTAGGA AGACTACCAA AGAATCAGCC TTACTAAACA

GAGGAGCCTG AATTTTAGGA AGACTACCAA AGAATCAGCC TTACTAAACA

GAGGAGCCTG AATTTTAGGA AGACTACCAA AGAATCAGCC TTACTAAACA

GAGGAGCCCG AATTTTAGGA AGCCTACCAA AGAATCAGCC TTACTAGACA

GAGGAGCATG AATTTTAGGA AGCCTACCAA AGAAGCAGCC TTATTGAACA

GAGGAGCCTG AGCTGTAGGA AGCCTACCAA AGAATCAGCC TTACTAGACA

GAGGAGCCGG AGTTGTAGGA AGCCTACCAA AGAATCAGCC TTACTAAGCA

GAGGAGCCTG AATTTTAGGA AGCCTACCAA AGAATCAGCC TTACTAAACA

GGGGGGCCTG AATGTGCGGA TGCCTACCAA AGAATTGGCC TTACTCAGCA

GAGGAGCCTG AATGTTAGGA AGCCTACCAA AGAATCAGCC TTACTCAACA

AAGGAGCCTG AATGTCAGGA CGCCTACCAA AGAATCGGCC TTACTCACCA

GAGGAGCCTG AATGTT---A AGCCTACCAA AGACTCAGCC TTACTCCGAA

GCGGAGCCTG AATTTTAGGA AGCCTACCAA AGAATTAGCC TTACTAAACA

GAGGAGCCTG AACTTCAGGA AGCCTACCAA AGAATCAGCG TTACTAACCA

---------- ---------- ---------- ---------- ----------

GAACAGCATG AGACTTCAGA AATCTACCAA AGACTCAGCT TTGCTAAACA

GGCCAGAAGG AGACACAGGG ACTCTACCAA AGAGCCGGCC CTGCTGAAGA

AGCTGTCCAT CCTTGCCTCC AAACTGGCCC CAGCCATGAA GACTCAGAAG

AGCTGTCCAT CCTTGCCTCC AAACTGGCCC CAGCCATGAA GACTCAGAAA

AGCTGTCCAT CCTTGCCTCC AAACTGGCCC CAGCCATGAA GACTCAGAAA

AGCTGTCCAT CCTTGCCTCC AAACTGGCCC CAGCCATGAA GACTCAGAAA

AACTGTCCAT CCTTGCCTCC AAACTGGCCC CAGCCATGAA GACTCAGAAA

AGCTGTCCAT CCTTGCCTCT AGACTGGCCC CAGCCGCAAA GATCCAGAAA

AGCTGTCTGT CCTGGCCTCC AAACTGACTC TGGCCGCAAA GCCCCAGAAA

AGCTGTCTGT CCTTGCCTCC AGACTGACAA CGGCAACAAA GCCCCAGGAA

AGCTGTCTGT CCTTGCCTCC AAACTCGCCC CGGCCACAAA GACCCAGAAA

AGCTGTCTAT CCTCGCCTCC AGACTGGTCC CCGCTGCCAG GACCCAGAAG

AGCTGTCCAT TCTTGCCTCC AAACTGGTCC CAGCCACAAA GACCCAGAAA

CGCTATCCAT CCTTGCCTCC AAACTGGCCC CAGCCGCGAA GACCCAGAAA

AGCTGTCCAT CCTCGCCTCC AAACTGGCCC CGGCCTCCAC GGCGCAGCAG

AGCTGTCTAT CCTTGCCTGT AAACTGGCCC CAGCCACAAA GACCCAGAAA

AGCTGTCCAT CCTCGCATGT AAACTGGTCC CCATCACAAA GACCCAGAAA

---------- ---------- ---------- ---------- ----------

AATTGTCTGT TCTGGCCAGG AAGCTGGCCC CGTCCATAGC AACCCAAGAA

AGTTGTCCGT CCTTGCCAGT AAACTGAATC CCGCCAAAAA CACCCACAAG

CTAAGATACC GACGGTGTTC CTCTGAACTT CTTCCAATGG CTAAAAGCTA

CTAAGATACC GACGGTGTTC CTCTGAACTT CTTCCAATGG CTAAAAGCTA

CTAAGATACC GACGGAGTTC CTCTGAACTT CTTCCAATGG CTAAAAGCTA

CTAAGATACC GACGGAGTTC CTCTGAACTT CTTCCAATGG CTAAAAGCTA

CTAAGATACC AACGGTGTTC CTCTGAACTT CTTCCAATGG CTAAAAGCTT

CTAAGATATC AGCGGTGTTC CTCTGAACTT CTTCCAGTGG CTAAAACCTA

CTCAGATGTC GGCGGTATTC CTCTTCCCTT GTTCCACTGG CTAAAAACTA

CTTAGATGTC GGCGGGATTC CTCTTCCCTT GTTCCATTGG CTAAAAACTA

CTTAGGTATC GGCGGTGTTC CTCTGAACTT GTTCCAGCGG CTAAAAGCTT

CTGCGGTCCC GGCGGTGCTC CTCGGACCTT CTCCCCGTGG CTAAAAGCTA

CTAAGATATC GGCGGTGTTC CTCTGAACTT CTTCCAGTGA CTAAAAGCTA

CTAAGGTATC GGCGGTGTTC CTCTGAACTT CTTCCAGTGG CTAAAAGCTA

CTAAGGTCCC AGCGGTGTTC CTCAGAACTC CTTCCCGTGG CCAAAAGCTG

CTAAGGTATC GGCGGTGTTC CTCTGAACTG CTTCCTGTGG CTAAAAGCTA

TCGAGATATT GGCATTGTTC CTCTGAACTC CTTCCAGTGG CTAAAAGCTA

---------- ---------- ---------- ---------- ----------

TTAAGCTCTC AGCCATGCTC TACTGAACTT CTTCCCGTGG CTGAATCCTA

GTAACGCCTT CGCAAAGTTC CTCCAGAGCT CTTCCAGTGG CCGAACGATA

CAAGCGCCTC AGATATAAAA GACTCCTGGA CGGATTTTCA TCCAGCACAG

CAAGCGCCTC AGATATAAAA GACTCCTGGA TGGATTTTCA TCCAGCACAG

CAAGCGCCTC AGATATAAAA GGCTCCTGGA TGGATTTTCA TCCAACACAG

CAAGCGCCTC AGATATAAAA GGCTCCTGGA TGGATTTTCA TCCAACACAG

TAAGCGCCTC AGATACAAAA GGCTCCTGGA TGGATTTTCA TACAACACAG

CAAGCGCCTC AGATATAAAC GGTTCCTGGA TGGATTTTCA TACAGTATGA

CAAGCGCCTC CGATACAAAA AGCTCCTAGA TGGATTTTCA CATAACGCAG

CAAGCGCCTC AGATATAAAA AGCTCCTAGA TGGATTTTCA CATAATGCAA

CAAGCGGCTC AGATATAAAC GGCTCCTCGA TGGGTTTTCA TACAGTACAA

CAAGCAGCTC CGATACCGGA GGCTCCTGGA CGGCTTCTCC TGCAGCATGA

TAAGCAGCAC AGATACAAAA GACTTCTGGA TGGATTTTCA TACAATCTAA

CAAGCGGCTC AGATATAAAA GGCTCCTGGA TGGATTTTCA CCCAATACAA

CAAGCGATTC AGATACACCA GGCTCCTGGA CGGGTTTTCT TCTGGTACGG

CAAAAGGTTC AGATACAGAA GGTTCTTGGA TGGATTTTCA TACAGTACAA

CAAGCGGCTC AGATATAAAA GATTTCTGGA TGGATTTTCA TATAATACAA

---------- ---------- ---------- ---------- ----------

CAAACGGCTC AGGTTTAAAA AGCTCCAGGA TGAATTTCCT AACAATATAA

CAACCAGCTC AGATTTAAAA ATCTTCTGGA TGTATTTTCG TGTATCAGTA

AGCAGCTGAA TCCATATTTG GCAGCTAGTG GATGGGATAA GAGGCCTAAC

AGCAGCTGAA TCCATATTTG GCAGCTAGTG GATGGGATAA GAGGCCTAAC

AACAGCTGAA TCCATATTTG GCAGCTAGTG GATGGGATAA GAGGCCTAAC

AACAGCTGAA TCCATATTTG GCAGCTAGTG GATGGGATAA GAGGCCTAAC

CACAGCTGAA TCCATATTTG GCAGCTAGTG GATGGGATAA GAGGCCTAAC

CACAGCTGAA TCCATATTTG GCAGCTAGTG GATGGGATAA GAGGCCTAAC

TGCAGCTGGA CCCATATTTG GCAGCTACTG GGTGGAGTAG GGGGTCTCAC

TGCAGCTGGA CCCATATTTG GCAGCTACTG GGTGGAATAG AGGGTCTAAC

TGCTGCTGAA CCCACATCTG GCAGCTAGCG CATGGGATAA AAGGCCCGAC

CGCAGCTGAA CCCGTGTCTG GCAGCTGGTG ACTGGGACAG AAGGCCTAGC

TGCAGCTGAA TCCATATTTG GCAGCTAGTG GATGGGACAA GAGGCTTAAC

TGCAGCTGAG TCCATATTTG ACAGCTACTG GATGGAATAA AAGGCCTGCT

TGCAGCTGAA TCCGCATTTG GCAGCTAGCG GGTGGGATAA GATGCCACAC

TGCAGCTGAA TCCATATTTG GCAGCTAGTG GTTGGGATAA GAGGCCTAAC

TGCAGCTGAA TCCATATTTG GCAGCTAGTA GATGGGATAA GAAGCCTAAC

---------- ---------- ---------- ---------- ---------C

TGCAGCTCAA CCTCCATTTT GGTGATTATG GGTGCAAGAA CATACCTGAC

TGCAGTTGGA CTCTCCTCGG GCTGATGAGA GGCGCAACAA AATGCCTGAC

AGTAAGCCCA TGGCACTTTA TTCTCTCGAA TCCATCAAGA TGACCTTCAT

AGTAAGCCCA TGGCACTTTA TTCTCTCGAA TCCATCAAGA TGACCTTCAT

AGTAAGCCTG TGGCACTTTA TTCTCTCGAA TCCATCAAGA TGACCTTCAT

AGTAAGCCCG TGGCACTTTA TTCTCTCGAA TCCATCAAGA TGACCTTCAT

AGTAAGCCCA TGGCACTTTA TTCTCTCGAA TCCATCAAGA TGAGCTTCAT

AGTAAGCCGT TGACATTTTA TTCTCTTGAA GCCATCAAAA TGAGCTTCAT

AGTAGGCCCT TGGCACTTTA TTCTCTTGAA GCTGTTAAAA TAAGCTTCAT

AGTAGGCCCT TGGCACTTTA TTCTCTTGAA GCTGTTAAAA TGAGCTTCAT

AGTAAGCCCC TGGCACTTTA TTCTCTTGAA GGCATCAAGA TGAGCTTCAT

AGTAAACCCC TGATGCTTTA TTCGCTCGAA ACTGTCAAAA TGAGCTTCAT

AGTAAGCCCT TGACACTTTA TTCGCTTGAA GCCATCAAAA TGAGCTTCAT

AGTAAACCCT TGACACTTTA CCCGCTGGAA GCCATCAAAA TGAGCTTCAT

AGTAAGCCCT GGACTCTTTA TTCGCTCGAA ACCATCAA-- ----------

AGTAAGCCCT CGGCACTTTA TTCTCTTGAG CCCATCAAAA TGAACTTCAT

AATAAGCCCG TGGCACTTTA TCCTCTTGAA GCCATCAAAA TGAGCTTCAT

AGTAAGCCTA TGGCACTTTA CTCTCTTGAA GCCATCAAA- TGAGCTTCAT

AGTCAGACTT TGGCACTTTA TCCCCTTGAA GCCCTCAGAA TAGGCTTCTT

TTTCAGCCTC TGGCACTTTA TCCCACCGAA GCCATCCAAA TACGCTTCTT

AGATTTGAGC AACAAGATGC CGTCCCTGCT GTTTGGTTCT GAAATCTTCC

AGATTTGAGC AACAAGATGC CGTCCCTGCT GTTTGGTTCT GAAATCTTCC

AGATTTGAGC AACAAGATGC CGTCCCTGCT GTTTGGTTCT GAAATCTTCC

AGATTTGAGC AACAAGATGC CGTCCCTGCT GTTTGGTTCT GAAATCTTCC

AGATTTGAGC AA---GATGC CGTCACTGCT GTTTGGTTCT GAAATCTTCC

AGATTTGAGC AACAAGATGC CATCGCTGCT CTTTGGTTCT GAAATCTTGC

AGATTTGAGC AACAAGATGC CGTCACTGCT GCTTGGTACC GAAAACGCCC

AGATTTGAGC AACAAGATGC CATCACTGCT GTTTGGTACG GAAAACGTCC

CGACCTGAGC AACAGGATGC CATCACTGCT GTTTGGTTCC AGAATCTTCC

AGATGTGAGT GACAAGATGC CATCGCTGCT GCTCGGTTCC GAAACCTTCC

AGATTTGAGC AACAAGGTGC CGTCACTGCT GTTTGGTTCT GAAATTTTCC

AGATTTGAGC AACAAGATGC CGTCCCTGCT GTTCGGTTCC GAAATCTTCC

---------- ---------- ---------- ---------- ----------

AGATTTGAGC AACAAGATGC CATCACTACT GTTTGGTTCT GAAATCTTCC

AGATCTGAGC AACAAGATGC CATCTCTGCT GTTTGGTACG GAAATCTTTC

AGATCTGAGC AACAAGATGC CGTCCCTGTT GGGTGGTGCC GAAATCCTTC

AGACTTGATT AGCAAAATGC CATCATTGCA GTTCAATGCC CAAATCTTCC

AGACTTGAAT AACAAAACAC CCTCGTTGCT TTTCGATACC CCGATCTTCC

CAGTATCCTT TCATGTGAAA TCAAGCTCAG ATTGCACGAC TGAGTCCTCA

CAGTATCCTT TCATGTGAAA TCAAGCTCAG ATTGCACGAC TGAGTCCTCA

CAGTATCCTT TCATGTAAAA TCAAGCTCAG ATTGCACGAC TGAGTCCTCA

CAGTATCCTT TCATGTAAAA TCAAGCTCAG ATTGCACGAC TGAGTCCTCA

CAGTATCTTT TCATGTGAAA TCAAGCTCAG ATTGCACGAC TGAGTCCTCA

CAGTATCCTT TCACATGAAA TCAGGCTCAG ATTGTGTGGC TGGGTCCTCC

CGTTTTCCTT TCATGTGAAG TCAGCATCCA GTTGCATGGC TGAGTCCTCC

CATTTTCCTT TCATGTGAAA TCAGTGTCCA GCTGCATGGC TGAGTCCTCC

CAGTGTCCTT CCCTGTGAAG TCGGGCTCAG ATGGCGCGAC TGAGTCCTCG

CGGTATCCTT CCGTGTGAAG CTGGGCCCTG AGCGCGTGGC CGAGTCCCCT

CGGTATCTTT TCACATGAAA TCGGGATCTG AGTGCATGAC CGAGTCTCCG

CGGTATCCTT TCATGTGAAA TCGGGCTCTG AGTGCACGGC GGAGTCCCTG

---------- ---------- ---------- ---------- ----------

CAGTGTCATT TCAGGTGAAA TCAGGCTCAG ACTGCATGGC TGAGTCCTCA

CGGTATCCTT TCACATGAAC TCAGGCTCAG ATTGCATGGC TGAGGCCTTT

CGATATACTT CCATGTGAAC TCAGGTTCGG ATTGCGTGGC CGAGGCCGCC

CAATATCCTT TCACATAAAC CTGGACTCAG AGCTCCCAAC TGATTCCACA

CAATTTCCTT TCATATAAAA CTGGAATCCG ATCCCGCGGC CGAGATTTCA

AGGACTTTTC CTGAGCACTG TGCTCCGGCA AGGCTTGCCT TAGGAGAGGC

AGGACTTTTC CTGAGCACTG TGCTCCGGCA AGGCTTGCCT TAGGAGAGGC

AGGACTTTTC CTGAGCACTG TGCTCCGGCA AGGCTTGCCT TAGGAGAGGC

AGGACTTTTC CTGAGCACTG TGCTCCGGCA AGGCTTGCCT TAGGAGAGGC

AGGACTTTTC CTGAGCACTG TGCTCCGGCA AGGCTTGCCT TAGGAGAGGC

AGGACTTTTC CCGAGCACTG TGCTCCAGCA AGGCTTGCCT TAGGAGAGGT

AGGACTTTTC CTGAGCACTG TGCCCCGTCG AGGCTTGCCT TAACAGAGGC

AGGACTTTTC CTGAGCACTG CGCTCCATCG AGGCTTGCCT TAACAGAGGC

CGGACTTTCC CTGAGCACTG TGCTCCCGCG AGGCTCGCCT TAGGAAAGGC

AGGACTTTCC CCGAGCACTG TGCGCCCGCG AGACTCGCCT TAGGAGAGGC

AGGACTTTTC CTGAGCACTG TGCTCCAGCC AGGCTCGCCT TAGGAGAGGC

AGGAGTTTTC CTGAGCACTG TGCTCCAGCA AGGCTTGCCT TAGGAGAGGC

---------- -----CACTG CGCTCCAGCA AGGCTCGCCT TACGGGGGGG

AGGACTTTTC CTGAGCACTG TGCTCCTGAG AGGCTTGCCT TAGGAGAGGC

AAGACTTTCC CCGAGCACTG TGCTCCAGCA AGGCTTGCCT TAGGAGAGGC

AGGACTTTCC CTGAGCACTG TGCCCCAACA AGGCTTGCCT TAGGAGAGGC

AGGATTTTTT CTGAGCACTG TTCTCCTCCT GAGAGTGCTC CAAGAGAGGA

AGGATTGTTT CCGAGCACTG TGCACCTGCT AGACCTGCCT TAGGAGAAAC

CCTCCAGTGC CCGTCTCAAC CTCCCAAGTG GACCTTTTCT TTCTTCTTGT

CCTCCAGTGC CCATCTCAAC CTCCCAAGTG GACCTTTTCT TTCTTCTTGT

CCTCCAGTGC CCGTCTCAAC CTCCCAAGTG GACCTTTTCT TTCTTCCTGT

CCTCCAGTGC CCATCTCAAC CTCCCAAGTG GACCTTTTCT TTCTTCCTGT

CCTCCAGTGC CCATCACAAC CTCCCAAGTG GACCTTTTCT TTCTTCTTGT

CCCCCGGTAC CAGTCACAGC CTTCCAAGTG GACCTTTTCT TTCTTCTTGT

CTCCCAGTGC TCACCTCCAT CTCCCAAGTG GACCTTCTCT TTCTTCTTGT

CTCCCAGTGC TCAGCTCCAG CTCCCAAGTG GACCTTCTCT TTTTTCTTGT

TGCCCAGTGT GCGTCTCAAC CTCCCAAGTG GACCTTCTCT TTCTTCCTGT

CCGCTGGGGC CGTTCTCCAC CTCCCAAGTG GACCTTCTCC TTCTTCCTGG

CCCCAGGTGC CTGTCTCAGC CTCCCAAGTG GACCTTTTCT TTCTTCTTGT

CGCCCGGTGC CCTTCTCAAC CTCCCAAGTG GACCTTTTCT GTTTTCTTGT

CCCCTGCTGC CCGTCTCCAC CTCCCAAGTG GGCCTTTTCT TTCTTTCTGT

CCCCAGGTGC CCATCTCAAC CTCCCAAGTG GACCTTTTCT TTCTTCTTGT

CCCCAGGTGC CCATCTCGAC CTCCCAAGTG GACCTTTTCT TTCTTCTTCT

CCCCAGGTGC CCATCTCAAC CTCCCAAGTG GACCTTCTCT TTCTTCTTCT

TCCCACACAC CCACCTCAGC CTC---AGTG GACCTTTTCC TTCCTCACGT

CCCTGCAAGT TCACCTCAGC CTCCAAAATG GAACTTTTCT TTTCTTATGT

CCCACGGTTG CCCTGGGATG GCAACATTCA GGGAAGACAC TGGCGTCCAT

CCCACGGTTG CCCTGGGATG GCCACATTCA GGGAAGACAC TGGCGTCCAT

CCCACGGTTG CCCTGGGATG GCCACATTCA GGGAAGACAC TGGCCTCCAT

CCCACGGTTG CCCTGGGATG GCCACATTCA GGGAAGACAC TGGTCTCCAT

CCCACGGTTG CCCTGGGATG GCCACATTCA GGGAAGACAC TGGCATCCAT

CCCACATTTG CCCTGGGATG GCCACATTCA GGGAAGACGC TGGCCTCCAT

CCCATGGTTG CCCTGGGGTG GCCACATTCA GGGAAGACAC TGGCCTCAGT

CCCATGGTTG CCCTGGGATG GCCACATTCA GGGAAGACAC TGGCCTCAGT

CCCATGGCTG CCCTGGGATG GCCGCATTCA GGGAAGACAC TGGCCTCCAC

CCCAAGGCTG CCCTGGAATG GCCACATTCA GGGAAGACCC TGGCCTCTGC

CTCACGGTTG CCCTGGGATG GCCACATTCA GGGAAGATAC TGGCCTCCGT

CTCATGGTTG CCCTGGGATG GCCACATTCA GGGAAGACAC TGGCCTCCAC

CTCACAGTTT CCCTGGCATG GCCTCGTTCA GGGACAGTCC TGACCTCCTC

CCCACGGTTG CTCTAGGATG GCCACATTCA GGGAAGATAC TGGCCTCCTT

CACATAGCGG TTCTGGGACG GCCACATTCA GGGAAGACAC TGACCTCCAG

CCCACGGTGG TTCTGGGACG GCCACATTCA GGGAAGACCC TGGCCTCCAG

CTCAGGGTTC TTCAGGGACA GCCACCTTCA GGGAAGACGC TGGGCTCAGT

CCCAAAGTTC TTCAGGGGCA GCCACTGTCA GGGAAGAAAT TGGCTTCGGC

AGTCAGACCC ACACTCAGGC TCCTCCAGCT CCTCTCCAAG ACTATGGAGG

AGTCAGACCC ACACTCAGGC TCCTCCAGCT CCTCTCCAAG ACTGTGGAGG

AGTCAGACCC ACACTCAGGC TCCTCCAGCT CCTCTACAAG ACTATGGAGG

AGTCAGACCC ACACTCAGGC TCCTCCAGCT CCTCTCCAAG ACTATGGAGG

AGTCAGACCC ACACTCAGGC TCCTCCAGCT CTTCTCCAAG ACTATGGAGG

AGTCAGGCAC ACACTCAGGC TCCTCCCGCT TCTCTCCAAG ACTACGGAGG

AGTCAGACAC ACACTCAGG- --------CT CCTCTCCAAG ATTATGGAGG

AGTCAGACAC ACACTCAGG- --------CT CCTCTCCAAG AATATGGAGG

AGTCAGGCCC ACACTCAGGC CCGGCCATCT CCTCTGCCAG ACTGCGGGGA

AGCCAGGCAG GCGCC----- -CCTCCAGTT CCCCTCCAGG ACCGCAGGGC

GGTCAGGCAG GTGCCCAGGC TCCTTCAGGT CCTCTTCAGG ACTATGGAGG

GGTCAGGCAC ATGCCCAGGC TCCTCCAGCT CCTCTCCAGG ACTACGGAGG

AGTCAGTCAC TTGCCCGGGC CCCTCCCGCG CCTCTCCCAG ACTGTGGGGG

CGTCAGGCAT GCTCCCAGGC TCCTCCAGCT CTGCTTCAAG ACTGTGGAAG

AGTCAGGCCC ACTCCCAGGC CCCTCCAGTT CCTGTCCAAG ACTCTGGCGG

AGTCAGGCCT GCTCCCAGGC TCCTCCAGCT CCGCTCCAAG ACTCTGGTGG

TGTGAGCTGC TCTCCCAAGC TACCCTCTCC CCTGAGCAAG CCCCTGGAAG

GGTGATTTGC AGAACCCTGC TCCCCCACTT CCTACTCAAG TCTGTAGAAG

CACTGCCATA GTCCAGACCA GAGCAGACTG CTCTGTCCTT GGCCTTCACA

CACTGCCATA GTCCAGACCA GAGCAGACTG CTCTGTCCTT GGCCTTCACA

CACTGCCATA GTCCAGACCA GAGCAGACTG CTCTGTCCTT GGCCTTCACA

CACTGCCATA GTCCAGACCA GAGCAGACTG CTCTGTCCTT GGCCTTCACA

CACTGCCATA GTCCAGACCA GAGCCAACTG CTCTGTCCTT GGCCTTCACA

TACTGCCATA GTCCAGACCA GAGCAGACTG CTCTGTCCTT GGCCTTCACA

CACTGCCATA GTTCAGACCA GAGCAGACTG CTCTGGCCTT GGCCTTCACA

CACTGCCATA GTTCAGACCA GGGCAGACCG CTCTGGCCTT GGCCTTCACA

CACTGCCGTG GTCCAGCCCA GAGCAGACTG CTCTGTCCTT GGCCTTCACA

CACCGCCCTA GTCCAGACCA GAGGGGGCTG TTCCGTCCTC GGGCTCCACA

CACTGCCATA GTTCAGACCA GAGCAGGCTG CTCTGTCCTT GGCCTTCACA

CACCGCCATC GTCCAGACCA GAGCAGGCCG CTCTGTCCTT GGCCTTCACA

CGCCGCCATC GTGCAGACCA GAGCAGGCTG CTCCATCCTG GGCCTTCATA

CACTGCCATC ATCCAGACTA GAGCAGGTCG CTCTGTCCTT GGCCTTCACA

CACAGCCACA GTCCAGACCA GAGCAGGTTT CTCTGTCCTC GGCCTTCAAA

CACTGCCATT GTCCAGACCA GAGCAGGTTT CTCAGTCTTT GGCCTTCATA

CAATGCTATA ATGAAGGTCA GAGCTGGTTG CTCTGTCCTT GGCCTTCATA

AAACACCATA GCAACAAACA GAGCAACTTG CTCTGTGCTT GGCCTTCATA

CACTTCTAGC ACTTTGTTCC CCAGGATGTT ACCGAATCTG GACAAAAAAA

CACTTCTAGC ACTTTGTTCC CCAGGATGTT ACCGAATCTG GACAAAAAAA

CACTTCTAGC ACTTTGTTCC CCAGGATGTT ACCGAATCTG GACAAAAAAA

CACTTCTAGC ACTTTGTTCC CCAGGATGTT ACCGAATCTG GACAAAAAAA

CACTTTTAGC ACTTTGTTCC CCGGGATGTT ACCGAATCTG GACAAAAAAA

CGCTTCTAGC ACTTTGTTCC CCAGGATGTT ACCGAATCTG GACAAAAAAA

CACTTCTAGC ACTTTGTTCA CCCGGATGTT ATCGAATCTG GACAAAAAGA

CACTTCTAGC ACTTTGTTCA CCCGGATGTT ACCGAATCTG GACAAAAAGA

CGCTTCTCGC ACTTTGTTCA CCAGGATGTT ATCGAATCTG GACAAAAAAA

CGCTCCTAGC ACTCTGCTCC CCGGGATGCT ACCGGATCTG GACGAAGAAA

CACTTCTAGC ACTTTGTTCT CCTGGATGTT ACCGAATCTG GACAAAAAAA

CACTGCTAGC ACTTTGTTCC CCTGGATGTT ACCGAATCTG GACAAAAAAA

CACTGCTAGC ACTTTGTTCC CCTGGATGCT ACCGAATCTG GACAAAGAAA

CGCTTCTCGC ACTTTGTTCC CCCGGGTGTT ACCGAATCTG GACAAAAAAA

CGCTGCTAGC ACTTTGTTCC CCTGGATGTT ACCGGATCTG GACAAAAAAA

CACTGTTAGC ACTTTGTTCC CCTGGATGTT ACCGGATCTG GACAAAAAAA

CAGTTTTAGC ACTTTCTTCC CCTGGATGTT ATAGGATCTG GACGAGAAGA

CCTTTTTAGC ACTTTCCTCC CCTGGATGTT ACAGGATCTG GACAAGAAGA

CGGAGCTTCT CCAGCCACAT GCCTACCATG CAGAGGCTCT TCATGACCCA

CGGAGCTTCT CCAGCCACAT GCCTACCATG CAGAGGCTCT TCATGACCCA

CGGAGCTTCT CCAGCCACAT GCCTACCATG CAGAGGCTCT TCATGACCCA

CGGAGCTTCT CCAGCCACAT GCCTACCATG CAGAGGCTCT TCATGACCCA

CGGAACTTCT CCAGCCACAT GCCTACTCTG CAGAGGCTCT TCATGACCCA

CGGAGCTTCT CCAGTCACAT GCCTTCCATG CAGAGGCTCT TCATGACCCA

CGGAACTTCT CCAGTCATAT GCCTACCATG CAGAGGCTCC TCTTGACCCA

CGGAACTTCT CCAGTAATAT GCCTATCATG CAGAGGCTCT TCCTGACCCA

CGGAGCTTCT CCAGCCACAT GCCTACTGTG CAGAGGCTTT TCCTGACCCA

CGGAGCTGCG TCAGCCACAC GCCTGCCATG CAGAGGCTCT TCATGACCCA

CGGAACTTCT CCAGTCACAT GCCTACCATG CAGAGGTTCT TCATGACCCA

CGGAGCTTCT CCAGTCACAT GCCTACCATG CAGAGGCTTT TCATGACCCA

CGGAGCTTCT CCAACCACAT GCCTACCGTG CAGAGGCTCT TCATGACCCA

CGGAGTTTCT CCAGTCACAT GCCCACCATA CAGAGGCTCT TCATGACGCA

TGGAGCTTCT CTAACCACAT GCCTACCATA CAGAGGCTCT TCATGACACA

CGGAACTTCT CCAGCCACAT GCCTACCATA CAAAGGTTCT TCATGACACA

AGGAGCTTAT CCAGTCATTT GCCTACCATC CAGAGGCTCT TCATGACTCA

AGGAACTTCT CCAGTCACAC CCCTTCCATC CAGAGGCTCT TCTTGACACA

GTTTACACAG GGCCTGAAAG GGTTACGGTC TCCAGCCTCC ATAGCAGACA

GTTTACACAG GGCCTGAAAG GGTTACGGTC TCCAGCCTCC ATAGCAGACA

GTTTACACAG GGCTTGAAAG GGTTACGGTC TCCAGCCTCC ATAGCGGACA

GTTTACACAA GGCTTGAAAG GGTTACGGTC TCCAGCCTCC ATAGCGGACA

GTTTACACAG GGTTTGAAAG GGTTGCGCTC TCCATCTTCC ATAGCAGACA

GTTTACACAG GGCCTGAAAG GGTTGAGGTC TCCAGCCTCC ATAGCAGACA

GTTTACACAG GGCCTAAAAG GGTTAAGGTC TCCAGCCTCT ATAGCCGATA

GTTTACACAG GGCCTAAAAG GGTTAAGGTC TCCAGCCTCT ATAGCAGATA

GTTTACACAG GGCTTAAAGG GGTTGAGGTC TCCAGCCTCC ATAGCAGACA

GTTAACACAG GGCCTCAAAG GGCTGCGGTC TCCCGCCTCC ATAGCTGACA

GTTTACACAG GGCTTAAAAG GGTTAAGATC TCCAGCCTCC ATAGCAGACA

GTTTACACAG GGCTTGAAAG GGTTAAGGTC TCCAGCCTCC ATAGCTGACA

GTTTACACAG GGCTTGAAAG GCTTAAGGTC CCCTGCCTCC ATCGCCGACA

GTTTACCCAG GGCTTGAAAG GGTTAAGGTC TCCAGCATCC ATAGCAGACA

GTTTACACAG GGCTTGAAAG GATTAAAGCC TCCAGCATCC ATAGCAGACA

GTTTACACAG GGCTTGAAGG GGTTAAGGTC TCCTGCATCC ATAGCAGACA

GTTTACACAG GGCTTCAAAG GGTTAAGGCT TCCAGCATCT CTGTCCAACA

GTTTTCACAG GGCTTAAAGG GGTTAAGATC TCCTACTTCC ATATCAGATG

AGGTCTTCTG TTCTCTGCCC TACTCGGTGG GCAGAGTCCT ATCCATTTGG

AGGTCTTCTG TTCTCTGCCC TACTCGGTGG GCAGAGTCCT ATCCATTTGG

AGGTCTTCTG TTCTCTGCCC TACTCGGTGG GCAGAGTCCT ATCCATTTGG

AGGTCTTCTG TTCTCTGCCC TACTCGGTGG GCAGAGTCCT ATCCATTTGG

AGGTCTTCTG TTCTCTTCCC TACTCGGTGG GCAGAGTGCT GTCCATTTGG

AGGTCTTCTG TTCTCTGCCC TACTCAGTGG GCAGAGTGCT GTCCATTTGG

GGGTCTTCTC TTCTCTGCCC TACTCGGTGG GCCGAGTGTT GTCCATTTGG

AGGTCTTCTG TTCTCTGCCC TACTCGGTGG GCCGGGTGTT GTCCCTTTGG

AGATCTTTTG TTCTCTGCCC TACTCGGTGG GCAGAGTGCT GTCCATTTGG

AGGTCTTCTG CTCCCTGCCC TACTCGGTGG GCCGGGTGCT GTCCATCTGG

AGGTCTTCTG TTCTCTTCCC TACTCGGTGG GCCGAGTGCT ATCCATTTGG

AGGTCTTCTG TTCTCTGCCC TACTCGGTGG GCCGAGTGCT CTCCATTTGG

AGATCTTCTG CTCTCTGCCC TACTCCGTGG GCCGAGTGCT GTCCATTTGG

AGGTCTTCTG TTCTCTGCCC TACTCTGTGG GCAGAGTGCT GTCCATTTGG

AAGTCTTCTG TTCTCTGCCC TACTCAGTGG GTAGGGTGCT GTCCATTTGG

AAGTCTTCTG TTCCCTGCCC TACTCAGTGG GCAGGGTGCT GTCCATTTGG

GCCTCTTCCC TTCTCTGCCC TACTCAGTGG GCAGGGTACT GTCCATATGG

ATCTCTTCTC TTCACTGCCC TACTCCCTGG GAAGGGTCCT CTCCATATGG

AGCCAGCATG GTCCTTCTGT CTCCTTCGAA ATCTCTTCTC TTCATTCCCC

AGCCAGCATG GTCCTTCTGT CTCCTTCGAA ATCTCTTCTC TTCATTCCCC

AGCCAGCATG GGCCTTCTGC CTCCTTCGAA ATCTCTTCTC TTCATTCCAC

AGCCAGCATG GGCCTTCTGC CTCCTTCGAA ATCTCTTCTC TTCATTCCAC

AGCCACCATG GGCCTTCTGC ATCCTTCGAA ATCTCTTCTC TTCATTCCAC

AGTCAGCATG GGCCTTCTGC CTCCTTCGAA ATCTCTGCTC TTCATTCTAC

AGCCAGCACG GACCCTCT-- -ACCTTCAAA TTGCCAGCTC TTCATTCTCC

AGCCAGCACG GGCCCTCT-- -ACCTTCAAA TTGCCAGCTC TTCATTCCAC

AGCCAGCATG GACCTTCTGC CTCCTTTGAA ATCTCTGCTT TTCAGTCCAC

AGCCAGCATG GGCCTTCCGC CCCCTTGGAG ATCTCTGCCC TTCCACCCTC

AGCCAGCATG GGCCTTCTGC CCCCTTCGAA ATCTCTACTC TTCATTCCGC

AGCCAGCATG GACCTTCTGC CCCCTTCGAA ATCTCTGCTC TTCATTCCAC

AGCCAGCATG GACCTTCTTC CCCCTTCAAA ATCTCTTCTC TCCATTCCAC

AGCCAGCATG GGCCCTCTG- --CCTTCGAA GTCACTGCTC TTCATTCCAA

AGCCAGCATG GTCCTTCTAC CCCCCTCGAA ATTTCTGCTC TTCATTCCAG

AGCCAGCATG GCCCTTCTGC CCCCCTGGAA ATCTCTGCTC TTCATTCTAA

AGCCAGCATG GTCCTTCTGC CCCCTTCGAA ATCACTACTC TCCATTCCAA

AGCCAGCATG GTCCTCCTAC CCCCTTCAAA ATCACTACTC TCCATTCCAA

TCACTGCAAG CGGCAACCAA GTCTGGGCAC CACAAGCAGC CACACCATGT

TCACTGCAAG CGGCAACCAA GTCTGGGCAC CACAAGCAGC CACACCATGT

TCACTGCAAG CGGCAACCAA GTCTGGGTAC CACAAGCAGC CACACCATGT

TCACTGCAAG CGGCAACCAA GTCTGGGTAC CACAAGCA-- ----------

TCACAGCAAG CGGCAATCAA GTCTGGGCAC CACAAGCAGC CACACCATGT

TCCCAGCAAG CAGCAAACAA GTTTGGGCAC TGTGAGCAGC CACACCATAT

TCATAGCAAG CAGCAGGGGA GCCTGAGCGC CCTGAGCAGC CACACCACCA

CCCTAGCAAG CAGCAGGGGA GCCTGAACGC CCTGAGCAGC CACACCACCA

TCGCAGCAAG GAGCAGCCAA GTCTGGACAC CACAAGCAGC CACACCATGT

TCACAGCAAG CGGCAGTCAC CTCTGGGCGC CATGAGCAGC CGCACCGTGT

TCACAGCAAG CGGCAGCCAG CTCTAAGCAC CATAAGCAGC CACACCATGT

TCACAGCAAG CGGCAGCCAA CTCTGGGCAC CACAAGCAGC CACACCATGT

TCACAGCGAG GAGCAGCCAC CTCTGAGCAC CACAAGCAGC CACACCATGT

TCACAGCACG CGGCAGCCAA GTCTGGGCAC CACAAGCAGC CACGCCATGT

TCACAGCAAG TGgcagccaa gcctgGGCAC AACAAGCAGC CACAACATGT

TTACAGCAAG TGGCAGCCAA GTCTGGGCCC TGCAAGCAGC CACACCCTGT

ACACAGCAAG TGGCCGCCAA CTCTGGGCAT CAGGAACAGC CATGCCATCT

TCGCAGCGAG TGGCAGCCAA GTCTGACCGC CGAGAACAGC TTTGTTGTGT

TACCATATGT GCCTCTTCCA GGCATGGAAG CTACATATAA CACCAGCGGC

TACCATATGT GCCTCTTCCA GGCATGGAAG CTACATATAA CACCAGCGGC

TACCATATGT GCCTCTTCCA GGCATGGAAG CTACATATAA CACCAGCGGC

---------- ---------- ---------- ---------- ----------

TACCATATGT GCCTCTTCCA GGCATGGAAG CTACATATAA CACCAGCGGC

TACCATATGT GCCTCTCCCA GGCGTGGAAG CTTCATATAA CACCAGAGGC

TACCACATGT GCCTCTTCCA GGCATGGAGG CTGCCCACAA CACCAACAGC

TACCAAATGT GCCTCTTCCA GGCATGGGAG CTGCCCACAC CACCAACAGC

TACCATATGT GCCTCTTCCA GGCGTGGAAG CTCCATACAG CACCAGCGGC

TACCGTCCAT GCCTCTTCCG GGCCTGGAAG CTGCTTACAG TGCCGGCGGC

TACCATATGT GCCTCTTCCA GGCCTGGAAG CTACTTACAA CACCAGTGGC

TACCATATGT GCCTCTTCCG GGCATGGAAG CTGCTTACAA CACCAGCGGC

TACCATATGT GCCTCTTCCA GGCATGGAAG CTACTTTCAC CACCAGCGGC

TACCATATGT GCCTCTTCCA GGCATGGAAG CTACTTGTAG CGCCAGAGGC

TACCATATGT GCCTCTTCCA GGCATGGAAG CTGCATATAG CACCAGAGGC

TACCATATGT GCCTCTTCCA GGCATAGAAG CTGCATGTAG CACCAGAAGC

TACCACATGT GCCTCTGTTG GGCATGGAAG CTACCACTAG GACCAGAGGC

TACCACATGT GCCTCCCCCA GGCATAGATG CTTC---CAG CACCGAAGGC

AGTCAGACGA GGCTGGAGCC TCCATTCCCT GCCTTGGTAC CAAAGTCTTG

AGTCAGACAA GGCTGGAGCC TCCATTCCCT GCCTTGGTAC CAAAGTCTTG

AGTCAAACAA GGCTGGAGCC TCCATTCCCT GCCTTGGTAC CAAAGTCTTG

---------- ---------- ---------- ---------- ----------

AGTCAGACGA GGTTGGAGCC TCCATTCCCT GCCTTGGTAC CAAAGTCTTG

AGTCAGATGA GGTTAGAACC TACATTCTCT GCCTTGGTAC CAAGGTCTTG

AGTCACCTGA GGCTAGAGCC TGTGTTTCCT GCCTTGGTGC CAAAGTCTTG

AGTCACCTGA GGCTAGAGCC TGTGTTTCCT GCCTTGGTGC CAAAGTCTTG

AACCAGATGA GGCTGGAGCC TTCGTTTGCT GCCTTGGTAC CAAAGTCTTG

AGTCACATGA GGCTAGAGCC TCCATTCCCT GCCTTGGTAC CAAAGTCTCA

AATCAGATGA GACTGGAGCC TCCATTCCCT GCCTTGGTAC CAAAGTCTTG

AGTCAGATGA GGCTTGAACC TCCATTTCCT GCCTTGGTAC CAAAGTCTTG

AGTCAGATGA GGTTAGAGTC TCCATTTCCT GCCTTGGTAC CAAAGTCTTG

AGTCAGACGA GACTAGAGCC TGCCCTCTCT GCCTTGGTAC CAAAGTCCTG

AGTGAGATGA GGCTTGAGCC TCCATTCCCT ACCTTGGTAC CAAAGTCTTG

AACCCGATAA GGCTAGAGCC TTCCTTCTCT GCCTTGGTAC CAAAGCCTTG

AGTCCAGTTA GGCCGGGACC TCCTTGCTCT GCTTTGGCAC CAAAGTCCTG

AATAACATGA GGCTGGAACC TCCATTCTCT GCTCTGCTAC CAAAGTCGTG

CTTGGTAGCA GAATCAGCTG TCAGCAAGCT CCTGCTTTCA GCCTCTGAGT

CTTGGTAGCA GAATCAGCTG TCAGCAAGCT CCTGCTTTCA GCCTCTGAGT

CTTGGTAGCA GAATCAGCTG TCAGCAAGCT CCTGCTTTCA GCCTCTGAGT

---------- ---------- ---------- ---------- ----------

CTTGGTAGCC GAATCAGCTG TCAGCAAGCT CCTGCTTTCC GCCTCTGAGT

CTTGGTAACA GAATCAGCTG TGAGCAAGCT CCTGCTTTCA GCCTCTGACT

CTTGGTAACA GAACCCTCTG TCAGCACGCT CCTGCTCTCG GCCTCGGAGC

CTTGGTAACA GAAGCCGCTG TCAGCACACT GCTGCTCTCT GCCTCTGAGC

CTTGGTCACG GAGCCAGCTG TCAGCCAACT CCTGCTTTCA GCCTCCGAGC

CCTGGTAACA GACTCAGCCG TCAGCAAGCT CCTGTTCTCA GCCTCAGAGT

CTTGGTAGCA GATTCAGCTG TCAGCAAGCT CCTGCTTTCA GCCTCCGAGT

CTTGGTAGCA GACTCAGCTG TCAGCAAGCT CCTGCTGTCA GCCTCTGAAT

CTTGGTAACA GACTCGACTG TCAGCAAACT CCTGCTTTCA ACCTCTGAGT

TTTGGTAACA GAATCAGCTG TCAGCAAGCT CCTCCTTTCA GCCTCTGAGT

CTTGGTAACA GAAACAGCTG TCAGCAAGCT CCTGCTTTCA GCCTCTGAGT

CGTGGTAACA GAGACAGCTG TCAGCAACCT ACTGCTTTCA GCCTCTGCGT

CTTAGCCTTT GAACCAGCGG TCAGTGTGCT CCGACTTTCA GCCTCTGACT

TTTAGCTCCA GAACCAACTT TAATAACACA CAGACTTTCA GCCTCCAACT

TCCAGGTTCG TGGATTGGAT GAGGATGGTG TGAAAGCAGC ATGCCCCTGC

TCCAGGTTCC TGGATTGGAT GAGGATGGTG TGAAAGCAGC ATGCCCCTGC

TCCAGGTTCC TGGATTGGAT GAGGATGGTG TGAAAGCAGC ATGCCCCTGC

---------- ---------- ---------- ---------- ----------

TCCAAGTTCC TGGGTTGGAT GAGGATGATG TGACAGCAGC ATGCCCCTGC

TCCAAGTTCC TGGATTTGAT GAGGATGGTG TGACAGTAGC ATGTCCCTGC

TCCAGGTTCC TGCGTTTGAT GAGGATGGTG TGTCAGCAGC TTGCCCCCGA

TCGCAGTTCC TGGCTGTGAT GAGGATGGTG TGTCAGCAGC CTGCCCCCGA

TCCAAGTCCC TGCATTTGCC GAGGATGGGG TGACAGCAGC GTGCCCACGA

TCCCGGTTCC CGGGTTTGAC GAGGACGGTG TGACGGCCGC CTGCCCCCGA

TCCAGGTTCC TGAATTTGAT GAGGATAGTG TGGCAGCAGC ATGCCCCCAT

TCCAGGTTCC TGGGTTTGAT GAAGATGGTG TGACAGCAGC ATGCCCCCAT

TCCAGGTTCC TGAGCTTGAT GAGGATGGGG TGAAAGCAGC ATGCCCCCGA

TCCAGGTTCC TGGGTTTGAT GA---TGATG TTACACAAGT ATGTCCCCGA

TCCAGGTTCC TGGATTTGAT GAGGATGGCG TGACAGCAGT GTGCCCCCGA

TCCAGGTCTC TGGATTCGAT GAGGATGGTA TGACAGCAGT GTGCCCCCGA

ACCAGATTCC TGCTTTAAAA GAGGGGAGAG TCCCCAGTGT GTGCTCCAAT

TCCAGGTTCC TCCATTTGAC GAGGATGTCT CCACTCCTGA GTGCCCCGTG

CCACAGAGCA GCCCCCCAGA ACAGAAAGAG GCTGAGCCAG AGAAGAGGCC

CCACAGAGCA GCCCCCCAGA ACAGAAAGAG GCTGAGCCAG AGAAGAGGCC

CCACAGAGCA GCCCTCCAGA ACAGAAAGAG GCTGAGCCAG AGAAGAGGCC

---------- ---------- ---------- ---------- ----------

CCACAGAGCA GCCCTCCAGA ACAGAAAGAG GCTGAGCCAG AGAAGAGGCC

CCACAGAGCA GCCCTCCAGA ACAGAAAGAG GCTGAGCCAG AGAAGAGGCC

CCACAGAGCA GTCCGGCACA GCAGGAGGAG GCCGAGCCAG AGAAGAGACC

CCACAGAGCA GCCCTGCACA GCAGAAAGAG GCTGAGCCAG AGAAGAGACC

CCGCAGAGCA GCCCTCCAGA GCAGAAGGAG ---------- ----------

CCACAGGGCA GCCCTCCAGA CCAGAAAGAG GCTGAGCTGG AGAAGAGGCC

CCACAGAGCA GCCCTCCAGA ACAGAAGAAG GCTGAGCCAG AGAAGAGGCC

CCACAGAGTA GCCCTCCAGA ACAGAAAGAG GCTGAGCCAG AGAAGAGGCC

CCACAGAGCA CCCCTCCGGA GCAGAAAGAG GCAGAGCCAG AGAAGAGGCC

CCACAGAGCA GCCCTGCAGA ACAGAAAGAG CCTGAGCCAG AGAAGAGGCC

CCACAGAGCA GCCATCCAGA GCAGAAAGAG GCTGAGCCAG AGAAGAGGCC

CCCCAGAGCA GTCCTCCAGA ACAGAAAGAG ---------- ----------

CAGCATAGTA GTGCCAC--- TCAGAAAGAG ACTGAACCAG AGAAGCGGCC

CCACAAAATG GTACCC---A ACAGAAAGAG ATGGAGCCCA AGAAGAAGCC

AAAGAAAGTC TCACAGATTC GCATCCGGAA AACCATTCCT AGGCCAGATC

AAAGAAAGTC TCACAGATTC GCATCCGGAA AACCATTCCT AGGCCAGATC

AAAGAAAGTC TCACAGATTC GCATCCGGAA AACCATTCCT AGACCAGATC

---------- ---------- ---------- ---------- ----------

AAAGAAAGTC TCACAGATTC GCATCCGGAA AACTATTCCT AGGCCAGATC

AAAGAAAGTC TCGCAGATTC GCATCCGGAA AACCATTCCT AGGCCAGATC

AAAGAAAGTC TCACAGATCC GCATCCGGAA AACCATTCCT AAACCAGATC

AAAGAAAGTC TCACAGATCC GCATCCGGAA AACCATTCCT AAACCAGATC

---------- ---------- ---------- ---------- ----------

GAAGAAAGTC TCACAGATTC GCATCCGGAA AACCATTCCT AAGCCAGACC

AAAGAAAGTC TCACAGATTC GTATCCGGAA AACCATTCCT AAGCCAGACC

AAAGAAAGTC TCACAGATTC GCATCCGGAA AACCATTCCT AAGCCAGACC

AAAGAAAGTG TCACAGATTC GAATCCGGAA AACCATCCCT AAGCCAGACC

AAAGAAAGTC TCACAGATAC GCATCCGGAA AACCATTCCT AAGCCTGACC

AAAGAAAGTC TCACAGATTC GCATCCGGAA AACTATTCCT AAGCCAGACC

---------- ---------- ---------- ---------- ----------

AAAAAAAGTC TCACAGATTC GAATCCGGAA AACTATTCCT AGGCCAGATC

CAAGAAGGTC TCTCAAATCC GGATCCGGAA GACCGTTCCT AAGCCAGACC

CTAATCTTAC CCCCATGGGC CTTCCTCGAC CCAAAAGGAG AAGGTATTAC

CTAATCTTAC CCCCATGGGC CTTCCTCGAC CCAAAAGGAG AAGGTATTAC

CTAATCTTAC CCCCATGGGC CTTCCTCGAC CCAAAAGGAG aAGGTATTAC

---------- ---------- ---------- -------GAG ----------

CTAATCTTAC CCCCATGGGC CTTCCTCGAC CCAAAAGGAG AAGGTATTAC

CTAATCTTAC TCCCATGGGC CTTCCTCGAC CCAAAAG--- ----------

CCAACCTTAC CCCAATGGGT CTGCCTCGGC CCAAAAGGAG GAGATACTAC

CCAACCTTAC CCCAATGGGC CTGCCTCGGC CCAAAAGGAG GAGATACTAC

---------- ---------- ---------- -------GAG ---GTACTAC

CTAACCTCAC GCCCATGGGC CTTCCTCGAC CCAAAAGGAG GAGGTATTAC

CTAACCTTAC CCCCATGGGC CTTCCTCGAC CCAAAAGGAG GAGATATTAC

CTAATCTTAC CCCCATGGGC CTTCCTCGAC CCAAAAGGAG GAGATATTAC

CTAACCTTAC CCCGATGGGC CTTCCACGAC CTAAAAGG-- GAGATATTAC

CTAATCTTAC CCCCATGGGC CTTCCTCGAC CCAAAAG-AG AAGATACTAC

CTAACCTTAC CCCCATGGGC CTCCCTCGAC CCAAAAGGAG ---ATACTAC

---------- ---------- ---------- -------GAG ---ATATTAC

CCAACCTTAC CCCTATGGGA CTGCCTCGAC CTAAAAGGAG AAGATATTTC

CGAACCTCAC CCCCATGGGG CTGCCTCGAC CCAAAAGG-- AAGATATTTC

CTGTACAACC TGATGTTCCA GACGTGGAAG ACCTATGTGC GTCAGCAGCA

CTGTATAACC TGATGTTCCA GACCTGGAAG ACCTACGTGC GTCAGCAGCA

CTGTATAACC TGATGTTCCA GACCTGGAAG ACCTACGTGC GTCAGCAGCA

---------- ---------- ---------- ---------- ----------

CTGTACAACC TGATGTTCCA GACCTGGAAG ACCTACGTGC ATCAGCAGCA

---------- ---------- ---------- ---------- ----------

TTGTACAACC TGATTTTCCA GAACTGGAAG TCCTTTGTGT ACCAGCAACG

TTGTACAACC TGATTTTCCA GAACTGGAAG ACCTTTGTGC ACCAGCAACG

TTGTACAACC TGATGTTCCA GAACTGGAAG ACCTATGTGC ACCAGCAGCA

TTGTACAGCC TGATGTTCCA GACCTGGAAG ACCTACGTGT ATCAGCAACG

TTGTACAACC TGATGTTCCA GACTTGGAGG ACCTATGTGT ATCAGCAGCA

TTGTACAATC TGATGTTCCA GACCTGGAAG ACCTTCGTGC ATCAGCAGCA

TTGTACAACC TGATGTTCCA GACTTGGAAG CTCTATGTGC TGCAGCAGCA

TTGTACAACC TGATGTTCCA GACCTGGAAG ATGTTTGTGA ATGAGCAGCG

TTGTACAGTT TGATGTTCCA GACCTGGAAG ACCTACGTGC ATCAGCGGCA

CTGTACAACC TGATGTTCCA GACCTGGCAG ACATATGTCC GTCAGCAGCA

TTGTACAACC TGATGTTCCA AACCTGGCAG ACCTACGTTC ATCAGCGACG

TTGTACAACC TGATGTTCCA GTCCTGGAAG AAGTATATCC ACCAGCGGCG

GGAGATGAGG AACAAGTACA TTAGAGCCGA GGTTCATGAT GCAAAGCAAA

GGAGATGAGG AACAAGTACA TTAGAGCCGA GGTTCATGAT GCAAAGCAAA

GGAGATGAGG AACAAGTACA CTAGAGCCGA GGTTCATGAT GCAAAGCAAA

---------- ---------- ---------- --------AT GCAAAGCAAA

GGAGATGAGG AGCAAGTACA TTAGAGCCGA GGTTCACGAT GCAAAGCAAA

---------- ---------- ---------- ---------- ----------

GGAGATGAGG AAGAGGTTCC AAAGAGCCGA GCATCATGAT ATGAAACAGA

GGAGATGAGG AAGAGGTTCC GAATAGCTGA GCATCATGAT ACGAAGCAGA

GGAGATGAGG AACAAGTACA TCAGAGCAGA GGATCATGAT GCAAAGCAAA

GGAGATGAGG AGCAAGTACC TGAGAGCCGA GAATCATGAT GCAAAGCGAA

GGAGATGAGG AACAAGTACA TTAGAGCTGA GAATCATGAT GCAAAGCAAA

GGAGACAAGG AACAAGTACT TGAAAGCCGA GAATCATGAT GTAAAGCAAA

GGAGACGAGG AACAAATACG TGAGAGCTGA GGACCATGAT GCAAAACAGA

GGAGATGAGG AACAAGTACC TGAGAGCCGA GGCATACGAT GCAAAGCAAA

GGAGATGAGG AACAAGTACA TGAGAGCTGA GGATTATGAT GCAAAGCGAA

AGAGAGAAGG AGGAAGTACT TGAGGCCCGA GGAGT----- ----------

GGCCAAGAAG AACAAGTACC GCAGAGCCGA GGACCACGCG GCCAAGCATA

AGCGAAGAGG AGCAAGTGCC ACAGAGCAGA AAGCCACGCG GCAAAACAGA

AGATGCGACA GGCCTGGAAG TCCTGGTTGA TCTACGTGGT TGTTCGTAGG

AGATGCGACA GGCCTGGAAG TCCTGGTTGA TCTACGTGGT TGTTCGTAGG

AGATGCGACA GGCCTGGAAG TCCTGGTTGA TCTACGTGGT TGTTCGTAGG

AGATGCGACA GGCCTGGAAG TCCTGGTTGA TCTACGTGGT TGTTCGTAGG

AGATGCGACA AGCCTGGAAG TCTTGGTTGA TCTACGTGGT TTTTCGTAGG

---------- ---------- ---------- ---------- ----------

AGATGCGCCA CGCCTGGAAG TCCTGGTTAA TATACATGGT TGCTCGTAGG

AGATGTGCCA AGCCTGGAAG TCCTGGTTGA TATACATGGT TTCTCGTAGG

AGATGCGACA GGCCTGGAAG TCCTGGTTGA TCTATGTGAT TTTTCGGCAA

ATATGCGACG GGCCTGGAAG TCCTGGTTGG TCTACGTGGT TTTCCGTAGG

AGATGCGGCA GGCCTGGAAG TCCTGGCTGA TCTACGTGGT TTTTCGTAGA

AGATGCGACA GGCCTGGAAG TCCTGGTTGA TCTATGTGGT TTTTCGAAGA

AGATGCGACA CGCCTGGAAA TCCTGGTTGA TCTACGTGGT TTTTCGTAGA

AGATGCGCTG GGCCTGGAAG TCCTGGTTGA TCTACGTGGT TGTTGGCAGG

AGATTCGCCA AGCCTGGAAG TCCTGGTTGA TCTACGTGGT GGTTCGTAGG

---------- ---------- ---------- ---------- ----------

AGCTGCAGCT GGCCTGGAAA CACTGGCTCA TCTACGTGGA CGTTCGTCGG

GGATTCACCG GGCATGGCAG CACTGGCTGA TCTATGTGGA AGTTCGTAGG

ACCAAACTTC AGATGCAGAC TACAGCTCTG GAGTTTAGGC AACGGATTAT

ACCAAACTTC AGATGCAGAC TACGGCTCTG GAGTTTAGGC AACGGATTAT

ACCAAACTTC AGATGCAGAC TACGGCTCTG GAGTTTAGGC AACGGAGTAT

ACCAAACTTC AGATGCAGAC TACGGCTCTG GAGTTTAGGC AACGGAGTAT

ACCAAACTTC AGATGCAGAC TATGGCTCTG GAGTTTAGGC AACGGAGTAT

---------- ---------- ---------- ---------- ----------

ACCAAATTTC GCATGAAGAG CACTGCTCTA GAGTTTCGGA GGCGGAGTGT

ACCAAACTTC ACATGAAGAC TACTGCTCTA GAGTTTAGGA GGCAGAGTGT

ACCAAACTTG AGATGCAGAG CACGGCTGTG GAGTTCAGGC AGCAGAGTAT

ACCAAACTTG AGATGCAGAC CACTGCCCTG GAGTTCAGGC AGCGGAGTAT

ACCAAACTTC ATATGCAGAC CACCGCCCTG GAGTTTAGGC AGCAGAGTAT

ACCAAACTTC AGATGCAGAC CAGCGCCCTG GAGTTTAGAC AGCAGAGAAT

ACCAAACATG AGATGCAGAA CACCGCCCTA GAGTTTAGGC AGCAGAATAT

ACCAAACTTC AGATGCAGGC CACCGCCCTG GAGTTCAGGC AGCGGAGTAT

ACCAAACTTC AGATGCAGAC TGCCGCCCTG GAGTTCCGGC AGCGAAATAT

---------- ---------- ---------- ---------- ----------

ACCAAACGGG GGATGCGTGC CTTGGCGCTG GAGTTCCAGG AGCAGAGCAT

ACCAAAGCTC AGATGCAGAC CGCTTCACTG GCATTCAGGA AGCAGAGCAC

CTTACGGGTG TGGTGGAGCA CGTGGAGGCA GCGACTAGGA CAGGTCCGTG

CTTACGGGTG TGGTGGAGCA CGTGGAGGCA GCGACTAGGA CAGGTCCGTG

CTTAAGGGTG TGGTGGAGCA TGTGGAGGCA GCAACTAGGA CAGGTCCGTG

CTTAAGGGTG TGGTGGAGCA TGTGGAGGCA GCAACTAGGA CAGGTCCGTG

CTTACG---- ---------- ---------- ---------- ----------

---------- ---------- ---------- ---------- ----------

CTTATGTTTC TGGTGGAACA AGTGGAGGTG GCGACTAGGA CGAGTCCACG

CTTATGTTTC TGGTGGAGCA AGTGGAGGTG GCGACTAGGA CAAGCCCATG

TTTACGGGTG TGCTGGACAG AGTGGAGGCG GCGACTGGGA CAGGCCTGCC

TCTGTGGGTG TGGTGGAGTG CGTGGAGATG GCGACTGGGC CAGGTCCGCC

TTTATGGCTC TGGTGGAGGC GGTGGAGGCA ACAACTGGGA CAGGTCTGTG

TTTGTGGGTG TGGTGGAGTG AGTGGAGGCG GCGACTGGGA CAGGCCCGCG

TTTGTG---- ---------- ---------- ---------- ----------

CCTGTGGATG TGGTGGAGTG CGTGGAGGCG GCGACTGGGG TGGGTCCGCG

CTTGTGGGTA TGGTGGAGCG AGTGGAGGAA GTGCCTACAG CAGGTCCGTG

---------- ---------- ---------- ---------- ----------

GCTGCGAGTG CCGTGGAGAG TGTGGAGGCA GCAGGTGCAG AAGGTCCGTG

TCTACGGGTG GCATGGAGTG TCTGGACGCG GCAACTGCAA GAGACCCAAA

TGAGCCGTGC CCTCCATGCC TCTGCTTTGA AGCACAGGGC CCTGAGCCTC

TGAGCCGTGC CCTCCATGCC TCTGCTTTGA AGCACAGGGC CCTGAGCCTC

TGAGCCGTGC CCTCCATGCC TCTGCTGTGA AGCACAGGGC CCTGAGCCTC

TGAGCCGTGC CCTCCATGCC TCTGCTGTGA AGCACAGGGC CCTGAGCCTC

---------- ---------- ---------- ---------- ----------

---------- ---------- ---------- ---------- ----------

CAGAGCAAGC TCTCCACGCA GCGGCCGTGA AGCACAGGGC CCTGAGCCTC

CAGAGCACGC TCTCCATGCA GTGGCTGTGA AGCACAGGGC CCTGAGCCTC

TGAGCCGTGC CCTCCATGCG TCAGCTGTGA AGCACAGGGC CCTGAGCCTC

TAGGCCGAGC CCTCCAGGCC ACAGCCGTGA AGCGCTGGGC CCAGAGCCTC

TGGGCCGTGC CCTGCATGCG TCAGCCGTGA AGCACAGGGC CCTGAGCCTC

TGGGCCGTGC TCTCCATGCC TCAGCTGTCA GGCACAGGGC CCTGAGCCTC

---------- ---------- ---------- ---------- ----------

TGGGCCGTGC CCTCCACGTC TCAGCGGTGA AGCACAGGGC CTTGGCCCTC

TGGGCCGTGC CCTCTACGCC TCAGCTATGA AGCACAGAGC CGTGAGCCTC

---------- ---------- ---------- ---------- ----------

TTGCCCATGT GATGGACACC TTGGCCTTGC AGCACTGGGC CATCAGCCTG

CAAGGTATAA ACTGGATGCC TCGGCTCTCC AGCACTGGGC CCTGAGCCTT

CAGGTGCAGG CTTGGTCACA GTGGCGGGAA CAGCTCCTGT ATGTCCAGAA

CAGGTGCAGG CTTGGTCACA GTGGCGGGAA CAGCTCCTGT ATGTCCAGAA

CAGCTGCAGG CTTGGTCACA GTGGCGGGAA CAGCTCCTGT ATGTCCAGAA

CAGCGGCAGG CTTGGTCACA GTGGCGGGAA CAGCTCCTGT ATGTCCAGAA

---------G CTTGGTCACA ATGGTGGGAG CAGCTCCTGT ATGCCCAGAA

---------- ---------- ---------- ---------- ----------

CAGCTGCAGG CTTGGCTGCG ATGGCAAGAA CAGCTTCTCA TTAGCCAGCG

CAGCTGCAGG GTTGGCTGCG ATGGCAAGAA CAGCTTCTAA TTAGCCAGCG

CAGCTGCAGG CTTGGTGTCA GTGGCAAGAC CAGCTCCTGC GTAGCCAGAG

CAGCTACAGG CTTGGTCACG GTGGCAGGAG CAGCTCCTCT TTGTCCAGAG

CAGCAGCAGG CTTGGTCACA GTGGTGGGAA CAGCTCCTGC ATGTCCAGAG

CAGCTGCAGG CTTGGTCACG GTGGCGAGAA CAGCTCCTGT GTGTCCAGAG

---------G TTTGGTCCCG GTGGCAGGAA CAACTGCTGC ATGTCCGGAG

CAGCTGCAGG CTTGGTCCCG GTGGCGGGGA CAGCTCCTGA ATGCCCAGAG

CAGCTGCAGG CTTGGTCGCG GTGGCAGGAA CAGCTCCTGT GTATGCAGAG

---------- ---------- ---------- ---------- ----------

AAGTTCTGGG CCTGGGCTCG GTGGCAGGAG CAGTTCCTAA GCGCCCAGAT

CAGTTTCGGG CGTTTTTCCA GTGGCGGCAC CTGTTCCTGC ACGTGCAAGC

GGAGAAACAA AAGGTTGTCT CTGCAGTGAA ACATCATCAG CACTGGCAAA

GGAGAAACAG AAGGTTGTCT CTGCAGTGAA ACATCATCAG CACTGGCAAA

GGAGAAACAG AAGGTCGTCT CCGCGGTGAA ACACCATCGG CACTGGCAAA

GGAGAAACAG AAGGTCGTCT CCGCGGTGAA ACACCATCGG CACTGGCAAA

GGAGAAACAG AAGGTTGCCT CTGCAGTGAA ACACCGTCAG CACTGGCAAA

---------- ---------- ---------- ---------- ----------

AGAGAGAAGG AAGGTGGCCA CTGCTGTGAG ACACCATCAG CGCTGGCAGA

AGATAGGAGG AAGGAGGCCA CTGCTGTGCA ACACTATCAG CACTGGCAGA

GGAGAGACAG AAGGCAGTCT TCGCAGTGAA GCACCATCAG GATCAACAAA

GGAGAGATGG AAGACGGTCT CCGCAGTGAC ACACCTTCAG CGTCGGCAGC

GGAAAGACAG AAGATGGTCT CTGCAGTGAA ACACCATCAG CACTGGCAAA

GGAGAGATGG CAGGTCGTCT CTGCAGTGAA ACACCATCAG CACTGGCAAA

GCAGAGGCGA AAGGTGATCT TGGCAGTGAA GCACCATCAG CACTGGCTAA

GGACAGACAG AAAATGGTCT CTGCCAAGGA ACACCAGGAG CACTGGCAAA

GGACAGACAT AAGGTGGCCT GTGCGTTGAA GCACCATGAG TACTGGCAAA

---------- ---------- ---------- ---------- ----------

TGCTCGGAAG AAGGAAACCA AGGCAGTGAG GCACTGCGAA GGCCGGGAGC

TAATAGGCAG AAGGAGGTCC AGGCAGTGAG ACACCATCAG CATGGGGAGA

AACGGAGATT TCTAAAGGCC TGGCTTGAAT ACCTGCAAGT CCGCAGAGTG

AACGGAGATT TCTAAAGGCC TGGCTTGAAT ACCTGCAAGT CCGCAGAGTG

AACAGAGATT TCTGAAGGCC TGGCTTGAAT ACCTGCAAGT TCGCAGAGTG

AACAGAGATT TCTGAAGGCC TGGCTTGAAT ACCTGCAAGT TCGCAGAGTG

AGCGGAGGTT TCTAAAGGCC TGGCTTGCAT ACCTGCAAAT CCGCAGAGTG

---------- ---------- ---------- ---------- ----------

AGCAGAGAAC TTTGAAGGCT TGGCTTAAAT ACCTCCGTAT CTGCAGGGTG

AGCAGAGATC TCTGAAGGCT TGGCTTAAAT ACCTCCAAAT CTGCAGGGTG

AGCGGAGATT CTTAAAGGCC TGGCTTGAAT ACCTGGATGT CCGCAGAGCC

AGTGGAGAGC CCTCAGGGCC TGGCTTGAGT ACCTGCAGCT GCGCAGAGAG

AGTGGAAATC CCTGAAGGCC TGGCTCAAAT ATGTGCAGGT CCGCAGAGTG

AGTGGAGATC CCTGAAGGCC TGGCTTGAAT ACCTGCAGGG CCGTCGAGTG

AGTGGAAATC CCTGAAGGCC TGGCTGCAGC ACCTGCGGAT CCGCAGGGTG

AGCGGAGATC CCTGCAGGCC TGGCTGGAAT ATCTGCAACT CCGCAGAACG

AGCGGAGCTC CCTGAGGGCT TGGTTTGAAT ATCTGCAGCT CCGAAGAGTG

---------- ---------- ---------- ---------- ----------

GATGGAGGGC CCTGCGGGCC TGGCGGGGCT ACCTGCAGGG CCGCAGAGAG

GGCGAAAGTT CATGAAGACC TGGCTGGTAT ATCTGCATCA CCGCAGAGAG

AAGAGACAGC AGAATGAGTA CATGCTGCTG TGTGCAGAAG AAGCTGCCCA

AAGAGACAGC AGGATGAGTA CATGCTGCTG TGTGCAGAAG AAGCTGCCCA

AAGAGACAGC AGAATGAGTA TATGCTGCTG TGTGCAGAAG AAGCTGCCCA

AAGAGACAGC AGAATGAGTA TATGCTGCTG TGTGCAGAAG AAGCTGCCCG

AAGAGACAGC AGAACGAGTA CATGCTGCTG TGTGCAGAAG AAGCTGCCCA

---------- ---------- ---------- ---------- ----------

AAGAGATGGC AGAATGAGTA CATGCTGCTA CAGGAAGAAG AGGCTGCCCA

AAGAGATGGC AGAATGAGTA CATGTTGCTA CAGGCAGAAG AGGCTGCCCA

AAGCGACAGA AGAATG--TA CGTGCTGCTC TGTGCAGAGG AAGCAGCCCT

AAGAGGCTGC GGAACCAGTA CATGCTGCTG TGCGCAGAGG ACGCTGAGCG

AAGAGGCAGC TGAACGAGTA TGTGCTGCTG TGTGCCGAAG AAGCGGCCCA

AAGAGACAGC GGAATGAGTA TGTGCTGCTG TGTGTCGAAG GAGCTGCCCA

AAGAGGCAGT GGAGAGAGT- ---------- ---------- ----------

AGGAGGCAGC AGAAAGCGTA TGTGCTGCTG TGCGCAGAAG CAGCTGCCCA

AAGAGACAGA GGAACG---A TGTGCTGATA CGTGCAGAAG AAGCTGCCCA

---------- ---------- ---------- ---------- ----------

AAGCGCCGCC AGGACCAATA CATGCTGCTC CGGGCCGAGC AGGCGGCTTT

AAGAGACGTC AAGGCCAGTA TATTGCACTT TGTGCCGAAG AAGCTGCCCG

GTTTGAGATG GCAGAAGAGC ACCACAGGCA CAGCCAGCTG CTGCTGCACA

GTTTGAGATG GCAGAAGAGC ACCACAGGCA CAGCCAGCTG CTGCTGCACA

GTGTGAGATG GCAGAAGAGC ACCACAGGCA CAGCCAGCTG CTGCTGCACA

GTGTGAGATG GCAGAAGAGC ACCACAGGCA CAGCCAGCTG ----------

GTGTGAGATG GCGGAAGAGC ACCACAGGCA CAGGCGGCTG CTGCTACACA

---------- ---------- ---------- ---------- ----------

GCGTGAGGCA GCGGCAGAGC ACCACCAACA CTGCCTGCTG CTGCTCCACA

GCGCGAGGCA GCAGCTGAAC ACCGCCAGCA CTACCTGCTG TTGCTTCACA

GGCGGAGGTG GCAGAAGAGC ACCACAGGCT CAGCCTGCTG CTGATGCGCA

GTGGAAGGCG GCAGGAGAAC ACTACAGGCG AGGCCTGCTG CTGCTTCACA

GTGGAAGGTG GCAGAAGATC ACAGTAGGTG CAGCTTGTTG CTACTACACA

GTGGAAGGTG GCGGAAGAGC ACTATAGGCG CAGCTTGCTG CTGCTGCACA

---------- ---------- ---------- ---------- CTGCTGCGTA

ACGGAGGATG GCGGACGAGT ACCACAGGCA CCGCCTCCTG GTGCAGCGCA

GTATAAGATG GCGGAGGAGC ACTGCCGGCA CCGCCTGCTG CTGCTGCGCC

---------- ---------- ---------- ---------- ----------

GCAGGCCATG GCCGAGGAGC ACCACAGGCA GCGGCTGGTG CTTTTGCAGA

ATATACCCTA GCAGAACAGC ATCACAGGCA CCGTTTGCTG TTGTTGCAGA

GGTTCTGGAA CCTCTGGCGG TCTCAGATTG AGCAGAAAAA GGAAAGAGAG

GGTTCTGGAA CCTCTGGCGG TCTCAGATTG AGCAGAAAAA GGAAAGAGAG

GGTTCTGGAA CCTCTGGCAG TCTCAGATTG AGGAGAAAAA GGAAAGAGAG

---------- ---------- ---------- ---------- ----------

GGTTCTGGAA CCTCTGGTGG TCTCAGATTG AGAAGAAAAA GGAAAGAGAG

---------- ---------- ---------- ---------- ----------

GGTTCTGGAA CCTCTGGCAG TCTCGAGTTG AACAGAGGGA GGAGAGGGTA

GGTTCTGGAA CCTCTGGCAG TCTCGAATTG AGCAGAGGGA GGAGAGGGTA

GGTTCTGGGA CCTATGGCAG TGTCGGATTG AGCAGAAGGA GGAAAGGAGA

GGGTCTGGAA CCTCTGGCAG TCGCGGCTGG AGCAGAGGGA GGAAAGAGAG

GGTTCTGGAA CCTCTGGCAG TCTCGGATTG AGCAGAGGGA AGAGAGAGAG

GGTTCTGGAA CCTCTGGCAG TCTCGGATGG AGCAGAGGGA GGAAAGGGAG

GTTTCTGGAA CCTCTGGCAA TCTCGAATTG AGCAGAAGGA GGAGAGAGCG

GGTTCTGGAA CCTCTGGCAG TCTCGGATTG AGCAGAGTGA GGAAAGAGAG

GGTTCTGGGG CTCCTGGCAG TCTCTGCTTG AGCAGAGGGA GGAACGGGAG

---------- ---------- ---------- ---------- ----------

GGTTCTGGCA CCGCTGGTGT GCTCGGGCTG ACCAGAGAGA GGACGAGAAG

GGTTCTGGAA TTGTTGGCAA TATCGGGTAG AACAGAAGGA AGAAGAGAAG

CTGCTCCCCT TACTGCATGC TGCCTGGGAC CACTACAGAA TAGCACTGCT

CTGCTCCCCT TACTGCATGC TGCCTGGGAC CACTACAGGA TAGCACTGCT

CAGCTGCCCT TACTGCATGC TGCCTGGGAC CACTACAGGA TAGCACTGCT

---------- ---------- ---------- --------GA TAGCACTGCT

CAGCTCCCCT TACTGCATGC TGCCTGGGAC CACCACAGGA TGGCATTGCT

---------- ---------- ---------- ---------- ----------

CAGCCCCCCT CACTGCTTGC AGCCCAGAGC CACTACAGGA TGACAGTCCT

CAGACCCCCT CATTGCATGC AGCCCTGAGC CACTACAGGG TGACAGTCCT

CAGCTCCCCG TGGTGTGCGC CGCCAGGGAC CACTACAGGA AGACATTGCA

CAGCTGCCCT TCCTGCGTGC TGCCTGGGAC CACGGCAGAA TAACATTGCT

CAGCTTCCCT CACTGTGTGC TGCCTGGGCC CACTGCAGAA TGACATTGCT

CAGCTCCCCT GCCTGCGAGC TGCCTGGGAG CACTACAGAA TAACATTGCT

CAGCTTTCCT TATTGAGTGC GGCTGAGGAC CACTACAGAA TAACATTGCT

CGGCTCTCCT TGCTGCGTGC TGCCTGGGAT CACTACAGGA CAACATTGCT

CAGCTGTCCT TACTGCGTGC TGCCTGGAAT CACTACAGGA TAACATTGCT

---------- ---------- ---------- ---------- ----------

CAGCTGCCGC AGCTCCTCCA GGCTCACAGC CACTACCGGC AAGCCTTGCT

CAGAGACCCT TGACCTCAGT GGCCCAAAAT CATTTCAGAA AAAAGCTGCT

GTGCAAATGT ATCGAATTGT GGCTACAGTA TACTCAGAAG AGGCGGTACA

GTGCAAATGT ATCAAATTGT GGGTACAGTA TACTCAGAAG AGGCGGTACA

GTGCAAGTGT ATCAAATTGT GGCTACAGTA TACTCAGAAG AGGCGGTACA

GTGCAAGTGT ATCGAATTGT GGCTACAGTA TACTCAGAAG AGGCGGTACA

GTGCAAGTGT GTCAAATGGT GGCTACAGTA TACCCAGAAG AGGCGGTACA

---------- ---------- ---------- ---------- ----------

ACGCAAGTGT GTCAGGGTGT GGCTACAGTA TGTGCACAAG AGAAGATGCC

ACACAAGTGT GTCAGGGTGT GGCTACGGTA TGTGCACAAG AGACAATGGC

ACACAAGTGT ATCAAGTCGT GGCTACAGTA TTCCCAGAAG AGGCGATACA

TCGCAAGTGT TTCCAGCTGT GGTCACAGTC CACCCAGAAG AGGCGATCCC

ACGCAAGTGT ATCAAATCAT GGTTACAGTA TACTCAGAAG AGGCGGTACA

ACACAAGTGT ATCAAATTGT GGCTACAGCA TACTCAGAAG AGGCGATACA

GCACAAGTGT ATCCAGTCGT GGTTACAATA TGCGCAGAAG AAGAGATACA

ACACAAGTGT ATCAAATTGT GGTTAAAGTA TACTCAGAAG CAGCAACACA

ACACAGGTGT TTCAAATTGT GGTTACAGTA CACTCAGAAG AGACGCTACA

---------- ---------- ---------- ---------- ----------

GCAGAAATGC CTTCAGCGGT GGGCACTCAA CGCCCGGGGG AGTCGGCACC

GCATAAGATT TTTGAAGGCT GGTTCCAGAA TGCTCAGAGG AGAAAGTCCA

AGCAGCTGCT ACAGGCCAGA GCGGATGGTC ATTTCCAGCA GAGAGCCCTG

AGCAGCTGCT ACAGGCCAGA GCGGATGGTC ATTTCCAGCA GAGAGCCCTG

AGCAGCGGCT ACAGGCCAGA GCGGATGGTC ATTTCCAGCA GAGAACCCTG

AGCAGCGGCT ACAGGCCAGA GCGGATGGTC ATTTCCAGCA GAGAGCCCTG

AGCAGCTGCT ACAGGCCAGA GCAGATGGGC ATTTCCAGCA GAGAGCCCTG

---------- ---------- ---------- ---------- ----------

TGCAGTCACT GCAGGCCAGA GCACTCGGTC ATTTTCAGCA GCGAGCACTG

AGCAGTTATT GCGGGCCAGA GCAGATGGTC ATTTCCAGCA GCGAGCACTG

AGCAG----- ---------- ---------- ---------- ----------

AGCAGCTGTT GCAGGCCAGA GCAGACGGCC ACTTCCAGCA GAGAGCCCTG

AGCAGCGGCT GCGGTCCAGA GCAGACAGTC ACTTCCAACA GAGAGCCCTG

AGCAGCTGCT GCAGGCCAGA GCAGACAGTC ACTTCCAGCA GAGAGTCCTA

AGCAGGTGCT GCGGAGCCGA GCAGACAAGC ATTTTCAGCA GAGAGCCCTG

AGCAGCTGCT GCAGGCCAGA GCAGATGGTC ATTTCCAGCA GCGAGCCCTG

AGCAGCTGCT GCTGGCCAGA GCGGATGGCC ATTTCCAGCA GAGAGCCCTG

---------- ---------- ---------- ---------- ----------

AACGGATGCA GTACGCCAGA GCCGAAGGTC ACTATCGGGG CAGGAGCCTG

GGCAGCTGCA GTATGCAAAA GCTGAGGCTC ATTTCCAGAG GAGAATCCTG

CCTGCTGCCT TCCACACATG GAACAGACTC TGGCGATGGC GCCACCAGGA

CCTGCTGCCT TCCACACATG GAACAGACTC TGGCGATGGT GCCACCAGGA

CCTGCTGCCT TCCACACGTG GAAGAGACTC TGGCGATGGC GCCAGCAGGA

CCTGCTGCCT TCCACACATG GAAGAGACTC TGGCGATGGC GCCAGCAGGA

CCTGCTGCCT TCCACACATG GAATAGACTC TGGCGAAGCC ACCAGCAGGA

---------- ---------- ---------- ---------- ----------

CCTGCTGCCT TCTACACGTG GTACAGAGTC TGGCGATGGC ACCAGCAGAG

CCTGCTGCCT TCTACACGTG GTACAGAGGC TGGCTATGGC ACCAGCAGAG

---------- ---------- ---------- ---------- ----------

CCTGCAGCCT TCCAGGCGTG GAGGAGACTC TGGCGGTGGC GCCAGCAGCA

CCTGCGGCCT TCAACACATG GAGAAGATTC TGGCGATGGC ACCAGCAGGA

CCTGCGGCTT TCCAGGCATG GAGAAGACTC TGGCAGTGGC GGCAGCAAGA

CCTGCCGCCT TCCAGACATG GAGAATGCTC TGGCGCTGGC ACCAGCAGGA

CCGGCTGCCT TCCGGGCATG GAGAAGACTC TGGCAACGGC ACCAGCAGGA

CCTGCTGCCT TCCAGGCATG GAGGAGACTC TGGCGATGGC ACCAGCAGGA

---------- ---------- ---------- ---------- ----------

TCCGTCCTCT TCGAGGCCTG GAGGAGCTTC AGCCGCCGGC AGCGGGAGCG

CCTTCCAGCT TCCAAGCCTG GAGAAGATTC AAGCAACAGC AGCAGCAGCA

AAATGTCCTC AGTGCAAGAG CAACACGTTT CCACAGGGAG ACATTAGAGA

AAATGTCCTC AGTGCAAGAG CAACACGTTT CCACAGGGAG ACATTAGAGA

CAATGTCCTC AATGCAAGAG CAACACGTTT CCACAGGGAG ACATTAGAGA

CAATGTCCTC AATGCAAGAG CAACACGTTT CCACAGGGAG ACATTAGAGA

AAACGTCCTC AGTGCAAGAG CTACACATTT TCACAGGGAG AAAGTAGAGA

---------- ---------- ---------- ---------- ----------

ACGGACCCTC CACACCAGAG CAGTGCGTTT TCACAGAGAG GCATTAGAGA

ACGGATCCTG CACACAAAAG CAGTGCGTTT CCACAGAGGG ACATTAGAGA

---------- ---------- ---------- ---------- ----------

GCGAGCCCTC GAGGCCCGGG CAGCGTGCTT TCACAGGGAG GTGTTAGAGA

AAGTGTTCTC AATGCAAGAG CAGCACGCTT TCACAGGGAG ACGTTGGAGA

AAGTGTTCTT CATGCGAGAG CGGCACGCTT TCACAGGGAG ACATTAGAGA

GAGCGTTCTC AGCGAGAGCG CAGCGCGTTT TCACAGGAAG ACAGTAAAGA

GAGCGTCCTT AAAGCAAGAG CAACTTGTTT TCACAGGGAG ATGGTAGGGA

AAATGCCCTT AATGCAAGAG CAATACGTTT TCACAGGGAG TTGTTAGGGA

---------- ---------- ---------- ------GGAG ACCCTAGGGA

GAGGGCACGG CAGGAAGAGG CTGTGAACTT CCGCAGGGAT CTGGTGAAGA

AAGAGAAATG AAGGAAACAG CACTGCATTT CCACAGGGAG GTGGTGGAGA

AGCAAGTATT TTCTCTCTGG AGGCAGAAGA TGTTTCAGCA TCGAGAAAAC

AGCAAGTATT TTCTCTCTGG AGGCAGAAGA TGTTTCAGCA TCGAGAAAAC

AGCAAGTATT TTCTATCTGG AGGCAGAAGA CGTTTCAGCA TCAAGAAAAC

AGCAAGTATT TTCTATCTGG AGGCAGAAGA CGTTTCAGCA TCAAGAAAAC

AGCAAGTATT TTCTATCTGG TGGCAGAAGA TGGTACAGCA TCGAGAAAAC

---------- ---------- ---------- ---------- ----------

AGCAAGTATT TGCTCTCTGG AGGCAGAAGG TGTCTCAGCA TCGAGAAATC

AGCAAGTATT TGCTCTCTGG AGGCAGAAGA TGTCTCAGCA TCGAGAAAAC

---------- ---------- ---------- ---------- ----------

AGCAGGTGTT TGCCATCTGG TGTCAGAAGA TGTCTCAGCA TCGAGAACAC

AGCAAGTATT TGCTATTTGG TGGCAGAAGA TGTTTCAGCA TCGAGAAAAC

AACAAGTATT TACTGTTTGG TGGCAGAAGA TGTTTCAGCA TCGAGAAAAC

AGCGAGTATT TTCTAGTTGG TGTCAGAAGA TGTTTCAGCA TCAAGAAAAC

AGCAAGTATT TGCTGTTTGG TGGCAGAAGA TGTTTCAACG TCGAGAGAAC

GGCAGGTATT TGCCATTTGG TGGCAGAAGA TGTCTCAGCA TCGAGAAAAC

GGCAAATGTT TGCTATTTGG CAGCAGAAGA TGCATCAGCA CCGAGAGAGC

GACGGGCGTT CGATACGTGG TGGCAGAAGG CGTATCGGCA GCGAGAAGTG

GGCAGGTATT TGACATATGG TGGCAGAGGA TGCGTCAGCA GCAAGAAAAC

CGCCTGGCAG AGAGAATGGC CATCCTTCAC GCAGAGCGAC AGCTTCTGTA

CACCTGGCAG AGAGAATGGC CATCCTTCAC GCAGAGCGAC AGCTTCTGTA

CGCCTGGCAG AGAGAATGGC CATCCTCCAC GCAGAGCGAC AGCTTCTGCA

CGCCTGGCAG AGAGAATGGC CATCCTCCAC GCAGAGCGAC AGCTTCTGCA

CGCTTGGCAG AGAGAATGGC CATCCTTCAC GCAGAGCGAC AGCTTCTGCA

---------- ---------- ---------- ---------- ----------

CGCTTGGCTG AGAGAATGGC TATGCTGCAG GCAGAGCAGC AGCTCTTGCG

TGCTTGGCAG AGAGAATGGC TATACTGCAG GCAGAGCAGC AGCTTTTGCG

---------- ---------- ---------- ---------- ----------

CGCTTGGCAG AGAGGATGGC CGTGCTCCAT GCAGAGCAGC ACCTTCTGCA

CGCTTGGCAG AGAGAATGGC CATCCTTCAT TCAGAGCGTC AGCTTCTGCA

CGCTTGGCAG AGAGAATGGC CATCCTTCAT GCAGAGCGGC AGCTTCTGCG

CGTTTGGCAG AGAGAATGGC CATCCTGCAT GCCGAGCGGC AGCTTCTCCA

CGCTTGGCAG AGCGAATGGC CATCCTCCAC GCAGAGCGGC AGATCCTGCA

CGCTTGGCAG AGAGAGTGGC CATCCTTCAT GCCGAGCAGT GGCTTCTGCA

CGCTTGGTAG AGAGAGTGGC CATTCTGCAT GCCGAGAGGC AACTTCTGCT

CGATTGTCTG AGAGGATGGC CGTCCTCCAC GCTGAGCAGC AGGTGCTCCG

CATTTGGCAG AGAGAATGGC TATTCTTCAC TCTGAACAGC TGCTGTTGCA

TAGGTCTTGG TTCATGTGGC ACCAGCAGGC AGCAGCACGT CACCAGGAGC

TAGGTCTTGG TTCATGTGGC ACCAGCAGGC AGCAGCACGT CACCAGGAGC

TAGGTCTTGG TTCACGTGGC ACCAGCAGGC AGCAGCACGT CACCAGGAGC

TAGGTCTTGG TTCACGTGGC ACCAGCAGGC AGCAGCACGT CACCAGGAGC

TAGGTCCTGG TTCACGTGGC ACCAGCAGGC AGCAGCACAT CACCAGGAGC

---------- ---------- ---------- ---------- ----------

TAGGTTCTGG TTCGTGTGGC ACCAGCGGGC AGCGGCGTGT CACCAGGAGC

TAGGTTCTGG TTCGTGTGGC ACCAGCAGGC AGCAGTGTGT CAGCTGGAGC

---------- ---------- ---------- ---------- ----------

GAAATCATGG TCCTTGTGGC GCCAGCAGGC AGCGGCACAC CGCCTGGAGC

AAGGTCCTGG TCCATGTGGC ATCAGCAGGC AGCAGCACGT CACCGGGAAT

AAGGTCCTGG TCCACGTGGC AGCAGCAGGC GGCTGCACGT CGGCAGGAGC

AAGGTCCTGG TGCACGTGGC ACCAGCAGAC AGTGGC---- --------GC

GAGGTCCTGG GCTGCGTGGG GCCAACAGGC GGCCACCCGT CACCAGGAGC

CAGGGCCTGG TCCACATGGC AGCAGCAGGT GGCAGCATGT CACCAGGAGC

CAGGGCCTGG TCCACGTGGC ACCAGCAGGC AGCAGCGTGT CACCAGGAGC

GCACTACTGG TCCAGGTGGC GCCGGCGGGC AGCCGAGCAG TCCGTGGAAA

TCGTTTCTGG TCTGTGTGGC TCACCCAGGT GGCAGTGCAT ACTAAGGAGC

AGGAGTGGCA AACAGTGGCC TGTGCCCACC ACCGCCACGG GCGGCTCAAG

AGGAGTGGCA AACAGTGGCC TGTGCCCACC ACCGCCACGG GCGGCTCAAG

AGGAGTGGCA AACAGCGGCC TGTGCTCACC ACCGCCATGG GCGGCTCAAG

AGGAGTGGCA AACAGTGGCC TGTGCTCACC ACCGCCATGG GCGGCTCAAG

AGGAGTGGCA AACAGTGGCC CGTGCCCACC ACCGCTGCAG GCGGCTCAGG

---------- ---------- ---------- ---------- ----------

GACAGCGGCA AGCCATGGCC ATTGCCCACC ACCACAGCGG GCTGCTCAGG

GACAGCAGCA AGCCATGGCC ATTGCCCACC ACCACAGTGG ACTGCTCAGG

---------- ---------- ---------- ---------- ----------

AGCAGCAGCA TGCACTGGCC TGTGCCCACC ACCACCTCGG GCGGCTCAGG

GGCAGCGGCA AGCAGTGGCC TGTGCCCACC ACCGCCACAG ACAGCTCAGG

GGCAGTGGCA ATCAGTGGCC TGTGCCCACC ACGGCCAGCG GCGGCTCAGG

GGCAGTGGCA AGCTGTGGCC TGTGCCCATC ACCGCCGTGG GCAGCTCAGG

GGCGACAGCA AGCAGCGGCC TGTGCCCACC ACCGCCGCTG GCGGCTCAGA

GACAGAAGCA AGCAGTGGCC TGTGCCCACC ACCGCCACTG GCGGGTCAGG

GACAGAGACA AGCAGTGGCG TGTGCCCACC ACCGCCAGTG GGAGCTCCAG

GAGGGAGCCA GGCCACAGCG TGCGCCCATC GCCGCCGCAG GCAGCTGCAC

GGGAGGGAGA AGTCGCAGCT GATAATCATC ACCGTCACAG GCAACTGCTG

AAAGCTTTCT GCCTCTGGAG GGAAAGTGCC CAAGGGCTCA GAACAGAGAG

AAAGCTTTCT GCCTCTGGAG GGAAAGTGCC CAAGGGCTCA GAACAGAGAG

AAAGCTTTCT GCCTCTGGAG GGAAAGTGCC CAAGGGCTCA GAGCAGAGAG

AAAGCTTTCT GCCTCTGGAG GGAAAGTGCC CAAGGGCTCC GAGCAGAGAG

AAAGCTTTCT GCCTCTGGAG GGAAAGTGCT CGAGGGCTCA GAACAGAGAG

---------- ---------- ---------- ---------- ----------

AGGGCTTTCT GCATCTGGAG GGAAAGCACC CAAGGATTTA GGATGGAGAG

AGGGCTTTCT GCATCTGGAA GGAAAGCACC CAAGGATTTA GGATAGAGAG

---------- ---------- ---------- ---------- ----------

AAGGCCTTCT GTGTCTGGAG GGAGGGCTCC CGAGGGCTCA GAACAGAGAG

AAGGCTTTCT GCATCTGGAG GGAGACTGCC CGAGGGCTCA GAACTGAGAA

AAGGCTTTCT GTGTCTGGAG GGAGAAAGCC CGAGGGCTCA GATCGGAGAA

AAGGCTTTCA ACGTCTGGAC AGAGAGTGCC CGAGGGCTCA GCACAGAGAA

AGAACTTTCC GTACCTGGAA GGAGAGAGCT CAGGGGCTCA GGAAAGAGAG

AGAACCTTTC ATGTCTGGAG GGAGAGTGCT CGAGGGCTCA GAACAGAGAA

AGAGCCTTCC ATGTGTGGAG GGAGAGTGCC CAAGGGCGGA GAACCGAGAA

GGGGCCTTCC GGGTCTGGAA GGAGAAC-CT CGAGGCCTCC GAGCTCAGCG

AAGGCTTTCC AGCTTTGGAA GGAGAACGTT CAGGAGCTCC GAACAGAGAG

GACGGGCAGG GTGCGGGCAG CAGAATTCCA CATGGCCCAG CTCCTGCGTT

GACGGGCAGG GTGCGGGCAG CAGAATTCCA CATGGCCCAG CTCCTGCGTT

GACGGGCAGG GTGCGGGCAG CAGAATTCCA CGTGGCCCAG CTCCTGCGCC

GACGGGCAGG GTGCGGGCAG CAGAATTCCA CGTGGCCCAG CTCCTGCGCC

GATGGGCCGG GTGCAGGCAG CAGAATTCAA TGCAGCCCAG CTCCTGCGTT

---------- ---------- ---------- ---------- ----------

GATGGGCAGG GCACAGGCTT CACACTTCCA CTCAGTACGG CTCCTGTGTT

GATGGGCAGG GCACAGGCTG CGCACTTCCA CTCTGCACAG CTCTTGAGTC

---------- ---------- ---------- ---------- ----------

GACGGGCACT ATGCGGGCTG CACGGTTCCA CGCGGCGTGG CTTCTGCGCC

GATGGGGCTA GTGCTGGCTG CAGAGTTCCA AGCGGCCCGG CTCCTGCACT

GATGGGCAGG GTGTTGGCTG CAGAGTTCCA CTCGGCACGG CTCCTGCGTT

GAAGGGCCAC GTGCTAGCTG CGGAATTCCA CTCAGCACGA CTCCTCCATT

GATGGGGGCA GCGCAGGCTG CGGAGTTCCA CTCGGCGCAG CTCCTGCGTT

GCTGGGAAAG GCGCGGGCTG TGGAGTTCCA CCGGGTGCAG CTCCTGCTTT

GCTGGGAAAG GCCTGGGCTG CGGAGTTCCA CGCGGTGCAG CTCCTGCGTT

GGCCGGGGAG GCGAGGGCTG CAGCCTTCCA CTCGTGTCTG CTCGTGCGTT

GATGGGCTTG AGGACGGCTA TGCAATTCCA CTCTCAGCAG GTCCTGTGGT

GGGCCTGGAG CCAGTGGAGG GAGTGCCTGG CCCTGCGGGG AGCGGAGCGG

GGGCCTGGAG CCAGTGGAGG GAGTGCCTGG CCCTGCGGGG AGCGGAGCGG

GGGCCTGGAG CCAGTGGAGG GAGTGCCTGG CCGTGCGGGG GGCGGAGCGG

GGGCCTGGAG CCAGTGGAGG GAGTGCCTGG CCGTGCGCGG GGCGGAGCGG

GGGCCTGGAG CCAGTGGAGG GAGTGCCTGG CCCTGCGGGG AGCGGAGCGG

---------- ---------- ---------- ---------- ----------

GGGCCTGGAG CAAGTGGAGG GAGGGCCTAG CTCTGAGGAT GGAGGAGCAG

GGGCCTGGAG CATGTGGAGG GAGTGCCTGG CCCTGAGGCT GGAAGAGCAG

---------- ---------- ---agcctgg ccctGCGGGG AGCCGAGCGC

GGGCCTGGAC CAGGTGGCGG GAGCGCCGGG CTCTGCGCAG TGCCGAGTGG

GGGCCTGGAG CCGGTGGAGG GAGTGCCTGG CCCTGCACGC TGCTGAGCAG

GGGCCTGGAA CAGGTGGAGG GAGTGCCTGG CCCTGCGGGG AGCCGAGCAG

GGGCCTGTAA CAGATGGATG GAGTGCCTGG CCCTGCGGGG CGCAGAGTGG

GGGCCTGGAA CGGGTGGAGG GAGTACCTGG CCCTGCGGGA CGCCGAGCGA

GGGCCTGGAA GAGGTGGAGG GAGtgcctgG CCCTGTGCGA TGCTGAGCGG

GGGCCTGGAA CAGGTGGCGG GAG------- ---------- ----------

TGACCTGGAG CAAGTGGCGA CAGTATGTGG CCCTGCAGAA CGTGAAGTGG

ATTCTTGGAG CAAGTGGAGG CAGTATGTGG TCCAGCGGAG TGCAAAATGG

CAGAAGCTGA TGCGAGCAGA CCTGCACCAC CAGCACAGCG TGCTGCACAG

CAGAAGCTGA TGCGAGCAGA CCTGCACCAC CAGCACAGCG TGCTGCACAG

CGGAAGCTGA TGCGAGCAGA TCGGCACCAC CAGCAGAGAG TGCGGCACAG

CGGAAGCTGA TGCGAGCAGA TCGGCACCAG CAGCAGAGAG CGCGGCTCAG

CGGAAGCTGA TGCGAGCGGA GCTGCACCAC CAGCACACGG TGCTGCTCAG

---------- ---------- ---------- ---------- ----------

CAAAAGCTGA AGCGTGCAGC CCTGCACAGC CAGCGCACCC TGCTCCACAG

CAGAAACTGA AGTGTGCGGC CCTGCACAGC CAGTGCATCC TGCTTCGCAG

TGGAAGCTGC TGCGAGCAGA GCTGCATCAC CAGCGTGCCG TGCTGCACCG

CGGAAGCTGG CGCAAGCCGA CCTGCACCAC CAGCACAGCT TGCTGCACAG

CGGAAGCTGA TGCGCGCCAG CCTGCACCAC CAGCAGGCCT TGCTGCATGG

CAGAAGCTGA CACGTGCAGA CCTGCATCAC CAGCACACCT CGCTGCACAG

CAGAAGCGGC TTGGAGCTGA GCTGCATTAC CAGCACACCC TGCTGCGCAA

CAGAAGCAGC TCCGAGCCGA CAGACATTAC CAGCGTGCCC TGCTGCACAG

CAGAAACTGG TGCGAGCTGA CTTACATCAC CAGCACTCCT TGCTGCACAG

---------- ---------- ---------- -AGTGCACCC TGCTGCGCAG

GAGAAGGTGA TCCGCGCCGA CTGCCATCAC CACCAGGCGC TGCTGCGGAG

CAGAAGTTGG TGTGTGCAGA CCTGCACTAC CGACACGCAT TGCTGAGCAG

GGCGCTGCAG GCATGGGTGA CTTACCAGGG CAGGGTGCGA AGCATCCTCC

GGCGCTGCAG GCATGGGTGA CTTACCAGGG CAGGGTGCGA AGCATCCTCC

GGCGCTGCAG GCGTGGGTGA CTTACCAGGG CAGGGTACGG AGCATCCTCC

GGCGCTGCAG GCGTGGGTGA CTTACCAGGG CAGGGTACGG AGCATCCTCC

GGCGCTGCAG GCGTGGGTGA CTTATCAGGG CAGGGTGCAG AGCATCCTCC

---------- ---------- ---------- ---------- ----------

GGCTCTGCAG AAGTGGCTGG TTTACCAGGA CAGGATGAGG AGTGTTCTTC

GGCTCTGCAG AAGTGGCTGG TTTACCAGAA TAGGGTGAGG AGTGTTCTGC

GGCGCTGCGG AAGTGGCTGA TTTACCGGGA CCAGGTGCGG AGCATCCTGC

GGCGCTGCAG ACCTGGGGGG TTTATCAGAG CCGGGTGCGG AGCGTGCTGC

GACACTGCAG ACCTGGGCGA CTTACCAGAG CCAGGTGCGG AGCATCCTCC

GACGCTGCAG ACCTGGCGGA CTTACCAGAG CCAGGTGCAG AGTGTCCTAC

GGCGCTCCAC GCCTGGGGG- ---------- ---------- ----------

GCTGCTGCGG AGATGGCTGG CTCACCAGAG TGGCGTGCGG AGCGTCCTGC

GGTGCTGCGG AGATGGATGA CTTACCAGGA CAGGGTGCGG CATGTCCTAC

AGTGTGGCGG GGATGGGTGA CTTACCAGGA CAGGGGGCGG CAGGTCCTGC

GAGCCTGACC GGCTGGATGA CGTACCAGGG CAGAGTTCAA GCTGTGCTGC

GGTCCTGAAG GCCTGGATGA CCTACCAGCA CAGAGTACAG CTTGTTCTGG

GGGAGGTGGC AGCCAGGGAG AGCCAGCACA ACAGGCAGCT GCTGCGCGGG

GGGAGGTGGC AGCCAGGGAG AGCCAGCACA ACAGGCAGCT GCTGCGCGGG

AGGAGGTGGC AGCCAGGGAG AGCCAGCACA ACAGGCAGCT GCTGCGAGGG

AGGAGGTGGC AGCCAGGGAG AGCCAGCACA ACAGGCAGCT GCTGCGAGGG

AGGAGGTGGC AGCCAAGGAG AGCCAGCACA ACAGGCAGCT GCTGCGGGGG

---------- ---------- ---------- ---------- ----------

AGGAGGTTGC AGCCAGGGAA AGACAGCACA ACAGGCAGCT GCTGTGGTGG

GGGAGGTGGC GGCCAGGGAG AGACAGCACA ACAGGCAGCT GCTGTGGTGG

AGGAGGTGAC AGCCAGGGAG AGGCGGCACC ACAGGCAGCT GCTGCGGTGG

AGGAGGCGGC TGCCAGGGAG AGCCGGCACA AGAGGCGGCT GCTGCGGCAT

AGGAGGTGGC AGCCAGGGAG AGCCAGCACA AGAGGCGGCT GCTGCGGAGT

AAGAGGTGGC AGCCAGGGAG AACCAGCACA AGAGGCAGCT GCTGCGGGAT

---------- ---------- ---------- ---------- ------AGCC

AGGACGTGGC AGCCAGGGAG AGCCGGCACG CCAGGCAGCT GCTGCGGGGT

AAGAGGTGGC AGCCAGGGAG AGCCAGCACA AAAGGCAGCT GCTACGGGGT

GAGAGGTGGC GGCCAGGGAG AGCCAGCACA ACAGGCGCTT GCTGCG----

GCCACGTGGC AGAGAGAGAA GAGTGGCATC GCCGGGAGCT CCTGCGGTGG

AACATGTAGC TGAGAAGGAG AAACAGCATA ATAGGCAACT GTTAAGGCGG

GCATTACGTC GCTGGAAAGA GAACACCATG GCCCGAGTGG ATGAAGCCAA

GCATTACGTC GCTGGAAAGA GAACACCATG GCCCGAGTGG ATGAAGCCAA

GCATTGCGTC GCTGGAAAGA GAACACCATG GCCCGAGTGG ATGAAGCCAA

GCATTGCGTC GCTGGAAAGA GAACACCATG GCCCGAGTGG ATGAAGCCAA

GTGTTACGTC GCTGGAAAGA GAACACCATG GCCCGCATGA ATGAAGCCAA

---------- ---------- ---------- ---------- ----------

GTGTTACATC GCTGGAGAGA AAACACCATG GCCCGTTTGG ATGGGGCCAA

GCGTTACATC TCTGGAGAGA AAACACCATG GCCCGTTTGG ATGGAGCCAA

GTGTTACGCC ACTGGAGACA GAACACCGTG GCCTGTGTGG ATGAAGCCAG

GTGTTACATC GCTGGAGAGA GAACGCCATG GCCCAAGCAG ACGAAGCAAA

GTGTTCCGTC GCTGGAGAGA GAACACCCTG GCCCGAGTGA ATGAAGCAAA

GTATTACATC GCTGGAGAGA GAACACCGTG GCTCGTGTAG ATGAAGCAAA

GTGTTACATC GCTGGAGAGA GAACACGCTG GCCCGCGTGG AAGAGGCGAG

GTGTTCCTGC GCTGGCGAGA GAACACCATG GCCCGTGCGG CTGAAGCTGA

GTGTTACATC GCTGGCGAGA GAACACCATA GCCTGTGTGG ATGAAGCTCA

---------- ---------- ---------- ---------- ----------

GTGTTTCAAC TCTGGAGGGA GAATGCCACG GCCCAGGTGG AGGAGGCCAG

GTATTTCACA CCTGGAAGAA ACATTCCACT GAATGTGTGA TTGAAGCAGA

AAAAACCTTT CAAGCAAGTA CTCATTACAG AAGGACCATA TGTTCCAAGG

AAAAACCTTT CAAGCAAGTA CTCATTACAG AAGGACCATA TGTTCCAAGG

AAAAACCTTT CAAGCAAGTG CTCATTACAG AAGGACCATA TGTTCCAAGG

AAAAACCTTT CAAGCAAGTG CTCATTACAG AAGGACCATA TGTTTCAAGG

AAAAACCTTT CAAGCAAGTA CTCATTACAG AAAGACCATA TGTTCCAAG-

---------- ---------- ---------- ---------- ----------

AAAAACCTCT CAAGCTAGAG TCCACTACAA CAGGACTCTG TGCTCCAAGG

AAAAACCTCT CAAGCAAGAG TTCACTACAG CAGGACTCTG TGCTCCAAGG

GAAAACTTCC CAAGCATCTG CTCACTACAG AAGGACCCTC TGTTCCAAGG

CAAGATGTCC CGGGCGGCAG CTCACTACAG GAGGACTCTG TGCTCCAAGG

GCAAACCTCC CGAGCAAGCA CTCACTATAG GCGAACCTTG TGTTCCAAGG

GAAAACCTCT CGAGCATGTG CTCACTACAG AAGGACCGTG TGTTTCAAGG

GAAGGTCTCC AGAGCCAAAG CACACTACGA AAGGGCCGTG TGTTCCAAGG

GAGAGTCTGC CAAGCTTGGG ACCACTACAG AAGGAGCCTG GGTTCCAAGG

AAAGGCCTCT CAAGCACGTG ATCACTACAG AAGGACCATG TGTTCAAAGG

---------- ---------- ---------- ---------- ---------G

GAAAGCCGTC CAGGCTGAGG GGCACTACAG AAGAACCGTC CTCTGGAAG-

GAAAACAGCT GAAGCGGTTG AGCATCTCAG GAGAAGAGTG GTGTCAAAGG

GCTGTCTGCG GACCTGGTTT CAGCGCTGGT GGGACTGCAG CCGGAGGTCA

GCTGTCTGCG GACCTGGTTT CAGCGCTGGT GGGACTGCAG CCGGAGGTCA

GCTGTCTGCG GACCTGGTTT CAGCGCTGGC GGGACTGCAG CCGGAGGTCA

GCTGTCTGCG GACCTGGTTT CAGCGCTGGC GGGACTGCAG CCGGAGGTCA

GTTGTCTCCG GACCTGGTTT CAGCGCTGGC GGGACTGCAG CCGGAGGTCA

---------- ---------- ---------- ---------- ----------

GTCGTCTCCG GACCTGGCTT CAGCGGTGGC AGGTCTGCAG GCAGAGGGAG

GTCGTCTCCG GAACTGGTTT CAACAC---- GGTTCTGCAG CCAGAGGGTG

GCTGTCTGCG GACCTGGTTG CGGCGCTGGC GGGACTGCAG CCGGAGAGCA

GCCGTCTCAG GACCTGGTTT CGGCTCTGGC GGGACCGAAG CCGGAGGGCT

GCTGTCTCAG GACGTGGTTT CGGCACTGGC GGGACCAAGG CCAGAGGGCG

GCCGTCTCAG GACATGGTTT CGGCGCTGGC GGGACCTTGG CCGGAGGGCA

GCCGTCTCCG GGGCTGGCTG AGGCACTGGC GGGCCCG--- ---------G

GCCGCCTCCG GACTGTGTTT CGGCGCTGGC GAGCCCGCAG CCAGAGAGCA

GCTGTGTCCA GGCTGTGTTT CAGCGCTGGC GGGACCGCAG CCAGAGCATG

GCTGCGTCCA GATTATATTC CGGCGCTGGC GGAGCCGCAG CCAGAGTGTG

GCCGTCTGCG AGTCCTGTTC CAGCGCTGGC GGGAGCGCGG CCGGAGCTCT

GCCTTCTTCG GTCTGTGTTT TTATACTGGA GGGAATTTAG CCAGAGGATT

GCCCAGCAGA GACTGCAGCT GGAGAGGGCA GTGCAACACC ACCACCGGCA

GCCCAGCAGA GACTGCAGCT GGAGAGGGCA GTCCAACACC ACCGCCGGCA

GCCCAGCAGA GACTGCAGCT GGAGAGGGCA GTCCAACACC ACCGCCGACA

GCCCAGCAGA GACTGCAGCT GGAGAGGGCA GTCCAACACC ACCGCCGACA

GCCCAGCAGA GACTGCAGCT GGAGAGGGCA GCCCAGCACC ACCACCGGCA

---------- ---------- ---------- ---------- ----------

GCTCAGCAGA CATTCCGGCT GCAACAGGCG GCTCAGCATC ACCGCCGGCA

GCCCAGCAGA GATTCCAGCT GGGACAGGCG GGTCAGCATC ACCGCTGGCA

GCCCAGCAGA GGGCCCAGCT GCAGAGGGCG GCCCAGCACC GCCGCCGGCA

GCCCAGCAGA GGGTGCAGAT GGAGCGGGCA GTGCGACATT GCTGCCGGAA

GCCCAGCAGA GAGTCCAGCT TAGGCGGGCG GCTGGGCATC ACGGCCGGCG

GCCCAGCAGA GAGCCCAGCT GCAGAGGGCA GCACAGCATT ACCAGCGGCA

GCACAGCAGA GGCTGCAGCT GGCGCGCGCG GAGCAGCATC AGCGACAGCG

GCCAAGCAGA GGGTGCAGAT GGAGGCGGCC GCCCTGCGCC AGCGCCGGCA

GCCCAGCAGA GAGTCCAGAT GGAGAGAGCG GCCCAGCATC ACCGGCTCCG

GCCCAGCAGC GGGTCCAGAT GGAAAGGGCA GCCCAGC--- ----------

TCCCAGCAGA GAGCGCAGGA GCTGCGGGCC GCGCGGCACC ACAGGGGGAG

TCGCTACAGA GAGCCCAGCT GGATCGTGCG GCCCAGCATC ACGGGAAGAG

GCTGCTGCTG GAGGGGCTGG CCCGGTGGAA GACGCACCAT CTGCAGTGTG

GCTGCTGCTG GAGGGGCTGG CCCGGTGGAA GATGCACCAT CTGCAGTGTG

GCTGCTGCTG GAGGGGCTGG CCCGGTGGAA GATGCACCAT CTGGAATGTG

GCTGCTGCTG GAGGGGCTGG CCCGGTGGAA GATGCACCAT CTGGAATGTG

GCTGCTACTG GAGGGGCTGG CCCGATGGAA GACACATCAT CTGGGGTGTG

---------- ---------- ---------- ---------- ----------

GCTGCTAGCG GGAGCAATGG CCAGGTGGAA GGCGTACCAT CTGTGTTGTG

GCTGCTGATG GAAGCAATGG CCAGGTGGAA GGCACACCAT CTGGGGTGTA

GCTGCTGCAG GACGGCATGG CCCGGTGGAA GAGGCACCAC CTGGGCTGTG

GCTGCTGCAG GAGGCCCTGG CCCGGTGGAA GGCGCACCAT CAGGGCTGCG

GCTGCTGCTG CAGACCATGG CCCGGTGGAA GGCGCACCAT TTCGGCTGTG

GCTGCTGCTA CAGGGCCTGG CACGGTGGAA GGTGCACCAT CTGGGCTGCG

GGCGCTGCGG CGGGCCCTGG GCGCGTGGAA GGCCCACCAC CGCCACTGTG

GCTGCTGCTG GAGGCCATGG CCCGCTGGAA GGCATACCAC CTGCAGTGCA

ACTGCTTCAG GAGATGATGG CGCAGTGGAA GGCCCACCAT CTGGGGTTTA

---------- ---------- ---------- ---------- ----------

GCTGCTCGGG GCCTGCATGG CGCGCTGGAA GAGCTACCAC TCGCGGCACG

GCTGCTGCAG GAGTGTATGA CCCGGTGGAA GCAACACCAT CTCTGCTGCA

TCAGGAAGAG GCTCCTGCAC AGGCAGAGCA CCCAACTGCT GGCACAGAGA

TCAGGAAGAG GCTCCTGCAC AGGCAGAGCA CCCAACTGCT GGCACAGAGA

TCAGGAAGAG GCTCCTGCAC AGGCAGAGTA CCCAACTGCT GGCACAGAGA

TCAGGAAGAG GCTCCTGCAC AGGCAGAGTA CCCAACTGCT GGCACAGAGA

TCAGGAAGAG GCTCCTGCAC AGGCAGAGCA CCCAACTGCT GGCACAGAGA

---------- ---------- ---------- ---------- ----------

TCAGGAAAAA GTTTCTGCAG AGGCAAGCTG CCCAGCTCCT GGCACAGAGA

TCAGAAAGAA GTTTCTGCAG AGGCAAGCTG CCCAACTCCT GGCACAGAGA

TCAGGAAACA GCTGCTGCAG AGGCAGGCCA CCCAGTTCCG GGCACAGAGG

TCAGGAAGAG GCTGCGGCAG AGGCAGGCCG CCCGACTCCG GGCCCAGACG

TCAGGAAGCG GCTCCTACAG AGGCAGGGCG CCCAGCTCCT GGCCCAGACT

TCAGGAAGAG GCTCCTGCAG AGGCGGGGTG CCCAGTTCCT GGCCCAGACA

CCAGGAGGAA GCACCTCCAG AGCCTGAGCA CCCAGCTGCT GGCCCAGACC

TCAGGAACAC GCTCCTGCGG CGTTTAGGCA CCCAGCTCCT GGCCCAGAGA

TCCGGAAAAT GCTCCTGCAG AGGCAGGGTA CCCGGTTCTT GGCCCAGAGA

---------- ---------- ---------- ---------- ----------

TCCGGAAGAT GCTCCTGCAG CGGCTCGGCG CCCGGCTGAT GGCCCAGAGA

TCAGAAAAAT GCTCTTGCAG AGGCAGGGAG TTCAGCTGAT GGCTCGAAGA

CTCAGCCGGA CCTGCTTCCG CCAGTGGAGA CAACAGCTGG CAGCCAGGAG

CTCAGCCGGA CCTGCTTCCG CCAGTGGAGA CAACAGCTGG CAGCCAGGAG

CTCAGCCGGA CCTGCTTCCG CCAGTGGAGA CAACAGCTGG CAGCCAGGAG

CTCAACCGGA CCTGCTTCCG CCAGTGGAGA CAACAGCTGG CAGCCAGGAG

CTCAGCCAGA CCTGCTTCCA CCAGTGGAGA CAACAGCTGG CAGCCAGGAG

---------- ---------- ---------- ---------- ----------

CTCGGTCGGG CCTGTTTCTG CCAGTGGAGG AAGCAGCTGG CAGTCAGGAA

CTCAGTCGGG CCTGCTTCTG CCAGTGGAGG AAGCAGCTGG CAGTCCGGAA

CTCAGCCGGG CCTGCTTCCA GCAGTGGAGA CAGCAG---- ----------

CTCAGCCGGG CCTACTTCCA CCAGTGGAGA CGACAGCTGC TGGACAGGAG

CGCAGCCGGG CCTGCTTCCA CCAATGGAAA CAACAGCTGG CAAACAGGAG

CTCAGCCAGT CCTACTTCCG CCTGTGGACA CAACAGCTGG TGAACAGGAG

CTCAGCCGCG CCTGCTTCCA CCAGTGGAGA CAACACTTGG CAGCCCGGAG

CTGAGCCGCT CGTGCTTTCA CCGGTGGAGA CAGCAGCTGG CAGCCAGGAA

CTCAGCCAGT CCTGCTTCTA TCATTGGAGA CGGCAGCTGG TGGCCCGGAG

---------- ---------- ---------- ------CTGG TGGCCCAGAG

CTCGGCCGCT CCTGCTTCTC CACGTGGAAG TGCCAGCTGG GGGAGAGGCA

CTCAGTCAGT CTTGTTTCTC CCAGTGGAAA CATCAGCTGG CGGAAAAGCA

GCAGGAGCAG CGGGCGACAG TGCGGGCCCT GTGGTTCTGG GCCTTCTCGC

GCAGGAGCAG CGGGCGACGG TGCGGGCCCT GTGGTTCTGG GCCTTCTCGC

GCAGGAGCAG CAGGCGACAG TGCGGGCCCT GTGGTTCTGG GCCTTTTCGC

GCAGGAGCAG CAGGCGACAG TGCGGGCCCT GTGGTTCTGG GCCTTTTCGC

GCAGGAGCAG CAGGCGTCAG TGCGGGCCCT GTGGTTCTGG GCCTTCTCAC

---------- ---------- ---------- ---------- ----------

GCAAGAACAG TGGGGCACAG CCAGGGCCCT GTGGTTCTGG GCCTTCTCCC

GCAAGAACAG TGGGGCACAG CCCGGGCCCT GTGGCTCTGG GCCTTCTCAC

---------- ---------- ---------- ---------- ----------

GCACGAGCAG CAGCGCACGG CGCAGGCCCT GTGGTTCTGG GCCTTCTCCC

GCGGGAGCAG CAGGGCACAG CACGGGCCCT GTGGTTCTGG TCCTTCTCAC

GCAGGAGCAG CAAGGCACAG CCCGGGCCCT GTGGTTCTGG GCCTTCTCCC

GCGGGAGCAG CAGGGCACGG CACGGGCGCT GTGGTTCTGG GCCTTCTCAC

GCAGGAGCAG CAGGGTACGG CGCGCGCTCT GTGGTTCTGG TCCTTCTCCC

GGAGGAGCAG CAGGGCACAG CGCGGGCCCT GTGGTTCTGG TCCTTCTCAC

GGAGGAGCAG CAGGGCACAG CGAAGGCCCT GTGGTTTTGG TCCATCTCGC

GCGGGAGCAG CAGGCCACCG TCCGCGCGCT CTGGTTTTGG TCCTTCACGC

GCGGGAACAC CAGGCAACGG TGCAAGCCTT GTGGTTCTGG TCCTTTTCCC

TGCAGGCAAA GGTGTGGGCC ACGTGGCTGG CCTTTGTACT GGAAAGGAGG

TGCAGGCAAA GGTGTGGGCC ACGTGGCTGG CCTTTGTACT GGAAAGGAGG

TGCAGGCAAA GGTGTGGGCC ACGTGGCTGG CGTTTGTACT GGAAAGGAGG

TGCAGGCAAA GGTGTGGGCC ACGTGGCTGG CCTTTGTACT GGAAAGGAGG

TGCAGGCAAA GGTGTGGGCC ACGTGGCTGG CCTTTGTGCT GGAAAGGAGG

---------- ---------- ---------- ---------- ----------

TACAGGCAAA GGTGTGGGCT GCATGGCTGG GCTTCGTACT TGAGAAGAGG

TACAAGCAAA GGTGTGGACT GCATGGCTGG GCTTCGTACT TGAGAGGAGG

---------- ---------- ---------- ---------- ----------

TGCAGGCGAA GGCCTGGGCC GCCTGGCGGG GCTTCGTGCA GGAGGCGAGG

TACAGGCAAA GGTGTGGGCC GCGTGGCTGG GCTTTGTGCT GGAAAGGCGG

TACAGGCCAA GGCGTGGGCC GCGTGGCTGG GCTTCGTGCT GGAGAGGAGG

TGCAGGCGAA GGTGTGGGCC GCGTGGCTGG ACTTTGTGAT GGAGAGGAGG

TACAGGCTAA GGCCTGGCAC GCGTGGCTGC TGTTTGTGCT GGAGCGGCGG

TGCAGGCAAA GGCCTGGGAC GCCTGGCTGG GTTTTGTGCT GGAAAGGAGA

TACAGGCAAA GGCCTGGGAT GCCTGGCAGC GCTACATGCT GGAGAGGAAG

TGCAGGGAAA GGTGTGGGAC GCCTGGCTGG CCTTTGTGCT GGAGCGGAGG

TGAAGGGGAA GGTTTTTGAT GCCTGGGTGG GATTTGTGCT GGAGCAGAGA

AGAAAGAAGG CGCGGCTGCA GTGGGCGCTC CAGGCCTACC AGGGGCAGCT

AGAAAGAAGG CGCGGCTGCA GCGGGCGCTC CAGGCCTACC AGGGGCAGCT

AGGAAGAAGG CGCGGCTGCA GCGGGCACTC CAGGCCTACC AGGGGCAGCT

AGGAAGAAGG CGCGGCTGCA GCGCGCGCTC CAGGCCTACC AGGGGCAGCT

AGGAAGAAGG CACGGATGCA GCAGGCGTTC CAGGCCTACC AGGGGCAGCT

---------- ---------- ---------- ---------- ----------

AGGAAGAAGG CACGGCTGGA GCAGGCTATG CAAGCCTACC ACCAGCAGCT

AGGAAGAAGG CACGGCTGGA ACGGGCTATG CAAGCCTACC AGCAGCAGCT

---------- ---------- ---------- ---------- ----------

AGGAAGAAGG CGCGGGAGGA GCAGGCAGTG CAGGTCTACC AGCTGCAGCT

AGGAAGAAGG TGCGGCTGGA GCGGGCCGGG CAGGCCTACC ACCAGCAGCT

AGAAAGAAGG CGCGGCTGGA GCAGGCGGTG CAGACCTACC ACGAGCAGCT

AGAAAGAAGG CGCGGCTGGA GCGCGCGGCC CAGGTCTACC ACCAGCAGCT

CGGAAGAAGG CGCGGCTGGA GCAGGCGGCA CAGGCCTTCC ACCACCAGCT

AGAAAGAAGG CGCGGCTGGA GCAGGCAATG CAGGCCTATC ATCACCAGCT

AGGAAGAAGG CTCGGCTAGA GCGAGCCGCG CAGGCCCACC AGCAGCAGCT

AGGAAGAGAG CCCGGCTGGA GCGGGCCGCG CTGGCCTACC GCGACGGCCT

AGGAAGAGAG CCAGGATTGA GCAAGCAGTG CAGGCCTATC ATGCTGATCT

CCTCCAGGAG GGTGCCACGC GGCTCCTGCG CTTTGCAGCC AGCATGAAGG

CCTCCAGGAG GGTGCCACGC GGCTCCTGCG CTTTGCAGCC AGCATGAAGG

CCTCCAGGAG GGTGCCACAC GGCTCCTGCG CTTTGCAGCC AGCATGAAGG

CCTCCAGGAG GGTGCCACAC GGCTCCTGCG CTTTGCAGCC AGCATGAAGG

CCTCCAGGAG GGTGCCATGC GGCTCCTGCG CTTTGCAGCC GGGATGAAGG

---------- ---------- ---------- ---------- ----------

CCTGCAGGAG GGGGCCACGC GGCTTCTGCG CTTCACGGCT GGCATGAAGG

CCTACAGGAG GGTGCCACAC GGCTTCTGCG CTTCACAGCT GGTACGAAGG

---------- ---------- ---------- ---------- ----------

CCTCAGGGAG GGCGTTGAGC GCCTCCTGCG CTTTGCGGCC GGCATGAAGG

CCTCCGGGAG GGTGCCACTC GCCTCTTGCG GTTCGCAGGC AGCATGAAGG

CCTCCGGGAG GGCGTCACGC GCCTCCTGCG GTTCACAGCT GGCATGAAGG

GCTCCAGGAG GGCGTCCCGC GCCTCCTGCG CTTCGCTGCC GGGATGAAGG

CCTCCGGGAG GGCGTCACCC GCCTCCTGAG GTTCGCAGCC GGCGTGAGGG

CCTCCAGGAG GGTGTCACCC ATCTCCTGCG GTTTGCAGCA GGTGTGAAGA

CCTCCAGGAG GGCGTCACCC AGCTCCTGCA ATTTGCAGCA GGCCAGAAGG

GGTCCACGCG GGCGTCACCC GCCTCCTGAG GTTCACGACG GGCATGAAGA

GCTCCAAGAG GGAGTCACCC GCCTTCTGAG ATTCATGGCT GGCATGAAAG

CCTCCCGGCA GCAGCTGCAG GCCCAGCAGC AGGTCCAGGC GGCTCACAGT

CCTCCCGGCA GCAGCTGCAG GCCCAGCAGC AGGTCCAGGC GGCTCACAGT

CCTCCCGGCA GCAGCTGCAG GCCCAGCAGC AGGTCCAGGC GGCTCACAGT

CCTCCCGGCA GCAGCTGCAG GCCCAGCAGC AGGTCCAGGC GGCTCACAGT

CCTCTCGGCA GCAGCTGCAG GCCCAGCAGC AGGTCCAGGC GGCTCACAGT

---------- ---------- ---------- ---------- ----------

CGCTCCGGCA ACAGCTCCAG GCCCAGCAGC AGGTCCAGGC AGCCCACAGC

CCTTCCGGCA GCAGCTCCAG GCCCAGCAGC AGGTCCAGGC AGCCCACAGC

---------- ---------- ---------- ---------- ----------

CCTTCCGGCA GCAGCTGCAC GCCCAGCAGC AGGTGCAGGC GGCCCACAGT

CCTTCAGGCA GCAGCTGCAC GCCCAGCAGC AGGAGCAGGC GGCTCACAGT

CCTTCCGGCA GCAGCTGCGC ACCCAGCAGC AGATGCAGGC GGCGCACAGT

CCCAGCGGCA GCGGCTGCGC GCCCAGCAGC AGGCCCAGGC AGCGCACAGT

CCTTCCGGCA GCAGCTGCAG GCCCAGCAGC AGGTCCAGGC TGCCCACAGC

CTTCTCGGCA GCAGCTGCAA GCCCAGAAGC AGGCGCAGGC GGCCCACAGC

CCTCCCGGCA GCAGTGGCAC ACCCAGCAGC AGGCTCAAGT GGCTCATGGC

GCTTCCGCGG GCAGCTCCAC GCCCAGCGGC AGGCCCAGGC GGCCCACAGT

GCTTCCGCAG GCAGCTCCAG GCCCATCAAC AGGCCCAGGT GGCTGACCGA

CTCCATCGTG CCGTCCGCCG CTGTGCCACG CTCTGGAAAC AGAAAGTGCT

CTACATCGTG CCGTCCACCG CTGTGCCACG CTCTGGAAAC AGAAAGTGCT

CTCCATCGTG CTGTCCACCG CTGTGCCACG CTCTGGAAAC AGAAAGTGCT

CTCCATCGTG CCGTCCGCCG CTGTGCCACG CTCTGGAAAC AGAAAGTGCT

CTCCACCGTG CTGTCCGCCA CTGTGCCACA CTCTGGAAAC AGAAGGTGCT

---------- ---------- ---------- ---------- ----------

CTCCACTGTG CAGTCCGCCA CTGTGCTGAG CTCTGGAAGA AGAAGGTACT

CTCCACTGTG CAGTCCGCCA CTGTGCTGAG CTCTGGAAAA AGAAGGTGCT

---------- ---------- ---------- ---------- ----------

CTCCACCGCG CAGTGCGTCG TTGTGCCACG CTCTGGAAAC AGAAGGCGCT

CTCCACCGTG CAGTCCATCG CTGTGCCATG CTCTGGAAAC AGAAGGTTCT

CTCCACCGGG CAGTCCGTCG CTGTGCCATG CTCTGGAAAC AGAAGGTGCT

CTCCAGCGGT CGGTCCGGCG CTGCGCCACG CTCTGGAAGG AGAAGGTGCT

CGGCACCGGG CGGTCCGTCG CTGTGCTGCG CTCTGGAAGC AGAAGGCGCT

CTCCACCGCG CTGTCCGCCG CTGCGCCATG CTCTGGAAGC AGAAGGTGCT

CTCCACCGTG CTGTCCGCCA CTGCGCCATG CTCTGGAAGC AGAAAGCCCT

GTCCACCAGG TCGTCCGGCG CTGCGCCACC CTCTGGAAGC ACAAGGC-CT

CTCCACCAGA CTGTGCACCG TTGCGCCATG CTCTGGAAGC AGAAAGCTCT

GGGCCGGGGC GGGATCGCAC CCAGCAGGAA AGTGACGTTT GAGGCTGCTG

GGGCCGGGGC GGGATCGCAC CCAGCAGGAA AGTGACGTTT GAGGCTGCTG

GGGCCGGGGC GGGGTCGCAC CCAGCAGGAA AGTGACGTTT GAGGCCGCTG

GGGCCGGGGC GGGGTCGCAC CCAGCAGGAA GGTGACATTT GAGGCCACTG

GGGCCGGGGC AGGATCACAC CCAGCAGAAA AGTGACATTT GAGGCTGCTG

---------- ---------- ---------- ---------- ----------

CGGTCCAGAC AAGATCGCAT TCAGCAGAAG AGTGACGTTT AAGGCTGCTG

TGGTCCAGGC AAGACCACAT TCAGCAAGAG AGTGACATTT AAGGCTGCGG

---------- ---------- ---------- ---------- ----------

GGGTCGGGGC AGGACTGAGC CCAGCAGAAG AGTGACGTTT GAGGCCGCCG

GGGCCAGGGC AAGACCATGC CC-GCAGGAG AGTGACATTT GACGCTGCTG

GGGCCCAGGC AGG------- ----CAGGAG AGTGACATTC GAAAGTGCTG

GGGT--GGGC CGC------- ----CAGGAG AGTGACTTTC GAGTCTGCTG

GGGCACGGGC AGG-CCACCC CCAGCAGGAG AGTGACATTT GAGGCCGCTG

GGGCCCAGCC AGGGCCCTGC CCAACAGGAG AGTGACATTT GAGACTGCTG

GGGCCCAACC AAGATCACAG CCAGCCACAG AGTGACGTTC GAGTCTGCTG

TGGCCAAGGC CGC-CCACGC CCAG-AGGAG AGTGACGTTC GAGTCCCCTG

CGGCAAAGGC AGA--CGCCC CCAGGAAGAG AGTGACATTC AA-GCCCGTA

GGGCTGATGG CACCCTTGAG ACCAAGAGGC CACAGGCTTC TCGGCCTCTG

GGGCTGATGC CACCCTTGAG ACCAAGAGGC CACAGGCTTC TCGGCCTCCG

GGGCTGATGC CACCGTGGGG ACGAAGAGGC CACAGGCTCC TCTGCTTCAG

GGGCTGATGC CACCGTGGGG ACCAAGAGGC CACAGGCTCC TCTGCTTCAG

GGGCTGATGC CACCCTGGAG ACTAAGAGGC CACAGGCTCC TTGGTCTCAG

---------- ---------- ---------- ---------- ----------

AGGCTGATGC T--------- ---------- --------CC CCTGTCTCAG

AGGCTGATGC TACCCAGGAG ACAAAGAAGC TTCGAGCTCC TCCATCTCAG

---------- ---------- ---------- ---------- ----------

GGGCTGATGC CCCCCTGGAG ACCAAAAGGC TACCAGCTCC TCGGCTTTGG

GGGCTGATGC CACCCTGGAG ACCAGGAGGC CAGGAGATCC TCTGCCCTGG

GGGCTGATGC CACCCTGGAG ACCAGGAGGC CACGAGCCAC TCGGCCTCGG

GGGATGCCGC CACCTCGCAG ACCAAGAGGC CACGAGCTCC CCAGCCTTGG

GAGACGCCAC CAGCCTGGAG ACCCAGAGGC CGCCAGCGCC CCGGCCTGAG

GGGCTGATGC CGCCCTGGAG ACCAAGAGGC CGGAAGCGA- --GGCCTGAG

GGGCTGATGC CGCTCTGGAG ACCAAGAGGC CGAGAGCCA- --AGCCTGAG

CTGCTGAGGC TGCTGTGGGC CCGGAGCCGT CGCGAGTGCC TCGGCCCCAG

GGAGTGAAGC CTCTTTGGAG TCAAAAAGGC CGAGACCCTG GTTTTCTGGG

GGAGCTCTGG GCCGCCTGGC TGCTGAGGAG CCCCACGCCC TGGAGCTCAA

GGAGCTCTGG GCCGCCTGGC TGCTGAGGAG CCCCACGCCC TGGAGCTCAA

GGAGCTCTGG GCTGCCTGGC TGCTGAGGAG CCCCACGCCC GGGAACTCAA

GGAGCTCTGG GCCGCCTGGC TGCTGAGGAG CCCCACGCCC AGGAACTCAA

GGAGCTCTGG GCCGCCTGGC TGCTAGGGAG CCCCATGCCC TGGAATTCAA

---------- ---------- ---------- ---------- ----------

GGCGTCCTGG GAAGTTTGGC TGCTGGAGAG CCCTTCATCC CAGAACTCAA

GGAGTCCTGG GTAGTTTGGC TGCTGGGGAG CCCTGCCACC TAGACCTCAA

---------- ---------- ---------- ---------- ----------

GGAGCTTTGG ATGGCCTGGC CCCTGGGGAC CCCCAGCTCC TGGAGCTCAA

GGGGCTCTGG GCAGCCTGTC TGCCGGGGAC CCCCAACTTC TAGAGCTAAA

GGAGCTCTGG GCAGCCTGGC TGCCGGGGAC CCGGAACTCT TGGAGCTCAA

GAGACGCTAG GCAGCCTGAG TGCCTCGGAC CCTCGGCATC TGGAGCTTAA

GGCGGCCTGG GCGGCCTACT TGCTGGGGAC CCCCAACTCC TGGAGCTGAG

CGAGGTCTGG GCAGCCTAGC CGCTGGGGAT CCCCAGCTCC TGGAACTGAA

CCATGTTTGG GCAGCCTGGC TGCTGGGGAC TCCCAGCTCC TGGATCTGAG

GTGGGGTTGG GCCCTTCAGC TTCTGGGGAC CCCTACCTCC CCGACCCGCA

AAGGCACAAG GTTTGCCCAT TAGTCTGGAG TCTCCATTTT CTGATCTGAA

CACTGCCCAC TCAGCGAGGA AGCAGCCGCG ACGCCCACAC TTCCTGTTGG

CACTGCCCAC TCAGCGAGGA AGCAGCCGCG ACGCCCACAC TTCCTGTTGG

CACTGCCCAC TCAGCGAGGA AGCAGCCGCG ACGCCCACAC TTCCTGCTGG

CACTGCCCAC TCAGCGAGGA AGCAGCCGCG ACGCCCACAC TTCCTGCTGG

CACTGCCCAC TCATCGAGGA AGCAGCCGCG ACGCCCACAC TTCCTGCTGG

---------- ---------- ---------- ---------- ----------

TGAGGCCCGC CCATCACGGA AACAGCCTCG ACGTCCGTCC TTCCTGCTGG

TGCAGCCCGC TCATCACGGA AACAGCCCCG ACGTCCATCC TTCCTGCTTG

---------- ---------- ---------- ---------- ----------

TGCTGCCCGC TGGGCACGGA AGCAGCCGCG ACGCCCAAGC TTCCTGGTGG

TGCTACCCGC TTGGCAAGAA AGCAGCCTCG ACGTCCACAT TTCCTGCTGG

TGCTGTCCGC TTGGCAAGGA AGCAGCCGCG ACGCCCAGAC TTTCTCCTGG

TGTTGCCC-- ---------- ---------- ---------- ----------

CACCACCCGC TCGGCGAGGA AGCAGCCCCG ACGCCCCGAC TTCTTGCTGG

CACAGCCCGC TCCGCGAGAA AGCAGCCCCG ATGCCCTGAC TTCCTGCTGG

CGCTGCCCGC CCGGTGAGGA GGCAGCCCCG ATGCCCTGAT TTCCTGCTGG

CGCTGTCC-C CCGCCCAGGA GCCCCCCAAA AGGCGGCAGC CTCCCCTCGG

TCCCATCTAT CCTGTGAGAA AGCAGCCTCG TTGCCCTGAC TTTCTGATAG

AGCCTGCGCA GAGCCAGAGG CCTGCCCCTG GCCCGAAGCA GCCCCCGACG

AGCCTGCGCA GAGCCAGAGG CCTGCCCCTG GCCCGAAGCA GCCCCCGACG

AGCCTGCGCG GAGCCAGAGG CCTGCCCCTG GCCCAAAGCA GATCCCGACG

AGCCTGCGCA GAGCCAGAGG CCTACCTCTG GCCCAAAGCA GATCCCGACG

AACCTGCGCA GGGCCAGAGG CCTGCCCCTG GCCCAAAGCA GGCCCCGACA

---------- ---------- ---------- ---------- ----------

AGCGTGTGCA GAGCCAGAGG TTAGTTTCTG GTCCGAAGCT GCCTCCTACA

AGCGTTTGGG GAGCCAG--G TTAGTTCCTG ATCCGAAGCT GCCTCCTATG

---------- ---------- ---------- ---------- ----------

AGCCCTTGCA GAGCCAGGTG TCCGCCCCTG GCCTGAAGCT ACCACCCACA

AGCCTGTGCA GAGCCAGAGG TCTCGCCCTG GCCTGAAGCT GCCCCCCACA

AGCCCTTGCA GAACCAGAGG CCTccccccg gccTGAAGCT GCCCCCCACA

---------- -----AGAGG CCTTTCCGTG ACCCAGAGCT CATTCTCGAG

AGCCCGTACA GAGCCAGAGG TCACCCCCTG GCCCAAAGCT GCCCCCCGTG

ATCCCAAGCA GAGCCAGAGG CCTCCCCGTG GCCGAAGGCT GCCCTCTACG

AACCCGAGCA GAGCC---GG CCTCCCCCTG GCCTAAAGGA GCCCCCCACC

AGGGTGCGCT GGGACAGAGG CC-GGACTCG CCCCCCAGAA GGCCCCCA--

CGTCTCTGCA GAGAGAGAAA CCA--TCCAG GCCTAATGCG TCCTCCGG--

CTGCTGCCTC TTTCCTCCTT CATGCCTGGA CTTTCAACTG CAGGCAGCGA

CTGCTGCCTC CTTCCTCCTT CATGCCTGGA CTTTCAACTG CAGGCAGCGA

CTGCTGCCTC CTTCCTCCTT CATGCCTGGG CTTTCAACTA CAGGCAGCGA

CTGCTGCCTC CTTCCTCCTT CGTGCCTGGG CTTTCAACTA CAGGCAGCGA

CTGCTGCCTC CTTCCTCCTT CATGCCTGGG CTTTCAACTG CAGGCTGCGA

---------- ---------- ---------- ---------- ----------

CTGTTGCCTC CTTCCTCATT CATGCCTGGT TCTGAGACTA CAGGCCACGA

CTGCTGCCTC CCTCCTCCAT CATGCCTGTT TCCGAGGCTA CAGGCCACGA

---------- ---------- ----TCTGGT GCTGAAGCTG CAGGCCACGA

CTGCTGCCCC CATCCTCCTT TGCGCCTGGC TCTGACCCTG AAGGCCACGA

CTGCTGCCCC CTTCATCCTT CATGCGCAGC TCTGGAACCA TGGGCCACGA

CTGCTGCCTC CTTCCTCCTT CACGCTGGGC TCCGACACTA CAGGCCACAA

CTGCTGCCCC CTTCCTCC-- -AGGCCTGGC TCAGAAACTG TGGGCCACGA

CTGCTGCCCC CTTCCTCCTT CCTGCGTGGC TGTCAAACCG CAGGCTGCGA

CTATTGCCTC CTTCCTCCTT CATGCTTGGC TCTGAAGTGG CAGGTGGCGA

CTGCTGCCTC CCTCCTCCTT CGTGCTGGCC CCCGCATCGG CAGGCAGCCC

CCGGCGCCCC CCTGCTTCTG TAG-TTCTGG CCTAGGATCG TGGGGGATCA

CTCCTGCCTC CCTCCTCCTT CTTACCAGAA TGCTCATTCC CAGAGGGAGA

CCTTGAGGCT GAACTTGAGG AGATCCAGCA GCAACTACTG CACTACCAGA

CCTTGAGGCT GAACTTGAGG AGATCCAGCA GCAACTACTG CACTACCAGA

CCTTGAGGCT GAACTTGAGG GGATCCAGCA GCAACTACTG CACTACCAGA

CCTTGAGGCT GAACTTGAGG GGATCCAGCA GCAACTACTG CACTACCAGA

CCTTGAGGCT GAACTTGAGG GGATCCAGCA ACAACTACTG CACTACCAGA

---------- ---------- ---------- ---------- ----------

GCTTGAGGCC GAGTTGGAGG GCATCCAGCA GCAGTTACAA CACTACCAAA

GCTTGAGGCT GAATTGGAGG GGATCCAGCA GCAGTTACAG CACTACCAAA

CCTGGAGGCT GAGCTCGAGG GGATCCAGCA GCAGCTGCAG CACTACCAGA

CCTGGAGGCT GAGCTTGAGG GCATCCGGCA GCAGCTGCAG GACTACCAGA

CCTGGAGGCT GAGCTTGAGA GCATCCAGCA GCAACTGCAG GACTACCAGA

CCTGGAGGCT GAGCTTGAGG GCATCCAGCA GCAGCTGCAG GACTACCAGA

CCTGGAGGCC GAGCTAGAGG GCATCCAGCA GCAGCTGCGG CACTGCCAGA

ACTCAAGGCT GAGCTTGAGG GGATCCGCCA GCAATTTGAG CACTACCAGA

ACTTGAGGTT GAGCTCGAGG GGATCCGCCA GCAATTACAG CACTACCAGG

ACTGCAGGCT GAGCTCGAGG GGATCCGCCA GCAGTTACAG CACTACCAGA

GCTGGAGGCT GAGCTGGAGG AGATCCGGGA GAGGCTGTGC AGTTACCAGG

ACTAGAGGCT GAGCTGCAAG AGATCCAACA GAACCTACAA CAGTATCATG

CCACCAAGCA GAACCTCTGG TCCTGTCGGC GGCAAGCGAG CAGCCTGCGC

CCACCAAGCA GAACCTCTGG TCCTGTCGGC GGCAAGCGAG CAGCCTGCGC

CCACCAAGCA GAACCTCTGG TCCTGTCAGC GGCAAGCGAG CAGCCTGCGC

CCACCAAGCA GAACCTCTGG TCCTGTCAGC GGCAAGCGAG CAGCCTGCGC

CCACCAAGCA GAACCTCTG- ---------- ---------- ----------

---------- ---------- ---------- ---------- ----------

CCACCAAGCA GAACCTTTGG TCATGCCAGC GACAAGCCAA CAGCCTGCGT

CCACCAAGCA GGACCTTTGG TCATGCCAGC AACAAGCCAA CAGCCTGCGT

GCACCAAGCA GAACCTCAG- ---------- ---------- ----------

CCACAAGGCA GAACCTCAGG TCCTGCCAGC GGCAAGCACA CAGCCTGAGG

CCATGAAGCA GAATCTCTCG TCCTGCCAGC GGCAAGCAAG AAGCCTGCGC

CCACGAAGCA GAACCTCCAG TCCTACCAGC GGCAAGCGAG CAGCCTGCGC

TCACGAAGGA GAACCTCTG- ---------- ---------- ----------

CCAACCAGCA GAACCTCCGG TCCTGCGAGC GGCAAGCCAG CAGCCTGCGC

CCACCAAGCA GAACCTCTGG TCCTGCCAGC GGCAAGCGAG CAGCCTGCGC

CCACCAAGCA GAACCTCTGC TCCTGCCAGC GGCAAGCCAG CATCCTGCGT

CCAACAAGCA GACCCTCAAG TCCTGGCAGA GACAGGCGGA CAGCCTGAGG

CCAACAGGCA GAATCTCAAG TCTTGGAAAA GGCAGGCAAA TAGCTTGCGC

AGGTGGCTGG AGCTGAACAG AGAGGAGCCG GGGCCTGAGG ACCAGGAAGT

AGGTGGCTGG AGCTGAACAG AGAGGAGCCG GGGCCTGAGG ACCAGGAAGT

AGGTGGCTGG AGCTGAGCAG AGAGGAGCCT GGACCTGAGG ACCAGGAAGT

AGGTGGCTGG AGCTGAGCAG AGAGGAGCCC GGACCTGAGG ACCAGGAAGT

---------- ---------- ---------- ---------- ----------

---------- ---------- ---------- ---------- ----------

AGGTGGCTGG AGCTAAGCCA GGAGGAGCCA AGCTGTGAGG ACCTCCATCT

AGGTGGCTGG AGCTAAGCCA GGAGGTGCCG AGATCTAAGG ACCTTCATCT

---------- ---------- ---------- ---------- ----------

CGGTGGCTAG AGCTGAGCAG GGCGGAGCCC CGGGCTGAGG ATCAGGAAGC

AGGTGGCTGG AGCTGAGCCA GGAGGAGCCC AGGCCTGAGG ACCAGGAAGC

CGGTGGCTGG AGCTGAGCCG GGAGGAGCCC AGGTCTGAAG ACCAGGAAGC

---------- ---------- ---------- ---------- ----------

AGGTGGCTGG CGCTGAGCCA GGAGGAGCCC CGGCCTGAGG CCCAGGCAGC

AGGTGGTTGG AGCTGAGCCG GGAGGAGCCC AGGCCTGAGG ACCAGGAAGC

AGGTGGCTGG AGCTGAGCCG GGAGGAGCCC CGACCAGAGG ACCAGGAAGC

AGGTGGTTGG CGCTGAGCAC GGAGGACCCC AGGCCAGACG AGGAGGAAGC

AGATGGCTGG AACTGAGCAC GGCGGATTCA AGGCCCGAGG AAGAAGAAAC

AGAGCAGCAG GTGCAGAAAG AGCTGGAAGT GGAAATGCAG ATCCAGCTGC

AGAGCAGCAG GTGCAGAAAG AGCTGGAAGT GGAAATGCAG ATCCAGCTGC

AGAGCAGCAG GTGCAGAAAG AGCTGCAAGT GGAACTGCAG ATCCAGCAGC

AGAGCAGCAG GTGCAGAAAG AGCTGCAAGT GGAACTGCAG ATCCAGCAGC

---------- ---------- ------AAGT GGAACTGCAG ATCCAGCAGC

---------- ---------- ---------- ---------- ----------

AGAAGAGCAG GTGAAAACAG AACTGG--GT GGAACTACAG ATCCAGCAGC

AGAAGAGCAG GTGAAAACCG AACTGG--GT GGAACTGCAG ATCCAGCAGC

---------- ---------- ---------- ---------- ----------

TGAGCAGCAG GTTGAGGAGG AGCTGCAGGT GGAGGTGCAG ATCCAGCAGC

AGAGCAGCAG GTGCAGGAAG AACTACAGGT GGAGCTGCAG ATCCAGCAGC

GGAGCAGCAG GTGCAGGAAG AACTGCAGGT GGAACTGCAC ATCCAGCAGC

---------- ---------- ---------- ---------- ----------

GGAGCAGCAG GTGCGGGAAG AGCTGGAAGT GGAAGCGCAG ATTCAGCGCC

AGAACAGCAG ATGCAGAAAG AGCTGGAGGT GGAAGCACAG ATCCAGCACC

AGAGCAGCAG GTGCAGAAGG AGCTGGAGGT GGAAGCACAG ATCCAGCACC

AGGCCACCAG GTGCAGCAAG AGCCGGggGT GGAGAAGCAG ATCACGCACC

AGTGCAGCAA GTTCAAAAAG AACTCC--AC TGAACTGCAG ATCAGCCGCC

TGGCAGAGGA GCTCCAGGCT CAGCGCCAGC CCATTGGCGC CTGCGTTGCC

TGGCAGAGGA GCTCCAGGCT CAGCGCCAGC CCATTGGCGC CTGCGTTGCC

TGGCAGAGGA GCTCCAGGCT CAGCGCCAGC CCATTGGCAC CTGCATCGCT

TGGCAGAGGA GCTCCAGGCT CAGCGCCAGC CCATTGGCAC CTGCATTGCT

TGGCAGAGGA GCTCCAGGCC CAGCGCCAGC CTACTGGCGC CTGCATTGCC

---------- ---------- ---------- ---------- ----------

TGGCTAAGGA GCTCGAGGCC CAGCGGCAGC CTGTCGGTAC ATGCATTGCT

TGGCTAAGGA GCTCGAGGCC CAGTGTCAGC CTG------- ----------

---------- ---------- ---------- ---------- ----------

TGGCCCATGA GCTGCAGGCC CGGCGCCAGC CCGTCCACAC CTGCATCTCT

TGGCCTCTGA GCTCCAGGCC CAGCGCCAGC CCATCCGCGC CTGCATCGCC

TGGCTGCACA GCTCCAGGCC TGGCGCCGGC CCGTGGACGC CTGCATTGCC

---------- ---------- ---------- ---------- ----------

TGGCCGCAGA GCTCCAGGCC CAGCGCCAGC CCATCCGCGC CTGCATCGCA

TGGCTGCCGA GCTCCAGGCC CAACGCCAGC CTGTCAGTGC CTGCATAGCC

TGGCGGCCAT GCTCCGGGCC CAGCACCAGC CCATTGGCGC CTGCATTGCC

TGGCGGAGGC ACTGCGGATG GAGAGGCAGC AGGTGCTGCG GGACACGGCT

TCTCCGAGGA GCTGCGAGAA GAGAGGCCTC GAGTTCAGCA TTACATCAGC

CGCATCCAGG CCCTGCGGCA GGCCCTGTGC ATGGACAGTA GCACCACCAC

CGCATCCAGG CCCTGCGGCA GGCCCTGTGC ATGGACAGTA GCACCACCAC

CGCATCCAGG CCCTGCGGCA GGCCCTGCGC ATGGACAGTA GCACCACCAC

CGCATCCAGG CCCTGCGGCA GGCCCTGCAC ATGGACAGTA GCACCACCAC

CGAATCCAGG CCCTGCGGCA GGCCCTGGGC ATGGCCAGCA GCACCGCCAT

---------- ---------- ---------- ATGGAAAGCA GTACCGCCAC

CGTGTTCGGG CCCTGAGACG GGCTCTGTGC ATGGACAGTA GCACCGTTAC

---------- -----AGACG GGCTCTGTGC ATGGACAGTA GCACCGTTAC

---------- ---------- ---------- ATGGACAGCA GCACTGCCAC

CGGGTCCAGG CCCTGCGGCG GGCTCTGTGC ATGGACAGTA GCACCGCCAC

CGTGTCCAGG CCCTGCGGCA GGCTCTGTGC ATGGACAGTA CCACCACCAA

CGTGTCCAGG CCCTGCGGCG AGCTCTGTGC ATGGACAGTA GCACCACCCC

---------- ---------- ---------- ATGGACAGTA CCACCGCTGA

CGCGTCCAGG CCCTGCGGCA GGCTCTGTGC ATGGACAGCA GCACCACCAC

CGTGTCCAGG CCCTGCGGCA GGCCCTGTGC ATGGACAGCA GCACCGTCAC

CGCGTCCGGG CCCTGCGGCA GGCCCTGGGC ATGGACCGCA GCACCGTCAC

CGCATCCAGG TCCTGCGGGC TGCCTTCTGT ATGGA---TA TCACTGATCC

CGCGTCCAGG ACCTTCGGGT AGCTTTCCGT ATGGACCTGA CGA---CCAC

CGCGGTCACC CGGCCTGTTG AGACCCACGA GCTCATTCGC AATGCAGCCG

CGCGGTCACC CGGCCTGTTG AGACCCACGA GCTCATTCGC AATGCAGCCG

CTCGGTCACC CGGCCTGTTG AGACCCACGA GCTCATTCGC AATGCAGCTG

CGCGGTCACC CGGCCTGTTG AGACCCACGA GCTCATTCGC AATGCAGCTG

CTCCAT---- ---------- ---------- ----ATCCAA AATGCTGCTG

CACCACCGCC GCGCCCCTGG AGTCCTACGA ACGCATCCGC AATGCGGCCG

TGCCACCGAT GCACCCATCC AGTCCTATGA GCGCATTCGC AATGCGGCTG

TGCCACCGAT GCACCCATCC CGTCCTACGA GCGCATTCGC AATGCGGCTG

CTCCACCGCG GCCCCCCTTG AGTCCTATGA GCGCATCCGC AATGCGGCCG

GGCCACCGCC GAGCCCCTCC CGCCCTACGA GCGCATCCGC AATGCAGCTG

CGCCACTGCC TCACCCCTCG CGCCCCACGA GCGCATCCGC AATGCCGCCG

TGTCACCGCC GCGCCCCTCG CGTCCCACGA GCGCATCCGC AATGCGGCCG

CCCCAAGTTT GAGGATCTTT C------CCA GTACATCCGC AATGCGGCCG

TGCCACCGCC GCGCCCCTCG CGCCCCACGA GCGCATCCGC AATGCGGCCG

CACCGCCGCC ---GCGCCCG AGTCCTACGA ACGCATCCGC AATGCCGCCG

CACTGTCGCC CCAACTCTCG CGCCCCATGA GCGCATCCGC AATGCCGCCG

TGCTACCCCC ACACCGCCCT ACCCATCTTA TAGAATCAAG AATGCCGCAG

TGCCCTCCCC CACCCCCCCC AGGCCTCCAA CCACATCAGG AACCCCGCCA

ATATCTCCAT CATCGTTATC TACTTCGTGG TAGTGATGGC CGTCGGAGCT

ATATCTCCAT CATCGTTATC TACTTCGTGG TAGTGATGGC CGTCGGAGCT

ACATCTCTGT CATCGTTATC TACTTCGTGG TGGTGATGGC CGTCGGAGCT

ACATCTCTGT CATCGTTATC TACTTCCTGG TGGTGATGGC CGTCGGAGCT

ACATCTCCGT CATTGTCATC TATTTTCTGG TGGTGATGGC TGTTGGGGCA

ATATCTCCGT GATTGTCATC TACTTCGTGG TGGTGATGGC CGTCGGGGCT

ACATATCAGT CATCGTCATC TACTTCGTGG TGGTGATGGC TGTCGGGGCC

ACATCTCAGT CATCGTCATC TACTTCGTGG TGGTGATGGC TGTCGGGGCC

ACATCTCCGT CATCGTCATC TACTTCTTGG TGGTGATGGC CGTCGGGGCA

ACATCTCTGT CATCGTCATC TACTTCGTGG TGGTGATGGC TGTCGGGGCC

ACATCTCTGT CATCGTCATC TACTTTGTGG TGGTGATGGC TGTGGGGGCT

ACATCTCTGT CATCGTTATC TACTTCGTGG TGGTGATGGC TGTCGGGGCT

ACATCTCCGT CATCGTCATC TACTTCGTGG TGGTCATGGC TGTGGGAGCT

ACATCTCCGT CATCGTTGTC TACTTCGTGG TGGTGATGGC TGTCGGGGCT

ACATCTCGGT CATCGTCATC TACTTCTTAG TGGTGATGGC CGTCGGGGCT

ACATCTCGGT CATCGTCGTT TACTTCGTGG TGGTGATGGC TGTCGGGGCT

ACATCTTTGT AATTGTCATC CACTTCTTGG TGGTCTTGGC TGTAGG-GCC

ACATTGCCGT CATTGTCATC TACTTCTTTG TCATCATGGC CACGGGGGCC

ATGTTTTCCA CCAATCGTGG GACTGTTGGA GGCTTCTTCC TGGCAGGCCG

ATGTTTTCCA CCAATCGTGG AACTGTTGGA GGCTTCTTCC TGGCAGGCCG

ATGTTTTCCA CCAATCGTGG AACTGTTGGA GGCTTCTTCC TGGCAGGCCG

ATGTTTTCCA CCAATCGTGG AACTGTTGGA GGCTTCTTCC TGGCAGGCCG

ATGCTGAGGA CCAACCGAGG CACTATAGGA GGCTTCTTCC TCGCTGGTCG

ATGTTTTCTA CTAATCGTGG CACGGTTGGC GGCTTTTTCC TGGCAGGCCG

ATGTTTTCCA CTAATCGTGG GACTGTTGGA GGCTTCTTCC TGGCAGGCCG

ATGTTTTCCA CTAATCGTGG TACCGTTGGA GGCTTCTTCC TGGCAGGCCG

ACACTGAAGA CAAACCGCAG CACTGTAAGA GAATTCTTCT TGGCTGGTCG

ATGTTTTCCA CTAACCGTGG GACCGTTGGA GGCTTCTTCC TGGCAGGTCG

ATGTTTTCCA CTAATCGTGG GACTGTTGGA GGCTTCTTCT TGGCAGGACG

ATGTTTTCCA CTAATCGTGG GACCGTTGGA GGATTCTTCC TGGCAGGACG

ATGTTTTCCA CCAACCGTGG GACTGTTGGA GGTTTCTTCC TGGCAGGCCG

ATGTTTTCCA CTAATCGTGG GACAGTTGGA GGCTTCTTCC TGGCAGGCCG

ATGTTTTCCA CTAATCGTGG GACTGTTGGA GGCTTCTTCC TGGCAGGTCG

ATGTTTTCCA CGAATCGTGG GACCGTTGGA GGCTTCTTTC TGGCAGGCCG

TTGCTGAAAA CAAGTCGAGG AACAGTGGGA GGCTTTTTCC TGGCTGGCCA

ATGCTGTCGA CCAATCGGGG AACAGTTGGA GGCTTCTTCC TGGCTGGCAG

AAGTATGGTG TGGTGGCCGA TGGCCACCGC TGAGACCCCA CCACCTCCAT

AAGTATGGTG TGGTGGCCGA TGGCCACCGC TGAGACCCCA CCACCTCCAT

AAGTATGGTG TGGTGGCCGA TGGCCACCAC TGAGACCCCA CCACCTCCAT

AAGTATGGTG TGGTGGCCGA TGGCCACCAC TGAGACCCCA CCACCTCCAT

TGATGTGGCC TGGTGGCCGA TGGCCACCGC TGGGGCCCCA CCATCTCCAT

AAGTATGGTG TGGTGGCCGA TGGCCACCAC TGGGACCCCA GCACCCGCAT

AAGTATGGTG TGGTGGCCGA TGGCCACCAC CGGAAGCTCA CTGTCTTCAC

AAGTATGGTG TGGTGGCCGA TGGCCACCAC TGCAAGCTCA CTGTCTTCAC

AGACGTGGCC TGGTGGCCGA TGGCCACCGC TGGGACCCCA CCACCTCCAC

AAGTATGGTG TGGTGGCCGA TGGCCGCTAC TGAGACCCCC CCTCCTGAGA

GAGTATGGTG TGGTGGCCGA TGGCTACCAC TGGGACCCTT CCTCCTGAAA

AAGTATGGTG TGGTGGCCGA TGGCC---AC TGGGACCCCA CCTCCTGAGA

AAGTATGGTG TGGTGGCCGA TGGAC----- -----CCCCA CTGACCCCAC

AAGTATGGTG TGGTGGCCGA TGGACACCAC TGCCACCGCC CCCCTCGCGC

AAGCATGGTG TGGTGGCCGA TGGACGTCAC CGCCGCC--- GCGCCCGAGT

GAGTATGGTG TGGTGGCCGA TGGACGTCAC TGTCGCCCCA ACTCTCGCGC

GGACATGGTG TGGTGGCCAA TGGATGATGC TGGAACCCCC CCGCCCTACC

GAGTATGGTG TGGTGGCCGc gggc---TAC TGCCCTCCCC CCCCCCCAGG

TGTCTGACCA CATCCGAAAT GCTGCTGACA TCTCAGTCAT TGTCATCTAT

TGTCTGACCG CATCCGAAAT GCTGCTGACA TCTCAGTCAT TGTCATCTAT

TGTCTGATCG CATCCAAAAT GCTGCTGACA TCTCAGTCAT TGTCATCTAT

TGTCTGATCG CATCCGAAAT GCTGCTGACG TCTCAGTCAT TGTCATCTAT

TGTCTGATCG AATCCAAAAT GCTGCTGACA TCTCCGTCAT TGTCATCTAT

TGTCGGAGCG TATCCAAAAC CCTGCAGACA TCTCTGTGAT TGTCATCTAT

TGTCAGACAA CATCAATAAT CCTGCTGATA TCTCTGTCAT TGTCATCTAC

TGTCAAATAA CATCAATAAT GCTGCTGACA TCTCTGTCAT CGTCATCTAT

TGTCAGAGCG TCTCCAAAAT GCTGCTGACA TCTCTGTCCT TGTCGTCTAC

CATCAGAGCG TATACAAAAC GCTGCTGACA TCTCCGTCAT TGTCATCTAT

TGTTGGACCG CATCCACAAT GCTGCTGACA TCTCTGTCAT TGTCATCTAT

TGTCAGAGCG CATCCGAAAC GCTGCTGACA TCTCCGTCAT TGTTGTCTAT

TTTCCCAGTA CATCCGCAAT GCGGCCGACA TCTCCGTCAT CGTCATCTAC

CCCACGAGCG CATCCGCAAT GCGGCCGACA TCTCCGTCAT CGTTGTCTAC

CCTACGAACG CATCCGCAAT GCCGCCGACA TCTCGGTCAT CGTCATCTAC

CCCATGAGCG CATCCGCAAT GCCGCCGACA TCTCGGTCAT CGTCGTTTAC

CATCTTATAG AATCAAGAAT GCCGCAGACA TCTTTGTAAT TGTCATCCAC

CCTCCAACCA CATCAGGAAC CCCGCCAACA TTGCCGTCAT TGTCATCTAC

TTTCTGGTGG TGATGGCTGT TGGGGCGATG CTGAAGACCA ACCGAGGTAC

TTTCTGGTGG TGATGGCTGT TGGGGCGATG CTGAAGACCA ACCGAGGTAC

TTTCTGGTGG TGATGGCTGT TGGGGCGATG CTGAGGACCA ACCGAGGTAC

TTTCTGGTGG TGATGGCTGT TGGGGCGATG CTGAGGACCA ACCGAGGTAC

TTTCTGGTGG TGATGGCTGT TGGGGCAATG CTGAGGACCA ACCGAGGCAC

TTTGTGGCAG TAATGGCTGT CGGGGCAATG CTGAGAACCA ACCGGGGCTC

TTTGTGGTGG TGATGGCTGT TGGAGCAATG GTGAAGACAA ACCGAAGCAC

TTTGTGGTGG TCATGGCTGT TGGAGCAATG CTGAAGACGA ACCGAAGCAC

TTCATAGTGG TGATGACTGT TGGGGCAACA CTGAAGACAA ACCGCAGCAC

TTCGTGTTGG TGATGGCCGT TGGAGCAATG CTGAGGACCA ACCGGGGTAC

TTCGTGGTGG TGATGGCTGT TGGGGCAATG CTGAAGACCA ACCGGGGCAC

TTCGTGGTGG TGATGGCTGT CGGGGCCATG CTGAAGACCA ACCGGGGCAC

TTCGTGGTGG TCATGGCTGT GGGAGCTATG TTTTCCACCA ACCGTGGGAC

TTCGTGGTGG TGATGGCTGT CGGGGCTATG TTTTCCACTA ATCGTGGGAC

TTCTTAGTGG TGATGGCCGT CGGGGCTATG TTTTCCACTA ATCGTGGGAC

TTCGTGGTGG TGATGGCTGT CGGGGCTATG TTTTCCACGA ATCGTGGGAC

TTCTTGGTGG TCTTGGCTGT AGG-GCCTTG CTGAAAACAA GTCGAGGAAC

TTCTTTGTCA TCATGGCCAC GGGGGTTATG CTGAAAATCA GTCAAGGAAC

TATAGGAGGC TTCTTCCTCG CTGGTCGTAT GAATTTCCTC CGGCGACGTC

TATAGGAGGC TTCTTCCTCG CTGGTCGTAT GAATTTCCTC CGGCGACGTC

TATAGGAGGC TTCTTCCTCG CTGGTCGTAT GAATTTCCTC CGGCGACGTC

TATAGGAGGC TTCTTCCTCG CTGGTCGT-- ---------- ----------

TATAGGAGGC TTCTTCCTCG CTGGTCGTAT GAATTTCCTC CGTCGACGTC

CGTGGGAGGC TTCTTCCTGG CTGGCCGAAT GAATTTCCTC CGGCGGCGTC

TGTTGGAGGA TTCTTTCTGG CTGGTCGAAT GAACTTCCTC CGGAGGCGGC

TGTTGGAGGC TTCTTTCTGG CAGGTCGAAT GAACTTCCTC CGGAGGCGGC

TGTAAGAGAA TTCTTCTTGG CTGGTCGAAT GAACTTCCTC CGGCGGCGTC

CGTTGGAGGC TTCTTTCTGG CTGGTCGAAT GAATTTCCTC CGGCGGCGCC

CATTGGAGGT TTCTTTTTGG CTGGTCGAAT GAATTTCCTC CGGCGGCGCC

TGTTGGAGGC TTCTTCCTGG CTGGTAGAAT GAATTTCCTC CGGCGGCGGC

TGTTGGAGGT TTCTTCCTGG CAGGCCGA-- ---------- ----------

AGTTGGAGGC TTCTTCCTGG CAGGCCGAAT GAATTTCCTC CGGCGGCGCC

TGTTGGAGGC TTCTTCCTGG CAGGTCGAAT GAATTTCCTC CGGCGGCGCC

CGTTGGAGGC TTCTTTCTGG CAGGCCGG-- ---------- ----------

AGTGGGAGGC TTTTTCCTGG CTGGCCAGAT GAACTTCCTC CGGCGCCGCC

AGTTGGAGGC TGCTTCTTAG CTGGCCGGAT GAATTTCCTC AGACGACGTC

TCTCTGACAG CAGCTTCATG GCCAACCTGC CTAATGGCTA TATGACGGAC

TCTCTGACAG CAGCTTCGTG GCCAACCTGC CTAATGGCTA TATGACGGAC

TCTCTGACAG CAGCTTCGTG GCCAACCTGC CTAATGGCTA TATGACGGAC

---------- ---------- ---------- ---------- ----------

TCTCTGACAG CAGCTTCGTG GCCAACCTGC CTAATGGCTA CATGACGGAC

TCTCCGACAG CAGCTTCGTG ACCAACCTGC CCAATGGCTA CATGCCGGAC

TGTCAGACAG CAGCTTTGTG GCCAACCTGC CCAATGGCTA CATGCCGGAC

TGTCTGACAG CAGCTTTGTG GCCAACCTGC CCAATGGCTA CATGCCGGAC

TCTCAGACAG CAGCTTTGTG GCCAACCTGC CCAATGGCTA CATGGCGGAC

TCTCTGACAG CAGCTTTGTG GCCAACCTGC CTAACGGCTA CATGACGGAC

TCTCTGACAG CAGTTTTGTG GCCAACCTGC CTAATGGCTA CATGACAGAC

TCTCTGACAG CAGCTTCGTG GCCAACCTGC CTAATGGCTA CATGACGGAC

---------- ---------- ---------- ---------- ----------

TCTCCGACAG CAGCTTCGTG GCCAACCTGC CTAATGGCTA CATGACAGAC

TGTCTGACAG CAGCTTCGTG GCCAACCTGC CTAATGGCTA CATGACAGAC

---------- ---------- ---------- ---------- ----------

TCTCTGACAG CAGCTTCGTG GCCAACTTGC CCAACGGCTA CATGATGGAC

TGTCGGACAG CAGCTTCGTG GCCAACTTGC CCAATGGCTA CATGATGGAC

CTGCAACGCC CAGATAGCTC CACCAGCTCA CCTGCTTCCC CCGCCATGGA

CTGCAACGCC CAGATAGCTC CACCAGCTCA CCTGCTTCCC CGGCCATGGA

CTGCAGCGCC CAGATAGCTC CACCAGCTCA CCTGCTTCCC CGGCCATGGA

---------- ---------- ---------- ---------- ----------

CTGCAACGCC CAGATAGCTC GTCCAGCTCA CCTGCTTCCC CGGCCATGGA

TTGCAGCGCC CAGACAGCTC CACCAGCTCC CCTGCCTCCC CGGCCATGGA

CTGCAGCGCC CAGAGAGCTC CAGTAGCTCC CCAGCATCCC CAGCCACAGA

CTGCAGCGCC CAGAGAGCTC CAGTAGCTCT CCAGCATCCC CGGCCACAGA

CTTCAACGCC CAGATAGCTC CACCAGCTCC CCGGCCTCCC CAGCCATGGA

CTGCAGCGCC CAGACAGCTC CACCAGCTCA CCTGCATCCC CCGCCATGGA

CTGCAGCGCC CAGACAGCTC CACCAGCTCC CCTGCCTCCC CAGCCATGGA

CTGCAGCGCC CAGATAGCTC CACCAGCTCC CCTGCTTCCC CGGCCATGGA

---------- ---------- ---------- ---------- ----------

CTGCAGCGCC CAGAAAGCTC CACCAGCTCC CCCGCCTCCC CAGCCATGGA

CTGCAGCGCC CAGATAGCTC CACCAGCTCC CCTGCCTCCC CGGCCATGGA

---------- ---------- ---------- ---------- ----------

CTGCAACGTC CCAGCAGTTG CACCAGCTCC CCCGTTTCCC CAGCGATGGA

CTGCAACGTC CCGACAGCTC CACCAGCTCT CCGGTTTCCC CGGCAACTGA

GAGGAGGCAC CCCCAGCCCC TGGCTGCCTC CTTCTCCTCT CCAGGATCCA

GAGGAGGCAC CCCCAGCCCC TGGCTGCCTC CTTCTCCTCT CCAGGATCCA

GAGGAGGCAC CCCCAGCCCC TGGCTGCCTC CTTCTCCTCT CCAGGATCCA

---------- ---------- ---------- ---------- ----------

GAGGAGGCAC CCCCAGCCCC TGGTTGCCTC CTTCTCCTCT CCAGGATCCA

GAGGAGGCAC CCGCAGCCCC TGGCCGCCTC CTTCTCCTCT CCGGGATCCA

GAGAAGGCAT CCCCAGCCCC TGGCTGCCTC CTTCTCTTCT CCAGGATCCA

GAGGAGGCAC CCCCAGCCCC TGGCTGCCTC CTTCTCATCT CCAGGATCCA

GAGGAGGCAC CCCCAGCCCC TGGCAGCCTC CTTCTCCTCA CCAGGATCCA

GAGGAGGCAC CCTCAGCCCC TGGCAGCCTC CTTCTCCTCT CCAGGATCCA

AAGGAGGCAC CCCCAGCCCC TGGCAGCCTC CTTCTCCTCT CCAGGATCCA

GCGGAGGCAC CCCCAGCCCC TGGCAGCCTC CTTCTCCTCT CCAGGATCCA

---------- ---------- ---------- ---------- ----------

GAGGAGGCAC CCCCAGCCCC TGGCAGCCTC CTTCTCTTCT CCAGGATCCA

GAGAAGAG-- -CCCAGCCCC TAGCCGCCTC TTTCTCTTCC CCAGGATCCA

---------- ---------- ---------- ---------- ----------

GAGAAGGCAT CCACAGCCTT TGTCTGCCTC CTCCTCCTCC TCTGGCTCCA

GAGGAGGCAT CTTCAGCCTA CCACACCGTC TTCATCCTCC TCGGGGCCCA

GCCTTTTTAG CTCCCTCTCC AGTGCCATGA AGCAGGCCCC TCAGGCCACC

GCCTTTTTAG CTCCCTCTCC AGTGCCATGA AGCAGGCCCC TCAGGCCACC

GCCTTTTTAG CTCCCTCTCC AGTGCCATGA AGCAGGCCCC TCAGGCCACC

---------- ---------- ---------- ---------- ----------

GCATTTTTAG CTCCCTCTCC AGTGCCATGA AGCAGGCCCC TCAGGCTTCC

GCCTGTTTAG CTCCCTCTCC AGTGCCATGA AGCAGACCCC ACAGACCCCC

GCCTGTTCAG CTCCTTCTCC AGTGCCATGA AACAGACCCC ACAGGCCCCG

GCCTGTTCAG CTCCTTCTCC GGTGCCATGA AACAGACCCC ACAGGCTCCC

GCCTTTTTAG CTCCTTCTCC AGTGCTGTGA AGCAGACCCC TCAGACCCCG

GCCTTTTTAG CTCCTTCTCC AGTGCCATGA AGCAGACCTC ACAGGCCCCC

GTCTCTTTAG CTCCTTCTCC AGTGCCATGA AGCAGACCTC ACAAGCCCCC

GCCTTTTTAG CTCCTTCTCC AGTGCCATGA AGCAGACCTC GCAGGCCCCC

---------- ---------- ---------- ---------- ----------

GCCTTTTTAG CTCCTTCTCC AGTGCCATGA AGCCAACCTC ACAAGTTCCA

GCCTTTTTAG CTCCTTCTCC AGTGCTATGA AGCCGACCTC ACAGGCTCCC

---------- ---------- ---------- ---------- ----------

GCCTCTTCAG TTCCCTCTCT AGTGCCATGA AGCAGACCAC ACAGGCAGCC

GTCTCTTCAG CTCATTCTCG AGCGCCATGA AACAGACCGC ACAAGCCACC

TCAGGACTGA TGGAGCCTCC AGGTCCCTCC ACGCCCATTG TTCAAAGACC

TCAGGACTGA TGGAGCCTCC AGGTCCCTCC ACGCCCATTG TTCAAAGACC

TCAGGACTGA TGGAGCCTCC AGGTCCCTCC ACGCCCATTG TTCAAAGACC

---------- ---------- ---------- ---------- ----------

TCAGGGCTGA TGGAGCCTCC TGGTCCCTCC ACGCCTGTTG TTCAAAGACC

TCAGGGCTCA TGGAGCCTCT GGGTCCCTCC ACACCTGTTG TTCAAAAACC

ACAGGGCTAA TGGAACCACC AACTCCTGTC ACACCTGTGG TTCAAAGACC

TCAGGGCTGA TGGAACCACC CACTCCTGTC ACACCCGTGG TTCAGAGACC

GTGGGCGTGG TGGAGCCTCC GGTTCCCTCC ACACCGGTTG TTCAAAGACC

TCGGGGCTGA TGGAGCCTCC GGGTCCCTCC ACGCCTGTTG TTCAGAGGCC

GCAGGGCTGA TGGAGCCTCC AGGTCCCTCT GCTCCCATTA TTCAAAGGCC

TCGGGGCTGA TGGAGCCTCT GGGTCCCTCC ACACCTGTCG TTCAAAAGCC

---------- ---------- ---------- ---------- ----------

TCAGGGTTGA TGGAACCTCC AGGCCCCTCC ACGCCTGTTG TTCAAAGGCC

TCAGGTTTGA TGGAGCCTCC AGCTCCCTCC ACACCTGTTG TTCAAAGGCC

---------- ---------- ---------- ---------- ----------

TCGGGGATGA TGGAGCCCCC AGCACCCCCT ACACCCATCA TCCAGAAACC

TCGGGGCTGT CGGAACCAGC GGGAAGCCCG GCACCCATAC CTCAGAAACC

CAGGATCCTG TTGGTGATCG ATGATGCCCA TACAGACTGG TCGAAGTATT

CAGGATCCTG TTGGTGATCG ATGATGCCCA TACAGACTGG TCAAAGTATT

CAGGATCCTG TTGGTGATCG ATGATGCCCA TACAGACTGG TCAAAGTATT

---------- ---------- ---------- ---------- ----------

CAGGATCCTG TTGGTAATTG ATGATGCCCA TACAGACTGG TCCAAGTATT

CAGGATCCTG TTGGTGATTG ATGACGCACA CACAGACTGG TCAAAGTATT

CAGGATCTTG TTGGTCATTG ATGATGCCCA CACAGACTGG TCGAAATATT

CAGGATCTTG TTGGTTATCG ATGACGCCCA TACAGACTGG TCAAAGTATT

CAGGATCTTG CTGGTGATTG ACGACACCCA CACAGACTGG TCTAAGTATT

CAGGATCCTG CTGGTGATCG ACGATGCCCA CACAGACTGG GCCAAGTATT

CAGGATCTTG TTGGTGATCG ATGATGCCCA CACAGATTGG ACCAAGTATT

CAGGATCCTG TTGGTGATCG ATGATGCCCA CACAGACTGG GCCAAGTATT

---------- ---------- ---------- ---------- ----------

CAGGATCCTG CTGGTGATCG ACGATGCCCA TACAGACTGG ACCAAGTACT

TAGGATCCTA CTGGTGATTG ATGATGCCCA TACGGACTGG GCCAAATATT

-------CTC CTGGTGATTG ATGATGCCCA CACCGACTGG GCCAAGTATT

CAAGATCCTG TTGGTGGTGG ATGATGCGCA TACAGACTGG GCCAAATATT

TAAGATCCTG TTGGTAGTGG ATGATGCCCA CACAGACTGG GCCAAGTATT

TCCATGGGAA GAAGGTGAAT GGAGAGATTG AGATCCGAGT GGAGCAGGCT

TCCATGGGAA GAAGGTGAAT GGAGAGATTG AGATCCGAGT GGAGCAGGCT

TCCATGGGAA GAAGGTGAAT GGAGAGATTG AGATCCGAGT GGAGCAGGCT

---------- ---------- ---------- ---------- -------GCT

TCCATGGGAA GAAGGTGAAT GGAGAGATTG AGATCCGAGT AGAGCAGGCT

TCCAGGGAAA GAAGGTGAAC GGGGAGATCG AGATTCGAGT GGAGCAGGCT

TTCATGGGAA GAAAGTGAAT GGCGACATTG AGATCCGGGT GGAACAGGCT

TTCATGGGAA GAAAGTGAAT GGCGACATTG AGATCCGAGT GGAACAGGCT

TCCAGGGCAA GAAGCTGAAT GGAGAGATTG AGATCCGAGT GGAACAGGCT

TCCATGGAAA GAAGGTGAAT GGAGAGATTG AGATCCGAGT GGAACAGGCC

TTCATGGAAA GAAGGTGAAT GGAGAGATTG AGATCCGAGT GGAGCAGGCT

TCCATGGAAA GAAGGTGAAT GGAGAGATTG AGATCCGAGT GGAGCAGGCT

---------- ---------- ---------- ---------- -------GCA

TCCATGGCAA GAAGGTTAAT GGAGACATTG AGATCCGAGT GGAACAGGCT

TCCATGGAAA GAAAGTGAAT GGAGAGATTG AGATTCGAGT GGAACAGGCT

TTCATGGGAA GAAGGTGAAT GGAGAGGTTG AGATCCGAGT GGAACAG---

TCTATGGAAA GAAGGTGAAC GGAGAGATTG AGATTCGAGT GGAACAGGCT

TCCACGCAAA GAGGACGAAT GGAGAGCTGG AGATCCGAGT GGAACAGGCT

GAATTCTCAG AGTTGAACCT AGCTGCCTAT GTGACCGGGG GCTGCATGGT

GAATTCTCAG AGTTGAACCT AGCTGCCTAT GTGACCGGGG GCTGCATGGT

GAATTCTCAG AGTTGAACCT AGCAGCCTAT GTTACCGGGG GCTGCATGGT

GAATTCTCAG AGTTGAACCT AGCAGCCTAT GTTACCGGGG GCTGCATGGT

GAATTCTCAG AGTTGAACCT AGCAGCCTAT GTGACCGGGG GCTGCATGGT

GAATTCTCAG AATTGAACCT TGCCGCCTAT GTGACCGGGG GCTGTATGGT

GAGTTCTCTG AGCTGAACCT GGCTGCTTAT GTCACCGGAG GCTGCATGGT

GAGTTCTCTG AGCTGAACCT GGCTGCTTAT GTTACCGGAG GCTGCATGGT

GAATTCTCCG AGTTGAACCT GGCTGCCTAT GTGACCGGGG GCTGTGTGGT

GAATTCTCAG AGTTGAATCT AGCCGCCTAT GTTACTGGGG GCTGTATGGT

GAATTCTCAG AGTTGAACCT AGCTGCCTAT GTTACTGGGG GCTGTATGGT

GAATTCTCAG AGTTGAACCT CGCCGCCTAT GTTACTGGGG GCTGCATGGT

GAATTCTCTG AGTTGAATCT GGCCGCTTAT GTCACGGGGG GCTGTATGGT

GAATTCTCAG AGTTGAACCT AGCTGCCTAT GTTACTGGAG GCTGTATGGT

GAATTCTCAG AGTTAAACCT AGCTGCCTAT GTTACTGGGG GCTGTATGGT

---------- ---------- ---------- ---------- ----------

GAATTCTCAG AGCTGAACCT GGCCTCCTAT GTCACCGGGG GCTGTATGGT

GAATTTTCAG AGTTGAACCT GGCGGCCTAT GTCACGGGGG GCTGTATGGT

GGACATGCAG GTCGTGAGAA ATGGGACCAA AGTGGTGAGC AGATCCTTCA

AGACATGCAG GTCGTGAGAA ATGGGACCAA AGTGGTGAGC AGATCCTTCA

GGACATGCAG GTCGTGAGAA ATGGGGCCAA AGTGGTGAGC AGATCCTTCA

GGACATGCAG GTCGTGAGAA ATGGGGCCAA AGTGGTGAG- ----------

GGACATGCAG GTTGTGAGAA ATGGGACCAA AGTGGTGAGC AGATCCTTCA

GGACATGCAG GTCGTGAGGA ACGGGACCAA GGTTGTGAGC AGATCCTTCA

GGACATGCAA GTTGTGAGAA ATGGCACCAA AATTGTGCGC AGGTCCTTCA

GGACATGCAA GTTGTGAGAA ATGGCACCAA AATTGTGCGC AGGTCCTTCA

GGACATGCAG GTCATCAGAA ACGGGACCAA GGTTGTGAGC AGATCCTTCA

GGACATGCAA GTCATGAGAA ATGGAACCAA GGTTGTGAGC AGATCCTTCA

GGACATGCAA GTCATGAGAA ATGGGACCAA GGTTGTGAGC AGATCCTTCA

GGACATGCAG GTCGTGAGAA ATGGGACCAA GGTTGTGAGC AGATCCTTCA

GGACATGCAA GTCGTGAGAA -TGGGACCAA GGTGGTAAGC AGATCCTTCA

GGACATGCAA GCCATGAGAA ATGGAACCAA GGTTGTGAGT AGGTCCTTCA

GGACATACAA GTCATGAGAA ATGGAACCAA GGTTGTGAGC AGGTCCTTCA

---------- ---------- ---------- ---------C AGGTCATTCA

GGACATGCAG GTCATGAGGA ATGGGACAAA AGTTGTGAGC AGATCATTCA

GGATGTGCAG GTCATCAGGA ACGGGACCAA AGTTGTGAAC AGATCCTTCA

AGCCAGACTT CATCCTGGTC CGCCAGCATG CCTACAGCAT GGCCCTGGGG

AGCCAGACTT CATCCTGGTC CGCCAGCATG CCTACAGCAT GGCCCTGGGG

AGCCAGACTT CATCCTGGTC CGCCAGCACG CCTACAGCAT GGCCCTGGGG

---------- ---------- ---------- ---------- ----------

AGCCAGACTT CATCTTGGTC CGCCAGCACG CCTACAGCAT GGCCCTGGGG

AACCAGACTT CATCCTGGTC CGCCAGCATG CCTACAGCAT GGCCCTGGGC

AACCTGACTT CATTCTGGTT AGACAGCATG CCTATAGCAT GGCCCTGGCT

AACCTGACTT CATCCTGGTC CGACAGCATG CCTACAGCAT GGCCCTGGCT

AACCAGACTT CATCCTGGTC CGCCAGCACG CCTACAGCAT GGCCCTGGGC

AGCCGGACTT CATCCTTGTC CGCCAGCACG CCTACAGCAT GGCCCTGGGC

AGCCAGACTT CATCCTAGTC CGTCAGCATG CCTACAGCAT GGCTCTGGGT

AGCCGGACTT CATCCTGGTC CGTCAGCATG CCTACAGCAT GGCCCTGGGA

AGCCGGACTT CATCCTGGTC CGCCAGCACG CCTACAGCAT GGCACTGGGG

AGCCAGACTT CGTCCTGGTG CGCCAGCACG CCCATAGCAT GGCCCTGGGC

AGCCAGACTT CATCCTGGTG CGCCAGCACG CCTACAGCAT GGCCCTGGGC

AGCCCGATTT CATCCTGGTG CGCCAGCACG CCTACAGCAT GGCCCTGGGC

AACCTGACTT TGTCTTGGTT CGGCAGCATG CTTACAGCAT GACCCTGGCA

AGCCCGACTT TGTCCTGGTC AGACAACATG CCTACAGCAT GACTCTTGGG

GAAGACTACC GCAGCCTGGT CATCGGCCTG CAGTATGGAG GGCTGCCTGC

GAGGACTACC GCAGCCTGGT CATCGGCCTG CAGTATGGAG GGCTGCCTGC

GAAGATTACC GCAGCCTGGT CATCGGCCTG CAGTATGGAG GGCTGCCTGC

---------- ---------- ---------- ---------- ----------

GAAGACTACC GCAGCTTGGT CATCGGCCTG CAGTATGGAG GACTGCCTGC

GAGGACTACC GCAGCCTGGT CATCGGCCTG CAGTACGGAG GGCTCCCCGC

GAGGACTACC GAAGCCTGGT CATCGGCCTC CAGTATGGAG GGCTTCCTGC

GAGGACTACC GGAGCCTGGT CATCGGCCTC CAGTATGGAG GGCTTCCTGC

GAAGACTACC GCAGCCTGGT CATCGGCCTG CAGTACGGAG GGCTGCCCGC

GAAGACTACC GCAGCCTGGT CATCGGCCTC CAGTATGGAG GGCTGCCTGC

GAAGACTACC GAAGCCTGGT CATTGGCCTT CAGTATGGAG GGTTGCCTGC

GAAGATTACC GCAGCCTGGT CATTGGCCTC CAGTACGGAG GGCTGCCCGC

GAGGACTATC GCAGCCTGCT CATCGGCCTG CAGTACGGGG GGCTGCCTGC

GAAGACCATC GCAGCCTGGT CATCGGCCTC CAGTACGGAG GGCTGCCTGC

GAAGATTACC GCAGCCTGGT CATCGGCCTC CAGTATGGAG GGCTGCCCGC

GAAGACTACC GCAGCCTGGT CATCGGCCTC CAGTATGGAG GGCTGCCTGC

GAGGATTACC GAAGCCTGGT CATTGGTCTC CAATATGGCG GACTCCCTAG

GAAGACTTCC GCAGCCTGAT CATCGGCCTC CAGTATGGCG GCCTGCCCAG

TGTCAACTCT CTCTACTCCG TCTACAACTT CTGCAGCAAG CCCTGGGTGT

TGTCAACTCT CTCTACTCCG TCTACAACTT CTGCAGCAAG CCCTGGGTGT

TGTCAACTCT CTCTACTCTG TCTACAACTT CTGCAGCAAG CCCTGGGTGT

---------- ---------- ---------- ---------- ---------T

TGTCAACTCT CTCTACTCTG TCTACAACTT CTGCAGCAAG CCCTGGGTGT

CGTCAACTCT CTCTACTCCG TCTACAACTT CTGCAGCAAG CCCTGGGTGT

TGTCAACTCT CTCTACTCCG TATACAACTT CTGCAGCAAG CCTTGGGTGT

TGTCAACTCT CTCTACTCCG TATACAACTT CTGCAGCAAG CCTTGGGTGT

CGTCAACTCC CTCTACTCTG TCTACAACTT CTGCAGCAAG CCCTGGGTGT

CGTCAACTCC CTCTACTCTG TCTACAATTT CTGCAGCAAG CCCTGGGTGT

CGTCAACTCC CTCTACTCTG TCTACAACTT CTGCAGCAAG CCCTGGGTGT

CGTCAACTCC CTCTACTCCG TCTATAACTT CTGCAGCAAG CCCTGGGTGT

CGTCAACTCC CTCTACTCTG TCTACAACTT CTGCAGCAAG CCCTGGGTGT

CGTCAACTCC CTCTACTCCG TCTACAACTT CTGCAGCAAG CCCTGGGTGT

TGTCAACTCC CTCTACTCCG TCTACAACTT CTGCAGCAAG CCCTGGGTGT

TGTCAACTCC CTCTACTCCG TCTACAACTT CTGCAGCAAG CCCTGGGTGT

TGTGAACTCC TTGTATTCCA TTTACAACTT CTGCAGCAAG CCCTGGGTGT

TGTGAACTCT CTGTACTCCA TCTACAACTT CTGCAGCAAA CCCTGGGTGT

TCTCTCAGCT CATTAAGATC TTCCATTCCC TGGGTCCTGA GAAGTTCCCG

TCTCTCAGCT CATTAAGATC TTCCATTCCC TGGGTCCTGA GAAGTTCCCG

TCTCTCAACT CATTAAGATC TTCCATTCCC TGGGTCCTGA GAAGTTCCCG

TCTCTCAACT CATTAAGATC TTCCATTCCC TGGGTCCTGA GAAGTTCCCG

TCTCTCAACT CATTAAGATC TTCCATTCCC TGGGTCCTGA GAAGTTCCCA

TCTCTCAACT CATTAAGATC TTCCATTCCC TGGGTCCTGA GAAGTTCCCG

TCTCTCAGCT CATTAAGATC TTCCATTCCT TGGGTCCTGA GAAGTTTCCA

TTTCTCAGCT CATTAAGATC TTCCATTCCT TGGGTCCTGA GAAATTTCCA

TCTCTCAGCT CATTAAGATC TTTCACTCCT TGGGCCCTGA GAAGTTCCCG

TCTCTCAGCT CATTAAGATC TTCCATTCCC TGGGTCCTGA GAAGTTCCCG

TCTCTCAACT CATCAAGATC TTCCATTCCC TGGGTCCTGA GAAGTTCCCA

TCTCTCAGCT CATTAAGATC TTACATTCCC TGGGTCCTGA GAAGTTCCCG

TCTCGCAGCT CATCAAGATC TTCCACTCCC TGGGTCCTGA GAAGTTTCCG

TCTCGCAGCT CATTAAGATC TTTCATTCCC TGGGTCCTGA GAAGTTCCCG

TCTCTCAGCT TATTAAGATC TTCCATTTCC TGGGTCCTGA GAAGTTCCCG

TCTCACAGCT CATCAAGATC TTCCATGCCC TGGGGCCCGA GAAGTTCCCG

TCTCCCAGTT AATTAAGATC TTCAATTCAT TGGGACCAGA GAAGTTTCCA

TCTCCCAACT CATTAAGATC TTCAATTCGC TGGGTCCAGA GAAGTTTCCT

CTTGTGGAGC AAACATTTTT CCCCAACCAT AAGCCAATGG TCACAGCCCC

CTTGTGGAGC AAACATTTTT CCCCAACCAT AAGCCAATGG TGACAGCCCC

CTTGTGGAGC AAACATTTTT CCCCAACCAT AAGCCAATGG TCACAGCCCC

CTTGTGGAGC AAACATTTTT CCCCAACCAT AAGCCAATG- ----------

CTTGTGGAGC AAACATTTTT CCCCAACCAT AAGCCAATGG TCACAGCCCC

CTTGTGGAGC AAACATTTTT TCCCAACCAT AAGCCAATG- ----------

CTAGTGGAGC AAACATTTTT CCCCAACCAC AAGCCAATG- ----------

CTAGTGGAGC AAACATTTTT CCCCAACCAC AAGCCAATGC TCACAGCCCC

CTGGTGGAGC AAACGTTTTT TGCCAACCAC AAGCCAATGC TCACCGCCCC

CTTGTGGAGC AAACATTTTT CCCCAATCAC AAGCCAATGC TCACAGCCCC

CTTGTGGAGC AAACATTCTT CCCCAACCAT AAGCCAATGC TCACAGTGCC

CTTGTGGAGC AAACGTTTTT CCCCAACCAT AAGCCAATG- ----------

CTGGTGGAAC AGACCTTCTT CCCCAACCAC AAGCCCATGC TCACGGCCCC

CTTGTGGAGC AAACATTTTT CCCCAACCAT AAGCCAATGC TCACAGCCGC

CTTGTGGAGC AGACATTTTT CCCCAACCAT AAGCCAATGC TCACAGCCCC

CTGGTGGAGC AAACGTTCTT CCCCAATCAC AAGCCAATG- ----------

CTTGTAGAAC AAACATTTTT CCCCAACCAC AAGCAAATGC TCACAACCCC

CTTGTGGAGC AGACGTTTTT CCCCAACCAT AAACAGATGC TTACAAGCCC

ACACTTCCCG GTGGTAGTCA AGCTGGGACA TGCCCACGCT GGAATGGGAA

ACACTTCCCG GTGGTAGTCA AGCTGGGACA TGCCCACGCT GGAATGGGAA

ACATTTCCCG GTGGTGGTCA AGCTGGGACA TGCCCACGCT GGAATGGGAA

---------- ---------- ---------- ---------- ----------

ACACTTCCCG GTGGTGGTCA AGCTGGGACA TGCCCACGCT GGAATGGGAA

---------- ---------- ---------- ---------- ----------

---------- ---------- ---------- ---------- ----------

GAACTTCCCC GTGGTGATCA AGCTGGGACA TGCCCATGCT GGCATGGGAA

ACACTTCCCC GTGGTGGTCA AGCTGGGACA CGCCCACGCC GGGATGGGAA

TCACTTCCCC GTGGTGGTTA AGCTGGGACA TGCCCACGCC GGAATGGGAA

ACACTTCCCT GTGGTGGTCA AGCTGGGACA CGCCCATGCC GGAATGGGAA

---------- ---------- ---------- ---------- ----------

GCATTTCCCC GTGGTGGTGA AGCTGGGTCA TGCCCATGCC GGCATGGGAA

ACACTTCCCC GTGGTGGTCA AGCTGGGGCA TGCCCACGCT GGAATGGGAA

ACACTTCCCT GTGGTGGTCA AGTTGGGACA TGCCCACGCT GGAATGGGAA

---------- ---------- ---------- ---------- ----------

GACTTTTCCA GTGGTAGTTA AGATGGGACA TGCTCATGCT GGGATGGGGA

GGACTTCCCT GTGGTGGTCA AACTAGGACA TGCTCACGCT GGAATGGGAA

AGATCAAAGT GGAAAACCAG CTTGACTTCC AGGACATCAC CAGCGTGGTC

AGATCAAAGT GGAAAACCAG CTTGACTTCC AGGACATCAC CAGCGTGGTC

AGATCAAAGT GGAAAACCAG CTTGACTTCC AGGACATCAC CAGCGTGGTC

--ATCAAAGT GGAAAACCAG CTTGACTTCC AGGACATCAC CAGCGTGGTC

AGATCAAAGT GGAAAACCAG CTTGACTTCC AGGACATCAC CAGTGTGGTC

---------- ---------- ---------- ---------- ----------

--ATCAAAGT AGAAAACCAG CACGACTACC AGGACATCAC CAGCGTGGTG

AGATCAAAGT AGAAAACCAG CACGACTACC AGGACATCAC CAGCGTGGTG

AGATCAAAGT GGAAAACCAG CACGACTACC AGGACATCAC GAGCGTGGTG

AGATCAAAGT AGAAAACCAG CACGACTACC AGGACGTCAC CAGCATGGTG

AGATCAAAGT AGAAAACCAG CATGACTACC AGGACATCAC CAGCATGGTG

---------- ---------- ---------- ---------- ----------

AGATCAAGGT GGAAAACCAG CACGACTATC TGGACATCAC CAGCATCGTG

AGATCAAAGT GGAAAACCAG CATGACTATC AGGACATCAC CAGCGTGGTC

AG-------- ---------- ---------- ---------- ----------

--ATCAAGGT GGAAAACCAG CATGACTACC AGGACATCAC CAGCGTGGTC

AGGTGAAAGT GGAAAACCAG TACGATTACC AGGACATCAC CAGCATTGTG

AGGTAAAAGT GGAAAACCAG TATGACTTCC AGGACATCGC CAGCGTGGTG

GCCATGGCCA AAACCTACGC CACCACCGAG GCCTTCATCG ACTCCAAGTA

GCCATGGCCA AAACCTACGC CACCACCGAG GCCTTCATCG ACTCCAAGTA

GCCATGGCCA AAACCTACGC CACCACCGAG GCCTTCATCG ACTCCAAGTA

GCCATGGCCA AAACCTACGC CACCACCGAG GCCTTCATCG ACTCCAAGTA

GCCATGGCCA AAACCTATGC CACCACCGAG GCCTTCATCG ACTCCAAGTA

---------- ---------- ---------- ---------- ----------

GCCATGGCCA AAACCTATGC CACCACGGAG GCCTTTATTG ACTCCAAGTA

GCCATGGCCA AAACCTATGC CACCACGGAG GCCTTTATTG ACTCCAAGTA

GCCATGGCCA AGACCTACGC CACCACCGAG GCCTTCATCG ACTCCAAGTA

GCTATGGCCA AAACCTATGC CACCACCGAG GCCTTCATCG ACTCCAAGTA

GCTATGGCCA AAACCTATGC TACCACTGAG GCCTTCATTG ACTCCAAGTA

---------- ---------- ---------- ---------- ----------

GCCATGGCCA AGACCTACGC CACCACCGAA GCCTTCATTG ACTCCAAGTA

GCCATGGCCA AAACCTACGC CACCACCGAG GCCTTCATCG ACTCCAAGTA

---------- ---------- ---------- ---------- ----------

GCCATGGCCC AAACCTACGC CACCACTGAG GCCTTCATTG ACTCCAAGTA

GCTATGGCCA AGACTTACGC CACCACAGAA AGCTTCATCG ATTCCAAGTA

GCCATGGCCA AGACTTATGC CACCACAGAG ACCTTTGTTG ACTCCAAATA

CGACATCCGC ATCCAGAAAA TTGGATCCAA CTACAAGGCT TACATGATGA

CGACATCCGC GTCCAGAAAA TTGGATCCAA CTACAAGGCT TACATGATGA

CGACATCCGC GTCCAGAAAA TTGGATCCAA CTACAAGGCT TACATGATGA

CGACATCCGC GTCCAGAAAA TTGGATCCAA CTACAAGGCT TACATGATGA

TGACATCCGT GTCCAGAAAA TTGGATCCAA CTACAAGGCG TACATGATGA

---------- ---------- ---------- ---------- -----GATGA

TGACATCCGC ATACAGAAGA TCGGATCCAA CTACAAGGCA TACATGATGA

TGACATCCGC ATACAGAAGA TTGGATCCAA CTACAAGGCA TACATGATGA

CGACATCCGC ATCCAGAAAA TCGGGGCCAA CTACAAGGCC TACATGATGA

TGACATCCGC ATCCAGAAGA TCGGGTCCAA CTACAAGGCC TACATGATGA

CGACATCCGC ATCCAGAAAA TCGGATCCAA CTACAAGGCT TACATGATGA

---------- ---------- ---------- ---------- ------ATGA

TGACATCCGC ATCCAGAAAA TCGGATCCAA CTACAAAGCC TACATG----

CGATATCCGC ATTCAGAAAA TCGGATCCAA CTACAAAGCT TACATGATGA

---------- ---------- ---------- ---------- ----------

CGACGTCCGC ATCCAGAAAA TCGGCTGCAA CTACAAGGCT TACAT-ATGA

CGACATTCGG ATCCAGAAAA TTGGCAACAA TTACAAGGCC TACATGATGA

TGACATTCGG ATCCAGAAAA TTGGCAGTAA CTACAAGGCC TACATGATGG

CCCCTTGGCT CGGGCTCATC GTGCTCCTGG GCAGCTGGAG CCTGGGGGAC

CTCCTTGGCT CGGGCTCATC GTGCTCCTGG GCAGCTGGAG CCTGGGGGAC

CTCCTTGGCT CGGGCTCATC GTGCTCCTGG GCAGCTGGAG CCTGGGGGAC

CTCCTTGGCT CGGGCTCATC GTGCTCCTGG GCAGCTGGAG CCTGGGGGAC

CTCCCTGGCT CGGGCTCGTC GTCCTCCTGG GCAGCTGGAG CCTGGGGGAC

CTCCCTGGCT CGGGCTGGTC GTGCTCCTGG GCATCTGGAG CCTGGGGGAC

CTCCCTGGCT TGGGCTTGTC CTGCTCCTGA GCTGCTGGAG CCTTGGGCAC

CTCCCTGGCT TGGGCTTGTC GTGCTCCTGA GCTGCTGGAG CCTTGGGCAC

CTCTGTGGCT CGGCCTCGTG GTGCTCCTGG GCAGCTGGGG ACTGGGGGAC

CTCCCTGGCT CGGGCTCGTC GTGCTCCTGG GCAGCTGGAG CCTGGGGGAC

CTCCCTGGCT CGGGCTAGTG GTGCTTCTGG GCAGCTGGAG CCTGGGGCAC

CTCCGTGGCT CGGGCTCGTC GTGCTCCTGG GCAGCTGGAG CTTGGGGGAC

---------- ---------- ---------- ---------- ----------

CGCCCTGGCT CGGGCTCGTC GTGCTCCTGG GCAGCTGGAG CCTGGGGCAC

---------- ---------- ---------- ---------- ----------

CTCCCTGGCT GGGACTCGTA GTGCTCCTGG GCAGCTGGAG CCTGGGCGAC

GCCCGTGGCT CAGTCTCGTG GTGCTTCTCA GCAGCTGGAG CCTGAGGGAG

CCCTCTGGCT CAGCCTCGTC ATGCTCCTGG GCAGCTGGAC CCTCGACGAC

TGGGGCGCCG AGGCGTGCAC ATGCGTGATC CGGGCCAAGG TGGTGGGGAA

TGGGGCGCCG AGGCGTGCAC ATGCGTGATC CGGGCCAAGG TGGTGGGGAA

TGGGGCGCCG AGGCGTGCAC ATGCGTGATC CGGGCCAAGG TGGTGGGGAA

TGGGGCGCCG AGGCGTGCAC ATGCGTGATC CGGGCCAAGG TGGTGGGGAA

TGGGGCGCCG AGGCGTGCAC ATGCGTGATC CGGGCCAAGG TGGTGGGGAA

TGGGGCGCTG AGGCGTGCAC ATGCGTGATC CGGGCCAAGG TGGTGGGGAA

TGGGGAACGG AAGCGTGCAC ATGCGTGATC CGGGCCAAAG TGGTGGGAAA

TGGGGCGCGG AAGCGTGCAC ATGCGTGATC CGGGCCAAAG TGGTGGGAAA

TGGGGCGCCG AGGCGTGCAC ATGCGTGATC CGGGCCAAGG TGGTGGGGAA

TGGGGCGCTG AGGCGTGCAC ATGCGTGATC CGAGCCAAGG TGGTAGGGAA

TGGGGCGCCG AGGCGTGCAC ATGCGTGATC CGGGCCAAGG TGGTAGGGAA

TGGGGTGCCG AGGCGTGCAC GTGCGTGATC CGGGCCAGGG TGGTGGGAAA

---------- ---------- -----TGATC CGGGCCAAGG TGGTGGGGAA

TGGGGCGCCG AGGCGTGCAC ATGCGTGATC CGGGCCAAGG TGGTGGGGAA

---------- ---------- -----TGATC CGGGCCAAGG TAGTGGGGAA

TGGGGCGCCG AGGCGTGCAC ATGC-TGATC CGGGCCAAGG TGGTGGGGAA

CTGGGGGTAG ACGCGTGCAC TTGTGTCATC CGTGCCAAGG TGGTGGGAAA

TCGGGGGCGG AGGCCTGCAC TTGCGTGATA CGTGCCAAGG TGGTGGGGAA

GAAGCTGGTA AAGGAGGGGC CCTTCGGCAC GCTGGTCTAC ACCATCAAGC

GAAGCTGGTA AAGGAGGGGC CCTTCGGCAC GCTGGTCTAC ACCATCAAGC

GAAGCTGGTA AAGGAGGGGC CCTTCGGCAC GCTGGTCTAC ACCATCAAGC

GAAGCTGGTA AAGGAGGGGC CCTTCGGCAC GCTGGTCTAC ACCATCAAGC

GAAGTTGGTA AAGGAGGGGC CTTTTGGCAC ACTGGTCTAC ACCATCAAGC

GAAGCTGGTG AAGGAGGGGC CCTTTGGCAC ACTGGTCTAC ACCATCAAGC

GAAGCTGGTG AAGGAAGGGC CCTTTGGCAC TCTGGTCTAC ACTATTAAGC

GAAGCTGGTG AAGGAGGGGC CCTTTGGCAC TCTGGTCTAC ACTATTAAGC

GAAGCTGGTG AAGGAAGGGC CCTTCGGCAC GATGGTCTAC ACCGTCAAGC

GAAACTGCTG AAGGAGGGGC CCTTTGGCAC GATGGTCTAC ACCATCAAGC

GAAGCTGGTG AAGGAGGGGC CCTTTGGCAC GCTGGTCTAT ACCATCAAGC

GAAGCTGGTG AAGGAGGGGC CGTTTGGTAC GCTGGTCTAC ACCATTAAGC

GAAGCTGGTG AAGGAGGGAC CCTTCGGCAC GATGGTCTAC ACCATCAAAC

GAAGCTGGTG AAGGAGGGGC CCTTTGGCAC ACTGGTCTAC ACCATCAAGC

GAAGCTGGTG AAGGATGGGC CCTTTGGCAC GCTGGTCTAC ACCGTCAAGC

GAAGCTGGTG AAGGAAGGGC CCTTTGGCAC GCTGGTCTAC ACCATCAAGC

GAAGCTGGTG AAGGAGGGAC CCTTTGGGAC ACTGATTTAC ACCATCAAGC

AAAGCTTGTG AAGGAGGGGC CTTTTGGAAC CCTGAGATAC ACCATCAAGC

AGATGAAGAT GTACCGAGGC TTCACCAAGA TGCCCCATGT GCAGTACATC

AGATGAAGAT GTACCGAGGC TTCACCAAGA TGCCCCATGT GCAGTACATC

AGATGAAGAT GTACCGAGGC TTCACCAAGA TGCCCCACGT GCAGTACATC

AGATGAAGAT GTACCGAGGC TTCACCAAGA TGCCCCACGT GCAGTACATC

AGATGAAGAT GTACCGAGGC TTCACCAAGA TGCCCCATGT GCAGTACATC

AGATGAAGAT GTACCGAGGC TTCACCAAGA TGCCCCATGT GCAGTACATC

AAATGAAGAT GTACCGAGGA TTCAGTAAGA TGCCCCATGT GCAGTACATT

AGATGAAGAT GTACCGAGGC TTCAGTAAGA TGCCCCACGT GCAGTACATT

AGATGAAG-- ---------- ---------- ---------- ----------

AGATGAAGAT GTACCGAGGA TTCACCAAGA TGCCCCATGT GCAGTACATC

AGATGAAGAT GTACCGAGGC TTCACCAAGA TGCCCCACGT GCAGTACATC

AGATGAAGAT GTACCGAGGC TTCACCAAGA TGCCCCACGT GCAGTACATC

AGATGAAGAT GTACCGAGGC TTCACCAAGA TGCCCCACGT GCAGTACATC

AGATGAAGAT GTACCGCGGC TTCACCAAGA TGCCCCATGT GCAGTACATC

AGATGAAGAT GTACCGAGGC TTCAGCAAGA TGCCCCATGT ACAATACATC

AGATGAAGAT GTACCGAGGC TTCACCAAGA TGCCCCACGT GCAGTACATC

AGATGAAGAT GTACAGGGGC TTTAGCAAGA TGCCCCATGT GCAGTATATC

AAATGAAGAT GTACCGAGGC TTCAGCAAGA TGCCTCACGT GCAGTACATC

CATACGGAAG CTTCCGAGAG TCTCTGTGGC CTTAAGCTGG AGGTCAACAA

CACACGGAAG CTTCCGAGAG TCTCTGTGGC CTTAAGCTGG AGGTCAACAA

CACACGGAAG CTTCCGAGAG TCTCTGTGGC CTTAAGCTGG AGGTCAACAA

CACACGGAAG CTTCCGAGAG TCTCTGTGGC CTTAAGCTGG AGGTCAACAA

CACACAGAGG CTTCTGAGAG TCTCTGTGGC CTTAAGCTGG AGGTCAACAA

CACACAGAAG CTTCCGAGAG TCTCTGTGGC CTTAAGCTGG AGGTCAACAA

CACACAGAAG CCTCTGAAAG TCTCTGTGGC CTTAAGCTAG AAGTCAACAA

CACACGGAAG CCTCTGAAAG TCTTTGTGGC CTCAAGCTAG AAGTCAACAA

---------- ---------- ---------- ---------- ----------

CACACAGAAG CTTCTGAAAG TCTCTGTGGC CTTAAGCTTG AGGTCAACAA

CACACAGAAG CTTCTGAAAG TCTCTGTGGC CTTAAGCTAG AGGTCAACAA

CACACGGAAG CTTCCGAAAG TCTCTGTGGC GTTAAGCTGG AGGTCAACAA

CACACGGAAG CTTCCGAGAG CCTCTGTGGC CTGAAGCTGG AGGTGAACAA

CACACAGAAG CTTCTGAAAG TCTCTGTGGC CTTAAGCTGG AGGTCAACAA

TACACGGAAG CTTCCGAAAG TCTCTGTGGC CTTAAGCTGG AGGTCAACAA

TACACGGAAG CCTCTGAGAG TCTGTGCGGC CTGAAGCTGG AAGTGAACAA

CACACAGAAG CTTCCGAGAG CCTTTGTGGG CTGAAGTTGG AAGTCAACAA

TACACGGAAG CTTCTGAAAG TCTTTGTGGG CTGAAGCTGG AGGTGAACAA

GTACCAGTAC CTGCTGACAG GTCGCGTCTA TGATGGCAAG ATGTACACGG

GTACCAGTAC CTGCTGACAG GTCGCGTCTA TGATGGCAAG ATGTACACAG

GTACCAGTAC CTGCTGACAG GTCGCGTCTA CGATGGCAAG ATGTACACAG

GTACCAGTAC CTGCTGACAG GTCGCGTCTA CGATGGCAAG ATGTACACAG

GTACCAGTAC CTGCTGACAG GTCGTGTCTA TGATGGCAAG ATGTACACAG

GTACCAGTAC CTACTGACAG GCCGCGTCTA TGATGGAAAG ATGTACACAG

ATACCAGTAC CTGCTGACAG GGCGCGTGTA TGAAGGCAAG ATGTACACAG

ATACCAGTAC CTGCTGACAG GGCGCGTGTA TGAAGGCAAG ATGTACACAG

---------- ---------- ---------- ---------- ----------

GTACCAGTAC CTGCTGACAG GCCGAGTCTA TGATGGCAAG ATGTACACAG

GTACCAGTAC CTGCTGACAG GCCGCGTCTA TGATGGTAAG ATGTACACAG

GTACCAGTAC CTGCTGACAG GCCGTATCTA CGATGGTAAG GTGTACACAG

GTACCAGTAC CTGCTCACAG GTCGTGTCTA TGATGGGAAG ATGTACACGG

GTACCAGTAC CTACTGACAG GCCGTGTCTA CGATGGCAAG GTATACACCG

GTACCAGTAC CTGCTGACAG GTCGTGTCTA TGATGGCAAG GTATATACAG

GTACCAGTAC CTGCTGACAG ---------- ---------- ----------

GTACCAGTAT CTGCTGACAG GTCGGGTATA TGATGGCAAG ATGTACACAG

GTACCAGTAC CTGCTGACAG GCCGTGTTTA TGACGGGAAG GTGTACACTG

GGCTGTGCAA CTTCGTGGAG AGGTGGGACC AGCTCACCCT CTCCCAGCGC

GGCTGTGCAA CTTCGTGGAG AGGTGGGACC AGCTCACCCT CTCCCAGCGC

GGCTGTGCAA CTTTGTGGAG AGGTGGGACC AGCTCACCCT CTCCCAGCGC

GGCTGTGCAA CTTTGTGGAG AGGTGGGACC AGCTCACCCT CTCCCAGCGC

GACTGTGCAA CTTTGTGGAG AGGTGGGACC AGCTCACCCT CTCCCAGCGC

GACTGTGCAA CTTTGTGGAG CGGTGGGACC AGCTCACCCT CTCCCAGCGC

GGCTGTGCAA CTTTGTGGAG AGGTGGGACC ACCTCACACT GTCCCAGCGC

GACTGTGCAA CTTTGTGGAG AGGTGGGACC ACCTCACACT GTCCCAGCGC

---------- ---------- ---------- ---------- ----------

GACTGTGTAA CTTTGTAGAG AGGTGGGACC AGCTCACCCT CTCCCAGCGC

GACTATGCAA CTTTGTGGAG AGGTGGGACC AGCTCACCCT CTCCCAGCGC

GACTCTGCAA CTTCGTGGAG CGGTGGGACC AGCTCACCAT CTCACAGCGC

GCCTGTGTAA CTTCGTCGAG AGATGGGACC AGCTCACCCT CTCGCAGCGC

GGCTGTGCAA CTTTGTGGAG AGATGGGACC AGCTCACGCT CTCCCAGCGC

GGCTGTGCAA CTTTGTGGAG AGATGGGACC AGCTCACCCT CTCCCAGCGC

---------- ---------- ---------- ---------- ----------

GCTTGTGTAA CTTTGTGGAG CGGTGGGACC GGCTCACCCT CTCCCAGCGC

GCCTGTGTAA CTTCGTTGAG CGATGGGACC AGCTGACCCT CTCCCAGCGC

AAGGGGCTGA ACTATCGGTA TCACCTGGGT TGTAACTGCA AGATCAAGTC

AAGGGGCTGA ACTATCGGTA CCACCTGGGT TGTAACTGCA AGATCAAGTC

AAGGGGCTGA ACTATCGGTA TCACCTGGGT TGTAACTGCA AGATCAAGTC

AAGGGGCTGA ACTATCGGTA TCACCTGGGT TGTAACTGCA AGATCAAGTC

AAGGGGCTGA ACTATCGGTA CCATCTGGGT TGTAACTGCA AGATCAAGTC

AAGGGCCTGA ACTATCGGTA TCACCTGGGC TGTAACTGCA AGATCAAATC

AAGGGCCTCA ATTACCGCTA CCACCTGGGT TGCAATTGCA AGATCAAGTC

AAGGGCCTCA ATTACCGCTA CCACCTGGGT TGCAATTGCA AGATCAAGTC

---------- ---------- ---------- ---------- --ATCAAGTC

AAGGGGCTGA ACTATCGATA TCACCTGGGC TGTAACTGCA AGATCAAATC

AAGGGGCTCA ACTACAGGTA TCACCTGGGC TGTAACTGCA AGATCAAATC

AAGGGGCTGA ACTATCGGTA TCACCTGGGT TGTAATTGCA AGATCAAGTC

AAGGGGCTAA ACTACCGCTA CCACCTGGGC TGTAACTGCA AGATCAAGTC

AAGGGGCTGA ACTATCGTTA TCACCTGGGT TGCAACTGCA AGATCAAGTC

AAGGGGCTGA ATTATCGGTA TCATCTGGGC TGTAACTGCA AGATCAAGTC

---------- ---------- ---------- ---------- ----------

AAGGGTCTAA ATTATCGGTA CCACTTGGGC TGCAACTGCA AGATTAAGTC

AAAGGCCTGA ATTATCGTTA CCATCTGGGC TGCAACTGCA AGATCAAATC

CTGCTACTAC CTGCCTTGCT TTGTGACTTC CAAGAACGAG TGTCTCTGGA

CTGCTACTAC CTGCCTTGCT TTGTGACTTC CAAGAACGAG TGTCTCTGGA

CTGCTACTAC CTGCCTTGCT TTGTTACCTC CAAGAACGAG TGTCTCTGGA

CTGCTACTAC CTGCCTTGCT TTGTTACTTC CAAGAACGAG TGTCTCTGGA

CTGCTACTAC CTGCCTTGCT TTGTGACTTC CAAGAACGAG TGTCTCTGGA

CTGCTACTAC CTGCCTTGCT TTGTGACCTC CAAGAACGAG TGTCTCTGGA

CTGCTACTAC TTGCCTTGCT TTGTGACCTC CAAGAATGAA TGTCTCTGGA

CTGCTACTAC TTGCCTTGTT TTGTGACCTC CAAGAATGAG TGTCTCTGGA

CTGCTACTAC CTGCCCTGCT TCGTGACCTC CAAGAATGAG TGCCTTTGGA

CTGCTACTAC CTGCCTTGCT TTGTAACCTC CAAGAACGAG TGTCTCTGGA

CTGCTACTAC CTGCCTTGCT TTGTGACCTC CAAGAACGAG TGCCTCTGGA

CTGCTACTAC CTGCCTTGCT TTGTGAGCTC CAAGAATGAG TGTCTCTGGA

CTGCT----- ---------- ---------- ---------- ----------

CTGCTACTAT CTGCCTTGCT TTGTGACCTC CAAGAATGAG TGTCTTTGGA

CTGCTACTAC CTGCCTTGCT TTGTGTCCGC CAAGAACGAG TGTCTCTGGA

---------- ---------- ---------- ---------- ----------

ATGTTATTAC CTGCCTTGCT TTGTGACTTC CAAGAATGAG TGTCTGTGGA

CTGCTACTAC CTGCCTTGTT TCGTGACCTC CAAGAACGAG TGCCTCTGGA

CCGACATGCT CTCCAATTTC GGTTACCCTG GCTACCAGTC CAAACACTAC

CCGACATGCT CTCCAATTTC GGTTACCCTG GCTACCAGTC CAAACACTAC

CCGACATGCT CTCCAATTTC GGCTACCCTG GCTACCAGTC CAAACACTAT

CCGACATGCT CTCCAATTTC GGCTACCCTG GCTACCAGTC CAAACACTAT

CCGACATGCT GTCCAATTTC GGCTACCCTG GCTACCAGTC CAAACATTAT

CCGACATGCT GTCCAACTTC GGCTACCCCG GCTATCAGTC CAAACACTAC

CCGACATGCT CTCCAATTTC GGGTACCCTG GCTATCAGTC CAAACACTAC

CCGACATGCT CTCCAATTTT GGGTACCCTG GCTATCAGTC CAAACACTAC

CCGACATGCT CTCCAACTTC GGCTACCCCG GCTACCAGTC CAAACACTAT

CCGACATGCT CTCCAATTTC GGCTACCCTG GCTACCAGTC CAAACACTAC

CCGACATGCT CTCCAATTTT GGCTACCCTG GCTACCAGTC CAAACATTAT

CCGACATGCT CTCCAATTTC GGCTACCCCG GCTACCAGTC CAAACACTAC

---------- ---------- ---------- ---------- ----------

CCGACATGCT TTCCAACTTC GGATACCCTG GCTACCAGTC CAAACACTAT

CCGACATGCT ATCCAGCTTC GGCCACTCTG GCTACCAGTC CAAACATTAT

---------- ---------- ---------- ---------- ----------

CGGACATGCT CTCCAACTTT GGCTACCCTG GGTACCAGTC GAAGCATTAT

CGGACATGCT CTCCAACTTC GGCTATCCCG GATACCAGTC CAAGCACTAT

GCCTGCATCG TGACAGAGCT GAATGAACCT CTCTCCAATG AAGATCGAAA

GCCTGCATCG TGACAGAGCT GAATGAACCT CTCTCCAATG AAGATCGAAA

GCCTGCATCG TGACAGAGCT GAATGAACCT CTCTCCAATG AAGATCGAAA

GCCTGCATCG TGACAGAGCT GAATGAACCT CTCTCCAATG AAGATCGAAA

GCCTGCATCG TGACAGAGCT GAATGAACCT CTCTCCAATG AAGATCGAAA

GCCTGCATCG TGACAGAGTT GAATGAACCT CTCTCCAATG AAGATCGAAA

GCCTGCATCG TGACAGAGCT GAATGAACCT CTATCTAATG AAGATAGAAA

GCCTGCATCG TGACAGAGCT GAATGAACCA CTATCTAACG AAGATCGAAA

GCCTGCATCG TGACCGAGCT GAATGAACCC CTCTCCAATG AAGATCGAAA

GCTTGCATC- ---------- ---------- ---------- ----------

GCCTGCATCG TGACAGAGCT CAATGAACCT CTTTCCAATG AAGATCGAAA

GCATGCATCG TGACAGAGCT CAATGAACCT CTCTCCAATG AAGATCGAAA

---------- ---------- ---------- ---------- ----------

GCCTGCATCG TGACGGAGCT CAATGAACCT CTCTCCAATG AAGATCGCAA

GCCTGCATCG TGACAGAGCT CAATGAACCT CTTTCCAATG AAGATAGAAA

---------G TGACAGAGCT CAACGAACCT CTCTCCAACG AAGATCGAAA

GCCTGCATTG TGACTGAGCT CAATGAGCCA CTGTCCAACG AGGACCGCAA

GCTTGCAT-G TGACCGAACT GAATGAGCCC CTCTCAAATG AAGATCGAAA

TCTCCTCTCT GTGGCCTACA AGAATGTGGT TGGTGCCAGG CGATCTTCCT

TCTCCTCTCT GTGGCCTACA AGAATGTGGT TGGTGCCAGG CGATCTTCCT

TCTCCTCTCT GTGGCCTACA AGAATGTGGT TGGTGCCAGG CGATCTTCCT

TCTCCTCTCT GTGGCCTACA AGAATGTGGT TGGTGCCAGG CGATCTTCCT

TCTCCTCTCT GTGGCTTACA AGAATGTAGT TGGTGCCAGG CGATCTTCCT

TCTCCTCTCT GTGGCCTACA AGAATGTAGT TGGTGCCAGG CGATCTTCCT

TCTCCTCTCT GTGGCCTACA AGAATGTAGT TGGTGCCAGG CGATCTTCTT

TCTCCTCTCT GTGGCCTACA AGAATGTAGT TGGTGCCAGG CGATCTTCTT

CCTCCTGTCC GTGGCCTACA AGAACGTGGT TGGTGCCAGG CGATCCTCTT

---------- ---------- ---------- ---------- ----------

CCTCCTCTCT GTGGCCTACA AGAATGTGGT TGGTGCCAGG CGATCTTCCT

CCTCCTCTCC GTGGCTTACA AGAATGTGGT TGGTGCCCGG CGATCTTCCT

---------- ---------- ---------- ---------- ----------

TCTTCTCTCT GTGGCCTACA AGAATGTGGT TGGTGCCAGG CGATCTTCCT

TCTCCTCTCT GTGGCCTACA AGAATGTGGT TGGTGCCAGG CGATCTTCCT

TCTGCTCTCC GTGGCCTACA AGAATGTAGT TGGTGCCCGG CGGTCTTCTT

TCTCCTGTCT GTCGCCTACA AGAACGTGGT CGGGGCTAGG CGGTCCTCCT

CCTGCTCTCT GTTGCTTACA AGAATGTGGT TGGTGCTAGG CGATCTTCTT

GGAGGGTCAT TAGCAGCATT GAGCAGAAAA CCATGGCTGA TGGAAACGAA

GGAGGGTCAT TAGCAGCATT GAGCAGAAAA CCATGGCTGA TGGAAACGAA

GGAGGGTCAT TAGCAGCATT GAGCAGAAAA CCATGGCTGA TGGAAACGAA

GGAGGGTCAT TAGCAGCATT GAGCAGAAAA CCATGGCTGA TGGAAACGAA

GGAGGGTCAT TAGCAGCATT GAGCAGAAAA CCATGGCTGA TGGAAACGAA

GGAGGGTCAT TAGCAGCATT GAGCAGAAAA CCATGGCTGA TGGAAATGAA

GGAGGGTTAT TAGTAGCATT GAGCAGAAAA CCATGGCAGA TGGGAATGAG

GGAGGGTTAT TAGTAGCATT GAGCAGAAAA CCATGGCAGA TGGGAATGAG

GGCGGGTCAT CAGCAGCATT GAGCAGAAAA CCATGGCGGA TGGAAATGAA

---------- ---------- ---------- ---------- ----------

GGAGGGTCAT CAGCAGCATT GAGCAGAAAA CCATGGCTGA TGGAAACGAA

GGAGGGTCAT CAGCAGCATT GAGCAGAAAA CCATGGCTGA TGGGAATGAA

---------- ---------- ---------- ---------- ----------

GGAGGGTGAT TAGCAGCATT GAACAGAAAA CCATGGCTGA TGGAAATGAA

GGAGGGTTAT CAGCAGCATT GAGCAGAAGA CAATGGCTGA TGGAAATGAA

GGAGGGTCAT CAGCAGCATT GAGCAAAAAA CAATGGCTGA TGGAAATGAG

GGCGAGTCAT CAGCAGCATC GAGCAGAAGA CCATGGCGGA CGGGAATGAG

GGAGAGTCAT CAGTAGTATT GAGCAGAAAA CCATGGCTGA TGGCAATGAG

AAGAAATTGG AGAAAGTTAA AGCTTACCGG GAGAAGATTG AGAAGGAGCT

AAGAAATTGG AGAAAGTTAA AGCTTACCGG GAGAAGATTG AGAAGGAGCT

AAGAAATTGG AGAAAGTGAA AGCTTACCGG GAGAAGATTG AGAAGGAGCT

AAGAAATTGG AGAAAGTGAA AGCTTACCGG GAGAAGATTG AGAAGGAGCT

AAGAAATTGG AGAAAGTT-A AGCTTACCGG GAGAAGATTG AGAAGGAGCT

AAGAAATTGG AGAAAGTTAA AGCTTACCGG GAGAAGATTG AGAAGGAGCT

AAGAAGCTGG AGAAAGTCAA AGCCTATCGG GAGAAGATTG AGAAGGAGCT

AAGAAGTTGG AGAAAGTTAA AGCCTACCGG GAGAAGATTG AAAAGGAGCT

AAGAAGCTGG AGAAAGTGAA AGCCTACCGG GAGAAGATCG AGAAGGAGCT

---------- ---------- ---------- ---------- ----------

AAGAAATTGG AGAAAGTTAA AGCTTACCGG GAGAAGATTG AGAAGGAGCT

AAGAAATTGG AGAAGGTTAA AGCTTACCGG GAGAAGATTG AGAAGGAGCT

---------- ---------- ---------- ---------- ----------

AAGAAATTGG AGAAGGTTAA AGCTTACCGG GAGAAGATTG AGAAGGAGTT

AAGAAATTGG AGAAGGTTAA AGCTTACCGG GAGAAGATTG AGAAAGAGCT

AAGAAGTTGG AGAAGGTAAA GGCTTACCGG GAGAAGATTG AGAAAGAGCT

AAGAAGCTGG AGAAGGTGAA GGCCTACCGG GAGAAGATCG AGAAGGAGCT

AAGAAGCTGG AAAAGGTCAA GGCCTATAGG GAGAAGATAG AAAAGGAGCT

GGAGACAGTT TGCAATGATG TCCTGTCTCT GCTTGACAAG TTCCTGATCA

GGAGACAGTT TGCAATGATG TCCTGTCTCT GCTTGACAAG TTCCTGATCA

GGAGACAGTT TGCAATGATG TCCTGTCTCT GCTTGACAAG TTCCTGATCA

GGAGACAGTT TGCAATGATG TCCTGTCTCT GCTTGACAAG TTCCTGATCA

GGAGACATTT TGCAATGATG TTCTGTCTCT GCTTGACAAG TTCCTGATCA

GGAGACAGTT TGCAATGATG TCCTGGCTCT GCTTGACAAG TTCCTCATCA

GGAGACAGTT TGCAATGATG TCTTGGCTCT GCTCGACAAG TTCCTTATCA

GGAGACAGTT TGCAATGATG TCTTGGCTCT GCTTGACAAG TTCCTTATCA

GGAGACCGTC TGCAACGATG TCCTGGCTCT GCTCGACAAG TTCCTCATCA

---------- ---------- ---------- ---------- ----------

GGAAACAGTG TGCAATGATG TCCTGGCTCT GCTTGACAAG TTCCTCATCA

GGAAACAGTG TGCAATGATG TGCTGGCTCT GCTTGACAAG TTCCTCATCA

---------- ---------- ---------- ---------- ----------

GGAGACAGTT TGCAATGATG TCCTGGCTCT GCTTGACAAG TTCCTCATCA

GGAGACAGTT TGCAATGATG TCCTGGCTCT GCTTGACAAG TTCCTCATCA

GGAGACCGTT TGCAATGATG TCCTGGCTCT GCTCGACAAA TTCCTCATCA

GGAGACCGTC TGCAACGACG TGCTGGCGCT GCTCGACAAG TTCCTCATTA

GGAGACAGTC TGCAACGACG TCCTGGCTCT GCTGGATAAA TTCCTAATCA

AGAACTGCAA TGATTTCCAG TATGAGAGCA AGGTGTTTTA CCTGAAAATG

AGAACTGCAA TGATTTCCAG TATGAGAGCA AGGTGTTTTA CCTGAAAATG

AGAACTGCAA TGATTTCCAG TATGAGAGCA AGGTGTTTTA CCTGAAAATG

AGAACTGCAA TGATTTCCAG TATGAGAGCA AGGTGTTTTA CCTGAAAATG

AGAACTGCAA TGATTTCCAG TATGAGAGCA AGGTGTTTTA CCTGAAAATG

AGAACTGCAA TGATTTCCAG TACGAGAGCA AGGTATTTTA CCTGAAAATG

AGAACTGCAA TGATTTTCAG TACGAGAGCA AGGTGTTCTA CCTGAAAATG

AGAACTGCAA TGATTTTCAG TATGAGAGCA AGGTGTTCTA CCTGAAAATG

AGAACTGCAA TGATTTCCAG TATGAGAGCA AGGTGTTTTA CCTGAAAATG

---------- ---------- ---------- ---------- ----------

AGAACTGCAA TGATTTCCAG TATGAGAGCA AGGTATTTTA TCTGAAAATG

AGAACTGCAA TGATTTCCAG TATGAGAGCA AGGTATTTTA CCTGAAAATG

---------- ---------- ---------- ---------- ----------

AGAACTGCAA TGATTTCCAA TATGAGAGCA AGGTGTTTTA CCTGAAAATG

AGAACTGCAA TGATTTCCAG TACGAGAGCA AGGTGTTTTA CCTGAAAATG

AGAACTGCAA TGATTTCCAG TATGAGAGCA AGGTGTTTTA CCTGAAGATG

AGAACTGCAA CGACTTCCAG TACGAGAGCA AAGTGTTCTA CCTGAAGATG

AGAATTGCAA TGACTTCCAG TATGAGAGCA AGGTCTTCTA CCTGAAAATG

AAGGGTGATT ACTACCGCTA CTTAGCAGAG GTCGCTTCTG GGGAGAAGAA

AAAGGTGATT ACTACCGCTA CTTAGCAGAG GTCGCTTCTG GGGAGAAGAA

AAAGGTGATT ACTACCGCTA CTTAGCAGAG GTCGCTTCTG GGGAGAAGAA

AAAGGTGATT ACTACCGCTA CTTAGCAGAG GTCGCTTCTG GGGAGAAGAA

AAAGGTGATT ACTACCGCTA CTTAGCAGAG GTCGCTTCTG GGGAGAAGAA

AAAGGTGATT ACTATCGCTA CTTGGCAGAG GTAGCTTCTG GGGAGAAGAA

AAGGGCGATT ACTACCGCTA CCTGGCAGAG GTGGCTTCTG GGGAGAAGAA

AAGGGCGATT ACTACCGCTA CCTGGCAGAG GTGGCTTCTG GGGAGAAGAA

AAGGGCGATT ACTACCGCTA CCTGGCCGAG GTGGCTTCCG GGGAGAAGAA

---------- ---------- ---------- ---------- ----------

AAAGGCGATT ACTACCGCTA CTTGGCAGAG GTAGCTTCCG GGGAGAAGAA

AAGGGTGATT ACTACCGCTA CTTGGCGGAG GTAGCTTCCG GGGAGAAGAA

---------- ---------- ---------- ---------- ----------

AAAGGCGATT ACTACCGCTA CTTGGCGGAG GTAGCTTCTG GGGAGAAGAA

AAAGGTGATT ACTACCGCTA CTTGGCAGAG GTAGCTTCTG GGGAGAAGAA

AAAGGCGATT ACTACCGCTA CTTGGCAGAG GTAGCTTCGG GGGAGAAGAA

AAGGGCGACT ACTACCGCTA CCTGGCCGAG GTGGCCGCCG GCGAGAAGAA

AAAGGCGATT ACTACCGTTA CCTGGCAGAA GTTGCTGCAG GTGAGAAGAA

AAACAGTGTG GTCGAAGCTT CTGAAGCTGC CTACAAGGAA GCCTTTGAAA

AAACAGTGTG GTCGAAGCTT CTGAAGCTGC CTACAAGGAA GCCTTTGAAA

AAACAGTGTG GTCGAAGCTT CTGAAGCTGC CTACAAGGAA GCCTTTGAAA

AAACAGTGTG GTCGAAGCTT CTGAAGCTGC CTACAAGGAA GCCTTTGAAA

AAACAGTGTG GTCGAAGCTT CTGAAGCTGC CTACAAGGAA GCCTTTGAAA

AAACAGTGTG GTGGAAGCTT CTGAAGCTGC CTACAAGGAA GCCTTTGAAA

AAACAGTGTG GTTGAAGCTT CTGAGGCAGC GTATAAGGAA GCCTTCGAAA

AAACAGTGTG GTTGAAGCTT CTGAGGCCGC GTATAAGGAA GCCTTCGAAA

GAACAGCGTG GTCGAAGCAT CTGAGGCCGC CTACAAGGAA GCCTTTGAGA

---------- ---------- ---------- ---------- ----------

AAACAGTGTG GTCGAAGCTT CTGAGGCTGC CTACAAAGAA GCCTTTGAAA

AAACAGTGTG GTCGAAGCTT CTGAGGCGGC CTATAAGGAA GCCTTTGAAA

---------- ---------- ---------- ---------- ----------

AAACAGTGTG GTTGAAGCTT CTGAGGCCGC TTACAAGGAA GCCTTTGAAA

AAACAGTGTG GTCGAAGCTT CTGAGGCTGC CTATAAGGAA GCTTTTGAAA

AAACAATGTG GTTGAAGCTT CTGAGGCTGC CTATAAAGAA GCCTTTGAAA

GAGCAGCGTG GTGGAGTCCT CCGAGGCCGC CTACAAGGAG GCCTTCGAGA

GAACAGTGTC GTGGAAGCTT CCGAGGCCGC CTACAAAGAG GCCTTTGAAA

TCAGCAAAGA GCAGATGCAA CCCACGCATC CCATCCGGCT GGGCCTGGCC

TCAGCAAAGA GCAGATGCAA CCCACGCATC CCATCCGGCT GGGGCTGGCC

TCAGCAAAGA GCAGATGCAG CCCACGCACC CCATCCGGCT GGGCCTGGCC

TCAGCAAAGA GCAGATGCAG CCCACGCACC CCATCCGGCT GGGCCTGGCC

TCAGCAAAGA GCAGATGCAG CCCACGCACC CTATTCGGCT GGGCCTGGCC

TCAGCAAAGA GCAAATGCAG CCCACACATC CCATCCGGCT GGGCCTGGCC

TCAGCAAAGA GCACATGCAG CCAACACACC CCATCCGGCT TGGCCTGGCC

TCAGCAAAGA GCACATGCAG CCAACACACC CCATCCGGCT TGGCCTGGCC

TCAGCAAAGA GCACATGCAG CCGACGCACC CCATCCGGCT GGGCCTGGCC

---------- ---------- ---------- ---------- ----------

TCAGCAAAGA GCACATGCAG CCAACGCACC CCATCCGGCT GGGTCTGGCC

TCAGCAAGGA GCACATGCAG CCGACGCACC CCATCCGCCT GGGCCTGGCC

---------- ---------- ---------- ---------- ----------

TCAGCAAAGA GCATATGCAG CCAACACATC CCATTCGGCT GGGTCTGGCC

TCAGCAAAGA GCACATGCAA CCGACGCATC CTATCCGGCT GGGCCTGGCC

TCAGCAAAGA GCACATGCAG CCTACACATC CCATCCGGCT GGGCCTGGCC

TCAGCAAGGA GCACATGCAG CCCACCCACC CCATCCGGCT GGGGCTGGCG

TCAGCAAAGA GCACATGCAG CCGACTCACC CAATTAGGCT GGGCTTGGCG

CTCAACTTCT CCGTGTTCTA CTATGAGATC CAGAATGCAC CTGAGCAAGC

CTCAACTTCT CCGTGTTCTA CTATGAGATC CAGAATGCAC CTGAGCAAGC

CTCAACTTCT CCGTGTTCTA CTATGAGATC CAGAATGCAC CCGAGCAAGC

CTCAACTTCT CCGTGTTCTA CTATGAGATC CAGAATGCAC CCGAGCAAGC

CTCAACTTCT CTGTGTTCTA CTATGAGATC CAGAATGCAC CTGAGCAAGC

CTCAACTTCT CTGTGTTCTA CTATGAGATC CAGAATGCAC CTGAGCAAGC

CTCAATTTTT CTGTGTTCTA CTATGAGATC CAGAATGCAC CAGAGCAGGC

CTCAATTTTT CTGTGTTCTA CTATGAGATC CAGAATGCAC CAGAGCAAGC

CTCAACTTCT CGGTGTTCTA CTACGAGATC CAGAATGCTC CGGAGCAGGC

---------- ---------- ---------- ---------- ----------

CTCAATTTCT CCGTGTTCTA CTATGAGATC CAGAATGCAC CCGAGCAGGC

CTCAACTTCT CTGTGTTCTA CTACGAGATC CAGAATGCAC CCGAGCAGGC

---------- ---------- ---------- ---------- ----------

CTCAACTTCT CTGTGTTCTA CTATGAGATC CAGAATGCAC CTGAGCAGGC

CTCAACTTCT CCGTGTTTTA CTACGAGATC CAGAATGCGC CTGAACAGGC

CTCAACTTCT CCGTGTTCTA CTATGAGATC CAGAATGCGC CTGAGCAAGC

CTCAACTTCT CCGTCTTCTA CTACGAGATC CAGAACGCTC CCGAGCAGGC

CTCAATTTCT CCGTGTTCTA CTACGAAATC CAGAATGCCC CCGAGCAGGC

CTGCCTCTTA GCCAAACAAG CCTTCGATGA TGCCATAGCT GAGCTGGACA

CTGCCTCTTA GCCAAACAAG CCTTCGATGA TGCCATAGCT GAGCTGGACA

CTGCCTCTTA GCCAAACAAG CCTTCGATGA TGCCATAGCT GAGCTGGACA

CTGCCTCTTA GCCAAACAAG CCTTCGATGA TGCCATAGCT GAGCTGGACA

CTGCCTCTTA GCCAAACAAG CCTTCGATGA TGCCATAGCT GAGCTGGACA

CTGCCTCTTA GCCAAACAAG CCTTCGATGA TGCGATAGCT GAGCTGGACA

CTGCCTCTTA GCCAAACAAG CCTTCGATGA TGCTATAGCT GAGCTGGACA

CTGCCTCTTA GCCAAACAAG CCTTCGATGA TGCTATAGCT GAGCTGGACA

CTGCCTCTTA GCCAAACAAG CCTTTGATGA CGCCATAGCT GAGCTGGACA

---------- ---------- ---------- ---------- ----------

CTGCCTCTTA GCCAAACAAG CCTTCGATGA TGCCATAGCT GAGCTGGACA

CTGCCTCTTA GCCAAACAAG CCTTTGATGA TGCCATAGCT GAGCTGGACA

---------- ---------- ---------- ---------- ----------

CTGCCTCTTA GCCAAACAAG CCTTTGATGA TGCCATAGCT GAGCTGGACA

GTGCCTCCTA GCCAAACAAG CCTTTGATGA TGCCATAGCA GAGCTGGACA

TTGCCTCCTA GCCAAACAAG CCTTTGATGA TGCCATAGCA GAGCTGGACA

CTGCCTCCTG GCCAAGCAAG CCTTCGACGA CGCCATCGCC GAGCTGGACA

CTGCCTTTTA GCCAAACAAG CCTTCGATGA CGCCATAGCA GAGCTGGACA

CACTAAACGA GGATTCCTAT AAGGACTCCA CGCTGATCAT GCAGTTGCTG

CACTAAACGA GGATTCCTAT AAGGACTCCA CGCTGATCAT GCAGTTGCTG

CACTAAACGA GGATTCCTAT AAGGACTCCA CGCTCATCAT GCAGTTGCTG

CACTAAACGA GGATTCCTAT AAGGACTCCA CGCTCATCAT GCAGTTGCTG

CACTAAATGA GGATTCCTAT AAGGACTCCA CGCTCATCAT GCAGTTGCTG

CACTAAACGA GGATTCCTAT AAGGACTCCA CACTCATCAT GCAATTGCTG

CATTAAACGA GGATTCCTAT AAGGACTCCA CTCTCATCAT GCAGTTGCTG

CATTAAACGA GGATTCCTAT AAGGACTCCA CTCTCATCAT GCAGTTGCTG

CGCTAAACGA GGATTCCTAT AAGGACTCCA CGCTCATCAT GCAGTTGCTG

---------- ---------- ---------- ---------- ----------

CACTAAACGA AGATTCCTAT AAGGACTCCA CGCTCATCAT GCAGTTGCTG

CTCTAAACGA GGATTCCTAT AAGGACTCCA CACTCATCAT GCAGTTGCTG

---------- ---------- ---------- ---------- ----------

CACTAAATGA GGATTCCTAT AAGGACTCCA CACTCATCAT GCAGTTGCTG

CACTAAACGA GGATTCCTAT AAGGACTCCA CACTCATCAT GCAGTTGCTG

CACTAAACGA GGATTCCTAT AAGGACTCCA CGCTCATCAT GCAGTTGCTG

CGCTGAACGA GGATTCCTAT AAAGACTCCA CTCTGATCAT GCAGCTGCTG

CACTAAATGA GGATTCCTAT AAGGACTCCA CTCTTATCAT GCAGTTACTG

CGAGACAACC TCACCCTCTG GACGAGCGAC CAGCAGGATG AAGAAGCAGG

CGAGACAACC TCACCCTCTG GACGAGCGAC CAGCAGGATG AAGAAGCAGG

CGAGACAACC TCACCCTCTG GACGAGCGAC CAGCAGGATG AAGAAGCAGG

CGAGACAACC TCACCCTCTG GACGAGCGAC CAGCAGGATG AAGAAGCAGG

CGAGACAACC TCACCCTCTG GACGAGCGAC CAGCAGGATG AAGAAGCAGG

CGAGACAACC TCACCCTCTG GACAAGCGAC CAGCAGGATG AAGAAGCAGG

CGAGACAACC TCACCCTCTG GACGAGCGAC CAGCAGGATG AAGAAGCCGG

CGAGACAACC TCACCCTCTG GACGAGCGAC CAGCAGGATG AAGAAGCAGG

CGAGACAACC TCACCCTCTG GACGAGCGAC CAGCAGGATG AAGACGCCGG

---------- ---------- ---------- ---------- ----------

CGAGACAACC TCACCCTCTG GACGAGCGAC CAGCAGGATG AAGAAGCAGG

CGAGACAACC TCACCCTCTG GACAAGCGAC CAGCAGGATG AAGAAGCAGG

---------- ---------- ---------- ---------- ----------

CGAGACAACC TCACCCTCTG GACGAGCGAC CAGCAGGATG AAGAAGCAGG

CGAGACAATC TCACCCTCTG GACAAGCGAC CAGCAGGATG AAGAAGCAGG

CGAGACAACC TCACTCTCTG GACCAGTGAC CAGCAGGATG AAGAAGCGGG

CGAGACAACC TCACTCTCTG GACGAGCGAC CAACAGGACG AGGAAGCAGG

CGAGACAACC TCACTCTCTG GACAAGCGAC CAGCAGGATG AGGAAGCAGG

AGAAGGCAAC ATGGCTGCCC CAGCAGTGTC CGGGCGGCAG GTGCGATGCT

AGAAGGCAAC ---------- ---------- ---------- GTGCGATGCT

AGAAGGCAAC ATGGCCGCCC CAGCAGCGTC CGGTCGGCAG GTGCGATGTT

AGAAGGCAAC ATGGCCGCCC CAGGAGTGTC CGGTCGGCAG GTGCGATGTT

AGAAGGCAAC ATGGCTGCCC CAGCAGTGTC CGGGCAGCAG GTGCGATACT

AGAGGGCAAC ATGGCAGCCC CAGCAGTGTC CGGGCGGCAG GTGCGATGCT

AGAAGGCAAC ATGGCCGCAC CAGCAACATC CGTGCGACAG GTGCGGAGTT

AGAAGGCAAC ATGGCCGCGC CTGCAGCGTC CGGACGACAG GTTCGGAGCT

AGAAGGCAAC ---------- ---------- ---------- GTGCGAAGCT

---------- ATGGCCGCGC TAGCAGTGTC CGGGCAGCAG GTGCGATGCT

AGAAGGCAAC ATGGCCGCCC CAGCGGTGTC CGGGCGGCAG GTGCGGTGCT

AGAAGGCAAC ATGGCAGCCC CAGCAGTGTC CGGGCGGCAG GTGCGATGCT

---------- ATGGCCTCGA GGGCAGTGTC CGGGCAACAG GTGCGATCCT

AGAAGGCAAC ATGGCCGCCC CAGCAGCGTC AGGCCGGCAG GTACGATGCT

AGAAGGCAAC ATGGCCGGCC CAGCGGTGAC CGGGCGCCAG GTGCGGTACT

AGATGGCAAT ATGGCTGGCC CAGCAGTGAC CCGG------ ----------

AGAAGGCAAC ATGGCGGTGG CCGCTACCTC TGGGCAGCAG ATACGCCGAT

AGAGGGCAAC ATGGCGGCGG TGGCGGCCTC GATGCGGCAG GTGCGGCAGT

TCAGTACCTC TGTGGTCAGA CCATTTGCCA AGCTTGTGAG GCAAATCCTG

TCAGTACCTC TGTGGTCAGA CCATTTGCCA AGCTTGTGAG GCAAATCCTG

TCAGTACCTC TGTGGTCAGA CCGTTTGCCA AGCTTGTGAG GCAAATTCTG

TCAGTACCTC TGTGGTCAGA CCGTTTGCCA AGCTTGTGAG GCAAATCCTG

TCGGTACATC TGTGGTCAGA CCATTTGCCA AGCTTGTGAG GCAAATCCTG

TCAGTACCTC TGTGGCTAGG CCATTCACCA AGCTTGTGCG GCAACTCCTG

TCAGCACATC TGTGGTCAGG CCCTTTTCGA AGCTTGTAAG GCAACTCTTG

TCAGTACATC TGTGGTCAGG CCCTTTGCCA AGCTTGTAAG GCAACTCCTG

TCAGTACATC TGTGGTCCGG CCGTTTGCCA AGCTCGTGAG GCAACTGCTG

TCAGTACATC TGTGGTCAGG CCATTTGCCA AGCTTGTGAG GCAAATCTTG

TTAGCACATC TGTGGTCAGG CCGTTTTCCA AGCTCGTGAG GCAAATCTTG

TCAGTACATC TGTGGTCAGG CCATTTACCA AGCTTGTGAG GCAACTCTTG

TCAGTACCTC TGTGGCCAGG CCCTTCGCCA AGCTTGTGAG GCAACTGCTG

TCAGTACATC TGTGGTCAGG CCATTTGCCA AGCTTGTGAG GCAACTTCTG

TCAGTACATC TGTGGCCAGG CCATTTGCCA AGCTTGTGAG GCAACTTCTG

---------- ---------- ---------- ---------- -CAACTTCTG

TCACCACTTC AGTGGCCAGA CCATTTGCCA AACTTGTCAG GAAACTTCTG

TCAGCACATC TGTGGCCCGA CCATTTGCCA AACTAGTCCG GCAACTTCTG

AAGGAACCCA AAGTGGCTGC TTCTGTTTTG AATCCCTATG TGAAGCGTTC

AAGGAACCCA AAGTGGCTGC TTCTGTTTTG AATCCCTATG TGAAGCGTTC

AAGGAACCCA AAGTGGCTGC TTCTGTTTTG AATCCCTATG TGAAGCGTTC

AAGGAACCCA AAGTGGCTGC TTCTGTTTTG AATCCCTATG TGAAGCGTTC

AAGGAACCCA AAGTGGCTGC TTCTGTTTTG AATCCCTATG TGAAGCATTC

AAGGAACCTA AAATGGCTGC TTCTATTTTG AATCCCTACG TGAAACGTTC

AAGGACCCCA AGGTGTCCCT TGCTGTTCTG AATCCCTACA TTAAGCGCTC

AAGGACCCCA AAGTGTCTCT GGCTGTTCTG AATCCCTACA TCAAGCGCAC

AAGGAACCCA AATTGGCGGC TTCTATTCTG AATCCGTATG TCAAGCGTGC

AAGGAACCCA AAATGGCTGC TTCTCTTTTG AATCCCTATG TAAAGCGTTC

AAGGAACCTA AAATGGCTGC TTCCATTATG AATCCCTACA TAAAGCGTTC

AAGGAACCCA AAATGGCTGC CTCAATTATG AATCCCTATA TAAAGCGATC

AAGGAGCCCA AGCTAGCGTC CTCCATCCTG AACCCACACG TGAAGCACAC

AAGGATCCTA AAGTGGCTGT GTCTATCCTG AATCCCTATG TAAAGCGATC

AAGGACCCCA AAGTGGCTCT TGCTCTTCTG AATCCCCATG TGAAGCGTTC

AAGGACCCCA AAGTGGCTCT TGCAATTCTG AATCCCCATG TGAAGCGCTC

AAGGAACCCA AAACAGTTGC CTCTTTTATG AATCCCCATA TCAAGCGTAC

AAGGAACCCA AAATGGCTGC TTCTATTCTC AACCCCCATG TCAAGCGTGC

CATTAAAGTG AAAAGCCTAA ATGACATCAC AGCAAAAGAG AGGTTCTCTC

CATTAAAGTG AAAAGCCTAA ATGACATCAC AGCCAAAGAG AGGTTCTCTC

TATTAAAGTG AAAAGCCTAA ATGACATCAC AGCAAAAGAG AGGTTCTCTC

TATTAAAGTG AAAAGCCTAA ATGACATCAC AGCAAAAGAG AGGTTCTCTC

CATTAAAGTG AAAAGCCTAA GTGACATCAT AGCAAAAGAG AGGTTCTCTC

CGTTAAAGTG AAAAGCCTAA ATGACATCAC GGCAAAAGAG CGGTTTTCTC

CATCAAAGTG AAAAGCCTGA AGGACATCAC TACAAAGGAA AAATTCTCTC

CGTCAAAGTG AAAAGCCTAA ATGACATCAC GAAAAGGGAA AAGTTCTCCC

AGTCAAGATG AAGAGCCTGA ATGACATCAC AGCAAAAGAG AGGTTTTCTC

AGTTAAAGTG AAAAGCCTAA GTGACATGAC GGCAAAGGAG AAGTTCTCTC

CGTGAAAGTG AAAAGCCTAA ATGACATGAC AGCAAAAGAG AGGTTCTCTC

CATTAAAGTG AAAAGCCTCA ATGACATGAC CACAAAAGAG AAGTTTTCTC

CATCAAGGTG AAGAGCATCG GCAACCTGAC AGCCCGGGAG AAGCTGTCCC

CCTTAAAGTG AAAAGCCTAA ATGACATCAC AGCAAAAGAG AGGTTTTCTC

CATTAAAGTT AAAAGCCTAA ATGACATCAC AGCAAAAGAG AGGTTTTCTC

GATTAAGGTG AAAAGCCTGA GTGACATCAC CGCCAAAGAG AGGCTTTCTC

CATTAAGATG GCAGCCCTAA ATGACTTAAT AGCCAAAGAA AAGTTTTCCC

CGTGAAGATG AAAAACCTGA ATGACATTAC TGCAAAAGAG AAGTTTTCCC

CCCTCACTAC CAATCTGATC AATTTGCTTG CTGAAAATGG TCGATTAAGC

CCCTCACCAC CAACCTGATC AATTTGCTTG CTGAAAATGG TCGATTAAGC

CCCTCACCAC CAACCTGATC AATTTGCTTG CGGAAAATGG TCGCTTAAGC

CCCTCACCAC CAACCTGATC AATTTGCTTG CGGAAAATGG TCGCTTAAGC

CCCTCACGAC GAACCTGATC AATTTGCTTG CTGAAAATGG TCGCTTAAGC

CCCTCACAGC CAACCTGATG AATTTGCTTG CTGAAAATGG CCGCTTGAGC

CGCTGACGGC CAACCTCATG AATTTACTTG CTGAAAATGG TCGCCTAGGC

CGCTGACGGC CAACCTCATG AATTTACTTG CTGAAAATGG TCGCCTAGGC

CCCTCACGGC CAACCTCATG AATCTGCTTG CTGAGAACGG TCGCCTGCGC

CTCTCACGTC CAACCTGATC AATTTGCTTG CTGAAAATGG TCGCTTGACT

CTATCACATC CAACCTGATC AATTTGCTTG CTGAGAACGG TCGCTTGAAC

CCCTCACATC CAACCTGATG AATTTGCTTG CTGAAAATGG TCGCCTGAAC

CAATCACCAC CAACCTCATC AATCTGCTTG CTGAAAATGG GTGTTTGAAC

CCCTCACATC CAATCTGATC AATTTGCTTG CTGAAAATGG TCGCTTGAGC

CCCTCACGTC CAACCTGATC AATTTGCTTG CTGAGAACGG CCGCTTGAAC

CCATCACCAC CAACCTGATC AATTTGCTTG CTGAAAATGG TCGCTTGAAC

CCATCACAAC CAACTTCATG AAATTGCTTG CTGAAAATGG CCGTTTAAAC

CCATCACCAC CAACTTTATC AGTCTGCTGG CTGAAAATGG CCGCTTGAAC

AATACCCAAG GAGTCGTTTC TGCCTTTTCT ACCATGATGA GTGTCCATCG

AATACCCAAG GAGTCGTTTC TGCCTTTTCT ACCATGATGA GTGTCCATCG

AATGCCCAGG GAGTCGTTTC TGCCTTTTCT ACCATGATGA GTGTCCATCG

AATGCCCAGG GAGTCGTTTC TGCCTTTTCT ACCATGATGA GTGTCCATCG

AATACCCAAG GAGTTGTTTC TGCCTTTTCT ACCATGATGA GTGTCCACCG

AACACCCAAG GAGTCATTTC TGCCTTTTCC ACCATGATGA GCGTTCACCG

AACACCCAGG GCGTCATCTC TGCCTTCTCC ACCATCATGA GTGTCCACCG

AACACCCAGG GTATCATCTC TGCCTTTTCC ACCATCATGA GTGTCCACCG

AATGCCCAGG GAGTCATCTC CGCCTTTTCT ACCATGATGA GCGTCCACCG

AATACCCCTG CGGTGATTTC CGCATTTTCT ACCATGATGA GTGTCCACCG

AATACCCCTG GAGTCATTTC TGCCTTTTCC ACCATGATGA GTGTGCACCG

AATACCCCTG GAGTCATTTC CGCCTTTTCC ACCATAATGA GCGTGCACCG

AACACCCCTG AGATCATTTC AGCCTTTTCC ACCATGATGA GCGTCCACCG

AATGCCCCAG GAGTCATTTC TGCCTTTTCT ACCATGATGA GTGTCCACCG

AGTACCCCGG CAGTCATTTC TGCATTCTCG ACCATCATGA GTGTCCACCG

AACGCCCCGG GAGTCATTTC GGCCTTTTCG ACCATCATGA GTGTCCACCG

AACACCCCAG GAATTATTTC AGCATTTTCT ACTATAATGA GTGTGCATCG

AACACCCCTG GTGTCATTTC CGCCTTCGCC ACCATGATGA GCGTGCACCG

CGGAGAGGTA CCTTGCACAG TGACCTCTGC ATCTCCTTTA GAAGAAGCCA

CGGAGAGGTA CCTTGCACAG TGACCTCTGC ATCTCCTTTA GAAGAAGCCA

CGGAGAGGTG CTTTGCACGG TGACCACTGC ATCTCCTTTA GAAGAAGCCA

CGGAGAGGTG CCTTGCACGG TGACCACTGC ATCTCCTTTA GAAGAAGCCA

CGGAGAGGTT CCCTGCACAG TGACCACCGC ATCTCCTTTA GAAGAAGCCA

TGGAGAGGTG CCGTGCACAG TGACCACTGC GTCTCCTCTG GAAGAAGCTA

TGGAGAAGTG CCGTGCACAG TGACCACAGC ATTTCCTTTA GATGAAGCTG

CGGAGAAGTG CCATGCACAG TGACCACAGC ATCTCCTCTA GATGACGCTG

TGGAGAAGTC CCCTGCACAG TGACCACCGC GTCT------ ----------

TGGAGAAGTA CCATGCACAG TTACCACTGC ATCTGCTTTA GATGAAGCCA

TGGAGAAGTG CCGTGCACAG TGACCACCGC GTCTCCTTTG GATGAAGCCA

TGGGGAAGTA CCTTGCTCAG TTACCACCGC ATCTCCTTTA GATGAGGCCA

CGGCGAAGTC CCCTGCACAG TGACCACAGC CTCT------ ---GAGGCCA

TGGAGAAGTA CCTTGCACAG TTACCACTGC CTCTCCTTTA GATGAAGCTA

TGGAGAAATT CCTTGCACAG TTACCACCGC ATCTCCCTTA GACAACACCT

TGGAGAGGTT CCTTGCACAG TCACCACTGC GTCTTCCTTA GACAACACCT

TGGAGAAGTT CCTTGTTCAG TTACTACTGC GTCT-CTTTA GATAGCGCAA

GGGAGAGGTC CAGTGCTCCG TGACCACGGC TTCTGCCCTA GACAGTGCCA

CACTCTCTGA ATTAAAAACT GTCCTCAAGA GCTTCCTAAG TCAAGGCCAA

CACTCTCTGA ATTAAAAACT GTCCTCAAGA GCTTCCTAAG TCAAGGCCAA

CACTCTCTGA ATTAAAAACT GTCCTCAAGA GCTTCCTGAG TCAAGGCCAA

CACTCTCTGA ATTAAAAACT GTCCTCAAGA GCTTCCTGAG TCAAGGCCAA

CACTCTCTGA ATTAAAAACA GTCCTGAAGA GCTTCCTAAG TCAAGGCCAG

CTCTCACTGA ATTAAAAACA GTCCTGAAAA GCTTCCTAGG TCAAGGCCAA

TTCTCTCTGA GTTAAAGACA GTGCTGAATA GCTTCCTGAG TAAAGGCCAG

TTCTCTCTGA GTTAAAGACG GTGCTGAAGA GCTTCCTGAG TCCAAACCAA

---------- ---------- ---------- ---------- ----------

CTCTGACTGA ATTGAAAACG GTCCTGAAGA GCTTCCTAAG TAAAGGCCAG

CTCTTACTGA ACTAAAAACG GTCCTGAAGA GCTTCCTAAG CAAAGGCCAA

CTCTTTCTGA ATTAAAAACG GTGCTGCAGA GCTTCCTAAG TAAAAATCAG

CGCTGTCTGA GCTCAAAACA GTCCTGCAGA GCTTCCTGAG CAAAAACCAA

CTCTTTCTGA ATTAAAAACA GTCCTGAAGA GTTTCTTAAG TAAAGGCCAG

CTCTTTCTGA ATTAAAAACC GTCCTGAACA GCTTCCTAAG ACAAGGCCAG

CTCTCTCTGA ATTGAAAACG GTCCTGAACA GCTTCCTAAG CCAAGGCCAG

CTCTTTCAGA ATTAGAAGCA GTCCTGAACA GCTTCCTACG TAAAGGTCAA

CGCTGACAGA GCTCAAGACT GTCCTCAACA GCTTCCTGAC TAAAGGCCAA

GTATTGAAAT TGGAGGCTAA GACTGATCCG TCAATCTTGG GTGGAATGAT

GTATTGAAAT TGGAGGCTAA GACTGATCCG TCAATCTTGG GTGGAATGAT

GTATTGAAAT TGGAGGCTAA GACTGATCCG TCAATCATGG GTGGAATGAT

GTATTGAAAT TGGAGGCTAA GACTGATCCG TCAATCATGG GTGGAATGAT

ATACTGAAAT TGGAGGTTAA GACTGATCCA TCAATCATGG GTGGAATGAT

ATATTGAAAT TGGAGGTTAA GACTGATCCA GCGATCATGG GTGGAATGAT

ATACTGAACC TGGAGGTCAA GACTGACCCA TCAATCATGG GTGGGATGAT

ATACTGAAAC TGGAGATCAA GACTGACCCG TCAATCATGG GCGGGATGAT

---------- ---------- -ACGGACCCG TCGATCATGG GTGGGATGAT

GTTTTGAAGT TAGAAGTTAA GATTGATCCG TCGATCATGG GTGGAATGAT

GTATTGAAAT TGGAAGTTAA GGTTGATCCG TCAATCATGG GGGGAATGAT

ATACTGAAAT TGGAAGTTAA GACTGATCCA TCAATCATGG GTGGAATGAT

GTGTTGAAGC TGGAGGTGAA GACCGACCCG TCCATCATGG GGGGGATGAT

GTATTGAAAT TGGAGATTAA GACTGATCCG TCAATCATGG GTGGGATGAT

GTGTTGAAAT TGGAGGTTAA GACGGATCCA TCAATCATGG GCGGAATGAT

GTGTTGAAAT TGGAGGTGAA GACTGACCCA TCAATCATGG GTGGAATGAT

GTCCTGAAAA TGGAAGCCAA GACTGATCCT TCAATCATGG GAGGAATGAT

GTCTTGAAAC TGGAGGTTAA GACGGACCCG TCAATCATGG GAGGGATGAT

TGTGCGCATT GGCGAGAAAT ATGTTGACAT GTCTGTCAAG ACCAAGATTC

TGTGCGCATT GGCGAGAAAT ATGTTGACAT GTCTGTCAAG ACCAAGATTC

TGTGCGCATT GGAGAGAAGT ATGTTGACAT GTCTGTCAAG ACCAAGATTC

TGTGCGCATT GGAGAGAAGT ATGTTGACAT GTCTGTCAGG ACCAAGATTC

TGTGCGTATT GGAGAGAAAT ACGTGGACAT GTCTGCCAAG ACCAAGATTC

TGTTCGAATT GGAGAGAAAT ATGTGGACAT GTCTGCAAAA ACAAAGATTC

TGTCCGAATT GGAGAGAAAT ACGTTGATAT GTCTGCAAAG AGCAAGATTC

TGTCCGAATT GGGGAAAAGT ACGTTGATAT GTCTGCAAAG AGCAAGATTC

TGTCCGCATT GGAGAGAAGT ACGTCGATAT GTCTGCAAAA ACCAAGATTC

TGTCCGTATT GGAGAGAAAT ACGTTGATAT GTCTGCAAAA ACCAAGATTC

TGTCCGTATT GGAGAGAAAT ATGCTGATAT GTCTGCAAGG ACCAAGATTC

TATCCGTATT GGAGAGAAAT ATGTTGATAT GTCTGCAAAA ACCAAGATTC

CGTCCGCATC GGAGAGAAGT ACGTGGACAT GTCTGCCAGG ACCAAGATCC

TGTCCGTATT GGAGAGAAGT ATGTTGATAT GTCTGCAAAA ACCAAGATTC

TGTCCGTATT GGCGAGAGAT ATGTTGATAT GTCTGCAAAA TCCAAGATTG

TGTCCGCATT GGCGAGAAGT ACGTGGATAT GTCGGCCAAG TCCAAGATTC

TGTTCGTATA GGAGAGAAAT ATGTGGATAT GTCTGCAAGA ACCAAGATCC

TGTCCGCATT GGGGAGAAAT TTGTCGATAT GTCTGCAAGA ACCAAGATTC

AGAAGCTGGG CAGGGCTATG CGGGAGATTG TCGGCGTGCG GTTCAAGGCT

AGAAGCTGGG CAGGGCTATG CGGGAGATTG TCGGCGTGCG GTTCAAGGCT

AGAAGCTGGG CAGGGCTATG CGGGAGACTC TCGGCGTGCG GTTCAAGGCT

AGAAGCTGGG CAGGGCTATG CGGGAGGCTG TCGGCGTGCG GTTCAAGGCT

AGAAGCTGAG CAAGGCTATG CGGGAGGTTA TCGGCGTGCG GTTCAAGGCT

AGAAGCTGAC CAGGGCCATG CAGGAGATAA TC-------- ----------

AGAAGCTCAG CAAGGCCATG CGGGATCTGC TCGGCGTGCG GTTCAAAACC

AGAAGCTCAG CAAGGCCATG CGGGAGATGC TCGGCGTGCG GTTCAAGACC

AGAAGCTGAG CAAGGCCATG CGAGAGATTA TG-------- ----------

AGAAGCTGAG CAGAGCAATG CGGGAGATTC TGGGCGTGCG CTTCAAGGCT

AGAAGCTGAG CAGGGCCATG CGGGAGGTTT TCGGCGTGCG CTTCAAGGCT

AGAAGCTGAG TAGGGTTATG CGGGAGGTTT TCGGCGTGCG CTTCAAGGCT

AGAAGCTGAG CAAGGCCATG CGCGAGGCGA TGGGCGTGCG CTTTAAGGCG

AGAAGCTGAG CAGGGCTATG CGGGAGATAT TCGGCGTGCG GTTCAAGGCT

AGAAGCTGAA GAGGGTTATG CTGGAGACTG TGGGCGTGCA GTTCAAGACT

AGAAGCTGAG CAGGATTATA CGGGAGACGG TG-------- ----------

AGAAGCTAAG CAGGATTATG AGGGAAGCTG TTGGGCAGCA GTTTAAGGTT

AGAAGCTGAG CAGAGTGATG AAAGAGGCTA TCggcgtGCA GCTGAAGACG

CAGCTGAAGG ACCCGCAGGG CCCCGGGCCA GGCTTGGAAG CGTTTGTGTC

CAGCTGAAGG ACCCGCAGGG CCCCGGGCCA GGCTTGGAAG CGTTTGTGTC

CAGCTGAAGG ACCCGCAGGG CCCCGGGCCA GGCTTGGAAG CGTTTGTGTC

CGGCTGAAGG ACCCGCAGGG CCCCGGGCCA GGCCTGGAAG CGTTTGTGTC

CACTTGAAGG ACCCACAGGG CCCTGGGCCT GGCTTGGAAG CGTTTGTGTC

CAGCTGAAGG ACCCGCAGGG CATCGGGCCA GCCTTGGAAG CATTTGTCTC

CAGCTGAAGG ATGCGCAGGG CCCTGGACCC GCTTTGGAAG CCTTTGTCTC

CTGCTGAAGG ATGCGCAGGG CCCAGGACCG GCTTTGGAAG CCTTCGTCTC

---------- ---------- ---------- -CCTTGGAGG CGTTCGTGTC

CAGCTGAGGG ACCCGCAGGG CGCGGGGCCG GCCCTGGAGG CATTTGTTTC

CAGCTGCGGG AGCCGCAGGG CGCGGAGCCG GCCTTAGAAG CGTTTGTCGC

CAGCTGAGGG ACCCGCAGGG AGCCGGGCAG GCCTTGGAAG CGTTTGTCTC

CAGCTCCGGG AACCGCAGGG CGCGGGAGCC GCCTTGGAAG CCTTCGTGTC

CTGCTGAAGG ATGCGCAGGG TGCTGCGCCG GCCTTGGAAG CATTTGTCTC

CAGCTGAGAG ACTCGCAGGA CGCGGGCCCA GCCTTGGAAG CGTTTGTCTC

---------- ---------- ---------- ---------- ----------

CAGCTTAAAG ACCCTCAAAC TATGGTATCA GCTTTGGAAA CTTTCATCTC

CAGCTGCGGA CCCGGCAGGA CGTCGGCCAG GCACTGGAGA CTTTCGTGTC

TGCTGCCAAG AAGCTACCAC GAGAAGATGT GTATGATGTT GTGGAAGGGT

TGCCGCCAAG AAGCTACCAC GAGAAGATGT GTATGATGTC GTGGAAGGGT

TGCTGCCAAG AAGCTACCAC GAGAAGATGT GTATGATGTT GTGGAAGGGT

TGCTGCCAAG AAGCTACCAC GAGAAGATGT GTATGATGTT GTGGAAGGGT

TGCTGCCAAG AAGCTACCAC AAGAAGATGT ATATGATGTT GTGGAAGGTT

TGCTGCCAAG AAGCTACCAC GAGAAGACAT GTATGATGTT GTGGAAGGTT

TGCTGCCAAG CAGCTGCCAC AGGAAGACAT GTGCGATGTT GTCGAGGGCT

TGCTGCCAAG AAGCTACCGC AGGAAGACAT GTGTGATGTT GTCGAGGGCT

CACTGCCAGG AAGCTGCCGA GCGAGGACGT GTATGATGTG GTGGAAGGGT

CGCTGCTAAG AAGCTCCCCC GAGAAGATGT GTACGATGTT GTGGAGGGGT

TGCTGCCAAG AAGTTACCTC GAGAAGACGT ATATGATGTT GTGGAAGGGT

TGCTGCCAAG AAGCTTCCGC GAGAAGATGT GTATGATGTC GTGGAAGGGT

GGCCGCCAAG AAGCTCCCAT GCGAAGATGT GTACGACATT GTGGAGGGCT

TGCTGCCAAG AAGCTGCCAC AAGAAGGTGT GTATGATGTT GTGGAAGGAT

TGCTGCCAAG AAGCTACCCC AAGAAGGGGT GTATGATGTC GTGGAAGGAT

---------- ---------- ---------- ---------- ----------

CATTGCCAAA ATGTTACCAC AAACTGGGCT ATATGATGTC GTGGAAGGTT

CAGGGCCAGG AAGTTACCTC CAGCTGGCCC GTGTGATGTC GTGGAAGAGT

ATATAAAGAT TTCTGTTGAG TGTGTCGAAA TTTTCCAGCT CCTAAGTGGA

ATATAAAGAT TTCTGTTGAG TGTGTCGAAA TTTTCCAGCT CCTAAGTGGA

ATATAAAGAT TTCTGTTGAG TGTGCAGAAA TTTTCCAGCT CCTAAGTGGA

ATATAAAGAT TTCTGTTGAG TGTGCAGAAA TTTTCCAGCT CCTAAGTGGA

ACATAAAGAT TTCTGTTGAG TGTGCCGAAA TTTTCCAGCT CCTGAGTGGA

ATATAAAGAT TTCTGTTGAG TGTGCTGAAA TTTTCCAGCT TCTGAGTGGA

ACATCAAGAT TTCCATGGAG TGTGCAGAAA TTTTCCAGCT CCTGAGTGGA

ACATCAAGAT TTCCATGGAA TGTGCAGAGA TTTTCCAGCT CCTGAGTGGG

ACATAAAGAT CTCCGTCGAG TGCGCCGAGG TCTTCCAGCT CCTGAGTGGG

ACATCAAGAT TTCTGTGGAG TGTGCCGAGA TTTTCCAGCT CCTGAGTGGG

ACATAAAGAT TTCTGTTGAG TGTGCAGAGA TCTTCCAACT CCTGAATGGG

ACATCAAGAT TTCTGTGGAG TGTGCAGAGA TTTTCCAGCT CCTGAGTGGG

ACATCAAGAT CTCCGTGGAG TGCACCGAGA TCTTCCAGCT CCTGAGTGGG

ATATAAAGAT TTCTGTGGAG TGTGCAGAAA TTTTCCAGTT CCTGAGTGCA

ACATAAAGAT TTCCGTTGAG TGTGCAGAAA TTTTCCAGCT CCTGAGTGGC

---------- ---------- ---------- ---------- ----------

ATATAAAGAT TTCTGTTGAG TGTTCAGAAA TTTTCCAGCT TCTAAGTGGA

ACGTCAAGAT CTCGGTGGAG TGCGCGGAGA TCTTCCAGCT GTTCGGTGGA

GAGAAACGAC CTGAAAGTGA AACGATGTTA ATATTTCAAG TTTTCGAGGC

GAGAAACGAC CTGAAAGTGA AACGATGTTA ATATTTCAAG TTTTCGAGGC

GAGAAACGAC CTGAAAGTGA AACGGTGTTA ATATTTCAAG TTTTTGAGGC

GAGAAACGAC CTGAAAGTGA AACGGTGTTA ATATTTCAAG TTTTTGAGTC

GACAAACGAC CTGAAAGTGA AATGATATTA ATATTTCAAG TTTTCGAGGC

GAGAAGCGAC CTGAAAGTGA AATGCTATTA ATATTTCAAG TTTTCGAAGC

GAGAAGCGAC CCGAGAGTGA AGTGCTGTTA ATATTTCAAG CTTTTGAGGC

GAGAAGCGGC CCGAGAGTGA AATGCTGTTA ATATTTCAAG CTTTCGAGGC

GAGAAGCGGC CCGAAAGTGA A--------- -TATTTCAAG TTTTGGAAGC

GAGAAGCGCC CTGAAAGTGA AATGCTTTTA GTATTTCAAG TTTTCGAGGC

GAGAAGCGAC CTGAAAGTGA AATGCTGTTA ATATTTCAAG TTTTTGAGGC

GAGAAGCGAC CTGAAAGTGA GATGCTATTA ATATTTCAAG TTTTTGAGGC

GAGAAGCGGC CTGAAAGCGA GATGCTGCTG GTCTTCCAGG CGCTGGAGGC

GAGAAGCGAC CAGAAAGTGA GATGCTGTCA ACATTTCAAG CATTGGAGGC

GAGAAGCGAC CTGAAAGTGA GATACTATTA ATATTTCAAG CTTTCGAGGC

---------- ---------- ---------- ---------- ----------

GAGAAAAGAA CTGAACATGA ACGGCTATTA ATATTTAAAG CTTTAGAAGC

GAGAAGAGGC CGGAGAGGGA GGTGATGCTG ATCCTGCAGG CCCTGGAAGC

CATATTATTG CGGACAGCAA GTGATCTTTC ACATTTCCAT GTTGTGGGAA

CATATTATTG CGGACAGCAA GTGATCTTTC ACATTTCCAT GTTGTGGGAA

CATATTATTG CGGACAGCAA GTGATCTTTC ACATTTCCAT GTTGTGGGAA

CATATTATTG CGGACAGCAA GTGATCTTTC ACATTTCCAT GTTGTGGGAA

CATATTGTTG CGGACAGCAA GTGATCTGTC ACATTTTCAT GTTGTGGGAA

CATATTGTTG AGGACAGCAA GTGATCTTTC CCATTTTCAT GTTGTGGGAA

CATATTACTG AGGACAGCCA GTGATCTTAC ACATTTCCAC GTGGTGGGCA

CATATTACTG AGGACAGCCA GTGATCTTAC GCATTTCCAC GTGGTGGGTG

CATCCTGCTG AGGACAGCCA GCGATCTGTC GCATTATCAT GTCGCGGGCA

CATATTATTG CGGACAGCCA GTGACCTTTC CCATTTTCAC GTGGTGGGGA

CATACTGTTG CGGACTGCGA GCGATCTTTC CCATTTTCAC GTTGTAGGGA

CATATTATTA CGGACCGCAA GTGATCTTTC CCATTTTCAT GTTGTGGGAA

CATCCTGCTG CGGACGGCCA GCGACCTGGC ACACCTGCAC ATGGCGGGCA

CATCTTATTG CGAACAGCTA GTGACCTGTC ACATTTTCAC ATTGTGGGAA

CATATTGTTG CGGACGGCAA GCGATCTTTC CCATTTTCAC GCTGTGGGAA

---------- ---------- ---------- ---------- ----------

CATATTACTT CGAACAGCAG GTGACCTTTC TCATTTTCAT GTTGTGGGAA

CATCCTGCTG CGCACGGCCA GCGACCTGGC CCACCTGCGC ATCGTGGGGA

CCAACATTGT GAAAAAGCTG ATGAACAACC ACATGAAGCT CATCTGTGAG

CCAACATTGT GAAAAAGCTG ATGAACAACC ACATGAAGCT CATCTGTGAG

CCAACATTGT GAAAAAGCTG ATGAACAACC ACATGAAGCT CATCTGTGAG

CCAACATTGT GAAAAAGCTG ATGAACAACC ACATGAAGCT CATCTGTGAG

CCAACATCGT GAAAAAGCTG ATGAACAACC ACATGAAGCT CATCTGTGAG

CCAACATCAT GAAAAAGCTG ATGAACAACC ACATGAAGCT TATTTGTGAG

CCAACATTGT GAAGAAGCTT CTGAACAACC ACATGAAGCT CCTCTGTGAA

CCAACATTGT GAAGAAGCTG CTGTACAACC ACATGAAGCT CCTCTGTGAG

CCCACATCGT GAAGAAGCTG CTGAACAACC ACATGAAGCT CATCTGTGAG

CCAACATCGT GAAGAAGCTA ATGAATAACC ACATGAAGCT CATCTGTGAG

CCAACATCGT GAAAAAGCTA ATAAATAACC ACATGAAGCT CATCTGTGAG

CGAATATCGT GAAAAAGCTC ATGAATAACC ACATGAAGCT CATCTGTGAG

GCGGCATCGT GAAGAAGCTG CTCAACAGCC ACATGAAGCT GCTCTGCGAG

CCAACATCGT GAAGAAGCTC ATGAATAACC ACATGAAGCT CATCTGCGAG

CCAACATCGT GAAAAAGCTC CTGAATAACC ACATGAAGCT CATCTGCGAG

---------- ---------- ---------- ---------- ----------

CCAATATCAT AAAAAAAGTT TTGAATAGCC ATATGAAGCT GATTTATGAA

CCAACATCGT CAAAACCCTC ATCAACAATC ACATGAAGGT GCTCTACGAG

TCCCTGTATG CCTCAGGTTA CAGGTTGGCT CGCGCCTGCC TGAGCCTGAT

TCCCTGTATG CCTCAGGTTA CAGGTTGGCT CGCGCCTGCC TGAGCCTGAT

TCCCTGTATG CCTCGGGTTA CAGGTTGGCT CGCGCCTGCC TGAGCCTGAT

TCCCTGTATG CCTCGGGTTA CAGGTTGGCT CGCGCCTGCC TGAGCCTGAT

TCCCTGTATG CCTCGGGTTA CAGGTTGGCT CGTGCCTGCC TGAGCCTGAT

GCCCTCTATG CCTCGGGGTA TAGGTTGGCT CGAGCCTGCC TGAATCTGAT

TCCCTGTATG CATCTGGTTA CAGGATGGCT CGAGCCTGCC TGGACCTGAT

TCCCTGTATG CGTCTGGTTA CAGGATGGCT CGAGCCTGCC TGGACCTGAT

TCCCTGTACG CCTCGGGGTA CAGGATGGCT CGAGCCTGCC TCAACATGAT

TCCCTGTACG CCTCGGGTTA CAGGATGGCT CGAGCCTGCC TGAACCTGAT

TCCCTGTATG CCTCGGGCTA CAGGATGGCT CGAGCCTGCC TGAACCTGTT

TCCCTGTATG CCTCGGGTTA CAAGATGGCT CGAGCCTGCC TGAACCTGAT

GCCCTCTACG CCTCGGGCTA CAGGATGGCT CGCGCCTGCC TCAACCTGCT

GCCCTGTATG CCTCCGGCTA CAGGACAGCT CGAGCCTGCC TGAACCTGAT

TCCCTGTATG CCTCAGGCTA CAGGTTGGCT CGAGCCTGCC TGAACCTGAT

---------- ---------- ---------- ---------- ----------

TCATTGAATC CTTCAAGCTA CAAAATGACT CAAACCTGCT TGAACCTCAT

TCGCTGTTCG CCTCGGGCTA CCAGATGTCC CGGGCCTGCC TGAACCTCAT

GACCGCCATG GTGACCCAGG GTCCGGAAGC TGCCAGGGAC GTCTGCAGCC

GACCGCCATG GTGACCCAGG GTCCGGAAGC TGCCAGGGAC GTCTGCAGCC

GGCCGCCATG GTGACCCAGG GTCCGGAAGC TGCCAGGGAT GTCTGCAGCC

GGCCGCCATG GTGACCCAGG GTCCGGAAGC TGCCAGGGAT GTCTGCAGCC

GGCTGCCCTG GTGACCCAGG GTCCGGAAGC TGCCAGGGAC GTCTGCAGCC

GGCTGCCATG GTGACTCAGG GTCCCGAAGC TGCCAGAGAT GTTTGCAGCC

GACTGCTGTG GTGACCCAGG GCCCTGAAGC TGCCAGAGAT GTGTGCAGCT

GACTGCCATG GTGACCCAGG GTCCTGAAGC TGCCAGAGAT GTGTGCAGCT

GGCCGCCATG GTGACCCAGG GTCCTGAAGC TGCCAGGGAC GTCTGCGGCC

GGCTGCCATG GTGACCCAGG GTCCCGAGGC CGCCAGGGAC GTCTGCAGCC

GGCCGCCATG GTGACCCAGG GCCCTGAGGC TGCCAGGGAT GTCTGCAGTC

GGCCGCCATG GTGACCCAGG GTCCTGAGGC CGCCAGGGAT GTCTGCAGCC

GACCGCCATG GTGGCCCAGG GGGCCGAGGC CGCCAGGGAC GTCTCTAGCC

GTCCGCCCTG GTGGCCCAGG GTCCCGAAGC GGCCAGGGAC GTCTGCAGTC

GGCGGCCCTG GTGGCCCAGG GAGCCGAAGC TACCAGGGAT GTCTGCAGCC

---------- ---------- ---------- ---------- ----------

GTCAGCTATG GTGGCACAGG GTCCAGATGC TGCCAGGGAT TTCTATAGTC

GGCGGCGATG GTGGCCCAGG GGCCGGATGC CGCCCGCGAC GTCTACGGCC

ATTTTGATTT GAATAAAAAG ACCCTGTACA CCCTGGTGAC CAAGAGGGAT

ATTTTGATTT GAATAAAAAG ACCTTGTACA CCCTGGTGAC CAAGAGGGAT

ATTTTGATTT GAATAAAAAG ACCTTGTACA CCTTGGTAAT CAAGAGGGAT

ATTTTGATTT GAATAAAAAG ACCTTGTACA CCTTGGTGAT GAAGAGGGAT

GTTTTGATTT GAATAAAAAG ACCTTGTACA CCCTGGTGAC CAAGAGGGAT

ATTTTGATTT GAATAAAAAG ACCTTGTATG CCCTGGTGAC CAAGAGGGAT

GTTTTGATTT GAATAAAAAA GCCTTGTATG CCCTGGTAAC CAAGAGGGAT

CTTTGGATTT GAATAAAAAA GCCTTGTTTG CCTTGGTGAC CAAGAGGGAT

ATTTTGATTT GAATAAAAAG ACCTTGTACA CCCTGGTGAC CAAGAGGGAT

ATTTTGATTT GAATAAGAAG ACCTTGTACA CCCTGGTGAC CAAGAGGGAT

ATTTTGATTT GAATAAAAAG ACCTTGTACA CCCTGGCCAC CAAGAGGGAT

ATTTTGATTT GCATAAGAAA ACCTTGTACA CCCTGGTGAC CAAGAGGGAT

TCTTCGATTT GAACAAGAAA ACCTTGTATG GCCTGGTGAC CAAGCGGGAC

ATTTTGATTT GAATACAAAA CCCTTGTATG CCCTGGTGAC CAAGAGAGAC

ACTTTGATCT GAATAAAAAG CCCTTGTACG CCCTGGTGAC CAAGCGGGAT

---------- ---------- ---------- ---------- ----------

ATTTTGACTT CAATAAGAAA TCGTTGTATG GTTTGGTGAA AATAAGAAGT

ATTTCGACTT CAACAAGAAG CCCCTCCGGG CTCTGGTGAC CAGACGGAGC

TCAAAGGGAG TGTACGACGT TCGGCAGGCC TACGTTCAGT TTGCTCTCTC

TCAAAGGGAG TGGACGACGT TCGGCAGGCG GAGGGGGAGT ATGCTCTCTC

TCAAAGGGAG TGTACGACGT TCGGCAGGCC TACATTCAGT TTGCTCTCTC

TCAAAGGGAG TGTACGACGT TCGGCAGGCC TACATTCAGT TTGCTCTCTC

TCAAAGGGAG TGCACGACAT CCGGCAGGCC TATGTTCAGT TTGCTCTCTC

TCAAAGGGAG TGCATGATGT CCGGCTGGCC TACATTCAGT TTGCCCTTTC

TCAAAGGGAG TGCATGACGT ACGGCTGGCC TATATCCAGT TTGCCCTTTC

TCAAAGGGAG TGCATGACGT CCGGCTGGCC TACATCCAGT TTGCCCTTTC

TCAAAGGGAG TGCCCGACGT CCGGCTGGCC TACATCCAGT TTGCTCTGTC

CCAAAGGGAG TACATGATGT CCGGCTGGCC TACATTCAGT TCGCCCTTTC

TCAAAGGGTG TGCATGATGT CCGGCTGGCC TACATTCAGT TTGCCCTTTC

TCTAAGGGCG TGCCTGACGT CCGGCTGGCC TACATCCAGT TCGCCCTCTC

ACGAAGGGGG CACCCGACGT CCGGCAGGCC TATATCCAGT TCGCGCTCTC

TCAAAGGGGG CGCCCGACGT CAGGCTGGCC TACATCCAGT TCGCCATTTC

TCAAAGGGCG TGCCCGACGT CCGGCTGGCC TACATCCGGT TTGCCCTTTC

---------- ---------- ---------- ---------- ----------

CCAAAGGGGC TACCAGATAT TCGATTGGCA TATATCCAGT TTGCACTTTC

CCCAAGGGCC TGCCCGACGT CCGGATGGCG TACGTTCAGT TCGCCCTCTC

CTTTTTAATT GCGGGTGATG ACAGCACTAT AGTGCAGGTG TTGGAAGTGA

CTTTTTAATG GCGGGTGATG ACAGCACTAT AGTGCAGGTG TTGGAAGAGA

CTTTCTAATT GCGGGTGATG ACAGCACTAT AGTGCAGGTG TTGGAAGTGA

CTTTCTAATT GCGGGTGATG ACAGCACTAT AGTGCAGGTG TTGGAAGTGA

CTTTCTAATT GCGGGTGATG ACAGCACTAT AGTGCAAGTG TTGGAAGTGA

CTTTTTAATT GCTGGTGATG ATAGCACTAT AGTGCAGGTG CTGGAACTGA

CTTCCTCATT GCAGGTGATG ACAACACTAT AGGGCAAGTG CTGGAGATAA

CTTCCTAATT GCGGGTGATG ACAACACCAT TGGACAAGTC CTGGAGATAA

CTTCTTGATT GCTGGTGACG ACAGCACTAT AGCGCAGGTG CTGGAGCTGA

CTTTCTGATT GCCGGTGATG ACAGCACGAT CACGCAAGTG CTGGAGTTGA

CTTTCTGATT GCCGGCGATG ACAGCACGAT CATGCAAGTG CTGGAGTTGA

CTTTCTGATT GCCGGTGATG ACAGCACCAT AGTGCAGGTG CTGGAGGTGA

CTTCCTTGTC ACGGGTGACA CCAGCACAGT GGTGCAGGTG CTGGAGCTGA

CTTTTTAATC GCAGGTGACG ACAGCACCAT CGTGCAAGTG CTGGAGTTGA

CTTCTTGATT GCCGGTGACG ACAGCACCAT CGTGCAAGTG CTCGAGTTGA

---------- ---------- ---------- ---------- ----------

CTTCTTAATT GCTGGTGATG ATAATACAAT TGCACGAGTA TTGGAAATGA

CTTCCTCATC GCCGGGGACG ACAACACCAT CGCGCAGGTG CTGGAGCTCA

AAGAATTTAT TCCTTGCATT TTTAGCTCAG GGATAAAGGA AGATAGGATC

AAGAATTTAT TCCTTGCATT TTTAGCTCAG GGATAAAGGA AGATAGGATC

AAGAATTTAT TCCTTGCATT TTTAGCTCAG GGATAAAGGA CGATAGGATC

AAGAATTTAT TCCT-GCATT TT-AGCTCAG GGATAAAGGA AGATAGGATC

AAGAATTTAT TCCTTGCATT TTTAGCTCAG GGATAAAGGA AGATAGGATC

AAGAATTTAT TCCTTGCATC TTTAGCTCAG GCATAAAGGA GGATAGGATT

AAGAGTTCAT CCCCTGCATT TTCAGCTCAG GCATAAAGGA AGATAAGATT

AAGAATTTAT CCCCTGCATC TTTAGCTCAG GTATAAAGGA GGATAAGATT

AAGAGTTTAT TCCTTGCATT TTTAGCTCAG GCATAAAGGA AGATAGGATT

AAGAATTTAT TCCTTGCATT TTTAGCTCAG GCATCAAGGA AGATAGGATC

AAGAATTTAT TCCTTGCATC TTTAGCTCAG GCATAAAGGA AGACAGGATT

AAGAATTTAT TCCTTGCATT TTTGGCTCAG GCATAAAGGA AGATCGGATT

A--AGTTCAT CCCCTGCATC TTCAGCTCGG GCATCAAGGA GGACAGGATC

AAGAATTTAT TCCTTGCATT TTTAGCTCAG GCATAAAGGA AGATAGGATT

AAGAGTTCAT ACCTTGCATC TTCAGCTCAG GCGTAAAGGA AGACAGGATT

---------- ---------- ---------- ---------- ----------

AAGAATTCCT CCCTGTCATT TTTAGTACTG GCTTGAAGGA AGATAGAATT

AAGACTTCAT CCCGGCCATT TTCAGCACCG GCTTGAGAGA GGACAGAATC

TCTACCATCA ATATTTTATT ATCCACACTG AAAACAAAGG GTGCTCGAGT

TCTACCATCA ATATTTTATT ATCCACACTG AAAACAAAGG GTGCTCGAGT

TCTACCATCA ATATTTTATT ATCCACACTG AAAACAAAGG GTGCTCGAGT

TCTACCATCA ATATTTTATT ATCCACACTG AAAACAAAGG GTGCTCGAGT

TCTACCGTCA ATATTTTGTT ATCCACACTG AGAACAAAGG GTGCTCGAGT

TCTACTGTCA ATATTTTGTT ATCCACACTG AAAACAAAGG GTGCTCGAGT

TCTACTATCA ACATTTTGTT GTCCACTCTG AAAACAAAGG GAGCTCGGGT

TCTACCATCA ACATTTTATT GTCCACACTG AAAACAAAGG GTGCTCGGGT

TCTACCATCA ATATTTTGTT GTCCACATTG AAAACAAAGG GTGCTCGGAT

TCCACCATCA GTATCCTGTT GTCCACACTG AAAACAAAGG GTGCTCGAGT

TCTACCATCA ATATTTTGTT ATCCACGCTG AAAACCAAGG GTGCTCGAGT

TCTACCATCA ATATTTTGTT ATCCACCCTG CAAACAAAGG GTGCTCGGGT

TCTACCGTCA ATATGTTGCT GTCCACGCTG AAAACAAAGG GTGCTCGAGT

TCTACCGTCA ATCTTTTGTT ATCTACACTG AAAACAAAG- GTGCTCGAGT

TCTACCGTCA GTCTCTTGTT ATCCACTCTG AAAACAAAGG GTGCTCGAGT

---------- ---------- ---------- ---------G GCGCTCGAGT

TCTACTATCA ATCTTTTGTT GTCCACCCTG GGAACCAAGG GTGCTCGAGT

TCCACCATCG ACCTCCTGCT GTCCACCCTG AGATCCAAGG GGGCCCGTGT

TTGGCTTTTC ATTGGTTTCA TGTTGATGTT TGGGTCACTT ATTGCTTCCA

TTGGCTTTTC ATTGGTTTCA TGTTGATGTT TGGGTCACTT ATTGCTTCCA

TTGGCTTTTC ATTGGTTTCA TGTTGATGTT TGGGTCACTC ATTGCTTCCA

TTGGCTTTTC ATTGGTTTCA TGTTGATGTT TGGGTCACTT ATTGCTTCCA

TTGGCTTTTC ATTGGTTTCA TGTTGATGTT TGGGTCACTT ATTGCTTCCA

TTGGCTTTTT ATTGGTTTCA TGTTGATGTT TGGGTCACTT ATTGCTTCCA

CTGGCTCTTC ATTGGTTTCA TGCTGATGTT CGGATCGCTT ATCGCCTCCA

CTGGCTCTTC ATTGGGTTCA TGCTGATGTT CGGGTCACTC ATCGCCTCCA

TTGGCTCTTC ATTGGTTTCA TGCTGATGTT TGGGTCACTC ATTGCTTCCA

TTGGCTCTTC ATTGGTTTCA TGTTGATGTT TGGGTCACTG ATTGCTTCTA

TTGGCTCTTC ATTGGTTTCA TGTTGATGTT TGGATCACTT ATTGCTTCCA

TTGGCTCTTC ATTGGTTTCA TGTTGATGTT TGGGTCACTT ATTGCTTCCA

GTGGCTCTTC ATCGGTTTCA TGTTGATGTT CGGGTCACTA ATTGCCTCCA

TTGGCTTTTC ATTGGTTTCA TGTTGATGTT TGGGTCACTT ATTGCTTCCA

TTGGCTTTTC ATTGGTTTCA TGTTGATGTT TGGGTCACTT ATTGCTTCCA

TTGGCTTTTC ATTGGTTTCA TGTTGATGTT TGGGTCACTT ATTGCTTCCA

TTGGCTCTTC ATTGGTTTCA TGTTGATGTT TGGGTCGCTT ATCGCTTCCA

CTGGCTCTTC ATTGGTTTCA TGCTGATGTT TGGTTCCCTC ATCGCTTCAA

TGTGGATTCT TTTTGGTGCA TATGTTACCC AAAATACTGA TGTTTATCCG

TGTGGATTCT TTTTGGTGCA TATGTTACCC AAAATACTGA TGTTTATCCG

TGTGGATTCT TTTTGGTGCA TATGTTACCC AAAATACTGA TGTTTATCCG

TGTGGATTCT TTTTGGTGCA TATGTTACCC AAAATACTGA TGTTTATCCG

TGTGGATTCT TTTTGGTGCA TATGTTACTC AAAATACTGA TGTTTATCCG

TGTGGATTCT TTTTGGTGCA TATGTTACCC AAAATACTGA TGTTTATCCA

TGTGGATCCT TTTTGGTGCA TATGTTACTC AAAATATTGA TGTTTACCCT

TGTGGATCCT TTTTGGTGCA TATGTTACTC AAAATATTGA TGTTTACCCT

TGTGGATTCT TTTTGGTGCA TACGTTACCC AGAATACTGA TGTTTATCCA

TGTGGATTCT CTTTGGTGCC TATGTTACCC AGAATACTGA TGTTTATCCA

TGTGGATTCT CTTTGGTGCA TATGTTACCC AAAATACTGA TGTTTATCCA

TGTGGATTCT TTTTGGTGCA TATGTTACCC AAAATACTGA TGTTTATCCA

TGTGGATTCT CTTTGGTGCA TATGTTACCC AAAATACCGA TGTTTATCCA

TGTGGATTCT TTTTGGTGCC TATGTTACCC AAAATATTGA TGTGTATCCA

TGTGGATCCT TTTTGGTGCA TATGTTACCC AAAATACGGA TGTGTATCCA

TGTGGATTCT TTTTGGTGCG TATGTTACCC AAA------- ----------

TGTGGATTCT TTTTGGTGCC TATGTTACTC AAAATACTAA TGTTTATCCA

TGTGGATCCT TTTTGGCGCC TATGTAACCC AAAATATAAG CGTCTATCCA

GGACTAGCTG TGTTTTTTCA AAATGCACTT ATATTTTTTA GCACTCTGAT

GGACTAGCTG TGTTTTTTCA AAATGCACTT ATATTTTTTA GCACTCTGAT

GGTCTAGCTG TGTTTTTTCA AAATGCGCTT ATATTTTTTA GCACTCTGAT

GGTCTAGCTG TGTTTTTTCA AAATGCACTT ATATTTTTTA GCACTCTGAT

GGACTAGCTG TGTTTTTTCA AAATGCACTT ATATTTTTTA GCACTCTGAT

GGACTAGCTG TGTTTTTTCA AAATGCACTT ATATTTTTTA GCACTCTGAT

GGACTAGCTG TGTTCTTTCA AAATGCACTT ATATTTTTTA GCACTCTGAT

GGACTAGCTG TGTTCTTTCA AAATGCTCTT ATATTTTTTA GCACTCTGAT

GGACTAGCTG TGTTTTTTCA AAATGCACTC ATATTTTTTA GCACTCTGAT

GGACTAGCTG TGTTTTTTCA AAATGCACTT ATATTTTTTA GCACTCTGAT

GGATTAGCTG TGTTTTTTCA AAATGCACTT ATATTTTTTA GCACTCTGAT

GGACTAGCTG TGTTTTTTCA AAATGCACTT ATATTTTTTA GCACTCTGAT

GGACTAGCTG TGTTCTTTCA AAATGCGCTG ATTTTTTTTA GCACTCTGAT

GGACTAGCTG TGTTTTTTCA AAATGCTCTT ATATTTTTTA GCACTCTGAT

GGACTAGCTG TGTTTTTTCA AAATGCACTT ATATTTTTTA GCACTCTGAT

---------- ---------- -AATGCACTG ATATTTTTTA G---------

GGACTAGCTG TATTTTTTCA GAATGCACTT ATATTTTTTA GCACCCTCAT

GGCCTGGCTG TGTTTTTCCA AAATGCGCTG ATATTTTTTA GTACTCTAAT

CTACAAATTT GGAAGAACCG AAGAGCTAAC CATGGCACCA CCTTCCAGGC

CTACAAATTT GGAAGAACCG AAGAGCTAAC CATGGCACCA CCTCCCAGGC

CTACAAATTT GGAAGAACTG AAGAGCTAAC CATGGCACCA CCTCGCAGGC

CTACAAATTT GGAAGAACTG AAGAGCTAAC CATGGCACCA CCTCGCAGGC

CTACAAATTT GGAAGAACCG AAGAGTTAAC CATGGCCCCA CCTCCCAGGC

CTATAAATTT GGAAGAACTG AAGAGCTAAC CATGGCGCGT CCTCCCGGGC

CTACAAATTC GGAAGAACTG AAGAGCTCGC CATGACAGCG CCTTCCAGCT

CTACAAATTT GGAAGAACTG AAGAGCTCGC CATGACATCA CCTTCCAGCT

CTACAAGTTT GGAAGAACTG AAGAGCTAAC CATGGGGCCG CCGTCCGGGC

CTACAAATTT GGAAGAACTG AAGAGCTGAC CATGGCACCT CCTTCTGGGC

CTACAAATTT GGAAGAACTG AAGAGCTTAC CATGGCACCA CCTTCTGGGC

CTACAAATTT GGAAGAACTG AAGAACTAAC CATGGCACCA CCTTCTGGGC

CTACAAATTC GGAAGGACGG AGGAGCTGAG C--------- ----------

CTACAAATTT GGAAGAACTG AGGAGCTGAC CATGGCACCA CCTTCCAGGC

CTACAAGTTT GGAAGAACTG AGGAGCTAAC CATGGCACCA CCTTCCAGGC

---------- ---------- ---------- -ATGACCTCA CCCTCTGGGC

CTACAAATTT GGACGAACAG AAGAATTAGG AATGGCCCTG CCCTTCGGTC

CTACAAGTTT GGAAGACTAG AAGAATTAGC GATGGCCCGT CCCGCCAATT

ACTGTCTTCT TCTGATCAGC ACTCTGGGTG TCTTTGCACT TAACTGCTTC

ACTGTCTTCT TCTGATCAGC GCTCTGGGTG TCTTTGCACT TAACTGCTTC

ACTGTCTCCT TCTGATCAGC GCTCTGGGCG TCTTTGCACT TAACTGCTTC

ACTGTCTCCT TCTGATCAGC GCTCTGGGCA TCTTTGCACT TAACTGCTTC

CCTGTCTCCT TCTCATCAGC GCTCTGGGCG TCTTTGCACT TGACAGCTTC

ACTGCCTCCT CCTGATCTGC GCGCTGGGCA TCTTTGTGGG CAACCACTTT

TCTGCCT--- TCTGCTAGGA GCGCTAGGTA TCTTTGCCCT TGGCCGCTTC

TCTGCCTCCT TCTGCTCCAA GCGCTAGGCA TCGTTGCCCT TGGCCACTTC

ACTGCCTCCT TCTGCTCGGC ACCCTGGGGG TCTTCGTCCT GCAGCGCGGC

ACAGCTTCTT TCTGATCGGT GCGCTGGGCG TCTTTGCACT CAACTGCTTC

ACAGCCTCCT TCTGACCAGT GCACTGGGCA TCTTCATACT CCACTGCTTC

ATAGCTTTCG TCTGATCAGC CTGCTGGGCA TCTTTGCATT CCACTGCTTC

---------- ---------- ---------- ---------- ----------

ACTGCCTCCT TCTGATCAGC ACGCTGGGCG TCTTTGCCTT TGACAGCTTC

GCTGTCTCCT GCTGGTCATT GCCCTGGGCA TCCTT----- ----------

ACTGCCTCCT TCTGATCATC GCCCTGGGCA TCTTTGCCTT CAGCCGCTTC

AACACCTCCT TCTAATTAGC TTCTTTGGCT TCTTTGCATT TAATAGCTTT

Ccctccttct cctcctcctg ctcctgctcc tgctcctcct cAGCTTCTTC

ACCAAAGGTC AGAAGAACAG CACGCTCATC TTCACAAGGG AAAACACCAT

ACCAAAGGTC AGAAGAACAG CACGCTCATC TTCACAAGGG AAAACACCAT

ACCAGAGGTC AGAAGAACAG CACGCTCATC TTCACAAAGG AAAACACCAT

ACCAAAGGTC AGAAGAACAG CACGCTTATC TTCACAAAGG AAAACACCAT

ACCAAAGGTC AGAAGAACAG CACGCTCATC TTCACAAAGG AAAACACCAT

GCTGCAGGTC AGAGGAACAG CACGCTCATC TTCACAAGGG AAAACACCAT

GCGGAGGGTC AGAG---CAG GACACTGATT TTCACAAAGG GAAACACCAT

ACAAAAGCTC AGAA---CAA CACACTGATT TTCACAAAAG GAAATACCAT

ACCGCGGGCC AGAAGAACAG CACCCTGATT CTCGCCCGGG AAAACGCCAT

ACCAGAGCTC AGAGGAACGG CACGCTCATT TTCACCAAGG AAAACACCAT

ACACGAGGGC AGAAGAACAG CACACTCATT TTCACAAAGG AAAACATCAT

ACAAGAGGTC AGAAGAACAG CACACTAATT TTCACAAAGG AACACACCAT

---------- ---------- ---------- ---------- ----------

ACTCAAGGTC AGAAGAACGG TACCCTAATT TTCACCACGG AAAACACCAT

---------- ---------- ---------- ---------- ----------

TCAGAGGCTC AGATGAACAG CACGCTGATT TTCACAAAGG AACACACCAT

GTAAGAGGTC AAAAGAACAA CACGTTGATT TTTACCAAGG AAAACACCAT

GCCCACGGTC CGGGAAACGG CACGCTGATC TTCACCAGGG AGGACACCGT

TCGGAACTGC AGCTGTTCTG CGGACATCCG GGATTGTGAC TACAGTTTGG

TCGGAACTGC AGCTGTTCTG CGGACATCCG GGATTGTGAC TACAGTTTGG

TCGGAACTGC AGCTGTTCTG CCGACATCCG GGATTGTGAC TACAGTTTGG

TCGGAACTGC AGCTGTTCTG CGGACATCCG GGATTGTGAC TACAGTTTGG

TCGGAACTGC AGCTGCTCTG TAGACATCCG GGATTGTGAC TACAGTTTGG

TCGGAACTGC AGCTGCTCTG CTGACATCCG GGACTGTGAC TACAGTTTGG

TCGTAACTGC AGCTGCCCCG CAGACATCCG GGACTGTGAC TACAGTTTGG

TCGCAACTGC AGCTGCCCAG TAGACATCAG GGACTGTGAC TACAGTTTGG

TCGGAACTGC ACCTGCTCCG CCGACATCCG GGACTGTGAC TACAGTTTGG

TCGGAAGTGC AGCTGCTCAG CAGACATCCG CGACTGTGAC TACAGTTTGG

TCGGAACTGC AGTTGCTCTG CAGATGTCCA GGACTGTGAC TACAGTTTGG

TCGGAACTGC AGCTGCTCTG CAGACATCTG GGACTGTGAT TACAGTCTGG

---------- ---------- ---------- ---------- ----------

TCGGAACTGC AGCTGCTCTA CAGACATCCA GGACTGTGAC TACAGTTTGG

---------- ---------- ---------- ---------- ----------

TCGGAACTGT AGCTGCCCTG CAGACATCCA AGACTGTGAC TACAGCTTGG

TCGAAACTGC AGCTGCTCTG CTGATATACG GGATTGTGAT TACAGTTTGG

CCGCAACTGC AGCTGCCCCG TGGCCGTCCG GGACTGTGAC TACGGCCTGG

CCAACCTGAT GTGCAACTGT AAAACCGTCC TGCCCCTTGC AGTAGAGCGA

CCAACCTGAT GTGCAACTGT AAAACCGTAC TGCCCCTTGC GGTAGAGCGA

CCAACCTGAT GTGCAGCTGT AAAACTGTCC TGCCCCTTGC GGTGGGGCGA

CCAACCTGAT GTGCAGCTGT AAAACTGTCC TGCCCCTTGC GGTGGGGCGA

CCAACCTGAT GTGCAGCTGT AAAACTGTCC TGCCCCTCAC TGTGGAGCGA

CCAACCTGAT GTGCAGCTGT AAAACCGTCC TGCCCTTCGC CGTTGAACAA

CCAACCTGAT GTGCAGCTGT AAGTCTGTCC TGCCTCTGGC CATGGAGCAA

CTAACTTGAT ATGCAGCTGT AAGTCTATCC TGCCTTCTGC CATGGAGCAA

CCAACCTGAT GTGCAGCTGC AAAACGGCCC TGCCTCTGGC GGTGGAGCGA

CCAACCTGAT GTGCAGCTGT AAAACCATGC TGCCTCTTGC CGTTGAGCAA

CCAACTTGAT GTGCAGCTGC AAAACTGTCC TGCCTTTTGC AATAGAGCAA

CCAACCTAAT GTGCAGCTGT AAAACCGTCC TGCCCTTTGC AATAGAGCGA

---------- ---------- ---------- ---------- ----------

CCAACCTGAT GTGCAGCTGC AAAACTGTCC TGCCCTTTGC AACCATGCGA

---------- ---------- ---------- ---------- ----------

CCAACCTGGT GTGCAGCTGT AAAACCGTCC TTCCATACTC GGTAGAGCAG

CCAGTCTGAT GTGCACTTGT AAAACTGTTT TGCCCTACAC AATAGAGCAA

CCAACCTGCT GTGCTCCTGC CGGACGGTCT TCCCGTCGGC CGCGGGCCCG

ACCAGCTACA ATGGCCATCT GACCATCTGG TTCACGGACA CATCTGCGCT

ACCAGCTACA ATGGCCATCT GACCATCTGG TTCACGGACA CATCTGTGCT

ACCAGCTACA ATGGCCATCT GACCATCTGG TTCACGGACA CATCTGCACT

ACCAGCTACA ATGGCCATCT GACCATCTGG TTCACGGACA CATCTGCACT

ACCAGCTACA ATGGCCGTCT GACCATCTGG TTCACGGACA CGTCAGTGCT

ACCAGCTACA GTGGCCATCT GACCATCTGG TTCACAGACA CGTCTGTGCT

ACCAGCTATC ATGGCCACCT CACCATCTGG TTCACAGATA TATCCACGTT

ACCAGCTATC ATGGCCATCT GACCATCTGG TTCACAGATA TATCCACATT

GCCAGCTACC ACGGCCACCT GACCGTCTGG TTCACGGACA CGTCCGCCCT

ACGAGCTACA GTGACCGTCT GACCATCTGG TTCACAGACA CGTCTGCGCT

ACCAGCTACG ATGGCCATCT GACCATCTGG TTCACGGATA CATCTGCACT

ACCAACTACA GTGGCCATCT AACCATCTGG TTCACAGACA CATCTGCACT

---------- ---------- ---------- ---------- ----------

ACCGGCTACA GTGGCCGTCT GACCATCTGG TTCACAGACG CATCTGCGCT

---------- ---------- ---------- ---------- ----------

ACCAGCTATC ATGGCCATCT CACCATCTGG TTCACAGACA TATCTGCACT

GCCAGCTATG ACAGTGATCT GACTATCTGG TTTACAGATA TCACTGCACT

GCCAGCTACA GCAGCGGCCT GACCATCTGG TTCACCGACG CCACTCTGTT

GGGCCACCTG CTGAACTTCA CGCTGGTCCA AGACCTGAAG CTTTCCCTGT

GGGCCACCTG CTGAACTTCA CGCTGGTCCA AGATCTGAAG CTTTCCCTGT

GGGCCGCCTG CTGAACTTCA CGCTGGTCCA AGACCTGAAG CTTTCCCTGT

GGGCCGCCTG CTGAACTTCA CGCTGGTCCA AGACCTGAAG CTTTCCCTGT

GGGCCGCCTG TTGAATTTCA CACTGGTCCA AGACCTGAAG CTTTCCTTGT

GGGCCGCCTG TTGAACTTCA CATTGGTCCA GGACCTGAAG CTTTCCCTGT

GGGCCACCTG CTGAAGTTCA CTCTGGTCCA AGACTTGAAG CTTTCCCTGT

GGGCCACGTG CTGAAGTTCA CTCTGGTCCA AGACTTGAAG CTTTCCCTAT

GGGCCGCCTG TTGAACTTCA CGCTGGTCCA GGACCTGAAG CTCGCCCTGT

GGGGCTCCTG CTGAACTTCA CGCTGGTCCG GGACCTGAAG CTTTCCCTGT

GGGCCTCCTA TTGAACTTCA CACTGGTCCG GGACCTGAAG CTTTCTCTGT

GGGCCTCCTG CTGAACTTCA CGCTGGTCCA GGACCTGAAG CTTTCTCTGT

---------- ---------- ---------- ---------- ----------

GGGCCTCCTA CTGAACTTCA CGCTGATCCA GGACCTGAAG CTTTCGCTGT

---------- ---------- ---------- ---------- ----------

GGGCCTCCTG CTGAATTTCA CCCTGGTCTG GGACCTCAAG CTCTCTCTGT

GGGCTTCCTC CTGAACTATA CCTTAGTTAA GGACCTGAAG CTTTCCCTAT

GGCGGCGCTG CTGAATCTCA CGGCGGTGCG GGACCTGAAG CTCTCCCTGT

GCAGCACCAA CACTCTCCCC ACTGAATACC TGGCTATTTG TGGTCTGAAG

GCAGCACCAA CACTCTCCCC ACTGAATACC TGGCTATTTG TGGTCTGAAG

GCAGCGCCAA CACTCTCCCC ACTGAATACC TGGCTATCTG TGGTCTGCAG

GCAGCGCCAA CACTCTCCCC ACTGAATACC TGGCTATCTG TGGTCTGCAG

GCAGTACCAA CACTCTCCCC ACTGAATACC TGGCTATTTG TGGTCTGAAG

GCAGCACGAA CACTCTCCCC ACTGAATACC TAGCTATCTG TGGTCTGAAG

GTGGTTCCAA CACCTTCCCC ACCAAGTACC TGGCTGTCTG TGGCCTGAAG

GTGGTTCCAG CACCTTCCCC ACCAAGTACC TGGCTATCTG TGGGCTGCAG

GCAGCACCAA CACCCTCCCC GCCGCGTACC TGGCCATCTG TGGGCTGAGG

GCAGTACCAA CACTCTCCCC ACTGAGTACC TGGCTATTTG CGGTCTGAAG

GTGGTACCAA CACTCTCCCC ACTGAATACC TGGCTATCTG TGGTCTGAAG

GCGGTACAAA CACTCTCCCC ACTGAGTACC TGGCCATTTG GGGTCTGAAG

---------- ---------- ---------- ---------- ----------

GCGGTACGAA CACTCTCCCC GCTGAATATC TGGCTATTTG GGGTCTGAAG

---------- ---------- ---------- ---------- ----------

GCAGTGCAAA CACTCTCCCC GAGGAGTACC TGGCTATTTG GGGTCTGAAG

GTAGCAGCAC TACGCTCCCA ACAGAATATC TGGCTATTTG GGGGCTGAAA

GCGGCCCGTC CACCCTCCCG GCGGAGTTCC TGGCCATCCG GGGCCTTCGA

AGGCTGCGCA TCTTCCCAGA GCAGAGCTTA CTCATCCATA GCGGTGGGGA

AGGCTGCGCA TCTTCCCAGA GCAGAGCTTA CTCATCCATA GCGGTGGGGA

AGGCTGCGCA TCTTCCCAGA GCAGAGCCTA CTCATCCATA GTGCTGGGGA

AGGCTGCGCA TCTTCCCAGA GCAGAGCCTA CTCATCCATA GTGCTGGGGA

AGGCTGCGCA TCTTCCCAGA GCAGAGCTTA TTCATCCATA GCGGTGGGGA

AGGCTTCGTG TCTTCCCAGA GCAGAGCTTA CTCGTCCACA GTGGTGCAGA

AGGCTTCACA TCTCCGGGGA GCAGAGCATA CTCATCCACA ACGGGAGGGA

AGGCTTCGCA TCTCCCGGGG GCAGAGTTTG CTCATCCACA GCAGAAGGGA

CGGCTCCGCA TCTCGGCCGA GCAGAGCTTA CTGATCCGCG GCGGCGGGGA

AGGCTTCGGG TCTCCCCTGA ACAGAGTTTG CTCATCCACG ACGGTGGGGA

AGGCTTCGTA TCTCACTGGA GCAGAGCTTA CTCATCTACA ACAATGGGGA

AGACTTCGCA TCTCACCAGA GCAGAGCTTA CTCGTCCACA GCGCTGGGGA

---------- ---------- ---------- ---------- ----------

AGGCTTCGCA TCTTTCCAGA GCAGAGCTTA CTCATCCA-- -CGGTGGGGA

---------- ---------- GCAGAGTTTA CTCATCCACA GCGGTGGAGA

AGGCTTCGCA TCTTCCCGGA GCAGAGCTTA CTCATCCATG GCGGGGACGA

AGACTTCGAA TCTTCCCAGA GCAAAGCTTG ATGATACACA GCAGTGGTGA

AGCCTCCGCG T--TCCCGGA ACAGAGCCTG CTGATCCACG GCAGCGGCGA

CAGTGACTCC TTACACAAAG GCTGGATGTA TATCTCATTC TTAGATATGG

CAGTGACTCC TTACACAAAG GCTGGATGTA TATCTCATTC TTAGATATGG

CAGTGACTCC TTACACAAAG GCTGGATGTA TATCTCATTC TTAGATATGG

CAGTGACTCC TTACACAAAG GCTGGATGTA TATCTCATTC TTAGATATGG

CAGTGACTCC TTACACAAAG GCTGGATGTA CATTTCATTC TTAGATATGG

CAGTGACTCC CTAGGCAAAG GCTGGATGTA TGTCACGTTC TTAGATATGG

AGGCAGTTCC TTGTACAAGG GCTGGGTGTT CATCTCACTC TTAGACGTGG

AGGCAGTTCC TTGTACAAAG GCTGGATGTT CATCTCATTC TTAGATGTGG

CGGTGACACC ------GAAG GCTGGCTGCA CATCTCCTTC TTGGACGTGG

GAGTGAACCC TTACAGCGAG GCTGGATGTA TCTCTCCTTC TTAGACATGG

AAGTGAACCC TTACACAAAG GCTGGATGTA CATCTCCTTC TTGGATATGG

GGCAGAGCCC TCACACACAG GCTGGACGTA TGTCTCATTC TTGGACATGG

---------- ---------- ------TGTC CGTCTCCTTC CTGGACACGG

CACAGAATCT TTACACGAAG GCTGG-CTTA TATCTCATTC TTAGATATGG

GAGCGAATCC TTGCACAAAG ACTGGATGTA TATCTCATTC TTAGACATGG

GAGTGAATCC AGGCACAAAG ACTGGATGTA TGTCTCATTC TTAGATATGG

CAGTAGTTTT TTGCCCACTA GTAGGTTTTA TGTTTCATTC TTGGATACAG

CCGCATCGCG AGAGAGAGGA GAAGG-ccta catttccttc ctggacacgg

CTCTTTTCAA CAGGGACTCA GCCTTAAATC ATATAGTATT GAAAACGTTA

CTCTTTTCAA CAGGGACTCA GCCTTAAATC ATATAGTATT GAAAACGTTA

CTCTTTTCAA CAGGGACTCA TCCTTAAATC ATATAGTATT GAAAACGTTA

CTCTTTTCAA CAGGGACTCG TCCTTAAATC ATATAGTATT GAAAACGTTA

CTCTTTTCAA CAGGGACTCG TCCCTAAATC GTATAGTATT GAAAATGTTA

CTCTTTTCAA CAGGGACTCA TCCTTAGAGC GTATAGTATT GAAAACGTGG

CTCTTTTCAA CAGAGACTCT TCTCTAAGTC ATATAGTATT GATGACATTT

CTCTTTTCAA CGGGGACTCA TCTTTAAGTC ATACAGTATT GACAACATTT

CTCTCTTCAA CAGGGACTCG TCCTTAAATC ATACAGTGTG GAGAACGTCA

CACTCTTCAA CAGGGAGTCT GCCTTAAATC ATACAGTGTT GCAAACTCTG

CACTTTTCAA CAGGAAGTCA TCCTTGAGGC ATACAGTATT GAAAACGTTG

CACTTTTCAA CAAGGAGTCA TCCCTAAATC ATTTAGCATT GAAAACGTTG

CCCTGTTCAA CCGGGACTCG GCCCTCAGTC CTACAGCGTG GAGAGCGTGG

CCCTTTTCAA CAGGGAATCG TCCTTAAATC ATACAGTATT GAAAATGTGG

CTCTTTTCAA CAGGGAATCG TCCTTAAGTC ATACAGTATT GCAAACGTTG

CTCTTTTCAA CAGGGAATCG TCCTTAGATC ATACAGTATT GAAAATGTCA

CTCTTTTCAA TGGGGAATCT TCTTTGAATC ATACAGCATT GAAAACGTCA

ctctgttcaa cgggaactcg tccttgaatc gtacagcgtc gaaaacgtcg

CCAGCATTGC CAACAACTTT CCTGACTTTT CTTACTGAAC CTTCCCAATG

CCAGCATTGC CAACAACTTT CCTGACTTTT CTTACTGAAG CTTCCCAATG

CCAGCATTGC CAACAACTTT CCTGACTTTT CTTATTAAAC CTTCCCAATG

CCAGCATTGC CAACAACTTT CCTGACTTTT CTTATTAAAC CTTCCCAATG

CCAGCATTGC CAACAACTTT CCTGATTTTT CTTACCAAAC CTTCCCAATG

CCAGCATTGC CAACAACTTT CCTGATTTTT CTTACTAAAC CTTCCCAATG

CTAGCCTCAC CGGTGACTTT CCTGACTTTT CTTACT---C TTTCCCAGTG

CTAGCCTCGC CAGTGACTTT CCTGACTTTT CTTACTAAAC GTCCCCAATG

CCAGCACCGC CCACGACTTC CCGGATTTCT CTTACTAGAC GTTCCCAGTG

CCAGCGTGGC CAACAACTTC CCCTACTTTT CCTACCAAAC CTTCCCAGTT

CCAGCATTGC CAACACTTTT CCTTACTTTT CTTACTAAAC TATCCCAATT

CCGGCATGGC CAACAACTTT CCCTACTTTC CTTACTACAC CTTCCCAATT

CCGGCCCCGC CGACGGCTTT CCGCGCCTCT CCCTCCACGC GCTCCCCGCG

CCAGCATCGC CAATAACTTC CCCTACTTTT CTTACTAAAC CTTCCCAATT

CCAGCACCGC CAACAACTTT CCCTACTTTC CTTACTAATC CGTCCCAATT

TCAGCATCAC CAACAGCTTT CCCTACCTCG CTTACTAAAC GATCCCACCT

CCAGTATCAC AAACAATTTC CCTTATCTTT CTTACTAAAT GTTTTCCATC

ctggcatccc cggctccttt cctCACCTTC CTTACCAGGC GCTCTCCGAG

CCAAGCAACA AAAGCTATGT CACATTTATT TACTGGTTAC CTAACCAAAC

CCAAGCAACA AAAGCTATGT CACATTTATT TACTGGTTAC CTAACCAAAC

CCAAGCCACA AAAGCTATGT CACATTTATT TACTGGTTAC CTAACCAAAC

CCAAGCCACA AAAGCTATGT CACATTTATT TACTGGTTAC CTAACCAAAC

CCAAGCAACA AAAGCTATGT CACATTTATT TACTGGTTAC CTAACCAAAC

CCAAGCAACC AAAGTTATGT TACGTTTATT TACTGGTTAC CTAACCAAAC

CCAAACAACC GAAGCTACCT CACCGTTATT TATTGGTTAC TTAACCAAAC

CCAAGCAACA GAAGCTATGT CACAGTTATT TACTGGTTAC TTAACCAAAC

GCAAGCAACG CAAGCTGTGT CACGGTCATT TACTGGTTAC CTAACCAAAC

C---ACAACA AAAGCTATGT CACCTTCATT TACTGGTTAC CTAACCAAAC

T---GCAACA AAAGCTATGT CACATTCATT TACTGGTTAT CTAACCAAAC

C---GCAACA AAAACTACGT CACGTTCATT TACTGGTTAC CTAACCAAAC

CCCGGCAACC AAAGCTACGT CACCTTCATC TACT------ ----------

CCAAGCAACG AAAGCTATGT CACATTCATT TACTGGTTAC CTAACGAAGC

CCAAGCAACA AAAGCTATGT CACCTTCATT TACTGGTTAC CTAACCAAAC

GCGAGCAACA AAAGCTACGT CACCTTCATT TACTG----- ----------

CCAAGCAACA AAAGCTATAT TACGTTCATT TACTGGTTAC CTAACCAAAC

CCGAGCGACC GAAGTTACGT CACCTTCATT TACT-GCTAC CTCAGCAAGC

TCCTGCAAAA CCACACCACC TATGCCTGTG ATGGGGACTA TTTGAATCTA

TCCTGCAAAA CCACACCACC TATGCCTGTG ATGGGGACTA TTTGAATCTA

TCCTGCAAAA CCACACCACC TATGCCTGTG ATGGGGACTA TTTGAATCTA

TCCTGCAAAA CCACACCACC TATGCCTGTG ATGGGGACTA TTTGAATCTA

TCCTGCAAAA CCACACCGCC TATGCCTGTG ATGGGGACTA TTTGAATCTA

TCCTGCAAAA CCACACCGCC TATGCCTGTG ACGGGGACCA TTTGAGCCTC

TCCTGCAAAA CCACACCGCC TATGCCTGTG ACGGGGACTA TTTGAATCTG

TCCTGCAAAA CCACACTGCC TATGCCTGTG ACGGGGACTA TCTGAATCTA

TCCTGCAGAA CCACACGGCC TATGCATGTG ACGGGGACCA CTTGAACCTG

TCCTGCAAAA CCACACCGCC TATGCCTGTG ACGGGGACCA TTTGAATCTG

TCCTGCAAAA CCACACCGCC TATGCCTGTG ACGGGGACCA TTTGAATCTG

TCCTGCAAAA CCACACCGCC TATGCCTGTG ATGGGGACCA TTTGAATCTA

---------- ---------- ---------- ---------- ----------

TCCTGCAGAA CCACACCGCC CACGCCTGCG ACGGGGACCG TTTGCTTCTG

TCCTGCAAAA CCACACCGCC TATGCCTGCG ATGGGGACCA CTTGAATCTC

---------- ---------- ---------- ---------- ----------

TTCTGCAAAA CCACACCGTG TACGCTTGTG ACGGTAACCG TTTGAGTCTT

TGCTGCAGAA CCACACGGCC CTGGCGTGTG ACGGGGACCG CCTGAGCCTG

CAGTGCCCTC GGCATTCTAC GATAAGTGTC CAATCGGCAT TTTATGGGCA

CAGTGCCCTC GGCATTCTAC GATAAGTGTC CAATCGGCAT TTTATGGGCA

CAGTGCCCTC GGCATTCTAC GATAAGTGTC CAATCGGCAT TTTATGGGCA

CAGTGCCCTC GGCATTCTAC GATAAGTGTC CAATCGGCAT TTTATGGGCA

CAGTGCCCTC GGCATTCTAC GATAAGTGTC CAATCAGCAT TTTATGGGCA

CAGTGCCCTC GGCATTCTAC GATAAGTGTT CAATCGGCAT TTTATGGGCA

CAGTGTCCTC GGCATTCAAC AATAAGTGTG CAGTCGGCAT TTTATGGGCA

CAGTGCCCTC GGCATTCAAC AATAAGTGTC CAGTCGGCAT TTTATGGGCA

CAGTGCCCTC GGCATTCCAC CATCAGTGTC CAATCGGCGT TTTACGGGCA

CAGTGTCCTC GGCATTCCAC GATAAGTGTC CAGTCGGCAT TTTATGGGCA

CAGTGTCCTC GGCATTCCAC GATAAGTGTC CAATCGGCAT TTTATGGGCA

CAGTGCCCTC GGCATTCTAC AATAAGTGTG CAATCGGCAT TTTACGGGCA

---------- ---------- ---------- ---------- ----------

CAGTGCCCTC GGCACTCCAC CATCAGCGTC CAGTCAGCCT TTTACGGGCA

CAGTGTCCAC GGCATTCTAC GATAAGTGTC CAGTCGGCAT TTTATGGGCA

---------- ---------- ---------- ---------- ----------

CAGTGCCCTC GACATTCGAC AATAAGTGTC CAGTCAGCCT TTTATGGTCA

CAGTGCCCAC GACACTCCTC CATCAGCGTC CAGGCGGCCT TCTACGGGCC

AGATTACCAA ATGTGTAGTT CCCAGAAGCC TGCCTCCCAG AGGGAAGACA

AGATTACCAA ATGTGTAGTT CCCAGAAGCC TGCCTCCCAG AGGGAAGACA

AGATTACCAA ATGTGTAGTT CCCAGAAGCC CGCCTCCCAG AGGGAAGACA

AGATTACCAA ATGTGTAGTT CCCAGAAGCC CGCCTCCCAG AGGGAAGACA

AGATTACCAA ATGTGTAGCT CCCAGAAGCC TGCCTCCCAG AGGGAAGACA

AGATTACCAA ATGTGTAATT CCCAGCAGCC TGCCTCCCAG AGGGAAGACA

AGATTTCCAA AGGTGTAGCC CCCAGGAGCC CATCTCCCAG AGGGAAGACA

AGATTACCAA ATGTGTAGTT CCCAGGAGCC CATCTCCCAG AGGGAAGACA

AGATTTCCGA ATGTGTAGCC CCCAGCAGCC AGCCCCCGAG AGGGAAGACA

AGATTACCAA AAGTGTAGTC CGCAGCAGCC TGCCTCCCAG AGGGAAGACA

AGATTACCAA ATGTGTAACA CCCAGCAGCC TGCCTCCCAG AGGGAAGACA

AGATTACCGA GCATGTAGTA CCGAGCAGCC TGCCTCCCAG AGGGAAGATG

---------- ---------- ---------- ---------- ----------

AGACAACCAC ACGTGTGGCC CCCGGCAGCC TGCACCCCAG AGGACAGCCG

AGATGATCAA ATGTGTAACT CCCAGCAGCC TGCATCCCAG AGGAAAGACA

---------- ---------- ---------- ---------- ----------

AGATTACCAA AGGTGTAATT CACAGCAGCC TGCAACCATG ATGAAAGACC

CGACTCCAAG AGGTGTGGCT CTCGGCCACC TGACGTGGAG AGGGCCGACG

GCTTAACCTG TGTGGCAGCC ACCACCTTCC AGAAGGTGCT GGACGAATGC

GCTTAACCTG TGTGGCAGCC ACCACCTTCC AGAAGGTGCT GGACGAATGC

GCTTAACCTG TGTGGCACCC ACCACCTTCC AGAAGGTGCT GGACGAATGC

GCTTAACCTG TGTGGCACCC ACCACCTTCC AGAAGGTGCT GGACGAATGC

GCTTAACCTG TGTGGCACCC ACCACTTTTC AGAAGGTGCT GGACGAATGC

GCTTAACCTG TGTGGCACCC ACCACTTTCC AGAAGGTGCT GGATGAATGC

ACTTGACCTG TGTGGCATCC ACCGCTTTGC AGAAGGTGCT GGACGAGTGC

ACCTGACCTG TGTGGCATCC ACCACTTTGC AGAAGGTGCT GGACGAGTGC

GCTTAGCCTG TGTGGCACCC ACCACCTTGC AG-------- ----------

GCCTAACCTG TGTGGTGCCC ACCACACTGC AGAAGGTGCT GGACGAGTGT

GCTTAGCCTG TGTGGCTCCC ACCACATTGC AGAAAGTGCT GGACGAATGC

GCTCAACCTG TGTGGCATCC ACCACGTTGC AG-------- ----------

---------- ---------- ---------- ---------- ----------

GCCTGACCTG TGCGGCGCCC ACCACGTTGC AGAAGGTGCT GGACGAGTGC

GCCTAACCTG TGTAGCATCC ACCACGTTGC AGAAGGTCCT GGATGAGTGC

---------- ---------- ---------- --AAGGTGCT GGATGAGTGC

ATTTAACCTG CGTGGCACCC ACGGCTTTGC AGAAAGTTTT GGATGAGTGC

GGCCGGCCTG CGCGGCCCCC ACCACCCTGC AGAAAGTGTT GGACGAGTGC

CAGAACCAGC GGGCCTGCCA CCTCCTGGTC AATAGCCGTG TTTTTGGACC

CAGAACCAGC GGGCCTGCCA CCTCCTGGTC AATAGCCGTG TTTTTGGACC

CAGAACCAGC GGGCCTGCCA CCTCCTGGTC AATAGCCGTG TTTTTGGACC

CAGAACCAGC GGGCCTGCCA CCTCCTGGTC AATAGCCGTG TTTTTGGACC

CAGAACCAGC GGGCCTGCCA CCTCCTGGTC AATAGCCGTG TTTTTGGACC

CAGAACCAGC GGGCCTGCCA CCTCCTGGTC AATAGCCGTG TATTTGGACC

CAGAACCAGC GGGCCTGCCA CCTCCTGGTC AATAGCCGAG TCTTCGGACC

CAGAACCAGC GGGCCTGCCA CCTCCTGGTC AATAGCCGAG TCTTCGGACC

---------- ---------- ---------- ---------- ----------

CAGAACCAGC GGGCCTGCCA CCTCCTGGTC AATAGCCGTG TTTTTGGACC

CAGAACCAGC GGGCCTGCCA CCTCCTGGTC AATAGCCGTG TTTTTGGACC

---------- ---------- ---------- ---------- ----------

---------- ---------- ---------- ---------- ----------

CAGAACCAAC GGGCCTGCCA CTTCCTGGTC AATAGCCGTG TTTTTGGACC

CAGAACCAGC GGGCCTGCCA CCTCCTGGTC AATAGCCGTG TTTTTGGACC

CAGAACCAGC GGGCCTGCCA CCTCCTGGTC AATAGCCGTG TCTTTGGACC

CAGAACCAGA GAGCCTGCCA ACTTCTTGTC AATAGTCGTG TTTTTGGACC

CAGGACCGGA GGGCCTGCCA GCTGCCCGTC AACAGCCGCC TCTTCGGACC

TGACCTTTGT CCAGGAAGCA GTAAATACCT CCTGGTCTCC TTTAAATGCC

TGACCTTTGT CCAGGAAGCA GTAAATACCT CCTGGTCTCC TTTAAATGCC

TGACCTTTGT CCAGGAAGCA GTAAATACCT CCTGGTCTCC TTTAAATGCC

TGACCTTTGT CCAGGAAGCA GTAAATACCT CCTGGTCTCC TTTAAATGCC

TGACCTTTGT CCAGGAAGCA GTAAATACCT CCTGGTCTCC TTTAAATGCC

TGACCTTTGT CCAGGAAGCA GTAAATACCT CCTGGTCTCC TTTAAATGCC

TGACCTTTGT CCAGGAAGCA GTAAATACCT CCTGGTCTCC TTTAAATGCC

TGACCTTTGT CCAGGAAGCA GTAAATACCT CCTGGTCTCC TTTAAATGCC

---------- ---------- ---------- ---------- ----------

TGACCTTTGT CCAGGAAGCA GTAAATACCT CCTGGTCTCC TTTAAATGCC

TGACCTTTGT CCAGGAAGCA GTAAATACCT CCTGGTCTCC TTTAAATGCC

---------- ---------- ---------- ---------- ----------

---------- ---------- ---------- ---------- ----------

TGACCTTTGT CCTGGAAGCA GTAAATACCT CCTGGTCTCC TTTAAATGCC

TGACCTTTGC CCAGGAAGCA GTAAATACCT CCTGGTCTCC TTTAAGTGCC

TGACCTTTGT CCAGGAAGCA GTAAATACCT CCTGGTCTCC TTTAAATGCC

TGACCTTTGT CCAGGAACGA CTAAATATCT CCTGGTCTCC TTTAAATGCA

GGACCCTTGT CCAGGCGTGG CCAAGTCCCT CCTGGTGTCC TTCAAATGCA

AACCTAATGA TTGCTTGTCT TACTCAGCTT TGCAAGTCCT ATCCCGAAGG

AACCTAATGA TTGCTTGTCT TACTCAGCTT TGCAAGTCCT ATCCCGAAGG

AACCTAATGA TTGCTTGTCT TACTCAGCTT CGCAAGTCCT GTCCCGAAGG

AACCTA---- ---------- ---------- ---------- ----------

AACCTAATGA TTGCTTGTCT TACTCAGCTT TGCAAGTCCT ATCCCGAAGG

AGCCTAATGA TTGCTTGTCT TACTCAGCTC TGCAAGTACT ATCCAGAAGG

AACCTAATGA CTGCCTGTCT TACACGGCTT CACAAGTCCT GTCCAGAAGG

AACCTAATGA CTGCCTGTCT TACACGGCTT TACAAGTCCT GTCCAGGAGG

---------A CTGCTTGTCT CACTCAGCTT TACCCGTATT GTCCAGAAGG

AACCTAATGA TTGTCTGTCT TACTCGGCTC TGCACGTGTT ATCCAGGAGA

AGCCTAATGA CTGCTTGTCT TACTCAGCTT TGCAAGTATT ATCCAGGAGG

------ATGA CTGCTTGTCC TACTCAGCTT TGCAAGTATT GTCCAGGAGG

---------A CTGCTTGTCC TACTCGGCGC TGCAAGTCTT ATCCCGGAGG

AACCTAATGA CTGCTTGTCT TATTCAGCTT TACAAGTGCT ATCCCGGAGG

AACCTA---A CTGCTTGTCT TATTCCCCGT TGCAAGTTTT ATCCCAGAGA

AACCTA---A CTGCCTGTCC TATTCCGCTT T--------- ----------

AACCTAATGA TTGCTTATCT TATTCAGCCT TAGATGTGTT ATCCAGGAGA

AGCCCGATGA TTGTTTATCT TATTCAGCCT TAGAAGTGTT GTCTCAGAGG

TGCTATGGGA AGCAGAGATG CAAAATCATC GTCAACAATC ACCATTTTGG

TGCTATGGGA AGCAGAGATG CAAAATCATC GTCAACAATC ACCATTTTGG

TGCTATGGGA AGCAGAGATG CAAAATCATC GTCAACAATC ACCATTTTGG

---------- ---------- ---------- ---------- ----------

TGCTATGGGA AGCAGAGATG CAAAATTGTC GTCAACAATC ACCATTTTGG

TGCTATGGGA AACAGAGATG CAAAGTCATT GTCAACAATC AGCACTTCGG

TGCTACGGGA AGCAGAGATG TAAGGTCCTC GTCGATAATT ACCACTTCGG

TGCTATGGGA AGCAGAGATG TAAGGTCCTC GTCGATAATT ACCACTTCGG

TGCTACGGGA AGCAGAGATG CAAAATCCTT GTCAACAACC ACCATTTCGG

TGTTACGGGA AGCAGAGGTG CAAAGTCCTC GTCAACAACC ACCATTTCGG

TGCTATGGGA AGCAGAGATG CAAAATCACG GTCAACAATC ACCATTTCGG

TGCTACGGGA AGCAGAGATG CAAAATTCTT GTCAACAATC ACCATTTTGG

TGCTACGGGA AGCAGCGATG CACAATCATC GTCAACAACC ACCATTTCGG

TGCTACGGGA GGCAGAGATG CAAAATCATT GTCAACGACC ACCATTTTGG

TGCTACGGAA AGCAGAGATG CAAAATCATT GCCAACAATC ACCACTTCGG

---------- ---------- ---------- --------TC ACCATTTCGG

TGCTATGGAA AGCAGAGATG CAAAATCATT GTCAACAATC ACCATTTTGG

TGTTACGGGA AACAGAGATG CAAAATCATG GTGAGCGATC ACCAGTTCGG

AAGCCCCTGT TTGCCAGGCG TGAAAAAATA CCTCACTGTG ACCTACGCAT

AAGCCCCTGT TTGCCAGGCG TGAAAAAATA CCTCACTGTG ACCTACGCAT

AAGCCCCTGT TTGCCAGGTG TGAAAAAATA CCTCACTGTG ACCTACGCAT

---------- ---------- ---------- ---------- ----------

AAGCCCCTGT TTGCCAGGCG TGAAAAAATA CCTCACTGTG ACCTACGCAT

AAGCCCCTGT CTGCCAGGAG TGAAAAAATA CCTCACTGTG ACCTACGCCT

AAGCCCATGT CTTCCCGGAG TGAAAAAATA CCTCACTGTT GCCTACGCGT

AAGCCCCTGT CTTCCAGGAG TGAAAAAATA CCTCACTGTT GCCTACGCAT

AAGCCCCTGT CTGCCAGGAG TGAAAAAATA CCTCACTGTC ACCTACGCCT

AAGCCCCTGT CTGCCAGGAG TGAAAAAATA TCTCTCGGTC TTCTACGCGT

GAGCCCCTGT CTGCCAGGAG TGAAAAAATA CCTCACTGTC ACCTATGCCT

AAGCCCCTGT CCGCCAGGAG TGAAAAAATA CCTCACCGTC ACCTATGCAT

GAGCCCCTGC CTGCCAGGAG TGAAGAAATA CCTCACAGTC AACTACGCGT

AAGCCCCTGT CTGCCAGGAG TGAAAAAACA CCTCACTGTT GCCTATGCAT

AAGCCCCTGT CTGCCAGGAG TGAAAAAATA CCTCACCGTT ACCTACGCAT

AAGCCCCTGT CTGCCCGGAG TGAAAAA-TA CCTCACAGTC ACCTACGCAT

AAGTCCTTGT CTGCCAGGAG TGAAAAAATA CCTCACTGTG GGCTATGCCT

AAGCCCGTGT CTGCCGGGAG TGAAGAAGTA CCTCACAGTG GGCTACGCTT

GTGTTCCCAA GAACATACTC ACAGCGATTG ATCCAGCCAT TGCTAATCTA

GTGTTCCCAA GAACATACTC ACAGCGATTG ATCCAGCCAT TGCTAATCTA

GTGTTCCCAA GAACATACTC ACAGCGATTG ATCCAGCCAT TGCTAATCTA

---TTCCCAA GAACATACTC ACAGCGATTG ATCCAGCCAT TGCTAATCTA

GTGTTCCCAA GAACATACTC ACAGCGATCG ATCCAGGCAT TGCTAATCTA

GTGTTCCAAA GAACATCCTT GCAGCGATTG ATCCAGCTGT TGCTAATCTA

GTGTTCCCAA GAACATACTC ACGGCGGTTG ACCCAGCCGT TGCTAACCTA

GTGTTCCCAA GAACATACTC ACGGCAGTGG ATCCGGCTGT TGCTAATCTA

GTGTTCCCAA GAGCATACTC TCAGCGATCG ACCCAGCCGT CGTCGACCTC

GTGTTCCCAA GAATATACTC ACCGCAATTG ACCCATCAGT TGCTAATCTA

GTGTTCCCAA GAACATACTC ACAGCGATCG ATCCAGCCAT TGTTAATCTA

GCGTTCCCAA GAACATACTC ACAGCAATTG ATCCAGCTAT TGCTAATCTG

GTG------- ---------- ---------- ---------- ----------

GTGTTCCCAA GAACATACTT ACAGCAATTG ATCCAGCTGT TGCTAATCTA

GTGTTCCCAA GAACATCCTC GCAGCAATTG ATCCAGCTGT TGCTAATCTA

GCGTTACCCA ATCCATACAC ACATCAATTG ---------- ----------

GTGTTCCCAA AAACATACTT ACAGCGGTTG ATCCAATGGT CTCTAATCTA

GCGTTCCCAA ACACATACTT TCTGCTATCG ATCCAATGGT TTCCCTGCTA

AAACCTTCTT TGAAGCAGAA AGATGGTGAA TATGGTATAA ACTTCGACCC

AAACCTTCTT TGAAGCAGAA AGATGGTGAA TATGGTATGA ACTTCGACCC

AAACCTTCTT TGAAGCAGAA AGATGGTGAA TATGGTATAA ACTTTGACCC

AAACCTTCTT TGAAGCAGAA AGATGGTGAA TATGGTATAA ACTTCGACCC

AAACCTTCTT TGAAGCAGAA AGATGGTGAA TATGATATAA ACTTCGACCC

AAACCTTCTT CGAAGCAGAA AGACACTGAA TATGGTATAA ACTTTGACCC

AATCCCTCTC TGA---AGAA AGACGATGAA TACGGTATAA AATTCAACCC

AATCCTTCTC TGA---AGAA AGATGATGAA CACGGTATAA CATTCAACCC

AGACCCTCTC TGGAGCAGGA AGATGGTGCG CACGGTGTCA CCTTTGACCC

AAACCTTCTG TGAAGCAGAA AGATGGCGAC TATGGCGTAG ACCTTGACCC

AAACCTTCTT TGAAGCAGAA AGATGGTGAA TATGGTATAA ACTTTGACCC

AAGCCTTCTC TGAAGCAGAA AGATGATGAA TATG------ ----------

---------- ---------- ---------- ----GAATAA ACTCAGACCC

AAACCTTCTT TGAAGCAGAG AGATGGTGAA CATGGTATAA ACTTTGACCC

GAACCTTCTT TGAAGAAGAA AAATGGGGAA TATGGTATAA ACTTTCACCC

---------- ---------- ---------- ---------- ----------

AACCCTTCTT TGAAGCAGAA AGATGGTGAC TCTGATATAA ACTTTGATCC

AATCCTACTT GGAAGCAGAA AGACGGTGAC TACGGCACCA AGCTGGGTCC

AAGCGGATCG AAGGTTCTGA GGAAAGATGG AATTCTTGTT AGCAACTCTC

AAGCGAATCA AAGTTTCTGA GGAAAGATGG AATTCTTGTT AGCGGCTCTC

AAGCGAATCG AAGATTCTGA GGAAAGATGG AATTCTTGTT AGCAACTCTC

AAGCGAATCA AAGGTTCTGA GGAAAGATGG AATTCTTGTT AGCAACTCTC

AAGCGGATCG AGGGTTCTGA GGAAAGATGG AATTCTTGTT AGCAACTCTC

AAGCGAATCG AGGGTTCTGA GGAAAGATGG AGTTATTGTT AGCAACTCCC

AAGCGGATCG AGGGTTGTGC GGAAAGATGG CGTTATTGTC AGCAACTCTC

AAGCGGGTCG AGGGTTGTGC GGAAAGATGG CGTTATCGTC AGCAACTCTC

CAGTGAGTCG AAGATTCTGC GGAAGGATGG CGTGATTGTC AGCAACTCTC

AAGGGAACCG AGGGTTCTGA GGAAAGATGG TGTGATTGTC AGCCACTCGT

AAGGGAACCG ACGCTTCTGA GGAAAGATGG AGTTATTGTT AGCAATTCTC

---------- ---------- ---------- ---------- ----------

CAGCGGAACG AGGGTGTTGA GGAAGGACGG TGCCCTGGTG AGCAGCTCGC

AAGCGAAACG AGGGTGCTGA GGAAAGACGG AGTTATCTTT AGCAACTCTC

AAGTGAATCC AGCGTTCTGA GGAAAGACGG GGTCATTGTT AGCAACTCCC

---------- ---------- ---------- ---------- ----------

AAGCGAATCA AAAGCTCTGG CGAAAGATGG AGTTATTGTC AGCAACTCTT

GGGCGGATCC CGAGGCCTGA AGACGGAGGG CGTCATTGTC AGCAGCTCGT

TGGCAGCCTT TGCTTACATT AGAGCCCACC CGGAGAGAGC TGCCCTGCTG

TGGCAGCCTT TGCTTACATT AGAGCCCACC CGGAGAGAGC TGCCCTGCTG

TGGCAGCCTT TGCTTACATT AGAGCCCACC CGGAGAGAGC TGCCCTGCTG

TGGCAGCCTT TGCTTACATT AGAG------ ---------- ----------

TGGCAGCCTT TGCTTACATT AGAGCCCACC CCGAGAGAGC TGCCCTGCTG

TGGCAGCATT TGCTTACATT AGAGCCCATC CCGAGAGAGC TGCCCTGCTG

TGGCTGCGTT TGCTTACATT CGAGCCCACC CCGAGAGAGC TGCTCTGCTG

TGGCCGCGTT CGCTTACATT CGAGCCCACC CAGAGAGAGC CGCTCTGCTG

TGGCGGCGTT TGCTTACATC CGGGCGCACC CCGAGAGAGC CGCCCTGCTG

TGGCAGCATT TGCTTACATT AGAG------ ---------- ----------

TGGCAGCATT TGCTTACATT AGAGCCCACC CAGAGAGAGC AGCCCTGCTC

---------- ---------- ----CCCACC CTGAGAGAGC TGCCCTGCTG

TGGCGGCCTT CGCTTACATT AGAGCCCACC CGGAGAGAGC GGCCCTGCTG

TGGCAGCATT TGCTTACATC AGAGCCCACC CCGAGAGGGC CGCCCTGCTG

TGGCAGCATT TGCTTACATT AGAGCCCACC CCGAGAGAGC CGCCCTGCTG

---------- ---------- ----CCCACC CCGAGAGAGC CGCCCTGCTG

TGGCCACATT TGCTTACATT AGAGCTCACT CAGAAAGGGC TGCTCTGCTG

TGGCCGTGTT CGCTTACGTT CGAGCACACC CCGAAAGGGC GGCCCTGCTG

TTCGTGTCCA GTGTCTGCAT CGGCCTGGCC CTCACACTGT GCGCCCTGGT

TTCGTGTCCA GTGTCTGCAT CGGCCTGGCC CTCACACTGT GCGCCCTGGT

TTCGTGTCCA GTGTCGGCAT CGGCCTGGCC CTCACATTGT GTGCCCTGGT

---------- ---------- ---------- ---------- ----------

TTTGTGTCCA GCGTCTGCAT CGGCCTGGCC CTCACATTGT GTGCCTTGGT

TTCGTGTCCA GTGTTTGCAT TGGCTTGGCC CTCACACTGT GTGCCTTGGT

TTCGTGTCTA GTGTCTGCAT CGGCCTGTTC CTCACACTGT GTGCCCTGGT

TTCATGTCCA GTGTCTGCAT CGGCCTGCTC CTCACACTGT GTGCCCTGGT

TTCGTGTCCA GCGTGTGCGT CGGCCTGGTG CTCACCCTGT GCGCCCTGGT

---------- ---------- ---------- ---------- ----------

TTCGTGTCCA GTGTCTGCAT CGGTCTGGCG CTCACGTTGT GTGCCTTGGT

TTCGTGTCCA GTGTCTGCAT CGGTCTGGCA CTCACGTTGT GCGCCTTGGT

TTCGTGTCCA GCGTCTGCGT GGGCCTGGCG CTCACGCTGT GCGCCTTGGT

TTCGTGTCCA GCGTCTGCCT CGGCCTGGTG CTCACCCTGG GTGCCCTGGT

TTCATGTCCA GTGTCTGCAT CGGCCTGGCG CTCACGATGT GTGCCTTGGT

TTTGTGTCCA GTGTCTGCAT TGGCCTGGTC CTCACCTTGT GCGCCCTGGT

TTTGTGTCCA GTGTTTGTAT TGGCCTAATC GTCACCCTGT TTGCTGTGGT

TTCGTGTCCA GCGTCTGCCT CGGGTTGGTC GTCACGCTGG GCGCGGTGGT

CATCAGAGAG TCCTGTGCCA AGGACTTCCG CGACTTGCAG CTGGGGAGGG

CATCAGAGAG TCCTGTGCCA AGGACTTCCG CGACTTGCAG CTGGGGAGGG

CATCAGAGAG TCCTGTGCCA AGGACTTCCG CAAGTTGCAG CTGGGGAGGG

---------- ---------- ---------- --------AG CTGGGA--GG

TATCAGAGAG TCCTGCACCA AGGATGTCCG CGAGATGCAG CTGGGGAGGG

CATCAAAGTG TCCTGCACCA AAGACTTCCG GGAGCTGCAT TTGGGGAGGG

CATCAGAGTG TCCTGCACCA AGGACTTCCA GGAGCTGAGG CAGGGGAGGG

CATCAGGGTG TCCTGTACGA AGGACTTCCG GGAGCTGAGG CAGGGGAGGG

CATCCAGCTG TCCTGCGCCA AGGACTTCCG GGAGCTGCGG CTGGGGAGGC

---------- ---------- ---------- ---------- ----------

CATCAGAGTG TCCTGCGTCA AGGACCTCAG GGATTTGCAG CTGGCGAGGG

TGTCAGAGGG TCCTGCACCA AAGACTTCCG GGAGCTGCAG CTGGCGAGAG

CATGCGTGTG TCCTGCGCCC GGGACCTCCG AGAGCTGCAG CTGGCCCAGG

CGTCAGAGTG TCCTGCACCA AGGACTTGCG GGAGCTGCAG CTGGGGAGGG

CATCAGAGTG TCCTGCACCA AGGACTTCCA GGAGCTGCAG CTGGGGAGGG

CGTCAAAGTG TCCTGCACCA AGGACGTCCG GGAGCTGCAG CGGCGGAGGG

GATCAGAGTA TCATGCACCA AAGACTTCCA GAAACTAAGC CAGGGCAAGA

GGCCCGAGGG TCGTGCCCCA AAGACTGCCG GGAACTGCGG CCCGCCCAAG

AGCAGCTGGT GCCAGGAAGT GACAAGGTCG AGGAGGACAG CGAGGATGAA

AGCAGCTGGT GCCAGGAAGT GACAAGGTCG AGGAGGACAG CGAGGATGAA

AGCAGCTGGT GCCAGGAAGT GACAAGGCCG AGGAGGACAG CGAGGATGAA

AGCAGCTG-T GCCAGGAAGT GACAAG-CCG AGGAG-ACAG CGAGGATGAA

AGCAGCTGGT GCCAGGAAGT GACAGGGCTG AGGAGGACAG TGAGGATGAA

AGCACCTGGT GCCAGGAATT GACCAGGCTG AGGAGGACAG TGAGGATGAA

AGCACCTGGT GCAAGGGAGT GACAAGGCGG AGGAGGACAG TGAGGAGGAT

AGCACCTGGT GCTAGGGAGC GACAAGGCGG AGGAGGACAG CGAGGAAGAC

AGCACCTGGT GCCGGGCAGT GACAAGGCGG AGGACGACAG CGAGGAGGGC

---------- ---------- ---------- ---------- ----------

AGCCCTTGGT GCCAGAAAGT GACAAGGCTG AAGAAGACAG CGAGGATGAG

AGCAACTGGT GCCAGGAAGT GACAAGGCCG AGGAGGACAG TGAGAATGAA

AGCGCCTGGT CCCGGGAAGT GACCGGGTGG AGGAGGACAG CGAGGAGGAG

AGCACCTGGT GCCAGCAAGC GACAAGGCCG AGGAGGACAG CGACGACAAA

AGCATCTGGT GCCAGGGAGT GACAAGGCTG AGGAGGACAG CGAGGACAAA

AGCATCTGGT GGCGGGGAGT GACAaggcgg aggaggacag tgatgacgag

AACGCTTAGT GCCGGAAGCG GAGAAAACAG AGAATGACAG TGAGGATGGA

AGCCCTTGAT GCTGGAGAGC GGCGGGGCGC GGGAGGGCAG CGGCGACGAA

GAGGAGGACC CCTCTGAGTC TGATTTCCCA GGGGAACTGT CGGGGTTCTG

GAGGAGGACT CCTCTGAGTC TGATTTCCCA GGGGAACTGT CGGGGTTCTG

GAGGAGGACT CCTCTGAGTC TGATTTCCCA GGGGAACTGT CGGGGTTCTG

GAGGAGGACT CCTCCGAGTC TGATT-CCCA GGGGAACTGT CGGGGTTCTG

GAGGAGGACT CCTCTGACTC TGACTTCCCA GGGGAACTGT CAGGGTTCCA

GAGGAGGACT CCTCTGACCC AGATTTTCCA GGAGAACTGT CTGGGTTTTG

GAGGAGGACT CCTCAGATTC TGAGTTTCCT GTGGAATTGT CCAGGTTTTG

GAGGAGGACT CCTCGGATTC GCAGTTTCCC GAGGAGTTGT CCAGGTTTTG

GAGGAGCACT CCTCCGACTC GGATTTCCCG GGGGAGCTGG CGACGTTCTG

---------- ---------- ---------- ---------- ----------

GAGGAGGACT CCTCGGACTC TGACTTTCCA GGGGAACTGT CTGGGTTTTG

GAGGAGGACT CTTCTAGCTC CGATTTTCCG GGGGAGCTGT CAGGGTTCTG

GAGGAGCACT CCTCGGACTC GGATATTCAG GGGCAGCTGT CGGGCTTCAG

GAGGAGGACT CCTCTGACTC CGATTTCCCG GGGGAGCGGC CGGCGTTTTG

GAGGAGAGCT CCTCAGACTC TGATTTTCCA GGAGAACTGT CAGGGTTTTG

gaggaggGCT CCTCAGACTC GGATCTTCCC GGGGAGCGGT CAGCGTTTCG

GAGGAAGATT CTTCAGACTC GGACTTTCCG GATGAAATGA CGGAATTTTT

GACGACGACT CCTCCGGCTC CGACTTCCCG GACGAATGGC CCGATTTCCA

TAGGACTTCA TATCCTATAT ACAGTTCCAT AGAAGCTGCA GAGCTCGCAG

TAGGACTTCA TATCCTGTAT ACAGTTCCAT AGAAGCTGCA GAGCTCGCAG

TAGGACTTCA TATCCTGTCT ACAGTTCCAT AGAAGCTGCA GAGCTCGCAG

TAGGACTTCA TATCCTGTCT ACAGTTCCAT AGAAGCTGCA GAGCTCGCAG

TAGGACTTCA TATCCTGTCT ACAGTTCCAT AGAAGCTGCA GAGCTCGCAG

TAGGACTTCA TATCCTGTCT ACAGTTCCAC AGAGGCTGCA GAGCTTGCAG

TAGGACTACG TATCCAGCCT ATAGCTCTAT AGAAGCCGCT GAGCTGGCAG

TAGGACTTCA TATCCAGCCT ATAGCTCTAT AGAAGCCGCC GAGCTGGCGG

TAGGACTTCC TATCCCGTCT ACAGCTCCAT AGAGGCCGCA GAACTGGCCG

---------- ---------- -----TCCAT AGAGGCTGCA GAGTTGGCAG

TAGGACTTCG TATCCCGTCT ACAGTTCCAT AGAAGCTGCG GAGCTGGCAG

TCGGACTTCA TATCCCATCT ACAGTTCCAT AGAGGCTGCA GAGCTGGCAG

CAGGACTTCC TACCCCGGCT ACACGTCCAT CGAGGCCGCG GAGCTGGCGG

TAGGACTTCG TATCCCGTCT ACAGTTCCAT CGAGGCTGCG GAGCTGGCGG

TAGGACTTCC TATCCCATCT ACAGTTCCAT AGAGGCTGCA GAGCTAGCAG

CAGGACTTCC TATCCCGTCT ACAGCTCCTT AGAGGCCGCG GAGCTTGCAG

CCGGACTTCT TACCCAGTCT ACAGCTCCAC GGAAGCTGCT GAGATGGCCG

CCGGTCGCCC CCTCCGCCCC GCGGAGCCCC GGAAGCGGCC GACCTGGCCG

AAAGGATTGA GCGCAGGGAG CAAATCATTC AGGAAATATG GATGAACAGT

AAAGGATTGA GCGCAGGGAG CAAATCATTC AGGAAATATG GATGAACAGT

AAAGGATTGA ACGCAGGGAG CAAATCATTC AGGAAATATG GATGAACAGT

AAAGGATTGA ACGCAGGGAG CAAATCATTC AGGAAATATG GATGAACAGT

AAAGGATTGA GCGCAGGGAG CAAATAATCC AGGAAATATG GATGAACAGT

AAAGGATTGA GCGCAGGGAA CAAATCATTC AGGAAATATG GATGAACAGT

AAAGGATTGA GCGCAGGGAG CAAATCATTC AAGAAATATG GATGAACAGT

AAAGGATTGA ACGCAGGGAG CAAGTCATTC AAGAGATATG GATGAACAGT

AGAGGATCGA GCGCAGGGAA CAGATCATCC AGGAGATATG GATGAACAGC

AAAGGATTGA GCGCAGGGAG CAGATCATCC AGGAAATATG GATGAACAGT

AAAGGATTGA GCGCAGGGAG CAAATCATCC AGGAAATATG GATGAACAGT

AGAGGATTGA GCGCAGGGAG CAAATCATCC AGGAAATATG GATGAACAGT

AAAGGATTGA GCGGCGGGAA CAAATCATCC AGGAAATCTG GCTGAACAGC

AGAGGATCGA GCGCAGGGAG CAGATCATTC AGGAAATATG GATGAACAGT

AAAGGATTGA GCGCAGAGAG CAAATCATTC AGGAAATATG GATGAACAGT

AGAGGATCGA GCGCAGAGAG CAAATCATTC AGGAAATATG GCTGAACAGT

AAAGGATAGA GCGTAGAGAT CAGATCATTC AGGAAATTTG GATGAACAGT

AGAGGCTGGA GTGGCGGGAC CAGATCATCC AGGAGATATG GCTGAGCAGT

GGTTTGGACA CCTCGCTCCC AAGAAACATG GGCCAGTTCT ACATGAAAGG

GGTTTGGACA CCTCGCTCCC GAGAAACATG GGCCAGTTCT ACATGAAAGG

GGTTTGGACA CCTCACTCCC GAGAAACATG GGCCAGTTCT ACATGAAAGG

GGTTTGGACA CCTCACTCCC GAGAAACATG GGCCAGTTCT ACATGAAAGG

GGTTTAGACA CCTCACTCCC CAGAAACATG GGCCAGTTCT ACATGAAAGG

GGTTTGGACG CCTCACTCCC GAGAAACATG GGCCAGTTCT ACATGAAAGG

GGCCTAGACT CCTCGCTCCC CAGGAACATG GGCCATTTCT ACATGAAAGG

GGCCTGGATT CCTCACTCCC CAGGAACGTC GGCCACTTCT ACATGAAAGG

GGTCTGGACA CCTCGCTCCC GAGACACGTG GGCCAGTTCT AC--------

GGCTTGGACA CTTCACTCCC GAGAAACATG GGCCAGTTCT ACATGAAAGG

GGTTTGGACA CTTCACTCCC AAGAAATTTC GGCCAGTTCT ATATGAAAGG

GGTTTGGACA CGTCACTCCC ACGAAACATG GGCCAGTTCT ACATGAAAGG

GGCTTGGACA CGTCACTCCC GAGGAACATG GGGCAGTTTT ACATGAAAGG

GGCTTGGACA CGTCACTCCC GAGAAACATG AGTCAGTTCT ACATGAAAGG

GGTTTGGACA GCTCGCTCCC GAGAAACATG AGCCAGTTCT AC--------

GGCTTGGACA GCTCGCTCCC ACGAAACATG AGCCAGTTCT ACATGAAAGG

GGTTTGGATA CCTCTTCTCC AAGAAATCTG GATCAATATT ATATGAAAGG

GGCTTGGACG CCTCCCCGCC GAGGAACTTG GGCCAGGACT ACATGAAAGG

CTTATATTTC CAACAGAGTT CCACAGATGA AGAAATAACA TTTGTATTTC

CTTATATTTC CAACAGAGTT CCACAGATGA AGAAATAACA TTTGTATTTC

CTTATATTTC CAACAGAGTT CCACAGATGA AGAAATAACA TTTGTATTTC

CTTATATTTC CAACAGAGTT CCACAGATGA AGAAATAACA TTTGTATTTC

CTTATATTTC CAGCAGAGTT CCACAGATGA AGAAATAACA TTTGTATTTC

CTTATATTTT CAACAAAGTT CCACAAATGA AGAAATAACA TTTGTATTTC

CTTGTATTTT CAACAGAGTT CTACAAATGA AGAAGTAACA TTTGTATTTC

CTTGTATTTT CAACAGAGTT CTACAAATGA AGAAGTAACA TTTGTATTTC

---------- ---------- ---------- ---------- ----------

CTTGTATTTT CAACAAAGTT CTACAAATGA AGAAATAACA TTTGTGTTCC

CTTATATTTT CAAAAGAGTT CTACAAATGA AGAAATAACA TTTGTATTTC

CTTATATTTT CAACAGAGTT CTACAAATAA AGAAATAACA TTTGTATTTC

ATTGTATTTT CAACAGAGTT CCGCAAATGA AGAAATCACA TTTGTATTTC

CTTATATTTT CAACAGACTT CCACAAATGA AGAGATAACA TTTGTATTTC

---------- ---------- ---------- ---------- ----------

CTTATATTTT CA-CAGAATT CTACAAATGA GGAAATGACA TTTATATTTT

CTTATATTTT CAGCAGAGTT CACCAGGAGA AGAAATTTCA TTTGTATTTC

CTTATATGTC CAGCGAGCTT CTTCAGAAGA TGAAGTGAAA TTTGTGTATC

AAGAAAAGGA AGATCTTCCT GTTACAGAGG ATAACTTTGT GAAACTTCAA

AAGAAAAGGA AGATCTTCCT GTTACAGAGG ATAACTTTGT GAAACTTCAA

AAGAAAGGGA AAATCTTCCT GTTACAGAGG ATAACTTTGT AAAACTTCAA

AAGAAAGGGA AAATCTTCCT GTTACAGAGG ATAACTTTGT AAAACTTCAA

AAGAAAGGGA GAATCTTCTG GTTACAGAGG ACAACTTTGT GAAACTTCAA

AAGAAAGGGA AAATCTTCCT GTTATAGAAG ATAATTTCGT GAAACTTCAA

AAGAAAAGGA AAATGTCTCT GTCACAGAGG ATAACTTTGT GAGAGTGCAA

AAGAAAAGGA AAATGTCCCT GTCACAGAGG ATAACTTTGT GAGAGTTCAA

--------GA AAATCTTCCT GTTACAGAGG ATAACTATGT GAAACTTCAA

AAGAAAGGGA AAATCTTCCT GTTACAGAAG ATAACTATGT GAAACTTCAA

AAGAAAGGGA AAATCTTCCT GTTATAGAGG ATAACTATGT GAGACTTCAA

AAGAAAGGGA AAATCTTCCT GTTACAGAGG ATAACTATGT GAAACTTCAA

AAGAAAGG-- ---------- ---------- ---------- ----------

AAGAAAGGGA AAATCTTCCT GTTACAAAGG ATAACTTTGT GAAACTTCAA

---------- ---------- ---------- ---------- ----------

CAG-AAGGGA AAACCTTCCT GTCACAGAAG ATAACTTTGT GAAACTTCAA

AAGAAAGGGA AAATCTTCCT GATGTAAAGG ATAACTATGT GAAGCTTCAA

AAGAACGGAA AAATATTCCT GTGGCAAGTG ACAATTATGT GACACTTCAA

GTTAAAGCTT GTGCTCTGAG CCAGATAAAT ACAAAGATGA AAAAGGATTT

GTTAAAGCTT GTGCTCTGAG TCAGATAAAT ACAAAGATGA AAAAGGATTT

GTTAAAGCTT GTGCTCTCAG CCAGATAAAT ACAAAGATGA AAAAGGATTT

GTTAAAGCTT GTGCCCTCAG CCAGATAAAT ACAAAGATGA AAAAGGATTT

GTTAAAGCTT GCGCTCTGAG CCAGATAAAT ACAAAGATGA AAAAGGATTT

GTTAAAGCTT GTGCTCTGAG CCAGATAAAT ACGAAGATGA AGAAGGAATT

GTTAAAGCCT GTGCCCTGAG CCACATAAAC ACAAAGATGG AAAAGGAATT

GTTAAAGCTT GTGCCCTGAG CCACATAAAC ACAAAGATGG AAAAGGATTT

GTAAAAGCTT GTGCTCTAAG CCAGATAAAT ACGAAGATGG AAAAGGAGTT

GTTAAAGCTT GTGCTCTGAG CCAGATAAAT ACAAAGATGG AAAAGGAATT

GTTAAAGCTT GTGCTCTAAG CCAGATAAAT ACAAAGATGG AGAAAGATTT

GTTAAAGCCT GTGCTCTGAG CCAGATAAAT ACAAAGATGG AGAAGGATTT

---------- ---------- ---------- ---------- ----------

GTTAAAGCTT GTGCTCTGAG CCAGATAAAT ACAAAGATGG AGAAGGATTT

---------- ---------- ---------- ---------- ----------

ATTAAAACTT GT-CTCTGAG CCAGATAAAC ACAAA---GG AAAAAGATTT

GTAAAAGCTT GTGCTCTAAG CTGGATAAAT ACAAAGCTGG AGAAGGAATT

GTTAAAGCCT GTGCTCTAAG CTGGATAAAT GCAGAGCCGG AGGAGGATTT

ATTTCCTGTT GGGAGAGAAA TTGCTGGAAT TGTATTAGAT GTTGGAAGCA

ATTTCCTGTT GGGAGAGAAA TTGCTGGAAT TGTATTAGAT GTTGGAAGCA

ATTTCCTGTT GGGAGAGAAA TTGCTGGAAT TGTATTAGAT GTTGGAAGCA

ATTTCCTGTT GGGAGAGAAA TTGCTGGAAT TGTATTAGAT GTTGGAAGCA

CTTTCCTGTT GGGAGAGAAA TTGCTGGAAT TGTATTAGAT GTTGGAAGCA

CTTTCCTGTT GGGAGAGAAA TTGCTGGAAT TGTGTTAGAA GTTGGAAGCA

CTTCCCTGTT GGGAGAGAAG TCGCTGGGAT TGTATTAGAT GTTGGCAAGA

CTTCCCTGTT GGAAGAGAAG TTTCTGGAAT TGTATTAGAG GTTGGCAGGA

CTTTCCTGTT GGCAGAGAAA TTGCTGGAGT TGTGTTAGAT GTTGGGAAAA

CTTTCCTGTT GGGAGAGAAG TTGCTGGAAT TGTGTTAGAT GTTGGAAGCA

CTTTCCTGTT GGGAGAGAAA TTGCTGGAAT TGTGTTAGAT GTTGGAAGCA

CTTTCCTGTT GGGAGAGAAA TTGCTGGAAT TGTGTTAGAT GTTGGAAGCA

---------- ---------- ---------- ---------- ----------

CTTTCCTGTT GGAAGAGAGG TTGCTGGAAT TGTGTTAGAT GTTGGAAGCA

---------- ---------- ---------- ---------- ----------

TATTCCTGTT GGGAGAGAAG TTGCTGGAAC TGTGTTAGAT GTTGGACGCA

TTTTCCTGTT GGGAGAGAAA TTGCTGGAGT TGTACTAGAG GTTGGAAGCA

TTTCCCTGTG GGGAGAGAAA TTGCAGGAAT AGTTTTGGAA GTTGGAAATA

AGGTATCATT CTTTCAACCA GATGATGAAG TAGTTGGAAT TTTGCCCCTG

AGGTATCATT CTTTCAACCA GATGATGAAG TAGTTGGAAT TTTGCCCCTG

AGGTATCATT CTTTCAACCA GATGATGAAG TAGTCGGAAT TTTGCCCCTG

AGGTATCATT CTTTCAACCA GATGATGAAG TAGTCG---- ----------

AGGTATCATT CTTTCAACCA GATGATGAAG TAGTCGGAAT TTTGCCTCTG

AGGTATCATT CTTTCAACCA GATGATGAAG TAGTCGGAAT TTTGCCATTG

AGGTATCGTT CTTTCAGCCA GATGATGAAG TCGTCGGGAT TTTGCCTTTG

AGGTAACGTT CTTTCAGCCA GACGACGAAG TGGTCGGAAT TCTGCCTTTG

AGGTGTCATT CTTTCAGCCA GATGATGAAG TAGTTG---- ----------

AGGTATCATT CTTTCAACCA GATGATGAAG TAGTGGGAAT TCTGCCCCTG

AGGTATCATT CTTTCAACCA GATGATGAAG TCGTCGGAAT TCTGCCCCTG

AGGTATCATT CTTTCAACCA GATGATGAAG TAGTCGGAAT TTTACCCCTG

---------- ---------- ---------- ---------- ----------

AGGTATCGTT CTTTCAACCA GATGATGAAG TAGTTGGAAT TTTACCCCTG

---------- ---------- ---------- ---------- ----------

AAGTCTCATT CTTTCAACCA GATGATGAAG TAGTAG--AT TGTACCCCTG

AAGTATCATT CTTTCATCCA GATGATGAAG TAGTAGGAAT TTTACCACTG

AAGTATCCTT CTTTCAACCA AATGATGAAG TGGTTGGAAT TTTACCGATG

GACTCTGAAG ACCCTGGACT TTGTGAAGTT GTTAGAGTAC ATGAGCATTA

GACTCTGAAG ACCCTGGACT TTGTGAAGTT GTTAGAGTAC ATGAGCATTA

GACTCTGAAG ACCCTGGACT TTGTGAAGTT GTTAGAGTAC ATGAGCATTA

---------- ---------- ---------- ---------- ----------

GATTCTGAAG ACCCTGGATT TTGTGAAGTT GTTAGAGTAC ATGAGCATTA

GACTCTGAAG ACTCTGGACT TTGTGAAGTT GTTAGAGTAC ATGAGCATTA

GATTCGGAAG ACCCCGGACT CTGCGAAGTT ATCCGAGTAC AGGAGCATTA

GATTCCGAAG ACCCCGGGCT CTGTGAGGTT ATTCGAGTAC ATGAGCATTA

---------- ---------- ---------- ---------- ----------

GATTCTGAAG ACCCTGGACT TTGTGAGGCT GTTCGAGTCC ATGAGCATTA

GATTCTGAAG ACCCTGGACT TTGTGAAGTT GTTAGAGTGC ACGAGCATTA

GATTCTGAAG ACCCTGGACT TTGTGAAGTT GTTAGAGTAC ATGAGCATTA

---------- ---------- ---------- ---------- ----------

GATTCCGAAG ACCCTGGACT CTGTGAAGTG CTTAGGGTAC ATGAGCACTG

---------- ---------- ---------- ---------- ----------

GGATCTGAAG ACTCAG--CT TCATGGTGCC GATAGAGTCC ACGAGCATTA

GATTCTGAAG AATCTGGTCT CTGTGAAATT GTTAGAATAC ATGAGCATTA

GATTCTGAAG AATCTGGCCT TTGTGAAGTG ATCAGAGTTC ACGAGCATTA

CTTGGTTCAT AAACCAGAAA AGGTCACATG GACGGAAGCA GCAGGAAGCA

CTTGGTTCAT AAACCAGAAA AGGTCACATG GACGGAAGCA GCAGGAAGCA

CTTGGTTCAT AAACCAGAAA AGGTCACATG GACGGAAGCA GCAGGAAGCA

-----TTCAT AAACCAGAAA AGGTCACATG GACGGAAGCA GCAGGAAGCA

CTTGGTTCAT AAACCAGAAA AGGTTACATG GACAGAAGCA GCAGGAACCA

CTTGGTTCAT AAACCAGAAA AGGTCACTTG GACAGAAGCA GCTGGAACCA

CCTGGTTCAT AAGCCAGAAA AGGTGCCATG GACGGAAGCC GCTGGAGTCA

CCTGGTTCAT AAGCCAGAAA AGGTGTCATG GACGGAAGCT GCTGGCGTCA

-----TTCAT AAGCCGGAAA AGGTCACGTG GACAGAAGCA GCTGGAACCA

CTTGGTTCAC AAACCAGAAA AAGTCACGTG GACAGAAGCT GCTGGAACCA

CTTGGTTCAC AAACCAGAAA AAGTTACATG GACAGAAGCT GCTGGAACCA

CTTGGTTCAC AAGCCAGAAA AAGTCACATG GACAGAAGCT GCTGGAACCA

---------- ---------- ---------- ---------- ----------

TTTGGTTCAC AAACCAGAAA AGGTTACATG GACAGAAGCA GCTGGAACCA

-----TTCAT AAACCAGAAA AGGTTACATG GACAGAAGCG GCTGGAACCA

CTT--TTCAT AAACCAGAAA AGGTTACGTG GGCAGAAGCG GCTGGAACCA

CTTGGTTCAT AAACCAGAAA AGGTCACGTG GGCCGACGCA GCAGGAGTCA

CTTGGTTCAT AAGCCAGAAA AAGTCACTTG GGTCGAAGCA GCGGGAACGA

TTCGGGATGG AGTGCGTGCC TATACAGCTC TGCATTATCT TTCTCATCTC

TTCGGGATGG AGTGCGTGCC TATACAGCTC TGCATTATCT TTCTCATCTC

TTCGCGATGG AGTGCGCGCC TATACAGCTC TGCATTATCT TTCTCATGTC

TTCGGGATGG AGTGCGCGCC TATACAGCTC TGCATTATCT TTCTCATGTC

TTCGGGATGG AGTGCGTGCC TATACAGCTC TGCATTATCT TTCTCATCTC

TTCGGGATGG AGTTCGAGCC TACACAGCAC TGCATTACCT TTCTCAGCTC

TCCGGGATGG AGTCCGAGCC TACACAGCCC TCTATTACCT TTCTCAGCTC

TCCGGGACGG AGTCCGAGCC TGCACAGCTC TGTATTATCT TTCTCAACTC

TCCGAGACGG AGTGCGAGCC TACACCGCTC TGCATTATCT TTCTCATCTC

TCCGGGATGG ATTACGAGTC TATACAGCCC TGCATTATCT TTCTCATCTC

TTCGGGATGG AGTACGAGCC TATACAGCTC TGCATTATCT TTCTCATCTC

TTCGGGATGG AGTACGAGCC TACACAGCTC TGCATTATCT TTCTCAGCTC

---------- ---------- ---------- ---------- ----------

TTCGGGATGG AGTACGAGCC TATACAGCTC TGCATTATCT TTCTCATCTC

TTCGGGATGG AATGCGGGCC TATACTGCTC TGCATTACCT TTCTCATCTC

TTCGGGATGG ACTACGAGCC TACACAGCTC TCCATTCCCT TTCTCATCTT

TCCGCGATGG GCTGCGTTCC TATACAGCTT TTCATTCTCT TTCCCATCTC

TCCGTGATGG AGTTCGTGCC TATACAGCTC TGCATTATCT TTCTCATATT

TCTCCTGGAA AATCAGTGCT GATAATGGAT GGAGCAAGTG CATTTGGTAC

TCTCCTGGAA AATCAGTGCT GATAATGGAT GGAGCAAGTG CATTTGGTAC

TCTCCTGGAA AATCAGTGCT GATAATGGAT GGAGCAAGTG CATTTGGTAC

TCTCCTGGAA AATCAGTGCT GATAATGGAT GGAGCAAGTG CATTTGGTAC

TCTGCTGGGA AATCAGTGCT GATAATGGAT GGAGCAAGTG CATTTGGTAC

TCTCCAGGAA AGTCAGTGCT GATCATGGAT GGAGCGAGTG CATTTGGTAC

TCTCCCGGGA AGTCCGTGCT GATCATGGAT GGGGCAAGTG CATTTGGCAC

TCTCCTGGAA AGTCTGTGCT GATCATGGAC GGGGCAAGTG CATTTGGCAC

TCTCCAGGAA AATCAGTGCT GATCATGGAT GGAGCAAGTG CGTTTGGCAC

TCGCCTGGAA AATCCGTGCT GATAATGGAC GGAGCAAGTG CACTTGGTAC

TCTCCTGGAA AATCAGTGCT GATAATGGAT GGAGCCAGTG CACTTGGTAT

TCTCCAGGAA AATCAGTGCT GATAATGGAT GGAGCAAGTG CACTTGGTAC

---------- ---------- ---------- ---------- ----------

TCTCCTGGAA AATCAGTGCT GATAATGGAT GGAGCAAGTG CCTTTGGTAC

TCGCCAGGAA AGTCGGTGCT GATAATGGAC GGAGCAAGTG CATTTGGAAC

GTTCCTGGGA ACTCGGTGCT GATCATGGAT GGAACAAGTG CTTTGGAGAT

TGTCCTGGAA AATCCGTGCT TATAATGGAC GGAGCAAGTG CATTTGGTAC

TCTCCTGGAA AATCAGTGCT GATAATGGAC GGAGCCAGTT CGCTTGGTAT

AATAGCTATT CAGTTAGCAC ATCATAGAGG AGCCAAAGTG ATTTCAACAG

AATAGCTATT CAGTTAGCAC ATCATAGAGG AGCCAAAGTG ATTTCAACAG

AATAGCTATT CAGTTAGCAC ATCATAGAGG AGCCAAAGTG ATTTCAACAG

AATAGCTATT CAGTTAGCAC ATCATAGAGG AGCCAAAGTG ATTTCAACAG

CATAGCTATT CAGTTAGCAC ATCATAGAGG AGCCAAAGTG ATTTCAACAG

AATAGCTATT CAGTTAGCAC ACCATAGGGG AGCCAAGGTG ATTTCAACAG

AATAGCCATT CAGTTAGCAC ACCACAGAGG AGCCAAGGTG ATCTCAACAG

AATAGCCATT CAGTTAGCCC ACCACAGAGG AGCCAAGGTT ATCTCAACAG

AATAGCTATT CAGTTAGCAC ACCATAGAGG AGCCAAAGTG ATTTCAACAG

AATAGCCATT CAGTTAGCAC ACCACAGAGG AGCCAAAGTG ATTTCAACAG

GATAGCTATT CAGTTAGCAC ACCATAGAGG AGCCAAAGTG ATTTCAACAG

GATAGCTATT CAGTTAGCAC ACCATAGAGG AGCCAAAGTG ATTGCGACAG

---------- ---------- ---------- ---------- ----------

AATAGCCATT CAGTTAGCAC ACCATAGAGG AGCCAAAGTG ATTTCAACAG

AATAGCTATT CAGTTAGCAC ACCACAGAGG AGCCAAGGTG ATTTCCACAG

GATAGCTATT CAGTTAGCCC ACCACAGAGG AGCCCCAGTG ATTTCAACAG

AATAGCTATT CAGTTGGCAC TCCATAGAGG GGCCAAAGTT ATTTCAACAG

AATAGCTATT CAGTTAGCAC ACCATAGAGG AGCTAAAGTT ATTTCTACAG

CATGCAGCCT TGAAGATAAG CAGTGCCTTG AAAGATTCAG ACCTCCCATA

CATGCAGCCT TGAAGATAAG CAGTGCCTTG AAAGATTCAG ACCTCCCATA

CATGCAGCCT TGAAGATAAG CAGTGCCTTG AAAGATTCAG ACCTCCCATA

CATGCAGCCT TGAAGATAAG CAGTGCCTTG AAAGATTCAG ACCTCCCATA

CATGCAGCCT TGAAGATAAG CAGTGCCTTG AAAGATTCAG ACCTCCCATA

CATGCAGCCT TGAAGACAAG CAGTTCCTTG AAAGACTTAG GCCTTCTATA

CACACAGCCT GGAGGACAAG CAACATCTTG AAAGGCTTAG GCCTTCTCTA

CACACAGCCT GGAGGACAAG CAACATCTTG AAAGGCTTAG GCCTTCTATA

CATGCAGCCT GGAAGACAAG CAGTGCCTCG AAAGACTTAG GCCTCCTGTA

CCTGCAGCCT TGAAGACAAG CAGTTTCTTG AAAGATTTAG GCCTCCTATA

CAGGCAGCCT TGAAGACAAG CAATGCCTTG AAAGACTTAG GCCTTCTATA

CGTGCAGCCT TGAAGACAAG CAGTACCTGG AAAGACTTAG GCCTTCTATA

---------- ---------- ---------- ---------- ----------

CGTGCAGCCT TGAAGACAAG CAGCACCTTG AAAGATTTAG GCCTGCTATA

CGTGCAGCCT GGAAGACAAG CAGTGCCTTG AAAGCCTTAG GCCTACTATA

CATGCAGCCT GGAAGACAAG CAGTTCCTTG CAAGAGGTAG GCCTACTATA

CCTACAGTCT GGAAGATAAA CAGTATCTTG AAAGACTTAG GCCTGCAATA

CATACAGTCC AGAAGACAAA GAGTACCTTG AAAAGCTGAG GCCAACTGTT

GCCCGAGTGA TTGATGTATC TAATGGGAAA GTTCATGTTG CTGAAAGCTG

GCCCGAGTGA TTGATGTATC TAATGGGAAA GTTCATGTTG CTGAAAGCTG

GCCCGAGTGA TTGATGTATC TAATGGGAAA GTTCATGTTG CTGAAAGCTG

GCCCGAGTGA TTGATGTATC TAATGGGAAA GTTCATGTTG CTGAAAGCTG

GCCCGAGTGA TTGATGTATC TAATGGGAAA GTTCTTGTTG CTGAAAGCTG

GCCCGAGTGA TTGATGTATC TAATGGTAAA GTCCATGTTG CTGAAAGCTG

GCCCGAGTGA TTGATGTGTC TAGTGGGAAA GTCCATGTTG CTGAAAGGTG

GCCCGTGTGA TTGATGTGTC TAATGGGAAA GTCCATGTTG CTGAAAGCTG

GCCCGAGTAA TTGATGTATC TAATGGGAAA GTCCATGTTG CTGAAAGCTG

GCTCGAGTGA TCGATGTTTC TAACGGGAAA GCCCATGTGG CTGAAAGCTG

GCCCGAGTCA TCGACGTTTC TAGTGGGAAA GCCCATGTTG CTGAAAGCTG

GCCCGAGTGA TCGATTTATC TAATGGGAAA GCCCATGTTG CTGAAAGCTG

-CCCGCGTGG TGGATATTTC TAACGGGAAA GCCCATATTG CTGAAAGCTG

GCCCGTGTGA TTGATGTATC TAATGGAAAG ATTCATGTTG CCGAAAGCTG

GCCCGAGTGA TTGATGTATC TAATGGGAAA GTCCATGTTG CTGAAAGCTG

GCCCGAGAGA TCGACATCTC TACTGGGAAA GTCCACGTGG CTGAAAGCTG

GTTCGAGTGA TAGATGTATC TAATGGAAAA GTTGACATTG CTGAAAGCTG

GCTCGAGTGA TAGATGTGTC TAACGGGAAG GTTGCCGTTG CTGAAAGCTG

TTTGGAAGAA ACAGGTGGCC TGGGAGTAGA TATTGTCCTA GATGCTGGAG

TTTGGAAGAA ACAGGTGGCC TGGGAGTAGA TATTGTCCTA GATGCTGGAG

TTTGGAAGAA ACAGGTGGCC TGGGAGTAGA TATTGTCCTA GATGCTGGAG

TTTGGAAGAA ACAGGTGGCC TGGGAGTAGA TATTGTCCTA GATGCTGGAG

TTTGGAAGAA ACAGGTGGCC TGGGAGTAGA TATTGTCCTA GATGCTGGAG

TTTGGAAGAA ACAGGTGGCC TAGGAGTAGA TATTGTCCTA GATACTGGAG

CTTGGAAGAA ACAGGTGGCC TGGGAGTAGA TATTGTCATa gatgcaggag

TTTGGAGGAA ACAGGTGGCC TGGGAGTAGA TATTGTCATA GATGCAGGAG

TTTGGAAGAA ACGGGTGGCC TGGGAGTCGA CATTGTCCTA GATGCTGGAG

TTTAGAAGAA ACAGGTGGCC TCGGAGTGGA CATTGTCCTA GATGCTGGAG

TTTAGAGGAA ACAGGTGGCC TGGGAGTAGA TATTGTCCTA GATGCTGGAG

TTTAGAAGAA ACAGGTGGCC TGGGAGTAGA TATTGTCCTG GATGCCGGAG

TTTAGAAGAA ACAGGTGGCT TGGGGGTAGA TATCGTCCTA GATGCTGGAG

TTTAGAAGAA ACAGGTGGCC TGGGAGTAGA TATTGTCCTA GATGCTGGAG

TTTGGAAGAA ACAGGTGGCC TGGGAGTAGA TATTGTCCTA GATGCTGGAG

TTTGGAAGAG ACAGGTGGCC TAGGAGTTGA TACTGTCCTA GATGCTGGAG

TTTGGAAGAA ACAGGTGGCA TGGGAGTAGA TATTATCCTA GATGCTGGAG

TTTGGAAGAG ACGGGCGGCC TGGGAGTCGA TATCGTTCTA GATGCTGGCG

TGAGATTATA TAGTAAAGAT GATGAACCAG CTGTAAAACT ACAACTACCA

TGAGATTATA TAGTAAAGAT GATGAACCAG CTGTAAAACT ACAACTACCA

TGAGATTGTA TAGTAAAGAT GATGAACCAG CTGTAAAACT ACAACTACCA

TGAGATTGTA TAGTAAAGAT GATGAACCAG CTGTAAAACT ACAACTACCA

TGAGATTATA TAGTAAAGAT GATGAACCAG ATGTAAAATT CCAACTGCCA

TGAGATTGTA TAGTAAAGAT GATGAACCAG CTGCAAAATT ACAACTACCA

TGAGATTATA TAGTAAAGAT GATGAACCAG CTGTAAAACA ACAACTACCA

TGAGATTATA TAGTAAAGAT GATGAACCAG CTGTAAAGTT ACATCTGCCA

TGAGATTGTA TAATAAAGAT GATGAGCCAT CCATGAAGTT ACAGCTACCA

TGAGGTTATA TAGTAAAGAT GACGAACCAG CTGAAAAATT ACAACTACCA

TGAGATTATA TAGTAAAGAT GATGAACCAG CTGTAAAATT ACACCTACCT

TGAGATTATA TAGTAAAGGT GATGAACCAG ATGTAAAATT ACAACTACCA

---------- ---------- ---------- ---------- ----------

TGAGATTATA TAGTAAAGAT GATGAACCAG CTATAAAATT ACAACTGCCT

TGAGATTATA TAGTAAAGTT GATGAACCCA CTGTCAAATT ACAAC---CA

TGAGATTGTA CAGTAAAGAT GATGAGCCTG CTTTAAAACC GCAACTGCCG

TGAGATTATA TAGTAAAGAT GATGAGCCTG CTTTAAAATT ACAACTACCA

TGAGACTGTA TAGTAAAGAA GATGAGTCAC CTTCAAAGTT ACAACTTCCA

CATAAACATG ATATCATCAC ACTTCTTGGT GTTGGAGGCC ACTGGGTAAC

CATAAACATG ATATCATCAC ACTTCTTGGT GTTGGAGGCC ACTGGGTAAC

CATAAACATG ATATCATCAC ACTTCTTGGT GTTGGAGGCC ACTGGGTAAC

CATAAACATG ATATCATCAC ACTTCTTGGT GTTGGAGGCC ACTGGGTAAC

CATAAACATG ATATCATCAC ACTTCTTGGT GTTGGAGGCC ACTGGATAAC

CATAAACATG ATGTTATCAC ACTTCTTGGT GTTGGAGGCC ATTGGGTAAC

CATAAGCATG ACATCATCAC ACTTCTCAGT GTTGGAGGCC ATTGGATAAC

CATAAGCATG ACATCATCAC ACTTCTTGGT GTCGGAGGCC ATTGGGTAAC

CATAAACATG ATATCATCAC ACTTCTTGGT GTTGGAGGCC ATTGGGTAAC

CATAAACATG ATATCATCAC CCTTCTTGGT GTTGGAGGCC ACTGGGTAAC

CATAAACATG ATATCATCAC ACTTCTTGGT GTTGGAGGCC ATTGGGTAAC

CATAAACATG ATATCATCAC ATTGCTTGGT GTCGGAGGCC ATTGGGTAAC

---------- ---------- ---------- ---------- ----------

CATAAGCATG ATATCATCAC ACTTCTTGGT GTTGGAGGTC ATTGGGTAAC

CATAAGCATG ACATCGTCAC ACTTCTAGGT GTTGGAGGCC ACTGGGTAAC

CATAAGCATG ATATCATCGC ACTTCTGGCT GTCGGAGGCC ACTGGGTGAC

CACAAGCATG ATATCATCAC ACTTCTTGGT GTTGGGGGTC ACTGGGTCAC

CACAAGCATG ATATCATCCT GCTTTTGAGT GTCGGTGGGC ACTGGATCAC

AACAGAAGAA AACCTTCAGT TGGATCCTCC AGATAGCCAC TGCCTTTTCC

AACAGAAGAA AACCTTCAGT TGGATCCTCC AGACAGCCAC TGCCTTTTCC

AACAGAAGAA AACCTTCAGC TGGATCCTCC AGATAGCCAT TGCCTTTTCC

AACAGAAGAA AACCTTCAGC TGGATCCTCC AGATAGCCAT TGCCTTTTCC

AACAGAAGAA AATCTTCAGT TGGATCCTCC AGACAGCCAT TGCCTTTTCC

AACAGAAGAA AACCTTCAGT TGGATCCTCC AGACAGCCAT TGCCTTTTCC

GACAGAAGAA AACCTTCAGT TGGATCCTCC AGATAGCCAT TGCCTTTTCC

AACAGAAGAA AACCTTCAGT TGGATCCTCC AGATAGCCAC TGCCTTTTCC

AACAGAAGAA AACCTTCAGT TGGATCCTCC CGATAGTCAT TGCCTTTTCC

AACAGAAGAA AACCTGCAGC TGGATCCTCC AGATAGTCAT TGCCTTTTCC

AACAGAAGAA AACCTGCAGT TGGATCCTCC AGATAGCCAT TGCCTTTTCC

AACAGAAGAA AAACTGCAGT TGGATCCTCC TGACAGCTAC TGCCTTTTCC

---------- ---------C TGGATCCCCC AGACAGCCAT TGCCTTTTCC

AACAGAAGAA AACCTTCAGT TGGATCCTCC AGATAGCCAT TGCCTTTTCC

AAGAGAAGAA AACCTTCAGT TGGATCCTCC AGATAGCCAC TGCCTTTTCC

AAGAGAAGAA AGCCTTCAGT TGGACCCTCC AGACAGCCAC TGCCTTTTGC

CACAGAAGAA AATCTTCAGT TGGATCCTCC GGATAGTCAC TGCCTTTTCC

CTCAGAAAAA AACCTTCAGT TGGACCCTCC AGACAGCAAC TGCCTGTTCC

TCAAGGGAGC AACGTTAGCT TTCCTGAATG ATGAAGTTTG GAATTTGTCA

TCAAGGGAGC AACGTTAGCT TTCCTGAATG ATGAAGTTTG GAATTTGTCA

TCAAGGGAGC AACGTTAGCT TTCCTGAATG ATGAAGTTTG GAATTTGTCA

TCAAGGGAGC AACGTTAGCT TTCCTGAATG ATGAAGTTTG GAATTTGTCA

TCAAGGGAGC AACGGTAGCT TTCCTGAATG ATGAAGTTTG GAATTTGTCA

TCAAGGGAGC AACAGTGGCT TTCCTTAATG ATGAAGTTTG GAATTTGTCA

TCAAGGGAGC AACAGTGGCT TTCCTTAATG ATGAAGTTTG GAATTTGTCA

TCAAGGGAGC AACGGTGGCT TTCCTTAATG ATGAAGTTTG GAATTTGTCA

TCAAGGGAGC AACAGTGGCT TTCCTTAACG ATGAAGTCTG GAATTTGTCC

TCAAGGGAGC AGCAGTGGCT TTCCTTAATG ATGAAGTTTG GAATCTGTCA

TCAAAGGAGC AACGGTGGCT TTCCTTAATG ACGAAGTTTG GAATTTGTCA

TTAAGGGAGC AACGGTGGCT TTCCTGAACG ATGAAGTTTG GAATTTGTCA

TGAAGGGAGC AACTGTGGCT TTCCTTAACG AGGAGGTGTG GAATTTGTCA

TCAAGGGAGC AACACTGTCT TTCCTTAATG ATGAAGTTTG GAATTTGTCA

TCAAGGGAGC AACAGTGTCT TTCCTTAATG ATGAAGTTTG GAATCTGTCA

TCAAGGGAGC AACTGTGTCT TTCCTTAACG AAGAAGTGTG GAATCTTCCA

TTAAGGGAGC CACGGTGTCT TTCCTTAATG ATGAAGTGTG GAATTTGTCG

TCAAGGGAGC CTCCATCTCT TTCCTCAATG ATGAAGTGTG GAACTTGTCA

AATGTACAAC AGGGAAAATA TCTTTGTATC TTAAAGGATG TGATGGAGAA

AATGTACAAC AGGGAAAATA TCTTTGTATC TTAAAGGATG TGATGGAGAA

AATGTACAAC AGGGAAAATA TCTTTGTATC TTAAAGGATG TGATGGAGAA

AATGTACAAC AGGGAAAATA TCTTTGTATC TTAAAGGATG TGATGGAGAA

AATGTACAAC AGGGAAAATA TCTTTGTATC TTAAAGGATG TGATGGAGAA

AATGTACAAC AGGGAAAATA TCTTTGTATC TTAAAAGATG TGATGGAGAA

AATGCACAAC AGGGAAAATA TCTTTGCATC TTAAAAGACG TGATGGAGAA

AATGCACAAC AGGGAAAATA TCTTTGTATC TTAAAAGATG TGATGGAGAA

AACGTTCAAC AGGGAAAATA TCTTT----- ---------- ----------

AATGTACAAC AGGGAAAGTA TCTTTGTATC TTAAAAGATG TGATGGAGAA

AATGTGCAAC AGGGAAAATA TCTTTGTATC CTAAAAGATG TGATGGAGAA

AATGTACAGC AAGGAAAATA TCTTTGTATC TTAAAAGATG TGATGGAGAA

AATGCACAAC AGGGGAAATA CCTTT----- ---------- ----------

AATGTACAAC AGGGGAAATA TCTTTGTATC TTAAAGGATG TGATGGAGAA

AATGTAAAAC AGGGAAAATA CCTTTGTATC TTAAGAGATG TGATGGAGAA

AATGCACAAC AGGGACCCTA CCTCTGTATC TTAAAAGATG TGATGGAGAA

AATGTGCAAC AAGGAAAGTA TCTTTGCATC TTAAAAGATG TAATGGAGAA

AATGTGCAGC AGGGCAAGTA TCTTTGTATC TTAAAAGATG TAATGGAAAA

GTTATCAACT GGTGTTTTCA GACCTCAGTT GGATGAACCC ATTCCACTGT

GTTATCAACT GGTGTTTTCA GACCTCAGTT GGATGAACCC ATTCCACTGT

GTTATCAACT GGTGTTTTCA GACCTCAGTT GGATGAACCC ATTCCACTGT

GTTATCAACT GGTGTTTTCA GACCTCAGTT GGATGAACCC ATTCCACTGT

GTTATCAACT GGTGTTTTCA GACCTCAGTT GGATGAACCC ATTCCACTGT

GTTATCAAAT GGTGTTTTTA Gacctcatat ggatgaagcc attccactgt

GCTATCAGCT GGTGTGTTCA GACCTCTGCT TGATGAACCC ATTCCACTGT

GTTATCAGCT GGTGTTTTCA GGCCTCTGCT CGATGAGCCC ATCCCACTGT

---------- ---------- -ACCACAGTT GGAAGAACCC ATCCCACTCT

GTTATCAACT GGTGTTTTTA GACCTCAGTT GGATGAACCC ATTCCACTAT

GCTATCAACT GGAGTTTTTA GGCCTCAGTT GGATGAACCC ATTCCATTGT

GTTATCGACT GGTGTTTTTA GGCCTCAGTT GGATGAACCC ATTCCACTAT

---------- ---------- ---------- ---------- ----------

GTTATCAACT GGTGTTTTTA Gacctcagct ggatgaacct attccactat

GCTATCAACG GGTGTTTTTA GACCTCAGCT AGATGAACCC ATTCCACTAT

GCTATCAAAT GGTGTTTTCA GACCTCAGTT GGATGAACCC ATTCCACTGT

GCTATCAACT GGTATTTTCA GACCTCTTTT GGATGAACCT GTGCCACCAT

CTTATCAAAT GGCATCTTCA GGCCTATGTT GGATGAACCT GTTCCTCTGT

ATGAGGCAAA AGTTTCCATG GAAGCTGTTC AGAAAAATCA AGGAAGAAAA

ATGAGGCAAA AGTTTCCATG GAAGCTGTTC AGAAAAATCA AGGAAGAAAA

ATGAGGCAAA AGTTTCCATG GAAGTTGTTC AGAAACATCA AGGAAGAAAA

ATGAGGCAAA AGTTTCCATG GAAGCTGTTC AGAAACATCA AGGAAGAAAA

ATGAGGCAAA AGTTTCCATG GAAGCTGTTC AGAAAAATCA AGGAAGAAAA

acgaggcaaa agtttccatg gaaattgttc agaaaaatca aggaagaaaa

ATGAGGCCAA AGTTTCCATG GAAGTTGTTC AGAAAAGTCA AGAAAGAAAA

ATGAGGCAAA GGTTTCCATG GAAGTTGTTC AGAAAAATCA AGAAAGAAAA

ATGAAGCCAA AGTTTCCATG GAAGTTGTTC AGAAAAATCA AGGAAGAAAA

ACGAGGCAAA AGTTACCATG GAAGTTGTTC AGAAAAATCA AGGAAGAAAG

ATGAAGCGAA AGTTTCCATG GAAGTTGTTC AGAAAAATCC AGGAAGAAAA

ATGAAGCAAA AGTTTCTATG GAAGTTGTTC AGAAAAATCA AGGAAGAAAA

---------- ---------- ---------- ---------- ----------

atgaggccaa agtttccatg gaggttattc agaaaaatca aggaagaaaa

ATGAGGCCAA AGTTTCCATG GAAGTTGTTC AAAAAAATGA AGGAAGAAAA

ATGAAGCCAA AGTTTCCATG GAAGTTGTTC AAAAAAATCA AGAAAGAAAA

ATGATGCCAA AGTTTCCATG GAAATTGTTC AGAAAAGTCA AGCAAGAAAA

ATGAAGCCAA AGGGACCATG GAAGTTGTTC AGAGCAATCA AGCAAGAAAA

AAGCAAGTTG TTCAATTTTT TTTAGATTCT AATCAAGAAA ATGATTTGCT

AAGCAAGTTG TTCAATTTTT TTTAGATTCT AATCAAGAAA ATGATTTGCT

AAGCAAGTTG TTCAATTTTT TTTAGATTCT AATCAAGAAA ATGATTTGCT

AAGCAAGTTG TTCAATTTTT TTTAGATTCT AATCAAGAAA ATGATTTGCT

AAGCAAGTTG TTCAGTTTCC TTTAGAGTCT AATCAAGAAA ATGATGTGCT

aagcaagttg ttcaatttTT TTTAGATTCT CATCAAGAAA ATGATTTGCT

AAGCAAGTTG TTCAATTTCT TCTAGATTCT AATGGAGAAA ATAACTTGCT

AAGCAAGTCG TTCAATTTCT TCTAGATTCT AATGAAGAAA ATAACTTGCT

AAGCAAGTTG TTCAATTT-- ---------- ---------- ATGATTTGCA

AAGCAAGTTG TTCAGTTTTT TTTAGATTCT AATCAAGAAA ATGATTTGCT

AAGCAAGTTG TTCAATTTTT TGTAGATTCT AATCAAGAAA ATGATTCGCT

AAGCAAGTTG TTCAATTTTT TTTAGATTCC AATCAAGAGA ATGACTTGCT

---------- ---------- ---------- ---------- ----------

aaacaagttg ttcaatttCT TGTAGATTCT AATCAAGAAA ATGATTTGTT

AAACAGGTTG TTCAATTTGT TTTGGATTCT AATCAAGAAA ATGATTTGCT

AAACAAGTTG TTCAGTTT-- ---------- ---------- ----------

AAACAAGTTA TCCAGTTTTT TTTAGATTTT AATCAAGAAA ATAATGTGAC

AGACAAGTTA TTCAGTTGAA TTCAGATTCT AATCAAGAAA ATGATTTTGC

ATGGGAAGAG AAGTTTCCTG AAAGAACAAC TGTTACTGAA TTACCTCAGA

ATGGGAAGAG AAGTTTCCTG AAAGAACAAC TGTTACTGAA TTACCCCAGA

ATGGGAAGAG AAGTTCCCTG AAAGAACAAC TGTTACTGAA TTACCCCAGA

ATGGGAAGAG AAGTTCCCTG AAAGAACAAC TGTTACTGAA TTACCCCAGA

ACGGGAAGAG AAGGTTCCTG AAAGAACAGC TGTTACTGAA TTACCCGAGA

ATGGGAAGAG ACATTGCCTG AAAGACAAAC TGTTACTCAG TTACCCCAGA

GTGGGAGGAG GCATCTCATG AAAGA---AC TGTCACGGAA CTGTCCCAGA

GTGGGAGGAA TCATCTCATG AAAGA---AC TGGCACGGAA CTGTCCCAGA

GTGGGATGAG ATGAGTCCTG AAAGAACGGC TGTTACTGCC CTTCCCCAGA

CTGGGAAGAG AAGGTTCCTG AAAGAACAGC CATTACAGAA TTACACCAGA

GTGGGAGGAG AAGTTTCCTG AAAGAACAGA CGTTACTGAA TTACTGCAGA

TTGGGAAGAG AAGTTTCCTG AAAGAACAGC CGTTAATGAG TTACTCCAGA

---------- ---------- ---------- ---------- ---------A

GTGGGACGAG AAGTTGCCTG AAAGAACGAC CGTTACTGAG TTACCCAAGA

ATGGGAAGAG AAGTTTCCTG AAA---CAAC CATTACTGAA TTATCCAAGa

---------- ---------- ---------- ---------- ---------G

CTGGAAAGAA GAGTTTTCCG AAAGAACAAC ATCTAAGCAA TTATCCCCGT

CTGGGAAGAA GAGCTTTCAG AAAGAATACC TGCTATAAAG CAATTCAAGC

CTTCACATGT ATCATTCTCC GAGCCTGATA TTCCGTCCTC AAAAAGTACT

CTTCACATGT ATCATTCTCC GAGCCTGATA TTCCGTCCTC AAAAATTACT

CTTCGCATGT ATCGTTCTCC GAGTCTGATA TTCCGTCCTC AGAAAATACT

CTTCACATGT ATCGTTCTCC GAGTCTGATA TTCCGTCCTC AGAAAATACT

CTCCACAAGT ATCATTCTCC GAGTCTGATA TTTCATCCTC AAAAAGTACT

CTCCCCACAT AGTATTCTCC GATTCTGATA TTCCATCCTC AGAAAATACT

GTCAACAGGT AACCTTGTCT GAATCGGACA CTTGGTCTTC AGATGGTACT

GTCAACGTGT ATCGTTGTCT GAATCGGACA CTTGGTCGTC AGATGGTACT

CTCCACAagt agcct---ac aaatctgatg gtccatcttc agaaggtact

CTCCTCATGT ATCATTCTCC GAATCTGATA TTCCACCCTC AGAAAGTACT

CTCCACAAGT ATCATTCTCC GAATCTGATA CTCCATCCTC AGAAAGTACT

CTCCACACGT ATCATTCTCC GAATCTGATA TCCCATCCTC AGAAAGTACT

CTCCACACGT ATCATTTTCT GAATCTGACC TCCCCTCCTC AGGAAGTGCC

CTCTACATGT ATCATTGTCC GAATCTGATA TTCCATCCTC AGAAAGTACT

ctccacctgt gtc---ctcc gaaactgata ttccatcctc agaaaGTGCT

GTTCAGCAGG ATCTTTCTCC GAAATGGATG TTCCATCCTC AGAAAGCACC

CTTCTCATCA ACAGCACTCC ACGTATGATA TTCCATCCTC AGAAAGTGTT

ATCCTGACTC ACCCCTCTCC AAATCTGATA CTTTATCCTC AAGAAGTGCA

GAGTTACCTG TGGACTGGAG TATTAAAACG CGACTCCTTT TCACCTCTTC

GAGTTACCTG TGGACTGGAG TATTAAAACG CGACTCCTTT TCACCTCTTC

GAGTTACCTG TGGACTGGAG TATTAAAACC CGACTCCTTT TCACCTCTTC

GAGTTACCTG TGGACTGGAG TATTAAAACC CGACTCCTTT TCACCTCTTC

GAGTTACCTG TGGACTGGAG TATTAAAACC CGACTCCTTT TCACCTCTTC

GAGTTACCTG TGGACTGGAG TATTAAAACT CGGCTCCTTT TCACCTCTTC

GAGTTACCTG TGGACTGGAG TATTAAAACC CGCCTCCTTT TCACCTCTTC

GAGTTACCTG TGGACTGGAG TATTAAAACC CGACTCCTTT TCACCTCTTC

gagttacctg tggactggag tattaaaacT CGACTCCTCT TCACCTCTTC

GAGTTACCTG TGGACTGGAG CATTAAAACA CGACTCCTTT TCACCTCTTC

GAGTTACCTG TGGACTGGAG TATTAAAACT CGACTCCTTT TCACCTCTTC

GAGTTACCTG TAGACTGGAG TATTAAAACT CGACTCCTTT TCACCTCTTC

GAGTTACCGG CAGACTGGAG TATTAAAACC CGACTCCTTT TCACCTCTTC

GAGTTACCTG TGGACTGGAG TATTAAAACT CGACTCCTTT TTACCTCTTC

GAGTTACCTG TGGACTGGAG TATTAAAACT CGACTCCTTG TCACCTCTTC

AAATTACCCG TGGACTGGAG TATTAAAACT AGACTTCTTT TCACCTCCTC

GAGTTTCCTG TGGATTGGAG TATTAAAACG CGCCTCCTTT TTACCTCTTC

GAGCTTCCGG TAGACTGGAG TATTAAAACT CGCCTCCTTT TCACCTCTTC

TCAACCCTTT ACCTGGGCAG ATCATTTGAA AGCACAGGAA GAAGCTCAAG

TCAACCCTTT ACCTGGGCAG ATCATTTGAA AGCACAGGAA GAAGCTCAAG

TCAACCCTTT ACCTGGGCAG ATCATTTGAA AGCACAGGAA GAAGCTCAAG

TCAACCCTTT ACCTGGGCAG ATCATTTGAA AGCACAGGAA GAAGCTCAAG

TGAACCCTTT ACCTGGGCAG ATCATTTGAA AGCACAGGAA GAGGCTCAAG

TCAACCCTTT ACCTGGGCAG AGCATTTGAA AGCACAGGAA GAGGCCCAAG

TCAACCCTTT TCCTGGGCAG AACATTTGAA AGCACAGGAA GAAGCCCAAG

TCAACCCTTT TCCTGGGCAG ATCATCTGAA AGCACAGGAA GAAGCCCAAG

TCAACCCTTT GCGTGGGCGG ATCATTTGAA AGCACAGGAA GAAGCCCAGG

TCAACCCTTT ACCTGGGCAG ATCATTTGAA AGCCCAGGAA GAGGCTCAAG

TCAACCCTTT ACCTGGGCAG ATCACTTGAA AGCACAAGAA GAGGCCCAAG

TCAACCCTTT ACCTGGGCAG ATCATTTGAA AGCCCAGGAA GAGGCTCAAG

TCACCCCTTC ACCTGGGCAG AGCATTTGAA AGCCCAGGAG GAGGCCCAGG

TCAACCCTTT ACCTGGGCAG ATCATTTGAA AGCACAGGAA GAGGCTCAAG

TCAGCCTTTT GCCTGGGCAG ATCATCTGAA AGCACATGAA GAGGCTCAAG

TCAACCCTTT GCCTGGGCAG ATCATTTGAA AGCACACGAA GAGGCTCAAG

CCAATCTTTT ACCTGGGCAG ATCATTTGAA GGCTCAAGAA GAAGCACAAG

CCAACCTTTC ACCTGGGCAG ATCATTTGAA AGCACAAGAA GAAGCTAGAG

GTCTTGTCCA GCATTGTAGG GCAACAGAAG TTACTTTGCC TAAAAGTATA

GTCTTGTCCA GCATTGTAGG GCAACAGAAG TTACTTTGCC TAAAAGTATA

GTCTTGTCCA GCATTGTAGG GCAACAGAAG TTACTTTGCC TAAAAGTATA

GTCTTGTCCA GCATTGTAGG GCAACAGAAG TTACTTTGCC TAAAAGTATA

GTCTTGTCCA GCATTGTAGG GCAATAGAAG TTACATTGCC TAAAAGTATA

GTCTCATCCA ACATTGTAGG GCAACTGAAG TTACTTTGCC TCAAAGTATA

GACTTGTCCA GCATTGTAGG GCAACAGAAG TTACATTACC ACAGAGTATA

GACTTGTCCA GCATTGTAGG GCAACAGAAG TGACTTTACC CCAGAGTATA

GTCTTGTCCA GCATTGTAGG GCAACAGAAG TTACTTTGCC TCAGAGTATA

GTATCATCCA GCATTGCAGG GCAACAGAAG TTACTTTGCC TCAAAGTATA

GTGTCATCCA GCATTGTAGG GCAACAGAAG TTACTTTGCC TCAAAGTATA

GTGTCGTCCA GCATTGTAGG TCAACAGAAG TTACTCTGCC TCAAAGTATA

GACTTGTCCA GCACTGCCGG GCCACAGAAG TGAGTTTACC CCTGAGTATA

GTCTTGTCCA GCACTGTAGG GCAACGGAAG TTACTTTGCC TCAAAGAATA

GTCTTGTCCA GCACTGTAGG GGGACAGAAG TCACCTTACC TGAACATATT

GTCTCGTACA GCACTGCCGG GCAACAGAAG TTACCTTACC TCAGAGGATA

GCTTTGTCCA ACACTGCAGA GCAACAGAAG TTGATCTGCC TGAGAATATA

GATTTATCCA GCACTGTAGA GCAACAGCAA TTAGTTTGCC TCATAGCATA

CAGGATCCCA AACTCTCCTC TGAGCTCCGT TGTACCTTCC AGCAGAGCCT

CAGGATCCCA AACTCTCCAC TGAGCTCCGT TGTACCTTCC AGCAGAGCCT

CAGGATCCCA AACTCTCCAC TGAGCTCCGT TGTGCCTTCC AGCAGAGCCT

CAGGATCCCA AACTCTCCAC TGAGCTCCGT TGTGCCTTCC AGCAGAGCCT

CAGGATCCCA AACTCTCCAC TGAGCTCCGT TGTACCTTCC AGCAGAGCCT

CAGGATCCCA AACTCTCCAC TGAACTCCGG TGTGCCTTCC AACAGAGCCT

CCGGATCCCA AACTGTCCAC TGTGCTACGT TGTGCCTTCC AGCAGACCCT

CAGGATCCCA AACTGTCCAC TGCGCTGCGC TGCGCCTTCC AGCAGGCTCT

CAGGATCCCA AGCTATCCAC TGAGCTCCGC TGTGCCTTCC AACAGAGCCT

CAGGATCCCA AACTCTCCAC TGAGCTCCGC TGTGCTTTCC AGCGGAGCCT

CAGGATCCCA GACTCTCCAC TCAGCTCCGT TGTGCCTTTC AGCAGAGCCT

CAGGACCCCA AACTCTGCAC TGAGCTTCGT TGTGCCTTCC AACAGAGCCT

CAGGACCCCA AGCTGTGCAC AGAGCTGCGC TGCGCCTTCC AGCAGAGCCT

CAGGATCCTA AATTGTCTAC TGAGCTCCGT TGTGCCTTCC AGCAGAGTCT

CAGGATACCA GGCTCTCCAC CGAGCTCCGC TGTGCCTTCC AGCAGAGCCT

CAG------- ---------- ---------- ---------- ----------

CAGGAGCCAC GACTCTCTGC TGAACTTCGT TGTGCGTTCC AGCAGAACCT

CAGGACCCCA AACTCTCCAC CGAGCTTCGC TGTGCCTTCC AGCAGAGCCT

TATCTATTGG CTCCACCCTG CTTTGTCTTG GCTACCACTG TTCCCTCGTA

TATCTATTGG CTCCACCCTG CTTTGTCTTG GCTACCACTG TTCCCTCGTA

TATCTATTGG CTCCACCCTG CTTTGTCTTG GCTACCACTG TTCCCTCGTA

TATCTATTGG CTCCACCCTG CTTTGTCTTG GCTACCACTG TTCCCTCGTA

TGTCTATTGG CTCCACCCTG CTTTGTCTTG GCTACCACTG TTCCCTCGTA

TATTTATTGG CTCCACCCTG CCTTCCCTTG GCTACCACTG TTCCCTCGTA

GGTCTACTGG CTTCACCCTG CCTTTTCATG GCTGCCACTC TTCCCTCGAA

GGTCTACTGG CTTCACCCTG CCTTTTCGTG GCTGCCACTC TTCCCTAGAA

TATCTATTGG CTCCACCCTG CCTTGTCTTG GCTACCACTG TTTCCTCGCA

CATCTATTGG CTCCACCCTG CCTTGTCTTG GCTGCCATTG TTCCCTCGTA

AATCTATTGG TTGCACCCTG CCTTTCCCTG GCTACCACTG TTCCCTCGCA

TATCTATTGG CTCCACCCTG CCTTTCCGTG GCTGTCAATG TTCCCTCGGA

CGTGTACTGG CTGCACCCGG CCACACCCTG GCTCCCGCTC TTCCCACGGA

TATCTATTGG CTCCATCCTG CCTTGCCATG GCTACCTCTG TTCCCTCGTA

CTTCTATTGG CTTCACCCTG CCTTGCCTTG GCTGCCACTA TTCCCTCGTA

---------- ---------- ---------- ---------- ----------

TATTTATTGG CTTCATCCCT CTCTGCCATG GTTACAGCTT TTCCCACGCA

CGTGTATTGG ATCCACCCGT CACTGCCCTG GATGCATTTG TTTCCACGGA

TTGGAGCTGA TAGAAAAATG GCTGGAAAGA CAAGTCCTTG GTCAAATGAT

TTGGAGCTGA TAGAAAAATG GCTGGAAAGA CAAGTCCTTG GTCAAATGAT

TTGGAGCTGA TAGAAAAATG GCTGGAAAGA CAAGTCCTTG GTCAAATGAT

TTGGAGCTGA TAGAAAAATG GCTGGAAAGA CAAGTCCTTG TTCAAATGAT

TTGGAGCTGA TAGAAAAATG ACTGGAAAGA CAAGTCCTTG GTCAAATGAT

TTGGAGCTGA TAGGAAAATG ACTGGAAAGA CAAGCCCATG GTCAAATGAT

TTGGAGCTGA TAGAAAAATG GCTGCAAAGA CAAGTCCATG GTCAACGGAT

TTGGAGCTGA TAGGAAAATG GCTGCAAAGA CAAGTCCGTG GTCAGCTGAT

TTGGAGCTGA TAGGAAAATG GCTGGAAAGA CAAGTCCTTG GTCAAATGAT

TCGGAGCTGA CAGAAAAATG GCTGGAAAGA CAACCCCGTG GTCAAATGAT

TTGGAGCTGA TAGAAAAATG GCTGGAAAGA TAAGCCCTTG GTCAAATGAT

TTGGAGCTGA TAGAAAAATG GCTGGAAAGG CAAGCCCTTG GTCAAACGAT

TTGGTGCCGA GAGGAAGATG GCCGGCAAGA ACAGCCCCTG GGCCACTGAC

TTGGAGCTGA TAGAAAAATG ACTGGAAAGA CAAGCCCTTG GTCAAATGAT

TAGGAGCGGA TAGAAGAATG GCTGGAAAGA CCAGCCCATG GTCAAATGAT

---------- ---------- ---------- ---------- ----------

TTGGAGCTGA CAGAAAGATG GCTGGAAAAA CAAGTCCTTG GTCAAATGAT

TTGGGGCCGA TAGGAAAATG GTCGGGAAGA ACAGTCCTTG GTCCCAGGAT

GCAACCCTGC AGCATGTTTT AATGAGTGAC TGGTCTGTGA GCTTTACTTC

GCAACCCTGC AGCATGTTTT AATGAGTGAC TGGTCTGTGA GCTTTACTTC

GCAACCCTGC AGCATGTTTT AATGAGTGAC TGGTCCGTGA GCTTTACTTC

GCAACCCTGC AGCATGTTTT AATGAGTGAC TGGTCCGTGA GCTTTACTTC

GCAACCCTGC AGCATGTTTT GATGAGTGAC TGGTCTGTGA GCTTTACTTC

GAAACCCTAC AACAAATTTT AATGAGTGAC TGGTCTGTGA GCTTTACTTC

GAAAGCCTGC AGCATGCTTT AATGAGTGAC TGGTCTGTGA GTTTTACGTC

GAAACCCTGC AGCATGCTTT AATGAGCGAC TGGTCTGTGA GTTTCACATC

GAAACCCTAC AACACATTTT AATGAGTGAC TGGTCTGTGA GCTTTACTTC

GAAACCCTGC AGCATGTGTT AATGAGTGAC TGGTCTGTGA GCTTTACTTC

GAAACCTTGC AACACATTTT AATGAGTGAC TGGTCTGTGA GCTTCACTTC

GAAACCCTGC AACACGTTTT AATGAGTGAC TGGTCTGTGA GCTTCACTTC

GAAGAGCTGC AGCATATTTT AATGAGTGAC TGGTCTGTGA GCTTTACCTC

GAAACCCTAC AACATATTTT AATGAGTGAC TGGTCTGTGA GTTTTACTTC

GAAACCCTAC AACACGTTTT AATGAGCGAC TGGTCTGTGA GCTTTACTTC

---------- ---------- ---------- ---------- ----------

GAGGAACTAC AACAAATTTT AATGAGTGAA TGGTCCATCA GCTTCACTTC

GAGACGCTGC AGCAAGTGCT GATGAGCGAA TGGTCCATCA GTTTCACTTC

TCTATATAAT TTGCTGAAGA CAAAACTTTG CCCCTATTTC TACGTTTGTA

TCTATATAAT TTGCTGAAGA CAAAACTTTG CCCCTATTTC TACGTTTGTA

TCTATATAAT TTGCTGAAGA CAAAACTTTG CCCCTATTTC TACGTTTGTA

TCTATATAAT TTGCTGAAGA CAAAACTTTG CCCCTATTTC TACGTTTGTA

TCTATATAAT CTTCTGAAGA CAAAACTTTG CCCCTATTTC TACGTTTGTA

TCTCTATAAT CTGCTGAAGA CAAAACTTTG CCCCTATTTC TACGTTTGTA

TTTATATAAC CTGCTGAAGA CGAAGCTTTG CCCCTATTTC TACGTTTGTT

TCTGTATAAC CTGCTGAAGA CAAAGCTTTG TCCCTATTTC TATGTCTGCT

TCTGTACAGC CTACTGAAGA CCAAGCTCTG CCCCTATTTC TACGTTTGCA

TCTATATAAC CTGCTGAAGA CAAAACTTTG CCCTTATTTC TACGTTTGTA

TCTCTATAAT CTACTGAAGA CAAAGCTTTG CCCATATTTC TACGTTTGTA

TCTATATAAT CTGCTGAAGA CAAAACTTTG CCCCTATTTC TACATTTGTA

TCTTTATAAT CTGGTGAAGA CCAAACTCTG CCCCTATTTC TACGTTTGCA

TCTATATAAT CTACTGAAGA CAAAACTTTG CCCCTATTTC TACGTTTGTA

TCTGTATAAT CTGCTGAAAA CAAAGCTTTG CCCCTATTTC TACGTTTGTA

---------- ---------- ---------- ---------- ----------

TTTGTATAAT CTACTGAAGA CAAAACTTTG TCCCTATTTC TATGTTTGTA

TCTCTATAAC CTGCTGAAGA CAAAGCTTTG CCCCTATTTT TATGTGTGCA

CCTATCAGTT TACTGTCCTG TTCCGAGCAG CAGGATTAGC TGGAAGTGAC

CCTATCAGTT TACTGTCCTG TTCCGAGCAG CAGGATTAGC TGGAAGTGAC

CCTATCAGTT TACTGTCCTC TTCCGAGCAG CAGGATTAGC TGGAAGTGAC

CCTATCAGTT TACTGTCCTC TTCCGAGCAG CAGGATTAGC TGGAAGTGAC

CCTATCAGTT TACTGTCCTG TTCCGAGCAG CAGGATTAGC TGGAAATGAC

CCTATCAGTT TACTATCCTA TTCCGAGCAG CAGGATTAGC AGGAAGTGAT

CCTATCAGTT TACGGTCCTG TTCCGGGCAG CAGGATTAGC AGGAAGCAGT

CCTATCAGTT CACCGTCCTG TTCCGGGCAG CAGGACTGGC CGGAAGCAGT

CTTACCAGTT CACGGTCCTG TTTCGAGCAG CAGGGTTAGC AGGAAGTGAT

CCTATCAGTT TACTATCCTG TTCCGTGCAG CAGGGTTAGC AGGAGATGAT

CCTATCAGTT TACGATCTTG TTCCGAGCAG CAGGATTAGC AGGAAATGAT

CCTATCAGTT TACTGTCCTG TTCCGTGCAG CAGGATTAGG AGGAAATGAT

CGTATCAGTT TACGATCCTG TTCCGTGCAG CAGGACTGGC CGGCAGTGAC

CCTATCAGTT TACTGTCCTC TTCCGTGCAG CAGGATTAGC AGGAAATGAT

CTTACCAGTT CACTGTCCTT TTCCGGGCAG CAGGATTTGC AGGAAGTGAT

---------- ---------- ---------- ---------- ----------

CCTACCAATT CACTGTGCTG TTTCGTGCAG CAGGGCTAGC AGGAAGTGAT

CCTATCAATT TACCGTGCTG TTCCGTGCTG CTGGATTAGG AGGATGTGAT

TTAATCACAG CTCTCATATC TCCAACAACT CGAGGTTTAA GAGAAGCTAT

TTAATCACTG CTCTCATATC TCCAACAACT CGAGGTTTAA GAGAAGCTAT

TTAATCACAG CTCTCATATC TCCTACAACT CGAGGTTTAA GAGAAGCTAT

TTAATCACAG CTCTCATATC TCCTACAACT CGAGGTTTAA GAGAAGCTAT

TTAATCACAG CTCTTGTATC TCCTACAACT CGAGGTTTAA GAGAAGCTAT

GTGATCACAG CTCTCATATC TCCTACAACT CGAGGTTTAA GAGAAGCTAT

GTGATCACAG CGCTCATCAC CCCTACAACT AGAGGCTTAA GAGAAGCTAT

GTGATCACAG CGCTCATATC CCCTACGACA AGAGGCTTAA GAGAAGCTAT

GCAGTCACGG CTCTTATTTC TCCTACAACT AGAGGTCTGA GAGAAGCCAT

GTAATCACGG CTCTCATATC TCCTACAACT AGAGGTATAA GAGAAGCTAT

GTAATCACAG CTCTCATCTC TCCTACAACT AGAGGCATAA GAGAAGCTAT

GTAATCACAG CTCTCATATC TCCTACAACT AGAGGTATAA GAGAAGCTAT

GTCCTCACTG CTCTCATCTC TCCTACCACC AGAGGAATCA GAGAAGCGAT

GTAATCACAG CTATCCTATC TCCCACAACT AGAGGTTTAA GAGAAGCTAT

GTCATCACAG CTGTCATATC TCCCACAACT AGAGGTTTAA GGGAAGCTAT

---------- ---------- ---------- ---------- ----------

GTAATTACAG CTGTCTTGTC ACCCACTACC AGAGGCTTAA GAGAAGCTAT

GTAATCACAG CTGTCATGTC CCCCACTACC AGAGGTTTAA GAGAAGCTAT

GAGAAATGAA GGTATTGAAT TTTCTCTGCC TTTAATAAAA GAAAGTGGCC

GAGAAATGAA GGTATTGAAT TTTCTCTGCC TTTAATAAAA GAAAGTGGCC

GAGAAATGAA GGTATTGAAT TTTCTCTGCC TTTAATAAAA GAAAGTGGCT

GAGAAATGAA GGTATTGAAT TTTCTCTGCC TTTAATAAAA GAAAGCGGCT

GAGAAATGAA GGTATTGAAT TTTCTCTGCC TTTAATAAAA GAAAGTGGCC

GAAAAATGAA GGTATTGAAT TTTCTCTGCC TTTAATAAAA GAAGGTGGCC

GAAAAGTGAA GGTATTGAAT TTTCTCTGCC TTTACTAGAA GAGAGTGGTC

GAGAAACGAA GGTATTGAAT TTTCTCTGCC TTTACTAGAA GAAATTGGCC

GAAAAATGAA GGTATTGAAT TTTCTCTGCC TTTAATAAAA GAAAATGGCC

GAAAAACGAA GGTATTGAGT TTTCTCTGCC TTTAATAAAA GAAAATGGCC

GAAAAATGAA GGTATTGAAT TTTCTCTGCC GCTAATAAAA GAAAATGGCC

GAAAAATGAA GGTATTGAAT TTTCTCTGCC TTTGATAAAA GCAAATGGCC

GAAAAATGAA GGTATTGAAT TTTCTTTGCC TTTAATAAAA GAAAACGGCC

GAAAAATGAA GGTATTGAAT TTTCTCTGCC TTTAATAAAA GAAAATGGCC

GAAAAATGAA GGTATTGAAT TTTCTCTGCC TTTAATTAAA GAAAATACCC

---------- ---------- ---------- ---------- ----------

GAAAAATGAA GGAATTGAAT TTTCTTTGCC TTTATTAAAA GAAAATGGTC

GAAGAACGAA GGTATTGAAT TTTCCTTGCC TTTAATAAAA GAACATGGGT

ATAAGAAGAC AGCATCTGGA ACAAGCTTGG GATATGGGGA GGAGCAAGCC

ATAAGAAGAC AGCATCTGGA ACAAGCTTGG GATATGGGGA GGAGCAAGCC

ATAAGAAGAC AGCATCTGGA ACAAGCTTGG GATATGGGGA GGAGCAAGCC

ATAAGAAGAC AGCATCTGGA TCAAGCTTGG GATATGGGGA GGAGCAAGCC

ATAAAAAGAC AGCATCTGGA ACAAGCCTGG GATATGGGGA GGAGCAAACC

GTAAGAAGAG AGCATCTGGA ACAAGCTTGG AATATGGGGA GGAGCAAGCA

ATAAGGAGAA AGTTCGAGAA CCAAGCCTGG AATCTGAGGA GGAGCAGGCA

ATAAGAAGAA AGTTCGAGAC CCAAGCCTGG AATCTGAGGA GGAGCAGGCA

GAAAGAAGGA AACATCTGGA ACAAGCTTGG GACATGCGGA AGAGCAAGCA

CTGAGAAGAA AGCGTCTGGA ACAGGCTTGC GACACGGGGA GGAGCAAGCC

ATGAGAAGAA GGCATCTGGA ACAAGCCTGG GACATGGGGA GGAACAAGTA

ATAAGAAG-A AGCATCTGGA ACAAGCTTGG GACATGGGGA GGAGCAAGCA

GCCCGAAG-A AGCCCCTGAA GCAAGCTCGG AGCATGGAGA GGTGCAAGCG

ATAAGAAGAA AGCATCTGGA ACAAGCTTGG GACATGGGGA GGAACAAGCA

ATAAGAAGAA AACATCTGGA GCAAGCTTGG GACATGGGGA GGAGCAACCA

---------- ---------- ---------- ---------- ----------

AACGGAAGAA AGCATCTGAG ACAAATTTAG AACCAGAAGA GGAACAACTT

CTAGGAAGAA GGCACCCGAG ACAAGCTTAG AGCCAGAAGA GGAACAAGCA

ATCAGTGATG AGGATGAAGA GGAAAGTTTT TCCTGGCTGG AAGAGATGGG

ATCAGTGATG AGGATGAAGA GGAAAGTTTT TCCTGGCTGG AAGAGATGGG

ATCAGTGATG AGGACGAAGA GGAAAGTTTT TCCTGGCTGG AAGAGATGGG

ATCAGTGATG AGGACGAAGA GGAAAGTTTT TCCTGGCTGG AAGAGATGGG

ATCAGTGATG AGGACGAAGA GGAAAGTTTT TCCTGGCTGG AAGAGATGGG

ATCAGTGATG AAGATGAAGA GGAAAGTTTT TCCTGGCTGG AAGAGATGGG

GTCAGTGACG AAGATGAAGA GGAAAGTTTG TCTTGGCTGG AAGAGATCGG

GTCAGTGACG AAGATGAAGA GGAGAGTTTT TCTTGGCTGG AAGAGATAGG

ATCAGTGATG AGGATGAAGA GGAAAGTTTT TCCTGGCTGG AAGAAATGGG

ATCAGTGATG AGGATGAAGA AGAAAGTTTT TCCTGGCTGG AAGAGATGGG

ATCAGTGATG AGGATGAAGA TGAGAGCTTT TCCTGGCTGG AAGAGATGGG

ATCAGTGATG AGGATGAAGA AGAAAGTTTT TCCTGGCTAG AAGAGATGGG

ATTAGTGATG AGGATGAAGA TGAAAGTTTT TCCTGGCTGG AAGAGATGGG

ATCAGTGATG AAGATGAAGA AGAAAGTTTT TCCTGGCTGG AAGAGATGGG

ATCAGTGATG AGGATGAAGA GGAAAGTTTT TCCTGGCTGG AAGAGATGGG

---------- ---------- ---------- ---------- ----------

ATTAGTGATG AGGATGAAGA GGAAAGTTTT TCCTGGTTGG AAGAGATGGG

ATAAGTGATG AAGATGAAGA GGAAAGTTAT TCTTGGCTCG AAGAGATCGG

TGTGCAAGAT AAAATTAAAA AGCCAGACAT ACTTTCTATC AAGCTGCGTA

TGTGCAAGAT AAAATTAAAA AGCCAGACAT ACTTTCTATC AAGCTGCGTA

TGTGCAAGAT AAAATTAAAA AGCCAGACAT ACTTTCTATC AAGCTTCGTA

TGTGCAAGAT AAAATTAAAA AGCCAGACAT ACTTTCTATC AAGCTTCGTA

AGTGCAAGAT GAAATTAAAA AGCCAGACAT ACTTTCTATC AAGCTTCGTA

TGTGCAAGAT AAAATTAAAA AACCAGACAC ACTCTCTATC AAGCTACGGA

TGTGCAGGAT CAAATTAAGA AGCCAGACTT GATCTCTGTC AAGCTGCGTA

CGTGCAAGAT CAAATTAAGA AGCCAGACGT GATCTCTATC AAGCTACGTA

TGTGCAAGAT AAAATTAAAA AGCCAGACAC ACTATCAATC AAGCTCCGTA

TGTCCAAGAT AAAATTAAAA AACCAGACGT GCTTTCTATC AAGCTGCGTA

TGTGCAAGAT AAAATTAAAA AACCAGACAT ACTCTCTATC AAGCTACGAA

TGTGCAAGAT AAAATTAAAA AACCAGATAT ACTGTCTATC AAGCTGCGTA

TGTGCAAGAT AAAATTAAAA AGCCAGACAG ACTTTCTATC AAATTACGGA

TGTGCAAGAT AAAATTAAAA AACCAGACAT ACTCTCTATC AAGCTGCGGA

TGTGCAAGAT AAAATTAAAA AACCTGACAT ACACTCTATC AAGTTACGTA

---------- ---------- ---------- ---------- ----------

TGTACAGGAT AAGATAAGAA AACCAGACA- --TCACTCTC AAACTTCGAA

AGTAAAAGAT AAAATTAAGA AACCGGACCT GGTCTCTGTT AAGCTCCACA

AAGAGAAACA TGAAGTACAA ATGGATCACA GACCTGAATC TGTTGTGTTG

AAGAGAAACA TGAAGTACAA ATGGATCACA GACCTGAATC TGTTGTGTTG

AAGAGAAACA TGAAGTACAG ATGGATCACA GACCTGAGTC TGTTGTGTTG

AAGAGAAACA TGAAGTACAG ATGGATCACA GACCTGAGTC TGTTGTGTTG

AAGAGAAACA TGAAGTACAA ATGGATCACA GACCTGAGTC TGTTGTGTTC

AAGAGAAACA TGAAGTACAA ATGGATCATA GACCTGAGTC TGTTGTGTTG

AAGAAAAACA TGAAGTACAG ATGGATCACC GACCAGAGTC TGTCGTACTG

AAGAAAAACA TGAAGTACAA ATGGACCACA GACCTGAGTC TGTCGTACTG

AAGAGAAGCA TGAAGTACAA ATGGACCACA GGCCTGAGTC TGTTGTGTTG

AAGAGATACA TGAAGTACAA ATGGATCACA GACCAGAGTC TGTTGTGTTG

AAGAGAAACA CGAAGTGCAA ATGGATCACA GACCTGAGTC TGTTGTGTTG

AAGAGAAACA TGAAGTACAA ATGGATCACA GACCTGAGTC TGTTGTGCTG

AAGAAAAACA TGAAGTGCAA ATGGATCATC GACCTGAGTC TGTCGTGTTG

AAGAGAAACA TGAAGTACAA ATGGATCACA GACCTGAGTC AGTTGTATTG

AAGAGAAACA CGAAGTACAA ATGGATCACA GGCCTGAGTC TGTTGTATTG

---------- ---------- ---------- ---------- ----------

AAGAGAAAAA GGAAGTGCAG CTGGATCACA AGCCCGAGTC TGTTGTGCTG

GAGAGAAACA CGAGGTGCAG ATGGATCACA AACCCGAGTC TGTCGTCCTG

GTAAAAGGAA TCAACACCTT TACATTGCTC AATTTTTTGA TTAACTCTAA

GTAAAAGGAA TCAACACCTT TACATTGCTC AATTTTTTGA TTAACTCTAA

GTAAAAGGAA TCAACACCTT TACATTGCTC AATTTTTTGA TTAACTGTAA

GTAAAAGGAA TCAACACCTT TACATTGCTC AATTTTTTGA TTAACTGTAA

ATGAAAGGAA TCAACACCTT TACATTGCTC AATTTTTTGA TCAACTCTAA

GTGAAAGGAA TCAACACCTT CACGTTGCTC AATTTTTTGA TGAATTGTAA

GTGAAAGGAC TCAATACCTT CAAACTGCTC AACTTTTTGA TCAATTGCAA

GTGAAAGGAC TCAACACCTT CAAACTGCTC AATTTTTTGA TCAACTGCAA

GTGAAAGGAA TCAACACTTT TACGTTACTC AATTTTTTGA TCAACTGCAA

GTGAAGGGAA TGAATACCTT TACACTGCTA AATTTTTTGA TCAACTGTAA

GTGAAGGGAA TGAATACCCT TACATTGCTG AATTTCTTGA TCAACTCTAA

GTGAAGGGAA CGAACACCTT TACATTGCTC AATTTTTTGA TCAACTGTAA

GTGAAGGGAA TGAACACTTT GATGTTGCTG AATTTTTTGA TCAACTGTAA

GTGAAAGGAA TCAACACCTT TACCTTGCTC AATTTCTTGA TCAACTGTAA

GTGAAAGGAA TCAACACCTT TACGTTGCTC AATTTTTTGA TCAACTGTAA

---------- ---------- ---------- ---------- ----------

GTGAAAGGAA TCAACACCTT TACCCTGCTG AACTTCCTGA TAAATTGCAA

GTGAAAGGAA TGAACACTTT CCGGTTGTTG AACTTCTTAA TAAACTGTAA

GAGTTTAGTT GCTACCTCAG GTCCACAGGC AGGACTTCCT CCAACCCTCT

GAGTTTAGTT GCTACCTCAG GTCCACAGGC AGGACTTCCT CCAACCCTCT

GAGTTTAGTT GCTACCTCAG GTCCACAGGC AGGACTTCCT CCAACCCTCT

GAGTTTAGTT GCTACCTCAG GTCCACAGGC AGGACTTCCT CCAACCCTCT

GAGTTTAGTT GCTACCTCAG GTCCACAGGC AGGCCTTCCT CCAACCCTCT

GAGTTTAGTT GCTACCTCAG GTCCACAGGC AGGACTTCCA CCAACCATCT

GAGTTTAGTT GCTACCTCAG GCGCACAGGC AGGTCTCCCA CCCACTCTCT

GAGTTTAGTT GCTACCTCAG GTGCACAGGC AGGTCTCCCA CCCACTCTCT

GAGTTTAGTT GCAACCTCAG GCCCGCAGGC AGGACTTCCA CCAACCCTGC

GAGTTTAATT GCTACCTCAG GTCCACAGGC AGGACTTCCA CCAACCCTCT

GAGTTTAATT GCTAGTTCAG GTCCGCAGGC AGGACTTCCA CCAACCCTCT

GAGTTTAATT GCTACTTCAG GTCCACAGGC AGGACTTCCA CCAACCCTCT

GAGTTTAGTT GCTAGCTCTG GTCCTCAGGC AGGACTGCCA CCAACCCTCT

AAGTTTAGTT GCTACCTCTG GTCCACAGGC AGGACTTCCA CCAACATTGT

AAGTTTAGTT GCCACCTCAG GTCCACAAGC AGGACTTCCA CCCACCCTGT

---------- ---------- ---------- ---------- ----------

GAGTGTAGTT GCTACGTCGG GGCCTCAGGC AGGCCTCCCC CCAACACTAT

GAGCCTAGTA GCCTCTGCGG GTCCTCAGGC AGGACTCCCA CCAACCCTGC

TGTCCCCTGT TGCTTTCCGA GGTGCCACAA TGCAAATGCT TAAGGCACGG

TGTCCCCTGT TGCTTTCCGA GGTGCCACAA TGCAAATGCT TAAGGCACGG

TGTCCCCTGT TGCTTTCCGA GGTGCCACAA TGCAAATACT TAAGGCACGA

TGTCCCCTGT TGCTTTCCGA GGTGCCACAA TGCAAATACT TAAGGCACGA

TATCCCCTGT TGCTTTCCGA GGTGCCACAA TGCAAATGCT TAAGGCACGA

TAGCCCCTGT GGCTTTCCGA GGTGCCACAA TGCAAATGCT TAAGGCACGA

TATCCCCAGT TGCCTTTCGA GGTGCCTCAA TGCAAATGCT TAAGGCACGA

TATCCCCAAT AGCCTTCCGA GGTGCCTCAA TGCAAATGCT TAAGGCACGG

TGTCCCCTAT AGCTTTCCGA GGTGCCACGA TGCAAATGCT TAAGGCACGA

TATCCCCAGT AGCTTTCCGA GGTGCCACAA TGCAAATGCT TAAGGCTCGA

TATCACCTGT AGCTTTCCGA GGTGCCACAA TGCAAATGCT AAAGGCAAGA

TATCCCCAGT AGCTTTCCGA GGAGCCACAA TGCAAATGCT TAAGGCACGA

TATCCCCTGT AGCCTTTCGA GGTGCCACAA TGCAAATGCT AAAGGCACGA

TATCCCCTAT AGCTTTTCGA GGTGCCACAA TGCAAATGCT TAAGGCACGA

TATCCCCTGT AGCTTTCAGA GGTGCCACAA TGCAAATGCT TAAGGCACGA

---------- ---------- ---------- ---------- ----------

TGTCTCCAGC AGCATTCCGG GGTGCCACCA TGCAAATGCT CAAGGCACGA

TGTCCCCTAC TGCTTTCCGA GGAGCCACGA TGCAGATGCT CAAGGCTCGA

AGTGTGAATG TGAAGACACA AGCTCTTTCT GGATACAGAG ACCAATTTAG

AGTGTAAATG TGAAGACACA AGCTCTTTCT GGATACAGAG ACCAATTTAG

AGTGTAAATG TGAAGACACA AGCTCTTTCT GGATACAGAG ACCAGTTTAG

AGTGTAAATG TGAAGACACA AGCTCTTTCT GGATACAGAG ACCAGTTTAG

AGTGTAAATG TGAAGACACA AGCTCTTTCT GGATACAGAG ACCAATTTAG

AGTGTAAATG TGAAGACACA AGCTCTTTCT GGATATAAAG CTCAGTTTAG

AGCATTAATG TAAAGACACA AGCTCTTTTT GGATACAGAG ATAAGTTTAG

AGCAGTAATG TAAAGACACA AGCTCTTTCT GGATACAGAG ATAAGTTTAG

AGTGTGAATG TGAAGAGCCA AGCTCCTTCT GGATACAAAG ATCAGTTTAG

AGTGTGAATG TGAAGACACA AGCTCTTTCT GGATACAGAA ATCAGTTTAG

AGTGTAAATG TGAAGACACA AGCTGTTTCT GGATACAAAG ATCAATTTAG

AGTATAAATG TGAAGGCACA AGGTCTTTCT GAATACAAAG ATCAATTTAG

AGTGTGAATG TGAAGGCACA TGCCCACGCT GGTTATAAAG ATCAGTTTAG

AGTATAAATG TGAAGACACA AGTTCATTCT GGATACAAGG ATCAATTTAG

AGTGTCAATG TGAAGACGCA AGCTCTTTCT GGATACAAAG ATCAATTTAG

---------- ---------- ---------- ---------- ----------

ACGGTGAATG TGAAGACACC AGTTTATTCT GGTTATAAAG ATCAGTTTAG

ACTATAAATG TGAAAAGACA AGTAGATGCT GGTTTTAAAG ATCAATTTAG

TTTGGAGATT ACAGGTCCTA TCATGCCTCA TTCTCTGCAT TCACTGACCA

TTTGGAGATT ACAGGTCCTA TCATGCCTCA TTCTCTGCAT TCACTGACCA

TTTGGAGATT ACAGGTCCTA TCATGCCTCA TTCTCTGCAT TCACTGACCA

TTTGGAGATT ACAGGTCCTA TCATGCCTCA TTCTCTGCAT TCACTGACCA

TTTGGAGATT ACAGGTCCTA TCATGCCTCA TTGCCTGCAC TCACTGACCA

TTTGGAAATC ACAGGTCCTG TCATGCCCCA TTCTCTACAT TCTGTAACCA

TTTGGAGATC ACAGGTCCTG TCATGCCTCA CGCTTTGCAT TCTGTGGCCA

TTTGGATATC ACAGGGCCTG TCATGCCTCA TGCATTGCAT TCTATGAGCA

TTTGGAGATT ACAGGTCCTA TCCTGCCTCA TTGTGTGCAT TCCGTCACCA

CTTGGAGATT ACAGGCCCCA TTATGCCTCA TTCTCTACAC TCTGTGACGA

TTTGGAGATT ACGGGTCCTA TCATGCCTCA TTCTCTACAT TCAGTAACCA

TTTGGAGATT ACAGGACCTA TCATGCCTCA TTGTCTATAT TCTATGACCA

TTTGGAGATC ACCGGTCCTG TCCTGCCTCA CTGTCTCCAT TCTGTGATGA

TTTGGAGATT ACAGGGCCTG TCATGCCTCA TTCTCTACAT TCTGTGACAA

TTTGGAGATT ACAGGTCCTG TCATGCCACA TTCTCTCCAT TCTGTGACCA

---------- ---------- ---------- ---------- ----------

TTTGGAGATT ACAGGCCCTG TCATGCCTCA TTCTCTGCAC TCATTGACTA

TTTGGAGATT ACAGGCCCCA TCATGCCTCA CTCTCTGCAT TCACTGACTA

TGCTGCTCAA ATCTTCACAG AGTGGATCTT TCTCTGCAGT ACTGTATCCA

TGCTGCTCAA ATCTTCACAG AGTGGATCTT TCTCTGCAGT ACTGTATCCA

TGCTGCTCAA ATCTTCACAG AGTGGGTCTT TCTCTGCAGT ACTATATCCA

TGCTGCTCAA ATCTTCACAG AGTGGGTCTT TCTCTGCAGT ACTATATCCA

TGCTGCTCAA ATCTTCACAG AGCGGGTCTT TCTCTGCACT ACTATATCCA

TGCTACTGAG ATCTTCACAG AGTGGGGCTT TCTGTGTCGG ACTGTATCCA

TGCTACTCCG ATCTTCACAG AGAGGCTCAT TCTCTGCGGG ATTGTATGCA

TGCTACTCCG ATCTTCACAG AGAGGGTCCT TCTCTGCAGG ACTGTATGCA

TGCTGCTCCA GTCTTCACAA AGCGGGTCCT TCTCGGCAGG ACTCTATCCA

TGCTACTCCA GTCTTCACAG AATGGGTCGT TTTCTGCAGG ACTGTATACA

TGCTACTCAG ATCTTCACAG AATGGGTCAT TTTCTGCTGG ACTATATACA

CGCTACTCAA ATCTTCACAG AGTGGGTCAT TTTCTGCAGG GCTGTATACC

TGCTTCTCAG ATCTGCACAG AGTGGGTCAT TTACTGCAGG ACTGTATACA

CACTACTCAG ATCTTCGCAG AGTGGATCAT TTTCTTCAGG ATTGTATACT

TGCTACTCAA ATCTTCACAG GCTGGGTCTT TTTCAGCAGG ACTGTATACA

---------- ---------- ---------- ---------- ----------

TGCTGCTCAA ATCTGCACAA AAAGGATCTT TTTCGGCAAG ATTATATACT

TGCTACTCAA GTCTGCACAG AAGGGCTCTT TCTCTGCCGG ACTGTATTCT

CACGAGCCAA CTGCTGTATT TAACATCTGC CTGCAAATGG ACAAAGTACT

CACGAGCCAA CTGCTGTATT TAACATCTGC CTGCAAATGG ACAAAGTACT

CACGAGCCAA CTGCTGTATT TAACATCTGC CTGCCAGTGG ACAAACTACT

CACGAGCCAA CTGCTGTATT TAACATCTGC CTGCCAGTGG ACAAAGTACT

CATGAACCAA CTGCTGTATT TAACATCTGC CTGCCAGTGG ACAAAGTACT

CATGAACCAA CTGCTGTATT TAATATTTGC CTACCAAAGG ACAAAGTACC

CATGAGCCAA CTGCCGTATT CAATGTTGGC CTACCACTGG ATAAGGAACT

CACGAGCCAA CTGCAGTTTT CAATGTTGGC CTGTCTCTGG ATAAAGAACT

CATGAGCCAA CTACTGTATT TAATATATGC CTGCCTATGG ATAATGTACT

CATGAGCCAA CTGCTGTATT TAATATCTGC CCACCAAAGG ATAATGTACT

CATGAGCCAA CTGCTGTATT TAATATCTGC CTGCCAGTGA ATAAAGTATT

CATGAGCCAA CTGTTGTATT TAATATCTGC CCACCAATGG ATAAAGTACT

CATGAGCCAA CTGCTGTGTT CAATATCAAC CCTCCAATAG CTAAAGGACA

CATGAGCCAA CTGCTGTATT TAATATCCAC CCTCCGGTGG ACAGTGTACT

CATGAGCCAA CTGCTGTATT TAATATCTGC CCACCAGTGG ATAAAGAACT

---------- ---------- ---------- ---------- ----------

CATGAACCAA CATCTGTATT TAACACATGT ACTCAAGTGG AAAAAATACC

CATGAACCGA CCAACGTATT TAACGTCCGT ACGCAGGAGG AGGAGGTCTT

TGATATGGAG GTTGTTCATA AGGAGCTTAC TAACTGTGGT TTGCACCCTA

TGATATGGAG GTTGTTCATA AAGAGCTTAC TAACTGTGGT TTGCACCCTA

TGATATGGAG GTTGTTCATA AGGAGCTTAC TAACTGTGGT TTGCACCCTA

TGATATGGAG GTTGTTCATA AGGAGCTTAC TAACTGTGGT TTGCACCCTA

TAATACGGAG GTTGTTCATA AGGAGCTTAC TAACTGTGGT TTGCACCCTA

AGATAAGGAG GCTGTACATA AGGAGCTTGC TAACTGTGGT TTGCACCATA

GGATAGAAAA GGTGCTCATA AGGACCTTGC TAACTGTGGG CTGCACCCTA

GGACAGAAAA GTTGCTCGTG AGGACCTTGC TAATTGTGGG CTGCATCCTA

GGATAAGGAG GTGGTACACA AAGAGCTTGC TAACTGTGGG TTGCATCCTA

GGATAAGGAG GCTGTTCACG AGGAGCTTGC CAATTGTGGA TTGCACCCTA

GGATAAGGAA ACTGTTCTTG AGGAGCTTGC TAACTGTGGA TTGCACCCTA

GGATAAGGCG ACTGTTCATG AGGAGCTTGC TAACTGTGGG TTGCACCCTA

GTTGAAGGAA GTTGTCCATG AGGAGCTTGC TAACTGTGGA TTGCACCCAA

GGTTAAGGAG AGTGTTCTTA AGGAACTTGC TAACTGTGGG TTGCATCCCA

GGATAAAGAG ACTGTTCGTA AGGAGCTTGC CAACTGTGGG TTGCATCCTA

---------- ---------- ---------- ---------- ----------

AAATAAGGAA GTCGTTCAAA AGGAGCTTGC TAATTGTGGA CTACATCTTA

AAATAAGGAG ACGCTTCACC TAGACCTCGC TGACTGCGGG CTACATCTCA

ACACTCTGGA GCAACTTAGT CAAATACCGT TACTTGGGAA ATCATCTTTA

ACACTCTGGA GCAACTTAGT CAAATACCGT TACTTGGGAA ATCATCTTTA

ACACTCTGGA GCAACTTAGT CAAATACCAT TACTTGGGAA ATCATCTTTA

ACACTCTGGA GCAACTTAGT CAAATACCAT TACTTGGGAA ATCATCTTTA

AGACTCTGGA GCAACTTAGT GAAATACCGT TACTTGGGAA ATCGTCTTTA

AGACTCTGGA GCAACTTAGT CAAATACCAT CACTGGGGAA GTCATCTTTA

AAACCCTGGA GCAGCTCAGT CAAATGCCAG TGCTGGGGAA GTCATCCTTG

AAACCCTGGA GCAGCTCAGT CAGAGGCCAG TGCTGGGGAA GTCATCCCTG

AGACTCTGGA CCAACTCAGT CAAATACCGT TACTGGGGAA ATCATCTTTA

AAACTCTGGA CCACCTTTGT CGAACACCAG TACTGGGAAA ATTGTCCTTA

AGACCCTGGA TCAACTTAGT CAAACACCAA TACTGGGAAA ATCATCTTTA

AGACTCTGCA CCAAATCAGT CAAACACCAG TACTGGGGAA GTCATCTTTA

AGACTCTGAA TCTCCTTAGC CAAGTACCGT TATTGGGGAA ATCGTCACTG

AGACTCTGGA CCAGCTTATT CAAATACCAT TACTGGGAAA ATCATCTTTA

AGACTCTGGA CCAACTTAGT CAAGTACCAT TATTGGGAAA GTCATCTTTA

---------- ---------- ---------- ---------- ----------

AAACTCTAAG TCAACTTACT CAGTTACCAA AACTAGAAAA ATCATCTTTA

AAACCCTGGA TCAGTTGATC CGGTCACCAG AGCTAGGAAA ATCTTCTATC

CGGAATGTGG TGCTGAGAGA CTACATTTAT AATTGGAGAT CCGGTGGAAA

CGGAATGTGG TGCTGAGAGA CTACATTTAT AATTGGAGAT CCGGTGGAAA

CGGAATGTGG TGCTGAGAGA CTACATTTAT AATTGGAGAT CCGGTGGAAA

CGGAATGTGG TGCTGAGAGA CTACATTTAT AATTGGAGAT CCGGTGGAAA

CGGAATGTGG TGATGAGAGA CTACATTTAT AACTGGAGAT CC--------

CGGAATGTTG AAATGAGAGA CTACGTTTAT AATTGGACAT CCGGTGGAAT

CGGAATGTGG AAATGAGTGA CTACATTGTA AATTGGAGAT CCGGTGAAGA

CGGAGTGTGG AAATGAGTGA CTACATTTTA AGTTGGAGAT CCGGTGGAGA

CGGAATGTGG AAATGAGTGA CTACGTTTAT AATTGGAGAT CC--------

CGGCATGTGG AAATGAGTGA CTACATTTAT AATTGGAGAT CCGGGGAAGC

AGACATGTGG AAATGAATGA CTACGTTTAT AATTGGAGAT CCGGAGGAAC

CGGAATGTGG AAATGAGTGG CTACATTTAT AATTGGAGAT CC--------

AGACATGTGG AAATGAATGA CTACATCTAT AATTGGAGGT CC--------

CGGCATGTGG AAATGAGTGA CTACATTTAT AATTGGAGAT CAGGTGGAGA

CGGCATGTCG AAATGAGTGA CTACACTTAT AATTGGAGAT CC--------

---------- ---------- ---------- ---------- --GGTGGAAA

CGATTCATGG AAATGGATAA TTACATTTGT AATTGGAGGT CT-GTGAAAA

CGGCTCCTCC AAGTGGACAA TTACAACTAC AGCTGGAGAG CCGGTGGAAA

AAATCTAAAA TCTCCTCAAA AAGTAGAGGT CGACATCATA GATGACAACT

AAATCTAAAA TCTCCTCAAA AAGTAGAGGT CGACATCATA GATGACAACT

AAATCTAAAA TCTCCTCAAA AAGTAGAGGT TGACGTCATA GATGACAACT

AAATCTAAAA TCTCCTCAAA AAGTAGAGGT TGACGTCATA GATGACAACT

---------- ---------- ---------- ---------- ----------

AAATCTAAAC TGCTCTCAAA ATTTAGAGGT CAACATCATA GATGACAATG

CAATCTAAGG CCTCCTGAGG ATGTAGCCAT CTACATTATA GACGACAACT

AAATCTGAAA CCTCCTGAGA ATATAGACGT CTACATTATA GATGACAACT

---------- ---------- ---------- ---------- ----------

AAATCTGAAG ---CCTGAAA ATGTCGAGAT CCACATCATT GATGACAATT

AACTCTAAAA TCTCCTCAAA ATGTAGAGGT CTACATCGTT GATGATACCT

---------- ---------- ---------- ---------- ----------

---------- ---------- ---------- ---------- ----------

AAATCTAAAA TCTCCTCAGA ACGTCGAGGT TGATATCATA GATGACAGCT

---------- ---------- ---------- ---------- ----------

TACTCTCCAG TCTCCAGAAA ACGTGGAGGT GCACATTATA GATGACACTT

GAATTTAAAG ACTCCTCAAA ATGTAACTGT TGAGATTGTA GATCATAATT

GAATCTGGAA ATTCCTCAAG ATGTCCAAGT CTCTATCATT GATAGCACCT

TTATCCTGAG GTGGAACAGG AGCGATGAGT CTGTCGGGAA TGTGACTTTT

TTATCCTGAG GTGGAACAGG AGCGATGAGT CTGTCGGGAA TGTGACTTTT

TTATCCTGAG GTGGAACAGG AGTGAAGAAT CTGTCGGGAA TGTGACTTTT

TTATCCTGAG GTGGAACAGG AGTGAAGAAT CTGTCGGGAA TGTGACTTTT

---------- ---------- ---------- ---------- ----------

TTACCCTAAA GTGGAACAGG AGAGATGAGT CTGTCAGGAA CACGACTTTT

ATACCCTAAA GTGGAACCAC CACAGAGAAT CAATGGGCAA CGTGACTTTT

ACACCCTAAA GTGGAGCAGC CACGGAGAGT CAATGGGCAG TGTGACCTTT

---------- ---------- ---------- ---------- ----------

TTTTCCTGAA GTGGAACAGC AGCAGTGAGT CTGTCAAGAA TGTGACTTTT

TTATCCTAAG GTGGAACAGG AGCAACCCGT CTATCAGGAA TGTGACCTTT

---------- ---------- ---------- ---------- ----------

---------- ---------- ---------- ---------- ----------

TTATCCTGAA GTGGAAAAGG AGCGGGGATT CTGTCAAGAA TGTGACTTTT

---------- ---------- ---------- ---------- ----------

TTACCCTGAA GTGGAGTCGG AGCGATGACT CTGTTGGGAA CGTGACTTTT

TTATCCTGAA ATGGAATTGG GATAATGAAC CTAACAGCAA TACTACTTTT

TTATCCTTCA TTGGAGCGTA GCTAACGGAT TTGATACCGA TGTGACTTTT

TCATTCGATT ATCAAAAAAC TGGGATGGAT AATTGGATAA AATTGTCTGG

TCATTCGATT ATCAAAAACC TGGGATGGAT AATTGGATAA AATTGTCTGG

TCATTCGATT GTCAAAAACC TGAGATGGAT AATTGGATAA AATTGCCTGG

TCATTCGATT ATCAAAAACC TGAGATGGAT AATTGGATAA AATTGCCTGG

---------- ---------- ---------- ---------- ----------

TCAGCAGATT ATGAAAAACC TGAGACGGAT AATTGGATAA GATTGCCCGG

TCAGCAGAAT ATCAAAAAGG AGAAGGAAGG ATCTGGTTAA AAGTGCCCAA

TCAGCAGAAT ATCGAACAAA AGACGAGGCG AAGTGGTTAA AAGTGCCTGA

---------- ---------- ---------- ---------- ----------

TCAGCAGATT ATCAAATACT AGGAACGGAT AACTGGAAGA AATTGTCTGG

TCAGCATATT ATCAAACACT TGGAAATGAT ACTTGGTTAA AATTGCCTGG

---------- ---------- ---------- ---------- ----------

---------- ---------- ---------- ---------- ----------

TCAGCAGATT ATCAAATCTC TGGGATGGAT AATTGGATAA AATTGCCTGG

---------- ---------- ---------- ---------- ----------

TCAGCTGAGT ACCAAATGTC TGGAATGGAT AATTGGATAA ACTTGCCTGG

TCAGCATATT ACCAAAAAGT AAAGATGAAC AATTGGGTTG AATTGATTGG

TCAGCGTATT ACCAAGAGGT TGAAGAGGAC ACTTGGAAGC CATTACCGGG

GTGTCAGAAT ATTACTAGTA CCAAATGCAA CTTTTCTTAT GAAGAAATTA

GTGTCAGAAT ATTACTAGTA CCAAATGCAA CTTTTCTTAT GAAGAAATTA

ATGTCAGAAT ATTACTAGTA CCAAATGCAA CTTTTCTTAT GAAGAAATTA

ATGTCAGAAT ATGACTAGTA CCAAATGCAA CTTTTCTTAT GATGAAATTA

---------- ---------- ---------- ---------- ----------

GTGTCAACAT GTCACAGGTA CCGAATGCAG CTTTTCTTTT AAAGAAATTA

ATGTCAACAT ATTACAAGGA CCAAATGTGA ATTCTCTTAC AGCAAGACAC

ATGTCAACAT ACTACAACGA CCAAGTGTGA ATTCTCTTAT ATCAAAACAC

---------- ---------- ---------- ---------- ----------

GTGTCAACAT ATTACTAGTA CCAAATGCAA CTTTTCTTTT GAAAAAATTG

GTGTCAGTCT GTTGCCAACA CCACGTGCGA CTTTTCTTAC GACAAAGTTA

---------- ---------- ---------- ---------- ----------

---------- ---------- ---------- ---------- ----------

ATGTCAATCT GTTACTAGTA CCGAATGCAA CTTTTCTTAT GAAAAAATTA

---------- ---------- ---------- ---------- ----------

GTGTCAGTCT ATTACTGGTA CCAGATGCGA CTTTTCTTTT GAACAAGTTA

ATGTCACAAG GTCACTGGCA CAAAATGCGA CTTTACTTTT GAAAAAATAA

ATGCCAGAAT GTCACCGAAA CAAATTGTGA CTTCTC-TTT CAAAAGCTTA

AATTGCGTAT AAGAGCAGAA AAAGAAAACA CTTCTTCATG GTATGAGGTT

AATTGCGTAT AAGAGCAGAA AAAGAAAACA CTTCTTCATG GTATGAGGTT

AATTGCGTAT AAGAGCAGAA AAAGAAAACA CTTCTTCATG GTATGAGGTT

AATTGCGTAT AAGAGCAGAA AAAGAAAACA CTTCTTCATG GTGTGAGGTT

---------- ---------- ---------- ---------- ----------

AATTTCGTGT AAGAGCAGAA CAAGGAATCA CTTCTTCATG GTGTGAGGTT

AGTTTCGTGT AAGAGCAGAA GAAGGGAACA CTTCTTCATG GAATGAGGCT

AGTTTCGTGT CAGAGCAGAG GAAGGGAACA CATCTTCGTG GAATGAGGTT

---------- ---------- ---------- ---------- ----------

AATTGCGCAT AAGAGCAGAA GAAGGAAACA CTTCCACATG GTATGAGGTT

AATTGCGTAT AAGAGCAGAG GAAGGAAACA CTTCTCCGTG GCATAATATT

---------- ---------- ---------- ---------- ----------

---------- ---------- ---------A CTTCTCCATG GCATGAAGTT

AATTGCGTAT AAGAGCAGAA AAAGGAAACA CTTCTCCATG GTATGAGGTA

---------- ---------- ---------- ---------- ----------

CATTACGTAT AAGAGCAGAA AAGGGAAATA CTTCTCCGTG GCAAGAAGTT

AAGTGCGTAT AAGATCTGAA AAAGGACAAA CATCACCATG GTATGACATT

ATGTGCGCGT CCGAGCCGAA AGGGGGAGAG CTTCTCCCTG GTCTAAGGTT

GACTCATTTA CACCATTTCG CAAAGCTCAG ATTGGTCCTC CAGAAGTACA

GACTCATTTA CACCATTTCG CAAAGCTCAA ATTGGTCCTC CAGAAGTACA

GACTCATTTA TACCATTTCG CAAAGCTCAA ATTGGTCCTC CAGAAGTACA

GACTCGTTTA CACCATTTCG CAAAGCTCAA ATTGGTCCTC CAGAAGTACA

---------- ---------- ---------- ---------- ----------

ATTTCAATTA CACCATTTGC AAAAGCTCAG ATTGGTCCTC CAGGAGTACA

GATTCATTTA TTCCATTCTA CAAAGCTCAC GTCAGCCCCC CAAGAGTACA

GATCCGTTTA TTCCATTCTA CACAGCTCAC ATGAGCCCCC CAGAAGTACG

---------- ---------- ---------- ---------- ----------

GAGCCATTTG TACCATTTCT AGAAGCTCAG ATTGGTCCCC CAGATGTGCA

GACTCATTCA CCCTATTTGA AAAAGCTCAG ATTGGTCCCC CAAAAGTACA

---------- ---------- ---------- ---------- ----------

GATTCATTTG TACCATATAA TGAAGCTCAG ATTGGTCCTC CAGAACTACG

GACTCATTTA TACCATTTCA AAAAGCTCAG ATTGGCCCTC CAGAAGTACA

---------- ---------- ---------- ---------- ----------

GAATCATTTG TCCCATTTCA AAAAGCTCAG ATTGGCCCTC CAAAAGTGCA

ACCCCATTTG TACCATTTCA AATAGCACAA ATTGGTCCTC CAGAAGTACA

ATTGGGTTTG TTCCATTTCT CATAGCTCAT ATTGGACCGC CAGAAGTCGA

TTTAGAAGCT GAAGATAAGG CAATAGTGAT ACACATCTCT CCTGGAACAA

TTTAGAAGCT GAAGATAAGG CAATAGTGAT ACACATCTCT CCTGGAACAA

TTTAGAAGCT GAAGATAAGG CAATAGTGAT ATACATCTCT CCTGGAACAG

TTTAGAAGCT GAAGATAAGG CAATAGTGAT ATACATCTCT CCTGGAACAG

---------- ---------- ---------- ---------- ----------

TTTAGAAGCT GAAGATACAG CAATAATAGT AAAGCTCTCT TCTGGAACAA

TTTAAAAGCT GAAGATAAAG CCATACTAGT CTACATCTCT CCTGG---AG

TTTAGAAGCT GAAGATAAAG CCATACTAGT CCACATCTCT CCTGG---AC

---------- ---------- ---------- ---------- ----------

TTTAGAAGCT GAAGATAAGG CAATAATACT GAGTATCTCT CCCGGAACAA

TTTAGAAGCC GAAGATAAGA CAATCATAAT AAACATCTCC CTGGGAGCGA

---------- ---------- ---------- ---------- ----------

TTTAGAAGCT GAAGATAAAG CGATAGTAAT TAACATCTCT CCTGGAACCG

TTTAGAAGCT GAAGATAAGG CAATAGTAAT CAACATCTCC CCTGGGACAG

---------- ---------- ---------- ---------- ----------

TTTGGAAGCT GAAGATAAAG CCATCGTAAT TAGCATCTTG CCTGGGACAA

GTTAGAAGCT GAAGATAAAT CAATAATAAT AAATATTTCT CCTGGAACAA

GTTGGAATCT CGGGACGGGG CAGTATTCAT CAGTGTTTCT CC---GAAAA

AAGATAGTGT TATGTGGGCT TTGGATGGTT TAAGCTTTAC ATATAGCTTA

AAGATAGTGT TATGTGGGCT TTGGATGGTT TAAGCTTTAC ATATAGCTTA

AAGATAGTGT CATGTGGGCT TTGGATCGTT CAAGCTTTAC ATATAGCTTA

AAGATAGTGT CATGTGGGCT TTGGATCGTT CAAGCTTTAC ATATAGCTTA

---------- ---------- ---------- ---------- ----------

AAGATAGTAT CATATGGGCT TTGGAGGGTT CAAGCTTTCT ATATAGCTTA

AAGACGGAAC CATGTGGGCA CAGGAGAAAC GCTGCTTCAA TTACACGATA

AAGACGGGAA CATGTGGGCA CTGGAGAAAC CTTCCTTCAG TTACACCATA

---------- ---------- ---------- ---------- ----------

AAGATAGTAT CATGTGGGCT ATGGATCGTT CAAGCTTTAG GTATAGCGTC

GAGACAGCGT CATGTGGGCT CTGGATAGCT CCAGCTTTAC CTACAGCTTA

---------- ---------- ---------- ---------- ----------

AAGATGATAA CATGTGGGCT ACAGAACGTT TAATGTTTAA ATATAACATA

AAGATAGTGT CATGTGGGCT ATGGATAGGT CACGCTTCAC TTACATTTTA

---------- ---------- ---------- ---------- ----------

AAGAGAGCCT CATGTGGGCT ATGGATAGTT CGAGCTTCAA GTACCGCTTG

GTAATAGTAA CATGTGGGCT ACTGATAATC AAAGGTTTAC CTACAGTTTA

GTAATCAGAT CTTGTTGGAT AATGAAATCT TGAGTTTCAC CTATGAGTTG

GTTATCTGGA AAAACTCTTC AGGTGTAGAA GAAAGGATTG AAAATATTTA

GTTATCTGGA AAAACTCTTC AGGTGTAGAA GAAAGGATTG AAAATATTTA

GTTATCTGGA AAAACTCTTC AAGTGTAGAA GAAAGGATTG TAAATATTTA

GTTATCTGGA AAAACTCTTC AAGTGTAGAA GAAAGGATTG AAAATATTTA

---------- ---------- ---------- ---------- ----------

GTTATCTGGA AAAATTCTTC AAGTGTAGAG AGAAGGACTA AAAATGTTTA

CGGATCTGGG GGAGGTCTTT TCCTGTCAGA GAA---ATTA AACCTATGTA

CGAATCTGGC AGAAGTCTTC CAGTGACAAA AAAACTATTA ACTCTACGTA

---------- ---------- ---------- ---------- ----------

GTTATCTGGA AAAACTCTTC AAGTCTAGAA GAAAGAACGG AAACTGTTTA

GATATCTGGA AAAACTCTTC AAGTGGAGAA AGAAGGACTG AAACTGTTTA

---------- ---------- ---------- ---------- ----------

GTCATCTGGA ACAGTTCG-- GGGTATGGAG ---------- ----------

GTTATCTGGA AAAATTCTTC AAGTGTAGAA AAAAGGACTG AAACTGTTGA

---------- ---------- ---------- ---------- ----------

GTTATCTGGA CGAATTCCTC TGGTGCAGAC AAAAGGATTG AGACTGTTTA

GTTATTTGGA AAAACTCTTC AAGTATAAAA GAAACCCATC ATACTGTTTA

ATCATCTGGA AGAACTCTTC AAGTGAAGAA AAAACCAGTC TATTTATCCA

TTCCAGACAT AAAATTTATA AACTCTCACC AGAGACTACT TATTGTCTAA

TTCCAGACAT AAAATTTATA AACTCTCACC AGAGACTACT TATTGTCTAA

TTCCAGACAT AAAATTTCTA AACTCTCACC AGAGACGACT TATTGTTTAA

TTCCAGACAT AAAATTTCTA AACTCTCACC AGAGACTACT TATTGTTTAA

---------- ---------- ---------- ---------- ----------

TCCCAGAGAT ACAATTTATG AACTCTCACC AGAGACTACT TATTGTTTAA

TTTTCCAAAA AAGATATCAA AGCTCTTGCC AGAGACTACC TACTGTTTAG

TTATGTAGAA AAGATACCAG AACTCTTGCC AGAGACTACT TACTGTTTAG

---------- ---------- ---------- ---------- ----------

TCCCGAAGAT AAAATTTATA AACTGTCACC AGAGATTACT TATTGTTTAA

CTCCAGAGAT AAAATTTATA ATCTCTCACC AGAGACCACT TACTGTTTAA

---------- ---------- ---------- ---------- ----------

---------- ---------- ---------- ---------- ----------

TTTCAGACAT AAGATTCATA AGCTTTCACC AGAGACTATG TATTGTTTAA

---------- ---------- ---------- ---------- ----------

TCCCAGAAGT AAAATTTATA AACTCTCACC TGAAACTACT TACTGTTTAC

TTCCAGAGAT AAAATTAATG ATCTCACACC AGAAACTACT TATTGTTTAA

TCCCAGAGAT GAAATTCATA ATCTGGCACC GGAGACCACT TACTGTTTGA

AAGTTGCAGC ACTACTTACG TCATGGAAAA TTGGTGTCTA TAGTCCAGTA

AAGTTGCAGC ACTACTTACG TCATGGAAAA TTGGTGTCTA TAGTCCAGTA

AAGTTGCAGC ACTACTTACG TCACGGAAAA TTGGTGTCTA CAGTCCAGTG

AAGTTGCAGC ACTACTTACG TCACGGAAAA TTGGTGTCTA TGGTCCAGTG

---------- ---------- ---------- ---------- ----------

AAGTTGCAGA AATACTTGTG CTACGGAAAG TTGGGGTCTA CAGTCCGGTG

AAGTCGCAGT GCACACATCG CTTCAGAGAC ACAGCAACTA TAGCTCAGCG

AAGTTGCAAT ACATCCGTCA CTTAAGAAAC ACAGCAATTA CAGCACTGTG

---------- ---------- ---------- ---------- ----------

AAGTTGCAGA ACTGCGTTTA CAAAGTAGAG TTGGTTGCTA TAGTCCAGTG

AAGTTGCAAG ACT---TTTG CCGAGAAAAA ACGGTATCTA TAGTCCTGTG

---------- ---------- ---------- ---------- ----------

---------- ---------- ---------- ---------- ----------

AAGTTGCAAG ACTACGTTTG CAGAGAAAAG TTGGTTTCTA TAGTCCAGTG

---------- ---------- ---------- ---------- ----------

AAGTGGCATA CCTCCGTGTG CCGAGAAAGA ACGGCATGTA CAGTCCCGTG

AAGTTGCAAG ATTACCTATT CTGAGAAAAA ATGGTTCCTA CAGTCCAGTA

AAGT---AAG AATGCTGCGG AGGAAAAAAA CCATTTTCTT CAGCCCGGTG

CATTGTATAA AGACCACAGT TGAAAATCTA CCTCCACCAG AAAATATAGA

CATTGTATAA AGACCACAGT TGAAAATCTA CCTCCACCAG AAAATATAGA

CATTGTATAA AGACCACAGT TGAAAATCTA CCTCCACCAG AAAATATAGA

CATTGTATAA AGACCACAGT TGAAAATCTA CCTCCACCAG AAAATATAGA

---------- ---------- ---------- ---------- ----------

TATTGTATAA ATACCACAGT TGAAAATATT CCTCGACCAG AAAATTTAGA

CGGTGTATAA ACACCACAGT GGCAAATATA CCTGTGCCAG AAAATCTAGA

CAGTGTATAA GCACCACAGT GGCAAATATG CCTGTGCCAG GAAATCTCCA

---------- ---------- ---------- ---------- ----------

TATTGTATAA ATACCACAGA GAGACATGTA CCTTCACCAG AAAATATACA

TACTGTATAA AGACCCCAGT TGCAAATCTG CCTCCACCAG AAAACATCCG

---------- ---------- ---------- ---------- ----------

---------- ---------T GGAAAATCTG CCTCCACCCG AAAATGTACA

TATTGTATAA ATACCACAGT TGAAAATCTG CCTGCACCAG AAAATATAGA

---------- ---------- ---------- ---------- ----------

TATTGTATCA GCACCACAGT TGAAAGTCTG CCTCCACCAG AAAATCTAAA

TTTTGTATAA ATACTACAGC TGAACATCTA CCTCGTCCAG AAAATGTAGA

TTTTGCATAA ATACTACAGT TGAAAA---G CCTCGTCCGA GAAACTTAGA

AGTCAGTGTC CAAAATCAGA ACTATGTTCT TAAATGGGAT TATACATATG

AGTCAGTGTC CAAAATCAGA ACTATGTTCT TAAATGGGAT TATACATATG

AGTCATTGTT CAAAATCAGA ACTATGTTCT TAAGTGGGAT TATACGTATG

AGTCATTGTT CAAAATCAGA ACTATGTTCT TAAGTGGGAT TATACGTATG

---------- ---------- ---------- ---------- ----------

CGTCAGTGTT AAAAATCAGA GATATGTTCT TAAATGGGAT CATGCATTTG

AGTGGATGTC CAAGGTGACA GCTATGTTCT GAAATGGGAC CACGCATTTG

AGTGGATGCC CAAGGCAAGA GCTATGTCCT GAAATGGGAC TACGCGTCTG

---------- ---------- ---------- ---------- ----------

AATCAATGCT GACAACCAGA TCTATGTTCT TAAGTGGGAT TACCCATATG

GCTTGATTCT AAAAACAACA TCTTTGTTCT TAGATGGGAT TACCCCTATG

---------- ---------- ---------- ---------- ----------

ACTTGAGGTT CTAAATGAGA ACTATGTGCT TAAGTGGGAT TACCCCTATG

AGTCAGTGCT AAAAATCAGA GCTATGTTCT TAAATGGGAC TACTCCTATG

---------- ---------- ---------- ---------- ----------

AGTCGATTCA AAAAAGTCGA TCTGTAGTCT TAAATGGGAT ACTGCAGATG

AGTTGATGCT GTAAATAATA ACTATGTTCT TAAATGGAAT TATCCATATG

TATTTCCGCT ATAAACACAA ACTACATCCT TAAATGGGAT TACCCGAACG

CAAACATGAC CTTTCAAGTT CAGTGGCTCC ACGCCTTTTT AAAAAGGAAT

CAAACATGAC CTTTCAAGTT CAGTGGCTCC ACGCCTTTTT AAAAAGGAAT

CAAACATGAC CTTTCAAGTT CAGTGGCTCC ATGCCTTTTT AAAAAGGAAT

CAAACATGAC CTTTCAAGTT CAGTGGCTCC ATGCCTTTTT AAAAAGGAAA

---------- ---------- ---------- ---------- ----------

CAAACGTGAC CTTTCGAGTT CAGTGGCTCC ATGCCTTTGC CAAAAGGAAT

CAAACATGAC CTTCAAAGCA CAGTGGATCC CTGGCTATTT AAAAAATATT

CAGACGTGCT CTTCAGGGCA CAGTGGCTTC CTGGCTATTC AAAAAGTAGT

---------- ---------- ---------- ---------- ----------

AAAACGCAAC TTTTCAAGCT CAGTGGCTCC GTGCCTTTTT TAAAAAGATT

GAAACGCAAC TTTCCAAGCT CAGTGGCTCT ATGCCTATTT AAAAAAGATT

---------- ---------- ---------- -TGCCTATTT AAAACAGATT

AAAAGGTGAC CTTTCGGGCG CAGTGGCTCT CCGCCTATGT AAAAATGCTT

AAAACGTGAC CTTTCAAGTT CAGTGGCTTC ATGCTTATTT TAAAAAGATT

---------- ---------- ---------- ---------- ----------

AAAATACGAC CTTTCAAGTT CAGTGGCTCC ATGCATATTT AAAAAAAATT

AAAATATGAA CTTCGAAGTT CAATATCTCC TTGGCTTTTA TAAAAGAATT

AGGACGGAAG CTTTCGTGTT CAGTGGCTCT ATGGTTTTTA CAAGAAATAT

CCTGGAAACC ATTTGTATAA ATGGAAACAA ATACCTGACT GTGAAAATGT

CCTGGAAACC ATTTGTATAA ATGGAAACAA ATACCTGACT GTGAAAATGT

CCTGGAAACC ATTTGTATAA ATGGAAACAA ATACCTGACT GCGAAAATGT

CCTGGAAACC ATTTGTATAA ATGGAAACAA ATACCTGACT GCAAAAATGT

---------- ---------- ---------- ---------- ----------

CCTGGGAGTC ATTTATATCA ATGGAAACAA ATAACTGACT GTGAAAACAT

CCTGGAAGCC ATTCAGATAA ATGGAAGCCA ATACTTAACT GTGCAAATGT

TCTTTT-TCC ATTCAGATAA ATGGAAACCA ATACCAACCT GTGCAGATGT

---------- ---------- ---------- ---------- ----------

CCTGGGAATC ATTCAGACAA ATGGAAACAA ATACCGAACT GTGAAAATGT

CCTGGGGACA GTTCAGGTAA ATGGAAACAA ATACCAGGCT GTAAAAATGT

CCTGAGAACC ATTCAGGAAA ATGGAAACAA ATACCAAACT GTGAAAGTGT

CCTGGGAACC ACGCAGAGAA ATGGAAAAAT ATCCAGGACT GTGAAAATGT

TTTGGAGACC ATTCAGAAAA ATGGAAACAA ATACCTGAGT GTAACAATGT

---------- ---------- ---------- ---------- ----------

CCTGGGAACC ATTCAGATAA GTGGAAAACA ATACCTGGCT GTGAAACTCT

CTAAGTGACT ATTCAGATAA ATGGAAAGTT ATATCTGGTT GTAAAAATAT

CTGAAGGACT ACACGGATAA ATGGAAGACG GTATCTAGCT GTGAAAACAT

CAAAACTACC CAGTGTGTCT TTCCTCAAAA CGTTTTCCAA AAAGGAATTT

CAAAACTACC CAGTGTGTCT TTCCTCAAAA CGTTTTCCAA AAAGGAATTT

CACAACTACC CAGTGTGTCT TTCCTCAAAA CACTTTCCAA AAAGGAATTT

CACAACTACC CAGTGTGTCT TTCCTCAAAA CACTTTCCAA AAAGGAATTT

---------- ---------- ---------- ---------- ----------

CACAACTACC CAGTGTGTCT TTCCTCAAAA TCTTTTCCTA AGAGGAATTT

CAGAACTACA CACTATGTCT TTCCT---G- ----TTCTAC ACAGGAATTT

CCAGACTACG CACTGTGTCT TTTCTCAAGA TACTGTCTAC ACGGGAACGT

---------- ---------- ---------- ---------- ----------

CACAAGTACC CACTGTGTCT TTCCTCGAGA GGTTTCCTCA AGGGGAATTT

CCACACAACC CAATGTGTCT TTCTTCAAAA TACTTTCCGA AAGGGAATTT

CAGAACTACC CAGTGTGTCT TTCCTCAAAA TCGTTTTCAG AAAGGAATTT

CACTACTACC TATTGTGTCA TCCCACAAGC CGTGTTCTTC AAAGGAATTC

CACAACCACC CAGTGTGTCT TCCCTCAGAA GTTTTTCCAA AAAGGAATTT

---------- ---------- ---------- ---------- ----------

GGAAACCACA CAGTGTGTCT TTCCTCCAAA TGTTTTCCCG AAAGGAATTT

CACAAGTAAA CATTGTGACT TCTCAAAAGA CATCACCACA GATGGAATTT

TAAAGCAACC AAGTGTGACT TCTCAGGAGA AATTTCCGAA ACAGGGATTT

ACCTTCTCCG CGTACAAGCA TCTGATGGAA ATAACACATC TTTTTGGTCT

ACCTTCTCCG CGTACAAGCA TCTGATGGAA ATAACACATC TTTTTGGTCT

ACCTTCTCCG CGTACAAGCA TCTGATGGAA ATAACACGTC TTTTTGGTCT

ACCTTCTCCG CGTACAAGCA TCTGATGGAA ATAACACGTC TTTTTGGTCT

---------- ---------- ---------- ---------- ----------

ACTATCTCCG TGTACAAGCA TCTGATGGAA ATAACTCATC CTTTTGGTCT

TCTTTCCCTG TGTACAAACC TCAGACGGAA ATAGCATATC CTTTTGGTCT

TCTTTCTCCG TGTACAAGCC TCAGAGGGAA ATCACACATC CTTTTGGTCT

---------- ---------- ---------- ---------- ----------

ACTATGTCCG TGTACGAGCA TCTAATGGAA ATGGTACCTC TTTTTGGTCT

ACCTTATCCG TGTTCAGGCA TCTGATGGAA ATACCACATC CGTTTGGTCT

ACTTTATCCG TGTACAAGCA TCCGATGGAA ATAACACATC ATTTTGGTCT

ACTTTATCCG TGTCCAAGCA TCTGAAGGAA ATGCTACATC TTTTTGGTCT

ACTTCCTCCG TATACAAGCA TCTAGCAGAA ATAACATCTC TCTTTGGTCT

---------- ---------- ---------- ---------- ----------

ACTTTTTCCG GGTGCAGGCA TCTAAGGGAA ATAGCACTTC TCGCTGGTCT

ATTATCTACG TGTACAAGCT TCAAATGGAA GTATCACATC TTTTTGGTCT

ACTATCTCCG GGTACAAGCG ATAGATGGAA AAAATTCATC ACTTTGGTCC

GAAGAGATAA AGTTTGATAC TGAAATACAA GCTTTCCTAC TTCCTCCAGT

GAAGAGATAA AGTTTGATAC TGAAATACAA GCTTTCCTAC TTCCTCCAGT

GAAGAGATAA AGTTTGATAC TGAAATACAA GCTTCCCTGC TTCCTCCAGT

GAAGAGATAA AGTTTGATAC TGAAATACAA GCTTCCCTGC TTCCTCCAGT

---------- ---------- ---------- ---------- ----------

GAAGAGAAGA GATTTGACAT TGAAATAATA AGTATCATAC ATCCTCCCAT

GAAGAGAAAT GTACTAATTC TCAAAAATAC ACTCTCCTTC CTCCTCCGGT

GAAGAGAAGT TTATTGATTC TCAAAAACAC ATTCTCCCTC CTCCTCCGGT

---------- ---------- ---------- ---------- ----------

GAAGAGAAGG AATTTAATAC TGAAATGAAA ACTATCATAT TTCCTCCAGT

AAAGAAGAAA AATTTGATGC TCAGTTACAA ATTACCCTGT TGCCCCCGGT

GAAGAGGAAA AATTTGATAC TGACACACAA ACTCTCATAC CACCTCCAGT

GAAGAAAAAA AATTTGACAC TGTCATGCAA ACTGTAATAC TTCCACCAGT

GAAGAGAAAA AACTTGATAC TGAAATACGA ACCATCATAT TTCCTCCAGT

---------- ---------- ---------- ---------- ----------

GAAGAGAAAG AATTTTACAT TGATACGCAA ACGGTCGTCC TTCCTCCCGT

ACTGAGAAAA AATTTGATAC TATGATAAAA ACCAATATAG CCCCTCCAAG

CAGGAAAGAA AATTTAATCC TTCTGTGGAA GCTAAACTAG GCCCTCCAAG

CTTTAACATT AGATCCCTTA GTGATTCATT CCATATCTAT ATCGGTGATT

CTTTAACATT AGATCCCTTA GTGATTCATT CCATATCTAT ATTGGTGATT

CTTTAACATT AGATCCCTTA GTGATTCATT ACATATCTCT ATCGGTGATT

CTTTAACATT AGATCCCTTA GTGATTCATT ACATATCTCT ATCGGTGATT

---------- ---------- ---------- ---------- ----------

CATTAATGTG AAATCCACCA GTGACTCACT GCGCATCTAC ATGGACCACG

CATTGCTGTC ACCCCCACGG GGGACTCCTT GCTTGTTTAT GTCAGC--TA

CATTACTGTC ACCGCCATGA GTGACACCTT GCTTGTTTAT GTCAAC--TG

---------- ---------- ---------- ---------- ----------

CATCAGCGTG AAATCCGTTA CTGATTCACT GCATGTCAGT GTTGGTCTCT

CATTACCATG AAATCCACCA ATGATTCCCT GCGGGTCTAT ATCAGTAATT

CATTAATCTG AAGCCCATTA ATAACTCCCT GCGTGTCTAT ATTGGCCATT

CATTAACATG AAACCCATTG ATGATTCAAT GCGTGTCTCT ATTGGTGATT

CATCACCGTG AAACCTCTTA GCGATTCCCT GCGCATCTAT CTCAGTCGTT

---------- ---------- ---------- ---------- ----------

CATTCGTATG AAATCCATCA ATGGCTCGCT GAACGTCTAT ATCGGTAATT

CATAAAGGTG ATACCGAGTG GTGATTCATT CTCTATCTAC ATTAGTAATT

CCTAAAGGTC GAATCCCGTG ATACTACGCT CCACGTCTAC ATCGCTTATC

ATCCACTGAT TTATGAAATT ATTTTTTGGG AAAACACTTC AAATGCTGAG

ATCCACTGAT TTATGAAATT ATTTTTTGGG AAAACACTTC AAATGCTGAG

ATCCACTGAT TTATGAAATT CTTTTTTGGG AAAACACTTC AAAGGCTGAG

ATCCACTGAT TTATGAAATT CTTTTTTGGG AAAACACTTC AAAGGCTGAG

---------- ---------- ---------- ---------- ----------

TCCCACTAAG CTATGAAATT ATTCTGTGGA AAAACACTTC AAATGCTGAG

ATGGACTCAT TTACGAAGTC ATCTTTTGGG AAAACACTTC CAATACTGAG

ATGGACTCAA TTACGAAATC ATCTTTTGGG AAAACACTTC CAATACTAAG

---------- ---------- ---------- ---------- ----------

ACCCGCTAAT TTATGAAGTA ATTTTTTGGG AAAACACTTC AAATGCTGAG

TCCCACTAAT TTATGAAATT GTATTTTGGG AAAACACTTC AAATGCTGAG

ACCCACTAAT TTATGAAATT ACTTTTTGGG AAAACACTTC AAACGCTGAG

ACCCCCTAAT TTATGAAATT GTTTTTTGGG AAAACTCTAC AAATGCTGAG

ACCCGTTAAT TTATGAAATT GTTTTTTGGG AGAACACATC AAGTGCTGAG

---------- ---------- ---------- ---------- ----------

ACCCACTTAC TTATGAAGCT CACTTGTGGA AGAACACTTC CAATGCTCAG

ACCCATTAAT CTATGAAGTT ATTTACTGGG AAAATAATTC AAATATTGAG

ATTCCTTAAT GTATGAAGTT ATCGTCATGG ACAACTCTTC AAAACTTGAG

AGAAAAATCG AGAAAAAAAC TGATGTTACA GTTCCTAATT TGAAACCACT

AGAAAAATCG AGAAAAAAAC TGATGTTACA GTTCCTAATT TGAAACCACT

AGAAAAATCA AGAAAAAAAC TGATGTTACT ATTCCTAATT TGAAACCACT

AGAAAAATCA AGAAAAAAAC TGATGTTACT ATTCCTAATT TGAAACCACT

---------- ---------- ---------- ---------- ----------

AGAAAACTAG AGAACAATCG TGATTTCACT ATTCCTCACT TGCAACCGCT

AAAAGCATTA AAGAACACCC AGAGTTCAAC GTTGTGAACC TGCGGCCGCT

ATAAGCGAGA AGGATGGCCC AGAGTTCACC CTCAAGAACC TGCAGCCGCT

---------- ---------- ---------- ---------- ----------

AGAAAACTAG AGAAAAGAAC CAATTTTATC TTTCCTGACT TGAAACCGCT

AGAAAACGGG AGAAAAAAAC TGATTTTACT TTTTCTAAAT TGAAACCGCT

AGGAAAGTAA AGAAAAGAAC TGATTTTACT TTTCCTGACT TGAAACCATT

ATGGTGGTCG AGAA---AAC TGATTTTACT ATTGACAAGT TGAAAGCACT

AGCAAACTTG AGAATAAAAT GGATTTTACT CTTCCTAACT TGGAACCTCT

---------- ---------- ---------- ---------- ----------

ATCAAAATAC AAACAAAAAC GGATTTTATT ATTCCTAATG TGGAACCACG

AACAAA-TTG AGAGTAAAAC AGAAATTGCT GTTTCTGATT TGCAGCCCCT

AAGAAAGTAG AATCCAAGAA GTCATTTAGA GTGCCAGACT TGAAGCCCTT

GACTGTATAT TGTGTGAAAG CCAGAGCACA CACCATGGAT GAAAAGCTGA

GACTGTATAT TGTGTGAAAG CCAGAGCACA CACCATGGAT GAAAAGCTGA

GACTGTATAT TGTGTGAAAG CCAGAGCACA CAGCGTGGAT GAAAAGCTGA

GACTGTATAT TGTGTGAAAG CCAGAGCACA CAGCATGGAT GAAAAGCTGA

---------- ---------- ---------- ---------- ----------

GACTGTATAT TGTCTCAAAG CCAGAGCACA CCTTGTGGAT AAAAAACAGA

GACTGTGTAC TGTGTCCAGG CTAGAGTGCT CTCCACTGGC G---TGTGGA

GACTGTGTAC TGTGTCCAGG CCAGAGTGCT CTTCAGGGCC C---TGCTGA

---------- ---------- ---------- ---------- ----------

GACTGTGTAT TGTGTCAAAG CCAGAGCACT CATTGAAAAT GACAGGCGGA

GACTGAATAT TGTGTCAAAG CCAGAGCACT CATCGAGGAT GACAAGTGGA

GACTGTATAT TGTGTCAAAG TCAGAGCCGT CATTGAGGAT GCAAAGTGGA

GACAGTCTAT TGTGTGAAAG CCAGAGCCCT CGTGGAGGAT GAGAAATGGA

GACTGTGTAT TGTGTCAAAG CCAGAGCACT TGTTGAGGAT GAAAAATGGA

---------- ---------- ---------- ---------- ----------

GACTGTATAT TGCATTAAAG CCAGAGCACT TATTGAGGAT GAAAAATGGA

GACTACGTAT TGTTTCAAAG CCAAAGCATT TCTTCAGGAT AATAAAAGCA

GACTGACTAC TGTTTCAAAG CCAAAGTGTA TATTAAGGAT GACAAAAACA

ATAAAAGCAG TGTTTTTAGT GACGCTGTAT GTGAGAAAAC AAAACCAGGA

ATAAAAGCAG TGTTTTTAGT GACGTTGTGT GTGAGAAGAC AAAACCAGGA

ATAAAAGCAG TGTTTTTAGT GGTGTTGTGT GTGAGGAAAC AAAATCAGGA

ATAAAAGCAG TGTTTTTAGT GATGTTGTGT GTGAGGAAAC AAAATCAGGA

---------- ---------- ---------- ---------- ----------

ATAAAAGCAG TATTTTTAGT GATGCTGTGT GTGAGAAAAC AAAACCAGGA

ATAAGACCAG TAAATTCAGT GATAAGCTGT GTAAGAAAAC ACATCCAGGA

ATAAGACCAG CAACTTCAGT GAAAAGCTGT GTGAGAAAAC ACGTCCAGGA

---------- ---------- ---------- ---------- ----------

ATAAAGGGAG CAGTTTTAGT GATACTGTGT GTGAGAAAAC AAAACCAGGA

ATAAAAGCAG TGGTTTTAGT GATACTGTGT GCGAGAAAAC AAAACCAGGA

ATAACAGCAG TGTTTTTAGT GATACTGTAT GCGAGAAGAC AAAACCAGGA

ACCTGAGCAG TGTGTTCAGT GACAGCCAGT GCCAGGAAAC CAAGCCAGAA

ATAAAAGCAG TGGTTTTAGT GACACTGTGT GTGAGAAAAC AAAACCAGGA

---------- ---------- ---------- ---------- ----------

ATACAAGCAG TGTTTTCAGT GAAATTGTGT GTGAGGAAAC AAAACCAG--

ATAAAAGCAG TCAATTTAGT GATGTTGTGT GCAGTAAAAT GACACCAGGT

ACAGGAGCAG TAATTTTAGT AATGTTGTTT GTGCCCAGAC AAAACCAGGA

GTCATTTATG CTGCGAAAGT CTTCTTGAGA TGCATCAATT ATGTCTTCTT

GTCATTTATG CTGCGAAAGT CTTCTTGAGA TGCATCAATT ATGTCTTCTT

GTCATTTATG CTGTGAAAGT CCTCTTGAGA TGCATCAATT ATGTCTTCTT

GTCATTTATG CTGTGAAAGT CCTCTTGAGA TGCATCAATT ATGTCTTCTT

---------- ---------- ---------- ---------- ----------

GTCGTGTATT TTGTGAAAGT CCTCCGGAAA GCCATCACTT ATGTGTTCTT

GCCTTTTATG CTGGGAGGAG CCTCCAAAAA TACCTGTATT ACGTGTTCTC

GTCCTTTATG CTTTGAGGAG CGTCTGGAAA TACCTGTGTC ATGTGTGCTT

---------- ---------- ---------- ---------- ----------

GTCATTTATG TTGTGAGCGT CTTCTTGAGA TGTGTCAAGT ATGTGTTCTT

GTCATTTATG TTGTGAAACT TCTCTTGAGA TGTATCAGTT ATGTGTTCTT

GTCCTTTACG TTGTGAAAGT TCTCTGGAAA TTTATCCTTT ATGTGTTCTT

CTTACTTTGG TTGTGAAAAT CTTCTGGAAA TATATCACTT TTGTTTATTG

GTCATTTATG CTGGGAAAAG CCTCTTGAAG TGCATCAATT ATGTGTTTTT

---------- ---------- ---------- ---------- ----------

GTCTTCTATA GCCTGAAAGC CCTCTTGAGG TGTATCAATT ATGTAGTCCC

GTATTTTATT GTTCAAAAGT TCTATTAAAA CAGGTAAAAC ATATATTCTC

GCCTTCTTTA GTGGAATATT CTTATGGCGA AGTATCAAGT ATGTGTTCTA

TCCACTTAAA CCTTCTTCCA GTATAGATGA GTATTTCTCT GAACAGCCAT

TCCACTTAAA CCTTCTTCCA ATATAGATGA GTATTTCTCT GAACAGCCAT

TCCACTTAAA CCTTCTTCCA ATATAGATGA GTATTTCTCT GAACAGTCAT

TCCACTTAAA CCTTCTTCCA ATATAGATGA GTATTTCTCT GAACAGTCAT

---------- ---------- ---------- ---------- ----------

CCCACTTAAA CCTTCCTCCA ACATAGATAA GTATTTCTCT GAACGGCCGT

CCCACTCAAG CCTCCCTGCA GTATCGACGA GTTTTTCTCA GAGCTGCCTT

CCCACTCAAG CCTCCCCGCA GTATTGATGA GTTTTTCTCT GAGCCGCCTT

---------- ---------- ---------- ---------- ----------

TCCAAGTAAA CCTCCTTCCT CTGTTGACGA GTATTTCTCC GATCAGCCAC

TCCAAGTAAA CCTCCATCCA CTATAGATGA GTATTTTTCT GAGCCACCGC

TCCAAGTAAA CCTCCTTCCA CTATAGATCA GTATTTCTCC GAACAGCCAC

TCCAAGTAAG CCTCCTTCCA CCGTAAATGA GGATTTGTCC GAACAGCCGC

TCCACGTAAA CCTCCTTCCA GTATAGATGA GTATTTCTTT GAACAGCCGA

---------- ---------- ---------- ---------- ----------

CCTTGAGAAA CCGCCGCCCT GTATCCAGGA GTATTTATCA GAACATTCGC

TCCATGGAAA CCTCCTTCAA GTATGAGTGA GAGGTTCTCT GATCAACCAT

TCCATGCAAA CCCCCTTCAA TTATAGGGGA GAGTCTCTAT GACCATCAGT

TGAAGAATCT TCTGCTTTCA ACTTCTGAGG AACAAATCGA AAAATGTATA

TGAAGAATCT TCTGCTTTCA ACTTCTGAGG AACAAATCGA AAAATGTATA

TGAAGAATCT TCTGCTTTCA ACTTCTGAGG AACAAATCGA AAAATGTATA

TGAAGAATCT TCTGCTTTCA ACTTCTGAGG AACAAATCGA AAAATGTATA

---------- ---------- ---------- ---------- ----------

TAGAGAATCT TCTGTTTTCA CCTTGTGAGG AACAAACTGA AAAATGTATA

CCAAAAGCCT TCTGCTTCTG ACTGCTGAAG AGCACACCGA GAGGTGCGTC

CAAAAAACCT TGTACTTCTG ACGGCTGAGG AGCACACGGA AAGATGCATC

---------- ---------- ---------- ---------- ----------

TAAGGAATCT ACTCCTTTCC ACTTCTGAGG AACAAACGGA AAGATGTATA

TCTGGAATCT CCTCCTTTTG ACATCCGAGG AACAAACGGA AAGGTGTATC

TAAAGAATCT ATTTCTTTCC ACTTCTGAGG AACAAATTGA AAGATGTATA

TCAAGAACCT ACTCCTTTTC ACTTCGGAGG AGCAAACTGA ACGGTGTATA

TAAAGAATCT CCTTCTCTCC ACTTCTGAGG AACAAACCGA AAGATGTATA

---------- ---------- ---------- ---------- ----------

TGAAGAACCT GCTCCTTTTG GCGTCTGAGG AACAAACCGA GAGATGTATC

TGAAAAATC- --TGCTTGTC GCTTCAGAAG AACAAACAGA AAGTTGTATA

TGAAAAATGT ATTCCTCATC ACCTCGGAGG AACAGACGGA GAGTTGTATC

ATTGAAAATA TAAGCACAAT TGCTGTAGAA GAAACTAATC AAACTGATGA

ATTGAAAATA TAAGCACAAT TGCTGTAGAA GAAACTAATC AAACTGATGA

ATTGAAAATA TAAGCACAAT TGCTGTAGAA GAAACTAATC AGACTGATGA

ATTGAAAATA TAAGCACAAT TGCTGTAGAA GAAACTAATC AGACTGATGA

---------- ---------- ---------- ---------- ----------

ATTAAAAATG AAAACACAGT TACTGTAGAA GAAACTAGTC CAATTGATGA

ATTGAGAACA CAGGTGTGGT TGCTGGAGAG GAGAATCGCG CACCTGAGGA

ATTGAGAATA CAGACACGGT CGCTGTAGAA GTAAAGCACG CGCCTGAGGA

---------- ---------- ---------- ---------- ----------

ATTGAAAATG CAAGCATTAT TACTATAGAA GAGACTGATG AAATTGATGA

ATTGAAAATA CGAACACTAT CTCTGTAGAA GAAACTAATC AAACTGATGA

ATTGAAAATA TAGACACTAT TTCTATAGGA ---ATTAATC AAATTGATGC

ATTGAAAATG TAAACAGCGT TACCGTAGAA GATGTTCGTC AAGTGGATGA

ATTGAAGATA CAATCACTAT TGCTGTAGAA GAAACTCATC AGATTGACGA

---------- ---------- ---------- ---------- ----------

ATTGAAAGGG TGAACCCTCT TCCCGAAGAA GAAACCAATC AAATGGACGA

ATTGAAGAAA CAAATA---T TGTTTTAGAA GAAACAGATC AAAATGGGAA

ATCGAAAACG CCAGCACCTC TGATCCTAAA GAAACAAGCG TGACTGATAA

AGATAAAAAA TACAGTTCCC AAACTAGCCA AGATTCAGGA AATTATTCTA

AGATAAAAAA TACAGTTCCC AAACTAGCCA AGATTCAGGA AATTATTCTA

AGATAAAAAA TACAGTTCCC AAACTAGCCA AGATTCAGGA AATTATTCTA

AGATAAAAAA TACAGTTCCC AAACTAGCGA AGATTCAGGA AATTATTCTA

---------- ---------- ---------- ---------- ----------

TGATAAAACA TACAGTTCCC AATCTAGTCA AGATTCAGGA AACTATTCTA

GGAGAGGAAG TACAGTTCCC AGAGCAGCCA GGACTCAGGG AACTACTCCA

GGACAGGAAG TACAGCTCAC AGACCAGCCA GGACTCGGGC AACTATTCCA

---------- ---------- ---------- ---------- ----------

AGTTAAAAAG TACAGTTCCC AAACTAGTCA AGATTCAGGA AACTATTCAA

AGATAAAAAA TACAGTTCCC AAACGAGTCA GGATTCGGGA AACTATTCGA

AAATAAAAAA TTTAATTCCC AAACTAGTCA AGATTCAGGA AACTACTCAT

GAATAAACAG TACAGTTCCC AGACAAGCGA AGATTCTGGC AACTATTCTA

AGATAAAAAA TACAATTCCC AGATTAGCCA AGACTCGGGA AACTATTCTA

---------- ---------- ---------- ---------- ----------

AGATAAAAAG TACAATTCCC AAACGAGTCG AGATTCAGGG AACTATTCTA

AAGTGTTATT TGTAGTAAAC AAGACAATCA AGATTCAGGA AACTTTTCCA

AAACAGCACC TGCAGCACGC AGAGCAGTCA AGATTCAGGA AACTACTCAT

ATGAAGATGA AAGCGTGTAT ATCAGCCTCG TGTTTTCATA TGATTCGCCT

ATGAAGATGA AAGCGTGTAT ATCAGCCTCG TGTTTTCACA TGATTCGCCT

ATGAAGATGA AAGTGTGTGT ATCAGCCTCG TGTTTTCACA TGATTTGCCT

ATGAAGATGA AAGTGTGTGT ATCAGCCTCG TGTTTTCACA TGATTTGCCT

---------- ---------- ---------- ---------- ----------

ATGAAGACGA AAGTGTGGTC ATCAGCCTAG TATTTTCACC GGATTGGACT

ACGAAGAGGA GGA------- ---------- ---------- ----------

ACGAAGAGGA GGA-GTGTCT GCGAGCCTAG AGACTCCGTC TGCTTTTGAT

---------- ----GTGTGC ATCAGCCTGG TGTTTTCACA TGCTTGGCTT

ACGAAGATGA AAGTGTGCAC ATCAGCCTCG TGTTTTCGTA TGTTGCGCCT

ATGAAGATGA AGGTGTGTGT ATCAGCTTCG TGTTTTCATC TGCGTTTCCT

ATGAAGATGA AAGTGTGTAC ATCGGCCTCG TGTTTTTACA GGCTTTGTCT

ATGAAGATGA AAAT------ ---------- ---------- ----------

ACGAAGATGA AAGCGTGTAT ATCAGCCTTC TGTTTTCACA TGCTTTGACT

---------- ----GTGTAC ATCAGCCTTG TGTTCTCACA TGCTTTGCCT

ACGAGGACGA AAGCG----- ---------- ---------- ----------

ACAAGGATGA AAGTGAGCTG ACCCACCTTA TGTTTTCACA TGCCTTGCTG

ATGACAGTGA GCCT-TGTAC ATCAGCCTGG TGATTTCATC CAACTCGACC

GATTACACAG ATGAATCTTG CACTTTCAAG ATATCATTGC GAAATTTCCG

GATTACACAG ATGAATCTTG CACTTTCAAG ATATCATTGC GAAATTTCCG

GATTACACAA GTGAATCTTG CACTTTTAAG ATATCATTGC GAAATTTCCG

GATTACACAA GTGAATCTTG CACTTTTAAG ATCTCATTGC GAAATTTCCG

---------- ---------- ---------- ---------- ----------

GAGTCTTCAA ATGAAGCTTG CATTTCAAAG ATGACATTAC GAAATTTCCG

---------- ---------- ---------- ---------- ----------

GGGTATCCAG ATGAACCTTG CACTATAAAC ATAACAATAC GAAATTCCCG

GATTTCACAG ATCAACCTTG TGTTTTTAAC ATGACGTTAC GAAGTCTGCG

GTTCTTTCAG ATGAACATTG CACTTTAAAG ATGAGATTCC GAAATTTCCA

GATTTGTCGG ATAAATTTTG CACTTTCAAG GTGACTTTTC GAAATTTCCG

GATTTTTCAG AGGACTTCTG CATTTTCAGG ATGACATTAA GAAATTTCCG

---------- ---------- ---------- ---------- ----------

GGTTTTTCAG ATGAACCTTG CATTTTAAAG ATGACCCTAC AAAATTTCCG

GATTTTTTAG AGAAACCTTG CACTTTAAAG ATAACGCTAC GAAACTTCCA

---------- ---------- ---------- ---------- ----------

GAAATTCCAG TGCAGTCTTG CAATTTAACG ATGACATCTC ATAATTTTCA

GAAACCCCAG CATCCTCCTT CACATTAAAG ATGGAATCTC AGAACTTCCA

GTCCATCTTA TCATGGGAAT TAAAAAACCA CTCCATTGTA CCAACTCACT

GTCCATCTTA TCATGGGAAT TAAAAAACCA CTCCATTGTA CCAACTCACT

GTCCATCTTA TCATGGGAAT TAAAAAACCA CTCCATTGTG GCAACTCGCT

GTCCATCTTA TCATGGGAAT TAAAAAACCA CTCCATTGTG GCAACTCACT

---------- ---------- ---------- ---------- ----------

GTCAATCTTA TCGTGGGAAT TAAAAAATCT TTCCATTGTC CCAACTCACT

---------- ---------- ---------- ---------- ----------

GCTAATTTTA TCCTGGGAAT TAGAGAACAA GTCTGGCCCA CCCGCTAACT

GCCAATTTTG TCATGGAAAT TAAAAAACCA TCCCATTGTA CCAACTTACT

GTCAGTCTTA TCATGGGAAT TAAAAAACCA TTCAATTGTA CCAACTCACT

GCTTATCTTA TCATGGGAAT TAAAAAACCA TTCGGTTGTG CCAGATCGCT

GGTAATTTTA TCATGGGAAT TAAGAAATCA TTCCATTGTA CCAACTCACT

---------- ---------- ---------- ---------- ----------

GCCAGTCCTA TCATGGGAGT TAAAAAACCA TTCCATTGTA CCAACTCACT

GTCAATCTTC TCATGGGAAT TAAAAAACCA TTCCATTGTA CCAACACACT

---------- ---------- ---------- ---------- ----------

GCACATTTTA TCTTGGAAAT CAAACAGCAT TGCCACTACG CCAATTTACT

TCACAACCTG ACGTGGGGAT TAAACAACTG TCCTATTACG CCCACTAGAT

ATACATTGCT GTATACAATC ATGAGTAAAC CAGAAGATTT GAAGGTGGTT

ATACATTGCT GTATACAATC ATGAGTAAAC CAGAAGATTT GAAGGTGGTT

ATACATTGCT GTATACAATC ATGAGTAAAC CAGAAGATTG GAAGATAGTT

ATAAGTTGCT GTATACAATC ATGAGTAAAC CAGAAGATTT GAAGATAGTT

---------- ---------- ---------- ---------- ----------

ATTCATTATC GTACACAGTC ATGAGTAGAG ACGAAGACAT GAAGACTGTG

---------- ---------- ---------- ---------- ----------

ACACCCTCTG GTACACAGTC ATGAGCAAAG ACGAAAATCT GACGAAGGTT

ACAAAGTACA GCACACAATT ATGAGTAAAC TAGACACTAT GAAAATAGTG

ATACATTATG GTACACAATC ATGAGTAAAC GAGAAGATAT GAAGGTCGTC

ATACATTACG GTACACGGTC ATAAGTAGAC CAGACCAGGT GAAGATTGTT

ATACACTGTG GTACACAATC ATGAGTAAAC CAGAGGATAT GAAGGTTGTG

---------- ---------- -----CAAAA AGAAGACCAT GACGCTGGTG

ATACATTATG GTACACAATC ATGAGTAAAT CAGAAGATAT GAAGATCGTT

ATACATTATG GTACACGATC ATGAGTAAAC CAGAAGGCAT GAAGATGGTT

---------- ---------- ---------- ---------- ----------

ATACTGTATG GTATAAAATT TGGAGTACCG TTGAAGAATG GAAGGCTGTT

TCACGGTGCA ATATACAGTC ATCAGTCAAG GCGACAATTT CACCGAGGTC

TGTGACGAGG CCTATGTCAC CGTCCTAGAA GGATTCAGCG GGAACAATTT

TGTGACGAGG CCTATGTCAC CATCCTAGAA GGATTCAGCG GGAACAATTT

TGTGACGAGG CCTATGTCAC CCTCCTAGAA GGATTCAGCG GGAACAATTT

TGTGACGAGG CCTATGTCAC CAGCCTAGAA GGATTCAGCG GGAACAATTT

---------- ---------- ---------- ---------- ----------

TGTGACGAGA TCTACCTCAC GCTAGTGGAA GGATTCAGAG GGAACCCTTT

---------- ---------- ---------- ---------- ----------

TGTGACGAGA GCTACGTCGC CATCGTCATA GTGCACAGAG GGGACTACAT

T--------- ---------- ---------- ---------- ----------

TGTGACGACA TGTACATCCC CCAGGTGGTT GGATTCAGAG AGAATTCTTT

TGTGACGAGA CCTACTCCAT CAGAGTTGAT GGATTCAGAG GGAACTATTT

TGTGACGAGA CGTATGTCCC CAGAGTTGTC GCATTCCGAG GGAACAGCAT

TGTGACGAGA CCTACATCCC CATGGTGTCC CGGTTCCACG GGAACGACAT

TGTGACGAGA CCTACATCCC CAAAGTTGTA GGATTCCGAG GGAGCCACTT

TGTGACGAGA CCTACATCCC AAGAGTCGTG GCATTCAAAG GGAACAGTAT

---------- ---------- ---------- ---------- ----------

TGTG--GAAT GCTACTTTAT CAATGTAACA GGCTTCAGTG GAGAGAATTT

--CGACGAGA GCTACATCAC CCAAGTGTGG GGCTTCGCCG GGGA-GAATT

CTGGCTGGCC ATAGACATGT CTTTTGAACC ACCAGAGTTT GAGATTGTTG

CTGGCTGGCC ATAGACA--- ---------- ---------- ----------

CTGGCTGGAC ATAGACATGT CTTTTGAACC GCCAGAGTTT GAGATTGTTG

CTGGCTGGAC ATAGACATGT CTTTTGAACC GCCAGAGTTT GAGATTGTTG

---------- ---------- ---------- ---------- ----------

CATGCCAGCC ATGGACATTT CTTTTGAACC ACCGGAGTTT GAGATTGTTG

---------- ---------- ---------- ---------- ----------

CGTGCCTGC- --AAACGCTC CTCTTGAGCC GCCAGAATTT GAGATCGTTG

---------- ---------- ---------- ---------- ----------

CTTCCTGGTG CCAGACAAGC CTTTGGACCC ACCAGAGTTT GAGATTGTTG

ATTCCTGGTA ACACACATGT CTTTGGAACC ACCAGAGTTT GATATTGTTG

CTTCCCAGGA ATAAACATGT CGTTGGAACC ACCAAACTTT GAGATTGTAG

CTGGCTGACC ACCAACA--- ---------- ---------- ----------

ATTCCTGGCA ACCGACATGA CTTTTGAACC ACCAGAGTTT GAGATTGTTG

CTTCCTGCCC TTAAACATGA GTTTTGACCC TCCGGACTTT GAGATTGTTG

---------- -------TAT CCTTTGAACC ACCAGACTTT GATATTCTTG

CTTTCCCATA TCAGAGACAA TTTTAGATCC ACCAGAAATT AGTATTTTTA

CATGCCGATA AAGGACACAA TTCTAGGCCC GCCTGAGGTT CACATCGCCG

GTTTTACCAA CCACATTAAT GTGATGGTGA AAAAAGGAAA CATGAGTGGA

---------- ---------- ---------- --AAAGGGAA CCTGAGTGGA

GTTTTACCAA CCACATTAAT GTGATCGTGA AAAAAGGAAA CATGAGTGGA

GTTTTACCAA CCACATTAAT GTGATCGTGA AA-------- ----------

---------- ---------- ---------- ---------- ----------

GTTTTACAGA CCATATTAAT GTGCTGGTGA AAAAAGAAAA CACCAGTGGA

---------- ---------- ---------- ---------- ----------

GCTTTACAGA CCACATAAAC GTGACGATGG AAAA---TAA TGTCACTGGG

---------- ---------- ---------- ---------- ----------

GTTTTACAAA CCACATCAGT GTGAATGTGA AAACTGGAAA CATCACCAAA

GCTTTACCGA CCACATTAAT GTCCTAGTGA AAAATGAAAA CATCACTGGA

GTTTTACTGA CCACATTAAA GTGATAGTGA AAAATGCAAA CATCACTGGA

---------- ---------- ---------- --AGTGGGAA CATGACTGGA

GTTATACAGA CCACATGAAT GTGATAGTGA AAAATAGGAA CATCACTGGA

ACTCTATGGG CCACATTAAT GTGATAGTGA AAACTGGAAA CGTCACTGGA

GTTTCAAGGA TCATATGAAG GTGTTGGTGA AGCACGGAAA CCTCACGGGA

ATTTCAAGGA TGCAGTTAAT GTCACTGTGC A-GAAATGGC TGACAAAGAA

CTCTGGAGGA TCAGATTAAT GTCACGGTGC ACATCGAGGA CGTCCCTGAA

AATTTCACCT ATATCATTGA CAAGTTAATT CCAAACACGA ACTACTGTGT

AATTTCACCT ATATCATTGA CAAGTTAATT CCAAACACGA ACTACTGTGT

AATTTCACCT ATATCATTGA CAAGTTAATT CCAAACACAA ACTACTGTGT

---------- ---------- ---------- ---------- ----------

---------- ---------- ---------- ---------- ----------

GATATCACCT ATGTCATTGA CAAGTTAATT CCAAACACAA ACTACTGTGT

---------- ---------- ---------- ---------- ----------

AACTTCACAT TTGTCCTTAG AGACTTACTT CCAAAGACAA ACTACTGTGT

---------- ---------- ---------- ---------- ----------

AATTTCAATT ATGTCATCGA CAAGTTAATT CCAAACACAA ACTACTGTAT

AATTTCACTT ACGTCATCGA CAAGTTACTT CCAAACACCA ACTACTGTGT

AATTTCACTT ATGTCATCGA CAACTTAATT CCAAACACAA ACCACTGTGT

GATTTCTCTT ACGTCATCGA CAAGTTAATT CCGAACACGA ACCACTGCGT

AATTTCAAGT ATGTCGTGGA CAAGTTACTT CCAAACACAA ACTACTGTGT

GATTTCGTTT ATGTCCTGGA CAAGTTGATT CCAAACACAA ACTACTGTGT

ACTCTCACCT ATGGCATTGA CAAGTTAATT CCAAACACAA ACCACTGCGT

AATGTAACTG TCACAACTGA TCCTCTGATT CCAAATTCAA ATTACTGTAT

GTCTTCAGCT CTGTCGTAGA AGGTTTGTTT CCGAACACCA ATTACTGCGT

ATCTGTTTAT TTATCTCCCT TAAAATGCAC CCTCCTTCCT GGCCAGGAAT

TTCTGTTTAT TTATCTCCCT TAAAATGCAC CCTCCTTCCT GGCCAGGAAT

ATCTGTTTAT TTCTCTCCCT TAAAATGCAC CCTCCTTCCT GGCCAGGAAT

---------- ---------- ---------- ---------- ----------

---------- ---------- ---------- ---------- ----------

ATCTGTATAT TTATCTCCTG TAAAATGCAC CTTCCTTCCT GGCCAAGAAT

---------- ---------- ---------- ---------- ----------

ATCTCTTTAT TT-TCTCCCT TAAAATGCAT CGTCCTTCCT GGCCAGGAAT

---------- ---------- ---------- ---------- ----------

ATCCGTTTAT TTTTCTCCCT TAAAATGTAC CCTCTTTCCC AGACGAGAAT

ATCTGTTTAC TTTTCTCCAG TAAAATGTAT CCTCCTTCCT GGACAAGAAA

ATCTGTTTAT TTTTCTCCCA CAAAATGTAC TCTGCTTCCT GGACAGGAAT

ATCAGTGTAT TTTCCAGCAA TTAAGTGCAC CCTCCT-CCT GCAGAGAATC

GTCTGTTTAT TTTTCCCCAT TAAAATGTAC TCTCCTTCCT GGCCAGGAAT

GTCTGTCTAT TTTTCCCCCC TAAAATGTAC CCTCCTTCCA GGCCTGGAAC

ATCGGTTTAT TTTCCCAGCT TGAAATGTGT CCTCCTTCCT GGCCAGGAAT

ATCTGCTCAG TGGTCATCTT CAAAATGTTT CACTTTTCCT AGCCAAGAAT

AACTGCTGAT CTTTCGGACT TAAAGTGCAT CTTCCTCTCC ACGGTGAAGT

CAGAATCAGC AGAATCTGCC AAAATAGGAG GAATAATTAC TGTGTTTTTG

CAGAATCAGC AGAATCTGCC AAAATAGGAG GAATAATTAC TGTGTTTTTG

CAGAATCAGC AGAATCTGCC AAAGTAGGAG GAATAATTAC TGTGTTTTTG

---AATCAGC AGAATCTGCC AAAGTAGGAG GAATAATTAC TGTGTTTTTG

---------- ---------- ---------- ---------- ----------

CAGAAGCATC AGAATCTGCC AAAATAGGAG GAATAATTAT CTTGTTTTTG

---------- ---------- ---------- ---------- ----------

CAGGATTATC AGAATCTGCT ---ATAGTAG GAATAACTAC TTCGTGTTTG

---AATCATC AGAATCTGCC AAAATAGGAG GAATAATTAC TGTGTCTGTG

CAGAGTCATC AGAATCTGCT ACAATAGGAG GAATAATTAC TCTGTTTTTA

CAGAGTCATC AGAATCTGCC AAAATAGGAG GAATAATTAC TATGTTTGTA

CAGAGTCATC AGAATCTGCC AAAATAGGAG GATTGATTAC TATGTTTTTG

CAGAGTCATC GGTATATGGC AAGATAGGAG TACTGGTTTT TTCAGCTTTA

CAGAGTCATC TGAATCCTAC AAAAT---AG GAATAATTGC TTTGTTTTTA

CAGAGTCATC GGAATCTGCC ATAATAGGAG GAATAGTCAC TGTGTTTTTA

CAGAGTCATC AGAATCTGCC AAAGTGGAAG GAATAATCAC TGCGTTTTTA

CAGTTTCAGA AAAAAGCACT TTAATACCAG TAGTATTTGT TGTTTGTTTC

CAGAATCCAT AAAATCACAA ATAGCAGGAG GGACGTTTGT GTTTGCTTTT

ATAGCATTGG TCTTGACAAG CACCATAGTG ACACTGAAAT GGATTGGTTA

ATAGCATTGG TCTTGACAAG CACCATAGTG ACACTGAAAT GGATTGGTTA

ATAGCGTTGG TCTTGATAAG CACCATAGTG ACACTGAAAT GGATTGGTTA

ATAGCGTTGG TCTTGATAAG CACCATAGTG ACACTGAAAT GGATTGGTTA

---------- ---------- ---------- ---------- ----------

ATGGCCGCGT TCTTGACAAG CACCATAGTA ATACTGAAAC GGATTGGCTA

---------- ---------- ---------- ---------- ----------

GTAGTGATGG TTTTCGTGAG CACTATCGTA ATGCTGAAAC GGATTGGTTA

GTAGCAGTTG TCTTCATCGC CACCGTCCTG ACACTGAAAC GGGTTGGTTA

ATAACTGCTG TCTTCATAAG CACTGTAATG ATACTGAAAC GGATTGGCTA

ATAGCGGCTG TCATCGTAAT CACCATAATA ATACTGAAAC GTGTTGGTTA

ATACTAGTTG TCTTCATAAG CACCATAGTA ATACTGCAAC ACACTGGTTA

ATGTTGTCTG CCATCTTCAG CGTGCTGACT ATTATGAGAC GAACGGGTTA

ATAGCAACTG CCATCATAAG CAGCACGATA ATACTGAAAC GGACTGGTTA

ATAGCAACTG TCTTCATAAG CAGCATAGTA ACACTGAAGC GGATTGGTTA

ATAGCAGCTT TCTTCATTAG CGGCATAATA ATACTGAAAC AGATGGGCTA

ATCATCATCC TTCTAATAAC AACCTTTGTG AAACTGAAAA ACACAGGATA

CTGTTGGTCG TCGGCCTCAC CTGTCTCACG GCCCTGAAAA TCGGAGGCTA

TATATGCTTA AGAAATAGCC TCCCCAAAGT CTTGgtcagg cctctgggtc

TATATGCTTA AGAAACAGCC TCCCCAAAGT CTTGgtcagg cctctgggtc

TATATGCTTA AGAAACAGCC TCCCCAAAGT CTTGGTGAGG CCTCTTGGTC

TATATGCTTA AGAAACAGCC TCCCCAAAGT CTTGGTGAGG CCTCTGGGTC

---------- ---------- ---------- ---------- ----------

TATATGCTTA CGAAACAATT TCCCCAAAGC CTTGGGCAGG CCTCTGAGCC

---------- ---------- ---------- ---------- ----------

TATATGCCTA AAAGACAATT TGCCCAATGT CTTGGGCAAG CCTCTGCAAC

CATATGCTTA AAGAACAAGT TCCCCAAAGC CTTG------ ----------

TATATGCTTA AGAAATGATT TCCCCAAAGT TTTGGGCAAG CT---GTGTC

TATATGCTTA AGAAATCATT TCCCCAAAGT TTTGGGCAGG CTTCTGGGTC

TATATGCTTA AAAAATAAGT TCCCCAAAGT GCTGGGCAGG CCTCTGTGTC

TATTTGTTTA AGTAACGAGT CCCCCAAAGT TTTGGGCCGG CTGCTGTCTT

CATATGCTTA AGAAAGGATT TCCCAAAAGT TTGGGGCAGG CCTCTGGGTC

TATATACCTA AGATATAACC TCCCCGATGC ATTGACCAAG CTCCTGACTC

TATATGCTTA AAAAAGTATT TCCCCAAAAC GTTGGGCAAG CCCCTGACTC

TATATGCAGA AAAAATACCT TCCCCAAAGC ATTGTCTAGA TCCCTAAGTC

CATTTGCTTA AGAAGAAATC TCCCCAAAGT TTTG-GTAGG CTACTGAGAC

aggcctctgC CACCTCTACA GAATCCCAGT TGATAGACCC GGAGTCCGAG

aggcctctgC CACCTCTACA GAATCCCAGT TGATAGACCC GGAGTCCGAG

AGACCTCCGC CACCTCTACA GAATCCCAGT TTACAGACCC AGACTCCGAG

AGACCTCCGC CACCTCTACA GAATCCCAGT TTACAGACCC AGACTCCGAG

---------- ---------- ---------- ---------- ----------

AGGCCTCTGC CACATCTGTG GAGTCCCAGT CCACAGACCC AGACTCCTCT

---------- ---------- ---------- ---------- ----------

AAACCTCTGA CACCTCAGCA GGATCCCATG AAGAAGATTC AGGCGCTGAG

---------- ---------- ---------- ---------- ----------

CAACTTCCAC CACTTCACCa ggactgcagc ttgtgGAGCC CAGTGCTGAG

CAGCCTCTGC CTCCTCGGCA GGACTGCA-- ---TGGAGCC GGACGCTGAG

CGGCCTCTGG CTCCTCCTCA GGACTGC--- ---TGGACCC AGATGCTGAG

TGGCCTCCTC CTCCTCCTCA CGGCCCTG-- ---AGTACCG GGGCTCTGAG

CAGCCTCTGC CTCCTCATCG AAGTCCCAGT TCTCAGACCC AGACACTGGG

AGGCTTCGTC CACCTCCAAG GAGTCACAGC TCATAGACCT AGATGTGGGT

GGGCCTCGGC CACCTCTGAG GAGTTGTCCT TGGTAGGCCC AGGTGTGGAT

AGGACTCCAC CACCCAGAAA GAAGGTAATC CCACTGGTTC GGGTCCAAAG

AGGTCTCAGC TGCCTCCACG GAGTCCCGGT TGGGAGGGCT GGCTTCCGGG

GAGCCTCTGA TGCTATCGTC TGACCCTCTT TCCCAGCTGC CCGCTCCTCA

GAGCCTCTGA TGCTATCGTC TGACCCTCTT TCCCAGCTGC CCGCTCCTCA

GAGCCTCTGA TGATATCATC T-ACCCTCTT TCCCAGCTGC CCACGCCTCA

GAGCCTCTGA TGGTATCATC T-ACCCTCTT TCCCAGCTGC CCACGCCTCA

---------- ---------- -GACTCTCTT TCCCAGCTGC CCACTCCTCA

GACCCTCTGA TGTTGTTATC T-ATTCTCTT TCCCAGCTGC CTGCCCCTCA

---------- ---------- --ACTCCTTG TCCCAGCTTG CTGCCCCTAG

GAGCCTCAGA -GACATTATC T-ACTCGTTT TCCCAGCTTG CGGCCCCTCT

---------- ---------- ---------- ---------- ----------

GA-ACTCTAA TGTCCCCATC T-ACTCCCTG GCCCAGCTGC CTGCTCCTGG

GA-CCTCCAG GGCTACCAAG T-ACTCCCTT TCCCAGCTCC CTGCTCCTCG

GA-CCTCCAG AGTT---ATC T-ACTCTCTT TCCCAGCTGC CTGCCCCTCA

GG-------- ---------- --ACCCTCTT GCCCAGCTGC CAGCTCCTCA

GA-CCGCCAA CGTTACCGTC T-accctcct gctcagctgc ccgctcctcA

GA-TCACCGA TGCTTCCA-- --ACCCTCTT TCCCAGCTGC CCCCTCCTCA

GA-GCTCCGA TGCTTCCATA T-ACACTCTC CCCCAGCTGC CCGCTCCTCA

GAAGCTCAGA TATTATTGCC T-GACCGCGC ACCCAGCTAC CAGTTCCACA

CA-CCTCCAG GTGTGCTATT T-AGCCGCTG TCTCAGCTAC CGGCCCCGCG

GCACCCGAAG ATTCGCCTGT ACAACGCAGA GCAGGTCCTG AGTTGGGAGC

GCACCCGAAG ATTCGCCTGT ACAACGCAGA GCAGGTCCTG AGTTGGGAGC

GCACCCGAAG ATTCGCCTGT ACAATGCAGA GCAGGTCCTG AGTTGGGAGC

GCACCCGAAG ATTCGCCTGT ACAACGCAGA GCAGGTCCTG AGTTGGGAGC

GCACCCGAGG ATTCACCTAT ACAACGAAGA ACAGGTCCTG AATTGGGAGC

ACACCCGAGG ATTCACCTGT ACAACGCCAA GCAGGTTCTG AGTTGGGAGC

GAAGCTGAAG CTTCACCTGT ACAATGACGA GCAGATGCTA AGCTGGGAGC

GAACCCAAGG CTTCACCTGT ACAATGATGA GCAGATTCTA ACTTGGGAGC

---------- ---------- ---------- ---------- ----------

AAACCTGAAG GTTCACCTGT ACAACGCCCA GCAGGCGCTG AGCTGGGAGC

AAACCCGAAG GTTCACCTGT ACAACGCCGA GCGGGTTCTG AGCTGGGAGC

GAACCCGAAG ATTCACCTGC ACAAAACCAA GCAGGTACTG AGTTGGGAGC

GAACCTGAAG ATTCGCCTCT ACAACGCGCA GCAGGTTATG AGCTGGGATC

AAGGCCGAAG CTCCACCTGT ACAACGCGAA GCAGCGCCTG AGCTGGGAGC

GAACCCGAAG ATTCGCCTGT ACAACGCGGA GCAGGTTCTG AGTTGGGAGC

AAACCTGAAG ATTCACCTGT ACAACGCGGA GCAGGTTCTG AGTTGGGAGC

GAACCAAAGA ATCCACCTGT ATAATTTAGA GCAAGTTCTG AGCTGGGATC

GAACGCCACG CTTCACCTGT ACAACCAGGA GCAGGTCCTG CGATGGCAGC

CAGTGGCCCT GAGCAATAGC ACGAGGCCTG TTGTCTACCA AGTGCAGTTT

CAGTGGCCCT GAGCAATAGC ACGAGGCCTG TTGTCTACCG AGTGCAGTTT

CTGTGGCCCT GAGCAATAGC ACAAGGCCTG TTGTCTACCG AGTGCAGTTT

CGGTGGCCCT GAGCAATAGC ACAAGGCCTG TTGTCTACCG AGTGCAGTTT

CAGTGACCCT GAGCAATGGC ACGAGGCCTG TGGTCTACCG GGTGCAGTTT

CAGTGGCCCT GAGCAATGAC ACAAGGCCGG TGGTCTACCG GGTACAGTAT

CTTCGCCCTC CAGCAACGAC ACGAGACCAG TGGTCTACCA GGTGGAGTAC

CGTCACCTTC CAGCAATGAC CCAAGACCAG TGGTCTACCA GGTGGAATAT

---------- ---------- ---------- ---------- ----------

CGGTGTCCCT GGACAGCGAC CCGAGGCCGG TGGTCTACCA GGTGCAGTAT

CAGTGTCCCT GAGCAATGAC TCGAGGCCCG TGGTCTACCA GGTGCAGTAT

CAGTGTCCCT GGACAATGAC ACAAGGCCAG TGGTCTACCG GGTGCAGTAC

CCGTCCCCCC GCCCGACGAC ACGGGGCCGG TGGTCTACCA GGTGCAGTAC

CCGTGTCCCT GGGCAATGGA ACGAGGCCAG TGCTCTACCA CGTGCAGTAT

CGGTGTTCCT GAGCAATGAC ACGAGGCCGG TGACCTACCG GGTGCAGTAC

CGGTGACCCT GAGCAACGAC TCCAGCTCGG TGGCCTACCG GGTGCAGTAT

CCGTCCCCAT AAGCAACCTG AAAAGCCCCG TGTTATACAG TGTGGAGTAT

CGGTCAGCCT GAGCGATCTC CCCGGCGCCG TGACCTACAG CGTGGAGTAC

AAATACTTCC CAATGGATTT CAATGTCACT CTACGCCTTC GAGCTGAGCT

AAATACTTCC CAATGGATTT CAATGTCACT CTACGCCTTC GAGCTGAGCT

AAATACTTCC CAATGGATTT CAATGTCACT CTACGTCTTC GAGCTGAGCT

AAATACTTCC CAATGGATTT CAATGTCACT CTGCGTCTTC GAGCTGAGCT

AAATACTTCC CAATGGATTT CAATGTCACT CTACGCCTTC GAGCAGAGCT

AAATACTTCC CAATGGATTT TAATGTCACT CTGCGCCTTC AAGCTGAGCT

AGCTACTTCC CATACTCATA CACAGTCTTC CTGCGCGTGC GAGCCAGGCT

AGCTTCTTCC CACACCCATT CACAGTCTTC CTGCGGGTGC GAGCCAAGCG

---------- ---------- ---------- ---------- ----------

AAATACTTCC CACGGCGTTT CAACATCTCT TTACGTGTTC GAGCTAAGCT

AAATACTTCC TACCACACTT AAACGTCTCC TTGCGCGTCC GAGCTAAGGT

AAATACTTCC CACCACATTT TAACGTCTCT TTACGTGTCC GCGCTGAGCT

AAATA----- ---------- ---------- ---------- ----------

AAATATTTCA CACCGTATTT CAATATCACT TTACGCCTTC GAGCTGAGCT

AAATACTTCC CACGGCACTT CAATGTCTCT TTGCGTGTTC AAGCTGAGCT

AAATA-TTTC CCAGGCACTG ---------- ---------- ----------

CATTACTTCC AGCCTCATTT TAACGTGACT TTACGTGTTA GAGCGGAGCG

AGATA-TTCA TGCCCAACTT AAACGTGACC CTGCGGGTCC GCGCCCAGCT

GGGAGCACTC CATTCTGCCT GGGTGACAAT GCCTTGGTTT CAACACTATC

GGGAGCACTC CATTCTGCCT GGGTGACAAT GCCTTGGTTT CAACACTATC

GGGAGCACTG CATTCTGCCT GGGTGACAAT GCCTTGGTTT CAACACTATC

GGGAGCACTG CATTCTGCCT GGGTGACAAT GCCTTGGTTT CAACACTATC

GGGAGCACTC CATTCTGCCT GGGTGACAAT GCCTTGGTTT CAGCACTATC

GGGAGAACTG CACTCTGCTT GGGTGACAAT GCCTTGGTTT CTACACTATC

CGGAAAGCAC ACTTCCCACT GGGTGAAGCT GGATCCATTT CAACACTATG

AGGGAACCTC ACTTCCAAGT GGGTGGGGCT GGAGCCATTT CAACACTATG

---------- ---------- ---------- ---------- ----------

CGGGGGCCTC GTTTCTGCCT GGGCAACAGC ACCTTGGTTC GAACACTATC

GGGAGACCTG GTTTCCACCT GGGTCAGAGT GCCATGGTTC CAGTACCTTA

GAAAGAACGT GTTTCTGACT GGGTGACAGT GCCTTGGTTC CAGCACTTTC

---------- ---------- ---------- ---------- ----------

GGGAGAACTC GTTTCTGCCT GGGTGGTAGT ACCTTGGTTT CAACACTACC

CGGAGAACGT GTCTCTGCCT GGGCGACGGT GCCTTGGTTT CAACACTACC

-GGAGAGCTT GTTTCCGCCT GGGCAGTGGT ACCTTGGTTT GAACACTACC

AGGAGCACTG GTTTCTGATT GGGTGGTGAT GCCTTGGTTT CAGCAATACC

CGGGGCCCTG TCGTCCCCCT GGCTCCAGCT GCCGTCCTTC AAGCTGTACT

GGAATGTGAC TGTCGGGCCT CCAGAAAACA TTGAGGTGAC CCCAGGAGAA

GGAATGTGAC TGTCGGGCCT CCAGAAAACA TTGAGGTGAC CCCAGGAGAA

GGAATGTGAC TGTCGGGCCT CCAGAAAACG TCGAGGTGAC CCCAGGAGAA

G-----TGAC TGTCGGGCCT CCAGAAAACA TTGAGGTGAC CCCAGGAGAA

GGAATGTGAC TGTTGGACCT CCAAAAAACA TTGAGGTGAC CCCAGGAGAA

TGAATGTTAC TATTGGGCCT CCAAAAGATG TTGGGGTGAC TCCAGGAGAA

AGAATGTTAC TGTTGGACCT CCAAAAAACA TCTCGGTGAC CCCAGGGAAA

AGAATGTTAC TGTTGGACCT CCGAAAAACA TCTCGGTGAC CCCAGGAAAA

------TGAC CGTCGGGCCT CCCCGGAACG TGCGGGTGAC CCCTGGAGAA

GGAATGCTAC CATTGGGCCT CCAGAAAACA TCCGGGTGAC CCCAGAAGAA

GGAATGTGAC CGTTGGGCCT CCTGAGAACG TTTGGGTGAC TCCAGGAGAA

GGAACGTTAC CATCGGGCCT CCAGAAAACA TTTGGGTGAC CCCAGGAGAC

------TTAC CATCGGGCCT CCAGAAAACC TGTCGGTGAA GCCTGGAGGA

GAAATGTTAC CATTGGGCCT CCAGAAAACA TTTCGGTGAC CCCAGGAGAA

GGAATGTTAC TGTTGGGCCT CCAGAAAACA TTGAGGTGGC TCCAGGAGAC

GCAATGTGAC CATCGGGCAT CCAGAAGACA TCAGGGTGTC AACAGAAGAA

TAAATGCAAC AATTGGACCT CCATTAGATG TAGTTGTGAC GCCACTTAAT

ACAACGTCAC TATCGGGCCA CCTAAGAACA TCCAGGTGAC TTCTGAGGAC

GGCTCCCTCA TCATCAGGTT CTCCTCTCCC TTTGACATCT TTTGTTATTA

GGCTCCCTCA TCATCAGGTT CTCCTCTCCC TTTGACATCT TTTGTTATTA

GGCTCCCTCA TCATCAGGTT CTCCTCTCCC TTTGACATCT TTTGGTATTA

GGCTCCCTCA TCATCAGGTT CTCCTCTCCC TTTGACATCT TTTGGTATTA

GGCTCCCTCG TCGTCAGGTT CTCCCCTCCC TTTGACATCT TTTGGTACTA

GGCTCCCTCA TCATTAACTT CTCCCCTCCC TTTGATATCT TTGAGTATTA

GGCTCCCTCG TCATACACTT CTCCCCTCCC TTTGACGT-T TTCAGTATTT

GGTTCCCTCG TCATACACTT CTCCCCTCCC TTTGATGT-T TTCAGTATCT

GGCTCCCTCA TCGTCATGTG CGCCCCTCCC TTTGATATCT TTCAATATTA

GGCTCCCTTA TCATCAGGCT TTCTGCTCCC TTCGATGTCT TTGTGTATTG

GGCTCCCTTG TCATCAGGTT TTCCTCTCCC TTTGACATCT TTTTGTATTA

GGCTCCCTTA TCATCAGGTT CTACCCTCCC TTTGACATCT TTGTGTACTA

GGCTCCCTCA TCGTCTCCTT CTCGGACCCC TTCGAAATAC GTGTGTATTT

GGCTCACTGA TCATCATGTT CTCCTCTCCC TTTGACGTCC TTGAGTATTA

GGCTCACTGA TCATCAGGTT CTCTGCCCCC TTTGACGTCT TTCAGTATTA

GGTTCACTTA TCATCGGGAT TTCCACTCCC TTTGACAT-T TTCAGTATTA

GACTCTCTTG GCATCAGATT CAAGCCTCCC TTCAATGTG- -TAAGTATTA

GGGACTCTCC GCCTGACCTT TTCTCCGCCT TTTGAGGACT TCCAGTATTA

TGTCCATTAC TGGGAAAAAG GAGGAATCCA ACAGGTCAAA GGCCCTTTCA

TGTCCATTAC TGGGAAAAAG GAGGAATCCA ACAGGTCAAA GGCCCTTTCA

TGTCCATTAC TGGGAAAAAG GAGGAATCCA ACAGGTCAAA GGCCCTTTCA

TGTCCGTTAC TGGGAAAAAG GAGGACTCCA GCAGGTCAAA GGCCCTTTCA

TGTCTATTAC TGGGAAGAAG CTGGAGTCCA AAAGGTCAAA GGCCCTTTCA

TGTCCATTAC TGGGAAAAAG CAGGAATCCA AAAGGTGAAA GGCCCTTATA

TGTCCACTAC TGGGAAAAGA CAGAAACCCA AAAGGTTAAA GGCCCTTACA

TGTCCACTAC TGGGAAAAGT CAGAAACCCA ACAGGTTGAA GGCCCTTTCA

CGTCCACTAC TGGGAAAAAG CAGGAATCCA TCAGGTGAGA GGTCCCTTCA

TGTCTACTAC TGGGAGAAGG CAGGAGCTAA ACAGGTGACA CGCCCGTTCA

CGTCCATTAC TGGGAAAAGG GCGGAATCCA AAAGGTTAAA GGCCCTTTCA

TGTCCGTTAC TGGGAAAGGG AAGGAATCCA ACAGGTTAAA GGCCCTTTCA

TGTGTGCTAC TGGGAGGAGG GAGGAGGCCA GAAG------ ----------

TATCCATTAT TGGGAAAAAG AAGGAATCCA AGAGGTGAAA GGCCCTTTCA

CGCCCATTAC TGGGAAAAGG CCGGAACCCA AAAGGTTAAA GGCCCTGTCA

CGTCTATTAC TGGGAAGAGG CCGGAACCCA AAAGGTGAAA GGTCCCATC-

TGTCCATTAC TGGAAGGATG AAGGAAAACC AATGGTCCAG GGCCCTTTCC

CGTCCACTAC TGGCCCAAAA CGGGGATCAA AAAGACCCTA GGTCCCGTCA

GAAGCAACTC CATTTCATTG GATAACTTAA AACCCTCCAG AGTGTACTGT

GAAGCAACTC CATTTCATTG GATAACTTAA AACCCTTCAG AGCGTACTGT

GAAGCAACTC CATTTCATTG GATAACTTAA AACCCTTCAG AGTGTACTGT

GAAGCAACTC CATTTCATTG GATAACTTAA AACCCTTCAG AGTGTACTGT

GAAGCAACTC CATTTCATTG GATAACTTAA AACCCTTCAG AGTATACTGT

AGAGCAACTT CATTTTGTTG GATGGTTTAA AACCCTTCAG AGTATACTGC

AGAGCAACTC CATTGTGCTG GATGATTTGA AGCCATTCAG AGTATATTGT

AGAGCAACTC CATTGTGCTG GGCAATCTGA AGCCATACAG AGTATATTGT

AGAGCAGCTC CATTTTGCTG GAGGATTTGA AACCCTTCAG AGTCTACTGT

GGAGTAACTT CATCACATTG AATGATTTAA AACCCTTAAG AGTATACTGT

GGAGCAACTC CATTGTGTTG AATGACCTGA AGCCCTTAAG AGAATATTGT

GAAGCAACGT CATTGTATTG AAGGATTTAA AACCCCTGAG AACATACTGT

---------- ---------- ---------- ---------- ----------

TAGGCAACTC CATTGTGCTG GGCGGTTTGA GACCCTTAAG AGAATACTGT

AGAGCAATTT CATTTTGTTG GATGGCTTAA AACCCTTACG TGTATACTGT

--AACAACTT CATTTTGCTG GATGGCTTAA AACCCTTAAG TGTATACTGT

CTGACTCGGC CATCCATCTG AAAGACTTCG ACAGCCCTGG GAACTACTGC

GCAACAAGTG GCTGCTGTTG GAAGACGTCC GTCCGGTGAC GGAATACTGT

TTACAAGTCC AGGCACAACT GCTTAGTAAC ATCTTTAGAG TCGGGCATTT

TTACAAGTCC AGGCACAACT GCTTAGTAAC ATCTTTAGAG TCGGGCATTT

TTACAAGTCC AGGCACAACT GCTTAGTAAC ATCTTTAGAC TGGGGCATTT

TTACAAGTCC AGGCACAACT GCTTAGTAAC ATCTTTAGAC TGGGGCATTT

TTACAAGTCC AGGCACAACT GCTTAGTAAC GT------AA ACGGGCACTT

TTACAAGTCA AGGCACAACT GTTT---GGC ACCATCAGAT CTGGGCATTT

TTACAAACCA AGGCCCAACT GATTAGAAAT ATCGGACCGC ACGGGCTCCT

TTACAAACTG AGGCACAACT GATTAAAAAA ATCCGACCAC ATGGGCTCTT

TTGCAAGTCG AGGCGCAACT GCTCCAGAAC GTCATCCGGC CCGGGCATTT

TTTCAAGTCA AGGCAGAACT GTTTGAAAAC ATCTCTCGAC CTGGACATTT

TTACAAGTCA AGGCGCAACT GGTTTCAAAG CTCTCTAGAC TCGGACATTT

TTACAAGTCA ATGCACAACT GTCTCCAGAC ATCTCCAGAG TCGGGCATTG

---------- ---------- ---------- ---------- ----------

TTACAAGTCA GGGCAGAACT GATTCAAAAC ATCTGCAGAC CCGGGCAGTT

TTACAAGTCA AGGCAGAACT TGTTCAAGGC ATCGTTCGAC CTGGGCACTT

TTACAAGTCA AGGCAGAACT GGTGAAACGT ATCTTTCAAC CTGGGTGTTT

TTACAAGTGC AAGCGGAACT GACTGTGAAG CCGCCTCGCA GAGGGCTGCT

CTGCAGGTGC AGGCCAGCCT GC---AGAGC CCCTTCAGGA TCGGCCTCCT

AAGCAACATA TCTTGCTACG AAACAATGGC AGATGCCTCC ACTGAGCTTC

AAGCAACATA TCTTGCTACG AAACAATGGC AGATGCCTCC ACTGAGCTTC

AAGCAACACA TCTTGCTACG AAACGATGGC AGATGCCTCC ACCGAGCTTC

AAGCAACACA TCTTGCTACG AAACGATGGC AGATGCCTCC ACCGAGCTTC

AAGCAACACA TCTTGCTACA AAATCATGGC AGATGCCTCC ACCAAGCTTC

AAGCAACACA TCTTGCTACG AAACAATGGC CGATGCCTCC ACCAAGCTTC

GAGCAATGTA TCTTGTCACG AAACAACAGC GGATGCCTCC ACCAGCCTGC

GAGCAATGTA TCCTGTCACG AAACAACAGC AAATGCCTCC GCCAGGCTGC

AAGCAACGCA TCTTGCCTGG AAACAACAGC AGATGCCTCC ACCAAACATC

AAGCAACATA TCTTGCTGTG AAACGGCAGC AGATGCTTCT GTCAAGCTTC

AAGCAACGTA TCTTGCTACG AAACAGCAGC AGATGCCACT ACCAAGCTTC

GAGCAACATA ACTTGTCACG AAACAATAGT AGATGCCTCT ACCGAGCTTC

---------- ---------- ---------- -----CCTCC ACCGAGCTGC

AAGCAACACA TCGTGCTGTG AAACAGCCGC CAATGCCTCG GCCAAGCTTC

AAGCAACATA TCTTGCTCTG AGACAACAAC TGATGCTGCT ACTGAACTTG

AAGCAACCCA TCTTGCTATA AGATATCAGC AAATGCTGCC ACTCGGCTCC

AAGTCCCATC TCTTGTCACA AAATCTACGC CAAGGCATTA ACTAAAGTTC

GAGCAATACG ACTTGCCTCG AGACCACGGC TGACGCTGTG ACCAGAGCCC

AGCAAGTCAT CCTGATCTCC GTGGGAACAT TTTCGTTGCT GTCGGTGCTG

AGCAAGTCAT CCTGATCTCC GTGGGAACAT TTTCGTTGCT GTCGGTGCTG

AGCAAGTCAT CCTGATCTCC GTGGGAACAT TTTCGTTGCT GTCGGTGCTG

AGCAAGTCAT CCTGATCTCC GTGGGAACAT TTTCGTTGCT GTCGGTGCTG

AGCAAGTCAT CCTGATCTCT GTGGGAACAT TTTCGTTGAT GTCGGTGCTG

AGCAAGTCAT CCTGATCTCT GTGGGAACCT TCTCATCACT GTTGGTCCTG

AGCAAGTCAT CCTGATCCTT TTTGGCATCT TCATGTTGCT GCTGGCACTG

AGCAAGTCAT CCTGATTCCG TTGGGCATCT TCGCATTGCT GCTCGGCCTG

AGCAAATAAT CCTGATCTCG CTGGGAGTCT TTTCATTGCT GTTGGTACTG

AACAAGACTT CCTGGCTGCC GGAACCACCT TTTTGGTGCT GTCAGTGGTG

AGCAAATCAT CATAATTGCC GTGGGAATCT TTCTGTTGCT GTTGGCACTG

AACAAATCAT CCTGATTGCT GTGGGAACCT TTTTGTTGCT GACAGTGCTG

AGCAGGCCAT CGGCATCGCC GTGGGCGTCT TCCTGGCGCT GGCCATCCCG

AGCAGGCCAT CCCGATCTTC GCGGGAACCT TCCTGGTAGT GCTGGCGCTG

AACAAGCCAT CCTGATCTTT GTGGGAACCT TTCTGTCAGT GGTGTTGCCA

AACAAGCCAT CCTGATCTTT GTGGGAATCT TCCTGTcggt gacgCTGCTA

AACAAACTAT CATAATTTCC GT---ATTCT TTACATCACT GTTTTTTCTT

AGTATGCCTT CTTAATCTTT CTGGGGGCCC TGTTAGCCAT GGTGACCCTG

GCAGGAGCCT GTTTCTTCCT GGTCCTGAAA TATAGAGGCC TGATTAAATA

GCAGGAGCCT GTTTCTTCCT GGTCCTGAAA TACAGAGGCC TGATTAAATA

GCAGGGGCCT GTTGCTTCCT GGTCCTGAAA TACAGAGGCC TGATTAAATA

GCAGGGGCCT GTTTCTTCCT GGTCCTGAAA TACAGAGGCC TGATTAAATA

GCAGGGGCCT GTTTCTTCCT GGTCCTGAAA TACAGAGGCC TGATCAAATA

GCAGGGGCCT GTTTCTTTCT GGTCCTGAAA TACGGAGGCC TGATTAAATA

ACGGGAGCCT GCTTCATCCT GTTCCTCAGA TACCAAAGCC GGGTGAAATA

ACGGGCGCCT GCTTCACCCT GTTCCTCAAA TACCAAAGCC GAGTGAAGTA

GCAGGGACCT GTTACTTTCT GGTCCTCAAA TATAGAGGCC TCGTCAAATA

GTAGGGTCCT GTCTCTTCCT GGTTCTGAGA TACAGAGGCC TGGTTAAACA

GCAGGGGCCT GTTTCTTCCT GGTCCTGAAA TACAAAGGCC TGATAAAATA

GCAGGCGGCT GTTTCTTCCT GGTCCTGAAA TATAGAAGCC TGATTAAACA

GTGGGGGCCT GCCTCTTCCT CGTCCTGAGA TACAAGGACC TCATCAAGGC

GCAGGGGCCT GTTACTTCCT GATGCTGAGA TACAGGGGCC TGATTAAATA

ATGGGAGCCT GTTTCTTCCT GGTCCTGAAA TACAGAGGCC TGGTTAAATA

ATAACGGTCT GCTTCTTCCT GACCCTGAAG TACAGAGGCC TGATTAAGTA

GTCCTGGGTT GCTACTTTGT GGCACAGAAA TTCCAAGGCC CAATAAAGCA

GCCTTCGGCT GCTTCCTGCT GGTGCGGTAT TCGCGCGGCC TCATCAAGTA

CTGGTTTCAC ACTCCACCAA GCATCCCATT ACAGATAGAA GAGTATTTAA

CTGGTTTCAC ACTCCACCAA GCATCCCATT ACAGATAGAA GAGTATTTAA

CTGGTTTCAC ACTCCACCAA GCATTCCATT ACAGATAGAA GAGTATTTAA

CTGGTTTCAC ACTCCACCAA GCATTCCGTT ACAGATAGAA GAGTATTTAA

CTGGTTTCAC ACTCCACCAA GCATCCCGTT ACAGATAGAA GAGTATTTAA

CTGGTTTCAC ACTCCACCGA GGATCCCATC ACAAATAGAA GAGTATTTAA

CTGGTTTCAA GCTCCACCAG ACATCCCAGA ACAAATAAAA GAGTATTTAA

CTGGTTTCAG GCTCCGCCAA ACATCCCGGA ACAAATCGAA GAGTATCTAA

CTGGTTCCAC TCTCCGCCAA ACATCCCACT GCAAATAGAA GAGTATTTAA

CTGGTTCCAC TCTCCGCCAA GCATTCCATC ACAGATAGAG GAGTATTTAA

CTGGTTTCAC TCTCCGCCAA CCATCCCATT ACAAATAGAA GAGTATTTAA

CTGGTTTCAG TCTCCACCAA GCATCCCATT ACAAATAGAA GAGTATTTAA

CTGGTTCCAC ACGCCGCCCC GCATCCCCTT ACAGATCGAA GAG-------

CTGGTTCCAC TCTCCGCCAA GGATCCCATC CCAGATAGAA GAGTATTTAA

CTGGTTTCAT TCTCCACCCA AGATCCCATT ACAAATAGAA GAGTATTTAA

CTGGGTTCA- -CTCCACCCA AGATCCCGTT ACAGATAGAA GAGTTTTTAA

TGTTTTCCAG TCACCGCCGG GAATCCCGCC TCAGATCAAA GAGTATTTAA

CTGGTTCCAG AAGCCCCCCA CCATCCCCTC CCAGATCGGA GAGTACCTGG

AAGACCCAAC TCAGCCCATC TTAGAGGCCT TGGACAAGGA CAGCTCACCA

AGGACCCAAC TCAGCCCATC TTAGAGGCCT TGGACAAGGA CAGCTCACCA

AGGACCCAAC CCAGCCCATC TTAGAGGTCT TGGACAAGGA CAGCTCACCA

AGGACCCAAC CCAGCCCATC TTAGAGGTCT TGGACAAGGA CAGCTCACCA

AGGACCCTGC TCAGCCCATC TTAGATGCCT TGGACAACGA CAGTTCACCA

AGGACCCAGC TCAGCCCATC TTAGAGGTCT TGGACAAAAA CAACTCACCG

AGGACCCAGA TCAGCTCATC CTGGAGGCCT TGGACAAGGA CGGTTCGCCG

AGGACCCAGA CCAATTCATC TTAGAGGTCT TGGACAAGGA CGGTTCACCG

AGGACCCGGA TCAGCCCATC TTGGAGGCCC TGGACAACGA CAGCTCACCC

AGGATCCGGC TCAGCCCATC TTAGATGCCC TGGACAAGGA CAGCTCGCCA

AGGACCCAAG CCAGCCTATC TTAGAGGCCC TGGACAAGGA CAGCTCGCCA

AGGACCCGGC CCAGCCGATT TTAGAGGCTT TGGAGCAGGA CAGCTCGCCA

---------- ---------- ---------- ---------- ----------

GGAACCCGGA TCAGCCTATC TTAGAGGCCT TGAGCAAGGA CAGCTCGCCA

AGGACCCAGC TCAACCTATT TTGGAGGCCT TGGACAAGGA CAGGTCACCG

ATCACCCAGC TCATCCTGTG TTGGAGGCCC TGGATAAGGA GAGTTCGCCA

GTGACCCCGA TGAGCCTATC CTAGAAGCCC TGGACAAGAA AAGCTCACCA

AGGACCCCAA GCTGCCCATG TGGGAGGAGC TGATGCAGAA CCCTCCGCCG

AAGGATGTCT GGGACTCTGT GTCCATTATC TCGTTTCCGG AAAAGGAGCA

AAGGATGTCT GGGACTCTGT GTCCATTATC TCGTTTCCGG AAAAGGAGCA

AAGGATGTCT GGGACTCTGT GTCCATCATT TCCTTTCCAG AAAAGGAGCA

AAGGATGTCT GGGACTCTGT GTCCATCATT TCCTTTCCAG AAAAGGAGCA

AAGGATGTCT GGGACTCTGT GTCCATCATT TCATTTCCAG AAAAGGGGCA

AAGGACGCCT GGGACTCTGT GTTCATTGTT TCATTTCCAG AAAAGGAGCA

AAGGATGCCT GGGACTCCGT ATCAATCATT TCGTCTCCAG AAAAGGAGCA

AAGGAGTCCT GGGACTCCGT GTCAATTATT TCTTCTCCAG AAAAGGAGCG

AAGGATACCT GGGACTGTGT GTCCGTCATT TCATTCCCAG CAAAGGAGCC

AAGGATACTT GGGACTCTGT GTCGGTCGTG ACGTTTCCAG AGAATGAGCA

AAGGATGCCT GGGACTCTGT GTCCATAGTT TCGTTTCCAG AGAAGGAGCG

AAGGATGCCT GGGACTCCGT GTCCATCGTT TCGTTTCCAG GGAAGGAG--

---------- ---------- ---------- ---------- ----------

GAGGAGGCCT ATGACTCTCT GTCCATCGTC GCCTTTCCAG AAGGGGAGAG

AAGGAAGCCT GGGACTCTGT GTCCATCGTT TCACTTCCAG AAAAGGAGCG

AAGGAGTCCT GGGTCTCTGT GTCCATCCTG TTGCTTCCAG AAAAGGAACC

GAAGAT--CT GGGACTCCTT ATCTGTTATT TCCTGTCCAG AAGAGGATCA

AAGGGT-CCT GGGATTCGGT GTCGGTCCTG TCGTGGGCTG ATGACCACCA

AGAAGCATTG GGAATGGTAC CACCTCCCGA AAATGTCAGA ATGAATTCTG

AGAAGCATTG GGAATGGTAC CACCTCCCGA AAATGTCAGA ATGAATTCTG

AGAAGCATTG GGAATGGTAC CACCTCCTGA AAATGTCAGA ATGAATTCTG

AGAAGCATTA GGAATGGTAC CACCTCCTGA AAATGTCAGA ATGAATTCTG

AGAA------ ---------- ---------- ---------- ----------

CGAAGCCTTG GGAATGGTGC CACCTCCTGA AAATGTCAGA ATGAATTCAG

GGAT------ ---------- ---------- ---------- ----------

AGATGCTCTA GGAATGATTC CACCCCCTGA GAAGGTCAGA ATGAATTCAG

CGAGG----- ---------- ---------- ---------- ----------

AGAAGCATTA GGAATGGTTC CACCTCCTGA AAATGTCAGA ATGAATTCAG

AGAAGCATTA GGGCTGGTGC CACCTCCTAA AAACGTCAGA ATGAAGTCAG

-GGAGCGTTA GGAATGGTGC CTCCTCCTGA AAACGTCAGA ATGAATTCAG

-----CCTCA GCAGTGGTGC CACCCCCTGA AAATCTCCAC ATGCATTCAC

AGAAGCCTTA GGAATGGTGC CGCCTCCTGA AAATGTCAGG ATGAACTCAG

GGAAGGGTTG GGAATGGTGC CTCCTCCGGA AAACGTCAAA ATGAATTCAG

AGAAGCATTA GGCATGGTGC CTCCTCCCCA AAATGTCAGA ATGAATTCTG

GACCGTGCTA GGAATTCTTC CCAAGCCCCG GAACGTAAGA ATGAATTCAG

AG---TGCTG GGAGCAGTGC CAAAGCCCGA AAATGTCAGG ATGAATTCGG

TTAATTTCAA GAACATTCTA CAGTGGGAGT CACCTGCTTT TGCCAAAGGG

TTAATTTCAA GAACATTCTA CAGTGGGAGT CACCTGCTTT TGCCAAAGGG

TTAATTTCAA GAACATTCTA CAGTGGGAGT CACCTGCTTT TGCCAAAGGG

TTAATTTCAA GAACATTCTA CAGTGGGAGT CACCTGCTTT TGCCAAAGGG

---------- ---------- ---------- ---------- ----------

TTAATTTCAA GAACATTCTA CAGTGGAAGT CACCTGCTTT TCCCAAAGGG

---------- ---------- ---------- ---------- ----------

TTAATTTCAA GAACATTCTA CAGTGGGAGG TACCTGCTTT CCCCAAAACG

---------- ---------- ---------- ---------- ----------

TTAATTTCAA GAATATTCTA CGATGGGAGT CACCTGCTTT TCCCAAGGGG

TTAATTTCAA GAACATTCTC CAGTGGGAGT TGCCTGCTTT TCCCAAAGGG

TTAATTTCCG GAATATTCTA CAATGGGAGT CGCCTGCTTC TCCCAAAGGG

TTAACTTCAA GAACATTCTC CGGTGGAAGT CACCTGCTTT TCCCAGAGGG

TTAATTTCAA GAACATTCTA CAGTGGGAGT CACCTGCTTT TCCCAAAGAG

TTAATCTCAA GAACGTTCTT CAGTGGGAGC CTCCTGCCTT CCCCAAAGGG

TTAACTTCAA GAACATCCTA CAGTGGGAGC CGCCAGCTTT TGCCAAAGGG

TTAATTTGAA GAACATTCTG CAGTGGGACC CACCTGCTTT CTATCAAGGA

TGAATTTGAA GAACATCTTA GAGTGGGAGT TGGCCCCTTT ACCCTATGAA

AACCTGACTT TCACAGCTCA GTACCTAAGT TATAGGATAT TCCAAGATAA

AACCTGACTT TCACAGCTCA GTACCTAAGT TATAGGATAT TCCAAGATAA

AACCTGACTT TCACAGCTCA GTACCTAAGT TATAGGATAT TCCAAGATAG

AACCTGACTT TCACAGCTCA GTACCTAAGT TATAGGATAT TCCAAGATAA

---------- ---------- ---------- ---------- ----------

AATCTGACTT TCACAGCTCA GTATCAAAGT TATAGGATAT TCCAAGATGT

---------- ---------- ---------T TACTGGTACT TCCAAGACCG

AACCTGACTT TCACAGCTCA GTATGAAAGT TACAGGTCTT TCCAAGATCA

---------- ---------- ---------T TACAGGAGAT TCCAAGACAT

AATCTGACGT TCACAGCTCA GTACCAAAG- ---------- ----------

ACTCTGACTT TCACAGCCCA GTACCAAAGT TATAGGAAAT TCCAGGATCT

AATCTGACTT TCACAGCTCA GCACCTAAGT TATAGGAACT TCCAGGATAC

AACCTCACTT TCACAGTTCA GTACCACAGC TATAAGTCCT TTCAAGACCA

AATCTGACTT ACACAGCTCA GTATCAAAGC TATAGGGGAT TTCAAGATAC

AATCTGACTT ACACAGCTCA GTTCCAAAGT TATAACAGCT TCCAAGATAA

AACCTGACTT ACACAGCTCA GTTCCAGAGT TATAACAGCT TCCAAGATGT

AATGTGACTT ACAGAGCCCA GTATGAGAGT TATAGGTCAT TTGAAGATGT

AATGTGACCT TCACAGCTCA ATCGATGAGC TACAGAGTCT TTGAAGACGT

ATGCATGAAT ACTACCTTGA CGGAATGTGA TTTCTCAAGT CTTTCCAAGT

ATGCATGAAT ACTACCTTGA CGGAATGTGA TTTCTCAAGT CTTTCCAAGT

ATGCACGAGT ACTACCTTGA CGGAATGTGA TTTCTCAAGT CTTTCCAAGT

ATGCACGAGT ACTACCTTGA CGGAATGTGA TTTCTCAAGT CTTTCCAAGT

---------- ---------- ---------- ---------- ----------

ATGCACGAGA GCTGCCTCGA CGGAATGTGA TTTCTCAAGT CTTTCCAAGT

CTGCAAGAAC ACGGCCTCCA CCCACTGCGA CTTCTCGGTT CTTTCTAAAT

CTGCAAGCGC ACTGCCTCGA CTCAGTGCGA CTTCTCTCAT CTTTCTAAAT

GTGCAAGGGG ACTGCCAAGA CGGAATGCAA TTTCTCCAGC CTCTCCAAGT

---------- ---------- ---------- ---------- ----------

ATGCACAAGT ACTGCCTTCA CGGAATGTGA TTTCTCAAGT CTTTCCAAGT

GTGCACAAGT ACTGCCTTGC TGGAATGTGA CTTCTCAAAC CTTTCCAAGT

GTGCAGTCAT ATTAACTTGA CAGAGTGTGA TTTCTCGAAC CTTTCCAAGT

ATGCAAGAGT ACTGCCTTGA CAGAATGTGA CTTCTCAAGT CTTTCCAAAT

ATGCAAGTGT ACTACCTTGA CGGAATGTGA TTTCTCAAGT CTTTCCAAGT

ATGCAACAGT ACTGCGCTGA CAGAATGCCA TTTCTCAAGC GTTTCCAAGT

GTGTACAAAT ATTATCATCA CTGAATGTAA TTTCTCAAAT ATTTCCAAGT

GTGCAAGAAC ATCGTGCTCA GGCAGTGTGA CTTCTCGCAT CTGGCCAAGT

ATGGTGACCA CACCTTGAGA GTCAGGGCTG AATTTGCAGA TGAGCATTCA

ATGGTGACCA CACCTTGAGA GTCAGGGCTG AATCTGCAGA TGAGCATTCA

ATGGTGACCA CACCTTGAGA GTCAGGGCTG AATTTGCAGA TGAGCATTCA

ATGGTGACCA CACCTTGAGA GTCAGGGCTG AATTTGCAGA TGAGCATTCA

---------- ---------- ---------- ---------- ----------

ATGGGGACCA CACCTTGAGA GTCAGAGCTG AATTTGCAGA TGAGCATTCA

ATGGAGACCA CACCGTGAGG GTCAGGACTG AGCTTGCAGA TGAACATTCG

ACGGAGACTA CACTGTGAGA GTCAGGGCTG AATTGGCGGA TGAACATTCG

ATGGGGACCA CACCCTGAGA GTCAGAGCTG AATTCGCAGA GGAGCACTCG

---------- ---------- ---------- ---------- ----------

ATGGTGATCA CACCCTGAGA GTCAGGGCTG AGTTTGCAGA TGAGCATTCT

ATGGTGACCA CACCTTGCGA GTCAGGGCTG AACTTGCAGA TGAATATTCA

ACGGCAACTA CACGTTGAGA GTCCGAGCGG AGTTCCAGGC TGAGCACTCA

ATGGTGACCA CACCTTGAGA GTCAGGACTG AATCTGCAGA CGAGCACTCA

ATGGTGACCA CACCTTGCGA GTCAGGACTG AATTTGCAGA TGAGCATTCA

ACGGTTACCA CATCTTTCGA GTCAGGGCTG AATTTGCAGA TGAATATTCA

ATGGTGAGTC CAAGATGCGT GTCAGGACTG AATCTGTAGA TGAACAGTCA

ACGGCGACCA CACCTTGCGG ATTTGGATGG AGACGGAGGG AGGCCATTCC

GACTGGGTAA ACATCACCTT CTGTCCTGTG GATGACACCA TTATTGGACC

GACTGGGTAA ACATCACCTT CTGTCCTGTG GATGACACCA TTATTGGACC

GACTGGGTAA ACATCACCTT CTGTCCTGTG GATGACACCA TTATTGGACC

GACTGGGTAA ACATCACCTT CTGTCCTGTG GATGACACCA TTATTGGACC

---------- ---------- ---------- ---------- ----------

GACTGGGTAA ACATCACCTT CTGTCCTGTG GATGACACTG TCATTGGGCC

GAGTGGGTAA ACGTCACCTT CTGTCCCGTG GAAGACACCA TTATCGGACC

GAGTGGGTCA ATGTCACCTT CTGCCCCGTG GAAGACACCA TCATTGGACC

GACTGGGTGC AGGTCGGCTT CTGCCCTGTG GATGATACTA TTATTGGGCC

---------- ---------- ---------- ---------- ----------

GACTGGGTAA ACATCACCTT TTGTCCTGTG GATGACACCA TGATTGGACC

GACTGGGTAA ACATCACCTT CTGTCCTGTG GATGACACCA TTATTGGACC

GAATGGGTCA CCACCGTCTT CTGTCCCGTG GATGACACTG TTATCGGGCC

GACTGGGTAA ACATCACCTT TTGTCCTGTG GATGACACTG TTATTGGTCC

GACTGGGTAA ACATTACCTT TTGTCCTGTG GATGACACCA TTATCGGACC

GACTGGATAA ACATTACCTT TTGCCCTATG GATGACACTG CCATTGGACC

GAGTGGGTAA ACCTCACCTT TTGTCCTTTG GAAGACACCA TCATTGGACC

GACTGGGTGA ACGTCACCTT CCACCCGGTG GAGGACACTG TCATCGGACC

CCCTGGAATG CAAGTAGAAG TACTTGCTGA TTCTTTACAT ATGCGTTTCT

CCCTGGAATG CAAGTAGAAG TACTTGCTGA TTCTTTACAT ATGCGTTTCT

CCCTGGAATG CAAGTAGAAG TACTTGCTGA TTCTTTACAT ATGCGTTTCT

CCCTGGAATG CAAGTAGAAG TACTTGCTGA TTCTTTACAT ATGCGTTTCT

---------- ---------- ---------- ---------- ----------

TCCTGGAATA CAAGTAGAAG TGCATGCTGA TTTTTTATAT GTGCGTTTCT

TCCTGAGATG CAGACAGAAT CCCTTGCTGA TTCGATACAC ATGCGTTTCT

TCCTGAGATG CAGATAGAAT CCCTTGCTGA GTCTTTACAC CTGCGTTTCT

GCCTCGGATG CAAGTGGAAG CCCTCGCTGA TTCTTTACAT TTGCGTTTCT

---------- ---------- ---------- ---------- ----------

ACCTGGAATG CAAGTAGAAG CACTTGCTAA CTCTCTCCAT CTGCGTTTCT

TCCTGGAATG CAAGTAGAAG CACTTGCTAA TTCTTTACAT ATACGTTTCT

TCCTGGATTG AAATTAAAGG CCCTGGGCAA TTCCTTGCAT GTGCTCTTCT

TCCTGGAATG CAAGTAGAAA CGCTTGCTGA TTCTTTATAT ATGCATTTCT

TCCTGGAATA CAAGTAGAAG CACTTGCTGA TTCTTTACAT ATGCGATTCT

ACCTGGGATG CAAGTAAAAG TCCTTGCTGA GTCGTTACAT ATG-------

TCCTGGAATA CAAGTAGAAC CATTGGCTGG TTCTTTACAC TTATATTTCT

GCCCAACCTG CAAGTGAAAC CCGTGTCCAG CTCCCTGCAC GTCCACTTCT

TAGCCCCTAA AATTGAGAAT GAATACGAAA CTTGGACTAT GAAGAATGTG

TAGCCCCTAA AATTGAGAAT GAATACGAAA CTTGGACTAT GAAGAATGGG

TAGCCCCTAA AATTGAAAAT GAATACGAAA CTTGGACTAT GAAGAATGTG

TAGCCCCTAA AATTGAAAAT GAATACGAAA CTTGGACTAT GAAGAATGTG

---------- ---------- ---------- ---------- ----------

CAGCCCCCCC AATTGAGAAC GAGCCTGAAA CATGGACCAT GAAGAATATT

CAGCCCCACA AATAGAGAAT GAGCCTGAGA CATGGACCAT GAAGAACATT

CAGCCCCACA AATTGAGAAT GAGCCTGAGA CGTGGACCTT GAAGAACATT

CGGCGCCCCC GATTGAGAAT GAACCTGAAA CATGGACCAT GAGGAATATT

---------- ---------- ---------- ---------- ----------

TAGCCCCTAA AATTGAGAAT GAACCTGAAA CATGGACCAT GAGAAATATT

TAGCCCCTAA AATTGCGAAG GAACATGAAA CATGGACCAT GAGGAATATT

TAGCCCCCCA AGTTGAGAAC GAGCCAGACT CATGGAGCAT GAAAAACTTT

CAGCCCCCAA AATTGAAAAT GAACCTGAAA CATGGACCAT GAAGAATATT

TAGCCCCCAA AGTTGTGGAT GAACCTGAAA CGTGGACCAT GAAGAATTTT

---------- ---------- ---------- ---------- ----------

CATATCCAAA GATTGAAAAT GAACCTAATA TATGGACATT AAGGGATTAT

CGTTCCCGAA GATTGAAAAC GAACCCGAAA CGTGGACGTT GAAGGACTAT

TATAACTCAT GGACTTATAA TGTGCAATAC TGGAAAAACG GTACTGATGA

TATAACTCAT GGACTTATAA TGTGCAATAC TGGAAAAACG GTACTGATGA

TATAATTCAT GGACTTATAA TGTGCAATAT TGGAAAAATG GTACTGATGA

TATAATTCAT GGACTTATAA TGTGCAATAT TGGAAAAATG GTACTGATGA

---------- ---------- ---------- ---------- ----------

TATGACTCAT GGGTTTACAG TGTGCAATAC TGGAAAAATG GTACTGATGA

TATAACTCAT GGGCTTACAG GGTACAATAC TGGAAAAACG GGACTAAGGA

TATGACTCAT GGGCTTACAG AGTGCAATAC TGGAAAAATG GGACTAATGA

TATAACTCAT GGGCTTATCA TGTGCAATAT TGGAAAAATG GCACTGACGA

---------- ---------- ---------- ---------- ----------

TATAACTCGT GGGCTTATAA TGTGCAATAT TGGAAAAACG GCTCTGATGA

TATAACTCAT GGGTTTATAA TGTGCAATAT TGGAAGAATG GCTCTGATGA

TACAACGACT GGGCTTACAA CTTGGAGTAC TGGAGGAACG GCTCGGATGA

TATAACTCAT GGACTTATAA TGTGCAATAC TGGAAAAATG GCACTGACAA

TATACCTCGT GGGTTTATAA CGTACAGTAT TGGAAAAATG GCACTGATGA

---------- ---------- ---------- ---------- ----------

TATTACTCAT GGACTTACAG AGTGATTTAT TGGAAAAATG GCACCAGTCA

TACGGCTCGT GGGAGTATAA GGTGCTCTAC TGGAAAAGGG GCACTTCCCA

AAAGTTTCAA ATTACTCCCC AGTATGACTT TGAGGTCCTC AGAAACCTGG

AAAGTTTCAA ATTACTCCCC AGTATGACTT TGAGGTCCTC AGAAACCTGG

AAAGTTTCAA ATTACTCCTC AGTATGACTT TGAGGTCCTC AGAAACCTGG

AAAGTTTCAA ATTACTCCTC AGTATGACTT TGAGGTCCTC AAAAACCTGG

---------- ---------- ---------- ---------- ----------

AAAGTTCCAG ATCACTCTTC AGTATGACTT TGAGGTCCTC AGAAACCTAG

GAAGTTTCAA GTCACATCTC AGTATGACTC TGAGGTTCTC AGAGACCTGG

GAAGTTTCAA GTTGTGTCTC CGTACGACTC TGAGGTCCTC CGGAACCTGG

AAAG------ ---------- ---------- ---------- ----------

---------- ---------- ---------- ---------- ----------

AAAGTGGCCG ATTACCTGTC AGTATGACTT TGAGGTCCTC CGAGATCTGG

AAAGCATTCA GTCAGTACTC AGTATGACTT TGAGGTCCTC AGAAATCTTG

GAAG------ ---------- ---------- ---------- ----------

AAAGTTTCAA GTTACTTCCC AGTatgactt tgaagccgta agaaacctgg

AAAGTTTCAA ATTACTGCTC CATATGATTT TGAGGTCCTC CAAAATCTGG

---------- ---------- ---------- ---------- ----------

TAAGTTTGAA AAAAATGCTC CCTATGACTC TGAAGTCCTT ACTGATCTGG

GAAGCTCGAG ATGGTCACCC CGTTTGAATC GGACATGCTC CCCGATCTGG

AGCCATGGAC AACTTATTGT GTTCAAGTTC GAGGGTTTCT TCCTGATCGG

AGCCATGGAC AACTTATTGT GTTCAAGTTC GAGGGTTTCT TCCTGATCGG

AGCCATGGAC AACTTATTGT GTTCAAGTTC AAGGGTTTCT TCCTGATCGG

AGCCATGGAC AACTTATTGT GTTC------ ---------- ----------

---------- ---------- ---------- ---------- ----------

AACCATGGAC AACTTATTGT GTTCAAGTTC AAGGGTTTCT TCCTGATCGC

AGCCATGGAC AACTTACTGC ATTCAAGTTC AAGGGTTTCT TCTCGACCAG

AGCCGTGGAC AACTTACTGC ATTCAAGTTC AAGGGTTTCT TCTCGACCAG

---------- ---------- ---------- ---------- ----------

---------- ---------- ---------- ---------- ----------

AGCCGTGGAC AACTTACTGC GTTCGAGTTC AAGGGTTTCT TCCCGAACGG

AGCCATGGAC AACTTACTGT GTTCAAGTTC AAGGGTTTCT TACTGATCGA

---------- ---------- ---------- ---------- ----------

agccatggac aacatattgt gttcaagttc aaggatttct tcctGATCGC

AGCCGTGGAC AACTTACTGT GTTCAAGTTC AAGGATTTCT TCCTGATAGG

---------- ---------- ---------- ---------- ----------

ATCCATGGAC GGTTTATTGT ATTCAAGCTC AAGGGTTTAT AACTAGTAAG

ATCCTCGGAC CACCTACTGC CTTCAAGCCC AAGGGCTCGT CCCCGCACTG

AACAAAGCTG GGGAATGGAG TGAGCCTGTC TGTGAGCAAA CAACCCATGA

AACAAAGCTG GGGAATGGAG TGAGCCTGTC TGTGAGCAAA CAACCCATGA

AACAAAACTG GGGAATGGAG TGAGCCTGTC TGTGAGAAAA CAACCAGTGA

---------- ---------- ---------- ---------- ----------

---------- ---------- ---------- ---------- ----------

AACAAAACCG GCCAGTGGAG TGACCCTGTC TGTGAGCAAA CAACTAATGA

AACAGAACGG GAGAGTGGAG CAAACCCGTC TGTGAACGGA CAACCAGTGA

AACAGAACAG GAGAGTGGAG TGAACCCATC TGTGAACGGA CAGGCAATGA

---------- ---------- ---------- ---------- ----------

---------- ---------- ---------- ---------- ----------

AACAAAACTG GGGAATGGAG CAAGCCTGTC TGTGAGCAAA CAGCCAATGA

AACAAACCCG GGGAATGGGG TGCGCCTGTC TGTGAGCAGA CCATCAATGA

---------- ---------- ---------- ---------- ----------

AACAAAAGTG GAGGATGGAG TCAGCCCGTC TGTGAGCAGA CAGCCGACGA

AACAAAACTG GGGAATGGAG TCAGCCTGTC TGTGAGCAAA CAACCAACGA

---------- ---------- ---------- ---------- ----------

AACAAAAGTG GAGAATGGAG TCAGCCAATC TGTGTCCAAA CTACTGACAA

AACAAGTCGG GCCTCTGGAG CCAGCCAATC TGCGAGACCA CGGCCGACGA

CGAAACGGTC CCCTCCTGGA TGGTGGCCGT CATCCTCATG GCCTCGGTCT

CGAAACGGTC CCCTCCTGGA TGGTGGCCGT CATCCTCATG GCCTCGGTCT

CGAGACGGTC CCCTCCTGGA TGGTGGCCAT CATCCTCATG GCCTCGGTCT

---------- ---------- ---------- ---------- ----------

---------- ---------- ---------- ---------- ----------

CGAGACGGTC CCCTCCTGGA TAGTCGCCTT GGTCCTCGTT GGGTGTGTCT

TGAAACAACC CCTCCCTGGA TCGTGGCCAT CATCCTTATA GCCTCCGTCT

CGAAATAACC CCTTCCTGGA TTGTGGCCAT CATCCTCATA GTCTCCGTCC

--ACACCACC CCCTCCTGGA TGGTGGCCGT CATCCTCATC GCCTCGATCT

---------- ---------- ---------- ---------- ----------

TGACACAGCC CCGTCCTGGA CTGTCGCCAT CGTCCTGGCA GCCTCGGTGT

CGACTCATCC CTGTCCTGGA TCGTCATCAC CGTCCTCACA GCCTCGGTTT

--CCA---GC GTGTCCTGGA TCATCGCACT CGTCATCGGG GCCTCCGTGT

CGAAATGACC CCCTCCTGGA TCGTTGCTGT TGTCCTCATA GCCTCGGTGC

CGAAACAACA CCCTCCTGGA TTATTACCAT CATCCTCATA GCCTCGGTTT

---------- ---------- ---------- ---------- ----------

TGAGTCACCT CCATACTGGA TTACTATTAT TGTTCTTATT GTATCCATGA

CGGCAAAACG CCCGTGTGGG TTATCGCCGT CATGCTGCTG GCGTCTCTGG

TCATGGTCTG CCTGGCACTC CTCGGCTGCT TCGCCTTGCT GTGGTGCGTT

TCATGGTCTG CCTGGCACTC CTCGGCTGCT TCGCCTTGCT GTGGTGCGTT

TCGTGGTCTG CCTGGCACTC CTTGGCTGCT TCGCCTTGCT GTGGTGCATT

---------- ---------- ---------- ---------- ----------

---------- ---------- ---------- ---------- ----------

TTGTGGCCGT CCTGCTGCTC CTGGGCTGTT TTGCTCTGCT GTGGTGCATT

TGATAGTCTT CCTCTTCCTA CTGGGCTGCT TCAGCATGGT GTGGTTCATT

TGGTGGTCTT CCTCTTCCTC CTGGGCTGCT TTGTCGTGCT GTGGCTCATT

TTGTGGTCTT CCTGGTGCTG TTGGCCTGCT TCGCCTTGCT GTGGTGCATC

---------- ---------- ---------- ---------- ----------

GCGTGGCCCT CCTGCTGCTC ---GGCTGCT CCGCCCTGCT GTGGTGCCTG

GCGCGGCCTG CCTGCTTCTC CTTGGCTGCT TCGCCCTGCT GTGGTACATC

GCGTGGCCCT CCTGCTCATG ---GGCTGCT TCGCCCTGCT CTGGTTTGTC

TCCTGGTCTG CCTGCTGCTC CTGGGCTGCT TTGCCTTAAT ATGGTGCATT

TCACTGTCTT CCTCCTGCTC CTCGGCTGCT TTGCCTTGAT GTGGTACATT

---------- ---------- ---------- ---------- ----------

TAGTGGTTTT ACTGACGGCG CTTGGTTGTT TTTCTCTACT GTGGTATTTC

TAGCGGTTCT CCTGGTGGTC CTCTGCAGCT TCTCCGTGCT CTGGTACTTA

TACAAGAAGA CAAAGTACGC CTTCTCCCCT AGGAATTCTC TTCCACAGCA

TATAAGAAGA CAAAGTACGC CTTCTCCCCT AGGAATTCTC TTCCACAGCA

TATAAGAAGA CAAAGTACAC CTTTTCCCCT GGGAATTCTC TTCCACAGCA

---------- ---------- ---------- ---------- ----------

---------- ---------- ---------- ---------- ----------

TACAAGAAGA CAAAGTATGC CTTCTCCCCT GGGAACTCCC TCCCGCAGCA

TACAAGAAGA CCAAGTACAC CTTCCATTCT GGGACGTCTC TTCCACAGCA

TATAAGAAGA CCAAGCATAC CTTCCGTTCT GGGACGTCTC TTCCACAGCA

TACAAGAAGA CCAAGTACAC CTTCTCCCCC GGGAACGCTC TGCCACAGCA

---------- ---------- ---------- ---------- ----------

TACAAGAGGA CCAAGCACGT CTTCTCCCCC AGGAACTGTC TGCCTCAGCA

TACAAGAAGA CCAAGTACAC CTTCTGCCCT AGGAACTCTC TTCCCCAGCA

TACAAGAAGA CCAAGTACGC CTTCTGCCCC GGGAACACGC TGCCCCAGCA

TATAAGAAGA CAAAGTACGC CTTCGCGCCT GGGAGCTCTC TGCCGCAGCA

TACAAGAAGG CAAAGTACAC CTTCGCCCCT GGGAATTCTC TTCCACAGCA

---------- ---------- ---------- ---------- ----------

TACAGAAAAG CAAAGTGTAT ATTCTTTCCT GGGTATTCTT TTCCTCAGCC

TACCGCCAAG CCAGATACGT CTTCTTCCCC GGCTATTCCC TCCCGAAGCT

CCTGAAAGAG TTTCTTCTGT TTTTCTCCTT TCCATTGTCG GATGAGAATG

CCTGAAAGAG TTTCTTCTGT TTTTCTCCTT TCCATTGTCG GATGAGAATG

CCTGAAAGAG TTTCTTCTGT TTTTCTCCTT TCCATTCTCG GATGAGAATG

---------- TTTCTTCTGT TTTTCTCCTT TCCATTCTCG GATGAGAATG

---------- ---------- ---------- ---------- ----------

TCTGAAAGAG TTTCTTCTGT TTTTCTCCTT CCCACTGTCT GATGAGAATG

CCTCAAGGAG TTTTTTCTGC TGTTCTCCTT CCCTCCCCCT GACGAAACCG

CCTGAAGGAG TTTTTTCTGC TGTTCTCCTT CCCTCCCCCC GAGGAGGCCG

CCTGAAGGAG TTCCGTCTGT TTTTCTCCTT CCCACTGCTG GACGAGAATG

---------- ---------- ---------- ---------- ----------

TCTGAAAGAG TTTCTTCTGT TTTTCTCCTT CCCGCCCTCT GATGAGAATG

CCTGAAAGAG TTTCTTCTGT TTTTCTCCTT CCCGCTCTCT GATGAGAGTG

CATGAAGGAG TTTTTCCTGT TCTTCACCTT CATTCCCTCC GACGAGAGCG

CCTGAAAGAG TTTCTGCTGA TTTTTAACTT CCCATCCTCT GAGGAGAATG

CCTGAAAGAG TTTCTGCTGT TTTTCTCCCC GCCGCCCCCT GAGGAGGATG

---------- TTTTTGCTGT TTTACTCCCT CCCGCCTCCT GATGAGAAGG

TCTGAAAGAG TAT-TTTCAT TTCTCTCACC AGTACCTTCC AGTGAGAGTG

TCTGAAAGAG T----CCCGT TTCTCTCCTC TCCGCCCTC- --CGAGAAGG

ATGTTTTTGA CAAGCTAAGT GTCATTATGG CTCAGTTTCC AACACCTTTT

ATGTTTTCGA CAAGCTAAGT GTCATTATGG CTCAGTTTCC AACACCTTTT

ATGTTTTTGA CAAGCTAAGT GTCATTATGG CTCAGTTTCC AACACCTTTT

ATGTTTTTGA CAAGCTAAGT GTCATT---- ---------- ----------

---------- ---------- ------ATGG CTCAGTTTCC AACACCTTTT

AGGTTTTTGA CAAACTAAGT GTCATTATGG CTCAGTTTCC AACACCTTTT

AAGTGTTCGA CAAACTGAGC ATCATCATGG CTCAGTTTCC GACACCTTTT

AGGTGTTCGA CAAACTAAGC ATCATCATGG CTCAGTTTCC CACACCTTTC

AAGTTTTTGA CAAACTCAGT GTCGTTATGG CTCAGTTTCC AACACCTTTT

---------- ---------- ------ATGG CTCAGTTTCC CACACCCTTT

AAGTCTTTGA CAAACTGAGT GTCATTATGG CTCAGTTTCC AACACCTTTT

AAGTTTTTGA CAAACTGAGT GTCATCATGG CTCAGTTTCC AACACCTTTT

AGGCCTTCGA CAAGCTGAGT GTGGTGATGG CTCAGTTCCC AGCACCTTTT

AAGTTTTTGA CAAGCTGAGC GTCGTGATGG CTCAGTTTCC AACACCTTTT

AAGTGTTTGA CAAACTAAGC GTCATT---- ---------- ----------

AAGTGTTCGA CAAGCTGAGC GTTGTCATGG CTCAATTTCC AACACCTTTT

AATCCTTTGA TAAATTAAGT ATCAT-ATGG CTCAGTTTCC AACATCTTTT

AACCCTGTGA CAAACTGAGC GTCGTCATGG CTCAGTTTCC GGCTCCTTTC

GGTGGCAGCC TGGATATCTG GGCCATAACT GTAGAGGAAA GAGCGAAGCA

GGTGGCAGCC TGGATATCTG GGCCATAACT GTAGAGGAAA GAGCGAAGCA

GGTGGCAGCC TGGATATCTG GGCCATAACT GTAGAGGAAA GAGCGAAGCA

----GCAGCC TGGATATCTG GGCCATAACT GTAGAGGAAA GAGCGAAGCA

GGTGGCAGCC TGGATGTCTG GGCCATAACT GTAGAGGAAA GAGCGAAGCA

GGTGGCAGCC TGGATATCTG GGCCATAACT GTAGAGGAAA GAGCGAAGCA

GGTGGGAGCC TGGACATCTG GGCCATAACC GTGGAGGAAA GAGCCAAGCA

GGTGGTAGCC TGGATGTCTG GGCCATAACT GTGGAGGAAA GGGCCAAGCA

GGTG------ ---------- GGCCATCACT GTGGAGGAAA GGGCGAAGCA

GGTGGCAGCC TGGATATTTG GGCCATAACT GTAGAGGAAA GAGCAAAGCA

GGAGGCAGCC TGGATATCTG GGCCATAACT GTAGAAGAAA GAGCAAAGCA

GGTGGCAGCC TGGATATCTG GGCCATAACT GTAGAGGAAA GAGCGAAGCA

GGAGGCAGCC TGGACATCTG GGCCATCACC GTAGAGGAGA GAGCGAAGCA

GGTGGCAGCC TGGATATCTG GGCCATAACT GTGGAGGAAA GAGCAAAGCA

---------- ---------- ---------- ---------- ----------

GTGGGTAGCC TGGACATCTG GCCCATAACT GTAGAAGAAA GAGCAAAGCA

GGTGGCAGCC TGGATATTTG GGCCATCACT GTAGAAGAAC GAGCAAAGCA

GGGGGCACCC TGGATATCTG GGCCATAACC GTGGAGGAAA GAGCAAAGCA

TGATCAGCAG TTCCATAGTT TAAAGCCAAT ATCTGGATTC ATTACTGGTG

TGATCAGCAG TTCCATAGTT TAAAGCCAAT ATCTGGATTC ATTACTGGTG

TGATCAGCAG TTCCATAGTT TAAAGCCAAT ATCTGGATTC ATTACTGGTG

TGATCAGCAG TTCCATAGTT TAAAGCCAAT ATCTGGATTC ATTACTGGTG

TGATCAGCAG TTCCATAGTT TAAAGCCAAT ATCTGGATTT ATTACTGGTG

TGATCAGCAG TTCCATAGTT TAAAGCCAAT ATCTGGATTT ATTACTG---

TGACCAGCAG TTCCAGAGTT TGAAGCCGAT ATCTGGATTC ATCACTGGTG

TGACCAGCAG TTCCTTAGCC TGAAGCCGAT AGCGGGATTT ATTACTGGTG

TGACCAGCAG TTCCATAGCC TAAAGCCAAG ATCGGGATTC ATTACTGGTG

TGATCAACAG TTCCATAGTT TAAAGCCAAT ATCTGGATTT ATTACTGGTG

TGATCAACAG TTCCATAGTT TAAAGCCAAT ATCTGGATTT ATTACTGGTG

TGATCAACAG TTCCATAGTT TAAAGCCAAT ATCTGGATTT ATTACTGGTG

CGATCAGCAG TTCCACAGTT TAAAGCCAAT ATCTGGATTT ATTACTGGTG

TGATCAGCAG TTCCACGGTT TAAAGCCAAT ATCTGGATTT ATTACTGGTG

---------- ---------- ---------- ---------- ----------

TGATCAACAA TTCCACAGTT TAAAGCCAAT ATCTGGATTT ATTACTGGTG

TGATCAGCAG TTCCATAGTC TCAAGCCAAC ATCGGGGTTC ATTACTGGTG

CGATCAGCAG TTTCACAGTC TCAAACCCAC ATCTGGATTT ATCACCGGTG

ATCAAGCTAG AAACTTTTTT TTTCAATCTG GGTTACCTCA ACCTGTTTTA

ATCAAGCTAG AAACTTTTTT TTTCAATCTG GGTTACCTCA ACCTGTTTTA

ATCAAGCTAG AAACTTTTTT TTTCAATCTG GGTTACCTCA ACCTGTTTTA

ATCAAGCTAG AAACTTTTTT TTTCAATCTG GGTTACCTCA ACCTGTTTTA

ATCAAGCTAG AAACTTTTTT TTTCAATCTG GGTTACCTCA ACCTGTTTTA

---------- ---------- ---------- ---------- ----------

ATCAAGCAAG GAACTTCTTT TTCCAATCTG GGTTACCTCA GCCCGTCTTA

ATCAAGCGAG GAACTTTTTT TTCCAATCTG GGTTACCTCA GCCTGTCTTA

ATCAAGCAAG AAACTTTTTT TTTCAATCTG GGTTACCTCA ACCTGTTTTA

ATCAAGCTAG AAACTTTTTT TTTCAATCTG GGTTACCTCA GCCTGTTTTA

ATCAAGCTAG AAACTTTTTT TTTCAATCTG GGTTACCTCA ACCTGTTTTA

ATCAAGCTAG AAACTTTTTT TTTCAATCTG GGTTACCTCA ACCTGTTTTA

ATCAAGCTAG AAACTTTTTT TTTCAATCTG GGTTACCTCA GCCTGTTTTA

ATCAAGCTAG AAACTTTTTT TTTCAGTCTG GGTTACCTCA ACCTGTTTTA

---------- ---------- ---------- ---------- ----------

ATCAAGCTAG AAACTTTTTT TTTCAATCTG GGTTACCGCA ACCTGTTTTA

ATCAAGCCAG AAACTTTTTT TTCCAATCTG GGTTACCTCA GCCTGTTTTA

ATCAAGCTAG GAACTTTTTT TTTCAATCTG GGTTACCTCA GCCCGTTTTA

GCACAGATAT GGGCACTAGC TGACATGAAT AATGATGGAA GAATGGATCA

GCACAGATAT GGGCACTAGC TGACATGAAT AATGATGGAA GAATGGATCA

GCACAGATAT GGGCACTAGC TGACATGAAT AATGATGGAA GAATGGATCA

GCACAGATAT GGGCACTAGC TGACATGAAT AATGATGGAA GAATGGATCA

GCACAGATAT GGGCACTAGC TGACATGAAT AACGATGGAA GAATGGATCA

---------- -GGCACTAGC TGACATGAAT AATGATGGAA GAATGGATCA

GCACAAATAT GGGCGCTGGC TGACATGAAT AAGGACGGAA GGATGGATCA

GCACAAATAT GGGCGCTAGC GGACATGAAT AACGATGGAA GGATGGATCA

GCACAGATAT GGGCACTAGC TGACATGAAT AACGATGGAA GGATGGATCA

GCACAGATAT GGGCGCTCGC TGATATGAAT AATGATGGAA GGATGGATCA

GCACAGATAT GGGCGCTAGC TGACATGAAC AATGATGGAC GAATGGATCA

GCACAGATAT GGGCACTGGC TGATATGAAT AATGATGGAA GAATGGATCA

GCACAGATAT GGGCACTGGC CGACATGAAT AACGACGGCA GGATGGATCA

GCACAGATAT GGGCACTAGC TGACATGAAT AATGATGGAA GAATGGATCA

---------- ---------- ---------- ---------- ----------

GCACAGATAT GGGCGCTAGC TGACATGAAT AATGATGGAA GAATGGATCA

GCACAGATAT GGGCATTAGC TGACATGAAC AATGATGGAA GAATGGATCA

GCACAGATAT GGGCCCTGGC TGACATGAAC AATGATGGTA GAATGGATCA

AGTGGAGTTT TCCATAGCTA TGAAACTTAT CAAACTGAAG CTACAAGGAT

AGTGGAGTTT TCCATAGCTA TGAAACTTAT CAAACTGAAG CTACAAGGAT

AGTGGAGTTT TCCATAGCTA TGAAACTTAT CAAACTGAAG CTACAAGGAT

AGTGGAGTTT TCCATAGCTA TGAAACTTAT CAAACTGAAG CTACAAGGAT

AGTGGAGTTT TCCATAGCTA TGAAACTTAT CAAACTCAAG CTACAAGGAT

AGTGGAGTTT TCCATAGCTA TGAAACTTAT CAAACTGAAG CTGCAAGGAT

GGTGGAGTTT TCCATAGCCA TGAAGCTCAT CAAACTGAAG CTGCAAGGAT

AGTGGAATTT TCCATAGCCA TGAAGCTTAT CAAACTGAAG CTACAAGGAT

AGTGGAGTTT TCCATAGCTA TGAAACTTAT CAAACTGAAG CTGCAAGGCT

AGTGGAGTTT TCCATTGCTA TGAAACTCAT CAAGCTGAAG CTCCAAGGAT

AGTGGAGTTT TCCATAGCTA TGAAACTCAT CAAACTCAAG CTGCAAGGAT

AGTGGAGTTT TCCATAGCTA TGAAACTTAT CAAACTGAAG CTACAAGGCT

GGTGGAGTTC TCCATCGCCA TGAAGCTCAT CAAGCTGAAG CTGCAGGGCT

AATGGAGTTT TCCATTGCTA TGAAACTTAT TAAATTGAAG CTGCAAGGAT

---------- ---------- ---------- ---------- ----------

AGTGGAGTTT TCCATCGCCA TGAAACTTAT CAAATTGAAG CTACAAGGCT

GATAGAATTT TCCATAGCAA TGAAACTTAT CAAACTGAAG CTCCAAGGTT

GATGGAGTTT TCCATTGCGA TGAAACTGAT CAAACTGAAG CTCCAAGGCT

ATCAGCTACC CTCTGCACTT CCCCCTGTCA TGAAACAGCA ACCAGTTGCT

ATCAGCTACC CTCTGCACTT CCCCCTGTCA TGAAACAGCA ACCAGTTGCT

ATCAACTACC CTCTGCACTT CCCCCTGTCA TGAAACAGCA ACCA---GCT

ATCAACTACC CTCTGCACTT CCCCCTGTCA TGAAACAGCA ACCA---GCT

ATCAGCTACC CTCTACACTT CCCCCTGTCA TGAAACAGCA ACCAGTTGCT

ATCAGCTCCC CTCCACACTT CCGCCTGTCA TGAAACAGCA ACCAGTTGCT

ATCAGCTCCC CCCCGCACTT CCCCCTGTCA TGAAGCAGCA GCCAGCGGCC

ATCAGCTCCC CTCCACACTT CCCCCTGTCA TGAAACAGCA ACCAGTGGCT

ATCAGCTCCC CTCTGCACTT CCCCCTGTCA TGAAACAGCA GCCAGTTGCG

ATCAGCTCCC CTCTGCACTT CCCCCTGTCA TGAAACAGCA ACCAGTTGCT

ATCAACTTCC TTCTGCACTT CCCCCCGTCA TGAAACAGCA ACCAGTTGCT

ATCAACTCCC CTCTGCGCTC CCCCCTGTCA TGAAACAGCA ACCAGTTGCT

ACCAGCTGCC CGCCACGCTG CCCCCCGCCA TGAAGCAGCA GCCCGTCGCT

ATCAGCTCCC CTCCGCTCTT CCCCCTGTCA TGAAACAGCA GCCAGTTGCT

---------- ---------- ---------- ---------- ----------

ATCAGCTCCC CTCTGCACTT CCCCCTGTCA TGAAACAGCA GCCAGTTGCT

ATCAGTTACC TGCTGCACTT CCTCCTGTCA TGAAACAGCA ACCTGTTGCT

ATCAGCTTCC CTCTGCACTT CCTCCCATCA TGAAACAGCA ACCCATTGCT

ATTTCTAGCG CACCAGCATT TGGTATGGGA GGTATCGCCA GCATGCCACC

ATTTCTAGCG CACCAGCATT TGGTATGGGA GGTATCGCCA GCATGCCACC

ATTTCTAGCG CACCAGCATT TGGTATGGGA GGTATCGCCA GCATGCCACC

ATTTCTAGCG CACCAGCATT TGGTATGGGA GGTATCGCCA GCATGCCACC

ATTTCTAGTG CACCAGCATT TGGTATGGGA GGTATTGCCA GCATGCCACC

ATTTCCAGTG CACCAGCATT TGGTATGGGA GGTATGGGCA GCATGCCACC

ATCTCTAGTG CACCAGCGTT TGGTATAGGA GGGATGGCTG GAATGCCACC

ATTTCCAGTG CACCAGCATT TGGTATAGGA GGGATTGCTA GCATGCCACC

ATTTCTAGTG CACCAGCATT TG-------- ---------- ----------

ATTTCTAGTG CACCAGCCTT TGGTATGGGA GGTATTGCCA GCATGCCGCC

ATTTCTAGCG CACCAGCCTT TGGTATAGGA GGTATTGCCA GCATGCCACC

ATTTCTAGTG CACCAGCGTT TGGTATAGGA GGTATTGCCA GCATGCCACC

CTTCCCACCG CGCCCACGTT CGATATTGGT GGCATTGCCA GCATGCCGCC

ATTTCTAGTG CCCCAGCGTT TGGTATAGGA GGTATCACCA GCATACCACC

---------- ---------- ---------- ---------- ----------

ATTTCTACTG CACCAGCATT TGGTATGGGA GGCATTGCCA GCATGCCACC

ATTTCTGGTG CACCCACATT TGGTATAGGA GGCATTGCCA GCATGCCACC

ATTTCCGGTG CACCAGCATT TGGTATAGGA GGCATCGCCA GCATGCCGTC

GCTTACAGCT GTTGCTCCAG TGCCAATGGG ATCCATTCCA GTTGTTGGAA

GCTTACAGCT GTTGCTCCAG TGCCAATGGG ATCCATTCCA GTTGTTGGAA

GCTTACAGCT GTTGCTCCAG TGCCAATGGG ATCCATTCCA GTTGTTGGAA

GCTTACAGCT GTTGCTCCAG TGCCAATGGG ATCCATTCCA GTTGTTGGAA

GCTTACAGCT GTTGCTCCAG TGCCAATGGG ATCCATTCCA GTTGTCGGAA

ACTTACAGCT GTTGCTCCAG TGCCAATGGG ATCCATTCCA GTTGTTGGAA

ACTGACAGCT GTTGCTCCCG TGCCAATGGG CTCCATCCCA GTTGTTGGAA

ACTCACAGCT GTTGCTCCTG TGCCAATGGG CTCCATTCCA GTTGTTGGAA

---------- ---------- ---------- ---------- ----------

GCTGACCGCT GTTGCTCCTG TACCAATGGG CTCCATTCCC GTTGTTGGGA

ACTTACAGCT GTTGCTCCAG TGCCAATGGG ATCCATTCCA GTTGTCGGAA

ACTCACAGCT GTTGCTCCAG TGCCAATGGG ATCCATTCCA GTTGTCGGAA

GCTCACAGCC GTAGCTCCCG TGCCAATGAG CTCCATCCCG GTCGTAGGAA

GCTTACAGCT GTTGCTTCTG TGCCAATGGG ATCCATTCCA GTTGTTGGAA

---------- ---------- ---------- ---------- ----------

CCTCACAGCA GTTGCTCCAG TGCCAATGGG GTCCATTCCC GTGGTCGGGA

TCTCACAGCT GTTGCTCCAG TCCCAATGGC ATCAATTCCA GTAGTAGGAA

TCTCACAGCT GTTGCCCCAG TGCCAATGCC ATCCATTCCA GTAGTCGGAA

TGTCTCCAAC CCTAGTATCT TCTGTTCCCA CAGCAGCTGT GCCCCCCCTG

TGTCTCCAAC CCTAGTATCT TCTGTTCCCA CAGCAGCTGT GCCCCCCCTG

TGTCTCCACC CCTAGTATCT TCTGTTCCCA CAGCAGCTGT GCCCCCCCTG

TGTCTCCACC CCTAGTATCT TCTGTTCCCA CAGCAGCTGT GCCCCCCTTG

TGTCTCCACC CCTAGTATCT TCTGTTCCTA CAGCAGCTGT GCCCCCGCTG

TGTCTCCACC CCTAGTATCT TCTGTTCCAA CAGCAGCAGT GCCTCCCCTG

TGTCTCCGCC CTTAGTATCT TCTGTCCCTC AAGCAGCAGT GCCTCCCCTG

TGTCTCCACC CTTAGTATCT TCTGTCCCTC CAGCAGCAGT GCCTCCCCTG

---------- ---------- ---------- ---------- ----------

TGTCTCCCCC ACTAGTGTCA TCTGTTCCTG CAGCAGGGGT GCCCCCCCTG

TGTCTCCACC TCTAGTATCT TCTGTTCCTA CAGCAGCAGT GCCTCCCCTG

TGTCTCCACC CCTAGTATCT TCTGTTCCTC CAGCAGCAGT GCCTCCCCTG

TGTCCCCACC CCTCGTCTCC TCCGTTCCTC CAGCAGCAGT GCCTCCCTTG

TGTCTCCACC CCTAGTATCT TCAGTTCCAA CGGCAGCAGT GCCTCCCATG

---------- ---------- ---------- ---------- ----------

TGTCTCCACC CCTCGTTTCT TCTGTTCCTC CAGCAGCAGT GGCTCCCCTG

TGTCCCCACC TCTCGTATCT TCTGTTTCCA CAGCAGCAGT TCCTCCCCTG

TGTCCCCGCC TCTAGTATCT TCCGTCCCTT CAGCAGCAGC GCCTTCCCTG

GCTAACGGGG CTCCCCCTGT TATACAACCT CTGCCTGCAT TTGCTCATCC

ACTAACGGGG CTCCCCCTGT TATACAACCT CTGCCTGCAT TTGCTCATCC

GCTAACGGGG CTCCCCCTGT TATACAACCT CTGCCTGCAT TTGCTCATCC

GCTAACGGGG CTCCCCCTGT TATACAACCT CTGCCTGCAT TTGCTCATCC

GCTAACGGGG CTCCCCCTGT TATACAACCT CTGCCTGCAT TTGCTCATCC

GCTAACGGGG CTCCCCCTGT CATACAACCT CTGCCTGCAT TTGCTCATCC

GCTAACGGGG CCCCTCCTGT CATACAGCCT CTGCCTGCAT TTGCTCATCC

GCTAACGGGG CTCCTCCCGT CATACAGCCT CTGCCTGCGT TTGCGCATCC

---------- ---------- ---------- ---------- ----------

GCCAATGGGG CGCCCCCGGT CATACAGCCT CTGCCTGCAT TTGCTCATCC

GCTAACGGGG CTCCCCCTGT TATACAACCT CTGCCTGCAT TTGCTCATCC

GCTAACGGGG CCCCCCCTGT TATACAACCT CTGCCTGCAT TTGCTCATCC

GCCAACGGGG CGCCCCCTGT GATCCAGCCC CTCACGGCCT TCGCTCACCC

GCTAACGGAG CCCCCCCAGT CATACAACCT CTGCCTGCAT TTGCACATCC

---------- ---------- ---------- ---------- ----------

GCGAATGGGG CTCCCCCTGT CATACAACCT CTGCCTGCAT TTGCTCATCC

GCTAATGGCA CTCCTTCTGT CATACAACCT CTGCCTGCTT TTGCTCATCC

GCTAACGGCA CGTCTTCGGT CATACAGCCT CTGCCTGCTT TTGCTCATCC

TGCAAGTGTC CCACCAGTGG CAGAGTGGGC TGTTCCTCAG TCATCAAGAC

TGCAAGTGTC CCACCAGTGG CAGAATGGGC TGTTCCTCAG TCATCAAGAC

TGCCAGTGTC CCACCAGTGG CAGAATGGGC TGTTCCTCAG TCATCAAGAC

TGCCAGTGTC CCACCAGTGG CAGAATGGGC TGTTCCTCAG TCATCAAGAC

TGCAAGTGTC CCTCCAGTGG CAGAATGGGC TGTTCCTCAG TCATCAAGAC

CGCAAGTGCC CCTCCAGTGG CAGAATGGGC TGTTCCTCAG TCATCGAGAC

TGCAAGCGCC CCTGCAGCGG CAGAATGGGC TGTGCCTCAG TCGTCAAGAC

TGCAAGCGCC CCTCCAGCAG CAGAATGGGC TGTGCCTCAG TCATCAAGGC

---------- ---------- ---------- ---------- ----------

TGCTAGTGTC CCTCCTACAG CTGAGTGGGC TGTTCCTCAG TCATCCAGGC

TGCAAGTGTC CCTCCCGTGG CAGAATGGGC TGTCCCTCAG TCATCCAGAC

TGCAAGTGTC CCACCCGTAG CAGAATGGGC CGTTCCTCAG TCATCCAGAC

CGCAAGTGGC GCTCCCGGGG CAGAATGGGC CGTCCCGCAG TCATCCAGGC

TGCAAGTGTA CCTCCAGTGG CAGAATGGGC TGTTCCTCAG TCATCAAGAC

---------- ---------- ---------- ---------- ----------

TG----TGCC CCTCCTGTGG CAGAATGGGC TGTTCCCCAG TCATCGAGAC

TGCAAGTGCC TCTCCTGTGG CAGAATGGGC TGTCCCTCAG TCATCAAGAC

CGCAAGTGTA CCTCCCGCTG CGGAATGGGC TGTCCCTCAG TCATCTCGGT

TGAAATACAG GCAATTATTC AATAGTCATG ACAAAACTAT GAGTGGACAC

TGAAATACAG GCAATTATTC AATAGTCATG ACAAAACTAT GAGTGGACAC

TGAAATACAG GCAATTATTC AATAGTCATG ACAAAACTAT GAGTGGACAC

TGAAATACAG GCAATTATTC AATAGTCATG ACAAAACTAT GAGTGGACAC

TTAAATATAG GCAGTTATTC AATAGTCATG ACAAAACTAT GAGTGGACAC

TGAAATACAG ACAATTATTC AATAGTCATG ACAAAACTAT GAGTGGACAC

TGAAATACAG GCAGTTATTC AACAGTCACG ACAAGACCAT GAGTGGACAC

TGAAATACAG GCAGTTATTC AACAGCCACG ACAAAACTAT GAGTGGACAC

---------- ---------- ---------- ---------- ----------

TGAAATACAG ACAGCTCTTC AATAGTCATG ACAAAACCAT GAGTGGACAC

TTAAATACAG ACAGTTATTC AACAGTCATG ACAAAACTAT GAGTGGACAC

TGAAATACAG ACAGTTGTTC AATAGTCATG ACAAAACTAT GAGTGGACAC

TCAAGTACCG GCAGTTGTTC AACAGCCACG ACAAGACCAT GAGCGGACAC

TGAAATACAG GCAATTATTC AATAGTCATG ACAAAACTAT GAGTGGACAC

---------- ---------- ---------- ---------- ----------

TGAAATACAG ACAGTTATTC AATAGTCATG ACAAAACTAT GAGTGGACAT

TGAAATATAG GCAACTGTTC AATAGCCACG ACAAAACAAT GAGTGGACAT

TGAAGTATAG GCAGCTGTTC AATAGCCATG ACAAAACGAT GAGCGGCCAC

TTAACAGGTC CCCAAGCAAG AACTATTCTT ATGCAGTCAA GTTTACCACA

TTAACAGGTC CCCAAGCAAG AACTATTCTT ATGCAATCAA GTTTACCACA

TTAACAGGTC CCCAAGCAAG AACTATTCTT ATGCAATCAA GTTTACCACA

TTAACAGGTC CCCAAGCAAG AACTATTCTT ATGCAATCAA GTTTACCACA

TTAACAGGTC CCCAAGCAAG AACTATTCTT ATGCAATCAA GTTTACCACA

TTAACAGGTC CCCAAGCAAG AACTATTCTT ATGCAATCAA GTTTACCACA

TTAACAGGTC CCCAGGCAAG AACTATTCTC ATGCAATCAA GTTTACCCCA

TTAACAGGTC CCCAGGCAAG AACTATTCTC ATGCAATCAA GTTTACCCCA

-------GTC CACAAGCAAG AACTATTCTC ATGCAATCAA GTTTACCACA

CTAACAGGTC CCCAAGCAAG AACTATTCTT ATGCAATCAA GTTTACCACA

TTAACAGGTC CACAAGCAAG GACTATTCTT ATGCAATCAA GTTTGCCACA

TTAACAGGTC CCCAAGCAAG AACTATTCTT ATGCAATCAA GTTTACCGCA

CTGACAGGCC CCCAAGCAAG AACTATCCTT ATGCAATCAA GTCTACCGCA

TTAACAGGTC CCCAAGCAAG AACAATTCTT ATGCAATCAA GTTTACCACA

---------- ---------- ---------- ---------- ----------

CTCACAGGTC CCCAAGCAAG AACTATTCTT ATGCAATCAA GTTTACCACA

TTAACAGGTC CTCAAGCAAG AACTATTCTT ATGCAATCAA GTTTACCACA

CTCACAGGTC CCCAAGCAAG AACTATCCTT ATGCAATCAA GTTTACCCCA

GGCTCAGCTG GCTTCAATAT GGAATCTTTC TGACATTGAT CAAGATGGAA

GGCTCAGCTG GCTTCAATAT GGAATCTTTC TGACATTGAT CAAGATGGAA

GGCTCAGCTG GCTTCAATAT GGAATCTTTC TGACATTGAT CAAGATGGAA

GGCTCAGCTG GCTTCAATAT GGAATCTTTC TGACATTGAT CAAGATGGAA

GGCTCAGCTG GCTTCAATAT GGAATCTTTC TGACATTGAT CAAGATGGAA

GGCTCAGCTG GCTTCAATAT GGAATCTTTC TGACATTGAT CAAGATGGAA

GGCTCAGCTG GCTTCAATCT GGAATCTTTC CGACATTGAT CAAGATGGAA

GGCTCAGCTG GCTTCAATAT GGAATCTTTC TGACATTGAT CAAGATGGAA

GGCTCAGTTG GCTTCAATCT GGAATCTTTC TGACATTGAT CAAGATGGAA

GGCTCAGCTG GCTTCAATAT GGAATCTTTC TGACATTGAT CAAGATGGAA

GGCTCAGCTG GCTTCAATAT GGAATCTTTC TGACATTGAT CAAGATGGAA

GGCTCAGCTG GCTTCAATAT GGAATCTTTC TGACATTGAT CAAGATGGAA

GGCTCAGCTG GCTTCAATAT GGAACCTCTC CGACATCGAC CAGGACGGGA

GGCTCAACTG GCTTCAATAT GGAATCTTTC TGACATTGAT CAAGACGGAA

---------- ---------- ---------- ---------- ----------

GGCTCAGCTG GCTTCAATAT GGAATCTTTC CGACATTGAC CAAGACGGAA

AACTCAGCTG GCTACAATAT GGAATCTTTC AGATATTGAT CAAGATGGGA

GGCTCAGCTG GCTACGATAT GGAATCTTTC GGACATCGAT CAAGATGGAA

AACTTACAGC AGAGGAATTT ATCCTGGCAA TGCACCTCAT TGATGTAGCT

AACTTACAGC AGAAGAATTT ATCCTGGCAA TGCACCTCAT TGATGTAGCT

AACTTACAGC AGAAGAATTT ATCCTGGCAA TGCACCTCAT TGATGTAGCT

AACTTACAGC AGAAGAATTT ATCCTGGCAA TGCACCTCAT TGATGTAGCT

AACTTACAGC AGAAGAATTT ATCCTGGCTA TGCACCTCAT TGATGTAGCT

AACTCACAGC AGAAGAATTT ATCCTGGCTA TGCACCTAAT CGATGTGGCT

AGCTCACCGC AGAAGAATTC ATCCTAGCGA TGCACCTGAT TGATGTTGCT

AACTCACTGC AGAAGAATTT ATCCTAGCTA TGCACCTAAT TGATGTTGCC

AACTCACAGC AGAAGAATTT ATCCTGGCTA TGCACCTAAT AGATGTAGCT

AACTCACAGC AGAAGAATTT ATCCTGGCTA TGCACCTCAT TGATGTAGCT

AACTCACAGC AGAAGAATTT ATCTTGGCTA TGCACCTAAT TGATGTAGCT

AACTTACAGC AGAAGAATTT ATCCTGGCTA TGCACCTAAT TGATGTAGCT

AGCTCACGGC CGAAGAGTTC ATCCTGGCCA TGCACCTCAT CGACGTGGCC

AACTCACAGC AGAAGAATTT ATCCTGGCTA TGCACTTAAT TGATGTAGCT

---------- ---------- ---------- ---------- ----------

AGCTCACAGC AGAAGAATTT ATCCTGGCTA TGCACCTAAT TGATGTGGCT

AACTAACAGC AGAAGAATTT ATTCTGGCAA TGCACTTAAT TGATGTGGCT

AACTCACAGC AGAAGAATTT ATCCTGGCTA TGCACTTAAT CGACGTGGCC

ATGTCTGGCC AACCACTGCC ACCTGTCCTG CCTCCAGAAT ACATTCCACC

ATGTCTGGCC AACCACTGCC ACCTGTCCTG CCTCCAGAAT ATATTCCACC

ATGTCTGGCC AACCACTGCC ACCTGTCCTG CCTCCAGAAT ACATTCCACC

ATGTCTGGCC AACCACTGCC ACCTGTCCTG CCTCCAGAAT ACATTCCACC

ATGTCTGGCC AACCACTGCC ACCCGTCCTG CCTCCAGAAT ACATTCCACC

ATGTCTGGCC AACCACTGCC ACCTGTCCTT CCTCCTGAAT ACATTCCACC

ATGTCTGGTC AGCCACTGCC GCCTGTCCTG CCTCCAGAAT ACATCCCTCC

ATGTCTGGTC AGCCACTGCC GCCCGTCCTG CCTCCAGAAT ACATCCCTCC

ATGTCTGGCC AACCGCTGCC ACCTGTCCTG CCTCCGGAAT ACATTCCACC

ATGTCTGGTC AACCACTGCC ACCGGTCCTG CCCCCAGAAT ACATTCCACC

ATGTCTGGCC AACCACTGCC ACCTGTCCTG CCTCCAGAGT ACATTCCACC

ATGTCTGGCC AACCACTGCC ACCTGTCCTG CCTCCAGAAT ACATTCCACC

ATGTCGGGCC AGCCGCTGCC CCCTGCCCTG CCCCCAGAAT ACATCCCGCC

ATGTCTGGCC AACCACTGCC ACCTGTCCTG CCTCCAGAAT ATATTCCACC

---------- ---------- ---------- ---------- ----------

ATGTCTGGCC AGCCCCTGCC ACCGGCTCTG CCTCCAGAGT ATATCCCACC

ATGTCTGGCC AGCCCCTACC ACCAGCACTG CCTCCAGAAT ATATTCCTCC

ATGTCCGGCC AACCCCTGCC TCCAGTATTG CCTCCAGAGT ATATTCCACC

TTCTTTTAGA AGAGTTCGAT CTGGCAGTGG TATATCTGTC ATAAGCTCAA

TTCTTTTAGA AGAGTTCGAT CTGGCAGTGG TATATCTGTC ATAAGCTCAA

TTCCTTTAGA AGAGTTCGAT CTGGCAGTGG TATATCTGTC ATAAGCTCAA

TTCCTTTAGA AGAGTTCGAT CTGGCAGTGG TATATCTGTC ATAAGCTCAA

TTCCTTTAGA AGAGTTCGAT CTGGCAGTGG TATATCTGTC GTAAGCTCAA

TTCCTTTCGA AGAGTTCGAT CTGGCAGTGG TATATCTGTC ATAAGCTCAA

TTCCTTTAGA AGAGTTCGCT CTGGCAGTGG GATGTCCGTC ATAAGCTCTT

TTCCTTCAGA AGAGTTCGCT CCGGCAGTGG GATGTCCGTC ATAAGCTCTT

TTCCTTTAGG AGAGTTCGGT CTGGCAGCGG TATATCTGTC ATAAGCTCAG

TTCCTTTAGA AGAGTTCGAT CTGGTAGTGG TATATCTGTC ATAACCTCAA

TTCCTTTAGA CGAGTTCGAT CTGGCAGTGG TATATCAGTC ATCAGCTCAA

TTCCTTTAGA AGAGTTCGAT CTGGCAGTGG TATATCTGTC ATAAGTTCAA

TTCCTTCCGG AGAGTCCGCT CTGGCAGTGG GGTGTCGGTC GTCAGCGCCG

TTCATTTAGA AGAGTTCGAT CTGGCAGTGG CATGTCTGTT ATAAGCTCAG

---------- ---------- ---------- ---------- ----------

TTCCTTTAGA AGAGTCCGCT CTGGCAGTGG CATATCTTCC ATAAGCTCCT

TTCATTTAGA AGAGTTCGTT CTGGCAGTGG TATATCTGTT ATAAGCTCTG

TTCATTTAGG AGAGTTCGCT CTGGCAGTGG CCTTTCCGTT ATGAGTTCAG

CATCTGTAGA TCAGAGGCTA CCAGAGGAAC CAGTTTTAGA AGATGAACAA

CATCTGTAGA TCAGAGGCTA CCAGAGGAAC CAGTTTTAGA AGATGAACAA

CATCTGTAGA TCAGAGGCTA CCAGAGGAAC CAGTTTTAGA AGATGAACAA

CATCTGTAGA TCAGAGGCTA CCAGAGGAAC CAGTTTTAGA AGATGAACAA

CATCCGTAGA TCAGAGGCTA CCAGAGGAAC CAGTTTTAGA AGATGAACAG

CATCTGCAGA TCAGAGGCTA CCAGAGGAAC CAGTTTTGGA AGATGAACAG

CGTCTGCAGA CCAGCGGCTG CCCGAGGAGC CATCGTCAGA GGATGAGCAG

CTTCTGTGGA TCAGAGGCTG CCTGAGGAGC CGTCGTCAGA GGATGAGCAG

CCTCTGTAGA CCAGAGGTTA CCAGAGGAAC CAGTGTTAGA AGACGAACAG

CTTCGGTGGA TCAGAGGTTG CCAGAGGAAC CAGTTTTGGA AGATGAACAG

CAGCAGTAGA TCAGAGGTTA CCAGAGGAAC CAGTTTTAGA AGATGAACAA

CGTCAGTAGA TCAGAGGTTA CCAGAGGAAC CAGCCTTAGA AGACGAACAA

GCTCCGTGGA CCAGCGGCTG CCGGAGGAGC CTGCACTGGA GGATGAACAG

CATCTGTAGA TCAAAGGTTA CCAGAAGAAC CAGTTTTAGA AGATGAACAG

---------- ---------- ---------- ---------- ----------

CCTCTGTCGA TCAGAGGTTA CCAGAGGAAC CAGTTTTAGA AGACGAACAG

TATCTGTAGA CCAAAGGTTA CCAGAGGAAC CAGTGTTAGA AGAGGAGCAG

GGTCTGTGGA TCAGAGGTTA CCAGAGGAAC CAGCATTAGA AGAAGAGCAG

CAACAATTAG AAAAGAAATT ACCTGTAGAA TCTCAGCAAA GAGATTCACT

CAACAATTAG AAAAGAAATT ACCTGTAGAA TCTCAGCAAA GAGATTCACT

CAACAATTAG AAAAGAAATT ACCTGTAGAA TCTCAGCAAA GAGATTCACT

CAACAATTAG AAAAGAAATT ACCTGTAGAA TCTCAGCAAA GAGATTCACT

CAACAATTAG AAAAGAAATT ACCTGTAGAA TCTCAGCAAA GAGATTCACT

CAACAATTAG AAAAGAAATT ACCTGTAGAA TCTCAGCAGA GAGATTCACT

CAGG---TGG AAAAGAAGCT GCCTGTGGAA TCTCAGCAGA GAGATTCACT

CAGC---CAG AGAAGAAACT GCCTGTGGAA TCTCAGCAAA GAGACTCGCT

CAACAATTGG AAAAGAAATT ACCTGTCGAG TCTCAGCAGA GAGATTCACT

CAACAACTAG AAAAGAAACT ACCTGTAGAG TCCCAGCAAA GGGATTCACT

CAACAACTAG AAAAGAAATT ACCTGTAGAA GCCCAGCAAA GAGATTCACT

CAACAAATAG AAAAAAAATT GCCTGTAGAG TCTCAGCAAA GAGATTCACT

CAGCAGCTGG AAAAGAAACT GCCGGTCGAG TCCCAGCAG- GCGATTCCCT

CAACAGCTAG AGAAGAAATT ACCTGTAGAG TCCCAGCAA- GAGATTCACT

---------- ---------- ---------- ---------- ----------

CAGCAACTGG AAAAGAAGCT ACCTG--GAA TCTCAGCAAA GAGATTCACT

CAACAGCTGG AAAAGAAGTT ACCTGTGGAA TCTCAGCAAA GAGATTCTCT

CAGCAGCTGG AAAAGAAATT GCCAGTGGAG TCTCAGCAGA GAGATTCACT

TGTTACACTT AAAAGAGCCT TAGAAGCAAA AGAACTAGCT CGGCAGCACC

TGTTACACTT AAAAGAGCCT TAGAAGCAAA AGAACTAGCT CGGCAGCAGC

TGTTACACTT AAAAGAGCCT TAGAAGCAAA AGAACTAGCT CGGCAGCAGC

TGTTACACTT AAAAGAGCCT TAGAAGCAAA AGAACTAGCT CGGCAGCAGC

TGTTACACTT AAAAGAGCCT TAGAAGCAAA AGAAATAGCT CGACAGCAGC

TCTTACACTT AAAAGAGCCT TAGAAGCAAA AGAACTAGCT CGGCAACAGC

TCTTACCCTC AAAAGAGCCT TGGAAGCAAA GGAACTGGCC CGGCAGCAGC

TCTTACCCTC AAAAGAGCCT TGGAAGCAAA GGAGCTGGCC CGGCAGCAGC

TCTTACACTC AAAAGAGCCC TAGAAGCGAA AGAACTAGCT CGGCAACAGC

TCTCACACTC AAAAGAGCCT TAGAAGCGAA AGAACTGGCT CGGCAGCAGC

GCTTACACTC AAAAGAGCGT TAGAAGCAAA AGAACTAGCT CGGCAGCAGC

TCTTACACTC AAAAGAGCCT TAGAAGCAAA AGAACTAGCT CGGCAGCAGC

CCTCACACTC AAAAGAGCAC TAGAGGCCAA GGAGATCCTC CGGCAGCAGC

TCTTACACTC AAAAGAGCCT TAGAAGCAAA AGAACTAGCT CGGCAACAGC

---------- ---------- ---------- ---------- ----------

TCTTACGGTC AAAAGAGCCC TAGAAGCAAA AGAACTGGCT CGGCAGCAGC

TCTTACACTT AAAAGAGCCT TAGAAGCAAA AGAACTTGCT CGTCAGCAAC

TCTCACCCTG AAGAGAGCTT TAGAAGCAAA AGAAGTAGCC CGTCAGCAGC

TACGAGACCA ACTGGATGAA GTGGAGAAAG AAACTAGATC AAAACTACAG

TACGAGACCA ACTGGATGAA GTGGAGAAAG AAACTAGATC AAAACTACAG

TACGAGACCA ACTGGATGAA GTGGAGAAAG AAACTAGATC AAAACTACAG

TACGAGACCA ACTGGATGAA GTGGAGAAAG AAACTAGATC AAAACTACAG

TACGAGACCA ACTGGATGAA GTGGAGAAAG AAACTAGATC AAAACTACAG

TACGAGACCA GCTGGATGAA GTGGAAAAAG AAACAAGATC AAAGCTACAG

TTCGAGAGCA GCTGGACGAG GTGGAGAAAG AGACCAGGTC AAAGCTGCAG

TCCGGGAGCA GCTGGACGAG GTGGAGAGAG AGACCAGGTC AAAGCTGCAG

TTCGAGACCA ACTAGATGAA GTGGAAAAAG AAACGAGATC AAAACTGCAG

TACGAGACCA ACTGGATGAA GTGGAGAAAG AAACTAGGTC AAAATTACAG

TACGCGATCA ACTGGATGAA GTTGAGAAAG AAACCAGATC AAAACTACAG

TACGAGACCA ATTAGATGAA GTTGAGAAAG AAACTAGATC AAAACTACAG

TGCGAGACCA GCTGGACGAC GTGGAGAAGG AGACGAGATC CAAACTCCAG

TACGAGACCA ACTGGATGAA GTTGAGAAAG AAACTAGATC AAAACTACAG

---------- ---------- ---------- ---------- ----------

TCCGGGACCA GCTGGATGAG GTTGAGAGAG AAACTAGATC AAAGCTGCAG

TGAGAGACCA GCTGGATGAG GTTGAGAAGG AAACTAGATC AAAGCTACAG

TTCGAGACCA GCTGGATGAA GTTGAGAAAG AAACCAGATC CAAGTTGCAG

GAGATTGATA TTTTCAATAA TCAGCTGAAG GAACTAAGAG AAATACACAA

GAGATTGATA TTTTCAATAA TCAGCTGAAG GAACTAAGAG AAATACACAA

GAGATTGATA TTTTCAATAA TCAGCTGAAG GAACTAAGAG AAATACATAA

GAGATTGATA TTTTCAATAA TCAGCTGAAG GAACTAAGAG AAATACATAA

GAGATTGATA TTTTCAATAA TCAGCTGAAG GAACTAAGAG AAATACATAG

GAGATTGATA TTTTCAATAA TCAGCTAAAG GAACTAAGAG AAATACATAA

GAGATTGATG TTTTCAACAA CCAGCTGAAG GAACTGAGAG AGATACACAG

GAGATTGATG TTTTCAACAA CCAGCTGAAG GAACTGAGAG AGATACATAG

GAGATTGATA TTTTCAATAA TCAGCTGAAG GAACTAAGAG AAATGCA---

GAGATTGATA TTTTCAACAA TCAGCTGAAG GAACTTAGAG AAATACATAA

GAGATTGATA TTTTCAATAA TCAGCTGAAG GAACTAAGAG AAATACATAG

GAGATTGATA TTTTCAATAA TCAACTGAAG GAACTAAGAG AAATACATAA

GAGATCGATA TTTTCAACAA CCAGCTGAAG ---------- ----------

GAGATTGATA TTTTCAATAA TCAGCTGAAG GAACTAAGAG AAATACACAA

---------- ---------- ---------- ---------- ----------

GAGATTGATA TTTTCAATAA TCAGCTGAAG GAACTCAGAG AAATACATAA

GAGATTGATA TTTTCAATAA TCAGCTAAAG GAACTAAGAG AAATACATAA

GAGATTGATA TTTTCAACAA TCAGCTGAAG GAACTAAGAG AAATCCATAG

TAAGCAACAA CTCCAGAAGC AAAAGTCCAT GGAGGCTGAA CGACTGAAAC

TAAGCAACAA CTCCAGAAGC AAAAGTCCAT GGAGGCTGAA CGACTGAAAC

TAAGCAGCAA CTCCAGAAGC AAAAGTCGAT GGAGGCTGAA CGACTGAAAC

TAAGCAGCAA CTCCAGAAGC AAAAGTCGAT GGAGGCTGAA CGACTGAAAC

TAAGCAGCAA CTCCAGAAGC AAAAGTCCCT GGAGGCGGAA CGACTGAAAC

TAAGCAGCAA CTCCAGAAGC AAAAGTCCAT AGAGGCTGAA CGACTGAAAC

TAAACAGCAG CTCCAGAAGC AGAGGTCCAT CGAGGCGGAG AGGCTAAAGC

CAAACAGCAA CTCCAGAAGC AGAGGTCCCT GGAGGCAGCG CGACTGAAGC

---------- ---------- ---------- ---------- ----------

TAAGCAGCAG CTCCAGAAAC AGAAGTCCAT AGAGGCTGAA CGACTGAAAC

TAAGCAACAA CTCCAGAAAC AAAAGTCCAT AGAGGCCGAA CGTCTGAAAC

TAAGCAGCAA CTCCAGAAAC AAAAATCCAT AGAAGCTGAA CGACTAAAAC

---------- ---------- ---------- ---------- ----------

TAAGCAGCAG CTCCAGAAAC AAAAGTCTAT AGAGGCTGAA CGACTGAAAC

---------- ---------- ---------- ---------- ----------

TAAGCAACAG CTCCAGAAAC AAAAATCCAT AGAGGCCGAA CGCCTGAAAC

CAAGCAACAG CTTCAaaaac aaaagaattt agaagcggaa aggttgaaac

CAAGCAACAA CTCCAGAAAC AGAAGAACTT AGAAGCTGAA AGATTGAAAC

AGAAAGAACA AGAACGAAAG ATCATAGAAT TAGAAAAACA AAAAGAAGAA

AGAAAGAACA AGAACGAAAG ATCATAGAAT TAGAAAAACA AAAAGAAGAA

AGAAAGAACA AGAGCGAAAG ATCATAGAAT TAGAAAAACA AAAAGAAGAA

AGAAAGAACA AGAGCGAAAG ATCATAGAAT TAGAAAAACA AAAAGAAGAA

AGAAAGAACA AGAGAGAAAG ATCGTAGAAT TAGAAAAACA AAAAGAAGAA

AGAAAGAACA AGAGCGAAAG ATCATAGAAT TAGAAAAGCA AAAAGAAGAA

AGAAAGAGCA GGAGAGGAAG AGCCTGGAGT TGGAGAAGCA AAAGGAAGAA

AGAAAGAGCA GGAGAGGAAG AGCCTGGAGT TAGAGAAGCA AAAGGAAGAC

---------- ---------- ---------- ---------- ----------

AGAAGGAACA AGAACGAAAG ATCCTGGAAT TAGAAAAACA AAAAGAAGAA

AGAAAGAACA AGAGCGAAAA ACCATAGAAT TAGAAAAACA AAAAGAAGAA

AGAAAGAGCA AGAGCGAAAG ATCATAGAAT TAGAAAAACA AAAAGAAGAA

---------- ---------- ---------- ---------- ----------

AGAAAGAACA AGAGAGAAAG ATCATAGAAT TAGAAAAACA AAAAGAGGAA

---------- ---------- ---------- ---------- ----------

AGAAAGAACA AGAGCGAAAG ATCCTAGAAT TAGAAAAACA AAAAGAGGAA

agaaagaaaa agagaggaaa acagtagaac tagaaaagca aaaagaggaa

AGAAAGAGCA AGAGAAAAAG ACAGTAGAAT TAGAGAAGCA GAAAGAAGAC

GCCCAAAGAC GAGCTCAGAG GGACAAGCAG TGGCTGGAGC ATGTGCAGCA

GCCCAAAGAC GAGCTCAGAG GGACAAGCAG TGGCTGGAGC ATGTGCAGCA

GCCCAAAGAC GAGCTCAGAG GGACAAGCAG TGGCTGGAGC ATGTGCAGCA

GCCCAAAGAC GAGCTCAGAG GGACAAGCAG TGGCTGGAGC ATGTGCAGCA

GCCCAAAGAC GAGCTCAGAG GGACAAGCAG TGGCTGGAGC ATGTGCAGCA

GCCCAAAGAC GCGCACAGAG GGACAGGCAG TGGCTGGAGC ACGTGCAGCA

GGTCAGAGAC GAGTTCAGAG GGACAAGCAA TGGCAGGAGC ATGTGCAGCA

GCTCAGAGAC GAGTTCAGAG GGACAAGCAA TGGCTGGAGC ATGTGCAGCA

---------- ---------- ---------- ---------- ----------

GCCCAAAGAC GAGCTCAGAG GGACCAGCAG TGGCTGGAGC GCGTGCATCA

GCCCAAAGAC GAGCTCAGAG GGACAAGCAG TGGATTGAGC ATGTGCAGCA

GCCCAAAGAC GAGCTCAGAG GGATAAGCAG TGGCCTGAGC ATGTGCCACA

--------AC GAGCTCAGCG GGACAAGCCG TGGCTGGAGC GCGTGCAGCG

GCCCAAAGAC GCGCTCAGAG GGACAAGCAG TGGCTTGAGC ATGTGCAGCA

---------- ---------- ---------- ---------- ----------

GCCCAAAGAC GAGCTCAGAG GGACAAACAG TGGCTGGAGC ATGTGCAGCA

agccaaagAC GTTCCCA--- GGACAAGCAG TGGCTTGACA GAGTTCAACA

GTCCAAAGGC GAGCGCACAG AGAGAAACCG TGGCCGGACC GGGCGCAGCA

GGAGGAGCAT CAGAGACCAA GAAAACTCCA CGAAGAGGAA AAACTGAAAG

GGAGGAGCAT CAGAGACCAA GAAAACTCCA CGAAGAGGAA AAACTGAAAG

GGAGGAGCAT CAGCGACCAA GAAAACTCCA CGAGGAGGAA AAACTGAAAG

GGAGGAGCAT CAGCGACCAA GAAAACTCCA CGAGGAGGAA AAACTGAAAG

GGAGGAGCAT CAGCGACCAA GAAAACTCCA CGAGGAAGAA AAACTGAAAG

GGATGAGCAT CAGCGGCCGA GAAAACTCCA CGATGAGGAA AGACAGAAGG

GGAGGAGCAG CAGCGCCCTC GGAAACCCCA CGAGGAGGAC AAACTGAAAG

GGAGGAGCAG CCACGCCCCC GGAAACCCCA CGAGGAGGAC AGACTGAAGG

---------- ---------- ---------- ---------- ----------

GGAGGAGCAG CAGCGGCCAC GAAAACCCCA CGAggaggag aagctcaaaG

GGACGAGCAT CAGCGGCCAA GGAAGATCCA TGATGAAGAA AAACTCCGAG

GGACGAGCCT CAGCAGCCAA GAAAACTCCA CGATGATGAA AAACTCAAAG

GGACGAGCTT CCGAGGCCCA GGAAAGCTGT GGATGAGGAG AGCCTCAAGG

GGAAGACCAT CAGCGACCAA GAAAGCTCCA TGATGAAGAG AAACTGAAAG

---------- ---------- ---------- ---------- ----------

GGAGGAACAC CAGCGGCCTA GAAAGTTCCA CGATGAAGAG AGACTGAGAG

AGATGAGTAT CAACGGCCAA AAAAACTCCA TGATGAAGAG AAACTAAA-G

GGACGACTAC CACCGGCCTA AAAAGCCACA CGAGGAGGAG AGGCTAAAGG

TTAAAGGGGA ATGGAGCATG GATTCTGGTT CTTCAGAGAG TCCTGCTAGT

TTAAAGGGGA ATGGAGCATG GATTCTG--- ---------- ----------

TTAAAGGGGA ATGGAGCATG GAGTCTGGTT CTTCAGAGAG TCCTGCTAGT

TTAAAGGGGA ATGGAGCATG GAGTCTGGTT CTTCAGAGAG TCCTGCTAGT

TTAAAGGGGA ATGGAGCATG GAGTCTGGTT CCTCCGAGAG TCCTGCTAGT

TTAAAGGGGA ATGGAGCATG GAGTCTGGTT CTTCAGAAAG TCCTGCTAGT

TTAAAGGGGA ATGGAGCATC GATACTGGCC CTACGGAAGC TCCCTCTAGT

TTAAAGGGGA ATGGAGCATC GATACTGGCC CTACTGAAAG TCCTGCTAGT

---------- ----AGCGTT ACTAGAGGTC CTTCAGATAG TCCCGCCAGC

TTAAAGGGGA ATGGAGCATG GATTCTGGTC CCTCAGAAAG TCCTGCAAGT

TTAAAGGGGA ATGGAGCATG GATTCTGGTT CTTCAGAAAG TCCCGCTAGT

TTAAAGGGGA ATGGAGCATG GATTCTGGTT CTTCAGAAAG TCCGGCTAGT

TTAAAGGGGA ATGGAG---- ---------- ---------- ----------

TTAAAGGGGA ATGG--CATG GATTCGGGTT CTTCAGAAAG TCCTGCAAGT

---------- ----AGCGTG GAGCCCGGCT CTTCAGAAAG TCCTGGGAGT

TTAAAGGGGA ATGG------ ---------- ---------- ----------

TTAAAGGGGA ATGGAGCATG GATTCAGGTT CTTCAGAAAG TCCTGCTGTT

TAAAAGGGGT CTGGAGCATG GATTCAGGTT CTTCAGAAAG CCCCGCTAGT

CTAAAGCGAG TAGCCTCTCC AGCAGCCAAG CCGGTCGTTT CGGGAGAAGA

---------- ---------- ---------- ---------- ---------A

CTAAAGCGAG TAGCCTCTCC AGCAGCCAAG CCGGTCGTTT CTGGAGAAGA

CTAAAGCGAG TAGCCTCTCC AGCAGCCAAG CCGGTCGTTT CGGGAGAAGA

CTAAAGCGAG TAGCCTCTCC AGCAGCCAAG CCGGCCGTTT CGGGAGAAGA

CTAAAGAGAG TGGCTTCCCC AGCAGCCAAG CCAGTCGTTT CCGGAGAAGA

CTAAAGAGAG TGGCTTCCCC AGCCGCCAAG CCAGCCATTC CTGGAGAAGA

CTAAAGAGAG TGGCTTCCCC GGCCGCCAAG CCAGCCATTC CCGGAGAAGA

CGAAAAAGAG -GCCTTCCGC GAAGGCCA-- -CGGCCGCTT CGGGAGAAGA

CTAAAGAGGG TAGCTTCCCC AGCAGCCAAG CCAGCCGTGT CGGGAGAAGA

CTAAAGAGAG TAGCTTCCCC AGCAGCCAAG CCGGCCATTT CGGGAGAAGA

CTAAAGAGAG TAGCTTCCCC AGCAGCCAAG CCAGCCGTTT CGGGAGAAGA

---------- ---------- ---------- ---------- ---------A

CTAAAGAGAG TAGCTTCCCC AGTAGCCAAG CCAGCTGTGT CGGGCGAAGA

CTGAAGAGAG TAGCTTCCCC AGCAGCCAAA CCGGCCGCTT CAGGAGAAGA

---------- ---------- ---------- ---------- ----------

CTAAAGAGAG TAGCATCACC AGCAGCTAAA CTTTCGATAC CAGGAGAAGA

CTAAAGAGGG TGTCATCACC AGCTTCCAAA CTTCCCGTAC CAGGGGAAGA

ATTTATTGCC ATGTACACTT ACGAGAGTTC TGAGCAAGGA GATTTAACCT

ATTTATTGCC ATGTACACTT ACGAGAGTTC TGAGCAAGGA GATTTAACCT

ATTTATTGCC ATGTACACTT ACGAGAGTTC TGAGCAAGGA GATTTAACCT

ATTTATTGCC ATGTACACTT ACGAGAGTTC TGAGCAAGGA GATTTAACCT

ATTTATTGCC ATGTACACTT ACGAGAGTTC TGAGCAAGGA GATTTAACCT

ATTTATTGCC ATGTACACTT ACGAGAGTTC TGAGCAAGGA GATTTAACCT

ATTTGTTGCT ATGTACACTT ACGAGAGTTC TGAGCATGGA GATTTAACCT

GTTTATTGCC ATGTACACAT ACGAGAGTTC TGAGCAAGGA GATTTAACCT

GTTTATTGCC ATGTACACTT ACGAGAGTTC TGAGCAAGGA GACTTAACCT

ATTTATTGCC ATGTACACTT ACGAGAGTTC TGAGCAAGGC GATTTAACCT

ATTTATTGCC ATGTACACTT ACGAGAGTTC TGAGCAAGGC GATTTAACGT

ATTTATTGCC ATGTACACTT ACGAGAGTTC TGAGCAAGGC GATTTAACCT

GTTTATTGCC ATGTACACTT ACGAGAGTTC TGAGCAAGGC GATTTAACCT

ATTTATTGCC ATGTACACTT ACGAGAGTTC TGAGCAAGGA GATTTAACCT

ATTTATTGCC ATGTACACTT ACGAGAGTTC TGAGCAAGGA GATTTGACCT

---------- ---------- ---------- ---------- ----------

ATATATTGCC ATGTACACTT ATGAGAGTTC TGAACAAGGC GACTTAACCT

ATTTATCGCC ATGTACACCT ACGAAAGCTC CGAACAGGGA GATTTAACCT

TTCAGCAAGG GGATGTGATT TTGGTTACCA AGAAAGATGG TGACTGGTGG

TTCAGCAAGG GGATGTGATT TTGGTTACCA AGAAAGATGG TGACTGGTGG

TTCAGCAAGG GGATGTGATT TTGGTTACCA AGAAAGATGG TGACTGGTGG

TTCAGCAAGG GGATGTGATT TTGGTTACCA AGAAAGATGG TGACTGGTGG

TTCAGCAAGG GGATGTGATT TTGGTTACCA AGAAAGATGG TGACTGGTGG

TTCAGCAAGG GGATGTGATT TTGGTTACCA AGAAAGATGG TGACTGGTGG

TTCAGCAAGG GGATGTGATT GTGGTTACCA AGAAGGATGG TGACTGGTGG

TTCAGCAAGG GGATGTGATT GTGGTTACCA AGAAAGATGG TGACTGGTGG

TTCAGCAAGG GGATGTGATT TTGGTTACCA AGAAAGATGG TGACTGGTGG

TTCAGCAAGG GGATGTGATT TTGGTTACCA AGAAAGATGG TGACTGGTGG

TTCAGCAAGG GGATGTGATT TTGGTTACCA AGAAAGATGG TGACTGGTGG

TTCAGCAAGG GGATGTGATT TTGGTTACCA AGAAAGATGG TGACTGGTGG

TTCAGCAAGG GGACGTGATT CTGGTCACCA AGAAGGATGG TGACTGGTGG

TTCAGCAAGG GGAAGTGATT TTGGTTACCA AGAAAGATGG TGACTGGTGG

TTCAGCAAGG GGATGTGCTT TTGGTTACCA AGAAAGACGG TGACTGGTGG

---------- ---------- ---------- ---------- ----------

TTCAGCAAGG AGATGTGATT TTGGTGACAA AGAAGGATGG TGATTGGTGG

TTCAGCAAGG GGACGTGATC TTGGTCACAA AGAAAGATGG CGACTGGTGG

ACAGGAACAG TGGGCGACAA GGCCGGAGTC TTCCCTTCTA ACTATGTGAG

ACAGGAACAG TGGGCGACAA GGCCGGAGTC TTCCCTTCTA ACTATGTGAG

ACAGGAACAG TGGGCGACAA GTCCGGAGTC TTCCCTTCTA ACTATGTGAG

ACAGGAACAG TGGGCGACAA GTCCGGAGTC TTCCCTTCTA ACTATGTGAG

ACAGGAACAG TGGGCGACAA GTCCGGAGTC TTCCCTTCTA ACTATGTGAG

ACAGGAACAG TGGGCGACAA GTCCGGAGTC TTCCCTTCTA ACTATGTGAG

ACGGGCACAG TGGGCGAGAC GTCCGGAGTC TTCCCTTCTA ACTATGTGAG

ACGGGAACGG TGGGCGACAA GTCCGGAGTC TTCCCTTCTA ACTATGTGAG

ACGGGAACAG TGGGCGACAA GTCCGGAGTC TTCCCTTCTA ACTATGTGAG

ACAGGAACAG TGGGCGACAA GTCCGGCGTC TTCCCTTCTA ACTATGTGAG

ACAGGAGCAG TGGGCGACAA GTCCGGAGTC TTCCCTTCTA ACTATGTGAG

ACAGGAACAG TGGGCGACAA GTCCGGAGTC TTCCCATCTA ACTATGTGAG

ACAGGAGCGG TGGGCGCCAA GACCGGAGTC TTCCCTTCTA ACTATGTGAG

ACAGGAACAG TGGGCGACAA ATCCGGAGTC TTCCCTTCTA ACTATGTGAG

ACAGGAACAG TGGGCGACAG ATCCGGAGTC TTCCCTTCTA ACTATGTGAG

---------- ---------- ---------- ---------- ----------

ACAGGCACTG TGGCTGACAA ATCTGGAGTC TTTCCTTCTA ACTACGTGAG

ACAGGAACAG CGGGTGACAA ATCCGGAGTC TTCCCTTCTA ACTACGTGCG

GCTTAAAGAT TCAGAGGGCT CTGGAACTGC TGGGAAAACA GGGAGTTTAG

GCTTAAAGAT TCAGAGGGCT CTGGAACTGC TGGGAAAACA GGGAGTTTAG

GCTTAAAGAT TCAGAGGGCT CTGGAACTGC TGGGAAAACA GGGAGTTTAG

GCTTAAAGAT TCAGAGGGCT CTGGAACTGC TGGGAAAACA GGGAGTTTAG

GCTTAAAGAT TCAGAGGGCT CTGGAACTGC TGGGAAAACA GGGAGTTTAG

GCTTAAAGAT TCAGAGGGCT CTGGAACTGC TGGGAAAACA GGGAGTTTAG

GCTTAAAGAC TCAGAGGGCT CTGGAACTGC GGGGAAAACA GGGAGTTTAG

GCTTAAAGAT TCAGAGGGCT CTGGAACTGC TGGGAAAACA GGGAGTTTAG

ACTTAAAGAT TCAGAG---- ---------- ---------- ----------

GCTTAAAGAT TCGGAGGGCT CTGGAACTGC TGGGAAAACA GGGAGTTTAG

GCTTAAAGAT TCAGAGGGCT CTGGAACTGC TGGGAAAACA GGGAGTTTAG

GCTTAAAGAT TCAGAGGGCT CTGGAACTGC TGGGAAAACA GGGAGTTTAG

GCTTAAGGAC GCCGAGGGTG CCGGGACCTC CGGGAAAGCC GGGAGTTTGG

GCTTAAAGAT TCAGAGGGCT CTGGAACTGC TGGGAAAACA GGGAGTTTAG

GCTGAAAGAC TCAGAGGGCT CTGGAACCGC TGGGAAAACA GGGAGTTTAG

---------- ---------- ---------- ---------- ----------

GCTTAAAGAC TCCGAGGTCC CTGGAGCTTC TGGGAAAGCA GGGAGTTTAG

GCTCAAGGAT TCCGAGGTCC CCGGAACCGC TGGGAAAACA GGAAGTTTGG

GAAAAAAACC TGAAATTGCC CAGGTTATTG CCTCATACAC CGCCACCGGC

GAAAAAAACC TGAAATTGCC CAGGTTATTG CCTCATACAC CGCCACCGGC

GAAAAAAACC TGAAATTGCC CAGGTTATTG CCTCATACAC CGCCACCGGC

GAAAAAAACC TGAAATTGCC CAGGTTATTG CCTCATACAC CGCCACCGGC

GAAAAAAACC TGAAATTGCC CAGGTTATTG CCTCATACAC CGCCACCGGC

GAAAAAAACC TGAGATTGCC CAGGTTATTG CATCATACAC TGCTACTGGT

GAAAAAAACC TGAAATCGCC CAGGTCATTG CTTCCTACAC TGCTACGGGT

GAAAAAAACC TGAAATTGCC CAGGTTATTG CTTCCTACGC TGCTACTGGT

---------- ---------- ---------- ---------- ----------

GAAAAAAACC TGAAATCGCG CAGGTCATTG CCTCCTACAC AGCTACGGGT

GAAAAAAACC TGAAATTGCG CAGGTTATCG CCTGCTACAC AGCTACTGGT

GAAAAAAACC TGAAATCGCC CAGGTTATTG CTTCGTACAC CGCGACGGGT

GGAAGAAGCC GG-------- ---------- ---------- ----------

GAAAAAAACC TGAAATTGCC CAGGTTATCG CCTCATACAC CGCTACCGGT

GAAAGAAACC TGAAATTGCC CAGGTCATTG CCTCGTACAC TGCTACCGGC

---------- ---------- ---------- ---------- ----------

GAAAGAAACC TGAAATTGCC CAGGTTATTG CATCTTATAC TGCTACTGGA

GAAAGAAACC AGAAATTGCC CAGGTTATTG CCTCTTACAC AGCCACCGGC

CCCGAGCAGC TCACTCTCGC CCCTGGTCAG CTGATTTTGA TCCGAAAAAA

CCCGAGCAGC TCACTCTCGC CCCTGGTCAG CTGATTTTGA TCCGAAAAAA

CCCGAGCAGC TCACTCTCGC CCCTGGTCAG CTGATTTTGA TCCGAAAAAA

CCCGAGCAGC TCACTCTCGC CCCTGGTCAG CTGATTTTGA TCCGAAAAAA

CCCGAGCAGC TCACTCTTGC CCCTGGTCAG CTGATTTTGA TCCGAAAAAA

CCTGAGCAGC TCACTCTGGC CCCTGGTCAG CTGATTTTGA TCCGAAAAAA

CCTGAACAGC TCACCCTGGC TCCTGGTCAG CTGATTTTGA TCCGAAAAAA

CCCGAACAAC TCACCCTGGC TCCTGGGCAG CTGATTCTGA TCCGGAAAAA

---------- ---------- ---------- ---------- ----------

CCTGAACAGC TTACCCTGGC CCCCGGTCAG CTGATCCTGA TCCGGAAAAA

CCCGAGCAGC TCACCCTGGC CCCTGGTCAG CTGATTTTGA TCCGAAAAAA

CCCGAGCAGC TTACCCTGGC CCCTGGTCAG CTGATTTTGA TCCGAAAAAA

---------- ---------- ---------- ---------- ----------

CCTGAGCAGC TGACTCTGGC CCCTGGTCAG CTGATTTTGA TCCGAAAAAA

CCTGAGCAGC TCACCCTGGC CCCTGGTCAG CTGATTTTGA TCCGAAAAAA

---------- ---------- ---------- ---------- ----------

CCTGAACAGC TTACCTTGGC ACCTGGACAA CTCATTTTGA TCCGGAAGAA

CCCGAACAAC TCACCCTGGC ACCCGGTCAA CTGATTCTGA TCCGCAAGAA

GAACCCAGGT GGATGGTGGG AAGGAGAGCT GCAAGCACGT GGGAAAAAGC

GAACCCAGGT GGATGGTGGG AAGGAGAGCT GCAA------ ----------

GAACCCAGGT GGATGGTGGG AAGGAGAGCT GCAAGCACGT GGGAAAAAGC

GAACCCAGGT GGATGGTGGG AAGGAGAGCT GCAA------ ----------

GAACCCAGGT GGATGGTGGG AAGGAGAGCT GCAAGCCCGT GGGAAAAAGC

GAACCCAGGC GGATGGTGGG AAGGAGAGCT GCAAGCACGT GGGAAAAAAC

GAACCCCGGT GGATGGTGGG AAGGAGAACT ACAAGCTCGA GGGAAAAAGC

GAACCCAGGT GGATGGTGGG AAGGAGAACT GCAAGCTCGA GGGAAAAAGC

---------- ---------- ---------- ----GCACGT GGAAAAAAGC

GAACCCAGGT GGATGGTGGG AAGGAGAGCT ACAGGCACGT GGGAAAAAGC

GAACCCAGGC GGTTGGTGGG AAGGAGAGCT GCAAGCCCGT GGGAAGAAGC

GAACCCAGGT GGATGGTGGG AAGGAGAGCT GCAAGCACGT GGGAAAAAGC

---------- ---------- ---------- ---------- ----------

GAATCCAGGT GGATGGTGGG AAGGAGAGCT GCAAGCACGT GGAAAGAAGC

GAATCCAGGA GGATGGTGGG AAGGAGAGCT GCAGGCACGT GGGAAAAAGC

---------- ---------- ---------- ---------- ----------

GAATCCAGGA GGATGGTGGG AAGGAGAACT TCAAGCTCGT GGGAAAAAGC

GAATCCCGGA GGATGGTGGG AAGGAGAGCT TCAAGCACGT GGGAAAAAAC

GCCAGATAGG CTGGTTCCCA GCTAATTATG TAAAGCTTCT AAGCCCTGGG

---------- ---------- ---------- ---------- ----------

GCCAGATAGG CTGGTTCCCA GCTAATTATG TAAAACTTCT AAGCCCTGGG

---------- ---------- ---------- ---------- ----------

GCCAAATAGG CTGGTTCCCA GCTAATTATG TAAAACTTCT AAGCCCTGGG

GCCAGATAGG CTGGTTCCCA GCTAATTATG TAAAACTTCT GAGCCCTGGG

GCCAGATAGG GTGGTTCCCA GCAAATTATG TCAAACTTCT AAGCCCTGGA

GCCAGATAGG GTGGTTTCCA GCAAATTATG TCAAACTTCT AAGCCCCGGA

GCCAGATAGG CTGGTTCCCA GCTAATTATG TAAAACTTCT AAGTCCTGGG

GCCAGATAGG CTGGTTTCCT GCTAATTACG TCAAACTTTT AAGCCCTGGG

GCCAGATAGG CTGGTTCCCC GCGAATTATG TAAAACTTTT GAGCCCTGGC

GCCAGATAGG CTGGTTTCCT GCTAATTATG TAAAACTTTT AAGCCCTGGG

---------- ---------- ---------- ---------- ----------

GCCAGATAGG CTGGTTCCCA GCTAATTATG TAAAACTTTT AAGCCCTGGG

GCCAGATAGG CTGGTTCCCA GCTAATTATG TCAAACTTTT AAGCCCTGGG

---------- ---------- ---------- ---------- ----------

GTCAGATAGG CTGGTTCCCA GCTAATTATG TAAAACTTTT AAGTCCTGGC

GACAGATCGG CTGGTTCCCG GCAAATTACG TCAAACTCCT GAGCCCCGGC

ACGAGCAAAA TCACTCCAAC AGAGCCACCT AAGTCAACAG CATTAGCGGC

---------- ---------- ---------- ---------- ----------

ACGAGCAAAA TCACTCCAAC AGAGCCACCT AAGTCAACAG CGTTAGCGGC

---------- ---------- ---------- ---------- ----------

ACGAGCAAAA TCACTCCAAC GGAGCCACCT AAGTCAACAG CATTAGCAGC

ACGAGCAAAA TCACTCCAAC AGAGCCACCC AAGTCAGCAG CATTACCAGC

ACAAGCAAAA TCACCCCAAC TGAGCTACCC AAGACCGCAG TGCAGCCAGC

ACAAGCAAAA TCACCCCAAC TGAGCTACCC AAGACCGCAG TGCAGCCAGC

ACGAGCAAAA TCACTCCAAC GGAGCCACCC AAGTCCGCGG CGTTGCCCGC

ACAAGCAAAA TCACTCCAAC AGACCCACCG AAGCCAACAG CTTTCCCAAC

ACGAGCAAAA TCACTCCAAC GGAGCCACCT AAGCCCACGG CGTTCCCGGC

ACGAGCAAAA TCACTCCAAC CGAGCCCCCT AAGCCAACGG CACTCCCGGC

---------- ---------- ---------- ---------- ----------

TCGAGCAAAA TCACTCCAAC AGAGCCACCT AAGTCAGCGG CCTTGCCTGC

ACGAGCAAAA TCACCCCAAC AGAGCCACCG AAGTCCACGG CCCTAACTGC

---------- ---------- ---------- ---------- ----------

ACCAGCAAAG TCACTCCAAC GGAGCCACCG AAGTCAATAG CACTACCTGC

ACCAGCAAGA TCACACCAAC CGAGCCCCCT AAGGCAACAG CAATACCTTC

AGTGGCCTTC AACAAGGGCC AGATCATCAA CCTCAACAAG GAGGACCCTG

---------- ---------- ---------- --TTACCAAG AAAGATGGTG

AGTGGCCTTC AACAAGGGCC AGATCATCAA CCTCAACAAG GAGGACCCCG

---------- ---------- ---------- --TTACCAAG AAAGATGGTG

AGTGGCCTTC AACAAGGGCC AGATCATCAA TCTCAACAAG GAGGACCCCG

AGTGGCCTTC AACAAGGGCC AGATCATCAA CCTCAACAAG GAGGACCCCG

AGTGGCCTTC AGCAAAGGCC AGATCATCAA CCTCAGCAAG GAGGACCCGG

AGTGGCCTTC AGCAAAGGCC AGATCATCAA CCTCAACAAG GAGGACCCGG

AG-------- ---------- ---------- ---------- ----------

AGTGGCCTTC AGCAAGGGCC AGGTCATCAA TCTCAACAAG GAGGACCCCG

AGTGGCCTTC AGCAAGGGCC AGATCATCAA CCTCAACAAA GACGACCCGG

AGTGGCCTTC AACAAGGGCC AGCTCATCAA CCTCAACAGG GAGGACCCTG

-----CTTTC AGCAAGGGCG TGATTCTGG- --TCACCAAG AAGGATGGTG

AG---CTTTC AGCAAGGGGA AGATTTTGG- --TTACCAAG AAAGATGGTG

AGtgGCCTTC AGCAAGGGCC AGATCATCAA TCTCAACAAG GAAGACCCCG

---------- ---------- ---------- ---------- ----------

AGTGGCATTC AACAAAGGCC AGATCATCAA TCTTAATAAG GAAGATCCTG

GGTGGCGTTC GGCAAGGGCC AGATCATCAA CCTCAACAAG GAAGACCCCG

ACTGGTGGAA AGGAGAAGTC AATGGACAAG TGGGGCTCTT CCCATCCAAT

ACTGGTGGAC AGGAACAGTG GGCGACAAGG CCGGAGTCTT CCCTTCTAAC

ACTGGTGGAA GGGGGAAGTC AATGGACAAG TGGGGCTCTT CCCATCCAAT

ACTGGTGGAC AGGAACAGTG GGCGACAAGT CCGGAGTCTT CCCTTCTAAC

ACTGGTGGAA AGGAGAAGTC AATGGACAAG TGGGACTCTT CCCATCCAAT

ACTGGTGGAA AGGAGAAGTG AATGGACAAG TGGGGCTCTT CCCGTCCAAC

ACTGGTGGAA AGGAGAAGTC AGTGGGCAGG TTGGGCTCTT CCCGTCCAAT

ACTGGTGGAA AGGAGAAGTC AGTGGGCAAG TTGGGCTCTT CCCATCCAAT

---------- ---------- ---------- ---------- ----------

ACTGGTGGAA GGGTGAAGTC CACGGGCAGG TGGGGCTCTT CCCGTCCAAC

ACTGGTGGAA GGGCGAGGTC AACGGGCAAG TGGGGCTCTT CCCCTCCAAC

ATTGGTGGAA AGGAGAAGTC AGTGGCCAAG TGGGGCTCTT CCCATCCAAT

ACTGGTGGAC AGGAGCGGTG GGCGCCAAGA CCGGAGTCTT CCCTTCTAAC

ACTGGTGGAC AGGAACAGTG GGCGACAAAT CCGGAGTCTT CCCTTCTAAC

ACTGGTGGAA AGGAGAAGTC AGCGGACAAG TGGGGCTCTT CCCGTCCAAT

---------- ---------- ---------- ---------- ----------

ATTGGTGGAA AGGAGAAGTC AATGGACAAG TGGGGCTCTT CCCATCCAAT

ACTGGTGGAA AGGGGAGGCG AACGGGCAAG TGGGCCTCTT CCCCTCCAAC

TATGTGAAGC TGTGGTGTTC AGACTTACAT CTCTTGGATA TGTTGACCCC

TATGTGAGGC T--GGTGTTC AGACTTACAT CTCTTGGATA TGTTGACCCC

TATGTGAAGC TATGGTGTTC AGACTTACAT CTCTTGGATA TGTTGACCCC

TATGTGAGGC T--GGTGTTC AGACTTACAT CTCTTGGATA TGTTGACCCC

TATGTGAAGC TGTGGTGTTC AGACTTACAT CTCTTGGATA TGTTGACCCC

TATGTGAAGC TGTGGTGTTC AGACTTGCAT CTCTTGGATA TGTTGACCCC

TATGTGAAGC TGTGGTGCTC AGACTTACAT CTCTTAGATA TGTTGACCCC

TATGTAAAGC TGTGGTGCTC AGACCTGCAT CTCTTAGATA TGCTGACCCC

---------- ---GGTGTTC AGACTTGCAT CTCCTGGATA TGTTGACCCC

TACGTGAAGT TGTGGTGCTC AGACTTACAC CTCTTGGATA TGCTGACCCC

TACGTGAAGC TGTGGTGCTC AGACTTACAC CTCTTGGATA TGTTGACCCC

TATGTGAAGC TGTGGTGTTC AGACTTACAT CTCTTGGATA TGTTGACCCC

TATGTGAGGC T--GGTGCTC CGACCTCCAC CTCCTGGACA TGCTGACGCC

TATGTGAGGC T--GGTGTTC CGACTTACAT CTCTTGGATA TGCTGACCCC

TATGTGAAGC TGTGGTGTTC AGACTTACAT CTCTTGGATA TGTTGACCCC

---------- ---GGTGTTC AGACCTACAT CTCTTGGATA TATTGGCCCC

TATGTGAAAA TGTGGTGTGC AGATTTACAT CTTTTGGATA TGTTGACACC

TACGTGAAGC TGTGGTGCGC GGACTTGCAT CTGCTGGACA TGCTGACCCC

AACTGAAAGA AAGCGACAAG GATACATCCA CGAGCTCATT GTCACCGAGG

AACTGAAAGA AAGCTACAAG GATACATCCA CGACCTCATT GTCACCGAGG

AACTGAAAGA AAGCGACAAG GATACATCCA CGAGCTCATT GTCACAGAGG

AACTGAAAGA AAGCGACAAG GATACATCCA CGAGCTCATT GTCACAGAGG

AACTGAAAGA AAACGACAAG GGTACATCCA CGAGCTCATT GTCACGGAGG

AACTGAGAGG AAGAGACAAG GGTACATCCA CGAACTCATT GTCACGGAGG

GACTGAGAGG AAGAGGCAAG GCTACATCCA TGAACTCATT GTCACAGAGG

GACTGAGAGG AAGCGGCAAG GCTACATCCA TGAACTCATT GTCACGGAGG

GACGGAGAGA AAGCGACAAG GCTACATCCA TGAGCTCATT GTCACCGAGG

AACTGAAAGA AAGCGACAGG GGTACATCCA CGAGCTCATC GTCACGGAGG

AACGGAGAGA AAGCGACAAG GATACATCCA TGAGCTCATT GTCACAGAGG

AACTGAAAGA AAGCGACAAG GGTACATCCA CGAACTCATT GTCACGGAGG

CACCGAGCGG AAGCGGCAGG GCTACATCCA CGAGCTCATC GTCACCGAGG

AACTGAAAGA AAGCGGCAAG GCTACATCCA CGAGCTCATC GTCACGGAGG

AACGGAGAGA AAACGACAAG GGTACATCCA CGAACTCCTT GTCACCGAGG

CACGGAAAGA AAGCGACAAG GATACATCCA CGAACTCATC GTCACGGAGG

AACAGAAAGA AAACGACAGG GATATATCCA TGAGCTACTT GTTACAGAAG

TACGGAAAGG AAGCGCCAGG GCTACATCCA CGAGCTGATC CTGACCGAAG

AGAACTATGT GAATGACCTG CAGCTGGTCA CAGAGATTTT TCAAAAACCC

AGAACTATGT GAATGACCTG CAGCTGGTCA CAGAGATTTT TCAAAAACCC

AGAACTACGT GAACGACCTG CAGCTGGTCA CAGAGATCTT TCAAAAACCC

AGAACTACGT GAACGACCTG CAGCTGGTCA CAGAGATCTT TCAAAAACCC

AGAACTATGT GAATGACCTG Cagctggtca cagagATCTT TCAAAAACCC

AGAACTATGT GAATGACCTA CAGCTGGTCA CAGAGATCTT TCAGAAACCC

AGAACTATGT GAACGACCTG CAGCTGGTCA CAGAGATCTT TCAGAAACCC

AGAACTACGT GAACGACTTG CAGCTGGTCA CAGAGATCTT TCAGAAACCC

AGAACTACGT GAATGATCTG CAGCTGGTCA CAGAGATCTT TCAGAAACTC

AGAACTACGT GAATGACCTG CAGCTGGTCA CAGAGATCTT TCAGAAACCC

AGAACTATGT GAATGACCTG CAGCTGGTCA CAGAGATCTT TCAGAAACCC

AGAACTATGT GAACGACCTG CAGCTGGTCA CAGAGATCTT TCAGAAACCC

AGAACTACGT CAACGACCTG CAGCTCGTCA CGGAGATCTT TCAGAAACCC

AGAACTACGT CAATGACCTG CAGCTTGTCA CTGAGATCTT TCAGAAACCC

AGAATTATGT CAATGACCTC CAGCTGGTCA CAGAGATCTT TCAGAAACCC

AGAACTACGT CAACGACCTG CAGCTGGTCA CAGAGATCTT TCAGAAACCC

AGAATTATGT CAATGACCTG CAGTTAGTCA CAGAGATCTT TCAGAAACCG

AGAATTACGT TAATGACCTG CAGCTAGTCA CAGAGGTCTT TCAGAAACCC

CTGATGGAGT CTGAGCTGCT GACAGAAAAA GAGGTTGCTA TGATTTTTGT

CTGATGGAGT CTGAGCTGCT GACAGAAAAA GAGGTTGCTA TGATTTTTGT

CTGATGGAGT CTGAGCTGCT GACAGAGAAA GAGGTTGCTA TGATTTTTGT

CTGATGGAGT CTGAGCTGCT GACAGAGAAA GAGGTTGCTA TGATTTTTGT

CTGATGGAGT CTGAGCTGCT GACAGAAAAA GAGGTTGCTA TGATTTTTGT

CTGATGGAGT CTGAGCTGCT GACAGAGAAA GAGGTTGCTA TGATTTTTGT

CTGACGGAGT CTGAGCTGCT GACAGAAAAA GAGGTTGCTA TGATTTTTGT

CTGACGGAGT CTGAGCTGCT GACAGAAAAA GAGGTTGCTA TGATTTTTGT

CTGATGGAGT CTGAGCTGCT GACAGAAAAA GAGGTTGCTA TGATTTTTGT

CTGATGGAGT CTGAGCTGCT GACAGAAAAA GAGGGTGCTA TGATTTTTGT

CTGATGGAGT CTGAGCTGCT GACAGAAAAA GAGGGTGCTA TGATTTTTGT

CTGATGGAGT CTGAGCTGCT GACAGAAAAA GAGGGTGCTA TGATTTTTGT

CTGATGGATT CTGAGCTGCT GACAGAGAAA GAGGGTGCTA TGATTTTTGT

CTGATGGAGT CTGAGCTGCT GACAGAAAAA GAGGTTGCTA TGATTTTTGT

CTGATGGAAT CTGAGCTGCT GACAGAAAAA GAGGTTGCTA TGATTTTTGT

CTGATGGAAT CTGAGCTGCT GACAGAAAAA GAGGTTGCTA TGATTTTTGT

CTGATGGAGT CTGAGCTGCT GACAGAAAAA GAGGTGGCTA TGATTTTTGT

CTAATGGAAT CCGAGCTGCT GACGGAAAAG GAGGTGGCCA TGATTTTTGT

GAACTGGAAG GAGCTGATTA TGTGTAATAT CAAACTACTA AAAGCGCTGA

GAACTGGAAG GAGCTGATTA TGTGTAATAT CAAACTACTG AAAGCGCTGA

GAACTGGAAG GAGCTGATTA TGTGTAATAT CAAACTACTG AAAGCGTTGA

GAACTGGAAG GAGCTGATTA TGTGTAATAT CAAACTACTG AAAGCGCTGA

GAACTGGAAG GAGCTGATTA TGTGTAATAT CAAACTACTG AAAGCGCTGA

CAACTGGAAG GAGCTGATTA TGTGTAATAT CAAACTGCTG AAAGCACTGC

TAACTGGAAG GAGCTGATTA TGTGTAATAT CAAACTGCTG AAGGCGCTGA

TAACTGGAAG GAGCTGATCA TGTGTAATAT CAAACTGCTG AAAGCGCTGA

TAACTGGAAG GAGCTGATTA TGTGTAATAT CAAACTACTG AAGGCGCTGC

TAACTGGAAG GAGCTGATTA TGTGTAATAT CAAACTACTG AAAGCGCTGA

TAACTGGAAG GAGCTGATTA TGTGTAATAT CAAACTATTG AAAGCGCTGA

TAACTGGAAG GAGCTGACTA TGTGTAATGT CAAACTGCTG AAAGCGCTGA

TAACTGGAAG GAGCTGATTA TGTGTAATAT CAAACTACTG AAGGCGCTGA

TAACTGGAAG GAGCTGATTA TGTGTAATAT CAAACTACTG AAAGCGCTGA

TAACTGGAAG GAGCTGATTA TGTGTAATAT CAAGCTACTG AAGGCACTGA

TAACTGGAAG GAGCTGATTA TGTGTAATAT CAAACTACTG AAAGCGCTGA

TAACTGGAAG GAGCTGATAA TGTGTAATAT CAAACTGCTG AAAGCCCTGA

CAACTGGAAG GAGCTGATAA TGTGTAACAT CAAACTACTG AAGGCCCTGA

GAGTCCGCAA GAAGATGTCC GGGGAGAAGA TGCCTGTGAA GATGATTGGA

GAGTCCGCAA GAAGATGTCC GGGGAGAAGA TGCCTGTGAA GATGATTGGA

GAGTCCGTAA GAAGATGTCC GGGGAGAAGA TGCCCGTGAA GATGATTGGT

GAGTCCGTAA GAAGATGTCC GGGGAGAAGA TGCCCGTGAA GATGATTGGT

GAGTCCGCAA GAAGATGTCC GGGGAGAAGA TGCCCGTGAA GATGATTGGT

GAGTGCGCAA GAAGATGTCG GGGGAGAAGA TGCCAGTGAA GATGATCGGG

GAGTCCGCAA GAAGATGTCC GGAGAGAAGA TGCCGGTGAA GATGATCGGT

GAGTCCGCAA GAAGATGTCT GGGGAGAAGA TGCCGGTGAA GATGATTGGC

GGGTCCGCAA GAAGATGTCT GGGGAGAAGA TGCCGGTGAA GATGATCGGC

GGGTCCGCAA GAAGATGTCT GGGGAGAAGA TGCCCGTGAA GATGATCGGA

GGGTCCGCAA GAAGATGTCG GGGGAGAAGA TGCCCGTGAA GATGATCGGC

GAGTTCGCAA GAAGATGTCT GGAGAGAAGA TGCCTGTGAA GATGATCGGT

GGGTCCGCAA GAAGATGTCG GGGGAGAAGA TGCCCGTGAA GATGATCGGG

GGGTCCGCAA GAAGATGTCC GGGGAGAAGA TGCCCGTGAA GATGATCGGC

GGGTCCGCAA GAAGATGTCT GGGGAGAAAA TGCCGGTGAA GATGATCGGG

GGGTTCGCAA GAAGATGTCT GGGGAGAAAA TGCCCGTGAA GATGATCGGG

GAGTTCGAAA GAAGATGTCT GGTGAGAGGA TGCCCGTGAA GATGATCGGC

GGGTGCGGAA GAAGATGTCG GGGGAGAGGA TGCCCGTGAA AATGATCGGC

GACATCCTGA GCGCACAGCT GCCGCACATG CAGCCCTACA TCCGCTTCTG

GACATCCTGA GCGCACAGCT GCCGCACATG CAGCCCTACA TCCGCTTCTG

GACATCTTGA GCGCCCAGCT GCCGCACATG CAACCCTACA TCCGCTTCTG

GACATCTTGA GCGCCCAGCT GCCGCACATG CAACCCTACA TCCGCTTCTG

GACATCCTGA GCGCCCAGCT GCCGCACATG CAGCCCTACA TCCGCTTCTG

GACATCCTGA GCGCTCAGCT GCCACACATG CAGCCCTACA TCCGCTTCTG

GACATCCTGA GCGCCCAGCT GCCCCACATG CAGCCTTACA TCCGCTTCTG

GACATCCTGA GCGCCCAGCT GCCGCACATG CAGCCTTACA TCCGCTTCTG

GACATCTTGA GCGCCCAGCT GCCGCATATG CAGCCGTACA TTCGCTTCTG

GACATCCTGA CGGCCCAGCT GCCACACATG CAGCCCTACA TCCGCTTCTG

GACATCCTGA CGGCGCAGCT GCCGCACATG CAGCCTTACA TCCGCTTCTG

GACATCCTGA CGGCCCAGCT GCCACATATG CAACCCTACA TCCGCTTCTG

GACATCCTCA CGGCCCAGCT GCCGCACATG CAGCCCTACA TCCGCTTCTG

GACATCCTGA CGGCCCAGCT GCCCCACATG CAGCCCTACA TTCGCTTCTG

GACATCCTGA GCGCCCAGCT GCCCCACATG CAGCCATACA TCCGCTTTTG

GACATCCTGA CCGCCCAGCT GCCCCACATG CAGCCATATA TCCGCTTCTG

GACATCTTGA CTGCCCAGTT GCCCCACATG CAGCCTTACA TCCGGTTCTG

GACATCCTCA CGGCCCAGCT GCCGCACATG CAGCCGTACA TCCGCTTCTG

CAGCCGCCAG CTCAACGGGG CTGCCCTGAT CCAGCAGAAG ACGGATGAGG

CAGCCGCCAG CTCAACGGGG CTGCCCTGAT CCAGCAGAAG ACGGACGAGG

CAGCCGCCAG CTCAACGGGG CTGCCCTGAT CCAGCAGAAG ACAGACGAGG

CAGCCGCCAG CTCAACGGGG CTGCCCTGAT CCAGCAGAAG ACAGACGAGG

CAGCCGCCAG CTCAACGGGG CTGCCCTGAT CCAGCAGAAG ACAGACGAGG

CAGCTGCCAG CTCAACGGGG CTGCCCTGAT CCAGCAGAAG ACGGACGAGG

CAGCTGCCAG CTCAATGGGG CCGCCCTCAT CCAGCAGAAG ACAGATGAGG

CAGCTGCCAG CTCAATGGGG CTGCCCTCAT CCAGCAGAAG ACGGACGAGG

CAGCTGCCAG CTCAACGGAG CCGCCCTGAT CCAGCAGAAG ACGGACGAGG

CAGCTGCCAG CTCAACGGGG CCGCCTTGAT CCAGCAGAAG ACTGACGAGG

CAGCTGTCAG CTCAACGGGG CTGCCCTCAT CCAGCAGAAG ACCGACGAGG

CAGCTGCCAG CTGAACGGGG CAGCCCTGAT CCAGCAGAAG ACGGATGAAG

CAGCTGCCAG CTCAACGGGG CCGCCCTCAT CCAGCAGAAG ACTGACGAGG

CAGCTGCCAG CTCAACGGGG CGGCCCTGAT CCAGCAAAAG ACAGACGAGG

TAGCTGCCAG CTAAATGGGG CAGCCCTGAT CCAGCAGAAG ACGGACGAAG

CAGCTGTCAG CTCAACGGGG CAGCCCTGAT CCAGCAGAAG ACGGACGAGG

TAGCTGCCAG CTAAACGGGG CAGCCCTCAT CCAGCAGAAG ACGGATGAAG

CAGCTGCCAG CTCAACGGGG CCGCCCTCAT CCAGCAGAAG ACCGACGAAG

CCCCAGACTT CAAGGAGTTC GTCAAAAGAT TGGCAATGGA TCCTCGGTGT

CCCCAGACTT CAAGGAGTTT GTCAAAAGAT TGGCAATGGA TCCTCGGTGT

CCCCAGACTT CAAGGAGTTC GTCAAAAGAT TGGCAATGGA TCCTCGGTGT

CCCCAGACTT CAAGGAGTTC GTCAAAAGAT TGGCAATGGA TCCTCGGTGT

CCCCAGACTT CAAGGAGTTT GTCAAAAGAC TGGCAATGGA CCCTCGGTGT

CCCCCGACTT TAAGGAGTTT GTCAAAAGAT TGGCAATGGA CCTGCGCTGT

CTCCAGACTT CAAGGAGTTC GTCAAAAGAC TGGCAATGGA CCCTCGGTGC

CTCCAGACTT CAAGGAGTTC GTCAAAAGAC TGGCAATGGA CCCTCGGTGC

CTCCGGACTT CAAGGAGTTT GTCAAGAGAC TGGCAATGGA GCCCAGATGT

CCCCAGACTT CAAGGAGTTT GTCAAAAGAC TGGCCATGGA CCCACGCTGT

CCCCGGACTT CAAGGAGTTT GTCAAAAGGC TGGCAATGGA CCCTCGATGT

CCCCGGACTT CAAGGAGTTT GTCAAAAGAT TGGCAATGGA CCCTCGATGT

CCCCTGACTT CAAGGAGTTC GTCAAGAGAC TCGCCATGGA CCCCCGCTGC

CCCTGGACTT CAAGGAGTTT GTCAAGAGAT TGGCAATGGA CCCTCGATGT

CTCCAGACTT CAAGGACTTT GTCAAAAGGT TGGCAATGGA CCCTCGATGT

CCCCGGACTT CAAGGAATTT GTCAAA---- ---------- ----------

TCCCCGAGTT CAAGGATTTT GTCAAAAGAT TAGCTATGGA TCCTCGATGT

TCCCCGAGTT CAAGGAGTTC GTCAAGAGAC TGGCCATGGA CCCCCGCTGT

AAAGGGATGC CACTCTCTAG TTTTATACTG AAGCCTATGC AACGGGTAAC

AAAGGGATGC CACTCTCTAG TTTTATACTG AAGCCTATGC AACGGGTAAC

AAAGGGATGC CACTCTCTAG TTTTATACTG AAGCCTATGC AACGGGTAAC

AAAGGGATGC CACTCTCTAG TTTTATACTG AAGCCTATGC AACGGGTAAC

AAAGGGATGC CACTCTCTAG TTTTATACTG AAGCCTATGC AACGGGTAAC

AAAGGGATGC CACTCTCTAG CTTTATTCTC AAGCCTATGC AACGGGTAAC

AAAGGCATGC CACTGTCCAG CTTCATACTG AAACCTATGC AGCGTGTCAC

AAAGGAATGC CTCTGTCCAG CTTTATACTG AAGCCTATGC AGCGTGTCAC

AAAGGGATGC CTCTTTCCAG TTTTATACTG AAGCCCATGC AGCGGGTGAC

AAGGGAATGC CCCTGTCTAG TTTTATACTG AAGCCTATGC AACGAGTAAC

AAAGGGATGC CACTCTCTAG TTTTATCCTG AAGCCTATGC AACGCGTAAC

AAAGGGATGC CTCTCTCTAG TTTTATACTG AAGCCTATGC AACGGGTAAC

AAAGGCATGC CGCTCTCCAG CTTCATCCTG AAGCCCATGC AGCGCGTCAC

AAAGGGATGC CACTCTCTAG TTTTATACTG AAGCCGATGC AACGGGTAAC

AAAGGGATGC CGCTCTCTAG CTTTATACTG AAGCCTATGC AGCGGGTGAC

---------- ---------- ---------- ---------- ----------

AAAGGGATGC CACTCTCTAG TTTTATATTG AAGCCTATGC AACGGGTAAC

AAAGGGATGC CCCTGTCCAG CTTCATCCTG AAGCCCATGC AGCGGGTGAC

AAGATACCCA CTGATCATTA AAAATATCCT GGAAAACACC CCTGAAAACC

AAGATACCCA CTGATCATTA AAAATATCCT GGAAAACACC CCTGAAAACC

AAGATACCCA CTGATCATTA AAAATATCCT GGAAAACACC CCTGAAAACC

AAGATACCCA CTGATCATTA AAAATATCCT GGAAAACACC CCTGAAAACC

AAGATACCCA CTGATCATTA AAAATATCCT GGAGAACACC CCTGAAAACC

AAGATACCCA CTGATCATTA AAAATATCCT GGAAAACACT CCTGAAAACC

AAGATACCCA CTGATCATTA AAAACATCCT GGAAAACACT CCTGAAAACC

AAGATACCCG CTGATCATTA AAAACATCCT GGAAAACACT CCCGAGAACC

AAGATACCCC CTGATCATTA AAAAT----- ---------- ----------

GAGATACCCA CTGATCATTA AAAATATCCT GGAAAACACT CCTGAAAACC

GAGATACCCA CTGATCATTA AAAATATCCT GGAAAACACT CCTGAGAACC

AAGATACCCA CTGATCATTA AAAATATCCT GGAAAACACT CCTGAAAATC

CAGATACCCC CTCATCatca agaacATTCT CGAGAACACC CTGGAAAACC

CAGATATCCA CTGATCATTA AAAATATCCT GGAAAACACT CCTGAAAACC

AAGATACcca ctgatcatca aaaacATCCT GGAAAACACT CCTGAAAACC

---------- ---------- -----ATCCT GGAAAACACA CCTGAAAACC

AAGATACCCA CTAATTATTA AAAATATTTT AGAAAACACT CCTGAAAACC

CAGATACCCC CTCATCATTA AAAACATCCT GGAAAACACG CCCGAAAACC

ACCCGGACCA CAGCCACTTG AAGCACGCCC TGGAGAAGGC GGAAGAGCTC

ACCCGGACCA CAGCCACTTG AAGCACGCCC TGGAGAAGGC GGAAGAGCTC

ACCCGGACCA CAGCCACTTG AAGCACGCTC TGGAGAAGGC AGAGGAGCTC

ACCCGGACCA CAGCCACTTG AAGCACGCTC TGGAGAAGGC AGAGGAGCTC

ACCCGGACCA CAGCCACTTG AAGCACGCCC TGGAGAAGGC GGAAGAGCTC

ACCCCGACCA CAGCCACTTG AAGCATGCCC TGGAGAAGGC GGAAGAGCTG

ACCCCGACCA CAGCCACCTG AAGCACGCCC TGGAGAAAGC AGAAGAGCTG

ATCCAGACCA CAGCCACCTG AAGCATGCCC TGGAAAAGGC GGAGGAGCTA

---------- ---------- ---------- ---------- ----------

ACCCAGACCA CAGCCACCTG AAGCACGCCC TGGAGAAGGC GGAGGAGCTG

ACCCCGACCA CAGCCACCTG AAGCACGCCC TGGAGAAGGC CGAAGAGCTG

ACCCCGACCA TAGCCACCTG AAGCACGCCC TGGAGAAGGC GGAAGAGCTG

ACCCTGACCA CAGCCACCTG AAGCACGCCC TGGAGAAGGC CGAAGAGCTG

ACCCTGACCA CAGTCACCTA AAACATGCCT TGGAGAAAGC AGAAGAGTTG

ACCCTGACCA CAGCCACTTG AAACACGCCC TGGAAAAGGC AGAAGAGCTG

ACCCCGACCA CAGCCACTTA CAACACGCCC TGGAGAAGGC CGAAGAGCTG

ACCCTGATTA CAGCCACTTG AAGCATGCTC TCGAGAAGGC TGAAGAATTG

ACCCCGACCA CTGCCACCTG AAGCTGGCCC TGGAGAAGGC GGAGGAGCTG

TGTTCCCAGG TGAACGAAGG GGTGCGGGAG AAGGAGAACT CTGACCGGCT

TGTTCCCAGG TGAACGAAGG GGTGCGGGAG AAGGAGAACT CTGACCGGCT

TGTTCCCAGG TGAACGAAGG GGTGCGGGAG AAGGAGAACT CCGACCGGCT

TGTTCCCAGG TGAACGAAGG GGTGCGGGAG AAGGAGAACT CCGACCGGCT

TGCTCCCAGG TGAACGAAGG GGTGCGGGAG AAGGAGAACT CCGACCGGCT

TGTTCCCAGG TGAACGAAGG TGTTCGTGAG AAGGAGAACT CAGACCGGCT

TGCTCCCAAG TGAACGAGGG AGTTCGAGAG AAGGAGAACT CTGACCGGCT

TGCTCCCAGG TGAACGAGGG AGTTCGAGAG AAGGAGAACT CAGACCGGCT

---------- ---------- ---------- ---------- ----------

TGCTCCCAGG TGAATGAAGG GGTGCGGGAG AAGGAGAACT CGGACCGACT

TGTTCACAGG TGAACGAAGG GGTGCGCGAG AAGGAGAACT CCGACCGCCT

TGTTCCCAGG TGAATGAAGG GGTGCGGGAG AAGGAGAACT CCGACCGGCT

TGTTCCCAGG TGAACGAAGG AGTGCGGGAG AAGGAGAACT CAGACCGGCT

TGTTCTCAGG TGAACGAAGG GGTACGCGAG AAGGAAAACT CTGACCGGCT

TGTTCCCAGG TGAATGAAGG AGTGCGCGAG AAGGAGAACT CTGACCGGCT

TGTTCCCAGG TGAACGAAGG AGTGCGTGAG AAGGAGAACT CGGACCGACT

TGTTCCCAAG TCAACGAAGG TGTGCGTGAG AAGGAGAATT CGGACCGGTT

TGCTCCCAAG TCAACGAGGG CGTGCGGGAG AAGGAGAACT CGGACCGGCT

GGAGTGGATC CAGGCCCACG TGCAGTGTGA AGGCCTGTCT GAGCAACTTG

GGAGTGGATC CAGGCCCACG TGCAGTGTGA AGGCCTGTCT GAGCAACTTG

GGAGTGGATC CAGGCCCATG TGCAGTGTGA AGGCCTGTCT GAGCAACTTG

GGAGTGGATC CAGGCCCATG TGCAGTGTGA AGGCCTGTCT GAGCAACTTG

GGAGTGGATC CAGGCCCATG TGCAGTGCGA AGGCCTGTCT GAGCAACTTG

GGAGTGGATC CAGGCCCATG TGCAGTGCGA GGGCCTGTCT GAGCAACTTG

GGAGTGGATC CAGGCGCATG TGCAGTGCGA AGGCCTTTCT GAGCAACTGG

GGAGTGGATC CAAGCCCACG TGCAGTGTGA AGGCCTTTCT GAGCAACTGG

---------- ---------- ---------- ---------- ---CAACTTG

GGAGTGGATC CAGGCCCACG TGCTGTGTGA AGGCTTGTCA GAGCAACTTG

CGAGTGGATC CAGGCGCACG TGCAGTGCGA AGGCCTGTCC GAGCAACTTG

GGAGTGGATC CAAGCCCACG TGCAGTGTGA AGGCCTGTCT GAGCAACTTG

-GAGTGGATC CAAGCCCACG TGCAGTGTGA AGGCCTGTCG GAGCAACTTG

GGAGTGGATC CAAGCTCACG TGCAGTGTGA AGGCTTGTCT GAGCAACTTG

GGAGTGGATC CAAGCCCATG TGCAGTGTGA AGGCCTGTCT GAGCAACTTG

GGAGTGGATC CAAGCCCACG TGCAGTGCGA AGGCCTGTCG GAGCAACTTG

GGAGTGGATC CAAGCCCATG TACAGTGTGA AGGCCTGTCT GAGCAACTTG

GGAGTGGATC CAGGCCCACG TACAGTGTGA AGGCCTCTCG GAGCAACTGG

TGTTCAATTC AGTGACCAAT TGCTTGGGGC CGCGCAAATT TCTGCACAGT

TGTTCAATTC AGTGACCAAT TGCTTGGGGC CACGCAAATT TCTGCACAGT

TGTTCAATTC AGTGACCAAC TGCTTGGGGC CGCGCAAATT TCTGCACAGC

TGTTCAATTC AGTGACCAAC TGCTTGGGGC CGCGCAAATT TCTGCACAGT

TGTTCAATTC TGTGACCAAT TGCTTGGGGC CACGCAAATT TCTGCACAGT

TGTTCAATTC AGTGACCAAT TGCCTGGGGC CAAGGAAGTT TCTGCACAGT

TGTTCAATTC GGTGACCAAC TGCTTGGGAC CACGCAAGTT TCTGCACAGC

TGTTCAATTC AGTGACCAAC TGCTTGGGAC CACGCAAGTT TCTGCACAGC

TGTTCAATTC AGTGACCAAC TGCTTGGGGC CACGCAAGTT TCTGCACAGC

TGTTCAATTC CGTGACCAAC TGCCTGGGGC CGCGCAAGTT CCTGCACAGT

TGTTCAATTC AGTGACCAAT TGCTTGGGGC CACGCAAGTT TCTGCACAGT

TGTTCAATTC AGTGACCAAT TGCTTGGGGC CACGCAAGTT TCTGCACAGT

TGTTCAATTC AGTGACCAAC TGCTTGGGGC CGCGCAAGTT CCTGCACAGC

TGTTCAATTC CGTGACCAAC TGCTTGGGGC CACGCAAGTT TCTGCACAGT

TGTTCAATTC AGTGACCAAT TGCTTGGGGC CACGCAAGTT TCTGCACAGT

TGTTCAATTC AGTGACCAAT TGCTTGGGGC CTCGCAAGTT TCTGCACAGT

TCTTCAACTC AGTCACCAAT TGCTTGGGAC CCCGGAAGTT TCTGCACAGT

TCTTCAACTC GGTGACCAAC TGCCTGGGGC CCCGCAAGTT CCTGCACAGT

GGGAAGCTCT ACAAGGCCAA GAGCAACAAG GAGCTGTATG GCTTCCTTTT

GGGAAGCTCT ACAAGGCCAA GAGCAACAAG GAGCTGTATG GCTTCCTTTT

GGGAAGCTCT ACAAGGCCAA GAGCAACAAG GAACTGTATG GTTTCCTTTT

GGGAAGCTCT ACAAGGCCAA GAGCAACAAG GAACTGTATG GTTTCCTTTT

GGGAAGCTCT ACAAGGCCAA GAGCAACAAG GAGCTGTACG GTTTCCTTTT

GGGAAACTCT ACAAGGCCAA GAGCAACAAG GAGCTGTACG GTTTCCTCTT

GGGAAGCTCT ACAAGGCCAA GAGCAACAAA GAGCTGTATG GCTTCCTCTT

GGGAAGCTCT ACAAGGCCAA GAGCAACAAA GAACTGTATG GCTTCCTCTT

GGGAAACTCT ACAAGGCCAA GAGCAACAAG GAGCTCTACG GTTTCCTGTT

GGGAAGCTCT ACAAGGCCAA GAGCAACAAG GAGCTGTATG GCTTCCTCTT

GGGAAACTCT ACAAGGCCAA GAGCAACAAG GAGCTGTATG GCTTCCTCTT

GGGAAACTCT ACAAGGCCAA GAGCAACAAG GAGCTGTACG GCTTCCTCTT

GGCAAGCTCT ACAAGGCCAA GAGCAACAAG GAGCTGTACG GCTTCCTCTT

GGGAAACTCT ACAAAGCCAA GAGCAACAAG GAGCTGTATG GCTTCCTCTT

GGGAAACTCT ACAAAGCCAA GAGCAACAAG GAACTGTATG GCTTCCTCTT

GGGAAACTCT ACAAAGCCAA GAGCAACAAG GAGCTGTACG GCTTCCTCTT

GGGAAATTGT ACAAAGCCAA GAGCAACAAG GAACTGTATG GCTTCCTTTT

GGGAAGCTGT ACAAGGCCAA GAGCAACAAG GAGCTGTACG GCTTCCTCTT

CAACGACTTC CTCCTGCTGA CTCAGATCAC GAAGCCTTTG GGGTCTTCTG

CAACGACTTC CTCCTGCTGA CTCAGATCAC GAAGCCCTTG GGGTCTTCTG

CAACGACTTC CTCCTGCTGA CTCAGATCAC AAAGCCCTTG GGATCTTCTG

CAACGACTTC CTCCTGCTGA CTCAGATCAC GAAGCCCTTG GGATCTTCTG

CAACGACTTC CTCCTGCTGA CTCAGATCAC CAAGCCCTTG GGGTCATCTG

CAACGACTTC CTCCTGCTGA CTCAAATCAC AAAGCCCTTG GGGTCTTCTG

CAATGACTTC CTCCTGCTGA CCCAAATCAC AAAGCCCTTA GGCTCTTCCA

CAACGACTTC CTCCTGCTGA CCCAAATCAC AAAGCCCTTA GGCTCTTCCG

CAACGACTTC CTCCTGCTGA CCCAGATCAC CAAGCCTTTG GGGTCTTCCG

CAACGACTTC CTGCTGCTGA CCCAAATCAT CAAGCCCCTG GGCTCCTCGG

CAACGACTTC CTCCTGCTGA CCCAAATCAT AAAGCCCTTG GGCTCTTCCG

CAACGATTTC CTCCTGCTGA CCCAAATCAT AAAGCCCCTG GGCTCTTCTG

CAACGACTTC CTGCTGCTCA CCCAGATCAT CAAGCCGCTG GGCTCCTCGG

CAACGACTTC CTCCTGCTGA CTCAGATCAT AAAGCCCTTG GGGTCTTCTG

CAACGACTTC CTCCTGCTGA CCCAAATCAT AAAGCCCTTG GGGTCTTCTG

CAACGACTTC CTCCTGCTCA CCCAAATCAT AAAGCCCCTC GGGTCTTCCG

CAATGACTTT CTCCTTCTGA CCCAGATAAT AAAGCCTTTG GGTTCTTCTG

CAACGACTTC CTCCTGCTGA CCCAGATCAT CAAGCCGCTC GGCTCCTCGG

GCACCGACAA AGTCTTCAGC CCCAAATCAA ACCTGCAGTA TAAAATGTAT

GCACCGACAA AGTCTTCAGC CCCAAATCAA ACCTGCAGTA TAAAATGTAT

GCACCGACAA AGTCTTCAGC CCCAAATCAA ACCTACAGTA TAAAATGTAC

GCACCGACAA AGTCTTCAGC CCCAAATCAA ACCTGCAGTA TAAAATGTAC

GCACCGACAA AGTCTTCAGC CCCAAATCAA ACCTGCAGTA TAAAATGTAC

GCACCGACAA AGTCTTCAGC CCCAAATCTA ACCTGCAGTA CAAAATGTAC

GCACTGACAA AGTCTTCAGC CCCAAATCTA ACCTGCAGTA TAAAATGTAC

GCACCGACAA AGTCTTCAGC CCCAAATCTA ACCTTCAGTA TAAAATGTAC

GCACCGACAA AGTCTTCAGC CCCAAATCCA ACCTGCAGTA TAAAATGTAC

GCACCGACAA AGTCTTCAGC CCCAAATCAA ACCTGCAGTA TAAAATGTAC

GCACCGACAA AGTCTTCAGT CCCAAATCTA ACCTGCAGTA TAAAATGTAC

GCACTGACAA AGTCTTCAGC CCCAAATCTA ACCTGCAGTA TAAAATGTAC

GCACCGACAA GGTCTTCAGC CCCAAGTCCA ACCTGCAGTA CAAGATGTAC

GCACCGACAA AGTCTTCAGC CCTAAATCTA ACCTGCAGTA TAAAATGTAC

GCACCGACAA GGTCTTCAGT CCCAAATCTA ACCTGCAGTA TAAAATGTAC

GCACCGACAA GGTCTTCAGC CCCAAATCTA ACCTGCAGTA TAAAATGTAC

GGACTGACAA GGTCTTCAGC CCCAAATCGA ATCTGCAGTA TAAAATGTAT

GCACCGACAA AGTCTTCAGC CCCAAGTCGA ACCTGCAGTA CAAGATGTAC

AAAACACCTA TTTTCCTAAA TGAGGTTCTA GTAAAATTAC CCACCGACCC

AAAACACCTA TTTTCCTAAA TGAGGTTCTA GTAAAATTAC CCACCGACCC

AAAACTCCTA TTTTCCTAAA TGAGGTTCTA GTAAAATTAC CCACTGACCC

AAAACTCCTA TTTTCCTAAA TGAGGTTCTA GTAAAATTAC CCACCGACCC

AAAACACCTA TTTTTCTAAA TGAGGTTCTA GTAAAATTAC CCACGGATCC

AAAACACCTA TTTTCCTAAA CGAGGTTCTA GTAAAGTTAC CCACTGACCC

AAAACGCCCA TTTTCTTAAA TGAGGTTCTA GTAAAATTGC CCACGGACCC

AAAACGCCCA TTTTCTTAAA TGAGGTTCTA GTAAAATTGC CCACGGACCC

AAAACCCCTA TTTTCCTAAA CGAGGTTCTA GTGAAATTAC CCACGGACCC

AAAACACCTA TTTTCCTAAA TGAGGTTCTA GTGAAGTTGC CCACTGACCC

AAAACACCTA TTTTCCTGAA TGAGGTTCTA GTAAAATTAC CCACTGACCC

AAAACACCTA TTTTCCTCAA TGAGGTCTTA GTAAAATTAC CCACTGACCC

AAGACGCCCA TTTTCCTCAA TGAGGTTCTG GTGAAGCTCC CCACCGACCC

AAAACACCCA TTTTCCTAAA TGAGGTTCTC GTAAAATTAC CCACTGACCC

AAAACA---- ---------- ---------- ---------- ----------

AAAACACCGA TTTTCCTCAA TGAGGTTCTA GTAAAATTGC CCACCGACCC

AAGACGCCCA TTTTCCTCAA TGAAGTCCTA GTAAAATTAC CCACTGACCC

AAAACGCCCA TTTTCCTGAA CGAGGTCCTG GTCAAGCTGC CGACCGACCC

TTCTGGAGAC GAGCCCATCT TCCACATCTC CCACATTGAC CGCGTCGTCC

TTCTGGAGAC GAGCCCATCT TCCACATCTC CCACATTGAC CGCGTCGTCC

TTCTGGAGAC GAGCCCATCT TCCACATCTC CCACATTGAC CGCGTCGTCC

TTCTGGAGAC GAGCCCATCT TCCACATCTC CCACATTGAC CGCGTCGTCC

TTCTGGAGAT GAGCCCATCT TCCACATTTC CCACATTGAC CGAGTCGTCC

TTCTGGAGAT GAACCCATCT TCCACATTTC CCACATTGAT CGAGTCGTGC

TTCTGGAGAT GAACCTATCT TTCACATTTC GCACATTGAC CGAGTCGTCC

TTCTGGAGAT GAGCCTATCT TCCACATTTC CCACATCGAC CGGGTCGTCC

TTCTGGAGAT GAACCCATCT TCCACATTTC CCACATCGAC CGGGTCGTGC

TTCTGGGGAT GAGCCCATCT TCCACATTTC CCACATTGAC CGAGTCGTCC

TTCTGGAGAT GAGCCCATCT TCCACATCTC CCACATTGAC CGAGTCGTCC

CTCTGGAGAT GAACCCGTCT TCCACATTTC CCACATCGAC CGCGTCGTCC

CTCTGGTGAC GAGCCCATCT TCCACATCTC ACACATCGAC CGGGTCGTTC

TTCTGGAGAT GAACCCATCT TCCACATTTC CCACATCGAC AGGGTCGTCC

---------- ---------- ---------- ---------- ------GTCC

TTCTGGAGAT GAGCCCATCT TTCACATTTC CCACATCGAC CGAGTCGTTT

TTCTGGAGAT GAGCCCATCT TCCACATTTC CCACATCGAT AGAGTCGTGC

CTCCGGAGAC GAGCCCATCT TCCACATCTC CCACATCGAC CGAG---TGC

GTTCCCAAAG GGCAACAGGC ATTGGAAGGT TGATGGTGAA CGTGGTTGAA

GTTCCCAAAG GGCAACAGGC ATTGGAAGGT TGATGGTGAA CGTGGTTGAA

GTTCCCAAAG GGCAACAGGC ATTGGAAGGT TGATGGTGAA CGTGGTTGAA

GTTCCCAAAG GGCAACAGGC ATTGGAAGGT TGATGGTGAA CGTGGTTGAA

GTTCCCAGAG GGCAACAGGC ATTGGAAGGT TGATGGTGAA CGTGGTCGAA

GTTCCCAAAG GGCCACAGGC ATTGGAAGGT TGATGGTGAA CGTGGTTGAA

GTTCCCAGAG AGCGACCGGT ATTGGAAGAT TGATGGTGAA CGTAGTAGAA

GTTCCCAGCG GGCGACCGGT ATTGGAAGGT TGATGGTGAA CGTGGTAGAA

GGTCCCAGAG GGCAACGGGC ATTGGAAGGC TGATGGTGAA CGTGGTAGAG

GCTCCCAAAG AGCCACAGGC ATCGGAAGGT TGATGGTGAA CGTGGTTGAA

GTTCCCAAAG GGCGACAGGT ATTGGAAGGT TGATGGTGAA CGTCGTTGAG

GTTCCCAAAG GGCAACAGGG ATTGGAAGGT TGATGGTGAA TGTTGTTGAA

GCTCCCAGAG GGCCACCGGC ATCGGGAGGC TGATGGTGAA CGTGGTGGAG

GTTCCCAGAG GGCAACAGGT ATCGGCAGGT TGATGGTGAA CGTCGTCGAA

GTTCCCAAAG GGCAACAGGT ATTGGAAGGT TGATGGTGAA CATTGTTGAA

GCCCCCAAAG GGCAACAGGT ATTGGGAGGT TGATGGTGAA CGTGGTTGAA

GCTCCCAAAG AGCAACAGGT ATCGGGAGGT TGATGGTGAA CATTGTTGAA

GCTCCCAGAG GGCCACAGGC ATTGGCAGGC TGATGGTGAA CGTGGTGGAA

GGCATCGAGT TGAAACCCTG TCGGTCACAT GGAAAGAGCA ACCCGTACTG

GGCATTGAGT TGAAACCCTG TCGGTCACAT GGAAAGAGCA ACCCGTACTG

GGCATTGAGT TGAAACCCTG TCGGTCACAT GGAAAGAGCA ACCCCTACTG

GGCATTGAGT TGAAACCCTG TCGGTCACAT GGAAAGAGCA ACCCCTACTG

GGCATTGAGC TGAAACCCTG TCGGTCACAT GGGAAGAGCA ACCCATACTG

GGCATTGAGC TGAAGCCCTG CCGGTCACAC GGAAAGAGCA ACCCGTACTG

GGCATTGAGC TGAAGCCCTG TCGGTCACAT GGAAAGAGCA ACCCGTACTG

GGCATTGAGC TGAAGCCCTG TCGGTCACAT GGAAAGAGCA ACCCGTACTG

GGCATTGAGC TGAAGCCCTG CCGGTCACAC GGAAAGAGCA ACCCCTACTG

GGCATTGAGC TGAAACCCTG TCGCTCCCAT GGAAAGAGTA ACCCGTACTG

GGCATTGAGC TGAAACCCTG TCGATCACAT GGAAAGAGTA ACCCGTACTG

GGCATTGAGC TGAAACCCTG TCGGTCACAC GGGAAGAGTA ACCCGTACTG

GGGATCGAGC TGAAGCCGTG TCGCTCTCAT GGGAAGAGCA ACCCATACTG

GGCATCGAGC TGAAGCCTTG TCGCTCACAT GGAAAGAGCA ACCCATACTG

GGCATTGAGC TGAAGCCCTG CCGCTCACAC GGAAAGAGCA ACCCTTACTG

GGCATTGAGC TGAAGCGTGG ----TCACGT GGAAAGAGTA ACCCATACTG

GGCATTGAAT TAAAGCCCTG TTGGTCACAT GGTAAGAGTA ACCCCTATTG

GGAATTGAGT TAAAGCCCTG CCGCTCACAC GGAAAGAGCA ACCCTTACTG

TGAGGTGACC ATGGGTTCCC AGTGCCACAT CACCAAGACG ATCCAGGACA

TGAGGTGACC ATGGGTTCCC AGTGCCACAT CACCAAGACG ATCCAGGACA

CGAGGTGACC ATGGGTTCCC AGTGCCACAT CACCAAGACG ATCCAGGACA

CGAGGTGACC ATGGGTTCCC AGTGCCACAT CACCAAGACG ATCCAGGACA

CGAGGTGACC ATGGGTTCCC AATGCCACAT CACTAAGACA ATCCAGGACA

TGAAGTGACC ATGGGCTCCC AGTGCCACAT CACCAAGACA ATCCAGGACA

TGAGGTGACC ATGGGCTCTC AGTGCCACAT CACCAAGACA ATCCAGGACA

TGAGGTGACC ATGGGCTCTC AGTGCCACAT CACCAAGACA ATCCAGGACA

CGAGGTGACC ATGGGCTCCC AGTGCCACAT CACCAAGACG ATCCAGGACA

TGAGGTGACC ATGGGCTCCC AGTGTCACAT CACCAAGACG ATCCAGGACA

TGAGGTGACC ATGGGTTCCC AGTGTCACAT CACCAAGACG ATCCAGGACA

CGAGGTGACC ATGGGCTCCC AGTGTCACAT CACCAAGACG ATCCAGGACA

CGAGGTGACC ATGGGCTCAC AATGCCACAT CACTAAGACC ATCCAGGACA

TGAGGTGACC ATGGGCTCCC AGTGCCACAT CACCAAGACC ATCCAGGACA

CGAGGTGACC ATGGGCTCCC AGTGTCACAT CACCAAGACC ATCCAGGACA

CGAGGTGACC ATGGGCTCCC AGTGCCACAT CACCAAGACC ATCCAGGACA

TGAAGTGACA ATGGGCTCCC AGTGTCATAT TACCAAGACG ATACAGGACA

CGAGGTGACC ATGGGCTCCC AGTGCCACAT CACCAAGACC ATCCAGGACA

CTCTGAACCC CAAGTGGAAT TCCAACTGCC AGTTCTTCAT CCGAGACCTG

CTCTGAACCC CAAGTGGAAT TCCAACTGCC AGTTCTTCAT CCGAGACCTG

CTCTGAACCC CAAGTGGAAT TCCAACTGCC AGTTCTTCAT CCGAGACCTG

CTCTGAACCC CAAGTGGAAT TCCAACTGCC AGTTCTTCAT CCGAGACCTG

CTCTAAACCC CAAGTGGAAC TCCAACTGCC AGTTTTTCAT CCGAGACCTG

CGCTGAACCC CAAGTGGAAT TCCAACTGCC AGTTCTTCAT CAGAGACCTG

CGCTGAACCC TAAGTGGAAT TCTAACTGCC AGTTCTTCAT CAGAGACCTG

CGCTAAACCC CAAGTGGAAT TCTAACTGCC AGTTCTTCAT CAGAGACCTG

CCCTGAACCC CAAGTGGAAT TCCAACTGCC AGTTCTTCAT CAGAGACCTG

CTCTGAATCC CAAGTGGAAT TCCAACTGCC AGTTCTTCAT CAGAGACCTG

CTCTGAATCC CAAGTGGAAT TCCAACTGCC AGTTCTTCAT CAAAGACCTG

CTCTGAATCC CAAGTGGAAT TCCAACTGCC AGTTCTTCAT CAAAGACCTG

CCTTGAACCC CAAGTGGAAC TCCAACTGCC AGTTCTTCAT CAGAGACCTG

CGCTGAACCC CAAGTGGAAC TCCAACTGCC AGTTCTTTAT CAAGGACCTG

CGCTCAACCC CAAGTGGAAC TCCAATTGCC AGTTCTTCAT CAGAGACCTG

CGCTCAACCC CAAGTGGAAT TCCAACTGCC AGTTCTTCAT CCGAGACCTG

CGCTGAATCC TAAGTGGAAT TCCAACTGCC AGTTCTTTAT CAAGGACCTA

CGCTGAGCCC CAAGTGGAAC TCCAACTGCC AGTTCTTCAT CAAAGACCTG

GAGCAGGAAG TCCTCTGCAT CACTGTGTTC GAGAGGGACC AGTTCTCACC

GAGCAGGAAG TCCTCTGCAT CACTGTGTTC GAGAGGGACC AGTTCTCACC

GAGCAGGAAG TCCTCTGCAT CACTGTGTTC GAGAGGGACC AGTTCTCACC

GAGCAGGAAG TCCTCTGCAT CACTGTGTTC GAGAGGGACC AGTTCTCACC

GAGCAGGAAG TCCTCTGCAT CACTGTGTTT GAGAGGGACC AGTTTTCACC

GAGCAGGAGG TTCTTTGTGT CACTGTGTTT GAGAGAGACC AGTTCTCACC

GAACAGGAGG TTCTCTGCAT CACTGTGTTT GAGAGGGACC AATTCTCGCC

GAGCAGGAGG TTCTCTGCAT CACAGTGTTT GAGAGGGACC AGTTCTCGCC

GAGCAGGAAG TCCTGTGCAT CACCGTGTTC GAGAGGGACC AGTTCTCACC

GAGCAGGAGG TGCTGTGCAT CACTGTGTTT GAGAGGGACC AGTTCTCGCC

GAGCAGGAGG TCCTGTGCAT CACCGTGTTT GAGAGGGACC AGTTCTCTCC

GAGCAGGAGG TCCTCTGCAT CACGGTGTTT GAGAGGGACC AGTTCTCTCC

GAGCAGGAAG TCCTGTGTAT CACCGTATTT GAGAGGGACC AGTTCTCTCC

GAGCAGGAAG TCCTCTGTAT CACTGTGTTT GAGAGGGACC AGTTCTCCCC

GAGCAGGAAG TCCTCTGCAT CACAGTGTTT GAGAGGGACC AGTTCTCCCC

GAGCAGGAAG TGCTCTGCGT CACGGTCTTT GAGAGGGACC AGTTCTCCCC

GAGCAGGATG TTCTCTGCAT AACGGTGTTT GAGAGGGACC AGTTCTCCCC

GAGCAGGACG TGCTGTGCAT CACCGTCTTC GAGCGGGACC AGTTCTCCCC

AGATGATTTT TTGGGTCGGA CGGAGATCCG TGTGGCGGAC ATCAAGAAAG

AGATGATTTT TTGGGTCGGA CGGAGATCCG CGTGGCGGAC ATCAAGAAAG

AGATGATTTT TTGGGTCGGA CGGAGATCCG TGTGGCGGAC ATCAAAAAGG

AGATGATTTT TTGGGTCGGA CGGAGATCCG TGTGGCGGAC ATCAAAAAGG

AGATGATTTT TTGGGCCGGA CGGAGATCCG CGTGGCGGAC ATCAAGAAAG

AGACGATTTT TTGGGGCGGA CGGAGATCCG TGTGGCCGAC ATCAAGAAAG

AGACGATTTT TTGGGTCGGA CAGAGATCCG AGTGGCCGAC ATCAAGAAAG

TGATGATTTT TTGGGTCGGA CAGAGATCCG AGTGGCCGAC ATCAAGAAAG

CGATGATTTC TTGGGTCGAA CAGAGATCCG TGTGGCTGAC ATCAAGAAAG

AGATGATTTT TTGGGTCGGA CGGAGATCCG CGTGGCCGAC ATCAAGAAGG

AGACGATTTT TTGGGTCGGA CAGAGATCCG TGTGGCCGAC ATCAAGAAGG

AGATGATTTT TTAGGTCGGA CGGAGATCCG CGTAGCAGAC ATCAAGAAGG

TGATG----- ---------- ---------- ---------- ----------

GGATGATTTT TTGGGTCGGA CAGAGATCCG CGTGGCTGAC ATCAAGAAAG

CGATGATTTT TTGGGTCGGA CAGAGATCCG TGTGGCCGAC ATCAAAAAAG

CGACG----- ---------- ---------- ---------- ----------

TGACGATTTT TTGGGTCGGA CAGAAATTCG TGTGGCCGAC ATCAAGAAAG

GGACGACTTT CTGGGTCGGA CGGAGATCCG TGTGGCCGAC ATCAAGAAGG

ACCAGGGCTC CAAAGGTCCA GGCAAACATC CTGTGTCTGC TTTGATGGAG

ACCAGGGCTC CAAAGGTCCA GGCAAACATC CTGTGTCTGC TTTGATGGAG

ACCAGGGCTC CAAGGGTCCA GGCAAACATC CTGTGTCTGC TTTGATGGAA

ACCAGGGCTC CAAGGGTCCA GGCAAACATC CTGTGTCTGC TTTGATGGAA

ACCAGGGCTC CAAGGGTCCC GGCAAACATC CTGTGTCTGC TTTGATGGAA

ACCAGGGCTC CAAGGGGCCA GGCAAGCATC CTGTTTCTGC CTTGATGGAA

ACCAGGGCTC CAAGGGGCCA GGCAAACATC CTGTGTCTGC CTTGATGGAA

ACCAGGGCTC CAAGGGGCCG GGCAAACATC CTGTGTCTGC CTTGATGGAA

ACCAGGGCTC CAAGGGGCCA GGCAAACATC CTGTGTCTGC CTTGATGGAA

ACCAGGGCTC CAAGGGTCCC GGCAAACATC CTGTGTCTGC CTTGATGGAA

ACCAGGGTTC CAAGGGTCCA GGCAAACATC CTGTGTCTGC TTTGATGGAA

ACCAGGGTTC CAAGGGTCCA GGCAAACATC CTGTGTCTGC CTTGATGGAA

---------- ---------- GGCAAACACC CTGTGTCTGC TTTGATGGAA

ATCAAGGTTC CAAAGGTCCA -GCAAACATC CTGTGTCTGC CTTGATGGAA

ATCAGGGTTC CAAGGGCCCA GGCAAACATC CTGTGTCTGC CTTGATGGAA

---------- ---------- ---------- ---------- ----------

ACCAGGGGTC CAAGGGGCCA GGTAAACATC CTGTATCTGC ATTGATGGAG

ACCAGGTCTC CAAGGGCCC- GGTAAACATC CCGTATCCGC ACTGATGGAG

ATCTGTAATA AAAGAAGGTG GCAACCACCT GAATTTCTAT TGGTCCATGA

ATCTGTAATA AAAGAAGGTG GCAACCACCT GAATTTCTAT TGGTCCATGA

ATCTGTAATA AGAGAAGGTG GCAACCACCT GAATTTCTGT TGGTCCATGA

ATCTGTAATA AGAGAAGGTG GCAACCACCT GAATTTCTGT TGGTCCATGA

ATCTGTAATA AGAGAAGGTG GCAACCACCT GAATTTCTAT TGGTCCATGA

ATCTGTAATA AGAGAAGGTG GCAACCACCA GAATTTCTCT TGGTCCATGA

ATCTGTAATA AGAGAAGGTG GCAACCACCT GAATTTCTCT TGGTCCATGA

ATCTGTAATA AGAGAAGGTG GCAACCACCT GAATTTCTCT TGGTCCATGA

ATCTGTAACA AGAGAAGGTG GCAACCACCT GAATTTCTTT TGGTCCATGA

ATCTGTAATA AGAGAAGGTG GCAACCACCT GAATTTCTCT TGGTCCATGA

ATCTGTAATA AGAGAAGGTG GCAACCACCA GAATTTCTCT TGGTCCATGA

ATCTGTAATA AGAGAAGGTG GCAACCACCT GAATTTCTCT TGGTCCATGA

ATCTGTAATA AGAGAAGGTG GCAACCACCA GAATTTCTCT TGGTCCATGA

ATCTGTAATA AGAGAAGGTG GCAACCACCT GAATTTCTCT TGGTCCATGA

ATCTGTAATA AGAGAAGGTG GCAACCACCT GAATTTCTCT TGGTCCATGA

---------- ---------- ---------- ---------- ----------

ATCTGTAATA AAAGAAGGTG GCAACCACCT GAATTTCTCT TGGTTCATGA

ATCTGTAATA AAAGGAGATG GCAGCCGCCT GAATTCCTCT TGGTGCACGA

TAGTGGCCCT GATCATCGCA AACATTTTCT CTTTAGGgta ttgagaaatg

TAGTGGCCCT GATCATCGCA AACATTTTCT CTTTAGGgta ttgagaaatg

TAGTGGCCCT GATCATCGCA AACATTTTCT CTTTAGGgta ttgagaaatg

TAGTGGCCCT GATCATCGCA AACATTTTCT TTTTAGGgta ttgagaaatg

TAGTGGCCCT GATCATCGCA AACATTTTCT CTTTAGGgta ttgagaaatg

TAGTGGCCCT GATCATCGCA AACATTTTCT CTTTAGGgta ttgagaaatg

CAGTGGCCCT GACCATCGCA AACACTTCCT CTTTAGGgta ttgagaaatg

CAGTGGCCCT GACCATCGCA AACATTTCCT CTTTAGGgta ttgagaaatg

TAGTGGCCCT GATCATCGCA AACATTTTCT CTTTAGGgta ttgagaaatg

TAGTGGCCCT GATCATCGCA AACATTTTCT CTTTAGGgta ttgagaaatg

TAGTGGCCCA GATCATCGCA AACATTTTCT CTTTAGGgta ttgagaaatg

TAGTGGCCCT GATCATCGCA AACATTTTCT CTTTAGGgta ttgagaaatg

TAGTGGCCCT GATCACCGCA AACATTTTCT CTTTAGGgta ttgagaaatg

TAGTGGCCCT GATCATCGCA AACATTTTCT CTTTAGGgta ttgagaaatg

TAGTGGCCCT GATCATCGCA AACATTTTCT CTTTAGGgta ttgagaaatg

---------- ---------- ---------- ---------- ----------

TAGTGGCCCT GATCATCGCA AACACTTTCT CTTTAGGgta ttgagaaatg

CAGTGGCCCC GATCACCGCA AACATTTCCT CTTTAGGgta ttgagaaatg

gagcccttac cagacccaat tgtatgtttt tcttgaatag gtatATGCTA

gagcccttac cag-cccaat tgtatgtttt tcttgaatag gtatATGCTA

gagcccttac cag-cccaat tgtatgtttt tcttgaatag gtatATGCTA

gagcccttac cag-cccaat tgtatgtttt tcttgaatag gtatATGCTA

gagcccttac cag-cccaat tgtatgtttt tcttgaatag gtatATGCTA

gagcccttac cag-cccaat tgtatgtttt tcttgaatag gtatATGCTA

gagcccttac cag-cccaat tgtatgtttt tcttgaatag gtatATGCTA

gagcccttac cag-cccaat tgtatgtttt tcttgaatag gtatATGCTC

gagcccttac cag-cccaat tgtatgtttt tcttgaatag gtat------

gagcccttac cag-cccaat tgtatgtttt tcttgaatag gtatATGCTG

gagcccttac cag-cccaat tgtatgtttt tcttgaatag gtatATGCTA

gagcccttac cag-cccaat tgtatgtttt tcttgaatag gtatATGCTC

gagcccttac cag-cccaat tgtatgtttt tcttgaatag gtatATGCTA

gagcccttac cag-cccaat tgtatgtttt tcttgaatag gtatATACTA

gagcccttac cag-cccaat tgtatgtttt tcttgaatag gtatATGCTA

---------- ---------- ---------- ---------- ----ATGCTA

gagcccttac cag-cccaat tgtatgtttt tcttgaatag gtatGTTCTA

gagcccttac cag-cccaat tgtatgtttt tcttgaatag gtatATGCTG

GCTAAACAGT TGGAAGCTCT TGGTTTAGCT GAAAAGCCTC AGTTGGTGAC

GCTAAACAGT TGGAAGCTCT TGGTTTAGCT GAAAAGCCTC AGTTGGTGAC

GCTAAACAGT TGGAAGCTCT TGGTTTAGCT GAAAAGCCTC AGTTGGTGAC

GCTAAACAGT TGGAAGCTCT TGGTTTAGCT GAAAAGCCTC AGTTGGTGAC

GCAAAACAGT TGGAAGCTCT TGGTTTAGCT GAAAAGCCTC AGTTGGTGAC

ACTAAACAGT TGGAAGCTCT CAGCTTAGCT GAAAAGCCTC AGTTGGTGAC

GCTAAACAGC TGGAAGCTCT GGGCTTGGCT GAAAAGCCTC AGTTGGTGAC

GCTAAGCAGC TGGAAGCTCT GGGCTTGGCT GAGAAGCCTC AGCTGGTGAC

---------- ---------- ---------- ---------- ----------

ACTAAACAGT TGGAAGCTCT TGGTTTAGCT GAAAAGCCTC AGTTGGTGAC

GCTAAACAGC TGGAAGCTCT CGGCTTGGCT GAAAAGCCTC AGTTGGTGAC

GCTAAACAGT TGGAAGCTCT TGGCTTGGCT GAAAAGCCTC AGTTAGTGAC

GCTAGACAGT TGGAAGCTCT GGGCTTAGCT GAGAAACCTC AGCTGGTGAC

GCTAAACAGT TGGAAGGCCT TGGGTTGGCT GAAAAGCCTC AGTTGGTGAC

GCTAAGCAGT TGGAAGCTTT TGGCTTAGCT GAAAAGCCTC AGTTGGTGAC

GCTAAACAGT TAGAAGCGCT TGGCTTAGCT GAAAAGCCTC AGTTGGTGAC

CCCAAACAAT TAGAAGCCCT TGGCTTAGCT GAAAAGCCTC AGTTGATGAC

GCCAAACAAC TGGAGGCCCT GGGTTTAGCT GAGAAGCCGC AGTTGATCAC

TCGCTTTCAA GAAGTTTTTC GGTCAATGTG GTCCGTGAAT GGTGATTCAA

TCGCTTTCAA GAAGTTTTTC GGTCAATGTG GTCCGTGAAT GGTGATTCAA

TCGTTTTCAA GAAGTTTTTC GGTCAATGTG GTCCGTGAAT GGTGATTCAA

TCGCTTTCAA GAAGTTTTTC GGTCAATGTG GTCTGTGAAT GGTGATTCAA

TCGCTTTCAA GAAGTTTTTC GGTCAATGTG GTCTGTGAAT GGTGATTCAA

TCGCTTTCAA GAAGTTTTTC GGTCAATGTG GTCTGTGAAT GGTGATTCCA

TCGCTTTCAA GAAGTTTTCC GGTCCATGTG GTCTGTGAAT GGCGATTCAA

CCGCTTCCAA GAAGTTTTCC GGTCCATGTG GTCTGTGAAT GGCGACTCAA

---------- ---------- ---------- ---------- ----------

TCGCTTTCAA GAAGTTTTTC GATCAATGTG GTCTGTGAAT GGTGATTCAA

TCGCTTTCAA GAAGTTTTTC GGTCAATGTG GTCTGTGAAT GGTGATTCCA

TCGCTTTCAA GAAGTTTTTC GATCAATGTG GTCTGTGAAT GGTGATTCAA

TCGCTTTCAA GAAGTTTTTC GGTCCATGTG GTCTGTGAAT GGTGATTCGA

TCGCTTTCAA GAAGTTTTTC GATCAATGTG GTCTGTAAAT GGCGATTCAA

CCGCTTTCAA GAAGTCTTCC GGTCAATGTG GTCTGTGAAT GGTGATTCCA

CCGCTTTCAA GAAGTCTTCA GGTCAATGTG GTCTGTGAAT GGTGACTCCA

TCGTTTCCTA GAAGTTTTCC GATCAATGTG GTCTATGAAT GGTGACTCAA

TCGCTTCCAG GAGGTTTTCC GGTCGATGTG GTCTATGAAT GGAGATTCCA

TCAGTAAGAT ATATGCAGGA ACTGGAGCTC TTGAAGGGAA AGCGAAGTTA

TCAGTAAGAT ATATGCAGGA ACTGGAGCTC TTGAAGGGAA AGCGAAGTTA

TCAGTAAGAT ATATGCAGGA ACTGGAGCTC TTGAAGGGAA AGCGAAGTTA

TCAGTAAGAT ATATGCAGGA ACTGGAGCTC TTGAAGGGAA AGCGAAGTTA

TCAGTAAGAT ATATGCAGGA ACTGGAGCTC TTGAAGGGAA AGCGAAGTTA

TCAGTAAGAT ATATGCAGGG ACTGGTGCTC TTGAAGGAAA GGCTAAGTTA

TCAGTAAAAT ATATGCAGGC ACCGGAGCCC TGGAAGGGAA GGCCAAGTTA

TCAGTAAAAT ATATGCAGGC ACCGGGGCCC TGGAAGGGAA GGCCAAGTTA

---------- ---------- ---------- ---------- ----------

TCAGTAAGAT TTATGCAGGA ACTGGAGCCC TTGAAGGAAA GGCTAAGTTA

TCAGTAAGAT ATACGCAGGG ACTGGAGCTC TTGAAGGAAA GGCTAAGTTA

TCAGTAAGAT ATATGCCGGA ACTGGGGCTC TGGAAGGAAA GGCTAAGTTA

TCAGTAAGAT ATATGCGGGA ACTGGCGCTC TCGAAGGAAA GGCAAAGTTA

TCAGTAAGAT TTATGCAGGA ACCGGAGCTC TTGAAGGGAA GGCCAAGTTA

TCAGTAAGAT ATACGCAGGA ACTGGAGCGC TTGAAGGGAA GGCCAAGTTA

TCAGTAAGAT ATATGCAGGG ACTGGAGCCC TTGAAGGGAA GGCCAAG---

TCAGCAAAAT TTATGCAGGC ACTGGAGCTT TGGAAGGCAA AGCAAAGTTA

TCAGCAAAAT CTACGCGGGA ACCGGAGCTC TTGAAGGCAA GGCCAAGCTG

AAAGATGGTG CTCGCTCTGT TACCCGAACA ATTCAGAATA ACTTCTTTGA

AAAGATGGTG CTCGTTCTGT TACCCGAACA ATTCAGAATA ACTTCTTTGA

AAAGATGGTG CTCGGTCTGT TACCCGGACA ATTCAGAATA ACTTCTTTGA

AAAGATGGTG CTCGGTCTGT CACCCGGACA ATTCAGAATA ACTTCTTTGA

AAAGATGGTG CTCGTTCTGT TACCAGAACA ATTCAGAATA ACTTCTTTGA

AAAGATGGTG CTCGTTCTGT AACCAGAACA ATTCAAAATA ACTTCTTTGA

AAAGATGGTG CGCGATCTGT CACCAGAACC ATTCAGAATA ACTTCTTCGA

AAAGATGGTG CGCGATCTGT CACCAGAACC ATCCAGAATA ACTTCTTCGA

---------- ---------- ---------- ---------- ----------

AAAGATGGTG CTCGTTCAGT TAGTAGAACA ATTCAGAATA ACTTCTTTGA

AAAGATGGTG CTCGTTCTGT TACTAGAACG ATTCAGAATA ACTTCTTTGA

AAAGATGGCG CTCGTTCTGT TACTAGAACG ATTCAAAATA ACTTCTTTGA

AAAGATGGTG CTCGTTCTGT GACTCGAACA ATTCAGAATA ACTTCTTTGA

AAAGATGGTG CTCGTTCTGT TACTAGAACA ATTCAGAATA ATTTCTTTGA

AAAGATGGAG CTCGCTCTGT TACCAGAACA ATTCAGAACA ACTTCTTTGA

---------- ---------- ---------- ---------- ----------

AAAGATGGTG CTCGTTCTGT AACCAGAACA ATTCAGAACA ACTTCTTTGA

AAGGATGGCG CTCGCTCCGT AACCAGAACG ATTCAGAATA ACTTCTTTGA

CAGCTCCAAG CAAGAGGCCA TTGATGTTTT GCTACTGGGA AATACTCTGA

CAGCTCCAAG CAAGAGGCCA TTGATGTTTT GCTACTGGGA AATACTCTGA

CAGCTCCAAG CAAGAGGCCA TTGATGTTTT GCTGCTGGGA AATACTCTGA

CAGCTCCAAG CAAGAGGCCA TTGATGTTTT GCTGCTGGGA AATACTCTGA

CAGCTCTAAG CAAGAGGCCA TTGATGTTTT GCTACTGGGA AATACTCTGA

CAGCTCCAAG CAAGAGGCAA TTGATGTTTT GCTACTGGGA AATACTCTAA

CAGCTCCAAG CAAGAAGCCA TTGATGTTCT GCTTCTGGGA AACACTCTGA

CAGCTCCAAG CAGGAAGCCA TTGATGTCCT GCTCCTGGGA AATACTCTCA

---------- ---------- ---------- ---------- ----------

CAGCTCCAAG CAAGAAGCAA TTGATGTTTT GCTCCTGGGA AATACTCTAA

CAGCTCCAAG CAAGAGGCCA TAGATGTTTT ACTACTGGGA AATACTCTAA

CAGCTCCAAG CAAGAGGCCA TTGACATTTT GCTCCTGGGA AATACTCTAA

CAGCTCCAAG CAAGAGGCCA TTGATGTTTT GCTTCTGGGA AATACTCTAA

CAGCTCCAAG CAAGAGGCCA TTGATGTTTT GCTGCTTGGA AATACTCTAA

CAGCTCCAAG CAAGAGGCCA TTGACATTTT GCTGCTGGGA AACACTCTGA

---------- ---------- ---------- ---------- ----------

CAGCTCCAAG CAAGAAGCCA TTGATATTTT GCTGTTGGGG AGTACTTTAA

CAGCTCCAAG CAAGAGGCCA TCGACGTTTT GCTTCTGGGG AATACGCTCA

ATAGTGATTT AGCTGACAAA GCTCGAGCAC TTTTAACTAC TGGAAGTTTG

ATAGTGATTT AGCTGACAAA GCTCGAGCAC TTTTAACTAC TGGAAGTTTG

ATAGTGATTT AGCTGACAAA GCTCGAGCAC TTTTAACTAC TGGAAGTTTG

ATAGTGATTT AGCTGACAAA GCTCGAGCAC TTTTAACTAC TGGAAGTTTG

ATAGTGATTT AGCTGACAAA GCTCGAGCAC TTTTAACTAC TGGAAGTTTG

ATAGTGACTT AGCTGACAAA GCTCGAGCTC TTTTAACTAC TGGAAGTTTG

ACAGCGACTT AGCCGACAAA GCCCGGGCAC TTCTAACTAC TGGGAGTTTG

ACAGCGATTT AGCTGACAAA GCCCGAGCGC TTCTAACTAC TGGAAGTTTG

---------- ---------- ---------- ---------- ----------

ATAGTGATTT AGCTGACAAA GCTCGAGCCC TTTTAACTAC TGGAAGTTTG

ATAGTGATTT AGCTGACAAA GCTCGAGCAC TTCTAACTAC AGGAAGTTTG

ATAGTGATTT AGCTGACAAA GCTCGAGCAC TTTTAACAAC TGGAAGTTTG

ATAGTGACTT AGCTGACAAA GCTAGAGCAC TTTTAACTAC TGGAAGTTTG

ATAGCGATTT AGCTGACAAA GCTCGAGCAC TCTTAACTAC TGGAAGTTTG

ACAGTGATCT AGCTGACAAA GCTCGAGCGC TCCTAACTAC TGGAAGTCTG

---------- ---------- ---------- ---------- ----------

ATAGTGACTT GGCAGATAAA GCTCGAGCAC TCTTAACTAC TGGAGGCTTA

ACAGCGACCT GGCCGATAAA GCCCGAGCGC TCCTAACCAC CGGAAGCCTC

CGTGTTTCTG AGCAGACATT ACAGTCAGCA TCTTCTAAAG TACTAAAGAG

CGTGTTTCTG AACAGACATT ACAGTCAGCA TCTTCTAAAG TACTAAAGAG

CGTGTTTCTG AACAGACATT ACAGTCAGCA TCTTCTAAAG TACTAAAGAG

CGTGTTTCTG AACAGACATT ACAGTCAGCA TCTTCTAAAG TACTAAAGAG

CGTGTTTCTG AACAGACATT ACAGTCAGCA TCTTCTAAAG TACTAAAGAG

CGTGTTTCTG AACAGACATT ACAGTCAGCA TCTTCTAAAG TACTAAAGAG

CGTGTGTCTG AACAGACATT ACAGTCAGCA TCTTCCAAAG TCTTAAAGAA

CGTGTTTCTG AACAGACATT ACAGTCAGCA TCTTCCAAAG TCTTAAAGAA

----TTTCTG AACAGACATT ACAGTCAG-- ---------- ----------

CGTGTTTCTG AACAGACGTT ACAGTCAGCA TCTTCTAAAG TACTAAAGAG

CGTGTTTCTG AACAGACATT ACAGTCAGCA TCTTCCAAAG TACTAAAGAG

CGAGTTTCTG AACAGACATT ACAGTCAGCC TCTTCTAAAG TACTAAAGAA

CGTGTTTCTG AACAGACATT ACAGTCAGCG TCGTCTAAAG TCCTAAAGAG

CGTGTTTCTG AACAGACATT GCAGTCAGCA TCTTCTAAAG TACTAAAGAG

CGAGTTTCTG AACAGACGTT ACAGTCAGCA TCTTCTAAAG TCCTAAAGAG

----TTTCTG AACAGACATT ACAGTCAG-- ---------- ----------

AGAGTTTCTG AGCAGACATT GCAGTCAGCA TCTCCAAAGA TACTAAAGAG

CGTGTTTCTG AGCAGACGTT ACAGTCAGCA TCCACAAAAG TACTGAAGAG

CATGTGTGAG AATTTCTACA AATATTCAAA GCCTAAGAAA ATTCGAGTAT

CATGTGTGAG AATTTCTACA AATATTCAAA GCCCAAGAAA ATTCGAGTAT

CATGTGTGAG AATTTCTACA AATATTCAAA GCCCAAGAAA ATTCGAGTAT

CATGTGTGAG AATTTCTACA AATATTCAAA GCCCAAGAAA ATTCGAGTAT

CATGTGTGAG AATTTCTACA AATATTCAAA GCCGAAGAAA ATTCGAGTAT

CATGTGTGAG AATTTTTACA AGTATTCAAA GCCCAGGAAA ATTCGAGTAT

CATGTGTGAG AACTTCTACA AGTACTCAAA GCCCAAGAAG ATCCGAGTGT

CATGTGTGAG AACTTCTACA AATACTCAAA GCCCAAGAAG ATCCGAGTGT

---------- ---------- ---------- ---------- ----------

TATGTGTGAG AATTTCTACA AGTATTCAAA GCCCAAGAAA ATTCGAGTAT

CATGTGTGAG AATTTCTACA AGTATTCAAA GCCCAAGAAA ATTCGAGTAT

CATGTGTGAG AATTTCTACA AGTATTCAAA GCCCAAGAAA ATCCGAGTAT

CATGTGTGAG AATTTCTACA AGTATTCGAA GCCCAAGAAA ATCCGTGTGT

CATGTGTGAG AATTTCTATA AGTATTCAAA ACCCAAGAAA ATTCGAGTAT

CATGTGTGAG AATTTCTACA AGTATTCAAA GCCCAAGAAA ATCCGAGTGT

---------- ---------- ---------- ---------- ----------

TATGTGTGAG AACTTCTATA AATATACAGA ACCCAAGAAA ATTCGAGTGT

TATGTGTGAG AACTTCTACA AGTATTCCAA GCCTAGGAAG ATCCGAGTGT

GTGTCGGAAC CTGGAATGTG AATGGTGGGA AGCAATTTCG CAGCATAGCT

GTGTCGGAAC CTGGAATGTG AATGGTGGGA AGCAATTTCG CAGCATAGCT

GTGTCGGAAC CTGGAATGTG AATGGTGGGA AGCAGTTTCG CAGCATAGCT

GTGTCGGAAC CTGGAATGTG AATGGTGGGA AGCAGTTTCG CAGCATAGCT

GTGTTGGAAC CTGGAACGTG AATGGTGGGA AACAATTTCG CAGCATAGCT

GTGTCGGAAC CTGGAATGTG AATGGTGGGA AGCAGTTTCG CAGTATAGCT

GTGTTGGAAC CTGGAATGTG AACGGCGGGA AACAGTTCCG CAGCATAGCA

GTGTTGGCAC CTGGAATGTG AACGGCGGGA AGCAGTTCCG CAGCATAGCG

---------- ---------- ---------- ---------- ----------

GTGTGGGCAC ATGGAACGTG AATGGCGGGA AGCAGTTTCG CAGCATAGCT

GTGTTGGAAC CTGGAACGTG AATGGTGGGA AGCAGTTTCG CAGTATAGCT

GTGTTGGAAC CTGGAATGTG AACGGTGGGA AACAGTTTCG CAGTATAGCT

GTGTTGGCAC CTGGAACGTG AATGGCGGGA AGCAGTTTCG CAGCATAGCT

GTGTTGGAAC CTGGAATGTG AATGGAGGGA AGCAGTTTCG CAGCATAGCT

GTGTTGGGAC CTGGAACGTG AACGGAGGGA AGCAGTTTCG CAGCATAGCT

---------- ---------- ---------- ---------- ----------

GTATTGGAAC CTGGAATGTG AATGGTGGGA AACAGTTTCG AAGCATTGCC

GCGCCGGAAC GTGGAACGTC AACGGGGGCA AGCAGTTCCG CAGCATCGCT

TTTAAGAATC AGACACTCAC TGACTGGCTT CTTGATGCAC CCAAGTTAGC

TTTAAGAATC AGACACTCAC TGACTGGCTT CTTGATGCAC CCAAGTTAGC

TTTAAAAATC AGACACTCAC TGACTGGCTT CTTGATGCAC CCAAGTTAGC

TTTAAAAATC AGACACTCAC TGACTGGCTT CTTGATGCAC CCAAGTTAGC

TTCAAGAATC AGACACTCAC TGACTGGCTT CTTGATGCAC CCAAGTTAGC

TTTAAGAATC AGACGCTGAC TGACTGGCTT CTTGATGCAC CCAAGTTAGC

TTCAAGAACC AGACCCTCAC CGACTGGCTT CTTGATGCAC CCAAGTTAGC

TTCAAGAACC AGACGCTCAC AGACTGGCTT CTGGACGCTC CCAAGTTAGC

---------- ---------- ---------- ---------- ----------

TTTAAGAATC AGACCCTTAC GGACTGGCTT CTTGATGCCC CCAAGTTAGC

TTTAAGAATC AGACACTCAC CGACTGGCTT CTTGATGCAC CTAAGTTAGC

TTTAAGAATC AGACACTCAC CGACTGGCTT CTTGATGCAC CCAAGTTAGC

TTTAAGAACC AAACGCTCAC TGACTGGCTT CTGGACGCAC CCAAGTTGGC

TTTAAGAATC AGACACTCAC TGACTGGCTG CTTGATGCAC CCAAATTAGC

TTCAAGAATC AGACGCTCAC TGACTGGCTG CTGGATGCGC CCAAGCTAGC

---------- ---------- ---------- ---------- ----------

TTTAAGAATC AGACACTTAC TGATTGGCTA CTAGAGACTC CAGTGTTAAC

TTTAAGAACC AGACCCTCAC CGACTGGCTG CTCGACGCCC CCAAACTGGC

TGGCATCCAG GAGTTTCAAG ATAAAAGAAG TAAGCCAACT GATATATTTG

TGGCATCCAG GAGTTTCAAG ATAAAAGAAG TAAGCCAACT GATATATTTG

TGGCATCCAG GAGTTTCAAG ATAAAAGAAG TAAGCCAACT GATATATTTG

TGGCATCCAG GAGTTTCAAG ATAAAAGAAG TAAGCCAACT GATATATTTG

TGGCATCCAG GAGTTTCAAG ATAAAAGAAG TAAACCAACT GATATATTTG

TGGCATCCAG GAGTTTCAAG ATAAAAGAAG TAAGCCAACT GATATATTTG

TGGCATCCAG GAGTTTCAAG ATAAGAGAAG TAAGCCAACT GATATATTTG

CGGCATCCAG GAGTTTCAAG ATAAGAGAAG TAAGCCAACT GATATATTTG

---------- ---------- ---------- ---------- ----------

TGGCATCCAG GAGTTTCAAG ATAAAAGAAG TAAACCAATG GATATATTTG

TGGCATCCAG GAATTTCAAG ATAAAAGAAG TAAGCCAACA GATATATTTG

TGGCATCCAG GAGTTTCAAG ATAAAAGAAG TAAGCCAACG GATATATTTG

TGGCATCCAG GAATTTCAAG ACAAACGAAG TAAGCCAACG GATATATTTG

TGGCATCCAA GAGTTTCAAG ATAAAAGAAG TAAGCCAACC GATATATTTG

TGGCATCCAG GGATTCCAAG ATAAAAGAAG TAAGCCAACT GATATATTTG

---------- ---------- ATAAAAGAAG TAAGCCAACC GATATATTTG

TAGTATCCAA GAACTTCAAG ATCAAAGAGA TAAGCCTACT GACATATTTG

CGGGGTCCAG GAGTTCCAAG ATCAGAGAAG CAAGCCTACG GACATATTTG

CAATTGGTTT TGAAGAAATG GTAGAATTGA ATGCTGGAAA CATTGTGAGT

CAATTGGTTT TGAAGAAATG GTAGAATTGA ATGCTGGAAA CATTGTGAGT

CAATTGGTTT TGAAGAAATG GTAGAATTGA ATGCTGGAAA CATTGTGAAT

CAATTGGTTT TGAAGAAATG GTAGAATTGA ATGCTGGAAA CATTGTGAAT

CAATTGGTTT TGAAGAAATG GTAGAACTGA ATGCAGGAAA CATTGTGAAT

CAATTGGTTT TGAAGAAATG GTAGAGTTGA ATGCTGGAAA CATTGTGAAT

CAATTGGCTT TGAAGAAATG GTGGAGCTGA ATGCTGGGAA CATTGTGAAT

CAATTGGCTT TGAAGAAATG GTGGAGCTGA ATGCTGGGAA CATTGTGAAT

---------- ---------- ---------- ---------- ----------

CAATTGGTTT TGAGGAAATG GTAGAGCTGA ATGCTGGAAA CATTGTGAAT

CAATTGGTTT TGAAGAAATG GTTGAGTTAA ATGCTGGAAA CATCGTGAAT

CAATTGGTTT TGAAGAAATG GTAGAGTTGA ATGCTGGAAA CATTGTGAAC

CAATTGGCTT TGAAGAAATG GTAGAACTGA ATGCTGGAAA CATTGTGAAT

CAATTGGTTT TGAAGAAATG GTAGAGTTGA ATGCTGGAAA CATAGTGAAT

CAATTGGTTT TGAAGAAATG GTGGAGTTGA ATGCTGGAAA CATTGTGAAT

CAATTGGTTT TGAAGAAATG GTAGAGTTGA ATGCTGGAAA CATTGTGAAT

CAATTGGTTT TGAAGAAATG GTAGAATTAC TTGCTGGAAA CATTGTGAG-

CAATCGGCTT TGAAGAAATG GTGGAATTAA ATGCAGGAAA TATAGTGAAT

AGCACAACAA ATCAGAAGCT CTGGGCTGTA GAACTTCAGA AGACAATCTC

AGCACAACAA ATCAGAAGCT CTGGGCTGTA GAACTTCAGA AGACAATCTC

AGCACAACAA ATCAGAAGCT CTGGGCTGTA GAACTTCAGA AGACAATCTC

AGCACAACAA ATCAGAAGCT CTGGGCTGTA GAACTTCAGA AGACAATCTC

AGCACAACAA ATCAGAAGCT CTGGGCTGTA GAACTTCAGA AGACAATCTC

AGCACAACAA ATCAGAAGCT CTGGGCTGTG GAACTTCAGA AGACAATCTC

AGCACAACAA ACCAGAAGCT GTGGGCAGTG GAACTGCAGA AGACCATCTC

AGCACAACCA ACCAGAAGCT GTGGGCAGTG GAGCTGCAGA AGACCATCTC

---------- ---------- ---------- ---------- ----------

AGCACAACAA ATCAGAAGCT CTGGGCTGCG GAACTTCAGA AGACCATCTC

AGCACAACCA ATCAGAAGCT CTGGGCTGTG GAACTGCAGA AGACCATCTC

AGCACAACAA ATCAGAAGCT CTGGGCTGTG GAACTTCAGA AGACCGTCTC

AGCACCACAA ATCAGAAGCT CTGGGCTGTG GAACTTCAGA AGACCATCTC

AGTACAACTA ATCAGAAGCT CTGGGCTGTG GAACTTCAAA AGACTATCTC

AGCACAACTA ATCAGAAGCT CTGGGCCGTG GAACTTCAGA AGACAGTCTC

AG-------- ---------- ---------- ---------- ----------

ATTACAACTA ATCAGAAATT ATGGGCTGTT GAACTACAGA AGATTCTCTC

AGCACGACGA ACCAGAAGCT CTGGGCTGCC GAACTGCAGA AGACCATCTC

CAGAGACAAC AAGTATGTGC TGCTGGCTTC TGAACAGTTG GTGGGCGTCT

CAGAGACAAC AAGTATGTGC TGCTGGCTTC TGAACAGTTG GTGGGCGTCT

CAGAGACAAC AAGTATGTGC TGCTGGCTTC TGAACAGTTG GTGGGCGTCT

CAGAGACAAC AAGTATGTGC TGCTGGCTTC TGAACAGTTG GTGGGCGTCT

CAGAGACAAC AAGTATGTGC TGCTGGCTTC TGAGCAGTTG GTGGGCGTCT

CAGAGACAAT AAGTATGTGC TGCTGGCTTC TGAACAGTTG GTGGGCGTCT

CAGAGACAAC AAGTATGTTC TCCTGGCCTC GGAGCAGTTG GTGGGTGTCT

CAGGGACAAC AAGTATGTCC TGCTGGCCTC GGAGCAGCTG GTGGGTGTCT

---------- ---------- ---------- ---------- ----------

CAGAGACAAC AAGTATGTGC TGCTGGCCTC TGAGCAGTTG GTGGGCGTCT

CAGAGACAAC AAGTATGTGC TGCTGGCTTC TGAGCAGCTG GTGGGCGTCT

CAGAGACAAC AAGTACGTGC TGCTGGCTTC TGAGCAGCTG GTGGGCGTGT

CAGGGACAAC AAGTATGTGC TGCTGGCATC CGAGCAGTTG GTAGGCGTCT

CAGAGACAAC AAATATGTGC TGCTGGCTTC TGAGCAATTG GTGGGCGTCT

CAGAGACAAC AAGTATGTGC TGCTGGCCTC TGAGCAGCTG GTGGGCGTCT

---------- ---------- ---------- ---------- ----------

CAGAGATAGA AAGTATGTGC TGCTGGCTTC AGAGCAATTG GTAGGCATCT

CAGAGATAAC AAGTACGTGC TGCTGGCGTC CGAGCAGCTG GTGGGGGTCT

GTTTGTTTGT TTTTATCAGA CCACAGCATG CTCCTTTTAT CAGGGATGTT

GTTTGTTTGT TTTTATCAGA CCACAGCATG CTCCTTTTAT CAGGGATGTT

GTTTGTTTGT TTTTATCAGA CCACAGCATG CTCCTTTTAT CAGGGATGTT

GTTTGTTTGT TTTTATCAGA CCACAGCATG CTCCTTTTAT CAGGGATGTT

GTTTGTTTGT TTTTATCAGA CCACAGCATG CTCCTTTTAT CAGGGATGTT

GTCTGTTTGT TTTTATCAGA CCACAGCATG CTCCTTTTAT CAGGGATGTT

GCCTGTTTGT CTTTATCAGA CCGCAGCATG CTCCCTTTAT CAGGGATGTT

GCCTGTTTGT CTTTATCAGA CCACAGCACG CTCCTTTTAT CAGGGATGTT

---------- ---------- ---------- ---------- ----------

GTCTGTTTGT TTTTATCAGA CCACAGCACG CTCCCTTCAT CAGGGATGTT

GCCTGTTTGT TTTTATCAGA CCACAGCATG CTCCTTTCAT CAGGGATGTT

GCCTGTTTGT TTTCATCAGA CCTCAGCATG CTCCTTTCAT CAGGGATGTC

GCCTGTTCGT TTTTATCAGG CCCCAGCATG CTCCGTTCAT CAGGGATGTT

GTCTCTTTGT TTTTATCAGA CCACAGCATG CTCCCTTCAT CAGGGATGTT

GCCTCTTTGT TTTTATCAGA CCACAGCATG CTCCTTTTAT CAGGGATGTT

---------- ---------- ---------- ---------- ----------

GTCTCTACGT TTTTATCAGA CCCCAACATG CTCCTTTCAT CAGGGATGTT

GCCTCTTCGT CTTCATCCGC CCCCAGCACG CCCCCTTTAT CAGGGATGTT

GCAGTTGATA CTGTGAAGAC TGGAATGGGA GGTGCAACTG GAAATAAGGG

GCAGTTGATA CTGTGAAGAC TGGAATGGGA GGTGCAACTG GAAATAAGGG

GCAGTTGATA CTGTGAAGAC TGGAATGGGA GGTGCAACTG GAAATAAGGG

GCAGTTGATA CTGTGAAGAC TGGAATGGGA GGTGCAACTG GAAATAAGGG

GCAGTTGATA CTGTGAAGAC TGGAATGGGA GGTGCAACTG GAAATAAGGG

GCAGTTGATA CAGTGAAGAC TGGAATGGGA GGTGCAACTG GCAATAAGGG

GCAGTCGATA CTGTGAAAAC TGGGATGGGA GGCGCGACTG GAAATAAGGG

GCAGTTGATA CTGTGAAAAC TGGCATGGGA GGTGCAACTG GAAACAAGGG

---------- ---------- ---------- ---------- ----------

GCAGTTGATA CTGTGAAAAC TGGAATGGGA GGCGCAACTG GAAATAAGGG

GCAGTTGATA CTGTGAAGAC TGGAATGGGA GGTGCCACTG GAAATAAGGG

GCAGTTGATA CTGTGAAGAC CGGAATGGGA GGTGCCACTG GAAATAAGGG

GCAGTCGATA CCGTGAAGAC TGGGATGGGC GGCGCCACTG GAAATAAGGG

GCAGTTGATA CTGTAAAGAC TGGAATGGGA GGTGCAACTG GAAATAAGGG

GCAGTTGATA CTGTGAAGAC TGGGATGGGA GGTGCAACTG GGAATAAGGG

---------- ---------- ---------- ---------- ----------

GGATTTGATA CCGTTAAAAC TGGAATGGGA GGTGCTACTG GAAATAAAGG

GCAGTTGATA CGGTGAAAAC TGGCATGGGA GGCGCGACAG GGAATAAAGG

AGCAGTTGCA ATCCGAATGC TCTTCCATAC AACCAGCCTT TGCTTCGTCT

AGCAGTTGCA ATCCGAATGC TCTTCCATAC AACCAGCCTT TGCTTCGTCT

AGCAGTTGCA ATCCGAATGC TCTTCCATAC AACCAGCCTT TGCTTTGTCT

AGCAGTTGCA ATCCGAATGC TCTTCCATAC AACCAGCCTT TGCTTTGTCT

AGCAGTTGCA ATCCGAATGC TGTTCCATAC AACCAGCCTT TGCTTCGTCT

AGCAGTTGCC ATCCGAATGC TGTTCCATAC AACCAGCCTT TGCTTCGTCT

CGCTGTTGCA ATCCGGATGC TCTTCCACAC CACCAGCCTT TGCTTTGTCT

CGCCGTTGCA ATTCGGATGC TCTTCCACAC CACCAGCCTT TGCTTCGTCT

---------- ---------- ---------- ---------- ----------

AGCAGTTGCA ATACGAATGC TGTTCCACAC CACAAGCCTC TGCTTTGTCT

AGCAGTTGCA ATCCGAATGC TATTCCATAC AACAAGCCTC TGCTTCGTCT

AGCGGTCGCA ATCCGAATGT TATTCCACAC GACAAGTCTT TGCTTCGTCT

AGCAGTGGCA ATCCGCATGC TGTTCCATAC CACGAGCCTG TGCTTTGTCT

AGCAGTTGCA ATTCGAATGT TATTCCACAC AACAAGCCTT TGCTTCGTCT

GGCAGTTGCC ATCCGGATGC TGTTCCACAC CACCAGCCTC TGCTTCGTGT

---------- ---------- ---------- ---------- ----------

AGCAGTTGCA ATACGGATGT TATTCCACAC CACTAGCCTT TGCTTTGTCT

GGCAGTCGCC ATACGGATGC TGTTCCATAC GACCAGCCTT TGTTTCGTCT

GTAGCCACTT TGCTGCAGGG CAGTCACAAG TCAAAGAAAG AAATGAAGAT

GTAGCCACTT TGCTGCAGGG CAGTCACAAG TCAAAGAAAG AAATGAAGAT

GTAGCCACTT TGCTGCAGGG CAGTCACAAG TCAAAGAAAG AAATGAAGAT

GTAGCCACTT TGCTGCAGGA CAGTCACAAG TCAAAGAAAG AAATGAAGAT

GTAGTCACTT TGCTGCAGGG CAGTCACAAG TCAAAGAAAG AAATGAAGAT

GTAGCCACTT TGCCGCAGGG CAGTCACAAG TCAAAGAAAG AAATGAAGAT

GCAGTCACTT TGCTGCAGGC CAGTCCCAAG TCAAAGAGAG AAATGAAGAT

GCAGCCACTT TGCTGCAGGG CAGTCCCAAG TCAAAGAGAG AAATGAAGAT

---------- ---------- ---------- ---------- ----------

GCAGCCACTT CGCTGCGGGA CAATCCCAAG TCAAAGAACG AAATGATGAT

GTAGCCACTT TGCTGCAGGG CAATCACAAG TCAAAGAAAG AAATGAAGAT

GCAGCCACTT TGCTGCGGGA CAATCACAAG TCAAAGAAAG AAATGAAGAC

GTAGCCACTT TGCCGCGGGC CAGTCACAGG TCAAAGAAAG AAATGAAGAC

GTAGCCACTT TGCTGCAGGG CAATCACAAG TTAAAGAAAG AAATGAAGAT

GCAGCCACTT CGCTGCGGGG CAGTCACAGG TGAAAGAGAG AAATGAGGAT

---------- ---------- ---------- ---------- ----------

GTAGTCACTT TGCTGCAGGC CAATCACAAG TCAAAGAAAG GAATGAAGAT

GTAGTCACTT CGCCGCGGGG CAGTCGCAGG TCAAGGAGAG AAATGAAGAC

TTTATAGAAA TAGCACGAAA ATTGAGTTTT CCTATGGGAA GGATGCTATT

TTTATAGAAA TAGCACGAAA ATTGAGTTTT CCTATGGGAA GGATGCTGTT

TTTGTAGAAA TAGCACGAAA ATTGAGTTTT CCTATGGGAA GGATGCTGTT

TTTGTAGAAA TAGCACGAAA ATTGAGTTTT CCTATGGGAA GGATGCTGTT

TTTGTAGAAA TAGCACGAAA GTTGAGTTTT CCTATGGGAA GAATGCTGTT

TTTGTAGAAA TAGCACGAAA ATTGAGTTTT CCTATGGGAA GGATGCTTTT

TTCGTAGAAA TAGCACGAAA ATTGAGTTTC CCCATGGGAA GAATGCTCTT

TTTGTAGAAA TAGCACGAAA ACTGAGTTTC CCCATGGGAC GAATGCTCTT

---------- ---------- ---------- ---------- ----------

TTTGTAGAAA TAGCGCGGAA GTTGAGTTTT CCAATGGGAA GGCTGCTCTT

TTTGTAGAAA TTGCACGGAA GTTGAGTTTT CCAATGGGAA GGATGCTGTT

TTTGTAGAAA TAGTACGGAA ACTGAGTTTC CCTATGGGAA GGATGCTGTT

TTTGTTGAAA TAGCACGCAA GCTGAGTTTT CCCATGGGAA GGATGCTGTT

TTTGTAGAAA TAGCTCGCAA ATTGAGTTTT CCTATGGGAA GGATGCTGTT

TTCGTAGAGA TAGCGCGGAA ACTGAGTTTT CCCATGGGAA GGATGCTGTT

---------- ------GAAA ATTGAGTTTT CCCATGGGGA GGCTGCTGTT

TTTGTAGAAA TTGCCAGAAG ATTGAGCTTT CCTATGGGGA GGATGTTGTT

TTTGGGGAAA TAGCTCGAAA ACTAACATTT CCCATGGGAC GGATGCTCTT

TTCCCATGAC TATGTATTTT GGTGTGGTGA TTTCAACTAT CGAATCGATC

TTCCCATGAC TATGTATTTT GGTGTGGTGA TTTCAACTAT CGAATCGATC

TTCCCATGAC TATGTATTTT GGTGTGGTGA TTTCAACTAT CGAATTGATC

TTCCCATGAC TATGTATTTT GGTGTGGTGA TTTCAACTAT CGAATTGATC

TTCCCATGAC TATGTATTTT GGTGTGGTGA TTTCAACTAT CGAATCGATC

TTCCCATGAC TATGTATTTT GGTGTGGCGA TTTCAACTAT CGAATCGATC

CTCCCATGAT TACGTATTCT GGTGTGGTGA TTTCAACTAT CGAATCGATC

CTCCCATGAC TATGTATTTT GGTGTGGTGA TTTCAACTAT CGAATTGACC

---------- ---------- ---------- ---------- ----------

CTCCCATGAC TATGTGTTTT GGTGTGGCGA TTTCAACTAC CGAATCGATC

TTCCCATGAC TACGTATTTT GGTGTGGTGA TTTCAACTAT CGGATTGATC

TTCCCATGAC TATGTATTTT GGTGTGGTGA TTTCAACTAT CGAATTGATC

T--------- ---------- ---------- ---------- ----------

TTCTCATGAT TATGTGTTTT GGTGTGGCGA TTTCAACTAT CGAATCGATC

TTCCCACGAT TATGTGTTTT GGTGTGGAGA TTTCAACTAC CGAATCGATC

TTCCCATGAT TATGTATTTT GGTGTGGCGA TTTCAACTAT CGAATTGATC

ATCACATGAC TATGTATTTT GGTGTGGTGA TTTTAATTAT CGTATTGATC

CTGCCACGAC TACGTGTTTT GGTGCGGTGA CTTTAATTAC CGTATCGATC

TCCCTAACGA AGAAGTTAAA GAGCTCATAA GACAGCAAAA TTGGGATTCT

TCCCTAACGA AGAAGTTAAA GAGCTCATAA GACAGCAAAA TTGGGATTCT

TCCCTAATGA AGAAGTTAAA GAGCTCATAA GACAGCAAAA TTGGGATTCT

TCCCTAATGA AGAAGTTAAA GAGCTCATAA GACAGCAAAA TTGGGATTCT

TGCCTAACGA AGAAGTTAAA GAGCTCATAA GACAGCAAAA CTGGGATTCT

TCCCTAATGA AGAAGTTAAA GAGCTTATAA GACAGCAAAA TTGGGATTCT

TTCCTAATGA AGAAGTTAAA GAGCTCATAA GACAGCAAAA TTGGGATTCT

TTCCGAATGA AGAGGTTAAA GAGCTCATAA GACAACAAAA CTGGGATTCT

---------- ---------- ---------- ---------- ----------

TCCCTAATGA GGAAGTGAAA GAGCTTATCA GACAGCAAAA CTGGGATTCT

TCCCTAATGA AGAAGTTAAA GAGCTCATAA GACAGCAAAA TTGGGATTCT

TCCCTAATGA AGAAGTTAAA GAGCTCATAA GACAGCAAAA TTGGGATTCT

---------- ---------- ---------- ---------- ----------

TCCCTAATGA AGAAGTTAAA GAGCTCATAA GACAACAAAA TTGGGATTCT

TCCCTAACGA AGAAGTTAAA GAGCTTATAA GACAGCAAAA TTGGGATTCT

TCCCTAATGA AGAAGTCAAA GAGCTCATAA GACAGCAAAA TTGGGATTCT

TACCAAATGA AGAAGTTAGG GAATTAATAA AACACCAGAA CTGGGATTCA

TCCCCAACGA AGAAGTGAAG GAGCTGATAC GGCAGCAGAA CTGGGATTCC

CTTATAGCAG GAGATCAACT TATCAATCAG AAAAATGCTG GACAGGTTTT

CTTATAGCAG GAGATCAACT TATCAATCAG AAAAATGCTG GACAGATTTT

CTTATAGCAG GAGATCAACT TATCAATCAG AAAAATGCTG GACAGATTTT

CTTATAGCAG GAGATCAACT TATCAATCAG AAAAATGCTG GACAGATTTT

CTTATAGCAG GAGATCAACT TATCAATCAG AAAAATGCCG GACAGATTTT

CTTATAGCAG GAGATCAACT TATCAATCAG AAAAATGCTG GACAG-----

CTTATAGCTG GAGATCAACT CATCAATCAG AAAAATGCAG GACAGATCTT

CTGATCGCTG GGGATCAGCT CATCAACCAG AAAAATGCAG GACAGATCTT

---------- ---------- ---------- ---------- -----ATTTT

CTTATCGCAG GAGATCAGCT TATCAATCAG AAAAATGCTG GACAGATTTT

CTTATAGCAG GAGATCAGCT TATCAATCAA AAAAATGCTG GACAGATTTT

CTTATAGCAG GAGATCAACT TATCAACCAG AAAAATGCTG GACAGATTTT

---------- ---------- ---------- ---------- -----ATTTT

CTTATAGCAG GAGATCAGCT TATCAATCAG AAAAATGCTG GACAGATTTT

CTTATAGCAG GAGATCAGCT AATCAATCAG AAGAATGCTG GACAGATTTT

CTTATAGCAG GAGATCAACT AATCAATCAG AAAAATACTG GACAGATCTT

CTTATAGCAG GAGATCAGCT TATCAATCAG AAAAATGCTG GACAGATCTT

CTTATCGCGG GAGACCAACT TATCAATCAG AAAAATGCTG GCCAGATTTT

TAGAGGATTT TTAGAAGGAA AGGTAACCTT TGCTCCGACA TATAAGTATG

TAGAGGATTT TTAGAAGGAA AGGTAACCTT TGCTCCGACA TATAAGTATG

TAGAGGATTT TTAGAAGGAA AGGTAACCTT TGCTCCGACA TATAAGTATG

TAGAGGATTT TTAGAAGGAA AGGTAACCTT TGCTCCGACA TATAAGTATG

TAGAGGATTT TTAGAGGGAA AGGTGACCTT TGCTCCAACA TATAAGTATG

---------- ---------- ---------- ---------- ----------

TAGAGGATTT TTAGAGGGAA AAGTAACGTT TGCTCCAACC TATAAATATG

TAGAGGATTC TTAGAGGGAA AAGTAACGTT TGCTCCAACC TATAAATATG

CAGAGGATTT TTAGAGGGAA AAGTTACCTT TGCTCCGACG TACAAATATG

TAGAGGATTT TTAGAAGGAA AAGTGACCTT TGCTCCAACG TATAAATACG

TAGAGGATTT TTAGAAGGAA AAGTGACCTT TGCTCCGACA TACAAATATG

TAGAGGATTT TTAGAAGGAA AAGTAACCTT TGCTCCGACA TATAAATATG

TAGAGGATTC TTGGAGGGAA AAGTAACCTT TGCTCCAACA TACAAATATG

TAGAGGATTT TTAGAAGGAA AAATGACTTT TGCTCCTACA TATAAATATG

TAGAGGATTT TTAGAAGGGA AAGTAACTTT TGCTCCGACA TATAAGTATG

TCGAGGGTTT TT-------- ---------- ---------- ----------

TAGAGGATTT TTAGAAGGAA AAATAACTTT TGCTCCTACC TACAAGTATG

TAGAGGATTC TTAGAGGGAA AAGTAACTTT CGCTCCTACG TACAAATATG

ACTTGTTTTC TGACGACTAT GACACCAGTG AAAAGTGCCG CACCCCTGCC

ACTTGTTTTC TGACGACTAT GACACCAGTG AAAAGTGCCG CACCCCTGCA

ACTTGTTTTC TGACGACTAT GACACCAGTG AAAAGTGCCG CACCCCTGCA

ACTTGTTTTC TGACGACTAT GACACCAGTG AAAAGTGCCG CACCCCTGCA

ACTTGTTTTC TGATGACTAT GACACCAGTG AAAAGTGCCG CACCCCTGCG

---------- ---------- ---------- ---------- ----------

ACTTGTTTTC CGAAGACTAT GACACCAGTG AAAAGTGCCG CACCCCTGCT

ACTTGTTTTC TGAAGACTAT GACACCAGTG AGAAGTGTCG CACCCCTGCT

ATTTGTTTTC TGATGACTAT GATACCAGTG AGAAGTGCCG TACCCCTGCG

ACTTGTTTTC TGATGACTAC GACACTAGTG AAAAGTGCCG CACCCCTGCA

ACTTGTTTTC TGACGACTAT GACACCAGTG AAAAGTGCCG CACCCCTGCT

ATTTGTTTTC TGAAGACTAT GACACGAGTG AAAAGTGCCG CACCCCTGCA

ATTTATTTTC GGACGACTAT GATACCAGTG AGAAATGCCG CACCCCTGCC

ATTTGTTTTC TGATGATTAT GACACCAGTG AAAAGTGCCG CACCCCTGCG

ATTTGTTTTC TGATGACTAT GACACCAGTG AAAAGTGCCG CACCCCAGCC

---------- ---------- ---------- ---------- ----------

ATTTGTTTTC TGATGACTAT GATACCAGTG AGAAATGTCG CACTCCAGCC

ACTTGTTTTC TGATGACTAC GATACCAGTG AAAAATGCCG CACCCCGGCG

TGGACAGACC GTGTCCTTTG GAGAAGGAGG AAATGGCCTT TTGATAGATC

TGGACAGACC GTGTCCTTTG GAGAAGGAGG AAATGGCCTT TTGATAGATC

TGGACAGACC GTGTCCTTTG GAGAAGGAGG AAATGGCCTT TTGATAGATC

TGGACAGACC GTGTCCTTTG GAGAAGGAGA AAATGGCCTT TTGATAGATC

TGGACAGACC GTGTCCTTTG GAGAAGAAGG AAATGGCCTT TTGATAGATC

---------- ---------- ---------- ---------- ----------

TGGACAGACC GAGTCCTCTG GAGAAGGAGG AAGTGGCCTT TTGACAGATC

TGGACAGACC GTGTCCTCTG GAGAAGGAGG AAGTGGCCTT TTGACAGATC

TGGACAGATC GTGTCCTCTG GAGAAGGAGG AAGTGGCCTT TTGATAGATC

TGGACAGACC GTGTCCTCTG GAGAAGACGG AAGTGGCCTT TTGATAGATC

TGGACAGACC GTGTCCTCTG GAGAAGAAGG AAGTGGCCTT TTGACAGATC

TGGACAGACC GTGTCCTCTG GAGAAGAAGG AAATGGCCTT TTGACAGATC

TGGACAGACC GTGTCCTCTG GAGAAGGAGG AAATGGCCTT TTGATAGATC

TGGACAGATC GTGTCCTCTG GAGAAGAAGG AAATGGCCTT TTGATAGATC

TGGACGGACC GTGTCCTCTG GAGAAGAAGG AAGTGGCCTT TTGATAGATC

---------- ---------- ---------- ---------- ----------

TGGACAGATC GCATTCTTTG GAGAAGAAGG AAATGGCCCT TTGATAGATC

TGGACGGATC GCATCCTCTG GAGGAGAAGG AAATGGCCAT TTGATAGATC

AGCTGAAGAT CTAGATCTTC TAAATGCTAG TTTTCAAGAT GAAAGCAAAA

AGCTGAAGAT CTAGATCTTC TAAATGCTAG TTTTCAAGAT GAAAGCAAAA

AGCTGAAGAT CTAGATCTTC TAAATGCTAG TTTTCAAGAT GAAAGCAAAA

AGCTGAAGAT CTAGATCTTC TAAATGCTAG TTTTCAAGAT GAAAGCAAAA

AGCTGAAGAT CTAGATCTTC TAAATGCTAG TTTTCAAGAT GACAGCAAAA

---------- ---------- ---------- ---------- ----------

AGCTGAAGAC TTAGATCTTC TGAATGCTAG TTTCCAAGAT GAAAGTAAAA

AGCTGAAGAT TTAGATCTCC TGAATGCTAG TTTCCAAGAT GAAAGTAAAA

AG-------- ---------- ---------- ---------- ----------

AGCTGAAGAT TTAGATCTCC TAAATGCTAG TTTTCAAGAT GAAAGCAAAA

AGCTGAAGAT TTAGATCTTC TAAATGCTAG TTTTCAAGAT GGAAGCAAAA

AGCTGAAGAT TTAGATCTTC TAAATGCTAG TTTTCAAGAT GGAAGCAAAA

AGCTGAAGAC TTAGATCTTC TCAATGCGAG CTTTCAGGAT GGAAGCAAAA

AGCTGAAGAT TTAGATCTTC TAAATGCTAG TTTTCAAGAT GAAAGCAAAA

AGCTGAAGAT TTAGATCTTC TAAGTGCGAG TTTTCAGGAT GAAAGCAAAA

--CTGAAGAC TTAGACCTTC TGAATGCTAG TTTTCAAGAT GGAAGCAAAA

AGCTGAAGAT TTAGATCTTC TGAATGCTAG CTTTCAAGAT GACAGCAAAG

AGCCGAAGAC CTCGATCTTC TGAATGCCGG CTTTCAAGAT GAAAGTAAAA

TTCTGTACAC GTGGACTCCA GGCACTTTGC TGCACTATGG AAGAGCTGAG

TTCTGTACAC GTGGACTCCA GGCACTTTGC TGCACTATGG AAGAGCTGAG

TCCTGTACAC GTGGACTCCA GGCACTTTGC TGCACTATGG AAGAGCTGAG

TCCTGTACAC GTGGACTCCA GGCACTTTGC TGCACTATGG AAGAGCTGAG

TCCTCTACAC GTGGACTCCA GGCACTTTGT TGCACTATGG AAGAGCTGAG

---------- ---------- ---------- ---------- ----------

TCCTGTATAC ATGGACTCCT GGCACCTTGC TGCACTATGG AAGAGCCGAG

TTCTTTATAC ATGGACCCCT GGCACCTTGC TGCACTATGG AAGAGCCGAG

---------- ---------- ---------- ---------- ----------

TCCTCTACAC ATGGACTCCT GGCACTTTGC TGCACTACGG AAGGGCTGAG

TCCTGTACAC ATGGACTCCT GGCACTTTGC TGCACTATGG AAGAGCTGAG

TCCTCTACAC CTGGACTCCG GGTACTTTGC TGCACTATGG CAGAGCGGAG

TCCTTTACAC GTGGACTCCT GGCACGCTTC TGCACTATGG GCGCGCAGAG

TCCTCTACAC CTGGACTCCT GGCACTTTGC TCCATTATGG AAGAGCCGAG

TCCTGTACAC CTGGACTCCT GGCACGCTGC TGCACTACGG ACGGGCTGAG

TCCTCTACAC TTGGACCCCT GGCACTTTGC TGCACTATGG AAGGGCCGAG

TCCTTTACAC TTGGAATCCT GGCACTTTGC TGCATTATGG AAGAGCTGAG

TCCTGTACAC TTGGACGCCC GGCACTCTGT TGCACTACGG AAGAGCCGAG

CTGAAGACTT CTGACCACAG GCCTGTCGTT GCCCTGATTG ATATAGATAT

CTGAAGACTT CTGACCACAG GCCTGTCGTT GCCCTGATTG ATATAGATAT

CTGAAGACTT CTGACCATAG GCCTGTCGTT GCCCTGATTG ATATAGATAT

CTGAAGACTT CTGACCATAG GCCTGTCGTT GCCCTGATTG ATATAGATAT

CTGAAGACCT CTGACCATAG GCCTGTCGTT GCCCTGATTG ATATAGATAT

---------- ---------- ---------- ---------- ----------

CTGAAGACTT CTGACCACAG GCCCGTTGTC GCCCTGATCG ATATAGATAT

CTGAAGACTT CTGACCATAG GCCCGTCGTT GCCTTGATTG ACATAGATAT

---------- ---------- ---------- ---------- ----------

CTGAAGACTT CTGACCATAG GCCTGTTGTT GCCCTGATCG ATATTGATAT

CTGAAGACTT CTGACCATAG GCCTGTCGTT GCCCTGATTG ATATAGATAT

CTGAAGACCT CCGACCATAG GCCTGTCGTT GCCTTGATTG ATATAGATAT

CTGAAGACTT CAGACCACAG GCCTGTCATT GCCCTGATCG ATATCGATAT

CTGAAGACTT CTGATCATAG GCCTGTCATT GCCATGATTG ATATAGATAT

CTGAAGACTT CGGACCATAG GCCTGTCGTT GCCCTGATTG ATATAGATAT

CTAAAGACGT CTGATCACAG GCCAGTCATT GCGCTGATTG ATATAGATAT

CTGAAGACTT CTGATCACAG ACCTGTTGTT GCACTGATCG ATATAGATAT

TTGAAGATCT CGGATCACAG GCCTGTGGTG GCCCTGATGG ATGTAGATAT

ATTTGAAGTT GAAGCTGAAG AGAGGCAAAA CATTTATAAA GAAGTAATTG

ATTTGAAGTT GAAGCTGAAG AGAGGCAAAA CATTTATAAA GAAGTAATTG

ATTTGAAGTT GAAGCTGAAG AGAGGCAAAA CATTTATAAA GAAGTCATTG

ATTTGAAGTT GAAGCTGAAG AGAGGCAAAA CATTTATAAA GAAGTAATTG

ATTTGAAGTT GAAGCTGAAG AGAGGCAAAA CATTTATAAA GAAGTAATTG

---------- ---------- ---------- ---------- ----------

ATTTGAAGTT GAAGCTGAAG AGAGACAAAA AATTTATAAA GAAGTTATTG

ATTTGAAGTT GAAGCTGAAG AGAGACAAAA AATTTATAAA GAAGTTATTG

---------- ---------- ---------- ---------- ----------

ATTTGAAGTT GAAGCTGAAG AGAGGCAAAA CATTTATAAA GAAGTAATTG

ATTTGAAGTT GAAGCTGAAG AGAGGCAAAA CATTTATAAA GAAGTAATCG

ATTTGAAGTT GAAGCTGAAG AGAGACAAAA CATTTATAAA GAAGTAATTG

CTTTGAAGTT GAAGCGGAAG AGAGGCAGAA CATTTATAAG GAGGTCATTG

ATTTGAAGTT GAAGCTGAAG AAAGGCAAAA TATTTATAAA GAAGTAATTG

ATTTGAAGTT GAAGCTGAAG AAAGGCAAAA CATTTATAAG GAAGTGATTG

ATTTGAAGTT GAAGCTGAAG AAAGGCAAAA CATTTATAAA GAAGTGATTG

ATTTGAAGTG GAAGCTAAAA CACGACAAAT TGTTTATAAG GAAGTCATTG

ATTTGAAGTG GAAGCTGAAG AGAGACAGAA CGTTTATAAA GAAGTGATCG

CAGTTCAGGG TCCACCAGAT GGTACAGTAT TGGTCTCAAT CAAAAGTTCT

CAGTTCAGGG TCCACCAGAT GGTACAGTAT TGGTCTCAAT CAAAAGTTCT

CAGTTCAGGG TCCACCAGAT GGTACGGTAT TGGTCTCAAT CAAAAGTTCT

CAGTTCAGGG TCCACCAGAT GGTACGGTAT TGGTCTCAAT CAAAAGTTCT

CAGTTCAGGG TCCACCAGAT GGTACGGTAT TGGTCTCAAT CAAAAGTTCT

---------- ---------- ---------- ---------- ----------

CAGTCCAGGG CCCACCGGAC GGCACAGTAC TGGTCTCAAT CAAAAGTTCT

CCGTCCAGGG CCCACCAGAT GGCACAGTGC TGGTCTCAAT CAAAAGTTCT

---------- ---------- ---------- ---------- ----------

CAGTTCAGGG TCCACCAGAT GGTACGGTGT TGGTCTCAAT CAAAAGCTCT

CAGTTCAGGG CCCACCAGAT GGTACAGTAT TGGTCTCAAT CAAAAGTTCT

CAGTTCAGGG TCCACCAGAT GGTACGGTGT TGGTCTCAAT CAGAAGTTCT

CCGTGCAGGG GCCACCCGAC GGGACCGTGT TGGTCTCAAT CAAAACTTCC

CAGTACAGGG CCCACCAGAT GGTACAGTAT TGGTCTCAAT CAGAAGTTCT

CAGTTCAGGG TCCCCCAGAT GGCACAGTAC TGGTCTCAAT CAAAAGTTCT

CGGTGCAGGG TCCACCTGAT GGCACAGTAT TGGTCTCAAT CAAAAGTTCT

CCACTCAAGG ACCAACAGAT GGCACAGTAT TGGTCACCAT CAAAAGGTCA

CTCTCCAGGG CCCCCCAGAT GGGACGGTGT TGGTCTCTAT CAAAAGTTCT

TTACCAGAAA ATAATTTTTT TGATGATGCC TTGATTGATG AGCTTCTGCA

TTACCAGAAA ATAATTTTTT TGATGATGCC TTGATTGATG AGCTTCTGCA

TTACCAGAAA ATAATTTTTT TGATGATGCC TTGATTGATG AGCTTCTGCA

TTACCAGAAA ATAATTTTTT TGATGATGCC TTGATTGATG AGCTTCTGCA

TTACCAGAAA ATAATTTTTT CGATGATGCC TTGATTGATG AGCTTCTGCA

---------- ---------- ---------- ---------- ----------

GCACAAGAGA ATACTTTCTT TGACGATGCT TTGATCGATG AGCTTCTGCA

GCACAAGAAA GTACTTTTTT TGATGATGCT TTGATTGATG AGCTTCTGCG

--------AA ATAATTTCTT TGATGATGCT TTGATTGATG AACC-CTAGG

TTACCAGAAA ATAATTTTTT CAACGATGCT TTGATTGATG AGCTTTTACA

GTCCAAGAAA ATAATTTTTT CGATGATGCT TTGATTGATG AGCTTCTGCA

GTACCAGAAA ATAATTTCTT TGATGATACT TTGATCGATG AGCTTCTGCA

TCCCCAGAAA GTAACTTTTT TGATGATGCT CTGATTGATG AGCTTCTGCA

TTACCAGAAA ATAATTTTTT CGATGATGGT CTGATTGATG AGCTTCTCCA

TTACCAGAAA ATAATTTTTT TGATGATGCT TTGATTGATG AGCTTCTCCA

CTGTCAGATA ATAACTTTTT TGATGATGCT TTGATTGATG AGCTTCTCCA

TCATCAGAAA ATAATTTTTT CGACGATAGC TTGATTAATG AGCTTCTTCA

TCTGTGGAAG ATCATTTCTT TGATGACATC TTGATCGATG AACTTCTCCA

GCAGTTTGCA AGTTTTGGTG AAGTTATACT TATAAGATTT GTAGAAGATA

GCAGTTTGCA AGTTTTGGTG AAGTTATACT TATAAGATTT GTAGAAGATA

GCAGTTTGCA AGTTTTGGTG AAGTTATACT TATAAGATTT GTAGAAGATA

GCAGTTTGCA AGTTTTGGTG AAGTTATACT TATAAGATTT GTAGAAGATA

GCAGTTTGCA AGTTTTGGTG AAGTTATACT TATAAGATTT GTAGAAGATA

---------- ---------- ---------- ---------- ----------

GCAGTTTGCA CACTTCGGCG AAGTTATACT TATAAGATTC GTAGAAGATA

GCAGTTTGCA CACTTTGGTG AAGTTATACT CATAAGATTT GTAGAAGATA

GCCAGTTGCA AATTTTGGTG AAGTTATACT CATAACATTT GTAGAAGATA

GCAGTTTACA AATTTCGGTG AAGTTATACT CATAAGATTT GTGGAAGATA

GCAGTTTACA AATTTTGGTG AAGTTATCCT CATAAGATTT GTGGAAGATA

GCAGTTTACA AATTTTGGTG AAGTTATACT CATAAGATTT GTAGAAGATA

GCAGTTTACA AATTATGGCG AAGTCATACT CATAAGATTT GTAGAAAATA

GCAGTTTGCA AATTTTGGGG AAGTTATACT CATAAGATTT GTGGAAGATA

GCAGTTTGCA AGTTTCGGTG AAGTTATACT CATAAGGTTT GTAGAAGATA

GCAATTTGCA AATTTTGGGG AAGTTATACT TATAAG---- ----------

GGAGTTTGGA AACCATGGTG AAGTTATCCT CATAAGGTTT GTAGAAGACC

GCAATTTGCC AACTATGGTG AAGTCATACT CATCAGGTTT GTAGAAGACA

AAATGTGGGT TACATTTTTG GAGGGAAGCT CTGCCTTGAA TGTTCTGAGC

AAATGTGGGT TACATTTTTG GAGGGAAGCT CTGCCTTGAA TGTTCTGAGC

AAATGTGGGT TACATTTTTG GAGGGAAGCT CTGCCTTGAA TGTTCTGAGC

AAATGTGGGT TACATTTTTG GAGGGAAGCT CTGCCTTGAA TGTTCTGAGC

AAATGTGGGT TACATTTTTG GAAGGAAGCT CTGCCTTGAA TGTTCTGAGC

---------- ---------- ---------- ---------- ----------

AAATGTGGGT TACATTTTTG GAGGGGAGCT CTGCCTTGAA TGTTCTGAGC

AAATGTGGGT TACATTTTTG GAGGGAAGCT CTGCCTTGAA TGCTCTGAGC

AGATGTGGGT TACGTTTCTG GAGGGAAGTT CTGCCTTGAG TGCTCTGAGC

AAATGTGGGT TACGTTTTTA GAGGGAAGCT CTGCCTTGAA TGTTCTGAAC

AAATGTGGGT CACATTTTTG GAGGGAAGCT CTGCCTTGAG TGTTCTGAAC

AAATGTGGGT TACATTTTTG GAGGGAAGTT CTGCCTTGAG TGTTCTAAGC

AAATGTGGGT TACATTTTTG GAGGGCAGCT CTGCCTTGAG TGTTCTCAGC

AAATGTGGGT TACATTTTTG GAAGGAAGCT CTGCCTTGAA TGCTCTGTGC

AAATGTGGGT TACATTTTTG GAGGGAAGCT CTGCCTTGAA TGTTCTGAAC

---------- ---------- ---------- ---------- ----------

AGATGTGGGT AACATTTTTG GAAGGAAGCT CTGCCCTGAA AGTTCTGAAC

AGATGTGGGT AACGTTTCTG GAAGGAAGCT CGGCCTTGAG CGTTCTGAGT

CTAAATGGTA AAGAGTTATT GAATCGGACT ATAACTATTG CTTTAAAAAG

CTAAATGGTA AAGAGTTATT GAATCGGACT ATAACTATTG CTTTAAAAAG

CTAAATGGTA AAGAGTTATT GAATCGGACT ATAACTATTG CTTTAAAAAG

CTAAATGGTA AAGAGTTATT GAATCGGACT ATAACTATTG CTTTAAAAAG

CTAAATGGTA AAGAGTTATT GAATCGGACT ATAACTATTG CTTTAAAAAG

---------- -----TTATT GAATCGGACT ATAACTATTA CTTTAAAAAG

CTAAATGGTA AAGAGTTATT AAATCGGACT ATAACAATTA CTTTAAAAAG

CTAAATGGGA AAGAGCTATT AAATCGGACT ATAACAATTA CTTTAAAAAG

TTAAATGGGA AAGAGTTATT GAATCGGACT ATAACTATTA CTTTAAAAAG

CTGAATGGGA AAGAGTTACT GGGTAGGACA ATAACAATTA CTTTAAAAAG

CTAAATGGTA AAGAGTTATT GGGTCGGACT ATAACTATTA CTTTAAAAAG

CTAAATGGTA AAGAGTTATT GGGTCGGACT ATAACTATCA CTTTAAAAAG

CTGAATGGAA CACAG----- ---------- ---------- ----------

CTAAATGGTA AAGAGTTATT GGGTCGAGCT ATTACCATTA CTTTAAAAAG

CTAAATGGTA AAGAGTTACT GGGTCGGACT ATAACTATCA CTTTAAGAAG

---------- -----TTACT GGGTCGGACT ATAACTATTA CTTTAAAAAG

CTAAATGGTA AAGAGTTTTT AGATCGACTC ATAAGTATTA CCTTAAAAAA

CTACACGGAA CTGAGCTGTT GGGCAGAGTT ATAAATATTA CCTTAAAAAG

TCCAGACTGG ATCAAAAATT TGGAAGAAGA AATGAGTTTA GAGAAAATTA

TCCAGACTGG ATCAAAAATT TGGAAGAAGA AATGAGTTTA GAGAAAATTA

TCCAGACTGG ATCAAAAATT TGGAAGAAGA AATGAGTTTA GAGAAAATTA

TCCAGACTGG ATCAAAAATT TGGAAGAAGA AATGAGTTTA GAGAAAATTA

TCCAGACTGG ATCAAAAATT TGGAAGAAGA AATGAGTTTA GAGAAAATTA

TCCAGACTGG ATCAAAAATT TGGAAGAAGA AATGAGTTTA GAGAAAATTA

TCCAGACTGG ATCAAAACTT TGGAAGAAGA GATGAGTTTA GAGAAAATCA

TCCAGACTGG ATCAAACATT TGGAAGAAGA GATGAGTTTA GAGAAAATCA

TCCAGACTGG ATCAAAAATT TGGAAGAAGA AATGAGTCTA GAGAAAATTA

TCCAGACTGG ATCAAAACTT TGGAAGAAGA AATGAGCTTA GAGAAAATCA

TCCAGACTGG ATCAAAAATT TGGAGGAAGA AATGAGTTTA GAGAAAATTA

TCCAGACTGG ATCAAAACTT TGGAAGAAGA AATGAGTTTA GAGAAAATTA

---------- ---------- ---------- ---------- ----------

TCCAGACTGG ATCAAAACTT TGGAAGAAGA AATGAGTTTA GAGAAAATTA

CCCAGACTGG ATCAAAACTT TGGAGGAAGA AATGAGTTTG GAGAAAATGA

TCCAGACTGG ATCAAGACTT TGGAAGAAGA AATGAGTTTG GAGAAAATTA

CCCAAACTGG ATTGCTGATT TGGAGAAAGA AATAAATTTG GAGAAAATTA

CCCGGACTGG ATCGCCAATC TGGAAGAAGA AATGAGTCTG GAGAAGATCA

GCATTGCATT GCCATCATCA ACAAGCTCTA CCCTGCTTGG TGAAGATGCA

GCATTGCATT GCCATCATCA ACAAGCTCTA CCCTGCTTGG TGAAGATGCA

GCATTGCATT GCCATCGTCA ACAAGCTCTA CCCTGCTTGG TGAAGATGCA

GCATTGCATT GCCATCGTCA ACAAGCTCTA CCCTGCTTGG TGAAGATGCA

GCATTGCATT GCCATCATCA ACCAGCTCTA CCCTGCTTGG TGAAGATGCA

GCATTGCGTT ACCATCATCA ACAAGCTCTA CCCTGCTTGG TGAAGATGCC

GTGTCACATT GCCCTCATCA ACCAGTTCCA CCCTGCTCGG TGAAGATGCA

GTGTCACGTT GCCCTCATCA GCAAGCTCCA CCCTGCTTGG TGAAGACGCA

GCATTGCTTT GCCAACATCA ACAAGTTCTA CCCTGCTTGG TGAAGATGCA

ACGTCCCCTT GCCATCATCA ACCAGCTCCA CCCTCCTAGG TGAAGACGCG

ATGTTGCATT GCCATCATCG ACAAGCTCCA CCCTGCTTGG TGAAGATGCG

GTGTTCCGTT GCCGTCATCA ACAAGCTCTA CCCTGCTCGG TGAAGATGCG

---------- ---------- ---------- ---------- ----------

GTGTTCCGTT GCCATCATCA ACAAGCTCTA CCCTGCTGGG TGAAGATGCA

GCGTACCATT GCCGTTGTCA ACGAGCTCCA CCCTGCTTGG CGAAGATGCC

GTGTGTCATT ACCATCATCA ACAAGCTCTA CTCTGCTTGG CGAAGATGCG

GTATTACAGT GCCACCATCA GCTAGTTCAA CTTTGCTTGG TGAAGATGCA

GCGTCACGTT GCCCGCCTCC TCCAGCTCCA CTCTGCTGGG CGAAGACACC

GAGGTTGCAG CAGATTTTGA TATGGAAGGT GATGTTGATG ACTATAGTGC

GAGGTTGCAG CAGATTTTGA TATGGAAGGT GATGTTGATG ACTATAGTGC

GAGGTTGCAG CAGATTTTGA TATGGAAGGT GATGTTGATG ACTATAGTGC

GAGGTTGCAG CAGATTTTGA TATGGAAGGT GATGTTGATG ACTATAGTGC

GAGGTTGCCG CAGATTTTGA TATGGAAGGT GATGTTGATG ACTACAGTGC

GAGGTCACAG CAGATTTTGA TATGGAAGGT GATGTTGATG ACTACAGTGC

GAGGTCTCAG CGGACTTTGA CATGGAAGGT GATGTCGACG ACTACAGTGC

GAGGTCGCAG CAGACTTTGA CATGGAAGGT GACGTCGATG ACTACAGCGC

GAGGTCACAA CAGATTTCGA CATGGAAGGT GATGTTGATG ACTACAGTGC

GAGGTCACGG CCGACTTCGA TATGGAAGGT GATGTGGATG ACTATAGTGC

GAGGTCACTG CAGATTTTGA TATGGAAGGT GATGTCGATG ACTACAGTGC

GAGGTCGCAG CAGATTTTGA TATGGAAGGG GACGTTGATG ACTATAATGC

---------- ---------- --------GC GACGTCGATG ACTATAGTGC

GAGGTCTCAG CAGATTTCGA CATGGAAGGT GATGTCGATG ACTATAGTGC

GAGGTGACGG CAGATTTCGA CATGGAAGGT GATGTTGATG ACTACAGTGC

GAGGTCGCAG CAGATTTTGA TATGGAAG-- ---------- ----------

GAAGTTGCTA CAGATTTTGT TATGGAAGGT GATGTTGATG ATGAGAGTGC

GAAGTGGCTA CGGATTTCGA TATGGAAGGA GACATCGATG ACGACAATGT

TGAAGTGGAG GAACTTCTTC CTCAGCATCT CCAGCCATCT TCAAGTTCCG

TGAAGTGGAG GAACTTCTTC CTCAGCATCT CCAGCCATCT TCAAGTTCCG

CGAAGTGGAG GAACTTCTTC CTCAGCATCT CCAGCCATCT TCAAGTTCCG

CGAAGTGGAG GAACTTCTTC CTCAGCATCT CCAGCCATCT TCAAGTTCCG

TGAAGTGGAG GAGCTTCTTC CCCAGCATCT CCAGCCATCT TCAAGTTCCG

AGAAGTGGAG GAAATTCTTC CTCAGCATCT CCAGCCATCA TCAAGTTCTG

TGAGGTGGAG GAGCTTCTTC CTCAGCACCT CCAGCCATCC TCCAGCTCTG

TGAAGTAGAG GAGCTTCTTC CTCAGCATCT GCAGCCGTCC TCAAGTTCTG

AGAAGTAGAG GAGCTGCTTC CTCAGCATCT CCAGCCCTCC TCAAGTTCTG

TGAGGTAGAG GAGATCCTTC CTCAGCATCT CCAGCCGTCT TCCAGTTCTG

TGAAGTGGAG GAAATTCTTC CCCAGCACCT CCAGCCATCT TCAAGTTCTG

TGAAGTAGAG GAAATTCTTC CTCAGCATCT CCAGCCATCT TCAAGTTCTG

CGAAGTGGAG GAAATTCTTC CTCAGCATCT CCAGCCCTCC TCCAGCTCTG

TGAAGTAGAG GAAATTCTTC CCCAGCATCT CCAGCCATCT TCAAGTTCTG

AGAAGTAGAG GAAATCCTTC CTCAGCATCT CCAGCCGTCT TCAAGCTCTG

---------- ---------- ---------- ---------- ----------

AGAAGTAGAA GAAATTCTTC CTCAGCATCT ACAGCCAACT TCAAGCTGTG

GGAAGTTGAA GAGAGTCTAC CTCAGCACCT GCAGCCGTCC CCCGGCTCTG

GCCTTGGTAC TTCCCCCAGC TCTTCACCCC GAACTAGTCC CTGCCAGTCA

GCCTTGGTAC TTCCCCCAGC TCTTCACCCC GAACTAGTCC CTGCCAGTCA

GCCTTGGTAC TTCCCCCAGC TCTTCACCCC GAACTAGTCC CTGCCAGTCA

GCCTTGGTAC TTCCCCCAGC TCTTCACCGC GAACTAGTCC CTGCCAGTCA

GTCTTGGCAC TTCCCCCAGC TCTTCACCCC GGACTAGTCC CTGCCAGTCA

GCCTTGGCAC TTCCCCAGGC TCCTCACCCC GGACTAGTCC CTGCCAATCA

GCCTCGGCAC CTCTCCAAGC TCTTCGCCCC GGACCAGTCC CTGCCAGTCA

GCCTGGGCAC TTCTCCAAGT TCTTCACCCC GGACCAGTCC CTGCCAGTCC

GCCTCGGCAC TCCCC----- ---------- ---------- ----------

GCCTTGGCAC GTCCCCCGGT TCTTCACCCC GGACCAGTCC CTGCCAGTCA

GCCTGGGCAC TTCCCCAGGC TCTTCACCCC GGACCAGTCC CTGCCAGTCA

GCCTCGGCAC TTCCCCGAGC TCTTCACCCC GTACGAGTCC CTGCCAGTCA

GCCTTGGTAC CTCGCCGAGC TCTTCA-CCC GGACCAGCCC CTGCCAGTC-

GCCTTGGCAC TTCCCCAAGC TCTTCACCCC GGACCAGTCC CTGCCAGTCT

GCCTGGGTGC TTCGCCAAGC TCTTCACCCC GGACCAGTCC CTGCCAGTCA

---------- ---------- ---------- ---------- ----------

GTCTTGGAAC TTCCCCGAGC TGTTCACCTC GGTCCAGTCC CTGCCAGTCC

GCCCCGGGAC CTCGCCGAGC TCGTCGCCTC GCTCCAGTCC CTGCCAGTCG

CCTACAATAT CAGAGGGTCC TGTACCTTCC CTTCCCATCA GACCAAGCCG

CCTACGATAT CAGAGGGTCC TGTACCTTCC CTTCCCATCA GACCAAGCCG

CCTACGATAT CAGAGGGTCC TGTACCTTCC CTTCCCATCA GACCAAGCCG

CCTACGATAT CAGAGGGTCC GGTACCTTCC CTTCCCATCA GACCAAGCCG

CCTACAATAT CAGAGGGTCC TGTACCTTCC CTTCCCATAA GACCAAGCCG

CCTACTGTGC CAGAGGTTCC TGTACCTTCT CTTCCCGTCA GACCAAGCCG

CCCACAGCAC CAGAGTACTC CGCACCTTCT CTTCCCATCA GACCTAGTCG

CCCACAGTAC CAGAGTACTC CGCCCCTTCT CTTCCCATCA GACCTAGCCG

---------- ---------- ---------- ---------- ----------

CCTACCATAT CGGAGGGGCC TGTGCCTTCC CTCCCAGTGA GACCAAGTCG

CCGACCATAT CGGAGGGCCC TGTACCTTCC CTTCCTGTAA GACCAAGCCG

CCTACAACTT CAGAGGGTCC TG------CC CTCCCCATAA GACCAAGTCG

CCCACAATA- ---------- ---------- ---------- ----------

CCTACCATAA CTGAGGGGCC TGTGCCTTCC CTTCCTGTAA GGCCAAGTCG

CCTACCATAG CAGAGGCTCC TGTACCGCCC CCTCCTGTAA GACCAAGCAG

---------- ---------- ---------- ---------- ----------

CCTACGATAG CTGATGTTCT GCCCCCTCCT CTTCCTGCCA GACCAACCCG

CCTACGCTGT CGGACGGTCC CGCGCCGCCC CTCCCGGTCA GGCCGAGCCG

ATCAAGAACT CCTGGGCCTC CCAGTGCACA GAGTTCTCCT ATTGACGCGC

ATCAAGAACT CCTGGGCCTC CCAGTGCACA GAGTTCTCCT ATTGACGCGC

ATCAAGAACT CCTGGGCCTC CCAGCGCACA GAGTTCACCT ATTGACGCGC

ATCAAGAACT CCTGGGCCCC CCAGTGCACA GAGTTCTCCT ATTGACGCGC

ATCAAGAACT CCGGGGCCTC CAAGTGCACA GAGTTCTCCT ATTGACGCAC

ATCAAGAACT CCTGGACCTC CAAGTGCACA GAGTTCTCCT GTTGACGTTC

ATCAAGAACT CCAGGACCTC TAAGTTCACA GGGTGCTCCA GTTGACACCC

ATCAAGAACT CCAGGACCTC CAAGTTCGCA GGGCTCTCCA GTTGACACTC

---------- ---------- ---------- ---------- ----------

ATCCAGAACG CCCGGGCCCC CTGCTTCACA AAGTTCTCCT GTTGACACTC

ATCAAGAACT CCTGGGCACC CGAGCTCACA AAGTTCTCCT GTCGACACTC

ATCAAGAGTT CCTGGGCACC CAACTTCACA AAGTTCTCCT AGTGACACTC

---------- ---------- ---------- --GTTCGCCT GTAGACAGTG

ATCAAGAATG CCTGGGCCTC CAAGTGCACA AAGTTCTACT GTTGACATTC

ATCAAGAACT CCGGGACCTC CAAGTTCACA AAGTTCTCCT GCCGACTCTC

---------- ---------- ---------- ---------- ----------

ATCAAAAACT TCTACACTTC CACCTTCACA GAATTCTCCT AGTGACACTC

ATCGAGAACC CCCGGCCCTC CAGCTTCTCA GACCTCCCCC GCAGACCTCC

AGCCAGCACA GAAAGACCCC GCCCAGCCCT TGGAGCCCAA gcggccgccg

AGCCAGCACA GAAAGACCCC GCCCAGCCCT TGGAGCCGAA gcggccgccg

AGCCAGCACA GAAGGACCCC GCCCAACCCT TGGAGCCCAA GCGGCCGCCA

AGCCAGCACA GAAGGACCCC GCCCAACCCT TGGAGCCCAA GCGGCCGCCA

AGCCAGCACA GAAAGACCCC GCCCAGCCCT TGGAGCCCAA GCGGCCACCG

AACCAGCACA GAAAGATCCT GCCCAGCCCC TGGAGCCCAA GAGACCACCA

AGCCAG--CA GAAGGAGTCT TCCCAGACCA TAGAGCCCAA GAGGCCACCC

AGCCAG--CA GAAGGATTCT TCCCAGACTC TAGAGCCCAA GAGACCACCC

---------- ---------- ---------- ---------- ----------

TGCCGGCACA GAAAGATTCT TCCCAGACCC TGGAACCCAA GCGGCCTCCC

TCCCAGCACA AAGAGATTC- --CCAGCCCC TTGAGCCCAA ACGGCCACCT

TGCCAGCCCA AAAAGATTCT TCCCAGATCC TGGAGCCCAA GCGGCCACCT

TGCCAGGACA GAAGGATGCT GCCCAGGCTC TGGAGCCCAA GCGTCCCCCT

AGTCAGCCCA GAAAGACTCT TCTCAGACCC TCGAGCCCAA GCGGCCTCCT

AGCCAGCACA AAAAGATTCT TCTCAGACCT TAGAGCCCAA ACGGCCTCCT

---------- ---------- ---------- ---------- ----------

TACCAGTAAC GAAAGAAGCT GGCTTTGTTT TGGAGCCTAA ACGTCCTCCT

AGCAAGTGCA GAAAGATCTC ACCCCGTCCT TAGAGCCTAA GCGCCCTCCT

ccgccccgcc cggTCGCCCC TCCCACACGC CCGGCTCCCC CACAGAGACC

ccgccccgcc cggTCGCCCC TCCCACACGC CCGGCTCCCC CACAGAGACC

CCGCCCCGCC CTGTTGCCCC TCCCACACGC CCGGCTCCCC CACAGAGACC

CCACCCCGCC CTGTTGCCCC TCCCACACGC CCGGCTCCCC CACAGAGACC

CCGCCCCGCC CAGTTGCTCC TCCCACACGC CCCGCTCCCC CACAGAGACC

CCTCCCCGCC CAGTGGCTCC TCCCACCCGT CCTGCTCCCC CACAGAGGCC

CCTCCCCGCC CAGTTGCTCC TCCAGCACGT CCTGCCCCAC CACAGAGGCC

CCTCCCCGCC CAGTCGCTCC TCCGGCACGT CCTGCCCCAC CACAGAGACC

---------- ---------- ---------- CCAGCGCCCC CACAGAGACC

CCTCCCCGCC CGGTTGCTCC TCCTGCACGT CCAGCTCCTC CACAACGACC

CCTCCCCGCC CGATTGCTCC CCCCGCACGC CCTGCGCCCC CCCAGAGACC

CCTCCCCGCC CAGTCGCTCC TCCCACACGT CCAGCTCCCC CACAAAGACC

CCTCCCCGCC CGGTTGCTCC TCCAGCCCGG CCTGCTCCCC CGCAGAGACC

CCTCCCCGCC CGATTGCTCC TCCAGCACGT CCAGCTCCCC CCCAGAGACC

CCTCCCCGCC CAGTTGCTCC TCCTACGCGT CCAGCTCCCC CACAAAGACC

---------- ---------- ---------- ---------- ----------

CCTCCTCGAC CAGTTGCCCC ACCTGCACGT CCAGCTCCAC CACAAAGACC

CCTCCTCGTC CGGGAGCTcc gcccgcccgc ccggccccgc cgcAGAGGCC

TCCTCCGCCT TCAGGGGCTA GGAGTCCTGC ACCCACTAGA AAGGAATTTG

TCCTCCGCCT TCAGGGGCTA GGAGTCCTGC ACCCACTAGA AAGGAATTTG

TCCTCCGCCT TCAGGGGCCA GGAGTCCTGC ACCCGCTAGA AAGGAATTTG

TCCTCCGCCT TCAGGGGCCA GGAGTCCTGC ACCCGCTAGA AAGGAATTTG

TCCTCCGCCT TCAGGGGCTA GAAGTCCTGC ACCCGCTAGA AAAGAATTTG

ACCTCCACCT TCAGGGGCTA GGAGTCCTGC ACCTGCTAGA AAAGAATTTG

ACCTCCACCT TCAGGGGCTA GGAGTCCGGC ACCTGCTAGA AAAGAATTTG

ACCTCCACCT TCAGGGGCTA GGAGTCCTGC GCCTGCTAGA AAAGAATTTG

ACCTCCGCCT TCAGGGGCTA GGAGTCCTGC ACCTGCTAGA AAAGAATTTG

ACCTCCGCCT TCAGGGGCTA GGAGTCCTGC ACCTGCTAGA AAAGAGTTTG

ACCTCCACCT TCAGGGGCTA GGAGTCCTGC ACCTGCTAGA AAAGAATTCG

ACCTCCACCT TCAGGGGCTA GGAGTCCTGC ACCTGCTAGA AAAGAATTTG

ACCTCCGCCT TCAGGGGCTA GGAGTCCTGC ACCTGCTAGA AAAGAATTTG

ACCTCCGCCT TCAGGGGCTA GGAGTCCTGC ACCTGCTAGA AAAGAATTTG

ACCTCCACCG TCAGGGGCTA GGAGTCCTGC ACCTGCTAGA AAAGAACTTG

---------- ----GGGCTA GGAGTCCTGC ACCTGCTAGA AAAGAATTTG

ACCCCCTCCT TCAGGGAATA GGAGTCCTGC ACCTTCTAGA AAGGAATTTG

GCCTCCGCCT TCAGGAGGTA GGAGTCCTGC CCCCGCTAGA AAAGAATTTG

GAGGTATTGG AGCCCCTCCC AGTCCTGGGG TAGCTAGGAG AGAGATGGAA

GAGGTATTGG AGCCCCTCCC AGTCCTGGGG TAGCTAGGAG AGAGATGGAA

GAGGTATTGG AGCCCCTCCC AGTCCTGGGG TAGCTAGGAG AGAGATGGAA

GAG------- ---------- ---------- ---------- ----------

CAGGTATTGG AGCCCCTCCC AGTCCTGGGG TAGCTAGGAG AGAGATGGAA

GAGGTACAGG AGCCCCTCCC AGTCCTGGGG TGGCTAGGAG AGAGATGGAA

GAGGTGTCGG AGCCCCTCCC AGTCCCGGGG TTACTAGGAG AGAGATGGAA

GAGGTGTTGG AGCCCCTCCC AGTCCCGGGG TTGCTAGGAG AGAGATAGAA

GAGGTATTGG AGCCCCTCCC AGTCCTGGGG TGGCTAGGAG AGAGATGGAA

GAGGTGTTGG AGCCCCTCCC AGTCCGGGGG TAGCTAGGCG AGAGATGGAA

GAGGTGTTGG AGCCCCTCCC AGTCCTGGGG CAGCTAGGCG AGAGATGGAA

GAGGTATTGG AGCCCCTCCC AGTCCTGGGG TAGCTAGGCG AGAGATGGAA

GAGGTGTTGG AGCCCCTCCC AGTCCCGGGG TAGCTAGGCG AGAGATGGAA

GAGGTGTTGG AGCCCCTCCC AGTCCTGGGG TAGCTAGGAG AGAGATGGAA

GAGGTGTAGG GGCCCCTCCC AGTCCTTGGG TAGCTAGGAG ACAGATGGAG

GAGGTGCTGG AGCCCCCCCC AGTCCTGGGG TAGCTAGGAG AGAGATGGAA

GAGGTCTTGG AGGCCCTCCC AGCCCTGGGG TATCTAAGAA AGATATAGCA

GAGGTCTGGG AGCCCCACCG AGCCCTGGGG CGGCTAGGAG AGAGATGGAA

GCACCCAAAA GCCCTGGAAC AACAAGGAAA GATAATATAG GACGCAGTCA

GCACCCAAAA GCCCTGGAAC AACAAGGAAA GATAATATAG GACGCAGTCA

GCACCCAAAA GCCCTGGAAC AACACGGAAA GATAACATAG GACGCAGTCA

-CACCCAAAA GCCCTGGAAC AACACGGAAA GATAACATAG GACGCAGTCA

GCACCCAAAA GCCCTGGAAC AACACGGAAA GATA---TAG GACGCAGTCA

GCACCCAAAA GCCCTGGAAC AACACGGAAA GATAATATAG GACGCAATCA

GCACCCAAAA GCCCGGGAAC AGCACGGAAA GATAATATAG GGCGTAATCA

GCACCCAAAA GCCCTGGAAC AGCACGGAAA GATAATATAG GGCGTAATCA

GCACCCAAAA GCCCTGGGAC AACACGGAAG GATAATATAG GACGCAATCA

GCACCCAAAA GCCCAGGAAC GACCCGGAGA GATAATCTAG GACGCAGCCA

GCACCCAAAA GCCCTGGAAC AACACGGAAA GATACTATAG GACGCAATCA

GCACCCAAAA GCCCTGGAAC AACACGGAAA GATAATATAG CCCGCAATCA

GCTCCCAAAA GCCCCGGAGT GACGCGGAAA GATAATATAG GACGCGGTCA

GCACCCAAAA GCCCTGGAAT GACACGGAAA GATAATATAG GACGTAATCA

GCACCCAAAA GCCCTGGAAT GACACGGAAA GATA---TAG GACGCAGTCA

GCACCCAAAA GCCCTGGAAT GTCACGGAAA GATAATATAG GGCGTAATCA

GCACCAAAAA GCCCTGGAAC AATAAAAAAA GATAGTTCAG GACAAAATGA

GCCCCCAAAA GCCCTGGGAC TTCAAGGAAA GAGAATTTAG GTCGAAATCA

GCCTTCACCT CAAGCAGGAC TTGCAGGCCC AGGACCTGCT GGATACAGTA

GCCTTCACCT CAAGCAGGAC TTGCAGGCCC AGGACCTGCT GGATACAGTA

GCCTTCACCT CAAGGAGGAC TTGCAGGCCC AGGACCTGCT GGATacagta

GCCTTCACCT CAAGGAGGAC TTGCAGGCCC AGGACCTGCT GGATacagta

GCCTTCACCT CAAGCAGGAC TTGCAGGCCC AGGACCTGCT GGATACAGTA

GCCTTCACCT CAAGCAGTAC TTGCAGGCCC GGGACCTGCT GGATATAGTg

GCCTTCCCCT CAAGCCGGAC TTGCAGGCCC AGGACCTTCT GGATATGGTG

GCCTTCCCCT CAAGCCGGAC TTGCAGGCCC AGGACCCGCT GGATACGGTG

GCCTTCCCCT CAAGCAGGAC CCCCAGGGCC AGCACCTGCT GGGTACAGTA

GCTTCCACCT CAAGCCGGAC TGCCAGGCCC GGGACTTGCT GGACACAGTG

GCCTTCACCT CAAGCAGGAT TCACAGGCCC AGGACCTGCT GGATACAGTG

GCCTTCACCC CAAGCAGGAC TCACAGCCCC AGGACCTACT GGATACAGTG

GCCTTCCCCT CAAGCAGGAC TTGCGGGCCC AGGATCTGCT GGATACACTA

GCCTTCACCT CAAGCTGGAA CCATAGGCCC AGGATCTACT GGATACAGTC

GCCTTCACCT CAGGCAGGAC TTGCAGGCCC AGCCCCTGCT GGATACAGTG

GCCTTCCCCT CAAGCAGGAC TCTCAGGCCC AGTGCCTGCT GGAATCAGTG

GTCCTTTCCT CAAGGAGGAT CAGTAAGCCC AGGAACTGTT GGAAGCAGTG

GCCTTCACCC ACAGGAGGAT ACCCTAGCGC AGGAGCCACC GGCTACGGTG

cagccagacc gACGATTCCT CCTCGTGCTG GAGTTATCAG TGCCCCACAG

cagccagacc gACGATTCCT CCTCGTGCTG GAGTTATCAG TGCCCCACAG

cagccagacc gATGATTCCT CCTCGTGCTG GAGTTATCAG TGCCCCACAG

cagccagacc gATGATTCCT CCTCGTGCTG GAGTTATCAG TGCCCCACAG

cagccagacc gACGATTCCT CCTCGTGCTG GAGTTATCAG TGCCCCACAG

cagccagacc aACAATTCCA CCCCGTGCTG GAGTTATCAG TGCCCCCCAG

CAGCTAGACC GACAATTCCA GCTCGTGCTG GAGTGATCAG TGCCCCTCAG

CGGCTAGACC GACAATTCCA GCTCGTGCTG GAGTGATCAG CGCCCCTCAG

cagccagacc gACAATTCCT CCTCGTGCTG GAGTCATCAG TGCCCCCCAG

CAGCCAGACC GATTATTCCG CCCCGTGCTG GAGTCATCAG CGCCCCCCAG

CAGCCAGACC GACTGTTCCA CCCCGTGCAG GAGTGATCAG TGCCCCCCAG

CAGCCAGACC GACCATTCCG CCCCGCGCTG GCGTGATCAG CGCTCCGCAG

TGGCCAGACC GACTATTCCA CCCCGTGCTG GTGTGATCAG CGCCCCCCAG

CAGCCAGACC GATTATTCCA CCCCGTGCTG GAGTTATTAG CGCCCCACAG

CAGCCAGACC GACGATTCCG CCCCGTGCTG GAGTTATTAG TGCCCCACAG

CCCCCAGACC G--------- ---------- ---------- ----------

TAGCCAAACC GGCTATCCCA CCCCGTGTTG GAATTATTAG CGCACCACAA

CAGCACGGCC GATCATTCCA CCTCGGGCCG GAGTTATTAG CGCGCCCCAA

AGCCACGCGC GGGCATCTGC TGGAAGACTG ACTCCTGAAA GCCAAAGCAA

AGCCACGCGC GGGCATCTGC TGGAAGACTG ACTCCTGAAA GCCAAAGCAA

AGCCATGCGC GGGCATCTGC TGGAAGACTG ACTCCTGAAA GCCAAAGCAA

AGCCATGCGC GGGCATCTGC TGGAAGACTG ACTCCTGAAA GCCAAAGCAA

AGCCAGGCAC GGGCATCTGC TGGAAGACTG ACTCCTGAAA GCCAAAGCAA

AGCCATGCTC GGGCAGCTGC TGGAAGACTG ACTCCTGAAA GCCAAAGCAA

AGCCAGGCTC GGGTGTCTGC TGGAAGGCTG ACTCCTGAAA GCCAAAGCAA

AGCCAGGCTC GGGTATGTGC TGGAAGGCCG ACTCCTGACA GCCAAAGCAA

AGCCATGCAC GGGCTGCAGC TGGAAGATTG ACTCCTGAAA GCCAAAGCAA

AGCCACGCAC GGGTGTCTGC TGGGAGACTG ACTCCTGAAA GCCAAAGCAA

AGCCATGCTC GGGCATCTGC TGGAAGATTG ACTCCTGAAA GCCAAAGCAA

AGCCATGCTC GGGCATCTGC TGGAAGACTG ACTCCTGAAA GCCAAAGCAA

AGCCAGGCTC GAGCATCTGC CGGTCGACTC ACCCCCGAAA GCCAAAGCAA

AGCTATGCTC GGGCATCTGC TGGAAGACTG ACTCCTGAAA GCCAAAGCAA

AGCCACGCCC GGGCATCTGC TGGAAGACAG ACTCCCGAAA GCCAAAGCAA

---------- ---------- ---------- ---------- ----------

ACCCTTTCCC GTGCTACTAC TGGAAAGATC GCCACTGAAG CCCCAAATAA

AGCCAAGCCC GGGTCTCCGC CGGAAGGTTG ACTCCTGAAA ACCAAAGCAA

AACATCAGAA ACGTCGAAAG GTTCAACTTT CCTTCCTGAA CCACTGAAGC

AACATCAGAA ACGTCGAAAG GTTCAACTTT CCTTCCTGAA CCACTGAAGC

AACATCAGAA ACGTCGAAAG GTTCAACTTT CCTTCCTGAA CCACTGAAGC

AACATCAGAA ACGTCGAAAG GTTCAACTTT CCTTCCTGAA CCACTGAAGC

AACATCAGAA ACATCAAAAG GTTCAACTTT CCTTCCTGAA CCACTGAAGC

GCCGGCAGAA GCAGCCAAAA GCTCAGCTTT CCTTCCCGAA CCACTGAAGC

ACCCTTGGAG ACATCGAAAG GTCCTGCTGT CCTTCCAGAA CCACTGAAGC

GCCCTCGGAG ACATTGAAAG GTCCTGCTGT CCTTCCAGAA CCACTGAAGC

GCCGTCAGAA GCCCCAAAAG GTTCCTCTCT CCTTCCAGAA CCGCTGAAGC

AACCTCAGAA GTACTGAAAG GGCCAGCTCT TCTTCCCGAG CCCCTGAAGC

ACCATCAGAA GTCCCAAAAG GTCCAGTTCT TCTTCCTGAA CCACTGAAGC

ACCATCAGAA GTACTGAAAG GTTCACCTCT TCTTCCTGAA CCCCTGAAGC

AGCCTCAGAA GGACCGAAAG GTTCAGCTTT CCTTCCTGAA CCGCTGAAGC

ACCATCTGAA GCACCAAAAG GTTCAGCACT CCTTCCAGAA CCACTGAAGC

ACCCTCCGAA GCACCAAGAG GTTCAGCTTA CCTTCCTGAA CCACTGAAGC

---------- ---------- GTTCGGCATA CCTTCCTGAA CCACTGAAGC

ATCATCTCAA GCACCAAAAG GTTCATCTTT ACTCCCTGAA CCATTAAAGC

ATCGTCAGAA CCGCTGAGAG GGTCATTTTC CCTTCCCGAA CCACTGAAGC

CTCAGGCTGC TTTTCCTCCG CAGTCTTCTT TGCCCCCGCC TGCTCAAAGG

CTCAGGCTGC TTTTCCTCCG CAGTCTTCTT TGCCCCCGCC TGCTCAAAGG

CTCAGGCTGC TTTTCCTCCA CAGTCTTCTT TGGCCCCGCC TGCTCAAAGG

CTCAGGCTGC TTTTCCTCCA CAGTCTTCTT TGGCCCCACC TGCTCAAAGG

CTCAGGCTGC TTTTCCTCCA CAGTCTTCTT TGCCCTCACC TGCTCAAAGG

CTCAGGCTGC TTTTCCTCCT CAGTCTTCTC TGCCCCTACC TGCTCAAAGG

CTCAGGCTGC ATTTCCTCCG CAGCCTTCTC TGCCCACACC TGCTCAAAAG

CTCAGGCTGC ATTTCCTCAG CAGCCTTCTC TGCCCACACC TGCTCAAAAG

CACAGGCTGC CTCTCCTG-- ---------- TGCCCCCACC TGCTCAGAAG

CTCAGGCCGC CCTTCCTGTG CCGccttctc tgccaccacc tGTTCAGAAG

CTCAGGCTGC CCTTCCTGTG CAGGCTTCTC TGCCCCCACC TGTTCAGAAG

CTCAGGCTGC CCTGCCTGTG CAGCCTACTC TGCCCCCACC TGTTCAAAAG

CTCAGGCAGC CGTTCCTGTG CAGCCTTCTC TCCCACCGCC GGGCCAGAAG

CTCAGACTCC TCTTCCTGTG CAGCCTTCTC TGCCTCCACC TGTTCAAAAG

CTCAGGCTGC CCTTCCCGGG CAGCCTTCTC TGCCCCAACC TGTTCAAAAG

CTCAGACTGC CCCTCCTGTG CAGCCTTCTC TGCCCGCACC TGTTCAGAAG

CTCAGACCGT TGCTCCTGCC CCACCTTCCT CTTCTTCATC TATTCAGAAG

CCCAGGCCGC GCCTGCCGTC CAATCCACCG CTCCTCCGGC GGCCCAGAAG

TTGCAAGAGC CTCTTGTCCC TGTGGCAGCA CCTATGCCTC AGTCTGGCCC

TTGCAAGAGC CTCTTGTCCC TGTGGCAGCA CCTATGCCTC AGTCTGGCCC

TTGCAGGAGC CTCTTGTCCC TATGGCAGCA CCTATGCCTC AGTCTGGCCC

TTGCAGGAGC CTCTTGTCCC TATGGCAGCA CCTATGCCTC AGTCTGGCCC

TTGCAGGAGC CCCTTGTCCC TACGGCAGCA CCTATGCCTC AGTCTGGCCC

TTGCAGGAGC CACTTGTCCC TGTGACAGCA CCTATGCCTC AATCTGCCCC

TTACAGGACC CACTTGTCCC CATAGCAGCA CCTATGCCTC CCTCCATTCC

TTACAGGACC CGCTTGTCCC CATAGCAGCA CCTATGCCTC CCTCTGGCCC

TTGCAGGAGC CTCTCATCCC TGTGGCAGCA CCTATGCCTC CATCTGGCCC

ATGCAGGAGC CTCTCATCCC GGTGGCCGCA CCTCTGGCCC AGGCTGCCCT

ATGCAGGAGC CTCTTGTCCC TGTGGCAGCA CCTTTGCCTC AGTCTGCCCC

ATGCAGGAGC CTCTTGTCCC TACTGCAGCG CCTATGCCTC CGGCTGCCCC

ATGCAGGAGC CCCTGGTCCC CACGGCAG-- ---------C AGCA------

ATGCAGGAGC CTCTTGTTCC TATGACAGCA CCTCTGCCTC AGTCTGCCCC

TTGCAGGAGC CTCTTGTCCC TGTGACAGCA CCTCTGCCTC AGTCTGTACC

ATGCAGGAGC CTCTTGTCCC GATGACAGCA CCTTTGCCTC AGTCGGCCCC

ATGCAGGAGC CTCTCATTCC TATGACTTCA TCCATCCCTC AACCCACTTC

ATGCAGGAGC CCATGGTCCC GACGGCCGCG TGTCCGCACC AGTCCGGCCC

CCAGCCAAAT TTGGAAACCC CACCACAACC ACCACCTCGA AGCAGGTCAT

CCAGCCAAAT TTGGAAACCC CACCACAACC ACCACCTCGA AGCAGGTCAT

CCAGCCAAAT TTGGAAACCC CACCACAACC ACCACCTCGA AGCAGGTCAT

CCAGCCAAAT TTGGAAACCC CACCACAACC ACCACCTCGA AGCAGGTCAT

CCAGACAAAT TTGGAAACCC CACCACAACC ACCACCTCGA AGCAGGTCAT

CCAGCCAAAT TTGGAAACCC CGCCACAACC ACCTCCTCGG AGCAGGTCAT

CCAATCGAAT TTGGAAACTC CACCCCTGCC CCCACCTCGG AGCAGGTCAT

CCAACCAAAT TTGGAAACCC CCCCACAGCC CCCACCTCGG AGCAGGTCAT

CCAGCCAAAC TTGGAAGCCC CTCCACAGCC ACCACCGCGG AGCAGGTCGT

GCAGCCCAGC CTGGAAACGC CCCCGCAGCC ACCCCCTCGG AGCAGGTCGT

CCAGCCGAAC TTGGAAACCC CGCCACAGCC ACCCCCTCGG AGCCGGTCGT

TCAGCCAAAC TTGGACACCC CCCCACAAGC GCCACCTCGG AGCAGGTCGT

-CAGCCTAAC CTGGACACGC CCCCTCAGCC GCCCCCTCGG AGCAGGTCCT

CCAGCCAAAT TTGGAAACCC CACCACAACC ACCACCTCGT AGCAGGTCAT

CCAGCCAAGT TTGGAAACTC CCCCTCAACC ACCGCCTCGA AGCAGGTCAT

ACAGCCAGTT TTGGAAACTC CACCCCAGCC ACCGCCTCGA AGCAGGTCAT

TCAACAAACC CTTGAAGCCC CACCACAGCC ACCACCTCGT AGCAGGTCAT

CGCCGCCAAC CTGGACGCGC CCCCTCTGCC GCCGCCCCGC AGCCGATCCT

CCCATAGCTT GCCTTCAGAA GCTTCCTCAC AACCGCAAGT AAAAACAAAT

CCCATAGCTT GCCTTCAGAA GCTTCCTCAC AACCGCAAGT AAAAACAAAT

CCCATAGCTT GCCTTCAGAA GCTTCCTCAC AACCGCAAGT AAAAACAAAT

CCCATAGCTT GCCTTCAGAA GCTTCCTCAC AACCGCAAGT AAAAACAAAT

CTCATAGCTT GCCCTCAGAA GCTTCCTCAC AACCACAGGT AAAAACAGAT

CCCATAGCTT GCCTTCAGAA GCTTCTTCAC AGCTGCAAGT AAAAACAAAT

CTCAAAGCTT GCCTTCAGAC TCCTCACCAC AGCTACAAGT AAAAATAAAT

CTCAAAGCTT GCCTTCAGAC TCCTCACCAC AGCTGCAGGT AAAAATAAAT

CCCACAGCTT GCCTTCAGAC TCTTCGCCAC AACTGCAGAT AAAAACAAAT

CCCACAGCTT GCCTTCTGAG CCTCCGGCGC AGCCGCAGGT GAAAACAAAC

CCCACAGCTT GCCTTCAGAG CCTTCATCAC AACCACAGGT AAAAACAAAT

CCCACAGCTT GCCTTCAGAA CCTTCACCGC AGCCGCAAGT GAAAACAAAT

CGCACAACCT GCCTTCCGAG CCCTCGGCCC AGCCACAGGC AAAGACAAAC

CCCATAGCTT GCCTTCAGAA TCTTCACCAC AGCAACAAGC AAAAACCAAT

CCCATAACTT GCCTTCAGAG TCTTCCTCAC AACAGCAAGT AAAAATAAAT

CCCATAACCT CCCTTCAGAA ACTTCCTCAC AACAGCAA-- ----------

CCCACAATTT GCCCCCAGAA TCTTCTTCGC AGCAGCAAAT AAAAACAGAT

CCCACAGCCT GCCGGCCGAG CCCACCCCTC ACCAGCAGGT TAAAGCCAAT

GGAATCTCTG ATGGCAAAAG AGAATCACCA TTAAAGATTG ACCCACTGTC

GGAATCTCTG ATGGCAAAAG AGAATCACCA TTAAAGATTG ACCCACTGTC

GGAATCTCTG GTGTCAAACG AGAATCACCG TTAAAGATTG ACCCACTGTC

GGAATCTCTG GTGTCAAACG AGAATCACCG TTAAAGATTG ACCCACTGTC

GGAATCTCTG GTGTCACACG AGAATCACCA TTAAAGATTG ACCCACTGTC

GGAGTCTCTG ATGTCAAAGG AGAATCACCA TTAAAGAGTG ACCCACTATC

GGAGCTTGTG GTGTCAAACA AGAGCCAACA TTGAAGAGTG ACCCACTCTC

GGCATTTCTG GTGTCAAACA AGAGCCAACA TTGAAGAGTG ACCCACTCTC

GGAGTCTCTG GTGTGAGACA AGACTCTACA TTAAAGAGTG ACCCACTGTC

GGAGTCTCCG CCGTCAGACT GGACTCGCCA TTAAAGAGTG ACCCATTGTC

GGAATCTCTG CTGTCAGACT GGAATCACCA TTAAAGAGTG ACCCACTGTC

GGAGTTTCCG CTGTCAGACT GGAATCACCA TTAAAGAGTG ACCTACTGTC

GGAGTGGCCG CGGTCAGACT GGACTCTCCG TTCAAGAGTG ACCCACTGTC

GGAGTCTCTG GTGTCAAACT AGAGTCAACA TTAAAGAGTG ACCCACTGTC

GGAGTCTCTG ATGTCAGATC AGAATCACCA TTAAAGAGTG ACCCACTGTC

---------- ---------- ---------- ---------- ----------

GAAACATATG ATAT---GCT TAGAGGACAA CTAAGCAGTG ACC---TTTC

GGCTCCTGTG GCCTTCCTTT CGAACCCCAA TTAAACCACG TCCTCCTGTC

ATTTAATCTG CTTGCTGTAT CAAAGGCTCA GCTATCTGTT CAAACGTCAC

ATTTAATCTG CTTGCTGTAT CAAAGGCTCA GCTATCTGTT CAAACGTCAC

ATTTAATCTG CTTGCTGTAT CAAAGGCTCA GCTATCTGTT CAAACGTCAC

ATTTAATCTG CTTGCTGTAT CAAAGGCTCA GCTATCTGTT CAAACGTCAC

ATTTAATCTG CTTGCTGTAT CAAAGGCTCA GCTATCTGTT CAAATGTCAC

ACTTAATCTG CTTGCTGTAT CAAAGGCTCA GCTATCTGTT CAAATGTCAC

ACTTAGTGTG CTTGCTGTAT CCAAGGCTCA GCCTTCTGCT CAGATTTCAC

ACTTAGTGTG CTTGCTGTAT CAAAGGCTCA GCCTTCTGTT CAGATTTCAC

ATTTAATCTG CTTGCTGTAT CAAAGGCTCA GCTCTGTGTT CAAACATCAC

ATTGAACCTG CTTGCTGTAT CAAAGGCTCA GCCATCTGTT CACAC-----

ATTTAATCTG CTTGCTGTAT CAAAGGCTCA GTTATCTGTT CAAACATCAT

ATTTAATCTG CTTGCTGTAT CAAAGGCTCA GTTATCTGTT CAAGCGTCAC

ATTGAACCTG CTTGCGGTCT CCAAGACTCA GTTCTCTGTT CCAACATCAC

ATTTAATCTG CTTGCTGTAT CAAAGACTCA GTTATCTGTC CAAACATTGC

ATTTAATCTG CTTG---TAT CAAAGGCTCA GTTATCTGTC GAAGCATCAC

---------- ---------- ---------- ---------- ----------

ATTTAATCCG ATTGTTCCAT CAAAGCCTCA GACATCTGTT CTATCCTCAC

TCTTAAGCCG CTTTCTGTAT CGAAGTGTCA CGTGTCCCCT CGACCGACCC

CTGTTCAGTT GCCTTCTGCA ACGCAAAGTA ATGTTTTGAG TTCTGTAAGT

CTGTTCAGTT GCCTTCTGCA ACGCAAAGTA ATGTTTTGAG TTCTGTAAGT

CTGTTCAGTT GCCTTCTGTA ACGCAAAGTA ATGTTTTGAG TTCTGTAAGT

CTGTTCAGTT GCCTTCTGTA ACGCAAAGTA ATGTTTTGAG TTCTGTGAGT

CTGTTCAGTT GCCTTCTGCA ACACAAAGTA ATGTTTTGAG TTCTGTAAGT

CTGTTCAGTT GCCTTCTGCA ACACAAAGTA ATGTTTTGAG TTCTACAAGT

CTGTTCAGTT GCCTTCTGCA TCGCAAAGTA AAGTTTTGAG TTCTGTAAGT

CTGTTCAGTT GCCTTCTGCA TCGCAAAGTC AAGTTTTGAG TTCTGTAAGT

CTGTTCAGTT GCCTTCTGCA ACACAAAGTA CTGTTGTGGG TTCTGTAAGT

-----CCGTT GCCTTCTGCA GCCCCAAGTA ACACCCTGAG TTCTGTAAGC

CTGTTCAGTT GCCTTCTGCA ACACAAAGTA ACATGTTGAG TTCTGTAAGT

CGGTTCAGTT CCCTTCTGTG ACACAAAGTA ACATTTTGAG TTCTGTAAGT

TGGCCCAGTT GCCCTCCGCG ACACCAAGTA ACATGTTGAG TTCTGCAAGT

CTGTTCAGTT GCCTTCTGCA ATGCAAAGTA ATGTTCTGAG TTCTGTAAGC

CTGCTCAGTT GCCTTCTACA GCACCAAGTC GTGTTCTGAG TTCTCTAACT

---------- ---------- ---------- ---------- ----------

AAGCTAAGTT GCCTTCCAAA TCACAAAGCT TTGCTATGAA TGATGAAAAT

CGGTTTGGCT GCCGCCCGTG GCCCCTCAGG CCGTCCCGAG GACCTTGAGT

TGCATGCCAA CAATGCCTCC AATTCCAGCT CGGAGTCAAT CCCAGGAAAA

TGCATGCCAA CAATGCCTCC AATTCCAGCT CGGAGTCAAT CCCAGGAAAA

TGCATGCCAA CAATGCCTCT AATTCCAGCT CGGAGTCAGT CCCAGGAAAA

TGCATGCCAA CAATGCCTCC AATTCCAGCT CGGAGTCAGT CCCAGGAAAA

TGCATGCCAA CAATGCCTCC AATTCCAGCT CGGAGTCAAT CCCAGGAAAA

TGCATGCTGG CAATACCTCC AATTCCAGCT CGGAGTAAAT CCCAGGAAAA

TGCATGCTAA CCATGCCTCC AGTTCCAGAG CAAAGCAAGT CACAGGAAAG

TGCATGCCAA CCAGGCCTCC AGGTCCAGAG GAAAGCAAGT CACAGGAGAG

TGCATGCCGA CGATGCCTCC AATTCCAGCC CGGAGTAAAT CCCAGGAAAA

TGCATGCCGA CAATGCCTCC AATTCCAGCC CGGAGTAAAT CCCAGGAAAA

TGCATGCC-- -------TCC AATTCCAGCC CGGAGTAAGT CCCAGGAAAA

TGCATGCCAA CATTGCCTCC CATTCCAGCC CGGAGTAAAT CCCAGGAAAA

TGCATGCCTC CACTGCTTCC CATTCCAGCC CGCAGTCAGC CCCAGGATAG

TGCATGCCAA CAATGCCTCC AATCCCAGCC CGGAGTAAAT CCCAGGAAAA

TGCATGCCGG CAACGCCTCC AGTTCCAGCG CGGAGTAAAT TCCAGGAGAA

---------- ---------- ---------- ---------- ----------

TACTTACAAG CAATGCCAGC TGTCTCAGCC GAAAGCATAT CCCAGGAAGA

CGCACTCCGT CGATGCCGCC CGTGCCGGCC CGGAGCCAGC GCCGGGACGA

TATGCGAAGT TCTCCAAACC CATTTATTAC TGGCTTGACC AGGACAAATC

TATGCGAAGT TCTCCAAACC CATTTATTAC TGGCTTGATC AGGACAAATC

TATGCGAAGT TCTCCAAACC CATTTATTAC CGGCTTGACC AGGACAAATC

TATGCGAAGT TCTCCAAACC CGTTTATTAC CGGCTTGACC AGGACAAATC

TATGCGAAGT TCTCCAAATC CATTTATTAC TGGCTTGATC AGGACAAATC

TATGCGAAGT TCTTCAAACC CATTTATTAC TAGCTTGACC AGCACAGATC

TGTGGGGAGT TCTGCAAACC CATTTC---C CAGCCTGCCT ACCAGGAACC

TATGGGCAGT TCTGCAAACC CGTTTC---C CAGCCTGCCC TGCCGGAACC

TATGCGGAGT TCTCCAAACC CATTTATTAC CAGCTTGACC AGCACAAACC

CACGCGATGT TCCCCAAACC CATTCATCCC AAGCTCAAGC AGCACAAATC

TATGCGATGT TCTCCAAACC CATTTATTAC GAGCTTGACC AGCACAAATC

CATGCGGTGT TCTCCAAACC CATTCACTAC GAGCTTGACC AGCACAAATC

CGTGCGCACT GCCTCAGACC CTGTCACCGC CGGCTTGTCC AGTACCAACC

TATGGAAAGT TCTCCAAATC CATTTATTCC TAGCTTGACC AGCACAAATT

CATGCGGAGT TCTCCAAACC CATTTATCAC TAGCTTGACC AGCACAAATC

---------- ---------- ---------- ---------- ----------

CAGAAAAAGT TCCTTAAACC CTTTTGTTCC TGGCTTAAAC AACACAAATC

CGCCCGCAGT TCCCCGAACC CTCCCGGCCC CGGCTCCGGC GGCCCCGAGC

CTTTCAGTGA CAGGACTGCT CCTGGAAACC CATTTAGAGC CAAGTCTGCT

CTTTCAGTGA CAGGACTGCT CCTGGAAACC CATTTAGAGC CAAGTCTGCT

CTTTCAGTGA CAGGACTGCT CCTGGAAACC CATTTAAAGC CGAGTCTGCT

CTTTCAGTGA CAGGACTGCT CCTGGAAACC CATTTAAAGC CGAGTCTGCT

CTTTCAGTGA CAGTACTGCT CCTGGAAACC CATTTAGAGC CGAGTCTGCT

CTTCCAGTGA CAGGACTGTT ---GGAAACC CATTTAGAAC TGAATCTCCT

CTTTTACAGA CAGGACTGCC CCTGGAAACC CATTTAGAGT TCAGTCTCCT

CTTTTACAGA CAGGACTGCC CCTGGAAACC CATTTAGAGT TCAGTCCGCT

CCTTCACTGA CAGAACTGCC CCTGGAAACC CCTTTAGAGC CCACTCTCCT

CTTTCACCGA CAGGACCGCT CCTGGAAACC CCTTTCGAGC TGAGTCTCCT

CTTTTACGGA CAGGACTGCT CCTGGAAACC CATTTAGAGC TGAGTCTCCT

CTTTCACAGA CAGGACTACT CCTGGAAACC CATTTAGAGC CAAGTCTCCT

CCTTCGTGGA TAGGACTGTG CCCAGAAACC CCTTCCGGGC TGAGTTTCCC

CTTTCATGGA CAGGACTACT GCTGGAAACC CATTTAGAGT TGAGTCT-CT

CTTTCACTGA CAGGACTGCT CCTGGAAACC CATTTAGAGC TGATTCTCCT

---------- ---------- ---------- ---------- ----------

CTTTCACTGT TAGAACTACT TTTGGAAATC CATTTCGAAG TGAATCTGCT

CCTTCACCCA CCGGCCCGGT CCCGGAAACC CATTTAAAAC TGAATGGCCC

TCATCTTCAC TTGATGGCTT TAAGGACAGT TTTGATCTAC AGGGCCAGTC

TCATCTTCAC TTGATGGCTT TAAGGACAGT TTTGATCTAC AGGACCAGTC

TCATCTTCAC TTGATGGCTT TAAGGAAAGT TTTGATCTAC AGGGGCAGTC

TCATCTTCAC TTGATGGCTT TAAGGAAAGT TTTGATCTAC AGGGGCAGTC

TCATCTTCAC TTGATGGGTT TATGGACAGT TTTGATCTAC AGGGCCAGTC

TCATCTTCAC TTGATGGTTT TAAGGACAGT TTTGATCTGC AGTGCCAGTC

TCAAGTTCAC TAGATGGTTT TGAGGACAAT TTTGATCTGC AGAGCCAGTC

TCAAGTTCGC TTGGTGGCTT TGAGGACAAT TTTGATCTGC AGAGCCAGTC

TCCTCTTCAC GTGACGGCTT CAAGGACAGC TTTGATCTGC AGGGCCAGTC

TCATCCTCCC TGGATGGGTT TAAGGACAGT TTTGATCCAC AGGGCCTGTC

TCATCTTCAT TTGATGGTTT TAAGGACAAT TTTGATCTGC AGGGCCAGTC

TCATTTTCAC TTGATGGCAT TAAGGACAGT TTTGATCTGC AGGGCCAATC

GCTCCATCCT TCGATGGCTT TCAGGACAGT TTCAAGCCGC CAGGCCAGTT

TCACCTTCAC CTGAAGACTC TGAGGACAGT TTTGATCTGC AGGGTCAATC

TCATCTTCCC TTGATGGCTT TGTGGACGGT TTTGATATGC AGAGCCAAAC

---------- ---------- ---------- ---------- ----------

TTGTTGCCCA GGGATGGATT TGAGGACAGT TTTGATTTGC AGAACTTCTC

GCACTTTCTG TCCGTACGTC GGAGGACGGT TTTGGTCCGC GGGGACAGGT

TTTAAAACCG AAAGGATGGG TAACCTTCGA GGAAGAAGAG GATTTTGTGA

TTTAAAACCA AAAGGATGGG TAACCTTCGA GGAAGAAGAG GATTTTGTGA

TTTAAAACCA AAAGGATGGG TAACCTTTGA GGAAGAAGAG GACTTTGTGA

TTTAAAACCA AAAGGATGGG TAACCTTTGA GGAAGAAGAG GACTTTGTGA

TTTAAAACCA AAAGGATGGG TAACCTTCGA GGAAGAAGAG GACTTTGTGA

TGTAAAACCA AAGGGATGGG TAACCTTTGA AGAAGAAGAC GACTTTGTAA

TGTAAAACCC AAAGGATGGG TAACATTTGA CGAAGATGAA GACTTTACAA

TGTAAAACCC AAAGGATGGG TAACCTTTGA CGAAGACGAC AACTTTACAA

TGGAAAACCG AAAGAGTGGG TGACCTTTGA GGACGACGAC GACTTTGTGG

TCTAACACCC AAAGGATGGG TAACCTTCGA GGAAGAAGAG GACTTTGTGA

TGCAAAACCA AAAGGATGGG TAACGTTCGA GGAAGAAGAG GACTTTGTGA

TGTACAGCCA AAAGGATGGG TGACCTTTGA GGAAGAAGAG GACC--GTGA

GGTCAATCCC AAAGGCTGGG TGACCTTTGA GGATGAAGA- ----------

TGTTAAACCA AAAGGATGGG TAACCTTTGA GGAAGAAGAG GACTTTGTGA

CGATAAACCA AAAGGATGGG TAACCTTCGA GGATGAAGAG GACTTTGTGA

---------- ---------- ---------- ---------- ----------

TGCAAACCCA AAAGGCTGGG TAACTTTTGA GGAAGATGA- --TTTCGTCA

T-TAAAACCC AAAGGCTGGG TGACGTTTGA GGAAGAGGAC GATG--GCGG

AAGGGAAGTC AAAGTCATCA GACTTACTGG GTAATCAGCC AAGTTCATTT

AAGGGAAGTC AAAGTCATCA GACTTACTGG GTTATCAGCC AAGTTCATTT

AAGGGAAGTC AAAGTCATCA GATTTACTGG GTAATCAGCC AAGTTCATTT

AAGGGAAGTC AAAGTCATCA GATTTACTGG GTAATCTGCC AAGTTCATTT

AAGGGAAGTC AAAGTCATCA GACTTACTGG GTAATCAGCC AGGTTCATTT

AAGGGAAATC AAAGTCACCA GACTTACTGG GTAATCAGCC ATCTTCATTT

AAGGAAAGTC AAGGTCGCCA GACTCCCTGG GTAATACAGC AGCTTCATTT

CAGGAAAGTC AAAGTCACCA GACTTAGTGG GTAACGCGCC AGCTTCATTT

CAGGGAAGTC GGAGTCATCA GCCTCCCTGG GTCATCAGCC AGGTTGGTTT

CAGGGAAGTC AGGGTCCCCA GACGTCCTGG GTAAGCAGCT GAGCTCGTCT

AAGGAAAGTC AAAGTCGTTA GACATACTGG GTCATCAGCC AAGTTCACTT

AAGGGAAGTC CACGTCACCA GACGTACTGG GTAACCAACC AGGTTCGTCT

---------- ---------- ---------- ---------- ----------

CACAGAAGTC AAAGT---CA GATTTACTGG CTAATCAACC AAGTTCGTTT

AGCAGAAGTC AAAATCACCA GGCTCGCTGG GTAACCAGCC AGGCTCACTT

---------- ---------- ---------G GTAGCCAGCC AGGTTCACTT

AAGCGAAATC AAAGTCACCA GATTTACTCA ACTCTCGATC AACTTCGCTT

AGCGGAGGCC AAGGTCATCA GGCTCGCTGG GCGCTCAGCC CGACTCCTTc

TCTTGGAATA AAGGTACAAA TGTCTCCTTC TGTGTGTTGC CGTCAAGAAG

TCTTGGAATA AAGGTACAAA TGTCTCTTTC TGTGTGTTGC CGTCAAGAAG

TCCTGGAATA AAGGTACAAA CGTCTCTTTC TGTGTGTTGC CATCAAGAAG

TCCTGGAATA AAGGTACAAA TGTCTCTTTC TGTGTGTTGC CATCAAGAAG

TCTTGGAATA AAGGTACAAG CATCTCTTTT TGTGTGGTGC CATCAAGAAG

TCTTGGAATA AAGATAAAAA TGTCTCTTTT TGCGTGTTGC CATCAAGAAG

---TGGAGTA AAGGTACAAA CGTTTCTTTC TGTGTGCTGC CAGCCAGAAG

---TGGAGTA AAGGTGCGAG CGTTTCTTTC TGTGTGTTGC CAGCCAGAAG

TCTCAAAACA AAGGTACAGA TGTTTCTTTT TGTGTCTTAC CATCGAGAAG

CCTTGGGGTA GGAGTACCAA TGTGTCTCTC TGTGTGTTGC CAGCAAGACC

TCTTGGAATA AAGGTACAGA TGTTCCTCTC TGTGTGCAGC CATCAAGAAG

TCTTGGGATA AAGGTACAGA GGTTTCTTTC TGTGCGTTGC CCTCAAGAAG

--------TG AAGGGCAAAA CCCATTTGCC TGGAAGACTA TATCCCCGTG

TCTTATAATA AAGGTACAAA TGTTCCTTTC TGTGTGTTGC CATCAAGAAG

TCTTGGAATA AGGGTACAAA TATTTCTTTC TGTGTGTTGC CATCAAGAAG

TCTTGGAAGA AAGGTATAAA TGCTTCCTTC TGTGTGCTGC CATCAAGAAG

CCTTGGAATA AAAATTCCAC TGTTTCTTTC TGTATGTTGC CATCAAGAAA

c---AGAACC AAAGCCCGGC CGTGGCCCTC TGCGTCTGGC CGGCGAGGAG

ACCTCCTCCA CCTCCTGTCC CTCCGCCCGG CACCAGCCCT CCAGTAGATC

ACCTCCTCCA CCTCCTGTCC CTCCGCCCGG CACCAGCCCT CCAGTAGATC

ACCTCCTCCA CCTCCTGTCC CTCTGCCTGG CACCAGCCCT CCAGTAGATC

ACCTCCTCCA CCTCCTGTCC CTCTGCCTGG CACCAGCCCT CCAGTAGATC

ACCTCCTCCA CCTCCTGTCC CTCCACCCAG CACCAGCCCT CCCGTAGATC

ACCACCTCCA CCTCCTGTCT CTCCACCTGG CACCAGCCCT CCAGTAGATC

GCCACCACCA CCTCCTGTCC CTCCACCTGG CACCACCTCA TCTGCAGGAC

GCCACCACCA CCTCCTGTCC CTCCACCTGG CACAACCTCA TCTGCAGGAC

GCCACCTCCA CCTCCTGTCC CACAACCCAG CACCAACCCT CCGGCGGATC

ACCACCCCCA CCGCCTGTCC CTCCACCTGG CACCACCCCT CCCGCAGACG

ACCACCTCCA CCTCCTGTCA CTCCATCTGG CACCACCCCT TCCGTAGCTC

ACCACCTCCA CCTCCTGTCC GTCCACCTGC CACTGCACTT CCAGTTGATC

GCCCTGGCTT CCCGATG--- ---------- ---------- ----------

ACCACCTCCA CCTCCTGTCC CTCCATCCAC CACCACCCCG CCAGTGGATC

ACCACCCCCA CCTCCGCTCC TGCCACCCGG TCCTGCCCCT CCAGGAGATC

ACCACCCCCA CCGCCTGTCC CTCCACCTGG CCCTGCCTCC CCAGGAGATC

GCCACCTAAA CCTCCTGTTC CAACATCTAA CACTACAGCT CCTATAGAGT

GCCGCCTCCT CCGCCAGGCC CCTCCTCCGG TGCCACCCTC GGAGACCCCT

CTTTCACGAC CTTGGCCTCT AAGGCTTCAC CCACACTGGA CTTTACAGAA

CTTTCACGAC CTTGGCCTCT AAGGCTTCAC CCACACTGGA CTTTACAGAA

CTTTCACGAC CTTGGCCTCT AAGGCTTCGC CCACACTGGA CTTTACAGAA

CTTTCACGAC CTTGGCCTCT AAGGCTTCGC CCACACTGGA CTTTACAGAA

CTTTCACTAC CTTGGCCTCT AAGGCATCAC CCACACTGGA CTTTACAGAA

CTTTCACAAC CTTGGCATCT AAGGCATCAC CCACCCTAGA CTTCACTGAA

CTAGCACAAC CCTGTCATCT AAGGCATCTC CCACATTGGA CTTCACAGAA

CTAGCACAAC CCTGCCATCT AAGGCACCTT CCACATTGGA CTTCACGGAA

CTTTCACAGC CTTGGCGTCT AAGGCGTCTC CCACACTAGA CTTCACGGAA

CTTTCACAGC CCTGGCCTCT AAGGCATCAC CCACGCTAGA CTTGACAGAA

CTTTCACAAC CTTGGCATCT AAGGCATCAC CCACACTAGA CTTCACAGAA

CTTTCGCAGC TTTGGCATCT AAGGCATCAC CCATACTAGA CTTCACAGAA

---------- ---------- ---------- ---------- ---CACAG-C

CTTTCACAAC CTTGGCATCT AAGGCTTCAC CCACACCAGA TTTTACAGAA

CTTGCACAGC TATGGCGTCG AAGGCATCAC CCATCCTGGG CTTTACAGAA

CTTTCACAGC CTTGGCATCT AAGGCATCAC CCACTCTGGA CTTTACAGAA

CTTTGACCTC TTTGGCATCT AAGACTTCAT CAACAGTAGA TTTTACAGAA

CTTTCGGCTC GCTGGCCCCC GGGGTGTCCC CACCCTCACA TTACACAGAT

AGACAGCAAC AAAACCTGCT GTTGCAGAAG GTAAAGGCAG AGCTGGAAGA

AGACAGCAAC AAAACCTGCT GTTGCAGAAG GTAAAGGCAG AGCTGGAAGA

AGA------- ---------- ---------- ---------- ----------

AGACGGCAAC AAAACCTGAT ATTGCAGAAG GTAAAGGAAG AGCTGGAAGA

AGACAGCAAC AAAAGCTGCT GTTGCAGAAG GTGAAGGAAG AGCTGGAAGA

AGA------- ---------- ---------- ---------- ----------

AGACAGCAAC AAAAACTACT TTTGCAGAAG ATGAAAGAAG AACTGACAGA

AGACAGCAAC AAAAGCTGCT TTTGCAGAAG ATGAAGGAAG AACTGACAGA

AGA------- ---------- ---------- ---------- ----------

AGA------- ---------- ---------- ---------- ----------

AGACCACAGC GAAACCTGCT GTTGCAGAAA ATGAAGGAAG ACCTGAAAGA

AGACAGCAGC GAAACCTGCC GTTGCAGAAG GTGAAGGAAG AGCTGAAAGA

AGATGGCGG- GACGGCAGAT GCTGAAGAAG ATGGAGGATG AACTGAAGGA

AGACAGCAAC GCCACCTGCT GCTGCAGAAG ATGAAGGAGG AGCTGGAAGA

AGA------- ---------- ---------- ---------- ----------

AGACAGCAAC AGGGCCCCCT GCTGCAGAAG ATGAAGGAGG AGCTGGCAGA

AGAGGGCAGC AGGACCAACT GTTCCTGATG ATAAAGAAGG AGCTGGAGGA

AGAAGACAGG ATGACTTGGC CGTCCAGAAA ATGAATGACC AGCTGGATGT

AGCCAAGAGG TTCCACATCA GCCTCCAGGA GTCCTATCAC AGCCTAAAGG

AGCCAAGAGG TTCCACATCA GCCTCCAGGA GTCCTATCAC AGCCTAAAGG

---------- ---------- ---------- ---------- ----------

AGCCAAGAGG TTCCACATCA GCCTCCAGGC GTCCTATCAC AGCCTAAAGG

AGTCAAGAAG TTGCATGCCA GCCTCCAGGT GGCACGTCAC AGCCTAAAGG

---------- ---------- ---------- ---------- ----------

AGCCAAGAAG GTTCATGCTA GCCTTCAGCT CTCCCATCAA AACCTCAAGG

AGCCAAGAAG GTCCATGCTA GCTTTCAGGT ATCCCATCAA AGCCTCAAAG

---------- ---------- ---------- ---------- ----------

---------- ---------- ---------- ---------- ----------

AGTCACGGAT GTGCACCTCA ACCTCCAGGT GTCCTATCAG AGCCTAAAGG

AGTAGAGAAG TTACACCACA GCCTCCAGAT GTCCTATCAG AGTCTAAAGG

GGTCAGAAAG CTGCACTTTG ACCTGCAGGC ATCCTACCAA AGCCT-----

GGCCAAGGCC ATCCACATCA GCCTCCAGGG GTCCTACTGG AGCCTAAAGG

---------- ---------- ---------- ---------- ----------

GATCAAGAAG GAGCACCTCT GCCTCCAGGT GTCCTGTGAG CGCCTCAAGG

GATTAAAAAA TCCCAGAGCA AGCTCGAGCT GTCCTATCGT ACCCTGAAGG

TGTAAAAATG TTCTATTCTA GCTTGGAAAC AGACTTTCAG AATCTAAAGG

AGAGGTCTCT GACTGACCTG CAGCTGTTGC TGGAGTCCCT TCAGGAGGAG

AGAGGTCTCT GACTGACCTG CAGCTGTTGC TGGAGTCCCT TCAGGAGGAG

---------- ---------- ---------- ---------- ----------

AGAGGTCTCT GACTGACCTG CAGCTGTTGC TGGAGTCCCT TCAGGAGGAG

AGAGGTCTCT GACCGACCTG GAGCTGCTGC TGGAGTCCCT TCAGGAGGAG

---------- ---------- ---------- ---------- ----------

AAAGTCATAT GATTGATGTG CGGAGGATAC TGGAGTCACT CCAGGAAAAG

AAAGTCATAT GATTGATGTG CAGAGGATAC TGGAGTCACT CCAGGAAAAG

---------- ---------- ---------- ---------- ----------

---------- ---------- ---------- ---------- ----------

ACAGGCATCT GATGGACCTG CAGGTGTCAC TAGAGGCCCT TCAGGAAGAG

AGAAGTATCT GACAGACCTG CAGGTGTTGG TGGAGTCCCT TCAGGAGGAG

---------- GTCAGACCTA TACCTGTCAC TGAAGTCCAG GCAGGAGG--

AGCAGTACCT GAGCGACCTG CAGCTGTGGC TGGAGTCCCT GGAGGAGGGC

---------- ---------- ---------- ---------- ----------

AGCAGCGCCA GGCCAACCTG CAGCGGGCAC TGAAGACCCT GCAGGAGA--

AGAGGTACCT GGTTGATCTG AAGCTGATTA TTGAGTCTCT TCAAGAGGAG

AAAAGTACCT GTCTGAATTG GAACAGATAG TGGAATGCCT ACAGGAAGAA

AGGGAGATCA CGGAGACCTT CAAGAGCCGA ATATCCAAGC TGGAGATGCT

AGGGAGATCA CGGAGACCTT CAAGAGTCGA ATATCCAAGC TGGAGATGCT

---------- ---------- ---------- ---------- ----------

AGAGAGATCA TGGAGACCTT CAAGAGCCGA ATATCCAAGC TGGAGATGCT

AGAGAGATCA TGGAGACCTT CGAGAGCCGA ATATCCAAGC TGGAGACACT

---------- ---------- ---------- ---------- ----------

AGAGATGTAA TGGAGACTTT CAAGAACAGA ATAACAAAGC TAGAAACTCT

AAAGATGTAA TGGAGATTTT CAAGAGCAGA ATAACCAAGC TAGAAACTCT

--AGAAGTCA TGGAAACTTT CAAGAGCCGA ATCTCCAGAC TAGAAGCTCT

---------- ---------- ---------- ---------- ----------

AGGGAGGTCA TGGAGACTTT CAAGAACCGT ATAGCTAAGC TTGAAACTCT

AAAGAGGTCA TGGAGACTTT CAAGAGTCGT ATATCCAAGC TCGAAACGCT

---------- ---------- ---------- ---------- ----------

AGAGAGGTCA TGGAGACTTT CAAGAGCCAG ATAGCCAAGC TGGAGACACT

---------- ---------- ---------- ---------- ----------

--AGAGGTCA TGGAGACGGT CCAGAGCCGG GTCACTAAGC TGGAGAACCT

AG-GAGGTGA TGAGGACCTT CCAGAGTCGA ATTTCCAAGC TGGAGACCCA

AGAGAGGTGA TGGAAACCTT TCAGACCCGC ATTTCCAAAC TAGAGGCTCT

ACAGCAAGTC ACCCAACTGG AGGCAGCGGA GCACCTCCAA AGCCGTCCCC

ACAGCAAGTC ACCCAACTGG AGGCAGTGGA GCACCTCCAA AGCCGTCCCC

---------- ---------- ---------- ---------- ----------

ACAGCAAGTC ACCCGACTGG AGGCAGCAGA GCACCTCCAA AGCCGTCCTC

ACCGCAAGTC ACCCAGCTGG AGGCAGCGGA GCACCTCCGA AGCCGTCCCC

---------- ---------- ---------- ---------- ----------

ACAGCAAGCT ACCCAACTGG AGATGATGGC CAGCCTCAGA ACCCGCCCCA

ACAGCAAGCT ACCCAACTGG AGATGATGGC CAGCCTCAGA ACCCGTCCCA

GGAGCGAGTC GCTCAGCTGG AGATGACGGA GAACCTCCGA AGCCGCCCCA

---------- ---------- ---------- ---------- ----------

GCAACAAGTG ACACAACTGG AGGTGACGCA CAGGTTCCGG ATCCGTCCCC

ACAGCAAGCG ACCCAACTGG AGATGACAGA GAGCCTCCGT AGCCGTCCCC

---------- ---------- ---------- ---------- ----------

ACAGCAAGTG TCCCAGCTGG AGATGATGCG GAAGCTCAGA CACGGGCCGC

---------- ---------- ---------- ---------- ----------

CCAGCAGGTG GCCCAGCGGG AGCTAGCGAA GCAGTTCTGG AGCCGGCCAA

ACAGCAAAGA GCCCAGTTAG AAATGACCGA GAAGCCCCAA AGCCACTCCC

GCAGCAAACA GCGCAGCCGG AGATGGTGGA GAAATGTAAA ACCCAGGCCC

CGCAGATGTT GTTCAAGTTC CTGAGTCCGC GCCTCTCACT GGCAACCGTC

CGCAGATGTT GTTCAAGTTC CTGAGTCCGC GCCTCTCACT GGCAACTGTC

---------- ---------- ---------- ---------- ----------

CGAAGATGTT GTTCAAGTTC GTGAGTCTGC TCTTCTCACT GGCAACCGTC

TACGGGTGGT GTTCAAGTTC CTGAGACCGC TCCTCTCACT GACCACCGTC

---------- ---------- ---------- ---------- ----------

AGGACTTTTT CTTCAGATTC ATAAGCCTGC TTCTCACTCT GACCACTATC

AGGACTTTTT GTTCAGATTC ATAAGCCTGC TTCTCACTCT GACCACTATC

GGGAGTTTCT GTTCATCTTC ATGAGCCTGC TCCTCACGCT GGCCACTGTC

---------- ---------- ---------- ---------- ----------

AGGAGTTTAT GTTCCGGCTC ATGAGCCTGC TCCTCACACT GGCTACCATC

AG---TTCCT GTTCCGATTC GGGAACCTAC TCCTTACACT GGCCACCATC

---------- ---------- ---------- ---------- ----------

AGGAGCTCTT GTTCAAGTTC ATGAGCCTGC TCCTCACGCT GGCCACCATC

---------- ---------- ---------- ---------- ----------

GGGAGTTCCT GCTCAAGTTC ATCAGCCTGC TCCTCACTCT GGCCACCATC

AAGTCTTTTT AGTTAGGTTC ATGAGCTTGC TCCTAACTTT GACCACCATC

AGGTGTTCCT GGACAAGTTC ATGAACCTAC TCGTCACTTT GACCAACATC

CTCTTGGTCT TTGTCTCCAC CTTGTGTGCC TGCCCCTCGT CACTGATCAG

CTCTTGGTCT TTGTCTCCAC CTTGTGTGCC TGCCCCTCGT CACTGATCAG

---------- ---------- ---------- ---------- ----------

CTCTTGGTCT TTATCTCCAC CTTGTGTGCC TGCCCCTCAT CACTGATCAC

CTCTTGGTCT TTGTCTCCAC CTTGTGTGCC TGCCCCTTGT CACTTGTCAA

---------- ---------- ---------- ---------- ----------

CTCTTGGTCG TGGTCTCTAC ATTGTGTTCT TGCCCTTTGC CCTTGCTCAA

CTCTTGGTCG TGGTCTCTAC ATTGTGTTCT TGCCCCTTGC CCTTGCTCAG

CTCCTGGTCC TCGTCTCCAC CGTGTGTGCC TGCCCCTTGT CCCTGGTCAA

---------- ---------- ---------- ---------- ----------

CTCCTCGTCT TCATCTCCAC TGTGTGCTCC TGCCCCTTGC CACTGCTCAA

CTCCTCGTCT TCATCGCCAC TGTGTGCACC TGCCCCTTGC TGCTGGTGAA

---------- ---------- ---------- ---------- ----------

CTCTTGGTCT CCGTGTCCAT CGTGTGCGCC TGCCCCTTGC CCCTCATCAA

---------- ---------- ---------- ---------- ----------

CTCTTGGTCT GCATCTCCAC CAAGTGCGCT TTCCCCTGGC CCTTCTTCAA

GTCCTCATAT GCGTGTCTAC CATCTGTAGC TGCCACTTGC CTTTCTTCAA

CTCCTGGTCT GCATCACTAG CATGTCCTGC TGCCCCTTGT CTTTGATCAG

CTCACGCCTG TGCACCTGCA CCATGCTGAT GCTGATCGGG CTTGGGGTCC

CTCACGCCTG TGCACCTGCA CCATGCTGAT GCTGATCGGG CTTGGGGTCC

---------- ---------- ---------- ---------- ----------

CTCACGCCTG TGCACTTGCA CCACCCTGAT GCTGATTGGG CTTGGGGTAC

CTCGCACCTG CGCACTTGCA GCATGCTGAT GCTGATTGGG CTTGGGGTCC

---------- ---------- ---------- ---------- ----------

CTCACGCCTG CGCATCTTCA TTGTATTTAT GATCATTGGG CTTGGGACCT

CTCACGCCTG CGCATCTTCA TTGTATTTAT GATCATTGGG CTTGGGACTT

CTCCCGCCTG CGCACGTGCA CCATCCTCGT CCTGCTCGGG CTCGGGGCGC

---------- ---------- ---------- ---------- ----------

TTCCCGCCTG CGCACGTGCA CCACACTCCT GCTGATCGGC CTCGTGGCCC

TTCGCATCTG CGCATGTGCA CCATACTCAT GCTGATCGGT CTCGGGGTCC

---------- ---------- ---------- ---------- ----------

GACGCGCCTG CGGACGTGCG CCACGCTCAT GCTGATCGGC CTCGGGGCCC

---------- ---------- ---------- ---------- ----------

GACGCACCTG CGCATGTGCG CCTCCCTCAT ACTCATAGGG CTCGGGGTCC

GTACCGTCTA CGTACTTGTA TCATCCTCTT GCTCATTGCC CTGGGGATGG

GAGCCCTCCG CGCATTTTAG CCACCCTCAT GGTGATTATC CTGGGAGCAG

TGGCCTGGCA GAGGTGGCGC GCCATCCCTG CCACAGACTG GCAGGAATGG

TGGCCTGGCA GAGGTGGCGC GCCATCCCTG CCACAGACTG GCAGGAATGG

---------- ---------- ---------- ---------- ----------

TGGCCTGGCA GAGGCGGCAC GCCATCCCTG CCACAGACTG GCCAGAATGG

TGGCCTGGCA GAGGTGGCAC ACCATCCCTG CCATGGACTG GCAGGCATGG

---------- ---------- ---------- ---------- ----------

TGGCCTGGCA GAAGCGGCAT GTCATCTCTA TTATTGACTG GCAGGCCTGG

TGGCCTGGCA GAAGCGCCAT GTCATCTCTA TTATTGACTG GCAGGCCTGG

TGGTGTGGCA GAAGCGACAC GCCATCCCCA CCGTGGCCTG GCAGGCGTGG

---------- ---------- ---------- ---------- ----------

TGGCCTGGCA GAAACGGCAT GCCTTCCCTA CCACCGACTG GCAGGCCTGG

TGGCCTGGCA GAAGTGGCAT GCCACCCTTG CCCTGCACTG GCAGGCCTGG

---------- ---------- ---------- ---------- ----------

TGGCCTGGCA GAGGTGGCCG GCCATCTCCA CCGTGGACTG GCAGGCGTGG

---------- ---------- ---------- ---------- ----------

TGATTTGGGA GAAGCAGTAT GCCATCCCTA CCGCTGACTG GCAAGCTTGG

TCGCCTGGCA GGAGTGGTAC AATATCCCAT ACATACATTG GCATGAATGG

TCGCCTGGCA GAACTGGCAG GCCAGACCAG ACATGGACTG GCATGCTTGG

GTCCCCTCCA GGTGTAGACT GCCTGAGATG CGCTGGCAGC TGAGCGAGCA

GTCCCCTCCA GGTGTAGACT GCCTG----- ---------- ----------

---------- ---------- ----GAGATG CGCTGGCAGC TGAGCGAGCA

GTCTCCTCCA GGTGTAGACT GCCTGAGATG CGCTGGCAGC TGAGCGAGCA

GTCCCCTCTA GGTGGAGACT GCCTGAGATG CGCTGGCAGC TGAGCGAACA

---------- ---------- ----GAGATG CGCTGGCAGC TGAGTGAGCA

GTCCCCTTTA AGTGGAGATC ACACGTGATG CGCGGGCAGT TGAGTGAGCA

GTCCCCTTTA AGTGGAGACA ACACGAGATG CGCTGGCAGT TGAGTGAGCA

ATCCCCTCCC GATGGAGACT GTCTGAGATG CGCGGGCAGC TGGGCGAGCA

---------- ---------- ---------- ---------- ----------

GTCCCCTCCA GATGGAGACT GCTT-AGATG CGCTGGCAGC TGAGCGAGCA

GACCCCTCTG GG---AGACT GCCTGAGATG CGCTGGCAGC TGAGCGAGCA

---------- ---------- ----GAGATG CGCTCCCAGC TGAGCGAGCA

GTCACCTCCA AGTGGAGACT CACTGAGATG CGCTGGCAGC TGAGCGAGCA

---------- ---------- ---------- ---------- ----------

GTTGCCTCCA AGTGGAGACT CACGGAGATC CGCTGGCAGC TGAGTGAGCA

CTCTTAACGA GGTGGAAATT G-CTGAGATC CGATGTCAGT TGGCAGAACA

CTTTCCTCCA AGTGGAAAGA G-GT-AGATC CGTTCTCAGC TGGGAGAGCA

GCTGCGCTGC CTGGAGCTGC AGGGCGAGCT GCGGCGGGAG TTGCTGCAGG

---------- ---------- ---------- ---------- ----------

GCTGCGCTGC CTGGAGCTGC AGGGTGAGCT GCGGCGGGAG CTGCTGCAGG

GCTGCGCTGC CTGGAGCTGC AGGGTGAGCT GCGGCGGGAG CTGCTGCAGG

GCTACGCTGC CTGGAGCTGC AGGGCGAGCT GCGTCGGGAG CTGCTGCAGG

GCTGCGCTGC CTAGAGCTAC AGGGTGAGCT GCGGCGGGAG TTGCTGCAGG

GCTTCATTGC CTGGAGCTTC AGGGAGAGCT GCGGCGGGAT TTGCTGCTGG

GCTTCATTGC CTGGAGCTTC AGGGAGAGCT TCGGCGGGAT TTGCTGCAGG

GCTGCGCTGC CTGGAGCTGC AGGGCGAGCT TCGGCGGGAG CTGCTGCAGG

---------- ---------- ---------- ---------- ----------

GCTGCGCTGC CTGGAGCTGC AGGGCGAGCT GCGGCGGGAG CTGCTGCAGG

ACTGCGCTGC CTGGAGTTGC AGGGTGAGCT GCGGCGGGAG CTGCTGCAGG

GCTGCGCTGC CTGGAGGTGC AGGGGGAGCT GCGGCGGGAA CTGCTGCAGG

GCTGCGCTGC CTGGAGCTGC AGGGCGACAT CCGGCGGGAG CTGCTGCAGG

---------- ---------- ---------- ---------- ----------

GCTGCGCTGC CTGGAGCTGC AGGGGGACCT ACGACGGGAG CTGGTGCAGG

GCTCCGATGC CTGGATGGTC AAGGGGAGCT TCGGCGAGAA CTGCTCCAGG

ATTGCGCTGC CTGGAGGTGC AGGGAGAACT GCGCCGGGAA TTGCTGCAGG

AGCTGGCAGA GTTCATGCGG CGCCGCGCTG AGGTGGAGCT GGAATACTCC

---------- ---------- ---------G AGGTGGAGCT GGAATACTCC

AACTGGCAGA GTTCATGCGG CGCCGCGCTG AGGTGGAGCT GGAGTACTCC

AACTGGCAGA GTTCATGCGG CGCCGCGCTG AGGTGGAGCT GGAGTACTCC

AGTTGGCAGA GTTCATGCGG CGACGCGCCG AGGTGGAGCT GGAGTACTCT

AGCTGGCTGA ATTCATGCGG CGGCGCGCTG AGGTGGAGCT GGAGTACTCC

AGCTAGCTGA GTTCATGCGG CGTCGAGCAG AGGTGGAGCT GGAGTATTCT

AGCTAGCTGA GTTCATGCGG CGCCGGGCGG AGGTAGAACT GGAGTATTCT

AGCTAGCCGA GTTCATGCGT CGCCGCGCCG AGGTGGAGCT CGAGTACTCC

---------- ---------- ---------- ---------- ----------

AGCTGGCCGA GTTCATGCGG CGGCGCGCCG AGGTGGAGCT GGAGTACTCC

AGCTGGCTGA GTTCATGCGG CGCCGCGCTG ATGTGGAGCT GGAGTACTCC

AGCTGGCCGA GTTCATGCGG CGGCGTGCCG ACGTGGAGCT CGAGTACTCC

AGCTGGCCGA GTTCATGCGG CGCCGCGCCG AGGTGGAGCA GGAGTACTCC

---------- ---------- ---------- ---------- ----------

AGCTGGCGGA GTTCATGAGG AGACGCGCAG AGGTGGAGCT CGAGTACTCG

ATCTGGGCGA GTTCATGCGG CGCAGGGCTG AGGTGGAGCT GGAATACTCC

ATCTGAGCGA ATTCCTGCGT CGCCGAGCAG AAGTGGAGCT GGAGTACTCG

CGGGGCCTGG AAAAGCTGGC CGAGCGCTTC TCCAGCCGTG GAAGCAGCCG

CGGGGCCTGG AAAAGCTGGC CGAGCGCTTC TCCAGCCGTG GAAGCAGCCG

CGGGGCCTGG AAAAGCTGGC CGAGCGCTTC TCCAGCCGTG GAAGCAGCCG

CGGGGCCTGG AAAAGCTGGT CGAGCGCTTC TCCAGCCGTG GAAGCAGCCG

CGGGGCCTGG AAAAGCTGGC TGAGCGTTTC TCCAGCCGCG GAAGCAGCCG

CGTGGCCTGG ACAAGCTAGC CGAGCGCTTC TCTGGCCGTG GAGGCAGCCG

CGGGGCCTGG ACAAGTTGGC TGAACGCTTT ACTAGCCGCA GTGGCAGCCG

CGGGGCCTGG ACAAGTTGGC TGAACGCTTT ACCAGCCGCA GTGGCAGCCA

CGGGGCCTGG AGAAGTTGGC AGAGCGCTTC TCCAGCCGCG GGGGCAGCCG

---------- ---------- ---------- ---------- ----------

CGGGGCTTGG ACAAGCTGGC CGAGCGCTTC TCCGGCCGGG GCGGCAGCCG

CGTGGCCTGG ACAAGCTGGC CGAGCGCTTC TCCAGCCGAG GAGGCAGCCG

CGGGGCCTGG AGCGCCTGGC TGAGCGCTTC TCCAGCCGCA GTAGCAGCCG

CGGGGCCTGG AGAAGCTGGC CGAACGCTTC TCCAGCCGAG GGGGCGGCCG

---------- ---------- ---------- ---------- ----------

CGAGGCCTGG ACAAGCTGGC TGAGCGCTTC TCCAGCCGAG GAGGCAGCCG

AAGGGACTGG AGAAGCTCAG CGACAGGTTC CTGGGCCGAG GAGCCAGGCG

CGTGGACTGG AAAGGCTGGC TGAACGCTTC TCCTCCAGGG ----CAACAA

GGAGCACCAA AGCTTCCGGA AGGAGCCGTC CCTCCTGCAG GATGAGCTCC

GGAGCACCAA AGCTTCCGGA AGGAGCCGTC CCTCCTGCAG GATGAGCTCC

GGAGCACCAA AGCTTCCGGA AGGAGCCGTC ACTCCTGCAG GACGAGCTCC

GGAGCACCAA AGCTTCCGGA AGGAGCCGTC ACTCCTGCAG GACGAGCTCC

GGAGCACCAA AGCTTCAGGA AGGAGCCGTG CCTCCTGCAG GACGAGCTCC

GGAGCATCAA AGCTTCCGGA AGGAGCCATC CCTCCTGCAG GATGAGCTCA

GGAGCAGCAA AGTTTCCGGA AGGAGCCAAC TCTCCTGCAG GATGAGCTCC

GGAGCAGCAA AGCTTCCGGA AGGAGCCGAC TCTCCTGCAG GCTGAGCTCC

GGAGCACCAA AGCTTCCGGA AGGAGCCTTC CCTCTTGCAG GAGGAGCTCC

---------- ---------- ---------- ---------- ----------

CGAGCACCAG AGCTTCCGGA AGGAGCCGTC CCTCCTGCAG GACGAGCTGC

GGAGCACCAA AGCTTCCGGA AGGAGCCGTC CCTCCTGCAG GAGGAGCTCC

GGAGCACCAG AGCTTCCGGA AGGAACCGTT GCTCCTGCAG GAGGAACTCC

GGAGCACCAA AGCTTCCGGA AGGAGCCGTC CCTCCTGCAG GAGGAACTGC

---------- ---------- ---------- ---------- ----------

AGAACTCCAA AACTTCCGGA AGGAGCCGTC CCTCCTGCAG GAGGAGCTCC

AGAGCAGCAG AACTTTCGGA AGGAGCCATC CCTTCTGtat gccaaggtAG

GGAGCTTCAG AGCTTCAGGG AGAAGACACC TCTCC---AG GGGGATCACG

TGGAGGTGGT CTCAGAGCTC CAGACGGCCA AGAAGACGTA CCAGGCATAT

TGGAGGTGGT CTCAGAGCTC CAGACGGCCA AGAAGACGTA CCAGGCATAT

TGGAGGTGGT CTCAGAGCTC CAGACGGCCA AGAAGACGTA CCAGGCATAT

TGGAGGTGGT CTCAGAGCTC CAGACGGCCA AGAAGACGTA CCAGGCATAT

TGGAGGTGGT CTCAGAGCTA CAGACGGCCA AGAAGACATA CCAGGCGTAT

TGGAGGTGGT CTCAGAGCTC CAGACGGCCA AGAAGACGTA TCAGGTGTAC

TGGAGGTGGT CTCAGAACTC CAGACAACCA AGAAGACGTA CCATGTATAC

TGGAAGTGGT CTCAGAACTC CAGACAACCA AGAAGATGTA CCATGTGTAC

TGGAGGTGGT GTCAGAGCTG CAGACGGCCA AGAAGACCTA TCACGCGTAT

---------- ---------- ---------- ---------- ----------

TGGAGGTGGT GTCGGAGCTT CAGACGGCCA AGAAAACGTA CCAGGGGTAC

TGGAGGTGGT GTCAGAGCTT CAGACGGTCA AGAAGACATA CCAGGTGTAC

TGGAGGTGGT GTCGGAGCTG CAGACGGCCA AGAAGACGTA CCACGCGTAT

TGGTGGTGGT GTCGGAGCTC CAGACGGCCA GGAAGACCTA CCAGGTGTAC

---------- ---------- ---------- ---------- ----------

TGGAGGTGGT CTCGGAGCTG CAGACGGCCA GGAAGACCTA CCAGGTGTAC

CTAGGATGGT ATTTGAGCCC CCAGGAGCCA TGAAAACCTA CCAGACATAT

GGGTGGTTCT GTCTGGGGTC C--ATGGCCA TGAAGACCTA TCAGGCATAC

CACATGGAGA GCGTGAATGC CGAGGCCAAG CTCCGGGAGG CCGAGCGGCA

CACATGGAGA GCGTGAATGC CGAGGCCAAG CTCCGGGAGG CCGAGCGGCA

CACATGGAGA GCGTGAATGC TGAGGCCAAG CTCCGGGAGG CTGAGCGGCA

CACATGGAGA GCGTGAATGC TGAGGCCAAG CTCCGGGAGG CCGAGCGGCA

CACACAGAGA GTGTGAATGC TGAGGTCAAG CTCCGGGAGG CCGAGCGGCA

CATACGGAGA GCATGAATGC TGAGAACAAG CTCCGTGAAG CTGAGCGACA

CACTTGGAGA GCATGAATGC TGAGACTAAA CTTCGGGAAG CTGAGAGGCA

CACTCAGAGA GCATGAATGC TGAGACTAAA CTTCGGGAAG CGGAGCGGCA

CACGTGGAGA GCGTGAACGC CGAGGCCAAG CTCCGAGAAG CTGAGCGACA

---------- ---------- ---------- ---------- ----------

CCCATGGAGA GCGTGAATGC TGAGGCCAAG CTCCCGGAAG CCCAACGGCA

CACACAGAGA GCATGAGTGC TGAGGCCAAG CTCCGGGAAG CCGAGCGGCA

CACACCGAGA GCCTGAATGC TGAGGCCAAG CTCCGCGAAG CCGAACGGCA

CGCACGGAGA GCGTGAACGC CGAGACCAAG CTCCGGGAAG CCGAGAGGCA

---------- ---------- ---------- ---------- ----------

CGCTCAGAAA GCGTGAACGC CAAGACCAAG CTCCGAGAAG CCGAGCGCCA

CGGGCAGAGA GCGTAAGCTC TGAGGGCAAG CTGAGAGAAG CTGAAAGGCA

CGTGCCGAGA GCGTGAATGC CGAGAACAAG CTGCGTGAGG CAGAACGGCA

GGAGGAGAAG CGGGCAGGCC GGAGTGTCCC CACCACCACC GCCACTGAGG

GGAGGAGAAG CGGGCAGGCC GGAGTGTCCC CACCACCACC GCCACTGAGG

GGAGGAGAAG CGGGCAGGCC GGAGTGTCCC CACCACCACT GCCACTGAGG

GGAGGAGAAG CGGGCAGGCC GGAGTGTCCC CACCACCACT GCCACTGAGG

GGAGGAGAAG CGGGCAGGCC GGAGTGCACC CACTGCCACC GCCACTGAGG

GGAGGAGAAG CGGGCTGGCC GGAGTGCCCC CGCCACCACT ACAGCTGAGG

AGAGGAGAAG CGGTCAGGCA AGAGTGCCCc caccactact accatTGAGA

AGAGGAGAAG CGGTCAGGCA GGAGTGCCTc caccaccact accacTGAGA

AGAGGAGAAG CGGGCAGGGC GCAGtgcccc tgctgcccct GCCACTGAGG

---------- ---------- ---------- ---------- ----------

GGAGGAAAAA CGGGCGGGCC GCAGTgccgc tgccgccgcc gccaccgAGG

GGAAGAAAAG CGGACAGGCC GGAGTgccgc caccgccact gccacTGAGG

GGAGGAGAAG CGGGCAGGCC GGACCGTGGC CACCGCC--- ACCACTGAGG

GGAGGAGAAg cgggcgggcc ggagcgctcc cgccgccgcc gcccccgACG

---------- ---------- ---------- ---------- ----------

GGAGGAGAAG AGAGCAGGCC GGAGCACCAC AGCCAGCACC GTCAGCGAGG

AGAAGAGAAG CGGGCCGGCC GATGTGGGGA GGCCGGCACC GCTACAGAGG

GGAGGAGAAG AGAGGTGGGA AGAGCGGCGC TAGGGCCACC GGCACAGATG

CAGGGCCCCT CCGCAAGAGC TCCCTCAAGA AGGGAGGGAG GCTGGTGGAG

CAGGGCCCCT CCGCAAGAGC TCCCTCAAGA AGGGAGGGAG GCTGGTGGAG

CAGGGCCCCT CCGCAAGAGC TCCCTCAAGA AGGGAGGGCG GCTGGTGGAG

CAGGGCCCCT CCGCAAGAGC TCCCTCAGGA AGGGAGGGCG GCTGGTGGAG

CAGGGCCCCT CCGCAGGACC TCCCTGAAGA AGGGTGGGCG GCTGGTGGAG

CAGGGCCCCT CCGCAAGAGC TCCCTCAAAA AAGGAGGGCG GCTGGTGGAG

CAGAGCTCCA CCGAAAGGGC TCTCTCAAGA AAGGAGGGCG GCTAGTGGAG

CAGGGCCCCA CCGAAAGAGC TCTCTCAAGA AAGGAGGGCG GCTAGTGGAG

CAGGGCCCCT CCGCAGGAGC TCCGTGAAGA AGGGTGGGCG GCTGGTGGAA

---------- ---------- ---------- ---------- ----------

CGGGGCCCCT GCGCAAGAGC TCCCTCAAGA AGGGAGGACG GCTGGTGGAA

CAGGGCCCCT CCGCAAGACC TCCCTCAAGA AGGGATCGCG GCTGGTGCAA

CAGGGCCTCT CCGAAAGGGC TCTGTCAAGA AGGGCGGGCG GCTGGTGGAG

CAGGGCCCCT GCGCAAGGGC TCCATCAAGA AGGGCGGGCG GCTGGTGGAG

---------- ---------- ---------- ---------- ----------

CAGGGCCGTG CCGGAAAACC TCCTTCAAGA AGGGCGGGCG GCTGGTGGAG

CTGGGCCTCT CCGAAGGACT TCGCTCAAGA AAGGAGGGCG CCTAGTAGAG

CTAAGACACC CCGTCGCACC TCCTCGAAGA AGGTGGGACG GCTGGTGGAG

AAGCGGCAGG CCAAGTTCAT GGAGCACAAA CTCAAGTGCA CAAAGGCGCG

AAGCGGCAGG CCAAGTTCAT GGAGCACAAA CTCAAGTGCA CAAAGGCGCG

AAGCGGCAGG CCAAGTTCAT GGAGCACAAA CTCAAGTGCA CAAAGGCGCG

AAGCGGCAGG CCAAGTTCAT GGAGCACAAA CTCAAGTGCA CAAAGGCGCG

AAGCGGCAGG CCAAGTTCAT GGAGCACAAA CTCAAGTGCA CAAAGGCACG

AAGCGTCAGG CCAAGTTCTT GGAGCACAAA CTCAAGTGCA CAAAAGCACG

AAGCGTCAGG CCAAGTTCTT GGAACATAAA CTTAAATGCA CCAAGGCCCG

AAGCGTCAGG CCAAGTACTT GGAGCATAAA CTTAAGTGCA CCAAGGCCCG

AAGCGCCACG CCAAGTTCCT GGAGCACAAA CTCCAGTGCA CAAAGGCACG

---------- ---------- ---------- ---------- ----------

AAGCGTCAGG CCAAGTTCTT GGAGCACAAA CTCAAGTGCA CAAAGGCGCG

AAGCGGCAGG CCAAGTTCTT GGAGCACAAA CTGAAGTGCA CGAAAGCGCG

AAGCGTCAGG CCAAGTTCTT GGAACACAAG CTCAAGTGCA CGAAGGCGCG

AAGCGCCAGG CCAAATTCCT GGAGCACAAA CTCAAGTGCA CGAAGGCGCG

---------- ---------- ---------- ---------- ----------

AAGCGGCAGG CCAAGTACCT GGAGCACAAG GTCAAGTGCA CAAAGGCTCG

AAGCGCCAGG TCAAGTACTT CGAGCACAAA CTGAAGTGCA CCAAGGCCCG

AAGCGCCAGG CCAAATACCT GGAGCACAAG CTGAAGTGCA CCAAGGCCCG

CAACGAGTAC CTGCTTAGCC TGGCTAGTGT CAACGCTGCT GTCAGTAACT

CAACGAGTAC CTGCTTAGCC TGGCCAGTGT CAACGCTGCT GTCAGTAACT

CAATGAGTAC CTGCTTAGCC TGGCCAGCGT CAACGCCGCT GTCAGTAACT

CAACGAGTAC CTGCTTAGCC TGGCCAGCGT CAACGCCGCT GTCAGTAACT

CAACGAGTAC CTGCTGAGCC TGGCCAGCGC CAATGCTGCT GTCAGGAACT

CAATGAGTAC CTGCTCAGCC TGGCCAGTGT CAATGCCGCT GTCAGCAACT

AAATGAGTAC CTGCTCAGCC TGGCCAGTGT CAATGCTGCT GTCAGCAACT

AAATGAGTAC CTGCTCAGCC TGGCCAGTGT CAATGCCGCC ATCAGCAACT

GAATGAATAC CTGCTGAGCC TGGCCAGCGT CAACGCTGCT GTCAGCAACT

---------- ---------- ---------- ---------- ----------

CAACGAGTAC CTGCTCAGCC TGGCCAGTGT CAATGCTGCT GTCAGCAACT

CAACGAGTAC CTGCTCAGCC TGGCCAGTGT CAACGCTGCG GTCAGCAACT

GAACGAATAC CTGCTGAGCC TGGCGAGCGT CAACGCTGCT GTCAGCAACT

GAACGAGTAC CTGCTCAGCC TGGCCAGCGT CAACGCCGCC GTCAGCAACT

---------- ---------- ---------- ---------- ----------

GAACGAGTAC CTGCTCAGCC TGGCCAGTGT GAACGCAGCC GTTAGAAACT

GAATGAGTAC TTGCTCAGTC TGCCCAGTGC AAACGCAGCC GTCAGCAACT

GAACGAGTAC CTGCTCAGCT TGGCCAGCGT TAATGCCGCC GTCAACAACT

ACTACCTGCA TGACGTCTTG GACCTCATGG ACTGCTGTGA CACAGGGTTC

ACTACCTGCA TGACGTCTTG GACCTCATGG ACTGCTGTGA CACAGGGTTC

ACTACCTGCA TGACGTCTTG GACCTCATGG ATTGCTGCGA CACAGGGTTC

ACTACCTGCA TGACGTCTTG GACCTCATGG ATTGCTGCGA CACAGGGTTC

ACTACTTGTG TGACGTCTTG GACCTCATGG ACTGCTGCGA CACAGGGTTC

ACTACTTGCA TGATGTCTTG GACCTGATGG ATTGCTGTGA CATGGGGTTC

ACTACTTACA CGATATCTTG GACCTCATGG ATTGCTGTGA CACAGGTTTC

ACTACTTACA CGATATCTTG GATCTCATGG ATTGCTGTGA CACAGGTTTC

ACTACCTGTG CGACATCTTG GACCTCATGG ACTGCTGTGA CACAGGCTTC

---------- ---------- ---------- ---------- ----------

ACTACCTGCG TGACGTCTTG GACCTCATGG ACTGCTGCGA CACAGGATTC

ACTACTTGCA TGATGTCTTG GACCTCATGG ACTGCTGTGA CACGGGCTTC

ACTACTTGTG TGACGCCATG GACCTCATGG ATTGCTGCGA CACGGGCTTC

ACTACCTGCG GGACGTCTTG GACCTCATGG ACTGCTGTGA CACCGGCTTC

---------- ---------- ---------- ---------- ----------

ACTACTTGCA GGACGTGCTG GACCTCATGG ATTGCTGCGG CACAGGGTTC

ACTATTCGAG GGACACCATG GACATCATGG ACTGCAATGA TCTGGGTTTC

ACTATGTCCA CGACGTGTCC GACCTTCTGG ATTGCTGTGA CGTGGGATTC

CACCTGGCCC TGGGGCAGGT GCTCCGGAGC TACACGGCCG CTGAGAGCCG

CACCTGGCCC TGGGGCAGGT GCTCCGGAGC TACACGGCCG CTGAGAGCCG

CACCTGGCCC TGGGGCAGGT GCTCCGAAGC TACATGGCTG CTGAGAGCCG

CACCTGGCCC TGGGGCAGGT GCTCCGAAGC TACATGGCTG CTGAGAGCCG

CACTTGGCCC TAGGGCAGGT GCTCCGGAGC TACACGGCTG CTGAGAGCCG

CACCTGGCTC TGGGGCAGGT GCTTCGGAGC TATGCAGTTG CTGAGAGCCG

CACTTGGCCC TGGAGCAGGC CCTCCGAAGC TACACAGCCG CTGAAAGCCG

CATTTGGCCC TGGAGCAGGC CCTCCGGAGC TACACAGCTG CTGAGAGCCG

CACTTGGCCC TGGGGCAGGC GCTCCGGAGC TACGTGGCTG CTGAGAGCCG

---------- ---------- ---------- ---------- ----------

CACTTAGCCC TGGGGCAGGT GCTCCGGAGC TACACGGCTG CTGAGAGCCG

CACTTGGCCC TGGGGCAGGT GCTCCGCAGC TACACAGCTG CCGAGAGCCG

CACTTGGCCC TGGGCCAGGC GCTGAGGAGC TACGTGGCTG CTGAGAGCCG

CACTTGGCTG TGGGCCAGGT GCTCCGGAGC TACGCGGCGG CCGAGAGCCG

---------- ---------- ---------- ---------- ----------

CACCTGGCCC TAGGCCAGCT GCTCCGGAGC TACACGGTCG CCGAGAGCCG

CACCTGGCCT TGAGCAGGTT TCTTGGTGGC TACACAGTGG TT-ATGGCCG

CACCTGGCAT TGGGCAAGGT GCTGAAGAGC TACGTGGCGG CAGAGACACG

CACCCAAGCC TCCCAAGTGC AGGGCCTGGG CAGCCTGGAA GAAGCTGTGG

CACCCAAGCC TCCCAAGTGC AGGGCCTGGG CAGCCTGGAA GAAGCTGTGG

CACCCAAGCC TCCCAAGTGC AGGGCCTGGG CAGCCTGGAA GAAGCCATGG

CACCCAAGCC TCCCAAGTGC AGGGCCTGGG CAGCCTGGAA GAAGCCGTGG

CGCCCAGGCC TCTCAAGTGC AGGGCCTGGG CAGCCTGGAA GAAGCCGTGG

TACCCAAGCC TCCCAGAGGC AGGGCCTATG CAGCTTGGAG GAAGCTCTGG

CACCCAAACC TCTCACATGC AGGGTTTAGG CAGTCTGGAA GAGGCTCTAG

TACCCAAACC TCCCAGATGC AGGGCTTAGG CAGCCTGGAA GAGGCTCTGG

CACCCAGGCC TCTCACATGC AGGGCCTGTG CAGCCTGGAA GAGGCCCTGG

---------- ---------- ---------- ---------- ----------

GACCCAAGCC TCCCAGATGC AGGGCCTGGG CAGCCTGGAA GAGGCCGTGG

CACCCAAGCC TCCCAAATGC AGGGCCTGGG CAGCCTGGAA GAGGCAGTGG

CACACAGGCC TCCCAGATGC AGGGTCTGGG CAGCTTGGAA GAGGCCGTGG

CACGCAGGCG TCCCAGATGC AGGGCCTGGG CAGCCTGGAG GAGGCGGTGG

---------- ---------- ---------- ---------- ----------

CACGCAGGCC TCCCAACTGC AGGGCTTGGG CAGCCTGGAA GAGGCTGTGG

GGCCCAGGCT TCCCCTGGGC AGAGCCCCAG CAAACTGGAG ATACCTTTGG

GGCCCAAACA TCGCGAATGC AGGGCCTGGC CGTCCTGGAG GGGGCCGTGG

AGGCCCTGGA TCCTCCAGGG GACAAAGCCA AGGTTCTCGA GGTGCATGCT

AGGCCCTGGA TCCTCCAGGG GACAAAGCCA AGGTTCTCGA GGTGCATGCT

AGGCCCTGGA TCCTTCAGGG GACAAGGCCA AGGTTCTCGA GGTGCATGCT

AGGCCCTGGA TCCTTCAGGG GACAAGGCCA AGGTTCTCGA GGTGCATGCT

AGTCCCTGGA TCCTCCAGGA GACAAGGCCA AGGTGCTCGA GGTGCACGCT

AGGCCCTGGA TCCCCCAGGG GACAAAGCCA AGGTGCTCGA GGTGCATGCT

AGGCCTTGGA TCCTCCAGGG GACAAAGCCA AAGTGCTTGA GGTTCATGCC

AGGCTTTGGA TCCTCCAGGG GACAAGGCCA AGGTGCTTGA GGTTCATGCC

AGGCCCTGGA TCCCCCAGGG GACAAGGCCA AGGTGCTTGA GGTGCATGCG

---------- ---------- ---------- ---------- ----------

AGGCCCTGGA TCCTCCAGGG GACAAGGCCA AGGTGTTGGA GGTGCATGCA

AGGCCCTAGA CCCTCCAGGC GACAAGGCCA AGGTGCTGGA GGTGCATGCA

AGGCCCTGGA CCCACCAGGC GACAGGGCCA AGGTGCTGGA GGTGCACGCG

AGGCCCTCGA CCCTCCAGGG GACAAGGCCA AGGTGCTGGA GCTGCACGCG

---------- ---------- ---------- ---------- ----------

AGGCACTGGA CCCCCCAGGG GACAAGGCCA AGGTGCTGGA GCTGCATGCC

ACGCCCTGGA CCCCGAGGGA GACAAAAGCA AAGTGCGGGA GTTCCACGCT

AGGGGCTGGA CCCTCTCGGG GACAAGGCCA AGGTGCTGGA GTTCAACGCC

ACCGTCTTCT GTCCCCCGCT GCGCTTTGAC TACCACCCCC ATGATGGGGA

ACCGTCTTCT GTCCCCCGCT GCGCTTTGAC TACCACCCCC ATGATGGGGA

GCCATCTTCT GTCCCCCGCT GCGCTTTGAC TACCACCCCC ATGATGGGGA

ACCATCTTCT GTCCCCCGCT GCGCTTTGAC TACCACCCCC ATGATGGGGA

ATCGCCTTCT GTCCCCCACT GCGCTTTGAT TACCAGCCCC ACGACGGGGA

ACTGCCTTCT GTCCTCCACT GCGTTTTGAC TACCAGCCCC ATGAGGGGGA

ATGGCCTTCT GTCCCCCTCT ACGTTTTGAC TACCAGCCCC ATGAAGGTGA

ATGGCCTTCT GTCCCCCTCT ACGTTTTGAA TACCAGCCCC ATGAAGATGA

ACGGCCTTCT GCCCCCCGCT GCGTTTTGAC TACCAGCCCC ATGAGGGCGA

---------- ---------- ---------- ---------- ----------

GTCGCCTTCT GCCCCCCACT GCGTTTTGAC TACCACCCCC ACGAAGGAGA

GTTGCCTTCT GTCCCCCACT TCGTTTTGAC TACCAGCCCC ACGAGGGGGA

GGGGCCTTCT GCCTTCCACC ACGCTTCGAT TACCACCCCC ATGAGGGGGA

GCTGCCTTCT GCCCCCCGCA GCGTTTCGAC TACCAGCCCC ATGATGGGGA

---------- ---------- ---------- ---------- ----------

ACTGCCTTCT GTCCCCCACT GCGCTTCGAC TACCAGCCCC ACGAAGGGGA

TTTCCTTTTT GCCCGCCCTT TGGATTTGAT TCCCACCCTT ATGAAGGGGA

ACGGCCTTCT GCCCGCCTCT GCGCTTCGAC TACCAGCCCT ATGATGGGGA

TGAGCCCCCC GAGCCAGGTG GGGAAGACGG GCCGGTCAAC CTGGCCCAGC

TGAGCCCCCC GAGCCAGGTG GGGAAGACGG GCCGGTCAAC CTGGCCCAGC

TGAG------ ---------- ---------- ---------- ----------

TGAGCCCCCC ATGCCGAGTG GGGAAGACAG GCCCGTCAAC CTGGCCCGGC

TGAGTGCCCC AAGCCGGGTG GGGAAGACGG GCCCGCCAAC TTGGCCCAGC

TGAG------ ---------- ---------- ---------- ----------

TGAGTTGTCC AAGCCAGATG GGGAAGATAG GCCCATCAAC GTTGCCCAGC

TGAGTTGTCC AAGCCAGGTG GGGAAGATAG GCCCATCAAC GTTGCCCAGC

TGAG------ ---------- ---------- ---------- ----------

---------- ---------- ---------- ---------- ----------

TGAGCCCCCC AAGCCGGGTG GGGAAGACTG GTCCACCGAC CTCGCCCCAC

TGAGCCCCAC AAACCAGGTG GGGAATACTG GTCCACCAAC CTGGCCCAGC

CGAG------ ---------- ---------- ---------- ---GCCTGGC

CGAGGCCCCC AAGCCGCAGG GGGAGGACAG GTCGGCCGAT CTGGCCCAGC

---------- ---------- ---------- ---------- ----------

CGAG-CCCAC AACCCAGCCA GGAAAGAGCA GTCCACCAGA CGGGCCCAGC

TGAGCACCCA AAGGCCAGAG CGGAGGAAGC ATCTGCCAAT G---CCGGGC

CGAGTACCCC GAGCCCGAGG AGACGGGACC GCCAGCCAAC GTCAACAGAC

AACAAAACCT GCTGTTGCAG AAGGTAAAGG CAGAGCTGGA AGAAGCCAAG

AACAAAACCT GCTGTTGCAG AAGGTAAAGG CAGAGCTGGA AGAAGCCAAG

---------- ---------- ---------- ---------- ----------

AACAAAACCT GATATTGCAG AAGGTAAAGG AAGAGCTGGA AGAAGCCAAG

AACAAAAGCT GCTGTTGCAG AAGGTGAAGG AAGAGCTGGA AGAAGTCAAG

---------- ---------- ---------- ---------- ----------

AACAAAAACT ACTTTTGCAG AAGATGAAAG AAGAACTGAC AGAAGCCAAG

AACAAAAGCT GCTTTTGCAG AAGATGAAGG AAGAACTGAC AGAAGCCAAG

---------- ---------- ---------- ---------- ----------

---------- ---------- ---------- ---------- ----------

AGCGAAACCT GCTGTTGCAG AAAATGAAGG AAGACCTGAA AGAAGTCACG

AGCGAAACCT GCCGTTGCAG AAGGTGAAGG AAGAGCTGAA AGAAGTAGAG

GG-GACGGCA GATGCTGAAG AAGATGGAGG ATGAACTGAA GGAGGTCAGA

AACGCCACCT GCTGCTGCAG AAGATGAAGG AGGAGCTGGA AGAGGCCAAG

---------- ---------- ---------- ---------- ----------

AACAGGGCCC CCTGCTGCAG AAGATGAAGG AGGAGCTGGC AGAGATCAAG

AGCAGGACCA ACTGTTCCTG ATGATAAAGA AGGAGCTGGA GGAGATTAAA

AGGATGACTT GGCCGTCCAG AAAATGAATG ACCAGCTGGA TGTTGTAAAA

AGGTTCCACA TCAGCCTCCA GGAGTCCTAT CACAGCCTAA AGGAGAGGTC

AGGTTCCACA TCAGCCTCCA GGAGTCCTAT CACAGCCTAA AGGAGAGGTC

---------- ---------- ---------- ---------- ----------

AGGTTCCACA TCAGCCTCCA GGCGTCCTAT CACAGCCTAA AGGAGAGGTC

AAGTTGCATG CCAGCCTCCA GGTGGCACGT CACAGCCTAA AGGAGAGGTC

---------- ---------- ---------- ---------- ----------

AAGGTTCATG CTAGCCTTCA GCTCTCCCAT CAAAACCTCA AGGAAAGTCA

AAGGTCCATG CTAGCTTTCA GGTATCCCAT CAAAGCCTCA AAGAAAGTCA

---------- ---------- ---------- ---------- ----------

---------- ---------- ---------- ---------- ----------

GATGTGCACC TCAACCTCCA GGTGTCCTAT CAGAGCCTAA AGGACAGGCA

AAGTTACACC ACAGCCTCCA GATGTCCTAT CAGAGTCTAA AGGAGAAGTA

AAGCTGCACT TTGACCTGCA GGCATCCTAC CAAAGCCT-- ----------

GCCATCCACA TCAGCCTCCA GGGGTCCTAC TGGAGCCTAA AGGAGCAGTA

---------- ---------- ---------- ---------- ----------

AAGGAGCACC TCTGCCTCCA GGTGTCCTGT GAGCGCCTCA AGGAGCAGCG

AAATCCCAGA GCAAGCTCGA GCTGTCCTAT CGTACCCTGA AGGAGAGGTA

ATGTTCTATT CTAGCTTGGA AACAGACTTT CAGAATCTAA AGGAAAAGTA

TCTGACTGAC CTGCAGCTGT TGCTGGAGTC CCTTCAGGAG GAGAGGGAGA

TCTGACTGAC CTGCAGCTGT TGCTGGAGTC CCTTCAGGAG GAGAGGGAGA

---------- ---------- ---------- ---------- ----------

TCTGACTGAC CTGCAGCTGT TGCTGGAGTC CCTTCAGGAG GAGAGAGAGA

TCTGACCGAC CTGGAGCTGC TGCTGGAGTC CCTTCAGGAG GAGAGAGAGA

---------- ---------- ---------- ---------- ----------

TATGATTGAT GTGCGGAGGA TACTGGAGTC ACTCCAGGAA AAGAGAGATG

TATGATTGAT GTGCAGAGGA TACTGGAGTC ACTCCAGGAA AAGAAAGATG

---------- ---------- ---------- ---------- -----AGAAG

---------- ---------- ---------- ---------- ----------

TCTGATGGAC CTGCAGGTGT CACTAGAGGC CCTTCAGGAA GAGAGGGAGG

TCTGACAGAC CTGCAGGTGT TGGTGGAGTC CCTTCAGGAG GAGAAAGAGG

---GTCAGAC CTATACCTGT CACTGAAGTC CAGGCAGGAG G---------

CCTGAGCGAC CTGCAGCTGT GGCTGGAGTC CCTGGAGGAG GGCAGAGAGG

---------- ---------- ---------- ---------- ----------

CCAGGCCAAC CTGCAGCGGG CACTGAAGAC CCTGCAGGAG A----AGAGG

CCTGGTTGAT CTGAAGCTGA TTATTGAGTC TCTTCAAGAG GAGAG-GAGG

CCTGTCTGAA TTGGAACAGA TAGTGGAATG CCTACAGGAA GAAAGAGAGG

TCACGGAGAC CTTCAAGAGC CGAATATCCA AGCTGGAGAT GCTACAGCAA

TCACGGAGAC CTTCAAGAGT CGAATATCCA AGCTGGAGAT GCTACAGCAA

---------- ---------- ---------- ---------- ----------

TCATGGAGAC CTTCAAGAGC CGAATATCCA AGCTGGAGAT GCTACAGCAA

TCATGGAGAC CTTCGAGAGC CGAATATCCA AGCTGGAGAC ACTACCGCAA

---------- ---------- ---------- ---------- ----------

TAATGGAGAC TTTCAAGAAC AGAATAACAA AGCTAGAAAC TCTACAGCAA

TAATGGAGAT TTTCAAGAGC AGAATAACCA AGCTAGAAAC TCTACAGCAA

TCATGGAAAC TTTCAAGAGC CGAATCTCCA GACTAGAAGC TCTGGAGCGA

---------- ---------- ---------- ---------- ----------

TCATGGAGAC TTTCAAGAAC CGTATAGCTA AGCTTGAAAC TCTGCAACAA

TCATGGAGAC TTTCAAGAGT CGTATATCCA AGCTCGAAAC GCTACAGCAA

---------- ---------- ---------- ---------- ----------

TCATGGAGAC TTTCAAGAGC CAGATAGCCA AGCTGGAGAC ACTACAGCAA

---------- ---------- ---------- ---------- ----------

TCATGGAGAC GGTCCAGAGC CGGGTCACTA AGCTGGAGAA CCTCCAGCAG

TGATGAGGAC CTTCCAGAGT CGAATTTCCA AGCTGGAGAC CCAACAGCAA

TGATGGAAAC CTTTCAGACC CGCATTTCCA AACTAGAGGC TCTGCAGCAA

GTCACCCAAC TGGAGGCAGC GGAGCACCTC CAAAGCCGTC CCCCGCAGAT

GTCACCCAAC TGGAGGCAGT GGAGCACCTC CAAAGCCGTC CCCCGCAGAT

---------- ---------- ---------- ---------- ----------

GTCACCCGAC TGGAGGCAGC AGAGCACCTC CAAAGCCGTC CTCCGAAGAT

GTCACCCAGC TGGAGGCAGC GGAGCACCTC CGAAGCCGTC CCCTACGGGT

---------- ---------- ---------- ---------- ----------

GCTACCCAAC TGGAGATGAT GGCCAGCCTC AGAACCCGCC CCAAGGACTT

GCTACCCAAC TGGAGATGAT GGCCAGCCTC AGAACCCGTC CCAAGGACTT

GTCGCTCAGC TGGAGATGAC GGAGAACCTC CGAAGCCGCC CCAGGGAGTT

---------- ---------- ---------- ---------- ----------

GTGACACAAC TGGAGGTGAC GCACAGGTTC CGGATCCGTC CCCAGGAGTT

GCGACCCAAC TGGAGATGAC AGAGAGCCTC CGTAGCCGTC CCCAG---TT

---------- ---------- ---------- ---------- ----------

GTGTCCCAGC TGGAGATGAT GCGGAAGCTC AGACACGGGC CGCAGGAGCT

---------- ---------- ---------- ---------- ----------

GTGGCCCAGC GGGAGCTAGC GAAGCAGTTC TGGAGCCGGC CAAGGGAGTT

AGAGCCCAGT TAGAAATGAC CGAGAAGCCC CAAAGCCACT CCCAAGTCTT

ACAGCGCAGC CGGAGATGGT GGAGAAATGT AAAACCCAGG CCCAGGTGTT

GTTGTTCAAG TTCCTGAGTC CGCGCCTCTC ACTGGCAACC GTCCTCTTGG

GTTGTTCAAG TTCCTGAGTC CGCGCCTCTC ACTGGCAACT GTCCTCTTGG

---------- ---------- ---------- ---------- ----------

GTTGTTCAAG TTCGTGAGTC TGCTCTTCTC ACTGGCAACC GTCCTCTTGG

GGTGTTCAAG TTCCTGAGAC CGCTCCTCTC ACTGACCACC GTCCTCTTGG

---------- ---------- ---------- ---------- ----------

TTTCTTCAGA TTCATAAGCC TGCTTCTCAC TCTGACCACT ATCCTCTTGG

TTTGTTCAGA TTCATAAGCC TGCTTCTCAC TCTGACCACT ATCCTCTTGG

TCTGTTCATC TTCATGAGCC TGCTCCTCAC GCTGGCCACT GTCCTCCTGG

---------- ---------- ---------- ---------- ----------

TATGTTCCGG CTCATGAGCC TGCTCCTCAC ACTGGCTACC ATCCTCCTCG

CCTGTTCCGA TTCGGGAACC TACTCCTTAC ACTGGCCACC ATCCTCCTCG

---------- ---------- ---------- ---------- ----------

CTTGTTCAAG TTCATGAGCC TGCTCCTCAC GCTGGCCACC ATCCTCTTGG

---------- ---------- ---------- ---------- ----------

CCTGCTCAAG TTCATCAGCC TGCTCCTCAC TCTGGCCACC ATCCTCTTGG

TTTAGTTAGG TTCATGAGCT TGCTCCTAAC TTTGACCACC ATCGTCCTCA

CCTGGACAAG TTCATGAACC TACTCGTCAC TTTGACCAAC ATCCTCCTGG

TCTTTGTCTC CACCTTGTGT GCCTGCCCCT CGTCACTGAT CAGCTCACGC

TCTTTGTCTC CACCTTGTGT GCCTGCCCCT CGTCACTGAT CAGCTCACGC

---------- ---------- ---------- ---------- ----------

TCTTTATCTC CACCTTGTGT GCCTGCCCCT CATCACTGAT CACCTCACGC

TCTTTGTCTC CACCTTGTGT GCCTGCCCCT TGTCACTTGT CAACTCGCAC

---------- ---------- ---------- ---------- ----------

TCGTGGTCTC TACATTGTGT TCTTGCCCTT TGCCCTTGCT CAACTCACGC

TCGTGGTCTC TACATTGTGT TCTTGCCCCT TGCCCTTGCT CAGCTCACGC

TCCTCGTCTC CACCGTGTGT GCCTGCCCCT TGTCCCTGGT CAACTCCCGC

---------- ---------- ---------- ---------- ----------

TCTTCATCTC CACTGTGTGC TCCTGCCCCT TGCCACTGCT CAATTCCCGC

TCTTCATCGC CACTGTGTGC ACCTGCCCCT TGCTGCTGGT GAATTCGCAT

---------- ---------- ---------- ---------- ----------

TCTCCGTGTC CATCGTGTGC GCCTGCCCCT TGCCCCTCAT CAAGACGCGC

---------- ---------- ---------- ---------- ----------

TCTGCATCTC CACCAAGTGC GCTTTCCCCT GGCCCTTCTT CAAGACGCAC

TATGCGTGTC TACCATCTGT AGCTGCCACT TGCCTTTCTT CAAGTACCGT

TCTGCATCAC TAGCATGTCC TGCTGCCCCT TGTCTTTGAT CAGGAGCCCT

CTGTGCACCT GCACCATGCT GATGCTGATC GGGCTTGGGG TCCTGGCCTG

CTGTGCACCT GCACCATGCT GATGCTGATC GGGCTTGGGG TCCTGGCCTG

---------- ---------- ---------- ---------- ----------

CTGTGCACTT GCACCACCCT GATGCTGATT GGGCTTGGGG TACTGGCCTG

CTGCGCACTT GCAGCATGCT GATGCTGATT GGGCTTGGGG TCCTGGCCTG

---------- ---------- ---------- ---------- ----------

CTGCGCATCT TCATTGTATT TATGATCATT GGGCTTGGGA CCTTGGCCTG

CTGCGCATCT TCATTGTATT TATGATCATT GGGCTTGGGA CTTTGGCCTG

CTGCGCACGT GCACCATCCT CGTCCTGCTC GGGCTCGGGG CGCTGGTGTG

---------- ---------- ---------- ---------- ----------

CTGCGCACGT GCACCACACT CCTGCTGATC GGCCTCGTGG CCCTGGCCTG

CTGCGCATGT GCACCATACT CATGCTGATC GGTCTCGGGG TCCTGGCCTG

---------- ---------- ---------- ---------- ----------

CTGCGGACGT GCGCCACGCT CATGCTGATC GGCCTCGGGG CCCTGGCCTG

---------- ---------- ---------- ---------- ----------

CTGCGCATGT GCGCCTCCCT CATACTCATA GGGCTCGGGG TCCTGATTTG

CTACGTACTT GTATCATCCT CTTGCTCATT GCCCTGGGGA TGGTCGCCTG

CCGCGCATTT TAGCCACCCT CATGGTGATT ATCCTGGGAG CAGTCGCCTG

GCAGAGGTGG CGCGCCATCC CTGCCACAGA CTGGCAGGAA TGGGTCCCCT

GCAGAGGTGG CGCGCCATCC CTGCCACAGA CTGGCAGGAA TGGGTCCCCT

---------- ---------- ---------- ---------- ----------

GCAGAGGCGG CACGCCATCC CTGCCACAGA CTGGCCAGAA TGGGTCTCCT

GCAGAGGTGG CACACCATCC CTGCCATGGA CTGGCAGGCA TGGGTCCCCT

---------- ---------- ---------- ---------- ----------

GCAGAAGCGG CATGTCATCT CTATTATTGA CTGGCAGGCC TGGGTCCCCT

GCAGAAGCGC CATGTCATCT CTATTATTGA CTGGCAGGCC TGGGTCCCCT

GCAGAAGCGA CACGCCATCC CCACCGTGGC CTGGCAGGCG TGGATCCCCT

---------- ---------- ---------- ---------- ----------

GCAGAAACGG CATGCCTTCC CTACCACCGA CTGGCAGGCC TGGGTCCCCT

GCAGAAGTGG CATGCCACCC TTGCCCTGCA CTGGCAGGCC TGGGACCCCT

---------- ---------- ---------- ---------- ----------

GCAGAGGTGG CCGGCCATCT CCACCGTGGA CTGGCAGGCG TGGGTCACCT

---------- ---------- ---------- ---------- ----------

GGAGAAGCAG TATGCCATCC CTACCGCTGA CTGGCAAGCT TGGGTTGCCT

GCAGGAGTGG TACAATATCC CATACATACA TTGGCATGAA TGGCTCTTAA

GCAGAACTGG CAGGCCAGAC CAGACATGGA CTGGCATGCT TGGCTTTCCT

CCAGGTGTAG ACTGTCCAAG GACTCTGGGC CTCCAGCCCA GCAACAAAAC

CCAGGTGTAG ACTGTCCAAG GACTCTGGGC CTCCAGCCCA GCAACAAAAC

---------- ---------- ---------- ---------- ----------

CCAGGTGTAG ACTGTCCAAG AACTCTGGGC CTCCAGCCCG GCAACAAAAC

CTAGGTGGAG ACTGTCCAAG GACTCTGAGC CTCCAGCCCA GCAACAAAAG

---------- ---------- ---------- ---------- ----------

TTAAGTGGAG ATCATTAAAG GATGCTAAAC CTCCAGCCCA GCAACAAAAA

TTAAGTGGAG ACAATTAAAG GATGCTAAGC CTCCAGCCCA GCAACAAAAG

CCCGATGGAG ACTGGCCAAG GACTCCAAGC CCCCG----- ----------

---------- ---------- ---------- ---------- ----------

CCAGATGGAG ACTGCCCAAG GATTCCAAGC CTCTAGCCCC ACAGCGAAAC

CTGGG---AG ACTGTCCAAG GCCTCTAATC CACTAGCCCA GCAGCGAAAC

---------- ---------- ---------- -----GCCTG GCGG-GACGG

CCAAGTGGAG ACTCTCCAAG GTCTTCAGCC CTCTAGCCCA GCAACGCCAC

---------- ---------- ---------- ---------- ----------

CCAAGTGGAG ACTCACCAGG GCCCCCAAGC CTCTCGCCCA GCAACAGGGC

CGAGGTGGAA ATTGTACAAA GACTCCAGGC CTGT--CCGG GCAGCAGGAC

CCAAGTGGAA AGAGTGTAAG GAGTCATGGG CGCGGAACAG ACAGGATGAC

CTGCTGTTGC AGAAGGTAAA GGCAGAGCTG GAAGAAGCCA AGAGGTTCCA

CTGCTGTTGC AGAAGGTAAA GGCAGAGCTG GAAGAAGCCA AGAGGTTCCA

---------- ---------- ---------- ---------- ----------

CTGATATTGC AGAAGGTAAA GGAAGAGCTG GAAGAAGCCA AGAGGTTCCA

CTGCTGTTGC AGAAGGTGAA GGAAGAGCTG GAAGAAGTCA AGAAGTTGCA

---------- ---------- ---------- ---------- ----------

CTACTTTTGC AGAAGATGAA AGAAGAACTG ACAGAAGCCA AGAAGGTTCA

CTGCTTTTGC AGAAGATGAA GGAAGAACTG ACAGAAGCCA AGAAGGTCCA

---------- ---------- ---------- ---------- ----------

---------- ---------- ---------- ---------- ----------

CTGCTGTTGC AGAAAATGAA GGAAGACCTG AAAGAAGTCA CGGATGTGCA

CTGCCGTTGC AGAAGGTGAA GGAAGAGCTG AAAGAAGTAG AGAAGTTACA

CAGATGCTGA AGAAGATGGA GGATGAACTG AAGGAGGTCA GAAAGCTGCA

CTGCTGCTGC AGAAGATGAA GGAGGAGCTG GAAGAGGCCA AGGCCATCCA

---------- ---------- ---------- ---------- ----------

CCCCTGCTGC AGAAGATGAA GGAGGAGCTG GCAGAGATCA AGAAGGAGCA

CAACTGTTCC TGATGATAAA GAAGGAGCTG GAGGAGATTA AAAAATCCCA

TTGGCCGTCC AGAAAATGAA TGACCAGCTG GATGTTGTAA AAATGTTCTA

CATCAGCCTC CAGGAGTCCT ATCACAGCCT AAAGGAGAGG TCTCTGACTG

CATCAGCCTC CAGGAGTCCT ATCACAGCCT AAAGGAGAGG TCTCTGACTG

---------- ---------- ---------- ---------- ----------

CATCAGCCTC CAGGCGTCCT ATCACAGCCT AAAGGAGAGG TCTCTGACTG

TGCCAGCCTC CAGGTGGCAC GTCACAGCCT AAAGGAGAGG TCTCTGACCG

---------- ---------- ---------- ---------- ----------

TGCTAGCCTT CAGCTCTCCC ATCAAAACCT CAAGGAAAGT CATATGATTG

TGCTAGCTTT CAGGTATCCC ATCAAAGCCT CAAAGAAAGT CATATGATTG

---------- ---------- ---------- ---------- ----------

---------- ---------- ---------- ---------- ----------

CCTCAACCTC CAGGTGTCCT ATCAGAGCCT AAAGGACAGG CATCTGATGG

CCACAGCCTC CAGATGTCCT ATCAGAGTCT AAAGGAGAAG TATCTGACAG

CTTTGACCTG CAGGCATCCT ACCAAAGCCT ---------- -----GTCAG

CATCAGCCTC CAGGGGTCCT ACTGGAGCCT AAAGGAGCAG TACCTGAGCG

---------- ---------- ---------- ---------- ----------

CCTCTGCCTC CAGGTGTCCT GTGAGCGCCT CAAGGAGCAG CGCCAGGCCA

GAGCAAGCTC GAGCTGTCCT ATCGTACCCT GAAGGAGAGG TACCTGGTTG

TTCTAGCTTG GAAACAGACT TTCAGAATCT AAAGGAAAAG TACCTGTCTG

ACCTGCAGCT GTTGCTGGAG TCCCTTCAGG AGGAGAGGGA GATCACGGAG

ACCTGCAGCT GTTGCTGGAG TCCCTTCAGG AGGAGAGGGA GATCACGGAG

---------- ---------- ---------- ---------- ----------

ACCTGCAGCT GTTGCTGGAG TCCCTTCAGG AGGAGAGAGA GATCATGGAG

ACCTGGAGCT GCTGCTGGAG TCCCTTCAGG AGGAGAGAGA GATCATGGAG

---------- ---------- ---------- ---------- ----------

ATGTGCGGAG GATACTGGAG TCACTCCAGG AAAAGAGAGA TGTAATGGAG

ATGTGCAGAG GATACTGGAG TCACTCCAGG AAAAGAAAGA TGTAATGGAG

---------- ---------- ---------- -------AGA AGTCATGGAA

---------- ---------- ---------- ---------- ----------

ACCTGCAGGT GTCACTAGAG GCCCTTCAGG AAGAGAGGGA GGTCATGGAG

ACCTGCAGGT GTTGGTGGAG TCCCTTCAGG AGGAGAAAGA GGTCATGGAG

ACCTATACCT GTCACTGAAG TCCAGGCAGG AGG------- ----------

ACCTGCAGCT GTGGCTGGAG TCCCTGGAGG AGGGCAGAGA GGTCATGGAG

---------- ---------- ---------- ---------- ----------

ACCTGCAGCG GGCACTGAAG ACCCTGCAGG AGA----AGA GGTCATGGAG

ATCTGAAGCT GATTATTGAG TCTCTTCAAG AGGAGAG-GA GGTGATGAGG

AATTGGAACA GATAGTGGAA TGCCTACAGG AAGAAAGAGA GGTGATGGAA

ACCTTCAAGA GCCGAATATC CAAGCTGGAG ATGCTACAGC AAGTCACCCA

ACCTTCAAGA GTCGAATATC CAAGCTGGAG ATGCTACAGC AAGTCACCCA

---------- ---------- ---------- ---------- ----------

ACCTTCAAGA GCCGAATATC CAAGCTGGAG ATGCTACAGC AAGTCACCCG

ACCTTCGAGA GCCGAATATC CAAGCTGGAG ACACTACCGC AAGTCACCCA

---------- ---------- ---------- ---------- ----------

ACTTTCAAGA ACAGAATAAC AAAGCTAGAA ACTCTACAGC AAGCTACCCA

ATTTTCAAGA GCAGAATAAC CAAGCTAGAA ACTCTACAGC AAGCTACCCA

ACTTTCAAGA GCCGAATCTC CAGACTAGAA GCTCTGGAGC GAGTCGCTCA

---------- ---------- ---------- ---------- ----------

ACTTTCAAGA ACCGTATAGC TAAGCTTGAA ACTCTGCAAC AAGTGACACA

ACTTTCAAGA GTCGTATATC CAAGCTCGAA ACGCTACAGC AAGCGACCCA

---------- ---------- ---------- ---------- ----------

ACTTTCAAGA GCCAGATAGC CAAGCTGGAG ACACTACAGC AAGTGTCCCA

---------- ---------- ---------- ---------- ----------

ACGGTCCAGA GCCGGGTCAC TAAGCTGGAG AACCTCCAGC AGGTGGCCCA

ACCTTCCAGA GTCGAATTTC CAAGCTGGAG ACCCAACAGC AAAGAGCCCA

ACCTTTCAGA CCCGCATTTC CAAACTAGAG GCTCTGCAGC AAACAGCGCA

ACTGGAGGCA GCGGAGCACC TCCAAAGCCG TCCCCCGCAG ATGTTGTTCA

ACTGGAGGCA GTGGAGCACC TCCAAAGCCG TCCCCCGCAG ATGTTGTTCA

---------- ---------- ---------- ---------- ----------

ACTGGAGGCA GCAGAGCACC TCCAAAGCCG TCCTCCGAAG ATGTTGTTCA

GCTGGAGGCA GCGGAGCACC TCCGAAGCCG TCCCCTACGG GTGGTGTTCA

---------- ---------- ---------- ---------- ----------

ACTGGAGATG ATGGCCAGCC TCAGAACCCG CCCCAAGGAC TTTTTCTTCA

ACTGGAGATG ATGGCCAGCC TCAGAACCCG TCCCAAGGAC TTTTTGTTCA

GCTGGAGATG ACGGAGAACC TCCGAAGCCG CCCCAGGGAG TTTCTGTTCA

---------- ---------- ---------- ---------- ----------

ACTGGAGGTG ACGCACAGGT TCCGGATCCG TCCCCAGGAG TTTATGTTCC

ACTGGAGATG ACAGAGAGCC TCCGTAGCCG TCCCCAG--- TTCCTGTTCC

---------- ---------- ---------- ---------- ----------

GCTGGAGATG ATGCGGAAGC TCAGACACGG GCCGCAGGAG CTCTTGTTCA

---------- ---------- ---------- ---------- ----------

GCGGGAGCTA GCGAAGCAGT TCTGGAGCCG GCCAAGGGAG TTCCTGCTCA

GTTAGAAATG ACCGAGAAGC CCCAAAGCCA CTCCCAAGTC TTTTTAGTTA

GCCGGAGATG GTGGAGAAAT GTAAAACCCA GGCCCAGGTG TTCCTGGACA

AGTTCCTGAG TCCGCGCCTC TCACTGGCAA CCGTCCTCTT GGTCTTTGTC

AGTTCCTGAG TCCGCGCCTC TCACTGGCAA CTGTCCTCTT GGTCTTTGTC

---------- ---------- ---------- ---------- ----------

AGTTCGTGAG TCTGCTCTTC TCACTGGCAA CCGTCCTCTT GGTCTTTATC

AGTTCCTGAG ACCGCTCCTC TCACTGACCA CCGTCCTCTT GGTCTTTGTC

---------- ---------- ---------- ---------- ----------

GATTCATAAG CCTGCTTCTC ACTCTGACCA CTATCCTCTT GGTCGTGGTC

GATTCATAAG CCTGCTTCTC ACTCTGACCA CTATCCTCTT GGTCGTGGTC

TCTTCATGAG CCTGCTCCTC ACGCTGGCCA CTGTCCTCCT GGTCCTCGTC

---------- ---------- ---------- ---------- ----------

GGCTCATGAG CCTGCTCCTC ACACTGGCTA CCATCCTCCT CGTCTTCATC

GATTCGGGAA CCTACTCCTT ACACTGGCCA CCATCCTCCT CGTCTTCATC

---------- ---------- ---------- ---------- ----------

AGTTCATGAG CCTGCTCCTC ACGCTGGCCA CCATCCTCTT GGTCTCCGTG

---------- ---------- ---------- ---------- ----------

AGTTCATCAG CCTGCTCCTC ACTCTGGCCA CCATCCTCTT GGTCTGCATC

GGTTCATGAG CTTGCTCCTA ACTTTGACCA CCATCGTCCT CATATGCGTG

AGTTCATGAA CCTACTCGTC ACTTTGACCA ACATCCTCCT GGTCTGCATC

TCCACCTTGT GTGCCTGCCC CTCGTCACTG ATCAGCTCAC GCCTGTGCAC

TCCACCTTGT GTGCCTGCCC CTCGTCACTG ATCAGCTCAC GCCTGTGCAC

---------- ---------- ---------- ---------- ----------

TCCACCTTGT GTGCCTGCCC CTCATCACTG ATCACCTCAC GCCTGTGCAC

TCCACCTTGT GTGCCTGCCC CTTGTCACTT GTCAACTCGC ACCTGCGCAC

---------- ---------- ---------- ---------- ----------

TCTACATTGT GTTCTTGCCC TTTGCCCTTG CTCAACTCAC GCCTGCGCAT

TCTACATTGT GTTCTTGCCC CTTGCCCTTG CTCAGCTCAC GCCTGCGCAT

TCCACCGTGT GTGCCTGCCC CTTGTCCCTG GTCAACTCCC GCCTGCGCAC

---------- ---------- ---------- ---------- ----------

TCCACTGTGT GCTCCTGCCC CTTGCCACTG CTCAATTCCC GCCTGCGCAC

GCCACTGTGT GCACCTGCCC CTTGCTGCTG GTGAATTCGC ATCTGCGCAT

---------- ---------- ---------- ---------- ----------

TCCATCGTGT GCGCCTGCCC CTTGCCCCTC ATCAAGACGC GCCTGCGGAC

---------- ---------- ---------- ---------- ----------

TCCACCAAGT GCGCTTTCCC CTGGCCCTTC TTCAAGACGC ACCTGCGCAT

TCTACCATCT GTAGCTGCCA CTTGCCTTTC TTCAAGTACC GTCTACGTAC

ACTAGCATGT CCTGCTGCCC CTTGTCTTTG ATCAGGAGCC CTCCGCGCAT

CTGCACCATG CTGATGCTGA TCGGGCTTGG GGTCCTGGCC TGGCAGAGGT

CTGCACCATG CTGATGCTGA TCGGGCTTGG GGTCCTGGCC TGGCAGAGGT

---------- ---------- ---------- ---------- ----------

TTGCACCACC CTGATGCTGA TTGGGCTTGG GGTACTGGCC TGGCAGAGGC

TTGCAGCATG CTGATGCTGA TTGGGCTTGG GGTCCTGGCC TGGCAGAGGT

---------- ---------- ---------- ---------- ----------

CTTCATTGTA TTTATGATCA TTGGGCTTGG GACCTTGGCC TGGCAGAAGC

CTTCATTGTA TTTATGATCA TTGGGCTTGG GACTTTGGCC TGGCAGAAGC

GTGCACCATC CTCGTCCTGC TCGGGCTCGG GGCGCTGGTG TGGCAGAAGC

---------- ---------- ---------- ---------- ----------

GTGCACCACA CTCCTGCTGA TCGGCCTCGT GGCCCTGGCC TGGCAGAAAC

GTGCACCATA CTCATGCTGA TCGGTCTCGG GGTCCTGGCC TGGCAGAAGT

---------- ---------- ---------- ---------- ----------

GTGCGCCACG CTCATGCTGA TCGGCCTCGG GGCCCTGGCC TGGCAGAGGT

---------- ---------- ---------- ---------- ----------

GTGCGCCTCC CTCATACTCA TAGGGCTCGG GGTCCTGATT TGGGAGAAGC

TTGTATCATC CTCTTGCTCA TTGCCCTGGG GATGGTCGCC TGGCAGGAGT

TTTAGCCACC CTCATGGTGA TTATCCTGGG AGCAGTCGCC TGGCAGAACT

GGCGCGCCAT CCCTGCCACA GACTGGCAGG AATGGGTCCC CTCCAGGTGT

GGCGCGCCAT CCCTGCCACA GACTGGCAGG AATGGGTCCC CTCCAGGTGT

---------- ---------- ---------- ---------- ----------

GGCACGCCAT CCCTGCCACA GACTGGCCAG AATGGGTCTC CTCCAGGTGT

GGCACACCAT CCCTGCCATG GACTGGCAGG CATGGGTCCC CTCTAGGTGG

---------- ---------- ---------- ---------- ----------

GGCATGTCAT CTCTATTATT GACTGGCAGG CCTGGGTCCC CTTTAAGTGG

GCCATGTCAT CTCTATTATT GACTGGCAGG CCTGGGTCCC CTTTAAGTGG

GACACGCCAT CCCCACCGTG GCCTGGCAGG CGTGGATCCC CTCCCGATGG

---------- ---------- ---------- ---------- ----------

GGCATGCCTT CCCTACCACC GACTGGCAGG CCTGGGTCCC CTCCAGATGG

GGCATGCCAC CCTTGCCCTG CACTGGCAGG CCTGGGACCC CTCTGGG---

---------- ---------- ---------- ---------- ----------

GGCCGGCCAT CTCCACCGTG GACTGGCAGG CGTGGGTCAC CTCCAAGTGG

---------- ---------- ---------- ---------- ----------

AGTATGCCAT CCCTACCGCT GACTGGCAAG CTTGGGTTGC CTCCAAGTGG

GGTACAATAT CCCATACATA CATTGGCATG AATGGCTCTT AACGAGGTGG

GGCAGGCCAG ACCAGACATG GACTGGCATG CTTGGCTTTC CTCCAAGTGG

AGACTGTCCA AGGACTCTGG GCCTCCAGCA GATCCTGTAA TTATTTTGCC

AGACTGTCCA AGGACTCTGG GCCTCCAGCA GATCCTGTAA TTATTTTGCC

---------- ---------- ---------- ---------- ----------

AGACTGTCCA AGAACTCTGG GCCTCCAGCA CATCCTGTAA TTATTTTGCC

AGACTGTCCA AGGACTCTGA GCCTCCAGCC GATCCTGTGA TTATTTTACC

---------- ---------- ---------- ---------- ----------

AGATCATTAA AGGATGCTAA ACCTCCATCA GCTCAC-TAA TTACTTTCCC

AGACAATTAA AGGATGCTAA GCCTCCATCA GATCACGTAA GTTCTCTCCC

AGACTGGCCA AGGACTCCAA GCCCCCGTCA GATTCTGTAG TTATTTTGTC

---------- ---------- ---------- ---------- ----------

AGACTGCCCA AGGATTCCAA GCCTCTATCA GATCTTGTAT TAATTTTGCC

AGACTGTCCA AGGCCTCTAA TCCACTATCA GACCCTGTGC TTATTTTGCC

---------- ---------- ---------- -------TCT CTGTCGTGCC

AGACTCTCCA AGGTCTTCAG CCCTCTAGCC GATACTGTAG TTATCTTGCC

---------- ---------- ---------- ------GTAG TTGTTTTGTT

AGACTCACCA GGGCCCCCAA GCCTCTCGCA GAAACGGTAG TTGTTCAGCT

AAATTGTACA AAGACTCCAG GCCTGTGCCG TGCGCT-AAA GTGGTCGGCC

AAAGAGTGTA AGGAGTCATG GGCGCGGTCA GTTTGT-TAG TTTCTCTAGC

AAAGAAACAT AAGAAGAAAA AGGAGCGGAA GTCATTGCCA GAAGAAGATG

AAAGAAACAT AAGAAGAAAA AGGAGCGGAA GTCATTGCCA GAAGAAGATG

---------- ---------- ---------- ---------- ----------

AAAGAAACAT AAGAAGAAAA AGGAGCGGAA GTCATTGCCA GAAGAAGATG

AAAGAAACAT AAGAAGAAAA AGGAGCGGAA GTCATTGCCA GAAGAAGATG

---------- ---------- ---------- ---------- ----------

AAAGAAGCAT AAGAAGGAAA AAGACTGGAA GTCATTCCAA GAAGAAGATG

AAAGAAGCAT AAGAAGAAAA AAGACCGGAA GCCATTACAA GAAGATGATG

AAAGAAGCAT AAGAAGAAAA AAGACCGGAA GTCGTTGCCA GCAGATGATA

---------- ---------- ---------- ---------- ----------

GAAGAAACAT AAGAAGAAAA AGGAGCGGAA GTCCTTACCA GAAGAAGATG

GAAGAAGCAT AAGAAGAAAA AAGGGGTGAA GTCATTACCA GAAGAAGATG

GAAGAAGCAC AAGAAGAAGA AGGAGCGGAA GTCTGTAGCT GAGGAAGACG

GAAGAAGCAT AAGAAGAAGA AAGACCGAAA GTCACTAGCA GAAGAAGATG

GAAGAAACAT AAGAAGAAGA AGGACCGGAA GTCATTAGCA GAAGATGATG

GAAGAAGCAT AAGAAGAAAA AGGACCGAAA GTCGCTACCT GAAGAAGATG

CAAGAAGCAC AAGAAAAAGA AGGAGCGCAA AGTCCTGGGA GACGCGGCTG

GAAGAAGCAC AAGAAAAAAA AAGACCGGAA AGAATTAGCA GATGAAGATG

TAGCCGAAAT ACAACACGCT GAAGAATTTC TTATCAAACC TGAATCCAAA

TAGCCGAAAT ACAACATGCT GAAGAATTTC TTATCAAACC TGAATCCAAA

---------- ---------- ---------- ---------- ----------

TAGCTGAAAT ACAACACGCT GAAGAATTTC TTATCAAACC CGAATCCAAA

TAGCCGAAAT ACAACACGTT GAAGAATTTC TTATCAAACC TGAATCCAAA

---------- ---------- ---------- ---------- ----------

TAGCTGAAAT CCAACATGCT GAAGAATTTC TTATCAAACC AGAATCCAAA

TTGCC-AAAT ACAACATGCT GAAGAATTTC TTATCAAACC AGAATCTAAA

TAGCT-AAAT ACAACATGCT GAAGAATTTC TTATCAAACC TGAATCCAAA

---------- ---------- ---------- ---------- ----------

TAGCGGAGAT ACAACATGCT GAGGAATTTC TTATCAAACC TGAATCCAAA

TAGCAGAAAT ACAACACGTT GAAGAATTTC TTATCAAACC TGAATCCAAA

TTGCCGAAAT ACAACTTGCT GAAGATTTTC TTATCAAGCC CGAATCCAAA

TAGCCGAAAT TCAGCACATT GAAGAATTTC TTATCAAACC TGAATCCAAA

TAGCCGAAAT ACAACATGCT GAAGAATTTC TTATAAAACC TGAATCCAAA

TAGCTGAAAT ACAACATGCT GAGGAATTCC TTATAAAACC TGAATCCAAA

TGGCGGAGCT CCAGCACGAA GGGGAATTCT TCATCAAGCC CGAGTCCAAA

TTGCTGAAAT ACAGCACATG GGTGAATTCT TCATCAAGCC TGAATCCAAA

GTTGCTAAGT TGGACACGTC TCAGTGGCCC CTTTTGCTAA AGAATTTTGA

GTTGCTAAGT TGGACACGTC TCAGTGGCCC CTTTTGCTAA AGAATTTTGA

---------- ---------- ---------- ---------- ----------

GTTGCTAAGT TGGACACGTC TCAGTGGCCC CTTCTGCTAA AGAATTTTGA

GTTGCTAAGT TGGACACGTC TCAGTGGCCC CTTTTGCTAA AGAATTTTGA

---------- ---------- ---------- ---------- ----------

GTGGCTCAAT TAGACACTTC TCAGT-GTCC TTGTTACTAA AGAATTTTGA

GTGGCTCAAT TAGACACTTC TCAGTGGCCC TTGTTACTAA AGAATTTTGA

GTGGCTCAGT TGGACACTTC TCAGTGGCCC TTGTTGCTAA --AATTTTGA

---------- ---------- ---------- ---------- ----------

GTGGCTCAGT TGGACACATC TCAATGGCCC CTGTTGCTCA AGAATTTTGA

GTGGCTCAGC TGGACACATC CCAGTGGCCC CTGTTGCTAA AGAACTTTGA

GTGGCTCAGT TGGACACGTC TCACTGGCCT CTGTTGCTAA AGAACTTTGA

GTGGCTCAGT TGGACACATC TCAGTGGCCC CTGTTGTTGA AGAATTTTGA

GTGGCTCAGT TGGACACATC TCAGTGGCCC TTGTTATTAA AGAATTTCGA

GTGGCTCTAT TGGACACCTC ACAGTGGCCC CTCCTGTTAA AGAATTTCGA

GTAGTTCAGT TGGACACGTC TCAGTGGCCC TTGCTGCTGA AGAACTTTGA

GTGGCTCAGT TGGACACATC TCAGTGGCCC TTATTGCTAA AGAATTTTGA

TAAGCTGAAT GTAAGGACAA CACACTATAC ACCTCTTGCA TGTGGTTCAA

TAAGCTGAAT GTAAGGACAA CACACTATAC ACCTCTTGCA TGTGGTTCAA

---------- ---------- ---------- ---------- ----------

TAAGCTGAAT GTAAGGACAA CACACTATAC ACCTCTTGCA TGTGGTTCAA

TAAGCTGAAT GTAAGGACAA CTCACTATAC ACCTCTTGCA TGTGGTTCAA

---------- ---------- ---------- ---------- ----------

TAAGCTAAAT GTAAGAACAA TACACTATAC ACCTATTCCT TGTAGTTCAA

TAAGCTAAAT GTAAGGACAG CACACTATAC ACCTCTTCCT TGTGGTTCAA

CAAACTAAAT GTCAGGACAA CACATTATAC ACCTCTTCCT TGTGGCTCAA

---------- ---------- ---------- ---------- ----------

TAAGCTAAAT GTAAGGACAA CGCACTATAC ACCTCTTCCT TGTGGTTCAA

TAAGCTAAAT GTAAGGACGA CACACTATAC ACCTCTTCCT TGTGGTTCAA

TAAGCTAAAT ATAAGAACAA CGCACTATAC CCCTCTTCCC TGTGGTTCAA

TAAACTAAAT GTAAGGACAA CACACTATAC TCCTCTTCCT TCTGGTTCAA

TAAGCTAAAT GTAAGGACAA CACACTATAC TCCTCTTCCT TCTGGTTCAA

TAAGCTGAAT GTCAGGACAA CACACTACAC CCCTCTTCCT TCTGGTTCAA

CAAGCTGAAC GTAAGGACCA CCCACTATAC TCCCCTCCCT TCCGGATCGA

TAAGCTAAAT GTACGAACAA CGCATTACAC TCCCCTTCCA TCTGGATCCA

ATCCTCTGAA GAGAGAGATT GGGGACTATA TCAGGACAGG TTTCATTAAT

ATCCTCTGAA GAGAGAGATT GGGGACTATA TCAGGACAGG TTTCATTAAT

---------- ---------- ---------- ---------- ----------

ATCCTCTGAA GAGAGAGATT GGGGACTATA TCAGGACAGG TTTCATTAAT

ATCCTCTGAA GAGAGAGATT GGGGACTATA TCAGGACAGG TTTCATTAAT

---------- ---------- ---------- ---------- ----------

ATCCTCTGAA GAGGGAGATT GAGGACTGTA TCAGGACAGG TTTCATTAAT

ATCCTCTGAA GAGGGAGATT GGGGACTATA TCAG-ACCGG TTTCATTAAT

ATCCTCTGAA GAGGGAGATT GGGGACTATA TTAG-ACAGG TTTCATTAAT

---------- ---------- ---------- ---------- ----------

ATCCTTTGAA GAGGGAGATT GGGGACTATA TCAGGACAGG TTTTATTAAT

GTCCTCTGAA GAGGGAGATT GGAGATTATA TCAGGACAGG TTTCATTAAT

ACCCTCTGAA GAGGGAGATT GGGGACTACA TCAGAACAGG TTTCATTAAT

ATCCCCTGAA GAGGGAGATT GCAGACTACA TCAGGACAGG TTTCATTAAC

ATCCTCTCAA GAGGGAGATT GCCGACTATA TTAGGACAGG TTTCATTAAC

ACCCTCTGAA GAGGGAAATC GCCGACTACA TCAGGACAGG TTTCATTAAC

ACCCTCTCAA GAGAGAGATC ACGGACTACA TCAGGACAGG TTTCATTAAC

ATCCTCTCAA GAGAGAAATT GCTGAGTATG TCAGGACCGG CTTCATTAAC

CTTGACAAGC CCTCTAACCC CTCTTCCCAT GAGGTGGTAG CCTGGATTCG

CTTGACAAGC CCTCTAACCC CTCTTCCCAT GAGGTGGTAG CCTGGATTCG

---------- ---------- ---------- ---------- ----------

CTTGACAAGC CCTCTAACCC CTCTTCCCAT GAGGTGGTAG CCTGGATTCG

CTCGACAAGC CCTCTAACCC CTCTTCCCAT GAGGTGGTAG CCTGGATTCG

---------- ---------- ---------- ---------- ----------

CTTGTCAAGC CCTATAACCC CTCTTCCCAT GAGGTGGCAG CCTGGATCCA

CTTGACAAGC CCTCTAACCC CTCTTCCCAT GAGGTGGTAG CCTGGATCCG

CTTGACAAGC CCTCCAACCC CTCTTCCCAT GAGGTGGTAG CCTGGATCCG

---------- ---------- ---------- ---------- ----------

CTTGACAAGC CCTCCAACCC CTCTTCCCAT GAGGTGGTAG CCTGGATCCG

CTTGACAAGC CCTCCAACCC CTCTTCCCAT GAGGTGGTAG CCTGGATCCG

CTCGACAAGC CCTCCAATCC CTCTTCCCAT GAGGTGGTAG CCTGGATTCG

CTTGACAAGC CATCCAACCC CTCTTCCCAT GAGGTGGTAG CCTGGATCCG

CTTGACAAGC CTTCTAACCC CTCTTCCCAT GAGGTGGTAG CCTGGATCCG

CTGGACAAGC CCTCCAACCC GTCTTCACAT GAAGTAGTGG CTTGGATCCG

CTTGACAAAC CCTCCAATCC CTCTTCCCAT GAGGTAGTTG CATGGATCCG

CTCGACAAGC CCTCCAACCC CTCTTCCCAT GAAGTGGTCG CTTGGATCCG

ACGGATACTT CGGGTGGAGA AGACAGGGCA CAGTGGTACG CTGGATCCCA

ACGGATACTT CGGGTGGAGA AGACAGGGCA CAGTGGTACT CTGGATCCCA

---------- ---------- ---------- ---------- ----------

ACGGATACTT AGGGTGGAGA AGACAGGGCA CAGTGGTACT CTGGATCCCA

ACGGATACTT AGGGTGGAGA AGACAGGGCA CAGTGGTACT CTGGATCCCA

---------- ---------- ---------- ---------- ----------

TCGAATACTT CAGGTAGAGA AGACTGACCA CAGTGGCACA CAGGATTCCA

ACGAATACTT CGGGTAGAGA AGACTGGCCA CAGTGGCACA CTAGATCCCA

ACGAATCCTT CGGGTGGAGA AGACAGGACA TAGCGGTACT CTGGATCCCA

---------- ---------- ---------- ---------- ----------

CCGAATACTT CGGGTGGAGA AGACGGGGCA CAGTGGTACT CTGGATCCCA

ACGGATACTT CGGGTGGAGA AGACAGGACA CAGTGGTACT CTAGACCCTA

ACGGATACTT CGGGTAGAAA AGACTGGACA CAGTGGTACT CTGGATCCCA

ACGAATACTT CGGGTTGAGA AGACAGGACA CAGTGGTACC TTGGATCCCA

GCGAATACTC CGTGTTGAGA AGACAGGACA CAGCGGTACT CTGGATCCCA

GCGGATACTT CGTGTAGAGA AAACAGGACA CAGTGGGACA CTGGACCCCA

GCGAATACTC CGGGTTGAGA AGACGGGCCA CAGTGGTACT TTGGATCCGA

GCGAATCCTG CGGGTGGAGA AGACAGGACA CAGTGGAACT CTAGATCCCA

AGGTGACTGG TTGTTTAATC GTGTGCATAG AACGAGCCAC TCGCTTGGTG

AGGTGACTGG TTGTTTAATC GTGTGCATAG AACGAGCCAC TCGCTTGGTG

---------- ---------- ---------- ---------- ----------

AGGTGACTGG TTGTTTAATC GTGTGCATAG AACGAGCCAC TCGCTTGGTG

AGGTGACTGG TTGTTTAATC GTGTGCATAG AACGAGCCAC TCGGTTGGTG

---------- ---------- ---------- ---------- ----------

AAGTGACTGA TTGTTTAATT GTGTGCATTG AACGAGTCAC TTGTTTGGTA

AAGTGACTGG TTGTTTAATT GTGTGCATTG AACGAGCCAC TCGTTTGGTG

AGGTGACTGG CTGTTTAATC GTGTGCATAG AACGAGCCAC TCGCTTGGTG

---------- ---------- ---------- ---------- ----------

AGGTGACTGG TTGTTTAATC GTGTGCATAG AACGAGCCAC TCGATTGGTG

AGGTGACTGG CTGTTTAATC GTGTGCATAG AACGAGCCAC TCGCTTGGTA

AGGTGACCGG CTGTTTAATT GTGTGCATAG AACGAGCCAC TCGCTTAGTG

AGGTGACAGG CTGTCTAATT GTGTGCATAG AAAGAGCCAC TCGCTTGGTA

AGGTGACTGG CTGTCTGATC GTGTGCATAG AACGAGCCAC CCGCTTGGTG

AGGTGACCGG CTGTCTGATC GTGTGCATCG AGCGAGCCAC CCGCCTGGTG

AGGTGACTGG CTGTTTAATT GTGTGTATAG AACGAGCTAC CCGCTTGGTC

AAGTGACGGG CTGCTTGATT GTCTGCATAG AACGAGCTAC CCGCTTGGTC

AAGTCACAAC AGAGTGCAGG CAAAGAGTAT GTGGGGATTG TCCGGCTGCA

AAGTCACAAC AGAGTGCAGG CAAAGAGTAT GTGGGGATTG TCCGGCTGCA

---------- ---------- ---------- ---------- ----------

AAGTCACAAC AGAGTGCAGG CAAAGAGTAT GTGGGGATTG TCCGGCTGCA

AAGTCACAAC AGAGTGCAGG CAAAGAGTAT GTGGGGATTG TCCGGCTGCA

---------- ---------- ---------- ---------- ----------

AAATCACAGC AGAGTGTAG- ---------- --TAGAATTG TCTGGCTGAA

AAATCACAAC AGAGTGCAGG CAAAGAGTAT GTTGGAATTG TCCGGCTGCA

AAATCACAGC AGAGTGCAGG CAAAGAGTAT GTGGGAATTG TTCGGCTGCA

---------- ---------- ---------- ---------- ----------

AAATCTCAAC AGAGTGCAGG CAAAGAGTAT GTGGGAATTG TCCGGCTACA

AAATCACAGC AAAGTGCAGG CAAAGAGTAT GTGGGAATTG TCCGGCTACA

AAGTCACAGC AGAGTGCAGG CAAAGAGTAT GT-GGGATTG TC---CTGCA

AAATCACAAC AGAGTGCAGG CAAAGAGTAT GTTGGAATTG TCCGGCTGCA

AAATCACAAC AGAGTGCAGG CAAAGAGTAT GTGGGAATTG TCCGGCTGCA

AAATCTCAGC AGAGCGCAGG CAAAGAGTAT GTGGGGATTG TTCGGCTGCA

AAATCTCAGC AGAGTGCAGG CAAAGAGTAT GTGGGAGTTA TCCGACTGCA

AAATCCCAAC AGAGTGCAGG CAAAGAGTAT GTGGGAATTG TTCGGCTGCA

CAATGCTATT GAAGGGGGGA CCCAGCTTTC TAGGGGAATC TTTTGGGTGA

CAATGCTATT GAAGGGGGGA CCCAGCTTTC TAGGGGAATC TTTTGGGTGA

---------- ---------- ---------- ---------- ----------

CAATGCTATT GAAGGGGGGA CCCAGCTTTC TAGGGGAATC TTTTGGGTGA

CAATGCTATT GAAGGGGGGA CCCAGCTTTC TAGGGGAATC TTTTGGGTGA

---------- ---------- ---------- ---------- ----------

CAAT-----T GCAGGAGGTA CTCAGCT--- ----GGAATC TTTTGCGTGA

CAATGCTATT GAAGGGGGTA CTCAGCTTTC TAGGG-AATC TTTTGGGTAA

CAATGCTATT GAAGGGGGTA CCCAGCTTTC TAGGG-CATA TTTTGGGTGA

---------- ---------- ---------- ---------- ----------

CAATGCTATT GAAGGGGGGA CTCAACTTTC TAGGGGAATC TTTTGGGTGA

CAATGCTATT GAAGGGGGGA CTCAACTTTT TAGGGGAATC TTTTGGGTGA

CATTGCTATT GAAGGAGAGA GCCAGCTTTC TAGGGGGATC TTCTGGGTGA

CAATGCTATT GAAGGGGGGA CCCAGCTTTC TAGGGGAATC TTTTGGGTAA

CGATGCCATT GAAGGGGGGA CCCAGCTTTC TAGGGGAATC TTTTGGGTGA

CAATGCTATT GAAGGGGGTG CCCAGCTTTC TAGGGGGATC TTTTGGGTGA

CAATGCTATT GAAAGTGAGG TTCAGCTTTC CAGGGGTATC TTCTGGGTGA

CAATGCCATT GAAGGCGAGA TTCAGCTTTC CAGG-GTATC TTCTGGGTGA

GTTGTGAGGC TGGCACCTAC ATTCGGACAT TATGTGTGCA CCTTGGTTTG

GTTGTGAGGC TGGCACCTAC ATTCGGACAT TATGTGTGCA CCTTGGTTTG

---------- ---------- ---------- ---------- ----------

GTTGTGAGGC CGGCACCTAC ATTCGGACAT TATGTGTGCA CCTTGGTTTG

GTTGTGAGGC CGGCACTTAC ATTCGGACAT TATGTGTGCA TCTTGGTTTG

---------- ---------- ---------- ---------- ----------

GCTGTGAAGC TGGCACCTAC ATTCGGACAC TATGTGTACA CCGTGGCTTG

GCTGTGAAGC TGGCACCTAC ATTCGGACAC TATGCGTACA CCTTGGCTTG

GTTGCGAGGC TGGCACCTAC ATTCGGACAC TGTGTGTGCA CCTTGGTTTG

---------- ---------- ---------- ---------- ----------

GTTGTGAGGC CGGTACCTAC ATTCGGACGC TGTGTGTGCA CCTTGGCTTG

GTTGTGAGGC CGGCACCTAC ATTCGGACGC TGTGTGTGCA CCTTGGTTTA

GCTGTGAGGC CGGCACCTAC ATTCGCACAC TGTGTGTGCA CCTGGGGTTG

GTTGTGAGGC TGGCACCTAC ATTCGGACGC TGTGTGTGCA CCTTGGTCTG

GCTGTGAGGC TGGCACCTAC ATTCGTACAC TTTGCGTGCA CCTTGGTTTG

GCTGTGAGGC TGGCACCTAC ATCCGGACAC TGTGTGTGCA CCTTGGCTTG

GCTGTGAGGC TGGAACATAC ATTCGCACAT TATGCGTCCA CGTGGGGCTG

GTTGTGAGGC GGGTACGTAC ATTAGAACCC TGTGCGTCCA CCTTGGCTTG

TTATTGGGAG TTGGTGGTCA GATGCAGGAG CTTCGGAGGG TTCGTTCTGG

TTATTGGGAG TTGGTGGTCA GATGCAGGAG CTTCGGAGGG TTCGTTCTGG

---------- ---------- ---------- ---------- ----------

TTATTGGGAG TTGGTGGTCA GATGCAGGAG CTTCGGAGGG TTCGTTCTGG

TTATTGGGAG TTGGCGGTCA AATGCAGGAG CTTCGGAGGG TTCGTTCTGG

---------- ---------- ---------- ---------- ----------

TTACTTGGAG TTGGTGGTCA GATGCAGGAA CTTTGGAGGG TGCGTTCTGG

TTACTGGGAG TTGGTGGTCA GATGCAGGAA CTTCGGAGGG TGCGTTCTGG

TTATTGGGAG TTGGCGGTCA GATGCAAGAA CTTCGAAGAG TTCGTTCTGG

---------- ---------- ---------- ---------- ----------

TTACTGGGAG TTGGCGGTCA GATGCAAGAA CTTCGGAGGG TTCGTTCTGG

TTATTGGGAG TTGGCGGTCA GATGCAGGAA CTTCGGAGAA TTCGTTCTGG

TTGCTTGGCG TCGGTGGTCA GATGCAGGAG CTTAGGAGGG TCCGCTCCGG

CTACTGGGAG TTGGAGGACA GATGCAGGAA CTTCGGAGGG TTCGTTCTGG

TTACTGGGAG TTGGAGGTCA GATGCAAGAA CTTCGGAGGG TTCGTTCTGG

TTACTGGGAG TTGGAGGGCA GATGCAGGAG CTCCGCCGGG TTCGTTCTGG

TTACTGGGAG TTGGAGGCCA AATGCAGGAG CTCCGTCGGA TCCGCTCTGG

CTGCTGGGAG TTGGAGGTCA GATGCAAGAA CTCCGAAGGG TCCGCTCCGG

AGTCATGAGT GAAAAGGACC ACATGGTGAC AATGCATGAT GTGCTTGATG

AGTCATGAGT GAAAAGGACC ACATGGTGAC AATGCATGAT GTGCTTGATG

---------- ---------- ---------- ---------- ----------

AGTCATGAGT GAAAAGGACC ACATGGTGAC AATGCACGAT GTGCTTGATG

AGTCATGAGT GAAAAGGACC ACATGGTGAC AATGCACGAT GTGCTTGATG

---------- ---------- ---------- ---------- ----------

AGTCATGAGT GAAAAGGACC ACACGGTGAT GATGCATGAA GTACTTGATG

AGTCATGAGT GAAAAG-ACC ACATGGTGAC AATGCATGAT GTACTCGATG

AGTCATGAGT GAAAAG-ACC ACATGGTGAC AATGCACGAT GTGCTTGATG

---------- ---------- ---------- ---------- ----------

AGTCATGAGT GAAAAGGACC ACATGGTGAC AATGCACGAC GTGCTTGATG

AGTCATGAGC GAAAAGGACC ACATGGTGAC AATGCATGAC GTGCTTGATG

AGTCATGAAT GAAAAGGACC ACATGGTGAC CATGCATGAT GTGCTCGATG

GGTTATGAGT GAAAAGGACC ACATGGTGAC TATGCATGAT GTGCTAGATG

AGTTATGAGT GAAAAGGACC ACATGGTGAC AATGCATGAC GTCCTGGATG

TGTGATGAGT GAAAAGGATC ACATGGTGAC GATGCACGAT GTCCTGGACG

AGTCATGGGG GAGAAGGACC ACATGGTGAC CATGCACGAT GTATTGGACG

AGTTATGGGA GAAAAGGATC ACATGGTCAC CATGCATGAT GTTCTAGATG

CTCAGTGGCT GTATGATAAC CACAAGGATG AGAGTTACCT GCGGCGAGTT

CTCAGTGGCT GTATGATAAC CACAAGGATG AGAGTTACCT GCGGCGAGTT

---------- ---------- ---------- ---------- ----------

CTCAGTGGCT ATATGATAAC CACAAGGATG AGAGTTACCT GCGGCGAGTT

CTCAGTGGCT GTATGATAAC CACAAGGATG AGAGTTACCT GCGACGAGTT

---------- ---------- ---------- ---------- ----------

CTCAGTGGCT GTATGATAAC CGTAAGGATG AGAGTTACTT GCAGC---TT

CTCAGTGGCT GTATGATAAC CATAAGGATG AGAGTTACTT GCGGCGTGTT

CTCAGTGGCT GTATGACAAC CATAAGGACG AAAGCTACCT GCGCCGAGTT

---------- ---------- ---------- ---------- ----------

CCCAGTGGCT GTATGACAAC CACAAGGATG AGAGTTACCT GCGGCGAGTT

CCCAGTGGCT GTACGACAAC CACAAGGATG AGAGTTACTT GCGGCGAGTT

CACAGTGGCT GTACGACAAC CACAAGGACG AGAGCTACCT GCGGCGAGTC

CGCAGTGGCT ATATGATAAT CACAAGGACG AGAGTTACCT GCGGCGAGTT

CCCAGTGGCT GTATGATAAT CACAAGGATG AGAGTTACCT GCGCCGAGTT

CCCAGTGGCT GTATGATAAT CACAAGGATG AGAGCTACCT GCGGCGAGTT

CCCAGTGGCA ATATGACAAT TACAAGGATG AGGGCTATCT GCGGCGGGTC

CTCAATGGCA ATATGACAAT AACAAGGATG AGAGTTACCT GAGGCGGGTC

GTTTACCCTT TGGAAAAGCT GTTGACATCT CATAAACGGC TGGTTATGAA

GTTTACCCTT TGGAAAAGCT GTTGACATCT CATAAACGGC TGGTTATGAA

---------- ---------- ---------- ---------- ----------

GTTTACCCTT TGGAAAAGTT GTTGACATCT CATAAACGGC TGGTTATGAA

GTTTACCCTT TGGAAAAGCT GTTGACATCT CATAAACGGC TGGTTATGAA

---------- ---------- ---------- ---------- ----------

GTTTACCATT TGGAAAAGCT GTTGACATCA CATAAACATC TGGCTATGAA

GTTTACCCTT TGGAAAAGCT GTTGACATCT CATAAACGTC TGGTTATGAA

GTTTACCCTT TGGAAAAGCT GCTGACATCT CATAAACGGC TGGTTATGAA

---------- ---------- ---------- ---------- ----------

GTTTACCCTT TGGAAAAGCT GTTGACTTCT CATAAAAGGC TGGTGATGAA

GTGTACCCTT TGGAAAAGCT GTTGACATCT CATAAACGGC TGGTTATGAA

ATTTTCCCCT TGGAGAAACT GCTGACGTCG CATAAGCGGC TGGTCATGAA

ATATACCCGT TGGAAAAGCT GCTGACATCT CATAAACGGC TGGTTATGAA

ATTTACCCTC TGGAAAAGCT GCTGACATCA CATAAGCGGC TGGTTATGAA

GTATACCCGC TGGAAAAGCT GTTAACGTCT CACAAGAGGA TGGTTATGAA

ATTTATCCTC TTGAGAAGCT GTTAACATCC CACAAGCGGT TGGTCATGAA

ATCTACCCTC TGGAGAAACT CCTGACCTCC CACAAGCGAC TAGTCATGAA

AGACAGTGCA GTAAATGCCA TCTGCTATGG GGCCAAGATT ATGCTTCCAG

AGACAGTGCA GTAAATGCCA TCTGCTATGG GGCCAAGATC ATGCTTCCAG

---------- ---------- ---------- ---------- ----------

AGACAGTGCA GTAAATGCTA TCTGCTATGG GGCCAAGATC ATGCTTCCAG

AGACAGTGCA ---------- ---------- ---------- ----------

---------- ---------- ---------- ---------- ----------

AGATAATGCA GTGGATGCAA TCTGC---GG GGCAGAGATC ATGCTTCCTG

AGATAGTGCA GTGAATGCAA TCTGCTATGG GGCCAAGATC ATGCTTCCTG

AGACAGTGCA GTGAATGCCA TCTGCTATGG GGCCAAGATC ATGCTTCCAG

---------- ---------- ---------- ---------- ----------

AGACAGTGCA GTGAACGCAA TCTGCTATGG GGCAAAGATC ATGCTCCCGG

AGACAGTGCA GTGAATGCGA TCTGCTATGG GGCGAAGATC ATGCTTCCGG

GGATAGCGCG GTGAACGCCA TCTGCTATGG AGCCAAGATC ATGCTTCCAG

AGACAGTGCG GTGAATGCAA TCTGCTATGG AGCCAAGATT ATGCTTCCAG

AGACAGCGCG GTGAATGCGA TCTGCTATGG AGCAAAGATC CTGCTTCCCG

GGACAGTGCG GTGAACGCAA TTTGCTACGG AGCCAAGATC CTGCTGCCAG

GGACAGTGCG GTCAATGCCA TCTGCTATGG AGCAAAGATC ATGCTTCCTG

AGACAGTGCG GTCAATGCAA TCTGCTATGG GGCCAAAATT ATGCTTCCTG

GTGTTCTTCG ATATGAGGAC GGCATTGAGG TCAATCAGGA GATTGTGGTT

GTGTTCTTCG ATATGAGGAC GGCATTGAGG TCAATCAGGA GATTGTGGTT

---------- ---------- ---------- ---------- ----------

GTGTTCTTCG ATATGAGGAT GGCATTGAGG TCAATCAGGA GATTGTGGTT

---------- ---------- ---------- ---------- ----------

---------- ---------- ---------- ---------- ----------

GTGTTCTTCA ATATGAGGAT GACATTAAGG TCAACCAGGA GATTGTGGTC

GTCTTCTTCG ATATGAAGAT GGCATTGAGG TCAACCAGGA GATTGTGGTC

GTGTTCTCCG ATATGAGGAT GGCATTGAAG TCAATCAGGA GATTGTGGTC

---------- ---------- ---------- ---------- ----------

GCGTTCTCCG ATACGAGGAT GGCATCGAGG TCAATGAGGA GATCGTCGTC

GTGTTCTCCG ATACGAGGAC GGCATTGAAG TTAATCAGGA GATTGTGGTC

GTGTTCTTCG CTATGAGGAT GGCATCGAGG TCAATCAGGA GATCGTGGTC

GTGTTCTTCG ATATGAAGAT GGCATTGAAG TCAATCAAGA GATTGTGGTC

GTGTTCTTCG GTACGAGGAC GGCATTGAAG TCAATCAGGA GATCGTAGTC

GTGTCCTGCG GTACGAGGAT GGCATTGAAC TCAACCAGGA GATTGTTGTC

GGGTTCTGCG CTATGAGGAC GGCATTGAAG TCAATCAAGA AGTTGTAGTC

GAGTTCTGAG GTATGAGGAT GGAATTGAAG TCAATCAGGA GATTGTGGTC

ATCACCACCA AAGGAGAAGC AATCTGCATG GCTATTGCAT TAATGACCAC

ATCACCACCA AAGGAGAAGC AATCTGCATG GCTATTGCAT TAATGACCAC

---------- ---------- ---------- ---------- ----------

ATCACCACCA AAGGAGAAGC AATCTGCATG GCTATTGCGT TAATGACCAC

---------- ---------- ---------- -CTATTGCGT TAATGACCAC

---------- ---------- ---------- ---------- ----------

ATTACCACTA AGGGGGAAGT TATCTGCATG GCTATCACAT TGATGACTAC

ATTACTACTA AGGGGGAAGC TATCTGCATG G---TTGCAT TGATGACTAC

ATCACCACCA AAGGGGAAGC CATCTGCATG G---TTGCAC TAATGACCAC

---------- ---------- ---------- ---------- ----------

ATCACCACCA AAGGGGAAGC AATCTGCATG GCCATTGCAT TAATGACCAC

ATCACCACCA AAGGGGAAGC AATCTGCATA GCAATTGCAT TAATGACCAC

ATTACCACCA AGGGGGAGGC CATCTGCATG GCCATTGCAC TCATGACCAC

ATCACCACCA AAGGGGAAGC CATTTGTATG GCTATTGCAT TAATGACCAC

ATCACCACCA AAGGGGAGGC GATCTGTATG GCTATTGCAT TAATGACCAC

ATCACCACCA AGGGGGAAGC TATCTGTATG GCCATTGCCT TGATGACCAC

ATCACGACCA AAGGGGAAGC CATTTGTGTG GCCATTGCCT TGATGACGAC

ATTACTACCA AAGGCGAGGC CATCTGTATG GCCATCGCCT TGATGACCAC

AGCGGTCATC TCTACCTGCG ACCATGGTAT AGTAGCCAAG ATCAAGAGAG

AGCGGTCATC TCTACCTGCG ACCATGGTAT AGTACCCAAG ATCAAGAGAG

---------- ---------- ---------- ---------- ----------

AGCAGTCATC TCTACCTGCG ATCATGGTAT AGTAGCAAAG ATCAAGAGAG

AGCAGTCATC TCTACCTGCG ACCATGGTAT AGTAGCCAAG ATCAAGAGAG

---------- ---------- ---------- ---------- ----------

AGCAGTGATT TCTACCTGTG ACCATGGTAT AATAGGCAAG ATAAAGAGGG

AGCAGTGATT TCTACCTGTG ACCATGGTAT AGTAGCCAAA ATAAAGAGGG

CGCAGTGATC TCTACATGTG ACCATGGTAT AGTAGCAAAG ATCAAGAGAG

---------- ---------- ---------- ---------- ----------

AGCAGTGATC TCTACCTGTG ACCATGGTAT CGTAGCCAAG ATTAAGAGGG

AGCAGTGATC TCTACCTGCG ACCATGGCAT AGTAGCCAAG ATTAAGAGAG

GGCAGTGATA TCTACCTGTG ACCATGGCGT CGTGGCCAAG ATCAAGCGAG

AGCAGTAATC TCTACCTGTG ACCATGGTAT AGTAGCTAAG ATCAAAAGAG

AGCAGTGATC TCTACCTGTG ACCATGGGAT AGTAGCTAAG ATCAAGAGAG

TGCCGTCATC TCCACCTGTG ACCATGGCAT AGTGGCCAAG ATCAAAAGGG

CGCAGTGATC TCTACCTGTG ACCATGGTAT CGTGGCCAAG ATTAAGAGAG

AGCCGTTATC TCTACCTGTG ACCATGGCAT AGTGGCCAAG ATTAAAAGAG

TGATCATGGA GAGAGACACT TACCCTCGGA AGTGGGGTTT AGGTCCAAAG

TGATCATGGA GAGAGACACT TACCCTCGG- ---------- ----------

---------- ---------- ---------- ---------- ----------

TGATCATGGA GAGAGACACT TACCCTCGGA AGTGGGGTTT AGGTCCAAAG

TGATCATGGA GAGAGACACT TACCCTCGGA AGTGGGGTTT AGGGCCAAAG

---------- ---------- ---------- ---------- ----------

TGATCATGGT AAGAGACACT TATCCT---A AGTGGGGTTT AGGTCCAAAG

TGATCATGGA GAGAGACACT TATCCTCGCA AGTGGGGTTT AGGTCCAA--

TGATCATGGA GAGAGACACT TACCCTCGGA AGTGGGGTTT AGGCCCAA--

---------- ---------- ---------- ---------- ----------

TAATCATGGA GAGAGACACT TATCCTCGAA AGTGGGGTTT AGGTCCAAAG

TGATCATGGA GAGAGACACT TACCCTCGGA AGTGGGGCTT AGGTCCCAAG

TGATCATGGA GAGAGACACT TACTCTCGGA AGTGGGGCTT AGGGCCAAAG

TGATCATGGA GAGAGATACT TACCCTCGGA AGTGGGGTTT AGGTCCGAAG

TGATCATGGA GAGAGATACA TACCCTCGGA AGTGGGGTTT AGGTCCAAAG

TTATCATGGA GCGAGACACT TACCCTCGGA AGTGGGGTCT TGGGCCCAAG

TTATCATGGA GAGAGACACT TACCCTCGGA AGTGGGGTCT GGGCCCAAAG

TGATCATGGA GAGGGACACA TATCCTCGCA AGTGGGGCCT GGGGCCAAAG

GCAAGTCAGA AGAAGCTGAT GATCAAGCAG GGCCTTCTGG ACAAGCATGG

GCAAGTCAGA AGAAGCTGAT GATCAAGCAG GGCCTTCTGG ACAAGCATGG

---------- ---------- ---------- ---------- ----------

GCAAGTCAGA AGAAGCTGAT GATCAAGCAG GGTCTTCTGG ACAAGTACGG

GCCAGTCAGA AGAAGCTGAT GATCAAGCAG GGCCTTCTGG ACAAGCATGG

---------- ---------- ---------- ---------- ----------

GCAAGTCAGA AGAGGATGAT GACCAAGCAA GGCCTTCTGG ACAAGCATGG

GCAAGTCAGA AGAAGATGAT GATCAAGCAG GGCCTTCTGG ACAAGCATGG

GCAAGTCAGA AGAGGCTGAT GATCAAGCAA GGCCTTCTGG ACAAGCATGG

---------- ---------- ---------- ---------- ----------

GCAAGTCAGA AGAGGCTGAT GATCAAGCAG GGCCTTCTGG ATAAGCACGG

GCAAGTCAGA AGAGGTTGAT GATCAAGCAG GGCCTTCTGG ATAAGCACGG

GCGAGTCAGA AGAAGCTTCT GATCAAGCAG GGGCTCTTGG ATAAGCACGG

GCAAGTCAGA AGAAGATGAT GATCAAGCAG GGCCTTCTGG ATAAGCATGG

GCCAGTCAGA AGAGGATGAT GATCAAGCAG GGCCTTCTGG ACAAGCATGG

GCAACTGAGA AGAAGATGAT GATCAAGCAG GGCCTCCTGG ACAAGCACGG

GCAAGTCAAA AGAAGATGAT GATCAAGCAG GGTCTTCTGG ACAAGCATGG

GCAAGTCAGA AGAAACTGAT GATCAAGCAA GGTCTTTTGG ACAAGCACGG

GAAGCCCACA GACAGCACAC CTGCCACCTG GAAGCAGGAG TATGTTGACT

GAAGCCCACA GACAGCACAC CTGCCACCTG GAAGCAGGAG TATGTTGACT

---------- ---------- ---------- ---------- ----------

GAAGCCCACA GAGAGTACGC CTGCCACCTG GAAGCAGGAG TATGTTGACT

GAAACCAACA GACAGCACGC CTGCCACCTG GAAGCAGGAG TATGTTGACT

---------- ---------- ---------- ---------- ----------

CAAACCCACA AACAGTACCC CTGCCACA-- -AAAAAGGAT TTCATTGCCT

CAAACCCACA GACAATACCC CTGCCACATG GAAACAGGAT TACATTGACT

CAAACCCACA GATAGCACCC CTCCCTCCTG GAAGCAGGAG TATGTCGACT

---------- ---------- ---------- ---------- ----------

CAAGCCCACA GATAGCACAC CTGCCACCTG GATGCAGGAG TATGTGGACT

CAAGCCCACG GACAGTACAC CTGCCACCTG GACGCAGGAG TATGTCGACT

CAAGCCCACA GACAGCACTC CTGCCTCATG GACCCAGGAC TACGTGGACT

CAAGCCCACA GATAGCACAC CTACTGCTTG GATGCAGGAA TACGTTGACT

CAAGCCCACG GACAGCACAC CCGCTGCTTG GGTACAGCAA TATGTGGACT

GAAGGCCACG GCCAGCACGC CCTCGGCATG GGTGCGGGAC TACGTGGACT

AAAGCCCAAC GACAGCACGC CTACAGCCTG GAAGGACGAC TATATTGACT

CAAGCCCAAC GAGAGCACCC CAACAACTTG GAAGCAGGAC TACGTGGACT

ACAGTGAGTC TGCCAAAAAA GAGGTGGTTG CTGCCGAAGC AGCAAAAACT

ACAGTGAGTC TGCCAAAAAA GAGGTGGTTG CTGCCGAAGC AGCAAAAACT

---------- ---------- ---------- ---------- ----------

ACAGTGAGAC TGCCAAAAAA GAGGTGGTTG CTGCCGAAGC AGCAAAAACT

ACAGTGAGTC TGCCAAGAAA GAGGTGGCTG CTGCTGGAGC AGCAAAGACT

---------- ---------- ---------- ---------- ----------

AT---GATTC TGGCAAGAAA GCACTGGTTG CTGCTGAAGC AGTGAA-CCT

ATAGTGATTC TGGCAAGAAC ACACTGGTTA CCGCTGAAGC AGTAAA-TGT

ACAGCGATTC TGGCAAGAAA GAGGTGGTTG CTGCCGAAGC AGTGAA-GTC

---------- ---------- ---------- ---------- ----------

ACAGCGACTC TGCAAAGAAA GAGGTGGCCG CCGCCGAAGT GGTGAAAGCC

ACAGT----- TGTGAAGAAA GAGGT-GCTG C--CCGAAGC TGTAAAAGCC

ACAGTGACTC AGGGAAGAAA GAGGCAGCAC CCCCTGGTGA TGGGAAGGTC

ACAGTGATAC TTCGAAGAGA GAAGTGGTTG CTGCTGAAGC AGCAAAAGCC

ACAGTGATTC TGTGAAAAAA GAGGTGGTTG CTGCTGAAAC TGTGGAAAGT

ACAG------ ---------- ---------- ---------- ----------

ACAGTGTCAC CGTGAAGACG GAGCCTGTCT C-ATGAAGAC CGAAAAGACC

ATAGCGCAAC TGTGAAGAAA GAACCAGTAA C---TGAGGT GGCCAAGGTA

GCGAAGCGGA AGCGAGAGAG TGAGAGTGAC GAGCAGTTGA TCaagaagga

GCGAAGCGGA AGCGAGAGAG TGAGAGTGAC GAGCAGTTGA TCaagaagga

---------- ---------- ---------- ---------- ----------

GCGAAGCGGA AGCGAGAGAG TGAGAGTGAC GAGCAGTTGA TCaagaagga

GCGAAGCGGA AGCGAGAGAG TGAGAGTGAT GAGCAGTTGA TCaagaagga

---------- ---------- ---------- ---------- ----------

CATA--CGGA AGCTGGATAG TGAGAGTGGT GAGCAGTTG- --AAGAAGGA

CATAAAAGGA AGCGGGATAG TGAGAGTGAT GAGCAGTT-- ----gaagga

CCCAAGCGGA AGCGCGAGAG TGAGAGTGAT GAACAGTTGG TCaagaagga

---------- ---------- ---------- ---------- ----------

CCGAAGCGGA AGCGAGACAG TGAGAGCGAT GAGCAGCTGC TCaagaagga

GAAAAGCGGA GGCGACAGAG TGAAAGTGAC GAGCAGTTGA TCaagaaggg

CAGAAGCGGA AGCGCGAGAG TGAAAGTGAT GA-CCAATGG TTAAAAAAGA

CCGAAGCGGA AGCGAGAGAG TGAAGGTGAC GAGCAATTGA GCaagaagga

CAAAAGcgga aacgagagag tgacagtgac gaacagttga ccaagaagga

------CGGA AGCGAGAGAG TGAGAGTGAC GAGCAGCTGC TCAAGAAAGA

AGGAAGtgga agaaagagag tgaggagggg ga-caggtgg ccaggaggag

CAGAAGAGAA AGAGGGAGAG CGGAAGTGAT GAGAAATTGA GTaagaagga

aaagaagaag agtaagaagg acaagaagGC CAAAGCTGGT CTGGAGAGCG

aaagaagaag agtaagaagg acaagaagGC CAAAGCTGGT CTGGAGAGCG

---------- ---------- ---------- ---------- ----------

aaagaagaag agtaagaagg acaagaagGC CAAGGCTGGT CTGGAGAGCG

aaagaagaag agtaagaagg acaagaagGC CAAAGCTGGT CTGGAGAGTG

---------- ---------- ---------- ---------- ----------

AAAGGAGAAG A---AGAAGG ATAAAAAGTC CAAAATGCTG CTAGAAAGTG

aaagaagaag a---agaagg ataagaagCC CAAAACTGTG CTAGAAAGTG

aaagaagaag a---agaaga a------gTC CAAAGCTGCT GCAGAGAGTG

---------- ---------- ---------- ---------- ----------

gaagaagaag a---agaaga aggagaagGC CAAAGCTGCC GCGGAGGGCG

aaagaagaag a---agaagg acaagaagGC CAAAGCTGCT GAGGAGAGTG

AAAGAAGAAG A---AGAAGG ACAAGAAGGC CAGGGAAGCT GTGGAGACTG

aaagaagaag aacaaaaagg acaaaaagGC CAAAGCTGTT GTAGAGAGTG

gaagaagaag aataaaaagg acagaaaggc caaagctgct ctagagagtg

AAAGAAGAAG AGTAAAAAGG ACAAAAAGGC CAAAGCTGCT GCTCCAAGTG

gaggaggagg a---agaCAG ACTGGAAGTC CAAAAAAGGC aagaagaaga

aaagaaaaag aagaaaaagg aaaagaagGC TAAAGAAGCA GTAGAGAGTG

GGGCCGAGCC TGGAGATGGG GACAGTGATA CCACCaagaa gaagaagaag

GGGCCGAGCC TGGAGATGGG GACAGTGATA CCACCaagaa gaagaagaag

---------- ---------- ---------- ---------- ----------

GGGCCGAGCC TGGAGATGGG GACAGTGATA CCACCaagaa gaagaagaag

GGGCCGAGCC TGGAGATGGG GACAGTGATA CTACCaaaaa gaagaagaag

---------- ---------- ---------- ---------- ----------

GTGGCGATGC TGGAGATGGG GACAGCCATA CTACCAAAAA GA---AAA--

GGGGCGAAAC TGGAGATGG- GACAACGACA CCACCAAAAA GA---AAAAG

GGGCAGAGCC TGCAGATGGG -ACAGTGACA CCACCaagaa gaagaagaag

---------- ---------- ---------- ---------- ----------

CGAGTGAGCC CGCAGATGGG GACAGTGACA GCGCCAGgaa gaagaagaag

CGGACGAGCC AGGAGACGGG GACAGTGACA CCACCaagaa gaagaagaag

TAGAAGAGCC AGGAGATGGG GACAGCGATG GCGTAaagaa gaagaagaag

CGGACGAGCC AGGAGATGTG GGCAACGACA GCACCAAAAA GA---AGAAG

cgggcgagct gggagatgtg GACAGTGACA CTACCAAAAA GA---AGAAG

AAAGCGAGCT GGGCCAGGTA GAAAGTGACA CAACCAAAAA GA---AGAAA

agaacaaga- aagaga---a GGCACTGACA GCTCCaagaa gaagaagaag

GAGGCGAGTC GATGGATGTG GGCAGCGATA GTACCAAAAA GAAGAAAAAA

aagaagaaAG CAAAAGAGGA GAATAATCTG ATTTCAACAA TTCCATCAGA

aagaagaaAG CAAAAGAGGA GAATAATCTG ATTTCAACAA TTCCATCAGA

---------- ---------- -AATAATCTG ATTTCAACAA TTCCGTCAGA

aagaagaaAG CAAAAGAGGA GAATAATCTG ATTTCAACAA TTCCGTCAGA

aagaagaaAG CAAAAGAGGA GAATAATCTG ACTTCAACAA TTCCATCAGA

---------- ---------- -AATAATCTA ATGCCAACAA TTCCATCAGA

--GAAGCAGG TAAAAGTGGA A--------- ---------- ----------

AAGAAGAAAG TAAAAGTGGA AAGTAATCTA ATGACAACAA TTCTGTCAGA

aagaagaaAG CTAAAGTCGA GCATAACCTA ATGCCAACAA TTCCATCAGA

---------- ---------- ---------- ---------- ----------

aagaagaaAA TAAAAGCGGA GGACAGTCTA ATGCCAACAA TTCCATCAGA

aagaagaaAA TAAAACTGGG GAACTATCTA ATGCCAACAA TTCCATCAGA

aagaagaaAA TCAAA---AA GAATAATCTA ATACCACAAA TTCCATCAGG

AAGAAGAAAA CAAAAGTGGA GAATAATGTA ATGCCAACAA TTTCATCAGA

AAGAAGAAAG CAAAAG---- ---------- ---------- ----------

AAGAAGAAAG CCAA-----A GAATAATGCA ATTCCAGTAA ATTCAACAAA

aagaagaaAG CCAGACTAGA GGAGAGCGTT ACTCCAGACG TTTCATCTAA

AAGAAGAGAG CAAAAGAAGT TGAGAATTTA GTGCAAAGAA CTACTTCACC

CAATTTGGCA GCAGGTACTG ATAATACAAG TTCCTTAGGA CCCCCAAGTA

CAATTTGGCA GCAGGTACTG ATAATACAAG TTCCTTAGGA CCCCCAAGTA

CAATTTGGCA GCAGGTAATG ATAATACAAG TTCCTTAGGA CCCCCAAATA

CAATTTGGTA GCAGGTAGTG ATAATACAAG TTCCTTAGGA CCCCCAAATA

CAATGTGGCA GTAGGTACTG GTAATACAAG TTCCTTAGGA TCCCAAAATA

CAATCTAACA GCAAGTACTG AAAAGATGGG TTCTGTAGGA CCCCCACGTA

---------- ---------- ---------- ---------- ----------

CAATTTGAAA GCAACTTTTG AAAAGACAGA TTCTTCAGGA TTTCCAGATA

CAACTTAGCA GGAAC---TG AAAAGTCAGG TTCCTTAGGA TCCCCAAATA

---------- ---------- ---------- ---------- ----------

TAAGTTGGCA GCAGCTACTG AAAAGACAGG TTCCTTAGGA CCCCCAAATA

AAAGTTGGCA GCAGGTACTG AAAAGACAGG TTCCTTACGA CCCTCAAATA

CACATTGGCA ACAAATCTGG AAAAGGAAAG TTCCTTAGGT CCCCCAAAGA

CAACTTGGCA GCAGACACTG AAAAGACAGG TCCCTTAGGA TCCTTGAATA

---------- ---------- ---------- ---------- ----------

CAACTTGCCA GCAGACACTG AAACGACAGA CTCCTTAGGA TCCCTCAGGA

CGTAGTGACA GCAGACCCAA AAGGGACAGG TCTCTTCTGT TCAGTGGAAA

CAATTCTGGA GCAGACCGCA GAGAGAAAGC TCCCTTAGGA CAATTGAAGG

TGCCAGTTCA TAAGTCATCT CCCCTTACTG AGGGACCTCT GAGCTTGAGT

TGCCAGTTCA TAAGTCATCT CCCCTTACTG AGAGACCTCT GAGCTTGAGT

TGCCAGTTCA TAAGTCATCT CCCCTTATTG AGGGACCTCT GAGTTTGAGT

TACCAGTTCA TAAGTCATCT CCCCTTATTG AGGGACCTCT GAGTTTGAGT

TGCCAGCTCA TAAGTCATCT CCCATTATTG AGGTACCTCT GAGCTTGAGT

TGCTAGTTCA CAATTTATTT GCCCTTATTG GGGTACCTTT GATCTTGAGT

---------- ---------- ---------- ---------- ----------

TGCCAGTTCA CAAAGCATAT TCCCTTGTTG GGGTACCTTT AAACGTGAGT

TGCCAGTTCA TAAACTCTCC ATCGTTATTG ATATACCTCT GAATTTGAAT

---------- ---------- ---------- ---------- ----------

TGTCAGTTCA CAATTCATCC CACCTTATTC AGGTACCTTT GGAATTGAGT

TGCCAACTCA CAATTCATCT CACCTTATTG AGGTACCTTT GGGCTTGAGG

TATCAGCTCA CAACTCATCT CGTCTTATTG GGGTATCTAC TGGCTTGGAT

TGCCAGTTCA TAAGTCATCT TCACTTACTG GGGTACCTTT ATACTTGAGT

---------- ---------- ---------- ---------- ----------

CACCAGTTCA CAAGTCCTCT CCTCTTATTG GGGTACCTTT GCGTGTGAGT

AGTCAGGGCC TAAGCCACCT CCCCTATCTG AGACACCCAT GCACCTAAGC

AGCCGGCAC- -ATGGGATCG CCTCCTACCC ---TATCGAT GCAGTTACAA

GAAGAAAATA ATGATTCAAA GTTGTTAGAA TCAGGTTTAA TGAATCAAGA

GAAGAAAATA ATGATTCAAA GTTGTTAGAA TCAGGTTTAA TGAATCAAGA

GAAGAAAATA ATGATTCAAA GTTGTTAGAA TCAGGTTTAA TGAATCAAGA

GAAGAAAATA ATGATTCAAA GTTGTTAGAA TCAGGTTTAA TGAATCAAGA

GAAGAAAATA ATGATTCAAA GTTGTTAGAA TCAGGTTTAA TGGATCAAGA

GAGGGGAATA ATGCTGACAA GACATTGGAA ACTGCTTTAA AAAATCAAGA

---------- ---------- ---------- ---------- ----------

GAAGAAAATA GTGATTCCAA CATATTGGAT TCAACTTTAA TGTATCAAGA

GAAAGAAATA ATAATCCCAT GTTATTAGAG GTAGATTTAA TGAACCAAGA

---------- ---------- ---------- ---------- ----------

GAAGAAGATA ATGATTCCAA GTTGTTAGAA GCACCTTTAA TGAATCAAGA

AAAGGAGATG ACGAATCCAA GTTGTTAGAA GTAGCTTTAA TGAATCAAGA

GAAGGAGATA ATAATCCCAG ATTTTTAGAA GAAACTTTAA TGAACCAAGA

AAAGGAGATA AAGATTCCAA GTTGTTAGAA GCAGCTTTAA TGGATCAAGA

---------- ---------- ---------- ---------- ----------

GAAAGAGATA ATGATTCTAC GTTGTTACAA GTAGCTTTCG GGAGTCTAGA

AACCCTAATG CTGACTCCAA AGCTTTAAAA GTGGCTTTGA TGG---AAGA

GAAACCGACA GCAGAACCAA ATCTTCAAAT GCCACATCAT TGGATAATGA

AAGTTCATGG GGAAAAAATG TATCGTCAAC AGAGAGTGGT AGGTTATTTA

AAGTTCATGG GGAAAAAATG TATCGTCAAC AGAGAGTGGT AGGTTATTTA

AAGTTCATGG GGAAAAAATG TATGGTCAAC AGAGAGTGGT AGGTTTTTTA

AAGTTCATGG GGAAAAAATG TATGGTCAAC AGAGAGTGGT AGGTTATTTA

AAGCTCATTT GGAAAAAATG TATCATCAAC AGAGACTGGT AGGTTATTAA

AAGTTCACTG GGAAAAAAAT TATCATCAGT GGAGAAAGAT AGGTTATTTA

---------- ---------- ---------- ---------- ----------

AAGTTTACCA AGAGATAATA TATTATCAAT GGAGAATGAT AGATTACTCA

AAGTTCACTG GGGAAAAATA TATCA---AT GGACAGTGAT AAGTTATTTA

---------- ---------- ---------- ---------- ----------

AAGTTCACTG AGAGAAAATG TATTATCAAT GGAGAGTAAT AGGTTATTTA

AAGTTCACCG GGAGAAAATA TATTGTCAAT GAAGAGTGAC AGGTTATTTA

AAGATCACTG GGTGGAAATG TCTTATCACC AGAGAGAAAT GGGCTACTTG

AAGTTTACAG GGAAAAAAGG TATCATCAAT AGAGAGTGAT AGGTTAATTA

---------- ---------- ---------- ---------- ----------

AAGTTCACCT GGAAAAAAGA TATCACCAAT GGAGAGTAGT AACTTATTTA

TCGTTCCCCT AGAAAGAAAA TGTCTTCCCT GCTCAGGGAC GGGGGATTCC

AACTGCTTTG GAAAGGGAGG AGATACCGCT GAAGAGGGAT GGGCCAACCA

AAGGGAAAAG AGCTCATGGA CCTGCTAAGG ATGGCCCATC ATTATTAATT

AAGGGAAAAG AGCTCATGGA CCTGCTAAGG ATGGCCCATC ATTATTAATT

AAGAGAAAAG AGCTCATGGA CCTGCTAAGG ATGGCCCATC ATTATTAGTT

AAGAGAAAAG AGCTCATGGA CCTGCTAAGG ATGGCCCATC ATTATTAGTT

AAGAAAAAAC AGCTCA---A C-----AAGG ATGGCCCAAC ATTATTAATT

AAGAGAAAGT AGCTCAGGAA CCTGCTAAGG ATAGCCCAGC ATTATTAAAT

---------- ---------- ---------- ---------- -------GTT

GAGAGAAGAG GTTTCATGGA ATTGCTAAAG AGAGCCCAAC ATCA---ATT

AAGAGAAAAG AGTTCATGAA TCTGCTAAGG ATGGCCCAAC ATTATTTATT

---------- ---------- ---------- ---------- ----------

AAGAAGAAAG AATTCGTGGA CCTGCTAAGG CTATCCCAAC ATTATTAATT

AAGAGGAAAG AGCTCATGGA TCTGCTAAGG ATGGCCCAAC ATTATTAATT

AAAAGAGAAG AAGTACTTTA CCGGCTAAGG AGGAGCCAAT ATTATTAACT

AAGAGAAAAG AATTCATGGA TCTTCTAAGG ATGAACCAAC ATTATTAAAT

---------- ---------- ---------- ---------- ----------

AAGAGAATGA AGGTCATGGA TATGCTAAGG ATGACCCGAC ATTAGTAACT

AAGAAATGAG TGCTCAAGGA GCTGT-A--- ATGCCCCGAT GCTTT---CC

ATGAAACTAC GGCTCTGGGT CCTGCT-AGA ATGACCCAAC ATTACAGAAT

GAGAATAGTC CATCAGTCAT ATTAGAAAGT GACACTGAGT TTAAAAAAGT

CAGAGTAGTC CATCAGTCAT ATTAGAAAGT GACACTGAGT TTAAAAAAGT

GAGAATAGTC CATCAGTCAT ATTAGAAAGT GATACTGAGT TTCAAAAAGT

GAGAATAGTC CATCAGTCAT ATTAGAAAGT GATACTGAAT TTCAAAAAGT

GAGAATAGTA CATTAGTCAT ATTAGAAAGT GATACTGAGT TTCAAAAAGT

GAGAATAGTA CCTCAGTC-- ----GAAAAT GATACTGAGT TTCAAGAACT

GGGAACAGCA CAGCAGCTAT ATTAGAGATC TGCAGTGAGA TTCTCGAGGT

GAGAATAGTA CAACAGACAT ATTAAAGGTC AATAGTGAGA TTCAAGAAGT

GAGAATAGTA CATCAGTCAT ATTAGAAAAT GATACTGAGT TTCAAGAAGT

---------- ---------- ---------- ---------- ----------

GAGAACAGTA CCTCAGTCAT GTTAGAAAGG AATACTGAGT TTAAAGAAGT

GAGAATAATA CATCGGTCGT ATTAAAAAGT AATGCTGAGT TTCAAGAAGT

GAGAATAAAA CATCAATCAT ATTAACAAAT AGTACTGAGT TTCAAGAAGG

GAAAATAATA CACCAATCAT ATTAGAACAT AATACTGAAT TTCCAGATAT

---------- ---------- ---------- ---------- ----------

AAGAATCTTA CATCAGGAAT ATTAGAAAAT AATACTGAGT TTCAAGAAGT

CAAAACAAGT CGTTGATCGT GTCAGTAAAA GATACAGAGC CTCACAAAAT

GAGAATGTAA CATTGGTCAT GATAGGAAAT GATACAGAGT CTCAAGAAAT

GACACCTTTG ATTCATGACA GAATGCTTAT GGACAAAAAT GCTACAGCTT

GACACCTTTG ATTCATGACA GAATGCAGAT GGACAAAAAT GCTACAGCTT

GACACCTTTG ATTCATGACA GAATGCTTAC AGACAAAAAT ACTACAGCTT

GACACCTTTG ATTCATGACA GAATGCTTAC AGACAAAAAT ACTACAGCTT

GACACCTTTG ATTCATGACA GAATGCTTAC AGACAAAAAT ACTACAGCTC

AGTGTCCTTG GTTCATGATA AAATATTTAT GGACAAAAAT AGTATGGCTT

CACACCTCCG ATTCATGACA GGATACTTTC AGACAAAAGT GCTACATATT

AACAGCTTTG ATTCATGATG GAACACTTTT AGGCAAAAAT TCTACATATT

GACATCTTTG ATTTATGATG AAATGCTTAT GAGTAAAAAT ACTACAGCTT

---------- ---------- ---------- ---------- ----------

AACTTCTTTG ATTCATAATG AAACGTTTAT GGACAGAAAT ACTACAGCTC

GACTCCTTTG ATTCATGATG AAATGTTTAT GGACAAAAAC ACTACAGCTT

TACTTCTTTG ATTCATAATG AAACATTTAT GAACAATGAC ACCACAGGGT

GACTTCTTTG ATTCATGATG AAACATTTAT GGACAAAAAT ACTACAGCTC

---------- ---------- ---------- ---------- ----------

GACTGCTTTG ATTCATAATG AAATGTTTAT AGATAGAAAT ACAACCTATT

GCCTTCTCGT AGTTATGGCA GAACTCTTCC AGACAGGAAC GCCTCAGCTG

GGAGACTCTG GATGACAGTG AAACTTTTGG AGACATTAGT ACCACTGCTC

TGAGGAATCA TATGTCAAAT AAAACTACTT CATCAAAAAA CATGGAAATG

TGAGGAATCA TTTA-AAGAT AAAACTAAGG -ATCAAAAAA CAGGAAA---

TGAGGAATCA TATGTCAAAT AAAACTACTT CATCGAAAAA CATGGAAATG

TGAGGAATCA TATGTCAAAT AAAACTACTT CATCGAAAAA CATGGAAATG

TGAGGAATCA TATGTCAAAT AAAACTACTT CATCAAAAAA CATGGAAATA

TGAGGAATCA TGTGTCAAAT AAAACTATAT CATCAAAATA TGTAGAAATG

TGAGACACCG TACGCCCGAC AGAATCACCT CGACGGAAAG GAAAGACATA

TGAGAAACCA TATGCTAAAT AGAACTACCT CAACAAAAAA TAAAGACATA

TGAGGAATCA TGAGTTAAAT AAAAATACTT CATCAGAAGA TGTGAAAATG

---------- ---------- ---------- ---------- ----------

TGGGGAATCA TGTGTCAAAT AAAACTACTT TATCAAAAAA TGTGGAAATG

TGGGGAATCA TGTGTCAAAT AAAACTATGT CATCAAAAAA TATGGAAATG

TGAGGACTCC TATGTCAAAT GAAAGTTCTT CCTCAAAAAA TATGGAAATG

TGGAAAATCA GGCATTAAAT ATAACTATTT TACCAAAAAA TGTGGAAATG

---------- ---------- ---------- ---------- ----------

TGGGGAATCA TGTATCAAAT AAAACCATTT CGTCAAAAGA TGAGGAAACA

CCAGCTGTCA TGGGCCTGAG AAAAGAGCTT CCCTGAGAAA AATAGACACT

CGGCTAGCCA TGTGTCACGT GAAGATATTC CAACAGAAAA AGAAAAAATG

GTCCAGAAAA AAGAGGGCCC CATTCCACCA CCAGAATCAG CAAGGGAGAA

---------- ---------- ---------- ---------- ----------

GTCCAAAAAA TAGAGGGCCC CATACTACCA CCAGAATCAG CAAATGAGAA

GTCCAAAAAA TAGAGGGCCC CATACTACCA CCAGAATCAG CAAATGAGAA

GTCCAAAAAA AAGAGGGCCC TATGCCACCA TCAGAATCAG CAAATGAGGA

GTCCAAACAA AAGATGGCCC CATGTCACCA CCACAATCAG CAAATGAGAA

CGTAAAAAAG ATGGAGATCT TGTTCCGCAG TCAGAATCCA CAGATGAGAA

TTTAGAAAAG ATGAAGATCC TATTCCACAA TCAGAATCTT CAAATGAGAA

GTCCAAAAAA AAGAAGGTCT TGTGACTCCA CCAGACTCAC CAAATGAGAA

---------- ---------- ---------- ---------- ----------

GCCCAAAAAA AAGAAGACCC TGTGCCACTA CCAGATT--- -----GAGGA

GTCCAAGAAA AAGAGGGTCC TGTACCAGTA CCAGATTCAG CAAATGAGAA

GTTCAAGGAA AAGAGGGCCC TGTACCACAA CAAGGGTCAG AAAATGAAAA

GTCCATAAAA AAGAGGGTTC TGTACCACAA ACAGATTCAG CAAATGAGAA

---------- ---------- ---------- ---------- ----------

GTTCACATGA AAAGGGCGCT TGCGTCGCCA CCAGATTCAG CACATGAGAC

GGCAGACAAT GTGAGGGCTT CGTCACCTCA GCAGATTCGG GGAATCAAAA

GCTAAAAGGA AAGTGAGAGC TTTAACGCTT TCAACATTAG CAAA--TAAA

AAACAAAGTG GTAGTAGGAA AGGGTGAATT TACAAAGGAC GTAGGACTCA

---------- ---------- ---------- ---------- ----------

AAATAAAGTG GTAGTAGGAA AAGGTGAACT TACAAAGGAT ATAGGACTCA

AAATAAAGTG GTAGTAGGAA AGGGTGAACT TACAAAGGAT ATAGGACTCA

AAATCAAGTG GTAGTAGGAA AGGACGAATT TACAAAGGAC TCAGGACTCA

GAATAAAATG GTAGTAGGAG AGGATGAATT TACAAAGGAC ACAAGACTCA

GAATAAAATG ATAGCAGGGC AAGGTGGATT TACCAAGAAC ACAGGACTTG

AAATAAAGTC ACAGTAGAAC AGGATGGATT TACAAAGAAC ATAGGACTTA

AAATAAAGTA GCAGTGGGAG AGGATGCATT TACAAAAGAC ACAGGACTCA

---------- ---------- ---------- ---------- ----------

GAA---GGTG GTAGTAGGAG AGGATGAATT TACGAAGGAC ACAGAACTCC

CAATACGGTG TTTGTAGAAG AGGATGAATT TACAAGGGAC ACAGGCCTCA

GAGTAAGGAA AAAGGGACAG AGGATGAGCT TACAAAGGAT ACAGGATTCA

GAATATGAAG GCAGTAGGAG AGAAGGAATT TACAAAGTAC AAAGGACTCA

---------- ---------- ---------- ---------- ----------

AAGTAAGGTG GCAGCAGGAA AGGATGAATT TTCAAAAGAC ACAGGATTCC

GAGCATGATG GCCCCAACAG AGGAGACATT CACAAATGGG CCAGACCTCA

GAATGAGATG ACACCAGAAG TGGGTGAATT TACTAATGGC ACAGAACTCA

AAGAGGTTTT TCCAAGCAGC CTATTTCTTA CTAACTTGGA TAATTTACAT

---------- ---------- ---------- ---------- ----------

AAGAGGTTTT TCCAAGCAGC CTATTTCTTA CTAACTTGGA TAATTTACAT

AAGAGGTTTT TCCAAGCAGC CTATTTCTTA CTAACTTGGA TAATTTACAT

AAGAGATTTT TCCAAGCAGC CTGTTTCTTA CTAACTTGGA TAATTTACAT

AAGAGGCTTT TCCAAGTAGC ATATTTCTTA CTAACTTGGC TAATGTACAC

AAGACGTTTT TCCCAGAAAG GTGTTTCTCA CCACTGTGGC TAAGCGGCAA

AAGACGCTTT TCCACATAAT ATATTTCTTA CCACTTTGTC TAACGTACAT

AAGAGATTCT TCCAAGCAAC ATGTTTGTTA CTAACTTGAC TAATGTCTGG

---------- ---------- ---------- ---------- ----------

AAGAGATTTT TCCAAACAAC ATATTTTTTG CTAACTTGGC TAATGTCCAA

AAGAGATTTC TCCAAAGAGC ATATTTCTTA CTAACTTGAC TAATGTCCAA

AAGAAACTTT TCCACACAGC ATATTTATTG GTAACTTGGA AAATGTCCAA

AAGAGATTTT TCCAAATATC ATATTTCTTA GCAACTGGAC TAATGTACAA

---------- ---------- ---------- ---------- ----------

AAGAGATTTT TCCAAACAGT TTATTTTTTA CAAATTTGAC TAACGTGCAA

GAGGAAGAAT GACAGGCACC CTGTGGCCTA CTCCCTCCGT CCCCGGCTCA

AAGAAGCATT TAAGGACAGC CCTCTTCTAT CTGGTTTGGT GACTAATCAC

GAAAATAATA CACACAATCA AAAGAAGACT GGCACTAAGA ATTTCATGAA

---------- ---------- ---------- ---------- ----------

GAAAATAATA CACACAATCA AAGAAAGACT GGCACTAAGA ATTTCATGAA

GAAAATAATA CACACAATCA AAGAAAGACT GGCACTAAGA ATTTCATGAA

GAAAATAGTA CACACAATCA AAGGAAGACT GGCACTAAGA ATTTCATGAA

AAAAATGATA CAAGCAATCA AAGGAAGACT GACACTAAGA ATTTTATGAA

GAAAGTGGGA GGCACAACCA A-AGAAGACT GGCTCTAAGA ACTTCCCGAG

GAAAATGGTA GGCACAATCA A-AGAAGACT GGCTCTAAGA ATTTCTTGAA

GAAAATGATA CCCACAATCA AAAGAAGAAT GGCACCAAGA ATTTTCTGAA

---------- ---------- ---------- ---------- ----------

GAAAATGATA CATACAATCA AAGAAAGATT GGCACTAAGA ACTTCCTGAA

GAAAATGATA CATACAACCA AAGGAAG--- GGCACTAAGA ATTTCCTGAA

GAAAATGATG CACAGAATCA AAAAAAAACT GGTACTAACA GTTTTCTGAA

GAAAATGATA CACAAAATCA AAGAAAGACC AGCACTAAGA ATGTACTGAA

---------- ---------- ---------- ---------- ----------

AAAAATGAAA CCCCAAATC- --GTAACTCT GGTGCTAAGA ATTTCCTGAA

GGGACACATG CCCCCAGACA AAGGA---CC AACAGAGAGA AACTCCTGAA

GAAGTTGACA AACTGAGGCA AAGCAAGTCC ACCCCCGACA ACCTTGTGCA

GAACCTTTTC TTACTGAGCA CTAGGAATGT AAGGTCATTA AATGATTCAA

---------- ---------- ---------- ---------- ----------

GAACCTTTTC TTACTGAGCA CTAGGAATGT AAGGTCATTA AGTGATTCAA

GAACCTTTTC TTACTGAGCA CTAGGAATGT AAGGTCATTA AGTGATTCAA

GAACCTTTTC TTACTGAGCA CTAGACACGT A-------TA AATGATTCAA

GAATCTTTTC TTACAAAGCA CTAGGAATGT AAGGTCATTA AATGTTTCAG

AGACATGTTT GTACTCGGTA CTGGGAATAT -CCATGGACA AGAAACCCAA

AGACATATTG ATACTAGGCA CTAGGAATAT -ACATCAATA AACAATTCAA

AAAACTTTTC TTGGTGAGCA CTAGTAACAT -AGGCCATTA AATGATTCAG

---------- ---------- ---------- ---------- ----------

GAACCTTTTC TTACTAAGCA CTAAGAATGT AAGGTCATTA AATGATTCGC

GAATCTTTTC TTACTAAGCT CTAAGAATGT Aaggtcatta aatgattcag

GGCCCTTTTC TTACCAAGCT CAAGGAATAT AAGGTCATCA ATTGATTCGG

GACCCTCTTC TTACTGAGAA ATAGGAATGT AAGATCATTA AATAATTCAT

---------- ---------- ---------- ---------- ----------

GACACTCCTC TTACTAAGCA CTAGGAATGT ---GTCATTG ACTAATTCAG

GTCCCTTTTC TTGTCAAACA TGAAGCACAT AAGTACAGTG AGTGGTCCAA

GCCCTCTTCC TTACTAAGCA GAGAAGATGT GAGGAAATTA GATGAGCAAG

CAAATAGAAC AAAGAAACAC ACAGCTCATT CAACCCAGTG GTCCAAAAAC

---------- ---------- ---------- ---------- ----------

CAAATAGAAC AAAGAACCAC ATGGCTCATT CAACCCAGTG GTCCAAAAAC

CAAATAGAAC AAAGAACCAC ATGGCTCATT CAACCCAGTG GTCCAAAAAC

CACATAGAAC AAAGAA---- ---------T CAACCCGATG GTCTGAAAAC

CAAATAGAAC AGAGACTCAC ATGGCCCATT CAACCCAGTG GTCCAAAAAT

CAGACACAAG ACAGATTCCC ATGGTTCATT CCGCTCGTTG GCTTCCAGAC

CAAATACAGT ACAGATTCAC ATGGAGCATT CAACTCAATG GCTTAAAACC

TCTATCGAGC AGATATTCAG ATCACTCAC- ---------- ----------

---------- ---------- ---------- ---------- ----------

CACATAGTGA AGGGATTCAT ATGGCCAATT CAACCCAGTG GTCCAAAAAC

caaatagagc agggaatcac atggcccatT CAACCCAGTG GTCCAAAAAC

CCAATACAGC AGGGTATCAC ATAGACTGGT CGACCCAGTG GCCTCATAAC

CCAATAGAAC GGGGCCTCAC ATAGCCCATT CAACCAAGTG GTCCAGAAAC

---------- ---------- ---------- ---------- ----------

CAAACAGATC CGGGCTTCCC AATGCCCACT CAACCCAGCA TTCAAGAAGC

CCAGTGACGC AGGGCTCCAG AAGATGCA-- CAAGCCAATG GTCAAAAAGA

CAAACAATTC AAGGCTTCAG AAGGACCGTA CATTTCAGCA AATAGGAAAA

ATGAAACATT TGACCCCGAG CACCCTCACA CAGATAGACG AGAAAGGGGC

---------- ---------- ---------- ---------- ----------

ATAAAACATT TGACCCCAAG CACCCTCACA CAGATAGACG AGAAAGGAGC

ATAAAACATT TGACCCCAAG CACCCTCACA CAGATAGACG AGAAAGGAGC

ATGAAACATT TCACCCCGAG CACACTCACA AAGATAGATG ACAAAGGGGC

AGGAAATATA TGACACAGGG CACCCTCACA CAGGTAGACG AGAAAAAGGA

GTAAACCGTT CAATCCAGAG TCTCCTTAAA CAGATTGACA GGAAAAAGTT

ATAAACTGTT CAACACAGTG TATCATTAAA CAGATAGACA TGAAAAAGTT

---------- ---------- ---------- ---------- ----------

---------- ---------- ---------- ---------- ----------

ATGAACTATT TGGCCCAGGG AACCCTCACA CAGATAGAGG AAAAAAGGGC

ATGAACTATC TGACCCAGGG CACTCTCACA CCGATAGAGG AGACAGGGGC

ATGACATCTC TGATC---AT TTCTTTCAAA CAAACAGAGA ATAAAAGAGG

ATGAAAAATT TGACCCTGGG CACCCTCATA CAGATAGACG AGAAAAGGGC

---------- ---------- ---------- ---------- ----------

GTGAAGAATT TGACCCAAGG CCCCCTCACA AAGGTACACG AGAAAATGAC

AGGCAATTTC TACTCCAAGG TAGCACCCTC CAGATACA-- -GAAAGATGT

ATGAAATCTA GGCTCCAAGA CACTACCATC AAAATGGGCG GGGAAAATTC

CATTTCAAAT AGATCTCCAT TACCCATTTC AGCATCTTAT AGAAAGAAAG

---------- ---------- ---------- ---------- ----------

CATTTCAAAT AGATCTCCAT TACCCATTTC ACCATCTTAC AGAAAGAAAG

CATTTCAAAT AGATCTCCAT TACCCATTTC ACCACCTTAC AGAAAGAAAG

CGTTTCAAA- ---------- --------TC TCTATCTTAT AGAAAGAAAA

CATTTTA-AT AACTCTGCAT TAGCCATTTC ATCTGCCTAT AGGAAGAAAA

CATTGCAAAT CATTCTCCCT CACACGTTTC ATCGTCCTAT GAGACAGGAA

CATTTCAAAT AGTTCTGACT CACACATTTC ATCATCCTAT AAGACAAAAA

---------- ---------- ---------- ---------- ----------

---------- ---------- ---------- ---------- ----------

CATTTCAAAT GACTCTGCAT TACCCGTTTC ATCAGCCTGT AGAGAGAGGA

CATT---GAT GGCTCTGCAT TACCTGTTTC ATCAGCCTAC AGGGAGAGAA

CATTCCAAAT GAGACGGTGT TACCGGTTTT GACAGCCTGT AGAGAGAGGA

CATTTCAAAT GATTCTGCAT TATCTATTTT GTCAGCCTGT AGGGAGAGAA

---------- ---------- ---------- ---------- ----------

CA---C--AT GACTCCGCAT TACCCAT--- GTCTGCCTAT AGCGAGAAGA

CACTACAGAT GACTCCACAT TGCCTACTCA G-CATTACAC AAGGAAAGAA

CACTCCCGAC ATTACCTCAG TGCCTACTTC ---AGCTCAG AAAAAGAGGA

ATTCTGGGGT CCAAGAAAGC AGTCATTTCT TAGCCATTCT AACCTTGGAG

---------- ---------- ---------- ---------- ----------

ATTCTGGGGT CCAAGAAAGC AGTCATTTCT TAGCTATTCT AACCTTGGAG

ATTCTGGGGT CCAAGAAAGC AGTCATTTCT TAGCTATTCT AACCTTGGAG

ATTCTGGGGT CCAAGAAAGC AGTCGTATCT TAGCCATTCT AACCTTGGAG

GTTCTGGAGT CCAAGAAAGC AGCCATTTTT TAGCTGTCCT AACTTTGGAT

GTTCTAGAAT TCGAGAGAGC AGTCCTTTCC TGGCCGTGCC ACCGCGGGAG

GTTCAAGAAT TCAAGAAAGC AATAATTTCT TAGCCATTCT ACCATGGAAT

---------- ---------- ---------- ---------- ----------

---------- ---------- ---------- ---------- ----------

CTTCTGGAGT CCAAGAAGGC AGTCATTTCT TAGCCTTTGT AACCTTAGGA

GTTCTGGGGC CCAAGAAAGC AATCATTTCT TAGCCTTCCT AACCTTGGAA

ATCCTGGGAT CCAAGAACAC AGTCATTTCT TACTCATGCT AAAATTGGAG

GTTCTGGGTT CCAAGAAAGC AGTCATTTAT TAGCCATCCT AATGGCAGAA

---------- ---------- ---------- ---------- ----------

GTTCTAGTCT CCAACAAAGT AATAACTTAT TAGCTATCCT CACTTTGGAG

GCTGGGCAAC TGAGGAAGGC TATCGGGCTA TGGACACCTT TGACTTAGAG

AGCCTGGAG- -GAGGTTGGA AGTCATTTCT T-GCCTCTCT GAACATGGAG

ATGACTGGTG ATCAAAATTC AGTCACATAC AAGAAAAAAG TTGAATTCCC

---------- ---------- ---------- ---------- ----------

ATGATTGGTG ATCAAAATTC AGTCACGTAC AAGAAAAAAG TTGAATTCCC

ATGATTGGTG ATCAAAATTC AGTCACGTAC AAGAAAAAAG TTGAATTCCC

ATGATTGGTG ATCAAAATTC AGTCATGTAC GAGAAAAAAG TTGAATTGCC

ATGATTGGTA ATCAAAACTC AGCTACATAC AAGAAAAAAG ATGAATTCCC

AGGTTCGTGC GTCGACACTC AGCGCCGTGT GACAAAACAG TCGCATTGCC

ATGTTCATAG ATCAAAACTC AGTCACATAT AAGAAAAAAA TTGAATTACC

---------- -----AACTC AGTCATGTAC AAGAAAAATG TTGAGTTCTC

---------- ---------- ---------- ---------- ----------

ATAACTGAAG GGCAAAACCA ACCCATGTAC AAGAAAAAAG TTGAATTCCC

ATGACTGGTG GTCAAAACCC ACTCATACAC AAGAAACAAG TTGAATTCCC

ATGATCAGAG GTGAAAGTTC AGTCACATAC AAGAAACAAG TTGAATTCCC

ATGATCAAAG GTCAAAACCC ACTCATGTAC AAGAAAAAAG TTGAATTCCC

---------- ---------- ---------- ---------- ----------

ACGATTAGAG GCCAAGACCC ACACAAGCAT GAGAAAAAAG TTGAATTCCC

CTTTCAGAAG GCCAA-AGGC GACCTCTCTC AAAAAACAAC TTGGA--CGC

GAAGCAGAAG GTCAG-TTCC ATTCACACAG AAAAAG-AGG CTGAACTCAA

TACGGGGTCT CCTGGCCATC TGGATCTCGT GGAAGGGAGC CTTCTTCAGG

---------- ---------- ---------- ---------- ----------

TACAGGGTCT CCTGGCCATC TGGATCTCAT GGAAGGGAGC CTTCTTCAGG

TACAGGGTCT CCTGGCCATC TGGATCTCAT GGAAGGGAGC CTTCTTCAGG

TGCAAGGTCT CCTGGCCATC TAGATCTCAT GGAAGGAAGC CTTCTTCAGG

TATGGGGTCT CCTGGCCGTC TGAATCTCAT GGAAGATATT TTTTTTCAGA

TGCAGAGCGT CGCGGACATG TGGATCTCTT TCTCGAGATC TCTCTCCAGA

TACAGGATCT CCTGGACACT TGAATCTCAT GAAAGAGGTC TTTCTTCAGA

TGAAGGGTCT CCTGATCACC TAGATCTAGT GGAAGAGAGC --CCTTCAGA

---------- ---------- ---------- ---------- ----------

TACAGATTCT CCTGGCCACC TGGATCTCAT GGGAAAGATC TTCCTTCAGA

TACAGCTTCT CCTGGCTACG TGGATCTCAT CGAAGAGATC TTCCTTCAGA

TACAGGTTTT CCTGACCACC AGAATATCAT GAAAAAACTC TTCATTCAGA

TTCAGGTTCT CCTGGCCACA TAGTTCTCAT GGAAGAGAAA CACCTTCAGA

---------- ---------- ---------- ---------- ----------

CAC-GGTTCT CCTGGCCACC TGAGTCTCAT GCAAGACATC TCCCGTAAAA

AAGTAAGTGT CCTGAGCACC TGCCTCACTT AGCAGAGGCT CTCTCTCTTA

AGCAGAGTCC CCCGGTCACC GGAATCTCGT AGAAGAGTCC ATCCATCCCA

GAACAGAGGG AGCGATTAAG TGGTGGGATA ACCACTATGG TACTCAGATA

---------- ---------- ---------- ---------- ----------

AAACAGAGGG AGCAATTAAG TGGTGGGATA AACACTATGG TACTCAGATA

AAACAGAGGG AGCAATTAAG TGGTGGGATA ACCACTATGG TACTCAGATA

AAACAGAAGG AGCTAATAAG TGGTGGGATA ACTACTATGG TACTCAAATA

AAACACAGAG AGCTATTAAA TGGTGGAATA AC-------- ----CAGATG

GAACACGGGG GCCCGTTGAA CGG------- ---------- ----------

AAATACAGGG GCCTACTAAA TGGTGGGATT ATCATTATGC TGCACAGATA

AAACACAGGG AGCTGTTAAA TGGTGGGACA ACCAATATGT TACTCAGACA

---------- ---------- ---------- ---------- ----------

AAACACAGGG ACCTGTTAAA ATGTGGGATA ACCACTATGA TACCCAGATA

CAACACAGGA ACCTGTTCAA TTGTGGGACA GTCAATATGC TACCCAGATA

AAACACAGGG GCCTGACAAA TCATGGGATG ACCAGTATGC TACCCAGACA

AAACACAGGA ATCTGTTAAA TTGTGGGATA ACCAATATTC TATGCAGATA

---------- ---------- ---------- ---------- ----------

AAACACAGGG GGCTGTTACA TTGTTGGATA ACCAGTATGC TGGCCAGACA

GAGCACAGGC AGCTACTAAA TCTTGGGCTA GTCACACTGC CAAGCAGATG

AAATCCAGGT AGAGGTTGCA TCC-GGGCAG GCCATTATGG CCAACAGA--

AAAGAAGAGT GGAAATCCCA AGAGAAGTCA GATACCATTT TGTCCCTGAA

---------- ---------- ---------- ---------- ----------

AAAAAAGAGT GGAAATCCCA AGAGAAGTCA GATACCATTT TGCCCCTGAA

AAAGAAGAGT GGAAATCCCA AGAGAAGTCA GATACCATTT TGCCCCTGAA

AAAGAAGAGT GGAAATCCCA AGAGAAGTCG GATACCATTT TGTCCCTGAA

AAAGAAAAGT GGAAATCCCA AGAGAAGTCA GACATCATTT TGACCCTGAA

---------- ---------- ---------- ---------- ----------

AAAGATATGT GGAAATCCAA AGAGAAGTCA GACACCATTT TGTCTCTGAG

AAAAAAGAGT GTAAATCCCA AGAGAAGTCA GACAGCATTT TGTCCCTGAC

---------- ---------- ---------- ---------- ----------

AGTGAAGAGT GGAAATCCCA AAAAAAGTCA GACACCATTT TGCCCCTGGG

AGAGAAGAGT GGAAATCCCA AGAGAAGTCA GACACCATTT CGCCCCTAGA

ACAGAAGCAT GGAAATCCCA AGTGTTACCA GACCCCATTT TGACCCTGGA

AGAGAAGAAT GGAAATCCCA AGAGAAGTCA GACACTACAT CCCCCCTGAC

---------- ---------- ---------- ---------- ----------

AAAGAAGAGT GGCAATCCCA AGAGAAGTCA GACACCCTTT GTCCCCTGAA

ATAGAAGATC ATCAGTC--- AGACAAGCAA GAGACCATTC CCGACCCGAG

ATGGAGGAGT GGAGGTCACA AGACAGTCA- --CCCCATTC TCACCCCCAA

CGCTTGTGAA AGCATAAATG AGGGACAAAA TAAGCCCGAA ATAGAAGTCA

---------- ---------- ---------- ---------- ----------

CCCTTGTGAA AGCATAAATG AGGAACAAAA TGAGCCCCAA ATAGAAGTCA

CCCTTGTGAA AGCATAAATG AGGAACAAAA TGAGCCCCAA ATAGAAGTCA

CCCTTGTGAA AGCATAAATG AGGGACAAAA TAAGCCCCAA ATAGAAGTCA

CATACATGAA AGCGTAAACA AGGGACAAGA TAGACCCCAA AGAGAAGCCA

---------- ---------- ---------- ---------- ----------

GCCTCATGGA AAC---AATG AGAAACAAAA TTGGCCTCAA AGAGAAACCA

TCCTTGCGAA AACATGAATG AGGGACAAGA TAGGCTCCCA AGAGAAGCCA

---------- ---------- ---------- ---------- ----------

CCCTTGTGAA AATATAAATG AAGGACAAGA TAAGCCCCAA AGAGAAGCTA

CCCCCGTGAA AACATAAATG AAGGACAAGA TAAGGCCCAA AGAGAAGCCA

TCCTTGGGAA GACGTAAATG AAAGACAAGA CAAGATTCAG AGAGAAGCCA

CCCTTGTGAA ATCATAAATG AGGGACAAAA GAGTCCCCAA AGAGAAGCCA

---------- ---------- ---------- ---------- ----------

CCATTGTGAC AGCATCAATG AGGGACAAGA TAGGCTTCAA ACAGAAGCCG

CCTTGCCAAG AGAACAAGTC CGAAACGAGC TGAGCTCCGG GCAGCAGTTC

GGAAGGAGAT AGAACAGCTG GGGAAAAAGA TGGGCGTCAA GTAGAAATCC

CCTGGGCACT GTGCTCTCAA AACCCACCAG TCTTGAAACT TCAGTCAGAT

---------- ---------- ---------- ---------- ----------

CCTGGGCACT GTGCTCTCAA AACCCACCAG TCTTGAAACT TCAGTCAGAT

CCTGGGCACT GTGCTCTCAA AACCCACCAG TCTTGAAACT TCAGTCAGAT

CCTGGGCACT GTGTTCTCAA CACCCGCCAG TGTTGAAA-- ----------

CTTGGGCATT GTGCTCTCAA AACCCACTAG TCTTGACACT TCCGCCAGAT

---------- ---------- ---------- ---------- ----------

CTTGGGTAAC ATGCTCTCAA ATCCCACCAG TGTTGAAATT TCAATCAGAA

GCTGGGCATT GTGCTCTCAA AACCCATCAG ATTTGATAAT TCAATCACAG

---------- ---------- ---------- ---------- ----------

TGTGGGCATT GTGCTCTCAA AACCCACCAG TCTCAAAACT TCAGCCAGAG

CCTGGGCATT GTCCTTTCAG AGCCCACCAG TCTTGACACC TCAGCCAGAC

ACTGGGCACT GTACTTTCAA AACCCGTCC- -----AAACT TCCACCAGAG

CTTGGGCATT GGGCTCTCAA AACCCACCAG TCTTGAAACT TCCACCAGAG

---------- ---------- ---------- ---------- ----------

CTTGGCCATT AGACTCTGAA GACCCGCTGG CCATGAAATT TCTACAAGAG

TTTTGG---- GAGCCCTCAA CAACCGTTTG CCTCAA---T CCAGCCAGAG

CAAGGGCACT GGACTCTCCA GAGACATTAA CCTTGAATCT CCTCCTGGCC

CAAGAGTATG ATGATACCAT ATCAGAAATG AAGAAGGAAG ATTTTGACAT

---------- ---------- ---------- ---------- ----------

CAAGAGTATG ATGATACCAT CTCAGAAATG AAGAAGGAAG ATTTTGACAT

CAAGAGTATG ATGATACCAT CTCAGAAATG AAGAAGGAAG ATTTTGACAT

---GAGTATG ATGATACCAT CTCAGAAATG AAGAAGGAAG ATTTTGACAT

CAAGAATATG ATGACACCAT CTCAGAAACA AAAAGGGAAG ATTTTGACAT

---------- ---------- ---------- -------AGG ATTTCGACAT

CAAGAATATG ATGATGCCAT CACCGAAAC- --AATCGAGG ATTTTGACAT

CAAGAATATG ATGATACCAT CTCAGAAATA AAGAGGGAAG ATTTTGACAT

---------- ---------- ---------- ---------- ----------

GAAGACTATG ATGACACCTT CTCAGAAATG AAGAGAGAAG ATTTTGACAT

GAAGACTATG ATGATACCTT CTCAGAAATG AAGAGAGAAG ATTTTGACAT

GAAGATTATG ATGATGACAT CCCAGAAAGC AAGAGTGAAG ACTTTGACAT

CAAGAATATG ATGATACTCT TGCAGAAACT AAGAGGGAAG ATTTTGATAT

---------- ---------- ---------- ---------- ----------

CAAGACTATG ATGATTACCT CTCAGAAATG AAGACCGAAG ACTTTGACAT

ACAGAATATG ATGACAGCAG CCTGGAAATT CAAGTGGAAG ATTTTGACAT

CAAGA---TG AAGACGACTA CCAGGAAACG ATACCGGAAG ATTTCGACAT

TTATGATGAG GATGAAAATC AGAGCCCCCG CAGCTTTCAA AAGAAAACAC

---------- ---------- ---------- ---------- ----------

TTATGGTGAG GATGAAAATC AGAGCCCCCG CAGCTTTCAA AAGAAAACAC

TTATGGTGAG GATGAAAATC AGAGCCCCCG CAGCTTTCAA AAGAAAACAC

TTATGGTGAG GATGAAAATC AGAGCCCCCG CAACTTTCAA AAGAAAACAC

TTATGATGAA GATGAAAATC AGAGCCCCCG CAGCTTTCAA AAGAAAACAC

CTATGGCGAG GAGGCAGGGC AGGGACCCCG CGGCTTTCAG CAGAAAACGC

TTACAGTGAG GACATAAAGC AAGGTCCCCG CAGCTTTCAA CAGAAAACAA

TTATGGTGAG GATGAAATCC AGGGTCCCCG CAGCTTTCAA AAGAGAACAC

---------- ---------- ---------- ---------- ----------

CTACGGCGAC TATGAAAATC AGGGCCTCCG CAGCTTTCAA AAGAAAACAC

TTATGGAGAG GATGAAAACC AGGGCCCCCG CAGCTTTCAA AAGAGAACAC

TTATGGTGAT GACGAAAATC AAGGTCCACG CAGCTTCCAA AAGAGAACCC

TTATGGTGAG GATGAAAATC AGGGGCCTCG CAGCTTTCAA AAGAGAACAC

---------- ---------- ---------- ---------- ----------

TTATGACGAG GATGAAAAGC AAGGTCCTCG CAGCTTTAAA AAGGAACCAC

CTATGAAGAT GGTGAGAGCC AAGGACCTCG CAGTTTCCAG AAGCGCACTC

CTATGGGGAG AATGAGGAGC AAGGACCCCG GGGCTTTAAT CAGATAACCC

GACACTATTT TATTGCTGCA GTGGAGAGGC TCTGGGATTA TGGGATGAGT

---------- ---------- ---------- ---------- ----------

GACACTATTT TATTGCTGCA GTGGAGCGGC TCTGGGATTA TGGGATGAGT

GACACTATTT TATTGCTGCA GTGGAGCGGC TCTGGGATTA TGGGATGAGT

GACACTATTT TATTGCTGCA GTGGAGCGGC TCTGGGATTA TGGTTTGAGT

GACACTATTT TATTGCTGCA ATAGAGCGGC TCTGGGATTA TGGAATTAGT

GGCACTATTT CATCGCGGCC GTGGAGGAGC TTTGGGACTA CGGGGTGCGT

GGCACTATTT TATTGCAGCT GTGGAACGAC TCTGGGACTA TGGGATGAGT

GACACTATTT TATTGCTGCA GTGGAGCGGC TTTGGGATTA TGGAATGAGT

---------- ---------- ---------- ---------- ----------

GACACTATTT CATTGCTGCA GTGGAGCGTC TCTGGGATTA TGGGATGAGT

GACACTATTT CATTGCTGCA GTGGAGCGGC TCTGGGATTA TGGAATGGGT

GGCACTATTT TATTGCTGCA GTGGAGCGGC TCTGGGATTA TGGGAAGAGT

GACACTATTT TATTGCTGCA GTGGAGCGGT TCTGGGATTA TGGAATGAGT

---------- ---------- ---------- ---------- ----------

GACACTATTA TGTCGCCGCA GTGGAGCAAC TCTGGGATTA TGGGATGAGT

GCCACTACTT CATTGCTGCC CAGGAAAAAA TCTGGGACTA TGGGCTCAAC

GACACTACTT CTTGGCTGCG GTTGAGGTGC TCTGGAACTA CGGGAAGCAG

AGCTCCCCAC ATGTTCTAAG AAACAGGGCT CAGAGTGGCA GTGTCCCTCA

---------- ---------- ---------- ---------- ----------

AGTTCCCCGC ATGTTCTAAG AAACAGGGCT CAGAGTGGCA GTGTCCCTCA

AGTTCCCCGC ATGTTCTAAG AAACAGGGCT CAGAGTGGCA GTGTCCCTCA

AGCTCCCTGC ATGTTCTAAG AAACAGGGCT CAGAGTGGCA GTGTCCCTCA

GGATTCCCCC ATGCTGTAGG AGACAGGGCT CAGAGTGGCA GAGTCCCTCA

GTGTCGCCTG GAGCTCTGGG AGACAGGGCG TGGAGTGGGG ACGCAGCTCG

ACATCTCAT- --GTTCTACG AAATAGGTAT CAAAGTGACA ATGTACCTCA

ACATCCCCCC ATGCTCTAAG AAACAGTGA- ---------- -------TCA

---------- ---------- ---------- ---------- ----------

AGATCTCCCC ATATACTAAG AAACAGGGCT CAAAGTGGGG ATGTCCAGCA

AAATCCCTCT ATGCACTAAG GAACAGGGCT CAGAGTGGAA GTGTCCCTCA

AACTCACCCT ATTCAGCAAG AAACAGAGTT GAGAGTGGAG GCGCCCCCCA

AGATCTCCCC ATGCTCTAAG AAACAGGGCT CAGAGTGACA GTGGCCCTCA

---------- ---------- ---------- ---------- ----------

AAATCTCCCC AAGCTCTAAA AAACAGGGCT CAGGATGGCA ATGTCCCTCA

CAATCTCCCC ACTTCCTAGG AGGCCGGGTC CAGAATACCA GTGCCTGGGA

AAATCCCCCC ACTTTCTTAA TGACGGGGAG CAGGATGCTG GAAGCTGGCA

GTTCAAGAAA GTTGTTTTCC AGGAATTTAC TGATGGCTCC TTTACTCAGC

---------- ---------- ---------- ---------- ----------

GTTCAAGAAA GTTGTTTTCC AGGAATTTAC TGATGGCTCC TTTACTCAGC

GTTCAAGAAA GTTGTTTTCC AGGAATTTAC TGATGGCTCC TTTACTCAGC

GTTCAAAAAA GTGGTTTTCC AGGAATTTAC TGATGGATCC TTTACTCAGC

GTTCAAGAAG GCGGTTTTCC AGGAATTTAC TGATGGCTCC TTTACTCAGC

GTTCCGGAAA GTGGTTTTCC GTGAGTTCAC GGACGGGTCG TTCACGCAGC

GTTCAAGAAA GTAGTTTTCC AGGAATTTAC TGATGGCTCC TTTAGTCAGC

GTTCAAGAAG GTGGTTTTCC AGGAATTTAC TGATGGCTCA GTCACTCAGC

---------- ---------- ---------- ---------- ----------

GTTCAAGAAG GTGGTTTTCC AGGAATTTAC TGATGGATCC TTTACTCAGC

GTTCAAGAAG GTGGTTTTCC AGGAATTCAC TGATGGCTCC TTCACTCAGC

GTTCAAAAAG GTGGTTTTCC AGGAATTCAC TGATGGCTCC TTTACTCAGC

GTTCAAAAAG GTGGTTTTCC AGGAATTTAC TGATGGCTCC TTTACTCAGC

---------- ---------- ---------- ---------- ----------

GTTCAAGAAG GTGGTTTTCC AGGAATTTAC TGATGGCTCC TTTACTCAAC

ATATAAGAAA GTGGTTTTCC AGGAGTTTGC AGATGGTTCC TTTACAGAGC

GTATAAGAAA GTGATCTTCC AGGAATTTGC AGACAGCTCT TTCACTCAAC

CCTTATACCG TGGAGAACTA AATGAACATT TGGGACTCCT GGGGCCATAT

---------- ---------- ---------- ---------- ----------

CCTTATACCG TGGAGAACTA AATGAACATT TGGGACTCCT AGGGCCATAT

CCTTATACCG TGGAGAACTA AATGAACATT TGGGACTCCT GGGGCCATAT

CCTTATACCG TGGAGAACTA AATGAACATT TGGGACTCCT GGGGCCATAT

CATTATACCG TGGAGAACTA AATGAACACT TAGGACTCTT GGGGCCATAT

GTGTGCACCG TGGGGAGCTG GACGCACACC TGGGGCTATT GGGGCCATAC

CCTTATATCG TGGAGAATTA AATGAACACC TGGGGTTGTT GGGCCCATAT

CCTTATATCG TGGAGAACTA AATGAACGCT TAGGAATCTT GGGGCCATAT

---------- ---------- ---------- ---------- ----------

CCTTATACCG TGGAGAACTG AACGAACACT TGGGACTCTT GGGGCCATAT

CCTTATATCG TGGAGAACTA AATGAGCACC TGGGACTCTT GGGGCCCTGT

CCTTGTACCG TGGAGAACTA AACGAACACT TGGGACTCTT GGGGCCCTAC

CCTTATACCG TGGAGAACTG AATGAACACT TGGGACTCTT GGGGCCATAT

---------- ---------- ---------- ---------- ----------

CTGTATATCA TGGAGAAATA AATGAACACT TGGGACTTTT GGGGCCATAT

CTCTGGTTCA TGGGGAGCTG ACTGAACACC TGGGACTCCT GGGGCCTTAT

CATTACATCG TGGAGAGTTA GAGGAGCACC TAGGGATTCT CGGGCCGTAC

ATAAGAGCAG AAGTTGAAGA TAATATCATG CAGGCCTCTC GTCCCTATTC

---------- ---------- ---------- CAGGCCTCTC GTCCCTATTC

ATAAGAGCAG AAGTTGAAGA TAATATCATG CAGGCCTCTC GTCCCTATTC

ATAAGAGCAG AAGTTGAAGA TAATATCATG CAGGCCTCTC GTCCCTATTC

ATAAGAGCAG AAGTTGAAGA TAATATCATG CAGGCCTCTC GTCCCTATTC

ATAAGAGCAG AAGTTGAAGA CAATATTGTG CAGGCCTCTC GTCCCTATTC

ATACGGGCGG AAGTGGAGGA CAACATCGTG CAGGCCTCCC GCCCCTACTC

ATAAGAGCAG AAGTTGAAGA CAACATTATG CAGGCCTCCC GTCCCTACTC

ATAAGAGCAG AAGTTGAAGA TAATATTGTG ATGGCTTCTC GTCCCTACTC

---------- ---------- ---------- ---------- ----------

ATAAGAGCAG AAGTTGAAGA CAATATCGTG CAGGCCTCTC GTCCCTACTC

ATCAGAGCAG AAGTCGAAGA CAATATCATG GAGGCCTCTC GTCCCTACTC

ATACGAGCAG AAGTTGAAGA CAATATCATG CAGGCTTCTC GTCCCTACTC

ATAAGAGCAG AAGTCGAAGA TAATATCATG CAAGCCTCTC GTCCTTACTC

---------- ---------- ---------- ---------- ----------

ATAAGAGCAG AAGTTGAAGA CTTTATCACG CAAGCCTCTC GTCCTTACTC

ATCAGAGCAG AAGTTGAAGA CTATATTGTG -AGGCTTCTC GGCCATACTC

ATCAGGGCAG AGGTTAATGA CAACATCATG CGGGCATCTC GGCCTTACTC

CTTCTATTCT AGCCTTATTT CTTATGAGGA AGATCAGAGG CAAGGAGCAG

CTTCTATTCT AGCCTTATTT CTTATGAGGA AGATCAGAGG CAAGGAGCAG

CTTCTATTCT AGCCTTATTT CTTATGAGGA AGATCAGAGG CAAGGAGCAG

CTTCTATTCT AGCCTTATTT CTTATGAGGA AGATCAGAGG CAAGGAGCAG

CTTCTATTCT AGCCTTATTT CTTATGAAGA AGATCAGAGG CAAGGAGCAG

CTTCTATTCT AGCCTTATTT CTTATGAGGA AGATCAGAGA CAAGGAGCAG

CTTCTACTCC AGCCTCGTGT CCTACCCGGA AGCCGACGG- --TGGAGCTG

CTTCTATTCT AGCCTCATTT CTTATAAAGA AGATCAGAG- --AGGAGAAG

CTTCTACTCT AGCCTCATTT CTTATAAGGA TGATCAGAGA CAAGGAGCCG

---------- ---------- ---------- ---------- ----------

CTTCTATTCT AGTCTTATTT CTTATGACGA AGATGAGGGA CAAGGAGCAG

CTTCTATTCT AGCCTTATTT CTTATGAGGA AGATGAGAGC CAAGGAGCAC

CTTCTATTCT AGTCTCATTT CTTATGAGGA AGATCAGCAG CAAGGAGCTG

CTTTTATTCT AGCCTTATTT CTTATGAAGA AACTCAAGAG CAAGGAGCAG

---------- ---------- ---------- ---------- ----------

CTTCTATTCT AGTCTTATTT CTTATAAGGA AGATCAGAGG CCAGGATCAG

CTTCTATTCC AGCCTCATTT CCTATGAGGA ACATCAGGGG CCTGAAAGTG

CTTCTATTCT AGCCTCATCT CCTACGAGGG GGATGGGGGC CAGGAAGCCA

AACCTAGAAA AAACTTTGTC AAGCCTAATG AAACCAAAAC TTACTTTTGG

AACCTAGGAA AAACTTTGTC AAGCCTAATG AAACCAAAAC TTACTTTTGG

AACCTAGAAA AAATTTTGTC AAGCCTAATG AAACCAAAAC GTACTTTTGG

AACCTAGAAA AAATTTTGTC AAGCCTAATG AAACCAAAAC TTACTTTTGG

AACCTAGACA AACCTTTGTC AAGCCTAATG AGACCAAAAC GTACTTTTGG

AACCTAGAAA AAACTTTGCC AAGCCTAATG AAACCAAAAC TTACTTTTGG

CACCAAGGAG CAACTTTGTG CGGCCCAACG AGACCAAAAC TTACTTCTGG

AACCTAGAAG AAACTTTGTC AAGCCTAATG AAACCAAAAT TTATTTTTGG

AACCTAGGAA AAACTTTGTC AAGCCTAATG AAACCAAAAC TTACTTTTGG

---------- ---------- ---------- ---------- ----------

AACCTAGAAG AAAGTTTGTC AACCCTAATG AAACCAAAAT TTACTTTTGG

AACCTAGACA AAAGTTTGTC AAACCTAATG AAACGAAAAT TTACTTCTGG

AACCTAGGAA AAAGTTTGTC AAGCCCAATG AGACCAAAAC TTACTTTTGG

AACCTAGAAA AAAGTTAGTG AAGCCTAATG AAACCAAAAC TTACTTTTGG

---------- ---------- ---------- ---------- ----------

AACCCGGAAA AAATTCAGTG ATGCCGAATG AAACCAAAAG CTACAGTTGG

CCCTCAGAAA GAAAGAAGTG AGACCTAAGG AGACCGCAAC ATATTCTTGG

AGCCCAAGAG GAAGGAAGTG AAGCCCAATG AAATCCGGCA CTACTTCTGG

AAAGTGCAAC ATCATATGGC ACCCACTAAA GATGAGTTTG ACTGCAAAGC

AAAGTGCAAC ATCATATGGC GCCCACTAAA GATGAGTTTG ACTGCAAAGC

AAAGTGCAAC ATCATATGGC GCCCACTAAA GATGAGTTTG ACTGCAAAGC

AAAGTGCAAC ATCATATGGC GCCCACTAAA GATGAGTTTG ACTGCAAAGC

AAAGTGCAGC ATCATATGGC ACCCACTAAA GATGAGTTTG ACTGCAAAGC

AAAGTGCAAC ATCATATGGC ACCCACTAAT GATGAGTTTG ACTGCAAAGC

AGAGTCCGGC CCCACATGGC GCCCACGGAC GGCGAGTTTG ACTGCAAGGC

AAAGTACAAC ATCATATGGC ACCCACAGAA GATGAGTTTG ACTGCAAGGC

GAAGTGCAAC ATCATATGGC ACCCACTAAA GATGAGTTTG ACTGCAAGGC

---------- ---------- ---------- ---------- ----------

AAAGTGCAGC ATCATATGGC ACCCACTAAA GATGAGTTTG ACTGCAAAGC

AAAGTACAAC ATCATATGGC ACCTACTAAA GATGAGTTTG ACTGCAAAGC

AAAGTGCAAC ATCATATGGC ACCCACTAAA GATGAGTTTG ACTGCAAAGC

AAAGTGCAAC ATCATATGGC ACCCTCTAAA GATGAGTTTG ACTGCAGAGC

---------- ---------- ---------- ---------- ----------

CAAGTGCAGC ATCATATGGC ACCCACGGAA GATGAGTTTG ACTGCAAAGC

AAGGTAGAGC CCCACATGGC CCCTACTGAC AAGGAGTTTG ACTGTAAGGC

AAGGTTCATC ACCAGATGGC ACCCACCGAG AACGAGTTTG ACTGCAAGGC

CTGGGCTTAT TTCTCTGATG TTGACCTGGA AAAAGATGTG CACTCAGGCC

CTGGGCTTAT TTCTCTGATG TTGACCTGGA AAAAGATGTG CACTCAGGCC

CTGGGCTTAT TTCTCTGATG TTGACCTGGA AAAAGATGTG CACTCAGGCC

CTGGGCTTAT TTCTCTGATG TTGACCTGGA AAAAGATGTG CACTCAGGCC

CTGGGCTTAT TTCTCTGATG TTGATCTGGA AAAAGATGTG CATTCAGGCC

CTGGGCGTAT TTCTCTGATG TTGACTTGGA AAAAGATATA CACTCAGGCT

CTGGGCCTAT TTCTCCGACG TGGACCTCGA GCGAGACCTG CACTCGGGGC

CTGGGCTTAT TTCTCTGATG TTGATCTTGA AAGAGATATG CACTCGGGAT

CTGGGCTTAT TTCTCTGATG TTGATCTGGA AAAAGATATG CACTCAGGCT

---------- ---------- ---------- ---------- ----------

CTGGGCTTAT TTTTCTGATG TTGATCTGGA GAAAGATGTG CACTCAGGCT

CTGGGCTTAT TTTTCTGATG TTGATCTGGA ACGAGACATG CACTCAGGCC

CTGGCCCTAT TTTTCAGATG TTGATCTG-- ---------- ----------

CTGGGCTTAT TTCTCTGATA TTGATCTGGA AAAAGATCTT CACTCAGGCT

---------- ---------- ---------- ---------- ----------

CTGGGCGTAT TTCTCGGACG TGGACCTGGA AAAAGACCTG CACTCAGGCT

CTGGGCTTAT TTCTCTGATG TCGATATGGA AAAAGATCTG CACTCAGGGT

CTGGGCTTAT TTCTCGGACG TTGACCTGGA AAAAGATCTG CATTCAGGCT

TGATTGGACC CCTTCTGGTC TGCCACACTA ACACACTGAA CCCTGCTCAT

TGATTGGACC CCTTCTGGTC TGCCACACTA ACACACTGAA CCCTGCTCAT

TGATTGGACC CCTTCTGGTC TGCCACACTA ACACACTGAA CCCTGCTCAT

TGATTGGACC CCTTCTGGTC TGCCACACTA ACACACTGAA CCCTGCTCAT

TGATTGGACC CCTTCTGGTC TGCCACACTA ACACACTGAA CCCTGCTCAT

TGATTGGACC TATTCTGATC TGCCACTCTA ACACACTGAA CCCTGCTCAT

TGATTGGGCC ACTGCTCGTG TGCCACGCCT CCACCCTGCA CCCCTCGCAC

TAATTGGACC CCTTCTGATT TGCCACGCGA ACACACTGAA TCCTGCTCAT

TGATTGGGCC CCTTCTCATC TGTCATACTA ACACACTGAA CCCTGCTCAT

---------- ---------- ---------- ---------- ----------

TGATTGGACC CCTTCTGATC TGCCGCAGTA ACACACTGAA CCCTGCTCAT

TGATTGGACC TCTTCTGATC TGCCGCACGA ACACACTGAA CCCTTCTCAT

---------- ---------- ---------- ---------- ----------

TGATTGGACC CCTTCTGATC TGCCACACTA ATACCCTGAA CCCTGTTCAT

---------- ---------- ---------- ---------- ----------

TGATTGGACC CATTCTGATT TGCCATGCCA ACACACTGAG TGCTGCCTAT

TGATTGGGCC AATATTGATC TGTCACCCCA ACAAGCTGAA CCCATCTTAT

TGATTGGCCC CATGCTGATT TGCCATACCA ATAAACTGAG CTCTTCCTAT

GGGAGACAAG TGACAGTACA GGAATTTGCT CTGTTTTTCA CCATCTTTGA

GGGAGACAAG TGACAGTACA GGAATTTGCT CTGTTTTTCA CCATCTTTGA

GGGAGACAAG TGACAGTACA GGAATTTGCT CTGTTTTTCA CTATCTTTGA

GGGAGACAAG TGACAGTACA GGAATTTGCT CTGTTTTTCA CTATCTTTGA

GGGAGACAAG TGACAGTACA AGAATTTGCT CTGTTTTTCA CTATCTTTGA

GGGAGACAAG TGACAGTACA GGAATTTGCT CTGTTTTTCA CTATCTTTGA

GCGCGGCCGC TCGCAGTGCA GGAGTTCGCG CTGCTCTTCG CGGTGTTCGA

GGGAGACAAG TGTCAGTACA GGAATTTGCT CTGCTTTTCA CTATCTTTGA

GGAAGACAAG TGACTGTGCA GGAATTTGCT CTGTTTTTCA CTATCTTTGA

---------- ---------- ---------- ---------- ----------

GGGAGACAAG TGACAGTGCA GGAGTTTGCC CTGGTTTTCA CTATATTCGA

GGGAGACAAG TGACAGTGCG GGAATTTGCT CTGTTTTTCA CTATTTTTGA

---------- ---------- ---------- ---------- ----------

GGGAGACAAC TGATGGTGCA GGAATTTGCT CTGTTTTTCA CTATCTTTGA

---------- ---------- ---------- ---------- ----------

GGAAGACAAC TGACAGTCCA GGAATTTGCT CTGTTTTTCA CTATCTTTGA

GGCCGACAGT TGACTGTCCA GGAATTCACT CTGTTCTTCA CCATCTTTGA

GGGAGGCAAC TGACAGTGCA GGAATTCACT CTCTTCTTCA CCATCTTCGA

TGAGACCAAA AGCTGGTACT TCACTGAAAA TATGGAAAGA AACTGCAGGG

TGAGACCAAA AGCTGGTACT TCACTGAAAA CATGGAAAGA AACTGCAGGG

TGAGACCAAA AGCTGGTACT TCACTGAAAA CACAGAAAGA AACTGCAGGG

TGAGACCAAA AGCTGGTACT TCACTGAAAA CACAGAAAGA AACTGCAGGG

TGAGACCAAA AGCTGGTACT TCACTGAAAA CATGGAAAGA AACTGCAGGC

TGAGACCAAG AGCTGGTACT TCACTGAAAA CATTGAAAGG AACTGCAGGG

TGAGACCAAG AGCTGGTACT GCGCTGAGAA CCTGGACCGG AAGTGCAGGC

TGAGACCAAG AGCTGGTACT TCACTGAAAA CGTGAAAAGG AACTGCAAGA

TGAGACTAAG AGCTGGTACT TCACTGAAAA CATGGAAAGG AACTGCAGGG

---------- ---------- ---------- ---------- ----------

TGAGACTAAG AGCTGGTACT TCACTGAAAA CCTGGAAAGG AACTGCAGAG

TGAGACCAAG AGCTGGTACT TCACTGAAAA CATGGAAAGG AACTGCAGGG

---------- ---------- ---------- ---------- ----------

TGAGACAAAG AGCTGGTACT TCACTGAAAA CATGGAAAGA AACTGCAGGG

---------- ---------- ---------- ---------- ----------

TGAAACCAAG AGCTGGTACT TCACTGAAAA CAGAGAAAGG AACTGCAAGT

TGAGACCAAG AGCTGGTATT TTGATGAGAA CATGGAGAGG AATTGCAAGC

TGAAACCAAG AGCTGGTACA TCTCTGACAA CATAAAGAGG AACTGTAGGC

CTCCCTGCAA TATCCAGATG GAAGATCCCA CTTTTAAAGA GAATTATCGC

CTCCCTGCAA TATCCAGATG GAAGATCCCA CTTTTAAAGA GAATTATCGC

CTCCCTGCAA TATCCAGATG GAAGATCCCA CTTTTAAAGA GAATTATCGC

CTCCCTGCAA TATCCAGATG GAAGATCCCA CTTTTAAAGA GAATTATCGC

CTCCCTGCAA TGTCCAGATG GAAGATCCCA CTTTTAAAGA GAATTATCGC

CTCCCTGCAA C------ATG GAAGACCCAA CTTTTAAAGA GAATTATCGC

CGCCCCGTGG CACCCCCGCG GGAGACCCCG CCTGGAGAGA AGAGTACCGC

CACCCTGCAA TTTCCAGATG GAAGACCCCA CTTTGAAAGA GAATTATCGC

CCCCCTGCAA CATCCAGACA GAAGACCCTA CTTTTAAAGA GAATTATCGC

---------- ---------- ---------- ---------- ----------

CTCCCTGCAA TGTCCAGAAG GAGGACCCTA CTCTAAAAGA AAACTTCCGC

CTCCCTGCAA TATCCAGATG GAGGACCCTG CTTTTAAACA AAATTATCGC

---------- ---------- ---------- ---------- ----------

CTCCTTGCAA TGTTCAGATG GAAGACCCCA GTTTTAAAAA GAATTACCGC

---------- ---------- ---------- ---------- ----------

CTCCTTGCAA AAGCCACATA GAGGACCCTG ATTTTAAAAA GAACTATCGC

CTCCATGCAA GGTCCAGGTG GATGACCCTG GTTTTCAAAG GAACAACCGC

CACCTTGTGC CGTCCAGATG GAAGACCCTG TCTTTAAAAT GAACCAT-GC

TTCCATGCAA TCAATGGCTA CATAATGGAT ACACTACCTG GCTTAGTAAT

TTCCATGCAA TCAATGGCTA CATAATGGAT ACACTACCTG GCTTAGTAAT

TTCCATGCAA TCAATGGCTA CATAATGGAT ACACTACCTG GCTTAGTAAT

TTCCATGCAA TCAATGGCTA CATAATGGAT ACACTACCTG GCTTAGTAAT

TTCCATGCAA TCAATGGCTA ---------- ---------- ----------

TTCCATGCAA TCAATGGTTA TGTGATGGAT ACACTACCTG GCTTAGTGAT

TTCCATGCCG TGAATGGTTA CGTCGCAGAT GCTCTGCTTG GCCTGGTGAT

TTCCATGCAA TCAATGGTTA TGTAATGGAT ACCCTACCAG GCTTAGTAAT

TTCCATGCAA TTAATGGCTA TGTGATGGAT ACACTCCCTG GCTTAGTGAT

---------- ---------- ---------- ---------- ----------

TTCCATGCAA TCAACGGCTA TGTGAAGGAT ACACTCCCTG GCTTAGTAAT

TTCCATGCAA TCAATGGCTA TGTGATGGAT ACTCTACCTG GTTTAGTAAT

-------CAA TCAATGGCTA TGTGATGGAT TCACTCCCTG GCTTAGTCAT

TTCCATGCAA TCAACGGCTA TGTAATGGAT ACACTACCTG GCTTAGTAAT

---------- ---------- ---------- ---------- ----------

TTCCATGCAA TCAATGGCTA TGTGATGGAT ACTCTGCCTG GCTTAGTCAT

TTTCATGCAA TCAATGGTTA TATGAAGGAT ACCTTGCCTG GATTGGTGAT

TTCCACGCAA TCAATGGCTA TGTGATGGAC ACTCTGCCTG GCTTGGTAAT

GGCTCAGGAT CAAAGGATTC GATGGTATCT GCTCAGCATG GGCAGCAATG

GGCTCAGGAT CAAAGGATTC GATGGTATCT GCTCAGCATG GGCAGCAATG

GGCTCAGGAT CAAAGGATTC GATGGTATCT GCTCAGCATG GGCAGCAATG

GGCTCAGGAT CAAAGGATTC GATGGTATCT GCTCAGCATG GGCAGCAATG

---------- ---------- ---------- ---------- ----------

GGCTGAGGAT CAAAGGATCC GATGGTATCT GCTCAGCATG GGCAGCAATG

GGCGGAAGGT CGGAGGACGA GGTGGCATCT GCTCAGCATG GGCGACCCCG

GGCTCAAGAT CAAAGGATTC GATGGTATCT TCTCAGCATG GGCAACAATG

GGCTCAGGAT CAAAGGATCC GATGGTATCT GCTCAGCATG GGCAGCAATG

---------- ---------- ---------- ---------- ----------

GGCTCAGGAT CAAAAGGTTC GATGGTATCT GCTCAGCATG GGCAGCAACG

GGCTCAGGAT CAAAGGATTC GATGGTATCT GCTCAGCATG GGCAGCAACG

GGCTCAGGGT CAAAGGATCC GGTGGTACCT GCTGAGCATG GGCAGCAATG

GGCTCAGGAA CAAAAGATCC GATGGTATCT GCTCAGCATG GGCAGCAATG

---------- ---------- ---------- ---------- ----------

GGCTCAGGAT CAAAGAATTC GGTGGTATCT GCTCAGCATG GGCAGCAATG

GGCTCAGCAC CAAAGGGTCC GATGGTATCT GCTTAGCATG GGTAGCAATG

GGCTCAGCAT CAGAGGATAC GCTGGTACCT CCTCACCATG GGGAGCAATG

AAAACATCCA TTCTATTCAT TTCAGTGGAC ATGTGTTCAC TGTACGAAAA

AAAACATCCA TTCTATTCAT TTCAGTGGAC ATGTGTTCAC TGTACGAAAA

AAAACATCCA TTCTATTCAT TTCAGTGGAC ATGTGTTCAC TGTACGAAAA

AAAACATCCA TTCTATTCAT TTTAGTGGAC ATGTGTTCAC TGTACGAAAA

---------- ---------- ------GGAC ATGTGTTCAC TGTACGGAAA

AAAACATCCA TTCTATTCAC TTTAGTGGAC ATGTGTTTAC TGTACGGAAA

GGCACGCACA GTCCGTGCAT TTCAGCGCGC ACAGCGTCAC CGTGCGGGAC

AGAACATCCA ATCTATTCAT TTCAGTGGAC ATGTTTTCAC TGTACGGAAA

AAAACATCCA TTCTATTCAT TTCAGTGGGC ATGTGTTCAC TGTTCGGAAA

---------- ---------- ---------- ---------- ----------

AAAACATTCA TTCCATTCAC TTCAGTGGAC ATGTGTTCAC TGTACGGAAA

AAAACATCCA TTCCATTCAT TTCAGTGGAC ACGTATTCAC TGTACGGAAG

AGAACACACA TTCTATTCAC TTCAGTGGAC ATGTGTTCAC TGTGCGGAAG

AAAACATCCA TTCTATTCAC TTCAGTGGGC ACGTGTTCAC TGTACGGAAA

---------- ---------- ---------- ---------- ----------

AAAATATCCA TTCTATTCAT TTCAGTGGAC ACGTGTTCAC TATACGGAAA

AAAATATCCA CTCCATTCAC TTCAGTGGCC AAGTGTTCAC TGTGCGGACA

AAAACATCCA CTCCATTCAG TTCCACGGGC AGGTTTTCAC CGTGCGATCC

AAAGAGGAGT ATAAAATGGC ACTGTACAAT CTCTATCCAG GTAAGTGTCA

AAAGAGGAGT ATAAAATGGC ACTGTACAAT CTCTATCCAG GTAAGTGTCA

AAAGAGGAGT ATAAAATGGC AGTGTACAAT CTCTATCCAG GTAAGTGTCA

AAAGAGGAGT ATAAAATGGC AGTGTACAAT CTCTATCCAG GTAAGTGTCA

AAAGAGGAAT ATAAAATGGC AGTGTACAAT CTCTATCCAG GTAAGTGTCA

AAAGAAGAGT ATAAAATGGC AGTGTACAAC CTCTATCCAG GTCAGTGTCA

GGGGGCGAGC ACAGGACGGC GGTGTGCAAC CTGTACCCAG GCCGCTGCCA

AAAGAGGAGT ATAAAATGGC AGTGTACAAC CTCTACCCAG GTCAGTGTCA

AAAGAAGAAT ATAAAATGGC AGTGTACAAC CTCTACCCAG GTAATTGTCA

---------- ---------- ---------- ---------- ----------

AAAGAGGAAT ATAAAATGGC AGTCTACAAC CTCTATCCAG GTAAGTGTCA

AAAGAGGAGT ATAAAATGGC GGTCTACAAC CTCTATCCAG GTAAGTGTCA

ACGGAGGAGT ACAAAATGGC AGTGTATAAT CTCTACCCAG GCGAGTGTCA

GAACAGGAGC ATAAAATGGC AGTCTATAAC CTCTATCCAG GTGAGTGTCA

---------- ---------- ---------- ---------- ----------

AAAGACGAGT ATAAAATGGC AGTCTACAGC CTTTATCCAG GTGAGTGTCA

AGGAAGGAAT ACAAAATGGC GGTCTACAAT CTCTATCCCG ---AATGTCA

AACAAAGAGT ACAAAATGGG AGTCTACAAC CTCTACCCTG GTGAGTGTCT

GACTCCCCTG GGAATGGCTT CTGGACACAT TAGAGATTTT CAGATTACAG

GACTCCCCTG GGAATGGCTT CTGGACACAT TAGAGATTTT CAGATTACAG

GACTCCCCTG GGAATGGCTT CTGGACGCAT TAGAGATTTT CAGATTACAG

GACTCCCCTG GGAATGGCTT CTGGACGCAT TAGAGATTTT CAGATTACAG

GACTCCCTTG GGAATGGCTT CTGGACGCAT TAGAGATTTT CAAATTACAG

AAATCCACTG GGAATGGCTT CTGGACACAT TCGAGATTTT CAGATTACAG

GGTCCCGCTT GGCATGGCTT CCGGTTACAT CCGGGACTCG CAGATCACGG

GATTCCTCTT GGAATGGCTT CTGGAAGCAT CCGTGATTTC CAGATTACAG

GATTCCCCTG GGGATGGCTT CTGGACACAT TCAAGATTTT CAGATTACAG

---------- ---------- ---------- ---------- ----------

GACTCCACTG GGGATGGCTT CCGGACACAT TAGAGATTTT CAGATTACAG

GACTCCTCTG GGAATGGCTT CTGGACGCAT TAGAGATTTT CAGATTACAG

GGCTCCCTTG GGATTCACTT CTGGGCGCAT TAAAGATTTC CAGATTACAG

GATTCCTCTG GGAATGGCTT CTGGACATAT TAAAGATTCT CAGATTACAG

---------- ---------- ---------- ---------- ----------

GACTCCCCTG GGAATGGCTT CTGGACACAT TAGAGATTCT CAGATTACAG

GAATGCTTTG GGGATGGCAT CGGGACAGAT CGCTGATCCT CAGATCACAG

GAATCCCCTG GGAATGGCCT CCGGTCGAAT TGCAGATTCC CAGATCACCG

CTTCAGGACA ATATGGAGTT TTTCAGGGAA ATCAAGACTC CTTCACACCT

CTTCAGGACA ATATGGAGTT TTTCAGGGAA ATCAAGACTC CTTCACACCT

CTTCAGGACA ATATGGAGTT TTTCAGGGAA ATCAAGACTC CTTCACACCT

CTTCAGGACA ATATGGAGTT TTTCAGGGAA ATCAAGACTC CTTCACACCT

CTTCAGGACA ATATGGAATT TTTCAGGGAA ATCAAGACTC CTTCACACCT

CTTCAGGACA ATATGGAGTT TTTCAGGGAA ATCAAGACTC CTTCACACCT

CTTCCGGCTA CCACGGGGTT TTTCAGGGTA ACCGGGATGC GTCCACGCCC

CTTCAGGACA TTATGGAGTT TTTCAGGGGA ATCAGGACTC ATCCACACCT

CTTCAGGACA ATATG--GTT TTTAAAGGAA ATCAAGACTC CTTCACACCT

---------- ---------- ---------- ---------- ----------

CTTCAGGACA ATATGGAGTC TTCCAGGGAA ACCGGGACTC CTCCACGCCT

CTTCAGGACA ATATGGA--- ---------- ---------- ----------

CTTCAGGACA ATATGGAGTT TTTCAGGGCA ATGACGACGC CACCACGGCC

CTTCAGGACA ATATGGGGTT TTTCAAGGAA ATCAAGACTC CTTCACACCT

---------- ---------- ---------- ---------- ----------

CTTCAGGATA TCATG--GTT TTTCAGGGAA ATCAGGACTC TTTCACACCG

CTTCTGGACA ATATGGCATC TTCAAGGGAA ATCGAGATCA CTCCAGCCCG

CCTCTGGGCA ATATGGAATT TTCCAAGGAA ACCAAGATCA TGACACACCC

GTGGTGAACT CTCTAGACCC ACCGTTACTG ACTCGCTACC TTCGAATTCA

GTGGTGAACT CTCTAGACCC ACCGTTACTG ACTCGCTACC TTCGAATTCA

GTGGTGAACT CTCTAGACTC ACCGTTACTG ACTCGCTACC TTCGAATTCA

GTGGTGAACT CTCTAGACTC ACCGTTACTG ACTCGCTACC TTCGAATTCA

GTGGTGAACT CTCTAGACCC ACCGTTATTG ACACGCTACC TTCGAATTCA

GTGGTGAACT CTCTAGACCC ACCTCTACTG ACCCGCTATA TTAGAATTCA

ATGGTGAACT CTCTGCACCC CCCGCGCTTC ACTCGATACC TTCGGATTCA

ATGATGAATT CTCTAGACCC ACCATTACTC ACTCGCTATC TTCGAATTCA

GTATTGAACT CTCTAGACCC ACCACTGTTG ACTCGTTATC TCAGAATTCA

---------- ---------- ---------- ---------- ----------

GTGCGGAACG CTCTCGAACC CCCGCTGGTG GCTCGCTACG TGCGCCTGCA

---------- ---------- ---------- ---------- ----------

GTGGTGAACT CTCTGGAGCC GCCGCTTCTG ACCCGCTTCG TCCGTGTGCA

GTGGTGAACT CTCTAGACCC ACCACTGCTG ACTCGATACC TTAGAATCCA

---------- ---------- ---------- ---------- ----------

GTGGTAAACT CTTTAGACCC ACCACTAGTG ACGTCCTACC TGAGAATTTA

GTGGTGAACC CCGTGGAGCC GCCCCTGTTT GCCAGATTCC TGAGGATCCA

GTGGTGAACT CTGTGGATCC TCCCCTGTTT GTGCGGTATC TGAGGATTCA

CCCCCAGAGT TGGGTGCACC AGATTGCCCT GAGGATGGAG GTTCTGGGCT

CCCCCAGAGT TGGGTGCACC AGATTGCCCT GAGGATGGAG GTTCTGGGCT

TCCCCAGAGT TGGGTGCACC AGATTGCCCT GAGGATAGAG GTTCTGGGCT

TCCCCAGAGT TGGGTGCACC AGATTGCCCT GAGGATAGAG GTTCTGGGCT

CCCCCAGAGT TGGGTGCACC AGATTGCCCT GAGGATGGAG GTTCTGGGCT

CCCTCAGAGC TGGGTGAACC AGATTGCCCT GAGACTAGAG GTTCTGGGTT

CCCCCAAGTG TGGGAGCGGC AAATTGCTCT GCGGCTCGAG ATCCTGGGGT

CCCCCAGATC TGGGAGCACC AAATTGCTCT GAGGCTTGAG ATTCTAGGAT

CCCCAAGAGC TGGGTCCACC AGATTGCCCT GAGGCTGGAG GTTCTGGGCT

---------- ---------- ---------- ---------- ----------

CCCGCAGAGC TGGGCGCACC ACATCGCCCT GAGGCTGGAG GTCCTGGGCT

---------- ---------- ---------- ---------- ----------

CCCTCAGAAC TGGGCTCAGC ACATCGCTCT GAGGCTGGAG CTGCTGGGCT

CCCCCAGAGC TGGGTGCACC AAATTGCCTT GAGGCTGGAG GTTCTGGGCT

---------- ---------- ---------- ---------- ----------

CCCGAAGAAA TGGATGCACC ACATTGCCCT GCGGCTGGAG GTTCTGGGCT

CCCCTGGAGC TGGGCTAACC ACATTGCCCT GAGGGTGGAA GTTCTGGGCT

TCCCCACAGC TGGGAAAAGA ACATTGCCCT GAGGACTGAG TTCCTAGGCT

GCGAGGCACA GGACCTCTAC ATGGTGGAGT ATGGGAAATA CAGCAATGAC

GCGAGGCACA GGACCTCTAC ATGGTGGAGT ATGGGAAATA CAGCAATGAC

GTGAGGCACA GGAGCTCTAC ATGGTGGAGT ATGGGAAATA CAGCAATGAC

GCGAGGCACA GGAGCTCTAC ATGGTGGAGT ATGGAAAATA CAGCAATGAC

GCGAGGCACA GGAGCTCTAC ATGGTGGAGT ATGGGAAATA CAGCAATGAC

GTGAGGCGCA GCCACTCTAC ATGGTGGAAT ATGGGAAATA CAGCAATGAC

GTGAGGCTCA GCAGCTGGAC ATGGTAGAGT ATGGAAAATA CAGCAACGAC

GTGAGGCCCA GCAGCAATAC ATGGTAGAGT ATGGAAAATA CAGCAACGAC

GTGAAGCACA GCAGCTCTAC ATGGTGGAAT ATGGGAAATA CAGCAACGAT

---------- ---------- ---------- ---------- ----------

GCGACACCCA GCAGCCCGCC ATGGTGGAGT ATGGGAAATA CAGCAACGAC

---------- ---------- ATGGTCGAGT ATGGGAAATA CAGCAATGAC

GTG------- ---------- ATGGTGGAGT ACGGCAAGTA CAGCAACGAC

GTGAGGCACA ACAGCTCTAC ATGGTGGAGT ATGGGAAATA CAGCAACGAC

---------- ---------- ---------- ---------- ----------

GCCAATCCCA ACAGCACTAC ATGGTGGAAT ATGGAAAATA CAGCAACGAC

GTGACACTCA GCAGGTGGCC ATGGTGGAAT ATGGGAAGTA CAGCAACGAC

GTGACACCCA ACAGGTGTAC ATGGTGGAGT ATGGAAAATA CAGCAACGAC

CTCTACGAAC TCCAGGCGAG CCGGTGGGAG TGGAAGAGAC TCAAAGCAAA

CTCTATGAAC TCCAGGCGAG CCGGTGGGAG TGGAAGAGAC TCAAAGCAAA

CTCTACGAGC TCCAGGCGAG CCGGTGGGAG TGGAAGAGAC TCAAAGCAAA

CTCTACGAGC TCCAGGCGAG CCGGTGGGAG TGGAAGAGAC TCAAAGCAAA

CTCTACGAGC TGCAGGCGAG CCGGTGGGAG TGGAAGAGAC TCAAAGCAAA

CTCTATGAGC TCCAGGCCAG CCGGTGGGAG TGGAAGAGAC TCAAAGCAAA

CTCTATGAGC TCCAGGCAAG TCGCTGGGAA TGGAAGAGAC TGAAGGCAAA

CTCTATGAAC TCCAGGCAAG TCGTTGGGAA TGGAAGAGAC TGAAGGCAAA

CTCTACGAGC TCCAGGCAAG CCGATGGGAG TGGAAGAGAC TCAAAGCAAA

---------- ---------- ---------- ---------- ----------

CTCTATGAGC TCCAGGCAAG CCGGTGGGAA TGGAAGAGAC TCAAAGCCAA

CTCTATGAGC TCCAGGCAAG CCGGTGGGAG TGGAAGAGAC TCAAAGCAAA

CTCTACGAGC TGCAGGCCAG CCGCTGGGAG TGGAAGAAAC TCAAAGCAAA

CTCTACGAGC TTCAGGCGAG TCGTTGGGAG TGGAAGAGGC TCAAAGCAAA

---------- ---------- ---------- ---------- ----------

CTCTATGAGC TCCAGGCCAG TCGCTGGGAG TGGAAGAGAC TCAAAGCAAA

CTCTACGAGT TGCAGGCAAG CAGGTGGGAA TGGAAAAGGC TCAAAGCAAA

CTGTATGAAC TGCAGGCAAG TCGGTGGGAA TGGAAGAGAC TCAAAGCCAG

GACGCCCAAA AACGGGCCCC CTCCGTGTCC TCGACTCGGG CACAGCTTCT

GACGCCCAAA AACGGGCCCC CTCCGTGTCC TCGACTCGGG CACAGCTTCT

GACGCCCAAA AACGGGCCCC CTCCATGTCC TCGGCTCGGG CACAGCTTCT

GACGCCCAAA AACGGGCCCC CTCCATGTCC TCGGCTCGGG CACAGCTTCT

GACACCCAAA AATGGGCCCC CTCCATGTCC TCGGCTCGGG CACAGCTTCT

GACACCCAAA AATGGGCCCC CTCCATGTCC TCGGCTTGGG CACAGCTTCT

GACACCCAAA AACGGGCCTC CTCCGTGTCC TCGGCTTGGA CACAGTTTCT

GACACCCAAA AATGGGCCTC CTCCATGTCC TCGGCTTGGA CATAGCTTCT

GACGCCCAAA AATGGGCCCC CTCCGTGTCC TCGACTTGGC CACAGCTTCT

---------- ---------- ---------- ---------- ----------

GACGCCCAAA AATGGGCCCC CTCCATGTCC TCGGCTTGGG CACAGCTTCT

GACGCCCAAA AATGGGCCCC CTCCATGTCC TCGGCTCGGG CACAGCTTCT

AACGCCCCTA ACCGGGCCCC CTCCGTGCCC TCGACTGGGC CACAGCTTCT

GACGCCCAAG AACGGGCCCC CTCCGTGTCC TCGGCTCGGG CATAGCTTCT

---------- ---------- ---------- ---------- ----------

GACCCCCAAA AATGGACCCC CTCCCTGTCC TCGGCTTGGC CACAGCTTCT

AGCCCCCAAA AATGGGCCGC CTCCATGCCC CCGACTTGGG CACAGCTTTT

GACACCCAAA AATGGGCCAC CTCCCTGCCC CAGGCTTGGT CACAGCTTCT

CCCTTGTGGG CAACAGGTAC CTGAATGACT TATATATCCT GGAATTACGG

CCCTTGTGGG CAACAGGTAC CTGAATGACT TATATATCCT GGAATTACGG

CCCTTGTGGG CAACAGGTAC CTGAATGACT TATATATCCT GGAATTACGG

CCCTCGTGGG CAACAGGTAC CTGAATGACT TATATATCCT GGAATTACGG

CCCTTGTGGG CAACAGGTAC CTGAATGACT TATACATCCT GGAATTACGG

CCCTTGTTGG CAACAGGTAC CTGAATGACT TATACATCCT GGAATTGCGG

CCCTTGTGGG CAACAGGTAC CTGAATGACT TATATATTCT CGAACTACGG

CCCTTGTGGG CAACAGGTAC CTGAATGACT TATATATTCT CGAACTACGG

CCCTTGTGGG CAACAGGTAC CTCAATGACT TGTATATCCT GGAATTACGC

---------- ---------- ---------- ---------- ----------

CCCTTGTGGG CAACAGGTAC CTGAATGACT TATATATCCT GGAATTGCGG

CCCTTGTGGG CAATAGGTAC CTGAATGACT TATACATCCT GGAGTTGCGG

CCCTGGTGGA CAACAGGTAC CTGAATGACT TGTACATCCT AGAAATGCGG

CCCTCGTGGG CAACCGGTAC CTGAATGACT TATATATCCT GGAATTGCGA

---------- ---------- ---------- ---------- ----------

CCCTTGTGGG CAA---GTAC CTGAATGACC TGTACATCCT GGAATTACGG

CTCTTGTGGG CAACAGGTAC CTGAATGACC TATATATTCT GGAGTTACGA

CCTTAGTAGG CAACAGGTAC CTGAATGACC TGTACATCCT GGAATTGCGC

CCAGGCTCTG GAGTGGTAGC CTGGGACATT CCCATCACTT ACGGGGTCCT

CCAGGCTCCG GAGTGGTAGC CTGGGACATT CCCATCACTT ACGGGGTCCT

CCAGGCTCCG GAGTGGTAGC CTGGGATATC CCCATCACTT ATGGGGTCCT

CCAGGCTCCG GAGTGGTAGC CTGGGATATC CCCATCACTT ATGGGGTCCT

CCAGGCTCCG GAGTGGTAGC CTGGGACATC CCTATCACTT ACGGGGTCCT

CCAGGCTCCG GAGTGGTAGC CTGGGACATC CCCATCACTT ATGGGGTCCT

CCAGGCTCTG GAGTGGTAGC TTGGGACATT CCCATCACTT ATGGTGTCCT

CCAGGCTCTG GAGTGGTAGC TTGGGACATC CCCATCACTT ACGGTGTCCT

CCAGGCTCTG GAGTGGTAGC CTGGGACATC CCCATCACTT ATGGGGTGCT

---------- ---------- ---------- ---------- ----------

CCAGGCTCCG GAGTCGTAGC CTGGGACATC CCCATCACTT ACGGCGTCCT

CCCGGCTCCG GAGTGGTAGC CTGGGACATT CCCATTACCT ACGGCGTCCT

CCGGGCTCAG GAGTGGTCGC CTGGGACATC CCCATCACTT ATGGGGTCCT

CCAGGCTCCG GGGTGGTAGC CTGGGACATC CCCATCACTT ACGGGGTCCT

---------- ---------- ---------- ---------- ----------

CCAGGCTCCG GTGTGGTGGC CTGGGACATC CCCATCACTT ACGGGGTTCT

CCAGGCTCTG GTGTGGTAGC CTGGGACATC CCCATCACTT ATGGGGTCCT

CCAGGCTCCG GAGTAGTCGC CTGGGACATC CCTATCACTT ATGGCGTCCT

ACCACCACCC CGGGAGTCAC ATACTGCCGT GGTCTACACC GAAAAAGACA

ACCACCACCC CGGGAGTCAC ATACTGCCGT GGTCTACACC GAAAAAGACA

ACCACCACCC CGGGAGTCAC ATACTGCCGT GGTCTACACC GAGAAAGACA

ACCACCACCC CGGGAGTCAC ATACTGCCGT GGTCTACACC GAGAAAGACA

ACCACCACCT CGGGAGTCAC ATACTGCCGT GGTCTACACC GAAAAAGACA

GCCTCCACCC CGGGAGTCAC ATACTGCTGT GGTCTACACT GAAAGAGATA

TCCTCCACCC CGGGAGTCAC ATACTGCTGT GGTCTACACT GAAAAAGATA

GCCTCCACCC CGGGAGTCAC ATACTGCTGT GGTCTACACT GAAAAAGATA

GCCCCCACCC CGGGAGTCGC ATACTGCTGT GGTCTACACT GAAAGGGATA

---------- ---------- ---------- ---------- ----------

GCCTCCACCC CGGGAGTCAC ATACGGCTGT GGTCTATACT GAGAAAGACA

GCCCCCACCC CGGGAGTCGC ACACTGCCGT GGTGTACACG GAGAAAGACA

GCCCCCACCC CGGGAGTCGC ATACGGCAGT AGTCTACACG GAAAAGGACA

GCCCCCTCCG CGGGAGTCAC ACACGGCTGT GGTCTACACT GAGAAAGACA

---------- ---------- ---------- ---------- ----------

GCCCCCACCA CGAGAGTCCC ATACTGCTGT GGTCTACACT GAGAAAGACA

GCCGCCCCCC CGGGAGTCCC ACACGGCTGT AGTCTATACA GAAAAAGACA

CCCGCCGCCC CGGGAATCAC ACACCGCAGT GGTCTACACC GAGAAGGACA

ATAAGAAGTC CAAGCTGGTG ATCTACGGCG GGATGAGTGG CTGCAGGCTG

ATAAGAAGTC CAAGCTGGTG ATCTACGGCG GGATGAGTGG CTGCAGGCTG

ACAAGAAGTC CAAGCTGGTG ATCTACGGCG GGATGAGTGG CTGCAGGCTG

ACAAGAAGTC CAAGCTGGTG ATCTACGGCG GGATGAGTGG CTGCAGGCTG

ACAAGAAGTC CAAGCTGGTG ATCTACGGCG GGATGAGTGG CTGCAGGCTG

ACAAAAAATC CAAGCTGGTG ATCTACGGAG GGATGAGTGG CTGCAGGCTG

ACAAGAAATC CAAGCTGGTG ATCTATGGAG GGATGAGTGG CTGCAGGCTA

ACAAGAAATC CAAGCTGGTG ATCTATGGAG GGATGAGTGG CTGCAGGCTA

ACAAGAAATC CAAGCTGGTG ATTTATGGAG GGATGAGTGG CTGCAGGCTG

---------- ---------- ---------- ---------- ----------

ATAAGAAGTC CAAGCTGGTG ATCTACGGAG GGATGAGTGG CTGCAGGCTG

ACAAGAAGTC CAAGCTGGTC ATCTATGGAG GGATGAGTGG CTGCCGGCTC

GGAAGAAGTC CAAGCTGGTG ATCTATGGAG GGATGAGTGG CTGCAGGCTA

ACAAGAAATC CAAGCTGGTC ATCTACGGCG GGATGAGTGG CTGCAGGTTG

---------- ---------- ---------- ---------- ----------

GTAAGAAATC CAAGCTGGTC ATCTATGGAG GGATGAGTGG CTGCAGGCTG

ACAAGAAATC CAAGTTGGTT ATCTATGGGG GAATGAGTGG CTGCAGGCTT

ATAAGAAATC CAAGTTGGTC ATCTACGGAG GCATGAGTGG CTGCAGGTTG

GGGGACCTGT GGACCCTAGA TATTGACACC CTGACGTGGA ATAAGCCCAG

GGGGACCTGT GGACCCTAGA TATTGACACC CTGACGTGGA ATAAGCCCAG

GGGGACCTGT GGACCCTAGA TATTGACACA CTGACGTGGA ATAAGCCCAG

GGGGACCTGT GGACCCTAGA TATTGACACA CTGACGTGGA ATAAGCCCAG

GGAGACCTGT GGACCCTAGA TATCGATACA CTGACATGGA ATAAGCCCAG

GGGGACCTCT GGACCCTTGA CATTGAGACT CTGACGTGGA ACAAGCCCAG

GGGGACCTTT GGACCCTGGA CATTGAGACA CTGACATGGA ATAAGCCCAG

GGGGACCTTT GGACCCTGGA CATTGAGACA CTGACATGGA ATAAGCCCAG

GGCGACCTCT GGACCCTGGA TATTGAAACC CTGACATGGA ATAAGCCCAG

---------- ---------- ---------- ---------- ----------

GGGGACCTCT GGACCCTGGA TATTGAGACC CTGACGTGGA ATAAACCCAG

GGGGACCTGT GGACCCTGGA TATTGAGACT CTGACATGGA ATAAGCCCAG

GGAGATCTCT GGACCCTGGA CATTGAGACT TTGACGTGGA ATAAGCCCAC

GGGGACCTCT GGACCCTCGA TATCGAGACT CTGACGTGGA ACAAGCCCAG

---------- ---------- ---------- ---------- ----------

GGGGACCTCT GGACCCTTGA TATTGACACA CTGACATGGA ACAAGCCCAG

GGGGACCTCT GGACCCTAGA TATTGAGACC CTGACCTGGA ATAAACCTAC

GGAGATCTCT GGACGCTGGA CATTGAAACG CTCACCTGGA ATAAACCCAG

TCTCAGCGGG GTGGCGCCTC TTCCTCGCAG TCTCCACTCG GCAACCACCA

TCTCAGCGGA GTGGCGCCTC TTCCTCGCAG TCTCCACTCG GCAACCACCA

TCTCAGCGGG GTGGCGCCTC TTCCTCGCAG TCTCCACTCG GCAACCACCA

TCTCAGCGGG GTGGCGCCTC TTCCTCGCAG TCTCCACTCG GCAACCACCA

TCTCAGCGGG GTGGCGCCTC TTCCTCGCAG TCTCCACTCA GCAACCACCA

TCTCAGCGGG GTGGCACCTC TTCCTCGTAG TCTTCACTCT GCAACCACCA

CCTTAGTGGG GTGGCACCCC TTCCTCGCAG CCTCCACTCT GCAACCACCA

CCTTAGTGGG GTGGCACCCC TTCCTCGCAG CCTCCACTCT GCAACCACCA

TCTCAGTGGG GTGGCGCCTC TTCCTCGCAG TCTCCACTCG GCAACTACAA

---------- ---------- ---------- ---------- ----------

TCTCAGTGGG GTGGCACCTC TTCCTCGAAG TCTTCACTCA GCCACGACCA

TCTCAGCGGG GTGGCACCTC TTCCTCGCAG CCTCCACTCG GCCACGACCA

TCTCAGTGGG CTAGCACCTT TGCCTCGGAG TCTCCATTCA GCCACCACCA

CCTCAGCGGG GTAGCCCCTC TCCCTCGCAG TCTCCACTCG GCCACGACGA

---------- ---------- ---------- ---------- ----------

CCTCAGCGGC GTGGCACCGT TGCCTCGCAG TCTTCACTCA GCCACGACCA

TCTCAGTGGG GTGGCTCCTC TCCCTCGAAG TCTTCATTCT GCCACCACCA

TCTCAGTGGC GTGGCTCCTC TCCCCCGCAG CCTTCACTCT GCTACAACCA

TCGGAAATAA AGCTCGGGCT GGCCACTGCG CAGTCGCCAT CAACACCCGC

TCGGAAATAA AGCTCGGGCT GGCCACTGCG CAGTCGCCAT CAACACCCGC

TCGGAAATAA AGCTCGGGCT GGCCACTGCG CAGTCGCCAT CAACACTCGC

TCGGAAATAA AGCTCGGGCT GGCCACTGCG CAGTCGCCAT CAACACTCGC

TTGGAAATAA AGCTCGGGCC GGCCACTGCG CAGTCGCCAT CAACACCCGC

TTGGAAACAA AGCTCGAGCT GGCCACTGTG CAGTTGCCAT CAACACCCGT

TAGGAAACAA AGCTCGAGCA GGCCACTGTG CTGTTGCCAT CAATACTCGC

TAGGAAACAA AGCTCGAGCA GGCCACTGTG CTGTTGCCAT CAATACTCGT

TAGGAAACAA AGCTCGAGCC GGCCACTGTG CTGTTGCCAT CAACACCCGC

---------- ---------- ---------- ---------- ----------

TAGGAAACAA AGCCCGAGCT GGCCACTGTG CCGTAGCCAT CAATACCCGC

TCGGCAACAA AGCCCGAGCC GGCCACTGCG CTGTAGCAAT CAATACCCGC

TAGGAAACAA AGCCCGGGCT GGCCACTGTG CTGTCGCGAT CAATACCCGT

TTGGAAACAA AGCTCGAGCC GGCCACTGCG CCGTGGCCAT CAACACACGC

---------- ---------- ---------- ---------- ----------

TTGGAAATAA ---TCGAGCT GGGCACTGTG CCGTCGCCAT CAACACCCGC

TAGGAAACAA AGCTCGAGCT GGCCACTGTG CAGTGGCCAT TAACACCCGA

TAGGAAACAA GGCAAGGGCC GGGCACTGCG CCGTGGCCAT CAACACCCGC

CTGTACATTT GGAGTGGGCG TGACGGCTAC CGCAAGGCCT GGAACAACCA

CTGTACATTT GGAGTGGGCG TGACGGCTAC CGCAAGGCCT GGAACAACCA

TTGTACATTT GGAGTGGGCG TGACGGCTAC CGCAAGGCCT GGAACAACCA

TTGTACATTT GGAGTGGGCG TGACGGCTAC CGCAAGGCCT GGAACAACCA

CTGTACATTT GGAGTGGGCG CGATGGCTAC CGCAAGGCCT GGAACAACCA

CTCTACATTT GGAGTGGGCG CGATGGCTAC CGCAAGGCCT GGAACAACCA

CTGTATATTT GGAGTGGCCG TGATGGCTAT CGCAAGGCCT GGAACAATCA

CTGTATATTT GGAGTGGCCG TGATGGCTAC CGCAAGGCCT GGAACAACCA

TTGTACATTT GGAGTGGGCG CGATGGCTAC CGCAAGGCCT GGAACAACCA

---------- ---------- ---------- ---------- ----------

CTATACATAT GGAGTGGGCG CGACGGCTAC CGAAAGGCCT GGAACAACCA

CTATACATTT GGAGTGGGCG TGATGGCTAC CGAAAGGCCT GGAACAACCA

TTGTACATTT GGAGTGGGCG TGATGGCTAT CGAAAAGCCT GGAACAACCA

CTATACATTT GGAGCGGCCG CGACGGCTAC CGCAAAGCCT GGAACAACCA

---------- ---------- ---------- ---------- ----------

CTGTACATCT GGAGCGGGCG GGACGGCTAC CGGAAAGCCT GGAACAACCA

CTCTATATCT GGAGTGGGCG AGACGGTTAC CGAAAAGCAT GGAATAACCA

CTGTACATCT GGAGCGGGCG GGATGGCTAC CGCAAAGCGT GGAACAATCA

GGTCTGCTGC AAGGACCTCT GGTACCTAGA GACAGAAAAG CCACCACCCC

GGTCTGCTGC AAGGACCTCT GGTACCTAGA GACAGAAAAG CCACCACCCC

GGTCTGCTGC AAGGACCTCT GGTACCTAGA GACAGAAAAG CCACCACCCC

GGTCTGCTGC AAGGACCTCT GGTACCTAGA GACAGAAAAG CCACCACCCC

GGTCTGCTGC AAGGACCTCT GGTACCTGGA GACAGAAAAG CCACCACCCC

GGTGTGCTGC AAGGACCTCT GGTACCTGGA GACAGAAAAG CCACCACCCC

GGTCTGCTGC AAGGACCTGT GGTATCTGGA GACAGAAAAG CCACCACCCC

GGTCTGCTGC AAGGACTTGT GGTATTTGGA GACAGAAAAG CCACCACCCC

AGTATGCTGT AAGGACCTGT GGTACCTGGA GACAGAAAAG CCACCTCCTT

---------- ---------- ---------- ---------- ----------

GGTCTGCTGC AAGGATCTCT GGTACCTGGA AACAGAAAAG CCACCGCCCC

GGTGTGCTGT AAGGACCTCT GGTACCTGGA AACAGAAAAG CCACCGCCCC

GGTCTGTTGC AAGGACCTCT GGTACCT--- -----AAAAA CCGCCACCCC

GGTCTGCTGC AAGGACCTCT GGTACCTCGA AACAGAAAAG CCACCAGCCC

---------- ---------- ---------- ---------- ----------

GGTTTGCTGC AAGGATCTCT GGTACTTGGA AACAGAAAAG CCGCCACCGC

AGTCTGCTGT AAGGACCTTT GGTACCTAGA GACAGAAAAG CCTCCTGCCC

GGTCTGCTGC AAGGACCTGT GGTACCTGGA GACAGAAAAA CCGCCTGCCC

CAGCCCGAGT ACAACTGGTA CGCGCCAACA CCAACTCCCT GGAGGTGAGC

CAGCCCGAGT ACAACTGGTA CGCGCCAACA CCAACTCCCT GGAGGTGAGC

CAGCCCGAGT ACAGCTGGTA CGCGCCAACA CCAACTCCCT GGAGGTGAGC

CAGCCCGAGT ACAGCTGGTA CGCGCCAACA CCAACTCCCT GGAGGTGAGC

CAGCCCGAGT ACAACTGGTA CGCGCCAACA CCAACTCCCT GGAGGTGAGC

CAGCCCGGGT ACAGCTGGTA CGAGCCAACA CCAACTCCCT GGAGGTGAGC

CAGCCCGAGT TCAACTAGTA CGAGCCAACA CCAACTCACT GGAGGTTAGC

CAGCCCGAGT ACAACTAGTA CGAGCCAACA CCAACTCACT GGAGGTTAGC

TAGCCCGGGT GCAACTGGTA CGAGCCAACA CCAACTCCCT GGAGGTGAGC

---------- ---------- ---------- ---------- ----------

CCGCCCGGGT ACAGCTGGTG CGAGCCAACA CCAACTCCCT GGAGGTGAGC

CGGCCAGAGT ACAACTGGTA CGAGCCAACA CCAATTCCCT GGAGGTGAGC

CAGCCCGGGT GCAGCTCGTT CGAGCCAACA CCAGTTCGTT AGAGGTGAGC

CGTCCCGGGT CCAGCTGGTC CGCGCCAACA CCAACTCCCT GGAAGTGAGC

---------- ---------- ---------- ---------- ----------

CCTCCCGGGT GCAGCTGGTG CGCGCCAACA CCACTTCCCT GGAGGTCAGC

CATCCCGGGT GCAGCTGGTC CGAGCCAACA CCAACTCCCT GGAGGTGAGC

CATCCCGGGT GCAGCTAGTG CGAGCCAACA CCAACTCGCT GGAGGTGAGC

TGGGGGGCAG TGGCAACAGC CGACAGCTAC CTTCTCCAGC TCCAGAAATA

TGGGGGGCAG TGGCAACAGC CGACAGCTAC CTTCTCCAGC TCCAGAAATA

TGGGGGGCAG TGGCAACAGC CGACAGCTAC CTTCTCCAGC TCCAGAAATA

TGGGGGGCAG TGGCAACAGC CGACAGCTAC CTTCTCCAGC TCCAGAAATA

TGGGGGGCAG TGGCAACAGC CGACAGCTAC CTTCTCCAGC TCCAGAAATA

TGGGGGGCAG TGGCGACAGC CGACAGTTAC CTTCTGCAGC TCCAGAAATA

TGGGGTGCAG TGGCAACAGC CGACAGTTAC CTTCTACAAC TCCAGAAATA

TGGGGTGCAG TGGCAACAGC TGACAGTTAC CTTCTACAAC TCCAGAAATA

TGGGGGGCAG TGGCAACAGC CGACAGTTAC CTTCTACAGC TCCAGAAATA

---------- ---------- ---------- ---------- ----------

TGGGGGGCAG TGGCGACAGC CGACAGTTAC CTTCTGCAGC TCCAGAAATA

TGGGGGGCAG TGGCAACTGC CGACAGTTAC CTTCTGCAGC TCCAGAAATA

TGGGGGGCGG TGGCAACCGC TGACAGTTAC CTTCTGCAGC TTCAGAAATA

TGGGGGGCGG TGGCCACAGC CGACAGTTAC CTGCTGCAGC TCCAGAAGTA

---------- ---------- ---------- ---------- ----------

TGGGGGGCCG TGGCCACAGC CGACAGTTAC CTCCTGCAGC TCCAGAAGTA

TGGGGGGCAG TGCCAACGGC TGACAGTTAT CTCCTGCAGC TCCAAAAGTA

TGGGGAGCAG TGCCAACAGC CGACAGCTAC CTGCTCCAGC TCCAGAAGTA

TGACATTCCT GCCACGGCTG CTACTGCCAC CTCCCCTACA CCCAATCCGG

TGACATTCCT GCCACGGCTG CTACTGCCAC CTCCCCTACA CCCAATCCGG

TGACATTCCT GCCACGGCCG CTACTGCCAC CTCCCCTACG CCCAATCCGG

TGACATTCCT GCCACGGCCG CTACTGCCAC CTCCCCTACG CCCAATCCGG

TGACATTCCT GCCACGGCCG CTACTGCCAC CTCCCCTACA CCCAATCCAG

TGACATTCCT GCCACGGCTG CTACTGCCAC CTCCCCCACA CCCAATCCAG

TGACATTCCT GCCACAGCTG CTACGGCTAC CTCCCCCACT CCCAATCCAG

TGACATTCCT GCCACAGCTG CTACGGCTAC CTCCCCCACT CCCAATCCAG

TGACATTCCT GCCACGGCTG CTACTGCCAC CTCCCCCACA CCCAATCCAG

---------- ---------- ---------- ---------- ----------

TGACATTCCT GCCACGGCTG CTACTGCCAC CTCCCCCACA CCCAATCCAG

CGACATTCCT GCCACGGCTG CTACTGCCAC CTCCCCCACA CCCAATCCAG

CGACCTACAT CCAACCACTA CTCCGGACAG CAGCCCTCCA TCCAACCTCG

TGACATTCCT GCCGCCGCAG CCACTGCCGC CTCCCCCACG CCCAACCCCG

---------- ---------- ---------- ---------- ----------

TGACATTCGC -CCAGTGCTG CCACTGTCAC CTCTTCTACC CCCAACCCGG

TGATATTCCT gccgccgctg ccactgccac cTCACCTGCA GCCAACCCTG

CGACATTC-- -CCGCTGCTG CTGCTGCCGC CTCTCCTGCA ACCAACCCCG

TCCCATCTGT GCCTGCCAAC CCTCCCAAGA GCCCTGCCCC AGCAGCAGCC

TCCCATCTGT GCCTGCCAAC CCTCCCAAGA GCCCTGCCCC AGCAGCAGCC

TCCCATCTGT GCCTGCCAAC CCTCCCAAGA GCCCTGCCCC AGCAGCAGCC

TCCCATCTGT GCCTGCCAAC CCTCCCAAGA GCCCTGCCCC AGCAGCAGCC

TCCCATCTGT GCCTGCCAAC CCTCCCAAGA GCCCTGCCCC GGCAGCAGCC

TCCCATCTGT GCCTGCCAAC CCTCCCAAGA GCCCTGCCCC AGCAGCAGCT

TCCCGTCTGT GCCTGCCAAC CCTCCCAAGA GCCCTGCGCC AGCAGCAGCT

TCCCGTCTGT GCCTGCCAAC CCTCCCAAGA GCCCTGCGCC AGCAGCAGCT

TCCCGTCTGT GCCCGCCAAC CCTCCCAAGA GCCCTGCCCC AGCAGCAGCT

---------- ---------- ---------- ---------- ----------

TCCCGTCTGT GCCTGCCAAC CCTCCCAAGA GCCCTGCCCC AGCAGCAGCC

TCCCATCTGT GCCTACCAAC CCTCCCAAGA GCCCTGCCCC GGCAGCAGCC

TTCCTTCTGT GCCTGC---- ---------- ---------- ----------

TCCCGTCGGT GCCTGCCAAC CCTCCCAAGA GCCCCGCCCC TGCGGCAGCG

---------- ---------- ---------- ---------- ----------

TCCCGTCGGC GCCTGCCAAT CCTCCCAAGA GTCCTGCACC TGCAGCAGCG

TTCCATCTGT GCCTATCAAT CCCCCCAAGA GCCCTGCCCC AGCTGCAGCA

TCCCGTCAGT GCCAGTCAAC CCCCCAAAGA GTCCCGCTCC GGCGGCTGCC

GCACCTGCTG TGCAGCCGCT GACCCAAGTA GGCATCACGC TCCTGCCCCA

GCACCTGCTG TGCAGCCGCT GACCCAAGTA GGCATCACGC TCCTGCCCCA

GCACCTGCTG TGCAGCCGCT AACCCAAGTA GGCATCACGC TCCTGCCCCA

GCACCTGCTG TGCAGCCGCT GACCCAAGTA GGCATCACGC TCCTGCCCCA

GCACCCGCTG TGCAGCCACT GACCCAAGTA GGCATCACGC TCCTGCCCCA

GCACCTGCTG TGCAGCCGCT AACCCAAGTA GGCATCACGC TCCTGCCCCA

GCACCTGCTG TACAACCACT GACCCAAGTA GGCATCACAC TTGTGCCCCA

GCACCTGCTG TACAGCCACT GACCCAAGTA GGCATCACAC TTGTGCCCCA

GCGCCTGCTG TGCAGCCGCT AACCCAAGTA GGCATCACGC TCCTGCCCCA

---------- ---------- ---------- ---------- ----------

GCACCTGCTG TGCAGCCGCT GACCCAAGTA GGCATCACGC TCCTGCCCCA

GCACCTGCTG TGCAGCCGCT GACCCAAGTA GGCATCACGC TCCTGCCCCA

---------- ---------- ---------- ---------- ----------

GCCCCTGCTG TACAGCCACT GACCCAAGTA GGCATCACGC TCCTGCCCCA

---------- ---------- ---------- ---------- ----------

GCTCCGGCTG TGCAGCCCCT GACCCAGGTG GGCATCACAC TTCTGCCCCC

GCCCCTGCTG TGCAGCCACT GGCCCAGGTT GGCATCACAC TGCTCCCACA

GCCCCTGCCG TGCAGCCACT TGCCCAGGTG GGCATTACGC TCCTCCCACA

GGCTGCCCCC GCACCCCCGA CCACCACCAC CATCCAGGTC TTGCCAACGG

GGCTGCCCCC GCACCCCCGA CCACCACCAC CATCCAGGTC TTGCCAACGG

GGCTGCCCCC GCGCCCCCGA CCACCACCAC CATCCAGGTC TTGCCGACGG

GGCTGCCCCT GCGCCCCCGA CCACCACCAC CATCCAGGTC TTGCCGACGG

GGCTGCCCCC GCGCCCCCGA CCACCACCAC CATCCAGGTC TTGCCGACGG

GGCTGCCGCC GCGCCCCCGA CCACCACCAC CATCCAGGTC TTGCCGACGG

GGCTGCCGCT GCACCCCCAA GCACAACCAC CATCCAGGTC TTGCCGACAG

GGCTGCCACT GCACCCCCAA GCACAACCAC CATCCAGGTC TTGCCGACAG

GGCTGCTGCT GCGCCCCCAA CCACCACCAC CATCCAGGTC TTGCCGACGG

---------- ---------- ---------- ---------- ----------

GGCTGCCACC GCGCCCCCGA CCACCACCAC CATCCAGGTC TTGCCGACAG

GGCTGCTGCC GCGCCTCCGA CCACCACCAC CATCCAGGTG TTGCCACCAG

---------- ---------- ---------- ---------- ----------

GGCTGCCGCC GCGCCCCCGA CCACCACCAC CATCCAGGTC TTGCCGACGG

---------- ---------- ---------- ---------- ----------

AGCTGCCACC GCACCCCCGA CCACCACCAC GATCCAGGTC TTGCCAACAG

GGCAGCTGCA ACCCCCCCAA CCACCACCAC CATCCAGGTC TTGCCGACAG

Ggccgcggcc acccctcctg ccgccaccac cATCCAGGTC TTGCCGACAG

TGCCTGGCAG CTCCATTTCT GTGCCCACCG CAGCCAGGAC TCAAGGTGTC

TGCCTGGCAG CTCCATTTCT GTGCCCACCG CAGCCAGGAC TCAAGGTGTC

TGCCTGGCAG CTCCATTTCT GTGCCCACTG CAGCCAGGAC TCAAGGTGTC

TGCCTGGCAG CTCCATTTCT GTGCCCACTG CAGCCAGGAC TCAAGGTGTC

TGCCTGGCAG CTCCATTTCT GTGCCCACTG CAGCCCGGAC TCAAGGTGTC

TGCCCGGCAG CTCCATTTCC GTGCCCACAG CAGCCAGGAC TCAAGGTGTC

TGCCAGGCAG CTCTATTTCT GTGCCCACTG CAGCCAGGAC TCAAGGTGTC

TGCCAGGCAG CTCCATTTCT GTGCCCACTG CAGCCAGGAC TCAAGGTGTC

TGCCAGGCAG CTCCATATCT GTGCCCACTG CAGCCAGGAC CCAAGGTGTT

---------- ---------- ---------- ---------- ----------

TGCCTGGCAG CTCGATTTCC GTGCCCGCTG CAGCCAGGAC TCAAGGTGTC

TGCCCGGCAG CTCGATTTCA GTGCCCGCTG CCGCCCGGAC TCAAGGTGTC

---------- ---------- ---------- ---------- ----------

TGCCCGGCAG TTCGATTTCC GTGCCCGCCG CAGCCAGGAC TCAAGGTGTC

---------- ---------- ---------- ---------- ----------

TGCCAGGCAG CTCCATCTCG GTGCCCACTG CAACAAGGAC ACAAGGTGTC

TGCCTGGCAG CCCCATCCCT GTGCCCACCA CAGGTCGGAC TCAAGGTGTC

TGCCTGGGAG CCCGATGCCT GTCCCCACAG CTGCTCGTGC TCAAGGTGTT

CCTGCTGTTC TCAAAGTGAC CGGTCCTCAG GCTACAACAG GAACTCCATT

CCTGCTGTTC TCAAAGTGAC CGGTCCTCAG GCTACAACAG GAACTCCATT

CCTGCTGTTC TCAAAGTGAC CGGTCCTCAG GCTACAACAG GAACTCCATT

CCTGCTGTTC TCAAAGTGAC CGGTCCTCAG GCTACAACAG GAACTCCATT

CCTGCTGTTC TCAAAGTGAC CGGTCCTCAG GCTACAACAG GAACACCATT

CCTGCTGTTC TCAAGGTAAC CGGTCCTCAG GCTACAACAG GAACCCCGTT

CCTGCTGTTC TCAAAGTGAC TGGTCCTCAA GCTACAACAG GAACACCACT

CCTGCTGTTC TCAAAGTGAC TGGTCCTCAA GCTACAACAG GAACACCACT

CCTGCTGTTC TCAAAGTGAC TGGACCTCAG GCTACAACAG GAACCCCATT

---------- ---------- ---------- ---------- ----------

CCTGCTGTTC TCAAAGTCAC CGGTCCTCAG GCTACAACAG GAACCCCATT

CCTGCTGTTC TCAAAGTTAC CGGTCCTCAG GCTACAACAG GAACCCCGTT

---------- ---------- ---------- ---------- ----------

CCTGCTGTCC TCAAAGTGAC TGGTCCTCAG GCTACCACAG GAACCCCGCT

---------- ---------- ---------- ---------- ----------

CCTGCTGTGC TCAAAGTCAC CGGGCCTCAG GCTACCACAG GAACCCCCCT

CCTGCTGTTC TCAAAGTGAC TGGCCCCCAG GCTCCAGCAG GAACTCCCCT

CCCGCTGTCC TCAAAGTGAC CGGTCCCCAG GCGGCCACAG GAGCCCCCCT

GGTCACCATG CGACCTGCCA GCCAGGCTGG GAAAGCCCCT GTCACCGTGA

GGTCACCATG CGACCTGCCA GCCAGGCTGG GAAAGCCCCT GTCACCGTGA

GGTCACCATG CGACCTACCA GCCAGGCCGG GAAAGCCCCT GTCACCGTGA

GGTCACCATG CGACCTACCA GCCAGGCCGG GAAAGCCCCT GTCACCGTGA

GGTCACCATG CGACCTGCCA GCCAGGCCGG GAAAGCCCCC GTCACCGTGA

GGTCACCATG CGACCTGCCA GCCAGGCCGG GAAAGCCCCT GTCACTGTGA

GGTCACCATG AGACCTGCAA GCCAGGCTGG AAAAGCTCCT GTCACTGTGA

GGTTACCATG AGACCTGCAA GCCAGGCTGG AAAAGCTCCT GTCACTGTGA

GGTCACTATG CGACCTGCCA GCCAACCTGG GAAAGCCCCT GTCACTGTGA

---------- ---------- ---------- ---------- ----------

GGTCACCATG CGACCTGCCA GCCAGGCTGG GAAAGCCCCC GTCACCGTGA

GGTCACAATG CGACCTGCCA GCCAGGCTGG GAAAGCCCCC GTCACCATGA

---------- ---------- ---------- ---------- ----------

GGTCACTATG CGGCCCGCCA GCCAGGCTGG GAAAGCCCCC GTCACTGTGA

---------- ---------- ---------- ---------- ----------

GGTCACTATG CGCCCTGCCA GTCAGGCTGG GAAAGCTCCT GTCACTGTGA

AGTCACTGTT CGACCAGCCA GCCAACCTGG AAAAGCTCCA GTTACTGTGA

GGTGACGGTG AGGCCAGCTA GCCAGGCTGG CAAAGCCCCG GTTACTGTGA

CCTCCCTTCC CGCCGGAGTG CGGATGGTTG TGCCAACACA GAGTGCCCAG

CCTCCCTTCC CGCCGGAGTG CGGATGGTTG TGCCAACACA GAGTGCCCAG

CCTCCCTGCC CGCTGGAGTG CGGATGGTTG TGCCAACACA GAGTGCCCAG

CCTCCCTGCC TGCTGGAGTG CGGATGGTTG TGCCAACGCA GAGTGCCCAG

CCTCCCTGCC TGCCGGAGTA CGGATGGTTG TGCCAACACA GAGTGCCCAG

CCTCCCTGCC CGCTGGTGTG AGAATGGTTG TGCCCACACA GAGTGCCCAG

CTTCCCTGCC TGCCAGTGTG AGAATGGTTG TACCCACTCA GAGTGCCCAG

CTTCCCTGCC TGCCAGTGTT CGAATGGTTG TACCCACACA GAGTGCCCAG

CCTCCCTTCC CGCTGGCGTG CGAATGGTTG TGCCAACGCA GAGTGCCCAA

---------- ---------- ---------- ---------- ----------

CCTCCCTTCC TGCAGGCGTG CGAATGGTTG TGCCAACGCA GAGCGCCCAG

CCTCCCTTCC TGCAGGCGTG CGGATGGTCG TGCCGACTCA GAGTGCCCAG

---------- ---------- ---------- ---------- ----------

CCTCCCTGCC TGCGGGGGTG CGAATGGTTG TGCCAACCCA GAGTGCCCAG

---------- ---------- ---------- ---------- ----------

CCTCCCTGCC TGCGGGGGTG CGAATGGTCG TGCCCACACA GAGTGCCCAG

CTTCGCTGCC CGCCGGTGTC CGAATGGTTG TACCGACACA AAGTGCCCAG

CCTCGCTGCC CGCCGGCGTA CGGATGGTTG TGCCGACGCA GAGTGCCCAG

GGAACGGTGA TTGGCAGTAG CCCACAGATG AGTGGGATGG CCGCACTGGC

GGAACGGTGA TTGGCAGTAG CCCACAGATG AGTGGGATGG CCGCGCTGGC

GGGACGGTGA TTGGCAGTAG CCCACAGATG AGTGGGATGG CCGCACTGGC

GGGACGGTGA TTGGCAGTAG CCCACAGATG AGTGGGATGG CCGCACTGGC

GGGACGGTGA TTGGCAGTAG CCCACAGATG AGTGGGATGG CCGCACTGGC

GGGACGGTGA TCGGTAGCAG CCCGCAGATG AGTGGGATGG CTGCGCTGGC

GGCACGGTGA TTGGCAGCAA CCCACAGATG AGTGGGATGG CTGCATTGGC

GGGACGGTGA TCGGCAGCAA CCCACAGATG AGTGGGATGG CCGCATTGGC

GGGACGGTCA TCGGCAGTAA CCCCCAGATG AGCGGGATGG CTGCGCTGGC

---------- ---------- ---------- ---------- ----------

GGGACGGTGA TTGGCAGCAG CCCACAGATG AGTGGCATGG CTGCGCTGGC

GGCACGGTGA TTGGCAGCAA CCCACAGATG AGTGGCATGG CTGCGTTGGC

------GTGA TTGGCAGCAA CCCACAGATG AGTGGGATGG CTGCCCTGGC

GGCACGGTGA TCGGCAGCAG CCCGCAGATG AGTGGGATGG CTGCACTGGC

---------- ---------- ---------- ---------- ----------

GGCACGGTGA TTGGCAGCAA CCCACAGATG AGCGGAATGG CAGCGCTGGC

GGAACAGTGA TTGGCAGCAA CCCTCAGATG AGTGGCATGG CAGCCCTGGC

GGAACTGTGA TTGGCAGCAG CCCTCAAATG AGTGGCATGG CAGCTCTGGC

CGCTGCGGCC GCTGCCACCC AGAAGATCCC CCCTTCCTCG GCACCCACGG

CGCTGCGGCC GCTGCCACCC AGAAGATCCC CCCTTCCTCG GCACCCACGG

CGCTGCGGCT GCTGCCACCC AGAAGATCCC CCCTTCCTCG GCACCCACGG

CGCTGCGGCT GCTGCCACCC AGAAGATCCC CCCTTCCTCG GCACCCACTG

TGCTGCGGCT GCTGCCACCC AGAAGATACC CCCTTCCTCG GCACCCACAG

AGCTGCAGCT GCTGCCACCC AGAAGATCCC GCCTTCCTCA GCGCCCACAG

TGCTGCTGCT GCTGCCACAC AGAAAATCCC TCCTTCCTCA GCACCCACAG

TGCTGCTGCT GCTGCCACAC AGAAAATCCC TCCATCCTCA GCACCCACGG

AGCTGCTGCC GCTGCCACCC AGAAGATCCC TCCTTCCTCG GCGCCCACAG

---------- ---------- ---------- ---------- ----------

AGCTGCAGCT GCCGCCACCC AGAAGATCCC TCCTTCCTCA GCACCCACTG

GGCTGCAGCA GCCGCCACTC AGAAGATCCC TCCTTCCTCG GCGCCCACAG

GGCTGCTGCT GCGGCCACCC AGAAGATCCC TCCGTCCTCG GCTCCCACAG

GGCGGCCGCG GCTGCCACAC AGAAGATCCC TCCTTCCTCG GCCCCCACGG

---------- ---------- ---------- ---------- ----------

AGCCGCAGCA GCGGCCACCC AGAAGATCCC GCCCTCCTCA GCACCCACCG

CGCAGCAGCT GCAGCCACCC AGAAGATCCC TCCTTCCTCA GCTCCCACAG

AGCAGCAGCA GCTGCCACCC AAAAAATCCC TCCTTCCTCA GCCCCCACTG

TGCTGAGTGT CCCAGCGGGT ACCACCATCG TGAAGACCAT GGCTGTGACA

TGCTGAGTGT CCCAGCGGGT ACCACCATCG TGAAGACCAT GGCTGTGACA

TGCTGAGTGT CCCAGCGGGC ACCACCATCG TGAAGACCAT GGCTGTGACA

TGCTGAGTGT CCCAGCGGGC ACCACCATCG TGAAGACCAT GGCTGTGACA

TTCTGAGTGT CCCCGCGGGC ACCACCATCG TGAAGACCAT GGCTGTGACA

TGCTGAGTGT CCCAGCAGGT ACCACCATTG TCAAGACTGT GGCTGTGACA

TGCTAAGTGT CCCAGCAGGC ACCACCATTG TCAAGACAGT GGCTGTGACA

TGCTGAGTGT CCCAGCAGGG ACCACCATCG TCAAGACAGT GGCTGTGACA

TGCTGAGTGT CCCAGCAGGC ACCACCATTG TCAAAACCGT GGCCGTGACA

---------- ---------- ---------- ---------- ----------

TGCTGAGCGT CCCGGCGGGC ACCACCATCG TCAAGACCGT GGCCGTGACA

TGCTGAGTGT CCCAGCAGGC ACCACCATTG TGAAAACCGT GGCCGTAACA

TGCTGAGCGT CCCGGCAGGC ACCACGATCG TTAAGACTGT AGCCGTGACG

TGCTGAGCGT GCCAGCGGGC ACCACCATCG TCAAAACCGT GGCAGTGACC

---------- ---------- ---------- ---------- ----------

TGCTTAGTGT GCCTGCCGGC ACTACCATCG TGAAAACTGT GGCTGTGACT

TACTGAGTGT CCCTGCAGGG ACCACCATTG TCAAAACTGT AGCAGTGTCT

TGTTGAGTGT ATCTGCAGGC ACCACCATCG TCAAAACTGT GGCAGTGTCG

CCTGGCACTA CCACCCTCCC AGCCACTGTG AAGGTGGCCT CCTCGCCAGT

CCTGGCACTA CCACCCTCCC AGCCACTGTG AAGGTGGCCT CCTCGCCAGT

CCTGGCACTA CCACCCTCCC AGCCACTGTG AAGGTGGCCT CCTCGCCAGT

CCTGGCACTA CCACCCTCCC AGCCACTGTG AAGGTGGCCT CCTCGCCAGT

CCTGGCACTA CCACCCTCCC AGCCACTGTG AAGGTGGCCT CCTCGCCAGT

CCTGGCACTA CTACCCTCCC AGCCACTGTG AAGGTGGCCT CCTCACCAGT

CCTGGTACAA CCACTCTTCC AGCCACTGTG AAGGTGGCCT CCTCACCTGT

CCTGGCACGA CCACTCTTCC AGCCACTGTG AAGGTGGCCT CCTCCCCTGT

CCCGGCACTA CCACCCTCCC GGCTACTGTG AAGGTGGCCT CCTCGCCAGT

---------- ---------- ---------- ---------- ----------

CCTGGCACCA CGACCCTCCC GGCTACTGTA AAGGTGGCCT CCTCACCAGT

CCTGGCACTA CCACCCTCCC GGCCACTGTG AAGGTGGCCT CCTCACCAGT

CCTGGCACCA CTACCCTCCC CGCCACGGTG AAGGTGGCCT CCTCACCTGT

CCCGGCACCA CCACACTCCC GGCCACGGTG AAAGTGGCCT CCTCTCCCGT

---------- ---------- ---------- ---------- ----------

CCCGGCACCA CCACCCTGCC AGCCACGGTG AAGGTGGCCT CCTCACCCGT

CCTGGCACCT CCACACTTCC AGCCACAGTG AAGGTGGCCT CCTCCCCAGT

CCTGGAACCA CCACGCTCCC AACCACCGTT AAGGTGGCCT CCTCTCCCGT

CATGGTGAGC AACCCTGCCA CTCGCATGCT GAAGACTGCA GCCGCCCAGG

CATGGTGAGC AACCCTGCCA CTCGCATGCT GAAGACTGCA GCCGCCCAGG

CATGGTGAGC AACCCTGCCA CTCGCATGCT GAAGACTGCA GCTGCCCAGG

CATGGTGAGC AACCCTGCCA CTCGCATGCT GAAGACTGCA GCTGCCCAGG

CATGGTGAGC AACCCTGCCA CTCGCATGCT GAAGACAGCA GCTGCCCAGG

CATGGTGAGC AACCCAGCCA CCCGAATGCT GAAGACTGCA GCCGCCCAGG

TATGGTGAGC AACCCAGCTA CTCGAATGCT AAAGACTGCA GCTGCCCAAG

CATGGTGAGC AACCCAGCCA CTCGAATGCT AAAGACTGCA GCTGCCCAAG

CATGGTGAGC AACCCGGCCA CCCGCATGCT GAAGACTGCA GCTGCCCAGG

---------- ---------- ---------- ---------- ----------

CATGGTGAGC AACCCAGCCA CTCGAATGCT GAAGACTGCA GCCGCCCAGG

CATGGTGAGC AACCCAGCCA CTCGCATGCT AAAGACTGCA GCCGCCCAGG

CATGGTGAGC AACCCGGCCA CACGCATGCT GAAGACTGCC GCCGCCCAGG

CATGGTGAGC AACCCGGCCA CACGCATGCT CAAGACCGCT GCCGCCCAGG

---------- ---------- ---------- ---------- ----------

CATGGTGAGC AACCCTGCCA CCCGCATGCT GAAGACTGCT GCCGCCCAGG

CATGGTGAGC AACCCAGCCA CCAGAATGTT GAAGACAGCA GCAGCCCAAG

CATGGTGAGC AACCCAGCCA CCCGGATGTT GAAGACAGCA GCGGCTCAGG

TGGGGACATC GGTTTCCTCC GCCACCAACA CGTCTACCCG CCCTATCATC

TGGGGACATC GGTTTCCTCC GCCACCAACA CGTCTACCCG CCCTATCATC

TGGGGACATC GGTTTCCTCC GCCACCAACA CGTCTACCCG CCCTATCATC

TGGGGACATC GGTTTCCTCC GCCACCAACA CGTCTACCCG CCCTATCATC

TGGGAACATC GGTTTCTTCT GCCACCAACA CATCTACCCG CCCTATCATC

TGGGAACATC TGTCTCCTCT GCCACCAACA CGTCTACCCG CCCTATCATC

TGGGGACATC TGTGTCCTCT GCTGCCAACA CATCTACCCG CCCTATCATC

TGGGGACATC TGTGTCCTCT GCTGCCAACA CATCTACTCG CCCTATCATC

TGGGGACATC TGTCTCCTCT GCTGCCAACA CATCTACCCG CCCTATCATC

---------- ---------- ---------- ---------- ----------

TGGGGACATC CGTCTCCTCT GCTGCCAACA CATCCACTCG CCCCATCATC

TGGGGACATC CGTCTCCTCT GCTGCCAACA CATCCACTCG CCCCATCATC

TGGGGACCTC TGTCTCCTCG GCCGCCAACA CATCCACGCG CCCTATCATC

TGGGCACGTC CGTCTCCTCA GCCGCCAACA CGTCCACCCG CCCCATCATC

---------- ---------- ---------- ---------- ----------

TGGGGACATC CGTGTCCTCG GCTGCCAACA CGTCCACTCG ACCTATCATC

TAGGCACATC AGTATCATCA GCTGCTAATA CTCCCACCCG CCCCATCATC

TGGGCACGTC GGTCTCGTCA GCTACTAATA CGCCCACGCG ACCCATCATC

ACAGTGCACA AGTCAGGCAC TGTGACAGTG GCCCAGCAAG CCCAGGTGGT

ACAGTGCACA AGTCAGGCAC TGTGACAGTG GCCCAGCAAG CCCAGGTGGT

ACAGTGCACA AGTCAGGCAC TGTGACGGTG GCCCAGCAAG CCCAGGTGGT

ACAGTGCACA AGTCAGGCAC TGTGACAGTG GCCCAGCAAG CCCAGGTGGT

ACGGTGCACA AGTCAGGCAC TGTGACAGTG GCCCAGCAAG CCCAGGTGGT

ACGGTGCACA AGTCAGGGAC TGTGACAGTG GCCCAGCAAG CCCAGGTGGT

ACAGTACATA AATCAGGGAC TGTAACAGTG GCCCAGCAAG CCCAGGTGGT

ACAGTACACA AATCAGGAAC TGTAACAGTG GCCCAGCAAG CCCAGGTGGT

ACGGTGCACA AGTCGGGGAC TGTGACAGTG GCCCAGCAAG CCCAGGTGGT

---------- ---------- ---------- ---------- ----------

ACAGTCCACA AATCAGGGAC TGTGACTGTG GCCCAGCAAG CCCAGGTGGT

ACAGTGCACA AGTCCGGGAC TGTGACTGTG GCCCAGCAAG CCCAGGTGGT

ACGGTGCACA AGTCCGGGAC CGTAACCGTA GCCCAGCAAG CCCAGGTCGT

ACGGTGCACA AGTCGGGGAC GGTGACGGTC GCCCAGCAAG CCCAGGTGGT

---------- ---------- ---------- ---------- ----------

ACTGTGCACA AATCCGGGAC AGTCACCGTG GCACAGCAAG CCCAGGTGGT

ACCGTCCATA AATCTGGCAC TGTGACTGTG GCCCAGCAAG CCCAGGTGGT

ACCGTGCACA AGTCGGGGAC CGTGACAGTG GCCCAGCAAG CCCAGGTGGT

GACCACAGTT GTGGGCGGGG TCACCAAGAC CATCACCCTG GTGAAGAGCC

GACCACAGTT GTGGGCGGGG TCACCAAGAC CATCACCCTG GTGAAGAGCC

GACCACAGTC GTGGGCGGGG TCACCAAGAC CATCACCCTG GTGAAGAGCC

GACCACAGTC GTGGGCGGGG TCACCAAGAC CATCACCCTG GTGAAGAGCC

GACCACAGTC GTGGGTGGGG TCACCAAGAC CATCACTCTG GTGAAGAGCC

CACCACGGTG GTGGGCGGGG TCACCAAGAC CATCACCTTG GTGAAAAGCC

GACCACGGTG GTAGGCGGAG TCACCAAGAC CATCACTCTA GTGAAGAGCC

GACCACGGTG GTAGGTGGAG TCACCAAGAC CATCACCCTA GTGAAGAGCC

GACCACAGTG GTGGGTGGGG TCACCAAGAC CATCACCCTC GTGAAGAGCC

---------- ---------- ---------- ---------- ----------

GACCACGGTG GTGGGTGGAG TCACCAAGAC CATCACCCTG GTGAAGAGCC

GACCACAGTC GTGGGTGGGG TCACCAAGAC CATCACCCTG GTGAAGAGCC

GACCACAGTG GTGGGTGGAG TCACCAAGAC CATCACCCTG GTGAAGAGTC

GACCACCGTG GTGGGTGGTG TCACCAAGAC CATCACCCTG GTGAAGAGCC

---------- ---------- ---------- ---------- ----------

GACCACTGTG GTGGGTGGCG TGACCAAGAC CATCACTTTG GTGAAGAGCC

GACCACCGTG GTCGGTGGGG TCACCAAAAC CATCACTCTG GTCAAGAGCC

GACCACTGTA GTGGGCGGAG TCACCAAAAC CATCACGCTA GTGAAGAGCC

CCATCTCTGT CCCAGGAGGC AGTGCTCTGA TTTCCAATCT GGGCAAAGTG

CCATCTCTGT CCCAGGAGGC AGTGCTCTGA TTTCCAATCT GGGCAAAGTG

CCATCTCTGT CCCAGGAGGC AGCGCTCTGA TTTCCAATCT GGGCAAAGTG

CCATCTCTGT CCCAGGAGGC AGCGCTCTAA TTTCCAATCT GGGCAAAGTG

CCATCTCTGT CCCAGGAGGC AGCGCTCTGA TTTCCAATCT TGGCAAAGTG

CCATCTCTGT CCCAGGAGGC AGTGCTTTGA TTTCTAATCT GGGCAAAGTG

CCATCTCTGT CCCGGGAGGC AGTGCTCTGA TTTCCAATCT TGGAAAAGTG

CCATCTCTGT CCCAGGAGGC AGTGCTCTGA TTTCCAATCT GGGAAAAGTG

CCATCTCTGT CCCAGGAGGC AGTGCTCTGA TTTCCAATCT GGGCAAAGTG

---------- ---------- ---------- ---------- ----------

CCATCTCTGT CCCAGGAGGC AGCGCTCTGA TTTCCAACCT TGGCAAAGTG

CCATTTCCGT CCCAGGAGGC AGTGCTCTGA TTTCCAACCT GGGCAAAGTG

CCATCTCTGT CCCCGGAGGC AGTGCTTTGA TTTCTAATCT CGGCAAGGTG

CCATCTCGGT CCCGGGAGGC AGCGCCCTGA TCTCTAACCT CGGCAAGGTG

---------- ---------- ---------- ---------- ----------

CCATCTCGGT TCCCGGAGGC AGTGCTCTGA TCTCCAACCT CGGCAAAGTC

CCATCTCTGT CCCAGGGGGC AGTGCACTGA TTTCTAATCT GGGCAAAGTG

CCATCTCCGT CCCAGGGGGC AGTGCTCTGA TTTCCAATCT GGGCAAAGTG

ATGTCGGTGG TCCAGACCAA ACCAGTTCAG ACTTCAGCAG TCACAGGCCA

ATGTCGGTGG TCCAGACCAA ACCAGTTCAG ACTTCAGCAG TCACAGGCCA

ATGTCGGTGG TCCAGACCAA ACCAGTTCAG ACTTCAGCAG TCACAGGCCA

ATGTCGGTGG TCCAGACCAA ACCAGTTCAG ACTTCAGCAG TCACAGGCCA

ATGTCAGTGG TCCAGACCAA ACCAGTTCAG ACTTCAGCAG TCACAGGCCA

ATGTCAGTGG TCCAGACCAA GCCAGTTCAG ACTTCAGCAG TCACTGGCCA

ATGTCAGTGG TCCAGACCAA ACCAGTTCAG ACTTCAGCAG TGACAGGCCA

ATGTCGGTGG TCCAGACCAA ACCAGTTCAG ACATCAGCAG TGACAGGCCA

ATGTCAGTAG TCCAGACCAA ACCAGTTCAG ACTTCAGCGG TTACAGGTCA

---------- ---------- ---------- ---------- ----------

ATGTCAGTGG TTCAGACCAA ACCAGTTCAA ACGTCAGCGG TCACAGGCCA

ATGTCAGTGG TGCAAACCAA ACCAGTTCAG ACTTCGGCAG TCACAGGCCA

ATGTCTGTGG TCCAGACCAA ACCTGTTCAG ACATCCGCAG TCACGGGCCA

ATGTCAGTGG TGCAGACCAA GCCTGTTCAG ACCTCAGCGG TCACGGGCCA

---------- ---------- ---------- ---------- ----------

ATGTCTGTTG TGCAGACCAA GCCCGTGCAG ACATCCGCCG TAACGGGCCA

ATGTCAGTTG TTCAGACCAA ACCGGTTCAG ACCTCAGCAG TCACAGGCCA

ATGTCGGTTG TTCAGACCAA GCCGGTCCAG ACCTCTGCAG TCACGGGCCA

GGCGTCCACG GGTCCTGTGA CTCAGATCAT CCAGAAAGGC ACCACAGGTG

GGCGTCCACG GGTCCTGTGA CTCAGATCAT CCAGAAAGGC ACCACAGGTG

GGCGTCCACG GGTCCTGTGA CTCAGATCAT CCAGAAAGGC ACCACAGGTG

GGCGTCCACG GGTCCTGTGA CTCAGATCAT CCAGAAAGGC ACCACAGGTG

GGCGTCCACA GGTCCTGTGA CTCAGATCAT CCAGAAAGGC ACCACAGGGG

GGCATCCACA GGCCCTGTGA CTCAGATCAT CCAGAAGGGC ACCACAGGTG

AGCATCTACA GGTCCTGTGA CTCAGATCAT CCAGAAGGGC ACCACAGGTG

AGCATCTACA GGTCCTGTGA CTCAGATCAT CCAGAAGGGT ACCACAGGTG

GGCATCTACG GGCCCCGTGA CTCAGATCAT CCAGAAGAGC ACCACAGGTG

---------- ---------- ---------- ---------- ----------

GGCATCTACG GGCCCGGTGA CTCAGATCAT CCAGAAGGGC ACCACAGGTG

GGCATCTACA GGCCCGGTGA CCCAGATCAT CCAGAAGGGC ACCACAGGTG

GGCATCCACA GGCCCAGTGA CCCAGATCAT TCAGAAGGGC ACTACAGGCA

GGCCTCGACG GGCCCCGTGA CGCAGATCAT CCAGAAGGGC ACCACAGGTG

---------- ---------- ---------- ---------- ----------

GGCCTCAACT GGCCCAGTGA CACAGATCAT CCAGAAGGGC ACTACAGGCG

GGCTTCTACT GGCCCTGTGA CTCAGATCAT CCAGAAGGGT ACCACAGGAG

GGCTTCGACC GGCCCCGTGA CTCAAATCAT CCAG-AGTGC ACGACCTGCG

TCACGACCCT AGGCACAGTG ACAGGCACCG TCTCCACCAG CCTTGCCGGG

TCACGACCCT AGGCACAGTG ACAGGCACCG TCTCCACCAG CCTTGCCGGG

TCACGACCCT AGGCACAGTG ACAGGCACGG TTTCCACCAG CCTTGCCGGG

TCACGACCCT AGGCACAGTG ACAGGCACGG TCTCCACCAG CCTTGCCGGG

TCACAACCCT AGGCACAGTG ACAGGCACAG TCTCCACCAG CCTTGCTGGG

TCACGACGTT GGGCACAGTC ACAGGCACAG TGTCCACCAG CCTAGCTGGA

TTACAACACT AGGCACAGTG ACAGGCACTG TCTCCACCAG CCTTGCCGGA

TTACAACGCT AGGCACAGTG ACAGGCACTG TCTCCACCAG CCTGGCCGGA

TCACAACTTT AGGCACGGTG ACAGGCACTG TCTCTACCAG CCTCGCTGGA

---------- ---------- ---------- ---------- ----------

TCACAACCCT CGGTACAGTG ACAGGCACCG TTTCCACCAG CCTGGCCGGA

TCACAACCCT GGGCACCGTG ACAGGTACCG TTTCCACCAG CCTTGCTGGA

CAGTGACC-- -GGCACCGTT TC-------- ---------- ----------

TCACAACCCT GGGCACGGTG ACCGGGACTG TTTCTACCAG CCTCGCTGGA

---------- ---------- ---------- ---------- ----------

TCACCACCTT GGGCACTGTG ACAGGCACTG TCTCTACCAG CCTGGCTGGT

TTACAACTCT TGGCACTGTG ACAGGCACAG TGTCTACCAG CCTTGCTGGA

TAACTACCCT GGGCACTGTG ACTGGCACCG TGTCCACCAG CCTCGCTGGC

GCGGGGGGCC ACAGCACTAG TGCTTCCCTG GCCACGCCCA TCACCACCTT

GCGGGGGGCC ACAGCACTAG TGCTTCCCTG GCCACGCCCA TCACCACCTT

GCAGGGGGCC ACAGCACCAG TGCTTCCCTG GCCACGCCCA TCACCACCTT

GCAGGGGGCC ACAGCACCAG TGCTTCCCTG GCCACGCCCA TCACCACCTT

GCGGGGGGCC ACAGCACCAG TGCGTCCCTG GCCACGCCCA TCACCACCTT

GCCGGGGGCC ACAGCACTAG TGCCTCCCTG GCCACGCCCA TCACCACCTT

GCTGGGGCAC ATAGCACCAG TGCTTCTCTG GCTACACCTA TCACTACTTT

GCTGGGGCAC ATAGCACCAG TGCTTCCCTG GCTACACCTA TCACTACCTT

GCTGGGGGCC ACAGTACCAG CGCCTCTCTG GCCACGCCCA TCACCACTTT

---------- ---------- ---------- ---------- ----------

GCTGGGGGCC ACAGTACCAG CGCCTCCCTG GCCACACCCA TCACCACCTT

GCTGGGGGCC ACAGTACCAG TGCTTCCCTG GCCACACCCA TCACCACCTT

-------GCC ACAGCACCAG TGCCTCCCTG GCCACGCCCA TCACCACCCT

GCAGGGGGGC ACAGCACCAG CGCCTCCCTG GCCACGCCTA TCACCACCCT

---------- ---------- ---------- ---------- ----------

GCTGGCGGTC ATAGCACCAA CGCCTCACTG GCCACGCCTA TCACCACTTT

GCAGGCGGGC ATAACACCAA TGCCTCTCTG GCCACTCCTA TCACCACCCT

GCTGGCGGCC ATAACACCAA TGCCTCTCTG GCCACTCCCA TCACCACCCT

GGGCACCATT GCCACCCTCT CAAGCCAGGT GATCAACCCC ACTGCCATCA

GGGCACCATT GCCACCCTCT CAAGCCAGGT GATCAACCCC ACTGCCATCA

GGGCACCATC GCCACCCTCT CAAGCCAGGT GATCAACCCC ACTGCCATCA

GGGCACCATC GCCACCCTCT CAAGCCAGGT GATCAACCCC ACTGCCATCA

GGGCACCATC GCCACCCTCT CCAGCCAGGT GATCAACCCC ACTGCCATCA

GGGTACTATC GCCACCCTCT CAAGCCAGGT GATCAACCCC ACTGCTATCA

GGGCACCATT GCTACTCTCT CAAGTCAGGT GATCAACCCT ACTGCTATCA

GGGCACTATT GCTACGCTCT CAAGCCAGGT GATCAACCCT ACTGCTATCA

GGGCACCATT GCCACCCTGT CGAGCCAAGT GATCAACCCC ACTGCCATCA

---------- ---------- ---------- ---------- ----------

GGGTACCATT GCCACCCTCT CAAGCCAAGT GATCAACCCC ACTGCCATCA

AGGCACCATT GCCACCCTCT CGAGCCAGGT GATCAACCCC ACTGCCATCA

GGGCACCATT GCCACTCTCT CAAGCCAGGT GATCAACCCC ACGGCCATCA

GGGGACCATC GCCACCCTCT CCAGCCAAGT GATCAACCCC ACAGCCATCA

---------- ---------- ---------- ---------- ----------

GGGGACCATC GCCACCCTCT CAAGCCAGGT GATCAATCCC ACGGCCATCA

GGGGACCATT GCCACTCTTT CGAGCCAGGT GATCAATCCT ACAGCCATTA

GGGTACCATT GCCACCCTCT CAAGTCAGGT GATCAACCCG ACCGCCATCA

CTGTGTCGGC CGCACAGACC ACGCTGACAG CGGCAGGCGG GCTCACAACC

CTGTGTCGGC CGCACAGACC ACGCTGACAG CGGCAGGCGG GCTCACAACC

CTGTGTCTGC CGCACAGACC ACGCTGACAG CGGCAGGCGG GCTCACGACC

CTGTGTCTGC CGCACAGACC ACGCTGACAG CGGCAGGCGG GCTCACGACC

CCGTGTCAGC TGCACAGACC ACGCTGACAG CGGCAGGCGG GCTCACGACC

CCGTGTCAGC TGCACAGACC ACACTGACAG CGGCTGGCGG ACTCAGCACT

CAGTGTCAGC TGCACAGACT ACACTAACAG CTGCTGGTGG ACTCACCACA

CAGTGTCAGC TGCACAGACT ACACTAACAG CTGCTGGTGG GCTTACCACA

CAGTGTCGGC TGCACAGACC ACGCTGACGG CAGCTGGTGG GCTCAGCACC

---------- ---------- ---------- ---------- ----------

CAGTTTCGGC CGCGCAGACC ACACTGACGG CGGCCGGTGG GCTGACCACC

CCGTGTCGGC CGCACAGACC ACGCTGACAG CGGCCAGTGG TCTCACCACC

CCGTGTCGGC CGCGCAGACC ACGCTCACGG CGGCCGGCGC TCTCACCACC

CCGTCTCGGC CGCCCAGACC ACGCTGACCG CGGCCAGCGG GCTCACCACC

---------- ---------- ---------- ---------- ----------

CCGTCTCCGC TGCTCAGACT ACGCTGACTG CCGCTGGTGG GCTCACCACA

CTGTGTCAGC TGCCCAGACC ACTCTGACAG CGGCAGGAGG GCTCACCACC

CGGTCTCAGC TGCCCAAACT ACCCTGACGG CAGCTGGGGG CCTCACCACC

CCAACCATCA CCATGCAGAC TGTCACCTTG GTGTGCTCCA ACCCACCCTG

CCGACCATCA CCATGCAGAC TGTCACCTTG GTGTGCTCCA ACCCACCCTG

CCAACCATCA CCATGCAGAC TGTCACCTTG GTGTGCTCCA ACCCACCCTG

CCAACCATCA CCATGCAGAC TGTCACCTTG GTGTGCTCCA ACCCACCCTG

CCTACCATCA CCATGCAGAC CGTGACCTTG GTGTGCTCCA ACCCACCCTG

CCCACCATCA CCATGCAGAC TGTGACACTG GTATGCTCCA ACCCACCATG

CCTACAATCA CAATGCAGAC TGTGACACTG GTGTGTTCCA ACCCACCCTG

CCCACAATCA CAATGCAGAC TGTGACACTG GTGTGTTCCA ACCCACCCTG

CCCACCATCA CCATGCAGAC TGTGACACTA GTGTGCTCCA ACCCACCCTG

---------- ---------- ---------- ---------- ----------

CCCACCATCA CCATGCAGAC CGTGACGCTG GTGTGCTCCA ACCCGCCCTG

CCCACCATCA CCATGCAGAC CGTGACGCTG GTCTGCTCCA ACCCGCCTTG

CCCACCATTA CCATGCAAGT GATGACGTTG ATGTGCTCTA GCCTGCCCGG

CCCACCATCA CCGTGCAGAC AGTGACGCTG GTCTGCTCCA ACCCGCCCTG

---------- ---------- ---------- ---------- ----------

CCAACCATCA CCATGCAGAC TGTGCCGCAG GTCTGCTCCA ACCCGCCGTG

CCGACCATTA CCATGCAGAC TGTGACCCTA GTGTGCTCCA ACCCCCCCTG

CCCACCATCA CCATGCAGAC CGTGACCCTG GTGTGCTCCA ACCCGCCCTG

TGAGACCCAC GAGACTGGCA CCACCAACAC GGCCACCACT GTCTGTTCGA

TGAGACCCAC GAGACTGGCA CCACCAACAC GGCCACCACC ----GCTCTA

TGAGACCCAC GAGACTGGCA CCACCAACAC AGCCACCACC GTCTGCTCAA

TGAGACCCAC GAGACTGGCA CCACCAACAC AGCCACCACC GTCTGCTCAA

TGAGACCCAC GAGACTGGCA CCACCAACAC GGCCACCACC GTCTGCTCGA

TGAGACCCAT GAGACGGGCA CCACCAACAC AGCTACCACC GTCTGCTCGA

TGAAACCCAT GAAACAGGCA CCACCAACAC AGCTACCACC GTCTGTTCAA

TGAAACCCAT GAAACAGGCA CCACCAACAC AGCTACCACC GTCTGTTCAA

TGAGACTCAC GAGACTGGCA CCACCAACAC AGCCACCACC GTCTGCTCCA

---------- ---------- ---------- ---------- ----------

CGAGACCCAC GAGACGGGCA CCACCAACAC GGCCACCACC GTCTGCTCCA

TGAGACCCAC GAGACAGGCA CCACCAACAC GGCCACCACC GTCTGCTCCA

TGAGACCCAG GAGATGGGGA C--------- ---------- ----------

TGAGACTCAC GAGACGGGCA CCACCAATAC GGCCACCACC gtctgctcca

---------- ---------- ---------- ---------- ----------

CGAGACCCAC GAGACAGGGA CTACCAATAC CGCCACCACC GCCTGCTCCA

CGAGACCCAT GAGACGGGCA CTACTAATAC AGCCACAACA GTCTGTTCAA

TGAGACCCAT GAGACAGGCA CCACCAATAC GGCCACCACC GTCTGCTCTA

ACCCGCCCTG CGAGACCCAC GAGACGGGca ccaccaacac cgccaccacc

AGCCCCAGTG CCAAACCCGC CAGACCAGCG CGACCAGCAC CACCATGACT

ACCCACCCTG CGAGACCCAT GAGACGGGca ccaccaccac cgccaccacc

ACCCACCCTG CGAGACCCAT GAGACGGGca ccaccaacac cgccaccacc

ACCCACCCTG CGAGACCCAT GAGACGGGca ccaccaacac cgccaccacc

ACCCTCCTTG CGAGACCCAT GAAACGGGCA CCACCAATAC TGCCACCACT

ACCCCCCCTG TGAAACCCAT GAGACAGGCA CCACCAACAC TGCCACAACA

ACCCCCCCTG TGAGACCCAT GAGACGGGCA CTACCAACAC TGCCACAACA

ACCCGCCCTG CGAGACCCAC GAGACTGGca ccaccaacac cgccaccacc

---------- ---------- ---------- ---------- ----------

ACCCGCCGTG CGAGACCCAT GAGACGGGCA CCACGCACAC GGCCACCACC

ACCCACCGTG CGAGACCCAT GAGACAGGCA CCACCAATAC CGCCACCACT

---------- ---------- ---------- ---------- ----------

accccccctg tgagacccac gagacgggca ccacgcacac ggccaccacc

---------- ---------- ---------- ---------- ----------

ACCCGCCCTG TGAGACCCAC GAGACCGGCA CCACGCACAC GGCCACCACT

ACCCCCCCTG TGAGATCCAT GAGACAGGCA CCACCAACAC TCCCACTGTG

ATCCACCATG TGAGACCCAT GAGACGGGCA CCACCAACAC GCCCACAGTG

gccacctccG CCGGGCAGCA TGGCTGCTCA AACCCACCCT GCGAGACCCA

GTGATGGCCG CCGGGCAGCA TGGCTGCTCA A-CCCACCCT GCGAGACCCA

gccacctccG CCGGGCAGCA TGGCTGCTCA AACCCACCCT GCGAGACCCA

gccacctccG CCGGGCAGCA TGGCTGCTCA AACCCACCCT GCGAGACCCA

gccacctccG CCGGGCAACA CGGCTGCTCA AACCCGCCCT GCGAGACCCA

GCCACCTCCG CAGGGCAACA TAGCTGCTCA GACCCACCCT GTGAGACCCA

GCCACCTCAG CTGGGCAGCA TGGCTGCTCG AATCCTCCCT GCGAGACTCA

GCCACCTCCG CTGGGCAGCA TGGCTGCTCG AACCCCCCCT GTGAGACTCA

gccacctccG CCGGGCAGCA CGGCTGCTCG AACCCGCCCT GTGAGACCCA

---------- ---------- ---------- ---------- ----------

GCCACGTCCG CCGGCCAGCA CAGCTGCTCC AACCCGCCGT GCGAGACCCA

GCCACCTCTG CTGGCCAGCA CGGCTGCTCC AACCCGCCCT GTGAAACCCA

---------G CTGGCCAGCT TGGCTGCTCC AACCCACCGT GCGAGACCCA

gccatgtccg ccggccagac cgtctgctcc aacccgccct gcgagaccca

---------- ---------- ---------- ---------- ----------

GCCACCG--A CTGTGCCGCA GGTCTGCTCC AACCCGCCGT GCGAGACCCA

GTCAGTGCCA CAGGGCAGTT TGTGtgctcc aaccctccct gtgagaccca

GTCAGCGCCC CGGGCCAGAT CGTGTGCTCC AACCCACCCT GCGAGGTGCA

CGAGACGGGC ACCACCAACA CTGCCACTAC AGCCATGAAG TCCCAGTGCC

CGAGACGGGC ACCACCAACA CTGCCACTAC AGCCATGAAG CCCCAGTGCC

TGAGACGGGC ACCACCAACA CTGCCACTAC AGCCATGAAG CCCCAGTGCC

TGAGACGGGC ACCACCAACA CTGCCACTAC AGCCATGAAG CCCCAGTGCC

CGAGACGGGC ACCACCAACA CTGCCACCAC AGCCATGAAG CCCCAGTGCC

TGAGACAGGC ACCACCAACA CTGCCACCAC GGCCATGAAG CCCCAGTGCC

TGAGACAGGT ACCACCAGCA CTGCCACTAC AGCAATGAAG CCTCCGTGCC

TGAGACAGGC ACCACCAGCA CTGCCACTAC AGCAATGAAG CCTCAGTGCC

CGAGACCGGC ACCACCAGCA CCGCCACCAC CGCCGTGAAG CCCCAATGCC

---------- ---------- ---------- ---------- ----------

CGAGACGGGC ACCACCAGCA CCGCCACCAC CGCTATGAAG CCCTTGTGCC

CGAGACGGGC ACCACCAACA CCGCCTCCAC TGCCATGAAA GCCTCATGCC

CGAGACGGGC ACCACCAGCA CTGCCACTAC GGCGGCGAAC CCTCCGTGTG

cgagacgggc accaccagca cggccaccac cgccatgAAG CCCCCGTGCC

---------- ---------- ---------- ---------- ----------

CGAGACAGGG ACTACCAATA CCGCCACCAC GGGAGTG-GC CGCCCCTGCG

tgagacaggt accaccaaca cagccacttc tagta---at cccccatgtg

CGAGACGGGC ACCACCAACA CGGCCACCGT TGCCCGG-AC CCACCCTGCG

AAACCCGCCA GACCAGCGCG ACCAGCACCA CCATGACTGT GGCCACCGGG

AAACCCGCCA GACCAGCGCG ACCAGCACCA CCATGACTGT GGCCACCGGG

AAACCCGCCA GACCAGCACG ACCAGCACCA CCATGACTGT GGCCACTGGG

AAACCCGCCA GACCAGCACG ACCAGCACCA CCATGACTGT GGCCACCGGG

AAACCCGCCA GACCAGCGCG ACCAGCACCA CCATGACTGT GGCCACTGGG

AAACCCGCCA GACCAGTGCA ACCAGCACCA CCATGACTGT GGCCACTGGG

AAACCCAGCA GACCAACATG ACCAGCACCA CCATGACTGT GGCCACTGGA

AAACCCAGCA GACCAACATG ACCACCACCA CCATGACTGT GGCCACTGGA

AAACCCGCCA GACCAGTGCA ACCAGTACCA CCATGACTGT GGCAACTGGG

---------- ---------- ---------- ---------- ----------

AAACCCGCCA GACCAGTGTG ACCAGCACCA CAATGACTGT GGCCACGGGA

AAACCCGCCA GACCAGTGCA ACCAGCACCA CCATGACTGT AGCCACTGGG

AGACCCAT-- -ACAGGCACC ACCAACACGG CCACCACCGC CTCCAACATG

AGACCCGCCA GACCAGCTCC ACCAGCACCA CCATGACTGT GGCCGGCGGG

---------- ---------- ---------- ---------- ----------

AGACACACCA GACCACCACC ACTGGCACCA CCATGACTGC GGCAGCCTGC

agacccatga aacgggtacc accaacacgg ccacCATGGC CTCCAACATG

AGACCCACGA GACGGGCACC ACCAGCACGG CCACCACGG- --CCACCGCG

GCCCCGTGCC CGAGCATGGC ACCTGTGTGC GAGAGCGGCT CGCCCAGCAC

GCCCCGTGCC CGAGCATGGC ACCTGTGTGC GAGAG----- ----------

GCCCCGTGCC CAAGCACGGC ACCTGCGTGC GAGAGCGGCT CGCCTAGCAC

GCCCCGTGCC CAAGCACGGC ACCTGCGTGC GAGAGCGGCT CGCCTAGCAC

GCCCCATGCC CGAGCGCGGC ACCTGTGTGT GAGAGCGGCT CGCCCAGCAC

GCCCCACGC- -----GCAGC ACCTGCATAC GAGAG-AGCC CACCTAGCAC

GCTCCATGCC CAAGTACAGC TCCTCAATAT GATTGCAGCT CTCCCAGTAG

GCTCCATGCC CAAGTGCAGC TCCTACATAT GAGAGCAGCT CTCCTAGCAG

GTCCCTTACC CGAGC----- --CTGTGTAC GACAGCAGCT CGCCCAGCAC

---------- ---------- ---------- ---------- ----------

GCCCCATGCC CAGGTGAGGC CCCCGCGTAT GAGAG---CT CACCTGGTCC

GCCCCGTGCC CGGGCGAAAT GCATGCGTAC GAGAGC--CT CAGCCAGCGC

GCTCCA-GCC CGTGCGGCGC TCCCGCCCCT GCGAG----T CCACTGGTAC

CCGCCGTGCC CGTGCGCGTC ACCCTCACGC GACCG---CT -GGCAAGCAC

---------- ---------- ---------- ---------- ----------

ACCCTGAGCG CTGG-GAGAC CCCTGCATGC GAGAG---GT CAACAGGCAC

ACCGCGTGTC CGTGTAAAGC ACCCACATGC CAAACCAGCG CAACCGGTAC

AACATGGGCC CTTGT--GGC GCCCGGCTCT GTGAG--ACA CGACTAACAC

CACAGTGACT GTGACACAAG TCTGCTCCAA CCCACCATGT GAGACCCACG

---------- ---------G TGTGCTCCAA CCCACCCTGT GAGACCCACG

CACAGTGACT GTGACGCAAG TCTGCTCCAA CCCACCATGT GAGACCCACG

CACAGTGACT GTGACGCAAG TCTGCTCCAA CCCACCATGT GAGACCCACG

CACAGTGACT GTGACGCAAG TCTGTTCCAA CCCACCATGT GAGACCCATG

CACTGTGACT GTGACACAAG TCTGCTCCAA CCCACCATGT GAGACCCACG

CACCATGACT ATGACACAAG TCTGCTCCAA CCCACCATGT GAGACCCATG

CACCATGACT ATGACACAAG TCTGCTCCAA CCCGCCATGT GAGACCCATG

CACCGTGACT GTGACGCAAG T--------- ---------- -AGACCCATG

---------- ---------- ---------- ---------- ----------

TGCTGTGACT GTGACACAAG TCTGCTCCAA CCCACCCTGC GAGACCCACG

CCCTGTGACT GTGACACAGG TCTGCTCCAA CCCGCCGTGC GAGACCCACG

CACCATGTCG GT--CACTGA TCTGCTCCAA CCCACCGTGC GAGACCCACG

TGCCGTGACG GTGATGCGGG TCTGCTCCAA CCCCCCGTGC GAGACCCACG

---------- ---------- ---------- ---------- ----------

CATCATGACT GTGCCGCAGG TCTGCTCCAA CCCGCCGTGC GAGACCCACG

CACCATGACT GTGAGACGGG TCTGCCCCAG TCCATCCTGT GAGACCACCG

CCCCACCACA GCGTCACGAG TCCCCTCCAA GCCCCCATGC CAAACCCACC

AGACAGGCAC CACCAACACC GCCACTACCT CGAATGCAGG CAGCGCCCAG

AGACTGGCAC CACCAACACG GCCACCACC- ---------- ----------

AGACAGGCAC CACCAACACC GCCACTACCT CGAATGCAGG CAGCGCCCAG

AGACAGGCAC CACCAACACC GCCACTACCT CGAATGCAGG CAGCGCCCAG

AGACAGGCAC TACCAACACT GCCACTACCT CGAATGCAGG CAGCGCCCAG

AGACAGGCAC CACCCACACG GCCACTACCT CGCATGCAGG CAGCTCCCAG

AGACGGGTAC CACCAACACC GCCACTACCT CCAATGCGGG CAGTGCTCAG

AGACGGGTAC CACCAACACC GCCACTACCT CCAATGCGGG CAGTGCTCAG

AGACGGGCAC CACCAATACC GCCACTACCT CCAATGCAGG CAGCGCCCAG

---------- ---------- ---------- ---------- ----------

AGACGGGCAC CACCAACACC GCCACTACCT CGAACGCAGG CAGCACCCAG

AGACAGGCAC CACCAACACG GCCACTACCT CGAATGCGGG CAGCGCCCAG

AGACGGGCAC CACCAGCACT GCCACTACCT CGAATGCAGG CGGCGCCCAG

AGACGGGCAC CACGCACACG GCCACCACCT CGCCTGCGGG CAGCATGCAG

---------- ---------- ---------- ---------- ----------

AGACAGGGAC TACCAATACC GCCACCACCT CAAATGCAGG GAGTGCCCAG

CCACAGGTTC TACGGGCCCC GTGACCACAG CTAGTGGGGG TAGTACCCAA

AAACTAGCTC AACCAGCACC ACCATGACTG TGAAGGCAGG GGGCCCCAAG

GTGTGCTCCA ACCCGCCATG CGAGACCCAC GAGACGGGCA CCACCCACAC

GACACCCCCA GCCCACCCTG TGAGACCCAC GAGACGGGCA CCACTCACAC

GTGTGCTCCA ACCCACCATG CGAAACCCAC GAGACAGGCA CCACCCACAC

GTGTGCTCCA ACCCACCATG CGAAACCCAC GAGACAGGCA CCACCCACAC

GTGTGCTCCA ATCCGCCGTG TGAGACCCAT GAGACGGGCA CCACCCACAC

GTGTGCTCCA ACCCGCCCTG TGAGACCCAT GAGACAGGCA CCACCCACAC

GTATGCTCTA ACCCACCTTG TGAGACTCAT GAGACGGGCA CCACACACAC

GTATGCTCCA ACCCGCCTTG TGAGACTCAT GAGACGGGCA CCACACACAC

GTGTGCTCCA ACCCGCCTTG TGAGACCCAC GAGACAGGCA CCACGCATAC

---------- ---------- ---------- ---------- ----------

GTCTGCTCCA ACCCACCCTG CGAGACCCAT GAGACAGGCA CCACCCATAC

GTCTGCTCCA ACCCGCCCTG CGAGACCCAT GAGACGGGCA CCACCCATAC

GTCTGCTCCA ACCCGCCATG CGAGACCCAC GAGACGGGCA CCACCCACAC

GTCTGCTCCA ACCCCCCGTG CGAGACCCAC GAGACGGGCA CCACGCACAC

---------- ---------- ---------- ---------- ----------

GCCTGCTCCA ACCCGCCCTG TGAGACCCAC GAGACCGGCA CCACGCACAC

GCCTTCATCA GCCGGCAGTC TGAGGTACAG CATACCCATA CAACAAACAC

GCCTGTTCCG ACCCACCCTG TGAGACCCGT GAGGCGGCGG CCACCACCAC

GGCCACCACC GCTACGGGCC AGCCCGAGGG TGGGCAGCAG CCCCCTGCTC

GGCCACCACT GTC------- ---------- ---------- ----------

AGCCACCACC GCCACGGGCC AGCCCGAGGG TGGGCAACAG CCCCCTGCTC

GGCCACCACC GCCACGGGCC AGCCCGAGGG TGGGCAACAG CCCCCTGCTC

GGCCACCACA GCCACAGGCC AGGCCGAGGG TGGGCAGCAA CCCCCTGCTC

AGCCACCACC GCCACAGGTC ACCCTGAAGG TGGGCAGCAG GCTCCTGCTC

AGCTACCACT GCCGCAGGCC AGCCTGAGGG TGGACAACAG CCTGCCAGTC

AGCTACCACT GCCGCAGGCC AGCCTGAGGG TGGACAACAG CCTGCCAGTC

GGCCACCACT GCCGCAGGCC AGCCTGAGGG TGGGCAGCAG CCTCCCACGC

---------- ---------- ---------- ---------- ----------

ACCCACCACC GCCGCGGGCC AGCCCGAGGG TGGGCAGCAG CCCCCTGCCC

ACCCACCACC GCCGCAGGCC AGCCCGAGCA TGGGCAGCAG CCCCCTGCCC

GCCCACCACG GCCGCTGGCC AGCCCGAGGG CGCTCAGCAG CCTGCCGCCC

GGCCACCACC GCCGCGGGCC AGCCCGAGGG CAGCCAGCAG GCCCCCGCCC

---------- ---------- ---------- ---------- ----------

GGCCACCACT GCCTCGGGCC AGCCTGATGG TGGGCAACAA ACCACCACCC

CCCAACTACA GCCGCTGGGG AGGCCAATGG GGTACAGGGG CCCACCA---

GGACAgcccg ggcgcGGGCC GGCCCGAGAC CGGG-AGCAC CCAGCGGCC-

CCTGTGAGAC ACACCAGACC ACTTCCACTG GCACCACCAT GTCGGTCAGC

---------- ---------- ---------- ---------- ----------

CCTGTGAGAC ACACCAGACC ACTTCCACCG GCACCACCAT GTCAGTCAGC

CCTGTGAGAC ACACCAGACC ACTTCCACCG GCACCACCAT GTCAGTCAGC

CCTGTGAGAC ACACCAGACC ACTTCCACCG GCACCACCAT GTCCATTGGC

CCTGTGAGAC TCACCAGACT ACTTCCACTG GCACCACCAT GTCAGTCAGT

CCTGCGAGAC ACACCAGACC ACTTCCACTG GCACCACTAT GTCAGTCAGT

CCTGCGAGAC ACACCAGACC ACTTCCACTG GCACCACTAT GTCAGTCAGT

CCTGCGAGAC GCACCAGACC ACTTCCACTG GCACCACCAT GTCGGTCAGC

---------- ---------- ---------- ---------- ----------

CCTGTGAAAC ACACCAGACC ACTTCCACCG GTACCACCAT GTCAGTCAGT

CCTGCGAGAC ACACCAGACC ACTTCCACTG GCACCACCAT GTCGGTCAGC

CCTGCGAGAC GCACCAGACC ACTTCCACTG GTACCACCAT GTCGGTCAGC

CCTGTGAGAC GCACCAGACC ACCTCCACCG GCACCACGAT GACCGTCAGT

---------- ---------- ---------- ---------- ----------

CCTGCGAGAC ACACCAGACC ACCACCACTG GCACCACCAT GACTATCAGC

-ATGCCAAAC ACATCAAACC AGTTCCACCA GCACCACTAT GACTGTGAAA

-CTGCGAGAC GCACCTCACC CACACGACCA ACACCCCCAC CACGGCTAGA

GTGGGTGCCC TGCTTCCCGA CGCCCAGGCT GGCACCGCGC TGCTGGCTCC

--GGGTGCCC TGCTTCCCGA CGCCCAGGCT GGCACCGCGC TGCTGGCTCC

ATGGGCGCCC TGCTTCCCGA CGCCCAGGCT GGCACCGCAC TGCTGGCTCC

ATGGGCGCCC TGCTTCCCGA CGCCCAGGCT GGCACCGCAC TGCTGGCTCC

GCGGGCGCCC TGCTTCCCGA TGCCCAGGCT GGCACCGTTC TGCTGGCTCC

GTGGGTGCCC TGCTCCCCGA TGCTCAGGCC AGCACAGCAC TGCTGGCTCC

GTGGGTGCCC TGATTCCTGA TGCCCAGGCT GGTGCCACAT TGCTGGCCTC

GTGGGTACCC TGATTCCTGA TGCTCAGGCT GGTTCCACAT TGCTGGCCTC

GTGGGTGCCC TGCTCCCTGA TGCCCAGGCT AGTGCCACGT TGCTTGCTCC

---------- ---------- ---------- ---------- ----------

GTGGGTGCCC TGCTCCCCGA TACCCAGGCT GGTGCTTCGT TGCTGGCTCC

ATGGGCACCC TGCTCCCCGA CAGTCAGGCG GGTGCTTCAT TGCTGACTCC

GTTGGGGCCC TGCTCCCTGA TGCCACGGTG GGTGCGGCCC TGCTGGCCCC

GTGGGCACCC TGCTCCCAGA TGACCAGGCT GGCTCGGCCT TGCTGGCGCC

---------- ---------- ---------- ---------- ----------

GTCGGCAGCC TGCACCCTGA TGACCAGGCT GGCCCCACAC TTCAGGCACC

GCAGGTGCTC TGCCTGCCGC TGCCGAGACT GGTACCACCA ACACAGCCAC

TCGGGCGCCG GGGAGCCCGA CGG-ATGACT GTGAAAGCCA CAGAGGCGTT

TTTCCCAACA CAGAGGGTGT GCTCCAACCC CCCCTGTGAG ACCCACGAGA

TTTCCCAACA CAGAGGGTGT GCTCCAACCC CCCCTGTGAG ACCCACGAGA

TTTCCCAACG CAGAGGGTGT GCTCCAACCC CCCCTGTGAG ACCCACGAGA

TTTCCCAACG CAGAGGGTGT GCTCCAACCC CCCCTGTGAG ACCCACGAGA

TTTTCCAACA CAGAGGGTAT GCTCCAACCC CCCCTGTGAG ACCCACGAGA

TTTCCCAACA CAGAGGGTGT GCTCCAATCC TCCCTGTGAG ACCCATGAGA

TTTCTCAACA CAGAGGGTAT GCTCCAATCC TCCTTGCGAG ACCCACGAGA

TTTCCCAACA CAGAGGGTAT GCTCCAACCC TCCTTGCGAG ACCCACGAGA

GTTCCCAACG CAAAGGGTGT GCTCCAATCC CCCGTGTGAG ACCCACGAGA

---------- ---------- ---------- ---------- ----------

TTTCCCGACA CAGAGGGTCT GCTCCAACCC CCCTTGTGAG ACGCACGAGA

TTTTCCAACG CAGAGGGTGT GCTCCAACCC TCCCTGTGAG ACCCACGAGA

CTTCCCCACA CAGCGGGTCT GTTCCAACCC GCCGTGTGAG ACCCACGAGA

TTTCCCAACC CAGCGGGTCT GCTCCAACCC CCCCTGTGAG ACCCACGAGA

---------- ---------- ---------- ---------- ----------

TTTTCCCACC CAGCGGGTCT GCTCCAACCC ACCTTGTGAG ACCCATGAGA

TACCTCGATC CAGCAGGTGT GCTCCAACCC ACCCTGTGAG ACCCACGAGA

GTGCCCAACC CCTCGGGTCT GCTCCAATCC TCCCTGTGAA ACCCACGAGA

CGGGCACCAC TCACACGGCC ACCACTGTCA CTTCCAACAT GAGTTCAAAC

CGGGCACCAC TCACACGGCC ACCACTGTCA CTTCCAACAT GAGTTCAAAC

CGGGCACCAC TCACACGGCC ACCACTGTCA CTTCCAACAT GAGCTCAAAC

CGGGCACCAC TCACACGGCC ACCACTGTCA CTTCCAACAT GAGCTCAAAC

CAGGCACCAC TCACACAGCT ACCACTGTCA CTTCCAACAT GAGCTCAAAC

CGGGCACTAC ACACACGGCT ACCACTGTCA CTTCCAACAT GAGTTCAAAC

CAGGTACAAC GCACACAGCC ACCACTGTCA CCTCTAACAT GAGCTCAAAC

CAGGTACCAC GCACACAGCC ACCACTGTCA CCTCTAACAT GAGCTCAAAC

CGGGCACCAC ACACACAGCC ACCACTGTCA CCTCCAACAT GAGCTCAAAC

---------- ---------- ---------- ---------- ----------

CGGGCACCAC GCACACAGCC ACCACAGTCA CCTCCAACAT GAGCTCAAAC

CCGGCACCAC GCACACGGCC ACTACTGTCA CCTCCAACAT GAGTTCCAAC

CGGGCACCAC GCACACGGCC ACCACTGTCA CCTCCAACAT GAGCTCCAGC

CGGGCACCAC GCACACAGCC ACCACCGTCA CCTCGAACAT GAGCTCCAAC

---------- ---------- ---------- ---------- ----------

CGGGCACCAC GCACACGGCC ACCACCGTCA CCTCCAATAT GAGCTCAAAC

CTGGCACCAC ACATACGGCC ACCACTGCCA CCTCCAATGT AGGCTCCAGT

CGGGCACCAC CAACACCGCC ACCACCGCCA CCTCGAGCAT GGGCGCCGG-

AGCCTGGCTC CATCCAACAC ATTTGTGGCC CCCCAGCCGG TGGTGGCCAG

AGCCTGGCTC CATCCAACAC ATTTGTGGCC CCCCAGCCGG TGGTGGCCAG

AGCCTGGCCC CATCCAACAC ATTTGTGGCC CCCCAGCCGG TGGTGGCCAG

AGCCTGGCCC CATCCAACAC ATTTGTGGCC CCCCAGCCGG TGGTGGCCAG

AGCCTGGCTC CATCCAACAC ATTTGTGGCC CCCCAGCCGG TGGTGGCCAG

AGCCTGGCTC CATCCAACAC ATTTGTGGCC CCCCAGCCAG TGGTAGCCAG

AGTTTGGCTC CATCTAACAC ATTTGTGGCT CCTCAGCCTG TTGTAGCCAG

AGCCTGGCTC CATCTAACAC ATTTGTGGCT CCCCAGCC-G TTGTAGCTAG

AGCCTGGCCC CATCCAACAC GTTTGTGGCC CCCCAGCCGG TGGTAGCCAG

---------- ---------- ---------- ---------- ----------

AGCCTGGCCC CCTCCAACAC CTTTGTGGCC CCCCAGCCCG TGGTAGCAAG

AGCCTGGCTC CTTCCAACAC GTTTGTGGCC CCCCAGCCAG TGGGAGCCAG

---------- ---------- ---------- ---------- ----------

AGTTTGGCAC CGTCGAACAC GTTTGTGGCC CCCCAGCCAG TGGTCGCCAG

---------- ---------- ---------- ---------- ----------

-GCCTGGCCC CATCTAACAC ATTCGTGGCC CCACAGCCGG TGGTTGCCAG

-GTTTGGCAC CATCCAACAC ATTTGTGGCT CCTCAGCCAG TGGCGCCCAG

AGCCTAGCTC CGTCCAGTAC GTTTGTGGCT CCCCAGCCGG TGGTGGCCAG

CCCAGCCAAG CTGCAGGCTG CAGCTACCCT GACCGAAGTG GCCAATGGCA

CCCAGCCAAG CTGCAGGCTG CAGCTACCCT GACCGAAGTG GCCAATGGCA

CCCAGCCAAG CTGCAGGCTG CAGCTACCCT GACTGAAGTG GCCAATGGCA

CCCAGCCAAG CTGCAGGCTG CAGCTACCCT GACTGAAGTG GCCAATGGCA

CCCAGCCAAG CTGCAGGCTG CAGCTACCTT GACTGAAGTG GCCAATGGCA

CCCAGCGAAG CTGCAGGCTG CAGCAACCCT AACCGAAGTG GCCAATGGCA

TCCAGCAAAG ATGCAGGCTG CAGCAACCCT TACTGAAGTG GCAAATGGCA

TCCAGCAAAG ATGCAGGCTG CAGCTACCCT TACTGAAGTG GCCAATGGCA

CCCAGCCAAG CTGCAGGCTG CAGCTACTCT GACTGAAGTA GCCAATGGCA

---------- ---------- ---------- ---------- ----------

CCCCGCAAAG CTGCAGGCTG CAGCCACCCT GACCGAGGTG GCCAACGGCA

TCCCGCAAAG CTGCAGGCCG CCGCTACCCT GACTGAGGTG GCCAATGGCA

---------- ---------- ---------- ---------- ----------

CCCGGCAAAG CTGCAGGCTG CGGCCACCCT GACGGAAGTG GCCAACGGCA

---------- ---------- ---------- ---------- ----------

CCCGGCGAAG CTGCAGGCAG CAGCCACCCT GACCGAAGTA GCCAACGGCA

TCCGGCGAAG CTCCAGGCAG CTGCTGCCCT GGCTGAGGTA GCTAATGGCA

TCCCGCCAAG CTGCAGGCTG CTGCCACACT AGCTGAGGTG GCCAACGGCC

TCGAGTCCCT GGGTGTGAAG CCAGACCTGC CGCCCCCACC CAGCAAAGCC

TCGAGTCCCT GGGTGTGAAG CCAGACCTGC CGCCCCCACC CAGCAAAGCC

TCGAGTCCCT GGGTGTGAAG CCTGACCTGC CACCCCCACC CAGCAAAGCC

TCGAGTCCCT GGGTGTGAAG CCGGACCTGC CACCCCCACC CAGCAAAGCC

TCGAGTCCCT GGGTGTGAAG CCGGACCTGC CGCCCCCACC CAGCAAAGCC

TTGAGTCCCT GGGTGTGAAG CAAGACCTGC CACCCCCACC TAGCAAAGCA

TTGAGTCCCT GGGTGTGAAA CCGGACTTGC CACCCCCACC CAGCAAAGCC

TTGAGTCCCT GGGTGTGAAA CCGGACTTGC CACCCCCACC CAGCAAAGCC

TCGAGTCCCT CGGTGTGAAG CCGGATCTGC CGCCCCCACC CAGCAAAGCT

---------- ---------- ---------- ---------- ----------

TTGAGTCTCT GGGCGTGAAG CCAGACCTAC CGCCCCCGCC CAGCAAAGCC

TCGAGTCCCT GGGTGTGAAG CCGGACCTAC CGCCCCCGCC CAGCAAAGCC

---------- ---------- ---------- ---------- ----------

TCGAGTCTAT CGGGGTGAAG CCTGATCTTC CGCCCCCACC CAGCAAAGCC

---------- ---------- ---------- ---------- ----------

TTGAAACCAT TGGGGTGAAG CCCGATCTTC CTCCTCCGCC TAGCAAAGCC

TCGAGTCACC AGTGGGGAAA CCTGATCCCC TGCCTCCACC CATGAAAGCT

TTGAGTCCAC AGCAGCGAAA CCCGACCCAC CTCCCCCGCC TGTGAAAGTT

CCCATGAAGA AGGAAAACCA GTGGTTTGAT GTGGGAGTCA TTAAGGGCAC

CCCATGAAGA AGGAAAACCA GTGGTTTGAT GTGGGAGTCA TTAAGGGCAC

CCCATGAAGA AGGAGAACCA GTGGTTTGAT GTAGGGGTCA TTAAGGGCAC

CCCATGAAGA AGGAGAACCA GTGGTTTGAT GTCGGGGTCA TTAAGGGCAC

CCCATGAAGA AGGAAAACCA GTGGTTTGAT GTGGGGGTCA TTAAGGGCAC

CCAGTAAAGA AGGAGAACCA ATGGTTTGAT GTGGGAGTCA TTAAGGGCAC

CCTATAAAAA AGGAGAACCA GTGGTTTGAT GTGGGGGTCA TTAAAGGTAC

CCTGTGAAAA AGGAGAACCA GTGGTTTGAT GTGGGGGTCA TTAAGGGTAC

CCCGTGAAGA AGGAGAATCA GTGGTTTGAC GTGGGGGTCA TCAAGGGCAC

---------- ---------- ---------- ---------- ----------

CCCGTGAAGA AGGAGAATCA GTGGTTTGAT GTGGGGGTCA TTAAGGGTAC

CCTGTGAAGA AAGAGAACCA GTGGTTTGAT GTGGGAGTGA TCAAGGGCAC

---------- ---------- -TGGTTCGAC GTGGGGGTCG TGAAAGGCAC

CCCGTGAAGA AGGAGAACCA GTGGTTCGAC GTGGGAGTCA TCAAGGGCAC

---------- ---------- ---------- ---------- ----------

CCTGTGAAAA AGGAGAACCA GTGGTTTGAC GTGGGAGTCA TTAAGGGCAC

ACTGCCAAGA AGGAAAACCA GTGGTTCGAT GTTGGGGTCA TCAAAGGCAC

CCGGTTAAGA AGGAGAACCA GTGGTTCGAC GTGGGCGTCA TCAAAGGCAC

CAATGTAATG GTGACACACT ATTTCCTGCC ACCAGATGAT GCTGTCCCAT

CAACGTAATG GTGACACACT ATTTCCTGCC ACCAGATGAT GCTGTCCCAT

CAATGTAATG GTGACACACT ATTTCCTGCC ACCAGATGAT GCTGTCCCAT

CAATGTAATG GTGACACACT ATTTCCTGCC ACCAGATGAT GCTGTCCCAT

CAATGTAATG GTGACACACT ATTTTCTGCC ACCAGACGAT GCTGTCCCAT

CAATGTAATG GTGACACACT ATTTCCTGCC ACCAGATGAT GCCGTCCCAT

CAGTGTAATG GTGACACACT ATTTTCTGCC ACCAGATGAT GCTGTTCAGT

CAGTGTAATG GTGACACACT ATTTTCTGCC ACCAGATGAT GCTGTTCAGT

CAATGTAATG GTGACACACT ATTTCCTGCC GCCAGATGAC GCTGTCCCGT

---------- ---------- ---------- ---------- ----------

CAACGTAATG GTGACACACT ATTTCCTGCC ACCAGATGAT GCTGTTCCTT

TAATGTAATG GTGACACACT ATTTCCTGCC ACCAGATGAC GCTGTCCCGT

CAATGTCACG GTGACCCACT ACTTCCTGCC GCCGGACGAG GCGG------

CAACGTGATG GTGACACACT ATTTCCTGCC GCCAGACGAT GCCGCCCAGT

---------- ---------- ---------- ---------- ----------

CAATGTAATG GTGACACACT ATTTTCTGCC CCCTGATGAC GCTGTCCCTT

CAACATGATG GTCATGCACT ATTTTCTGCC CCCAGACGAT GCCC---CAA

CAATATGATG GTGACCCACT ACTTCCTACC ACCTGACGAT GCCTCCCCGA

CAGACGATGA TTTGGGCACC GTCCCTGACT ATAACCAGCT GAAGAAGCAG

CAGAC----- ---------- ------GACT ATAACCAGCT GAAGAAGCAG

CAGACGATGA TTCGGGCACC GTCCCTGACT ATAACCAGCT GAAGAAGCAG

CAGACGATGA TTCGGGCACC GTCCCCGACT ATAACCAGCT GAAGAAGCAG

CAGACGACGA CTCGGGCAAT GTCCCTGACT ATAACCAGCT GAAGAAGCAG

CGGATGATGA CTCAGGCACT GTCCCTGACT ATAACCAGCT GAAGAAGCAG

CAGATGATGA CTCAGGCACC GTTCCAGACT ATAACCAGCT AAAGAAGCAG

CAGATGATGA CTCAGGCACG GTCCCAGACT ATAACCAGCT AAAGAAGCAG

CAGATGATGA CTCGGGCACT GTCCCTGACT ATAACCAACT GAAGAAGCAG

---------- ---------- ---------- ---------- ----------

CAGACGATGA CTCGGGCACC GTCCCCGACT ACAACCAGCT AAAGAAGCAG

CTGATGACGA CTCGGGCACT GTCCCAGACT ATACGCAGCT GAAGAAGCAG

-----GACGC CGCGGGCCCC GCTTCCGACT ACCACGAGCT GCAGAAGCAA

CTGACGATGA CTCAGGGACC GTTCCAGACT ACAACCAGCT GAAGAAGCAA

---------- ---------- ---------- ---------- ----------

CTGAAGATGA CTCCAGCGCG ATCCCTGACT ACACCCAGCT GAAGAAGCAG

GTGATGATGA CTCTGGCACC ATCCCTGACT ACAACCAGCT AAAGAAGCAA

CGGACGATGA CTCCGGCACC ATCCCCGACT ACAACCAGCT AAAGAAGCAA

GAGCTGCAGC CAGGCACAGC CTATAAGTTT CGTGTTGCCG GAATCAATGC

GAGCTGCAGC CAGGCACAGC CTATAAGTTT CGTGTGCCAG GAATCAATGC

GAGCTGCAGC CAGGCACAGC CTATAAGTTT CGTGTTGCCG GAATCAATGC

GAGCTGCAGC CAGGCACAGC CTATAAGTTT CGTGTTGCCG GAATCAATGC

GAGCTGCAGC CAGGCACAGC CTATAAGTTT CGTGTTGCCG GAATCAACGC

GAACTGCAGC CAGGCACCGC CTATAAATTT CGTGTTGCCG GGATCAACGC

GAGCTGCAGC CAGGCACAGC TTATAAATTT CGAGTTGCTG GAATCAATGC

GAGCTACAGC CAGGCACGGC TTACAAATTT CGAGTTGCTG GAATCAATGC

GAGCTGCAGC CGGGCACAGC CTATAAGTTT CGTGTGGCTG GGATCAACGC

---------- ---------- ---------- ---------- ----------

GAGCTGCAGC CGGGCACAGC CTATAAGTTC CGTGTTGCCG GGATCAATGC

GAACTGCAGC CCGGCACAGC CTATAAGTTT CGTGTTGCCG GGATCAATGC

GAGCTGCAGC CGGGCACCGC CTACAAGGTT CGCGTGGCGG GCATCAACGC

GAGCTGCAGC CGGGCACGGC CTATAAGTTC CGGGTGGCTG GGATCAACGC

---------- ---------- ---------- ---------- ----------

GAGCTGCAGT CAGGCACAGC CTATAAGTTC CGTGTAGCAG GGGTCAATGC

GAGCTCCAGC CGGGCACAGC CTACAAGTTC CGTGTCGCAG GCATCAATGC

GAACTGCAGC CCGGAACAGC GTACAAGTTC CGTGTGGCTG GGATCAATGC

CTGTGGCCGG GGGCCCTTCA GCGAAATCTC AGCCTTTAAG ACGTGCCTGC

CTGTGGCCGG AGGCCCTTCA GCGAAATCTC AGCCTTTAAG ACGTGCCTGC

CTGTGGCCGG GGGCCCTTCA GCGAAATCTC AGCCTTTAAG ACGTGTCTGC

CTGTGGCCGG GGGCCCTTCA GCGAAATCTC AGCCTTTAAG ACGTGTCTGC

CTGCGGCCGG GGGCCTTTCA GTGAAATCTC AGCCTTTAAG ACATGTCTGC

CTGTGGCCGG GGGCCCTTCA GTGAGATCTC AGCATTTAAG ACGTGTCTAC

TTGTGGCCGG GGCCCCTTCA GTGAGATCTC AGCCTTTAAG ACTTGTCTGC

TTGTGGCCGG GGACCCTTCA GTGAGATCTC AGCCTTTAAG ACTTGTCTGC

CTGTGGCCGG GGGCCGTTCA GCGAGATCTC AGCCTTTAAG ACGTGTCTGC

---------- ---------- ---------- ---------- ----------

CTGTGGCCGA GGGCCCTTCA GCGAGATCTC GGCCTTTAAG ACGTGTCTGC

CTGTGGCAGG GGGCCCTTCA GCGAGATCTC AGCTTTTAAG ACGTGTCTGC

CTGTGGCCGG GGCCCGTTCA GCGAGATCTC GGCCTTCAAG ACGTGCCTGC

CTGTGGCCGG GGGCCCTTCA GTGAGATTTC CGCCTTTAAG ACGTGTCTGC

---------- ---------- ---------- ---------- ----------

CTGCGGCCGG GGACCCTTCA GCGAGATCTC AGCCTTCAAG ACATGCTTGC

CTGTGGCCGG GGGCCCTTCA GCGAGATCTC TGCCTTTAAG ACTTGTCTTC

CTGTGGGCGG GGCCCCTTCA GTGAAATCTC AGCTTTCAAG ACGTGTCTGC

CTGGTTTCCC AGGGGCCCCT TGTGCCATTA AAATCAGCAA AGAAACCAGT

CTGGTTTCCC AGGGGCCCCT TGTGCCATTA AAATCAGCAA AG--------

CTGGTTTTCC AGGGGCCCCT TGTGCCATTA AAATCAGCAA AGAAACCAGT

CTGGTTTCCC AGGGGCCCCT TGTGCCATTA AAATCAGCAA AGAAACCAGT

CTGGTTTCCC AGGGGCCCCT TGTGCCATTA AAATCAGCAA AGAAACCAGT

CTGGTTTCCC AGGGGCCCCT TGTGCCATTA AAATCAGCAA AGAAACCAGC

CTGGTTTTCC AGGGGCTCCT TGTGCTATTA AAATCAGCAA GGAAACCAGT

CTGGGTTCCC AGGGGCTCCT TGTGCTATTA AAATCAGCAA GGAAACTAGT

CTGGTTTCCC AGGGGCCCCT TGTGCCATTA AAATCAGCAA AGAAACCAGT

---------- ---------- ---------- ---------- ----------

CTGGTTTCCC AGGGGCCCCC TGTGCCATTA AAATCAGCAA AGAAACCAGT

CTGGTTTCCC AGGGGCCCCC TGTGCCATTA AAATCAGCAA AGAAACCAGT

CCGGTTTCCC GGGGGCCCCC TGCACCATTA AAATCAGCAA AGAAACCAAC

CTGGCTTCCC AGGGGCGCCT TGTGCCATTA AAATCAGCAA AGAGACCAGT

---------- ---------- ---------- ---------- ----------

CCGGCTTCTC GGGGGCTCCT TGTGCCATTA AGATAAGCAA AGAAACCAGC

CTGGCTTTCC TGGGGCTCCT TGTGCCATTA AAATCAGTAA AGAATCCAGC

CAGGCTTCCC CGGAGCCCCC TGTGCCATAA AAATCAGCAA G-AGTCAAGT

AAAGACAGCT CTGGCACCAA GCCAGCCAAC AAGCGGCCCA TGTCCTCTCC

---------- ---------- ---------- ---------- ----------

AAAGACAGCT CTGGCACCAA GCCGGCCAAC AAGCGGCCCA TGTCCTCTCC

AAAGACAGCT CTGGCACCAA GCCGGCCAAC AAGCGGCCCA TGTCCTCTCC

AAAGACAGCT CTGGCACCAA GCCGGCCAGC AAACGGCCCA TGTCCTCTCC

AAAGACAGCT CTGGCACCAA GCCTGCCAGC AAGCGGCCCA TGTCCTCTCC

AAAGACAGCT CTGGCACCAA GCCAGCTAGC AAGCGGCCCA TGTCGTCTCC

AAAGACAGCT CTGGCACCAA GCCGGCCAGC AAGCGGCCCA TGTCGTCTCC

AAAGACGGCT CTGGCAGCAA GCCGGCCAGC AAGCGGCCCA TGTCTTCTCC

---------- ---------- ---------- ---------- ----------

AAAGACAGCT CTGGCGCCAA GCCAGCCAGC AAGCGGCCCA TGTCCTCTCC

AAAGACAACT CTGGCACCAA GCCAGCCAGC AAGCGGCCCA TGTCCTCTCC

AAGGACAGCT CTGGCACCAG GCCGGCCAGC AAGCGGCCCA TGGCCTCGCA

AAAGACAGTT CTGGCACCAA GCCGGCCAGT AAGCGGCCCA TGTCCTCCCC

---------- ---------- ---------- ---------- ----------

AAAGACAGCT CTGGCGCCAA GCCAGCCAGC AAACGGCCCA TGTCCTCTCC

AAAGACAGCT CCACTTCAAA ACCTGCCAGC AAGCGTCCCT TGTCCTCGCC

AAAGACGGCT CTGGAACAAA GCCTGCGAGC AAGAGGCCCT TCTCCTCCCC

AGAAATGAAA TCTGCTCCAA AGAAATCTAA GGCCGATGGT CAGATGACCC

------GAAA TCTGCTCCAA AGAAATCTAA GGCCGATGGT CAGATGACCC

AGAAATGAAA TCTGCTCCAA AGAAATCTAA GGCCGATGGT CAG-------

AGAAATGAAA TCTGCTCCAA AGAAATCTAA CGCCGATGGT CAGATGACGC

AGAAATGAAA TCTGCTCCAA AGAAATCTAA GGCCGATGGT CAGATGACGC

AGAAATGAAA TCTGCTCCAA AGAAATCTAA GGCAGATGGT CAAATGACGC

AGAAATGAAA TCTGCTCCAA AGAAGTCTAA GGCTGATGGT CAG-------

AGAAATGAAA TCTGCTCCAA AGAAGTCTAA GGCTGATGGT CAGATGACGC

AGAAATGAAA TCCGCTCCAA AGAAATCTAA GGCAGATGGT CAG-------

---------- ---------- ---------- ---------- ----------

GGAAATGAAA TCCGCTCCAA AGAAATCTAA GGCAGATGGT CAG-------

GGAAATAAAA TCCGCTCCAA AGAAATCTAA GGCAGATGGT CAGATGACGC

AGAAATGAAA TCTGCTCCAA AGAAATCCAA GGCCGACGGG CAG-------

TGAAATGAAA TCCGCTCCAA AGAAGTCTAA GGCCGATGGG CAGATGACGC

---------- ---------- ---------- ---------- ----------

GGAAATAAAA TCCGCTCCAA AGAAATCGAA GGCAGACGGG CAGATGACGC

AGAAATGAAA TCCGCTCCAA AGAAATCCAA GGCAGATGGC CAGATGACGC

GGAAATGAAA TCAGCTCCAA AGAAGTCCAA GGCAGATGGC CAGATGACGC

TCAAGGCGAG CGAGGGCGAG AGTGGGGGCA GCATGCACAC GGCGCTCTCC

TCAAGGCGAG CGAGGGCGAG AGTGGGGGCA GCATGCACAC GGCGCTCTCC

---------- ---------- ---------- ---------- ----------

TCAAGGCGAG CGAGGGCGAG AGTGGAGGCA GCATGCACAC GGCGCTCTCC

TCAAGGCGAG CGAAGGCGAG AGTGGGGGCA GCATGCGCAC GGCGCTCTCC

TCAAGGCCAG CGAGGGCGAG GGTGGGGGCA GCATGCGCAC GGCCCTCTCT

---------- ---------- ---------- ---------- ----------

TCAAGTCGAG CGAAGGCGAG GGAGGGAACA GCATGCGCAC CGCACTTTCG

---------- ---------- ---------- ---------- ----------

---------- ---------- ---------- ---------- ----------

---------- -GAGGGCGAG GGCGGGGGCA GCATGCGCAC GGCGCTCTCC

TCAAGGCGAG CGAGAGCGAG AGCGGGGGCA GTATGCGTAC GGCGCTCTCC

---------- ---------- ---------- ---------- ----------

TCAAGGCGAG CGAAGGCGAT GGCGGGGTCA GCATGCGCAC GGCGCTCTCC

---------- ---------- ---------- ---------- ----------

TGAAGGCGAG CGAGGGCGAG GGCGGGGGCG GCATGCGCAC GGCGCTCTCC

TCAAGTCGAG CCGGAGGGAG AGCGTGAGCA GCATGCGGAC GGCCCTGTCC

TGAAGTGCAG CCGGAGCGAG AGCGCCAGCA GTCTGCGGAC CGCTCTGTCC

GACCTCTACC TGGAGCATTT GCTGCAGAAG CGTAGTCGGC CAGAGGCTGT

GACCTCTACC TGGAGCACTT GCTGCAGAAG CGTAGTCGGC CAGAGGCTGT

---------- ---------- ---------- ---------- ----------

GACCTCTACC TGGAGCACTT GC-------- ---------- -----GCTGT

GACCTCTACC TAGAGCACTT GCTGCAGAAG CGTAGTCGGC CAGAGGCTGT

GACCTCTACC TGGAGCACTT GCTGCAGAAA CGTAGCCGGC CGGAGGCTGT

---------- ---------- ---------- ---------- ----------

GACCTCTACC TGGAGCACTT ACTACAGAAG CGTAACCGAC CTGAGACTTC

---------- ---------- ---------- ---------- -----GCTGT

---------- ---------- ---------- ---------- ----------

GACCTCTACC TGGAGCACTT GCTGCAGAAG CGCAGCCGGC CCGAGGCTGC

GACCTCTACC TGGAGCACTT GCTGCAGAAG CGCAGCCGGC CGGAGGCCAT

---------- ---------- ---------- ---------- -----GCTGT

GACCTCTACC TGGAGCACTT GCTGCAGAAG CGCAGCCGGC CCGAGGCTGT

---------- ---------- ---------- ---------- ----------

GACCTGTACC TGGAGCACTT GCTGCAGAAg cgcagccggc cggagACAGC

GACCTGTACC TGGAGCACTT GCTGCAGAAC CGCGCCAAGC CCGAGGTTGC

GACCTCTACC TGGACCACTT GCTGCACAAC CGCGCCAAGC CCGAGACCAT

ATCGCATCCA TTGAATACTG TGACCGAGGA CATGTACACC AACGGGTCTC

ATCGCATCCA TTGAATACTG TGACCGAGGA CATGTACACC AACGGGTCTC

---------- ---------- ---------- ---------- ----------

ATCGCATCCA TTGAATACTG TGACCGAGGA CATGTACACC AACGGGTCTC

CTCACATCCA TTGAATACTG TGACCGAGGA CATGTACACC AACGGGTCTC

GTCACACCAG TTGAATGCTG TAACTGAGGA CATGTACACC AATGGTTCTG

---------- ---------- ---------- ---------- ----------

ATTGAACCAG TCAAATGTTA CTACTGAGGA CATGTACACC AATGGGTCCC

GTCACATCAG TTGAATGCTG TGACTGAAGA CATGTACACC AATGGGTCTA

---------- ---------- ---------- ---------- ----------

GTCACACCAG GTGAACGCCG TGACTGAGGA CATGTACACC AACGGTTCCG

GTCGCACCAG TTGAATGCCA TGACCGAGGA CATGTATACC AATGGGTCTG

GTCACACCAG TTGAATGCTG CAACTGAGGA CATGTATACC AACGGATCTG

GTTACACCAG TTGAATGCCG TGACTGAAGA CATGTACACC AATGGGTCTG

---------- ---------- ---------- ---------- ----------

GGCGCACCAG CTGAATGCTG TGACGGAGGA CATGTGCTCC AATGGGTCTG

TTCCTTCCCC TCAAATTCTG TGACTGAGGA CATATACACC AATGGATCTG

TTCGTATCCA TCCAGCGCCA TGAGCGAGGA CATATACACC AACGGGTCGG

CTGCCCCAGG TAGCCCTGCC AAGGGACAGG AGGTGCGGAA AGTGCGACTC

CTGCCCCAGG TAGCCCTGCC AAGGGACAGG AGGTGCGGAA AGTGCGACTC

---------- ---------- ---------- ---------- ----------

CTGCCCCAGG TAGCCCTGCC AAGGGACAGG AGGTGCGGAA AGTGCGACTC

CTGTCCCGGG TAGCCCTGCC AAGGGGCAGG AGGTGCGGAA AGTGCGACTC

CGGCCCCAGG TAGCCCTGCC AAGGGGCAGG AGGTGCGGAA AGTGCGACTC

---------- ---------- ---------- ---------- ----------

CTGCTCCAGG TAGCCCTGCC AAAGGTCAGG AGGCTCGGAG AGTTCGTCTC

ATGCCCCAGG TCCTCTCTCT AAGGGACAGG AGGTGTGGAA AGAG-GACTC

---------- ---------- ---------- ---------- ----------

CTGCCCCAGG GAGCCCTACC AAAGGGCAGG AGGTGCGGAA GGTGCGACTC

CTGCCCCAGG TAGCCCTACC AAGGGGCAGG AGGTGCGGAA GGTGCGACTC

CTGCCCCCGG GAGCCCAAGC AAGGA----- -CATGCGGAA GGTGCGACTC

CTGCCCCAGG TAGCCCTGCC AAGGGGCAGG AGGTGCGGAA AGTGCGACTC

---------- ---------- ---------- ---------- ----------

CTGCCCCAGA CAGCCCTGCC ACGAGGCAGG AGATGCGGAA AGTGCGCGTC

CTGCACCAGC TAGTCCTGGC AAGGCACGGG AGCCCCGGAA AGTACGCTTC

CCGAGCCAGG CAGCCCGGCC AACGGACGGG AAGTGCGGAA AGTGCGGCTC

ATACAGTTTG AGAAGGTCAC AGAAGAGCCC ATGGGAATCA CGCTGAAGCT

ATACAGTTTG AGAAGGTCAC AGAAGAGCCC ATGGGAATCA CTCTGAAGCT

---------- ---------- ---------- ---------- ----------

ATACAGATTG AGAAGGTCAC AGAGGAGCCC ATGGGAATCA CTCTGAAGCT

ATACAGTTTG AGAAGGTCAC AGAAGAGCCC ATGGGAATCA CTCTAAAGCT

ATACAGTTTG AGAAGATTAC AGAGGAGCCC ATGGGTATCA CTCTGAAGAT

---------- ---------- ---------- ---------- ----------

ATACAATTTG AGAAGATCAC AGAAGAGCCC ATGGGGATCA CTTTGAAGCT

ATGCAGTTTG AGAAAGCCAC AGAGGTGCCC TTGGGAATCA CTCTGAAGCT

---------- ---------- ---------- ---------- ----------

ATCCAGTTTG AGAAGGTCAC CGAGGAGCCC ATGGGAATCA CCCTGAAGCT

ATACAGTTCG AGAAGGTCAC AGAGGAGCCC ATGGGAATCA CTCTGAAGCT

ATCCAGTTTG AGAAAGTCAC CGAGGAGCCC ATGGGAATCA CCTTGAAACT

ATACAGTTTG AGAAGGTCAC AGAGGAGCCC ATGGGAATCA CTCTGAAGCT

---------- ---------- ---------- ---------- ----------

ATACAGTTTG AGAAGGTCAC CGAGGAGCCC ATGGGAATCA CTCTGAAGCT

ATCCAGTTTG AGAAGGTCAC AGAAGAGCCC ATGGGAATTA CTCTGAAGCT

ATCCAGTTTG AGAAGGTCAC GGAGGAGCCC ATGGGGATTA CTCTGAAGCT

GAATGAAAAA CAGTCCTGTA CGGTGGCCAG AATTCTTCAT GGTGGCATGA

GAATGAAAAA CAGTCCTGTA CGGTGGCCAG AATTCTTCAT GGTGGCATGA

---------- ---------- ---------- ---------- ----------

GAATGAAAAA CAGTCCTGTA CAGTGGCCAG AATTCTTCAC GGTGGCATGA

GAATGAAAAA CAGTCCTGTA CAGTGGCCAG AATTCTTCAC GGCGGCATGA

GAATGAAAAA AAATCCTGTA CAGTGGCCAG AATTCTTCAT GGCGGCATGA

---------- ---------- ---------- ---------- ----------

GAATGAAAAA CAGTCGTGTA CCGTGGCCAG AATTCTCCAT GGCGGCATGA

GAATGAAAAG CAGTCCTGTA CTGTAGCCAG AATTCTTCAT GGTGGCATGA

---------- ---------- ---------- ---------- ----------

GAACGAAAAG CAGTCTTGTA CGGTGGCAAG GATTCTCCAC GGCGGCATGA

GAATGAGAAG CAGTCCTGTA CAGTGGCAAG AATTCTTCAT GGTGGCATGA

CAATGACAAG CAGTCCTGCA CTGTGGCCCG AATTCTGCAC GGTGGCATGA

AAATGAAAAA CAGTCCTGTA CAGTGGCCAG AATTCTTCAT GGGGGCATGA

---------- ---------- ---------- -----TTCAT GGAGGCATGA

GAATGAAAAA CAGTCCTGCA CGGTGGCCAG GATCC-TCAT GGAGGCATGA

GAATGAGAAA CAGTCGTGCA TGGTGGCCAG AATTCTCCAT GGGGGCATGA

GAATGAGAAA CAGTCGTGCA TGGTGGCCAG GATTCTACAT GGAGGCATGA

TCCATAGACA AGGCTCCCTT CACGTGGGGG ATGAGATCCT AGAAATCAAT

TCCATAGACA AGGCTCCCTT CACGTGGGGG ATGAGATCCT AGAAATCAAT

---------- ---------- ---------- ---------- ----------

TCCATAGACA AGGCTCTCTT CACGTGGGGG ATGAGATCCT AGAAATCAAT

TCCATAGACA AGGCTCCCTT CATGTGGGGG ATGAGATCCT AGAAATCAAT

TTCACAGACA AGGCTCACTT CACGTGGGGG ATGAGATCCT AGAGATCAAT

---------- ---------- ---------- ---------- ----------

TTCATAGACA AGGCTCCCTT CATGTTGGGG ATGAGATCCT AGAAATCAAT

TTCATAGACA AGGCTCCCTT CCTGTGGGGG ATGAGATCCT AGAAAGCAGT

---------- ---------- ---------- ---------- ----------

TTCACCGACA AGGCTCCCTT CACGTCGGGG ATGAGATCCT AGAAATCAAT

TCCATAGACA AGGCTCCCTT CACGTCGGGG ATGAGATCCT AGAAATCAAT

TCCACCGACA AGGTTCCCTT CATGTGGGGG ATGAGATCCT TGAGATCAAT

TTCATAGACA AGGCTCCCTT CATGTGGGGG ATGAGATTCT AGAAATCAAT

TTCATAGACA AGGCTCACTT CACGTGGGGG ATGAGATCCT AGAAATCAAT

TTCACAGACA AGGTTCACTT CACGTGGGGG ATGAAATCCT AGAGATCAAT

TTCACAGACA AGGCTCCCTT CACGTGGGAG ATGAGATCTT GGAAATCAAT

TTCATAGACA AGGCTCCCTT CACGTGGGGG ATGAGATCCT AGAGATAAAT

GGCACAAATG TGACAAATCA TTCAGTGGAT CAGCTGCAGA AGGCGATGAA

GGCACAAATG TGACAAATCA TTCAGTGGAT CAGCTGCAGA AGGCGATGAA

---------- ---------- ---------- ---------- ----------

GGCACAAATG TGACTAATCA TTCAGTGGAT CAGCTGCAGA AGGCGATGAA

GGCACGAACG TGACTAATCA TTCAGTGGAT CAGCTGCAGA AGGCGATGAA

GGCACAAATG TGACTAATCA TTCAGTAGAT CAGCTACAGA AGGCAATGAA

---------- ---------- ---------- ---------- ----------

GGCACAAATG TGACTAATCA CTCAGTAGAT CAGTTGCAGA AGGCAATGAA

GGCACACACA TGACCAGTCA CTCAGCGGAT CAGT-GCAGA AGGCAGT-AA

---------- ---------- ---------- ---------- ----------

GGTACAAACG TGACAAATCA CTCAGTAGAT CAGCTGCAGA AGGCGATGAA

GGCACAAACG TGACTAATCA CTCAGTAGAC CAGCTGCAGA AGGCGATGAA

GGCACGAATG TGACCAACCA CTCAGTGGAC CAGCTGCAGA AGGCAATGAA

GGCACAAATG TGACTAATCA CTCCGTAGAT CAGCTACAGA AGGCGATGAA

GGTACAAATG TGACTAATCA CTCTGTTGAT CAGCTACAGA AGGCAATGAA

GGTACAAATG TGACAAATCA CTCAGTTGAT CAGCTCCAGA AGGCAATGAA

GGCATGAGTG TGACTAACCA CTCTGTTGAT CAGCTACAGA AGGTGATGAA

GGCACCAGTG TGACCAACCA CTCAGTCGAC CAGCTGCAGA AGGTGATGAA

AGAAACCAAA GGAATGATCT CATTAAAAGT AATTCCCAAC CAGCAAAGCC

AGAAACCAAA GGAATGATCT CATTAAAAGT AATTCCCAAC CAGCAAAGCC

---------- ---------- ---------- ---------- ----------

AGAAACCAAA GGAATGATCT CATTAAAAGT AATTCCCAAC CAGCAAAGCC

AGAAACCAAA GGAATGATCT CATTAAAAGT AATTCCCAAC CAGCAAAGCC

AGAAACAAAA GGAATGATCT CATTAAAAGT AATTCCCAGC CAGCTAAGTC

---------- ---------- ---------- ---------- ----------

GGAAACCAAA GGAATGATCT CATTAAAAGT CATTGCTAAT CAGCAGAGTC

AGAAACCAAA GGAATTATCT CATTAAAAGT AATTCCTAAC CAACAAAGTC

---------- ---------- ---------- ---------- ----------

AGAAACCAAA GGAATGATCT CCTTAAAAGT AATTCCCAAC CAGCAGAATC

AGAAACCAAA GGAATTATCT CATTAAAAGT AATTCCCAAC CAGCAAAATC

AGAAACTAAA GGAATCATCT CATTGAAAGT CATTCCCAGT CAGCACAATC

GGAAACCAAA GGAATAATTT CATTAAAAGT AATTCCCAAC CAGCAAAATC

AGAAACCAAA GGAATGATCT CATTAAAAGT GATTCCCAAC CAGCAAAACC

AGAAACGAAA GGAATAATCT CATTGAAAGT GATTCCCAAC CAGCAAAATC

AGAAACCAAA GGAACGATCT CCTTAAAGGT GATTCCCAAC CAGCAGAGCC

AGAAACCATG GGAACAATTT CGTTAAAAGT AATCCCCAAC CAGCAGAGCC

GTCTTCCTGC ACTACAGATG TTCATGAGAG CGCAGTTTGA CTATGATCCC

GTCTTCCTGC ACTACAGATG TTCATGAGAG CGCAGTTTGA CTATGATCCC

---------- ---------- ---------- ---------- ----------

GTCTTCCTGC ACTACAGATG TTCATGAGAG CGCAGTTTGA CTATGATCCC

GTCTTCCTGC ACTACAGATG TTCATGAGAG CGCAGTTTGA CTATGATCCC

GTCTTCCTGC ACTACAGATG TTCATGAGAG CTCAGTTTGA CTATGATCCC

---------- ---------- ---------- ---------- ----------

GCCTTCCTGC GCTACAGATG TTCATGAGAG CACAGTTTGA CTATGATCCC

GTCTTCCTGC ACTACAGGTG CCCGTTCAAG TTCAGATTGA CTATGATCCT

---------- ---------- ---------- ---------- ----------

GTCTTCCTGC GCTACAGATG TTCATGAGAG CACAGTTTGA CTATGATCCC

GCCTCCCTGC ACTACAGATG TTCATGAGAG CGCAATTTGA CTATGATCCC

GTCTTCCTGC GCTGCAGATG TTCATGCGGG CGCAGTTTGA CTATGATCCC

GTCTTCCTGC ACTACAGATG TTCATGAGAG CGCAGTTTGA CTATGATCCC

GGCTTCCTGC ACTACAGATG TTTATGAGAG CACAGTTTGA CTATGATCCC

GGCTTCCTGT ACTACAG--- ---------- ---------- ----------

GTCTCCCTGC TCTCCAGATG TTCATGAGGG CCCAGTTTGA CTACGATCCC

GTCTCCCTGC GCTTCAGATG TTCATGCGGG CGCAGTTTGA CTACGACCCC

AAAAAGGACA ATCTGATCCC TTGCAAGGAG GCGGGACTGA AGTTTGCTAC

AAAAAGGACA ATCTGATCCC TTGCAAGGAG GCGGGACTGA AGTTTGCTAC

---------- ---------- ---------- ---------- ----------

AAAAAGGACA ACCTGATCCC TTGCAAGGAG GCGGGACTGA AGTTTGCTAC

AAAAAGGACA ATCTGATCCC TTGCAAGGAA GCGGGACTGA AGTTTGCTAC

AAAAAGGACA ATCTGATCCC TTGCAAGGAG GCGGGACTGA AGTTTGTTAC

---------- ---------- ---------- ---------- ----------

CAAAAGGACA ACCTTATTCC TTGCAAGGAG GCAGGACTGA AGTTTGTTAC

AAAAAAGACA ATCTGATTCC CTGCAAGGAG GCAGGCCTGA AGTTTGTCAC

---------- ---------- ---------- ---------- ----------

AGAAAGGACC ACCTGATCCC TTGCAAGGAG GCGGGACTGA AGTTTGTTAC

AAAAAGGACA ATCTGATTCC CTGCAAGGAG GCGGGACTGA AGTTTGTCAC

AAGAAGGACA ACCTGATCCC CTGTAAGGAG GCAGGGCTGA AATTTGTGAC

AAAAAGGACC ATCTTATCCC TTGCAAGGAG GCCGGACTGA AGTTTGTTAC

AAAAAGGACA ATCTGATCCC TTGCAAGGAA GCAGGACTGA AGTTTGTTAC

---------- ---------- ---------- ---------- ----------

CAGAAGGACC CCCTGATCCC CTGCAAGGAG GCGGGCCTGA AGTTTGCCAC

AAGAAGGACC ACTTGATTCC CTGCAAGGAG GCCGGCCTGA AGTTCAACAC

TGGGGACATT ATCCAGATTA TCAACAAGGA TGACAGCAAT TGGTGGCAGG

TGGGGACATT ATCCAGATTA TCAACAAGGA TGACAGCAAT TGGTGGCAGG

---------- ---------- ---------- ---------- ----------

TGGGGACATT ATCCAGATTA TCAACAAGGA TGACAGCAAT TGGTGGCAGG

TGGGGACATT ATCCAGATCA TCAACAAGGA TGACAGCAAT TGGTGGCAGG

TGGGGACATA ATTCAGATCA TCAACAAGGA TGACAGCAAC TGGTGGCAGG

---------- ---------- ---------- ---------- ----------

TGGAGACATT ATCCAGATAA TCAACAAAGA TGACAGCAAT TGGTGGCAGG

TGGGGACATT ATCCAGATCA TCAACAAGGA TGATAGCAAC TGGTGGCAGG

---------- ---------- ---------- ---------- ----------

TGGGGACATC ATCAAGATCA TCAACAAGGA TGACAGTAAC TGGTGGCAGG

TGGGGACGTT ATCCAGATCA TCAACAAGGA TGACAGTAAC TGGTGGCAAG

TGGAGACATC ATCCAGATCA TCAACAAGGA CGACAGCAAC TGGTGGCAGG

TGGGGACATT ATCCAGATCA TCAACAAGGA TGACAGCAAC TGGTGGCAGG

TGGGGACATC ATTCAGATCA TCAACAAGGA TGATAGCAAC TGGTGGCAGG

---------- ---------- ---------- ---------- ----------

TGGGGACATC ATCCAGATCA TCAACAAGGA TGACAGCAAC TGGTGGCAGG

CGGGGACGTC ATCCAGATCA TCAACAAGGA TGACAGCAAC TGGTGGCAGG

GACGGGTGGA AGGCTCCTCC AAGGAGTCAG CAGGATTGAT CCCTTCCCCT

GACGGGTGGA AGGCTCCTCC AAGGAGTCAG CAGGATTGAT CCCTTCCCCT

---------- ---------- ---------- ---------- ----------

GACGGGTGGA AGGCTCCTCC AAGGAGTCAG CAGGATTGAT CCCTTCCCCT

GACGGGTGGA AGGCTCCTCC AAGGAGTCAG CAGGATTGAT CCCTTCCCCT

GGCGGGTGGA AGGCTCCTCC AAGGAGTCAG CAGGATTGAT TCCTTCCCCA

---------- ---------- ---------- ---------- ----------

GGCGGGTGGA GGGCTCCTCC AAAGAGTCTG CAGGATTAAT CCCTTCTCCT

GGCGGGTGGA AGGCTCTTCC AAGGAGTCAG CAGGATTGAT CCCTTCCCCT

---------- ---------- ---------- ---------- ----------

GGCGGGTGGA AGGCTCTTCC AAGGAATCCG CAGGATTGAT CCCGTCTCCT

GGCGGGTGGA AGGCTCCTCC AAGGAGTCAG CAGGACTGAT TCCTTCTCCT

GGCTGGTGGA GGGCTCCTCA GCCGAGTCAG CGGGCCTCAT CCCCTCTCCT

GACGGGTTGA AGGCTCATCC AAGGAATCAG CAGGACTGAT CCCTTCCCCT

GGCGGGTAGA AGGTTCCTCC AAGGAGTCAG CAGGATTGAT TCCTTCCCCT

---------- ---------- ---------- ---------- ----------

GCCGGGTGGA AGGCTCGGCC AGGGAGGCCG CGGGCTTGAT CCCCTCTCCG

GCAAGGTGGA AAGCCCCAAC GCGGAGTCGG CGGGGCTGAT CCCGTCACCT

GAGCTGCAGG AATGGCGAGT GGCAAGTATG GCTCAGTCAG CTCCTAGCGA

GAGCTGCAGG AATG------ ---------- ---------- ----------

---------- ---------- ---------- ---------- ----------

GAACTGCAGG AATG------ ---------- ---------- ----------

GAGCTGCAGG AATGGCGAGT TGCAAGTGTG GCTCAGTCAG CTCCTAGCGA

GAGCTGCAAG AATGGCGAGT GGCAAGTGTG GCTCAGTCAG CGCCACCTGA

---------- ---------- ---------- ---------- ----------

GAGCTACAGG AATGGAGAGT GGCAAGCGTG GCTCACTCTG CTCCAAGTGA

GAGCTGCAAG AATGGCGAGT GGCAAGTGTG GCACAGTGTG CCCCAAGTGA

---------- ---------- ---------- ---------- ----------

GAGCTGCAGG AGTGGCGGGT GGCGAGCGCC GCGCAGTCCG CGCCGAGCGA

GAGCTGCAGG AATGGCGAGT GGCAAGTGTG GCTCAGTCTG CTCCGAGCGC

GAGCTGCAAG AGTGGCGAGT GGCAAGCATG GCGCAGTCTG CTCCAAGTGA

GAGCTGCAAG AATGGCGAGT GGCGAGTGTG GCCCAATCTG CTCCGAGTGA

GAGTTGCAGG AATGGCGAGT GGCAAGTATG GCTCAGTCAG CTCCAAGTGA

---------- ----GCGAGT GGCAAGTGTG GCTCAGTCGG CTCCAAGTGA

GAGCTGCAAG AGTGGCGAGT GGCGAGTGTG GCCCAGTCAG CTCGGGCCGA

GAGCTGCAAG AGTGGCGAGT GGCGAGCATG GCCCAGTCCG CTCCCAGCGA

AGCCCCGAGC TGCAGTCCCT TTGGGAAGAA GAAGAAGTAC AAAGACAAAT

---------- ---------- ---------- ---------- ----------

---------- ---------- ---------- ---------- ----------

---------- ---------- ---------- ---------- ----------

AGCCCCGAGC TGCAGTCCCT TTGGGAAGAA GAAGAAGTAC AAAGACAAAT

GGCCCCAAGT TGCAGTCCCT TTGGGAAGAA GAAGAAGTAC AAAGACAAGT

---------- ---------- ---------- ---------- ----------

AGCACCAAGT TGCAGTCCCT TTGGGAAGAA GAAGAAGTGT AAAGACAAGT

AGCCCCAAGC TGCAGTCCCT TTGGGAAGAA GAAGAAGTAC AAAGACAAGT

---------- ---------- ---------- ---------- ----------

GGCCCCGAGC TGCAGCCCGT TCGGGAAGAA GAAGAAGTAC AAGGACAAGT

AGCCCCGAGC TGCAGTCCCT TCGGAAAGAA GAAGAAGTAC AAAGACAAGT

AGCGCCAAGC TGCAGCCCCT TCGGGAAGAA GAAGAAGTAC AAGGACAAGT

AGCCCCAAGC TGTAGTCCCT TTGGAAAGAA GAAGAAGTAC AAAGACAAGT

AGCCCCAAGT TGCAGTCCCT TTGGGAAGAA GAAGAAGTAC AAAGACAAGT

AGCTCCGAGC TGCAGCCCCT TTGGGAAGAA GAAGAAGTAC AAAGACAAGT

GTCCCCGAGC TGCAGCCCCT TTGGGAAGAA GAAGAAGTAC AAAGACAAGT

AGCTCCGAGC TGCAGCCCTT TTGGGAAGAA GAAGAAATAC AAGGACAAGT

ATCTGGCCAA GCACAGCTCG ATTTTTGATC AGTTGGATGT TGTTTCCTAC

---------- ---------- -TTTTTGATC AGTTGGATGT TGTTTCCTAC

---------- ---------- ---------- ---------- ----------

---------- ---------- -TTTTTGATC AGTTGGATGT TGTTTCCTAC

ATCTGGCCAA GCACAGCTCA ATTTTTGACC AGTTGGATGT TGTTTCCTAT

ACCTGGCCAA GCACAGCTCA ATTTTTGACC AGTTGGATGT TGTTTCATAC

---------- ---------- ---------- ---------- ----------

ACCTGGCTAA GCACAGTTCC ATTTTTGACC AGTTGGATGT TGTTTCCTAT

ACCTGGCGAA GCACAGCTCA A-TTTTGACC AGTTGGATGT TGTTT-----

---------- ---------- ---------- ---------- ----------

ACCTGGCCAA GCACAGCTCC ATCTTTGACC AGCTGGACGT GGTGTCCTAC

ACCTTGCCAA GCACAGCTCA ATTTTTGACC AACTGGATGT CGTTTCCTAC

ACCTGGCCAA GCACAGCTCA ATTTTTGACC AGTTGGATGT GGTTTCCTAC

ATCTGGCAAA ACACAGCTCC ATTTTTGACC AATTGGATGT TGTTTCCTAT

ATCTGGCAAA GCACAGCTCA ATTTTTGACC AGTTGGATGT TGTTTCCTAT

ATCTGGCAAA ACACAGTGCA ATTTTTGACC AGTTGGATGT TGTTTCCTAT

ACCTGGCGAA GCACAGCTCA ATTTTCGATC AGTTGGACGT TGTTTCCTAT

ACCTGGCCAA GCACAGCTCA ATTTTTGACC AGCTGGACGT GGTTTCCTAC

GAGGAAGTCG TTCGGCTCCC TGCATTCAAG AGGAAGACCC TGGTGCTGAT

GAGGAAGTCG TTCGGCTCCC TGCATTCAAG AGGAAGACCC TGGTGCTGAT

---------- ---------- ---------- ---------- ----------

GAGGAAGTCG TTCGGCTCCC TGCATTCAAG AGGAAGACCC TGGTGCTGAT

GAAGAAGTCG TTCGGCTCCC TGCATTCAAG AGGAAGACCC TGGTGCTGAT

GAGGAAGTTG TTCGGCTGCC TGCATTCAAG AGGAAGACCC TGGTGCTGAT

---------- ---------- ---------- ---------- ----------

GAGGAAGTTG TTCGGCTCCC TGCATTCAAG AGGAAGACCT TGGTGTTGAT

---------- ---GACTTCC TACATTCAGG AGGAAGACTT GGGCACTACT

---------- ---------- ---------- ---------- ----------

GAGGAAGTGG TCCGGCTCCC TGCGTTCAAG CGGAAGACCC TGGTGCTGAT

GAGGAAGTTG TTCGGCTCCC TGCGTTCAAG AGGAAGACCC TGGTGCTGAT

GAAGAAGTTG TCCGCCTGCC TGCATTCAAG AGGAAGACTC TGGTGCTGAT

GAGGAAGTTG TCCGGCTCCC TGCGTTCAAG AGGAAGACCC TGGTGCTGAT

GAAGAAGTTG TTCGACTCCC TGCATTCAAG AGGAAGACCC TGGTGCTGAT

GAAGAAGTTG TTCGACTCCC TGCCTTCAAG AGGAAGACGC TGGTGCTGAT

GAAGAAGTCG TGCAGCTCCC AGCATTCAAG AGGAAGACCC TGGTGCTGAT

GAAGAGGTGG TGCGGCTGCC TGCCTTCAAG AGGAAGACCC TGGTGCTGAT

CGGAGCCAGT GGGGTGGGTC GCAGCCACAT TAAGAATGCC CTGCTCAGCC

CGGAGCCAGT GGGGTGGGTC GCAGCCACAT TAAGAATGCC CTGCTCAGCC

---------- ---------- ---------- ---------- ----------

CGGAGCCAGT GGGGTGGGTC GCAGCCACAT TAAGAATGCC CTGCTCAGCC

CGGAGCCAGT GGGGTAGGTC GCAGCCACAT TAAGAATGCC TTGATCAGCC

TGGAGCCAGT GGGGTAGGTC GCAGCCATAT TAAGAATGCC CTGCTCAGCC

---------- ---------- ---------- ---------- ----------

CGGAGCCAGT GGGGTGGGTC GTAGCCATAT TAAGAATGGT CTGCTCAGTC

TGGAGCCAGC GGGGTGGGTC GCAGCCACAT TAAGAATGCA CTGCTCAGCC

---------- ---------- ---------- ---------- ----------

CGGGGCCAGT GGCGTGGGCC GCAGCCACAT TAAGAACGCC CTGCTCACCC

CGGAGCAAGT GGGGTGGGTC GCAGCCACAT TAAAAATACC CTGCTTAGCC

AGGTGCCAGT GGTGTGGGGC GTAGCCACAT TAAGAACGCC CTGATCCGCC

AGGAGCCAGT GGGGTGGGTC GTAGCCACAT AAAGAATGCC CTGCTCAGCC

AGGAGCCAGT GGGGTGGGTC GCAGCCACAT TAAGAATGCC CTGCTCAGCC

AGGAGCCAGT GGGGTAGGTC GCAGCCACAT TAAGAATGCC CTGCTCAGCC

AGGAGCCAGT GGGGTAGGCC GGAGCCATAT CAAGAATGCC CTGGTCAGCA

CGGTGCCAAC GGAGTGGGTC GCAGCCACAT TAAGAACGCT CTGCTCAGCA

AGAATCCGGA GAAGTTTGTG TACCCTGTCC CATATACAAC ACGGCCGCCA

AGAATCCGGA GAAGTTTGTG TACCCTGTCC CATATACAAC ACGGCCACCA

---------- ---------- ---------- ---------- ----------

AGAATCCGGA GAAGTTTGTG TACCCTGCCC CATATACAAC ACGGCCGCCA

AGAATCCGGA GAAGTTTGTA TACCCTACCC CATATACAAC GCGGCCACCA

AGAATCCAGA AAAGTTCGTG TACCCTGCCC CGTATACAAC ACGGCCGCCA

---------- ---------- ---------- ---------- ----------

ACAATCCAGA GAAGTTTGCG TACCCTGCCC CATATACAAC ACGGCCACCA

AGAATCCAGA GAAGTTTGTC TACCCTGCCC CGTATACAAC ACGGCCGCCA

---------- ---------- ---------- ---------- ----------

AGAACCCGGA CAAGTTCGCG TACCCGGCCC CGTACACGAC GCGGCCGCCC

AGGATCCGGA GAAGTTCGCG TACCCTGCCC CGTATACAAC ACGGCAGCCC

AGAACCCCGA GAAGTTTGTG TACCCTGCCC CGTACACCAC GCGACCACCC

AGAATCCAGA GAAGTTTGTG TACCCCACCC CGTATACGAC CCGGCTCCCA

AGAATCCAGA GAAGTTTGTG TACCCTGCCC CATATACAAC ACGGCCACCC

AGCAGCCGGA GAAGTTTGTG TACCCTGCTC CACATACAAC TCGGCCCCCT

AGAACCCAGA GAAGTTTGTG TACCCAGCTC CATATACCAC CCGACCCCCC

ACAACCCGGA GAAGTTTGTG TACCCCACAC CGTACACCAC CCGACCGCCA

AGGAAGAGTG AGGAAGATGG GAAGGAGTAC CACTTTATCT CAACGGAGGA

AGGAAGAGTG AGGAAGATGG GAAGGAGTAC CACTTTATCT CAACGGAGGA

---------- ---------- ---------- ---------- ----------

AGGAAGAGTG AGGAAGATGG GAAGGAGTAC CACTTTATCT CAACAGAGGA

AGAAAGAGTG AGGAGGATGG GAAGGAGTAC CACTTCATCT CAACAGAGGA

AGGAAAAGCG AGGAAGATGG GAAGGAATAC CACTTCATCT CAACAGAGGA

---------- ---------- ---------- ---------- ----------

AAGAAGAGTG AGGAAGATGG GAAGGAGTAT CATTTCATCT CAACAGAGGA

AGGAAGAGCG AGGAAGATGG GAAGGAGTAC CACTTCATCT CAACGGAGGA

---------- ---------- ---------- ---------- ----------

AAGAAGGGGG AGGAGGACGG CAAGGAGTAC CACTTCATCT CCACCGAGGA

AAGAAGAATG AGGAAGACGG GAAGGAGTAC CACTTCATCT CCACGGAGGA

AAGAAGGGAG AGGAGGACGG GAAGGACTAC CACTTCATCT CCACAGAGGA

AAGAAGAGCG AGGAGGATGG GAAGGAGTAC CACTTCATCT CAACTGAAGA

AAGAAGAGTG AGGAAGATGG GAAGGACTAC CACTTCATCT CGACTGAGGA

AAGAAGAATG AGGAAGACGG GAAGGACTAC CACTTCATCT CGACCGAGGA

AAGAAGAGCG AGGAAAATGG GAAGGAGTAC CACTTCATCT CCACTGAGGA

AAGAAGAACG AGGTGGACGG GAAGGAATAC CACTTCATCT CCACCGAAGA

GATGACGAGG AACATCTCTG CCAATGAGTT CTTGGAGTTT GGCAGCTACC

GATGACGAGG AACATCTCTG CCAATGAGTT CTTGGAGTTT GGCAGCTACC

---------- ---------- ---------- ---------- ----------

GATGACAAGG AACATCTCTG CCAATGAGTT CTTGGAGTTT GGCAGCTACC

GATGACGAGA AACATCTCTG CCAATGAGTT CTTGGAGTTT GGCAGCTACC

GATGACAAGG AACATCTCTG CCAACGAGTT CTTAGAGTTT GGCAGCTACC

---------- ---------- ---------- ---------- ----------

GATGACGAAG AACATCTCTG CCAATGAGTT CTTGGAGTTT GGCAGCTATC

GATGACTAGG AACATCTCTG CCAACGAGTT CTTGGAGTTC GGCAGTTACC

---------- ---------- ---------- ---------- ----------

GATGACGAAG AACATCTCCG CCAACGAGTT CCTAGAGTTC GGCAGCTACC

GATGACCAGG GGCATCTCTG CCAACGAGTT CCTGGAGTTT GGCAGCTACC

GATGACCAGG AGCATTTCTG CCAACGAGTT CTTGGAGTTT GGCAGCTACC

GATGACAAGG AACATCTCTG CCAATGAGTT CTTGGAGTTT GGCAGCTACC

GATGACAAAG AATATCTCTG CCAATGAATT TTTGGAGTTT GGCAGCTACC

TATGACCAAG AGTATCTCTG CCAATGAGTT CTTGGAGTTT GGCAGTTACC

GATGACCAAG AATATCTCGG CCAACGAGTT CTTGGAATTT GGCAGCTACC

GATGACCAAG AACATCTCCA CCAACAAGTT CTTGGAGTTC GGCAGCTACC

AAGGCAACAT GTTTGGCACC AAATTTGAAA CAGTGCACCA GATCCATAAG

AAGGCAACAT GTTTGGCACC AAATTTGAAA CAGTGCACCA GATTCATAAG

---------- ---------- ---------- ---------- ----------

AAGGCAACAT GTTTGGCACC AAATTTGAAA CAGTGCACCA GATTCATAAG

AGGGCAACAT GTTTGGCACC AAATTTGAAA CAGTGCACCA GATTCATAAG

AGGGCAATAT GTTTGGTACC AAGTTTGAAA CAGTACACCA GATTCATAAG

---------- ---------- ---------- ---------- ----------

AGGGCAACAT GTTTGGCACC AAATTTGAAA CAGTGCACCA GATTCATAAG

AGGGCAACAT GTTTGGCACA AAATTTGAAA CAGTGCACCA GATTCATAAG

---------- ---------- ---------- ---------- ----------

AGGGCAACAT GTTCGGCACC AAGTTCGAGA CCGTGCACCA GATTCACGAG

AGGGCAACAT GTTTGGCACC AAATTTGAAA CAGTGCACCA GATTCATCAG

AGGGCAACAT GTTCGGCACC AAATTCGCCA CAGTGCTCCA GATCCACGAA

AGGGCAACAT GTTTGGCACC AAATTTGAAA CAGTGCATGA GATTCATAGG

AGGGCAACAT GTTTGGTACC AAATTTGAAA CAGTACATCA GATTCATAAG

AGGGCAACAT GTTTGGCACC AAATTTGAAA CGGTGCATCA GATTCACAAG

AGGGCAACAT GTTTGGTACC AAATTTGAGA CGCTGCACCA GATCCACCAG

AGGGCAACAT GTTTGGCACC AAGTTTGAGA CGGTGCATCA AATCCACGAG

CAGAACAAGA TTGCCATCCT TGACATTGAG CCCCAGACCC TGAAAATTGT

CAGAACAAGA TTGCCATCCT TGACATTGAG CCCCAGACCC TGAAAATTGT

---------- ---------- ---------- ---------- ----------

CAAGACAAGA TTGCCATCCT TGACATTGAG CCCCAGACCC TGAAAATTGT

CAGGACAAGA TTGCCATCCT TGACATCGAG CCACAGACCC TGAAGACTGT

CAGGACAAGA TTGCCATCCT TGACATTGAG CCCCAGACCC TGAAAGTCAT

---------- ---------- ---------- ---------- ----------

CAGGACAAGA TTGCCATCCT TGACATTGAG CCCCAGACCC TAAAGACTGT

CAGGACAAGA TTGCCATCCT TGACATTGAG CCCCAAACCT TGAAGATTGT

---------- ---------- ---------- ---------- ----------

CAGGACAAGA TCGCCATCCT GGACATCGAG CCCCAGACCC TGAAGATCGT

CAGGACAAGA TTGCCATCCT TGACATCGAG CCCCAGACCC TGAAGATTGT

CAGGGCCAGA TCGCTATCCT GGACGTGGAG CCCCAGACGC TAAAGATCGT

CAGGACAAAA TTGCCATCCT TGACATTGAG CCTCAGACCC TGAAGATTGT

CAGGACAAAA TTGCCATCCT TGACATTGAG CCCCAGACCC TGAAGATTGT

CAAGACAAAA TCGCCATCCT TGACATTGAG CCCCAGACCC TGAAGGTGGT

CAGGACAAAG TGGCCATCCT GGACATCGAG CCCCAGACCC TGAAGATCAT

CACGACAAGA TCGCCATCTT GGACATCGAG CCCCAGACCC TGAAGATTAT

TCGGACAGCA GAACTTTCGC CTTTCATTGT GTTCATTGCA CCTACTGACC

TCGGACAGCA AAACTTTCAC CTTTCATTGT GTTCATTGCA CCTACTGACC

---------- ---------- ---------- ---------- ----------

TCGGACAGCA GAACTTTCAC CTTTCATTGT ATTCATTGCA CCTACTGACC

TCGGACAGCA GAACTTTCAC CTTTCATTGT GTTCATTGCA CCTACTGATC

TCGGACAGCA GAACTTTCGC CTTTCATTGT GTTCATTGCA CCTACTGACC

---------- ---------- ---------- ---------- ----------

TCGGACAGCT GAGCTTTCAC CTTTCATTGT GTTCATCGCA CCTACTGACC

TCGGACAGCA GAACTTTCAC CTTTCATTGT GTTCATTGCA CCAACTGACC

---------- ---------- ---------- ---------- ----------

CCGCACGGCC GAGCTGTCCC CTTTCATCGT GTTCATCGCA CCTACTGACC

TCGGACAGCA GAACTTTCAC CTTTCATTGT GTTCATCGCA CCTACTGACC

CCGGACGGCT GAACTCTCAC CTTTCATCGT GTTTATTGCC CCCACAGACC

TCGGACAGCA GAACTTTCAC CTTTCATTGT GTTCATTGCA CCTACTGACC

TCGGACGGCA GAACTTTCAC CATTCATTGT GTTCATCGCA CCTACTGACC

TCGGACCGCA GAACTCTCGC CTTTCATTGT GTTCATTGCA CCTACTGACC

CCGGACAGCA GAACTGTCCC CCTTCATCGT GTTCGTTGCT CCGACCAACA

TCGGACAGCA GAGCTTTCTC CTTTCATCGT GTTCATTGCT CCGACAGACA

AGGGCACTCA GACAGAAGCC CTGCAGCAGC TGCAGAAGGA CTCTGAGGCC

AGGGCACTCA GACAGAAGCC CTGCAGCAGC TGCAGAAGGA CTCTGAGGCC

---------- ---------- ---------- ---------- ----------

AGGGCACTCA GACAGAAGCC CTGCAGCAGC TGCAGAAGGA CTCTGAGGCC

AGGGCAGTCA GACAGAAGCC CTGCAGCAGC TGCAGAAGGA TTCTGAGGCC

AGGGCACTCA GACGGATGCC CTGCAGCAAC TGCAGAGGGA TTCTGAAGCC

---------- ---------- ---------- ---------- ----------

AGGGCACTCA GACTGAAGCC CTGCAGCAGC TGCAGAAGGA CTCTGAGGCC

AGGGCACTCA GACAGAAGCC CTGCAGCAGC TGCAGAAGGA CTCCGAGGCC

---------- ---------- ---------- ---------- ----------

AGGGCGCTCA GACGGAGGCC CTGCAGCAGC TGCAGAAGGA CTCGGAGGCC

AGGGCACTCA GACGGAAGCC CTTCAGCAGC TGCAGAAGGA CTCAGAAGCC

AGGGCACTCA GACGGAAAGC CTGCAGCGG- TGCAGAAGGA CTCGGAGGCC

AGGGCACTCA GACGGAAGCC CTGCAGCAGC TGCAGAAGGA CTCTGAGGCC

AGGGCCCTCA GACTGAAGCC CTGCAGCAGC TGCAGAAGGA CTCTGAGGCC

AGGGCACTCA GACGGAAGCC CTGCAGCAGC TGCAGAAGGA CTCCGAAGCC

AGGCCCAGCA GTCGGAAGCT CTGCAGCAGC TCCAGAAGGA CTCCGAGTCT

AGGCTGACAA GTCAGAAGCT CTGCAGCAGC TGCAGAAGGA CTCGGAGATG

ATCCGCAGCC AGTACGCTCA CTACTTTGAC CTCTCACTGG TCAATAATGG

ATCCGCAGCC AGTACGCTCA CTACTTTGAC CTCTCACTGG TCAATAATGG

---------- ---------- ---------- ---------- ----------

ATCCGCAGCC AGTACGCTCA CTACTTTGAC CTCTCACTGG TCAATAATAG

ATCCGCAGCC AGTACGCTCA CTACTTTGAC CTATCACTGG TCAATAATGG

ATCCGCAGCC AGTATGCTCA CTACTTTGAC CTGTCTCTGG TCAACAATGG

---------- ---------- ---------- ---------- ----------

ATCCGTAGTC AGTATGCTCA TTACTTTGAC CTCTCTTTGG TGAATAATAG

ATCCGCAGCC AGTATGCTCA CTACTTTGAC CTCTCGCTGG TCAATAACAG

---------- ---------- ---------- ---------- ----------

ATCCGCAGCC AGTACGCGCA CTACTTCGAC CTGTCCCTGG TCAACAACGG

ATTCGCAGCC AGTATGCACA TTACTTCGAC CTCTCACTGG TCAATAATGG

GTCCGCAGCC AGTATGCACA CTACTTCGAC CTGTCGCTGG TCAATAATGG

ATTCGCAGCC AGTACGCACA CTACTTTGAC CTCTCCTTGG TTAATAATGG

ATCCGCAGCC AGTATGCTCA CTACTTTGAC CTCTCGCTGG TCAATAATGG

ATCCGCAGCC AGTACGCACA CTACTTTGAC CTCTCGCTGG TCAACAACGG

ATCCACAACA GATACGCACA CTATTTCGAT CTCACGCTCG TAAACAACGG

ATCCGCAGCC GATACGCTCA TTACTTTGAC CTCTCTCTGG TGAACAATGG

TGTTGATGAA ACCCTTAAGA AATTACAAGA AGCCTTCGAC CAAGCGTGCA

TGTTGATGAA ACCCTTAAAA AATTACAAGA AGCCTTCGAC CAAGCGTGCA

---------- ---------- ---------- ---------- ----------

TGTTGATGAA ACCCTTAAGA AATTACAAGA AGCCTTCGAC CAAGCGTGCA

TGTTGATGAA ACCCTAAAGA AATTACAAGA AGCCTTCGAC CAAGCATGCA

CGTTGATGAA ACCCTCAAGA AATTGCAAGA AGCCTTTGAC CACGCATGCA

---------- ---------- ---------- ---------- ----------

TGTTGATGAA ACTCTTAAGA AATTGCAAGA AGCCTTTGAC CAGGCTTGCA

CGTTGATGAA ACCCTTAAGA AGTTGCAAGA AGCATTTGAC CAAGCATGCA

---------- ---------- ---------- ---------- ----------

CGTGGAGGAG ACGCTGACGA AGCTGCAGGA GGCCTTCGAG CAGGCGTGCA

CGTCGATGAA ACCCTTCAGA AATTGCAAGA TGCCTTTGAT CAGGCATGCA

CGTGGATGAA ACCCTGACCA AAGTGCAGGA GGCCTTTGAG CGGGCATGCA

CGTTGATGAA ACCCTCAAGA AATTGCAAGA AGCCTTTGAC CAGGCATGCA

TGTTGATGAG ACCCTCAAGA AATTGCAAGA AGCCTTTGAC CAGGCATGCA

TGTGAACGAA ATCCTCCAGA AACTGCAAGA AGCCTTTGAC CAGGCATGCA

TATCGACGAG AGCCTCCAGA TCCTGCAGGA GGCCTTCGAG CTGGCTTGTA

CATCGAGGAA ACCCTCAAGA TGTTGCAGGA CGCCTTCGAA CAGGCCTGCA

GTTCTCCACA GTGGGTGCCT GTCTCCTGGG TTTACATGGA GAAACGAGAG

GTTCTCCACA GTGGGTGCCT GTCTCCTGGG TTTACATGGA GAAACGAGAG

---------- ---------- ---------- -----ATGGA GAAACGAGAG

GTTCTCCACA GTGGGTGCCT GTCTCCTGGG TTTACATGGA GAAACGAGAG

CTTCTCCGCA GTGGGTGCCT GTTTCCTGGG TTTACATGGA GAAACGGGAG

GTTCTCCACA GTGGGTGCCT GTCTCCTGGG TTTACTTGGA GAAGCGGAAG

---------- ---------- ---------- -----ATGGA GAAGCGGGAG

GTTCTCCACA GTGGGTGCCT GTCTCCTGGG TTTACATGGA GAAGCGGGAG

GGTCTCCACA GTGGGTCCCT GTCTCCTGGG TTTACATGGA GAAGCGGGCC

---------- ---------- ---------- ---------- ----------

GCTCTCCGCA GTGGGTGCCC GTGTCCTGGG TCTACATGGA CAAGCGGGAG

GTTCTCCACA GTGGGTGCCT GTCTCCTGGG TTTACATGGA GAAGCGGGAG

GCTCTCCACA GTGGGTGCCT GTATCCTGGG TTTAC----- ----------

GTTCTCCACA GTGGGTGCCT GTCTCCTGGG TTTACCTGGA GAAGCGGGAG

GCTCTCCACA GTGGGTGCCT GTCTCCTGGG TGTAC----- ----------

GCTCCCCCCA GTGGGTGCCT GTCTCCTGGG TCTACATGGA CAAACGGGAG

GCTCTCCCCA GTGGGTCCCC GTCTCCTGGG TCTACATGGA GA---GGGCC

GCTCCCCACA GTGGGTTCCC GTCTCTTGGG TCTACGAGGA AGGAAGGG--

TTCACGTGCC TTGGCCGCGA GGGGCGGGTG TATGATGACC TCAAGTATGT

TTCACGTGCC TTGGCCGCGA GGGGCGGGTG TATGATGACC TCAAGTATGT

TTCACGTGCC TTGGCCGCGA GGGACGGGTG TATGATGACC TCAAGTACGT

TTCACGTGCC TTGGCCGCGA GGGACGGGTG TATGATGACC TCAAGTACGT

TTCACGTGCC TTGGCCGGGA TGGGCAGGTG TATGATGACC TCAAGTATGT

TTCACGTGTC TCGGCCACGA CGGGCAGGTG TATGATGACC TCAAGTATGT

TTCACATGCC TTGGCCGCGA TGGGCAGGTA TATGATCACC TCAAATATGT

TTCACATGTC TTGGCCGTGA TGGGAAGGTA TATGATCACC TCAAATATGT

---------- ---------- ---------- ---------- ----------

---------- ---------- ---------- ---------- ----------

TTCACGTGCC TGGGTCGAGA CGGGCAGGTG TATGATGATC TCAAGTACGT

TTCACGTGCC TTGGCCGAGA TGGGCGGGTG TATGACGACC TCAAGTATGT

---------- ---------- ---------- ---------- ----------

TTCACGTGCC TCGGCCGGGA TGGACAGAGG TATGATGACC TCAAATACGT

---------- ---------- ---------- ---------- ----------

CTCACGTGCC TGGGTCAGGA TGGGCAAGTG TATGACGACC TCAAGTACGT

TTCACCTGCC TCACTAGGGA GGGCCAAGTG TACGACGACC TCAAGTATGT

--TACCTGTC TGGGTCGGGA TGGCCAGGTG TATGATGACC TCAAGTATGT

GTGGCTGCAG GGGAGGCAGG TATGGATGTA TTGTCGCCTG TACCGCACTT

GTGGCTGCAG GGGAGGCAGG TATGGATGTA TTGTCGCCTG TACCGCACTT

GTGGCTGCAG GGGAGGCAGG TGTGGATGTA TTGTCGCCTG TACCGCACTT

GTGGCTGCAG GGGAGGCAGG TGTGGATGTA TTGTCGCCTG TACCGCACTT

GTGGCTACAG GGGAGGCAGG TCTGGATGTA TTGCCGCCTG TACCGCACTT

CTGGCTTCAG GGGAGGCAGG TGTGGATGTA CTGTTACCTG TACCGCAAAT

CTGGCTGCAG GGGAGGCAGG TATGGATGTA TTGTCGCTTA TACCGCACTT

TTGGCTGCAG GGGAGGCAGG TATGGATGTA TTGTCGCTTA TACCGCAGTT

---------- ---------- ---------- ---------- ----------

---------- ---------- ---------- ---------- ----------

CTGGCTGCAG GGGAGGCAGG TGTGGATGTA CTGTCGCCTG TACAGAAAAT

CTGGCTGCAG GGGAGGCAGG TGTGGATGTA TTGTCACCTG TACCGCAAGT

---------- ---------- ---------- ---------- ----------

CTGGCTGCAG GGCAGGCAGG TGTGGATGTA TTGTCGCCTG TACCGCAAGG

---------- ---------- ---------- ---------- ----------

GTGGCTGCAG GGGAGGCAGG TTTGGATGTA CTGTCGCCTG TACCGCAAGA

CTGGCTGCAG GGGAGGCAGG TTTGGATGTA CTGCCGGCTC TACCGAAAAT

GTGGTTGCAA GGCCGGCAGc acttagtaca ttggcacatc tatcaatcaa

TCGAGCGCTT CCGCCATGCT CAGCTTCTGG ACGCAGCAAA AGCAGGTGGT

TTGAGCGCTT CCGCCATGCT CAGCTTCTGG ACGCAGCAAA AGCAGGTGGT

TCCAGCGCTT CCACCACTCT CAGCTTCTGA ATGCAGCAAA AGCAGGTGGT

TCCAGCGCTT CCACCACTCT CAGCTTCTGA ATGCAGCAAA AGCAGGTGGT

TTGAGCGCTT CCACCATGCG CAGCTCCTGG ATGCAGCAAA AGCAGGTGGT

TCCAGCGCTT CCACCACTCT GGGCTTCTGG ATGCAGCAAA AGCAGGTGGT

TTGAGCGCTT CCGCCGTGTC GAGCTTCTGG ATGCAGCAAA AGCAGGTGGT

TTGAGCGCTT CCGCCGTGTT GAGCTTCTGG ATGCAGCAAG AGCAGGTGGT

---------- ---------- ---------- ---------- ----------

---------- ---------- ---------- ---------- ----------

TTGAGCGTTT CCGCAGCCCC GAACTTCTGG ATTCAGCTAA AGCAGGTGGC

TCGAGCGTTT CCGCCGCCCT GAACTTCTAG AGTCAGCTAA AGCAGGTGGC

---------- ---------- ---------- ---------- ----------

TCCCTCGCTT CCGCCGCTCT GAGCTCCTGG ATGCAGCCAA AGCAGGCGGT

---------- ---------- ---------- ---------- ----------

TAGAACGTTT CCACCGCCCT GAGCTGCTGG ATGCGGCCAA AGCAGGTGGG

TGCCCCGCTT CTGCTGGCCA GAGATCCTGG ATGCAGCCAA AGCAGGAGGG

tctattgctt ggcgcatagt aagcacttag atacctcaaa attaTGTGGT

GAGTTCTTGC TGCGGTATGC CCGGGTGGCA CCTCCTGGCA AGAAGTGTGC

GAGTTCTTGC TGCGGTATGC CCGGGTGGCA CCTCCTGGCA AGAAGTGTGC

GAGTTCTTGC TGCGTTATGC CCGGCTGGCA CCTCCTGGCA AGAAGTGTGC

GAGTTCTTGC TGCGTTATGC CCGGCTGGCA CCTCCTGGCA AGAAGTGTGC

GAGTTCTTGC TGCGTTACGC CCGGGTGGCA CCTCCTGGCA AGAAGTGTGC

GAATTTTTGC TGCGTTATGC CCGGGTGGCA CCTCCTGGCA AAAAGTGTGC

GAATTTTTGT TGAGTTATGC CCGGGTGGCA CCGCCTGGCA AGAAATGTGC

GAATTTTTGC TGCGTTATGC CCGGGTGGCA CCACCTGGCA AGAAATGTGC

---------- ---------- ---------- ---------- ----------

---------- ---------- ---------- ---------- ----------

GAATTTTTGC TGCGTTTTGC CCGAGTGGCA CCTCCTGGCA AGAAGTGCGC

GAATTTTTGC TCCGTTCTGC CCGAGTGGCG CCTCCTGGGA AAAAGTGTGC

---------- ---------- ---------- ---------- ----------

GAGTTTTTGC TGCGTTGTGC CCGGGTGGCG CCTCCTGAGA AGAAGTGCGC

---------- ---------- ---------- ---------- ----------

GACTTTCTGC TTCGCCATGC CCGGGTGACA CCTGCAGAGA AGAAATGTGC

GAGTTCCTCA TCCGCCATGC CCGAGTGGCC CCCCCAGCGA AAAAGTGCGC

GAGTTCCTGC TGAAACATGC CCGCGTGTGC CCCCCTGGAA ATAAGTGTGC

CTTTGTGCTG ACTCGGGACG GCCGCCCGGT CAAGGTGCAG CGAACCATCT

CTTTGTGCTG ACTCGGGACG GCCGCCCGGT CAAGGTGCAG CGAACCATCT

CTTTGTGCTG ACTCGGGACG GCCGTCCGGT CAAGGTGCAG CGGACCATCT

CTTTGTGCTG ACTCGGGACG GCCGTCCGGT CAAGGTGCAG CGGACCATCT

CTTTGTGCTG ACTCGCGACG GCCGCCCGGT CAAGGTGCAG CGGACCATCT

CTTTGTGCTA ACTCGGGATG GCCGCCCAGT CAAGGTGCAG CGGACCATCT

TTTTGTGCTG ACCCAGGACG GCCGTCCAGT GAAGGTGCAG CGGACCATTT

TTTTGTGCTG ACTCGGGATG GCCGGCCAGT GAAGGTGCAG CGGACCATTT

---------- ---------- ---------- ---------- ----------

---------- ---------- ---------- ---------- ----------

CTTTGTGCTG ACGCGGGACG GCCAGCCCAT CAAGGTGCAG CGAACCATCT

CTTTGTGCTG ACGCGGGATG GCCGCCCTGT CAAGGTGCAG CGAACCATCT

---------- ---------- ---------- ---------- ----------

CTTCGTGCTG ACGCGGGACG GCCGCCCGGT CAAGGTGCAG CGGACCATCT

---------- ---------- ---------- ---------- ----------

TTTCGTGCTG ACTCGAGATG GCTCCCCAGT CAAGGTGCAG CGCACCATCT

CTTCGTACTG ACTCGGGACG GCCGCCCCAT CAAAATTCAG CGAACCATTT

CTTTGTGCTG ACTCGGGATG GCCGGCCCGT CAAAGTCCAG CGTACCATCT

TCAGTGAGTG TTTCTACACC ATGGCCATGA ACGAGCTGTG GAGAGCCACA

TCAGTGAGTG TTTCTACACC ATGGCCATGA ACGAGCTGTG GAGGGCCACA

TCAGCGAGTG TTTCTACACC ATGGCCATGA ACGAGCTGTG GAGGGCCACA

TCAGCGAGTG TTTCTACACC ATGGCCATGA ACGAGCTGTG GAGGGCCACA

TCAGCGAGTG TTTCTACACT ATGGCCATGG ACGAGCTGTG GAGGACCACA

TCAGCGAGTG CTTCTACACC ATGGCCATGA ATGAGCTGTG GAGGGTGACA

TCAGCGAGTG CTTTTACACC ATGGCCATGA ATGAACTGTG GAAAGTAACA

TCAGCGAGTG CTTCTACACC ATGGCCATGA ATGAACTGTG GAAAGTAACG

---------- ---------- ---------- ---------- ----------

---------- ---------- ---------- ---------- ----------

TCAGCGAGTG TTTCTACACC ATGGCCATGA ACGAGCTGTG GAGGGTGACT

TTAGTGAATG TTTCTACACT ATGGCCATGA ACGAGCTGTG GAGAGTGACA

---------- ---------- ---------- ---------- ----------

TCAGCGAGTG TTTCTACACC ATGGCCATGG ACGAGCTGGG CAGGGTCACG

---------- ---------- ---------- ---------- ----------

TCAGCGAGTG TTTTTACACC ATGGCCATGG ACGAGCTATG GCGGGCCACA

TCAGCGAGTG CTTCTACACT ATGGCCATGG ATGAGCTGTG GAAGGCCACC

TCAGCGAGTG CTTCTATGTC ATGGCCATGG ATGAACTCTG GAGGACCACG

GGGGAAGTGC GGTACCAGAC GGAAGCGGTG GAGATGATGG ATCAGATCGT

GGGGAAGTGC GGTACCAG-- ---------- ---------- ----------

GGGGAAGTAC GGTACCAGAC GGAAGCCGTG GAGATGATGG ATCAGATCGT

GGGGAAGTAC AGTACCAGAC GGAAGCCGTG GAGATGATGG ATCAGATTGT

GGGGAAGCAC GGTACCAGAC GGAAGCGGTG GAGATGATGG ATCAGATTGT

GGAGAAGGGC ATTACCAGAG GGAAGCAGTG GAGATGATGG ATCAGATTGT

GGGGAAATGC ATTATCAGAG AGAAGCCGTA GAGATGATGG ATCAGATCAT

GGGGAAGTGC GTTATCAGAG TGAAGCCATA GAGATGATGG ATCAGATCAT

---------- ---------- ---------- ---------- ----------

---------- ---------- ---------- ---------- ----------

GGGGAGGCGC GTTACCAGAA GGAAGCGGTG GAGATGATGG ----------

GGGGAAGCGC ATTACCAGAT CGAAGCGGTG GAAATGATGG ATCAGATCGT

---------- --------GG TGAGGCGACG TCCATGATGG AGCAGATCGT

GGGGACGCGC GCTTCCAGAA CGAGGCGGTG GAGATGATGG ATCAGATCGT

---------- ---------- ---------- ---------- ----------

GGGGACACCT GCTACCGGAG GGAGGCGCTG GCGATGATGG AGCAGCTGAC

GGCGACCCCC GATACCAGAG GGAAGCCTTG GAGATGATGG ATCAGATTAT

GGGGAGACCC GTTACCAGAG GAGAGTTTTG GGGAAAGTAG ATCAGATTGT

CCACTGGGTG CAGGAGGACG CGTCGGGACT GGGCCGGCCC CAGCTCCAGG

---------- ---------G CGTCGGGACT GGGCCGGCCC CAGCTCCAGG

CCACTGGGTG CGGGAGGACG CGTCGGGACT GGGCCGGCCC CAGCTCCAGG

CCACTGGGTG CGGGAGGACG CGTCGGGACT GGGCCGGCCC CAGCTCCAGG

CCACTGGGTA CGGGAGGACG CGTCGGAACT GGGCCGGCCC CAGCTCCAGG

CCGCTGGGTG CGTGAGGACC AGTCAGGATT GGGCCAGCCC CGGTTGGCAG

CCACTGGGTA AGGGAGGACC CAGCTGGGCT GGGCCGGCCT CAGCTCTCGG

CCACTGGGTA CGGGAGGACC CGGCTGGGTT GGGCCGGCCT CAGCTCTCAG

---------- ---------- ---------- ---------- ----------

---------- ---------- ---------- ---------- ----------

---------- ---------- ---------- ---------- ----------

ACACTGGGTG CGTGAAGACC CGTCGGGGCT GGGCCGACCC CAGCTCCCGG

GCACTGGGTG CGGGAGGACC CAGCGGACCT GGGCCGGCCC CAGCTTCCAG

CCACTGGGTG CGGGAGGACC CGTCGGGGCT gggccggccc cagctcccgg

---------- ---------- ---------- ---------- ----------

CCACTGGGTA CGGGAAGACC CTTCGGGGCT GGGGAGACCG CAGCTACCGG

ATACTGGGTT CGGGTGGACC CCACTGGGCT GGGCCAGTCG CAGCTCTCAG

GTTCTGGGTC CGGGAGGACC CGTCGGGGCT GGGCAGACCC AAGCTGTCCG

GGGCCCCGGC TGCGGAGCCC ATGGCGGTGC CCATGATGCT ACTGAACCTG

GGGCCCCGGC TGCGGAGCCC ATGGCGGTGC CCATGATGCT ACTGAACCTG

GGGCCCCGAC TGCGGAGCCC ATGGCGGTGC CCATGATGCT GCTGAACCTG

GGGCCCCGAC TGCGGAGCCC ATGGCGGTGC CCATGATGCT GCTGAACCTG

GGGCCCCAGC CGTGGAGCCC ATGGCAGTGC CCATGATGCT GCTGAACCTG

GGGCCCCAGC CGTAGAGCCC ATGGCCGTTC CCATGATGCT GCTCAACCTG

GAACACTGGC CACAGAGCCC ATGGCAGTGC CCATGATGCT GCTCAACCTG

GGGCACTGGC CACAGAGCCC ATGGCAGTGC CCATGATGCT GCTCAGCCTG

---------- ---------- ---------- ---------- ----------

---------- ---------- ---------- ---------- ----------

---------- ---------- ---------- ---------- ----------

GGGCCCCGGC CTCAGAGCCC ATGGCAGTGC CCATGATGCT GCTCAACCTG

GCACCCCGGC CGCAGAGTCC ATGGCTGTGC CCATGATGGT GCTCTGGCTG

gggcgccccg cgcggAGCCC ATGGCCGTGC CCATGATGCT GCTGGCCCTG

---------- ---------- ---------- ---------- ----------

GGGCCACCAG GGCTGAGCCC CTGGCAGTAC CCATGATGCT GCTCTGCCTG

GATCTCCAAT GGCTGACTCG ATGGCTGTGC CCATGATGGT GCTCAACTTG

GTGCCCCTGC GGCGGAGGCC ATGGCCGTTC CCATGATGCT GCTGAACCTC

GTGGAGCAGC TCGGGGAGGC AGATGAGGAG CTGGCGGGCA AATACGCAGA

GTGGAGCAGC TCGGGGAGGC AGATGAGGAG CTGGCGGGCA AATACGCAGA

GTAGAGCAGC TTGGGGAGGC AGATGAGGAG CTGGCAGGCA AATACACAGA

GTAGAGCAGC TTGGGGAGGC AGATGAGGAG CTGGCAGGCA AATACACAGA

GTGGAGCAGC TCGGGGAGGC GGACAAGGAG CTGGCGGACA AATATGCAGA

GTGGAACAGC TCTCCGAGGC GGATATGGAG CTGGCAGCCC AATATGCGGA

GTGGAGCAGC TTGGAGAAGA AGATGAGGAG ATGACCGACA AGTATGCAGA

GTGGAGCAGC TTGGAGAAGA AGATGAGGAG CTGACCAACA TGTATGCAGA

---------- ---------- ---------- ---------- ----------

---------- ---------- ---------- ---------- ----------

---------- ---------- -----AGGAC CGGGGAGCCC CCTCCCCGGA

GTGGAGCAGC TCGGGGAGTC GGATGAGGAG CTGGCGGGCA ACTACGCGGA

GTGCTGCAGC TCGGGGAGGC AGATGAGGAA CTGGCGGACC AGTATGCGGA

GTGGAGCAGC TCGGGGAGGC GCGCGAGGAG CTGGCGTGCA AGTACGCGCA

---------- ---------- ---------- ---------- ----------

GTGGAGCAGT TCTCGGAGGC TGATGCTGAG CTGGCCACCA GATACGCAGA

GTGGACCAGC TTGGGGAGGA CGATGACAAG CTGACCCGCA AGTTTGCTGA

GTGGACCAGC TCGGGGAGGC AGATGAAGTC CTAACGGGGA AGTACAAGGA

GCTGGGGGAC TGGTGCGCCC GGAGGATTCT GCAGCACGTG CAGCTGAACA

GCTGGGGGAC TGGTGCGCCC GGAGGATTCT GCAGCACGTG CAGCTGAACA

GCTGGGGGAC TGGTGCGCCC AGAGGATTCT GCAGCACGTG CAGCTGAACA

GCTGGGGGAC TGGTGCGCCC AGAGGATTCT GCAGCACGTG CAGCTGAACA

GCTGGGTGAC TGGTGCGCCC GGAGGGTTCT GCAACACGTG CAGCTGAAGA

GCTGGGGAAC TGGTGCACCC AGAGGCTGCT GCAGCATGTG CAGCTGAAAG

ACTAGGGGAC TGGTGTGCCC ATAGGATTCT TCAGCATGTC CAG-------

ACTAGGGGAC TGGTGTGTCC ACAGGATTCT TCAGCATGTC CAGATGACCA

---------- ---------- ---------- ---------- ----------

---------- ---------- ---------- ---------- ----------

GTTGGGGGAT TGCCGTGCCC AGAGGACCCG CGAGCATCTC CGG-------

GCTGGGGGAC TGGTGCGCCC AGAGGATTCT GCAGCACGTC CAG-------

GCTGGGGGAC TGGTGTGCTG CCAGGGTGCT GCAGCACGTC CAG-TGAAAG

GCTGGGGGAC TGGTGCGCCC GCAGGATGCT GCAGCACGTC CAG-TAGAAA

---------- ---------- ---------- ---------- ----------

GCTGGGGGAC TGGTGTGTCC ATCGGATTCT ACAGCATGTC CAGCTGAGCA

GTTGGGAGAG TGGTGTACTC AGGACATCTT GAAGCATCTC CAGCTGAACA

GCTGGGAGAT TGGTGCGCCC AGAGGATCCT GCGGCACCTC CAGGTAGAAA

ACTTCAGGGA TTACTTATGG CAACTTTTAC CTCCAGCAGC CATTGTTGCT

ACTTCAGGGA TTACTTATGG CAACTTTTAC CTCCAGCAGC CATTGTTGCT

ACTTCAGGGA TTACTTATGG CAACTTTTAC CTCCAGCAGC CATTGTTGCT

ACTTCAGGGA TTACTTATGG CAACTTTTAC CTCCAGCAGC CATTGTTGCT

ACTTCAGGGA TTCCTTACGG GATCTTTTAC CTCCAGCAGG CATTGTTGCT

ACTTCAAGGA TTACTTGTGG GATCTCCTAC CTCCAGCAGC CATTTTCGCT

---------- ---------- ------GTAC CTCCAGCAGT CATGTTCGCT

AGTTTAAAAA TCACCTGTCA GATTTCATCC CTCCAGCAGC TATTTTTGCT

---------- ---------- ------ATAC CTCCAGCAGC CATTTTTGCT

---------- ---------- ---------- ---------- ----------

---------- ---------- ---------- ---------- ----------

---------- ---------- ---------- ---------- ----------

ACTTTAAGGA GTACATGTGG GATCTCATTC CTCCAGTAGC CATTTTGGCA

ACTTCAGGGA TTACTTGTGG GATCTCATAC CTCCAGCAGC CATTTTGGCT

---------- ---------- ---------- ---------- ----------

ACTTTGGGAA TTATGTGTGG GAGCTTATAC CTCCAGCAGC AATTTTCGCT

ACTTCTGGAT CAACATGTCG GATTTGCTGC CCCGGTTGGC CATTTACGCT

ACTTCTTGGA TGATCTCTGG GATCATATCC CACGGTCAGC TCTGTATGCC

TTTCTTCTCA CCTCAGCACT AATGGGGATC CTCTGCTGCT TCACCCCCAA

TTTCTTCTCA CCTCAGCACT AATGGGGATG CTCTGCTGCT TCACCCCCAA

TTTCTTCTCA CCTCAGCACT AATGGGGATC CTCTGCTGCT TCACCCCCAA

TTTCTTCTCA CCTCAGCACT AATGGGGATC CTCTGCTGCT TCACCCCCAA

TTTTTTCTCA CCTCAGCACT AATAGCGATC CTCTGCTGCT TCAGCTCCAA

TTTCTTATCA CCACAGCACT AATGGGCACT CTCTGTTGCC TCACCCCCAA

CTTTTTGTCA TCGTGGCCCT CACGGGTGTC CTCTGTGGCC CCAC-CCCAG

CTTTTTGTCA CCACAGCAAT AATGAGTATT CTTTGCTGCC TCACC-----

TTTCTTATCA CCACAGCAAT AATGGGGACC CTCTGCTGCC TCACTCCCAA

---------- ---------- ---------- ---------- ----------

---------- ---------- ---------- ---------- ----TCCCAA

---------- ---------- ---------- ---------- ----TCCCAA

TTTGTCATCT CCACCGTGTT TTTGGGAAGC ATCTGCTGCC TCACCCCTGT

TTTCTTATCA CCACAGCACT AATGGGGACC CTCTGTTGCC TCACTCCCAA

---------- ---------- ---------- ---------- -----CCCAA

TTCCTCGTCT GCGCATCACT AATGGCGATT GTCTGCTGCT TCAC-CCCAA

TTTGTGGTCT CCTCTGCAGT AATATCTCTG CTATTCTGTC TCAC-CCCAG

ATTCCCGTCA CTTCCGGCAT AATGTTCCTT CTCTGCTGCT TCACT--CAA

GCCCTCCCTC CACGCCTGGC CCAGCTCGGT GGTTGAAGCC GAGAGCAATG

GCCCTCCCTC CACGCCTGGC CCAGCTCGGT GGTTGAAGCC GAGAGCAATG

GCCCTCCCTC CACGCCCGGC CTAGCTCAGT GGTTGAAGCC CGGAGCAACG

GCCCACTCTC TGGGCTGAGC CAGGGTCTAT GATCAGCGAG GGGAGTCCTG

GCCCTCCCTC CACGCCTGGC CCAGCTCGGT GGCTGAAGCC AAGAGCAACG

GCCTGCCTTC AGTGCCTGGC CCAGTTCGGT GGTTGGATGT GCCAGCAACG

GCCTTCCATC AGTGCCTGGC CCAGCTCTGT GGTCAAGTGC AGGAGTAACG

---------- ---------- ---------- ---------- ----------

GCCCTCTCTG AGCGCTTGGC CCAGCTGGGT GGTTGAGCCT GGCGGCAACG

---------- ---------- ---------- ---------- ----------

ACCCACGCTC AGTGCCTTGC CCAGCAACAT GGTTGAACAC AGAGGCAACG

ACCTACCGCC TGGGCTGAGC CAGGCTCTGT GATCCCCTGG GGGAGCTCTG

GCCCGTCCTT GAGGTCTGGC CTGGCCCGGT GGTGGGGCCG CAAGGGAACG

GCCCCTCCTC AGTGCCTGGC CCAGCTCGGT GGTCCCTCTC AAGAGTCATG

GCCCTTCTTC AGTGCCTTGC CCAGCTCAGT GGTTGAAAGC AAAAGCAACG

GCCCCTCCTC AGGGCCTGGC CCAGCTCGGT GGCTGAAAGC AGAAGCAACG

ACCCTCCCTC GGGGCAGAAG ATGGCTCTTT GGTGCCCCAA GGGAGAACTG

ACCCTCCCTG CGGGCCTGGC CCGGCCCACT GCTGATCCCT GGGACGAATG

TGACCCTGAA GTGTCAGGCT CATTCCCAGA ATGTGACATT TGTGCTGCGC

TGACCCTGAA GTGTCAGGCT CATTCCCAGA ATGTGACATT TGTGCTGCGC

TGACCCTGAA GTGTCAGGCT CCTTCCAAGA ATGTGACATT CATTCTGCGC

TGACCCTCAG GTGTCAGGTT CAGGTTCAGG A-GTATCGTC TATA---AGA

TGACCCTGAA GTGTCGGGCT CCTTCCCAGA ATGTGACATT CACGCTGCGC

TGACTCTGAG GTGCCAGGCT CCTTTCCACA ATGTGACATT CGTGCTTCAG

TCACTCTCCG ATGCGGGACT CATTTCCAGA ATGTTACAGT CACTCTGGGG

---------- ---------- ---------- ---------- ----------

TGACCCTGAG GTGCCGGGTT CCCTTCCAGA ACGTCTCAGT CATGCTGGGC

---------- ---------- ---------- ---------- ----------

TGAGCCTCAG GTGCCAGTCT CACTTCCAGA ATGTGACATT CATGCTGGGG

TGACCATCTG GTGTCAGGCT GGGGGCCAAG GAGTTCCGCC TATAGAAAGG

TGACCTTCAG GTGCTTGTCT GCTATCCCGA ACGTGACCTT CATGCTGGGA

TGATTCTGCG ATGCTACACT AACAAAAGTG ATGTAAACTT TGCTCTCAAA

TGACTCTGAA GTGCTGGTCT ACGTTCCAGA ATGCTACCTT CAGGCTGGGG

TGACTCTGAC GTGCAGGTCT CCATTCCGGA ACTCGACCTT CTGTCTGGGG

TGACCCTCAG GTGCAGGG-- -GGTCACAGG AGGCTG-ATC TGTACCGCAA

TGACGCTCCG GTGCCAGGCG TGGGTGGGCG GCCTGACGTT CTCCCTGCTC

AAGGTGAACG ACTCTGGGTA CAAGCAGGAA GAAAGCAGTG AACACTTGCA

AAGGTGAACG ACTCTGGGTA CAAGCAGGAA GAAAGCAGTG AACACTTGCA

AAGGTGAATG ATTCTGGGTA CAAGCAGGAA GAAAGCAGTG AACACTTGCA

AAAAAAACCA ATCCTGGGTA CGAATACAAC GAGCCCAGTG ACCCCCTGGA

AAGGTGAACG ACTCTGCATT CCAGCGCGAA GAAAGCAGTG AGCACGTGCA

AAGCCACAGG ACTCAGAGTA CCAGCAAGAG GACAACAGTG AGCCCCTATG

AGGTTGCACA ACTCTCAGTA CCAGCAACAG GGACAGAGTC AGCACTTACA

---------- ---------- ---------- ---------- ----------

AGGTTGGGAG ACCCCAGGTA CAGGCAGGAG GCGCACAGCG AGTATGTGCA

---------- ---------- ---------- ---------- ----------

AAGCTGCAGG ACTCTGGGTA CAGGCAGGAG GAAGAGAGTG AACACCTGTG

GAATCC---G ATCCTGGG-A CAGAAGGAAG GAGAGCAGTG ACCCCCTGGA

AAGCTGCAGG ATGCCGGGTA CAGGCCAGAG GAGACGAATC CACATGTGCA

AAGGATGGGA AACCTTTGGA CCAGCATTCA CAGCCCAGTG AACCCCTTCG

AAGTTGAACA ACACTGGGTC CGAGGAACAG GAG---AGTG AACATTTACA

AAGCTGAACA GCACTGTTTC CCCACGGTCA CCACTAAGTG ATGTGGTTCT

AAGCTAGGAA ATTCTGATCA CAGATATGAC GAGCCCAGTG AGCCCCTGCA

CGGGCTCAGA CCTGGGACCT CCTGCAGCG- -CGCCCAGTG ATGTCCTGGA

GCTGGTGGTC ACAGATATGG CGGACAAGCG CAAACTCCAA GGTGAGATTG

GCTGGTGGTC ACAGATATGG CGGACAAGCG CAAACTCCAA GGTGAGATTG

GCTGGTGACC ACAG--ATGG CGGACAAGCG CAAACTCCAA GGTGAGATTG

GCTGGTGGTG ACAG--ATGG CGGACAAGCG CAAACTCCAA GGTGAGATTG

GCTGGTGGTC ACAGATATGG CGGACAAGCG CAAACTCCAA GGTGAGATTG

GCTGGTGGTT ACAGATATGG CGGATAAGCG CAAACTCCAA GGTGAGATTG

GTTGGTGGTC ACAG--ATGG CGGACAAGCG CAAACTCCAA GGTGAGATTG

---------- ------ATGG CGGACAAGCG CAAACTCCAA GGTGAGATTG

GCTGGTGGTC ACAG--ATGG CGGACAAGCG CAAACTCCAA GGTGAGATTG

---------- ---------- ---------- ---------- ----------

GCTGCAGGTC AAAGATATGG CGGACAAGCG CAAACTCCAA GGTGAGATTG

GCTGGTGGTG ACAG------ ---------- ---------- ----------

GCTGCAGGTG ACAGGT---- ---------- ---------- ----------

ACTTGTGGTT ACCA--ATGG CGGACAAGCG CAAACTCCAA GGTGAGATTG

GCTGGTGGTC ACAGATATGG CGGACAAGCA CAAACTCCAA GGTGAGATTG

CCTTCTGGTT ACAGGT---- ---------- ---------- ----------

GCTGGTGGTG ACAGATATGG CGGATAAGCG AAAACTCCAA GGTGAGATTG

CCTGGTGCTG ATAG--ATGG CGGACAAGCG CAAACTTCAA GGAGAGATCG

ATCGCTGCCT CAAGAAGGTG TCCGAGGGCG TGGAGCAGTT TGAAGATATT

ATCGCTGCCT CAAGAAGGTG TCCGAGGGCG TGGAGCAGTT TGAAGATATT

ATCGCTGCCT CAAGAAGGTG TCCGAGGGCG TGGAGCAGTT TGAAGATATT

ATCGCTGCCT CAAGAAGGTG TCCGAGGGCG TGGAGCAGTT TGAAGATATT

ATCGCTGCCT CAAGAAGGTG TCCGAGGGCG TGGAGCAGTT TGAAGATATT

ATCGCTGCCT CAAGAAGGTG TCTGAGGGTG TGGAGCAGTT TGAAGATATT

ATCGCTGCCT CAAGAAGGTG TCCGAAGGTG TGGAGCAGTT TGAAGATATT

ATCGCTGCCT CAAGAAGGTG TCCGAAGGGG TGGAGCAGTT TGAAGATATT

ACCGCTGCCT CAAGAAGGTG TCCGAGGGCG TGGAGCAGTT TGAAGACATC

---------- ---------- ---------- ---------- ----------

ATCGCTGCCT CAAGAAGGTG TCCGAGGGCG TGGAACAGTT TGAGGATATT

---------- ---------- ---------- ---------- ----------

---------- ---------- ---------- ---------- ----------

ATCGCTGCCT CAAGAAGGTG TCCGAGGGTG TGGAGCAGTT TGAAGATATC

ATCGCTGCCT CAAGAAGGTG TCCGAGGGCG TGGAGCAGTT TGAGGATATT

---------- ---------- ---------- ---------- ----------

ATCGCTGCCT CAAGAAGGTG TCCGAAGGTG TTGAGCAGTT CGAGGACATT

ACCGCTGCCT CAAGAAAGTG TCCGAGGGCG TCGAGCAGTT TGAGGATATC

TGGCAGAAGT GGTCGGAACC CAGTGACCCC CTGCAGCTAC TGGTGTCAGG

TGGCAGAAGT GGTCGGAACC CAGTGACCCC CTGCAGCTAC TGGTGTCAGG

TGGCAGAAGT GGTCGGAACC CAGTGACCCC CTGCAGCTAC TGGTGTCAGG

TGGCAGAAGt ggtcagaccc cggcaactcc ctggggctcc tgttctcagG

TGGCAGAAGT GGTCTTCACC CAGTGATCTC CTGGAGCTCC TGGTCCCAGG

TGGCAGAAGT GGTCAGAGCC CAGTGACCCC CTGGAGCTCC TGATCTCAGG

TGGCAAAAGT GGTCAGAACC CAGCGATCTC TTGGAAATAC ATGTGTCAGG

TGGCAAAAGT GGTCGGAACC CAGCGATCTC TTGGAAATAC ATGTGTCAGG

TGGCAGAAGT GGTCAGAGCC CAGTGACGCC CTGGAGCTGG GGGTGACAG-

---------- ---------- ---------- ---------- ----------

TGGCAGAAGT GGTCACTCCC CAGTGACCCT CTGGAGCTGC TGGTCTCAGG

---------T GGTCACACCC CAGTGACCTC CTGGAGCTCC TGGTCTCAGG

---------- ---------C CAGCGACCCC CTACAGCTCC TGCTCTCAGA

TGGCAGAAGT GGTCCCAGCC CAGTGATGCC CTGCAACTGC TGGTGACAG-

TGGCAGAAGT GGTCACACCC CAGTGACCCT CTGGATCTCA ACGTCACAGG

---------T GGTCACAGCC CAGTAACCCT GTGGATCTGA TGGTCACAGG

TGGCAGAAGT GGTCATCCCC CAGCGCCCCC CTGGTGCTCA GGGTCTCAGG

TGGCAGAAG- -GTCGCAGAT CAGCGAGACC CTGGAGATCT GGGTGATGG-

CCAGGCTTTC CCCATGGCGG GGACCTACAG GTGCTACGGC TCACGCAGCT

CCAGGCTTTC CCCATGGCGG GGACCTACAG GTGCTACGGC TCACGCAGCT

CCAGGCTTTC CCCATGGCGG GGACCTACAG GTGCTACGGC TCACGCAGCT

TCAGGCTTTC CCCATGGCGG GGACCTACAG GTGCTACGGC TCACGCAGCT

TCAGGCTTTC CCCATGGCGG GGACCTACAG GTGCTACGGC TCACGCAGCT

TCTGGCTTTC TCCATGAAGG GGACCTACAG GTGCTATGGC TCACACAGCT

GCAGGCATTC TCTAGGAGGG ACACC---AA GTG---TAGC TGT---AACT

GCAGGCATTC TCTAGGGGGG ACACT---AG GTG---TAGC TGT---AACT

-CAGGCCTTC ATGCTGTGGG GCACCTACAG GTGCTACAGC GCCCACAGCA

---------- ---------- ---------- ---------- ----------

TGAGGCTTTC TCCATGGGGG GGACCTACAG GTGCTACGGC TCTATCAAAA

CCAGGCCTTC CGCATCGGGG GCACTTACAG GTGCTACGGT TCACGCGGCA

TCGGGTCTTC TCTATCGGTG GCACTCACC- GTGCTACAGC TCAGACAGCA

-TGGGTGTTC CCGC----GG GCACCTACAG CTGCTACTAC -CACACGCCT

GCAGCAC-TC A-CATGGGAG GGACCTACAG ATGCTACAGC TCACACAGCA

GCAGGCCTTC ACCATGGGGG GCACCTACAG ATGCTACACG TCATTCAGGA

CCAGGCTTTC CTCTTCGAAG GCACTTACCG ATGCTTCGGC TTTTATAGTT

-CAGTC--TC CCCCTGAGTG GGGGAGGCAG AGGAGGTGGC TTGTGTCTCT

CCAACCCCTA CCTGCTGTCT GGTGGTCAGG ACAAACCCTT CCTGTCTGCC

CCAACCCCTA CCTGCTGTCT GGTGGTCACG ACAAACCCTT CCTGTCTGCC

CCGACCCCTA CCTGCTGTCT GGTGGTCAGG ACAAGCCCTT GCTGTCGGCC

CCGACCCCTA CCTGCTGTCT GGTGGTCAGG ACAACACCTT CCTGTCTGCC

CCAACCTCTA CCCGCTGTCT GGTGCTCTGT ACAAACTCTC CCTGTCAGCC

CTTCTCCCTA CCTACTGTCA GGTGGTCATG ACAAGTCCTC CCTGTCTGCC

CA---CCCTA TCTGTT---- GGAAGTTATG ATAAGCCTTT TCTCTGTGCC

TA---CCCTA TTTGTTGTCA GGAAGCCATG ATAAACCATT CCTATCTGCC

CCGACCCCTA CCTGTGGTCA GGAGCCTACG GCAAGCCCAG CCTCTCGGCC

---------- ---------- ---------- ---------- ----------

CCTCTCCCTA CCTGTTGTCA ---------- -TACACCCTC CTTACGGGTG

CCTCCCCCTA CCTGCTGTCA C--------- ---------- ----------

TCG-CCCCCA CCTGCTGTCA GGTGGTCAGC ACAAGCCCAT CTTATCAGCA

CCGCGCCCTA CGTGCTGTCG -TATCACTTC CCAAGCCCCT CCTCAGTGCC

CCTCCCCTTA CCTGTTGTCA GGCTATCACA ACAAGCCCTC CCTGACTGCC

------AGTA CCTGTTGTCC ---------- ---CGCCTTC CCTCTCGGTG

CTTTCCCCTC TCTGTGGTCC GGCTACTACA GTCCGCCCCA GCTGTCAGCA

CCTTCTCATT TCTGCTCTCC -GAGTCTATA CCAAACCCTC CCTGCGGGCC

TGGCCCAGCG CTGTGGTGCC TCGAGGAGGA CACGTGACTC TTCGGTGTCA

TGGTCCAGTG CTGTGGTGCC TCGAGGAGGA CATGTGACTC TTCGGTGTCA

TGGCCCAGCC CTGT------ ---------- -ATGTGACTC TTTGGTGTCA

CGGCCCAGCG CTCTGGTGCC TCAGGGAGGA CACGTGACTC TTCAGTGTCA

CGGCCGAGCC CTGTGGTTCC CCAGGGAGGA CGTGTGACTC TCTCCTGTGA

TGGCCAAGCC CTGTGGTGCC TCAAGGAAGA TACGTGACTC TTCAGTGTCA

TCGCCAAGTT ATATTGTTCC TCAGGGACAG AATGTGACTC TTATTTGTGA

TGGCCAAGTT ATGTTGTTCC TCTGGGACAG AATGTGACTC TTACATGTGA

CTGCCCAGCC CCGTGGTGAC CTCAGGGGAC AATGTGACCC TCCAGTGCCA

---------- ---------- ---------- ---------- ----------

CATCCAGGAC CCACGGTGGC CCCAGGAGAG AATGTGACCC TGCTGTGTCA

---------- ---------- ---------- ---------- ----------

TGGCCAAGTC CCATGGCTCA ACTCAGAAGT CACGTGAATA TTCAGTGTCA

TGGCCCAGCT CGGTGGTCCC TCTCAAGAGT CATGTGATTC TGCGATGCTA

TGGCCAAGTC ATGTGGTTCC TGTAGGACAG CATGTGCATC TTCAGTGTCA

TGGCCAGGCC CTGCAGTGGT CTCAGGAGGA AAAGTGACCC TGCAGTGTGC

GAGCCAAGCC AACACGTGGC CTGGGGACAC AATGTGACCC TCCGGTGTCG

TGGCCCGGCC CACTGCTGAT CCCTGGGACG AATGTGACGC TCCGGTGCCA

CGCCCGTACA GAATTCCTAC GGAAGAAAGC CAGACATCAG GGCCCTGTGG

CGCCCGTACA GAATTCCTAC GGAAGAAAGC CAGACATCAG GGCCCTGTGG

CGCCCGTACA GAATTCCTAC GAAAGAAAGC CAGACATCAG GGCCCTGTGG

CGCCCGTACA GAATTCCTAC GAAAGAAAGC CAGACATCAG GGCCCTGTGG

TGCCCGTACA GAATTCCTAC GGAAGAAAGC CAGACATCAG GGCCCTGTGG

CGCCCGCACA GAATTTCTAC GGAAGAAAGC TAGACATCAG GGCCCTGTGG

CGCCCGTACG GAATTCCTAC GGAAGAAAGC CAGGGGCCGA GGCCCTGTGG

C--------- ---------- ---------- ---------- ----------

C--------- ---------- ---------- ---------- ----------

---------- ---------- ---------- ---------- ----------

GGCCCGCACA GAATTCCTAC GGAAGAAAGC CAGGCACCAG GGCCCTGTGG

---------- ---------- ---------- ---------- ----------

A--------- ---------- ---------- ---------- ----------

CGCCCGCACA GAATTCCTAC GTAAGAAAGC CAGGTATCAG GGCCCCGTGG

CGCCCGCACA GAATTCCTAC GCAAGAAGGC CAGGCGCCAG GGCCCTGTGG

A--------- ---------- ---------- ---------- ----------

CGCCCGGACA GATCTCCTTC GGAAAAGAGC CCGGAGCC-- GGCCCTGTGG

GGCCCGCACT GACTTCCTGC GGAAGAAGGC CAGAGGCCCG GGCCACGTGG

ACCTGTTTCG GGAGCTGCTG GAGGAAGGGA AAGGAGTGAT CAGAGGCAAT

ACCTGTTTCG GGAGCTGCTG GAGGAAGGGA AAGGAGTGAT CAGAGGCAAT

ACCTGTTTCG GGAGCTGCTG GAGGAAGGGA AAGGAGTGAT CAGAGGCAAT

ACCTGTTTCG GGAGCTGCTG GAGGAAGGGA AAGGAGTGAT CAGAGGCAAT

ACCTGTTTCG AGAGCTGCTG GAGGAGGGGA AAGGCCTGAT CAGAGGCAAT

ACTTGTTTCG GGAGCTTCTG GAGGAAGGGA AAGGGGTGAC CGGAGGCAAT

ACCTGTTTCG GGAGCTGCTG GAAGAAGGAA AAGGAGTGCC CAGAGGCAAT

---------- ---------- ---------- ---------- ----------

---------- ---------- ---------- ---------- ----------

---------- ---------- ---------- ---------- ----------

ACCTATTTCG GGAGCTGCTG GAGGAAGGGA AAGGGGCGAC CAGAGGCAAT

---------- ---------- ---------- ---------- ----------

---------- ---------- ---------- ---------- ----------

ACCTGTTTCG GGAGCTGCTG GAAGAAGGGA AAGGGGTGAC CAGAGGCAAT

ACCTGTTTCG AGAGCTGCTA GAAGAAGGGA AAGGGGTGAC CAGAAGCAAT

---------- ---------- ---------- ---------- ----------

ACCTGTTCCG GGAGCTGGTG GAGGAAGGAA AGGGAGCCGG CGGAGGGAAC

ACCTGTTTCG GGGGCTG--G GAGGACGGGG GGGGGGCGCT GAGCGGCAAC

AAAGAGTACG AGGAAGAAAA GCGACAGGAG AAAGCTGTGG GCCTGGGGCT

AAAGAGTACG AGGAAGAAAA GCGACAGGAG AAAGCTGTGG GCCTGGGGCT

AAAGAGTACG AGGAAGAAAA GCGACAGGAG AAAGCTGTGG GCCTGGGGCT

AAAGAGTACG AGGAAGAAAA GCGACAGGAG AAA------- ----------

AAAGAGCACG AGGAAGAAAA GCGACAGGAG AAAGCTGTGG GCCTGGGGCA

AAAGAGTATG AGGAAGAAAA GCGACAGGAG AAAGCTGTGG GCCTGGGGCT

AAAGAGCATG AGGAAGAGAA ACGACAGGAA AAAGCTGTGG GCCTGGGGCT

---------- ---------- ---------- ---------- ----------

---------- ---------- ---------- ---------- ----------

---------- ---------- ---------- ---------- ----------

AGAGAGTATG AGGAAGAAAA GCGACAGGAG AAAGCCGTGG GCCTGGGGCT

---------- ---------- ---------- ---GCTGTGG GCCTGGGGCT

---------- ---------- ---------- ---------- ----------

AAAGAATATG AGGAAGAAAA GCGACAGGAA AAAGCGGTGG GCCTGGGGCT

AAAGAGTATG AGGAGGAAAA ACGACAGGAG AAG------- ----------

---------- ---------- ---------- ----CTGTGG GCCTGGGGCT

AGGGAGTATG AGGAAGAAAA GAGGCAGGAG CAGGGGGTGG GGCTGGCCCT

AAGGAGCACG AGGAGGAAAA GCGACAGGAG AAGGCTGTAG GGCTGGGCTT

CACCCTGTTC ACCTGTGGCC CCCACACTTT GCATTCTCTG GTCACCATCC

CACCCTGTTC ACCTGTGGCC CCCACACTTT GCATTCTCTG GTCACCATCC

CACCCTGTTC ACCTGTGGCC CCCACACTTT GCATTCTCTG ATCACCATCC

---------- ---------- ---------- ---------- ----------

CACCCTATTC ACCTGTGGCC CCCACACTTT GCATTCTCTG ATCACCATCC

CACCTTGTTC ACCTGTGGCC CCCACACTTT GCATTCTCTG ATCACCATCC

CACCTTATTC ACCTGTGGCC CCCACAGTTT GCATTCTCTG ATCACCATCT

---------- ---------- ---------- ---------- ----------

---------- ---------- ---------- ---------- ----------

---------- ---------- ---------- ---------- ----------

CACCTTGTTC ACCTGCGGCC CCCACACTTT GCATTCTCTG ATCACCATCC

CACCTTGTTC ACCTGTGGCC CCCACACTTT GCATTCTCTG GTCACCATCC

---------- ---------- ---------- ---------- ----------

GACCCTGTTC ACCTGTGGCC CCCACACCTT GCATTCTCTG GTGACCATCC

---------- ---------- ---------- ---------- ----------

CACCTTGTTC ACCTGCGGAC CTCACAGCTT GCATTCTTTG GTCACCATCC

GACGCTGCTC ACCTGTAGAG CCCACATCCT CCACTCGCTG GTCACCATCC

GACGTTGCTG ACCTGCGGCC CTCACGTCCT GCACTCCCTG GTCACCATCC

TCGGGACCTG GGCCCTCATT CAGGCCCAGC CCTGCTCCTG CCACGCCCTG

TCGGGACCTG GGCCCTCATT CAGGCCCAGC CCTGCTCCTG CCACGCCCTG

TTGGGACCTG GGCCCTCATT CAGGCCCAGC CGTGCTCCTG CCACGCCCTG

---------- ---------- ---------- ---------- ----------

TCGGGACCTG GGCCCTCATT CAGGCCCAGC CCTGCTCCTG CCACGCTCTG

TGGGGACCTG GGCCCTCATT CAGGCCCAGC CCTGCTCCTG CCACGCCCTG

TGGGAACCTG GGCCCTCATA CAGGCCCAGC CCTGCTCCTG CCACGCGCTG

---------- ---------- ---------- ---------- ----------

---------- ---------- ---------- ---------- ----------

---------- ---------- ---------- ---------- ----------

TTGGGACCTG GGCCCTCATC CAGGCCCAGC CCTGCTCCTG CCACGCCCTC

TTGGGACCTG GGCCCTCATT CAGGCCCAGC CCTG------ ----------

---------- ---------- ---------- ---------- ----------

TCGGGACCTG GGCCCTGGTC CAAGCACAGC CCTGCTCCTG CCACGCCCTG

---------- ---------- ---------- ----CTCCTG CCACGCCTTG

TCGGGACCTG GGCCCTTATT CAGGCCCAAC CCTGCTCCTG CCACGCC---

TTGGCACCTG GATCATCATG GAGATCCGGC CCAGGGCCTG CCATGCCCTG

TGGGGACGTG GGGCATCATG AAGGTCCAGC CCAGGTCCTG CCACTTCCTG

GCTCTGGCCA CTTTCTCCTA TCTCCTGTTC TTCCGAGCCC TCAGCCTCCT

GCTCTGGCCA CTTTCTCCTA TCTCCTGTTC TTCCGAGCCC TCAGCCTCCT

GCTCTGGCCA CCTTCTCCTA TCTCCTGTTC TTCCGAGCCC TCAGCCTGCT

---------- ---------- ---------- ---------- ----------

GCCCTGGCCA CTTTCTCCTA TCTCCTGTTC TTCCGAGCCC TCAGCCTGCT

GCTCTAGCCA CCTTCTCCTA TCTCCTGTTC TTCCGAGCCC TCAGCCTGCT

GCTCTTGCCA CCTTCTCCTA TCTTCTCTTC TTCCGAGCCC TCAGCCTGCT

---------- ---------- ---------- ---------- ----------

---------- ---------- ---------- ---------- ----------

---------- ---------- ---------- ---------- ----------

GCCCTGGCCA CCTTCTCGTA CCTGCTGTTC TTCCGTGCGC TCGGCCTGCT

---------- ---------- ---------- ---------- ----------

---------- ---------- ---------- ---------- ----------

GCCCTGGCCA CCTTCTCCTA TCTTCTGTTC TTCCGCGCGC TCGGCCTGCT

GCACTAGACA CCTTCTCCTA TCTACTGTTC TTCCGAGCCC TCAGCCTGCT

---------- ---------- ---------- ---------- ----------

GCACTGGCCA CCTTCCTCTA CCTCCTCTTC TTCCGAATGG TCACTGTATT

GCCTTGGGCA CGTTCTCTTA TCTGCTCTTC TTTCGCACGG TCACGGCCTT

GGGCCTGCCC ACTCCCACGC CCTTCACCAA TGCCGTCCAG CTGCTGCTGA

GGGCCTGCCC ACTCCCACGC CCTTCACCAA TGCCGTCCAG CTGCTGCTGA

GGGCCTGCCC ACTCCCACGC CCTTCACCAA TGCCGTCCAG CTGCTACTGA

---------- ---------- ---------- ---------- ----------

GGGCCTGCCT ACTCCCACGC CCTTCACCAA TGCCGTCCAG CTGCTGCTCA

GGGCCTGCCC ACTCCCACGC CCTTCACCAA TGCTGTCCAG CTGCTGCTAA

GGGCCTGCCC ACTCCCACAC CCTTCACCAA TGCTGTCCAG CTGCTGTTGA

---------- ---------- ---------- ---------- ----------

---------- ---------- ---------- ---------- ----------

---------- ---------- ---------- ---------- ----------

GGGCCTCCCC ACCCCCACAC CCTTCACCAA TGCGGTTCAG CTGCTGCTCA

---------- ---------- -------CAA TGCGGTCCAG CTGCTGCTGA

---------- ---------- ---------- ---------- ----------

GGGCCTGCCC ACCCCGACGC CCTTCACCAA TGCCGTCCAG CTGCTGCTGA

AGACCTGCCC ACACCCACAC CGTTCACCAA TGCCGTACAG CTGCTGCTCA

---------- ---------- ---------- ---------- ----------

TGGGCTCCCG GCCCCCACGC CTTTCACCAA TGCCGTGCAG CTGCTGCTCA

TGGCCTGCCG GCCCCCACGC CCTTCACCAA TGCCATTCAG CTGCTGCTCA

CGCTGAAGCC GCTGGAGGCC GTGCGCGAGG ACGCCTTCTA CGCCCGCCCG

CGCTGAAG-- ---------- ---------- ---------- ----------

CGCTGAAGCC GCTGGAGGCC GTGCGCGAGG ACGCCTTCTA CGCCCGCCCG

---------- ---------- ---------- ---------- ----------

CGCTGAAGCC GCTGGAGGCC GTGCGCGAGG ACGCCTTCTA CGCCCGCCCG

CATTGAAGCC TCTGGAGGCC GTGCGTGAGG ACGCCTTCTA CGCCCGCCCG

CACTGAAGCC ACTGGAAGCT GTGCGTGAGG ATGCCTTCTA CGCCCGCCCG

--------CC ACTGGAAGCT GTGCGTGAGG ACGCCTTCTA CGCCCGCCCG

---------- -------GCC GTGCGCGAGG ACGCCTTCTA CGCCCGCCCG

---------- ---------- ---------- ---------- ----------

CACTCAAGCC GCTGGAGGCC GTGCGCGAGG ACGCCTTCTA CGCCCGCCCG

CACTGAAGCC GCTGGAGGCC GTGCGCGAGG ACGCCTTCTA CGCCCGCCCG

---------- -------GCC GTGCGAGAGG ACGCCTTCTA CGCCCGCCCG

CACTGAAGCC GCTGGAGGCC GTGCGCGAGG ACGCCTTCTA CGCCCGCCCG

CACTGAAG-- ---------- ---------- ---------- ----------

--------CC GCTGGAGGCC GGGAGCGAGG ACGCCTTCTA CGCCCGCCTG

CGCTCAAGCC GCTGGAGGCC GTGCGGGAAG ATGCCTTCTA CAGCCGCAGC

CGCTCAAGCC GCTGGACTAC GTGCGGGAGG AGGCCTTCTA CGCCCGCTCC

CTGCCCGCCC GCCTCTTCTA CATGATCCCC GTCTTCTTCG CCTTCCGCAT

---------- ---------- ---------- ---------- ----------

CTGCCCGCCC GCCTCTTCTA CATAAAGGCC GTCTTCTTCG CCTTCCGCAT

---------- ---------- ---------- ---------- ----------

CTGCCCGCCC GCCTGTTCTA CATGATCCCC GTCTTCTTCG CCTTCCGCAT

CTGCCTGCCC GCCTATTCTA CATGATCCCC GTCTTCTTCG CCTTCCGCAT

CTGCCCACCC GCCTCTTCTA CATGATCCCG GTCTTCTTCG CCTTCCGCAT

CTGCCCACCC GCCTCTTCTA CATGATCCCG GTCTTCTTCG CCTTCCGCAT

CTGCCCGCCC GCCTCTTCTA CATGATCCCG GTCTTCTTCG CCTTCCGCAT

---------- ---------- ---------- ---------- ----------

CTGCCCGCCC GCCTCTTCTA CATGATTCCC GTCTTCTTCG CCTTCCGCAT

CTGCCTGCCC GCCTCTTCTA CATGATCCCC GTCTTCTTCG CCTTCCGCAT

CTGCCTGCCC GCCTCTTCTA CATGATCCCC GTCTTCTTCG CCTTCCGCAT

CTGCCCGCGC GCCTCTTCTA CATGGTCCCC GTCTTCTTCG CCTTCCGCAT

---------- ---------- ---------- ---------- ----------

CTGCCCGCGC GCCTCTTCTA CATGATCCCC GTCTTCTTCG CCTTCCGCAT

CTGGCCTTCC GCCTCTTCTA CATGCTGCCC ATCTTCTTTG CCTTCCGGAT

TTCGCCTTCC GGCTCTTCTA CATGACCCCC GTCTTCTTCA CCTTCCGCAT

GCGCTTCTAC GTGGCCTGGA TTGCCGCCGA GTGCGGCTGC ATTGCCGCCG

---------- ---------- ---------- ---------- ----------

GCGCTTCTAC GTGTACGGGA TTGCCGCCGA GTGCGGCTGC ATTGCCGCCG

---------- ---------- ---------- ---------- ----------

GCGCTTCTAC GTGGCCTGGA TTGCCGCCGA GTGCGGCTGC ATTGCCGCCG

GCGCTTCTAC GTGGCCTGGA TTGCAGCTGA GTGCGGCTGC ATTGCCGCTG

GCGCTTCTAC GTGGCCTGGA TTGCGGCCGA GTGCGGTTGC ATTGCCGCGG

GCGCTTCTAC GTTGCCTGGA TTGCGGCCGA GTGCGGTTGC ATTGCCGCGG

GCGCTTCTAC GTGGCCTGGA TCGCCGCCGA GTGCGGCTGC ATTGCCGCCG

---------- ---------- ---------- ---------- ----------

GCGCTTCTAC GTGGCCTGGA TTGCCGCTGA GTGCGGCTGC ATTGCTGCCG

GCGCTTCTAC GTGGCCTGGA TTGCGGCCGA GTGCGGCTGC ATTGCCGCCG

GCGCTTCTAC GTGGCCTGGA TTGCCGCCGA GTGCGGCTGC ATTGCCGCGG

GCGCTTCTAC GTGGCCTGGA TTGCCGCCGA GTGCGGCTGC ATTGCCGCCG

---------- ---------- ---------- ---------- ----------

GCGCTTCTAC GTGGCCTGGA TTGCAGCCGA GTGCGGCTGC ATTGCCGCTG

GCGCTTCTAC GTCGCCTGGA TCGCAGCCGA GTGTGGCTGC ATAGCAGCTG

GCGCTTCTAC GTGGCCTGGA TCATGGCCGA GTGCGGCTGC ATCGCGGCCG

GCTTTGGGGC CTACCCCGTG GCCGCCAAAG CCCGGGCCGG AGGCGGCCCC

---------- ---------- ---------- ---------- ----------

GCTTCGGTTC CTACCCCGTG GCCGCCAAAg cccgggccgg gggcggcccc

---------- ---------- ---------- ---------- ----------

GCTTCGGGGC CTACCCCGTG GCCGCCAAAG CCCGGGCCGG GGGTGGCCCC

GCTTCGGGGC TTACCCCGTG GCCGCCAAAg cccgggccgg gggcggcccc

GCTTCGGGGC CTACCCTGTG GCTGCCAAAG CCCGGGCCGG TGGCGGCCCC

GCTTCGGGGC CTACCCTGTG GCTGCCAAAg cccgggccgg gggcggcccc

GCTTCGGGGC CTACCCCGTG GCCGCCAAAg cccgggccgg gggcggcccc

---------- ---------- ---------- ---------- ----------

GCTTCGGGGC CTACCCTGTG GCCGCCAAgg cccgggccgg gggcggcccc

GCTTCGGGGC CTACCCGGTG GCCGCCAAAg cccgggccgg gggcggcccc

GCTTCGGGGC CTACCCCGTG GCCGCCAAAg cccgggccgg gggcggcccc

GCTTCGGGGC CTACCCCGTG GCCGCCAAAg cccgggccgg gggcggcccc

---------- ---------- ---------- ---------- ----------

GCTTTGGGGC CTACCCGGTG GCCGCCAAAg cccgggccgg gggcggcccc

GCTTCGGGGC CTACCCTGTG GGGGCCAAGT CGAGGGCGGG AGGCGGCCCC

GCTTCGGGGC CTACCCCGTC GGGGCCAAGG CCAGGGCCGG GGGCGGCCCG

ACCCTCCAAT GCCCACCCCC CAGCAGTCCG GAGAAGTCCT TGGAGTATGA

---------- ---------- ------TCCG GAGAAGTCCT TGGAGTATGA

ACCCTCCAAT GCCCACCCCC CAGCAGTCCG GAGAAGTCCC TGGAGTATGA

---------- ---------- ---------- ---------- ----------

ACCCTCCAAT GCCCACCCCC CAGCAGTCCG GAGAAGTCCC TGGAGTATGA

ACCCTCCAAT GCCCACCCCC CAGCAGTCTG GAGAAGTCCT TGGAGTACGA

ACCCTCCAAT GCCCACCCCC TAGCAGTCCG GAGATTTCCC TGGAGTATGA

ACCCTCCAAT GCCCACCCCC TAGCAGTCCG GAGATTTCCC TGGAGTATGA

ACCCTCCAAT GCCCACCCCC CAGCAG---- ---------- ----------

---------- ---------- ---------- ---------- ----------

ACCCTCCAAT GCCCACCCCC CAGCAGTCCG GAGAAGTCCC TGGAGTACGA

ACCCTCCAAT GCCCACCCCC CAGCAGTCCG GAAGAGGCCC TGGAGTACGA

ACCCTCCAAT GCCCACCCCC CAGCAG---- ---------- ----------

ACCCTCCAAT GCCCACCCCC CAGCAGTCTG GAGAAGTCCC TGGAGTACGA

---------- ---------- ---------- ---------- ----------

ACCCTCCAAT GCCCACCCCC CAGCAGTCCG GAGAAAGCCC TGGAGTACGA

ACCCGGCCCT GCCCCGCCCC CGACAGGCCG GGGCAG-CCA CCAAGTATGA

ACCGTCGAAT GTCCTCCCCC CGACAGTCCG GAGCGGGCCG TGGAGTACGA

CTATGAGACC ATCCGCAACA TCGACTGCTA CAGCACAGAT TTCTGCGTGC

CTATGAGACC ATCCGCAACA TCGACTGCTA CAGCACAGAT TTCTGCGTGC

CTATGAGACC ATCCGCAACA TCGACTGCTA CGGCACAGAT TTCTGCGTGC

---------- ---------- ---------- ---------- ----------

CTATGAGACC ATCCGCAACA TCGACTGCTA TGGCACGGAT TTCTGCACGC

CTATGAGACC ATCCGCAACA TCGACTGCTA TGGCACAGAC TTCTGCGTGC

CTATGAGGCC ATCCGTAACA TCGACTGCTA TGGCACAGAC TTCTGCGTGC

CTATGAGACC ATCCGTAACA TCGACTGCTA TGGCACAGAC TTCTGCGTGC

---------- ---------- ---------- ---------- ----------

---------- ---------- ---------- ---------- ----------

CTATGAGACC ATCCGCAACA TCGACTGCTA TGGCACTGAC TTCTGCGTGC

CTATGAGACC ATTCGCAACA TCGACTGCTA CGGCACCGAC TTCTGCGTGC

---------- ---------- ---------- ---------- ----------

CTACGAGACC ATCCGCAACA TCGACTGCTA CGGCACGGAC TTCTGTGTGC

---------- ---------- ---------- ---------- ----------

CTATGAGACC ATCCGCAACA TCGACTGCTA CGGCACAGAC TTCTGCGTGC

CTACGAGACC ATCCGCAACA TCGACTGTCA CGGGACCGAC TTCTGCGTCC

TTACGAGACG ATCCGGAACA TCAACTGCCA CGGCACCGAC TTCTGCGTCC

GGGTGCGCGA TGGCATGCGG TACTGGAACA TGACGGTGCA GTGGTGGCTG

GGGTGCGCGA TGGCATGCGG TACTGGAACA TGACGGTGCA GTGGTGGCTG

GGGTGCGCGA TGGCATGCGG TACTGGAACA TGACGGTGCA GTGGTGGCTG

---------- ---------- ---------- ---------- ----------

GGGTGCGCGA GGGCATGCGG TACTGGAACA TGACGGTGCA GTGGTGGCTG

GGGTGCGGGA TGGCATGCGG TACTGGAACA TGACGGTGCA ATGGTGGCTG

GTGTGCGGGA TGGCATGCGG TACTGGAACA TGACCGTGCA GTGGTGGCTG

GTGTGCGGGA TGGCATGCGA TACTGGAACA TGACCGTGCA GTGGTGGCTG

---------- ---------- ---------- ---------- ----------

---------- ---------- ---------- ---------- ----------

GGGTGCGGGA TGGCATGCGG TACTGGAACA TGACAGTGCA GTGGTGGCTG

GGGTGCGAGA AGGCATGCGG TACTGGAACA TGACGGTGCA GTGGTGGCTG

---------- ---------- ---------- ---------- ----------

GGGTGCGGGA TGGCATGCGC TACTGGAACA TGACGGTGCA GTGGTGGCTG

---------- ---------- ---------- ---------- ----------

GCGTGCGGGA TGGCATGCGG TACTGGAACA TGACGGTGCA GTGGTGGCTG

GGGTCCGAGA CGGGATGCGG TACTGGAACA TGACGGTGCA GTGGTGGCTG

GGGTGCGGGA CGGGATGCGC TACTGGAACA TGACGGTGCA GTGGTGGCTG

GCGCAGTATA TCTACAAGAG CGCACCTGCC CGTTCCTATG TCCTGCGGCT

GCGCAATATA TCTACAAGAG CGCACCTACC C-TTCCTATG TCCCGCG-CT

GCGCAGTATA TCTACAAGAG CGCACCTGCC CGTTCCTATG TCCTGCGGCT

---------- ---------- ---------- ---------- --------CT

GCGCAGTACA TCTACAAGAG CGCACCGACC CGCTCCTATG TGCTGCGGCT

GCACATTATA TCTACAAGAG CGCACCTTTC CGCTCCTATG TTTTAAG-CT

GCACAGTACA TCTACAAGAG TGCACCTTTC CGCTCCTACG TATTGAGGCT

GCACAGTACA TCTACAAGAG CGCACCTTTC CGCTCCTACG TTTTGAGG--

---------- ---------- ---------- ---------- -------GCT

---------- ---------- ---------- ---------- ----------

GCACAGTATA TCTACAAGAG CGCACCTGCC CGCTCCTATG TTCTCAGGCT

GCGCAGTACG TCTACAAGAG CGCGCCCGCC CGCTCCTACG TCCTCAGGCT

---------- ---------- ---------- ---------- -------GCT

GCCCAGTACG TCTACAAGAG CGCGCCCACC CGCTCCTACG TGCTGAGGCT

---------- ---------- ---------- ---------- --------CT

GCGCAGTATA TCTACAAGAG CGCACCCGTG CGCTCCTACG TGCTGAG---

GCCCAATACA TCTACAAGAC GGCACCGGCC CGCTCCTACG TGCTTCGGAT

GCCCAGTACA TCTACAAGAG CGCTCCGGCC CGCTCCTACG TGCTGCGGAT

CCTTGTCCAG CTGGCCACTG CCAGGCCTCG ACCGGTCAAG GCCAGGCCCC

CCTTGTCCAG CTGGCCACTG CCAGGCCTCG ACCGGTCAAG GCCAGGCCCC

CCTTGTCCAG CTGGCCACTG CCAGGCCTCG ACCTGTCAAG GCCAGGCCCC

CCTTGTCCAG CTGGCCACTG CCAGGCCTCG ACCTGTCAAG GCCAGGCCCC

CCTTGTCCAG CTGGCCACTG CAAGGCCTCG ACCTGTCAAG GCCAGGCCCC

ACTTGTTCAG CTGGCCACTG CCAGACCTCG ACCTGTCAAG GCTAGGCCAC

GCTTATCCAG CTGGCCACTG CCAGGCCTCG GCCTGTAAAA GCTAAGCCTC

---------- ---------- ---------- ---------- ----------

GCTCGTCCAG CTGGCCACCG CCAGGCCGCG ACCGGTCAAG GCCAGGCCGC

---------- ---------- ---------- ---------- ----------

GCTGGTCCAG CTGGCCACTG CTAGGCCTCG ACCTATCAAG GCTAGGCCCC

GCTCATCCAG CTGGCTACTG CTAGGCCTCG ACCTGTCAAG GCTAGGCCCT

GCTTGTTCAG CTGGCCACCG CTCGGCCTCG GCCCATCAAA GCTAGGCCCC

TCTTGTTCAG CTGGCCACTG CCAGGCCTCG TCCCATCAAG GCTAGGCCCC

GCTTGTTCAG CTGGCCACTG CCAGGCCTCG GCCCATCAAG GCTAGACCCC

---------- ---------- ---------- ---------- ----------

GCTAGTCCAG CTGCCCACAG CCTGCCCCAG ACCCACCTGG GCCAGGCCCT

GCTGCTGCAG CTGCCCAcgg cccggccccg gccccggctc gccccgcTGC

TGGACTGGCG TGTCCAGTCT AAAGACTGGC CCCACGCCGG CCGCCCTGCC

TGGACTGGCG TGTCCAGTCT AAAGACTGGC CCCACGCTGG CCGCCCTGCC

TGGACTGGCG TGTCCAGTCT AAAGACTGGC CCCACGCCGG CCGCCCTGCC

TGGACTGGCG TGTCCAGTCT AAAGACTGGC CCCACGCCGG CCGCCCTGCC

TGGACTGGCG TGTCCAGTCT AAAGACTGGC CCCACGCCGG CCGCCCTGCC

TGGACTGGCG TGTCCAGTCC AAAGACTGGC CCCACGCTGG CCGGCCTGCT

TGGACTGGCG TGTGCAGTCC AAAGACTGGC CCCATGCTGG CCGTCCTGCT

---------- ---------- ---------- ---------- ----------

TGGACTGGCG CGTGCAGTCC GAGGACTGGC CGCACGCCGG CCGGCCTGCC

---------- ---------- ---------- ---------- ----------

TGGATTGGCG TGTCCAGTCC AAAGACTGGC CGCATGCTGG CCGTCCTGCC

TAGACTGGCG TGTCCAGTCC AAAGACTGGC CCCACGCTGG CCGTCCAGCC

TAGACTGGCG TGTCCAGTCC CAAGACTGGC CTCATGCTGG CCGGCCCGCC

TGGACTGGCG AGTCCAGTCC AAAGACTGGC CCCACGCTGG CCGTCCAGCC

TGGACTGGCG TGTCCAGTCC AAAGACTGGC CCCATGCTGG CCGTCCCACC

---------- ---------- ---------- ---------- ----------

TGGACTGGCA CCAGCCATCA CCAGATTGGC CCTACGCTGG AGACCCAAGG

TGGACTGGCG CGTCCCCTCC CCGGACTGGC CCCACGCCGG CCGTCCCTCC

CACGAGCTGC GCTACAGTAT CTACAGAGAC CTGTGGGAGC GAGGCTTCTT

CACGAGCTGC GCTACAGTAT CTACAGAGAC CTGTGGGAGC GAGGCTTCTT

CATGAGCTGC GCTACAGCAT TTACAGAGAC CTGTGGGAGA GAGGCTTCTT

CATGAGCTGC GCTACAGCAT TTACAGAGAT CTGTGGGAGA GAGGGTTCTT

CATGAGCTGC GCTACAGCAT CTACAGAGAC CTGTGGGAGC GGGGCTTCTT

CATGAGCTGC GCTACAGCGT CTACAGAGAC CTGTGGGAGC GGGGCTTCTT

CATGAGCTTC GCTACAGCAT CTACAGAGAC CTGTGGGAGA GAGGCTTCTT

---------- ---------- ---------- ---------- ----------

CACGAGCTGC GCTACAGCAT CTACAGGGAC CTGTGGGAGC GCGGCTTCTT

---------- ---------- ---------- ---------- ----------

CATGAGCTTC GCTATAGCAT CTACAGAGAC CTGTGGGAAC GAGGCTTCTT

CATGAGCTAC GCTACAGCAT CTACAGAGAC CTGTGGGAAC GAGGCTTCTT

CATGAGCTGC GCTACAGCAT CTACAGAGAC CTGTGGGAGC GAGGCTTCTT

CATGAGCTGC GCTACAGCAT CTACAGAGAC CTGTGGGAGC GAGGCTTCTT

CACGAGCTGC GCTATAGCAT TTACAGAGAC CTATGGGAGC GAGGCTTCTT

---------- ---------- ---------- ---------- ----------

CATGAGCTGC GGTACCGAGT CTACAAGGAC CTGTGGGAGA GGGGTTACTT

CACGAGCTCC GATACCGCGT CTACCGTGAC CTGTGGCAGC GGGGCTACTT

CCTCAGTGCG GCTGGCAAGT TCGGAGGTGA CTTCCTGGTC TATCCTGGTG

CCTCAGTGCG GCTGGCAAGT TCGGAGGTGA CTTCCTGGTC TATCCTGGTG

CCTCAGTGCG GCTGGCAAGT TCGGAGGTGA CTTCCTGGTC TATCCTGGTG

CCTCAGTGCG GCTGGCAAGT TCGGAGGTGA CTTCCTGGTC TATCCTGGTG

CCTCAGCGCG GCTGGGAAGT TTGGAGGTGA CTTCCTGGTC TATCCGGGTG

CCTCAGTGCC GCTGGCAAGT TCGGGGGGGA CTTCTTGGTC TATCCTGGTG

CCTCAGCGCA GCTGGGAAGT TTGGTGGTGA CTTCCTGGTC TATCCTGGTG

---------- ---------- ---------- ---------- ----------

CCTCAGCGCC GCGGGCAAGT TCGGCGGCGA CTTCCTGGTC TACCCAGGCG

---------- ---------- ---------- ---------- ----------

CCTCAGTGCA GCTGGCAAGT TCGGGGGTGA CTTCCTGGTC TATCCTGGTG

CCTCAGTGCT GCTGGCAAGT TCGGGGGCGA CTTCCTGGTC TATCCCGGTG

CCTCAGTGCC GCGGGCAAGT TTGGGGGCGA CTTCCTGGTC TATCCTG---

CTTAAGTGCC GCTGGCAAGT TTGGGGGAGA CTTCCTGGTC TATCCTGGGG

CCTCAGTGCT GCTGGCAAGT TCGGGGGCGA CTTCCTGGTC TATCCTGGTG

---------- ---------- ---------- ---------- -------GTG

CCTCACCTCT GGGGGCAAGT TTGGGGGTGA CTTTCTGGTC TATCCAGGTG

CCTCACGGCC GGGGGCAAGT TCGGCGGGGA CTTCCTGGTC TACCCCGGCG

ACCCCCTCCG CTTCCACGCC CATTATATCG CTCAGTGCTG GGCCCCCGAG

ACCCCCTCCG CTTCCACGCC CATTATATCG CTCAGTGCTG GGCCCCCGAG

ACCCCCTCCG CTTCCATGCC CACTACATCG CTCAGTGCTG GGCCCCGGAG

ACCCCCTCCG CTTCCATGCC CACTACATCG CTCAGTGCTG GGCCCCGGAG

ACCCACTCCG CTTCCACGCC CACTACATCG CTCAGTGCTG GGCCCCCGAG

ACCCGCTCCG CTTCCATGCT CACTATATTG CTCAGTGTTG GGCCCCTGAG

ATCCGCTGCG TTTCCATGCT CACTACATTG CTCAGTGCTG GTCTGCTGAG

---------- ---------- ---------- ---------- ----------

ACCCGCTCCG TTTCCACGCG CACTACATCG CCCAGTGCTG GGCCCCCGAC

---------- ---------- ---------- ---------- ----------

ACCCACTTCG TTTCCACGCC CACTACATTG CTCAGTGCTG GGCTCCTGGG

ACCCGCTCCG TTTCCATGCC CACTACATCG CCCAGTGCTG GGCTCCTGGG

---------- ---------- ---------- ---------- ----------

ATCCGCTGCG TTTCCATGCC CACTACATTG CTCAGTGCTG GGCTGCTGGG

ACCCGCTACG TTTCCATGCT CACTACATTG CTCAGTGCTG GGGTGCTGAG

ATCCGCTCCG TTTCCACGCC CACTACATCG CGCAGTGCTG GGCGGCTGGG

ACCCTCTTCG CTTCCACGCC CACTTTGTGG CTCAGTGCTG CACCCCTGGG

ACCCCCTGCG CTTCCACGCT CACTTCGTCG CCCTGTGCTG GCAGCCCGAG

GACACCATCC CACTCCAAGA CCTGGTTGCT GCTGGGCGCC TTGGAACCAG

GACACCATCC CACTCCAAGA CCTGGTTGCT GCTGGGCGCC TTGGAACCAG

GACCCCATCC CACTCCAGGA CCTGGTTGCT GCTGGGCGCC TTGGAACCAG

GACCCCATCC CACTCCAGGA CCTGGTTGCT GCTGGGCGCC TTGGAACCAG

GACCCCATCC CACTCCAGGA CCTGGTTGCT GCTGGGCGCC TTGGAACCAG

GACCCCATCC CACTCCAGGA CCTGGTCTCT GCTGGCCGCC TTGGGACCAG

GACCCCATCC CACTTCAGGA CCTGGTCTCT GCAGGCCGCC TGGGAACCAG

---------- ---------- ---------- ---------- ----------

GACCCCATCC CCCTGCAGGA CCTGGTCTCT GCCGGCCGCC TGGGCACCAG

---------- ---------- ---------- ---------- ----------

GACCCCATTC CACTCCAGGA CCTGGTTTCT GCTGGCCGCC TGGGGACCAG

GACCCCATCC CACTCCAGGA CCTGGTTTCT GCTGGACGCC TCGGAACCAG

---------- ---------- ---------- ---------- ----------

GACCCGATCC CCCTCCAGGA CCTGGTTTCT GCTGGCCGCC TCGGAACCAG

GACCCAATCC CACTCCAGGA CCTGGTTTCT GCTGGCCGCC TCGGAACCAG

GACTCCATCC CACTCCAGGA CCTGGTCTCA GCCGGCCGCC TCGGAACCAG

GACCCCCTCC CACTGCCAGA CCTTGTCTCT GCTGGCCGCC TGGGGACCAA

GAGCCCCTTC CGCTCCGGGA CCTGGTCTCC GCCGGACGAT TGGGCACCAA

CGTCAGAAAG ACCCTGCTCC TCTGTTCTCC GCAGCCTGAT GGTAAGGTGG

CGTCAGAAAG ACCCTGCTCC TCTGTTCTCC GCAGCCTGAT GGTAAGGTGG

CGTCAGAAAG ACCCTGCTCC TCTGTTCTCC GCAGCCTGAT GGTAAGGTGG

CGTCAGAAAG ACCCTGCTCC TCTGTTCTCC GCAGCCTGAT GGTAAGGTGG

CGTCAGAAAG ACCCTGCTCC TCTGCTCTCC GCAGCCTGAT GGTAAGGTGG

CGTCAGAAAG ACCCTGCTCC TGTGTTCACC GCAGCCTGAT GGTAAGGTGG

TGTCAGAAAG ACTCTGCTTC TCTGCTCCCC TCAGCCTGAT GGGAAGGTGG

---------- ---------- ---------- ---------- ----------

CGTGAGGAAG ACCGTGCTGC TCTGCTCCCC ACAGCCCGGC GGCAAGGTGG

---------- ---------- ---------- ---------- ----------

CGTCAGAAAG ACGCTGCTGC TCTGTTCCCC TCAGCCTGAT GGTAAAGTGG

TGTCAGGAAG ACGCTGCTCC TCTGTTCTCC GCAGCCTGAT GGTAAGGTGG

---------- ---------- ---------- ---------- ----------

CGTCAGAAAG ACACTGCTGC TCTGTTCCCC ACAGCCTGAC GGTAAGGTGG

CGTCAGAAAG ACCCTGCTCC TTTGTTCACC GCAGCCTGAT GGTAAGGTGG

TGTCAGAAAG ACCCTGCTGC TGTGTTCCCC GCAGCCGGAC GGGAAGGTGG

CGTTCGGAAG ACTCTGCTTC TGTGCTCCCC TCTGCCCGAC GGCTCCGTGG

CGTCCGCAAG ACGGTGCTCC TGTGCTCGGC CCGCCCCGAC GGCTCCGTCT

TCTACACCTC CCTGCAATGG GCCAGCCTGC AGGGGAGATA CCGCTGTTGG

TCTACACCTC CCTGCAATGG GCCAGCCTGC AGGGGAGATA CCGCTGTTGG

TCTACACCTC CCTGCAATGG GCCAGCCTGC AGGGGAGATA CCGCTGTTGG

TCTACACCTC CCTGCAATGG GCCAGCCTGC AGGGGAGATA CCGCTGTTGG

TCTACACCTC CCTGCAGTGG GCCAGCCTGC AGGGGCGGTA TCGCTGTTGG

TCTACACCTC CCTGCAGTGG GCCAACCTGC AAATGGGATA TCACTGTTGG

TTTATACTTC CCTGCAGTGG GCCAGCCTGC AGGGATTATA TTACTGCTTG

---------- ---------- ---------- --GGTCAATA TCGATGTTCA

TCTACACCTC CCTGCAGTGG GCCGGCCTGC AGGGGCGCTA CAGCTGTTGG

---------- ---------- ---------- ---------- ----------

TCTACACCTC CCTGCAGTGG GCCAGCCTAC AGGGACAATA TTACTGTTGG

TCTACACCTC CCTTCAGTGG GCCAGCCTGC AGGGGAGATA TCGCTGTTGG

---------- ---------- ---------- --GGGAGATA TCACTGTTGG

TCTACACCTC CCTGCAGTGG GCCAGCCTGC AGGGCAGTTA CCACTGCTGG

TGTACACCTC CCTGCAGTGG GCCAGCCTGC AGGGGCAATA TCACTGTTAT

TGTACACCTC CCTGCAGTGG GCCAGCCTGC AGGGGCGAGC TCACTGTGCG

CCTACACGTC TCTGCAGTGG GCTGGCCTGC CGGAGAGGCT CAGCTT-CAG

GCTACACCTC GCTGGAGTGG GCCGGGCTGG CC-AGGGCTA CCGCTGTTGG

TCACAGCCCA GTGACCCCCT GGAGCTGGTG ATGACAGGAG CCCTGCCGAG

TCACAGCCCA GCAACCCCCT GGAGCTGGTG ATGACAGGAG CCCTGCCGAG

TCAGAGGACA GCGACCCCCT GGACCTGGTG ATGACAGGAG TCCTGCCGAG

TCAGAGGACA GCGACCCCCT GGACCTGGTG ATGACAGGAG TCCTGCCGAG

TCAGAGCCCA GTGACCCCCT GGAGCTGGTG ATGACAGGAG CCCTGCCCAG

TCAAACCACA GTGACACCCT GGAGCTGGTG GTGACAGGAG TGCAGCCAGG

TCAGAATACA GTAACATCTT AGAGATGGTA GTGACAGGAG TGATGCCCAA

TCAGGCTACA GTGACCTCCT GGAGCTGGTG GTGACAGGAG TGAAGCCTAA

TCAGAGCCCA GTGATGCCCT GGAGCTGCGG GTGTCAGGAG CCCAGGCAAG

---------- ---------- ---------- ---------- ----------

TCAGCCTCCA GTGGCCCCCT GGACATCCTG GTCGCAGGAG TGCAGCCAGG

TCAGAGCGCA GTGACCCCCT GGAGCTGGTG GTGACAGGAG CCCTGCCGAG

TCAGAGCCCA GTGA-CCCCT GGACATTCTG GTGGCAGGAG AGCAGCCGGG

TCCCAGCCCA GTGATGCCCT GCAACTGCTG GTGACAG--G CGCTGCCGGG

TCAGCACACA GCGTCATCCT GGAGCTGGTG GTAACAGGAG CCCATCCTGG

TCAGGACACA GTGACCCCCT GGACCTGGTG ATGATAGGAG CCCACCCGGG

ACAGAGCTGG GGGCTCCCTT GAAGCTGCTG CTGCCA-GCG CCCTGCCCAG

TCAACGCCCA GTGATGTCCT GGACCTGGTG CTGATAGGAG CCTGGCCCGG

TCCTCTTGTG ACCTCAGGAA AGAGCGTGAC CCTGCTGTGT CAGTCACGGA

TCCTCTTGTG ACCTCAGGAA AGAGCGTGAC CCTGCTGTGT CAGTCACGGA

TCCTCTTGTG ACCTCAGGAG AGAGCGTGAC CCTGCTGTGT CAGTCACGGA

TCCTCTTGTG ACCTCAGGAG AGAGCGTGAC CCTGCTGTGT CAGTCACAGA

CCCTGTGGTG ACCTCAGGAG GGAACGTGAC CCTCCAGTGT GGCTCACAG-

CCCCATGGTG GCCTCAGGAG AGGATGTGAC CTTTGTGTGC CAGTCACAGA

CTCCACAGTA CACTCAGGAG AGAATGTGAC CCTGATGTGT TGGTCAACGG

CTACACAGTG CACTCAGGAG AGAACGTGAG CCTGCTGTGT TGGTCAATGG

CCCCTCGGTG CCCTGGGGAG TGACGGTGGC CCTGCGCTGT GGCTCTGAGA

---------- ---------- ---------- ---------- ----------

ACCCACTGTG GCCCCAGGAG AGAATGTGAC CCTGCTGTGT CAGTCACTGG

CCCTGTTGTG ACCTCAGGAG GGAACGTGAC CCTCCTGTGT GGCTCACGGT

CCTCATAGTG TCCCCAGGAG AGAATGTTTC CCTGAGGTGT TTGTCAC-AA

TCCCGCGGTG GCGCCCGCGG CCAACGTGAG CCTGCGCTGC G---CGGGCC

CCCCTCAGTG ACCTCAGGAG ATAGCGTGAC CCTGCAGTGT CGCTCAGACT

CCCCTCGGTG GCCCCAGGAC AGAACGTGAC CCTGCAGTGT CAATCAAGTT

CTCTGAGGTG GCCTCAGGAC AGAACGTGAC CCTGCAGTGT CGGTCAGAGT

CCCACTGCTG ATCCCTGGGA CGAATGTGAC GCTCCGGTGC CAGGCGTGGG

TGGACACTTT CCTTCTGATC AAGGAGCGGG CAGCCCATCC CCTACTGCAT

TGGACACTTT CCTTCTGATC AAGGAGCGGG CAGCCCATCC CCTACTGCAT

TGGACACTTT CCTTCTGTTT AAGGAGGGGG CAGCCCATCC CCTGCCGCGT

TGGACACTTT CCTTCTGTTT AAGGAGGGGG CAGCCCATCC CCTGCCGCGT

---------- ---------- ---------- ---------- ----------

CAGACACTTT CTTTCTGGCC AAGGATGGGG CAGTTGATCC CCCACTGAGA

TGGACACTTT CATTCTGTCC AAGGAGGGAT CAGGCCAGCC ACCCCTTCGA

TGGATACTTT CATTCTGTCC AAGGAGGGAT CGGCCCAACA ACCCCTGCGA

TGGACACCTT TCTCCTGCAC AAAGTGGGCT CAGGCACCCC TCCCCAGCGC

---------- ---------- ---------- ---------- ----------

TGGATATTTT CCTTCTGTCC AAGGAGGGGG CAGCCAATCC CCCACTGCGT

TTGATGGGTT CATTCTGACT AGGGAAGGAG AACACGGGCC TCCCTGGACC

GGAAGATTTT CCTTCTGGCC AAGGAGAAGA CAGCTGACAC CCCATTGCCT

GGGGCATCTT CGCGCTGTAC CGCGAGGGCC TGGCGGCGCC GCTGCAGTAT

TTGATACATT CCATCTGTTT AAGGAGGGCT TGATTGCCCC TCCCCAGCAA

TTGACTCCGT CTATTGGTTC AAGGAGGACA TCAATCACCC TCCTCAGCTC

CAGACTGGTG CGTCCTGTGC AAGGAGGGAG AAGATCAGCC GCTGCAGGAC

TGGGCACGTT CTCCCTGCTC CGGGCTCAGA CCTGGGACCT CCTGCAGCGC

CTGCACCAGG CTGAATTCCC CATGAGTCCT GTGACCTCAG GGGGGACCTA

CTGCACCAGG GTGAATTCCC CATGAGTCCT GTGACCTCAG GGGGGACCTA

CTGCACCGGG CTGAATTCCC CATGGGTCCT GCGACCTCAG GGGGGACCTA

CTGCACTGGG CTGAATTCCC CATGGGTCCT GTGACCTCAG GGGGGACCTA

---------- ---------- ---------- ---------- ----------

CTCTACCAGG CTGAATTCTC CATGGGTCCT GTGACCTTAG ----------

CTATACCAGT CAGAATTCTC CATGAGTGGT GTGACATCAA GG--CACCTA

CTATCCCAGG CAGAATTCTC CATGAGTGCT GTGACCTCCT CAGGCACCTA

CTGTGTCAGG CCACCTTCAC GCTGGGTCCT GTGACCTCGG GGGCCACCTA

---------- ---------- ---------- ---------- ----------

CTTTACCAGG CGGAATTTTC CATGAGTCCT GTGACCTCAA GGGGCACCTA

CTCAGCCAGG CCCTGTTCCC TGTGGGCCCC GTGACCCCCA GGTGGTCGTT

ATCACCCGGG TCGAATTCTC TATCCACCCT GTGACCTCAG GTGGCACTCA

C----CTGGG TGGACTTCCC GCTGGGCGCC GACGCCCT-- -GGGCACCTA

CTGTTTCAGA CCAACTTCAC CATGAGTCCT GTGACCTCAG GAGGGACCTA

ATTTTCCAGG CCAACTTCAC CATGAGCGCT GCGACCTCAG GGGGCACCTA

CCG--TCAGG CTGACTTCTT CTTCCCTGCT GTGACTCCGG ATGGCACTTA

C----CCGGG CCGACTTCCT GCTCCCGGCT GTGGGCGCGA GGGCTACCGC

CAGGTGCCTG TCACACCCCA GTGACCCCCT GGAGCTCATA GTCTCAGGAA

CAGGTGCCTG TCACACCCCA GTGACCCCCT GGAGCTCATA GTCTCAGGA-

CAGGTGCCTG TCACGCCCCA GTGACCCTGT GGAGCTCACT GTCTTAGGAA

CAGGTGCCTG TCACGCCCCA GTGACCCCGT GGAGCTCACA GTCTTAGGA-

-------TGG TCACACCCCA GTGACCCCCT GGAGATTCTG AGCTCAGGAA

-------TTG TCACTGCCCA GTCACCCCCA GGAGCTCATG GTCTTAGGAA

CAGGTGCCTG TCATTTGCCA GTGCCCCTGT GGAGCTCATA GTCTCAGGAA

CAGGTGCTTG TCATCTGCCA GTGCCCCTGT GGAGCTCACA GTCTCAGGAA

CAGGTGCTGG TCATGGCCCA GGGACCCCCT GCAGCTCGAG GTGTCAGGAA

---------- ---------- ---------- ---------- ----------

CAGGTGCTTA TCACACCCAA GTGTCCCCGT GAAGATCCTG GTCTCAGGAA

CAGATGCTGG TCACACCCCA GTGACCTCCT GGAGCTCCTG GTCTCAGGA-

CC-GTGCCTG TCACAGTCCA ATGTCCCCCT GCAGCGCTGG G--TCAG-AA

CAGCTGCCTG TCGCAGCGCA GCGAGCCGCT GGTCATCAGC TGGGAAG--A

CAGGTGTTTC TCACAACCCA GTGACCCCCT GGAGCTGGTG GTCTCAGT-A

CAGATGCTTG TCCCAGCCCA GTGACCCTCT GGAGCTGGTG CTCTCAG--A

CCGATGCTGG TCCTCCCCCA GCGCCCCCCT GCTGCTCCGG GTCTCAG--A

TGTGTG---G TCAACGCCCA GTGATGTCCT GGACCTGGTG CTGATAG--A

TGGCTGCGAG AGTCGGCGCC TTCCTCAAGA ATGCCTGGGA CAAGGAGCCA

---------G AGTCGGCGCC TTCCTCAAGA ATGCCTGGGA CAAGGAGCCA

TGGCTGCGA- ---------- ---------- ---------- ----------

---------- ---------- ---------- ---------- ----------

TGGCCGCGAG ACTCGGCGCC TTCCTCAAGA ATGCTTGGGC CAAGGAGCCA

TGGCTGCTAG ACTCGCCGCC TTCCTCAAGA ATGCTTGGGC CAACGAGCCA

TGGCTGCTAG AATCTCCGCC TTCCTTAAGA ATGCCTGGGC AAAGGAGCCG

TGGCCGGGAG AATCTCTGCC TTCCTCAAGA ATGCCTGGGC GAAGGAGCCG

TGGCTGGGAC CGGCTCCGCC CTCCTC---- --TCCCAGGG CCAGGACCCA

---------- ---------- ---------- ---------- ----------

TGGCCGGGAG ACTCGCCGCC TTCCTCAAGG ATGCCTGGGC CAAAGAGCCG

---------- ---------- ---------- ---------- ----------

TGGCCGGGA- ---------- ---------- ---------- ----------

TGGCTGCGAA AGCCGCCGCC TTCCTCAAGA ATGCCTGGGC CAAGGAGCCG

TGGCGGCTAG ACTCGCCGCC TACCTCAAGA ATGCCTGGGG CAAGGAGCCG

TGGCGACGAA ATTCACCACC TTCCTCAAGA ATGCCTGGGC CAAGGAGCCG

TGGCAGGCAG AATCTTCGGG TTTCTGAAGG AGGCCTGGGC CAAGGAACCC

TGGCGGCGAG GTTCGCCAAC TTCCTGAAGG AGTCCTGGGC CAAGGAGCCG

GTGCTGGTCG TGTCCTTCGT CGTCGGGGGC CTCGCTATGT CTCTGGCAGA

GTGCTGGTCG TGTCCTTCGT CGTCGGGGGC CTCGCTATGT CTCTGGCAGA

---------- ---------- ---------- ------ATGT CTCTGGCAGA

---------- ---------- ---------- ------ATGT CTCTGGCAGA

GTACTGGTCG TGTCCTTCTT CATCGGGACC CTCGCTATGT CTCTAGCAGA

GTGCTGGTGG TGTCCTTCAC CGTCGCGGGC CTCGCTATGT CTCTAGCAGA

GTGCTGGTGG TGTCCTTCAC TGTCTGGGGC CTCGCTATGT CGCTGGCAGA

GTGCTGGTGG TGTCCTTCTC TGTCTGGGGC CTCGCTATGT CTCTGGCTGA

GCAC------ ---------- ---------- ---------- ----------

---------- ---------- ---------- ---------- ----------

GTGCTGGTCG CGTCCTTCAC CATCGGGGGC CTCGCTATGT CTCTGGCAGA

---------- ---------- ---------- ---------- ----------

---------- ---------- ---------- ---------- ----------

GTGCTGGTTG CGTCCTTCAC CATCGCGGGC CTCGCTATGT CTCTGGCAGA

GTGCTGGTCG CATCCTTCAC CATCACGGGA CTCG--ATGT CTCTGGCAGA

GTGCTGGTCG CGTCCTTCAC CATCGCTGGC CTCG------ ----------

GTGCTGACCG TGTCCTTCGC CATCGGCTCC TTGGCTATGT CGCTTGCGGA

GTCCTTACCG TGTCGGCGGT CATCGGAAGC CTGG--ATGT CCCTGGCAGA

TGAGCTCTTA GCTGATCTCg aagaggcagc agaagaggag gaaggaggaa

TGAGCTCTTA GCTGATCTCg aagaggcagc agaagaggag gaaggaggaa

TGAGCTCTTA GCTGATCTCg aagaggcagc agaagaggag gaaggaggaa

CGAGCTCTTA GCTGATCTCg aagaggcagc agaagaggag gaaggaggaa

TGAGCTCTTA GCTGATCTCg aagaggcagc agaagaggag gaaggaggaa

TGAGCTCTTA GCTGACCTag aagaggcagc agaagaggaa gaaggaggaa

TGAGCTCTTG GCTGACCTTg aggaggcagc agaagaagaa gaaggaggaa

TGAGCTCTTG GCTGACCTCG AGGAGGCAGC AGAAGAAGAG GAAGGAGGAA

---------- ---------- ---------- ---------- ----------

---------- ---------- ---------- ---------- ----------

TGAGCTTCTG GCCGACCTTg aggaggcggc agaggaggaa gaaggaggaa

---------- ---------- ---------- ---------- ----------

---------- ---------- ---------- ---------- ----------

TGAGCTCTTG GCTGACCTCg aggaggcggc tgaagaggag gaaggaggaa

CGAGCTCTTG GCTGACCTCg aggaggcggc tgaagaggag gaaggaggaa

---------- ---------- ---------- ---------- ----------

TGAGCTCCTG GCTGACCTGG AGGAGGCAGC TGAGGAGGAA GAGGGTGGGA

TGAGCTCCTG GCAGACCTgg aggaggcggc ggaggaggag gagggaggca

gctatgggga ggaagaagag gagccagcga tcgaggatgt gcaggaggag

gctatgggga ggaagaagag gagccagcga tcgaggatgt gcaggaggag

gctatgggga ggaagaggag gagCCAGCCA TCGAGGATGT GCAGGAGGAG

gctatgggga ggaagaggag gagCCAGCCA TCGAGGATGT GCAGGAGGAG

gctatgggga ggaggaagag gagccagcga tcgaggatgt gcaggaggag

gctatgggga ggaagaagag gaGCCAGCGA TTGAGGATGT TCAGGAAGAG

gctatggaga agaagaggag gaGCCAGCAA TTGAGGATGT ACAAGAGGAG

GCTATGGAGA AGAGGAGGAG GAGCCAGCAA TTGAGGATGT ACAGGAGGAG

---------- ---------- ---------- ---------- ----------

---------- ---------- ---------- ---------- ----------

gctatgggga ggaagaagag gagccagcaa tcgaggatgt gcaggaggag

---------- ---------- ---------- ---------- ----------

---------- ---------- ---------- ---------- ----------

gctatgggga ggaagaagag gagccagcga tcgaggatgt gcaggaggaA

gctatgggga ggaagaagag gagCCAGCAA TTGAGGATGT GCAGGAGGAA

---------- ---------- ---------- ---------- ----------

GCTTTGGGGA GGAGGAAGAA GAACCTCCCA TTGAAGATGT CCAGGAGGAG

gctacgggga ggaagaagag gaACCGGCCA TCGAGGATGT GCAGGAGGAA

ACACAGCTGG ATCTTTCCGG GGATTCAGTC AAGACCATCG CCAAGCTATG

ACACAGCTGG ATCTTTCCGG GGATTCAGTC AAGACCATCG CCAAGCTATG

ACACAGCTGG ATCTTTCCGG GGATTCGGTC AAGACCATCG CCAAGCTATG

ACACAGCTGG ATCTTTCCGG GGATTCGGTC AAGACCATCG CCAAGCTATG

ACACAGCTGG ATCTTTCCGG GGATTCGGTC AAGACCATCG CCAAGCTATG

ACACAACTGG ATCTTTCTGG GGATTCAGTC AAGAGCATCG CCAAGCTATG

ACACAACTGG ATCTTTCTGG AGATTCAGTC AAGAGCATTG CCAAGCTATG

ACCCAGCTGG ATCTTTCTGG AGATTCAGTC AAGAGCATTG CCAAGCTATG

---------- ---------- ---------- ---------- ---------G

---------- ---------- ---------- ---------- ----------

ACGCAGCTGG ATCTTTCTGG GGATTCAGTC AAGAGTATCG CCAAGCTGTG

---------- ---------- ---------- ---------- ----------

---------- ---------- ---------- ---------- ----------

ACACAGCTGG ATCTTTCTGG GGATTCGGTC AAGAGCATTG CCAAACTATG

ACACAGCTGG ACCTTTCTGG GGACTCAGTC AAGAGCATCG CCAAGCTATG

---------- ---------- ---------- ---------- ----------

ATGCAGCTGG ACCTCTCCGG GGACTCGGTC AAGAGCATTG CCAAGTTGTG

ATGCAGCTGG ACCTGAGCGG GGACTCGGTC AAGAGCATCG CCAAGCTGTG

GGATAGTAAG ATGTTTGCTG AGATTATGAT GAAGATTGAG GAGTATATCA

GGACAGTAAG ATGTTTGCTG AGATTATGAT GAAGATTGAG GAGTATATCA

GGACAGTAAG ATGTTTGCTG AGATTATGAT GAAGATTGAG GAGTATATCA

GGACAGTAAG ATGTTTGCTG AGATTATGAT GAAGATTGAG GAGTATATCA

GGACAGTAAG ATGTTTGCTG AGATCATGAT GAAGATTGAG GAGTATATCA

GGACAGCAAG ATGTTTGCTG AGATTATGAT GAAGATTGAG GAGTATATCA

GGATAGCAAG ATGTTTGCTG AGATCATGAT GAAGATTGAG GAGTATATCA

GGACAGCAAG ATGTTTGCTG AGATCATGAT GAAGATTGAG GAGTATATCA

GGACAGCAAG ATGTTCGCGG AGATCATGAT GAAGATCGAG GAGTACATCA

---------- ---------- ---------- ---------- ----------

GGACAGCAAG ATGTTTGCTG AGATCATGAT GAAGATTGAG GAGTATATCA

---------- ---------- ---------- ---------- ----------

---------- ---------- ---------- ---------- ----------

GGACAGCAAG ATGTTTGCTG AAATTATGAT GAAGATTGAG GAGTATATCA

GGACAGCAAG ATGTTTGCTG AGATCATGAT GAAGATCGAG GAGTATATAA

---------- ---------- ---------- ---------- ----------

GGACAGCAAG ATGTTTGCTG AGATCATGGT GAAGATTGAA GAGTATGTCA

GGACAGCAAG ATGTTCGCGG AGATCATGGT GAAGATCGAA GAGTACATCA

GCAAGCAAGC CAAAGCTTCA GAAGTGATGG GACCAGTGGA GGCCGCGCCT

GCAAGCAAGC CAAAGCTTCA GAAGTGATGG GACCAGTGGA GGCAGCACCT

GCAAGCAAGC CAAAGCTTCA GAAGTGATGG GACCGGTGGA GGCAGCGCCT

GCAAGCAAGC CAAAGCTTCA GAAGTGATGG GACCGGTGGA GGCAGCGCCT

GCAAGCAAGC CAAAGCTTCC GAAGTGATGG GGCCAGTGGA GGCAGCCCCT

GCAAGCAAGC CAAAGCTTCA GAAGTGATGG GGCCAGTGGA GGCAGCCCCT

GCAAGCAAGC CAAAGCTTCA GAAGTGATGG GACCGGTGGA GGCAGCCCCT

GCAAGCAAGC CAACGTGTCG GAAGTGATGG GACCAGTGGA GGCAGCTCCT

GCAAGCAGGC GAAGGCGGCG GAAGTGATGG GACCGGTGGA GGCGGCCCCT

---------- ---------- ---------- ---------- ----------

GCAAGCAGGC CAAGGCCTCG GAAGTGATGG GACCTGTTGA GGCGGCCCCT

---------- ---------- ---------- ---------- ----------

---------- ---------- ----TGATGG GGCCGGTGGA GGCGGCCCCC

GCAAGCAAGC CAAAGCTTCA GAAGTGATGG GCCCGGTGGA GGCGGCCCCT

GCAAGCAAGC CAAAGCTTCA GAAGTGATGG GCCCAGTAGA GGCGGCCCCT

---------- ---------- ----TGATGG GCCCGGTAGA GGCGGCCCCT

GTAAACAGGC CAAGGCTTCT GAAGTGACGG GGCCAGTGGA GGCAGCCCCT

GTAAGCAAGC CAAGGCTTCC GAAGTGCTGG GCCCTGTGGA AGCCGCCCCT

GAATACCGCG TCATCGTGGA TGCCAACAAC CTGACCGTGG AGATCGAAAA

GAATACCGCG TCATCGTGGA TGCCAACAAC CTGACCGTGG AGATCGAAAA

GAATACCGCG TCATCGTGGA TGCCAACAAC CTGACCGTGG AGATCGAAAA

GAATACCGCG TCATCGTGGA TGCCAACAAC CTGACCGTGG AGATCGAAAA

GAATACCGCG TCATTGTGGA TGCCAACAAC CTGACCGTGG AGATCGAAAA

GAGTACCGGG TCATTGTGGA TGCCAACAAC CTGACTGTGG AGATCGAGAA

GAGTACCGAG TCATTGTGGA TGCCAACAAC CTGACTGTGG AGATAGAAAA

GAGTACCGAG TCATTGTGGA TGCCAACAAC CTGACTGTGG AGATAGAAAA

GAGTACCGTG TCATCGTGGA CGCCAACAAC CTGACCGTGG AGATCGAGAA

---------- ---------- ---------- ---------- ----------

GAATACCGAG TCATCGTGGA TGCTAACAAC CTGACCGTGG AGATCGAGAA

---------- ---------- ---------- ---------- ----------

GAGTACCGGG TCATCGTGGA CGCCAACAAC CTGACGGTGG AGATCGAGAA

GAGTACCGCG TCATCGTGGA TGCCAACAAC CTGACCGTGG AGATTGAGAA

GAATACCGGG TCATCGTGGA CGCCAACAAC CTGACCGT-G AGATCGAGAA

GAATACCGGG TCATCGTGGA TGCCAACAAC CTGACCGTGG AGATCGAGAA

GAGTATCGGG TCATCGTGGA TGCCAACAAC CTGACTGTGG AGATTGAgaa

GAATATCGAG TCATCGTGGA CGCCAACAAC CTGACCGTGG AGATTGAGAA

CGAGCTGAAC GGTGTGGCCG GCGGCCTGAC CCTGCCACCG ATCGAGGAGG

TGAGCTGAAC GGTGTGGCCG GCGGCCTGAC GCTGCCACCG ATCGAGGAGG

CGAGCTAAAC GGTGTGGCCG GCGGCCTGAC CCTGCCCCCG ATTGAGGAGG

CGAGCTGAAC GGCGTGGCCG GCGGCCTGAC CCTGCCCCCG ----------

CGAGCTGAAC GGTGTGGCCG GCGGCCTGAC CCTGCCCCCG ATCGAGGAGG

TGAGCTGAAC GGTGTGGCTG GCGGCCTGAC CCTGCCCCCG ATTGAGGAGG

CGAGCTGAAC GGTGTGGCTG GAGGGCTGAC CCTGCCCCCA ATTGAGGAGG

TGAGCTGAAC GGTGTGGCTG GAGGGCTCAC CCTGCCCCCA ATTGAGGAGG

CGAGCTGAAC G--------- ---------- ---------- ----------

---------- ---------- ---------- ---------- ----------

TGAGCTGAAC GGGGTGGCTG GGGGCCTGAC CCTGCCCCCG ATCGAGGAAG

---------- ---------- ---------- ---------- ----------

TGAGTTGAAC GGGGTGGCCG GCGGCCTCAC CCTGCCCCCG ----------

CGAACTGAAC GGGGTGGCCG GCGGCCTGAC CCTGCCTCCG ATCGAGGAGG

TGAACTGAAC GGAGTAGCTG GAGACCTGAC CCTGCCCCCA ----------

CGAGCTTAAC GGCGTGGCTG GGGGCCTGAC CCTGCCCCCG ATCGAGGAGG

tgagctgaAT GGGGTGGGGT GGGGGTTAA- -CCGCCGCCC ATTGAGGAGG

TGAGCTGA-- -GCGTGGCAG GGGGCCTGAC CCTGCCCCCG ATCGAGGAGG

ACGCCTACCA GGAGGACCTG GGATTCAGCC TGGGCCACCT GGGCAAGTCG

ACGCCTACCA GGAGGACCTG GGATTCAGCC TGGGCCACCT GGGCAAGTCG

ACGCCTACCA GGAGGACCTG GGATTCAGCC TGGGCCACCT GGGCAAGTCG

---------- ---------- ---------- ---------- ----------

ACGCCTACCA GGAGGACCTG GGATTCAGCC TGGGCCACCT GGGCAAGTCG

ATGCCTACCA GGAGGACCTG GGCTTCAGTC TGGGCCACCT GGGCAAGTCA

ATGCCTATCA AGAAGACCTG GGCTTTAGCC TGGGCCACTT GGGCAAGTCA

ATGCCTATCA GGAAGACCTG GGCTTTAGCC TGGGCCACTT GGGCAAGTCA

---------- ---------- ---------- ---------- ----------

---------- ---------- ---------- ---------- ----------

ACGCCTACCA AGAGGACCTG GGCTTCAGCC TGGGCCATCT GGGCAAATCA

---------- ---------- ---------- ---------- ----------

---------- ---------- ---------- ---------- ----------

ACGCCTACCA GGAGGACCTG GGCTTCAGCC TGGGCCACCT GGGCAAATCA

---------- ---------- ---------- ---------- ----------

ACGCCTACCA GGAGGACCTG GGCTTCAGCC TGGGGCACCT GGGCAAGTCG

ACGCCTACCA GGAGGATTTG GGCTTCAGCC TGGGCCACCT GGGCAAGTCG

ACGCCTACCA GGAGGACCTC GGCTTCAGCC TGGGCCACCT GGGCAAGTCG

GGCAGTGGGC GTGTGCGGCA GACACAGGTA AACGAGGCCA CCAAGGCCAG

GGCAGTGGGC GTGTGCGGCA GACGCAGGTA AACGAGGCCA CCAAGGCCAG

GGCAGTGGGC GCGTGCGGCA GACGCAGGTC AACGAGGCCA CCAAGGCGAG

---------- ---------- ---------- ---------- ----------

GGCAGTGGGC GCGTGCGGCA GACACAGGTC AACGAGGCCA CCAAGGCCAG

GGGAGTGGGC GTGTGCGGCA GACACAGGTG AACGAGGCCA CCAAGGCCAG

GGCAGTGGGC GTGTGCGGCA GACACAGGTG AATGAGGCCA CCAAGGCTAG

GGCAGTGGGC GTGTGCGGCA GACCCAGGTG AATGAGGCCA CCAAGGCCAG

---------- ---------- ---------- ---------- ----------

---------- ---------- ---------- ---------- ----------

GGCAGCGGGC GGGTACGGCA GACACAGGTG AACGAGGCCA CCAAAGCCAG

---------- ---------- ---------- ---------- ----------

---------- ---------- ---------- ---------- ----------

GGCAGTGGGC GTGTCCGGCA GACGCAAGTG AATGAGGCCA CCAAAGCCAG

---------- ---------- ---------- ---------- ----------

GGCAGCGGGC GAGTGCGGCA GACGCAGGTG AACGAGGCCA CCAAAGCCAG

GGCAGCGGGC GCGTGCGGCA GACCCAAGTC AACGAAGCCA CCAAGGCCCG

GGGAGCGGGC GGGTGCGGCA GACACAGGTC AACGAGGCCA CTAAAGCCCG

GATCTCCAAG ACGCTGCAGG GCCTGGAGAT TGTGAACCCA CAGGCGGCAG

GATCTCCAAG ACGCTGCAGG GCCTGGAGAT TGTGAACCCA CAGGCGGCAG

GATCTCCAAG ACGCTGCAGG GCCTGGAGAT TGTGAACCCG CAGGCGGCAG

---------- ---------- ---------- ---------- ----------

GATCTCCAAG ACGCTGCAGG GCCTGGAGAT TGTGAACCCA CAGGCCGCGG

GATCTCTAAG ACATTGCAGG GCTTGGAGAT TGTGAACCCA CAAGCAGCAG

GATCTCCAAG ACACTACAGG GTCTGGAGAT TGTGAACCCA CAAGCAGCCG

GATCTCCAAG ACACTGCAGG GTCTAGAGAT TGTGAACCCA CAAGCAGCCG

---------- ---------G GCCTGGAGAT CGTGAACCCA CAGGCAGCCG

---------- ---------- ---------- ---------- ----------

GATCTCTAAG ACACTGCAGG GCCTAGAGAT TGTGAACCCA CAAGCAGCTG

---------- ---------- ---------- ---------- ----------

---------- ---------- ---------- ---------- ----------

GATCTCCAAG ACGCTACAGG GCCTGGAGAT CGTGAACCCA CAAGCTGCCG

---------- ---------G GCTTGGAGAT TGTGAACCCA CAGGCGGCCG

GATCTCCAAG ACGCTGCAGG GCCTGGAGAT CGTAAACCCG CAGGCGGCGG

CATCTCCAAG ACGCTGCAGG GCTTGGAGAT CGTGAACCCA CAGGCGGCTG

GATCTCCAAG ACCTTACAGG GCTTGGAGAT CGTGAATCCG CAGGCAGCGG

AGAAGAAGGT GGCTGAGGCC AACCAGAAGT ATTTCTCCAG CATGGCTGAG

AGAAGAAGGT GGCTGAGGCC AACCAGAAGT ATTTCTCCAG CATGGCTGAG

AGAAGAAGGT GGCTGAGGCC AACCAGAAGT ATTTCTCTAG CATGGCTGAG

---------- ---------- ---------- ---------- ----------

AGAAGAAGGT GGCTGAGGCC AATCAGAAGT ATTTCTCCAG CATGGCAGAG

AGAAAAAGGT GGCTGAGGCC AACCAGAAGT ACTTCTCCAG CATGGCCGAG

AGAAGAAGGT GGCAGAGGCC AATCAGAAGT ATTTCTCTAG CATGGCCGAG

AGAAGAAGGT GGCAGAGGCC AACCAGAAGT ATTTCTCTAG CATGGCCGAG

AGAAGAAGGT GGCGGAGGCC AACCAGAAGT ACTTCTCCAG CATGGCCGAG

---------- ---------- ---------- ---------- ----------

AGAAGAAGGT GGCTGAAGCC AACCAGAAGT ATTTCTCCAG CATGGCCGAG

---------- ---------- ---------- ---------- ----------

---------- ---------- ---------- ---------- ----------

AGAAGAAGGT GGCCGAGGCC AACCAGAAGT ACTTCTCCAG CATGGCCGAG

AGAAGAAGGT GGCTGAGGCC AACCAGAAGT ACTTCTCCAG CATGGCCGAG

AGAAGAAGGT GGCCGAGGCT AACCAGAAGT ACTTCTCCAG CATGGCCGAG

AGAAGAAGGT GGCTGAGGCC AACCAGAAGT ACTTTTCCAG CATGGCCGAG

AGAAGAAGGT AGCCGAGGCC AATCAGAAAT ACTTCTCCAG CATGGCCGAG

TTCCTCAAGG TCAAGGGCGA GAAGAGTGGC CTTATGTCCA CCAGGTTCCT

TTCCTCAAGG TCAAGGGCGA GAAGAGTGGC CTTATGTCTA CCAGGTTCCT

TTCCTCAAGG TCAAGGGCGA GAAGAGTGGC CTCATGTCCA CCAGGTTCCT

---------- ---------- ---------- ---------- --AG------

TTCCTCAAGG TCAAGGGCGA GAAGAGTGGC CTCATGTCTA CCAGGTTCCT

TTCCTCAAGG TCAAGGGTGA GAAGAGTGGC ATCATGTCCA CCAGGTTCCT

TTCCTCAAGG TCAAGGGTGA GAAAAGTGGC ACCATGTCTA CTAGATTCCT

TTCCTCAAGG TCAAGGGTGA GAAAAGTGGC ACCATGTCTA CCAGGTTCCT

TTCCTCAAGG TCAAGGGCGA CAAGAGCGGC ATCATGTCCA CCAG------

---------- ---------- ---------- ---------- ----------

TTCCTCAAGG TCAAGGGCGA GAAAAGCGGC ATCATGTCCA CCAGGTTCCT

---------- ---------- ---------- ---------- ----------

---------- ---------- ---------- ---------- ----------

TTCCTCAAGG TCAAGGGCGA GAAGAGCGGC CTCATGTCCA CCAGGTTCCT

TTCCTCAAGG TCAAGAGCGA GAAGAGTGGC ATCATGTCTA -T--GTTCCT

TTCCTCAAGG TGAAGAGCGA GAAGAGCGGC ATCATGTCCA CC--------

TTCCTCAAGG TCAAGGGGGA GAAGAGTGGG GTGCTGTCCA CGAGGTTTCT

TTCCTTAAAG TCAAGGGCGA GAAGAGCGGC ATCATGTCCA CCAGGTTCCT

CATGAGAGTG CTGGACTCCT ACGGGGATGA CTACCGGGCC AGCCAGTTCA

CATGAGAGTG CTGGACTCCT ACGGGGATGA CTACCGGGCC AGCCAGTTCA

CATGAGAGTG CTGGACTCCT ATGGGGATGA CTACCGGGCC AGCCAGTTCA

---------- ---------- ---------- ---------- ----------

CATGAGAGTG CTGGACTCCT ACGGGGATGA CTACCGGGCC AGCCAGTTCA

CATGAGAGTG CTCGACTCCT ATGGGGATGA TTACCGGGCC AGCCAGTTCA

CATGCGAGTG CTGGACTCAT ACGGGGATGA CTACCGGGAT AGCCAGTTCA

CATGAGGGTG CTGGACTCCT ACGGGGATGA CTACCGAGAT AGCCAGTTCA

---------- ---------- ---------- ---------- ----------

---------- ---------- ---------- ---------- ----------

CATGAGAGTG CTGGATGCTT ATGGCGATGA CTACCGTGCC AGCCAGCTCA

---------- ---------- ---------- ---------- ----------

---------- ---------- ---------- ---------- ----------

CATGAGAGTG CTGGACTCTT ACGGGGACGA CTACCGAACC AGCCAGCTCA

CATGCGAGTG CTGGACTCCT ACGGGGATGA CTACCGGGCC GGCCAGTTCA

---------- ---------- ---------- ---------- ----------

CATGCGAGTC CTGGATTCCT ATGGAGATGA CTACAGGAAG AGCCAGTTCA

CATGAGAGTC CTCGACTCTT ATGGTGACGA CTACAGGAAA AGTCAGTTCA

CCATTGTGCT GGAGGATGAG GGCAGCCAGG GCACGGATGC CCCCACCCCA

CCATTGTGCT GGAGGATGAG GGCAGCCAGG GCACGGATGC CCCCACCCCA

CCATTGTGCT AGAGGATGAG GGCAGCCAGG GCACGGATGC TCCCACCCCA

---------- ---------- ---------- ---------- ----------

CCATTGTGCT GGAGGATGAG GGCAGCCAGG GCACAGATGC TCCCACCCCA

CCATCATGCT GGAGGATGAG GGCAGCCAAG GCACAGATGC CCCCACCCCA

CCATTGTGTT GGAGGATGAT GGCAGCCAAG GCACTGATGT CCCTACCCCA

CCATTGTGTT GGAGGATGAT GGCAGCCAAG GCACAGATGT CCCTACCCCA

---------- ---------- ---------- ---------- ----------

---------- ---------- ---------- ---------- ----------

CCATAGTCCT GGAGGACGAG GGCAGCCAGG GCACAGATGC TCCCACTCCC

---------- ---------- ---------- ---------- ----------

---------- ---------- ---------- ---------- ----------

CCATTGTGCT GGAGGATGAG GGCAGCCAGG GCACAGATGC CCCCACCCCA

CTATCGTGCT GGAGGACGAG GGCAGCCAGG GCACTGATGC CCCCACCCCA

---------- ---------- ---------- ---------- ----------

CTATTGTCCT GGAGGATGAG GGGAGCCAAG GCCCTGATGC CCCCACCCCG

CCATCGTCCT GGAGGATGAG AGCAGTCACG GTCCTGACGC CCCAACCCCA

GGCAATGCGG AGAATGAGCC TCCAGAGAAA GAGACACTGT CCCCGCCCAG

GGCAATGCTG AGAATGAGCC TCCAGAGAAA GAGACATTGT CCCCGCCCAG

GGCAATGCTG AGAATGAGCC TCCAGAGAAA GAGGCACTGT CCCCGCCCAG

---------- ---------- -----AGAAA GAGGCACTGT CCCCGCCCAG

GGTAATGCTG AGAATGAGCC TCCAGAGAAA GAGGCACTGT CCCCGCCGAG

GGCAATGCCG AGAATGAACC CCCAGAGAAA GAGGGGCTGT CCCCACCCAG

GGCAATGTTG AGAATGAACC TCCAGAGAAA GAGGGACTTT CCCCACCACA

GGCAATGCTG AGAATGAACC TCCAGAGAAA GAGGGGCTTT CCCCATCCCA

---------- ---------- ---------- ---------- ----------

---------- ---------- ---------- ---------- ----------

GGCAATGCTG AGAATGAGCC CCCAGAGAAA GAGGGGCTGT CACCACCCCG

---------- ---------- ---------- ---------- ----------

---------- ---------- ---------- ---------- ----------

GGCAATGCTG AGAATGAGCC TCCAGAGAAA GAAGGGCTGT CCCCACCCAG

GGCAATGCTG AGAACGAGCC TCCTGAGAAA GAGGGACTGT CCCCGCCCAG

---------- ---------- ---------- ---------- ----------

GGAAATGCAG AGAATGAGCC CCCTGAAAAG GAAAGTCTGT CACCTACCCC

GGCAACGCAG AGAATGAGCC GCCTGAGCGG GAGGGGCTGT CCCCGCCGCG

AAGGCCTGCA CCCCCAGAAC CCGGCAGCCC AGCCCCCGGT GAGGGGCCCA

AAGGCCTGCA CCCCCAGAAC CCGGCAGCCC AGCCCCCGGC GAGGGGCCCA

AAGGCCTGCA CCCCCAGAAC CTGGCAGCCC AGCGCCCGGC GAGGGGCCCA

AAGGCCTGCA CCCCCGGAAC CTGGCAGCCC AGCGCCCGGC GAGGGGCCCA

AAGGCCTGCA CCCCCAGAAC CTGGCAGCCC AGTCCCCGTC GAGGGGCCCA

AAGGCCGGTA CCCCCAGAAC CCAGCAGCCC AGCCCCAGGC GAGGGGCCCA

AAGGACTGCA ACCCTAGACC CCAGCAGCCC AGCCCCTGGC GAGGGGCCCA

AAGGACTGCA ACCTTAGACC CCACCAGCCC AGCCCCTGGC GAGGGGCCCA

---------- ---------- ---------- ---------- ----------

---------- ---------- ---------- ---------- ----------

AAGGCCCGCA CCTCCAGAAC CCAGCAGCCC AACCCCCGGG GAGGGGCCCA

---------- ---------- ---------- ---------- ----------

---------- ---------- ---------- ---------- ----------

ACGGCCTGCA CCCCCAGAAT CCAGCAGCCC GGTCCCTGGC GAGGGGCCCA

AAGGCCGGTA CCCCCCGAGC CCAGCAGCCC GGCTGCTGGG GAGGGGCCCA

---------- ---------- ---------- ---------- ----------

ACGG-CGGC- -CTCTTGAGC CCAGCAGTCC TCCCCCTGGG GAAGGTCCCA

GAGACCCGGA CCCCTGGAAC CCAGTAGCCC TACTCCAGGG GAGGGCCCCA

GTGGGCGGAA GAGGCGGCGA GTGCCACGGG CAGGAAATGC GCTGACTCCA

GTGGGCGGAA GAGGCGGCGA GTGCCACGGG CAGGAAATGC ACTGACTCCA

GTGGGCGGAA GAGGCGGCGA GTGCCGCGGG CAGGGACTGC GCTGACTCCA

GTGGGCGGAA GAGGCGGCGA GTGCCGCGGG TGGGAACTGC GCTTACTCCA

GTGGGCGGAA GAGGAGGCGA GTACCACGGG CAGGAACTGC GCTGACTCCA

GTGGGCGGAA GAGGCGGCGA GTACAACGGG CAGGAGCTGC ACTGACTCCA

GTGGGCGGAA GAGGCGGCGG GCACCACGAG CAGCATCTTC ATTGACTCCA

GTGGGCGGAA GAGGCGGCGG GCGCCACGGG TGGGGGCTTC ATTGACTCCA

---------- ---------- ---------- ---------- ----------

---------- ---------- ---------- ---------- ----------

GTGGACGCAA GAGGCGGCGA GCACCCCGGG CGGGAGCTGT GCTCACTCCA

---------- ---------- ---------- ---------- ----------

---------- ---------- ---------- ---------- ----------

GTGGGCGGAA GAGGCGGCGA GCACCCCGGG CGGGGGCTGC CCTGACTCCA

GTGGGCGGAA GAGGCGGCGG GCACCCCGGG CAGGAACTGC GCTGACCCCA

---------- ---------- ---------- ---------- ----------

GTGGAAGAAA AAGGCGGAGA GCGCCCCGGG GTGGGGCACT GCTAACCCCA

GTGGGAGGAA GAGGCGCCGA ACCCCCCGAA CAGGAGCCGG GCTGCCACCG

GAGCTGGCCC CGGTGCAGAT TAAGGTTGAG GAAGACTTTG GCTTTGAAGC

GAGCTGGCCC CGGTGCAGAT TAAGGTTGAG GAAGACTTTG GCTTTGAAGC

GAGCTGGCTT CAGTGCAGAT TAAGGTCGAG GAAGACTTTG GCTTTGAAGC

GAGCTGGCTT CAGTGCAGAT TAAGGTCGAG GAAGACTTTG GCTTTGAAGC

GAGCTGGCAC CAGTGCAGAT TAAGGTCGAG GAAGACTTTG GCTTTGAAGC

GAGCTGGCCC CAGTACAGAT TAAGGTTGAG GAAGACTTTG CCTTTGAAGC

GAACTGGCCC CAGTGCAGAT TAAGGTCGAG GAAGACTTTG GCTTTGAAGC

GAACTGGCCC CAGTGCAGAT TAAGGTTGAG GAAGACTTTG GCTTTGAAGC

---------- ---------- ---------- ---------- ----------

---------- ---------- ---------- ---------- ----------

GAACTAGCCC CGGTACAGAT TAAGGTCGAG GAAGACTTTG GCTTCGAAGC

---------- ---------- ---------- ---------- ----------

---------- ---------- ---------- ---------- ----------

GAACTGGCCC CAGTGCAAAT TAAGGTCGAG GAAGACTTTG GCTTTGAAGC

GAGCTGGCCC CAGTGCAGAT CAAGGTCGAG GAAGACTTTG GCTTCGAAGC

---------- ---------- ---------- ---------- ----------

GAGCTAGGCC CAAGCCAGAT CAAAGTTGAG GATGACTTCA GCTTCGAGCC

GAGCTCGGCC CCGGTCAGAT CAAAACCGAA GACGATTTTG GCTTTGAACC

AGATGAGGCC CTGGATTCCA GTTGGGTTTC TCGGGGTCCA GACAAACTGC

AGATGAGGCC CTGGATTCCA GTTGGGTTTC TCGGGGTCCA GACAAACTGC

AGATGAGGCC CTGGATTCCA GCTGGGTTTC TCGGGGTCCA GATAAACTGC

AGATGAGGCC CTGGATTCCA GCTGGGTTTC TCGGGGTCCA GATAAACTGC

AGACGAGGCC CTGGATTCCA GTTGGGTGTC TCGGGGTCCA GACAAACTGC

AGATGAGGCT CTGGATTCCA GTTGGGTTTC TCGGGGGCCA GATAAACTGC

AGATGAGGCC TTGGATTCAA GTTGGGTTTC CCGAGGGCCA GACAAATTGC

AGATGAGGCC TTGGATTCAA GTTGGGTTTC CCGAGAGCCA GACAAATTGC

---------- ---------- ---------- ---------- ----------

---------- ---------- ---------- ---------- ----------

AGATGAGGCC CTGGACTCCA GTTGGGTTTC TCGGGGGCCA GACAAACTGC

---------- ---------- ---------- ---------- ----------

---------- ---------- ---------- ---------- ----------

AGACGAGGCT CTGGATTCAA GCTGGGTTTC TCGGGGGCCA GACAAACTCC

GGATGAGGCC CTGGATTCAA GCTGGGTGTC TCGGGGGCCA GACAAACTGC

---------- ---------- ---------- ---------- ----------

AGATGAGGCC CTCACCACAG GCTGGGCAGC CCGAGGCCCT GACAAACTGC

CGAAGAAACG CTGGGCTCGA GCTGGGCCTC CCGGGGCTCT GACAAGCTGC

TGCCCTACCC GACCCTGGCC AGCCCAGCCT CTGACCGGGA GGCCAGAGGA

TGCCCTACCC GACCCTGGCC AGCCCAGCCT CTGACCAGGA GGCCAGAGGA

TGCCCTACCC AACTCTGGCC AGCCCAGCCT CTGACCGGGA GGCCAGAGGA

TGCCCTACCC AACTCTGGCC AGCCCAGCCT CTGAC----- ----------

TGCCCTACCC GACCCTAGCC AGCCCACCCT CTGAT----- ----------

TGCCCTACCC TACCCTAGCC AGCCCACCCT TTGAT----- ----------

TACCCTACCC TACCCTAGCT AGCCCACCCT TTGACCGGAA AGCCAGAACA

TACCCTACCC TACCCTGGCC AGCCCACCCT TTGACCGGAA AGCCAGAACA

---------- ---------- ---------- ---------- ----------

---------- ---------- ---------- ---------- ----------

TGCCCTACCC CACCCTAGCC AGTCCCCCAT TTGACCGGGA GGCCAGAGCA

---------- ---------- ---------- -----CGAGA GTCCAGAGCA

---------- ---------- ---------- -----CGGAA GGCCAGAGCT

TACCATACCC CACCCTAGCC AGCCCCCCCT TTGACCGGAA GGCCCGAGGA

TGCCCTACCC CACCTTGGCC AGCTCCCCCT TCGACCGGGA GGCCAGACCA

---------- ---------- ---------- ---------- ----------

TCTCCTACCC CACTCTGGCC AGCCCTCCCT TTGACGAAGA TGCGGGCCTA

TCCAGTATCC CGGCCTGGTC AGTCCTCCCT TTGAC--GCT TGTGTCACCC

GCCCGCTCGG TGTCTGGGCT GAAGCAGACA CTGCTGGCGG AGTCCGAGGC

G--CGCTCGG TGTCTGGGCT GAAGCAGACA CTGCTGGCGG AGTCCGAGGC

GCCCGCTCGG TGTCCGGGCT GAAGCAGACA CTGCTGGCGG AGTCCGGGGA

---------- ---------- ---------- ---------- ----------

---CGTTCCG TGTCCGGGCT GAAGCAGACA CTGCTGGCAG AGTCCGGGGC

---CGCTCAG TGTACGGGCT GAAGCAGACA CTGTTGGCCG AATCGGGGGC

GTCCGTTCGG TGTCCGGGTT GAAAGAGACA CTGTTGGCCG AGTCAGATGT

GCCCGTTCGG TGTCCGGGTT GAAGGAGACA CTGTTGGCGG AGTCAGACAT

---------- ---------- ---------- ---------- ----------

---------- ---------- ---------- ---------- ----------

GCCCGCTCCG TGTCAGGACT GAAACAGACG CTGTTGGCTG AGTCGGGGGC

GCCCGCTCGG TGTCCGGCCT GAAGCAGACG TTGTTGGCTG AATCTGGGGT

GAT--CTCCG TGTCTGGACT AAAGCAGACG TTACTGGCCG AGTCAGAAGT

GCCCGCGCTG CGTCCGGGAT GAAGCAGACG CTGCTGGCCG AGTCGGGGGC

G----CTCAG TGTCCGGCAT GAAACAGACG TTGTTGGCCG AGTCGGGGAC

---------- ---------- ---------- ---------- ----------

TCCAGGTCGG TGTCAGGCCT CAAGCAGACC CTGCTCAGCG AATCCGGGGC

GCCCGGTCGG TGGGGGGCCT GCGGCAGACC CTGCTGGCCG AGTCGGGGGC

TCTGACCAGC TACAGCCACC GGGTGTTCTC GGCCTGGGAC TTCGGTCTCT

TCTGACCAGC TACAGCCACC GGGTGTTCTC GGCCTGGGAC TTCGGTCTCT

TCTGACCAGC TACAGCCACC GGGTGTTCTC AGCCTGGAAC TTCGGCCTCT

---------- ---------- ---------- ---------- ----------

TCTGACCAGC TACAGCCACC GGGTGTTCTC GGCCTGGGAT TTCGGCCTCA

TCTGACCAAC TACAGCTACC AGGTGTTCTC AGCCTGGGAC TTTGGCCTTT

TCTGACCAGC TACAGTCATC GAGTGTTCTC AGCTTGGAAT TTTGGCCTCT

TCTGACCAGC TACAGTCACC GAGTGTTCTC AGCTTGGAAT TTTGGCAAA-

---------- ---------- ---------- ---------- ----------

---------- ---------- ---------- ---------- ----------

CCTGACCAGC TACAGCCATC GGGTGTTCAC CGCCTGGGAC TTCGGCCTCT

CCTGACCAGC TACAGCCACC GGGTATTCTC CGCCTGGGAC TTTGGCCTCT

CCTGACCAGC TACAGCCACC GGGTCTTCTC CGCCTGGGAC TTTGGCCTCT

TCTGACCAGA TATAGTCACC GCGTGTTCTC CGCCTGGAAC TTCGGCCTCC

TCTGACGAGC TACAGCCATC GCGTGTTCTC GGCCTGGAAC TTCGGCCTCT

---------- ---------- ---------- ---------- ----------

TCTCACCAGC TACAGCCACC GGATCTTCTC CGCCTGGGAC TTCAGGCTCC

ACTCATCCGC TACAGCGACC GCATCTTCTG CGGTTGGGAC TTCTGCCTGT

GCGGGGACGT CCACGTGCGG CTGCGCCAGC GCATCATCTT GTACGAATTA

GCGGGGACGT CCACGTGCGG CTGCGCCAGC GCATCATCTT GTACGAATTA

GCGGGGACGT CCACGTGCGG CTGCGCCAGC GCATCATCTT GTACGAATTA

---------- ---------- ---------- ---------- ----------

GCGGGGACGT CCACGTGCGG CTGCGCCAGC GCATCATCTT GTACGAATTA

GCGGGGACGT CCACGTGCGA CTGCGCCAGC GCATCCTCCT GTATGAGTTG

GCGGGGACAT GCACGTGCGG CTGCGCCAGC GCATCATTTT GTATGAACTG

ACGGGGACGT GCACGTGCGG CTGCGCCAGC GCATCATTTT GTATGAACTA

---------- ---------- ---------- ---------- ----------

---------- ---------- ---------- ---------- ----------

GTGGGGATGT CCACGTGCGG CTGCGCCAGC GCAACATCCT GTATGAGCTG

GCGGGGATGT CCACGTTCGG CTGCGCCAGC GCACCATCCT ATACGAGTTG

GTGGAGATGT CCACGTACAG CTGCGACAGC GCCACCTCTT CTACGAGCTG

ACGGGGATGA GCACGTGCGG CTGCGCCAGC GCATCATCCG TTACGAGCTG

GTGGCGATGT CCACGTGCGG CTGCGCCAGC GTGTCATCTG CTACGAGTTG

---------- ---------- ---------- ---------- ----------

AGGGCGCCGG CCCGGCGGAG CTCCGCAGGC GAGCGCTCCG CTACGAGCTG

CAGACCCCAA GGCCGTCCGG CTACGACACA ACACCGTGCG CTTCGAGATC

AAGGTGGAGC TGGAGGAGAC AGTGGTGCGG CGCCAGGCTG CGGTGCGGAC

AAGGTGGAGC TGGAAGAGAC AGTGGTGCGG CGCCAGGCTG CGGTGCGGAC

CAGGTGGAGC TGGAGGAGAC AGTGGTGCGG CGCCAGGCTG CTGTGCGGAC

---------- ---------- ---------- ---------- ----------

CAGGTGGAGC TGGAGGAGAC GATGCTGCGT CGCCAGGCTG CGGTGCGGAC

CAGGTGGAGC TGGAGGAAGC AGACGTGAGG CGCCGAGCTG CAGTGCGGAC

CAGGTGGACC TAGAGGAAGC AGCAGTCCGG CGCCGTGCTA CGGAGCAGAC

CAGGTGGACC TAGAGGAAGC AGTAGTCCGG CGCCGTGCTG CGGAGCAGAC

---------- ---------- ---------- ---------- ----------

---------- ---------- ---------- ---------- ----------

CAGGTGGAGC TGGAGGAAGC TGTGGTGCGG CGCCAGGCTG CAGCGCGGAC

CAGGTGGAGC TGGAGGAAGC GGTGGTGCGG CGGCAGGCTG CGGTGCGGAC

AAGGTAGAGC TGGAGGAAGC AGAAGTGCGG CGTCAGGCTG CAGTGCGGAC

CAGGTGGAGC TGGAGGAGGA AGCCGTGCGG CGCCGGGCTG CGATGCGGAC

CAGGTGGAGC TGGAGGAGGC AGTGGTGCGG CACCAGGCTG CAGCGCGGAC

---------- ---------- ---------- ---------- ----------

CAGGTGGAAC TGGAGGAGGC CGCAGCGAGG AAGAGCGCGG AGGCCCGGAG

CAGGTGGAGC TGGAGGAGGA GAACGTGCGG CAGTGGGAGG CGGCCTGGAC

GCTGGGCCAG CAAGCCAGGG TTTGGTTGGT GCGGGTGCTG CTCAACCTGC

GCTGGGCCAG CAAGCCAGGG TTTGGTTGGT GCGGGTGCTG CTCAACCTGC

TCTGGGCCAG GAAGCCAGGG TTTGGTTGGT GCGGGTGCTG CTCAACCTGC

---------- ---------- ---------- ---------- ----------

GCTGGGCCAG CAAGCCAGGG TTTGGTCGGT GCGGGTGCTG CTCAACCTGC

TCTGGGCCAG AAAGTCAAGA TTTGGTTGGT GCGGGCGCTG CTCAACTTGC

GCTGGGCCAA CGAGTCAAGG TGTGGTCGAT GAGGGCGCTG CTCAACTTGC

TCTGAGCCAA CGAGCCAAGG TGTGGTCGAT GAGGGCGCTG CTCAACGTGC

---------- ---------- ---------- ---------- ----------

---------- ---------- ---------- ---------- ----------

CCTGGGCCAG CAAGCCAGGG TGTGGTCGGT GCGGATCCTG CTCAATCTGC

CCTGCACCAG CAAGCCAGGG TGTGGTCAGT GAGGGTGCTG CTCAACCTAC

CCTGGGTCAG CACGTCAGGG TGTGGTTGGT GCGAGCACTG CTCAACC---

GCTGGGCCAG CAGATCAGGG TGTGGTCGGT GCGGGCGCTG CTCAACCTGC

CCTGGGTCAG CAAGCCAGGG TGTGGTTGGT GCGGGGGCTG CTCAACCTGC

---------- ---------- ---------- ---------- ----------

CCGGGGGCAG TGGGCTGCGC TGTGGGGGGC TCGGACCCTC CTTAACCTGC

GCTGCCGAAG CGCATGGCCA TGTGGGGGCT GCGGGGGCTG CTCAACCTGC

TGGTGGTCGC GCTCCTGGGG GCAGCCTTCT ATGGCGTCTA CTGGGCTACG

TGGTGGTCGC GCTCCTGGGG GCAGCCTTCT ACGGCGTCTA CTGGGCTACG

TGGTGATCGC ACTCCTTGGG GGAGCCTTCT ATGGCATCTA CTGGGCTACA

---------- ---------- ---------- ---------- ----------

TGGTGGCCGC GCTCCTGGGG GCAGCGTTCT ATGGCGTCTA CTGGGCCACA

TGGTGGTCGC GCTCCTGGGG GCAGCCTTCT ATGGCGTCTA CTGGGCGACA

TAGTCCTCGC GCTCCTGGGG GCTGCCTTCT ATGGCATCTA CTGGGCCACA

TGGTCCTCGC GCTGCTCGGG GCGGCCTTCT ATGGCATCTA CTGGGCCACC

---------- ---------- ---------- ---------- ----------

---------- ---------- ---------- ---------- ----------

TGGTGATTGC ACTCCTGGGA GCGGCCTTCT ACGGAATCTA CTGGGCTACA

TGGTGGTTGC ACTCCTGGGG GCGGCCTTCT ACGGCGTCTT CTGGGCTACA

---------- ---------- ---------- ---------- ----------

TGGTTTTCGC GCTCCTGGGG GCGGCCTTCT ACGGCGTGTA CTGGGCTACG

TGGTGATTGC GCTTCTGGGT GCAGCCCTCT GTGGAATTTA CTGGGCTACG

---------- ---------- ---------- ---------- ----------

TTATGCTGGC CTTGCTCGGG GCAGCCTTCT ACGGGATCTA CTGGGCCACG

TGGTCCTGGG GCTGCTGGGG GCGGCCTTCT TTGGGGTCTA CTGGGCCACC

GGGTGCACCG TGGAGCTGCA GGAGATGCCC CTGCTGAAGC TTGGGGTGAA

GGGTGCACCG TGGAGCTGCA GGAGATGCCC CTGCTGAAGC TTGGGGTGAA

GAGACAACCG TGAAGCTGCA GGAGACACCC CTGCTGAAGC TTGTGGTGAA

---------- ---------- -GAGACGTCC CTGCTGAAGC TTGGGGTGAA

GAGTCCACGG TGGAGCTACA GGAGACGCCC CTGCTGAAGC TTGGGGTGAA

GGATACGCCG TGAAGCTGCA GGAGAAGACC CTGTTAAGGC TGGGGGTGAA

GAGTACACCG TGACACTGCA GGAGACACCA CTACTCAAGC TTCTGGTGAA

GAGTACACCT TGACACTACA GGAGACGCCC CTATTCAAGC TTCTGGTGGA

---------- ---------- ---------- ---------- ----------

---------- ---------- ---------- ---------- ----------

GGGGCCACCG TGGAGCTTCA GGAGACGCCC CTGCTAAAGC TCCTGGTGGA

GAGTCCACTG TGAATCTGCA GAAGAGGCCC CTGTTAAAGC TCGGGGTGGA

---------- ---------- ---------- ---------- ----------

ACTTTCACGG TGGAGTCGCA GGAGACGCCC CTGCTAAAGC TCGTGGTGGA

GAGTACACCG TGGAGCTGCA GGAGAATTCC CTGCTAAAGC TGCTGGTGGA

---------- ---------- ---------- ---------- ----------

GACTCCGACG GGAGGCTGCA GAACGACCGT ATTCTGAAGC TGCTGGTCGC

CAGGAGTCCA TCCAGCTGCA GAAAGACCCC TTCCTGAAGC TCCTAGTGGA

TTACCTTCCG TCCATCTTCA TCGCTGGGGT CAATTTTGTG CTGCCGCCCG

TTACCTTCCG TCCATCTTCA TCGCTGGGGT CAATTTTGTG CTGCCGCCCG

TTACCTTCCG TCCATCTTCA TCGCTGTGGT CAACTTTGTG CTGCCGCCCG

TTACCTTCCG TCCATCTTCA TCGCTGTGGT CAACTTTGTG CTGCCGCCCG

TTACCTTCCG TCCATCTTCA TCGCCGGGGT TAACTTTGTG CTGCCGCCGG

TTACCTTCCG TCCATCTTCA TCTCTGTGGT CAACTTTGTG CTCCCGCCCG

TTATCTTCCA TCTATCTTCA TTTCCGTGTT CAACTTTGTG CTGCCTCCTG

TTATCTACCA TCCATCTTCA TTTCTCTGTT CAACTTTGTG CTGCCTCCTG

---------- ---------- ---------- ---------- ----------

---------- ---------- ---------- ---------- ----------

TTACCTTCCA TCCATCTTCA TCTCTGGGGT TAACTTCTTG CTGCCACCTG

TTACCTTCCA TCCATTTTCA TCGCCGGGGT CAACTTTGTG CTGCCACCAG

---------- ---------- ---------- ---------- ----------

TTACCTTCCG TCCATCTTCA TCTCCGTGGT CAACTTCCTG CTGCCGTTTG

TTACCTTCCA TCCATCTTCA TTGCCGTATT CAACTTCGTG CTGCCACCCG

---------- ---------- ---------- ---------- ----------

CTATCTGCCG TCCATCTTCA TGTCCGGTGC TAACCTGATT CTGCCGCCCG

CTTCCTCCCG TCCATCTTCA TCTCCGCGGT CAACCTGATC CTGCCGCCCG

TGTTCAAGCT CATTGCTCCA CTGGAGGGCT ACACTCGGAG TCGCCAGATC

TGTTCAAGCT CATTGCTCCA CTGGAGGGCT ACACTCGGAG TCGCCAGATC

TGTTCAAGCT CATTGCTCCA CTGGAAGGCT ACACTAGAAG TCGCCAGATC

TGTTCAAGCT CATTGCTCCA CTGGAAGGCT ACACTAGAAG TCTCCAGATC

TGTTCACGCT GATTGCTCCA CTGGAGGGCT ACACCCGGAG TCGCCAGATC

TGTTCGCCGT CATTGCCCCC CTGGAGGGCT ATACTCGGAG CCGCCAGATC

CGTTCGCGTT CATTGCCTCA CTAGAGGGCT ACACTCACAG CCGTCAGATC

TGTTCAAGTT CATTGCCTCA CTAGAGGGCT ACACTCAAAG CCGACAGATC

---------- ---------- ---------- ---------- ----------

---------- ---------- ---------- ---------- ----------

TGTTCAAGCT CATCGCCCTG CTGGAGGGCT ACACCAGGAG CCACCAGATT

TGTTCAAGTT CATTGCCCCA CTGGAGGGCT ATACCAGGAG CCGCCAGATC

---------- ---------- ---------- ---------- ----------

TGTTCAACCT CATTGCCCCG CTGGAGGGCT ACACTCGGAG CCGCCAGATA

TGTTCACGTT CATTGCCTCG CTGGAGGGCT ACACCCGAAG CCGCCAGATC

---------- ---------- ---------- ---------- ----------

TCTTCTTTCT CACTGGGCCT C--GAGGGCT ACACTCGGAG CCGCCAGGTC

TCTTCACCCT GCTGACCAAG TTGGAGGGGT ATACCCTGAG CCGCCAGGTC

GTTTTTATCC TGCTCAGGAC CGTGTTTCTT CGCCTCGCCT CCCTGGTGGT

GTTTTTATCC TGCTCAGGAC CGTGTTTCTT CGCCTCGCCT CCCTGGTGGT

GTTTTTATCC TGCTCAGGAC CGTGTTTCTT CGCCTGGCCT CCCTGGTGGT

GTTTTTATCC TGCTCAGGAC CGTGTTTCTT CGCCTGGCCT CCCTGGTGGT

ATTTTAATCT TGCTGAGGAC CCTGTTTCTT CGCCTGGCCT CCCTGGTGGT

GTTTTTATTT TGCTCAGGAC TGTGTTTCTC CGTCTCACCT CCTTGGTGGT

GTCTTGATCC TGCTTAGGAC TGTGTTTTTG CGTCTGGCCT CCCTGGTGTT

GTCTTGATCC TGCTTAGGAC TGTGTTTTTG CGTCTGGCCT CGCTGGTGTT

---------- ---------- ---------- ---------- ----------

---------- ---------- ---------- ---------- ----------

GCTTTTATCC TGCTCAGGAC CGTGTTTCTC CGCCTGGCAT CCCTGCTAGT

GTTTTAATAC TGCTCAGGAC TATATTTCTC CGCCTGGCCT CCCTGCTGGT

---------- -------GAC TGTATTCCTC CGCCTGGCTT CCCTGGTGGT

GTTTTAGTCC TGCTCAGGAC CGTGTTTCTA CGCCTCATCT CCCTGCTGGT

GTTTTAATCC TGCTCAGGAC TGTGTTTCTC CGCCTTTTCT CTCTCCTGGT

---------- ---------- ---------- ---------- ----------

TTCATCCTTC TTCTAGTGAC CGTGTTTCTC CGGCTGGCCT CGCTCGTGGT

CTGTTCATCC TGCTCAGGAC CGTGTTCCTC CGACTCGCTT CCCTGGTGGT

CCTGCTCTTC TCTCTCTGGA ATCAGATCAC TTGTGGGGGC GACTCCGAGG

CCTGCTCTTC TCTCTCTGGA ATCAGATCAC TTGTGGGGGC GACTCCGAGG

CCTGCTCTTC TCTCTCTGGA AACAGATCAC TTGTGGGGGC GACTCCGAGG

CCTGCTCTTC TCTCTCTGGA AACAGATCAC TTGTGGGGGC GACTCCGAGG

CCTGCTCTTC TCTCTCTGGA ATCAGATCAC TTGTGGGGGC GACGCCGAGG

CCTGCTCTTC TCCCTCTGGA ATCAGATTAC CTGTGGGGGC GATGGCCAGG

TCTTCTGTTC TCTCTGTGGA ATCAGATCAC GTGTGGAGGA AACATGGAGG

CCTTCTGGTC TCTCTGTGGA GTCAGATCAC GTGTGGGGGA AACATGGAGG

---------- ---------- ---------- ---------- ----------

---------- ---------- ---------- ---------- ----------

CCTGCTCTTC TCCCTCTGGA ATCAGATCAC TTGTGGGGGC AAGGCTGAGG

CCTGATCTTC TCTCTCTGGA AGCAGATCAC TTGCGGGGGC AATGCAGAGG

CCTCATCATC TCCCTCTGGA ATAAGATCAC TTGTGGGGGA AATGCTGAGA

TCTGCTCTTC TCCCTCTGGA GTAAGATCAC TTGCTGGGGC GACGCCGAGG

CCTGCTCATC TCTCTCTGGT ATCAGATCAC TTGCGGGGGC AATGCCGAGG

---------- ---------- ---------- ---------- ----------

CCTGCTCGGG TCCCTCTGGA AGCAGATCAC CTGTGGGGGC AACCCCGAGG

CCTCCTCCTC ACCCTGTGGG CCAAGATCAC CTGTAAGGGG GACCCAAATG

CTGAGGACTG CAAAACCTGT GGCTACAATT ACAAACAACT TCCGTGCGAG

CTGAGGACTG CAAAACCTGT GGCTACAATT ACAAACAACT TCCGTGCGAG

CTAAGGACTG CAAAACCTGT GGCTACAATT ACAAAGAACT TCCGTGCGAG

CTAAGGACTG CAAAACCTGT GGCTACAATT ACAGAGAACT TCCGTGCGAG

CTGAGGACTG CAAAACCTGT GGCTACAATT ACAAAGAACT TCCGTGCGAG

ATGAAGAGTG CAAAACTTGT GGCTACAATT ACAAAGAGCT TCCGTGCGAG

CAGAAGAATG CAAGGCTTGT GGCTACAATT ACAAAGAAAT TCCGTGCGAG

CAGAAGGATG CAAGGCCTGC GGCTATAATT ACAAAGAAAT TCCGTGCGAG

---------- ---------- ---------- ---------- ----------

---------- ---------- ---------- ---------- ----------

CTGAAGAGTG TAAGACCTGT GGCTACAATT ACAAGGAACT ACCGTGCGAG

CTGAGAAGTG CAAAACCTGC GGCTACAATT AC---GAACT TCCGTGCGAG

ATGACAACTG CAAAGCCTGT GGCTACAACT ACAAAGACCT TCCGTGCGAG

CCGAGGATTG CAAAGGCTGT GGCTACAATT ACAGAGAACT TCCGTGCGAG

CTGAGGATTG CAAAGCCTGT GGCTACAATT ACAAAGAACT TCCGTGCGAG

---------- ---------- ---------- ---------- ----------

CTGAGGCCTG CGCCACCTGC GGCTACGCCT ACAGGGAACT ACCGTGTGAG

CCCCGAGCTG CGAGGGCTGT GGCTACAACA GCAAGGAGCT GCCTTGCGAG

ACTGTCCTGG GCCAGGAAAT GTACAAACTT CTGCTCTTTG ATCTGCTGAC

ACTGTCCTGG GCCAGGAAAT GTACAAACTT CTGCTCTTTG ATCTGCTGAC

ACTGTCCTGG GCCAGGAAAT GTACAAACTT CTGCTCTTTG ATCTGCTGAC

ACTGTCCTGG GCCAGGAAAT GTACAAACTT CTGCTCTTTG ATCTGCTGAC

ACTCTCGTGG GCCAGGAAAT GTACAAGCTC CTACTCTTTG ATCTGCTGAC

ACTCGGCTTG GCCAAGAGAT GTACAAGCTT CTGCTTTTTG ACCTGCTGAC

ACTCGACTGG GCCAGGAGAT GTACAAACTT GTGCTCTTTG ATCTGCTGAT

ACTCGGCTGG GCCAGGAGAT GTACAAACTT GTACTCTTTG ATCTGCTGAT

---------- ---------- ---------- ---------- ----------

---------- ---------- ---------- ---------- ----------

ACCCGGCTGG GGCAGGAGAT GTACAAACTC CTGCTCTTTG ATCTGCTGAC

ACTCGGCTGG GGCAAGAGAT GTACAAACTC CTGCTTTTTG ATCTGCTGAC

ACACGTATGG GACAGGAGAT GTACAAACTT CTGATCTTTG ATCTGCTGAC

ACTCGGCTGG GGCAGGAGAT GTACAAACTC CTGCTGTTTG ACCTGCTGAC

ACCCGGCTAG AGCAGGAGAT GTACAAACTC ATGCTTTTTG ATCTGCTGAC

---------- ---------- ---------- ---------- ----------

ACTCGGCTAG GGCAGGAGAT GTACAAGCTG CTGCTGTTTG ACCTGCTGAC

ACCATGCTGG GCCAGGAGAT GTATAAACTG CTGCTGTTTG ACCTGCTGAC

TGTCTTGGCA GTCGCGCTGC TCATCCAGTT TCCTAGAAAG CTGATCCCGT

TGTCTTGGCA GTCGCGCTGC TCATCCAGTT TCCTAGAAAG ----------

TGCCTTGGCA GTCGCGCTGC TCATCCAGTT TCCTAGAAAG CTGATCCCGT

TGCCTTGGCA ATCGCGCTGC TCATCCAGTT TCCTAGAAAG CTGATCCCGT

TGGCTTGGCA GTCTCGCTGC TCATCCAGTT TCCTCGAAAG CTGATCCCAT

TAGCTTGGCA GTAATGCTGC TCATCCAGTT TCCTCGAAAG CTGATCCCAT

GGGCCTGTTG GTCACGCTGT TGATCCAGTT TCCTAGGAAG CTGATCCCAT

GGGCCTGTTG GTCACGCTGT TGGTCCAGTT TCCTAGGAA- ----------

---------- ---------- ---------- ---------- ----------

---------- ---------- ---------- ---------- ----------

TGGCGTGGCT GTCATGCTGC TCATCCAGTT TCCTCGGAAG CTGATCCCAT

TGGCATAGCA GTCATGCTGC TCTTCCAGTT CCCTCGGAAG ----------

TGGTCTCACT GTTACACTGC TCATTCAATT TCCTCGGAAG CGGATTCCAT

TGGCTTGGCA GTCGTGCTGC TCGTCCAGTA TCCTCGAAAG CTGATATCAT

CGGCTTGGCA GTCACGCTAC TCATCCAGTT TCCTCGAAAG CTGATCCCGT

---------- ---------- ---------- ---------- ----------

CTGCCTGGCC GTGCCTCTGC TGGTTCAGTT CCCACGCAAA GTGATCCC--

CGTTCTGGCA GTCATCCTGC TGGTGCGCTA CCCCCGCAA- --GATCCAAT

CTAAGCTTTG TGGTCCATTC CGGGGGCAGT CGTCCATCTG GGCCCAGATC

---------- ---------- ---------- ---------- ----------

CTAAGCCGTG TGGTCCATTC CGGGGGCAGT CGTCCATCTG GGCCCAGATC

CTAAGCCGTG TGGTCCATTC CGGGGGCAGT CGTCCATCTG GGCCCAGATC

CTAAGCTATG TGGTCCATTC CGGGGGCAGT CGTCCATCTG GGCCCAGATC

CTAAGCCGTG TGGTCCATTT CGGGGTCAGT CATCCATCTG GGCCCAGATC

CTAAGCTGTG TGGCCCATTC CAAGGGAAGT TGTCCATTTG GGCCCAGATC

---------- ---------- ---------- ---------- ----------

---------- ---------- ---------- ---------- ----------

---------- ---------- ---------- ---------- ----------

CTAAGCTGTG TGGTCCATTC CAAGGGCAGT CGTCTATCTG GGCCCAGATC

---------- ---------- ---------- ---------- ----------

CGAAGCTGTG TGGTCCATTT CGGGGACAGA CGTCCATCTG GACCCAGATC

CCCAGCTGTG TGGCCCGTTC CGGGGTCAGA AGACCATCTG GGTCCTGATC

CCAAGCTGTG TGGTCCATTC CAGGGACAGC CATCCATCTG GGCCCAGATC

---------- ---------- ---------- ---------- ----------

-TAAGCCGCT TGGTGCACAC CGGAGTCAGA CTTCAATCAG GCCCCGAAGC

CTAAGTTGTG CGGGCCGTTC CGAAACAAGA CAAGCATCTG GGATGTGGTG

CCTGAGTCTA TTTCCAGCCT CCCTGAGACC ACCCAGAATT TCCTCTTCTT

---------- ---------- ---------- ---------- ----------

CCTGAGTCTA TTTCCAGCCT CCCTGAGACC ACCCAGAACT TCCTCTTCTT

CCTGAGTCTA TTTCCAGCCT CCCTGAGACC ACCCAGAACT TCCTCTTCTT

CCTGAGTCTA TTTCCAGCCT CCCTGAGACC GCCCAGAACT TCCTCTTCTT

CCAGAATCTA TTTCCAGCCT CCCTCAGACC GCCCAGAACT TTTTCTTCTT

CCTGAGTCCA TTGAGAGCCT CCCTCAGACT GCCCAGAATT TTCTCTACTT

---------- ---------- ---------- ---------- ----------

---------- ---------- ---------- ---------- ----------

---------- ---------- ---------- ---------- ----------

CCTGAGTCTA TTTCCAACCT CCCTCAGACC GTCCAGAACT TTCTCTTTTT

---------- ---------- ---------- ---------- ----------

CCGGACTCTA TTCACAACCT CCCTCAGTCT ACCCAGAGTT TTCTCTTCTT

CCTAACTCCA CTGACAGCCT CCCTCAGACC GCCCGGGACT TCGTCCTCTT

CCTGAGTCTA TTTATGGCCT CCCTCGGGCC GCTCAGAACT TTCTCTTCTT

---------- ---------- ---------- ---------- ----------

CCCGAGGGCA GCAACAGAA- --CTGGGACC ACCCAAGCCC TGCACCTCCT

CCACAGGCCA TCGAGGAACT GCCCCTGCCC GCCCAGAACA TGTTTTTCTT

CCTGGGGACC CAGGCTTTTG CTGTGCCCCT TCTGCTGATC TCCAGCATCC

---------- ---------- ---------- ---------- -----CATCC

CCTGGGGACC CAGGCTTTTG CTGCGCCCCT TCTGCTGATC TCCAGCATCC

CCTGGGGACC CAGGCTTTTG CTGCGCCCCT TCTGCTGATC TCCAGCATCC

CCTGGGGACT CAGGCTTTTG CTGTACCCCT TCTGCTGATT TCCAGCATCC

CCTGGGAACC CAGGCTTTTG CTGTGCCCCT TCTGCTGATC TCCAGCATCC

TCTGGGAACC CAGGCTTTTA CTGTACCCCT TCTGATCGTC TCTAGCATCC

---------- ---------- ---------- ---------- ----------

---------- ---------- ---------- ---------- -----CATCC

---------- ---------- ---------- ---------- ----------

CCTGGGGACC CAGGCTTTTG CTGTGCCTCT TCTCTTAATC TCCAGCATCC

---------- ---------- ---------- ---------- ----------

CTTGGGCACC CAGGCCTTTG CTG---CCCT TCTGCTGATC TCCAGCATCT

CCTGGGGACC CAGTCTTTTG CTGTGCCCCT TCTGCTGATC TCCAGCATCC

CCTGGGGACC CAGGCCTTTG CTGTGCCCCT TCTGCTGTTC TCCAGCATCC

---------- ---------- ---------- ---------- ----------

CCAAGGCCCT CAGCTCTCTA ACTCCCCGCC ACAATTGATC TCCCTCATTC

CCTGGGGACC CAGACGTTTG CGGTCCCGCT ATTCCTCCTG GCCAGCCTCC

TGATGGCGTA CACTGTGGCT CTGGCTAACT CCTACGGACG CCTCATCTCT

TGATGGCGTA CACTGTGGCT CTGGCTAACT CCTACGGACG CCTCATCTCT

TGATGGCGTA CACTGTGGCT CGGGCTAATT CCTACGGACG CCTCATCTCT

TGATGGCGTA CACTGTGGCT CGGGCTAACT CCTACGGACG CCTCATCTCT

TGATGGCGTA CACTGTGGCT ctggctaact cctacggtcg tctcatctct

TGATGGCATA TACTGTGGCT CTGGCCAACT CCTATGGACG CCTCATCTCT

TGATGATGTA TACAGTAGCC CTGGCTAACT GTTATGGACG CCTCATCTCT

---------- ---------- ---------- ---------- ----------

TGCTGGCCTA CACCGTGGCC CTGGCCCACT CGTACGGACG CGTCATCTCC

---------- ---------- ---------- ---------- ----------

TGATGGCATA TACCCTGGCC TTGGCTAACT CCTATGGACG TCTGGTCTCT

---------- ---------- ---------- ---------- ----------

TGATGGCCTA TACCGTGACC CTAGCTAACT CCTACGGACG CCTCGTCTCT

TGATGGCATA TATCCTGGCC CTGGCTAACT CCTACGGACG CCTCATCTCT

TCATGGCATA TACAGTGTCC GTGGCCAACT CATATGGGCG CCTCATCTCT

---------- ---------- ---------- ---------- ----------

TGATGGTCTA CACCTTAACG CTGGCCAGCT CCTACGGCCG CCTGGTCTCC

TGATGTTCTA CACGATAGCT CTTGCCAGTT CCTACAACCG GCTCATTCAC

GAGCTCAAAC GTCAGAGACA GACGGAGGCG CAGAATAAAG TCTTCCTGGC

GAGCTCAAAC GTCAGAGAGA GACGGAGGCG CAGAATAAAG TCTTCCTGGC

GAGCTCAAAC GTCAGAGAGA GACGGAGGCG CAGAATAAAG TCTTCCTGGC

GAGCTCAAAC GTCAGAGGGA GACGGAGGCG CAGAATAAAG TCTTCCTGGC

cagctcaaac gtcaGAGAGA GACGGAGGCG CAGAACAAAG TCTTCCTCGC

GAGCTTAAAC GTCAGATAGA GACGGAGTCG CAGAATAAAG TCTTCCTGGC

GAGCTCAAAC GCCAGATAGA GACGGAAGTA CAAAATAAAG TCTTCCTAGC

---------- ---------- ---------- ---------- ----------

GCGCTGAGAC GCCAGATGGA GACGGAGGCG CAGAACAAGA CGTTCCTGGC

---------- ---------- ---------- ---------- ----------

GAGCTCAAAC GCCAGATAGA GACGGAGGCG CAGAATAAGG TCTTCCTGGC

---------- ---------- ---------- ---------- ----------

GAACTCAAAC AGCAAA---- GAAGGAGTCT CAGAACAAAG TCTTCCTGGC

GAGCTCAAAC GCCAGATACA GAAGGAAGTG CAGAATAAGG TCTTCCTGGC

GAGCTCAAGC GCCAGATAGA GACGGAGGCG CAGAATAAAG TCTTCCTGGC

---------- ---------- ---------- ---------- ----------

ACGCTGAAGG GACACATCAA GATGGAGAGC GAGAGTAAAG TGCTGTTGGC

AAGCTCCAGA AGCAGATAGA GTTGGAGAGC CAAAGCAAGA GGTTCCTGGT

ACGGCGCGCT GTGGCGCTGA CCAGGTTCCT CATGAGAGTG CTGGACTCCT

ACGGCGCGCT GTGGCGCTGA CCAGGTTCCT CATGAGAGTG CTGGACTCCT

ACGGCGCGCT GTGGCGCTGA CCAGGTTCCT CATGAGAGTG CTGGACTCCT

ACGGCGCGCT GTGGCGCTGA CCAG------ ---------- ----------

ACGGCGCGCT GTGGCGCTGA GCAGGTTCCT CATGAGAGTG CTGGACTCCT

ACAGCGCGCT GTGGCACTGA GCAGGTTCCT CATGAGAGTG CTCGACTCCT

CCAGCGAGCT GTAGCTCTGA GCAGATTCCT CATGCGAGTG CTGGACTCAT

---------- ---------- --AGGTTCCT CATGAGGGTG CTGGACTCCT

CCAGCGCGCC GTGGCGCTGA gcAG------ ---------- ----------

---------- ---------- ---------- ---------- ----------

ACAGCGCGCT GTGGCACTGA GCAGGTTCCT CATGAGAGTG CTGGATGCTT

---------- ---------- ---------- ---------- ----------

ACAGCGTACT GTGGCGTTAA GT-------- ---------- ----------

ACAGCGCGCT GTGGAACTGA GCAGGTTCCT CATGAGAGTG CTGGACTCTT

ACAGCGCGTT GTGGCGCTGA GC--GTTCCT CATGCGAGTG CTGGACTCCT

---------- ---------- ---------- ---------- ----------

CAAGAGGATT GTGGAGCTCG GCAGGTTTCT CATGCGAGTC CTGGATTCCT

GCAGCAGACG GTGGAACTGA GCAGGTTCCT CATGAGAGTC CTCGACTCTT

ACGGGGATGA CTACCGGGCC AGCCAGTTCA CCATTGTGCT GGAGGATGAG

ACGGGGATGA CTACCGGGCC AGCCAGTTCA CCATTGTGCT GGAGGATGAG

ATGGGGATGA CTACCGGGCC AGCCAGTTCA CCATTGTGCT AGAGGATGAG

---------- ---------- ---------- ---------- ----------

ACGGGGATGA CTACCGGGCC AGCCAGTTCA CCATTGTGCT GGAGGATGAG

ATGGGGATGA TTACCGGGCC AGCCAGTTCA CCATCATGCT GGAGGATGAG

ACGGGGATGA CTACCGGGAT AGCCAGTTCA CCATTGTGTT GGAGGATGAT

ACGGGGATGA CTACCGAGAT AGCCAGTTCA CCATTGTGTT GGAGGATGAT

---------- ---------- ---------- ---------- ----------

---------- ---------- ---------- ---------- ----------

ATGGCGATGA CTACCGTGCC AGCCAGCTCA CCATAGTCCT GGAGGACGAG

---------- ---------- ---------- ---------- ----------

---------- ---------- ---------- ---------- ----------

ACGGGGACGA CTACCGAACC AGCCAGCTCA CCATTGTGCT GGAGGATGAG

ACGGGGATGA CTACCGGGCC GGCCAGTTCA CTATCGTGCT GGAGGACGAG

---------- ---------- ---------- ---------- ----------

ATGGAGATGA CTACAGGAAG AGCCAGTTCA CTATTGTCCT GGAGGATGAG

ATGGTGACGA CTACAGGAAA AGTCAGTTCA CCATCGTCCT GGAGGATGAG

GGCAGCCAGG GCACGGATGC CCCCACCCCA GGCAATGCGG AGAATGAGCC

GGCAGCCAGG GCACGGATGC CCCCACCCCA GGCAATGCTG AGAATGAGCC

GGCAGCCAGG GCACGGATGC TCCCACCCCA GGCAATGCTG AGAATGAGCC

---------- ---------- ---------- ---------- ----------

GGCAGCCAGG GCACAGATGC TCCCACCCCA GGTAATGCTG AGAATGAGCC

GGCAGCCAAG GCACAGATGC CCCCACCCCA GGCAATGCCG AGAATGAACC

GGCAGCCAAG GCACTGATGT CCCTACCCCA GGCAATGTTG AGAATGAACC

GGCAGCCAAG GCACAGATGT CCCTACCCCA GGCAATGCTG AGAATGAACC

---------- ---------- ---------- ---------- ----------

---------- ---------- ---------- ---------- ----------

GGCAGCCAGG GCACAGATGC TCCCACTCCC GGCAATGCTG AGAATGAGCC

---------- ---------- ---------- ---------- ----------

---------- ---------- ---------- ---------- ----------

GGCAGCCAGG GCACAGATGC CCCCACCCCA GGCAATGCTG AGAATGAGCC

GGCAGCCAGG GCACTGATGC CCCCACCCCA GGCAATGCTG AGAACGAGCC

---------- ---------- ---------- ---------- ----------

GGGAGCCAAG GCCCTGATGC CCCCACCCCG GGAAATGCAG AGAATGAGCC

AGCAGTCACG GTCCTGACGC CCCAACCCCA GGCAACGCAG AGAATGAGCC

TCCAGAGAAA GAGACACTGT CCCCGCCCAG AAGGCCTGCA CCCCCAGAAC

TCCAGAGAAA GAGACATTGT CCCCGCCCAG AAGGCCTGCA CCCCCAGAAC

TCCAGAGAAA GAGGCACTGT CCCCGCCCAG AAGGCCTGCA CCCCCAGAAC

-----AGAAA GAGGCACTGT CCCCGCCCAG AAGGCCTGCA CCCCCGGAAC

TCCAGAGAAA GAGGCACTGT CCCCGCCGAG AAGGCCTGCA CCCCCAGAAC

CCCAGAGAAA GAGGGGCTGT CCCCACCCAG AAGGCCGGTA CCCCCAGAAC

TCCAGAGAAA GAGGGACTTT CCCCACCACA AAGGACTGCA ACCCTAGACC

TCCAGAGAAA GAGGGGCTTT CCCCATCCCA AAGGACTGCA ACCTTAGACC

---------- ---------- ---------- ---------- ----------

---------- ---------- ---------- ---------- ----------

CCCAGAGAAA GAGGGGCTGT CACCACCCCG AAGGCCCGCA CCTCCAGAAC

---------- ---------- ---------- ---------- ----------

---------- ---------- ---------- ---------- ----------

TCCAGAGAAA GAAGGGCTGT CCCCACCCAG ACGGCCTGCA CCCCCAGAAT

TCCTGAGAAA GAGGGACTGT CCCCGCCCAG AAGGCCGGTA CCCCCCGAGC

---------- ---------- ---------- ---------- ----------

CCCTGAAAAG GAAAGTCTGT CACCTACCCC ACGG-CGGC- -CTCTTGAGC

GCCTGAGCGG GAGGGGCTGT CCCCGCCGCG GAGACCCGGA CCCCTGGAAC

CCGGCAGCCC AGCCCCCGGT GAGGGGCCCA GTGGGCGGAA GAGGCGGCGA

CCGGCAGCCC AGCCCCCGGC GAGGGGCCCA GTGGGCGGAA GAGGCGGCGA

CTGGCAGCCC AGCGCCCGGC GAGGGGCCCA GTGGGCGGAA GAGGCGGCGA

CTGGCAGCCC AGCGCCCGGC GAGGGGCCCA GTGGGCGGAA GAGGCGGCGA

CTGGCAGCCC AGTCCCCGTC GAGGGGCCCA GTGGGCGGAA GAGGAGGCGA

CCAGCAGCCC AGCCCCAGGC GAGGGGCCCA GTGGGCGGAA GAGGCGGCGA

CCAGCAGCCC AGCCCCTGGC GAGGGGCCCA GTGGGCGGAA GAGGCGGCGG

CCACCAGCCC AGCCCCTGGC GAGGGGCCCA GTGGGCGGAA GAGGCGGCGG

---------- ---------- ---------- ---------- ----------

---------- ---------- ---------- ---------- ----------

CCAGCAGCCC AACCCCCGGG GAGGGGCCCA GTGGACGCAA GAGGCGGCGA

---------- ---------- ---------- ---------- ----------

---------- ---------- ---------- ---------- ----------

CCAGCAGCCC GGTCCCTGGC GAGGGGCCCA GTGGGCGGAA GAGGCGGCGA

CCAGCAGCCC GGCTGCTGGG GAGGGGCCCA GTGGGCGGAA GAGGCGGCGG

---------- ---------- ---------- ---------- ----------

CCAGCAGTCC TCCCCCTGGG GAAGGTCCCA GTGGAAGAAA AAGGCGGAGA

CCAGTAGCCC TACTCCAGGG GAGGGCCCCA GTGGGAGGAA GAGGCGCCGA

GTGCCACGGG CAGGAAATGC GCTGACTCCA GAGCTGGCCC CGGTGCAGAT

GTGCCACGGG CAGGAAATGC ACTGACTCCA GAGCTGGCCC CGGTGCAGAT

GTGCCGCGGG CAGGGACTGC GCTGACTCCA GAGCTGGCTT CAGTGCAGAT

GTGCCGCGGG TGGGAACTGC GCTTACTCCA GAGCTGGCTT CAGTGCAGAT

GTACCACGGG CAGGAACTGC GCTGACTCCA GAGCTGGCAC CAGTGCAGAT

GTACAACGGG CAGGAGCTGC ACTGACTCCA GAGCTGGCCC CAGTACAGAT

GCACCACGAG CAGCATCTTC ATTGACTCCA GAACTGGCCC CAGTGCAGAT

GCGCCACGGG TGGGGGCTTC ATTGACTCCA GAACTGGCCC CAGTGCAGAT

---------- ---------- ---------- ---------- ----------

---------- ---------- ---------- ---------- ----------

GCACCCCGGG CGGGAGCTGT GCTCACTCCA GAACTAGCCC CGGTACAGAT

---------- ---------- ---------- ---------- ----------

---------- ---------- ---------- ---------- ----------

GCACCCCGGG CGGGGGCTGC CCTGACTCCA GAACTGGCCC CAGTGCAAAT

GCACCCCGGG CAGGAACTGC GCTGACCCCA GAGCTGGCCC CAGTGCAGAT

---------- ---------- ---------- ---------- ----------

GCGCCCCGGG GTGGGGCACT GCTAACCCCA GAGCTAGGCC CAAGCCAGAT

ACCCCCCGAA CAGGAGCCGG GCTGCCACCG GAGCTCGGCC CCGGTCAGAT

TAAGGTTGAG GAAGACTTTG GCTTTGAAGC AGATGAGGCC CTGGATTCCA

TAAGGTTGAG GAAGACTTTG GCTTTGAAGC AGATGAGGCC CTGGATTCCA

TAAGGTCGAG GAAGACTTTG GCTTTGAAGC AGATGAGGCC CTGGATTCCA

TAAGGTCGAG GAAGACTTTG GCTTTGAAGC AGATGAGGCC CTGGATTCCA

TAAGGTCGAG GAAGACTTTG GCTTTGAAGC AGACGAGGCC CTGGATTCCA

TAAGGTTGAG GAAGACTTTG CCTTTGAAGC AGATGAGGCT CTGGATTCCA

TAAGGTCGAG GAAGACTTTG GCTTTGAAGC AGATGAGGCC TTGGATTCAA

TAAGGTTGAG GAAGACTTTG GCTTTGAAGC AGATGAGGCC TTGGATTCAA

---------- ---------- ---------- ---------- ----------

---------- ---------- ---------- ---------- ----------

TAAGGTCGAG GAAGACTTTG GCTTCGAAGC AGATGAGGCC CTGGACTCCA

---------- ---------- ---------- ---------- ----------

---------- ---------- ---------- ---------- ----------

TAAGGTCGAG GAAGACTTTG GCTTTGAAGC AGACGAGGCT CTGGATTCAA

CAAGGTCGAG GAAGACTTTG GCTTCGAAGC GGATGAGGCC CTGGATTCAA

---------- ---------- ---------- ---------- ----------

CAAAGTTGAG GATGACTTCA GCTTCGAGCC AGATGAGGCC CTCACCACAG

CAAAACCGAA GACGATTTTG GCTTTGAACC CGAAGAAACG CTGGGCTCGA

GTTGGGTTTC TCGGGGTCCA GACAAACTGC TGCCCTACCC GACCCTGGCC

GTTGGGTTTC TCGGGGTCCA GACAAACTGC TGCCCTACCC GACCCTGGCC

GCTGGGTTTC TCGGGGTCCA GATAAACTGC TGCCCTACCC AACTCTGGCC

GCTGGGTTTC TCGGGGTCCA GATAAACTGC TGCCCTACCC AACTCTGGCC

GTTGGGTGTC TCGGGGTCCA GACAAACTGC TGCCCTACCC GACCCTAGCC

GTTGGGTTTC TCGGGGGCCA GATAAACTGC TGCCCTACCC TACCCTAGCC

GTTGGGTTTC CCGAGGGCCA GACAAATTGC TACCCTACCC TACCCTAGCT

GTTGGGTTTC CCGAGAGCCA GACAAATTGC TACCCTACCC TACCCTGGCC

---------- ---------- ---------- ---------- ----------

---------- ---------- ---------- ---------- ----------

GTTGGGTTTC TCGGGGGCCA GACAAACTGC TGCCCTACCC CACCCTAGCC

---------- ---------- ---------- ---------- ----------

---------- ---------- ---------- ---------- ----------

GCTGGGTTTC TCGGGGGCCA GACAAACTCC TACCATACCC CACCCTAGCC

GCTGGGTGTC TCGGGGGCCA GACAAACTGC TGCCCTACCC CACCTTGGCC

---------- ---------- ---------- ---------- ----------

GCTGGGCAGC CCGAGGCCCT GACAAACTGC TCTCCTACCC CACTCTGGCC

GCTGGGCCTC CCGGGGCTCT GACAAGCTGC TCCAGTATCC CGGCCTGGTC

AGCCCAGCCT CTGACGACTA CGGCATCCGG AATCGAGACG AGGTTTCCTT

AGCCCAGCCT CTGACGACTA TGGCATCCGG AATCGAGACG AGGTTTCCTT

AGCCCAGCCT CTGACGATTA TGGCATCCGG AATCGAGATG AGGTTTCCTT

AGCCCAGCCT CTGACGATTA TGGCATCCGG AATCGAGATG AGGTTTCCTT

AGCCCACCCT CTGATGACTA CGGCATCCGG AATCGAGACG AGGTTTCCTT

AGCCCACCCT TTGATGATTA TGGTATCCGG AATCGGGATG AGGTTTCTTT

AGCCCACCCT TTGACGACTA TGGCATCCGG AACCGAGATG AGGTTTCCTT

AGCCCACCCT TTGACGATTA TGGCATCCGG AACCGAGATG AGGTTTCCTT

---------- -----GATTA CGGGATCCGC AATCGGGACG AGGTTTCCTT

---------- -----GATTA TGGTATCCGG AATCGGGATG AGGTGTCCTT

AGTCCCCCAT TTGACGATTA CGGTATCCGG AACCGGGATG AGGTGTCCTT

---------- -----GATTA TGGTATCCGG AATCGGGACG AGGTGTCTTT

---------- -----GATTA CGGGATCCGC AATCGGGACG AGGTGTCCTT

AGCCCCCCCT TTGACGATTA CGGCATCCGG AACCGGGATG AGGTCTCCTT

AGCTCCCCCT TCGAC----- ---------- ---------- ----------

---------- -------TTA CGGCATCCGG AATCGGGATG AAGTCTCCTT

AGCCCTCCCT TTGACGAGTA CGGCATCCGA AACAGAGATG AGGTCTCCTT

AGTCCTCCCT TTGACGAGTA CGGCATCCGG AACCGTGACC AAGTTACCTT

CATCAAAAAG CTGAGGCAAA AGATGCGGGA CAACACCAGC CCCATCAGCG

CATCAAAAAG CTGAGGCAAA AGATGCGGGA CAACACCACC CCCATCAGCG

CATCAAAAAG CTGAGGCAAA AGATGCGGGA CAACACCAGC CCCATCAGCG

CATCAAAAAG CTGAGGCAAA AG-------- ---------- ----------

CATCAAAAAG CTGAGGCAAA AGATGGGGGA CGACACCAGC CCCATCAGCG

CATCAAAAAG CTGAGGCAAA AGATGGGGGA CAACACCAGT CCCATCAGCG

TATCAAGAAG CTTAGGCAAA AGATGGGGGA CAACACTAGC CCCATCAGCG

TATCAAGAAG CTTAGGCAAA AGATGGGGGA CAATACCAGC CCCATCAGCG

CATCAAAAAG CTGAGGCAGA AG-------- ---------- ----------

CATCAAAAAG CTGAGACAGA AAATGGGGGA CAACACCAGC CCCATCAGCG

CATCAAAAAG CTGAGGCAGA AA-------- ---------- ----------

CATCAAAAAG CTGAGGCAAA AAATGGGGGA CAACACCAGC CCCATCAGCG

CATCAAGAAG CTGCGGCAGA AG-------- ---------- ----------

CATCAAGAAG CTGAGGCAAA AGATGGGGGA CAACACCAGC CCCATCAGCG

---------- ---------- ---------- ---------- ----------

CATCAAAAAA CTGAGACAAA AGATGGGGGA CAACACGAGC CCCATCAGCG

TATCAAGAAG CTGAGGAACA AAATGGGGGA GAACACCAGC CCCATCAGTG

CAGCAAAAAA CTGAGGAACA AAATGGGGGA CAACAGCAGC CCCATCAGCG

TGATTCTGGT GAGCTCGGGG AGCAGGGGCA ATAAGCTGCT GTTCAGGTAC

TGATTCTGGT GAGCTCGGGG AGCAGGGGCA ATAAGCTGCT GTTCAGGTAC

TGATTCTGGT GAGCTCGGGG AGCAGGGGCA ATAAGCTGCT GTTCAGGTAC

---------- ---------- ---------- ---------- ----------

TGATTCTGGT GAGCTCGGGG AGCAGGGGCA ATAAGCTGCT GTTCCGGTAC

TGATTCTGGT GAGCTCGGGG AGCAGGGGCA ATAAGCTGCT GTTCAGGTAC

TGATCCTGGT GAGCTCGGGC AGCAGGGGCA ATAAGCTGCT CTTCAGGTAT

TGATCCTGGT GAGCTCGGGC AGCAGGGGCA ATAAGCTGCT CTTCAGGTAC

---------- ---------- ---------- ---------- ----------

TGATTCTTGT GAGCTCGGGG AGCCGGGGCA ATAAGCTGCT GTTCAGGTAC

---------- ---------- ---------- ---------- ----------

TGATTCTGGT GAGCTCGGGG AGCAGGGGCA ATAAACTGCT GTTCAGGTAC

---------- ---------- ---------- ---------- ----------

TGATTCTGGT GAGCTCGGGC AGCCGGGGCA ATAAGCTGCT GTTCAGGTAC

---------- ---------- ---------- ---------- ----------

TGATTCTGGT GAGCTCGGGG AGCAGGGGCA ATAAGCTGCT GTTCAGGTAC

TGATCCTGGT CAGTTCAGGG AGCAGGGGCA ATAAGTTACT ATTCAGGTAC

TCATCCTGGT CAGCTCGGGG AGCAGGGGCA ACAAGCTGCT CTTCAGATAC

CCCTTCCAGA GAAGCCAGGA GCACCCGGCG TCCCAGACAA GTAAGCCGCG

CCCTTCCAGA GAAGCCAGGA GCACCCGGCG TCCCAGACAA GTAAGCCGCG

CCCTTCCAGA GAAGCCAGGA GCACCCGGCG TCCCAGACAA ATAAGCCACG

---------- ---------- ---------- ---------- ----------

CCCTTCCAGA GGAGCCCGGA GCACCCGGCG TCCCAGACAA GTAAGCCCCG

CCCTTCCAGA GAAGCCAGGA GCACCCGGCG TCCCAGACAA ATAAGCCACG

CCCTTCCAGA GGAGCCAGGA GCACCCCGCG TCCCAGACAA ATAAACCACG

CCCTTCCAGA GGAGCCAGGA GCACCCCGCG TCCCAGACAA ATAAACCACG

---------- --AGCCCGGA GCACCCCGCG TCGCAGTCAA GTCAGCCACG

CCTTTCCAGA GGAGCCAGGA GCATTCGGCG TCCCAGAAGA GTAAGCCTCG

---------- ---------- ---------- ---------- ATAAGCCTCG

CCCTTCCAGA GAAGCCAGGA GCACCCGGCG TCCCAGACAA GTAAGCCTCG

---------- ---------- ---------- ---------- GCAAGCCTCG

CCCTTCCAGC GGAGCCAGGA GCACCCCGCG TCCCAGACAA ACAAACCACG

---------- ---------- ---------- ---------- ----------

CCCTTCCAGA GAAGCCAGGA GCACCCCGCG TCCCAGACAA ATAAGCCGAG

CCATTTCAGA GAAACCAGGA ACACCCTGCA TCCCAGACAA ACAAGCCACG

CCCTTCCAGC GGCCGCAGGA GCACCCCGCC GCCCAGCCCA GCCAGCCGAG

TAGCAGATAC GCTGCCAGCA ACACGGGCGA CCATGCTGAT GAGCAGGACG

TAGCAGATAC GCTGCCAGCA ACACGGGCGA CCATGCTGAT GAGCAGGACG

TAGCAGATAT GCTGTTAACA ACACGGGCGA CCATGCTGAT GACCAGGATG

---------- ---------- ---------- ---------- ----------

TAGCCGATAC GCTGTTAACA ACACGGGCGA CCATGCTGAT GACCAGGATG

TAGCAGATAT GCTGTTAACA ACATGGGCGA CCATACTGAT GACCAGGATG

TAGCAGATAC GCTGTCAACA ACACGGGAGA GCATGTTGAT GACCAGGATG

GAGCAGATAT GCTGTCAACA ACACTGGCGA ACATGCTGAT GACCAGGACG

TAGCAGGTAT GCTGTTAACC ACACCGGGGA CCACGTTGAT GACCAGGACG

TAGCAGATAC GCTGTCAACA GTGCCGGAGA GCATGCTGAA GACCAGGATG

TAGCAGATAT GCCGTCAACA ACACTGGAGA CCACGCTGAC GACCAGGACG

TAGCAGATAT GCCGTCAACA ACACTGCAGA CCAGGCCGAG GATCTGGATG

TAGCAGATAT GCTGTGAACA GTACAGGCGA TCATGCTGAT GACCAGGATG

AAGCAGATAT GCAGTGCACA GCACGGGGGA CCACTCTGAC GACCAGGATG

---------- ---------- ---------- ---------- ----------

GAGCAGGTAT GCTGTTAACA GCACCGGGGA CCACTCTGAG GACCAGGACG

TAGCCGATAT GCTGTTAACA ACACAGGAGA CATTACAGAT GACCAAGATG

GAGCCGATAC GCCGTGAACA GCAGCGGGGA TGTCTCCGAG GACCAGGACG

GCGATTCCAG GCCGCCCGAC GTGGAGGGGG ACGACTGTCT CCCCGCGTAC

GCGATTCCAG GCCGCCCGAC GTGGAGGGGG ACGACTGTCT CCCCGCGTAC

GTGATGCCAG G--------- ---------- ---------- ----------

---------- GCCGCCTGAC GTGGAGGGGG ACGACTGTCT CCCCGCGTAC

GCGATTCCAG G--------- ---------- ---------- ----------

GCGACTCCAG G--------- ---------- ---------- ----------

GTGACTCCAG GCCGCCTGAC GTTGAGGAGG ATGACTGTCT TTCTGAATAC

GTGACTCCAG GCCGCCCGAC GTTGAGGGGG ATGACTGTCT TCCTGAATAC

GCGACTCCAG G--------- ---------- ---------- ----------

GGGACCCCAG GCCGGCCGAC GTCAGCGAGG ACGATTGTCT CCCGGAGTAC

GTGATTCCAG G--------- ---------- ---------- ----------

GCGACTCCAG GCCGCCCGAT GTCCCAGAGG ACGATTGTCT ACCTGAGTAC

GAGACTCCAG G--------- ---------- ---------- ----------

GTGACTCCAG GCAGCCCGAC GTCAGCGAGG ACGACTGCCT CCATGAGTAC

---------- ---------- ---------- ---------- ----------

GAGACTCCAG -CCGCCTGAC GTGGATGGGG ACGACTGTCT CCTCGAATAC

GGGATTCCAG ACCCCCCGAC GTCATCTCTG ATGACTGTCT CCGGAGTTAT

GGGACTCCAG ACCGCCGGAC GTGGCCACCG ATGACTGCCT GCAGGAATAT

CGCCACCTCT TCTGCCCGGA CCTGCTGCGG GACAAAGTGG CCTTCATCAC

CGCCACCTCT TCTGCCCGGA CCTGCTGCGG GACAAAGTGG CCTTCATCAC

---------- ---------- ---------G GACAAAGTGG CCTTCATCAC

CGTCACCTCT TCTGCCCGGA CCTGCTGCGG GACAAAGTGG CCTTCATCAC

---------- ---------- ---------G GACAAAGTGG CCTTCATCAC

---------- ---------- ---------- ---------- ----------

CACCACCTCT TCTGCCCGGA CCTTCTCCAG GACAAAGTGG CTTTTATCAC

CACCATCTCT TCTGCCCGGA TCTTCTCCAG GACAAAGTGG CCTTTATCAC

---------- ---------- ---------- ---------- ----------

CGCCACCTCT TCCATCCGGA CTTGCTCCAG GACAAGGTGG CTTTCATCAC

---------- ---------- ---------G GACAAAGTGG CATTCATCAC

CGCCACCTCT TTAGCCCCGA CCTACTCCAG GACAAAGTCG CCTTCATCAC

---------- ---------- ---------- ---------- ----------

TGCCACCTCT TCAGCCCGGA CCTGCTCCGG GACAAAGTGG CCTTTATCAC

---------- ---------- ---------- ---------- ----------

CGCCACCTCT TCTGCCCAGA CCTCCTCCGG AACAAGGTGG CCTTTATCAC

AGCTACCTCT TCTGCCCAGA TATTCTCCGT GAAAAAGTGG CCTTTATCAC

TCGTGCCTCT TCAGCCCGGA CATCCTCCGG GGCAAGGTGG CCTTCATCAC

AGGAGGCGGC TCTGGGATTG GGTTCCGGAT TGCTGAGATT TTCATGCGGC

AGGAGGCGGC TCTGGGATTG GGTTCCGGAT TGCTGAGATT TTCATGCGGC

AGGTGGCGGC TCTGGGATTG GGTTCCGGAT TGCTGAGATT TTCATGCGGC

AGGTGGCGGC TCTGGGATTG GGTTCCGGAT TGCTGAGATT TTCATGCGGC

AGGTGGCGGC TCTGGGATCG GGTTCCGGAT TGCTGAGATT TTCATGCGGC

---------- ---------- ---------- ---------- ----------

TGGTGGTGGT TCTGGGATTG GCTTCCGGAT CGCCGAGATT TTCATGAGGC

AGGTGGTGGT TCTGGGATTG GCTTCCGGAT CGCAGAGATT TTCATGAGGC

---------- ---------- ---------- ---------- ----------

GGGTGGTGGC TCTGGAATTG GGTTCCGGAT TGCTGAGATT TTCATGCGGC

CGGTGGTGGT TCTGGCATCG GGTTCCGGAT TGCTGAGATT TTTATGCGGC

AGGCGGTGGC TCTGGGATCG GGTTCCGGAT AGCTGAGATA TTTATGCGGC

---------- ---------- ---------- ---------- ----------

TGGTGGAGGC TCGGGTATCG GGTTCCGGAT CGCCGAGGTC TTCATGCGGC

---------- ---------- ---------- ---------- ----------

TGGTGGGGGC TCAGGGATCG GATTCCGGAT TGCTGAGATC TTCATGCGGC

TGGAGGAGGC TCAGGAATTG GGTTCCGAAT TGCTGAAATT TTCATGAGGC

CGGAGGAGGC TCAGGAATCG GCTTTCGCAT TGCTGAGATT TTCATGAGAC

ACGGCTGCCA TACGGTGATT GCCAGTAGGA GCCTGCCGCG AGTGCTGACG

ACGGCTGCCA TACGGTGATT GCCAGTAGGA GCCTGCCGCG AGTGCTGACG

ACGGCTGTCA CACGGTGATC GCCAGCAGGA GCCTGCCACG AGTTCTGATG

ACGGCTGCCA CACGGTGATC GCCAGCAGGA GCCTGCCACG AGTGCTAATG

ATGGCTGCCA CACGGTGATC GCCAGCAGGA GCCTGCCCCG ACTGTTGACG

---------- ---------- ---------- ---------- ----------

ATGGCTGCCA CACTGTCATC GTCAGCAGGA GTCTGCCGAG AGTGTCCGAG

ATGGCTGCCA CACTGTCATC GTCGGCAGGA GTCTGCAGAA AGTGACCACG

---------- ---------- ---------- ---------- ----------

ACGGCTGTCA CACAGTCATC GCCAGCAGAA GTCTTCCCAG AGTATCGATG

ACGGCTGCCA CACAGTCATC GCCAGCAGAA GCTTTCCAAG AGTGTCTAAG

ACGGCTGCCA TGTGGTCATC GCCAGCAGAA GCCTTCCGAG AGTGTCAACG

---------- ---------- ---------- ---------- ----------

ATGGCTGCCA CACGGTCATC GCCAGTAGGA GCCTGCCGAG ATTGTCAGCG

---------- ---------- ---------- ---------- ----------

ATGGTTGCCA CACTGTCATC GCCAGCAGGA GCCTGCCCAG ACTGCTGACG

ACGGCTGCAA CACAGTCATT GCTAGCAGAA ACCAGCAAAG GGTGACTGAT

ATGGCTGTCA CGCCGCCATC TCCAGCAGGA GCCTGCTGGG AGTGTTTGAG

GCCGCCAGGA AGCTGGCTGG GGCCACCGGC CGGCGCTGCC TCCCTCTCTC

GCCGCCAGGA AGCTGGCTGG GGCAACCG-- ---------- ----------

GCCGCCAGGA AGCTGGCTGG GGCCACTGGC CGGCGCTGCC TCCCTCTCTG

---------- ---------- ---------- ---------- ----------

GCCGCCAGGA AGCTGGCTGC TGCCACCGGC CGGCGCTGCC TCCCTCTCTC

---------- ---------- ---------- ---------- ----------

GCTGCTAAGA AGTTGGTTGC TGCCACTGGA AAGCGGTGTC TCCCTCTGTC

GCTGCTAAGA AGTTGGTTGC TGCCACCGGA AAGCGGTGCC TCCCTCTGTC

---------- ---------- ---------- ---------- ----------

GCTGCCAGAA AGCTGGCCGC TGCCACCGGC CAGAGGTGCC TCCCTCTGTC

GCTGCCAGGA AGCTGGCTGC TGCCACTGGC CAGCGGTGCC TCCCCTTATC

GCTGCCAGAA AGCTAACTGC TGCCACTGGC CGGCGGTGCC TCCCTTTATC

---------- ---------- ---------- ---------- ----------

GCTGCCAAGA AGCTGGCTGC CGCCACCGGC CAGCGGTGCC TGCCCGTGTC

---------- ---------- ---------- ---------- ----------

GCTGCCAAGA AGCTGACTGA GGCCACGGGC CAGCAGTGTC TCCCCCTCTC

GCCGCTAGGA AACTCATTGC TGCTACTGGT CAGCAGTGCC TTGCCCTGTC

GCTGCTGAGA AACTGAGTGT GGCCACAGGC CAGCGTTGCC TGCCTTTGTC

TATGGACGTC CGAGCGCCCC CAGCTGTCAT GGCCGCCGTG GACCAGGCTC

---------C CGAGCGCCCC -AGCTGTCAT GGCCGCCGTG GACCAGGCTC

TATGGACGTC CGAGCACCCC CAGCTGTGAT GGCTGCCGTG GACCAGGCTC

---------- ---------- ---------- ---------- ----------

TATGGACGTC CGAGTACCCC CGGCTGTCAT GGCCGCTGTG GACCAGGCTC

---------- ---------- ---------- ---------- ----------

TATGGATGTC CGAGTTCCCC CAGCTGTCAT GGCTGCTGTG GACCAAGCGC

TATGGATGTC CGAGTTCCCC CAGAGGTCAT GACTGCTGTG GATCAAGCGC

---------- ---------- ---------- ---------- ----------

TCTGGACGTC CGAGCTCCCC TGGCCATCGC GGCAGCTGTA GAGCAGGCCC

TCTGGACGTT CGAGCTTTTC CAGCCATCAC AGCTGCCGTG GACCAGGCAC

TCTGGATGTC CGAGCTCCCC CAGCCATCAT AGCCGCCGTG GAGCAGGCAC

---------- ---------- ---------- ---------- ----------

TCTGGACGTG CGTGACCCCC TGACCATCAT GGCCGCTGTG GATGTGGCGC

---------- ---------- ---------- ---------- ----------

TCTGGATGTC CGAAATCCCC CAACCATCTT GGCTGCAGTG GACAAGGCAC

TCTGGATGTC CGAGAGCCTC AGGCCATTGC TGCAGCGGTA GATCAGGCTC

TCTGGACGTG CGGCAGCCCC AGACGATCGC CGTGGCAGTC GACACAGCCC

TGAAGGAGTT TGGCAGAATC GACATTCTCA TTAACTGTGC GGCCGGGAAC

TGAAGGAGTT TGGCAGAATC GACATTCTCA TTAACTGTGC GGCCGGGAAC

TGAAGGAGTT TGGCAGAATT GACATTCTCA TTAACT---- ----------

---------- ---------- ---------- ---------- ----------

TGAAGGAGTT CGGGAAAATT GACATTCTCA TTAACTGTGC GGCTGGAAAC

---------- ---------- ---------- ---------- ----------

TGAAAGAATT TGGCAAAATC GACATCCTCA TTAACTGTGC TGCAGGGAAC

TGCAAGAATT TGGCAAAATC AACATCCTCA TTAACTGTGC AGCTGGAAAC

---------- ---------- ---------- ---------- ----------

TGAAGGAGTT CGGGAAGATC GACATTCTCA TCAACTGTGC AGCCGGGAAC

TGAAGGAGTT TGGGAAGATC GACATTCTGG TTAACTGTGC AGCGGGAAAC

TGAAGGAGTT TGGGAAAATC GACATTCTCA TAAACTGTGC GGCTGGAAAC

---------- ---------- ---------- ------CTGC GGCTGGGAAC

TGACGGAGTT TGGGAAAATT GACATCCTCA TTAATGGTGC AGCTTTGAAC

---------- ---------- ---------- ---------- ----------

TGGAGGAGTT TGGGAAGATT GACATTCTCA TTAACGGTGC AGCTGGGAAC

TGAAGAAGTT TGGCAAGATT GACATTCTCA TCAATGGTGC AGCAGGTAAT

TGAAGGAGTT GGGCCGGGTG GACATTCTCA TCAATGGTGC GGCAGGTAAC

TTCCTGTGCC CCGCTGGCGC CTTGTCCTTC AACGCCTTCA AGACCGTGAT

TTCCTGTGCC CCGCTGGCGC CTTGTCCTTC AACGCCTTCA AGACCGTGAT

---------- ---------- ---------- ---------- ----------

---------- ---------- ---------- ---------- ----------

TTCCTGTGCC CTGCCGGCGC CTTGTCCTTC AACGCCTTCA AGACCGTGTT

---------- ---------- ---------- ---------- ----------

TTTTTATGCC CTGCCAGTGC ATTGTCTTTC AATGCCTTTA AGACTGTGGT

TTCCTATGCC CTGCCAGTGC TTTGTCTTTC AATGCCTTTA AGACCGTGGT

---------- ---------- ---------- ---------- ----------

TTCCTGTGCC CGGCCAGCGC CTTGTCCTCC AACGCCTTCA AGACTGTGAT

TTCCTGTGCC CTGCCAGTGC ACTGTCCTTC AACGCCTTTA AGACCGTGAT

TTCCTGTGTC CGGCCGGCGC GTTGTCCTTC AATGCCTTCA AGACCGTGAT

TTCCTGTGTC CTGCCAGTGC ACTGTCCTTC AATGCCTTCA AGACCGTGCT

TTCCTGTGTC CGGCCGGCGC GTTGTCCTTC AGAGCCTTCA AGACCGTGGT

---------- ---------- ---------- ---------- ----------

TTCCTGTGCC CCGCTGGGGC CCTGTCCTTC AACGCCTTCA AGACCGTGCT

TTTTTGTGTG CAGCCAGCAC CATTTCCTTC AATGCTTACA AAACTGTGAT

TTCCTGTGCC CAGCCAAGGC TCTGTCTTTC AACGCCTACA AGACGGTGAT

GGACATCGAT ACCAGCGGCA CCTTCAATGT GTctcgtgtg ctcGACCACG

GGACATCGAC ACCAGCGGCA CCTTCAACGT GTCTCGTGTG CTCGACCATG

---------- ---------- ---------- ---------- ---GACCATG

---------- ---------- ---------- ---------- ----------

GGACATAGAC ACCGGTGGCA CCTTCAACGT GTCTCGCGTA CTTGACCACG

---------- ---------- ---------- ---------- ----------

GGACATTGAC ACCCTTGGCA CCTTCAATGT GTCTCGTGTG CTTGACCATG

TGACATTGAC ACCATCGGTA CCTTCAATGT GTCTAGTGTG CTTGACCATG

---------- ---------- ---------- ---------- ----------

GGACATCGAC ACCTTGGGCA CCTTCAACGT GTCTCGCGTG CTCGACCATG

GGACATCGAC AGCTTGGGCA CCTTCAACAT GTCTCGCGTG CTCGACCACG

GGACATCGAC ACCTTGGGGA CCTTCAATGT GTCTCGCGTG GTCGACCATG

GGACATCGAC ACTTTGGGCA CCTTCAACGT GTCTCGCGTG CTGGACCATG

GGACATCGAC ACCATCGGCA CCTTCAACGT GTGCCGTGTG CTCGACCACG

---------- ---------- ---------- ---------- ----------

GGACATTGAT GCTGCCGGCA CCTTCAACGT GTCCCGTGTG CTCGACCATG

CGAAATTGAT ACAATTGGCA CCTTCAATGT CTCCAAAATC GTCAACCATG

TGACATAGAC ACCATCGGCA CCTTCAACGC CTCCAAAGTG CTCGAACCCA

GAGGGGTGAT CGTGAACATC ACTGCCACCC TGGGGAACCG GGGGCAGGCG

GAGGGGTGAT CGTGAACATC AGTGCCACCC TGGGGAACCG GGGGCAGGCG

GAGGGGTGAT CGTGAACATC ACTGCCACCC TGGGGAACCG GGGGCAGGCG

---------- ---------- ---------- ---------- ----------

GAGGGGTGAT TGTGAACATC ACTGCCACCC TGGGACAGCG GGGGCAGGTG

---------- ---------- ---------- ---------- ----------

GAGGAGTGAT CGTGAACATT ACCGCCACCC TCAGTATGCG GGGGCAGGTG

GAGGAGTGAT TGTGAACATT ACTGCCACCC TCAGTATGAG GGGGCAGGTG

---------- ---------- ---------- ---------- ----------

GAGGGGTGAT CGTGAACATC ACTGCAACCC TGGGCACCCG GGGGCAGGTG

GAGGGGTGAT CGTGAACATC ACTGCGACTC TGGGTACCCG GGGGCAGGTG

GAGGAGTGAT TGTAAACATC ACTGCGACTC TCGGTGCCCG AGGGCAGGTG

GAGGGGTGAT CGTGAACATC ACCGCCACCC TCGGTGCCCG GGGTCAGGTG

GAGGCGTGAT CGTCAACATC ACCGCAACTC TGAGCCACCG GGGGCAGATG

---------- ---------- ---------- ---------- ----------

GAGGGGTGAT CTTAAACATC ACTGCCACGC TGAGCACACG TGGACAGGTG

GTGGTGTGAT CATTAATATC ACTGCTACCC TGGACTACCG GGGGCAGGCG

GTGGGATGTG CCTGGTCAGT GGGATGGTCC CATCTCCTCG AGGGCAGAGC

CTCCAGCATG CAGGCTCCGC CAAGGCCGCT GTGGACGCGA TGACGCGGCA

CTCCAGCATG CAGGCTCCGC CAAGGCCGCT GTGGACGCGA TGACGCGGCA

CTCCAGCATG CAGGCTCTGC CAAGGCTGCT GTGGACGCGA TGACACGGCA

---------- ---------- ---------- ---------- ----------

CTCCAGCATG CGGGCTCTGC CAAGGCTGCT GTGGACGCGA TGACACGGCA

---------- ---------- ---------- ---------- ----------

CTGCAGCATG CAGGCGCTGC CAAGGCGGCT GTGGATGCTA TGACGCGACA

CTACAGCATG CAGGCGCTGC CAAAGCAGCT GTGGATGCTA TGACGCGACA

---------- ---------- ---------- ---------- ----------

CTCCAACACG CAGGCTCTGC CAAGGCGGCC GTGGATGCGA TGACGCGGCA

CTTCAACATG CGGGCTCTGC CAAGGCAGCT GTGGATGCGA TGACACGGCA

CTCCAACACG CAGGCTCTGC CAAGGCGGCT GTGGATGCAA TGACGCGGCA

CTGCAGCATG CTGGCTCTGC CAAGGCGGCT GTGGATGCGA TGACCCGGCA

CTGCAGCACG CGGGCTCCGC CAAGGCGGCC GTGGATGCGA TGACACGTCA

---------- ---------- ---------- ---------- ----------

TTCCAGCATG CAGGTGCCGC CAAGGCTGCC GTTGACGCCA TGACGCGGCA

CTCCAGCATG CAGGCACTGC CAAGGCAGCT GTAGATGCAA TGACAAGGCA

CTCC--CTCT CCCGCTCTAA TGAAGCTGCT TTGGATGCCA TGACCCGGCA

CTTGGCTGTG GAGTGGGGTC CCCAAAACAT CCGCGTCAAC AGCCTCGCCC

CTTGGCTGTG GAGTGGGGTC CCCAAAAAAT CCGCGTCAAC AGCCTCCCCC

CTTGGCTGTG GAGTGGGGTC CCCAGAACAT CCGTGTCAAC AGCCTCGCCC

---------- ---------- ---------- ---------- ----------

CCTGGCTGTG GAGTGGGGTC CGCAGAATAT CCGTGTCAAC AGCCTCGCCC

---------- ---------- ---------- ---------- ----------

CTTGGCTGTG GAGTGGGGTC CCCAGAATAT CCGTGTCAAC AGCCTGGCTC

CTTGGCTGTG GAATGGGGTC CCCAGAATAT CCGTGTCAAC AGCCTGGCTC

---------- ---------- ---------- ---------- ----------

CCTGGCTGTG GAGTGGGGCC CCCAGAACAT CCGCGTCAAC AGCCTTGCCC

CTTGGCCGTG GAGTGGGGTC CCCAGAACAT CCGCATCAAC AGCCTCGCCC

TCTGGCTGTG GAGTGGGGTC CCCAAAACAT CCGTGTCAAC AGCCTTGCCC

CTTGGCTGTG GAATGGGGGC CCCAGAACAT CCGCGTCAAC AGCCTGGCTC

TCTGGCTGTG GAATGGGGTC CCCAGAACAT CCGCGTCAAC AGCCTGGCCC

---------- ---------- ---------- ---------- ----------

CCTGGCCGTA GAGTGGGGGA AGCAGAACAT CCGGGTCAAC AGCCTGGCTC

TTTGGCTGTG GAATGGGGTC ACAACAACGT TCGAGTTAAT AGCCTGGCCC

CCTGGCCGTG GAGTGGGGCC CCTATAATGT CCGGGTCAAC AGCCTGGCCC

CTGGCCCCAT CAGTGGCACA GAGGGGCTCC GGCGACTGGG TGGCCCTCAG

CCGGCCCCAT CAATGGCACG GAGGGGCTCC GGCGACTGGG TGGCCCTCAG

CTGGCCCCAT CAGTGGTACG GAGGGGCTCC GGCGACTGGG TGGCCCCCAG

---------- ---------- ---------- ---------- ----------

CCGGCCCCAT CAGTGGCACG GAGGGGCTCC GGCGACTGGG TGGCCCCCAG

---------- ---------- ---------- ---------- ----------

CTGGTGCCAT CAGCGGCACT GAGGGTCTGC GGAGATTAGG AGGCCCCAAG

CTGGTGCCAT CAGTGGCACT GAGGGTTTAA GGCGATTAAG AGGCTCCAAT

---------- ---------- ---------- ---------- ----------

CCGGCCCCAT CAGTGGGACA GAGGGGTTGC GGCGTCTGGG TGCCCCGCAG

CTGGCCCCAT CAGTGGCACG GAGGGGTTCC GGCGGCTGGG TGGCCACCAG

CTGGTCCCAT CAGTGGCACG GAGGGGTTCC GGCGGCTGGG TGGTCCCCAG

CTGGCCCCAT TGGTGGCACA GAGGGATTCC GGCGGCTGGG TGGTCCACTG

CCGGGGCCAT CGGCGGCACG GAGGGCTTCC GGAGGCTGAT TGCTTCCTCG

---------- ---------- ---------- ---------- ----------

CTGGTCCTAT CGCTGGCACT GAGGGCTTCC GGCGTTTGGG TGGTCCCCAG

CAGGCCCCAT TAGTGGCACT GAAGGGATGC GTCGACTTGG TACCCATCAA

CCGGCCCCAT CAGCGGCACT GAGGGCATGC GGCGTCTCGG GGGTTCCCAG

GCCAGCCTGA GCACCAAGGT CACTGCCAGC CCGCTGCAGA GGCTGGGGAA

GCCAGCCTGA GCACCAAGGT CACTGCCAGC CCGCTGCAGA GGCTGGGGAA

GCCGGCCTGA GCACCAAGGT CACTGCGAGC CCGCTGCAGA GGCTAGGGAA

---------- ---------- ---------- ---------- ----------

GCCAGCCTGA GCACCAGGGT GGCTGTGAGC CCGCTGCAGA GGCTGGGGAA

---------- ---------- ---------- ---------- ----------

GCCAGTTCGA AATTTAAGTA TCTTTCAAGT CCTATTCCAA GACTCGGAAC

GCCAGTTCGA AATTAAAGCA TTTTTCAAAT CCTATTCCAA GACTTGGGAC

---------- ---------- ---------- ---------- ----------

GCCGGCCTGA GGGCGAAGGT CCTGGCCAGC CCCCTGCAGA GGCTGGGGAA

GCCAGTGTCA GCACCAAGGT TCTGGACATC CCCCTGCAGA GGCTGGGCAA

GCCAGCATGA GTGGGAAGTT CCTGGCCAGC CCCCTGCAGA GGCTGGGGAA

GCCAGTGCAC ACATGAAGGC TCAGGCCAGC CCCCTGCAGA GGCTGGGGAA

GACATCGCGA GGACCTACGT CCAGGAGACA CCCCTGCAGA GGCTGGGGTT

---------- ---------- ---------- ---------- ----------

GCCAGCAGAA GGCTGCACCT CTTGGGGATG CCACTCCAGA GGCTGGGGAA

CCCAACTGGG ATAGAAATAT TTTGGGAAGC CCTCTCCAGA GAGTGGGGAA

ACCCACTTGA GCAGAGCCAT CCTGGGGATC CCCCTCCAGA GGCTGGGGAA

CAAGACCGAG ATCGCCCACA GCGTGCTCTA CCTGGCCAGC CCTCTGGCTT

CAAGACCGAG ATCGCCCACA GCGTGCTCTA CCTGGCCAGC CCTCTGGCTT

CAAGACCGAG ATTGCCCACA GCGTGCTCTA CCTGGCCAGC CCTCTGGCTT

---------- ---------- ---------- ---------- ----------

CAAGACGGAG ATTGCCCACT GCGTGCTCTA CCTGGCCAGC CCTCTGGCCT

---------- ---------- ---------- ---------- ----------

CAAGACAGAA ATCGCCCACA GCGTGCTGTA CCTAGCCAGC CCTCTGGCTT

CAAGACAGAG ATCGCTCACA GCGTGCTGTA CCTGGCCAGC CCTCTGGCTT

---------- ---------- ---------- ---------- ----------

CAAGACAGAA ATCGCCCACA GCGCCCTCTT CCTGGCCAGC CCGCTGGCGT

CAAGACCGAG GTGGCCCACG GCGCGCTGTA CCTGGCCAGC CCCTTGACAT

CAAGACGGAG ATCGCCCACA GTGTGCTGTA CCTGGCTAGT CCTTTGGCGT

CAAGACGGAA GTGGCCCACT GCGCCCTTTT CCTGGCCAGC CCACTGGCTT

CAAGACGGAG GTGGCCCACA GCGCGCTCTT CCTGGCCAGC CCCCTCGCGT

---------- ---------- ---------- ---------- ----------

CAAGACCGAG ATGGCCCACA GCGCGCTCTT CCTGGCCAGC CCCGCGGCAT

TAAGACCGAG ATTGCCCACA GTGCACTGTT TTTGGCTAGC CCCTTATCAT

CAAGACAGAG ATCGCCCACA GTGCCCTGTA CCTGGCCAGT CCCTTGGCAT

CCTACGTGAC GGGGGCCGTG CTGGTGGCCG ATGGCGGGGC ATGGTTGACG

CCTACGTGAC GGGGGCCGTG CTGGTGGCCG ATGGCGGGGC ATGGCTGACG

CCTACGTGAC GGGGGCTGTG CTGGTGGCCG ATGGTGGGGC ATGGTTGACG

---------- ---------- ---------- ---------- ----------

CCTGCGTGAC GGGGGCCATG CTGGTGGCTG ATGGCGGGTC ATGGTTGACA

---------- ---------- ---------- ---------- ----------

CCTATGTCTC AGGGATTGTG TTGGTGGTTG ATGGTGGTAG CTGGATGACG

CCTATGTCTC AGGTATCGTG CTGGTGGTTG ATGGTGGAAG CTGGATGACA

---------- ---------- ---------- ---------- ----------

CCTTCGTGAC GGGGGCCCTG CTGGTGGTTG ACGGGGGCGC CTGGCTAACA

CCTATATGAC AGGCGCTGTG CTGGTGGTTG ACGGCGGGGC ATGGCTGACC

CCTACGTGAC GGGTGCCCTG CTGGTGGTTG ACGGCGGAGC GTGGCTGACG

CCTATGTGAC GGGGACTGTG CTGGTAGTGG ATGGTGGCTC CTGGCTGACC

CCTTCGTGAC CGGGATCGTG CTGGTGGTGG ACGGTGGGGC GTGGATGACC

---------- ---------- ---------- ---------- ----------

CCTTTGTGAC AGGCGCCGTG CTGGTGGCCG ACGGTGGCCT GTGGCTGACC

CCTTTGTGAC AGGCACAATC CTGGTGGTGG ATGGTGGGAG CTGGCTGACT

CCTATGTCAC GGGGGCCTCC CTGGTGGTGG ATGGCGGCAG CTGGCTGACA

TTCCCAAACG GTGTCAAAGG GCTGCCGGAT TTCGCATCCT TCTCTGCTAA

TTCCCAAACG GTGTCAAAGG GCTGTCGGAT GTCGCATCCT TCTCTGCTAA

TTCCCAAACG ATGTCAAAGG GCTGGCGGAT TTCGCATCCT TCTCTTCTAA

---------- ---------- ---------- ---------- ----------

TTGCCAAACG ATGTGAAGGG ACTGGCAGAT TTTGCATCCT TCTCTGCTAA

---------- ---------- ---------- ---------- ----------

CTCCCAAATG ACATTGGGCG ACTGCTAGAG TTTGAATCCT CCTCTGCTAA

TTCCCCAATG GCATCAAGCA ACTGCTAGAG TTTGAATCCT TCTCTGCTAA

---------- ---------- ---------- ---------- ----------

TTCCCTAATG ACGTCCAGTC GCTGGCAGAT TTCTCACC-- -CTCCTCTAA

TTCCCTAATG ACCTCAAGCT GCTGGCAGAT TTCACTGC-- -CTCTGCTAA

TTCCCTAATG ACATCAAGTT GCTGGCGGAT TTCGCATCCT TCTCTGCTAA

TTTCCGAATG ACATCGGCCG TCTGGCCCAT GTGTCCTC-- -CCCTGCTAA

CTCCCCAGCG ACGCCCGGAA GCTGATGGAC T---CGGCCA TGTCTGCGAA

---------- ---------- ---------- ---------- ----------

TCCCCCAACA GCATGGAGAC GCTGGCAGGG TCCCAGTCCT TCCCGGCAAA

TATGCCAACG ATTTCATGAA GTTGTTGGCG ATTGAAACCT TCCCTTCCAA

TCCCCCAACG GCTCCTTGGG TCGGTCGGCG TCCGCGTCCT TCTCTGCTAA

GCTCATGGTG CTGTCTCCTG CCGACAAGAC CAACGTCAAG GCCGCCTGGG

GCTCATGG-- ---------- ---------- ---------- ----------

GCTCATGGTG CTGTCTCCTG ACGACAAGAA ACACGTCAAG GCCGCCTGGG

---------- ---------- ---------- ---------- ----------

GCTCATGGTG CTGTCTCCCG CCGACAAGAG CAACGTCAAG GCCGCCTGGG

----ATGGTG CTGTCTCCCA CGGACAAGAG CAACGTCAAG GCTGCCTGGG

GCTGATGGTG CTCTCTGCAG ATGACAAAAC CAACATCAAG AACTGCTGGG

GCTGATGGTG CTCTCTGGGG AAGACAAAAG CAACATCAAG GCTGCCTGGG

----ATGGTG CTGTCTCCCG CTGACAAGAC CAACATCAAG ACTGCCTGGG

GCTTATGGTG CTGTCTGCCG CCGACAAGGG CAATGTCAAG GCCGCCTGGG

GCTCATGGTG CTGTCTCCCG CCGACAAGAC CAACATCAAG TCCACTTGGG

ACTCATGGTG CTTTCCCCCA GCGACAAAAG CAACGTCAAG GCCGCCTGGG

GCTC------ ---------- ---------- ---------- ----------

GCTCATGGTG CTCTCTGCCG CCGACAAGGC CCACGTCAAG GCCTTCTGGG

----ATGGTG CTGTCTGATA ACGACAAGAC CAACGTCAAG GCCACCTGGA

GCTCATGGTG CTGTCTGCTG CTGATAAGGC CAACGTGAAG ACCGTCTGGG

ACTCATGGCC TTGTCTGCCG AAGACAAGTG CAACGTGAAA GAATTCTGGG

ACTCATCTTT CTCTCTCCCG GACACCAGAC CCGCTCCTCA CCCGCCCTGG

GTAAGGTCGG CGCGCACGCT GGCGAGTATG GTGCGGAGGC CCTGGAGAGG

---------- ---------- ---------- ---------- ----------

GTAAGGTCGG CGAGCACGCT GGCGAGTATG GTGCGGAGGC CCTGGAGAGG

---------- ---------- ---------- ---------- ----------

GTAAGGTCGG CAGCCACGCT GGCGACTATG GTGCCGAGGC CCTGGAGAGG

AAAAGGTTGG CGGCCATGCT GGCGACTACG GCGCGGAGGC CCTGGAGAGG

GGAAGATTGG TGGCCATGGT GGTGAATATG GCGAGGAGGC CCTACAGAGG

GGAAGATTGG TGGCCATGGT GCTGAATATG GAGCTGAAGC CCTGGAAAGG

AAAAGATCGG CAGCCACGGT GGCGAGTATG GCGCCGAGGC CGTGGAGAGG

GCAAGGTTGG CGGCCACGCT GCAGAGTATG GCGCAGAGGC CCTGGAGAGG

ATAAGATTGG TGGCCACGCT GGCGACTACG GCGGAGAGGC GCTGGACAGG

ATAAGGTCGG CGGCAATGCT GCGGGGCTCG GCGCGGGGGC TCCGACCCAG

---------- ---------- ---------- ---------- ---------G

GGAAGGTCGG CGGCCATGCC GCTGAGTTCG GCGCCGAGGC CCTGGAGAGG

GCAAGGTTGG CGACCACGCT GGCCGGTGTC TAGCCAAGGC CCCGATGCCG

AGAAGGCCGG CGGCAACGTG GGCCGGTACC CA-CCCAGGC CCC------G

GAAAATTGGG TGAGAACACA CACGTCTACG GCACTGAAGC CCTGGTGAGG

CACCGGCCCG CAGCCACCCA GATCTCTCGG GTGCTCCAAC CCCGATGGGG

ATGTTCCTGT CCTTCCCCAC CACCAAGACC TACTTCCCGC ACTTCGACCT

---------- ---------- ---------- ---------- ----------

ATGTTCCTGT CCTTCCCCAC CACCAAGACC TACTTCCCCC ACTTCGACCT

---------- ---------- ---------- ---------- ----------

ATGTTCCTGT CCTTCCCCAC CACCAAGACC TACTTCCCCC ACTTCGACCT

ATGTTCCTGT CCTTCCCCAC CACCAAGACC TACTTCCCCC ACTTCGACCT

ATGTTCGCTG CCTTCCCCAC CACCAAGACC TACTTCTCTC ACATTGATGT

ATGTTTGCTA GCTTCCCCAC CACCAAGACC TACTTCCCTC ACTTTGATGT

ATGTTCCTGG GCTTCCCCAC CACCAAGACC TACTTCCCCC ACTTCGACTT

ATGTTCCTGA GCTTCCCCAC CACCAAGACC TACTTCCCCC ACTTCGACCT

ACCTTCCAGT CCTTCCCCGC CACCAAGACT TACTTGCAGC AGTTCGACCT

ATGTTCCTGA GCTTCCCCAC CACCAAGACC TACTTCCCCC ACTTCGACCT

CTCTTCACCA GCTACCCGCA GACCAAGACC TACTTCCCGC ACTTCGACCT

CTCTTCGCAG CGTACCCCAG CACCAAGGCC TACTTCCCGC ACCTGGGTGT

ATGTTCTTCT CCTTTCCCAC CACCAAGACC TACTTTCCTC ACTTCGACCT

ACCTTCCTCT CCTTCCCCAC CACCAAGACC TACTTCCCTC ACATGGACCT

ACCTTCCTCT CTTTCCCCCC CACCAAGACC TACTTCCCGC ACTTCGATTT

CTGTTCCTCT CTTTCCCCCC CACTAAGACC TACTTCCCCC ACTTCGAGCT

GAGCCACGGC TCTGCCCAGG TTAAGGGCCA CGGCAAGAAG GTGGCCGACG

---------- ---------- ---------- ---------- ----------

GAGCCACGGC TCTGACCAGG TTAACAAACA CGGCAAGAAG GTGGCCGACG

---------- ---------- ---------- ---------- ----------

GAGCCACGGC TCTGCCCAGG TCAAGGGCCA CGGCAAGAAG GTGGCCGACG

GAGCCACGGC TCCGCCCAGG TCAAGGCCCA TGGCAAGAAG GTGGCAGATG

AAGCCCCGGC TCTGCCCAGG TCAAGGCTCA CGGCAAGAAG GTTGCTGATG

AAGCCACGGC TCTGCCCAGG TCAAGGGTCA CGGCAAGAAG GTCGCCGATG

CACCCACGGC TCTGAGCAGA TCAAAGCCCA CGGCAAGAAG GTGTCCGAAG

GAGCCACGGC TCCGCGCAGG TCAAGGGCCA CGGCGCGAAG GTGGCCGCCG

GAGCCCCGGC TCCGCCCAGG TCAAGGCCCA GTGCAAGAAG GTGGCTGATG

GTCCCACGGC TCCGCCCAGA TTAAGGGCCA CGGCAAGAAG GTGGGCGACG

G---CACGGC TCGGCGCAGC TGCGCGCGCA CGGCTCCAAG GTGGTGGCCG

CTGCCCCGAC GAGGCGCAGC TGCTGCGCCA AGGACAGCGC GTGGCTGACG

GGGCCATGGC TCTGGCCAGG TCAAGGCACA TGGCAAGAAG GTGGGGGAAG

GACCCCGGGC TCCGCTGACA TCATGGCCCA TGGCAAGAAG GTGGCGGATG

T-TCCGCGGC TCGGCTCAAA TCAAGGGTCA AGGCCAGAAA ATCGCCGACG

GAGCCAGGGC TCGGCTCAGG TCAAGGCCCA TGGCAAGAAG GTGGCCGACG

CGCTGACCAA CGCCGTGGCG CACGTGGACG ACATGCCCAA CGCGCTGTCC

---------- ---------- ---------- ---------- ----------

CGCTGACCCT CGCCGTGGGG CACGTGGACG ACATGCCCCA GGCGCTGTCC

---------- ---------- ---------- ---------- ----------

CGCTCACCAA CGCCGTGGCG CACGTGGACG ACATGCCCAA CGCGCTGTCT

CGCTGACCAA CGCCGTGTCC CATGTGGACG ACATGCCCTC TGCCCTGTCT

CCTTGGCCAA AGCTGCAGAC CACGTCGAAG ACCTGCCTGG TGCCCTGTCC

CTCTGGCCAA TGCTGCAGGC CACCTCGATG ACCTGCCCGG TGCCCTGTCT

CCCTGACCAA GGCCGTGGGC CACCTGGACG ACCTGCCCGG CGCCCTGTCT

CGCTGACCAA AGCGGTGGAA CACCTGGACG ACCTGCCCGG TGCCCTGTCT

CGCTGACCAC CGCCGTGGCC CACCTGGACG ACCTGCCCGG TGCCCTGTCC

CTCTGACCAA AGCCGTGGGC AGCATTGACG ACCTGGCCGG CGCCCTGTCC

CCGTGGGCGA CGCGGTCAAG AGCATCGACA ACATCCCCAG CGCGCTGGCC

CCCTGACCCT CGCCGCCGGC CACCTGGATG ACCTGCCCGG CGCCCTGTCG

CACTGACCCA AGCTGTTGGC CACCTGGATG ACCTGCCTAG CGCCCTGTCT

CGCTGACCCT CGCCGTCGGC CACATGGACG ACCTGCCCGG CGCCCTGTCC

CCGTGTCCTT GGCCGTGGCG CACATGGATG ACCTGGCCAC CGCCCTGTCC

CCCTGACCAC GGCCGTGGCC CACCTCGATG ACCTGGACAG CACCCTGTCG

GCCCTGAGCG ACCTGCACGC GCACAAGCTT CGGGTGGACC CGGTCAACTT

---------- ---------- ---------- ---------- ----------

AAGCTGAGCG ACCTGCACGC GCACAAGCTT CGGGTGGACC CGGTCAACTT

---------- ---------- ---------- ---------- ----------

GCGCTCAGCG ACCTGCACGC GCACAAGCTT CGGGTGGACC CGGTCAACTT

GCTCTGAGTG ACCTGCATGC GCACAAGCTG AGAGTGGACC CTGTCAACTT

ACTCTGAGCG ACCTGCATGC CCACAAACTG CGTGTGGATC CTGTCAACTT

GCTCTGAGCG ACCTGCATGC CCACAAGCTG CGTGTGGATC CCGTCAACTT

ACTCTCAGCG ACCTGCACGC GCACAAGCTG CGGGTGGACC CGGTGAATTT

GAACTGAGTG ACCTGCACGC TCACAAGCTG CGTGTGGACC CGGTCAACTT

GCCCTCAGCG ACCTGCACGC CTACAAGCTG CGCGTGGACC CCGTCAACTT

GCTCTGAGCG ACCTGCACGC CCACAAGCTG CGTGTGGACC CCGTCAACTT

AAGCTGAGCG AGCTGCACGC CTACGTCCTG CGCGTGGACC CGGTCAACTT

GCCCTGAGCG ACCTGCACGC CCACAAGCTG CGGGTGGACC CGGTCAACTT

GCACTCAGCG ACCTGCACGC TCACAAGCTG AGGGTGGACC CTGTCAACTT

AAGCTGAGTG ACCTGCACGC CTACAAGCTG AGGGTGGACC CTGTCAACTT

GCCCTGAGCG ATCTGCACGC CCACAACCTG AAGGTGGATC CCGTCAACTT

GCGCTGAGCG ACCTGCACGC CCACAAGCTC AGAGTGGACC CGATCAACTT

CAAGCTCCTA AGCCACTGCC TGCTGGTGAC CCTGGCCGCC CACCTCCCCG

---GC----- ---------- ---------- ---------- ----------

CAAGCTCCTG AGCCACTGCC TGCTGGTGAC TCTGGCCGCT CACCTCCCCG

---------- ---------- ---------- ---------- ----------

CAAGCTCCTG AGCCACTGCC TGCTGGTGAC CCTGGCCGCC CACCACCCGG

CAAGCTCCTG AGCCACTGCC TGCTGGTGAC CCTGGCCTGT CACCACCCAG

CAAGTTCCTG AGCCACTGCC TGCTGGTGAC CTTGGCTTGC CACCACCCTG

CAAGCTCCTG AGCCACTGCC TGCTGGTGAC CTTGGCTAGC CACCACCCTG

CAAGCTCCTG TCCCACTGCC TGCTGGTGAC CATGGCCAAC CACCACCCCA

CAAGCTTCTG AGCCACTCCC TGCTGGTGAC CCTGGCCTCC CACCTCCCCA

CAAGCTCCTG AGCCACTGCC TGCTGGTGAC CCTGGCCTGC CACCACCCCA

CAAGCTCCTG AGCCACTGCC TGCTGGTGAC TCTGGCCTGC CACAACCCCG

CAAGCTCCTG AGCCACTGCC TGCTGGTGAC CCTGGCCAGC CACCACCCCT

CAAGTTCCTG GGCCACTGCC TGCTGGTCAC CCTGGCCTGC CACCTCCCGG

CAAGCTCCTG AGCCACTGCC TGCTGGTGAC TCTGAGCAGC CACCAACCCA

CAAGCTCCTG AGCCACTGCC TGCTGGTGAC TCTGGCCTGC CACCTGGGCG

CAAGCTCCTG TGCCACAATG TGCTCGTGAC TCTGGCCGGT CACCTCGGCA

CAAACTGCTG GCTCACTGCA TCCTGGTCGT GGTGGCCAGA CACCTTCCCG

CCGAGTTCAC CCCTGCGGTG CACGCCTCCC TGGACAAGTT CCTGGCTTCT

---------- ---------- ---------- ---------- ----------

CCGAGTTCAC CCCTGCGGTG CACGCCTCCC TGGACAAGTT CCTGGCTTCT

---------- ---------- ---------- ---------- ----------

CCGAATTCAC CCCTGCGGTG CACGCCTCTC TGGACAAGTT CCTGGCTTCT

CAGAACTCAC TCCTGCGGTC CACGCCTCCT TCGACAAGTT CATGGCTTCT

GAGATTTCAC ACCCGCCATG CACGCCTCTC TGGACAAATT CCTTGCCTCT

CCGATTTCAC CCCCGCGGTG CATGCCTCTC TGGACAAATT CCTTGCCTCT

GTGAATTCAC CCCTGCGGTG CACGCCTCCC TGGACAAGTT CCTGGCCAAC

GTGATTTCAC CCCCGCGGTC CACGCCTCCC TGGACAAGTT CTTGGCCAAC

CCGAATTCAC CCCTGCCGTC CACGCCTCCC TGGACAAGTT CTTCGCCGCC

GCGAGTTCAC CCCTGCTATC CACGCCTCCC TGGACAAGTT CCTGGCCTCC

CGGAATTCAC TCCCGCCGTC CACGCCTCCC TGGACAAGTT CTTGGCCTCC

ACGACTTCAC CCCCGCCGTC CACGCCTCCA TGGACAAGTT CATGGCCGGC

CGGAGTTCAC CCCTGAGGTC CATGCCTCCC TGGACAAGTT CCTCAGCAAC

GCGATTTCAC CCCTGCCGCC CACGCCTCCC TGGACAAGTT CCTCAGCTCT

AGGATTTCAC TCCCGAGATC CATGCCTCCT TGGACAAGTT CTTGGCTCTT

CCGAGTTCAC CCCCTCCGCC CACGCCGCCA TGGACAAGTT CCTGAGCAAA

GTGAGCACCG TGCTGACCTC CAAATACCGT ATGGTGCTGT CTCCTGCCGA

---------- ---------- ---------- ---------- ----------

GTGAGCACCG TGCTGACCTC CAAATACCGT ATGGTGCTGT CTCCTGACGA

---------- ---------- ---------- ---------- ----------

GTGAGCACCG TGCTGACCTC CAAATACCGT ATGGTGCTGT CTCCCGCCGA

GTGAGCACCG TCCTGACCTC CAAATACCGT ATGGTGCTGT CTCCCACGGA

GTGAGCACTG TGCTGACCTC CAAGTACCGT ATGGTGCTCT CTGCAGATGA

GTGAGCACCG TGCTGACCTC CAAGTACCGT ATGGTGCTCT CTGGGGAAGA

GTGAGCACCG TGCTGACCTC CAAATATCGT ATGGTGCTGT CTCCCGCTGA

GTGAGCACCG TGCTGACCTC CAAATACCGT ATGGTGCTGT CTGCCGCCGA

GTGAGCACCG TGCTGACCTC CAAGTACCGT ATGGTGCTGT CTCCCGCCGA

GTGAGCACCG TGCTGACCTC CAAGTACCGT ATGGTGCTTT CCCCCAGCGA

GTGAGCACCG TGCTGACCTC CAAGTACCGT ---------- ----------

GTGAGCACCG TGCTCGTCTC CAAGTACCGC ATGGTGCTCT CTGCCGCCGA

GTGAGCACCG TGCTGACCTC CAAATATCGT ATGGTGCTGT CTGATAACGA

GTGAGCACTG TGCTGACCTC CAAGTACCGT ATGGTGCTGT CTGCTGCTGA

CTGTCCACCG TGCTGACCTC GAAGTACCGT ATGGCCTTGT CTGCCGAAGA

GTGGCCACCG TGCTGACCTC CAAGTACCGC ATCTTTCTCT CTCCCGGACA

CAAGACCAAC GTCAAGGCCG CCTGGGGTAA GGTCGGCGCG CACGCTGGCG

---------- ---------- ---------- ---------- ----------

CAAGAAACAC GTCAAGGCCG CCTGGGGTAA GGTCGGCGAG CACGCTGGCG

---------- ---------- ---------- ---------- ----------

CAAGAGCAAC GTCAAGGCCG CCTGGGGTAA GGTCGGCAGC CACGCTGGCG

CAAGAGCAAC GTCAAGGCTG CCTGGGAAAA GGTTGGCGGC CATGCTGGCG

CAAAACCAAC ATCAAGAACT GCTGGGGGAA GATTGGTGGC CATGGTGGTG

CAAAAGCAAC ATCAAGGCTG CCTGGGGGAA GATTGGTGGC CATGGTGCTG

CAAGACCAAC ATCAAGACTG CCTGGGAAAA GATCGGCAGC CACGGTGGCG

CAAGGGCAAT GTCAAGGCCG CCTGGGGCAA GGTTGGCGGC CACGCTGCAG

CAAGACCAAC ATCAAGTCCA CTTGGGATAA GATTGGTGGC CACGCTGGCG

CAAAAGCAAC GTCAAGGCCG CCTGGGATAA GGTCGGCGGC AATGCTGCGG

---------- ---------- ---------- ---------- ----------

CAAGGCCCAC GTCAAGGCCT TCTGGGGGAA GGTCGGCGGC CATGCCGCTG

CAAGACCAAC GTCAAGGCCA CCTGGAGCAA GGTTGGCGAC CACGCTGGCC

TAAGGCCAAC GTGAAGACCG TCTGGGAGAA GGCCGGCGGC AACGTGGGCC

CAAGTGCAAC GTGAAAGAAT TCTGGGGAAA ATTGGGTGAG AACACACACG

CCAGACCCGC TCCTCACCCG CCCTGGCACC GGCCCGCAGC CACCCAGATC

AGTATGGTGC GGAGGCCCTG GAGAGGATGT TCCTGTCCTT CCCCACCACC

---------- ---------- ---------- ---------- ----------

AGTATGGTGC GGAGGCCCTG GAGAGGATGT TCCTGTCCTT CCCCACCACC

---------- ---------- ---------- ---------- ----------

ACTATGGTGC CGAGGCCCTG GAGAGGATGT TCCTGTCCTT CCCCACCACC

ACTACGGCGC GGAGGCCCTG GAGAGGATGT TCCTGTCCTT CCCCACCACC

AATATGGCGA GGAGGCCCTA CAGAGGATGT TCGCTGCCTT CCCCACCACC

AATATGGAGC TGAAGCCCTG GAAAGGATGT TTGCTAGCTT CCCCACCACC

AGTATGGCGC CGAGGCCGTG GAGAGGATGT TCCTGGGCTT CCCCACCACC

AGTATGGCGC AGAGGCCCTG GAGAGGATGT TCCTGAGCTT CCCCACCACC

ACTACGGCGG AGAGGCGCTG GACAGGACCT TCCAGTCCTT CCCCGCCACC

GGCTCGGCGC GGGGGCTCCG ACCCAGATGT TCCTGAGCTT CCCCACCACC

---------- ---------- -----GCTCT TCACCAGCTA CCCGCAGACC

AGTTCGGCGC CGAGGCCCTG GAGAGGCTCT TCGCCAGCCA CCCGCAGACC

GGTGTCTAGC CAAGGCCCCG ATGCCGATGT TCTTCTCCTT TCCCACCACC

GGTACCCA-C CCAGGCCCC- -----GACCT TCCTCTCCTT CCCCACCACC

TCTACGGCAC TGAAGCCCTG GTGAGGACCT TCCTCTCTTT CCCCCCCACC

TCTCGGGTGC TCCAACCCCG ATGGGGCTGT TCCTCTCTTT CCCCCCCACT

AAGACCTACT TCCCGCACTT CGACCTGAGC CACGGCTCTG CCCAGGTTAA

---------- ---------- ---------- ---------- ----------

AAGACCTACT TCCCCCACTT CGACCTGAGC CACGGCTCTG ACCAGGTTAA

---------- ---------- ---------- ---------- ----------

AAGACCTACT TCCCCCACTT CGACCTGAGC CACGGCTCTG CCCAGGTCAA

AAGACCTACT TCCCCCACTT CGACCTGAGC CACGGCTCCG CCCAGGTCAA

AAGACCTACT TCTCTCACAT TGATGTAAGC CCCGGCTCTG CCCAGGTCAA

AAGACCTACT TCCCTCACTT TGATGTAAGC CACGGCTCTG CCCAGGTCAA

AAGACCTACT TCCCCCACTT CGACTTCACC CACGGCTCTG AGCAGATCAA

AAGACCTACT TCCCCCACTT CGACCTGAGC CACGGCTCCG CGCAGGTCAA

AAGACTTACT TGCAGCAGTT CGACCTGAGC CCCGGCTCCG CCCAGGTCAA

AAGACCTACT TCCCCCACTT CGACCTGTCC CACGGCTCCG CCCAGATTAA

AAGACCTACT TCCCGCACTT CGACCTG--- CACGGCTCGG CGCAGCTGCG

AAGACCTACT TCCCGCACTT CGAGCTGCGC GCGGGCTCGG CGCAGCTGCG

AAGACCTACT TTCCTCACTT CGACCTGGGC CATGGCTCTG GCCAGGTCAA

AAGACCTACT TCCCTCACAT GGACCTGACC CCGGGCTCCG CTGACATCAT

AAGACCTACT TCCCGCACTT CGATTTTTCC GCGGGCTCGG CTCAAATCAA

AAGACCTACT TCCCCCACTT CGAGCTGAGC CAGGGCTCGG CTCAGGTCAA

GGGCCACGGC AAGAAGGTGG CCGACGCGCT GACCAACGCC GTGGCGCACG

---------- ---------- ---------- ---------- ----------

CAAACACGGC AAGAAGGTGG CCGACGCGCT GACCCTCGCC GTGGGGCACG

---------- ---------- ---------- ---------- ----------

GGGCCACGGC AAGAAGGTGG CCGACGCGCT CACCAACGCC GTGGCGCACG

GGCCCATGGC AAGAAGGTGG CAGATGCGCT GACCAACGCC GTGTCCCATG

GGCTCACGGC AAGAAGGTTG CTGATGCCTT GGCCAAAGCT GCAGACCACG

GGGTCACGGC AAGAAGGTCG CCGATGCTCT GGCCAATGCT GCAGGCCACC

AGCCCACGGC AAGAAGGTGT CCGAAGCCCT GACCAAGGCC GTGGGCCACC

GGGCCACGGC GCGAAGGTGG CCGCCGCGCT GACCAAAGCG GTGGAACACC

GGCCCAGTGC AAGAAGGTGG CTGATGCGCT GACCACCGCC GTGGCCCACC

GGGCCACGGC AAGAAGGTGG GCGACGCTCT GACCAAAGCC GTGGGCAGCA

CGCGCACGGC TCCAAGGTGG TGGCCGCCGT GGGCGACGCG GTCAAGAGCA

GGCGCACGGC TCCAAGGTGG CTGACGCCCT GACCCTCGCC GCCGGCCACC

GGCACATGGC AAGAAGGTGG GGGAAGCACT GACCCAAGCT GTTGGCCACC

GGCCCATGGC AAGAAGGTGG CGGATGCGCT GACCCTCGCC GTCGGCCACA

GGGTCAAGGC CAGAAAATCG CCGACGCCGT GTCCTTGGCC GTGGCGCACA

GGCCCATGGC AAGAAGGTGG CCGACGCCCT GACCACGGCC GTGGCCCACC

TGGACGACAT GCCCAACGCG CTGTCCGCCC TGAGCGACCT GCACGCGCAC

---------- ---------- ---------- ---------- ----------

TGGACGACAT GCCCCAGGCG CTGTCCAAGC TGAGCGACCT GCACGCGCAC

---------- ---------- ---------- ---------- ----------

TGGACGACAT GCCCAACGCG CTGTCTGCGC TCAGCGACCT GCACGCGCAC

TGGACGACAT GCCCTCTGCC CTGTCTGCTC TGAGTGACCT GCATGCGCAC

TCGAAGACCT GCCTGGTGCC CTGTCCACTC TGAGCGACCT GCATGCCCAC

TCGATGACCT GCCCGGTGCC CTGTCTGCTC TGAGCGACCT GCATGCCCAC

TGGACGACCT GCCCGGCGCC CTGTCTACTC TCAGCGACCT GCACGCGCAC

TGGACGACCT GCCCGGTGCC CTGTCTGAAC TGAGTGACCT GCACGCTCAC

TGGACGACCT GCCCGGTGCC CTGTCCGCCC TCAGCGACCT GCACGCCTAC

TTGACGACCT GGCCGGCGCC CTGTCCGCTC TGAGCGACCT GCACGCCCAC

TCGACAACAT CCCCAGCGCG CTGGCCAAGC TGAGCGAGCT GCACGCCTAC

TGGATGACCT GCCCGGCGCC CTGTCGGCCC TGAGCGACCT GCACGCCCAC

TGGATGACCT GCCTAGCGCC CTGTCTGCAC TCAGCGACCT GCACGCTCAC

TGGACGACCT GCCCGGCGCC CTGTCCAAGC TGAGTGACCT GCACGCCTAC

TGGATGACCT GGCCACCGCC CTGTCCGCCC TGAGCGATCT GCACGCCCAC

TCGATGACCT GGACAGCACC CTGTCGGCGC TGAGCGACCT GCACGCCCAC

AAGCTTCGGG TGGACCCGGT CAACTTCAAG CTCCTAAGCC ACTGCCTGCT

---------- ---------- ---------- ---------- ----------

AAGCTTCGGG TGGACCCGGT CAACTTCAAG CTCCTGAGCC ACTGCCTGCT

---------- ---------- ---------- ---------- ----------

AAGCTTCGGG TGGACCCGGT CAACTTCAAG CTCCTGAGCC ACTGCCTGCT

AAGCTGAGAG TGGACCCTGT CAACTTCAAG CTCCTGAGCC ACTGCCTGCT

AAACTGCGTG TGGATCCTGT CAACTTCAAG TTCCTGAGCC ACTGCCTGCT

AAGCTGCGTG TGGATCCCGT CAACTTCAAG CTCCTGAGCC ACTGCCTGCT

AAGCTGCGGG TGGACCCGGT GAATTTCAAG CTCCTGTCCC ACTGCCTGCT

AAGCTGCGTG TGGACCCGGT CAACTTCAAG CTTCTGAGCC ACTCCCTGCT

AAGCTGCGCG TGGACCCCGT CAACTTCAAG CTCCTGAGCC ACTGCCTGCT

AAGCTGCGTG TGGACCCCGT CAACTTCAAG CTCCTGAGCC ACTGCCTGCT

GTCCTGCGCG TGGACCCGGT CAACTTCAAG CTCCTGAGCC ACTGCCTGCT

AAGCTGCGGG TGGACCCGGT CAACTTCAAG TTCCTGGGCC ACTGCCTGCT

AAGCTGAGGG TGGACCCTGT CAACTTCAAG CTCCTGAGCC ACTGCCTGCT

AAGCTGAGGG TGGACCCTGT CAACTTCAAG CTCCTGAGCC ACTGCCTGCT

AACCTGAAGG TGGATCCCGT CAACTTCAAG CTCCTGTGCC ACAATGTGCT

AAGCTCAGAG TGGACCCGAT CAACTTCAAA CTGCTGGCTC ACTGCATCCT

GGTGACCCTG GCCGCCCACC TCCCCGCCGA GTTCACCCCT GCGGTGCACG

---------- ---------- TCCCCGCCGA CTTCACGGCC GAGGCCCACG

GGTGACTCTG GCCGCTCACC TCCCCGCCGA GTTCACCCCT GCGGTGCACG

---------- ---------- TCCCCGCCGA CTTCACTGCC GAGGCCCACG

GGTGACCCTG GCCGCCCACC ACCCGGCCGA ATTCACCCCT GCGGTGCACG

GGTGACCCTG GCCTGTCACC ACCCAGCAGA ACTCACTCCT GCGGTCCACG

GGTGACCTTG GCTTGCCACC ACCCTGGAGA TTTCACACCC GCCATGCACG

GGTGACCTTG GCTAGCCACC ACCCTGCCGA TTTCACCCCC GCGGTGCATG

GGTGACCATG GCCAACCACC ACCCCAGTGA ATTCACCCCT GCGGTGCACG

GGTGACCCTG GCCTCCCACC TCCCCAGTGA TTTCACCCCC GCGGTCCACG

GGTGACCCTG GCCTGCCACC ACCCCACCGA ATTCACCCCT GCCGTCCACG

GGTGACTCTG GCCTGCCACA ACCCCGGCGA GTTCACCCCT GCTATCCACG

GGTGACCCTG GCCAGCCACC ACCCCTCGGA ATTCACTCCC GCCGTCCACG

GGTCACCCTG GCCTGCCACC TCCCGGACGA CTTCACCCCC GCCGTCCACG

GGTGACTCTG AGCAGCCACC AACCCACGGA GTTCACCCCT GAGGTCCATG

GGTGACTCTG GCCTGCCACC TGGGCGGCGA TTTCACCCCT GCCGCCCACG

CGTGACTCTG GCCGGTCACC TCGGCAAGGA TTTCACTCCC GAGATCCATG

GGTCGTGGTG GCCAGACACC TTCCCGCCGA GTTCACCCCC TCCGCCCACG

CCTCCCTGGA CAAGTTCCTG GCTTCTGTGA GCACCGTGCT GACCTCCAAA

CCGCCTGGGA CAAGTTCCTA TCGGTCGTAT CCTCTGTCCT GACCGAGAAG

CCTCCCTGGA CAAGTTC--- ---------- ---------- ----------

CCGCCTGGGA CAAGTTCCTA TCGGTCGTAT CCTCTGTCCT GACCGAGAAG

CCTCCCTGGA CAAGTTCCTG GCTTCTGTGA GCACCGTGCT GACCTCCAAA

CCTCCTTCGA CAAGTTCATG GCTTCTGTGA GCACCGTCCT GACCTCCAAA

CCTCTCTGGA CAAATTCCTT GCCTCTGTGA GCACTGTGCT GACCTCCAAG

CCTCTCTGGA CAAATTCCTT GCCTCTGTGA GCACCGTGCT GACCTCCAAG

CCTCCCTGGA CAAGTTCCTG GCCAACGTGA GCACCGTGCT GACCTCCAAA

CCTCCCTGGA CAAGTTCTTG GCCAACGTGA GCACCGTGCT GACCTCCAAA

CCTCCCTGGA CAAGTTCTTC GCCGCCGTGA GCACCGTGCT GACCTCCAAG

CCTCCCTGGA CAAGTTCCTG GCCTCCGTGA GCACCGTGCT GACCTCCAAG

CCTCCCTGGA CAAGTTCTTG GCCTCCGTGA GCACCGTGCT GACCTCCAAG

CCTCCATGGA CAAGTTCATG GCCGGCGTGA GCACCGTGCT CGTCTCCAAG

CCTCCCTGGA CAAGTTCCTC AGCAACGTGA GCACCGTGCT GACCTCCAAA

CCTCCCTGGA CAAGTTCCTC AGCTCTGTGA GCACTGTGCT GACCTCCAAG

CCTCCTTGGA CAAGTTCTTG GCTCTTCTGT CCACCGTGCT GACCTCGAAG

CCGCCATGGA CAAGTTCCTG AGCAAAGTGG CCACCGTGCT GACCTCCAAG

TACCGTAGGA CCTTCCTGGC TTTCCCCGCC ACGAAGACCT ACTTCTCCCA

TACCGCAGGA CCTTCCTGGC CTTCCCCGCC ACGAAGACCT ACTTCTCCCA

------AGGA CCTTCCTGGC TTTCCCCGCC ACGAAGACCT ACTTCTCCCA

TACCGC---- ---------- ---------- ---------- ----------

TACCGTAGGA CCTTCCTGGC CTTCCCCGCC ACCAAGACCT ACTTCTGTCA

TACCGTAGGA TGTTCCTGGC CTTTCCCCTC ACCAAGACCT ACTTCCCCCA

TACCGTAGGA CCTTCCTGAC -TTCCCATCC ACCAAAACCT ACTTCCCACA

TACCGTAGGA CCTTCGTGGC TTTCCCCTCC ACCAAAACCT ACTTCCCACA

TATCGTAGAA CCTTGGAGGC CTTCCCGCGC ACCAAGATCT ACTTCTCCCA

TACCGTAGGA CCTTCGTGGC CTTCCCTTCC TCCAAGACCT ATTTCCTCCA

TACCGTAGGA CCTTCCAGTC CTTCCCCGCC ACCAAGACTT ACTTGCAGCA

TACCGTAGGA CCTTCCTGGC CTTCCCCTCC ACCAAGACTT ACTTCTTCCA

TACCGT--GC TCTTCACCAG CTACCCGCAG ACCAAGACCT ACTTCCCGCA

TACCGCG-GC TCTTCGCCAG CCACCCGCAG ACCAAGACCT ACTTCCCGCA

TATCGTAGGA CCTTCTTCGT CTTCTCCTCC ACCAAGACCT ACTTCCCCCA

TACCGTAGGA CTTTCGCTGC CTTCCCCTCC ACCAAGACCT ACTTCCCCCA

TACCGT--GA CCTTCCTCTC TTTCCCCCCC ACCAAGACCT ACTTCCCGCA

TACCGC--GC TGTTCCAGGC TTTCCCCACC ACCAAGACCT ACTTCTCCCA

CCTGGACCTG AGCCCCGGCT CCTCACAAGT CAGAGCCCAC GGCCAGAAGG

CCTGGACCTG TGCC------ ---------- ---------- ----------

CCTAGACCTG AGCCCCGGCT CCGCCCAGGT TAGAGCACAC GGCCAGAAGG

---------- ---------- ---------- ---------- ----------

CCTGGACCTG AGCCCCGGCT CCGCGCAGGT CAGAGCGCAC GGCCAGAAGG

CCTGAACCTG AGCCCTGGCT CTGCCCAGGT CAAAGCCCAC GGCCAGAAGG

CTTGGACCTG AAACCAGGTT CCAGCCAGGT TAAAGCCCAT GCCCA-AATG

CTTGGACCTG AGGCCAGGCT CTAGCCAGGT TAAAGCCCAT GCCCAAAAGG

CATGGACCTG AGCCCGGGCT CCGCCCAGGT CAGAGCCCAC GGCCGCAAGG

CCTAAACTTG AGCCCCGGCT CCGCCCAGGT CGCAGCCCAC GGCCAGAAGG

GTTCGACCTG AGCCCCGGCT CCGCCCAGGT CAAGGCCCAG TGCAAGAAGG

CCTGGACCTG AGCCCCGGCT CCGCGCAGGT CAAAGCCCAC GGCAAGAAGG

CTTCGACCTG CACGCGGGCT CGGCGCAGCT GCGCGCGCAC GGCTCCAAGG

CTTCGAGCTG CGCGCGGGCT CGGCGCAGCT GCGGGCGCAC GGCTCCAAGG

CTTCGACCTA AGCCCGGGTT CAGCCCAGGT CAAGGCCCAC AGCAAGAAGG

CTTCAACCTG AGGCCGGGCT CAGCCCAGGT CAAGGCGCAC GGCAAAAAGG

CTTCGATTTT TCCGCGGGCT CGGCTCAAAT CAAGGGTCAA GGCCAGAAAA

CTTCGACCTG AGCCACGGCT CCGCTCAGAT CAAGGCTCAT GGCAAGAAGG

TGGCGGACGC GCTGAGCCTC GCCGTGGAGC GCCTGGACGA CCTACCCCAC

---------- ---------- ---------- ---------- ----------

TGGCGGACGC GCTGAGCCTC GCCGTGGAGC GCCTAGACGA CCTACCCCGC

---------- ---------- ---------- ---------- ----------

TGGCGGACGC GCTGAGCCTC GCGGTGGAAC GCCTGGACGA CCTACCCCGC

TGGCCGCTGC GCTGACCCTC GCAGTCTACC ACCTCGACGA CTTGCCTGAC

TGGCCGATGC ACTGACGCTC GCTGCCCACC ACTTGCACGA CCTGTCTGGT

TGGCCGATGC ACTGACTCTC GCTACCCAGC ACCTGGACGA CCTGCCTGCT

TGGCCGACGC GCTGACCCTC GCCGCAGACC ACCTGGACGA CCTGCCCGGC

TGGCCGACGC GCTGAGCTTG GCCGTGAACC ACCTGGACGA CCTGCCGGGC

TGGCTGATGC GCTGACCACC GCCGTGGCCC ACCTGGACGA CCTGCCCGGT

TCGCCGACGC ACTGACTCTG GCCGTGGACC ACATGGACGA CTTGCCCCGA

TGGTGGCCGC CGTGGGCGAC GCGGTCAAGA GCATCGACAA CATCCCCAGC

TGGCTGACGC CCTGACCCTC GCCGCCGGCC ACCTGGATGA CCTGCCCGGC

TGGCAGACGT GCTGACCCAC GCCGTCCACC ACCTGGACGA CCTGCCCAGG

TGGCCGCCGC ACTGACCCAA GCGGTCGCCC ACCTGGACAA CGTGCCCGGT

TCGCCGACGC CGTGTCCTTG GCCGTGGCGC ACATGGATGA CCTGGCCACC

TGGCCGACGC TCTGTCCACG GCCGCGGGGC ACTTCGATGA CATGGACAGC

GCGCTGTCCG CGCTGAGCCA CCTGCACGCG TGCCAGCTGC GAGTGGACCC

---------- ---------- ---------- ---------- ----------

GCGCTGTCCG CTCTGAGCCA TCTGCACGCT TGCCAGCTGC GAGTGGACCC

---------- ---------- ---------- ---------- ----------

GCGCTGTCCG CGCTGAGCCA CCTGCACGCG CGCCAGCTGC GAGTGGACCC

ACTTTGTCGG CTCTGAGCGA CTTGCACGCG TGTAAGCTGA GAGTGGACCC

TCTCTGTCTG CTCTCAGTGA TCTACACGCA CATGAGCTCC GCGTGGACCC

TCTCTGTCTG CTCTCAGTGA TCTACATGCG CACAAGCTCT GTGTGGACCC

GCCCTGTCCG CTCTGAGCGA CCTGCACGTG CGCACGCTGC GCGTGGACCC

ACCCTGTCGT ACCTGCGCGA GCTGCACACG CACAAGCTGC GCGTGGACCC

GCCCTGTCCG CCCTCAGCGA CCTGCACGCC TACAAGCTGC GCGTGGACCC

GCCCTGTCGG CTCTGAGCGA CCTGCACGCG CATAAGCTGC GAGTGGACCC

GCGCTGGCCA AGCTGAGCGA GCTGCACGCC TACGTCCTGC GCGTGGACCC

GCCCTGTCGG CCCTGAGCGA CCTGCACGCC CACAAGCTGC GGGTGGACCC

GCGCTGTCCG CGCTGAGCGA CCTGCACGCG CACAAGTTGA GGGTGGATCC

GCGTTGTCCG CGCTGAGTGA CCTACACGCG CACAGGCTCC GAGTGGATCC

GCCCTGTCCG CCCTGAGCGA TCTGCACGCC CACAACCTGA AGGTGGATCC

GCCCTGTCCG CTCTGAGCGA CCTGCACGCC CACAAGCTGA GAGTGGACCC

GGCCAGCTTC CAGCTCCTGG GCCACTGCCT GCTGGTAACC CTCGCCCGGC

---------- ---------- ---------- ---------- ----------

AGCTAACTTC CCGCTCCTGG GCCACTGCCT GCTGGTGACC CTCGCCCGGC

---------- ---------- ---------- ---------- ----------

GGCCAGCTTC CAGCTCCTCG GCCACTGCCT GCTGGTGACC CTCGCCCGGC

GGCCAACTTC AAGCTCCTGG GCCACTGTCT GCTGGTGACC CTCGCCCGGC

TGCTAACTTT CAGTTCTTAG -CCACTGTCT GCTGGTGACT CTTGCCCGGC

TGCTAACTTC CAGTTCTTCA GCCACTGTCT GCTGGTGACT CTCGCCAGGC

CCACCACTTC GGGCTGCTGG GCCACTGTCT GCTGGTGACC CTCGCCCGGC

GGTCTTTTTC AAGCTGCTGT GCCACTGCCT GCTGGTGACC CTCGCCCGGC

CGTCAACTTC AAGCTCCTGA GCCACTGCCT GCTGGTGACC CTGGCCTGCC

CGTCAACTTC AAGCTGCTGA CCCACTGCCT GCAGGTGACC CTCGCCCGGC

GGTCAACTTC AAGCTCCTGA GCCACTGCCT GCTGGTGACC CTGGCCAGCC

GGTCAACTTC AAGTTCCTGG GCCACTGCCT GCTGGTCACC CTGGCCTGCC

AGTCAACTTC AAGCTCCTTG GCCACTGCCT GCTGGTAACC CTGGCCCGGC

GGTCAACTTC CAGCTCCTGG CCCACTGCCT GCTGGTGACC CTGGCCCGCC

CGTCAACTTC AAGCTCCTGT GCCACAATGT GCTCGTGACT CTGGCCGGTC

GGTCAACTTC AAACTGCTGG CTCACTGCAT CCTGGTTGTC CTGGCCAGGC

ACTACCCCGG AGACTTCAGC CCCGCGCTGC AGGCGTCGCT GGACAAGTTC

---------- ---------- ---------- ---------- ----------

ACTACCCCGG AGACTTCAGC CCGGCGCTGC AGGCGTCGCT GGACAAGTTC

---------- ---------- ---------- ---------- ----------

ATTACCCCGG AGACTTCAGC CCCGCGCTGC AGGCGTCGCT GCACAAGTTC

ACTACCCCGG AGACTTCAGC CCTGCTCTGC AGGCATCGCT GGACAAGTTT

ACCATCCCAG AGACTTCAGC CCGGAGATGC ATGCCTCCTT GGACAAGTTT

ACTATCCTGG AGACTTCAGC CCAGAGATGC ATGCCTCCTT GGACAAGTTT

ACTACCCTGG AGACTTCGGC CCCGCCATGC ACGCCTCGGT GGACAAATTC

ACTACCCCGG AGACTTCAGC CCCAACATGC ACGCCTCGCT GGTCAAGTTT

ACCACCCCAC CGAATTCACC CCTGCCGTCC ACGCCTCCCT GGACAAGTTC

ACTTCCCTGG AGACTTCAGC CCCACCCTGC AGGCCTCGCT GGACAAATTT

ACCACCCCTC GGAATTCACT CCCGCCGTCC ACGCCTCCCT GGACAAGTTC

ACCTCCCGGA CGACTTCACC CCCGCCGTCC ACGCCTCCAT GGACAAGTTC

ACTACCCGGG AGACTTCAGT GCCACCATGC AGGCCTCGCT GGACAAGTTT

ACTACCCTGG GGACTTCAGC CCCACCATGC ATGCTTCGCT AGACAAGTTC

ACCTCGGCAA GGATTTCACT CCCGAGATCC ATGCCTCCTT GGACAAGTTC

ATTGCCCGGG CGAGTTCACC CCCTCCGCCC ACGCCGCCAT GGACAAGTTC

CTGAGCCACG TTATCTCGGC GCTGGTTTCC GAGTACCGCA TGTCTCTGAC

---------- ---------- CCTGACCGAG AAGTACCGCA TGTCTCTGAC

CTGAGCCACG TGATCTCTGC GCTGGCTTCT GAGTACCGCA TGTCTCTGAC

---------- ---------- CCTGACCGAG AAGTACCGCA TGTCTCTGAC

CTGAGCCACG TGGCCTCGGC GCTGGCTTCC GAGTACC--- ----------

CTGAGTCATG TGATCTTGGC ACTGGCCTCC AAGTATCGCA TGTCTCTGAC

CTGGGTCACG TGACTTCGGC ACTGGTCTCC AAGGATCGCA TGTCTCTGAT

CTGGGTCACG TGACTTCGGC ACTGGTCTCC AAATATCGCA TGTCTCTGAT

CTGCACCACG TGATCTCGGC GCTGACCTCC AAGTACCGCA TGTCTCTGAC

CTGAACCACG TGATCTCGGC GCTGGCCCCC AGCAGTGGCA TGTCTCTGAC

TTCGCCGCCG TGAGCACCGT GCTGACCTCC AAGTACCGTA TGTCTCTGAC

CTGAGCGACG TGGTTTTGGC GCTGGCCTCC AGCTATCACA TGTCTCTGAC

TTGGCCTCCG TGAGCACCGT GCTGACCTCC AAGTACCGTA TGTCGCTGAC

ATGGCCGGCG TGAGCACCGT GCTCGTCTCC AAGTACCGCA TGTCGCTGAC

CTGAGCCACG TGATCTCCGC GCTGGTCTCC AAGTACCGCA TGTCTCTGAC

CTGAGCCACG TAAACTCAGT GCTGGTGTCC AAGTACCGC- ----------

TTGGCTCTTC TGTCCACCGT GCTGACCTCG AAGTACCGTA TGTCTCTGAC

CTGAGCAAAG TGGCCACCGT GCTGACCTCC AAGTACCGCA TGTCTCTGAC

CAAGACTGAG AGGACCATCA TTGTGTCCAT GTGGGCCAAG ATCTCCACGC

CAAGACTGAG GGGACCATCA TTGTGTCCAT GTGGGCCAAG ATCTCCACGC

CAAGACTGAG AGGACCCTCA TTGTGTCCAT GTGGGCCAAG ATCTCCACCC

CAAGACTGAT AGGACCCTCA TTGTGTCCAT GTGGGCCAAG ATCTCCACTC

---------- ---------- ---------- ---------- ----------

CAAGACTGAG AGGGCCATCA TTGTGTCCAT GTGGGGCAAG ATCTCCACAC

GAAGAATGAG AGAGCTATCA TCATGTCCAT GTGGGACAAA ATGGCGCCTC

GAAGAATGAG AGAGCTATCA TCATGTCCAT GTGGGAGAAG ATGGCTGCTC

CAAGAGCCAG AGGACCATCA TCATGTCCCT CTGGGACAAG GTCTCCTCCC

CAGAGCTGAG AGGACCATCA TCGTGTCCAT GTGGAGCAAG ATCTCCACAC

CAAGGCCGAG AGGACCATCA TCCTGTCCAT GTGGGGCAAG ATCTCCACCC

CAAGGCTGAG AGGACCATCA TCACATCCAT GTGGAACAAG ATCTCCACAC

CAAGGCCGAG AGGACCATCA TCCTGTCCAT GTGGGGCAAG ATCTCCACAC

CAAGGCCGAG AGGACCATCA TCGTGTCCAT GTGGGGCAAG ATCGCCTCGC

CAAGGTGGAG AGGACCATCA TCACGTCCAT GTGGGGCAAG GTCTCCACAC

---------- ---------- ---------- ---------- ----------

CAAGACCGAC AAGGCTATCA TCCTCACCAT CTGGAACAAG ATCGCCACTC

CAAGGCCGAC AAGACCGCAG TCATCTCCCT CTGGGGAAAG GCGGCCACCC

AGGCCGACAC CATCGGCACC GAGACTCTGG AGAGGCTCTT CCTCAGCCAC

AGGCCGACAC CATCGGCACC GAGACTCTGG AGAG------ ----------

AGGCCGACAC CATTGGCACC GAGACTCTGG AGAGGCTCTT CCTCAGCTAC

AGGCCGACAC CATCGGCACC GAGACTCTGG AGAG------ ----------

---------- ---------- ---------- ---------- ----------

AGGCCGACAC CATTGGCACT GAGACCCTGG AGAGGCTCTT CATCACCTAC

ATGCAGAGCC CATTGGCACT GAGACTCTGG AGAGGCTCTT CAGCAGCTAC

AGGCCGAGCC CATTGGCACT GAGACTCTAG AGAGGCTCTT CTGCAGCTAC

AGTCTGAGGA CATTGGCGCA GAGGCCCTGG AGAGGCTCTT CTACAGCTTC

AGGCGGACCT CATTGGCACC GAGACCCTGG AGAGGCTCTT CTCCTGCTAC

AGGCGGATGC TATTGGCACC GAGGCCCTGG AGAGG----- ---------C

AGGCGGACGC CATCGGCACC GAGGCCCTGG AGAGGCTCTT TGCCAGCTAC

AGGCGGATGT CATC------ ---------- ----GCTCTT CACCAGCTAC

AGGCCGACGC CATCGGCACC GAGGCCCTGG AGCGGCTCTT CGCCAGCCAC

AGGCTGACAG CATCGGCACC GAGGCCCTGG AGAGGCTCTT CGCCAGCTAC

---------- ---------- ---------- ----GACTTT CGCTGCCTTC

AGGCTGACTC TGTTGGTACT GAGGCCTTGG AACGGACCTT CCTCTCTTTC

AGGCTGACGC CATCGGGGCC GAGTCTCTGG AGAGGCTGTT CCAGGCTTTC

CCGCAGACCA AGACCTACTT CCCGCACTTC GACCTGCACC CGGGGTCCGC

---------- ---------- ---------- ---------- ----------

CCGCAGACCA AGACCTACTT CCCGCACTTC GACCTGCACC CGGGGTCCGC

---------- ---------- ---------- ---------- ----------

------GCCA AGACCTACTT CTGTCACCTG GACCTGAGCC CCGGCTCCGC

CCTCAAACGA AGACCTACTT CCCGCACTTC GACCTGCACG CGGGGTCGGC

CCCCAGACGA AGACCTACTT CCCACACTTC GACCTGCACC ATGGGTCTCA

CCCCAGACGA AGACCTACTT CCCCCACTTC GACCTGCACC ATGGGTCTCA

CCGCAGACCA AGACCTACTT CCCGCACTTC GACCTGCGCC CGGGCTCCGC

CCGCAGG-CA AGACCTACTT CCCGCACTTC GACCTGCACA CGGGCTCCGC

CCGCAGGCCA AGACCTACTT CCCGCACTTC GAGCTGCGCG CGGGCTCGGC

CCCCAGACCA AGACCTATTT CCCGCACTTC GACCTGAACC CCGGCTCCGC

CCGCAGACCA AGACCTACTT CCCGCACTTC GACCTGCACG CGGGCTCGGC

CCGCAGACCA AGACCTACTT CCCGCACTTC GAGCTGCGCG CGGGCTCGGC

CCCCAGACCA AGACTTACTT CCCGCACTTC GACCTGCACC CTGGCTCGGC

CCCTCCACCA AGACCTACTT CCCCCACTTC AACCTGAGGC CGGGCTCAGC

CCCCCCACCA AGACCTACTT CCCGCACTTC GATTTTTCCG CGGGCTCGGC

CCCACCACCA AGACCTACTT CTCCCACTTC GACCTGAGCC ACGGCTCCGC

GCAGTTGCGC GCGCACGGCT CCAAGGTGGT GGCCGCCGTG GGCGACGCGG

---------- ---------- ---------- ---------- ----------

GCAGCTGCAA GCGCACGGCT CCAAGGTGGT GGCCGCCGTG GGCGACGCAG

---------- ---------- ---------- ---------- ----------

GCAGGTCAGA GCGCACGGCC AGAAGGTGGC GGACGCGCTG AGCCTCGCGG

GCAGCTGCGT GCGCACGGCT CAAAGGTGGT GGCCGCCTTG GGCGACGCTG

GCAGTTGCGT GCCCACGGCT CCAAGATCTT GGCCGCTGTG GGTGATGCGG

GCAGTTGCGG GCCCACGGCT TCAAGATCAT GACCGCCGTA GGGGATGCGG

GCAGCTGCGC GCTCACGGCG CCAAGGTGGC GGCCGCGCTG GACAAAGCGG

GCAGCTGCGC GCGCACGGCT CCAGGGTGGT GGCCGCCGTG GGCGACGCGG

GCACCTGAGG GCGCACGGCG CCAAGGTGGT GGCCG-CGCT AACGACGCGG

GCAGCTGCGC GCGCATGGCT CCAAGGTGGT GGCCGCCGTG GGCGACGCGG

GCAGCTGCGC GCGCACGGCT CCAAGGTGGT GGCCGCCGTG GGCGACGCGG

GCAGCTGCGG GCGCACGGCT CCAAGGTGGC GGCCGCCGTG GGCGAGGCGG

GCAGCTGCGC GCGCACGGCT CCAAGGTGGT GGCCGCTGTG GGTGACGCGG

CCAGGTCAAG GCGCACGGCA AAAAGGTGGC CGCCGCACTG ACCCAAGCGG

TCAAATCAAG GGTCAAGGCC AGAAAATCGC CGACGCCGTG TCCTTGGCCG

TCAGATCAAG GCTCATGGCA AGAAGGTGGC CGACGCTCTG TCCACGGCCG

TGAAGAGCAT CGACGACATC GGCGGCGCCC TGTCCAAGCT GAGCGAGCTG

---------- ---------- ---------- ---------- ----------

CGAAGAACAT CGACAACATC GGCGCCGCCC TGTCCAAGCT GAGCGAGCTG

---------- ---------- ---------- ---------- ----------

TGGAACGCCT GGACGACCTA CCCCGCGCGC TGTCCGCGCT GAGCCACCTG

TCAAGAACAT CGACAACATC ACCGGCGCCC TGGCCAAGCT GAGCGAGCTG

TTAAGAACAT CGACAACCTC TCTGGTGCTT TGACTAAGTT GAGCGAGCTA

TTAAGAGCAT CGACAACCTC TCTAGTGCTT TGACTAAGCT GAGCGAGCTG

TCAAGAACAT CGACAACTTG GCCGGCGCAC TGTCCAAGCT GAGCGAGCTG

TCAAGAGCAT TGACAACGTG ACGAGCGCGT TGTCCAAGCT GAGCGAGCTG

TGCGCAGCCT CGACGACGTG GCGGGTGCCC TGTCCGCCCT CAGCGACCTG

TCAAGAGCAT CGATAACATC GCGGGCGCCC TGTCCAAGCT GAGCGAGCTG

TCAAGAGCAT CGACAACATC CCCAGCGCGC TGGCCAAGCT GAGCGAGCTG

CGGGCAGCGT GGACGACGTA GCGGGCGCCC TGGCCAGGCT GAGCGAGCTG

TCAAGAGCAT CGACAATATC TCGGGCGCGC TGTCCAAGCT GAGCGAGCTG

TCGCCCACCT GGACAACGTG CCCGGTGCGT TGTCCGCGCT GAGTGACCTA

TGGCGCACAT GGATGACCTG GCCACCGCCC TGTCCGCCCT GAGCGATCTG

CGGGGCACTT CGATGACATG GACAGCGCCC TGTCCGCTCT GAGCGACCTG

CACGCCTACA TCCTGCGCGT GGACCCGGTC AACTTCAAGC TCCTGTCCCA

---------- ---------- ---------- --------GC TCCTGTCCCA

CACGCCTACG TCCTGCGCGT GGACCCGGTC AACTTCAAGC TCCTGTCC-A

---------- ---------- ---------- ---------C TCCTGTCCCA

CACGCGCGCC AGCTGCGAGT GGACCCGGCC AGCTTCCAGC TCCTCGGCCA

CATGCCTACA TCCTGCGGGT GGACCCGGTC AACTTCAAgC TGCTGTCCCA

CACGCCTACA TCCTGCGTGT GGACCCGGTC AACTTCAAGC TCCTGTCTCA

CATGCCTACA TCCTGCGTGT GGATCCGGTC AACTTCAAGC TCCTGTCCCA

CACGCCTACG TGTTGCGTGT GGACCCGGTC AACTTCAAGC TCCTGTCCCA

CACGCCTACG TGCTGCGCGT GGACCCGGTC AACTTCAAGT TCCTGTCTCA

CACGCCTACA AGCTGCGCGT GGACCCCGTC AACTTCAAGC TCCTGAGCCA

CACGCCTACA TCCTGCGCGT GGACCCGGTC AACTTCAAGC TCCTGTCCCA

CACGCCTACG TCCTGCGCGT GGACCCGGTC AACTTCAAGC TCCTGTCACA

CACGCCTACG TGCTGCGCGT GGACCCCGTC AACTTCAAGC TCCTGTCCCA

CACGCCTACA TCCTGCGCGT GGACCCGGTC AACTTCAAGC TCCTGTCACA

CACGCGCACA GGCTCCGAGT GGATCCGGTC AACTTCCAGC TCCTGGCCCA

CACGCCCACA ACCTGAAGGT GGATCCCGTC AACTTCAAGC TCCTGTGCCA

CACGCCCACA AGCTGAGAGT GGACCCGGTC AACTTCAAAC TGCTGGCTCA

CTGCCTGCTG GTCACCCTGG CCGCGCGCTT CCCCGCCGAC TTCACGGCCG

CTGCCTGCTG GTCACCCTGG CCGCGCGCTT CCCCGCCGAC TTCACGGCCG

CTGCCTGCTG GTC-CCCTGG CCGCGCGCTT CCCCGCCGAC TTCACGGCCG

CTGCCTGCTG GTCACCCTGG CCGCGCGCTT CCCCGCCGAC TTCACGGCCG

CTGCCTGCTG GTGACCCTCG CCCGGCATTA CCCCGGAGAC TTCAGCCCCG

CTGCCTGCTG GTCACCGTGG CCGCGCACTT CCCTGCCGAC TTCACGGCCG

CTGTCTGCTG GTCACACTGG CTGCACACTT CCCCGCCGAC TTTACCCCTG

CTGTCTGCTG GTCACAATGG CCGCACGCTT TCCCGCCGAC TTCACCCCTG

CTGCCTGCTG GTCTCCCTGG CCGTGCACCT GCCCGCGGAC TTCACCGCCG

CTGCCTGCTG GTCACGTTGG CCTCGCACTT CCCCGCCGAC TTCACGGCCG

CTGCCTGCTG GTGACCCTGG CCTGCCACCA CCCCACCGAA TTCACCCCTG

CTGTCTGCTG GTCACGGTGG CCTTGCACTT CCCCGCCGAG TTCACGGCCG

CTGTCTGCTG GTGACCCTGG CATCGCACTT CCCGGCCGAC TTCTCGGCCG

CTGCCTGCTG GTGACCGTGG CCGCGCGCTT CCCCGCCGAC TTCACGGCCG

CTGTCTGCTG GTGACCTTGG GCACGCGCTT CCCCGCTGAC TTCACGGCCG

CTGCCTGCTG GTGACCCTGG CCCGCCACTA CCCTGGGGAC TTCAGCCCCA

CAATGTGCTC GTGACTCTGG CCGGTCACCT CGGCAAGGAT TTCACTCCCG

CTGCATCCTG GTTGTCCTGG CCAGGCATTG CCCGGGCGAG TTCACCCCCT

AGGCCCACGC CGCCTGGGAC AAGTTCCTAT CGGTCGTATC CTCTGTCCTG

AGGCCCACGC CGCCTGGGAC AAGTTCCTAT CGGTCGTATC CTCTGTCCTG

AGGCCCACGC CGCCTGGGAC AAGTTCCTAG CGGTCGTATC CTCTGTCCTG

AGGCCCACGC CGCCTGGGAC AAGTTCCTAT CGGTCGTATC CTCTGTCCTG

CGCTGCAGGC GTCGCTGCAC AAGTTCCTGA GCCACGTGGC CTCGGCGCTG

AAGCCCACGC CGCCTGGGAC AAGTTTATGT CGGTTGTGTC CTCTGTCCTG

AGGTCCACGA AGCTTGGGAC AAGTTCATGT CCATCCTGTC TTCTGTCCTG

AGGTCCACGA AGCCTGGGAC AAGTTCATGT CTATCCTGTC TTCTATCCTG

AGACCCACAT CGCCTGGGAC AAGTTCCTGA GCCAAGTGTC CGCCATCCTG

ACGTGCACGC CGCCTGGGAC AAGTTCCTGT CGATTGTATC CGGCGTCCTG

CCGTCCACGC CTCCCTGGAC AAGTTCTTCG CCGCCGTGAG CACCGTGCTG

ATGCCCACGC CGCTTGGGAC AAGTTCCTGT CCGTCGTGTC CAACGTACTG

ACACCCACGC TGCCTGGGAC AAGTTCCTGA CCGTCGTGTC TTCCGTCCTG

AGGCGCACGC CGCCTGGGAC AAGTTCATGT CCATCGTGTC CTCCGTGCTG

AGGCCCACGC CGCCTGGGAC AAATTCCTGT CCGTTGTGTC TTCCGTCCTG

CCATGCATGC TTCGCTAGAC AAGTTCCTGA GCCACGTAAA CTCAGTGCTG

AGATCCATGC CTCCTTGGAC AAGTTCTTGG CTCTTCTGTC CACCGTGCTG

CCGCCCACGC CGCCATGGAC AAGTTCCTGA GCAAAGTGGC CACCGTGCTG

ACCGAGAAGT ACCGCGCAGA AAAAGTACAT GAGTTAAATG AAGAAATAGG

ACCGAGAAGT ACCGCGCAGA AAAAGTACAT GAGTTAAATG AAGAAATAGG

ACCGAGAAGT ACCGCGCAGA AAAAGTACAT GAGTTAAATG AAGAAATAGG

ACCGAGAAGT ACCGCGCAGA AAAAGTACAT GAGTTAAATG AAGAAATAGG

GCTTCCGAGT ACCAGGCAGA AAAAGTACAT GAGTTAAATG AAGAAATAGG

ACCGAGAAGT ACCGCGCAGA AAAAGTACAT GAGTTAAATG AAGAAATAGG

ACTGAGAAGT ACCGCGCAGA AAAAGTACAT GAATTAAATG AAGAAATAGG

ACTGAGAAGT ACCGCGCAGA AAAGGTACAT GAATTAAATG AAGAAATAGG

ACTGAGAAGT ACCGC----- ---------- ---------- ----------

ACGGAGAAGT ACCGCGCAGA AAAAGTACAT GAATTGAATG AAGAAATAGG

ACCTCCAAGT ACCGTGCAGA AAAAGTACAT GAGTTGAATG AAGAAATAGG

ACCGAGAAGT ACCGCGCAGA AAAGGTACAT GAGTTGAATG AAGAAATAGG

ACCGAGAAGT ACCGCGCAGA AAAAGTACAC GAATTAAATG AAGAAATAGG

ACCGAGAAGT ACCGAGCAGA GAAAGTACAT GAGTTGAATG AAGAAATAGG

ACCGAGAAGT ACCGCGCAGA AAAAGTACAT GAGTTGAACG AAGAAATAGG

GTGTCCAAGT ACCGCGCTGA GAAGGTGCAC GAGTTGAATG AAGAGATAGG

ACCTCGAAGT ACCGTGCAGA AAAAGTACAT GAATTGAATG AAGAAATTGG

ACCTCCAAGT ACCGCGCGGA GAAAGTGCAC GAGCTGAATG AGGAAATCGG

AAAACTCCTT GCTAAAGCCG AACAGCTAGG GGCTGAAGGT AATGTGGATG

AAAACTCCTT GCTAAAGCCG AACAGCTAGG GGCTGAAGGT AATGTGGATG

AAAACTCCTT GCTAAAGCCG AACAGCTAGG GGCTGAAGGT AATGTGGATG

AAAACTCCTT GCTAAAGCCG AACAGCTGGG GGCTGAAGGT AATGTGGATG

AAAACTCCTT GCTAAAGCTG AACAGCTGGG GGCTGAAGGT AATGTGGATG

AAAACTCCTT GCTAAAGCTG AGCAGCTAGG TGCTGAAGGA AATGTGGATG

AAAACTTCTT GCTAAAGCTG AGCAGTTAGG AGCTGAAGGA AATGTGGATG

AAAACTTCTT GCTAAAGCTG AGCAGCTAGG AGCTGAAGGA AATGTGGACG

---------- ---------- ---------- ---------- ----------

AAAGCTTCTT GCTAAAGCTG AGCAGCTGGG AGCTGAAGGA AATGTGGATG

AAAACTACTT GCTAAAGCTG AGCAACTGGG AGCTGAAGGA AATGTGGATG

AAAACTTCTT GCTAAAGCTG AGCAGCTGGG AGCCGAAGGA AATGTGGATG

AAAACTTCTT GCTAAAGCTG AGCAGCTGGG AGCTGAAGGA AATGTGGATG

AAAGCTCCTT GCTAAAGCCG AGCAGCTGGG AGCAGAAGGG AACGTGGATG

AAAACTCCTT GCTAAAGCAG AGCAGCTAGG AGCAGAAGGG AATGTGGATG

GAAGCTGCTG GCGAAGGCGG AGCAGCTGGG CGCTGAGGGC AATGTGGAGG

AAAACTGCTT GCTAAAGCTG AACAGCTGGG AGCTGAAGGA AATGTGGATG

GAAGCTGCTG GCCAAGGCTG AGCAGCTAGG CGCCGAGGGC AACGTGGACG

AATCCCAGAA GATTCTTATG GAAGTGGAAA AAGTTCGTGC GAAGAAAAAA

AATCCCAGAA GATTCTTATG GAAGTGGAAA AAGTTCGTGC GAAGAAAAAA

AATCCCAGAA GATTCTTATG GAGGTGGAAA AAGTTCGTGC GAAGAAAAAA

AATCCCAGAA GATTCTTATG GAGGTGGAAA AAGTTCGTGC GAAGAAAAAA

AATCCCAGAA GATTCTTATG GAAGTGGAAA AAGTTCGTGC AAAGAAAAAA

AATCCCAGAA GATTCTTATG GAAGTGGAGA AAGTTCGTGC AAAAAAAAAA

AATCCCAGAA GATTCTTATG GAAGTGGAGA AGGTTCGCTC AAAGAAAAAA

AATCCCAGAA GATTCTTATG GAAGTGGAGA AAGTTCGTGC AAAGAAAAAA

---------- ---------- ---------- ---------- ----------

AATCACAGAA GATTCTCATG GAAGTGGAGA AAGTCCGTGC AAAGAAAAAA

AATCCCAGAA GATTCTTATG GAAGTGGAGA AAGTCCGTGC AAAGAAAAAA

AATCCCAGAA GATTCTTATG GAAGTGGAGA AAGTCCGTGC AAAGAAAAAA

AATCCCAGAA GATTCTTATG GAAGTAGAGA AAGTTCGTGC AAAGAAAAAA

AATCGCAGAA GATCCTTACG GAAGTGGAGA AAGTTCGTGC AAAAAAAAAA

AATCCCAGAA GATCCTCACG GAAGTAGAGA AAGTCCGTAC AAAGAAGAAA

AGTCGCAGAA GACCCTCATG GAGGTGGAGA AGGTCCGCAC CCGGAAGAAA

AATCCCAGAA GATTCTTATG GAAGTGGAGA AAGTTCGAGC AAAGAAAAAA

AGTCCCAGAA GGTCCTCATG GAGGTGGAGA AGGTCCGGGC CAAGAAGAAA

GAAGCTGAGG AAGAATACAG AAATTCCATG CCTGCATCCA GTTTTCAGCA

GAAGCTGAGG AAGAATACAG AAATTCCATG CCTGCATCCA GTTTTCAGCA

GAAGCTGAGG AAGAATACAG AAATTCCATG CCTGCGTCCA GTTTTCAGCA

GAAGCTGAGG AAGAATACAG AAATTCCATG CCTGCGTCCA GTTTTCAGCA

GAAGCTGAGG AAGAATATAG AAATTCTATG CCTGCTTCCA GTTTTCAGCA

GAAGCTGAGG AAGAATACAG AAATTCCATG CCTGCATCCA GTTTTCAACA

GAAGCAGAGG AAGAATACAG AAATTCCATG CCTGCATCCA GTTTTCAACA

GAAGCAGAGG AAGAATATAG AAATTCCATG CCTGCATCCA GTTTTCAACA

---------- ---------- ------CATG CCTGCATCCA GCTTCCAGCA

GAAGCTGAGG AAGAATACAG AAATTCCATG CCTGCGTCCA GTTTTCAGCA

GAAGCTGAGG AAGAATACAG AAATTCCATG CCTGCATCCA GTTTCCAACA

GAAGCTGAGG AAGAATACAG AAATTCCATG CCTGCGTCCA GTTTTCAGCA

GAAGCCGAGG AAGAATACAG AAACTCCATG CCTGCGTCTA GTTTCCAGCA

GAAGCTGAGG AGGAATACAG AAACTCCATG CCTGCATCTA GTTTCCAGCA

GAAGCTGAGG AAGAATACAG AAATTCCATG CCTGCATCCA GTTTTCAGCA

GAAGCCGAG- ---------- ---------- ---------- ----------

GAAGCTGAGG AAGAATATAG AAATTCTATG CCAGCATCCA GTTTCCAGCA

GAGGCCGAGG AAGAATATCG AAATTCCATG CCCGCCTCCA GCTTCCAGCA

GCAAAAGCTG CGTGTCTGCG AGGTCTGTTC AGCCTACCTT GGTCTCCATG

GCAAAAGCTG CGTGTCTGCG AGGTCTGTTC AGCCTACCTT GGTCTCCATG

GCAAAAGCTG CGTGTCTGCG AGGTCTGTTC AGCCTACCTT GGTCTCCATG

GCAAAAGCTG CGTGTCTGCG AGGTCTGTTC AGCCTACCTT GGTCTCCATG

GCAAAAGCTC CGTGTCTGTG AGGTCTGTTC AGCCTACCTT GGTCTCCATG

GCAAAAGCTG CGTGTCTGTG AGGTCTGTTC AGCCTACCTT GGTCTCCATG

GCAAAAACTC CGTGTTTGCG AGGTTTGTTC AGCTTATCTT GGTCTCCATG

GCAAAAACTC CGTGTTTGCG AGGTTTGTTC AGCTTACCTT GGTCTCCATG

GCAAAAGCTG CGTGTGTGTG AGGTCTGCTC AGCCTACCTC GGTCTCCATG

ACAGAAGCTT CGTGTCTGTG AGGTCTGTTC GGCCTACCTT GGCCTCCATG

GCAGAAGCTG CGTGTCTGTG AGGTCTGTTC AGCCTACCTT GGGCTCCATG

GCAGAAGTTG CGTGTCTGTG AAGTCTGCTC GGCCTACCTT GGTCTCCACG

GCAGAAGTTG CGTGTCTGTG AGGTCTGCTC TGCCTACCTT GGTCTCCATG

GCAGAAGCTG CGTGTCTGTG AGGTCTGTTC AGCCTATCTT GGCCTCCATG

GCAGAAGCTG CGTGTCTGTG AGGTCTGCTC AGCCTACCTT GGTCTCCATG

---------- ---------- ---------- ---------- ----------

GCAGAAACTA CGGGTCTGTG AGGTGTGCTC TGCGTACCTT GGTCTCCATG

GCAGAAATTG CGGGTCTGTG AAGTCTGTTC GGCGTACCTG GGTCTCCATG

ACAATGACCG TCGCCTGGCA GACCACTTCG GTGGCAAGTT ACACTTGGGG

ACAATGACCG TCGCCTGGCA GACCACTTCG GTGGCAAGTT ACACTTGGGG

ACAATGACCG TCGCCTGGCA GACCACTTCG GTGGCAAGTT ACACTTGGGG

ACAATGACCG TCGCCTGGCA GACCACTTCG GTGGCAAGTT ACACTTGGGG

ACAATGACCG TCGCCTGGCA GACCACTTTG GTGGCAAGTT ACACTTGGGG

ATAATGACCG ACGTCTGGCA GACCACTTCG GGGGCAAGTT ACACTTGGGG

ACAATGACCG TCGTCTTGCA GACCACTTCG GAGGCAAATT ACACTTGGGG

ACAATGACCG TCGGCTTGCA GACCACTTCG GGGGCAAGTT ACACCTGGGG

ACAACGACCG CCGCCTGGCA GACCACTTCG GGGGCAAGTT ACACCTGGGC

ACAATGACCG TCGTCTTGCA GACCACTTCG GGGGCAAGTT ACACTTGGGA

ACAATGACCG TCGTCTTGCA GACCACTTCG GGGGCAAGTT ACACTTGGGG

ACAACGACCG CCGTCTCGCA GACCATTTCG GGGGCAAGTT GCACTTGGGG

ACAATGACCG TCGTCTTGCA GATCACTTTG GGGGAAAGTT ACACCTGGGG

ACAATGACCG TCGCCTTGCA GACCACTTTG GGGGCAAGTT ACACTTGGGA

ACAATGACCG GCGCCTCGCA GACCACTTTG GGGGCAAGTT ACACTTGGGG

---------- ---------- ---------- ---------- ---------G

ATAATGATCG TCGTCTTGCA GATCACTTCG GGGGCAAATT ACACTTGGGG

ACAACGATCG CCGACTGGCC GACCACTTTG GAGGCAAATT ACACTTGGGC

TTCATTCAGA TCCGAGAGAA GCTTGATCAG TTGAGGAAAA CTGTCGCTGA

TTCATTCAGA TCCGAGAGAA GCTTGATCAG TTGAGGAAAA CTGTCGCTGA

TTCATTCAGA TCCGAGAGAA GCTTGATCAG TTGAGGAAAA CTGTCGCTGA

TTCATTCAGA TCCGAGAGAA GCTTGATCAG TTGAGGAAAA CTGTCGCTGA

TTCATTCAGA TCCGAGAGAA GCTTGATCAG TTAAGGAAAA CTGTTGCTGA

TTCATTCAGA TCCGAGAAAA GCTTGATCAG TTGAGGAAAA CTGTGGCTGA

TTCATTCAGA TCCGAGAGAA GCTTGATCAG TTGAGGAAAA CTGTGGCTGA

TTCATTCAGA TCCGGGAGAA GCTTGATCAG TTGAGGAAAA CTGTGGCTGA

TTCATCCAGA TTCGAGAGAA GCTCGACCAG TTGAGGAAAA CTGTGGCTGA

TTCATTCAGA TCCGTGAGAA GCTCGATCAG TTAAGGAAAA CTGTGGCTGA

TTCATTCAGA TCCGAGAGAA GCTTGATCAG TTGAGGAAAA CTGTGGCTGA

TTCATTCAGA TCCGGGATAA GCTTGACCAG TTGAGGAAAA CTGTGGCTGA

TTTATTCAGA TCCGTGAGAA GCTTGATCAG TTGAGGAAAA CTGTGGCAGA

TTCATTCAGA TCCGAGAGAA GCTTGACCAG TTGAGGAAAA CTGTTGCTGA

TTCATTCAGA TCCGGGAAAA GCTGGACCAG CTGAGGAAAA CAGTGACTGA

TTCATTCAGA TCCGGGAGAA GCTGGACCAG CTGCGGAAAA CTGTGGCTGA

TTTATTCAGA TTCGTGAGAA ACTGGATCAG TTGAGGAAAA CAGTGGCAGA

TTCATTCAGA TCCGGGAGAA GCTGGACCAG TTGAGGAAAA CCGTGGCGGA

AAAGCAGGAG AAGAGAAATC AGGATCGCTT GAGGAGGAGA GAGGAGAGGG

AAAGCAGGAG AAGAGAAATC AGGATCGCTT GAGGAGGAGA GAGGAGAGGG

AAAGCAGGAG AAGAGAAATC AGGATCGCTT GAGGAGGAGA GAGGAGAGGG

AAAGCAGGAG AAGAGAAATC AGGATCGCTT GAGGAGGAGA GAGGAGAGGG

AAAGCAGGAG AAGAGAAATC AGGATCGCTT GAGGAGGAGA GAGGAGAGGG

AAAGCAAGAG AAGAGAAATC AAGATCGCTT GAGGAGGAGA GAGGAAAGGG

AAAACAGGAA AAAAGAAATC AGGATCGATT GAGGAGAAGA GAGGAGAGAG

AAAACAGGAA AAAAGAAATC AGGATCGATT GAGGAGAAGA GAGGAGAGAG

AAAGCAGGAG AAGAGAAATC AGGATCGCCT GCGGAGGAGA GAAGAGAGGG

GAAGCAGGAG AAGAGAAATC AGGATCGGCT GAGGAGGCGA GAGGAGAGAG

AAAGCAAGAG AAGAGAAATC AGGATCGTCT GAGGAGGAGA GAGGAGAGAG

AAAGCAGGAG AAGAGAAACC TGGATCGCTT GAGGAGGAGA GAGGAGAGGG

AAAGCAGGAC AAGAGAAATC AGGACCGCCT GAGGAGGAGA GAGGAGAGGG

AAAGCAGGAG AAGAGGAATC AGGACCGCCT GAGGAGGAGA GAGGAGAGGG

AAAGCAGGAG AAGAGAAACC AGGATCGCTT gaggaggaga gaggagagag

GAAGCAGGAG AAGAGAAACC AGGACCGCCT CAGGCGGCGG GAAGAACGGG

AAAACAAGAG AAAAGAAATC AAGATCGTTT GAGGAGGAGA GAAGAGAGAG

GAAACAGGAG AAAAGAAACC AGGATCGCCT GAGGCGGCGA GAAGAGCGCG

AACGGGAGGA GCGTCTGAGC AGGAGGTCGG GATCAAGAAC CAGAGATCGC

AACGGGAGGA GCGTCTGAGC AGGAGGTCGG GATCAAGAAC CAGAGATCGC

AGCGGGAGGA GCGTCTGAGC AGGAGGTCGG GATCAAGAAC CAGAGATCGC

AGCGGGAGGA GCGTCTGAGC AGGAGGTCGG GATCAAGAAC CAGAGATCGC

AGCGAGAGGA GCGTCTGAGC AGGAGGTCAG GATCAAGAAC CAGAGATCGC

AACGAGAAGA GCGGCTGAGT AGAAGGTCTG GATCAAGAAC CAGAGATCGC

AGCGAGAAGA GCGCCTGGGC AGGCGGTCTG GATCAAGAAC CCGAGATCGG

AGCGAGAAGA GCGCCTGGGC AGGAGGTCTG GATCAAGAAC CCGAGATCGC

AGCGGGAGGA GCGGCTGAGC AGGAGGTCTG CATCGAGAAC CAGAGATCGC

AGCGGGAGGA GCGGCTGAGC AGAAGGTCTG GATCAAGAAC AAGAGATCGC

AGCGGGAGGA GCGGCTGAGC AGGAGGTCTG GATCAAGAAC CAGAGATCGA

AGCGAGAGGA GTGGCTGAGC AGGAGGTCTG GATCAAGAAC CAGAGATCGC

AACGGGAGGA GCGGCTGAGT AGGAGATCTG GATCAAGAAC CAGAGATCGT

AGCGCGAGGA GCGGCTGAGC AGGAGGTCTG GATCGAGAAC CAGAGACCGC

agagagagga gaggCTGAGC AGACGGTCTG GATCAAGGAC CAGAGATCGC

AACGTGAGGA GAGACTGAGC AGGCGGTCTG GATCGAGGAC CAGAGACCGC

AACGAGAAGA AAGGATGGGT CGAAGGTCTG GATCAAGAAC TAGAGATCGA

AACGCGAGGA AAGGCTGGGC AGGAGGTCCG GATCAAGAAC CAGGGATCGC

AGGAGGTCAC GCTCCCGGGA TCGGCGTCGG AGGCGGTCAA GATCTACCTC

AGGAGGTCAC GCTCCCGGGA TCGGCGTCGG AGGCGGTCAA GATCTACCTC

AGGAGGTCAC GCTCCCGGGA TCGGCGTCGG AGGCGGTCAA GATCTACCTC

AGGAGGTCAC GCTCCCGGGA TCGGCGTCGG AGGCGGTCAA GATCTACCTC

AGGAGGTCAC GCTCCCGGGA TCGGCGTCGG AGGCGGTCAA GATCTACCTC

AGGAGGTCAC GCTCCCGGGA CCGGCGTCGG AGGCGGTCAA GGTCTACATC

AGGAGGTCAC GTTCCCGGGA TCGTCGAAGG AGGCGGTCTA GGTCTACCTC

AGAAGGTCAC GCTCCCGGGA TCGTCGACGG AGGCGGTCTA GGTCTACCTC

AGGAGGTCAC GTTCCCGGGA GCGGCGTCGA AGGCGGTCAA GGTCTGCCTC

AGGAGGTCGC GCTCCCGGGA CCGACGTCGC AGGCGATCGA GGTCAACTTC

AGGAGGTCAC GCTCCCGGGA CCGGCGTCGG AGGAGGTCCA GATCTACCTC

AGGAGGTCGC GTTCCCGGGA CCGGCGTCGG CGGCGGTCAA GATCTACCTC

AGGAGGTCAC GTTCCAGGGA GAGACGTCGG AGACGGTCCA GATCTGCCTC

AGGAGGTCCC GTTCTCGAGA CCGGCGCCGT AGGCGGTCCA GGTCCACCTC

AGGAGGTCGC GCTCTCGGGA CCGGCGTCGG AGGCGGTCAA GATCTACCTC

AGGAGGTCTC GCTCTCGGGA CCGGCGAAGG AGGCGGTCAA GGTCAGCCTC

AGAAGGTCAC GATCTCGGGA CAGACGACGA AGGCGGTCAA GGTCAGGCTC

AGAAGGTCAC GATCTCGGGA CAGGCGGCGG AGGCGGTCGA GGTCGGCCTC

CCGAGAGCGA CGGAAATTGT CCCGGTCCCG GTCCCGAGAT AGACATCGGC

CCGAGAGCGA CGGAAATTGT CCCGGTCCCG GTCCCGAGAT AGACATCGGC

CCGAGAGCGA CGGAAGTTGT CCCGGTCCCG GTCGCGAGAT AGACATCGGC

CCGAGAGCGA CGAAAGTTGT CCCGGTCCCG GTCCCGAGAT AGACATCGGC

CCGAGAGCGA CGGAAGTTGT CCCGGTCCCG GTCCCGAGAT AGACATAGGC

CCGGGAGCGA CGGAAGTCTT CCCGGTCCCG GTCCCGAGAC AGACACCGGC

CCGAGAGCGG CGGAAGTTTT CCCGTTCCAG GTCCCGAGAT AGATACCGGC

CAGAGAGCGG CGGAAGTTTT CCCGTTCCAG ATCCCGAGAT AGATACAGGC

CCGAGAGCGG AGGAAGTCTT CCCGGTCCCG GTCCCGAGAC AGACACCGAC

CAGAGAGCGG CGGAAGTCGT CCCGCTCCCG GTCCCGAGAC AGACACCGGC

CAGGGAGCGA CGAAAGTCAT CCCGGTCCCG GTCCCGAGAC AGACACCGGC

CAGAGAAAGA CGAAAATCAT CCAGGTCCCG CTCCCAAGAC AGACACCGGC

CAGAGAGCGA CGGAAGTCTT CCCGGTCTCG GTCTCGAGAC AGACACCGGC

CCGGGAGCGG CGGAAGTCTT CCCGGTCCCG GTCCCGAGAC AGACACCGGC

CCGTGAGCGA CGCAAGTCTT CCCGGTCCTG GTCCCGAGAC AGACACCGGC

CAGAGAGCGG CGGAAATCCT CGAGATCTAG GTCCCGAGAC AGACACCGGC

TCGTGAGAGG CGAAAGT--- CCCGTTCCCG ATCTCGGGAC AGACATAGGC

CCGCGAGCGG CGGAAGT--- CCCGCTCCCG CTCCCGCGAG CGCCACAGGC

GCCACCGCAG CCGTTCCCGG AGCCACAGCC GGGGACATCG TCGGGCTTCC

GCCACCGCAG CCGTTCCCGG AGCCACAGCC GGGGACATCG TCGGGCTTCC

GCCACCGCAG CCGTTCCCGG AGCCACAGCC GGGGACATCG TCGGGCTTCC

GCCACCGCAG CCGTTCCCGG AGCCACAGCC GGGGACATCG TCGGGCTTCC

GCCACCGCAG CCGTTCCCGG AGCCACAGCC GGGGACATCG TCGGGCTTCC

GCCACCGTAG CCGTTCCCGG AGTCACAGCC GGGGACACCG CCGGGCTTCC

GACATCGCAG CCGTTCCCGG AGCCACAGCC GGGGTCACCG ACGGGCTTCC

GCCATCGCAG CCGTTCCCGG AGCCACAGCC GGGGTCACCG CCGGGCTTCC

GCCACCGCAG CCGTTCGCGG AGCCACAGCC GGGGTCACCG CCGGGTTTCC

GCCACCGAAG CCGCTCCCGG AGCCACAGCC GTGGCCATCG ACGGGCATCC

GCCACCGCAG CCGTTCCCGG AGCCACAGCC GGGGCCACCG CCGGGCTTCC

GCCACCGCAG TCGTTCCCGG AGCCACAGCC GGGGCCACCG CTGGGCTTCC

GCCACCGAAG CCGTTCCCGT AGCCACAGCA GGGGCCACCG CCGGGCCTCC

GCCACCGGAG CCGCTCCCGG AGCCACAGCC GGGGCCACCG CCGGGCTTCC

GCCACCGGAG CCGTTCCCGG AGCCACAGTC GGGGCCACCG CCGGGCCTCC

GACACCGCAG CCGGTCCAGA AGCCACAGCC GGGGCCACCG CCGGGCCTCC

GCCACCGCAG CCGCTCACGT AGCCATAGCA GGGGCCATCG CCGAGGTTCC

GCCATCGGAC CCGCTCGCGC AGCCCCAGCC GGGGCCACCG CCGGGGCTCC

CGGGACCGAA GTGCGAAATA CAAGTTCTCC AGAGAGCGGG CATCCAGAGA

CGGGACCGGA GTGCGAAATA CAAGTTCTCC AGAGAGCGGG CATCCAGAGA

CGGGACCGGA GTGCGAAATA CAAGTTCTCC AGAGAGCGGG CATCCAGAGA

CGGGACCGGA GTGCGAAATA CAAGTTCTCC AGAGAGCGGG CATCCAGAGA

CGGGACCGGA GTGCGAAATA CAAGTTCTCC AGAGAACGGG CATCTAGAGA

CGGGACCGGA GTGCGAAATA CAAGTTCTCC AGAGAGCGGG TAGCAAGAGA

AGGGACCGGA GTACAAAATA CAAGTTCTCC AGGGAGCGCT CATTGAGAGA

AGGGACCGGA GTACAAAATA CAAGTTCTCC AGGGAGCGCT CATTGAGAGA

CGGGACGGGA GTGCGAAATA CAAGTTCTCC AGAGAACGGG CTTCGAGGGA

AGGGACCGGA GCTCGAAATA CAAGTTCTCC CGAGAGCGGA CTTCAAGGGA

AGGGACCGGA GTGCGAAATA CAAGTTCTCC AGAGAGCGGA CATCGAGAGA

AGGGACCAGA GTGCGAAATA CAAGTTCTCC AGAGAGCGGG CGTCGAGGGA

AGGGACCGGA GTTCGAAATA CAA------- ---------- ----------

AGAGACCGGA GTTCGAAATA CAAGTTCTCC AGAGAGCGGA CGTCGAGAGA

AGGGATCGGA GCTCGAAATA CAAGTTCTCC CGAGAGCGAG CATCGAGAGA

AGAGACCGCA GCTCGAGACA CAAGTTCTCC CGAGAAGGAG CAGCCCGAGA

AGAGACAGAA GTTCGAAACA CAAGATCT-- AGAGATCAAT CTTCAAGAGA

AGAGACCGCA GTTCCAAACA CAAGTCACCC AGAGATCGGT CTACAAGAGA

GGAGTCCTGG GAGAGCGGGC GGAGCGAGCG AGGGCCCCCG GACTGGAGGC

GGAGTCCTGG GAGAGCGGGC GGAGCGAGCG AGGGCCCCCG GACTGGAGGC

GGAGTCCTGG GAGAGCGGGC GGAGCGAGCG AGGGCCCCCG GACTGGAGGC

GGAGTCCTGG GAGAGCGGGC GGAGCGAGCG AGGGCCCCCG GACTGGAGGC

GGCATCCTGG GACAGCGGGC GGAGTGAGCG GGGGCCCCCG GACTGGAGGC

GGAGTCCTGG GAGTGTGGGC GGAGCGAGCG TGGGTCCATT GACTGGAGGC

GGAATCCTGG GAGTACAGGC GGAATGAACG AGGGCCCACT GACTGGAGGC

GGAATCCTGG GAGTACGGGC GGAATGAACG AGGGCCCACT GACTGGAGGC

GGAGTCCTGG GAGTGTGGGC GCAGCGAGCG GGGGGCCATC GACTGGCGGC

GGAGTCCTGG GAGCGCGGGC GGAGTGAGCG GGGGGCCACC GACTGGAGGC

GGAGTCCTGG GAGCACGGGC GCAGTGAGCG AGGGGCCGCT GATTGGAGGC

GGAGTCCTGG GAGCGCGGGC GGAGTGAGCG GGGGGCCACC GACTGGAGGC

---------- ---------- ---------- ---------- ----------

GGAGTCCTGG GAGAGAGGAC GGAGCGAGCG GGGTGCTGCC GACTGGAGGC

GGAGTCCTGG GAGCAAGGGC GGAGTGAGAG GGGCGCTGCA GAGTGGAGGC

GGAATCTTGG G------GGC GGAGTGAGAG AGGCCCCTTC GACTGGAGGC

GAAGTCCCAG GATAAAGAGA GGAGGGAAAA GAGCTTTGCT GAAAAAAGGC

AAAGTCCCGG GACGGAGAGA GGAAGGAGAA GAG---TTTG GAGAAGCGGC

TTGAGAGCTC CAACGGGAAG ATGGCTTCAC GGAGGTCAGA AGAGAAGGAG

TTGAGAGCTC CAACGGGAAG ATGGCTTCAC GGAGGTCAGA AGAGAAGGAG

TTGAGAGCTC CAACGGGAAG ATGGCTTCAC GGAGGTCAGA AGAGAAGGAG

TTGAGAGCTC CAACGGGAAG ATGGCTTCAC GGAGGTCAGA AGAGAAGGAG

TCGAGAGCTC CAACGGGAAG ATGGCTTCAC GGAGGTCTGA AGAGAAGGAG

TTGAGAGCTC CAACGGGAAG GCAGCTTCAC GGAAGTCAGA GGAGAAGGAG

TGGAGAACTC CAATGGCAAG ACTGCATCGC GGAGGTCGGA GGAGAAGGAG

TGGAGAATTC CAATGGCAAG ACTGCATCGC GGAGGTCGGA GGAGAAGGAG

TGGAGAGCTC CAACGGGAAG TCGGCGTCCC GGAAGTCGGA GGAGAAGGAG

TTGAGAGTGC CAACGGGAAG GCCGCATCGC GGAGGTCGGA GGAGAAGGAG

TGGAGAGCTC CAACGGGAAG ATGGCTTCGC GGAGGTCGGA GGAGAAGGAG

TCGAGAGCTC CAACGGGAAG ACGGCTTTGC GGAGGTCGGA GGAGAAGGAG

---------- ---------- ---------- ---------- ----------

TCGAGAGCTC TAATGGGAAG GCAGCGCCAC GGAGGTCGGA GGAGAAGGAG

TCGAGGGCTC CAACGGAAAG GCAGCCTCAC GGAGGTCGGA GGAGAAGGAG

TGGAGGGCCC GAACGGA--- ---------- --AGGTCTGA AGAGAAAGAG

TTGAGGGTAC AAATGGCAAC GCTGGTTCAA AGAGATCAGA AGAAAGAGAA

ACGAGGGCAC CAATGACAAA CCACGGCCCA GGCGGTCGGA GGAAAGGGAA

GCCGGCGAGA TCCTTGCGGG TGATGTTGTG CACGTAGGGG CACGTGTTGC

GCCGGCGAGA TCCTTGCGGG TGATGTTGTG CACGTAGGGA CACGTGTTGC

GCCGGCGAGA TCCTTGCGGG TGATGTTGTG CACGTAGGGG CACGTGTTGC

GCCGGCGAGA TCCTTGCGGG TGATGTTGTG CACGTAGGGG CACGTGTTGC

GCCGGCGAGA TCCTTGCGGG TGATGTTGTG CACGTAGGGG CACGTATTGC

GCCGGCGAGA TCCTTGCGGG TGATGTTGTG CACATAGGGG CAGGTGTTGC

GCTGGCGAGA TCCTTGCGGG TGATGTTATG GACGTACGGG CACGTGTTGC

GCTGGCGAGA TCCTTACGGG TGATATTATG GACGTAGGGG CAAGTGTTGC

GCTGGGGAGA TCCTTGCGGG TGATGTTGTG CACGTAGGGG CAGGTGTTGC

GCCGGCGAGA TCCTTGCGCG TGACGTTGTG CACGTAGGGG CAGGTGTTGC

GCTGGCGAGA TCCTTGCGCG TGATGTTGTG CACGTAGGGG CAGGTGTTGC

GCTGGCGAGA TCCTTGCGGG TGATGTTGTG CACGTAGGGG CAGGTGTTGC

---------- --CTTACGGG TGATATTGTG CACGTAGGGG CAGGTATTGC

GCCGGCGAGC TCCTTGCGGG TGATGTTGTG CACGTAAGGG CAGGTGTTGC

GCTGGCGAGA TA-------- ---------- ---------- ----------

GCAGGCGAGA TTCTTTCGGG TGATGTTGTG CACGTAGGGA CAGGTGTTGC

GCTGGCGAGA TCCTTCCGGG TGATGTTGTG CACGTAAGGG CACGTGTTGC

GCTGGCGAGA TCCTTGCGCG TGATGTTGTG CACGTAGGGG CAGGTGTTGC

AGGAGAAGCG GTGGCAGCGT TGTCCCTCCT CCACGATCAG CCCGTTCCCG

AGGCGAAGCG GTGGCAGCGT TGTCCCTCCT CCACGATCAG CCCGTTCCCG

AGGCGAAGCG GTGGCAGCGT TGTCCCTCCT CCACGATCAG CCCGTTCCCG

AGGCGAAGCG GTGGCAGCGT TGCCCCTCCT CCACGATCAG CCCGTTCCCG

AGGCGAAGCG GTGGCAGCGC TGTCCCTCCT CCACGATCAG CCCGTTCCCG

AGGCGAAGCG ATAGCAACGC TGTCCCTCCT CCACAATCAG CCCATTCCCG

AGGCGAAGCG GTGACAGCGC TGCCCCTCCT CCACAATGAG GCCGTTCCCG

AGGCGAAGCG ATGGCAACGC TGCCCCTCCT CCACAATGAG TCCGTTCCCG

AGGCGAAGCG GTGGCAGCGC TGGCCCTCCT CCACGATCAG CCCGTTCCCG

ATGCGAAGCG GTGGCAGCGC TGGCCCTCCT CGACGATCAG CCCGTTCCCG

AGGCGAAGCG GTGGCAGCGC TGTCCCTCCT CCACGATCAG CCCGTTCCCG

AGGCGAAACG GTGGCAGCGC TGTCCTTCCT CCACGATCAG CCCGTTCCCG

AGGCGAAGCG GTGGCAGCGC TGGCCCTCCT CCACGATGAG TCCGTTCCCG

AGGCGAAGCG GTGGCAGCGC TGTCCCTCCT CCACGATGAG GCCGTTGCCG

---------- ---------- ---------- ---------- ----------

AGGCGAAGCG GTGACAGCGC TGGCCCTCCT CCACGATCAG TCCGTTGCCA

AAGCGAAGCG GTGGCAGCGT TGTCCCTCCT CCACGATCAA CCCATTCCCG

AGGCGAAACG ATGGCAGCGT TGGCCCTCCT CCACGATCAG CCCGTTCCCG

CAGCCGGGGC AGAACAGCAT AACTGGTACC TCTCCCTGCA GCTCATGTGC

CAGCCGGGGC AGAACAGCAT AACTGGTACC TCTCCCTGCA GCTCATGTGC

CAGCCGGGGC AGAACAGCAT AACTGGTACC TCTCTCTGCA GCTCATGTGC

CAGCCGGGGC AGAA---CAT AACTGGTACC TCTCTCTACA GCTCATGTGC

CAGCCGGGGC AGAACAGCAT AACTGGTACC TCTCCCTGCA GCTCATGTGC

CAGCCCGGGC AGAACAGCAT ---------- ---------- ----------

CAGCCCGGGC AGAACAGCAT AACTGGTACC TCTCCTTGCA GCTTGTGTGC

CAGCCCGGGC AGAACAACAT AACTGGTACC TCTCCCTGCA GCTTGTGTGC

CAGCCGGGGC AGAACAGCAT ---------- ---------- ----------

CAGCCCGGGC AGAAGAGCAT AACTGGTACC TCTCCCTGCA GGTCCTCTGC

CAGCCCGGAC AGAAGAGCAT AACTGGTACC TCTCCCTGCA GCTGGTGTGC

CAGCCGGGAC AGAAAAGCAT AACTGGTACC TCTCCCTACA GCTTGTGTGC

CAGCTCGGGC AGAAGAGCAT ---------- ---------- ----------

CAGCCCGGAC AGAAAAGCAT AGCTGGTACA TCTCCCTGCA GCTCGTCTGC

---------- ---------- ---------- ---------- ----------

CAGCCCGGGC AGAAGAGCAT AACTGGTACC TCTCCCTGCA GCTCCTGTGC

CAGCCTGGAC AGAAAAGCAT AATTGGTACC TCTCCTTACA GCTCATCTGC

CAGCTCGGAC AGAAAAGCAT --CTGGTTCC TCAGCCTTCA GCTGCTCTGC

CCTGAGGCTG AGAGGCTCTT CACGGTGAGC ACCAAGGTCT ACTTTCCGCT

CCTGAGGCTG AGAGGCTCTT CACAGTGAGC ACCAAGGTCT ACTTTCCGCT

CCTGAGGCTG AGAGGCTCTT CACAGTAAGC ACCAAGGTCT ACTTTCCGCT

CCTGAGGCTG AGAG------ ---------- ---------- --------CT

TCTGACGAGG AG--GACCTT CCTGGCCGCC ACCAAGACCT ACTTCCAGCT

---------- --AGGCTCTT CACAGTGAGC ACTAAGGCCT ACTTTCAGCT

CCTGAGCCTG AGGC---ATT CACA---AAC ACCAAGAA-- --TTTCCTCT

CCTGAGCCTG AGGCGCAATT CACA---AAC ACCAAGGT-- --TTTCCTCT

---------- ----GCTCTT CACAGCTAG- ACCAAGACCT ACTTCAAGCT

CCCGAGCCTG AGAGGCTCTT CACTGTGAGC ACCAAGGTCT ACTTCCCACT

CCTGAACCCG AG--GACCTT CCAGTCCAGC ACCAAGACCT ACTTCCCGCT

CCTCAGCCTG AGAGGCTCTT CACCGTGAGC ACCAAGACCT ACTTCCCACT

---------- --AGGCTCTT CACGGTAAGC ACCAAGATCT ACTTCCCGCT

CCCAAG--TG AGAGGCTCTT CGCAGCGAGC ACCAAGGCCT ACTTCCCGAT

---------- --A-GCTCTT CACGGTGAGC ACCAAGGCCT ACTTCCTGCT

CTCAACCCTG A---GACCTT CCTCTCCACC ACCAAGACCT ACTTCAAGCT

CCCAAGGTGA GT--GTACTT CAAGGTGAGC TCCAGGGACT --TTAGAGCT

CCCGAGCTGA GT--GCTGTT CCAGGCTCAG ACCAAGACCT ACTTCAAGCT

GCTAATCCAG TGTTTCCACG TCGTGCTGGC CTCCCACCTG CAGGACGAGT

GCTAATCCAG TGTTTCCACG TCGTGCTGGC CTCCCACCTG CAGGACGAGT

GCTAATCCAG TGTTTCCACG TCGTGCTGGC CTCCCACCTG CAGGACGAGT

CCTGTCCCAC TGCCTGCTGG TCACCCTGGC CGCGCGCTTC CCCGCCGACT

CCTCGGCCAC TGCCTGCTGG TGACCCTCGC CCGGCATTAC CCCGGAGACT

GCTGATCCAG TGTTTCCAGG TCGTGCTGGC TTCCCACTTG CAGGACGAGT

GCT--TCCCG TGTTCCAGGG TGGTACTGGC CTCCCATCTG CAGGGTGAAT

GCT--TCCCA TGTTCCAGGA CGGTACTGGC CTCCCAGCTG CAGGGTGAGT

CCTGTCCCAC TGCCTGCTGG TCTCCCTGGC CGTGCACCTG CCCGCGGACT

GGTGATCCAG TGTTTCCAGG TGGTGCTGGC CTCCCACCTG CAGGGCGAGT

GCTGATCCAG TGTTTCCAGG TGGTGCTGGC CTCCCACCTG CAGGACGAGT

GCTGATCCAG TGTTTCCAGG TGGTGCTGGC CTCCCACCTG CAGGGCGAGT

GCTGATGCAG TGTTTCCAGG TGGTGCTGGC CTCACACCTG CAGGACGAGT

GCTGATCCAC TGCTTCCAGG TGGTGCTGGC CTCCCACCTG CAGGGCGAGT

GCTGTTTCAG TGCTTCCAGG TGGTGCTGGC CTCCCACTTG CAGGGCGAGT

CCTGAGCCAC TGCCTGCTGG TGACTCTGGC CTGCCACCTG GGCGGCGATT

CCTGTGCCAC AATGTGCTCG TGACTCTGGC CGGTCACCTC GGCAAGGATT

CCTCTCCCAC TGCATCCTGG TGTCCTTGGC CTCTCGCTTC CCGGCCGACT

TCACCGTGCA AATGCAAGCG GCGTGGGACA AGTTCCTGAC TGGTGTGGCC

TCACCGTGCA AATGCAAGCG GCGTGGGACA AGTTCCTGAC TGGTGTGGCC

TCACCGTGGA AATGCAAGCG GCGTGGGACA AGTTCCTGAC TGGTGTGGCC

TCACTGCCGA GGCCCACGCC GCCTGGGACA AGTTCCTATC GGTCGTATCC

TCAGCCCCGC GCTGCAGGCG TCGCTGCACA AGTTCCTGAG CCACGTGGCC

TCACAGTGGA GATGCATGCT GCATGGGACA AGTTCCTGAC AGGCGTGGCC

TCACTGTGGA GATGCAGGTG GAGGAGGATA AGTTCTC--- ----------

TCACTGTTGA GATGCAGGCA GAGGGGTACA AATTTTC--- ----------

TCACCGCCGA GACCCACATC GCCTGGGACA AGTTCCTGAG CCAAGTGTCC

TCACGGTGGA GATGCAGGCG GCGTGGGATA AGTTCCTGAC GGGCGTGGCC

TCACCGTGGA GATGCAGGCG GCGTGGGACA AGTTCCTGAC GGGCGTGGCG

ACACGGTGGA GATGCAAGCG GCCTGGGATA AGTTCCTGAC CGGCGTGGCG

TCACAGTGGA GATGCACGCG GCGTGGGACA AGTTCCTGAC CGCCTTGGCG

TCACCGTGGA GAT-CAGGCG GCGTGGGAGA AGTTCCCGAC GGCCGTGGCC

TCATGGTAGA GATGCAGGCG GCATGTGACA AGTACCTGAC GGGCGTGGCT

TCACCCCTGC CGCCCACGCC TCCCTGGACA AGTTCCTCAG CTCTGTGAGC

TCACTCCCGA GATCCATGCC TCCTTGGACA AGTTCTTGGC TCTTCTGTCC

TCACGGCCGA GGCCCACGCC GCCTGGGACA AGTTCCTGTC CATTGTGTCC

GTGGTGCTGA CCGAAAAATA CCGCATGGTG CATCTGACTC CTGAGGAGAA

GTGGTGCTGA CCGAAAAATA CCGCATGGTG CACCTGACTC CTGAGGAGAA

GTGGTGTTGA CGGAAAAGTA CCGCATGGTG CATCTGACTC CTGAGGAGAA

TCTGTCCTGA CCGAGAAGTA CCGCATGGTG CATCTGACTC CTGAGGAGAA

TCGGCGCTGG CTTCCGAGTA CCAGATGGTG CATCTGACTG GTGAAGAAAA

GTGGTGCTGA CTGAAAAGTA TCGCATGGTG CACCTGACTC CTGATGAGAA

-TGGTCCTGG GAGAGAAATA CTGCATGGTG CACCTGACTG ATGCTGAGAA

-TGGTGCTGG GAGAGAAATC CTGCATGGTG CACCTGACTG ATGCTGAGAA

GCCATCCTGA CTGAGAAGTA CCGCATGGTG CATCTCTCTG GTGAAGAGAA

GTGGTGCTGA CGGAGAAGTA CCGC------ ---------- ----------

GTGGTGCTGA CGGAGAAGTA CCGCATGGTG CATCTGACTG CTGAAGAGAA

GTGGTGCTGA CGGAGAAGTA CCGCATGGTG CATCTGACTG GTGAAGAGAA

GTGGTGCTGA CAGAGAAGTA CCGCATGGTG CATCTGTCTG GAGAGGAGAA

GTGGCGCTGA CCGAGAGCCA CCGCATGGTG AACCTGACCT CTGACGAGAA

ATGGCGCTGA CTGAGAAGCA CCGCATGGTG AATCTGACTG CTGCTGAGAA

ACTGTGCTGA CCTCCAAGTA CCGTATGGTG CATATGACTG ATGCTGAGAA

ACCGTGCTGA CCTCGAAGTA CCGTATGGTG CATTTCACAC CTGAGGAGAA

TCCATCCTGA CTGAGAAATA CAGAATGGTG CGTCTGAGCG CTGAGGAGAA

GTCTGCCGTT ACTGCCCTGT GGGGCAAGGT GAACGTGGAT GAAGTTGGTG

GTCTGCCGTT ACTGCCCTGT GGGGCAAGGT GAACGTGGAT GAAGTTGGTG

GAATGCCGTT ACCGCCCTGT GGGGCAAAGT GAACGTGGAT GAAGTTGGTG

GAATGCCGTC ACCACCCTGT GGGGCAAGGT GAACGTGGAT GAAGTTGGTG

ATCTGCCGTT ACTGCCCTGT GGGGCAAGGT GAATGTGGAC GAAGTTGGTG

GAATGCCGTT TGTGCCCTGT GGGGCAAGGT GAATGTGGAA GAAGTTGGTG

GGCTGCTGTT AATGGCCTGT GGGGAAAGGT GAACCCTGAT GATGTTGGTG

GGCTGCTGTC TCTGGCCTGT GGGGAAAGGT GAACGCCGAT GAAGTTGGTG

GGATACATTC ACTGCCCTGT GGGGCAAGGT GAATGTGGAA GAAGTTGGTG

---------- ---------- ---------- ---------- ----------

GAGTCTTATC TCCAGCATGT GGGGCAAGGT GAATGTGGAT GAAGTTGGCG

GGGTATCGTC ACTGGCCTGT GGGGCAAGGT GAACGTGGAT GAAGTTGGTG

GGGCGTCGTC ACCGGCCTGT GGGGCAAGGT GAACGTGGAT GAAGTCGGTG

GACTGCCGTC CTTGCCCTGT GGAACAAGGT GGACGTGGAA GACTGTGGTG

GACACAAGTC ACCAACCTGT GGGGCAAGGT GAATGTGAAA GAGCTTGGTG

GAAACTTGTC ACCACTATGT GGGGCAAGTT AGACGTGGAT GCTGCTGGTG

GTCCGCTATC ACTTCCATAT GGGGCAAGGT CGACGTGGAA GCAATTGGTG

GTCTGCCATC ACCACCCTTT GGGGCAAGGT GAACCCAGAG GAGCACGGAG

GTGAGGCCCT GGGCAGGCTG CTGGTGGTCT ACCCTTGGAC CCAGAGGTTC

GTGAGGCCCT GGGCAGGCTG CTGGTGGTCT ACCCTTGGAC CCAGAGGTTC

GTGAGGCCCT GGGCAGGTTG CTGGTGGTCT ACCCTTGGAC CCAGAGGTTC

GTGAGGCCCT GGGCAGGCTG CTGGTGGTCT ACCCTTGGAC CCAGAGGTTC

GTGAGGCCCT GGGCAGGCTG CTGGTGGTTT ACCCTTGGAC CCAGAGGTTC

GTGAGGCCCT GGGCAGGCTG CTGGTTGTCT ACCCATGGAC CCAGAGGTTC

GCGAGGCCCT GGGCAGGCTG CTGGTTGTCT ACCCTTGGAC CCAGAGGTAC

GTGAGGCCCT GGGCAGGCTG CTGGTTGTCT ACCCTTGGAC CCAGCGGTAC

GTGAGGCCCT GGGCAGGCTG CTGGTTGTCT ACCCATGGAC CCAT---CTC

---------- ---------- ---------- ---------- ----------

GTGAGGCCCT GGGCAGGCTG CTGATTGTCT ACCCCTGGAC TCAGAGGTTC

GTGAGGCCCT GGGCAGGCTG CTGGTGGTCT ACCCATGGAC TCAGAGGTTC

GTGAGGCCCT GGGCAGGCTG CTGGTGGTGT ACCCCTGGAC CCAGAGGTTC

GTGAGGCCCT GGGCAGGCTG CTGGTCGTGT ATCCCTGGAC CCAGAGGTTC

GTGAGGCCCT GAGCAGGCTG CTGGTGGTCT ACCCATGGAC CCGGAGGTTC

CTGAGACCCT GGGCAGGGTT CTGGTTGTC- ACCCATGGAC CCAGAGGTTC

GGGAAGCTTT GGGAAGGCTT CTGGTGGTCT ATCCCTGGAC CCAAAGGTTC

GCGAAGCCCT GGCCAGGCTG TTGGTTGTCT ACCCCTGGAC CCAGAGGTTC

TTTGAGTCCT TTGGGGATCT GTCCACTCCT GATGCTGTTA TGGGCAACCC

TTTGAGTCCT TTGGGGATCT GTCCACTCCT GATGCTGTTA TGGGCAACCC

TTTGATTCCT TTGGGGATCT GTCCTCTCCT GCTGCTGTTA TGGGCAACCC

TTTGAGTCCT TTGGGGATCT GTCCTCTCCT GATGCTGTTA TGGGCAACCC

TTTGAGTCCT TTGGGGATCT GTCCACTCCT GATGCTGTTA TGAACAACCC

TTTGACTCCT TTGGGGACCT GTCCTCTCCT TCTGCTGTTA TGGGCAACCC

TTTGATAGCT TTGGGGACCT GTCCTCTGCC TCTGCTATCA TGGGTAACCC

TTTGATAGCT TTGGAGACCT ATCCTCTGCC TCTGCTATCA TGGGTAATGC

TTCGAGTCCT TTGG--ACCT GTCCTCTGCA CATGCTGTTA TGAGTAATGT

---------- ---------- ---------- ---------- ----------

TTTGACTCCT TTGGGGACCT GTCCACTCCT GATGCTGTTA TGAGCAATGC

TTTGACTCCT TTGGGGACCT GTCCTCTGCT GCTGCTGTTA TGGGAAATGC

TTTGACAGCT TTGGTGACCT GTCCTCTGCC TCTGCCATCA TGGGCAATCC

TTTGAAAGCT TTGGGGACTT GTCCACTCCT GCTGCTGTGT TCGCAAATGC

TTTGAACACT TTGGGGACCT GTCCACTGCT GAAGCTGTCC TGCACAACGC

TTTGGCCACT TTGGGGACCT GTCCTCTGCT TGTGCTGTCA TGGACAATCC

TTTGATTCCT TTGGCAACCT ATCTTCTCCA TCTGCTATTA TGGGCAACCC

TTTGACTCCT TCGGCAACCT GTCCTCCGCC TCCGCTGTGC TGGGAAACCC

TAAGGTGAAG GCTCATGGCA AGAAAGTGCT CGGTGCCTTT AGTGATGGCC

TAAGGTGAAG GCTCATGGCA AGAAAGTGCT CGGTGCCTTT AGTGATGGCC

TAAGGTGAAG GCTCATGGCA AGAAAGTGCT TGGTGCCTTT AGTGATGGCC

TAAGGTGAAG GCTCATGGCA AGAAAGTGCT TGGTGCCTTT AGTGATGGCC

TAAGGTGAAG GCTCATGGCA AGAAGGTGCT AGGTGCCTTT AGTGACGGCC

TAAAGTGAAG GCCCACGGCA AGAAGGTGCT GAGTGCCTTT AGCGAGGGCC

TAAGGTGAAG GCCCATGGCA AGAAGGTGAT AAACGCCTTC AATGATGGCC

CAAAGTGAAG GCCCATGGCA AGAAAGTGAT AACTGCCTTT AACGATGGCC

CAAGGTGACG GCCCATTGCA AGAAGGTGCT GGCTACCTTC AGTGAGGGTC

---------- ---------- ---------- ---------- ----------

TAAAGTGAAG GCCCATGGCA AGAAGGTGCT GAACTCCTTT AGTGATGGCC

TAAGGTGAAG GCTCATGGCA AGAAAGTGCT AGACTCCTTC AGTGAGGGTC

TAAAGTGAAG GCCCATGGCA AGAAGGTGCT GCAGTCCCTG GGTGATGGTA

TAAGGTAAAA GCCCATGGCA AGAAGGTGCT AACTTCCTTT GGTGAAGGTA

TAAAGTGCTG GCCCATGGCG AGAAAGTGTT GACCTCCTTT GGTGAGGGCT

TAAGGTGCAG GCTCATGGCA AGAAGGTGCT GCACTCTCTC GGTGATGGCC

CAAAGTCAAG ACCCATGGCA AGAAGGTGCT GACCTCCCTT GGTGATGCTG

CAAGGTCAAG GCCCATGGGA AGAAGGTGCT GACTTCCTTT GGCGATGCCG

TGGCTCACCT GGACAACCTC AAGGGCACCT TTGCCACACT GAGTGAGCTG

TGGCTCACCT GGACAACCTC AAGGGCACCT TTGCCACACT GAGTGAGCTG

TGAATCACCT GGACAACCTC AAGGGCACCT TTGCCCAGCT CAGTGAGCTG

TGAATCACCT GGACAACCTC AAGGGTACCT TTGCCCAGCT CAGTGAGCTG

TGACTCACCT GGACAACCTC AAGGGCACAT TTGCCCATCT GAGTGAGCTG

TGAATCACCT GGACAACCTC AAGGGCACCT TTGCTAAGCT GAGTGAGCTG

TGAAACACTT GGACAACCTC AAGGGCACCT TTGCTCATCT GAGTGAACTC

TGAATCACTT GGACAGCCTC AAGGGCACCT TTGCCAGCCT CAGTGAGCTC

TGAATCACCT GGACAACCTC GGGGAAAATT TTGTCCAGCT GAGTGAGCTG

---------- ---------- ---------- ---------- ----------

TGAAAAACCT GGACAACCTC AAGGGCACCT TTGCTAAGCT CAGTGAGCTT

TGAAGAATCT CGACAACCTC AAGGGCACCT TTGCTAAGCT GAGCGAGCTG

TCAACAACCT GGACAACCTG AAGGGCACCT TTGCTAAGCT GAGTGAGCTG

TGAATCACCT GGACAACCTC AAGGGCACCT TTGCTAAACT GAGTGAGCTG

TGAAGCACCT GGACAACCTC AAGGGCACCT TTGCCGATCT GAGCGAGCTG

TGAAGCACCT GGACGACCTC AAGCATTTCT ATGCTGCCCT GAGTGAGCTG

TCAAGAACCT GGACAACCTC AAGGGTACCT TCTCCAAGCT GAGTGAGCTG

TCAAGAACCT CGACAACCTC AAGGGCACCT TCTCCAAGCT GAGTGAATTG

CACTGTGACA AGCTGCACGT GGATCCTGAG AACTTCAGGC TCCTGGGCAA

CACTGTGACA AGCTGCACGT GGATCCTGAG AACTTCAGGC TCCTGGGCAA

CACTGTGACA AGCTGCATGT GGATCCTGAG AACTTCAAGC TCCTGGGCAA

CACTGTGACA AGCTGCATGT GGATCCTGAG AACTTCAAGC TCCTGGGCAA

CACTGTGACA AGTTGCACGT GGATCCTGAG AATTTCAGGC TCCTGGGCAA

CATTGTGACA AGCTGCACGT GGACCCTGAG AACTTCAGGC TCCTGGGCAA

CACTGTGACA AGCTGCATGT GGATCCTGAG AACTTCAGGC TCCTGGGCAA

CACTGTGACA AGCTGCATGT GGATCCTGAG AACTTCAGGC TCCTGGGCAA

CACTGTCACA AGCTCCATGT GGATCCTGAG AACTTCAGTC TCCTGGGCAA

---------- ---------- ---------- ---------- ----------

CACTGTGACA AGCTGCACGT GGATCCCGAG AACTTCAAGC TGCTGGGCAA

CACTGTGACA AGCTTCACGT GGATCCTGAG AACTTCAGGC TCCTGGGCAA

CACTGTGACA AGCTGCACGT GGATCCTGAG AACTTCAGGC TCCTGGGCAA

CACTGTGACA AGCTGCACGT GGATCCTGAG AATTTCAGGC TCCTGGGCAA

CACTGTGACA AGCTGCACGT GGATCCTGAG AATTTCAGGC TCCTGGGCAA

CACTGTGACA AGCTGCACGT GGATCCTGAG AACTTCAGGC TCCTGGGCAA

CACTGTGACA AACTGCATGT AGACCCTGAG AACTTCAGGC TCCTAGAGAA

-ACTGCGACA AGCTCCACGT GGACCCTGAG AACTTCAGGC TCCTGGGGAA

CGTGCTGGTC TGTGTGCTGG CCCATCACTT TGGCAAAGAA TTCGCCTATC

CGTGCTGGTC TGTGTGCTGG CCCATCACTT TGGCAAAGAA TTCGCCTATC

CGTGCTGGTG TGTGTGCTGG CCCATCACTT TGGCAAAGAA TTCGCCTATC

CGTGCTGGTG TGTGTGCTGG CCCATCACTT TGGCAAAGAA TTCGCCTATC

CGTGCTGGTG TGTGTGCTGG CCCATCACTT CGGCAAAGAA TTCGCCTATC

CGTGCTGGTG GTTGTGCTGG CTCACCACTT TGGCAAGGAT TTCGCCTATC

TATGATTGTG ATTATGATGG GCCACCACCT GGGCAAAGAA TTCGCCTTCC

TATGATCGTG ATTGTGCTGG GCCACCACCT GGGCAAGGAT TTCGCCTTCC

CGTGCTGGTT GTTGTGCTGT CTCATCATTT TGGCAAAGAA TTCGCCTATC

---------- ---------- ---------- ---------- ----------

CGTGTTGGTG TGTGTGCTGG CCCGCCACTT TGGCAGGGAA TTCGCCTACC

CGTGCTGGTG TGTGTGCTGG CTCGCAACTT TGGCAAGGAT TTCGCCTATC

TGTGCTGGTG TGTGTGCTGG CGCGCCACTT TGGCAAGGAA TTCGCCTTCC

TATGCTGGTG GTTGTGATGG CTCGCCACTT TGGCAAGGAA TTCTGTTTTC

GGAGCTGGTG TGTGTGATAG CTCACCACCT GGGCAAGGAA TTCGCCTATC

CGTGCTGGTG TGTGTGATGT CCCGCCACCT TGGCGATGAG TTCGCCTATC

TGTGCTGGTG ATTATACTGT CAAAGTACCT CAGCAAAAAG TT---CTGGC

CGTGCTGGTC GTGGTCCTGG CCCGTCACTT CGGCAAGGAC TTCGCCTGGC

AGAAAGTGGT GGCTGGTGTG GCTAATGCCC TGGCCCACAA GCACATGTCC

AGAAAGTGGT GGCTGGTGTG GCTAATGCCC TGGCCCACAA GCACATGTCC

AGAAAGTGGT GGCTGGTGTG GCTAATGCCC TGGCCCACAA GCACATGCAC

AGAAAGTGGT GGCTGGTGTG GCTAATGCCC TGGCCCACAA GCACATGCAC

AGAAAGTGGT GGCTGGTGTG GCTAATGCCC TGGCTCACAA GCACATGTCC

AGAAGGTGGT GGCTGGTGTG GCTACTGCCC TGGCTCACAA ACACATGTCC

AGAAGGTGGT GGCTGGAGTG GCCAGTGCCC TGGCTCACAA GCAC------

AGAAGGTGGT GGCTGGAGTG GCCGCTGCCC TGGCTCACAA GCACATGTCC

AGAAGGTGGT GGCTGGTGTG GCCAATGCCC TGGCTCACAA ACAC------

---------- ---------- ---------- ---------- ----ATGCTT

AGAAGGTGGT GGCTGGTGTG GCCAATGCCC TGGCCCACAA ACACATGTTT

AGAAGGTGGT GGCTGGTGTG GCCACTGCCC TGGCTCACAA GCACATGTTT

AGAAGATGGT GGCTGGTGTG GCCGCTGCCC TGGCTCACAA GCACATGCTA

AGAGGGTGGT GGCTGGTGTG GCTAATGCCC TGGCTCACAA GCATAGTCCT

AGAAGTTGGT GGCTGGTGTG GCCACTGCCC TGGCTCACAA GCACATGCAC

AGAAGGTGGT GGCCGGTGTG GCTAACGCTC TGGCTCACAA GCACATGCAC

AGAAATCTGT GGCTGGTGTG GCCAATGTTT TGATCCACAA ACTCATGCTT

AGAAGTTGGT GGCCGGTGTG GCCAGTGCCC TGGCCCACAA GCAC-TGCTT

ATTATCAACA CATCATATGT TGAAATCACC ACCTTCTTCT TGGTTGGGAT

ATTATCAACA CATCATATGT TGAAATCACC ACCTTCTTCT TGGTTGGGAT

CTTCCCAATT CTTCTGAAAT TGAGATTACC ACCTTCTTTC TGATTGGAAT

CTTCCCAATT CTTCTGAAAT TGAGATTGCC ACCTTCTTTC TGTTTGGAAT

ATTATCAACA CATCATACGT TGAAATCACC GCCTTCTTC- --GTTGGGAT

ATCATCAACA CATCACATTT TGAAATCACC ACTTTCTTCT TGGTTGGGAT

---------- CTTCAGAAAT TGAGATCACC ACCTTCTTCC TGATTGGAAT

GTTCTCAATT CTTCAGAAAT TGAGATCACC ACCTTCTTCC TGATTGGAAT

---------- ---------- ---------- ---------- ----------

CCTCTCAATG CCTCAGAAGC TGCAGTCTCC ACATTCCTGT TGATTGGGAT

GCTCTCAATA CCTCAGAAGT TGAAATCTCC ACCTTCCTGT TGATTGGAAT

CCTCTCAATA CGTCAGAAGT TGACATCTCC ACATTCCTGT TGATTGGGAT

ACTCAAAATA GATCCAGTCT TCAACCTACC ACCTTCCTAC TACTTGGCAT

CTGCTCTACA GATCACATGA TGGAATCTCC ATCTTCTTCC TGATTGGGAT

ACTCTCAATT CTTCTGAAAT TGAAATCTCC ACCTTCATCC TGGTTGGAAT

ACTCTCAATT CTTCTGACAT TGAAATCACC ACCTTCTTCC TCATTGGAAT

ACCCTGAATG TCTCTCACAT C--AATCATC ACTTTTATAC TGACAGGTAT

CCTCCCAATC ACACCCAGTT CCACCCCTCC TCCTTCTTCT TGCTGGGATT

GCCAGGGCTA GAATATGCAC ACATCTGGAT CTCTATCCCC ATCTGCAGCA

GCCAGGGCTA GAATATGCAC ACATCTGGAT CTCTATCCCC ATCTGCAGCA

GCCAGGGTTG GAGCATGCTC ATGTATGGAT CTCTGTCCCC ATCTGCCTCA

GCCAGGGTTG GAGCATGCTC ATGTATGGAT CTCTGTCCCC ATCTGCCTCA

GCCAGGGCTA GAATATGCAC ACAGCTGGAT CTCTATCCCC ATCTGCAGCG

GCCAGGGCTA GAATATGCAC ACGTCTGGAT CTCTATTTCC ATCTGCATCA

CCCAGGCCTG GAGTATGCTC ACATGTGGAT CTCCATTCCC ATCTGCCTCA

CCCAGGCCTG GAGTATGCTC ATGCGTGGAT CTCCATTCCC ATCTGCCTCA

---------- ---------- ---------- ---------- ----------

CCCAGGGCTT GAGGGTGTGC ACATTTGGAT CTCCATCCCT ATCTGCCTCA

CCCAGGGCTT GAGCATGTGC ACATTTGGAT CTCCATCCCC ATCTGCTTCA

CCCAGGGCTT GAGCACGTGT ACGTTTGGAT CTCCATCCAT ATCTGCCTCA

TCCAGGATTG GAAGCTTGCC ACATATGGAT TTCCATCCCT TTCTGCCTGG

ACCAGGGCTA GAGTATACAC ACATCTGGAT GTCTATTTCT ATCTGTAGCA

CCCAGGATTG GAACATATTC ATGTCTGGAT CTCCATCCCT ATCTGCCTCA

CCCTGGGCTG GAGCATATGC ACATCTGGAT CGCCATCCCT ATCTGCCTCA

ACCAGGAATG CCAGAGAAGA ATCCTTGGAT GGCATTCCCT CTGGGACTCC

GCCGGGCCTG GAAGCCGCAC ACATCTGGAT TGGTTTGCCC ATCTGTGCTG

TGTATCTTAT TGCTATTCTA GGAAATGGCA CCATTTTTAT CATCAAGACA

TGTATCTTAT TGCTATTCTA GGAAATTGCA CCATTTTTAT CATCAAGACA

TGTATTTGGT AGCCATCCTA GGCAATTGCA CAATCTTTGT TATCAGGACT

TGTATTTGGT AGCCATCCTA GGCAATTGCA CAATCTTTGT TATCAGGACT

TTTATCTTAT TGCTATTCTA GGAAATTGCA CCATTTTTAT CATCAGGACA

TGTATCTCAT TGCTATTCTA GGAAACTGCA CCATCTCTAT TATCAAGATG

TGTACCTGGT GGCCATCTTG GGCAACTGTA CCATCTTTGT GATCAGGACT

TGTACCTGGT GGCCATCCTG GGCAACTGCA CCATCTTTGT GATCAGGACT

---------- ---------- ---------- ---------- ----------

TGTACCTCAT GGCTATCCTG GGAAAC--CA CCATTTTTGT CATCAGGACA

TGTACCTCCT GGCCATCCTG GGCAACTGCA CCATCTTTGT TATCAGGACA

TGTATCTCCT GGCCATCCTG GGCAACTGCA CCATTTTTGT CATTAGGACA

TATACCTACT GTCACTGATA GGAAATGTTG GCCTTATAAT TATCAAGACA

TGTACCTTGT TATCATTCTT GGAAACTGCA TAGTTTTTGT CATCAAGACA

TGTACCTAGT GGCCATTCTG GGCAATTGCG CCATCTTTGT GATCAGAACA

TGTACCTAGT GGCCATCTTG GGTAATTGCA CCATCTTTGT GATCAGGACA

TCTACACTCT CACTCTTCTT GGCAATTGCA CCATCTCCAT TATTAAGATG

CCTACTTGAT CTCGTTTGTG GGGAACTGCA CCATCTTTGT GATCAAGACC

GAGCCCTCCT TGCATGGGCC CATGTACTAT TTTCTTTCCA TGTTGGCTAT

GAGCCCTCCT TGCATGAGCC CATGTACTAT TTTCTTTCCA TGTTGGCTAT

GAGCCCTCAC TCCATGCACC CATGTACTAT TTCCTTTCCA TGTTGGCTGT

GAGCCCTCAC TCCATGCACC CATGTACTAT TTCCTTTCCA TGTTGGCTGT

GAGCCCTCTT TGCATGAGCC CATGTACTAT TTT-TTTGCA TGTTGGCTAC

GAGCCCTCCT TGCATGAGCC CATGTACTAT TTCCTTTCCA TGTTGGCTAT

GAACCCTCAC TCCATGCTCC CATGTACTAT TTCCTTTCTA TGCTGGCCAT

GAACCCTCAC TCCATGCTCC CATGTACTAT TTCCTTTCCA TGTTGGCCAT

---------- ---------- ---------- ---------- ----------

GAGCCTTCTC TGCATGAGCC CATGTACCAT TTCCTCTCCA TGCTGGCATT

GAGCCTTCTC TGCATGAGCC TATGTACTAT TTCCTCTCTA TGCTGGCCCT

GAGCCTTCCC TTCAGGAGCC CATGTACTAT TTCCTCTCCA TGCTGGCCAT

GACCACAAAC TGCATGAACC TATGTACCTC TTTCTATGCA TGCTCTCTGT

GACGCTTCCC CCTATGAGCC CATGTCCTAT TACCTTTCCA TGTTTGTCCT

GAGCCCTCAC TCCGTGCACC CATGTACTAT TTCCTTTCCA TGTTGGCGGT

GAGCCCTCGC TCCATGCCCC CATGTACTAT TTCCTCTCCA TGCTGGCCTT

GATAGAAGCC TTCATGAGCC CATGTACTAC TTTCTCTCCA TCTTGGCACT

GAGCAGAGCC TCCACCAGCC CATGTTCTAC TTCCTGGCCA TGCTGGCTAC

GTCAGACTTG GGTTTGTCTT TATCATCTCT GCCCACTGTG TTAAGCATCT

GTCAGACTTG GGTTTGTCTT TATCATCTCT GCCCACTATG TTAAGCATCT

CTCTGACCTG GGCCTGTCCT TCTCCTCCCT ACCCACTATG CTAAGGATCT

CTCTGACCTG GGCCTGTCCT CCTCCTCCCT ACCCACTATG CTAACGATCT

GTCAGACTTG GGTTTGTCTT TATCTTCTCT CCCCACTATG TTAAGGATCT

GTCTGACTTG GGTTTGTCTT TATCTTCTCT GCCTACCATG TTGAAGATCT

CTCTGACTTG GGCCTGTCCC TCTCTTCCCT CCCCACCATG CTCCAGATCT

CTCTGACTTG GGCCTGTCCC TTTCCTCCCT CCCCACCATG CTCCGGATCT

---------- ---------- ---------- ---------- ----------

ATCGGACCTG GGCCTGTCTT TCTCTTCTCT TCCCACCATG CTTAGGATTT

ATCTGACCTG GGCTTGTCTT TCTCTTCTCT TCCCTCCATG ATGAGAATCT

CTCTGACCTG GGTTTGTCTT TCTCCTGTCT TCCCACCATG CTGAGAATCT

GGTTGATTTG ATGCTTACTT CTTCAACGCT TCCCAAGATA CTCAGCCTTT

GTCTGATTTG GGGTTGTCCT TATCCTCCCT TCCTACCATG CTAAGAATCT

CTCTGACCTG GGCCTTTCCC TCTCCTCCCT CCCGACTATG CTAAGGGTCT

TTCGGACCTG GGCCTGTCCC TCTCCTCACT CCCCACTATG CTGAGGATCT

GTCTGATGTG GGTCTCTCCA TGTCCACCCT GCCCTCCATG CTCAGCATTT

CGTTGACCTG GGCTTGTCCA CAACCACCAT CCCCAAGATG CTAGGCACCT

TCCTGTTCAA TGCCCCTGAA ACTTCTTCTA GTGCCTGCTT TGCCCAGGAA

TTCTGTTCAA TGCTCCTGAA ATTTCATCCA ATGCCTGCTT TGCCCAGGAA

TTGTATTCAA TGCCACGGGA ATCTCCCCAA ATGCTTGCTT TGCTCAAGAA

TTGTATTCAA TGCCACGGGA ATCTCCCCAA ATGCTTGCTT TGCTCAAGAA

TCCTGTTCAA TGCTCCACAA ATTTCTTCCA ATGCCTGCTT TGCCCAGGAG

TCTTGCTCAA TGCTCCTGAA ATTTCTACCA ATGCCTGCTT TGCCCAGGAA

TTGTTTTCAA ------GAGA ATCTCTCCAA ATGCCTGCTT TGCTCAAGAA

TTGTGTTCAA TGCCACAGGA ATCTCCCCAA ATGCCTGCTT TGCTCAAGAA

---------- ---------- ---------- ---------- ----------

TTTTGTTCAA TGCCATGGGG ATTTCCGTTG ATGCCTGCAT TGCTCAGGAA

TCTTGTTCAA TGCCATGGAG ATTTCTGCTG ATGCATGTAT TGCCCAGGAA

TCTTGTTCAA TGCCATGGGG ATTTCTGCGG ATGCCTGCTT TGCCCAGGAA

TCTGGTTCAA TGATAGAGAG ATCTACTTCG AAGCCTGCCT CACTCAAATG

TCCTGTTCAA TGCTCCTAGA ATTTCTGCCA ATGCCTGCTT TGCCCAAGAA

TCTTGTTCAA TGCCACAGGA ATTTCTCCAA ATGCCTGCTT TGCTCAAGAA

TCTTGTTCAA TGCCACAGGC ATTTCTCCAA ATGCCTGCTT TGCTCAGGAA

TCTGGTTCAA TGCCCCTGAA ATCCCCTTTG ACGCCTGTAT CACTCAGATG

TCTGCTTCAA CGTCAGGGAG GTTAGCTTCG ATGCCTGCAT GACCCAGATG

TTCTTCATTC ATGGATTCTC AGTACTGGAG TCCTCAGTCC TCCTGATCAT

TTCTTCATTC ATGGATTCTC AGTACTGGAG TCCTCAGTCC TCCTGATCAT

TTCTTTATTC ATGGATTCAC AGATATGGAG TCCTCAGTGC TTCTGGTCAT

TTCTTTATTC ATGGATTCAC AGATATGGAG TCCTCAGTGC TTCTAGTCAT

TTCTTCAT-C ATGGGTTCTC AGTCCTGGAG TCCTCAGTCC TCCTGATCAT

TTCTTCATCC ATGGATTCAC AGTACTG--- TCCTCAGTCC TCCTGATCAT

TTCTTTATCC ACGGCTTCAC AGACATGGAG TCCTCAGTGC TCCTGATCAT

TTCTTTATCC ATGGCTTCAC TGACATGGAG TCCTCAGTGC TTCTGATTAT

---------- ---------- ---------- ---------- ----------

TTTTTCATCC ATGGATTCAC AGACTTGGAG TCCTCGGTAC TCCTGATCAT

TTTTTCATCC ATGGATTCAC AGACATGGAA TCCTCAGTGC TTCTAATCAT

TTTTTTATCC ACAGATTAAC AGACATGGAG TCCTCAGTGC TCCTCATCAT

TATTTTATCC ATTCTCTGTC TACTATGGAA TCTGGATTTA TTTTGGCCAT

TTATTCATCC ATAAATTCAC TGCCCTGGAA TCTTCAGTGT TCCTGATCAT

TTCTTCATCC ATGGATTCAC AGATATGGAA TCCTCAGTTC TTCTGCTCAT

TTCTTCATCC ATGGATTCAC CGATATGGAG TCCTCGGTTC TTCTGCTTAT

TTCTTTATCC ATGGATTTGG AGTGGTGGAA TCTGGGGTGT TGGTATCCAT

TTCTTCATCC ACTCATTCTC CTTAACGGAG TCAGGCATCC TGCTGGCCAT

GTCATTTGAT AGATTCCTAG CCATCCACAA TCCTCTGAGA TACACCTCAA

GTCCTTTGAT AGATTCCTAG CCATCCACAA CCCTCTGAGA TACACCTCAA

GTCTTTTGAC CGCTTTTTGG CCATACGCAA CCCTCTGAGG TACGGCTCTA

GTCTTTTGAC CGCTTTTTGG CCATACGCAA CTCTCTGAGG TACAGCTCTA

GTCATTTGAT AGATTCCTAG CCATCCACAA CCCTCTGAGA TACAGCTCAA

GTCATTTGAC CACTTCCTTG CCATCCACAA TCCTCTAAGG TACAGTTCCA

GTCTTTTGAC CACTTTTTGG CCATACGCAT CTCCCTTAGA TACAGCTCTA

GTCTTTTGAC CGCTTCTTGG CCATACGCAA CCCCCTCAGA TACAGCTCTA

---------- ---------- --------AA CCCTCTGAGA TACAATTCTG

GTCTTTTGAT CGCTTTGTAG CAATTTGCAA CCCCCTGAGA TACCGCTCCA

GTCCTTTGAT CGCTTTCTAG CCATTCGCAA CCCCCTGAGG TATAGCTCTA

GTCCTTTGCC CACTTTGTAG CCATTCGCAA CCCCCTGAGA TATAGCTCCA

GGCGTTTGAC CGCTACATAG CCATCTGCCT CCCGCTAAGA CATTCTACTA

GCTCTCTGAC TACTTCTTCA CCTTTCACAA GTCTTTGAGA AACAATTTTA

GTCCTTTGAC CGCTTTTTAG CCATACACAA CCCTCTGAGA TACAGCTGTA

GTCCTTTGAT CGCTTTTTGG TCATACACAA CCCACTGAGA TACAATTCTA

GGCCTTTGAT CGCTTTGTGG CTATTCGAGA TCCTCTGCGT TATGCTTCAG

GAGCTTTGAC CGCTATGTGG CCATCTGTAA CCCACTCAGA TACTCAGCGA

TCCTGACAAC TGTCAGAGTT GCCCAAATAG GGATAGTATT CTCCTTTAAG

TCCTGACAAC TGTCAGAGTT GCCCAAATAG GGATAGTATT CTCCTTTAAG

TCCTCACCAG TGCCAGAGTT GCCAAAATGG GGCTGGTGTT TCTCATTAAA

TCCTCACCAG TGCCAGAGTT GCCAAAATGG GGCTGGTGTT TCTCATTAAA

TTCTTACAAC TGTCAGAGTT GCCCAAATAG GAATAGTATT CTCCTTTAAG

TTCTTACAAC TGTCCGAGTT GCCCAAATAG GACTGGTACT CTCCTTCAAA

TCCTCACCAG TGCAAGAGTT GCCAAAATGG GACAGGTGTT TCTCATTAAA

TCCTCACCAG TGCAAGAGTC GCCAAAATGG GACTGGTGTT TCTCATTAAA

TCCTCACCAG TGCCAGAGCT GACAAAATGG GATCGCTCTT TTTCATGAAA

TCCTTACTAG CTCCAAAGTT TTGTACATTG GGTTAGCATT TGCTTTTAAA

TTCTTACCAG CTCCAGAGTT TTGCATATTG GACTGACATT TGCTATTAAA

TCCTTACCAG CTCTAGACTG TTGCAAATTA GGCTGGCATT TGCTGTTAAA

TCTTAACTTC CACAGTGATC TTGGGTTTGG GTTTGGCTGT TGCTTTCAGA

GCCTTACTAG TGCTAGGGTG GCCCAAATAG GAATGATATT TTTCCTTAAG

TCCTGACCAG TGCTAGAGTT GCCAAAATAG GTCTGGTATT TCTCATTAAA

TCCTGACCAG TGCCAGAGTG GCCAAAATAG GGCTGGTATT TCTTATTAAA

TCCTTACCTC TGGGGTCATT GGCAAGATAG GATTAGGTGT CATTGCCCGA

TACTGACCGG CAGTAGCATT GTCAAGATGG GTTTGGCCAT TTTAGTACGC

AGCATGCTCC TGGTTCTTCC CTTCCCTTTC ACTTTAAGAA GCTTGAGATA

AGCATGATCC TGGTTCTTCC CTTCCCTTTC ACTTTAAGAA GCTTGAGATA

AGCATGCTCT TAGTACTCCC ATTTCCTTTC ACTCTTAAAA GGTTGACATA

AGCATGCTCT TAGTACTCCT ATTTCCTTTC ACTCTTAAAA GGTTGACATA

AGCATGCTCC TGGTTCTTCC CTTCCCTTTC ACTTTAAGAA GCTTGAAATA

AGCATGCTCC TGGTTCTTCC CTTTCCCGTC ACTTTGAGAA GCTTGAGATA

AGCATGCTCC TAGTGCTCCC ATTCCCTTTC ATTCTGAAGA GACTGGCATA

AGCATGCTCC TAGTGCTCCC ATTTCCTTTC ACTCTGAAAA GACTGGCATA

AGCATGTTGT TAGGGTCTCC ATTTCCTTTT ACTCCTAGAA GATTAACATA

AGCATTCTCC TAGTTCTTCC CCTCCCTTTC ACTTTAAAGA GACTCAAATA

AGCATTCTAC TAGTTCTTCC TCTTCCTTTC ACTTTAAAGA GACTCCAATA

AGCATTCTGC TAGTTCTTCC TCTTCCTTTC ACTTTAAAGA GACTCAGA--

GGTGCTGTAC TCCTAAGTCC ACACCCCTTT TTACTGAGGT GGCTTTCTTA

AGCATTCTTT TGGTGCTGTC CTTTTCTTTC ACATTGCAAA TGTTGAAATA

AGTATGTTCT TAGTGCTTCC ATTTCCTTTC ACTCTGAAAA GGTTGACATA

AGTATGCTAT TGGTGCTCCC ATTTCCTTTC ACTCTGAGAA GATTAATATA

ACTGTCTGTG TGGTCTTCCC TGTGCCATTT CTGATCAAGA GGCTGCCTTT

AGCTTCATTT TGATCCTTCC TTTACCCTTC CTCCTGAGGA GACTGCCATT

TTGCAAGAAA AACCAATTAT CCCATTCCTA CTGTCTCCAC CAGGATGTCA

TTGTAAGAAA AACCAATTAT CCCATTCCTA CTGTCTCCAC CAGGATGTCA

CTGTAGGAAA AGCCTACTCT CTCATTCCTA TTGTCTCCAT CAGGATGTCA

CTGTAGGAAA AGCCTACTCT CTCATTCCTA TTGTCTCCAT CAGGATGTCA

TTGTAAGAAA AACCAATTAT CTCATTCCCA CTGTCTCCAC CAGGATGTGA

CTGTAAGAAA AGCCAGTTGT CCCATTCCTA TTGTCTACAT CAGGATGTCA

CTGCCGGAAA AGCCTCCTTT CTCACTCCTA TTGTGTCCAT CACGATGTCA

TTGTCAGAAA AGCCTCCTTT CTCACTCCTA TTGTCTCCAT CAGGATGTCA

---------- ------CTTT CTCACTCTTA TTGTCTCCAG CAGCATGGCA

CTGTAATAAA CGCCTGTTAT CCCACTCCTA CTGCCTCCAC CAGGACATCA

CTGTAATAAA CGCCTGCTTT CACACTCCTA CTGCCTCCAC CAGGATGTGA

-TGTAATAAA AGGCTGTTAT CACACT---- ---CCTGCAC CAGGACCTAC

CTGCAAAACA AATATCATCT CCCATACTTA TTGCGAGTTT ATGGCTCTGA

TTGTAAGAAA AGCTATTTAT CTCATTCTTA TTG--TCTAC CAAGATGTCC

TTGTAGGAAA AGCCTACTTT CTCACTGTTA CTGCTACCAC CCCGATGTTA

TTGTAGGAAA AGCCTACTTT CCCATTGTTA CTGCTACCAT CCTGATGTTA

CTGCAGTTCT AATGTCCTCT CCCACTCTTA CTGCCTCCAC CAGGATGCCA

TTGCAAGGTC AACATCCTGC ATCACGCTTA CTGTCTCCAT CCCGACCTCA

TGAAGTTGGC CTGTTCTATG TCTGTTCTCA ATAACTCCGA AGTCAAGCTT

TGAAGTTGGC CTGCTCTATG TCTGTTCTCA ATAACTCCAA AGTCAAGCTT

GGAAGCTGGC CTGCTCCATG TCTGTTCTCA ATTACTCCAA AGTCAAGCTT

GGAAGCTGGC CTGCTCCATG TCTGTTCTCA ATGACTCCAA AGTGAAGCTT

TGAGGTTGGC CTGCTCTATG TCTGATCTCA ATAACTCTGA AGTCAAGCTT

TGAAGTTGGC CTGCTCTATG TCTGTTCTCA ATAACTCCGA AGTCAAGCTT

TGAAGCTGGC CTGCTCTATG TCTGTTTTCA ACAACTCTGA GGTCAAGTAT

TGAAGCTGGC CTGCTCTATG TCTGTTTTCA ACAACTCCGA GGTCATGTAT

TGAAGCTGGC CTCCGGTATG TCTGTTTTTA ATGACTCTGA GGTCAAATTT

TGAAGCTGGC GTGCTCTATG GCTGTTCTCA ATAACTCTGA GGTCCAACTT

TGAAACTGGC CTGCTCTATG TCTGCTTCCA ATAGTTCTGA GGTCCAGCTT

TCCGGCTGGT CTGCTCTATG TCTGTTTTCA ATAACTCTGA GATCCAGCTT

TAAAGCTAGT TTGCAGTATG TCAATCATAA ACACTTCTGA TGTGGAAATT

TGAAGCTCCC CTGCTCTATG TCTGTTCTCA ATCGCTCTGA GGTTCAGCTT

TGAAGTTGGC TTGTGGCATG ACTGTTCTCA ATAATTCCGA GGTCCAGCTT

TGAAATTGGC CTGTGGGATG CACACTCTCA ATTCTTCTGA CATTGAAATC

TGCGACTTGC TTGTGCTATG CCAGTCATCA CTAACTCTGA AG---AACCC

TCCACCTGCC CTGTGGGATG TCTGCTCTCC ACCCTTCCAA CTTCAACTTC

TTCCTTCTGA TTGGGATCCC AGGACTGGAA CATGCCCACA TTTGGTTCTC

TTCCTTCTGA TTGGGATCCC AGGACTGGAA CATGCCCACA TTTGGTTCTC

TTCCTTCTGA TTGGGATCCC AGGACTG--- CATGCCCACA TTTGGTTCTC

TTCCTTCTGA TTGGGATCCC AGGACTG--- CATGCCCACA TTTGGTTCTC

TTCCTTCTGA TTGGGATCCC AGGACTGGAA CATGTCCACA TTTGGTTCTC

TTTCTTCTGA TTGGGATCCC AGGACTGGAA CATGCCCACA TTTGGATCTC

TTTCTTCTAA TTGGGATCCC AGGACTGGAA TATGCCCATG CATGGATCTC

TTTCTTCTAA TTGGGATACC AGGACTGGAA TATGCCCACG AGTGGATCTC

TTTCTTCTGA TTGGGCTTCC AGGGCTGGAA CATGCCCACA TTTGGATCTC

TTCCTTCTGA TTGGAATTCC AGGATTGGAA CATGCCCACG AGTGGATCTC

TTTCTTCTGA TTGGAATCCC AGGTCTGGAA CATGCTCACA TCTGGATCTC

TTTCTTCTGA TTGGAATCCC AGGCCTGGAA CATGCCTACC TCTGGATCGC

TTCTTCCTCA TTGGGATTCC AGGGATGGAG CATGCTCACC TTTGGATTTC

TTTCTTCTGA TTGGGATCCC AGGACTGGAA CATGCCCACA TCTGGATTTC

TTTCTTCTGA TTGGAATCCC AGAGCTGGAA TACACCCACA TCTGGATCTC

TTCTTCCTCA TTGGAATCCC TGGGCTGGAG CATATGCACA TCTGGATCGC

TTCATCCTTA CGGGCATCCC GGGTTTGGAG GCCATGCATA TTTGGATCTC

CTCCTTCTGT CTGGCTTCCC AGGGCTGGAA TCAGCTTATC CGTGGATTGC

CATCCCCATT TGCCTCATGT ACCTGCTTGC CATCATGGGC AACTGCACCA

CATCCCCATT TGCCTCATGT ACCTGCTTGC CATCATGGGC AACTGCACCA

CATCCCCATT TGCCTCATGT ACCTGGTTGC CATCATGGGC AACTGCACCA

CATCCCCATT TGCCTCATGT ACCTGGTTGC CATCATGGGC AACTGCACCA

CATCCCCATT TGCCTCATGT ACCTGATTGC CATCATGGGC AACTGCACCA

CATCCCCATT TGCCTCATGT ACCTGGTTGC CATCATGGGC AACTGTGCCA

CATCCCCATC TGCCTCATGT ACCTTATTGC CATCGTGGGT AACTGCACTA

CATCCCTATC TTCCTCATGT ACCTTATTGC CATCATGGGT AACTGCATTA

TATTCCCATT TGCCTCATGT ACCTGGTTGC TATCATAGGC AACTCTACTA

CATTCCCATT TGCCTCCTAT ATCTGGTTGC CATCATGGGC AACAGTACCA

CATTCCCATT TGCCTCATGT ACCTGGTTGC CATCATGGGC AACAGCACCA

CATTCCCATC TGCCTCATGT ACACGGTTGC CATCATGGGA AACAGCACCA

CTTCCCAATT TGCTTCATGT ACCTCATTGC CATCCTGGGG AACTGCACCA

CATTCCCATC TGCCTCATGT ACCTTGTTGC CATCATGGGC AACTGTACCA

CACCCCAATC TGCCTCATGT ACTTGGTTGC CATCATAGGC AACTGCACAA

CATCCCTATC TGCCTCATGT ACCTAGTGGC CATCTTGGGT AATTGCACCA

CATCCCCTTC TGCTGCCTAT ACATGATCTC CATCTTGGGT AACACCACCA

CCTCCTCTTG TCCACCATCT ACACCATAAT GCTCCTGGGG AACAGCATCA

TTCTCTTTAT TATAAAGACA GAGCCCTCGC TTCATGAGCC CATGTATTAT

TTCTCTTTAT CATAAAGACA GAGCCCTTGC TTCATGAGCC CATGTATTAT

TTCTCTTTAT CATAAAGACA GAGCCCTTGC TTCATGAGCC CATGTATTAT

TTCTCTTTAT CATAAAGACA GAGCCCTTGC TTCATGAGCC CATGTATTAT

TTCTCTTTAT CATAAAGGCA GAGCCCTCGC TTCATGAGCC CATGTATTAT

TTCTCTTTAT TATAAAGACA GAGTCCTCAC TTCATGAGCC CATGTATTAT

TCATTTTTGT CATCAAGACA GAGCCTTCTC TTCATGAACC CATGTACTAT

TCATTTTTGT CATCAAGACA GAACCCTCTC TTCACGAGCC CATGTACTAT

TCCTCTTCAT CATAAAGACA GAGCCCTCAC TTCATGAACC TATGTATTAT

TTCTCTTTAT CATAAAGACA GAGCCCTCAC TTCATGAACC CATGTATTAT

TTCTTTTTAT CATTAGGACA GAACCCTCAC TTCATGAGCC CATGTACTAT

TCCTTTTTAT CATAAAGACA GAGCCCTCAC TTCACGAGCC CATGTATTAT

TTCTTTTCTT CATAAAAACA GAGACCGCGC TGCATGAACC TATGTATTAT

TTCTCTTTTT CATAAAGACA GAGCCCTCAC TTCATGAGCC CATGTATTAT

TTCTTGTTAT CATAAAGGCA GAGCCCTCAC TCCATGAGCC TATGTATTAT

TCCTCTTTGT GATCAGGACA GAGCCCTCGC TCCATGCCCC CATGTACTAT

TCCTGGCTGT CATTCAGACA GAGTCCTTGC TCCACCAACC CATGTACCTG

TCCTCTATGT ACTCTGGATA GAATGGAGTC TCCACAAGCC CATGTACTAC

TTCCTTGCCA TGTTGGCTGT CTCTGACATG GGCCTGTCCC TCTCCTCCCT

TTCCTTGCCA TGTTGGCTGT CTCTGACATG GGCCTGTCCC TCTCCTCCCT

TTCCTTACCA TGTTGGCTGT CTCTGACATG GGCCTGTCCC TGTCCTCTCT

TTCCTTACCA TGTTGGCTGT CTCTGACATG GGCCTGTCCC TCTCCTCTCT

TTCCTTGCCA TGTTGGCTGT CTCTGACATG GGCCTGTCCC TCTCCTCCCT

TTCCTTGCCA TGTTGGCTGT CTCTGATATG GGCCTATCTC TCTCCTCCCT

TTCCTTACCA TGTTGGCTGT CTCTGATATG GGTTTGTCCT TCTCTTCCCT

TTCCTTACCA TGTTGGCTGT CTCTGATATG GGCTTGTCCT TCTCTTCTCT

TTCCTTGCCA TGTTGGCTGT CTCTGATATG AGGCTGTCCC TCTCCTCCCT

TTTCTTGCCA TGTTGGCTAT CTCTGACCTG GGCCTGTCCT TCTCCTCTCT

TTCCTTGCCC TGTTGGCCAT CTCTGACCTG GGCCTATCCT TCTCCTCCCT

TTCCTTGCCA TGTTGGCCAC CTCTGACCTG GGTCTGTCCT TTTCCTCCCT

TTCCTCTCCC TGCTGGCCTT CTCTGACCTG GGACTGTCCC TCTCCTCCCT

TTCCTTGCCA TGTTGTCTGT CTCTGACCTG GGCCTGTCCC TCTCTTCCCT

TTCCTTGCCA TGTTGGCTGT CTCTGACCTG GGCCTGTCCC TCTCCTCGTT

TTCCTCTCCA TGCTGGCCTT TTCGGACCTG GGCCTGTCCC TCTCCTCACT

TTCCTCTCCA TGCTGGCACT AACTGACCTG GGCCTCACCC TCACCACTCT

CTTTTGGCCC TGCTGACCAC CACAGACTTA GGCATGGGAC TCTCGACGGT

TCCTACCATG TTGAGGGTCT TCTTGTTCAA TGCCATGGGA ATTTCACCTA

TCCTACCATG TTGAGGATCT TCTTGTTCAA TGCCATGGGA ATTTCACCTA

TCCTACCATG TTGAGGATCT TCTTGTTCAA TGCCATGGGA ATTTCACCTG

TCCTATCATG TTGAGGATCT TCTTGTTCAA TGCCATGGGA ATTTCACCTG

TCCTACCATG ATGAGGATCT TCTTGTTCAA TGCCATGAGA ATTTCACCAA

TCCTACCATG CTGAGGATCT TCTTGTTCAA TGCCATGGGA ATTTCACCTA

TCCTACCATG CTTAAGATCT TCTGTTTCAA TGCCATGCAA ATCTCACCCA

TCCTACCATG CTTAAAATCT TCTTTTTCAA TGCCATGGCA ATCTCACCCA

TCCTACAATG TTGAGGATAT TCTTGTTCAG TGCCATGGAA ATTTCACCTA

CCCTACCATG CTGAGAATCT TCTTGTTCAA TGCCATGGAA ATTTCACCCA

TCCTACTATG CTGAGAATCT TCTTGTTCAA TTCCATGGGA ATTTCACCCA

CCCTACTATG CTGAGAATCT TCTTGTTCAA CGCCATGGGA ATTTCACCCA

TCCAACCATG CTGAGAATCT TCTTGTTCAA TGCCACTGGC ATTTCTGCTG

TCCTACCATG CTGAAGATCT TCTTGTTCAA TGACATGGAA ATTTCACCCA

TCCTACCATG CTGAGGGTTT TCTTGTTCAA TGCCATGAGA ATTTCACCCA

CCCCACTATG CTGAGGATCT TCTTGTTCAA TGCCACAGGC ATTTCTCCAA

GCCCACAGTC ATGCAACTCC TCTGGTTCAA TGTCCAGGAA ATTAGCTTTG

GCCCACGGTT CTGGGCGTCC TGTGGAGTCT TCACCAGGAG ATAAGCCTCA

ATGCCTGCTT TGCTCAAGAA TTCTTCATTC ATGGATTCAC TGTCATGGAA

ATGCCTGCTT TGCTCAAGAA TTCTTCATTC ATGGATTCAC TGTCATGGAA

ATGCCTGCTT TGTTCAAGAA TTCTTCATTC ATGGATCCAC TCTCATGGAA

ATGCCTGCTT TGCTCAAGAA TTCTTCATTC ATGGATTCAC TCTCATGGAA

ATGCCTGCTT TGCTCAAGAA TTCTTCATTC ATGGATTCAC TGTCATGGAA

ATGCCTGCTT TGCTCAAGAA TTCTTCATCC ATGGATTCAC TGTCATGGAA

ATGCCTGTTT TGCTCAAGAG TTCTTTATCC ATGTATTCAC TGCCATGGAG

ATGCCTGTTT TGCCCAAGAG TTCTTTATCC ATGGCTTCAC TGTCATGGAG

ATGCCTGCTT CGCCCAAGAA TTTTTCATCC ATGGATTCAC AGTCATGGAA

ATGCCTGCTT TGCTCAAGAA TTTTTCATCC ATGGATTCAC AGTCATGGAG

ATGCTTGCTT TGCTCAAGAA TTCTTCATCC ATGGATTCAC TGTCATGGAA

ATGCCTGTTT TGCTCAAGAA TTCTTCATCC ATGGATTCAC CGTCATGGAA

ATGCCTGCTT TGCTCAAGAG TTTTTCATTC ATGGATTCTC AGCCATGGAG

ACGCCTGCTT TGCTCAAGAA TTCTTCATTC ATGGATTCAC GGTCATGGAA

ATGCGTGCTT TGCTCAAGAG TTCTTCATTC ATGGATTCAC TGTCATGGAA

ATGCCTGCTT TGCTCAGGAA TTCTTCATCC ATGGATTCAC CGATATGGAG

AGGCCTGCTT TGCCCAGTTT TTCTTCCTTC ATGGTTTCTC CTTTATGGAA

ACTCCTGCAT CGCCCAAGCA TACTTCATCC ACGGACTATC CAGCATAGAG

TCCTCAGTAC TTCTAATTAT GTCTTTGGAC CGCTTTCTTG CCATTCACAA

TCTTCAGTAC TTCTAATTAT GTCTTTGGAC CGCTTTCTTG CCATTCACAA

TCTTCAGTAC TTCTAATTAT GCCTTTGGAC CGCTTTATTG CCATTTACAA

TCCTCAGTAC TTCTAATTAT GCCTTTGGAC CGCTTTATTG CCATTTACAA

TCCTCAGTAC TTCTAATTAT ATCTTTGGAC CGCGTTCTTG CTATTCGCAA

TCATCAGTGC TTCTAATTAT GTCTCTGGAC CGCTTTCTTG CCATTCACAA

TCCTCTGTGC TTCTGATTAT GTCTTTGGAC CGCTTTCTTG CCATTCACAA

TCCTCTGTGC TTCTGATTAT GTCTTTGGAC CGCTTTCTTG CCATTCACAA

TCTTCAGTAC TTCTAATTAT GTCTTTGGAC CGGTTTCTTG CTATTCACAA

TCCTCAGTAC TGCTGGTTAT GTCTTTGGAT CGCTTTCTTG CCATTCACAA

TCCTCCATGC TTCTAGCTAT GTCTTTGGAT CGCTTTCTTG CCATTCATAA

TCCTCCGTAC TTCTAGTTAT GTCTTTGGAT CGCTATCTTG CCATTCACAA

TCGTCAGTTC TTCTTATCAT GTCCGTGGAT CGTTTTATAG CCATCTACAA

TCCTCAGTGC TTCTAATTAT GTCTGTGGAT CGCTTTCTTG CCATTCACAA

TCCTCAGTTC TTCTAATTAT GTCCTTGGAT CGCTTTCTTG CCATTCATAA

TCCTCGGTTC TTCTAATTAT GTCTTTGGAT CGGTTTCTTG CCATTCATAA

TCTTCAGTAT TATTGGCCAT GGCTTATGAC CGTTTTGTTG CTATCTGCCG

TCCGGTGTCC TCCTGGCCAT GTCTTGGGAC CGCTTCGTCG CCATCTGCAA

TCCCTTAAGA TACAGTTCTA TCCTCACTAG CAACAGGGTT GCTAAAATGG

TCCCTTAAGA TACAGTTCTA TCCTCACTAG CAACAGGGTT GCTAAAATGG

TCCCTTAAGA TACAGTTCTA TCCTCACTAG CAACAGGCTT GCTAAAATGG

TCCCTTAAGA TACAGTTCTA TCCTCACTAG CAACAGGCTT GCTAAAATGG

CCCCTTAAGA TACAGTTCTA TCCTCACTAG CAACAGGGTT GCTAAAATGG

TCCCCTAAGA TATAGTTCTA TCCTTACTAG CAACCGGGTT GCTAAGATAG

CCCCTTAAGG TACAGTTCTA TCCTCACTGG CAGTAGGGTT GCTAAAATAG

CCCTTTAAGA TACAGTTCTA TCCTCAATGG CCGCAGAGTT GCTAAAATAG

TCCACTAAGG TATAGTTCTA TTCTCACTAG CAAAACAGTT GCTAAAGTGG

TCCCCTGAGA TATAATTCTC TCCTCACTAG CAACAGAGTT GCTAAAATGG

TCCCCTGAGA TACAGTTCTC TCCTCACTAG TGACAGAGTT GCTAAAATGT

TCCCCTGAGA TATAGTTCTC TTCTCACTAG CAAAAGAGTT GCTAAAATGG

TCCTCTGAGA TATACTTCTA TCCTGACCAC TGGTAGAGTT GTTAAAGTCT

TCCCCTGAGA TACAGTTCTA GTCTTACCAG CAACAGGGCT GTTAAAATGG

TCCCCTGAGA TATAATTCTA TCCTCACTAG CAACAGGGTT GCTAAAATGG

TCCCCTAAGA TACAGCTCTG TCCTCACTAG CGATCGGGTT TATAAAATGG

CCCACTCCAC TATGCCTCTA TTCTCACAGA GACTGTCATT GGCAGAATTG

CCCCTTGCGC TATGCTTCCC TAGTGACCGG CTCAACTATC ATCAAAATCG

GACTTATTTT AGCCATTAGG AGCATTCTCT TAGTGATTCC ATTTCCCTTC

GACTTATTTT AGCCATTAGG AGCATTCTCT TAGTGATTCC ATTTCCCTTC

GACTTATTTT AGCCATTAGG AGCATTCTCT TAATGCTTCC ATTTCCCTTC

GACTTATTTT AGCCATTAGG AGCATTCTCT TAATGCTTCC ATTTCCCTTC

GACTTATTTT AGCCATTAGG AGCATTCTCT TAGTGCTTCC ATTTCCCATC

GACTAACCTT AGCCATTAGG AGCATTCTTT TAGTGCTTCC ATTTCCTTTC

GGTTAATTTT AGCCATCAGG AGCACAGCTT TGGTGCTTCC ATTCCCTTTC

GGCTAATTTT AGCCTTCAGG AGCACAGTTT TGGTGCTTCC ATTCCCTTTC

GACTAATCTT AGCCATTAGG AGCATTATTT TAGTGCTTCC ATTCCCTTTC

GACCGATTTT AGCCACCAGG AGCCTTCTCT TAGTGCTTCC ATTTCCTTTC

GCCTGATCTT AGCCATCAGG AGCATTCTCT TAGTGCTTCC ATTCCCCTTC

GACTGATCTT AGCCATCAGG AGCATTCTCT TAGTGCTTCC ATTCCCATTC

GCCTTATATT CACAATCAAA AGTATCTTAT TAGTTTTTCC TTTCCCTTTC

GACTGATCTT AACTATTAGG AGCATTCTCC TAGTTCTCCC ACTCCCCTTC

GACTGATCTT GGCTTTCAGG AGCATTCTTT TAGTGCTCCC ATTCCCCTTC

GCCTCGCCAT GGCCTTCAGG AGCGTCCTCT TAGTGCTGCC ATTCCCCTTC

GGATAGCCAT CGTTATCCGA TGTGTTTTAC CCGTCCTGCC CTCTCTCTTC

GAGCAATCGT TTTAACCAGG AGCTTGCTAC TGATCCTTCC CGCGATCATC

ACCTTAAGGA GATTAAAATA TTGTCAAAAG AATCTTCTTT CTCACTCATA

ACCTTAAGGA GATTAAAATA TTGTCAAAAG AATCTTCTTT CTCACTCATA

ACTCTAAGGA GATTAAAATA TTGTCAAAAG AATCTTCTTT TGA---CATA

ACTCTAAGGA GATTAAAATA TTGTCAAAAG AATCTTCTTT CTCACTCATA

ACCCTAAGGA GATTAAAATA TTGTCAAAAG AATCTTCTTT CTCACTCATA

ACTCTAAGGA GGTTAAAATA TTGTAAAAAG AATCTTCTTT CCCACTCTTA

ACACTAAGGA AACTGAAATA CTGTCAAAAG AATCTGCTCT CTCACTTATA

ACACTAAGGA AACTGAAATA CTGTCACAAG AATCTCCTCT CTCACTCATA

ACCTTAAGAA GATTAAAATA TTGTCAAAAG AATATCCTTT CTCACTCCTA

ATGCTAAGGA GATTGAAATA TTGTCAGAAG AATGTCCTTT CTTACTCATA

ACTCTAAGGA GATTAAAATA TTGTCAGAAG AATCTCCTTT CTCACTCATA

ACTCTGAAGA GATTAAAATA TTGTCAGAAG AATCTCCTTT CTCACTCATA

ATTCTGAAGA GGCTGAAATA CTGTAAGAGA AGTCTGCTGT CCCACTCCTA

ATTCTCAGGA GGTTAAAATA TTGTCAGAAG AATCTTCTTT CTCACTCATA

ACTTTAAGGG GACTAAAATA CTGTCAGAAG AATCTCCTTT CTCACTCATA

ATTCTAAGGA GACTGAAATA CTGCCAGAAG CATGTCCTTT CTCACTCATA

CTGCTCAAAC GTCTGCCCTT TTGTCACTCT CATCTCCTCT CCCACTCCTA

CGCCTGAGAT TCTTCTTCTA TTGCCGTCCC CATGTCCTCT CTCACTCTTT

CTGTCTTCAT CAGGATACCA TGAAGCTGGC CTGCTCTGAC AACAAGACCA

CTGTCTTCAT CAGGATACCA TGAAGCTGGC CTGCTCTGAC AACAAGACCA

CTGTCTTCAT CAGGATACCA TGAAGCTGGC CTGCTCTGAC AACAAGACCA

CTGTCTTCAT CAGGATACCA TGAAGCTGGC CTGCTCTGAC AACAAGACCA

CTGTCTTCAT CAGGATACCA TGAGGCTGGC CTGCTCTGAC AACAAGATCA

CTGTCTTCAT CAAGATACCA TGAAGCTGGC CTGCTCTGAC AACAAGATTA

CTGCCTTCAT CAGGACGTCA TGAAACTGGC CTGCTCTGAT AACAAGATCA

CTGCCTTCAT CAAGATGTCA TGAAACTGGC CTGCTCTGAT AACAAGATCA

CTGTCTTCAC CAGGATACTA TGAAGTTGGC CTGCTCTGAT AATAGGATCA

CTGTCTTCAT CAGGATACCA TGAAGCTGGC CTGCTCTGAC AACAAGATCA

CTGTCTGCAT CAGGATACCA TGAAGCTAGC CTGCTCTGAC AACAAAATCA

CTGTCTTCAT CAGGATACCA TGAAGCTGGC CTGCTCTGAC AACAAGATCA

CTGCCTCCAC CAGGATGTCA TGAAGCTGGC CTGTTCTGAC AACAGGGTCA

CTGTCTTCAT CAGGATACCA TGAAACTGGC TTGCTCTGAC ATCAAGATTA

TTGTCTTCAT CAGGATACCA TGAAGCTGGC CTGCTCTGAC AACAAGATCG

CTGCTTTCAT CAGGACACCA TGAAGCTGGC CTGCTCTGAC AACAAGATCA

TTGCCTTCAT CAGGACATGA TCCGCTTGGT ATGTGCTGAC ACTCGAATCA

CTGTCTGCAC CAGGATCTGC TCCGCTTGGC TTGTTCTGAC ATTCGGTTCA

ATGTCATCTA TGGCTTCTTC ATTGCTCTCT GTACTATGCT GGACTTGGCA

ATGTCATCTA TGGCTTCTTC GTTGCTCTCT GTACTATGCT GGACTTGGCA

ATGTCATCTA TGGCTTTTTC GTTGCTCTCT GTACTATGCT GGACTTGGCA

ATGTCATCTA TGGCTTTTTC GCTGCTCTCT GTACTATGCT GGACTTGGCA

ATGTCATCTA TGGCTTCTTC ATTGCTCTCT GTACCATGCT GGACTTGGCA

ATGTCATCTA TGGTTTCTTT GTTGCTCTCT GTACTATGCT AGACTTGGCA

ATTTCATCTA TGGTTTCTTT GCGGCTCTCT GTACTATGCT GGACTTTGCC

ATTTCATCTA TGGCTTCTTT GTGGCTCTCT GTACCATGCT GGACTTTGCC

ACTTCATCTA TGGCTTCTTT GTTGCTCTCT GTACCATGCT GGACTTCATG

ATGTTATCTA TGGTTTCTTT GTTGCTCTCT GTACTATGCT GGACTTGGCT

ATGTTATCTA TGGCTTCTTT GTTGCTCTCT GTACTATGCT GGACTTGGCA

ATGTTATCTA TGGCTTCTTT GTTGCTCTCT GTACCATGCT GGACTTGGCA

ATATCATCTA CGGCATGTGT GTGGCGCTAA CAGCTTTTTT AGATTTGTGC

ATGTCATCTA TGGCTTTTTT GTTGCTCTCT GTACTATGCT GGACTTGGCA

ATGTCGTCTA TGGCTTCTTT GTTGCTCTCT GTACTATGCT GGACTTGGCA

ATGTGGTTTA TGGCTTCTTT GTTGCACTCT GTACTATGCT GGACTTAACT

ATAGTTGGTA TGGTTTTGTT ATTGCTCAT- -CATCGTGCT TGACCCTCTC

ACAGTTTCTA TGCCCTTGGG CTCGTGATCA GCACTATCTT GGATTCGGTG

CTGATTGTTT TGTCTTATGT GCTGATCTTG AAGACTATAC TCAGCATTGC

CTGATTGTTT TGTCTTATGT GCTGATCTTG AAGACGATAC TCAGCATTGC

TTGATTGTTT TGTCTTATGT GCTGATCTTG AAGACTATAC TCAGCATTGC

TTGATTGTTT TGTCTTATGT GCTGATCTTG AAGACTATAC TCAGCATTGC

TTGATTCTTT TGTCTTATGT GCTGATCTTG AAGACTGTAC TCAGCATTGC

TTGATTGTTT TGTCTTATGT GCTGATCCTG AAGATGGTAC TCAGCATTGC

TTGATTCTTA TGTCTTACAT GCTGATCTTG AAGACAGTTC TCAGTATTGC

TTGATTCTTA TGTCTTATGT GCTGATCTTG AAGACAGTTC TCAGTATTGC

TTGATTGCTC TGTCTTATGT GATGATCTTG AGGACTGTTC TCAGCATTGC

TTCATTATTC TTTCTTATAC ACTGATCCTG AAGACTGTAC TCAGCATTGC

TTAATTTTTC TGTCATATAT GCTCATCTTG AAGACTGTAT TCAGCATTGC

TTAATTGTTT TGTCTTACAT GCTTATCTTG AAGACCGTAC TCAGCATTGC

TGCATTTCTC TCTCTTACAT GTTGATTCTA AAAACTGTTC TAGGAATTGC

TTGATTGTGC TGTCATATAT GCTAATTTTG AGGACTGTAC TTAGCATTGC

CTGATTTTTC TGTCATATGT ACTGATCTTG AGGACTGTAC TCAGCATTGG

TTGATTTTTG TGTCGTATGT TCTGATCTTG AGGACTGTAA TGAATATTGG

CTCATTCTGG TCTCCTATGG TTTGATCTTG CACAGTGTCC TTGGCACTGC

TTCATTCTGG CCTCCTATGT CCTGATCCTC CACACCGTGC TGGCTGTGGC

ATCTTTGGCA GAGAGGCTTA AGGCCCTAAA TACCTGTGTC TCCCACATCT

ATCTTTGGCA GAGAGGCTTA AGGCCCTAAA TACTTGTGTC TCCCACATCT

ATCTTTGGCA GAGAGGCTTA AAGCCCTAAA TACCTGTGTC TTCCACATCT

ATCTTTGGCA GAGAGGCTTA AAGCCCTAAA TACCTGTGTC TTCCACATCT

ATCTTTGGCA GAGAGGTTTA AGGCCCTAAA TACCTGTGTC TCCCACATCT

ATCTCTAGCA GAGAGGCTCA AGGCCCTAAA CACCTGTGTC TCCCACATCT

ATCTCTGGTA GAAAGGCTAA AAGCCCTAAA TACCTGTGTC TCCCACATCT

ATCTCTAGCA GAAAGGCTTA AGGCTCTAAA TACCTGTGTC TCCCACATCT

ATCTCTGGCA GAGAGGTTAA AGGCCCTCAA TACCTGTGTC TCCCATATCT

ATCTTTGGCA GAGAGACATA AGACCCTGAA CACCTGTGTC TCCCATTTCT

ATCTCTGGCA GAGAGGCTCA GGGCTCTCAA TACCTGTGTC TCCCACATCT

ATCTCTGGCA GAGAGACTCA AGGCCCTGAA TACCTGTGTC TCCCATATCT

CTCACAAAAG GGTCGCCTCA AGGTTTTCAA CACTTGCATT TCCCACATCT

ATCTCTGGCA GAGAGACTCA AGGCTCTCAA CACCTGTGTG TCCCACATCT

ATCTTTGGAA GAGCGGCTCA AAGCTCTCAA CACCTGTGTC TCCCACGTCT

CTCTCTGGAA GAAAGACTCA AAGCCCTCAA CACGTGTGTG TCTCACTTTT

CTCCCACGCT GAGAGAATTA AGGCGCTCAA CAACTGCCTA TCCCACATTC

TGCCCGCGGG GAGAGGCTGA AGTCTCTCCA AACCTGCACC TCCCACATCC

GTGCTGTGCT CACCTTCTAT GTGCCCATCA TCACCCTGGC TGCCATGCAT

GTGCTGTGCT CACCTTCTAT GTGCCCATCA TCACCCTGTC TGCCATGCAT

GTGCTGTGCT CACCTTCTAT GTGCCCATCA TCACCCTGGC TGCCATACAT

GTGCTGTGCT CACCTTCTAT GTGCCCATCA TCACCCTGGC TGCCATGCAT

GTGCTGTGCT CACCTTCTAT GTGCCCATCA TCACCCTGGC TGCCATGCAC

GTGCCGTGCT CATCTTCTAT CTGCCCATCA TCACGCTGGC TGCCATGCAT

GTGCTGTGCT CATCTTCTAT GTCCCCATCA TCACCTTGGC TGCCATACAT

GTGCTGTGCT CATCTTCTAT GTGCCCATCA TCACCCTGGC TGCCATACAT

GTGCTGTGCT TATCTTCTAT GTGCCCATCA TTCTTCTGGC TGCTATGCAT

GTGCTGTACT CATTTTCTAT GTGCCCATCA TTACTCTGGC TGCCATGCAT

GTGCTGTGCT CATTTTCTAT GTACCCATCA TCACCCTTGC TGCCATGCAC

GTGCTGTGCT CATTTTCTAT GTACCCATCA TCACCCTGGC TGCCATGCAC

GTGCTGTACT GATATTTTAT GTGCCTATAG TCGCTCTGGC AATTATTCAC

GTGCTGTGCT CATCTTCTAC GTACCCATCA CCACTCTGGC TGCCTTGCAT

GTGCTGTGCT CATCTTCTAT GTGCCCATCA TCACCCTGTC TGCCATGCAT

GTGCTGTACT TATCTTCTAT GTGCCCATCA TCACCCTGTC TGCCATGCAT

TAGCTGTACT CATCCTTTAC GTGCCCATGG TGGGGTTGTC AATGGTCCAC

TCGCCACCAT GGTCTTTTAC GTTCCCATCA TCGGCCTGAC CATGGTTCAC

CACTTTGCCA AGCACAAAAG CCCTCTTGTT GTGATCCTTA TTGCAGATAT

CGCTTTGCCA AGCACAAAAG CCCTCTTGTT GTGATCCTTA TTGCAGATAT

CGCTTTGCCA AGCACAAAAG CCCTCTTGTT GTGATCCTTA TTGTAGATAT

CGCTTTGCCA AGCACAAAAG CCCTCTTGTT GTGATCCTTA TTGCAGATAT

CGCTTTGCCA AGGACAAAAG CCCTTTTGCT GTGATCCTTA TTGCAGATAT

CGCTTTGCCA AGCACAAACG CCCCCTAGTG GTAATCCTCA TTGCAGATAT

CGCTTTGCCA AGCACAAAAG CCCCCTACTT GTAATCCTTA TTGCAGATAT

CGCTTTGCCA AACACAAAAG TCCCCTACTT GTAATCCTTA TTGCAGATAT

CGCTTTGCAA AACACAAAAG CCCCCTTGTT GTAATCCTCA TTGCAGATAT

CGCTTTGCCA AGCACAAAAG CCCCCTCGCT ATAATCCTTA TTGCAGACAT

CGCTTTGCCA AGCACAAAAG CCCTCTAGTT GTAATCCTTG TTGCAGATAT

CGCTTTGCCA AGCACAAAAG CCCCCTCATT GTAATTCTTA TTGCAGATAC

CGCTTTGCCA AACATGTTTC TCCAGTTATT AAGGCAATAA TAGCTGATAC

CGCTTTGCCA AGCACAAAAA CCCCCTTGTT TTGCTTTTGA TTGCAGATAC

CGCTTTGCCA AACACAAAAG CCCCCTTATT ATGATCCTTA TTGCAGATGT

CGCTTTGCCA AGCACAAAAG CCCCCTCGTT ATGATCCTTA TTGCCGATGT

CGCTTTGCCC GGCATGCACC ACCACTGGTT CATGTGGTCA TGGCCAATGT

CGTTTCGGAA AGCATCTGAG TCCTCTGGTC CATGTGCTCA TGGGCCACAT

GTTCTTGTTG GTGCCGCCCC TTATGAACCC CATTGTGTAC TGTGTAAAGA

GTTCTTGTTG GTGCCGCCCC TTATGAACCC CATTGTGTAC TGTGTAAAGA

GTTCTTGTTG GTGCCGTCCC TTATGAATCT CATTGTGTAC TGTGTAAAGA

GTTCTTGTTG GTGCCGTCCC TTATTAATCT CATTGTGTAC TGTGTAAAGA

GTTCTTTTTG GTGCCACCCC TTATGAACCC CATTGTGTAC TGTGTAAAGA

GTTCTTGCTG GTGCCACCCT TGATGAACCC CATTGTGTAT TGTGTAAAGA

GTTTTTGTTG GTGCCACCCC TGATGAACCC CATTGTGTAC TGTATAAAGA

GTTCTTGTTG GTGCCACCCC TGATGAACCC CATTGTGTAC TGTATAAAGA

GTTCTTATTG CTGCCACCCT TGATGAACCC CATTGTGTAC TGTGTAAAGA

ATTCTTGTTG TTGCCACCCC TAATGAACCC CATTGTGTAT TGTGTAAAGA

ATTCTTGTTG TTCCCGCCTC TAATGAACCC CATTGTGTAT TGTGTAAAAA

ATTCTTGTTG TTGCCTCCCT TAATGAACCC CATTGTATAT TGTGCAAAGA

TTTTCTTCTG GTTCCCCCTT TAATGAATCC AATTGTGTAC TCTGTGAAAA

ATTCTTGTTG GTGCCGCCCC TGATGAACCC CGTTGTGTAC TCCATTAAGA

GTTCTTGTTA GTGCCACCCC TGATGAACCC CATTGTGTAC TGTGTGAAGA

CTTCTTGTTA GTGCCACCCG TGATGAACCC TATTGTGTAT TGTGTAAAAA

CTACTTGCTA TCACCCCCTG TGATGAACCC CATTATCTAC AGTGTGAAAA

CTACATTCTT TTCCCTCCCC TGATGAACCC CATCATCTAC AGTATCAAAA

CTCGACAAAT CTGGGAGAAG ATCTTGGGGA AGTTGCTTAA TTGGTATAAC

CTCGACAAAT CTGGGAGAAG ATCTTGGGGA AGTTGCTTAA TTGGTATAAC

CT---CAAAT CTGGGAGAAG ATCTTGGGGA AGTTGCTTAA T---TCTGAC

CT------AT CCGGGAGAAG ATCTTGGGGA AGTTGCTTAA TTGGTCCAAC

CTCGACAAAT CTGGGACAAG ATCTTGGGGA AGTTGCTTAA CTTGTCTAAC

CTCGACAAAT CCGGGAGAAG ATTCTGGGGA AGTTGCTTAA CTGGTCCAAT

CTCGACAAAT CCGGGAGAAG GTCTTGGGAA AGTTGGTTAA CTGGTCCAAC

CTCGGCAAAT CCGGGAGAAG GTCTTGGGAA AGTTGGTTAA CTGGCCCAAC

CTCGACAAAT ACGGGAGAAG GTCCTGGGGA AGCTGCTTCA CTGGTCCAAT

CTCAACAAAT CTGGGAGAAG GTCTTGGGGA AGCTATCTAA CTGGCTCAAC

CTCGACAAAT CCGGGAAAAG GTCTTGGGAA AGTTGTTTAA TTTGCTCAAT

CTCGACAAAT CCGAG---AG GTCCTGGGGA AATTGTTTAG CTTGCTCAAT

GTCAGCATAT TAGAAATCTG ATCCTTGCAA AATTATGTGA -TTGCTCAAT

CCAGACAGAT CCGGGAGAAA ATCTTGGGGA AGTGGCTTCA CTGGTCCAAC

CCCAGCAGAT CCGGGAGAAG GTCTTAGCGA AGTGGCTTAA CTGGTCCAAC

CCCGGCAAAT CCGGGAGAAG GTCTTGGGGA AGTGGCTGAA CTGGTCCAAC

CTAAGCAGAT TCGCCAGGGT ATCCAGGAAA AGTGCACTA- GTGGCCCAAC

CCCAGCAGAT CCGCAAAAGG ATCCAAGGAA TGTTCTATAT ---TTCCAAC

AACAGTGCTG GCCCCTTCTT GCTGACTGGC TTCTTGGGCT CAGAGGCAGT

AACAGTGCTG GCCCCTTCTT GCTGACTGGC TTCTTGGGCT CAGAGGCAGT

AACAGTGCTG CCCCCTTCCT GCTGACTGGT TTCCTGGGCT CAGAGGCAAT

GGCAGCTCGC ATCCCTTCCT GTTGACTGGT TTTCCAGGCT TGGAGGCAGC

GACAGTGCTG CCCCCTTCTT GCTGACTGGC TTCCTGGGCT CAGAGACAAT

GACACTGCAG CCCCTTTCCT GCTGACTGGC TTCCTGGGCT CAGAGGGCAC

AACAGTGATG CTCCTTTCCT ACTGACTGGC TTTCTGGGCT TGGAAATGAT

AGCAGTGATG CTCCTTTCCT ACTGACGGGC TTTCTGGGCT TGGAAATGAT

TACAGTGCTG CTCCCTTCTT ACTGACTGGC TTCCTGGGCT CAGAGACAAT

AATACCAGTG CTCCCTTCCT CTTGACGGGT ATTCCAGGCC TGAGAGCGGC

AATACCAATG CTCCCTTCTT CCTGACGGGT ATCCCAGGCC TGAGAGCAGC

AATACCAATG ATCCATTCCT CCTGACGGGT ATCCCAGGCC TGGCAGCAGC

AATACCAATG CTCCCTTCTT CCTGACGGGT ATCCCAGGCC TGAGAGCAGC

AACAGTGCTG CTCCCTTCTT GCTGACCGGC TTTCTGGGCT CAGAGGCAGC

AATAGTGCTG CCCCCTTTTT GCTGACTGGA TTCCTGGGCT TAGAGACAAT

ATTAGTGCTG ACTCCTTTCT GTTGACTGGA TTCCCAGGTC TCGAGGCAGC

AGCAACACAG CCCCTTTCTT GCTGACTGGC TTCCCAGGCT TGGAGGTAGC

TTCAGCACTT CCTCCTTCTG TCTCCTGGGC TTCCCAGGGC TGGAATCAGC

TCACTACCGG ATCTCTATGT CCTTCTTTGT CATCTACTTC TCCGTCCTTT

TCACTACCGG ACCTCTGTGT CCTTCTTTGT CATCTACTTC TCCATCCTTT

TCACCACTGG ATCTCTATTC CCTTCTTTGT CATCTACTTC TGCATCCTTT

TCATCACTGG ATTTCCTTAT TTTTCTTGTT CATCTATATG TCTGTCCTTT

TCACCGTTGG ATCTCTATTC CCTTCTTTGT CATCTACTTC TCCATCCTTT

TCACCACTGG ATCGCCATTC CCTTCTTTGC CATCTATCTG TCCATTCTAT

TCACCACTGG ATCTCTATTC CTTTTTTTGT CATCTACTTC TCCATTATTT

TCACCACTGG ATCTCTATCC CTTTCTTTGT CATCTACTTC TCCATCATTT

ACACCACTGG ATCTCTATTC CCTTCTTTGT TATCTACTTC TCCATCCTTT

CAAGATCTGG GTCTCCATCC CTGTTTGCCT CCTGTATGCC GGCGCCCTCT

CCAGGTTTGG ATCTCCATCC CTTTTTGTCT CCTGTATGTC ATCGCCCTCT

CCAGGTGTGG ATCTCCATTC CTATTTGTCT CCTGTATGTC ACTGCCCTGT

CCAGGTCTGG ATCTCCATCC CGTTCTGCCT CCTATATGGC ATTGCCCTCA

TCACCACTGG ATAGGTATTG CCTTTTTCAT CATCTACCTC TCCATCCTTT

TCACCACTGG ATTGCTATTC CTTTCTTTCT CATCTACCTC TCTATTATCT

GCATCACTGG ATCTCCATCC CTTTCTGTAC AGTCTACTTC TCTGTGCTCC

TCATCGCTGG ATCTCCATTC CCTTTTTTGT GGTGTATCTT TCTGTTATTC

TTATCCGTGG ATTGCCCTCC TCTTGTCCAC CATCTACACC ATAATGCTCC

TTGGAAATGG CACTCTTCTT GTCCTCATTT GGAATGATCA CAGCCTCCAT

TTGGAAATGG CACTCTTCTT GTCCTCATTT GGAATGATCA CAGCCTCCAT

TTAGAAATGG CACACTTCTT GTCCTCATTT GGAATGACCA CAGCCTCCAT

TTGGCAATGG CACCCTCCTT CTTCTCATTA AGGAAGATCA CAATCTTCAT

TTGGGAATGG CACACTTCTT GTCCTCATTT GGAATGACCA CAGCCTTCAT

CTGGAAATGG AACACTTCTC ATCCTCATAT TGAAGGACCG CATCCTTCAT

TAGGGAATGG TACCCTTCTC TTCATCATTT GGAATGACCA CAGCCTTCAT

TAGGGAATGG TACCCTTCTC TTCATCATTT GGAGTGACCA CAGTCTTCAT

TTGGAAATGG TACACTTCTT CTTCTCATTT GGAGTGACCA CGGTCTTCAT

CTGGGAACAG TGTGATCCTG TTTGTGGTCC TCCGTGAACA GAGCCTTCAT

CTGGGAACAG CATGATCCTA TTTGTGGTCT TCCGTGAGCA GAATCTCCAT

CTGGGAACAG CATGATCCTG TTTATGGTCA TTCATGAGCA GAGCCTCCAT

CTGGAAACAG CATGATCCTG TTTGTGGTCC TCCGTGAGCA GAGTCTCCAT

TTGGCAATGG CACACTTCTT TTCCTCATTA GGAATGACCA CAGCCTTCAC

TTGGCAACAG CATACTTCTC TTCCTCATTC TGAGTGACCA CAGTCTTCAT

TTGGAAACAG CACCCTCCTC TACCTCATTA AAGACGACCA CACTCTCCAT

TTGGCAATAG TGGTCTCCTC TTCCTCATCA AGAATGAACA CAGCCTCCAT

TGGGGAACAG CATCATCCTC TATGTACTCT GGATAGAATG GAGTCTCCAC

GAGCCCATGT ACTACTTCCT GGCTATGCTG GCAGACACGG ACCTTGGGAT

GAGCCCATGT ACTACTTCCT GGCTATGCTG GCAGACACGG ACCTTGGGAT

GAGCCCATGT ACTACTTCCT GGCTATGCTG GCAGGCACGG ACCTTGGGAT

GAGCCCATGT ACTTCTTTCT GTCCATGCTG GCTGCCACAG ACCTGGGGCT

GAGCCAATGT ACTACTTCCT GGCTATGCTG GCAGGCACGG ACCTTGGGAT

GACCCCATGT ACTACTTCCT GGCTATGCTG GCAGGGACAG ACCTTGGGGT

GAGCCCATGT ACTACTTCTT GGCTGTGCTG GCAAGTACAG ACCTTGGGAT

GAGCCCATGT ACTACTTCTT GGCTGTGCTG GCAAGTATGG ACCTTGGGAT

GAGCCCATGT ACTACTTCCT TGCCATGCTG GCAGGTACAG ACCTGGGAAT

GAGCCCATGT ATTATTTCCT CTCTATGCTT TCAGCCACAG ACCTGAGCTT

GAACCAATGT ATTATTTCCT CTCTATGCTT TCAGCCACAG ACCTGAGCTT

GAGACCATGT ATTATTTCCT TTCTATGCTG TCAGCCACAG ACCTGAGCTT

GAGCCCATGT ATTATTTCCT CTCTATGCTT TCAACCACAG ATCTGAGCTT

GAACCCATGT ACTACTTCCT GGCTATGCTG GCAGGCACAG ACCTTGGGAT

GAACCCATGT ACTACTTCCT GGCAATGTTG GCAGGCACAG ACCTGGGGAT

GAACCCATGT ACTACTTCCT GGCCATGCTG GCAGGCACAG ACCTCATGGT

GAGCCCATGT ACTATTTCCT GGCCATGCTG GCAGCCACAG ACCTTGGTGT

AAGCCCATGT ACTACCTTTT GGCCCTGCTG ACCACCACAG ACTTAGGCAT

GACATTCACT ACAATGCCCA CAGTCCTGGG TGTCCTGCTG CTAGACCAGA

GACATTCACT ACAATGCCCA CAGTCCTGGG TGTCCTGCTG CTAGACCAGA

GACACTCACT ACGATGCCCA CTGTCCTGGG TGTCCTGCTG CTAGACCAGA

GACCCTGACC ACAATGCCCA CGGTGCTGGG AGTCCTCTGG CTGGATTACA

GACACTCACT ACAATGCCCA CAGTCCTAGG TGTCCTGCTG CTAGAACAGA

AACACTAACT ACAATGCCCA CAGTCCTGGG TGTCCTGTTG CTGGACCAGA

GACACTAACG ACGATGCCCA CAGTGCTGGG TGTTCTGGTG CTGAACCAGA

GACACTAACC ACGATGCCCA CAGTGCTGGG TGTCCTGGTG CTAAACCAGA

GACATTAACA ACAATGCCCA CAGTCCTGGG TGTTCTACTG CTAGACCACA

ATCCCTGTGC ACACTTTCCA CTACCCTTGG TGTCTTCTGG TTTGAAGCCC

ATCCCTGTGC ACACTTTCTA CTACCCTTGG TGTCTTCTGG TTTGAAGCTC

ATCCCTGTGC ACACTTGCCA CTACTCTTGG TGTGTTCTGG TTTGAAGCCC

GTGTCTGTGC ACGCTTTCCA CTACTCTGGG CGTCTTCTGG TTTGAAGCTA

GACATTAACC ACGATGCCCA CAGTCCTGGG TGTCCTATTG CTGGGCCAGA

TACACTAACT ACAATGCCCA CAGTCCTGGG TGTCCTAATG TTGAACCAGA

GACCCTGACC ACGATGCCCA CTGTCATGGG CGTATTATGG ATGAATTACA

CACTCTAACC ACAATGCCCA CTGTTCTGGG GGTCTTATGG CTGGACCAGA

GGGACTCTCG ACGGTGCCCA CGGTTCTGGG CGTCCTGTGG AGTCTTCACC

GGGAGATTGC CCATGCTGCC TGTTTCACCC AATCCTTCAT TCATTCACTG

GGGAGATTGC CCATGCTGCC TGTTTCACCC AATCCTTCAT TCATTCACTG

GGGAGATTGC CCAGGCTGCC TGTTTCACTC AGTCCTTCAT TCATTCACTG

GGGAGATTGG AAGCGCAGCC TGCTTTTCCC AGGCCTACAT ACACTCACTT

GGGAGATTGC ACAGGCTGCC TGTTTCACTC AGTCCTTCAT TCATTCACTG

GGGAGATCAC CCAGGGAGCC TGCTTCACTC AGTCCTATAT TCACACACTG

GGGAGATTGC ACACGGAGCC TGTTTCATTC AGTCCTACAT CCATTCCCTA

GGGAGATTGC ACAAGGGGCC TGTTTCATTC AGTCCTACAT TCACTCACTA

GGGAGATTGC CCAGAGTGCC TGTTTCACTC AGTCCTACAT TCACTCACTG

GAGAGATCAA CTTAAATGCC TGTGTTGCTC AGATGTTCCT CCATGGATTT

GAGAAATCAC CTTAAGTGCC TGCATTGCCC AGATGTTCCT CCATGGATTT

GAAAGATCAA CTTAAATGCT TGTATTACGC AGATGTTCCT CCATGCATTT

AAGCGATCAA CATCAATTCC TGCATTGCTC AGCTCTTCAT CCACACTTTG

GAGAGATTGC CCACAGTACC TGCTTCACTC AGTCCTACAT TCACACACTG

GGAAGATTGC ACAGAGCGCC TGCTTCACTC AGTCCTACAT TCACTCACTG

GGAAGATATA TCCTGGGGCT TGTTTCATAC AAGCCTACAT CCACTCCCTT

GGAAAATTGG TCATGGCATA TGCTTCTCCC AGGCCTATAT CCACACTCTC

AGGAGATAAG CCTCAACTCC TGCATCGCCC AAGCATACAT CCACGGACTA

GCCATTGTAG AATCAGGTAT CTTGCTTGTT TTGGCCTATG ACTGTTTCAT

GCCATTGTAG AATCAGGTAT CTTGCTTGTT TTGGCCTATG ACTGTTTCAT

GCCATAGTAG AATCAGGTAT CTTGCTTTTT ATGGCCTATG ACCATTTCAT

TCCTTTGTCG AGTCTGGCAT TCTGCTTGCC ATGGCCTATG ACCGTTTTAT

GCAATTGTAG AATCGGGTGT TTTGCTTGCC ATGGCCTATG ACCGTTTCAT

GCCATTGTGG AATCTGGTAT CTTGCTTGCC ATGGCCTATG ACCGATTTAT

GCCATTGTAG AATCAGGGGT CTTGCTTGCC ATGTCTTATG ATCGATTTGT

GCCATTGTAG AATCGGGGGT CTTGCTTGCC ATGTCTTATG ATCGATTTGT

GCTTTTGTAG AATCAGGTGT CTTACTTGCC ATGGCCTATG ACCGTTTTAT

ACTTTCATGG AGTCTGGGGT TTTGCTGGCC ATGGCCTTTG ACCGTTTTGT

ACTTTCATGG AGTCTGGGGT TCTGCTGGCC ATGGCCTTTG ATCGTTTTGT

ACTTTCATGG AGTCTGGGGT TCTGCTGGCC ATGGCCTTTG ATCGTTTTGT

TCTCTTGTGG AGTCTTCGGT TCTATTATCC ATGTCTATCG ACCGCTATGT

GCCATTGTAG AATCAGGTAT TTTGCTGGCC ATGGCTTACG ACCGTTTTAT

GCCATTGTAG AGTCAGGTGT CTTGCTTGCC ATGGCCTATG ATCGTTTTAT

TCCATTGTCG AATCAGGTGT TTTGCTTGCC ATGGCCTATG ACCGTTTTAT

TCTATTGTAG AGTCAGGGGT CTTGCTCGCC ATGGCCTATG ACCGTTTCAT

TCCAGCATAG AGTCCGGTGT CCTCCTGGCC ATGTCTTGGG ACCGCTTCGT

TGCCATCCGC ACACCACTGA GGTACAACTG CATTCTTACC AATTCCCGAG

TGCCATCCGC ACACCACTGA GGTACAACTC CATTCTTACC AATTCCCGAG

TGCCATCCGC ACACCACTGA GGTACAACTC CATTCTTACC AATTTCCGAG

TGCCATCTGC AACCCTCTTA GATATACCTC CATACTTACT ACTACTCGAA

TGCCATCCGC ACACCACTGA GGTACAGCTC TACTCTTACC AATTCACAAG

TGCCATTCGC TCCCCTCTCA GGTACAACTC CATTCTCACC AATTCCCAAG

GGCCATCTGT ACCCCTCTGC ATTATAACTC TATCCTTACC AATTCTAGGG

TGCCATCTGC ACACCACTGC ATTACAACTC TATCCTTACC AATTCCAGGG

TGCCATCCGT GCCCCTCTTA GGTACAACTC AATTCTTACC AACTCTCGAG

GGCCATCTGT GATCCACTGA GATACACCAC CATCCTCACC AATGCCAGGA

AGCCATCTGT AATCCACTGA GATACACCAC CATCCTCACC AATGCCAGAA

GGCCATCTGT GACCCACTGA GATATACCAC AATCCTCACC AATGCCAGGA

TGCCATCTGC AACCCGCTGC GTTATTCCAC CGTCTTGACC AATGCCAGGA

TGCTATCCGC TCCCCACTGA GGTACAACAC CATCCTTACC AATTCCCGGG

TGCCATTTGC TCCCCTCTGA GGTACAACTC CATTCTTATC AGTTCCCGGG

TGCTATCCGC AATCCCTTGA GATACACTTC CGTTCTCACC AATACTAGGG

TGCCATTCGC AACCCACTCA GATATACCGC TATCCTCACC AATTCCCAGG

CGCCATCTGC AACCCCTTGC GCTATGCTTC CCTAGTGACC GGCTCAACTA

TGATGAACAT AGGACTGGGG GTACTGATGA GAGGTTTTAT GTCCATTTTG

TGATGAACAT AGGACTGGGA TTACTGATGA GAGGTTTTAT GTCTATTTTG

TGATGAACAT AGGACTGGGG GTACTGATGA GAGGTTTTAT GTCCATTTTG

TAGTGAAGAT TGGGCTGGGA GTTCTGATTA GGGGATTTGT ATCTGTTGTT

TGATGAACGT AGGACTAGGG GTTGTGATGA GAGGCTTTGT GTCCATTGTG

TGATGAAGGT AGGACTAGGG GTACTGTTGA GAGGCTTTTT GTCCATTGTG

TGATGAAGAT GGCGCTGGGG TTACTGCTCA GAGGTTTTGT ATCTATTGTG

TGATGAAGAT GGCACTGGGA GCACTGCTCA GAGGATTTGT GTCCATCGTG

TGATGAAGAT AGGACTTGGA GTACTGATGA GGGGCTTTGT GTCCCTTGTG

TCGCCCAGAT TGGGATGAGC ATGTTGACAA GAAATGTTGC TGTGATGTTG

TTACCCAGAT TGGGGTGAGC ATGTTGATAA GGAACGTTGC TGTCATGTTA

TTGCCCAGAT TGGGGTGAGC ATATTGATAA GGAATGTTGC TGTCATGCTG

TCATCCAGAT CGGGCTGACC ATGTTGATAA GGAATGTTGC TGTCATGCTA

TGGTGAAGCT AGGACTGGTA GTACTGATGA GGGGCTTTGT GTCCATTGTG

TGATGAAGGT AGGGCTAGGG GTACTGATGA GGGGCTTTGT GTTCATTGTG

TGGTTAAGTT GGGAGTAGGG ATATTTCTGA GGGGTTTTAT GTCCATCGTG

TGATGAAGAT TGGCATTGGG GTACTGATGA GAGCTGCTTT GTCTATTATG

TCATCAAAAT CGGAGCAATC GTTTTAACCA GGAGCTTGCT ACTGATCCTT

CCCATAATTC TTTCACTCTA CTGCTACCCA TATTGTGGTT CCCGTGCCCT

CCCATAATTC TTGCACTCTA CTGCTACCCA TATTGTGGTT CCCGTGTCCT

CCCCTAATTC TTTCACTCTA CTGCTACCCA ---TGTGGTT CCCGTGTTCT

CCCCCAATCA TGCCCCTCTA TTTTTTTCTC TATTGTCACT CCCATGTTCT

CCCCTAATTC TTTCACTCTA CTGCTACCCA TATTGTCATT CCCGTGTTCT

CCCCCAATCC TGCCACTCTA TTGGTTCCCA TACTGTCGGT CCCACGTTCT

CCCCCAATTC TACCTCTCTT TTGGTTCCCA TACTGCCATT CCCATGTCCT

CCCCCAATCA TGCCTCTCTT TTGGTTCCCA TACTGCCATT CCCATGTTCT

CCCCCAATTC TACCTCTCTA TTGGTTCCCA TATTGTCGTT CTCATGTTCT

CCAGTTGTGC TTTTTGTCAA GAGGCTGTCC TTCTGCAGTT CTATGGTCCT

CCAGTGGTGC TCTTTGTTAA GAGGCTGTCC TTCTGCGGTG CAGTGGTCCT

CCGGTTGTGC TGTTTGTCAA GAGGCTGTCC TTCTGCAATT CTATGGTCCT

CCAGTTGTGC TTTTTGTCAA GAGGCTGGTC TTCTGCAGGT CCTTGGTTCT

CCCCCAATTC TGCCACTTTA TTGGTTCCAA TACTGCCATT CCCATGTTCT

CCCCCAATTG TGCCACTCTA TTGGTTTCCA TATTGTCGTT CTCATGTTCT

CCTTTAATTG TGCGCCTCTT TTCATTTTCA TATTGCCATT CTCATACTCT

CCCATAATTG TGAGACTTCA TTGGCTCCCT TACTGCCATT CTCATGTCCT

CCCGCGATCA TCCGCCTGAG ATTCTTCTTC TATTGCCGTC CCCATGTCCT

CTTGCACACA TTTTGCCTCC ATCAAGATGT CATAAAACTC GCCTGTGCTG

CTTGCACACA TTTTGCCTCC ATCAAGATGT CATAAAACTC GCCTGTGCTG

CTTGCGCACA TTTTGCCTTC ACCAAGATGT CATAAAATTT GCCTGTGCTG

TTCACATGCA TTCTGCCTTC ACCAGGATGT CATTAAACTG GCCTGTGCTG

CTTGCACACA TTTTGCCTCC ACCAAGATGT CATGAAACTC GCCTGTGCTG

TTCCCACGCC TTTTGCCTCC ACCAAGATGT CATGAAACTT GCCTGTGCTG

TTCTCATGCC TTCTGCCTCC ACCAAGATGT CATGAAACTC GCATGTGCTG

TTCTCATGCC TTCTGCCTCC ACCAAGATGT CATGAAACTG GCATGTGCTG

TTCCCATGCT TTTTGCCTCC ACCAGGATGT CATGAAACTC GCCTGTGCTG

CTCACATTCT TACTGCTACC ATGTTGATCT CATTCAACTC TCATGCACAG

TTCACATTCT TACTGTTACC ATGTTGACCT CATTCAACTC TCATGCACAG

TTCACATTCT TACTGCTACC ATGTTGATCT CATTCAACTC TCATGCACAG

TTCACATTCC TACTGCTACC ATGTCGATCT TATTCAACTC TCATGCACAG

TTCCCATGCC TTTTGCCTCC ACCAAGATGT CATGAAACTC GCCTGTGCTG

CTCCCATGCT TTCTGTCTCC ACCAAGATGT CATGAAACTT GCTTGTGCTG

CAATCATGCT TTTTGTCTTC ACCAAGAAGT CATGAAACTG GCCTGTGCTG

CTCCCATGCC TTCTGCCTCC ACCAGGATGT CATCAAGTTG GCTTGTGCCA

CTCTCACTCT TTCTGTCTGC ACCAGGATCT GCTCCGCTTG GCTTGTTCTG

ATATCACGTT TAATCACATA TATATTATTC AGACTTCTAC TGTCTTTTTA

ATATCACGTT TAATCACATA TATATTATTC AGACTTCTAC TATCTTTTTA

ATATTACGTT TAATCACATA TATATTATTC TGACTGCTAC TGTGTTTTTA

ATACCACCTT CAACCAACTG TACGTTGTGC TTGTGGTCAT ATTTGTGCTA

ATATTACATT TAATCGCTTA TACGTTATTC TGGTTGCTAC TGTCTTTTTA

ATATTACATT TAATCGCTTA TACGTTATTC TGGTTGCTAC TTTCTTCCTT

ACATTACCTT TAACCGAATA TACGTTGTTT TGGTTGCCAC TTTCTTCCTT

ACATTACTTT TAACCTCATA TATGTTGTTT TGGTTGCTAC TTTCTTCCTC

ATATAACATT TAACCGCATA TACGTGGTTC TGGTTGCTAC TTTCTTCCTA

ACAACAGAAT CAACAGCATC C---TTGGTC TGTTTGC-AC TCTTCTCCTT

ACAACAGAAT CAACAGCATC C---TCGGTC TGTTTGC-AC TTTTCTCCTT

ACAACAGAAT CAACAGCATC C---TTGGTC TGTTTGC-AC TCTTCTCCTT

ATAACAGAAT CAACAGCATT C---TGGGTC TGATTGC-AC TCTTCTCCTT

ATATCACATT TAATAGCTTG TACGTAATTC TGGTTGCTAC TTTCTTCTTA

ATATTACATT TAATCGCCTG TATGTAGCTC TGGTCGCTAC TTTCTTCCTG

ACATAACCTT CAACAGAATC TACGTGATTC TGGTCTCCAC ATTCTTCCTA

ACATCACCTT CAATCGCCTA TACGTAGTTG TGTTTGCCGT TCTAA---TG

ACATTCGGTT CAACAGTTTC TATCTTGGGC TCGTGATCAC TCTCATCTTG

GATGCTCTAA TCATCATCTT TTCTTATATA CTAATCCTCA AGACAGTGAT

GATGCTCTAA TCCTCATCTT TTCTTATATA CTAATCCTCA AGACAGTGAT

GATGCTCTAA TA-------- ----TATATA CTAATCCTTA AGACAGTGAT

GATTCTCTGA TTATCTTCAT CTCCTATGTG TTGATACTCA AGACTGTCCT

GATGCTCTGA TCATTTTCTT TTCTTATGTT CTCATCCTTA AAACAGTTAT

GATGCTCTGA TCATTGTCTT TTCTTATATC TTAATCCTTA AGACAGTTAT

GATGCACTCA TCATTGTCTT TTCCTATGTC CTTATTCTGA AGACAGTTAT

GATGCGCTCA TCATTATCTT TTCCTATGTC CTCATTCTGA AGACAGTTAT

GATGTTCTGA TCATCATCTT TTCTTATATC TTGATCCTTA AGACAGTTAT

GACTGTCCTT GTATCTTGCT CTCCTATGTC CTGATCATCC GATCTGTCCT

GATTGTCCTT GCATCTTGCT CTCCTATGTA CTGATCATCC GGTCTGTTCT

GACTGTCCTT GCATCTTGCT CTCCTATGTT CTGATCATCC AATCTGTCCT

GACTGTCCTT GCATCTTGAT CTCCTATGTC CTTATTATTC GTTCCGTCCT

GATGCGCTGA TTATCCTCTT TTCTTACATT CTAATTCTTA ATACAGTTAT

GATGCTCTAA TTATCCTCAT CTCTTATATT CTAATCCTGA AGCGAGTTAT

GACTCTCTGA TAATTCTCTT CTCCTATATC CTGATTCTTA AGACTGTCAT

GACTTCTTAA TCATCGTCTT CTCTTATGTT CTGATACTCA AGACAGTCAT

GATTCGGTGT TCATTCTGGC CTCCTATGTC CTGATCCTCC ACACCGTGCT

GGGCATTGCG TCTGGACAAG AGGAAGCTAA ATCTCTCAAC ACTTGTGTCT

GGGCATTGCG TCTGGACAAC AGGAAGCTAA ATCTCTCAAC ACTTGTGTCT

GGGCATTGTG TCTGGCCAAG AGCAAGCTAA AGCTCTCAAC ACTCGTGTCT

GAGCATTGCC TCCAGAGAGG AGACGGCTAA GGCTCTCAAT ACCTGTGTCT

AGGCATTGCC TCCAGAGGGG AACAAACTAA GGCTCTCAGC ACTTGTGTCT

GGGCATTGCC TCTGGAGAGG AGCAAGCTAA AGCTCTCAAC ACTTGTGTTT

GGGCATCGCC TCTGGAGAGG AGAGAGCAAA GGCTCTCAAC ACTTGTGTCT

GGGCATTGCC TCTGGAGAGG AGAGAAAAAA GTCTCTCAAC ACCTGTGTTT

GGGCATTGCC TCTGGAGAGG AGCGAGCAAA GGCTCTCAAC ACATGTGTCT

CAGCATCACC TCCATAGAGG GCAGGCAGAA AGCCTTCAAC ACCTGCATTT

CAGCATTGCC TCCTCAGAGG GGCGGCAAAA AGCCTTCAAC ACCTGCATAT

CAACATTGCT TCCTCAGAGG GACGGCAGAA AGCCTTCAAT ACTTGCATAT

TGGCATTGCT TCCTCAGAGG GACGGCGGAA AGCCTTCAAC ACTTGCATAT

GGGCATTGCC TCTGGAGAGG AGCGAGCTAA GGCTCTCAAC ACCTGTGTCT

GGGCATTGCC TCTGGAGAGG AACAAGCCAA GGCCCTCAAC ACCTGTGTCT

GGGGATTGCC TCTGCTGAAG AGAGAGCCAA GGCTCTCAAT ACCTGCATGT

GGGCATTGCC TCTGGGGAGG AACGAGCCAA GGCCCTCAAT ACCTGTGTGT

GGCTGTGGCT GCCCGCGGGG AGAGGCTGAA GTCTCTCCAA ACCTGCACCT

CCCATATTAG CTGTGTCCTA GTATTTCACA TCACTGTGAT GGGACTGTCA

CCCATATTAG CTGTGTCCTA GTATTTCACA TCACTGTGAT GGGACTGTCA

CCCATATTGA CTGT--CCTA GTATTTCACA TCACTGTGAT GGGACTGTCA

CCCATATCTG CTGTGTCCTG GTTTTCTATG TTGCAGTGAT TGGATTATCT

CTCATATTAG CTGTGTCCTG GTATTTTACA TCACTGTGAT CAGCCTGACC

CCCATATTAG CTGTGTCCTG GTGTTTTACA TCACTGTGAT TGGCCTGACT

CCCACATTAG CTGTGTCCTG GTCTTTTACA TCACAGTGAT TGGCCTGACC

CACACATTAG CTGTGTCCTG GTCTTTTACA TCACTGTGAT TGGCCTGACC

CCCATATTAG CTCTGTCCTG GTATTTTATA TCACTGTGAT TGGCCTGAGC

CCCACATCAG TGCTGTTGCC ATCTTCTACA TCCCTCTCAT CAGCCTGTCT

CCCACATCAG TGCTGTTGCC ATCTTTTACA TCCCCCTTGT CAGCTTGTCT

CGCACATCAG TGCTGTTGCC ATCTTCTACA TCCCTCTCAT CAGCTTGTCT

CTCACATCAG TGCTGTTGCT ATCTTCTACA TCCCTCTCAT CAGCTTGTCT

CGCATATTAG CTGTGTCCTA GTCTTTTACA TCACAGTGAT TGGCCTGAGT

CCCATATTAG CTGTGTTCTA GTGTTTTATA TCACTGTGAT TGGCTTGACC

CCCACATATG CTGTGTTCTC GTCTTCTATG TTACTGTGAT TGGTCTGACA

CCCACATCTG CTGCATCCTT GTCTTCTATG TCACTGTGGT TGGCCTGACT

CCCACATCCT CGCCACCATG GTCTTTTACG TTCCCATCAT CGGCCTGACC

TTCATTCACA GGTTTGGGAA ACATGCACCT CATGTGGTCC CCATTACCAT

TTCATTCACA GGTTTGGGAA ACATGCACCT CATGTGGTCC CCATTACCAT

TTCATTCACA GGTTTGG-AA ACATGCACCT CATATGGTCC TCATTACCAT

CTGATCCATC GATTTGGAAA AAATGTTCCA CATGTAGCTC ACATTATCAT

TTCATTCACA GGTTTGGAAA ACATACACCA CATGTGGCCC ACATAACCAT

TTCATCCACA GGTTTGGGAA ACATGCCCCA CATGTGGTTC ATATTACAAT

TTCATTCACA GGTTTGGAAA GCATGCCCCC CATGTGGTGC ACATCACCAT

TTCATTCACA GGTTTGGAAA GCATGCACCC CATGTGGTGC ACATTACCAT

TTCATCCATA GGTTTGGGAA ACATGTGCCA CATGTGGTCC ACATTACCAT

CTTGTCCATC GCTATGGCCA TTCAGCACCT CCCTTTGTCC ACACCATCAT

CTTGTCCATC GCTATGGCCA TTCAGCACCT CCATTTGTCC ACACCATCAT

CTTGTCCATC GCTATGGACA TTCAGCACCT CCATTTGTCC ACACCATCAT

CTTGTCCATC GCTATGGCCA TTCGGCACCT GCATTTGTCC ACACTATTAT

TTCATCCATC GCTTTGGGAA ACATGTACCA CATGTGGTCC ACATTACCAT

TTTATCCACC GGTTTGGAAA ACATGTACCA CGTGTAGTCC ACCTTACCAT

TTCATTCACA GGTTTGGGAA GAATGTGCCA CATGTGGTCC ACATTATCAT

TTCATCCATC GGTTTGGAAA GCATGTGCCA CATGTGGTCC ATATCACAAT

ATGGTTCACC GTTTCGGAAA GCATCTGAGT CCTCTGGTCC ATGTGCTCAT

GAGCTATGTC CATTTTCTCT TTCCTCCATT CGTGAATCCT ATCATTTATA

GAGCTATGTC CATTTTCTCT TTCCTCCATT CGTGAATCCT ATCATTTATA

GAGCTATGTC CATTTTCTCT TTCCTCCATT CATGAATCCT ATCATTTACA

GAGCTATGTC TACTTTGTCT TCCTCCCATT TATAAATCCA ATAATCTATA

GAGCTATGTC CACTTTCTCT TCCCTCCCTT CGTGAATCCC ATTATTTACA

GAGCTATGTT TATTTTCTAT TTCCTCCATT CATGAACCCC ATTATATACA

GAGCTATGTC TACTTTCTCT TTCCTCCATT TATGAACCCC ATCATTTATA

GAGCTATGTC TACTTTCTCT TTCCTCCATT TATGAACCCC ATCATTTACA

GAGCTATGTC TACTTTCTCT TTCCTCCATT CATGAACCCC ATTATATACA

GGCCAATATC TTCCTGCTTA TTCCTCCTGT ACTCAACCCT ATCATCTACA

GGCCAATGTC TTCCTTCTCA TCCCTCCCGT GCTCAACCCC ATCATCTATA

GGCCAATGTC TTCCTGCTCA TCCCTCCTGT GCTCAACCCT ATCATCTACA

GGCCAATGTT TTCCTGCTTA TTCCTCCTGT GCTCAACCCT ATAATCTATA

GAGTTATGTC TACTTTCTTT TTCCTCCATT CATGAATCCT ATTATATACA

GAGCTATTTC TACCTTCTCT TTCCTCCATT CATGAACCCT ATTATATACA

GAGCTATGTC TACTTCCTCT TTCCGCCCTT AATGAATCCT ATCATCTATA

GAGCTATGTC TACTTCCTTT TCCCCCCATT AATGAATCCC GTTATCTATA

GGGCCACATC TACATTCTTT TCCCTCCCCT GATGAACCCC ATCATCTACA

GCATCAAGAC CAAGCAGATT CAAAGAAGCA TTAGCAACTC AACATCTAAA

GCATCAAGAC CAAGCAGATT CAAAGAAGCA TTAGCAACTC AACATCTAAA

GCATCAAGAC CAAGCAGATT CAAAGAAGCA TTGGAAATGC AGCAGTCACG

GTGTTAAGAC CAAGCAGATA CAGAATGGCA TCGGAAACGC AGCAGCCACG

GCATCAAGAC CAAGCAGATT CAAGGAAGCA TTAGCAACTT GACATCTAAA

GCATCAAGAC CAAGCAGATT CGAAAAAGCC TCAGCAATTT GACATCTAAA

GCATCAAGAC CAAGCAGATT CAGAGGAGCA TTAGCAACTT CACATCGAAA

GCATCAAGAC CAAGCAAATT CAGAGGAGTG TTAGCAACTT CACATCGCAA

GCATCAAGAC GAAACAGATT CAGAAAAGCA TCAATAACAC CACATCATCT

GTGTGAAGAC TAAGCAGATT CAAAAGGCTG TTAACAATAC CAGTGCTCAG

GTGTGAAGAC TAAGCAGATT CGAAAGGCTA TTAATAATAC CAATGCTCAA

GTGTGAAGAC GAAGCAGATT CGAAAGGTTA TTAATAATAC CAATGATCAG

GTGTGAAGAC TAAACAGATT CGAAAGGCGA TTAATAATAC CAATGCTCAG

GCATCAAGAC CAAGCAGATT CAGAGGAGCA TCATCAACTT GACAGCTACA

GTATCAAGAC CAAGCAGATT CAGAGAAGCA TTAGCAACTT GACATCTCAA

GCATCAAGAC CAAGCAAATT CAAAGTGGCA TTAACAACTT GACATCTCCA

GTGTCAAGAC TAAGCAAATA CAGACTGGTT TTAGCAATCT GACATCCCCA

GTATCAAAAC CCAGCAGATC CGCAAAAGGA T-AACACCAC CCAATCCCAC

TTTCCAACCT TCTTGTTGAC CGGCATTCCT GGCCTATCTG CCCATGTCTG

TTTCCAACCT TCTTGTTGAC CGGCATTCCT GGCCTATCGG CCCATGTCTG

TTTCCTACCT TCTTGCTGAG TGGCATTCCT GGGTTGCACG TGCACATCTG

TTTCCTACCT TCTTGCTGAG TGGCATTCCT GGGTTGCACG TGCACATCTG

TTTCCAACCT TCTTGTTGAC CAGCATTCCT GGCCTATCTG CCCATGCCTG

TTTCCAACCT TCTTGTTGAC TTGCATTCCT GGCCTATCTG TCCATGCCTG

TTGACAACCT TCTTGTTGAC TGGCTTTCCT GGCTTATCTG CTCATGGCTG

TTACCAACCT TCTTGTTGAC TGGCATTCCT GGCCTATCTG CTCACAGCTG

TCCTCAAACT TCCTCCTCAC TGCCTTCCCT GGGCTGCTTG CTCATGTTTG

CCTCTGACCT TCCTCTTGAC GGGTATTCCA GGCCTGGCGG CCAAGATCTG

CCTCTGACCT TCTTCCTGAC GGGTATCCCA GGCCTGGCAG CCCAGGTTTG

CCTCTGACAT TCCTCCTGAC GGGTATCCCA GGCCTGGCAG CCCAGGTGTG

ACTCTGACCT TCTTCCTGAC GGGTATCCCA GGCCTGGCAG CCCAGGTCTG

TTTCCAAGCT TCCTGTTGAC TGGCATTCCT GGCCTATCTG TCCATGCCCT

TTTTCAACCT TCTTGCTGAC CAGTATTCCT GGCCTATCTC TCCATTCTTG

TTTCCAACCT TCTTGCTGAC TGGCATTCCT GGTCTATCTG TCCAAGCCTG

TTTTCAACTT TTCATCTGAC AGGCATTCCA GGAATTGAGG CACATGCCTG

CCCTCCTCCT TCCTCCTGCT GGGGGTACCA GATCTGGCTG CACACAACTG

GATCTCCATT CCTTTCTGTT GTTTTTATGC CATTGCCCTC TCTGGGAACA

GATCTCCATT CCTTTCTGTT GTTTTTATGC CATTGCCCTC TCTGGGAACA

GATCTCCATC CCACTGTGCT TCATGTACCT GGTTTCCATC CTGGGCAACT

GATCTCCATC CCACTGTGCT TCATGTACCT GGTTTCCATC CTGGGCAACT

GATCTCCATT CCTTTCTGTT GTTTTTATGC CATTGCCCTC TCTGGGAACA

GATGTCCATT CCCTTCTGTT GTCTTTATGC CATTGCCCTC TCTGGGAACA

GATCTCCATC CCTTTCTGTT GTCTTTATGC CACTGCCCTC TCTGGCAACA

GATCTCCATC CCTTTCTGTT GTCTTTATGC CACTGCCCTC TCTGGCAACA

GATTTCCATT CCTGTCTGCT GTCTCTACAC CATTGCCCTC TTGGGAAACA

GGTCTCCATC CCTGTTTGCC TCCTGTATGC CGGCGCCCTC TCTGGGAACA

GATCTCCATC CCTTTTTGTC TCCTGTATGT CATCGCCCTC TCTGGGAACA

GATCTCCATT CCTATTTGTC TCCTGTATGT CACTGCCCTG TCTGGGAACA

GATCTCCATC CCGTTCTGCC TCCTATATGG CATTGCCCTC ACTGGAAACA

GATCTCCATT CCTTTCTGCT GTGTCTATGC CATTGCCCTC ACAGGGAATG

GCTCTCCATT CCATTTTGTT GTCTCTATGG CATTGCCCTG TCTGGGAACA

GTTCTCCATC CCATTCTGTT GTCTCTACAC CATTGCCCTC TCGGGGAACA

GATCTCCATT CCTTTCTGCT GTCTCTATAC CATTGCCCTA TTGGGAAATA

GCTGAGCTTC CCCTTCTGCT CCGTGTACCT GATCGCTCTC ATGGGTAACT

GCGTGATCCT GTTTGTCATC ATTACCCAGC AGAGTCTCCA TGAACCCATG

GTGTGATCCT GTTTGTCATC ATTACCCAGC AGAGTCTCCA TGAACCCATG

GCACAATTCT TTTCATCATT A-----AAAC AGAGTCTCCA TGAACCTATG

GCACAATTCT TTTCATCATT A-----AAAC AGAGTCTCCA TGAACCTATG

CTATGATCCT GTTCATCATC ATTACCCAGC AGAGTCTCCA TGAACCCATG

GCATGATCCT GTTTGTCATC ATTACCCAGC AGAGTCTCCG TGAACCCATG

GCATGATCCT CTTTATCATT GTGACCCAGC ACAGTCTGCA TGAACCGATG

GCATGATCCT CTTCATCATT GTGACCCAGC ATAGTCTGCA TGAACCTATG

GCATGATCTT GTTTGTCATC ATCACTGAGC GGAGACTGCA CAAGCCCATG

GTGTGATCCT GTTTGTGGTC CTCCGTGAAC AGAGCCTTCA TGAGCCCATG

GCATGATCCT ATTTGTGGTC TTCCGTGAGC AGAATCTCCA TGAACCAATG

GCATGATCCT GTTTATGGTC ATTCATGAGC AGAGCCTCCA TGAGACCATG

GCATGATCCT GTTTGTGGTC CTCCGTGAGC AGAGTCTCCA TGAGCCCATG

GCATGATCCT GTTTGTCATC ATTACTCAGC AGAGTCTCCA TGAGCCCATG

GCATGGTCCT GTTTGTCATC CTCACTCAGC GGAGTCTGCA TGAGCCCATG

GCATGATCCT GTATGTCATC ATCACCCAGA AGAGTCTCCA CGAGCCCATG

GCATGATACT GTTTGTCATT ATCACAGAAC AGAGCCTCCA TGAGCCAATG

GCACCATCCT GTTTATGATC AAGACTGAGC AGAGCCTCCA CCAACCCATG

TATTATTTCC TCTTCAGGCT ATCAGCCACT GATCTGGGCT TGACTGTTTC

TATTATTTCC TCTCCATGCT ATCAGCCACT GATCTGGGCT TGACTGTTTC

TATCTCTTCT TGTCCATGCT GGCTCTGATT GACCTGGGTC TGTCCCTTTG

TATCTCTTCT TGTCCATGCT GGCTCTGATT GACCTGGGTC TGTCCCTTTG

TATTATTTTC TCTCCATGCT GTCAGCCACT GATCTGGGCT TGACTGTTTC

TACTATTTCC TCTCCATGCT GTCAGCCACT GATCTGGGCC TGACTGTTTC

TACTATTTCC TCTCTATGCT CTCTGCCACT GACCTGGGTT TGACTATTTC

TACTATTTCC TCTCTGTGCT CTCTGCCACT GACCTGGGTT TGACTTTTTC

TACTATTTCC TGTCCATGCT CTCAGCTGTT GATCTGTGTC TGACCATCAC

TATTATTTCC TCTCTATGCT TTCAGCCACA GACCTGAGCT TATCCCTGTG

TATTATTTCC TCTCTATGCT TTCAGCCACA GACCTGAGCT TATCCCTGTG

TATTATTTCC TTTCTATGCT GTCAGCCACA GACCTGAGCT TATCCCTGTG

TATTATTTCC TCTCTATGCT TTCAACCACA GATCTGAGCT TGTGTCTGTG

TACTATTTCC TCTCCATACT GTCAGCTGCT GACCTGGGCT TGACTGTTTC

TACTATTTCC TCTCCATACT GTCAACTACT GACCTGGGCC TGACTGTTTC

TACTATTTCC TCTCCATGCT GTCGACTACT GACCTGGGCT TGACAGTTTC

TACTTTTTCC TTTCTATGCT GTCAGCTACT GACTTGGGCT TGACTGTTTC

TTCTACTTCC TGGCCATGCT GGCCACCGTT GATCTGGGCT TGTCCTCGGC

TTCATTGTCA ACAACATTAG GTATCCTCTG GTTTGAGGCA CGTGAAATCA

TTCATTGTCA ACAACATTAG GTATCCTCTG GTTTGAGGCA CGTGAAATCA

CACTCTCCCT ACAGTCCTCA GCATCTTTTG GGTTGGGGCA CGAGAAATTA

CACTCTCCCT ACAGTCCTCA GCATCTTTTG GGTTGGGGCA CGAGAAATTA

TACATTGTCA ACAACATTAG GTATCCTTTG GTTTGATGCC CGTGAAATTA

TACAATGTCA ACAACACTAG GTATCCTGTG GTTTGATGCA CGTGAGATCA

TACAATGTCA ACCACCTTGA GAATCCTGTG GTTTCAAGCA AACGAAATCA

TACAATGTCA ACTACCTTGA GAATCCTGTG GTTTCAGGCA AATGAAATCA

AACCCTTCCC ACGGTGCTTG GGGTTCTCTG GTTTCATGCC AGGGAGATCA

CACACTTTCC ACTACCCTTG GTGTCTTCTG GTTTGAAGCC CGAGAGATCA

CACACTTTCT ACTACCCTTG GTGTCTTCTG GTTTGAAGCT CGAGAAATCA

CACACTTGCC ACTACTCTTG GTGTGTTCTG GTTTGAAGCC CGAAAGATCA

CACGCTTTCC ACTACTCTGG GCGTCTTCTG GTTTGAAGCT AAAGCGATCA

TACAATGTCA ACAACATTAG GCATCTTCTG GTTTGATGCA AGTGAAATCA

TACCATGTCA ACAACACTAG GTGTCCTATG GTTTAATGCA AGCGAAATCA

TACAATGTCG ACAACATTAT CAATTCTGTG GTTTGAGGCA AATCAAATTA

CACAATGTCA ACAACACTAA GTGTCCTTTG GTTTGATGCC AGAGATATCA

CACCATCCCC AAGATGTTGG GCATCTTCTG GCTCCACCTG CAGGAGATCA

GTAGCTGCAT TGTCCAGATG TTTTTTCTTC ATGGATTCAC TTTTATGGAA

GTAGCTGCAT TGTCCAGATG -TTTTTAATC ATGGATTCAC TTTTATGGAA

GCGCCTGCTT TGCTCAGCTC TTTTTCATTC ACTGTTTATC CTTCCTCGAG

GCGCCTGCTT TGCTCAGCTC TTTTTCATTC ACTGCTTATC CTTCCTCGAG

GTAGCTGCAT TGTCCAGATG TTTTTTCTTC ATGGATTCGC TTTTATGGAA

GTAGCTGCAT TGTCCAGATG TTTTTTCTTC ATGGATTCAC TTTCCTGGAA

GTTT-TGCAT TGTTCAGATG TTTTTTCTCC ATGGATTCAC ATGTATCGAA

GTTT-TGCAT TGTTCAGATG TTTTTTCTCC ATGGATTCAC ATTTATAGAA

GTCTCTGTGT GTTTCAAATG TTTCTAATTC ACACTTTTTC CTTCACGGAA

ACGCCTGTGT TGCTCAGATG TTCTTTCTCC ATGGATTTAC TTTCATGGAG

CCGCCTGCAT TGCCCAGATG TTCTTTCTCC ATGGATTTAC TTTCATGGAG

ACGCTTGTAT TACGCAGATG TTCTTTCTCC ATGCATTTAC TTTCATGGAG

ACTC------ -------CTC TTCTTCATCC ACACTTTGTC TCTTGTGGAG

GTACCTGTAT TATTCAGATG TTTTTTCTTC ATGGATTCAC TGTCATGGAG

GT--CTGCTT TGCTCAAGAA TTCTTCATCC ATGGATTCAC AGATATGGAA

GT--CTGCTT TGCTCAGGAA TTCTTCATCC ATGGATTCAC CGATATGGAG

CCGGCTGTAT TGTCCAGATG TTCTTCCTTC ATGGGTTCAC TTTCATGGAG

GC--CTGCCT AACCCAGATG TTCTTCATCC ACATGTTCAC CGGCATGGAG

TCTGGAGTGC TGGTGGCTAC AGCCTTTGAC CGTTATGTGG CCATCTGTGA

TCTGGAGTGC TGGTGGCTAC AGCTTTTGAC CGCTTTGTTG CCATTTGTGA

TCCTCTGTGC TACTGTCTAT GGCCTTTGAC CGCTTTGTGG CTATCTGCTG

TCCTCTGTGC TACTGTCTAT GGCCTTTGAC CGCTTTGTGG CTATCTGCTG

TCTGGGGTGC TGGTGGCTAT GGCCTTTGAC CGTTATGTGG CTATTTGTGA

TCTGGGGTGC TGGTGGCCAT GGCCTTTGAC TGTTATGTGG CAATCTGTGA

TCTGGGGTGC TAGTGGCTAT GGCTTTTGAC CGTTATGTAG CAATCTGCAA

TCTGGAGTGC TAGTGGCTAT GGCTTTTGAT CGTTATGTAG CGATCTGCAA

TCATCTGTGC TCTTGGCCAT GGCTCTTGAC CGGTATGTGG CTATCTGTCA

TCTGGGGTTT TGCTGGCCAT GGCCTTTGAC CGTTTTGTGG CCATCTGTGA

TCTGGGGTTC TGCTGGCCAT GGCCTTTGAT CGTTTTGTAG CCATCTGTAA

TCTGGGGTTC TGCTGGCCAT GGCCTTTGAT CGTTTTGTGG CCATCTGTGA

TCTTCGGTTC TATTATCCAT GTCTATCGAC CGCTATGTTG CCATCTGCAA

TCTGGGGTGC TCGTGGCCAT GGCCTTTGAC CGATATGTGG CAATCTGTGA

TCCTCAGTTC TTCTGCTCAT GTCCTTTGAC CGCTTTTTAG CCATACACAA

TCCTCGGTTC TTCTGCTTAT GTCCTTTGAT CGCTTTTTGG TCATACACAA

TCTGGGGTCC TGGTGGCCAT GGCCTTTGAT CGATTTGTGG CTATCTGTGA

AGCATGATGC TCGTGGCCAT GGGCTATGAC CGCTACGTGG CCATCTGCAA

CCCTCTGAGG TACACTACCA TTCTCACTAA TTCCAGAATC ATTCAAATGG

TCCTCTGAGG TACACTACCA TTCTCACTAA TTCCAGAATC ATTCAAATGG

CCCCTTGCAC TATGCTTCCA TTCTCACCAA ---CACAGTC ATTGGCAGGA

CCCCTTGCAC TATGCTTCCA TTCTCACCAA ---CACAGTC ATTGGCAGGA

TCCTCTGAGG TACACTACCA TTCTCACTAA TTCCAGAATC ATTCAGATGG

TCCTCTGAGG TACTCTGCCA TTCTCACGAA TTCCAGAATC ATTCAGATGG

CCCTCTTAGA TATACCATGA TTCTTACTAA TTCTAGAATC ATTCAGATGG

CCCTCTTAGA TATACCATGA TTCTTACTAA TTCTAGAATC ATTCAGATGG

CCCTCTGCGC TATGCTACCA TTCTCAC--- -TCCAAAATC ATCAAGATTG

TCCACTGAGA TACACCACCA TCCTCACCAA TGCCAGGATC GCCCAGATTG

TCCACTGAGA TACACCACCA TCCTCACCAA TGCCAGAATT ACCCAGATTG

CCCACTGAGA TATACCACAA TCCTCACCAA TGCCAGGATT GCCCAGATTG

CCCGCTGCGT TATTCCACCG TCTTGACCAA TGCCAGGATC ATCCAGATCG

TCCCCTGAGG TACGCCAATA TCCTCACAAA TTCCAGAATC ATTCAGATGG

CCCTCTGAGA TACAGCTGTA TCCTGACCAG TGCTAGAGTT GCCAAAATAG

CCCACTGAGA TACAATTCTA TCCTGACCAG TGCCAGAGTG GCCAAAATAG

CCCTTTGAGA TATACCACTA TCCTCACCAA TGCTAGGATT ACACAATTAG

GCCTCTCCGC TACTGCACCA TCCTTACCAA TATTAGGGTT ATCC---TAG

GTCTTCTGAT GATTACACGT GCTATAGTAC TAATATTGCC ACTACTTTTG

GTCTTCTGAT GATTACACGT GCTATAGTAC TAATATTACC ACTACTTTTG

TTGGCCTGAT CTCTCTGCGT AGTGTAGCAC TTATTTTTCC ATTGCCTTTT

TTGGCCTGAT CTCTCTGCGT AGTGTAGCAC TTATTTTTCC ATTGCCTTTT

GTCTCCTAAT TATTACACGT GCAATAGTAC TAATGGTGCC ACTACTTTTG

GCATCGTGAT GATTTTACGT ACTGTGGTAC TAATAGTTCC ATTGCTCTTG

GTTTCCTAGT GCTGATGCGC CCTCTACTAT TAATAGTTCC CCTACTTTTG

GTTTCCTAGT GATAATGCGT GCTCTGTTAT TAATAGTCCC ACTGCTTCTG

GGATTGCAGT GCTCAGGAGT GCTATTCCAC TAGT------ CCCACTTCTG

GGATGAGCAT GTTGACAAGA AATGTTGCTG TGATGTTGCC AGTTGTGCTT

GGGTGAGCAT GTTGATAAGG AACGTTGCTG TCATGTTACC AGTGGTGCTC

GGGTGAGCAT ATTGATAAGG AATGTTGCTG TCATGCTGCC GGTTGTGCTG

GGCTGACCAT GTTGATAAGG AATGTTGCTG TCATGCTACC AGTTGTGCTT

GTATCTTGAT GATTATACGT ACTGTGCTTT TAATAGTACC ACTTCTTTTG

GTCTGGTATT TCTCATTAAA AGTATGTTCT TAGTGCTTCC ATTTCCTTTC

GGCTGGTATT TCTTATTAAA AGTATGCTAT TGGTGCTCCC ATTTCCTTTC

GTGTGACAAT GGTCATTCGT ATGATAGTTT TAATGTTGCC CTTGCTCTTA

GGTT---AGC TGTCCTGAGG AATTTCTCTC TGATAATTCC AATGGTGTTT

CTCCTTAAGC CTCTCTATTT CTGTAGAATG AATGCCCTTT CTCACTCCTA

CTCCTTAAGC CTCTCTATTT CTGTAGAATG AATGCCCTTT CTCACTCCTA

ATGCTCAAAA GATTCACCTA TTGTGGCTCC CCAGTTCTCT CACATTCTTA

ATGCTCAAAA GATTCACCTA TTGTGGCTCC CCAGTTCTCT CACATTCTTA

CTCTTTAAGC CTCTCTATTT CTGTAGAATG AATGCCCTTT CCCACTCCTA

CTGCTTAAGC CTCTCTATTT CTGCAAAATG AATGTCCTGT CCCACTCCTA

CTCCTTACAC CTATCTATTT CTGTAAAAGG AAGGCCCTTT CCCACTCCTA

CTCCTTAAAC CTGTCTCTTT CTGTAAAAGG AATACCCTCT CCCACTCCTA

GTCCGCTTGA CCTTCTTTTT TTGTCACTCT CATGTCCTTT CCCATTCCTA

TTTGTCAAGA GGCTGTCCTT CTGCAGTTCT ATGGTCCTCT CACATTCTTA

TTTGTTAAGA GGCTGTCCTT CTGCGGTGCA GTGGTCCTTT CACATTCTTA

TTTGTCAAGA GGCTGTCCTT CTGCAATTCT ATGGTCCTTT CACATTCTTA

TTTGTCAAGA GGCTGGTCTT CTGCAGGTCC TTGGTTCTTT CACATTCCTA

CTCCTTAAGC AACTCTCTTT CTGTAGAGCT AAGGTCCTTT CCCACTCCTA

ACTCTGAAAA GGTTGACATA TTGTAGGAAA AGCCTACTTT CTCACTGTTA

ACTCTGAGAA GATTAATATA TTGTAGGAAA AGCCTACTTT CCCATTGTTA

TTATTTAAGA AGCTGTCATT CTGTGGTCCT AATGGCCTTT CTCACTCCTA

TTTCTCTTGA GGTTGCCCT- --GTGGGCAC CGAATCATCC CCCACACCTA

TTGTTACCAT CCAGATGTGA TTCAATTAGC ATGTTCAGAC ATTCGGGCAA

TTGTTACCAT CCAGATGTGA TTCAATTAGC ATGTTCAGAC ATTCGGGCAA

TTGTCTCCAC CAAGAAGTGA TGAAATTGGC CTGTGCCAAT ATGAAGGCCA

TTGTCTCCAC CAAGAAGTGA TGAAATTGGC CTGTGCCAAT ATGAAGGCCA

CTGTTACCAT CCAGACGTGA TTCAATTAGC ATGTTCAGAC ATTCGGGCTA

CTGTTACCAT CCAGATGTGA TTAAATTGGC ATGTTCAGAC ATTCGGGCTA

CTGTTATCAT CCAGATGTGA TTAAGTTAGC ATGTTCAGAC ACTTGGGCCA

CTGTTATCAT CCAGATGTGA TTAAGTTAGC ATGTTCAGAC ACTCGAGCCA

CTGTCTGCAC CAGGACATTA TCCACCTTGC CTGTGCTGAC ATTAAGTTTA

CTGCTACCAT GTTGATCTCA TTCAACTCTC ATGCACAGAC AACAGAATCA

CTGTTACCAT GTTGACCTCA TTCAACTCTC ATGCACAGAC AACAGAATCA

CTGCTACCAT GTTGATCTCA TTCAACTCTC ATGCACAGAC AACAGAATCA

CTGCTACCAT GTCGATCTTA TTCAACTCTC ATGCACAGAT AACAGAATCA

CTGTTACCAT CCAGATGTAA TTAAATTATC ATGTTCAGAC ACTCGGGCCA

CTGCTACCAC CCCGATGTTA TGAAGTTGGC TTGTGGCCCT GTCAGAGTCA

CTGCTACCAT CCTGATGTTA TGAAATTGGC CTGTGGGCCT GTAAGAATCA

CTGTTACCAC CCTGATGTGA TTAAGTTGTC ATGCTCAGAT ACCAAAGTCA

TTGTGAGCAC ATGGGCATTG CCCGGTTGGC ATGTGCAAGT ATCAAACTCG

ATAGCATCTG TGGATTAATT GATCTCATCC TGACCACTAT AGATACACCA

ATAGCATCTG TGGATTAATT GATCTCATCC TGACCACTAT AGATACACCA

ACAGCATCTA TGGCATGTTT GTCATCGTCT CTACAGTGAT AGACTCACTG

ACAGCATCTA TGGCATGTTT GTCATCGTCT CTACAGTGAT AGACTCACTG

ACAGCATCTG TGGATTAATT GATCTTATCC TGACCACTAT AGATATGCCA

ACAGCATCTG TGGGTTAATC GATCTCATTC TGGCCACAAT AGATACACCA

ATAGCATCTG TGGACTAGTT GATCTCATTC TGACCACAGT AGATACTCCA

ATAGCATCTG TGGACTAGTT GATCTCATTC TGACCACAAT AGATACTCCA

ATGTTATATA CGGGCTGGTT GTGATCATTA TGTTGTGGTT GGACTCTTTG

ACAGCATCCT TGGTCTGTTT GCACTCTTCT CCACGACATT TGACTGTCCT

ACAGCATCCT CGGTCTGTTT GCACTTTTCT CCACAACATT TGATTGTCCT

ACAGCATCCT TGGTCTGTTT GCACTCTTCT CCACAACATT TGACTGTCCT

ACAGCATTCT GGGTCTGATT GCACTCTTCT CCACAACATT TGACTGTCCT

ACAGCATCTG TGGATTAATT GGGCTCGTCC TGACCACAAT AGATACGCCA

ATGGCATCTA TGGCTTATGC CTTGTCCTCT GCTCCTTTGT TGACTCTACA

ATATAATCTA TGGGTTGTGC CTTGTTCTCT GCTCCTTTGT TGACTCTGTG

ATAGTATATG TGGGTTAGCA GCTCTCATCC TGAGCACAAT TGACACACCA

ACATCATTTA TGGTTTATGT GTCATCTGCC TTATTCTTTT GGACATACTG

TGCATTGTCC TGTCATATAT CTTAATTATT CACTCTGTCC TCAGAATTGC

TGCATTGTCC TGTCATATAT CTTAATTATT CACTCTGTCC TCAGAATTGC

CTCATCCTCT TCTCTTACGC TCTGATCCTG CACACCGTGC TGTCCATCGC

CTCATCCTCT TCTCTTACGC TCTGATCCTG CACACCGTGC TGTCCGTCAC

TGCATTGTCC TGTCGTATAT CTTAATTATT TACTCTGTCC TCAGAATTGC

TGCATTGTTC TGTCTTATAT CTTAATTATT CACTCTGTCC TGAGTATTGC

TGCATTGTCT TGTCTTATGT ACTGATCATT CGCTCTGTCT TCACTATTGC

TGCATTGTCT TATCTTATAT ACTGATCATT CGCTCTGTCC TCAGTATCGC

GGTATTTTTG TGTCTTATGT TTTTATCCTT CACTCAGTGT TAAGAATTGC

TGTATCTTGC TCTCCTATGT CCTGATCATC CGATCTGTCC TCAGCATCAC

TGCATCTTGC TCTCCTATGT ACTGATCATC CGGTCTGTTC TCAGCATTGC

TGCATCTTGC TCTCCTATGT TCTGATCATC CAATCTGTCC TCAACATTGC

TGCATCTTGA TCTCCTATGT CCTTATTATT CGTTCCGTCC TTGGCATTGC

TGCATTGTCC TGTCTTACAT TCTGATTATT CGCTCTGTCC TCAATATCAC

TTCATTATAA TTTCATACAT CCTAATTTTG AAAACAGTGC TGGGTATTGC

TTCATTATAA TTTCATACAT TCTGATTTTG AAAACAGTGC TGGGTATTGC

TGCATTGTTC TGTCTTACAT CCTGATCATT CGCTCTGTCC TCAGTATAGC

CTGATTGCCC TCTCCTACAT CAGGATCCTC CACACCATCT TCAGACTGCC

CTCCCCTGAA GAATGGCACA AGGTCTTCAG CACCTGTGTC TCCCATGTGG

CTCCCCTGAA GAATGGCACA AGGTCTTCAG CACCTGTGTC TCCCACGTGG

CTCCAGGGCT GAGAGACTCA AGGCTCTTAA CACCTGTGTT TCCCACATCT

CTCCAGGGCT GAGAGACTCA AGGCTCTTAA CACCTGTGTT TCCCACATCT

CTCCCCTGAA GAACGGCACA AGGTCTTCAG CACCTGTGTC TCCCACATGG

CTCCCCTGAA GAACGGCATA AGGTCCTCAG CACCTGTGTC TCCCACATTG

CTCCTCTGAA GGACGGCACA AGGCCTTCAG CACCTGTGTG TCCCACATTG

CTCCTCTGAA GAAAGGCACA AGACCTTCAG CACCTGTGTG TCCCACATTG

ATCCCGTGAG GGGAGGCTCA AGGCCCTCAA CACGTGTGCA TGCCACATCT

CTCCATAGAG GGCAGGCAGA AAGCCTTCAA CACCTGCATT TCCCACATCA

CTCCTCAGAG GGGCGGCAAA AAGCCTTCAA CACCTGCATA TCCCACATCA

TTCCTCAGAG GGACGGCAGA AAGCCTTCAA TACTTGCATA TCGCACATCA

TTCCTCAGAG GGACGGCGGA AAGCCTTCAA CACTTGCATA TCTCACATCA

CTCCCCTGAA GAGCGGCACA AGGTCTTTAG CACCTGTATC TCCCACATTG

CTCAAAAAGT GGTCAGCTCA AGGCACTTAA TACTTGTGTT TCCCACATC-

CTCAAAAGAT GGTCAGGTCA AGGCACTTAA TACGTGTGTT TCCCACATCT

ATCTCCCACA GAGCAGAAGA AGGCATTCAG CACCTGTGGG TCTCACATAG

CTCTCAGGAC GCACGACTCA AAGCCATAAA CACTTGCATC TCCCACATCT

GAGCAGTTGC TTTCTTCTAC ATCCACATGC TGAGCCTGTC CTTGGTGTAT

GAGCAGTTGC TTTCTTCTAC ATCCACATGC TGAGCCTGTC CTTGGTGTAT

GTGCTGTGCT GCTCTTCTAC GCTCCCATGA TTGGCCTCTC TGTCATCCAT

GTGCTGTGCT ACTCTTCTAC ACTCCCATGA TTGGCCTCTC TGTCATCCAT

GAGCAGTTGC TATCTTCTAC ATCCCCATGC TGAGCCTGTC CTTGGTGCAT

GAGCAGTTGC TATTTTCTAT ATTCCCATGT TGAGCTTGTC CTTGGTACAT

GAGCAGTTGC TGTTTTCTAC ATCCCGATGT TTAGCCTGTC TCTGGTGCAT

GAGCAGTTGC AGTTTTCTAC ATCCCAATGT TTAGCCTGTC TCTGGTACAT

GTGCTGTTCT CATTTTATAT GTGCCTATGA TAGGAGTATC TCTTGTCCAT

GTGCTGTTGC CATCTTCTAC ATCCCTCTCA TCAGCCTGTC TCTTGTCCAT

GTGCTGTTGC CATCTTTTAC ATCCCCCTTG TCAGCTTGTC TCTTGTCCAT

GTGCTGTTGC CATCTTCTAC ATCCCTCTCA TCAGCTTGTC TCTTGTCCAT

GTGCTGTTGC TATCTTCTAC ATCCCTCTCA TCAGCTTGTC TCTTGTCCAT

GAGCAGTTGC TGTTTTCTAC ATCCCCATGA TGAGCCTGTC CTTGCTGCAT

TTACTGTCTT CATCTTTTAT GTGC------ TGAGTGTGTC TATGACTCAC

TTACTGTCTT CATCTTCTAT GTGCCCCTCA TCAGTCTGTC TCTTGTTCAT

GGGCTGTTTC CATCTTTTAT ATCCCCATGA TTAGCCTATC CCTAACACAT

GTGTCATCCT GGCCTTCTTC ACCCCCC--- TCTTCTCCTT CCTCACCCAC

CGCTATGGTC GGTCAGCCCC CAGAGTAGTC CATTCAGTGA TGGCTAATGT

CGCTATGGTC TGTCAGCCCC CAGAGTAGTC CATTCAGTGA TGGCTAATGT

CGCTTTGGAA AGCAGGCACC CACCCTGGTC CAGGTGGTCA TGGGTTTCAT

CGCTTTGGAA AGCAGGCACC CACCCTGGTC CAGGTGGTCA TGGGTTTCAT

CGCTATGGTC ACTCAGCCCC CAGAGCGGTC CATTCAGTGA TGGCCAATGT

CGCTATGGTC GGTCAGCTCC CAAAGTGGTC CATTCAATGA TGGCCAATGT

CGCTACGGTC GCTCAGCGCC CAAAGTAGTC CATGCAATGA TGGCCAATGT

CGATATGGTC GGTCAGCACC CAAGGTAGTC CATACAATGA TGGCCAATGT

CGTTTTGCCA AACACTCTTC CCCGTTTGTC CATATCTTTA TGGCACATAT

CGCTATGGCC ATTCAGCACC TCCCTTTGTC CACACCATCA TGGCCAATAT

CGCTATGGCC ATTCAGCACC TCCATTTGTC CACACCATCA TGGCCAATGT

CGCTATGGAC ATTCAGCACC TCCATTTGTC CACACCATCA TGGCCAATGT

CGCTATGGCC ATTCGGCACC TGCATTTGTC CACACTATTA TGGCCAATGT

CGCTATGGTC AGTCAGTCCC CAAAGTAGTC CACTCAATGA TGGCTAATAT

CGCTTTGCCA AGCATGCCTC TCCCCTTGTT CATGTCATGA TGTCTACTGT

CGTTATGGTC ATTCTGCGCC TCCCTTTGTT CACACCCTCA TGGCCAATGT

CGCTATGGTC AGATGGCTCC TAAGATTGTT CATACAATGA TGGCCAACAT

CGCTTTGGAC AACAAAATCC CTGGCTAGTC CACATCCTCC TGGCCAACCT

ATACCTGCTT TTACCCCCTG TGCTCAACCC CATCATCGAC AGTGTAAAAA

ATACCTGCTT TTACCCCCTG TGCTCAACCC CATCATCTAC AGTGTAAAAA

GTATCTTCTC TTTCCTCCTG TGATGAATCC CATTGTCTAC AGTGTGAAGA

GTATCTTCTC TTTCCTCCCA TGATGAATCC CATTGTCTAC AGTGTGAAGA

ATACCTGCTT TTGCCCCCTG TGCTCAACCC CATCATCTAC AGTGTAAAAA

GTACTTGCTT TTGCCCCCTG TGCTCAACCC CATCATCTAT AGTGTAAAAA

TTACCTTCTT TTACCCCCTG TGCTCAACCC CATCATCTAT AGTGTGAAAA

TTACCTTCTT CTACCCCCTG TGCTCAACCC CATCATCTAC AGTGTGAAAA

CTACTTGTTG GTTCCACCAG TGCTCAATCC AGTCATCTAT AGTGTGAAGA

CTTCCTGCTT ATTCCTCCTG TACTCAACCC TATCATCTAC AGTGTGAAGA

CTTCCTTCTC ATCCCTCCCG TGCTCAACCC CATCATCTAT AGTGTGAAGA

CTTCCTGCTC ATCCCTCCTG TGCTCAACCC TATCATCTAC AGTGTGAAGA

TTTCCTGCTT ATTCCTCCTG TGCTCAACCC TATAATCTAT AGTGTGAAGA

TTACCTACTT CTGCCCCCTG CTCTTAATCC CATCATCTAT AGTGTAAAAT

CTACCTGTTT GTGCCTCCCA TGCTCAACCC TATCATCTAC AGTGTGAAGA

CTTCCTGCTA ATCCCTCCCG TGCTCAACCC CATCATCTAC AGTGTGAAGA

CTACCTGATG CTTCCACCTG TGCTGAACCC CATTATATAC AGTGTGAAAA

GTACGTGGTC GTCCCGCCCG CCCTCAACCC CATCATCTAT GGGGTGAGGA

CAAAACAAAT CCGCAAGATG TCGGTCCTCA ATAATACCAT TGCTGAGCCT

CAAAACAAAT CCGCAAGATG TCGGTCCTCA ATAATACCAA TGCTCAGCCT

CTAAACAGAT CCG----ATG TTGGTCCTCA ATAATAGCAA TGCTCAGCCT

CGAAACAGAT CCG----ATG TTGGTCCTCA ATAATAGCAA TGCTCAGCCT

CAAAACAGAT CCGTAAGATG TCAGTCTTCA ATAATACCAA TGTGCAGCCT

CAAAACAGAT CCGTAAGATG TTGGTCCTCA ACAATACCAA CACTCAGCCT

CAAAGCAAAT CAGAAAGATG CTCATCCTTA ATAATACCAA TCCCCATCCT

CAAAACAAAT CAGAAAGATG CTCATCCTTA ATAATACCCA TTCCCAGCTT

CCAAGCAGAT CCG----ATG TTGGTCCTCA ATCATACCAA TGCTGAGCTT

CTAAGCAGAT TCAAAAGATG TGGGTCCTCA ACAATACCAG TGCTCAGCCT

CTAAGCAGAT TCGAAAGATG TTGGTCCTCA ATAATACCAA TGCTCAACCT

CGAAGCAGAT TCGAAAGATG TTGGAGCTCA ATAATACCAA TGATCAGCCT

CTAAACAGAT TCGAAAGATG TTGGTCCTCA ATAATACCAA TGCTCAGACT

CCAAACATAT CCGCAAGATG GAGGTCTTAA TCAACTTGAC AGCTACATTT

CAAAGAAGAT TCACAGGATG TCCTCTTTCA ATAACACCTG CTCCATTTTC

CAAAGCAGAT TCGAATGATG TTGGCCCTCA ATAACACCAA TGTTCAGCCT

CCAAACAGAT TCG-AAGATG TCAACTCTCA ATAGTAGCAC TTTGCAGCCT

CCAAGCAGAT CCGTGA-ATG TTGATTTCCA ACACTACCAG CTTCCATCCT

CTGATCTTCC TCCTGATGGG CATTCCAGGC CTGAAAGCCA CCCAGTACTG

CTGATCTTCT TCCTGATGGG CATTCCAGGC CTGAAAGCCA CCCAGTACTG

CTGATCTTTT TCCTGATGGG CATTCCAGGC CTGAAAGCCA CCCAGTACTG

CTGATCTTTT TCCTGACTGG CATTCCAGGC CTGAAAGCCA CCCAGTACTG

CTGACTTTCC TCCTGATGGG CATTCCAGGT CTGAAAGCTG CCCAGTTCTG

CTGGTCTTCT TCCTGACGGG CATTCCAGGC CTGAAAGCAG CCCAGGTCTG

CCAACCTTCC TCCTCACTGG TATCCCAGGC CTGAGAGCAG CTCAGGTCTG

CCAACCTTCC TCCTCACTGG TATCCCAGGC CTGAGAGCAG CTCAGGTCTG

CTGACCTTCT TCCTGACGGG TATCCCAGGC TTGAGAGCAT CCCAGGTATG

CTGACCTTCC TCTTGACGGG TATTCCAGGC CTGAGAGCGG CCAAGATCTG

CTGACCTTCT TCCTGACGGG TATCCCAGGC CTGAGAGCAG CCCAGGTTTG

CTGACATTCC TCCTGACGGG TATCCCAGGC CTGGCAGCAG CCCAGGTGTG

CTGACCTTCT TCCTGACGGG TATCCCAGGC CTGAGAGCAG CCCAGGTCTG

CCAAGCTTCC TGTTGACTGG CATTCCTGGC CTACAGTCTG TCCATGCCCT

TCCACCTTCC TGCTGACAGG CATCCCTGGA CTGGAGGCTC TTCACATCTG

GTGATCTTCT TCCTGACGGG TATTCCAGGG ATGAAAGCAG CCCAGGCCTG

CTGACCTTCC TTCTGACGGG TATTCCTGGA ATGAGTGCTA CCCAAGTCTG

TCTGCATTCC TCCTGTTAGG CATCCCTGGG ATGGAGGATC AGCACCTCTG

GATCTCCATC CCTTTTTGTC TCCTATATGT TGTTGCCGTC TCTGGAAATA

GATCTCCATC CCTTTTTGTC TCCTATATGT TGTTGCCATC TGTGGAAATA

GATCTCTATC CCTTTTTGTC TCCTGTATGT TGTCGCCCTC TCTGGAAATA

GATCTCTATC CCTTTTTGTC TCCTGTATGT TGTCGCCCTC TCTGGAAATA

GATCTCCATC CCTTTTTGTC TCCTATATGC TGTTTCCCTC TCTGGAAATA

GATCTCCATC CCCTTTTGTC TTTTGTATGT CATCGCCCTC TCTGGAAACA

GATCTCCATT CCCTTTTGTC TCCTTTATGT AATCGCCCTC TCTGGGAACA

GATCTCCATT CCCTTTTGCC TCCTTTA--- ------CCTT TCTGGGAACA

GATCTCCATT CCCTTTTGTC TCCTGTATGC TGTTGCCCTC TCTGGAAACA

GGTCTCCATC CCTGTTTGCC TCCTGTATGC CGGCGCCCTC TCTGGGAACA

GATCTCCATC CCTTTTTGTC TCCTGTATGT CATCGCCCTC TCTGGGAACA

GATCTCCATT CCTATTTGTC TCCTGTATGT CACTGCCCTG TCTGGGAACA

GATCTCCATC CCGTTCTGCC TCCTATATGG CATTGCCCTC ACTGGAAACA

GATCTCCATT CCTTTCTGCT GTGTCTATGC CATTGCCCTC ACAGGGAATG

GATCTCCATA CCCTTCTGTG CCATGTTCTT CATTACCTTG GTGGGCAACA

GATATCTATC CCGGTTTGCC TCCTGTATGT GCTCGCCATC TCTGGGAACA

GATTTCTATT CCTTTTTGTA TCCTTTATGC CATTGCCCTC ACTGGAAATA

GATCTCCATC CCCTTCTGCT CCGTGTACAT CATAGCTCTG CTGGGCAATG

GCATGATCCT GTTTGTGGTC CTCTGTGAAC GGAGCCTCCA TAAGCCTATG

GCATGATCCT GTTTGTGGTC CTCTGTGAAC GGAGCCTCCA TAAGCCTATG

GCATGATCCT GTTTGTAGTC CTCTGTGAAC AGAGCCTCCA TAAGCCTATG

GCATGATCCT GTTTGTGGTC CTCTGTGAAC AGAGCCTCCA TAAGCCTATG

GCATGATCCT GTTTGTGGTC CTCAGTGAAC ATAGCCTCCA CCAGCCTATG

GCATGATCCT GTTTGTGGTC TTCCGTGAGC AGAGCCTCCA TGAGCCTATG

GCATGATCCT GTTGGTGATC CTTCGTGAGC AGAGCCTCCA TGAGCCTATG

GCATGATCCT GCTGGTGATC GTTCGTGAGC AGAGCCTCCA TGAGCCTATG

GCATGATCCT GTTGGTAGTA CTCCGTGAAC AGAGCCTCCA TGAGCCTATG

GTGTGATCCT GTTTGTGGTC CTCCGTGAAC AGAGCCTTCA TGAGCCCATG

GCATGATCCT ATTTGTGGTC TTCCGTGAGC AGAATCTCCA TGAACCAATG

GCATGATCCT GTTTATGGTC ATTCATGAGC AGAGCCTCCA TGAGACCATG

GCATGATCCT GTTTGTGGTC CTCCGTGAGC AGAGTCTCCA TGAGCCCATG

GCATGATCCT GTTTGTCATC ATTACTCAGC AGAGTCTCCA TGAGCCCATG

TGACCATCAT GACAGTTATC TGGAGGGAGC AGACCCTCCA TGTGCCTATG

GCTTGATCCT CACTGTCATC CACAAAGAGT CGTCTCTCCA CCAGCCCATG

GCATGATATT ATTTGTAGTA ATCCATGAGA GGAGTCTCCA TGAGCCCATG

CCACCATCCT GTTGGTTGTT GCCTCTGACA AGACCCTCCA CAAGCCCATG

TACTATTTCC TCTCTATGCT TTCAGCCACA GACCTGAGCT TGTCCCTGTG

TACTATTTCC TCTCTATGCT TTCAGCCACA GACCTGAGCT TGTCCCTGTG

TATTGTTTCC TCTCTATGCT TTCAGTCACA GACCTGAACT TGTCCCTGTG

TATTGTTTCC TCTCTATGCT TTCAGTCACA GACCTGAGCT TGTCCCTGTG

TACTATTTCC TATCTATGCT TTCAGCCACA GACCTGAGCT TGTCCCTGTG

TATTATTTCC TCTCTATGCT TTCAGCCACA GACCTGAGCT TGTCCTTGTG

TACTATTTCC TGTCTATGCT TTCAGTCACA GACCTGAGCT TGTCTCTCTG

TACTACTTTC TCTCTATGCT CTCCATCACA GACTTGAGCT TGTCTCTCTG

TATTATTTTC TCTCCATGCT CTCAGCCACG GACCTGAGCT TGTCTCTCTG

TATTATTTCC TCTCTATGCT TTCAGCCACA GACCTGAGCT TATCCCTGTG

TATTATTTCC TCTCTATGCT TTCAGCCACA GACCTGAGCT TATCCCTGTG

TATTATTTCC TTTCTATGCT GTCAGCCACA GACCTGAGCT TATCCCTGTG

TATTATTTCC TCTCTATGCT TTCAACCACA GATCTGAGCT TGTGTCTGTG

TACTATTTCC TCTCCATACT GTCAGCTGCT GACCTGGGCT TGACTGTTTC

TACCTCTTCC TGGCCATGCT GGCTGCCTCT GATCTGGGTC TGTCCCTCTT

TACTTATTTC TCTCCATGCT GGCCCTGACT GACCTGGGTC TCACCCTCAC

TACTACTTCC TCTCCATGTT GTCAGCCACT GACTTGGGTT TGTCCCTCTG

TACCTCTTCC TCTGCATCCT CTCGCTCACA GACCTGGTGC TTTGCTCCGC

TACACTTTCT ACTACCCTTG GTGTCTTCTG GTTTGAAGCC CGAGAAATCA

TACACTTTCT ACTACCCTTG GTGTCTTCTG GTTTGAAGCC CGAGAAATCA

TACACTTTCT ACTACCCTTG GTGTCTTCTG GTTTGAAGCC CAAGAAATCA

TACACTTTCT ACTACCTTTG GTGTCTTCTG GTTTGAAGCC CAAGAAATCA

TACACTTTCT ACTACCCTTG GTGTCTTCTG GTTTGAAGCC CAAGAAATCA

CACACTTTCT ACTACGCTTG GTGTCTTCTG GTTTGAAGCC CGAGAGATCA

TACACTTTCC ACTACCCTTG GTGTCCTCTG GTTTGAAGCC CGGGAAATCA

CACACTTTCC ACTACCCTTG GTGTCCTCTG GTTTGAAGCT CGAGAGATCA

CACACTCTCC ACTACCCTTG GTGTCTTCTG GTTTGAGGCC CGAGAGATCA

CACACTTTCC ACTACCCTTG GTGTCTTCTG GTTTGAAGCC CGAGAGATCA

CACACTTTCT ACTACCCTTG GTGTCTTCTG GTTTGAAGCT CGAGAAATCA

CACACTTGCC ACTACTCTTG GTGTGTTCTG GTTTGAAGCC CGAAAGATCA

CACGCTTTCC ACTACTCTGG GCGTCTTCTG GTTTGAAGCT AAAGCGATCA

TACAATGTCA ACAACATTAG GCATCTTCTG GTTTGATGCA AGTGAAATCA

CACCTTCCCC ACAATACTGA GGATCTTCTG GCTAGATGCT CGAGAGATGA

CACCCTGCCC ACAGTCATGC AGCTCCTCTG GTTCAACAAT AATAAGATCA

CACTCTCTCC ACAACACTGG GAGTTTTGTG GTTTGATGCC CGAGAGATCA

CACATTACCA AAGATGCTGG TCATCTTCTG GTTTGGGGCA CAGCACATCT

ACCTAAATGC CTGCATTGCC CAGATGTTCT TTCTACACGG ATTTACTTTC

ACCTAAATGC CTGCATTGCC CAGATGTTCT TTCTACACGG ATTTACTTTC

GCCTAAATAC CTGCATTGCT CAGATGTTCT TTCTACATGG ATTTACTTTC

GCCTAAATAC CTGCATTGCT CAGATGTTCT TTCTACATGG ATTTACTTTC

ACGTAAATGC CTGCATTGCC CAGATGTTCT TTCTCCATGG ATTTACTTTC

ACTTAAATGC CTGCATTTCC CAGATGTTCT TTCTCCATGG ATTTACTTTC

ACTTAAATGC GTGCATTGCC CAGATGTTCT TTCTCCATGG GTTTACTTTC

ATTTAAATGC GTGTATTGCC CAGATGTTCT TTCTCCATGG GTTTACTTTC

GTTTAAATGC CTGCATTGCC CAGATGTTCT TTCTCCATGG ATTTACTTTC

ACTTAAATGC CTGTGTTGCT CAGATGTTCT TTCTCCATGG ATTTACTTTC

CCTTAAGTGC CTGCATTGCC CAGATGTTCT TTCTCCATGG ATTTACTTTC

ACTTAAATGC TTGTATTACG CAGATGTTCT TTCTCCATGC ATTTACTTTC

ACATCAATTC CTGCATTGCT CAGCTCTTCT TCATCCACAC TTTGTCTCTT

GTTTAAATAC CTGTATTATT CAGATGTTTT TTCTTCATGG ATTCACTGTC

CTTACTCTTC TTGTTTCACC CAAATGTTCT TTACTCATAC CTTCCAAAAA

GATTTGAAGC CTGTTTTGCT CAGTTATTTT TCCTGCATGT GTTTTCATTC

CTCTTGATGC CTGCATGGCC CAGATGTTTT TCCTTCACAG TTTCACTTTC

CCTATCATGG CTGCCTCACC CAAATGTTCT TCATCCACAT GATCTTTGGC

ATGGAGTCTG GGGTTCTACT GGCCATGGCC TTTGATCGTT TTGTGGCCAT

ATGGAGTCTG GGGTTCTACT GGCCATGGCC TTTGATCGTT TTGTGGCCAT

ATAGAGTCTG GGGTTCTACT AGCCATGGCC TTTGATCGTT TTGTGACCAT

ATAGAGTCTG GGGTTCTACT AGCCATGGCC TTTGATCGTT TTGTGACCAT

ATGGAGTCTG GGGTTCTACT GGCCATGGCC TTTGATCGTT TTGTGGCCAT

ACGGAGTCTG GCGTTCTGCT GGCCATGGCC TTTGATCGTT TTGTGGCCAT

ATGGAGTCTG GAGTTCTGCT GGCCATGGCC TTTGATCGTT TTGTGGCCAT

ATGGAATCCG GAGTTCTGCT GGCCATGGCC TTTGATCGTT TTGTGGCTAT

ATGGAGTCTG GGGTTCTTCT GGACATGGCC TTTGATCGTT TTGTGGCCAT

ATGGAGTCTG GGGTTTTGCT GGCCATGGCC TTTGACCGTT TTGTGGCCAT

ATGGAGTCTG GGGTTCTGCT GGCCATGGCC TTTGATCGTT TTGTAGCCAT

ATGGAGTCTG GGGTTCTGCT GGCCATGGCC TTTGATCGTT TTGTGGCCAT

GTGGAGTCTT CGGTTCTATT ATCCATGTCT ATCGACCGCT ATGTTGCCAT

ATGGAGTCTG GGGTGCTCGT GGCCATGGCC TTTGACCGAT ATGTGGCAAT

TTTGAGTCTC TGATTATTCT GGTGATGGCC TTTGACCGCT ATGTGGCTAT

ATGGAATCCT CTGTCTTGCT GGCAATGTCC TTTGACCGTT ATGTGGCCAT

ATGGAGTCTG GGGTGCTGCT GGCCATGGCC TTCGACCGTT TCATCGCCAT

ACTGAGTCAG CTGTCCTGCT GGCCATGGCT TTTGACCGCT TCGTGGCCAT

CTGTTACCCA CTGAGATACA CTACCATCCT TACCATTGGG ATGAGCATGT

CTGTTACCCA CTGAGATACA CTACCATCCT TACCATTGGG ATGAGCATGT

CTGTGACCCA CTGAGATACA CTACCATTTT TACCATTGGG ATAAGCATGT

CTGTGACCCA CTGAGATACA CTACCATTTT TACCATTGGG ATAAGCATGT

CTGCGACCCA CTGAGGTACA CTACCATCCT TACCATTGGG ATGAGCATGT

TTGTGACCCA CTGAGATACA CCACTGTCCT CACCATAGGG GTGAGCATGC

CTGTGATCCA CTAAGATATA CCACTATTCT TACCATTGGC ATAATTGTAC

CTGTGATCCA CTAAGATATA CCACTATTCT TACCATTGGC ACAATTGTGT

CTGTGACCCA CTGAGATACA ACAGCATCCT CACAATTGGG GTGAGTGTGT

CTGTGATCCA CTGAGATACA CCACCATCCT CACCATTGGG ATGAGCATGT

CTGTAATCCA CTGAGATACA CCACCATCCT CACCATTGGG GTGAGCATGT

CTGTGACCCA CTGAGATATA CCACAATCCT CACCATTGGG GTGAGCATAT

CTGCAACCCG CTGCGTTATT CCACCGTCTT GACCATCGGG CTGACCATGT

CTGTGATCCC CTGAGGTACG CCAATATCCT CACAATGGGT ATCTTGATGA

ATCTTGCCCA TTGCACTACT CTTCCATCCT CACTATAGGT TTGGCCATTT

CTGTCGCCCC CTCCATTACG CCTCCATCCT CACCATTGGC ATAGCCATCA

CTGTGATCCA CTGAGGTATG CTACCATACT CACAATTGGC TTGGCTGTAG

CTGCCGCCCA CTACACTACT CGTCCATCCT CA---TTGTA ATGGCCTGCA

TGATAAGAAA TGTTGCCGTC ATGTTGCCAG TCATGCTCTT TGTCAAGAGG

TGATAAGAAA TGTTGCCGTC ATGTTGCCAG TCATGCTCTT TGTCAAGAGG

TGATAAGAAA TGTTGCTGTC ATGTTGCCAG TTGTGCTCTC TGTGAAGAGG

TGATAAGAAA TGTTGCCGTC AGGTTGCCAG TTGTGGTCTT TGTGAAGAGG

TGATAAGAAA TGTTGCTGTC ATGTTGCCGG TTGTGCTCTT TGTCAAGAGG

TGATACGGAA CATTGCTGTC ATGTTGCCTG TCGTGTTCTT TGTCAGGAGG

TGATAAGGAA TGTTGCTGTC ATGTTGCCCG TTGTGCTTTT TGTCAAGAGG

TGATAAGGAA TGTTGCCGTC ATGTTGCCAG TTGTGCTCTT TGTCAAGAGG

TGATAAGGAA TGTTGCTGTC ATGTTGCCAG TTGTGCTTTT TGTCAAGAGG

TGACAAGAAA TGTTGCTGTG ATGTTGCCAG TTGTGCTTTT TGTCAAGAGG

TGATAAGGAA CGTTGCTGTC ATGTTACCAG TGGTGCTCTT TGTTAAGAGG

TGATAAGGAA TGTTGCTGTC ATGCTGCCGG TTGTGCTGTT TGTCAAGAGG

TGATAAGGAA TGTTGCTGTC ATGCTACCAG TTGTGCTTTT TGTCAAGAGG

TTATACGTAC TGTGCTTTTA ATAGTACCAC TTCTTTTGCT CCTTAAGCAA

TTGTGCGAAC TTTGTCTGTA GAGGTGCCCG CTCCCATCCT CTTGAGGAGG

TTTGTCGCTG TGTTCTCGCT GTTCTTCCCC CTCTTTTCCT GCTCAAGCGC

GGGTGAGAGT AATTGCAGTC ATTACACCTA TGGTGTTGCT TGTCAAGAGG

TAGTCCGAGG CCTCTTCTTT GTCTTCCCAT TCATCATCCT GATCCAGAGA

TTGTCCTTCT GCAGTTCTAT GGTCCTTTCA CATTCTTACT GCTACCATGT

TTGTCCTTCT GCAGTTCTAT GGTCCTTTCA CAT------- ----------

TTGTCCTTCT GCAGTTCTAT GGTCCTTTCA CATTCTTACT GCTACCATGT

TTGTCCTTCT GCAGTTCTAT GGTCCTTTCA CATTCTTACT GCTACCATCT

TTGTCCTTCT GCGGTTCTAT GGTCCTTTCA CATTCTTACT GCTACCATGT

CTGTCCTTCT GCAGTTCCAT GGTCCTTTCA CACTCTTACT GCTACCATGT

TTGTCCTTCT GCAGTTCTCT GGTTCTTTCA CATTCATACT GTTACCATGT

TTGTCCTTCT GCAGTTCTCT GGTTCTTTCA CATTCATACT GCTACCATGT

CTGTCCTTCT GCAGTACTCT TGTCCTTTCA CATTCTTACT GCTACCACGT

CTGTCCTTCT GCAGTTCTAT GGTCCTCTCA CATTCTTACT GCTACCATGT

CTGTCCTTCT GCGGTGCAGT GGTCCTTTCA CATTCTTACT GTTACCATGT

CTGTCCTTCT GCAATTCTAT GGTCCTTTCA CATTCTTACT GCTACCATGT

CTGGTCTTCT GCAGGTCCTT GGTTCTTTCA CATTCCTACT GCTACCATGT

CTCTCTTTCT GTAGAGCTAA GGTCCTTTCC CACTCCTACT GTTACCATCC

CTGTGCTTCT GCCACTCCAA TGTACTTTCC CATTCCTACT GCTTGCATCC

CTGCCCTTCT GCCGCTCACA CCTTCTCTCT CACTCCTACT GTCTCCACCA

CTCTCATTCT GCCAGAGAAA AGTTCTTTCT CACTCCTACT GTTACCATAT

TTACCATTCT GTGGGCATCA TGTGATCCCC CACACCTACT GTGACCACAT

TGATCTCATC CAACTCTCCT GCACAGACAA TAGGATCAAC AGCATCCTTG

---------- ---------- ---------- ---------- ----------

TGATCTCATC CAACTCTCCT GCACAGACAA TAGGATCAAC AGCATCCTTG

TGATCTCATC CAACTCTCCT GCACAGACAA CAGGATCAAC AGCATCCTTG

TGATCTCATC CAACGCTCTT GCACAGACAT TCGGGTCAAC AGCATCCTTG

TGATCTCATC CAACTCTCTT GCACAGACAA CAGAATCAAC AGCATCTTAG

AGATCTCATC CAGCTCTCAT GTACAGACAA CAGAATCAAC AGCATTCTTG

AGATGTCATC CAGCTCTCAT GTACAGACAA CAGAATCAAT AGCGTTCTTG

TGATCTCATC CAACTCTCAT GCACAGACAA CAGAATCAAC AGCATCCTTG

TGATCTCATT CAACTCTCAT GCACAGACAA CAGAATCAAC AGCATCCTTG

TGACCTCATT CAACTCTCAT GCACAGACAA CAGAATCAAC AGCATCCTCG

TGATCTCATT CAACTCTCAT GCACAGACAA CAGAATCAAC AGCATCCTTG

CGATCTTATT CAACTCTCAT GCACAGATAA CAGAATCAAC AGCATTCTGG

AGATGTAATT AAATTATCAT GTTCAGACAC TCGGGCCAAC AGCATCTGTG

TGACATCATA AAGCTCTCCT GTTCCAACAC TAGGATCAAT AGCAGCTTCG

GGATATGATT CGTCTGGTCT GTGCTGACAT CCGAGTCAAC AGCTGGTATG

TGACTTCATC AAGTTGTCAT GCACAGATAA CCGAATCAAT AGCATCATGG

GGGCATTGCC AAGCTGGCAT GTGCCAACAT CAAGATCAAC ATCATCTACG

GTCTGTTTGC GCTTTTGTCC ACTACAGGGT TTGACTGCCC TTGCATCCTG

---------- ---------- ---------- ---------- ----------

GTCTGTTTGC GCTTTTCTCC ACTACAGGGT TTGACTGCCC TTGTATCCTG

GTCTGTTTGC GCTTTTCTCC ACTACAGGGT TTGACTGCCC TTGTATCCTG

GTCTGTTTGC ACTCTTCTCC ACTGCAGGGT TTGACTGCCC TTGCATCCTG

GTCTGTTTGC ACTCTTCTCC ACTACAGGCT TTGACTGCCC TTGCATCTTG

GTCTGTTTGC ACTATTCTCT ACTACAGGGT TTGACTGCCC CTGCATCTTG

GTCTGTTTGC ACTATTCTCT ACTACAGGGT TTGACTGCCC TTGCATCTTG

GTCTGTTTGC ACTCTTCTCC ACTACAGGGT TTGACTGCCC TTGCATCTTG

GTCTGTTTGC ACTCTTCTCC ACGACAGGGT TTGACTGTCC TTGTATCTTG

GTCTGTTTGC ACTTTTCTCC ACAACAGGAT TTGATTGTCC TTGCATCTTG

GTCTGTTTGC ACTCTTCTCC ACAACAGGAT TTGACTGTCC TTGCATCTTG

GTCTGATTGC ACTCTTCTCC ACAACAGGAT TTGACTGTCC TTGCATCTTG

GATTAATTGG GCTCGTCCTG ACCACAGGGA TAGATACGCC ATGCATTGTC

GATTGTTTGT GGTGCTCTCC ACCATGGGAC TTGACTTTTT ACTTATTCTC

GATTTGCTCT TATCATTCTC ATTGTTGTGT TGGACCCATT GCTTGTTGTG

GGCTGTTTGT TCTCTTTTCA ACATCAGGCA TTGACTGTCC GTGCATCCTC

GCCTGACGGT TGCACTCTTG GTCACAGGGA TGGATGTGGT GCTCATTGGC

CTCTCCTATA TCCTGATCAT TCGATCTGTC CTCAGCATTG CTTCCTCAGA

---------- ---------- ---------- ---------- ----------

CTCTCCTATA TCCTGATCAT TCGATCTGTC CTCAGCATCG CTTCCTCAGA

CTCTCCTATA TCCTGATCAT TCGATCTGTC CTCAGCATCG CTTCCTCAGA

CTCTCCTATA TCCTGATAAT TAGATCTGTC CTCAGAATTG CTTCCTCAGA

CTCTCCTACA TCCTGATCAT CCGATCTGTC CTCCGCATTG CTTCCTCAGA

CTCTCCTATG TACTGATCAT CCGATCTGTG CTCAGCATTG CTTCCTCTGA

CTGTCCTATG TACTGATCAT CCGATCTGTC CTCAGCATTG CTTCCTCTGA

CTGTCCTATA TCCTGATCAT CCGATCCGTT CTCAGCATTG CTTCCTCAGA

CTCTCCTATG TCCTGATCAT CCGATCTGTC CTCAGCATCA CCTCCATAGA

CTCTCCTATG TACTGATCAT CCGGTCTGTT CTCAGCATTG CCTCCTCAGA

CTCTCCTATG TTCTGATCAT CCAATCTGTC CTCAACATTG CTTCCTCAGA

ATCTCCTATG TCCTTATTAT TCGTTCCGTC CTTGGCATTG CTTCCTCAGA

CTGTCTTACA TTCTGATTAT TCGCTCTGTC CTCAATATCA CCTCCCCTGA

CTGTCTTATG TGCTGATTCT GAAAACTGTA CTGAGCATCA CTTCACATGG

CTTTCCTATT GCCTCATCCT GAGAAGTATC TTAGGGACTG CCATCTGGGC

ATTTCCTATG TTCTGATAAT CCATTCAGTG CTGAGCATTG CATCCCCCGA

TTTTCTTATG GCTTCATCCT CCATACTGTG CTCCACCTGC CCTCCCAGGA

AGAGAGGCGG AAAGCCTTCA ACACCTGCAC ATCCCACATC AGTGCTGTTT

---------- ---------- ---------- ---------- ----------

GGAGAGGCAG AAAGCCTTCA ACACCTGCAC ATCCCACATC AATGCTGCTG

GGAGAGGCAG AAAGCCTTCA ACACCTGCAC ATCCCACATC AGTGCTGTTG

GGAGCGGCAC AAAGCCTTCA ACACCTGTGT ATCCCACGTC AGTGCTGTTG

AAAGCGGCAC AAAGCCTTCA ACACCTGCAT ATCCCACATC AGTGCTGTTG

TGAGCGACAG AAAGCCTTCA ATACATGCAT ATCCCACATC AGTGCTGTTG

TGAGCGACAG AAAGCCTTCA ATACATGCAT ATCCCACATC AGTGCTGTTG

GGAACGGCAG AAAGCCTTCA ATACCTGCAT ATCACACATC AGTGCTGTGG

GGGCAGGCAG AAAGCCTTCA ACACCTGCAT TTCCCACATC AGTGCTGTTG

GGGGCGGCAA AAAGCCTTCA ACACCTGCAT ATCCCACATC AGTGCTGTTG

GGGACGGCAG AAAGCCTTCA ATACTTGCAT ATCGCACATC AGTGCTGTTG

GGGACGGCGG AAAGCCTTCA ACACTTGCAT ATCTCACATC AGTGCTGTTG

AGAGCGGCAC AAGGTCTTTA GCACCTGTAT CTCCCACATT GGAGCAGTTG

TGGCCGCCTG AAGGCCCTCA ACACTTGCAT TTCCCACCTC TCTGCTGTGG

TGAGCGCCTA CGGGCCCTCA ATAACTGTCT GTCTCACTTT CTGGCTGTTC

GGAGCGGAAG AAAGCCTTCA ACACATGCAT TTCTCATATT AGTGCTGTCG

TGCACAGGTC AAGGCCTTCA GCACCTGTGG GGCCCACATA TGTGTCATCC

CCATCTTCTA CCTCCCTCTC ATCAGTTTGT CTCTTGTCCA TCGCTATGGC

---------- ----CCTCTC ATTAGTTTGT CTCTTGTCCA TCGCTATGGC

CCATCTTCTA CATCCCTCTC ATCAGTTTGT CTCTTGTCCA TCACTATGGC

CCATCTTCTA CATCCCTCTC ATCAGTTTGT CTCTTGTCCA TCACTATGGC

CCATCTTCTA CATCCCTCTC ATCAGCTTGT CTCTTGTCCA TCGCTATGGC

CCATCTTCTA CATCCCTCTC ATCAGCTTGT CTCTCGTCCA TCGCTATGGC

CCATCTTCTA CATTCCTCTC ATCAGTTTGT CTCTTGTACA CCGCTATGGT

CCATCTTCTA CATTCCTCTC ATCAGCTTGT CTCTTGTACA CCGCTACGGC

CCATCTTCTA CATCCCTCTC ATCAGCTTGT CTCTTGTCCA TCGCTACGGC

CCATCTTCTA CATCCCTCTC ATCAGCCTGT CTCTTGTCCA TCGCTATGGC

CCATCTTTTA CATCCCCCTT GTCAGCTTGT CTCTTGTCCA TCGCTATGGC

CCATCTTCTA CATCCCTCTC ATCAGCTTGT CTCTTGTCCA TCGCTATGGA

CTATCTTCTA CATCCCTCTC ATCAGCTTGT CTCTTGTCCA TCGCTATGGC

CTGTTTTCTA CATCCCCATG ATGAGCCTGT CCTTGCTGCA TCGCTATGGT

TCCTCTTCTT TACTCCCATG ATCTGCCTGT CTATGTTGCA CCGCTTTGGC

TAGTGCTCTA TGTCCCCATG GTGAGTTTGT CCATGACTCA TCGCTTTGCA

CCATATTCTT CATTCCTCTC ATCAGTTTAT CTCTAGTGCA TCGTTATAGC

TGGTCTTCTA CATCCCGGCT TTCTTCTCCT TCTTCACCCA CCGCTTTGGT

CATTCAGCAC CTCCATTTGT CCACATCATC ATGGCCAATG TCTTTCTGCT

CATTCAGCAC CTCCATTTGT CCACATCATC ATGGCCAATG TCTTTCTGCT

CATTCAACAC CTCCATTTGT CCACACCATC ATGGCCAATG TCTTTCTGCT

CATTCAGCAC CTCCATTTGT CCACACCATC ATGGCCAATG TATTTCTGCT

CATTCAGCAC CTCCATTTGT CCACAGCGTC ATGGCTAACG TCTTTCTGCT

CATTCAGCGC CTCCATTTGT CCACACTCTC ATGGCCAACG TCTTCCTGCT

CATTCAGCCC CCGCCTTCGT CCACACCATC ATGGCCAATG TCTTCCTGCT

CATTCAGCAC CCGCCTTTGT CCACACCGTC ATGGCCAACG TCTTCCTGCT

CATTCAGCAC CTCCATTTGT GCACACCATC ATGGCCAACG TCTTCCTACT

CATTCAGCAC CTCCCTTTGT CCACACCATC ATGGCCAATA TCTTCCTGCT

CATTCAGCAC CTCCATTTGT CCACACCATC ATGGCCAATG TCTTCCTTCT

CATTCAGCAC CTCCATTTGT CCACACCATC ATGGCCAATG TCTTCCTGCT

CATTCGGCAC CTGCATTTGT CCACACTATT ATGGCCAATG TTTTCCTGCT

CAGTCAGTCC CCAAAGTAGT CCACTCAATG ATGGCTAATA TTTACCTACT

CCACGGCTTC CCTCACATAT CTATGTGCTT ATGGCCAACG TGCATTTCCT

AAACATACTT CACCACTGGT CCATGTGACT ATGGCCAATA TCTACTTGCT

CACAATGCCC CACCATTTGT GCACACTATG ATGGCCAATA TCTTTCTCCT

CACTGGATGC CACCTCAGGT TCACATTTTC GTGGCCAATC TCTACCTCCT

AATCCCTCCT GTGCTCAACC CTATTATTTA CAGTGTAAAG ATTAAGCAGA

A-TCCCTCCT GTGCTCAACC CTATTATTTA CAGTGTAAAG ACTAAGCAGA

AATCCCTCCT GTGCTCAACC CTATTATCTA CAGTGTGAAG ACTAAGCAGA

AATCCCTCCT GTGCTCAACC CTATTAT-TA CAGTGTGAAG ACTAAGCAGA

AATCCCTCCT GTGCTCAACC CTATTATCTA CAGTGTGAAG ACTAAGCAGA

CATCCCTCCT GTGCTCAACC CTATCATCTA TAGTGTAAAG ACTAAGCAGA

CATCCCTCCT GTGCTCAACC CTATAATCTA CAGTGTGAAG ACAAAGCAGA

CATCCCTCCT GTGCTCAACC CTATAATCTA CAGTGTGAAG ACGAAGCAGA

TACACCTCCT GTGCTCAACC CTATCATCTA TAGTGTGAAG ACTAAACAGA

TATTCCTCCT GTACTCAACC CTATCATCTA CAGTGTGAAG ACTAAGCAGA

CATCCCTCCC GTGCTCAACC CCATCATCTA TAGTGTGAAG ACTAAGCAGA

CATCCCTCCT GTGCTCAACC CTATCATCTA CAGTGTGAAG ACGAAGCAGA

TATTCCTCCT GTGCTCAACC CTATAATCTA TAGTGTGAAG ACTAAACAGA

TCTGCCCCCT GCTCTTAATC CCATCATCTA TAGTGTAAAA TCCAAACATA

CATTCCTCCT GTGATGAATC CTATTGTCTA TGTGGTGAAA ACCAAGCAGA

GGCTCCTCCT GTGATGAACC CCATCATTTA TAGTATAAAG ACTAAGCAGA

TGTCCCTCCT GTACTCAATC CCATTATCTA CAGTGTGAAG ACCAAACAGA

GGTGCCCCCC ATGCTCAACC CACTGGTGTA TGGCATTAAC TCCAAGCATA

TTCAAGCCAC TTTCTTCCTG ACGGGCTTCC AAGGTCTAGA AGGTCTCCAT

TTCAAGCCAC TTTCTTCCTG ACGGGCTTCC AAGGTCTAGA AGGTCTCCAT

TT---GCCAC TTTCTTCCTG ATGGGCTTCC AAGGTCTAGA AGGTCTCCAT

TTCGAGCCGC TTTCTTCCTG ATGGGCTTCC AAGGTCTAGA AGGTCTCCAT

TTCAAGCCAC TTTCTTTCTG ACGGGCTTCC AAGGTCTAGA AGATCTCCAT

TTCGGGCCAC TTTCTTCCTG ACCGGCTTTC AAGGTTTGGA AGATCTCAAT

TCCGAGCCAC TTTCTTCCTG ACAGGCTTTC AAGATCTGGA AAAGTTCCAT

TTCGAGCCAC TTTCTTCCTG ACAGGCTTTC AAGGTCTGGA AGAATTCCAT

TTCGAGCCAC TTTCTTCCTG ACAGGCTTCC AAGGTCTGGA AGATCTCCAT

TTCAATCAAC CTTCCTCCTC ACTGCATTCC CTGGGCTGGA ACTCGCTCAT

TTCGACCAAA CTTCCTACTC ACTGCATTTC CTGGACTGGA ACTTGCTCAC

TTCGATTGAA CTTCTTCCTC ACTGCATTCC CTGGGCTGGA ACTGGTTCAT

TTCGATCTAA CTTCTTCCTC ACTGCACTCC CTGGAATGGA AACTGCCACT

TCCGCGCCAC TTTCTTCTTG ACAGGCTTCC AGGGTGTGGA ACATCTCCAT

TGCGG----T TTTTCTTCTG ATTGGAATCC CAGAGCTGGA ATACACCCAC

TTCGAACCAC CTTCTTCCTC ATTGGAATCC CTGGGCTGGA GCATATGCAC

TACGATCCAC TTTCCTGGTG ACAGGAATCC CTGGACTAGA GACTATGCAT

TCC---TCTC CATCATCCTG ACTGGCTTCC CAGGATTGGA AGATTCCTTG

GGCTGGATCT CTATTCCCTT CTGCTTCATC TACCTGACAG TTATCTTGGG

GGCTGGATCT CTATTCCCTT CTGCTTCATC TACCTGACAG TTATCTTGGG

GGCTGGATGT CTATTCCCTT CTGCTTCATC TACCTGACAG TTATCTTGGG

GGCTGGATCT CTATTCCCTT CTGCTTCATC TACCTGACAG TTATCTTGGG

GGCTGGATCT CTATTCCCTT CTGCTTCATC TACTTGACAG TTATCTTGGG

GGCTGGATCT CTATTCCCTT CTGCTCCATC TACTTGACAG TTATCTTGGG

GGCTGGATCT CCATTCCCTT CTGCTCCATC TACTTAATAG TGATTTTAGG

GGCTGGATCT CCATTCCCTT CTGCTCCATC TACTTAATAG TCATTTTAGG

GGCTGGATCT CTATTCCCTT CTGCTCCATC TACTTGACAG TTATTTTGGG

GTCTGGATCT CCATCCCTGT CTGCTGTCTC TACATCATTG CTCTCTTGGG

ATCTGGATCT CCATCCCTGT CTGCTGTCTC TACACCATTG CCCTCTTGGG

GTCTGGATCT CTATCCCTGT CTGCTGTCTC TATACGATTG CCCTCTTGGG

GTCTGGATCT CCATCCCCAT CTGCTGTCTC TATTCCATTG CCCTCCTGGG

GGTTGGATCT CTATTCCCTT CTGCTCCATC TACTTGACAG TTATTGTAGG

ATCTGGATCT CCACCCCAAT CTGCCTCATG TACTTGGTTG CCATCATAGG

ATCTGGATCG CCATCCCTAT CTGCCTCATG TACCTAGTGG CCATCTTGGG

ATGTGGCTTT CTATTCCATT TAGCATGATG TTCTTTGTGA CCTTGGCAGG

TATTGGATGT TTATTCTGCT TGGAGCCCTC TACTCTGTTT CCATCCTAGG

GAACCTCACC ATTCTCCACG TCATTTGTAC TGATGCCACT CTCCATGGAC

GAACCTCACC ATTCTCCACG TCATTTGTAC TGATGCCACT CTCCATGGAC

GAACCTCACC ATTCTCCACG TCATCCGTAC TGATGCTACT CTCCATGAAC

GAACCTCACC ATTCTCCACG TCATCCGTAC TGATGCTACT CTCCATGAAC

GAACCTCACC ATCCTCCACG TCATCCATAC TGATGCCACT CTTCATGAAC

GAACCTTACC ATTCTCCACG TCATCCGTAC TGACGCCACC CTCCATGAAC

GAACCTTACC ATTCTCCATG TCATCCGAAC TGACGCCACT CTCCATGAAC

AAACCTCACT ATTCTCCATG TCATCCGGAC TGATGCCACT CTCCATGAAC

GAACCTTACC ATCCTCCATG TCATCCGTAC TGATGCCACC CTCCATGAAC

AAATACTATG ATCCTGTTTG TCATCTTTGT TAAGCAGCGT CTCCACAAGC

AAACAGCATG ATTCTGTTTG TCATCATCAT TGAGAGAAGT CTCCACAAGC

AAACGGCATG ATCCTGTTTG TCATCATTGT TGAGCAGAGT CTCCACAAGC

AAACAGCTTG ATCTTGTATA TCATCATTAT TGAGAGGCGT CTCCACAAGC

GAACCTTACC ATCCTCCACG TCATCCGTAC TGATGCCACC CTCCATGAAC

CAACTGCACA ATTCTTGTTA TCATAAAGGC AGAGCCCTCA CTCCATGAGC

TAATTGCACC ATCCTCTTTG TGATCAGGAC AGAGCCCTCG CTCCATGCCC

CAACATCACC ATAATGTGGA TTATCTGGAC TGAGAGAAGC CTCCATGCTC

AAACTCCCTG ATCCTGGTCA TCCTCAAGGA GGAGCAGAGC CTGCACCAAC

CCATGTACTA TTTCTTGGGC ATGCTAGCTG TCACAGACTT AGGCCTTTGC

CCATGTACTA TTTCTTGGGC ATGCTAGCTG TCACAGACTT AGGCCTTTGC

CCATGTACTA TTTCTTGGGC ATGCTAGCTC TCACAGACTT AGGCCTTTGC

CCATGTACTA TTTCTTGGGC ATGCTAGCTC TCACAGACTT AGGCCTTTGC

CCATGTACTA TTTCTTGGCC ATGCTAGCTC TCACAGACTT AGGCCTTTGC

CCATGTACTA TTTCTTGGCC ATGCTGGCGC TCACAGACTT GGGACTTTGC

CCATGTACTA TTTCTTGGCT ATGCTGGCCC TAACAGACTT GGGACTCTGC

CTATGTACTA TTTCTTGGCT ATGCTGGCCC TCACAGACTT GGGACTCTGC

CCATGTACTA TTTCTTGGCC ATGCTTGCCC TCACAGACTT GGGCCTTTGC

CCATGTACTA TTTCCTCTTT ATGCTGTCAG CTGCTGATTT GTGTCTGACC

CCATGTACTA TTTCCTCTCT ATGCTGTCAG CTGTTGATCT ATGTCTGACC

CCATGTACTA TTTCCTGTCC ATGTTGTCAG TTGTTGATTT ATGTCTGACC

CCATGTACTA TTTCATTTCT ATGCTGTCAG CTGTCGATTT GTGTCTGACC

CCATGTACTA TTTTTTGGCC ATGCTGGCCC TCACGGACTT GGGCCTTTGC

CTATGTATTA TTTCCTTGCC ATGTTGGCTG TCTCTGACCT GGGCCTGTCC

CCATGTACTA TTTCCTCTCC ATGCTGGCCT TTTCGGACCT GGGCCTGTCC

CCATGTATCT CTTTCTGGCA ATGCTGACAG CCTCAGACCT GGGTTTGTCT

CCATGTATTA TTTCCTGGCC ATGCTGGCCA TCAGTGACCT GGGTGTGAAC

CTTTCCACAC TGCCCACTGT GCTGGGCATT TTCTGGTTTG ATACCAGAGA

CTTTCCACAC TGCCCACTGT GCTGGGCATT TTCTGGTTTG ATACCAGAGA

CTTTCCACAC AGCCCACTGT CCTGGGCATT TTCTGGTTTG ATGCCAGAGA

CTTTCCACAC AGCCCACTGT CCTGGGCATT TTCTGGTTTG ATGCCAGAGA

CTTTCCACAC TGCCCACTGT GCTGGGCATT TTTTGGTTTG ATGCAAGAGA

CTTTCCACGT TGCCCACTGT GCTGGGCATT TTCTGGTTTG ATGCTAGAGA

CTCTCTACAC TGCCCACTGT GCTGGGAATC TTCTGGTTTG ATGCCAGAGA

CTCTCCACAC TGCCCACTGT GCTGGGAATC TTCTGGTTTG ATGCCAGAGA

CTTTCCACTC TGCCCACTGT GCTGGGCATC TTTTGGTTTG ATGCCAGAGA

ATCACGACCC TCCCCACTGT GCTTGGGGTT CTCTGGTTTC ATGCCCGGGA

ATCTCAACCC TTCCCACTGT GCTTGGGGTT CTCTGGTTTC ATGCCCGGGA

ATCTCAACCC TCCCCACTGT GCTTGGGGTG CTCTGGTTTC ATGCCCGGGA

ATCTCAACTC TCCCCACTGT GCTTGGCGTT CTTTGGTTTC ACGTCTTGGA

CTTTCCACAC TTCCCACTGT TCTGGGCATC TTCTGGTTTG ATGCCCGAGA

CTCTCCTCGT TTCCTACCAT GCTGAGGGTT TTCTTGTTCA ATGCCATGAG

CTCTCCTCAC TCCCCACTAT GCTGAGGATC TTCTTGTTCA ATGCCACAGG

CTCTCTGCAT TCCCTACACT TATAAGGATC TTATGGCTGG AGGCCCGAGA

TGCTCAACTC TGCCCACAGT CCTAGGCACC TTCTGCTTCA ACGTCAGGGA

GATTGGCATC CCTGCCTGTT TCACTCAGCT CTTCTTCATC CACACCTTGT

GATTGGCATC CCTGCCTGTT TCACTCAGCT CTTCTTCATC CACACGTTGT

GATTGGCATC CCTGCCTGTT TCACTCAGCT CTTCTTCATT CACACCTTGT

GATTGGCATC CCTGCCTGTT TCACTCAGCT CTTCTTCATT CACACCTTGT

GATTGGCATG CCTGCCTGTT TCACCCAGCT CTACTTCATC CACACCTTGT

AATTGGCATC CCTGCCTGCT TCACTCAGCT CTTCTTCATC CATACCTTGT

GATTGGTATT CCTGCCTGCT TTACTCAACT ATTCTTCATC CACACCTTGT

GATTGGTATT CCTGCCTGCT TTACTCAGCT ATTCTTCATC CACACCTTGT

AATTGGCATC CCTGCCTGCT TTACTCAGCT CTTCTTCATC CATACCTTGT

AATCAGCTTT AAAGCTTGCC TCATTCAAAT GTTCTTTGTA CATGCTTTCT

GATTAGCCTG AAAGCTTGCC TCATTCAAAT GTTCTTTGTG CATGGCTTCT

GATCAGCTTC AAAGCGTGCT TCATTCAAAT GGCCTTTGTA CATGTCTTCT

GATCAGCTTA AAAGCTTGTC TCATTCAAAT GTTCTTTGTC CATGCCTTCT

GATTGGCATC CCTGCTTGCT TCACTCAGCT GTTCTTCATC CATACATTGT

AATTTCACCC AATGCGTGCT TTGCTCAAGA GTTCTTCATT CATGGATTCA

CATTTCTCCA AATGCCTGCT TTGCTCAGGA ATTCTTCATC CATGGATTCA

AATGAGTTTC TCTGCTTGTT TCACCCAGAT GTTCTTCATC CACACCTTCC

GGTTAGCTTC GATGCCTGCA TGACCCAGAT GTTCTTCATC CACTCATTCT

CTTCAATGGA GTCATCAGTT CTGTTATCCA TGTCCATTGA CCGCTACGTG

CTTTAGTGGA GTCATCAGTT CTGTTATCCA TGTCCATTGA CCGCTATGTG

CTCTAGTGGA GTCATCAGTT CTGTTATCCA TGTCCATTGA CCGCTACGTG

CTCTAGTGGA GTCATCAGTT CTGTTATCCA TGTCCATTGA CCGCTACATG

CTCTAGTGGA GTCATCTGTT CTATTATCTA TGTCCATTGA CCGCTACGTG

CTCTGCTGGA GTCATCCGTT CTATTGTCCA TGTCCTTTGA CCGTTATGTG

CTTTAGTGGA GTCATCAGTC CTACTGTCGA TGTCCTTCGA CCGCTATGTA

CTTTAGTGGA GTCATCGGTT CTACTGTCTA TGTCCTTCGA CCGCTATGTA

CTCTAGTGGA ATCATCAGTT CTGTTGTCCA TGTCCATTGA CCGCTATGTG

CCTTCCTGGA GTCCTCAGTG CTGGCAATTA TGGCTTTTGA CCGCTTCATG

CCTTCCTGGA GTCCTCAGTG CTGGTAGCCA TGGCCTTTGA TCGCTTTATG

CCTTCCTGGA GTCCTCGGTG CTGGTAGCCA TGGCCTTTGA CCGCTTCATG

CCTTCCTGGA GTCCTCAGTG CTGGTAGCCA TGGCCTTTGA CCGCTTCATG

CTCTGGTGGA ATCTTCAGTT CTATTGTCCA TGTCCATTGA CCGCTATGTG

CTGTCATGGA ATCCTCAGTT CTTCTAATTA TGTCCTTGGA TCGCTTTCTT

CCGATATGGA GTCCTCGGTT CTTCTAATTA TGTCTTTGGA TCGGTTTCTT

AGAATTTTGA GTCAGCTGTG ATTCTGGTCA TGGCCTTTGA TCGCTATGTG

CCTTAACGGA GTCAGGCATC CTGCTGGCCA TGAGCTTTGA CCGCTATGTG

GCCGTCTGCA ACCCACTGCA TGACTCCACC GTCCTGACAC CTGCATGTAT

GCCATCTGCA ACCCACTGCA TTACTCCACC GTCCTGACAC CTGCATGTAT

GCCATCTGCA ACCCACTGCA TTACTCCACC GTCCTGATAC CTGTACATAT

GCCATCTGCA ACCCACTGCG TTACTCCACC ACCCTGATAC CTGTACATAT

GCCATCTGCA ACCCACTGCG TTACTCCACT GTCCTGACAC CTGCACGTAT

GCCATTTGCA ACCCCCTGCG TTACTCCACC GTCCTGAGGC CGGCACGCAT

GCAATTTGCA ACCCACTGCG TTATTCCACC ATCCTGACAT CCAGAAGGAT

GCAATTTGCA ACCCACTGCG TTATTCTACC ATCCTGACGC CCAGAAGGAT

GCCATCTGTA ACCCACTACA CTACTCCACC ATCCTGACAC CTGCTCGTAT

GCTATCTGTA ACCCACTGAA ATATGCCACT GTCCTCAAAG ACATGATGAT

GCTATCTGTA ACCCAATGAA GTATGCCATT ATCTTCACAG ACATGGTAAT

GCCATCTGTA ACCCACTGAA GTATGCCACT GTCCTCACAG ACACGATGAT

GCTATCTGTA ACCCACTACA ATATGCCACC ATCCTCACAG ACGTGATCAT

GCCATTTGCA ACCCACTACG TTACTCCACC ATCCTCACAC CTGCACGCAT

GCCATTCATA ATCCCCTGAG ATATAATTCT ATCCTCACTA GCAACAGGGT

GCCATTCATA ATCCCCTAAG ATACAGCTCT GTCCTCACTA GCGATCGGGT

GCAATTTCTA AACCCTTGCA CTATGCCTCC ATCCTCACCA GCAGTGTAAT

GCCATCTGTA ACCCACTCAG ATACTCAGCG ATACTGACCG GCAGTAGCAT

TGTCAAGATG GGGCTAAGCT CAGTGCTTAG AAGTGCTCTC CTCATCCTCC

TGTCAAGATG GGGCTAAGCT CAGTGCTTAG AAGTTCTCTC CTCATCCTCC

TGTCAAGATG GGGCTAAGCT CAGTGCTTAG AAGTGCCCTC CTCATCCTCC

TGTCAAGATG GGGCTAAGCT CAGTGCTTAG AAGTGCCCTC CTCATCCTCC

TGTCAAAATG GGGTTAAGCT CCATGCTTAG AAGTGCCCTC CTCATCCTCC

AGTCAAGATG GGGCTGGGCT CAGTGCTTCG GAGTGCCCTC CTTATCCTCC

TGTGAAGATG GGGCTGAGCT CAGTGCTCAG AAGCGCACTC CTCATTCTCC

TGTGAAGATG GGGCTGAGCT CAGTACTCAG AAGTGCACTC CTCATTCTCC

TGTCAAGATG GGACTGAGTT CAGTGATTAG AAGCTCCCTC CTCATCCTCC

CATGGTGATC GGACTATTCA TTTGCGTACG ACAACTAATT TTCATCTTTC

CTTGGTGATT GGACTGGTCA TCTGCCTACG GCAAGTTATT TTCACCTTTC

CATGGTGATT GGACTGGTCG TCTGTATACG ACAAGTCATT TTCATTCTTT

CATGGTCATT GGACTAGTCA TCTGCATACG GCAAGTAGTT TTTATGTTTC

TGTTAAGATG GGGCTGAGCT CAGTGTTTAG AAGTGCCCTT CTCATCCTCC

TGCTAAAATG GGACTGATCT TGGCTTTCAG GAGCATTCTT TTAGTGCTCC

TTATAAAATG GGCCTCGCCA TGGCCTTCAG GAGCGTCCTC TTAGTGCTGC

TGCCAAGATT GGTTTGGCCA TTTTATTACG AACACTGACA GTTGAGGTAC

TGTCAAGATG GGTTTGGCCA TTTTAGTACG CAGCTTCATT TTGATCCTTC

CCTTGCCATT CCTCCTGAAG CGCTTCCAAT ACTGCCACTC CCATGTGCTG

CCTTGCCATT CCTCCTGAAG CGCTTCCAAT ACTGCCACTC CCATGTGCTG

CCTTGCCATT CCTCCTGAAG CGCTTCCAGT ACTGCTGCTC CCATGTGCTG

CCTTGCCATT CCTCCTGAAG CGCTTCCAGT ACTGCTGCTC CCATGTGCTG

CCTTGCCATT CCTCCTGAAG CGCTTCCAAT ACTGCTGCTC CCATGTGCTG

CCCTGCCATT TCTCCTTAAG CGCTTCCAGT ACTGCCGCTC CCATGTGCTG

CATTACCATT CCTCCTTAAA CGCTTCCATT ATTGCCGCTC CCATGTGTTA

CATTACCTTT CCTCCTTAAA CGCTTCCATT ATTGCCGATC CCACGTGTTA

CCTTGCCCTT CCTCCTTAAG CGCTTCCAGT ACTGCCGCTC CCATGTGCTG

CCATGCTTCT AGCCTTGAAG ACTGTATCTT TCCAGGAAAG AAAGGAGCTT

CCATGGTTCT AACCTTGAAG AATGTATCTT TCTATGCAGG CAAAGAACTT

CCATGTTTCT AGCTTTGAAG AGTACATCTT TCCACGGGGG CCAGGAACTT

CCATGATTCT AGCTTTAAAG AATGTATCTT TCCATGGAGG CAAAGAGCTT

CCCTGCCATT CCTTCTTAAG CGCTTCCAGT ACTGCCACTC CCATGTGCTG

CATTCCCCTT CACTTTAAGG GGACTAAAAT ACTGTCAGAA GAATCTCCTT

CATTCCCCTT CATTCTAAGG AGACTGAAAT ACTGCCAGAA GCATGTCCTT

CCCTTCCCAT CTTGCTTAGG AGGCTACACT TCTGTTGCTC TAATGTGCTC

CTTTACCCTT CCTCCTGAGG AGACTGCCAT TTTGCAAGGT CAACATCCTG

GCTCATGCTT ATTGTCTTCA CCTGGAGATC ATGAAGCTGG CCTGCTCTAG

GCTCATGCTT ATTGTCTTCA CCTGGAGATC ATGAAGCTGG CCTGCTCTAG

GCTCATGCTT ATTGTCTTCA CCTGGAGATC ATGAAGCTGG CCTGCTCTGG

GCTCATGCTT ATTGTCTTCT CCTGGAGATC ATGAAGCTGG CCTGCTCTGG

GCCCATGCTT ATTGTCTCCA CCTGGAGATC ATGAAGCTGG CCTGCTCAAG

GCCCATGCCT ATTGTCTGCA CCTAGAGATT ATGAAGCTGG CCTGCTCTAG

GCTCATGCCT ACTGTCTGCA TCTGGAGATC ATGAAACTAG CTTGCTCCAG

GCCCATGCCT ATTGTCTACA TCTGGAGATC ATGAAACTAG CCTGCTCCAG

GCTCATGCCT ATTGTTTGCA CCTAGAGGTC ATGAAGCTGG CCTGCTCTAG

TCCCATCCAT TTTGTTACCA CCCAGATATA ATCAGAGATA CATATTCCAA

TCCCACCCAT TTTGCTACCA CCCAGATATG ATCAAATACT CATATTCCAA

TCCCACCCAT TTTGCTACCA CCCAGATGTG ATCAAATACT CATATTCCAA

TCCCACCCAT TTTGCTACCA TCCAGATATG ATCAAATACA CACACTCTAA

GCCCATGCCT ACTGCCTGCA CCTAGAGATC ATGAAGCTGG CCTGTTCAAG

TCTCACTCAT ATTGTCTTCA TCAGGATACC ATGAAGCTGG CCTGCTCTGA

TCTCACTCAT ACTGCTTTCA TCAGGACACC ATGAAGCTGG CCTGCTCTGA

TCTCACTCCT ACTGCCTACA TCCAGACATC ATCAAACTCT CCTGCTCAGA

CATCACGCTT ACTGTCTCCA TCCCGACCTC ATCCACCTGC CCTGTGGGGA

CATCATTGTC AATCACATCT ATGGGCTCTT TGTTGTGGCC TGCACCGTGG

CATCATTGTC AATCACATCT ATGGGCTCTT TGTTGTGGCC TGCACCGTGG

CATCACTGTC AATCACATCT ACGGGCTCTT TGTTGTGGCC TGCACTGTGA

CATCACTGTC AATCACATCT ACGGGCTCTT TGTTGTGGCC TGCACTGTGA

CATCATTTTC AATCACATCT ATGGGCTCTT TGTTGTGACT TGCACTGTGG

CATCATAGTC AACCACATCT ACGGGCTCTT TGTTGTGGCC TGCACAGTGG

CATCATCGTC AATCATATCT ATGGGCTCTT TGTTGTGGCC TGTACTGTGG

CATCATTGTC AATCATATCT ATGGGCTCTT TGTTGTGGCC TGCACTGTGG

CATCATGGTG AATCACATCT ATGGGCTCTT CGTTGTGGCG TGCACTGTGG

CCCCTGGATC AACAGCTTTT TGGGCTTGTT TCTTCAGCTC TACATGACTA

CCCCTGGATC AGCAGTTTTT TGGGCATGTT TCTTCAGCTC TACCTGACTA

CCCCTGGATC AGCAGCTTTT TGGGCATGTT TCTTCAGCTC TACATGACTA

TCCCTGGATC AGCAGTTTTT TTGGCATGTT CCTTCAGCTG TATCTCACTA

CATCGTTGTC AACCACATCT ATGGGCTCTT TGTTGTGGCC TGCACCGTGG

CAACAAGATC GATGTCGTCT ATGGCTTCTT TGTTGCTCTC TGTACTATGC

CAACAAGATC AATGTGGTTT ATGGCTTCTT TGTTGCACTC TGTACTATGC

TACTAGGATA AATAGCATCT TTGGCTTGTT CACTGTCCTT TCCACCATGT

CACCACCTTC AACAGCTTAT TTGGCCTATT CATCGTGCTC TCCACATTCC

TGGACTCCCT GCTCATCTTT CTCTCATACG CCCTCATCCT TCGCACCGTG

TGGACTCCCT GCTCATCTTT CTCTCATACG CCCTCATCCT TCGCACCGTG

TGGACTCCCT GCTCATGTTT CTCTCATACG CCGCCATCCT TCGCACCGTG

TGGACTCCCT GCTCATGTTT CTCTCATACG CCGCCAATCT TCACACCGTG

TGGACTCCCT GCCCATCTTC CTCTCATACG CCCTCATTCT TTGCACCGTG

TGGACTCCCT GCTCATCTTC CTCTCGTACA CCGTTATCCT TCGCACTGTG

TAGACTCCCT ACTCATATTC CTCTCATATA CTCTCATCCT CCATGCTGTA

TAGACTCCCT GCTTATATTC CTCTCATACA CCCTCATCCT CCATGCTGTA

TGGACTCCCT CCTCATCTTC CTCTCGTATG CCCTTATCCT CCGCACCGTG

CTGACTTAAT GTTCATTCTT GTCTCCTATG TCCTGATAAT CCGTGCTGTC

CTGACTTACT CTTCATTCTT TTCTCCTACA TTCTGATTCT CCGAACTGTC

CTGACTTATT GTTCATTCTT TTCTCCTATG TCCTAATTCT TCGTACTGTC

CTGACTTACT GTTCATTCTT TTCTCCTATG CCCTGATTCT CCGTACTGTC

TGGACTCCCT ACTCATCTTC CTCTCCTATA CACTTATCCT CCGCACTGTG

TGGACTTGGC ACTGATTTTT CTGTCATATG TACTGATCTT GAGGACTGTA

TGGACTTAAC TTTGATTTTT GTGTCGTATG TTCTGATCTT GAGGACTGTA

TTGACTTTCT CCTCATTCTC TTCTCCTATG TCCTGATCCT AAAGACTGTG

TGGACTCCGT GCTCATCTTC CTTTCCTACG TCCTGATCCT CAGGTCCGTC

CTCAGCATTG CCTCCCACCA GGAGCGACTC CGAGCCCTCA ACACCTGTGT

CTCAGCATTG CCTCCCACCA GGAGCGACTC CGAGCCCTCA ACACCTGTGT

CTCAGCTTCA CATCCTGCCA GGAGCGACTC ---GCCCTCA ACACCTGTAT

CTCAGCTTCA CATCCTGCCA GGAGCGACTC ---GCCCTCA ACACCTGTAT

CTCAGCATTG CCTCCCGCCA GCAGCGACTC CGAGCCCTCA ACACCTGTGT

CTGAGTATCG CCTCCCGCCA AGAGCGACTC CGAGCCCTCA ACACCTGCGT

CTAGGCAAAG CCTCCCGTCA GGAGCGCCTC CGTGCTCTCA ATACCTGTAT

CTAGGCAAAG CCTCCCGTCA GGAGCGCCTC CGTGCTCTCA ATACCTGTAT

CTGAGCATCG CCTCCCGCCA GGAGCGACTT CGAGCCCTCA ACACCTGTGT

CTGAGCATCA TGACCCCCAA GAAGCAAAAG AAAGCTCTCA ACACTTGTGT

TTGAGCATTG TGGCCCCTAA GAAGCAACAA AAAGCTCTCA GCACTTGTGT

CTGAGCACTG TGGCCCCTAA GAAGCAGCAA AAAGCTTTCA GCACTTGCAT

CTGAGCATTG TAGCCCCCAA AAAGCAGCAG AAAGCTCTCA GTACCTGTGT

CTGAGCATCG CCTCCCGCCA GGAGCGACTT CGGGCCCTCA ACACCTGTGT

CTCAGCATTG GATCTTTGGA AGAGCGGCTC AAAGCTCTCA ACACCTGTGT

ATGAATATTG GCTCTCTGGA AGAAAGACTC AAAGCCCTCA ACACGTGTGT

CTGAGCATTG CATCCCATGG GGGACGCCTC AAAGCTCTCA ACACCTGCAT

CTGACCATCG TGTCCCAGTC AGGGCAGTCC AAGGCCCTCA ACACCTGTAT

CTCTCATATC TGTGCTGTAC TGCTCTTCTA CATCCCCATG ATTGGCTTGT

CTCTCATATC TGTGCTGTAC GGCTCTTCTA CATCCCCATG ATTGGCTTGT

CTCTCATATC TGTGCTGTAC TGCTCTTCTA CATCCCCATG ATTGGCTTAT

CTCTCATATC TGTGCTGTAC TGCTCTTCTA CATCCCCATG ATTGGCTTAT

CTCACATGTC TGTGCTGTGC TACTCTTCTA TATTCCCATG ATTGGGTTGT

CTCCCATATC TGTGCTGTGC TGCTCTTCTA CATCCCCATG ATTGGTTTGT

CTCTCATATC TGTGCAGTAC TGCTATTCTA CATCCCCATG ATTGGCTTAT

CTCTCATATC TGTGCAGTAC TGCTTTTCTA CATCCCCATG ATTGGCTTAT

CTCCCACATC TGTGCTGTGC TGCTCTTCTA CATCCCCATG ATTGGTTTGT

GTGTCACATC TGTGCTGTCA CTATTTTCTA TGTGCCAATG ATCAGCCTGT

CTGTCATATC TGTGCTGTCA CCATTTTCTA TGTGCCAATG ATCAGCCTGT

CTGTCACCTC TGTGCTGTTA CTATTTTCTA CGTTCCAATG ATCAGCTTGT

CTGTCACATC AGTGCTGTCA CCATTTTTTA TGTGCCAATG ATTAGCCTGT

CTCCCATATT TGTGCTGTGC TACTTTTTTA CATACCCATG ATTGGCTTGT

CTCCCACGTC TGTGCTGTGC TCATCTTCTA TGTGCCCATC ATCACCCTGT

GTCTCACTTT TGTGCTGTAC TTATCTTCTA TGTGCCCATC ATCACCCTGT

CTCCCATCTC TGTGCTGTGG TGCTTTTCTT CACTCCCATG GTCAGTCTAT

CTCCCACATC TGTGCGGTGC TCATCTTCTA CATCCCCATG CTGGGCGTGT

CTCTTGTGCA TCGCTTTGGT GAACATCTGC CCCGCGTTGT ACTCTTCATG

CTCTTGTGCA TCGCTTTGGT GAACATCTGC CCCGCGTTGT ACTCTTCATG

CTCTTGTGCA TCGTTTTGGT GAACATCTGC CCCGCGTTGT ACTCCTCATG

CTCTTGTACA TTGTTTTGGT GAACATCTGC CCCGCGTTGT ACTCCTCATG

CTCTTGTTCA TCGCTTCGGT GAACATCTGC CCCACGCTGT GCTCCTCATG

CTCTTGTGCA TCGCTTTGGC GAGCATCTGC CCCGCATTGT ACTCCTCATG

CCCTTGTACA TCGCTTTGGT GAACACCTTC CCCGCATTGT AATTCTGATG

CCCTTGTGCA TCGCTTTGGT GAGCACCTTC CCCGCATTGT ACTTCTGATG

CTCTCGTGCA CCGCTTTGGT GAACATCTGC CCCGTATTGT ACTCCTCATG

CCTTTACACA TCGCCTCCTT AGCTCTACTC CGAGGGTGAT CAGTATTTTG

CCCTTACACA TCGCCTTCTC AGCTCCACCC CAAGAGTGGT CAGCATTTTG

CCTTTACACA CCGCCTCCTG AACTCCACCT CCAGGGTAGT CAGCACTCTG

CTCTGACACA CCGTCTCCTC AACTCAACCC CAAGGGTGAT CAGCATTTTG

CTCTCATACA CCGCTTTGGT GAACATCTGC CCCATATCAT ACTCCTTATG

CTGCCATGCA TCGCTTTGCC AAACACAGCC CCCTTATTAT GATCCTTATT

CTGCCATGCA TCGCTTTGCC AAGCACAGCC CCCTCGTTAT GATCCTTATT

CTATGCTGCA CAGGTTTGGA CCAAAGCTTC CCTCCCATGT TGTGCTCTTG

CCATGATTCA CCGGTATGGG AAACGGGCCC CTCCATTCAT CACATTGATG

TCCTATGTGT ATCTGCTGGT ACCACCCCTT ATGAACCCCA TCATCTACAG

TCCTATGTGT ATCTGCTGGT ACCACCCCTT ATGAACCCCA TCATCTACAG

TCCTATGTGT ATCTGCTGGT ACCACCCCTC ATGAACCCCA TTGTCTACAG

TCCTATGTGT ATCTGCTGGT ACCACCCCTC ATGAACCCCA TTGTCTACAG

TCCTATGTAT ATCTGCTGGT ACCACCACTC ATGAACCCCA TTGTCTACAG

TCTTATGTGT ATCTGCTGGC CCCACCCCTC CTGAACCCCA TTGTCTACAG

TCTTATGTGT ATCTGTTGGT GCCACCCCTC ATGAACCCCA TTGTCTACAG

TCATATGTGT ATCTATTAGT ACCACCCCTC ATGAACCCCA TTGTCTACAG

TCTTACGTAT ATCTGCTGGT ACCACCCCTC ATGAACCCCA TTGTCTACAG

GCCAATGTGT ATTTGCTCTT ACCACCTGTA GTGAACCCTA TCATTTACAG

GCCAATGTTT ATTTGCTCTT ACCGCCTGTA CTAAACCCTA TCATTTACAG

GCCAACGTTT ATTTGCTCTT ACCACCTGTG CTGAACCCTA TCATTTACAG

GGCAATACTT ACTTGCTCTT ACCACCGGTA CTGAACCCTG TCATTTACAG

TCTTATGTGT ATCTGCTGGT GCCACCCCTC ATGAACCCCA TTGTCTACAG

GCAGATGTGT TCTTGTTAGT GCCACCCCTG ATGAACCCCA TTGTGTACTG

GCCGATGTCT TCTTGTTAGT GCCACCCGTG ATGAACCCTA TTGTGTATTG

GCTAACATGC ACTTTCTTAT CCCTCCTGTA ATGAACCCTA TTGTTTATGT

TCCATCGTCT ATCTCTTCGT GCCACCCATG CTCAACCCCA TCATCTATAG

CATCAAGACC AAGCAAATTC GCCAGCGCAT CATTAAGAAG TTTAACAGCA

CATCAAGACC AAGCAAATTC GCCAGCGCAT CATTAAGAAG TTTAACAGCA

CATCAAGACC AAGCAGATTC GCCAGTGCAT CATTAAGAAG TTTAATGGCA

CATCAAGACC AAGCAAATTC GCCAGTGCAT CATTAAGAAG TTTAACGGCA

CATCAAGACC AAGCAAATTC GCCAGCGCAT CATTAAGAAG TTTAACAGCA

CATCAAGACA AAGCAAATTC GCCAACGCAT CATTAAGAAG TTGAACAGCA

CATCAAGACC AAGCAAATAC GCCAACGTAT TATCAAGAAG TTTAATGAGA

CATCAAGACC AAGCAAATAC GACAGCGTAT CATCAAGAAG TTTAATGGAA

CATCAAGACC AAGCAAATCC GCCTCCGTAT TATTAAGAAG TTTAACAGCA

CTTGAAGACC AGGACGATCC GCCAGGCTAT GCTCCAGCTA CTTAACAATA

CTTGAAGACC AAGACGATCC GCCACGCTAT GCTCCAGCTG TTCAATAATA

CTTGAAGACC AAGACCATCC GCCAGGCTAT GCTCCAGCTG CTCAATAATA

TCTGAAGACC AAGACAATTC GTCGAGCTAT GTTGCAATTC TTCAATAATA

CATCAAAACC AAGCAAATCC GCCTACGCAT TGTTAAGAAG TTAAATGGCA

TGTGAAGACC CAGCAGATCC GGGAGAAGGT CTTAGCGAAG TTC-------

TGTAAAAACC CGGCAAATCC GGGAGAAGGT CTTGGGGAAG TTT--TAATA

AGTAAAAACC AAACAAATTC GAGAAAGGAT CATTAGGATC CTTAATATCA

CATCAAGACC AAGGATATCT ACAAAAAGAT GGACAAGATG C--AACACCA

GCAGCAGCGT TTCTGCTACC TTCCTGCTGA GTGGCATCCC TGGGCTGCGC

GCAGCAGCGT TTCTGCTACC TTCCTGCTGA GTGGCATCCC TGGGCTGCGC

GCAGTCACGT TTCTGCTACC TTCTTGCTGA GTGGCATTCC TGGGTTGCAC

GCAGCCACGT TTCTGCTACC TTCTTGCTGA GTGGCATTCC TGGGTTGCAC

GCAGCATTTC CTCCTCTACC TTCCTGCTGA GTGGCATTTC TGGGCTGCAC

GCAGCA---T TTCCTCTACC TTCCTGCTGA GCGGCATCCC CGGGCTGCAC

GCA------T GTCTTCTACC TTTTTGCTGA GTGGCATCCC TGGGCTGCAC

GCA------T GTCTTCTACC TTCTTGCTGA GTGGCATCCC TGGGCTGCAC

GCAGCC---T TTCCTCTACC TTTCTGCTGA CTGGCATTCC TGGGCTGCAC

CCAGTGCTCA GCCTCTGACC TTCCTCTTGA CGGGTATTCC AGGCCTGGCG

CCAATGCTCA ACCTCTGACC TTCTTCCTGA CGGGTATCCC AGGCCTGGCA

CCAATGATCA GCCTCTGACA TTCCTCCTGA CGGGTATCCC AGGCCTGGCA

CCAATGCTCA GACTCTGACC TTCTTCCTGA CGGGTATCCC AGGCCTGGCA

ACAGCAGCAT TCCCTCTACA TTCCTGCTGA GTGGCATTAC AGGTCAGCAC

-CAACAGTGT TCCATCCATC TTTTTCCTTT TAGGGATCCC AGGACTTACT

CCAGCCATGA TCCCTTCTTC TTCATCCTCA CGGGCATCCC TGGATTTGCC

CTGATGCCCG CCTGTCTGGC TTTATACTGA CAGGTATACC AGGAATGGAG

CCCAATCCCA CCCCTCCTCC TTCCTCCTGC TGGGGGTACC AGATCTGGCT

ATGCACATCT GGATCTCCAT CCCACTGTGC TTCATGTATC TGGTTTCCAT

ATGCACAGCT GGATCTCCAT CCCACTGTGC TTCACGTATC TGGTTTCCAT

GTGCACATCT GGATCTCCAT CCCACTGTGC TTCATGTACC TGGTTTCCAT

GTGCACATCT GGATCTCCAT CCCACTGTGC TTCATGTACC TGGTTTCCAT

ATGCACACCT GGATCTCCAT CCCACTGTGC TTCATGTACC TGGTCTCAAT

ATGCACGTCT GGATCTCCAT CCCGCTATGC TTCATGTACC TGGTTTCCAT

ATGCATATCT GGATCTCCCT CCCACTATGC CTCATGTACC TGGTTTCCAT

ATGCATATCT GGATCTCCCT TCCACTCTGT CTCATGTACC TGGTTTCCAT

ACGCACGTCT GGATCTCCAT CCCTCTGTGC TTCCTGTACC TAGTTTCCAT

GCCAAGATCT GGGTCTCCAT CCCTGTTTGC CTCCTGTATG CCGGCGCCCT

GCCCAGGTTT GGATCTCCAT CCCTTTTTGT CTCCTGTATG TCATCGCCCT

GCCCAGGTGT GGATCTCCAT TCCTATTTGT CTCCTGTATG TCACTGCCCT

GCCCAGGTCT GGATCTCCAT CCCGTTCTGC CTCCTATATG GCATTGCCCT

ATTCACATCT GGATCTCTAT CCCATTGTGC TTCACATACC TGGCTTCCAT

GCCCATGTCT GGATTTCTTT GCCCTTCTGT TTTGTTTACA TTATGGCTCT

TATCACATCT GGATCTCCAT CCCCTTCTGC TGTTTTTACA CCATCTCCAT

AAGAATTCTT GGATGGCATT CCCACTGGGA CTCCTCTACA CCCTCACTCT

GCACACAACT GGCTGAGCTT CCCCTTCTGC TCCGTGTACC TGATCGCTCT

CCCGGGCAAC TGCACAATTC TTTTTATCAT TAAAACAGAG CGCCTTCATG

CCTGGGCAAC TGCACAATTC TTTTTATCAT TAAAACAGAG CGCCTTCATG

CCTGGGCAAC TGCACAATTC TTTTCATCAT TAAAACAGAG TGTCTCCATG

CCTGGGCAAC TGCACAATTC TTTTCATCAT TAAAACAGAG TGCCTCCATG

CCTGGGCAAC TGCACAATTC TTTTTATCAT TAAAACAGAG CGCCTCCATG

CCTGGGCAAC TGCACGATCC TTTTTATCAT TAAAACAGAG CCCCTGCATG

CCTGGGCAAC TGCACAATTC TTTTTATCAT TAAGACAGAG CCCCTCCATG

CCTGGGTAAC TGCACAATCC TTTTTATAAT TAAGACAGAG CCCCTCCATG

CCTGGGAAAC TGCACCATTC TTTTTATCAT CAAAACAGAG CCCCTTCATG

CTCTGGGAAC AGTGTGATCC TGTTTGTGGT CCGAACAGAG C--CTTCATG

CTCTGGGAAC AGCATGATCC TATTTGTGGT C---GCAGAA T--CTCCATG

GTCTGGGAAC AGCATGATCC TGTTTATGGT CAGAGCAGAG C--CTCCATG

CACTGGAAAC AGCATGATCC TGTTTGTGGT CCGAGCAGAG T--CTCCATG

CCTGGGCAAC TGCATGGTTC TTTTTATCAT TAAAGCAGAG CCCCTCCATG

TATTGGCAAC GCCACCATTT TGATAGTCAT ATGGACTGAG AAACTCCGAG

CATGGGCAAC ACTACCATCC TCACTGTAAT TCAGACTGAT AGTCTCCATC

TCTTGGCAAT TGCACCATCT TGTCCATCAT TAAGATGGAT AGACTCCATG

CATGGGTAAC TGCACCATCC TGTTTATGAT CAAGACTGAG CAGCTCCACC

AACCTATGTA TCTCTTCCTG TCCATGCTGG CTCTGATTGA CCTGGGTCTC

AACCTATGTA TCTCTTCCTG TCCATGCTGG CTCTGATTGA CCTGGGTCTG

AACCTATGTA TCTCTTCTTG TCCATGCTGG CTCTGATTGA CCTGGGTCTG

AACCTATGTA TCTCTTCTTG TCCATGCTGG CTCTGATTGA CCTGGGTCTG

AGCCTATGTA TCTCTTCCTG TCCATGCTGG CTCTGATTGA CCTGGGTCTG

AGCCCATGTA CCTCTTCCTG TCCATGCTGG CTCTGACTGA CCTGGGTCTG

AACCCATGTA CCTCTTCCTG TCCATGCTTG CTTTGACTGA CCTGGGTCTC

AGCCCATGTA CCTCTTCCTG TCCATGCTCG CTCTGACTGA CCTTGGTCTC

AGCCCATGTA CCTCTTCCTA TCCATGCTGG CTCTCACAGA TCTGGGTCTG

AGCCCATGTA TTATTTCCTC TCTATGCTTT CAGCCACAGA CCTGAGCTTA

AACCAATGTA TTATTTCCTC TCTATGCTTT CAGCCACAGA CCTGAGCTTA

AGACCATGTA TTATTTCCTT TCTATGCTGT CAGCCACAGA CCTGAGCTTA

AGCCCATGTA TTATTTCCTC TCTATGCTTT CAACCACAGA TCTGAGCTTG

AGCCCATGTA CCTCTTCCTC TGCATGCTGG CTCTGACTGA CCTGGGTCTG

ATCCAATGTT CTTTTTCCTG GCTATACTGT CAGCCATAGA CGTGACTCTC

AGCCCATGTT CTACTTCTTG GCCATTCTGT CATCAATTGA TCTGGGACTA

AGCCCATGTA CTACTTTCTC TCCATCTTGG CACTGACTGA TGTGGGTCTC

AACCCATGTT CTACTTCCTG GCCATGCTGG CCACCGTTGA TCTGGGCTTG

TCCCTTTGCA CTCTCCCTAC AGTCCTGGGC ATCTTTTGGG TTGGAGCACG

TCCCTTTGCA CTCTCCCTAC AGTCCTGGGC ATCTTTTGGG TTGGAGCA--

TCCCTTTGCA CTCTCCCTAC AGTCCTCAGC ATCTTTTGGG TTGGGGCACG

TCCCTTTGCA CTCTCCCTAC AGTCCTCAGC ATCTTTTGGG TTGGGGCACG

TCCCTTTGCA CTCTGCCTAC AGTCCTGGGC ATCTTTTGGG TTGGAGCACG

TCCCTGTGCA CCCTCCCTAC AGTGCTGGGC ATCTTTTGGG TTGGGACACG

TCTCTTTGTA CCCTTCCTAC AGTGCTGGGC ATCTTTTGGG TGGGAGCACG

TCTCTTTGTA CCCTCCCTAC AGTGCTAGGC ATCTTTTGGG TGGGAGCACG

TCCCTCTGCA CCATCCCTAT GGTGCTGGGC ATCTTTTGGG TTGGGGCACA

TCCCTGTGCA CACTTTCCAC TACCCTTGGT GTCTTCTGGT TTGAAGCCCG

TCCCTGTGCA CACTTTCTAC TACCCTTGGT GTCTTCTGGT TTGAAGCTCG

TCCCTGTGCA CACTTGCCAC TACTCTTGGT GTGTTCTGGT TTGAAGCCCG

TGTCTGTGCA CGCTTTCCAC TACTCTGGGC GTCTTCTGGT TTGAAGCTAA

TCCCTTTGCA CACTCCCTAC AGTACTTGGT ATCTTTTGGG TTGGAGCACA

TCTACATCCT CAGTGCCCCG AATGTTGGGT ATCTTCTGGC TTGATGCTCA

TCCACATCAA CCATCCCTAA GATGCTTGGA ATCTTCTGGC TCAACCTAAG

TCTATATCCA CCCTGCCTTC CATGCTCAGC ATTTTCTGGT TCAATGCCCC

TCCTCGGCCA CCATCCCCAA GATGTTGGGC ATCTTCTGGC TCCACCTGCA

AGAAATTAGC CATGATGCCT GCTTTGCTCA GCTCTTTTTC ATTCACTGCT

-GAAATTAGC CATGATGCCT GCTTTGCTCA GCTCTTTTTC ATTCACTGCT

AGAAATTAGC CACGATGCCT GCTTTGCTCA GCTCTTTTTC ATTCACTGTT

AGAAATTAGC CACGATGCCT GCTTTGCTCA GCTCTTTTTC ATTCACTGCT

AGAAATAAGC CATGATGCCT GTTTTGCTCA GCTCTTCTTC ATTCACTGCT

GGAAATCAGT CATGATGCCT GTTTTGCCCA GCTCTTTTTC ATTCACTGTT

AGACATTGGT CATGATGCTT GCTTTACCCA GCTCTTTTTC ATCCACTGCT

AGACATTAGC CATGATGCTT GCTTTACCCA GCTCTTTTTC ATCCACTGCT

AGATATCGGT CATGACGCCT GTTTTGCCCA GCTCTTCTTC ATTCACTGCT

AGAGATCAAC TTAAATGCCT GTGTTGCTCA GATGTTCTTT CTCCATGGAT

AGAAATCACC TTAAGTGCCT GCATTGCCCA GATGTTCTTT CTCCATGGAT

AAAGATCAAC TTAAATGCTT GTATTACGCA GATGTTCTTT CTCCATGCAT

AGCGATCAAC ATCAATTCCT GCATTGCTCA GCTCTTCTTC ATCCACACTT

AGAAATTCAT CGTGATGCCT GCTTTGCTCA GCTCTTTTTC ATTCACAGCT

CGAGATTGGC TTTGGAGCTT GTGTGGCCCA GATGGTTCTG ATACACACCT

AGGAATCTCC TTTGAGGGCT GTCTCATCCA GATGTTTTTC ATTCACCTAT

TGAAATCCCC TTTGATGCCT GCATCACTCA GATGTTTTTC ATCCATGTGT

GGAGATCAGC TTTGGGAGCT GCCTAACCCA GATGTTCTTC ATCCACATGT

TCTCCTTCCT CGAGTCCTCT GTGCTACTGT CTATGGCCTT TGACCGCTTT

TCTCCTTCCT CGAGTCCTCT GTGCTACTGT CTATGGCCTT TGACCGCTTT

TATCCTTCCT CGAGTCCTCT GTGCTACTGT CTATGGCCTT TGACCGCTTT

TATCCTTCCT CGAGTCCTCT GTGCTACTGT CTATGGCCTT TGACCGCTTT

TGTCCTTCCT CGAGTCCTCT GTGCTACTGT CTATGGCCTT TGACCGCTTT

TGTCCTTCCT AGAGTCTTCC GTGCTGCTAT CTATGGCCTT TGACCGCTTT

TGTCCTTCTT GGAATCCTCT GTGCTTCTCT CTATGGCCTT TGATCGTTTT

TGTCCTTCTT AGAATCCTCT GTGCTTCTCT CTATGGCCTT TGATCGCTTT

TGTCCTTCTT GGAGTCCTCC GTGCTGCTGT CCATGGCCTT TGATCGCTTT

TTACTTTCAT GGAGTCTGGG GTTTTGCTGG CCATGGCCTT TGACCGTTTT

TTACTTTCAT GGAGTCTGGG GTTCTGCTGG CCATGGCCTT TGATCGTTTT

TTACTTTCAT GGAGTCTGGG GTTCTGCTGG CCATGGCCTT TGATCGTTTT

TGTCTCTTGT GGAGTCTTCG GTTCTATTAT CCATGTCTAT CGACCGCTAT

TATCCTTTCT GGAGTCCTCT GTGCTACTGT CTATGGCCTT TGACCGCTTT

TCTCAGGAAT GGAGTCTGCA GTCCTACTGG CTATGGCCTT TGACCGCTAT

GCACTGGCAT GGAGTCATCT GTACTTGTGG CCATGGCCTA TGATCGTTAT

TTGGAATCAC AGAATCTGGG GTGTTGGTAT CCATGGCCTT TGATCGCTTT

TCACCGGCAT GGAGAGCATG ATGCTCGTGG CCATGGGCTA TGACCGCTAC

GTGGCTATCT GCCACCCCTT GCACTATGTT TCCATTCTCA CCAACACAGT

GTGGCTATCT GCCACCCCTT GCACTATGTT TCCATTCTCA CCAACACAGT

GTGGCTATCT GCTGCCCCTT GCACTATGCT TCCATTCTCA CCAACACAGT

GTGGCTATCT GCTGCCCCTT GCACTATGCT TCCATTCTCA CCAACACAGT

GTGGCTATCT GCCGCCCCTT GCACTATGCT TCCATTCTCA CCAACACAGT

GTAGCCATCT GCCGTCCCTT GCACTACGCT TCAATCCTCA CCAACACGGC

GTGGCCATCT GTCGCCCTTT GCACTATGCT TCTATTCTCA CCCATACGGT

GTGGCTATCT GTCGTCCTTT GCACTATGCT TCCATCCTCA CCCACACAGT

GTGGCCATTT GCCGCCCCTT GCACTATGCT TCCATCCTCA CCAACACGGT

GTGGCCATCT GTGATCCACT GAGATACACC ACCATCCTCA CCAATGCCAG

GTAGCCATCT GTAATCCACT GAGATACACC ACCATCCTCA CCAATGCCAG

GTGGCCATCT GTGACCCACT GAGATATACC ACAATCCTCA CCAATGCCAG

GTTGCCATCT GCAACCCGCT GCGTTATTCC ACCGTCTTGA CCAATGCCAG

GTGGCCATTT GCCGCCCCTT GCACTACGCT TCCATCCTAA CCAACACAGT

GTGGCCATCT GTGAACCACT CCACTATACA ACCATTCTGA -TGTTTGGGT

GTGGCCATCT GTAACCCTCT TCATTACACA TTAGTGCTGA -AAACAAGGT

GTGGCTATTC GAGATCCACT GCGTTATTCT TCCATCCTCA CCTATGAGGT

GTGGCCATCT GCAAGCCTCT CCGCTACTGC ACCATCCTTA CCAATAAATC

CATTGGCAGG ATTGGCCTGG TCTCTCTGGG TCGTAGTGTA GCACTCATTT

CATTGGCAGG ATTGGCCTGG TCTCTCTGGG TCGTAGTGTA GCACTCATTT

CATTGGCAGG ATTGGCCTGA TCTCTCTGGG TCGTAGTGTA GCACTTATTT

CATTGGCAGG ATTGGCCTGA TCTCTCTGGG TCGTAGTGTA GCACTTATTT

CATTGGCAGG ATTGGCCTGG TCTCTCTGGG TCGTAGTATA GCGCTTATTT

CATTGGCAGG ATTGGCCTCG CCTCTCTGGG CCGTAGCGTA GTGCTTATTT

GATTGGCAGA ATAGGTCTGG CCTCTCTGGG CCGTAGTGTG GCACTTATTT

GATTGTCAGA ATAGGTCTGG CCTCTCTGGG CCGTAGTGTT GCACTCATTT

TATTGGCAGG ATTGGCCTGG CTTCCCTGGG CCGTAGTGTG GCACTCATTT

GATCGCCCAG ATTGGGATGA GCATGTTGAC AAGAAATGTT GCTGTGATGT

AATTACCCAG ATTGGGGTGA GCATGTTGAT AAGGAACGTT GCTGTCATGT

GATTGCCCAG ATTGGGGTGA GCATATTGAT AAGGAATGTT GCTGTCATGC

GATCATCCAG ATCGGGCTGA CCATGTTGAT AAGGAATGTT GCTGTCATGC

TGTTGGCAGG ATTGGTCTGG CCTCCTTGGG CCGCAGTGTA GCACTCATTT

GCTGGCAGGA ATTGGTCTGG GTATCATATT GAGTGCAGTT GTGCTCACAC

GGTATTCATC ATTGCGTTAA TCATTCTACT AAGACCTTTA TCGTTCGCCA

CATTGGCAAG ATTGGATTAG TTGTCCTTGC CCGTGCTGTC TTTGTGGTCC

CATTAGGGTT ATCGGGTTAG CTGTCCTGA- --GGAATTTC TCTCTGATAA

TTCCATTACC TTTTATGCTC AAAAGATTCC CCTATTGTGG CTCCCCAGTT

TTCCATTACC TTTTATGCTC AAAAGATTCC CCTATTGTGG CTCCCCAGTT

TTCCATTGCC TTTTATGCTC AAAAGATTCA CCTATTGTGG CTCCCCAGTT

TTCCATTGCC TTTTATGCTC AAAAGATTCA CCTATTGTGG CTCCCCAGTT

TTCCATTGCC TTTTATGCTC AAAAGATTCT CCTATTGTGG CTCCCCAGTT

TTCCATTGCC TTTTATGCTC AAAAGATTCC CCTACTGTGG CTCCCCAGTC

TCCCATTGCC TTTCATGCTC AAACGGTTCC CTTATTGTGG CTCCCTAGTT

TCCCATTGCC TTTCATGCTC AAACGGTTCC CTTACTGTGG CTCCCTAGTT

TCCCCTTGCC TTTCATGCTC AAAAGATTCC CCTACTGTGG CTCCCCTGTC

TGCCAGTTGT GCTTTTTGTC AAGAGGCTGT CCTTCTGCAG TTCTATGGTC

TACCAGTGGT GCTCTTTGTT AAGAGGCTGT CCTTCTGCGG TGCAGTGGTC

TGCCGGTTGT GCTGTTTGTC AAGAGGCTGT CCTTCTGCAA TTCTATGGTC

TACCAGTTGT GCTTTTTGTC AAGAGGCTGG TCTTCTGCAG GTCCTTGGTT

TCCCACTGCC CTTTATGCTC AAGAGATTCC CCTATTGTGG CTCCAGGATC

TACCCTTTAT CTACCTAATC CACCGTTTGC CCTTTTGTAA GGCCTGCATT

TACCTTTTGT TCTGCTTATC CTACGGTTGC CATTTTGTGG ACATCAAATT

TCCCTGCACC CTTGCTTATC AAGAGGCTGC CTTTCTACCA CTCCAATGTT

TTCCAATGGT GTTTTTTCTC TTGAGGTTGC CCTACTGTGG GCACCGAATC

CTCTCACATT CTTATTGTCT CCACCAAGAA GTGATGAAAT TGGCCTGTGC

CTCTCACATT CTTATTGTCT CCACCAAGAA GTGATGAAAT TGGCCTGTGC

CTCTCACATT CTTATTGTCT CCACCAAGAA GTGATGAAAT TGGCCTGTGC

CTCTCACATT CTTATTGTCT CCACCAAGAA GTGATGAAAT TGGCCTGTGC

CTCTCACATT CTTACTGTCT CCACCAAGAA GTGATGAAAT TGGCCTGTGC

CTCTCCCATT CCTACTGTCT CCACCAAGAA GTGATGAAAT TGGCCTGTGC

CTCTCACATT CCTATTGTCT CCACCAAGAA GTGATGAAAC TGGCCTGTGC

CTCTCACATT CCTATTGTCT CCATCAAGAA GTGATGAAAC TGGCCTGTGC

CTTTCACACT CCTATTGTCT CCACCAAGAA GTAATGAAAT TGGCCTGTGG

CTCTCACATT CTTACTGCTA CCATGTTGAT CTCATTCAAC TCTCATGCAC

CTTTCACATT CTTACTGTTA CCATGTTGAC CTCATTCAAC TCTCATGCAC

CTTTCACATT CTTACTGCTA CCATGTTGAT CTCATTCAAC TCTCATGCAC

CTTTCACATT CCTACTGCTA CCATGTCGAT CTTATTCAAC TCTCATGCAC

CTCTCACATT CCTACTGTCT CCACCAGGAA GTGATGAAAT TGGCCTGTGC

CTTGCCCATA CCTATTGTGA GCACATGGGA ATTGCCAAGT TGGCTTGTGC

ATCCTTCATA CTTATTGTGA GCATATGGGC ATTGCCCGCC TATCCTGTGC

CTCTCTCACT CTTACTGCCT CCATCAGGAT GTCATGCGAC TTGCTTCTGC

ATCCCCCACA CCTATTGTGA GCACATGGGC ATTGCCCGGT TGGCATGTGC

CGACATGAAG GCCAACAGCA TCTACGGCAT GTTTGTCATC GTCTCTACAG

CGACATGAAG GCCAACAGCA TCTACGGCAT GTTTGTCATC GTCTCTACAG

CAATATGAAG GCCAACAGCA TCTATGGCAT GTTTGTCATC GTCTCTACAG

CAATATGAAG GCCAACAGCA TCTATGGCAT GTTTGTCATC GTCTCTACAG

TGACATCAAG GCCAACAGCA TCTACGGCAT GTTTGTCATT GTCTCCACAG

AGACATTGAG GCCAACAGCA TCTATGGCAT GTTTGTCATC GTTTCCACGG

AGACATCAAG GCAAACAGCA TTTATGGTAT GTTCGTCATT GTTTCTACAG

AGACATCAAG GCAAACAGCA TTTATGGCAT GTTCGTCATT GTTTCTACAG

AGACATCAAG GCAAATAGCG TCTATGGCAT GTTTGTCATT GTTTCCACGG

AGACAACAGA ATCAACAGCA TCCTTGGTCT GTTTGCACTC TTCTCCACGA

AGACAACAGA ATCAACAGCA TCCTCGGTCT GTTTGCACTT TTCTCCACAA

AGACAACAGA ATCAACAGCA TCCTTGGTCT GTTTGCACTC TTCTCCACAA

AGATAACAGA ATCAACAGCA TTCTGGGTCT GATTGCACTC TTCTCCACAA

AGACATCAAG GCCAACAGTA TCTATGGCAT GTTTGTCATT GTTTCCACAG

AGACATACAA ATCAACACCA TTTATGGGCT CTTTGT---T GCTTCCTTCC

AAGCATA-AG GTCAACATCA TCTATGGCTT ATGTGC---C ATTTCCATCC

CAGCACACGT GTCAACATAC TTTATGGTCT TATTGCTGTC ATCTGTACTC

AAGTATCAAA CTCGACATCA TTTATGGTTT ATGTGTCATC TGCCTTATTC

TGGGTATAGA CTCACTGCTC ATCCTCTTCT CTTATGCTCT GATCCTGCGC

TGGGTATAGA CTCACTGCTC ATCCTCTTCT CTTATGCTCT GATCCTGCGC

TGGGTATAGA CTCACTGCTC ATCCTCTTCT CTTACGCTCT GATCCTGCAC

TGGGTATAGA CTCACTGCTC ATCCTCTTCT CTTACGCTCT GATCCTGCAC

TGGGTATAGA CTCCCTGCTC ATCCTCTTCT CTTACTCCCT GATCCTGCGC

TGGGTGTAGA CTCGGTCCTC ATCCTCTTCT CTTACGCCCT GATCCTACGC

TGGGGGTGGA CTCTCTGCTC ATCCTCTTCT CCTATGCACT TATCCTGCGC

TGGGAGTGGA CTCTTTGCTC ATCCTCTTCT CCTATGCACT TATTCTGCGC

TGGGTGTGGA CTCACTGCTC ATTCTCTTCT CTTACGCCAT GATCCTGCGC

CAGGGTTTGA CTGTCCTTGT ATCTTGCTCT CCTATGTCCT GATCATCCGA

CAGGATTTGA TTGTCCTTGC ATCTTGCTCT CCTATGTACT GATCATCCGG

CAGGATTTGA CTGTCCTTGC ATCTTGCTCT CCTATGTTCT GATCATCCAA

CAGGATTTGA CTGTCCTTGC ATCTTGATCT CCTATGTCCT TATTATTCGT

TGGGTATAGA CTCACTGCTC ATCCTCTTCT CCTACGCCCT GATTCTGCGC

TTGTCTTTGA TCTCATACTG GTTGGGATCT CCTATGGCTA TATTATCCAG

TGGTATTTGA CATCATAGCC ATTATCCTCT CCTATGTCCT GATCCTTCGT

TGGGATTGGA TGTTCTTGTC ATTCTTTTCT CTTATATTCT CATCTTGAAG

T---TTTGGA CATACTGCTG ATTGCCCTCT CCTACATCAG GATCCTCCAC

ACCGTGCTGT CCATCGCCTC CAGGGCTGAG AGATTCAAGG CCCTTAACAC

ACCGTGCTGT CCATCGCCTC CAGGGCTGAG AGATTCAAGG CCCTTAACAC

ACCGTGCTGT CCATCGCCTC CAGGGCTGAG AGACTCAAGG CTCTTAACAC

ACCGTGCTGT CCGTCACCTC CAGGGCTGAG AGACTCAAGG CTCTTAACAC

ACTGTGCTTT CCATCGCCTC CAGGGCTGAG AGACTCAAGG CTTTTAACAC

ACTGTGCTGT GCATTGCCTC CAGGACAGAG AGACTCAAAG CTCTTAACAC

ACTGTGTTAT CCATTGCCTC AAGGGCTGAA AGACTCAAAG CTCTCAATAC

ACCGTATTGT CCATTGCCTC AAGGGCTGAA AGACTCAAAG CTCTCAATAC

ACTGTGCTCT CCATTGCCTC CAGGGCCGAG AGACTCAA-G CTCTTAACAC

TCTGTCCTCA GCATCACCTC CATAGAGGGC AGGCAGAAAG CCTTCAACAC

TCTGTTCTCA GCATTGCCTC CTCAGAGGGG CGGCAAAAAG CCTTCAACAC

TCTGTCCTCA ACATTGCTTC CTCAGAGGGA CGGCAGAAAG CCTTCAATAC

TCCGTCCTTG GCATTGCTTC CTCAGAGGGA CGGCGGAAAG CCTTCAACAC

ACCGTGCTGA CCATTGCCTC CAGGGCCGAG AGACTCAAAG CTCTTAACAC

GCTGTATTCC GCCTCAAATC TCAAGGTGCC CGTCTCAAAA CACTGAGTAC

GCTGTCTTTC GACTCCCTTC CCATGATGCT CGGATCAAAG CTCTCAGCAC

ACTGTACTGG GCATCGCCTC ATGGGCTGAG AGGTTCAAAG CCTTCAATAC

ACCATCTTCA GACTGCCCTC TCAGGACGCA CGACTCAAAG CCATAAACAC

CTGTGTTTCC CACATCTGTG CTGTGCTGCT CTTCTACACT CCCATGATTG

CTGTGTTTCC CACATCTGTG CTGTGCTGCT CTTCTACACT CCCATGATTG

CTGTGTTTCC CACATCTGTG CTGTGCTGCT CTTCTACGCT CCCATGATTG

CTGTGTTTCC CACATCTGTG CTGTGCTACT CTTCTACACT CCCATGATTG

CTGTGTCTCC CACATCTGTG CTGTGCTGCT CTTCTACACT CCCATGATTG

CTGTGTTTCC CACGTTTGCG CTGTGCTCCT CTTCTACACG CCCATGATTG

ATGTGTATCA CACATCTCTG CTGTGCTTCT GTTCTATACT CCCATGATTG

ATGCGTTTCA CACATCTCTG CTGTGCTTCT GTTCTATACT CCCATGATAG

GTGTGTTTCC CACATCTGTG CTGTTCT-CT CTTCTACATC CCCATGATTG

CTGCATTTCC CACATCAGTG CTGTTGCCAT CTTCTACATC CCTCTCATCA

CTGCATATCC CACATCAGTG CTGTTGCCAT CTTTTACATC CCCCTTGTCA

TTGCATATCG CACATCAGTG CTGTTGCCAT CTTCTACATC CCTCTCATCA

TTGCATATCT CACATCAGTG CTGTTGCTAT CTTCTACATC CCTCTCATCA

ATGTGTCTCC CATGTCTGTG CCGTACTCCT CTTCTATACT CCCATGATTG

TTGTGGCTCC CATGCTCTAG TTATGTTTGT TTTCTATACC CCTGCCCTCT

CTGTGGCTCT CATGTATGTG TCATTCTAGT GCTCTATGTC CCCATGGTGA

CTGCCTCTCT CACATCTGTG CTTGTCTGCT CTTCTACATA CCTTTTATTG

TTGCATCTCC CACATCTGTG TCATCCTGGC CTTCTTCACC CCGGCCCTCT

GCCTCTCTGT CATCCATCGC TTTGGAAAGC AGGCACCCCA CCTGGTCCAG

GCCTCTCTGT CATCCATCGC TTTGGAAAGC AGGCACCCCA CCTGGTCCAG

GCCTCTCTGT CATCCATCGC TTTGGAAAGC AGGCACCCAC CCTGGTCCAG

GCCTCTCTGT CATCCATCGC TTTGGAAAGC AGGCACCCAC CCTGGTCCAG

GCCTCTCTGT CATCCATCGC TTTGGAAAGC AGGTACCCCA CCTGGTCCAG

GCTTGTCTGT TATCCACCGC TTTGGACAGC AGATGCCCCA CCTGGTGCAG

GATTGTCTGT AATCCACCGC TTTGGGAAGC AGGCGCCCCA TCTAGTTCAG

GATTGTCTGT AATCCACCGC TTTGGGAAAC AGGCCCCCCA TCTAGTTCAG

GTTTGTCAAT GACTCATCGT TTTGCCAAAC ATGCCTCTCC ACTGGTCCAT

GCCTGTCTCT TGTCCATCGC TATGGCCATT CAGCACCTCC CTTTGTCCAC

GCTTGTCTCT TGTCCATCGC TATGGCCATT CAGCACCTCC ATTTGTCCAC

GCTTGTCTCT TGTCCATCGC TATGGACATT CAGCACCTCC ATTTGTCCAC

GCTTGTCTCT TGTCCATCGC TATGGCCATT CGGCACCTGC ATTTGTCCAC

GCTTGTCCAT CATCCACCGT TTTGGGAAGC AGGCACCCCA TCTGGTCCAG

TCTCCTTCCT CACGCACAGG TTTGTCCAAA ACATACCCCC TTATATCCAC

GTTTGTCCAT GACTCATCGC TTTGCAAAAC ATACTTCACC ACTGGTCCAT

GTGCCACGAT GGTCCACCGT TTTGGGAAAC ACCTCTCACC CATTGTGCAC

TCTCCTTCCT CACCCACCGC TTTGGACACA AAATCCCTGG CTACGTCCAC

GTGGTCATGG GTTTCATGTA TCTTCTCTTT CCTCCTGTGA TGAATCCCAT

GTGGTCATGG GTTTCATGTA TCTTCTCTTT CCTCCTGTGA TGAATCCCAT

GTGGTCATGG GTTTCATGTA TCTTCTCTTT CCTCCTGTGA TGAATCCCAT

GTGGTCATGG GTTTCATGTA TCTTCTCTTT CCTCCCATGA TGAATCCCAT

GTGGTCATGG GTTTCATGTA TCTTCTCTTC CCTCCTGTGA TGAATCCCAT

GTGCTCATGG GTTTTGTGTA TCTTCTTTTC CCGCCCCTGA TGAATCCCAT

GTGGTCATGG GCTTTGTGTA TCTCCTCTTC CCTCCTGTGA TGAACCCCAT

GTGGTCATGG GCTTTGTGTA TCTCCTCTTC CCTCCTGTGA TGAACCCTAT

GTTATCATGG CCAACGTCTA CCTGTTGGCA CCTCCCGTGA TGAACCCTAT

ACCATCATGG CCAATATCTT CCTGCTTATT CCTCCTGTAC TCAACCCTAT

ACCATCATGG CCAATGTCTT CCTTCTCATC CCTCCCGTGC TCAACCCCAT

ACCATCATGG CCAATGTCTT CCTGCTCATC CCTCCTGTGC TCAACCCTAT

ACTATTATGG CCAATGTTTT CCTGCTTATT CCTCCTGTGC TCAACCCTAT

GTGCTCATGG GCTTCGTGTA TCTTCTTTTC CCTCCCCTGC TGAACCCCAT

ATCCTTGTTG CCAACATCTA TGTCGTCATC CCACCTGCAC TCAACCCTGT

GTGACTATGG CCAATATCTA CTTGCTGGCT CCTCCTGTGA TGAACCCCAT

ACAGTGATGG CCAATGTGTA TCTTTTACTC CCACCTGTAC TTAACCCCCT

ATCCTCCTGG CCAACCTGTA CGTGGTCGTC CCGCCCGCCC TCAACCCCAT

TGTCTACAGT GTGAAGACCA AACAGATCCG GGATCGAGTG ACGCATGCCT

TGTCTACAGT GTGAAGACCA AACAGATCCG GGATCGAGTG ACGCATACC-

TGTCTACAGT GTGAAGACTA AACAGATCCG GGATCGAGTG ACCCATGCCT

TGTCTACAGT GTGAAGACGA AACAGATCCG GGATCGAGTG ACCCATGCCT

TGTCTACAGT GTGAAGACCA AACAGATCCG GGATCGAGTG ACCCATGCCT

TGTCTACAGT GTGAAGACAA AACAGATCCG AGATCGGGTG ACCCATGCCT

AGTCTATAGT GTCAAGACCA AACAGATCCG GGATAGAGTA ACCAATGCCT

TGTCTATAGT GTCAAGACCA AACAGATACG GGATAGGGTA GCCCATGCCT

CATTTACAGT GCAAAGACTA AGCAGATCCG CCAGCGGATT ACCCACCTCC

CATCTACAGT GTGAAGACTA AGCAGATTC- ---GAGGATC ATCCATGTGT

CATCTATAGT GTGAAGACTA AGCAGATTC- ---GCGAATC ATACACATGT

CATCTACAGT GTGAAGACGA AGCAGATTC- ---GCGGATC ATCCGCGTGT

AATCTATAGT GTGAAGACTA AACAGATTC- ---GCGGATT ATCTATGTCT

TGTCTACAGT GTGAAGACCA AGCAGATCCG AGATCGGGTG GCCCGAGCCT

TATTTATGGG ATAATGACCA AGCAAATCAG AGAGTATGTG ATCCGAGTAT

CATTTATAGT ATAAAGACTA AGCAGATCCG ---------- ----------

CATCTACAGC ATAAAGACCA AACAGATTCG TAAGCGGATA GTCCAACTAT

CATCTATGGG GTGAGGACCA AGCAGATCCG TGAACGTGTA GCCAGCATTT

TTACTCACGA TGTTATTCGA AAGACTGGAA TAGCTGTCCT CACCCGGGCA

--AATCACGA TGTTATTCGA AAGATTGGAA TAGCTATCCT CACCCGGGCA

TTACTCACAG TGTTATTGGA AAGATTGGAA TAGCTGTCCT CATCCGGGCA

TT-------- ---------- ---------- ---------- ----------

TTACTCATGG TGTTATTGGA AAGATTGGAA TAGCTATCTT CACCCGGGCA

TTACCCATGA TGTTATTGTA AAGATTGGAA TAGCTGCCTT CACCCGGGCA

TTAC------ ---AGTCAGG AGGGTCTTTA AAACTGGCCT AATATTTGCT

TTAC------ ---AGTCAGA AGGGTCTTTA AAACTGGCCT AATATTTGCT

TCACTCATGG CATCATTGGA AAGATTGGGA TAATTGTGCT CACAAGAGCA

T-ACCCATGG CATCATTGGC AAGATTGGAA TAGCTGTCAT CATCCGGGCA

T-ACCCATGG CATCATTGGA AAGATTGGAA TAGTTGTCCT CACCCGGGCA

T-ACCCACAC TATCATTGGC AAGATCGGAA TAGCTGTCCT CACCCGGGCA

T-ACCCATGG TGTCATTGGC AAGATTGGAA TAGTTGTTCT CACTCGGGCA

TTACAAATGG TGTCATCATT GGGATTGGGT TGGCAATTGC TGGAAGGGCC

--ACCCATGG TGTCATTGGC AAGATAGGTG CAGCAGTCCT TCTGAGAAGT

--ACCAATGC GAGAATTGTC CACATGGGT- --GCA-TGCT GATCAGGAGT

TTACCTCTGG GGTCATTGGC AAGATAGGAT TAGGTGTCAT TGCCCGAACT

T--CCAATGC CAGGATCACT AAGATGGGTC TGGCCATTTT CTCCCGCACC

GTCTGTGTGG TATTCCCTGT GCCCTTCCTT ATAAAGTGCC TACCCTTCTG

GTCTGTGTGG TATTCCCTGT GCCCTTCCTT ATAAAGTGCC TACCCTTCTG

ATCTGTGTGG TCTTCCCTGT GCCCTTCCTT ATAAAGCATC TACCCTTCTG

---------- ---------- ---------- ---------- -------C--

GTCTGTGTGG TCTTCCCTGT GCCCTTCCTT ATAAAACGCC TACCTTTCTG

ATCTGTGTAG TCTTCCCTGC ACCCTTCCTT ATAAAGCGGC TACCCTTCTG

ATCTGTGTTA TTCTACCATT TCCCTTTACA CTAAAGAGGT TGAAATTCTG

ATCTGCGTTG TTCTGCCATT TCCCTTTATA CTAAAGAGGT TAAAGTTCTG

GTCTGTGTGG TCTTCCCTGT GCCTTTCCTG ATAAAGAGGC TACCCTTCTG

GTCTGCGTGG TCTTCCCTGT GCCCTTTCTT ATAAAGCGGC TACCCTTCTG

GTCTGTGTGG TGTTCCCTGT GCCCTTCCTT ATAAAGCGGC TACCTTTTTG

GTCTGTGTCG TCTTCCCCGT GCCCTTCCTT ATAAAGCGGC TGCCCTTTTG

TTCTCTGTGG TCTTCCCCGT GCCTTTTCTT ATAAAGAGGC TGCCCTTCTG

CTGGCTCTGG TTTTTCCAGC TTCCTTCCTC CTAAAGAGGC TTCAGTATCG

GTAGGAGCTG TGTTACCAGT GCCTTTTCTT ATCAAAAGGC TACCTTTCTG

GTTGCTGTCA TGTTGCCAGT TGTGCTCTTC GTCAAGAGAT TGTCCTTCTG

GTCTGTGTGG TCTTCCCTGT GCCATTTCTG ATCAAGAGGC TGCCTTTCTG

TTTGGGGCCG TCTTCCCCCT GCCCGTCCTC CTGAAACGCC TGCCCTTCTG

CCATTCCAAT GTCTTGTCTC ATTCATACTG TCTTCACCAA AACATGATGC

CCATTCCAAT GTCTTGTCTC ATTCATACTG TCTTCACCAA AACATGATGC

CCATTCCAAT GTCTTGTCTC ATTCATATCA TCTTCACCAA GATATGATGT

---------- ---------- ---------- ---------- ----------

CCATTCCAAT GTCTTGTCTC ATTCATATTG TCTTCACCAA GACATGATGC

CCAATCCAAT ATCTTGTCTC ATTCATACTG TCTTCACCAA GATGCAATGA

TAAGAAAAGT CTGTTATCTC ATTCTTACTG CCTTCACCAG GACGTTATGA

TAAGAAAAGT CTCTTATCTC ATTCTTACTG CCTTCACCAG GATGTTATGA

CCATTCCAAT GTCTTGTCTC ATTCATATTG TCTTCATCAG GATGCAATGA

CCATTCCAAT GTCTTGTCTC ATTCGTACTG TCTCCACCAA GATGCAATGC

CCATTCCAAT GTCTTGTCTC ATTCTTATTG TCTCCACCAA GATGCAATGA

CAGATCCAAC ATCTTGTCTC ATTCATACTG TCTCCACCAA GATGCAATGC

CCGATCCAAT GTTTTGTCTC ATTCATACTG TCTCCACCAA GATGCCATGC

TGCTGTCAAC ATTCTCTCCT ATCCCTTCTG CCTTCACCAG GACCTCATAA

CAACTCCAAT GTCCTTTCCC ACGCGTACTG CCTCCATCAG GATGCCATGA

TAGGTCTATG GTCCTCTCCC TTTCTTACTG CTACCATGTT GATCTCATTC

CAGTTCTAAT GTCCTCTCCC ACTCTTACTG CCTCCACCAG GATGCCATGC

CCACCAAAAT GTGCTCTCCC ACTCCTACTG TCTCCACTCC AACCTCATCA

GGCTAGCTTG TGCCAGCACC CGCATCAACA GCCTCTACGG CCTCATCGTC

GGCTAGCTTG TGCCAGCACC CGCGTCAACA GCCTCTACGG CCTCATCGTC

GGCTAGCTTG TGCCAGCACC CATGTCAACA GCCTCTATGG CTTCAT-GTC

---------- ---------- ---------- ---------- ----------

GGCTAGCCTG TGGCAGCACC CACATCAACA GCTTCTATGG CCTCATCGTC

GGCTAGCCTG TGCCAGCACC CGCATCAACA TCCTATATGG CCTCATTGTT

AAGTAGCTTG TTCTGACAAC AGAATCAATG TGATCTATGG GTTTTTTGTA

AGGTAGCTTG TTCCAACAAC AGAATCAACG TAATCTATGG ATTTTTTGTG

GGCTAGCCTG TGCCAGCACA CGCATTAACA ACCTCTATGG CCTCATCACT

GGTTAGCCTG TGCCAGCACT CGTGTCAACA GCCTCTATGG CCTCATAGTA

GGCTAGCCTG TGCCAGTACC CGCATCAACA GTCTCTACGG CCTCATCATA

GGCTAGCCTG TGCCAGCACC CGCGTCAACA GCCTCTACGG CCTCCTCATA

GACTAGCTTG TGCCAGTACC CGCGTCAACA GCCTCTATGG TCTCATCATA

AGACAACTGT ATCCAGTCGT CGGGTTAGCA GTATCTATGG CCTCATGGTG

GGCTAGCCTG TGCTGACACC CGTGTCAATA GCATCTATGG CTTACTGGCT

ATCTCTCATG TTCCAACAAC AGAATCAACA GCATTCTAGG CCTGCTTGCA

GACTTGCTTG TGCTAGCATA CGTGTCAATA TCCTTTATGG CCTAATCACT

GCCTCCCGTG CACCGACACG ACCCTCAACA ACATCTATGG CCTCTTCGTC

GTCATCTTCA CACTGGGGCT CGATGTTCTC CTCACTCTAC TGTCTTATGT

GTCATCTTCA CACTGGGGCT CGATGTTCTC CTCACTCTAC TGTCTTATGT

GTCATCTTCA CACTGGGGCT CGATGCCCTC CTCATTCTAA TGTCTTATGT

---------- ---------- ---------- ---------- ----------

GTCATCTTCA CACTGGGGCT CGATGCCCTC CTCATTCTAA TGTCTTATGT

GTCATCCTCA CTCTGGGGCT TGATGCCATT GTTATCCTCT TGTCCTATGT

GCTATTTTAT CTCTGATACT TGGTGT---- -GCATTTCTG TGTCTTACAT

GCTACTTCAT CTCTGATACT TGGTGT---- -TCATTTCTG TGTCTTACAT

GTCATCTTAA CATTAGGGCT TGATGCCTTC ATCATTCTCT TTTCTTACAT

GTCATCTTCA CTTTGGGGTT TGATGCCTTC ATCATTCTCT TTTCTTATGT

GTCATCTTCA CATTGGGGCT GGATGCTATT ATCATTCTCT TTTCTTATGT

GTCATCTTCA CATTGGGGCT GGATGCCCTC GTCATTCTCT TTTCTTACGT

GTCATCCTCA CCTTGGGACT GGATGCCCTC ATGATTCTTT TTTCTTATGT

GTCATCTGCT CTATGGGACT TGATTCTGTG CTTCTCCTGC TCTCCTATCT

GTGATCTTCA CCATTGTGCT AGACGCCTTG ATTCTTCTGG TCTCTTATAT

CTCTTCTCCA CAACAGGATT TGATTCCTTG CATC--CTAC TCTCCTATGT

GTCATTTGTA CTCTGGGACT GGATGCTCTT ATTATTCTCT TCTCTTATAT

GTCCTCTCCA CCTTCGGGCT CGACTCTGTG CTCATCGCCC TCTCCTACGC

ACTCACCCTG AAGACTGTGC TGGGCATTGT CTCCAGAGGT GAAAGGCTGA

ACTCACCCTG AAGACTGTGC TGGGCATTGT CTCCAGAGGT GAAAGGCTGA

ACTCATCCTG AAGACTGTGC TGGGCATTGC CTCCAGAGGT GAAAGGCTCA

---------- ---------- ---------- ---------- ----------

ACTCATCCTG AAAACTGTGC TGGGCATTGC CTCCAGTGGT GAAAGGCTCA

CCTTATCCTA AAGACTGTGC TGGGCATTGC CTCCAATGCT GAAAGGCTCA

GCTGATCCTT AAAATTGTCC TGGGCATTAC TTCACAGAAG GGTCGCCTCA

GCTGATCCTT AAAATTGTCC TGGGCATTAC TTCCCATAAG GGTCGCCTCA

ACTCATCCTG AAGACTGTGC TGGGCATTGC CTCCAGAGCT GAAAGGCTCA

GCTCATCCTG AAGACAGTAC TGGATATCGC TTCCAGAGCT GAAAGGCTCA

GCTCATCCTG AAGACTGTGC TGGGCATTGC TTCCAGAGAT GAAAGGCTCA

GCTCATCCTG AAGACTGTGC TGGGCATTGC TTCCAGAGCT GAAAGGCTCA

TCTCATCCTG AACACAGTGC TGGGAATTGC CTCCAGGGAT GAAAGGCTCA

CCTCATCCTT GGCACAGTGT TGAGGATAGC CTCCAAAGCA GAGAGGGTCA

TCTAATCTTT CATGCAGTCT TGGGCATTGC TTCCTGGGAA GAGAGGCTCA

CTTGATCATT CGGTCTGTTC TCCGCATTGC TTCCTCAGAG GAACAGCAGA

GCTTATCTTG AAGACTGTAC TAGGTATTGC CTCATGGGCT GAGAGGCTCA

CTTGATCCTC AAGACCGTGC TGGGTATCGT GTCAGGGGAT GGGTGGCTCA

AAACCCTCAG CACATGCCTC TCTCACATGT CTACCGTGCT CCTCTTCTAT

AAACCCTCAG CACATGCCTC TCTCACATGT CTGCCGTGCT CCTCTTCTAT

CAACCCTCAG CACCTGCCTC TCTCACATGT CTGCCATGTT CCTCTTCTAT

---------- ---------- ---------- ---------- ----------

AAACCCTCAG CACCTGCCTC TCTCACATGT TCGCTGTGCT CCTCTTCTAT

AAGCCCTCAA CACCTGCTTC GCTCACATTT GTGCTATGCT CCTCTTCTAT

AGTTCTTTAA CACTTGTGTC TCTCATATCT GTGCTGTGCT CATCTTCTAC

AGTTCTTTAA CACTTGCATC TCTCATATCT GTGCTGTGCT CATCTTCTAT

AATCCCTCAA CACCTGTCTC TCTCACATCT GTGCTGTTCT CCTTTTCTAT

AAGCGCTCAA CACCTGCCTC TCCCACATCT GTGCTGTGCT CCTTTTTTAT

AAGCTCTCAA CACCTGCCTC TCTCACATCT GTGCTGTGCT CCTCTTCTAT

AAGCCCTCAA CACCTGTCTC TCTCACATCT GTGCTGTGCT CCTCTTCTAT

AAGCTCTCAA TACATGTCTC TCTCACATCT GTGCTGTTCT TCTCTTCTAT

AAGCCCTCAA CACCTGCATC TCCCACATCT GTGCTGTACT CACCTTCTAT

AGGCTCTCAA TACTTGCCTC TCTCATATTT TTGCAGTGCT GCTTTTCTAT

AAGCCTTCAA CACCTGCATA TCCCATATTG GTGCAGTTGC CATCTTCTAC

AGGCCTTCAA TACCTGCCTC TCCCATATCT GTGCTGCATT GCTCTTCTAT

AGGCCCTCAA CACTTGTGTC TCCCACATGT GTGCTGTTCT GGTCTACTAC

GTTCCTTTTA TGGGTGCTGC CTCCATGATC CACAGATTTT GGGAGCATTT

GTTCCTTTTA TGGG---TGC CTCCATGATC CACAGATTTT GGGAGCATTT

GTTCCTTTTA TTGG---TGC CTCCATGATT CACAGATATG GGGAACATTT

---------- ---------- ---------- ---------- ----------

GTTCCTTTTA TTGG---TTC CTCCATGATT CACAGATTTG GTAAGCATTT

GTTCCTCTTA TTGG---TGC CACCATGATC CACAGATTTG GGAAGCATTT

GTGCCCATCA TCAC---CTT GGCAGCCCTT CATCGTTTTG CTAAAAACGT

GTGCCTATCA TCAC---CTT GGCAGCCCTT CACCGTTTTG CTAAAAACAT

GTTCCTCTCA TTGG---GGT CACCATAGTC CACAGATTTG GGAAGCATTT

GTTCCTCTCA TTGG---TGT CACCATGATC CACAGGTTTG GGAAACATCT

ATTCCTCTCA TTGG---AGT CACCATGATC CACAGATTTG GAAAGCATTT

GTTCCTCTCA TTGG---CGT CACTATGATC CACAGATTTG GGAAGCATTT

GTTCCCCTCA TTGG---TAT TACCATGGTC CATAGATTTG GAGAGCATTT

ACACCAATGA TTGG---GTT ATCTATGATC CGTCGCTATG GGCAGAATGC

GTGCCTCTCA TTGG---CAT GACGCTGATT CACCGGTATG GGAAGCATTT

GTGCCCCTCA TCAG---TCT GTCTCTTGTT CATCGTTATG GTCATTCTGC

GTACCCCTCA TTGG---TGT CACCATGGCC CACCGTTTTG GGAAGCATCT

GTGCCCATGA TTGG---GCT GGCCATGATG CACCGCTTTG GACAGCACCC

ATCACCAGTA GTGCACATGG TCATGGCTGA TATATACCTA CTGCTCCCGC

ATCACCAGTA GTGCACATGG TCATGGCTGA TATATACCTA CTGCTCCTGC

ATCACCAGTA GTGCACATGC TCATGGCCAA TATATACCTA CTGCTCCCCG

-TCAGCAGTG GTCCATTCAG TGACGGCCAA TGTATACCTG CTTTTACCCC

ACCACCAGTA GTGCACATGC TCATGGCCAA CATATACCTG CTGCTCCCCC

GTCACCAGTC GTGCACACGC TCATGGCCAA TATCTACCTG CTACTCCCGC

TTCTCCAGTT GTTGGAGTCA TAATAGCTGA TATTTTCCTT CTGGTTCCAC

TTCGCCAGTC ATTGGAGTCA TAATAGCTGA TATCTTCCTT CTGGTTCCAC

ATCACCAGTA GTGCACATGC TGATGGCCAA TATCTATCTA CTACTACCCC

GTCACCAGTA GTACACACAT TCATGGCCAA TACCTATCTG TTACTGCCTC

ATCACCAGTG GTGCACACAC TCATGGCCAA TATCTATCTG TTACTCCCCC

ATCACCAGTA GTGCACATGC TCATGGCCAA TATATATCTG TTACTCCCCC

ACCACCAGTG GTACATACGC TAATGGGCAA TGTCTATCTG CTACTTCCCC

TTCCCCAATT GTCCATGTGA TCATGGCCAA TGTCTATTTG TTAGTCCCAC

GTCACCTGTA GTACACACAC TCATGGCCAA TATCTACCTG CTGCTCCC-C

GCCTCCCTTT GTTCACACCC TCATGGCCAA TGTCTTCCTG CTAATCCCTC

GTCACCCATT GTGCACACAC TGATGGCCAA TGTGTATCTA TTACTCCCAC

TTCTCCTCTG CTCCAGGTCA TGATGGCCAA TGCCTACCTC TTCATCCCAC

CTGTGCTAAA CCCCATTGTC TACAGTGTGA AGACCAAGCA AATTATGAAA

CTGTGCTAAA CTCCACTGTC TACAGTGTGA AGACCAAGCA GATTATGAAA

CTGTGCTAAA CCCCATTGCC TACAGTGTGA AGACCAAGCA GATT------

CTGTGTTCAG CCCCATCGTC TACAGTGTAA AACCAAAACA GATC------

CTGTGCTAAA CCCCATTGTC TACAGTGTGA AGACCAAGCA GATTATGAAG

CTGTGCTAAA CCCCATTGTC TATAGTGTGA AGACCAAACA GATAATGAAA

CTCTAATGAA TCCCATTGTA TATTCTGTGA AGAGTCAGCA GATTATGAAG

CTCTAATGAA TCCTATTGTA TATTCTGTGA AGAGTCAGCA TATTATGAAG

CAGTGCTAAA CCCTATTGTC TACAGTGTGA AGACCAAGCA GATAATGGAA

CTTTACTAAA CCCTGTTGTC TATAGTGTGA AAACCAAGCA GATAATGATG

CTGTGCTCAA TCCCATTGTC TACAGTGTGA AGACCAAGCA GATAATGATA

CTGTTCTAAA CCCCCTTGTC TATAGTGTGA AGACCAAGCA GATAATGGTA

CTGTACTAAA CCCCATTGTC TATAGTGTGA AAACCAAGCA AATCATGGTC

CCCTAATGAA TCCTATTGTC TACAGTGTCA AGACCAAGCA GATTATGGAG

CTGTGCTCAA TCCCATTGTG TACAGTGTCA GAATTAAGCA GA-TATGGCA

CCGTGCTCAA CCCCATCATC TACAGTGTGA AGACAAAGCA GATTATGCTC

CTGTGCTTAA CCCCATCATC TACAGTATAA AGACCAAACA GATTTTGAAT

CGGTGGTCAA CCCGATCGTC TACAGCCTGA AGACCAAGGA AATCATGTTG

ATTTCTAATA ACTCTGTTAA CAATATCTAT GGGATTATTG TTACCTCTAC

ATTTCTAATA ACTCTGTTAA CAATATCTAT GGGATTATTG TTACCTCTAC

-TCTTCAATA GTCAAGTCAG CAGCATCTAT GGCCTCGTGG TCATCTGTTC

-TCTTCAATA GTCAAGTCAG CAGCATCTAT GGCCTCGTGG TCATCTGTTC

ACTTCTAACA ACTCTGTTAA CAATATCTAT GGAATTATTG TTACCTCCAC

AATTCCAATA GCTCTATTAA CAATATCTAT GGGCTTATTG TTATTTCCAC

AATTCCAGTA GCTCTATCAA CAATATCTAT GGACTTGTTG TTACCTCCAC

AACTACAATA GCTCTATTGA CAATATCTAT GGACTTATTG TGACCTCCAC

G---CTAAGA GCAAACTCAA CAGCATCCTG GGGTTGATTG TCCTGGCCAC

ATTTTCAATA ATAGAATCAA CAGCATCCTT GGTCTGGCAC TCTTCTCCAC

ATTTTCAATA ACAGAATCAA CAGCATCCTC GGTCTGGCAC TTTTCTCCAC

ATTTTCAATA ACAGAATCAA CAGCATCCTT GGTCTGGCAC TCTTCTCCAC

ATTTCCAATA ACAGAATCAA CAGCATTCTG GGTCTGGCAC TCTTCTCCAC

GTCTTAATCA ACCGGGCCAA CAGCATCTGT GGATTAGGGC TCGTCCTGAC

TTGTCCAATA ATAGAGTCAA TGGCATCTAT GGCTTACTTG TCCTCTGCTC

ATTTCCAATT CCAGAATCAA TATAATCTAT GGGTTGCTTG TTCTCTGCTC

ATCTCTCACA --CGTGTCAA CATACTTTAT GGTCTTGCTG TCATCTGTAC

ATTTCCAACA ---GAACCAA CATTATCTAC GGCCT---TG TAGCTTTATC

TTTTGGGCTG GATTCGTTGC TGATTGTGAT CTCCTATGGG CTCATACTCC

TTTTGGGCTG GATTCGTTGC TGATTGTGAT CTCCTATGGG CTCATACTCC

CATGGGACTT GATTCAGTGC TTCTCCTCCT ATCCTATGTC CTCATCCTGG

CATGGGACTT GATTCAGTGC TTCTCCTCCT ATCCTATGTC CTCATCCTGG

TTTTGGGCTG GATTCATTGC TGATTGTGAT CTCCTATGTG CTCATACTCC

TTTCGGGTTG GATTCGTTGC TGATTGTGGT CTCCTATGGA CTCATACTCC

TTTTGGGATA GATTCATTGC TCATCGTGGT GTCCTATGGG CTTATACTCC

TTTTGGGCTA GATTCATTGC TCATTGTGGT GTCCTATGGG CTTATACTCC

TTTTGGGCTG GACTCACTGC TCATCATGGT CTCATATGTG CTGATTCTTT

GACAGGGTTT GACTGTCCTT GTATCTTGCT CTCCTATGTC CTGATCATCC

AACAGGATTT GATTGTCCTT GCATCTTGCT CTCCTATGTA CTGATCATCC

AACAGGATTT GACTGTCCTT GCATCTTGCT CTCCTATGTT CTGATCATCC

AACAGGATTT GACTGTCCTT GCATCTTGAT CTCCTATGTC CTTATTATTC

CACAGGGATA GATACGCCAT GCATTGTCCT GTCTTACATT CTGATTATTC

CTTTGGAGTT GACTCTACAT TCATTATAAT TTCATACATC CTAATTTTGA

CTTTGGAGTT GACTCTGTGT TCATTATAAT TTCATACATT CTGATTTTGA

TCTGGGATTG GATGTTCTTG TCATTCTTTT CTCTTATATT CTCATCTTGA

CATGGGATTT GACACCTCTG TGATAGGTTT CTCCTACATC CAGATTCTCC

ACACTGTATT GGGTATTGCC ACTGGAGAAG GGAGGAAGAA GGCACTCAAC

ACACCGTATT GGGTATTGCC ACTGGAGAAG GGCGGAAGAA GGCACTCAAC

GCACAGTGTT GAGTATAGCC TCCAAGGCAG AGAGAGTGAG AGCCCTCAAT

GCACAGTGTT GAGTATAGCC TCCAAGGCAG AGAGAGTGAG AGCCCTCAAT

ACACTGTACT GGGTATTGCC ACTGGAGAAG GGCGGAAGAA GGCGCTCAAC

ACACTGTGCT CGGTATTGCC ACTGGTGAAG GGAGGAAGAA GGCACTCAGC

ATACTGTACT GGGAATTACC ACTGGAGATG GGAGGAAGAA AGCACTCAAC

ATACTGTACT TGGAATTGCC ACTGGAGATG GGAGGAGGAA AGCACTCAAC

GTACAGTGCT GAGCATTTCT TCTGGGGAGG GGCGGTGGAA GGCTCTCAAT

GATCTGTCCT CAGCATCACC TCCATAGAGG GCAGGCAGAA AGCCTTCAAC

GGTCTGTTCT CAGCATTGCC TCCTCAGAGG GGCGGCAAAA AGCCTTCAAC

AATCTGTCCT CAACATTGCT TCCTCAGAGG GACGGCAGAA AGCCTTCAAT

GTTCCGTCCT TGGCATTGCT TCCTCAGAGG GACGGCGGAA AGCCTTCAAC

GCTCTGTCCT CAATATCACC TCCCCTGAAG AGCGGCACAA GGTCTTTAGC

AAACAGTGCT GGGTATTGCC TCAAAAAGTG GTCAGCTCAA GGCACTTAAT

AAACAGTGCT GGGTATTGCC TCAAAAGATG GTCAGGTCAA GGCACTTAAT

AGACTGTACT GGGCATTGCC TCATGGGCTG AGAGGTTAAA GGCCTTCAAT

GGGCCGTCTT CCACCTCTCA TCCTGGAATG CCCGGTTCAA GGCTCTCAAC

ACATGTGGCT CACACGTCTG TGCTGTGCTT GCTTACTATG TGCCTATGAT

ACATGTGGCT CACACGTCTG TGCTGTGCTT GCTTACTATG TGCCTATGAT

ACTTGCATCT CCCACATCTA TGCTGTACTC ACCTTCTATA CACCAATGAT

ACTTGCATCT CTCACATCTG TGCTGTACTC ACCTTCTATA CACCAATGAT

ACATGTGGCT CACACATCTG TGCTGTGCTT GCTTACTATG TGCCTATGAT

ACATGTGGCT CACATGTCTG TGCCGTGCTC GCCTACTACG TGCCTATGAT

ACATGTGGTT CACATATCTG TGCTGTGCTT GCTTATTATG TACCAATGAT

ACATGTGGCT CACATGTCTG TGCTGTGCTT GCTTATTATG TACCAATGAT

ACATGTGTGT CACACATCTG TGCTGTGCTT GCTTACTATG TGCCAATGAT

ACCTGCATTT CCCACATCAG TGCTGTTGCC ATCTTCTACA TCCCTCTCAT

ACCTGCATAT CCCACATCAG TGCTGTTGCC ATCTTTTACA TCCCCCTTGT

ACTTGCATAT CGCACATCAG TGCTGTTGCC ATCTTCTACA TCCCTCTCAT

ACTTGCATAT CTCACATCAG TGCTGTTGCT ATCTTCTACA TCCCTCTCAT

ACCTGTATCT CCCACATTGG AGCAGTTGCT GTTTTCTACA TCCCCATGAT

ACTTGTGTTT CCCACATCT- TACTGTCTTC ATCTTTTATG TGCCACTCAT

ACGTGTGTTT CCCACATCT- TACTGTCTTC ATCTTCTATG TGCCACTCAT

ACCTGCCTCT CTCACATCTG TGCTTGTCTG CTCTTCTACA TACCTTTTAT

ACTTGTGGCT CCCACATCTG TGTCATTTTA GCCTTCTACA TCCCAGCCTT

TGGCTTGTCT ATAGTGCACC GCCTTGGACA TCGTGTGTCC CCTCTGCTGC

TGGCTTGTCT ATAGTGCACC GCCTTGGACA TCGTGTGTCC CCTCTGCTGC

TGGGCTATCT ATGATCCACC GCTATGGACA GAATGCTTCC CCAATTGTCC

TGGGCTATCT ATGATCCACC GCTATGGACA GAATGCTTCC CCAATTGTCC

TGGCTTGTCT ATAGTGCACC GCCTTGGACA TCATGTGTCC CATGTGCTGC

TGGCTTGTCT ATGGTGCACC GCTTTGGAAA TCACGTGTCT CCTCTGGTGC

TGGCTTGTCT ATGGTACATC GCTTTGCACA TCATGTGTCT CCTCTGCTAC

TGGCTTGTCT ATGGTACATC GCTTTGCACA TCATGTATCT CCTCTGCTTC

TGGCCTGTCT ATGGTGCATC GCTTTGGACA TGATGTGTCT CCTCTGCTGC

CAGCCTGTCT CTTGTCCATC GCTATGGCCA TTCAGCACCT CCCTTTGTCC

CAGCTTGTCT CTTGTCCATC GCTATGGCCA TTCAGCACCT CCATTTGTCC

CAGCTTGTCT CTTGTCCATC GCTATGGACA TTCAGCACCT CCATTTGTCC

CAGCTTGTCT CTTGTCCATC GCTATGGCCA TTCGGCACCT GCATTTGTCC

GAGCCTGTCC TTGCTGCATC GCTATGGTCA GTCAGTCCCC AAAGTAGTCC

CGTGCTTGCC T--AGTCATA GG-TTGGTAC ATTTACATCT CTCTTTCTGC

TGTTCTGGCC TTAATTCATA GATTTGGCAC GTTCACATCT CCCCTTCTCC

TGGTGCCACG ATGGTCCACC GTTTTGGGAA ACACCTCTCA CCCATTGTGC

TTTCTCCTTC CTCACCCACC GCTTTGGCCA CCAGAAAATT CCTCTCATCC

AAGCCATGAT GGCCAATGCC TACCTCTTCT TCCCACCTGT TGTCAATCCT

AAGCCATGAT GGCCAATGCC TACCTCTTCT TCCCACCTGT TGTCAATCCT

ATGTGCTGAT GGCCAATGTC TACTTGCTGG TTCCACCTCT CATGAACCCC

ATGTGCTGAT GGCCAATGTC TACTTGCTGG TTCCACCTCT TATGAACCCC

AAGCCATGAT GGCCAATGCC TACCTTTTCT TTCCCCCTGT GGTCAACCCC

AAGTCATGAT GGCCAATGCT TATCTCTTTT TCCCACCTGT TGTCAACCCC

AGACCATGAT GGCCAATGCT TATCTCTTCT TCCCCCCTGT CATCAACCCC

AAACCATGAT GGCCAATGCT TATCTCTTCT TCCCCCCAGT CATCAACCCC

AAATCATGAT GGCCAATGCT TACCTCTTCT TTCCACCTGT TGTCAACCCC

ACACCATCAT GGCCAATATC TTCCTGCTTA TTCCTCCTGT ACTCAACCCT

ACACCATCAT GGCCAATGTC TTCCTTCTCA TCCCTCCCGT GCTCAACCCC

ACACCATCAT GGCCAATGTC TTCCTGCTCA TCCCTCCTGT GCTCAACCCT

ACACTATTAT GGCCAATGTT TTCCTGCTTA TTCCTCCTGT GCTCAACCCT

ACTCAATGAT GGCTAATATT TACCTACTTC TGCCCCCTGC TCTTAATCCC

ATGTAACCAT GGCAAATGTA TACCTCCTTT TCCCACCCTT TTTAAACCCT

ATGTAACCAT GGCAAATGTC TACTTTCTTT TTCCACCCTT TATGAACCCT

ACACAGTGAT GGCCAATGTG TATCTTTTAC TCCCACCTGT ACTTAACCCC

ACATCCTCCT GGCCAACCTC TACGTGATTG TCCCACCTGC CCTCAACCCT

ATTGTCTACA GCATTAAGAC CAAGGAGATC CATGGTGCCA TTGTCCGAAT

ATTGTCTACA GCATTAAGAC CAAGGAGATC CATGGTGCCA TTGTCCGAAT

ATTGTCTACA GTGTTAAGTC CAAGCAGATT C---GTGACA GAATCCTCAA

ATTGTCTACA GTGTTAAGAC CAAGCAGATT C---GTGACA GAATCCTCAA

ATTGTCTATA GCATCAAAAC CAAAGAAATT C--------- ----------

ATTGTTTACA GCATTAAGAC CAAAGAGATA TGTCATGGCA TTGTCCAAAT

ATTGTCTACA GCATTAAGAC CAAGGAGATT CGTCGTGTCA TCATTCGAAT

ATTGTCTACA GCATCAAAAC CAAGGAGATC CGTCGTGGCA TTGTTCGAAT

ATTGTCTATA GCATTAAGAC CAAGGAGATC CGTCGTGGCA TTGTCCGAAT

ATCATCTACA GTGTGAAGAC TAAGCAGATT CAAAAGGCTG TTATCAAGGT

ATCATCTATA GTGTGAAGAC TAAGCAGATT CGAAAGGCTA TTGTCAAGGT

ATCATCTACA GTGTGAAGAC GAAGCAGATT CGAAAGGTTA TTGTCAAGAT

ATAATCTATA GTGTGAAGAC TAAACAGATT CGAAAGGCGA TTGTTAAGGT

ATCATCTATA GTGTAAAATC CAAACATATC CGCAAGGTTA TACTCAATCT

GTTATCTATA GCATTAAGAC AAAAGAAATG CATCATGGCA TATTGCAAAT

GTCATATACA GCATCAAAAC CAAAGAAATT CGCAGTAGTA TTGTTCGTGT

CTCATCTACA GCATAAAGAC CAAACAGATT CGTAAGCGGA TAGTCCAACT

GTCATCTATG GGGTCAGGAC CAGGCAGATC TTGGAGCAGG TGCTGAGAAT

GCTAATGGGA GACTGGAATT CCTCAGTGTT GCTGGCCATG GCCTTTGACC

GCTAATGGGA GACTGGAATT CCTCAGTGTT GCTGGCCATG GCCTTTGACC

GAAAATGGTA GACTGGAATT CCTCAGTGTT GCTGGCCATG GCCTTTGACC

GAAAATGGTA GACTGGAATT CCTCAGTGTT GCTGGCCATG GCCTTTGACC

---------- ---------- ---------- ---------- ----------

GCTAATGGTG GTGTGCAATT CCTCTGTGCT GCTGGCCATG GCTTTTGACC

GCT-ATGCTT TCCTGGAATT CCTCAGTGCT TCTGGCTATG GCCTTTGACC

GCTGATGCTT GCCTGGAATT CCTCAGTGCT TCTGGCTATG GCCTTTGACC

GCTGATGGTG ATGTGGAATT CCTCAGTGCT GCTTGCCATG GCCTTTGACC

CTTAATGTGG GTCCTCAACT CCGGATTGCT CATGGCTATG GCCTTGGACT

CTTAATGTTG GTCCTCAATT CCAGGTTGTT CATGGCTATG GCCTTGGACA

CTTAATGTTG GAGCTCAATT CTTCGGTGCT CCTGGCTATG GCCTTTGATC

TTTAATGTTG GTCCTCAATT CAGGGGTGCT CATGGCTATG GCTTTGGACC

GCT-ATGGTG GTCTGCAATT CTTCAGTGTT GCTGGCCATG GCCTTTGATC

GCTAATGGTA GTCTGGAATT CCTCAGTGCT GCTGGTCATG GCCTTTGACC

ACTAATGGTG GTCTGGAATT CAGGTGTTTT GCTTGCCATG GCCTTTGACC

ATTTATGCTT TCCTTGAATT CTGGGGTGTT GGTATCCATG GCCTTTGATC

GCT-ATGTTG ATTTCCAACT CCGCCGTGTT GGTGGCCATG GCCTTCGACC

GTTTTGTTGC TATCTGCCAT CCACTGCACT ACCCCACCAT CCTCACCAAC

GTTTTGTTGC TATCTGCCAT CCACTGCACT ACCCCACCAT CCTCACCAAC

ATTTTGTTGC TATCTGCCAT CCACTGCATT ACCCCACCAT CCTCACCAAC

GTTTTGTTGC TATCTGCCAT CCACTGCATT ACCCCACCAT CCTCACCAAC

---------- ---------- ---------- ---------- ----------

GTTTTGTTGC TATCTGCCGT CCCCTGCACT ACCCCACCAT CCTCACCAAC

GTTTCATAGC AATCTGTCGT CCACTGCACT ATTCTACCAT CCTCACCAAC

GCTTCATAGC AATCTGCCGT CCACTGCACT ATTCTACCAT CCTCACCAAC

GTTTTGTTGC TATCTGCCGT CCACTGCACT ATGCCTCTAT CCTCACCAAC

GTTATGTGGC TGTCTGCTTC CCACTGCATC ACTCTAGTAT CCTGACCCCA

ATTATGTGGC TATCTGTTTC CCACTCCACC ACTCTACTTT CCTGACCCCA

GTTTTGTGGC CATTTCCAAC CCCTTGAGAT ATGCTACTAT TCTAACTC--

GTTATGTGGC CATCTGCTTC CCACTGCGTC ACTCTAGCAT CCTGACTCCA

GTTTTGTTGC TATCTGCTAT CCACTGCACT ATTCCACCAT CCTCACCAAT

GTTTTGTTGC CATCTGCCAT CCACTGCACT ATTCCACCAT CCTCACCAAC

GTTTTGTTGC CATATGTCGT CCACTGCACT ATTCCACCAT TCTTACCAAC

GCTTTGTGGC TATTCGAGAT CCACTGCGTT ATTCTTCCAT CCTCACCTAT

GCTACGTGGC CATTTGCGAC CCCCTGCGCT ACACAACTGT GCTTACCGGC

AGTGTAATTG GCAAAATTGG TTTGGCCTGT TTGCTACGAA GCTTGGGAGT

AGTGTAATTG GCAAAATTGG TTTGGCCTGT TTGCTACGAA GCTTGGGAGT

AGTGTAATTG GCAAAATTGG TTTGGCCTGT CTGCTGCGAA GCTTGGGAGT

AGTGTAATTG GCAAAATTGG TTTGGCCTGT CTGCTGCGAA GCTTGGGAGT

---------- ---------- ---------- ---------- ----------

AGTGCAATTG GCAAGATTGG TTTGGCCTGC TTGCTCCGAA GTACGGGCGT

AGTGTGATTG GCAAAATTGG TATGGCCTGC TTGCTTCGAA GCATGGGGGT

AGTGTGATTG GCAAGATTGG TATGGCCTGC TTGCTACGGA GCATGGGAGT

AGCGTGATTG GCAAGATTGG TTTGGTTTGC TTGCTACGAA GCATGGGAGT

TCTGTTGTGG GTAAACTGGG GGAAGGTATG ATGATGAAAG GGCTGGGG--

GCTGA-GTGG GTAAACTGGG AGTAGCTGTG ATGATGAGAG GATTGGGG--

---------- ---------- ---------- ---------- --TTAGGGGA

TCTGTGGTGG GCAAATTGGG CGCAGCAGTG ATGATACGTG GGCTGGGG--

GGTGTAATTG GCAAGATTGG TTTGATCTGC TTGCTGAGAA GCATGGGAGT

AGTGTAATTG GCAAGACTGG TTTGGTCTGC TTGCTGCGAA GCATGGGAGT

AGTATAATCG GCAAGATTGG TTTGGCTTGC TTGCTGCGGA GCATGGCAGT

GAGGTCATTG GCAAGATTGG ATTAGTTGTC CTTGCCCGTG CTGTCTTTGT

TCCTTGGTGT GCCGTCTGGG GCTGATGGCT CTGGCCAAAG GGATGGTTTT

TGTACTTCCC ACACCTTTGC TACTGAGACA CTATCACTAC TGCCATGGCG

TGTACTTCCC ACACCTTTGC TACTGAGACA CTATCACTAC TGCCATGGCG

TGTACTTCCC ACACCTTTGC TACTGAGACA CTATCAATAC TGCCATGGCG

TGTACTTCCC ACACCTTTGC TACTGAGACA CTATCAATAC TGCCATGGCG

---------- ---------- ---------- ---------- ----------

TGTACTGCCC ACACCTTTGC TACTGAGGCG CTATCACTAC TGTCATGTCG

TGTTCTGCCT ACACCTTTGC TACTGAGACA GTATCACTAT TGCCACATCG

TGTTCTGCCT ACACCTTTGC TACTGAGACG CTATCACTAT TGCCGGGTCG

TGTACTGCCA ACACCTTTGT TACTGAGACG CTATCACTAC TGCCGCATCC

-GTCCTTTCT GTTTCATGGT GTCTAGGATG CCCTT----C TGCCCCAATA

-GCCCCTTCT GCATTATGGT CTCCAGGATG CCCTT----C TGCCCCAAAA

CTGTCATTCT GTGTCCTACT TCTTAGTCTA TCATT----C TGCCACAGCG

-GCCCCTTCT GTTTCA---T CTCCAGAATG CCTTT----C TGCCCCAACA

TGTAATGCCC ACGCCGTTGC TACTGAGACA CTATCTGTAC CATCACGTCA

TGTACTGCCT ACACCTTTGC TACTGAGACG CTATCACTAC TGCCATAAAG

TGTACTACCC ACACCTGTGC TACTGGGACG CTACGACTAC TGCCATATCG

GGTCCTCCCT GCACCCTTGC TATCAAGAGG CTGCCTTT-C TACCACTCCG

GATTCTGCCC ATTCCCCTGC TGCTCAGACA GCTGACCTTC TGCCGGACAG

CCCTCTCTCA CGCCTTCTGT TTGCACCAGG ATGTTCTAAG ATTATCCTGT

CCCTCTCTCA CGCCTTCTGT TTGCACCAGG ATGTTCTAAG ATTATCCTGT

CCCTCTCTCA TGCCTTCTGT TTGCACCAGG ATGTTTTGAG ATTATCCTGT

CCCTCTCTCA TGCCTTCTGT TTGCACCAGG ATGTTTTGAG ATTATCCTGT

---------- ---------- ---------- ---------- ----------

CCCTCTCCCA TGCTTTTTGC TTGCACCAGG ATGTTCTTAA ATTATCATGT

CCCTCTCCCA TGCCTTCTGC TTACACCAAG ATGTTCTGAG GCTATCCTGC

CCCTCTCCCA TGCCTTCTGC TTACACCAAG ATGTTCTGAG GCTGTCCTGC

ACCTGTCCCA TGCCTTCTGC CTGCACCAGG ATGTTCTGAA ACTCTCCTGT

TCATC-CCCA GTCATACTGT GAGCACATGG CTGTGCTGAA GTTGATGTGT

TCATTGTCCA GTCATACTGT GAGCACATGG CTGTGTTAAA GCTGGTGTGT

TACTCCATCA CTCCTACTGC TTCCACCCTG ATGTGATGAA GCTCTCATGT

TCATTCCCCA GTCCTACTGT GAACACATGG CTGTGCTGAA GTTGGTGTGT

CCCTTTCCCA TGCCTTCTGC TTGCACCAGG ATCTTCTGAA ATTATCATGT

TTCTCTCCCA TGCCTTCTGC TTGCACCAGG ATGTTCTGAA ATTATCCTGT

CCCTTTCCCA TGCCTTCTGC TTGCACCAAG ATGTCCTAAA ATTAGCCTGT

TTCTCTCTCA CTCTTACTGC CTCCATCAGG ATGTCATGCG ACTTGCTTCT

TCATCGCCCA CACCTACTGC GACCACATGG CGGTGGTCAA GATGGCCTGT

ACAGATGCCA GGACCAACAG TATTTATGGG CTTTGTGTAG TCATTGCCAC

ACAGATGCCA GGATCAACAG TATTTATGGG CTTTGTGTAG TCATTGCCAC

ACAGATGCCA GGATCAATAG CATTTATGGG CTTTGTGTAG TCATTGCCAC

ACAGATGCCA GGATCAATAA CATTTATGGG CTTTGTGTAG TCATTGCCAC

---------- ---------- ---------- ---------- ----------

TCAGATGCCA AGATCAACAG CCTTTACGGA CTGTGTGTAG TCATTGTCAC

TCTGATGCCA GGATCAACAG TGTCTATGGA CTATGTGTAG TTATTGCTAC

TCTGATGCCA GGATCAACAG CGTCTATGGA CTATGTGTAG TTATTGCTAC

TCTGATGCCA GAATCAACAG CGTTTATGGA CTGTGTGTAG TCATTGCCAC

GCTGACACTA GAGTTAATCG TGCATATGGG CTTTTTGCGG CCTTCTCTGT

GCAGACACTA GAGTAAACCG TGCCTATGGG CTCTTTCCGG CTTTCTCGGT

TCAGATACAA AGATCAATAG TGCATTTGGA CTGACTGCAA TTATTTCTAC

GCTGACACTA GAGTAAATCG AGCTTATGGG CTCTTTGTAG CATTCTCAGT

TCAGATGCCA GGATAAATAG TGTTTATGGA CTTTGTGTCG TCATTGCCAC

TCAGATGCCA GGATCAATAG CAATTATGGG TTATGTGTAG TTCTCATCAC

TCCGATGCTA GGGTCAATAG CATTTATGGA CTGTGTGTAG TTATTGCCAC

GCCAGCACAC GTGTCAACAT ACTTTATGGT CTTATTGCTG TCATCTGTAC

GGGCACACCG GGCCCAACCG TATCTACGGC CTCTTCTTGG TCGTCCTGGT

ACTAGGTGTG GATTCAATCT TCATACTTCT TTCTTATGTT CTGATTCTTA

ACTAGGTGTG GATTCAATCT TCATACTTCT TTCTTATGTT CTGATTCTTA

ACTAGGTGTG GATTCAATCT TCATACTTCT TTCTTATGTT CTGATTCTTA

ACCAGGTGTG GATTCAATCT TCATCCTTCT TTCTTATGTT CTGATTCTTA

---------- ---------- ---------- ---------- ----------

ACTGGGAGTG GATTCAGTCT TCATACTTCT TTCTTATATT CTGATTCTGA

ACTAGGTGTG GATTCAGTCT TCATACTTAT TTCTTATGTT CTTATTCTGA

ACTAGGTGTG GATTCAGTCT TTATACTTAT TTCTTACGTT CTTATTCTGA

ACTAGGTGTG GATTCAGTCT TCATCCTTCT TTCCTATGTT CTGATTCTGA

GGTTGGCTTT GACATTATTG TCATCAGTGT ATCCTATGCA ATGATTCTGA

GGTTGGCTTT GATATAATTG TCATCAGTAC ATCCCATGTG ATGATTCTGA

CGCTGGAGTG GACTCTTTCT TTATCCTGCT TTCCTATGTC TTGATCATCC

GGTGGGCTTT GACATGATGA TCATCAGTAT ATCTTATGTG ATGATTCTGA

ACTAGGTGTG GATTCCCTCT TCATACTTCT TTCATATGTC CTGATTCTGA

ACTGGGTGTG GATTCAGTCT TCATACTTCT TTCTTATGTC CTGATTCTGA

ACTGGGTGTG GATTCAGTCT TCATACTTCT TTCCTATGTC CTGATTCTGC

TCTGGGATTG GATGTTCTTG TCATTCTTTT CTCTTATATT CTCATCTTGA

GGTGGGCCTT GACTGTCCGC TCATCGGGGC CTCCTATGCC CTGATCCTGT

ATACTGTGCT GGATATTGCA TCTCGTGAAG AGCAGCTAAA GGCACTCAAC

ATACTGTGCT GGATATTGCA TCTTGTGAAG AGCAGCTAAA GGCACTCAAC

ATACTGTGCT GGGTATTGCA TCTCGGGAAG AGCAGCTAAA GGCACTCAAC

ATACTGTGCT GGGTATTGCA TCTTGGGAAG AGCAGCTAAA GGCACTCAAC

---------- ---------- ---------- ---------- ----------

ATGCTGTGCT GGGTATTGCA TCTCGTGAAG AGCGGCTAAA GGCACTCAAC

ACGCCGTGCT GAGTATCGCA TCCCGTGAAG AGCGACTAAA AGCTCTGAAC

ATGCAGTGTT GAGTATTGCA TCCCGTGAAG AGCGACTAAA AGCTCTGAAT

ATGCTGTGCT GGGTATAGCA TCCCGTGAAG AGCGGCTAAA GGCACTCAAC

GAACAGTGCT ACAGTTACCC TCAGGTGAAG CCCGGCTCAA GGCTTTTGGC

GAACTGTTCT GGGGTTGCCC TCTGATGAAG CCCGGTTCAA GGCTTTTGGC

ATGCTGTCTT TCGTCTTCCA TCTCGCGATG CCCAACACAA AGCTCTGAGT

AAGCTGTGCT GGCATTACCC TCTGGAGAAG CCAGGATGAA GGCTTTTGGC

ATGCTGTGCT GGGCATTGCG TCTCATGAAG AGCAGCTAAA GGCACTCAAC

ATGCTGCTCT GGGCATTGCA TCTCGTGAAG AGAAGCTAAA GGCACTCAAC

ATGCTGTGCT GGGCATTGCA TCACGTGAAG AGCGGCTAAA AGCACTGAAC

AGACTGTACT GGGCATTGCC TCATGGGCTG AGAGGTTAAA GGCCTTCAAT

GGGCCGTGTT GCACTTGTCT TCCCGTGGGG CCCGCCTCCG TGCCCTCAGC

ACATGTGTAT CCCATATCTG TGTGGTGCTT ATCTTCTTTG TGCCAGTTAT

ACATGTGTAT CCCATATCTG TGTGGTGCTT ATCTTCTTTG TGCCAGTTAT

ACATGTGTAT CCCATGTCTG TGTGGTGCTC ATCTTCTTTG TGCCAGTTAT

ACATGTGTAT CCCATATCTG TGTGGTGCTC ATCTTCTTTG TGCCAGTTAT

---------- ---------- ---------- ---------- ----------

ACGTGTGTAT CCCACATCTG TGTGGTGCTC ATCTTCTTTG TACCGGTTAT

ACCTGTGTTT CCCATGTCTG TGTGGTGCTC ATCTTCTTTG TACCAGTAAT

ACCTGTGTTT CCCATGTCTG TGTGGTACTC ATTTTCTTTG TACCAGTAAT

ACGTGTGTAT CTCATATCTG TGTGGTGGTC ATCTTCTTTG TGCCAGTCAT

ACATGTGCCT CCCGTATCTG TGTCATCTTG GCTTTCTATA TCCCAGCTCT

ACATGTGCTT CCCTTATCTG TATCATCTTG GCTTTTTATA TCCCAGCCCT

ACCTGTGGCT CCCACCTTGC GGTCATCCTG GTCTTCTACA TTCCTGCCTT

ACCTGTGCTT CCCATATCGG AGTTATCTTG GCTTTTTATG TCCCAGCCCT

ACATGTATAT CTCATGTCTG TGCGGTGCTC ATCTTCTTTG TGCCAGTTAT

ACATGTGTAT CTCATATCTG TGTGGTGCTC ATCTTCTTTG TGCCAGTCAT

ACATGTGTGT CTCATATCTG CGTGGTGCTC ATCTTCTTTG TGCCAGTCAT

ACCTGCCTCT CTCACATCTG TGCTTGTCTG CTCTTCTACA TACCTTTTAT

ACCTGCTCTG CCCAACTGTC GGTCGTTCTC GTCACTTATG GCCCTGCCCT

TGGGGTGTCA ATGGTCCATC GCTTTGGGAA GCATCTGTCT CCCATAGTCC

TGGGGTGTCA ATGGTCCATC GCTTTGGGAA GCATCTGTCT CCCATAGTCC

TGGGGTGTCA ATGGTCCATC GCTTTGGGAA GCATCCATCT CCCATAGTCC

TGGGGTGTCA ATGGTCCATC GCTTTGGGAA GCATCCATCT CCCATAGTCC

---------- ---------- ---------- ---------- ----------

TGGGGTGTCA ATGGTCCATC GCTTTGGGAA GCACCTTTCT CCTATAGTCC

TGGGGTGTCA ATGGTCCATC GCTTCGGGAA ACATCTCTCT CCCATAGTCC

TGGGGTGTCA ATGGTTCATC GCTTTGGGAA ACATCTCTCT CCCATAGTCC

TGGGGTGTCA ATGGTCCATC GCTTTGGGAA GAATTTGTCT CCTACAGTTC

CTTTACTTT- CTTACCCACC GCTTTGGACA TCATGTGCCC CGCGTGGTAC

CTTTACTTT- CTCACTCACC GCTTTGGACA CCATGTGCCT CAAGTGGTAC

CTTCTCTTT- CTCACCCACC GTTTTGGACA CCAAGTCCCC AAGCATGTGC

CTTCACTTT- CTTACCCATC GCTTTGGACA TCATGTGCCT CGGGTGGTTC

TGGAGTGTCA ATGGCCCATC GCTTTGGGAA GCATCTGTCT CCCATCGTCC

TGGAGTATCA ATGGTCCATC GCTTTGGGAA GCATCTGTCT CCCATAGTTC

TGGTGTATCG ATGGCACATC GCTTTGGGAA GCATCTGTCA CCCACCATTC

TGGTGCCACG ATGGTCCACC GTTTTGGGAA ACACCTCTCA CCCATTGTGC

CATCTCTGCA ATCATCCACC GCTTTGGCCG CTCCATCCCA GTCCACAGCC

ACATCCTCAT GGCAGACATC TACCTTCTTC TTCCCCCAGT CCTTAACCCT

ACATCCTCAT GGCAGACATC TACCTTCTTC TTCCCCCAGT CCTTAACCCT

ACGTCTTCAT GGCAGACATC TACCTTCTTC TTCCCCCAGT CCTTAACCCT

ACGTCTTCAT GGCAGACATC TACCTTCTTC TTCCCCCAGT CCTTAACCCT

---------- ---------- ---------- ---------- ----------

ATGTCCTTAT GGCTGACATC TACCTGCTTC TCCCCCCAGT ACTTAACCCT

ACATCCTCAT GGCTGACATC TACCTGCTTC TCCCCCCAGT GCTTAACCCA

ACATCCTCAT GGCTGACATC TACCTGCTTC TACCCCCAGT GCTGAACCCA

ACATGCTCAT GGCTGACATC TACTTGCTTC TTCCCCCTGT ACTTAACCCC

ACATCATGTT GGCTATTCTC TATCTCCTGG TACCTCCCAT GCTCAACCCC

ATATCATGTT TACCAATTTC TATCTCCTGG TACCTCCCAT GCTCAACCCC

ACATCTTTCT GGCTAATCTC TATGTGCTGG TGCCTCCTGT GCTCAACCCA

ACATCATGTT GGCTATTCTC TATCTACTGG TACCTCCCAT GCTCAACCCC

ACGTCCTCAT GGCTGACATC TACTTGCTTC TTCCCCCGGT GCTTAACCCA

ACGTCCTCAT GGCTGACATC TATCTGTTTC TTCCCCCAGT GCTTAACCCT

ACATCCTCAT GGCTGACATC TACCTGCTGC TGCCCCCAGT GCTCAACCCT

ACACAGTGAT GGCCAATGTG TATCTTTTAC TCCCACCTGT ACTTAACCCC

ACATCCTCCT GGCCAACCTC TACCTCCTTA TCCCCTCTCT GTTCAACCCA

ATTGTCTATA GTGTCAGAAC AAAGCAGATT CGTCTCCACA AGTTTGTCCT

ATTGTCTATA GTGTCAGAAC AAAGCAGATT CGTCTCCGCA AGTTTGTCCT

ATTGTCTATA GTGTCAGAAC AAAGCAGATT CATCTCTGCA AATTTGTCCT

ATTGTCTATA GTGTCAGAAC AAAGCAGATT CATCTCTGCA AATTTGTCCT

---------- ---------- ---------- ---------- ----------

GTTGTCTACA GTGTCAGGAC AAAGCAGATT CGTCTCAGAA AGTTTGGAAT

GTTGTCTACA GTGTGAGGAC AAAACAGATC CGCCTCCGCA AGTTTAGATT

GTTGTCTACA GTGTGAGGAC AAAACAGATT CGTCTCCGCA AGTTTAGGTT

ATTGTCTACA GTGTCCGGAC AAAGCAGATT CGTCTCCGCA AATTTGGACT

AT-ATCTATG GAGTTAGAAC CAAACAGAT- -GACACAAGA GATTCAGCCC

ATCATCTATG GAGTCAGTAC CAAGCAGAT- -GACTCAAGA AGTTCAGCCC

ATCATCTACG GGGCTAGAAC CAAGGAGAT- -GACTCAAGA GGTTCAGCCC

ATTATTTATG GAGTTAAAAC CAAACAGAT- -GACTCAAGA GGTTCAGCCC

GTCATCTATA GTATCAAGAC AAAGCAGATT CGTCTCCGCA AATTTGGATT

GTTGTCTACA GTGTCAGGAC AAAGCAGATT CGTCTCCGCA AGTTTGGAAT

GTTGTCTATA GTGTCAGAAC AAAACAAATT CGACTCCGGA AGTTTGGAAT

CTCATCTACA GCATAAAGAC CAAACAGATT CGTGTCCAAC TATTT--TTT

GTCATCTTTG GGGTAAGGAC AAAGGAGATT C----GAAGA TATTTCTCCT

AAGGAGGAGG TTTCAATTCA TGCTATCCAA CATTACTCAG ATATTCTATC

AAGGAGGAGG TTTCAATTCA TGCTATCCAA CATTACTCAG ATATTCTATC

AAGGAGGAGG TTTCAATTCA TGCTGTCCAA CATTACTCAG ATGTTCTATC

AAGGAGGAGG TTTCAATTCA TGCTGTCCAA CATTACTCAG ATGTTCTATC

---------- ---CAATTCA TGTTATCCAA CATCACTCAG ATGTTCTATC

GGGAAGGAGG GTTCAACTGA TGCCACCGAA CATTACTCAG ATGTTCTACC

AAGTAGAAGG TTTCAATTCA TGCTGTCAAA CATAACTCAT ATGTTTTATC

AAGTAGAAGG TTTCAATTCA TGCTATCCAA CATTACTCAT ATGTTCTACC

AGGAGAGAGG TTTCAGGTCA TGCTGTCCAA CATTACTCAG ATATTCTACC

AGGAAGGTGG GTTTCATT-A TGGTCCTCAA CAATACCAGG ACCTTCCTCT

AGGAAGGTGG GTTTCATT-A TGGTCCTCAA TAATACCAAG ACCTTCTTCC

AGAAAGGTGG GTTTCATT-A TGGAGCTCAA TAATACCAAG ACATTCCTCC

AGGAAAGTGG GTTTCCTT-A TGGTCCTCAA TAATACCAAG ACCTTCTTCC

AAGGAGAAAA CTTCAATT-- TGCTTTCCAA AATACCACA- ACTTTCTTCT

AGGGAGGAGG CTT------- ---AGACCAA CATCACTTGG ATGTTCTATC

AAGGAGGAGG CCT--ATCCA TGGCCCTCAA TAACACCAAG ATCTTCTTCC

AGGAGAAAGG GCACAACCCA TATTGCCAAA TAATACTC-- ACATTTCTGC

TAGGAGGGGG TTCCACTTCA TGATTTCCAA CACTACCAAC ACGTTCCTCC

TCACCAGCTT TCCTGGATTG GAAGGCATCA AACACTGGAT TTTCATCCCC

TGACCAGCTT TCCTGGATTG GAAGGCATCA AACACTGGAT TTTCATCCCC

TCACCAGCTT TCCTGGATTG GAAGCCATCA AACACTGGAT TTTCATTCCT

TCACCAGCTT TCCTGGATTG GAAGCCATCA AACACTGGAT TTTCATCCCC

TCTCCAGCTT TCCTGGACTG GAAGCCATCA AACACTGGAT TTTCATACCC

TCACTGGCTT TCCTGGATTG GAAGCCATTG AACACTGGAT TTTCATCCCC

TCAGTGGCTT TCCTGGTTTG GAAGCCTTTG AACACTGGAT TTTCATCCCT

TCAGTGGCTT TCCTGGTTTG GAAGCCTTTG AACACTGGAT CTTCATCCCC

TCACTGGCTT TCCAGGACTG GAAACCATTG AGCACTGGAT TTTCATCCCC

TGACGGGTAT TCCAGGCCTG AGAGCGGCCA AGATCTGGGT CTCCATCCCT

TGACGGGTAT CCCAGGCCTG AGAGCAGCCC AGGTTTGGAT CTCCATCCCT

TGACGGGTAT CCCAGGCCTG GCAGCAGCCC AGGTGTGGAT CTCCATTCCT

TGACGGGTAT CCCAGGCCTG AGAGCAGCCC AGGTCTGGAT CTCCATCCCG

TGACAGGCTT CCAGGGTGTG GAACATCTCC ATGGTTGGAT CTCTATTCCC

TCACTGGCTT TCCTGGATTG GAAGTCATGG AACACTGGAT TTTCATTCCA

TGACGGGTAT TCCAGGGATG AAAGCAGCCC AGGCCTGGAT ATCTATCCCG

TAACAGTCAT TCCTGGCCTG GAAGCTGCCC ACATATGGAT TTCTATACCC

TGTTAGGTAT CCCTGGGATG GAGCATCAGC ACATCTGGAT CTCCATCCCC

TTTTTCTTTA TGTACATGGT TGCCATCTCA GGCAATTGTT TCATTCTGAT

TTTTTCTTTA TGTACATGGT TGCCATCTCA AGCAATTGTT TCATTCTGAT

TTTTTCTTTA TGTACATGGT GGCCATCTCA GGCAATTGTT TCATTCTGAT

TTTTTCTTTA TGTACATGGT GGCCATCTCA GGCAATTGTT TCATTCTGAT

TTTTTCTTTA TGTACATGGT GGCCATCTCA GGCAACTGTT TCATTCTGAT

TTTTTCTTTA TGTACATAGT GGCCATCTCA GGAAATTGTT TAATTCTGAT

TTTTTCTTGA TGTACCTGGT GGCCATCTCA GGCAATTGTC TCATTCTGAT

TTTTTCTTGA TGTACCTGGT GGCCATTTCA GGCAATTGTC TCATTCTGAT

CTTTTCTTTA TGTATCTTAT GGCCATCTCA GGTAATTGTT TCATTCTAAT

GTTTGCCTCC TGTATGCCGG CGCCCTCTCT GGGAACAGTG TGATCCTGTT

TTTTGTCTCC TGTATGTCAT CGCCCTCTCT GGGAACAGCA TGATCCTATT

ATTTGTCTCC TGTATGTCAC TGCCCTGTCT GGGAACAGCA TGATCCTGTT

TTCTGCCTCC TATATGGCAT TGCCCTCACT GGAAACAGCA TGATCCTGTT

TTCTGCTCCA TCTACTTGAC AGTTATTGTA GGGAACCTTA CCATCCTCCA

TTTGTTTTTA TGTTCCTGGG GGCCACCTCA GGCAATTGTT TCACCCTGAT

GTTTGCCTCC TGTATGTGCT CGCCATCTCT GGGAACAGCT TGATCCTCTT

TTTTACTTCC TCTATGCCAT TGCCATCTCT GGGAATAGCA TGATCTTGTT

TTCTGCTCCA TGTACATCAT GGCTCTGCTG GGCAATGCCA CCATCCTGTT

CATTATTAAG ACCAACCCTC GTCTGCACAC ACCCATGTAC TATCTACTAT

CATTATTAAG ACCAACCCTC GTCTGCACAC ACCCATGTAC TATCTACTAT

CATTATTAAG ACCAGCCCTC GTCTGCACAC ACCCATGTAC TATCTACTGT

CATTATTAAG ACCAGCCCTC GTCTGCACAC ACCCATGTAC TATCTACTGT

CATTATTAAG ACCAACCCTC GTCTGCACAC ACCCATGTAC TACCTACTAT

AATCATTAAG ACCAGCCCCC ACCTGCACAC ACCCATGTAC TGTCTACTGT

GATTATTAAG ACCAGCCCTC GTCTGCACAC ACCCATGTAC TATCTGCTCT

AATTATTAAG ACCAACCCTC GTCTGCACAC ACCCATGTAC TATCTACTTT

AATTATTAAG AGCAACTCTC GTCTCCACAC ACCCATGTAC TATCTACTGT

TGTGGTCCTC CGTGAACAGA GCCTTCATGA GCCCATGTAT TATTTCCTCT

TGTGGTCTTC CGTGAGCAGA ATCTCCATGA ACCAATGTAT TATTTCCTCT

TATGGTCATT CATGAGCAGA GCCTCCATGA GACCATGTAT TATTTCCTTT

TGTGGTCCTC CGTGAGCAGA GTCTCCATGA GCCCATGTAT TATTTCCTCT

CGTCATCCGT ACTGATGCCA CCCTCCATGA ACCCATGTAC TATTTTTTGG

AATCATTAAG ACCAACCCTC ATCTACACAC ACCCATGTAC TATTTGCTGT

TGTGGTCTTC CATGAACAGA GCCTCCATGG ACCCATGTAT TATTTCCTCT

TGTCATCCTC ACCAATCAAA GCCTCCATGA ACCCATGTAC TATTTGCTCT

GGTTGTTGCC TGTGACAAGA CCCTTCATGA GCCCATGTAC TTCTTCCTCT

CCTTGCTGGC CCTCACTGAC CTGGGGCTGT GTGTGTCCAC GTTGCCCACC

CCTTGCTGGC CCTCACTGAC CTGGGGCTGT GTGTGTCCAC GTTGCCCACC

CCTTGCTGGC CCTCACTGAC CTGGGGCTGT GTGTGTCCAC CTTGCCCACC

CCTTGCTGGC CCTCACTGAC CTGGGGCTGT GTGTGTCCAC CTTGCCCACC

CCTTGCTGGC CCTCACCGAC CTGGGGCTGT CTGTGTCCAC CTTGCCCACC

CCTTGCTGGC CCTCGTTGAC CTGGGACTGT CATTGTCCAC CTTGCCAACC

CCTTGCTGGC GCTCACTGAC CTGGGGCTGT CAGTGTCTAC CTTGCCCACC

CCTTGCTGGC CCTCACTGAC CTGGGGCTGT CAGTGTCTAC CTTGCCCACC

CCTTGTTAGC CCTCACTGAT CTGGAACTGT CAGTGTCCAC CTTGCCTACC

CTATGCTTTC AGCCACAGAC CTGAGCTTAT CCCTGTGCAC ACTTTCCACT

CTATGCTTTC AGCCACAGAC CTGAGCTTAT CCCTGTGCAC ACTTTCTACT

CTATGCTGTC AGCCACAGAC CTGAGCTTAT CCCTGTGCAC ACTTGCCACT

CTATGCTTTC AACCACAGAT CTGAGCTTGT GTCTGTGCAC GCTTTCCACT

CCATGCTGGC CCTCACGGAC TTGGGCCTTT GCCTTTCCAC ACTTCCCACT

CTTTGCTGGC CCTCTCTGAC GTGGGACTGT CAGAGTCCAC CTTGCCCAAA

CGATGCTTTC AGCCACAGAC TTGGGCTTGT CCTTGTGCAC CCTGCCCAAG

TCATGCTATC AACCACCGAC ATGTGCCTGT CCCTCTCCAC TATGCCCACT

GCATCCTCTC GCTCACAGAC CTGGTGCTTT GCTCCACCAC ATTACCTAAG

ACTATGGGGA TCTTCTGGTT TAACTCCCAT AGTATCTACT TTGGAGCGTG

ACTATGGGGA TCTTCTGGTT TAACTCCCAT AGTATCTACT TTGGAGCGTG

ACTATGGGGA TCTTCTGGTT TAACTCCCAT CATATCTACT TTGGAGCATG

ACTATGGGGA TCTTCTGGTT TAACTCCCAT CATATCTACT TTGGAGCATG

ACTATGGGGA TCTTCTGGTT TAACTCCCAC AGTATCTACT TTGGAGCGTG

ACCGTGGGGA TCTTTTGGTT CGACTTCCGC AGCATCTACT TTGGAGCCTG

ATGGTGGGTA TTTTTTGGTT CAATTGCCAT AGCATTTATT TTGGAGCCTG

ATGGTGGGTA TTTTTTGGTT CAATTACCAT GGCATTTATT TTGGAGCCTG

ACAATGGGGA TTTTTTGGTT CAAATCCCAC AGGATCCACT TTGAAGCCTG

ACCCTTGGTG TCTTCTGGTT TGAAGCCCGA GAGATCAACT TAAATGCCTG

ACCCTTGGTG TCTTCTGGTT TGAAGCTCGA GAAATCACCT TAAGTGCCTG

ACTCTTGGTG TGTTCTGGTT TGAAGCCCGA AAGATCAACT TAAATGCTTG

ACTCTGGGCG TCTTCTGGTT TGAAGCTAAA GCGATCAACA TCAATTCCTG

GTTCTGGGCA TCTTCTGGTT TGATGCCCGA GAGATTGGCA TCCCTGCTTG

ACACTGGCTA TTTTGTGGCT CCATGCTGGT GAGATTTCCT TTGATGCCTG

ACACTAACCA TTTTGTGGCT CCAGGATGGT GAGATTTCCT TTGCTGCCTG

ACTCTAGGTG TCCTCTGTTT TAATATACAA GAAATTGGTT GGAATGCTTG

ATGCTGGCTA TCTTCTGGTT TGGGGCCCAG CACATCTCCT ACATTGGCTG

TCAAATCCAG ATGTTCTGCA TCCACTCTTT TTCCTTCATG GAGTCCTCAG

TCAAATCCAG ATGTTCTGCA TCCACTCTTT TTCCTTCATG GAGTCCTCAG

TCAAATCCAG ATGTTCTGCA TCCACTCATT TTCCTTCATG GAGTCCTCGG

TCAAATCCAG ATGTTCTGCA TCCACTCATT TTCCTTCATG GAGTCTTCGG

CCAAATCCAG ATGTTCTGCA TCCATTCATT TTCCTTCATG GAGTCCTCAG

CCAGATCCAG ATGTTCTGCA TCCATTCATT TTCATTCATG GAGTCATCAG

CCAAATCCAG ATGTTCTGCA TCCACTCTTT TTCCTTCATG GAGTCTGCGG

CCAAATCCAG ATGTTCTGCA TCCACTCTTT TTCCTTCATG GAGTCTGCAG

TCAAATCCAG ATGTTCTGTA TCCACTCCTT TTCCTTCATG GAGTCCTCCG

TGTTGCTCAG ATGTTCTTTC TCCATGGATT TACTTTCATG GAGTCTGGGG

CATTGCCCAG ATGTTCTTTC TCCATGGATT TACTTTCATG GAGTCTGGGG

TATTACGCAG ATGTTCTTTC TCCATGCATT TACTTTCATG GAGTCTGGGG

CATTGCTCAG CTCTTCTTCA TCCACACTTT GTCTCTTGTG GAGTCTTCGG

CTTCACTCAG CTGTTCTTCA TCCATACATT GTCTCTGGTG GAATCTTCAG

TCTAGCCCAA ATGTTTTGCA TCCATTCTAT CTATAGTCTA GAGTCCTCAG

CTTAGCCCAG ATGTTTTGTG TCCACGCCAT CTATGCGCTG GAGTCCTCAG

TATTAGCCAA ATGTTCTTCA TTCATTTCCT TACTGTCATG GAGTCATCAG

CCTCACTCAA ATGTTCTTCA TCCACATGGT CTTTGCCACC GAGTCAGCTG

TGCTCCTCAT GATGTCCTTT GACCGCCTTG TGGCCATCTG CCACCCTCTG

TGCTCCTCAT GATGTCCTTT AACT---TTG TGGCCATCTG CTACCCTCTG

TGCTCCTCAT CATGTCCTTT GACCGCTTTG TGGCCATCTG CCACCCTCTG

TGCTCCTCAT CATGTCCTTT GACCGCTTTG TGGCCATCTG CCACCCTCTG

TGCTCCTCAT TATGTCCTTT GACCGCTTTG TGGCTATCTG CCACCCTCTG

TGCTGCTCAT CATGTCCTTT GATCGCTTTG TGGCCATCTG CCACCCCCTA

TGCTCCTTGT CATGTCATTT GATCGTTTTG TGGCCATCTG CTACCCACTG

TGCTCCTCGT CATGTCCTTT GATCGTTTTG TGGCCATCTG CCACCCACTG

TGCTCTTGGT CATGTCCTTT GACCGCTTTG TGGCTATCTG CCACCCACTG

TTTTGCTGGC CATGGCCTTT GACCGTTTTG TGGCCATCTG TGATCCACTG

TTCTGCTGGC CATGGCCTTT GATCGTTTTG TAGCCATCTG TAATCCACTG

TTCTGCTGGC CATGGCCTTT GATCGTTTTG TGGCCATCTG TGACCCACTG

TTCTATTATC CATGTCTATC GACCGCTATG TTGCCATCTG CAACCCGCTG

TTCTATTGTC CATGTCCATT GACCGCTATG TGGCCATTTG CAACCCACTA

TTCTTCTTGC CATGGCCTTT GAT---TATG TGGCTGTCTG CAACCCTCTC

TTCTTCTTGC CATGGCCTTT GATCGATATG TGGCTATCTG CCAGCCTCTG

TGCTCTTGGC CATGGCCTTT GATCGCTATA TTGCTATATG TAACCCATTG

TCCTGCTGGC CATGGCTTTT GACCGTTTCG TGGCCATCTG CCGCCCACTA

AGGTATTCGG TCATTATCAC TGGCCAGCAA GTGGTCAGAG CAGGCCTAAT

AGGTATTCGG TCATTATCAC TGGCCAGCGA GTGGTCAGGG CAGGCCTAAT

AGGTATTCGG TCATTATCAC TGGCCAGCAA GTGGTCAGGG CAGGTCTGAT

AGGTATTCGG TCATTATCAC TGGCCAGCAA GTGGTCAGGG CAGGCCTGAT

AGGTATTCGG TCATTGTCAC TGGCCAACGA ATGGTCAGGG CAGGCCTAAT

AGGTACTTGA CCATTATCAC TGGCCAGCGA ATGGTGCGGG CAGGCCTGAT

AGGTACTCGG TCATTATCAC CATTGAGAGG GTGATGAGGG CAGGCCTATG

AGGTACTCCT CCATTATTAC CGTTCAGAGG GTAATGAGGG CAGGCCTATG

CGGTACTCGG TCATTATCAC TGGCCAGAGA GTAATCAGGG CAGGCTTGCT

AGATACACCA CCATCCTCAC CAATGCCAGG ATCGCCCAGA TTGGGATGAG

AGATACACCA CCATCCTCAC CAATGCCAGA ATTACCCAGA TTGGGGTGAG

AGATATACCA CAATCCTCAC CAATGCCAGG ATTGCCCAGA TTGGGGTGAG

CGTTATTCCA CCGTCTTGA- -AATGCCAGG ATCATCCAGA TCGGGCTGAC

CGTTACTCCA CCATCCTCAC ACCTGCACGC ATTGTTAAGA TGGGGCTGAG

AGATGTACAA CCATTCTCA- -CACGGCATC ATAGGCAGAA TCACCCTTGT

AGGTACAGCA CCATTCTCA- -CACAGTGTC ATAGGCAGAA TCACATGTGC

AGATACACCA CCATCCTCAC TCCAACCAAG ATTATCCATA TTGGAATGAT

CATTACTCGT CCATCCTCA- -CCTGGTGTC ATTGGGAAAA TTGTAATGGC

TGTCATCTTC CGGGGACCTG TGGCCACTAT CCCTATTGTC CTCCTCCTGA

TGTCATCTTC CGGGGACCTG TGGCCACTAT CCCTATTGTC CTCCTCCTGA

TGTCATTTTC CGGGGACCTG TGGCCACTAT CCCTATTGTC CTCCTCCTGA

TGTCATTTTC CGGGGACCTG TGGCCACTAT CCCTATTGTC CTCCTCCTGA

TGTCATCTTC CGGGGACCTG TGGCCACTAT CCCTATTGTG CTCCTCCTGA

TGTTATCCTC CGAGGACCTG TGGCCCTTAT CCCCCTTGTC CTCCTGCTGA

TGTCATCCTC AGGGGGCCTG TAGCCCTTAT CCCAATTGTC CTCCTCCTGA

TGTCATCCTC AGGGGGCCTG TAGCCCTTAT CCCAATTGTG CTCCTCCTGA

TGTCATCCTC CGGGGACCTC TGGCCCTTAT CCCTATTGTT CTCCTCCTGA

CATGTTGACA AGAAATGTTG CTGTGATGTT GCCAGTTGTG CTTTTTGTCA

CATGTTGATA AGGAACGTTG CTGTCATGTT ACCAGTGGTG CTCTTTGTTA

CATATTGATA AGGAATGTTG CTGTCATGCT GCCGGTTGTG CTGTTTGTCA

CATGTTGATA AGGAATGTTG CTGTCATGCT ACCAGTTGTG CTTTTTGTCA

CTCAGTGTTT AGAAGTGCCC TTCTCATCCT CCCCCTGCCA TTCCTTCTTA

TGGAATATTC CGTAGTGTAG CTGTTGTTTC CCCTTTTATC TTCTTGCTGA

TGGGATATTT CGTAGTACAT CCATGGTTTC CCCCTTCATC TTCTTGCTGA

TATAATGGCA AGAGGGACTA TACTCATGAC TCCTCTCCTT GTTCTTCTTA

CTGCGTAGTC CGAGGCCTCT TCTTTGTCTT CCCATTCGTC ATCCTGATCC

AGGCTTTTCC CTACTGTGGA TCTGTGGTCC TCTCCCACTC ATTTTGCCTG

AGGCTTTTCC CTACTGTGGA TCTGTGGTCC TCTCCCACTC ATTTTGCCTG

AGGCTTTTCC CTACTGTGGA TCTGTGGTCC TCTCCCACTC GTTTTGCCTG

AGGCTTTTCC CTACTGTGGA TCTGTGGTCC TCTCCCACTC GTTTTGCCTG

AGGCTTTTCC CTACTGTGGA TCTGTGGTTC TCTTCCACTC ATTTTGCCTG

AGACTTTTCC TTACTGCAGA CCTCCAGTCC TCTCTCACTC ATTTTGTCTG

AGAATTTTCC CTACTGTGGA CCTCTTGTTC TCTCCCATTC ATTTTGTCTA

AGGATTTTCC CTACTGTGGA CCTCTTGTCC TCTCCCACTC ATTTTGTCTA

AAGTCTTTCC CTATTGTGGG CCTCTAGTTC TCTCCCACTC ATTTTGCCTA

AGAGGCTGTC CTTCTGCAGT TCTATGGTCC TCTCACATTC TTACTGCTAC

AGAGGCTGTC CTTCTGCGGT GCAGTGGTCC TTTCACATTC TTACTGTTAC

AGAGGCTGTC CTTCTGCAAT TCTATGGTCC TTTCACATTC TTACTGCTAC

AGAGGCTGGT CTTCTGCAGG TCCTTGGTTC TTTCACATTC CTACTGCTAC

AGCGCTTCCA GTACTGCCAC TCCCATGTGC TGGCCCATGC CTACTGCCTG

AGCAACTACC CTACTGTGGG CACCGTGTCA TGGCACATAC ATACTGTGAG

AGCGACTGCC CTACTGTGGG CATCGGGTCA TGGCACATAC ATACTGTGAG

AGCGTCTTTC TTTCTGCAGG GATAATATTC TCTCTCACTC CTACTGTTAC

AGAGATTGCC ATTCTGTGGG CATCATGTGA TCCCCCACAC CTACTGTGAG

CACCAGGAAG TGATACAGCT GGCCTGCACA GATATCACCT TCAATAATCT

CACCAGGAAG TGATACAGCT GGCCTGCACA GATACCACCT TCAATAATCT

CACCAGGAAG TGATACAGCT AGCCTGCACA GATACCACCT TCAATAACCT

CACCAGGAAG TGATACAGCT AGCCTGCACA GATACCACCT TCAATAACCT

CACCAGGAAG TGATACAGCT GGCCTGTACA GATACCACTT TCAATAATCT

CACCAGGAAG TGATACACCT GGCCTGCACA GACACCACCT TTAACAACAT

CACCAGGAGG TGATACATCT GGCATGTGTG GACACCACCT TCAACAACTT

CACCAGGAAG TGATACACCT GGCATGTGTA GACACCACCT TCAACAACCT

CACCAGGACA TGATACATCT GGCCTGCACA GACACCACCT TCAACAGCAT

CATGTTGATC TCATTCAACT CTCATGCACA GACAACAGAA TCAACAGCAT

CATGTTGACC TCATTCAACT CTCATGCACA GACAACAGAA TCAACAGCAT

CATGTTGATC TCATTCAACT CTCATGCACA GACAACAGAA TCAACAGCAT

CATGTCGATC TTATTCAACT CTCATGCACA GATAACAGAA TCAACAGCAT

CACCTAGAGA TCATGAAGCT GGCCTGTTCA AGCATCGTTG TCAACCACAT

CACATGGGCA TTGCTCAACT GGCCTGTGCC AGTATAACTG TCAATGTTGT

CACATGGGCA TTGCTAGCTT GGCCTGTGCC AACATCACGG TCAATGTTAT

CATCCTGATG TGATCAAATG TTCCTGCTCT GATATCAGGG TTAACAGCCT

CACATGGGCA TTGCCAAGCT GGCATGTGCC AACATCAAGA TCAACATCAT

GTATGGACTG ATGGTGGTAG TTTTCACTGT GATGCTGGAC CTGGTGCTCA

GTATGGACTG ATGGTGGTAG TTTTCATTGT GATGCTGGAC CTGGTGCTCA

GTATGGACTG ATGGTAGTAG TTTTCACTGT AATGCTGGAC CTGGTGCTCA

GTATGGACTG ATGGTAGTAG TTTTCACTGT GATGCTGGAC CTGGTGCTCA

GTATGGACTG ATGGTGGTAG TTTTCACTGT GATGCTGGAC CTGGTGCTCA

GTACGGGCTG GCAGTGGTAG TATTCACCAT GATGCTGGAC TTGGTGCTCA

ATATGGACTG TCACTGGTGG TGTTCACCGT GATGTTAGAC CTTGTGCTCA

ATATGGACTG TCACTGGTGG TGTTCACCGT GATGCTAGAC CTCGTGCTCA

CTATGGACTC TTTGTACTTG TATCCACCTT TGGTATGGAT CTACTGTTCA

CCTTGGTCTG TTTGCACTCT TCTCCACGAC AGGGTTTGAC TGTCCTTGTA

CCTCGGTCTG TTTGCACTTT TCTCCACAAC AGGATTTGAT TGTCCTTGCA

CCTTGGTCTG TTTGCACTCT TCTCCACAAC AGGATTTGAC TGTCCTTGCA

TCTGGGTCTG ATTGCACTCT TCTCCACAAC AGGATTTGAC TGTCCTTGCA

CTATGGGCTC TTTGTTGTGG CCTGCACCGT GGGTGTGGAC TCCCTACTCA

CTATGGGCTG ACTGTAGCGC TTCTGGCCAT GGGTCTGGAT TCCATCCTCA

TTATGGGCTG ACTGCAGCCC TACTGGCCAT GGGATTGGAT TCCATCCTCA

CTATGGACTT ATTGCTGTCC TTTGTTCCTT TGGCTTGGAT GCTCCTTTGA

CTATGGTCTG ACGGTTGCAC TCTCGGTCAC AGGGATGGAT GTGGTGCTCA

TCGCACTGTC CTATGGACTC ATCCTGCACA CAGTAGCAGG CCTGGCCTCC

TCGCACTGTC CTATGGACTG ATCCTGCACA CAGCAGCAGG CCTGGCCTCC

TCGCACTGTC CTATGGACTC ATCCTGCACA CAGTAGCAGG CCTGGCCTCC

TCGCACTGTC CTATGGACTC ATCCTGCACA CAGTAGCAGG CCTGGCCTCC

TCGCACTGTC CTATGGGTTC ATCCTGCACA CGGTAGCAGC CCTGGCCTCC

TTGGACTGTC ATATGGCTTC ATCCTGCACA CAGTGGCGGG CCTGGCCTCC

TTGCACTGTC CTATGGATTT ATCCTACACA CAGTGGCAGG TCTGGCTTCC

TTGCGCTGTC CTATGGATTC ATCCTATACA CGGTGGCTGG TCTGGCTTCC

TCTTCCTCTC CTACATGCTC ATTCTTCGTT CTGTTTTGGC CATTGCTTCC

TCTTGCTCTC CTATGTCCTG ATCATCCGAT CTGTCCTCAG CATCACCTCC

TCTTGCTCTC CTATGTACTG ATCATCCGGT CTGTTCTCAG CATTGCCTCC

TCTTGCTCTC CTATGTTCTG ATCATCCAAT CTGTCCTCAA CATTGCTTCC

TCTTGATCTC CTATGTCCTT ATTATTCGTT CCGTCCTTGG CATTGCTTCC

TCTTCCTCTC CTATACACTT ATCCTCCGCA CTGTGCTGAG CATCGCCTCC

ATGCCATCTC CTATGTCTTT ATCCTCCAAG CCGTCTTTCA TCTACCATCT

TTGCCATCTC GTATGTCTTT ATCCTCCAAG CAGTCTTTCG CCTGCCATTT

TTGGCATCTC CTATGTGTTG ATCCTTCTTT CTGTGTTCAA CATTGCATCC

TTGGCTTTTC TTATGGCTTC ATCCTCCATA CTGTGCTCCA CCTGCCTTCC

CAAGAGGAGC AGCGCCGTGC CTTTCAGACA TGCACCGCTC CTCTCTGTGC

CAAGAGGAGC AGCGCCGTGC CTTTCAGACA TGCACCGCTC ATCTCTGTGC

CAGGAGGACC AGCACCGTGC CTTTCAGACA TGCACTGCTC ATCTCTGTGC

CAGGAGGACC AGCACCGTGC CTTTCAGACA TGCACTGCTC ATCTCTGTGC

CCAGAGGAGC AGCGCCGTGC CTTTCAGACA TGCACGGCTC ATCTCTGTGC

CGAGAGGAGC AACTCCGAGC CTTCCAAACA TGTACTTCTC ATCTCTGTGC

CAAGAGGAGC AGATCCGAGC CTTCCAAACA TGTACTTCAC A--TCTGTGC

CAAGAGGAGC AGATCCGAGC CTTCCAAACA TGTACTTCAC ATCTCTGTGC

CGTGAGGAAC GCCTCAAAGC TCTGAACACA TGTGTGTCAC ATATCCTGGC

ATAGAGGGCA GGCAGAAAGC CTTCAACACC TGCATTTCCC ACATCAGTGC

TCAGAGGGGC GGCAAAAAGC CTTCAACACC TGCATATCCC ACATCAGTGC

TCAGAGGGAC GGCAGAAAGC CTTCAATACT TGCATATCGC ACATCAGTGC

TCAGAGGGAC GGCGGAAAGC CTTCAACACT TGCATATCTC ACATCAGTGC

CGCCAGGAGC GACTTCGGGC CCTCAACACC TGTGTCTCCC ATATTTGTGC

CATGATGCTT GGCGCAAGGC TCTAAGTACC TGTGGCTTTC ACCTTACAGT

CGTGAAGCCC GGCACAAAGC TCTGAGTACC TGTGGCTCCC ACCTTGCAGT

CCAGAGGAAC GATACAAAGC ATTTAGCACC TGCATCTCCC ATATTGGTGC

CAGGATGCAC AGCTCAAGGC CTTCAGCACC TGTGGGGCTC ACGTATGTGT

TGTGCTAGTA TTCTTTGTGC CCATGATGGG GCTGTCCCTG GTGCACCGTT

TGTGCTAGTA TTCTTTGTGC CCATGATGGG GCTGTCCCTG GTGCACCGTT

TGTGCTAGTA TTCTTTGTGC CCATGATGGG GCTGTCCCTG GTGCACCGTT

TGTGCTAGTA TTCTTTGTGC CCATGATGGG GTTGTCCCTG GTGTACCGTT

TGTGCTAGTA TTCTTTGTGC CCATGGTGGG GCTGTCCCTG GTTCACCGTT

TGTGCTGATA TTCTTTGTGC CCATGGTAGG GCTGTCTCTG GTGCACCGTT

TGTGCTTGTG TTCTTTGTGC CCATGACGGG GCTGTCTCTT GTACACCGCT

TGTGCTTGTG TTCTTTGTGC CCATGATGGG ACTGTCCCTG GTACACCGCT

TGTATTGGCA TTTTATGTAC CAATGATTGG GGTCTCCACA GTGCACCGCT

TGTTGCCATC TTCTACATCC CTCTCATCAG CCTGTCTCTT GTCCATCGCT

TGTTGCCATC TTTTACATCC CCCTTGTCAG CTTGTCTCTT GTCCATCGCT

TGTTGCCATC TTCTACATCC CTCTCATCAG CTTGTCTCTT GTCCATCGCT

TGTTGCTATC TTCTACATCC CTCTCATCAG CTTGTCTCTT GTCCATCGCT

TGTGCTACTT TTTTACATAC CCATGATTGG CTTGTCTCTC ATACACCGCT

CATCCTGATT TTCTACATCC CTGCCTTTTT CTCCTTCTTC ACTTACCGCT

TATCCTAGTT TTCTACATCC CTGCCTTCTT CTCCTTCCTT ACTCACTGCT

TGTGGCTATT TTCTACATTC CCCTCATTGG ACTCTCTGCT GTTCATAGAT

CATCCTGGTC TTCTACATTC CGGCTTTCTT CTCCTTCTTC ACACACCGCT

TTGGGAAGCA TGCCCCACCT GCTATTCATC TTCTTATGGC CAATGTCTAC

TTGGGAAGCA TGCCCCACCT GCTATTCATC TTCTTATGGC CAATGTCTAC

TTGGGAAGCA TGCCCCACCT GCTTTTCATC TTCTTATGGC CAATGTCTAC

TTGGGAAGCA TGCCCCACCT GCTTTTCATC TTCTTATGGC CAATGTCTAC

TTGGGAAGCA TGCCCCACCT GCTATTCATC TTCTTATGGC TAATGTCTAC

TTGGGAAGCA TGTTCCACGT GCTGTGCACC TTCTTATGGC GAATGTCTAC

TTGGGAAACA TGCTCCACCT GCTGTACATC TTCTCATGGC CAACATCTAC

TTGGGAAACA TGCTCCACCT GCTGTGCATC TTCTCATGGC CAACATCTAC

TTGGGAAGGA TGCTCCACGC TACCTACATG TCCTCATGTC CAACATCTAC

ATGGCCATTC AGCACCTCCC TTTGTCCACA CCATCATGGC CAATATCTTC

ATGGCCATTC AGCACCTCCA TTTGTCCACA CCATCATGGC CAATGTCTTC

ATGGACATTC AGCACCTCCA TTTGTCCACA CCATCATGGC CAATGTCTTC

ATGGCCATTC GGCACCTGCA TTTGTCCACA CTATTATGGC CAATGTTTTC

TTGGTGAACA TCTGCCCCAT ATCATACACC TCCTTATGTC TTATGTGTAT

TTGGACACCG TGTCCCCAAG CACGTGCACA TTTTTCTGGC TAATCTCTAT

TTGGCAACCG TGTCCCCAAG AGTGTGCACA TATTTCTGGC CAATCTCTAT

GGGGAAAGAA GGCACCTCCA TTTGCTCACA CTTTGATGTC TAATGCTTTC

TTGGTCACTG GATGCCTCCT CAGGTTCACA TTTTCGTGGC CAATCTCTAC

CTTTTTGTGC CTCCCATGCT TAACCCAATC ATATACAGCA TTAAGACCAA

CTTTTTGTGC CTCCCATGCT TAACCCAATC ATATACAGTA TTAAGACCAA

CTTTTTGTGC CTCCCATGCT TAACCCAATC ATATACAGTA TTAAGACCAA

CTTTTTGTGC CTCCCATGCT TAACCCAATC ATATACAGTA TTAAGACCAA

CTTTTTGTGC CTCCCATGCT TAACCCAATC ATATACAGTA TTAAGACCAA

CTTTTTGTGC CGCCCATGCT TAACCCAATC ATATACAGTA TTAAGACCAA

CTCTTCGTAC CTCCCATGCT TAACCCAGTC ATATATAGTA TTAAGACAAA

CTCTTTGTAC CACCCATGCT TAACCCAGTC ATATATAGTA TTAAGACAAA

CTCTTTGTCC CCCCTGTGCT CAACCCTCTC ATTTATAGTG CCAAGACAAA

CTGCTTATTC CTCCTGTACT CAACCCTATC ATCTACAGTG TGAAGACTAA

CTTCTCATCC CTCCCGTGCT CAACCCCATC ATCTATAGTG TGAAGACTAA

CTGCTCATCC CTCCTGTGCT CAACCCTATC ATCTACAGTG TGAAGACGAA

CTGCTTATTC CTCCTGTGCT CAACCCTATA ATCTATAGTG TGAAGACTAA

CTGCTGGTGC CACCCCTCAT GAACCCCATT GTCTACAGCA TCAAAACCAA

GTGCTCGTGC CTCCTGTACT CAACCCAATC ATCTATGGGG TCAAGACCAA

GTGCTGGTGC CTCCTGTGCT CAACCCAATC ATCTATGGGG TGAAGACAAA

CTCATTCTTC CCCCTGTGCT AAACCCAATT ATCTATAGTG TGAAGACCAA

CTCCTGGTAC CCCCTGTGCT CAACCCGCTG GTGTATGGCA TTAATACCAA

GGAGATCCAC CGTGCCATTT TCATCCTCAC GGACATCCCT GGATTTGAGG

GGAGATCCAC TGTGCCATTT TCATCCTCAC GGGCATCCCT GGATTTGAGG

GGAGATCCGC CGTGTCATTT TTCTTCTCAC TGGATTTTCT GGCATGGAGC

GGAGATCCGC CATGTCATTT TCACACTGAC TGGGATTCCA GGAATGCCAG

GGAGATTCGC CGTGCCATTT GCTTTCTGAT TGGGATACCA GGGCTGGAGC

GGAAATACGC CGTGCTATCT TCATTTTGGT TGGCATCCCA GGGCTAGAGG

GGAAATCCGA AGAGCAATCT TCATCCTCAC GGGCATCCCA GGATTTGAGG

GGAAATCCGA AAAGCAATCT TCATCCTCAC CGGCATCCCA GGATTTGAGG

AGAGATCCGC CGAGCCATTT TCATTCTCAT GGGCATCCCT GGATTTGAGG

GCAGATTCAA AAGGCTGTTT TCATCCTCCT TGGGATCCCA GGATTGGAGA

GCAGATTCGA AAGGCTATTT TTATCCTCAC GGGCATCCCT GGATTTGAGG

GCAGATTCGA AAGGTTATTT G-ACCTTGGT TGGAATCACT GGACTGGAAA

ACAGATTCGA AAGGCGATTT TCATCCTCAC TGGCATCCCA GGATTTGAAG

GCAAATCCGC CTACGCATTT TCCTCCTCAC TGGATTTTCT GGCTTAGAGG

AGAGATCCGC AGTGCCACCT TCATCCTAAC AGGCATCCCT GGATTTGAGG

GCAGATTCGC CGTGCCATCT TCATCCTCAC GGGCATCCCT GGATTTGAGG

ACAGATTCGA AGGGCTAT-T TTATACTGAC AGGTATACCA GGAATGCCAG

GCATATCCGG AGGATTCTGT TTATCCTGAC AGGCTTTTGG GGACTGGAGG

CCTCCCACAT CTGGATCTCC ATCCCCGTCT GCTGTCTCTA CACCATCTCC

CCTCCCACGT CTGGATCTCC ATCCCCGTCT GCTGTCTCTA CACCATCTCC

AGCAATACCC CTGGTTCTCC ATCCCCTTCT CCTCTATCTA TGCCATGGTG

AGAAAGACTT ATGGATGGCC TTGCCCCTCT GTCTTCTTTA CAGCACCACG

ATGCTCAAGT ATGGATCTCT GTCCCCATCT GCCTCATGTA CTTGGTGGCC

CAGAGCACCA C----TCTTC ATGATCATCT TATGTATCTA CCTGGTGACC

CTATCCATCT CTGGATCTCC ATCCCCTTCT TCTGCCTCTA CACCATTTCC

ATATCCATCT CTGGATCTCC ATCCCCTTCT TCTGCCTCTA CACCATATCC

CCTCCCACAT CTGGATCTCC ATCCCCTTCT TCTCTCTCTA CACTGTCTCC

ATTCC---TT T-GGTTTGCC TTTCCATTCT GTGCCATGTA TGTTGTGGCT

CTTTCCACAT CTGGATCTCC ATCCCCTTCT GCTGCCTCTA TACCATCTCC

CTACACAGTG T-GGATTGGG ATTCCATTCT GTGCTATGTA TATCATTGCT

CTTACCACAT CTGGATCTCC ATCCCCTTCT GCTGTCTCTA CACCATCTTT

AGCAATATCC CTGGGTTTCT ATCCCCTTCT CCTTCATCTA TGCCATGGTG

TCTCTCACAT CTGGATCTCC GTCCCCTTCT GCTGCTTATA CATAACCTCC

CCTATCACAT CTGGATCTCC ATCCCCTTCT GCTGTTTTTA CACCATCTCC

AGAAGAATTC TTGGATGGCA TTCCCACTGG GACTCCTCTA CACCCTCACT

ATTCCTTGCA CTGGATCTTT ATCCTGCTAG GGGTGCTCTA CTGTGTCGCC

ATCATGGGCA ATACCACCAT CCTCGTCATT CGCACAGAGC CATCTGTCCA

ATCATGGGCA ATACCACCAT CCTCGTCATT CGCACAGAGC CATCTCTCCA

CTTTTGGGAA ATTGCATGGT TCTCGTGATA TGGACTGAGC CAAGCCTGCA

ATCTTGGGAA ATGTCACCAT CCTTGTCCTC AAAGTTGAGC AAAGTCTCCA

ATCATAGGCA ATTGCACAAT CCTCGTGATC AGAACTGAGC CCTCACTCCA

ATATCTGGCA ACCTCAGCAC AATCCTAATC AGAATCTCCT CTCAGCTCCA

ATCATGGGCA ACACCACCAT CCTCGTCATC CGCACAGAGC CATCTCTCCA

ATCATGGGCA ACACCACCAT CCTAGTCATC CGCACAGAGC CATCTCTCCA

ATCGTGGGCA ACACCACCAT CCTCGCCATC CACACACCAC CAGGCCTCCA

CTAGTCGGCA ATATCACTCT CCTTGTAATC CGGAATGATC CCACCTTGCA

ATCGTGGGGA ACACCACCAT TCTCGTCATC CGCACAGAGC CATCCCTCCA

TTGATAGGAA AT-TCTCTAC TTTTATCATC AAATCTGAAC CCAGTCTCCA

TTCCTGGGAA ACTGCATGGT GCTCGTGATC CGGACTGAGC CAACACTGCA

CTTTTAGGCA ATTTCATGGT GCTAGTGATA TGGACTGAGC CAAGCCTACA

ATCATGGGCA ACAACGCCAT CCT-GTTATA TGGAAGGAGC AGACCCTCCA

ATCATGGGCA ACACTACCAT CCTCATCATC AGAACTGAGT CTTCTCTCCA

CTTCTTGGCA ATTGCACCAT CTT-ATCATT AAGATGGATA GAAGCCTCCA

ATCCTGGGAA ATTCCCTGAT CCT-ATCCTC AAGGAGAAGC AGAGCCTGCA

CCAGCGCATG TATCTGTTTC TCTCCATGCT GGCCCTGACG GACCTGGGTC

CCAGCCCATG TATCTGTTTC TCTCCATGCT GGCCCTGACA GACCTGGGTC

CCAGCCCATG CTTTACTTCC TGTCCATGCT GGCCCT--CT GACCTGTGCA

TGAGCCCATG TGTTATTTTC TAGCCATGTT AGCTGCCACT GACCTCAGCC

TGCACCTATG TACTATTTCC TTTCCATGTT GGCTGTCTCT GACCTGGGCT

TCACCCTATG TATTTTTTTC TGAGCCACTT GGCTTTTGCT GACATGGGCT

CCAGCCCATG TACCTCTTCC TCTCCATGCT GGCCCTCACT GACCTGGGCC

CGAGCCCATG TACCTCTTCC TCTCTATGCT GGCCCTCACT GACCTGGGTC

CCAGCCCATG TACCTGTTTC TCTCCATGTT GGCAGTGACT GACCTGGGCC

TGAGCCCATG TACCTCTTTC TGGCCATGCT GGCTACCACT GACCTGGTCC

CCAGCCCATG TACCTATTTC TCTCCATGCT GGCCCTGACT GACCTGGGCC

TGAATGTATA TATATCTTCC TGGCGATGTT GGGAGCCACA GACATTGGAA

CCAACCCATG TTCTATTTTC TGGGGTTGCT GGCCCTTACG GACCTATGCA

TCAGCCCATG TTCTACTTCC TGGCCATGCT GGCCCTCACA GACCTGTGCA

TGGTCCTATG TATATCTTCT TGGCCATGCT GGCTGCCTCT GATCTGGGTC

CCAACCAATG TACTACTTTC TATCCATGCT GGCATTCACT GACTTGGGTC

TGAGCCCATG TACTACTTTC TCTCCATCTT GGCACTGACT GATGTGGGTC

CCAACCCATG TACTACTTCC TGGCCATGCT GTCTGTCAAC GACCTAGGCG

TCACCCTCAC CACCCTACCC ACAGTCATGC AGCTTCTCTG GTTCAACGTT

TCACCCTCAC CACCCTACCC ACAGTCATGC AGCTTCTCTG GTTCAACGTT

TGGGGCTCTC CACTGTGTAC ACAGTGCTGG GGATCCTGTG GGGGATCATT

TTTCACTGTC TTCCATGCCT ACCATGGTCA GTGTTCACTG GTTCAACTGG

TGTCCCTCTC CTCCCTACCC ACTATGCTGA GGATCTTTGT ATTCAATGCC

GTTCCTCTTC TGTCACCCCC AACATGCTTG TAAACTTCCT GGTGGAGAGA

TCACTCTTAC CACACTCCCT ACTGTCATGC AGCTTCTCTG GTTCAACATT

TAACTCTCAC CACACTCCCT ACAGTCATGC AGGTCCTCTG GTTCAACATT

TCACCCTCAC CACCCTGCCC ACGGTCATGC AACTCTTCTG GTTCAACATT

TTACTTCCA- --CACAACCT AAAATGCTGG CCATATTCTG GTTTCACAAT

TCACCCTCAC CACCTTGCCC ACAGTCATGC AGCTTCTCTG GTTCAACATT

TTATCACCAG TATTGTCCCC AAAATGCTTG GAATGTTTTG GTCCCACTTG

TTGGACTGTC CACTGTACAC ACAGTGCTGG GCATCCTGTG GGGACTTAGC

TGGGGCTGTC AACAGTGCAC ACTGTGCTGG GGATTATGTG GGGACTCATT

TGTCCCTCTT CACTTTCCCC ACAATGCTAA GGATCTTCTG GCTGGATGCC

TGTCCACTAC TACCTGGCCT ACCATGTTCA GTGTCTTCTG GTTTCACGTC

TCTCTATATC CACCCTGCCT TCCATGCTCA GCATTTTCTG GTTCAATGCC

TGTCCTGCTC TACTCTGCCC ACAGTCCTGG GCACCTTCTG CTTCAACGTC

CGTAGAATCA GCTCTGAGGC CTGTTTTGCT CAGTTTTTCT TCCTTCATGG

CGTAGAATCA GCTCTGAGGC CTGTTTTGCT CAGTTCTTCT TCCTTCATGG

CGAGAGATCA ACTTGGATTC CTGCATTACC CAGTCCTATT TCATCCATGG

CATTCAATAA CTTTTAGTGG CTGCCTTATT CAGATGTTCT TCATCCACAC

ACAGGAGTCT CCCCAAATGC TTGCTTCGCT CAAGAATTCT TTATTCATGG

AATACAATCT CCTATCATGC ATGCGCCATC CAGCATGGTT CGGCTAATTT

CGGGAAATCA GCTTTGAGGC CTGCTTTGCA CAGTTCTTCT TCCTCCATGG

CGGGAAATCA GCTTTGAGGC CTGCTTTGCA CAGGTATTCT TCCTCCATGG

CAGAAAATTA GCTTTGAAGC CTGCTTTGCC CAGTTCTTCT TCCTCCATGG

CATGAGATTG AATACCATGC CTGCCTCATG CGGTTGTTCT TCATCCATGC

CCCAAGATCA GCTTTGAAGC TTGCTTTGCA CAGGTATTTT TCATTCATAC

CCAGAGATCT ATTTTGATGC TTCCCTTTTC CAGATGTGGC TCATCCA--C

CAGGAGATTG GCCTGGATGC CTGTATCTCC CAGACTTATT TTGTCCATGG

CAAGAGATCA GCTTGGATTC TTGTATCACC CAGTCCTATT TTATCCACGG

CGAGAGCTGA CCCTGTCTGC TTGCTTTACT CAAATGTTCT TCATTCACAC

CGGGATATTC CCTTTGATGC CTGTCTGGTC CAAATGTACT TCATTCATGT

CCTGAAATCC CCTTTGATGC CTGCATCACT CAGATGTTTT TCATCCATGT

AGAGAGGTTG CCTTCAACGC CTGCATGGCC CAGATGTTCT TCATCCACCT

ATTCTCCTTT ATGGAGTCTT CTGTCCTCCT GGCTATGTCC GTTGACTGCT

ATTCTCCTTT ATGGAGTCTT CTGTCCTCCT GGCTATGTCT GTTGACCGCT

TGTGTCCTTC ATGGAGTCCT CTGTCCTCCT TGCTATGGCC TTTGACCGGT

ATTTAGGGGA GTAGAATCAG GTGTTCTCGT GGCCATGGCC TTTGATCGCT

ATTCACAGAT ATGGAGTCCT CAGTGCTTCT GGTCATGTCT TTGGACCACT

CTTTGGGACA GCTGAATGCT TCCTTCTGGC TGCCATGGCA TATGATCGTT

GTTCTCCTTC ATGGAGTCAT CTGTTTTATT GGCCATGTCC TTTGACCGTT

GTTCTCATTC ATGGAATCAT CTGTTCTATT GGCCATGTCC TTTGACCGTT

ATTCTCCTTC ATGGAATCAT CTGTCCTATT GGCCATGTCC TTTGATCGCT

CTTTTCTTCT GTGGAGTCTG GATTGCTCAT GGCTATGGCC TTGGACGGTT

ATTCTCTTTC ATGGAATCTT CAGTCCTATT GGCCATGTCC TTTGATCGCT

ATTTCAGGGT ATTGAATCAG GAGTCCTGCT GGTCGTGGCT CTGGACCACT

ACTTTCCTGC ACCGAGTCTG GAGTTCTTCT TGTCATGGCC TTTGATCGCT

TCTGTCCTTC ATGGAATCCT CTGTTCTCCT CCCTATGTCC TTTGACCGTT

CTTCCAGGAT TTTGAATCAG CTGTCATCCT GGCGATGGCC TTTGACCGCT

TTTCTCCATT ATTGAATCAG CTGTGTTGTT GGCCATGGCC TTTGATCGAT

GTTTGGAATC ACAGAATCTG GGGTGTTGGT ATCCATGGCC TTTGATCGCT

GTTCTCCTTC ATGGAGTCCG GCATCCTGCT GGCCATGAGC TTTGACCGCT

ATGTGGCCAT CTGCTGTCCC CTCCATTATG CCTCCATCCT CACCAATGAA

ATGTGGCCAT CTGCTGTCCC CTCCATTATG CCTCCATCCT CACCAATGAA

ACATTGCAAT TTGCAATCCA CTGCGTTATT CCTCCATCCT GACTAATTCC

TTGTGGCCAT CGGCTTTCCT TTGCACTATG CTACCATTCT CACTCACAGT

TCTTGGCCAT ACGCAACTCT CTGAGATACA GCTCCATCCT CACCAGTGCC

TTGTGGCAAT CTGCAACCCT CTGCTTTATC CAACTAAAAT GTCCACACAA

ATGTGGCCAT CTGTCGCCCA CTCCACTATG CCTCCATCCT CACCAGTGAG

ATGTGGCCAT CTGTCGCCCA CTCCACTATG CCTCCATCCT CACCAGTGAG

ATGTGGCTAT CTGCCGTCCC CTACATTATG CCACCATCCT CACCAATGAA

ACGTGGCCAT CTGCTTCCCA TTGCATCACT CTAGTATCCT GACCCCATCC

ATGTAGCCAT CTGTCGCCCC CTCCATTATG CCACCATCCT CACCAGCGAA

ATATAGCAAT TTGTTATCCT CTGAGGCATG CTACTATATT CACTCAACTA

TTACTGCAAT CTGCAATCCT CTGAGATATA GCTCCATCCT AACTAATAGC

ATATTGCAAT TTCCAAACCA CTGCACTATT CTTCCATCCT GACTAATTCT

ATGTGGCCAT TTCTCGTCCA TTGCACTATT CCTCCATCCT CACTAACAGC

TTATAGCAAT CAGAGAGCCC CTGCGATATG CAACCATTCT AACCAATAAT

TTGTGGCTAT TCGAGATCCA CTGCGTTATT CTTCCATCCT CACCTATGAG

ATGTGGCCAT CTGTAATCCT CTCAGATACT CACTGATATT AACCAATGCC

GTCATTGGTA GAACTGGGTT AGCCATCATT TGCTGCTGTG TTCTGGCGGT

GTCATTGGTA GAATTGGGTT AGCCATTATT TGCCGCTGTG TTCTGGCTGT

AGAATTAGCA AAATTGGGCT CACTATAATA GGTAGGAGTT TTTTCTTTAT

GTCATCAGCA AGATTGCAGC AGCAGTCCTG CTACGGAGTA TGGGGACTGT

AGAGTTGCCA AAATGGGGAT GGTGTTTCTC ATTAAAAGCA TGGTC---GT

GTCTGTGTCC AGTTACTTGT AGTGGCTTAT ATAGGTGGTT TTCTTAATGC

GTCATTGGTA GAATAGGGTT GGCCATCATT TGCCGCTGTG TCCTGGCTGT

GTCATTGCTA GAATAGGGTT GGCCATCATT TGCCGCTGTG TCTTGGCTGT

GTCATTGGTA GAATTGGGCT TGCCATCACT TGCCGCTGTG TTCTGGCTGT

GTCATGGGTA AACTGGGGGC AGGTATGATA AT-------- ----------

GTCATTGGCA GGATTGGGTT GGCCATCATT GGCCGCTGTA TCCTGGCTGT

GTCACTCATA ---TTGGAGT TGGGGTGACA TTGAGGGCCA TTCTG---GT

AGAGTCATTC AGCTCATGGT GACCATTTTG ATGAGAAGTG CTTTGTCTAT

AGGATTGCTA AAATAGGGCT TGCCATATTA GGTAGGAGTT TCTTCTTTAT

ATAATTGCCA GGATAGGTTT GGCCATTTTT GTAAGAACCT TGACTGTACA

GTAATCATTG GCATTGGGTT GTTAATTGCT GGAAGGGCCC TGGCTCTGGT

GTCATTGGCA AGATTGGATT AGTTGTCCTT GCCCGTGCTG TCTTTGTGGT

AGGATCACTA AGATGGGTCT GGCCATTTTC TCCCGCACCT TTGGGGCCGT

TCTTCCCTCC CTTTTCCATG CCTCTCCACT GGTCCATGTT ATCATGGCCA

TCTTCCCTCC CTTTTCCATG CCTCTCCACT GGTCCATGTT ATCATGGCCA

TATACCGCCC ATCATCCACC TTTCCCCTGT GGCCCACGTT CTCATTGGTA

GCTCCCTGTG CCTTTGCATT TGTCACCACC AATACACACA TTCATGGCCA

ACTTCCATTT CCTTTTCACA AGTCCCCAGT GGCCATAAAC CTCATTGCTG

TTCTTTCTTT ACCTTT---- ---------- -GACCAGAAC ATGATGTCTG

TCTTCCCTCC CTTTTCCATG CCTCTCCACT GGTCCATGTT CTCATGGCCA

TCTTCCCTCC CTTTTCCATG CTTCTCCACT GGTCCATGTT CTCATGGCCA

TCTTCCCTCC CTTTTTCATG CCTCTCCACT GGTCCATGTT ATCATGGCCA

-------TCC CTTTTGCAAG TGCCC---AT GGTACATGTC ATGTTAGCTA

TCTCCCTTCC CTTTTCCATG CCTCTCCACT GGTCCATGTT ATCATGGCCA

AATCCCATGC CTACTGTGTA TCCCATCATA TATACATATC ATCTTGCCAA

TCTTCCTGTC ATCATCCATA CGTCTCCACT GGTCCATGCC ATAATAGCCA

TACACCCCCT ATTATCCACC TCTCCCCAGT GGTCCATGTC CTTATGGGCA

GGTGCCCCTT CCCATCAGGC TTCCCTCACA CATCTATGTG CTCATGGCCA

TTTTCCAGCT TCCTTTAATG CTTCTCCAAT TGTCCATGTA CTCATGGCCA

CCTCCCTGCA CCCTTGCACC TCTCACCCAT TGTGCACACA GTGATGGCCA

CTTCCCCCTG CCCT----TA TCCCTCCATC AGTCCACACC ATCATAGGCA

ATATCTACCT GCTGGCACCC CCGGTGATGA ACCCCATCAT TTACAGTGTA

ATATCTACCT GCTGGTACCC CCGGTGATGA ACCCCATCAT TTACAGTGTA

ACATCTACAT CCTTTTCCCA CCTTTAATGA ATCCCATCAT CTACAGTGTC

ATATCTACCT GCTTCTCCCT CCTGTGCTCA ATCCCATTGT GTACAGTGTT

ATGTTTTCTT GCTAGTGCCA CCTCTTATGA ACCCCATTGT TTATTGTGTG

TGTTCTACAT GGTGGTGATC CCCATGTTGA ACCCCCTCAT TTACAGTCTC

ATATCTACCT GCTGGCACCT CCTGTGATGA ATCCCATCAT TTACAGTGCA

ATATCTATCT GCTGGCACCT CCTGTGATGA ACCCCATCAT TTACAGTGTA

ACGTCTACCT GTTGGCACCT CCCGTGATGA ACCCTATCAT TTACAGTGC-

TTCTCTGTCT CCTGGTACCT CCCATGCTCA ACCCCATCAT CTACAGAGTT

ATATCTACCT GTTGGCACCT CCTGTGATGA ACCCCATCAT TTACAGTGTC

ACCTTTACCT ACTGGTTCCT CCTTTACTCA ATCCCTTTGT CTGTGGGGTG

ATATCTATCT GTTGGCACCC CCTGTGATGA ATCCTATCAT TTATAGTGTC

ACATCTATAT CCTTTTCCCA CCTTTGATGA ACCCCATCAT CTACAGTGTC

ACATACACTT GCTGGCCCCT CCTGTGATGA ATCCCATTAT TTACAGTGTC

ATGTTTACTT GCTGGCTCCT CCTGTGATGA ACCCCATCAT TTATAGTATA

ATGTGTATCT TTTACTCCCA CCTGTACTTA ACCCCCTCAT CTACAGCATA

ACCTCTACTT CCTGTTCCCA CCCATGTTGA ACCCCATCAT TTATGGTGTC

AAGAACAAGC AGATCCTTTC CCTCAAAAAT AATAGCAGCA CTTCAATGGC

AAGAACAAGC AGATCCTTTC CCTCAAAAAT AATAGCAGCA CTTCAATGGC

AAGACCCAAC AGATTTTTTC TCTGAAAA-- AATAGCAGCA CTTCAATGGC

ACGACCAAGC AGATCCTAGG TCTTAAAAAT AATAGCAGCA CTTCAATGGC

AAGACACGGC AAATTTTCTG CTCCAAAAAC AATAGCAGCA CTTCAATGGC

AGGAACAAGG AGAT------ ---------- AATGACAGCA CTCCAATGGC

AAGACCAAAC AGATTCTCTT TCAAAGAAA- AATAGCAGCA CATTAATGGC

AAGACCAAGC AGATCCTGTT GCAAAGAAA- AACATCAGCT CATTAATGGC

---------- -----CTCGC CCCCAAAA-- ---------- ----------

AGAACGAAAC AGATC----- ---------- AATGCCAGCA CTTCGATGGC

AAGACTAAGC AGATCATTTC CCAAAGA--- AAT---AACA CTTCAATGGC

AAGCCCAAAT ACATCCTCTT CCAGAGGA-- AATAGCAGCA CTTCAATGGC

AAGACTAAGC AAATCTTTTC CAAAAAAAA- AATAGTAGCA CTTCAATGGC

AAGACCCAAC AGATTTTTTC TCTGAAAAG- AATAGCAGTG ATGCTGTGGA

AAGACTAAGC AGATACTCTT CCACAGGAA- AACAGCACCA AATTCCAGGT

AAGACTAAGC AGATCCTCTT CCAGAGAAA- AACAGTTCTA AATTGCAGGT

AAGACCAAAC AGATTTTTTT TAGGAGAAA- AATGGCACAA TCTTCATGCC

AAGACCAAGG AGTTC-TTTT TCTAACAA-- AATCACACCC AGTTCCACCC

CCCCACCTTC TTGCTGGTGG GCATGCCAGG CCTATCAGGT GCACCCTCCT

CCCCACCTTC TTGCTGGTGG GCATGCCAGG CCTATCAGGT GCACCCTCCT

CCCCACCTTC TTGCTTGTGG GCATGCCAGG CCTATCAGGT GTACCCTCCT

CCCCACCTTC TTGCTTGTGG GCATGCCAGG CCTATCAAGT GTACCCTCCT

CCCCACCTTC CTGTTAGTGG GCATGCCAGG CCTGTCAAGT GCACCCTCCT

TCCCACCTTT TTGTTGGTGG GCATGCCAGG CCTGGCAGCT GTACCCTCTT

TCCCACTTTC CTGTTAGTTG GCCTTCCTGG CCTGGAAGCT ACACCGTCCT

CCCCACTTTT CTATTAGTGG GCCTGCCTGG CCTGGAAGCT GCACCATCCT

---------- ---------- ---------- ---------- ----------

CCCCACCTTC CTGTTGGTGG GCCTGCCAGG CCTATCAGCT GTACCCTCCT

CCCCACCTTC CTGTTGGTGG GCATGCCAGG TCTGTCAGCT GTACCCTCCT

CCCCACCTTC CTGTTGGTGG GCCTGCCAGG CCTGTCAGCT GTACCCTCCT

CCCTATGTTC CTGCTGGTAG GCATGCCAGG CCTGTCAAGC GTACCCTCCT

GCCCATATTT ATCCTGAGAG GTTTCCCCGG ACCAGAGTAT ATTCTTCCTT

CTCTGAGTTC ATCCTGATGG GATTCCCAGG CATTCACAGC TGGCAACATT

TTCAGAGTTC ATCTTGATGG GAATTCCAGG CATTCACAAC TGGCAGCACT

ATCGCTGCTT TTTCTGATTG GGATACCTGG CCTAGAGACT GTGCAGTGCT

CTCCTCCTTC TTCTTGCTGG GATTGCCGGG CCTGGAAGCC GCACACATCT

GGTGGACATT GCCCCTCATT GCTGTCTACC TTCTCTCTGC ACTGGGAAAT

GGTGGACATT GCCCCTTATT GCTGTCTACC TTCTCTCTGC ACTGGGAAAT

GGTGGACGAT GCCCCTCATT GCTGTCTACC TTCTCTCTGC ACTGGGAAAT

GGTGGACGAT GCCCCTCATT GCTGTCTACC TTCTCTCTGC ACTGGGAAAT

GGTGGACAAT GCCCCTCATT GCTGTCTACC TTCTCTCTGC GCTGGGCAAT

GGTGGACCAT GCCCCTCGTC ATTGTCTATT TGCTTTCTGC CCTGGGCAAT

GGTGGTCAGT TCCTCTCATC ACTATCTACC TTCTCTCTGC TCTGGGCAAT

GGTGGTCAGT GCCTCTCATC ACTATCTACC TTCTCTCTGC CTTTGGCAAT

---------- ---------- ---------- ---------- ----------

GGTGGGCAGT ACCCCTCATC ACTGTCTACC TTCTGTCTGC CCTGGGCAAT

GGTGGACAAT ACCACTCATC ACCATCTACC TTCTCTCTGC CCTGGGCAAT

GGTGGACAAT ACTCCTCATC ACTGTCTACA GTCTCTCTGT TTTGGGCAAT

GGTGGACGAT ACCCCTCATC ACCGTCTACC TTCTTTCTGC CCTGGGCAAC

GGCTCTCAAT CCCATTCTGC CTGGCATACT TAGTAGCATT TTTTGGTAAT

GGCTCTCCTT GCCGTTGGCA CTACTCTACC TCTCAGCAAT TGGTGCTAAC

GGCTTTCTTT GCCCTTGGCA CCACTCTACC TTGTGGCAAT TGGTGCCAAC

GGTGG--AAT TCCATTCTGT GCCATGTATG TCGTTGCCAT GGTTGGAAAT

GGATTGGTTT GCCCATCTGT GCTGCCTACT TGATCTCGTT TGTGGGGAAC

GGCACCATCC TCTGGATCAT TGCCCTGCAG CCCGCCCTGC ACCGCCCAAT

GGCACCATCC TCTGGATCAT TGCCCTGGAG CCCGCCCTGC ACCGCCCAAT

GGTACCATCC TCTGGATCAT TGCCCTGGAG CCTGCCCTGC ACCGCCCAAT

GGTACCATCC TCTGGATCAT TGCCCTGGAG CCTGCCCTGC ACCGCCCAAT

GGTACTATCC TCTGGATCAT TGCACTGGAG CCTGCCCTGC ACCGCCCAAT

GGTACTGTCC TCTGGATCAT TGCTCTGGAA CCTGCCCTGC GTCGACCAAT

GGTACAGTCC TCTGGATCAT TGCCCTGGAG CCCACACTGC ATCGACCAAT

GGTACAATCC TCTGGATCAT TGCCCTGGAG CCCACATTGC ATCGACCAAT

---------- ---------- ---------- ---------- ----------

GGTGCTATCC TCTGGATCAT TGCCCTGGAG CCCACACTGC ACCGCCCAAT

GGTACTATCC TCTGGATCAT TGCCCTGGAT CCCACCCTGC ACCGCCCAAT

GGTACTATCC TCTGGGTCCT TGCCCTGGAG CCCACTCTGC ATCGCCCAAT

AGTACCATCC TGTGGATTAT TGCCATGGAG CCCACCCTGC ACCGCCCCAT

TGCATCATCC TCTCTGTCGT TTGGATTGAG TCTTCCCTCC ACCAGCCCAT

ACACTCATCC TCATCACCAT TTGCCAAAAC TCTACTCTGC AGCAGCCTAT

CTCCTCATTC TCATCACCAT CTGCCAGGAC TCTGCTCTGC AACAACCCAT

TACCTGCTCC TGTTCATCAT TCGATCTGAA AGCAGTCTCC ACAAGCCCAT

TGCACCATCC TGTTTGTGAT CAAGACCGAG CAGAGCCTCC ACCAGCCCAT

GCACTTCTTC CTCTTCTTGC TTAGTGTGTC TGATATTGGA TTGGTCACTG

GCACTTCTTC CTCTTCTTGC TTAGTGTGTC TGATATTGGA TTGGTCACTG

GCACTTCTTC CTCTTCTTGC TTAGTGTGTC TGATATTGGC TTGGTTACCG

GCACTTCTTC CTCTTCTTGC TTAGTGTGTC TGATATTGGC TTGGTTACCG

GCACTTCTTC CTCTTCTTAC TTAGTGTGTC TGATATTGGC TTGGTCACTG

GCACTTCTTC CTCTTCTTGC TCAGTGTGTC TGATGCTGGC TTAGCCACAT

GTACTTCTTC CTCTTCTTGC TTAGTGTGTC TGATGTTGGC TTGGCCACAG

GTACTTCTTC CTCTTCTTGC TTAGTGTGTC TGATGTTGGC TTGGCCACCG

---------- ---------- ---------- ---------- ----------

GTACTTCTTC CTCTTCCTGC TCAGCGTGTC TGATGTTGGC TTGTCCACAG

GTACTTCTTC CTCTTCCTGC TGAGTGTGTC TGATGTTGGC TTGGCCACAG

GTACTTCTTC CTCTCCCTGC TTAGTGTGTC TGATGTTGGC CTGGCCACAG

GTACTTCTTC CTCTTCCTGC TTAGTGTGTC TGATGTTGGT TTGGCTACAG

GTACTACTTC CTTTCCATCC TGGCACTGAC TGACCTGGGT ATGTCCCTGT

GTACTATTTC CTAGGCATCC TCTCTGTTGT CGACATGGGC TTGGCCACCA

GTACTATTTC CTAGGCATCC TTTCTGTAGT AGACATGGGC CTGGCCACCA

GTACCTCTTC TTGGCTATGT TGGGGGTCAC TGATATTGCC CTCAGTACTT

GTTCTACTTC CTGGCCATGC TGGCTACCGT TGACCTGGGC TTGTCCACAA

CCCTGATGCC CACACTGCTG ATTAGCAAAA TCAGCATTTC TTTTCGATGC

CCCTGATGCC CACACTGCTG ATTAGCAAAA TCAGCATTTC TTTTCGATGC

CCCTGACGCC CACACTGCTG ATTAGCAAAA TCAGCATTGC TTTTAGATGC

CCCTGATGCC CACACTGCTG ATTAGCAAAA TCAGCATTGC TTTTAGATGC

CCCTGATGCC CACACTGCTG ATTAGCAAAA TCAGCATTGT TTTTCGATGC

CCCTGATGCC CACACTGTTG ATCCAAAAGA TCAGCATTGC TTTTCGATGC

TGCTAATGCC CACCCTGCTA ATTAGCAAGA TTGCCATCGC TTTCCGATGC

TGTTAATGCC CACCCTGCTA ATTAGCAAGA TTGCCATTGC TTTCCGATGC

---------- ---------- ---------- ---------- ----------

CCCTGATGCC CACCCTGCTG ATCAGCAAGA TCTGTATTGC TTTCCGATGC

CCCTGATGCC CACCCTGCTG ATTAGCAAGA TTGGCATAGC CTTCCGATGC

CCCTGATGCC CACTCTGCTG ATCAGTAAGA TCAGCATTGT TTTCCGAAGC

CCATCATGCC CACTCTGCTG GTTGGGAAAA TTGGTATTAT TTTCCGATGT

CTACACTTCC CACCATGCT- ATTGGCAAGA TTGGTTGCTT GCTGAGAAGC

CCATCATGCC CAAGATCCTG ATAGTAAAAG CCACCATCTT GCTGCGAAGC

CCATCGTGCC CAAGATTCTG GTCATAAAAG TTACCATCTT GCTGCGGAGC

GCATCCTCCC CAAGATGTTG CTCACTCAGA TTGG---TGA CCTTAAGGGC

CCACCATCCC CAAGATGCTG ATTGTCAAGA TGGGTATTTT AGTACGCAGC

CTGGGTCTCC ATCTGCCCCT GCCATTCCTG CTGGCCTACA TGCCCTACTG

CTGGGTCTCC ATCTGCCCCT GCCATTCCTG CTGGCCTACA TGCCCTACTG

CTGGGTCTCC ATCTGCCCCT GCCATTTCTG CTAGCCTGCA TGCCCTACTG

CTGGGTCTCC ATCTGCCCCT GCCATTTCTG CTGGCCTGCA TGCCCTACTG

CTGGGTCTCC ATCTGCCCCT GCCATTTCTG CTGGCCCACA TGCCCTACTG

CTGGGTCTCC ATCTGCCCCT GCCATTTCTC CTGGCCCACA TGCCCTACTG

TTGAGTCTCC ATCTGCCCCT GCCATTCCTG TTGGCTCACA TGCCATATTG

TTGAGTCTCC ATCTGCCCCT GCCAATCCTG TTGGCTCGCA TGCCATATTG

---------- -----CCTGT GCCTTTCCTT ATCAAACGGC TCCCCTTCTA

CTGGGTCTCC ACCTGCCCCT GCCATTCCTC CTGGCCCACA TGCCTTACTG

TTGGGTCTCC ACCTGCCCCT GCCATTCCTC CTGGCTCACA TGCCCTACTG

CTGGGTCTCC ATCTGCCCCT GCCATTTCTA CTGGCCCACA TGCCCTACTG

CTGGGTCTCC ACCTGCCCCT GCCGTTCCTC CTGGCCCGCA TGTCCTATTG

ATGGGAGTTG TAATGCCCAC GCCGTTGCTA CTGAGACACT ATCTGTACCA

ATGGGAGTTG TACTGCCTAC ACCTTTGCTA CTGAGACGCT ATCACTACTG

ATGGCAGTTG TACTACCCAC ACCTGTGCTA CTGGGACGCT ACGACTACTG

T--GCTATCC TGGTGGCCCC ATGCCTTGTG CTTATCCGCC TGAAACATTA

TTCATTTTGA TCCTTCCTTT ACCCTTCCTC CTGAGGAGAC TGCCATTTTG

CCTCCCACAG GTCCTAACCC ATTCTTATTG CTTGCATCCA GATGTGGCTC

CCACCCACAG GTCCTAACCC ATTCTTTTTG CTTGCATCCA GATGTGGCTC

CCGCCCACAG GTCCTAACCC ATTCTTATTG CTTGCATCCA GATGTGGTTC

CCGCCCACAG GTCCTAACCC ATTCTTATTG CTTGCATCCA GATGTGGCTC

CCGCCCACAG GTCCTAACCC ATTCTTACTG CTTGCATCCA GATGTGGCCC

CCACCCACAA GTCCTGACCC ATTCTTATTG CTTGCACCCT GATGTGGCCC

CCGACCACAG GTCCTGACAC ACTCTTACTG CTTACACCCA GATATGGTTC

CCGTCCACAG GTCCTGACAC ATTCTTACTG CCTGCACCCA GATATGGCTC

CCGCTCCAAC ATCCTCTCCC ATTCTTTCTG TCTCCACCAA GATGTCATGC

CCGTCCACAG GTCCTGACCC ATTCTTACTG CTTGCACCCG GATATAGCCC

CCACCCACAG GTTCTGACCC ATTCTTACTG CTTGCACCCA GATATAGCCC

CCACCCACAG GTCCTGACCC ATTCTTATTG CTTGCATCCA GATATGACCC

CCGGCCGCAG GTCCTGACCC ACTCATACTG CTTACATCCC GATATGACTC

TCACGTCAAT ACCCTTTCCC ATGCCTTCTG CTTGCACCAG GATCTTCTGA

CCATAAAAAT GTTCTCTCCC ATGCCTTCTG CTTGCACCAG GATGTTCTGA

CCATATCAAT GCCCTTTCCC ATGCCTTCTG CTTGCACCAA GATGTCCTAA

CCGAACCACA GTCATCTCCC ACACTTACTG TGAGCACATG GCCATTGTAA

CAAGGTCAAC ATCCTGCATC ACGCTTACTG TCTCCATCCC GACCTCATCC

GTTTGGCCTG CCCAGAAGCT TGGGGTGCAG CCTACAGCCT ATTTGTGGTT

GTTTGGCCTG CCCAGAAGCT TGGGGTGCAG CCTACAGCCT ATTTGTGGTT

GTTTGGCCTG CCCAGGAGCT TGGGGTGCAG CCTATAGCCT ATTTGTGGTT

GTTTGGCCTG CCCAGGAGCT TGGGGTGCAG CCTATAGCCT ATCTGTGGTT

GTTTGGCCTG CCCAGGAGCT TGGGGTGAAG TCTACAGCCT ATTTGTGGTT

GTTTGGCCTG CCCAGGGGCT TGGGGTGCAC TCTACAGCTT CTTTGTGGTC

GCCTAGCTTG CCCAGGAACA TGGGGTGCAT TCTATAGCCT GGTTGTGGTA

GCCTAGCTTG CCCAGAAGCA TGGGGTGCGG TCTACAGCCT GGTTGTGGTA

GCCTTGCCTG TCCAGCACCC GTGTCAACAG TCTATGGCCT TATTGCTGTC

GTTTGGCCTG CCCCAGAGGT GGGGGAGCAG TCTACAGCCT CTTTGTGGTC

ATTTGGCCTG TCCAGGAGCT TGGGGTGCAT TCTATAGCCT CTTTGTGGTT

GATTGGCCTG CCCAGGAGCT TGGGGTGCTG TCTATAGCCT CTTTGTGGTC

GGTTGGCCTG TCCAGGAGCT GGGGGTGCAG TCTATAGCCT CTTTGTAGTC

AATTATCATG TTCAGATGCC AGGAATAGTG TTTATGGACT TTGTGTCGTC

AATTATCCTG TTCAGATGCC AGGAATAGCA ATTATGGGTT ATGTGTAGTT

AATTAGCCTG TTCCGATGCT AGGGATAGCA TTTATGGACT GTGTGTAGTT

AGCTGGCTGC TGATGACATC CGGGACAAGG CCTATGGTCT GTTTGTGGCC

ACCTGCCCTG TGGGGACACC ACCTACAGCT TATTTGGCCT ATTCATCGTG

CTTTCAGCCA TGGGTTTGGA CCCCCTGCTT ATTTTCTTCT CCTATGGCCT

CTTTCAGCCA TGGGTTTGGA CCCCCTGCTT ATTTTCTTCT CCTATGGCCT

CTTTCAGTCA TGGGTTTGGA CCCCCTGCTG ATTTTCTTTT CCTATGGCCT

CTTTCAGTCA TGGGTTTGGA CCCCCTGCTG ATTTTCTTTT CCTATGGCCT

TTTTCAGCCA TGGGTTTGGA CCCCCTGCTT ATTTTCTTCT CCTATGGCCT

CTGTCTGCTA TGGGATTGGA CCCCCTGCTT ATTTTCCTCT GCTATGGCCT

CTGTCAGCGA TGGCACTAGA CCCTCTGCTT ATCTTCTTCT CATATGGCCT

CTGTCAGCAA TGGTACTAGA CCCTCTGCTG ATTTTCTTCT CCTATGGGCT

ATCTTCACCA AGGGCTCTGA CTCCCTCTCC ATCCTTCTCT CCTATGTGTT

CTGTCTGCCA TGGGCTTGGA TCCTCTGCTT ATTTTATTCT CCTATGGCCT

CTGTCAGCCA TGGGATTAGA CCCCCTGCTT ATTTTCTTCT CCTATGGCCT

CTGTCAGCTA TGGTATTGGA CCCCTTGGTT ATTTTCTTCT CTTATGGTCT

CTGTCGGCCA TGGGGTTGGA CCCTCTCCTT ATTTTCTTTT CCTACGGCCT

ATTGCCACAC TAGGTGTGGA TTCCCTCTTC ATACTTCTTT CATATGTCCT

CTCATCACAC TGGGTGTGGA TTCAGTCTTC ATACTTCTTT CTTATGTCCT

ATTGCCACAC TGGGTGTGGA TTCAGTCTTC ATACTTCTTT CCTATGTCCT

TTTACTATCC TGGGCTTTGA CATTATCTTC ATTACCCTGT CCTACATTTT

CTCTCCACAT TCGGACTGGA CTCCGTGCTC ATCTTCCTTT CCTACGTCCT

GATTGGCAAG GTGTTGCAAG GTGTGGAGTC CAGAGAGGAT CGCTGGAAGG

GATTGGCAAG GTGTTGCAAG GTGTGGAGTC CAGAGAGGAT CGCTGGAAGG

GATTGGCAAG GTGTTGCAAG GTGTGGAGTC CAGGGAAGAT CGCTGGAAGG

GATTGGCAAG GTGTTGCAAG GTGTGGAGTC CAGGGAAGAT CGCTGGAAGG

GATTGGTAAG GTGTTGCAAG GTTTGGAGTC CAGGGAGGAT CGCTGGAAGG

GATTGGCAGA ATGTTGCAAG GTGTGGGGTC TAAGGAAGAT CGCTGGAAGG

GATTGGCAGA GCATTGCAAG GTGTGGGGTC TACCGAGGAT CGCTGGAAGG

GATTGGGAGA GCACTGCAAG GTGTGGGATC TGCTGAGGAC CGCTGGAAGG

CATACTCAAG ACAGTGATGG CTATTGCCTC AGGAGAGGGC AGGCTGAAGG

AATTGGCAGG GTGTTGCAAG GTTTGGGATC CTGTGAGGAT CGCTGGAAGG

AATTGGCAGG GTGTTGCAAG GTTTGGGATC CAGTGAGGAT CGCTGGAAGG

AATTGGCAGG GTATTGCGAG GTTTGGGATC CAGTGAGAAT CGCTGGAAGG

AATTGGCCGG GTGTTGCAGG GCTTGGGATC CAGTGAGGAT CGCTGGAAGG

GATTCTGAAT GCTGTGCTGG GCATTGCGTC TCATGAAGAG CAGCTAAAGG

GATTCTGAAT GCTGCTCTGG GCATTGCATC TCGTGAAGAG AAGCTAAAGG

GATTCTGCAT GCTGTGCTGG GCATTGCATC ACGTGAAGAG CGGCTAAAAG

GATCTTCATA GCAGTGTTCC GTTTACCCCA GAAAGAGGCC CGCCTCAAAG

GATCCTCAGG TCCGTCCTGA CCATCGTGTC CCAGTCAGGG CAGTCCAAGG

CTGGTCAAAC CTGTGCTGCC CACCTCTCTG CAGTGCTCCT CTTCTATATC

CTGGTCAAAC CTGTGCTGCC CACCTCTCTG CAGTGCTCCT CTTCTATATC

CTGGTCAAAC CTGTGTTGCC CACCTTTCTG CCGTGCTCCT CTTCTATATT

CTGGTCAAAC CTGTGTTGCC CAACTTTCTG CCGTGCTCCT CTTCTATATT

CTGGTCAAAC CTGTGTTGCC CACCTCTCTG CCGTGCTCCT CTTCTATTTC

CTGGCCAAGC CTGTGCTGCT CACCTTTGTG CTGTGCTCCT CTTCTATGTC

CTGGTCAAAC GTGTGCTGCT CACCTCTCTG CTGTGCTCCT CTTCTACACA

CTGGGCAAAC GTGTGCTGCT CACCTCTCTG CTGTGCTTCT CTTCTACATA

CACTCAACAC CTGTGTTTCT CACATTTGCG CTGTACTCAT CTTCTATGTG

CTGGCCAAAC CTGTGCTGCC CACCTCTCTG CTGTGCTTCT CTTCTATGTG

CTGGTCAAAC CTGTGCTGCC CATCTCTCTG CTGTGCTCCT CTTTTACATG

CTGGCCAAAC CTGTGCTGCC CATCTCTCTG CTGTGCTCTT CTTCTACGTG

CGGGCCAGAC CTGTGCTACT CACCTCTGTG CTGTGCTGCT CTTCTATGTT

CACTCAACAC ATGTATATCT CATGTCTGTG CGGTGCTCAT CTTCTTTGTG

CACTCAACAC ATGTGTATCT CATATCTGTG TGGTGCTCAT CTTCTTTGTG

CACTGAACAC ATGTGTGTCT CATATCTGCG TGGTGCTCAT CTTCTTTGTG

CCTTCAACAC CTGCATTGCT CACATCTTTG TTTTCCTTGA ATTCTATCTC

CCCTCAACAC CTGTATCTCC CACATCTGTG CGGTGCTCAT CTTCTACATC

CCTATGATCC TCCTGGCACT GATTAACCAT CCTGAGCTGC CAATCACTCA

CCTATGATCC TCCTGGCACT GATTAACCAT CCTGAGCTGC CAATCACTCA

CCTATGATCC TCCTGGCACT GATTAGCCAT CTTGAGCTGC CAATCACTCA

CCTATGATCC TCCTGGCACT GATTAGCCAT CTTGAGCTGC CAATCACTCA

CCTATGATCC TCCTGGCACT GATTAATCAT CTTGAGCTGC CAATCACTCA

CCCATGATCC TGCTGGCACT GATTAGCCAT CTGAAGCTGC CCATCCCTCA

CCCATGATTC TCTTGGCACT CATTAACCGA TTCAAGCTGC CACTCCCTCT

CCCATGATTC TCTTGGCACT CATTGACAGA TTCAAGCTGC CACTCCCTCC

CCGCTCATTG GAGTGTCTGT CATTCATCGT TTTGGGAAAC ATCTGTCACC

CCAATGGTCC TCCTGGCTCT CATTGATCAT CTCAGGATGC CAATCCCTCA

CCCATGATCT TTCTGGCTCT CATTGACCGT CTCAGGGTGC CAATACCTCA

CCCATGATCC TCCTGGCACT CATTGACCAT CTTAAGGTGC CAATCCCTCA

CCCATGATTC TCCTGGCCCT CATCGACCGC CTCCGGTTGC CAATTCCTCA

CCAGTTATTG GAGTGTCAAT GGCCCATCGC TTTGGGAAGC ATCTGTCTCC

CCAGTCATTG GAGTATCAAT GGTCCATCGC TTTGGGAAGC ATCTGTCTCC

CCAGTCATTG GTGTATCGAT GGCACATCGC TTTGGGAAGC ATCTGTCACC

CTTGCTTTCT TCTCTTTTTT CACTCATCAG GTTTGGATTC CAATCCCCCC

CCCATGCTGG GCGTGTCCAT GATTCACCGG TATGGGAAAC GGGCCCCTCC

GCATACCCAT ACTCTTCTAT CCTATGTCCA TTTCCTTCTT CCTCCATTGA

GCATACCCAT ACTCTTCTAT CCTATGTCCA TTTCCTGCTT CCTCCATTGA

GCATACCCAT ACTCTTCTCT CCTATGTCCA TTTCCTTCTT CCTCCATTGA

GCATACCCAT ACTCTTCTCT CCTATGTCCA TTTCCTTCTT CCTCCATTGA

GCATACTCAT ACTCTTCTCT CCTATATCCA TTTCCTGCTT CCTCCATTGA

GCATGCTCAT ACACTTCTCT CCTATATCCA CTTCCTGCTC CCTCCACTTA

GCCTGCCCAT ACTCTCCTCT CCTATGTCCA CTTCCTGCTC CCTCCACTGA

GCCTGCTCAC ACGCTCCTCT CCTATGTCCA CTTCCTGCTC CCTCCACTGA

ACTGACTCAT GCACTCATGG CAAATGCCTA CCTTCTTGTA CCTCCTGTGC

GCCTGCCCGT ACTCTTCTCT CCTATGTCCA CTTCCTGCTT CCCCCATTGA

GCCTGCCCAT ACTCTTCTCT CCTATATCCA CTTCCTGCTT CCTCCATTGA

GCCTGCCCAT ACTCTTCTCT CCTATGTCCA CTTCCTGCTT CCTCCATTGA

GTCTACCCAT ACCCTTCTCT CCTATGTCCA TTTCCTGCTT CCTCCATTGA

CATCGTCCAC GTCCTCATGG CTGACATCTA CTTGCTTCTT CCCCCGGTGC

CATAGTTCAC GTCCTCATGG CTGACATCTA TCTGTTTCTT CCCCCAGTGC

CACCATTCAC ATCCTCATGG CTGACATCTA CCTGCTGCTG CCCCCAGTGC

CTATGTCCAC ATCCTCCTCT CTAACCTGTA TCTATTGGTT CCACCTCTTC

ATTCATCCAC ACATTGATGT CCATCGTCTA TCTCTTCGTG CCACCCATGC

TAAACCCTAT TCTCTATAGT GTCAAGATGA AGGAGATTAG AAAGAGAATA

TAAACCCTAT TCTCTATAGT GTCAAGATGA AGGAGATT-G AAAGAGAATA

TAAACCCTAT TCTCTATAGT GTCAAGATGA AGGAGATTAG AGAAAGAATA

TAAACCCTAT TCTCTATAGT GTCAAGATGA AGGAGATTAG AGAAAGAATA

TAAACCCTAT TCTCTATAGT GTCAAGATGA AGGAGATTAG AGAGAGAATA

TAAACCCTGT TCTCTACAGT GTCAAAATGA AAGAGATTCA AGAGAAAATA

TAAACCCAGT TCTCTACAGT GTCAAGATGA AGGAGATTAG AGAAAAAATT

TGAACCCAGT TCTCTACAGT GTCAAGATGA AGGAGATTAG AGAAAAAATT

TGAACCCCAT TGTGTATACC ATAAAGACCA AAGAAATACG AAGGAAGAT-

TAAACCCTAT TCTCTATAGT GTCAAGATGA AGGAGATTAG AGAGAGAATC

TAAACCCCAT TCTCTATAGT GTCAAGATGA AGGAGATTAG AGAGAGAATA

TAAACCCTAT TCTCTATAGT GTCAAGATGA AGGAGATTAG AGAGAGAATA

TAAACCCTAT TCTCTATAGT GTAAAGATGA AAGAGATTAG AGATAAAATA

TTAACCCAGT CATCTATAGT ATCAAGACAA AGCAGATTCG TTTAGGAATT

TTAACCCTGT TGTCTACAGT GTCAGGACAA AGCAGATTCG TCTAGGCATT

TCAACCCTGT TGTCTATAGT GTCAGAACAA AACAAATTCG ACTGGGGATT

TCAACCCCAT TGTCTATGGA GTAAAGACCA AGGAAATTAG GAATCGCATC

TCAACCCCAT CATCTATAGC ATCAAGACCA AGGATATCTA CAAAAAGA--

TTCAATAACA CCACTTCGTC TTCCTCAAAC TTCCTCCTCA CTGCATTCCC

TTCAATAACA CCACTTCGTC TTCCTCAAAC TTCCTCCTCA CTGCATTCCC

TTCAATAACA CCACTTCGTC TTCCTCAACT TTCCTCCTCA CTGCATTCCC

TTCAATAACA CCACTTCGTC TTCCTCAACT TTCCTCCTCA CTGCATTCCC

TTCAATAACA CCACTTCGTC TTCCTCAAAC TTCCTCCTCA CTGCATACCC

TTCAATAATA CCACCTCCTC CTCCTCATAC TTTCTCCTCA CTGCATTTCC

-TTAATAATA CCAATCCCCA TCCTCCAACC TTCCTCCTCA CTGGTATCCC

-TTAATAATA CCCATTCCCA GCTTCCAACC TTCCTCCTCA CTGGTATCCC

-TCAATCATA CCAATGCTGA GCTTCTGACC TTCTTCCTGA CGGGTATCCC

TTCAATAATA CCACATCCTC CCCTTCAACC TTCCTCCTCA CTGCATTCCC

TTCAATAACA CCACATCATC TTCCCCAAAC TTCCTACTCA CTGCATTTCC

TTCAATAACA CCACATCCTC CTCCTTGAAC TTCTTCCTCA CTGCATTCCC

TCCAATAACA CTA---CCTC TTCCTCTAAC TTCTTCCTCA CTGCACTCCC

TTAATCAACT TGACAGCTAC ATTTCCAAGC TTCCTGTTGA CTGGCATTCC

-TCAATAGCA CCAGGTCAAT TTTCTCTACC -TCTTTCTAA CAGGCATCCC

-TCAATAACA CCAATGTTCA GCCTGTGATC TTCCTTCTAA CAGGTATCCC

ATCACTAACA TCTCTGAAGA ACCCTTCTAC TTCATCCTTA CGGGCATCCC

TCCAACACTA CCAACTTCCA TCCCTCCACG TTCCTCCTGT TAGGTATCCC

TGGGCTGGAA TGTGCTCATG TCTGGATCTC CATTCCAGTC TGCTGTCTCT

TGGGCTGGAA TGTGCTCATG TCTGGATCTC CATTCCAGTC TGCTGTCTCT

TGGGCTGGAA CGTGCTCATG TCTGGATCTC CATTCCAGTA TGCTGCCTCT

TGGGCTGGAA CGTGCTCATG TCTGGATCTC CATTCCAGTA TGCTGTCTCT

TGGGCTGGAA TGTGCTCATG TCTGGATCTC CATTCCAGTC TGCTGTCTCT

TGGGCTGGAA CGTGTTCATG TCTGGATCTC CATTCCTGTC TGCTGTCTCT

AGGCCTGAGA GCAGCTCAGG TCTGGATCTC CATTCCCTTT TGTCTCCTTT

AGGCCTGAGA GCAGCTCAGG TCTGGATCTC CATTCCCTTT TGCCTCCTTT

AGGCTTGAGA GCATCCCAGG TATGGATCTC CATTCCCTTT TGTCTCCTGT

TGGGCTGGAA CTCGCTCATG TCTGGATCTC CATCCCTGTC TGCTGTCTCT

TGGACTGGAA CTTGCTCACA TCTGGATCTC CATCCCTGTC TGCTGTCTCT

TGGGCTGGAA CTGGTTCATG TCTGGATCTC TATCCCTGTC TGCTGTCTCT

TGGAATGGAA ACTGCCACTG TCTGGATCTC CATCCCCATC TGCTGTCTCT

TGGCCTACAG TCTGTCCATG CCCTGATCTC CATTCCTTTC TGCTGTGTCT

TGGACTGGAG ACCGTACTAA TATGGATCTC TTTCCCACTG GGTGTCATAT

TGGTCTGGAG GCTGTACAAG AATGGATCTC GATTCCACTG GGTGTCATGT

GGGTTTGGAG GCCATGCATA TTTGGATCTC CATCCCCTTC TGCTGCCTAT

TGGGATGGAG CATCAGCACA TCTGGATCTC CATCCCCTTC TGCTCCATGT

ACACCATTGC CCTCTTGGGA AACAGTATGA TCTTTCTTGT CATCATTACT

ACACCATTGC CCTCTTGGGA AACAGTATGA TCTTTCTTGT CATCATTACT

ACACCATTGC CCTCTTGGGA AACAGTATGA TTTTTCTTGT CATCATTACT

ACACCATTGC CCTCTTGGGA AACAGTATGA TTTTTCTTGT CATCATTACT

ACACCATTGC CCTCTTGGGA AACAGTATGA TCTTTCTTGC CGTCATTACT

ACACCATTGC CCTCTTGGGA AATAGCATGA TCCTTTTTGT CATTGTTATT

ATGTAATCGC CCTCTCTGGG AACAGCATGA TCCTGTTGGT GATCCTTCGT

A--------- CCTTTCTGGG AACAGCATGA TCCTGCTGGT GATCGTTCGT

ATGCTGTTGC CCTCTCTGGA AACAGCATGA TCCTGTTGGT AGTACTCCGT

ACATCATTGC TCTCTTGGGA AATACTATGA TCCTGTTTGT CATCTTTGTT

ACACCATTGC CCTCTTGGGA AACAGCATGA TTCTGTTTGT CATCATCATT

ATACGATTGC CCTCTTGGGA AACGGCATGA TCCTGTTTGT CATCATTGTT

ATTCCATTGC CCTCCTGGGA AACAGCTTGA TCTTGTATAT CATCATTATT

ATGCCATTGC CCTCACAGGG AATGGCATGA TCCTGTTTGT CATCATTACT

ACCTAATTGC CCTCCTAGGG AACTGCACCA TCCTCTTTGT TATCAAAACC

ACCTCATTGC CCTGCTAGGG AACTGCACCA TCCTCTTTGT TATCAGAACC

ACATGATCTC CATCTTGGGT AACACCACCA TCCTGGCTGT CATTCAGACA

ACATCATGGC TCTGCTGGGC AATGCCACCA TCCTGTTGGT TGTTGCCTGT

AAGCGGAGAC TCCACAAACC CATGTATTAT TTCCTCTCCA TGCTGGCAGC

AAGCGGAGAC TCCACAAGCC CATGTATTAT TTCCTCTCCA TGTTGGCAGC

AAGCGGAGAC TCCACAAGCC CATGTATTAT TTCCTCTCCA TGCTGGCAGC

AAGCGGAGAC TCCACAAGCC CATGTATTAT TTCCTCTCCA TGCTGGCAGC

AAGCGGAGAC TCCACAAGCC CATGTATTAT TTCCTCTCCA TGCTGGCAGC

GAGCGGAGTC TGCACAAACC CATGTACTAT TTCCTGTCCA TGCTTTCGGT

GAGCAGAGCC TCCATGAGCC TATGTACTAT TTCCTGTCTA TGCTTTCAGT

GAGCAGAGCC TCCATGAGCC TATGTACTAC TTTCTCTCTA TGCTCTCCAT

GAACAGAGCC TCCATGAGCC TATGTATTAT TTTCTCTCCA TGCTCTCAGC

AAGCAGCGTC TCCACAAGCC CATGTACTAT TTCCTCTTTA TGCTGTCAGC

GAGAGAAGTC TCCACAAGCC CATGTACTAT TTCCTCTCTA TGCTGTCAGC

GAGCAGAGTC TCCACAAGCC CATGTACTAT TTCCTGTCCA TGTTGTCAGT

GAGAGGCGTC TCCACAAGCC CATGTACTAT TTCATTTCTA TGCTGTCAGC

CAGCAGAGTC TCCATGAGCC CATGTACTAT TTCCTCTCCA TACTGTCAGC

ACTTCCAGCC TTCATGAGCC TCAGTATATC TTCCTATCTA TGTTGGCAGC

ACTGCCAACC TTCATGAGCC TCAATATATC TTTCTGTCTA TGCTAGCAGC

GAGTCCTTGC TCCACCAACC CATGTACCTG TTCCTCTCCA TGCTGGCACT

GACAAGACCC TTCATGAGCC CATGTACTTC TTCCTCTGCA TCCTCTCGCT

TGTTGATCTA TGTCTGACCA TTACGACCCT TCCCACTGTG CTTGGTGTTC

TGTTGATCTA TGTCTGACCA TTACGACCCT TCCCACTGTG CTTGGTGTTC

TGTTGATCTA TGCCTGACCA TTACGACCCT TCCCACTGTG CTTGGTGTTC

TGTTGATCTA TGCCTGACCA TTACGACCCT TCCCACTGTG CTTGGTGTTC

TGTTGATCTA TGTCTGACCA TCACGACCCT TCCCACTGTG CTTGGTGTTC

TACTGACCTT TGTCTGACCA TCACAACCCT TCCCACTGTG CTTGGTGTTC

CACAGACCTG AGCTTGTCTC TCTGTACACT TTCCACTACC CTTGGTGTCC

CACAGACTTG AGCTTGTCTC TCTGCACACT TTCCACTACC CTTGGTGTCC

CACGGACCTG AGCTTGTCTC TCTGCACACT CTCCACTACC CTTGGTGTCT

TGCTGATTTG TGTCTGACCA TCACGACCCT CCCCACTGTG CTTGGGGTTC

TGTTGATCTA TGTCTGACCA TCTCAACCCT TCCCACTGTG CTTGGGGTTC

TGTTGATTTA TGTCTGACCA TCTCAACCCT CCCCACTGTG CTTGGGGTGC

TGTCGATTTG TGTCTGACCA TCTCAACTCT CCCCACTGTG CTTGGCGTTC

TGCTGACCTG GGCTTGACTG TTTCTACAAT GTCAACAACA TTAGGCATCT

TACAGATGTG GGTCTGTCTT TAACAACCCT CCCCACTGTG CTTGGGGTTC

TACAGATGTG GGTCTGTCAT TATGCACCCT TTCCACAACA CTTGGTGTCT

AACTGACCTG GGCCTCACCC TCACCACTCT GCCCACAGTC ATGCAACTCC

CACAGACCTG TGCTTTGCTC C-ACCACATT ACCTAAGATG CTGGCTATCT

TCTGGTTTCA TGCCCGGGAG ATCAGCTTTA AAGCTTGCTT CATTCAAATG

TCTGGTTTCA TGCCCGGGAG ATCAGCTTTA AAGCTTGCGT CATTCAAATG

TCTGGTTTCA TGCACGGGAG ATCAGCTTTA AAGCTTGCTT CATTCAAATG

TCTGGTTTCA TGCACGGGAG ATCAGCTTTA AAGCTTGCTT CATTCAAATG

TCTGGTTTCA TGCCCGGGAG ATCAGCTTTA AAGCTTGCTT CATTCAGATG

TCTGGTTTCA TGCCCGGGAG ATCAGTTTGA AAGCCTGCCT CCTTCAAATG

TCTGGTTTGA AGCCCGGGAA ATCAACTTAA ATGCGTGCCT CATTCAAATG

TCTGGTTTGA AGCTCGAGAG ATCAATTTAA ATGCGTGCCT CATTCAAATG

TCTGGTTTGA GGCCCGAGAG ATCAGTTTAA ATGCCTGCCT CATTCAAATG

TCTGGTTTCA TGCCCGGGAA ATCAGCTTTA AAGCTTGCCT CATTCAAATG

TCTGGTTTCA TGCCCGGGAG ATTAGCCTGA AAGCTTGCCT CATTCAAATG

TCTGGTTTCA TGCCCGGGAG ATCAGCTTCA AAGCGTGCTT CATTCAAATG

TTTGGTTTCA CGTCTTGGAG ATCAGCTTAA AAGCTTGTCT CATTCAAATG

TCTGGTTTGA TGCAAGTGAA ATCAGTTTAA ATACCTGTAT TATTCAGATG

TCTGGTTACA TTTCCAGGAG ATCAGCTTTA AAGCC----- -ATTCAAATG

TGTGGTTCGA AGCCCGAAAG ATCAACTTAA ACGCCTGCAT TGCACAGATG

TCTGGTTCAA TGTCCAGGAA ATTAGCTTTG AGGCCTGCTT TGCCCAGTTT

TCTGGTTTGG GGCCCAGCAC ATCTCCTACA TTGGCTGCCT CACTCAAATG

TTCTTTGTGC ATGCTTTCTC CTTGCTGGAG TCCTCGGTGC TGGTAGCCAT

TTCTTTGTGC ATGCCTTCTC CTTGCTGGAG TCCTCGGTGC TGGTAGCCAT

TTCTTTGTGC ATGCCTTCTC CTTGCTGGAG TCCTCGGTGC TGGTAACCAT

TTCTTTGTGC ATGCCTTCTC CTTGCTGGAG TCCTCGGTGC TGGTAACCAT

TTCTTTGTGC ATGCCTTCTC CTTGCTGGAG TCCTCAGTGC TGGGAGCCAT

TTCTTTGTGC ATGCCTTCTC CTGGCTGGAG TCCTCAGTGC TGGCAACCAT

TTCTTTGTGC ATAGCTTCTC ATTCCTGGAG TCTTCAGTGT TGGTGGCCAT

TTCTTTGTGC ATGGCTTCTC ATTCCTGGAG TCTTCAGTGT TGGTGGCCAT

TTCTTTTTGC ATGTCTTCTC CTTCCTGGAG TCCTCAGTGC TGGTAGCCAT

TTCTTTGTAC ATGCTTTCTC CTTCCTGGAG TCCTCAGTGC TGGCAATTAT

TTCTTTGTGC ATGGCTTCTC CTTCCTGGAG TCCTCAGTGC TGGTAGCCAT

GCCTTTGTAC ATGTCTTCTC CTTCCTGGAG TCCTCGGTGC TGGTAGCCAT

TTCTTTGTCC ATGCCTTCTC CTTCCTGGAG TCCTCAGTGC TGGTAGCCAT

TTTTTTCTTC ATGGATTCAC TGTCATGGAG TCTGGGGTGC TCGTGGCCAT

TTCTTTATAC ATACCTTCTC CTTCCTGGAG TCCTCAGTGC TGGTAGCCAT

TTCTTTCTTC ATGGATTTAC TTTCATGGAG TCTGGAGTTC TGCTGGCCAT

TTCTTCCTTC ATGGTTTCTC CTTTATGGAA TCTTCAGTAT TATTGGCCAT

TTCTTCATCC ACATGGTCTT TGCCACCGAG TCAGCTGTCC TGCTGGCCAT

GGCCTTTGAC CGCTTCGTGG CTATCTGTAA CCCACTGAAC TATGCTACTA

GGCCTTTGAC CGCTTCGTGG CTATCTGTAA CCCACTGAAC TATGCTACTA

GGCCTTTGAC CGCTACGTGG CTATCTGTAA CCCACTGAAC TATGCTGCTA

GGCCTTTGAC CGCTTCGTGG CTATCTGTAA CCCACTGAAC TATGCTGCTA

GGCCTTTGAC CGCTTCCTGG CTATCTGTAG CCCACTGAAC TATGCTACTA

GGCGTTTGAT CGCTGCCTGG CTATCTGTGA CCCGCTGAAG TATGCTACTG

GGCTTTTGAC CGCTACTTAG CCATCTGCAG CCCTCTCAAG TATGCTACTT

GGCTTTTGAC CGCTACTTGG CCATCTGCAG CCCTCTGAAG TATGCTACTT

GGCCTTTGAC CGTTTCATGG CTATCTGTAA CCCACTGGAA TATGCAACCG

GGCTTTTGAC CGCTTCATGG CTATCTGTAA CCCACTGAAA TATGCCACTG

GGCCTTTGAT CGCTTTATGG CTATCTGTAA CCCAATGAAG TATGCCATTA

GGCCTTTGAC CGCTTCATGG CCATCTGTAA CCCACTGAAG TATGCCACTG

GGCCTTTGAC CGCTTCATGG CTATCTGTAA CCCACTACAA TATGCCACCA

GGCCTTTGAC CGATATGTGG CAATCTGTGA TCCCCTGAGG TACGCCAATA

GGCCTTTGAC CGCTTCATGG CCATCTGTAA TCCACTGAAG TATGCCACTA

GGCCTTCGAT CGTTTTGTGG CCATCTGTGA CCCACTGCAT TATGCTACCA

GGCTTATGAC CGTTTTGTTG CTATCTGCCG CCCACTCCAC TATGCCTCTA

GGCTTTTGAC CGTTTCGTGG CCATCTGCCG CCCACTACAT TACTCGTCCA

TCCTCACAGA CAGGATGGTC CTGGTGATAG GGCTGGTCTG CATTAGACCA

TCCTCACAGA CAGGATGGTC CTGGTGATAG GGCTGGTCTG CATTAGACCA

CCCTCACGGA CAGGATGATC CTGGTGATAG GGGTGGTCTG CATTAGACCA

CCCTCACGGA CAGGATGATC CTGGTGATAG GGCTGGTCTG CATTAGACCA

TCCTCACAGA CAGGATGATC CTGGGGATAG GACTGGTCTG TTTTCGACCA

TCCTCACAGA TATGATGGTC GTGGTAATTG TACTGGTTTG CATACGCCAA

TCCTCACAGA CATGATGAGT TTTGTGATTG GATTGATCTG TATAAGACAG

TCCTGACAGA CATGATGAGC TTTGTGATCG GACTGATCTG TATAAGACAG

TGCTCACAGA CATGATGAGT CTGGTCATTG GACTGGTCTG CATACGAGAA

TCCTCAAAGA CATGATGATC ATGGTGATCG GACTATTCTG CGTACGACAA

TCTTCACAGA CATGGTAATC TTGGTGATTG GACTGGTCTG CCTACGGCAA

TCCTCACAGA CACGATGATC ATGGTGATTG GACTGGTCTG TATACGACAA

TCCTCACAGA CGTGATCATC ATGGTCATTG GACTAGTCTG CATACGGCAA

TCCTCACAAA TTCCAGAATC ATTCAGATGG GTATCTTGAT TATACGTACT

TCCTCACAGA CATGATGACC ACGGTGATTG GGCTGGTCTG CATCCGGCAA

TTCTAACAGA CTCATTGTCA A----GATTG GAGTTGCCCT GTTCAG--AA

TTCTCACAGA GACTGTCATT GGCAGAATTG GGATAGCCGT TATCCGAT--

TCCTCAGCCC TGGTGTCATT GGGAAAATTG TAATGGCCTG CGTAGTCCGA

GCAGTTTTCT TACTTCCCCT TCTTGTAGCC ATAAACACTG TGTCTTTTCA

GCAGTTTTCT TACTTCCCCT TCTTGTAGCC ATAAACACTG TGTCTTTTCA

GTAGTTTTCT TACTTCCCGT TGTTGTAGCC ATAAACACTG CATCTTTTCA

GTAGTTTTCT TACTTCCCGT TGTTGTAGCC ATAAACACTG CATCTTTTCA

GTAGTTTTCT TACTTCCTGT TGTTGTAGCC ATAAACACTG CGTCTTTTCA

CTACTTTTCT CAAGTTCTAT TATTGTAGCC TTACAGACTA TATCTTTTTA

GTGGTTTTCA TTTTCCCCTC AATTATGGCT GTGAAGAGTG TGTCCTTCCA

GTGTTTTTCA TGTTTCCTTC AGTTATGGCT GTGAAGAGTG TGTCCTTCCA

GTGATTTTCA TGTTCCCCTG TGTGCTAGCC GTGAAGAACA TGTCCTTCCA

CTAATTTTCA TCTTTCCCAT GCTTCTAGCC TTGAAGACTG TATCTTTCCA

GTTATTTTCA CCTTTCCCAT GGTTCTAACC TTGAAGAATG TATCTTTCTA

GTCATTTTCA TTCTTTCCAT GTTTCTAGCT TTGAAGAGTA CATCTTTCCA

GTAGTTTTTA TGTTTCCCAT GATTCTAGCT TTAAAGAATG TATCTTTCCA

GTGCTTTTAA TAGTACCACT TCTTTTGCTC CTTAAGCAAC TCTCTTTCTG

GTAATTATCA TGTTTTTCAT GTTTCTAGCT TTGAAGAACA TGTCTTTCCA

GTGCCTTCCC TC---TTC-C ACTTCTGGTC CTGACTTACT TTCCTTTCTG

GTGTTTTCCG TCCTGCCCTC TCTCTTCCTG CTCAAACGTC TGCCCTTTTG

GGCCTCTTCT TTGTCTTCCC ATTCGTCATC CTGAAGATTG --TTCTGCCA

TGGGGGTCAC GAGCTTTCCC ATCCATTTTG CTACCACCCA GAAGTGATCA

TGGGGGTCAC GAGCTTTCCC ATCCATTTTG CTACCACCCA GAAGTGATCA

TGGGGGTCAT GAGCTTTCCC ATCCATTTTG CTACCACCCA GAAGTGATCA

TGGGGGTCAT GAGCTTTCCC ATCCATTTTG CTACCACCCA GAAGTGATCA

TGGAGGTCAA GAGCTTTCCC ATCCATTTTG CTACCACCCA GAGGTGATCA

TGGGGGTCAA GAGCTTTCCC ACCCATTCTG TTACCACCCA GATGTCATCA

AGGAGGCCAA GAGCTTTCAC ATCCATTTTG CTTCCATCCA GATATAATCA

AGGAGGCCAA GAGCTTTCCC ATCCATTTTG CTTCCATCCA GATATAATCA

CGAAGGGCAA GAACTTTCCC ACCCATTTTG CTACCACCCA GATATGATCA

GGAAAGAAAG GAGCTTTCCC ATCCATTTTG TTACCACCCA GATATAATCA

TGCAGGCAAA GAACTTTCCC ACCCATTTTG CTACCACCCA GATATGATCA

CGGGGGCCAG GAACTTTCCC ACCCATTTTG CTACCACCCA GATGTGATCA

TGGAGGCAAA GAGCTTTCCC ACCCATTTTG CTACCATCCA GATATGATCA

TAGAGCTAAG GTCCTTTCCC ACTCCTACTG TTACCATCCA GATGTAATTA

GGGAGGTCAA GAGCTCTCCC ATCCATTTTG CTACTTCGCG GACATAATCA

CCGCTCCCAC ATCCTTTCTC ATTCGTATTG TCTGCACCAG GACATAATCC

TCACTCTCAT CTCCTCTCCC ACTCCTATTG CCTTCATCAG GACATGATCC

CCGGGAAAAT GTGCTCTCCC ACTCCTACTG TCTCCACTCC AACCTCATCA

AATACACATA TTCCAAACCT TGGATCAGCA GTTTTTGGGG ATTTCTTCAG

AATACACGTA TTCCAAACCT TGGATCAGCA GTTTTTGGGG ATTTCTTCAG

AATACACCTA TTCCAAACCT TGGATCAGCA GTTTTTGGGG ATTTCTTCAG

AATACACCTA TTCCAAACCT TGGATCAGCA GTTTTTGGGG ATTTCTTCAG

AATACACATA TTCCAAGCCT TGGATCAGCA GTTTTTGGGG ATTTCTTCAG

AATACACACA TTCCAACCCC TGGATCAGTA GTTTTTGTGG GTTTCTTCAG

AATTCTCATA CACCAACCCC TGGATCAGCA GTTTCTGGGG ATTTCTTCAG

AATACACATA CACCAACCCC TGGATCAGCA GCTTCTGGGG ATTTCTTCAG

AATACACACT TTCCAACAAC TGGATCAGCA GTTTCTGGGG TTTTATTCTG

GAGATACATA TTCCAACCCC TGGATCAACA GCTTTTTGGG CTTTCTTCAG

AATACTCATA TTCCAACCCC TGGATCAGCA GTTTTTTGGG CTTTCTTCAG

AATACTCATA TTCCAACCCC TGGATCAGCA GCTTTTTGGG CTTTCTTCAG

AATACACACA CTCTAATCCC TGGATCAGCA GTTTTTTTGG CTTCCTTCAG

AATTATCATG TTCAGACACT CGGGCCAACA GCATCTGTGG ATTAATTGGG

AATACGCATA TTCTAACCCT TGGGTGGCCA GTTTTGGGGG CTTTTTTCAG

GCCTTGCCTG CGCTGACATT AGCTTTAACG TTTATATGGG TTTGTTATCA

GCTTGGTATG TGCTGACACT CGAATCAATA GTTGGTATGG TGTACTGGTA

GCCTCCCGTG CACCGACACG ACCCTCAACA ACATCTATGG CTTCGTCGTC

CTCTACCTGA ATGGCACTGA CGTATTGTTT ATTCTTTTCT CCTATGTCCT

CTCTACCTGA ATGGCACTGA TGTATTGTTT ATTCTTTTCT CCTATGTCCT

CTATACCTGA ACGGCACTGA TGTATTGTTT ATTCTTTTCT CCTATGTCCT

CTATACCTGA ACGGCACTGA CGTATTGTTT ATTCTTTTCT CCTATGTCCT

TTCTACCTGA ATGGCACTGA CTTATTGTTT ATTCTTTTCT CCTATGTCCT

CTCTACCTTA ATGGCACTGA CTTGCTCTTT ATTCTTTTCT CCTATGTCCT

CTCTACCTGA ATGGTACTGA CTTATTGTTC ATTCTTTTCT CCTATGTCCT

CTCTACCTGA ACGGCACTGA CTTATTGTTC ATTCTTTTCT CCTACGTCCT

CTCTACCTGA ATGGCACAGA TCTATTGTTC ATTCTTTTCT CCTATGTCCT

CTCTACATGA CTGGTACTGA CTTAATGTTC ATTCTTGTCT CCTATGTCCT

CTCTACCTGA CTGGCACTGA CTTACTCTTC ATTCTTTTCT CCTACATTCT

CTCTACATGA CTGGCACTGA CTTATTGTTC ATTCTTTTCT CCTATGTCCT

CTGTATCTCA CTGGCACTGA CTTACTGTTC ATTCTTTTCT CCTATGCCCT

CTCGTCCTGA CCGGGATAGA TACGCCATGC ATTGTCCTGT CTTACATTCT

CTGTATCTGA CTGGCACTGA CTTATTGTTC ATCCTTTTAT CCTATGTCCT

CTGTG-CTGT GGGGCCTGGA CTCCATGGGT ATCTTTGTGT CCTATGTTTT

TTGCTCATCA TCGTGCTTGA CCCTCTCCTC ATTCTGGTCT CCTATGGTTT

CTCTCCACCT TCGGGCTCGA CTCTGTGCTC ATCGCCCTCT CCTACGCCTT

GATCCTCCGT ACTGTTCTGG GCATTGTGGC CCGAAAGAAG CAACAAAAAG

GATCCTCCGT ACTGTTCTGG GCATTGTGGC CCGAAAGAAG CAACAAAAAG

GATCCTCCGT ACTGTTCTGA GCATTGTGGC CCGAAAGAAG CAACAAAAAG

GATCCTCCGT ACTGTTCTGA GCATTGTGGC CCGAAAGAAG CAACAAAAAG

GATTCTCCGT ACTGTTCTGA GCATTGTGGC CCCAAAGAAG CAACAAAAAG

GATCCTCCGT ACTGTGCTGA GTACTGTGAC TCCAAAGAAA CAGCAGAAAG

GATCCTTCGC ACTGTTCTGA ACATCGTGGC CCCTCAAAAA CAACAGAAAG

GATCCTTCGC ACTGTTCTGA ACATCGTGGC TCCTCAAAAA CAGCAGAAAG

GATCCTACGT ACTGTTCTGA GTATTGTGGC CCCCAAGAAA CAGCAAAAAG

GATAATCCGT GCTGTCCTGA GCATCATGAC CCCCAAGAAG CAAAAGAAAG

GATTCTCCGA ACTGTCTTGA GCATTGTGGC CCCTAAGAAG CAACAAAAAG

AATTCTTCGT ACTGTCCTGA GCACTGTGGC CCCTAAGAAG CAGCAAAAAG

GATTCTCCGT ACTGTCCTGA GCATTGTAGC CCCCAAAAAG CAGCAGAAAG

GATTATTCGC TCTGTCCTCA ATATCACCTC CCCTGAAGAG CGGCACAAGG

CATCCTCCGT ACTGTTCTGA GCATCGTGGC ACCCAAGAAG CAACAAAAAG

TATCTTGCAC TCAGTGTTGA GAATTGCATC CTCAGAGGAA CAGCAGAAAG

GATCTTGCAC AGTGTCCTTG GCACTGCCTC CCACGCTGAG AGAATTAAGG

GATCCTCAAG ACCGTGCTGG GTATCGTGTC AGGGGATGGG TGGCTCAAGG

CTCTCAGCAC TTGTGTCTGT CACATCTGTG CAGTCACTAT TTTCTATGTG

CTCTCAGCAC TTGTGTCTGT CACATCTGTG CAGTCACTGT TTTCTATGTG

CTCTCAGCAC TTGTGTCTGT CACATCTGTG CGGTCACTGT TTTCTATGTG

CTCTCAGCAC TTGTGTCTGT CACATCTGTG CGGTCACTGT TTTCTATGTG

CTCTCAGCAC TTGTGTCTGT CACATCTGCG CAGTCACTGT TTTCTATGTA

CTCTCAGCAC TTGTGTCTGT CACATTTGTG CTGTCATTGT CTTCTATGTT

CTCTTAGTAC ATGTGTCTGT CACATCTGTG CTGTGACCAT TTTCTATGTA

CTCTTAGCAC ATGTGTTTGT CACATCTGTG CTGTGACCAT TTTCTATGTG

CACTGAGCAC CTGTGTCTGT CATATCTGTG CTGTTACTGT TTTCTATGTG

CTCTCAACAC TTGTGTGTGT CACATCTGTG CTGTCACTAT TTTCTATGTG

CTCTCAGCAC TTGTGTCTGT CATATCTGTG CTGTCACCAT TTTCTATGTG

CTTTCAGCAC TTGCATCTGT CACCTCTGTG CTGTTACTAT TTTCTACGTT

CTCTCAGTAC CTGTGTCTGT CACATCAGTG CTGTCACCAT TTTTTATGTG

TCTTTAGCAC CTGTATCTCC CACATTGGAG CAGTTGCTGT TTTCTACATC

CCCTTAGCAC TTGTGTCTGT CACATCTGTG CTGTCACTGT TTTCTACGTG

CCTTCAACAC CTGCATATCC CATATTGGTG CAGTTGCCAT CTTCTACGTG

CGCTCAACAA CTGCCTATCC CACATTCTAG CTGTACTCAT CCTTTACGTG

CCCTCAACAC TTGTGTCTCC CACATGTGTG CTGTTCTGGT CTACTACGTG

CCACTGATCA GCCTCTCTTT GGCACACCGC CTCTTCCACT CCACCCCAAG

CCACTGATCA GCCTCTCTTT GGCACACCGC CTCTTCCACT CCACCCCAAG

CCAATGATCA GCCTCTCTTT GGCACACCGT CTCTTCCACT CTACCCCAAG

CCAATGATCA GCCTCTCTTT GGCACACCGC CTCTTCCACT CTACCCCAAG

CCAATGATCA GCCTGTCCTT GGCACATCGC CTCTTCCAGT CCACCCCAAG

CCAATGATCA GCCTGTCTTT TGCACACCGT CTCTTCCGCT CCACCCCAAG

CCAATGATCA CCCTGTCTTT GGCACACCGT CTCTTCAGTT CTACGCCCAG

CCAATGATCA CCCTGTCTGT GGCACACCGT CTCTTCAGTT CTACTCCCAG

CCGATGATCA GCCTGTCTCT GGCGCATCGC TTCTTCAGCT CCACGCCAAG

CCAATGATCA GCCTGTCCTT TACACATCGC CTCCTTAGCT CTACTCCGAG

CCAATGATCA GCCTGTCCCT TACACATCGC CTTCTCAGCT CCACCCCAAG

CCAATGATCA GCTTGTCCTT TACACACCGC CTCCTGAACT CCACCTCCAG

CCAATGATTA GCCTGTCTCT GACACACCGT CTCCTCAACT CAACCCCAAG

CCCATGATGA GCCTGTCCTT GCTGCATCGC TATGGTCAGT CAGTCCCCAA

CCAATGATCA CCCTATCTAT GGCACATCGC CTCTACAGCT CTACTCCAAG

CCCCTCATCA GTCTGTCTCT TGTTCATCGT TATGGTCATT CTGCGCCTCC

CCCATGGTGG GGTTGTCAAT GGTCCACCGC TTTGCCCGGC ATGCACCACC

CCCATGATTG GGCTGGCCAT GATGCACCGC TTTGGACAGC ACCCTTCTCC

GGTGCTCTGT AGCACTTTGG CCAATATTTA TCTGCTCTTA CCACCTGTGC

GGTGCTCTGT AGCACTTTGG CCAATATTTA TCTGCTTTTA CCACCTGTGC

GGTGCTCTGT AGCACTTTGG CCAATATTTA TCTGCTCTTA CCACCTGTGC

GGTGCTCTGT AGCACTTTGG CCAATATTTA TCTGCTCTTA CCACCTGTGC

GGTGCTCTGT AGCACTTTGG CCAATATTTA TCTGCTCTTA CCACCTGTTC

GGTGCTGTGT AGCACTTTGG CCAATGTGTA TCTGCTCCTG CCACCTGTCC

GGTGATCTGT AGTATTTTGG CCAATATGTA TCTGCTCTTG CCACCAGTGC

GGTGATCTGT AGTATTTTGG GCAATATGTA TCTGCTCTTG CCGCCTGTGC

AGTGCTCTGC AGTGTTTTGG CCAATATTTA TCTGCTCCTA CCACCTGTAC

GGTGATCTGT AGTATTTTGG CCAATGTGTA TTTGCTCTTA CCACCTGTAG

AGTGGTCTGT AGCATTTTGG CCAATGTTTA TTTGCTCTTA CCGCCTGTAC

GGTAGTCTGC AGCACTCTGG CCAACGTTTA TTTGCTCTTA CCACCTGTGC

GGTGATCTGT AGCATTTTGG GCAATACTTA CTTGCTCTTA CCACCGGTAC

AGTAGTCCAC TCAATGATGG CTAATATTTA CCTACTTCTG CCCCCTGCTC

AGTGCTCTGT GGCATTTTGG CCAATGTTTA TCTGCTTGTA CCACCTGTAC

CTTTGTTCAC ACCCTCATGG CCAATGTCTT CCTGCTAATC CCTCCCGTGC

ACTGGTTCAT GTGGTCATGG CCAATGTCTA CTTGCTATCA CCCCCTGTGA

TCTGCTCCAG GTCATGATGG CCAATGCCTA CCTCTTCATC CCACCGGTGG

TGAACCCTAT CATTTACAGC TTGAAGACCA AGACAATCCG CCAGGCTATG

TGAACCCTAT CATTTACAGC TTGAAGACCA AGACAATCCG CCAGGCTATG

TGAACCCCAT CATTTACAGC TTGAAGACCA AGACAATCCG CCAGGCTATG

TGAACCCTAT CATTTACAGC TTGAAGACCA AGACAATCCG CCAGGCTATG

TGAATCCTAT CATTTACAGC TTGAAGACCA AGACAATCCG CCAGGCTATG

TGAACCCCAT CATTTACAGC TTGAAGACCA AGACAATCCG CCAGGCTGTG

TGAATCCTAT CATTTACAGC TTGAAGACCA AGGTTATTCG CCAAGCCATA

TGAATCCTAT CATTTACAGC TTGAAGACCA AGGTTATTCG CCAAGCCATA

TGAACCCTAT CATTTATAGT TTGAAGACAA AGACGATCCG CCAGGCGATG

TGAACCCTAT CATTTACAGC TTGAAGACCA GGACGATCCG CCAGGCTATG

TAAACCCTAT CATTTACAGC TTGAAGACCA AGACGATCCG CCACGCTATG

TGAACCCTAT CATTTACAGC TTGAAGACCA AGACCATCCG CCAGGCTATG

TGAACCCTGT CATTTACAGT CTGAAGACCA AGACAATTCG TCGAGCTATG

TTAATCCCAT CATCTATAGT GTAAAATCCA AACATATCCG CAAGGTTATA

TGAATCCTGT AATTTATAGC TTGAAGACCA AGACAATCCG CCAGACTATG

TCAACCCCAT CATCTACAGT GTGAAGACAA AGCAGATTCG AATGGCAATT

TGAACCCCAT TATCTACAGT GTGAAAACTA AGCAGATTCG CCAGGGTATC

TCAACCCGAT CGTCTACAGC CTGAAGACCA AGGAAATCCG CAAGGGGATT

TTCCAGTTCC TCACTGGCAT CCCTGGGCTG GAGCAACTAC ATATCTGGCT

TTCCAGTTCC TCACTGGCAT CCCTGGGCTG GAGCAACTAC ATATCTGGCT

TTCCAGTTCC TTACTGGCAT CCCTGGGCTG GAGCACCTAC ACATCTGGCT

TTCCAGTTCC TTACTGGCAT CCCTGGGCTG GAGCACCTAC ACATCTGGCT

TTCCAGTTCC TCACTGGCAT CCCTGGGCTG GAGCATCTAC ACGTCTTGCT

CTCCAGCTCC TCACTGGCAT CCCCGGGCTG GAGCACCTAC ACGTCTGGCT

TTCCGCCTCC TCACTGGCAT CCCTGGGCTG GAGCACCTAC ATGTGTGGCT

TTCCAGCTCC TCACCGGCAT CCCTGGGCTG GAGCACCTAC AAATGTGGCT

TTACAGTTGC TCACAGGCAT CCCTGGGCTG GAGTACCTAC ACATGTGGCT

CTCCAGATCC TCCTTGGGAT CCCAGGATTG GAGAATTCC- --TTTGGGTT

CTCCAGCTCC TCATTGGCAT CCCTGGGCTG GAGCATCTGC ACATCTGGCT

CTCCAGACCT TGGTTGGAAT CACTGGACTG GAAACTACAC AGTGTTGGAT

TTGCAA-TTC TCATTGGCAT CCCTGGCCTG AAGGAGCTGC ACCTGTGGAT

CTCAAT-TCC TATCAGAAAT CTCAGAACTG GAAAAGTTCC AAATGTGGGT

CTCCAGCTAC TGCTTGGGAT TCCAGGGCTG GAGACTGCCC ACGTCTGGAT

ATCAAGATAC TGCTTGGCAT TCCTGGGCTG GAGGCTGCCC ACATGTGGAT

CACCGATACC TTCTGGGCAT TCCTGGCTTG GAGGATCAGC ACATCTGGAT

TTCAGGTTCC TCCTGGGCAT CCCTGGCCTC GAGTCAGTCC CCTTCTGGTT

GTCCATCCCC TTCTGCATCA TGTACATCAC TGCCCTGGAA GGCAATGGCA

GTCCATCCCC TTCTGCATCA TGTACATCGC TGCCCTGGCA GGCAATGGCA

ATCCATCCCC TGCTGCATCA TGTACATTGC CGCCCTGGCA GGCAATGGCA

ATCCATCCCC TGCTGCATCA TGTACATTGC CGCCCTGGCA GGCAATGGCA

ATCCATCCCC TTCTGCATCA TGTACATAGC CACCCTGACA GGCAATGGCA

CTCCATCCCC TTCTGCACCA TGTACATTGC CTCCCTGGTA GGCAACAGCA

GTCCATTCCC TTCTGTACTA TGTACATAGC TGCCCTGATA GGCAATGGTG

GTCCATCCCC TTCTGTATTA TGTACATAGC TGCCCTGGTA GGCAATAGCG

CTCCATACCC TTTTTCACCA TGTACATAGC TGCACTGGCA GGCAACGGCA

TGCCTTTCCA TTCTGTGCCA TGTATGTTGT GGCTCTAGTC GGCAATATCA

CTCCATCCCC TTCTGTACTA TGTATGCAGC TGCCCTGGCA GGCAATGGCA

TGGGATTCCA TTCTGTGCTA TGTATATCAT TGCTTTGATA GGAAAT-TCT

CTCCATTCCC TTCTGTCTGA TGTACTTGGT GGCTGTGTCA GGAAATGGCA

TAAGATTCCA TTCTTCACTA TCTATCTCAT GGCCTTTGCA GGCAGAAGTG

CTCCATCCCT TTCTGCCTGG TCTATCTGCT GGCACTGATG GGAAATGCTG

CTCCATCCCC TTCTGTCTTG TCTACCTGTT GGCACTAATG GGAAATGTTG

TTCAATCCCC TTTTTTACCT CCTATATTAT TGCACTTCTT GGTAACAGCC

CTCCTTACCT GTGTGTTCCA TCTGCCTGGT GACAGTCCTG GGCAACTGTA

TCCTAATTTG TGTCATCCTC TCCCAGGCAA TCCTGCATGA GCCCATGTAC

TCCTAATTTG TGTCATCCTC TCCCAGGCAA TCCTGCATGA GCCCATGTAC

TCCTAATTTG TGTCATCCTC TCCCAGGCAA TCCTGTATGA GCCCATGTAC

TCCTAATTTG TGTCATCCTC TCCCAGGCAA TCCTGTATGA GCCCATGTAC

TCCTAATTTG TGTCATCCTC TCCCAAGCAA TTCTGCATGA GCCCATGTAC

TTCTAATTTG TGTCATCCTC TCCCAGCCAA GTCTGCATGA GCCCATGTAC

TTCTAATCTG TGTCGTCCTC TCCCAGCCAA GCCTCCACGA GCCCATGTAC

TTCTCGTCTG TGTCATCCTT TCCCAGTCAA GCCTCCATGA GCCTATGTAC

CCCTGATCTG CATCATTGTC TCCCAACCAA GCCTTCATGA GCCCATGTAC

CTCTCCTTCA TGTAATCCGG AATGATCCCA CCTTGCATGA GCCCATGTAC

TTTTAATTTG TGTCATCCTC TCCCAGCCAA GCCTGCATGA GCCTATGTAC

CTACTTTTGA TATCATCAAA TCTGAACCCA GTCTCCATGA ATGTATATAT

TGCTGATCTG TGTGGTGGCT ATGGAGCGCA GTCTTCATGA ACCTATGTAT

TCCTTCTCTA CCTTCTCTCC ATGGATTACA GACTCAGTGA GCACGTGTTC

CCCTTTTATT AATTGTCAAG TCAGACCCCA AGCTGCATGA ACCTATGTAC

CCCTTTTATT AATTGTCAAG TCAGATCCTA AGCTGCATGA ACCAATGTAC

TCCTCATCTT TATCATTGTC ACTGATCACA GCCTTCATGA ACCAATGTAT

CCACCCTGTT GGCCATCCTC TCAGAGCCTG CCCTACGCAA GCCCATGTAC

ATATTCTTAT CTATGGCCAG TGCTGATGTC TTGCTCTCTA CCACCACCAT

ATATTCTTAT CTATGGCCAG TGCTGATGTC TTGCTCTCTA CCACCACCAT

ATATTCTTAT CCATGGCCAG TGCTGATGTT TTGCTCTCTA CCACCACCAT

ATATTCTTAT CCCTGGCTAG TGCTGATGTT TTGCTCTCTA CCACCACCAT

GTATTCTTAT CCATGGCCAG TGCTGATGTC TTGCTCTCTA CTGCCACCAT

ATATTCCTGT CCATGGCCAG TGCTGATGTC TTGCTCTCCA CCTCTACCAT

ATTTTCCTGT CTATGGCCAG TGC------- -------CTA CCTCCACGAT

ATATTCCTGT CTATGGCCAG TGCTGATGTC CTGCTCTCTA CCTCCACCAT

ATCTTCCTGT CCATGGCCAG TGCTGACATC CTGCTCTCCA CCTCCACCAT

CTCTTTCTGG CCATGGCTAC CACTGACCTG GTCCTTACTT CCA---CACA

ATATTCCTGT CCATGGCCAG TGCGGATATC TTGCTCTCCA CTTCCACCAT

ATCTTCCTGG CGATGGGAGC CACAGACATT GGAATTATCA CCAGTATTGT

CTCTTCCTCT CCATGGCTTT TTGGGATCTG GTACTCTCTA CATCCACAGT

TTTTACTGCT CCATGGCAGT GGCAGATTTC ATCCTTTCCA ATACATTTGT

CTCTTTCTAT GCATGTCTGG GACTGACTTG ATGCTCACTT CTTCTACGCT

TTCTTTCTAT GCATGTCCAT GGCTGATTTG ATGCTCACTT CTTCGACACT

ATCTTCTTAT GCATGGCTGT ACTTGACATT GGACTCTCCA CCATTACAGT

CTCTTCTTCT GCATGTCCCT CATCGATCTG GTGGCCTGTG CCTCCATCCT

GCCTAAGGCC CTGGCCAATT TGTGGCTAGG TTATAGCATT TCCTTTGATG

GCCTAAGGCC CTGGCCAATT TGTGGCTAGG TTGTAGCATT TCCTTTGATG

GCCTAAGGCC CTGCCCAATT TGTGGCTAGG TTATAGCGTT TCCTTTGATG

GCCTAAGGCC CTGGCCAATT TGTGGCTAGG TTATAGCGTT TCCTTTGATG

GCCTAAGGCC CTGGCCAATT TCTGGCTAGG TTACAGCATT GCCTTTGATG

GCCTGAGGCT CTGGCCACCT TCTGGCGAGG TTCTAGCATT TCCTTTGATG

GCCCAAGACC CTGGCCAACT TCTGGCTAGG TTCTAGCATC TCCTTTGACG

GCCCAAGACC CTGGCCAACT TCTGGCTAGG TTCTAGCATC TCCTTTGACG

GCCCAAGACA CTGGCCAATT TCTGGTTTGG TTCCAGCATT TCCTTTGATG

ACCTAAAATG CTGGCCATAT TCTGGTTTCA CAATCATATT GAATACCATG

GCCCAAAGCA CTGGCCAACT TCTGGCTGGG TTCAAGCATT TCCTTCGATG

CCCCAAAATG CTTGGAATGT TTTGGTCCCA CTTGCCAATC TATTTTGATG

ACCCAAAGCC CTCAGTATCT TCTGGTTTGA TGATGTAATT TCTTTTGGTG

ACCCCAAACT TTCAGCATCT TCTGGCTGGG TCCTCAGATC ACCTTTATTG

TCCCAAGTTA CTCAGCCTCT TTTGGTTCAA TGACAGAATC TGTTTTGAAG

TCCCAAGATA CTCAGCCTCT TTTGGTTCAA TGATCGAATC TATTTTGAAG

TCCCAAAGCC TTAACCATTT TTTGGTTTAA TTATGGAATA TCTCTCGATG

ACCCAAAATG CTCTCCATCT TCTGGTTCAA CGCTGGCATC GACACCAATG

GCTGCCTCAC TCAGATGTTC TTCATTCACT CTGCTGTCCT GCTGGCCATG

GCTGCCTCAT TCAGGTGTTC TTCATTCACT CTGCTGTCCT GCTGGCCATG

GCTGCCTTAC TCAGATGTTC TTTGTTGACT CTGTTGTCCT GGCGGCCATG

GCTGCCTCAC TCAGATGTTC TTTGTTGACT CTGTTGTCCT GGCGGCCATG

GCTGCCTCAC TCAGATGTTC TTTGTCGACT CTGCTGTCCT GCTGGCCATG

GATGCCTCAC CCAGATGTTC TTTGTTGACT CTGCTATCCT GTTGGCTATG

GCTGCCTGAC TCAGATGTTC TTCGTTGACT CTGCTGTCCT GCTGGCCATG

GCTGCCTGAC GCAGATGTTC TTCGTTGACT CTGCTGTCCT GCTGGCCATG

GCTGCCTCAC ACAGATGTTC TTCGTTGATT CTGCAGTCCT GCTGGCCATG

CCTGCCTCAT GCGGTTGTTC TCTTCGGAGT CTGGATTGCT CATGGCTATG

GCTGCCTCAC CCAGATGTTC TTCGTTGACT CTGCGGTCCT GCTGGCCATG

CTTCCCTTTT CCAGATGTGG CAGGGTGAAT CAGGAGTCCT GCTGGTCGTG

GTTGTGTGAC CCAACTCTTC TTCGTGGAGT CAGGCATTCT CTTAGCCATG

GATGCCTTAC CCCGATGT-- TTTACAGATT ATGCCATCCT GTTCACCATG

CCTGCCTCAC TCAAATGTAC TCTGCGGAAT CTGGATTTAT GTTGGCCATG

CTTGCCTCAT TCAAATGTAC TCTACGGAAT CTGGATTTAT CTTGACTATG

GGTGTATTAC TCAGTTATTC TACATTGAAT CAGGAATCCT TCTGGTAATG

CCTGCTTGGT CCAGATGTTC TGCATGGAGT CCACCGTGCT TCTGGCCATG

GCCTTTGACC GCTATGTGGC CATCTGCTCC CCCCTGATCC TCACAAGCAA

GCCTTTGACC GCTATGTGGC CATCTGC-CC CCCCTGATCC TCACAAGCAA

GCCTTTGATC GCTACGTGGC CATCTGCTCC CCGCTGATCC TCACAAGCAA

GCCTTTGATC GCTACGTGGC CATCTGCTCC CCGCTGATCC TCACAAGCAA

GCCTTTGACC GCTATGTGGC CATCTGCTGC CCTCTGATCC TCACGAACGA

GCCTTTGACC GCTATGTGGC CATCTGCTCC CCTCTGATCC TCACAAGTGA

GCTTTTGACC GCTATGTGGC AATATGCTCT CCTCTGATCC TGACCAGGCA

GCTTTTGACT GCTATGTGGC AATATGCTCT CCTCTGATCC TAACAAGGCA

GCCTTTGACC GCTATGTGGC CATCTGCTCC CCTCTGATCC TCACAAGCAA

GCCTTGGACG GTTACGTGGC CATCTGCTTC CCATTGATCC TGACCCCATC

GCCTTTGACC GCTTTGTGGC TATCTGCTAC CCTCTGATCC TGACGAAGAC

GCTCTGGACC ACTATATAGC AATTTGTTAT CCTCTGATAT TCACTCAACT

GCTTTTGACC GCTTTGTGGC CATTTGCTAC CCATTGATTC TTAGTCATGG

GCATTCAATC ACTATGTTGC CATTTCCTTC CTCC----TC TCACTTGTCA

GCTTTTGACC GTTATGTGGC CATTTGCCAC CCACTGATGC TGACACCTGC

GCTTTTGACC GGTATGTGGC CATTTGCCAG CCCCTGGTTC TGACACCTAC

GCATTTGATC GCTATGTTGC AATTTGTTAC CCACTGATCC TTACCCCTTC

GCCTTTGACC GCTATGTGGC CATCTGCCAC CCATTGATCT TCACCAATGC

GGTCATTGGG AAGAGCTTCA TCATTATGTT TCCATCCATC TTTCTCCTTG

GGTCATTGGG AAGAGCTTCA TCATTATGTT TCCATCCATC TTTCTCCTTG

GGTCATTGGG AAGAGGTTCA TCATTATGTT TCCATCCATC TTTCTCCTCA

GGTCATTGGG AAGAGGTTCA TCGTTATATT TCCATCCATC TTTCTCCTCA

GGTCGTTGGG AAGAGCTTCA TCATCATGTT TCCATCCATC TTTCTCCTCA

GGTCATCGGG AAGAGCTTCA TCATCATGTT TCCATCACTC TTTCTCCTCA

GGTCATGGGA AAGAGTTTTA TAATCATGTT CCCATCCATC TTTCTCCTCA

GGTCATGGGA AAGAGTTTTA TAATCATGCT CCCATGTATC TTTCTCCTAA

GGCCATTGGG AAGAGCTTCA TCATCATGTT TCCATCCATC TTTCTCCTCA

CGTCAT---- ---------- ---------- ----TCCCTT TTGTTTCTCC

GGTCATTGGG AAGAGCTTCA TCATCATGTT TCCATCAGTC TTTCTTCTCA

AGTCACTCAT ATTGCCATTC TGG---TAAT CCCATGCCTA CTGCTCATC-

TGTCATTGGC AAAAGTTTTG CAACTGTTTT CCCCATTGTC TTCCTTGTAA

GATTATTACT AAAAGCTTTT TGATAATCTT CCCAAATGTG TTCTT--TAA

AGTCATTGTA GGCGGAGCTG TACTCCTCAG TCCACACCCC TTCCTTCTAA

AGTCATTGTA AACGGGGCGA TCCTCCTCAG TCCACATCCC TTTCTCCTGA

AGTGATTACA AAAAGTTTTT CTGCTGTATT CCCCTTAATA TTCCTTTTGA

CATCATGGTC AA-GGCTCCC TACTCATGTT CCCCTGCCCA TTTTTGATCA

AGCACCACTA TTGCCAGATC AATATCATTG CACACACATT TTGTGAGCAC

AGCACCACTA TTGCCAGATC AACATCATTG CACACACATT TTGTGAGCAT

AGTGCCACTA TTGCCGGATC AACATCATTG CACACACATT TTGTGAGCAC

AGCGCCACTA TTGCCGGATC AACATCATTG CACACACATT TTGTGAGCAC

AGCGCCACTA TTGCCGGATC AACATCATTG CACACACATT TTGTGAGCAC

AGCGCCGCTA CTGCCGCACC AACATCATTG CGCACACATT TTGTGAGCAC

AGAGGCACTA CTGCCGGATC AATATCATTC AACATACCTT CTGTGAGCAC

AGCGGCACTA CTGCCGAATC AATGTCATTG AGCACACATT TTGTGAGCAT

AGCGCCACTA TTGCAGGAAC AACATCATTG CACACACATT TTGTGAACAC

AGGACC--TT CTGCCCCATT TGGATCATCC CCCAGTCATA CTGTGAGCAC

AGCGCCACTA TTGCCGGATA AACATCATTG CACACACTTT TTGTGAGCAC

---------T TCACAGAACC AAGTTAATAT CCCACACTTA CTGTGAACAC

AGCGTCCCTT CTGCAGAACA AATATCATTG CCCATACATT CTGTGAACAT

AGCAACCTTT CTGCCCAACA CTCATCATTC CCCATAGATA CTGTGAGCAC

GGTGGTCCTA CTGCAAAACT AATCTCATTT CCCATACTTA CTGTGAGTTC

AGTGGTCCTA CTGCAAAACT AACATCATAG CCCACACTTA CTGTGAGTTC

AAAGGTCTTT CTGTAAAAAT AACCTTCTTC CACATACATA TTGTGAGCAC

AGAGGTCTTT CTGCGGGACC AA--TCATCT TCCACACCTA CTGTGAGCAT

ATGGGCATTG CCCATCTGTC CTGTTCTGAT ATCTCCATCA ATGTCTGGTA

ATGGGCATTG CCCATCTGTC CTGTTCTGAT ATGTCCATCA ATGTCTGGTA

ATGGGCATTG CCCACCTCTC CTGTTCTGAT ATCTCCACCA ATGTCTGGTA

ATGGGCATTG CCCACCTCTC CTGTTCTGAT ATCTCCATCA ATGTCTGGTA

ATGGGCATTG CCCATCTGTC CTGTTCTGAT ATCTCCATCA ATGTCTGGTA

ATGGGCATCG CCCGTCTGTC CTGTTCTGAT ATCTCCATCA ATGTCTGGTA

ATGGGTATCG CCCGGCTGTC CTGCTCTGAC ATTTCCATTA ACGTCTGGTA

ATGGGTATCG CCCGGCTGTC CTGTTCTGAC ATTTCCATTA ATGTCTGGTA

ATGGGCATTG CCCGTCTGTC CTGTTCTGAT ATCTCCATAA ATGTCTGGTA

ATGGCTGTAA TGAAGCTGGT GTGTGCTGAC ACTAGGGTTA ATCATGCATG

ATGGGCATCG CCCGTCTGTC CTGTTCTGAT ATCTCCATCA ATATCTGGTA

ATGGCCCTTG TGAAGCTTGC ATATGTTTAC A------TCA ATAAATTCTA

ATGGGGTTGG CAAAACTGGC TTGTGCTGAT ATCACCATCA ATATTTGGTA

ATAAGCATTG CCTGGCTTGC CCATATGGTT ATCTCCATCA GCTTCTGGTA

ATGGCCCTAA TAAAGCTGGT TTGTGATAAA ACCAAGATTC GCCAAGCCTA

ATGGCCTTGA TAAAGCTGGT TTGTGATCAA ACCAAGATCC GAAGAGCCTA

ATTGGTTTGG CCAAGTATGC TTGTTCCAGC ATTAGAGTGA ATATCTGGTA

ATGGCCGTGG TCAAGCTGGC CTGTGGCGAC ACCACCGCCA ACTGGGTATA

TGGGTTGGCA GCTGCTCTTC TCTCCACAGG CCTAGACATC ATGCTTATTA

TGGGTTGGCA GCTGCTCTTC TCTCCACAGG CCTGGACATC ATGCTTATTA

TGGGTTGGCA GCTGCTCTTC TCTCCACAGG CCTGGACATC ATCCTTATTA

TGGGTTGGCA GCTGCTCTTC TCTCCACAGG CCTGGACATC ATCCTTATTA

TGGGCTGGCA GCTGCTCTTC TCTCCACAGG CCTGGACATT ATCCTTATTA

TGGGTTGGCT GCTGCTCTTC TCTCCACTGG CCTGGACATC ATCCTTATTG

TGGACTGGCA GCTGCTCTTC TCTCTACAGG CCTGGACATC ATTCTCATCG

TGGGCTGACA GCTGCTCTTC TCTCTACAGG CCTGGACATC ATCCTCATCG

TGGGTTGGCA GCTGCTCTCC TCTCCACAGG CCTGGACATC ATCCTTATTG

TGGGCTCTTT GCAGCCTTCT CTGTGGTTGG CTTTGATGTT TTTGTCATCA

TGGGTTGGCA GCTGCTCTAC TCTCCACAGG CCTGGACATC ATCCTTATCA

TGATCTCTTT GGAGCTTTTA CTGTTGGTGG CCTTGACTTC GTTTTGATCA

TGGAATCTCT GTGCCTTTAC TCAGTGTTAT GCTAGATATG GTGACAATAG

TGACTTTGCT GTGCCAGTAA TGACTGTCAC CTCAGATCTC ATCTTCACTG

CAGCCTAATT GTGGCCTTCC TGACAGCGGG GCTAGACTTC ATCTTGATCA

TAGCCTTATG GTGGCCTTCC TGACAGGGGG ACTCGACTTC ATCTTGATCA

TGGTGTATTT GTTGGTTTGC TATCTGTGAT ATTAGACTTT ATCCTTGTTG

CGGCCTGGCC GCGGCCCTGC TGGTGATTGG GGTGGACCTG CTGTTCATCG

CTGTTTCCTA CATCCACATC CTCCAATTCC GCCTCCTTTC TCAAGATGCC

CCGTTTCCTA CATCCACATC CTGCAATTCC GTCTCCTTTC TCAAGATGCC

CTGTTTCCTA CATCCGCATC TTCCAATTCC ACCTCCCTTC TCAAGATGCC

CTGTTTCCTA CATCCACATC CTCCAATTCC ACCTCCCTTC TCAAGATGCC

CTGTTTCCTA CATCCACATC CTCCAATTCC GCCTCCCTTC TAAAGACGCC

CTGTTTCCTA TGCCCACATC CTCCGTTTCT ACCTTCCTTC TCGAGAGGCA

CTGTTTCCTA TGTCCACATC CTCCGATTCC GCCTCTCTTC CCAAGAAGCC

CTGTTTCCTA TGTTCACATC CTC---TTCC GCCTCTCTTC CCAAGAAGCC

CTGTTTCCTA TGTCCACATC CTCCAATTCC GCCTCTCTTC CAGAGAAGCC

GTGTATCCTC TGTGATGATT TGGAGACTGT GGTTACCTTC AGGTGAAGCC

CTGTTTCCTA CATTCACATC CTCCAATTCC ACCTCCCTTC TCAAAATGCC

CCCTCTCTTA TATACAAATA TCTATCCTCC ACCTGCCCCA GAAAAAGGCA

TCATCTCCTA CGGGCTGATT CTGAGATTCA GAATGCCATC CCATGATGCT

ACATCTCCTA CACTCTCATC CTTT--CTTC CCCTCCCATC CCAGGATGTC

TTTGCTCCTA TGTTCTTATC CTGCTCTTCC AACTCCCATC CAAGGCAGCC

TTTGCTCCTA CGTCCTTATT CTTTTCTTCC GTCTCCCATC CAAGGCCGCC

TGGCATCCTA CTGTGTGATT CTCTATTTTC GCATTCCCTC TCAGGATGCA

GCCTGTCCTA TTTCCTCATT GTGCGATTGC GTCTCTCCTC ACAGAAGGCC

CGCTCCAAGG CCCTGAGTAC CTGTGGATCC CATATCTGTG TCATCCTACT

CGCTCCAAGG CCCTGAGTAC CTGTGGATCC CATATCTGTG GCATCCTACT

TGCTCCAAGG CCCTGAGTAC CTGTGGATCC CATGTCTGTG TCATCCTACT

CGCTCCAAGG CCCTGAGTAC CTGTGGATCC CATGTCTGTG TCATCCTACT

CGCTCCAAGG CCCTGAGTAC CTGTGGATCC CATGTCTGTG TCATCCTACT

CGCTCCAAGG CCCTGAGCAC CTGTGGATCC CATGTCTGTG TCATCCTGCT

AGGTCCAAGG CCCTGAGTAC TTGTGGATCC CATGTCTGTG TCATCCTGCT

CGGTCCAAAG CCCTAAGCAC TTGTGGATCC CACGTCTGTG TCATCCTGCT

CGGTCCAAAG CCTTCAGCAC CTGTGGATCC CACGTCTGTG TCATTCTACT

CAGTTCAAAG CTTTTGGCAC ATGTGCCTCC CA-------- ----------

CGGTCCAAGG CCCTGAGCAC GTGTGGCTCC CATGTCTGTG TCATCCTACT

TGTCTTACGG TATTTAATAC ATTTATTCCC CACAGGTGTA TCTATTTCCA

CGGATGAAAG CACTCAGCAC CTGTGGCTCC CATGTCTGTG TTATCCTCAT

TGCCAGAAAG CTCTCAGCAC ATGTGCTTCC TATGCTAGTG TCATCCCTAT

CGCCTCAGGA CCTTAAGCAC CTGTGTCTCC CATATATGGG TCATTTTGAT

CGCCTCAAGT CTTTAAGCAC CTGTGTCTCC CACGTGTGGG TCATTTTGGT

CGTCACAAAG CTCTCCATAC CTGTGGTTCC CATATCTGTG TCACCTTCCT

CGGGCTAAGA CCTTCAGCAC CTGTGGCTCA CACATTTGCA TCATCCTCAT

CTTCTATGTC CCTGCGCTTT TTTCTGTCTT TGCCTACTTT GGTGTCCCAT

CTTCTATGTC CCTGCCCTTT TTTCTGTCTT TGCCTACTTT GGTATCCCAT

CTTCTATGTC CCTGCCCTTT TTTCTGTCTT TGCCTATTTT GGTATCCCAC

CTTCTATGTC CCTGCCCTTT TTTCTGTCTT TGCCTATTTT GGTATCCCAC

CTTCTATATC CCTGCCCTCT TTTCTGTCTT TGCCTACTTT GGTATCCCAC

TTTCTATATC CCAGCCCTCT TTTCCGTCTT TGCCTACTTT GGTATCCCAC

TTTCTATATC CCAGCCCTCT TCTCTGTCTT TGCCTACTTT GGTATCCCAC

GTTCTATATC CCAGCCCTCT TCTCTGTCTT TGCCTACTTT GGTATCCCAC

ATTCTATGTC CCTGCCCTCT TTTCCGTCTT TGCCTACTTT GGTATACCAG

--TTTATGTC CCTGCTTTCT TTACTTACCT CACCCACTTT GG-GTGCCC-

CTTCTATATC CCTGCCCTCT TCTCTGTCTT TGCCTACTTT GGTATCCCAC

ATTCTATCTC CTTGCTTTTT TT-------- --CTCACTCT GG-ATCCCAT

GTTCTACCTT CCTGGGATTT TTACTGTCAT TGCCCAGTTT GG-ATTCCCA

ATTCTACACA CCAGGAATGT TCTCTGTCTT GACTCACTTT TGTATTCTGT

GTTTTATACA CCAGCCTTTT TCTCCTTTCT TACCCACTTT GGCATTGCTC

ATTTTATACA CCAGCCTTTT TCTCGTTTCT AACTCACTTT GGTATTGCTC

CTTTTTCATC CCTGGAATCT TCACAGTTAT GGTCCAATTT GGCATCCCAC

TTCCTACATG CCGGCCCTCT TCTCCTTCTA CAGTCACTTT GG-GTGGCAC

GCTATGTCCA TATTCTCCTG GCCAGCCTCT ACGTTGTCAT TCCTCCTATG

GCTATGTCCA TATTCTCCTG GCCAACCTCT ACGTTGTCAT TCCTCCTATG

GCTATGTCCA TATTCTCCTG GCCAACCTCT ACATTGTCAT TCCTCCTATG

GCTATGTCCA TATTCTCCTG GCCAACCTCT ACGTTGTCAT TCCTCCTATG

GCTATGTTCA TATCCTCCTG GCCAACCTCT ATGTTGTCAT TCCTCCTATG

GCTATGTTCA TATCCTCCTG GCCAACCTCT ATGTGGTCAT CCCACCTATG

GTTATGTCCA CATCCTGCTG GCCAACCTCT ACGTGGTCAT CCCGCCTATG

GCTATGTCCA TATCCTGCTG GCCAACCTCT ATGTGGTCAT CCCGCCTATG

GCTATGTCCA TATCCTCCTG GCCAATCTCT ATGTGGTCAT CCCACCTATG

--ATGGTACA TGTCATGTTA GCTATTCTCT GTCTCCTGGT ACCTCCCATG

GCTATGTCCA TATCCTCTTG GCTAACCTCT ATGTGGTCAT CCCACCTATG

CATATATACA TATCATCTTG CCAAACCTTT ACCTACTGGT TCCTCCTTTA

AACATGTCCA CATCCTGCTA GCCAACCTTT ATGTTCTTGT TCCTCCCATG

ACTCCTACCA CAAAATATAT TCCAACCTCT GGGTAACCAT CTCTCCAGCA

CACATATCCA CATTTTCATA GCTAATGTAT ATCTCCTTAT TCCACCTATG

CACATATTCA CATTTTTATT GCTAATATAT ATCTCCTCAT TCCACCCATG

CGCATATCCA CATTCTAATG GCTAATGTCT GCATGCTGGT TCCTCCTGTG

CCCACATCCA CATCCTCCTG GCCAACCTCT ACCTCCTCTT CCCACCCATG

CTCAATCCCG TTATTTATGG AGTGAGGACT AAGCCAATAC TGGAAGGGGC

CTCAATCCCG TTATTTATGG AGTGAGGACT AAGCAGATAC TGGAAGGGGC

CTCAATCCCA TTATTTATGG AGTGAGGACC AAGCAAATAC TGGAAGGGGC

CTCAATCCCA TTATTTATGG AGTGAGGACC AAGCAAATAC TGGAAGGGGC

CTCAATCCCA TTATTTACGG AGTGAGAACC AAGCAAATAC TGGAAGGGGC

CTCAATCCTA TTATTTATGG AGTGAGGACC AAGCAAATTT TGGAAGGGGC

CTTAAGCCTG CGATTTATGG AGTAAGAACA AAGCAGATAC TGGAAGGAGC

CTTAATCCTG TGATTTATGG AGTAAGAACA AAGCAGATAC TGGAAGGAGC

CTTAACCCCA TTATTTATGG AATAAGAACC AAACAAATTT GGGAAGGGGC

CTCAACCCCA TCATCTACAG AGTTAGAACG AAACAGATCA TGGACAAGGT

CTCAATCCCA TTATTTATGG AGTGAGGACC AAGCAGATTT GGGAAGGGGT

CTCAATCCCT TTGTCTGTGG GGTGAAGCCC AAATACATCC TAGATAAGGG

ATGAACCCAA TTATCTATGG AGTAAAGACC AAACAGATCC GTGAACGTGT

CTCAATTCAG TTTTCTATGG A-TAAAGGCC AAGCATATTC CAGACAAGAT

ATGAACCCCA TTATTTATGG TGTTAAAACC AAGAGGATCA GGGAGGGATT

ATGAATCCCA TTATTTATGG CATTAAAACA AAAAGGATCA GGGATGGATT

ATGAACCCCA TCATATACGG GATCAAGACT AAACAGATCA AGAACCGTGC

CTCAACTCCA TGATCTATGG TGTGAAAATT AAGGAGGTAT GCAACAAGGT

TATGTCAGAT TCCACAGGGA TCCCAGGGCT GGAGGCTGCC CACTTCTGGA

TATGTCAGAT TCCACAGGGA TCCCAGGGCT GGAGGCTGCC CACTTCTGGA

TATGCCAGAT TCAACAGGGA TCCCAGGGCT GGAGGCTGCC CAATTCTGGA

TATGCCAGAT TCAACAGGGA TCCCAGGGCT GGAGGCTGCC CAATTCTGGA

TATGTCGGAT TCCACAGGGA TCCCAGGGCT AGAGGCTGCC CACTTCTGGA

TATGTCAGTT TCCACCGGGA TCCCAGGGAT GGAGTCTGCC CACTTCTGGA

TACAACAGGC TCCACTGGGA TCCCAGGGCT GGAGTTTGCC CACCTCTGGA

TACAACAGCC TCCACTGGGA TCCCAGGGCT GGAGTTCGCC CACCTCTGGA

TATGTCTGTT TCCACAGGGT TCCCAGGACT GGAGCCCTTC CACATTTGGA

T--------- ---ACTGGAA TTCCAGGGCT TGAACTAAAG TATTACTGGA

TATGTCAACC T--ACTGGAA TTCCAGGGCT TGAACAAAAG TATTATTGGA

AATGTCAGCT T--ACTGGAA TTCCAGGGCT TGAACAAAAG TACTACTGGA

GATGTCAGCT T--ACTGGAA TTCCAGGCCT TGAACAGCAG TATTACTGGA

TATGACTATT T----GGGAA TCCCAGGGAT GCAAGACCAG CATGTTTGGA

-ATGTCAAAC TCCACAGGGA TCCCAGTGCT TGAAGACAGT CACATCTGGA

TATGGCAGCT TCCACAGGGA TCCCAGGACT GGAGGCCAGT CATACCTGGA

AATGTCTGCT GCCACAGGTA TTCCAGGACT AGAGCCTTTC TATATGTGGA

GATGTTGATT TCCTTAGGTA TCCCTGGGAT GGAGCATCAG CACATCTGGA

TTGCCATCCC TTTCTGTGCC ATGTATCTTG TAGCACTGGT TGGAAATGCT

TTGCCATCCC TTTCTGTGCC ATGTATCTTG TAGCACTGGT TGGAAATGCT

TAGCCATCCC TTTCTGTGCT ATGTATCTTG TAGCACTGGC TGGAAATGCT

TAGCCATCCC TTTCTGTGGT ATGTATCTTG TAGCACTGGC TGGAAATTCT

TTGCCATCCC TTTTTGTGTC ATGTATCTTG TAGCATTGAC TGGAAATGCT

TAGCCATCCC TTTCTGTGCC ATGTATCTTG TAGCACTAGC TGGTAATTCT

TTGCTATCCC TTTCTGTGTC ATGTATCTCA TAGCTCTGCT TGGTAATGCT

TTGCCATCCC CTTCTGTGTC ATGTATGTGG TTGCTCTGCT TGGGAATGCT

TTGCCATCCC CTTTTGTGCT ATGTATCTCA CAGCACTGGC TGGGAATGCT

TGGCATTCCC ACTGGGTGCT ATATATGTCA TTGCCCTCTT TGGCAATGGT

TGGCATTCCC ACTGGGTGCT ATATATGTCA TCGCCCTTTT TGGCAATGGT

TGGCATTCCC TCTGGGTGCT ATATATGTCA TTGCCCTCTT TGGCAATGGT

TGGCACTTCC ACTGGGTGCT ATATATTTCA TTGCCCTCTT TGGTAACGGT

TTGCCATCCC CTTCTGCTCC ATGTATATCC TTGCTCTGGT TGGCAATGGC

TTGCTATCCC ATTTTGTGCC ATGTACCTGG TGGCACTGAC TGGAAATGTT

TAGCCATTCC CTTTTGTGCC ATGTACCTGG TGGCACTGGC TGGAAATGCT

TCTCCATTCC CTTCTGTGCC ATGTATTTGG TAGCTCTGAT TGGGAACTTA

TCTCCATCCC CTTCTGCTCC ATGTACATCA TGGCTCTGCT GGGCAATGCC

GCCCTCATCC TGGTCATTGC CATGGACAAT GCTCTTCATG CACCTATGTA

GCCCTCATCC TGGTCATTGC CATGGACAAT GCTCTTCATG CACCTATGTA

GCCCTCATCC TGGTCATTGC CTTGGACAAT GCTCTTCATG CACCTATGTA

GCCCTCATCC TGGTCATTGC CTTGGAAAAT GCTCTTCATG CACCTATGTA

GCCCTCATCC TGATCATTGT CAGGGACAAT GCTCTTCATG CGCCCATGTA

GCCCTCATCC TGGTTATTTC CCTGGACAAT GCCCTTCACA CTCCCATGTA

GCCCTCATCC TTATCATAGG GACAGAGAGT GCCCTTCATG CACCCATGTA

GCCCTCATCC TTATCATTGG GACAGAGAGT GTGCTTCACA CACCCATGTA

GCCCTCATTC TTGTCATTGT GACAGATAGA GCTCTTCATG CACCCATGTA

GTCATCATCT CTACCATCAA GTCTGAACTG TCCCTGCACA TGCCCATGTA

GTCATTATTT CTACCATCAA GTCTGAATCA TCCCTGCATA TCCCTATGTA

GTCATCATTT CTACCATCAA GTCTGAATTG TCCCTGCATA TCCCCATGTA

ATCATCATCT CCACCATCAA GTCAGAATCG TCCCTGCATA TCCCTATGTA

ACCATTCTCT ATATCATCAT GACGGACAGA GCTCTACATG AGCCAATGTA

GCCCTTATAA TGGTCATTGT GATGAACAAT ACTCTTCATA AACCCATGTA

CTCCTTATTC TTGTCATTGG GATGGACAAG GCTCTTCATG AACCCATGTA

ATCCTCATCT TGGTCATATG TACTGATCAC AGCCTCCACA CACCAATGTA

ACCATCCTGT TGGTTGTTGC CTGTGACAAG ACCCTTCATG AGCCCATGTA

CCTCTTCCTC TGCCTTCTCT CACTCACAGA CCTGGCTCTC AGTTCTACCA

CCTCTTCCTC TGCCTTCTCT CACTCACAGA CCTGGCTCTC AGTTCTACCA

CCTCTTCCTC TGCCTTCTCT CACTCACAGA CCTGGCTCTC AGTTCTACCA

CCTCTTCCTC TGCCTTCTCT CACTCACAGA CCTGGCTCTC AGTTCTACCA

CCTCTTCCTC TGCCTTCTCT CACTCACGGA CCTGGCTCTT AGTTCTACCA

CCTCTTCCTT TGTCTTCTGT CACTTACCGA CCTGGCTCTC AGTTCTACTA

CCTCTTCCTC TGCCTTCTCT CACTCACTGA CCTGGCTCTC AGCTCCACCA

CCTCTTCCTC TGCCTTCTCT CACTCACTGA CTTGGCTCTC AGCTCCACCA

TCTCTTCCTT TGCATTCTGT CACTCACTGA CCTGGCTCTC AGCTCTACCA

TTACTTTCTG TGTATGCTGG CACTGGCAGA CACAGGACTT GCCCTTTGTA

CTATTTTCTG TTCATGCTGG CATTGGCAGA CATGGGGCTT GCCCTTTGTA

TTACTTTCTG TGTATGCTGG CATTGGCAGA CATGGGACTT GCCCTTTGTA

CTACTTCCTC TGCATGCTGG CATTGGCCGA CATGGGACTT GCCCTTTGTA

CCTCTTCCTA TGCCTACTTT CCATCACTGA CCTGGTACTC TGCTCAACCA

CTTCTTCCTT TGCCTTCTCT CACTCACCGA CCTGGCCCTC AGCTCTACCA

CCTTTTCCTT TGTCTTCTCT CACTCACGGA CCTGGCCCTC AGTTCAACCA

CATCTTCCTT TGTCTACTCT CATTCTCAGA CTTAACCATC AGCTCTACAA

CTTCTTCCTC TGCATCCTCT CGCTCACAGA CCTGGTGCTT TGCTCCACCA

CTGTGCCCAA GATGCTGGCC ATTTTGTGGC TCCATGCTGG TGAGATTTCC

CTGTGCCCAA GATGCTGGCC ATTTTGTGGC TCCCTGCTGG TGAGATTTCC

CTGTGCCCAA GATTCTGGCC ATTTTGTGGC TTCATGCTGG TGAGATTTCC

CTGTGCCCAA GATTCTGGCC ATTTTGTGGC TTCATGCTGG TGAGATTTCC

CTGTGCCCAA GACTCTGGCC ATTTTGTGGC TTCATGCTGG TGAGATTTCC

CTGTGCCCAA GATGCTGGCC ATTTTGTGGT TCCACGCTGG TGAGATTTCC

CTGTACCTAA AATGTTGGCT ATTTTATGGC TCAATGCTAA TGAGATTTCC

CTGTACCTAA AATGTTGGCC ATTCTATGGC TCCATTCTAA TGAGATTTCC

CTGTGCCCAA GATGCTGGCC ATTTTATGGA TGCACGCTGG TAAGATTTCC

CTATGCCTTC CATGCTAGGC ATATTTTGGT TTAACTACAA GTCCATCGCC

CTTTGCCCTC TATGCTAGGC ATATTTTGGT TTAACTATAA GTCCATTGCT

CTTTGCCCTC TATGCTAGGT ATATTCTGGT TTAACTACAG GTCCATCACC

CTTTGCCCTC TATGCTGGGC ATATTCTGGT TTAACTACAA GTTTATTGCT

CATTGCCCAA AATGCTAACA ATCTTCTGGC TTAGGTCCCA CATAATTTCC

CCATGCCCAA AACACTGGCT ATTTTGTGGC TCCATGCTGG TGAGATTTCC

CCACGCCCAA GACACTAACC ATTTTGTGGC TCCAGGATGG TGAGATTTCC

CAGTACCAAA AATGTTAGCA ATCCTTTGGT TTCATGCTGG TCAGATGACT

CATTACCTAA GATGCTGGCT ATCTTCTGGT TTGGGGCCCA GCACATCTCC

TTTGGTGGAT GCCTGGCCCA GATGTTTTGT GTCCATTCTA TCTATGCTCT

TTTGGTGGAT GCCTGGCCCA GATGTTTTGT GTCCATTCTA TCTATGCTCT

TTTGGTGGAT GTCTGGCCCA GATGTTTTGT GTTCATTCTA TCTATGCTCT

TTTGGTAGAT GTCTGGCCCA GATGTTTTGT GTTCATTCTA TCTATGCTCT

TTTGGTGGAT GCCTGGCCCA GATGTTCTGT GTCCATTCTA TCTATGCTCT

TTTGGTGGAT GCCTCACCCA GATGTTTTGT ATTCATTCTA TCTATACACT

TTTGGTGGAT GCCTAGCTCA AATGTTTTGT GTCCACTCCA TCTATGCTCT

TTTGGTGGAT GCCTCGCCCA GATGTTTTGT GTCCACTCCA TCTATGCTCT

TTCAGTGGGT GCCTGGCCCA GATGTTTTGT GTCCATTCTA TCTATGCTCT

TTTGATGCCT GCCTTGTTCA GATGTACTTC ATCCATACCT TCTCAGCCAT

TTTGATGCCT GCCTTGTTCA GATGTACTTC ATTCATACCT TCTCTGCCAT

TTTGATGCCT GCCTTGTTCA GATGTACTTC ATCCACACCT TCTCAGCCAT

TTTGATGCTT GTCTTGTTCA AATGTACTTC ATCCACACCT TCTCAGCTAT

TTCTATGGCT GCCTCACCCA GATGTTTTTT GTTCACACCA TCTTTGCCAC

TTTGATGCCT GTCTAGCCCA AATGTTTTGC ATCCATTCTA TCTATAGTCT

TTTGCTGCCT GCTTAGCCCA GATGTTTTGT GTCCACGCCA TCTATGCGCT

TTTGCAGGCT GCCTGGCCCA GATGTTCTTT TTGCACTCTA TATTTGCTGT

TACATTGGCT GCCTCACTCA AATGTTCTTC ATCCACATGG TCTTTGCCAC

GGAGTCCTCA ATTCTACTTG CCATGGCCTT TGATAGGTAT GTGGCTATCT

GGAGTCCTCG ATTCTACTTG CCATGGCCTT TGATAGGTAT GTGGCTATCT

GGAGTCCTCG ATTCTACTTG CTATGGCCTT TGATAGATAT GTGGCTATCT

GGAGTCCTCG ATTCTACTTG CTATGGCCTT TGATAGATAT GTGGCTATCT

AGAGTCCTCG ATTCTACTTG CCATGGCCTT TGATAGATAT GTGGCTATCT

GGAATCTTTA ATTCTTCTTG CCATGGCCTT TGATGGATAT GTGGCCATCT

TGAGTCCTCG GTTCTCCTTG CCATGGCCTT TGATCGATAT GTTGCTATCT

TGAGTCCTCG GTTCTCCTTG CCATGGCCTT TGATCGATAT GTAGCTATCT

GGAGTCCTCA GTTCTTCTTG CCATGGCCTT TGATCGATAT GTGGCTATCT

TGAATCTGGC GTGCTGGTGG CCATGGCCTT TGATCGGGTT GTGGCAATCT

TGAATCTGGC GTGCTGGTGG CCATGGCTTT TGATCGGGTT GTAGCCATCT

TGAGTCTGGT GTCCTGGTGG CCATGGCCTT TGATCGGGTT GTAGCAATCT

TGAATCTGGA GTGCTAGTGG CCATGGCTTT TGATAGAGTT GTGGCTATCT

CGAGTCAGCT GTCCTGCTGG CCATGGCTTT TGACCGCTAT GTGGCTATCT

AGAGTCCTCA GTTCTTCTTG CCATGGCCTT TGAT---TAT GTGGCTGTCT

GGAGTCCTCA GTTCTTCTTG CCATGGCCTT TGATCGATAT GTGGCTATCT

AGAATCTTCT GTTCTCCTAG CTATGGCATT TGATCGCTAT GTGGCCATCT

CGAGTCAGCT GTCCTGCTGG CCATGGCTTT TGACCGTTTC GTGGCCATCT

GTAACCCATT AAGGTACACA ACCATTCTCA ACCATGCTGT CATAGGCAGA

GTAACCCATT AAGGTACACA ACCATTCTCA ACCATGCTGT CATAGGCAGA

GTAACCCATT AAGATATACA ACCATTCTCA ACCATGCTGT CATAGGCAGA

GTAACCCATT AAGATATACA ACCATTCTCA ACCATGCTGT CATAGGCAGC

GTAACCCATT AAGGTACACG GCCATTCTCA ATCATGCTGT CATAGGCAGA

GCAACCCATT AAGATATACA ACCATTCTCA ACCATACTGT CATAGGCAAA

GCAACCCACT GAGATATACA ACCATCCTCA ACCATACTGT CATAGCCCAA

GCAACCCACT GAGATATACA ACCATCCTCA ACCATACTGT CATAGCCCAA

GCAACCCCCT GAGATACACA GCAATTCTCA ACCATACCGT CATAGGAAGA

GGAAACCCCT CAGGTACGGC ACCATCCTCA CCAATAGCGT GGTCTGCAGA

GGGACCCCCT CAGGTATGGT ACCATCTTAA CCAATGGTGT GGTCGGCAGA

GGAGCCCCCT CAGATATAGC ACCATCCTAA CTAATGGTGT GGTCTGCAGA

GGAACCCTCT GAGGTATGGA ACCATCCTAA CCAATGGGGT GGTCTGCAGA

GCCACCCACT TCATTATATA TCCATCCTGA ACGCCACTGT GATTGGAAAG

GCAACCCTCT CAGATGTACA ACCATTCTCA ACCACGGCAT CATAGGCAGA

GCCAGCCTCT GAGGTACAGC ACCATTCTCA ACCACAGTGT CATAGGCAGA

GCAATCCACT GAGATATACA ACCATCCTCA ATCATGGTGT GATTGTCAGA

GCCGCCCACT ACATTACTCG TCCATCCTCA GCCCTGGTGT CATTGGGAAA

ATTGGCTTTG TTGGGCTATT CCGTATTGTC TCCCCCTTCA TCTTCTTGCT

ATTGGCTTTG TTGGGCTATT CCGTATTGTC TCCCCCTTCA TCTTCTTGCT

ATTGGCTTTG TTGGGATATT CCATATTGTT TCCCCCTTCA TCTTCTTGCT

ATTGGCTTTG TTGGGATATT CCATATTGTT TCCCCCTTCA TCTTCTTGCT

ATTGGCTTTG TTGGGATATT CCGTATTGTC TCTCCCTTCA TCTTCTTGCT

ATTGGCCTTG TTGGCATATT CTGTATTGTC TCTCCCTTCA TCTTCTTGCT

ATTATCTTTG CTGGCATAGT CCGTATTGTC TCCCCTTTCA TCTTTTTGCT

ATTATCTTTG CTGGGATAGT CCGTATTGTC TCCCCTTTCA TCTTTTTGCT

ATTGGGTTTG TTGGGATATT CCGTATTGTT TCCCCCTTCA TCTTCTTGCT

ATCATCTTGA TAAGGGCCAT CTGTGTGGTC TTCCCGGTGC CTTTCCTCAT

ATCATCCTGA TAAGAGCAGT CTTTGTGGTC TTCCCTGTGC CTTTCCTCAT

ATCATCTTGA CAAGGGCAGT CTGTGTGGTC TTCCCTGTGC CTTTTCTCAT

ATTATCTTGA CAAGGGCTGT CTGTGTGGTC TTCCCTGTGC CTTTCCTCAT

ATTGGTCTAG CATGTGTCTT CCGATTTGTT TTCCCCTTTG TCATCCTCAT

ATCACCCTTG TTGGAATATT CCGTGTTGTT TCCCCTTTTA TCTTCTTGCT

ATCACATGTG CTGGGATATT TCGTATGGTT TCCCCCTTCA TCTTCTTGCT

ATTTTCTTTG CTGGAATAAT TAGGTGTACT GCTTTTCTCA TCTTCCTGGT

ATTGTAATGG CCTGCGTAGT CCGATTTGTC TTCCCATTCG TCATCCTGAT

GAGGCGACTC CCCTACTGTG GTCACCGTGT CATGACACAC ACATACTGTG

GAGGCGACTC CCCTACTGTG GTCACCGTGT CATGACACAC ACATACTGTG

GAGGCAACTC CCTTACTGTG GTCACCGTGT CATGACACAC ACACACTGTG

GAGGCAACTC CCTTACTGTG GTCAACATGT CATGACACAC ACACACTGTG

GAGGCGACTC CCCTACTGTG GTCACCGTGT CATGACACAC ACATACTGTG

GAGGAGACTG CCCTACTGTG GTCACCATGT CATGGCACAC ACATACTGTG

GAGGCGACTG CCCTACTGTA GTCACCGTGT CATGGCTCAC ACATACTGTG

GAGGCGACTG CCCTACTGTG GTCACCGTGT CATGACTCAC ACATACTGTG

CAGACGACTG CCCTACTGTA GGCACCGTGT CATGGCACAC ACATACTGTG

CAAGCGGCTC CCCTTCTACC GCTCCAATAT CCTCTCCCAC TCCTTCTGCC

CAAGCGTCTT CCCTTCTACC GCTCCAACAT CCTCTCCCAC TCCTTCTGCC

CAAGCGGCTC CCCTTCTATG GCTCCAACAT TCTCTCCCAC TCCTTCTGCC

CAAGCGCCTT CCTTTCTACC GCTCCAATAG CCTCTCCCAC TCCTTTTGCC

TGAACGCTTA CCCTTCTGTG GACATCACAT TATCCCCCAC ACGTACTGTG

GAAGCAACTA CCCTACTGTG GGCACCGTGT CATGGCACAT ACATACTGTG

GAAGCGACTG CCCTACTGTG GGCATCGGGT CATGGCACAT ACATACTGTG

GTGGAGGCTC CCTTACTGTG GGCATCACAT CATGGCACAT ACCTACTGTG

CCAGAGATTG CCATTCTGTG GGCATCATGT GATCCCCCAC ACCTACTGTG

AGCATATGGG CATTGCCCGA CTGGCCTGTG CCAACATCAC TGTCAATATT

AGCATATGGG CATTGCCCGA CTAGCCTGTG CCAACATCAC TGTCAATATT

AGCATATGGG CATTGCC--- CTGGCCTATG CCAACATCAC CGTCAATATT

AGCATATGGG CATTGCC--- CTGGCCTATG CCAACATCAC CGTCAATATT

AGCACATGGG CATTGCCCGA CTCGCCTGTG CCAATATCAC TGTCAATGTT

AGCACATGGG CATTGCTCGA CTGGCCTGTG CCGACATCAC CGTCAATATT

AGCATATGGG CATTGCTAGG CTTGCCTGTG CCAACATCAC TGTCAACATT

AGCATATGGG CATTGCTAGG CTTGCCTGTG CCAATATTAC TGTTAATATT

AGCATATGGG CATTGCCCGA CTGGCTTGTG CTAACATCAC TGTCAATATT

TCCACCAAGA TGTCATGAGC CTTGCCTGTG CCAGCACCCG GGTCAACAGT

TCCACCAAGA CGTCATGCGA CTTGCCTGTG CCAGCACTCG TGTCAACAGT

TCCATCAAGA TGTCATGCGC CTCGCCTGTG CCAGCACTCG TGTCAACAGT

TCCACCAAGA TGTCATGCGC CTGGCCTGTG CCAGCACCCG TGTCAACAGT

AACACATGGG CATTGCCAAG CTGGCCTGTG CCAGCATCAA GTCCAACACT

AGCACATGGG CATTGCTCAA CTGGCCTGTG CCAGTATAAC TGTCAATGTT

AGCACATGGG CATTGCTAGC TTGGCCTGTG CCAACATCAC GGTCAATGTT

AACACATGGG CATTGCCCGG CTATCTTGTG GTGATATCAG ATTGAATATT

AGCACATGGG CATTGCCAAG CTGGCATGTG CCAACATCAA GATCAACATC

GTCTATGGGC TAACTGTGGC TCTGCTGGCC ATGGGACTGG ATTCCATTCT

GTCTATGGGC TAACTGTGGC TCTGCTGGCC ATGGGACTGG ATTCCATTCT

GTCTAT-GGC TGACTGTGGC TCTGCTGGCC ATGGGACTGG ATTCTATCCT

GTCTAT-GGC TGACTGTGGC TCTGCTGGCC ATGGGACTGG ATTCTATCCT

GTCTATGGGC TGACTGTGGC TCTGCTGGCC ATGGGACTGG ATTCTATTCT

GTCTACGGGC TGACAGTGGC TCTGCTGGCC ATGGGCCTGG ATGCTATCCT

GTCTATGGGC TGACTGTGGC TCTGCTGGCC ATGGGCCTGG ATTCCATCCT

GTCTATGGGT TGACTGTAGC TTTGCTGGCC ATGGGCCTGG ATTCCATCCT

GTCTATGGAT TGACTGTGGC TCTGCTAGCC ATGGGTCTGG ATTCCATCCT

CTCTACGGCC TTATTGCGGT TATCTTCACC AAGGGTTCTG ACTCCCTCTC

CTCTATGGAC TTATTGCTGT CATCTTCACC AAGGGTTCTG ACTCCCTCTC

CTCTACGGCC TCATTGCTGT CATCTTCACC AAGGGCTCTG ACTCCCTCTC

CTTTATGGGC TCATTGCTGT TATCTTTACC AAAGGTTCTG ACTCCCTCTC

ATTTATGGTC TCACGGTGGC ACTTTCAGTC ACTGGCGTGG ATGTGGTCCT

GTCTATGGGC TGACTGTAGC GCTTCTGGCC ATGGGTCTGG ATTCCATCCT

ATTTATGGGC TGACTGCAGC CCTACTGGCC ATGGGATTGG ATTCCATCCT

GTCTTTGGTC TGACTATGGC CCTCTTGGTT ATGGGACTGG ATGCTGTGCT

ATCTATGGTC TGACGGTTGC ACTCTCGGTC ACAGGGATGG ATGTGGTGCT

CATTGCCATT TCCTATGGCT TTATCCTCCA TGCAGTCTTT CATCTTCCAT

CGTTGCCATT TCCTATGGCT TTATCCTCCA TGCAGTCTTT CATCTTCCAT

CATTGCCATT TCCTATGGCT TTATTCTCCA TGCAGTCTTT CATCCTCCAT

CATTGCCATT TCCTATGGCT TTATCCTCCA TGTAGTCTTT CATCTTCCAT

CATTGCCGTC TCCTATGGCT TTATCCTCCA TGCGATCTTT CATCTGCCGT

CATCGCCATC TCCTATGGCT TTATCCTCCA TGTGGTCTTT TATCTCCCAA

CATTGCCATC TCCTATGGCT TCATCCTCCG GGCTGTCTTC CGTCTCCCAT

CATTGCCATC TCCTATGGCT TCATCCTCCG GGCTGTCTTC CGTCTTCCAT

CATTGCTGTC TCCTATGGTT TTATCCTCCG TGCTGTCTTT CGTCTTCCAT

CATCCTCCTC TCCTATGCAT TCATACTTCA AACAGTGATG GCCATTGCCT

CATCCTCTTC TCCTATGTGT TCATATTCCG AACAGTAATG GCCATTGCCT

CATCCTCCTT TCCTATGTGT TCATACTCCG AACAGTAATG TCCATTACCT

CATCCTCCTC TCCTATGCTT TTATTCTTCG AACAGTGATG GCCATTGCTT

CATTGCTACC TCCTATGGCC TGATCCTGAG GGATGTGCTG CGCTTGCCCT

CAATGCCATC TCCTATGTCT TTATCCTCCA AGCCGTCTTT CATCTACCAT

CATTGCCATC TCGTATGTCT TTATCCTCCA AGCAGTCTTT CGCCTGCCAT

CATAGCAGTA TCTTATTGTA TCATTCTTCA TGCTGTATTC CGTCTTCCAT

CATTGGCTTT TCTTATGGCT TCATCCTCCA TACTGTGCTC CACCTGCCTT

CTCATGATGC CCAGCACAAA GCTCTGAGTA CCTGTGGCTC CCACATTGGC

CTCATGATGC CCAGCACAAA GCTCTGAGTA CCTGTGGCTC CCACATTGGC

CTCGTGACGC CCAGCACAAA GTTCTAAGTA CCTGTGGCTC CCACATTGGA

CTCGTGATGC CCAGCACAAA GTTCTAAGTA CCTGTGGCTC CCACATTGGA

CTCATGATGC CCAGCACAAG GCTCTGAGTA CCTGTGGCTC CCACATTGGT

CTCGTGTTGC CCAGCACAAG GCTCTGAGCA CCTGTAGCTC CCACCTCGGT

CCCGTGACGC TCAACACAAG GCTCTGAGTA CCTGTGGATC TCACCTTGGT

CCCGTGATGC TCAACACAAG GCTCTGAGTA CCTGTGGATC TCACCTTGGT

CCCGTGATGC CCAGAACAAG GCTCTGAGTA CCTGTGGTTC CCACTTGGGT

CAGGGGAGGG CCAGCTGAAG GCGCTCAGCA CCTGTGTTTC CCACATCTGT

CAGGGAAGGG CCGGCTGAAG GCACTCAACA CCTGTGTTTC ACATATCTGT

CAGGGGAAGG CCGGCTAAAG GCACTCAACA CCTGTGTTTC ACACATCTGT

CAGGAGAGGG CAGGCTGAAG GCCCTCAACA CCTGTGTGTC ACACATTTGC

CTAAAGATGC TCAGTTCCGA GCATTTAGCA CATGTGGAGC TCACATTTGT

CTCATGATGC TTGGCGCAAG GCTCTAAGTA CCTGTGGCTT TCACCTTACA

TTCGTGAAGC CCGGCACAAA GCTCTGAGTA CCTGTGGCTC CCACCTTGCA

CCCGTGATGC ACGACGCAAG GCTCTAGGAA CATGTGGGTC ACACATTGGG

CCCAGGATGC ACAGCTCAAG GCCTTCAGCA CCTGTGGGGC TCACGTATGT

ATCATCCTGG TTTTCTACAT CCCTGCCTTC TTCTCCTTCC TCACCCACCG

ATCATCCTGG TTTTCTACAT CCCTGCCTTC TTCTCCTTCC TCACCCACCG

GTCATCCTGG TTTTCTACAT CCGTGCCTTC TTCTCCTTCC TCACCCACCG

GTCATCCTGG TTTTCTACAT CCCTGCTGCC TTCTCCTTCC TCACCCACCG

GTCATCCTGG TTTTCTACAT CCCTGCCTTC TTCTCCTTCC TTACCCACCG

GTCATTCTGG TGTTTTACAT CCCTGCCTTC TTCTCACTCC TCACACATCG

GTCATCTTGG TTTTCTATAT ACCTGCTTTC TTCTCCTTTC TTACCCACCG

GTCATTCTGG TTTTCTATAT ACCTGCTTTC TTCTCCTTCC TCACCCATCG

GTCATCCTGG TTTTCTACAT CCCTGCCTTC TTCTCCTTCC TCACCCACCG

GCGGTTCTCA TCTTCTACGT GCCGCTCATT GGGGTGTCGG TCATTCACCG

GCTGTACTTA TCTTCTATGT TCCACTCATT GGGGTATCTG TCATTCACCG

GCTGTACTCA TCTTCTACGT GCCACTCATC GGGGTGTCTG TCATTCATCG

GCCGTGCTCA TCTTCTATGT ACCACTCATT GGAGTGTCTG TCATTCATCG

GTGATTCTTG TTTTCTATAT CCCTGCCTTT TTTTCCTTCT TTACCCACCG

GTCATCCTGA TTTTCTACAT CCCTGCCTTT TTCTCCTTCT TCACTTACCG

GTTATCCTAG TTTTCTACAT CCCTGCCTTC TTCTCCTTCC TTACTCACTG

GTCATCCTTG TTTTCTATAT TCCTGCTTTC TTCTCTTTCC TCACCCACCG

GTCATCCTGG TCTTCTACAT TCCGGCTTTC TTCTCCTTCT TCACACACCG

CTTTGGTCAC CACGAAGTCC ACATCTTTCT GGCTAATCTC TATGTGCTGG

CTTTGGTCAC CACGAAGTCC ACATCTTTCT GGCTAATCTC TATGTGCTGG

CTTTGGACAC CACCAAGTCC ACATCTTTCT GGCTAATCTC TATGTGCTGG

CTTTGGTCAC CACCAAGTCC ACATCTTTCT GGCTAATCTC TATGTGCTGG

CTTTGGTCAC CACCAAGTCC ACATCTTTCT GGCTAATCTC TATGTGCTGG

CTTTGGTCAC CACCGAGTCC ACATCTTTCT AGCGAATCTG TATGTGCTGG

TTTTGGTCAC AACCGAGTTC ACATCTTCCT GGCTAACCTC TATGTGCTGG

CTTTGGTCAC AATCGAGTTC ATATCTTCCT GGCTAACCTC TATGTGCTGG

CTTTGGTCAC AACAGAGTTC ACATCTTTCT GGCAAATCTC TATGTGCTGG

TTTTGGTCAC CAC---CC-C ATGCCCTAAT GGCTAATGCC TATCTTCTTA

TTTTGGTCAC CAC---CT-C ATGCTCTCAT GGCTAATGCC TACCTTCTTG

TTTTGGTCAC CAC---CT-C ATGCTCTCAT GGCTAATGCC TACCTTATTG

TTTTGGTCAC CATTGTCT-C ACGCCCTCAT GGCCAATGCC TACCTTCTTG

CTTTGG---C CACAGAGTAC ACATTATACT TGCAAATCTT TATCTTCTTG

CTTTGGCAGA CACCGTGTCC ACATTTTTCT GGCTAATCTC TATGTGCTCG

CTTTGGCCAC AACCGTGTCC ACATATTTCT GGCCAATCTC TATGTGCTGG

TTTTGGATAC AACCGTGTCC ATATTTTTTT GGCCAACCTA TATGTGTTGT

CTTTGG---T CACTGGATGC ACATTTTCGT GGCCAATCTC TACCTCCTGG

TGCCTCCTGT ACTCAATCCT ATTCTCTATG GAGCTAGAAC CAAGGAGATT

TGCCTCCTGT ACTCAATCCT ATTCTCTATG GAGCTAGAAC CAAGGAGATT

TGCCTCCTGT GCTCAATCCT ATTATCTATG GAGCTAGAAC CAAGCAGATT

TGCCTCCTGT GCTCAATCCT ATTATCTATG GAGCTAGAAC CAAGCAGATT

TGCCTCCTGT ACTCAATCCT ATTATCTATG GAGCTAGAAC CAAGGAGATT

TGCCTCCTGT CCTCAATCCT ATTATCTATG GAGCCAGAAC CAGGGAGATT

TGCCTCCTGT ACTCAATCCC ATCATCTATG GAGCAAGAAC GAAGGAGATT

TGCCTCCCGT GCTCAATCCC ATCATCTACG GTGCAAGAAC CAAGGAGATT

TGCCTCCTGT GCTCAATCCT ATTATCTATG GAGCTAGAAC CAAGGAAATT

TATCCCCTGT GCTAAACCCC ATCGTCTATA CTATGAAGAC CAAAGAGATA

TACCCCCTGT GCTTAACCCT ATAGTCTATA CTGTGAAGAC CAAGGAGATA

TACCCCCTGT GCTAAACCCC ATAGTCTATA CTGTGAAGAC CAAGGAGATA

TGCCCCCTGT GTTAAACCCC ATAGTTTATA CTGTGAAGAC CAAGGAGATA

TGCCCCCTGT TCTCAACCCC CTGGTCTATG GCATTAATAC TAAACAGATA

TGCCTCCTGT ACTCAACCCA ATCATCTATG GGGCCAGGAC CAAGGAGATT

TGCCTCCTGT GCTCAACCCA ATCATCTATG GGGCCAGAAC CAAGGAGATT

TGCCACCAGT GCTCAACCCC ATCATCTATG GAGCTAAGAC CAAAGAGATC

TACCCCCTGT GCTCAACCCG CTGGTGTATG GCATTAATAC CAAGCATATC

CGGAGTCCCA ATGACACCCA GTTTCACCCC TCCTCCTTCC TGTTGCTGGG

CGGAGTCCTA ATGACACCCA GTTTCACCCC TCCTCCTTCC TGTTGCTGGG

CGGAGCTTCA ATAGTTCTGC CTTATACCCT CGCTTCCTCC ---TAATGGG

TGGAGCTTCA ATAGTTCTGC CTTATACCCT CGCTTCCTCC ---TAATGGG

CGGAGTCCGA ATGACACCCA GTTTCACCCC TCCTCCTTCC TGTTGCTGGG

CGGAGTCCTA ATGAAACCCA GTTTCACCCC TCCTCCTTCT TATTGCTGGG

CGGAGTCCCA ACGACACTCA GTTTCACCCT TCCACTTTCT TGTTGCTGGG

CGGAGTCCCA ATGACACTCA GTTTCACCCT TCCACTTTCT TGTTGCTGGG

CAGAGACCCA ATGACACTCA GTTTCACCCT TCCTCCTTCT TGTTGCTGGG

CGGAGGCTCA ACAATACCAG TGCTCAGCCT CTGACCTTCC TCTTGACGGG

CGAAAACTCA ATAATACCAA TGCTCAACCT CTGACCTTCT TCCTGACGGG

CGAAGCCTCA ATAATACCAA TGATCAGCCT CTGACATTCC TCCTGACGGG

AGAAGGCTCA ATAATACCAA TGCTCAGACT CTGACCTTCT TCCTGACGGG

CGGA--CTCA ATGACACTGG CTTCCACCCT TCTACGTTTA TTCTTCTGGG

AAAAGTATCA ATGACACTCA ATTCCACCCC TCGTCTTTCC TGCTGCTGGG

AAAAGTTTCA ACATTAGTCA GTCTCATCTG TCTTCCTTCC TACTTTTGGG

AGGA-ATTCA ACCTGAGCAG CTATAATCCC AGCTTCTTTA TCTTAGTGGG

CGCCA-CCCA ACACTACCAG CTTCCATCCC TCTATGTTCC TCCTGTTGGG

GATCCCAGGA CTAGAAACAC TTCACATCTG GATCGGCTTT CCCTTCTGTG

GATCCCAGGA CTAGAAACAC TTCACATCTG GATCGGCTTT CCCTTCTGTG

CCTCTCAGGC CTTGAAAGCA GATATGACTT GATTTCCCTC CCCATCTTCT

CCTCTCAGGC CTTGAAAGCA GATATGACTT GATTTCCCTC CCCATCTTCT

AATCCCAGGA CTAGAAACAC GTCACATCTG GATCGGCTTT CCCTTTTGTG

AATCCCAGGA CTAGAATCAC TTCACATCTG GATCGGTCTT CCCTTCTGTG

AATCCCAGGA CTAGAGTCTA TTCACATCTG GATTGGATTT CCTTTCTGTG

GATCCCAGGA CTAGAGTCTC TTCACATCTG GATTGGATTT CCTTTCTGTG

GATCCCAGGA CTAGAAGCTC TTCACATCTG GATTGGCTTT CCCTTTTGTG

TATTCCAGGC CTGAGAGCGG CCAAGATCTG GGTCTCCATC CCTGTTTGCC

TATCCCAGGC CTGAGAGCAG CCCAGGTTTG GATCTCCATC CCTTTTTGTC

TATCCCAGGC CTGGCAGCAG CCCAGGTGTG GATCTCCATT CCTATTTGTC

TATCCCAGGC CTGAGAGCAG CCCAGGTCTG GATCTCCATC CCGTTCTGCC

AATCCCAGGG ATGCAAGACC AGCATGTTTG GATTGCCATC CCCTTCTGCT

AATCCCAGGA ATGGAAACGC TCCACATCTG GATTGGTTTC CCTTTCTGTG

AATCACAGGA CAAAACATA- TCCAAGTATG GATTGGCTTC CCCTTCTGTG

GATCCCAGGG CTTGAGAAGT TCCACATCTG GATTGCGATT CCCTTCTGTG

TATCCCTGGG ATGGAGGATC AGCACATCTG GATCTCCATT CCCTTCTGCT

CTGTGTACAT GATCGCACTC ATAGGGAACT TCACTATTCT ACTTGTGATC

CTGTGTACAT GATCGCACTC ATAGGGAACT TCACTATTCT ACTTGTGATC

TGATTTATGC CACCTCAATT GCTGGGAACA TTACCATCCT CTTCATTATC

TGATTTATGC CACCTCAATT GCTGGGAACA TTACCATCCT CTTCATTATC

CTGTGTATAT GATTGCACTC ATAGGGAACT TCACTATTCT ACTGGTGATC

CTGTGTACAT GACAGCACTC ATAGGGAACT TCACTATTCT CTTGGTGATC

TTGTATACAT GATTGCTCTC TTAGGAAACT TAACCATATT GTCTGTGATC

TTGTATACAT GATTGCTCTC TTAGGAAACT TAACCATCTT GTTTGTGATC

CTGTGTACAT GATTGCACTC ATAGGCAACT TCACCATTCT CTTGGTAATA

TCCTGTATGC CGGCGCCCTC TCTGGGAACA GTGTGATCCT GTTTGTGGTC

TCCTGTATGT CATCGCCCTC TCTGGGAACA GCATGATCCT ATTTGTGGTC

TCCTGTATGT CACTGCCCTG TCTGGGAACA GCATGATCCT GTTTATGGTC

TCCTATATGG CATTGCCCTC ACTGGAAACA GCATGATCCT GTTTGTGGTC

CCATGTATAT CCTTGCTCTG GTTGGCAATG GCACCATTCT CTATATCATC

CTGTGTACCT GATGGCTGTC CTTGGGAATA TCTGCATCCT TTTTGTGATT

CTGTGTACTT GACAGCTGTC CTAGGAAATA TGTGCATACT CTATGTGATT

TTATCTACCT TGTGGCCATT GTGGGTAACT GTGTCCTGCT GTACCTCATT

CCATGTACAT CATGGCTCTG CTGGGGAATG GCACCATCCT ATTGGTCGTT

AAGACTGACA GCAGCCTACA CCAGCCCATG TTCTACTTCC TGGCCATGTT

AAGACTGACA GCAGCCTACA CCAGCCCATG TTCTACTTCC TGGCCATGTT

AGAACTGAGT CTTCCCTCCA TCAACCAATG TATTACTTTC TGTCAATGCT

AGAACTGAGT CTTCCCTCCA TCAACCAATG TATTACTTTT TGTCAATGCT

AAGACCGAGA GCAGCCTACA CCAGCCCATG TTCTACTTCC TGGCCATGCT

AAGACAGACA GCACCCTGCA CCAGCCCATG TTCTACTTCC TGGCCGTGTT

AAAACTGAGA GCAGCCTCCA CCAGCCCATG TTCTACTTTC TGGCTATGTT

AAAACTGAGA GCAGCCTCCA CCAACCCATG TTCTATTTTC TGGCTATGTT

AAGACTGAGA ACAGCCTACA CCAGCCCATG TTCTACTTCC TAGCCATGTT

CTCCGTGAAC AGAGCCTTCA TGAGCCCATG TATTATTTCC TCTCTATGCT

TTCCGTGAGC AGAATCTCCA TGAACCAATG TATTATTTCC TCTCTATGCT

ATTCATGAGC AGAGCCTCCA TGAGACCATG TATTATTTCC TTTCTATGCT

CTCCGTGAGC AGAGTCTCCA TGAGCCCATG TATTATTTCC TCTCTATGCT

ATGACGGACA GAGCTCTACA TGAGCCAATG TACCTCTTCC TATGCCTACT

CAGACTGAGA GTAGCCTCCA TCAGCCCATG TTCTACTTCT TGGCCATTCT

CAGATTGACA GAAGCCTCCA TCAGCCCATG TTCTACTTCT TGGCCATTCT

GCTGTGGAGC ACAGCCTCCA TGAGCCCATG TTCTTTTTCC TGTCCATGCT

GCCTCTGACA AGACCCTCCA CGAGCCCATG TACCACTTCC TCTGCATCCT

GGCCACCACT GATGTGGGTC TCTCAACAGC TACCATCCCT AAGATGCTTG

GGCCACCATT GATGTGGGTC TCTCAACAGC TACCATCCCT AAGATGCTTG

GGCATTCACT GACCTGGGCC TATCTACCAC TACCTTGCCT ACCATGTTCA

GGCATTCACT GACCTGGGCC TATCTACCAC TACCTTGCCT ACCATGTTCA

GGCTACCATT GACTTGGGCC TTTCAACAGC TACCATCCCT AAGATGCTTG

GGCTGCCATT GACCTGGGCC TCTCAACAGC TACTATCCCT AAGATGCTTG

GGCTGCCATT GATTTGGGTC TCTCAACAGC AACCATCCCC AAGATGCTTG

GGCTACCATT GACTTGGGCC TCTCAACAGC AACCATCCCT AAAATGCTTG

GGCAACCATT GACTTGGGAC TCTCAACAGC TACCATCCCT AAGATGCTTG

TTCAGCCACA GACCTGAGCT TATCCCTGTG CACACTTTCC ACTACCCTTG

TTCAGCCACA GACCTGAGCT TATCCCTGTG CACACTTTCT ACTACCCTTG

GTCAGCCACA GACCTGAGCT TATCCCTGTG CACACTTGCC ACTACTCTTG

TTCAACCACA GATCTGAGCT TGTGTCTGTG CACGCTTTCC ACTACTCTGG

TTCCATCACT GACCTGGTAC TCTGCTCAAC CACATTGCCC AAAATGCTAA

GGCATCTATT GATCTGGGGT TATCCACATC CACTATTCCC AAGATGCTTG

GTCATCGATT GATCTGGGAC TATCAACATC CACTATCCCC AAAATGCTTG

GGCCAAAACT GATCTCATTT TGTCATCTAC AACAATGCCC AAATTACTCA

CTCACTTACA GACCTGGTGC TTTGCTCCAC CACATTGCCC AAGATGCTGG

GAATCTTCTG GATCAACCTC AGAGGGATCA TCTTTGAAGC CTGCCTCACC

GAATCTTCTG GATCAACCTC AGAGGGATCA TCTTTGAAGC CTGCCTCACC

GTGTCTTCTG GTTCCATGTG CAGGAGATAT CCTTCAATGC TTGTCTGGTC

GTGTCTTCTG GTTCCATGTG CAGGAGATCT CCTTCAATGC TTGTCTGGTC

GGATTTTCTG GATCAACCTC AGAGAGATCA TCTTTGAAGC CTGCCTCACC

GGATTTTCTG GATCAACCTC AGAGAAATCA CCTTTGGAGG CTGCCTCATC

GGATCTTCTG GCTTAATCTC AGAGAGATAC TCTTTGAAGA CTGCCTTATA

GGATCTTCTG GATTAATCTT AGAGAAATAC TCTTTGAAGA CTGCCTGATA

GGATCTTCTG GTTTAGTCTC AGGGAGATTA TCTTTGAAGC TTGTCTCACA

GTGTCTTCTG GTTTGAAGCC CGAGAGATCA ACTTAAATGC CTGTGTTGCT

GTGTCTTCTG GTTTGAAGCT CGAGAAATCA CCTTAAGTGC CTGCATTGCC

GTGTGTTCTG GTTTGAAGCC CGAAAGATCA ACTTAAATGC TTGTATTACG

GCGTCTTCTG GTTTGAAGCT AAAGCGATCA ACATCAATTC CTGCATTGCT

CAATCTTCTG GCTTAGGTCC CACATAATTT CCTTCTATGG CTGCCTCACC

GCATCTTCTG GCACAAGCTG AGAGATATCT CCTTTGAGGG CTGTCTCATC

GCATCTTTTG GCTCAACCTA AGAGAAATCT CCTTTGAGGG CTGTCTCATC

GTAACCTCTG GTTTGGTGAC AAGAAAATCA CCTTCTTGGG CTGCCTCACC

CCATCTTCTG GTTTGGGGCC CAGCCCATCT CCTTCCATGG TTGCCTCACT

CAGATGTTTT TTATCCACAA CTTCACACTT ATGGAGTCAG CAGTCCTTGT

CAGATGTTTT TTATCCACAA CTTCACACTT ACGGAGTCAG CAGTCCTTGT

CAAATGTACT GCATTCATGT TTTCTCAATT ATCGAGTCAG CTGTACTCTT

CAAATGTACT GCATTCATGT TTTCTCAATT ATCGAGTCAG CTGTACTCTT

CAGATGTTTT TCATTCACAA CTTCACACTT ATGGAGTCAG CAGTACTTGT

CAGATGTTTT TCATTCACAA GTTCACACTT ATGGAGTCAG CAGTCCTCTT

CAGATGTTTT TCATCCACAA ATTCACACTT ATGGAGTCAT CAGTCCTCTT

CAGATGTTTT TCATCCACAA ATTCACACTT ATGGAGTCAA CAGTCCTCTT

CAGATGTTTT TCATCCACAA TTTCACACTT ATGGAGTCAG CAGTCCTCTT

CAGATGTTCT TTCTCCATGG ATTTACTTTC ATGGAGTCTG GGGTTTTGCT

CAGATGTTCT TTCTCCATGG ATTTACTTTC ATGGAGTCTG GGGTTCTGCT

CAGATGTTCT TTCTCCATGC ATTTACTTTC ATGGAGTCTG GGGTTCTGCT

CA-CTCTTCT TCATCCACAC TTTGTCTCTT GTGGAGTCTT CGGTTCTATT

CAGATGTTTT TTGTTCACAC CATCTTTGCC ACCGAGTCAG CTGTCCTGCT

--GATGTTTT TCATCCACAT CTTTACTGGC ATGGAATCTG TAGTCCTCCT

--TAAGCTTT ATGTTAGCAT CTTCACTGGT GTGGAGTCAG CAGTCCTTCT

CAGATGTTCT TCCTCTATTT TAGCTTTTTT GTAGATTCTG CAATACTGTT

CAAATGTTCT TCATCCATGC TGCTTTTGCC ACTGAGTCAG GTGTTCTGCT

GGCAATGGCT TATGACAGCT ATGTGGCCAT CTGCAATCCA CTCCAATATA

GGCAATGGCT TATGACAGCT ATGTGGCCAT CTGCAATCCA CTCCAATATA

GGCTATGGCC TTTGACCGCT TTATAGCAAT CCGAGAACCC TTGCGCTATG

GGCTATGGCC TTTGACCGCT TTATAGCAAT CCGAGAACCC TTGCGCTATG

GGCAATGGCT TATGACCGCT ATGTGGCCAT CTGCAACCCG CTCCAATACA

GGCAATGGCT TATGACCGCT ATGTAGCCAT CTGCAACCCA CTTCGATATA

GGCAATGGCT TATGACCGCT ATGTGGCCAT CTGTATCCCA CTTCGGTACA

GGCAATGGCT TATGACCACT ATGTGGCCAT CTGTATCCCA CTTCGGTACA

GGCCATGGCT T--------- ---------- ---------- ----------

GGCCATGGCC TTTGACCGTT TTGTGGCCAT CTGTGATCCA CTGAGATACA

GGCCATGGCC TTTGATCGTT TTGTAGCCAT CTGTAATCCA CTGAGATACA

GGCCATGGCC TTTGATCGTT TTGTGGCCAT CTGTGACCCA CTGAGATATA

ATCCATGTCT ATCGACCGCT ATGTTGCCAT CTGCAACCCG CTGCGTTATT

GGCCATGGCT TTTGACCGCT ATGTGGCTAT CTGCCACCCA CTTCATTATA

TGCCATGGCT TATGACCGCT ATGTAGCTGT TTGTAACCCT CTTCGCTATA

TGGCATGGCT TATCGCTGCC ATGTAGCTGT GTGTACAATC ATTCACTATA

GGCCATGGCC TATGACCGCT ATGTGGCCAT ATGCTCTCCA CTGAGATACA

GGCCATGGCC TTTGACCGCT TCGTGGCCAT CTGCCGCCCA CTACACTACT

GCGCCATCCT CACCAACAAG GTTGTTTCTG TGATTGGTCT TGGTGTGTTT

GCGCCATCCT CACCAACAAG GTTGTTTCTG TGATTGGTCT TGGTGTGTTT

TAGCCATCCT AACCAATGAT GTAATCATTG GGATTGGGTT GGCAATTGCT

CAGCCATCCT AACCAATGAT GTAATCACTG GGATTGGGTT GGCAATTGCT

GCACCATTCT CACCACCAAG GTCATTTCTG TGATTAGTCT TGGTGTGCTT

GTACCATTCT CACCAATAAG GTTGTTTCTG TGATTGGCCT TGGCGTATTA

GCACCATTCT CACCAACAAG GTGGTTTCTA TAATCGGCAT TGTTGTGCTA

GCACCATTCT CACCAATAAG GTCGTTTCTA TGATTGGCAT TGCTGTGCTA

---------- --CCAATAAG GTTGTTTCTG TGATTGGTCT TGGTGTGTTA

CCACCATCCT CACCAATGCC AGGATCGCCC AGATTGGGAT GAGCATGTTG

CCACCATCCT CACCAATGCC AGAATTACCC AGATTGGGGT GAGCATGTTG

CCACAATCCT CACCAATGCC AGGATTGCCC AGATTGGGGT GAGCATATTG

CCACCGTCTT GACCAATGCC AGGATCATCC AGATCGGGCT GACCATGTTG

TATCCATCCT GAACGCCACT GTGATTGGAA AGATTGGTCT AGCATGTGTC

GCACAATCCT TACTGACAAG ATAGTATCTC TCATTGGTTT TGGAGTAATG

ACACAATCCT TACTAACAAG ACTGTATCTT TCACTGG-TT TACAGTAATT

CCACCATTCT GACCCACCAG GTCATCACTA AGATTGTGGT GGGAATCATC

CGTCCATCCT CAGCCCCGGT GTCATTGGAA AGATCACAGT GGCCTGCATA

GTGAGGGCTT TAATTTTCGT CATTCCCTCT ATACTTCTTA TATTGCGGTT

GTGAGGGCTT TAATTTTCGT CATTCCCTCT ATACTTCTTA TATTGCGGTT

GGAAGGGCCT TGGCTCTGGT CTTTCCAGCT TCCTTGCTCT TGAAGAAGCT

GGAAGGGCCT TGGCTCTGGT CTTTCCAGCT TCCTTCCTCT TGAAGAAGCT

GTGAGGGCTT TTATTTTTGT CATTCCCTTT ATATTTCTCA TTTTGAGGTT

GTGAGGGCAT TTATTTTTGT CATCCCATTT GTATTTCTTA TTTTGCGACT

GTGAGGGCAA TTATCTTTGT TATTCCATTT ATATTTCTAA TTTTGAGACT

GTGAGGGCAA TTATCTTTGT TATTCCATTT ATATTTCTAA TTTTGAGACT

GTGAGAGCAA TTATTTTAGT CATTCCATTT GTATCTCTCA TACTGAGATT

ACAAGAAATG TTGCTGTGAT GTTGCCAGTT GTGCTTTTTG TCAAGAGGCT

ATAAGGAACG TTGCTGTCAT GTTACCAGTG GTGCTCTTTG TTAAGAGGCT

ATAAGGAATG TTGCTGTCAT GCTGCCGGTT GTGCTGTTTG TCAAGAGGCT

ATAAGGAATG TTGCTGTCAT GCTACCAGTT GTGCTTTTTG TCAAGAGGCT

TTCCGAGGCC TTCTCTTTGT TTTCCCCTTT GTCATCCTCA TTGAACGCTT

GGGAGATCTT TCCTCTGTGT ATTCCCCCTT GTATTTCTCA TCCTGAGATT

GAGCAATCTT CTGTCTCTCT AGTCCCATTG GTATTTCTAA TGCTG---TT

CTCAGGAGCT TCTGTGTCAT CTTGCCAATT GTTTTCTTGC TAAAACGACT

TCCCGAAGCC TAATCGTTGA CCTCCCGTTC ATCATCCTGA TCCGCAGATT

GCCCTTCTGT GGGAATCATG TAATTCCCCA CACCTACTGT GAGCACATGG

GCCCTTCTGG GGGAATCATG TCATTCCCCA CACCTACTGT GAGCACATGG

TCAATATCAT GATGTCAATA TTCTGTCCTA CCCTTTCTGC CTGCACCAGG

TCAATATCAT GATGTCAATA TTCTGTCCTA CCCTTTCTGC CTGCACCAGG

GCCCTTCTGT GGAAATCATA TCATTCCCCA CACCTATTGT GAGCACATGG

ACCCTTCTGT GGGAATCATG TCATTCCCCA TACCTACTGT GAGCACATGG

GCCTTTCTGT GGGCATCACA TCATTCCCCA TACATACTGT GAGCACATGG

GCCCTTCTGT GGGCATCACA TCATTCCCCA TACCTACTGT GAGCACATGG

ACCTTTCTGT GGGAATCACG TCATTCCCCA CACCTACTGT GAGCACATGG

GTCCTTCTGC AGTTCTATGG TCCTCTCACA TTCTTACTGC TACCATGTTG

GTCCTTCTGC GGTGCAGTGG TCCTTTCACA TTCTTACTGT TACCATGTTG

GTCCTTCTGC AATTCTATGG TCCTTTCACA TTCTTACTGC TACCATGTTG

GGTCTTCTGC AGGTCCTTGG TTCTTTCACA TTCCTACTGC TACCATGTCG

ACCCTTCTGT GGACATCACA TTATCCCCCA CACGTACTGT GAACACATGG

ACCTTTCTGT GGGCACTATG TCATCCCCCA CACCTACTGT GAGCACATGG

GCATGTCTGT GGC---GCTG TCAACCCCAA CACATATTGT GAACACTATG

ACCCTTCTGC CGAACACGCA TCATCCCCCA TACATACTGT GAACACATAG

ACCATTCTGT GGGCATCAAG TGATCTCCCA CACCTACTGT GAGCACATGG

GTCTTGCTCA TCTATCTTGT GCCAGCATCA AAATCAATAT TATTTATGGT

GTCTTGCTCA TCTATCTTGT GCCAGCATCA AAATCAATAT TATTTATGGT

ACCTCATAAA GACAACTGTA TCCAACCGTC AAGTCAGCAG CATCTATGGC

ACCTCATAAA GACAACTGTA TCCAACCGTC AAGTCAGCAG CATCTATGGC

GTCTTGCTCG TCTGTCTTGT GCCAGCATCA AAATCAATAT TATTTATGGT

GTCTTGCTCG TCTGTCTTGT GCTAGCATCA AGATCAATAT CATCTATGGC

GTCTTGCACG ACTGTCTTGT GCCAGTGTCA AAGCTAATGT AATTTATGGC

GTCTTGCACG GCTGTCTTGT GCAAGTGTCA AGGCCAATGT AATTTATGGC

GACTTGCTCG TCTGTCTTGT GCCAGTATCA AGATCAATAT TATTTATGGC

ATCTCATTCA ACTCTCATGC ACAGACAACA GAATCAACAG CATCCTTGGT

ACCTCATTCA ACTCTCATGC ACAGACAACA GAATCAACAG CATCCTCGGT

ATCTCATTCA ACTCTCATGC ACAGACAACA GAATCAACAG CATCCTTGGT

ATCTTATTCA ACTCTCATGC ACAGATAACA GAATCAACAG CATTCTGGGT

GCATTGCCAA GCTGGCCTGT GCCAGCATCA AGTCCAACAC TATTTATGGT

GTCTTTCTCG CCTGGCCTGT GCCAGTATCA GGGTCAACAT TATATATGGG

ATATTGCTCA TCTTCCCTGT GCCAGTATAA GGACACACGT CATTTCTGGG

GTGTAGCCCG ACTCTCTTGT GCTGACATCT CCATCAACAT TTGGTATGGT

GCATTGCTAA ACTGGCTTGT GTCAACATCA AGATCAACAT CATCTACGGC

TTATGTGCCA TTTGTAATCT AGTGTTTGAC ATCACAGTCA TTGCCCTTTC

TTATGTGCCA TTTGTAATCT AGTGTTTGAC ATCACAGTCA TTGCCCTTTC

TCATGGGTCA TCTGTTCCAT GGGACTTGAT TCAGTGCTTC TCCTCCTATC

TCATGGGTCA TCTGTTCCAT GGGACTTGAT TCAGTGCTTC TCCTCCTATC

TTATGTGCCA TTTGTAATCT AGTGTTTGAC ATCACAGCCA TTTCCCTTTC

TTGGGTGCCA TTTGTAATCT ACTGTTTGAC ATCGTAGCCA TTGCCCTTTC

TTGTGTGCCA TATGCAATCT ACTGTTTGAC ATAGTAGCAA TTGCTCTATC

TTATGTGCCA TATGTAATCT ACTGTTTGAC ATAGTAGCAA TTGTTCTTTC

TTATGTGCCA TCGGTAATCT GGTGTTTGAT ATTATAGCCA TTTTCCTTTC

CTGTTTGCCT TCTCCACGAC AGGGTTTGAC TGTCCTTGTA TCTTGCTCTC

CTGTTTGCTT TCTCCACAAC AGGATTTGAT TGTCCTTGCA TCTTGCTCTC

CTGTTTGCCT TCTCCACAAC AGGATTTGAC TGTCCTTGCA TCTTGCTCTC

CTGATTGCCT TCTCCACAAC AGGATTTGAC TGTCCTTGCA TCTTGATCTC

CTGGTGGCAC TTTCAGTCAC TGGCGTGGAT GTGGTCCTCA TTGCTACCTC

CTGGTAACCA TTGCTATCTT GGGGTTTGAC ATCACGGCCA TTGCCCTTTC

CTGGCAGCAA TTGCTCTTTC AGAAGTTAAT GTCATAGTCA TGATC-----

GTCTGTGCCA CTTATACTGT AGTCTCTGAC ATTGTCCTCA TTGCTGTCTC

CTGGTTGCAC TCTTGGTCAC GGGGGTTGAT GTGGTGCTCA TTGGCTTTTC

TTATGTGCAT ATTCTTTGTG CTGTTTTCCG TCTTCCTACT CATGAAGCCC

TTATGTGCAT ATTCTTCGTG CTGTTTTCCG TCTTCCTACT CATGAAGCCC

CTATGTCCTC ATCCTGGGCA CAGTGTTGAG TATAGCCTCC AAGGCAGAGA

CTATGTCCTC ATCCTGGGCA CAGTGTTGAG TATAGCCTCC AAGGCAGAGA

TTATGTGCAG ATACTTCATG CTGTTTTCCG TCTTCCTTCC CGTGAAGCCC

GTATGTGCAG ATACTCCAAG CAGTTTTCCG TCTTCCTTCC CATGAAGCCC

TTACATACAA ATATTACGTA TTGTGTTTCA TCTCCCTTCT CTGGAAGCCC

TTACATACAA ATATTACGTG TTGTGTTTCA TCTCCCTTCT CGGGAAGCCC

TTATGTTCAG ATCCTCCGTG CTGTTTTTCG TCTTCCTTCC CAGGAAGCGC

CTATGTCCTG ATCATCCGAT CTGTCCTCAG CATCACCTCC ATAGAGGGCA

CTATGTACTG ATCATCCGGT CTGTTCTCAG CATTGCCTCC TCAGAGGGGC

CTATGTTCTG ATCATCCAAT CTGTCCTCAA CATTGCTTCC TCAGAGGGAC

CTATGTCCTT ATTATTCGTT CCGTCCTTGG CATTGCTTCC TCAGAGGGAC

CTATGGCCTG ATCCTGAGGG ATGTGCTGCG CTTGCCCTCT AAAGATGCTC

CTATGTCCAG ATTCTCTATG CTGTTTTCCG TCTTCCCTCC TATGAGGCCA

--ACTTCCGG AGTGGC---A CAGTTGTCTG TCTTCCCTTC TTTGAGACCA

TTATACCATT ATCCTGAGTG CAGTCTTTCG CCTCCCTTCT CGTGGTGCCC

CTATGGCTTC ATCCTACATG CTGTGCTCCA CCTACCCACC CAGGATGCAC

GACTCAAGTC CCTCAGCACA TGTGGTTCAC ATGTGTGTGT AATCCTTGCC

GACTCAAGTC CCTCAGCACA TGTGGTTCAC ATGTGTGTGT AATCCTTGCC

GAGTGAGAGC CCTCAATACT TGCATCTCCC ACATCTATGC TGTACTCACC

GAGTGAGAGC CCTCAATACT TGCATCTCTC ACATCTGTGC TGTACTCACC

GACTCAAGTC CCTCAGCACA TGTGGTTCAC ACGTGTGTGT AATCCTTGCC

GACTCAAGTC CCTCAGCACA TGTGGTTCTC ACGTTTGTGT AATCCTTGCC

GGCTTAAGTC CCTCAGCACT TGTGGTTCTC ATGTCTGTGT CATCCTTGCC

GGCTCAAGTC CCTCAACACT TGTGGTTCTC ATGTCTGTGT CATCCTTGCC

GACTCAAGTC CCTCAGCACA TGTGGTTCTC ATGTTTGTGT AATCCTTGCC

GGCAGAAAGC CTTCAACACC TGCATTTCCC ACATCAGTGC TGTTGCCATC

GGCAAAAAGC CTTCAACACC TGCATATCCC ACATCAGTGC TGTTGCCATC

GGCAGAAAGC CTTCAATACT TGCATATCGC ACATCAGTGC TGTTGCCATC

GGCGGAAAGC CTTCAACACT TGCATATCTC ACATCAGTGC TGTTGCTATC

AGTTCCGAGC ATTTAGCACA TGTGGAGCTC ACATTTGTGT GATTCTTGTT

GACTCAAGTC TCCCGGTACC TGTGGTTCCC ATGTCTGTGT AATTTTAGCA

GACTCGAGTC TCTTCGTACT TGTGGTTCCC ATGTTTGTGT AATTTTGGCC

GCCAGAAGGC TTTGGGAACC TGTGGATCTC ATGTCTGTGT CATCCTCATT

AACGCAAGGC CTTCAGCACC TGTGGGGCCC ATGTATGTGT TATCTTGGTC

TTCTATACAC CAGCCCTCTT TTCCTTTATG ACTCATCGCT TTGGCCGAAA

TTCTATACAC CAGCCCTCTT TTCCTTTGTG ACTCATCGCT TTGGCCGAAA

TTCTATACAC CAATGATTGG GCTATCTATG ATCCACCGCT ATGGACAGAA

TTCTATACAC CAATGATTGG GCTATCTATG ATCCACCGCT ATGGACAGAA

TTCTATACCC CAGCCCTCTT TTCCTTTATG ACTCATCGCT TTGGCCAAAA

TTCTATACCC CAGCCCTCTT TTCCTTCATG ACACATCGCT TTGGTCGAAA

TTCTACACAC CAGCCCTCTT TTCCTTCATG ACTCACCGCT TTGGTAGGAA

TTCTACACAC CAGCCCTCTT TTCCTTCATG ACACATCGCT TTGGTAGGAA

TTCTATACAC CAGCCCTCTT CTCCTTTATG ACACATCGCT TTGGTAGAAA

TTCTACATCC CTCTCATCAG CCTGTCTCTT GTCCATCGCT ATGGCCATTC

TTTTACATCC CCCTTGTCAG CTTGTCTCTT GTCCATCGCT ATGGCCATTC

TTCTACATCC CTCTCATCAG CTTGTCTCTT GTCCATCGCT ATGGACATTC

TTCTACATCC CTCTCATCAG CTTGTCTCTT GTCCATCGCT ATGGCCATTC

TTCTATATCC CTGCCTTTTT TTCCTTCTTT ACCCACCGCT TTGGCCACAG

TTTTACACTC CAGCCTTCTT CTCTTTTATG ACTCACCGCT TTGGCCGAAA

ATTTACACTC GAATTCTCCT CTCATTTGTA ACCAACCTCT TGGGT---AA

TTCTATACCC CTGCCTTCTT CTCAGTCCTT GCTCATCGCT TTGGACATGA

TTCTATATCC CAGCTTTCTT CTCCTTCTTC ACCCACCGCT TTGGTCACTG

TGTGCCCCGC TATATCCATA TACTCCTAGC CAATCTCTAT GTTGTGGTGC

TGTGCCCCGC TATATCCATA TACTCCTAGC CAGTCTCTAT GTTGTGGTGC

TGCTTCCCCA ATTGTCCATG TGCTGATGGC CAATGTCTAC TTGCTGGTTC

TGCTTCCCCA ATTGTCCATG TGCTGATGGC CAATGTCTAC TTGCTGGTTC

TGTTCCCCAC TATATCCATA TACTCCTAGC CAATCTCTAT GTTGTGGTGC

TGTGCCCCGC TATATCCACA TACTCCTGGC CAATCTTTAT GTTGTAGTGC

TGTGCCTCGC TATATACACA TACTTCTGGC CAACCTTTAT GTTGTAGTGC

TGTACCTCGT TATATACATA TACTTCTGGC CAACCTTTAT GTTGTAGTGC

TGTTCCCCGC TATATCCATA TACTTTTAGC TAATCTTTAT GTTGTAGTGC

AGCACCTCCC TTTGTCCACA CCATCATGGC CAATATCTTC CTGCTTATTC

AGCACCTCCA TTTGTCCACA CCATCATGGC CAATGTCTTC CTTCTCATCC

AGCACCTCCA TTTGTCCACA CCATCATGGC CAATGTCTTC CTGCTCATCC

GGCACCTGCA TTTGTCCACA CTATTATGGC CAATGTTTTC CTGCTTATTC

AGTACCTCCC CAGGTCCACA TTATACTTGC AAATCTTTAT CTTCTTGTGC

TGTTCCCCAT TACATCCACA TCCTTCTGGC CAATCTGTAT GTAGTAGTGC

TATTCTATAT TACTGCTA-A TCCTTTGGGC CAATCCGTAT GTAGTGGTGC

TGTCCCACGT CATGTCCTCA TCCTATTTGC CAACTTTTAT GTTGCCATTC

GGTGCCACCT CAGGTTCACA TTTTCGTGGC CAACCTCTAC CTCCTGGTGC

CACCAATGCT CAATCCTGTC ATATATGGAG TCAGAACCAA GCAGATCAAA

CACCAATGCT CAATCCTGTC ATATATGGAG TCAGAACCAA GCCGATCAAA

CACCTCTCAT GAACCCCATT GTCTACAGTG TTAAGTCCAA GCAGATTAA-

CACCTCTTAT GAACCCCATT GTCTACAGTG TTAAGACCAA GCAGATTAA-

CACCAATGCT CAATCCTGTT ATATATGGAA TCAGAACCAA ACAGATCAAA

CACCAATGCT CAATCCTGTC ATCTATGGGG TCAGAACCAA ACAGATCAAA

CACCAATGCT CAATCCTGTC ATATACGGGG TGAGAACCAA ACAAATCAAA

CACCAATGCT GAATCCTGTC ATATATGGGG TGAGAACCAA ACAAATCAAA

CACCGATGCT CAATCCTGTC ATATATGGAG TCAGAACCAA GCAAATTAAA

CTCCTGTACT CAACCCTATC ATCTACAGTG TGAAGACTAA GCAGATTAAG

CTCCCGTGCT CAACCCCATC ATCTATAGTG TGAAGACTAA GCAGATTAAG

CTCCTGTGCT CAACCCTATC ATCTACAGTG TGAAGACGAA GCAGATTAAG

CTCCTGTGCT CAACCCTATA ATCTATAGTG TGAAGACTAA ACAGATTAAG

CCCCTGTTCT CAACCCCCTG GTCTATGGCA TTAATACTAA ACAGAT--AA

CACCGATGCT CAATCCTGTT ATCTATGGAG TCAGAACCAA GCAGATTAAA

CACCGGTGCT CCACTCTGCT ATCTATGAAG TCAGAG-CAA GCA----AAA

CTCCAGCTCT CAATCCTATT GTCTATGGAG TAAAGACAAA GCAAATTGAC

CCCCCGTGCT CAACCCGCTG GTGTATGGCA TTAACACAAA GCACATCACT

ATATTGCCCT TCATTCTGGT AGGGATCCCA GGCCTGGAGC AATTCCATGT

ATATTGCCCT TCATTCTGGT AGGGATCCCA GGCCTGGAGC AATTCCATGT

---TTCCCCT TCATTCTGGT AGGGATCCCA GGCCTGGAGC AATTCCACAT

---TTCCCCT TCATTCTGGT AGGGATCCCA GGCCTGGAGC AATTCCACAT

ATATTGACCT TCATTCTGGT AGGGATCCCA GGCCTGGAGC AATTCCACGT

ATGTTGCCCT TCATTCTGGT GGGGATCCCG GGCCTGGAGC AGTACCATGT

ATGTTACCCT TCCTTTTGGT GGGGATCCCA GGCTTGGAGC AATTCCATGT

ATATTGCCTT TCCTTTTGGT GGGGATCCCA GGCTTGGAGC ACTTCCACGT

TTAT-ACTGT TCACTCTTGT GGGTATCCCA GGCCTGGAAC AATTCCATGT

GTCATT-CAT TCCTGTTGAT TGGGATCCCA GGGCTTGAGG GTGTGCACAT

GTCATT-CCT TCCTGTTGAT TGGAATCCCA GGGCTTGAGC ATGTGCACAT

ATCATT-CAT TCCTGTTGAT TGGGATCCCA GGGCTTGAGC ACGTGTACGT

GTTATG-CCT TCCTACTACT TGGCATTCCA GGATTGGAAG CTTGCCACAT

ATAATGTCTT TTATCCTCAT TGGGATACCT GGCCTGGAGA AGTTCCACAT

ATAATGCCAT TCATCCTATT GGGAATCCCA GGGTTGGAGC ACTTCCACAT

ATAATAGCCT TCACCCTTTT GGGCATCCCT GGACTAGAAC TGTATCATAT

ATCCTATTCT TTATCTTAGT GGGGATCCCA GGGCTTGAGA AGTTCCACAT

GTATTG-AGT TCATCCTAAT GGGTTTCCCG GGCATTCACA GCTGGCAGCA

GTGGATTGGA ATTCCCTTCT GTATCATCTA CATTGTAGCT GTTGTGGGAA

GTGGATTGGA ATTCCCTTCT GTATCATCTA CATTGTAGCT GTTGTGGAAA

GTGGATTGGA ATTCCCTTCT GTATCATCTA CATTGTAGCT GTTGTGGGAA

GTGGATTGGA ATTTCCTTCT GTGTCATCTA CATTGTAGCT GTTGTGGGAA

GTGGATTGGG ATTCTTTTTT GTATCATCTA CATTGTAGCT GTTGTGGGAA

GTGGATTGGG ATTCTCTTCT GTATCATCTA CATTGTAGCT GTTATGGGAA

GTGGATTGGC ATTCCCTTCT GTGTGATCTA CGTCATAGCT ATGACGGGAA

GTGGATTGGC ATTCCCTTCT GTGTGATCTA CATCATAGCT GTGGTGGGAA

GTGGATTGGG ATTCCCTTCT GCATTGTCTA CATTGTAGCT GTTATGGGAA

TTGGATCTCC ATCCCTATCT GCCTCATGTA CCTCATGGCT ATCCTGGGAA

TTGGATCTCC ATCCCCATCT GCTTCATGTA CCTCCTGGCC ATCCTGGGCA

TTGGATCTCC ATCCATATCT GCCTCATGTA TCTCCTGGCC ATCCTGGGCA

ATGGATTTCC ATCCCTTTCT GCCTGGTATA CCTACTGTCA CTGATAGGAA

CTGGATTGGG ATTCCCTTTT GTGTCATCTA TATTGTGGCT ATGGTGGGGA

CTGGATCGGG ATTCCCTTCT TCATTATCTA CCTCGTGGCC CTTGCAGGCA

CTGGATCAGC ATCCCTTTCT GCTTAATCTA TCTTGTGGCC ATTGTGGGCA

CTGGATTGCA ATTCCCTTCT GCACTATCTA CCTTGTGGCC ATTGTGGGTA

CTGGCTCTCC CTTCCATTGG CCCTTCTCTA TCTCTCAGCC CTTTTGGCCA

ACTGCATCCT TCTCTACCTC ATTGTGGTGG AGCATAGTCT TCATGAACCC

ACTGCATCCT TCTCTACCTC ATTGTGGTGG AGCATAGTCT TCATGAACCC

ACTGCATCCT TCTCTACCTA ATTGTGGTGG AGCATAGTCT TCATGAACCC

ACTGCATCCT TCTCTACCTC ATTGTGGTGG AGCATAGTCT TCATGAACCC

ACTGCATCCT TCTCTACCTC ATTGCAGTGG AGCATAGTCT TCACGAACCC

ACTGCATCCT TCTCTACCTC CTTGCGGTGG AACGTAGCCT TCACGAACCC

ACTGTGTCCT TCTCTACCTC ATCGTAGCGG AGCGGAGCCT TCACGAGCCC

ACTGTATCCT TCTCTACCTC ATCACAGTGG AGCGGAGCCT TCATGAGCCC

ACTTCATCCT TCTTTACCTC ATTGCAGTGG AGTGCAGCCT TCATGAACCC

AC--CACCAT TCTATTTGTC ATCAGGACAG AGCCTTCTCT GCATGAGCCC

ACTGCACCAT CCTATTTGTT ATCAGGACAG AGCCTTCTCT GCATGAGCCT

ACTGCACCAT TTTATTTGTC ATTAGGACAG AGCCTTCCCT TCAGGAGCCC

ATGTTGGCCT TTTAATAATT ATCAAGACAG ACCACAAACT GCATGAACCT

ATTGCCTCCT CCTGTACCTC ATTGCAGTTG AGCGTAGCCT CCATGAACCC

ACAGTGTCCT TCTCTATCTT ATTTCCACGG AGCGCAGCCT CCATGAGCCC

ATAGTATCCT TCTCTACCTT ATTGCAGTGG AGCGCAGTCT TCATGTACCT

ACTGTGTCCT GCTGTACCTC ATTGCTGTGG AGCACAGCCT CCATGAGCCC

ATGCCATGAT CCTGCTCACT ATATGGCAGG AAATGGTTCT GCACCAGCCT

ATGTTCTTCT TTCTCTCCAT GCTGGCCATG ACTGACCTCA TCTTGTCCAC

ATGTTCTTCT TTCTCTCCAT GCTGGCCATG ACTGACCTCA TCTTGTCCAC

ATGTTCTTCT TTCTCTCCTT GCTG-CCATG ACTGACCTCA CCTTGTCCAC

ATGTTCTTCT TTCTCTCCTT GCTG-CCATG ACTGACCTCA CCTTGTCCAC

ATGTTCTTCT TTCTCTCCAT GCTGGCCATG ACTGACCTCA TCCTGTCCAC

ATGTTCTTCT TTCTCTCCAT GCTGGCCATG ACTGACCTCA TCTTATCCAC

ATGTTTTTTT TCCTGTCCAT GCTGGCATTG ACCGATCTCA TCTTGTCCAC

ATGTTTTTTT TTCTGTCCAT GCTGGCAATG ACTGATCTCA TCCTGTCTAC

ATGTTCTTCT TTCTCTCCAT GCTGGCCATG ACTGACCTCA TTTTGTCCAC

ATGTACCATT TCCTCTCCAT GCTGGCATTA TCGGACCTGG GCCTGTCTTT

ATGTACTATT TCCTCTCTAT GCTGGCCCTA TCTGACCTGG GCTTGTCTTT

ATGTACTATT TCCTCTCCAT GCTGGCCATC TCTGACCTGG GTTTGTCTTT

ATGTACCTCT TTCTATGCAT GCTCTCTGTG GTTGATTTGA TGCTTACTTC

ATGTTTTTTT TCCTCTCTAT GCTGGCCACA ACCGATCTCA TCTTGTCCAC

ATGTTCTTTT TCCTTTCTAT GCTGGCTGCT ACAGATCTCA TTCTGTCTAA

ATGTTCTTTT TCCTTTCCAT GCTGGCCATT ACTGACCTCA TATTATCTAC

ATGTTCTTTT TCCTGTCCAT GCTGGCCAAA ACTGATCTCA TTCTGTCATC
[truncated: 2,128,681 more chars]
