# Supplementary material for: Early History of Mammals Is Elucidated with the ENCODE Multiple Species Sequencing Data
Source: PLoS Genet. 2007 Jan 5;3(1):e2. doi: 10.1371/journal.pgen.0030002 (PMC1761045; doi:10.1371/journal.pgen.0030002)
Supplement: Supplementary file 2 [file pgen.0030002.sd002.doc]

18 429675

human	TGCCTAGTTA ATGAAGGAGT TCCCCCTTCT GAGTATGCAA TATCCATGAA

chimp	TGCCTAGTTA ATGAAGGAGT TCCCCCTTCT GAGTATGCAA TATCCATGAA

baboon	TGCCTAGTTA ATGAAGGAGT TCCCCCTTCT GAGCATGCAA TATCCATGAA

macaque	TGCCTAGTTA ATGAAGGAGT TCCCCCTTCT GAGCATGCAA TATCCATGAA

marmoset	TGCCTAGTTA ATGAAGGAGT TCCCTCTTCT GAGCATGCAA TATCCATGAA

galago	TGCTCAGTTA ATGAAGGAGT TCCCCCTTCT GAGCATGCAA TATCCATGAA

rat	TGCCTAGTTA ATGAAGGAGT TTCCCCTCTT AAGCATGCAA TATCCATGAA

mouse	TGCCTAGTTA ATGAAGGAGT TTCCCCTCCT AAGCATGCAA TATCCATGAG

rabbit	TGTCTAGTTG ATGAAGGAGT TCCCTCTGCT GAGCATGCAA CATCCATGAA

cow	TACCTAGTTA ATGAAGGAGT TCCCTCTCCT GAGCATGCAA CATCCATGAA

dog	TACCTAGTTA ATGAAGGAGT TCCCCTTGCT GAGCATGCAA CATCCATGAA

rfbat	TGCCTAGCTG ATGAAGGAGT TCCCCTTGCT GAGCATGCAA CATCCATGAG

shrew	TGCCTAGTTG ATGAAGGAGT TTCCCCTTCT GAGCATGCAA TATCCATGAA

armadillo	TTCCTAGTTA ATGAAGGAGT TCCCTCTCCT GAGCATGCAA TATCCATGAA

elephant	TTCCTAGTTA ATGAAGGAGT TCCCTCTCCT GAGCATGCAA CATCCACGAA

tenrec	TTCCCAGTTA ATGAAGGAAT TCCCTCTCCT GAGCATGCAA TATCCATGAA

monodelphi	TTCTTAGCTA ATGAAGGAAT TTCCTCTCCT GAGCATGCAA CATACATGAA

platypus	CGCCTAGTTA ATGAAGGAGT TTCCTCTGCT GAGTATGCAA CATTCATGAA

AACCTTTTAG AAGCCCTTCT GGAACTACAA GCATATGCTG ATGTTCAGGC

AACCTTTTAG AAGCCCTTCT GGAACTACAA GCATATGCTG ATGTTCAGGC

AACCTTTTAG AAGCCCTTCT GGAACTACAA GCATATGCTG ATGTTCAGGC

AACCTTTTAG AAGCCCTTCT GGAACTACAA GCATATGCTG ATGTTCAGGC

AACCTTTTAG AAGCTCTTTT GGAACTACAA GCATATGCTG ATGTTCAGGC

AACCTTCTGG AAGCCCTTCT GGAACTGCAA GCGTATGCTG ATGTTCAAGC

AACCTTCTAG AAGCTCTTCT GGAACTCCAA GCCTATGCGG ATGTTCAGGC

AACCTTCTAG AAGCTCTTCT GGAACTCCAA GCTTATGCTG ATGTTCAGGC

AACCTGTTAG AAGCGCTTCT GGAGCTACAA GCGTATGCTG ACGTCCAGGC

AACCTTTTAG AGGCTCTCCT GGAACTACAA GCATATGCTG ATGTTCAGGC

AACCTTTTAG AAGCCCTTCT GGAACTACAA GCATATGCTG ATGTTCAGGC

AACCTTCTGG AAGCCCTCCT GGAGCTGCAG GCGTACGCGG ATGTCCAGGC

AACCTTTTAG AAGCCCTTCT GGAACTCCAA GCATATGCTG ATGTTCAGGC

AACCTTTTAG AAGCCCTCTT GGAACTGCAG GCATATGCTG ATGTCCAGGC

AACCTTTTAG AAGCCCTCCT GGAACTGCAA GCATATGCTG ATGTTCAGGC

AATCTCTTAG AAGCCCTCCT GGAGCTCCAA GCATATGCCG ATGTCCAGGC

AACCTTTTAG AAGCTTTACT GGAACTGCAA GCGTATGCTG ATGTTCAAGC

AACCTGCTGG AGGCCCTGCT GGAACTGCAG GCCTACGCCG ACGTCCAGGC

AGTCTTAGCA AAGTATGATG GTAAGTTCTC CATTTGTTAC AGAAATCATC

AGTCTTAGCA AAGTATGATG GTAAGTTCTC CGTTTGTTAC AGAAATCATC

AGTCTTAGCT AAGTATGATG GTAAGTTCTC TGTTCATTAC AGAAATCATC

AGTCTTAGCT AAGTATGATG GTAAGTTCTC TGTTCATTAC AGAAATCATC

AGTCTTAGCA AAGTATGATG GTAAGTTCTC AGTTCATTAC AGAAATCATC

AGTCCTAGCA AAGTATGATG GTAAGTTCTC AGTTCATTAC AGAGCTCACC

AGTCTTAGCA AAGTATGATG GTAAGTTCTC AGTTCATTAC AGAAATCATC

AGTCTTAGCA AAGTATGATG GTAAGTCCTC AGTTCATTAC AGAAATCATC

CGTCTTAGCA AAGTATGATG GTAAGTTCTC AGTTCATTAC AGAAATCATC

AGTCTTAGCA AAGTATGACG GTAAGTTCTC AGTTCATTAC AGAAATCATC

AGTCTTAGCA AAGTATGATG GTAAGTTCTC AGCTCATTAC AGACATCATC

CGTGCTGGCG AAGTATGACG GTGAGTTCTC AGTTCATTAC AGAAATCATC

AGTCTTAGCA AAGTATGATG GTAAGT---- --TCCATTAA AGAAACCAAC

AGTCTTAGCC AAGTACGATG GTAAGTTCTC AGTTCATTAC AGAAATCATC

AGTCTTAGCA AAGTATGATG GTAAGCTCTC AGTTCATTAT AGAAATCATC

CGTCTTGGCC AAGTACGATG GTGAGTTCTC AGCTCATTGC AGAAATCATC

AGTTTTAGCA AAATATGATG GTAAGTTCTC AGCTCATTAT AAAACTCATC

CGTTTTAGCC AAATATGACG GTGAGTACCC AGCTCATTAA AGAGCTTGTC

AGCCTCGTGT TATAAGACTG ATTGTAAAAT AAAGAAATGG AGCTGATTTA

AGCCTTGTGT TATAAGACTG ATTGTAAAAT AAAGAAATGG AGCTGATTTA

AGCCCTGTGT TATAAGACTG ATTGTAAAAT AAAGAAATGG AGCTGATTTA

AGCCTTGTGT TATAAGACTG ATTGTAAAAT AAAGAAATGG AGCTGATTTA

AGCCTTATAT TATAAGACTG ATTGTAAAAT AAAGAAATGG AGCTGATTTA

GGCCTCGTGT TGTAAGACTG ATTGTAAAAT AAAGAAATGG AGCTGATTTA

AGCCTTGTGT TATAAAACTG ATTGCCAAGT AAAGAAATGG AACTGATTTA

AGCTTTGTGT TATAAAACGG ATTGCCAAGT AAAGAAATGG AACTGATTTA

AGCCTTGTGT TCTAAGACTG ATTGTAAAAT AAAGAAATGG AGCTGATTTA

AGCTTTGTGT TACAAGACTG ATTGTAAAAT AAAGCAGTGG AACTGATTTA

AGCTTTGTGT TATAAGACTG ATTGTAAAAT GGAGAAATGG AGCTGATTTA

AGCTTTGTAG TAT--GACAG ATTGTAAAAT AAAGAAATGG AGCTGATTTA

AGCCTTGTGT TCTAAGAATG ATTGTAAAAT AAAGAAATGG AGCTGATTTA

AAGCTTGTGT TATAAGACTC ATTGTAAAAT AAAGAAATGG AGCTGATTTA

AGCCTTGTGT TATAAGACTG ATTGTAAAAT AAAGAAATGG AGCTGATTTA

AGCCTTGTGT GCTCAGACTG ATTGTAAAAT AAAGAAATGG AGCTGATTTA

AGCTTTGTGT TGTAAGATTG ATTGTAATAT AAAGAAACAT AACTGATTTA

TGCTCTGTGC CATGAGATAA ATTGTAAAAT AAAGCAGtag gTTTGATTTA

TTAGATTCTG ATAACATATG TTCACATTAT TTGTTACAAC AGCGTCCCAC

TTAGATTCTG ATAACATATG TTCACATTAT TTGTTACAAC AGCGTCCCAC

TTAGATTCTG ATAACATATG TTCGCATCAT TTGTTACAAC AGCGTCCCAC

TTAGATTCTG ATAATATATG TTCGCATCAT TTGTTACAAC AGCGTCCCAC

TTAGATTCTG ATAACATATG TTCACATTAT TTGTTACAAC AGCGTCACAC

TTAGATTCTG ATAACATATG TTCACATTAT TTGTTACAAC AGCGTCCCAC

TTAAATTCTG ATAACATATG TTCACATTAC TTGTTATAAC AGCGTCCCAC

TTAAATTCTG ATAACATATG TTCGCATTAC TTGTTACAAC AGCGTCCCAC

TTAGATTCTG ATAACATATG TTCCCATTAT TTGTTACAAC AG-GTCCCAC

TTAGATTCTG ATAACATATG TTCACATTAT TTGTTACAAG AGCGTCCCAC

TTAGATTCTG ATAACATATG TTCACATTAT TTGTTACAAC AGCGTCCCAC

TTAGATTCCG ATAACATATG TTCCCATTAT TTGTTACAAT AGCGTCCCAC

TTAGATTCTG ATAACATATG TTCACATTAT TTGTTACAAC ATCGTCCCAC

TTAGATTCTG ATAACATATG TTTGCACGGT TTGTTACAAC AGCATTCCAC

TTAGATTCTG ATAACATATG TTCACATTAT TTGTTACAAC AGCGTCCCAC

TTAGATTCTG ATAACATATG TTCACATTAT TTGTTACAAA AGCGTCCCAC

TTGGATTCTG ATAACATATG TTCAAATTAT TCCTTAAAAC AGCGTCCCAC

TTGAATTCTG ATAACACATG TTCAAATTAT TCCATGCAAC AGCGCCCCGC

CCTCCACTGA AACTTGGCTC ACACTTTGAA AAATTCTTTT GTTGCAAATG

CCTCCACTGA AACTTGGCTC ACACTTTGAA AAATTCTTTT GTTGCAAATG

CCTCCACTGA AACTTGGCTC ACACTTTGAA AAATTCTTTT GTTGCAAATG

CCTCCACTGA AACTTGGCTC ACACTTTGAA AAATTCTTTT GTTGCAAATG

CCTCTGCTAA AACTTGGCTC ACACTTTGAA AAATTCCTTT GTTGCAAATG

CCTCCACTAA AACTTGGCTC ACACTTTGAA AAATTCCTTT GTCACAAATG

CCTCCACTAA AACTTGGCTT GCACTTTGAA AAATTCCTTT ATTTCAAATG

CCTCCACTAA AACTTGGCTT GCACTTTGAA AAATTCCTTT ATTTCAAATG

CCTCTACT-A AACTTGGCTC GCACTTTGAA AAATTCTTTT GTTGCAAATG

CCTCCACTAA AACTTGGCTC TCACTTTGCT AAATTCCTTT GTTGCAAATG

CCTCCGCTAA ATCTTGGCTC ACACTTTGCA AAATTCCTTC GTCGCGAATG

CCTCCACTAG AACTTGGCTC ACACGGTGCA CAATTCCTTT GTTGCAAATG

CCTCAACTAA AACTTGGCTC ACACTTTGCA AAATTCTTTT GTTGCAAATG

CCTCCACTGA AACTTGGCTT GCACTTTGAA AAATTCCTTT GTTTCAAGTG

CCTCCACTAA AACTTGGCTC ACACTTTGAG AAATTCCTTT GTTGCAAATG

CCTCCACTGA AACTTGGCTC GCACTTTGCG GAATTCCTTT GTTGCAAATG

CCTCTGCTAA AACTTGGCTC ACATTTTGAA AAATTCTGTT GTCGTAAGTG

CCGACACTAA GGCTTGGCTC GCCGCTCGAA AATCTCTGTT GTTGCGAAGG

GACAACTCAC CATACCAAGG CCCTGTGTTG AGCTTTGTTT ATGTTGTGTA

GACAACTCAC CATACCAAGG CCCTGTGTTG AGCTTTGTTT ATGTTGTGTA

GACAACTCAC CATACCAAGG CCCTGTGTTG AGCTTTGTTT ATGTTGTGTA

GACAACTCAC CATACCAAGG CCCTGTGTTG AGCTTTGTTT ATGTTGTGTA

GACAACTCAC CATACCAAGG CCCTGTGTTG AGCTTTGTTT ATGTTGTATA

GACAACTCAC CATACCAAGG CCCTGTGTTG AGCTTTGTTT ATGTTGTGTA

GACAGCTCGC CCTATCAAGG CCC-ATGTTG AGCTTTGTTT ATGTTGTGTA

GACAGCTCAC CCTATCAAGG CCC-ATGTTG AGCTTTGTTT ATGTTGTGTA

GACAACCCGC CATACCAAGG CCCTGTGTTG AGCTTTGTTT ATGTTGTATA

GACAACTCAC TATACCAAAG CCCTGTGTTG AGCTTTGTTT ATGTT-TGTA

GACAACTCAC CATACCAGGG CCCTGTGTTG AGCTTTGTTT ATGTTGTGTA

GACAAGTCGC CACACCAGGG CTCTGTGTTG GGTTTTGTTT ATGTTGTGTA

GACAACTCAC CATACCAAGG CCCTGTGTTG AGCTTTGTTT ATGTTGTGTA

AACAATTCAC CATACCAAGG CTCTGTGTTA ATCTTTGTTT ATGTTGTGTA

GACAATTCAC CATACCAAGT CCCTGTGTTG AGCTGTGTTT ATGTTGTGTA

GACAGTTCAC CCTACCAAGG CCCTGTGTTG AGCTCTGTTT ATGTTGCCTA

GACAACTCGC CATACCAAGT GCCTGTATTC AGTTTTGTTT ATATTGTGTA

GACAACTCAC CCTTCCACAG GCCTGTGTTG AGTTTTGTTT ACATTGTGTA

CCTGGCAACA GAGATGATGT TCTTGTTCAT TATTCATGGA AAAAGAGCCT

CCTGGCAACA GAGATGATGT TCTTGTTCAT TATTCATGGA AAAAGAGCCT

CCTGGCAACA GAGATGATGT TCTTGTTCAT TATTCATGGA AAAAGAGCCT

CCTGGCAACA GAGATGATGT TCTTGTTCAT TATTCATGGA AAAAGAGCCT

CCTGGCAACA GAGATGATGT TCTTGTTCAT TATTCATGGA AAAAGAGCCT

CCTGGCAACA GAGATGATGT TCTTGTTCAT TATTCATGGA AAAAGAGCCT

CCTGGCAACA GAAATGATGT TCTTGTTCAT TATTCATGGA AAAAGAGTCT

CCTGGCAACA GAAATGATGT TCTTGTTCAT TATTCATGGA AAAAGAGTCT

CCTGGCAACA GAGATGATGT TCTTGTTCTT TATTCATGGA AAAAGAGCCT

CCTGGCAACA GAGATGATGT TCTTTCTCAT TATTCATGGA AAAAGAGCCT

CCTGGCAACA GAGATGATGT TCTTATTCAT TATTCATGGA AAAAGAGCCT

CCTGGCAACA GACATGATGT TCTTATTCAT TATTCATGGA AAAAAAGCCT

CCTGGCAACA GAGACGATGT TCTTATTCAT TATTCATGGA AAAAGAGCCT

CCGGGCAACA GAGATGATTT TCTTATTCAT TATTCATGGA AAAAGAGCCT

CCTGGCAACA GAGATGATGT TCTTGTTCAT TATTCATGGA AAAAGAGCCT

CCTGGCAACA GAGAGGATAT TCTCGTTCGT TGTTCATGGA AAAAGGGGCT

CCTGGCAACA GAGATGATGT CCATGCTCAT TATTCATGGA AAAAGAGCCT

CCTGGCAACG GGGATGGTGT CCACAATCAG TTTTCATGGA AAAAGAGCCT

CATTAGTGGC AGGACACATA TTGTTGCTGA GATGCCTGTT AGGGGTTTTG

CATTAGTGGC AGGACACATA TTGTTGCTGA GATGCCTGTT AGGGTGTTTG

CATTAGTGGC AGGACACATA TTGTTGCTGA GATGCCTGTT AGGGGTTTTG

CATTAGTGGC AGGACACATA TTGTTGCTGA GATGCCTGTT AGGGGTTTTG

CATTAGTGGC AGGACACATC TTGTTGCTGA GATGCCTATT AGGGGTTTTG

CATTAGTGGC AGGACACATA CTCTTGCCGA GATGCCTGTT AGGGGTTTTG

CACTGGTGGC AGGACACATA GTGTTGCTGA ACTGCCTATT AGAGGCTTTG

CACTGGTGGC AGGACACAGA GTGTTGCTGG ACTGCCTATT AGAGGCTTTG

CATTAGTGGC AGGACACACG TTGTTGCTGA GATGCCTATT AGGGGTTTTG

CATTAGTGGC AGGACACATA CTGTTGCTGA GATGCCTGTT AGGGGTTTAG

CATTAGTGGC AGGACACAAA CTGTTGCTGA GATGCCTGGT AGGAGTTTTG

CATTAGTGGC AGGACACATA CTGTTGCTGA AATGCCTGTT AGGGGTTTTG

CATTAGTGGC AGGACACATT CTGTTGCTGA GATGCCTGTT AGGAGTTTTG

GATTAGTGTC AGAACACATA CTGTTGTTGA GATGCCTGTT ACGGGGTTTC

CATTAGTGGC AGGACACAGA CTGTTGCTGA GATGCCTGTT ATGGGTTTTA

CATTAGTGGC AGGACACAAA CTGTTGCTGA GATGCCTATT CTGGGTTTTG

CATTAGTGGC AGGACACATT CTGTTGCTGA GCTACCTATT ATAGCTTTAG

CATCTGTGGC AGGACCCGCA CAGTTGCCGA GATACCTATT AGGGTTTCAG

AGTTCAGCAG TTGTCAATAG AGTACAGATG ACCTAATGAA TTCTGAAGGG

AGTTCAGCAG TTGTCAATAG AGTACAGATG ACCTAATGAA TTCTGAAGGG

AGTTCAGCAG TTGTCAATAG AGTACAGATG ACCTAATGAA TTCTGAAGGG

AGTTCAGCAG TTGTCAATAG AGTACAGATG ACCTAATGAA TTCTGAAGGG

AGTTCAGCAG TTGTCAATAG AGTACAGATG ACCTAATGAA TTCTGAAGGG

AGTTCAGCAG TTGTCAATAG AGTGCAGATG ACCTAATGAA TTCTGAAGGG

AGTTCAGCCG TTGTCAATAG AGTGCAGCTG ACCTAATGAA CTCTGAGGGG

AGTTCAGCGG TTGTCAATAG AGTGAAGCTG ACCTAATGAG CTCTGCGGGG

AGTTCGGTAA TTGTCAATAG AGTGTGGATG ACCTAATGAA TTCTGAAGGG

AGTTCAGCAG TGGTCAATCC GCTGCAGATG ACCTAATGAA TCCTGGAGGG

AGTTCAGCAG TTGTCAATAG ACTGCAGATG ACCTAATGAA TTCTGGAGGG

AGTTCAGCAG TTGTCAATAG ACTGCAGATG ACCTAATAAA TTCTGGAGGG

AGATCAGC-- -TGTCAATAG ACTGCAGATG ACCTAATGAA TTCTGGAGGG

AGTTCAGCAG TTGTCAATAG AGTGCAGGTG ACTTAATGAA TTCTGAAGGG

AGTTCAGCAG TTGTCAATAG AGTGCAGATG ACCTAATGAA TTCAGAAAGG

--------AG TGGTCAATAG AGTGCAGATG ACCTAATGAA TTCCAGAGGG

AGTTCAGCAT TTGTCAATAG AGTGCAGATG ACCTAATGAA TTTGAAAGGG

AGTTCAGCGC TTGTCAGTAG AGCGCCGATG ACCTCACGAC TCCTACAGGG

AGTTTTGGTG CTGACTTGGC TCAGGCCTGT ATCATTTACA GTAGAATTAC

AGTTTTGGTG CTGACTTGGC TCAGGCCTGT ATCATTTACA GTAGAATTAC

AGTTTTGGTG CTGACTTGGC TCAGGCCTGT ATCATTTACA GTAGAATTAC

AGTTTTGGTG CTGACTTGGC TCAGGCCTGT ATCATTTACA GTAGAATTAC

AGCTTTGGTG CTGACTTGGC TCAGACCTGT ATCATTTACA GTAGAATTAC

AGTTTTGGCG CTGACTTGGC TCAGGCCTGT ATCATTTACA GTAGAATTAC

AGTTTTGGTG CTGACTTGGC TCGTGCTTGT CTCACTTACA GTAGAATTAC

AGTTTTGGTG CTGACTTGGC TCGTGCTTGT GTCACTTACA GTAGAATTAC

AGTTTTGGTG CTGACTTGGC CCAGGCCCGT ATCATTTACA GTAGAATTAG

TGTTTTGGCT CAGACTTGGC TCAGGCCTGT GTCATTTACA GTAGAATTAC

TGTTTTGGTG AGGACTTGGC CCAGGCCTGT GTCATTTACA GTAGAATTAT

TGTTTTTGTG TGGACTTGGC TCAGGCCTGT GTCACTTACA GTAGAATTAC

TGTTTTGGTG TGGACTTGGC ACAGGCCTGT ATCCTTTACA GTAGAATTAC

AATTTTGGTG CTGACTTGGC ACAGGCCTGT GTCATTTCCA GTAGTATTAC

AATTTTGGTT CTGACTTGGC ACAGGCTTGT GTCATTTACA GTAGAATGAC

CAGTTCGGTG CTGAGTTGGC CCAAGCCTGT GTCATTTACA GTGGAATGAC

GATTTTGGCT CTGACTTGGC ACAGGTCTGT GTTATTTACA GAAGAATTAC

ATCCTTGGCT CGAGCCGGGC ACCCTTCTGC GTTATTTACA GTAGAATCAT

TGTTCTTGAG GCACACCCAA CAGCAGGATT ATATGTCCTT TTTACTAATT

TGTTCTTGAG GCACACCCAA CAGCAGGATT ATATGTCCTT TTTACTAATT

TGTTCTTATG GCACACCCAA CAGCAGGATT ATATGTCCTT TTTACTAATT

TGCTCTTACG GCACACCCAA CAGCAGGATT ATATGTCCTT TTTACTAATT

TGTTCTTG-- -CCCACCCAA CAGCAGGATT ATATGTCATT TTTGCTAATT

TGTTCTTGAG GCACACCC-G CAGGAGAGCT GTACGTTATC TGTGCTAATT

TGTTCTTAA- GCACACCCAA CAACAGGAT- -TATGTTATC TCTCCAAGTT

TGTTCTTGA- GCACACCCAA CAACAGGAT- -TATGTTATC TCTCCAAGTT

TGTTCTTGAG GC-CCTCCAA CCGCAGGAT- -TATGTTGTC TTTGTGAACT

TGTTCTCGAG GCACACCCGA CAGCAGGATT AGATGTTATC TTTGCAAATC

TGTTCTTGTA GCACACCCGA CAGCAAGATT ATATGTTATC TTTGCAAATT

TGTTCTTGCG GCACACCCGA CAG-AGGATT GTATGTTATC TTTGCAAATC

TGTTCTTGAG GAACACCCGA CGGCAGGGTT ATATGTTATC TTTGCGAATT

TATTCTTGAG ACATACCTAA CAGTAAGAGT ATATGTTACC TTTGCAAGTT

TGTTCCTGAG GCACACCCGA CCGCAGGATT ATATGTTATC TTTGCAAATT

CGTTCTTGAG GCACACCCGA CAGCAGGAT- -TAGGATGTC TCGGCAAATT

TGTTCTTGAG GCACACCCAA CAGCAGGATT GTATGTTATC TTTGCAAAAT

TGCTCTGAAG GCACACCCTA CAGCCAGGGG CTCTATTATC TCCACACAGT

GGAATGCTCT GGTTGGGTAA AATTTGTTGG GTTTGGTAAT CCTATTGCCC

GGAATGCTCT GGTTGGGTAA AATTTGTTGG GTTTGGTAAT CCTATTGCCC

GGAATGCTCT GGTGGGGTAA AATTTGTTGG GTTTGGTAAT CCTATTGCCC

GGAATGCTCT GGTGGGGTAA AATTTGTTGG GTTTGGTAAT CCTATTGCCC

AGAATGCTCT GGTTGGGTAA AATTTGTTGG GTTTGGTAAT CCTATTGCCC

GGAATGCTCT GCTTGGGTAA AATTTGTTGT CTTTAGTAAT CCTATTACCC

GGAATGTTCT GGTTGGGTGG AATTTGTTGG CTTTGGTAAT CCTATTGCCC

GGAATGTTCT GGTTGGGTGA AATTTGTTGG CTTTGGTAAT CCTATTGCCC

GGAGTTCTCT GTTTGGGTAA AATTTGTTGG CTTTAGTAAT CCTACTGCCC

GGAATGCTCT GGTTGGGTAA AATTTGCTTG CTTTAGTAAT CCTCTGGTCC

GGAATGCTCT GGTTGGGTAA AATTTATTGG CTTTAGTAAT CCTATTGCTT

GGAATGTTCT GGTTGGGTAA AGTTTGTTGC CCTCAGTAAT CCTATCGCCC

GGAATTCTCT GGTTGGAGA- ---------- ---------- ----------

GGAATGCTCT GGTTGGGTAA AATTTGTTAG CTTTAGTAAT TCTATTGCCC

GGAATGCTCT GGTTGGGTAA AATTTATTGG CTTTAGTAAT CCTATTGTCC

GGAACGCTCT GGTTGAGTAA GATT-GTTGG CTTCAGT--- CTTATCGCCC

GGAATGTGCC CATTGGGTAA AACTCCTGGG TTTTATTAAT CTTGTCTGGA

GGAATGCTCT GTGTGGGGAA AATCCCTGGG TTTTATTTAA CGTACTGGAA

AGTTATCATA ATTTACTCCC TCTCCAAAAG TTGAAAATAA CAACTGGTGT

AGTTATCATA ATTTACTCCC TCTCCAAAAG TTGAAAATAA CAACTGGTGT

AGTTATCATA ATTTACTCCC TCTCCAAAAG TTGCAAGTAA CATCTGGTGT

AGTTATCATA ATTTACTCCC TCTCCAAAAG TTGCAAGTAA CATCTGGTGT

AGTTATCATA ATTTACTCCC TCTCCAAAAG TTGCAAATAA CATCTGGTGT

AGTTATCATA ATTTACTCCC TCCCCCAAAA CTGCAAATAA CATCTGGTGT

AGTTATCATA ATTTACTTCC TCTCCAAAAG TTGCAAATCA CATCTGGTGT

AGTTATCATA ATTTACTTCC TCTCCAAAAG TTGCAAATCA CATCTGGTGT

AGTTATCATA ATTTACTTCC TCTCCAAAAG TTGCAAATAA CATCTGGAAT

AGTTATCATA ATTTACTTTC TCTCCAGAAG CTGCAAATAA CATCTGGTGT

AGTTATCATA GTTTACTCCC TCTCCA-AAG TTGCAAATAA CATCTGGTGT

AGTTATCATA ATTTACTCCC TCTCCAAAAG TTGCACATGA CATCTGGTGT

---------- ---------- ---------- ---------- ----------

AGTTATCCTA ACTTACTCCC TCTCCAAAAG TTGCAAATAA CATCTGGTCT

AGCTATTGTA ACTTACTCCC TCTCAAAAAG TTGCAAATAA CACCTGGTCT

AGCTAATGCA ATTTGCTCCT TCTAAAAAAG CTGCACATAA CACCTGACCT

GACTGTTATA ATTTACTACC TCTCCAGAAA AAGCAAGTGA CATCTGGTCA

GATTGTCACA ATGTATTGCC TTTCCAGAAA CCGCAACTTG CATCAGGTCT

TTGGCTTTGA ACTTCCCAGT TGAATCAGAA CTCCCAAGTG GCACATTTTG

TTGGCTTTGG ACTTCCCAGT TGAATCAGAA CTCCCAAGTG GCACATTTTG

TTGGCTTTGA ACTTCCTAAT TGAATCAGAA CTCCCAGGCG GCACATTTTG

TTGGCTTTGA ACTTCCTAAT TGAATCAGAA CTCCCAGGCG GCACATTTTG

TTGGCTTTGA ATTTCCCAAT TGAATCAGAA CTCCCAGGTG GCACATTTTG

TTGGCTTTGA ATTTCCCGAT TGAATCAGAA GTCCCAGGTG GCACATTCTG

TTGGCTTTGA ATTTCCCGAT TGAATCAGAA ATCCCAGATG GCACATTTTG

TTGGCTTTGA ATTTCCCGAT TGAATCAGAA ATCCCAGATG GCACATTTTG

TTGGCTCTGA ATTTCCTGAT TGAATCAGAA CTCCCATGTG ACACATTTTG

TT-GCTTTGT GTTTCCTGAT TGAATCAGAC TTCCCAGGTG ACACACTCTG

TTGGCTTTGT ATTTCCCGAT TGAATCAGAA CTCCCAGGTG GCACATTTTG

TCGACTTTGT ATTTCCCGAT TGAAGCAGGA CTCCCAGGTG CTGCATTCTG

---------- ---------- ---------- ---------- ----------

TTGGCTGTGA ATTTCCTGCT TCAATATGAA ATCCCAGGTG GCACATTTAG

TTGGCTTTGA ATTTCCTAAT T-AATCAGAA CTCCCATATG GCACATTTTG

TCGACTTTGA ATTTCCTGAC TGAATCAGAA TTTCTAGTTG GCACTTTTTG

TTGCCTTTGA ACTTCCCAAG TGAATCAGTA CTCCTAGGTG TCTTGTTTTG

TTGTCCTTGA TGTTCCCGAT TGAATCGCAA CTCCCAGGAG TGAAATTCTG

AGCTTTCTTT TTTCCAACAG ATATAAGCTT ACCAAAGTCA GCAACAATAT

AGCTTTCTTT TTTCCAACAG ATATAAGCTT ACCAAAGTCA GCAACAATAT

AGCTTTCTTT TTTCCAACAG ATATAAGCTT ACCAAAGTCA GCAACAATAT

AGCTTTCTTT TTTCCAACAG ATATAAGCTT ACCAAAGTCA GCAACAATAT

AGCTTTCTTT TTTCCAACAG ATATAAGCTT ACCAAAGTCA GCAACAATAT

AGCTTTGTTT TCGTCTGCAG ATATAAGCTT ACCAAAGTCG GCCACCATAT

AGCTTTC--T TTCCTGACAG ATATAAGCTT ACCAAAGTCG GCAACAATAT

AGCTTTC--T TTCCTGACAG ATATAAGCTT ACCAAAGTCG GCGACAATAT

AGCTTTCTTT TTTCTAACAG ATATAAGCTT ACCAAAGTCG GCAACAATAT

AGCTTTTCTT TTTCTGACAG ATATAAGCTT ACCAAAGTCG GCAACAATAT

AACTTTCTTT TTTCTAACAG ATATAAGCTT ACCAAAGTCG GCAACAATAT

AGCTTTCTTT TTTCTAACAG ATATAAGCTT ACCAAAGTCG GCAACAATAT

--CTTTCTTT TTTCTGACAG ATATAAGTTT ACCAAAGTCG GCCACAATAT

AGCTTTCTTT TTTCTAACAG ATATAAGCTT ACCAAAGTCG GCAACAATAT

AGCTTCTTTT TTTCTAACAG ATATAAGCTT ACCGAAGTCG GCAACAATAT

AGCTTTCTCT TTTCGGACAG ACATCAGCTT ACCAAAGTCG GCGACCATAT

AACTTTCTTT TTCCCAACAG ATATAAGCTT ACCCAAATCG GCAACAATAT

GAGTTTCTTT TTCTCAACAG ATATAAGCTT ACCCAAATCG GCAACAATAT

GCTACACAGC TGCTTTGCTC AAAGCAAGAG CTGTCTCTGA CAAGTAAGTG

GCTACACAGC TGCTTTGCTC AAAGCAAGAG CTGTCTCTGA CAAGTAAGTG

GCTACACAGC TGCTTTGCTC AAAGCAAGAG CTGTCTCTGA CAAGTAAGTG

GCTACACAGC TGCTTTGCTC AAAGCAAGAG CTGTCTCTGA CAAGTAAGTG

GCTACACAGC TGCTTTGCTC AAAGCAAGGG CTGTTTCTGA CAAGTAAGTG

GCTACACAGC GGCCTTGCTC AAAGCAAGAG CTGTCTCTGA CAAGTAAGTG

GCTACACAGC GGCCCTGCTC AAAGCAAGAG CTGTCTCTGA CAAGTAAGTG

GCTACACAGC CGCCCTGCTC AAAGCAAGAG CTGTCTCTGA CAAGTAAGTG

GCTACACAGC TGCCTTGCTC AAAGCAAGAG CTGTCTCTGA CAAGTAAGTG

GCTACACAGC TGCCTTGCTC AAAGCAAGAG CTGTCTCTGA CAAGTAAGTG

GCTATACAGC TGCCTTGCTC AAAGCAAGAG CTGTCTCTGA CAAGTAAGTG

GCTACACAGC TGCCTTGCTC AAAGCAAGAG CTGTCTCTGA CAAGTAAGTG

GCTACACAGC TGCCTTGCTC AAAGCAAGAG CTGTCTCTGA CAAGTAAGTG

GCTACACAGC TGCTTTGCTC AAAGCAAGAG CTGTCTCTGA CAAGTAAGTG

GCTACACAGC TGCCTTGCTC AAAGCAAGAG CTGTCTCTGA CAAGTAAGTG

GCTACACAGC TGCCTTACTC AAAGCACGAG CTGTCTCCGA CAAGTAAGTG

GCTACACAGC TGCATTGCTC AAAGCAAGAG CTGTCTCTGA CAAGTAAGTG

GCTACACAGC GGCCCTGCTC AAAGCAAGAG CTGTCTCTGA CAAGTAAGTG

AGAGACCCCT GCTTATAAGC ATGCATTTTC CAAAACCTGC TGTTCTTTTC

AGAGACCCCT GCTTATAAGC ATGCATTTTC CAAAACCTGC TGTTCTTTTC

AGAGACCCCT GCTTATAAGC ATGCATTTTC CAAAACCTGC TGTTCTTTTC

AGAGACCCCT GCTTATAAGC ATGCATTTTC CAAAACCTGC TGTTCTTTTC

AGAGACCCCT GCTTATAAGC ATGCATTTTC CAAAACCTGC TGTTGTTTTC

AGAGACCCCT GCTTATAAGC ATGCATCTTC CAATACCTGC TGATCTTTTC

ATTGA-CCCT GCTTAAAAGC ATGCATTTCC CCAAAGCTGC TGATCTTTTA

ATTGA-CCCT GCTTTAAAGC ATGCATTTCC CCAAAGCTGC TGATCTTTTA

AGAGACCCCT GCTTATGAGC ATGCATTTTC CAAAACGTGC TGATCTTTTC

GGAGACCCCT GCTTATGAGC ATGCATTTTC CAAAACCTGC TGATCTTTCC

AGAGACCCCT GCTTATGAGC ATGCATTTTT CAAAACCTGC TGATCTTTTC

AGACACCCCT GCTTATGACC ATGCATTTTC CAAAATCTGC TGATCTTTTC

AGAGACCC-T GCTTGTGAGC ATGCATTTTC CAAAACCTGC TGATCTTTTC

AGAGATCCCT GCTTATAAGC ATGCATTTTC CAAAACCTGC TGATCTTTCC

AAAGATCCCT GCTTATGAGC ATGCATTTTC CAAAACCGGC TGATCTTTTC

AAAGACCCCT GCTTATGAGC ATGTGTTTTC CCACACCTGC TGATCTTTCC

AGAGATTGCT GCTTCTGAGC ATGCATTTTC CAAAGTCTGC TGATCTTTTC

CGGGACTCCT GCTTCTGAGC TTGCCTTCTC CAGAATCTGC TGATCCGTTC

TTTCACTATT GGAAGGACAG CCTCTGGTAT CAGACAGGTT TGCTGTTAAG

TTTCACTATT GGAAGGACAG CCTCTGGTAT CAGACAGGTT TGCTGTTAAG

TTTCACTATT AGAAGGACAG CCTCTGGTAT CAGACAGGTT TGCTATTAAG

TTTCACTATT AGAAGGACAG CCTCTGGTAT CAGACAGGTT TGCTGTTAAG

TTTCACTATT AGAAGGACAG CTTCTGGTGT CAGACAGGTT TGCTGTTAAG

TTTCACTACC AG---GACAG TCTCTGGTGT CAGACAGGTT TGCTGTTTAG

TTTCACTACT AGAAGGACAG CCTCTGGTGT CAGACAGGTT TGCTGTTAAG

CTTCACTA-T AGCAGGACAG CCTCTGGTAT CAGACAGGTT TGCTGTTAAG

TTTCAGTACT AAAAGGACAG CCTCTGGTGT CAGACAGGTT TGCTGTTAAG

TTTCACTACT AGAAGGACAG CCTCTGGTGT CAGACAGGTT TGCTGTTCAG

TTTCACTACT AGAAGGACAG CCTCTGATGT CAGACAGGTT TGCTGTTAAG

TTTCATTACT AGA---ACAG CCTCTGGTGT CAGCCAGGTT TGCTGTTAAG

TTTCTCTACT GGAAGGACAG CCTCTGATGT CAGACAGGTT TGCTGTTAAG

TTTTACTATT AGAAGGACAG CCTCTGATGT CAGACAGGTT TACTGTTAAG

TTTCACTACT AGAAGGACAG CCTCTGATGT CAGACAGGTT TGCTGTTAAG

TTTCACTGTT AGAAGGACGG CCTGTGGTGT CAGACAGGTT TGCTGTTCAG

TTTCACTGTT AGCAGAACTG CTTTTCGTCT CAGAAAGGTT TGCTTTTCAG

TCTCCCCATT AGCGGGATGC CTTCTGGTGT CAGGCAGGTT TGCTGTTCAG

TGTGTGTACA CTTCAAGACC AAAGTAATTT TCTTTCATTC TTTTTATCCT

TGTGTGTACA CTTCAAGACC AAAGTAATTT TCTTTCATTC TTTTTATCCT

TGTGTGTACA CTTCAAGACC AAAGTAATTT TCTTTCATTC TTTTTATCCT

TGTGTGTACA CTTCAAGACC AAAGTAATTT TCTTTCATTC TTTTTATCCT

TGTGTGTGCA CTTCAAGATC GAAGTAATTT TCTTTCATTC TTTTTATCCT

TGTGTGTGCA CTTCAAGACC GAAGTAATTT TCTTTCATTC TTTTTATCCT

TGTGTGTGCA CTTCAAAACT GGAGTAATTT TCTTTCATTC TTTTTATCCT

TGTGTGTGCA CTTCAAAACT GGAGTAATTT TCTTTCATTC TTTTTATCCT

TGTGTGTGCA CTTCAAGACC GAAGTAATTT TCTTTCATTC TTTTTATCCT

TGTGTGTGCA CTTCAAGACC GAAGTAATTT TCTTTCATTC TTTTTATCCT

TGTGTGTGCA CTTCAAGACC GAAGTAATTT TCTTTCATTC TTTTTATCCT

TGTGTGTGCA CTTCAAAATC GAAGTAATTT TCTTTCATTC TTTTTATTCT

TGTGTGTGCA CTTCAAGACC GAAGTAATTT TCTTTCATTC TTTTTATCCT

TGTGTGTGCA CTTCAAGACC GAAGTAATTT TCTTTCATTC TTTTTATCCT

TGTGTGTGCA CTTCAAGACC GAAGTAATTT TCTTTCATTC TTTTTATCCT

TGTGTGTGCA CTGCGAGACC GAAGTAATTT TCTTTCATTC TTTTTATCCT

TGTGTGTGCA CTTCAAGATC GAAGTAATTT TCTTTCATTC TTTTTATCCT

TGTATGTGCA CTTCAAGACC TAAGTAATTT TCTTTCGTTC TTTTTATCCT

GTAGATCGCC AGTACCTACT GCAACATCTT TTCTCCCTAC ACAGCGACTC

GTAGATCGCC AGTACCTACT GCAACATCTT TTCTCCCTAC ACAGCGACTC

GTAGATCGCC AGTACCTACT GCAACATCTT TTCTCCCTAC ACAGCGACTC

GTAGATCGCC AGTACCTACT GCAACATCTT TTCTCCCTAC ACAGCGACTC

GTAGATCGCC AGTACCTACT GCAACATCTT TTCTCCCTAC ACAGCGACTC

GTAGATCGCC AGTACCTACT GCAACATCTT TTCTCCCTAC ACAGCGACTC

GTAGATCGCC AGTACCTACT GCAACATCTT TTCTCCCTAC ACAGCGACTC

GTAGATCGCC AGTACCTACT GCAACATCTT TTCTCCCTAC ACAGCGACTC

GTAGATCGCC AGTACCTACT GCAACATCTT TTCTCCCTAC ACAGCGACTC

GTAGATCGCC AGTACCTACT GCAACATCTT TTCTCCCTAC ACAGCGACTC

GTAGATCGCC AGTACCTACT GCAACATCTT TTCTCCCTAC ACAGCGACTC

GTAGATCGCC AGTACCTACT GCAACATCTT TTCTCCCTAC ACAGCGACTC

GTAGATCGCC AGTACCTACT GCAACATCTT TTCTCCCTAC ACAGCGACTC

GTAGATCGCC AGTACCTACT GCAACATCTT TTCTCCCTAC ACAGCGACTC

GTAGATCGCC AGTACCTACT GCAACATCTT TTCTCCCTAC ACAGCGACTC

GTAGATCGCC AGTACCTACT GCAACATCTT TTCTCCCTAC ACAGCGACTC

GTAGATCGCC AGTACCTACT GCAACATCTT TTCTCCCTAC ACAGCGACTC

GTAGATCGCC AGTACCTACT GCAACATCTT TCCTCCCTAC ACAGTGACTC

CAGCTTGGGA GGGCAGGGCC AGGTTGTCAC AGCTTCCCCT GTGGTGTCTG

CAGCTTGGGA GGGCAGGGCC AGGTTGTCAC AGCTTCCCCT GTGGTGTCTG

CAGCTTGGGA GGGCAGGGCC AGGTTGTCAC AGCTTCCCCT GTGGTGTCTG

CAGCTTGGGA GGGCAGGGCC AGGTTGTCAC AGCTTCCCCT GTGGTGTCTG

CAGCTTGGGA GGGCAGGGCC AGGTTGTCAC AGCTTCCCCT GTGGTGTCTG

CAGCTTGGGA GGGCAGGGCC AGGTTGTCAC AGCTTCCCCT GTGGTGTCTG

CAGCTTGGGA GGGCAGGGCC AGGTTGCCAC AGCTTCCCCT GTGGTGTCTG

CAGCTTGGGA GGGCAGGGCC AGGTTGCCAC AGCTTCCCCT GTGGTGTCTG

CAGCTTGGGA GGGCAGGGCC AGGTTGTCAC AGCTTCCCCT GTGGTGTCTG

CAGCTTGGGA GGGCAGGGCC AGGTTGTCAC AGCTTCCCCT GTGGTGTCTG

CAGCTTGGGA GGGCAGGGCC AGGTTGTCAC AGCTTCCCCT GTGGTGTCTG

CAGCTTGGGA GGGCAGGGCC AGGTTGTCAC AGCTTCCCCT GTGGTGTCTG

CAGTTTGGGA GGGCAGGGCC AGGTTATCAC AGCTTCCCCT GTGGTGTCCG

CAGCTTGGGA GGGCAGGGCC AGGTTGTCAC AGCTTCCCCT GTGGTGTCTG

CAGCTTGGGA GGGCAGGGCC AGGTTGTCAC AGCTTCCCCT GTGGTGTCTG

CAGCTTGGGA GGGCAGGGCC AGGTTGCCAC AGCTTCCCCT GTGGTGTCTG

CAGCTTGGGA GGGCAGGGCT AGGTTGTCAC AGCTTTCCCT GTGGTGTCTG

CAGCTTGGGA GGGCAGGGCT AGGCTGAGCC AGCACTCCCT GTGGTGTCTG

CCTGCCAAGC ACAGCTCTGG AGTTAGCCCT GGGTGTGAGG TGAGAAAGAG

CCTGCCAAGC ACAGCTCTGG AGTTAGCCCT GGGTGTGAGG TGAGAAAGAG

CCTGCCAAGC ACAGCTCTGG AGTTAGCCCT GGGTGTGAGG TGAGAAAGAG

CCTGCCAAGC ACAGCTCTGG AGTTAGCCCT GGGTGTGAGG TGAGAAAGAG

CCTGCCAAGC ACAGCTCTGG AGTTAGCCCT GGGTGTGAGG TGAGAAAGAG

CCTGCCAAGC ACAGCTCTGG AGTTAGCCCT GGGTGTGAGG TGAGAAAGAG

CCTGCCAAGC ACAGCTCTGG AGTTAGCCCT GGGTGTGAGG TGAGAAAGAG

CCTGCCAAGC ACAGCTCTGG AGTTAGCCCT GGGTGTGAGG TGAGAAAGAG

CCTGCCAAGC ACAGCTCTGG AGTTAGCCCT GGGTGTGAGG TGAGAAAGAG

CCTGCCAAGC ACAGCTCTGG AGTTAGCCCT GGGTGTGAGG TGAGAAAGAG

CCTGCCAAGC ACAGCTCTGG AGTTAGCCCT GGGTGTGAGG TGAGAAAGAG

CCTGCCAAGC ACAGCTCTGG AGTTAGCCCT GGGTGTGAGG TGAGAAAGAG

CCTGCCGAGC ACAGCTCTGG AGTTAGCCCT GGGTGTGAGG TGAGAAAGAG

CCTGCCAAGC ACAGCTCTGG AGTTAGCCCT GGGTGTGAGG TGAGAAAGAG

CCTGCCAAGC ACAGCTCTGG AGTTAGCCCT GGGTGTGAGG TGAGAAAGAG

CCTGCCAAGC ACAGCTCTGG AGTTAGCCCT GGGTGTGAGG TGAGAAAGAG

CCTGCCAAGT ACAGCTCTGG AGTTAGCCGT GGGTGTGAGG TGAGAAAGAG

CCTGCCACGT GCAGCTCTGG AGTTAGCCGT GGGTGTGAGG TGAGAAAGAG

ATTGCATGGT CTGGTTTTTT TCATTCACTT GGGCAGGTGT CTTGCCCGCA

ATTGCATGGT CTGGTTTTTT TCATTCACTT GGGCAGGTGT CTTGCCCACA

ATTGCATGGT CTGGTTTTTT TCATTCACTC GGGCAGGTGT CTCGCCCGCA

ATTGCATGGT CTGGTTTTTT TCATTCACTC GGGCAGGTGT CTCGCCCGCA

ATTGCATGGT CTGGTTTTTT TCATTCACTC GGGCAGGTGT CTCGCCCGCA

ATTGCATGGT CTGGTTTTTT TCATTCACTT GGGCAGGTGT CTTGCCCGCA

ATTGCATGGT CTGGTCTTTT TCATTCCCTT GGGCAGGTGT CTTGCCCACA

ATTGCATGGT CTGGTCTTTT TCATTCCCTT GGGCAGGTGT CTTGCCCACA

ATTGCATGGT CTGGTTTTTT TCATTCACTT GGGCAGGTGT CTCACCCACA

ATTGCATGGT CTGGTTTTTT TCATTCACTC GGGCAGGTGT CTTGCCCGCA

ATTGCATGGT CTGGTTTTTT TCATTCACCT GGGCAGGTGT CTGGCCCGCA

ATTGCATGGT CTGG-TTTTC TCATTC-CCC GGGCACGTGT CTCGCCCGCG

ATTGCATGGT CTGGTTTTTT TCATTCACTT GGGCAGGCGT CTCGCCCACA

ATTGCATGGT CTGGTTTTTT TCATTCATTT GGGCAGGTGT CTCGCCCACA

ATTGCATGGT CTGGTTTTTT TCATTCACTT GGGCAGGTGT CTCGCCCGCA

ATTGCATGGT CTGGTTTTTT TCACTCACTC GGGCAGCTGT CT-GCCCGCA

ATTGCATGGT CTGGTTTTTT TCATTCACTG GGGCAGATGA TTTGCCCACA

ATTGCATGGT CTGGTTTTTT TTATTGGCTG GGGCGGCTGA GGCGCCCTCA

GTTTGGGCAT GCAAATCCTT CCCAGTTCTG TTTTTAAAAC CTAGTTTTCT

GTTTGGGCAT GCAAATCCTT CCCAGTTCTG TTTTTAAAAC CTAGTTTTCT

GTTTGGGCAT GCAAATCCTT CCCAGCTCTG TTTTTAAAAC CTAGTTTTCT

GTTTGGGCAT GCAAATCCTT CCCAGCTCTG TTTTTAAAAC CTAGTTTTCT

GTTTGGGCAT GCAAATCCTC CCCAGTTCTG TTTTTAAAAC CTAATTTTCT

GTTTGGGCGT GCAAATTCTC CCCAGGTCTG TTTTTAAAAC CTAGTTTTCT

GTTGGGGCAT GCAAATTCTC CCCAGTTCTG TTTTGAAAAC CTAGGATTCT

GTTGGGGCAT GCAAATTCTC CCCAGTTCTG TTTTGAAAAC CTAGGATTCT

GTTTCGGTGT GCAAATTCTC CCCAGTTCTG TTTTTAAAAC CTAGTTTTCT

GTTTGGGCAT GCAAATTCTC CCCAGTTCTG TTTTTAAAAC CTAGTTTTCT

GTTTGGGCAC GCAAATTATC CCCAGTTCTG TTTTTAAAAC CTAGTTTTCT

CTTTGGGCCT GCAAATTCTC CCCCGTTCTG TTTTTAAAAC TGAGTTTTCT

GTTCGGGCAT GCAAACTCTC CCCAGTTCGG TTTTTAAAAC CCGGACTCCT

GTTTGGGCAT GCAAATTCTC CCCAGTTCTG TTTTTAAAAC ATAGTTTTCT

GTTTGGGCAT GCAAATTCTC CCCAGTTCTG TTTTTAAAAC ATCGTTTTCT

GTTTGGGCAC GCAAACCCGC CCCAGTTCTG TTTTTAG--- --CGTTTTCT

GTTTGGGCAT GCAAATTTCC CCCCATTCTG TTTTTAAAAC CTAGTTTTCT

GTCTGGGCAT GCAAATGCTC GCCCCCTCCG CTTCGAGAAC GTGAACCTTT

CTTTTTCTAC ACTGTCTGCC CCCAAGCAGT GGTCCCACCT GGATGGTTTT

CTTTTTCTAC ACTGTCTGCC CCCAAGCAGT GGTCCCACCT GGATGGTTTT

CTTTTTCTAC ACTGTCTGCC CCCAAGCAGT GGTCCCAACT GGATGGTTTT

CTTTTTCTAC ACTGTCTGCC CCCAAGCAGT GGTCCCAACT GGATGGTTTT

CTTTTTCTAC ACTGTCTGCC CCCAAGCAGT GGTCCCAACT GGATGGTTTT

CTTTTTCTAC ACTGTCTGCC CCCCAGCCGT GGTCCCAACA GGATGGTGTT

CTTTTTCTAC ACTGTCTGCC CCCAGGCAGT GGTCCCAACT GCATGATGTT

CTTTTTCTAC GCTGTCTGCC CCCAAGCAGT GGTCCCAACT GCATGATGTT

CTTTTTCTAC ACCGTCTGCC CCCAAGCAGC GGTCCCAACT GGATGTTGTT

CTTTTTCTAC ACTGTCTGCC CCCAAGCAGT GGTCCCAACT GGATGGTGTT

CTTTTTCTAC ACTGTCCGCC CCCGAGCAGT GGTCCCAACT GGATGGTGTT

CTTTTTCTGT ACTGTCTGCC CCCAAGCAGT CGTCCCAACT GGAAGGTGTT

CTTTTTCTAC ACTCTCTGCC CCCAAACAGT GGTCCCCACT GCGTGGTGTT

CTTTTTCTAC ACTGTCTGCC CCCAAGCAGT GGTCCCAACT GGATGGTGTT

CTTTTTCTAC ACTGTCTGCC CCCAAGCAGT GGTCCCAACT GGATGGTGTT

CTTTTTCTGC ACTATCGGTC CCCACGCAGT GGTCCCAACC GGATGGTGTT

CTTTTTCTAC ATCGTCCGCC CTTACCCGAC AGCCCCAAGT GAATCATGTC

CTTTTTATAA ACCGCCCACC CCCAGCCGGT CACCCCGACT GCACGGCGTC

TTCTTTCTGC CAGATTCTCT CCTGAGGCTG CATCTCGGCG GGGGCTGAGC

TTCTTTCTGC CAGATTCTCT CCTGAGGCTG CATCTCGGCG GGGGCTGAGC

TTCTTTCTGC CAGATTCTCT CCTGAGGCTG CATCTCGGCG GGGGCTGAGC

TTCTTTCTGC CAGATTCTCT CCTGAGGCTG CATCTCGGCG GGGGCTGAGC

TTCTTTCTGC CAGGTTCTCT CCTGAGGCTG CATCTCGGCG GGGGCTGAGC

TTCTTTCTGC CAGATTCTCT CCTGAGGCTG CATCTCGACG GGGGCTGAGC

TTCTTTCTGC CAGATTCTCT CCAGAGGCTG CATCTCGGCG GGGGCTGAGC

TTCTTTCTGC CAGATTCTCT CCAGAGGCTG CGTCTCGGAG GGGGCTGAGC

TTCTTTCTGC CAGATTCTCT CCCGAGGCTG CCTCGCGGCG GGGCCTGAGC

TTCTTTCTGC CAGATTCTCT CCTGAGGCTG CATCTCGGCG GGGGCTGAGC

TTCTTTCTGC CAGATTCTCT CCCGAGGCTG CATCTCGGCG GGGGCTGAGC

TTCTTTCTAC CAGATTCTCT CCTGAGGCTG CGTCTCGGCG GGGGCTGAGC

TTTTTTTTGC CAGATTCTCT CCCGAGGCTG CATCTCGGCG GGGGCTGAGC

TTCTTTCTGC CAGATTCTCT CCAGAAGCGG CATCACGGCG GGGTCTGAGC

TTCTTTCTGC CAGATTCTCT CCCGAGGCTG CATCTCGGCG GGGGCTGAGC

TTCTTTCTGC CAGGTTCTCT CCCGAGGCTG CTTCGCGGCG GGGGCTGAGC

TTCCCTCTGC CAGATTTTCT CCAGAGGCCG CATCCCGACG AGGGCTGAGC

TTGACTCCAC CAGGTTCTCC CCGGAGGCGG CTTCCCGGAG GGGGCTGAGC

ACAGCAGAGA TGAATGCAGT AGAGGCCATT CATAGAGCTG TGGAATTCAA

ACAGCAGAGA TGAATGCAGT AGAGGCCATT CATAGAGCTG TGGAATTCAA

ACAGCAGAGA TGAATGCAGT AGAAGCCATT CATAGAGCTG TGGAATTCAA

ACAGCAGAGA TGAATGCAGT AGAAGCCATT CATAGAGCTG TGGAATTCAA

ACAGCAGAGA TGAATGCGGT AGAGGCCATT CATAGAGCTG TGGAATTCAA

ACAGCAGAGA TGAATGCGGT AGAGGCCATT CACAGAGCTG TGGAATTCAA

ACAGCAGAGA TGAATGCAGT AGAAGCCATT CACAGAGCTG TGGAATTTAA

ACAGCAGAGA TGAATGCAGT AGAAGCCATC CACAGAGCTG TGGAATTTAA

ACAGCCGAGA TGAACGCAGT GGAGGCCATT CACAGAGCTG TGGAGTTCAA

ACAGCAGAGA TGAATGCGGT AGAGGCCATT CATAGAGCTG TGGAATTCAA

ACAGCAGAGA TGAATGCAGT AGAGGCCATT CACAGAGCCG TGGAATTCAA

ACGGCAGAGA TGAACGCAGT GGAGGCCATT CACCGAGCTG TGGAATTCAA

ACAGCAGAGA TGAATGCGGT CGAGGCCATT CATAGAGCTG TGGAATTCAA

ACAGCAGAGA TGAATGCAGT AGAGGCCATT CATAGAGCTG TGGAATTCAA

ACAGCAGAGA TGAACGCGGT AGAGGCCATT CATAGAGCTG TGGAATTCAA

ACAGCAGAGA TGAACGCGGT GGAGGCCATT CACAGAGCTG TGGAGTTCAA

ACCGCGGAGA TGAATGCAGT GGAGGCCATT CACAGAGCCG TCGAGTTCAA

ACGGCCGAGA TGAACGCTGT GGAGGCTATT CACCGAGCGG TGGAATTCAA

TCCTCATGTG CCAAAAGTGA GTCTTAATTG ATTTCTGCAT ACTTGTGGAT

TCCTCATGTG CCAAAAGTGA GTCTTAATTG ATTTCTGCAT ACTTGCGGAT

TCCTCATGTG CCAAAAGTGA GTCTTAATTG ATTTCTGCAT ACTTGTGGAT

TCCTCATGTG CCAAAAGTGA GTCTTAATTG ATTTCTGCAT ACTTGTGGAT

TCCTCATGTG CCAAAAGTGA GTCTTAATTG ATTTCTGCAT ACTTGTGGAT

TCCTCATGTG CCAAAAGTGA GTC------- ---------- ----------

TCCACACGTG CCAAAAGTGA GTCTTAATTG ATTTCTGCAT ACTTGTGGAT

TCCACACGTG CCAAAAGTGA GTCTTAATTG ATTTCTGCAT ACTTGTGGAT

CCCTCACGTG CCAAAGGTGA GCCTTAATTG ATTTCTGCAT ACTTGCGGAT

TCCTCACGTG CCAAAAGTGA GTC-TAATTG ATTTCTGCAT AGTTGTGGAT

TCCTCACGTG CCAAAAGTGA GTCCTAATTG ATTTCTGCAT ACTTGTGGAT

CCCTCACGTT CCCAAGGTGG GTCTTAATTG ATTTCTGCAT ACTTGTGGAT

TCCTCACGTG CCAAAAGTGA GTCT------ ---------- ----------

TCCTCACGTG CCAAAAGTGA GTCTTAATTG ATTTCTGCAT ATTTGTGGGT

TCCTCACGTG CCAAAAGTGA ----TAATTG ATTTCTGCAT ACTTGTGGAT

CCCTCACGTG CCAAAAGTGA GTGT------ ---------- ----------

TCCTCACGTG CCAAAAGTAA GTCTTAATTG ATTTCTGCAT ACTTGTGGAT

CCCTCACGTG CCAAAAGTGA GTCTTGATTG ATTCTTGTAA CCTGGCGCAT

AGGGATCTGC CCTGTTCCAT TTATTTTTCT CTTGACAAGT AAGATGTGAA

AGGGATCTGC CCTCTTCCAT TTATTTTTCT CTTGACAAGT AAGATGTGAA

AGGGATCTGC CCTCTTCCAT TTATTTTTCT CCTGACAAGT AAGACGTGAA

AGGGATCTGC CCTCTTCCAT TTATTTTTCT CCTGACAAGT AAGACGTGAA

GGGGATCTGC CCTCTTCCAT TTATTTTTCT TTTGACAAGT AAGGCATGAA

---------- ---------- ---------- ---------- ----------

AGGGATCTGC CCTCTTCCAT TTATTTTCCT CTTGACAAGT AGGATGTGAA

AGGGATCTGC CCTCTTCCAT TTATTTTCCT CTTGACAAGT AGGATGTGAA

AGGAATCTGC CCTCTTCCAT TTATTTTTCT CCTGACAAGT AAGATGTGAA

AGGGATCTGC CCTCTTCCAT TTATTTTTCT CTCGACAAGT AAGATGTGAA

AGGGATCTGC CCTCTTCCAT TTATTTTTCT CTTGACAAGT AAGATGTGAA

AGGGATCTGC CCTCTTCCAT TTATACTTCT CTTGACGAGT AAGATGTGAA

---------- ---------- ---------- ---------- ----------

AGGGATCTGC CCTCTTCCAT TTATTTTTCT CTTGACAAGT AAGATGTGAA

AGGGATCTGC CCTCTTCCAT TTATTTTTCT CTTGACAAGT AAGATGTGAA

---------- ---------- ---------- ---------- ----------

AGGGATCTGC CCTCTTTCAT TTATTTTTCT CTTGACATGT AAGATGTGAA

AGACCTCTGG CCTCTTCTGT TGATCTTCCT CCTGGTAAGT GAAGTGTGAA

ATGTTGTTCC ATGCTTTTCT AATTTAAATG TTGCACATGT TGAATACTGA

ATGTTGTTCC ATGCTTTTCT AATTTAAATG TTGCACATGT TGAATACTGA

ATGTTGTTCC ATGCTTTTCT AATTTAAATG TTGTACATGT TGAATACTGA

ATGTTGTTCC ATGCTTTTCT AATTTAAATG TTGTACATGT TGAATACTGA

ATGTTGTTCC ATGCTTTTCT AATTTAAATG TTGCACATGC TGAATACTGA

---------- ---------- ---------- ---------- ---ATATTGA

ATGTTGTTCC ATGCCTTTCT AATTTAAATG TTGCACATGT TGAGTGTTGA

ATGTTGTTCC ATGCCTTTCT AATTTAAATG TTGCACATGT TGAGTGTTGA

ATGTTGTTCC ATGCCTTTCT AATTTAAATG TTGCACATGT TGAATATTGA

ATGTTGTTCC ATGCCTTTCT AATTTAAATG TTGCACATGT TGAATATTGA

ATGTTGTTCC ATGCCTTTCT AATTTAAACG TTGCACATGT TGAATATTGA

GTGTTGTTCC ATGCCTTTCT AATTTAAATG TTGCACATGC TGAATACTGA

---------- ---------- ---------- ---------- ----------

ATGTTGTTCC ATGCATTTCT AATTTAAGTG CTGCACATGT TGAATATTGA

ATGTTGTTCC ATGCCTTTCT AATTTAAACG TTGCACATGT TGAGTATTGA

---------- ---------- ---------- ---------- ----------

ATGTTGTTCC ATGCTTTTCT AATTTAAATG TTGCACATGT TGAGTGTTGA

ATGCCACCCC TCGCATTTGC ATTGTACAGG CTATATGTGC TGATTGTTGA

ATTCTAAATT ATTAGGTATT AACACAAGTG TGTCCTGCTT TTTTATTTCA

ATTCTAAATT ATTAGGTATT AACACAAGTG TGTCCTGCTT TTTTATTTCA

ATTCCAAATT ATTATGTATT AACACAACTG TGTCCTGCTT TTTTATTTCA

ATTCCAAATT ATTATGTATT AACACAACTG TGTCCTGCTT TTTTATTTCA

ATTCCAGATT ATTGTGTATT AACACAACTG TGTTCTGCTT TTTTATTTCA

ATTCCAAATT ACTATGTATT AACACATCTG TCTCCTGCTT TTTC-TTTCA

ATTCATAATC ACTGTACAGT AACACATCTG TCTCCTGTTT TTTAATTTCA

ATTCATAATC ACTGTATAGT AACACATCTG TCTCCTGTGT TTTAATTTCA

ATTCCAAATT ACCCTGCATT AACACATCTG TGTTCTGCTT TTTTGTTTCA

ATTCCAAATT GCTGTGTATT AATGCATCTG TCTCCTGCTT TTGTATTTCA

ATTCCAAATT ACTGTGTTTT AATGCATCTG TCTCCTGCTT TTTTATTTCA

ATTCCCAATT ACT-CGTATT AACACGTCTG TCTCCTGCTT TTTTATTTCA

---------- ---------- ---------- ---------- ----------

ATTCCAAATT GCTATGTATT AACACGTCTG TCTTCTGCTT TTTTATTTCA

ATTCCAAATT GCTATGTATT AACACATCTG TCTCCTGCTT TTTTATTTCA

---------- ---------- ---------- ---------- ----------

ATTTCAAATT GCTGTATATT AACACATCTG TCTCTTCTTT TTTTATTTCA

ATTCCAAAGA GCTGTATATT AACCCATCTG TCTCCTGCCT TTGTATTGCA

GTACCTACTA GAAATGAAAA GCTTAATCCT ACCCCCAGAA CATATCCTGA

GTACCTACTA GAAATGAAAA GCTTAATCCT ACCCCCAGAA CATATCCTGA

GTACCTACTA GAAATGAAAA GCTTAATCCT ACCCCCAGAA CATATCCTGA

GTACCTACTA GAAATGAAAA GCTTAATCCT ACCCCCAGAA CATATCCTGA

GTACCTACTA GAAATGAAAA GTTTAATCCT ACCCCCAGAA CACATCCTGA

GTACCTACTA GAAATGAAAA GCCTAATCCT ACCCCCAGAA CACATCCTGA

GTATCTGCTA GAAATGAAAA GCTTAATCCT CCCACCAGAG CACATCCTGA

GTATCTACTA GAAATGAAAA GCTTAATCCT CCCACCAGAA CACATCCTGA

GTACCTACTA GAAATGAAAA GCTTAATCCT ACCCCCAGAA CATATCCTGA

GTACCTACTA GAAATGAAAA GCTTAATCCT GCCCCCAGAA CACATCCTGA

GTACCTACTA GAAATGAAAA GCTTAATCCT ACCCCCAGAA CATATCCTGA

GTACCTCCTA GAAATGAAAA GCTTAATCCT GCCCCCAGAA CACATCCTGA

---------- ---------- ---------- ---------- ----------

GTACCTACTA GAAATGAAAA GCTTAATCCT TCCCCCAGAA CATATCCTGA

GTACCTACTA GAAATGAAAA GCTTAATCCT ACCCCCAGAA CATATCCTGA

---------- ---------- ---------- ---------- ----------

GTACCTACTA GAAATGAAAA GCTTAATCCT ACCCCCAGAA CATATTCTGA

GTACCTCCTA GAGATGAAAA GCTTAATCCT TCCCCCAGAA CACATCCTGA

AGAGAGGAGA CAGTGAAGCA ATAGCATATG CATTCTTTCA TCTTGCACAC

AGAGAGGAGA CAGTGAAGCA ATAGCATATG CATTCTTTCA TCTTGCACAC

AGAGAGGAGA CAGTGAAGCA ATAGCATATG CATTCTTTCA TCTTGCACAC

AGAGAGGAGA CAGTGAAGCA ATAGCATATG CATTCTTTCA TCTTGCACAC

AGAGAGGAGA CAGTGAAGCA ATAGCATATG CATTCTTTCA TCTTGCACAC

AGAGAGGAGA CAGTGAAGCA ATAGCGTACG CCTTCTTTCA TCTGGCGCAC

AGAGAGGAGA CAGTGAAGCA ATAGCATATG CATTCTTTCA TCTTGCACAC

AGAGAGGAGA CAGTGAAGCG ATAGCATATG CATTCTTTCA TCTTGCACAC

AGAGAGGAGA CAGTGAAGCA ATAGCATATG CATTCTTTCA TCTTGCCCAC

AGCGAGGGGA CAGTGAAGCA ATAGCATATG CATTCTTCCA TCTGGCGCAC

AGCGAGGGGA CAGTGAAGCG ATAGCATATG CGTTCTTTCA TCTGGCACAC

AGAGAGGGGA CAGTGAAGCC ATAGCCTATG CGTTCTTTCA CCTGGCTCAC

---------- ---------- ---------- ---------- ----------

AGAGAGGAGA CAGTGAAGCA ATAGCATATG CGTTCTTTCA TCTTGCGCAC

AGAGAGGAGA CAGTGAAGCA ATAGCATATG CGTTCTTCCA TCTTGCACAC

---------- ---------- ---------- ---------- ----------

AGAGAGGCGA CAGTGAAGCA ATAGCCTATG CTTTCTTTCA TCTTCAGCAC

AGCGAGGAGA CAGTGAAGCA ATAGCGTACG CCTTCTTTCA TCTTCAGCAC

TGGAAGAGAG TGGAAGGGGC TTTGAATCTT TTGCATTGTA CGTGGGAAGG

TGGAAGAGAG TGGAAGGGGC TTTGAATCTT TTGCATTGTA CGTGGGAAGG

TGGAAGAGAG TGGAAGGGGC TTTGAATCTT TTGCATTGTA CGTGGGAAGG

TGGAAGAGAG TGGAAGGGGC TTTGAATCTT TTGCATTGTA CGTGGGAAGG

TGGAAGAGGG TGGAAGGGGC TTTGAATCTT TTGCATTGTA CATGGGAAGG

TGGAAGAGGG TGGAAGGGGC TTTGAATCTT TTACACTGTA CGTGGGAAGG

TGGAAGAGGG TGGAAGGGGC TTTGAATCTC TTGCATTGTA CGTGGGAAGG

TGGAAGAGGG TGGAAGGGGC TTTGAATCTC TTGCATTGTA CGTGGGAAGG

TGGAAGAGGG TGGAAGGGGC TTTGAACCTT TTACATTGTA CGTGGGAAGG

TGGAAGAGGG TAGAAGGAGC TTTGAATCTT CTCCATTGTA CATGGGAAGG

TGGAAGAGGG TGGAAGGGGC TTTGAATCTT TTGCATTGTA CGTGGGAAGG

TGGAAGAGGG TCGAAGGGGC TTTGAACCTT TTGCATTGTA CCTGGGAAGG

---------- ---------- ---------- ---------- ----------

TGGAAGAGGG TGGAAGGGGC TTTGAATCTT TTGCATTGTA CATGGGAAGG

TGGAAGAGGG TGGAAGGGGC TTTGAACCTT TTGCATTGTA CGTGGGAAGG

---------- ---------- ---------- ---------- ----------

TGGAAGAGGG TGGAGGGGGC TTTGAATCTT TTACATTGTA CGTGGGAAGG

TGGAAGAGAG TGGAGGGCGC TTTGAACCTT TTACATTGTA CGTGGGAAGG

CAGTAAGTAA TTTTCTTTTT TTGAAACCAC AAATTCAAAG GTTTCCAAAT

CAGTAAGTAA TTTTCTTTTT TTGAAACCAC AAATTCAAAG GTTTCCAAAT

CAGTAAGTAC TCTTCTTTTT TTGAAACCAC AAATTCAAAG GTTTCCAAAT

CAGTAAGTAC TCTTCTTTTT TTGAAACCAC AAATTCAAAG GTTTCCAAAT

CAGTAAGTAA TTTTCTTTTT TTGAAACCAC AAATTCAAAG GTTTCCAAAT

CAGTAAGTAA TTTTCTTTCT TTGAAACCAC AAATTCAAAG GTTTCCAAAT

CAGTAAGTAA TTGTTTT-CT TTGAACCCAC AAATTCAAAG GTTTCCAAAT

CAGTAAGTAA TTTTTTTTCT TTGAACCCAC AAATTCAAAG CTTTCTAAAT

CAGTAAGTAA TTTTCTTTCT TTGAAACCAC AAATTCAAAG GTTTCCAAAT

CAGTAAGTAA TTTTCTTTCT TTGAAACCAC AAATTCAAAG GTTTGTAAAT

CAGTAAGTAA TTTTCTTTCT TTGAAACCAC AAATTCAAAG GTTTCTAAAT

CAGTAAGTAA TTTTCTTTCT TTTTAACCAC AAATTCAAAG GTTTCTAAAT

---------- ---------- ------CCAC AAATTCAAAG GTTTCTGAAT

CAGTAAGTAA TTTTCTTCCT TTGAAACCAC AAATTCAAAG GTTTCCAAAT

CAGTAAGTAA TTTTCTTTCT TTGAAACCAC AAATTCAAAG GTTTCCAAAT

---------- ---------- ---------- ---------- ----------

CAGTAAGTAA TATTCTTTCT TTGAAACCAC AAATTCAGAG GTTTCCAAAT

CAGTAAGTGT TTTCTTTTCT TGGAAACCAC AAACCCACAG TTTTCCCAGC

GTTTGATTCA TCACTGCCAG CCCCTGAAAT TACACAATAC GTTCCTCAAA

GTTTGATTCA TCACTGCCAG CCCCTGAAAT TACACAATAC GTTCCTCAAA

GTTTGATTCA TCACTGCCAG CCCCTGAAAG TACACAATAT GTTC------

GTTTGATTCA TCACTGCCAG CCCCTGAAAG TACACAATAT GTTC------

GTTTGATTCA TCACTGCCAG CCCCTGAAAT TACACCATAT ATTCCTCAAA

GTTTGATTCA TCACTGCCAG CCTCTGAGAT TACACATTAT GTTCCTCAAA

GTTTGATTCA TCACTGCCAG ACCCCAAAAT TACACAA--- -----TCAAA

GTTTGATTCA TCACTGCCAG CCCCCAAAAT TATACAA--- -----TCAAA

GTTTGATTCA TCACTGCCAG CCCTTGAGAT TAAACAATAT GTTCCTCAAA

GTTTGATTCA TCACTACCAA CCACTGAAAT TACACAATGT GTTTCTAAAA

GTTTGATTCA TCACTGCCGG CCCCTGAAAT TACACAATAT GTTCCTCAAA

GTTTGATTCA TCATTGCCAG CC-CTGAAAT TACACAATAT GTTCCTCAAA

GTTTGATTCA TCACTGCCAG CCCCTGAAAT TACACAACAT GTTCTTCAAA

GTTTGATTCA TCACTGCCAG CCCCTGAAAT TACACAATAT GTTCCTCAAA

GTTTGATTCA TCACTGCCAG CCCCTGAAAT TACACAATAT GTTCCTCAAA

---------- ---------- ---------- ---------- ----------

GTTTGATTCA TCACTACCAG CCACTGAAAT TACACAATAT GTTCCTCAAA

GTTTAATCCG CGCCTACCCC CCCCCGCAGT TACACAATCT GTTCCTCGGA

GTCAGAAAGA AGAGTTTTAA TCAAGATTTT ATAATATGTT TCCAGTTCAT

GTCA------ ---------- -TAAGATTTT ATAA-ATGTT TCCAGTTCAT

--CAGAAAGG AGGGTTTTAA GCAAGATTTT ATAATATGTT TCCAGTTCAT

--CAGAAAGG AGGGTTTTAA GCAAGATTTT ATAATATGTT TCCAGCTCAT

GTCAGAATGA AGAGTTTTAA TCAAGATTTT ATAATATATT TCTAGTTCAT

GTCACAAAGA AAAGTTTTAA TTAAGATTGT ATAGTATGTT TCTAGTTCAT

GTCCGAAGGA AGAGTTTTAA -TAAGACTTT ---CTATGTT TCTGGATTAT

GTCCTAAAAA AGAGTTTCTA TTAAGCCTTT -----GTGTT TCTGGACTGT

GTCAGAAAGA AAAGTCTTAA TCAAGGTTTT ATACTATGTT TCTAGTTCAT

GTCAGAAAGA AATGTTTTAA TTAAGATTTA AAAACATGTT CCAAGTTCAT

GTCAGAAAGA CAAGTTTTAA TCAAGATTTA AAAATACATT TCTAGTCCAT

GTCAGAAAGA AAAGTTTTAA TCAAGATTTA AAAATATGTT TCTAGTTCAT

GTCAGAAAGA AAAGTTTTCA TCAAGATTTA AAATTAAGTT TCTAGTTCAT

GTCAGAAAGG CAAGTTTTAT TCAAGATTTT ATAATATGTT TTTAGTTCAT

GTCAGAAAGA AAAGTTTTAA TCAAGATTTT ATGTTTTGCC CTTAGTTCAT

---------- ---------- ---------- ---------- ----------

GTCTGAAAGA AATGttttat taaaaatttt atGCTTTGTT TTTAGTTCAT

GTGTGAAAAA AATGCTTTAT TGAGAATTTT ACGCTCTGTT TTTGCTTTAT

TACATAGCTG CCACCTTATC TGAATCACAT TTATTGAAAC ACTGTTCTGG

TACATAGCTG CCACCTTATC TGAATCACAT TTATTGAAAC ACTGTTGTGG

TACATAGCTG CCACCTTATC TGAATCGTAT TTATTTAAAC ACTGTTCTGG

TACATAGCTG CCACCTTATC TGAATCGTAT TTCTTTAAAC ACTGTTCTGG

TACACAGCTG CCACCTTATC TGAATTATAT TTATTTGAAC ACAGTTCTGG

TTTGCAGCTG CCACCTTATC TGAATTATAT TTATTTGAAC AAGGTTCTGG

TACACAGCTG CCTCCTTGTC GTAATTATGT TTGTTTAAAC ATGCCTCTGG

TGCATGACTG CCACCATATC GGAATTATGT TTGCTTAAAC ATGGCCCTGG

TACACAGCTG CCACCTTATC TGAATTATAC TTATTTAAAC AAGTTTCTGG

TACACAACTG CCACATTATC TGAATTATAT TTATTTAAGC AAGGTTCTGG

CGTACAGCTG CCACCTTATC TGAATTATGC TTATTTAAAC AAGGTTCTGG

TACACAGCTG CCACCTTATC TGAATTATAC TTATTTAAAC AAGGTTCTGG

TACAGAGCTG CCACCTTATC TGAATTATAC ----TTAAAC AAGGTTCTGG

TACACAGCTG TCACCTTATC CAAATTATAC TCATTTAAAC AAGGTTCTGG

TACACAGCTA CCGCCTTATC TGAATTATAC ACATTTAAAC AAGGTTCTGG

---------- ---------- ---------- ---------- ----------

TAAGCAGCTG CCACCTTATC TGAATTATAC TCATTTAAAC AACGCTCTGG

GAAGCAGCTG CCACCTTATC TGAATTATAC CCTTTTAAAC ACGGCTCTGG

CGATCCTCAC CGATGAGATT CTGATTCTAA TTTATTCTCT TTGAACCTAA

CGATCCTCAC CGATGAGATT CTGATTCTAA TTTATTCTCT TTGAACCTAA

TGATCCTCAC CGATGAGATT CTGATTCTAA TTTATTCTCT TTGAACCTGA

TGATCCTCA- ---------- ---------- ---------- ----------

CTATCCTCAC CGCTGAGGTT CAGATTCTAA TTTATTCTCT TTGAACCTAA

TTAGCCTCAC CGATGAGATT ATGATTCTAA TGTATTCTCT TTGAACCTGA

TTCTCCTCAC CGGTCAGATT CTGATTCTAA TTTATTCTCT TTGAACCTAA

TTCTCCTCAC CGATAAGATT ATGATTCTAA TTTATTCTCT TTGAACCTGA

TTATCCTCAC CGATGAGATT GTGATTCTAA TTTATTCTCT TTGAACCTAA

TTATCTTCAC CGATGAGATT CTGATTCTAA TTTATTCTCT TTGAACCCGA

TTATCCTCAC CGATGAAATT CTGATTCTAA TTTATTCTCT TTGAACCTGA

TTATCCTcaC CGATGAAATT CTGATTCTAA TTTATTCTCT TTGAACCTAA

TTATCCTCAC CGATGAAATT CTGATTCTAA TTTATTCTCT TTGAACCTAA

TTATCCTCAC CGATGAGATT CCGATTCTAA TTTATTCTCT TTGAACTTAA

TTATCCTCAC CGATGAGATT CTGATTCTAA TTTATTCTCT TTGAACCTAA

---------- ---------- ---------- ---------- ----------

TTATCTTCAT CCATAAGATT TTGATTTGCA TTGGTTCTCT CTCAATTTGA

TTACCCTCAC CAATGAAATT CTGACTCCAC TCTTTTCTTC TTGGCTCCAA

TGGCAACTTA TCTCACACTT GACTGGGCCC TGCACTGCAG GGTGGGTAAA

TGGCAACTTA TCTCACACTT GACTGGGCCC TGCACTGCAG GGTGGGTAAA

TGGCAACTTA TCTCACACTT GACTAGGCCC TGCACTGCAG GGTGGGTAAA

------CTTA TCTCACACTT GACTGGGCCC TGCACTGCAG GGTGGGTAAA

TGGCAACTTA TCTCACACCC GACTGGGCCC TGCACTGCAG GGTGGGTTAA

TGGCAACTTA TCTCATACTT TTCTGGACCC TGCACAGCAG GGTGGGTAAA

TGTCAACTTA CCTCAGGCTT TGTTGGACCC CGCACAGCAG GGTGGGTGAA

TGTCAACTTA TCTCAGGCTC TGTTGGACCC TGCACAGCAG GGTGGGTGGA

TGGCAACATA TATCACACTT TGCTAGACCC TGCACAGCAG GGTGGGCAAG

TGGTAACTTA TCTCACACTT GGCTGGACCC CGCCCAGCAG GGTGGATAAA

TGGTAACTTA TCTCACAGTC CGCTGGACCC TGCACAGCAG GGTGGGTAAA

TGGTAACTTA TCTCACACTT TGTTGGACCC TGCACAGCAG GGTGGGTGAA

TGATAA---- ---------- ---------- ---------- ----------

AGGCAACTTA TCTCACACTT TGCTGGACCC TGCACAATAG AGTGGGTAAA

TGGTAACTTA TCTCACACTT TGCTGGACCC TGCTCAGCAG GGTGGGTGAA

------CTTA TCGCACACAT TGCTGAACCC TGCACAGCAG GCTGGGTGAG

TGGTAACTTA TCTCACACAT TTTTAAACCT AGCACAGCAG GGCGGATAAG

AAGCAACCAA TACCATCCAC TGTTAACCCC AGCACAACAG GGTGGGTGAG

GGATAGAATT GCAAGCAGAA ATTGTATTTG CTTTTGTTTA GTGAAGCCAA

GGATAGAATT GCAAGCAGAA ATTGTATTTG CTTTTGTTTA GTGAAGCCAA

GGATAGAATT GCAAGCAGAA ATTGTATTTG CTTTTGTTTA GTGAAGCCAA

GGATAGAATT GCAAGCAGAA ATTGTATTTG CTTTTGTTTA GTGAAGCCAA

GGATAGAATT GCAAGCAGAA ATTGTATTTG CTTTTGTTTA GTGAAGCCAA

GGATAGAATT GCAAGCAGAA ATTGTATTTG CTTTTGTTTA GTGAAGCCAA

GGATAGAATT GCAAGTAGAA ATTGTGTTTG CTTTTGTTTA GTGAAGCC--

GGATAGAATT GCAAGTGGAA ATTGTGTTTG CTTTTGTTTA GTGAAGCC--

GGAT------ ---------- ATTAAATTTG AT----TTTA GTGAAGCCAG

GGATAGAATT GCAAACAGAA ATTGTGTTTG CTCTTGTTTA GTGAAGCCAA

GGATAGAATT GCAAACAGAA ATCGTATTTG CTTTTGTTTG GCGATGCCAA

GGATAGAATT GCAAACAGAA ATTGTATTTG CTTTTGTTGA GTGAAGCCAA

---------- ---------- ---------- ---------- ----------

GGATAGTATT GCAACCAGAA ATTTTATTTT TCTTTGTTTA GGGGAGCCCA

AGATAGGATT ACAGGCAGAA ATTGTATTTG CTTTTGTTTA GTGAAGCCAA

AGATAGGATT CCGAGC-GAA ATAGTATTTG CTCTTGTTTA GGGAAGCCAC

GGATAGAATT CCAGTCGAAA ATTGTATTTG TTTTTGTTTG GCGAAGCCAA

GGAGACAATT CCAGGCAGAA AGCGTTTTTG CTCTTGTTTA GCATAGCCAA

AAGCCTTAAT ATTATTTTTT GAGCTCAATG CAAATGGCAA CTGACATGAG

AAGCCTTAAT ATTATTTTTT GAGCTCAATG CAAATGGCAA CTGACATGAG

AAGCCTTATT ATTATTTTAT GAGCTCAATG CAAATGACAG CTGACATGAG

AAGCCTTATT ATTATTTTAT GAGCTCAATG CAAATGACAG CTGACATGAG

AAGCCTTAAT ACTATTTTAT GAGCTCAATG CAAATGACAA GTGACATGGG

AGACCTTAAT ATTATTTTAT GAACTCAATG TAAATGACAA CTGACATGAG

AAATCTTAAT ATTATTTTAT GAACTTAATG TAAATGATAT CTGACATGAG

AAATCTTAAT ATTATTTTAT GAACTTAATG TAAATGATAT CTGACATGAG

AAGCCTTAAT ATGATTTTAT AAGCTCAGTG TAAATGACA- ATGACAT--G

AAGTCTTAAC ATTATTTTAT GAACTCGATG TAAATGACAA CTGACATGAG

AAAGCTTAAT CTTATTTTAT GAACTCGATA TAAATGACAA CTGACATGAG

AAGTCTTAAT ATTATTTTAT GCACTAGATG TAGATGACAA CTGACATGAG

---------- ---------- ---------- ---------- ----------

AAACCTTAAT ATTGTTTTAT GAACTCAGTG TAAATGACAA CTGATGTGAG

AGGCCTCAAT ATTATTTTAT GAACTCAGTG TAAATGACAA CTGACGTGAG

AAG-CTTGAT GTTATTTTCT GTGCTCAATG TCAATGACAA CCGATCGGAG

AAGCCTTAAT GTTATTTAAT GGACTTAAAG CAGATGACAA CTGACAAGAG

AAGTCTTAAC ATTATTTTAT GGGCTTAATG CCTATGACAA CTGACAAGAG

TATTTCATTT AAAAAATTAC TCCCTGAAGT GCAAACTGAC TTCAATTTTG

TATTTCATTT AAAAAATTAC TCCCTGAAGT GCAAACTGAC TTCAATTTTG

TATTTCATTT AAAAAATTAC TCCCTGAAGT GCAAACTGAC TTCAATTTTG

TATTTCATTT AAAAAATTAC TCCCTGAAGT GCAAACTGAC TTCAATTTTG

TATTTCATTT AAAAAATTAC TCCCTGAAGT GCAAACTGAC TTCAATTTTG

TATTTCATTT AAAAAATTGT TCCCTGAAGT GTACAATGAC TTCAATTTTG

TATTTCTCTT TAAAAATTCT TTCCTGAAGT TCAAACTGAC TTTCATTTTA

CATTTTTCTT TAAAAATCCT TTCATGAAGT TCAAACTGAC TTCAATTCTG

TATTTCACTG CAAAAATTGC TCCCTGAAGT GTGAACCAAC TTCAATTTTG

TATTTCATTT AAAAAATTAC TCCCTGAAGT GCAAACTGAC TTCAGTTTTG

TATTTCATTT AAAAAATTGC TCCCTGAAGT ACAAACTGAC TTCAATTTTG

TATTTCATTT AAAAAATTGC TCCCTGAAGT GCAAACTTAC TTCAATTTTG

---------- ---------- ---------- ---------- ----------

TATTTCATTT TAAAAATTGT TCCCTGAAGT GCAAACTCAC TTCAATTTTG

TATTTCACTT AAACAGTCGC TCCCTGCAGT GCATACTGAC TTCGATTTTG

---------- ---------- ---------- ---------- ----------

TGCTTCCTTT TTAAAATGGC TCCCTGGGTA GAAATCCAGT TTCAACTATT

TATTTAATTT TGAAAATTGC TCCCTGCAGT GCAAGCCAAC TTCATGAGGG

AATAAAATTT AGATTCAAAT TTATAGCCAC TTTGAAAAAG CCGCAGAGTT

AATAAAATTT AGATTCAAAT TTATAGCCAC TTTGAAAAAG CCGCAGAGTT

AATAAAATTT AGATTCAAAT TTATAGCCAC TTTGAAAAAG CCGCAGAGTT

AATAAAATTT AGATTCAAAT TTATAGCCAC TTTGAAAAAG CCGCAGAGTT

AATAAAATTT AGATTCAAAT TTATAGCCAC TTTGAAAAAG CTGCAGAGTT

AATAAAATTT AGATTCAAAT TTATAGCCAG TTTGAAAAAG CCGAAGAGTT

AATGAAATTT ACTTTCAAAA TTATAGCCAT TATGAAAAAA CCGAAGAGCT

AATGAAGTTT ACTTTCAAAA TTATAGCAAT TGTGAAAAAA CCGAAGAGCT

AATAAAATTT AGATTCAAAT TTATAGCCAC CTTGAAAAAG CCGAAGAGCT

AATAAAGTTT AGATTCAAAT TTATAGCCAC TTTGAAAAAG CCGAAGAGTT

AATAAAATTT AGATTCAAAT TTATAGCCAC TTTGAAAAAG CCGAAGAGTT

AATACAATCT AGATTCAAAT TTATAGCCAC TTTGAAAAAG CCGAAGAATT

---------- ---------T CTATAGCCAC TTTGAAAAAG CCGAAGAGCT

AGTAAAATTT AGATTCAAAT TTATAGCCAC TTTGAAAAAG CCAAAGAGTT

AGTAAAATTT AGATTCAAAT TTATAGCCAC TTTGAAAAAG CTGAAGAGTT

---------- ---------- ---------- --------aa tt--------

GATGAGATTT AAATTCATAT TTATAACCTC TTTGAATGAG CTGAAGCACT

GATGAAATCT TAATGTAAAT GTGTCCCGAC CTTGAAAATG CCGAAGAACT

AGTCTGGGTG TTTTAATGTT TTAATCTCTG TTTAAGAGGT TTCTGTTATG

AGTCTGGGTG TTTTAATGTT TTAATCTCTG TTTAAGAGGT TTCTGTTATG

AGTCTGGGTG TTTTAATGTT TTAATCTCTG TTTAAGAGGT TTCTGTTATG

AGTCTGGGTG TTTTAATGTT TTAATCTCTG TTTAAGAGGT TTCTGTTATG

AGTCTGGGTG TTTTAATGTT TTAATCTCTG TTTAAGAGGT TTCTGTTATG

AGTCTGGGTG TTTTAATGTT TTAATCTCTG TTTAAGAGGT TTCTGTTACG

AGTCTGGGTG TTTTAATGTT TTAA--TCTG TTTAAGAGGT TTCTGTTACA

AGTCTGGGTG TTTTAATGTT TTAA--TCTG TTTAAGAAGT TTCTGTTACA

AGTCTGGGCG TTTTAATGTT TTAATCTCTG TTTAAGAGGT TTCTGCTAC-

AGTCTGGGTG TTTTAATGTT TTAATCTCTG TTTAAGAGGT TTCTGTTACA

AGTCTGGGTG TTTTAATGTT TTAATCTCTG TTTAAGAGGT TTCTGTTACA

AGACTGGGTG TTTTAATGTT TTAATCTCTG TTTATGAGGT TTCTGTTACG

AGTCCGGGTG TTTTAATGTT TTAATCTCTG TTTAAGAGGT TTCTGTAACG

AGTCTGGGTG TTTTAATGTT TTAATCTCTG TTTAAGAGGT TTCTGTTACG

AGCCTGGGTG TTTTAATGTT TTAATCTCTG TTTAAGAGAT TTCTGCTACG

---------- ttttaatgtt ttaatCTCCG TTTAAGAGAT TTCTGTTCTG

AGCCTGAGTG TTTTAATGTT TTAATCTCTG TTTAAGAGGT TTCTGTTACT

GGCCTGAGTG TTTTAATATT TTAATCTCTG TTTAAGAGGT TTCTGTTTCT

AGAGGAAAAT TGATAACATT TTTACCTCAG CAAACTGGCT CTGCTCTAAT

AGAGGAAAAT TGATAACATT TTTACCTCAG CAAACTGGCT CTGCTCTAAT

AGAGGAAAAT TGATAACATT TTTACCTCAG CAAACTGGCT CTGCTCTAAT

AGAGGAAAAT TGATAACATT TTTACCTCAG CAAACTGGCT CTGCTCTAAT

AGAGGAAAAT TGATAACATT TTTACCTCAG CAAACTGGCT CTGCTCTAAT

AGAGGAAAAT TGATAACATT TTTACCTCAG CAAACTGGCT CTGCTCTAAT

AAAAGAAAAT TTGTAACATT TTTACCTCAG CAAACTGGCT CTGTTCTAAT

AAAAGAAAAT TTGTAACATT TTTACCTCAG CAAACTGGCT CTGTTCTAAT

--GAGAAAAT TGATAACATT TTTACCTCAG CACACTGGCT CTGCTCTAAT

AGAGGAAAAT TGATAACATT TTTACCTCAG CCAACTGGCT CTGCTCTAAT

AGAGGGAAAT TGATAACATT TTTACCTCAG CCAACTGGCT CTGCTCTAAT

AGAGGAAAAT TGATAACATT TTTACCTCAG CTAACGGGAC GTGCTCTAAT

AGAGGAAAAT TGATAACACT TTTACCTCAG CAAACTGGCT CTGCTCTAAT

AGGGGAAAAT TGATAACATT TTTACCTCAG CAAACTGGCT CTGCTCTAAT

AGAGGAAAAT TGATAACATT TTTACCTCAG CAAACTGGCT CTGCTCTAAT

AGAGGAAAAT TGATAACATT TCTACCTCAG CAAACTGGCG GCGCCCTAAT

AGAAGAAAAT TGATAACATT TTTACCTCAG CAAACTGGCT CTGTTCTAAT

AGAGGAAAAT TGATAACATT TTTACCTCAG CAGACTGGCT TTCTTCTAAT

CCCACATTTA TAGTGATTTA TATTCTCCGT GGCAATGTGC TGTAACCTCA

CCCACATTTA TAGTGATTTA TATTCTCCGT GGCAATGTGC TGTAACCTCA

CCCACATTTA TAGTGATTTA TATTCTCCGT GGCAATGTGC TGTAACCTCA

CCCACATTTA TAGTGATTTA TATTCTCCGT GGCAATGTGC TGTAACCTCA

CCCACATTTA TAGTGATTTA TATTCTCCGT GGCAATGTGC TGTAACCTCA

CCCACATTTA TAGTGATTTA TATTCTCCGT GGCAATGTGC TGTAACCTCA

CCCACGTTCA TCGTGATTTA TATTCTCCAT GGTAATGTGC TATAACCTCA

CCCACATTTA TAGTGATTTA TATTCTCCAT GGCAATGTGC TATAACCTCA

CCCACATTTA TAGTGATTTA TAGTCTCCGT GGCAATGTGC TGTAACCTCA

CCCACGTTTG TAGTGATTTA TATTCTCCGT GGCAACGCGC TGTAACCTCA

CCCACGTTCG TGGTGATTTA TATTCTCCAT GGCAATGTGC TGTAACCTCG

CCCACGTTTA CAGTGATTTA TATTCTCCGT GGCAGCGTGC GGTAACCCCA

CCCACGTTTC TAGTGATTTA TATTCTCCGT GGCAATGTGC TGTAACCTCA

CCTGCATTTA TAGTGATTTA TATTCTCCGT GGCAATGTGC TGTAACCTCA

CCCACGTTTA TAGTGATTTA TATTCTCCGT GGCAACGGGC TGTAACCTCA

CCCGCATTGA GAGTGATTTA TATTCTCCGT GGCAATGCGC TGTAACCCCA

CCTGAGTTTA TAGTGATTTA TATTCTCTGT GGCAATGGGC TGTAACCTCA

CCCGAGTTTA GAGTGATTTA TATTCTCTGT GGCAAGGTAC GGTAACCTCA

GAGTGATACC ACTGCGGTGT AGATTGACTG CATTCCAAAT GCTGGAATCA

GAGTGATACC ACTGCGGTGT AGATTGACTG CATTCCAAAT GCTGGAATCA

GAGTGATACC ACTGCGGTGT AGATTGACTG CATTCCAAAT GCTGGAATCA

GAGTGATACC ACTGCGGTGT AGATTGACTG CATTCCAAAT GCTGGAATCA

GAGTGATACC ACTGCGGTGT AGATTGACTG CATTCCAAAT GCTGGAATCA

GAGTGATACC ACTGTGGTGT AGATGGACTG CATTCCAAAT GCTGGGATCA

CAGTGATACC ACTGTGGTGT GGATTGACTG CATTCCAAGT GCTGGAATCA

CAGTGATACC ACTGTGGTGT AGATTGACTG CATTCCAAAT GCTGGAATCA

GAGTGATACC ACTGTGGTGT GGATTGACTG CATTCCAAAT GCTGGAATCA

GAGTGATAGC ACTGTGGTGC AGATTGACTG CATTCCAAAT GCTGGGATCA

GAGTGATACC ACTGTGGTGC GGATTGACTG CATTCCAAAT GCTGGACTCA

GAGTGATACC ACTGTGGTGT GGATTGACTG CACTCCAAAC GCTGGGATCA

GAGTGATACC GCTGTGGTGC AGATTGACTG CATTCCAAAT GCTGGAATCA

GAGTGATACC ACTGTGGTGT AGATTGACTG CATTCCAAAT GCTGGAATCA

GAGCGATACC ACTGCGGTGT AGATTGACTG CATTCCAAAT GCTGGAATCA

GAGCGATACC ACTGCGGTGT AGATTGACTG CATTCCAAAT GCTGGGATCC

GAGTGATACC ACTGTGGTGC AGATTGACTG CATTCCAAAT GCTGGAATCA

GTGTGATACC ACTGTGGTGA GGATTGACTG CATTCCAAAT GCTGGAATCA

CATACATAAT GTTTTTACTT TTAAATAATA TTCAGACACC AGAATAAATA

CATACATAAT GTTTTTACTT TTAAATAATA TTCAGACACC AGAATAAATA

CATACATAAT GTTTTTACTT TTAAATAATA TTCAGACACC AGAATAAATA

CATACATAAT GTTTTTACTT TTAAATAATA TTCAGACACC AGAATAAATA

CATACATAAT GTTTTTACTT TTAAATAATA TTCAGACACC AGAATAAATA

CATACATAAT GTTTTTACTT TTAAATAATA TTCAGACACC AGAATAAATA

CATACATGCT GTTTTTACTT TTAAATAATA TTCAGAGACC AGAATAAATA

CATACATACT GTTTTTACTT TTAAATAATA TTCAGACACC AGAATAAATA

CATACATAAT GTTTTTACTT TTAAATAATA TTCAGACACT AGAATAAATA

CATACATAAT GTTTTTACTT TTAAATAATA TTCAGACACC AGAATAAATA

CATACATAAT GTTTTTACTT TTAAATAATA TTCAGACACC AGAATAAATA

CATACATAAT GTTTGTACTT TTAAATAATA TTCAGACACC AGAATAAATA

CATACATAAT G-TTTTACTT TTAAATAATA TTCAGACACC AGAATAAATA

CATACATAAT GTTTTTACTT TTAAATAATA TTCAGACACC AGAATAAATA

CATACATAAC GTTTTTACTT TTAAATAATA TTCAGACACC AGAATAAATA

CATACAGAAT GTTTTTACTT TTAAATAATA TTCAGACACC AGAATAAATA

CATACATAAT GTTTTTACTT TTAAATAATA TTCAGACACC AGAATAAATA

CATACATAAT Gtttttattt ttaaataata ttCAGACACC AGAATAAATA

CCATATGTGC ACTGATGCTG GTTACAATGG AACCTTGTCA TTTCTTGTGA

CCATATGTGC ACTGATGCTG GTTACAATGG AACCTTGTCA TTTCTTGTGA

CCATATGTGC ACTGATGCTG GTTACAATGG AACCTTGTCA TTTCTTGTGA

CCATATGTGC ACTGATGCTG GTTACAATGG AACCTTGTCA TTTCTTGTGA

CCATATGTGC ACTGATGCTG GTTACAATGG AACCTTGTCA TTTCTTGTGA

CCATATGTGC ACTGATGCTG GTTACAATGG GACCTTGTCA TTTCTCATGA

CCATATGTGC TGTGATGCTG GTTACAATGG AACCTTGTCG TTTCTTGTGA

CCATATGTGC ACTGATGCTG GTTACAATGG GACCTTGTCA TTTCTTATGA

CCATATGTGC ACCGATGCTG GGTACAACAG AACCTTGTCA TATTTTGTGA

CCATATGTGC ACTGATGCTG GTTACAATGG AACCTTGTCA TGTCTCGTGA

CCATATGTGC ACGGATGCCG GTTACAATGG AACCTTGTCA TGTCTTGGGA

CCATATGTGC ACCGATGCTG GTTACAATGG AACCTTGTCA TTTCTTGTGA

CCATATGTGC ACTGATGCTG GTTACAATAG AACCTTGTCA TTTCTTGGGA

CCATATGTGC ACTGATGCTG GTTACAATGG AACCTTGTCA TTTCTTGTGA

CCATATGTGC ACTGATACTG GTTACAATGG AACCTTGTCA TTTCTTGTGA

CCATATGTGC ACCGACGTGG GTTACAATGG AGCCTTGTCA TTCCTCGTGA

CCATATGTGC ACTGAGGCTA GTTACAATGG AACCTTGTCA TTTCTTGTGA

CCATATGTTG ACCGAGGCTG GTTACAATGA AACCTTGTCA TCCTGTGTGG

GGGAGAACAT TCTTTGTTTC TGATTCCTGT TTGAAATAAG TATGCAAAAG

GGGAGAACAT TCTTTGTTTC TGATTCCTGT TTGAAATAAG TATGCAAAAG

GGGAGAACAT TCTTTGTTTC TGATTCCTGT TTGAAATAAG TATGCAAAAG

GGGAGAACAT TCTTTGTTTC TGATTCCTGT TTGAAATAAG TATGCAAAAG

GGGAGAACAT TCTTTGTTTC TGAATCCTGT TTGAAATAAG TATGCAAAAG

GGGAGAACAT TCTTTGTTTC TGATTCCTGT TTGAAATAAG TACACAAAAG

GGGAGAATGT TCTTTGTCTC TGATTCTTAT TTGAAATCAG TATGCAGAAG

GGGAGAATGT TCTTTGTTTC TGATTCTTAT TTGAAATCAG TTTGCAAAAG

GAGAGAACGT TCTTTGTTTC TGATTCCTGT TTGAAATAAG TATGCAAAAG

GGGAGAACAT TCTTTGTTTC TGATTTCTGT TTGAAATAAG TATGCAAAAG

GGGAGAACAT TCTTTGTCTC TGATTCCTGT TTGAAACGAG TACGCAGAAG

GGGAGAACAT TCTTTGTTTC TGATTCCTGT TTGAAATAAG TATGCAAAAG

GGGAGAACAT TCTTTGTTTC TGATTCCTGT TTGAAATGAG TATGCAAAAG

GGGAGAACAT TCTTTGTTTC TGATTCCTGT TTGAAATAAG TATGCAAAAG

AGGAGAACAT TCTTTGTTTC TGATTCCTGT TTGAAATAAG TATGCAAAAG

GGGAGAACAC TCTTTGTTTC TGATCCCTGT TTGAAAGAAG AATGCAAAAG

GGGAGAACAT TCTACATTGC ATAGTCCTGT TGGAAAGAAG TATGCAAAAG

AGGAGAACAT TCTGGGGTCC TTATTACTGT TTGAAATAAG TATGCGGAAG

ATAAATATGT ACATTGACAG CATTTTGTTC ATAGGAACAG TGAGACTAAC

ATAAATATGT ACATTGACAG CATTTTGTTC ATAGGAACAG TGAGACTAAC

ATAAATATGT ACATTGACAG CATTTTGTTC ATAGGAACAG TGAGACTAAC

ATAAATATGT ACATTGACAG CATTT----- ---------- ----------

ATAAATATGT ACATTGACAG CATTTTGTTC ATAGGAACAG TGAGACTAAC

ATAAATATGT ACATTGACAG CGTTTTGTTC ATA-GAACAG TGAGACTAAC

GCATACATGT ACATTGACAG CATTTTGCTC ATAGGAGCAG TGAGACTGAC

GCACATATGT ACATTGACAG CATTTTGCTC ATAGGAACAG TGAGACTAAC

ATAAACATGT ACATTGACAG CATTTTGTTC ATAGGAACAG TGAGAGTAAC

ACAAATATGT ACATTGACAG CATTTTGTTC ATAGGAACAG TGAGACTAAC

ATGAATACGC ACACCGATGG CATTTTGTTC CTAGCAACAG TGAGACTAAC

ATAAATATGT ACATTGACAG CATTTTGTTC ATAGGCACAG TGAGACTAAC

ATAAATATGT ACATTGACAG CATTTTGAGC ACA------- ------TTAC

ATAAATATGT ACATAGACAA CATTTTGTTC ATTGGAACAG TGAGACTAAC

ATAAATATGT ACAGTGACAG CATTTTGTTC ATAGGCACAG TGAGACTAAC

ATAAATACGT GCAGGGGGAG CGTTTTGTTC ATAGGCACAG TGAGACTAAC

ATAAATAGGA CCATTGACAG CATTTTGTTC ATAGGAACAG TGAGACTAAC

ATAAATATGT CCACTGACAG CATTTTGTTC ATAGGAACAG TGAGACTAAC

ATGCTGATGA CATTGTAACA GGGAGAGTAA GTCATCTGAT GCTACAAAAG

ATGCTGATGA CATTGTAACA GGGAGAGTAA GTCATCTGAT GCTACAAAAG

ATGCTGATGA CATTGAAACA GGGAGAGTAA GTCATCTGAT GCTACAAAAG

---------- -----AAACA GGGAGAGTAA GTCATCTGAT GCTACAAAAG

ATGCCGATGA CATTGTAACA GGGAGGGTAA GTCATCTGAT GCTACAAAAG

ACGCCGATGA CATTGGAACA GGAAGAATAA GTCATCTGAT GCCACAAAAG

ATGCTGCTGA CATTGGAACA GGGAGACTAG GTCATCTGAT GCCATAGACA

ATGCTGCTGA CATTGGAACA GGGAGAATAA GTCATCTGAT GCCATAGACA

ATGCTGATGA CATTGTAACA GGGAGAGTAA GTCATCCGAT GCCACAAAAG

ATGCTGTTGA CATTGTAACA GGGAGAGTAA GTTATCTGAT GCCCCAAAAG

ATGCTGATGA CATCGTAACA GGGAGGGTAA GTTATCTGAA GCCACAAAGG

ATGCTGATGA CATGGTAACA GGGAGAGTAA GTCATCTGAT GCCACAAAAG

ATGCTGATGA CATTGTACCA GGGCGAGTAA GTTATCTGAT GCCACAGAAG

ATGCTGATGA CATTGTAGCA GGTAGAGTAA GTTATCTGAA GCCACAAAAG

ATGCTGATGA CATCGTAACC GGGAGAGTAA GTTGTCTGAA GCCACAAGAG

ATGCTGACGA CATGGTCACC GGGAGAGTCA GCTGTCCGAA GCCACAAGAG

ATGTTGATGA CATTGCGACA GTGAGAATAA GTTATCATAA GCCACAAAAG

ATGTTGATGA CATCGGAACA GTGAGAGTAA GTTATCGTAA GGCACAAGAG

CAGAATTAGG TGGCCTAACA TCATCAAAAT ACAGATAATC TCTCGAATGT

CAGAATTAGG TGGCCTAACG TCATCAAAAT ACAGATAATC TCTCGAATGT

CAGAATTAGG TGACCTAATG TCATCAAAAT ACAGATAATC TCTCGAATGT

CAGAATTAGG TGACCTAATG TCATCAAAAT ACAGATAATC TCTCGAATGT

CAGAATTATG TGGCCTAACA TTACCAAAAT ACAGATAATC TCTCGAATGT

CAGAATTAGG TGGCCTAATA TCATCAAAAT ACAGATAATC TCTCTAATGT

CAGAATTAGG TGGCCTGACA TCATCAAAAT GTAGACAATC TCTCTAATGT

CAGAATTAGG TGGCCTGACA TCATCAAAAT GTAGACAATC TCTCTAATGT

CAGAGTTAGG TGGCCTGACA TCATCAAGAT ACAGATAATC TCTCTAATGT

CAAAGTTACG TGGCCTGACA TCCTCAAAAT ACAGATAATC TCTCTAATGT

CAGAATTAGG TGGCCTGACA TCATCAAAAC ACAGATCATC TCTCTAA-GT

CAGAATTAGG TGGCCTGACA TCATCAAAAT ACAGATAATC TCCCGAACGT

GAGAGTTCGG TGCTGTGACA CCATCAAAAT GCAGATAATC TCTCTAATGT

CAGAATTAGG TGGCCTGACA TCACTGAAAT ATAAATAATC TCTCTAATGT

CAGAATTAGG TGGCCTGACA TCATCAACAT ACAGATAATC TCTCTAATGC

CAGAATTAGG TGGCCTGACA GCCTCAGCAA ACAGATAATC TCTCTAATGT

CATAATCAGG TGGCCTGACA TCATCAAAAT GCAGATAATC TCTCGAATGC

CATAATCAGG TGGTCTGACA TCACCGGAAT ACAGATAATC TCTCGGATCC

GTGAGTATTT TCTCTTTTCA GCTTTTCGGA TGATCCCTTA TCCCTTGGAA

GTGAGTATTT TCTCTTTTCA GCTTTTCGGA TGATCCCTTA TCCCTTGGAA

GTGAGTATTT TCTCTTTTCA GCTTTTCGGA TGATCCCTTA TCCCTTGGAA

GTGAGTATTT TCTCTTTTCA GCTTTTCGGA TGATCCCTTA TCCCTTGGAA

GTGAGTATTT TCTCTTTTCA GCTTTCCGGA TGATCCCTTA TCCGTTGGAA

GTGAGTAGTC TCTCTTTTCA GCTTTTCGAA TGATCCCTTA TCCCTTGGAA

GTGAGTAGTC TTTCTTTGCA GCTTTCCGGA TGATCCCATA TCCTCTGGAG

GTGAGTAGTC TTTCTTTGTA GCTTTCCGGA TGATCCCGTA TCCCCTGGAG

GTGAGTAGTC TCTCTTTTCA GCTTTTCGGA TGATCCCTTA TCCCTTGGAA

GTGAGTCGCC TCTCTTTTCA GCTTTTCGGA TGATCCCTTA TCCCTTGGAA

GTGAGTCGTC TCTTTTTTCA GCTTTTCGGA TGATCCCTTA TCCCTTGGAA

GTGAGTCTTC TCTCTTTTCA GCTTTTCGGA TGATCCCGTA CCCCTTGGAA

GTGAGTCGTT TCTCTTTTCA GCTTTCCGGA TGATCCCGTA TCCCTTGGAA

GTGCATAGTG TATCTTTTCA GCTTTTCGGA TGATCCCTTA TCCCTTGGAA

ACGCGTAGTC TCTCTTTTCA GCTTTTCGGA TGATCCCTTA TCCCTTGGAA

GCACGTGGTC TCTCTCTTCA GCTTTCCGGA TGATCCCTTA TCCCTTGGAA

GTGAGTATTT GTTTTTTTCA GCTTTTAGGA TGATCCCTTA TCCCTTGGAA

CTAAGTCGTT TGTCTTTTCA GCTTTCAGGA TGATTCCCTA CCCGTTGGAG

AAGGGGCACC TATTTTATCC TTACCCAATC TGTACAGAAA CAGCAGACCG

AAGGGGCACC TATTTTATCC TTACCCAATC TGTACAGAAA CAGCAGACCG

AAGGGGCACC TATTTTATCC TTACCCAATC TGTACAGAAA CAGCAGACCG

AAGGGGCACC TATTTTATCC TTACCCAATC TGTACAGAAA CAGCAGACCG

AAGGGGCACC TATTTTATCC TTACCCAATT TGTACAGAAA CAGCAGACCG

AAGGGGCACC TATTTTATCC TTACCCAATC TGTACAGAAA CAGCAGACCG

AAGGGGCACC TATTTTATCC ATACCCAATC TGTACAGAGA CAGCTGACCG

AAGGGACACC TATTTTATCC ATACCCAATC TGTACAGAAA CAGCTGACCG

AAGGGGCACC TATTTTATCC TTACCCTATC TGTACAGAAA CAGCAGACCG

AAGGGGCATC TGTTTTATCC CTACCCCATC TGTACAGAGA CAGCAGATCG

AAGGGGCATC TGTTTTACCC TTACCCCATC TGTACAGAAA CAGCAGATCG

AAGGGGCATC TGTTTTACCC CTACCCAATC TGTACAGAAA CAGCAGACCG

AAGGGTCATC TTTTTTATCC TTACCCAATC TGCACAGAAA CAGCAGACCG

AAGGGACACC TATTTTATCC TTACCCAATC TGCACAGAAA CAGCAGATAG

AAGGGGCATC TATTTTACCC TTACCCAATC TGTACAGAAA CAGCAGACAG

AAGGGGCACC TATTTTACCC TTACCCCATC TGTACAGAAA CGGCAGACAG

AAAGGGCACC TTTTTTACCC CTACCCCATC TGTACAGAAA CGGCAGACAG

AAAGGGCACC TGTTTTACCC GTATCCGATC TGCACGGAAA CAGCGGACAG

AGAGCTGCTT CCATGTAAGT ACATGTGGAA AATATTACAA CATTGTTTTG

AGAGCTTCTT CCATGTAAGT ACATGTGGAA AATATTACAA CATTGTTTTG

AGAGCTGCTT CCATGTAAGT ACATGTGGAA AATATTACAA CATTGTTTTG

AGAGCTGCTT CCATGTAAGT ACATGTGGAA AATATTACAA CATTGTTTTG

AGAGCTGCTT CCATGTAAGT ACATGTGGAA AATATTACAA CATTGTTTTG

AGAGCTGCTT CCATGTAAGT ACATGTGGAA AATATTATAA CATTGTTTTG

GGAGCTACTT CCCTGTAAGT ACATGTGGAA TGGACTGTAA CATTGTTTTG

GGAGCTGCTT CCCTGTAAGT ACATGTGGAA TGGATTGCAA CATTGTTTTG

AGAGCTGCTC CCATGTAAGT ACATGTGGAA AATATTACAA CATTGTTTTG

TGAGCTGCTT CCATGTAAGT ACACGTGGAA AGTATGACAA CATTGTTTTG

AGAGCTGCTT CCGTGTAAGT ---------- ---------- ----------

GGAACTGCTT CCGTGTAAGT GC-CGTGGAA AGTATGACAG CATTGTTTTG

AGAGCTGCTT CCCTGTAAGT ---------- ---------- ----------

AGAGCTGCTT CCATGTAAGT GCATGTGGGA AATATTATAA CATTGTTTTG

AGAACTGCTT CCATGTAAGT ACATGTGGAA GATATTAGAA CATTGTTTTG

AGAACTGCTC CCATGTAAGT ACATGTGGGA AATATTACAA CATTGTTTTG

GGAGCTGCTT CCATGTAAGT ACATGTGGAA AGTATTACAA CATTGTTTTG

GGAGCTGCTT CCATGTAAGT ACATGTGGAA AATATTACAA CATTGTTTTG

ACAATTCAGG CTAAATGGTT ATTATCAAAT CATACTATGA CAAGGATCAT

ACAATTCAGG CTAAATGGTT ATTATCAAAT CATACTATGA CAAGGATCAT

ACAATTCAGG CTAAATGGTT ATTATCAAAT CATACTGTGA CAAGGATCAT

ACAATTCAGG CTAAATGGTT ATTATCAAAT CATACTGTGA CAAGGATCAT

ACAATTCAGG CTAAATGGTT ATTATCAAAT CATACTGTGA CAAGGATCAT

ACAATGCAGG GTAAATAGTT ATTATCAAAT CATACTGCGA CAAGGATCAT

ACAATCCAAG CTAAATAGCT GTTATCCAAT CAGACTTGGA CAGGGATCAT

ACAATCCAGG CTAAATAGCT ATTATCCAAT CAGACTGTGG CAGGGATCAT

ACAGTTCAGG CTAAATAGTT ATTATCAGAT CAGACTGTGG TAAGGCGCAG

ACAATTCCAG CTAAATAGTT ATTATCAAAT TATACTGTGA TAAGGATCGT

---------- ---------- ---------- ---------- ----------

ACAATCCTGG CTAACTAGTT ACTATCAAGT CATACTGTGA CAAGG-TCAT

--------AG CTGAATAGTC ATTATCAAAT CAGACTGTGG GAGGA-TCCT

ACTCTTCAGG CTAAATAGTT ATTATCAAAT TATCCTGTGA TAAGGATCAC

ACAATTCGGA CTAAATAGTT ATTATCGAAT CATACTGTGA CAAGGCTCAT

ACAGTTTGGG CTAAATAGTT ATTAGCGAAT CATACTGTGA CGTGGAGCAT

ACAATTTGGA CTAAATAATT GTTATCAAAT CAGACTGTGA CAAGTATCAC

ACAAACTGGG CTAACTAGTT ATTATCCAAT CAAGCTGTGA CAAGTCACAA

GTCAGGCTCT TCTCTTCTCC ACAGCTTTCC ATGAAGTCTC AGTTTACCCA

GTCAGGCTCT TCTCTTCTCC ACAGCTTTCC ATGAAGTCTC AGTTTACCCA

GTCAGGCTCT TCTCTTCTCC ACAGCTTTCC ATGAAGTCTC AGTTTACCCA

GTCAGGCTCT TCTCTTCTCC ACAGCTTTCC ATGAAGTCTC AGTTTACCCA

GTCAGGCTCT TCTCTTCTCT ACAGCTTTCC ATGAAGTCTC AGTTTACCCA

GTCAGGCTCT TCTCATCTCC ACAGCTTTCC ACGAAGTCTC AGTTTACCCA

ATTGAGCTCT TCTTTTCTCC TCAGCTTTCC ATGAAGTCTC AGTTTACCCA

AGTAAGCTCT TCTTTTCTCC TCAGCTTTCC ATGAAGTCTC AGTTTACCCA

GTCAGACTCT TCTCTTCTCC ACAGCTTTCC ATGAAGTCTC AGTTTACCCA

GTCAGGCCCT TCTCCTCTCC ACAGCTTTCC ATGAAGTCTC AGTTTACCCC

---------- --TCTCCCCC GCAGCTTTCC ACGAAGTCTC AGTTTACCCG

GTCAAGCTCT TCTCTTCTCC ACAGCTTTCC ATGAAGTCTC AGTTTACCCA

GTCAGGCTTT TCTC------ ACAGCTTTCC ACGAGGTCTC AGTTTACCCA

ATCAGGCTCT TCTCTTCTCC ACAGCTTTCC ATGAAGTCTC AGTTTACCCC

GTCAGGCTTT TCTCTTCTCC ACAGCTTTCC ATGAAGTCTC AGTTTACCCA

GTCAGGCCCT TCTCTTCTCC ACAGCTTTCC ATGAAGTCTC AGTTTACCCA

TTCAGGCTTT TTCCTTCTCT GCAGCTTTCC ATGAAGTATC AGTTTACCCA

TTAAGACTCC GTTCTCCCCT ACAGCTTTCC ATGACGTTTC CGTTTACCCG

AAGAAGGAGC TTCCCTTCTT TATTCTCTTT ACTGCTGGAT TATGTTCCTT

AAGAAGGAGC TTCCCTTCTT CATTCTCTTT ACTGCTGGAT TATGTTCCTT

AAGAAGGAGC TTCCCTTCTT TATTCTCTTT ACCGCTGGAT TATGTTCCTT

AAGAAGGAGC TTCCCTTCTT TATTCTCTTT ACTGCTGGAT TATGTTCCTT

AAGAAGGAGC TTCCCTTCTT TATTCTCTTT ACTGCTGGAT TATGTTCCTT

AAGAAGGAGC TTCCCTTCTT TATCCTCTTC ACTGCGGGAT TGTGTTCCTT

AAGAAGGAAC TTCCCTTCTT CATCCTCTTC ACTGCTGGAT TGTGTTCCTT

AAGAAGGAAC TTCCCTTCTT CATCCTCTTC ACTGCTGGAC TGTGCTCCTT

AAGAAGGAGC TTCCCTTCTT CATTCTCTTT ACTGCTGGAT TGTGTTCCTT

AAAAAGGAGC TTCCCTTCTT CATTCTCTTT ACTGCTGGAT TGTGTTCCTT

AAGAAGGAGC TCCCCCTCTT CATTCTCTTC ACTGCCGGGT TGTGCTCCTT

AAGAAGGAGC TTCCCTTCTT TATTCTGTTT ACTGCTGGAT TGTGTTCCTT

AAGAAGGAGC TTCCCTTCTT TATTCTCTTT ACTGCTGGAC TGTGTTCCTT

AAGAAGGAGC TTCCCTTCTT TATTCTCTTT ACTGCTGGAT TATGTTCCTT

AAGAAGGAGC TTCCCTTCTT TATTCTCTTT ACTGCTGGAT TGTGTTCCTT

AAGAAGGAGC TCCCCTTCTT TATCCTCTTC ACCGCTGGAC TGTGTTCCTT

AAGAAGGAGC TTCCTTTCTT TATTCTCTTC ACTGCTGGAT TATGTTCCTT

AAGAAGGAGC TTCCTTTCTT CATCCTCTTC ACCGCCGGGC TGTGTTCGTT

CACAGCCATG CTGGCCCTCC TGACACATCA GTTCCCGGAA CTTATGGGGG

CACAGCCATG CTGGCCCTCC TGACACATCA GTTCCCGGAA CTTATGGGGG

CACAGCCATG CTGGCCCTCC TGACACATCA GTTCCCGGAA CTTATGGGGG

CACAGCCATG CTGGCCCTCC TGACACATCA GTTCCCGGAA CTTATGGGGG

CACAGCCATG CTGGCCCTCC TGACACATCA GTTCCCGGAA CTTATGGGGG

CACAGCCATG CTGGCCCTCC TGACACACCA GTTCCCGGAA CTCATGGGGG

CACAGCCATG CTGGCCCTCC TGACACATCA GTTTCCGGAA CTTATGGGAG

CACAGCCATG CTGGCCCTCC TGACACATCA GTTTCCGGAA CTTATGGGAG

CACGGCCATG CTGGCTCTCC TGACACATCA GTTCCCGGAA CTCATGGGAG

CACAGCCATG CTGGCCCTCC TGACACATCA GTTCCCAGAA CTCATGGGGG

CACAGCCATG CTGGCCCTCC TGACGCACCA GTTCCCGGAG CTCATGGGGG

CACAGCCATG CTGGCCCTCC TGACACATCA GTTCCCGGAA CTTATGGGGG

CACAGCGATG CTGGCCCTCC TGACACACCA GTTCCCAGAA CTTATGGGGG

CACAGCCATG CTGGCCCTCC TGACACATCA GTTCCCGGAA CTTATGGGGG

CACAGCCATG CTGGCCCTCC TCACACATCA GTTCCCAGAA CTCATGGGGG

CACAGCCATG CTGGCCCTCC TGACCCATCA GTTCCCGGAG CTCATGGGGG

CACAGCCATG CTGGCCCTCC TGACCCATCA GTTCCCAGAG CTTATGGGGG

CACGGCCATG CTGGCCCTGC TGACGCATCA GTTCCCCGAG CTCATGGGTG

TCTTCGCAAA AGCTGTGAGT GAAAAGATAA GCTTGCACAT ATAAATACGT

TCTTCGCAAA AGCTGTGAGT GAAAAGATAA GCTTGCACGT ATAAATACGT

TCTTCGCAAA AGCTGTGAGT GAAAAGATAA GCTTGCACAT ATAACTACGT

TCTTCGCAAA AGCTGTGAGT G--AAGATAA GCTTGCACAT ATAACTACGT

TCTTCGCAAA AGCTGTGAGT GAAGAGATAA GGCTGCAT-- ATAAATACGT

TCTTCGCAAA AGCTGTGAGT GAAAAGATAA GCTTGCGCAT ATAAATAAGT

TCTTCGCAAA AGCTGTGAGT GAAAAGATAA GGCAGCTCAT ATAAGTAAGC

TCTTCGCAAA AGCTGTGAGT GAAAAGATAA GGCAGCTCAT ATAAGTAAGC

TCTTCGCGAA GGCTGTGAGT GCATAGATAA GGCTGCTCAT ATAAATAAGT

TCTTTGCAAA AGCTGTGAGT AAAAAGATAA GGTTGCTCAT ATAAATAAGT

TCTTCGCGAA AGCCGTGAGT AAA-AGATAA GGTCGCTCAT AGAAATAAGT

TCTTCGCAAA AGCTGTGAGT AAAAAGATAA GGATGCCCAT ATAAATAAGT

TCTTCGCGAA AGCTGTGAGT AAAAAGATAA GGTTGCTCAT ATAAATAAGT

TCTTTGCAAA AGCTGTGAGT AAATAGATAA GGTTGCTCAT ATAAATAAGT

TCTTCGCAAA AGCTGTGAGT AAAAAGATAA GGTTGCTCAT ATAAGTAAGT

TCTTTGCAAA AGCTGTGAGT A--------- ---------- ----------

TCTTCGCAAA AGCTGTGAGT AAATAGATAA GGGTCCTCGT CTAAATAAGT

TCTTCGCGAA AGCCGTGAGT GAAAAGATAA GGGTCCTCAT AGAAATAAGT

TATATTTCCT CTCATGTTCA CTTATAGCCT CGACCTCTTT GAAGCCTGAT

TATATTTCCT CTCATGTTCA CTTATAGCCT CGACCTCTTT GAAGCCTGAT

TATATTTCCT CTCATCTTCA CTTATAGCCT CGACCTCTTT GAAGCCTGAT

TATATTTCCT CTCATCTTCA CTTATAGCCT CGACCTCTTT GAAGCCTGAT

TATACTTCCT C---TCTTCA CTTATAGCCT CGACCTCTTT GAAGCCTGAT

TATACTTCCT CTCATCTTCA CTTATAGCCC TGACCTCTTT GAAGTCTGAT

TACACTTC-C CTCATCTTCA TTTATAG-CC TGGAGTCTTT GAAGTCTGAT

TATACTTC-T CTCATCTTCA TTTATAG-CC TGGACTCTTT GAAGTCTGAT

TACACTTCCT CTCATCTTCA CTTATAGCCC TGACCTCTTT GAAGTCTGAT

GATACTTCCT CTCGTCTTCA CTTATAGCCC TGACCTCTTT GAAGTCTGAT

CATGCCCCCT CCCGGCCTCA CTTACGGCCC TGACCCCTTT GAAGTCTGAC

TATACTCCCT CTCGTCTTCC CTTGGAGCCC TGGCCTCTTT GAAGTCTGAT

TATACTTCCT CTCATCTTCA CTTCTAGCCC TGACCCCTTT GAAGTCTGAT

TATACTTCCT CTCTTCTTCA CCTATAGCCC TGGCCTCTTT GAAGTCTGGT

GATACTCCCT CTCGTCTTCA CTTATAGCCC CGACCTCTTT GAAGTCTGAT

---------- ---------- ---------- ---------- ----------

TCTACCTCCT CCCGTCCTCA CTTGTAGCCA GAGCCCCTTT GAAGTCAGGT

TATACTTTCT CTCTTCTTTA GTTATAGTCC AAAGCTCTTT GAAGTCAGAT

ATTGATTACC GTGAAATGTC AATTGTCTGA TTGCCTGATG TCTTCTTTGC

ATTGATTACC GTGAAATGTC AATTGTCTGA TTGCCTGATG TCTTCTTTGC

ATTGATTACC GTGAAATGTC AATTGTCTGA TTGCCTGATG TCTTCTTTAC

ATTGATTACC GTGAAATGTC AATTGTCTGA TTGCCTGATG TCTTCTTTAC

ATTGATTACA GTGAAATGTC AATTGTCTGA TTGTCTGATG TCTTCTTTGC

ATTGATTACA GTGAAATGTC AATTGTCCAA T--CTTGATG TCTTCCTTGC

ATTGATTACA GTGGGATGTC AATTGTCTGC TTGCCTGA-- -TTTCCTTGC

ATTGATTACA GTGGGATGTC AATTGTCTGC TTGCCTGA-- TTTTCCTTGC

ATTGATTACA GTGAAATGTC AATTGTCTGA TGGCCTGA-- TCTTCTTTGC

ATTGATTACA GTGAAATGTC AATTGTCTGA TTGCCTGATG CTCTGTTCAC

ATTGATTACA GAGAAATGTC AATTGTCTGG TCGCCTGATG CCCTCCTTGC

ATTGATTACA GTGAAATGCC AATTGTCTGA TTGCCTGATG CCTTCTTTGC

ATTGATTACA GTGAAATGTC AATTGTCCAA TTGCCTGATG CtttttttgC

ATTGATTACA GTGAACTGTC AATTGTCTGA TTGCCTGATT TCTTCTTTGC

ATTGATTACA GTGAAATGTC AATTGTCTGA TTGCCTGATG TCTTCCTTGC

---------- ---------- ---------- ---------- ---TCCTTGC

ATTGATTACA CAGAAATGCC CACTGTCAGA TGGCCTGATG TCTTCTTTGC

ATTGATTACG GTGAAATGTC AATTGTTGGA TTGCCTGAtg tctCTTTTCC

AGTTCCTCAG CACTTTGTTT GCCCCCTTAA ACTTTGTCAT GGAGAAAGTG

AGTTCCTCAG CACTTTGTTT GCCCCCTTAA ACTTTGTCAT GGAGAAAGTG

AGTTCCTCAG CACTTTGTTT GCCCCCTTAA ACTTTGTCAT GGAGAAAGTG

AGTTCCTCAG CACTTTGTTT GCCCCCTTAA ACTTTGTCAT GGAGAAAGTG

AGTTCCTCAG CACTTTGTTT GCCCCCTTAA ACTTTGTCAT GGAGAAAGTG

AGTTCCTCAG CACGTTGTTT GCCCCCTTAA ACTTTGTCAT GGAGAAAGTG

AGTTCCTCAG CACTTTGTTT GCCCCCTTGA ACTTTGTTAT GGAGAAAGTG

AGTTCCTCAG CACTTTGTTT GCCCCCTTGA ACTTTGTTAT GGAGAAAGTG

AGTTCCTCAG CACTTTGTTT GCCCCCTTAA ACTTTGTCAT GGAGAAGGTG

AGTTCCTCAG CACTTTGTTT GCCCCCTTAA ACTTTGTCAT GGAAAAAGTG

AGTTCCTCAG CACTTTGTTT GCCCCCTTGA ACTTTGTCAT GGAGAAAGTG

AGTTCCTCAG CACTTTGTTT GCCCCCCTAA ACTTTGTTAT GGAGAAAGTG

AGTTTCTTAG CACTTTGTTT GCTCCCTTAA ACTTTGTCAT GGATAAAGTG

AGTTCCTCAG CACTTTGTTT GCTCCTTTAA ACTTTGTCAT GGAGAAAGTA

AGTTCCTCAG CACTTTGTTT GCCCCCTTAA ACTTTGTCAT GGAGAAAGTC

AGTTCCTCAG CACCTTGTTT GCCCCCTTAA ACTTCGTCAT GGAGAAAGTG

AGTTCCTCAG CACTTTGTTT GCCCCTTTAA ATTTCGTCAT GGAGAAAGTA

AGTTTCTCAG CACTTTGTTT GCCCCTTTAA ATTTTGTGAT GGAGAAGGTT

GAGAGCATCC TCCCATCCAG TCTGTGGCAC CAGCTAACAC GGATCTGAGC

GAGAGCATCC TCCCATCCAG TCTGTGGCAC CAGCTAACAC GGATCTGAGC

GAGAGCATCC TCCCATCCAG TCTGTGGCAC CAGCTAACAC GGATCTGAGC

GAGAGCATCC TCCCATCCAG TCTGTGGCAC CAGCTAACAC GGATCTGAGC

GAGAGCATCC TCCCATCCAG TTTGTGGCAC CAGCTAACAC GGATCTGAGC

GAGAGTGTCC TCCCGTCCAG TCTGTGGCAC CAGCTGACGC GGATCTGAGC

GAAAGCATCC TCCCCTCCAG TTTGTGGCAT CAGCTAACAC GGATCTGAGC

GAAAGCATCC TCCCCTCCAG TTTGTGGCAT CAGCTGACAC GGATCTGAGC

GAGAGCATCC TCCCATCCAG CCTGTGGCAC CAGCTCACAC GGATCTGAGC

GAGAGCATCC TGCCATCCAG TCTGTGGCAC CAGCTGACTC GGATCTAGGC

GAAAGCATCC TCCCATCCAG CCTGTGGCAC CAGCTGACGC GGATCTAGGC

GAAAGCATCC TCCCGTCCAG TCTGTGGCAC CAGCTGACAC GAATCTAGGC

GAAAGCATAC TACCGTCCAG TTTGTGGCAC CAGCTGACAC GGATCTAGGC

GAAAGCATCC TCCCATCCAG TCTGTGGCAC CAGCTAACGC GGATCTAAGC

GAGAGCATCC TCCCATCCAG TCTGTGGCAC CAGCTGACCC GGATCTGAGC

GAGAACATCT TGCCTTCCAG CCTGTGGCAC CAGCTGACAC GGGTCTAAG-

GAAAGTATCC TGCCATCCAG TCTGTGGCAC CAGCTGACAA GGATCTAAGA

GAAAGTATTC TGCCCTCCAG TCTGTGGCAC CAGCTGACGA GGATCTGAGA

TCCTCGTGCC AACTCCTTGT GGACCGCAAG AAAGCATGAC TTTGAAAAAG

TTCTCGTGCC AACTCCTTGT GGACCGCAAG AGAGCATGAC TTTGAAAAAG

TCCTCGTGCC AACTCCTTGT GGACCGCAAG AAAGCATGAC TTTGAAAAAG

TCCTCGTGCC AACTCCTTGT GGACCGCAAG AAAGCATGAC TTTGAAAAAG

TCCTCGTGCC AACTCCTTGT GGACCGCAAG AAAGCATGAC TTTGAAAAAG

TCCTCGTGCC AACTCCTTGT GGACTGCAAG AAAGCATGAC TTTGAAAAAG

TCTTCGTGCC AACTTCTCAT GGACCGCAAG AAAGCATGAC TTTGAAAAAG

TCTTCGTGCC AACCTCTCAT GGACCGCAAG AAAGCATGAC TTTGAAAAAG

TCTTCGTGCC CACTCCTCAC GGACGGCGGG AGAGCGGGAG GCTGGGGAGG

TCTTTGTGCC AACTCCTCGT GGACCGCAAG AAAGCATGAC TTTGAAAAAG

TCTTCGTGCC AACTCCTCGT GGACCGCAAG AAAGCATGAC TTTGAAAAAG

TCTCCGTGCC AATTCCTCCT GGACTGCAAG AAAGCATGAC TTTGGAAAAG

TCTTCGTGCC AATGCCTCAT GGA-CGCAAG AAAGCATGAC TCTGAAAAAG

TCCTCGTGCC AACTCCTTGT GGACTGCAAG AAAGCATGAC TTTGAAAAAG

TCTTCGTGCC AACTCCCCGT GGACCGCAAG AAAGCATGAC TTTGAAAAGG

-----GTGCC AACGCCCTGC CCTCCG-AGG CAAGCATGAC TTTGAAAAAG

TCTTCATGCC GACTCCTTGT TGACCGCGAG AAAGCATGAT TTTTTAAAAG

TCTTCGTGCC AACTCCTTGT TGACCGCAAG AAAGCATGAT TTTTAAAAAG

GGAAGCCATT CCGAGATTTT AAAATGTTCA TGGACTATTC CATATTAAAA

GGAAGTCATT CCGAGATTTT AAAATGTTCA TGGACTAT-- ----------

GGAAGCCATT CCGAGATTTT AAAATGTTCA TGGACTATTC CATATTAAAA

GGAAGCCATT CCGAGATTTT AAAATGTTCA TGGACTATTC CATATTAAAA

GGAAGCCATT CCGAGATTTT AAAATGTTCA TGGACTATTC CATATTAAAA

GGAAGCCATT CCG-GATTTT AAAATGTTCA TGGACTGTTC CATATTAAAA

GGAAACCATT CTGAGATTTT AAACTGTTCA TGGACTATTC CTTATTAAAG

GGAAACCATT CTGAGATTTT AAACTGCTCA TGGACTATTC CTTATTAAAG

GGAAGCCATC CCAAGACGGC AGAGTGTTCA CGGACTGTTC CGTATTAAAG

GGAAGCCATT CCAGGATTTT AAAACCTTCA TGAACTGTTC CATATTAAAA

GGAAGCCATT CCGAGATTTT AAATTGTTCA TGGACTGTTC CATATTAAAA

GGAAGCCATT CCAAGATTTT AAA-CATTCG TGGACTGTTC CATATTAAAA

GGAAGCCATT CCGAGGTTTT AAAATGTTCA TGGACAGTTC CATATTAAAA

GGAAGCCATT CCAAGATTTA AAAATGCCCA TGGACCGTTC CATATCAAAA

GGAAGCCATT CCGAGACTTA GAAACGTTCA TGGACTGTTC CATATTAAAA

GGAAGCCATT CCGAGACTCA GCACCATTGA TGGACTGTTC CACATTAAAA

GGAAGCCGTT CTAAGACTTA AAAATCTTCA TGGATTGTCT CATATTAAAA

GGAAGCCGTT CTGAGACTTA AAAATCTTCA TGGATTGTCT CATATTAAAA

GCTGTTTTTG TTGTACAAAA TTCACTGATG TTCAGTTCTA TTTTATTTTG

---------- ---------- ---------- ---------- ----------

GCTGTTTTTG TTGTACAAAA TTCATTGATG TTCAGTTCTA TTTTATTTTG

GCTGTTTTTG TTGTACAAAA TTCATTGATG TTCAGTTCTA TTTTATTTTG

GTTGTTTTTG TTGTACAAAA TTCATCAATG TTCAGTTCTA TTTTATTTTG

GCTG-TTTTG TTGTACAAAA TT-ACTGATG TTCAGTTCTA TTTTATTTTG

GCTGGTTTTG TTGTACAAAA TTTATTGATG TTCCGTTCTA TTTTATTTTG

GCTGGTTTTG TTGTACAAAA TTTATTGATG TTCAGTTCTA TTTTATTTTG

GCT-GTTTGG TTGTACACAA TTCATTCATG TTCAGTTCTA TTTTATTTTG

GCTGTTTTTG TTGTACAAAA TTCACCGATG TTCAGTTCTA TTATATTTTG

GCTGTTTTTG TTGTACAAAA TTCATTGATG TTCGGTTCTA TTATATTTTG

GCTGTTTTTG TTGTACAAAA TTTGTTGATG TTCATTTCTA TTATATTTTG

GCTGTTTTTG TTGTACAAAA TCCATTACTG TTCAGTTCTA TTATATTTTG

GCTGTTTTTG TTGTACAAAA TTCATTGATG TTCAGTTCTA TTCTATTTTG

GCTGTTTTTG TTGTACAAAA TTCATTGATG TTCAGTTC-- ---TATTTTG

GCCGTTTTTG TTGTACAAAA TCCATTGATG TTCAGTTTTA TTATATTTTG

GAAGTTTTTG TTGTACAAAA TTCATTGATG TTCAGTTCTA TTATATTTTG

ACTGTTTTTG TTGTACAAAA TACATTGATG TTCAGTTCTA TTATATTTTG

CCTTCAGAAA AGAAGAAATT TTATTCTTAA AAAATAGCTC CCCTGGTTGC

---------- --------TT TTATTCTTAA AAAATAGCTC CCCTGGTTGC

CCTTCAGAAA AGAAGAAATT TTATTCTTAA AAAATAGCTC CCCTGGTTGC

CCTTCAGAAA AGAAGAAATT TTATTCTTAA AAAATAGCTC CCCTGGTTGC

CCTTCAGAAA AGAAGAAATT TTATTCTTAA CAAATGGTTC CCCTGGTTGC

CCTTCAGAAA AGAAGAAATT TTATTCTTAA AAAATGGCTC CCCTGGTTGC

CCTTCAAAAA AAAAGTAATT CTTGTTTTTA AAAATGGCTC CCCTGGTTGC

CCTTCAAAAA AAAAGtaaTT CTCGTTTTTA AAAATGACTC CCCTGGTTGC

CCTTCAGAAA AGAAGAAATT TTATTCTTAA AAAACGGCTC CCCTGGTTGC

CCTTCAGAAA AG--AAAATT TTATTCCTAA AAAATGGCTC CCCTGGTTAC

CCTTCAGAAA AGaaaaaaTT TTACTTTTAA AAAGCAGTTC CCC-GGTTGC

CCTTCAGAAA AG--AAAATT TTATTCTTAA AAAATGGCTC CCCTGGTTGC

CCTTCAGAAA AGACAAAATT CTATTCTTAA AAAATGGCTC CCCTGGTTGC

CCTTCAGAAA AGAAAAAATT TTATTCTTTA AAAATAGCTC CCCTGATTGC

CCCTCAGAAA AGAAAAAATT TTATTCTTAA AAAATGGCTC CCCTGGTTGC

TCTTCAGAAA ATTTTAAATT TTATTCGTAA AAAACGGCTC CCCTGGTTGC

CCTTCAGAAA AGATAAAATT TTATTCCTTA AAAACAGCTC CCCTGCTTGC

CCTTCAGAAA AGGAAAAATT TTATTTGTAA GAAATCGCTC CCCTGACTGC

AATGTGATGT TAAGTATATT ACATGTCAGT TCATATATTA CCATATAAGG

AATGTGATGT TAAGTATATT ACATGTCAGT TCATATATTA CCATATAAGG

AATGTGATGT TGAGCATATT ACATGTCAGT TCATATATTA CCATATAAGG

AATGTGATGT TGAGCATATT ACATGTCAGT TCATATATTA CCATATAAGG

AATGTTATGT TAAGCATATT ACATGTCAGT TCATATATTA CCATATAAGG

AATGTTATGT TAAGCATATT ACATGTCAGC TCATTTATTA CCATATAAGG

AATGTTATGT TAAGCACATT ACATGCCAGC TCATATATCG CCATATAAGG

AATGTTATGT TAAGCATATT ACATGCCAGC TCATATATCG CCATATAAGG

AATGTTATGT TAAGCATATT ACATGTCAGT TCATATATTA CCATATAAGG

AGTGTTATGT TAAGCATATT ACATATCAGT TCATATATTA CCATATAAGG

AGGGTCAGGT CAGGCACCTC CCGT--TGGC TCACACGCTG CCATAAAAGG

AATGTTATGT TAAGCATATT ACATGTCAGT TCATATATTA CCATATAAGG

AATGTTATGT TAAGCATATT ACATGTCAGT TCATATATTA CCATATAAGG

AATGTTAGGT TAAGCATATT ACATGTCAGT TCATATATTA CTATATAAGG

AATGTTATGT TAAGCATATT ACATGTCAGT TCATATATTA CCATATAAGG

AATATTATGT TAAGCATATT ACATGTCAGT TCATCTATTA ----------

AGTGTTACAT TAAGCATATT ACATGTCAGT TCATATATTA CCATACAAGG

AATGTTACAT TAAGCATATT ACACGGCAGT TCATATATTA CTACAGAAGG

AAGAACCTAC AGGGCACTTC AATTAGCTGA GCAGTCCCCA GCTCTGATTT

AAGAACCTAC AGGGCACTTC AATTAGCTGA GCAGTCCCCA GCTCTGATTT

AAGAACCTAC AGGGCACTTC AATTAGCTGA GCAGTCCCCA GCTCTGATTT

AAGAACCTAC AGGGCACTTC AATTAGCTGA GCAGTCCCCA GCTCTGATTT

AAGAACCTAC ATGGCACTTC AATTAGCTGA GCAGTCCCCA GCTCTGATTT

AAGAACCTAC AGGGCACTTC AGTTAGCTGA GCTGTCCCCA GTTCTGATTG

AAGAACCTAC AGGATACTTC AATTAGCTGA GCAGTCCCCA GCTCTCATTC

AAGAACCTAC AGGATACTTC AATCAGTTGA GCAGTCCCCA GCTCTCATTA

AAGAACCCAC AGGTCACTTT AATTAGCTGA GCAGTCCCCA GCTCTGATTA

AAGAACCTAC G-GACACTTC AATTAGCTGA GCAGGCCCCA GCTCTGATTG

AAGAACCTAC GAGGCTCTTC GATTAGCCGA GC-GGTGGCA GCCCTGATTG

AAGAACCTAC AGGGCACTTC AATTAGCTGA GCAATCCCCA GCTTTCATTG

AAGGACCTAC GGGGCACTTC AATTAGCTGA GTAGTCCCCA GCTCTGATTG

AAGAACCTAC AGGGCACTTC AATTAGTCGA GCAATCCCTG GCTCTGATTG

AAGAACCTAC AAGGCACTTC GATTAGCTGA GCAGTCCCCA GCTCTGATTG

---------- ---------- ---------- ---------- -------TTG

AAAAACCCAT AAGCCACTTT AATTTGCTAA GCAATGTCTG GCTCTGATTG

GAGAACCTCC AAGTCACCTT AATGAATAAA GCAGCATCTT ACTCCGATTG

CCTTTGGCCA TCGTCTCACT CTATTTATTC TCCAAAAGAA TGAATTACTA

CCTTTGGCCA TCGTCTCACT CTATTTATTC TCCAAAAGAA TGAATTACTA

CCTTTGGCCA TCATCTCACT CTATTTATTC TCCAAAAGAA TGAATTACTA

CCTTTGGCCA TCATCTCACT CTATTTATTC TCCAAAAGAA TGAATTACTA

CTTTTGGCCA TCATCTCACT CTATTTATTC TCCAAAAGAA TGAATTACTA

CTTTTGGCCA TCATCTCACA CTATTATTTC TCCAAAAGAA TGAATTACTA

CTTTAGGATA TCATTTCACT CTGCTTACTC TCCAAAACAG TGAATTATTA

CTTTAGGATA TTATTTCACT CTGCTTATTC TCCAAAACAA TGAATTACTA

CTTTTGGACA TCATCTCACT CTATGCATTC TCCAAAAGAA TGAATTACTA

CTTTTGGACA TCATCTCACT CTATTTATTC TCCAAAAGAA TGAATTACTA

CTTTTGGACA TCATCTCACT CTATTTATTC TCCAAAAGAA TGAATTACTA

CTTTTGGACA TCATCTAACT CTATTTATTC TCCAAAAGAA TGAATTACTA

CCTTAGCTCA TTGTCACAGT CTATTTATTC TTCAAAAGAA TGACTTATTA

CTTTTGGACA TCATCTCACA CTATTTATTC TCCAAAAGAA TGAATTACTA

CTTTTGGACA TCATCTCACA CTATTCATTC TCCAAAAGGA TGAATTACTA

CTTTTGGATA TCATCTCACA CTATTTATTC TCCAAAAGGA TGAATTACTA

CCTTTGGACT CTATCTCATA CCACTTCTTC TCCAAAAGAA TGAAttggca

CAACTGATTG CCATCCCAAA CTTCTTCATA TCCAAAATCA GGAATTAATT

ATCCAAGGGC TGGTGTAAAT ATTTTAAAAA CTTATTATTT CATGAGAGTT

ATCCAAGGGC TGGTGTAAAT ATTTTAAAAA CTTATTATTT CATGAGAGTT

ATCCAAGGGC TGGTGTAAAT ATTTTAAAAA CTTATTATTT CATGAGAGTT

ATCCAAGGGC TGGTGTAAAT ATTTTAAAAA CTTATTATTT CATGAGAGTT

ATCCAAGGGC TGGTGTAAAT ATTTTAAAAA CTTATTATTT CATGAGAGTT

ATCCAAGGGC TGGTGTAAAT ATTTTAAAAA CTTATTATTT CATGAGAGTT

ATCTGAGGGC TGGTGTAAAT GTTTTAAAAA CTTAGTATGT CCTGTGAGTT

ATCTGAGGGC TGGTGTGAAT ATTTTGAAAA CTTATTATTT CATGTGAGTT

ATCCAAGGGC TGGTGTAAAT ATTTTAAAAA CTTATTATTT CATGCGAGTT

ATCTAAGGGC TGGTGTAAAT ATTTAAAAAA CTTGCTATTT CATGAGAGTT

ATCCAAGGGC TGGTGTAAAT ATC-ACAAAA CTTATTATTT CCCGAGAGTC

ATCCAAGGGC TGGTGTAAAT ATTTAAAAAA CTTATTATTT CATGAGAGTT

ATCCAAGGGC TGGTGTAAAT GTTTAAAAAA CTTATTATTT CATGAGAGTT

ATCCAAGGGC TGGTGTAAAT ATTTTAAAAA CTTATTATTT CATGAGAGTT

ATCCAAGGGC TGGTGTAAAT ATTTAAAAAA CTTATTATTT CACGAGAGTT

ATCCAAGGGC TGGTATAAAT ATTTTTAAAA ATTATTCTTT CACGAGAGTT

aTCCAAGGGC TGGTGTAAAT ATTTTAAATA CTTAATGTTT TATGATAGTT

ATCAAAGGGC TGGGGTAAAT ATCTTGGGCA TTGAGCATCT CATGGCATCT

GCTGAGAGCA TTGTTTATAG TTACAGCAAT ATATAGGAAA ATTTCCAAAA

GCTGAGAGCA TTGTTTATAG TTACAGCAAT ATATAGGGAA ATTTCCAAAA

GCTGAGAGCA TTGTTTATAG TTACAGCAAT ATATAGGAAA GTTTCCAAAA

GCTGAGAGCA TTGTTTATAG TTACAGTAAT ATATAGGAAA GTTTCCAAAA

GCTGAGAGCA TTGTTTATAG TTACAGCAAT ATATAGGAAA ATTTCCAAAA

GCTGAGAGCA TTGTTTATAG TTACAGCAAT ATATAGGAAA ATTTCCAGAA

GTTGAAAGCA TTGTTTATAG TTACAGCAAT GTATAGGAAA ATTTCCAAAG

GTTGAGAGCA TTGTTTATAG TTACAGAAAT GTATAGGAAA ATTTCCAAAG

GTTGAGGGCA TTGTTTATAG TTACAGCAAT ATATAGGAAA ATTTCCAAGA

ATTGAGAGCA TTGTTTATAG TTACGGCAAT ATATAAGAAA ATTTCAACAG

ACTGAGAGCA TTGTTTATAG TTACAGCAAT ATATAAGAAA ATTTCCACAA

GCTGAGAGCA TTGTTTATAG TTACAGCAAT ATATAGGAAA ATTTCCACAA

GCTGAGAGCA TTGTTTATAG TTACAGCAAT ATATAGGAAA ATTTCCACA-

GCTGAGAGCA TTGTTTATAG TTACAGCACT ATATAGGAAA ATATCTAAAA

ACTGGGAGCA CTGTTTATAG TTACAGCAAT ACGTAGGCAA ATATCCAAAA

ACTGAGAGCA GTGTTTATAG CTGCAGTAAG ACATAGGCAA ATACCCCAAG

GCTAAGAGCA TTGTTTATAG TTACAGCAAT GTATAGGAAA ATATCCAAGA

ACTGAGAGCG TTGTTTACAG CTTCTAAAAC ACTCAGGAAA ATACCCAAAA

TGACAACAGG GTTCGAGCTT TCATGAACTA TTGGAGAATA ATGAGGCAGA

TGACAACAGG GTTCAAGCTT TCATGAACTA TTGGAGAATA ATGAGGCAGA

TGACAACAGG GTTCAAGCTT TCACAAACCA TTGGAGAATA ATGA--CAGA

TGACAACAGG GTTCAAGCTT TCACAAACCA TTGGAGAATA ATGA--CAGA

TGACAACAGG GTTCAAGCTT TCACGAACTA TTGGAGAATA ATGAGGCAGA

TGACGACAGA GTTCAAGCTT TCATGAACTA TTGGAGAGTA ATGAGGCAGA

TAATGACAGA GTTTGAGTTT -------CTG TTGGAGAATA GTGGGGCAGG

GAACGACAGA GTTGGAGCTT -------CTG CTGGAGGGTA ATGAGGCAGA

TGACGACAGG GTTCAAGCTT TCACGAACTA TTGGAGAGTA ATGAGGCAGA

GGACGATAGA GTTCAAGCTT TCATGAACTG TTGGAGAAAA ATGGGGCAGA

TGACAACAGA GTTCAAGCTT TCATGAACTA TTGGAGAATA ATGAGGCAGA

TGACGACAGA GTTCAAGCTT TCATGAACTA TTGGAGAATA ATGAGGCAGA

TGACGACAGA GTTCAAGCT- ---TGAACTA CTGGAGAATA ATGAGGCAGA

TGACGACAGA GTTCAAGCTT TCACGAACTA TTGGAGAATA ATGAGGCAGA

TGATGACGGA GTTCAAGCTT TCATGAACTA TTGGAGAAAA ATGAGGCAGA

TGATGACAGA GTTCAAGCTT TCATGAATGA TCAGAGAATA ATGAGGCAGA

AGAAGACAGT CTTTGGACAT TCATGAACTA GTAGAGAATA ATGAGGCAGA

AGAAGACAAA AAGCCCGCTT CCATGAACTA GCTAAGAATA ATGAGGCAGA

TGCCAAATTC TTTTCACCAG AGGGTTGAAA ATTCACTTTA TTATGTTTTG

TGCCAAATTC TTTTCACCAG AGGGTTGAAA ATTCACTTTA TTATGTTTTG

TGCCAAATTC TTTTCACCAG AGGGTTGAAA ATTCACTTTA TTATGTTTTG

TGCCAAATTC TTTTCACCAG AGGGTTGAAA ATTCACTTTA TTATGTTTTG

TGCCAAAATC TTTTCACTAG AGGGTTGAAA ATTCACTTTA TTATGTTTTG

TGCCAAATTC TTTTCACCAG AGGGTTGAAA ATTCACTTTA TTATGTTTTG

TGCCAAATAT TTCTTAGCAG A-GTTCAAAA TTTCACTTTA TTATGTTTTG

TGCCGAATAT TTCTTAGCAG A-GTTCAAAA TCTCACTTTA TTATGTTTTG

TGCCAGATGC TCTTCACCAG AGGGTTGAAA ACTCACTTTA TTATGTTTTG

TGCCAAATTC TTTTCACCAG AGGGTCGAAA ATTCACTTTA ATATGTTTTG

TGCCAAATTC TTTTCACCAG AGGGTTGAAA ATTCACTTTA TTATGTTTTG

TGCCAAATTC TTTTCACCAG AGGGTTGAAA ATTCACTTTA TTATGTTTTG

TACTAAATTC TTTTCACCAG AGACTTGAAA ATTCACTTTA TTATGTTTTG

TGCCAAATTC TTTTCACCAG AGAATTGAAA ATTCACTTTA TTATGTTTTG

TGCCAATTTC TTTTCACCAG AGGGTTGAAA ATTCACTTTA TTATGTTTTG

TGCCAAA--- ---------- ---------- ---------- ----------

TGTCAAATTC TCCCCCCAGG AGGATTGAAA AATCATTTTT TAATGTTTCT

TGGTAGAttc cctgcccacg aggagTGATG TCTAAATTTA CTGTGTCTTG

ATTTCAAAGA AAACGTAGAC TTGTAAATAA CAATAGTTCT TTTGCTGATC

ATTTCAAAGA AAACGTAGAC TTGTAAATAA CAATAGTTCT TTTGCTGATC

ACTTCACAGA AAACGTAGAC TTGTAAATAA CAATAGTTCT TTTGCTGATC

ACTTCACAGA AAACGTAGAC TTGTAAATAA CAATAGTTCT TTTGCTGATC

ATTTCACAGA AAACGTGGAC TTGTAAATAA CAATAGTTCT TTTGCTGATC

ATTTCACAGA AAATGCAGAC TTGTAAATAA CAATAGTTCT TTTGCTGAGC

ATTTCACAGA AAATGCTGAC TTGTAAATAA CAATAGTTCT TTTGTTGATC

ACTTCACAGA AAATGCCGAC TTGTAAATAA CAATAGCTCT TTTGCTGATC

ATTTCACAGA AAATGTAGAC TTGTAAATAA CAATAGTTCT TTTGCTGATC

ATTTCACAGA AAATGTAGAC TTGTAAATAA CAATAGTTCT TTTGCTGATC

ATTTCACAGA AAATGCAGAC TTGTAAATAA CAATAGTTCT TTTGCTGATC

ATTTTACAGA AAATGTAGAC TTGTAAATAA CAATAGTTCT TTTGCTGATC

ATTTCACAGA AAATGTAGAC TTGTAAATAA CAATAGTTCT TTCACTGATC

ATTTCACAGA AAATGTAGAC TTGTAAATAA CAATAGTTCT TTTGCTGATC

ATTTCACAGA AAATGCAGAC TTGTAAATAA CAATAGTTCT TTTGTTGATC

---------- ---------- ---------- ---------- ----------

GATTCACAGA AAATGTGGTC TTGTAGACAC CAATCTTTCT CTCGCTGATC

ATTTCCCAGA AAATATAGTC CTATAGGTAA AAACCATTTT CTTGCTGATG

TGAAAATTTT CTCTGCCTAG ATAAGTCTTG GGGGCCTTGC ATGTGTGCAC

TGAAAATTTT CTCTGCCTAG ATAAGTCTTG GGGGCCTTGC ATGTGTGCAC

TGCAAATCTT CTCTGCCTAG GTAAGTCTTG GGGGCCTTGC ATGTGTGCAC

TGAAAATTTT CTCTGCCTAG GTAAGTCTTG GGGGCCTTGC ATGTGTGCAC

TGAAAATTTT CTCTGCCTAG GTAAGTCTTG GGTGCCTTGC ATGTGTGCAC

TGAAAATGTT CTCTACCTAG GTAAGTCTTG GGGGCCTTGC ATGTGTGCAC

TGAAACTTGT CTGTGCTTCT G-GAGTCTTG GGGGCCTTGC ATGTGTGCAC

TGAAACTTGT CTCTGCCTC- ---AGTCTTG GGGGCCTTGC ATGTGTGCAC

TGAGAATTTT CTCCGCCTAG GTAAGTCTTG GGAGCCTTGC ATGTGTGCAC

TGAAAGTTTT CTCTGCCTAG GTAAGTCTTC GGGGCCTTGC ACGTGTGCAC

TGAAAGTTTT CTCTGCGTAG GTAAATCGTG GGGGCCTTGC ACGTGTGCAC

TGAAAATTTT CTCTGCGTAG GTAA-TCTTC GGAGCCTTGC ATGTGTGTAC

TGAAAATTTT CTCTGCCTAG GTAAGTCTTG GGGGCCTTGC ACGTGTGCAC

TGAACATTTT TTCTGCCCAG GTAAGTCTTG GGGGCCTTGC ATGTGTGCAC

TGAAAATTTT CTCTGCCCAG GGAAGTCTTG GGGGCCTTGC ACGTGTGCAC

---------- ---------- -----TCTTG GGGGCTTTGC AGGTGTGCAC

TGGAATTTTC TGCTTCCCAG GTAGGTCTTG GGGGCTTTGC ACGTGTGCAC

CGGAGTTTCT CACTAACAAG GTAGGTGCTG GGGGCTTTGC ACGTGTGCAC

ATCCAGAGCT TCCAGGCAGT CCTGACAGCG CACGGCGCAG CACCAGTGGA

ATCCAGAGCT TCCAGGCAGT CCTGACAGCG CACGGCGCAG CACCAGTGGA

ATCCAGGGCC TCCAGGCAGT CCTGACAGCG CACGGCGCAG CACCAGTGGA

ATCCAGGGCC TCCAGGCAGT CCTGACAGCG CACGGCGCAG CACCAGTGGA

GTCCAGGGCT TCCAGGCAGT CCTGACAGCG CACGGCACAG CACCAGTGGA

ATCCAAGGCC TCCAGGCAGT CCTGACAGCG GACGGCGCAG CACCAGTGGA

GTCCAGGGCT TCCAGGCAGT CCTGACAGCG CACGGCACAG CACCAGTGGA

GTCCAGGGCC TCCAGGCAGT CCTGACAGCG CACGGCACAG CACCAGTGGA

GTCCAGGGCC TCCAGGCAGT CCTGGCAACG CACGGCACAG CACCAGTGGA

GTCCAGGGCC TCCACGCAGT CCTGGCAGCG CACGGCACAG CACCAGTGGA

GTCTAGGGCC TCCAGGCAGT CCTGACAGCG CACGGCGCAG CACCAGTGGA

GTCCAGGGCC TCCAGGCAGT CCTGACAACG CACAGCACAG CACCAGTGGA

ATCGAGGGCC TCCAGGCAGT CCTGACAGCG CACGGCGCAG CACCAGTGGA

GTCCAGAGCC TCCAGGCAGT CCTGACAGCG CACAGCACAG CACCAGTGGA

ATCCAGAGCC TCCAGGCAGT CCTGACAGCG CACGGCGCAG CACCAGTGGA

GTCCAGAGCC TCCAGGCAGT CCTGACAGCG CACGGCACAG CACCAGTGGA

GTCCACTACT TCCAAACAGT CCTGACAGCG CACAGCACAG CACCAATGGA

GTCGAGGGCT TCCAGGCAGT CCTGGCAGCG GACGGCGCAG CACCAGTGGA

ACTTACACCC ACACTTGGTC ATCCGGGTGA CATGGGAGGT GTCGTAGCCT

ACTTACACCC ACACTTGGTC ATCCGGGTGA CATGGGAGGT GTCGTAGCCT

ACTTACACCC ACACTTGGTC ATCCGGGTGA CATGGGAGGT GTCGTAGCCT

ACTTACACCC ACACTTGGTC ATCCGGGTGA CATGGGAGGT GTCGTAGCCT

ACTTACACCC ACACTTGATC ATCCGGGTGA CATGGGAGGT GTCGTAGCCT

ACTTACACTC GCACTTGGTC ATCCGGGTGA CACGGGAGGT GTCATAGCCT

ATTTACACTC ACACTTGGTC ATCCGGGTGA CATGGGATGT GTCATAGCCT

ATTTACACTC ACACTTGGTC ATCCGGGTGA CGTGGGATGT GTCATAGCCT

ACTTACACTC GCACTTGGTC ATCCGGGTGA CGCGGGAGGT GTCGTAGCCT

ACTTACACTC ACACTTGGTC TTCCGGGTAA TGTGGGAGGT GTCGTAGCCT

ACTTACACTC GCACTTGGTC ATCCGCGTGA CGCGGGACGT GTCGTAGCCT

ACTTACACTC ACACTTGGTC ATCCTCGTGA CGCGGGACGT GTCGTAGCCT

ACTTACACTC GCATTTGGTC ATCCGGGTGA CTCGGGAGGT GTCATAGCCT

ATTTACACTC ACATTTGGTC ATCCGGGTGA CATGGGAGGT GTCGTAGCCT

ACTTACACTC GCACTTGGTC ATCCGGGTGA TGCGAGAGGT GTCGTAGCCT

ACTTACACTC ACACTTGGTC ATGCGGGTGA CGCGGGAGGT GTCGTAGCCT

ACTTACACTC GCATTTGGTC ATTCGGGTGA CCCGGGAAGT GTCATAGCCC

ACTTGCACTC GCACTTGGTC ATCCTGGTGA CGCGGGCCGT GTCGTAGCCC

CTCCCACAGC ACATGACTTC ACAGCTGTCC ATGCCCCGGG AAGTCAGGTT

CTCCCACAGC ACATGACTTC ACAGCTGTCC ATGCCCCGGG AAGTCAGGTT

CTCCCACAGC ACATGACTTC GCAGCTGTCC ATGCCCCGGG AAGTCAGGTT

CTCCCACAGC ACATGACTTC GCAGCTGTCC ATGCCCCGGG AAGTCAGGTT

CTTCCACAGC ACATGACTTC ACAGCTGTCC ATGCCCCGGG AAGTCAGGTT

CTCCCGCAGC ACATGACTTC ACAGCTGTCC ATGCCCCGGG ACGTCAGGTT

CTCCCACAAC ACATAACTTC GCAGCTGTCC ATGCCTCGGG AAGTCAAGTT

CTCCCACAAC ACATAACTTC GCAGCTGTCC ATGCCTCGGG AAGTCAAGTT

CTCCCGCAGC ACATGACTTC ACAGCTGTCC ATGCCTCTGG ACGTCAGGTT

CTGCCACAGC ACATGACTTC GCAGCTGTCC ATGCCCCGGG AAGTCAGGTT

CTCCCGCAGC ACATGACCTC GCAGCTGTCC ATGCCGCGGG AAGTCAGGTT

CTCCCGCAGC ACATGACTTC ACAGCTGTCC ATGCCTCGGG AGGTCAGGTT

CTCCCGCAGC ACATGACTTC ACAGCTGTCC ATGCCTCGGG AAGTCAGGTT

CTCCCGCAGC ACATGACTTC GCAGCTGTCC ATGCCTCGGG AAGTCAGGTT

CTCCCGCAGC ACATGACTTC GCAGCTGTCC ATGCCTCGGG AAGTCAGGTT

CTCCCGCAGC ACATGACTTC GCAGCTGTCC ATGCCTCGGG AGGTCAGGTT

CTTCCGCAGC ACATGACTTC ACAGCTGTCC ATGCCACGGG AGGTCAGGTT

CTCCCGCAGC ACATGACCTC GCAGCTGTCC ATGCCGCGGG ACGTCAGGTT

GCACACACGG CCTGCTGTAC CCAGGGAGCC TGGAAGACAC TATCTGTACC

GCACACACGG CCTGCTGTAC CCAGGGAGCC TGGAAGACAC TATCTCTACC

GCACACACGG CCTGCTGTAC CCAGGGAGCC TGGAAGACAC TATCTCTACC

GCACACACGG CCTGCTGTAC CCAGGGAGCC TGGAAGACAC TATCTCTACC

GCACACACGG CCTGCTGTAC CCAGGGAGCC TAGAAGACAC TATCTCTAAC

GCACACACGG CCTGCTGTAC CCAGGGAGCC TAGAAGACAC TATCTCTAGC

GCACACACGG CCTGCTGTAC CCAGGGAGCC TGGAAGAGAC TATCTCTAAC

GCACACACGG CCCGCTGTAC CCAGGGAGCC TGGAAGACAC TATCTTTAAC

GCACACACGA CCTGCTGTAC CCGGGGAGCC TGGAAAACAC TATCTCTAAC

GCACACACGG CCCGCTGTAC CCAGGGAGCC TGGAAGACAC TATCTCTAAC

GCACACGCGG CCCGCCGTCC CCGGGGAGCC TGGAAGACAC TATCTCTAAC

GCACACACGT CCTGCTGTAC CCAGGGAGCC TGGAAGACAC TATCTCTAAC

GCACACACGG CCTGCTGTAC CCAAGGAGCC TGGAAAATAC TATCTCTAAG

GCACACACGG CCTGCTGTAC CCAGAGAGCC TGGAAGACAC TATCTCTAAG

GCACACTCGA CCTGCTGTAC CCGGGGAGCC TGGAAGA--C TATCTCTAAC

GCAGACTCGG CCTGCTGTAC CTGGGGAGCC TGGAAGACAC TATCTCTAAC

GCACACCCGA CCTGCAGTAC CCAGGGAACC TAGAAAGAAC TATCTCTAAC

GCACACCCGG CCCGCCGTCC CTAGGGAGCC TGGGAGAGAT GATCACCCAC

TTAAGGGCTT CTTTGATTTA CAGATGCCTG GAGCAGTAAC CAGAGTAGAT

TTAAGGGCTT CTTTGATTTA CAGATGCCTG GAGCAGTAAC CAGAGTAGAT

TTAAGGGCTC CTTTGATTTA CAGATGCCTG GAGCAGTGAC CAGAGTAGAT

TTAAGGGCTC CTTTGATTTA CAGATGCCTG GAGCAGTGAC CAGAGCAGAT

TTAAGGGCTT CTTTGATTTA CAGATGCCTG AAGCAATAAC CAGAGTAGAT

TCAAGGGCTT CTTTGATTTG CAGATGCCTG GGGCAATAAC CAAAGTAGAT

TGAAGGGTTG CTTTGATTTA CTGATGCCTG GGGCAATCGC CAGACTAGAT

TGAAGGGTTG CTTTGATTTA CAGATGCCTG GGGTAATCAC CAGACTAGAT

TGAAGGGCTC CTTTGATTTA CAGATGCCTG GGGCAATGAC CAGAGTAGAT

TTAAGGGCTT CTTTGATTTA CAGATGCCAG GGGCAATAAC CAGAGGAGAT

TTGAGGGCCT CTCTGACTTA CAGACGCCTG GGGCAACAGC CGCAGTAGAT

TTAAGGGTTT CTTTGATTTA TGGCTG-CTG GGGCAATAAG CAGAGTAGAT

TTAAGGACTT CTTTGATTTA CAGATGCCTG GGGCAATAAC CCGAGTAGAT

TTAAGGGCTT CTTTGATTTA CAGATGTCTG GGGCAACAGC CTGAGTAGAT

TTAAGGGCTT CTTTGATTTA CAGATGCCTG GGGCAATAAC CAGAATAGAT

GTAAGGGCTT CTTTGATTTA CAGATGCCTG GGGCAATCAC CAGAACAGAT

TTAAGGTTTC CCCTGATTTC CAGATGCCTG GCATAATAAC CACAGCAGAT

TTAAAAGGTT CCTTGATTGG CAGATGCCCG AGGCAATAAC CAAGGCAGAT

AACTTACCTG CCTCTCGGTC CCTGATACAG TAGTCTGGAG AATTCTCAAA

AACTTACCTG CTTCTCGGTC CCTGATACAG TAGTCTGGAG AATTCTCAAA

AACTTACCTG CCTCTCGGTC CCTGATACAG TAGTCTGGAG AATTCTCAAA

AACTTACCTG CCTCTCGGTC CCTGATACAG TAGTCTGGAG AATTCTCAAA

AACTTACCTG TCTCTCGGTC CCTGATACAG TAGTCTGGAG AATTCTCAAA

AACTTACCTG CCTCTCGGTC CCTGATACAG TAGTCTGGAG AATTCTCAAA

AACTTACCTG CCTCTCGGTC CCTGATACAG TAGTCTGGAG AATTCTCAAA

AGCTTACCTG CCTCTCGGTC CCTGATACAG TAGTCTGGAG AATTCTCAAA

AACTTACCTG CCTCTCGGTC CCTGATACAG TAGTCTGGAG AATTCTCAAA

AACTTACCTG CATCTCGGTC CCTGATACAG TAGTCTGGAG AATTCTCAAA

AACTCACCTG CGTCTCGGTC CCTGATACAA TAGTCTGGAG AATTCTCAAA

AACTTACCTG CATCTCGGTC CCTGATACAG TAGTCTGGAG AATTCTCAAA

AACTTACCTG CATCTCGGTC CCGGATACAG TAGTCAGGAG AATTCTCAAA

AACTTACCTG CATCTCGGTC CCTGATACAG TAGTCTGGAG AATTCTCAAA

AACTTACCTG CGTCTCGGTC CCTGATACAG TAGTCTGGA- ----------

AACATACCTG CATCCCGGTC CCTGATACAG TAGTCTGG-G AATTCTCAAA

AACTTACCTG CATCTCGGTC TCTGATACAA TAGTCTGGAG AATTCTCAAA

AACTTACCTG CATCCCGGTC CTTGATACAG TAGTCTGGAG AGTTCTCAAA

ATACACGAGG TCATTTTTCG TTGGCTTCTT AAACCTCTCG TTAGCCACAG

ATACACGAGG TCATTTTTTG TTGGCTTCTT AAACCTCTCG TTAGCCACAG

ATACACGAGG TCATTTTTGG TTGGCTTCTT AAACCTCTCG TTAGCCACAG

ATACACGAGG TCATTTTTTG TTGGCTTCTT AAACCTCTCG TTAGCCACAG

ATACACGAGG TCATTTTTTG TTGGCTTCTT AAACCTCTCG TTAGCCACAG

ATACACGAGG TCATTTTTTG TTGGTTTCTT AAACCTCTTG TTAGCTACTG

ATACACAAGG TCATTTTTCG TTGGCTTCTT AAACCTCTTA TTGGCTACAG

ATACACGAGG TCATTTTTCG TTGGCTTCTT AAACCTCTTA TTGGCTACAG

GTACACAAGG TCATTTTTCG TTGGCTTCTT AAACCTCTTG TTAGCCACAG

ATACACGAGG TCATTTTTCG TTGGCTTTTT AAACCTCTTG TTAGCCACAG

ATACACGAGG TCATTTTTGG TTGGCTTCTT GAACCTCTTG TTAGCCACCG

ATATACGAGG TCGTTTTTTG TTGGTTTCTT AAACCTCTTG TTAGCCACAG

GTACACAAGG TCATTTTTCG TGGGCTTCTT AAAGCGCTTG TTAGCCACAG

ATATACAAGG TCATTTTTCG TTGGTTTCTT AAACCTCTTG TTAGCCACAG

---------- ---------- ---------- ---------- ----------

GTACACAAGG TCATTTTTCG TTGGCTTCTT GAACCTCTTG TTGGCCACAG

ATAGACCAGG TCATTTTTGG TTGGCTTCTT AAATCTCTTG TTAGCCACAG

ATACACTAAG TCATTTTTGG TTGGTTTCTT GAACCTCTTG TTTGCCACGG

TGAAACCTGT GCCATCCTGG TTCATGACCA CCTGGATGGC CCCATTGTAC

TGAAACCTGT GCCATCCTGG TTCATGACCA CCTGGATGGC CCCATTGTAC

TGAAACCTGT GCCATCCTGG TTCATGACTA CCTGGATGGC CCCATTGTAC

TGAAACCTGT ACCATCCTGG TTCATGACCA CCTGGATGGC CCCATTGTAC

TGAAACCTGT GCCATCCTGG TTCATGACCA CCTGGATGGC CCCATTGTAC

TGAAGCCAGT GCCATCCTGG TTCATGACCA CCTGGATGGC TCCGTTGTAC

TGAAGCCAGT GCCGTCCTGG TTCATGACCA CCTGGATGGC CCCGTTGTAC

TGAAGCCAGT GCCATCCTGG TTCATGACTA CCTGGATGGC CCCATTGTAC

TGAAACCAGT GCCATCCTGG TTCATGACGA CCTGGATGGC TCCATTGTAC

TGAAACCAGT GCCATCCTGG TTCATGACAA CCTGGATGGC CCCATTGTAC

TGAACCCCGT GCCATCCTGG TTCATGACGA CCTGGATGGC CCCGTTGTAC

TGAAACCAGT GCCATCCTGA TTCATGACGA CCTGGATGGC GCCGTTGTAC

TGAAGCCGGT GCCATCCTGG TCCATGACGA CCTGGATGGC CCCGTTGTAC

TGAAACCAGT GCCATCTTGG TTCATGACGA CCTGGATGGC TCCATTGTAC

---------- ---------- ---------- ---------- ----------

TGAAACCGGT GCCGTCCTGG TTCATGACGA CCTGGATGGC CCCGTTGTAC

TGAAACCAGT GCCATCTTGA TTCATGACCA CTTGGATGGC CCCATTGTAC

TGAAGCCGGT CCCATCTTGA TTCATGACCA CCTGAATGGC CCCGTTGTAT

TTCCTCCAGA GATAATCGCC CGTTTTCCTG AAGTCGGCCA TGGCCAGCCA

TTCCTCCAGA GATAATCGCC CGTTTTCCTG AAGTCGGCCA TGGCCAGCCA

TTCCTCCAGA GATAATCGCC CGTTTTCCTG AAGTCGGCCA TGGCCAGCCA

TTCCTCCAGA GATAATCGCC CGTTTTCCTG AAGTCGGCCA TGGCCAGCCA

TTCCTCCAGA GATAATCTCC CGTTTTCCTG AAGTCGGCCA TGGCCAGCCA

TTCCTCCAGA GATAGGAGCC CGTCTTTCTG AAGTCGGCCA TGGCCAGCCA

TTCCTCCAGA GATAGTCGCC TGTTTTCCTG AAGTCAGCCA TGGCCAGCCA

TTCCTCCAGA GATAGTCGCC TGTTTTCCTG AAGTCAGCCA TGGCCAGCCA

TTCCTCCAGA GGTAATCGCC CGTTTTCCTG AAGTCGGCCA TGGCCAGCCA

TTCCTCCAGA GATAATTGCC CGTTTTCCTG AAGTCAGCCA TGGCCAGCCA

TTCCTCCAGA GATAATCGCC CGTTTTTCTG AAGTCCGCCA TGGCCAGCCA

CTCCTCCACA GGTAATCGCC CGTTTTCCTG AAGTCAGCCA TGGCCAGCCA

TTCCTCCAGA GATAATCGCC TGTTTTTCTG AAGTCAGCCA TGGCCAGCCA

TTCCTCCAGA GATAGTCACC CGTTTTCCTG AAGTCAGCCA TGGCCAGCCA

---------- ---------- ---------- ---------- ----------

TTCCTCCAGA GATAATCGCC CGTCTTTCTG AAGTCGGCCA TGGCCAGCCA

TTCTTCCAGA GATAATCTCC CGTCTTTCTA AAGTCAGCCA TGGCCAGCCA

TTTCTCCACA GATAATCTCC TGTTTTCCTA AAGTCCGCCA TGGCGAGCCA

GCATGTCCTG AGAGTACATG AGCGCTCACC CCGTGGCACT TGCACTCTTG

GCATGTCCTG AGAGTACATG AGCGCTCACC CCGTGGCACT TGCACTCTTG

GCATGTCCTG AGAGTACACG AGCGCTCACC CCATGGCATT TGCACTCTTG

GCATGTCCTG AGAGTACACG AGCGCTCACC CCATGGCATT TGCACTCTTG

GCATGTCCTG AGAGAACACG AGCGCTCACC CCGTGGCACT TGCACTCTTG

GCATGTCCTC AGGGTGCAGG AGCACTCACA CCGTGACACT TGCATTCTTG

GCATGTCCTC AGAGTACAGG AACGCTCACG CCATGACACT TGCATTCTTG

GCATGTCCTC AGAGTACAGG AGCACTCACA CCATGACACT TGCATTCTTG

GCACGTCCGC AGAGTACACG AGCGCTCACA CCGTGGCACT TGCACTCTTG

GCAGGTCCTC AGGGTACAGG AACGCTCACG CCATGACACT TGCACTCCTG

GCATGTCCTC AGCGTACACG AGCACTCACG CCGTGACACT TGCATTCCTG

GCAGGTCCTC AGCGTACATG AGCACTCACG CCATGACACT TGCACTCTTG

GCACGTCCTC AAAGTGCAGG AGCACTCACA CCGTGGCACT TGCACTCCTG

GCATGTCCTC AGAGTACAGG AACACTCACG CCGTGACACT TGCACTCTTG

---------- ---------- ---------- ---------- ----------

GCACGTCCTC AGAGTACAGG AGCGCTCACC CCGTGACATT TGCACTCTTG

ACAGGTCCTC AGGGTGCAGG ATCACTCACC CCATGGCATT TGCACTCTTG

ACAGGTCCTC AGAGTACAGG ATCACTCACC CCATGGCATT TACACTCTTG

TTTCAAGAAC CGCTTTACAG CCTTTTTAAA AAGTCAGTGC ACTTTTATTC

TTTCAAGAAC CGCTTTACAG CCTTTTTAAA AAGTCAGTGC ACTTTTATTC

TTTCAAGAAC CGCTTTACAG CCTTTTTAAA AAGTCAGTGC ACTTTTATTC

TTTCAAGAAC CGCTTTACAG CCTTTTTAAA AAGTCAGTGC ACTTTTATTC

TTTCAAGAAC CGCTTTACAG ACTTTTTAAA AAGTCAGTGC ACTTTTATTC

TTTCAAGAAC CGCTTTACAG CCTTTTTAAA AAGTCAGTGC ACTTTTATTC

TTTCAGGAAG CGCTTTACAG CCTTTTTTAA AAGTCAGTGC ACTTTTATCC

TTTCAAGAAG CGCTTTACAG CCTTTTTAAA AAATCAGTGC ACTTTTATCC

TTTCAAGAAC CGCTTTACAG CCTTTTTAAA AAGTCAGTGC ACTTTTATTC

TTTCAAGAAC CTCTTTACAG CCTTTTTAAA AAGTCAGTGC ACTTTTATTC

CTTCAAGAAC CGCTTCACCG CCTTTTTAAA AAGTCAGTGC ACTTTTATTC

TTTCAGGAAG CGCTTCACAG CCTTTTTAAA AAGTCAGTGC ACTTCTATTC

CTTCAAAAAG CGCTTGACAG CCTTTTTAAA AACTCAGTGC GCTTTTATTC

TTTCAAGAAC CGCTTTACAG CCTTTTTAAA AAGTCAGTGC ACTTTTATTC

---------- ---------- ---------- ---------- ----------

TTTCAAGAAG CGCTTGACCG CCTTTTAAAA AAGTCAGTGC ACTTTTATTC

TTTCAAGAAC CGCTTTACAG CCTtttttta aaGTTTGTTC ATTTTTATTA

CTTCAGGAAC CGCTTGACGG CCTTTTTTTA AAGCCAGTAC GTTTTCACTG

CAATCAAAAG TAGCCTCCTA AGTTTTGCCA GGCAGGAGAG CCTGACAGCT

CAATCAAAAG TAGCCTCCTA AGTTTTGCCA GGCAGGAGAG CCTGACAGCT

CAATCAAAAG TAGCCTCCTA AGTTTTGCCA GGCAGGAGAG CCTGACAGCT

CAATCAAAAG TAGCCTCCTA AGTTTTGCCA GGCAGGAGAG CCTGACAGCT

CAATCAAAAG TAGCCTCCTA AGTTTTGCCA GGCAGGAGAG CCTGACAGCT

CAATCAAAAG TAGCCTCCTA AGTTTTGCTA GGCAGGAGAG CCTGACAGCT

TAATCAGAAG TAGCCTCCGA AGTTTTGGTA GGCAGGAGAG CCTGACAGCT

CAATCAAAAG TAGCCTCGGA AGTTTTGATA GGCAGGAGAG CCCGACAGCT

CAATCAAAAG TAGCCTCCTA AGTTTTGCTA GGCAGGAGAG CCTGACAGCT

CGATCAAAAG TAGCCTCCTA AGTTTTGCTA GGCAGGAGAG TCTGACAGCT

CAATCAAAAG TAGCCTCCTA AGTTTTGCTA GGCAGGAGTG CCTGACAGCT

CAATGAAAAG TAGCCTCGTT GGTTTTGCTA GGCAGGAGAG CCTGACAG--

CAATCAAAAG TAGCCTCCTA AGTTTTGCTA GGCAGGGGAG CCTGACAGCT

CAATCAAAAG TAGCCTCCTA AGTTTTGCTA GGCAGGAGAG CCTGACAGCT

---------- ---------- ---------- ---------- ----------

CAATCAAAAG TAGCCTCCTA AATTCTACTG GGCAGGAGAG CCTGACAGCT

AGATCAGAAG TTGTCTACTA AAGTCTGCTG AGCAAGGCAT CCTAGCAGCT

AAGGATGAAC TAGACTCGTA AGCTTTGCTG GGAAGGGATG CCTGGCTGCC

GTTCACCTCT ACAGGTGAAG AATCATTGCA CAAACAGTAA TTACCAATAG

GTTCACCTCT ACAGGTGAAG AATCATTGCA CAAACAGTAA TTACCAGTAG

GTTCACCTCT ACAGGTGAAG AATCATTGCA CAAACGGTAA TTACCAATAG

GTTCACCTCT ACAGGTGAAG AATCATTGCA CAAACGGTAA TTACCAATAG

GTTCACCTCT ACAGGTGAAG AATCATTGCA TAAACAGTAA TTACCAATAG

GTTCACCTCT GCAAGTGAAG AATCATTGCA CAAACAGTAA TTACCAATAG

GTTCACCTCT AGGAGTGAAC CCTCATTGCA CAAACAACAG TTACTAAT--

GTTCACCTTT ATAAGTGAAC AATCATTGCA AAAACAACAG TTACCAAT--

GTTCACCTCT ACAAGGGAAG AATCATTGCA CAAACAGTAA TTTCCCATAG

GTTCACCTCT ACCAGTGAGG AATCATTGCA TAAACAGTAA TTACCAATAG

GTTCACCTCC ACGAGTGAAG AATCATTGCA CAAACAGCAA TTACCAATAG

------CTCT ACAAGTGAGG AATTATTGTA CAAACAGTAA TTACCAATAG

GTTCACCTCT ACAAGTGAGG AATCATTGCA CAAACAGTAA TTACCAATAG

GTTCACCTCT ACAAGTGAAG AATCATTGCA CAAACAGTAA TTACCAATAG

---------- ---------- ---------- ---------- ----------

GTTCACCTCC ACAAGTCAAG AATCATTGCA CAAACACTAA TTACCAATCG

GTTCATCTCT AAAAGTAAGA AGCCATTGCA CAACTAATAA TTGCCTGTAG

ATTCACATCT GTAAGTCAGG AGCTCTTGCA CAACTAGTAA TTGACAATAG

TTGTAGGATT GCACAACCAT AGTCTATTCT TGGCAGTAGT GACTGTGTTC

TTGTAGGATT GCACAACCAT AGTCTATTCT TGGCAGTAGT GACTGTGTTC

TTGTAGGATT GCAAAACCAT AGTCTATTCT TGGCAGTAGT GACTGTGTTC

TTGTAGGATT GCAAAACCAT AGTCTATTCT TGGCAGTAGT GACTGTGTTC

TTGTAGGATT GCACAACCAT AGTCTATTCT TGGCAATAGA GGCTATGTTC

TTGGAGGATT ACACAACCAT AGTCTATTCT TGGCAGTAGT CACTATGTTC

-TGTAGGATT GCATAACCAC TGTCTATTCT TGGCAGTGGT CACGATGTTC

-TGTAGCATT CCACAGCCAC TATCTATTCT TGGCAGTGGT CACGATGTTC

CTGTAGGATT GCACAACCAC AGTCTATCCT TGGCAGTAGT CAACCTCTTC

TTGTAGGATT GCACAACCGT AGTCTATTCT TGGCAGCAGT CACTGTGTTC

TTGGAGGATT ACACAACCAT AGGCTATTCT TGGCAGCAGT CACGATGTTC

TTGTAGGATT GCACAACTGA AGTCTATTCT TGGCAGTAGT CACTATATTC

CTGCTGGGTT GCACAATCAC AGTCTATTCT TGGCAGTAGT CATTATGTTC

TTGTAGGATT ACACAACCAT AGTCTATTCT TGGCAGTAAT CACTATGTCC

---------- ---------- ---------- ---------- ----------

TTGTAGGATT GCACAACCGT GGGCTATTCT TGGCAGTCGT CACTCTGTCC

CTGTAGAATT GCACAATGAT AGTCTATCTT TGGCAGTTAT CACCATGTCC

TTGCAGAATT GCACAACCAA TGATTATTCT TGGCAGCTGT TACTTTGGCT

ATAAATTCAT GAAAATAAGA AAGGTACTAT TTGTAAACAG TTCAGGTCAC

ATAAATTCAT GAAAATAAGA AAGGTACTAT TTGTAAACAG TTCAGGTCAC

ATAAATTCAT GAAAATAAGA AAGGTACTAT TTGTAAACAG TTCAGGTCAC

ATAAATTCAT GAAAATAAGA AAGGTACTAT TTGTAAACAG TTCAGGTCAC

ATAAATTCAT GAAAATAAGA AAGGTACTAT TTGTAAATAG TTCAGGTCAC

ATAAATTCAT GAAAATTAGA AAGGTATTAT TTGTAAACAG TTCAGGTCAC

ACGACTTCAT GCAAAT---A AAAGTACTAT TTGTAAATAA CTCAGAT--C

ACAACTTCAT GCAAAT---A AAAGTATGAT TTGTAAACAA CTCG-----C

ATAGATTCAT GAAAATAAGA AAGGTACTAT TTGTAAACAG TTCAGGTCAC

ATAAATTCAT GAAAATAAGA A-GGTACTGT TTGTAAACAG CTCAGGTCAC

ATAAATTCAT GAAAATAAGA AAGGTACTAT TTGTAAACAG TTCAGGTCAC

ATAAATTCAT GAAAATAGGA AAGGTACTAT TTGTAAACAG TTCAGGTCAC

ATAAATTCAT GCAAATAAGA AAGTTACTAT TTGTAAACAG TTCAGGTCAC

ATAAATTCAT GATGATAAGA AAGGTATTAT TTGTAAACAG TTCAGGTCAC

---------- ---------- ---------- ---------- ----------

ATACATTCAT GGAAACAAGG AAGGCACTAT TTGTAAATAG TTCAGGTCAC

ATAAATTAAT AAAAATAAGA AAGGTACTAT TTGTAAACAG TTCAGGTCAC

ATAAATGAGC AAAATTAAGG GAGGTACTAT TTGTAAACAG TTCAGATCAC

ACTCATGTTA TACCATGTGC TAAGCAACCA TACCTTCCTG CCAGCTCTGT

ACTCATGTTA TACCATGTGC TAAGCAACCA TACCTTCCTG CCAGCTCTGT

ACTCATGTTA TACCATGTGC TAAGCAACCG TACCTTCCTG CCAGCTCTGT

ACTCATGTTA TACCATGTGC TAAGCAACCG TACCTTCCTG CCAGCTCTGT

ACTCATCTTA TTCCATGAGC TAAGCAACCA TACCTTCCTG CCAGCTCTGT

ACTCATCTTA TTCCATGTGC TAAGCAACCA TACCTTCCTG CCAGCTCTGT

ACATGTTCTC TTTTATGTAC TAAGGAACCA TACCTTCCTT CCAGCTCTGT

ACTTATTCTA TTCTATGTGC TGGGTAACCA TACCTTCCTT CCAGCTCTGT

ACTCATCTTA GTCTATGTGC TAAGCAACCA TACCTTCCTG CCCGCTCTGT

ACTCATCTTA TTCCATGTGC TAAGCAACCA TACCTTCCTG CCGGCTCGGT

ACTCATTTTA TTCCATGTGT TAAGCAACCA TACCTTCCGG CCGGCTCTGT

ACTCATCTTA TTCCATGTGC TAAGCAACC- TACCTTCCGG CCGGCTCTGT

ACTCATCTTT TTCTGTGTGC TAAGCAACCA TACCTTCCTG CCGGCTCTGT

ACTCATCTTA TTCCATGTGC TAAGCAACCA TACCTTCCTC CCGGCTCTGT

---------- ---------- ---------- ---------- ----------

ACTCATCTCA CGCTGCGTGC TAAGCAACCC TACCTTCCTG CCGGCTCTGT

ACTCATCtta tTTTTTGTGC TGACCAAGCC TACCTTCCTC CCAGCTCTGT

ACTCATCTTA TTTTTTTTGC TGACCAACCC TACCTTTCTT CCTGCACGGT

TGTTGTGAAG ATTCATCAGG GCTCTGGCAt cctttccttt cctttccttt

TGTTGTGAAG ATTCATCAGG GCTCTGGCAt cctttccttt cctttccttt

TGTTATGAAG ATTCATCAGG GCTCTGGCAt cctttccttt cctttccttG

TGTTATGAAG ATTCATCAGG GCTCTGGCAt cctttccttt cctttccttG

TGTTATGAAG ATTCATCAGG GCTCTGGCAT CCTTTCCTTT CCTCTCCTTG

TGTTGTGAAG ATTCATCAGG GCTCTGGCGT CCTTTCCTTT TCTTTCCTTG

TGTTGTGAAG ATTCATCAGG GCTCTGGCAt cctttccttt cctttccttG

TGTTGTGAAG GTTCATCAGG GCTCTGGCAT CCTTGCCTTT CCTCTCCTTG

TGTTGTGAAG ATTCATCAGC GCTCTGGCAt cctttccttt cctttccttG

TGTTGTGAAG ATTCATCAGG GCTCTGGCAT CCTTGCCTTT CCTTTCCTTG

TGTTGTGAAG GTTCATCAGG GCTCTGGCAT CCTTTCCCTT CCTTTCCTTG

TGTTGTGCAC GTTCATCAGC GCTCTGGCGT CCTTGCCCTT CCGCTCCTTG

TGTTGTGGAG ATTCATCAGG GCCCTGGCAT CCTTTCCTTT CCTTTCCTTG

TGTTGTGAAG GTTCATCAGG GCTCTGGCGt cctttccttt cctttccttG

---------- ---------- ---------- ---------- ----------

TGTTGTGAAG GTTCATCAGC GCCCTGGCGT CCTTCCCTTT CTTCTCCTTG

TGTTGTGAAG GTTCATCAGT GCTCGGGCAT CTTTTCCTTT CCTTTCCTTG

TGTTGTGAAG ATTCATTAGG GCTCTGGCAT CTTTTCCTTT CCTCTCCTTG

GCATCCACAA AGCGCGGGCA AATTTGATCC CATAGTCAAT GTTATCACTG

GCATCCACAA AGCGCGGGCA AATTTGATCC CATAGTCAAT GTTATCACTG

GCATCCACGA AGCGCGGGCA AATTTGATCC CATAGTCAAT GTTATCACTG

GCATCCACGA AGCGCGGGCA AATTTGATCC CATAGTCAAT GTTATCACTG

GCATCCACAA AGCGCGGGCA AACTTGATCC CATAGTCAAT GTTATCACTG

GCATCCACAA AGCTCGGGCA AATTTGATCC CATAGTCAAT GTTGTCACTG

GCATCTACAA AGCACGGGCG AATTTGATCC CGTAGTCAAT GTTGTCACTA

GCATCTACAA AGCACGGGCA AACTTGATCC CGTAGTCAAT ATTGTCACTG

GCATCCACAA AGCTCGGGCA AACTTGATCC CATAGTCAAT GTTATCACTG

GCATCCACAA AGCTCTGGCA AACTTGATCC CATAGTCAAT GTTATCACTG

GCATCCACGA AGCTCGGGCA AATTTGATCC CATAGTCAAT GTTATCGCTG

GCGTCCACGA AGCCCGCGCA AACTTGATCC CATAGTCAAT GTTGTCACTG

GCATCCACAA AGCTCGGGCA AACTTGATCC CATAGTC-AT GTTATCACTG

GCATCCACAA AGCACGGGCA AATTTGATTC CATGGTCAAT ATTATCACTG

---------- ---------- ---------- ---------- ----------

GCATCCACGA AGCGCGGGCA AACTTGACCC CATAATCGAT GTTGTCGCTG

GCATCCACAA AGCTCGGGCA AATTTGATGC CATAGTCAAT GTTATCGCTG

GCGTCCACAA AGCACGGGCG AACTTGATGC CATAGTCAAT GTTATCGCTG

CAGCCACCCC AATCAAAAAT GCCTTTGCTG TCCTTGGCGC TTCCCATCTT

CAGCCACCCC AATCAAAAAT GCCTTTGCTG TCCTTGGCGC TTCCCATCTT

CAGCCACCCC AATCAAAAAT GCCCTTGCTG TCCTTGGCGC TTCCCATCTT

CAGCCACCCC AATCAAAAAT GCCCTTGCTG TCCTTGGCGC TTCCCATCTT

CAGCCACCCC AATCAAAAAC GCCCTTGCTG TCCTTGCCAC TTCCCATCTT

CAGCCACCCC AGTCGAAAGT GCCCTTGCTG TCCTTGGCGG TTCCCTTCTT

CAGCCGCCCC AGTCGAAGGT ACCTTTGCTG TCCTTGCCAC TCCCTTTCTT

CAGCCACCCC AGTCGAAGGT GCCTTTGCTG TCCTTGGCAC TTCCTTTCTT

CAGCCACCCC AGTCGAAGGT GCCCTTGCTG TCCTTGGCAG TTCCTTTCTT

CAGCCACCCC AGTCAAAAGT GCCCTTGTTA TCCTTGGCGG TTCCCTTCTT

CAGCCACCCC AGTCGAAACT GCCCTTGCTG TCCTTGGCGG TTCCCTTCTT

CAGCCGCCCC AGTCGAAAGT GCCCTTGCTG TCCTTGGCGG TCCCCTTCTT

CAGCCCCCCC AGTCGAAAGT GCCTTTGCTG TCCTTGGCTG TTCCC-TCTT

CAGCCACCCC AATCGAAAGT GCCCTTGCTG TCCTTGGCGG TTCCCTTCTT

---------- ---------- ---------- ---------- ----------

CAGCCACCCC AATCGAAAGT GCCCCTGCTG TCCTTGGCAG TACCCTTCTT

CATCCGCCCC AATCAAaagt gcctttgctg tctttggaag ttcctttctt

CATCCGCCCC AGTCGAAAGT GCCCTTGCTG TCCTTGGCAG AGCCTTTCTT

CTTTGGATCA CAGGAACAGG ATTTTACTTC TCCTTGGCTA CAGGCCCTGG

CTTTGGATCA CAGGAACAGG ATTTTACTTC TCCTTGGCTA CAGGCCCTGG

CTTTGGATCA CAGGAACAGG ATTTTACTTC TCCTTGGCTA CAGGCCCTGG

CTTTGGATCA CAGGAACAGG ATTTTACTTC TCCTTGGCTA CAGGCCCTGG

CTTTGGATCA CAGGAACAGG ATTTTACTTC TCCTTGGCTA CAGGCCCTGG

CTTTGGATCA CAGGAACAGG ATTTTAATTC TCCTTGGCTA CAGGCCCTGG

CTTTGGATCG CAGGAACAGG ACTTTAATTC TCCTTGGCTA CAGGCCCTGG

CTTTGGATCA CAGGAGCAGG ACTTTAATTC TCCTTGGCTA CAGGCCCTGG

CTTTGGATCA CAGGAACAGG ATTTTAATTC TCCTTGGCTA CAGGCCCTGG

CTTTGGGTCA CAGGAACAGG ATTTTAATTC TCCTTGGCTA CAGGCCCTGG

CTTTGGATCA CAGGAACAGG ATTTTAACTC TCCTTGGCTA CAGGCCCTGG

CTTGGGATCA CAGGAACAGG ATTTTAATTC TCCTTGGCTA CAGGCCCTGG

CTTCGGATCA CA-GAACAGG ATTTTA--TT CTCTTGGCTA CAGGCCCTGG

CTTTGGATCA CAGGAACAGG ATTTTAACTC TCCTTGGCTA CAGGCCCTGG

---------- ---------- ---------- ---------- ----------

CTTTGGATCA CAGGAACAGG ATTTTAACTC TCCTTGGCTA CAGGCCCTGG

ctttggGTCG CAGGAGCAAG ATTTTAGTTC TCCCTGGCTA CAGGCTCTGG

CTTGGGGTCA CAGGAACAGG ACTTGAGCTC CCCTTGGCTG CAGGCCCGGG

TGATGGCAAA TACAACTCCA GCTGAGGAGA TGGCATAAAC AAAGGCAGAT

TGATGGCAAA TACAACTCCA GCTGAGGAGA TGGCATAAAC AAAGGCAGAT

TGATGGCAAA TACAACTCCA GCTGAGGAGA TGGCATAAAC AAAGGCAGAT

TGATGGCAAA TACAACTCCA GCTGAGGAGA TGGCATAAAC AAAGGCAGAT

TGACGGCAAA TACGACTCCA GCTGAGGAGA TGGCATAAAC AAAGGCAGAT

TGATGGCAAA TACAACTCCA GCTGAGGAGA TGGCATAAAC AAAGGCAGAT

TGATGGCAAA TACAACGCCA GCTGAGGAGA TGGCGTAAAC AAAGGCCGAT

TGATGGCAAA TACAACGCCA GCTGAAGAGA TGGCGTAAAC AAAGGCCGAT

TGATGGCAAA TACAACTCCA GCTGAGGAGA TGGCGTATAC AAAGGCAGAT

TGATGGCAAA TACAACTCCA GCTGAAGAGA TGGCGTAAAC AAACGCAGAT

TGATGGCAAA CACAACTCCA GCTGAGGAGA CGGCGTAAAC AAAGGCAGAT

TGACGGCAAA TACAACTCCA GCTGAGGAGA TGGCGTAAAC AAAGGCAGAT

TGATGGCAAA TACAACTCCA GCTGAGGAGA TGGCGTAAAC AAAGGCAGAT

TGATGGCAAA TACAACTCCA GCTGAAGAGA TGGCGTAAAC AAAGGCAGAT

---------- ---------- ---------- ---------- ----------

TGATAGCAAA TACAACTCCG GCTGAGGAGA TGGCGTAAAC AAAGGCAGAT

TGATGGCAAA CACGACGCCG GCGGAGGAGA TGGCGTACAC GAAGGCGGCT

TGATGGCAAA GACAACTCCG GCAGAGGAGA TGGCATAGAC GAAAGCAGCC

TCCCGACTAC CTGAAACAAA AATGCTTTTC TATTTACTTT CTTCTGGAAC

TCCCGACTAC CTGAAACAAA AATGCTTTTC TATTTACTTT CTTCTGGAAC

TCCCGACTAC CTGAAACAAA AATGCTTTTC TATTTACTTT CTTCTGGAAC

TCCCGACTAC CTGAAACAAA AATGCTTTTC TATTTACTTT CTTCTGGAAC

TCCCGACTAC CTGAAACAAA AATGCTCTTC TATTTACTTT CTTCTGGAAC

TCACGACTAC CTGAAACAAA AATGCTTTGA TATTTACTT- ---CTGGAAC

TCCCGGCTAC CTGAAACAAA AACGCTTTTC TTTTTCCTTT CAG-TGGAAC

TCCCGACTAC CTGAAACAAA AATGCCTTTC TTTTTCCTTT CCTCTGGAAC

TCCCGACTAC CTGAAACAAA AATGCTTTTC TATGTACTTT CTTCTAGAAC

TCCCGGCTAC CTGTAACAAA GATG-TGTTC TATTTACTTT CTTCTGGAAC

TCCCGACTAC CTGCAACAGA AATGCTTTTC TATTTACTTT CTTCTGGAAC

TCCCGACTAC CTGAAACGAA AATGCTTTGC TATTTACTTT CTTCTGAAAC

TCCCGACTAC CTGAAACAAA AATGCTTTTC T--------- ----------

TCCCGACTAC CTGAAACAAA AATGCTTTTC TATTTACTTT CTTCTGGAAC

---------- ---------- ---------- ---------- ----------

TCCCGACTAC CTGAAACAAA AATGCTTTTC TATTTACTTT CTTCTGGAAC

TCTCGACTAC CTGTAATCAA AGCAATCATC TATTGACTTA ACTCTGGAAT

TCCCGGCTAC CTGCAACGAG AATGCCCGTC TACTGACTTC TTTCTGGGAT

ACTAATCACG GATTGGGTAA AGACAGTGAA AAGTTTTTTT TGGCTGGAAG

ACTAATCACG GATTGGGTAA AGACAGTGAA AAGTTTTTTT TGGCTGGAAG

ACTAATCACG GATTGGGTAA AGACAGTGAA AAGTTTTTTT TGGCTGGAAG

ACTAATCACG GATTGGGTAA AGACAGTGAA AAGTTTTTTT TGGCTGGAAG

ACTAATCACG GATTGGGTAA AGACAGTGAA AAGCTTTTTT TGGCTGGAAG

ACTAATCACA GATTGGGTAA AGACAGTGAA AAGCTTTTTT TGGCTGGAAG

ACTAGTCGTG GATCGGGTAG AGACAGTGAA AAGCTTTTTT TGGTTGGAAG

ACTAATCATG GATCGGGTAG AGACAGTGAA AAGCTTTTTT TGGCTGGAAG

ACTAATCACG CATTGGGTAA AGACAGTGAA AAGCTTTTTT TGGCTGGAAG

ACTAATCACG GATTGGGTAA AGACAGTGAA AAGCTTTTTT TGGCTGGAAG

ACTAATCACG GATTGGGTAA AGACAGCGAA AAGCTTTTTT TGGCTGGAAG

ACTAATCACG GATTGGGTAA AGACAGTGGA AAGCTTTTTT TGG-TGGAAG

---------- ---------- ---------- ---------- ----------

ACTAATCATT GATCGGGTAA AGACAGTGAA CAGCTTTTTT TGGCTGGAAG

---------- ---------- ---------- ---------- ----------

ACTAATCACG GATTGGGTAA AGACAGTGAA ATGCTTTTTT TGGCTGGAAG

GCTAACCACG CATGGGGAAA AGACAATTGA GAGGGTTTTG CGGCAGGAAG

GCTAATCACG GAGCGGTGAA AGAGAGTAAA AGGACATGTT TGGCTGGGAG

GAAACAAATT TACCGCCTAT AATTAGACTT GGGCAAGAAC CCTTTGGGGT

GAAACAAATT TACCGCCTAT AATTAGACTT GGGCAAGAAC CCTTTGGGGT

GAAACAAATT TACCGCCTAT AATTAGACTT GGGCAAGAAC CCTTTGGGGT

GAAACAAATT TACCGCCTAT AATTAGACTT GGGCAAGAAC CCTTTGGGGT

GAAACAAATT TACCGCCTAT AATTAGACTT GGGCAAGAAC CCTTTGGGGT

GAAACAAATT TACCGCCTAT AATTAGACTT GGGCAAGAAC CCTTTGGGGT

GAAACAAATT TACCGCCTCT AATTAGACTT GGGCAAGAAC CCTTTGGGG-

GAAACAAATT TACCGCCTAT AATTAGACTT GGGCAAGAAC CCTTTGGGG-

GAAGCAAATT TACCGCCTAT AATTAGACTT GGGCAAGAAC CCTTTGGGGT

GAAACAAATT TACCGCTTAT AATTAGACCT GGGCAAGAAC CCTTTGGGGT

GAAACAAATT TACCGCCTAT AATTAGACTT GGGCAAGGAC CCTTTGGGGT

GAAACAAATT TACCGCCTAT AATTAGACTT GGGCAAGAAC CCTTTGGGGT

---------- ---------- ---------- ---------- ----------

GAAACAAATT TACCGCCTAT AATTAGACTT GGGTAAGAAC CCTTTGGGGT

---------- ---------- ---------- ---------- ----------

GGAACGAATT TACCGCCTAT AATTAGGCTT GGGCAAGAAC CCTTTGGGGT

AAAACAAATT TGTCACCTAT GATTTGACTT GTGCACGAGC CATTTGTTGT

AAAACATGTT TTCTGACTCT AATTAGGCCT GCGTACAAAC CACTTGGACT

GCTTTCTTAG AATTTTCTTT ATTAGAGCAC AGGCTACTTT CACTTACAGG

GCTTTCTTAG AATTTTCTTT ATTAGAGCAC AGGCTACTTT CACTTACAGG

GCTTTCTTAG AATTTTCTTT ATTAGAGCAC AGGCTACTTT CACTTACAGG

GCTTTCTTAG AATTTTCTTT ATTAGAGCAC AGGCTACTTT CACTTACAGG

GCTTTCTTAG AATTTTCTTT ATTAGAGCAC AGGCTACTTT CACTTACAGG

GCTCTCTTAG AATTTTCTTT ATTAGAGCAC AGGCTACTTT CACTTACAGG

GCTTTCTTAG ACTTTTCTTT ATTAGAGAGC AGCCTACTTT CACTTACAGG

GCTTTCTTAG ACTTTTCTTT ATTAGAGAGC AGCGTATTTT CACTTACAGG

GCTTTCTTAG AATTTTCTTT ATTAGACCAC AGGCTACTTT CACTTACAGG

GCTTTCTTAG AATTTTCTTT ATTAGAGCAC AGGCTACTTT CACTTACAGA

GCTTTCTTAG AATTTTCTTT ATTAGAGCCC AGGCTACTTT CCCTTACAGG

GCTTTCTTAG AATTTTCTTT ATTAGTGCAT AGGCTCCTTT CACTTACAGG

---------- ---------- ---------- ---------- ----------

GCTTTCTTAG AATTTTCTTT ATTAGAGCAC AGCCTACTTT CACTTACAGG

---------- ---------- ---------- ---------- ----------

GCTTTCTTAG AATTTTCTTT ATTAGAGCGC AGGCTACTTT CACTTACAGG

GCTTTCTTAG AATTTTCTTT ATTAGAGCAA AGTCTACTTT CACTTACTGA

ACTTTCTTCA AAACCATTTT ATTAGCAAAC TGGCCATTTT TCCCTTCATG

CTGAAAGGTA CATATTTCAG TGCAATAACT ACATTAAATT ATTTTAATAA

CTGAAAGGTA CATATTTCAG TGCAATAACT ACATTAAATT ATTTTAATAA

CTGAAAGGTA CATATTTCAG TGCAATAACT ACATTAAATT ATTTTAATAA

CTGAAAGGTA CATATTTCAG TGCAATAACT ACATTAAATT ATTTTAATAA

CTGAAAGGTA CATATTTCAG TGCAATAACT ACATTAAATT ATTTTAATAA

CTGAAAGGTA CATATTTCAG TGCAATAACT ACATTAAATT ATTTTAATAA

CTGAGAGGAA CATATTTCAG TGCAATAACT ACATTAAATT ATTTTAATAA

CTGAGAGGGA CATATTTCAG TGCAATAACT ACATTAAATT ATTTTAATAA

CTGAAAGGTA CATATTTCAG TGCAATAACT ACATTAAATT ATTTTAATAA

CTGAAAGGTA CATATTTCAG TGCAATAACT ACATTAAATT ATTTTAATAA

CTGAAAGGTA CATATTTCAG GGCAATAACT ACATTAAATT ATTTTAATAA

CTGAAAGGTA CATATTTCAG TGCAATAACT ACATTAAATT ATTTTAATAA

---------- ---------- ---------- ---------- ----------

CTGAAAGGTA CATATTTCAG TGCAATAACT ACattaaatt attttaataa

---------- ---------- ---------- ---------- ----------

CTGAAAGGGG CATATTTCAG TGCAATAACT ACATTAAATT ATTTTAATAA

CTAAAAGGGA CATATTTCAG TGCAATAACT ACATTCAATT ATTTTAATAA

CTGAAAGGTC CATATGTCAG TGCAGTAACT CTGTTATATT ATTTTAATAA

TCTGTAGTAA CTCATAACAC GGTGTTGTGG TATAGTGACA GATATAAGGC

TCTGTAGTAA CTCATAACAC GGTGTTGTGG TATAGTGACA GATATAAGGC

TCTGTAGTAA CTCATAACAC GGTGTTGTGG TATAGTGACA GATATAAGGC

TCTGTAGTAA CTCATAACAC GGTGTTGTGG TATAGTGACA GATATAAGGC

TCTGTAGTAA CTCATAACAC GGTGTTGTGG TATAGTGACA GCTATAAGGC

TCTGTAGTAA CTCATAACAC GGTGTTGTGG TATAGTGACA GATATAAGGC

TCTGTAGTAA TTCATAACAC GGTGTTGTGG TGGAGTGACA GATATAAGGC

TCTGTAGTAA CTCATAACAC GGTGTTGTGG TGGAGTGACA GATATAAGGC

TCTGTAGTAA CTCATAACAC GGTGTTGCGG TATAGTGACA GATATAAGGC

TCTGTAGTAA CTCATAACAC GGTGTTGTGC TATAGTGACA GATATAAGGC

TCTGTAGTAA CTCATAACAC GGTGTTGCGG GATAGTGACA GATATAAGGC

TCTGCAGTAA CTCATAACAC GGTGTTGTGG TACAGTGACA GGTATAAGGC

---------- ---------- ---------- ---------- ----------

tttGTAGTAA TTCATAACAC GGTGTTGTGG TACAGTGACA GATATAAGGC

---------- ---------- ---------- ---------- ----------

TCTGTAGTAA CTCATAACAC GGTGTTGTGG TATAGTGACA GATATAAGGC

TCTGTAGTAA CTCATAATAC AGTGTTGTGG TTTAATGACA GATATAAGAC

TCTCCAGTAA GTCATAATAC AGCGCTGTGG TATAATGGCA GATATAAAAC

TCTTTAGGGT CGCACCTCAT TTTTCAAGTG GAGCTGTATT TCATTGTTAT

TCTTTAGGGT CGCACCTCAT TTTTCAAGTG GAGCTGTATT TCATTGTTAT

TCTTTAGGGT CGCACCTCAT TTTTCAAGTG GAGCTGTATT TCATTGTTAT

TCTTTAGGGT CGCACCTCAT TTTTCAAGTG GAGCTGTATT TCATTGTTAT

TCTTTAGGGT CGCACCTCAT TTTTCAAGTG GAGCTGTATT TCATTGTTAT

TCTTTAGGGT CGCACCTCGT TTTTCAAGTG GAGCTGTATT TCATTGTTAT

TCTTTAGGGT CGCACCTCAT TTTTCAAGTG GAACTGTATT TCATTGTTAC

TCTTTAGAGT CGCACCTCAT TTTTCAAGTG GAACTGTATT TCATTGTTAC

TCTTTAGGGT CGCACCTTGT TTCTCAAGTG GAGCTGTATT TCATTGTTAT

TCTTTAGGGT CGCACCTCAT TTTTCAAGTG GAGCTGTATT TCATTGTTAT

TCTTTAGGGT CGCACCTCAT TTTTCAAGTG GGGCTGTATT TCATTGTTAT

TCTTTAGGGT CGCACCTCAT TTTTCAAGTG GAGCTGTATT TCATTGTTAT

---------- ---------- ---------- ---------- ----------

TCTTTAGGGT CGCACCTCGT TTTTCAAATG GAGCTGTATT TCACTGTTAT

---------- ---------- ---------- ---------- ----------

TCTTTAGGGT CACACCTCGT TTTTCAAGTG AGGCTGTATT TCATTGTTAG

TCTTTAGGGT CGCACCTCAT TTTTCAAATG GTGCTGTATT TCATTGTTAT

TCTTTCGGGC CGCTTCTTGT TTTTCAGGCA ATGCAGTATT TCAGTGTCAT

TAGCATGCCC TTTTATTAAT GGTTGCTGCG GCAAATATAT TAACAATCCA

TAGCATGCCC TTTTATTAAT GGTTGCTGTG GCAAATATAT TAACAATCCA

TAGCATGCCC TTTTATTAAT GGTTGCTGCA GCAAATATAT TAACAATCAA

TAGCATGCCC TTTTATTAAT GGTTGCTGCA GCAAATATAT TAACAATCAA

TAGCATGCCC TTTTATTAAT GGTTGCTGCG GCAAACATAT TAACAATCAA

TAGCATGCCC TTTTATTAAT GGTTGCTGCA GCAAATATAT TAACAATCAA

CAGCATGGCC TTTTATTAAT GGTTGCTGCA GCGAATATAT TAACAATCAA

TAGCATGGCC TTTTATTAAT GGTTGCTGCA GCAAATATAT TAACAATCAA

TAGCATGCGT TGTTATTCAT GGTTGCTGCA GCAAATATAT TCACAATCAA

TAGCATGCCT TTTTATTAAT GGTTGCTGCA GCAAATATAT TAACAATCAA

TAGCGTGCCT CTTTATTAAT GGGTGCGGCG GCAAATATAT CAACAATCAA

TA----GCCT TTTTATTAAT GGTTGCTGCA GCAAATACAT TAACAATCAA

---------- ---------- ---------- ---------- ----------

TAGCATGTCT TTTTATTAAT GGTTGCTGCA GCAAATATAT TAACAATCAA

---------- ---------- ---------- ---------- ----------

TAGCACGCTT CTTTATTAAT GGTTGCCGCA GCAAATCTAT TCACAATCAA

TAGCATGCTT TTTTATTAAT GGTTGCCACA GTAAATATAT TAACAACCAG

TAGAAGGCCT CTTCCTGCAT GGTTGCCACT GTAAATATAT TTGCAAACAA

AATAGAAAAG GCAGAAAACA GAGGTGATTC AATATGTCAA GAGGAGTGTT

AATAGAAAAG GCAGAAAACA GAGGTGATTC AATATGTCAA GAGGAGTGTT

AATAGAAAAG GCAGAAAACA GAGGTGATTC AATATGTCAA GAGGAGTGTT

AATAGAAAAG GCAGAAAACA GAGGTGATTC AATATGTCAA GAGGAGTGTT

AATAGAAAAG GCCGAAAACA GAGGTGATTC AGTATGTCAA GAGGAGTGT-

AGTAGAAAAG GCAGAGAA-A GAGGCGATTC AGCATGTCAA GACGAGTGTT

AATAGAAAAG GCAGAAAGCA GAGGCGATTC AATCTGTCAG GACCAGTGTT

AATAGAAAAG GCAGAAAGCA GAGGCAATTC AATCTGTCAA GACCAGTGTT

AATAGAAAAG GCAGAA--CA CAGGTGAGTT AGGATGTCAA GCGCAGTGCT

AATAGAAAAG GAAGCAAATG GAGGTGATTC AATATGTCAA AACGAGTGTT

AATAGAAAAG GCAGAGAACG GAGGCGACTC GGTATGTCAA GACGAGTGTT

AATAGACAAG GCAGAGAACG GAGGTGATTC AATATGTCAA GACGAGTGTT

---------- ---------- ---------- ---------- ----------

AATAGAGAAG GCAGAAAACA GAAGTGATTC AATATGTCAA GACGAGTGTT

---------- ---------- ---------- ---------- ----------

AATAGGAGAG GCAGAACACA GTGGCGATTC GGTCCCTCAG GATGAGTGTG

AATAGAAAAG GCTGAAAACA GACGTGATCC AATAGGTCAA CAAGAGTGTT

TTGAGAAAAG ACTGAACTCA GAAGTGATTC AATATGTTAA GAAGAGTGTT

GAACCACACA GTGGGAGAAC CTTTTCACAT TGAAGCCCTT CCGTTTGTCA

GAACCACACA GTGGGAGAAC CTTTTCACAT TGAAGCCCTT CCGTTTGTCA

GAACCACACA GTGGGAGAAC CTTTTCGCAT TGAAGCCCTT CCGTTTGTCA

GAACCACACA GTGGGAGAAC CTTTTCGCAT TGAAGCCCTT CCGTTTGTCA

GAACCACACA GTGAGAGAAC CTTTTCACAT TGAAGTCCTT CCGTTTGTCA

GAACCACACA GTGGGAGAAG TTTTTCGCAT TGAAGCCCTT CCGTTTGTCA

GAACCACGCA GTGGGAGAAC TTTTCCGAAT CGAAGCCTTT CCATGTGTCA

GAACCACACA GTGGGAGAAC TTTTCCGCAT GGAAGCCTTT CCATGTGCCA

GAGCCACACA GTAGGAGCAC CTTTCTGAAT GGAAGCCCTT CCGTTTGTCA

GAACCACGCA GTGGGAGAAC CTTTTCGCAT CGAAGCCCTT CCGTTTGTCA

GAACCACACA GTGGGAGAAC CTTTTCGCCT TGAAGCCCTT CCGCCTGTCA

GAACCACACA GTGGGAGAAC CTTTTCGCAT CGAAGCCCTT CCGTTTGTCA

---------- ---------- ---------- ---------- ----------

GAACCAGACA GTGGGAGAAC CTTTTCGCAT TGAAGCCCTT CCGTTTGTCA

---------- ---------- ---------- ---------- ----------

GAAACACACA GTGGGAGGAC CTTTTTGCAT GGAAGTCCTG CCGCCTGTCA

GAACCATACA TTGGGAGAAC CATTTTGCAT TGCAGCACTC CCATTTGTCA

GAATCATATA TGGGGAGACC CATTTTGCAG GGAATTTCTT CGCTTTGCCG

TTCAGTGCTA AAATTTATTA TACATCCCTT GCAATATTTG CCTGCTAGTC

TTCAGTGTTA AAATTTATTA TACATCCCTT GCAATATTTG CCTGCTAGTC

TTCAGTGTTA AAATTTATTA TACGTCCCTT GCAATATTTG CCTGCTAGTC

TTCAGTGTTA AAATTTATTA TACGTCCCTT GCAATATTTG CCTGCTAGTC

TTCTGTGTTA AAATTTATTA TACATCCTTT GCAATATTTG CCTGCTAGTC

TTTAATGCTA AAATTTATTA TCCATCCTTT GCAATATTTG CCTGCTGGTC

CTCATGGCTA GAGCTTACTG TGCATCCCTT GCAAAGTCTG CTTGCTAGTC

CTCACGGCTA TAGCTTACTG TGCATCCCTT GC-------- -----TAGTC

TTCATGGCTA AAATTTATTA TACGTCCCTG GCAATATTTG CCTGCAAGTC

TTCAGTGCTA AAATTTATTA TACATCCCTT ACAATATCTG CCTGCTAGTC

TTCGGTGCTA AAATTTATTA TATGTCCCCT GCAATATTTG CCTGCTAGTC

TTCAGTGCTA AAATTTATTA TACACCCCTT GCAATATTTG CCTGCTAGTC

---------- ---------- ---------- ---------- ----------

TTCAGTGCTA AAATTTATTA TACATCCTTT GCAATATTTG CCTGCTAGTC

---------- ---------- ---------- ---------- ----------

CTCGGTGTTG AAATTTATTA TGCATCCCTT GCAATATTTG TCTGCTAGCC

TTCGGGGCTA AAATTTATTG TTTGTTCTTT ATAATATTTG CCTGCTAGCC

CTCAGGGCTA AATTTTGCAA TATAGTCTAT GCAATATTTT CCTGCTAGCT

TCATTATTAA AAATCAGGAA AACATATCTA CACCATATTA AATGGCACTT

TCATTATTAA AAATCAGGAA AACATATCTA CACCATATTA AATGGCACTT

TCATTATTAA AAATCAGGAA AACATATCTA CACCATATTA AATGGCACTT

TCATTATTAA AAATCAGGAA AACATATCTA CACCATATTA AATGGCACTT

TCATTATGAA AAATCAGGAA AACATATCTA CACCATATTA AATGGCACTT

TCATTATT-A AAATCAGG-A AACATATCTA CACCATATTA AATGACACTT

TCACTATTAA AAACCATTAT TGCAAGTCTA CACAATATTA AGTGGCACTT

TCATTATTAA AAA------- ------TCTA CACAATGTTA AATGGCACTT

TCATTATTAA AAATCAGGAA AACATATCTA CGCCCTATTA AATGGCACTT

TCATTATTAA AAATCAGGAA AACGTATCTA CACCATATTA AATGGCACTT

TCATTATTAA AAATCAGGAA AACATATCTA CACCATATTA AATGGCACTT

TCATTATTAA AAATCAGGAA AACATATCTA CACCATATTA AATGGCACTT

---------- ---------- ---------- ---------- ----------

TCATTATTAA AAATCAGGAA AACTTATCTA CACCATATTA AATGGCACTT

---------- ---------- ---------- ---------- ----------

ACATTATTAA AAATAGAGAA AATACTTCTA CATCATATTA AATGGCACTT

TCATTATTAA AAATCAGAAA AACATATCCT CACCATATTA AATGACACTT

TTATTATTAA AAATCAGAAA ACCATATCGA CACCCTATTA AATTACACTT

ACCTCAGCTT TCAGAAAAAC TTACTTCGGA GTAGGACCCT GCCAAAAAGG

ACCTCAGCCT TCAGAAAAAC TTACTTCGGA GTAGGACCCT GCCAAAGAGG

ACCTCAGCCT TCAGAAAAAC TTACTTCGGA GTAGGACCCT GCCAAAGAGG

ACCTCAGCCT TCAGAAAAAC TTACTTCGGA GTAGGACCCT GCCAAAGAGG

ACCTCAGCCC TCAGAAAAAC TTACTTCGGA GTAGGACCCT GCCAAAGAGG

ACCTCAGCCT TTAGAAAAAC TTACTTCGGA GCAGGACCCT GCCGAAGAGG

ACCTCAGCCA TTCAGGAAAC TCACTTCGGA GGAGGACCCG GCCAAAGAGG

ACCTCAGCTA TCCAGGAGAC TCACTTCGGA GGAGGACCCG GCCAAAGAGG

ACCTCAGCCT TCAGAAAAAC TTACTTCGGA GCAGAACCCT GCCAAAGAGG

ACCTCAGCCT TCAAAAAAAC TTACTTCGGA GCAGGACCCT GCCGAAGAGG

ACCTCAGCCT TCAAAAAAAC TTACTTCGGA GCAGGACCCT GCCAAAGAGG

ACCTCAGCCT TCAAAAAAAC TTACTTCGGA GCAGGACCCT GCCGAAGAGG

---------- ---------- ---------- ---------- ----------

ACCTCAGCCT TCAGAAAAAC TTACTTCGGA GCAGGACCCT GCCAAAGAGG

---------- ---------- ---------- ---------- ----------

ACCTCAGCCT TCAAAAAAAC TTACTTCGGA GCAGCAACCG GCCAAAGAGG

ACCTCAGCTT TTAAAAAAAC TTACTTCGGA GCAGAACTTT GCCGAAGAGA

ACCTCAGCCC TTAAAATAAC TTACTGCGCA GCAAGACTCG GCCGAACAGG

CTGTGATCCC TGTCCAGGGT GTTGCAATTC CAGCGGTGCT GGCGGAACTG

CTGTGATCCC TGTCCAGGGT GTTGCAATTC CAGCGGTGCT GGCGGAACTG

CTGTGATCCC TGTCCAGGGT GTTGCAATTC CAGCGGTGCT GGCGGAACTG

CTGTGATCCC TGTCCAGGGT GTTGCAATTC CAGCGGTGCT GGCGGAACTG

CTGTGATCCC TGTCCAGAGT GTTGCAATTC CAGCGGTGCT GGCGGAACTG

CTGTGATCCC TGTCTAGGGT GTTGCAGTTC CAGCGGTGCT GGCGGAACTG

CTGTGGTCTC TGTCCAGGGT GTTGCAGTTC CAGCGATGCT GGCGGAACTG

CTGTGATCTC TGTCCAGGGT GTTGCAGTTC CAGCGATGCT GGCGGAACTG

CCGTGATCCC TGTCCAGGGT GTTGCAGTTC CAGCGGTGCT GTCGGAACTG

CTGTGATCCC TGTCCAGGGT GTTGCAGTTC CAGCGGTGCT GGCGGAACTG

CTGTGATCCC TGTCCAGCGT GTTGCAGTTC CAGCGGTGCT GGCGGAACTG

CTGTGGTCGC GGTCGAGGGT GTTGCAGTTC CAGCGGTGCT GGCGGAACTG

---------- ---------- ---------- ---------- ----------

CTGTGGTCCC TGTCTAGGGT GTTGCAGTTC CAACGGTGCT GGCGGAACTG

---------- ---------- ---------- ---------- ----------

CTGTGGTCTC TGTCCAGGGT GTCACAGTTC CAGCGGTGCT GTCGGAACTG

TTGTGGTCCC TGTCCAGGGT GTGGCAGTTC CAGCGGTGCT GGCGAAACTG

CTGTGGTCCC GATCCAAGGT GTTGCAGTTC CATCGGTGTT GGCGGAACTG

GTGCTGGCAT TCCCCTGGCT AATGGCACGC ATCACATCTG GATGTCGGTG

GTGCTGGCAT TCCCCTGGCT AATGGCACGC ATCACATCTG GATGTCGGTG

GTGCTGGCAT TCCCCTGGCT AATGGCACGC ATCACATCTG GATGTCTGTG

GTGCTGGCAT TCCCCTGGCT AATGGCACGC ATCACATCTG GATGTCGGTG

GTACTGGCAT TCCCCAGGCC AATGGCACGC ATCACATCTG GGTGTCGGTG

GTGCTGGCAC TCCCCAGGCC AATGGCGCGC ATCACGTCCG GGTGCCGGTG

ATGCTGGCAC TCCCCAGGCC AATGGCACGC ATCACGTCTG GGTGTCGGTG

GTGTTGGCAC TCCCCAGGCC AATGGCACGC ATCACATCTG GGTGTCGGTG

ATACTGGCAC TCCCCAGGCC TATGGCACGC ATCACGTCCG GGTGTCGGTG

GTGCTGGCAC TCCCCAGGCC GATGGCACGC ATCACATCCG GGTGTCGGTG

GTGCTGGCAC TCCCCAGCCC AATGGCACGC ATCACGTCCG GGTGTCGGTG

GTGCTGGCAC TCCCCAGGCC AATGGCACGC ATCACGTCGG GGTGTCGGTG

---------- ---------- ---------- ---------- ----------

GTGCTGGCAC TCCCCAGGCC AATGGCTCGC ATCACATCTG GGTGGCGGTG

---------- ---------- ---------- ---------- ----------

GTGCTGGCAT TCCCCAGGCC AATGGCACGC ATTACATCCG GGTGTCGGTG

GTGCTGACAC TCCCCAGGCC GATGGAGCGC ATCACCTCTG GGTGCCGGTG

GTGCTGACAC TCCCCAGGCC GATGGAGCGC ATGACCTCCG GGTGCCGGTG

ACACAGCTGC CGCTGGCTGC TCACCAGGCC TGGCACATTA TCGCACATCA

ACACAGCTGC CGCTGGCTGC TCACCAGGCC TGGCACATTA TCGCACATCA

ACACAGCTGC CGCTGGCTGC TCACCAGGCC TGGCACATTA TCGCACATCA

ACACAGCTGC CGCTGGCTGC TCACCAGGCC TGGCACATTA TCGCACATCA

ACACAGCTGC CGCTGGCTGC TCACCAGGCC TGGCACATTA TCACACATCA

GCACAGCTGC CGCTGGCGGC TTACCAGGCC GGGCACATTG TCACACATCA

GCACAGCTGA CGCTGGCGGC TCACCAGACC TGGCACATTG TCACACATCA

GCACAGCTGA CGCTGCCGGC TCACCAGGCC TGGCACATTG TCACACATCA

GCACAGCTGC CGCTGGCGGC TCACCAGGCC TGGCACATTG TCACACATCA

GCACAGCTGC CGTTGGTGGC TCACCAGGCC TGGCACGTTG TCACACATCA

GCACAGCTGG CGCTGGCGGC TCACCAGGCC TGGCACATTG TCACACATCA

GCACAGCTGC CGCTGGCGGC TCACCAGGCC TGGCACATTG TCACACATCA

---------- ---------- ---------- ---------- ----------

GCACAGCTGA CGCTGGCGGC TCACCAGGCC TGGCACATTG TCACACATCA

---------- ---------- ---------- ---------- ----------

GCACAGTTGG CGCTGGCGGC TCACCAAGCC TGGCACATTG TCACACATCA

GCACAGCTGA CGCTGGCGGC TGACCAAGCC TGGCACGTTG TCACACATGA

GCACAGCTGC CGCTGGCGGC TGACCAGGCC TGGCACGTTG TCACACATCA

CCCTGGAGGA GCCTGTAGCT CTCATGTACC TAAAATCTTT CTATTGTTAT

CCCTGGAGGA GCCTGTAGCT CTCATGTACC TAAAATCTTT CTATTGTTAT

CCCTGGAGGA GCCTGTAGCT CTCATGTACC TAAAATCTTT CTATTGTTAT

CCCTGGAGGA GCCTGTAGCT TTCATGTACC TAAAATCTTT CTATTGTTAT

CCCTGGAGGA GCCTGTAGCT CCCATGTACC TAAAATCTTT CTATTGTTAT

CCCTGGAGGA GCCTGTAGCT CTCATGTACC TGAAATCTTT CTATTGTTAT

CCCTGGAGGA GCCTGTCGCT CTCATGTACC TGAAACT--- --GCTGCTAT

CCCTGGAGGA GCCTGTAGCT CTCATGTACC TGATACTTTT CTATTGTTAT

CCCTGGAGGA GTCTGTAGCT CTCATGTACC TGAAATCTTT CTATTGTTAT

CCCTGGAGGA GCCCGTAGCT CTCATGTACC TAAAATCTTT CTATTGTTAT

CCCTGGAGGA GCCTGTAGCT CTCATGTACC TGAAATCTTT CTATTGTTAT

CCCTGGAAGA GCCTGTAGCT TTCATGTACC TGAAATCTTT CTATTGTTAT

---------- ---------- ---------- --AAA---TT CTATTGTTAT

CCCTGGAGGA GCCTGTAGCC CTCATGTACC TGAAATCTTT CTATTGTTTT

---------- ---------- ---------- ---------- ----------

CCCTGGAGGT GCCTGTAGCT CTCATGTACC TGAAATATCT CTATTGTTAT

CCCTTGAGGA GCCCACAGCT CTCATGTACC TGAAACTTTT CTATTGTTTT

CCCTCGAGGA GCCCGCCGCT CTCATGTACC TGATATCGTT CTATTGTTTG

TAAGGACACT AAATGTTTGG AAGAATCAAA CAGCTATAAC TCCATAAAGT

TAAGGACACT AAATGTTTGG AAGAATCAAA CAGTTATAGC TCCATAAAGT

TAAGGACACT AAATGTTTGG AAGAATCAAA CAGTTATAGC TCCATAAAGT

TAAGGACACT AAATGTTTGG AAGAATCAAA CAGTTATAGC TCCATAAAGT

TAAGGACACT AAATGTTTGG AAGAATCAAA CAGTTATAGC TCCATAAAGT

TAAGGACACT AAATGTTTGG AAGAATCAAA CAGTTATAGC TCCATAAAGT

CACGAGCACT ACATATTTGG AAAAA----T CAGTTATAGT TCCATAAAGT

CACCAGCACT AAATATTTGA AAAAA--AAT CAGTTATAGC TCCATAAAGT

TAAGGACACT AAATGTTTGG AAGAATCAAA CAATTATAGC TCCATAAAGT

TAAGGATACT AAATGTTTGG AAGAATCAAA CAGTTATAGC TTCATAAAGT

TAAGGACACT AAATGTTTGG AAGAATCAAA CAGTTATAGC TCCATAAAGT

TAAGGACACT AAATGTTTGG AAGAATCAAA CAGTTATAGC TCCATAAAGT

TAAGGACACT AAATGTTTGG AAGAATCAAA CTGTTATAGC ACCATAAAGT

TAAGGACAGT AAATGTTTGG AAGAATCAAA CAGTTATAGC TCCATAAAGT

---------- ---------- ---------- ---------- ----------

TAAGGACACT AAATGCTTGG AAGAATCAAA TAGTTATAGC TCCATAAAGC

TAAGGACATT AAATGTTTGG GAGAATCAAA CAGTTATAGC TTCGTAAAGG

CAATGACATT AAGGGTTTGG AAGACTCAAA CCGTTACAGC TCCATAAAGT

ACTTTGAAAA GAAACTTAAA TTACATCATC ATCTCTACCC AGATGTTCTC

ACTTTGAAAA GAAACTTAAA TTACATCATC ATCTCTACCC AGATGTTCTC

ACTTTGAAAA GAAACTTAAA TTACATCATC ATCTCTACCC AGATGTTCTC

ACTTTGAAAA GAAACTTAAA TTACATCATC ATCTCTACCC AGATGTTCTC

ACTTTGAAAA GAAACTTAAA TTACATCATC ATCTCTACCC AGATGTTCTC

ACTTTGAAAA GAAACTTAAA TTACATCATC ATCTCTACCC AGATGTTCTC

ATTTTGAAAA GAAACTTAAA TTACAGCATT ATCTCTTTGC AGATGTTCTC

ATTTTGAAAA GAAACTTAAA TCACATTATC ATCTCTAGGC AGATGTTCTC

ACTTTGAAAA GAAACTTAAA TTACATCATC ATCCCTACCC AGATGTTCTC

ACTTTGAAAA GAAACTTAAA TTACATCATC ATCTCTACCC AGATGTTCTC

ACTTTGAAAA GAAACTTAAA TTACATCATC ATCTCTACCC AGATGTTCTC

ACTTTGAAAA GAAACTTAAA TTACATCATC ATCTCTGCCC AGATGTTCTC

ACTTTGAAAA GAAACTTAAA TTACATCATC ATCTCTACCC AGATGTTCTC

ACTTTGAAAA GAAACTTAAA TTACATCATC ATCTGTACCC AGATGTTCTC

---------- ---------- ---------- ---------- ----------

ACTTTGAAAA GAAACTTAAA TTACATCATC ATCTGTACCC AGATGTTCTC

ACTTTGAAAA GAAACTTAAA TTACATCATC ATCTGTACCC AGATGTTCTC

ACTTTAACCA AAAAGTTAAA TTACATCATC ATCTCCGTCC ATATGTTCTC

TATTAGTCGT GAAATGTTTA GAAAGGTACC TTTTATATAA CCCAAGCTGC

TATTAGTCGT GAAATGTTTA GAAAGGTACC TTTTATATAA CCCAAGCTGC

TATTAGTCGT GAAATGTTTA GAAAGGTACC TTTTATATAA CCCAAGCTGC

TATTAGTCGT GAAATGTTTA GAAAGGTACC TTTTATATAA CCCAAGCTGC

TATTAGTCGT GAAATGTTTA GAAAGGTACC TTTTATATAA CCCAAGCTGC

TATTAGTTGT GAAATGTTTA GAAAGGTACC TTTTATAGAA CCCAAGCCGC

CATTCGTG-T AAAATGTATA GAAAGGTACC TTTGACACAA TCCAAGTTGC

CATTCGTGGT GAGAGGTTTA GAAAGGCACC TTTTATACAA CCCAAGTTGC

TATTAGTTGT GAAATGTTTA GAAAGGTACC TTTTATATAA CCCAAGCTGC

TATTAGTTGT GAAATGTTTA GAAAGGTACC TTTTATATAA CCCAAGCTGC

TATTAGTCGT GAAATGTTTA GAAAGGTACC TTTTATATAA CCCAAGCTGC

TATTAGTTGT GAAATGTTTA GAAAGGTACC TTTTATATAA CCCAAGCTGC

TATTAGTTGT GAAATGTTTA GAAAGGTACC TTTTATATAA CCCAAGCTGC

TATTAGTTGT GAAATGTTTA GAAAGGTACC TTTTATATAA CCCAAGCTGC

---------- ---------- ---------- ---------- ----------

TATTAGTTGT GAAATGTTTA GAAAGGTACC TTTTATATAA CCCAAGCTGC

TATTAGCAGT GAACTGTTTA GAAAGGTACC TTTCATATAA CCCAAGCTGC

TATTAGTAGT GAAATGTTTA GAGAGGTACC TTTTATAAAA TCCAAGCTTC

CATAGGTGTC TTTTGAAGCG ATCAAACAAG GAGAATGTCT TTTTCTTGTA

CATAGGTGTC TTTTGAAGCG ATCAAACAAG GAGAATGTCT TTTTCTTGTA

CATAGGTGTC TTTTGAAGCG ATCAAACAAG GAGAATGTCT TTTTCTTGTA

CATAGGTGTC TTTTGAAGCG ATCAAACAAG GAGAATGTCT TTTTCTTGTA

CATAGGTGTC TTTTGAAGCG ATCAAACAAG GAGAATGTCT TTTTCTTGTA

CATAGGTGCC TTTTGAAGCG ATCAAACAAG GAGAATGTCT TTTTCTCGTA

CATAGGAATC TTTTGAAAGG ACCAAACAAG GAGAATATCT CTTTTT-GTT

CATAGGTATC TTTTGAAAGG GCCAAATAAG GAATATATCT TTTTTTTGCA

CATAGGTGTC TTTTGAAGGG ATCAAACAAG GAGAATGTCT TTTTCTTGTA

CATAGGTGTC TTTTGAAGCG ATCAAACAAG GAGAATGTCT TTTTCTTGTA

CATAGGTGTC TTTTGAAGCG ATCAAACAAG GAGAATGTCT TTTTCTTGTA

CATAGGTGTC TTTTGAAGCG ATCAAACAAG GAGAATGTCT TTTTCTTGTA

CATAGGTGTC TTTTGAAGCG ATCAAACAAG GAGAATGTCT TTTTCTTGTA

CATAGGTGTC TTTTGAAGCG ATCTAACAGG GAGAATGTAT TTTTCTTGTA

---------- ---------- ---------- ---------- ----------

CATAGGTGTC TTTTGAAGCG ATTGAACAGG GGGAATGTCT TTTTCTTATA

CATAGGTGTC TTTTGAAGCG ATCAAACAGG AAAAATGTCT TTTTCTTGTA

CATAGGTGTC TTTTGAAGCA ATCAAACACG GAAAACGTCT TTTTCCTGTG

TTTTCATTAT TTTATATGAT CATAAAATCG TTACAGCTGT GACTTCCAAA

TTTTCATTAT TTTATATGAT CATAAAATCG TTACAGCTGT GACTTCCAAA

TTTTCATTAT TTTATATGAT CATAAAATCG TTACAGCTGT GACTTCCAAA

TTTTCATTAT TTTATATGAT CATAAAATCG TTACAGCTGT GACTTCCAAA

TTTTCATTAT TTTATATGAT CATAAAATCG TTACAGCTGT GACTTCCAAA

TTTTCATTAT TTTATATGAT CATAAAATCG TTACAGCTGT GACTTCCAAA

TTTCTATTAT TTGATATGAT CATAACATCA TTG-AGGTGT GAC-TTACAA

TTCCCATTAT TTTATATAAT CATAAAATCA TTGCAGCTGT GAC-TCAAAA

TTTTCATTAT TTTATATGAT CATAAAATCG CTACAGCTGT GACTTCCAAA

TTTTCATTAT TTTATATGAT CATAAAATCG TTACAGCTGT GACTCCCCAA

TTTTCATTAT TTTATATGAT CATAAAATCG TTACAGCTGC GACTTCCAAA

TTTTCATTAT TTTATATGAT CATAAAATCG TTACAGCTGT GACTTCCAAA

TTTTCATTAT TTTATATGAT CATAAAATTG TTACAGCTGT GACTTCCAAA

TTTTCATTAT TTTATATGAT CATAAAATTG TTACAGCTGT GATTTCCAAA

---------- ---------- ---------- ---------- ----------

TTTTCATTAT TTTATACGAT CATAAAATTG TTACAGCTGT GACTTCCAAA

TTTTCATTAT TTTATATGAT CATAAAATCG TTAAAGCTGT GACTTCCAAA

TTTTCATTAT TTTATAGGAT CATAAAATCG TTAAAGCTGT GACTCCCCCA

AAATATATTT AAGTGACAGC TGAGAGACAA TGTGACAAAA AGCCGCATTT

AAATATATTT AAGTGACAGC TGAGAGACAA TGTGACAAAA AGCCGCATTT

AAATATATTT AAGTGACAGC TGAGAGACAA TGTGACAAAA AGCCGCATTT

AAATATATTT AAGTGACAGC TGAGAGACAA TGTGACAAAA AGCCGCATTT

AAATATATTT AAGTGACAGC TGAGAGACAA TGTGACAAAA AGCCGCATTT

AAATATATTT AAGTGACAGC TGAGAGACAA TGCGACAAAA AGCCGCCTTT

AAATATATTT AAGTGACAGC TGAGAGACAG TGTGACAAAA TGCCGCATTT

AAATATATTT AAGTGACAGC TGAGAGACAG TGTGACAAAA TGCCGCGTTT

AAATATATTT AAGTGACAGC TGAGAGACAA TGTGACAAAA AGCCGCATTT

AAATATATTT AAGTGACAGC TGAGAGACAA TGTGACAAAA AGCCGCATTT

AAATATATTT AAGTGACAGC TGAGAGACAA TGTGACAAAA AGCCGCATTT

AAATATATTT AAGTGACAGC TGAGAGACAA TGTGACAAAA AGCCGCATTT

AAATATACTT ACGTGACAGC TGAGAGACAA TGTGACAAAA AGCCGTATTT

AAATACATTT AAGTGACAGC TGAGAGACAA TGTGACAAAA AGTCGCATTT

---------- ---------- ---------- ---------- ----------

AAATACATTT ACATGACAGC TGAGAGACAA TGTGACAAAA AGTCGCATTT

AAATATGTTT AAGTGACAGC TAAGAGACAA TGTGACAAAA AGCTGCATTC

AAATACATTC AAGTGACAGC CGAGAGACAA CGTGACAAAA AGCCGCATTC

AGAGTTAGTT TTTAATAATT AATGAATGTG TCACATAGTG CAGTATAACA

AGAGTTAGTT TTTAATAATT AATGAATGCG TCACATAGTG CAGTATAACA

AGAGTTCGTT TTTAATAACT AATGAATGTG TCACATAGTG CAGTATAACG

AGAGTTTGTT TTTAATAACT AATGAATGTG TCACATAGTG CAGTATAACG

ATAATTAGTT TTTAATAACT AATGAATGTG TCACGTAGTG CAGTGTAACA

AGAATTAGTT TTTAATAACT AACAAATGTG TCAAATAGCC CAGTATAACA

AGAGTTGGTT TCTAATAGCT AACAAATGTA TCCCATAGTC CAGTATACCA

AGAATTAGTT TCTAATAACT AACAAATGCA TCACATAGTC CAGTCGAACA

AGAATTAATT TCT--CAAAT AACAAATGTG TCACATAGTC CAGTATAACA

AGAATTACTT TCTAATAACT AACAAATGTG TCACATAGTC CAGTATAACA

AGAATTAGTT TCTAATAACT AACAAATGTG TCACACAGTC CAGTATAACA

AGAATTAGTA TGTAATAACT AACAAATGTG TCACATAGTC CAGTATAACA

AGAATTAATT TCTAATAACT AACAAATGTG TCACATAGTC CAGTATAACA

AGAATTTGTT TCTAATAACT AACAAATGTG TCACATAGTC CAGTATAACA

---------- ---------- ---------- ---------- ----------

AGAACTAGTT TCCAATAACT AAGAAATGTG TCACGTAGTC CAGTATAACA

AAAAGTAGTT TTTAATAAGT AACAAATGTG TCATGTAGTG CAGTATAAAA

AGAGGTAGGC TCTAATAAGC AACAAATGCA TCAGGGCGTT CAGCATAAAA

TTTTTATTGG CACTTAAAAT AAATGTATTA CTCACTAGTG GATATGCCGA

TTTTTATTGG CACTTAAAAT AAATGTATTA CTCACTAGTG GATATGCCGA

TTTTTATTGG CACTTAAAAT AAATGTATTA CTCACTAGTG GATATGTCGA

TTTTTATTGG CACTTAAAAT AAATGCATTA CTCACTAGTG GATATGTCGA

TTTTTGTTGA CACTTAAAAT AAATGTATTA CTTACTAGTG GATATGCTAA

TTTTTATTGG CACTTGAAAT AAATGTATTA CTCACTAGTG GATATGCC-A

TTTTTGTTGG CATTTGAAGT GAATGCATTA CTCACTAGTG GACATGCCAA

TTTTTGTTGG CATTTGAAGT GAGTGCATTA CTCACTAGTG GACGTGCCAA

TTTTTATTGG CACTTGAAAT AAATGTATTA CTCACTAGTG AATGTGCCAA

TTTTTATTGG CACTTGAAAT AAATGTATTA CTCACTAGTG GATGTGCCAA

TTTTTATTGG CACTTGAAAT AAATGTATTA CTCACTAGTG GATGTGCCAG

TTTTCATTGG CACTTGAAAT AAATGTATTA CTCACTGGTG GATGTGCCAA

TTTTTATTGG CACTTGAAAT ACATGTATTA GTCACTGGTA GACGTGCCAA

TTTTTATTGG CAGTTGAGAT AAATGTATTA CTCACTAGTG AATGTGCCAA

---------- ---------- ---------- ---------- ----------

TTTTTATTGG CGCTTGATAT AAATGTATTA CTCACTGGTG GATGTGCCAG

ATTTTATTGG CATTTGAGAT AAATGTATTA CTCACCAGTG GACATGCCAA

AATTTATTGG CACCAGAGGT GAATGTATTA CTCACCAGAA GATATGCCAA

AAAAAATATC CACCAAAATT CCACCCCCAC AAAGCTAAAA ATGATTATTT

AAAAAATATC CACCAAAATT CCACCCCCAC AAAGCTAAAA ATGATTATTT

AAAGAATATC CACCCAAATT CCACCCCCAC AGAGCTAAAA ATGATTATTT

AAAAAATATC CACCCAAATT CTACCCCCAC AGAGCTAAAA ATGATTATTT

AAAAAATATC CACCCAAATT CCACCCTCAC AGAGCTAAAA ATGATTATTT

AAAAAATCCC CACCCTATTT CCACCCCCAC AGAGCTACAA ATGATTATTT

CAAATGTCTC CACCCTATTT TCACTCCCTC CGAGTCACAA GTTATTATTT

CAAAAATCCC CACCCTATTT CCACCCCCTC AGAGGCACAA ATTATTATTT

AAAAAATCCC CACCCTATTT CCACCCCCAC AGAGCTATGA ATGA----TT

AAAAAATCCC CACCCTATTT CCACCCCCAC AGAGCTACAA ATGATTATTT

AAAAAATCCC CACCCTACTT CCACCCCCAC AGAGCTACAA ATGATTATTT

GAAAAATCCC CACCCTATTC CCACCCCCAC AGAGCTACAA ATGATTAATT

AAAAAATCTC CACCCTATTT CCACCCCCAC AGAGCTACGA ATAATTATTT

AAAAAATCCC CACCCTATTA CCACCTCCTC AGAGCAACAA ATGATTATTT

---------- ---------- ---------- ---------- ----------

GAACTATCCC CACCCTGTTT CCACTCCCAC AAAGCCACGG ATGATGATTT

AAAAAATCCC CACCCTCTTT CCACCCCCAC AGTGCTAAAA ATTATTATTT

AAAAAATCCC CACCCTCTTC CCACCCCAAC AGGGCTACAA ATGATTATTT

ATTAATCAGT GACAGAGTGA CTTGGAGTGC AAAACAGAAA TCATCATGGT

ATTCATCAGT GACAGAGTGA CTTGGAGTGC AAAACAGAAA TCATCATGGT

ATTAATCAGT GAC--AGTGA CTTGGAGTGC AAAAAAGAAA TCATCATGGT

ATTAATCAGT GAC--AGTGA TTTGGAGTGC AAAAAAGAAA TCATCATGGT

ATTAATCAGT GAT--AGTGA CTTGGAGTGC AAAACAGATG TCATCGTGGT

ATTAAGAGGT GATAGAATGA CGTGGAGTGC AAAACAGAAG TCATCACAGT

ATTAAGCAGT GACAGAGGGA CTTGGAGTGC AAAACAGAAG TCATTATGGT

ATTAAGCAGT GACAGAGAGG ATTGGAGTGC ACAACAGGAG TCATCATGGT

ATTGAGCAGT GACAGAGTGA GTTGGAGTGC AAAACAGAAG TCATCATGGT

ATTAAGCAGT GACAGAGTGA CTTGGAGTGC AAATCAGAAG TCATCGTGGT

ATTAAGCAGT GACAGAGTGA CTTGGAGTGC AAAACAGAAG TCATCATGGT

ATTAAGTAGT ----CAGTGA CTTGCAGTGC AAA-CATTAG TCCTCATGGT

ATTAAGCAGT GACAGAGTGA CTTGGAGTGC AAAACAGAAG TCATCATGCA

TTTAAGCAGT GACAAAGTGA CATGGAGTAG AAAACAGAAG TCATCATGGT

---------- ---------- ---------- ---------- ----------

ATTAAGCAGT GACAGAGTGG TTTGGAGTAC GAGGCAGAAA TTGGTATGGT

ATTGAGCAGT GACAGAGCGC CTTGGAGTAC AAAACAGAAG TCTTCATGGC

ATTATGCGGT GACAGAGTGC TTTGGAGAAC AAAACAGAAG TCGTTGCGAT

TATCTGTCAG TGTGTTGGCT TGAAAGAGAG TGATGTGGTG TTAAAGTCTA

TATCTGTCAG TGTGTTGGCT TGAAAGAGAG TGATGTGGTG TTAAAGTCTA

TGTCTGTCAG TGTGTTGGCT TGAAAGAGAG TGATGTGGTG TTAAAGTCTA

TGTCTGTCAG TGTGTTGGCT TGAAAGAGAG TGATGTGGTG TTAAAGTCTA

TATCTGTCAG TGTGCTGGCT TGAAAGAGAG TGATGTGGTG TTAAAGTCTA

TATCTGTCAG TGTGTTGGTT TGAAAGAGAG TGATGTGGTG TTAAAGTCTA

TATCTGTCAG CGTGTTGGTT TGAAAGAGAA TGATGTGGTG TTAAAGGCTA

GATCTGTCAG TGTGTTGGTT TGAAAGAGAA TGATGTGGTG TTAAAGACAA

TATCTGTCAG TGTGTTGGTT TGAAAGAGAG TGATGTGGTG TTAAAGTCTA

TATCTGTCCG TGTGTTGGTT TGAAAGAGAA TGATGTGGTG TGAGAGTCTG

TATCTGTCAG TGTGTTGCTT TGAAAGAGAG TGATGTGGTG TTAAAGTCTA

TATCTGTCAG TGTGTTGGTT TGAAAGAGAG TGATGTGGTG CTAAAGTCTA

TATCTGTCAG TGTGTTGGTC TGAAAGAGAA TGATGTGGTG CTAAAGTCTA

TATCTGTCAG TGTGTTGGTT TGAAAAAGAG TGATGTAGTG TTAAAGTCTA

---------- ---------- ---------- ---------- ----------

AATCTGTCAG TGTGTTGGTT TGAAAGAGAG TGATGTGGTG TTAAAGTCTA

TATCTGTCAT TGTGTTGCTT TGAAAGTGAA TGATGTGGTG TTAACGTCTA

CATCTGTCAT TGTGTTGGTT TGAAAGTGAG TGATGTGGTG TTAGCGTCTA

GCCTTGACCT CTCCCACCCT CTCTCTGTCC TCCATACACT ATCTAAAACT

GCCTTGACCT CTCCCACCCT CTCTCTGTCC TCCATACACT ATCTAAAACT

GCCTTGACCT CTCCCACCCT CTCTCTGTCC TCCATACACT ATCTAAAACT

GCCTTGACCT CTCCCACCCT CTCTCTGTCC TCCATACACT ATCTAAAACT

GCCTTGACCT CTCCCACCCT CTTTCTGTCC TCCATACACT ATCTAAAACT

GCCTTGACCT CTCCCACCCT CTCTT--TCC TCCATATACT ATCTAAAAGC

GCCTTGACCT CTCCTATCCT CGCTTTGTCC TCAAT--GCT ATCTAGAAGC

GCCTTGACCT CTCCTAGCCT CGCTTGGGCC TCAGT--GCT ATCTAGAAGT

GCCTTGACCT CTCCCACCCT CTCTTTTTCC TCCAT--GTT CATTAAAAGT

GCCTTGACCT CTCCCACCCT CTCTCTGTCC TCCATATACT ATCTAAAAGT

GCCTTGACCT CTCCTACCCT CTCTCTGTCC TAC------- ATCT--AAGT

GCCTTGACCT CTCCCACCCT CTCCCTGTCC TCCATGCACT ATCTAAAAGT

GCCTTGACCT CTCCCACCCT CTCTCTGTCC TCTGTACACT ATCTAAAAGT

GCCTTGACCT TTCCCACCCT CTCTCTGTCC TCCACACACT CTCTAGAAGT

---------- ---------- ---------- ---------- ----------

GCCTTGACCT CTCCCACCCT CTCTCTGTCC TCCATACACT GTCTCAAAGT

GCCTTGACCT CTCCCACCCT CGTTCTGTCC TCTGTACACT ATCTGAAATT

GCCTTGACCT CTCCCACCCT CTCTCTGTCC TCTGTACACT ATCTAAAGAG

ATCTTCAAAT AAATCAGTAA ATTGTTGAAG TCACATTACC AGGAGATTAT

ATCTTCAAAT AAATCAGTAA ATTGTTGAAG TCACATTACC AGGAGATTAT

ATCTTAAAAT AAATCAGTAA ATTGTTGAAG TCACATTACT AGGAGATTAT

ATCTTAAAAT AAATCAGTAA ATTGTTGAAG TCACATTACT AGGAGATTAT

ATCT-----T AAATCAGTAA ATTGTTGAAG TTACATTACC AGGAGATTAT

ATCTTAAAAT AAATCAGTAA ATTGCTGAAG TCACATCATG AGGAGATTAC

ATCTTAAAAT AAATCAGTTA ATTGTTGAAA TAAAATTATC AGGAGATTAT

ATCTTAAAAT AAATCAGTTA ATTGTTGAAA TAAAATTATC AGGAGATTAT

ATCTTAAAAT AAATCAGTAT ATTTCTGAAG CCACATTACC AGGAGATTAT

ATTTTAAAAC AAATCAGTAA ATTGTTAAAG TTACATTACC AGAAGATTAT

ATCATAAAAT AAATCAGTAG ATTGCTGAAG TCACATTACC AGAAGATTAT

ATCTT-AAAT GAATCAGTAA ATTGTTGAAG TCAC------ -----ATTAT

ATCTTAAAAT ATATCAGTAA ACTACTGAAG TCACATTGCC AGAAGATTAC

ACCTTAAAAT AAATCAGTAA ATTGTTGAAG TCACATTACC AGGAGATTAT

---------- ---------- ---------- ---------- ----------

ATCTTGAAAT AAATCAGTAA ATTGTTGAAG TCACATTACC AGGAGATCAT

ATACTAAAAT AATTAGCTTA ATTGTCCAAG TAACATCACT AGGAGTTTAT

AGGTTAGAAT AAATCAGTAA ATTGTGCAGG ACACATCACT GGGAACTTAT

AATCAATCAG TTCAACTTTT CCTGCTGTTA GTTTTCTTAT GACTTAATGA

AATCAATCAG TTCAACTTTT CCTGCTGTTA GTTTTCTTAT GACTTAATGA

AATCAATCAG TTCAACTTTT CCTGCTGTTA GTTTTCTTAT GACTTAATGA

AATCAATCAG TTCAACTTTT CCTGCTGTTA GTTTTCTTAT GACTTAATGA

AATCAATCAG TTCAACTTTT CCTGCTGTTA GTTTTCTTAT GACTTAATGA

AATCAATCAG TTCAACTTTT CCTGCTGTTA GTTTTCTTAT GACTTAATGA

AATCAATCAA TTTAACTTTT TCTGCTGTTA GTTTTCTTAT GACTTCATGA

AATCAATCAA TTCAACTTTT TCTGCTGTTA GTTTTCTTAT GACTTAATGA

AATCAATCAA TTCAACTTTT CCTGCTGTTA GTTTTCTTAT GACTTAGTGA

AATCAATCAG TTCAACTTTT CCTGCTGTTA GTTTTCTTAT GACTTGATGA

AATCAATCAG TTCAACTTTT CTTGCTGTTA GTTTTCTTAT GACTTAATGA

AATCAATCAG TTCAACTTTT CTTGCTGTTA GTTTTCTTAT GACTTAATGA

AATCAATCAG TTAAACTTTT CTTGCTGTTA GTTTTCTTAT GACTTAATGA

AATCAATCAG TTCAACTTTT CCTGCTGTTA GTTTTCTTAT GACTTAATGA

---------- ---------- ---------- ---------- ----------

AATCAATCAG TTCAACTTTT CCTGCTGTTA GTTTTCTTAT GACTTAATGA

AATCAATCAG TTCAAGTTTT CCCTCTGTTA GTTTTTATAT GACTTAATGA

AATCAATCAG TTCAACTTTT TTCCCTGTTA GTTTTTGTAT GACTTAATGA

CTAATTTCTT CTTTAAAGAA TGACTTTGAA CAAATGAGCA TGTAAACAAA

CTAATTTCTT CTTTAAAGAA TGACTTTGAA CAAATGAGCA TGTAAACAAA

CTGATTTCTT CTTTAAAGGA TGACTTTGAA CAAATGAGCA TGTAAACAAA

CTGATTTCTT CTTTAAAGAA TGACTTTGAA CAAATGAGCA TGTAAACAAA

CTAATTTCTT CTTTAAAGAA TGACTTTGAA CAAATGAGCA TGTAAACAAA

CTAATTTCTT CTTTAAAGAA TGACTTTGAA CAAATGAGCA TGTAAACAAA

CTAATTTCTT CTCTAAAGGA TGACTTTGAA CAAATGATCA TATAAACACA

CTAATTTCTT CTCTAAAGGC TGACTCTGAA CAAATTATCA TATAAACAAA

CTAATTTCTT CTTTAAAGAA TGACTTTGTA CAAATGAACA TGTAAACAAA

CTAATTTCTT CTTTAAAGAA TGACTCTGAA CAAATGAGCG TGTAAACAAA

CTAATTTCTT CTTTAAAGAA TGACTTTGAA CAAATGAGCA TGTAAACAGA

CTAATTTCTT CTTTAAAGAA TGACTTTGAA CAAATGAGCA TGTAAACAAA

CTAATTTCTT CTTTAAAGAA TGACTTTGAA CAAATGAGCA TGTAAACAAA

CTAATTTCTT CTTTAAAGAA TGACATTGAA TAAATGAGCA TGTAAACAAA

---------- ---------- ---------- ---------- ----------

CTAATTTCTT CTTTAAAGAA TGACTTTGAG CAAATGAGCA TGTAAACAAA

CTAATTTCTT CCTTAAAAAA TGACTTTGAA CAAATGAGCA TGTAAACATA

CTAATTTCTT CCTCACAGAA TGACTCTGAA CAAATAAGCA TGTAAACATA

GtaaaaattC AGTAGTTTTA ATACGTGAAC TATTATCTTA CCTTTAGTAA

GtaaaaattC AGTAGTTTTA ATACGTGAAC TATTATCTTA CCTTTAGTAT

GtaaaaattC AGTAGTTTTT ATAAGTGAAC CATTATCTTA CCTTTAGTAA

GtaaaaattC AGTAGTTTTT ATAAGTGAAC CATTATCTTA CCTTTAGTAA

GtaaaaattC AGTAGTTTTA ATAAGTGAAC CATTATCTTA CCTTTAATAA

GTAAAAATTC AGCAGTTTTA ATAAGTGAAC CATTTTCGTA CCTTTAATAA

GTAAAGATTC AACAGTTTTA ATAAGTGAGC CATTACCTTA CCTTTAATAA

GTAAGGATTC AACGGTTTTA ATAAGTGAGC CATTATCTTA CCTTTAATGA

GTAAA-ATTC AGCACTTTTA ATAAGTGAAC CATTATCTTA CCTTTAATAA

Gtaaaaattt aGCAGTTGTA ATATGTGAAC CATTATCTTA CCTTCAATAA

GTAAAAATTC AGCAGTTTTA ATAAGTGAA- CATTATCTTA CCTTTAATAA

GTAAAAATTC AGCAGTTTTA ATAAGTAAAC CATTATCTTA CCTTTAATAA

GTAAAAATTT GACATTATTC ATAGGTGAAC CACT------ ----------

GTAAAAATTC AACAGTTTTA ATAAGTGAAC TATTATCTTA CCTTTAATAA

---------- ---------- ---------- ---------- ----------

GTNNNAATTC AGCAGTTTTA ATAAGTGAAC CATTATATTA CCTTTAATAA

ATAAAACTCT AACACTCCTA ATCATTGAAA CATTAACTTA CCTTTAGTAA

ATAAAAAGCC AACACTTCTA ATAATTGAAT CATCATCTTA CCTTTAGTAA

ATCTGTCATA TGTTCAAGTC CAAGACCATG TAAAAATACT TCCAGATCTC

ATCTGTCATA TGTTCAAGTC CAAGACCATG TAAAAATACT TCCAGATCTC

ATCTGTCATA TGTTCAAGTC CAATACCATG TAAAAATACT TCCAGATCTC

ATCTGTCATA TGTTCAAGTC CAATACCATG TAAAAATACT TCCAGATCTC

ATCTGTCATA TGTTCAAGTC CAAGACCATG TAAAAATACT TCCAGATCTC

ATCTGTCATG TGTTCAAGTC CAAGACCATG TAAAAATATT TCCAGATCTC

GTCTGTCATA TGTTCCAGTC CAAGGCCATG TAAAAATATC TCCAGATCTC

GTCTGTCATA TGTTCAAGTC CAAGGCCATG TAAAAATATT TCCAGATCTC

ATCTGTCATG TGTTCAAGTC CAAGACCATG TAAAAATATT TCCAGATCTC

ATCTGTCATA TGTTCAAGTC CAAGACCATG TAAAAATATT TCCAGATCTC

ATCCATCATA TGTTCAAGTC CAAGACCATG TAAAAATATT TCCAGATCTC

ATCTGTCATA TGTTCAAGTC CAAGACCGTG CAAAAATATT TCCAGATCTC

---------- ---------- ---------- ---------- ----------

ATCTGTCATA TGTTCAAGTC CAAGACCATG TAAAAATATT TCCAGATCTT

---------- ---------- ---------- ---------- ----------

ATCTGTCATA TGTTCAAGTC CAAGACCATG TAAAAATAAG TCCAACTCTC

TTCTGTCATA TGTTCAAGTC CAAGTCCATG TAAAAATAGT TCAAGATCAC

CTCTGTCAAG TGTTCAAGCT GAAGACCGTG CAAAAATATC TCCAGATCGC

CAAATGCTGT ATATGAACTA TACCTCATGA TGTTTGTTTC TTTTTGCAAT

CAAATGCTGT ATATGAACTA TACCTCATGA TGTTTGTTTC TTTTTGCAAT

CAAATGCTGT ATATGAACTA TACCTCATGA TGTTTGTTTC TTTTTGCAAT

CAAATGCTGT ATATGAACTA TACCTCATGA TGTTTGTTTC TTTTTGCAAT

CAAATGCTGC ATATGAACTA TACCTCATGA TGTTTGTTTC TTTTTGCAAT

CAAAAGCTGT ATATGAACTA TACCTCAAGA TGTTTATTTC TTTTTGCAAT

CAAACGCTGT ATATGGACTA TACCTCAAGA TGTTTATTTC TTTTTGCAAT

CAAACGCTGT ATACGGGCTA TACCTCAAGA TGTTTATTTC TTTTTGCAAT

CAAATGCTGT ATAAGAACTA TACCTCAAGA TGTTTATTTC TTTTTGCAAT

CAAATGCTGT ATATGAACTA TACCTCTAGA TGTTTATTTC TTTTTGCAAT

CAAATGCTGT ATATGAACTA TACCTCTAGA TGTTTATTTC TTTTTGCAAT

CAAGTGCTGT ATATGAACTA TACCTCTACA TGTTTATTTC TTTTTGCAAT

---------- ---------- ---------- ---------- ----------

CAAATGCTGC ATATGAACTA TACCTCTAGA TGTTTATTTC TTTTTGCAAT

---------- ---------- ---------- ---------- ----------

CAAATGCTGT GTATGAACTA TACCTCTGGA TGTTTATTTC TATTTGCAAT

CAAAAGCTGT ATATGAACTA TACCTCTGGA TGTTTATTTC TTTTGGCAAT

CAAAAGCGGA ATATGAACTA TACCTCTGGG TGTTTATTTC TTTTAGCAAT

CTCACTTGGC ATCTTTCCAT CTTTGGTTTG TAGCATTTTA TTAGCTCCAA

CTCACTTGGC ATCTTTCCAT CTTTGGTTTG TAGCATTTTA TTAGCTCCAA

CTCACTTGGC ATCTTTCCAT CTTTGGTTTG TAGCATTTTA TTAGCTCCAA

CTCACTTGGC ATCTTTCCAT CTTTGGTTTG TAGCATTTTA TTAGCTCCAA

CTCACTGGGC AGCTTCCCAT CTTTGGTTTG TAACATTTTA TTAGCTCCAA

TTCGCTTGGT ATCTTTCCAT CTTTGGTTTG TAGCATTTTA TTAGCTCCAA

CTCACTTGGT GTCCTGCCAT CTTTGGTCTG TAGCATTTTA TTAGCTCCGA

CTCGCTTGGT GTTCTGCCAT CTTTGGTCTG TAGCATTTTA TTAGCTCCCA

CTCACTTGGC GTCTTTCCAT CTTTGGTTTG CAGAGTTTTG TTAGCTCCAA

TTCACTTGGT GTCTTTCCAT CTTTGGTTTG TATCATTTTA TTAGCTCCAA

CTCACTTGGT GTCTTTCCAT CTTTGGTCTG TAGCATTTTA TTAGCTCCAA

CTCGCTTGGT GTCTTTCCAT CTTTGGTTTG TAGCATTTTA TTAGCTCCAA

---------- ---------- --TTAGTTTG CAGTGTTTTA TTAGCTCCAA

TTCACTGGGT GTCTTTCCAT CTTTGGTTTG TAGCATTTTA TCAGCTCCAA

---------- ---------- ---------- ---------- ----------

CTCACTTGGT GTCTTTCCAT CTTTGGTTTG TAGCATTTTA TTAGCTCCAA

CTCTCCCGCT GTCTTTCCAT CTTTGGTTTG TATTGTTTTA TTAGCTCCCA

CTCTGCTGGT GTCTTTCCAT CTTGGGTTTG TAGAGTTTTA TCAGCTCCAA

GTTCAAGCAA CTTCAAAACT ATATTTTTAT GACCCTGACG TGCTGCCCAC

GTTCAAGCAA CTTCAAAACT ATATTTTTAT GACCCTGACG TGCTGCCCAC

GTTCAAGCAA CTTCAAAACT ATATTTTTAT GACCCTGACG TGCTGCCCAT

GTTCAAGCAA CTTCAAAACT ATATTTTTAT GACCCTGACG TGCTGCCCAT

GTTCAAGCAA CTTCAAAACT ATACTTTTAT GACCCTGACG TGCTGCCCAC

GTTCAAGCAA CTTCAAAACT ACATTTTTAT GACCCTGACG CGCTGCCCAT

GTTCAAGCAA CTTCAGAATT ACATTTTTAT GACCCTGCCG TGCTGCCCAT

GTTCAAGCAA CTTCAGAATT ACATTTTTAT GACCCTGCCG TGCTGCCCAT

GTTCAAGCAA CTTCAAAACT ACACTTTTAT GTCCCTGACG TGCTGCCCAT

GTTCAAGCAA CTTCAAAACT ACATTTTTAT GACCCTGACG TGCTGCCCAT

GTTCAAGCAA CTTCAAAACT ACATTTTTAT GACCCTGCCG TGCTGCCCAT

GTTCAAGCAA CTTCAAAACT ACATTTTTAT GACCCTGACG TGCTGCCCAT

GTTCAAGCAA CTTTAAAACG ACGTTTTTAT GACCCTGACG TGCAGCCCAT

GTTCAAGTAA CTTCAAAATT ACATTTTTAT GACCCTGACG TGCTGCCCAT

---------- ---------- ---------- ---------- ----------

GTTCAAGCAA CTTCAAAATA ACATTTTTAT GACCCTGATA TGCTGCCCAC

GTTCAAGCAA CTTCAAAACT ACACTTTTAT GACCCTGACG TGCTGCCCAC

GTTCAAGCAA CTTTAAAACC GTAGTTTTAT GACCATGACG AGCTGCCCAT

GTTAAAGCCT CTCACAGTGT AACCATTCTC ATCCTGGGTA TTAACTTCTG

GTTAAAGCCT CTCACAGTGT AACCATTCTC ATCCTGGGTA TTAACTTCTG

GTTAAAGCCT CTCACAGTGT AACCATTCTC ATCCTGGGTA TTAACTTCTG

GTTAAAGCCT CTCACAGTGT AACCATTCTC ATCCTGGGTA TTAACTTCTG

GTTAAAGCCT CTCACAGTGT AACCATTCTC ATCCTGGGTA TTAACTTCTG

GTTAAAGCCT CTCACAGTGT AGCCATTCTC ATCCTGGGTA TTAACTTCTG

GTTAAGGCCT CTCACAGTAT AACCATTCTC ATCCTGGGCG TTGACTTCTG

GTTAAGGCCT CTCACAGTAT AACCATTCTC ATCCTGGGCG TTAACTTCGG

GTTAAAGCCT CTCACAGTAT AACCATTCTC ATCCTGGATA TTAACTTCTG

GTTAAAGCCT CTCACAGTAT AGCCGTTCTC GTCCTGGGTA TTCACTTCTG

GTTAAAGCCT CTTACAGTAT AACCATTCTC ATCCTGGGTA TTAACTTCTG

GTTAAAGCCT CTCACAGTGT AACCGTTCTC ATCCTGTGTA TTGACTTCTG

GTTAGAGCCT CTCACAGTAT AACCATTCTC ATCTTGAATA TTGACTTCTG

GTTAAAGCCT CTCACAGTAT AACCGTTCTC ATCCTGGGTA TTAACCTCTG

---------- ---------- ---------- ---------- ----------

GTTAAAGCCT CTCACAGTGT AGCCATTCTC ATCCTGGGCA TTAACTTCTG

ATTAAGGCCT CATACTGTAT AACCATTTTC ATCTTGGGCA TTTATTTCTG

GCAAGAGCCT CTTACTGTAT ACCCGTTATC GTCTTGGGCA TTTATCTCTG

CTCCATGAGC AACAAGGAGA GCAACAACCT GGGTGTGACC ATCTCGAGCA

CTCCATGAGC AACAAGGAGA GCAACAACCT GGGTGTGACC ATCTCGAGCA

CTCCATGAGC AACAAGGAGA GCAACAACCT GGGTGTGACC ATCTCGAGCA

CTCCATGAGC AACAAGGAGA GCAACAACCT GGGTGTGACC ATCTCGAGCA

CTCCACTAGC AACAAGGAGA GCAACAACCT GGGTGTGACC ATCTCGAGCA

CTCCATGAGC AACGAGGAGA GCAACGACCT GAGGGTGACC ATCCCGGGCA

CTCCATGAGC AACAAGCAGA GCAACGACCT GAGTATGACC ATCTCGGGCA

CTCCGTGAGC CACAAGCAGA GCCACAACCT GAGTATGACC GTCTCGGGCA

CTCCATGAGC AACAAGCAGA GCAACAACTT GAGGGTGACC ATCCCGAGCA

CTCCATGAGC AACAAGGAGA GCAACGACCT GAGGGTGGCC GTCACGTGCA

CTCCATGAGC AACAAGGAGA GCAACAACCT GAGGGTGACC ATCTCGTGCA

CTCCGTGAGC AACAAGGAGA GCGACAACCT GGGTGTGACC GTCGCGGGCA

CTCCATGAGC AACAAGGAGC GCAACCACCT GAGGGTGACC GTCCCGTGCA

CTCCATAAGC AACAAGGACA GCAACAACCT GAGGGTGACC AGCTCGTGCA

---------- ---------- ---------- ---------- ----------

CTCCATGCGC AACAAGAAGA GCAACAACCT GAGGGTGACC ACCTCGGGCA

CTCCATGAGC AACAAGGAGA GCAACAACCT GAGGATGGCC TTCTCGTGCT

CTCCGTGTCC AACGAGAAGA GCCACCAACT GAGAATGACC TTCTCGAGCT

GCATACATGA TTGGGGTCAT AAGTCTCCTC TCTTACCTAC AAGCAACATT

GCATACATGA TTGGGGTCAT AAGTCTCCTC TCTTACCTAC AAGCAACATT

GCATACATGA TTGGGGTCAT AAGTCTCCTC TCTTACCTAC AAGCAACATT

GCATACATGA TTGGGGTCAT AAGTCTCCTC TCTTACCTAC AAGCAACATT

GCATACATGA TTGGGGTCAT AAGTCTCCTC TCTTACCTAC AAGCAACATT

GCATACATGA TTGGAGTCAT AAGTCTCCTC TCTTACCTAC AAGCAACATT

GCATACATGA TTGGAGTCAT AAGTCTCCTC TCTTACCTAC AAGCAACATT

GCATACATGA TTGGCGTCAT AAGTCTCCTC TCTTACCTAC AAGCAGTATT

GCATACATGA TGGGAGTCAT AAGTCTCCTC TCTTACCTAC AAGCAACATT

GCGTACATGA TTGGTGTCAT AAGTCTCCTT TCTTACCTAC AAGCGACATT

GCATACATGA TTGGAGTCAT AAGTCTCCTC TCTTACCTAC AAGGAACATT

GCGTACATGA TTGGAGTCAT GAGTCTCCTC TCTTACCTAC AAGGAACATT

GCATACATGA TTGGAGTCAT AAATCTCCTC CCTTACCTAC AAGCAACATT

GCATACATGA CTGGAGTCAT AAGTCTCCTC TCTTACCTAC AAGCAACATT

---------- ---------- ---------T TCTCACCTAC AAGCAACATT

GCATACATGA TTGGAGTCAT CTGTCTCCTT TCTTACCTAC AAGCAACATT

GCATACATGA TTGGAGTCAT ACATTTTCTC TCTTACCTGC AAGCAACATT

GCATACATCA GTGGAGACAT GCGTTTCCTC CCATACCTGC AAGTAGCATT

TGGATCAGCA TTTCTTGAAA GTAGTAGTTC TACACACTTC AAGATCTGTT

TGGATCAGCA TTTCTTGAAA GTAGTAGTTC TACACACTTC AAGATCTGTT

TGGATCAGCA TTTCTTGAAA GTAGTAGCTC TACACACTTC AAGATCTGTT

TGGATCAGCA TTTCTTGAAA GTAGTAGCTC TACACACTTC AAGATCTGTT

TGGATCAGCA TTTCTTGAAA GTAGTAGCTC TACACATTTC AAGATCTGTT

TGGATCAGCA TTTCTTGAAA GTAACAGCTC TACACACTTT AAGATCTGTT

AGGATCCGCA TTTCGTGAAA GTAGTAACTC TACACACTTC AAGACTTGTT

TGGGTCCGCA TTTCTTGAAA GTAGTAACTC TACACATTTC AAGACTTGCT

TGGGTCAGCA TTTCTTGAAA GTAATAGCTC TACACACTTC AAGATCTGCT

TGGATCAGCA TTTCTTGAAA GTAGTAGCTC TATACACTTC AAGATCTTTT

TGGATCAGCA TTTCTTGAAA GTAGTAGCTC TACACACTTT AAGATCTGTT

TGGATCAGCA TTTCTTGAAA GTAACAGCTC CACACACTTC AAGATCTGTT

TGGATCAGCA TTTCTGGAAA GTAGTAGTTC TACACACTTC AAGATCTGTT

TGGATCAGCA TTTCTTGAAA GTAGTAGTTC TACACACTTC AAGATCTGTT

TGGATCAGCA TTTCTTGAAA GCAGTAGCTC CACACACTTC AAGATCTGTT

TGGGTCAGCA TTTCGGGAAA GTAGTAGCTC TACACACTTT ATGATCTGTT

TGGGTTAGCA TTTCTCGAAA GTAGCAATTC TACACTCTTT AAAATCTGAC

AGGGCTGGCA TTTCTTGAAA GAAGTAGCTC TGCAGTCTTC AAAATCCGTT

CCTCTGAGCC ATGAGCAGAA CATGCAGTTA TCAAAATACT TTGCTTATCT

CCTCTGAGCC ATGAGCAGAA CATGCAGTTA TCAAAATACT TTGCTTATCT

CCTCTGAGCC ATGAGCAGAA CATGCAGTTA TCAAAATAGT TTGCTTATCT

CCTCTGAGCC ATAAGCAGAA CATGCAGTTA TCAAAATAGT TTGCTTATCT

CCTCTGAGCC ATGAGCAGAA CATGCAGTTA TCAAAATAGT TTGCTTATCT

CCTCTGAGCC ACGAGCAGAA CACGCAGTTA TCAAAATAGT CTGCTTATCT

CCTCTGAGCC ACGAGCAGAG CAAGCAGTTA TCAAAACGGT TAACTTGTCT

CCTCTGAGCC ACGAGCAGAA CAAGCACTTA TCAAAATGGT TAACTTGTCT

CCTCTGAGCC ACGAGCAGAA CATGCAGTTA TCAAAATAGT TTGTTTATCT

CCTCTGAGCC ACGAGCAGAA CATGCAGTAA TCAAAACAGT TTGCTTATCT

CCTCTGAGCC ACATGCAGAA CACGCAGTTA TCAAAATAGT TTGCTTATCT

CTTCTGAGCC ACGAGCAGAA CATGCGGTTA TCAGAATCGT TTGCTTATCT

CTTCTGAGCC ACGAGCAGAA CATGCAGTTA TCAAAATTGT CTGCTTATCT

CCTCTGAGCC ACGAGCTGAA CATGCAGTTA TCAAAATAGT TTGCTTATCT

CCTGTGAGCC TCGAGCAGAA CACGCAGTTA TCAAAACAGT GTGCTTATCT

CCTGTGAGCC ACGAGCAGAA CACGCAGTCA TCAAAACAGT TTGCTTATCT

CCTCTGAAGC ACGTGCAGAA CATGCAGCCA TCAAAATAGT ATACTGATCT

CTTCTGGAAC TCGAGCTGAA CATGCAGCCA TCAGAACAGT GTGTTGATCT

AAACATCTTA CCCTTCTCAA AGCTTGCATT AGCACCTCTG TCCAAAAGGA

AAACATCTTA CCCTTCTCAA AGCTTGCATT AGCACCTCTG TCCAAAAGGA

AAACATCTTA CCCTTCTCAA AGCTTGCATT AGCACCTCTG TCCAGAAGGA

AAACATCTTA CCCTTCTCAA AGCTTGCATT AGCACCTCTG TCCAGAAGGA

AAACATCTTA CCCTTCTCAA AGCTTGCATT AGCACCTCTG TCCAAAAGGA

AGACATCTTA CCCTTATCAA AGCTTGCATT AGCACCTCTG TCCAAAAGGA

AAGCATCTTA CCCTTATCAA AGCTTGCATT AGCACCCCTG TCCAAAAGGA

AAGCATCTTA CCCTTATCAA AGCTGGCATT AGCACCTCTG TCCAAAAGGA

AAACATCTTA CCCTTATCAA AGCTTGCATT AGCACCTCTA TCCAAAAGAA

AAACATCTTA CCCTTATCAA AGCTTGCATT AGCACCTCTG TCCAGAAGGA

AAACATCTTA CCCTTATCAA AGCTTGCATT AGCACCTTTG TCCAAAAGGA

AAACATCTTA CCTTTATCAA AGCTTGCATT AGCACCTCTG TCCAAAAGGA

---------- ---------- ---------- ---------- ----------

AAACATCTCA CCCTTATCGA AGCTTGCATT GGCACCTCTG TCCAAAAGGA

ACACATCTTA CCCTTCTCAA AGCTTGCATT AGCACCTCTG TCCAAAAGGA

AAATATCTTA CCCTTTTCAA AGCTTGCATT AGCACCTCTG TTCAAAAGGA

AAATATCTTA CCCTTATCAA AGCTTGCATT AGCCCCTCTG TCCAACAAGA

AACCTTCTTA CCTTTTGAAA AGCTTGCATT TGCACCTCTG TCCAACAGGA

CCCGAACCAT CTCACCAGAA TCTAGGAGCT CCTGGACCAA TGAAACATCT

CCCGAACCAT CTCACCAGAA TCTAGGAGCT CCTGGACCAA TGAAACATCT

CCCGAACCAT CTCACCAGAA TCTAGGAGCT CCTGGACCAA TGAAACATCT

CTCGAACCAT CTCACCAGAA TCTAGGAGCT CCTGGACCAA TGAAACATCT

CCCGAACCAT CTTACCAGAA TCTAGGAGCT CCAGGACCAA TGAAACATCT

CCCGAACGAC CTCACCAGAA TCTAGGAGTT CCTGGACCAA CGAAATATCT

ACCGAACCAT CTCACCAGAA TCCAGGAGTT CCTTCACTAA GGAAATATCT

AGCGAACCAT CTCACCAGAA TCCAGGAGTT CTTTAACTAA AGAAATATCT

CCCGCACCAT CTCACCAGAA TCTAGGAGCT CCTTCACCAG AGAAGTATCT

CCCGAACCAT CTCACCAGAA TCTAGGAGCT CCTCGACCAA TGAAATATTG

CCCGAACCAT CTCACCGGAA TCCAGGAGCT CCTTGACCAA TGAAGTATCT

CCCGAACCAT CTCACCAGAA TTTAGGAGCT CCTTGACCAG TAAAATATCT

---------G CTTACCAGAA TCGAGGAGCT CCTGGACCAA CGAAATATCT

CCCGAACCAT CTCACCAGAA TCTAGGAGTT CCTCCACCAA TGAAATATCT

CCCGAACCAT CTCACCAGAA TCTAGGAGCT CCTCGACCAA CGAAATATCT

CCCGGACCAT CTCACCAGCG TTTAGGAGCT CTTCAACCAA CGAAGTATCT

TACGCACCAT CTCACCAGAA TTTAGAAGTT CTTCAACCAG TGAAATATCT

TACGCACCAT CTCACCAGAA TTTAGGAGCT CTTCAAGTAG TGAACCATCA

CCGATGGTCA TTGCTTTCTT AAATTTTTCT TTCTTTTCTT CTCGAGATAC

CCGATGGTCA TTGCTTTCTT AAATTTTTCT TTCTTTTCTT CTCGAGATAC

CCGATGGTCA TTGCTTTCTT AAATTTTTCT TTCTTTTCCT CTCGAGATAC

CCGATGGTCA TTGCTTTCTT AAATTTTTCT TTCTTTTCCT CTCGAGATAC

CCGGTGGTCA ATGCTTTCTT AAATTTTTCT TTCTTTTCTT CTCGAGATAC

CCTGTGGTCA ATGCTTTCTT AAAAGTTTCA TTCTTTTCTT CTCGAGATAG

CCGGTGGTCA GTGCTTTCTT GAATGTTTCG TTCTTCTCTT CTCGAGATAC

CCGGTGGTCA GTGCTTTCTT AAATGTCTCG TTCTTCTCTT CTCGAGATAC

CCTGTGGTCA ATGCTTTCTT AAATGTTTCA TTCTTCTCTT CTCGAGATAC

CCTGTGGTCA AAGCCTTCTT AAATGTTTCC TGCCTTTCTT CTCGAGATAC

CCTGAGGTCA GTGCTTTTTT AAACGTCTCA TTCTTTTCTT CTCGAGATAC

CCTGTGGTCA ATGCTTTCTT AAATGTTTCA TTCTTTTCCT CTCGAGATAC

CCCGTGGTCA ATGCTTTCTT AAATGTTTCA TTCTTGTCTT CTCCAGATAC

CCAGTGGTCA ATGCTTTCTT AAACGTTTCA TTCTTTTCTT CTCTAGATAC

CCTGTGGTCA ATGCTTTCTT AAATATTTCA TTCTTTTCTT CTCGAGATAG

CCTGTGGTCA ATGCTTTCTT AAACATTTCA TTCTTTTCTT CTCGAGATAC

CCTGAAGTCA AAGCTTTTTT AAAGGTTTCA TCTTTTTCTT CTCCATGTAC

CCCGTAGTCA AAGCTCTCTT CAATACCTCA TCCTTCTCTT CTCCGTGTAC

CCAATCTCCC AGCCATCATC CTCGCTCTCG CTACTCTCGC CTCCACCATG

CCAATCTCCC AGCCGTCATC CTCGCTCTCG CTACTCTCGC CTCCACCATG

CCAATCTCCC AGCCGTCATC CTCGCTCTCG CTACTCTCCC CTCCACCATG

CCAATCTCCC AGCCGTCATC CTCGCTCTCG CTACTCTCCC CTCCACCATG

CCAATCTCCC AGCCGTCATC CTCACTCTCG CTACTCTCGC CTCCACCATG

CCTATCTCCC AGCCGTCATC CTCGCTCTCG CTACTCTCGC CTCCACCATG

CCAATATCCC AGCCGTCATC CTCACTATCG CTGCTCTCGC CTCCATCATG

CCAATATCCC AGCCGTCATC CTCACTATCG CTGCTCTCGC CTCCATCATG

CCGATCTCCC AGCCATCATC CTCGCTGTCG CTACTCTCTC CTCCACCATG

CCAATCTCCC AGCCGTCATC CTCACTGTCA CTACTCTCGC CTCCACCATG

CCAATCTCCC AGCCGTCATC CTCGCTCTCG CTACTCTCGC CTCCACCATG

CCAATCTCCC AGCCGTCATC CTCGCTGTCG CTGGTCTCGC CTCCACCATG

CCGATCTCCC AGCCGTCATC CTCGCTCTCG CTGCTCTCGC CTCCATCATG

CCAATCTCCC AGCCGTCATC CTCGCTATCA GTACTCTCGC CTCCACCATG

CCAATCTCCC AGCCGTCATC CTCGCTCTCG CTACTCTCGC CTCCACCATG

CCAATTTCCC AGCCATCATC CTCACTCTCG CTACTCTCCC CTCC------

CCTATATCCC AGTAGTCTTC TTCGCTGTCG CTGCTCTCGC CTCCACCATG

CCGATGTCCC AGCCGTCCTC GTCGCTGTCG CTGCTCTcgc cgccACCATG

CAGAGGTCGC CTCTGGAAAA GGCCAGCGTT GTCTCCAAAC TTTTTTTCAG

CAGAGGTCGC CTCTGGAAAA GGCCAGCGTT GTCTCCAAAC TTTTTTTCAG

CAGAGGTCGC CTCTGGAAAA GGCCAGCGTT GTCTCCAAAC TTTTTTTCAG

CAGAGGTCGC CTCTGGAAAA GGCCAGCGTT GTCTCCAAAC TTTTTTTCAG

CAGAGGTCGC CTCTGGAAAA GGCCAGCGTT GTCTCCAAAC TTTTTTTCAG

CAGAGGTCGC CTCTGGAAAA GGCCAGCGTC TTCTCCAAAC TTTTTTTCAG

CAGAAGTCGC CTCTGGAGAA AGCCAGCTTT ATCTCCAAAC TCTTCTTCAG

CAGAAGTCGC CTTTGGAGAA AGCCAGCTTT ATCTCCAAAC TCTTCTTCAG

CAGAAGTCGC CTCTGGAGAA GGCCGGCGTC CTCTCCAAAC TTTTTTTCAG

CAGAGGTCGC CTCTGGAAAA GGCCAGCGTC GTCTCCAAAC TTTTTTTCAG

CAGAGGTCGC CCCTGGAAAA GGCCAGCGTC CTCTCCAAAC TTTTCTTCAG

CAGAGGTCGC CTCTGGAAAA GGCCAGCGTC ATCTCCAAAC TTTTTTTCAG

CAGAGGTCGC CCCTGGAAAA GGCCGGCGTC ATCTCCAAAC TTTTTTTCAG

CAGAGGTCGC CTCTGGAAAA AGCCAGCGTC ATCTCCAAAC TTTTCTTCAG

CAGAAGTCGC CTTTGGAAAG GGCCAGCGTC ATCTCCAAAC TTTTCTTCAG

---------- ---------- ---------- ---------- ----------

CAGAGATCAC CCCTGGAAAA GGCAAATCTC CTCTCGAAAC TTTTCTTCAG

CAGAGATCAC CGCTGGAGCG AGCCAACCTC TTCTCCAAAC TTTTCTTCAG

GTGAGAGAAA AGAGAGGAAA TACCAAATAA ATGGAATAAT TATTAAATAC

GTGAGAGAAA AGAGAGGAAA TACCAAATAA ATGGAATAAT TATTAAATAC

GTGAGAGAAA AAAGAGGAAA TACCAAATAA ATGGAATAAT TATCAAATAC

GTGAGAGAAA AAAGAGGAAA TACCAAATAA ATGGAATAAT TATCAAACAC

GTGAGAGAAA AGAGAGGAAA TACCAAATAA ATGGAATAAT TATCAAATAC

GTGAGAGAAA AGGGAGGAAA TACCAAATAA ATGGAATAAT TATCAAATAC

GTGAGAGGAA TGGGA-AAAA CACGAAATAA ACTGAATAAT TATAGACCAG

GTGAGAGGAA GGGGAGAAAA CACGAAATAA ATCGAATAAT TATAAAGCAG

GTGAGCGTAA AGGGAGAGAA CCC--AATAA TTTGAGCAAG TA-CAAATAC

GTGAGGTAAA AAGGAGGGAA TATCAAATAA GTGGGATAAT TATCAAATAC

GTGAGAGAAA AGAGGGGGAA TACTAGATAA ATGCGATAAC TATCAAATAC

GTGAGGGAAA A--GAGGGAA TACCAAATAA ATGGGATCGT TATCAAATCC

GTGAGAGGAA AGGGAGAGAA TACCAAATAA ATAGAATAAT TATCAAATAC

GTGAGAGAAA AGGGAGGGAA TACTGAATAA ATGGGATAAT TGTCAAACAT

GTGAGAGAAA AGGAAGGGGA TACAGAATAA ATGGGATAAT TATCAAACAC

-----GGGAA AG--AGGTGA TACAGACTA- ---------- ---------C

GTAAGAGAAA AGGAAGGGAA TAGCAAAGGA ATAAGATAAT TGTCAAACAA

GTAAGCGAAA AGGAAGGGAA TCCTAAAGAA ATAGGATAAT TGTCAAACAC

CAGAAAGTAA ATCAATATTT TCCTTATTTT CTTGGTTTCA AATATATTTT

CAGAAAGTAA ATCAATATCT TCCTTATTTT CTTGGTTTCA AATATATTTT

CAGAAAGTAA ATCAATATCT TCCTTATTTT TTTGGTTTCA AATATATTTT

CAGAAAGTAA ATCAATATCT TCCTTATTTT TTTGGTTTCA AATATATTTT

CAGAAAGTAA ATCAATATCT TCCTTATTTT CTTGGTTTCA AATATATTTT

CAGAAAGTAA ATCAATATCT TCCTTATTTT CTTGGTTTCA AATATATTTT

CAAG------ -TCCATATCT TCTGTATTAT TTTGGTTTCA AATATATTTT

CAAGAAAGCG TCCCATATCT TCCTTGTTCT TTTGGTTTCA AATATATTTT

CAGCACTTCA ATCAACATCT TCCTTATTGT CTGGGTTTCA AATGAATTTT

C----AGTAA ATCAATATCT TCCTTATTTT CTTGGTTTCA AATATGTTTT

CAGAAAGTAA ATCATTATCT TCCTTATTTT CTTGGTTCCA AATCTATTTT

CAGAAAGTAA ATAAGTATCT TCCTTATTTT CTTGGTTTCA AATATATTTT

TAGAAAGTAA ATCAATATCT TCCTTATTTT TTTGGTTTCA AATGTATTT-

GAGAAAGTAA ATCAATATCT TCCTTATTTT CTTTGTTTCA AATATATTTT

CAGAAAGTAA ATCAAAATCT TCCTTATTTT CTTGGTTTCA AATATATTTT

CAGAAAGTAA ATCGAAATCT TCCTTATTTT CTTGGTTTCA AATAGATTTT

CAGAAAGTAA ACCAATATCT TCCTTCTTTT CTTGGTTCCA AATATATTTT

GAGAAGGTAA GTCAATATCT TTCTTATTCT TCTAATTTCA AATAGATTTT

TTTTTTAGCT GGACCAGACC AATTTTGAGG AAAGGATACA GACAGCGCCT

TTTTTTAGCT GGACCAGACC AATTTTGAGG AAAGGATACA GACAGCGCCT

TTTTTTAGCT GGACCAGACC AATTTTGAGG AAAGGATACA GACAGCGCCT

TTTTTTAGCT GGACCAGACC AATTTTGAGG AAAGGATACA GACAGCGCCT

TTTTTTAGCT GGACCAGACC AATTTTGAAG AAAGGATACA GACAGCGCCT

GTTTTTAGCT GGACCAGACC AATTTTGAGG AAAGGGTACA GACAGCGCCT

TTTTTCAGCT GGACCACACC AATTTTGAGA AAAGGGTACA GACACCACTT

CTTTTTAGCT GGACCACACC AATTTTGAGG AAAGGGTACA GACACCACTT

CTTTCTAGCT GGACTAGACC GATTTTGAGA AAAGGATACA GACAACGCCT

TTTTTTAGCT GGACCAGACC AATTTTGAAG AAAGGATACA GACAGCGCTT

TTTTTCAGCT GGACCAGACC AATTTTGATA AAAGGCTATA GACAACGCCT

TTTTTTAGCT GGACCATACC AATTTTGAAG AAAGGGTACA GGCAGCGCCT

---------- ---------- ---------- ---------- ----------

TTTTTTAGCT GGACCAGACC AATTTTGAGG AAAGGGTACA GACAGCGCCT

TTTTTTAGCT GGCCCGGACC AATTTTGAGG AAAGGTTACA GACAGCACCT

TTTTTCAGCT GGACCAGACC AATTTTGCGC AAAGGGTACA GGCAGCGCCT

TTTTTTAGTT GGACTAGACC AATTTTGAGT AAAGGTTTCA GAAAACGCTT

CTTTTTAGCT GGACGAAACC GATTTTGAAG AAAGGATATA GACAACATCT

GGAATTGTCA GACATATACC AAATCCCTTC TGTTGATTCT GCTGACAATC

GGAATTGTCA GACATATACC AAATCCCTTC TGTTGATTCT GCTGACAATC

GGAATTGTCA GATATATACC AAATCCCTTC TGCTGATTCT GCTGACAATC

GGAATTGTCA GATATATACC AAATCCCTTC TGCTGATTCT GCTGACAATC

GGAATTGTCA GACATATACC AAATCCCTTC TGCTGATTCT GCTGACAATC

GGAATTGTCA GACATTTACC AGATTCCTTC TGCTGATTCT GCTGACAATC

GGAGCTGTCA GACATATACC AAGCCCCTTC TTCTGATTCA GCTGACCACC

GGAGTTGTCA GACATATACC AAGCCCCTTC TGCTGATTCA GCTGACCACT

GGAATTGTCA GACATTTACC AAATCCCTTC TGCTGATTCT GCAGACAACC

GGAATTGTCA GACATATACC ATATCTCTTC TTCTGATTCT GCTGACAATC

GGAATTATCA GACATATACC AAGTCCCTTC TACTGATTCT GCTGATCATC

GGAGTTGTCG GACATATACC AAATCTCTTC CGCTGATTCT GCTGACAATC

---------- ---------- ---------- ---------- ----------

GGAATTGTCA GACATATACC AAATACCTTC TGCTGATTCT GCTGACAATC

GAAATTATCA GACATATACC AAATCCCATC TGTTGATTCT GCTGACAATC

GGAACTATCA GACATATACC AAATTCCATC TGTGGATTCT GCTGACAATC

GGAATTGTCA GACATATATC AAATTCCCTC ATCTAACTCT GCAGACAATC

GGAATTGTCC GACATATATC AAATTCCCAC CGCTGATTCA GCTGACAACC

TATCTGAAAA ATTGGAAAGG TTCTCCTTGG ATATACTTGT GTGAATCAAA

TATCTGAAAA ATTGGAAAGG TTCTCCTTGG ATATACTTGT GTGAATCAAA

TATCTGAAAA ATTGGAAAGG TTCTCCTTGG ATATACTTGT GTGAATCAAA

TATCTGAAAA ATTGGAAAGG TTCTCCTTGG ATATACTTGT GTGAATCAAA

TATCTGAAAA ATTGGAAAGG TTCTCCTTGG ATATACTTGT GTGAATCAAA

TATCTGAAAA ATTGGAAAGG TTCTCCTTGG ATATACTTGA GTACATCCAG

TGTCTGAAAA GCTAGAAAGG TTCCCCTTG- --TCACTCAT GGGA--CAGA

TGTCTGAAAA ACTAGAAAGG TTCTCTTTG- --ACACTCAT GGGA--CAGA

TGTCTGAGAA GCTGGAGAGG TTCTCATTGG ATATACTTGT GTGAACCAAA

TATCTGAAAA ATTGGAAAGG TTCTCCATGG ATGTACTTGT GTGAATAAAA

TGTCTGAAAA ATTGGAAAGG TTCTCCTTGG ATGTCTTTGG GAGAGTAAAA

TCTCAGAAAA ATTGGAAAGG TTCTCCCTGG ATGTACTTGT ATGAATAAAA

---------- ---------- -TTTAGTTGG GTTCCATC-T GTAAATAAAA

TATCTGAAAA ATTGGAAAGG TTCTTCTTGG ATATTCTTGA GTGAATCAAG

TCTCTGAAAA ACTGGAAAGG TTCTCCTTGG ATATACTTCA GTGAATCAAG

TCTCTGAAAA ACTGGAAAGG TTTTCCTTGC ATATACCTGA GTGAATCATG

TATCTGAAAA GTTAGAAAGG TTCTCCTTGG ATATACTTGA GTGAATCAAA

TATCTGAAAA ACTAGAAAGG TTCTCCCGGG TTATACTTGT GCGACTCAAG

CTGTTAAGGA AATAGACAAC TAAAATATTT GTTTTTATTC TTTTGCAGAG

CTGTTAAGGA AATAGACAAC TAAAATATTT GTTTTTATTC TTTTGCAGAG

CTGTTAAGGA AACAGACAAC TGAAATATTT GTTTTTATTC TTTTGCAGAG

CTGTTAAGGA AACAGACAAC TGAAATATTT GTTTTTATTC TTTTGCAGAG

CTGTTAAGGA AATAGACAAG TAAAATATTT GTTTTTATTC TTTTGCAGAG

CTGTTAAGGA AATAGGCAAC TCAAATATTT GTTTTTATTC TTTTGCAGAG

GTGGGAAGGA AATAAGCAGC TCA------- GTTTCTTTTT CTTTGCAGAG

ATATGAAGGA GATAAGCAGC TCATATATTT GTTTCTTATT CCTTGCAGAG

CTATTAAGGA AACAGGCAAC TCAAATATTT GTTTTTGTTC TTTTGCAGAG

CTGTTAAGGA AATAAG-AAT TCAAATATTT GTTTTTATTC TTTTGCAGAG

CTATAAAGGA AATAAATAGC TCAAATATTT GTTTTTATTC TTTTGCAGAG

CGGTTAAGGA AATAAACAAC TCAAATATTT GTTTTTATTC TTTTGCAGAG

CTGTTGAGCA AACAAACAGC TCTAATATTT GTTTTAATTC TTTTCCAGAG

CTGTTAAGGA AATAAACAAC TCAAATATTT GTTTTTATTC TTTTGCAGAG

CCGTTAAGGA AATAAACAAC TCAAATATTT GTTTTTATTC TTTTGCAGAG

CTGTTAAGGA AACAAACAAC TCATATATTT GTTTTTATTC TTTTGCAGAG

CTGTTAAGTA AACAAACAAC TCAAACATTT GTTTTtgtTT TTTTTCAGGG

CTGTTAAGTC ACCCAGTGAC TCCAACATTT GTTTTGGTTT CCTTTCAGGG

AATGGGATAG AGAGCTGGCT TCAAAGAAAA ATCCTAAACT CATTAATGCC

AATGGGATAG AGAGCTGGCT TCAAAGAAAA ATCCTAAACT CATTAACGCC

AATGGGATAG AGAGCTGGCT TCAAAGAAAA ATCCCAAACT CATTAATGCC

AATGGGATAG AGAGCTGGCT TCAAAGAAAA ATCCCAAACT CATTAATGCC

AATGGGATAG AGAGCTGGCT TCAAAGAAAA ACCCCAAACT CATTAATGCC

AATGGGATAG AGAGCTGGCT TCAAAGAAAA ATCCCAAACT CATTAATGCC

AATGGGACAG GGAACAAGCT TCCAAAAAGA AGCCCCAGCT TATCCACGCC

AATGGGACAG AGAACAAGCT TCAAAAAAGA ATCCCCAGCT TATCCACGCC

AATGGGATAG AGAGCTGGCT TCAAAGAAAA AACCCAAGCT CATTAATGCC

AATGGGACAG AGAACTGGCT TCAAAGAAAA ATCCCAAACT CATAAATGCC

AATGGGACAG AGAGCTGGCA TCAAAGAAGA ACCCCAAACT TATTAATGCC

AATGGGACAG AGAGCTGGCA TCCAAGAAAA ATCCGAAACT CATTAATGCC

AATGGGACAG AGAGCTGGCT TCCAAAAAAA ATCCCAAACT AATTAATGCC

AATGGGACAG AGAGCTGGCT TCAAAGAAAA ATCCCAAACT CATTAATGCT

AATGGGACAG AGAGCTGGCT TCCAAGAAAA ATCCCAAACT CATTAATGCC

AATGGGACAG AGAGCTGGCT TCAAAGAAAA ATCCCACACT CATTAATGCC

AATGGGACAG AGAGCTGGCA TCAAAGAAAA AACCCAAACT CATTAATGCC

AATGGGACAG AGAGCTGGCT TCCAAGAAGA ACCCCAAACT CATTAATGCC

CTTCGGCGAT GTTTTTTCTG GAGATTTATG TTCTATGGAA TCTTTTTATA

CTTCGGCGAT GTTTTTTCTG GAGATTTATG TTCTATGGAA TCTTTTTATA

CTTCGGCGAT GCTTTTTCTG GAGATTTATG TTCTATGGAA TCTTGTTATA

CTTCGGCGAT GCTTTTTCTG GAGATTTATG TTCTATGGAA TCTTGTTATA

CTTCGGCGAT GCTTTTTCTG GAGATTTACA TTCTATGGAA TCTTGTTATA

CTTCGGCGAT GCTTTTTCTG GAGATTTATG TTCTATGGAA TCTTATTGTA

CTCCGGCGAT GCTTTGTCTG GAGATTCGTC TTCTACGGAG TCTTGTTATA

CTTCGGCGAT GCTTTTTCTG GAGATTCCTC TTCTATGGAA TTTTGCTATA

CTTCGGAGGT GCTTTTTCTG GAGATTTATG TTCTATGGAA TCTTATTATA

CTTCGACGAT GCTTTTTCTG GAGATTTATG TTCTATGGAA TCATATTATA

CTTCGGCGAT GCTTTTTCTG GAGATTTGCG TTCTATGGAA TCTTATTATA

CTTCAGCGAT GCTTTTTCTG GAGATTTACG TTCTATGGAA TCTTACTATA

CTTCGACGAT GCTTTTTCTG GAAATTTATG TTCTATGGAA TCTTATTATA

CTTCGACGAT GCTTTTTCTG GAGATTTACG TTCTATGGGA TTATATTGTA

CTTCGGCGAT GCTTTTTCTG GAGATTTGTG TTCTATGGAA TCTTGTTATA

CTTCGACGAT GTTTTTTCGG GAGATTTATG TTCTATGGAA TCTTGCTCTA

ATTAGACGAT GTTTTTTCTG GAGATTTGTG TTTCATGGAA TCATATTATA

CTTCGACGAT GCTTCTTCTG GAGATTCATG TTCTATGGAC TCTTGCTGTA

TTTAGGGGTA AGAAAAAAAT CAGTGATATC TGTAAATGGT TCCATTGTAA

TTTAGGGGTA AGAAAAAAAT CAGTGATATC TGTAAATGGT TCCATTGTAA

TTTAGGGGTA AGAAAAAAAT CAGTGATATC TATAAATGGT TCCATTGTAA

TTTAGGGGTA AGAAAAAA-T CAGTGATATC TATAAATGGT TCCATTGTAA

TTTAGGGGTA AGAAAGAAAT CAGTGATATC TGTAAATGGT TCCATTCTAA

TTTAGGGGTA AGAAAAAAAT CAGTGATATC TGTAAATGGC TCTATAGTAA

CCTCGGGGTG AGAAAAAAGT CATCGG-ATC TATGAATGGT TCCATTGTAA

CCTAGGGGTA AGAAAAAAGT CATTGG-ATC TATGAATGGT TCCATTGTAA

TTTAGGGGTA AGAAAAAAAT CAGTGATATC TGTAAATGGT TCCATTGTCA

TTTAGGGGTA AGTAAAA--T CAGTGATGTC TGTAAGTGCT TTCATTATAA

TTTAGGGGTA AGAAAAAAAT CAGTGCTATC TGTAAGTGGT TCCATTGTAA

TTTAGGGGTA AGAAAACAAT CGGTGATATC TGTAAATGGT TCCATTGTAA

TTTAGGGGTA AGAAAAAAAT CAGTGATATC TGTTAATGGT TCCATTGTAA

TTTAGGGGTA AGAAAAAAAC CAGTGATATG TGTAAATGGT TCCATTGTAA

TTTAGGGGTA AGAAAAAAAT CAGTGATACC TGTAAATGGT TCCATTGTAA

TTTAGGGGTA AGAAATAA-T CAGTGATACC TGTAAATGGT TCCATTGTAA

CTTAGGGGTA AGGGGAAATC TAATGCTGTC CATCAGTGAT TTCACAGTAA

TTTAGGGGTA AGaccaacat tattaTTATT ATTCAGTGAA TATCTTGAAA

ATATTAAAGA ACATGATGCT TAAAACAGAT TAGGGAAAAT AGGAAGTCAC

ATATTAAAGA ACATGATGCT TAAAACAGAT TAGGGAAAAC AGGAAGTCAC

ATATTAAAGA ACATGATGCT TAAAACAGAT TAGGGAAAAC AGGAAGTCAC

ATATTAAAGA ACAGGATGCT TAAAACAGAT TAGGGAAAAC AGGAAGTCAC

ATATTAAAGA ACATGCTTCT TAAAACAGAT TAAGGAAAAC AGGAAGTCAC

ATATTAGAGA ACATGTTGCT TAAAACAGGT TAGGGAAAAC AGGAGGTCAC

ATATTAGAGA ACATGATACA TACAAGAGAT TAGGGGAAAT AGGAAGTCAC

ATATTAGAGA ACATGATACA TAAAAGAGAT TAGGGGAAAC AGGAAGTCAC

ATATTAGAGA ACATGATGCT CAAAAGAGAT TAGGAAAAAC AGGAAGTCAC

ATATTAGAGA ACATGCTGCT TAAAACAGAG CATGGAAAAC AGGAAGTCAC

ATATTAGAGA ACACGCTGCT TCAAACAGAT TAGGGAAGAT AGGAAGTCAC

ATATTAGAGA ACA---TGCT TAAAACAGAG TAGGGAAAAC AGGAAGTCAC

ATATTTGAGG ACATGCTGCT TAAAAGAGAT TTGGGAAAAC AGGAAGTGAC

ATATTAGAGA ACATGCTGCT TAAAACAGAT TAGTGAAAAC AGGAAGTCAC

ATATTAGAGA ATATGCTGCT TAAAACAGAT TAGTGAAAAC AGGAAGTCAC

ATACTAGAGA ACAGGTTGCT TAAAGCGGAT TAGTGAACAC AGGAGGTGAC

ACATGAGAGA ACATACAGTT TAAAACAGTG AAAGAAGAAC AGGAAGTAAC

TCATTAGAGG ACCTCTCACC TGAGAAAGAT TAAGGAGAAC AGGAGGTGAC

CAAAGCAGTA CAGCCTCTCT TACTGGGAAG AATCATAGCT TCCTATGACC

CAAAGCAGTA CAGCCTCTCT TACTGGGAAG AATCATAGCT TCCTATGACC

CAAAGCAGTA CAGCCTCTCT TACTGGGAAG AATCATAGCT TCCTATGACC

CAAAGCAGTA CAGCCTCTCT TACTGGGAAG AATCATAGCT TCCTATGACC

CAAAGCAGTA CAACCTCTCT TACTAGGAAG AATCATAGCT TCCTATGACC

CAAAGCAGTA CAGCCTCTCT TACTGGGGAG AATCATAGCT TCCTACGATC

CAAGGCTGTC CAGCCTGTCT TGCTAGGAAG AATCATAGCA TCCTATGATC

CAAGGCTGTC CAGCCTGTCT TGCTAGGAAG AATCATAGCA TCCTATGATC

CAAAGCCGTA CAGCCTCTCC TATTGGGGCG AATCATAGCT TCCTATGATC

CAAAGCAGTC CAGCCTCTCT TACTGGGAAG AATCATAGCT TCCTATGACC

CAAAGCAGTC CAGCCTCTCT TACTTGGAAG AATCATAGCT TCCTATGATC

CAAAGCAATC CAGCCTCTCC TACTGGGAAG AATCATAGCT TCCTACGATC

CAAAGCAGTC CAGCCACTCT TACTCGGAAG AATCATAGCT TCCTACGATC

CAAAGCTGTG CAGCCTCTCT TACTGGGAAG AATTATAGCT TCCTATGATC

CAAGGCAGTC CAGCCACTCT TACTGGGAAG AATCATAGCT TCCTATGATC

CAAAGCAGTA CAGCCACTCC TACTGGGAAG AATTATAGCT TCCTATGATC

CAAAGCAGTA CAGCCTCTAT TACTGGGAAG AATCATAGCC TCCTATGATC

CAAGGCAGTG CAGCCTCTCC TCCTGGGGCG AATCATTGCC TCCTATGACC

CGGATAACAG AACGCTCTAT CGCGATTTAT CTAGGCATAG GCTTATGCCT

CGGATAACAG AACGCTCTAT CGCGATTTAT CTAGGCATAG GCTTATGCCT

CGGATAACAG AACGCTCTAT TGCGATTTAT CTAGGCATAG GCTTATGCCT

CGGATAACAG AACGCTCTAT TGCGATTTAT CTAGGCATAG GCTTATGCCT

CAGATAACAG AACGCTCTAT TGCGATTTAC CTGGGCATAG GCTTATGCCT

CAGACAACAG AGCGTTCCAT AGCGATCTAT CTAGGCATAG GCTTATGCCT

CAGACAACAG AGCGTTCCAT TGCCATTTAC CTAGGCATAG GCTTATGCCT

CAGAAAACAG AACGTTCCAT TGCCATTTAC CTTGGCATAG GCTTATGCCT

CAGACAACAG AGCGCTCCAT TGCCATTTAC CTGGGCATAG GTCTGTGCTT

CAGATAACAG AACGCTCCAT TGCCATTTAC TTAGGCATAG GCCTATGCCT

CAGATAACAG AACGCTCCAT TGCAATCTAC CTAGCCATTG GCTTATGCCT

CAGATAACAG AACGGTCCAT TGCGATTTAC CTAGGCATAG GCTTATGCCT

CAGATAACAG AACGCTCTAT TGCCATTTAT CTAGGCATAG GCTTATGCCT

CAGATAACAG AACGCTCCAT TGCAATTTAC CTTGCCGTTG GCTTGTGCCT

CAGATAACAG AACGCTCCAT TGCGATTTAC TTGGCCATAG GCCTGTGCCT

CAGATAACCG AACGCTCCAT TGCCTTGTAC CTGGCCGCAG GCTTGTGCCT

CTGACAACGG AACGTGCCAT TGCAATATAT CTAGGCATAG GCCTGTGCCT

CAGACAACGG AACGCTCTAT CGCGTACTAC CTGGGCATCG GCCTGTGTCT

TCTCTTTATT GTGAGGACAC TGCTCCTACA CCCAGCCATT TTTGGCCTTC

TCTCTTTA-- ---------- ---------- ---------- ----------

TCTCTTTATC GTGAGGACAC TGCTCTTACA CCCAGCCATT TTTGGCCTTC

TCTCTTTATC GTGAGGACAC TGCTCTTACA CCCAGCCATT TTTGGCCTTC

TCTCTTTATT GTGAGGACAC TGCTTCTACA CCCAGCCATT TTTGGCCTTC

TCTCTTTATC GTGAGGACAC TGCTTCTACA CCCAGCCATT TTTGGCCTTC

CCTCTTCATT GTCAGGACAC TGCTTCTGCA CCCAGCTATT TTTGGCCTTC

TCTCTTCATT GTCAGGACAC TGCTTCTTCA CCCAGCTATT TTTGGCCTTC

ACTCTTTGTT GTGAGGACCC TACTCCTACA CCCAGCAATT TTTGGTCTTC

TCTCTTTATC GTGAGGACAC TGCTCCTGCA CCCAGCCATT TTTGGTCTCC

TCTCTTTATC ATGAGGCCGC TGCTCCTGCA CCCAGCCATT TTTGGCCTTC

GCTCTTTATC ATGAGGACAC TGCTCTTGCA CCCCGCCATT TTTGGCCTTC

TCTCTTTATC GTGAGAACAC TGCTCCTGCA TCCAGCCATT TTTGGCCTTC

TCTGTTCGTT GTGAGGACGC TGCTCCTGCA CCCAGCCATT TTTGGACTTC

TCTCTTCATC GTGAGGACGC TCCTCCTACA CCCAGCCATT TTTGGCCTCC

TCTCTTCATT GTGAGGACAC TGCTCCTGCA CCCAGCCATT TTTGGCCTCC

TCTCTTTATA GTGAGGACAC TTCTTCTACA CCCTGCCATC TTTGGCCTTC

TCTCTTTATT GTGCGCACCC TGTTGCTGCA CCCTGCCGTT TTTGGCCTCC

ATCACATTGG AATGCAGATG AGAATAGCTA TGTTTAGTTT GATTTATAAG

---------- ---------- ---------- ---------- ----------

ATCACATTGG AATGCAGATG AGAATAGCTA TGTTTAGTTT GATTTATAAG

ATCACATTGG AATGCAGATG AGAATAGCTA TGTTTAGTTT GATTTATAAG

ATCACATTGG AATGCAGATG AGAATAGCTA TGTTTAGTTT GATTTATAAG

ATCACATTGG AATGCAGATG AGAATAGCTA TGTTTAGTTT GATTTATAAG

ATCACATTGG AATGCAGATG AGAATAGCCA TGTTTAGCTT GATTTATAAG

ATCGCATTGG AATGCAGATG AGAACAGCTA TGTTTAGCTT GATTTATAAG

ATCATATTGG AATGCAGATG AGAATAGCCA TGTTCAGTTT GATTTATAAG

ATCACATTGG AATGCAGATG AGAATAGCTA TGTTTAGTTT GATTTATAAG

ATCACATTGG AATGCAGATC AGAATAGCTA TGTTTAGTTT GATTTATAAG

ATCACATTGG GATGCAAATG AGGATAGCTT TGTTTAGTTT GATTTATAAG

ATCACATTGG AATGAAAATG AGAATAGCTA TGTTCAGTTT AATTTATAAG

ATCACATTGG AATGCAGATG AGAATAGCGA TGTTTAGCTT GATTTATAAG

ATCACATGGG AATGCAGATG AGAATAGCTA TGTTTAGTTT GATTTATAAG

ATCATATTGG AATGCAGATG AGAATAGCTA TGTTTAGTTT GATTTATAAG

ATCACATGGG AATGCAGATG AGAATAGCCC TGTTTAGTCT GATTTACAAA

ATCACATCGG AATGCAGATG AGGATCGCCC TGTTCAGTCT GATTTACAAA

AAGGTAATTT TTCTTTTAGA CTTTAAAGCT GTCAAGCCGT GTTCTAGATA

-------TTT TTCTTTTAGA CTTTAAAGCT GTCAAGCCGT GTTCTAGATA

AAGGTAATTT TTCTTTTAGA CTTTAAAGCT GTCAAGCCGT GTTCTAGATA

AAGGTAATTT TTCTTTTAGA CTTTAAAGCT GTCAAGCCGT GTTCTAGATA

AAGGTAATTT TTCTCTTAGA CTTTAAAGCT GTCGAGCCGT GTTCTAGATA

AAGGTAATTT TTCTTTTAGA TTTTAAAGCT GTCAAGCCGT GTTCTAGATA

AAGGTAATTT TTCCTTTAGA CTTTAAAGTT GTCAAGCCGT GTTCTTGATA

AAGGTAACTT TTCTTTTAGA CTTTAAAGTT GTCAAGCCGC GTTCTTGATA

AAGGTAATTT TTCTTTTAGA CTTTAAAGCT GTCAAGCCGT GTTCTAGATA

AAGGTAATTT -CCTTTTAGA CTTTGAAGCT ATCAAGCCGT GTTCTGGATA

AAGGTAATTT TCCTTTTAGA CTTTAAAGCT GTCAAGCCGT GTTCTGGATA

AAGGTAATTT TCCTTTCAGA CTTTAAAGCT GTCAAGCCGT GTTCTAGATA

AAGGTAA--- ---------- ---------- ---------- ----------

AAGGTAATTT TCCTTTTAGA CCTTAAAACT GTCAAGCCGT GTTTTAGATA

AAGGTAATTT TTCTTTTAGA CTTTAAAACT GTCCAGCCGT GTTCTTGATA

AAGGTAATTT TTCTTTTAGA CTTTAAAACT TTCGAGCCGG GTTCTAGATA

AAGGTAATTT TCCTTTCAGA CCTTAAAACT GTCAAGCCGT GTTCTAGATA

AAGGTAATTT TCCTCCTAGA CTTTAAAATT GTCAAGCCGA GTCCTGGATA

AAATAAGTAT TGGACAACTT GTTAGTCTCC TTTCCAACAA CCTGAACAAA

AAATAAGTAT TGGACAACTT G-TAGTCTCC TTTCCAACAA CC-GGACAGA

AAATAAGTAT TGGACAACTT GTTAGTCTCC TTTCCAACAA CCTGAACAAA

AAATAAGTAT TGGACAACTT GTTAGTCTCC TTTCCAACAA CCTGAACAAA

AAATAAGTAT TGGACAACTT GTTAGTCTCC TTTCCAACAA CCTGAACAAA

AAATAAGTAT TGGACAGCTT GTAAGTCTCC TTTCCAACAA CCTGAACAAA

AAATAAGTAT TGGACAACTT ATTAGTCTTC TTTCCAACAA CCTGAACAAA

AAATAAGTAT TGGACAACTT GTTAGTCTTC TTTCCAACAA CCTGAACAAA

AAATAAGTAT TGGGCAACTT ATTAGTCTCC TTTCCAACAA CCTGAACAAA

AAATAAGTAT TGGACAACTT GTTAGTCTCC TTTCCAACAA CCTGAACAAA

AAATAAGTAT TGGACAACTT GTTAGTCTCC TTTCCAACAA CCTGAACAAA

AAATAAGTAT TGGACAACTT GTTAGTCTCC TTTCCAACAA TCTGAACAAA

---------- ---------- ---------- ---------- ----------

AAATAAGTAT TGGACAACTT GTTAGTCTCC TTTCCAACAA CCTGAACAAA

AAATAAGTAT TGGACAACTT GTCAGTCTCC TTTCCAACAA CCTGAACAAA

AAATAAGTAT TGGACAACTT GTTAGTCTCC TTTCCAACAA CCTGAACAAA

AAATAAGCAC TGGACAGCTT ATCAGTCTCC TTTCCAACAA TCTGAACAAG

AAATAAGCAC CGGACAGCTC GTTAGCCTTC TTTCAAACAA CTTGAACAAA

TTTGATGAAG TATGTACCTA TTGATTTAAT CTTTTTTTTT TCAGGGACTT

ATTGATG-AG TATGTACCTA TGA--TTAAT C-TTTTTTTT TCAGGGACTT

TTTGATGAAG TATGTACCTA TTGATTTAAT CTTTTTTTTT TCAGGGACTT

TTTGATGAAG TATGTACCTA TTGATTTAAT CTTTTTTTTT TCAGGGACTT

TTTGATGAAG TATGTACCTG TTGATTTAAT ATTTTTTTTT TCAGGGACTT

TTTGATGAAG TATGTACCTA TTGATTTAAT CTCTTTTTTT TCAGGGACTT

TTTGATGAAG TATGTACCCA TTGATTTAAT GTTATTTTTT TCAGGGACTT

TTTGATGAAG TATGTACC-A TTGACTTAAT GTTTTTTTTT TCAGGGACTT

TTTGATGAAG TATGTACCTA CTGATTTAAT CTCTTTTCTT TCAGGGGCTT

TTTGATGAAG TATGTACCTA CTGATTTACT CTTTTTTTTT TCAGGGACTC

TTCGATGAAG TATGTACCTA CTGATTTAAT GTTTTTTTTT TCAGGGACTT

TTTGATGAAG TATGTACCTA CTAATTTAAT CTTTTTTTTT TCAGGGACTT

---------- ---------- ---------- ----TTTTTT TCAGGGACTT

TTTGATGAAG TATGTGCCTA CTGATTTGAT TTTCTGTCTT TCAGGGCCTA

TTTGATGAAG TATGTATCTA CCAATTTAAT CTTTTTTTTT TCAGGGACTT

TTTGATGAAG TATGTATCTA CCAATTTAAT CTTT------ ----------

TTTGATGAAG TATGTAGCTG CTCATTTAAT CTTTTTTTTT TCAGGGGCTG

TTTGATGAAG TATGTTTTTG CTGATTTTAT CTTTGTGTTC CCAGGGTCTG

GCATTGGCAC ATTTCGTGTG GATCGCTCCT TTGCAAGTGG CACTCCTCAT

GCATTGGCAC ATTTCGTGTG GATCGCTCCT TTGCAAGTGG CACTCCTCAT

GCATTGGCAC ATTTCGTGTG GATCGTTCCT CTGCAAGTGG CACTCCTCAT

GCATTGGCAC ATTTCGTGTG GATCGTTCCT CTGCAAGTGG CACTCCTCAT

GCATTGGCAC ATTTCGTGTG GATCGCTCCT TTGCAAGTGG CACTCCTCAT

GCTTTGGCTC ATTTCGTGTG GATCGCCCCT TTGCAAGTAA CGCTTCTGAT

GCCTTGGCAC ATTTTATATG GATTGCTCCT TTACAAGTGG TTCTTCTGAT

GCCTTGGCAC ATTTTATATG GATTGCTCCT TTACAAGTGA CTCTTCTGAT

GCGCTGGCCC ATTTCGTGTG GATTTCACCT CTGCAAGTGA CTCTGCTGAT

GCACTGGCAC ATTTTGTGTG GATCGCTCCT CTGCAAGTGA CGCTGCTCAT

GCACTGGCGC ATTTCGTGTG GATTGCTCCT CTACAAGTGA CGCTCCTGAT

GCGTTGGCCC ATTTCGTGTG GATTGCTCCT TTACAAGTGA TGCTGCTGAT

GCCTTGGCAC ATTTTGTGTG GATTGCTCCT TTACAAGTGA CACTTCTAAT

GCATTAGCAC ATTTCGTGTG GATCGCTCCT TTACAAGTGA CGCTGCTGAT

GCACTGGCAC ATTTTGTGTG GATTGCTCCT TTACAAGTGA CACTTCTGAT

---------- ---------- ---------- ---------- CGCTCTTGAT

GCCCTGGCAC ATTTTGTCTG GATTGTACCT TTACAAGTGG TGCTCCTGAT

GCGCTGGCCC ATTTTGTGTG GATCGCGCCT CTCCAGGTGA TGCTGCTGAT

GGGGCTAATC TGGGAttTAC AGAGATCAGA GAGCTGGGAA GATCAGTGAA

GGGGCTAATC TGGGATTTAC AGAGATCAGA GAGCTGGGAA GATCAGTGAA

GGGGCTAATC TGGGAttTAT AGAGATCAGA GAGCTGGGAA GATCAATGAA

GGGGCTAATC TGGGAttTAT AGAGATCAGA GAGCTGGGAA GATCAATGAA

GGGGCTAATC TGGGAttTAC AGAGATCAAA GAGCTGGAAA GATCAATGAA

GGGGCTGTTG TGGGATTTAC AGAGATCAGA GAGCAGGAAA GATCAATGAA

GGGGCTTCTC TGGGATTTAC AGAGATAAAA GAGCTGCAAA GATCAATGAA

GGGGCTTCTC TGGGATTTAC AGAGATCAGA GAGCTGCAAA GATCAATGAA

GGGGCTGCTC TGGGATTTAC AGGGATCAGA GAGCTGGAAA GATCAATGAG

GGGGCTGCTC TGGGATTTAC AGAGATCAGA GAGCTGGAAA GATCAATGAA

GGGGCTGCTC TGGGATTTAC AGAGATCAGA GAGCTGGAAA GATCAATGAA

GGGGCTGCTT TGGGATTTAT AGAGATCAGA GAGCTGGAAA GATCAATGAA

GGGGCTACTC TGGGATTTAT AGAGATAAGA GAGCTGGAAA GATCAATGAA

GGGACTGCTG TGGGATTTAC AGAGATCAGA GAGCTGGAAA GATCAATGAA

GGGGCTGATC TGGGATTTGT AGAGATAAGA GAGCTGGAAA GATCAATGAA

GGGGCTGCTC TGGGA----- ---------- ---------- ----------

GGGACTTCTT TGGGATTTTC AGAGAAAGGA GAGCAGGAAA GATAAATGAA

GGGGCTGCTG TGGGATTTAC AGAGACAGAA GAGCCGGAAA GATAAATGAG

AGACTTGTGA TTACCTCAGA AATGATTGAA AATATCCAAT CTGTTAAGGC

AGACTTGTGA TTACCTCAGA AATGATTGAA AATATCCAAT CTGTTAAGGC

AGACTTGTGA TTACCTCAGA AATGATTGAA AATATCCAAT CTGTGAAGGC

AGACTTGTGA TTACCTCAGA AATGATTGAA AATATCCAAT CTGTGAAGGC

AGACTTGTGA TTACATCAGA AATGATTGAA AATATCCAAT CTGTGAAGGC

AGACTTGTGA TCACCTCAGA AATGATTGAA AATATCCAGT CTGTGAAGGC

AGACTTGTGA TCACATCAGA AGTTATTGAC AATATCTATT CTGTTAAGGC

AGACTCGTGA TCACATCAGA AATTATTGAT AATATCTATT CTGTTAAGGC

AGACTAGTGA TCACTTCAGA AATGATTGAA AACATCCAAT CTGTTAAAGC

AGACTGGTGA TAACGTCAGA AATGATTGAA AATATCCAAT CAGTTAAGGC

AGACTCGTGA TCACCTCAGA AGTGATTGAA AATATCCAAT CTGTTAAGGC

AGACTTGTGA TCACCTCAGA AATGATTGAA AATATCCAAT CTGTTAAGGC

AGGCTTGTAA TCACCTCGGA AATGATTGAA AATATACAGT CTGTTAAGGC

AGACTTGTGA TCACCTCAGA AATGATTGAA AATATTCAGT CTGTCAAGGC

AGACTTGTGA TCACCTCTGA AATGATTGAA AATATCCAAT CTGTGAAGGC

---------- ---------- ---------- ---------- ----------

AGACTTGTGA TAACTTCAGA AATGATTGAC AATATACAGT CTGTTAAGGC

AGACTTGTGA TCACCGCAGA AATTATTGAG AATATCCAAT CCGTTAAAGC

ATACTGCTGG GAAGAAGCAA TGGAAAAAAT GATTGAAAAC TTAAGACAGT

ATACTGCTGG GAAGAAGCAA TGGAAAAAAT GATTGAAAAC CTAAGACAGT

ATACTGCTGG GAAGAAGCAA TGGAAAAAAT GATTGAAAAC CTAAGACAGT

ATACTGCTGG GAAGAAGCAA TGGAAAAAAT GATTGAAAAC CTAAGACAGT

ATACTGCTGG GAAGAAGCAA TGGAAAAAAT GATTGAAAAC CTAAGACAGT

ATACTGCTGG GAGGAAGCAA TGGAAAAAAT GATTGAAAAC CTAAGACAGT

ATATTGTTGG GAATCAGCGA TGGAGAAGAT AATTGAAAGC TTGAGAGAGT

ATATTGTTGG GAATCAGCGA TGGAGAAAAT GATTGAAAAC TTGAGAGAGT

ATACTGCTGG GAGGAAGCGA TGGAAAAAAT GATTGAAAAC CTAAGACAGT

ATACTGCTGG GAAGAAGCAA TGGAAAAAAT CATTGAAAAC CTAAGACAGT

ATACTGCTGG GAGGAAGCAA TGGAAAAAAT AATTGAAAAC ATAAGACAGT

ATACTGCTGG GAGGAAGCAA TGGAAAAAAT GATTGAAAAC CTAAGACAGT

ATACTGTTGG GAGGAAGCAA TGGAAAAAAT AATTGAAAAC CTCAGACAGT

ATACTGCTGG GAGGAGGCAC TGGAAAAAAT GATTGAAAAT TTCAGACAGT

ATACTGCTGG GAGGAAGCAA TGGAAAAAAT GATCGAAAAC CTAAGACAGT

---------- ---------- ---------- ---------- ----------

ATATTGCTGG GAGGAGGCAA TGGAAAATAT GATTGAAAAT ATAAGAGAGT

CTACTGTTGG GAGGAAGCAA TGGAAAACAT GATTGAAAGC CTCAGAGAGT

AAGTAACTGA GCTGCAGGTG TGTGATTGTA ACAACAAAGA AATGCTGAAA

AAGTAACTGA GCTGCAGGTG TGTGATTGTA ACAACAAAGA AATGCTGAAA

AAGTAACTGA GCTGCAGGTG TGTGATTGGA ACAACAAAGA AATGCTGAAA

AAGTAACTGA GCTGCAGGTG TGTGATTGGA ACAACAAAGA AATGCTGAAA

AAGTAACTGA GCTGCAGGTG TGTGATTGGA ACAACAAAGA AATGCTGAAA

AAGTAACTGA GCTGCAGGTG TGTGATTGGA ACAACAAAGA AATGCTGAAA

AAGTAACTGA GCCACAGGTG TGTGATTGGA ACAACAGGGA AATGCTGAAA

AAGTAACTGA GCCACAGCTG TGTGATTGGA ACAACAAGGA AATGCTTAAA

AAGTAGCTGA GCTGCAGGTG TGTGATTGGA ACAACAGAGA AATGCTGAAA

AAGTAACTGA GCTGCAGGTG TGTGATTGGA ACAACAAAGA GATGCTGAAA

AAGTAACTGA GCTGCAGGTG TGTGATTGGA ACAACAAAGA AATGCTGAAA

AAGTAATTGA GCTGCAGGTG TGTGATTGGA ACAACAAAGA AAGGCTGACA

AAGTAACTGA ACTGCAGGTG TGTGATTGGA ACAACAAAGA AATGCTGAAA

AAGTAACTCA GTTGCAGGTG TGTGATTGGA AAAACAAAGA AATGCTGAAA

AAGTAACTGA GTTGCAGGTG TGTGATTGGA ACAACAAAGA AATGCTGAAA

----ACCTGT GCTGCAGGTG TGCGATGGGA ACAACAAGGC AATGCTGAAA

AAGTAACTAA ACTCCAGGTG TGTGATTAGA ACAACAAAGA AAAGGTGAAA

AAGTAATGGA ACCCCAGCTG TGTGATTGGA ACAACAAAGA AAAGGAGAAA

TATTAAGTCC TTTGCCATGT AAATAGAAAA AGAGTATTAT TTCCCAAACA

TATTAAGTCC TTTGCCATGT AAATAGAAAA AGAGTATTAT TTCCCAAACA

TATTAAGTCC TTTGCCATGT AAACAGAAAA AGAGTATTAT TTCCCAAACA

TATTAAGTCC TTTGCCATGT AAACAGAAAA AGAGTATTAT TTCCCAAACA

TATTAAGTCC TTTGCCATGT AAATAGAAAA AGAGCATTAT TTCCCAAACA

TGCTCAGTCC TTTGCCATGT AAACAGAAAA AGAGGATTAT TTCCCAAACA

TGTTAAGTCC TTTGCCGTGT AAATAAAAAA AGAGTATTAT TTTTCAAATA

TGTTAAGTCC TTTGCCATGT AAATAGAAAA AGAGTGTTAT TTTCCAAATA

AGCTAAGCCC TTTGCCATGT AAATAAAAAA AGAGTGTTAT TTCCCCAACA

TGGTAAGTCC TTTGCCATGT AAATAGAAAA AGACTACTAT TGCCCAAACA

TATCAAGTCC TTTGCCATGT AAATAGAGAA AGAGTAATAT TTCCCAAACA

TGTTAAGTCC TTTGCCATGT AAATAGA--- ----TATTAT TTCCCAAACA

TATTAAGTCC TTTGCCATGT AAATAGAAAA AGAGTATTAT TTCCCAAACA

TGTTAAGTTC TTTGCCATGT AAATAGGAAA AGAATATTAT TTCCAAAACA

TGTTAAGTCC TTTGACATGT AAATAGAAAA AGAGTATTAT TTCCTAAACA

TGTTAAGTCC TTTGCCATGT AAACAGAAAA AGAGTATTAT TTCCCCAACA

TGTTAAGTCC TCTGCCATGT AAATAAAAAA AGTGTACTAT TTCCCAAACA

TGTTAAGTCC CCTGCCTGGT AAATAGAGAA GGGCTGCTAT TTCCCAAACA

TTATTGCTCA CCTGTTTTTG TTATGCCTTT CAAGATAAAT CCAGGAAAAA

TTATTGCTCA CCTGTTTTTG TTATGCCTTT CAAGATAAAT CCAGGAAAAA

TTATTGGTCA CCTGTTTTTG TTATGCACTT CAAGATAAAT CCAGGAAAAA

TTATTGGTCA CCTGTTTTTG TTATGCATTT CAAGATAAAT CCAGGAAAAA

TTATTGGTCA CCTGTTTTTG TTATGCCTTT CAAGATAAAT CCAGGAAAAA

TTATTGGTCA CCTGTTTTTG TTATGCTTAT CAAGACAAAT CCAGGAAAAA

TTATTGGTCA CCTGTTTTTG TTATGCTTTT CAAGGTAAAT CCAGGAAAAA

CTATTGCTCA CCTGTTTTTG TTATGCCTTT CAAGGTAAAT CTAGGAAAAA

TTTAGGGCCA CCTGTTTCTG TTATGCCTTT TAAGACAAAC CCAGGAAAAA

TGATTGGTCA CCTGTTTTTA TTATGCCTTT CAAGACAAAT CCAGGAAAAA

TTATTGGTCA CCTGTTTTTA TTATGCTTTT CAAAGCAAAT CCAGGAAAAA

TTATTGCTCA CCTGCTTTTA TTATGTCTTT CAATACAAAT CCAGGAAAAA

TTATTGGTCA TCTGTTTTTA TTATGCCTTT TAAGACAAAT ACAGGAAAAA

TTATTGGTCA TCTGTTTTTG TTATGCCTTT CAAGACAAAT CCGGGAAAAA

TCATTGATCA CCTGTTTTTG TTATGCCTTA CATGGCAAAT CCAGGAAAAA

TTACTGGTCA TCTGTTTTTG TTATGCCTTC CATGGCTAAT CCAGGAAAAA

TTATTGGTCA TTTCCCTTTA TTATGCCATT TAAGAAAAAT CCAGGAAACA

CTTGTGGTCA CCTAGTTTTG TTTTGCTTTT CAGGAAAAAT CCAGGAAATG

TCTGGCATGA AATTGTCACT CGAAAAGGCT AGAAGTGTTG ACATAAATAT

TCTGGCATGA AATTGTCACT CGAAAAGGCT AGAAGTGTTG ACGTAAATAT

TCTGGCATGA AATTGTCACT CGAAAAGGCT AGAAGTGTTG ACATAAATAT

TCTGGCATGA AATTGTCACT CGAAAAGGCT AGAAGTGTTG ACATAAATAT

TCTGGCATGA AATTGTCACT TGAAAAGGCT AGAAGTGCTG ATGTAAATAT

TCTGGCATGA AATTGTTACC TGAAAAGTCT GGAAGTGCTG ACGTAAATAT

TACAGCATGA AATTGTTACC TGAAAAGGCT GGGAGTTCTG ACGTAAAAA-

TACATCATGA AATTGTTACC TGAAAAAGCT GGGAGTTCTG ACGTAAAAA-

TCCGGCATGA AATTGTTACC CGAAAAGGCT GGAAGTACTG ACGTAAATAT

---------- ---------- ---------- ---------- ----------

TCTGGCATGA AATTGTTACC TGAAAAGGCT TGAAGTGCTG ACATAAATAT

TCTGGCATGA AATGGTTACC TGAAAAGGCT GGAAGTGCTG CCATAAATAT

TCTGGCATGA AATTGTTACC TGAAAAGGCT GGCAGTGCTG ACGTAAATAA

TCTGGCATGA AATTGTTACC CGAAGAAGCT GGAAGAGCTG ACGTAAATAT

TCGCGCATGA AATTGTTACC TGAAAAGGCA GGAAGTGCTG ACGTGAATAT

TCTGGCATGA AATTGTTACC AGAAGAGGCT GAAAGTGCTG ATGTAAATAT

TCTGGCATGA AATTGTTGCT TGAAAAGGTT GGAAGAACTG ACGTAAGTAT

CCCAGCTTCT TAGAGTTCTC CTAACGGGTC AGAAGAGTGA TGGGAAATGT

GGGAAAGAGT TGCTCCTACT CAAGAGAGAT AATGTTCTTT TTTTTATAGA

GGGAAAGAGT TGCTCCTACT CAAGAGAGAT AATGTTCTTT GTTTTATAGA

GGGAAAGAAT TGCCCCTACT CAAGAGAGAT AATGTTCTtt tttttataGA

GGGAAAGAAT TGCCCCTACT CAAGAGAGAT AATGTTCTtt tttttataGA

GGGAAAGAAT TGCTTCTACT CAAGAGAGAC AATGTTCTtt tttttataGA

GGGAAAGAAA TGCTCCTGCT CCAGAGAGAG AACATTCTtt tttttataGA

-GGAAAGAAT TGCTT-TACT CAGCAGAGAC AATGTTCTGT TTTCCACAGG

-GGAAGGAAT TGCTT-TACC CCGCAGAGAC AATGTTCTGT TTTGCACAGG

GGGGAAGAAT TGCCT---CT CAACAGAGAG ACTGTTCTTT TCTTAATAGA

---------- ---------- ---------- --------TT TTTTTTTAGA

GGGAAAGAAT TGCTTCTGCT CAAAAGAGAT AATGTCTTtt tttttataGA

GGGAAAGAAT TGCTTCTGCT CAAAAGAGAT ATTGTTCT-T TTTTTATAGA

GGGAAAGAAT TGCTTCTACT CAAAAAAGAC AATGTTCTTT TTTTTATAGA

GGGAAAGAAT TGCTTCTGAT CAAGAGAGAT AATGTTCTtt ttttaataGG

GGGAAAGAAT TGCTTCTATT CAAGAAAGAT AATGTTCTTT TTTTCATAGA

GGGAAGGAAT TGAGTCTACT CAAGAGAAAT AATGTTCT-- ----------

GGGAAATAGT TGCATGTACC AGAGAGATAA AATGTTCTGG TTTTTACAGA

GCGTAAGAGC TGCTTTGACC AGAGAAGGAT AGTGCTCTTT ATTTCACAGA

ACAGAACTGA AACTGACTCG GAAGGCAGCC TATGTGAGAT ACTTCAATAG

ACAGAACTGA AACTGACTCG GAAGGCAGCC TATGTGAGAT ACTTCAATAG

ACAGAACTGA AACTAACTCG GAAGGCAGCC TATGTGAGAT ACTTCAATAG

ACAGAACTGA AACTAACTCG GAAGGCAGCC TATGTGAGAT ACTTCAATAG

ACAGAACTGA AACTGACTCG GAAGGCAGCC TATGTGAGAT ACTTCAATAG

ACAGAACTGA AGCTGACCCG GAAGGCAGCC TATGTGAGAT ACTTCAATAG

GAGGAACTGA AAATGACCCG GAGGTCAGCC TATATGAGGT TCTTCACTAG

GTGGAGCTGA AAATGACCCG GAAGGCGGCC TATATGAGGT TCTTCACTAG

ACAGAACTGA AACTGACCCG GAAAGCAGCC TACGTGAGAT ACTTCAACAG

ACAGAACTGA AACTCACCCG GAAGGCAGCC TATGTGAGAT ACCTCAATAG

ACAGAGCTGA AACTGACCCG GAAGGCAGCC CATGTGAGAT ACTTCAATAG

ACAGAACTGA GACTGACCCG GAAGGCTGCC TATGTGAGAT ACGTCAACAG

ACAGAACTGA AACTGACCCG GAAGGCAGCC TATGTGAGAT ACTTCAATAG

TCGGAACTGA GACTGACTCG GAAGGCAGCC TATGTGAGAT ACTTCAACAG

ATAGAGCTGA GACTGACCCG GAAGGCAGCC TATGTGAGAT ACTTCAACAG

---------- ---TGACCCG GAAGGCAGCC TATGTGAGAT ACTTCAACAG

ACTGAACTGA AATTGACCCG AAAAGCCGCA TATGTGAGAT ACTTCAATAG

ACGGAACTGA AGCTGACCCG AAAGGCTGCT TATATGAGGT ACTTCAATAG

CTCAGCCTTC TTCTTCTCAG GGTTCTTTGT GGTGTTTTTA TCTGTGCTTC

CTCAGCCTTC TTCTTCTCAG GGTTCTTTGT GGTGTTTTTA TCTGTGCTTC

CTCAGCCTTC TTCTTCTCAG GGTTCTTTGT GGTGTTTTTA TCTGTGCTTC

CTCAGCCTTC TTCTTCTCAG GGTTCTTTGT GGTGTTTTTA TCTGTGCTTC

CTCAGCCTTC TTCTTCTCAG GGTTCTTTGT GGTGTTTTTA TCTGTGCTTC

CTCAGCCTTC TTCTTCTCAG GGTTCTTTGT GGTGTTTTTA TCGGTCCTTC

CTCTGCCTTC TTCTTTTCAG GGTTTTTTGT AGTCTTTTTA TCTGTGCTTC

CTCTGCCTTC TTCTTTTCAG GGTTCTTTGT AGTCTTTCTA TCTGTGCTTC

CTCAGCCTTC TTCTTCTCAG GGTTCTTTGT GGTGTTTTTA TCTGTGCTTC

CTCAGCCTTC TTCTTCTCAG GGTTCTTTGT GGTATTTTTA TCTGTGCTTC

CTCAGCCTTC TTCTTCTCAG GGTTCTTTGT GGTGTCTTTA TCTGTGCTTC

CTCAGCATTC TTCTTCTCGG GGTTCTTTGT GGTGTTTTTA TCTGTGCTTC

CTCAGCTTTC TTCTTCTCAG GCTTTTTCGT AGTGTTTTTG TCCGTGCTTC

CTCAGCTTTC TTCTTCTCAG GGTTTTTTGT AGTGTTTTTA TCTGTGCTTC

CGCAGCCTTC TTCTTCTCAG GGTTCTTCGT GGTGTTTTTA TCTGTGCTGC

CTCAGCCTTC TTCTTCTCAG GGTTCTTCGT GGTGTTTCTG TCTGTGCTAC

CTCAGCCTTC TTCTTCTCAG GTTTCTTTGT TGTATTTCTA GCTGTGCTTC

CTCAGCCTTC TTCTTCTCGG GGTTCTTCGT GGTGTTCCTG TCGGTGCTAC

CCTATGCACT AATCAAAGGA ATCATCCTCC GGAAAATATT CACCACCATC

CCTATGCACT AATCAAAGGA ATCATCCTCC GGAAAATATT CACCACCATC

CCTATGCACT AATCAAAGGA ATCGTCCTCC GAAAAATATT CACAACCATA

CCTATGCACT AATCAAAGGA ATCGTCCTCC GAAAAATATT CACAACCATA

CATATGCACT AATCAAAGGA ATCATCCTCC GAAAAATCTT TACAACCATC

CTTATGCACT GATCAAGGGA ATAATCCTTC GGAAAATATT CACAACCATC

CCTACACAGT CATCAATGGA ATCGTCCTCC GAAAAATATT CACAACCATT

CCTACACAGT CATCAACGGA ATCGTCCTAC GAAAAATATT CACAACCATT

CTTACGCACT TACCAAAGGA ATCATCCTGC GCAAAATATT CACCACCATC

CTTATGCACT GCTCAAAGGA ATCATCCTTC GAAAAATATT CACAACCATC

CCTATGCACT GATCAAAACA ATCATCCTTC GCAAAATATT CACAACCATC

CCTATGCACT GATCAAAGGA ATCATCCTCC GAAAAATATT CACAACCATC

CCTACGCGTT GCTCAAAGGC ATTATCCTTC GAAAAATATT CACCACCATC

CCTATGCACT GATCAAAGGA ATCATCCTTC GCAAAATATT CACAACCATC

CCTACGCAAT GCTCAAAGGA ATCATCCTTC GAAAAATATT CACTACCATC

CTTACGCACT GCTCAAGGGA ATCATCCTTC GAAAAATATT CACCACTATT

CTTATGCCTT GATGAAAGGA ATTATCCTGC GAAAAATATT TACAACCATC

CCTCCATGCT GACCAAGGGA ATTGTCCTCC GCAAGATATT CACCACCATC

TCATTCTGCA TTGTTCTGCG CATGGCGGTC ACTCGGCAAT TTCCCTGGGC

TCATTCTGCA TTGTTCTGCG CATGGCGGTC ACTCGGCAAT TTCCCTGGGC

TCATTCTGCA TTGTTCTGCG CATGGCGGTC ACTCGGCAAT TTCCCTGGGC

TCATTCTGCA TTGTTCTGCG CATGGCGGTC ACTCGGCAAT TTCCCTGGGC

TCATTCTGCA TTGTTCTGCG CATGGCAGTC ACTCGACAAT TTCCCTGGGC

TCATTCTGCA TCGTTCTGCG CATGGCTGTC ACTCGGCAGT TCCCCTGGGC

TCATTCTGCA TTGTCCTGCG TATGTCAGTC ACACGGCAAT TCCCCACTGC

TCATTCTGCA TTGTCCTACG TATGTCAGTC ACACGGCAGT TCCCCACTGC

TCATTCTGCA TCGTTCTGCG CATGGCCGTT ACTCGGCAGT TCCCCTGGGC

TCATTCTGCA TCGTTCTGCG CATGGCAGTT ACTCGGCAGT TCCCCTGGGC

TCATTCTGCA TCGTTTTGCG CATGGCAGTC ACTCGGCAAT TTCCCTGGGC

TCGTTCTGCA TCGTCCTGCG CATGGCAGTC ACTCGGCAAT TCCCCTGGGC

TCATTCTGCA TTGTTCTGCG CATGACAGTC ACACGGCAGT TTCCTTGGGC

TCATTCTGCA TTGTTCTGCG CATGGCAGTC ACACGGCAAT TCCCCTGGGC

TCATTCTGCA TCGTTCTGCG CATGGCTGTC ACTCGGCAGT TCCCCTGGGC

TCATTCTGCA TCGTTCTGCG CATGTCTGTC ACTCGGCAGT TCCCCTGGGC

TCATTCTGTA TTGTTCTTCG AATGGCAGTA ACTCGGCAGT TTCCCTGGGC

TCGTTCTGCA TTGTACTGCG GATGGCGGTC ACCCGGCAGT TCCCCTGGGC

TGTACAAACA TGGTATGACT CTCTTGGAGC AATAAACAAA ATACAGGTAT

TGTACAAACA TGGTATGACT CTCTTGGAGC AATAAACAAA ATACAGGTAT

TGTACAAACA TGGTATGACT CTCTTGGAGC AATAAACAAA ATACAGGTAT

TGTACAAACA TGGTATGACT CTCTTGGAGC AATAAACAAA ATACAGGTAT

TGTACAAACA TGGTATGACT CTCTTGGAGC AATAAACAAA ATACAGGTAT

TGTGCAAACA TGGTATGACT CTCTTGGAGC AATAAACAAA ATACAGGTAT

TGTACAGATA TGGTATGATT CTTTGGGAAT GATAAGAAAA ATACAGGTAT

CGTACAGATA TGGTATGATT CTTTTGGAAT GATAAGAAAA ATACAGGTAT

TGTGCAAACA TGGTATGACT CGCTTGGAGC AATAAACAAA ATCCAGGTAT

TGTACAAACT TGGTATGATT CTCTGGGAGC AATAAACAAA ATACAGGTAT

TGTACAAACA TGGTATGACT CTCTTGGAGC AATAAACAAA ATACAGGTAT

TGTGCAAACG TGGTATGACT CTCTTGGAGC AATAAATAAA ATACAGGTAT

TGTGCAAACA TGGTACGACT CTCTTGGAGC CATCAACAAA ATACAGGTAT

TGTTCAAACA TGGTATGACT CACTTGGAGC AATAAACAAA ATACAGGTAT

TGTGCAAACG TGGTATGACT CGCTCGGAGC AATAAACAAA ATACAGGTAT

TGTGCAAACC TGGTATGACT CATTTGCAGC AATAAATAAA ATACAGGTAT

TGTACAAACC TGGTATGATT CTCTTGGAGC AATAAACAAA ATACAGGTAT

CGTGCAGATG TGGTACGACT CCATCGGAGC GATTTACAAG ATACAGGTAT

GGAATATAAC TTAACGACTA CAGAAGTAGT GATGGAGAAT GTAACAGCCT

GGAATATAAC TTAACGACTA CAGAAGTAGT GATGGAGAAT GTAACAGCCT

GGAATATAAC TTAACAACTA CAGAAGTAGT GATGGAGAAT GTAACAGCCT

GGAATATAAC TTAACAACTA CAGAAGTAGT GATGGAGAAT GTAACAGCCT

GGAATATAAT TTAACAACTA CAGAAGTAGT GATGGAGAAT GTAACAGCCT

GGAATATAAC TTAACAACTA CAGAGGTAGT GATGGAGAAC GTAACAGCCT

GGAGTATAAC TTAATGTTCA CAGGCCTAGT CATGGAGAAT GTAACAGCAT

GGAGTATAAC TTAATGACCA CAGGCATAAT CATGGAAAAT GTAACAGCAT

GGAATACAAC TTAACAACTA CAGAGGTCGT GATGGATAAT GTAACAGCCT

GGAATATAAC TTAACAACTA CAGATGTAGT GATGGAGAAT GTAACAGCCT

GGAATATAAC TTAACAACTA CGGAAGTAAT GATGGAGAAT GTAACAGCCT

GGAATATAAC TTAACAACCA CAGAGGTAGT GATGGAGAAT GTAACAGCCT

GGAGTATAAC TTAACAACTA CAGAAGTAGA GATGGAGAAT GCAACAGCTT

GGAGTATAAC TTAACAACTA CAGATGTAGT GATGGAGACT GTAACAGCCT

GGAATATAAC TTAACAACTA CAGATGTGGT GATGGAAAAT GTGACAGCCT

GGAATACAAC TTGACTACAA CAGATGTTGT GATGGAAAAC GTAACAGCCT

GGAATATAAC TTAACAACCA CAGAAGTTAT GATGGAAAAT ATAACAGCAT

GGAATACAAC TTAACAACTA CAGATGTTGT GATGGAAAAC GTAACAGCTT

TCTGGGAGGA GGTCAGAATT CTCTTCTTCA GTAATTTCTC ACTTCTTGGT

TCTGGGAGGA GGTCAGAATT CTCTTCTTCA GTAATTTCTC ACTTCTTGGT

TCTGGGAGGA GGTCAGAATT CTCTTCTTCA GTAATTTCTC ACTTCTTGGT

TCTGGGAGGA GGTCAGAAAT CTCTTCTTCA GTAATTTCTC ACTTCTTGGT

TCTGGGAGGA GGTCAGAATT CTCTTCTTCA GTAATTTCTC ACTTCTTGGT

TCTGGGAGGA GGTTAGAGTT CTCTTCTTCA GTAATTTAGC ACTTCTTGGT

TTTGGGAGGA GGTGAGAATT CTCAGTTTTA GTCATCTCTG TCTAGTGGGA

TTTGGGAGGA GGTGAG-ATT GTCAGTTTCA GTCATCTCTG CCTTGTGGGA

TCTGGGAAGA GGTCAGAACT CTCTTCTTCA GTAATTTCTC ACTTCTTGGT

TCTGGGAGGA GGTCAGAATT CTCTTCTTCA GTAACTT--- ACTTCTTGGT

TCTGGGAGGA GGTCAGAATT CTCTTCTTCA GCAACTTCTC ACTTCTTGGT

TCTGGGAGGA GGTCAGAATT CTCTTCTTCA GTAATTTCGC AATTCTTAGT

ACTGGGAAGA GGTCAGaatt CTCTTCTTCA GCAACTTTTC ACTTCTTGGT

TCTGGGAGGA GGTCAGACTT CTCTTCTTCA GTAACTTCTC ACTTCTTGGC

TCTGGGAGGA GGTCAGaatt CTCTTCTTCA GTAACTTCTC ACTTCTTGGT

TCTGGGAAGA GGTCAGA-CT CTCTTCTTCA GTAACTTCGC ACTACTCGGC

TCTGGGATGA GGTCAGGATT CTCTTCTTTA GCAACTTCTC ACTTCTTGGT

TCTGGGACGA GGTCAGGATT CTCATCTTTA GTAACATCTC GCTCCTCGGT

ACTCCTGTCC TGAAAGATAT TAATTTCAAG ATAGAAAGAG GACAGTTGTT

ACTCCTGTCC TGAAAGATAT TAATTTCAAG ATAGAAAGAG GACAGTTGTT

ACTCCTGTCC TGAAAGATAT TAATTTCAAG ATAGAAAGAG GACAGTTGTT

ACTCCTGTCC TGAAAGATAT TAATTTCAAG ATAGAAAGAG GACAGTTGTT

ACTCCTGTCC TGAAAGATAT TAATTTCAAG ATAGAAAGAG GACAGTTGTT

GCTCCTGTCC TAAAAGACAT CAATTTCAAG ATAGAAAGGG GACAGTTGTT

AATCCTGTGT TGAAAAACAT CAATTTGAAT ATAAAGAAAG GAGAGATGTT

AATCCTGTGC TGAAAAACAT CAATTTGAAT ATAGAGAAAG GAGAGATGTT

GCTCCTGTCT TGAAAGATAT CAGCTTCAAG ATAGAAAGAG GGCAGCTGTT

ACTCCTGTCC TGAAAGATAT CAGTTTCAAG ATAGAAAGAG GACAGTTGTT

ACTCCTGTCC TAAAAGACAT CAATTTCAAG ATAGAAAGAG GACAGTTGTT

ACTCCTGTGC TGAGAGGTAT CAATTTCAAG GTAGAAAGAG GACAGTTGTT

ACTCCTGTCC TGAAAGATAT CAATTTTAAA ATAGAAAGAG GACAATTGTT

ACTCCTGTCC TTAAAGATAT TAATTTCAAG ATAGAAAGAG GACAATTATT

GCTCCTGTCC TGAAGGATAT CAATTTCAAG ATAGAAAGAG GACAGTTATT

ACTCCTGTGC TCAGGGACAT CAATTTCAAG ATCGAGAAAG GACAGCTGTT

ACTCCTGTCC TTAAAAATAT CAGTTTCAAG ATAGAAAAGG GACAATTGTT

CCCCCTGTCC TCAAAGACAT CAGTTTCAAG ATAGAAAAAG GACAGTTGTT

GGCGGTTGCT GGATCCACTG GAGCAGGCAA GGTATTCCAG ACTTCACTTC

GGCGGTTGCT GGATCCACTG GAGCAGGCAA GGTATTCCAG ACTTCACTTC

GGCGGTTGCT GGATCCACTG GAGCAGGCAA GGTATTCCAG ACTTCACTTC

GGCGGTTGCT GGATCCACTG GAGCAGGCAA GGTATTCCAG ACTTCACTTC

GGCGGTTGCT GGATCTACTG GAGCAGGCAA GGTATTCCAG ACTTCACTTC

GGCGGTGGCT GGATCTACTG GAGCAGGCAA GGTATTCCAG ACTTCACTTC

GGCTATTACT GGATCTACTG GAGCAGGAAA GGTATTGTAG ACATCACTCC

GGCTATTACT GGATCTACTG GATCAGGAAA GGTATTGTAG ACATCACTCC

GGCGGTGGCT GGATCTACTG GTGCTGGAAA GGTATTCCAG ACTTCGCTTC

GGCAGTTGCT GGATCTACGG GAGCAGGCAA GGTATTCCAG ACTTCACTTC

GGCGGTTGCT GGATCTACTG GAGCAGGCAA GGTATTCCAG ACTTCACTCC

GGCTGTTGCG GGATCTACTG GAGCCGGCAA GGTATTCTAG ACTTCACTTC

GGCAGTTGCA GGATCTACTG GAGCGGGCAA GGTATTTCAG ACCTCTCTTC

GGCAGTTGCT GGATCTACTG GAGCAGGCAA GGTATTCCAG ACTTCCCTTT

GGCGGTTGCT GGATCTACTG GAGCAGGCAA GGTTTTCCAG ACTTCACTTC

GGCAGTTGCT GGGTCTACTG GAGCAGGCAA GGTTTTGCAG ACCTCACTTC

GGCTGTTGCT GGATCTACTG GGGCAGGCAA GGTATTTCAG ACTTCACTTT

GGCTGTAGCT GGATCTACTG GGGCAGGCAA GGTATTGCAG ACCTCACTGC

TAATGGTGAT TATGGGAGAA CTGGAGCCTT CAGAGGGTAA AATTAAGCAC

TAATGATGAT TATGGGAGAA CTGGAGCCTT CAGAGGGTAA AATTAAGCAC

TAATGATGAT TATGGGAGAA CTGGAGCCTT CAGAGGGTAA AATTAAGCAC

TAATGATGAT TATGGGAGAA CTGGAGCCTT CAGAGGGTAA AATTAAGCAC

TAATGATGAT TATGGGAGAA CTGGAGCCTT CAGAGGGTAA AATTAAGCAC

TAATGATGAT TATGGGAGAA CTGGAGCCTT CAGAGGGTAA AATTAAGCAC

TGATGCTGAT ACTGGGAGAA CTGGAAGCGT CAGAGGGAAT TATTAAGCAC

TGATGTTGAT TTTGGGAGAA CTGGAAGCTT CAGAGGGAAT TATTAAGCAC

TGATGATGAT TATGGGAGAG TTGGAGCCTT CAGAGGGTAA AATTAAGCAC

TCATGATGAT TATGGGAGAA TTGGAACCTT CAGAGGGTAA AATTAAGCAC

TAATGATGAT TATGGGAGAA CTGGAGCCTT CAGAGGGTAA AATTAAGCAC

TGATGATGAT TATGGGAGAA CTGGAACCTT CAGAGGGCAA AATTAAGCAC

TAATGATGAT TATGGGAGAA CTGGAGCCTT CAGAAGGTAA AATTAAGCAC

TAATGATGAT TATGGGAGAA CTAGAGCCCT CAGAGGGTAA AATTAAGCAC

TAATGATGAT TATGGGAGAA CTGGAGCCCT CAGAGGGTAA AATTAAGCAC

TGATGATGAT TATGGGAGAA CTGGAGCCCT CGGAAGGCAC AATCAGGCAC

TAATGATGAT CATGGGAGAA TTGGAACCTT CAGAGGGCAA AATTAAGCAC

TGATGATGAT CATGGGTGAG CTGGAGCCTT TGGAAGGCAA AATCAAGCAC

AGTGGAAGAA TTTCATTCTG TTCTCAGTTT TCCTGGATTA TGCCTGGCAC

AGTGGAAGAA TTTCATTCTG TTCTCAGTTT TCCTGGATTA TGCCTGGCAC

AGTGGAAGAA TTTCATTCTG TTCTCAGTTT TCCTGGATTA TGCCTGGCAC

AGTGGAAGAA TTTCATTCTG TTCTCAGTTT TCCTGGATTA TGCCTGGCAC

AGTGGAAGAA TTTCATTCTG CTCTCAGTTC TCCTGGATTA TGCCTGGAAC

AGTGGAAGGA TTTCATTCTG CTCTCAGTTT TCCTGGATCA TGCCTGGCAC

AGTGGAAGAG TTTCATTCTC CTCTCAAATT TCTTGGATTA TGCCGGGTAC

AGTGGAAGAG TTTCATTCTG CTCTCAATTT TCTTGGATTA TGCCGGGTAC

AGTGGAAGAA TATCATTCTG CTCTCAGTTT TCCTGGATCA TGCCTGGCAC

AGTGGAAGAA TTTCATTCTG CTCTCAGTAT TCCTGGATCA TGCCTGGAAC

AGTGGAAGAA TTTCATTCTG CTCTCAGTTT TCTTGGATTA TGCCTGGCAC

AGTGGAAGAA TTTCCTTCTG CTCTCAGTTT TCATGGATTA TGCCTGGCAC

AGTGGAAGAA TTTCATTCTG CCCTCAGTTT TCCTGGATCA TGCCTGGAAC

AGTGGGAGAA TTTCGTTCTG CTCTCAGTTT TCCTGGATCA TGCCTGGTAC

AGTGGAAGAA TTTCATTCTG TTCTCAGTTC TCCTGGATCA TGCCTGGCAC

AGTGGGAGAA TTTCCTTCTG CTCCCAGTTC TCCTGGATCA TGCCCGGCAC

AGCGGAAGGA TTTCCTTTTG TTCTCAGTTC TCTTGGATCA TGCCTGGTAC

AGCGGAAGGA TCTCCTTCTC TCCCCAATTC TCGTGGATCA TGCCAGGAAC

CATTAAAGAA AATATCATCT TTGGTGTTTC CTATGATGAA TATAGATACA

CATTAAAGAA AATATCATCT TTGGTGTTTC CTATGATGAA TATAGATACA

CATTAAAGAA AATATCATCT TTGGTGTTTC CTATGATGAA TACAGATACA

CATTAAAGAA AATATCATCT TTGGTGTTTC CTATGATGAA TACAGATACA

CATTAAAGAA AATATCATCT TTGGTGTTTC CTATGATGAA TACAGATATA

CATTAAAGAA AACATCATCT TTGGTGTTTC CTATGATGAG TATAGATATA

TATCAAAGAA AATATCATCT TTGGTGTTTC CTATGATGAG TACAGATATA

TATCAAAGAA AATATCATCT TTGGTGTTTC CTATGATGAG TACAGATATA

CATTAAAGAG AACATCATCT TCGGTGTTTC CTACGATGAG TATAGATATA

CATTAAAGAT AACATCATCT TTGGTGTTTC CTATGATGAA TATAGATATA

CATTAAAGAA AACATCATCT TTGGTGTTTC CTATGATGAG TATAGATATA

CATTAAAGAA AACATCATCT TTGGTGTTTC CTATGATGAG TATAGATATA

TATAAAAGAA AATATCATCT TTGGTGTTTC CTATGATGAA TACAGATATA

CATTAAAGAA AACATCATCT TTGGTGTTTG TTATGATGAG TATAGATACA

CATTAAAGAA AACATCATCT TTGGTGTTTC CTATGATGAG TATAGATATA

CATTAAAGAG AACATCGTCT TCGGGGTTTC CTATGACGAG TACAGATACA

TATTAAAGAA AACATTATTT TTGGTGTTTC TTATGATGAA TACCGATATA

CATCAAGGAG AACATCGTCT TCGGGGTTTC TTTCGATGAG TACCGATACA

GAAGCGTCAT CAAAGCATGC CAACTAGAAG AGGTAGGTTG AATTTGACCT

GAAGCGTCAT CAAAGCATGC CAACTAGAAG AGGTGGGTTG AATTTGACCT

GAAGTGTCAT CAAAGCATGC CAACTAGAAG AGGTAGGTTG AATTTGACCT

GAAGTGTCAT CAAAGCATGC CAACTAGAAG AGGTAGGTTG AATTTGACCT

GGAGTGTCAT CAAAGCATGC CAACTAGAAG AGGTAGGTTG AATCTGACCT

GGAGTGTTAT CAAAGCATGC CAACTAGAAG AGGTAGGTTG AATTTGACCT

AGAGTGTTGT CAAAGCTTGC CAACTACAGG AGGTATGTTG AATTTGACCT

AGAGTGTTGT CAAAGCTTGC CAACTACAGC AGGTAGGTTG AATTTGACCT

AGAGTGTCAT CAAAGCTTGC CAACTAGAAG AGGTAGGTTG AATTTGACCT

GAAGTGTCAT CAAAGCATGC CAACTAGAAG AGGTAGGTTG AATTTGACCC

GGAGTGTCAT CAAAGCATGC CAACTAGAGG AGGTAGGTTG ATTTTGACCT

GGAGTGTCAT CAAAGCATGC CAACTAGAAG AGGTAGGTTG ATTTTGACCT

AGAGTGTCAT CAAAGCATGT CAATTAGAAG AGGTAGGTTG AATTTGACCT

GGAGTGTCAT CAAAGCCTGC CAACTAGTGG AGGTAGGTTG AATTTGACCT

GGAGTGTCAT CAAAGCCTGC CAGCTAGAAG AGGTAGGTTG AATTTGACCT

GGAGTGTCGT CAAAGCCTGC CAACTCGAAG AGGTA----- ----------

GAAGTGTCAT CAAAGCCTGC CAATTAGAAG AGGTAGGTTA ATTTTGACAT

GGAGTGTCAT CAAGGCCTGC CAATTGGAGG AGGTAGGTTG ATTCTGACAT

TTACCACAAG CATTATTGTC AAGGTTGTTA TAAAGAGTAA TAAGCCAGCC

TTACCACAAG AATTATTGTC AAGGTTGTTA TAAAGAGTAA TAAGCCAGCC

TTACCACAAG CATTATTGTC AAGGTTGTTA TAAAGAGTAA CAAGCCAGCC

---------- ---------- ---------- ---------- ----------

TTACCACAAG CATTG-TGTC AAGGTTGTTA TAAAGAGTGA TAAGCCAGCC

--ACCACAAG CATCATTGTC AAGGTTGTTC CAAAGATTGA TAAGCCAGCC

TTCCCACAAG CATTGTTGTG AAGGTTGTTC TAAAAA-CGA TAAGCCAGCC

TTCCCACAAG CGTTGTTGTC AAGGTTGTTC TAAAAA-TGA TAAGCCAGCC

TCAGCACAAG CGTTCTTGTC AAGGTTGTTT TAAAAAGTGA TAAGCCACCC

TTACCACAAG CAGTGTTGTC AAGGTTGTTC TGAAGAGTGA TAATACAGGT

TTACCACAAG CAGTATTGTC AAGGTTGTTC TAAAGAGTGA TAAGATAGCT

TTACCACAAG CAGTATTGTC AAGGTTGTCC TAAGGAGTGA TAAGGCAGCC

TTGCCACAAG AAATATTGTC AAGGTTGTTA TGAAGAATGA CAAGGCAGCC

TTACCACAGG CAGTGTTGTC AAGGTTGTTC TAAAGAGTAA TAAGCCAGCC

TTACCACAAG CAGTATTGTC AAGGTTGTTC TAAAGAGTGA TAACCCAGCT

TTGCCACAAG CAGTATCATC AACGTTGTAC TAAAGAGTGA TTAGCCAACC

TTATTCCAAG TAATGTTGTC AAAGTTTAAT CAGAGCTTGA TAAGACAACT

TTGTGGCCAG TGATGTTGTC ATGGCCTTTC AAAAGGCTGA TAAGCCAGTC

ATAAAAAAGG GTTTATGGTA TTTTCCTATC TACAAAGTCA CACTCAAATG

ATAAAAAAGG GCTTATGGTA TTTTCCTATC TACAAAGTCA CACTCAAATG

ACAGAAAAAG GTTTATGGTA TTTTCCTATC TACAAAGTCA CACTCAAATG

---------- ---------- ---------- ---------- ----------

ATAAAAAAAG TTTTATGGTA TTTTCCTATC TGCAAAGTCA CACTCAAATG

ATAAAACAGG GTTTATGGTA TTTTCCTATC TACAAAGTCA CACTCAAATG

ATATA--AAA GTTTATGGTA TTTTCCTATC TTCAAAGCTA CACTCAAATA

ATATA--AAA GTTTATGGTA TTTTCCTATC TTCAAAGCTA CACTCAAATA

ATAAACCAAG GTTTAT---- ---------- -----AGTCA CACTCAAATG

ATAAAACAAG GTTTATGGTA TTTTCCTATC TACAAAGTCA CACTCGAATG

GTAAAACAAG GTTTATGATA TTTTCCTATC TACAAAGCCA CACTCAAATG

ATAAAACAAG GCTGACACTA TGTTGCTATC TACAGAGTCA CACACAAATG

ATAAAACAAA GTTTATGATA TTTTCCTATC TAC-AAGTCA CACTCAAATG

ATAAAACAAG GTTTATGGTC TTTTCCTATC TACAAAGTCA CACTCAAATG

ATAAAACAAG GTTTATGGTA TTTTCCTATC TACAAAGTTA CACACAAATG

ATAAAAAAAG GTTTATGGGC TTTCCCTATC AGCAAAATCA CACTTAAATG

ATAAAAGAAG ATTTATGGTC TTTTTCTATC TCTAAAGTCA CACGAGCATG

ATAAACCAAG ATTTACAGTT TTTTTCTGTC TGCAAAGTCA CCCTAAAGTG

TACTCAGTAA ATATTGCAAA ATTACACATT AATAATAAAG TAAGTTAATC

TACTCAGTAA ATATTGCAAA ATTACACATT AATAATAAAG TAAGTTAATC

TACTCAGTAA ATATTGCAAA ATTACACATT AATAAT---- TAAGTTAATC

---------- ---------- ---------- ---------- ----------

TACTCAGTAA ATATTGCAAA ATTACACATT AATAATAAAG TAAATTAATC

TTCTCAGTAA ATATTGCAAA ATTTCACATT AATAATAAAA CAAGTTAATC

TACTCAGCAA ATATTGCTCA ATTACACATT AATGATAAAA TAAGTTAATC

AACTCAGCAA ATATTGCTCA ATTACACATT AATGATAAAG TAAGTTAATC

TACTCTGCAA ATATTGCAAA ATTACACATT AATAATAAAC TAAGTTAATC

TACTCAGCAA ATATTGCATA ATTACACATA AATAATAAAA TAAGTTAATC

TACTCAGCAA ATATTGCAAA ATTGCA-ATT AAT-----AA TAAGTTAATC

TGCTCAGCAA ATATTGCACA ATCACACATT AAT-----AA TAAGTTAATC

TACTCAGCAA ATATTGC--- --------TT AATAATATAA TAAGTTAATC

TGCTCAGCAA ATATTGCAAA ATTACACAGT AATAATAAAA TAAGTTAATC

TGCTCAGCAA ATATTGCAAA ATTACACATT ---------A TACGTTAATC

TGCTCAGCAA ATATTGCAAA ATTACCCATT AGTAATAAAA CAAGTTAATC

TGCTTAGCAA ATATTGCAAA ATTGCACATT AGTAATAAAA TGAGTTAATT

TGCCCGGTAA ATATTGCAAA ATTATCCGTT AGCAGTAAAG TAATATTATC

TTAAAACCAA CACCCTTAAG ATAAATATAT TACCAGATTT GAGCGCTCTT

TTAAAACCAA CACCCTTAAG ATAAATATAT TACCAGATTT GAGTGCTCTT

TTAAAACCAA CACCCTTAAG ATAAATATGT TACCAGATTT CAGCACCCTT

--AAAACCAA CACCCTTAAG ATAAATATGT TACCAGATTT CAGCACCCTT

TTAAAACCAA CATCCTTAAG ATGAATATAT TACCATATTT GAGTGCCCTT

TTAAGACCAA CACCCTTAAG ATAAATATAT TACCAGATTT GAGTGCCCTT

TT-AAACCAA CACCCTTAAG ATAAATATAT TACCAGATTT GAGTATCCTT

TT-AAACCAA CACCCTTAAG ATAAATATAT TACCAGATTT GAGTATCCTT

TTAAAACCAA CACCCCTAAG ATAAA----- ---------- ----------

TTAAAACCAA CACCCTTAAG ATAAATATAG CACCAGATTG GAGTGCCTTT

TTAAAACTAA CACCCTTAAG ATAAATATAT TACCAGATTT GGGTGCCCTT

TTAAAACCAA CACCCTTAAG ATAAATATAT TACCAGGTTT GAGTGCCCTT

TTAAAACCAA CACCCT-AAA ATAAATATAT TACCAGATTA AAGTGCTCTT

TTAAAACCAA CACCCTTAAG CGAAATCTAT TACCAGATTT GAGAGCCCTT

TTAAAAACAG CAGCCTTAAG ATAAATATAG CACCAGTTTT GAGAGCCCTT

TT-------- ---------- ---------- ---------- ----------

TTACAGCCAA CACCTTTAAA ATAAATATAT TACCTCATGT GATGTTTCTT

CTAGAACTGC CAAGGTTAAG ATAGTTACAT CACCTTGTTT GAGAGTCTCT

AGTAATCAGC AAAGATAAAT GTTTAAAAAA TTTTATGTTA AATCAAATAG

AGTAATCAGC AAAGATAAAT GTTTAAAAAA TTTTATGTTA AATCAAATAG

AGTAATCAGC AAAGATAAAT GTTTAAAAAA TTTTATGTTA AATCAGTTAG

AGTAATCAGC AAAGATAAAT GTTTAAAAAA TTTTATGTTA AATCAGTTAG

AGTAATGAGC AAAGATAAAT CTTTATAAAA TTTTATGTTA AA-----TAG

AGTAGTCAGC AAAGATAAAT GTTTAAAAAA CTTTATGTTA AATCAAATAG

AGTAGTCAGC AAAGGTCAAT GTTTAAAAAA CTTTGTTTTA AATCAAAC--

AGTAGTCAGC AAAGGTCAAT GTTTAAAAAA CTTTGTTTTA AATCAAAC--

---------- ---------- ---TAAAAAA CTTTATGTTA AATCAAATAG

AGTAGTCAGC AAAGATAAAT GTTTAAAAAA TTTTATGTTA AATCAAATAG

CGTAATCAGC AAAGATCAAT ATTTAAAAAA TTTTATGTTT AATCAAACAG

AGTAATCAGC AAAGATAAAT GTTTAAAAAA TTTTATGTTT AATCAAATAG

AGTAATCAGC AAAGATAAGT GCTAAAAAAC TTTTATGTTA A-TCAAATAG

AGTAATCAGC AAAGATAAAT GTTTAAAAAA TTTTATGGTA AATCAAATAC

AGTAATCAGC AAAGATAAAT GTTTAAA--- TTTTATGTTA AATCAAATAG

---------- ---------- ---TAAACAA TTTTATGATA GACCTAATAG

AGGAATCAAC AAAGACAAAT GTTTAAAAGA Gtttatttaa aataaaataa

AGCCATCACC AAACATAAAT GTTTTAAAAA TTTTATGACA AATCAAATAG

AGAAAGCCAA ACACTAATAA TTAATAGGAC ATCTCCAAGT TTGCAGAGAA

AGAAAGCCAA ACACTAATAA TTAATAGGAC ATCTCCAAGT TTGCAGAGAA

AGAAAGTCAA ACACTAATAA CTAATAGGAC ATCTCCAAGT TTGCAGAGAA

AGAAAGTCAA ACACTAATAA CTAATAGGAC ATCTCCAAGT TTGCAGAGAA

AGAAAGCCAA ACACTAA--- -TAATAGGAC ATCTCCAAGT TTGCAGAGAA

AGAAAGCCAA ACAC----AA ATAATAGGAT ATCTCCAAGT TTGCCGAGAA

AGAATGTCAA GTGCTCATAA ATAATAGGAC ATCACCAAGT TTGCAGAACA

AGAATGTTAA GTACTCATAA ATAATAGGAC ATCACCAAGT TTGCAGAACA

AGAAAGCCAA ATACCAGTAA ATAATAGGAC ATCTCCAAGT TTACAGAGAA

A---AGCTAA ATACTAATAA ATAATAGGAC ATCTCCAAGT TTGCAGAGAA

AAATAGCCAA ACACTAATAA ATAATAGGAC ATTTCCAAGT TTGCAGAGAA

AAAAAGCCAA ACACTAATAA ATAATAGGAC ATCTCCAAGT TTGCAGAGAA

AAAAAAATAA ACATTAATAA ATAATAGGAC ATCTCCAAAT TTGCTGAGAA

AGAAAGCCAA ACACTAATAA ATAATAGGAC ATCTCCAAGT TTGCAGAGAA

AGAAAGCCAA ACACTAATAA ATAACAGGAC ATTTCCAAGT TTGCAGAGAA

AGAAAGTCAA ACACTAATAA A--------- ---------- ----------

aaaaaGACAA AAAGTAATAA ATAATAGGAC ATATCAAAGT TTGCGGAAAA

AGAGAGTCAA GTACTAATAA ATTACAGGAA ATCTCCAAGT TTGCAGAAAA

AGACAATATA GTTCTTGGAG AAGGTGGAAT CACACTGAGT GGAGGTCAAC

AGACAATATA GTTCTTGGAG AAGGTGGAAT CACACTGAGT GGAGGTCAAC

AGACAATATA GTTCTTGGAG AAGGTGGAAT CACACTGAGT GGAGGTCAAC

AGACAATATA GTTCTTGGAG AAGGTGGAAT CACACTGAGT GGAGGTCAAC

AGACAATATA GTTCTTGGAG AAGGTGGAAT CACATTGAGT GGAGGTCAAC

AGACAACATA GTTCTTGGAG AAGGCGGAAT CACACTGAGT GGAGGTCAGC

AGACAACACA GTTCTTGGAG AAGGTGGAGT CACACTCAGT GGAGGTCAAC

AGACAACACA GTTCTTGGAG AAGGTGGAGT CACACTGAGT GGAGGTCAGC

AGACAATACA GTTCTTGGAG AAGGTGGAAT TACTCTGAGT GGAGGTCAAC

AGACAACGTA GTTCTCGGAG AAGGTGGAAT CACACTGAGT GGAGGTCAGC

GGACAACATC GTTCTTGGAG AAGGTGGCGT CACGCTGAGC GGAGGGCAAA

AGACAATATA GTTCTTGGAG AAGGTGGGAT CACACTGAGT GGAGGGCAGC

AGACAACATA GTTCTTGGAG AAGGTGGAAT CACCCTGAGT GGAGGTCAGC

AGACAATACA GTTCTTGGAG AAGGTGGGAT CACATTGAGT GGAGGTCAAC

AGACAATATA GTTCTTGGAG AGGGTGGAAT CACACTGAGT GGAGGGCAAC

---------- ---------- ---------- ---------- ----------

AGACAACACA GTTCTTGGAG AAGGTGGAAT CACTCTGAGT GGAGGTCAAC

AGATAATACA GTCCTGGGGG AAGGTGGCAT CACCTTGAGT GGAGGTCAGC

GAGCAAGAAT TTCTTTAGCA AGGTATTTTC TTTTTAGAGC AGTATACAAA

GAGCAAGAAT TTCTTTAGCA AGGTATTTTC TTTTTAGAGC AGTATACAAA

GAGCAAGAAT TTCTTTAGCA AGGTATTTTC TTTTTAGAGC AGTATACAAA

GAGCAAGAAT TTCTTTAGCA AGGTATTTTC TTTTTAGAGC AGTATACAAA

GAGCAAGAAT TTCTTTAGCA AGGTATTTTC CTTTTAGAGC TGTATACAAA

GAGCAAGAAT CTCTTTAGCA AGGTATTTTC TTTTTAGAGC AGTATATAAA

GTGCAAGAAT TTCTTTAGCA AGGTATTCTG TTTGTAGAGC AGTATATAAA

GTGCAAGGAT TTCTTTAGCA AGGTATTTTG TTTATAGAGC AGTATATAAA

GAGCAAGAAT TTCTTTAGCA AGGTATTTTC ATTTTAGAGC AGTATACAAA

GAGCGAGAAT TTCTTTAGCA AGGTATTTTT T----AGAGC AGTATACAAA

GAGCAAGAAT TTCCCTAGCA AGGTATTTTC TTTTCAGAGC AGTATATAAA

GAGCAAGAAT TTCTTTAGCA AGGTATTTTC TTTTTAGAGC AGTATATAAA

GAGCCAGAAT TTCTTTAGCA AGGTACCTTT TTTTTAGAGC AGTATACAAA

GAGCAAGAAT TTCTTTAGCA AGGTATTTTC TTTTTAGAGC AGTATACAAA

GAGCCAGAAT CTCGTTAGCA AGGTATTTTC TTTTTAGGGC AGTGTACAAA

---------- ---------- ---------- ---------- ----------

GAGCAAGAAT TTCTTTAGCA AGGTATTTCC TTTTTAGAGC AATATACAAA

GGGCCAGAAT TTCATTAGCC AGGTATTTTT CTTTCAGAGC AGTGTACAAA

GATGCTGATT TGTATTTATT AGACTCTCCT TTTGGATACC TAGATGTTTT

GATGCTGATT TGTATTTATT AGACTCTCCT TTTGGATACC TAGATGTTTT

GATGCTGATT TGTATTTATT AGACTCTCCT TTTGGATACC TAGATGTTTT

GATGCTGATT TGTATTTATT AGACTCTCCT TTTGGATACC TAGATGTTTT

GATGCTGATT TGTATTTATT AGACTCTCCT TTTGGATACC TAGATGTTTT

GATGCTGACT TGTACCTTTT AGACTCTCCT TTTGGATACT TAGATGTTTT

GATGCTGATT TGTACCTGTT AGATTCCCCC TTCGGATATC TAGATGTTTT

GATGCTGATT TGTACCTATT AGATTCCCCT TTTGGATATC TAGATGTTTT

GATGCTGATT TGTACCTATT GGACTCTCCT TTTGGATATC TAGATGTCTT

GATGCTGATT TGTACCTATT AGACTCTCCT TTTGGATATC TAGATGTTTT

GATGCTGATT TATATCTCTT AGACTCTCCT TTTGGATACC TGGATGTTTT

GATGCTGATT TGTACCTATT AGACTCTCCT TTTGGATACC TAGATGTTTT

GATGCTGATC TGTACCTATT GGACTCTCCT TTTGGTTACC TAGATGTTTT

GACGCTGATT TGTACCTCTT AGACTCTCCT TTTGGATACC TAGATGTTTT

GATGCTGATT TGTACCTATT AGACTCTCCT TTTGGATACC TAGATGTTTT

---------- ---------- ---------- ---------- ----------

GATGCTGATT TATACCTCTT AGATTCCCCA TTTGGCTATT TAGATATGTT

GATGCTGACT TATATCTCCT AGACTCTCCT TTTGGCTACT TGGATGTGCT

AACAGAAAAA GAAATATTTG AAAGGTATGT TTTTTTATAT CTTAAAGCTG

AACAGAAAAA GAAATATTTG AAAGGTATGT TTTTTTATAT CTTAAAGCTG

AACAGAAAAA GAAATATTTG AAAGGTATGT TtttttGTAT CTTAAAGCTG

AACAGAAAAA GAAATATTTG AAAGGTATGT T--------- ----------

AACAGAAAAA GAAATATTTG AAAGGTATGT Ttttttatat CTTAAAGCTG

AACAGAAAAA GAAATATTTG AAAGGTACGT TTTTTTATGT CCTCTAGCTG

AACTGAAGAA CAAATATTTG AAAGGTATGT TTTTTTATAT TCCAAAGTTG

TACTGAAGAA CAAGTATTTG AAAGGTATGT TTTTTTATAT TCCAAAGCTG

AACAGAAAAA GAAATATTTG AGAGGTATGT TTTTTTATAT CTTAAAGCTG

AACAGAGAAA GAAATATTTG AAAGGTATGT Ttttttatat CTTAAAGCTG

AACAGAAAAA GAAATATTTG AAAGGTATGT TTTTTTATAT CTTAAAGCTG

AACAGAAAAA GAAATATTTG AAAGGTATGT TTTTTTATTT CTTAAAGGTG

AACAGAAAAA GAAATATTTG AAAGGTAGGT -TTTTTATAT CTTAAAGCTG

AACAGAAAAA GAAATATTTG AAAGGTATGG TTTTTTCTAT CTTAAAGCTG

AACAGAAAAA GAAATTTTTG AAAGGTATGT TTTTTTCTGT CTCAAAGCTG

---------- ---------- ---------- -TTTTTCTGT CTTACAGCTG

AACTGAAAAG GAGATATTTG AAAGGTAAGT TTTTTTCTGT TTTGAAGCTG

TACGGAAAAG GAAATATTTG AAAGGTATGT TTTTTTCCTT CCTCAAGCTG

TGTCTGTAAA CTGATGGCTA ACAAAACTAG GATTTTGGTC ACTTCTAAAA

TGTCTGTAAA CTGATGGCTA ACAAAACTAG GATTTTGGTC ACTTCTAAAA

TGTCTGTAAA CTGATGGCTA ACAAAACTAG GATTCTGGTC ACTTCTAAAA

---------- ---------- ---------- ---------- ----------

TGTCTGTAAA CTGATGGCTA ACAAAACTAG GATTCTGGTC ACTTCCAAAA

TGTCTGTAAA CTGATGGCTA ACAAAACTAG GATTTTGGTA ACTTCTAAAA

TGTCTGTAAA TTGATGGCCA GCAAAACTAG GATTCTGGTC ACATCTAAAA

TGTTTGTAAA TTGATGGCCA ACAAAACTAG GATTTTGGTT ACATCTAAAA

TGTCTGTAAA TTGATGGCCA ACAAAACTAG AATTTTGGTC ACTTCTAAAA

TATCTGTAAA TTGATGGCTA ACAAAACTAG GATTTTGGTC ACTTCTAAAA

TGTCTGTAAA TTGATGGCTA ACAAAACTAG GATCTTGGTC ACTTCTAAAA

TGTCTGTAAA CTGATGGCTA ACAAAACTAG GATTTTAGTC ACTTCTAAAA

TGTCTGTAAA TTGATGGCTA ACAAAACTAG GATTTTGGTC ACTTCTAAAA

TGTCTGTAAA TTGATGGCTA ACAAAACTAG GATTTTGGTT ACTTCCAAAA

TGTCTGTAAA TTGATGGCGA ACAAAACTAG AATTTTGGTC ACTTCTAAAA

TGTTTGTAAA TTGATGGCGA ACAAAACTAG GATTTTGGTC ACTTCTAAAA

TGTTTGCAAG CTGATGGCAA ACAAGACCAG GATATTGATC ACTTCTAAAA

TGTCTGCAAG CTTATGGCCA ATAAAACGAG GATCCTCGTC ACTTCCAAAA

TGGAACATTT AAAGAAAGCT GACAAAATAT TAATTTTGCA TGAAGGTAGC

TGGAACATTT AAAGAAAGCT GACAAAATAT TAATTTTGCA TGAAGGTAGC

TGGAACATTT AAAGAAAGCT GACAAAATAT TAATTTTACA TGAAGGTAGC

---------- ---------- ---------- ---------- ----------

TGGAACATTT AAAGAAAGCT GACAAAATAC TAATTTTACA TGAAGGTAGC

TGGAACATTT AAAGAAAGCT GACAAAATAT TAATTTTACA TGAAGGTAGC

TGGAGCAGTT AAAGAAAGCT GACAAGATAC TCATTTTGCA TGAGGGCAGC

TGGAACACTT AAGGAAAGCT GACAAAATAC TAATTTTGCA TCAGGGCAGT

TGGAACATTT AAAGAAAGCT GACAAAATAT TAATTTTACA TGAAGGCAGC

TGGAGCACTT AAAGAAAGCT GACAAAATAT TAATTTTACA TGAAGGTAGC

TGGAACATTT AAAGAAAGCT GACAAAGTCT TAATTTTACA TGAAGGTAGC

TGGAACATTT AAAGAAAGCT GACAAAATAT TAATTTTACA TGAAGGTAGC

TGGAGCATTT AAAGAAAGCC GACAAAATAT TAATTTTACA TGAAGGGAGC

TGGAACATTT AAAGAAAGCT GACAAAATAC TAATTTTACA TGAAGGTAGC

TGGAACATTT AAAGAAAGCT GACAAAATAT TAATTTTACA TGAAGGTAGC

TGGAACATTT AAGGAAAGCC GACAAAATAC TAATTTTGCA TGAAGGTAGC

TGGAACATTT AAAGAAAGCT GACAAGATAT TAATATTACA TGAAGGCAGC

TGGAACACTT GAAGAAAGCC GACAAAATCT TAATTCTGCA CGAAGGCAGT

AGCTATTTTT ATGGGACATT TTCAGAACTC CAAAATCTAC AGCCAGACTT

AGCTATTTTT ATGGGACATT TTCAGAACTC CAAAATCTAC GGCCAGACTT

AGCTATTTTT ATGGAACATT TTCAGAACTC CAAAATCTAC GGCCAGACTT

---------- ---------- ---------- ---------- ----------

AGCTATTTTT ATGGGACATT TTCAGAACTA CAAAATCTAC GGCCAGACTT

AGCTATTTTT ATGGCACATT TTCTGAACTA CAAAATCTAC GGCCGGACTT

AGCTATTTTT ATGGGACGTT TTCTGAACTA CAAAGTCTAC GTCCAGACTT

AGCTATTTTT ATGGGACATT TTCTGAGCTA CAAAGTCTAC GTCCAGACTT

AGCTATTTTT ATGGTACTTT TTCTGAACTC CAAAGTCTAC GGCCGGATTT

ATCTATTTTT ATGGGACATT TTCTGAATTA CAAAATCAGC GGCCCGACTT

TGCTATTTTT ATGGGACATT TTCTGAATTA CAAAGTCTCC GGCCCGACTT

AGCTATTTTT ATGGGACGTT TTCTGAATTA CTAAATCTAC GGCCTGACTT

AGCTATTTTT ATGGAACGTT TTCTGAACTG CAAAACCTGC GGCCAGACTT

AGCTATTTTT ATGGAACATT TTCTGAACTA CAAAATCTAC GGCCAGACTT

AGCTATTTTT ATGGGACATT TTCTGAACTA CAAACTCTAC GGCCAGACTT

AGTTATTTTT ATGGGACATT TTCTGAACTA CAAAACCTGC GGCCAGACTT

TGCTATTTCT ATGGAGCCTT TTCTGATCTT CAAAATATGC GTCCTGACTT

TGCTATTTCT ACGGCGCGTT TTCAGAACTT CAGAACCTGC GGCCAGACTT

TAGCTCAAAA CTCATGGGAT GTGATTCTTT CGACCAATTT AGTGCAGAAA

TAGCTCAAAA CTCATGGGAT GTGATTCTTT CGACCAATTT AGTGCAGAAA

TAGCTCAAAA CTCATGGGAT ATGATTCTTT CGACCAATTT AGTGCAGAAA

---------- ---------- ---------- ---------- ----------

TAGCTCAAAA CTCATGGGAT ATGATTCTTT TGACCAATTT AGTTCAGAAA

TAGCTCAAAG CTCATGGGAT TTGATTCTTT CGACCAGTTT AGTGCAGAAA

CAGTTCCAAA CTCATGGGGT ATGATACTTT TGACCAGTTT ACTGAGGAAA

CAGTTCGAAA CTCATGGGGT ATGATACTTT TGACCAGTTT ACTGAGGAAA

TAGCTCAAAG CTCATGGGAT ATGATTCTTT TGACCAGTTT AGTGCAGAAA

CAGCTCGAAG CTCATGGGAT GTGACACTTT CGACCAGTTT ACTGCAGAAC

CAGCTCGAAG CTCATGGGCT ATGATTCTTT TGACCAGTTC AGTCCAGAAA

TAGCTCAAAG CTCATGGGAT ATGATTCTTT CGACCAGTTT AGTGCAGAAA

CAGCTCAAAG CTCATGGGAT ATGATTCTTT TGATCAGTTT AGTGCAGAAA

CAGCTCAAAG CTCATGGGAT ATGATTCTTT TGACCAGTTT AGTGCAGAAA

CAGCTCGGAG CTCATGGGAT ATGATTCTTT CGATCAGTTT AGTGCAGAAA

CAGCTCAAAG CTCATGGGCT ATGATTCTTT CGACCACTTC AGCGCAGACA

TAGCTCAAAA CTCATGGGAT ATGATTCTTT TGACCAATTC AGTGCAGAAA

CAGCTCCAAG CTCATGGGCT ACGACTCCTT CGACCAGTTC AGCGCCGAAA

GAAGAAATTC AATCCTAACT GAGACCTTAC ACCGTTTCTC ATTAGAAGGA

GAAGAAATTC AATCCTAACT GAGACCTTAC GCCGTTTCTC ATTAGAAGGA

GAAGAAATTC AATCCTAACT GAGACCTTGC GCCGTTTCTC ATTAGAAGGA

---------- ---------- ---------- ---------- ----------

GAAGAAATTC AATCCTCACT GAGACCTTAC GCCGTTTCTC TTTAGAAGGA

GAAGAAATTC CATCCTCACT GAGACTCTGC GCCGTTTCTC ATTAGAAGGA

GAAGGAGTTC AATTCTAACT GAGACCTTAC GCAGGTTCTC AGTGGA---C

GAAGAAGTTC AATTCTAACT GAGACCTTAC GCAGGTTCTC AGTAGA---C

GAAGAAATTC AATCCTAACT GAGACCTTAC GGCGTTTCTC ATTAGAAGGA

GAAGAAATTC AATCATAACT GAGACTTTAC GGCGTTTCTC ATTAGAAGGA

GAAGAAACTC CATCATCACT GAGACTTTAC GGCGTTTCTC CTTAGAAGGA

GAAGAAATTC AATCTTAACT GAGACTTTAC GGCGTTTCTC ATTTGAAGGA

GAAGAAATTC CATCCTAACG GAGACTTTGC GGCGTTTCTC ACTGGAAGGG

GAAGAAATTC AATTATAACT GAAACCCTGC GGCGTTTTTC ATTAGAAGGA

GAAGAAATTC AATCCTAACG GAGACCTTAC GGCGTTTCTC ATTAGAAGGA

GAAGAAACTC CATCATCACG GAGACCTTGC GGCGCTTCTC TCTAGAAGGA

GAAGAAGTTC AATCCTAACA GAAACCCTGA GACGTTTCTC AATAGAAGGT

GGAGAAACTC CATCCTGACG GAAACCCTGC GGCGTTTCTC CCTGGACGGC

GAGAACTGGA TATATATTCA AGAAGGTTAT CTCAAGAAAC TGGCTTGGAA

GAGAACTGGA TATATATTCA AGAAGGTTAT CTCAAGAAAC TGGCTTGGAA

GAGAACTGGA TATATATTCA AGAAGGTTAT CTCAAGAAAC TGGCTTGGAA

---------- ---------- ---------- ---------- ----------

GAGAGCTGGA TATATATTCA AGAAGGTTAT CTCAAGAAAC TGGCTTGGAA

GAGAAATGGA TATATATTCA AGACGGTTAT CTCAGGAAAG TAGCTTGGAA

GAGAAGAGGA TATATATTCA AGGCGATTAT CGCAAGATAG CACACTGAAC

GAGAAGTGGA TGTATATTCA AGGAGATTAT CGCAAGATAG CACACTGAAC

GAGAAATGGA CATATATTCA AGACGGTTAT CTCAAGAAAG TGGCTTGGAA

GAGAAATAGA TATATATTCA AGACGGTTAT CTCAAGATAC TGGCTTGGAA

GAGAAATGGA TATATATTCC AGACGGTTAT CTCAAGACAG TGGCTTGGAA

GAGAAATGGA TATATATTCA AGACGGTTAT CTCAAGACAA TGGCTTGGAA

GAGAGATGGA TATATACTCA AGAAGGTTAT CTCAAGACAG CATCTTGGAA

GAGAAATGGA CATATATTCA AGACGATTAT CTCAAGACAG TGGTTTGGAA

GAGAAATGGA TATATATTCA AGAAGATTAT CTCAAGACAG TGGCTTGGAA

GAGAGATGGA CATATATTCA AGAAGACTCT CGCAAGACAG CGGCTTAGAA

GAGAAATTGA TATTTACTCC CGACGTTTAT CTCAAGATAG TGGCATGGAT

GAGAGATGGA TATCTATTCC CGGCGCCTCT CAAAAGACAG CGGATTGGAA

ATAAGTGAAG AAATTAACGA AGAAGACTTA AAGGTAGTGA CTACATGGAA

ATAAGTGAAG AAATTAACGA AGAAGACTTA AAGGTAGTGA CTACATGGAA

ATAAGTGAAG AAATTAATGA AGAAGACTTA AAGGTAGTGA CTACATGGAA

---------- ---------- ---------- ---------- ----------

ATAAGTGAAG AAATTAATGA AGAAGACTTA AAGGTCGTGA CTACATGGAA

ATAAGTGAAG AAATTAATGA AGAATATTTA AAGGTAGTGA CTACCTGGAA

ATCACCGAAG AAATTAATGA AGAAGATTTA AAGGTAGTGA CAACATGGAA

ATCACTGAAG AAATTAACGA AGAAGATTTA AAGGTAGTGA CAACATGGAA

ATTAGTGAAG AGATTAATGA AGAAGACTTA AAGGTAGTGA CCACATGGAA

ATAAGTGAAG AAATTAATGA AGAAGATTTA AGGGTGGTGA CTACATGGAA

ATAAGTGAAG AAATTAATGA AGAGGATTTA AAGGTAGTGA CTACATGGAA

ATAAGTGAAG AAATAAATGA AGAAGATTTA AAGGTGGTGA CTACGTGGAA

ATTAGCGAAG AAATCAATGA GGAAGACCTA AAGGTAGTGA CCACATGGAA

ATAAGTGAAG AAATTAATGA AGAAGATTTA AAGGCAGTGA CCACATGGAA

ATAAGTGAAG AAATTAATGA AGAAGATTTA AAGGTAGTGA CCACATGGAA

ATAAGCGAAG AAATTAATGA AGAGGATCTA AAGGTAGTGA CCACGTGGAA

ATAAGTGATG AAATTAATGA AGAAGATTTA AAGGTAGTTA CCACATGGAA

ATCAGCGAGG AGATTAACGA GGAAGATTTA AAGGTAGTGA CCACATGGAA

CACATACCTT CGATATATTA CTGTCCACAA GAGCTTAATT TTTGTGCTAA

CACATACCTT CGATATATTA CTGTCCACAA GAGCTTAATT TTTGTGCTAA

CACATACCTT CGATATATTA CTGTCCACAA GAGCTTAATT TTTGTGCTAA

---------- ---------- ---------- ---------- ----------

CACATACCTT CGATACATTA CTCTCCACAA GAGCTTAATT TTTGTGCTAA

CACATACCTT AGATACCTAG CTGTCAACAA GAGCTTAAGT TTGGTACTAA

CACATACCTT CGATATTTCA CGCTCCACAG AGGCTTATTT GCAGTGCTGA

CACATACCTA CGATATTTTA CTCTCCATAA AGGCTTACTG CTAGTGCTGA

TACATACCTT CGGTATATTA CTGTCCACAG GAGCTTGATT TTTGTGCTAA

CACATACCTT CGATATATTA CTGTCCACAA GAGCTTAATG TTTGTGCTAA

CACATACCTT CGATATATTA CTGTCCACAA GAACTTAATT TTTGTGCTAA

CACATACCTT AGATATATTA CTGTCCACAA GAACTTAATT TTTGTGCTCA

CACATACCTT CGATATATTA CTGTTCATAA GAATTTAATT TTGGTGCTAA

CACATATCTT CGATACATTA CTGTCCATAA GAGATTAATT TTTGTGCTAA

CACATACCTT CGATATGTTA CTGTCCACAA GAGCTTAATT TTTGTGCTAA

CACGTACCTT CGCTACATTA CCGTCCACAA GAGCTTAATT GTTGTGCTCA

CACCTACCTC AGATATATCA CTATTCATAA GAACTTAGTT TTTGTCTTAA

TACATATCTC AGATACATGA CCATTCACAA GAAGTTGATT TTTGTGCTAA

TTTGGTGTAT TATGTGTTTT ACATTTACGT GGGAGTAGCC GACACTTTGC

TTTGGTGTAT TATGTGTTTT ACATTTACGT GGGAGTAGCC GACACTTTGC

TTTGGTGTAT TATGTGTTTT ACATTTACGT GGGAGTAGCC GACACTTTGC

-------TAT TATGTGTTTT ACATTTACGT GGGAGTAGCC GACACTTTGC

TTTGGTGTAT TATGTGTTTT ACATTTACGT GGGAGTTGCT GACACTTTGC

TTTGGTGTAC TACTTGTTCT ACATTTACGT GGGAGTAGCT GACACTTTGC

TTTGGTGTAT TACATTTTTT ACATTTACGT GGGAGTGGCT GACACTTTGC

TTTGGTGTAT TATATTTTTT ACATTTACGT GGGAGTGGCT GACACTTTGC

TTTGGTGTAC TACTTTTTTT ACATTTATGT GGGAGTAGCC GACACCTTGC

TTTGGTGTAT TATATTTTTT ACATTTATGT GGGAGTGGCT GACACTTTGC

TTTGGTGTAT TATATTTTCT ACATTTATGT GGGAGTAGCT GATACTTTGC

TTTGGTGTTC TATATGTTTT ACATTTACGT GGGAGTAGCT GACAGTTTGC

TTTGGTGTTT TATATTATTT ACATTTATGT GGGAGTCGCT GACACTTTGC

TTTGGTGTTT TACTTTTTGT ACATTTACGT GGGAGTAGCT GACACTTTCC

TTTGGTGTAT TATCTTTTGT ACATTTATGT GGGAATAGCT GACAGTTTGT

TTTGGTGTTT TATGTTTTCT ACATCTATGT GGGAGTAGCC GACACTCTGC

TTTGGTGTAC TACTTGTTTT ATATATATGT GGGAGTAGCT GACACATTCT

TGATGTGTAT TACATGTTTT ATATTTATGT GGGGGTAGCA GACACCTTGC

TTGCTATGGG ATTCTTCAGA GGTCTACCAC TGGTGCATAC TCTAATCACA

TTGCTATGGG ATTCTTCAGA GGTCTACCAC TGGTGCATAC TCTAATCACA

TTGCTATGGG ATTCTTCAGA GGTCTACCAC TGGTGCATAC TCTAATCACA

TTGCTATGGG ATTCTTCAGA GGTCTACCAC TGGTGCATAC TCTAATCACA

TTGCTCTGGG ATTCTTCAGA GGTCTACCAC TGGTGCATAC CCTAATCACA

TTGCTCTGGG CTTCTTCAGA GGTCTGCCCC TGGTGCATAC TCTCATCACA

TTGCTCTGAG CCTCTTCAGA GGTTTGCCCC TGGTGCATAC GTTAATCACA

TTGCCCTGAG CCTCTTCAGA GGTTTGCCGC TGGTGCATAC GTTAATCACA

TTGCATTGGG ATTATTCAGA GGTTTGCCAC TGGTGCATAC TCTAATCACA

TTGCTCTAGG ACTCTTCAGA GGTTTACCAC TGGTGCATAC TCTAATCACA

TTGCTCTGGG ACTCTTCAGA GGTTTACCAC TGGTGCATAC TCTAATCACA

TCGCTCTGGG ATTCCTCCGA GGCTTACCAC TGGTGCATAC GCTAATCACA

TTGCTCTGGG GATTTTCAGA GGTCTGCCAC TGGTACACAC TCTGATCACA

TCGCTCTGGG ACTCTTCAGG GGTTTACCAC TGGTGCACAC TCTAATTACA

TTGCCCTGGC AATCTTCAGA GGTTTACCAC TGGTGCATAC CCTAATCAAA

TGGCCCTGGG ATTCGCTAGG GGTTTGCCAC TGGTACATGC TCTCATCAAA

TTGCCCTAGG AATGCTTAGA GGCTTACCAC TGGTGCATAC ACTAATATCG

TTGCCCTGGG GTTCCTCCGT GGACTCCCCC TGGTGCACAG CCTGATATCC

GTGTCGAAAA TTTTACACCA CAAAATGTTA CATTCTGTTC TTCAAGCACC

GTGTCGAAAA TTTTACACCA CAAAATGTTA CATTCTGTTC TTCAAGCACC

GTGTCGAAAA TTTTACACCA CAAAATGTTA CATTCTGTTC TTCAAGCACC

GTGTCGAAAA TTTTACACCA CAAAATGTTA CATTCTGTTC TTCAAGCACC

GTGTCGAAAA TTTTACACCA CAAAATGTTA CATTCTGTTC TTCAAGCACC

GTGTCAAAAA TTTTACACCA CAGAATGTTG CATTCTGTTC TTCGAGCGCC

GCATCAAAAA TTTTGCACAG GAAAATGTTA CATTCTATTC TTCATGCCCC

GCATCAAAAA TTTTGCACAG GAAAATGTTA CACTCCATTC TTCACGCCCC

GTGTCGAAAA TTTTACACCA CAAAATGTTA CATTCTGTTC TTCAGGCGCC

GTGTCAAAAA CTTTACACCA CAAAATGCTA CAGTCTGTTC TTCAAGCCCC

GTGTCGAAAA TTTTACACCA CAAAATGTTA CATTCTGTTC TTCAAGCACC

GTATCGAAAA TCTTACACCA GAAAATGTTA CATTCTGTTC TTCATGCACC

GTGTCAAAAA TATTACACCA CAAAATGCTG CATTCTGTTC TTCATGCACC

GTGTCGAAAA TTTTACACCA CAAAATGTTA CATTCTGTTC TTCAAGCACC

GTGTCAAAAA CTTTACACCA CAAAATGTTA CGTTCTATTC TTCAAGCACC

GTGTCGAACA TTTTACACCG CAAAATGTTG CATTCTGTTC TTCAAGCACC

GTCTCCAAAA TTCTACATCA CAAAATGTTA TGCTCTGTTT TGAAAGCACC

GTTTCCAAAA TTCTCCACCA GAAGATGCTG CATTCGGTCC TGCAGGCGCC

TATGTCAACC CTCAACACGT TGAAAGCAGG TACTTAGGTG GGATTCTTAA

TATGTCAACC CTCAACACGT TGAAAGCAGG TACTTAGGTG GGATTCTTAA

TATGTCAACC CTCAACACGT TGAAAGCAGG TACTTAGGTG GGATTCTTAA

TATGTCAACC CTCAACACGT TGAAAGCAGG TACTTAGGTG GGATTCTTAA

TATGTCAACC CTCAACACAT TGAAAGCAGG TACTTAGGTG GGATTCTTAA

TATGTCAACC CTCAACATGT TAAAAGCAGG TATTTAGGTG GGATTCTGAA

TATGTCAACC TTCAACAAGC TGAAAGCAGG TACTCAGGTG GGATTCTTAA

TATGTCGACC ATCAGCAAGC TGAAAGCAGG TACTCAGGTG GGATTCTTAA

CATGTCAACA CTCAACACAT TGAAAGCAGG TACTTAGGTG GGATTCTTAA

TATGTCAACC CTCAACACGT TGAAAACAGG TACTCAGGTG GGATTCTTAA

TATGTCAACC CTCAACACAC TGAAAGCAGG TATTTAGGTG GGATTCTTAA

TATGTCGACC CTGAACACCT TGAAAGCAGG TACTTAGGTG CGATTCTTAA

TATGTCAACC CTC-ACGCAC TGAAAGCAGG TACTTAGGTG GGATTCTTAA

TATGTCAACC TTAAACACAT TAAAAGCAGG TACTTAGGTG GGATACTTAA

TATGTCAACG TTCAACACAT TGAAAGCAGG TACTCAGGTA GGATTCTTAA

TATGTCGACC TTCAACACGT TGAAAGCAGG TACT------ ----------

TATGTCAACC TTCAACACTT TGAAAGCAGG TAATTAGGGG GAATTCTGAA

CATGTCGACT TTCAACACTT TGAAAACAGG TACTAAGGTA GCATTCTCAA

TAGATTCTCC AAAGATATAG CAATTTTGGA TGACCTTCTG CCTCTTACCA

TAGATTCTCC AAAGATATAG CAATTTTGGA TGACCTTCTG CCTCTTACCA

TAGATTCTCC AAAGATATAG CAATTTTGGA TGATCTTCTG CCTCTTACCA

TAGATTCTCC AAAGATATAG CAATTTTGGA TGATCTTCTG CCTCTTACCA

TAGATTCTCC AAAGATATAG CAATTTTGGA TGATCTTCTG CCTCTTACCA

TAGATTCTCC AAAGACATAG CGATCTTGGA TGACCTCCTG CCTCTTACCA

CAGATTCTCC AAAGATATAG CAATTTTGGA TGACTTTCTG CCTCTTACCA

CAGATTCTCC AAAGATATAG CAATTTTGGA TGACTTTCTG CCTCTTACCA

TAGATTCTCC AAGGATATAG CCATTCTGGA TGATCTGCTG CCTCTTACCA

TAGATTCTCC AAAGATATAG CAGTTTTGGA TGATCTTCTG CCTCTTACCA

TAGATTCTCC AAAGATATAG CGATTTTGGA TGATCTTCTG CCCCTTACCA

TAGATTTTCC AAAGATATAG CAATTTTGGA TGATCTTCTG CCTCTTACCA

TAGATTCTCC AAAGATATAG CAATTTTGGA TGATCTTCTG CCTCTTACCA

TAGATTCTCC AAAGATATAG CAATTTTGGA TGATCTTCTG CCTCTTACCA

TAGATTCTCC AAAGATATAG CAATTTTGGA TGATCTTCTG CCTCTTACCA

---------- ---------- ---------- ---------- ----------

CCGTTTCTCC AAAGATATAG CAATTCTGGA TGACCTCCTG CCACTTACAA

CAGATTCTCC AAAGATATGG CCATTTTGGA TGACCTTCTG CCACTTACAA

TATTTGACTT CATCCAGGTA TGTAAAGTCA GTTTGCCTCA TCTGAAATGA

TATTTGACTT CATCCAGGTA TGTAAAGTCA GTTTGCCTCA TCTGAAATGA

TATTTGACTT CATCCAGGTA TGTAAAGTC- ---AGCCTCA TCTGAAATGA

TATTTGACTT CATCCAGGTA TGTAAAGTCA GTTAGCCTCA TCTGAAATGA

TATTTGACTT CATCCAGGTA TGTAAAGACA GTTTGGCTCA TCTGAAATGA

TATTTGACTT CGTCCAGGTT TGTAAAGTCC AGTTGCCCCT TCTGAAATGA

TATTTGACTT CATCCAGGTC TGTAAAGTTG GTTTACCTCC TCTGAAATGA

TTTTTGACTT CATTCAGGTT TGTAAAGTTG GTTCACCTCC TCTGAAATGA

TATTTGACTT CATCCAGGTT TGTAAAGTTG GT--GCCTCA TCTGAAATGA

TATTTGATTT TGTTCAGGTT TGTAAAGCCA GTTTGCCTCA TCTGAAATGA

TATTTGACTT CATCCAGGTT TGTAAAGTCA GTTTGCCTCA TCTGAAATGA

TATTTGACTT CATCCAGGTT TGTAAAGTCG GTTTGCCTCC TGTGGAATGA

TATTTGACTT CATTCAGGTT TGTAAAGTCA GTTTACTTCA TCTGAAACGA

TATTTGACTT CATCCAGGTC TGTAAAGTCA GTTTGCCTCA TCTGAAGTGA

TGTTTGACTT CATCCAGGTT TGTAAAGTCA GCTTGCTTCA CCTGAAACAA

---------- ---------- ---------- ---------- ----------

TATTTGACTT CATCCAGGTG TGTAAAGTCA GTTTGCCTCA CCTGAAACAA

TATTTGACTT CATTCAGGTT GGTAAAGTCA GCTCCCCTTA GCTTGAAGAA

TTGAGAGCAT ATTTCCTCCA AACCTCACAG CAACTCAAAC AACTGGAATC

TTGAGAGCAT ATTTCCTCCA AACCTCACAG CAACTCAAAC AACTGGAATC

TTGAGAGCAT ATTTTCTCCA AACCTCACAG CAACTCAAAC AACTGGAATC

TTGAGAGCAT ATTTTCTCCA AACCTCACAG CAACTCAAAC AACTGGAATC

CTGAGAGCAT ATTTCCTCCA AACCTCACAG CAACTCAAAC AACTAGAATC

TTGAGAGGAT ACTTCCTACA CACCTCACAG CAACTCAAAC AACTGGAATC

TTGAGGGCAT ACTTCCTTCA TACCTCCCAA CAGCTCAAAC AACTGGAATC

TTGAGGGCCT ACTTCCTTCA TACAGCACAG CAGCTCAAAC AACTGGAATC

CTGAGAGCGT ACTTCCTCCA TACCTCACAG CAACTCAAGC AACTGGAATC

TTGAGAGCCT ACTTCCTCCA CACCTCCCAG CAACTCAAGC AGCTGGAATC

CTGCGGGCCT ACTTCCTCCA CACCTCACAG CAACTCAAGC AACTGGAATC

TTGAGAGCCT ACTTCCTGCA GACCTCCCAG CAACTCAAAC AACTGGAATC

TTGAGAGCCT ACTTCCTCCA CACCTCACAG CAGCTCAAAC AACTAGAGTC

TTGAGAGCAT ACTTCCTTCA CACCTCACAG CAACTCAAAC AACTGGAATC

TTGAGAGCAT ACTTCCTCCA CACCTCACAG CAGCTCAAAC AATTGGAATC

---------- ---------- ---------- ---------- ----------

TTAAGGGCAT ATTTCCTCCA AACCTCCCAG CAGCTAAAAC AACTGGAATC

ATGAGAGCAT ACTTTCTCCG CACATCCCAG CAACTTAAAC AACTGGAATC

TGAAGGTATG TGTTTTCTAT GGAAATATTT CCAGGCAGGA GTCCAATTTT

TGAAGGTATG TGTTTTCTAT GGAAATATTT CCAGGCAGGA GTCCAATTTT

TGAAGGTATG TGTTTTCTAT GGAAATATTT CCAGGCAGGA GTCCAATTTT

TGAAGGTATG TGTTTTCTAT GGAAATATTT CCAGGCAGGA GTCCAATTTT

TGCAGGTATG TGTTTTCTAT GGGAATATTT CCAGGCAGGA GTCCAATTTT

TGAAGGTATG TGTTTTCTAT GGGAATATTT CCAGGCAGGA GTCCAATTTT

TGAAGGTACA -ATCTCCTAT GGGATGA-TT TCAGGCAGGA GTCCAATTTT

TGAAGGTACA TATCTCCTAT GGGATGA-TT TCAGGCAGGA GTCCAATTTT

TGAAGGTATG TGTTTTATAT GGAACA---T TCAGGTAGGA GTCCAATTTT

TGAAGGTATG TGTTCTCTAT GGAAATATTT CCAGGCAGGA GTCCAATTTT

TGAAGGTAGG TGTTTTCTAT GGGAATATTT TCAGGCAGGA GTCCAATTTT

AGAAGGTGTG TGTTTCTTAT GCGAATATTT CCAGGCAGGA GTCCAATTTT

TGAAGGTATG TATT-TCTGT GTGACTGTTT CCAGGCAGAA CTCCAATTTT

TGAAGGTATG TGTTTTCTAT GGGACTATTT CCAGCCAGAA GCCCAATTTT

TGAAGGTATG TGTTTTCTAT GGGGATAATT TCAGCCAGGA GTCCCATTTT

---------- ---------- ---------- ---------- ----------

TGAAGGTATG TGTTTTTAAT TTGAATACTT CCAGCCAGGA CTCCAATTTT

TGAAGGTATG TGCTTTTAAT TGAAATACTT CCAGCCCGGA GTCCAATATT

CACTCATCTT GTTACAAGCT TAAAAGGACT ATGGACACTT CGTGCCTTCG

CACTCATCTT GTTACAAGCT TAAAAGGACT ATGGACACTT CGTGCCTTCG

CACTCATCTT GTTACAAGCT TAAAAGGACT ATGGACACTT CGTGCCTTCG

CACTCATCTT GTTACAAGCT TAAAAGGACT ATGGACACTT CGTGCCTTCG

CACTCATCTT GTTACAAGTT TAAAAGGACT ATGGACACTT CGTGCCTTCG

CACTCATCTG GTTACAAGCT TAAAAGGACT ATGGACACTT CGTGCCTTCG

CACGCACCTT GTTACAAGCT TAAAAGGACT GTGGACACTT CGAGCCTTCC

CACCCACCTT GTGACAAGCT TAAAAGGACT CTGGACACTT CGAGCCTTCC

CACTCATCTT GTTACAAGCT TGAAAGGATT ATGGACACTT CGTGCCTTTG

CACTCATCTT GTTACAAGTT TAAAAGGACT ATGGACACTT CGTGCCTTTG

CACTCATCTT CTTACAAGTT TAAAAGGACT GTGGACACTT CGGGCCTTTG

CACTCATCTG GTTACAAGCT TAAAAGGCCT ATGGACACTG CGTGCCTTCG

CACTCATCTT GTTACAAGCT TAAAAGGACT GTGGACACTT CGTGCTTTTG

CACTCATCTT GTTACAAGTT TAAAAGGACT GTGGACACTT CGTGCCTTTG

CACTCATCTT GTTACAAGCG TAAAAGGACT ATGGACGCTC CGTGCCTTTG

---------- ---------- ---------- ---------- ----------

TACTCATCTG GTTACAAGTT TAAAGGGACT GTGGACTCTA CGAGCTTTCG

TACTCATCTT ATTACAAGCT TAAAGGGGCT ATGGACATTG CGTGCATTTG

GACGGCAGCC TTACTTTGAA ACTCTGTTCC ACAAAGCTCT GAATTTACAT

GACGGCAGCC TTACTTTGAA ACTCTGTTCC ACAAAGCTCT GAATTTACAT

GACGGCAGCC TTACTTTGAA ACTCTGTTCC ACAAAGCTCT GAATTTACAT

GACGGCAGCC TTACTTTGAA ACTCTGTTCC ACAAAGCTCT GAATTTACAT

GACGGCAGCC TTACTTTGAA ACTCTGTTCC ACAAAGCTCT GAATTTACAT

GTCGTCAGCC TTACTTTGAA ACTCTTTTCC ACAAAGCTCT GAATTTACAT

GACGCCAGAC TTACTTTGAA ACCCTATTCC ACAAAGCTCT GAATTTGCAC

GACGCCAGAC TTACTTTGAA ACTCTGTTCC ACAAAGCTCT GAATTTGCAC

GACGGCAGCC TTACTTTGAA ACTCTGTTCC ACAAAGCTCT GAATTTACAC

GACGGCAGCC TTACTTTGAA ACTTTATTCC ACAAAGCTCT GAATTTACAT

GACGCCAGCC TTACTTTGAG ACTTTGTTTC ACAAAGCTCT GAACTTACAT

GACGTCAGCC TTACTTTGAA ACTCTGTTCC ACAAAGCTTT GAATTTACAT

GGCGACAGCC TTACTTTGAA ACTCTGTTCC ATAAAGCGCT AAACTTACAT

GACGTCAGCC TTACTTTGAA GCTCTGTTCC ACAAAGCTCT GAATTTGCAT

GACGCCAGCC TTACTTTGAA ACTCTGTTCC ACAAAGCTCT GAATTTACAC

---------- ---------- ---------- ---------- ----------

GACGCCAACC GTACTTTGAG ACTTTGTTCC ATAAAGCTCT GAATTTACAC

GACGCCAAAC GTATTTTGAA GCGCTGTTCC ACAAGGCTCT GAATTTACAC

ACTGCCAACT GGTTCTTGTA CCTGTCAACA CTGCGCTGGT TCCAAATGAG

ACTGCCAACT GGTTCTTGTA CCTGTCAACA CTGCGCTGGT TCCAAATGAG

ACTGCCAACT GGTTCTTGTA CCTATCAACA CTGCGCTGGT TCCAAATGAG

ACTGCCAACT GGTTCTTGTA CCTATCAACA CTGCGCTGGT TCCAAATGAG

ACTGCCAACT GGTTCTTGTA CCTATCAACA CTGCGCTGGT TCCAAATGAG

ACTGCCAACT GGTTCTTGTA CCTGTCAACC CTGCGCTGGT TCCAAATGCG

ACTGCCAACT GGTTTATGTA TCTGGCAACC TTGCGCTGGT TCCAGATGAG

ACTGCCAACT GGTTTATGTA TCTGGCAACC TTGCGCTGGT TCCAAATGAG

ACTGCCAACT GGTTCTTGTA TCTGTCAACA CTACGCTGGT TCCAAATGAG

ACTGCCAACT GGTTCTTGTA TCTGTCAACA CTGCGCTGGT TCCAAATGAG

ACTGCCAACT GGTTCTTGTA CCTGTCAACG CTGCGCTGGT TCCAAATGAG

ACTGCCACCT GGTTCTTATA CCTGTCAACA CTGCGCTGGT TCCAAATGAG

ACAGCCAACT GGTTTTTGTA CCTATCAACT TTGCGCTGGT TCCAAATGAG

ACTGCCAACT GGTTCCTGTA CCTATCAACA CTACGCTGGT TCCAAATGAG

ACTGCCAACT GGTTCTTGTA CCTGTCGACA CTTCGCTGGT TCCAAATGAG

---------- ---------- ---------- ---------- ----------

ACAGCAAATT GGTTCCTATA CCTGTCCACA CTCCGCTGGT TCCAAATGAG

ACCGCCAACT GGTTCCTGTA TCTGTCCACT CTCCGCTGGT TCCAAATGAG

AATAGAAATG ATTTTTGTCA TCTTCTTCAT TGCTGTTACC TTCATTTCCA

AATAGAAATG ATTTTTGTCA TCTTCTTCAT TGCTGTTACC TTCATTTCCA

AATAGAAATG ATTTTTGTCA TCTTCTTCAT TGCTGTTACC TTCATTTCCA

AATAGAAATG ATTTTTGTCA TCTTCTTCAT TGCTGTTACC TTCATTTCCA

AATAGAAATG ATTTTTGTCA TCTTCTTCAT TGCTGTTACC TTCATTTCCA

AATAGAAATG ATATTTGTCG TCTTCTTCAT TGCTGTTACC TTCATTTCCA

AATAGACATG ATTTTCGTCC TCTTCTTCAT TGTTGTTACC TTCATCTCCA

AATAGACATG ATATTTGTCC TCTTCTTCAT TGTTGTTACC TTCATCTCCA

GATAGAAATG ATTTTTGTCC TCTTCTTCAT TGCCGTAGCC TTCATTTCCA

AATAGAAATG ATTTTTGTCA TCTTCTTCAT TGCTGTTACC TTCATTTCCA

AATGGAAATT ATTTTTGTCA TATTCTTCAT TGCTGTTACC TTCATCTCCA

AATAGAGATG ATTTTCGTCG TCTTCTTCGT TGCTGTTACC TTCATTTCTA

GATAGAAATT ATTTTTGTCC TCTTCTTCAT TGCCGTTACC TTCATTTCCA

GATAGAAATG ATTTTTGTCA TCTTCTTCAT TGCTGTTACC TTCATTTCCA

AATAGAAATT ATTTTTGTCA TCTTCTTCAT TGCTGTTACC TTCGTTTCCA

---------- ---------- ---------- ---------- ----------

AATAGAAATT GTTTTTGTCA TCTTCTTCAT TGCTGTGACC TTCATCTCCA

GATAGAAATG GTATTTGTCA TCTTCTTCAT TCTGGTGACC TTCATCTCCA

TTTTAACAAC AGGTATAGGA GAAGGAGAAG GAAGAGTTGG TATTATCCTG

TTTTAACAAC AGGTATAGGA GAAGGAGAAG GAAGAGTTGG TATTATCCTG

TTTTAACAAC AGGTATAGGA GAAGGAGAAG GAACAGTTGG TATTATCCTG

TTTTAACAAC AGGTATAGGA GAAGGAGAAG GAACAGTTGG TATTATCCTG

TTTTAACTAC AGGTATAGGA GAAGGAGAAG GAACAGTTGG TATTATACTG

TTTTAACAAC AGGTATAGGT GAAGGAGAAG GAACCGTTGG AATTATTCTG

TTTTAACAAC AGGTATAGGT GAAGGAGAAG GAACGACTGG TATTATTCTA

TTTTAACAAC AGGTATAGGT GAAGGAGAAG GAACAGCTGG TATTATTCTA

TTTTAACAAC AGGTATAGGT GAAGGAGAAG GAAGAGTTGG TATTATTCTA

TTTTAACAAC AGGTATAGGT GAAGGAGAAG GAAGAGTTGG GATTATTCTA

TTTTAACAAC AGGTGTAGGT GAAGGAGAAG GAACAGTTGG TATTATTCTA

TTTTAACAAC AGGTATAGGT GAAGGAGAAG GAACGGTTGG CATTATTCTA

TTTTAACAAC AGGTATAGGT GAAGGACAAG GACAAATTGG TATTGTACTA

TTTTAACAAC AGGTATAGGT GAAGGAGAAG GAACAGTTGG TATTATTCTA

TTTTAACAAC AGGTATAGGT GAAGGAGAAG GAACAATTGG CATTATTCTA

---------- ---------- ---------- ---------- ----------

TTTTAACTAC AGGTATAGGA AATGGAGAAG GAGAAATTGG AATTCTTCTA

TTTTAACCAC AGGTATAGGT GATGGAGAAG GGAAAGTTGG AATTGTTCTA

ACTTTAGCCA TGAATATCAT GAGTACATTG CAGTGGGCTG TAAACTCCAG

ACTTTAGCCA TGAATATCAT GAGTACATTG CAGTGGGCTG TAAACTCCAG

ACTTTAGCCA TGAATATCAT GAGTACATTG CAGTGGGCTG TAAACTCCAG

ACTTTAGCCA TGAATATCAT GAGTACATTG CAGTGGGCTG TAAACTCCAG

ACTTTAGCCA TGAATATCAT GAGTACATTG CAGTGGGCTG TAAACTCCAG

ACTTTAGCCA TGAATATCAT GGGGACATTG CAGTGGGCTG TGAACTCCAG

ACTTTAGCTA TGAATATTAT GAGTACTTTG CAATGGGCTG TGAACTCAAG

ACTTTAGCTA TGAATATCAT GAGTACTTTG CAGTGGGCTG TGAACTCAAG

ACTTTAGCCA TGAATATCAT GAGTACGTTG CAGTGGGCTG TGAACTCCAG

ACTTTAGCCA TGAATATCAT GGGTACATTG CAGTGGGCTG TAAACTCTAG

ACATTAGCCA TGAATATCAT GGGTACGTTG CAGTGGGCTG TAAACTCCAG

ACATTAGCCA TGAATATCAT GAGTACATTG CAGTGGGCTG TGAACTCCAG

ACTTTAGCCA TGAATATTAT GAATACATTG CAGTGGGCTG TCAATTCCAG

ACTTTAGCCA TGAATATCAT GAGTACATTG CAGTGGGCTG TGAACTCCAG

ACATTAGCCA TGAATATCAT GAACACATTG CAGTGGGCTG TAAACTCCAG

---------- ---------- ---------- ---------- ----------

ACTTTAGCCA TGAATATCAT GAGTACCTTG CAGTGGGCTG TGAACTCCAG

ACATTAGCCA TGAACATCAT GGGTACATTG CAGTGGGCAG TAAACGCGAG

CATAGATGTG GATAGCTTGG TAAGTCTAGA AAGTTCTTCC AAGATTGATG

CATAGATGTG GATAGCTTGG TAAGTCTAGA AAGTTCTTCC AAGATTGATG

CATAGATGTG GATAGCTTGG TAAGTCTAGA AAATTCTTCC AAGATTGATG

CATAGATGTG GATAGCTTGG TAAGTCTAGA AAATTCTTCC AAGATTGATG

CATAGATGTG GATAGCTTGG TAAGTCTAGA AAGTTCTTGC AAGATTGATG

CATAGACGTG GATAGCTTGG TAAGTCCAGA AAGTTCTTGC AAGATTGATG

CATTGATACA GATAGCTTGG TAAGTCCAGA AAGTTCTTGC CTGGTTGGTG

CATTGATACA GATAGCTTGG TAAGTCCAGA AAGTTCTTGC ATGGTTGGTG

CATAGATGTA GATAGCTTGG TAAGTTCAGA AAGTTATTGA AAGATTGATG

CATAGATGTG GATAGCTTGG TAAGTCCAGA AAGTTCTTAC AAGGTTGATG

CATAGAGGTG GACAGCTTGG TAAGTCCAGA AAGTTCTTGC CAGAGTGATG

CATAGATGTG GATAGCTTGG TAAGTCCAGA AAGTTCTTGC ACGATTGATG

CATAGATGTG GATAGCTTGG TAAGTCCAAA AAGTTCTTAA AAGATTGATG

CATAGATGTG GATAGTTTGG TAAGTCCAGA AGGTTCTTGC AAGATTAATA

CATAGATGTG GACAGTTTGG TAAGTCCAGA AAGTTCTTGC AAGATTGCTG

---------- ---------- ---------- ---------- ----------

CATAGATGTG GACAGTTTGG TAAGTTCAGA AGTGCTTAGA AATGTTGATG

CATTGATGTG GACAGTTTGG TAAGTATAGA ATGTTCTTAC TAGGTCAGAG

CAATCTGAGA ATTAGGTGAT CACAAAAATA AACAGGCCTA TACAGAGCCC

CAATCTGAGA ATTAGGTGAT CACAAAAATA AACAGGCCTA TACAGAGCCC

CAATCTGAGA ATTAGGTGAT CACAAAAATA AACAGGCCTA TACAGAGCCC

CAATCTGAGA ATTAGGTGAT CACAAAAATA AACAGGCCTA TACAGAGCCC

CAATCTGAGA ATTAGGTGAT CACAAAAATA AACAGGCCGA TACAGAGCCC

TAATCTGAGA ATTAAGTGAT CACAAAAACA AA-------- -ACAGAGCCC

CAATCTGAGA TTTAAGTAAT CACAAAAATA AACAGGTCTC TACAAAACCC

CAATCTGAGA TTTAAGCAAT CACAAAAATA AACAGGTCTC TACAGAACCC

CAATCTGAGA ATTAAGTGAT CACAAAAATA AACAGGTCTA TTCAGAACCC

TAATCTGAGA GTTAAGTGAT CACAAAAATA AACAGGTGTA TAAAGAGCCC

CAATCTGAGA ATTAAATGAT CACAAAAATA AACAGGTCTA TACAGAGCCC

CAATCTGAGA ATTAAGTGAT CACAAAAATA AACAGATCTA TCAAAAGCCC

CAATCTGAGA ATTATGTGAT CACAAAAATA AACAGGTCTA TCCAGAGCTC

CAATCTGAGA ATTAAGTGAT CACAAAAATA AACAGGTCTA TACAGAGCCC

CAACCTGAGA ATTCAGTGAT CACCAAAATG AAAAGGTCTA TACAGAGCCT

---------- ---------- ---------- ---------- ----------

CAACCTGCAA ATTTATCGAT CACAAAAGTC AACAGGTTAA TACAAAGCCC

CAGTCTTCAA ATTTAATGGT CATGAAAATC AACCTATTTG TCCAAAGTCC

ATTTATTGTT ATTTTTATTT CAGATGCGAT CTGTGAGCCG AGTCTTTAAG

ATTTATTGTT ATTTTTATTT CAGATGCGAT CTGTGAGCCG AGTCTTTAAG

ATTTATTGTT ATTTTTATTT CAGATGCGAT CTGTGAGCCG AGTCTTTAAG

ATTTATTGTT ATTTTTATTT CAGATGCGAT CTGTGAGCCG AGTCTTTAAG

ATTTATTGTT ATTTTTATTT CAGATGCGAT CTGTGAGCCG AGTCTTTAAG

ATTTATTGTT ATTTGTATTT CAGATGCGAT CTGTGAGCCG AGTCTTTAAG

AATTATTGTT ATTTTCATTT CAGATGCGAT CTGTGAGCAG AGTCTTTAAG

AATTATTGTT ATTTTCATTT CAGATGCGAT CTGTGAGCAG AGTGTTTAAG

ATTTATTGTT AATTTCCTTT CAGATGCGAT CTGTGAGTCG AGTCTTTAAG

ATTTATtatt ---tttattt CAGATGCGAT CTGTGAGCCG AGTCTTTAAG

ATTTATTGTT A-TTTCATTT CAGATGCGAT CGGTGAGCCG AGTCTTCAAG

ATTTATTGTT ATTTTCATTT CAGATGCGAT CTGTGAGCCG AGTCTTTAAA

ATTTGTTTTT TTTTTTATTT CAGATGCGAT CTGTGAGCCG AGTCTTCAAA

ATTTATTGTT ATTTTAATTT TAGATGCGAT CTGTGAGCCG AGTCTTTAAG

ATTTATTGTT ACTTTGATTT CAGATGAGAT CTGTGAGCCG AATCTTTAAG

---------- ---------- ---------- ---------- ----------

ATTTGTTGTT TATTCTGTTT CAGATGCGGT CTGTGAGCCG AGTATTTAAA

ATTGATTGTT TATTTTGTTC CAGATGCGGT CTGTGAGCAG AGTATTTAAA

TTCATTGACA TGCCAACAGA ACACGTGAAG AAAGATGACT GGCCCTCAGG

TTCATTGACA TGCCAACAGA ACACGTGAAG AAAGATTACT GGCCCTCAGG

TTCATTGACA TGCCAACAGA ACACGTGAAG AAAGATGACT GGCCCTCAGG

TTCATTGACA TGCCAACAGA ACACGTGAAG AAAGATGACT GGCCCTCGGG

TTCATTGACA TGCCAACAGA ACACGTGAAG AAAGATGACT GGCCCTCAGG

TTTATTGATA TGCCGACAGA ACATGTGAAG AAAGATGACT GGCCTTCAGG

TTTATTGATA TACAAACAGA ACATGTGAAG AAATGTGACT GGCCCTCTGG

TTTATTGATA TACAAACAGA ACATGTGAAG AAAAGTGACT GGCCCTCTGG

TTTATTGACA TGCCAACAGA ACATGTGAAG AAAGATGACT GGCCCTCAGG

TTTATTGATA TGCCAACAGA TCATGTGAAG AAAGATGACT GGCCCTCAGG

TTTATTGACA TGCCTACAGA ACATGTGAGG GAAGATGACT GGCCCTCAGG

TTTATTGACA TGCCAGCAGA ACATGTGAAG AAAGATGACT GGCCCTCAGG

TTTATTGATA TGCCAACAGA ACAT-CAAAA AAAGAGGACT GGCCCTCAGG

TTTGTTGACA TACCAACAGA ACACGTGAAG AAAGATGACT GGCCCTCTGG

TTTATCGACA TGCCAACAGA ACACGTGAAG AAAGATTACT GGCCCTCAGG

---------- ---------- ---------- ---------- ----------

TTCATTGATA TGCCAGCAGA ACACGTAAAT AAAGAAAACT GGCCCTCTGG

TTCATCGACA TGCCAACGGA GCATGTGAAG AGAGAGAACT GGCCTTCAGG

GGGCCAAATG ACTGTCAAAG ATCTCACAGC AAAATACACA GAAGGTGGAA

GGGCCAAATG ACTGTCAAAG ATCTCACAGC AAAATACACA GAAGGTGGAA

GGGCCAAATG ACCGTCAAAG ATCTCACAGC AAAATACACA GAAGGTGGAA

GGGCCAAATG ACGGTCAAAG ATCTCACAGC AAAATACACA GAAGGTGGAA

GGGTCAAATG ACCATCAAAG ACCTCACAGC AAAATACATA GAAGGTGGAA

AGGCCAAATG ACCGTCAAAG ACCTCACAGC AAGATATATA GACAGTGGGA

AGGTGAAATG GTTGTCAAAG ACCTTACTGT GAAATATGTG GATGATGGGA

AGGCGAAATG GTTGTCAAAG ACCTTACTGT GAAATACATG GATGATGGAA

AGGCCAAATG ACTGTCAAAG GTCTCACAGC AAAATATATA GACAGCGGAA

GGGCCAAATG ACTGTCAAAG ACCTCACAGC AAAGTATACA GATGGAGGGA

GGGCCAAATG ACTGTCAAAG ACCTCACAGC AAAGTATATA GATGGTGGGA

GGGCCAAATT ACCGTCAAAG ACCTCACAGC AAAATATATA GATGGTGGGA

TGGCCAAATG ACCATCAAAG ACCTCACTGC AAAATATATA GATGGAGGGA

GGGCCAAATG ACTGTCAAAG ACCTTACAGC AAAATATATA GATGGTGGGA

GGGTCACATG ACTGTCAAAG ACCTCACAGC AAAATATATA GATGGTGGCA

---------- ---------- ---------- ---------- ----------

AGGTCAGATG ATTGTCAAAG ACCTCACAGC CAAATATATA GATGGTGGGA

AGGCCAAATG ACCGTCCAGG ACCTCACAGC CAAGTACCTA GATGGAGGTC

ATGCCATATT AGAGAACATT TCCTTCTCAA TAAGTCCTGG CCAGAGGGTG

ATGCCATATT AGAGAACATT TCCTTCTCAA TAAGTCCTGG CCAGAGGGTG

ATCCCATATT AGAGAACATT TCCTTCTCAA TAAGTCCTGG CCAGAGGGTG

ATCCCATATT AGAGAACATT TCCTTCTCAA TAAGTCCTGG CCAGAGGGTG

ATGCCATATT AGAGAACATT TCCTTCTCAA TAAGTCCTGG CCAGAGGGTG

ATGCCATATT GGAGAACATT TCCTTCTCAA TAAGTCCCGG ACAGAGGGTG

ATGCCATATT AGAGAACATT TCTTTTTCAA TAAGTCCTGG CCAGAGGGTG

ATGCCGTATT AGAGAACATT TCTTTTTCAA TAAGTCCTGG ACAGAGGGTG

ATGCCATCCT GGAGAACATT TCCTTCTCAA TAAGTCCTGG CCAGAGGGTG

ATGCCATACT AGAGAACATA TCCTTCTCAA TAAGTCCCGG CCAGAGGGTA

ATGCCATATT AGAGAACATT TCCTTCTCAA TAAGTCCTGG CCAGAGGGTG

ATGCCATATT AGAGAACATT TCCTTCTCAA TAAGTCCTGG CCAAAGGGTG

ATGCCATACT AGAGAACATC TCCTTCTCTA TAAGTCCTGG CCAGAGGGTG

ATGCCATATT AGAGAACATT TCATTCTCAA TAAGTCCTGG CCAGAGGGTG

ATGCCATATT AGAGAACATT TCCTTCTCAA TAAGTCCTGG CCAGAGGGTG

---------- ---------- ---------- ---------- ----------

ATGCTGTATT AGAGAACATT TCCTTCTCAA TAAGTCCTGG GCAGAGGGTG

CGGCCATCTT GGACAATATC TCCTTTTCGA TAAGTTCCGG TCAGAGGGTG

AGATTTAGCT TTTTTGAGAC TACTGAACAC TGAAGGAGAA ATCCAGATCG

AGATTTAGCT TTTTTGAGAC TACTGAACAC TGAAGGAGAA ATCCAGATCG

AGATTTAGCT TTTTTGAGAC TACTGAACAC TGAAGGAGAA ATCCAGATTG

AGATTTAGCT TTTTTGAGAC TACTGAACAC TGAAGGAGAA ATCCAGATCG

AGATTTAGCT TTTTTGAGAC TACTGAACAC TGAAGGAGAA ATCCAGATTG

AGACTTAGCT TTTTTGAGAC TCCTGAACAC TGAAGGAGAT ATCCAGATCG

AGATTTGGCC TTTTTACGAA TGTTGAACAT TAAAGGCGAA ATACAGATCG

AGATTTGGCA TTTTTACGAA TGTTGAACAT TAAAGGTGAT ATAGAGATTG

AGATTTAGCA TTTTTGAGAC TACTGAACAC TGAAGGAGAA ATCCAGATTG

AGATTTAGCT TTTTTGAGAC TGCTGAATAC CAAAGGAGAA ATCCAAATAG

AGATTTAGCT CTTTTGAGAC TGGTGAACAC TGAGGGAGAA ATCCAAATAG

AGATTTAGCT CTTCTGAGAC TGCTGAACAC CGAGGGAGAG ATCCAAGTAG

AGTTTTGGCT CTTTTGAGAC TGCTGAATAC GGAAGGAGAA ATCCAAATTG

AGATTTAGCT TTTTTGAGAC TGCTAAACAC TAAGGGAGAA ATTCAGATTG

AGTATTAGCT TTTTTGAGGC TGCTGAACAC TGAAGGAGAA ATTCAGATTG

-------GCT TTCCTGAGGC TGCTGAACAC CGCCGGAGAA ATCCAGATTG

AGACTTAGCA TTTTTAAGAC TACTGAACAC AGAAGGAGAA ATGCAGATTG

AGGTCCAGCC TTTTTGAGGC TGCTGAACAC TGAAGGAGAT ATCCGGATCG

ATGGTGTGTC TTGGGATTCA ATAACTTTGC AACAGTGGAG GAAAGCCTTT

ATGGTGTGTC TTGGGATTCA ATAACTTTGC AACAGTGGAG GAAAGCCTTT

ATGGTGTGTC TTGGGATTCA ATAACTTTGC AACAGTGGAG GAAAGCCTTT

ATGGTGTGTC TTGGGATTCA ATAACTTTGC AACAGTGGAG GAAAGCCTTT

ATGGTGTGTC TTGGGATTCA ATAACTTTGC AACAGTGGAG GAAAGCCTTT

ATGGCGTGTC TTGGGATTCA ATAACTTTGC AACAGTGGAG GAAAGCCTTT

ATGGTGTCTC CTGGAACTCA ATGACCTTAC AAGAATGGAG AAAAGCTTTT

ATGGTGTCTC ATGGAATTCA GTGACCTTAC AAGAATGGAG GAAAGCTTTC

ATGGTGTGTC TTGGGATTCG ATCACTTTGC AGCAGTGGAG AAAAGCCTTT

ATGGTGTGTC TTGGGATTCA ATTACTTTGC AACAATGGAG GAAGGCCTTT

ATGGCGTGTC TTGGGACTCA ATCCCTTTGC AAGAATGGAG GAGAGCCTTT

ACGGAGTGTC TTGGGAGTCG GTGACGGTGC AGCAGTGGAG AAGAGCCTTT

ATGGTGTGTC TTGGGATTCA ATAACTGTAC AACAGTGGAG GAAAGCCTTT

ATGGCGTGTC TTGGGATTCA ATAACTTTGC AAGAGTGGAG GAAAGCCTTT

ATGGTGTGTC CTGGGATTCA ATAACTTTGC AACAGTGGAG GAAAGCCTTT

ATGGGGTGTC CTGGGACTCA ATCACTTTGC AGCAGTGGAG GAAAGCCTTT

ATGGTATATC TTGGAATTCA ATACCCTTGC AGCAGTGGAG AAAAGCATTT

ATGGCATATC CTGGGACGCA GTACCTGTGC AGCAGTGGAG GAAGGCGTTT

GGAGTGATAC CACAGGTGAG AGAAAGTATT TATTTTTTCT GGAACATTTA

GGAGTGATAC CGCAGGTGAG AGAAAGTATT TATCTTTTCT GGAACATTTA

GGAGTGATAC CACAGGTGAG AGAAAGTATT TATCTTTTCT GGAACATTTA

GGAGTGATAC CACAGGTGAG AGAAAGTATT TATCTTTTCT GGAACATTTA

GGAGTGATAC CACAGGTGAG AGAAAGTATT TATCTTTACT GGAACATTTA

GGAGTGATAC CACAGGTGAG AGAAAGTGTT TATCTTTTCT GGAACATTTA

GGAGTGATAA CACAGGTGAG AGAAAGTATT TATCTTTTCT GGAACATTCA

GGAGTGATAA CACAGGTGAG AGAAAGTATT TATCTTTTCT GGAACATTCA

GGAGTGATCC CACAGGTGAG AGAAAGTATT TATCTTTTCT GGAACATTTA

GGAGTCATAC CACAGGTAAG AGAAAGTATT CATCTTTTCT GGAACATTTA

GGGGTGATCC CACAGGTAAG AGAAAGTATT TATATTCTCT GGAACATTTA

GGAGTGATCC CGCAGGTGAG AGAAAGTATT TATCTTTTCT GGAACATTTA

GGAGTCATAC CACAGGTAAG AGAAAGTATT TATATTTAAT GGAACGTTTA

GGAGTGATAC CACAGGTGAG AGAAAGTATT TATCTTTTCT GGAACATTTA

GGAGTGATCC CGCAGGTGAG ---------- ---------- ----------

GGGGTGATCC CACAGGTGAG AGAAAGTATT CATCTTTTCT GGAACATTTA

GGAGTAATAC CACAGGTAAG AGAAAGTATT TGTATTTTCT GGGACATTTA

GGAGTAATAC CTCAGGTAAG AGAAAGTATT CATATTTTCC GGAAGTTTTC

GAAAAAACTT GGATCCCTAT GAACAGTGGA GTGATCAAGA AATATGGAAA

GAAAAAACTT GGATCCCTAT GAACAGTGGA GTGATCAAGA AATATGGAAA

GAAAAAACTT GGATCCCTAT GAACAGTGGA GTGATCAAGA AATATGGAAA

GAAAAAACTT GGATCCCTAT GAACAGTGGA GTGATCAAGA AATATGGAAA

GAAAAAACTT GGATCCCTAT GAACAGTGGA GTGATCAAGA AATATGGAAA

GAAAAAACTT GGATCCCTAT GAACAGTGGA GTGATCAAGA AATATGGAAA

GACAAAACTT GGATCCCAAT GGAAAATGGA GAGATGAAGA AATATGGAAA

GACAAAACCT GGATCCCAAT GGAAAATGGA AAGATGAAGA AATATGGAAA

GAAAAAACTT GGATCCCTAT GAACAATGGA GTGATCAAGA AATATGGAAA

GAAAAAACTT GGATCCCTAT GGACAGTGGA GTGATCAAGA AATATGGAAA

GAAAAAACTT GGATCCCTAT GGACAGTGGA ATGATCAAGA AATATGGAAA

GAAAAAACTT GGATCCCTAT GGACAATGGA ATGATCAAGA AATATGGAAA

GAAAAAACCT GGATCCCTAT GATCAGTGGA ATGATCAAGA AATCTGGAAA

GAAAAAACTT GGATCCCTAT GGACAATGGA GTGATCAAGA AATATGGAAA

---------- ---------- ---------- ---------- ----------

GAAAAAACTT GGATCCCTAT GAACAGTGGA GTGATGAAGA AATATGGAAA

GAAAAAATCT GGATCCTTAT GGGCAATGGA GCGATCATGA ATTATGGAAA

GAAAGAACCT GGATCCCTAT GGGCAGTGGA CAGACCAGGA ACTATGGAAA

GTTGCAGATG AGGTAAAGTG TTAAACACTT ACCCATTGAG GCCCAACCTT

GTTGCAGATG AGGTAAAGTG TTAAACACTT AACCGTTGAG GCCCAACCTT

GTTGCAGATG AGGTAAAGTG TTAAACACTT ACCCGTTGAG GCCCAACCTT

GTTGCAGATG AGGTAAAGTG TTAAACACTT ACCCGTTGAG GCCCAACCTT

GTTGCAGATG AGGTAAAGTG TTAAACACTT ACCCGTTGAG GCCCAACCTT

GTTGCAGATG AGGTAAAGTG TTAAACACTT ACCCGCTGAG GCTCAACCTT

GTTGCAGATC AGGTAAAGTG CTAAATGCTA ACCCCTTGAG AACCAACCTT

GTTGCAGATG AGGTAAAGTG CTAAATGCTA ACCCCTTGAG AACCAACCTT

GTTGCAGATG AGGTAAAGTG TTAAACACTT ACCTGTTGAG GCCCAACCTT

GTTGCAGATG AGGTAAAGTT T-AAACACTT ACCCATTGAA GTCTAACCTT

GTTGCGGATG AGGTAAAGGG TGAAACACTT ACCCCCTGAG GCCCAACCTT

GTTGCAGACG AGGTAAAGTG TTAAACACTT A-CCACTGAA GCCTAACCTT

GTTACAGAAG AGGTAAAGTG TTAAACACTC ACCCATTGAA GCCCAACCCT

GTTGCTGATG AGGTAAAGTG TTAAACTCTT ATACGTTGAG GCCCAACCCT

---------- ---------- ---------- ---------- ----------

GTTGCAGATC AGGTAAAGTG TTAAACACTT ACCCGTTGAG GCTCAACCCT

GTTGCAGATG AGGTAAAATG TTAAACACTT ACTTTTTGAG GCCCAACCTT

GTGGCAGAGG AGGTAAAGTG TTAAACACTT ACCTGTTGAG GCCCAACCTT

AACATGCCCC TAATAAAATG TACTTAGATT AACCGTTTTC ATTATCAAAG

AACATGCCCC TAATAAAATG TACTTAGATT AACCGTTTTC ATTATCAAAG

AACATGCCCC TAATAAAATG TACTTAGATT AACCGTTTTC ATTATCAAAG

AACATGCCCC TAATAAAATG TACTTAGATT AACCGTTTTC ATTATCAAAG

AACATGCCCC TAATAAAATG TACTTAGATT AACCGTTTTC ATTATCAAAG

AACTTGCCCC TAATAAAATA CACTTAGATT AACCGTTTTC ATTATCAAAG

AACCTTCTCC TAATAAAATG CACTGAGATT AACCGTTTTC ATTATCAGGG

AACCTTCTCC TAATAAAATG AACTGAGATT AACCGTTTTC ATTATCAGGG

AACATGCCCC TAATAAAATG CACTTAGATT AACCGTTTTC ATTATCAAAG

AACATGCCCC TAATAAAATG CACTTAGATT AACTGTCTTC ATTATCAAAG

AATATGCCCC TCATAAAATA CACTTAGATT AGCTATTTTT ATTATCAAAG

AACATGCCCC TAATAAAATG CGCTTAGATT AACTGTTTTC AGTATCAGAG

AACATGTCCC TAATAAAATA CACATAGATT AACCATTTTC ATTATCAAAG

GACATTCCCC TAATAAAATG CCCTTAGATT AACCATTTTC ATTATCAAAG

---------- ---------- ---------- ---------- ----------

AACATGCCTC TAATAAAATG CCTGCACATT AACTGTTGTC ATTACCAAAG

AATATAGACC TAATAAAGTA TACTTAGATT AACTATTTTC ATTACTCCAG

AAAATATCCC TAATAAAATA TAGTTAGATT AAGCCTTTTC AGCAGCCAAG

TTTCCTATTA CCCAACAAAA GACTTTCTTT TTAGAACATA CCAAATAATT

TTTCCTATTA CCCAACAAAA GACTTTCTTT TTAGAGCATA CCAAATAATT

TTTCCTATTA CCCAGCAAAA GACTTTCTTT TTAGAACATA CCAGATAATT

TTTCCTATTA CCCAACAAAA GACTTTCTTT TTAGAACATA CCAGATAATT

TTTCCTATTA CCCAACAAAA GACTTTCTTT TTAGAACATA CCAAATAATT

TTTCCTATTA CCCAACAAAA GACTTTCTCT TTAGAACATA CCAGATAATT

TTTCCTATTA CCCAGCAAAA GACTTGTTTT GTAGAACATA CCAAGTGATT

TTTCCTATTA CCCAGCAAAA AACTTGCTTT GTAGAACATA CCAAGTCATT

TTTCCTATTA CCCAACAAAA GACTTTCTTT TTAGAACATA CCAAATAATT

TTTCCTATTA CTGAACAAAA GAtttttttt tTAGAACATA CCAGATCATT

TTTCCTATTA CCCAACAAAA GATTTCCTTT TTAGAACATA CCAAATCATT

TTTCCTATTA CCCAACAAAA GCCTGTCTTT TTAGAACATA CCAAATCATT

TTTCCTATTA CCCAACAAAA GATTTTCTTT TTAGAACATA TCAAATCATT

TTTCCTATTA CCCAACAAAA GACTTTCTTT TCAGAACTTA CCAAATCATT

---------- ---------- ---------- ---------- ----------

TTTCCTATTA CCAAACCAGA GACTTCCTTT TTAGAACATA CCAAATCATC

TTTCCTATTA CCCAACAAAA GTTTTTCTTt ttAGAACATA TCAAGTCATT

TTTCCTATTA CCCAACAGAA GACTTTCTCC TTAGAACATA CCAGGTCATT

AGAAGAACTC TAAAACAAGC ATTTGCTGAT TGCACAGTAA TTCTCTGTGA

AGAAGAACTC TAAAACAAGC ATTTGCTGAT TGCACAGTAA TTCTCTGTGA

AGAAGAACTC TAAAACAAGC ATTTGCTGAC TGCACAGTAA TCCTTTGTGA

AGAAGAACTC TAAAACAAGC ATTTGCTGAC TGCACAGTAA TCCTCTGTGA

AGAAGAGCTC TAAAACAAGC GTTTGCTGAT TGCACAGTAA TCCTCTGTGA

AGAAGAACTC TAAAACAAGC ATTTGCTGAC TGCACAGTAA TCCTTTGTGA

CGACGAGTTC TAAGACAAGC CTTCGCTGGT TGCACAGTAG TCCTCTGTGA

CGACGAGTTC TAAAACAAGC CTTCGCTGGT TGCACAGTCA TCCTCTGTGA

CGGAGAACTC TGAAACAAGC ATTTGCTGAT TGCACAGTAA TCCTCTGTGA

CGAAGAACCC TAAAACAAGC ATTTGCTAAT TGCACAGTAA TCCTCTCTGA

CGAAGAACCC TAAAGCAAGC GTTTGCAGAT TGCACAGTGA TCCTTTCTGA

CGCAGAGCTC TAAAACAAGC GTTTGCTGAT TGCACCGTGA TCCTCTGTGA

CGAAGAACTC TAAAGCAAGC ATTTGCTGAT TGCACAGTAA TCCTCTGTGA

CGAAGAACGC TAAAACAAGC ATTTGCTGAT TGCACAGTAA TCCTCTGTGA

---------- ---------- ---------- ---------- ----------

CGAAGAACTC TAAAACAAGC ATTTGCTGAT TGCACAGTCA TCCTCTGTGA

CGGAGAACAC TGAAGAATGC ATTTGCCAAC TGCACTGTAA TCCTCTCTGA

CGCAAAACTC TAAAGCAAGC ATTTACCAAC TGCACAGTCA TCCTCTCAGA

ACACAGGATA GAAGCAATGC TGGAATGCCA ACAATTTTTG GTGAGCTTTA

ACACAGGATA GAAGCAATGC TGGAATGCCA ACAATTTTTG GTGAGTTTTA

ACACAGGATA GAAGCAATGC TGGAATGCCA ACAATTTTTG GTGAGCTTTA

ACACAGGATA GAAGCAATGC TGGAATGCCA ACAATTTTTG GTGAGCTTTA

ACACAGGATA GAAGCAATGC TGGAATGCCA GCAATTTTTG GTGAG----A

ACACAGGATA GAAGCAATGT TGGAATGTCA ACGATTTTTA GTGAGATTTG

ACACAGGATA GAAGCAATGT TGGACTGTCA GCGATTTTTG GTGAGCCTTG

ACACAGGATA GAAGCGATGT TGGATTGCCA GCGATTTTTG GTAAGACTTG

ACACAGGATA GAGGCAATGT TGGAATGCCA ACGATTTCTG GTGAGATTTA

ACACAGGATA GAAGCAATGT TGGAATGTCA ACGATTTTTG GTGAGATTTA

ACACAGGATA GAAGCGATGT TGGAATGTCA ACGATTCTTG GTGA-ATTTG

ACACAGGATA GAAGCAATGT TGGAATGCCA GCGATTTTTG GTGAGATTTA

GCACAGGATA GAGGCAATGT TGGAATGCCA ACGATTTTTA GTGAGAAATA

GCACAGGATA GAAGCAATGT TGGAATGTCA ACGATTTTTG GTGAGATTTA

---------- ---------- ---------- ---------- ----------

GCACAGGATA GAAGCGATGT TGGAATGTCA GCGCTTTTTG GTGAGATTCA

ACACAGGATA GAAGCAATGC TGGAGTGTCA GCGATTTTTG GTGACATTTA

ACACAGGATA GAAGCAATGC TCGAGTGTCA ACGCTTTCTG GTGAGTTTTA

CTAGGTCATA GAAGAGAACA AAGTGCGGCA GTACGATTCC ATCCAGAAAC

CTAGGTCATA GAAGAGAACA AAGTGCGGCA GTACGATTCC ATCCAGAAAC

CTAGGTCATA GAAGAGAACA AAGTGCGGCA GTATGATTCC ATCCAGAAAC

CTAGGTCATA GAAGAGAACA AAGTGCGGCA GTATGATTCC ATCCAGAAAC

CTAGGTCATA GAAGAGAACA AAGTGCGGCA GTATGATTCC ATCCAGAAAC

CTAGGTCATA GAAGAGAACA AAGTGCGGCA GTACGACTCC ATCCAGAAAC

TTAGGTCATA GAGCAGGGCA ATGTCTGGCA GTATGAATCC CTCCAGGCAC

ATAGGTCATA GAAGAGAGCA ATGTCTGGCA GTACGACTCC CTTCAGGCAC

CTAGGTCATA GAAGAGAACA CAGTGCGGCA GTACGAATCC ATCCAGAAAT

CTAGGTCATA GAGGAGAACA AGGTGCGGCA GTATGATTCC ATCCAGAGGA

CTAGGTCATC GAGGACAGCA GGCTGCGGCA GTTCGAGTCC ATCCAGAGGC

CTAGGTCATA GAGGAGAACA AAGTACGGCA GTACGATTCC ATGCAGAAGC

CTAGGTCATA GAGGAGAACA AAGTGCGGCA GTATGACTCC ATCCAGAAGC

CTAGGTCATA GAGGAGAACA AAGTGCGGCA GTACGATTCC ATCCAGAGGC

---------- ---------- ---------- ---------- ----------

CTAGGTCATA GAGGAGAACA AAGTGCGGCA GTACGATTCC ATCCAGAAGC

CTAGGTCATA GAAGAGAACA AAGTGCGTCA GTACGAGTCA ATCCAGAAGC

CCAGGTGATA GAGGAGAACA AGGTCCGGCA GTACGAGTCT ATCCAGAAGC

TGCTGAACGA GAGGAGCCTC TTCCGGCAAG CCATCAGCCC CTCCGATGAA

TGCTGAACGA GAGGAGCCTC TTCCGGCAAG CCATCAGCCC CTCCGATGAA

TGCTAAATGA GAGGAGCCTC TTCCGGCAAG CCATTAGCCC CTCCGATGAA

TGCTGAATGA GAGGAGCCTC TTCCGGCAAG CCATCAGCCC CTCCGATGAA

TGCTGAACGA GAAGAGCCTC TTCCGGCAAG CCATCAGCCA CTCAGATGAA

TGCTGAGCGA GAAGAGCCTC TTCCGACAGG CCATCAGCCC CTCAGATGAA

TTCTGAGCGA GAAGAGTGTC TTCCAGCGAG CCCTTAGCTC CTCAGATGAA

TTCTGAGTGA GAAGAGTATC TTCCAGCAGG CCATTAGCTC CTCGGATGAA

TGCTGAGTGA GAAGAGCCTC TTCCGGCAGG CCATCAGCTC CTCGGATGAA

TGCTGAGCGA GAAGAGCCTC TTCCGGCAGG CCATCAGCCC CGCAGA---G

TGCTGAGCGA GAGGAGCGCC TTCCGGCAGG CCATCGGGCC CCCCGA--GG

TTCTGAGCGA AAAGAGCCTC TTCCAGCAGG CCATCAGCCC CTCGGATGAA

TGCTGAGTGA GAAGAGCCTC TTCCGCCAGG CCATCAGCTC CTCTGATGAA

TGCTGAGCGA GAAGAGCCTC TTCCGGCAGG CTATCAGCCC CTCAGATGAA

---------- ---------- ---------- ---------- ----------

TGCTGAGCGA GAAGAGCCTC TTCCGGCAGG CCATCAGCCC TTCGGATAAA

TGGTGAATGA GAAGAGTCTC TACAGGCAAG CCATCAGTCA CTCAGATGAA

TGCTGAATGA GAAGAGCGTC TTCAAGCAGG CCATCAGCCA CTCGGATGAA

TTAGTTTTAT GAATTTATTT CTATATCCAC TTAGGTACAA AACTTTTGTA

TTAGTTTTAT GAATTTATTT CTATATCCAT GTAGGTACAA AACTTTTGTA

TTAGTTTTAT GAATTTATTT TTATATCCAC TTAGGTACAA AACTTTTGTA

TTAGTTTTAT GAATTTATTT TTATATCCAC TTAGGTACAA AACTTTTGTA

TTAGTTTTAT GAATTTCTTT TTATATCCAC TTAGGTACAA AACTTTTGTA

TTAGTTTTAT AAATTTATTT TTATATCCAC TTAAGTACAA AACTTTTGTA

TTAGTTTTAT ACATTTAATA CTATATCCAC TTAGGTACAA AAGTTTTGTA

TTAGTTTTAT ACATTTAATA CTATATCCAC TTAGGTACAA AAGTTTTGTA

TTAGTTTTAT AAACTTAATT TTATAGTCAC TTAGGTATAA AACTTTTGTA

TGAATTTTAT -TATTTATTT TTATATCTAC TTAGGTACAA AACTTTTGTA

TTCACTTTAT AAATTTATT- ----ATCCAC TTAGGTACAA AACTTTTGTA

TTCATTTTAG G-ATTTATTT TTATATCCAC TTAGGTACAA AACTTTTATA

TTAGTTTTAT GAACTTAATA TTATATCCAC TTAGGTACAA AACTTTTGTA

TTCACTTTAT GAATTTATTT TTATATCCAC TTAAGTACAA AATCTTTATA

---------- GAATTTATTT TTATATCCAT TTAGGTACAA AACTTTTGTA

CTTGTTTTAT AAATTTATTT TTATATCCAT TTAGGTACAA AACTTTTATA

TCAGTTTTTG ATATTTCTTT TTATATCCAT TTAAGAGCAG AAAAATCATA

TCAGTTTTTA AAATTCTCTT TTTCATCCAC TGAAGCAGAA AAGTTTTGTA

AGAAAGATAA CACTTTTATT GCATTATAAT TTCATATTTT ACAGGAGTCA

AGAAAGATAA CACTTTTATT GCATTATAAT TTCGTATTTT ACAGGAGTCA

AGAAAGATAA CACTTTTATT GCATTATAAT TTCATATTTT ACAGGAGTCA

AGAAAGATAA CACTTTTATT GCATTATAAT TTCATATTTT ACAGGAGTCA

AGAAAGATAA CACTTTTATT GCATTATAAT TTCATATTTT ACAGGAGTCA

AGAAAGATAA TACTTTTATT GCATTATAAT TTCATGTTTT ACAGGACTCA

AGAAAGATAA CACTTTTATT GCATTATAAT TTCATATTTT ACA--AGTCA

AGAAAGATAA CACTTTTATT GCAGTATAAT TTTATATTTT ACA--AGTCA

AGAAAGATAA CAC--TTATT GCATTATAAT TTCATCTTTT AAAGGAGTCA

AGAAAGATAA CACTTTTATT GCATTATAAG TTCATATTT- ACAGGAGTCA

AGAAAGATAA CACTTTTATT GCATTATAAT CTCATATTTT GCAGGAGTCA

AAAAAGATAA CA-TTTTATT GCattataat tttatatat- ----taGTCA

AGAAAGATAA CACTTTTATT GCATTATA-C TTCATATTTT ATAGGAGTCA

AGAAAGATAA CACTTTTATT GCATTATAAT TTCATATTTT ATAGGAGTCA

AGAAAGATAA CACTTTTATT TCATTATAAT TTCGTATTTT ATAGGATTTA

AGAAAGATAA CACTTTTATT GCATTATAAT TTCATATTTT ATAAGAGCTA

AAAAAGATAA CATATTTATT GCATTAGAAT TTCATTCTTT ACAGAAGTCA

AGAAAGATTC CATGTTTATT GCATTGTCAT TTTATCTGTA GCCAGAATCA

TAATGCAAAC TTATAGCATA AATATACATG ATGCTACCAA ATGGCAATGT

TAATGCAAAC TTATAGCATA AAtatacatg atgctaCCAA ATGGCAATGT

TAATGCAAAC TCATAGCATA AATATACATG ATGCTACCAA ATGGCAATGT

TAATGCAAAC TCATAGCATA AATATACATG ATGCTACCAA ATGGCAATGT

TAACGCAAAC TCATAGCATA AATATACATG ATGCTACAAA ATGGCAATGT

GAATGCAAAC TCATAGCATA AATAGTCATG ATGCAACCGA ATGATAATGT

TAATGCAAAT TAAGAGCGTA AATATACATG ATGCAACCAG ATGGCAATGT

TAATGCAAAC TCAGAGAGTA AATATACATG ATGCAACCAG ATGGCAATGT

TAATGCAAAC TCATAGCATA AATATACATG AT-CAACCAA ATGGTAACAT

TAACACAAAC CCATAGCATA AATATACATG ATGCAACCA- ----------

TAATGCAAAC TCATAGCATA AATATACATG ATGCAACCAA TCAGCAATGT

CAATGCAAAC TCATAGCATA AATATACATG ATGCAACCAA CCAGCAATGT

TAATGCAAAC TCATAGCATA AATATACATG ATACAACCAA TCAGCAATGT

TAATGCAAAC TCATAGCATA AATATACATG ATGCAACCAA ACAACAATGT

TAATGCAAAC TCGTAGCATA AATATACACG ATGCAACTAA ACAGCACTGT

GAATGCAACG TTATAGCATA AATATACATG ACGCAACCAA ACAACAATGT

TGATGCAAAC TCATAGCATT AATATACATG ATGTAACCAA TTAGCAATGT

TATTTCAAAC CCATCGCATT AATATACATG GTGCAGTTCA ACAGCTAGGT

AACCACTAAG AGAAAAACAT AAAACTAGAA TTTAACAAGC AAAATACTTA

AACCACTAAG AGAAAAACAT AAAACTAGAA TTTAACAAGC AAAATACTTA

AACTACTAAG AGAAAAACAT AAAACTAGAA TTTAACAAGC AAAATACTTA

AACTACTAAG AGAAAAACAT AAAACTAGAA TTTAACAAGC AAAATACTTA

AACCACTAAA AGAAAAACAT AAAATTAGAA TTTAACAGGC AAAATGCTTA

CACCACTAAA AGAAAAACAT AAAACTAGAA TTTAACAGGC AAAATACTTC

AACCACTAAA AGAAAAATGT AAAACTAGAA TTTAACAGGC AAAATACTTC

AACCACTAAA AGAAAAATGT AAAACTAGAA TTTAACAGGC AAAATACTTT

AGCCACTAAA AGAAAAATGT AAAAC----- TTTAGCAGGC AAAATACTTA

-----TTAAA AGTGAAACTG AAAACTAGAG TTAAACAGGC AAAATACTTA

AACCAGTAAG AGAAAGATGT AAAACTAGAA TTTAACAGGC AAAATACTTA

AACCATTAAA ATAAAAATGT AAAACTAGAA TTTAACAGGC AAAATACTTA

AACCATTAAA AGAAAAATGT AAAACTAGAA TTTAACAGGC AAAATACTAA

AGCaattaaa ataaaaatgt aaaattagaa tttaaCAGGC AAAATACTTA

AACCATTAAA AGAAAAATGT GAAACTAGAA TTTAACAGGC AAAATACTTA

AACCTTTAGA AGAAGAATGT AAAACTAGAA TTTAACAGGC AACATACTTA

AGGTAATATA AGAAAAACAC AAAACCAAAA TTTAACAGGC AAAATATTTA

AAGCATCTTA AGAAAAACAC AAAATTAGGG TTTGATAATC AAAGTGTTTA

ATATGGCTTT TAATGGAAAA TAACTGTTTA GAAATGATTT GTTATTGCCC

ATATGGCTTT TAATGGAAAA TAACTGTTTA GAAATGATTT GTTATTGCCC

ATATGGCTTT TAATGGAAAA TAACTGTTTA GAAATGATTT GTTATTGCCC

ATATGGCTTT TAATGGAAAA TAACTGTTTA GAAATGATTT GTTATTGCCC

ATATGGCTTT TAATGGAAAA TAACTGTTTA GAAATGATTT GTTATTGCCC

ATATGGCTTT TAATGGAAAA TAACTGTTTA GAAGTGATTT GTTATTGCCC

ATATGGCTTT TAATGGAAAA TAACTGTTTA GAAATGTTTT GTTATTGCCC

GTATGGCTTT TAATGGAAAA TAACTGTTTA GAAATGTCTT GTTATTGCCC

ATATGGCTTT TAATGGAAAA TAACTGTTAA GAAATAATTT ATTG----CC

ATATGGCTTT TAA-GAAAAG TAACTGTTTA GAAATGATTT GTTATTGCCC

ATATGGCTTT TAATGGAAAG TAACTGTTTA GAAATGATTG GTTATTGCCC

ATATGGCTTT TAATGGAAAG TAACTGTTTA GAAATGATTA ATTATTGCCT

ATATGGCTTT TAATGAAAAG TAACTGTTTA GGAAAGATTC ATTATTGCCA

ATATGGCTTA TAATGGAAAG TAACTGTTTA GAAATGATTT GTTATTGCCC

ATATGGCTTG TAATGAAAAG AAACTGTTTA GAAATGATTT ATTATTGCCC

ATATGGCTTG TAATGAAAAG AAATTGT--- --AATGATTT ATTATTGCCC

ATATGGCTTG TAATGGAAAG AAATTGTTGT AAAATAATTT GTTATTGCCA

ATATGGCTTC TAGCAAAAAG GAATCATTTT GCAATCATTT GTTATTGCCA

CATTTCAAGT GAACATAAAA TTATGTCCAT TTAAAACGGT ACAAGTTATC

CATTTCAAGT GAACATAAAA TTATGTCCAT TTAAAATGGT ACAAGTTATC

CATTTCAAGT GAACATAAAA TTATGTCCAT TTAAAATGGT ACATGTTATC

CATTTCAAGT GAACATAAAA TTATGTCCAT TTAAAATGGT ACATGTTATC

CATTTCAAGT GAACATAAAA TTATGTCCAT TTAAAATGGC AAATGTTATC

CATTTCAAAG GAACATAAAA TTAGGTCCAT TTAAAATGGC AAATGTTATC

CATTTCAGAT GAGCATAAAA TTATCTCCAT TCAAACTGGC ACATTTCATC

CATTTCAGAT GAACATAAAA TTATCTCCAT TCAAAATGGC ACATTTCATC

CATTTCAAGT GAACATAAAA TTATGTCCAT TTAAAATGGT ACATGTTATT

CGTTTCAAGT GAACATAAAA TTATGTCTTT AAAA--TGAT ACATGTTATC

CATT-CAAGT GAACATGAAG TCATGTCTAT TTAA------ ----------

CATTTCAAGT GAACAGAAAA TTATGCCCAT TTAAAATGGT GCATTTTATC

CATTTCAAGT GAACATAAAA TTATGTCCAT TTAAAATGGT ACATATTAGT

CATTTCAAGT GAAGATAAAA TTATGCCCAT TTAAAATGGT ACAGGTTATC

CATTTCAAGT GAACAGAGAA TTATGTCCAT TTAAAATGGT ACATGTTTTC

CATTCCAAGT GAACATCAAA TTATGTCCAT TTAAAATGGT ACAAATATTC

CATTTCAAGT AAAGATAAAA TCAAGTCTGT CTATAATGGT ACATTTTGTC

CATTTAGAGT GAAGATAAAA TCACGTTCCT CTAAAATGTT ACATATACTC

TAAGCCAATT CTGTTTTAAT ACCCCTGAAC ATCTTGATTA AACTATTACA

TAAGCCAATT CTGTTTTAAT ACCCCTGAAC ATCTTGATTA AACTATTACA

TAAGCCAATT CTGTTTTAAT ACCCCTGAAC ATCTTGATTA AACTATTACA

TAAGCCAATT CTGTTTTAAT ACCCCTGAAC ATCTTGATTA AACTATTACA

TAAACCAATT CTGTTTTAAT ACCCCTGAAC ATCTTGATTA AACTATTACA

TGAGCCAATT CTGTTTTAAT ACCCCTGAAC ATGTTGATTA AACTATTATA

TAAGCCAATT CTGCTTTAAT ACCCCTGAAC ATCTCCATTA AACGATTACA

TACACCAAAT CTGCTTTAAT ACCCCTGAAC ATCTTGGTTA AATTATTACA

TAAGCCAATT CTGCTTTAAT ACCCCTGAAC ATTTTGATGA AACTGTTACA

TAAGCCAA-- --GTTTTAAT ACCCCTGAAC ATCTTGAGTA AACTATTATG

-AAGCCAA-- --GTTCTAAT ACCCCTGAAC GTGTGGATTA AACTATTACA

TAAGCCAATT CTGTTTTAAC ACCCCTGAAC ATGTTGATTA AACTATTACA

TAAGCAAA-- -TTGTTTAAT ACCCCTGAAC ATGTTGGTTA AACTATTACA

TAAGCCAATT CTGTTTTGAT ACCCCTGAAC ATCTTGATTA AACTAGTACA

TAAGCCAACT CTGCTTTGAT ACCCCTGAAC ATCTTCATTA AACTATTATA

TAAGCCAATT CTGTTTTGAT ACCCCTGGGC ATTTTAACTA AACTATGATA

TATGCTAATT CTGTGGTGAT ACCCCTGAAT ATCTTAATTA AGCTATTACA

TAAGCCAATT CTGTGGTAAT ACCCCTGAAT AGTATCGTTA CACTAGAACA

ATTTTTCTAT TATAAAACTA CTTGAAAAGT AACAAAATGT TGGTTATAAA

ATTTTTCTAT TATAAAACTA CTTGAAAAGT AACAAAATGT TGGTTATAAA

ATTTTTCTAT TATAAAACTA CTTGAAAAGT AACAAAATGT TGGTTATAAA

ATTTTTCTAT TATAAAACTA CTTGAAAAGT AACAAAATGT TGGTTATAAA

ATTTTTCTAT TATAAAACTA CTTGAAAAGT AACAAAATGT TGGTTATAAA

ATTTTTCTAT TATAAAACTA CTTGAAAAGT AATAAAAGGT TGGTTATAAA

ATGTTTCTAT TATAAAACTA CTTGAAAGGT AATGTGA--- -GGTTATAAA

AGGTTTCTAT TATAAAACTA CTTGGAAAGT AATGTGA--- -GGTTATAAA

AGTTTTTTCT TATAAAACTA CTTGAAAAGT GACAAAA-GT TGGCTATAAA

ACTTTTCTAT TATAAAACTA CTTGAAAAGT ---------T TGGTTATAAA

GTATTTCTAT TATAAAACTA CTTGAAAAGT AACAAAATGT CGGTTATAAA

ATATTTCTAT TATAAAACTA CTTGAAAAGT AACAAAATGT TGGTTATAAA

ATTTTTCTAT TATAAAACTA CTCCAAAAGT AACAAAATGC TGGTTATAAA

ATTTTTCTAT TATAAAACTA CTTGAAAAGT AACAAATTGT TGGTTATAAA

ATTTTTCTAT TATAAAACTA CTTGAAAAGC AAAAAAAAGT TGGTTATAAA

ATTTTTCCAT TATAAAACTA CTTGAGAAGC AAGAAAATGT GGGCAATAAA

ATTTTCATAA TAGAAAACTA CTTGAAAAGT ATTAAAACAT TGGCTATAAA

AGTTTCATAT TAGAAAACAA TTTGAAAACT AACTCAAAGT TGGCTAGAAA

TATCTTTACA AAAAATTGAA TAGTGGTCCC GCACTCATAA TTTATATTAC

TATCTTTACA AAAAATTGAA TAGTGGTCCT GCACTCATAA TTTATATTAC

TATCTTTACA AAAAATTGAA TAGTGGTCCC GCACTCATAA CTTATATTAC

TATCTTTACA AAAAATTGAA TAGTGGTCCC GCACTCATAA CTTATATTAC

TATCTTTACA AAAAATTGAA TAGTGGTCCC GCACTCATAA TTTATATTAC

TATCTTTACA AAATATTGAA TAGTGGTCCC GCACTCATAC TTTATATTAC

TATCTTTACA AAAGGCTGAA TAGTGGTCCC GCACCTGTCC TTTATATTAC

TATCTTTACA AAAAGCTGAA TAGTGGTCCC GCACGCCTCC TTTATATTAC

TATCTTTACA AAAAA-TGAA TAGTGGTCCC GCACTCATAC TTTATATTAC

TATCTTTACA AAAAATTGAA TAGTGGTCCC GCACTCATCG TTTATATTGC

TATCTTTACA AAAAATTGAA TAGTGGTCCC GCACTCATAC TTTATGTTAC

TATCTTTACA AAAAATTGAA TAGTGGTCCC GCACTCATAC TTTATATTAC

TATCTTTACA AAAATTTGAA TAGTGGTCCC GCACTCATAC TTTATATTAC

TATCTTTACA AAAAATTGAA TAGTGGTCCC GCACTCATCC TTTATATTAC

TATCTCTACA AAAAATTGAA TAGTGGTCCC GCACTCATAC TTTATATCAC

TATCTTTACA AAGA-TTGAA TAGTGGTCCC GCA-TCACAC CTTATATTAC

CATCTTTACT CAAAATTGAA TCGTGGTCCC ATAAGCATAC AATATGTTAC

CAGCTTTACT AAAAACTGAA TTGTGGTCCT ATACACACAC ATCATAGTAT

AGTGAAAGTA GGCAGGCCTA TTTGTTAGGT TTTTCTAGTG TTCATTTTTG

AGTGAAAGTA GGCAGGCCTA TTTGTTAGGT TTTTCTAGTG TTCATTTTTG

AGTGAAAGTA GGCAGGCCTA TTTGTTATGT TTGTCTAGTG TTCATTTTTG

AGTGAAAGTA GGCAGGCCTA TTTGTTAGGT TTGTCTAGTG TTCATTTTTG

AGTGAAAGTA GGCAGGCCTA TTTGTTAGGT TTTTCTAGTG TTCATTTTTG

AGTGAAAGTA GGCAGGCCTA TTTGTTAGGT TTTTCTATTG TTCGTTTTTG

AGGAAAAGTA GGCAGGCCTA TTTGTTACGT TTTTCTAGTG TTCGTGTTTG

AGGAAAAGTA GGCAGGCCTA TTTGTTTGGT TTTTCTATTG TTCGTGTTTG

AGTGAAAGTA GGCAGGCCTA TGTGTTAGGT TTTTCTATTG TTCATGTTTG

AGTGAAAGTA GGCAGGCCTA TTTGCTAGAT TTTTCTATTG TTCATTTTTG

AGTGAGAGTA GGCAGGCCTG TTGGTTTGGT TTTGCGATTG TTCGTTTTTG

AGTGAAAGTA GGCAGGCCTA TTTGTTAGGT TTTTCTATTG TTCATTTTTG

AGTGAAAGTA GGCAGGCCTA TTTGTTAGGT TTTTCTA--- TTCGTTTTTG

AGTGAAAGTA GGCAGGCCTA TTTGTTAGGT TTTTGTATTG TTCATTTTTG

AATGAAAGTA GGCAGGCCTA TTTGTTAGGT TTTTCTATTG TTCATTTTTG

AGAGAAAGTA GGCAGGCCTA TTGGTTAGGT TTTTCTATTG TTCATTGTTG

AATAGCGGTA GGTAGATCTA TTTGTGTGTT TTTTCTATTG CACATTTTGG

AGTGAAAGGA GGCAGGCCTA TTGGTTAGGT ATACCTATTG TATGTTTTTG

TGTAAGTTCC AATTCACTTC TTTTGAGTTG TTGTTTTCTA TTTGCCTTGT

TGTAAGTTCC AATTCTCTTC TTTTGAGTTG TTGTTTTCTA TTTGCCTTGT

TGTAAGTTCC AATTCTCTTC TTTTGAGTTG TTGTTTTCTA TTTGCCTTGT

TGTAAGTTCC AATTCTCTTC TTTTGAGTTG TTGTTTTCTA TTTGCCTTGT

TGTAAGTTCC AATTCTCTTC TTTTGAGTTG TTGTTTTCTA TTTGCCTTGT

TGTAAGTTCC AATTCTCTTC TTTTGAGTTG TTGTTTTCTA TTTGCCTTGT

TCTACGTTCC AATTCTCTTC TTTGGAGTTG TTATTTTCTA GTTGCCTTGT

TCTAAGGTCC AATTCTCTTC TTTGGAGTTG TTGTTTTCTA GTTGCCTTGT

TGTAGGTTCC AATTCTCTTC TTTGGAGTTG TTGTTTTCTG GTTGCCTTGT

CGTAGGTTCC AAATCTCTT- ----GAGTTG TTGTTTTCTA TTTGCCTTGT

TGGAAGTTTC AAATCTCTTC TTTTGAGTTG TTGTTTTCGG TTTGCCTTGT

TGTAAGTTCC AAATCTCTTC TTTTGAGTTG TTGTTTTCTA TTTGCCTTGT

CGTAAATTCC AAATATCTTC TTTTGTGTTG TTGTTTTCTA TTTGCCTTGT

CATAAGTTCC AAATCTCTTC TTTTGAGTTG TTATTTTCTG TTTGCCTTGT

CGTAAGTTCC AAATCTCGTC TTTTGAGTTG TTATTTTCTA TTTGCCTTGT

CGTAAGTTCC AAATCTCGTC TTTTGAGTTG TTATTTTCTA TTTGCCTTGT

CGTGAGTTCC AAATCTCTTC TTTTGAGTTA TTTCCTTCTA TTTGCCTTGT

CGTAAGACCC AAAACTCTTC TTTTAAGTTG TTGTTTTCTC TTTGCCTTGT

ATTACTGCTG CTGCTGCTTC TTTTGGTGTT CTGGGAACAC TGGGTGACTT

ATTACTGCTG CTGCTGCTTC TTTTGGTGTT CTGGGAACAC TGGGTGACTT

ATTACTGCTG CTGCTGCTTC TTTTGGTGTT CTGGGAACAC AGGGTGACTT

ATTACTGCTG CTGCTGCTTC TTTTGGTGTT CTGGGAACAC AGGGTGACTT

ATTACTGCTG CTGCTGCTTC TTTTGGTGTT CTGGGAACAC TGGGTGACTT

ATTACTGCTG CTGCTGCTTC TTTTGGTGTT CTGGGAACAC TGGGTGGCTT

ATTGCTGCTG CTGCTGCTTC TTTTGGTGTT CTGGGAACAC TGGGCGACTT

ATTGCTGCTG CTGCTGTTTC TTTTGGTGTT CTGGGAACAC TGAGCGACTT

ATTACTGCTG CTGCTGCTTC TTTTGGTGTT CTGGGAACAC TGGGCGATTT

ATTACTGCTG CTGCTGCTTC TTTTGGTGTT CTGGGAACAC TGGGTGACTT

ATTACTGCTG CTGCTGCTTC TTTTGGTGTT CGGGGAACAC TGGGTGGCTT

ATTACTGCTG CTGCTGCTTC TTTTGGTGTT CTGGGAACAC AGGGTGGCTT

ATTACTGCTG CTGCTGCTTC TTTTGGTGTT CTGGGAACAC TGGGTGACTT

ATTACTGCTG CTGCTGCTTC TTTTGGTGTT CTGGGAACAC TGGGAGACTT

ATTACTGCTG CTGCTGCTTC TTCTGGTGTT CTGGGAACAC TGGGCGACTT

ATTACTGCTG CTGCTGCTTC GTTTGGTGTT CTGGGAGCAC TGGGTGATTT

ATTACTGCTG CTGCTGCTTC TTTTGGTGTT CGGAGAACAC TGGGTGACTT

ATTACTGCTG CTGCTGCTTC TTTTGGGGTT CGGAGAACAC TGTGCGACTT

TACTTCTAGG AACAGGAAGA AAAGATTTAA CTCTTGAAAC ACCCAACTCA

TACTTCTAGG AACAGGAAGA AAAGATTTAA CTCTTGAAAC ACCCAACTCA

TACTTCTAGG AACAGGAAGA AAAGATTTAA CTCTTGAAAC ACCCAACTCA

TACTTCTAGG AACAGGAAGA AAAGATTTAA CTCTTGAAAC ACCCAACTCA

TACTTCTAGG AACAGGAAGA AAAGATTTAA CTCTTGAAAC ACCCAACTCA

TACTTCTAGG AACAGGAAGA AAAGATTTAG CTCTTGAAAC ACCCGACTCA

TACTTCTAGG AACAGGAAGA AAAGATTTAA CTCTTGAGAC ACCCAACTCA

TGCTTCTAGG AACAGGAAGA AAAGATTTAA CGCTTGAGAC ACCCATCTCA

TACTTCTAGG AACAGGAAGA AAAGATTTAA CTCTTGAAAC ACCCAACTCA

TACTTCTAGG AACAGGAAGA AAAGATTTAA CTCTTGAAAC ACCCAACTCA

TACTTCTAGG AACAGGAAGA AAAGATTTAA CTCTCGAAAC ACCCAACTCC

TACTTTGAGG AACAGGAAGA AAAGATTTAA CTCTTGAAAC ACCCGACTCA

TACTTCTAGG AACAGGAAGA AAAGATTTAA CTCTTGATAC ACCCAACTCA

TACTTCTAGG AACAGGAAGA AAAGATTTAA CTCTTGAAAC ACTCAACTCA

TACTTCTAGG AACAGGAAGG AAAGATTTCA CTCTTGAAAC ACCCAACTCG

TACTTCTAGG AACAGGGAGA AAAGATTTAA CTCTTGAAAC GCCCAACTCG

TATTTCTAGG TACAGGGAGA AAAGATTTTA CTCTTGAAAC GCCCAACTCA

TATTCCTGGG CACAGGGAGG AAAGATTTCA CTCTCGAAAC CCCCGACTCG

GTCTTTGATT TACTGTTGCT GCATTCAGTA GTTTGATGGC TGCTGAGAGG

GTCTTTGATT TACTGTTGCT GCATTCAGTA GTTTGATGGC TGCTGAGAGG

GTCTTTGATT TACTGTTGCT GCATTCAGTA GTTTGATGGC TGCTGAGAGG

GTCTTTGATT TACTGTTGCT GCATTCAGTA GTTTGATGGC TGCTGAGAGG

GTCTTTGATT TACTGTTGCT GCATTCAGTA GTGTGATGGC TGCTGAGAGG

GGCTTGGATT TATTGTTGCT GCATTCGGTA GTTTGGTGGC TGCTGAGAGG

GTCTTGGATT TACTGTTGCT GCATTCGGTA GTTTGATGGC TGCTGAGAGG

GTCTTTGATT TACTGTTGCT GCATTCGGCA GTTTGACGGC TGCTGGGAGG

GTCTTTGATT TACTGGTGCT GCATTCGGTA GCTTGATGGC TGCTGAGAGG

GTCTTTGATT TACTGTTGCT GCATTCGGTT GTTTGATGGC TGCTGAGAGG

GTCTTGGATT GCCTGTTGCT GCATTCGGCC GCTTGGTGGC TGCTGAGAGG

GTCTTGGATT TACTGTCGTT GCATTCGGTA GTTTGATGGC TGCTGAGAGG

GTCTTTGATT TATTGTTGCT GCACTCGGTA GTTTGATGGC TGCTGAGAGG

GTCTTTGACG TACTGGTGCT GCGTTTTGTA GTTTGATGGC TGCTGACAGG

GTCTTTAATT TACTATTGCT GCATTCTGTA GTTTGATGGC TGCTGAGAGG

GTCTTTAGCG TCCTGTTGCT GCGTTCCATA GTTTGATGGC TGCTGAGAGG

ATTTTTGGTT TGCTGATACT GCATTCTACA GTTGGACTGC TGCTGAGAGG

GGTTTCGGTG TGTTGTTGCT GCATTCAAGA GTTGGATTGC TGCTAAGAGG

ACTGACCTCC TGCTCACCTT GCTGATATCA GACTCAGACT TGCTGGAGCA

ACTGACCTCC TGCTCACCTT GCTGATATCA GACTCAGACT TGCTGGAGCA

ACTGACCTCC TGCTCACCTT GCTGATATCA GACTCAGACT TGCTGGAGCA

ACTGACCTCC TGCTCACCTT GCTGATATCA GACTCAGACT TGCTGGAGCA

ACAGACCTCC TGCTCACCTT GCTGATATCA GACTCAGACT TGCTGGAGCA

ACTAACCTCC TGCTCACCTT GCTTATATCA GATTCAGACT TGCTGGAGCA

ACTGGCTTCC TACTTACCTT GCTTATATCA GACTCAGATT TGCTGGAACA

ACTGGCTTCC TACTTACCTT ACTTATATCA GACTCAGATT TGCTGGAACA

ACTGACCTCC TGCTCACCTT GCTTAGGTCA GATTCCGACT TGCTGGAGCA

ACTGACCTCC TGCTTACCTT GCTTATATCA GATTCAGATT TGCTAGAGCA

ACTGGGCTCC TGCTTACCTT GCTTATGTCA GATTCAGATT TGCTGGAGCA

ACTGACCTCC TGCTTACCTT GCTTATATCA GATTCGGATT TGCTGGAGCA

ACTGACCTCC TGCTTACCTT GCTTATGTCA GATTCTGATT TGCTGGAGCA

ACTGACCTCC TGCTTACCTT GCTTATATCA GATTCCGATT TGCTGGAACA

ACTGACCTCC TGCTCACCTT GCTTATATCA GATTCAGATT TGCTGGAACA

ACTGACCTCC TGCGCACCTT GCTTATATCA GACTCGGATT TGCTAGAGCA

ACAGACATCC TATTTACCTT GCTGATATCA GATTCAGATT TGCTGGAACA

ACAGACCTCC TGCTCACCTT GCTTATGTCA GATTCAGATT TACTGGAACA

CATGCTCTGA AGTTCCTTGA CAAGATCTGC TTCGTCATCT GAACCCAGAG

CATGCTCTGA AGTTCCTTGA CAAGATCTGC TTCGTCATCT GAACCCAGAG

CATGCTCTGA AGTTCCTTGA CAAGATCTGC TTCGTCATCC GAACCCAGAG

CATGCTCTGA AGTTCCTTGA CAAGATCTGC TTCGTCATCC GAACCCAGAG

CATGCTCTGA AGTTCCTTGA CAAGATCTGC TTCATCATCT GAACCCAGAG

CATACTCTGA AGTTCCTTCA CCAGATCCAC TTCATCATCT GAGCCCAGAG

CATACTCTGG AGTTCTTTGA CCAGATCTGC CTCATCATCT GAACCCAAAG

CATACTCTGG AGTTCTTTGA CCAGATCTGC TTCATCATCT GAACCCAAAG

CATGCTCTGA AGCTCCTTGA CGAGATCTGC TGCATCGTCT GAGCCCAGAG

CATGCTCTGA AGTTCCTTGA CAAGATCTGC TTCATCATCC GAACCCAGAG

CATACTCTGA AGCTCCTGGA CGAGGTCGGC TTCGTCGTCC GAACCCAGAG

CATACTCTGA AGTTCCTTGA TAAGGTCAGC TTCATCGTCT GAACCCAGAT

CATACTCTGA AGTTCTTTAA CAAGGTCCAT TTCATCATCG GAACACAGAG

CATACTCTGA AGTTCCTTAA CAAGGTCTGC TTCATCATCA GAACCTAGAG

CATGCTCTGA AGTTCCTTAA CAAGGTCGGC TTCATCATCT GAACCCAGAG

CATGCTCTGC AGTTCCTTAA CGAGGTCTGC TTCGTCATCG GAGCCCAGAG

CATACTCTGA AGTTCTTTCA CAAGGTCTAC CTCATCGTCT GAACCGAGAG

CATGCTCTGA AGTTCCCTCA CAAGGTCTAC CTCGTCATCG GAACCTAGAG

AGAGTCTCTG ATCCAAATTC AAGGCCAGAC TTCCTGCGAG GACTGGTGTT

AGAGTCTCTG ATCCAAATTC AAGGCCAGAC TTCCTGCGAG GACTGGTGTT

AGAGTCTCTG ATCCAAATTC AAGGCCAGAC TTCCTGCGAG GACTGGTGTT

AGAGTCTCTG ATCCAAATTC AAGGCCAGAC TTCCTGCGAG GACTGGTGTT

AGAGTCTCTG ATCCAAATTC AAGGCCAGAC TTCCTTCGAG GACTGGTGTT

AGAGTCTCTG ATCCAAATTC AAGGCCAGAT TTCCTGCGAG GACTGGTGTT

AGAGTCTGTG GTCCAAAGTC AAGCCCAGGT TTCTTGCGAG GACTGGTGTT

AGAGTCTGTG GTCCAAAGTC AAGCCCAGGC TTCTTTCGAG GACTGGTGTT

AGAACCTCTG ATCCAAATTC AAGGCCAGAC TTCTTGCGGG GACTGGTGCT

AGAGTCTTTG ATCCAGATTC AAGGCCAGAC TTCTTGCGAG GACTGGTGCT

AGAGCCTTTG TTCCAAATTC AAGGCCGGAC TTCTTGCGAG GACTGGTGCT

AGAGTTTTTG ATCCAAGGTC AAGGCCAGAC TTTTTGCGAG GACTCGTGCT

AGAGTCTTTG ATC------C AA-------- ---------- ----------

AGAGTCTTTG GTCCAGATTC AAGGCCTGAC TTCTTGCGAG GATTGGTGCT

AGAGTCTTTG ATCCAGATTC AAGGCCAGAC TTCTTGCGAG GACTGGTGCT

AGAGCCTTTG GTCCAGATTC AAAACCAGAC TTCTTGCGAG GACTGGTGCT

ACAATCTTTG CTCCAGATTC AAGACCAGAC TTCTTTCGGG GACTAGTGTT

ACAGCCTTTG ATCTAGATGT AAGGCCGGAT TTCTTTCGAG GGCTGGTGTT

CACCTTTCTC CAGGCACCAC TCTCTCCTTT CTTCTTGTAA CTGGAAACTA

CACCTTTCTC CAGGCACCAC TCTCTCCTTT CTTCTTGTAA CTGGAAACTA

CACCTTTCTC CAGGCACCAC TTTCTCCTTT CTTTTTGTAA CTGGAAACTA

CACCTTTCTC CAGGCACCAC TCTCTCCTTT CTTTTTGTAA CTGGAAACTA

CACCTTTCTC CAGGCACCAC TCTCTCCTTT CTTCTTGTAA CTGGAAACTA

CACCTTTCTC CAGGCACCAC TTT---CTTT CTTCTTGTAA CTGGAAACCA

CACCTTTCTC CAGGCTGCAC TCTCTCCTTT CTTCTTGTAA CTTGAAACTA

CACCTTTCTC CAGGCGCCGC TCTCCACTTT CTTTTTGTAA CTTGACACTA

CACCTTTCTC CAGGCCCCGC TCTCTCCTTT CTTCTTGTAA CTTGAAACAA

CACCTTTCTC CAGGCACCAC TCT---CTTT CTTCTTGTAA CTTGAAACTA

CACTCTTCTC CAGGCACCAC TCT---CTTT CTTTCTGTAA CTTGAGACTA

CACCTTTCGC CAGGTACCAT TCTCTCCTTT CTTCTTGTAA CTTGAAACTA

---------- ---------- ---------- ---------- ----------

CACCTTTCTC CAGGCACCGT TCTCTCCTTT TTTCTTATAA CTTGAAACTA

CACCTTTCTC CAGGCACCAT TCTCCCCTTT CTTCTTGTAA CTTGAAACTA

CACTTTTCTC CACGCGCCAT TCTCTCCTTT CTTTTTGTAA CTTGAAACGA

CACCTTTCTC CAGGAATTAT TATCCCCTTT CTTCTTGTAA CTTGAAACCA

GACTTTCCTC CAGGACCCAC TCTCTCCTTT CTTCCTGTAG CTGGAAACCA

TGGATAAAGG GAAACTTCCT CCTTTGAAAT CAGCTGTATG CTGGTCTAGC

TGGATAAAGG GAAACTTCCT CCTTTGAAAT CAGCTGTATG CTGGTCTAGC

TGGATAAAGG GAAACTTCCT CCTTTGAAAT CAGCTGTATG CTGGTCTAGT

TGGATAAAGG GAAACTTCCT CCTTTGAAAT CAGCTGTATG CTGGTCTAGT

TGGATAAAGG GAAACTTCCT CCTTTGAAAT CAGCTCTATG CTGTTCTAGC

AAGATAAAGG AAAACTTCCT CCCTTGAAAT CAGCTGTGTG CTGGTCAAGC

TTGACAGAGG GAAACTTCCC CCTTTGAAGT CTGCTATCTG CTGGTCCAGC

TTGACAGAGG AAAACTGCCC CCTTTGAAGT CTGCGATCTG CTGATCCAGC

TGGATAGAGG AAAACTTCCC CCTCTGAAAT CCGCTGTGTG CTGGTCTAGC

TGGACAGAGG GAAGCTTCCT CCTTTGAAAT CAGCTGTATG CTGGTCCAGC

TAGATAAAGG GAAACCTCCT CCTTTGAAAT CAGCTGTATG CTGGTCCAGC

TGGATAAAGG GAAACTTCCT CCTTTGAAAT CAGCTGTATG CTGGTCTAGC

---------- ---------- ---------- ---------- ----------

TGGATAAAGG GAAACTTCCT CCCTTGAAAT CAGTTATATA CTGGTCGAGC

TGGATAAAGG GAAACTTCCT CCTTTGAACT CAGCAATATA CTGGTCTAGC

GGGACAAAGG GAAACTTCCT CCTTTGAAGT CAGCCATAGA CTGGTCGAGC

TGGAGAGGGG GAAGCTTCCT CCTTTGAAAT CAGCTATATA CTGATCGAGC

TGGATAATGG GAAGCACCCA CTCCGGAACT CAGCCATATA CTGGTCCAAC

TCTGAAACAA CATACCTGCT CTGGGGAGGG GACAGCCATG CAGTACAGCT

TCTGAAACAA CATACCTGCT CTGGGGAGGG GACAGCCATG CAGTACAGCT

TCTAAAACAA CATACCTGCT CTGGGGAGGG GACAGCCATG CAGTACAGCT

TCTGAAACAA CATACCTGCT CTGGGGAGGG GACAGCCATG CAGTACAGCT

TCTGAAACAA CATACCTGCT CTAGGGAGGG GACAGCCATG CAGTACAGCT

TCTGAAATGA TATACCTGCT CTGGGGAGGG GACAGCCGTG TAGCACAGCT

TCTGAAACAA CATACCTGTC CTGGGAAGGG GACAGCCATG CAGAATGGCT

TCTGAAACGA CATACCTGCC CTGGGAAGGG GACAGCCATG CAGAACCGCT

TCTGAAACAA TGGACCTGCT CTCGGGAGGG GACAACCGTG CAGAACAGCT

TCTGAAACAA CATACCTGCT CTCTGGAGGG GACAGCCATG CAGAACAGCT

TCTGAAACAA CATACCTGGG GTCTGCAGGG GACAGCCGTG CAGCACGGCT

TCTGAAACAA CATACCTGCT CTCGGGAGGG GACAGCCGTG CAGGACAGCT

---------- --CACCTGCC CTCTGAAGGG GACAGCCATG CAGAACAGCT

TCTGAAACAA CATACCTGCT CTCAGGAGGG GACAGCCATG CAGAACAGCT

TCTGAAACAA CATACCTGCT CTTGGGAGGG GACAGCCGTG CAGAACAGCT

TCTGAAACAA CGTACCTGCT TGCGGGAGGG GGCAGCCGTG CAGCACAGCT

TCTGAAACAA CATACCTGCT CTGGGGAGAG GACAGCCATG CAGGACTGCC

TCTGAAATAA CATACCTGCT CTGGGGAGAG GACAGCCATG TAGAATGGCC

TTATTTAGCA AGATGCTGAG AGCAGCTTTG ACCACAGCCT GCTGTCCTTG

TTATTTAGCA AGATGCTGAG AGCAGCTTTG ACCACAGCCT GCTGTCCTTG

TTATTTAGCA AGATGCTGAG AGCAGCTTTG ACCACAGCCT GCTGTCCTTG

TTATTTAGCA AGATGCTGAG AGCAGCTTTG ACCACAGCCT GCTGTCCTTG

TTGTTTAGCA AGATGCTGAG AGCAGCTTTG ACCACAGCCT GCTGTCCTTG

TTATTAAGCA AGATGCTGAG AGCAGCTTTC ACCACCGCCT GCTGCCCTTG

TTATTAAGCA GAATGCTGAG AGCAGCCCTC ACCACAGCCT GCTGCCCTTG

TTATTAAGCA GAATGCTGAG GGCGGCCCTC ACCACAGCCT GCTGTCCTTG

TTATTGAGTA AGATGCTGAG AGCAGCTTTC ACCACAGCCT GCTGCCCGTG

TTATTAAGCA AAATGCTGAG AGCAGCTCTA ACTACAGCCT GCTGCCCTTG

TTATTGAGCA AGATGCTGAG CGCAGCTTTG ACCACGGCCT GCTGTCCTTG

TTATTGAGCA ATATGCTGAG AGCCGCCTTA ACCACAGCCT GCTGTCCTTG

TTATTGAGCA GGATGCTGAG GGCGGCTTTA ACCACAGCCT GCTGTCCTTG

TTATTGAGCA AGATGCTGAG AGCAGCTTTA ACCACAGCCT GCTGTCCTTG

TTATTAAGCA AGATGCTGAG AGCAGCTTTA ACGACAGCTT GCTGTCCTTG

TTGTGAAGCA AGATGCTGAG AGCAGCTTTC ACCACAGCCT GCTGCCCGGG

TTATTTAGCA AAATACTGAG AGCAGCCTTC ACCACTGCTT GCTGTCCTTG

TTGTTCAGCA AGATGCTGAG GGCTGCTTTG ACCACGGCTT GCTGCCCTTG

GCTAGGGGTC TTTTCACAGA GGCTCTTGAC AATATTGCTT CTTGAACTCT

GCTAGGGGTC TTTTCACAGA GGCTCTTGAC AATATTGCTT CTTGAACTCT

GCTAGGGGTC TTTTCACAGA GGCTCTTGAC AATATTGCTT CTTGAACTCT

GCTCGGGGTC TTTTCACAGA GGCTCTTGAC AATATTGCTT CTTGAACTCT

GCTAGGGGTC TTTTCACAGA AGCTCTTGAC AATATTGCTT CTTGAACTCT

GCTAGGGGTC TCTTCATAGA GGCTCTTGAC AATATTGCTT CTTGAACTCT

GCTAGGGGTT TGTTCATGGC GGCTCTTGAC AGTATTGCTT CTTGAACTCG

GCTAGGGGTT TGTTCATGGA GGCTCTTGAC AATATTGCTT CTTGAACTCT

GCTGGGGGTC TCTTCACTGA GGCTCGCGAC AGGATTGCTG CTTGAACTCT

GCTGGGGGTC TTTTCACAGA GGCTCTTGAC AATATTGCTT CTTGAACTCT

GCTGGGGGTC TTTTCACAGA GGCTCTTGAC AATATTGCTT CTTGAACCCT

GCTAGGGGTC TTTCCACAGA GGCTCTTGAC AAAATTGCTT CTTGAACTCT

ATTGGGGGTC TTTTCACAGA GGCTCTTGAT AATATTGCTT CTTGCACTCT

GCTAGGGGTC TTTTCACAGA GGCTCTTGAC AATATTGCTT CTTGAACTTT

GCTGGGGGTC TTTTCACAGA GGCTTTTGAC AATATTGCTT CTTGAACTCT

GCTGGGGGTC TGTTCACAGA GGCTCTTGAC AGTATGGCAT CTTGCACTCT

GCTGGGAGCC TTTTCACAGA TGCTCTCGAC AGTATCGCTT CTTGAACTCT

GCTGGGAGTC TCTTCACAGA GGCTCTCGAA AGTATGGCTT CTTGAACTTT

GGGTGCGATG ACGCCATTCC ACAGCTTAGA CATCCACCTA CTTACTTCAC

GGGTGCGATG ACGCCATTCC ACAGCTTAGA CATCCACCTA CTTACTTCAC

GGGCGCGATG ACGCCGTTCC ACAGCTTAGA CATCCACCTA CTTACTTCAC

GGGCGCGATG ACGCCGTTCC ACAGCTTAGA CATCCACCTA CTTACTTCAC

GGGTGTGATG ACGCCATTCC ACAGCTTAGA CATCCATCTA CTTACTTCAC

GGGTGCGATG ACTGCATTCC ACAGCTTAGA CATCCACCTA CTTACTTCAC

GGGAGCAATG ATGGCGTTCC ACAGCTTAGA CATCCACCTA CTTACTTCAC

GGGAGCAATG ACAGCATTCC ACAGCTTAGA CATCCACCTA CTTACTTCAC

GGGCGCGATG ACAGCGTTCC ACAGCTTGGA CATCCACCTA CTTACTTCAC

GGGTGCAATG ACAGCATTCC ATAGCTTAGA CATCCACCTA CTCACTTCAC

GGGCGCGATG ATGGCGTTCC ACAGCTTAGC CATCCACCTA CTTACTTCAC

GGGTGCAATG ACAGCGTTCC ACAGCTTACA CATCCACCTA CTTACTTCAC

GGGAGTGATC ACGGCATTCC ACAGTTGAGA CATCCACCTA CTTACTTCAC

GGGTGCAATG ATGGCGTTCC ACAGCTTAGA CATCCACCTA CTTACTTTAC

GGGTGCAATA ACGGCATTCC ACAGCTTGGA CATCCACCTA CTTACTTCAC

GGGGGCAATG ACGGCGTTCC ACAGCTTGGA CATCCACCTA CTTACTTCAC

GGGAGCAATG ACAGCATTCC ACAATTTGGA CATCCACCTA CTTACTTCAC

GGGAGCGATC ACGGCATTCC ATAATTTGGA CATCCACCTA CTTACTTCAC

TGTCACTTGG GCATGCCCAG GAACTACAGG ACAAGACAGG AAATATTTTG

TGTCACTTGG GCATGCCCAG GAACTACAGG ACAAGACAGG AAATATTTTG

TGTCACTTGG GCATGTCCAG GAACTACAGG ACAAGACAGG AAATATTTTG

TGTCACTTGG GCATGTCCAG GAACTACAGG ACAAGACAGG AAATATTCTG

TGTCACTTGG GCATGTCCAG GGACTACAGG ACAAGACAGG AAATACTTTG

TGTTGCTTGG GCATGTCCTG GGATTACAGG ACAAGACAGG AAATACTTCG

TGTTGCTTGG GCATGTCCCG GGACTACTGG ACAAGACAGG AAATACTTGG

TGTTGCCTGG GCATGTCCCG GGACTACTGG ACACGACAGG AAATACTTTG

TGTCGCTTGG GCACGTCCAG GGACCACAGG ACAAGACAGG AAATACTTTG

TGTTGCTTGG GCGTGCCCCG GGACTACAGG ACAAGACAGG AAATGCTGTG

TGTTGCTTGG GCATGTCCTG GGACTACAGG ACAAGACAGG AAATACTTTG

CGTCACTTGG GCATGCCCTG GGATGACAGG ACATGACAGG AAGTACTTTG

TGTTGCTTGG GCATGTCCTG GGACTACAGG ACAAGACAGG AAATACTTTG

TGTCACTTGG GCGTGTCCTG GGACTACAGG GCAAGACAGG AAATACTTTG

TGTTGCTTGG GCATGTCCTG GGACGACTGG ACAAGACAGG AAATACTTTG

TGTTGCTTGT GCATGTCCGG GGACTACCGG ACAAGACAGG AAACACTTCG

TGTCACCTGG GCATGTCCTG GAACTACCGG ACAGGAGAGG AAATATTTTG

AGTCACATGG GCATGTCCTG GAACCACTGG GCATGACAGG AAATACCTGG

GTCCAAGAAG TGCTTCAGGT GTGCCCAAGC GGGCCAGGCA GGAGTTAAGC

GTCCAAGAAG TGCTTCAGGT GTGCCCAAGC GGGCCAGGCA GGAGTTAAGC

GTCCAAGAAG TGCTTCAGGT GTGCCCAAGC GGGCCAGGCA GGAGTTAAGC

GTCCAAGAAG TGCTTCAGGT GTGCCCAAGC GGGCCAGGCA GGAGTTAAGC

GTCCAAGAAG TGCTTCAGGG GTGCCCAAGC GGGACAGGCA GGAGTTAAGC

GCCCAAGAAG AGCTTCAGGT GTACCCAAGC GGGCCAGGCA GGAGTTGAGC

GTCCAAGAAG TGCTTCCGGT GTACCCAAGC GGGCCAGGCA GGAGTTGAGC

GTCCAAGAAG TGCTTCTGGT GTACCCAAGC GGGCCAGACA GGAGTTGAGC

GGCCGAGAAG TGCTTCAGGT GTGCCCAGGT GGGCCAGGCA GGAGTTGAGC

GCCCGAGAAG TGCTTCCGGC GTGCCCAGGC GGGCCAGGCA CGAGTTGAGC

GTCCAAGAAG TGCTTCAGGT GTGCCCAGGC GGGCCAGGCA GGAGTTCAGC

GCCCCAGAAG TGCTTCAGGT GTGCCCAAGC GGGTCAGGCA GGAGTTGAGC

GCCCCAGGAG CGCTTCAGGG GTGCCCAAGC GGGCCAGGCA GGAGTTGAGC

GCCCAAGAAG CGCATCTGGG GTGCCCAAGC GGGCCAGGCA GGAGTTGAGT

GCCCAAGAAG CGCTTCAGGT GTGCCCAAGC GGGCCAGGCA GGAGTTGAGC

GTCCCAGCAG TGCTTCAGGT GTGCCCAGGT GCGAGAGGCA GGAGTTGAGC

GCCCAATCAG TGCTTCAGGT GTGCCTAACC GGGACAAGCA GGAGTTCAAC

GCCCTATAAG GGCCTCTGGA GTGCCCAGGC GGGACAAACA GGAGTTCAGC

TGACGCCAGA CCCTTGCATG GGCTCGCCAT CCCACCGCAG CTGCACCCAG

TGACGCCAGA CCCTTGCATG GGCTCGCCAT CCCACCGCAG CTGCACCCAG

TGACGCCAGA CCCTTGCATG GGCTCGCCAT CCCACCGCAG CTGCACCCAG

TGACGCCAGA CCCTTGCATG GGCTCGCCAT CCCACCGCAG CTGCACCCAG

TGACGCCAGA CCCTTGCATG GGCTCACCAT CCCACCGCAG CTGCACCCAG

TGACGCCAGA CCCTTGCATG GGCTCTCCAT CCCACCGCAG CTGCACCCAG

TGCCTCCAGA CCCTTGACTG GGCTCGCAAT CCCAGCGCAG CTGAACCCAG

TGCCTCCAGA CCCTTGAATG GGCTCGCAAT CCCAGCGCAG CTGAACCCAG

TGTCGCCAGA CCCTTGCATG GGCTCGCCAT CCCACCGCAG CTGTACCCAG

TGGCGCCACA CCCCTGCATG GGCTCGCCGT CCCAGCGCAG CTGCACCCAG

TGGCGCCAGA CCCCTGCATG GGCTCGCCGT CCCACCGCAG CTGAACCCAG

TGGCGCCAGA CCTCTGCATG GGCTCGCCGT CCCACCGCAG CTGCACCCAG

TGGCGCCAGA CCCCTGCATG GGCTCTCCAT CCCACCGCAG CTGAACCCAG

TGGTGCCAGA CCCGTGCATG GGCTCGCCAT CCCACCGTAG CTGAACCCAG

TGGCGCCAGA CCCCTGCATG GGCTCGCCAT CCCACCGCAG CTGGACCCAG

TGGCGCCAGA CCCCTGCATG GGCTCGCCAT CCCACCGCAA CTGGACCCAG

TGGTGCCAGA CCCATGCATT GGTTCTCCAT CCCATCTTAG CTGAACCCAG

TGGTGCCAGA CCCGTGGATG GGCTCTCCAT CCCATCTTAA CTGAACCCAG

CGGAAATGCT GCTGCACCAG CAAGTCGGAG CCCTGGAGAC AGGCCTTGGC

CGGAAATGCT GCTGCACCAG CAAGTCGGAG CCCTGGAGAC AGGCCTTGGC

CGGAAATGCT GCTGCACTAG CAAGTCAGAG CCCTGGAGAC AGGCCTTGGC

CGGAAATGCT GCTGCACCAG CAAGTCGGAG CCCTGGAGAC AGGCCTTGGC

CGGAAATGCT GCTGTACCAG CAAGTCAGAG CCCTGGAGAC AGGCCTTGGC

CGGAAATGCT GCTGCACCAG CAGCTCGGAG CCCTGGAGAC AGGCCTTGGC

CGGAAGTGTT GCTGCACCAG CAGGTCAGAG CCCTGGAGAC AAGCCTTGGC

CGGAAGTGTT GCTGTACCAG CAGGTCAGAG CCCTGGAGAC AGGCTTTGGC

CGGAAGTGCT GCTGCACCAG CAGGTCAGAG CCCTGCAGAC AGGCCTTGGC

CGGAAATGCT GCTGCACCAG CAAGTCGGAG CCCTGGAGAC AGGCCTTGGC

CGGAAATGCT GCTGCACCGG CAAGTCAGAG CCCTGGAGAC AGGCCTTGGC

CGGAAATGCT GCTGCACCAG CAGGTCAGAG CCCTGCAGGC AGGCCTTGGC

CGGAAATGCT GCTGCACCAG CAGGTCCGAG CCCTGGAGGC AGGCCTTGGC

CGGAAATGCT GCTGCACCAG CAAATCAGAG CCCTGGAGAC AGGTCTTGGC

CGGAAATGCT GCTGCACCAG TAGGTCGGAG CCCTGGAGAC ATGCCTTTGC

CGGAAGTGTT GCTGCACCAG CAGGTCGGGG CCCTGGAGAC AGGCCTTGGC

CGGAAATGCT GCTGGACCAC CAAATCAGAG CCCTGGAGAC GGGATTTGGC

CGGAAATGTT GCTGCACTAA GAGATCAGAT CCCTGGAGAC ACGACTTTGC

GATGGTTCCC ATCAGAAAGC AGTTCTCATG AAAGTAATAT TACCTTTTTG

GATGGTTCCC ATCAGAAAGC AGTTCTCATG AAAGTAATAT TACCTTTTTG

GATGGTTCCC ATCAGAAAGC AGTTCTCATG AAAGTAATAT TACCTTTTTG

GATGGTTCCC ATCAGAAAGC AGTTCTCATG AAAGTAGTAT TACCTTTTTG

GATGGTTCCC ATCAGAAAGC AGTTCTCATG AAAGTAATAT TACCTTTGTG

AATGGTTCCC ATCAGAAAGC AGTTCTCGTG AAAGTAGTAT TACCTTTCTG

GAGGGTTCCC AGCAGAAAGC AGTTCTCATG GAAATAATAT TACCTTTCTG

AATGGTTCCC ACCAGAAAGC AGTTCTCATG GAAATAATAT TACCTTTTTG

AATGGTCCCC ATCAGGAAGC AGTTCTCATG AAAATAATAT TACCTTTTTG

GATTGTTCCC ATCAGAAAGC AGTTCTCATG AAAGTAGTAT TACCTTTTTG

AATGGTTCCC ATGAGAAAGC AGTTTTCATG AAAGTAGTAT TACCTTTTTG

AACGGTGCCC ATCAGGAAGC AGTGCTCGTG AAAGTAGTAT TACCTTTATG

GATGGTTCCC ATCAGAAAGC AGTTCTCATG AAAATAGTAT TACCTCTTTG

AATGGTTCCC ATCAAAAAGC AGTTCTCATG AAAGTAATAT TACCTTTTTG

AATGGTTCCC ATCAGAAAAC AGTTCTCGTG GAAGTAATAT TACCTTTTTG

GATGGTTCCC ACCAGAAAGC AGTTCTCATG GAAGTAGTAT TACCTTTGTG

AATTGTCCCC ATCAGAAAGC AGTTCTCATG AAAGTAATAT TACCTTTTTG

AATGGTTCCC ACCAGAAAGC AGTTTTCATG GAAGTAATAT TACCTTTTTG

GAAAGTGCAG GGGCTTTCAG TGCTGCGATT TTCAAGAGGT GCCAAAAAGT

GAAAGTGCAG GGGCTTTCAG TGCTGCGATT TTCAAGAGGT GCCAAAAAGT

GAAAGTGCAG GGGCTTTCAG TGCTGCGATT TTCAAGAGGT GCCAAAAAGT

GAAAGTGCAG GGGCTTTCAG TGCTGCGATT TTCAAGAGGT GCCAAAAAGT

GAAAGTGCAG GGACTTTCAG TGCTGCGATT TTCAAGAGGT GCCAAAAAGT

GACAGTGCAG GGGCTCTCAG TGCTGCGATT TTCCAGAGGT GCCAGAAAAT

GAAAGTGCAT GGGCTTTCAG TGCTGCGATT CTCCAAAGGT GCCAAAAAGT

GAAAGTGCAG GGGCTTTCAG TGCTGCGATT TTCCAAAGGT GCCAGAAAGT

GAAAGTGCAT GGGCTTTCTG TGCTGCGATT TTCAAGAGGT GCCAAAAAGT

GAAGGTACAG GGGCTTTCAG TGCTGTGATT TTCCAGAGGT CCCAAAAAGT

AAAGGTCCAG GGGCTTTCAG TGCTGCGATT TTCCAGAGGT GCCAGGAAGT

AAAAGTACAG GGGCTTTCAG GGCTGCGATT CTCCAGAGGT GCCAAAAAGT

AAAAGTACAT GGGCTTTCAG TGCTGCGATT TTCCAGAGGT GCCAAAAAGT

AAAAGTACAG GGGCTTTCAG TGCTGCGATT TTCGAGAGGT GCCAAAAAGT

AAAAGTACAG GGGCTTTCAG TGCTGCGATT TTCAAGAGGC GCCAGAAAGT

AAACGTATAG GGGCTTTCAG GGCTGCGATT TTCCAGAGGT GCCAGAAAGT

AAAAGTACAG GGGTTTTCAG TGCTACGATT TTCTAGAGGT GCCAGAAAGT

AAAAGTATAG GGATTTTCAC TGCTACGGTT CTCAAGGGGA GCTAGAAAGT

CCCTCAATAA CTCCGACAGT GAAGATTTTT CTAAATTTTC TAAAATGATG

CCCTCAATAA CTCCGACAGT GAAGATTTTT CTAAATTTTC TAAAATGATG

CCCTCAATAA CTCAGACAGT GAAGATTTTT CCAAATTCTC TAAAATGATG

CCCTCAATAA CTCAGACAGT GAAGATTTTT CCAAATTCTC TAAAATGATG

CCCTCAATAA CTCAAACAAT GAAGATTTTT CCAAATTCTC TAAAATGATG

CCCCCAGTAA CTCAGACAAT GAAGATTTTT CCAGATTCTC TAAGATGATG

CCCCCAGTAA CTCAGACAAT GATGATTTTT CCAAATTTTC CAAGATGACG

CCCCCAGTAA CTCAGACAAT GAAGATTTTT CCAAATTTTC CAAGATGACG

CCCCCAATAA TTCAGACAAG GAAGAATTCT CCAAATTTTC TAAGATGATG

CCCCCAGTAA CTCAGACAAT GAAGATTTTT CCAAGTTCTC TAAGATGACG

CTCCCAGTAA CTCCGACAAC GAAGATTTTT CCAAATTCTC TAAGATGACG

CCCCCAATAA CTCAGACATG GAAGATTTTT CCAAATTCTC TAAGATGATG

CCCCCAATAA CTCTGACAAT GAACATTTTT CCAGATTCTC TAAGATAATG

CCCCCAATAA CTCAGACAAA GAGGATTTTT CCAAATTCTC TAAGATGATG

CCCCCAATAA CTCAGACAAT GACGATTTCT CCAAGTTCTC CAAGATGACG

CCCCCAAGAG TTCAGACAAC GACGCTCTCT CCAAATTCTC CAAGATGATG

CTCCTAATAG CTCAGATAAG GAAGCATTTT CCAACTTCTC CAGGATGATG

CTCCCAACAA CTCTGATAAT GAAGCTTTTT CCAAATTCTC TAGGATGACG

ATGATTTTCT TCTTACTGGG AGATTGTTTC ACTGGGATCA GACAAGCTAT

ATGATTTTCT TCTTACTGGG AGATTGTTTC ACTGGGATCA GACAAGCTAT

ATGATTTTCT TCTTACTGGG AGACTGTTTC ACTGGAATCA GACAAGCTAT

ATGATTTTCT TCTTACTGGG AGATTGTTTC ACTGGAATCA GACAAGCTAT

ATGATTTTCT TCTTAACAGG AGATTGTTTC ACTGGGATCA GACAAGCTGT

ATGATTTTCT TCTTAACAGG AGACTGTTTC ACTGGGATCA GACAAGCTGT

ATTATTTTCT TCTTAACAGG GAACTGCTTT ACTGGGATCA GACAGGCTGT

ATTATTTTCT TCTTAACAGG GAACTGCTTC ACTGGGATGA GACAAGCTGT

ATGATTTTCT TCTTTACAGG AGATTGTTTC ACTGGGATCA GACAAGCTGT

ATGATTTTCT TGTTAGCAGG AGATTGTTTC ACTGGGATCA GACACGCTGG

ATGACTTTCT TGTTAACAGG GGACTGTTTC ACTGGGATCA GACAAGCTGT

ATGATTTTCT TGTTCACAGG AGATTGCTTC ACTGGGATCA GACATGCTGT

ATAATTTTCT TGTTGACAGG AGCTTGTTTC ACTGGGATCA AACATGCTGT

ATGATTTTCT TGGTAACAGG AGACTGTTTC ACAGGGATCA GACAAGCTGT

ATGACTTTCT TGTTACCAGG AGATTGTTTC ACTGGGATCA GACAAGCTGT

ATGGTTTTCT TGCTAGCCGG CGATTGCTTC GCTGGGATCA GACAGGCTGT

ATGATTTTCT TACTTACAGG AGGCTGTTTC ACTGGGATGA GACAAGCTGA

ATGACTTTTT TGCTCACAGG AGGCTGTTTC ACAGGGATCA GACAAGCTGA

TCTTACCGCT ACTAATGAAC AGGTCTAGTA GCTGTTCCTT GGAAAAACCA

TCTTACCGCT ACTAATGAAC AGGTCTAGTA GCTGTTCCTT GGAAAAACCA

TCTTACCGCT ACTGATGAAC AGGTCTAGCA GCTGTTCCTT GGAAAAGCCA

TCTTACCGCT ACTGATGAAT AGGTCTAGCA GCTGTTCCTT GGAAAAGCCA

TCTTACCGCT ACTGATGAAC AGGTCTAGTA GCTGTTTCTT GGAAAAACCA

TCTTACCGCT ACTGATGAAC AGGTCTACCA GCTGTTCCTT GGAGAAACCA

TCTTACCATT ACTGATGAAC ACGTCTACTA GCTGTTCCTT GGAGAAACCT

TCTTACCATT CCTGATGAAC ACGTCTACTA GCTGCTCTTT GGAGAAACCC

TCTTACCACT ACTGATGAAC AGGTCTAATA GCTGCTCCTT GGAGAAACCT

TCTTACCACT ACTGATGAAC AGATCTACTA GCTGTTCCTT GGAGAAATCA

TCTTACCACT ACTGATGAAC AGGTCTACGA GCTGTTCCTT GGAGAAACCA

TCTTACCACT ACTGATGAAT AGGTCCGCAA GCTGTTCCTT GGAGAAACTC

TCTCACCGCT ACTGATAAAC AGGTCTACCA GCTGTTCCTT GGAGAAACCA

TCTTACCGCT ATTGATGAAG AGGTCTATTA GCTGTTCCTT GGAAAAACCA

TCTTACCGTT CCTGATAAAC AGGTCGACTA GCTGTTCCTT GCAGAAAGCA

TCTTACCGCT CTTGATGAAC AGGTCGACCA GCTGTTCTTT GGAGAAACCA

TCTTACCACT ACTGATGAAT AATTCGACTA GCTGATGCTT AGAGAACTCA

CCTTACCGCT ATTGATGAAT AACTCCACTA GCTGCTCCTT GGAGAAGTTG

GCATCTACTT CAGCTCTCAC TATTTCACAG GAGAATCCTG CAGCCATTTG

GCATCTACTT CAGCTCTCAC TATTTCACAG GAGAATCCTG CAGCCATTTG

GCATCTACTT CAGCTCTCAC TATTTCACAG GAGAATCCTG CAGCCATTTG

GCATCGACTT CAGCTCTCAC TATTTCACAG GAGAATCCCG CAGCCATTTG

GCATCTACTT CAGCTTTCAC TATTTCACAG GAGAATCCCG CAGCCATTTG

GCATCTACTT CAGCTGTCAC TATTTCACAG GAGAATCCTG CAGCCATTTG

GAATCCACCT CAGCTCTCAC TATTTCACAG GGGAATCCTG CAGCCATCTG

GAATCCACCT CGGCTCTCAC TATTTCACAG GGGAATCCTG CAGCCATCTG

GCATCCACTT CAGCTCTTAC TATTTCACAG GAAAACCCTG CAGCCATTTG

GCATCCACTT CAGCTCTCAC TATTTCACAG GGGAATCCTG CAGCCATTTG

GCATCCACTT CGGCTCTCAC TATTTCACAG GTGAATCCCG CAGCCATTTG

GCATCCACTT GGGCTCTCAC TATTTCACAG GAGAATCCTG CAGCAATCTG

GCATCCACTT CAACTCTCAC TATTTCACAG GGGAATCCTG CAGCCATTTG

GCATCCACTT CAGCTCTCAC TATCTCACAG GAGAATCCTG CAGCCATTTG

GCATCCACTT CAGCTCTCAC TATTTCACAG GAGAAACCCA CAGCCATTTG

GCATCAACAT CAGCTCTAAC TATATCACAT GAGAACCCCA CAGCCGCCTG

GCATCCACTT CAGCTCTCAC TATTTCACAG GTGAATCCTG CAGCTCTTTG

GTGTCCACTT CGGCTTTCAC TATTTCACAA GGGAATCCTG CAGCTGTCTG

TCTGTGCTGT GATAAGAAAA TATACCTTCA GGCAGAGTGC AAGCTGATGT

TCTGTGCTGT GATAAGAAAA TATACCTTCA GGCAGAGTGC AAGCTGATGT

TCTGTGCTGT GATAAGAAAA TTTACCTTCA GGCAGAGTGC AAGCTGATGT

TCTGTGCTGT GATAAGAAAA TTTACCTTCA GGCAGAGGGC AAGCTGATGT

TCTGTGCTGT GATAAGAAAA CATACCTTCA GGCAGAGTGC AAGCTGATGT

TCTATGCTAT GAAAAGAAAA CATACCTTCA GGCAGAGTGC AAGCTGATGT

TCTGTGCTGG GATAACAAAA CATACCTTCA TGCAGAGGGC AAGCTGATTT

TCTGTACTGT GATAACAAAA CATACCTTCA TGCAGAGGGC AAGCTGATTT

TCTGTGCTAG GAAAAGAAAA CATACCTTCA TGCAGAGTGC AAGCTGATGT

TCTGTGCTGT GATAAGAAGA --TACCTTCA GGCAGAGTGC AAGCTGATGG

TCTGTGCTGT GATAAGAAAT --TACCTTCA TGCAGAGTGC AAGCTGATGT

TCTGTGCTGT GATAAGGAAA --TACCTTCA TACAGAGTGC AAGCTGATGT

TCTGTGCTGT GATAAGAAAA --TACCTTCA TGCAGAGGGC AAGTTGATGC

CCTATGCTGG GATAAGGAAA CATACCTTCA TGCAGAGAGC AAGCTGATGG

TCTGTGCTGT GATGAGAAAA CATACCTTCA TGCAGAGTGC AAGCTGATGT

TCTGTGCTGC AGTAAGAAAA CATACCTTCA TGCAGAGTGC AAGCTGGAGC

TTTTTGCTAT GGTaagaaaa caTACCTTCA TGCAGAGTGC GAGCTGATGT

CTTGTGCTGT AATAAGAAGA TATACCTTCA GGCAGAGTGC TATCTGATGG

ACTATGTAGT CTTGCAAGCT TCCTTCTGGG CCGTGGAAAA TGACATTATG

ACTATGTAGT CTTGCAAGCT TCCTTCTGGG CCGTGGAAAA TGACATTATG

ACTATGTAGT CTTGCAAGCT TCCTTCTGGG CCGTGGAAAA TGACATTATG

ACTATGTAGT CTTGCAAGCT TCCTTCTGGG CCGTGGAAAA TGACATTATG

ACTATGTAGT CTTGCAAACT TCCTTCTGGG CCGTGGAAAA TGACATTATG

ACTATGTAGT CTTGCAAGCT TCCTTCTGGG CCGTGGAAAA TGACATTACG

GCTATGTAGT CTTGCAAGCT TCCTTCTGGG CCATGGAAAA TGACATTATG

GCTATGTAGT CTTGCAAGCT TCCTTCTGGT CCATGGAAAA TGACATTATG

GCTATGAAGT CTTGCAAGCT TCCTTCAGGG CCATGAAATA TGACATTATG

GCGATGTAGT CTTGCAAGCT CCCTTCTGGG CCATGGAAAA TGACATTATG

GCTATGTAGT CTTGCAAGCT TCCTTCTGGA CCATGGAAAA TGACATTATG

GCTATGTAGT TTTGCAAGCT TCCTTCTGGG CCATGGAAGA TGACGTTATG

GCTATGTAGT CTTGTAGGCT TCCTTCAGGG CCGTGAAAAA TGACATTATG

GCTATGTAGT CTTGCAAGCT TCCTTCTGGG CCATGGAAAA TGACATTATG

GCTATGTAGT CTTGCAAGCT TCCTTCTGGG CCATGGAAAA TGACGTTATG

GCTATGTAGT CTTGCAAGCT TCCTTCTGGA CCATGGAAAA TGACAGTGTG

GCTATGTAGT CTTGCAAGCT TCCTTCTGGG CCATGAAAGA TAACATTATG

GCTATATAGT CTTGCAAGCT TCCTTCAGGG CCATGGAAGA CCACGTTATG

ATATTGCTCA ACCTATTAAC AAACCAAGAC CTACCAGCCT GAGGTAGTTC

ATATTGCTCA ACCTATTAAC AAACCAAGAC CTACCAGCCT GAGGTAGTTC

ATATTGCTCA ACCTATTAAC AAACCAAGAC CTACCAGCCT GAGGTAGTTC

ATATTGCTCA ACCTATTAAC AAACCAAGAC CTACCAGCCT GAGGTAGTTC

ATATTGCTCA ACCTATTAAC AAACCAAGAC CTACCAGCCT GAGGTAGTTC

ATACTGCTCA ATCTATTCAC AAACCAAAAC CTACCAGCCT GAGGTAGTTC

ATATTGCTCA ACCTGTTCA- AAACCAAGAC ATACCAGCCT GAGGTAGTTC

ATATTGCTCA ACCTGTTCA- AAAGCAAGAC ATACCAGCCT GAGGTAGTTC

ATATTGCTCG ACCTGGAAC- AAACCAAGCC CTACCAGCCG GAGGTAGTTC

ATATTGCTCG ACCTATTAAT AAACCCAGAC CTACCAGTCT GAGGTAGTTC

GTATTGCTCG ACCTATTAAT AAACCAAGAC CTACCAGCCT GAGGTAGTTC

ATATTGCTCA ACCTAGTAAC AAACCAAGAC CTACCAGCCG GAGGTAGTTC

ATATTGTTCA ACCTATAAAT AAACCCAGAC CTACCAGCCT GAGGTAGTCC

ATATTGCTCG ACCTATTAAT AAACCAAGAC CTACCAGCCT GAGGTAGTTC

ATATTGCTCG ACCTATGGAT AAACCAAGAC CTACCAGCCT GAGGTAGTTC

ATATTGCTCG ACCTACGGAG AAAC---AAC TTACCAGCCT TAGGTAGTTC

ATATTGCTCA ACCTGTTGAG AAATGAAGAC CTACCAATCG GAGGTAGTTC

ATATTGCTCC ACCTGTAGAT AAACCAAGAC CTACCAGCCG GAGGTAGTTC

TGCATCATCT GGAGAGGGAT CATGGAGGCA TAAGTCACAC TACTGAGACA

TGCATCATCT GGAGAGGGAT CATGGAGGCA TAAGTCACAC TACTGAGGCA

TGCATCATCT GGAGAGGGAT CATGGAGGCA TAAGTCACAC TACTGAGGCA

TGCATCATCT GGAGAGGGAT CATGGAGGCA TAAGTCACAC TACTGAGGCA

TGCATCATCT TAAGAGGGAT CATGGAGGCA TAAGTCACAC TACTGAGGCA

TGCAGCATGT GAAGAGGGAT CATGGAAGCG TAAGTCACAC TACTGAGGCA

TGCAGCATCT GAAGTGGGAT CATGGATGTA TAAGTCACAC TACTGAGGCA

TGCAGCATCT GAAGTGGGAT CATGGATGCG TAAGTCACAC TACTGAGGCA

TGCAGCGTCT TCAGAGGAAT CATGGATGCG TACGTCACAC TACTAAGGCA

TGCAGCATCT GAAGAGGGAT CATAGACGCG TAGGTCACAC TACTGAGGCA

TGTAGCATCT GAAGAGGGAT CATTGACGCG TAAGTCACAC TACTGAGGCA

TGCAGCACCT GCAGAGGGAT CAGAGATGCG TAGGTCACAC TGCTGAGGCA

TGCAGCATCT GCAGAGGGAT CATGGATGTG TACGTCACAC TATAGAGGCA

TGCAGCATCT GAAGAGGAAT CATGGATGCA TAAGTCACAC TATTGAGGCA

TGCAGCATTG GAAGAGGGAT CATAGATGCG TAAGTCACAC TACTGAGGCA

TGCAGCACAG GAAGAGGGAT CATAGATGCG TACGTCACAC TACTGAGGCA

TGTAGCATCT GGAGAGGGAT CATGGATGCA TAAGTCACAC TGCTGAGGCA

TGTAGCATCT GAAGAGGGAT CATGGATGCG TAAGTCACAC TACTGAGGCA

GCCTTCTTGA GGACCTATTA TTTGGGGCAT AAAAGCATTT TATATTAACA

GCCTTCTTGA GGACCTATTA TTTGGGGCAT AAAAGCATTT TATATTAACA

GCCTTCTTGA GGACCTATTA TTTGAGGTAT AAAAGCATTT TATATTAACA

GCCTTCTTGA GGACCTATTA TTTGAGGTAT AAAAGCATTT TATATTAACA

GCCTTCTTGA GGACCTATTA TTTGAGGCAT AAAAGCATTT TATATTAACA

GCCTTCCTGA GGACCTATT- ---------- ---------- TATATTAACA

GCCTTCTTGG GGACCTATTA CTTGAGGCAC AAAAGCATTT TATAGCAACA

GCCTTCTTGG GGACCTATTA CTTGAGGCAT AAAAGCATTT TATAACAATA

GCCTTCTTGA GGGCCTATTA CTTGAGGCAT AAAAGCTGTT TAT---TATA

GCCCTCCTGA GGCCCTATTA TTTGAGGTGT AAAAGCATTT TATACCAACA

GCCTTCTTGA GGACCTATTA TCTGACACAT AAAAGCATTT TATACTAACA

GCCTTCTTGA GGACCTATTA TCTGAGGCAT AAAAGCATTT TACACTAACA

GCCTTCTCGA GGACCTATTA CTGGAGGCAT AACAGCATTT TAGACTAACA

GCCTTCTTGA GGACCTATTA TCTGAGGCAT AAAAGCATTT TATACTAACT

GCCTTCTTGA GGACCTATTA TTTGAGGCAT GAAAGC---- -----CAACA

GCCTTCTTGG GGACCTATGA TTGGAGGCAT CACAGCAATT TATACCAACA

GCCTTCTTGA GGACCTATTA TTTGAGGCAC AAAAAAGCAT CACATGAACA

GCCTTCTTGA GGTCCTACTG TTCCCAGCAT AAAAGCCTCT CATACTAGCA

GGCAACTCTT CCAGACTCCA CCATCCATCA GAAGAGATTG CCTGGAAATG

GGCAACTCTT CCAGACTCCA CCATCCATCA GAAGAGATTG CCTGGAAATG

GGCAACTCTT CCAGACTCCA CCATCCATCA GAAGAGATTG CTTGGAAATG

GGCAACTCTT CCAGACTCCA CCATCCATCA GAAGAGATTG CTTGGAAATG

GGCAACTCTT CCAGACTCCA CCATCCATCA GAAGAGATTG CCTGGAAATG

GGCAACTCTT CCAAACTCCA CCATCCATCA GAAGAGATGG CTTGGAAATG

GGCAACTCTT CCAGACCCCA ACATCCATCA GAAGAGATGG CTTGGAAGTG

GGCGCCTCTT CCAGACTCCA CCATCCATCA GAAGAGATGG CTTGGAAATG

GGCAACTCTT CCAGACTCCA CCACCCATCA GAAGAGATTG CTTGAAAATG

GGCAACTCTT CCAGGCTCCA CCATCCATCA GAAGAGATTG CTTGGAAATG

GGAAAATCCT CCAGACTCCG CCATCCATCA GATGAGATTG CTTGGAAATG

GGCAACTCTT CCAGACTCCA CCATCCATCA GAAGAGATGG CTTGGAAATG

GGGAGCTCTT CCAGGCTCCG CCATCCATCA GAAGAGATTG CCTGGAAATG

GGCAACTCTT CCAGACTCCA CCATCCATCA GAAGAGATTG CTTGGAAATG

GACAACTCTT CCAGGCTCCA CCATCCATCA GAAGAGATTG CTTGGAAATG

GCCGACTCCT CCAGACTCCA CCATCCATCA GAAGAGATCG CTTGGAAATG

GATGACTCTT CTGGATTTCT CCATCCATCA GAAGAGACTG CCTGGAAATG

CACAACTCTT CTAGTCGCTT CCATCCATCA GAAGAGATTG CTTGGAAATG

ATTTGTCAGA GCTTGACTCA CTGCTTTTGA AAAATCATCC CATGATGTCT

ATTTGTCAGA GCTTGACTCA CTGCTTTTGA AAAATCATCC CATGATGTCT

ATTTGTCAGA GCTTGACTCA CTGCTTTTGA AAAATCATCC CATGATGTCT

ATTTGTCAGA GCTTGACTCA CTGCTTTTGA AAAATCATCC CATGATGTCT

ATTTGTCAGA GCTTGACTCA CTGCTTTTGA AAAATCATCC CATGATGTCT

ATTTGTCAGA GCTTGACTCA CTGCTTTTGA AAAATCATCC CATGAAGTCT

ATTTGTCAGA GCCTGACTCA CTGCTTTTGA AAAATCTTCC CATGATGTCT

ATTTGTCAGA GCCTGACTCA CTGCTTTGGA AAAATCTTCC CATGATGTCT

ATTTGTCAGA GCTTGACTCA CTGCTTTTGA AAAATCATCC CACGATGTCT

GTTTGTCAGA GCTTGACTCA CTGCTTTTGA AAAATCATCC CATGATGTCT

ATTTGTCAGA GCTTGACTCA CTGCTTTTGA AAAATCATCC CATGATGTCT

ATTTGTCAGA GCTTGACTCA CTGCTTTTGA AAAATCATCC CATGATGTCT

ATTTGTCAGA GCTTGACTCA CTGCTTTTGA AAAATCATCC CATGATGTCT

ATTTGTCAGA GCTTGACTCA CTGCTTTTGA AAAATCATCC CATGATGTCT

ATTTGTCAGA GCTTGAATCA CTCCTTTTGA AAAATCATCC CAGGATGTCT

ACTGGTCAGA GCTTGACTCA CTGCTTTCGA AAAATCGTCC CATGATGTTG

GTTTGTCAAA GCTTGACTCA TTGCTTTTGA AAGATCATCC CAAGATGTCT

GTTCGTCACA GCCTGACTCA CAGCTTTGGA AAAATCATCC CACGATGTCT

GTTTGCGGAT ATTTAAAGCA CATATTGTGT TTTCACATTC CAAGTCATTC

GTTTGCGGAT ATTTAAAGCA CATATTGTGT TTTCACATTC CAAGTCATTC

GTTTGCGGAT ATTTAAAGCA CATATTGTGT TTTCGCATTC CAAGTCATTC

GTTTGCGGAT ATTTAAAGCA CATATTGTGT TTTCGCATTC CAAGTCATTC

GTTTGCGGAT ATTTAAAGCA CATATTGTGT TTTCGCATTC AAAGTCATTC

GTTTGCGGAT ATGTAAAGCA CATATTGTGT TTTCGCATTC AAAGTCATTC

GTTTGCGGAT ATTTAAAGTG CATATTGTGT GTTCACATTC AAAGTCGTTC

GTTTGCGGAT ATTTAAAGTG CATATCGTAT GTTCACATTC AAAGTCGTTC

GTTTGCGGAT ACTTAAAGTA CATATTATGT TTTCGCATTC AAAATCATTC

GTTTGCGAAT ATTTAAAGCA CATATTGTGC TTTCACATTC AAAGTCATTC

GTTTGCGGAT ATTTAAAGCG CATATTGTGT TTTCACATTC AAAGTCATTC

GTTTGCGGAT ATTTAAAATA CATATTGTGT TTTCACATTC AAAGTCATTC

GTTTGCGGAT ATTTAAAACA CATATTGTGT TCTCGCACTC GAAGTCATTC

GCTTGTGGAT ATTTAAAGCA CATATTGTGT TTTCACATTC AAAATCATTC

GTCTGCGGAT AGTTAAAGCC CATATTGTGT TTTCGCATTC AAAGTCATTC

GTTTGCGGAT ATTTAACGCG CATATTGTGT GTTCACTTTC AAACTCATTC

GTTTGCGGAT AATTAAAGCG CATATCGTGT TTTCAGTTTC AAAATCGTTC

GTTTACGGAT ATTTAAAGCA CATATTGTGT TTTCTGCTTC AAAATCATTC

ACCCACTGAA ATCCTTAAGG GTATTTTAAG AGCATCTGTT AGAAGAGTAA

ACCCACTGAA ATCCTTAAGG GTATTTTAAG AGCATCTGTT AGAAGAGTAA

ACCCACTGAA ATTCTTAAGG GTATTTTAAG AGCATCTGGT AGAAGAGTAA

ACCCACTGAA ATCCTTAAGG GTATTTTAAG AGCATCTGGT AGAAGAGT-A

ACCCACTGAA ATCCTTAAGG GTATTTTAAG AGCATCTGTT AGAAGAATAA

ACCCACTGAA ATCCTTACGG GTATTTTAAG AGCATCTGTT AGAAGAATAA

ACCCACGGAA ATCCTTAAGG GTATTTTAAG AGCATCTGTT AGAAAAACAA

ACCCACGGAA ATCCTTAAGG GTATTTTAAG AGCATCTGTT AGAAGAACAA

ACCTACGGAA ATCCTTAAGG GTATTTTAAG AGCATCTGTT AGAAGAATAA

ACCCACTGAA ATCCTTAAGG GTATTTTAAG AGCATCTGTT GGAAGAAAAA

ACCCACTGAA ATCCTTAAAG GTATTTTAAG AGCGTCTGTG GGAAGAATGA

ACCCACTGAA ATCCTTAAGG GTATTTTAAA AGCATCTGTT GGAAGAATAA

ACCCACTGAA ATCCTAAGGG GTATTTTCAG AGCATCTGTT GAAAGAATGA

ACCCACTGAT ATCCTTAAGG GTATTTTAAG AGCATCTGTT GGAGGAATAA

ACTCATTGAA ATCCTTAAGG GTATTTTAAG AGCATCTGTT GAAAGAATAA

GCCCACTGAG ATCCTTAAGG GTATTTTAAG AGCATCTATT GAA--AAGAA

ACTAATTGAA ATCCTTAAGG GTATTTTAAG AGCATCTGTT AAGACAATCA

ACCAACAGAA ATTCTTACGG ATATTTTCAA AGCATCTGGG AGAAGAATAA

AAGAATCACT CACTCAGATT CTCCAGCAAA TGCTTGCAGT CATCAGTGGC

AAGAATCACT CACTCAGATT CTCCAGCAAA TGCTTGCAGT CATCAGTGGC

AAGAATCACT CACTCAGATT CTCCAGCAAA TGCTTGCAGT CATCAGTGGC

AAGAATCACT CACTCAGATT CTCCAGCAAA TGCTTGCAGT CATCAGTGGC

AAGAATCACT CACTCAGATT CTCCAGCAAA TGCTTGCAGT CATCAGTGGC

AAGAATCACT CACTCAGATT CTCCAGCAAA TGTTTGCAGT CATCGGTGGC

AGAGAT---- ---------- ---------- ---------- ----------

AGAGATCACT CACTCAGATT CTCCAGCAAA TGCTTGCAGT CATCAGTGGC

AAGAATCACT CACTCAGATT CTCCAGCAAG TGCTTGCAGT CATCAGTAGC

AAAAATCACT CACTCAGATT CTCCAGCAGG TGCTTGCAGT CATCAGTAGC

AAGAATCACT CACTCAGGTT CTCCAGCAGA TGCTTGCAGT CATCGGTGGC

AAGAATCACT TACGCAGATT CTCCAGCAGG TGTTTGCAGT CGTCGGTGGC

AAGAATCACT CACGCAGATT CTCCAGCAAA TGTTTGCAGT CATCGGTGGC

AACAATCACT CACTCAGATT CTCCAATAGG TGCTTGCAGT CATCAGTGGC

AAGAATCACT CACTCAGATT CTCCAGCAGA TGCTTGCAGT CATCAGTGGC

AAGAATCACT CACTCCAATT CTCCAGCAGG TGTTTGCAGT CATCTGTGGC

GAGATTCACT CACTCAAGTT TTCTAGAAGA TGTTTGCAGT CATCAGTAGC

AGAGAACACT CACTGAGGTT CTCCAGAAGA TGCTTGCAGT CATCTGTTGC

AACATCATGC ACAGTCCGAT TGCACTTGTC TCTCCTTTCT GGCTCAAGCC

AACATCATGC ACAGTCCGAT TGCACTTGTC TCTCCTTTCT GGCTCAAGCC

AACATCATGC ACAGTCCGAT TGCACTTGTC TCTCCTTTCT GGCTCAAGCC

AACATCATGC ACAGTCCGAT TGCACTTGTC TCTCCTTTCT GGCTCAAGCC

AACATCATGC ACAGTCCGAT TGCACTTGTC TCTCCTCTCC GTCTCAAGCC

AACATCATGT ACAGTCCGAT TGCACTTGTC TCTCTTTTCT GGTTCAAGCC

---------- ---------- ---------- ---------- ----------

AACATCGTGC ACAGTCCGAT TGCACTTGTC TCTCCTTTCC GGCTCTAGTC

AACATCATGC ACAGTCCGAT TGCACTTGTC TCTCTTTTCT GGCTCCAGCC

AACATCGTGT GCGGTCCGAT TACACTTATC TCTTCTTTCC GGCTCCAGCC

GACATCATGC ACGGTCCTAT TGCACTTATC TCTCCTTTCT GGCTCAAGCC

CACATCGTGC ACAGTCCGAT TGCACTTCTC TCTCCTGTCC GGCTCCAGCC

CACATCATGC ACAGTCCGAT TGCATTTGTC TCTCCTTTCT GGCTCCAGCC

AACATCATGC ACAGTCCGAT TGCACTTATC TTTCCTTTCA GGCTCGAGCC

AACGTCGTGA ACAGTCCGAT TGCACTTATC GTTCCTTTCA GGCTCCAGCC

GACGTCATGT ACAGTCCGAT T--------- ---------- ----------

AACATCATGC ACAGTCCGAT TGCACTTATC TCTCTTTTCC GCCTCAAGTC

CACGTCATGC GCAGTTCGAT CGCACTTATC TTTCCTTTCA GCCTCCAGCC

CTCCGTGCCT ACACAAGATT TCTAGGCAGT TCTATAAAGA CAAGAGCTCT

CTCCGTGCCT ACACAAGATT TCTAGGCAGT TCTATAAAGA CAAGAGCTCT

CTCCGTGTCT ACACAAGATT TCTAGGCAGT TCTGTAAAGA CAAGAGCTCG

CTCCGTGTCT ACACAAGATT TCTAGGCAGT TCTGTAAAGA CAAGAGCTCG

CTCCGTGCCT ACACAAGATT TCTAGGCAGT TCTGTAAAGA CAAGGGCTCT

CTCTGTGTCT ACACAAGATT TCTAGGCAGT TCTGTAAAGA CAAGATCTCT

---------- ---------- ---------- ---------- ----------

CTCCATGCCT ACACAAGACT TCTAGGCAGT TCTGTAAAGA CAAGAACACT

CTCCGTGCCT ACATAAGATT TCTAGGCAGT TCTGTAAAGA CAAGAACTCC

CTCCGTGCCT ACACAAGACT TCTAGGCAGT TCTGTGAAGA CAAGAACTCC

CTCCGTGCTT ACACAAGATT TCCAGGCAGT TCTGCAAAGG CAAGGGCTCT

CTGCATGCCT ACACAAGATT TCTAGGCAGT TCTGTAAAGA CAAGAGCTCT

CTCCGTGCCT ACATAATATT TCAAGGCAGT TCTGTAAAGA CAAGAGCTCA

CTCCGTGCCT ACATAAGATT TCTAGGCAGT TCTGTAATGA CAAGAGCTCC

CTCCATGCTG ACACAAGATT TCTAAGCAGT TCTGTAAAGA CAAGAGCTCT

---------- ---------- ---------- ---------- ----------

CACCATGGCT ACACAAGATT TCTAGGCAGT TCTGGAATGA GAAGAGCTCT

TGCCGTGACC GCAGAGGATT TCTAGGCAAT TCTGGAGAGA TGAGAGAGGC

ATTAACCTTA AAACCTTTGG AAGCAGCAAT GTGGGCAGCA GTCCAGCCTT

ATTAACCTTA AAACCTTTGG AAGCAGCAAT GTGGGCAGCA GTCCAGCCTT

ATTAACCTTA AAACCTTTGG AAGCAGCAAT GTGGGCAGCA GTCCAGCCTT

ATTAACCTTA AAACCTTTGG AAGCAGCGAT GTGGGCAGCA GTCCAGCCTT

ATTAACCTTA AAGCCTTTGG AAGCAGCAAT GTGGGCAGCA GTCCAGCCTT

ATTAACCTTA AAACCTTTGG AAGCAGCAAT ATGGGCAGCA GTCCAGCCTT

----ACCTTG AAACCTTTGG AAGCAGCAAT ATGGGCAGCA GTCCAGCCTT

ATTAACCTTG AAACCTTTGG AGGCAGCAAT GTGGGCAGCA GTCCAGCCTT

ATTAACCTTA AAGCCTTTCG AAGCAGCGAT GTGGGCAGCA GTCCAGCCCT

ATTAACCTTA AAACCCTTTG AAGCAGCAAT ATGGGCAGCA GTCCAGCCTT

ATTAACCTTA AAACCTTTCG AAGCAGCAAT ATGGGCAGCG GTCCAGCCTT

ATTAACCTTA AAACCCTTTG AAGCAGCAAT ATGGGCAGCG GTCCAGCCTT

GTTAACCTTG AACCCTTTAG AAGCAGCGAT GTGGGCAGCG GTCCAGCCCT

ATTAACCTTA AAGCCTTTTG AGGCAGCAAT ATGAGCAGCA GTCCAGCCTT

GTCAACCTTA AAACCTTTTG AGGCAGCAAT GTGGGCAGCA GTCCAGCCTT

----ACCTTA AAACCTTTCG AAGCAGCAAT GTGGGCAGCC GTCCAGCCCT

ATTAACCTTA AGACCTTTTG ATGCGGCTAT GTGGGCAGCA GTCCAGCCTT

ATTAACCTTC AAACCTTTCG AAGCCGCAAT GTGGGCAGCC GTCCAACCCT

CTCTGTTGGC GTGGTTAATG AGGTCTGCAG GAACAACAGG CTTGTGGTAC

CTCTGTTGGC GTGGTTAATG AGGTCTGCAG GAACAACAGG CTTGTGGTAC

CTCTGTTGGC GTGGTTAATG AGGTCTGCAG GAACAACAGG CTTGTGGTAC

CTCTGTTGGC GTGGTTAATG AGGTCTGCAG GAACAACAGG CTTGTGGTAC

CTCTGTTGGC GTGGTTAATG AAGTCTGCAG TAACAACAGG CTTGTGGTAC

CTCTGTTGGC ATAGTTAATG AGGTCTGCAG GAATAACAGG CTTGTGATAC

CTTTGTCAGC GTGGTTAATG AGGTCCGCAG GAACCACAGG CTTATGGTAC

CTTTGTCAGC GTGGTTAATT AGGTCTGCAG GAACCACAGG CTTATGGTAC

CTCTATCGGC GTGGTTAATG AGATCTGCAG AAACAACGGG TTCGTAGTAC

CACTGTCAGC GTGGTTGATG AGGTCTGCAG GAACAACAGG CTTGTGGTAC

CTCTGTCAGC ATGGTTAACG AGGTCTGCAG GAACCACAGG CTTGTGGTAC

CTCTGTCGGC CTGGTTAATG AGGTCTGCAG GAACCACAGG CTTGTGGTAC

CTCTGTCAGC GTGGTTAATG AGCTCCTCGG GAACAATGGG CTTGTGGTAC

CTCTGTTGGC GTGGTTAATG AGGTCTGTGG GAACAACAGG CTTGTGGTAC

CTCTGTCAGC ATGGTTAATG AGGTCTGCAG GAACAACAGG CTTGTGGTAC

CCCTGTTGGC GTGGTTAATG AGGTCGGCGG GGATAACGGG CGTGTGGTAC

CCTTGTCAGC ATGGTTGATA AGATCTGTAG GAACAATAGG CTTGTAGTAC

CCTTGTCAGC CTGGTTGATG AGATCGGCCG AAATGACAGG CATGTAGTAC

ATAAGAAGCT TGAGGCTGTC CACATTACCA GTGTCCACAG CTGCGTGAAC

ATAAGAAGCT TGAGGCTGTC CACATTACCA GTGTCCACAG CTGCGTGAAC

ATAAGAAGCT TGAGGCTGTC CACATTACCA GTGTCCACAG CTGCATGAAC

ATAAGAAGCT TGAGGCTGTC CACATTACCA GTGTCCACAG CTGCATGAAC

ATAAGAAGCT TGAGGCTGTC CACATTACCA GTGTCCACAG CTGCGTGAAC

ATCAGAAGTT TGAGGCTGTC CACATCACCA GTGTCCACAA CTGCATGAAC

ATCAATAGCT TGAGGCTGTC CACATTACCA GTGTCCACGG CTGCATGAAT

ATCAGGAGCT TGAGGCTGTC CACATTACCA GTGTCCACAG CTGCGTGAAT

ATGAGGAGCT TCAGGCTGTC CACATTACCA GTGTCCACGG CTGCATGCAC

ATAAGAAGCT TGAGGCTGTC CACATTACCA GTGTCCACGG CTGCATGAAT

ATGAGAAGCT TGAGGCTGTC CACATTACCA GTGTCCACAG CTGCATGAAC

ATGAGAAGCT TGACGCTGTC CACATTACCA GTGTCCACAG CTGCGTGCAG

ATAAGAAGCT TGAGACTGTC CACATTGCCA GTGTCCACAG CTGCATGAAC

ATAAGAAGCT TGAGGCTGTC CACATTTCCA GTGTCCACAG CTGCATGAAC

ATAAGAAGCT TGAGGCTATC CACATTACCA GTGTCCACAG CTGCGTGAAC

AGGAGGAGCT TGAGGCCATC CACACTACCA GTGTCCACCG CAGCGTGAAC

ATAAGGAGCT TCAGACTGTC CACATTGCCA GAATCCACAG CAGCATGAAT

ATGAGGAGCG TGAGGCTGTC CACGTTTCCA GAATCCACAG CGGCGTGGAC

TGGTGTCCAG CCATCCTGAA AATAAATACT CACTGTGGTT TTTACACTTC

TGGTGTCCAG CCATCCTAAA AATAAATACT CACTGTGGTT TTTACACTTC

TGGTGTCCAG CCATCCTGAA AATAAATACT CACTGTGGTT TTTACACTTC

TGGTGTCCAG CCATCCTGAA AATAAATACT CACTGTGGTT TTTACACTTC

GGGTGTCCAG CCATCCTGAA AATAAATACT CACTGTGGTT TTTACACTTC

TGGTGTCCAG CCATCCTGTA AAGAAATACT CACTCTGGTT TTTACATTTC

TGGTGTCCAG CCATCCTGAA AATACATACT TACTCTGGTT TTTATACTCC

TGGCGTCCAG CCATCCTGAA AATACATACT TACTCTGGTT TTTATGCTCC

TGGTGTCCAG CCATCCTGAA AACAAATACT CACTCTGGTT TTTATACTTC

TGGTGTCCAG CCATCCTGAA AATAAATACT CACTCTGGTT TTTACACTTC

CGGTGTCCAG CCATCCTGAA GATAAATACT CACTCTGGTT TCCACACTTC

TGGTGTCCAG CCATCCTGAA AATAAATACT CACACTAGTT TTTACGCTTC

TGGTGTCCAG CCATCCTGGA AATAAATACT CACTCTGGTT TTTACACTCC

TGGTGTCCAG CCATCCTGAA AATAAATACT CACTCTGGTT TTTACACTTC

TGGTGTCCAG CCATCCTGAA AACAAATACT CACTCTGGTT TTTACACTTC

TGGTGTCCAG CCATCCTGAA AACAAATACT CACTCTGGTT TTTACACTTC

TGGGGTCCAA CCATCCTGCA AACAAATACT TACACTGGTT TTTACATTTC

TGGGGTCCAG CCATCCTGAA AGTCAATACT TACACTGGTT GTTATACTTC

GATTGGTTCC AGCTTCCAAC AAGAGTTTAA TACATTCTTT ATTTCCATTT

GATTGGTTCC AGCTTCCAAC AAGAGTTTAA TACATTCTTT ATTTCCATTT

GGTTGGTTCC GGCTTCCAAC AAGAGTTTAA TACATTCTTT ATTTTCATTT

GGTTGGTTCC GGCTTCCAAC AAGAGTTTAA TACATTCTTT ATTTTCATTT

GATCGGTTCC AGCTTCCAAC AAGAGTCTAA TACATTCTTT ATTTCCATTT

GGTTAGTTCC AGCTTCCAAC AAGAGTTTAA TACATTCTTT ATTTCCGTTT

GGTCAGTGCC AGCTTCCAGC AGGAGTTTGA TACATTCCTT ATTTCCAGTT

GGTCAGTGCC AGCTTCCAGC AGGAGTTTGA TACATTCCTT ATTTCCATTT

GGTCAGTTCC AGCTTCCAAC AAGAGTTTAA TACAGTCTGT ATTTCCATTT

GGTCAGTTCC AGCTTCCAAC AAGAGTTTAA TACATTCTTT ATTTCCATTT

GGTCAGTTCC GGCTTCCAGT AGGTATTTAA TACACTCCTT ATTTCCATTT

GGTCACTTCC AGCTTCCAAC AAGAGTTTAA TACATTCTTT ATTTCCATTT

GGTCAGCTCC AGCTTCTAAC AAGAGTTTGA TACATTCTTT GTTTCCATTT

GGTCTGTTCC AGCTTCCAGT AGGACTTTAA TACATTCTTT ATTTCCATTT

GGTCAGTTCC AGCTTCCAAC AAGAGTTTAA TACATTCTTT ATTTCCATTT

GGTCTGTTCC AGCTTCCAAT AAGAGTTTAA TACATTCTTT ATTTCCATTT

GGTCTGTTCC AGCTTCCAAC AATAGTTTAA TACATTCATT ATTTCCATTT

GGTCTGTTCC AGCTTCCAGC AGGAGTTTGA TACATTCGTT ATTTCCGTTT

TTACAGGCCA GGTATAGAGG TGTCTGTCCT CCATCAGCAG CATGATTAAT

TTACAGGCCA GGTATAGAGG TGTCTGTCCT CCATCAGCAG CATGATTAAT

TTACAGGCCA GGTATAGAGG TGTCTGTCCT CCATCAGCAG CGTGATTAAT

TTACAGGCCA GGTATAGAGG TGTCTGTCCT CCATCAGCAG CATGATTAAT

TTACAGGCCA GGTATAGAGG TGTCTGTCCT CCATCAGCAG CATGATTAAT

TTACAGGCCA GGTATAGAGG TGTCTGTCCT TCATCAGCAG CGTGATTAAT

TTACAGGCCA GGTACAGAGG TGTCTGTCCT CCAGCAGCAG AGTGATTAAT

TTACAGGCCA AGTACAAAGG TGTCTGTCCT CCAGCAGCAG AGTGATTAAT

TTACAGGCCA GGTATAGAGG TGTCTGTCCT CCATCAGCAG CATGATTGAC

TTACAGGCCA GGTATAGAGG TGTCTGTCCT CCATCAGCAG CATGATTAAT

TTACAGGCCA GGTATAGAGG TGTCTGTCCT TCATCAGCAG CATGATTAAT

TTACAGGCCA GGTATAGAGG TGTCTGTCCT CCATCAGCAG TATGATTAAT

TTACAGGCCA GGTATAGAGG TGTCTGTCCT CCATCAGCAG CATGATTAAT

TTACAGGCCA GGTACAGAGG TGTCTGTCCT CCATCAGCAG CATGATCAAT

TTACAGGCCA GGTATAGAGG TGTCTGTCCT CCATCAGCAG CATGGTTAAT

TTACAGGCCA GGTAGAGAGG TGTCTGTCCT TCAGCAGCAG CATGATTAAT

TTACAGGCCA GGTATAGAGG TGTTTGTCCT CCTTCAGCAG CATGGTTAAT

TTACAGGCCA GGTACAGAGG TGTCTGACCT CCTTCAGCAG CATGGTTAAT

GTTAGCATCA TATGAAATTA ATAATTCTAC ACACCTAGAA CTTACTCGAA

GTTAGCATCA TATGAAATTA ATAATTCTAC ACACCTAGAA CTTACTCGAA

GTTAGCATCA TATGCAATTA ATAATTCTAC ACACCTAGAA CTTACTCGAA

GTTAGCATCA TATGCAATTA ATAATTCTAC ACACCTAGAA CTTACTCGAA

ATTAGCATCA TATGCTATTA ATAATTCTAC ACACCTAGAA CTTACTCGAA

GTGAGCATCA TATGCAATCA ATAATTCTAC ACACCTAGAA CTTACTCGAA

GTTGGCATTG TATGCAGTTA GTAATTCTAT ACATCTATAA CTTACTCAAA

GTTGGCATTG TATGCAGTTA GTAATTCTAT ACATCTAGAA CTTACTCAAA

ATCAGCATCA GATGCAAGTA GTAATTCTAA ACACCTAGAA CTTACTCGAA

ATTAGCATCA TATGCAATTA ATAATTCTAC ACACCTAGAA CTTACTTGAA

GTTGGCATCA TATGCAATTA ATAATTCTAC ACACCTAGAA CTTACTTGAA

GTCAGCATCA TATGCAGTTA ATAATTCTAC ACACCTAGAA CTTACTTGAA

GTTAGCATCG TATGCAATTA GTAATTCTAC ACACCTAGCA CTTACTTGAA

GTTAGCATGA TATGCAATTA ATAATTCTAC ACACCTAGAA CTTACTCGAA

GTTAGCACGA TATGCAATTA GTAATTCTAC ACACCTAGAA CTTACTTGTC

GTTAGCATGG TATGCAACTA ATAATTCTAC ACATCT-GAA CTTACTTGAC

GTCAGCATGA TATGCAATTA GTAATTCTGC ACACCTAAAA CTTACTTGAA

GTCAGCGTGA TATGCAATTA GTAATTCTGC ACACCTATGA CTTACTTAAC

ATGTCCCTGA GCAGCTGCAG CACACAAGGG TGTGAAGCCA TTTTTATCAG

ATGTCCCTGA GCAGCTGCAG CACACAAGGG TGTGAAGCCA TTTTTATCAG

ATGTCCCTGA GCAGCTGCAG CACACAAGGG TGTGAAGCCA TTTTTATCAG

ATGTCCCTGA GCAGCTGCAG CACACAAGGG TGTGAAGCCA TTTTTATCAG

ATGTCCCTGA GCAGCTGCAG CACACAAGGG TGTGAAGCCA TTTTTATCAG

ATGTCCCTGA GCAGCTGCAG CACACAAGGG TGTGAAGCCA TTTTTATCAG

ATGTCCCTGA GCGGCTGCAA CGCACAAGGG TGTGAAGCCA TTTTTATCAG

ATGTCCCTGA GCAGCTGCGA CGCACAAGGG TGTGAAGCCA TTTTTATCAG

ATGTCCCTGA GCAGCTGCGG CACACAAGGG TGTGAAGCCG TTCTTCTCAG

ATGTCCCTGA GCAGCTGCAG CACACAAGGG TGTGAAGCCA TTTTTATCAG

ATGTCCCTGA GCAGCTGCAG CACACAAGGG TGTGAAGCCA TTTTTATCAG

ATGTCCCTGA GCAGCTGCAG CACACAAGGG TGTGAAGCCA TTTTTATCAG

GTGTCCCTGT GCAGCTGCAG CACACAAGGG TGTAAAGCCG TTTTTATCAG

ATGTCCCTGA GCAGCTGCAG CACACAAGGG TGTGAAGCCA TTTTTATCAG

ATGTCCCTGA GCAGCTGCGG CACACAAGGG TGTGAAGCCG TTTTTATCAG

GTGTCCCTGA GCAGCTGCAG CACACACGGG TGTGAAGCCA TCGTTATCAG

ATGTCCCTGA GCAGCTGCTG AACACAAGGG TGTGAAGCCA TTTTTATCAG

ATGTCCCTGA GCGGCTGCTG AACACAAGGG GGTGAAGCCA TTTTTATCGG

CAGCATTGAC TTGGGCTTCT GCACTCAGCA GCAATCTCAC ACAGTCTATT

CAGCATTGAC TTGGGCTTCT GCACTCAGCA GCAATCTCAC ACAGTCTATT

CAGCATTGAT TTGGGCTTCT GCACTCAGCA GCAATCTCAC ACAGTCTATT

CAGCATTGAT TTGGGCTTCT GCACTCAGCA GCAATCTCAC ACAGTCTATT

CAGCATTGAC TTGGGCTTCT GCACTCAGCA GCAATCTCAC ACAGTCTATT

CAGCATTGAC TTGGGCTTCT GCATTCAGCA GCAATCTCAC ACAGTCTATT

CAGCGTCGAC GCGGGCTTCT GCATTCAGCA GCAATCGCAC ACAGTCTATT

CAGCGTCGAC TCGGGCTTCT GCATTCAGCA GCAATCGCAC ACAGTCTATT

CAGCATTGAC TTGGGCTTCT GCATTCAGCA GCAATCTCAC ACAGTCTATT

CAGCATTGAC TTGGGCTTCT GCATTCAGCA GCAATCTCAC ACAGTCTATT

CAGCATTGAC TTGGGCTTCT GCATTCAGCA GCAATCTCAC ACAGTCTATT

CAGCATCGAC TTGGGCTTCT GCATTCAGCA GCAATCTCAC ACAATCTATT

CAGCGCTGAC TTGGGCTTCT GCATTCAGCA GCAATCTCAC ACAGTCTATT

CAGCATCAAT TTGGGCTTTT GCATTCAGCA GCAATCTCAC ACAGTCTATT

CAGCGTTGAC TTGGGCTTCT GCATTCAGCA GCAATCTCAC ACAGTCTATT

CAGCATTGAC TTGGGCTTCT GCATTCAGCA GCAATCTCAC ACAGTCTATT

CAGTATTAAC TTGTGCTTCT GCATTCAGCA GCAATCTCAC ACAGTCTATT

CATCATCAAC TTGCGCATTG GCATTCAGCA GCAATCTCAC ACAGTCTGTT

ACCTGTATGT CCATTCTTAG CAGCAGAATA CAAGGCAGAA TGGCCATCTT

ACCTGTATGT CCATTCTTAG CAGCAGAATA CAAGGCAGAA TGGCCATCTT

ACCTGTATGT CCATTCTTAG CAGCAGAATA CAAGGCAGAA TGGCCATCTT

ACCTGTATGT CCATTCTTAG CAGCAGAATA CAAGGCAGAA TGGCCATCTT

ACCTGTATGT CCATTCTTAG CAGCAGAATA CAAGGCAGAA TGGCCATCTT

ACCTGTATGT CCATTCTTAG CAGCAGAATA CAAGGCAGAA TGGCCATCTT

ACCTGTATGT CCATTCTTAG CAGCAGAATA CAAGGCAGAA TGGCTATCTT

ACCTGTATGT CCATTCTTAG CAGCAGAATA CAAGGCAGAA TGGCCATCTT

ACCTGCATGT CCATTCGTAG CAGCAGAATA CAAGGCAGAA TGGCCATCTT

ACCTGTATGT CCATTCTTAG CAGCAGAATA CAAGGCAGAA TGGCCATCTT

ACCTGTATGT CCATTCTTAG CAGCAGAATA CAAGGCAGAA TGGCCATCTT

ACCTGTATGT CCATTCTTAG CAGCAGAATA CAAGGCAGAA TGGCCATCTT

ACCTGTATGT CCATTCTTAG CAGCAGAATA CAAGGCAGAA TGGCCATCTT

ACCTGTATGT CCATTCTTAG CAGCAGAATA CAAGGCAGAA TGGCCATCTT

ACCTGTATGT CCATTCTTAG CAGCAGAATA CAAGGCAGAA TGGCCATCTT

ACCTGTATGT CCATTCTTAG CAGCAGAATA CAAGGCAGAA TGGCCATCTT

ACCTGTATGT CCATTCTTAG CAGCAGAATA CAAGGCAGAA GAGCCATCTT

ACCTGTATGT CCATTCTTAG CAGCAGAATA CAAGGCAGAA GAGCCATCTT

CACAGGAGTA ATTAATGTCC AGTCCTTCTT CATTAAGCAG CATTGATAAT

CACAGGAGTA ATTAATGTCC AGTCCTTCTT CATTAAGCAG CATTGATAAT

CACAGGAGTA ATTAATGTCC AGTCCTTCTT CATTAAGCAG CATTGATAAT

CACAGGAGTA ATTAATGTCC AGTCCTTCTT CATTAAGCAG CATTGATAAT

CACAGGAGTA ATTAATGTCC AGTCCTTCTT CATTAAGCAG CATTGATAAT

CACAGGAGTA ATTAATGTCC AGTCCTTCTT CATTAAGCAG CATTGATAAT

CACAGCAGTA ATTTATGTCC AGTCCTTCTT CATTAAGCAG CATTGATAAT

CGCAGGAGTA ATTTATGTCC AGTCCTTCTT CATTAAGCAG CATTGATAAT

CACAGGAGTA ATTAGTGTCC AGTCCTTCTT CATTAAGCAG CATTGATAAT

CACAGGAGTA ATTAATGTCC AGTCCTTCTT CATTAAGCAG CATTGATAAT

CACAGGAGTA ATTAATGTCC AGTCCTTCTT CATTAAGCAG CATTGATAAT

CACAGGAGTA ATTAATGTCC AGTCCTTCTT CATTAAGCAG CATTGACAAT

CACAGGAGTA ATTAATGTCC AGTCCTTCTT CATTAAGCAG CATTGATAAT

CACAGGAGTA ATTAATGTCC AGTCCTTCTT CATTAAGCAG CATTGACAAT

CACAGGAGTA ATTAATGTCC AGTCCTTCTT CATTAAGCAG CATTGATAAT

CGCAAGAGTG ATTGATGTCG AGTCCTTCTT CATTAAGCAG CATTGACAGT

CACAGGAGTA ATTAATGTCC AGTCCTTCTT CATTAAGCAG CATTGATAAT

CACAGGCGTG ATTAATGTCC AGTCCTTCTT CATTAAGCAG CACAGACAAT

AAAGTGACAT TTCCCTGGGC AGCAGCTTGC TGAAGAAGGG TGGGCCTGCC

AAAGTGACAT TTCCCTGGGC AGCAGCTTGC TGAAGAAGGG TGGGCCTGCC

AAAGTGACAT TTCCCTGGGC AGCAGCTTGC TGAAGAAGGG TGGGCCTGCC

AAAGTGACAT TTCCCTGGGC AGCAGCTTGC TGAAGAAGGG TGGGCCTGCC

AAAGTGACAT TTCCCTGGGC AGCAGCTTGC TGAAGAAGGG TGGGCCTGCC

AAAGTGACAT TTCCCTGGGC AGCAGCTTGC TGAAGAAGGG TGGGCCTGCC

AGAGTGACAT TTCCCTGGGC AGCAGCTTGC TGAAGAAGGG TGGGCCTGCC

AAAGTGACAT TTCCCTGGGC AGCAGCTTGC TGAAGAAGGG TGGGCCTGCC

AAAGTGACAT TTCCCTGGGC AGCAGCTTGC TGAAGAAGGG TGGGCCTGCC

AAAGTGACAT TTCCCTGGGC AGCAGCTTGC TGAAGAAGGG TGGGCCTGCC

AAAGTGACAT TTCCCTGGGC AGCAGCTTGC TGAAGAAGGG TGGGCCTGCC

AAAGTGACAT TTCCCTGGGC AGCAGCTTGC TGAAGAAGGG TTGGCCTGCC

AAAGTGACAT TTCCCTGGGC AGCAGCTTGC TGAAGAAGGG TGGGCCTGCC

AAAGTGACAT TTCCCTGGGC AGCAGCTTGC TGAAGAAGGG TGGGCCTGCC

AAAGTGACAT TTCCCTGGGC AGCAGCTTGC TGAAGAAGGG TGGGCCTGCC

AAAGTGACAT TTCCCTGGGC AGCAGCTTGC TGAAGAAGGG TGGGCCTGCC

AAAGTGACAT TTCCCTGGGC AGCAGCTTGC TGAAGAAGGG TGGACCTGCC

AAAGTGACAT TTCCCTGGGC AGCAGCTTGG TGAAGACGGG TGGGCCTGCC

AGCCAGGGGG GCAGGACCAC CACTCATTAG CAAAGGGGTT AGGGAGGGTG

AGCCAGGGGG GCAGGACCAC CACTCATTAG CAAAGGGGTT AGGGAGGGTG

AGCCAGGGGG GCAGGACCAC CACTCATTAG CAAAGGGGTT AGGGAGGGTG

AGCCAGGGGG GCAGGACCAC CACTCATTAG CAAAGGGGTT AGGGAGGGTG

AGCCAGGGGG GCAGGACCAC CACTCATTAG CAAAGGGGTT AGGGAGGGTG

AGCCAGGGGG GCAGGACCAC CACTCATTAG CAAAGGGGTT AGGGAGGGTG

AGCCAGGGGG GCAGGACCAC CACTCATTAG CAAAGGGGTT AGGGAGGGTG

AGCCAGGGGG GCAGGACCAC CACTCATTAG CAAAGGGGTT AGGGAGGGTG

AGCCAGGGGG GCAGGACCAC CACTCATTAG CAAAGGGGTT AGGGAGGGTG

AGCCAGGGGG GCAGGACCAC CACTCATTAG CAAAGGGGTT AGGGAGGGTG

AGCCAGGGGG GCAGGACCAC CACTCATTAG CAAAGGGGTT AGGGAGGGTG

AGCCAGGGGG GCAGGACCAC CACTCATTAG CAAAAAGGTT AGGGAGGGTG

AGCCAGGGGG GCAGGACCAC CACTCATTAG CAAAGGGGTT AGGGAGGGTG

AGCCAGGGGG GCAGGACCAC CACTCATTAG CAAAGGGGTT AGGGAGGGTG

AGCCAGGGGG GCAGGACCAC CACTCATTAG CAAAGGGGTT AGGGAGGGTG

AGCCAGGGGG GCAGGACCAC CACTCATTAG CAAAGGGGTT AGGGAGGGTG

AGCCAGGGAG GCAGGACCAC CACTCATTAG CAAAGGGGTT AGGGAGGGTG

ACCCAGGGAC ACAGGACCAC AACTTGTTAG CAAAGGGGTT AGGGAGGGGG

ACCAGCCTGT AGGTTAAAGT GACTAGAGGA TTATGTGGTT TCTAACATTA

ACCAGCCTGT AGGTTAAAGT GACTAGAGGA TTATGTGGTT TCTAACATTA

ACCAGCCTGT AGGTTAAAGT GACTAGAGGA TTATGTGGTT TCTAACATTA

ACCAGCCTGT AGGTTAAAGT GACTAGAGGA TTATGTGGTT TCTAACATTA

ACCAGCCTGT AGGTTAAAGT GACTAGAGGA TTATGTGGTG TCTAACATTA

ACCAGCCTGT AGGTTAAAGT GACTAGAGGA TTATGTAGTT TCTAACATTA

ACCAGCCTGT AGGTTAAAGT GACTAGAAGA TTACATGGTT TCTAACATTA

ACCAGCCTGT AGGTTAAAGT GACTAGAAGA TTACATGGTT TCTAACACTA

ACCAGCCTGT AGGTTAAAGT GACTAGAGGA TTATGTGTTT TCTAGAAGGT

ACCAGCCTGT GGGTTAAAGT GACTAGAGGA TTAAGTGGTT TCTAATATGA

ACCAGCCTGT GGGTTAAAGT GACTAGAGGA TTATGCAGTT TCTAACATTA

ACCAGCCTGT GGGTTAAAGT GACTAGAAGA TCATGTGGTT TCTAACATTA

ACCAGCCTGT GGGTTAAAGT GACTAGAAGA TTATATGGTT GCTGACATTA

ACCAGCCTGT GGGTTAAAGT AACCAGAGGA TTAGGTGGTT TCTAACATTA

ACCAGCCTGT GGGTTAAAGT GACCAGAGGA TTATGTGGTT TCTAACATTT

ACCAGCCTGT GGGTTAAAGC GACCAAAGGA TTATGTGGTT TCTAACATTA

ACCAGCCTGT GAGTTAAAGT GACCAAGGGG AAACACAATT TCTAACACTA

GCCACCCTGT GAGTTAAAGG GACcaGAGGT TCACGAGGCT TTTAACACCA

TCAATCTGTT AATATTTACT TAGCATGACT GTTATTTCAT TTAACAAAAA

TCAATCTGTT AATATTTACT TAGCATGACT GTTATTTCAT TTAACAAAAA

TCAATCTGTT AATATTTACT TAGCATAACT GTTATTTCAT TTAACAAAAA

TCAATCTGTT AATATTTACT TAGCATAACT GTTATTTCAT TTAACAAAAA

TCAATCTGTT AATATTTACT TAGCATGACT GTTATTTCAt taaaaaaaaa

TCAATCTGTT AATATTGACT TAGCATTACT GTTATTTCAA TTAACAAAAA

TCAATCTGTT AATACTGTCT TAGCATTACT GTTATTTCAT TTAACAAAGA

TCAATCTGTT AATACTTTCT TAGCACTACT GTTATTTCAT TTAACAAAGA

TCAATCTGTT AATATGTACT TAGGATTCCT GTTATTCCAT TCAACAAAGA

CCAATCTGTT AATATTTACT TAGCATTACT GTTATTTCAT TTAACAAAAG

TCAATCTGTT AATATTTACT TAGCACTACT GTTATTCCAT TTAACAAGAA

TCATTCTGTT AATATTTACT TAGCACTACT GTTATTTCAT TTAATAAAAA

TCAATCTGTT AATGTTTACT TAGAACTACT GTTATTTCAT TTAACAAAAG

TCAATCTGTT AATATTTACT TAGCACTACT GTTATTTCAT TTAACAAAAA

TCAATCTGTT AATATTGACT TAGCACTACT GTTATTTCAT TTAACAAAAA

TCAATCTGTT AATATTTACT TAGCACTACT GTTATTTCAT TTAATAAATA

TTGGTCTGTT AAGATTTATT TAGTCTCATT GTTATTTCAT TTAATAGAAA

CTGGCCCATT AAGACTGATT TAGCATTAGC AGTATTTTTA TTCATAGAGA

CAATGAGTTA GTTAGATATT TCTCATCAGT ATTTAGCAGC TTTAGAAGAG

CAATGAGTTA GTTAGATATT TCTCATCAGT ATTTAGCAGC TTTAGAAGAG

CAATGAGTTA GTTAGATATT TCTCATCAGT ATTTAGCAGC TTTAGAAGAG

CAATGAGTTA GTTAGATATT TCTCATCAGT ATTTAGCAGC TTTAGAAGAG

taatGAGTTA GTTAGATACT TCTCATCAGT ATTTAGCAGC TTTAGAAGAG

TCGTAAGTTA GTTAGGTATT TTTCATCAGT ATTTAGCAGC TTTGGAAGAG

TTGCAAATGA GTTAGTT--T TCTCATCAGT ATTTAGCAGT TTTAGAAGAG

CTGTAAGTTA GTTAGATAGT TCTCATCAGT GTTTAGCAGC TTTAGAAGAG

TCGTAAGTTA GTTAGAGATT TCTCATCAGT ATTTAGCAGC TTTAGAAGGG

TCATAAGTTA GTTAGG-ATT TCTCATCAGT ATTTAGCAGC TTTAGAAGAG

TCATAAGTTA GTTAGATTTC TCTCATCAGT ATTTAGCAGC TTTAGAAGAG

TCATAAGTTA GTTAGATATT TCTCATCAGA ATTTAGCAGC TTTAGAAGAG

TCATAAGTTA GTTAGATATT TCTCATCAGT ATTTAGCAGT TTTAGAAGAG

TTAAGAGTTA GTTAGATAAT TCTCATCAGT ATTTAGCAGC TTTAGAAGAG

TCATAAGTTA GTTAGATATT TCTCATCAGT ATTTAGCAGC TTTAGAAGAG

TCATAAGTTA GTTAGCTATT TCTCATCAGC ATTTAGCAGC TTTAGAAGAG

TTTCAAGTTT GTTAGACAGT CATTATCAGT ACTTTGCAGC CTTAGAAAAG

TCTTCAGTTA GTTAGAtatt tcttagaagt atttagcagC CTTAGAGGAT

AGTCTGTTAA GTGTGTGAAA TCGGCTGCAT ATAGCATGAA AAATTTTCTG

AGTCTGTTAA GTGTGTGAAT TCGGCTGCAT ATAGCATGAA AAATTTTCTG

AATCTGTTAA GTGTGTGAAT TCGGCTGCAT ATAGCATGAA AAACTTTCTG

AATCTGTTAA GTGTGTGAAT TCGGCTGCAT ATAGCATGAA AAACTTTCTG

AATCTGTTAA GTGTGTGAAG TCGGCTGCAT ATAGCATGAA AAATTTTCTG

AATCTGTTAA ATGTGTGAAG TCGGCTGCAT ATAGCATGGA CAATTTTCTG

AATCTGTTAA GAGTGTGAAG TCGGCTGCAT ATAGCCTGGA AAATTTTCTG

AATCTGTTAA GAGTGTGAAG TCGGCTGCAT ATAGCCTGGA AAATTTTCTG

AATCTGGTAA GTGTGTGAAG TCGGCTGTAT ATAGCATGGA AAATTTTCTG

AATCTGTTAA GTGTGTGAAG TCGGCTGCAT ATAGCATGGA AAATTTTCTG

AATCTGTTAA GTGTGTGAAG TCGGCTGCAT ATAGCATGGA AAATTTTCTG

AATCTGTTAA GTGTGTGAAG TCGGCTGCAT ATAGCATGGA AAATTTTCTG

AATCTGTTAA GTATGTGAAG TCGGCTGCAA ATAGCATGGA AAATTTTCTG

AATCTGTTAA GTGTGTGAAG TCGGCTGCAT ATAGCATGGA AAATTTTCTG

AATCTGTTAA GTGTGTGAAG TCGACTGCAT ATAGCATGGA AAATTTTCTG

AATCTGTTAA GTGTGTGAAG TCGACTGCAC AGAGCATGGA AAATTTTCTG

AATTTGTTGA GTGTGTGAAG CCTGCTATAA ATACTATGAA AAACTTTCTG

AATTTGTTAA GTGTGGAAAG TTTGCTGCAT ATAGTTTGGA AGATTTTGTG

AATTCTTCCT TGATCAAAAG GCTGTAAAAA CAGTAAAGTA AAAAGTATTT

AATTCTTCCT TGATCAAAAG GCTGTAAAAA CAGTAAAGTA AAAAGTATTT

AATTCTTCCT TGATCAAAAG GCTGTAAAAA CAGTAAAGTA AAAAGTATTT

AATTCTTCCT TGATCAAAAG GCTGTAAAAA CAGTAAAGTA AAAAGTATTT

AATTCTTCCT TGATCAAAAG GCTGTAAAAA CAGTAAAGTA AAAAATATTT

AATTCTTCCT TGATCAAAAG GCTGTGAAAA TAGTAAAGTA AAAAGTATTT

AATCCTTCCT TGATCAAAAG GCTGTAAAAA CAGTAAAGTA CACAGTATTT

AATCCTTCCT TGATCAAAAG GCTGTAAAAA CAGTAAAGTA CAAAGTATTT

AATTCTTCCT TGATCAAAAG GCTGTAAAAA CAGTAAAGTA AAAAATACTT

AATTCTTCCT TGATCAAAAG GCTGTAAAAA CAGTAAAGTA AAAA------

AATTCTTCCT TGGTCAAAAG GCTGTAAAAA CAGTAAAGTA AAAAGTATTT

AATTCTTCCC TGATCAAAAA GCTGTAAAAA CAGTAAAGTA AAAAGTATCT

AATTCTTCCT TGATCAAAAG GCTGTAAAAA CAGTAAAGTA AAGAGTATTT

AATTCTTCCT TGATCAAAAG GCTGTAAAAA CAGTAAAGTA AAAAGTATTT

AATTCTTCCT TGATAAAAAG GCTGTAAAAA CAGTAAAGTA AAAAGTATTT

AATTCTTCCT TGATCAAAAG GCTGCAAAAA CAGTAAAGTA AAAAGTATTT

AACTCTTCCT TGATCAAAAG GCTGTAAAAA AAGGAAAGTA TAAAGTTTAA

AATTGTTTCT TGACCAAAAG GCTGAAAAAG AAAAAGAGGA AAAAGCCTCT

TTACTTCATA CTTACAAAAT TTTCTTAAAA ACAAACAAAA AACAGGCCCA

TTACTTCATA CTTACAAAAT TTTCTTAAAA ACAAACAAAA AACAGGCCCA

TTACTTCATA CTTACAAAAT TTTCTTAAAA ACAAACAAAA AACAGGCCCA

TTACTTCATA CTTACAAAAT TTTCTTAAAA ACAAACAAAA AACAGGCCCA

TTACTTCATA CTTACAAAAT TTTCTTAAAA ACAAACAAAA AACAGGCCCA

TTACTTCATA CTTACAAAAT TTTCTTAAAA ACAAACAAAA AACAGGCCCA

TTACTTCATA CTTACAAAAT TTTCTTAAAA ACAAACAAAA AACAGGCCCA

TTACTTCATA CTTACAAAAT TTTCTTAAAA ACAAACAAAA AACAGGCCCA

TTACTTCATA CTTACAAAAT TTTCTTAAAA ACAAACAAAA AACAGGCCCA

---------- ---------- ---------- ---------- ----------

TTACTTCATA CTTACAAAAT TTTCTTAAAA ACAAACA-AA AACAAGCCCA

TTACTTCATA CTTACACAAT TGTCTTAAAA ACAAAC--AA AACAAGCCCA

TTACTTCATA CTTACAAAAT TTTCTTAAAA ACAAAC--AA AAGAAGCCCA

TTACTTCATA CTTACAAAAT ATTCTTAAAA ACAAACAAAA AACAAGCCCA

TTACTTCATA CTTACAAAAT TTTCTTAAAA ACAAACAAAA AACAAGCCCA

TTACTTCATA CTTACAACAT TTTCTTAAAA ACAAAC-AAA AACAAGCCCA

CCATTTTGCT CTTCCAAAAT TTTTTTAAAC ACAAATAAGA ACTAGATTCA

CTGCTTTATA TTTACAAAGC TCTATTAAAA AAAAATAATA TTTATATTCA

CAAAAATTAA AAGTACTTAT TAGAAATGGA GAAATTATAG AGAACAGCTT

CAAAAATTAA AAGTACTTAT TAGAAATGGA GAAATTATAG AGAACAGCTT

CAAAAATTAA AAGTACTTAT TAGAAATGGA GAAATTATAG AGAACAGCTT

CAAAAATTAA AAGTACTTAT TAGAAATGGA GAAATTATAG AGAACAGCTT

CAAAAATTAA AAGTACTTAT TAGAAATGGA GAAAGTATAG AGAACAGCTT

CAAAAATTAA AAGTACTTAT TAGAAATGGA GAAATTATAG AGAACAGCTT

CAAAAATTAA AAGTACTTAT TAGAAATGGA -AAATTATAG AGAACAGCTT

CAAAAATTAA AAGTACTTAT TAGAAATGGA -AAATTATAG AGAACAGCTT

CAAAAATTAA AAGTACTTGT TAGAAATGGA GAAATTATAG CAAACAGCTT

---AAATTAA AAGTACTTAT AGAAAATGGA GAAATTATAG AGAACAGCTT

CAAAAATTAA AAGTACTTAT TAGAAATGGA GAAATTATAG AGAACAGCTT

CAA-AATTAA AAGTACTTAT CAGAATTGGA GAAATTCTAG AGAACAGCTT

CAAAAATTAA AAGTACTTAT TAGAAATG-A GAAATTATAG AGAACAGCTT

CAAAAATTAA AAGTACTCAT TAGAAATGGA GAAGCTACAG AGAACAGCTT

CAAAAATTAA AAGTACTTAT TAGAAATGGA GAAGTTATAG AGAACAGCTT

CAAAAATTAA AAGTACTTAT ATGAAATCGA GAAATTATAG AGAACAGCTT

CAAAAATGAA AGTTACACAT TAGAGACAGA GACAGGATAG AAAACAGCTT

CTAAAGCTTA AAGTACTCAC CTCAAACCGA TCAAATGTTG AAAACAGCTT

CTGGCATCAG CTTCAGAGGG TGGAGAAGGT TAATGTCATA GCCCAGATCA

CCGGCATCAG CTTCAGAGGG TGGAGAAGGT TAATGTCATA GCCCAGATCA

CCGGCATCAG CTTCAGAGGG TGGAGAAGGT TAATGTCATA GCCCAGATCA

CCGGCATCAG CTTCAGAGGG TGGAGAAGGT TAATGTCATA GCCCAGATCA

CTGGCATCAG CTTCAGAGGG TGGAGCAGGT TAATGTCATA GCCCAGATCA

CCAGCATCAG CCTCAGAGGG TAGAGAAGGT TAATGTCATA GCCCAGATCA

CTGGCACCAG CTTCAGAGGG TGGAGAGGGT TAAGGTCACA GCCCAGACCA

CTGGCACCAG CTTCAGAGGG TGGAGAGGGT TAAGGGCACA GCCCAGACCA

CTGGCATCAG CTTCAGAGGG TGAAGAAGGT TAATGTCATA GCCCAGGTCA

CCGGCATCAG CTTCAGAGTG TGGAGAAGGT TAATTTCATA GCTCAGAGGG

CTGGCATCAG CTTCAGAGTG TGGAGAAGGT TAATGTCATA GCTCAGGTCA

CTGGCATCAG CTTCAGAGTG TGGAGAAGGT TAGCGTCATA GCTCAGATCA

CTGGCATCAG CTTCAGAGTG TAGAGAGGGT TAATGTCATA GCTCAGATCA

CTGGCATCAG CTTCAGAGTG TGGAAAAGGT TAATGTCACA GCCCTGATCT

CCGGCATCAG CTTCAGAGTG TGGAGAAGGT TAATGTCATA GCCCAGATCA

CCGGCATCAG CTTCAGAGTG T------GGT TAAGGTCACA GCCCCGATCA

CTGGCACCAG CTTCAGAATG TGGAGGTGGT TAATTTCATA GCCCACATCA

CTGGCATCAG CTTCAGAACA CAGAGATGGT TAATTTCAAA GCTCGGATCA

TTATCCCTTT ACCTGATGCT GTTACCAGGA GGCTGTCTGA GGCACCTGGT

TTATCCCTTT ACCTGATGCT GTTACCAGGA GGCTGTCTGA GGCACCTGGT

TTATCCCTTT ACCTGATGCT GTTACCAGGA GGCTGTCTGA GGCACCTGGT

TTATCCCTTT ACCTGATGCT GTTACCAGGA GGCTGTCTGA GGCACCTGGT

TTATCCCTTT ACCTGATGCT GTTACCAGGA GGCTGTCTGA GGCACCTGGT

TTATCCCTTT ACCTGATGCT GTTACCAGGA GGCTGTCTGA GGCACCTGGG

TTATCCCTTT ACCTGATGCT GTTACCAAGA GGCTGTCTGA GGCACCGGGG

TTATCCCTTT ACCTGATGCT GCTACCAAGA GGCTGTCTGA GGCACCGGGG

TTATCCCTTT ACCTGATGCT GTTACCAGGA GGCTGTCTGA GGCACCTGGC

ATATCCCTTT ACCTGATGCT GTTACCAGGA GGCTGTCAGA GGCACCTGCT

ATATCCCTTT ACCTGATGCT GTTACCAGGA GGCTGTCTGA GGCACCTGCT

ATATCCCTTT ACCTGATGCT GTTACCAGGA GGCTGTCTGA GGCACCTGCT

ATATCCCTTT ACCTGATGCT GTTACCAGGA GGCTGTCTGA GGCACCTGCT

ATATCCCTTT ACCTGATGCT GTTACCAGGA GGCTGTCTGA GGCGCCTGGT

ATATCCCTTT ACCTGATGCT GTTACCAGGA GGCTGTCTGA GGCACCTGGT

ATATCCCTTT ACCTGATGCT GTTACCAGGA GGCTGTCTGA GGCACCTGGT

TCATCCTTTT ACCTGATGCT GTTACCAGGA GGCTGTCTGA GGCACATGGT

ATATCCCTTT ACCTGAGGCT GCTACCAGGA GGCTGTGGGA GGCCCAGGGT

CTACAGGATG AGGCACTAAC GGGGTTTATG GAAGAGCAAA AGGCAATGGT

CTACAGGATG AGACACTAAC GGGGTTTATG GAAGAGCAAA AGGCAATGGT

CTACAGGATG AGGCACTAAC GGGGTTTATG GAAGAGCAAA AGGCAATGGT

CTACAGGATG AGGCACTAAC GGGGTTTATG GAAGAGCAAA AGGCAATGGT

CTACAGGATG AGGCACTAAC GGGGTTTATG GAAGAGCAAA AGGCAATGGT

CTACAGGATG AGGCACTAAC GGGGTTTATG GCAGAGCGAG AGGCAGTGGT

CTACGGGATG AGGCACTAAC AGGGTTTATG GAAGAGCGAA AGGCAACGGT

CTACGGGATG AGGCACTAAC AGGGTTTATG GAAGAGCAAA AGGCAACGGT

CTACTGGTTG AGGCACTAAC AGGGTTTATG GAAGAGCGAA AGGCAATGGT

CTACGGGATG AGGCACTAAC AGGGTTTATG GAAGAGCGAA AGGCAGTGGT

CTACAGGATG AGGCACTAAC AGGGTTTATG GAAGAGCAAA AGGCAATGGT

CTACAGGATG AGGCACTAAC AGGGTTTATG GAAGAGCAAA AGGCAATGGT

CTACAGGATG CGGCACTAAC AGGGTTTATG GGAGAGCGAA AGGCAATGGT

CTAGAGGCTG AAGCACTAAC AGGGTTTATG GAAGAGCGAA AGGCAATGGT

CTACAGGATG AGGCACTAAC AGGGTTTATG GAAGAGCAAA AGGCAATGGT

CTACAGGATG AGGCACTAAC AGGGTTTATG GGACAGCGGA AGGCAATGGT

CTACAGGATG AGGCACTAAC GGGGTTTATG GAGGAGCTAC AGGCAATGGT

GCACAGGATG AGGCACTAAC GGGGTTTATG GAGGAGCTAC AGGCAATGGC

GGTAGGAATG ACAAGGGAAC TGTCTGAACA TGCAGGTTGG TTCAGTCCGG

GGTAGGAATG ACAAGGGAAC TGTCTGAACA TGCAGGTTGG TTCAGTCCGG

GGTAGGAATG ACAAGGGAAC TGTCTGAACA TGCAGGTTGG TTCAGTCCGG

GGTAGGAATG ACAAGGGAAC TGTCTGAACA TGCAGGTTGG TTCAGTCCGG

GGTGGGAATG ATAAGGGAAC TGTCTGAACA TGCAGGTTGG TTCAGTCCGG

GGTGGGAATG ACAAGGGAAC TGTCTGAACA TGCAGGGTGG TTCAGTCCAG

GGTGGGAATG ACAAGGGAGC TGTCTGTACA TGCGGGTTGG TTCAGTCCAG

GGCGGGAATG ACAAGGGAGC TGTCTGAACA TGCGGGTTGG TTCAGTCCAG

GTTGGGAATG ACAAGGGAAC TGTCTGAACA TGCAGGTGGC TTCAGTCCAG

GGTGGGAATG ACAAGGGAAC TTTCTGAACA TGCGGGTTGG TTCAGTCCAG

GGTGGGAATG ACAAGGGAAC GCTCTGAACA TGCAGGTTGG TTCGGTCCAG

GGTGGGAATG ACAAGGGAAC GTTCTGAACA TGCAGGTTGG TTCAGTCCCG

GCTGGGGAGG ACAGGGGAAC GTTCTGAACA TGCAGGCTGG TTCAGTCCAG

GGTGGGAATG ACAAGGGAGC TGTCTGAACA TGCAGGTTGG GTCGGTCCAG

GGTGGGAATG ACAAGGGAAC TGTCTGAACA TGCAGGTTGG TTCAGTCCAG

GGTGGGAATG ACAAGGGGTC TGTCTGAACA TGCAGGTGGT TTCAGTCCAG

GGTGGGAATG ACAAGGGGAT TTTCGGAACA TGCAGGTTGG CTCAGTCCAG

GGTGGGGATG GCATGGCTAC TTTCTGAACA TGCAGGCTGG CTCGGTCCCG

GGTTGCAGCA GGCCAGGCAC CCACCTGAGA CGTGGCCAGG GCTGAAACGG

GGTTGCAGCA GGCCAGGCAC CCACCTGAGA CGTGGCCAGG GCTGAAACGG

GGTTGCAGCA GGCCAGGCAC CCACCTGAGA TGTGGCCAGG GCTGAAACGG

GGTTGCAGCA GGCCAGGCAC CCACCTGAGA TGTGGCCAGG GCTGAAACGG

GGTTGCAGCA GGCCAGGCAC CCACCTGAGA CGTGGCCAGG GCTGAAACGG

GGTTTCAGCA GGCCAGGCAC CCACCTGAGA CGTGGCCAGG GCTGAAACGG

GGTTTCAGCA GGCCAGGCAC CCACCTGAGA CGTGGCCAGG GCCGAAACGG

GGTTCCAGCA GGCCAGGCAC CCACCTGAGA CGTGGCCAGG GCCGAAACGG

GGTTTCAGCA GGCCAGGCAC CCACCTGAGA CGCAGCCAGG GCGGAAACGG

GGTTTCAGCA GGCCAGGCAC CCACCTGAGA TGTGGCCAGG GCTGAAACGG

GGTTTCAGCA GGCCGGGCAC CCACCTGAGA CGTGGCCAGG GCCGCAACGG

GGTTTCAGCA GGCCAGGCAC CCACCTGAGA CGTGGCCAGG GCTGACACAG

GGTTTCAGCA GGCCGGGCAC CCACCTGAGA CGTGGCCAGG GCTGAAACGG

GGTTTCAGCA GGCCGGGCAC CCACCTGAGA CGTGGCCAGG GCTGAAACGG

GGTTTCTGCA CGCCAGGCAC CCACCTGAGA CGTGGCCAGG GCCGAAACGG

GGTTTCAGCA CGCCAGGCAC CCACCTGAGA CGTGGCCAGG GCTGAAACGG

GGTTTCGGAA GGCCAGGCAC CCACCTGAGA AGTGGCCATG GCTGAAATGG

GGTTGCAGCC AGGCAGGCAC CCACCTGAGA CGCGGCCACG GCGGGCAGGG

CACAGCCTGC AGGTGCCACA GTTAAATCTA TGGATGGTTT TGGTGGCAGC

CACAGCCTGC AGGTGCCACA GTTAAATCTA TGGATGGTTT TGGTGGCAGC

TACAGCCTGC AGGTGCCACA GTTAAATCTA TGGATGGTTT TGGTGGCAGC

TACAGCCTGC AGGTGCCACA GTTAAATCTA TGGATGGTTT TGGTGGCAGC

CACAGCCTGC AGGTGCCACA GTTAAATCTA TGGATGGTTT TGGTGGCAGC

CACAGCCTGC AGGTGCCACA GTTAAATCTA TGGATGGTTT TGGTGGCAGC

GACAGCCTGC AGATGCCACA GTTAAATCTA TGGATGGTTT TGGTGGCAGC

GACAGCCTGC AGGTGCCACA GTTAAATCTA TGGACGGTTT TGGTGGCAGC

CACAGCCTGC AGGTGCCACA GTTAGCTCTA TGGACGGCTT TGGTGGCAGC

CACAGCCTGC GGGTGCCACA GTTAAATCTA TGGATGGTTT TGGTGGCAGC

CACAGCCTGC CGGTGCCACA GTTAAATCTA TGGACGGTTT TGGTGGCAGC

CACAGCCTGC CGGTGCCACA GTTAAATCTA TGGATGGTTT TGGTGGCAGC

CACAGCCGGC AGGTGCCACA GTTAAATCTA TGGACGGTTT TGGTGGCAGC

CACAGCCTGC AGGTGCCACA GTTAAATCTA TGGATGGTTT TGGTGGCAGC

CACAGCCTGC AGGTGCCACA GTTAAATCTA TGGATGGTTT TGGTGGCAGC

CACAGCCTGC GGGTGCCACA GTTAAATCTA TGGAGGGTTT TGGTGGCAGC

CACAGCCTGC AGGTGCCACA GTTAAATCTA TGGTTGGTTT TGGTGGCAGC

CGCAGCCCGC GGGCACCGCA GCTAAGTCTC CCGCCGGTTT GGGGGGCGGC

TGAGGGGAGG ATGACTTAGG AAGGTTCTCC TCATTGATCA CCCTGTTCCC

TGAGGGGAGG ATGACTTAGG AAGGTTCTCC TCATTGATCA CCCTGTTCCC

TGAGGGGAGG ATGACTTAGG AAGGTTGTCC TCATTGGTCA CCCTGTTCCC

TGAGGGGAGG ATGACTTAGG AAGGTTGTCC TCATTGGTCA CCCTGTTCCC

TGAGGGGAGG ATGACTTAGG AAGGTTCTCC TCATTGATCA CCCTGTTCCC

TGAGGGGAGG ACGATTTAGG GAGGTTCTCC TCATTTATTA CCCTGCTCCC

TGAGGGGAGG ATGACTTAGG GACGTTCTCC TCATTTACTA CCTTGGTCCC

TGAGGGGAGG ACGACTTAGG GACGTTCTCC TCATTTACTA CCTTGGTCCC

TGAGGGGAGG AAGACTTAGG GGGGTTCTCT TCCGTAATGA CCCTGCTCCC

TGAGGGGAGG AAGATTTAGA GAGATTCTCC TCATTTATTA CCCTGTTCCC

TGAGGGGAAG ATGACTTAGG GAGAGTCTCC TCACTTATTA CCCTGTTCCC

TGAGGGGTGG TTGGTTTGGG GAGGTTCTCC TCATTTATGA CCCTGTTCCC

TGAGGGGAAG ATGACTTAGT GAGATTCTCC TCTGTTACGA CCCTGCTCCC

TGAGGGGAGG AAGACTTAGG AAGGTTCTCC TCATTTATTA CCCTGTTCCC

TGAGGGGAGG ATGACTTAGG GAGGTTCTCC TCATTTATTA CCCTGTTCCC

TGGGGGGAGG ATGACTTAGG GAGGCTCTCC TCATTTATGA CCCTGTTCCC

TGAGGGGAGG GTGACTTAGA GAGGTTTTCC TCATTTATTA CCCTAATTCC

TGGGGGGAGG AAGACTTAGC GAGGTTTTCC TCGTTCATTA CCCTGATTCC

TTGTGGCAAA CTGGAAGGAG TCGAAGCCAC AGTTTTGTTA TCAACTTTGG

TTGTGGCAAA CTGGAAGGAG TCGAAGCCAC AGTTTTGTTA TCAACTTTGG

TTGTGGCAAA CTGGAAGGAG GCGAAGCCAC AGTTTTGTTA TCAACTTTGG

TTGTGGCAAA CTGGAAGGAG GCGAAGCCAC AGTTTTGTTA TCAACTTTGG

TTGTGGCAAA CTGGAAGGAG GCGAAGCCAC AGTTTTGTTA TCACCTTTGG

TTGTGGCAAG CTGGAAGGAG GTGAAGCCAC AGTTTTGTTA TCAACTTTGG

TTGTGGCAAT GTAGAGGGAG GTGAAGCCAC AATTTTGTTA TCAACTTTGG

TTGTGGCAAA GTAGAGGGAG GTGAAGCCAC AATTTTGTTA TCAACTTTGG

GGGGGGCAAG CTGGAAGGAG GCGAAGCCAC AGTTTTGTTT TCAACTTTGG

TTGTGGCAAA CTGGAAGGAG GCGAAGCCAC AGTTTTGTTA TCAGCTTTGA

TTGTGGCCCA CTGGAAGGAG GCGAAGCCAG AGTTTTGTTA TCAACTTTGG

TGGGGGCAAA CTGGAAGGAG GCGAAGCCAC AGTTTTGTTA TCCACTTTGG

TTGGGGCAGG GTGGAAGGAG GTGATGCCAC AGTTTTGTTA TCGACTTTGG

TTGTGGCAAA CTGGCAGGAG GTGAAGCTAC AGTTTTGTTA TCAACTTTGG

TTGTGGCAAA CTGGTAGGAG GCGAAGCCAC AGTTTTAT-- -CAACTTTGG

TTGTGGCACA CTGGTGGGAG GCAAAGCCAC CGTTTTATGA TCAACTTTGG

TTGTGGTGAA CTGGAAGGAG ATGAAGTCGT GGTTTTGTTA TCAACTTTGG

GTGGGGCGGG GCGGAAGGAG GCGGCACCCC GGTTTTGCTA TCGACTTTGG

CCCCTGTGTT CGAGGCCCTG CTGCTGTCTA ACCTTGAGTT GGGGGTGTGG

CCCCTGTGTT CGAGGCCCTG CTGCTGTCTA ACCTTGAGTT GGGGGTGTGG

CCCCTGTGTT CGAGGCCCTG CTGCTGTCTA ACCTTGAGTT GGGGGTGTGG

CCCCTGTGTT CGAGGCCCTG CTGCTGTCTA ACCTTGAGTT GGGGGTGTGG

CCCCTGTGTT CGAGGCCCTG CTGCTATCTA ACCTTGAGTT GGGGGTGTGG

CCCCTGCATT GGAGGCCCTG CTGCTATCCA ACCTTGAGTT GGGGGTGTGG

CCCCTGCATT GGAGGCCC-- ---------- ----TCAGTT GAGGGTGTGG

CCCCTGCATT GGAGGCCC-- ---------- ----TCAGTT GGGGGTGTGG

CCCCCGCACT GGAGGCCCTG CTGCTGTCCA ATCTTGAGTT GGGGGTGGGG

TCCCTGTGCT GGAGGCCCTG CTGCTATCCA ACCTTGAGTT GGGGGTGCGG

CCCCTGCGCT GGGGGCC--- CTGCTATCCA ACCTTGAGCT GGGGGTGTGG

CCCCTGCACT GGGGGCCCTG CTGCTATCCA ACCTTGAGTT GGGGGTGGGG

CCCCCGCACT GGAGGCCCTG CT---ATCCA GCCTTGAGTT GGGGGTGTGG

CCCCTGCATT GGAGGCCCTG CTGCTATCCA GCCTTGAGCT GGGGGTGTGG

CCCCTGCATT TGAGGCCCTG CTGCTATCCA ACCTTGAGTT GGGGGTGCGG

GCCCTGCGTT CGAGGCCCGG CTGCTATCCA ACTTTGAGTT GGGGGTGTGG

CCCCTGCATT AGAGGATCTG CTGCTATCCA ACTTTGATTT GAGGGTGTGG

CCCCCGCGTT GGCTGGGCGG CCGCTGTCCT ACCTTGAGCT GGGGGTGGGG

TGGAGAAGGA GTTTGGGAGA GCCCTGGCTT TTTTGGAGGG ATAGGAGGAG

TGGAGAAGGA GTTTGGGAGA GCCCTGGCTT TTTTGGAGGG ATAGGAGGAG

TGGAGAAGGA GTTTGGGAGA GCCCTGGCTT TTTTGGAGGG ATAGGAGGAG

TGGAGAAGGA GTTTGGGAGA GCCCTGGCTT TTTTGGAGGG ATAGGAGGAG

TGGAGAAGGC GTTTGGGAGA GCCCTGGCTT TTTTGGAGGG ATAGGAGGAG

TGGAGAAGGA GTTTGGGAGA GGCCTGGCTT TTTTGGAGGG ATAGGAGGAG

TGGAGAAGGA GTTTGGGAGA GCCCTGGCTT TTTGGGAGGG ATAGGAGGAG

CGGAGAAGGA GTTTGGGAGA GCCCTGGCTT TTTGGGAGGG ATAGGAGGAG

TGGAGAAGGC GTCTGGGACA GCCCTGGTTT TTTCGGGGGG ATGGGAGGAG

TGGAGAAGGA GTTTGGGAGA GCCCTGGCTT TTTTGGAGGG ATGGGAGGAG

CGGAGAAGGA GTCTGGGAGA GCCCTGGCTT TTTTGGAGGG ATGGGAGGAG

TGGAGAAGGA GCTTGGGACA GCCCTGGCTT TTTTGGCGGG ATAGGAGGAG

GGGAGAAGGG GCTTGGGAGA GCCCTGGCTT TTTGGGAGGG ATGGGAGGCG

TGGAGAAGGA GCTTGGGAGA GCCCTGGCTT TTTTGGAGGG ATAGGAGGAG

TGGAGAAGGA GTTTGGGAGA GCCCTGGCTT TTTTGGAGGG ATGGGAGGAG

TGGAGAAGGA GTCTGGGAGA GCCCTGGCTT TTTTGGAGGG ATGGGGGGCG

TGGTGAAGGA GTTTGGGAGA GCCCTGGCTT TTTTGGTGGG ATGGGAGGAG

AGGCGAAGGG GTCTGGGACA GCCCTGGCTT TTTCGGAGGG ATGGGCGGAG

GATTTCCTCT GTCAACTCGA GGGCTTGTAA ATCTTGACAG TGCTTGGGTC

GATTTCCTCT GTCAACTCGA GGGCTTGTAA ATCTTGACAG TGCTTGGGTC

GATTTCCTCT GTCAACTCGA GGGCTTGTAA ATCTTGACAG TGCTTGGGTC

GATTTCCTCT GTCAACTCGA GGGCTTGTAA ATCTTGACAG TGCTTGGGTC

GGTTTCCTCT GTCAACTCGA GGTCCTGTAA ATCTTGACAG TGCTTGGGTC

GATTTCCTCT GTCGACTCGA GGGCTTGTAA ATCTCGACAG TGCTTGGGTC

GATTTCCTCT GTCAACTCGA GGGCTGGTAA ATCTGGACAG CGCTTGGGTC

GATTTCCTCT GTCAACTCGA GGGCTGGTAA ATCTGGACAG CGCTTGGGTC

GATTTCCTCT ATCGACTCGA GGACTTGTGA ACCTCGACAG AGCTTGGGTC

GGTTTCCTCT GTCAACTCGG GGGCTTGTAA ATCTCGACAG TGCTTGGGTC

GGTTTCCTCT GTCGACTCGG GGGCCCGTGA ACCTCGACAG CGCCTGGGTC

GGTTTCCTCG GTCAACTCGA GGGCCTGTAA ATCTTGACAG TGCTTGGGTC

GGTTTCCTCG GTCAACTCGA GGGCTGGTGA ATCTTGACAG AGCCTGGGTC

GATTTCCTCG GTCAACTCGA GGGCTCGTAA ATCTTGACAG CGCTTGGGTC

GATTTCCTCT GTCAACTCGA GGGCCTGTAA ATCTTGACAG CGCTTGGGTC

GGTTTCCTCT GTCAACACGA GGGCTTGTAA ATCTTGACAG CGCTTGGGTC

GATTTCCTCG GTCAACTCGA GGGCTTGTAA ATCTTGACAG TGCTTGGGTG

GGTTCCCCCG GTCGACACGG GGGCCGGTGA ACCTGGAGAG CGCTTGGGTG

ACAGTATTCC GAGCTAGTTG TTTGGCCACT AGGTTGTCAC GACTTGTAGG

ACAGTATTCC GAGCTAGTTG TTTGGCCACT AGGTTGTCAC GACTTGTAGG

ACAGTATTCC GAGCTAGTTG TTTGGCCACT AGGTTGTCAC GACTTGTAGG

ACAGTATTCC GAGCTAGTTG TTTGGCCACT AGGTTGTCAC GACTTGTAGG

ACAGTATTCC GAGCCAGTTG TTTGGCCACT AGGTTGTCAC GACTTGTAGG

ACAGTATTCC GAGCTAGTTG TTTGGCCACT AGGTTGTCAC GACTTGTAGG

ACAGTATTCC GAGCTAGCTG CTTGGCTACT AGGTTGTCAC GACTCGTAGG

ACAGTATTCC GAGCTAGCTG CTTGGCTACT AGGTTGTCAC GACTCGTAGG

ACAGTGTTCC GAGCTAGCTG TTTGGCCACT AGGTTGTCGC GACTTGTAGG

ACAGTATTCC GAGCAAGCTG TTTGGCCACT AGGTTGTCAC GACTTGTAGG

ACCGTGTTCC GGGCCAGCTG TTTGGCCACG AGGTTGTCAC GACTCGTGGG

ACAGTATTCC GAGCTAGCTG TTTGGCCACT AGGTTGTCAC GACTTGTAGG

ACGGTATTGC GAGCCAGCTG TTTGGCCACC AGGTTGTCGC GACTCGTCGG

ACAGTATTCC GAGCCAGCTG TTTGGCCACT AGGTTGTCAC GGCTTGTAGG

ACAGTATTCC GAGCGAGCTG TTTGGCCACT AGGTTGTCAC GACTTGTAGG

ACGGTGTTCC GAGCGAGCTG TTTGGCCACT AGGTTGTCAC GACTCGTGGG

ACAGTATTCC GAGCTAGCTG TTTGGCTACT AAGTTGTCAC GACTCGTGGG

ACCGTGTTGC GGGCCAGCTG CCTGACGACC AAGTTGTCCC GGCTGGTGGG

CGAGACATCT CTTGACGGAG GACTTTGGGT AGTATTTCCA TTTTGGTCTG

CGAGACATCT CTTGACGGAG GACTTTGGGT AGTATTTCCA TTTTGGTCTG

TGACATATCT CTTGACGGAG GACTTTGGGT AGTATTTCCA TTTTGGTCTG

TGACATATCT CTTGACGGAG GACTTTGGGT AGTATTTCCA TTTTGGTCTG

CGAGACATCT CTTGACGGAG GACTTTGGGT AGTATTTCCA TTTTGGTCTG

AGAGACGTCT CTAGATGGAG GACTTGGGGT AGTATTTCCG TTTTGGTCTG

AGAAACATCT CTGGAGGGAG GACTTTGAGT GTTATTTCCA TTCTGGTCTG

AGAGACATCT CTCGAGGGAG GGCTTTGAGT GTTATTTCCA TTCTGGTCTG

AGAGACGTCT CTTGATGGAG GACTCTGGGT GGTGTTTCCA TTTTGGTCGG

AGAGACTTCT CTGGATGGAG GGCTTTGGGT GGTGTTTCCA TTTTGGTCTG

AGACACGTCC CTGGACGGGG GGCTCTGCGT GGTGTTCCCG TTCTGGTCCG

AGAGACATCT CTGGATGGAG GGCTTTGGGT AGTATTTCCA TTTTGGTCTG

GGACACATCC CTGGAAGGAG GACTCTGCGT GGTGTTTCCG TTTTGGTCGG

GGAAACATCC CTTGATGGAG GACTTTGAGT AGTGTTTCCA TTTTGGTCTT

AGACACATCT CTTGATGGAG GACTCTGCGT CGTATTTCCA TTTTGGTCTT

AGAGACATCT CTGGAAGGGG GACTTTGAGT GTTGTTTCCA TTTTGGTCCT

AGAGACATCT CTTGATGGAG GGCTTTGGGT AGTGTTTCCA TTTTGGTCTT

GGAGACTTCC CTGGATGGAG GGCTCTGGGT GGTGTTCCCA TTCTGGTCTT

GGTCGTTAGC ATTGCCCTGA AATCTAAATC TAGCTGCTTG GACCCCTTTT

GGTCGTTAGC ATTGCCCTGA AATCTAAATC TAGCTGCTTG GACCCCTTTT

GGTCGTTAGC ATTGCCCTGA AATCTAAATC TAGCTGCTTG GACCCCTTTT

GGTCGTTAGC ATTGCCCTGA AATCTAAATC TAGCTGCTTG GACCCCTTTT

GGTCATTAGC ATTGCCCTGA AATCTAAATC TAGCTGCTTG GACCCCTTTT

GGTCATTAGC ATTGCCCTGA AATCGGAATC TAGCGGCCTG GACCCCTTTT

GGTCATTTGC ATTGCCCTGA AATCTAAATC TAGCTGCTTG GACCTTTCTT

GGTCATTTGC ATTGCCCTGA AATCTGAATC TAGCTGCTTG GACCCTTCTT

GGTCGTTTGC GTTGCCTTGA AATCTAAACC TGGCTGCTTG GACCCCTTTT

GGTCATTAGC ATTGCCCTGA AATCGAAATC GAGCCGCTTG GACCCTTTTT

GGTC----GT GGTGCCT--G AATCGGAATC G--------- ----------

GGTCATTAGC ATTGCCCTGG AATCTAAACC TAGCTGCTTG GACCCCTTTT

GGTCATTGGC ATTGCCCTGG AATCGGAACC TCGCGGCCTG GACCCTTTTT

GGTCATTAGC ATTGCCCTGA AATCTAAATC TAGCTGCTTG GACCCCGCTT

GGTCATTAGC ATTGCCCTGA AATCTAAATC TAACTGCTTG GACCCCTTTT

GGTCATTAGC ATTGCCCTGA AATCTAAACC TCACTGCTTG GACCCCTTTT

GGTCATTGGC ATTGCCCTGA AATCTAAATC TAGCTGCTTG GACCCTTCTT

GGTCATTGGC GTTGCCCTGG AACCTGAACC GGGCCGCTTG GACCCTTCCT

CAGTTGCTCA ATCATTTTCC TCATCTTGTC TATCTCCTCT TTGAGGTCAG

CAGTTGCTCA ATCATTTTCC TCATCTTGTC TATCTCCTCT TTGAGGTCAG

CAGTTGCTCA ATCATTTTCT TCATCTTGTC TATCTCCTCT TTGAGGTCAG

CAGTTGCTCA ATCATTTTCT TCATCTTGTC TATCTCCTCT TTGAGGTCAG

CAGTTGCTCA ATCATTTTCT TCATCTTGTC TATCTCCTCT TTGAGGTCAG

CAGCTGCTCG ATCATTTTCT TCATCTTGTC GACCTCCTCC TTGAGGTCAG

CATTTGCTCC ATCATTTTCT TCATCTTGTC TATCTCCTCT TTGAGGTCGG

CATTTGCTCC ATCATTTTCT TCATCTTGTC TATCTCCTCT TTGAGGTCGG

CAGTTGCTCG ATCATCTTCT TCATCTTGTC TATCTCCTCT TTGAGGTCGG

CAGCTGCTCG ATCATCTTCT TCATCTTGTC TATCTCCTCT TTGAGGTCAG

CAGCTGCTCC AGCGCCCGGC GCAGCCGCTC CAGCTCGTCC CGCAGCCCCG

CAGCTGCTCA ATCATTTTCT TCATCTTGTC TATCTCCTCT TTGAGGTCTG

CAGCTGCTCG ATCATCTTCT TCAGCTTGTC GATCTCCTCC TTGAGGTCGG

CAGCTGCTCA ATCATTTTCT TCATCTTGTC TATCTCCTCT TTGAGGTCAG

CAGTTGCTCG ATCATCTTCT TCATCTTGTC TATCTCCTCT TTGAGGTCAG

CAGCTGCTCA GTCATCTTCT TCATCTTGTC GATCTCCCCT TTGAGGTCAG

CAGTTGCTCG ATCATTTTTT TCATCTTGTC TATCTCTTCT TTGAGGTCAG

CAGCTGCTCG ATCGTCTTTC TCATCTTGTC GATCTCCTCC CTGAGGTCGG

TGGTGTGTGC TTCTTCCCGG TTCAGCTTGG CACGAAGCTG TTCCCGCTCA

TGGTGTGTGC TTCTTCCCGG TTCAGCTTGG CACGAAGCTG TTCCCGCTCG

TGGTGTGTGC TTCTTCCCGG TTCAGCTTGG CACGAAGCTG TTCCCGCTCG

TGGTGTGTGC TTCTTCCCGG TTCAGCTTGG CACGAAGCTG TTCCCGCTCG

TGGTGTGTGC TTCTTCTCGG TTCAGCTTGG CACGAAGCTG TTCCCGCTCG

TGGTGTGCGC TTCTTCCCGG CTCAGCTTGG CACGGAGCTG CTCCCGTTCC

TGGTGTGTGC TTCTTCTCGG GTCAGCTTGG CCCGCAGCTG CTCCCGTTCC

TGGTGTGTGC TTCCTCTCGG CTCAGCTTGG CGCGCAGCTG CTCCCGTTCC

TGGTGTGGGC TTCCTCTCGG TTCAGCTTGG CACGCAGCTG CTCCCGTTCC

TGGTGTGTGC TTCTTCTCGG TGCAGCTTGG CACGAAGCTG TTCCCGCTCC

CGGCCAGCGC CTCCTCGCGG TGCAGCCGGG CGCGCAGCTG CTCGCGCTCC

CGGTGTGTGC TTCTTCTCGG TGCAGCTTGG CACGAAGCTG CTCCCGTTCG

CCGTGTGCGC CTCTTCCCGG TGCAGCTTGG CCCGCAGCTG CTCGCGCTCC

TGGTGTGTGC TTCCTCTCGG TTCAGCTTAG CACGAAGCTG CTCCCTTTCG

TCGTGTGTGC TTCCTCGCGG TTCAACTTGG CACGAAGCTG CTCCCTTTCG

TCGTGTGTGC TTCCTCGCGG CTCAGCTTGG CACGGAGCTG CTCCCGCTCG

TTGTGTGTGC TTCCTCTCTG TTAAGCTTGG CTCGGAGTTG CTCCCTCTCT

CCGTGAGCGC TTCCTCCCTG CTCAGCTTGG CCCGCAGCTG CTCCCGCTCC

GTGTCAAACT CAGAGAGTTG TTTTTCCATC TGAGCTTCCA TTTCGAGAGC

GTGTCAAACT CAGAGAGTTG TTTTTCCATC TGAGCTTCCA TTTCGAGAGC

GTGTCAAACT CAGAGAGCTG TTTTTCCATC TGAGCTTCCA TTTCGAGAGC

GTGTCAAACT CAGAGAGCTG TTTTTCCATC TGAGCTTCCA TTTCGAGAGC

GTATCAAACT CAGAGAGTTG TTTTTCCATC TGAGCTTCCA TTTCGAGAGC

GTATCGAACT CAGAGAGCTG TTTTTCCATC TGAGCTTCCA TTTCGAGAGC

GTATCAAACT CAAAGAGCTG TTTCTCCAGC TGAGCTTCCA TTCCGAGAGC

GTGTCAAACT CGGAGAGCTG TTTCTCCAAC TGAGCTTCCA TTCCGAGAGC

GTGTCGAACT CGGAGAGCTG CTTTTCCATC TGCGCTTCCA TCTCGAGAGC

GTGTCAAACT CCGAGAGCTG CTTTTCCATC TGAGCTTCCA TTTCGAGAGC

GTGTCCGAGT CCGACAGCTG CTTCTCCAGG TGCGCCTCCA GGTCGACAGC

GTGTCAAACT CAGAGAGCTG TTTTTCCATC TGAGCTTCCA TGTCGAGAGC

GTGTCGAACT CGGAGAGCTG CTTCTCCAGC TGCGCCTCCA TCTCGACAGC

ATATCGAACT CAGAGAGCTG TTTCTCCAGC TGAGCTTCCA TTTCGAGAGC

GTATCAAACT CAGAGAGCTG TTTTTCCATC TGAGCCTCCA TTTCGAGAGC

GTATCAAACT CGGACAGCTG CTTTTCCATC TGAGCTTCCA TCTCGAGAGC

GTATCAAATT CAGAGAGTTG TTTCTCCATC TGTGCTTCCA TTTCGACAAC

GTGTCGAATT CGGAGAGCTG CTTCTCCATG CGGGCTTCCA TCTGGAAAGC

TGCTTGCACT CTTTGACCAG CATCAGGACC ACCTGCTTGT TCTTAGCTTC

TGCTTGCACT CTTTGACCAG CATCAGGACC ACCTGCTTGT TCTTAGCTTC

TGCTTGCACT CTTTGACCAG CATCAAGACC ACCTGCTTGT TCTTAGCTTC

TGCTTGCACT CTTTGACCAG CATCAGGACC ACCTGCTTAT TCTTAGCTTC

TGCTTGCACT CTTTGACGAG CATCAGGACC ACCTGCTTGT TCTTAGCTTC

TGTTTACACT CCTTGACCAG CAGCAGGACC ACCTGCTTGT TTTTAGCTTC

TGCTTGCACT CCTTCACCAG CATCAGGACC ACGTGCTTGT TCTTAGCTTC

TGCTTACACT CCTTCACCAG CATCAGGACC ACGTGCTTGT TCTTAGCTTC

TGCTTACACT CCTTCACCAG CATCAGCACC ACGTGCCGGT TCTTAGCTTC

TGCTTGCACT CCTTGACCAG CATCAGGACC ACGTGCTTGT TCTTAACTTC

TGCTTGCACT CCTTGACCAG CATCAGCACC ACGTGCTTGT TCTTAGCTTC

TGTTTGCACT CCTTGACGAG CATCAGTACC ACGTGCTTGT TCTTAGCTTC

TGCTTACACT CCTTGACCAG CATGAGCACC ACGTGCTTGT TCTTAGCTTC

TGTTTACACT CCTTTACAAG CATCAGGACC ACGTGCTTGT TCTTAGCTTC

TGCTTGCACT CCTTCACCAG CATCAGGACA ACGTGCTTGT TCTTAGCTTC

TGCTTGCACT CCTTGACCAG CATCAGCACC ACGTGCTTGT TCTTAGCGTC

TGTTTGCACT CCTTGACCAG CATTAAGACA ACATGCTTAT TTTTAACTTC

TGCTTGCACT CTTTGACCAG CATGAGCACC ACCTGTTTGT TCTTAGCTTC

TTGTGCTCTT GCTCAAGGGC TTGTAGCTGA AGCTTCTCCA TTTCCAGCTG

TTGTGCTCTT GCTCAAGGGC TTGTAGCTGA AGCTTCTCCA TTTCCAGCTG

TTGTGCTCTT GCTCAAGGGC TTGTAGCTGA AGCTTCTCCA TTTCCAGCTG

TTGTGCTCTT GCTCAAGGGC TTGTAGCTGA AGCTTCTCCA TTTCCAGCTG

TTGTGCTCTT GCTCAAGGGC TTGTAGCTGA AGCTTTTCCA TTTCCAGCTG

TTGTGCTCTT GCTCCAGGGC TTGTAGCTGA AGCTTTTCCA TTTCCAGCTA

TTGTGCTCCT GTTCAAGGGC CTGAAGCTGG AGCTTTTCCA TCTCCAACTG

TTGTGCTCCT GTTCAAGGGC CTGGAGCTGG AGCTTTTCCA TCTCCAACTG

TGGTGCTCCT GGTCCAGGGC TTGCAGCTGA AGCTTTTCCA TTTCCAGCTG

TTGTGCTCCT GCTCAAGTGC TTGGAGCTGA AGCTTCTCCA TCTCCAGCTG

TTGTGCTCCT GCTCCAGCGC CTGCAGCTGC AGCTTCTCCA TCTCCAGCTG

TTGTGCTCTA ACTCAAGGGC TTGCAGCTGC AGCTTTTCCA TCTCCAGCTG

TTGTGCTCCT GCTCCAGTGC CTGTAACTGC AGCTTCTCCG TCTCCAGCTG

TTGTGCTCCT GCTCAAGGGC TTGTAGCTGA AGCTTTTCCA TTTCCAGCTG

TTGTGCTCCT GCTCAAGGGC CTGGAGCTGA AGCTTTTCCA TTTCCAGCTG

TTGTGCTCCT GCTCCAGGGT CTGGAGCTGC AGCTTCTCCA TTTCCAGCTG

TTGTGCTCCT GCTCCAATGT CTGTAATTGC AGCTTTTCCA TTTCCAGCTG

CTGTGCTCCT GCTGGAGGAC GTGCAACTGC GACTTCTCCA TTTCCAACTG

AAAGAAAGTG AGAAAGTGGG TGGCTGCCCA AGAATAGCAC CCAAACTGGA

AAAGAAAGTG AGAAAGTGGG TGGCTGCCCA AGAATAGCAC CCAAACTGGA

AAAGAAAGTG AGAAAGTGGG TGGTTGCCCA AGAATAGCAC CCAAACTGGA

AAAGAAAGTG ACAAAGTGGG TGGTTGCCCA AGAATAGCAC CCAAACTGGA

AA--AAAGTG AGAAAGTGGG TGGTTGCCCA AGAATAGCAC CCAAACTGAA

GAAGAAAGTG AGAAAGTGGG TGGTTGCCCA AGAATAGCAC CCAGATTGGG

GAAGATAGTG AGAAGGTGGG TGGTGCC--- AGAATAGCAC TCAGGCT-GG

GAAGATAGTG AGAAGGTGGG TCATGCCCCT AGAATAGCAC CCAGACT-GG

GAAGAAAGTG ACCAAGTGGG TGGTTGTCCA AGAATAGCAC CCAGACTGGG

AAAGAAAGTG AGAAAGTGGG TGGTTCCCCA AGAATAGCAC CCAGACTGGG

GAAGAGGGTG AGAAAGTGGG CGGTTGCCCA AGAATAGCAC CCAGACTGGA

AAAGAAA--G GGAAAGTGGG TGGCTGCCCA AGAATAGCAC CTAGACTAGA

TAAGAAA--- ---------- ---------- ---------- ----------

AAAGAAAGTG AGAAAGTGGG TGGTTTCCCA AGCATGGCAC TTAGACGGGG

AAAGACAGTG AGAAAGTGGG TGGTTGTCCA AGAATAGCAC CCAGACTAGG

AAAAGACGTG AGAAAGTAGG TGGCT-CCCA AGAATAGCAC CCAGACGAGG

AAAGAAAGGG ATAAAGTGGG TGGCTGTTCA AGAATAGCTC CCAAAGTGGG

GAAGAGAGGG AGGAAGTGGG GGGCTGCTCA AGAATAGCAC CCAACGTGGG

AGCTATGAAT GAAGAGGCAC ATACCTTCTT TTGTCTGCTC TCAGCAGCAG

AGCTATGAAT GAAGAGGCAC ATACCTTCTT TTGTCTGCTC TCAGCAGCAG

AGCTATGAAT GAAGAGGCAC ATACCTTCTT TTGTCTGCTC TCAGCAGCAG

AGCTATGAAT GAAGAGGCAC ATACCTTCTT TTGTCTGCTC TCAGCAGCAG

AGCTATGAAT GAAGAGGCAC ATACCTTCTT TTGTCTGCTC TCAGCAGCAG

AGCCATGAAT GAAGAGGCAC ATACCTTTTT TTGTCTGCTC TCAGCAGCAG

AGCCGTGAAT GAGGAGGCCC ATACCTTTTT CTGCCTACTC TCAGCGGCAG

AGCCATGAAT GAAGAGGCTC ATACCTTTTT TTGTCTGCTC TCAGCGGCCA

AGCCATGAAT GAAGAGGCAC ATACCTTCTT CTGTCTGCTC TCGGCGGCAG

AGCCATGAAT GAAGAGGCAC ATACCTTCTT TTGTCTGCTC TCAGCAGCAG

AGCCATGAAT GAAGAAACAC ATACCTTCTT TTGTCGGCTT TCAGCAGCAG

AGCCATGAAT GAAGAGGCAC ATACCTTTTT TTGTCTGCTC TCAGCAGCAG

---------- ---------- -TACCTTCTT TTGTCTGCTC TCGGCAGCAG

AGCCATGAAT GAAGAGGCAC ATACCTTCTT TTGTCTGCTC TCAGCAGCAG

AGACATGAAT GAAGAGTCAA ATACCTTCTT TTGCCTGCTC TCAGCAGCAG

AGTCATGAAT GAAGAGTCAC ATACCTTCTT CTGCCTGCTC TCAGCGGCAG

AGAAATGAAT GAAGAGGCAC ATACCTTCTT TTGTCTGTTT TCAGCAGCAG

AGAGATAAAG GATGGGACAC TTACCTTCTT CTGCCTGCTC TCTGCGGCGG

CCAGCTGTGC GGACATCCTT TCTTGCATTT TCTTGCAGTG GGCCATGACT

CCAGCTGTGC GGACATCCTT TCTTGCATTT TCTTGCAGTG GGCCATGACT

CCAGCTGTGC GGACATCCTT TCTTGCATTT TCTTGCAGTG GGCCATGACT

CCAGCTGTGC GGACATCCTT TCTTGCATTT TCTTGCAGTG GGCCATGACT

CCAGCTGTGC GGACATCCTT TCTTGCATTT TCCTGCAGTG GGCCATAACT

CCAGCTGTGT GGCCATCCTT TCCTGCATTT TTCTGCAGTG GGCCATGACT

CCAGCTGTGC GGACATTCTT TCTTGCATTT TTCTGCAGTG GGCCATGACG

CCAGCTGTGC GGACATCCTT TCTTGCATTT TTCTGCAGTG GGCCATGACA

CCAGCTGTGT GGACATCCGT TCCTGCATTT TCCTGCAGTG GGCCATGACT

CCAGCTGTGT GGACATCCTT TCTTGCATTT TTCTGCAGTG TGCCATGACT

CCAGCTGCGT GGACATCCTC TCCTGCATCT TTCTGCAGTG GGCCATGACC

CCAGCTGTGT GGACATCCTT TCTTGCATTT TTCTGCAGTG TGCCATGACT

CAAGCTGGGC GGCCATTCTC TCCTGCATTT TCCTGCAGTG GGCCATGACG

CCAGCTGTGT GGACATCCTC TCCTGCATTT TTCTGCAGTG GGCCATGACA

CCAGCTGTGT GGACATCCGC TCCTGCATTT TTCTGCAGTG GGCCATGACA

CCAGCTGTGT GGACATCCGC TCCTGCATCT TCCTGCAGTG GGCCATGACG

CCAGCTGTGT GGACATTCTC GCCTGCATTT TCCTGCAGTG AGCCATGACA

CCAGCTGGGC GGACATCCTC TCTTGCATTT TCCGGCAGTG AGCCATGACG

GCTTCAAGGA TGGAGAGGGG GTTGGTACAA ACTGGCTTCT CTTTGTCACC

GCTTCAAGGA TGGAGAGGGG GTTGGTACAA ACTGGCTTCT CTTTGTCACC

GCTTCAAGGA TGGAGAGGGG GTTGGTACAC ACTGGCTTCT CTTTGTCACC

GCTTCAAGGA TGGAGAGGGG GTTGGTACAC ACTGGCTTCT CTTTGTCACC

GCTTCAAGGA TGGAGAGGGG GTTGGTACAA ACTGGCTTCT CTTTGTCACC

GCTTCAAGGA TGGAGAGGGG GTTGGTACAA ACCGGCTTCT CTTTGTCCCC

GCTTCAAGAA TGGAGAGTGG GTTGGTGCAG ACTGGCTTCT CTTTGTCACC

GCTTCAAGGA TGGAGAGTGG ATTGGTGCAG ACTGGCTTCT CTTTGTCACC

GCTTCGAGGA TGGACAGGGG GTTGGTGCAA ACAGGCTTCT CTTTGTCACT

GCTTCGAGGA TGGAGAGGGG GTTGGTACAA ACTGGCTTCT CTTTATCGCT

GCTTCAAGGA TGGAGAGGGG GTTGGTACAA ACTGGCTTCT CTTTCTCGCC

GCTTCAAGGA TGGAGAGGGG GTTGGCGCAA ACTGGCTTCT CTTTATCACC

GCTTCAAGGA TCGAGAGTGG GTTGGTGCAA ACGGGCTTCT CTTTGTCACT

GCTTCAAGGA TGGAAAGGGG GTTGGTACAA ACTGGCTTCT CTTTCTCACC

GCTTCAAGGA TGGAGAGGGG GTTGGTACAA ACCGGTTTCT CTTTCTCCCT

GCTTCCAGGA TGGAGAGCGG GTTGGTACAA ACTGGCTT-T CTTTATCCCC

GCATCCAAAA TGGAGAGGGG GTTGGTACAA ACTGGCTTCT CTGCATCACC

GCCTCCAAAA TGGAGAGGGG GTTGGTACAG ACGGGCTTCT CTGCTTCACC

AGCACCTGCT TCATAGTCTC TCTGGAGTGC CAGGAACGGG TCATTTAGAT

AGCACCTGCT TCATAGTCTC TCTGGAGTGC CAGGAACGGG TCATTTAGAT

AGCACCTGCT TCATAGTCTC TCTGGAGTGC CAGGAACGGG TCATTTAGAT

AGCACCTGCT TCATAGTCTC TCTGGAGTGC CAGGAATGGG TCATTTAGAT

AGCACCTGCT TCATAGTCTC TCTGGAGTGC CAAGAACGGG TCATTTAGAT

AGCACCTGCT TCATAGTCTC GCTGGAGCGC CAAAAATGGG TCGTTTAGAT

AGCACCCGCT TCATAGTCTC TCTGGAGTGC CAGGAAAGGG TCGTTTAGAT

AGGACCTGCT TCATAGTCTC TCTGAAGTGC CAGGAAGGGG TCATTTAGAT

GGCACCTGCT TCATAGTCTC TTTGGAGTGC CAAGAACGGG TCGTTTAGAT

GGCACCTGCT TCATAGTCTC TCTGTAGTGC CAGGAATGGG TCATTTAAAT

GGCACCTGCC TCATAGTCTC TCTGCAGTGC CAAGAATGGG TCATTCAGAT

GGCCCCTGCT TCATAGTCTC TCTGCAGTGC CAAGAATGGG TCATTTAAAT

GGCACCTGCT TCATAGTCTC TCTGGAGTGC CAAGAATGGG TCGTTTAAAT

AGCACCTGCT TCATAGTCTC TCTGCAGTGC CAGGAATGGG TCATTTAGAT

GGCACCTGCT TCATAGTCCC TCTGCAGTGC CAGGAATGGG TCATTTAAGT

AGCACCTGCT TCATAGTCTC TCTGGAGCGC CAGGAATGGG TCATTTAAGT

AGCACCTGCT TCATAGTCCC TCTGCAGAGC CAGGAATGGG TCATTTAGGT

AGCACCTGCT TCGTAGTCTC TCTGCAGAGC CAGGAATGGG TCATTGAGGT

TAAATCTCCC ATACCGTTCC TGGATAAATA CCTCCTTCCT GCGAGCCTGG

TAAATCTCCC ATACCGTTCC TGGATAAATA CCTCCTTCCT GCGAGCCTGG

TAAATCTCCC ATACCGTTCC TGGATAAATA CCTCCTTCCT GCGAGCCTGG

TAAATCTCCC ATACCGTTCC TGGATAAATA CCTCCTTCCT GCGAGCCTGG

TAAATCTCCC ATACCGTTCC TGGATAAATA CCTCCTTCCT GCGAGCCTGG

TAAATCTCCC ATACCGTTCC TGGATAAATA CTTCCTTCCT GCGAGCCTAG

TAAATCTCCC ATAACGTTCC TGGATGAACA CCTCCTTCCT GCGAGCCTAC

TAAACCTCCC ATACCGTTCC TGGATGAACA CCTCCTTCCT GCGAGCCTAC

TAAATCTCCC ATACCGTTCC TGGATAAACA CCTCCTTCCT GCGAGCCTAG

TAAATCTCCC ATACCGTTCC TGGATAAATA CCTCCTTCCT GCGAGCCTAG

TAAATCTCCC ATACCGTTCC TGGATAAATA CCTCCTTCCT GCGAGCCTAG

TAAATCTTCC ATACCGTTCC TGGATAAATA CCTCCTTTCT GCGAGCCTAG

TAAATCTTCC ATACCGTTCC TGGATAAACA CCTCCTTCCG GCGTGCCTAA

TAAATCTTCC ATACCGCTCT TGGATAAATA CCTCTTTCCT GCGTGCCTGG

TAAATCTCCC ATACCGTTCC TGGATAAATA CCTCCTTCCG GCGAGCCTAA

TAAATCTCCC ATATCGTTCC TGGATAAATA CCTCCTTCCG CCGAGCCTAG

TAAACCTTCC ATATCGTTCT TGAACAAAGA CCTCCTTCCT TCGTGCCTGA

TAAACCTTCC ATATCGTTCC TGGATAAACA CCTCTTTTCT GCGAGCCTAG

AACAAAATTA GATTGAAAGC AGTCTTTATT CAAACCCTGC CAGTAATTTT

AACAAAATTA GATTGAAAGC AGTCTTTATT CAAACCCTGC CAGTAATTTT

AACAAAGTTA GATTGAAAGC AGCCTTTATT GAAACCCTGC CAGTAATTTT

AACAAAGTTA GATTGAAAGC AGCCTTTATT CAAACCCTGC CAGTAATTTT

AACAAAATTA GATTGAAAGC AGTCTTTATT CAAACCCTGC CAGTGATTTT

GACAAAATTA GATTGAAAGC AGTCTTTATT CAAACCCTGC CAGTAATTTT

AACAAAATTG GATTGGATAC ATCCTTTATT CAAACTCTGC CGGTGATTTT

AACAAAATTG GAtTGGATAC ATTCTTTACT CAAACTCTGC CAGTGATTTT

AACAAAAGTA GATTGAAAGC ACTCTTTATT CAAACCCTGC CAGTAATTTT

AACAAAATCA GATTGAAGGC AGTCTTCATT CAAACTCTAT CAGTAATTTG

AACGAAATCA GATTGAAAGC AGTCGTCACT CAAACCCTGC TAATAATTTT

AACAAAATCA GATTGAAAGC AGTCTTTATT CAAACCTTGC CAGTAATTTT

AACAAAATCA GATTGAAAGC CATCTTGATT CAAACCCTGC TAGGAATTTT

AAAAAAATCA GATTGAAAGC AGTCTTTATT CAAATCCTGC CAGTAATTTT

AACAAAACCA GA-------- ---------- ---------- ----------

GACAAAATCA GACTCAAAGC AGCCTTTATG CAAACCCTGC CAGTAATTTC

AAAAAAATCA AATTGAGCAC AGTTTTTATT CAAGCCCTGC CAGGGATTTT

GGAGAAAGGA GACCCAAAGA AGTCAAGATG GAAAAACTAT ATGGATTTTT

ACCACCATTA ATTTTCATAA ATTCAGCTCT GCTCAGCCCT TACCCGCAGG

ACCACCATTA ATTTTCATAA ATTCAGCTCT GCTCAGCCCT TACCCGCAGG

GCCACCATTA ATTTTCATAA AATCAGCTCT GCTCAGCTCT TACCCGCAGG

GCCACCATTA ATTTTCATAA ATTCAGCTCT GCTCAGCTCT TACCCGCAGG

ACAACCATTA ATTTTCATAA ATTTAGCTCT GCTCAGCTCT TACCCGCAGG

ACCACCATCA ATTTTCATGA ATTCAGCTCT GCTCAGCACT TACTCGGAGG

ACCACCATTA ATTTTCATAA ATTCAGTTCT GCTCACCTCT TACCCGCAGG

ACCACCATTA ATTTTCATAA ATTCAGCTCT GCTCACCTCT TACCCTCAGG

ATCACC--TA ACTTTCATGA ATTCAGCTCT ACTCAGCTCT TACCCGCAGG

ACCACCATTA ATCT-CATGA ATTCAGCTCT GCTCAGC--- ----------

AGCACCATTA ATTTTCATGA ATTCAGCTCT GCTCAGCTCT TACCCGCAGG

ACCACCATTA ATTT-CATGA ATTCAGCTCT GCTCAGCGCT TACCCGTAGG

ACCACCATGA ---------- ---------- -------CCT TACCCTCAGG

ACCACCATTA ATTTTCATGA ATTGAGCTCT GCTCAGCTCT TACCCGCAGG

---------- ---------- ---------- ---------- ----------

ACCACTGTGA ATCTTCATGG ATTCAGCTCT GCTCAGCTCT TACCCGCAGG

ATCATCACCA ATTTTCATGA ACATAATATT GCATAGTACT TACCCTCAAT

TCCCCCTTCA ATTTTCATGA ATTCAGCTCT GCTCAGCCCT CACCCTCAGG

GCCTCGATGA CAAGGTCTCT GGCCTCCAGC TCCCCTTCCA TCACGCTGAG

GCCTCGATGA CAAGGTCTCT GGCCTCCAGC TCCCCTTCCA TCACGCTGAG

GCCTCGATGA CGAGGTCTCT GGCCTCCAGC TCCCCTTCCA TCACGCTGAG

GCCTCGATGA CGAGGTCTCT GGCCTCCAGC TCCCCTTCCA TCACGCTGAG

GCCTCGATGA CGAGGTCTCT GGCCTCCAGC TCCCCTTCCA TCACGCTGAG

GCCTCGATGA CCAGGTCTCT GGCTTCCAGC TCCCCTTCCA TCACGCTCAG

GCCTCAATCA CGAGGTCTCT GGCTTCTAGT TCACCTTCCA TCACACTGAG

GCCTCAATCA CCAGGTCTCT GGCTTCTAGT TCACCTTCCA TCACGCTAAG

GCCTCGATGA CAAGGTCTCT GGCCTCCAGC TCCCCTTCCA TCACGCTGAG

---------- ---------- ---------- ---------- ----------

GCCTCGATGA CCAGGTCTCT GGCCTCCAGC TCCCCTTCCA TCACGCTGAG

GCCTCGATGA CCAGGTCTCT GGCCTCCAGC TCCCCTTCCA TCACACTGAG

GCCTCGATGA CCAGGTCTCT GGCCTCCAGC TCTCCCTCCA TCACGCTCAG

GCCTCAATAA CGAGGTCTCT GGCCTCCAGT TCCCCTTCCA TCACGCTTAG

---------- ---------- ---------- ---------- ----------

GCCTCGATGA CGAGATCTCT GGCCTCCAGT TCTCCTTCCA TCACGCTGAG

GCTTCTATGA CAAGGTCTCT CGCTTCCAGT TCTCCTTCCA TTACACTGAG

GCCTCAATGA CGACATCCCT GGCTTCCAGC TCCCCTTCCA TGACGCTCAG

GAGCATCCGC AGCTCGGATT TACTGAGAGT ATCCACATCA AACTCTTTTT

GAGCATCCGC AGCTCGGATT TACTGAGAGT ATCCACATCA AACTCTTTTT

GAGCATCCGC AGCTCGGATT TACTGAGAGT ATCCACATCA AACTCTTTTT

GAGCATCCGC AGCTCGGATT TACTGAGAGT ATCCACATCA AACTCTTTTT

GAGCATCCGC AGCTCCGATT TACTGAGAGT ATCCACATCA AACTCTTTTT

AAGCATCCGC AGCTCTGTCT TACTGAGGGT GTCCACATCA AACTCTTTTT

GAGCATCCGC AGCTCTGATT TGCTGAGGGT GTCCACATCA AACTCCTTTT

GAGCATCCGC AGCTCTGATT TGCTGAGCGT GTCCACATCA AATTCCTTTT

AAGCATCCGC AGCTCTGACT TACTCAGGGT GTCCACGTCA AACTCCTTTT

---------- ---------- ---------- ---------- ----------

GAGCATCCGG AGCTCTGATT TACTGAGGGT GTCCACATCA AACTCTTTTT

GAGCATGCGG AGCTCTGACT TACTGAGCGC ATCCACATCA AAATCTTGTT

GAGCATCCGA AGCTCTGGTT TGCTGAGAGT GTCCACATCA AATTCTTTTT

GAGCATCCTC AGCTCTGATT TACTGAGTGT GTCCACATCA AACTCTTTCG

---------- ---------- ---------- ---------- ----------

GAGCATCCGC AGCTCAGACT TACTGAGCGT GTCCACATCA AACTCTTTTC

GAGCATTCGC AGCTCTGGTT TACTGAGTGT GTCCACATCA AATTCTTTTT

GAGCATCCGC AGTTCAGCTT TGCTGAGGGT GTCCACGTCG AACTCCTTTT

TCTGTAACAA TGTTTTTCCC ACTGAGATGG GAAAGCTGCC ATGCTTTATT

TCTGTAACAA TGTTTTTCCC ACTGAGATGG GAACGCTGCC ATGCTTTATT

TCTGTAACAA TGTTTTTCCC ACTGAGATGG GAAAGCTGCC ATGTTTTATT

TCTGTAACAA TGTTTTTCCC ACTGAGATGG GAAAGCTGCC ATGTTTTATT

TCTGTAACAA TGTTTTTCCC ACTGAGATGG GAAAGCTGCC ATGTTTTATT

TCTGTAACAG TGTTTTTCCC ACTGAGATGG GAAAGCTGCC ATGTTTTATT

TCTGTAACAA TGTTTTCCCC ACTGAGA--- -----CTGCC ATGTTTTATT

TCTGTAACAA TGTTTTCCCC TCTGAGA--- -----CTGCC ATGTTTTATT

TCTGTAACAA TGTTTTGCCC ACTGAGATGG AAAAGCTGCC ATGTTTTATT

---------- ---------- ---------- ---------- ----------

TCTGTAACAA TCTTCTTCCC ACTGAGATGG GAAAGCTGCC ATGTTTTATT

TCTGTAACAA TGTTTTTCCC ACTGAGATGG CAAAGCTGCC ATGTTTTATT

TCTGCAA--- ---------- ---------- ---------- ----------

TCTGTAACAA TGTTTTTCCC ACTGAGATGG GAAAGTTGCC ATGTTTTATT

---------- ---------- ---------- ---------- ----------

TCTGTAACAA TGTTTTTCCC ACTGACATGG GACAGCTGAC ATGTTTTATT

TCTGTAACAA TGCTTTTCCC ATTGAACTGA GAAAGCTTTT atatttttta

TCTGCGACAA TGCCTTTCCA ATAGGAGCGG TGGAGCTACC ATGTTTTATT

AATGCTGCTG GTTGCTCTAA AGATTTTAAT TAAGCAAAGT GCTCCATTTT

AATGCTGCTG GTTGCTCTAA AGATTTTAAT TAAGCAAAGT GCTCCATTTT

AATGCTGCTG GTTGCTCTAA AGATTTTAAT TAAGCAAAGT GCTCCATTTT

AATGCTGCTG GTTGCTCTAA GGATTTTAAT TAAGCAAAGT GCTCCATTTT

AATGCTTCTC GTTGCTCTAA AGATTTTAAT TAAGCAAAGT GCTCCATTTT

AATGCTGCTG GTTGCTCTAA AGATTTTAAT TAAGCAAAGT GCTCCatttt

AATGCTGCTG CTCATTCTAA AGATTTTAAT CGGACAAAGC ACTC-----T

AATGCTGCTG CTCATTCTAA AGACTTTAAT TGAGCAAAGC ACTCTACTTT

AATGCTGCTG GTTGCTCTAA AGATTTTAAT TAAGCAAAGC ACTCTCTTTT

---------- ---------- ---------- ---------- ----------

AATGCTGCTG GTTGTTCTAA AGATTTTAAT TAAGCAAGGC ACTCTATTTT

AATGCTGCTG GTGG---TAA AGATTTTAAT TAAGCAAAGT ACTCCATTTT

--TGTTGCTG GTTGCTCTAA AGATTTTAAT TAAGCAGAGC ACTCCATTTT

AATGCTGCTG GTTGCCCTAA AGATTTTAAT TAAGCAATGC ACTGCATTTA

---------- ---------- ---------- ---------- ----------

CATGCTGCTG GTTGCTCTAA AAATTTTAAT TAAGCAAAGC ACTCCATTTG

aatattaatG TTTCTTTTAA AGGTTTTAAT AAAACCATAT GCTCCATCTT

AATGTTCCTG GTTGCTTCAA AAGTTTTAAT TGAGTAAAGC TCTTAATCTC

AAAAACTAAT AAATTATGAA GACATTGCCA AGATCCTGAT TCATATTAGA

AAAAACTAAT AAATTATGAA GACATTGCCA AGATCCTGAT TCATATTACA

AAAAACTAAT AAATTATGAA GACATTGCCA AGATCCTGAT TCATATTAGA

AAAAACTAAT AAATTATGAA GACATTGCCA AGATCCTGAT TCATATTAGA

AAAAACTAAT AAATTATGAA GACATTGCCA AGATCCTGAT TCATACTAGA

aaaaattaat aaattatGAA AGCATTGCCA AGATCCTGAT GCATATTAGA

AAGGACTAAT AAATTATAAA GACAGTGCCA ACATTCCGAT TCATGTAAGA

AAGGACTAAT AAATTATAAA GACAGTGCCG AGATTCTGAT TCATGTGAGA

AAAAACTAAT AAATTATGAA GACATTGCCA AGATTCTGAT TCAT------

---------- ---------- ---------- ---------- ----------

AAATACTAAT AAATTATGAA GACATTGCCA AGATTCTGAT TCATATTAGA

AAAAACTATT AAATTATGAA CACATTGCCA AACTTCTTAT TCATATTAGA

GAAAACTAAT AAATTATGAA GACATTGCCA AGATTCTAAC TCACATTAGA

AAAAACTAAT ACATTATGAA GATGTTGACA AGATTCTGAT TCATGTTAGA

---------- ---------- ---------- ---------- ----------

AAACACGAAT AAATTATGAA GGCCCGGCCA GCGCTCTGAT TTGTATTAAA

AAAAACTGAC AAATAATATT TGTACTGCAA AGATTCTGAT TTGTATTGAA

CAGGACTAAC ATTTAATCGA TGCCCTGCAA ATATTCTGAT TAGTGCTAGC

GTCATTTCCA TACTGCCTTA TCCATTAGGA GTTTTTTCAG AGTGGAACAG

GTCATTTCCA TACTGCCTTA TCCATTAGGA GTTTTTTCAG AGTGGAACAG

GTCATTTCCA TACTGCCTTA TCCATTAGGA GTTTTTTCAG AGTAGAACAG

GTCATTTCCA TACTGCCTTA TCCATTAGGA GTTTTTTCAG AGTAGAACAG

ATCATTTCCA TACTGCCTTA TCCATTAGGA GTTTTTTCAG AGTAGAACAG

GGCATTTCCA TACTGGCTTA TCCATTAGGA GTTTTCTCAG AGTCGAATGG

GTCATTTCCA TACTGCCT-- ACCAGAAAGA ATTTATTCTG AGCAGAATAG

GTCATTTCCA TACTGCCT-- ACCAGAAAGA GTTCATTCTG AGCAGAATAG

---------- ---------- ---------- ---------- ----------

---------- ---------- ---------- ---------- ----------

GTCATTTCCA TACTGCCTTA TCCATTAGGA GTTTTTTCAG AGTAGAATAG

GTCATTTCCA TACTG-CTTA TCCACTAGGA GCTTTTTCAG GCTACAATAG

GACATTTCCA TACTGCCATG TCCATTAGGA GCTTTTTCAG TGTAGAATAG

GTCATTTCCA TACTGCCTTA TCCATTAGGA GTTTTTTCAG AGAAGAATAG

---------- ---------- ---------- ---------- ----------

GTTCGTTCCA TCCTCCCGTA TCCACCAGGA GCTTTTTCAG AGTACAACAG

GTCATTTCCA TACTACTCTA TACATTAAGA GTTTTTCCAC AGTGGAATGA

AACACTTTCA TTCTTCCTta TCCAATTGGA GTTTTTCCAA AGTGGAAGAT

CAATATGATA AAAGTCATCA CATTTGGCTA AGGCACTGAA AATAGCCCGG

CAATATGATA AAAGTCATCA CATTTGGCTA AGGCACTGAA AATAGCCCGG

CAATATGATA AAAGTCATCA CATTTGGCCA GGGCACTGAA AATAACCCGG

CAATATGATA AAAGTCATCA CATTTGGCCA GGGCACTGAA AATAACCCGG

CAATATGATA AAAGTCATCA CATTTGGCTA AGGCACTGAA AATAACCCGG

CACTACGATA AAAGTCATCA CATTTGGCTA AGGCACCGAA AATAACCCGG

CAATATGATA AAAGTCATTG CATTTGGCTA AGGTACTGAA AATAACCCGG

CAATATGATA AAAGTCATCG CATTTGGCTG AGGTACTGAA AATAACCCGG

---------- ---------- ---------- ---------- ----------

---------- ---------- ---------- ---------- ----------

TAATATAATA AAAGTCATCA CATTTGGCTA AGGCACTGAA AATAACCCGG

CAACATAATA AAAGTCATCA CATTTGGCCG AGGCGCTGAA AATAACCCGG

CAATA-AATA AAAGTCATCA CATTTGGCTA ACACACTGAA AATAACCCGG

CAATATGATA AAAGTCATTA CATTAGGGTA AGGCACTGAA AATAACCCGA

---------- ---------- ---------- ---------- ----------

CAATGTGATC AGAGTGATCA CATTCCGCGA AGGGGCTGAA AATAAGCCGG

TAATATGCCC AGAGTCACAA TATTTGGCTA AGGAATTGAA AACACTACAG

CAATACGACA AAGGTCGCAA TACCAACCTG AGGAACCGAA AGTAAGCTGG

TTTCGAATAA TCTTTGCTGC CAATTTCCAG AATGTGAAAA TCTATCACCT

TTTCGAATAA TCTTTGCTGC CAATTTCCAG AATGTGAAAA TCTATCACCT

TTTCGAATAA TCTTTGCTGC CAATTTCCAG AATGTGAAAA TCTATCACCT

TTTCGAATAA TCTTTGCTGC CAATTTCCAG AATGTGAAAA TCTATCACCT

TTTCAAATAA TCTTTGCTGC CAATTTCCAG AATGTGAAAA TCTATCACCT

TCTCGAATAA CCTTTCTTGC CAATTCCCAG AATATGAAAA TCTATCACCT

-CTCGAATGA TCTTTCTGGC T--------G AATCTGAAAA TCTATCACGT

-CTCGAATAA TCTTTCCTGC CAAGTCCCAG AATCTGAAAA TCTATCACCT

---------- ---------- ---------- ---------- ----------

---------- ---------- ---------- ---------- ----------

TCTCGAATAA T--------- ---------- ---------- ----------

TCTCGAATAA TCTTTCCTGC CAATTTCCAG GATATGAAAA TCTATCACCT

TAT-GAATAA TCTTTCCTGC CAATTCCCAA GATGGAAAAA TCTATCGCCT

TCTCGAAAAA TCTTTCCTGC CAATTCCCAG GATGTAAAAA TGTATCACCT

---------- ---------- ---------- ---------- ----------

TCTCGAATCA TCCTTCTTGC CAATTCTCAG AATATGAAAA TCTATCACCT

ACTCGGCTCT CCTTTGCTGC CAACTGTCAT CTTGATGAAA TCTATCCCTT

TCTCGAATAT TCTTTCCTGC CAGCTCCCAT GCTGAGCTAC TCTATTACCT

CACCAAGACC ATATGGTACC CCTTCCCAAA TCACCAGATC CCAAAAATGT

CACCAAGACC ATATGGTACC CCTTCCCAAA TCACCAGATC CCAAAAATGT

CACCAAGACC ATATGGTACC CCTTGTGAAA TCACCAGATC CCAAAAATGT

CACCAAGACC ATATGGTACC CCTTGTGAAA TCACCAGATC CCAAAAATGT

TACCAAGACC ATATGGTACC CCTTCCCAAA TCACCAGATC CCAAAAATGT

TATCAAGACC ATATGGTACC CCTTCCCAAA TCACCAGATC CCATAAATGT

GATTGAGACC TTAGGGCAAA CCTTCCCAAA TCTCCATATC CCCAAAATGC

TATTGAGACC TTATGGCAAA CCTCCCCAAA TCTCCAGATC TCtaaaGTTC

---------- ---------- ---------- ---------- ----------

---------- ---------- ---------- ---------- ----------

---------- ---------- ---------- ---------- ----------

TATCAGGACC ATATGGTACG CCTTCCCAAA TCACCAGATC CCAAAAATGT

TATCAAGACC ATATGGTACA CGTTCCCAAA TCTCTAGATC CCTGAAATGT

TATCAAGACC ATATGGTACC CCTTCCCAAA TCACCAGATC CCAAAAATAT

---------- ---------- ---------- ---------- ----------

TATCAAGACC ATATGGTCCC CCCTCTCAAA TCACTAGATC CCAAAACTGT

TATCAGGAAC ATATGTTACC CCTTCCAGAA TTGCAAGATT CCAAAAATGT

CATCAGGACC ACATGGTTCC CCTTGTGGAA GAACTAGACG GCAGAGATGT

GTCTTTATTC AGCCTCTTTA GCAAACTCAC AAGCCTGCAT ATATAGTTAC

GTCTTTATTC AGCCTCTTTA GCAACCTCAC AAGCCTGCAT ATATAGTTAC

GTCTTTATTC AGCGTCTTTA GCAACCTCAC AAGCCTGCAT ATATAGTTAC

GTCTTTATTC AGCGTCTTTA GCAACCTCAC AAGCCTGCAT ATATAGTTAC

GTCTTTATTC AGCCTCTTTA GCAACTTCAC AAGCCTGCAT ATATAGTTAC

GTTTTTATTC ATCCTCTTTT GCAATCCCAC AAGCCTGCAT ATGTAGTTAC

TTTCTCATTC AGCCTCTTTA GCCATCCCAT AAGCTTGCAT AGGTAGTTAC

ATTCTTGTTC AGCCTCTTTA GCCACCCCAC TGGCTTGCAT ATGCAGTTAC

---------- ---------- ---------- ---------- ----------

---------- ---------- ---------- ---------- ----------

---TTTATTC AGCCTCATTA GCAACCCCAC AAGCCTGCAT ATGTAGTTAC

GTTTGTATTC AACATATTTA GCAACCCCAC AAGCC----T ATGTAGTTGC

AATTTTATTC AGCCTATTTT GTAACCACAC -AGTCTGTGT ATGTAGTTAC

GTTTTTATTC AGCCTCTTTA GCAACCCCAC AAGCCTGCAT ATGTGGTTAC

---------- ---------- ---------- ---------- ----------

CTTTTTATTC CGCCTCTTTA AAAACCCCAC AAGCCTGCAT ATGTAGTTAC

GTTTTTATTT GGTCTCTTTA GCAATCCAAC AGACTTGCAT ATGTAGTTGC

GTCTTTGTGC AGCCTCTTAG GCACTCAGAT GGGCCTATTT ACATAGTTGC

TAGGCAATCA TAAAGCAGGG TGTTTATACC ATTAGGCATT ACTCAGCTTT

TAGGCAATCA TAAAGCAGGG TGTTTATACC ATTAGGCATT ACTCAGCTTT

TAGGCAACCA TAAAGCAGGG TGTTTATACC ATTAGGCATT AATTAGCTTT

TAGGCAACCA TAAAGCAGGG TGTTTATACC ATTAGGCATT AATTAGCTTT

TAGGCAACCA TAAAGCAGAG TGTTTATACC ATTAGGCATT AATTAGCTTT

TAGGCAACCC GAAAGCAGGA TGCTTAAACC ATTAGGCATT AATTAGCTTT

TAGGCAACGG GAAGGCAAAG TATTTATACC ATTAGTCATT AATTAGCTTT

TAGGCAACGG GAAGGCAAAG TGTTTATACT GTTAGGCATT AATTAGCTTT

---------- ---------- ---------- ---------- ----------

---------- ---------- ---------- ---------- ----------

TAGGCAACCA TAAGGCAGGG TGTTTATACC ATTAGGCATC AATTAGCTTT

TAGGCAACCA TAATGTAGGG TGTTTATACC ATTAGGCATT AATTAGCTTT

TAGGCAACCA TAAAGCAGGG TGTTTATACC ACTAGGCATT AATTAGTTTT

TAGGCAACCA TAAAGCACGG TGTTTCTACC ATTAGGCATT AATTAGCTTT

---------- ---------- ---------- ---------- ----------

TAGGCAACTA TAACACAGGG TGTTTATCCC ATTAGGTATT AATTAATTTT

TAGGCAACTA TAAAACATAG TGTTGATACC ATTAGGCATT AATTACTTTT

TAGGCAACCA TAAAGCAAAG TGTTTTCACC ATTAGCCATT AATTACCTTT

TTCTTAGAAA GTTTAAAAAC TATATAAATA TGTTGTGAGA CCATACGTCA

TTCTTAGAAA GTTTAAAAAC TATATAAATA TGTTGTGAGA CCATACATCA

TTCTTAGAAA GTTTAAAAAC TATATAAATA GGTTGTGAGA ----------

TTCTTAGAAA GTTTAAAAAC TATATAAATA GGTTGTGAGA CCATACGTCA

TTCTTAGAAA GTTTAAAAAC TACATAAATA GGTTGTGAGA CCATACGTCA

TTCTTAGAAA GTTTAAAAAC TGTATAAATA GGTTGTGAGA CCATACGTCA

TTCTTAGAGA GTTTTAAAAC TATATAAATA GGTTGTGAGA CCATACGTCA

TTCTTAGAAA GTTTTAAAAC TATATAAATA GGTTGAGAGA CCATACGTCA

---------- ---------- ---------- ---------- CCATATGTCA

---------- ---------- ---------- ---------- CCATACGTCA

TTCTTAGAAA GTTTAAAAAC TATATAAATA GGTTGTGAGA CCATACGTCA

TTCTTAGAAA GTTTTAAAAC TATAGAAATA GGTTGTGACA CCATACGTCA

TTCTTAGAAA ATTTTAAAAC TATATAAATA AGTTGTGAGA ----------

TTCTTCGAAA GTTTAAAAAC TATAAAAATA GGTTGTGAGA CCATACGTCA

---------- ---------- ---------- ---------- ----------

TTCTTAGAAA GTTTTAAAAC TATATAAATA GGCTGTGCGT CCATACGTCA

TCCTTAGAAA GTTTAAAAAA TATATAAATA GGTCATAAGA CCATACGTCA

TTCTTAGAAA GTTTTAAAAA TAGGCAATTC GGTCATAAGA CCGTCCGTCA

GGCAGTGTTT GTATATATTT CAGCGTATCC AAGCAAGGCT TTAACTTTCC

GGCAGTGTTT GTATATATTT CAGCGTATCC AAGCAAGGCT TTAACTTTCC

---------- ---------- ---------- ---------- ----------

GGCAGTGTTT GTATATATTT CAGCGTATCC AAGCAAGGCT TTAACTTTCC

GGCAGTGTTT GTATATATTT CAGCGTATCC AAGCAAGGCT TTAACTTTCC

AGCAGTGTTT GTATATATTT CAGCGTATCC AAGCAAGGCT TTAACTTTCC

GGCAGTGTTT GTATATC-CT CGGCGTATCC AAGCAGAGCT TTAACTTTCC

GGCAGTGTTT GTATATA-CT CAGCGTATCC AAGCAGGGCT TTAACTTTCC

AGCAGTGTTT GTATGTATTT CAGCGTATCC AAGCAAGGCT TTAACTTTCC

AGCAGTGTTT GTATATATTT CAGCGTATCC AAGCAAGGCT TTAACTTTCC

AGCAGTGTTT GTATATATTT CAGTGTATCC AAGCAAGGCT TTAACTTTCC

AGCAGTGTTT GTATATATCT TAGCGTATCC AAGCAAGGCT TTAACTTTCC

---------- ---------- ---------- ---------- ----------

AGCAGTGTTT GTATATATTT CAGCGTATCC AAGCAAGGCT TTAACTTTCC

---------- ---------- ---------- ---------- ----------

ACCAGTGTTT GCATCTATTT CAGCGTATCC AAGCAAAGCT TCAACTTTCC

AGCAGTGTTT GTATATGTTT CAGCGTATCC AGGCAAAGTT TTAACTTTCC

AGCGATGTTT GTGTACGTCT CCGGGTATCC AGGCAGAGCT TGGACTTCCC

ATCCTACAGA CGAAGCTGTA CTTTCCTGAA TGGCAGCCAG GAAAAAACTT

ATCCTACAGA CGAAGCTGTA CTTTCCTGAA TGGCAGCCAG GAAAAAACTT

---------- ---------- ---------- ---------- ----------

ATCCTACAGA CGAAGCTGTA CTTTCCTGAA TGGCAGCCAG GAAAAAACTT

ATCCTACAGA CGAAGCTGTA CTTTCCTGAA TGGCAGCCAG GAAAAAACTT

ACCCTACGGA CGAAGCTGTA CTTTCCTGAA TGGCAGCCAG GAAAAAACGC

GTGCTACGGA CAGATCTGTA CTTTCCTGAA CGGCAGCCAG GAAAAATCTT

GTGCTGCGGA CAGATCTGTA CTTTCCTGAA CAGCAGCCAG GAAAAATCTT

ATCCTACGGA CGAAGCTGTA CTTTCCTGAA TGGCAGCCAG GAAAAAGCTT

ATCCTACGGA CGGAGCTGTA CTTTCCTGAA TAGCAGCCAG GAAAAAACTT

ATCCTACGAA CGGAGCTGTA CTTTCCTGAA TGGCAGCCAG GAAAAAACTT

ATCCTACGGA TGGAGCTGTA CTTTCCTGAA TGGCAGCCAG GAAAAAACTT

---------- ---------- ---------- ---------- ----------

ATCCTACGGA CGGAGCTGTA CTTTCCTGAA TGACAGCCAG GAAAAAGCTT

---------- ---------- ---------- ---------- ----------

ATCCTACGGA CGGAGCTGGA CTTTCCTGAA CGGCAGCCAG GAAAAAGCTT

ATCCTACGGG CGGAGCTGTA CTTTCCTGAA TGGCAGCCAG GAAAAATTTT

GTCCTGTGGC CCGAGCTGTT CTTTCCTGAC CGGCAGCCAG GAAAAATCTT

GGGGATTTGG CAGGAAGACT GTGGGAAGCC TGGTGTTCCA CCAGGCTGAG

GGGGATTTGG CAGGAAGACT GTGGGAAGCC TGGTGTTCCA CCAGGCTGAG

---------- ---------- ---------- ---------- ----------

GGGGATTTGG CAGGAAGACT GTGGGAAGCC TGGTGTTCCA CCAGGCTGAG

GGGGATTTGG CAGGAAGACT GTGGGAAGCC TGGTGCTGCA CCAGGCTGAG

GGAGATTTGG CAGGAAGACT GTGGGAAGCC TGGTGCAACA CCAGGCTGGG

GAAGATTTGG CAGGAACAGG GTGGGAAGCC TGGTGCAACA CCAAGCTGCA

GAAGATTTGG CAGGAACACG GTGGGAAGCC TGGTGCTGCA CCAAGCTGCA

GGAGGTTTGG CAGGAAGACT GTGGGAAGCC TGGTGCAACC CCAGGCTGAG

GGAGATTTGG CAGGAAGACT GTGGGAAGCC TGGTGCAACA CCAGGCTGAG

GGAGATTTGG CAGGAAGACT GTGGGAAGCC TGGTGCAACG CTAGGCTGAG

GGAGATTTGG CAGGAGGACT GTGGGAAGCC TGGTGCCACA GCAGGCTGAG

---------- ---------- ---------- ---------- ----------

GCAGATTTGG CAGGAAGACT GTGGGAAGCC TGGTGCAACA CCAGCCTGCG

---------- ---------- ---------- ---------- ----------

GCAGATTTGG CAGGAAGACT GTGGGAAGCC TGGTGCAACA CCAGGCTGAG

GTAGATTTGG CAGGAAGACT GTGGGAAGTC TGGTGCAAGA CCAGCCTGAG

GTCGGCTTGG CAGGCCGCCC CGGGAAGGCC TGCTGCAGGA GCCCCCCGAG

TGGAGCGGAG CTTTCTACAA AGACAAATGG AGCCCCAGAT TGTAATCAGG

TGGAGCAGAG CTTTCTACAA AGACAAATGG AGCCCCAGAT TGTAATCAGG

---------- ---------- ---------- ---------- ----------

TGGAGCGGAG CTTTCTA--- ---------- ---------- ----------

TGGAGCAGAG CTTTCTACAA AGACAAATAG AGCCCCAGAT TGTAATCAGG

TGGAGCAGAG CTTTCTACAA AGACAAATGG AGCCCCAGAT TGCAATCAGG

TAGAGCGGGG CTTTCTACAA AGACAAATAG AGCCCCAGAT TGCAATCAGG

TAGAGCCGGG CTTTCTACAG AGACAAATGG AGCCCCAGAT TGCTATCAGG

TGGAGCGAGG CCTTGTGCAG AGACAAATGG AGCCCCAGAT TGCAATCAGG

TGGAGCAGAG CTTTCTACAA AGACAAATGG AGCCCCAGAT TGCAATCAGG

TGGAGCAGAG CTTTCTACAA AGACAAATGG AGCCCCAGAT TGCAATCAGG

TGGAGCAGAG CTTTCTCCGC AGACAAACGG AGCCCCAGAT TGCAATCAGG

---------- ---------- ---------- ---------- ----------

TGGAGCAGAG CTTTCTACAA AGACAAATGG ATCCCAAGAT TGCAATCAGG

---------- ---------- ---------- ---------- ----------

TGGAGCAGAG CTTTCTGCTC AGACAAATGG AGCCCGAGAT TGCAATCAGC

TGGAGCAGGC CTTTCTAAAA GGATAATTGG AGCCCAAGAT CGCTATCAGG

CCAAGCCGGG CTCCCGACAC GGACAAATGG AACCCCGGAT CGCAATTAGC

CCCTATTGTA TAAACTTGGG GTTTTCAGGC CATACAGCAT TTTTGGCATT

CCCTATTGTA TAAACTTGGG GTTTTCAGGC CATACAGCAT TTTTGGCATT

---------- ---------- ---------- ---------- ----------

---------- ---------- ---------- ---------- ----------

CCCTATTGTA TAAACTTGGG GTTTCCAGGC CATACAGCAT TTTTGGCATT

TCCCATTGTA TAAACTTGGG GTTTCCATGC CATACAGCAT TTT-GGCATT

TTCTATTGTG TAAACTTGGG GTTTCCATCG AGCCTGGCCT TTTTGGCATC

TTCTATTGTG TAAACTCGGG GTTTCC---- ---------T TTTTGGCATT

CCCTATTGTA TAAACTTGGG GTTTCCATGC CATACAGC-T TTTTGGCATT

CCCTATTGTA TAAACATGGG GTTTCCATGC CATACAGCAT TTTTGGCATT

CCCTATTGTA TAAAGTTGGG GTTTCCATGC CATACAGCAT TTTTGGCATT

CCCTATTGTG TAAACTTGGG GTTTCCACGC CATACAGAAG TTTTGGCATT

---------- ---------- ---------- ---------- ----------

AACTATTGTA TAAACTTGGG GTTTCCATGC CATACAGCAT TTTTGGCATT

---------- ---------- ---------- ---------- ----------

CTCTATTGTA TCAACTTGGG GTTTCCACGC CATACAGCAT TTCTGGCATT

ACCTATTGTA TAAACTTGGG GTTTCCATGC CGGGTTACAT TTTTGGCATT

ACCCATTGTC TCGGTTCGGA GTTTCCATGC CATGATGCCC CTTTGGCATT

ATTATTTAGC TGGAAAACCA ATTAGCATTC TTTTTATATC TGGGTGTTAA

ATTATTTAGC TGGAAAACCA ATTAGCATTC TTTTTATATC TGGATGTTAA

---------- ---------- ---------- ---------- ----------

---------- ---------- ---------- ---------- ----------

ATTATTTAGC TGGAAAACCA ATTAGCATTC TTTTTATATC TGGATGTTAA

ATTATTTAGC TGGAAAACCA ATTAGCATTC TTTTTATATC TGGGTGTTAA

ATTCTTTAGC TGGAAAACCA ATTAGCATTC TTTTTATATT TAGGTGTTGG

ATTCTTTAGC TGGAAAACCA ATTAGCATTC TTTTTATATT TAGGTGTTGG

ATTATTTAGC TGGAAAACCA ATTAGCATTC TTTTTATATC TGGGTGTTAA

ATTATTTAGC TGGAAAACCA ATTAGCATTC TTTTTATATC TGAGTGTTAA

ATTATTTAGC TGGAAAACCA ATTAGCATTC TTTTTATATC TGGGTGTTAA

ATTATTTATC TGGAAAACCA ATTAGCATTC TTTTTATATC TGGGTGTTAA

---------- ---------- ---------- ---------- ----------

ATTATTTAGC TGTTAAACCA ATTAGCATTC TTTTTTTATC GGGGTGTTAA

---------- ---------- ---------- ---------- ----------

ATTATTTAGC TGGGAAATCA ATTAGCATTC TTTTTATATC TTGGTGTTAA

ATTATTTAGC TGGAAAACTA ATTAGAATTC TTTTTTTATC TGGGTGTTAA

GTTGTTTGGC TGGAAAGCCA ATTAGAATGT TTTTTTTATC TGGGTGTTAA

TACTTGGGCC TTAGAGCATT TTGGCCGTCT CTGGGCAATG CCTGACAAAT

TACTTGGGCC TTAGAGCATT TTGGCCGTCT CTGGGCAATG CCTGACAAAT

---------- ---------- ---------- ---------- ----------

---------- ---------- ---------- ---------- ----------

TACTTGGGCC TTAGAGCATT TTGGCCGTCT CTGGGCAATG CCTGACAAAT

TGCTTGGGCC TTAGAGCATT TTGGCCGTCT CTGGGCAATG CCTGACAAAT

TGCCTGGGCC TTAGAACATT TTGGCCGTCT CTGGGCCAGG CCTGACAAAT

TGCCTGGGCC TTAGAACATT TTGGCCGTCT CTGGGCAAGG CCTGACAAAT

TACTTGGGCC TTAGAGCATT TTGGCCGTCT CTGGGCAATG CCTGACAAAT

TACTTGGGAC TTAGAGCATT TTGGCCGTCT CTGGGCAATG CCTGACAAAT

TACTTGGGAC TTAGAGCGTT TTGGCCGTCT CTGGGCAAAG CCTGACAAAT

TACTTGGGAC TTAGAGCATT TTGGCCGTCT CTGGGCAATG CCTGACAAAT

---------- ---------- ---------- ---------- ----------

TAGTTGGGCC CTAGAGCATT TTGGCCGTCT CTGGGCACTG CCTGACAAAT

---------- ---------- ---------- ---------- ----------

GACTTGAGCC CTAGAGCGTT TTGGCCGTCC CCGGGCAATG CCTGACAAAT

TACTAGGGCT CTGGCCCGTT CTGGCCGTCC CCGGGCAATG CCTGACAAAT

TACTAGGCCC GCCGGACCTC CTGGATGTCC CCAGGCGCTG CCTGACAAAT

TGAGATAAAA CTGTTACTTC ATGTTTCCTA CAAAGACAAA ACAAAACTGT

TGAGATAAAA CTGTTACTTC ATGTTTCCTA CAAAGACAAA ACAAAACTGT

---------- ---------- ---------- ---------- ----------

---------- ---------- ---------- ---------- ----------

CGAGATAAAA CCGTTACTTC ATGTTTCCTA CAAAGACAAA ACAAAACTGT

TAAGATAAAA CCGTTACTTC ATGTTTCCTA CAAAGAGAAA ACAAAACTGC

TAAGATAAAA CCGTTACTTC ATGTTTCCTC CGAAGAGAAA ACAAAACTGT

TAAGATAAAA CCGTTACTTC ATGTTTCCTC CAATGAGAAA ACAAAACTGT

TAAGATAAAA CCGTTACTTC ATGTCTTCTA CAAAGAGAAA ACAAAACTGC

TAAGATAAAA CTGTTACTTC ATGTTTCCTA CAAAAAGAAA ACAAAACTGT

TAAGATAAAA CCGTTACTTC ATGTTTCCTA CAAAGAGAAA ACAAAACTGT

TAAGATAAAA CTGTTACTTC ATGTTTCCTA CAAAAAGAAA ACAAAACTAT

---------- ---------- ---------- ---------- ----------

TAAGATAAAA CTGTTACTTC ATGTTTCCTA CAAAGAGAAA ACAAAACTGC

---------- ---------- ---------- ---------- ----------

TAAGATAAAG CTGGTACGTC ATGATTCCTA CCAAGAGAAA ACAAAACTGC

TAAGATAAAA CTGTTACTTA ATGTTTCCTA CTAAGAGAAA ACAAAACTGT

TAAGATAAAG CCGTTACTTA ATGATTCCTA CAAAGGGCAA ACAAAACTGT

TTCCTGCTCT TATTAATTGG ATGGTCAATT TCCACATAAC AAATAAGAAG

TTCCTGCTCT TATTAATTGG ATGGTCAATT TCCACATAAC AAATAAAAAG

---------- ---------- ---------- ---------- ----------

---------- ---------- ---------- ------TAAC AAATAAAAAG

TTCCTGCTCT TATTAATTGG ATGGTCAATT TCCACATAAC CAATAAAAAG

TTCCTGTTCT TATTAATTGG ATGGTCAATT TCCACATAAC AAATAAAAAG

TTCCTGCTCT TATTAATTAG ATGGTCAATG TCCACACAAC AAATAGAAAG

TTCCTGCTCT TATTAATTAG ATGGTCGATG TCCACATAAC AAATAAAGAG

TTCCTGCTCT TATTAATTGG ATGGTCAATT TCCACATAAC AAATACAAAG

TTCCTGTTCT TATTAATTGG ATGGTCAATT TCCACATAAC AAATAAAAAG

TTCCTGCTCT TACTAATTGG ATGGTCAATT TCCACATAAT AAATAAAAAG

TTCCTGCTCT TATTAATTGG ATGGTCAATT TCCACATAAC AAATAAAAAG

---------- ---------- ---------- ---------- ----------

TTCCTGCGCT TATTAATTGG ATGGTCAATT TCCACATAAA AAATAAAAAG

---------- ---------- ---------- ---------- ----------

TTCCTGCTCT TATTAATTGA ATGGTCAATT TCCACACAAG AAATAAAAAG

TTCCTGTCCT TATTAATTGG ATGGTCAATT TCCAGGTAAA AAAAAGGGGG

TTCCTGCTCC CATTGATTGG GTGGTCAGTT GCCACAAAAA AGGAAAAAAA

GTAAGCATAA GATTAGTCAT TCAGAGGACA CTGCCCAGTT TGAGAGAATA

GTAAGCATAA GATTAGTCAT TCAGAGGACA CTGCCCAGTT TGAGAGAATA

---------- ---------- ---------- ---------- ----------

GTAAGCATAA GATTAGTCAT TCAGAGGACA CTGCCCAGTT TAAGAGAATA

GTAAGCATAA GATGAGTCAT TCAGAGGACA CTGCCCAGTT TGAGAGAATA

GTAAGCATAA GATTAGTCAG TCAGAGGGCA CTGCCCAGTT TGAAAGAATA

GTAGGCATAA GATTAGTCAT GCAGAGGGTA CTGCCCAGTT GGAACAAATA

GTAGGCATAA GATTAGTCAG GCAGAGGGCC CTGCCCATCT GGAACAAATA

GTAGGCACAA GATTAGTCAT TCAGAGGGCA CTGCCCAGTT TGAGAGAGTA

GTAGGCATAA AATTAGTCAT TCAGAGGGCA CTGCCCAGTT TGAAAGAATA

GTAGGCATAA GATTAGTCAT TCAGAGGGCA CTGCCCAGTT GGAGAGAATA

GTAGGTATAA GGTGAGTCAT TCAGAGAGCA CTGCCCAGTT TGAAAGCACA

---------- ---------- -------GCA CTGCCCAGTT TGAAAGAACA

GTAGGCACAA GATGAGTCAT TCAGGGGGCA CTGCCCAGTT TGAAAGAATA

---------- ---------- ---------- ---------- ----------

GTAAGCATAA GATTAGTCAC TCAGAGGGCA CTGCCCAGTT TGAAAGAATC

GGGGGCACAA GATTAGTCAT CCAGAAGGCA CTGCCCAGTT TGAAAGAATA

GAAGAAAGAA GATGACTCAG GGAGAGGGCC CTGCCTAGTT TGAGAGGGCG

AACAGCCAAA CATTTCCCTA TCTCGAAAGG GGAAAATCTC CAGGATGATG

AACAGCCAAA CATTTCCCTA TCTCGAAAGG GGAAAATCTC CAGGATGATG

---------- ---------- ---------- ---------- ----------

AACAGCCAAA CATTTCCCTA TCTCGAAAGG GGAAAATCTC CAGGATGATG

AACAGCCAAA CATTTCCCTA TCTCAAA--G GGAAAATCTC CAGGATGATG

AACAGCCAAA CATTTCCCTA TCTTGAAAGG GGAAAGTCTC CAGGATGATG

AACAGCCAAA TAGTTCTGTA TCTTAAAAGG GCAAAGTCTC CCAGATGATG

AACAACCAAA TACTTCTGTA TCTTGAAAGG GCAAAGTCTC CCAGATGATG

AACAGCCAAA TATTTCCCTA TCTTGAAAGG GGAAAATCTC CAGGATGATG

AACAGCCAAA AATTTCCCTA TCTTGAAAGG GGAAAATCTC CAGGATGATG

AACAGCCAAA TACTTCCCTA TCTTAAAAGG GGAAAATTTC CAGGATGATG

AACAGCCAAA TATTTCCCTA TCTTGAAAGG GGAAAATCCC C-GGATGATG

AACAGCCAAA TATTTCCCTA TCTTGAAAGG GGAAGTCATC CAAGATGACG

AACAGCCAAA TGTTTCCCTA TCTTGAAAGG GGAAAATCTC CAGGATGATG

---------- ---------- ---------- ---------- ----------

AACAGCCAAA TATTTCCTTA TCTTGAAAGG GGAAATTCTC CAGGATGATG

AACAGCCAAA TTTCCTCTGT TTTTGAAAAG GGAATCTATC CAGAATGATG

AGCCGCTGAG CACAGCCCCA GTGTGGGCGG GGAAAGTCTT CCGGTAGAGG

CAAAATATTT CAAAGGGAAG TGAACAAACA GAACGGAAAC ACAGTTATAA

CAAAATATTT CGAAGGGAAG TGAACAAACA GAACGGAAAC ACAGTTATAA

---------- ---------- ---------- ---------- ----------

CAAAATATTT CGAAGGGAAG TGAACAAACA GAAAGGAAAC ACAGTTATAA

CAAAATATTT AGAAGGGATA TGAACAAACA GAAAGGAAAC ACAGTTACAA

CAAAATATTT AGAAGGGAAG TGAACAAACA GAAAGGAAAC ACAGTTATAA

CAAAATATTT AGAGGGGAAG TGAACAAACA GAAAGGAAGC ACAGTTATAA

CAAAATATTT AGAAGGGAAA TGAACAAACA GAAAGGGAAC ACAGTTATAA

CAAAATATTT AGAAGGGAAG TGAACAAACA GAGAGGAAGC ACAGTTCTAA

CAAAATATTT AGAAGGGAAG TGAACAAACA GAAGGGAAAC ACTGTTATAA

CAAAATATTT AGAAGGGAAG TGAACAAACA GAAAGGAAAC A--GTTATAA

CAAAATATTT AGAAGGGAAG TGAACAAACA GAAAGGAAAC ACAGTTACAA

CAAAATATTT AGAAGGGAAG TGAACAAACA GAAAGGAAAG ACAGTTATAA

CGAAATGTTT AGAAGGGAAG TGAACAAACA GAAAGGAAAC ACAGTTATAA

---------- ---------- ---------- ---------- ----------

CAAAATATTT AGAAGGGAAG TGAACAAACA GAAAGGAAAC CCAGTTATAA

CAAAATATTT AGAGGGGAAG CCAGCAAACA AAAAAGGAAA GCAATCATAA

CAGAGTGTGC CGAGGACGAG GGAACTGACA GATGGGAAGC TCAATCATAA

AGGGCCAGTA TGAACCTTAT TTGGATTAAA ATTTGTACCA TTCACACAAC

AGGGCCAGTA TGAACCTTAT TTGGATTAAA ATTTGTACCA TTCACATAAC

---------- ---------- ---------- ---------- ----------

AGGGCCAGTA TGAACCTTAT TTGGATTAAA ATTCGTACCA TTCACACAAC

AGGGCCAATA TTAACCTTAT TTGGATTAAA ATTTATAGCA TTCACACAGC

AGGGCCAACC TTAACCTTAT TTGGATTAAA ATTTATACCA TTCACACAAT

AGGACCAATC TTAACCTTAT TTGGATTAAA TTTTATACCA TTCACATGAG

AGGTCCAATC TTAACCTTAT TTGGATTAAA TTTTATATCA TTCACATGAG

AAGACCAATC TTGACCTTAT TTGGATTAAA ACTTATAGCA GTCACACAAC

AGGGCCACTC TTAACCTTAT TTGGATTAAA ATTTATACCA TTCACACAAG

AGGGCCACTC TTAACCTTAT TTGGATTAAA ATTTATGCCA TTCACACAAC

AGAGCGACTC TTAACCTTAT TTGGATTAAA TTTTATATCA TTCACACAAC

AGGCCTACTC TTAACCTTAT TTGGAGTAAA ATTTATACCA TTCATACAAC

AGGGCCGCTC GTAACCTTAT TTGGCTTAAA ATGTATACCG TTCACACAAC

---------- ---------- ---------- ---------- ----------

AGGGCCACTC TTCACCTTAT TTGGATTAAA ATTCATACCA TTCACACAAC

AGGGAAACTC TTAACCTTAT TTGGATTAAA ACTTACACCA TTCACATAAC

AGGGCCACTC TTAACCTTAT TTGGATTAAA ATGTTCACCA TTCATACAAC

ACAGTTTCTT TTAAAAGTTT CATTTTTTTT CTCAGTGGAA AGTCTCTGAT

ACAGTTTCTT TTAAAAGTTT CATTTTTTTT CTCAGTGGAA AGTCTCTGAT

---------- ---------- ---------- ---------- ----------

ACAGTTTCTT TTAAAAGTTT CATTTTTTTT CTCAGTGGAA AGTCTCTGAT

ACAATTTCTT TTAAAAACTT CATTTTTTTT CTCAGTGGAA AGTCTCTGAT

AGAATTTCTT TCAAAATTTT CACTTTTTGT CTCAGTGGAA AGTCTCTGAT

AGGGCTGCCT TCAAAAGTTT CACTTTCTTT GTCCAGGTAA AGTCTCCAAT

AGGACTGCCT TCAAAAGTTT CACTTTCTTT TCCCAGGTAA AGTCTCCAAT

AGGATTGCTT TTAAAATT-- -------TTT CTTAGTAGAA AGTCTCTGAT

AGGGTTTCTT TTCAAATTTT CATTTTTTTT CTCGGTAGAA AGTCTCTGAT

AGCATTTTTT TTCAATTCTT CACTTTTCTT CTCAGTAAAA AGTCTCTGAT

AGGACTCTTT TTAAAATTCT CATTTTTTTT CTCAGTACAA AGTCTCTGAT

AGGGtttttt tttaaattTT CATTTATTTT CTTAGCAGAA AGTATGTGAA

AGGATTTCTT TTTAAATTTT CATTTTTTTT CTCAGTGGAA AGTCTCTGGT

---------- ---------- ---------- ---------- ----------

AGGATCTCTT TTTAAATGTC CACATTTTGT CCAAATGGAA AGCCTCTGAT

AGGGTATTTT TTAAAAATTT ATTTTTTTTT CTCAGTGGAA GGTCTCTGAT

AGGGtttttt ttttaatttt taattttTTT CTAAGTGGAA AGCATCTGAC

CCACACCATC TGTTTACCTC ATGCCAATAA AATTCCTGAG GTTGTCAAGG

CCACATCATC TGTTTACCTC ATGCCAATAA AATTCCTGAG GTTGTCAAGG

---------- ---------- ---------- ---------- ----------

CCACACCATC TGTTTGCCTC ATGCCAATAA AATTCCTGAG ATTGTCAAGA

CCACACCATC TGTTTGCCTC ATGCCAAATC AATCCCTGAG ATTGTCAAGA

CCGCACCATC TGTTTGCCTC ATGCCAATAA AATTCCTGAG ATTGTCAAGA

CCACACCATC TGTTTGCCTC AT--CAATAA AATTCCTGAG ATTGCCAAGA

CCACTCCATC TGTTTGCCTC AT--CAATAT AATTCCTGAG ACTGCCAAGA

CCACAACATC TGTTTGTCTC ATGCCAATAA AATTCCTGAG ATTGTCCAGA

CTACACCATC TGTTTGCCTC ATGCCAATAA AATTCCTGAG ATTGTCAAG-

CCACACCATC TGTTTGTCTC ATGCCAATAA AATTCCTGAG ATTGTCAAGA

CCATACCACC TGTTTGTCTC ATGCCAATAA AATTCCTGAG ATTGTCAAGG

CCACACCATC TGCTTGCTCT GTGCCAACAA AATTCCTGAG ATTGTCAAGG

CCACACCATC TGTTTATCTC ATGCCAATAA AATTCCTGAG ATTATCAAAA

---------- ---------- ---------- ---------- ----------

CCACACCATC TGTTTGTCTC ATGCCAAGAA AATTCCTGGG ATTGTCAAGG

CCACACCATC TGTTTGCCTC ATGCCAAGAA AATTCCAGGG ATTATAAAGT

CCACACCATC TGTTTGCCTC ATAGCAATAA AATTCCGGAG ATTGTAAAGT

CACAGGATAC TTGTGAAAAA GATTCAGATA AGATTTTTCA TGGTTAAATC

CACAGGATAC TTGTGAAAAA GATTCAGATA AGATTTTTCA TGGTTAAATC

---------- ---------- ---------- ---------- ----------

CACAGGATAC TTATGAAAAA GATTCAGATA AGATTTTTCA TGGTTAAATC

CATAGGATAC TTGTGAAAAA GATTAAGATA AGATTTTTTG TGGTTAAATC

CACAGGATAC TTGTGAAAAA GATTAAGATA AGATTTTTCA TGGTTGAATC

CACGGGACAC TTGGGTAAAA GATTAAGATA AGG-TTTGCA TGGTTAAATC

C--------C TTGGGTAAAA GATTAAGATA AGA-TCTTCA TGGTTAAATC

CACAGGATAT TTGTGAAAAT GATTATGATA AGGTTTTTCA TGCTGAAATC

-ACAGAATAC CTGTGAAAAA AATTAAGATG ACCTTTTTCA TGGTTAAATC

CACAGAGTAC TTGTGAAAAA GATTAAGATA AGATTTTTCA TGGTGAAATC

CACAGAATAC TTGCAAAAAA AATTAAGA-- ---TTTTTCA TGGTTAAGTC

GGCAGATTCC TTGTGAAAAA GATAAAGATA AGATTTTTCA TGGTTAAATC

TACAGGATAC TTGCTAAAAA GATTAAGATA AGATTTTTCA CGATTAAACC

---------- ---------- ---------- ---------- ----------

CACAGGATAC TTGCTAAAAA GATTAAGGCA AGATTTTTCA TGGCTACATC

GACGGGATAC TTGCTAAAAA GATTAAGATA AGGTTTTTCA TGTTTGGATa

GAGGGGTTAC TTGCAAAAAA GATTAAGATA AGGCTTTGTA ATTTTGTCTC

TTCAGGTCAG AGAGAGCTTT ATTTATTCCA TAAACAAGTT TGCTGTGTTG

CTCAGGTCAG AGAGAGCTTT ATTTATTCCA TAAACAAGTT TGCTGTGTTG

---------- ---------- ---------- ---------- ----------

TTCAGGTCAG AGAGAGCTTT ATTTATTCCA TAAACAAGTT TGCTGTGTTG

TTCAGGTCAG AGAGAGTTTT ATTTATTCCA TAAACAAGTT TGCTATGTTG

TTCAGGTCAG AGAG--CTTT ATTTATTTCA TAAACAAGTT TGCTGTGTTG

TTCAGGTCAG AGAGGGAATC AGTTATTACA TAAACAAG-C TGCCACACTG

TTCAGGTCAG AGCGGGAATC AGTTATTGCA TAAACAAG-C TGCCTCACTG

TTCAGGTCAG AGAGA----- GCTGATTCCA TAAACAGGTT TGTTGTGTTG

TTTAGGTCAC AGAGA-CCTT ATTTATTACA CAAACAAGTT TGCTGAGTTG

TTCAGGTCAG AGAGAGCTTT ATTTATTCCA TAAACAAGTT TGCTGGGTTG

ATCAGGTCAG GGAG--TTTT GTTTATTCCA TAAACAGGTT TGCTTGGTTG

TTCAGGTCAG AG----CTTT ATTTATTTCA TAAACAATTT TCCTGGATTG

TTCAGGTCAG AGAGAGCTTT ATTTATTCTA TAAACAAGTT TGCTGGGTTG

---------- ---------- ---------- ---------- ----------

TTCAGGTCAG AGAGAGCTTT GTTTATTCCA TAAACAAGTT TGCTGGCTTG

TTCAAGTCAG AGAGAACTTT ATTTATTTCA CAAAAAAGTT TGTTGTGCTG

TTCAGGTCAG AGAGGGCATT ATATATTCCA CAAAAATGTT GGTTGTGCTG

GAATCCCTGA CTCAAGATGT TTCCTTTTTG TAGTTTTTAT TGGCTCAAGG

GAATCCCTGA CTCAAGATGT TTCCTTTTTG TAGTTTTTAT TGGCTCAAGG

---------- ---------- ---------- ---------- ----------

GAATCCCTGA CTCAAGATGT TTCCTTTTTG TAGTTTTTAT TGGCTCAAGG

GAATCCCTGA CTCAAGATGT TTCCTTTTTG TAGTTTGTAT TGGCTCAAGG

GAATCCCTGG CTCAAGATGT TTCCTTCTTA GAGTTTTTAT TGGCTCAAGG

AAATCCCTGA CTCAAGATGT TTCCTTCTTG TAGTTTTTAT TGGCTCAACG

AAATCCCTGA CTCAAGATGT TTCCTTCTTG TAGTTTTTAT TGGCTCAAAG

GAATCCCTGA CTCAAGATGT TTCCTTCTTG TAGTTTTTAT CGCCTCAAAA

GAATCCGTGA GTCAAGAT-T TTCCTTCTTG TAATTTTTGT TCGCTCAAAG

GAATCCCTGA CTCAGGATGT TTCCTCCATG TAGTTTTTAT TGGCTCAAAG

GAATCCCTGA CTCAAGATGT TTCCTTCTTG CAGCTTTTAT TGGCTCAAAG

GAATCCCTGA GTCAAGATAT TTCCTTCTTG TAGTTTTTAT TGGCTCAAAG

AAATCTCTGA CTCAAGATGT TTCCTTCTTC TAGTTTTTAT TGGCTCAAAT

---------- ---------- ---------- ---------- ----------

GAATTCCTGA CTCAAGATGT TTCCTTCCTG TAGTTGTTAT TGGCTCAAAG

GAATCCCTGA TTGAAGCTGC TTCCTCTTCA CCATATTCAT TGGCTCCAAG

GAATCCCTGA CTCAAGCTGT TTCCTTCCCT CGTTTTTCAT TGACTGCAGG

TAATGATGAT TGGAAAGTAG GAAAAGGAAA GAATAACACA AGAAGTTTCC

TAATGATGAT TGGGAAGTAG GAAAAGGAAA GAATAACACA AGAAGTTTCC

---------- ---------- ---------- ---------- ----------

TAATGATGAT TGGAAAGTAT GAAAAGGAAA GAATAACACA AGAAGTTTCC

TAATGATGAT TGGAAAGTAT GAAAAGGAAA GAATAACACA AG-AGTTTCC

TAATGATGAT TAGGAAGTAT GAAAGGGAAA GAATAACACA AGAAGTTTCC

T-------AC TGgaaTGGAA GAAGAGGAAA GAATAACACA AGG-AGTTTC

TGATGATGAC TGGAAAGTAT GAAAAGGAAA GAATAACACA GGA-GTTTCC

TAATGGTGGT TGGGAAGTAT GAAAAGGAAG AAATAACACA AGGAGTTTCC

TAGTGATGAT TGGAAAATAT GAAAAAA-AA AAATAACAAA AGGAGT-TCC

-AATGGTAAT TGGAAAATAC GAAAATA-AA GAATAACACA AGAAGTCTCC

TAGTGATGAT TGGAAAGTAT GAAAACGAAA GAATAACACA AGAAGTTTCC

TAATGATGGT TGAAAGTTAT GTAAAGGAAA GAAATACACA AGAAGCTTCC

TAATAATGAT TGGAAAGTAT GAAAAGGAAA GAATAACACA AGAAATTTCC

---------- ---------- ---------- ---------- ----------

TAGTAATGAT TGGAAAGTAT GAGAAAGAAA GACTAACACA GGGGGTTTCC

TAATAATGAT TGAAAAAAAT GAAAAAGAAG GAATAACATA GGAATTTTCC

CAATACAGGC AAAAAAGAAG GGAAAAGGAA AAAGAAAGCA AAAAATTTCt

TGTTTTAATT TACGGGATAT TTTATTTAGA ATCACAATTG TTTTTATAAT

TGTTTTAATT TACGGGATAT TTTATTTAGA ATCACAATTG TTTTTATAAT

--TTTTGATT TATAGGATAT TTTATTTAGA ACCACAATTG TTTTTATAAT

TGTTTTGATT TATGGGATAT TTTATTTAGA ATCACAATTG TTTTTATAAT

TG-TTTAATT TATGGAATAT TTTATTTAGA ATCACAATTG TTTTTATAAT

TGTTTTAATT TATGAGAGAT TTTATTTAGA AACACAATTG TTTTTATAAT

CGTTTCCCTT GACTGGATAT TTTA-TTAGA ATCACAATTG TTTTTACAGT

TGTTTCACTT GATTGGATAG TTTA-TTAGA ATCACAGTTG TTTTTATAGT

TGTTTTAATT TATGGGATAT TTTATTTAGA ATCACAATTG TTTTTATAAT

TATTTTAATT TATGAGATAT TTTATTTGGA ATCACAATTG TTTTCATAAT

TGTTT-AATT TATGAGATAT TTTATTTAGA AACACAATTG TTTTCATAAT

TGTTTTAATT TATGAGATAT TTTATTTCAA ATCACAATTG TTTTCATAAT

G-GTTTAATT TATGAGATAT TTTACTTAGC ATTACAATGC GTTTCATAAT

TGTTTTAATT TATGTGATAT TTTATTTAGA ATCACAATTG TTTTTTTAAT

---------- ---------- ---------- ---------- ----------

TTTTTTAATT TATGTAGTTT TTTTTTCAGA ATCCCAATTG TTTTCATAAT

TGTTTTAGTT TATGTGATAT TATAGTTAAT GTCACTATTG TGTTCTTAAT

tgTTTACATC TGTGTGATAT GCTGCTTAGG TCTGCTATTG TTTGTAAAAC

ACAATAATAA CAAACGTCAC TCTGAATAAA CAGGAATATA TGTTTATGTT

ACAATAATAA CAAACGTCAC TCTGAATAAA CAGGAATATA TGTTTATGTT

ACAACAATAA CAAATGTCAC TCTGAATAAA CAGGAATATA TGTTTATGTT

ACAACAATAA CAAATGTCAC TCTGAATAAA CAGGAATATA TGTTTATGTT

ACAACAATAA CAAATGTAAC TCTGAATAAA CAGGAATATA TGTTTATGCT

ACAGCAATAA CAAAGGTCAC TCTGAATAAA CAGGAATATA TGTTTATGTT

GTAGCAACAA CAAGTGCTGC AG--AGTAAA CAGGAATACA TGTTTATGTT

GCAGCAATAA CAAATGCTAC AGTGAATAAA CAGGAATTCA TGTTTATGTT

ACAGCAATAA CAAATGTCAC TCTGAATAAA CAGGAATATA TGTTTATGTT

ACAGCAATAA CAAATGTCAC TCTGAATAAA CAGGAATATA TGTTTATGTT

ACAGCGATAA CAAATGTCAC TCTGAATAAA CAGGAATATA TGTTTATATT

ACAGCAATAA CAAATGTCAC TCTGAGTAAA CAGGAATATA TGTTTATGTT

ATTGTAATAA CAAATGTCAC TCTGAATAAA CAGGAATATA TG-------T

ACAGCAATAA CAAACGTCAC TCCAAGTAAA CAGGAATATA TGTTTATCTT

---------- ---------- ---------- ---------- ----------

ATAGCAATAA CAAACATCAC TCTGAATAAA CAGGAATACA TGTTTATACT

ATAATTATAA CAAATGTCAT ACAGAATAAA CAGGACCTAA AGTTTACATT

ACAGCAATAA CAGATGTACC ACCAAGTAAA CAGGAATGTG TGTTTATGTT

GTACAGTAAC TTGTTCCAAG GAGTTATTAA AACTGTAAAC CAGACTTTGC

GTACAGTAAC TTGTTCCAAG GAGTTATTAA AACTGTAAAC CAGACTTTGC

GTACAGTAAC TTGTTCCAAG GAGTTATTAA AACTGTAAAC CAGACTTTGC

GTACAGTAAC TTGTTCCAAG GAGTTATT-A AACTATAAAC CAGACTTTGC

GTACAGTAAC TTGTTCCAAG GAGTTATTAA AACTGTAAAC AAGATTTTGC

GTATAGACTC TTGTTCCAAA GAATTATTAA AACTGTAAAC CAGGCTTTGC

GAGCAGAAAC TTGTTCCAAG GCATTATTTA AACTGCAAAG CAATCTTTGC

GAGCAGAAAC TTGTTCCAAG GCATTATTTA AACTGCAAAC CAATCTTTGC

ATACAGTAAC TTGTTCCAAG GAGTTGCTAA AACTGTAAAC CAGGTCTTGC

GTACAGTTAC TTGTTCCAAG GAGTTATGGA AACTCTAAAC CAGGATTTAC

ATACAGTAAC TTGTTCCAAG GAGTTATTAA AACTGTAAAC TAGGCTTTGA

GTACAGTAAC ATGTTCCAAG GAGTTATGAA AACTGTAAAC GAGGCTTTGC

GTGCAGTGGC TTGTTCCCAG GAGTTCT-TA AACTGTAAAC CAGGCTTCGC

GTATAGTAGT TTATTCCAAG GCATTTTTAA AACTATAAAC CAGGATTTGC

---------- ---------- ---------- ---------- ----------

GTACAGTATC TTGTTCCACA GAGTTATTGA AATTGTAAAC CAAGCTTTGC

ATACAGGAAC TTGTTCCAAG GAATAATTAG GATTGTAAAC TAGTCTTCTC

GAATAGTAAC TTGTTCCAAG GGATACTTTG GATTGGGCCT GAAACTTTGC

AAAAGGAAAG CAAAACATTT AGCTATTTGT GCTCTACTCC TTCAGAAAAT

AAGAGGAAAG CAAAACATTT AGCTATTTGT GCTCTACTCC TTCAGAAAAT

AAGAGGAAAG CAAAACATTT AGCTATTTGT GCTCTACTCC TTCAGAAAAT

AAGAGGAAAG CAAAACATTT AGCTATTTGT GCTCTACTCC TTCAGAAAAT

AAAAGGAAAG CAAAACATTT AGCTATTTGT GCTCTACTCC TTCAGAAAAT

AAGAGGAAAG CAAGACATTT AGCTATTTGT GCTCTACTCC TTCAGAAAAT

AAGAGGAAGG CAAAACATTT AGCTAATTGT GCTCTACTCC TTCAGAAACT

AAGAGGAAGG CAAAACATTT AGCTAATTGT ACTCCACTCC TTCAGAAAAT

AAGAGGAAAG CAAAACATTT AGCTATTTGT GTTCTACTCC TTCAGAAAAT

AAGAGGAAAG CAAAACATTT AGCTATTTGT GCTCTACTCC TTCAGAAAAT

AAGAGGAAAG TAAAACATTT AGCTATTTGT GCTCTACTCC TTCAGAAAAT

AAGAGGAAAG CAAAACATTT AGCTATTTGT GCTCTACTCC TCCAGAAAAT

AAAAGGGAAG CAAAACATTT AGCTATTTGT GTTCTACTCC TTCAGAAAAT

AAGAGGAAAG CAAAACATTT AGCTATTTGT GCTCTACTCC TTCAGAAAAT

---------- ---------- ---------- ---------- ----------

GAGAGGAAAA CAAAACATTT AGCTATTTGT GCTCCACTCC TTCAGAAAAT

CAAAGGAAAG CAAAATGGTA AATTAAAGGA GAAATGCTTA TTAGTAACAT

AAAAGGAAAG TAAAACATTT GGCTATTTGT GATCTCCTCC TTCAGAAAAT

ACTTTGCTTT CATAGTAAAA GTCTTCAGAA CCAAGTAATT TGAGTAGCAT

ACTTTGCTTT CATAGTAAAA GTCTTCAGAA CCAAGTAATT TGAGTAGCAT

ACTTTGCTTT CATAGTAAAA GTCTTCAGAA CCAAGTAATT TGAGTAGCAT

ACTTTGCTTT CATAGTAAAA GTCTTCAGAA TCAAGTAATT TGAGTAGCAT

AGTTTGCTTT CATAGTAAAA GACTTCAGAA CCAAGTAATT TGAGTAGCAT

ACTTTGCTTT CATAGTAAAA GTCTTCAGAA CCGAGTAATT TGAGTAGCAT

ATTTTGCTTT CATAATAAAA GCCTTCAGAA CTAAGTCACT TACGTAGCGT

ACTTTGCTTT CATAATAAAA GCCTTCAGAA CGAAGTGTCT TACGTAGCAT

ACTTTG-TTT CATAATAAAA GTCTTTAGAA CCAAGTAATT TGAGTAGCAT

ACTTTGCTTT CATAGTAAGA GTCTTTAGAA CCAAGTAATT TGAGTAGCAT

ACTTTGCTTT CATAGTAAGA GTCTTCAGAA CCAAGTAATT TGAGTAACAT

ACTTTGCTTT CATAGTA--A GTCTTCAG-A CTAAGTAATT TGAGTAGCGT

ACTTTGCTTT CATAGT--GA GTCTTCAAAA CCAAGAAATT TGAGTAGCGT

ACTTTACTTT CATAGTACAA GTCTTCAGAA CCAAGTAATT TGAGTACTGT

---------- ---------- ---------- ---------- ----------

ACTTTGCGTT CATAGTAAGA GTCTTCAGAA CCATGTAATT TGAGTAGCAT

GTATTATTAT CATATGCTAA GACCGAAACA TGAAAAAAAT TGAATAACAT

ACTTTGTGTA CAAAGTAAGA CTCTTCGCAT CTAAGACATT CGAGTGGCAT

GCCCTTTAGC AACATTCTTT CTACTATTTC CAAATGTGTG CAGTTTTCAG

GCCCTTTAGC AACATTCTTT CTACTATTTC CAAATGTGTG CAGTTTTCAG

GCCCTTTAGC AACATTCTTT CTACTATTTC CAAATGTGTG CAGTTTTCAG

GCCCTTTAGC AACATTCTTT CTACTATTTC CAAATGTGTG CAGTTTTCAG

GCCCTTTAGC AACATTCTTT CTACTATTTC CAAATGTGTG CAGTTTTCAG

GCCCTTTAAC AACATTCTTT CTACTATTTC CAAATGTGTG CAGTTTTCAG

GCCCTTTAGC AACATTCTTT CTGCTGCTCC CAAATGTGTG CAGTTTTCAG

GCCCTTTCGC AACATTCTTT CTACTGCTTC CAAATGTGTG CAGTTTTCAG

GCCCTTTAGC TACATTCTTT CTACTATTTC CAAATGTGTG CAGTTTTCAG

GCCCTTTAGC GACATTCTTT CTACTATTTC CAAATGTGTG CAGTTTTCAG

GCCCTTTAGC AACATTCTTT CTACTATTTC CAAATGTGTG CAGTTTTCAG

GCCCTTTAGC AACATTGTTT CAACTATTTC CAAATGTGTG CAGTTTTCAG

GCCCTTTAGC AGCATTCTTT CAACTATTTC CAAATGTAAG CAGGTCTCGG

GCCCTTTAGC AACATTCTTT CTACTACTTC CAAATGTATG CAGTTTTCAG

---------- ---------- ---------- ---------- ----------

GCCCTTTGGA AACATTCTTT CTACTATTTC CAAATGTGTG CAGTTTTCAG

GTCTTTTGAG AGAATTCCTC CTGTTCTTTC CAAATGTATA AACTTTTTAT

GCCCTTTAGC AGCATTCTAT CTTTCATTTC CAAATATATC CATTTTTCTG

GACCACAATG TTTTACTATA AGTTTATTTG AAACAAAAAT CTTCCCAGAT

GACCAAAATG TTTTACTATA AGTTTATTTG AAACAAAAAT CTTCCCAGAT

GACCACAATG TTTTACTATA AGTTTATTTG AAACAAAAAT CTTCCCAGAT

GACCACAATG TTTTACTATA AGTTTATTTG AAACAAAAAT CTTCCCAGAT

GACCACAGCG TTTTACTATA AGTTTATTTG AAACAAAAAT CTTCCCAGAT

GACCACAGTG TTTTACTATA AGTTTATTTG AAACAAAATT CTTCCCAGAT

GACCACAGTG TTTTACTATA GGTTTATTTG AACCAAAATT CTTCCCTGAT

GACCACAGTG TTTTACTATA GGTTTATTTG GACCAAAATT CTTCCCAGAT

GACCACAGTA TTTTACTATA AGTTTATTTG AAACAAAATT CTCCCTAGAC

GACCACAGTA TTTTACTACA AGTTTATTTG AAACAAAATT CTTCCCAGAT

GGCCACAGTG TTTTACTATA AGTTGATTTG AAACACAATT CTTCCCACAT

GACCGCAGTG TTTTACTATA AGTTTATTTG AAACAAAATT CTTCCCAGAT

GACCACAGCG CTGTACTAT- -GTTTATTTG AAACTAAATT CTTTCCAGAT

GACCACAGTG TTTTACTATG AGTTTATTTG AAACAAAATT CTTCCCAGAT

---------- ---------- ---------- ---------- ----------

GATCACAGAG TTTTACTATG AGTTTATTTG AAAAAAAAAT CTTCCCTGGT

GATCAGAATA TTTGATTGTA AGATTCTTTG AAACTGGATT ATTTTTGGAT

TCCTGCTCTC TTTTACTCCA AGCTTCTTTG AAACTTGACT CTTTCCAGAT

ATTTATTGTT AAAAATTCTT CTGCCCAACC CTTGGCTGCA TCCCAGTCTT

ATTTATTGTT AAAAATTCTT CTGCCCAACC CTTGGCTGCA TCCCAGTCTT

ATTTATTGTT AAAAATTCTT CTGCCCAACC CTTGGCTGCA TCCCAGTCTT

ATTTATTGTT AAAAATTCTT CTGCCCAACC CTTGGCTGCA TCCCAGTCTT

ATTTATTGTT AAAAATTCTT CTGCCCAGCC CTTGGCAGCA TCCCAGTCTT

ATTTATTGAT AAAAATTCTT CTGCCCAGCC CTTGGCAGCA ACCCAGTCTT

ATTTATTGAT GAATATTCTT CTGCCCCCCC CTTGGCTACA TCCCAGTCCT

ATTGATTGAT ACAAACTCTT CTG----CCc cttagccaca tcccagtctt

ATTTGCTGAT AAAAATTCTC TTGCCAGC-C CTTGGCAGCA TCCCAGCCTT

ATTTATTGAT AAAAATTCTT CTGCCCAGGC CTTGGCAACC TCCCAGTCTT

ATTTATTGAA AAAAATTCTT CTGCCCAGCC CTTGGCGGCA TTCCAATCTT

ATTTATTGAT AAAAATTCTC CTGCCCAGCC GTTGGCAGCA CCC--GTGAT

ATTTATTGAT AAAAATTCTT CTGCTTGGCT TTTGGCAACA TCCCAGTCTC

ATTTATTGAT AAAAATTCTT CTGGCCAGCC CTTGGCAACA TCCCAGGCTT

---------- ---------- ---------- ---------- ----------

ATTTATTGAT AAAAATTCTT CTGGCCAACC CTTGGCAACC TCCCGGTCCT

ATTTATTAAT AAAAATGCCT TTGCCCTACC TTTGGCTACT CCCCTATTCT

ATTCATTGCT AAAAACCCCT TTGCTTAAAC CTTGGCAACT CCTCAGCCTC

CCATCTGCTA TGGGGCTGGA GACGGAGAAG GACGTACAGC TCTTCATGGA

CCATCTGCT- ---------- ---------- ---------- ----------

CCATCTGCTA TGGGACTGGA GACGGAGAAG GACGTGCAGC TCTTCATGGA

CCATCTGCT- ---------- ---------- ---------- ----------

CCATCTGCTA TGGGGCTGGA GACGGAGAAA GACGTGCAGC TCTTTATGGA

CCATCTGCTA TGGGGCTGGA GACGGAGAAG GACGTGCAGC TCTTCATGGA

CCATTTGCTA TGGGGCTGGA GACCGAGAAC GACGTGCAGC TCTTCATGGC

gcatTTGCTA TGGGGCTGGA GACCGAGAAC GATGTGCAGC TCTTCATGGC

CCATCTACAA TGGGGCTGGA GACCGAGAAA GACGTACAGC TCTTCATGGA

CCATCTGCTA TGGGGCTGGA GACCGAGAAG GATGTCCAGC TCTTCATGGA

CCATCTGCTA TGGGGCTGGA GACCGAGAAC GACGTCCAGC TCTGCATGGA

CCATCTGCTA TGGGGCTGGA GACCGAGAAG GACGTGCAGC TCTTCATGGA

CCAGCTGCT- ---------- ---------- ---------- ----------

CCATCTGCTA TGGGGCTGGA GACCGAGAAG GACGTCCAGC TCTTCGTGGA

---------- ---------- ---------- ---------- ----------

CCATCTGCTA TGGGCTTGGA AAGCGAGAAG GACGTCCAGC TCTTCATGGA

CCATCTGCTA CAGTGCCGGA GCCCGAGAAA TGCATGAAGT CCATCCAGGA

TCATCTGCTA TGGGGCTGCA GACGGAGCGG GGCAGCGGGG GCTTCCTGAG

CGACGATACG CACTCCTTTG ACAAAGTGTG GATCTGCAGC CATGCCCTCT

------GACA CACAGTTTTG ACGGCATTTG GAAGGCCAGC TTCACCACCT

CGACGATACG CACTCCTTTG ACAAAGTGTG GATCTGCAGC CATGCCCTCT

------TACG CACTCCTTTG ACAAAGTGTG GATCTGCAGC CATGCCCTCT

CGACGATACG CACTCCTTTG ACAAAGTGTG GATCTGCAGC CATGCCCTCT

CGACGATACG CACTCCTTTG ACAAAGTGTG GATCTGCAGC CATGCCCTCT

CGATGATACA CACTCCTTTG ACAAAGTGTG GATCTGCAGC CACGCGCTCT

CGATGATACA CACTCCTTTG ACAAAGTGTG GATCTGCAGC CATGCTCTCT

CGACGATACG CACTCCTTTG ACAAAGTGTG GATCTGCAGC CATGCCCTCT

CGACGATACG CACTCCTTTG ACAAAGTGTG GATCTGCAGC CACGCTCTAT

CGACGATACG CACTCCTTTG ACAAAGTGTG GATTTGCAGC CATGCCCTGT

CGACGATACT CACTCCTTAG ACAAAGTATG GATCTGCAGC CACGCCCTCT

---------- ---------- ---------- ---------- ----------

CGACGATACG CACTCCTTAG ACAAAGTGTG GATCTGCAGC CATGCCCTCT

------GACA CACAGTTTCG ATGGCATCTG GAAGGCCAGC TTCACCACCT

CGACGAGACT CACAGTTTCG ACGGCATCTG GAAGGCCAGC TTCACCACCT

TCGTGACACC CACAGTTTTG ATAAAGTTTG GATCTGCAGC CATGCCCTCT

CcagcaCACA CACAGCTTTG ACGGGATCTG GAAGGCCAGC TTCACCACCT

TTGAAATCAG CAAATACGTA ATGTACAAGT TCCTGACTTT TTAGCCACat

TCACTGTGAC GAAATACTGG TTTTACCGCT TGCTGTCTTT TTAGCCACat

TTGAAATCAG CAAATACGTA ATGTACAAGT TCCTGACTTT TTAGCCACAT

TTGAAATCAG CAAATACGTA ATGTACAAGT TCCTGACTTT TTAGCCACAT

TTGAAATCAG CAAATACGTA ATGTACAAGT TCCTGACTTC TTAGCTACAT

TTGAAATCAG CAAATACGTG CTGTACAAGT TCCTAACTTT T-AGCCACAT

TTGAAATCAG CAAATACGTG ATCTACAAGT TCCTGACCTT TTAGACACAG

TTGAAATCAG CAAATATGTG ATGTACAAGT TCCTGACCTT TTAGAGACAC

TTGAAATCAG CAAGTACGTG CTGTACAAGT TCCTGACTTT TTAGCCACAT

TTGAAATGAG CAAATATGTG ATCTACAAGT TCCTGACTTT CCAACCACAG

TTGAGGTCAG CAAGTACGTG ATCTACAAGT TCCTGACTTT TCAGCTACAT

TTGAAGTCAG CAAATATGTG ATTTACAAGT TCCTGACTTC TCAGCCACAT

---------- ---------- ---------- -------TTT TCAGCTACAT

TTGAAATCAG CAAGTACATA ATCTACAAGT TCCTGACTTT TTAGCCACAT

TCACTGTGAC AAAATACTGG TTTTATCGCT TGCTGTC--- ----------

TCACTGTGAC GAAATACTGG TTTTACCGTC TGCTCTCCTT TCAGCCACAG

TTGAGGTCAG CAAGTACCTG ATCTACAAGG TCCTAACTTT TTACCCATGT

TCACCGTGAC AAAATACTGG TTTTACCGCT TGCTCTCTCT TTAGCCCCAT

aaaatgttaa CTAGCTTGTT TTTTTAACTA TTCCAGGATT TTAATGCCTT

aaaatgttaa CTAGCTTGTT TTTTTATCTA TTCCAGGATT TTAATGCCTT

AAAAGGTTAA CTAGCTTGTT TTTTTAATTA CTCCAGGATT TTAATGCCTT

AAAAGGTTAA CTAGCTTGTT TTTTAAATTG CTCCAGGATT TTAATGCCTT

AAAATGTTAA CTAGCTTGTT TTTTTAATTA TTCCAGGATT ATAATGCCTT

AAGATGTTAA CTAGCTTGTT TTCCATATTA TGCCAGGATT ATAATGCCCT

AATATGTTAA CTAGCTCCTT TCTCAAACTG TTCCAGGATC CTAATGCCTT

AATATGTTAA CTAGCTCATT TCTCAAATTG TTCCAGGATC CTGATGCCTT

AAAATCTCAA CTAGCTTGTG TTTCAAATTA -CCCAGGATT TTGATGCCTT

AAACTGTTAA CTAACTTATT TTTCAAATTA TTCCAGGATT ATAATGCCTT

AAAATGTTAA CCAGCTTGTT TTTCAAATTA TTCCAGGATT ATAATGCCTT

AAAATGTTAA CTAGCTTGTT TTTCCAATTA TTCCAGGATT GTCATGCCAT

AAAATATCAA CTAGCTTGTT TTTCAAATTA TTCCAGGATT TTAATGCCTT

AAAATGTTAA CTCACTTGTG TTTCAAATTA TGCCAGGATT ACAATGCCTT

---------- ---------- ---------- ---------- ----------

AAAAGGTGAA CTCGCATGTT TTTCAAATTA TTCCAGGATT ATAATGCCTT

AATATGTTAA CTAGTCTGTT TTTCAAATTA TTCTAGGATT GTGGTGCCTT

AAAATGTTAA CTAGCCTATT TTACAAATTA CCCTAGGATC GTGATGCCTT

TTGTAAAGAC CTGCCTAATG GTTCTGCCTT CAGTGCAGAC AATATGGAAG

TTGTAAAGAC CTGCCTAATG GTTCTGCCTT CAGTGCAGAC AATATGGAAG

TTGTAAAGAC CTGCCTAATG GTTCTGCCTT CAGTGCAGAC AATATGGAAG

TTGTAAAGAC CTGCCTAATG GTTCTGCCTT CAGTGCAGAC AATATGGAAG

TTGTAAAGAC CTGCCTAATG GTTCTGCCTT CGGTGCAGAC AATATGGAAG

TTGTAAAGAC CTGCCTAATG GTTCTGCCTT CGGTGCAGAC AATATGGAAG

TTGTGAAGAC CTGCCTCATG GTCCTGCCTT CCGTGCAGAC GATATGGAAG

TTGTGAAGAC CTGCCTAATG GTCTTGCCTT CCGTGCAGAC AATATGGAAG

TCGTAAAGAC CTGCTTAATG GTCCTGCCTT CAGTGCAGAC GATATGGAGG

TTGTAAAGAC CTGCCTAATG GTCCTGCCTT CAGTGCAGAC AATATGGAAG

TCGTGAAGAC CTGCCTCATG GTCCTGCCTT CGGTGCAGAC CATATGGAAG

TTGTAAAGAC GTGCCTCATG GTCCTGCCTT CCGTACAGAC GATATGGAAG

TTGTAAAGAC CTGCCTAATG GTCCTACCTT CTGTGCAGAC AATATGGAGG

TTGTAAAGAC CTGCCTAATG GTCCTGCCTT CGGTGCAGAC AATATGGAAA

---------- ---------- ---------- ---------- ----------

TTGTAAAGAC CTGCCTAATG GTCTTGCCTT CAGTGCAGAC AATATGGAAG

TCGTAAAGAC CTGTTTAATG GTCTTGCCAT CAGTACAGAC GGTATGGCAC

TCGTAAAGAC CTGCCTCATG GTCCTGCCTT CCGTGCAGGC GGTGTGGAAG

AGTGTGACAG ATGTTATCAT TGCTCCATTG TGTTATTTAA CAAGATTTCA

AGTGTGACAG ATGTTATCAT TGCTCCATTG TGTTATTTAA CAAGATTTCA

AGTGTGACAG ATGTTTTCAT TGCTCCATTG TGTTATTTAA CAAGATTTCA

AGTGTGACAG ATGTTTTCAT TGCTCCATTG TGTTATTTAA CAAGAGTTCA

AGTGTGACAG ATGCTATCAT TGCTCCATTG TGTTATTTAA TAAGATTTCA

AGTGTGACCG ACGTTATCAT CGCCCCATTG TGTTATTTAA CAAGAATTAA

AGTGTGACAG ATGTTGTCAT TGGCCCATTG TGTTCTTTAA CAAGACTTCG

AGTGTGACAG ACGTTGTCAT TGGCCCATTG TGTTATTTAA CAAGATTTTT

AGTGTGACAG ATGTTGTCAT TGCGCCACTG TGTTATTTAA TAAGTTTTCA

AGTGTGACAG ATGTTGTCAT TGCCCCATTG TGTTATTTAA CAAGAGTTCA

AGTGTAACAG ATGCTGTCAT TGCCCCGTTG TGTTATTTAA TAAGATTTCA

AGCGTGACAG ATGTGGTTAT TGCCCCACTG TGTTATTTAA TAAGATTTCA

AGTGTGACAG ATGTTGTCAT TGCCCCTTTG TGTT-TTTAA CAAGGTTTCA

AGTGTGACAG ATGTTGCCAT TGCCCCATTG TGTTATTTAA CAAAATTTCA

---------- ---------- ---------- ---------- ----------

AGCGTGACAG ATGTTGTCAT TGCACCATTG TGCTATTTAA CAAGGTTTTA

AGCATTACAG ATGGCTTCAT TGCTCCATTG TATTATTTAA GAAGAGTTTA

AGCGGGACCA GTGTCCTGGT GGCCCCGCTG TGTTTTTTAA TCAAAACTCA

CTGTATATTT TATGTTGCTC TATCAGATTG CTTAAAAGCA TTTTTATTTA

CTGTATATTT TATGTTGCTC TATCAGATTG CTTAAAAGCA TTTTTATTTA

CTGTATATTT TATGTTGCTC TATCAGATTG CTTAAAAGCA TTTATATTTA

TTGTATATTT TATGTTGCTC TATCAGATTG CTTAAAAGCA TTTATATT--

CTGTATATTT TATGTTGCTC TATCAGATTG CTTAAA-GCA TTTTTATTTA

CTGTATATTT TGTGTTGCTC TATCAGATTG -----AGGCG TTTTTA----

CTGTATATCT TGTGTTACTC AATGCAATCG CTTTGGAGTG C-TTTCTTCG

CTGTATATTG TGTGTTACTC TATGAAATCG CTTTGGAGTG TGTTTCTTTG

TTGTATATTT TGTGTTGCTC TATCAGATTG CTCTAAAGAA GGTTTATTTA

CTGTATATTT TGTGTTGCCC TATCAGATTG TTTTAAAGCA CACT--TTTA

CTGTATATTT TGTGTTGCTC TATCAGATTG CTTTTAAGTA CTTTTATTTA

CTGTATATTT TATGTTGCTC TATCAGATTG CTTTAAAGCA CTTTTATTTA

CTGTATATTT TGTGTTGCTC TACCATATTG CTTTGAAGCA CTTTTATTTA

CTGTATATTT TGTGTTGCTC TATGCGATTT CTTTAAAGCA CTTTTATTTA

---------- ---------- ---------- ---------- ----------

CTGTATATTT TGTGTTGCTC TATCAGATTG CTCTAAAGCA CTTTTATTTA

TTGTATATTT TGTGTTGCTT TATCAGTTTT CCAAAAAGCA CCTTGACTTA

GTGAATATTT TCTGTGGCTT TCTCGGTTTT TCTGAAAGGA GTTCGAGTTG

TTTATGTTGT TGAACTAATA TATCTGCAGT CAGAGAAATC AGAGGACACC

TTTATGTTGT TGAACTAATA TATCTGCAGT CAGAGAAATC AGAGGACACC

TTTATGTTGT TGAACTAATA TATCTGCAGT CAGAGAAATC AGAGGACACC

--TATGTTGT TGAACTAATA TATCTGCAGT CAGAGAAATC AGAGGACACC

TTTATGTTGT TGAACTAATA TATCTGCAGT CAGAGAAATC AGAGGACACC

-TTATTATAT TGAACTAATA TATCAGCAGT CAGAGAAATC AGAGGACACC

TCTATCTTAT TGATCTCATA CACGAGCAGT CAGAGAAATC AGAGGACACC

TCTATTGTAC TAATCTCATA TATGAGCAGT CAGAGAAATC AGAGGACACC

TTTATCTTGT TGATATAATA TATCAACAGT CAGAGAAATC AGAGGACAAC

TTTATCTTGT ---ACTAATA TATCAGCAGT CAGAGAAATC AGAGGACACC

TTTATCTTGT TGAACTAATA TATCAGCAGT CAGAGAAATC AGAGGACACC

TTGATCTTGT TGAGCTAATA TATCAG---- CAGAGAAATC AGAGGATACC

TTTATCTTGT TAAATTAATA TATCAG---- CAGAGAAATC GGAGGACACC

TTTATCTTAT TGAACTAATA TATCAGCAGT CAGAAAAATC AGAGGACACC

---------- ---------- ---------- ---------- ----------

TATATCTTGT TGAAATAATA TATCAGCGGT CAAGAGAAAC GGAGGAGACT

TTAATCTTGT AGAGCTCTTA TGTCAGCAGT CAGAGGAATC AGAAGACTGC

TCGACCCCGC GGCCCTGTTA CATCAGCGGT CAGAGAAACC AGATGGTTGC

ACTTCAACAA TTAACCAAAT AGGACAGGGA CACCTATCCT GGAGAAATCA

ACTTCAACAA TTAACCAAAT AGGACAGGGA CACCTATCCT GGAGAAATCA

ACTTCAACAA TTAACCAAAT AGGACAGGGA CACCTATCCT GGAGAAATCA

ACTTCAACAA TTAACCAAAT AGGACAGGGA CACCTATCCT GGAGAAATCA

ACTTTAACAA TTATCCAAAT AGGACAGGGA CACCTATCCT GGAGAAATCA

ACTTCAACAA TTATCCAAAT AGGACATGGA CACCTATCCT GAAGAAGTGA

ACTCCAACAA TTACCCCAAT AGGACAGGGA CACCTATCCT GGAGAAGTCA

ACTCCAACAA TTACCCCAAT AGGACAGGGA CACCTATCCT GGAGAAGTTA

ACTTCAACAA -TATCCAAAT AGGACAGGGA CTCCTATCCT GGTCAAGTGA

ACTTCAACAT TTATCCAAAT AGGACAGA-- CACCTATCCT GGAGAAGTCG

ACTTCAACAA TTATCCAAAT AGGACAGAGA CACCTATCTT GGAGAAGTGG

ACTTCAACAA TTGTCCAAAT AGGACAGAGA CACCTATCCT GGAGAAGTCG

TCTCCAATCA CTC-CCAAAT AGGCCAGA-- CACCTATCCT GGAGAAGTCG

ACTTAAACAA CGACCTAAAT AGTTCAGGGA CACCTCTCCT GGAGAAGTCG

---------- ---------- ---------- ---------- ----------

ACTTCAACAA TGATCTGAAT AGGGCAGGGC CACCTCTCCT GGAGAAGTCG

ATTTAAGGGA TTCTTTAAAT AGACCAGGGA CACCTATTCT GTAGAAGTCA

CTTGAATCAA TTGGCTCCAT GAAGTAGGGA AACCTCTCCC ACTGAAGCCA

TTTTGCCTTT ATAGGAAAAG GTAGGGGTGT GGGAAGAGAA AGGAAGAGAC

TTTTGCCATT ATAGGAAAAG GTAGGGGTGT GGGAAGAGAA AGGAAGAGAC

TTTTGCCATT ATAGGAAAAG GTAGGGGTGT GGGAGGAGAA AGGAAGAGAC

TTTTGCCATT ATAGGAAAAG GTAGGGGTGT GGGAGGAGAA AGGAAGAGAC

TTTTGCCATT ATAGGAAAAG GTGGGAGTGT GGAAAGAGAA AGGAAGAGGC

TTTTGCCATT ATAGGAAAAG ATAGGGGTGT GAGAAGAGAA AGGAAGAGAC

TTTTGCCATT ATGGGAAGAG GTAGAGGTGG GGGAAGAGGA AGGAAGAGAC

TTTTGCCTTT ATGGGAAGAA GTAGAGGTGG GGGAAGAGGA AGGAAGAGAC

TTTTGCCATG ATAGGAAAAG GTAGGGGTGG CAGAAGAGAA AGGAAGTGAC

TTTTGCCATT ATAGGAAAAG GTAGGGGTGG GA--AGAGAA AGGAAGAGAC

TTTTGCCATT ATAGGAAAAG GTAGGGGTGG AAGAAGAGAA AGGAAGAGAC

TTTTGCCATT ATAGGAAAAG GTAAGGGTGG GAGAAGAGAA AGGAAGAGGC

TTTTGCCTTT ATAGGAAAAG GTAGGGGTGG GAGAAGA-AA AGGAAGCGAC

TTTTGCCATT ATAGGAAAAG GTAGGAGTGG TTGAAGAGAA AGGAAGAGGC

---------- ---------- ---------- ---------- ----------

TTTTGTCTTT ATAGGAAAAG GTAGGGGTGG GACAGGAGAA AGGAAGAGGC

TTTTGCCATT ATAAGAAAAG GTAGGGGTGG GTAAAGAAAG AGGAAGAGAC

TTCTGCCATT ATAGGAAAGG GGAGGGGCGG GAGACCAGAA AGGAAAAGAC

ATGACTCAGA GTGGGAAACA GCACGTAGCC AAGGGCAAAT ATTTCTCTCA

ATGACTCAGA GTGGGAAACA GCACGTAGCC AAGGGCAAAT ATTTCTCTCA

ATGACTCAGA GCGGGAAACA GCACAGAGCC AAGGACAAAT ATTTCTCTAA

ATGACTCAGA GCGGGAAACA GCACAGAGCC AAGGACAAAT ATTTCTCTAA

ATGACTCAGA GTGGGAAACA GCACATAGCC AAGGGCAAAT ATTTCTCTCA

GTGACTCAGA GTGGGAAACA GTGCAGAGCC AAGGGCAAAT ATTTCTCTCA

GTGACTCGG- -TGGGAAACA GCATGTCGCC AAGGGTAAAT ATTGCCCGCC

GGGACTCGG- -TGGGAAACA GCATGTCACC AAGGGCAAAT ATTGCTCGCC

ATGACTCAGA GGGGGATGCA GCACATAGCC AAGGGCAAG- ----------

ATGACTCATA GTGGGAAAGA GCACGTAACC AAGGGCAAAT ATTTCTCCCA

ATGACTCAGA GTGGGAAACA GCACGTAAGC AAGGGCAAAT ATTTCTCCCA

ATGAGTCAGA GTGGGAAACA GCACGTAACC AAGGGCAAAT ATTTCTCCCA

ATGACTCAGA GTGGGAAACA GCACGTAACC AAGGGCAAAT ATTTCACCCA

ATGACTCAGA GTGGGAAACA GCACATAGCC AAGGGCAAAT ATTTCTCTCA

---------- ---------- ---------- ---------- ----------

CGGATGCAGA CTGGGAAACA GCACGTAGCC AAGGGCAAAC ATTTCCCTCA

CCAGCTCAGA GTGGGAAACA GCACGTAGCC AAGGGCAAAT ATTTCCCTCC

TCATCTCAGG GAGGAAAACA TCACATTGCC AACCACATAT ATTTCTCTGA

GACATAAATG GATCAGGAAG AGGAGAGCAG CAGGTCTCTT TGGGCATTCC

GACATAAATG GATCAGGAAG AGGAGAGCAG CAGGTCTCTT TGGGCATTCC

GACATAAATG GATCAGGAAG AGGAGAGCAG CAGGTCTCTT TGGGCATTCC

GACACAAATG GATCGGGAAG AGGAGAGCAG CAGGTCTCTT TGGGCATTCC

GACATAAACG GATCAGGAAG AGGAGAGCAG TGGGTCTCTT TGGGCATTCC

GACATAAATG GATCAGGAAG ACAAGAGCAG CAGGTCTCTT TACACATTCC

GATATAAATG AATCCGGAAG AGGAGAGCAA CAGGTCTCCG TGGGCATTCC

AATATAAATG ATTCAGGAAG AGGAGAGCAA CAGGTCTCTG TGGGCATTCC

---------G GATCAGGAAA AGGAGAGCAA TAGGTCTCTT TGGGCATTCC

GACATAAACG GAGCAGGAAG AGAGGAGCTA CAGGTCTCTT TGGGCATTCC

GACATAAACG CATCAGGAAG AGCAGAGCAA AAGGTCTCTT TGGGCATTCC

GACTTAAATG GATCAGGAAG AGGGGAGCAA CAGGTCTCTT TGGACATTCC

GATATAAATG GAGCAGGAAG AGGGGAGCAA CAGGTCTCTC TGGGCATTCC

AATGTAAATG GATCAGGAAG AGGGTAGCAA CCGGTCTCTT AGCGCATTCC

---------- ---------- ---------- ---------- ----------

GACATAAAGG GATCAGGAAG AGGGGAGCAG CAGGTCTCCT GAGGCACTCC

AACAGAAACG AATCAGGAAG AAAGGAGCAA GAGGTCTCTT TGGGAATGAT

ACCAGCAATG GGTGAGGAAG GGGTGGGCTT CAGGCCTTTT TGGGGAGACC

TGCTAATTCT TGATTTCACA TTCCAAGAAG CCTTCAGTTT TAGAAATAGA

TGCTAATTCT TGATTTCACA TTCCAAGAAG CCTTCAGTTT TAGAAATAGA

TGCTAATTCT TGATTTCACA TTCCAAGAAG CCTTCAGTTT TAGAAATAGA

TGCTAATTCT TGATTTCACA TTCCAAGAAG CCTTCAGTTT TAGAAATAGA

TGCTAATTCT TGATTTCTCA TTCCAAGAAG CCTTCAGTTT TAGAAACAGA

TGCTAATTCT TGATTTCACA TTCCAAGAAG CCCTCAGTTT TAGAAATAGG

TGCTAATTTC TGATTTCACA TTCCAAGGAG ACTTCCGTTT TAGAGA--AA

TGC------- ---------- ---------- ---------- ----------

TGCTAATTCT TGACTTCACA TTCCAAGAAC CCTTCAGTTT CAGAAATGGG

TGCTAATTCT TGATTTCACA TTCCAAGAAG CCTTCAGTTT TAGAAATGGA

TGCTAATTCT TGATTTCACA TTCCAAGAAG CCTTCAGTTT TAGAAATGAA

TGCTAATTCT TCATTTCACA TTCCAAGAAG CCTTCAGTTT TAGAAATGAA

TGCTGATTCT TGATCTCACA TTCCAAGAAG CCTTCAGTTT TAGAAATGAG

TGCTAATTCT TGATCTCACA TTCCAAGAAA ACTTCAGTTT TAGAAATGGA

---------- ---------- ---------- ---------- ----------

TGCTAATTCC TGATTCCACA TTCCAAGAAG CCCTCAGTTT TAGAAATGAA

CTCTAATTCT CCAGCCTATG TTCCAAGAAA CATTCAGACT TAGAGAAGGA

AGCAAATTCT CCAGCTCCTG TTCCAAGAAA GCCTCAGTTT TGAAGAAGGG

AAGAACTGCC AGTTCCCTGC AAGCACCTTG GCCAAGTTTA TTTACTAGGG

AAGAACTGCC AGTTCCCTGC AAGCACCTTG GCCAAGTTTA TTTACTAGGG

AAGAACTGCC AGTTCCCTGC AAGCAGTTTG GCCAAGTTTA TTTACTAGGG

AAGAACTGCC AGTTCCCTGC AAGCAGCTTG GCCAAGTTTA TTTACTAGGG

AAGAACTGCC AGTTCCCTAC AAGCAGCTCG GCCAAGTTTA TTTACTAGGG

AAGAACTGCC AGTTCTCTGC AAGCAGCTTG GCCAAGTTTA TTTACTAGGG

AAATATTGTC ATTTCCCTAC AAGCAACTTA GACAAGTTTA TTTACTAGGA

---------- ---------- ---------- -----GCTTA TGTACTAGGG

AAGAACTGCC AGTTCCCTGC AAGCAACTTG GCCAAGTTTA TTTACTAGGG

AAGAACTGCC AGTTCCCTGC AAGCAACTTG GCAAAGTTTA TTTACTAGGG

AAGAACTGCC AGTTCCCTGC GAGCAACTTG GCCAAGTTTA TTTACTAGGG

AAGAACTGCC AGTTCCCTGC AAGCAACTTG GCCAAGTTTA TTTAGTAGGG

AAGAACTGCC AGTTCCCTGG AAGCAACTTG GCTGAGTTTA TTTACTAGGG

TAGAACTGCC AGTTCCCTGC AAGCAACTTG GCCAAGTTTA TTTACTAGGG

---------- ---------- ---------- ---------- ----------

AAGAACTGGC AGTTCCCTGC AAATGACTTG GCCAAGTTTA CTTATTAGGG

AAGAACTGCC AGTTCTCTTT AAGCTCCTTG GCCAAGTTTA TCATTTAGGG

AAGAGCTGCC AGTTCCCAGC AAGCCTTTTG GCTAAGTTTA TCGTTTATCG

AAAGGGCCAA AGGAAAGAGA GTTTCCTTTG TTAAATAATG GAGCCCTGAC

AAAGGGCCAA AGGAAAGAGA GTTTCCTTTG TTAAATAATG GAGCCCTGAC

AAAGGGCCAA AGGAAAGAGA GTTTCCTTTG TTAAATAATG GAGTCCTGAC

AAAGGGCCAA AGGAAAGAGA GTTTCCTTTG TTAAATAATG GAGTCCTGAC

AAAGGGCCAA AGGAAAGAG- -TTCCCTTTG TTGAATAATG GAGCCCTGAC

AAAAGGCCAA AGGAAAGAGA GTTTCCTTTG TTGAATAATA GAGCTCTGCC

AAAGCATCAA AGGAAGGAGA GTTCCCTTAG TTAAATAACG AAGCATTGAC

AAAGCATCAA ATGAAGGAGT GTTTCCTTTG TAAAATAATG AAGCATTG--

GAAGGACCAA AGGAAAGAGA GTTTCCTTTG TTAAAGAATG GGGCTCTGAC

AAAGGGCCAA AGGAAAGACA GTTTCCTTTG TTAAATAATG GAGCTCTGAC

AAAGGGCCAA AGGAAAGAGA GTTTCCGTTG TTATATAATG GAGCTCTGAC

AAAGGGCCAA AGGAAAGAGA GTTTCCTTTG TTAAATAATG GAGCTCTGAC

AAAGGGCCAA AGGAAAAGGA GTTTCCTTTG TTAAATAATA GAGCTCTGAC

AAAGGGCCAA AGGAAAGAGA GTTTCCTTTG TTAAATAATG GAGCTCTGAC

---------- ---------- ---------- ---------- ----------

ACAGGGCCAA AGGAAAGGGA ATTTCTGTTG TTAAATAATA GAGCATGGAG

GAAGAGCCAG AGGAAAGGGC ATTCTCTTTG TTAAATATTT CAGCTCTGAC

AAAGGCCCAA GGGAAAGAAA GCCTCCCCAA ATATATAATG GAGCTTTGAC

TTAAAGCCAT GTTCCTCTCA TTAGAATGCA TTTGGCATTG ACAACGCTTC

TTAAAGCCAT GTTCCTCTCA TTAGAATGCA TTTGGCATTG ACAACGCTTC

TTAAAGCCAT GTTCCTCTCA TTAGAATGCA TTTGGCTTTG ACAACGCTTC

TTAAAGCCAT GTTCCTCTCA TTAGAATGCA TTTGGCTTTG ACAACGCTTC

TTAAAGCCAT GTTCCTTTCA TTAGAATGCA TTTGGCATTG ACAACGCTTC

TTAAAGCCAT GTTCCTCTCA TTAGAATGCA TTTGGCATTG ACAACGCTTC

TTAGAGCCAT GTTCCATTCA TTAGAATGCA TTTGGCCTTG AGAACACTCC

------CCAT CTTCTAGTCA TTGGGATTCA TTTGGCATCC AGAGCACTCC

TGAAAGCCAT GTTCCTCTCA TTAGTGTTCC TTTCCCACTG ACAACGCTTC

T--AAGCCAT GTTCCTCTCA TTAGAATTCA TTTGGCCTTG ACAACATATC

TTAAAGCCAT GTTCCTCTCA TTAGAATTCA TTTGGCATTG ACAACGCTTC

TTAAAGCCAT GTTCCTCTCA TTAGAATTCA TTTGGCACTG ACAACGCTTC

TTAAAGCCAT GTTCCTCTCA TTAGAATTCA TTTGGCATTG ACAACGCTTC

TTAAAGCCAT GTTCCTCTCA TTAAAATGCA TTTGGCATTG ACAACGCTTC

---------T GTTCCTTTCA TTAGAATTCA -TTGGCATTG ACAACGCTTC

TTAAGGCCAT GTTCCTCTCA TTAGAATTCA TTTGGCATTG ACAATGCTCC

TTAAAGGAAT GTTCAACTCA TTTTAATTCA TCTGGCATTG ACAACATTTC

CCAAAGGAAC ATTCCTCTCA TTAGAATTCA TTCGGCATTG ACGGCGCATC

ACTATCATTA TGATTCAGCG ACTGAATAAC AATAATGGGA AGTGGTTTCC

ACTATCATTA TGATTCAGCG ACTGAATAAC AATAATGGGA AGTGGTTTCC

ACTATCATTA CGATTCAGCG ACTGAATAAC AATAATGGGA AGTGGTTTCC

ACTATCATTA CGATTCAGCG ACTGAATAAC AATAATGGGA AGTGGTTTCC

ACTATCACTA TGATTCAGCG ACTGAATAAC AATAATGGGA AGTGGTTTCC

ACTATCATTA TGATTCAGCG ACTGAATAAC AATAAGAGGA ACCAGTTTGC

CCTGTCTTTA TGATTCAAC- -----AGAAC AATAGTGGGA ACCAGCTTCT

CCTATCTTTA TGATTCAGC- -----GAAAC AATGGTGGGA ACCAGTTTCT

ACTCTCCTTA TGATTCAGCG ACTGAAGAAC AATAATGGGA AACGGTTTCC

ACTATCATTA TGATTCAGCG ACTGGATAAC AATAATGGGA AACAGCTTCC

ACTATCATTA TGATTCAGCG ACTGAACAAC AATAATGGGA AACGGTTTCC

ACTATCATTA TGATCCAGCG ACTGAATAAC AATAATGGGA AACAGTTTCC

ACTATCATTA CGATTCAGCG ACTGAATAAC AATAATGGGA AATGGTTTCC

ACTATCATTA TGATTGAGCG GCTGAATAAC AATGATGGGA AATGGTTTCA

ACTATCATCA CGATTCAGCG ACTGAGTAAC AATAATGGGA AATGCTTTGG

ACTATCATCA TGATTCAGC- ------TAAC AATAATGGGA AATGCTTTCG

ACTATCATTA TGATTCAGCG TTTGGGTAAC AATAATGGAA AATGGTTTCA

ACTATCGTTA GGATTCCGCA CCTGGATTAC ACCCAGGGTA AATGGCCTCA

ACATTTTTCC ATATACCCGG GCCAGCATGT CTGGGGGCAA ATACGTAGAC

ACATTTTTCC ATATACCCGG GCCAGCATGT CTGGGGGCAA ATACGTAGAC

ACATTTTTCC ATATACCCGG GCCAGCATGT CTGGGGGCAA ATACGTAGAC

ACATTTTTCC ATATACCCGG GCCAGCATGT CTGGGGGCAA ATACGTAGAC

ACATTTTTCC ATATACCCGG GCCAGCATGT CTGGGGGCAA ATACGTAGAC

ACATTTTTCC ATATGCCCAG GCCAGCATGT CTGGGGGCAA ATACGTAGAC

ACATTTTTCC CTATACCCGC GCCAGCATGT CTGGGGGTAA ATACGTAGAC

ACGTTTTTCC CTATACCCGC GCCAGCATGT CTGGGGGCAA ATACGTAGAC

ACATTTTTCC ATATACCCGC GCCAGCATGT CTGGGGGCAA ATACGTAGAC

ACATTTTTCC ATATACCCGA GCCAGAATGT CGGGGGGCAA ATACGTAGAC

ACATTTTTCC ATTCACCCCG GCCAGCATGT CGTGGGGCAA ATACGTAGAC

ACATTTTTCC ATATACTCGG GCCAGCATGT CTGGGGGCAA ATACGTAGAC

ACATTTTTCC ATA------- ---------- ---------- ----------

ACATTTTTCC ATATACCCCG GCCAGCATGT CTGGGGGCAA ATACGTAGAC

ACATTTTTCC ATATACCCCG GCCAGCATGT CTGGGGGCAA ATACGTAGAT

ACATTTTTCC ATATAGCCCG GCCAGCATGT CTGGGGGCAA ATACGTAGAC

ACATTTTTCC ATACACCCAA AGCAGAATGT CTGGTGGCAA ATACATAGAC

GCCTTTCCCC AGATACGCGG GGCAGCATGT CTGGGGGCAA GTACGTAGAC

TCGGAGGTAG GGCCCTCGCC CTGCAGAGTA CAGAGGGGTG TGGTGTCAGG

TCGGAGGTAG G--------- ---------- ---------- ------CAGG

TCGGAGGTAG GGCCCTCGCC CTGCAGGGTA CGGAGGGGTG TGGTGTCAGG

TCGGAGGTAG GGT------C CTGCGG---A CCGCGCGGCG GGGCCTCAGG

TCGGAGGTAG GGCCCTCGCC CCACCGAGTA CCGAGGGGTG TGGTGTCAGG

TCCGAGGTAG GGCCCTCTCC CTGCCTGGTA CCGAGGGGTG TGGTGTCAGG

TCCGAGGTAG GGCCCTCTCC CCGCCTGGTA CCGAGGGGTG TGGTGTCAGG

TCCGAGGTAG GGCCCTCTCT CTGCCTGGTA CCGAGGGGTG TGGTGTCAGG

TCCGAGGTAG GGCCCTCTCC CTGCCTGGTA CCGAGGGGTG TGGTGTCAGG

TCAGAGGTAC GGCCCTCTCC CAGCCTGGTA CCGAGGGGTG TGGTGTCAGG

TCCGAGGTAG Ggcgctctcc ccgc-gggcA CCGAGGGGTG TGGTGTCAGG

TCCGAGGTAG GGCCCTCTCC CTGCCTGGTA CGAAGGGGTG TGGTGTCAGG

---------- ---------- ---------- ---------- ----------

TCCGAGGTAG GGCCCTCTCC CTGCCTGGTA CCGAGGGGTG TGGTGTCAGG

TCCGAGGTAG GGCCCTCTCC CTGCCTGGTA CTGAGGGGTG TGGTGTCAGG

TCCGAGGTAG GGCCCTCTCC CTGCCTGGTA CCGAGGGGTG TGGTGTCAGG

TCAGAGGTAG GGCAATTTCT CTGCCTGGTA CGGAGGGGTG TGGTGTTAGG

GCGGAGGTAG GCCCCCCTGG GCGCCTGGTA CAGAGGGGGA GGGTGcCAGG

GACATCTCTA CACCGTTCCC ATCCGGGAAC AGGGCAACAT CTACAAGCCC

GACATCTCTA CACCGTTCCC ATCCGGGAAC AGGGCAACAT CTACAAGCCC

GACATCTCTA CACCGTTCCC ATCCGGGAAC AGGGCAACAT CTACAAGCCC

GACATCTCTA CACCGTTCCC ATCCGGGAAC AGGGCAACAT CTACAAGCCC

GACATCTCTA CACCGTTCCC ATCCGGGAGC AGGGCAACAT CTACAAGCCC

GACATCTCTA TACCGTTCCC ATCCGGGAAC AGGGCAACAT CTACAAGCCC

GACATCTCTA CACTGTTCCC ATCCGGGAAC AGGGCAACAT CTACAAGCCC

GACATCTCTA CACTGTTCCC ATCCGGGAAC AGGGCAACAT CTACAAGCCC

GACATCTCTA CACTGTTCCC ATCCGGGAAC AGGGCAACAT CTACAAGCCC

GACATCTCTA CACTGTTCCC ATCCGGGAAC AGGGCAACAT CTACAAGCCC

GGCACCTCTA CACCGTTCCC ATCCGGGAGC AGGGCAACAT CTACAAGCCC

GACATCTCTA CACTGTTCCC ATCCGGGAAC AGGGCAACAT CTACAAGCCC

---------- ---------- ---------- ---------- ----------

GACATCTCTA CACCGCTCCC ATCAGGGAAC AGGGCAACAT CTACAAGCCC

GACATCTCTA TACCGCTCCC ATCCGGGAAC AGGGCAACAT CTACAAGCCC

GACATCTCTA CACCCTTCCC ATCCGGGAGC AGGGCAACAT CTACAAGCCC

GGCTTCTCTA CTCGGCGCCC ATCCGAGAAC AGGGAAATAT CTACAAGCCC

GGCTGCTCTA CTCCGTGCCC AGCCGGGACC AGGGCAACAT CTACAAGCCG

AACAACAAGG CCATGGCAGA CGAGCTGAGC GAGAAGCAAG TGTACGACGC

AACAACAAGG CCATGGCAGA CGAGCTGAGC GAGAAGCAAG TGTACGACGC

AACAACAAGG CCATGGCAGA CGAGCTGAGC GAGAAGCAAG TGTACGACGC

AACAACAAGG CCATGGCAGA CGAGCTGAGC GAGAAGCAAG TGTACGACGC

AACAACAAGG CCATGGCAGA CGAACTGAGT GAGAAGCAAG TTTACGACGC

AACAACAAGG CCATGGCAGA GGAGGTGAAC GAGAAGCAAG TCTACGACGC

AACAACAAGG CCATGGCAGA CGAGGTGAAT GAGAAGCAAG TGTACGACGC

AACAACAAGG CCATGGCAGA CGAGGTGACT GAGAAGCAAG TGTATGACGC

AACAACAAGG CCATGGCCGA CGAGGTGAAC GAGAAGCAAG TGTACGACGC

AACAACAAGG CTATGGCAGA GGAAATGAAC GAGAAGCAAG TGTACGACGC

AACAACAAGG CCATGGCGGA GGAGATGAGC GAGAAGCAGG TGTACGACGC

AACAACAAGA CCATGGCAGA CGAAATGAAC GAAAAGCAAG TGTACGACGC

---------- ---------- ---------- ---------- ----------

AACAACAAGG CCATGGCAGA GGAGATGAAT GAAAAGCAAG TGTACGACGC

AACAACAGGG ACATGGCAGA GGAGATGCAC GAGAAGCAAG TGTACGACGC

AACAACAAGG CCATGGCCGA AGACATGAAC GAGAAGCAAG TGTACGACGC

AACAATAAGA CCATGGCCGA CGAAATGAAC GAGAAGCAAA TGTACGATGC

CACAACAAGA CCATGGCAGA CGACCTGCCC GAGAAGCAGC TGTATGACGC

GCACACCAAG GAGATCGACC TGGTCAACCG CGACCCTAAA CACCTCAACG

GCACACCAAG GAGATCGACC TGGTCAACCG CGACCCTAAA CACCTCAACG

GCACACCAAG GAGATCGACC TGGTCAACCG CGACCCTAAA CACCTCAACG

GCACACCAAG GAGATCGACC TGGTCAACCG CGACCCTAAA CACCTCAACG

GCACACCAAG GAGATCGACC TGGTCAACCG CGACCCCAAA CACCTCAACG

GCACACCAAA GAGATCGACC TGGTCAACCG CGACCCCAAG CATCTCAACG

GCACACCAAG GAGATTGATC TGGTCAACCG CGACCCCAAG CATCTCAACG

GCACACCAAG GAGATTGACC TGGTCAACCG CGACCCCAAG CATCTCAACG

GCACACCAAG GAAATCGACC TGGTCAACCG CGACCCCAAG CATCTCAACG

GCACACCAAG GAGATAGATC TGGTCAACCG CGACCCCAAG CATCTCAACG

GCACACCAAG GAAATCGACC TGGTCAACCG CGACCCCAAG CATCTCAACG

GCACACCAAG GAGATCGACC TGGTCAACCG CGACCCCAAG CATCTCAACG

---------- ---------- ---------- ---------- ----------

GCACACCAAG GAGATCGACC TGGTCAACCG CGACCCCAAG CATCTCAACG

ACACACCAAG GAGATCGACC TGGTCAATCG CGACCCCAAG CATCTCAACG

GCACACCAAG GAGATCGACC TGGTCAACCG CGACCCCAAG CATCTCAACG

ACACACCAAG GAGATCGACC TGGTCAACAG AGACCCCAAG CATCTCAATG

GCACACCAAG GAGATCGATC TGGTCAACCG GGACCCCAAG CACCTCAACG

ATGACGTGGT CAAGGTAAGC CAACAGATGT GGGTCTCCAC ACTAGCATGA

ATGACGTGGT CAAGGTAAGC CAACAGATGT GGGTCTCCAC ACTAGCATGA

ACGACGTGGT CAAGGTAAGC CAACAGATGT GGGTCTCCAC ACTAGCATGA

ACGACGTGGT CAAGGTAAGC CAACAGATGT GGGTCTCCAC ACTAGCATGA

ACGATGTGGT CAAGGTAGGC CAGCAGATGT GGGTCTCCAC ACTGGCATGA

ACGACGTGGT CAAGGTAAG- CCGCAGATGT GGATCTCCAC ACCAGCATGA

ACGACGTGGT CAAGGTAAAC CAGCAGATGT GGGTCTCCAC ACGAGCATGA

ACGACGTGGT CAAGGTAAGC CAGCAGATTT GGGTCCCCAC ACTAGCATGA

ACGACGTGGT CAAGGTAGGC CAGCAGATGT GGGTCTACAC ACCAGCATGA

ACGACGTGGT CAAGGTAAGC CAGTAGATGT GGGTCTCTAC ACTAGCATGA

ACGACGTGGT CAAGGTAGGC CAGCAGATGT GGGTCTCCAC ACTAGCATGA

ACGACGTGGT CAAGGTAAGC CAGCAGCTGT GGGTCTCCAC ACCAGCATGA

---------- ---------- ---------- ---------- ----------

ACGACGTGGT CAAGGTAAGC CAGCAGATTT GGGTCTCCAC ACCAGCATGA

ACGACGTGGT CAAGGTAAGC CAGCAGATCT GGGTCTCCCC ACTAGCATGA

ACGACGTGGT CAAGGTAAGC CAGCCGCTCT GGGTCTCCCC ACTAGCATGA

ACGATATGGT CAAGGTAAGC CAGCTGATTT GGGTCTCCAC ACCAGCATGA

ACGATGTGGT CAAGGTCGGC CGGCAAATTT GGGCCTCCCC ACCAGCGTGA

CTAAGTGGGA AAAGAGACTT AACACAGATG GAAAAAAAAC ATAACAGAGT

CTAAGTGGGA AAAGAGACTT AACACAGATG GAAAAAAAAC ATAACAGAGT

CTAAGTGGGA AAAGAGACTT AACACAGATG GAAAAAAAAC ATAACAGAGT

CTAAGTGGGA AAAGAGACTT AACACAGATG GAAAAAAAAC ATAACAGAGT

CTAAGTGGGA AAAGAGACTT AACACAGATG GAAAAAAAAC TTAACAGAGT

CTAAGTGGGA AAAAAGACCT AACACAGATG GAAAAAAAAC ATAACAGAGT

CTAAGTGGGA AAAGAAACTT GACACAGGTG GAAAAAAGGC ATAACGGAGG

CTAAGTGGGA AAAGAGACTT AACACAGATG GAAAAAAGGC ATAACAGAGT

CTCAGTGGGA AAAGA----- --------TG GAAAAAAGGC ATAGCCAAGT

CTAAGTGGGA AAAGAGACTT AACACAGATG GAAAAAAGGC ATAACAGAGT

CTAAGTGGGA AAAGAGACTT AACGCAGATG GAAAAAAGGC ATAACAGAGT

CTAAGTGGGA AAAGAGACTT AATGCAGATG GAAAAAAGGC ATAACAGAGT

---------- ---------- ---------- ---------- ----------

CTAAGTGGGA AAAGAGACTT AACGCAGATG GAAAAAAAGC CTAACAGAGC

CTAAGTGGGA AAAGAGACTT AACCCAAATG GAAAAAACGC ATAACAGAGT

CTAAGTGGGA AAAAAAACTG AACCCAGATG GAGAGAAAGC ATAGAGGAGT

CTAAATGGGA AAAGAGACTT AACATAGATG GAAAAAAAGC ATAACAGAGT

CTAAATAGCA AAAGAGACCA AACCCACATG GAAAAAGAGC ACGGCAGAGT

TTACAAAACA CATTTCAAAG AGCAAGTGTG GGGATCCTCA AATTAAAGAA

TTACAAAACA CATTTCAAAG AGCAAGTGTG GGGATCCTCA AATTAAAGAA

TTACAAAACA CATTTCAAAG AGCAAGTGTG GGGATCCTCA AATTAAAGAA

TTACAAAACA CATTTCAAAG AGCAAGTGTG GGGATCCTCA AATTAAATAA

TTACAAAACA CATTTCAAAG AGCAAATGTG GGGATCCTCA AATTAAAGAA

TTACAAAATA CATTTCAAAG AGCAAGTGTG GGGATCCTCA AATTAAAGGA

TTACAGACTA CATGTC-AAG CGTGACTGTG GGGATCCTCA AATTAGAGAA

TTACAAAATA TACGTC-AAG AGTGAGTGTG GAGCTCCTCA AATTAGAGAA

TTACAAAATA TGTGTCAAAG AGCAAGTATG GGGATCCTCA AATTAAAGAA

TTACAAAATA CATTTCAAAG AGCAAGTGTG GGGATCTTCA TATTAAATAA

TAACAGAATA CATTTCAAAG AGCAAGTGTG GGGATCCTCa tattaaaaaa

TTACCAAATG CATTTCAAAG AGCAAGGGTG AGGATCCTCA TATTAAAGAA

---------- ---------- ---------- ---------- ----------

TTACAAAATA TATTTCAAAG AGCAAATGTG GGGATCCTCA AATTAAAGAA

TTACAAAATA CATTTCAAAG AGCAAGTGTT GGGATCCTCA AATTAAAGAA

TTACAAAGTA CATTTCAAAG GGCAAGTGTG GGGATCCTGA CATTAAAGCA

TTACAAAACA AACTCCAAAG GGCAAGTATA GGGATCCTCA GACCAAATAA

TTACCCGACA CATGCAGGAG GCAAGGGATA GGAATTCTCC AACTGAAGCC

ATTAAAAGAA AAATAAATGT TTAAAATAGT TTAACTATTT TTCAGTTAGT

ATTAAAAGAA AAATAAATGT TTAAAATAGT TTAACTATTT TTCAGTTAGT

ATTAAAAGAA AAATAAATGT TTAAAATAGT TTAACTATTT TTCAGTTAGT

ATTAAAAGAA AAATAAATGT TTAAAATAGT TTAACTATTT TTCAGTTAGT

ATTAAAAGAA AAATAAATGT TTAAAATAGT TTAACTATTT TTCAGTTAGT

ACTAAAAGAA ACAAAAATGT TTAAAATA-- -------TTT TTCAATTGGT

AT----CAGA GGAAAAATGT TTAAAATCAC TTAACTATTT TTCAGTTAGT

AT----TAGA AGAAAAATGT TTAAAATCAC TTAACTATTT TTCAGTTAGT

ATTAAACAAA AAAAAAATGT TTAAAATAGT TTAAGTATTT TTCAGTTAGT

ATTAAAAGGA GGAAAAATGT TTAAAATAGT TTGATT-TTT TTTATTCAGT

attaaaaGGA AGAA-AATGT TTAAAATAGT TTAACTATTT TTCAGTTAGT

ATTAAGAGGA AGAAAAGTGT TTAAAATAGT TTAACTATTT TTCTATTAGT

---------- ---AAAATGT CTAAAATAGT TTTA---TTC TTTAGTTGGT

ATTAAAAGGA AGAAAAATGT TTAAAATAGT TTAACTATTT TCCGTTTAGT

ATTAGCAGGA AGAAAAATGT TTAAAATAGT TTAACTATTT TTCAGTTAGT

ACCAGAAGGA AGAAAACAGT TGAAAATAGT TTGACTGTTT TTCAGTTACT

ATTAAAAGGA AGTAAAAGGT TGaaaataat ttaaatatta ttcaattaat

ATGAAAAGGA AGGaaagtgc tttgaaTCAA CTAACTATTA ATGTGTTAGC

CCAGGAAACA TAAAATGGCA TGCTTGCACA TAAACCATTG TTTAGGGTGG

CCAGGAAACA TAAAATGGCA TGCTTGCACA TAAACCATTG TTTAGGGTGG

CCAGGAAACA TAAAGTGGCA TGCTTGCACA TAAACCATTG TTTAGGGTGA

CCAGGAAACA TAAAGTGGCA TGCTTGCACA TAAACCATTG TTTAGGGTGG

CCAGGAAACA TAAAATGGCA TGCTTTTACA TAAACCATTG TTTAGGGTGG

CCAGGAAATA TAAAATGGCA TGCTCTCACA TGAACTATTG TTTGGGGTGG

CCAGGAAACA TAAACCAGCA TACTTTCACA TGAACCATTG TTTAGGGAGG

CCAGGAAACA TAAAACAGCA TGCTTTCACA TGAGCCATTG TTTAGGGAGG

CCAGGAAACA TAAAACAGCA CGCTCTCAAA TGAACCATTG TTTAGGGTGG

CCAGGAAACA TAAAATGGCG TGCTTTCATA TGAGCCATTG TTTAGCGTGG

CCAGGAAACA TAAAATGGCA TGCTTTCACA CGAACCATTG TTTAGGGTGG

TCAGGAAACA AAAAATGGCA TGCTTTCACA -GAACCATTG TTTCAGGTGG

CCAGGAAACC TAAAATGGCA TGCTTTCACA CAAGCCATTG TTTAGG--GT

CCAGGAAACA TAAAATCTCA TGCTTTGACA CGAACCATTG TTTAGGGTGG

CCAGGAAACA TACAATGGTA TGCTTTCATA GGAGCTATTG TCTAGGGT-G

CCAGGAAACA TAAAATGGCA TGCTTTCATA CAACCCATTG TTTAGGGTTG

tCAGGAAACA TAAAATTGTA TGGTATTACA CAAACCATTG TTTAGGGTGG

TTGGGAAACC TGAAATAGTA GAGTTTTCCA CGAACCATTG TTTGGGGTGG

GGGAAGTGTT TTTAATTTTG CCTTAAAGGA AATCTGCATG ATCCACAGGC

GGGAAGTGTT TTTAATTTTG CCTTAAAGGA AATCTGCATG ATCCACAGGC

GGGAAGTGTT TTTAATTTTG CCTTAAAGGA AATCTGCATG ATCCACAGGC

GGGAAGTGTT TTTAATTTTG CCTTAAAGGA AATCTGCATG ATCCACAGGC

GGGAAGTGTT TTTAATGTTG CCTTAAAGGA AATCTGCATG ATCCACAGGC

GGAAAGTGTT TTTAATTTTG TCTTAAAGGA AATCTGCGTG AGCCATGTAC

GGGAAATGTT TTTAATTCTG TCTTAAAGGA AATCTGCATG ATCCATGTAC

GGGAAATGTT TTTAATTCTG TCTTAAAGGA AATCTGCATG ATCCATGTAG

GGGAAGTGTT TTTCATTTTA TCTTAAAGGA AATCTGCATG ATCCATGTAC

GGGAAGTGCT TTTAATTTTG TCCTAAAGGA AATCTGCATG ATCCATGTAC

GGGAAGTGTT TTTAATTTTG TCTTAAAGGA AATCTGCATG ATCCATGTAC

GGGAAGTATT TTTAATTTTG TCTTAAAGGA AATCTGCATG ACCCATGTAC

GGGAAGTATT TTCAATTTTG ------AGGA AATCTGCATG ACCCATATAC

GGGATGTGTT TGTAATTTTG TCTTAAAGGA AATCTGCATG ATCCATGTAC

GGGAAGTGTT TTTAATTTTG TCTTAAAGGA AATCTGCACG ATCCATGTAC

GGGAAATGTT TTTAATTTTG TCTTAAAGGA AATCTGCATG ATCCATGTAC

GGAAAATATT TTTAATTTTG TCTTAAAGGA AATCTGCATG ATTCGTGTTC

GGGAAATGGG TTGAATTTTT TCTTAAAGGA AATCTGCACA AGCAATCTAC

TATGAACTAC CAAGGGAATT AGTTGGTAGA ACAGAATTAC ACCTGCACAG

TATGAACTAC CAAGGGAATT AGTTGGTAGA ACAGAATTAC ACCTGCACAG

TATGAACTAC CAAGGGAATT AGTTGATAGA ACAGAATTAC ACCTGCACAG

TATGAACTAC CAAGGGAATT AGTTGATAGA ACAGAATTAC ACCTGCACAG

TATGAACCAC CAAGGGAATT AGTTGGTAGA ACAGAATTAC ACCTGCACAG

TATGAGCTAC CAAGGGAATT AGCTGGTAGA ACAGATTTAC ACCTGCACAG

TGGGGAGTAC CAAGGGATTT AGCCAATAGA ACAGAGTTTC ACCTGCAGAG

TGGGGAGTAC CAAGGGATTT AGCCAGTAGA ATAGAATTAC ACCTGCAGAG

TGTGAACTAC CCAGGGAATT AGCTGGTAGA ACAGACTGAC ACCTGCACAG

TGTGAACTAC CAAGGGAATT AATTGGTAGA ACAGATTTAC ACCTGCACAT

TGTGAACTAC CAGGGGAATT AATTGGTGGA ACAGATTTAC ACCTGCAAGG

TATGAAGTAC CAAGGGAATT AATTGGTACA ACAGATTTAC ACTTGCACAG

TGTGAAGTAT CAAGGGAATT AA----TAGA ATAGATTTAT ACCTGCAGAG

TATAAACTAC CGAGGGAATT AATTGGTAGA ACAGATTTAC ACCTGCACAG

TATGAACTAC CAAGGGAATA AATGGGTAGA AAAGATTGAC ACCTGCACAG

TATGAATTAC CAAGGGAATC AATGGGTAGA ACAGATTCAC ACCTGCACAG

TATGAAATAC CCAGGGAATT AACTGGTAGA ACAAATTTAC ATCTGCACAG

CATGAAACAT CCAGGGAATT AACTGGTAGA ACAGATTTGC ACCTGCACAG

AATACATTTC CTGCCTTTCA TGGGAACTAT GTTGATGTTT CAGATATGAA

AATACATTTC CTGCCTTTCA TGGGAACTAT GTTGATGTTT CAGATATGAA

AATACATTTC CTGCCTTTCA TGGGAACTAT GTTGGTGTTT CAGATATGAA

AATACATTTC CTGCCTTTCA TGGGAACTAT GTTGGTGTTT CAGATATGAA

AGTACATTTC CTGCCTTTCA TGGGAACTAT GTTGATG-TT CAGATATGAA

AATACATTTC CTGCCTCTCA TGGGAACTAT GTTGATGTTT TAGATATAAA

ACTACATTTC CTGCCTCTCA CGGAAACTGT GCTGATGGTT CAGATAGAAA

AACACATTTC CTGCCTCTCA GGGAAACTGT GTTGATGGTT CAGATATAAA

AATACACTTC CTGCCTCTCA TGGGAACTCT GTTGATGTTT CAGATATAAA

AATACATTTC CTGCCTCTC- ------CTAT GTTGATGTTT TAGAAATAAA

AATACATTTC CTGCCTCTCA TGGGAACTAT GTTGATGTTT CAGATATAAA

AGTACATTTC CTGCCTCTTA TGGGAACCAT GCTGATGTTT CAGATATAAA

GATATACTTC TTACCTCTGC TGGGAAC--T GTTGATGTTT CAGGTATAAA

AATACACTTC CTGCCTCTCA TGGGAAATAC GTTGATGTTT CAGTTATAAA

AATACATTTC CTGCCTCTCA CGGGAACTAC ATTGATGTTT CAGATATAAA

AACACACTTC CTGCCTTCCG TGGGAACTGT GTTGGTGTTT CAGATATAAA

AATACATTTC CTGTCTCCCA CAGGAACTAC ATCAATGTTT AGGATATGAA

AATGTACTTC CTGCTTTTCA CAGGAACTGC CCCAATCTTT CAAGTGAGAA

ATACATCTTG TTTTCTTTAT TGATCTGAGA CTTCCTCCCT AGTAATTACA

ATACATCTTG TTTTCTTTAT TGATCTGAGA CTTCCTCCCT AGTAATTACA

ATACATCTTG TTTTCTTTAT TGATCTGAGA CTTCCTCCCT AGTAATTACA

ATACATCTTG TTTTCTTTAT TGATCTGAGA CTTCCTCCCT AGTAATTACA

ATACATCTTG TTTTCTTCAT TGATCTGAGA CTTCCTCCCT AGTAATTACA

ATACATCTTG TTTTCTTTAC TGATCTGAGA CTTCCTCCCT TGTAATTACG

ATACATCTTG ATTTCTTTAT TGATCTGAGA CTTCCTCCCT GTGCATGCCA

GTACATCTTG CTTTCTTTAT TGATCTGAGA CTTCCTCCCT GGGCATTACA

ATACATCTTG TTTCCTTTAT TGATCAGAGA CTTCCTCCCT AGGCATTATA

ATACATTTTG TTTTCTTTAC TGATCTGAGA CTTCCTCCCT A-TAATTACG

ATACATCTTG TTTTCTTTAT TGATCTGAGA CTTCCTCCCT AGTAATTATG

ATACATCTTG TTTTCTTTAT CAATCTGAGA CTTCCTCCCT AGTAATTCTG

ATACATTATG CTTTTGTTAT tgaTCAGAGA CTTCCTCCCT AGTAATTACA

ATGCACTGTG TTTGCTTTGT TGTTGTAAGA CTTCCTCCCT AGTAATTACA

ATGCATCTTG TTTTCTTTAT TGTTCTGAGA CTTCCTCCCT AGTAATTACG

AGGTATCTTG TTTTCTTTCT TGTTCTGTGA CTTCCTCCTT AGTAATTATG

ATGTCCCTTG CTTTCATTAT TGTTCTAGCA TTTGATCTCC AGTAATCATG

ACGCCTCTTG TTTTCACTAT TGTTCCAGGA TTTCCTCTCC AGTGATTAGG

TGTTCTCAAA AACAAATGCC AGAAGGAAGA AGCAGATTTA ATAGTGCATT

TGTTCTCAAA AACAAATGCC AGAAGGAAGA AGCAGATTTA ATAGTGCATT

TGTTCTCAGA AACAAATGCC AGAAGGAAGA AACAGATTTA ATAGTGCATT

TGTTCTCAAA AACAAATGCC AGAAGGAAGA AACAGATTTA ATAGTGCATT

TGTTCTCAAA AACAAATGCC AGAAGCAAGA AACAGATTTA ACAGTGCATT

TGTTCTCAAA AACAAATGCC AGAAGCAAGG AACAGACTCA ATAGTGCATT

TGTTCTCAGA AACAAATGCC AGAAACAAAG -ACAGACTTA ATAGTGCATT

TGTTCTCA-A AACAAATGCC TGAAACAAAG -ACAGACTTA ATAGTGCATT

TGTTCTCAGA AACAAATGCC AGAAGCAAGG AATAGGCTTA ATAGTTCATT

TGTTCTCAAA AACAAATACC AGAAGTAAGG AACAGACTTA ATAGTGCATT

TGTTCTCAAA AACAAATGCC AGAAGTAAGG AACAGACTTA ATAGGGCATT

TGTTCTCAAA AACAAACGCC AGAAGTAGGG AACAGACTTA ACAGCACATG

TGTTCTCAAA AACAAATGCC AGAAGTAAGG AACAGACTTA ATAGGGCATT

TGTTCTCAAA AACAAACGCC AGAAGTAAGG AGCAAACTTA ATAGTGCATT

TGTTCTCAAA AACAAATGCC AGAAATAAAG AACAAACTTA ACAGTGTGTT

TGTTCTCAAA AACAAATGCC AGAAGTAAAG ACTAGCCATA GTGGTGCATT

TGTTCTCAAG CATGAATGCC AGAAGTAATG AACAAAACCA AAAGCACAAT

TGTTCCCTGA CACAAATACC AAGAGAAAGG AACAGACCCT CCAGTGCCTT

TTACAAGGCC CATTAATAAA GGTCAGCATG TTCAAACCTG CAGAGATTTA

TTACAAGGCC CATTAATAAA GGTCAGCATG TTCAAACCTG CAGAGATTTA

TTACAAGGCC CATTAGTAAA GGTCAGCATG TTCAAACCTG CAGAGATTTA

TTACAAGGCC CATTAGTAAA GGTCAGCATG TTCAAACCTG CAGAGATTTA

TTACAAGGCC CATTAATAAA GGTCAGCATG TTCAAACCTG CAGAGATTCA

TTACAAGACT CACTAGTAAA GGTCAACGTC TTCAAACCTG CAGGGATTTA

TTCCAAGGCG CATTAATAAA GGTCAGCATG TTCAGGCCTA CAGAGATTTA

TTATAAGGCG CATTAATAAA GGTCAGCATG TTCAGGCCTG CAGAGATTTA

TTACAAGGCC TATTCATAAA GGTCAGAATG ---------- ---AGATTAA

TTACAAGGCC CATTAATAAA GGTCAGTATG TTCAAACCTG CAGAGATTTA

TTACAAGGCC CATTAATAAA GGTCAGTATG TTCAGACCTG CAGAGATTTA

TTACAAGGCC CATTTATAAA GGTCAGCATG TTCAAACCTG CAGAGATTTA

TTACAAGGCC CATTCATAAA GGTCACTATG TTCCAACCTG CAGAGATTTA

TTTCAAGGCC CATTAATAAA GGTCAGTATG TTCAAACCTG CAAAGGTTTA

TTACAAGGCC CATTAACAAA GGTCAGTATG CTCAAACCTG CGGAGATGTA

TTACAAGGCT CATTAATAAA GGTCAGCGTG C--------- -GGAGCTGTA

TTACAAGCTC CATTAATAAA GGTCAGCATG TTCAAACTTG CAGAGATTTA

TCCCAAAGCC CATTAATAAA GGTCAACACG CTCCAACCTG TGGAGATCTG

TTTCATTCAC TGTGTGTTTA GTAAACAGAT TTCAAATATT GCCTAGATAC

TTTCATTCAC TGTGTGTTTA GTAAACAGAT TTCAAATATT GCCTAGATAC

TTTCATTCAC TGTGTGTTTA GAAAACGGAT TTCAAATATT GCCTAGATAC

TTTCATTCAC TGTGTGTTTA GTAAACGAAT TTCAAATATT GCCTAGATAC

TTTCATTCAC TATGTGTTTA ATAAACAGAT TTCAAATATT GACTAGATAC

TTTCATGCAC TATTTGTTTA ATAAACAGAT TTTAAATATT GCCTAGATAC

TTTAAATCAC TATTTGTTTA ATAAACAGA- ---------- ----------

TTTCATTTAC TATTTGTTTA ATAAACAGAT TTAAAATATT ACCTAGATGA

TTCCTTTCAC TGTGTGTTTA GTAAACAGAT TTAAAATATT GCCTAGATAC

TTTCATTCAC TATTTGTTTA ATAAACAGAT TTTTAATATT TCCAACATAC

TTTCGTTCAC TATGTGTTTA ATAAACAGAT TTTAAATATT GCTGACATAC

TTTCATTCAC TATCTGTTTA ATAAACAGAT TTAAAATATT GCCAAGATAC

TTTCATTCAT TATTTGTTTA ATAAACAAAT TTTAAATATT GTCTAGATAT

TTTCATTCAC TATTTGTTTA ATAAACAGAT TTAAAATATT GCCTGGATAC

TTTCATTCAC TATTTGTTTA ATAAACAGAT TTAAAATATT GCCTAGATAC

GTTCATTCAC TACTTGTTTA ATAAACAGAT ATAAATGATT GCCTAGTTAC

TTTCATTCAA TATTTGTTTA ATAAACAGAT TTAAAATATT GCCTAGATGT

TTTTCTTAAA TGGATATTAC ATGAATGAAT TAGAAATAAT GTCTAGATGT

ATTGAACAGA ATGAAATCTC TGAGATTTAA ATGTAAACTG TTAACTCCTT

ATTGAACAGA ATGAAATCTC TGAGATTTAA ATGTAAACTG TTAACTCCTT

ATTGAACAGA ATGAAA--TC TGAGATTTAA ATATAAACTG TTAACTCCTT

ATTGAACAGA ATGAAA--TC TGAGATTTAA ATATAAACTG TTAACTCCTT

ATTGAACAGA ATGAAATCTC TGAGATTTAA ATGTAAACTG TTGACTCTTT

ATTTAACAGG ATGAAATCTC TGAGATTTAA ATATAAACTG TTGGCTCCTT

---------- ---------- ---------- ---------- ----------

ATTTAACTGG ATGGGATATC TGAGATTTAA ATATAAGCTT TTGACTCCCG

ATTTAACAGG AAGAAATCTC TAAGATTTAT ATATAAACTG TTGACTCCTT

ATTTAACAAA ATGAC----- ---------A ACATAAACTC TTGACTCCTT

TTTTAACAAG ATGAAACCTC TTAGATTTAA ATATAAACTG TTGGTTCCTT

ATTTAACAAG ATGAAAGCTC TGAGATTTAA ATATAAACTG TTGACTCCTT

GTTTAACAAG ATGAAATTTC TGAGATTTAA ATATACACTG TCAACTTCTT

ATTTAATAGG ATGAAATCTC TGAGATTTAA ATATAAATAG TTGACTCCTT

ATTTAACAGG ATGAAATCAC TGAGATTTAA ATGTAAACTG TTGACTCCTT

AATGAACAG- ATGCC-TCCC TGAGCTTTAA ACATAAACGG TTGGCTCCTT

ATTTAACAGG ATGCAATCTC TGAGATTAGA GTATAAACTG TTGGTTCCTC

GTTTAACCAG ACCCAGTCCC TGAGACTGAA ACATCAACTG TTGACTCTTA

ATAAAGCATC TGTTGACACA CAAAAATATC ATTTTAGATT GACTTTGAAG

ATAAAGCATC TGTTGACACA CAAAAATATC ATTTTAGATT GACTTTGAAG

ATAAAGTGTC TGCTGAGACA CAAAAATATC ATTTTAGATT GACTTTGAAG

ATAAAGTGTC TGCTGAGACA CAAAAATATC ATGTCAGCTG GGCTTCGAGG

ATAAAGCATC TACTGAGACA CAAAAATATC ATTTTAGATT GACTTTGAAG

ATAAAGCATC TGTTAAGACA CAGAAATATC ATTTTAGATT GACTTTGAAG

---------- ---------- ---------- -TTTCAGATT GACTTTGAAG

ATAAATCATC CACTGAGACA CAGAAATATT ATTTCAGATT GACTTTGAAG

ATAAAATACC TCTTGAGACA CAGAAATATC ATTTTAGATC GACTTTGAAG

ATAAAGCATC TATTAAGACA TACAAAT--- ATTTCAGATT GATTTTGAAG

ATAAAGCATC TATTAAGACA TAGAAATATC ATTGCAGATT GATTTTGAAG

ATAAAGCATC TAATACGACA GAGAAATATC ATTTCAGATT GATTTTGAAG

ATAAAGCATC TGTTAGGACA CAGAAATATC ATTGTAGATT GATTTTGAAG

ATAAAGCATC TATTAAGACA CAGAAACATC ATTTTAGATT GATTTTGAAG

ATAAAGCATC TATTAAGACA CAGAAATATC ATTTTAGATT GATTTTGAAG

AT-------- ---TAAGACA CAGAACTATC ATTTTAGATT GACTTCGAAG

ATAAAGCATC TCTAAAGACA AAGAAATGTC ATTTTAGATT GATTTTGAAG

ATAAAGCATC CATGAAGACT GAGGAATGTC ATCTCAGATC GACTTCGAAG

ATGTGATTGC AGAACCAGAA GGGACACACA GTTTTGACGG CATTTGGAAG

ATGTGATTGC AGAACCAGAA GGGACACACA GTTTTGACGG CATTTGGAAG

ATGTGATTGC AGAACCAGAA GGGACACACA GTTTCGATGG CATTTGGAAG

ATGTGATCGC AGAGCCGGTG ACTACGCACT CCTTTGACAA AGTGTGGATC

ATGTGATTGC AGAACCAGAA GGGACACACA GTTTCGACGG CATTTGGAAG

ATGTGATTGC AGAACCAGAA GGGACACATA GTTTTGATGG CATTTGGAAG

ATGTGATTGC GGAACCAGAA GGGACACACA GTTTCGACGG CATCTGGAAG

ATGTGATTGC AGAACCAGAA GGGACACACA GTTTCGACGG CATCTGGAAG

ATGTGATTGC AGAACCAGAA GGGACACACA GTTTCGACGG CATCTGGAAG

ATGTGATTGC AGAACCAGAA GGAACACACA GTTTCGATGG CATCTGGAAG

ATGTGATTGC AGAACCAGAA GGAACACACA GTTTTGATGG CATCTGGAAG

ATGTGATTGC AGAGCCAGAA GGGACACACA GTTTTGACGG CATCTGGAAG

ATGTGATCGC TGAACCAGAA GGAACACACA GTTTTGATGG CATCTGGAAG

ATGTGATTGC AGAACCAGAA GGGACACACA GTTTTGATGG CATCTGGAAG

ATGTGATTGC AGAACCAGAA GGGACACACA GTTTCGATGG CATCTGGAAG

ATGTAATTGC AGAGCCAGAA GGGACTCACA GTTTCGACGG CATCTGGAAG

ATGTGATAGC AGAACCAGAA GGAACACACA GCTTTGATGG AATCTGGAAG

ACGTGATTGC CGAGCCCGAG GGCACACACA GCTTTGACGG GATCTGGAAG

GCCAGCTTCA CCACCTTCAC TGTGACGAAA TACTGGTTTT ACCGCTTGCT

GCCAGCTTCA CCACCTTCAC TGTGACGAAA TACTGGTTTT ACCGCTTGCT

GCCAGCTTCA CCACCTTCAC TGTGACGAAA TACTGGTTTT ACCGCTTGCT

TGCAGCCATG CCCTCTTTGA AATCAGCAAA TACGTAATGT ACAAGTTCCT

GCCAGCTTCA CCACCTTCAC TGTGACGAAA TACTGGTTTT ACCGCCTGCT

GCCAGCTTCA CCACCTTCAC TGTGACAAAA TACTGGTTTT ACCGCTTGCT

GCCAGCTTCA CCACCTTCAC TGTGACAAAA TATTGGTTTT ACCGCTTGCT

GCCAGCTTCA CCACCTTCAC TGTGACAAAA TATTGGTTTT ACCGCTTGTT

GCCAGCTTCA CCACCTTCAC TGTGACGAAA TACTGGTTTT ACCGCCTACT

GCCAGCTTCA CCACCTTCAC TGTGACAAAG TACTGGTTTT ACCGTTTGCT

GCCAGCTTCA CCACCTTCAC TGTGACAAAA TACTGGTTTT ACCGCTTGCT

GCCAGCTTCA CCACCTTCAC TGTGACAAAG TACTGGTTTT ACCGCCTGCT

GCCAGCTTCA CCACCTTCAC TGTGACGAAA TATTGGTTTT ACCGCTTGCT

GCCAGCTTCA CCACCTTCAC CGTGACGAAA TACTGGTTTT ACCGCTTGCT

GCCAGCTTCA CCACCTTCAC TGTGACAAAA TACTGGTTTT ATCGCTTGCT

GCCAGCTTCA CCACCTTCAC TGTGACGAAA TACTGGTTTT ACCGTCTGCT

GCAAGCTTTA CCACCTTCAC TGTGACGAAA TACTGGTTTT ACCGCTTGCT

GCCAGCTTCA CCACCTTCAC CGTGACAAAA TACTGGTTTT ACCGCTTGCT

GTCTCTTTGG CATCCCGATG GCACTCATCT GGGGCATTTA CTTCGCCATT

GTCTCTTTGG CATCCCGATG GCACTCATCT GGGGCATTTA CTTCGCCATT

GTCTCTTTGG CATCCCAATG GCGCTCGTCT GGGGCATTTA CTTCGCCATT

GACTCCTGGC CATTCCCCTG GCCTTCATTG CGGGAATTCT CTTTGCCACC

GTCTCTTTGG CATTCCGATG GCGCTCATCT GGGGTATTTA CTTTGCCATT

GTCTGTTCGG CATCCCAATG GCACTGATCT GGGGCATTTA CTTCGCCATT

GTCTCTTCGG CATCCCTATG GCGCTCATCT GGGGCATCTA CTTTGCCATC

GTCTCTTCGG CATCCCAATG GCACTCATCT GGGGCATTTA CTTTGCCATT

GTCTCTTTGG CATCCCAATG GCACTCATCT GGGGCATTTA CTTTGCCATT

GTCTCTTTGG CATCCCAATG GCACTCATCT GGGGCATTTA CTTTGCCATT

GTCTCTTTGG CATCCCAATG GCACTCATAT GGGGCATTTA CTTTGCCATT

GTCTCTTTGG CATCCCGATG GCCCTCATCT GGGGCATTTA CTTTGCCATC

GTCTCTTTGG CATCCCAATG GCACTCATCT GGGGCCTTTA CTTTGCCGTT

GTCTCTTTGG CATCCCAATG GCACTCATAT GGGGCATTTA CTTTGCCATT

GTCTCTTCGG CATCCCGATG GCGCTCATCT GGGGCATTTA CTTCGCCATT

CTCTCTTTGG CATCCCGATG GCACTCATCT GGGGCGTCTA CTTTGCCATC

CTCTCTTCGG CATACCAATG GCACTCATCT GGGGCATTTA CTTTGCCATC

CTCTCTTGGG CATCCCCTTG GCCCTCCTCT GGGGCATCTA CTTTGCCATC

CTCTCTTTCC TGCACATCTG GGCAGTTGTA CCATGCATTA AGAGCTTCCT

CTCTCTTTCC TGCACATCTG GGCAGTTGTA CCATGCATTA AGAGCTTCCT

CTCTCTTTCC TGCACATCTG GGCAGTTGTA CCATGCATTA AGAGCTTCCT

CTCAGCTGTC TGCACATCTG G--------- ---------- ----------

CTCTCTTTCC TGCACATCTG GGCAGTTGTC CCATGCATTA AGAGCTTCCT

CTCTCTTTCC TGCACATCTG GGCAGTTGTA CCGTGCATTA AGAGCTTCCT

CTCTCTTTCC TGCACATCTG GGCAGTTGTA CCGTGCATCA AGAGCTTCCT

CTCTCCTTCC TGCACATCTG GGCGGTTGTA CCGTGCATCA AGAGCTTCCT

CTCTCTTTCC TGCACATCTG GGCAGTTGTG CCTTGCATTA AGAGTTTCCT

CTCTCTTTCC TGCACATCTG GGCAGTTGTA CCATGCATTA AGAGTTTCCT

CTTTCTTTCC TGCACATCTG GGCAGTTGTG CCGTGCATTA AGAGTTTCCT

GTGTCCTTCC TGCACATCTG GGTGGTCGTG CCCTACATTA AGAGCTTCCT

CTCTCTTTCC TGCACATCTG GGCAGTCGTA CCATGTATTA AGAGTTTCCT

CTCTCTTTCC TGCACATCTG GGCAGTTGTA CCGTGCATTA AGAGTTTCCT

CTCTCTTTCC TGCACATCTG GGCAGTCGTA CCATGCATTA AGAGTTTCCT

CTCTCTTTCC TGCACATCTG GGCGGTTGTG CCATGCATCA AGAGCTTCCT

CTTTCCTTCT TGCACATCTG GGCAGTTGTG CCATGCATCA GGAGCTACCT

CTCTCCTTCC TGCACATCTG GGCCGTGGTG CCCTGCATCC GGAGCTACCT

GATTGAGATT CAGTGCATCA GCCGTGTCTA TTCCATCTAC GTCCACACCG

GATTGAGATT CAGTGCATCA GCCGTGTCTA TTCCATCTAC GTCCACACCG

GATTGAGATT CAGTGCATCA GCCGTGTCTA TTCCATCTAC GTCCACACCG

---------- ---------- ---------- ---------- ----------

GATTGAGATT CAGTGCATCA GCCGTGTCTA TTCCATCTAC ATCCACACCG

GATCGAGATT CAGTGCATCA GCCGCGTCTA TTCCATCTAC GTACACACCT

GATTGAGATT CAGTGCATCA GCCGTGTCTA TTCCATCTAC GTCCACACCT

GATTGAGATT CAGTGCATCA GCCGCGTCTA CTCCATCTAC GTCCATACCT

GATTGAGATT CAGTGCATCA GCCGAGTCTA TTCCATCTAC GTCCACACCT

GATTGAGATT CAGTGCATCA GCCGTGTCTA TTCCATCTAC GTCCACACCT

GATTGAGATT CAGTGCATCA GCCGTGTCTA TTCCATCTAC GTCCACACCT

GATTGAGATT CAGTGCATCA GCCGTGTCTA TTCCATCTAC ATCCACACCT

GATTGAGATT CAGTGCATCA GCCGTGTCTA TTCCATCTAC ATCCACACCT

GATTGAGATT CAGTGCATCG GCCGTGTCTA TTCCATCTAC ATCCACACCT

GATTGAGATC CAGTGCATCA GCCGTGTCTA TTCCATCTAC GTCCACACCT

GATCGAGATC CAGTGCATCA GCCGTGTCTA TTCCATCTAC GTCCACACCT

GATTGAAATC CAATGTATCA GTCGGATCTA TTCAATCTGC ATCCATACCT

GATAGAGATC CAGTGCATCA GCCGCGTCTA CTCCATCTGC ATCCACACCT

TCTGTGACCC ACTCTTTGAA GCTGTTGGGA AAATATTCAG CAATGTCCGC

TCTGTGACCC ACTCTTTGAA GCTGTTGGGA AAATATTCAG CAATGTCCGC

TCTGTGACCC ACTCTTTGAA GCTGTTGGGA AAATATTCAG CAATGTCCGC

---------- ---------- ---------- ---------- ----------

TCTGTGACCC ACTCTTCGAA GCTATTGGGA AAATATTCAG CAATGTCCGC

TCTGCGACCC ACTCTTTGAA GCTATTGGCA AAATATTCAG CAATATCCGC

TCTGTGATCC ACTCTTTGAA GCGATTGGCA AGATATTCAG CAATATCCGC

TCTGCGATCC ACTCTTTGAA GCTATTGGCA AGATATTCAG CAACATCCGC

TCTGTGATCC ACTCTTTGAA GCTATTGGCA AAATATTCAG CAATGTCCGC

TCTGTGACCC GCTGTTTGAG GCTATTGGCA AAATATTCAG CAATATCCGC

TCTGTGACCC GTTCTTTGAG GCTGTTGGCA AAATATTCAG CAATATCCGC

TCTGTGACCC GCTCTTTGAG GCTTTTGGCA AAGTATTCAG CAATATCCGC

TCTGTGACCC ACTCTTTGAG GCTATTGGCA AAATATTCAG CAATATCCAC

TCTGTGACCC ACTCTTCGAG GCTGTTGGCA AACTATTCAG CAATATCCGT

TCTGTGACCC GCTCTTTGAG GCCATTGGCA AAATATTCAG CAGTATCCGC

TCTGCGACCC GCTCTTTGAG GCCATTGGCA AAATATTCAG CAACATCCGC

TCTGTGACCC ACTCTTTGAG GCTATTGGCA AAATATTCAG TCATGTCCGG

TCTGCGACCC ACTCTTCGAG GCCATCGGCA AGGTGTTCAG CAACATCCGA

ATCCCTGGTC CAATTTCAAG TTCCAATTGC TCCTCTCATC AACTGAATGA

ATCCCTGGTC CAATTTCAAG TTCCAATTGC TCCTCTCATC AACTGAATGA

ATCCCTGGTC CAATTTCAAG TTTCAATTGC TCCTCTCATC AGCTGAATGA

---------- ---------- ---------- ---------- ----------

ATCCCTGGTC CAATTTCAAG TTTAAATTTC TCCTGTCATC AGCTGAATGA

ATCCTTGGTC CAATTTCAAG TTTCAATTGC TCCTCTCATC AGCTCAATGA

ATCCTTGGTC CAATTC---- ---CAATTGC TCCACTCAGC -ACTGAATGA

ATCCTTGGTC CAATTC---- ---CAATTGC TCCACTCAGC AACTGAATGA

ATCCTTGGTC CAATTCCAAG TCTCAATTGC TCCTCTCATC AGCTGAATGT

ATCCTCAGTC CGA------- TTTCAATTGC TCCTCTCACT AGCTGAATGA

ATCCCTGGTC CAATTTCAAG TTTCAATTGC TCCTCTCATC AGCTGAATGA

ATCCTTGGTC CAATTTCAAG TTTTATTTGC TCCTTTCATC AGCTGAGTGA

ATCCTTGGTC CAATTTCAAG TTTC-ATTGC TCCTCTTATC AGCTGGATGA

ATCCTTGGTC CAGCTTCAAG GTCCAATTGC TCCTCTTATC AGCTGCATGA

ATCCTTGGTC CAATTTCAGG TTTCAATTGC TCCTCTCATC AGCTGAATGA

ATCCTTGGTC C-------GG TTTCAATTGC TCCTCTCATC CGCTGAATGA

ATCCTTGGTC CGATTTCAAG TTTCAATTGC TTCCCTTACC AGCCAAGCAA

GCCCTTGATC TAGGCCCCCG CCTCAGCCTC TCCTTATTTC AGCCAAGGGA

GGTCAGCCTT CGTTTATTTT CAAGAATAAT CACGCTTTAT CCAAACTAAT

GGTCAGCCTT CGTTTATTTT CAAGAATAAT CACGCTTTAT CCAAACTAAT

GGTCAGCCTT CGTTTATTTT CAAGAATAAT CACGCTTTAT CCAAACTAAT

---------- ---------- ---------- -ACGCTTTAT C-AAACTAAT

GGTCAGCCTT CCTTTATTTT CAAGAATAAT CATGCTTTAT CCAAACTAAT

GGTCAGCCTT CGTTTATTTT CAAGAATAAT CACGCTTTAT CCAAACTAAT

GGCCCTCTTT CACTTATTTT CAAGAATAAT CACACATTCT CTACGCTAAC

GGCCAGCTTT CACTCATTTT CAAGAATAAT CACAGGTTCT CTAAGCCAGC

GGTCAGCCTC CGTTTATTTT CAAGAATAAT GACGCTTTAT CCACACTAAT

GGTCAGCCTT CGTTTATTTT CAAGAATAAT CACGCTTTCT CCAAACTAAT

GGTCAGCCTT CGTTTATTTT CAAGAATAAT CACGCTTTCT CCAAACTAAT

GGTCAGCCTT CGTTTATTTT CAAGAATAAT CACGCTTTCT CCAAACTAAT

CGTCAGCCTT CGTTTGTTTT CAAGAGTGAT CACGCTTTTT CCAAGCTAAT

GGTCAGCTTT CCTTTAGTTT CAGGAATAAT CACACTTTAT CCAAACTAAT

GATCAGCCTT CGTTTGTTTT CAAGAATAAT CACCCTTTAT CCAAACTAAT

GATCAACCTT TGCTTA-TTT CTAGAATAAC CACGCTTTAT CCAAACTAAT

GGTCAGCCTC AGTTTATTTT CAGGAATATT CACGCTTTGT CCAAACTAAT

GATCAGCCTG CAATTAACTC CCGGGCTATC TGCGAGTCAT CCAAACTAAC

CCATCACGGG GTGGTTTAAA AAGATTGAAG TATTTTGCTG GAATAAGTTC

CCATCACGGG GTGGTTTAAA AAGATTGAAG TATTTTGCTG GAATAAGTTC

CCATCACGGG GTGGTTTAAA AAGATTGAAG TATTTTGCTG GAATAAGTTC

CCATCACGGG GTGGTTTAAA AAGATTGAAG TATTTTGCTG GAATAAGTTC

CCATCATGGG GTGGTTTAAA AAGATTGAAG TATTTTGCTG GAATAAGTTC

CCGTCACGGG GTGGTTTAAA AAGATTGGAG TAATTTGCTG GAATAAGTTC

CCCTCacagg atggcttaAA AAGCTTGAGG TAATTTGCTG GAATAAGTTC

CCCTCACAGG ATGGTTTAAA AAGCTTGAGG TAATTTGCTG TAATAAATTC

CCATCACGGG GTGGTTTAAA AAGATTGAGC TAATTTGCTG GAATCAGTTC

CCATCACGGG GTGGTTTAAA AAGATGGAGG TATTTTGCTG GAATAAATTC

CCATCATGGG GTGGTTTAAA GAGATTGAGG TATTTTGCTG GAATAAATTC

CCATCACGGG GTGGTTTAAA AAGATTGAGG TCTTTTGCTA GAATAAATTT

CCATCATGGG GTGGTTTAAA AAGATTGAGG TATTTTGCTG GAACCAGTTC

CCATTATGGA GTGGTTTAAA AAGACGGAGG TATTTTGCTG GAATAAACTC

CCATCACGGG GTGGTTTAAA AAGATTAAGG TATTTTGCTG GAATAAGTTC

CCATCACAGG GTGGTTTAAA AAGATTGAGG TATTTTGCTG GAAGAAGTTC

TCATCAAAGG GTGATTTAAA GGCATCAAGG TATTTTGCTG GAATAAGCTC

CCATCGGGGA GAGGTTTCCA GGGAGCTAAG CCTTTTGCTG GCCTAAGTTC

AAATTCTTCT GAACTCAAAC TGAGGAATTT CACCGTAAAC CTGAGTCGTA

AAATTCTTCT GAACTCAAAC TGAGGAATTT CACCGTAAAC CTGAGTCGTA

AAATTCTTCT GACCTCAAAC TGAGGAATTT CACCGTAAAC CTGAGTCATA

AAATTCTTCT GAACTCAAAC TGAGGAATTT CACCGTAAAC CTGAGTCATA

AAATTCTTCT GAACTCAAAC TGAGGAATTT CACCGTAAAC CTGAGTCATA

AAATTCTTCT GAACTCAAAC TGAGGAATTT CACCGTAAAC CTGAGTCATG

AAGTCTTTCT GAACCCAAAC TGTGGAATTT CACCGTGTAC CTGAGTC-TC

AAGTTTTTCT GAACCCAAAC TGAGGAATTT CACCGTGTAC CTGAGTC-TC

AAATTCTTCT GAATTCAAGC TGAGGAATTT CACCGTAAAC CTGAGTCATG

AAATTCTTCT GAATTCAAAC TGAGGAATTT TACCGTAAAC CTGAGTCATA

AAATTCTTCT GAACTCAAGC TGAGGAATTT CACCGTAAAC CTGAGTCATA

GGATTCTTCT GAACTCAAAC TGAGGAATGT TACCGTAAAC CTGAGTCATA

AAAT-----T GAACACAAAC TGAGGAATTT TAACGTAAAC CTGAGTCATA

AAATTCTTCT GAACTCAAAC TGAGGAATTT TGCCGTAAAC CTGAGTCAAA

AAATTCTTCT AAACTCAAAC TGAGGAATTT TACCGTTAAC CTGAGTCATA

AAATTCTTCT GAACTCAAAC TGAGGAATTT TACCGTAAAC CTGAGTCATA

AGCGCCTTCT GAACCCAAAC TGAGGAATCT CACCATTAAC CTGAATTGTA

AGCTTCTCCT GAACCCAAAC TGAGGAATTT TGCCCTGAAC CTGAGCTGTA

CAGAAAGCCA TTTAAAACAG ACACTGGCAT GGATATAGTT TTACTTTTAA

CAGAAAGCCG TTTAAAACAG ACACTGGCAT GGATATAGTT TTACTTTTAA

CAGAAAGCCA TTTAAAACAG ACACTGGCAT GGATATAGTT TTACTTTTAA

CAGAAAGCCA TTTAAAACAG ACACTGGCAT GGATATAGTT TTACTTTTAA

GTGAACGCCA TTTAAAGCAG ACACTGGCAT GGACATAGTT TTACTTTTAA

CAGAAAGCCA TTTAAAACAG ACACTGGCAT GGACATAGTT TTACTTTTAA

CAGAATCGCA TTTGAAACAG ACACTGGCAT GGCCACAGTT TGACTTTTAA

CAGAAAGCCA TTTAAAACAG ACACTGGCAT GGCCACAGTT TGAATTTTAA

CAGAAAGCCA TTTAAAACAG ACACTGGCAT GGACATAGTT TTACTTTTAA

CAGAAAGCCA TT-AAAACAG ACACTGGCAT GGACATAGTT TTACTTTTAA

CAGAAAGCCA TTTAAAACAG ACACTGGCAT GGACATAGTT TTACTTTTAA

CAGAAAGCCA TTTAAAACAG ACACTGGCAT GAACATAGTT TTACTTTTAA

GAGAAAGCTA CTTAAAACAG ACACTGGCAT GGACATAGTT TGACTCTTAA

TAGAAAGCCA TTTAAGACAG ACACTGGCAT GGGCATAGTT TTACTTTTAA

TAGAAAGCCA -TTAAGACAG ACACTGGCAT GGACATAGTT TTACGTTTAA

CAGAAAACCA TTTAAGGCAG ACACTGACAT GGCCATAGTT TTACGTTTAA

CGGAAAGCCA TTTAAAACAG ACACTGGCAT GGATAGAGTT TTACTTTTTT

CAAAAAGTCA GTTAAGACAG ACACTGGCAT GGAAATCATT TTACTTTTTT

ACTGTGTACA TAACTGAAAA TGTGCTATAC TGCATACTTT TAAATGTAAA

ACTGTGTACA TAACTGAAAA TGTGCTATAC TGCATACTTT TAAATGTAAA

ACTGTGTACA TAACTGAAAA TGTGCTATAC TGCATACTTT TAAATGTAAA

ACTGTGTACA TAACTGAAAA TGTGCTATAC TGCATACTTT TAAATGTAAA

ACTATGTATA TAACTGAAAA TGTGCTATAC AGCATACTTT TAAATGTAAA

ACTGTGTACA TAACT-AAAA TGTGCTATGC TGCA-ACTTT TAAGTGTAAA

ACTGTGTACA TAACTGAAAA TGTACTAGAC TGTATACGTT TAAATGTAGA

ACTGTGCACA TAACTGAAAG TGTACTAGAC TGTATACTTT TACATGTAGA

ATTGTGTACA TAACTGAAAA TGTACTATA- ------CTTT TACGTGTAAA

ACTGTGTACA TAACTGAAAA TGTACTATAC TGAATAATTT TAAGTGAAAA

ACAGTGTACA TAACTGAAAA TATAGTATAT TGCATACTTT TAAGTGTAAA

ACTGTGTACA TAATTGAAAA TATACTATAC TGCATACTTT AAAGTGTAAA

ACTGTGTGCA TAACTGAACA TGTACTATAC TACATG-TTT TAAGTGTAAA

ACTGTGTACA TAATTTAAAA AGTACTATAT TACATACTTT AAAGTATAAA

ACTGTGTACA TAAGTGAAAA TGTACTATAT TACATACTTT TAAAAGTAAA

ACTGTGTACA TAACTGAAAA TGTACTATAT TACAGACTTT AAAGTGTAAA

ACTATGTACA TAATTGAAAG TGTACTATTT TAGATATTTG CAAATGTAAA

ACTGTGTACA TAACAGAAAA TGTACTATTT TCGATATTTT CAAATGTAAA

GATATTTTTA TCTTTATATG AAGAAAATCA CTTAGGAAAT GGCTTGATTC

GATATTTTTA TCTTTATATG AAGAAAATCA CTTAGGAAAT GGCTTGATTC

GATATTTTTA TCTTTATATG AAGAAAATCA CTTAGGAAAT GGCTTGATTC

GATATTTTTA TCTTTATATG AAGAAAATCA CTTAGGAAAT GGCTTGATTC

GATACTTTTA TCTTTATATG AGGAAAATCA CTTAGGAAAT GGCTTGATTC

GATA-TTTTA TCTTTATATA AGGAAAATCG CTTGGGAAAT GGCTTAATTC

GATATCTTTA TCTTTATATA AGGAAAATCA CTTGGGAAGT GGTTCGTTTC

GATATTTTTA TCTTTATATA AGGAGAATCA CTTGGGAAAT GATTCAATTC

GATATTTTTA TCTTTATATG ATGAACATCA CTTGGGAAAT GGCTTGATTC

G-TTTTTTTA TCTTTATATG AGGAAAATCA CTTGGGAAAT TGTTTGATTC

GATATTTTTA TCTTTATATG AGGAAAATCA CTTGGGAAAT TGCTTGACTC

GATATTTTTA TCTTTATATG AGGAAAATCA CTTGGGAAAT TGCTTGATTC

GATATTTTTA TCTTTATCGG AAGAAAATCA CTTGGGAAAT TGCTTGAATC

GGTATTTTTA TCTTTATATA AGGAAAATCA CATGGGACAT TGCTTGATTC

GATATTTTTA TCTTTAGAGG AAGAAAATCA CTTTGGAAAT TGCTTGATTC

GAGAGCTGTA TCTTTCAGGA AGGAAAACTA CTTTGGGAGT TGCTTGATTC

CATAATTTTA TCTTCGTATG ATGAAAATCA CTTTGAAAAT AGCCTGCTTG

AATAGCTTTA TCTTCCTCTG AGGAAAGTAA CTTTGGAATC ACCCTGATCC

AATCTGTAAA CTGTATTCCA AGACATGTCT GTTCACATAG ATGCTTAGTT

AATCTGTAAA CTGTATTCCA AGACATGTCT GTTCACATAG ATGCTTAGTT

AGTCTGTAAA CTGTATTCCA AAACATGTCT GTTCACATAG ATGCTTAGTT

AGTCTGTAAA CTGTATTCCA AGACATGTCT GTTCACATAG ATGCTTAGTT

AATATGCAAC CTGTATTCCA AGACATGTCT GTTCACATAG ATGCTTAGTT

AATCTGTAAA CTGCATTCCA AGACATGTCT GTTCACATAA ATGCTTAGTT

AGTCTTTAAA CTGTGTTCCA AGACATGTCT GTTCTCCTAG ATACTCAGTT

AGTCTGTAAA CTGTGTTCCA AGACATGTCT GTTCCCCTAG ATACTCAGTT

AATCTGTAAA CTGTATCCCA AGACATGTCT GTTCACATAG ATGCTCAGTT

AATCTGTAAA CTGTATCCCA AGACATGTCT GTTCATATAG ATGCTTATTT

AATCTGTAAA CTGTATCCCA AGACATGTCT GTTC------ -----TAGTT

AACCTGTAAA CTGCATCCCA AAACATGTCT GTTCACATAG ATGCTTAGTT

AGTCTGTAAA CTGTATCTCA AGACATGTCT GTTCTCGTAG ATGCTTAGT-

AATCTGTAAA CTGTATCCTA AGACATGTCT GTTCACATAG ATGCTTAGTT

AATCTGTAAA CTGTATCCTA AGACATGTCT GTTCACATAG ATGCTTAGTT

AATCTGTAAA CGGTATCCTG AGACATGCCT GTACACCTAG ATGCTTAGGT

AATGTGGAAA CCTTATCCTC AGAGATGTCT GTTTACATAG ATCTGTAGTT

CACAGGTAAA CTGTATCCTG AGCAATGTCT GTTCACTTAG AAATATAGAC

GGTCCAAAAG ATTTGAAATT TTATATGCTT ACTGATATAT TTTACAATTT

GGTCCAAAAG ATTTGAAATT TTATATGCTT ACTGATATAT TTTACAATTT

GGTCCAAAAG ATTTGAAATT TTATATGCTT ACTGATATAT TTTACAATTT

GGTCCAAAAG ATTTGAAATT TTATATGCTT ACTGATATAT TTTACAATTT

GGTCCAAAAG ATTTGAAATT TTATATGCTT ACTGATATAT TTTACAATTT

GGTCCAAAAG ATTTAAAATT TTATATGCTT ACTGATACAT TTTACACTTT

GATCCAAAAG GTTTGAAATT TTATATGCTT ACTG--ATAT TTTACACTTT

GATCCAAAAG GTTTGAAATT TTATATGCTT ACTGATATAT TTTACACTTT

GGTCCAAAAA ACTTGAGATT TTATATGCTT AATGATATAT TTTACACTTT

GGTCCAAAAG ACTTGAAATT TTATATGCTT ACTG--ATAT TTTACACTTT

GGTCCAAAAG ATTTGAAATT TTATATGCTT ACTGATATAT TTTACACTTT

GGTCCAAAAG ATTTGCAATT TTATATGCTT ACTGATGTAT TTTACACTTT

-GTCCAAAAG ATTTGAAATT TTATATGCTT ACTGATATAT TTTACACTTT

GGTCCAAAAG ATTGGAAGTT TTATATGCTT ACTGATATAT TTGGCACTTT

GGCCCAAAAG ACATGAGATT TTATAGGCTT ACTGATATAT TTTTCACTTT

GGTCCCAAAG ACTTGATATG TTATATGCTT ACTGATATAT TTTGCACTTC

GATCCAAGAC ATTTACAATA TTATATGCTT ATTGGTATAT TTTACACTTT

AATCCAGGGC ATTAGCCATA GTGTACGCTT ACTGATATAT TTGACACTTT

TTTATCATGC ATGTCCTGTA AAGGTTACAA GCCTGCACAA TAAAAATGTT

TTTATCATGC ATGTCCTGTA AAGGTTACAA GCCTGCACAA TAAAAATGTT

TTTATCATGC ATGTCCTATA AAGGTTACAA GCCTGCACAA TAAAAATGTT

TTTATCATGC ATGTCCTATA AAGGTTACAA GCCTGCACAA TAAAAATGTT

TTTATCATGC ATGTCCTATA AAGGTTACAA GCCTGCACAA TAAAAATGTT

TTTATCCTGC ATGTCTTATA AAGGTTACAA GTCTGCACAA TAAAAATGTT

TTTATGCTGC ATGTCCTATA AAGATTTCAA ATCTGCACAA TAAAATTGTT

TTTATGCTGC ATGTCCTATA AAGATTTCAA ATCTGCACAA TAAAATTGTT

TTTATCCTGC ATGTCCTATA AAGGTTACAA ATCTGCACAA TAAACAATTA

TTTATCTTGC ATGTCCTGTA ------ATAA GTCTACACAA TAAAAATGTT

TTTATCTTGC ATGTCCTGTA AAGGTTACAA GCCTGCACAA TAAAAATGTT

TATATCCTGC ATGTCCTGTA AAGGTTACAA GTCTACACAT TAAAAATGTT

TTTATCCTGC ATGTCCTATA ACCGTTACAA GTCTACACAA TAAAAATGTT

TTTATCTTGC ATGTCCTGTA AAGAGTACAC ATCTTCACAA TAAAAATGTT

TTCATCCTGC ATGTCCCGTA AAGATTACAA GTCTGCACAA TAAAAATGTT

TTAATCCTGC ATGTCCTATA AAGGCTACAA GTCTGCACAA TAAAAAAGTT

TTTATCCTGC ATGTACTGTA AAGGCTACAA TTCTGCACAA TAAAAATGTT

TTTATTCTGC ATGTACTGTA AATGCTGCAA TTCTGCACAA TAAAAATGTT

TAACGGTTAA GGGGAAGAGG GAGCAAGAGA AGGGAACAGG GGGCAGAGAT

TAACGGTTAA GGGGAAGAGG GAGCAAGAGA AGGGAACAGG GGGCAGAGAT

TAACAGTTAA GGGGAAGAGG GAGCAAGAGA AGGGAACAGG GGGCAGAGAT

TAACAGTTAA GGGGAAGAGG GAGCAAGAGA AGGGAACAGG GGGCAGAGAT

TAACAGTTAA GGGAAAGAGG GAGCAAGAGA AGGGAACAGG GGGCAGAGAT

TAACTGTTAG GGGGAAGAGG GAGCAAGAGG AGGGAACAGG GGGCAGAGAT

TAACAGTTAA GGGGAAGAAT GGGCAAGGGC AAGGAACAGG GTGTAAAGAT

TAACAGTTAA GGGGAAGAAT GGGCAAGGGC AGGGAACAGG GGGTAAAGAT

AAACTGTTAA GGGGAAGAGC GAGCAAGAGC AGGGAACAGG GGGTAGAGAT

TAACAGTTAA GGGGAAGAGG GAGCAAGAGA AGGAAACAGG AGGTAGAGAT

TAACAGTTAA GGGAAAGAGG GAGCAAGAGA AGGGAACAGG GGGTAGAGAT

TAACAGTTAC GGAGAAGAGG GAGC-AGAGA AGAGAACAGG GAGTAGAGAT

TAACAGTTAA GGGGAAGAAG AAGCAAGACA AGGAAACAGG GAGCAGAGAT

TAACAGTTAA GGGGATGGGG GAGCAAGAGA GCAGAACAGG GGGTAGAGAT

TAACAGTTAA GGGGAAGAGG GAGCACAAGA AGAGAACAGG GGGAAGAGAT

TAACAATTAA GGGGAAGAGG GA---TAGAG ATGGAACGGG GGGCAGAGAT

TAACAGTTCA AGGGAAGAGG GAGTTGGGGA AGGAAACAGG AGGTGGAGAT

TAACAGTTAA GGGGGAAAGG AAGCGGATGA AAGAAAGAGG GGGCAGAGGG

AAGTGTTCTA AACTCTCTTT GAATTTATTA CAGAGACCAT TAACTGCCAA

AAG------- ---------- ---------- ---------- ----------

AAGTATTCTA AACTCTCTTT GAATTTATTA CAGAGACCAT TAACTGCCAA

AAGTATTCTA AACTCTCTTT GAATTTATTA CAGAGACCAT TAACTGCCAA

AAGTGTTCTA AACTCTCTTT GAATTTATTA CAGAGACCGT TAACTGCCAA

AAGTGTTTTA ACCTGTCTTC AGATTTATTA CAGAGACCAT TAACTGCAAA

AAGGGGCTCA ACTTGACTTT GAATTTATTA CAGAGACCAT TAACTGCCAA

AAGTGGCTCA ACTTGAGTTT GAATTTATTA CAGAGCCCAT TAACTGCCAA

AAGTGTTTTA AAC--ATTCT GCTTTGATTA TAGAGACCAT TAATTTCTAA

AAGAGTTTTA AACTCTCTTC AAACTTATTA CAGAGACCAT TAACCACCAA

AAGTGTTTTA AACTCTCTTT GAATTTACGA CAGACACCAT TAACTGCCAA

AAGTGTTTTA AACTCTCTTT GAATTTATTA CAGAGACCAT TAACT-CCCG

AAGTGTTTTA A-CTCTCTTT GAATTTATTA CAGAGGCCAT TAACTACCAG

AAGTGTTTTA AATCCTCTTT GAATTTATTA CAGAGACCAT TAACTGCCAA

AAGTGTTTTA AACGCTCTTT GAATTTATTA CAGACACCAT TAACTGCCAC

AA-TGTTTCC AACGCTCTTT GAATTTAGTG CAGAGCCCAT TAACTGCCAG

AAGTGATTTA AAACCTCCCT GAATTTATTA CAGAGACCAT TAGCTGCTCA

AGGTGACTTA AAACCTTCCT GAATTTATTA CAGAGCTTTT TAGCTGCAGG

GCTCAGATGC AAAAAGCAAT AAGACAATAA AGGTAAAATT TAAGGCAACT

---------- ---------- ---------- ---------- ----------

GCTCAGATGC AAAAAGCAAT GAGACAATAA AGGTAAAATT TAAGGCAACT

GCTCAGATGC AAAAAGCAAT GAGACAATAA AGGTAAAATT TAAGGCAACT

GCTCCGATGC AAAAAGCAGT GAGACAATAA AAGTAAAATT TAAGGCAACT

GCTCAAATGC AAAAAGCAAT GAGACAATAA AGGTAAAATT TAAGGCAGCT

GCTCAGATGC AAAATGCATT GAGACACTAA AGGTAAAATT TAAGGCACGT

GCTCAGATGC AAAAAGCATT GAGACAATAA AGGTAGAATT TAAGGCACCT

GCTCAGATGC AAAAAGCTCT GAGACAACAA AGGTACAATT TAAGGCAGCT

GCTCAAATGC AAAAGGCTAT GAGACAACAA AGGTAAAATT TAAGGCAGCT

GCTCAGATGC AAAAGGCAAT GAGACAAGAA AAGTAAAATT TAAGACAGCT

GCTTAGATGC AAAAGGCAAT GAGACAATAA AGGTAAAATT TAAGGCAGCT

GCTCAGATGC AAAAGGCAAT GAGACAATAA AGGTAAAATG TAAGGCAGCT

GCTCAGATGC AAGAAGAAAT GAGACAATAA AGGTAAAATT TAAGGCAAGT

TCTCAGATAC AAAAAGCAAT GAGATAATAA AGGTAAAA-T TAAGGCAGCT

GCTCAGATAC AAAAATCCAT GAGACAACGG AGGTAAAATT TAAGGCAGTT

GCTCAAATGC AAAAGGCAAT GAGATCATTA AGGTAAATTC AAAGATGGCT

GCTCAAAGGC AAAAAGAAAT GAAACTGTTG ACGTCAAATC AAAGGCAGAT

AAACGGAACT TGGCTTCCAG TGTAACTCAT TAGGCATCTC AAAACACAAG

---------- ---------- -------CAT TAGGCATCTC AAAACACAAG

GAAGGGAACT TGGCTTCCAG TGTAACTCAT TAGGCATCTC AAAACACAAG

GAAGGGAACT TGGCTTCCAG TGTAACTCAT TAGGCATCTC AAAACACAAG

GAAGGGAACT TGGCTTCCAG TGTAACTCAT TAGGCATTTC AAAACGCAAG

GAAGAGAACT TGGCCACCAG TGTAACTCAT TAGGCGTTTC CAAACACAAG

GAAGGGAGCT TGGCTTCCAC ACTAACTCAC TAGGCATTTC CGAACACAAG

GAAGGGAATT TGGCTTACAC ACTAACTCAT TAGGCATTTC AAAACACAAG

GAAGGGAACT TGGCTTCCTT TGTTACTCAT TAGACATTTC AAAACACAAG

GAAGGGAACT CGGCTTCCAG TATAACTCAT TAGGCATTTC AAAACACAAG

GAAGGAAACT TGGCTTCCAG TGTAACTCAT TAGGCATTTC AAAACACAAG

GAAGTGAATT TGGCTTCCAG TGTAACTCAT TACGCATTTC AAAACACAAG

GAAGCGAACT TGGCTTCCAA TGTAACTTAT TAGGCATTTC AGAACACAAG

GAAGGGAACT TGGCTTCTAG TGTAACTCAT TAGGCATTTC AAAATGCAAG

GAACGGAACT TGGCTTGCAG TGTAACTCAT TAGGCATTTC AAAACACAAG

GAACGGAACT TGGCTGGCAG TGTTATTCAT TAGGCATTTC AAAACACAAG

CAAAAGAACT TGGCTTCCAT TGTAAATCAT TAGACATTTT CAAAATCAAG

GAAGGGAACT TGGCTTCCAG TCTACCTCGT GAGGTATTTC AAGAGCCAAA

GATGATTGCC TCGTCTGATC AAAGCAAATT TCATTAGGTG AAATAGCTTG

GATGATTGCC TCGTCTGATC AAAGCAAATT TCATTAGGTG AAATAGCTTG

GATGATTGCC TCCTCTGATC AAAGCAAATT TCATTAGGTT AAATAGCTTG

GATGATTGCC TCCTCTGATC AAAGCAAATT TCATTAGGTT AAATAGCTTG

GATGATTGCC TCCTCTGGTC AAAACAAATT TCATTAGGTG AAATAGCTTG

GACGATTGCC TCCTCTGATC AAAGCAAATT TCATTAGCTG AAATAGCTTG

GGTGATTGCC TCCTCTGATC AAAGTAAATT TCATTAGCTG AAGTAGCTTC

GATGATTGAC TCCACTGATC AAAGTCAATT TCATTAGCTG AACTAGCTTG

GATGATTGCC TCCTCTGATC AAAGCAAATT TCATTAGCTG AAATAGCTTG

GATGATTGCC TCCTCTGATC AAAGCAAATC TCATTAGCTG AAATAACTTG

GATGATTGCC TCCTCTGATC AAAGCAAATT TCATTAGCTG AAATAGCTTG

GATGATTGCC TTCTCTGATC AAAGCAAATT TCATTAGCTG AAATAGCTTG

GATGATTGCC TCCTCTGATC AAAGCAAATT TCATTAGCTA AAATAACTTG

GATGATTGCC TTCTCTGATC AAAGTAAATT TCATTAGATG AAATAGCTTG

GATGATTGCC TTCTCTGATC AAAGCAAATT TCATTAGATG AAATAGCTTG

GATGATTGCC TTCTCTGATC AAAGCAAATT TCATTAGATG AAATAACGTG

AAGAATAGCC TCCCCTGATC AAAGCAAATT TCATTAGGAG AAATTGCTTG

GCTGAACGCT TCTCCTGATC AAAGAAAATT GCATTAGAAG AAGTCACCTG

ACTTATAGCT GGCCTATAAA AAATGACAAC TAGTTTGAAA TTTGATCAGA

ACTTATAGCT GGCCTATAAA A--------- ---------- ----------

ACTTATAGCT GGCCTATAAA AAATGACAAC TAGTTTGAAA TTTGATCAGA

ACTTATAGCT GGCCTATAAA AAATGACAAC TAGTTTGAAA TTTGATCAGA

ACTTAGAGCT GGCCTATAAA AAATGACAAC TAGTTTGAAA TTTGATCAGA

ACGTACAGCT GGCCCATAAA AAACGACAAC TAGTTTGAAA TTTGATCAGA

ACTTCTGGCT GGTCTGCGAA AAATGACAAC TAGTTTGAAA TTTGGTCAGA

ACTTCTAGCT GGCCTATAAA AAACGACAAC TAGTTTGAAA TTTGGTCAGA

ACTTATAGCT GCCCTATAAA AAATGACAAC TAGTTTGAAA TTTGATCAGA

ACTTATAGCT GGCCTATAAT AAATGACAAT TAGATTGAAA TTTAATCTGA

ACTTCTAGCT GGCCTATAAT AAATGACAAC TAGATTGAAA TTTAATCAGA

ACTTAGAGCT GGCCTGT-AT AAACAACAAC TAGATTAAAA TTTCATCAGA

ACTTATAGCT GGCATATAAT AAATGACAAC TAGATTGAAA TTTAATCAGA

ACTTATAGCT GGCCTATAAT AAGTGACAAC TAGTTTCAAA TTTTATCAGA

ACTTATAGCT GGCCTATAAT AAATGACAAC TAGTTTGAAA TTTCATCAGA

ACTTATAGCT GGCTTGCCAT AAATGACAAC TAGTTTGAAC TTTCATCAGC

ACTTATAAGT GGACTGTAAT TAATTTTAAA GAGTTTGAAA TTTCATCACA

ACTTGGAAGT GGGCTATAAT TaATTAGAAC TAGTTAGGCA TTGCTTCTGA

ATGCAAAATT GAACAAGGTA GGTTCTAAAG CAGATTAACT AACTCTAATT

---------- ---------- ---------- ---------- --CTCTAATT

ATGCAAAATT GAACAAGGTA GATTCTAAAG CAGATTAACT AACTCTAATT

ATGCAAAATT GAACAAGGTA GATTCTAAAG CAGATTAACT AACTCTAATT

ATGCAAAATT GAACAAGGTA GGTTCTAAAG CAGATTAACT AACTCTAATT

ATGCAGAACT GAACAAGGTA GGTTCTGAAG ATGATTAACT AGCTTTAATT

ATGCAAGATT GAACAAAGTA CATTCTAGAG CTGATTAACT AGCTCTAATT

ATGCAGAATT GAACAAAGTA TGCTCTAAGG CTGATTAACT AGCTCTAATT

AAGCAAAATT GAACAAGGTA GGTTCTAAAG CTGATTAACC AGCTCTAATT

ATACAAAACT GAACACGGCA GGTTCCAAAG ATGATTAACT AGCTCTAATT

ATGTAAAATT GAACACTGTA GGTTCCAAAG CTGATTAACT AGCTCTAATT

ATGCAAAATT GAACACGGTA AGTTCCAGAG CTGATTAACT AGCTCTAATT

ATGCAAAATT GAACATGGTA GGTTCCAAAG CTGATTAACT AGCTCTAATT

ATGGAAAATT GAACGAGGTA GGTCCTGAAG CTGATTAACT AGCTCTAATT

ATGCAGAATT GAACATGGTA GGTTCTAAAG CTGATTAACT AGCTCTAATT

ATGCAAAATG GAATATGGTA GGTTTTAAAG CTGATTAACT AGCTCTAATT

ATGCCAAACT GAATTCAATA ATTTCTGAAG ATGACtaaac aacaCTAATT

ATATAAAATT GAATACAGAA AGTTTGGAAG CTGATTACCC AACTCTAATT

GAGAGTACAT GTGCTCATTC CTGGCATTGA TTTGCTGGCT TCTAATTCAG

GCGAGTACAT GTGCTCATTC CTGGCATTGA TTTGCTGGCT TCTAATTCAG

GCGAGTACAT GGGCTCGTTC CTGGCATTGA TTTGCTGGCT TCTAATTCAG

GCGAGTACAT GTGCTCGTTC CTGGCATTGA TTTGCTGGCT TCTAATTCAG

GCGAGTACAT GTGCCCATTC CTGGCATTGA TTTGCTGGCT TCTAATTCAG

GCAAGTACAT TTGCTCACTC CTGGCATTGA TTTGCTGGCT TCTAACTCAG

ACGAGTGTAT TCGCCGGCTC TCAGCATCGA TCTGCCAGCT TCTAATTCAG

ACAAGCAGAT TTGCTGGCTC CTAACATTGA TTTGCCAGCT TCTAATTCAG

GCAAGTACAT TTTCTGGCTC TTGGCATTGA TCTGCTGACT CCTAATTCAG

GCAAGTACAT TTGCTGGTTC CTGGCATTGA TTTGCTAGAT TCTAATTCAG

GCAAGTACAT TTGCTGGTTC CTGGCATTGA TTTGCTGGAT TCTAATTCAG

GCAAGTACAT TTTCTGGTTC CTGGCATTGA TTTGCTGGGT TCTAATTCAG

GCAAGTACAT TTGCTGGTTC CTGGCATTGA TTTGCTGGAT TCTAATTCAG

GCAAGTACAT TTGCTGGTTC CTGGCATTGA TTTGCTAGAT TCTAATTCAG

GCAAGTACAT TTGCTGGTTC CTGGCATTGA TTTGCTAGAT TCTAATTCAG

GCAAGTACTT TTGCTGGTTC CTGGCATTGA TTTGCTGGAT TCTAATTCAG

GCAAGCGCAT TACCTTGTTC CTGGTATTGA TTTGCTAAAT CTTAATTCAC

GCAGGTCCAT TTGCCAATTC CTGGCATTGA TTTGCTGAAT TCTCATTCTA

GATATGAGTT TAAAATATTA ATCCACAAAC TTCTCATTTT CTTTATTGGT

GATATGAGTT TAAAATATTA ATCCACAAAC TTCTCATTTT CTTTATTGGT

GGTATGGGTT TAAAATATTA ATCCACAAAC TTCTCATTTT CTTTATTGGT

GGTATGGGCT TAAAATATTA ATCCACAAAC TTCTCATTTT CTTTATTGGT

AGTATGAGTT TAAAATATTA ATCCACAAAC TTCTCATTTT CCTTATTGGT

GGTATGAGTC TAAAATATTA ATCCACAAAC TTCTCATTTT CTTTATTGGT

GGTATGGGTT TACGATATTA ATCCACAAAC TTTTCATTTT CTTTATTGGT

GGTATGAGTT TACGATATTA ATCCACAAAC TTTTCATTTT CTTTATTGGT

GGTATGAGTT TAAAATATTA ACCCACAAAC TTCTCATTTT CTTTATTGGT

GGTATGAGTT TAAAATATTA ATCCACAAAC TTCTCATTTT CTTTATTGGT

GGTATGAGTT TAAAATATTA ATCCACAAAC TTTTCATTTT CTTTATTGGT

GGTATGAGTT TAAAATATTA ATCCACAAAC TTCTCATTTT CTTTATTGGT

GGTATGAGTT TAAAATATTA ATCCAC-AAC TTCTCATTTT CTTTATTGGT

GGTATGAGTT TAAAATATTA ATCCCCTAAC TTCTCATTTT CTTTATTGGT

GGTATGAGTT TAAAATATTA ATCTACAAAC TTCTCATTTT CTTTATTGGT

GGCATGAGTT TAAAATATTA ATCCACAAAC TCCTCATTTT CTTTATTGGT

AATATGAACT AAAAATATCA ATCAACAAAT TTATCATTCT GTTTAATGGT

AATAGGAGCT AAAAATATCA GTCCACAAAT TTTCCAATTC ATTTATTGGT

TAATACAACT CATGACAAAT TGTACTGCCT AAATTCTTCA TCTTAATGCT

TAATACAACT CATGACAAAT TGTACTGCCT AAATTCTTCA TCTTAATGCT

TAATACAACT CATGACAAAT TGTACTGCAT AAATTCTTAA TCTTAATGCT

TAATACAACT CATGACAAAT TATACTGCAT AAATTCTTAA TCTTAATGCT

TAATACAACT CATGACAAAT TGTACTGCAT AAATTCTTCA TCTTAATGCT

CAATACAACT CATGACAAAT TGTACTACAT AAATTCTTCA TCTTAATGCT

CAGTACAATT CATGACAAAT TGTGCTACAT AAATTCTTCA TCTTAATGAT

CAGTACAACT CATGACAAAT TGTGCTACAT AAATTCTTCA TCTTAATGAT

CAATACAACT CATGACAAAT GATACAACAT AAATTCTTCA TCTTAATGCT

CAATACAACT CATGACAAAC TATACTCCAT AAATTCTTCA CCTTAATGCT

CAATACAACT CATGACAAAT TGTACTCCAT AAATTCTTCC TCTTAATGCT

CAATACAACT CATGACAAAT TGTACTCCAT AAATTCTTCG TCTTAATGCT

CAATACTACT CATGACAAAT TGTACTCCAT AAATTCTTCA TCTTAATACT

CAATACAACT CATGACAAAT TGTACTGCAT AAATTCTTCA TCTTAATGCA

CAATACAACC CATGACAAAT TCTACTACAT AAATTCTTCA TCTTAATGCT

CAATACAACC CATGACAAAT TCGACTACAT AAATTCTTCA TCTTAATGCT

CAATACAATT CTTGATGAAT TGTTCTATAG AAATTTTTCA TCTTAACATT

CAATACAGCC CTTGATAGAT GGCGCTGGAT AAATTCTTCA TCTTAATGTT

TCCACTCGAA TTTTCTGATT CTTCCATCTA TTTCATATAT TTATTTTTTT

TCCACTCGAA TTTTCTGATT CTTCCATCTA TTTCATATAT TTATTTTTTT

TCCACTCGAT TTTTCTGATT CTTCCATCTA TTTCATATAT TTATTTTTTT

TCCACTCGAT TTTTCTGATT CTTCCATCTA TTTCATATAT TTATTTTTTT

TCCACTCCTT TTTTCCAATT CTTCCATCTA TTTCATATAT TTATTTTTTT

TCCACTTGAT TTTTCTGATT CTTCCATCTA TTTCCTATAT TTATTTTTTA

TCGCTCTTGT TTTTCTTATT CTCCCATCTA TTTTATGCAT TTATTTTTTC

T--CCTTGGT TTTT-TTATT CTTCCATCTA TTTTATGCAT TTATTTTTTC

TCCCCTTGAT TTTTCTGATT CTTCCATCTA TTTCATATAC TTATTTTCTC

TCCACTTGAT TTTTCTGATT CTTCCAtc-- ---------- --ATTTTTT-

TCCATTTGAT TTTTCTGATT CTTCCAT--A TTTCATATAT TTATTTTTTC

TCCACTTGAT TTTTCTGATT CTTCCATCTA TTTCATACAT TTATTTTTTC

TCTACTTGAT TCCCCCAATT TCTCCATTTA TTTCATATTT TTATTTTTTC

TCCACTTGAT TTTTCTGATT CCTCCATCTA TTTCATATAT TTGTTTTTTC

TCCACTTGAT TTTTC-GATT CTTCCATCTA TTTCATACAT TTATTTTTTC

TCCACTTGAT TTTTCTGATG CTTCCAACTA TTTTATATAT TTATTTTTTC

TCTGCTTGGT TGATCAGATT CCTCCATCTC ATTCATATAT TTATTTTTCA

TCTGCTTGCT TTTCCTGGTT ATTCCATCTC TCCCCTGACC CAAGTTATCA

TTTCCTCTGC TAAATTTTCT TAGAGTTAGT CAGAAAAATG AGAATGATTA

TTTCCTCTGC TAAATTTTCT TAGAGTTAGT CAGAAAAATG AGAATGATTA

TTTCCTCTAC TAAATTTTCT TAGAGTTAGT CAGAAAAATG AGAATGATTA

TTTCCTCTAC TAAATTTTCT TAGAGTTAGT CAGAAAAATG AGAATGATTA

TTTCTTCTGC TAAATTTTTT TAGAGTTAGT CAGAAAAATG AGAATGATTA

TTTCTTCTGC TAAATTTTCT CAGAGTCAGT CAGAAGAAGG AGAATGATTA

TTTCTTCTAC TAAATGTTCT TAGAGTCAGT CAGAAAAATG AGAATGATTA

TTTCTTCTAC TAAATGTTCT TAGAGTCAGT CAGAAAAATG AGAATGATTA

TTCCTTCTGC TAAATGTTCT TAGAGTCAGA CAGAAAAATG AGAGAGATTA

TTTCTTCTGG TAAATTTCCT TAGTATCAGT TAGAAAAATG AGAATGATTA

TTTCTTCTGC TAAATTTCCT CAGAGTCAGT CAGAAAAATG AGAATGATTA

TTTCTTCTGC TAAATTTGCT TAGAGTCAGC TAGAAAAATG AGAATGATTA

TTTCTTCTGC TCAATTTTCT TAGAGTTAGT CAGAAAAATG AGAAGGATTA

ATTCTTCTGT TAAATTTCCT TAGAGTCAGT CAGAAAAATG AGAATGATTA

TTTCTTCTGC TAAATTTCCT TTGAGTCAGT CAGAAAAATG AGAATGATTA

TTTTGTCTAT TAAATGTCCT TTGAGTCAGT CAGGAAAATG ATCATGATTA

TTTCTTCTGA CAAACTTTCT AAGGTTCTGT CAGAGAAATG AGTCTGATGA

ACTCTCCCGC CAAACTTTCT TGGAGTCACT CAGAGAAATG AGAATGATTG

ATAGAGATTA TTTCTCAACT CTGGAAATAT TTGTGAGTAA TTTTGCCAAA

ATAGAGATTA TTTCTCAACT CTGGAAATAT TTGTGAGTAA TTTTGCCAAA

ATAGAGATTA TTTCTCAACT CTGGAAATAT TTGTGAGTAA TTTTGCCAAA

ATAGAGATTA TTTCTCAACT CTGGAAATAT TTGTGAGTAA TTTTGCCAAA

ATAGAGATTA TTTCTCAACT CTGGAAATAT TTGTGAGTAA TTTTGCCAAA

ATAGAGATTA TTTCTCAACT CTGGAAATAT TTGTGACTAA TTTTGCCAAA

ATAGAGACTG TTTCTCAACT CTGGAAATGT GTGTGAGTAA TTTTGCCAAA

ATAGAGACTA TTTCTCAGCT CTGGAAATGT TTGTGAGTAA TTTTGCCAAA

ATAGCGATTA TTTCTCAACT CTGGAAATAT TTGTGAGTAA TTCTGCCAAA

ATAGAGATTA TTTCTCAACT CTGGAAATAT TTGTGAGTTA TTTTGCCAAA

ATAGAGATTA TTTCTCAACT CTGGAAATAT TTGTGAGTCA TTTCACCAAA

ATAGAGATTA TTTCTCAACT CTGCAAGTAT TTGTGAGTAA TTTTGTCCAA

ATTGAGATTA TTTCTCAACT C--GAACTAT TAGT----AA TTTTACCAAA

ATAGAGATTG TTTCTCAACC CTGGAAATAT TTGTGAGTAA TTCTGCCAAA

ATAGAGATTA TTCCTCAACT CTGAAAATAT TTGTGAATAA TTTTGCCAAA

ATAGAGATTA TTTCTCCACT CTGAAAATAT TTGTGAGTAA TTCTGCCAAA

ATAAAGATCT TCTCTCAACT TTGAAAACAC TTTTGAGCAT TTTTACCAAA

CTGGTTCTCT CTTCCCAACT ACGGAAATAT TCCCAAATTT TTCCACCAGA

TTATTAGTCA GAAATGCTAA TTGAAAACTC CAGATGGCAG CATCACAGTC

GTATTAGTCA GAAATGCTAA TTGAAAACTC CAGATGGCAG CATCACAGTC

GTATTAGTCA GAAATGCTAA TTGAAAACTC CAGATGGCAG CATCACAGTC

GTATTAGTCA GAAATGCTAA TTGAAAACTC CAGATGGCAG CATCACAGTC

GTATTAGTCA GAAATGCTAA TTGAAAACTC CAGATGGCAG CATCACAGTC

GTATTAGTCA GAAATGCTAA TCGGAAACTC CAGATGGCAG CATCACAGTT

GTATTAGTCA GAAATGCTAA TTGAAGGTCC CAGCAAG--- -AGAGCGGCT

GTATTAGTCA GAAATGCTAA TTGAAAGCCC CAGCCAG--- CAGCTCGGTT

GTCTTAGTCA GAAATGATCA TTGAAAACTC CAGATGGCAT CAGCACAGTT

GTATTAGTCA GAAATGCTAA TTGAAAACTC CAGATGGCAG TATCACAGTT

CTATTAGTCA GAAATGCTAA TTGAAAACTC CAGATGGCAG TATCACAGTT

GTATTAGTCA GAAATGCTAA TTGAAAACTC CAGATGGCAG TATCACAGTT

GTATTAGTCA GAAATGCAAA TTGAAAACTC CAGATGGCAG TATCACAGTT

GTATTAGTCA GAAATGCTAA TTGAAATCTC CATATGGCAG TATCACAGAT

GTCTTAGTCA GAAATGCTAA TTGAAAACTC CAGATGGCAG TATCGCGGTT

GTCTTAGTCA GAAATGCTAA TTGAAAACTC CAGATGGCAG TATCAGAGTT

GTATTTGTCA AAAATGTTCA TTGAATACAC CAGAGGGCAG TCTTACAGTG

CTATTAGTCA GAAATGCTAA TTGAAAACAC CAGATGGCAG TATCACAATT

ACCTTGCTTT GCTAATTTTC TTTGAAATCT TGCCCTCCTA AGAAAACACA

ACCTTGCTTT GCTAATTTTC TTTGAAATCT TGCCCTCCTA AGAAAACACA

ACCTTGCTTT GCTAATTTTC TTTGAAATCT TGCCCTCCTA AGAAAACACA

ACCTTGCTTT GCTAATTTTC TTTGAAATCT TGCCCTCCTA AGAAAACACA

ACCTTGCTTT GCTAATTTTC TTTGAAATCT TGCCCTCCTA AGAAAACACA

ACCT----TT GCTAATTTTC TTTGAAATCT TGCCCTCCTA AGAAAACACA

ACCTTGCTTT GCTAGTTTTC TTTGAAATCT TGCCCTCCTA GGAAAACACA

ACCTTGCTTT GCTAGTTTTC TTTGAAATCT TGCCCTCTTA GGAAAACACA

ACCTTGCTTT GCTAATTTTC TTTGAAATCT TGCCTTACTA AGAAAACACA

ACCTTGCTTA GCTAGTTTTC TTTGAAATCT TGCCCTCCTG AAAAAACACA

ACCTTGCTTT GCTAATTTTC TTTGAAATCT TGCCCTCCTA AGAAAACACG

ACCTTGCTTT GCTAATTTTC TTTGAAATCT TGCCCTCCTA AGAAAACACA

ACCTTGCTTT GCTAATTTTC TTTGAAATCT TGCCCTCCTA AGAAAACATA

ATCTTGCTTT GCTAATTTCC TTTGAAATCT TGCCCTTCTA AGAAAACACA

ACCTTGCTTT GCTAATTTTC TTTGAAATCT TGCCCTCCTA AGAAAACACG

ACCTTGCTTT GCTAATTTTC TTTGAAATCC TGCCCTCCTA AGAAAACACG

ACCTTGCTTT GCTAATTCTC TTTGAAATCT TTCTTTCCTA AGAAAACACA

CCCTCGCTTT ACTAATTTCC TTTGAAATCT TCCTCCCCTA AGAAAACAGT

TTGAGAAATC TCTTTGAAAC ATTATCTTTT GTCAGCAGTA TATTAAAATT

TTGAGAAATC TCTTTGAAAC ATTATCTTTT GTCAGCAGTA TATTAAAATT

TTGAGAAATC TCTTTGAAAC ATTATCTTTT GTCAGCAGTA TGTTAAAATT

TTGAGAAATC TCTTTGAAAC ATTATCTTTT GTCAGCAGTA TGTTAAAATT

TTGAGAAATC TCTTTGAAAC ATTATCTTTT GTCAGCAGTA TATTAAAATT

TTGAGAAATC TCTTTGAAAC ATTATCTTTT GTCGGCAGTA TATTACAATT

-TGAGAAACC TCTTTGAAAC ACTAGCTTTC GTCAACAGTC TACTAAA-TC

-TGAGAAATC TCTTTGAAAC ACTAGCTTTT GTCAGCAGTC TATTAAAATG

TTGAGAAATC TCTTTGAAAC ATTATCTTTT GTCAGCAGTA TATTAAAGTC

TT--GAAATC TCTTTGAAAC ATTATCTTCT GTCAGCAGTA TATTAAAATC

TTGAGAAATC TCTTTGAAAC ATTATCTTTT GTCAGCAGTA TATTAAAATT

TTGAAAAATC TCTTTGAAAC ATTATCTTTT GTCAACAATA TATTAAAATT

TTGAAAAATC TCTTTGAAAC ACTATCTTTT GTCAGTAATA TATTAAAATT

TTGAGAAATC TCTTTGAAAC ATTATCTTTT ATCAGCAGTA TATTGTAATT

TTGAGAAATC TCTTTGAAAC ATTATCTTTT GTCAGTAGTA TAATAAAATT

TTGAGAAATC TCTTTGAAAC ATTATCTTTT GTCAGTAGTA TACTAACATT

TCAAGAAGCC CCTTTGAAAT GTGATCTGTT GTCAGCAGTA TTTTAAAATT

TGGAGAAATG TCTTTGAAAT CTTATCTTTT GCCGGTAGTA TATTACAGTT

ACAGCCCTTC CTGACTTTAT ATTGAGAATT TAAACTACAT GTGTTATGTA

ACAGCCCTTC CTGACTTTAT ATTGAGAATT TAAATTACAT GTGCTATGTA

ACAGCCCTTC TTGACTTTAT ATTGAGAATT TAAACTACAT GTGTTATTTA

ACAGCCCTTC TTGACTTTAT ATTGAGAATT TAAACTACAT GTGTTATTTA

ACAGCCCTTC CTGACTTTAT ATTGAGAATT TAAACTGCAT GTGTTATTTA

ACAGCCCATC ATGGCTTTCC ATTGAGAATG TAAACTACAC GTGTTATTTA

ATAGCCCTTC AGGAATCCAT ATCAAGAGTT TAAAGTACAT GTGTTATTTA

ATAGCCCTTC AGGAATTCAT ATTGAGAGTT TAAAGTACAT GTGTTATTTA

ACAGTCCTTC ATGACTTTAA ATTGAGAATT TAAACTACAT GTGTTATTTC

ACAGTCTTTC ATGGCTTTAT ATTGATAATT TAAACTACAT GTGTTATTTA

ATAGCCTTTC ATGACTTTAT ACTGAGAATT TAAACTGCAC ATGTTATTTA

ACAGCCCTTC GTGACTTTAT ATTGAGAACC TAAACTACGT ATGTTATTTA

ACAGCCCTTC ATGAC---AT ACCGAGAATT TAAACTACAC GTGTTATTTA

ATAGCCCTCC ATGACTTTAT ATTGAGAATT CAAACTACAT GTGCTATTTA

ACAGCCCTTC ATGGTTTTAT ATTGAGAATT AAAACTACAC GTG-------

ACAGCCCTTC ATGACTTTAT ACCGGGAATG TCAACTGCAT GTGTTAGTTA

ACAGCCCTGC ATGACTTGGC TTTGAGAATT TAAACTGCAT GTGTTGTTTA

ACAACTTTGA AAGCCTCTAA TTTCAAAATG TAAGCTGCAT GTGTTGTTTC

GCTCTAATCC CACCTCAGAT GTTATGTTTG TAAACTTACA CCTCCTGGTT

GCTCTAATCC CACCTCAGAT GTTATGTTTG TAAACTTACA CCTCCTGGTT

GCTCTAAGCC CACCTCAGAT GTTATGTTTG TAAACTTACA CCTCCTGGTT

GCTCTAAGCC CACCTCAGAT GTTATGTTTG TAAACTTACA CCTCCTGGTT

GCTCTAATCC CACCTCAGAT GTTATGTTTG TAAACTTATA CCTCCTGGTT

GCTCTAACCC CACCTCAGAT GTTATGTTTG TAAACTTATA CCTCCTGGTT

GCTCTAATCC CACCTCAGAT GTTATGTTTG TAAACTTATA CCTCCTGGTT

GCTCTAATCC CACCTCAGAT GTTATGTTTG TAAACTTATA CCTCCTGGCT

ACTCTAATCC CACCTCACAT GTTATGTTTG TAAACGTATA CCTCCTGGTT

GCTCTAATCC CACCTCAGAT GTTATGTTTG TAAACTTATA CCTCCTGGTT

GCTCTAATTC CACCTCAGAT GTTATGTTTG TAAACTTACA CCTCCTGGTT

GCTCTAATCC CACCTCAGAT GTTATGTTTG TAAACTTACA CCTCCTGGTT

GCTCTAATTC CACCTCAGAT GTTATGTTTG TAAACTTATA CCTCCTGGTT

GCTCTAATCC CACCTCAGAT GTTATGTTTG TAAACTTACA CCTCCTGGTT

---CTAATCC CACCTCAGAT GTTGTGTTTG TAAAGTTATA CCTCCTGGTT

GCTCTAAGCC CACCTCAGAT GTTGTGTTTG TAAAGTTATA CCTCCTGGTT

GCTCTGATCC CACCTCAGAT GTTATGTTTG TAAAGTTATC CCTCCTGCTT

ACTCTGATCC CACCTCAGAT GTTTGGTTTG TAAAGTTACA CCCCCTGGCT

CATTTTATAG GATGCAATTA AATGATAGTT GCAAATCGCT CTGACACAGT

CATTTTATAG GATGCAATTA AATGATAGTT GCAAATCGCT GTGACACAGT

CATTTTATAG GATGCAATTA AATGACAGTT GCAAATCGCT GTGACACAGT

CATTTTATAG GATGCAATTA AATGACAGTT GCAAATTGCT GTGACACAGT

CATTTTATAG GATGCAATTA AATGACAGTT GGAAATCACT GTGACACAGT

CATTTTATAG GATGCAATTA AACGATAACT GCAAATGGTT CGGACACAGT

CATTTTATAG GATGCAATTA AATGATGGCT GCAAATGCTT GTGGCTCAGC

CATTTTATAG GATGCAATTA AATGATGGCT GCAAATGGTT GTGGCTCAGT

CATTTTATAG GATGCAATTA AATGATAGCT GCAAATGGTT GTGACACAGT

CATTTTATAG GATGCAATTA AGTGATAGCT CAAAATGGTT GTGACACAGT

CATTTTATAG GATGCAATTA AGTGATAGCT GCACATGGTT GTGACACAGT

CATTTTATAG GATGCAATTA AGTGATAGCT GCAAATGGTC ATGACACAGT

CTTTTTATAG GCTGCAATTA AGTGAGAGCT GCAAATGGTT GTGACACCGT

CATTTTATAG GAAGCAATTA AATGATAGCT GCAAAAGGTT GTGACACAGT

CGTTTTATAG GGTGCAATTA AATGATAGCT GCTAAAGGCT GTGACCCAGT

GATTTTATAG GGTGCAATTT AATGATAGTT GCTAACGGTT GTGACACAGT

CTTTTTATAG GATGCAATTA AATGATAGCT GCAAATGGTA TTGATACAGC

CTTTTTACTG GAGGGAATCA ATTGATAGCC ACGCATGGGA ACAACAGAAC

AATGTATTTT AGCATGGTAG CTTGATTTAA GGCTACTGAC ACGTTTGTTC

AATGTATTTT AGCATGGTAG CTTGATTTAA GGCTACTGAC ACGTTCGTTC

AATGTATTTT AGCATGGTAG TTTGATTTAA GGCTACTGAC ATGTTTGTTC

AATGTATTTT AGCATGGTAG TTTGATTTAA GGCTACTGAC ATGTTTGTTC

AATATATTTT AGCATGGTAG TTTGATTTAA GGCTATTGAC ACGTTTGTTC

AATATATTTT AGCACGGTTG TTTGATTTAA GGCTATTGAC ATGTTTGTTC

CATATATTTT AACATGGTCA TTTGATTTAA AGCCATTGAC ATGTTGATTC

CATATATTTT AACATGGTCA TTTGATTTAA AGCCAGTGAC ATGTTGATTC

CATATATTTC AACAT-GTAG TTTGATTTAA AGCCATTGAA ATGTTTGTTC

AATATATTTC AGCATGGCAA TCTGATTTAA GGCTATTGAT ATGTTTGTTC

AATAAATTTT CGCATGGTAG TTTGATTTAA GGCTATTGAT ATGTTTGTTC

AATATATTTT AGCATGGTAG TTTGATTTAA AGCTATTGAT ATATTTGTTC

AATATATTTT AGCACTGTAG TTTGATTTAA GGCTATTGAT ATGTTTATTC

AATAAATTTT AACATGGTAG TTTGATTTAA GGCTATTGAC ATGTTTTTCC

AGTATATTTT AGCATGGTAG TTTGATTTAA AGCTATTGAC ATGTTTGTTC

AATATAATTT AGCATGGGGG TTTGATTTAA AGCTATTGAC ACATTTGTTC

TATATATTTT AACATGGAAG GTTGATTTAA AGCTATCAAC ATGTTTGTTC

CGCAGAATTT TACATGGTAT TTTGATTTAA GGCGTTCAGC GTGTTGACTC

AAAAGAAGTC ATCATTAGGT ACTAGCAAAA CACATTTTAT CTTTTGCAAG

AAAAGAAGTC ATCATTAGGT ACTAGCAAAA CACATTTTGT CTTTTGCAAG

AAAAGAAGTC ATCTTTAGGT ACTAGCAAAA CACATTTTAT CTTTTGCAAG

AAAAGAAGT- ---------- ---------- ---------- ----------

AAAAGAAGTC ATCTTTAGGT ATTAGCAAAG CACATTTTAT CTTTTGCAAG

AAAAGAAGCC ATCTTTATGC ATTAGCCAAA TACATTTTAT ATTTTCTAAG

AAAAGAAGCC ATCTTTATTC ACCAGCAAAA TACA-TTTAT CTCTCCTAAG

AAAAGAAGTC ATCTTTATTC ACCAGCAAAA TACA-TTTAT CTCTCCTAAG

AAAAGAAGTC ATCTTTATGT ACCAACAAAA TACAGTTTAT TTTTGCCATG

AAAAGAAGTC ATCTTTATGT ACTAGTAAAA TAGATTTTAT CTTTGCTAAG

CAAAGAAGTC ATCTTTATGT ACTAACAAAA TACATTTTAT CTTTGCTAAG

AAAAGAAGTC ATCTTTATGT ACTAACAAAA TACAATTTAT CTGTGCTAAG

-AAAGAACTC ATCTTTATGT ACTAGCAAAA TACCtttc-t ctttgcc-ag

AAAAGAAGTC ATCTTTGAAC ACTAGCAAAA TACATTTCAT CTCTTCCAAG

AAAGGAAGTC ATCATTATGC ACCAGCAAAA TACATTTCAT CTCTTCCAAG

AAAGGATGTC ATCTTTATGC ACTAACAACA TACATTTCAT CTTTTCCA--

AAAGTAGGTT ACCTTTATGC AATTGCGAAA TACATTTTAT CTTTCCCATG

AAAGTGGGTA GCCCATATTT ACTGTCAAAA TGCCCTTCAT CTATCTCCAG

ACAATTTCTA GAGTGCTCTA CATGTTTGCT TTTTACTCAA CTTTAACATG

ACAATTTCTA GAGTGCTCTA CATGTTTGCA TTTTACTCAA CTTTAACATG

ACAATTTCTA GAGTGCTCTA CATGTTTGCT TTTTATTCAA CTTTAACATG

---------- ---------- ---------- ---------- ----------

ACAATTTCTA ACGCGCTCTA CAAGTTTGCT TTTTATTCAA CTTTAACATG

ATAATTTCTA CAGTGCTCTA CATGTTTGCT TTTTATATAA TTTTAACATG

ACAATTTCTA GAGTGCCCTA CATGTTTGCT TTTTATACAA TTTTAACATG

ACAATTTCGA GAGTGCCCTA CATGTTTGCT TTTTATACAA TTTTAACATG

ACAATTTCTA GAGTGCTCTG CACATTTGCT TTCTATGTAA TTTTAGCATG

ACAATTTCTA GAGTGCTCTA CATGTTTGCT TTTTATATAA TTTTAACACA

ACAATTTCTA GAGTGCTCTA TATGTTTGCA TGTAATACAA TTTTAACATG

ACAATTTCTA GAGTGCTCTA CATGTTTGCT TTTTCTGTAA TTTTAACACA

acaatttcta gagtgctcta catgtTTGCT TTTTATATAA TTTTAACATG

ACGCTTTCTG GAGTGCTCTA CATGTTTGCT TTTTAAATAT CTTTAACATG

ACAATTTCTA GAGTGCTCTA CACGTTTGCT TTTTATACAA TTTTAATATG

---------- GAGTGCTCGA TGTGTTTGCT TTTTATACAA TTTTAACATG

GCATTTTGAA GAGCGCTATA CATATTTGCT ACTTACATAA TTTTCACACA

AGTAATTCTA AAGTGCAGTT CGAATTTGCT TTTCATATGA TTTTTACACA

TAACATCTTT CAGCAGAAAA TCTCAAGAAG CTTTTATAAA CGTTAATTAA

TAACATCTTT CAGCAGAAAA TCTCAAGAAG CTTTTATAAA CGTTAATTAA

TAACATCTTT CAGCAGAAAA TCTCAAGAAG CTTTTATAAA CGTTAATTAA

---------- ---------- ---------- ---------- ----------

TAACATCTTT CGACAGAAAA TCTCAAGAAG CTTTTATAAA CGTTAATTAA

TAATATCTTG CAGCAGACAA TCTCAAGAAG CTTTTATAAA CATTAATTAA

CAATATCTTT CCGTAGACAA TCTCAAGAAG CTTTTATAAG CATTAATTAA

CACTATCTTT CAGGAGACAA CCTCAAGAAG CTTTTATAAG CATTAATTAA

TACTATCTTT CAGCAGACAA TCTCAAGAAG CTTTTATAAA CATTAATTAA

TAATATCTTT CAGCAGACAA TCTCAAGAAG CTTTTATAAA CATTAATTAA

TAATATCTTT CAGCAGACAA TTTCAAGAAG CTTTTATAAA CATTAATTAA

TAGTATCTTT CAGTGGACAA TCTCAAGAAG CTTTTATAAA TGTTAATTAG

TAATAACTTT TAGCAGACAA TCTCAAGaag cttttataaa cattaattaa

CAATGTCTTT CAGCAGGCAA TCTCAAGAAG ATTTTATAAA CATTAATTAA

TAGTATCTTT CAGCAGACAA TCTCAAGAAG CTTTTATAAA CATTAATTAA

TCATATCTTT CAGCAGACAA TCTCAAGAAG CTTTTATAAA CATTAATTAA

GAATGTCTTT TAACAGACTA TATCAGAAAG CTTTTATAAA CATTAATTAA

TGGCCTCTTG TAGCACACGA GTCCAGAAAG CTTTTATAAA CATTAATTAA

CTGAACTTCA CAATACTCTC AAGAACTGAG TATTCATCCA TTTACATGGC

CTGAACTTCA CAACACTCTC AAGAACTGAG TATTCATCCA TTTACATGGC

CTGAACTTCA CAACGTTCTC AAGAACTGAG TATTCATCCA TTTACATGGC

---------- ---------- ---------- ---------- ----------

CTGAATTTCA CAACACTCTC AAGAACTGAG TATTCATCCA TTTACATGGC

CTGAACTTCA CAACACTCTC AAGAACTGAG TATTCATTCA TTTCCACGGC

GTAAACTTCA CAACACACTT GAGAGCTGTA TATTCATCCA TTTACATGGC

CTAAACTTCA CAACACACTT GAGAACTGTA TATTCATCCA CTTACATGGC

GTGAACTTCA CAACACTCTC GAGAACTGAG TATTCATCCA TCTACATGGC

CTGAACTTCA CACCACTCTT GAGAACTGAG AATTCATCCA TTTACATGGC

CTGAACTTCA CAACACTCTC AAGAACTGAG TATTCATCCA TTTACATGGC

CTGAACTTCA CAACACTCTT GAGAACT--- -ATTCATCCC CTTACATGGC

ctgaacctca caacaatctt gagaactaag tattcatcca tttATATGGC

TAGAACTTCA CAACACTCTC CAGAACTAAG TATTCATCCA TCTACATGGC

CTGAACTTCA CAACTCTCTC GAGAACTAAG TATTCATTCA TTTACATGGC

CCGAACTTCA CAACACTCTC GAGAACTAAG TATTCATCCA TTTCATGGGC

CTGAGCTTCA CAACACCCTC GAGTATTAAG TATTTATCCA TTTACATGGC

CTGAGCCTCA AAACACCCTT GCACATTAAG TATTCATCTC TTTCTGTGGC

AGGAAGCCAG ATGAACATTA CGGTTATAAA TCTAAAGATA ATAAAATTAA

AGGAAGCCAG ATGAACATTA CGGTTATAAA TCTAAAGATA ATAAAATTAA

AGGAAGCCAG AAGAACATTA CGGTTATAAA TCTAAAGATA ATAAAATTAA

---------- ---------- ---------- ---------- ----------

AGGAAGCCAG ATGAACATTA CAGATATAAA TCTAAAGATA ATAAAATTAA

AGGAAGCCAG ATGAATGTTA CAATTGTAAA TCGGAAGATA ATAAAATTAA

AGGAAGCCAG ATGAGCATTA CAATTATAAA TCAAAGAATC ATAAAATTAG

AGGAAGCCAG ATGAACATTA CAATTATAAA TCAAAAGATC ATAAAATTAA

TGGAAGCCAG ATGAACATTA CAATTTTAGA CCTACAGATA ACAAAATTAA

AGGAAGCCAG ATGAACATTA CAATtataaa tctaaataaa ataaaattAA

AGGAAGCCAG ATAAACATTA CAATTATAAA TCTAAATAAA ATAAAATTAA

AGGAAGCCAG GAGAACATTA CAATTATAAA TCTATATAAA ATAAAATTAA

AGGAAGCCAA ATGAACATTA CAACTATGAA TCTAAATAAA ATAAAATTAA

AGGAAGCCAG ATCAACATTA CAATTATAAA TCTGAAGATA ATAAAATTAA

AGGGAGCCAG ACGAATATTA CAATTATAAA TCTAAAGATA ATAAAATTAA

AGGCAGCCAG CGGAACATCA CAATTACAAA TCTAAAGATA ATAAAATTAA

AAGCAGCCAA ATGAATATAA TAGTCATAGC TCCAGAGATG ATAAAATTAC

ACACAGCCAG GCGAACATTA TAGTCATGGC TGTGGAGACT GCAAAATTAA

AGATTTTCAT CAGATAGACT TTATTCAATC CACGTGTCAG GAAAGGAAAG

AGATTTTCAT CAGATAGACT TTATTCAATC CACGTGTCAG GAAAGGAAAG

AGATTTTCAT CAGATAGACT TTATTCAATC CACGTGTCAG GAAAGGAAAG

---------- ---------- ---------- ---------- ----------

AGATTTTCAT CAGATAGACT TTATTCAATC CACGTGTCAG GAAAGGAAAG

AGATTTTCAT CAGATAGACT TTATTCAACT CACGTGTCAG GAGAGGAT-G

AGTTTTTCAT CACGGACATT TTATTCAGTT CATGTGTCCG GAAGGGAGAG

AGTTTTTCAT CATGGAGATT TTATTCAGTT TATGTGTCAG GAAGGGATAG

AGCTTTTCAT CAGATATACT TTATTCAATT CATATGTTAG GACAGGAAAG

CGATTCTCAC CAGATAGACT TTATTCAATT CATGTGTAAG GAAAAGATAG

AGATTCTCAT CAGATAGTCT TTATTCAATT CACATGTCAG AAAAGGATAG

AGATTCTCAT CAGATAGAGT TTATTCAATT CACGTGTCAG AAAAGAATAA

ATATTTTCAT CAGATAGACT TTATTTGATT C--GTGTCAG CAAAAGATA-

CAACTTTCAC TAGATAGAGT TTATTCAATT CATGTGTCAG GAAAGGATAA

TCATTTTCAT CAGATAGACT TTATTCAATT CATGTGTCAG GAAAGGATAA

TGATTTTCAT CAGGGAGACT TTCCTCAATT CATGTGCCAG GAAAGGCTGA

AGATTTTAAT CAGATAGACT TTATTCTGTT GATGCGTCAA TAAATGATAA

CCCTCTTCAA CAAATAGTCC TTGTTCATTC CATCTGTCAA GAAATAATGA

CATTTTGAAG AAAATAAATT ATATATTAAT GTATATTGTG TGTATTTTTG

CATTTTGAAG AAAATAAATT ATATATGAAT GTATATTGTG TGTATTTTTG

CATTTTGAAG AAAAGAAATT ATATATGAAT GTATATTATG TGTATTTTTG

---------- ---------- ---------- ---------- --------TG

CATTTTGAAG AAAATAAATT ATATGTGAAT GTATACTATG TGTATTTTTG

CATTTTGAAT AAAGGAAAGT ATATATGAAT ATATTTTATG TGTATTTTTG

TTTTTTGAAT GAAATAAACT ATGTATGAAT ATGCATTA-- TATGTTTTTG

TTTTTTGAAT GAAATAAACT ATGTATGAAT ATGCATTA-- TATGTTTTTG

CATCTCAAAG GAAATAAACT GTATAGGAAT GTATATTATG TATATTTTTG

TATTCTGAAT AAAATAAACT ATATACGAAT ACATGTTATG TGTACTTTTG

CATTTTGAAT GAAATAAATT ATTTATCCAT GTATGTAATG TATACTTTTG

CCTT-TGAAT GAAATAAATT ACATACAAAT GTATATTATG TGTATTATTG

CATTTTGAAT GAAATAAATT ATATATAAAT GTATATTGAG TATATTTTTG

CATTCTGAGT GAAACAAATA ATATATGAAT GAATATTTTG TGTATTTTTG

CATTTTGAAT AAAATAAATT ACATGTGAAT GTATATGATG TGTATTTTTG

CATTTGGAAT AAAATACATT ATATACGAAT GTATATTATG TGCATTTTTG

CATTTAGAAT CAAACAAATT ATGTATACAT GTATATTATG TGTATTTTTG

TGTTTGGAGA CAAGCAGATT ATGTATGTAT GCACATTCTA CGCATTTTTG

GCATTTCATC AGCCAGACAC ATGCAATTGC CATGCAAATG GATTCAGAAA

GCATTCCATC AGCCAGACAC ATGCAATTGC CATGCAAATG GATTCAGAAA

GCATTCCATC AGCCAGACAC ATGCAATTGC CATGCAAATG GATTCAGAAA

GCATTCCATC AGCCAGACAC ATGCAATTGC CATGCAAATA GATTCAGAAA

GCA------- -GCCAGACAC ATGCAATTGC CATGCAAATG AATTCAGAAA

GCATTTCATC AGCCAGACAC ATGCAATTGC CATGCAAATA GATTCAGGAA

GCATTCCAAC AGCcacacac acacaacTCC CATGCAAATG GATTCAGAAG

GCATTCCAGC AGCCACACAC ACACAATTGC CATGCAAATG GATTCAGCAG

GCATTCCATC AGCCAGACAC ATGCAATTGC CATGCAAATG GATTCAGAAA

GCATTCCATC AACCAGACAC ATGCAATTGC CATGCAAATG GATTCAGA--

GCATGCTTCC AGCCAGACAC ATGGAGTTGC CG-GCAGATG GATCCAGAAA

GCATTCCATC AGCTAGACAC ATGTAATTGC CATGCAAATG GATTCAGAAA

CCATTCCATC AGCCACTTAC CTACAATTGC CATGCAAATG GATTCAGAAA

GCATTCCATC AGCCAGACAC ATGCAATTGC CATGCAAATA GATTCAGGAA

GCATTCCATC AGCCAGACAC ATGCAATTGC CATGCAAATA GATTCAAGAA

GCATTCCCTC ACCCATACCC AGGCACTCGC CATGCCAGTG GATTCAGGAA

GCATTCTATC ATCCAGACAC ATGCAATTGC CATGCAAATG GATTCAATAA

GACTTCCATC AGCAGGGCTG AAGCCGGCCC CGTGCAGAGG AATTGGGCGC

AACATTTATT CCCTTACGCT AAACTATTAT AGCTGACTAA TGATTTTTTC

AACATTTATT CCCTTACGCT AAACTATTAT AGCTGACTAA TGATTTTTTC

AACATTTATC CCCTTACGCT AAACTATTAT AGCTGACTAA TGATTTTTTC

AACATTTATC CCCTTACGCT AAACTATTAT AGCTGACTAA TGATTTTTTC

AACATTTATT CCCTTACGCT AAACTATTAT AGCTGACTAA TGATATTTTC

AACATTTATT CCCGTACGCT AAACTATTAT AGCTGACTAA TGATTTTCTC

AACATTTTTT CCTTCACGCT CAACGGTTAT AGCTGACTAA TGATTTCTTC

AACGTTTTTT CCTTTATGCT CAACTATTAT AGCTGACTAA TGATTTCCTC

AACATTTTTG CCTTTACGCT AGACTATTAT AGCTGACTAA TGATTTTTTC

--CATTTTTT CCTTTACGCT AAACTATTAT AGCTGACTAA TGATTTTTTC

ACCAGTTTGT CCTTTAGGCT AAACTATTCT AGCTGACTAA TGATTTTTTC

AACATTTTCT CCTTTACGCT AAACTATTAT AGCTGACTAA TGTTTTTTTC

AACATTTTTT CCTTTACGCT AAACTATTAT AGCTGACTAA TGATTTTTTC

AACAATTTTT CCTTTACGCT AAACTGTTAT AGCTGACTAA TGTTTTTTTC

AACATATTTT CT-TTATGCT GAACTGCCAT AGCTGACTAA TGTTTTTTTC

AACATTTTTT CCATTACGTG AAGCTGTGAT AGCTGACTAA TGTTTTTTTC

AACATTTTTT CCTTTACGCA GAACTGTTAT GGCTGACTAA TGATTTTTTC

CACCTGTTTT CCCTGGCGCT AAAGTCCTGT CGCTGACTAA TGCCTTTTTC

TGCTGAAGAT GATCTGAAAA TGATCATCAT GTTTCTCTGT GCTACAGGGA

TGCTGAAAAT GATCTGAAAA TGATCATCAT GTTTCTCTGT GCTACAGGGA

TGCTGAAGAT GATCTGAAAA TGATCATCGT GTTTCTATGT GCTACAAGGA

TGCTGAAGAT GATCTGAAAA TGATCATCGT GTTTCTATGT GCTACAAGGA

TGCTGAAGAT GATCTGAAAA TGATCATCGT GTTCCTCTGT GCTATGGGGG

TGCTGAAGAT GATCTGAAAA TGATCATCAT GTTTCTCTCT GCTATTGGGA

TGCTGAAGAT GATCTGAAAA TGATCATGGT GTTTCCCTTT GCTACTGGGG

TGCTGGAGAT GATCTGAAAA CGATCACGGT GTTTCCCTTT GCTACTGGGA

TGCTTAGGAT GATCTGAAAA TGAGCATCAT GTTTTTCTGT GCTGCTGGGA

TGCTGAGGAT GATCTGAAAA TGATCATCAT GTTTCTGTGT GCTTCTAGGA

TGCTGAAGAT GATCTGAAAA TGTCCATCAT GTTTCTCTGC ACCACTGGGA

TGCTGAAGAT GATCTGAGAA TGATCATCGT GTTTCTCTGT GCTACTGGGG

TGCTGAAGAT GATCTGAAAA TGATCAGCGT GTATCTCTGT G-TACTGGAA

TGCTGAAGAT GATCTGAAAA TGATCATAAT GTTTCTCCTT GCCACTGGGT

TGCTGAAGAT GATCTGAATA TGATCCTCGT GTTTCTCTGT GCTACTGGGA

TGTTGAAGAT GATCTGAAAA TGATCATCAT GTTTCTCTGT GCTGCTGGGG

TGCGAGCGAT TATCTAAAAA TGATCATCAT GTTTCTCTGT GCTACTGGAA

TGCCGACGAT GATCCGAAAA TGCCCACCAC GTTTCTCTAG ACAACTGGCG

CATAAATTGC ATTTTTATGT CTCAGATATC TTTTCAGCTG TGTTTATATT

CATAAATTGC ATTTTTATGT CTCAGATATC TTTTCAGCTG TGTTTATATT

CATAAATTGC ATTTTTATGT CTCAGATATC TTTTCAGCTG TGTTTATATT

CATAAATTGC ATTTTTATGT CTCAGATATC TTTTCAGCTG TGTTTATATT

CATAAATTGC ATTTTTATGT CTCAGATATC TTTTCAGCTG TGTT----TT

CATAAATTGT GCTTTTATGT CTCAGATATC TCTTCAGCTG TGTTTATATT

CATAAAGTGT GCTTTTATGG CTCTGATATT TCTTCAGCTG TGTTTATATT

CATAAATTGT GCTTTTAT-G CTCAGATATT TCTTCAGCTG CGTTTATATT

CATAAATGGT GCTTTTATGT CTCAGATATC TCTTCAGCTG TGTTTATAGC

TATAAATTGT GCTTTTATGT CTCAGATATC TCTTCAGCTG TGTTTATATT

CATAAATTGT GTTTTTATGT CTCAGATATC TCCTCAGCTG TGTTTATATT

CATAAATTGT GCTTTTATGT CTCAGATCTC TCTTCAGCTC TGTTTATATT

CATAAATTGT GCTTCTATGT CTCAGATATC TCTTCAGCTG TGCTTATAGT

CATAAATTGT GCTTTTATGT CTCAGATATC TCTTCAGCTG TGTTTATATT

CATAAATTGT GCTTTTATGT CTCAGATATC TCTTCAGCTG TGTTTATATC

CATAAATTGT GCTTTTATGT CTCAGATATC TCTTCAGCTG TGTTTATATT

CATGAATTGT GCTCTTATGT CTCAGATATC TCTTTAGTTA TGTTTGTATC

CATGAATCGT GCCTCTCTGC ATCAAATATC TTTTAATCTC AGTTTTTATG

CCGCTTGGGA TTTGTTTCAA TAAAAACATC CAGTAAATCT AAAACTTATT

CCGCTTGGGA TTTGTTTCAA TAAAAACATC CAGTAAATCT AAAACTTATT

CCCCTTGGGA TTTGTTTCAA TAAAAACATC CAGTAAATCT AAAACTTATT

CCCCTTGGGA TTTGTTTCAA TAAAAACATC CAGTAAATCT AAAACTTATT

CCCCTTGGGA TTTGTTTCAA TAAAAACAGC CAGTAAATCT AAAACTTATT

CCCCTTGGGA TTTGTTTCAA TAAAAACATC CAGTAAATCT AAAACTTATT

CCCCTTGGGT TTTGTTTTAA TAAAAACATC CAGTAAATCT AAAACATATT

CCCCTTGGGT TTTGTTTTAA TAAAAACATC CAGTAAATCT AAAACATATT

CCCCTTGGGT TTTGTTTCAA TAAAAACATC CAGTAAATCT AAAATGTATT

CCCCCTGGGA TTTGTTTCAA TAAAAACATC CAGTAAATCT AAAATGTATT

CCCCTTGGGA TTTGTTTCAA TAAAAACATC CAGTAAATCT AAAATGTATT

CCCCTTGGGA TTTGTTTCAA TAAAAACATC CAGTAAATCT AAAACATATT

CCCCTTGGGA TTTGTCTCAA TAAAAACATC CAGTCAATCT AAAACATATT

CCCCTTGGGA TTTGTTTCAA TAAAAACATA CAGTAAATTT AAAATGTATT

CCCCATGGGA TTTGTTTCAA TAAAAACATA CAGTAAATCT AAAATGTACT

CCCCCTGGGA TTGGTTTCAA TAAAAACGTA CAGTAAATCT AAAATGTATT

CCAGTTGGGA CTTGTTGCAA TAAAAGTATA CAGTAAACCT AAAATTTATT

GAAACCAGGA TTTGTTTCCA TAAAGACATA CAGAGTGCCT AACATTTACT

GAGAGAAAAT CACGTGTGGC ATTTGATTTG TTTCCTTTAA TCCTAAAATC

GAGAGAAAAT CACATGTGGC ATTTGATTTG TTTCCTTTAA TCCTAAAATC

GAGAGAAAAT CACCTGTGGC ATTTGACTTA TTTCCTTTAA TCCTAAAATC

GAGAGAAAAT CACGTGTGGC ATTTGACTTA TTTCCTTTAA TCCTAAAATC

GAGAGAAAAT CACATGTGTC ATTTGACTTG TTTCCTTTAA TCCTAAAATC

GAGAGAAAAT CACGTGTGGC ATTTGACTTG TTTTCTTTAA TCCTAAAAAC

GAGAGAAAAT CACGTGTGGC ATTTGACCTG TTTGCTTTAG TCCGAAGAAC

GAGAGAAAAT CACGTGTGGC ATTTGACCTG TTTGCTTTAC TCCAAAAAAC

GAGAGAAAAT CACATGTGGC ATGTGACTTG TTTCCTTTAG TCCTAAAAAC

GAGAGAAAAT CACGTGTGGC ATTTGACTTG TTTCCTTTAA TCCTAAAAAC

GAGAGAAAAT CACGTGTGGC ATTTGACTTG TTTCCTTTAA TCCTAAAAAC

GAGAGAAAAT TGTGTGTGGC ATTTGACTTG TTTCCTTTAA TCCTAAAAAC

GAGGGGAAAA CATGTGTGGT ATTTGACTTG TTTCCTTTAA TCCTGAAA-C

GAGAGAAAAT CACATGTGGC ATTTGACTTG TTTCCTTTAA TCCTAAAAAT

GGGAGAGAAG CACATCTGGC ATTTGACTTG TTTCCTTTAA TCCTAAAAAC

GGGAGCAAAG CACCTGTGGC ATTTGACTTG TTTCCTTTAA TCCTAAAAAC

GAAAGAAAAT TATGTATGGG ATTTGACTTG TTTCCTTTAG TCTTAAAAAA

GAATGAAAAG CCTTTCTAAA CCCTGGCTTG TTTCCGTTAA TCTTTAAAAA

TCAGTGAAAT GCCCAAGCTG CCCTCCAGAA GGCCACTGTG TTAGAAAGGG

TCAGTGAAAT GCCCAAGCTG CCCTCCAGAA GGCCACTGTG TTAGAAAGGG

TCAGTGAAAT GCCGAAGCTG CCCTCCAGAA GGCCACTGTG TTAGAAAGGA

TCAGTGAAAT GCCGAAGCTG CCCTCCAGAA GGCCACTGTG TTAGAAAGGA

TCAGTGAAAT GCCCAAGCTG CCCTCCGTTA GACCATTGTG TTACAAAGGA

TCAGCGAAAT GCCCAGGCTG CCTTCCGGAA GGCCATTGTG TTAGAAAGGA

TAGGTAAAGC GCCTGG---- ----CTAGAA GGCCATTGTG TAAGAAAGGA

TAGATAAAGT GCCCAA---- ----CTAGAA GGCCATTGTG TAAGAAAAGA

TCAGTGAAAT GCCCAGGCCA GCTTCCAGAA GGCCATTGTG TTAAAAAGGA

TCAGTGAAAT ACCCAGGCTG CCTTCCGGAA GGCTATTGTG TTAGAACGGA

TCAGTGAAAT GCCCAGGCTG CCTTCCGGAA GGCCATTGTG TTAGAACGGA

TCGGTGAAAT GCCCAGGCTG CCTTCCAGAA -GCCATTGTG TGAGACTGGA

TCAGTGAAAT GCCCACGCTG CCTTCCGGAA GGCCATTGTG TTAGAAAGGA

TCTGTGAAAT GCCCAGGCTG CCTTCCGGAA GGCCATTGTG TTAGAAAGGA

TCTGTGAAAT GCCCAGGCTG CCTTCCGGAA GGCCATTGTG TTAGAAAGGA

TCTGTGAAAT GCCCATGCTG CCTTCCGGAA GGCCATTGTG TTGGAAAGGA

TCTTCAAAAT GTCCAAGCTG CCTTCAGGAA GACCATTGTG TTGGAGAGGA

TCTACCAAAT GCCCCATCTG CCTTCAGGAA GACCATTGTA TTCGAGAGGA

AGATTATGTA CTGTATTTCT TCCTACTATT TTAAATGCAA ATAAATAATA

AGATTATGTA CTGTATTTCT TCCTACTATT TTAAATGCAA ATAAATAATA

AGATTGTGTA CTGTATTTCT TCCTACTATT TTAAATGCAA ATAAATAATA

AGATTATGTA CTGTATTTCT TCCTACTATT TTAAATGCAA ATAAATAATA

AGATTATGTA CTATATTTCT TCTCACTATT TTAAATACAA ATGAATAATA

AGATTATGTA CTGTATTTCT TCCTAGTATT TTAAATGCAA ATAAATAATA

AGATTATGGG CTCTATGTCT TCCTACTATT TTAAACACAA ACAAATGATA

AGATTATGAG CTCTATGTCT TCCTACTATT TTAAATACAA ACAAATAATT

AGATTATGTG CTGTATTTCT TCCTACAATT TTAAATGCAA ATAAGTGATA

AGATTATGTA CTGTATTTCT TCCTACTATT TTAAATGCAA ATAAATAATA

AGACTATGTA CTGTATTTCT TCCTACTATT TTAAATGCAA ATAAATAATA

AGATTATGTG CTGTATTTCT TTCTATTGTT TTAAATGCAA ATAAATCATA

AGATTATGTA CAGTATTTCT TCCTACTATT TTAGAGGCAA ACAAATAATA

AGATTAGGCA AAGTATTTCT TCCTACtatt ttaaatacaa ataaataata

AGGTTATGTA CTATATTTCT TCCTACTATT TTAAATGCAA ATAAATAATG

AGATTATGTA CTGGATTTCT TCCTACTATT TTAAATGCAA ATAAATGACA

AGATTATGTA CTGTATTTCT TCCTACCATT TTAAATGCaa ataaataata

AGATTATGGG CTGTATTTCT TCCTACTATT TTAAATACAA ACAAATAATA

TATCATGAAc agcattttag ctattaaatt ttaaacctag gggaaattta

TATCATGAAc agcattttag ctattaaatt ttaaacctag gggaaattta

TATCATGAAc agcattttag ctattaaatt ttaaaactag gggaaattta

TATCATGAAc agcattttag ctattaaatt ttaaaactag gggaaattta

TACCATGAAt agcattttag ctcttaaatt ttaaacctgg gggaaattta

TATCATGAAc agcattttag ctcttaaatt ttaaacctag gagacattta

CTCCGTAAA- AGCATTTTCG CTCTTGAGTT TTAAACCTAG GGGAAATTTA

CTTCGTAAG- AGCATTTTTG CTCTTGAGTT TTAAACCTAG GGGAAATTTA

---TATGAAc agcacattca ctcttaaatt ttaaacctaa gggaaattta

TATCATGAAC agcattttgg ctcttaaaat ttaaacctag gggaaatcta

TATCATGAAc aacattttag ctcttaaaat ttaaacctcg gggaaattta

TATCATGAAc aacattttag ctcttaaaat ttaaacctag gagaaattta

CATCATGAAc aacattttag ctcttagact t-aaacctag aggaaattta

tatCATGAAc agcattttaa cccttgaatt ttaaacctag gggaaattta

TATCATGAAC agcattttag ctcttaaatt ttaaacttag gggaaattta

TATCGTGAAc agcactttag ctcttaaatt ttaaacctag gagcaactta

tataataaac agcactttag ttcttcaatt ttaaacctag gggaaattta

TTTCAAGAAC GGCACGCTAG CTCTTAAATT CAAAACCTTG GGGGACTTCT

atccatgtgt ctttgattca ctaaatctta aatcattact caactgtatg

atccatgtgt cttggattca ctaaatctta aatcattact caactgtatg

atccttgtgt ttttgattca ctaaatctta aatcattact caactgtatg

atccttgtgt ttttgattca ctagatctta aatcattact caactgtatg

atctgtgtgt ttttgattca ctaaatctta aatcattacc caactgtatg

atccatgtgt ttttgattca ctaaatctta aatcattacc caactgtatg

ATCCATGTGT TTTTGATTCG CTAAACCTTA AACCATTACC CAACTGGGTG

ATCCATGTGT TTTTGATTCA CTAAACCTTA AACCATTACC CAACTGGATG

atccatgtgt ttt-GATTCA ATGACTTTTA AGCCATTACA CAACTGTATG

atccttgtgt ttttgattca ctaaatccta aatcattacc caactgtatg

atccatgtgt ttttgattca ctaaatccta aatcattacc caactgtatg

atccatgtgt ttttgattca ctaaatccta aatcattacc caactgtatg

atccatgtgt ttttgattca ctaaatccta aatcattacc caactgtatg

atccatatgt ttttgattca caaaatctta aatcattacc caacagtatg

atttaaat-- t--tgattca ccaaatcttg actcattacc caactgtatg

atccatgtgt ttttcattca c-aaatctta actcattaCC CAACTGTATG

atacatgtgt ttctgatgca cttaaactta aatcattacc caactgtatg

TCACATGTGT TTCTGATCAG CTTCTCCTTA AATCATCCCC CAACTGTATG

tccctctaag ctctttgatg tcaagagatc tAGATCTATT CTAAGTCAGT

tccctctaag ctctttgatg tcaagaaatc tAGATCTATT CTAAGTCAGT

tccctctgag ctctttgatg tcaagaaatc tAGATCTATT CTGAGTCAGT

tccctctaag ctctttgatg tcaagaaatc tAGATCTATT CTGAGTCAGT

tccctctaag ctctttgatg tc-------- tAGATCTATT CTGAGTCAGT

tccctctgag ctctttgaag tcaagcaatc tAGATCTACT CTGAGTCAGT

TCCCTCCAAC CTCTTTGATG TCATAAAATC TAGATCTATT CTGAGTGGGT

TCCCTCCGAC CTCTTTGATG TCATAAAATC TAGATCCATT CTGAGTGGGT

TCACTCTAAG TTCTTTGATC TTAAGAAATC TAGATCTATT CTGAGCTAGT

tctctctaag ctctttgatg tcaagaaatc tAGATCTATT CTGAGTCAGT

tctctctaag ctctttgatg ttaagaaatc tA-ATCTATT CTAAGTCAGT

tctctctaag ctctttgatg tcaagaaatc tAGAGCTATT CTGAGTCAAT

tctctctaag ctctttgatg tcaagaaatc tAGATCTATT CTGAGTCAGT

tctctataag ctctttgatg tcaagaaatc tAGATCTCTC ATGAAGCAGT

tctctctaag cactctgatg tcaagaaatc tAGATCTATT CTGAGTCAGT

TCTCTCTTCA TGCT-TCATA GCAAGAAATC TAGATCTATT CTGAGTCAGT

tctctctcaa cactttgatg tcaagcagtc tAGTTCTTCT CTGAATCAAT

GCTTCCAAAG CACTTTATTG GCAAGAAATT GCATTCTCCT CCTAGTCATC

GACCTTGTTC GACAAATTTT TAAAATAAGC CATCTAACTA AATATATGAC

GACCTTGTTA GACAAATTTT TAAAATAAGC CATCTAACTA AATATATGAC

GACCTCGTTC GACAAATTTT TAAAATAAGC CGTCTTACTA AATATATGAC

GACCTCGTTC GACAAATTTT TAAAATAAGC CGTCTTACTA AATATATGAC

GACCCCGTTC AACAAATTTT TAAAAGAAGC CGTCTAACTA AATATATGAC

GACCTGGTTT GATAAATTTT AAAAATAAGC CATCTAACTA AATATATGAC

GACCTGTTTC GGCAAATTTC AAGAATAAGC CATCTAACTA AATATATGAC

GACCTGTTTC GGCAAATTTC AAGAATAAGC CATCTAACTA AATATATGAC

GACCTGGTTC GACAAATTTT AAAAATAAGC CATCTAACTA AATATATGAC

GACCTGATTC AACAAATTTT AAAAATAAAC CATATAACTA AACATATGAC

GACCTGGTTC GACAAATTTT AAAAATAAGC CATCAAACTA AATATATGAC

GACCTGGTTC AACAAATTTT TAAAATAAGC CATCTAACTA AATATATGAC

GACCTGGTTT GACAAATTTT AAAAATAAGC CATCCAAGTA AATATATGAC

GACCTGGTTT GACAAATTTT AAAAATAAGC CATCTAACTA AATATTTGAC

GACCTGGTTT GATAAATTTT AAAAATAAGG CATCTAACTA AATATATGAC

GACCTGGTTT GACAAATTTT TAAAATAAGC CATCTAACTA AATATATGAC

GACCTTATTT GACAAATTTC AAAAATTGGC CATATAACTT AGTGCAGGAC

AGCCAGGATG GACAAATTTG AAACGTCCAC TCTAGAACTG CAGATGGGAC

ATTTATTGAA ATTCATTTTG CACCATGAAG CAACTGCCTT GCAAAGAGTG

ATTTATTGAA ATTCATTTTG CACCATGAAG CAACTGCCTT GCAAAGAGTG

ATTTATTGAA ATTCATTTTG CACCATGAAG CAACTGCCTT GAACAGAGTG

ATTTATTGAA ATTCATTTTG CACCATGAAG CAACTGCCTT GAACAGAGTG

ATTTATTGAA ATTCATTTTG CACCATGAAG CCACTGCCTT GCAAAGAGTG

ATTTATTGAA ATTCATTTTG CAGCATGAAG CAACTGCTGA GCAGAGAGTG

ATTTATTGAA ATTCATTTTG TACCGTGGAG CAACTGCCTT GCAAAACGTG

ATTTATTGAA ATTCATTTTG TACCGTGAAG CAACTGCCTT GCAAAACGTG

ATTTATTGAA ATTCATTTTG TA-TGTGAAG CAACTGCCTT GCACAGAGTG

ACTTATTGAA ATTCATTCTG TACCATGAAG AAACAGCCTT ACAAAGAGTG

ATTTATTGAA ATTCATTCTG TACCACGAAG CAACTGCCTT GCAAAGAGTG

ATTTATTGAA ATTCATTCTG TACCATGAAG CAACTGCCTT GCACAGAGTG

ATTTATTGAA ATTCATTCTG TACCATGAAG CAACTGCCTT GCAAAGAGTG

ATTTATTGAA ATTCATTCTG TACCATGAAG CAACTGCCTT GCTAAGAGTG

ATTTATTGAA ATTTATCCTG CACTGTGAAG TAACTGCCTG GCAAAGAGTG

ATTTATTGAA ATTCATTCTG TACCATGAAG CAACTGCCTT GCAAAGAGTG

ATTTATAGAA ATTCATTCTT TGCCAAGGAG CTACTTTCTT ACAAAAAATA

ATTCACTGAA ATTCATTCCG GAGCCTGGAG CCGCTCGCTT GCAAGAATCT

CTGAAAATAG GAAGGAATTT CTAGACTAAT TTGATTAAAT ATTCAGTTGT

CTGAAAATAG GAAGGAATTT CTAGACTAAT TTGATTAAAT ATTCAGTTGT

CTGAAAATAG GAAGGAATTT CTAGACTAAT TTGATTAAAT ATTCAGTTGT

CTGAAAATAG GAAGGAATTT CTAGACTAAT TTGATTAAAT ATTCAGTTGT

CTGAAAATAG GAAGGAATTT CTAGACTAAT TTGATTAAAT ATTCAGTTGT

CTGAAAATAG GAAGGAATTT CTAGGCTAAT TTGATTAAAT ATTCAGTTGT

CTGAAAATAG GAAGGATTTT CTAAGCTAAT TTGATTAAAT ATTCTGTTGT

CTGAAAATAG GAAGGAATTT CTAGGCTAAT TTGATTAAAT ATTCTGTTGT

CTGAAAATAG GAAGGAATTT CTAGGCTAAT TTGATTAAAT ATTCAGTTGT

CTGAAAATAG GAAGGAATTT CTAGGCTAAT TTGATTAAAT ATTCAGTTGT

CTGAAAATAG GAAGGCATTT CTGGGCTAAT TAGATTAAAT ATTCAGTTGT

CTGAAAATAG GAAGGAATTT CTAGGCTAAT TTGATTAAGT ATTCAGTTGT

CTGAAAATAG GAAGGAATTT CTCGACTAAT TTGATTAAAT ATTCAGTTCC

CAGAAAATAG TAAGGAATTT CTAGGCTAAT TTGATTAAAT ATTCAGTTGT

CTGAAAATAG TAAGGAATTT CTAGGCTAAT TTGATTAAAT ATTCAGTTGT

CTGAAAATAG TAAGGAATTT CGAAGCTAAT TTGATTAAAT ATTCAGTTGT

CTAAGAATAG TAAGAGATTT CAAAAGCAAT TTGACTGACT CTGCTAATGT

CCGTGAATAG GAAGATATTT CAGATGCAAA ATGATTAAAT CTGCTATGTT

TGCCATATGC TTTCGAAAAT TGATTTATCC TTCCTCCCAC CTTGACATGC

TGCCATATGC TTTCGAAAAT TGATTTATCC TTCCTCCCAC CTTGACATGC

TGCCATATGC TTTCAAAAAT TGATTTATCC TTCCTCCCAC CTTGACATGC

TGCCATATGC TTTCAAAAAT TGATTTATCC TTCCTCCCAC CTTAACATGC

TGCCATATGC TTTCGAAAAT TGATTTATCC TTCCTCCCAC CTTGACACTC

TACCATATGC TTTCGAAAAT TGATTTATCC GTCCCCCCAC CTTGACATGC

TGCCATATGC TTTCGAAAAT TGATTTGTCC TTCCTCCCAC CTAGACACGT

TACCATATGC TTTCGAAAAT TGATTTGTCC TTCCTCCCAC CTAG--ACGT

TACCATATGC TTTCGAAAAT TGATTTATCC TTCTTCCCAC CTTGACATGC

TAACATATGC TTTCAAAAAT TGATTTATCC TTCTATCCAC CTTGACATGC

TACCATATGC TTTCGAAAAT TGATTTATCC TTCCTCCCAC CTTGACGTGC

TACCATATGC TTTCGAAAAT TGATTTATCC TTCCTCCCAC CTTGACATGC

TACCATATGC TTTCGAAAAT TGATTTATCC TTCCTCCCAC CTTGACATGC

TGCCATATGC TTCCAAAAAT TGATTTATCC TTTCTCCCAC CTTGACATGC

TAACATCTGC TTTCGAAAAT TGATTTATCC TTCCTCCCAC CTTGACGTGC

TACCATATGC TTTCGAAAAT TGATTTATCC TTCCTCCCAC CTTGACATGC

TACCGTTTGC TTTCAAAAAT TCATTTATCC CTTCTCCCAC CTTGCCATTC

TTCCGTACGC TTTCgccccc tgcctcctca ctcccccACG GTTGACATGC

TTACTGATTG AAAGCTATGC ACAGAAAAGG GCAGGTTATT GCTCCAAATT

TTACTGATTG AAAGCTATGC ACAGAAAAGG GCAGGTTATT GCTCCAAATT

TTACTGATTG AAAGCTATGC ACAGAAAAGG GCAGGTTATT GCTCCAAATT

TTACTGATTG AAAGCTATGC ACAGAAAAGG GCAGGTTATT GCTCCAAATT

TTACTGATTG AAAGCTATGC ACAGAAAAGG GCAGGTTATT GCTCCAAATT

CTTCTGATGG AAAGCTATGC ACAGAAAAGG GCAGGTTTTT GCTTCAAATT

TTACTGATTG GAAGCTATGC ACAGAAAAGG AAAGGTTCTT GATCCAAACA

TGACTGATTG AAAGCTATGC ACAGAAATGG AAAGGTTTTT GTTCCCAACA

TTACTGATTG AAAGCTAAGC ACAGAAAAGG GCAGGTTTTT GCTCCGAATT

TTACTGATTG AAAGCTTTGC ACAGAAAAGG GCAGGTTTTT GCTCCAAATT

TTACTGATTG AAAGCTTTGC ACAGAAAAGG GCAGGTTTTT GCTCCAGATT

TTACTGATTG AAAGCTATGT ACAGAAAAGG GCAGGTTTTT GCTCCAAATT

TTGCTGATTG AAAGCTATAC ATAGAAAAGG GCAGGTTTTT GCTCCAAATT

TTCATGATTG AAAGCTAAGT ACAGAAAAGG GCAGGTTTTT GCTCCAAATT

TTACTGATTG AAAGCTATGC ACAGGAAAGG GCAGGTTTTT GCTCTCGATT

TTAGGGACTG AAAGCTCAGC ACAGAGAAGG CCAGGCTTTT GCTCTAAATT

TGACTGTCAG AAAACTAGGA ACAGAAAGGG ACAGGTTTTT GCTCTAAATT

TCACTGATTG AAAACTATGC ACGAGAAACA ACAGGTATTT GCCTTCAATT

ACAGATGACA CATCTTTGAA CTCTATTGTG TCGGGGAAAA CACCTTCTTG

ACAGATGACA CATCTTTGAA CTCTATTGTG TCGGGGAAAA CACCTTCTTG

ACAGATGACA CATCTTTGAA CTCTATTGTG TGGGGGAAAA CACCTTCTTG

ACAGATGACA CATCTTTGAA CTCTATTGTG TGGGGGAAAA CACCTTCTTG

ACTGATGACA CAGCTTCGAA GTCTATTGTG TGGGGAAAAG CACCTTCTTG

AAAGATGACA CATCTCTGAA CTCTATTGTG TGGGAAAAAA CACCTTCTTA

ACAGATGACA CATCCTTGAC CTGCATTGTC TGAAGA-AAA CACCTTCTTG

ACAGATGACA TATCTTTGAA GTGCATTGTC TGGAGAGAAA CACCTTCTTG

ACAGATGACA CATCTTTGAA CTCTATTGTG TGGAGAAAAG CCCCTTCTTA

GTAGGTGTCA CATCTTTGAA CTCTATTGTG TGGGGGAAAA TACCTTCTTA

ACAGATGACA CATCTTTGAA CTCTATTGTG TGGAGAAAAA CACCTTCTTA

CCAGATGACA CATCTTTAAA CTCTATTGTG TGGAGAAAAA CAGTGTCTTA

ACAGATGACA CATGTTTGAA CTCTATTGTG TGTGGAAAAA CACCTTCTTA

ACAGATGACA CATCTTCGAA CTCTATTGTG TGGGGAAAAA CACCTTCTTA

ACAGATGACA CATCTCTGAA CTCTATTGTG TGGAGAAAAA CACCTTCTTA

ACTGGTGACA CATCTTTGAA CTCTATTGTG TAGAGAGAA- -ACCTTGTTA

ACAGATGACA CATCTTTGAA CTCTATTGTG TGAAGAAAAA CACCTTCTTA

ACTGATGACA CATCTTTGAA CTCCATTGTG TCAAGAAAAA CACCTTCTTA

CTGTAGTTAG ATTTCCTGTT TGTGAGTATT TTCTGGTAAC CAGACTTCCT

CTGTAGTTAG ATTTCCTGTT TGTGAGTATT TTCTGGTAAC CAGACTTCCT

CTGTAGTTAG ATTTCCTGTT TGTGAGTATT TTCTGGTAAC TAGACTTCCT

CTGTAGTTAG ATTTCCTGTT TGTGAGTATT TTCTGGTAAC TAGACTTCCT

CTGTAGTTAG ATTTCCTGTT TGTGAGTATT TTCTGGTAAC TAGACTTCCT

CTGTAGTTAG ATTTCCTGTT TGTGAGTATT TTCTGGTAAC TAGACTTCCC

CTGGAGCTGG ATTTCCTGTT TGTGT---GT TTCTGGTGAC TGTGTTTCCC

CTGTAGCTGG ATTTCCTGTT TGTGA---AT TTCTAGTAAC TCTATTTCCC

CTGTAGTTAG ATTTCCTGTT TGTGAGCATT TTCTGGTAAC TAGACTTCCC

CTGTAGTTAG ATTTCCTGTT TATGAGTATT TTCTGGTAAC TAGACTTCCC

CTGTAGTTAG ACTTCCTGTT TATGAGTATT TTCTGGTGAC TAGACTTCCC

CTGTAGTTAG ATTTCCTGTT TGTGACTATT TTCTGGTAAC TAGACTTTCC

CTGTAGTTAG ACTTCCTGTT TGT--GTATT TTCTGGTAAC TAGACTTCCC

CTGTAGTTAG ATTTCCTGTT TGTGACTATT TTCTGGTAAG TAGACTTCCC

CTCCAGCTAG ATTTCCTGTT TGGGAGTATT TTCTGGTAAC TAGACTTCCC

CTGTAGTTAG ATTTCCTGTT TGTGAGGATT GCCTGGTA-- ---ACTTCCC

CTGAAGCTAG ATTTCCTGTT TGTGCGTCTT TTCTGGTAAC TGAACTTCCC

CTGGAGATAG GTTTCCTGTT TGTGAGTGTT TGCTGGTAAC TGAACTTCCC

ATATTAAATC ACCAGAAAAG TGCAGATAAA ATCCCAGTTT CATTAATGAC

ATATTAAATC ACCAGAAAAG TGCAGATAAA ATCCCAGTTT CATTAATGAC

ATATTAAATC ACCAGAAAAG TGCAGATAAA ATCCCAGTTT CATTAATGAC

ATATTAAATC ACCAGAAAAG TGCAGATAAA ATCCCAGTTT CATTAATGAC

ATATTAAATC ACCAGAAAAG TACAGATAAA ATCCCAGTTT CATTAATGAC

CTATTAAATC ACCAGAAAAG TGCAGATAAC ACCCCAGCTT TATTAAGGAC

ATATTAAGTC ACCAGAGAAG TACAGATAAA ATCCCAGTCT CCCTAATGTC

ATATTATGTC ATCAGGAAAG TACAGAAAAA ATCCCAGTTA CCCTAATGTC

ATATTAAATC AGCAGAAAAG TACAGATAAA ACCCCAGTTT CATTAATGAC

ATATTAAATC ACTAGAAAAG TGCAGATATA ACCCCAGTTT CACTAATGAC

ATATTAGATC CCCAGAAGAG TACAGATAAA ACCCCAGTTT CATTAACCAC

ATATTAAATC ACCAGAAAAG TGCAGATGAA ACTCCAGTTT CATTA---AC

ATAT------ ---------- ---------- ---------- ----------

ATATTAAATC ACTGGAAAAG TGCAGATAAA ACCCCAGTTT CATTAACAAC

ATATTAA--- ---------G TGCAGATAAA AT--CAGTTT TATTAATGAC

CTATTAAATC ACCACAAAAG TGCAGATAAA ACCCCAGTTT CGTTAATGGT

ATATtatatC ATCAGAAAAG TGAAGATAAA ACCCCAATTT CATTAAAAAT

ATTTTAAATC ACTGAGAAAG TGCAGATAAA ACCCCAGTTT CATTAAAGAC

AATGAAAGGT GACAGCCCTT GAATCTGTCA TAAGTTGATT AATTACTTTC

AATGAAAGGT GACAGCCCTT GAATCTGTCA TAAGTTGATT AATTACTTTC

AATGAAAGGT GACAGCCCTT GAATCTGTCA TAAGTTGATT AATTACTTTC

AATGAAAGGT GACAGCCCTT GAATCTGTCA TAAGTTGATT CATTACTTTC

AATGAAAGGT GACAGCCATA GAATCTGTTG TAAGTTGATT AATTATTTTC

AATGAAAGGT GACAAGCTTC GAATCTGTCA TAAGCTGATT AATTACTTTC

G-TGAAAGGT GACAGCTCTT GAATCTGTCA TAAGTTGATT AATTACTTTC

GATGAAAGGT GAAAGATCTT GAATCTGTCA TAAGTTGATT AATTACTTTC

AATGAAAGGT GACAGCCCTT GAATC---CA TAAGTTGATT AATTACTTTC

AATGAAAGGT GACAGTCCTA GAATCTGTCA TAACTTGATT AATTGCTTTT

AATGAAAGGT GACAGCCCTT CAGTCTGTCA TAAGTTGATT AATTACTTTC

AATGAAAGGT GACAGCCCTT GAATCTGTCA TGAGTTGATT AATTACTTTC

---------- ---------- ---------- ---------- ----------

AATGAAAGGT GACAGCACTT GAATCTGTCA TAAGTTGATT AATTACTTTC

AATGAAAGGT GATAGCATTT GAATCTGTCA TAAGTTGATT AATTACTTTC

AATGAAAGGC GATAACATTT GAATCTGTCC TATGTTGATT AATTACTTTT

AATGAAAGGT GACAGCACTT AAAACTCTCA TGAATTGACT AACTATCTCT

AATGAAAGGT GACAGCACTT GAATACGTCA TAAACTGATT AATTACCTCC

CAAACATTAT TAAAGATATG ACGAATATCC TTCATAATCC CAAATGGGTT

CAAACATTAT TAAAGATATG ACGAATATCC TTCATAATCC CAAATGGGTT

CAAATGTTAT TAAAGATATG ACAAATATCC TTCATAATCC CAAATGGGTT

CAAATGTTAT TAAAGATATG ACAAATATCC TTCATAATCC CAAATGGGTT

CAAATGTTAT TAAAGATATG ACTAATTTCC TTCATAATCC CAAATGGGTT

CAAATGTTAT TAAAGATTCA AGGAATATCC CTCGCAATTC CAAATGGGTT

CAACTGCTAT TAAACACTCA GTGAATATCC CTCCCAATTC CAAA-GGGTT

CAACCGCTAT TAAACATTCA GTGAAGATCC CTCTCAATTC CAAA-GGGTT

TAAATGTTAT TAAAGGTTTA ATGAATATCC CTCACAATTC CAAATGGGTT

CAAATGTTAT TAAGGATTCA ATGAATATCC CTCACAATTC CAAATGGGTT

CAAATGTTAC TAAAGATTCA ATGAATATCC CTCACAATTC CAAATGGGTT

CAAACGTTAT TAAAGATTCA ATGAATATCC CTCACAATTC CAAATGGGTT

---------- ---------- ---------- ---------- -AAATGGGTT

CAACTGTTAT TAAAGATTCA ATGAACATCC TTCATAATTC CAAATGGGTT

CAAATGTTAT TAAAGATTCA ATGAATATCT CTCACAATTC CAAATGGGTT

TAAATGTTAT GAAAGATTTA ATGAATACCC CTCACAATTC CAAATGGGTT

TGAATATTAT CAAAATTCCA GTGAATTTTC CTTGCAACTC CATATGGCTT

CGGTTGCTAG TGAAGATTCA ATGAATTTTT CTGACAGTTT CGAATGGGCT

TTATTTTCAG AGTTAGATGA TCATTATATG TGAAATGTCC ATTTATTCTT

TTATTTTCAG AGTTAGATGA TCATTATATG TGAAATGTCC ATTTATTCTT

TTATTTTCAG AGTTAGATGA TCGTTATATG TGAAATGTCC ATTTATTCTT

TTATTTTCAG AGTTAGATGA TCGTTATATG TGAAATGTCC ATTTATTCTT

TTATTTTCAG AGTTAGATGA TCATTATATG TGAAATGTCC ATTTATTCTT

TTATTTTCAG AGTTAGATGA TCATTATATC TGAAATGGCC ATTTATTCTT

TTTTTTTCAG AGTTAGACGA TCATTATATC TGAAATGTCC ATTTATTCTT

TTGTTTTCAG AGTTAGAT-A TCATTATATC TGAAATGTCC ATTTATTCTT

TTATTTTCGG AGCTAGGTGA TCATTATAGC TGAAGTGTCC ATTTATTCTT

TTATTTTCAG AGTTAAATAA TCATTATATC TAAAATGTCC ATTTATTCTT

TTATTTTCAG AGTTAGATGA TCATTATATC TGAAATGTCC ATTTATTCTT

TTATTTTCAG AGTTAGATGA TCATTATATC TGAAATGTCC ATTTATTCTT

TTATTTTCAG AGTTAGATGA TCATTATATC TGAGATGTCC ATTTATTCT-

TTATTTTCAA AATTACATTA TTATTATATC TGAAATGACC GTTTATTCTT

TTATTTTCAG AGTTAGATGA TTATTACATT TGAAATGTCC ATTTATTCCT

TTATTTTCAG AGTTAGATGG TGATTATATT TG-------- ----------

TTATTTTCAG TAGTCTGTAA TTATTATATG AAAAATGGTC ACTAGATACC

CTCCTCTCAG CATTCTTCCG GCCTCCTGCG TGAATTGTCC TTTGGTTGCC

CTTTCCAAGA AGTTGAGTAC TTTCAACCAT TCTTAAAGGA CAAAGATGTT

CTTTCCCAGA AGTTGAGTAC TTTCAACCAT TCTTAAAGGA CAAAGATGTT

CTTTCCCAGA AGTTGAGTAC TTTCAACCAT TCTTAAAGGA CAAAGATGTT

CTTTCCCAGA AGTTGAGTAC TTTCAACCAT TCTTAAAGGA CAAAGATGTT

CTTTCCCACA AGTTGAGTAT TTTCAATCAT TCTTAAAGGA CAAAGATGTT

CCTTCCCAGA AGTGGAGTAC TTGCAACCAT TTTTAAAGGA CAAAGAGGTT

TTTTCCCAGA AGTCGCATGT TGTCAGCCAT TCTTAAAGAA CAAAGATGTT

TTTTCCCAGA AGTTGCATGT TGTCAGCCAT TCTTAAAGAA CAAAGATGTT

CCTTCCCAGA AGTTGAGTAC TTTCAACTAT TCTTAAAGGA CAGAGATGTT

AATTCCCAGA AGTTGAATAC TTTCAGCCAT TCTTAAAGGA CAAAGATATT

CTTTCCCACA AGTTGAATAC TTTCAGCAGT TCTTAAAGGA CAAAGATGTT

CTTTCCCAGA AGTTGAATAC TTTCAGCCAT TCTTAAAGGA CAAAGATGTT

--TTCACAGA AGTTAACTAG TCTCAGCAGT ACTTAAAGAA CAGAGATGTT

CTTGCCTAGA AAGTAAATAC TTTTAGCTAC TCTTAAAGGA CAGAGATGTT

CTTGCCTAGA AATTGAATAT TTCCAACCAT TCTTAAAGAA CAAAGATGTT

---------- ---------- ---------- ---------- ----------

GTTGCCTAGA AATGACATTT TTTTAACCAT TCTTAGAGGA CAATGATATT

GTTGCCCAGA AATGGAATTT TTTTGCCCGT TCCTTTGGGA CAGGGGTGGT

TGTTTGATTC ACTTTCACCT GATATGTGCA CAACATTTTA AGAGAATGAA

TGTTTGATTC ACTTTCACCT GATATGTGCA CAACATTTTA AGAGAATGAA

TGTTTGATTC ACTTTCACCT GATATGTGC- --ACATTTTA AGAGAATGAA

TGTTTGATTC ACTTTCACCT GATATGTGC- --ACATTTTA AGAGAATGAA

TGTTTGATTC ACTTTTACCT GATATGTGTA CAACATTTTA AGAGAATGAA

TGTTTGATTC ACTTTCACCT GATATGTGCA CAACATTTTA GAAGAATGAA

TGTTTGATTC GGTTTCATCT GCTGTGTGC- --ACATTTCA AGGGAATGAA

TGTTTGATTT GGTTTCATCT GCTATGTGC- --ACATTTCA AGGGAATGAA

TGTTTGATTC AATTTCACCT GATATATGCA CAACATTTTA AGAGAATGAA

TAATTGATTC AGTTTCACCT GCTATGTGCA TGGCATTTTA AGAGAATGAA

TAGTTGATTC AGTTTCACCT GCTATGTGTA CAACATTTTA AGAGAATGAA

TAGTTGACTC AGTTTCACTT GCTATGT--- ---------- -GAGAATTAA

TATTTGATTC ACTTTTACCT GCTATGTGCA CAACATTTTA AGAGAATGAA

TGTTTGATTC AGTTTCACCT GATATGTGCT CAACATTTTA AGAGAATGGA

TGTTTGATTC AGTTCCACCT GATATGTGTA CAACATTTTA AGAGAATGAA

----TGATTC AGTTTCACCT GATGTGTGCA CAGCCATGTA AGAGAATGAA

TGTTTGATTC AGTTTCACTT GACATGTGTA CAACATTTTA AGAGAATGAA

TGTTCAGCTC CATCTCACTT GATATGGGCA TGGCATCTGG GGAGAATTAA

TTAGTCTGTT TGAATCATTG TTTTTCCCTG AGAAGTAGTT GATCTTACAT

TTAGTCTGTT TGAATCATTG TTTTTCCCTG AGAAGTAGTT GATCTTACAT

TTAGTCCGTT TGAATCATTG TTTTTCCCTG AGAAGTAGTT GATCCTACAT

TTAGTCCGTT TGAATCATTG TTTTTCCCTG AGAAGTAGTT GATCCTACAT

TTAGTCTGTT TGAATCATTG TTTTT-CCTG AGAAGTAGTT GATCTTACAT

TTAGTCTGTT TGAATCATTG TTTTC-CCTG AGAAGTAGTT GATCTTACAT

TTAGTCCGTT TGAATCATTG TTTTTCCCTG AGAAATGCTT GATCTTGCAT

TCAGTCTGTT TGAATCATTG TTTTTTCCTG GGAAATAGTT GATCTTGCAT

TTA--CTGTT TGAATCATTG T--------- ---------- GATCTTACAT

TTAGTCTGTT TGAATCATTG TTTTTCCCTG AGAAATAATT GATCTTACAT

TTAATCTGCT TGAATCATTG TTTTTCCCCA AGAAATAATT GATCTTACAT

TTAGACTATT TGAATCATTG TTTTTCCCTG AGAAATAATT GATCTTACAT

TT--TCTGTT ATACTCATTG TTTTTCTCTG AGAAATAATT GATCTTACCT

TTAGCCTGTT TGAATCATTG TTTTTCCCTG AAAAATAGTT TTTCTTACAT

TTAGTCAGTT TGAATCATTG TTTTTCCCTA AGAAATAGTT GATCTTACAT

TTAGTTGGTT TGAATCA--G TTTCTCTCTA AGAAATAGCT GATCTCACGT

TTAATCTGCT TGAATCATGG CTTTTCCCTG AGAAATAATT GATCTAATGT

TTAGATTCTT TAATTCATTT GCTTTCCATG GGAAATAATT GATCTAATGC

TAAACATTTG TCTGTAACAA TAGGTTAACT ATAGCCAAAT TATAACCATA

TAAACATTTG TCTGTAACAA TAGGTTAACT ATAGCCAAAT TATAACCATA

TAAACATTTG TCTGTAACAA TAGGTTAACT GTAGCCAAAT TATAATCAAA

TAAACATTTG TCTGTAACAA TAGGTTAACT GTAGCCAAAT TATAATCAAA

TAAACATTTG TCTGTAACAA TAGGTTAACT ATAGCCAAAT CATAACCATA

TAAGCATTTG TCTGTAACAA TAGGTTAATC ATCGCCAAAT TATAACCATA

TAAGCATTTG TCTGTAACAA TAGGTTAAGT AGAGCCGAAT TATAACCATA

TAAGCATTTG TCTGTAACAA TAGGTTAACT ATAGCCAAAT TATTACCATA

TAAGCATTTG TCTATAACAA TAGGTTAACT ATAGCCAAAT TCTAACTGTA

TAAGCATTTG CCTATAACAA TAGGTCAATT ATAGCCAAAT TATAACCATA

TAAGCATTTG TCTATAACAA TAGGTCAGCT ATAGCCAAAT TATAACCATA

TAAGCATTTG TTTATAACAA TAGGTCAACT ATAGCCAAGT TATAACCATA

CAAGCATTTG TCTACAACAA TAGGTCAACT ATGGCCAAAT TGTAACCATA

TAAACATGTG TCTGTAACAA TAGGTTAACT ATAGCCAATT TATAACCATA

TAAATATTTG TCTATAATAA TAGCTTGACT ATTGCCAAAT CACAACCATA

GAAACATTTG CCTGTAACAA TAGCTTCACT ACTGCCAAAT CCTAACCATA

TAAGCATTTG TCTCTAACAA TAGGTTAACT ATAGCCAAAT TATAAGTAAA

TAAGCATTTG TTCGTAACAA TAGGTTAACT ATAGCCAAAT TATAGTCTCT

CCATTCACAC AAAATCCTAT AAACAGCATG TGATCATATT GCTTCTAGAA

CCATTCACAC AAAATCCTAT AAACAGCATG TGATCATATT GCTTCTAGAA

CCATTCACAC AAAATCCTAT AAACAGCATG TGATCATATT GCTTCTAGAA

CCATTCACAC AAAATCCTAT AAACAGCATG TGATCATATT GCTTCTAGAA

CCTTTCACAC AAAATCCTAT AAACAGCATG TGTTCATATT GCTTCTAGAA

CCTTTCACAC AAAATCCTAT AAACAGCATG TGATCATTTT GCTTCTAGAA

CCTTTcacac aacGTCCTAT AAACAGCACG TGATCAC-TA GCTTCTAGGA

CCTTTcacac aaAATCCTAT AAACAGCGTG TGATTGC-TT GCTTCTAGGA

CCTTTCACAC AAAATCCTAT AAACAGCATG TGATCATATT ACTTCTAGAA

CCTTTCACAC AAAATCCTAT AAACAGCATG TGATCATATT GCTTCTAGAA

CCTTTCACAC AAATTCCTAT AAACAGCATG TGATCATATT GCTTCTAGAA

CCTTTCACAC AGAATTCTAT AAACAGCATG TGATCATATT GCTTCTAGAA

CCTTTCACAC CAAATCCTAT AAACAGCATG TGATCATCTT GCCTCTAGAA

CCTTTCACAC AAAATCCTAT AAACAGCATG TGATCATTTT GCTTCTAGAA

CCTTTCACAC TAAATCCCAT AAACAGTATG TGATCGTATT GCTTCTAGAA

CCTTTCACAC GAGATCCTAT AAACAGCGTG TGATCATACT GCTTCTAGAA

CCTACCACAC AAAATCCTAT GAGCAGCACA TGATATTATT GCTTTTAGAA

TTTTTCCCAC AAAGCCCCAT AAACAGCACG CGATCATATT GCTTTTAGAA

TTTATGATTG TTTTCTTCCA AAAGGAAGCT AAATTTAGCC TAGTAATTCT

TTTATGATTG TTTTCTTCCA AAAGGAAGCT AAATTTAGCC TAGTAATTCT

TTTATGATTG TTTTCTTCCA AAAGGAAGCT AAATTTAGCC TAGTAATTCT

TTTATGATTG TTTTCTTCCA AAAGGAAGCT AAATTTAGCC TAGTAATTCT

TTTATGATTG TTTTCTTCCA AAAGGAAGCT AAATTTAGCC TAGTGATTCT

TTTATGATTG TTTTCTTCCA AAAGGAAGCT AAATTTAGCC CAGTAGTTCT

TTTATGATTG TTTTCTTCCA AAAGGAAGCT AAATTTAGCC TAGTAATTCT

TTTATGATTG TTTTCTTCCA AAAGGAAGCT AAATTTAGCC TAGTGATTCT

TTTATGATTG TTTTCTTCCA AAAGGAATCT AAATTTAGCC TGGTAA---T

TTTATGATTG TTTTCTTCCA AAAGGAAGCT AAATTTAGCT TAGTAATTCT

TTTATGATTG TTTTCTTCCA AAAGGAAGCT AAATTTAGCC TAGTAATTCT

TTTATGATTG TTTTCTTCCA AAAGGAAGCT AAATTTAGCC TAGTAATTCT

TTTATGATTG TTTTCTTCCA AAAGGAAGCT AAATTTAGCC TAGT-ATCCT

TTTATGATTG TTTTCTTCCA AAAGGAAGCT AAATTTAGCC TACTAATTCT

TTTATGATTG TTTTCTTCCA AAAGGAAGCT AAATTTAGCC TAGTAATTCT

TTTATGATTG TTTTCTTCCC AGAGGAAGCT AAATTTAGCC TCCTGGGTCG

TTTATGATTG TTTTCTTCCA AAAGAAAGCT AAATTTAGCA TAGTAATCCT

TTTATGATTG TTTTCTTTCA GAAGGAAGCT AAATTTAGCA TGGTAATTCC

AATGCGCTCA AGAAAACAAG CCTGCTGTGA TTTCTAGAAT AAATGAATGT

AATGCGCTCA AGAAAACAAG CCTGCTGTGA TTTCTAGAAT AAATGAATGT

AATGCTCCCA AGAAAACAAG CCTGCTGTGA TTTCTAGAAT AAATGAATCC

AATGCTCCCA AGAAAACAAG CCTGCTGTGA TTTCTAGAAT AAATGAATCC

AATGCTCCCA AGGAAACAAG CCTGCTGTGA TTTCTAGAAT AAATGAATCT

AGTGCTCCCA AGGAAACAAG CCTGCTGTGA TTTCTAGAAT AAATGCATCT

AATGCTCCCA AGGAAACAAG CCTGCTGTGA TCTCCAGAAT AAATGAATCT

AATGCTCCCA AGGAAACAAG CCTGCTGTGA TTTCTAGAAT AAATGAATCT

AATGCTCCCA AGGAAACAAG CCTGCTGTGA TTTCTAGAAT AAATGAATCT

AATGCTCCCA AGGAAACAAG CCTGCTGTGA TTTGTAGAGT AAATGAATCT

AATGCTCCCA AGGAAACAAG CCTGCTGTGA TTTCTAGAAT AAATGAATCT

GATGCTCCCA AGGAAACAAG CCTGCTGTGA TTTCTAGAAT AAATGAATCT

AATGCTCCCA AGGAAACAAG CCTGCTGTGA TTTCTAGAGT AAATGAATTT

AATACTTCCA GGGAAACAAG CCTGCTGTGA TTTCTAGAAT AAATGAATCT

AATGCTCCCA AGGAAACAAG CCTGCTGTGA TATCTAGAAT AAATTAATCT

AATGCTCCCA AGGAAACAAG CCTGCTGTGA TTTCTAGAAT ACATGGATCC

GAGACTTCCA AGAAAACAAG CATTCTGTGA CTTAGAACAT AAACCCAACT

AGCGCGCTGA AGAAAAAAAG TCTGGTTTGA TTTAGAGTAA AACTCAAAGT

AACCACAGTT CCTTTACTTG ACTGAGAAAG TTTAAATATC AACCTAGTCA

AACCACAGTT CCTTTACTTG ACTGAGAAAG TTTAAATATC AACCTAGTCA

AACCGCAGTT CCTTTACTTG ACTGAGAAAC TTTAAATATC AACCTAGTCA

AACCGCAGTT CCTTTACTTG ACTGAGAAAC TTTAAATATC AACCTAGTCA

AACCACAGTT CCTTTACTTG ACTGAGAAAG TTTAAATACC AACCTAGTCA

AGCCACAGTT CCTCTATTTG ACTGAGAAAG TTTAAATATC AGCCTAGTCA

AGCCACAGTT CCTTTACTTG GCTAAGAAAG TTTAAGTATC AACCTAGTCA

AGCCACAGTT CCTTTACTTG ACTAAGAAAG TTTAAGCATC AACCTAGTCA

AGCCACAGTT CCTTAACTTG ACTGAGAAAG TTTAAATATC AACATAGTCA

AACCACAGTT CCTTTACTTG ACTGAGAAAG TTTAAATATC ACCCTAGTCA

AACCACAGTT CCTTTACTTG ACTAAGAAAG TTTAAATATC AACCTAGTCA

AACCACAATT CCTTTACTTG ACTGAGAAAG TTTAAATATC AACCTAGTCA

AGCCACAAAT CCTTTACTGC AGTGTGAAAG TTTAAGTATC AACCTAGTCA

AACCACAATT CTTTCACTTG ACTGAAAAAG TTTAAATATC AACCTAGTCA

AACCGCAATT CCTTTACTTG ACTGAGAAAG TTTAAATATC AACATAGTCA

AACCACAGTT CATTTACTTG ACTGAGGAAG CTTAAATGCC AACATGGTCA

AACCACAATT CATTTACTTG ACTGAGAACA TTTAAATTTC AGTATAGTCA

AACCACAATT CATTTCCTCG ACTGAGAAAG TTTGAATATC AATCAACCCT

TTAACCACAG TTATTAAACC ACGTTAAACA ATTGTTTGCA TCTCAATTGG

TTAACCACAG TTATTAAACC ACGTTAAACA ATTGTTTGCA TCTCAATTGG

TTAGCCACAG TTATTAAACC ACATTAAACA ATTGTTTGCA TCTCAATTGG

TTAGCCACAG TTATTAAACC ACATTAAACA ATTGTTTGCA TCTCAAGGGG

TTAACTACAG TCATTAAACC ACATTAAACA ATTGTTTGCA TCTCAATTGC

TTAACCACAG ATATTAAACC ACATCAAACA ATTGTTTGCA TCTTAATTGG

TTAGCCACAG ATATGAAACC ACATTAAGCA ATTGTTTGCA TCACAATTGG

TTAGCCACAG ATAGAAACCC ACATTAAGCA ATTGTTTGCA TCACAATTGG

TTAGCCACAG ATCTGAAACC ACATTAAACA ATTGTTTGAA TCTCAATTGG

TTAACCACAG ATATTAAACC ACATGAAACA ATTGTTTGCA TCTTAATTGG

TTAACCACAG ATATTAAACC ACATTAAACA ATTGTTTGCA TCTCAATTGG

TTAGCCACCG ATATTAAACC ACATTACA-A ATTGTTTGCA CCTCAATTGG

CTAACCACAG TTATtaaacc acactaaacc aTTGTTTGCA TCTCAATTGG

TTAACCACAG ATATTAAACC ACATTAAACA ATTGTTTGCA TCGCAATTGG

TTAACCACAG ATACTATACC ACATTCAACA ATTGTTTGCA TCACAATTGG

TTAACCACAG ATAGTGAACC ACATGAAACA A--------- ----------

TTAACCACAG TTCTGAAGTC ACACGAAACA ATGATTTGTT TCTCAATTGG

GTGGGCAAAA CTACTCAACC ACATGAAGTA ACTAGTTTTG TCTCAGGTGG

CTTAAAATTA AACTATGTGG AGTCATGTCC AAGATGAATG TGAATATGAA

CTTAAAATTA CACTATGTGG AGTCATGTCC AAGATGAATG TGAATATGAA

CTTAAAATTA AGCTATGTGG AGTCATGTCC AAGATGAATG TGAATATGAA

CTTAAAATTA AGCTATGTGG AGTCATGTCC AAGATGAATG TGAATATGAA

CTTAAAATTA AACTATGTGG AGTCATGTCC GAGATGAATG TGAATATGAA

CTTAAAATTA AACTATGTGG AGTCATGTCC AAGATGAACG TGGATATGAA

CTTAAAATTA AACTGTGTGG ATTCATGTCC AAGATGAACG TGAACATGAA

CCTAAAATTA AACTATGTGG AGTCATGTCC AAGATGAACG TGAACATGAA

GTTAAAATTA AACTATGTGG AGTCATGTCC AAGATGAATG TGAACATGAA

CTTAAAATTA AACTATGTAA AGTCATGTCC AGGATGAATG TGAACATGCA

CTTAAAATTA AACTATGTGG AGTCATGTCC AAGATGAATG TGAACATGAA

TTTAAAATTA AACTATGTAG CGTCACGTCC ACGATGAACG TGGACATGAA

CTTAAAATTA AACTATGTAG AGTCATGTCC AAGATGAATG TGAACATGAA

CTTAAAATTA AACCATGTGG AGTCATGTCC AAGATGAATG TGAATATGAA

CTTAAATTTA AACTATGTGG AGTCATGTCC AAGATGAATG TGAACATGAA

---------- ---------- ---------- ---------- ----------

TACAAAATTA AGCTTTGCAC AGTCATGTCC AAGATGGACG TTAATAGGAA

ATTAAGATGA AACTGTAAAG CTTTGTGCCC AGGACAGATG TCAACGTGAG

GTATCAGCTT CCCAACTTCA CCGCGGAAAC ACCCATCCAG AATGTAACTA

GTATCAGCTT CCCAACTTCA CCGCGGAAAC ACCCATCCAG AATGTAACTA

GTATCAGCTT CCCAACTTCA CCGCGGAAAC AGCCATCCAG AATGTAACTA

GTATCAGCTT CCCAACTTCA CCGCGGAAAC AGCCATCCAG AATGTAACTA

ATATCAGCTT CCCAACTTCA CTGCAGAAAC ACCCATCCAG AATGTAACTA

GTATCAGCTT CCCAACTTCA CCGCGGAAAC ACCCATTCAG AATATAACTA

GTACCAGCTT CCCAACTTCA CCGCAGAAAC CCCCATCCAC AATGTAACTA

GTATCAGCTC CCCAACTTCA CGGCAGAAAC CCCCATCCAG AATGTAACTA

GTATCAGCTT CCCAACTTCA CCGCTGAAAC GCCCATCCAG AACGTAACCA

ATATCAGCTT CCTAACTTCA CTGCAGAAAC ATCCATTCAG AATGTAACTA

GTATCAGCTT CCCAACTTCA CTGCCGAAAC ACCCATCCAG AATGTAACTA

GTACCAGCTG CCCAACTTCA CCGCGGAAAC GCCCATCCAG AATGTAACTA

GTATCAGCTT CCCAACTTCA CTGCCGAGAC GCCCATCCAG CATGTAACTC

GTACCAGCTT CCCAACTTCA CTGCAGAAAC ACCCATCCAG AATGTAACTA

GTATCAGCTT CCCAACTTCA CTGCAGACAC ACCCATCCAG AACGTAACAA

---------- ---------- ---------- ---------- ----------

GTATCCACTT CCCAACTTTA TTGCAGAAAC TGAGATCCAA AACATAACAA

CTACTCGCTG CCCCACTTCA TCGCGAATAC CACCCTCCTA AATATAACAA

CATTTATGTT TTAGAAGACC TTCAGAAGGT TGCTGAGTAC AAGACTACTA

CATTTATGTT TTAGAAGACC TTCAAAAGGT TGCTGAGTAC AAGACTACTA

CATTTATGTT TTAGAAGACC TTCAGAAGGT TGCTGAGTAC AAGACTACTA

CATTTATGTT TTAGAAGACC TTCAGAAGGT TGCTGAGTAC AAGACTACTA

CATTTATGTT TTAGAAGACC TTCAGAAGGT TGCTGAGTAC AGGACTACTA

CATTTACGTG TTAGAAGACC TGCAGAAGGT TGCAGAATAC AAGACTACTA

CATTTATGTT TTAGAAGACC TTCAGAAGGT ATCTGAGTTC AAGACTACTA

CATTTATGTT TTAGAAGACC TTCAGAAGGT ATCCGAATTC AAGACTACTA

CATCTACGTT TTAGAAGACC TTCAGAAGGT TGCTGAATAC ACGACTACTA

CATTTACGTT TTAGAAGACC TTCAGAAGGT TGCTGAGTAC AAGACTACTA

TATTTACGTT TTAGAAGACC TTCAGAAGGT TGCTGAGTAC AAGACTACTA

CATCTACGTC TTGGAAGACC TTCAGAAGGT CGCTGAGTAC AGGACTACTA

CCTTTATGTT TTAGAAGACC TGCAGAAGGT TGCCGAATAC AAAACTACTA

CATTTACGTC TTAGAAGACC TTCAAAAGGT TGCTGAGTAT AAGACTACTA

CATTTATGTT CTAGAAGACC TCCAGAAGGT TGCTGAGTAC AAGACTACTA

---------- ---------- ---------- ---------- -----TACTC

GATTTATGTC TTAGAAGATC TCCATAATGT TTCTGAGTAC AAGACTACTA

GATCTACGTC TTAGAAAACC TCCAGGACTT CGTAGAGTAC CCGACTACTA

TGATGATCAA CTCATTAGCT GTGGCAGCGT CAACAGAGGG ACCTGCCAGC

TGATGATCAA CTCATTAGCT GTGGCAGCGT CAACAGAGGG ACCTGCCAGC

TGATGATCAA CTCATTAGCT GTGGCAGCGT CAACAGAGGG ACCTGCCAGC

TGATGATCAA CTCATTAGCT GTGGCAGCGT CAACAGAGGG ACCTGCCAGC

TGATGACCAA CTCATTAGCT GTGGCAGCGT CAACAGAGGA ACCTGCCAGC

CGATGACCAA CTCATCAGCT GTGGCAGCGT CAACAGAGGG GCCTGCCAGC

TGACGACCAG CTCATCAGCT GTGGCAGCGT CAACAGAGGG ACCTGCCAAA

TGATGATCAA CTCATTAGCT GTGGCAGTGT CAACAGAGGG ACTTGCCAGC

TGATGACCAA CTCATTAGCT GTGGCAGCGT CAACAGAGGG ACCTGCCAGC

TGATGATCAA CTCATTAGCT GTGGCAGTGT CCACAGAGGG ACCTGCCAGC

CGATGACCAA CTCATTAGCT GTGGCAGTGT CCACAGAGGG ACCTGCCAGC

CGACGACCAG CTCATCAGCT GCGGCAGCGT CCGCAGAGGC ACGTGCCGGC

CGATGACCAG CTCATTAGCT GTGGCAGCGT CCACAGAGGG ACCTGCCAGC

CGACGACCAG CTCATTAGCT GTGGCAGCGT CAACAGAGGG ACCTGCCAGC

TGATGACCAA CTCATTACCT GTGGCAGCGT CAACAGGGGG ACCTGCCAGC

TGACGACCGA CTCATCAGCT GCGGCAGCGT CCACCGGGGC ACCTGCCAGC

TGACGATCAG CTTATTAGTT GTGGTAGTGT TAATCGAGGG ACCTGCCAGC

CTATGAGCAA CTGATCATCT GCGGGAGTGT GGATGGAGGG GCCTGCTGGA

GACATGCATT CGATATCAGT GAGAAGGCTA AAGGAAACGA AAGATGGTTT

GACATGCATT CAATATCAGT GAGAAGGCTA AAGGAAACGA AAGATGGTTT

GACATGCATT CGATATCAGT GAGAAGGCTA AAGGAAACGA AAGATGGTTT

GACATGCATT CGATATCAGT GAGAAGGCTA AAGGAAACGA AAGATGGTTT

GACATGCATT CGATATCAGT GAGAAGGCTA AAGGAAACAA AAGATGGTTT

GCCATGCATT CAATATCAGT TAGAAGGCTA AAGGAAACAC AGGATGGTTT

GGCATGCATT CAATATCGGT GAGGCGGCTG AAGGAAACCC AGGACGGTTT

GGCATGCATT CGATATCGGT GAGACGGCTG AAGGAAACCC AAGATGGTTT

GACATGCATT CGATATCAGT GAGAAGGCTG AAGGAAACAC AGGATGGTTT

GACATGCACT CGATATCAGT GAGAAGGCTA AAGGAAACAC AAGATGGTTT

GACATGCATT CGATATCGGT GAGAAGGCTA AAGGAAACGC AAGATGGGTT

GGCATACATT CGATATCGGT GAGGAGGCTG AAGGAGACGC AAGACGGCTT

GACATGCATT CTATCTCCGT GAGACGGCTG AAGGAAACGC AAGATGGTTT

GGCATGCATT CAATATCAGT GAGAAGGCTC AAGGAAACAC AAGATGGTTT

GGCATGCATT CGATATCGGT GAGAAGGCTA AAGGAAACGC AAGATGGATT

GGCATGCATT CCATATCAGT GAGAAGGATC AAGGAGACCC AGGATGGCTT

GGCATACATT CAATATCTGT CAGGAGGTTA AAGGAAACAC AAGACGGTTT

GGTTTGCATT CCATCTCCGT GAGGCGGCTG AAGGAAACTC TGGATGGCTT

TATGTTTTTG ACGGACCAGT CCTACATTGA TGTTTTACCT GAGTTCAGAG

TATGTTTTTG ACAGACCAGT CCTACATTGA TGTTTTACCT GAGTTCAGAG

TATGTTTTTG ACGGACCAGT CCTACATTGA TGTTTTACCT GAGTTCAGAG

TATGTTTTTG ACGGACCAGT CCTACATTGA TGTTTTACCT GAGTTCAGAG

TATGTTTTTG ACGGACCAGT CCTACGTTGA TGTTTTACCT GAGTTCAGAG

TAAGTTTTTG ACGGACCAGT CCTATATTGA TGTTTTACCT GAGTTCCGAG

TAAGTTTTTG ACAGACCAGT CCTACATTGA TGTCCTGCCA GAATTCCGAG

TAAGTTTTTG ACAGACCAGT CCTATATTGA TGTCTTACCA GAATTCCAAG

TAAGTTTCTG ACGGATCAGT CCTATATTGA TGTCCTACCC GAGTTCCGAG

TAAGTTTTTG ACAGACCAAT CCTATATTGA TGTTCTACCT GAACTCCGAG

CAAGTTTTTG ACAGACCAGT CTTACATTGA TGTTCTACCG GAGTTCAGAG

TAAGTTTCTG ACAGACCAGT CCTACATTGA TGTTTTACCC GAGTTCCGAG

CAAGTTCTTG ACAGACCAGT CCTATATTGA TGTCCTGCCT GAGTTCCGAG

TAAGTTTTTG ACAGACCAGT CCTATATTGA TGTTCTACCT GAGTTCCGAG

TAAGTTTTTG ACGGACCAGT CCTATATTGA TGTTCTACCT GAGTTCCGAG

TAAGTTTCTG ACAGACAAGT CCTACATCGA CGTGCTCCCC GAGTTCCAAG

TAAATTTTTG ACAGACCAGT CCTATATTGA TGTTCTACCA GAATTCCAAG

TAAGTTCTTG ACGGATCAGT CTTACATCGA CGTTCTGCCG CAGTTCCGAG

ATTCTTACCC CATTAAGTAT CCATGCCTTT GAAAGCAACA ATTTTATTTA

ATTCTTACCC CATTAAGTAT CCATGCCTTT GAAAGCAACA ATTTTATTTA

ATTCTTACCC CATTAAGTAT CCATGCTTTT GAAAGCAACA ATTTTATTTA

ATTCTTACCC CATTAAGTAT CCATGCTTTT GAAAGCAACA ATTTTATTTA

ATTCTTACCC CATTAAGTAT CCATGCCTTT GAAAGCAACA ATTTTATTTA

ACTCTTACCC CATTAAGTAT CCATGCCTTT GAAAGCAACC ATTTTATTTA

ATTCCTACCC CATCAAGTAT ACATGCCTTC GAAAGCAACC ATTTTATCTA

ATTCCTACCC CATAAAGTAT ACATGCCTTC GAAAGCAACC ATTTTATTTA

ATTCCTACCC CATTAAGTAT CCACGCTTTT GAAAGCAACC ATTTTATTTA

ATTCCTACCC CATTAAGTAT CCACGCCTTT GAAAGCAACC ATTTTATTTA

ACTCCTACCC CATTAAATAT CCACGCCTTT GAAAGCAACC ACTTTATTTA

ATTCTTACCC CATTAAGTAT CCACGCCTTT GAAAGCAACC ATTTTATTTA

ACTCTTACCC CGTTAAATAT CCATGCCTTC GAGAGCAACC ATTTCATTTA

ATTCTTACCC CATTAAGTAT CCATGCCTTT GAAAGCAACC ATTTTATTTA

ATTCGTACCC CATTAAGTAT CCATGCCTTC AAACACAACC AGTTTATTTA

CCTCCTACCC CATTAAGTAT CCACGCCTTC GAAAGCAACC GCTTTATTTA

ATTCTTACCC CATTAAATAT ACATGCCTTT GAAAGCAGTG ACTTCATTTA

ATTCGTACCC CATCAAGTAT CCACGCTTTT GAGAGTAATC AGTTCATCTA

CTTCTTGACG GTCCAAAGGG AAACTCTAGA TGCTCAGACT TTTCACACAA

CTTCTTGACG GTCCAAAGGG AAACTCTAGA TGCTCAGACT TTTCACACAA

CTTCTTGACG GTCCAAAGGG AAACTCTAAA TGCTCAGACT TTTCACACAA

CTTCTTGACG GTCCAAAGGG AAACTCTAAA TGCTCAGACT TTTCACACAA

CTTCTTGACG GTCCAAAGGG AAACTCTAAA TGCTCAGACT TTTCACACAA

CTTTCTGACC GTCCAAAGGG AAACTCTGGA CTCTCAGACT TTTCATACTC

CTTTCTGACT GTCCAGAAGG AAACCCTAGA TGCTCAGACT TTCCATACAA

CTTTCTGACT GTCCAAAAGG AAACTCTAGA TGCTCAGACT TTTCATACAA

CTTCCTGACG GTCCAAAAGG AAACTCTAGA TGCTCAGACT TTCCACACAA

CTTTTTGACG GTCCAGCGGG AAACTCTAGA TGCTCAGACT TTTCACACAA

CTTTTTGACA GTCCAGCGAG AAACTCTAGA TGCTCAGACT TTTCACACGA

CTTCTTGACG GTCCAACGGG AAACTCTCGA TGCTCAGACT TTTCACACGA

CTTCCTGACA GTCCAGCGGG AAACGCTGGA TGCTCAGACT TTTCACACGA

CTTTTTGACA GTCCAACGGG AAACCCTAGA TTCTCAGACT TTTCACACAA

CTTTTTGACT GTCCAACGGG AAACTCTAGA ATCTCAGACC TTTCACACAA

CTTTCTCACG GTCCAGCGAG AAACGCTGGA CTCGCCGTCC TTCCACACGC

CTTTTTAACA GTCCAAAGAG AAACTCTGGA ATCTCAGACT TATCACACAA

TTTCCTGACC GTACAGAAAG AAACTGTGGA ATCCCAAACT TTCCACACAA

GAATAATCAG GTTCTGTTCA ACTCTGGATT GCATTCCTAC ATGGAAATGC

GAATAATCAG GTTCTGTTCA ACTCTGGATT GCATTCCTAC ATGGAAATGC

GAATAATCAG GTTCTGTTCA ACTCTGGATT GCACTCCTAC ATGGAAATGC

GAATAATCAG GTTCTGTTCA ACTCTGGATT GCACTCCTAC ATGGAAATGC

GAATAATCAG GTTCTGTTCA ACTCTGCATT GCATTCCTAC ATGGAAATGC

GAATAATCAG GTTCTGTTCG ACTCTGGGCT GCATTCGTAC ATGGAAATGC

GAATAATCAG GTTCTGTTCG ACTCTGGGTT GCACTCCTAC ATGGAAATGC

GAATAATCAG GTTCTGTTCG ACTCTGGGTT GCACTCCTAC ATGGAAATGC

GAATCATCAG GTTCTGTTCG ACTCCGGCTT GCATTCCTAC ATGGAAATGC

GAATAATCAG GTTCTGTTCG ACTCTGGATT GCATTCGTAC ATGGAAATGC

GAATAATCAG GTTCTGTTCG ACTCTGGATT GCATTCCTAC ATGGAAATGC

GAATCGTCAG GTTCTGCTCG ACTCGGGCTT GCATTCCTAC GTGGAAATGC

GAATAATCAG GTTCTGTTCG ACTCCGGGTT GCATTCTTAC ATGGAGATGC

GAATAATCAG ATTCTGTTCG ACTCTGGATT GCATTCCTAC ATGGAAATGC

GAATCATCAG GTTCTGTTCG ACTCTGGATT GCATTCCTAC ATGGAAATGC

GTATCATCAG GTTCTGCTCG ACTCGGGCTT GCACTCCTAC ATGGAAATGC

GAATAATCAG GTTCTGTTCG ACTCTGGATT GCATTCATAT ATGGAAATGC

GAATTATCCG GTTCTGTTCG ATTCGGAATT ACATTCCTAC ATGGAAATGC

CTCTGGAGTG TATTCTCACA GAAAAGAGAA AAAAGAGATC GAAGTGTTTA

CTCTGGAGTG TATTCTCACA GAAAAGAGAA AAAAGAGATC GAAGTGTTTA

CTCTAGAGTG TATTCTCACA GAAAAGAGAA AAAAGAGATC GAAGTGTTTA

CTCTAGAGTG TATTCTCACA GAAAAGAGAA AAAAGAGATC GAAGTGTTTA

CTCTGGAGTG TATTCTCACA GAAAAGCGAA AAAAGAGATC GAAGTGTTTA

CCCTGGAGTG TATTCTCACC GAAAAGAGAA GAAAGAGATC GAAGTATTCA

CTCTGGAGTG CATTCTGACG GAAAAAAGAA GAAAGAGATC GAAGTGTTTA

CCCTGGAATG CATCCTGACA GAAAAAAGAA GGAAGAGATC GAAGTGTTTA

CTCTGGAGTG TATTCTCACG GAGAAGAGGA GGAAGAGAGC GAAGTGTTTA

CTCTGGAGTg tattctgacg gaaaagagaa gaaagagatc gaagtgttta

CTCTGgagtg tattctcacg gaaaagagaa gaaagagatc gaagtgttta

CCCTGGAGTG TATTCTCACA GAAAAGAGGA GGAGGAGATC GAAGTGTTCA

CTCTGGAGTG TATTCTCACA GAGAAGAGAA GAAAGAGGGC GAAGTGTTCA

CTCTTGAGTG TATTCTCACT GAAAAGAGAA GAAAGAGATC GAGGTGTTTA

CTCTGGAGTG TATTCTCACT GAGAAGAGAA GGAAGAGGTC GAGGTGTTTA

CCCTGGAGTG CATCCTCACC GAGAAGCGCC GCAAGCGGGC GAGGTGTTCA

CTCTTGAATG TATTCTTACC GAAAAGAGAA GAAAAAGGTC GAAGTATTTA

CTCTCGAATG CATCCTCACA GAGAAGAGGA GGAGGAGGTC GAAGTGTTCA

ATATACTTCA GGCTGCGTAT GTCAGCAAGC CTGGGGCCCA GCTTGCTAGA

ATATCCTTCA GGCTGCGTAT GTCAGCAAGC CTGGGGCCCA GCTTGCTAGA

ATATCCTTCA GGCTGCATAT GTCAGCAAGC CCGGGGCCCA GCTTGCTAGA

ATATCCTTCA GGCTGCATAT GTCAGCAAGC CCGGGGCCCA GCTTGCTAGA

ATATCCTTCA GGCTGCATAT GTCAGCAAGC CTGGGGCCCA ACTTGCGAGG

ACATCCTCCA AGCTGCATAC GTCAGTAAGC CTGGGGCCCA CCTTGCCAAG

ATATCCTCCA AGCCGCATAT GTCAGTAAAC CGGGGGCCAA TCTTGCTAAG

ATATCCTCCA AGCCGCGTAT GTCAGTAAAC CAGGGGCCAA TCTTGCTAAG

ATATCCTTCA GGCTGCATAT GTCAGTAAGC CTGGGGCCCA TCTTGCTAGG

atattctcca agctgcatat gtcagtaagc ctggggctca gCTCGCTAGG

atattctcca agctgcatat gtcagtaagC CTGGGGCCCA TCTCGCTAAA

ATATCCTCCA AGCTGCCTAT GTCGGCAAGC CTGGGACCCA TCTCGCCAAC

ATATTCTCCA AGCTGCGTAT GTCAGTAAAC CCGGGGCCCA TCTCGCTAAG

ATATTCTTCA GGCTGCATAT GTCAGTAAGC CTGGGGCCCA TCTCGCTAAG

ATATCCTTCA GGCTGCCTAC GTCAGTAAGC CTGGGGCCTA TCTCGCTAAG

ACGTGCTGCA GGCCGCGTAC GTGGGCAAGC CCGGGGCGCA ACTGGCCAAG

ACATTCTACA AGCAGCATAT GTAAGTAAAC CTGGTGCACA TCTGGCTAAG

ATGTCCTCCA GGCTGCCTAT GTCAGTAAGC CAGGGGCCCA TCTGGCTCGG

CAAATAGGTG TGTGCATTCC CTATCAAATA TGTCAACGAC TTCTTCAACA

CAAATAGGTG TGTGCATTCC CTATCAAATA TGTCAACGAC TTCTTCAACA

CAAATAGGTG TGTGCATTCC CTATCAAATA TGTCAACGAC TTCTTCAACA

CAAATAGGTG TGTGCATTCC CTATCAAATA TGTCAACGAC TTCTTCAACA

CAAATAGGTG TGTGCATTCC CTATCAAATA TGTCAATGAC TTTTTCAATA

CAGATAGGTC TGTGCATTCC CTATCAAATA CGTCAATGAA TTCTTCAACA

CAAATAGGTC TGTGCATTCC CCATCAAATA TGTCAATGAC TTCTTCAACA

CAAATAGGTC TGTGCATTCC CCATCAAATA TGTCAATGAC TTCTTCAACA

CAGATTGGTC TGTGCATTCC CTATCAAATA TATCAATGAA TTCTTCAACA

CAAATAGGTC TGTGCATTCC CTGTCAAATA TGTCAATGAG TTCTTCAACA

CAAATAGGTC TGTGCGTTCC CTATCAAATA TGTCAATGAA TTCTTCAACA

CAAATAGGTC TGTGCGTTCC CGATCAAGTT GGTCAATGAA TTCTTCAACA

CAAATAGGTC TGTGCATTCC CTATCAAGTA TGTCAATGAA TTCTTCAACA

CAAATAGGTC TGTGCATTCC CGATCAAATA TGTCAACGAA TTCTTCAACA

CAAATAGGTC TGTGCATTCC CAATCAAATA TGTCAATGAC TTCTTCAACA

CAGATCGGTC TGTGCCTTTC CTGTCAAATA TGTCGATGAG TTCTTCCATA

CAAATAGGTC TGTGCATTTC CAATCAAGTT TGTCAACGAT TTCTTTAACA

CAAATAGGTC TGCGCATTCC CCATCAAATA CATCAATGAG TTCTTCAGCA

AGATCGTCAA CAAAAACAAT GTGAGATGTC TCCAGCATTT TTACGGACCC

AGATCGTCAA CAAAAACAAT GTGAGATGTC TCCAGCATTT TTACGGACCC

AGATCGTCAA CAAAAACAAT GTGAGATGTC TCCAGCATTT TTATGGACCC

AGATCGTCAA CAAAAACAAT GTGAGATGTC TCCAGCATTT TTATGGACCC

AGATCGTCAA CAAAAACAAT GTGAGATGTC TCCAGCATTT TTACGGACCC

AGATTGTCAA CAAAAACAAT GTGAGATGCC TCCAACATTT TTATGGACCA

AGATTGTCAA CAAAAACAAC GTACGGTGTC TCCAGCATTT TTATGGACCC

AGATTGTCAA CAAAAACAAC GTGAGATGTC TCCAGCATTT TTACGGACCC

AGATCGTCAA CAAAAACAAC GTGAAATGTC TCCAGCATTT TTATGGACCC

AAATCGTCAA CAAAAACAAT GTGAGATGTC TCCAGCACTT TTATGGCCCC

AGATCGTCAA CAAAAACAAT GTGAGATGTC TTCAGCACTT TTATGGACCC

AGATCGTCAA CAAAAACAAC GTGAGATGTC TCCAGCATTT TTATGGACCG

AGATTGTCAA CAAAAACAAT GTGAGGTGTC TCCAGCACTT CTATGGACCC

AGATCGTCAA CAAAAATAAC GTGAGATGTC TCCAGCATTT TTATGGGCCC

AGATTGTCAA CAAAAACAAT GTGAGATGTC TCCAGCATTT TTATGGACCC

GGATCGTCAA CAAGAACAAC GTCAGGTGTC TCCAGCACTT CTACGGGCCC

AAATTGTGAA CAAGAATAAC GTGAAATGTC TTCAGCATTT TTATGGGAAC

AGATTGTCAA CAAGAACAAC GTGAGGTGTC TGCAACACTT TTATGGGAAC

AATCATGAGC ACTGCTTTAA TAGGGTAAGT AGGAAAAATT CCCAAAACCT

AATCATGAGC ACTGTTTTAA TAGGGTAAGT AGGAAAAATT CCCAAAACCT

AATCATGAGC ACTGTTTTAA TAGGGTAAGT AGGAAAAATT CCCAAAACCT

AATCATGAGC ACTGTTTTAA TAGGGTAAGT AAGAAAAATT CCCAAAACCT

AATCATGAGC ACTGTTTCAA TAGGGTAAGT AGGAAAAATG CCCAAAACCT

AATCATGAGC ACTGCTTTAA CAGGGTAAGT AGGAAAAATG CCCAAAACCT

AACCACGAGC ACTGTTTCAA TAGGGTAAGT AGGAAAAATG CCCCAAACCT

AACCATGAGC ACTGTTTCAA TAGGGTAAGT AGGAAAAATG CCCCAAACCT

AATCATGAGC ACTGCTTCAA TAGGGTAAGT AGGAAAAATG CCCAAAACCT

AACCATGAAC ACTGCTTCAA TAGGGTAAGT AGGAAAAATG CCCAAAACCT

AACCACGAAC ACTGCTTTAA TAGGGTAAGT AGGAAAAATG CCCAAAACCT

AACCACGAGC CCTGCTTCAA TAGGGTAAGT AGGAAAAATG CCCAAAACCT

AACCACGAGC ACTGCTTTAA CAGAGTAAGT AGGAAAAATA CCCATAACAT

AACCATGAAC ACTGCTTTAA CAGGGTAAGT AGGAAAAATG CCCAAATCCT

AACCATGAAC ACTGCTTTAA TAGGGTAAGT AGAAAAAATA CCCAAAACCT

AACCACCTGC ACTGCTTTAA TCGGGTAAGT AAAAAAAATG CGCCCAACCT

AATCACAAGC ACTGTTTTAA TAGGGTAAGT AGGAAAAATT TCCAAAACCT

CACCACAGAC ACTGTGCGAA TAGGGTGAGT AGGGCAGAGT TCCAGAATCT

GAAACCATTA TACTCCCATC ACAAAAATTC TTGAAACAAG ATGGAACTTG

GAAACCATTA TACTCCCATC ACAAAAATTC TTGAAACAAG ATGGAACTTG

GAAACCATTA TACTCCCATC ACAAAAATTC TTGAAACAAG ATGGAACTTG

GAAACCATTA TACTCCCATC ACAAAAATTC TTGAAACAAG ATGGAACTTG

GAAACCATTA TACTCCCATC ACAAAAATTC TTGAAACAAG ATGGAACTTG

GAAACCATTA TACTCCCATC ACAGAAATTC TTGAAACAAG GTGGAACTTG

GAAACCATTA TACTCCCATC TCAAAAATTC TTGAAACAAG ATGTAACTTG

GAAATCATTA TACTCCCATC TCAAAAATTC TTGAAACAAG ATGTAACTTG

GAAACCATTA TACTCCCATT GCAGAAATTT CTGAAACAGG ATGGAACTTG

GAAACTATTA TACTCCCATC GCAAAAATTC TTGAAACAAG ATGAAACTTG

GAAACAGTTA TATTCCCATC GCAAAAATTC TTGAAACAAG ATGGAGCTTG

AAAACCATTA TACTCCCATC ACACAAATTC TTGAAACAAG ATGGAACTCG

GAAAACTTTA TACTCCCATT GCAAAAATTC TTGAAACAAG ATGGAACTTG

GAAACCATTA TACTCCCATC ACAAAAATTC TTGAACCAAG ATGGAACTTA

GAAACCATTA TACTCCCATC GCAAAAATTC TTGAAACAAG ATGGAAATTG

GAAAGCGTTA TACTCCCATC GCAAAAATTC TTCAAACAAG ATGGACCTTG

GAAAACATTA GACTTACGAT GCAGAATTCA TTGAAACAAG ATGGAGCTTG

GAAAGATTTG TACCCACAGT CCAGAGCTCC TGGGATCGGG GTGGAGTGTG

CCAACATAGT CTTACAAAGA GCCAACTTCC TTGTTTTTAA ATATCATTTA

CCAACATAGT CTTACAAAGA GCCAACTTCC TTGTTTTTAA ATATCATTTA

CCAACATAGT CTTACAAAGA GCCAACTTCC TTATTTTTAA ACATCATTTA

CCAACATAGT CTTACAAAGA GCCAACTTCC TTGTTTTTAA ACATCATTTA

CCAACTT--C CTTACAAAGA GCCAACTTCC TTGTTTTTAA ATATCGTTTA

CCAAAACGGT CTTACAAGGA GCCAACTTCC TTGCTTTAAA ATATTATTTA

CCA--ACAGT CTTGCAAGGA GCCAACTTCC TTGTTTTCAA ATACCACTTG

CCA--ACAGT CTTGCAAGGA GCCAACTTCC TTGTTTTCAA ATACCACTTG

CCAGTATAGT CTTACAAGGA GCTAACTTCC TTGTTTTTAA ATATCATTTA

CCAACATAGT CTTACAAAGA GCCAACTTCC TTGTTTTTAA ACATCATTTA

CCAACATAGT CCTACAAGGA GCCAACTTCC TTGTTTTTAA ATATCATTTA

CCAACGCAGT CTTACAA-GA GCCAACTTCC TTGTTTTTAA TTATCATTTA

CCAACA---- -ATACAAG-T GCCAACTTCC TTGTTTTTAA ATATCATTTG

CCAACATAGT CTTAGAGGAA GCCAACTTCC TTGTTTTTAA ATATCATTTA

CCAACATAGT CTTACAGGAA GCCAACTTCC TTCTTTTAAA ATATCATTTA

CCAACATAGT CTTACGAGAA GCCGACTTCC TTCTTTTTAA ATATCGTGTC

CCAACACAGT CTTACAAAAA GCCAAATTTC TTGTTTGCAA ATTCTGTTCA

CCAGTTCCTG CTTAGGATGA GTTAGCGTTC TTGCTCTCCA GTTGTGTTTC

TACTCAAATT CAAAGAGTCC AACTGCTTCC CAAAACAAGG TAGTAATTTT

TACTCAAATT CAAAGAGTCC AACTGCTTCC CAAAACAAGG TAGTAATTTT

TACTCAAATT CAAAGAGTCC AACTGCTTCC CAAAACAAGG TAGTAATTTT

TACTCAAATT CAAAGAGTCC AACTGCTTCC CAAAACAAGG TAGTAATTTT

CACTCAAATT CAAAGAGTCC AACTTCTTCA CAAAACAAGG TAGTAATTTT

TACTCAAATT CAAAGACTCC AACTGCTTCC CAAAACAAGG TAGTAATTTT

TACTCCAATT CAAAGAGTCC AACTGCTTCC CAAAACAAGG TAGTAATTTT

CACTCCAATT CAAAGAGTCC AACTGCTTCC CAAAACAAGG TAGTAATTTT

TACTGAAATT CAAAGAGACC AACTGCTTCC CAAAACAAGG TAGTAATTTT

TACTCAAATT CAAAGAGTCC AACTGCTTCC CAAAACAAAG TAGTAATTTT

TACTCAAATT CAAAGAGTCC AACCGCTTCC CAAAACAAAG TAGTAATTTT

TACTCAAATT CAAAG-GTCC AACCGCTTCC CCAAACAAAG TAGTAATTTT

TAC-CAAATT CAAAGAGTCC AACTGCTTCC CAAAACAAAG TAGTAATTTT

TCCTCAAATT CAAAAAGTCC AACTGCTTCC CAAAACAAAG TAGTAATTTT

TACTCAAATT CAAAGACTCC AACTGTTTCC CAAAAC-AAG TAGTAATTTT

TACTCAA-TT CAAAGA-GCC AACTGCTTCC CAAAATGAAG TCGTAATTTT

TGCTTTAATC CAAAGTGTCC AACCTTGTTC CAAGACAAAG TAGTATTTTC

TCCTTCAATG TGAAAAGGTC CAGTGCTTCC CCAAACAAGG CGGCCATCCC

CCATCTCCGT TCCATCAAAG GAAGAAAGCA AAGTGATTAG AACAATTTTA

CCATCTCTGT TCCATCAAAG GAAGAAAGCA AAGTGATTAG AACAATTTTA

CCATCTCCGT TCCATCAAAG GAAGAAAGCG AAGTGATTAG AACAGCTTTA

CCATCTCCGT TCCATCAAAG GAAGAAAGCG AAGTGATTAG AACAGCTTTA

CCATCTCCGT TCCATCAAAG GAATAAAGCG AAGTGATTAC AACAGTTTTT

CCATCTCCGT TCCATCAAAG GAAGAAAGCA GGGTGATTAG AACAATTTTA

CCATCTCCGT CCGGTCACAG GGAGAAAGGA AAGCAATTAG GACAATTTTC

CCATCTCCGT CCGGTCACAG AGAGAAAGGA AAGCAATTAG AACAATTTTC

CCATCTCCGT TCCATCAAAG GAAGAAAGCA AAGCGATTAG AGCAGTTTTG

CCATCTCCGT TGCATCAAAG GAAGAAAGCA AAATGATTAG AACAGTTTTG

CCATCTCCGT TCCATCAGAG GAAGAAAGCA AAGTGATTAG AACAATTTTG

CCATCTCC-- CCCATCAAAG GAAGAAAGCA AAGTGATTAG AACAATTTTG

CCACCTCCAT TCCATCAGAA GAAGAAAGCA AAGTGATTAG AACCATTTTG

CCATCTCCGT TCCATCAAAG AAAGAAAGCA AAGTGATTAG AACCATTTGG

CCATCTTTGT TCCATCAAAG GAGGAGAGAA AAGTGATTAG AACAGTTTTA

CCATCTCTGT TCCATCAGAG GAAGA-AGTC TAATGATCAG AGCCATTTTG

CTGCCACAGT TCTATCAAAG AAAAAAAATA AATAAATTTT AAAAGTTTTA

CACTGTCTAT TTTGTTGACT GAGGAAAACA AATGGATTAA AATAAGTTTG

AAGGGGACAA AATCAAAACA AGAGCAAACA AGGATGTTTC TTTATTATGA

AAGGGGACAA AATCAAAACA AGAGCAAACA AGGATGTTTC TTTATTATGA

AAGGGGACAA AATTAAAACA AGAGCAAACA AGGATGTTTC TTTAGTATGA

AAGGGGACAA AATTAAAACA AGAGCAAACA AGGATGTTTC TTTAGTATGA

AAGGGGACAA AATCAAAACA AGAGCAAACA AGGATGTTTC TTTATTATGA

AATGGGACAA AACGAAAACA AGAGCAAACA AGGATGTTTC TTCATTACAA

AATAGAACAA AATCAAAACA AGAGCAAACA AGGGGGTTTC -TTATCACTA

AATAGAGCAA AATCAAAACA AGAGCAAACA AGGATGTTTC -TTATCACTA

AATGGAGCAA AATCAAAAGA AAAGCAAACA AGAATGCTTC TTTATTACTG

AATGAAACAA AATCAAAACA AGAGCAAACA AGGATGTTTC TTTATTACTA

AATGAAACAA AATCAAAACA AGAGCAAACA AGGATGTTTC TTTATTAGTA

AATGAAACAA AAGCAAAACA AGAGCAAACA AGGATGTTTC TTTATTACTA

AATGAAACAA AATCAAAACA AGAGTAAACA GGGATGTTTC TATATTACTA

AATGGAACAA TATCAAAATA AGAGCAAACA AGGATGTTTC TTTATTACTA

AATGGAACTA AATCCAAACA AGAGCAAACA AGGATGTTTC TTTATTACTA

AAGGGAGCAG CATCAAAACA AGAGCGAGGA AGGAGGCTTC TCGATTACTA

GAGAGAATAT AATCAAAGCA GGGGTCAGCA AAGATGTTTC TTTATTACTA

GAGGGGATGA AATCCAAACA AGAGCCAGCA AAGATGTTTC TTTATTACTC

TTATAAAGAT GATAGATGAG TAAAATCGAG TTACATTTAA GTTGTAATGG

TTATAAAGAT GATAGATGAG TAAAATCAAG TTACATTCAA GTTGTAATGG

TTATAAAGAT GATAGATGAG TAAAATCGAG TTACATTTAA GTTGTAATGG

TTATAAAGAT GATAGATGAG TAAAATTGAG TTACATTTAA GTTGTAATGG

TTATAAAGAT GATAGATGAG TAAAATCAAG TTACATTTAA GTTGTAATGG

TTATAAAGAT GACAGATGAG TACAATTCGG TTACATTTAA GTTGTAATGG

CCATAAAGAT GACGGATGGA TAAAATTGAG TTACATTTAA GTTGTAATGA

CCATAAAGAT GATGGATGGA TAAAATTGAG TTACATTTAA GTTGTAAGGA

TTATAAGGAT GACAGATGAG GAAAATTGAG TTACATTTAA GTTGTAATGG

TTATAAAGAT GATAGATGAG TAAAATTGGG TTACATTTAA GTTGTAATGG

TTATAAAGAT GATAGATGAG TAAAATTGGG TTATATTTAA GTTGTAATGG

TTATAAAGAT GATAGATGAG TAAAATTGGG TTACATTTAA GTTGTAATGG

TTATAAATAT GACAGATGAG TCAAATTGGG TTATGTTTAA GTTGTAATGG

TTATAAAGAT GATAGATGAG TGAAATTGGG TTACATTTAA GTTGTAATGG

TTATAAAGAT GATAGATGAG TAAAATTGGG TTGCATTGAA GTTGTAATGG

CTATAAAGAT GATAGATGAG TAAAATTGGG TTGGATTTAA GTTGTAATGG

TTCTAAAAAT GATAGATGGG TAAAATTAGG TTATGTTTAA GTTGAAATGG

TTCTAAAGAT GATAGATGTT CCAAATTAGG TTAGGCTTAA GTAGTAATGG

AAGAAAATAA TTGCAGCCTT CCATCAGGAA ACCACAAGGA CCGTTATTGA

AAGAAAATAA TTGCAGCCTT CCATCAGGAA ACCACAAGGA CCGTTATTGA

AAGAAAATAA TTGCAGCCTT CCATCAGGAA ACCACAAGGG CCGTTATTGA

AAGAAAATAA TTGCAGCCTT CCATCAGGAA ACCACAAGGA CCGTTATTGA

AAGAAAATAA TTGCAGCCTT CCATCAGGAA ACCACAAGGA CCATTATTGA

AAGAAAATAA TTGCAGCCTT CCATCAGGAA ACCACAAGGA CCATTATTGA

AGGAAAATAA TTGCAGCATT CCATCAGGAA ACCACAAGGC CCATTATTGA

AAGAAAATAA TTGCAGCGTT CCATCAGGAA ACCACAAGGT CCATTATTGA

AAGAAAATAA TTGCAGCCTT CCATCAGGAA ACCACAAGGA CCATTATTGA

AAGAAAATAA TTGCAGCCTT CCATCAGGAA ACCACAAGGA CCATTATTGA

AAGAAAATAA TTGCAGCCTT CCATCAGGAA ACCGCAAGGA CCATTATTGA

AAGAAAATAA TTGCAGCCTT CCATCAGGAA ACCACAAGGA CCATTATTGA

AAGAAAATAA TTGCAGCCTT CCATCAGGAA ACCACA-GGA CCATTATTGA

AAGAAAATAA TTGCAGTCTT CCATCAGGAA ACCACAAGGA CCATTATTGA

AAGAAAATAA TTGCAGTCTT CCATCAGGAA ACCACAAGGA TCATTATTGA

AAGAAAATAA TTACAGTCTT CCATCAGGAG ACCGCAAGGA TCATTATTGA

AAGAAAATAA TTGCAGCCTT CCATCAGGAA ACCACAAGGT CCATTATTGA

AAGAAAATAA TTGCAGTCTT CCATCAGGAA ACCACAAGGA TCGTTATTGA

TCTGTCATCC TCAGGGCATG TCTCGTAATA TTTAATAAAA TAAACAAAGA

TCTGTCATCC TCAGGGCATG TCTCGTAATA TTTAATAAAA TAAACAAAGA

TCTGTCATCC TCAGGGCATG TCTCATAATA TTTAATAAAA TAAACAAAGA

TCTGTCATCC TCAGGGCATG TCTCATAATA TTTAATAAAA TAAACAAAGA

TCCGTCATCC TCAGGGCATG TCTAGTAATA TTTAATAAAA TAAACAAAGA

TCTCTCCTCC TCGGGGCATG TCTCGCATTA TTTAATAAAA TAAACAAAGA

TCTGTCCTCC CCGGGGCATG TCTTGTAATA TTTAATAAAA TAAACAGAGA

TCTGTCCTCT TCAGGGCATG TCTTGTAATA TTTAATAAAA TAAACAGAGA

TCTGTCATCC TCAGGGCATG TCTTGTAATA TTTAATAAAA TAAACAAAGA

TCTGTCATCC TCAGGGCATG TCTCATAATA TTTAATAAAA TAAACAAAGA

TCTGTCATCC TCAGGGCATG TCTCGTAATA TTTAATAAAA TAAACAAAGA

TCTGTCATCC TCAGGGCATG TCTCGTAATA TTTAATAAAA TAAACAAAGA

TCTCTCATCC TCAGGGCATG TCTTGtaata ttaaataaaa taaa----ga

TCTCATATCC TTAGGGCATG TCTCataata tttaataaaa taaacaaaga

TCTCTCATCC TCAGGGCATG TCTCGCAATA TTTAATAAAA TAAACAAAGA

TCTCTCCTCC TCAGGGCATG TCTTGCAATA TTTAATAAAA TAAACAAAGA

TCTCTTCTCT TAGGGGGATG TCTAGTAATA TTTAATAAAA TAAACAAAGA

TCGGCCATCT TCAGGCTATG TCGAGTGATA TTTAATAAAA TAAACAAAGA

TTTATAACTT TTATAGCCAT ATATCTATTC CCACACCACT TGAATTACAA

TTTATAACTT TTATAGCCAT ATATCTATTC CCACACCACT TGAATTACAA

TTTATAACTT TTATAGCCAT ATATCTATTC CCACACCACT TGAATTACAA

TTTATAACTT TTATAGCCAT ATATCTATTC CCACACCACT TGAATTACAA

TTTATAACTT TTCTAGCCAT ATATCTATTC CCACACCACT TGAATTACAA

TTTATAACTT TTATAGCCAT ACATCTATTC CCACACCGTC TGAATTATAA

TTTATAAGTT CTATAGCCGT GTATCTATTC CCACGCTATG CAAATTAAAA

TTTATAACTT CTATAGCCGT GTATCTATCC CCACACTATT CAAATTAGAA

TTTATAACTT TCATGGCCAT ATATCTATTC CCACACCATT TGAATTACGA

TTTATAACTT TTATAGCTAT ATATCTATTC CCACACCATT TGAATTACAA

TTTATAACTT TTATAGCCAT ATATCTGTTC CCACACCATT TGAATTACAA

TTTATAATTT TTATAGCCGT ATATCTATTC CCCCACCATT TGAATTACAA

tttataaCTT TTATAGCCAT ATATCTATTC CTACACCATT TGAAGTACAA

tttataattt tCATAGCCAT ATTTCTATTT CCACACCATT TGACTTACAG

TTTATAACTT TTATAGCCAT GTATCTATTC CCACACCATT TGAATTGCAA

TTTATAACTT TGATAGCCAT ATATCTATTC CCACACCATT TGAATTACAA

CAAATAACTC TTATAGTTGT ATAACTATGC TCACACCCTT TTGATTAAAG

CAAATAACTT TTAGAGCCTC ATAACTGTAC CCACACCATT TGAATTAAAA

ATAACATCCA TGTGATTAAC GGTTTGGCAT GCCTTATTCC AACAATCTAG

ATAACATCCA TGTGATTAAC GGTTTGGCAT GCCTTATTCA AACAATCTAG

ATAACATCCC TGTAATTAAC GGTTTGGCAT GCCTTATTTA AACAATCTAG

ATAACATCCC TGTAATTAAC GGTTTGGCAT GCCTTATTTA AACAATCTAG

ATAGCATCTG TGTAATTAAC GGCTTGGCAC GCCTTATTTA AACAATCTAG

ATAACACCCA TGTAATTAGC GGTTTGGCAT GCCTTAGTTA AACAATCTAA

ATAGCACCCA TGTCATTTAT GGTCCGGCGT GGCTTACTTA GACAG--CAG

ATAGAATCCA TGTCATTTAT GGTCTGGCAC GGCTTATTTA GACTG--CAG

ATAACGTCCA TGTAATTAAC GGTTTGGCAT GCCTTACTTA AACAATCTGG

GTAACATCCA TATAATTAAC GGTTTGGCAT G-CTTATTTA AACAGTCTAG

ATAACATCCA TGTAATTAAC GGTTTGGCAT GTCTTATTTA AACAATCTAG

ACAATATCCA TGTAATTAAC GGTTTGGCAT GTCTTATTTA AACAATCCAG

ATAACATCTG TGTAATTAGC GGTTTGACAT GTCTTGTTTA AACAATCTAA

ATAACAGCCA TGTAATTAAC GGTTTAGCAT GTCTTATTTA AACAGTCTAG

ATAACATCCA TGTAATTAAC GTTTTGGCAT GTCTTATTTA AACAATCTAG

ATAACATCCA TGTAATTAAC GGTTTGGCAT GTCTTATTTA AACAATCTAG

ATAGCATCCC TGTAATTAAA GGTTGGATTT AGCTCAGTTA AACGATCTGG

ATAGCATCTG GGTAATTAAA GGTTTGACAT GTCTTGTTTT AACACGCCCG

CACTATTTTG TATTAGGGAT GAAAAATGTG AAGCCAGATT ATAGAGTTTT

CACTATTTTG TATTAGGGAT GAAAAATGTG AAGCCAGATT ATAGAGTTTT

CACTATTTTG TATTAGGGAT GAAAAATGTG AAGTCAGATT GTAGAGTTTT

CACTATTTTG TATTAGGGAT GAAAAATGTG AAGTCAGATT ATAGAGTTTT

CACTATTTTG TATTAGGAAT GAAAAATGTG AACTCAGAGT ATAGAGTTTT

CACTATTTTG TATTAGGGGT GAAAAATGTG AAGTCAGATG ATAGAGTTTT

TGCTATTTTG TATTAGGAAT GATAAATGGA AAGTCAGATT GTAAAGTTTT

TGCTATTTTG TATTAGGAGT GATAAATGTA AAGTCAGATT ATAAAGTTTC

CATTATTTTG TATTGGGGAT GAAAAATGTG AAGTCAGATT ACAGAGTATT

CACTATTTCG TATTAGGGAT GAAAAATGTG AAGTCAGATT TCAGGGTTTT

CACT-TTTTG TATTAGGGAT GAAAAATGTG AGGTCAGACT TCAGGGTTTT

C-CTATTTTG TGTTAGGGAT GAAAAATGTG AAGTCAGATT TCAGGGTTTT

CACTATTTTA TATTAGGGCT GAAAAATATG AAGTCAGATC TCAGGGTATT

CATTACTTTG TGTTAAGGAT GAAAAATGTG AAGTCAGATT TCAGGGCTTT

CACTATTGGT TATTAGGGAT GAAAAATGTG AAGTCAGATT TCAAGGTTTT

CACTATTTGG TATTAGGGAT AAAAAATGTG AAGCCTGATT TCAGGGTTTT

CACTATTTTA TGTTAGCAAT GAAAAACGTG CATCATGCTT TCATTTCTTT

CACTATTTTA TATGGGTGGT GAAAAATGTG CAGAAAGATT TCAGACTTTT

TCTTATTGTG CTCAAAAATG AGAAAATGTT TCTAGGATCC TGGATACATT

TCTTATTGTG CTCAAAAATG AGAAAATGTT TCTAGGATCC TGGATACATT

TCTTATTGTG CTCAAAAATG AGAAAATGTT TCTAGGATCC TGGATACATT

TCTTATTGTG TTCAAAAATG AGAAAATGTT TCTAGGATCC TGGATACATT

TCCTATTGTG CTCAAAAATG AGAAAATGTT TCTAGGATCC TGGATATATT

TCTTCCTGTG CTCAGAAATG AGAGACTGTT TCTAGGATCC TGGATACGTT

TCTTCCTGTG TTCAGAAATG AGAGAATGTT TCTAGCATCC TGGATACGGG

TCTTCCTGTG TTCAAGAATG AGGGAACATT TCTAAGGTCC TGGATATGTT

TCCTCCTGTG CTCAGAAATG AGAGAATTTT TCTAGGATCC TGGATACATT

TCTTCCTGTG CTTCAAAATG AGAGAATGTT TCTAGGATCC TGGATACATT

TTTTCCTGTG CTCTGAAATG AGAGAATGTT TCTAGGATCC TGGATACATT

TCTTCCTGTG CTCCGAAATG AGAGAATGTT TCTATGATCC TAGATACATT

TCTGCCTGTG CTCAGAAATA AGAGAATGTT TCTAGAATTC TGGATATATT

TTTTCCTGTG CCCTGAAATG ATAAAATGTT TCTAGGATCC TGGCCTCATT

TGTCCCCCTG CCCAGAAATG AGAAAATATT TCTAGGATCC TGGCTACAGT

TCATCCTATG CCCAGAAATG AGAAAATATT TCTAGGATCC TGGCTGCATT

CTTTGCTGTG CCCAGGAACA AGAGAATATT TCCAGGATTC TGGCTATATT

TCTCATTGTG TCCAGAATTA AGAGCATATC tctgggttcc ttgttacatt

CCAGGAAATG GGGAAAATGC ACAGATTAAA TTGGTCTGTT AGCTCTGAAA

CCAGGAAATG GGGAAAATGC ACAGATTAAA TTGGTCTGTT AGCTCTGAAA

CCAGGAAATG GGGAAAATGC ACAGATTAAA TTGGTCTGTT AGCTCTGAAA

CCAGGAAATG GGGAAAATGC ACAGATTAAA TTGGTCTGTT AGCTCTGAAA

CCAGGAAATG AGGAAAATGC AAAGATGAAA TTGGTCTGTT AGCTCTGAAA

CCAGGAAATG GGGAAAATGC AAAGATTAAA TTGGTCTGTT AGCTCTGAAA

CCAGGATTTG GGGACAAAGC G-GGATTGA- -------ACC AGCTCTGAAA

CTAGGATTTG GGGACAAAGC A-GGATTGAA CTGGCTCACC AGCTCTGAAA

CCAGGAAATG GGGAATTACA A-AGATTAAA TTGGTCTGTT AGTTCTGAAA

CCAGGAAATG GGAAAAATAC AAAGATTAAA TTGGTATGTT AGCTCTGAAA

CCAGCATATG GGAAAAATGC AAAGATTAAA TTGGTCTGCG AG--CTGAAA

CCAAGACATG GGAAAAATGC AGAGGTTAAA TTGGTCTGTG AG--CCGAAA

CCAGGAAATG GAAAAAATGC TAAGACTAAA TTGATGTGTT AGCTCTGAGA

CCAGGAAATG GGAAAAAGCG GAAGATTAAA TTGGTTTGTT AGCTCTGGAA

CCAGGAAATG GGAAAAATGC AAAGATTAAA TTGGTACGTT AGCTCTGAAA

CCAGGAAATG GGGAAAATGT AAAGATTAAA TTGGTCTGTT CGCTCTGAAA

CTAGGAAATA GGAAAGATGC AAAGATTAAA TTGGTCTATT AGTTCTAAAA

cCAGGAAATG GGGAAAATGC AAAGATTCGA TTGGCCTAGG CTCTCTGAAA

TCAATAGTAT TTAGTGCTAA TGCACTAAGG AGATTACAAG CACTAATGCA

TCAATAGTAT TTAGTGCTAA TGCACTAAGG AGATTACAAG CACTAATGCA

TCAATAGTGT TTAGTGCTAA TGCACTAAGA AGATTACAAG CACTAATGCA

TCAATAGTGT TTAGTGCTAA TGCACTAAGA AGATTACAAG CACTAATGCA

TCAATAGTA- ---------- ---------- --------AG CACTAATGCA

TCAATAGTAT TTAGTGCTAA TGCACTAAGC GGATTACAAG CACTAATGCA

TCAATAGTGC TTACAGCTAA TGCACTAAAC AGGTTACAAG CACTAATGCG

TCAATAGTGC TTAGAGCTAA TGCACTAAAC AGGTTACAAG CACTAATGCA

TCAATAGTAT TTAGTGCTAA TGCACTAAGC AGATTACAAG CACTAATGCA

TCAATAGTAT TTAGTGCTAA TGCACTAAGC AGATTCCAAG CACTAATGCA

TCAATAGTAT TTAGTGCTAA TGCACTAAGC AGA-TACAAG CACTAATGCA

TCAATACTGT TTGGCGCTAA TGCACTAAGC AGATTACAAA CACTACTG-A

TCAATAGTAT TTAATGCTAA TGCACTAAGC AGATTACAAA CACTAATGCA

TCAATAGTAT TTACTGCTAA TGCACAAAGC AAATTACAAG CGCTAATGCA

TCAATAATAT TTAGCGCTAA TGCACTAAGC AGATTACAAG CACTAATGCA

TCAATAGTAT TTAGTGCTAA TGCACTAAGC AGATTACAAG CCCTAATGCA

TCAATACTAT TTAGTGCTAA TGCACTGAGC AGATTACAAG TACTAATGTA

TCAATAGTAT TTAGCGCTAA TGCACTAAGC AGATTACAAG TTCTAATGCA

GTAGCAGTCA ATAGCGAAAA CTAATATCTC TAAAAAATAT CTTGACAATA

GTAGCAGTCA ATAGCGAAAA CGAATATCTC TAAAAAATAT CTTGACAATA

GTAGCAGTCA ATAGCGAAAA CGAATATCTC TAAAAAATAT CTTGACAATA

GTAGCAGTCA ATAGCGAAAA CGAATATCTC TAAAAAATAT CTTGACAATA

GTAGCAGTCA ATAGTGAAAA CGAATATCTC TAAAACATAT CTTGACAATA

GTGGCAGTCA ATAGCAAAAA CGAATATCTC TAAAAATTAT CTTGACAATA

GTGGCAGTCA ATAGCAAAAG CCAATGCCGC TAAACATTAC CTCGACGATG

GTGGCAGTCA ATAGCAAAAG TGAGTGCCTC TAAGAATTAC CTTGACAATG

GTGGCAGTCA ATAGC-TAAG CGAATATCTC TAAAAATTAT CTTGACAATA

ATGGCAGTCA ATAGTAAAAA TGAATATCTC TAAAAATTAT CTTGGTAATA

GTGGCAGTCA ATAGCAAAAA CGGATATCTC TACGAGTTAT CTTGACAATA

GTGGCAGTCA ATAGCAAAAA CGAATATCTC TA-AAATTAT CTTGACAATA

ATGGCAGTCA ATAACAAAAG CGAATATCTC TAAAAATTAT CTTGATGATA

GTGGCCATCA GTAGTAAAAA CGAGTACCTC TAAAAATTAT CTTGACAATA

GTGGCAGTCA ATAGCAAAAA CGAATATCTC TAAAAATTAT CTTGACAATA

GTGGCAGTCA ATAGCAAAAA TGAATATCTC TAAAAATTAT CTTGACAATA

GTAGTAGTCA ATTGCAAAAA CAAATAACTC TTAGAATTAT CTCAGCAATG

GTCACAGTCA ATAGCAAAAA CAAATATCTC TCAGAATGAT CTTGACCATA

AAATGTGCAA TATTTTCCAT TTATCTTAAC TAATTACATT ATAAATCACA

AAATGTGCAA TATTTTCCAT TTATCTTAAC TAATTACATT ATAAATCGCA

AACTGTGCAA TATTTTCCAT TTATCTTAAC TAATTACATT ATAAATCACA

AACTGTGCAA TATTTTCCAT TTATCTTAAC TAATTACATT ATAAATCACA

AAAAGTGCAA TATTTTCCAT TTATCTTAAC TAATTACATT ATAAATCACA

AAATGTGCAA TATTTTCCAT TTATCTTAAC TAATTACATT ATAAATCACA

AAACGTGCAC TATTTTCCAT TTATTTTCAC TAATTGCATC GCAAATCACA

AAATGTGTAA TATTTTCCAT TTATTTTCAC TAATTACATC ACAAATCACA

AAATGTGCAA TATTTTCCAT TTATTTTAAC TAATTACATT ATAAATCACA

AAATGTGCAA CATTTTCCAT TTATTTTAAC TAATTACATT ATGAATCACA

AAATGTGCAA TATTTTCCAT TTATTTTAAC TAACCACATT ATAAATCACA

AAATGTGCAA TACTTTCCAT TTATTTTAAC TAATTACATT ATAAATCACA

AAATGTGTAA TATTTTCCAT TTATTTTAAC TAATTACATT ATATATCACA

AAATATGCAA TATTTTCCAT TTTATTTAAC TAATTGCATG ATTAATCACA

AAATGTGCAA GATTTTCCAT TTATTTTAAC TAATTACATT ATAGATCACA

GAATGTGC-A TATTTCCCAT ATATTTTAAC TAATGATGTT A---ATCAGA

AAATGTGAAA TATTTTCAAT TTGTATTAAC TAACTACAGT ATAAACCAAA

AAATGTGCCA TATTTTCAAT TGGCATTAAC TAATTCCATT ATAAACCAAA

AATTAAACTG AGCTGTTGTT TGTTTAATGC TGTGTTTCCT GCTAACCTTA

AATTAAACTG AGCTGTTGTT TGTTTAATGC TGTGTTTCCT GCTAACCTTA

AATTAAACTG AACTGTTGTT TGTTTAATGC TGTGTTTCCT GCTAACCTTA

AATTAAACTG AACTGTTGTT TGTTTAATGC TGTGTTTCCT GCTAACCTTA

AAT-----CG AACTGTTGTT TGCTTAATGC TGTGTTTACT ACTAACCTTA

GATTAAACTG AACTATTGTT TGTTTAATGC TCTGTTTCCT TCTAACCTTA

AAATAAATGG CACTATTGTT TGCTTAAGGC TCTGTTTCCT TCTAACCTTA

AAATAAATTG CACTATTGTT TGCTTAAGGC TCTCTTTCCT TCAAACCTTA

AATTAAACTG AGGTATTGTT TATTTAATA- -CTGACTCTT TCTAACCTTC

AGTTTAACTG AACTATTGTT TGTTTAATGT TCTGTTTCCT TCTAACCTTA

AGTTAAACTG AACTGTTGTT TGTTTAATGC CCTATTTCCT TTTAACCTTA

AGTGAAAATG AACTATTGCT TGTTTAATGC TCTGCTTCCT TCTCGCCTTG

AGTAAAATTA AGCTATCATT TGCTTAATGT TCTATTTCCT CCTAACTCCA

AGTTAAACTG AACTATTGTT TGTTTAATAC TCTGCTTCCT TCTTACCTTA

AATTAAACTG AACCATTGTT TGTTTAATAT TCTGTTTCCT TCTAACCTTA

AACTAAACCC AACTATTGTT TGTTTGATAT TCTGTTTCCT TCTAACCTTA

AGTTAAACTG AACTATTGTT TGTTTTATAA TCTATTTCCT TCTAACTTTA

AGTTAAACAC GGCTATTGTA TATGTAATCC TGCATTTCCT TCTAACCTTG

TTCTTGTCTA TAACCTGAAC GTAGTTCCCC ATGGCTCAGA AAACCCTCTG

TTCTTGTCTA TAACCTGAAC GTAGTTCCCC ATGGCTCAGA AAACCCTCTG

TTCTTGTCTA TAGTCTGAAC CCAGTTCCCC ATGGCTCA-A AAACCCTCTG

TTCTTGTCTA TAATCTGAAC CCAGTTCCCC ATGGCTCA-A AAACCCTCTG

TTCTTGTCTA TAACTTGAAC CCAGTTCCCC ATGGCTCAGA AAACCCTCTG

TTCCCGTCTA TAACCTCAAC CCAGCTCCCC ATGGCTCAGG AAACCCTCTG

TTCTTGTCTA TAACCTCACC CCAGTTCCCT ATAGCTCAGG AAAACCTCTG

TTCCTGTCTG TAACCTCAGC CCAGTTCCCC ATAGCTCAGG AAAACCTCTG

TTCCTGTCTG TAGCCTCAAC CCAGTTCCCC ATGGCTCAGG AAACTCTCTG

TTCTTGTCTA TAACCTCAGC TCAG-TCCCC GAGGCTCAGG AAACTCTCTG

TTTTTGTCTA TAACCTCAGC CCAGTTCCCC ATGGTTCAGG AAACCCTCTG

TTCTTGTCTA TAACCTCAAC CCAGTTCCCC ATGGCTCAGG AAACCCTCTG

TTCTTGTCTA TAACCTCAAT CCAGTTTCCC ATGACTCGGG AAGCCCTCTG

TACTTGTCTG TAACCTCAAC ACAGTTCCCT ATGGCTCAGG AAACCCTCTG

TTCTTGCCTG TAGCCTCAAC CCAGTTCCCC ATGGCTCAGA AAACCCTCTG

TTCTTGTCCA TAACCTCAGC CCAGTTCCCT ATGGCTCAGG AAAGCTTCTG

TTTTTACCCA TAGCCTCAAC CCAATTCCCT ATGCCTTGGG AAAGCCTCTG

TTCTTGACTA TAGCCACGAT CCTGCCCCCC ATGGCTCAGG AATCCCCCTA

GGATTAAGTA TAGTTTACAC AACCTTGCAG TCATTAACCT GTGTACTTTC

GGATTAAGTA TAGTTTACAC AACCTTGCAG TCATTAACCC GTGTACTTTC

GGATTAAGTA TAGTTTATAC AACCTTGCAG TCATTAACCC ATGTACTTTC

GGATTAAGTA TAGTTTATAC AACCTTGCAG TCATTAACCC ATGTACTTTC

GGATTAAGTA TAGTTTACAC AACCTTGCCA TCATTAACCC TTGTACTTTC

GGATTAAGTA TAGTTTACAC AACCCTCCAT TCATTAACCT GTGTACATTC

GGATTAAGTA TCGTTTACAC AACCTCTCAT TCATTAACCC ATGTACATTC

GGATTAAGTA TCATTTACAC AACCTCTCAT TCATTAACCC ATGTACATTC

GGATTAAGTA TAGTTTACAC AACCTCTCAT Tcatt----- ----------

AGATTAAGTA TAGTTTACAC AGCCTCTCAC TCATTAACCC ATGTACATTC

GGATTAAGTA TAGTTTACAC AACCTCTCAT TCATTAACCC AAGTACATTC

GGATTAAGCA CGGTTTACAC AACCTCTCAT TCATTAATAC ATGTACATTT

GGATTAA--- TAGTTTACAC GCCCTCTCAT TCATTAACTC ATGTACATTC

GGATTAAGTA TAGTTTACAC AACCTCTCAT TCATTAACCC ATGTACATTC

AGATTAAGTA TAGTTTACAC AACCTCTCAC TCATTAACCA ATGTA-----

GGATTACGTA TAGTTTACAC AACCTCTCAC TCATTAACCA ATGTACATTC

AGATGGAATA TAGTTTATAC AGTCTCTCAT TCATTACCAT ATGCACATTC

GACCCAACTG TAGATTGCAC CATCTTCCAG TCATTAACCC ATCCACATCC

TCATTCTAAA ACACATGGAC ACAGTCTGAG ATCTGATTAC CTTAGGAAAT

TCATTCTAAA ACACATGGAC ACAGTCTGAG ATCTGATTAC CTTAGGAAAT

TCATTCTAAA ACACATGGAC ACAGTCTGAG ATCTGATTAC CTTAGGAAAT

TCATTCTAAA ACACATGGAC ACAGTCTGAG ATCTGATTAC CTTAGGAAAT

TCATTCTAAA CCACATGGAC GCAGTCTGAG ATCTGATTAC CTTAGGAAAT

TCATTCTAAA CCACATGGAC CCCGCCTGAG ATCTGATTAC CTTAGGAAAT

TCCTTCTAAA CCACATGGGC TCAGCCTGAT ATCTGATTAC CTTAGGAA--

TCCTTCTAAA CCACATGGGC TCAGCCTGAT ATCTGATTAC CTTAGGAA--

-------AAA CCACATGGGC TCA-TCTGAG ACATGATTAC CTTAGGAAGT

TCGTTCAAAA CCACATGGCC TTGGTCTGAG ATCTGAATAC CTTAGGAAAC

TTATTCTAAA CCACACAGAC TTGGTCTGAG ATCTGATTAC CTTAGGAAAT

TCATTCTAAA CCACATGAAC TTGGTCTGAG ATCTGACTAG CTTAGCAAGT

CCATACCAAA CCACATGGAC TTGGTCTGCG ATCTGATTAC CTTAGGAAAT

TCATTCTCAG CCACGTGGAC TCAGTCAAAG GTCTGATTGT CTTAGGAAAT

-TGTTCTAAA CCACACGGAC GCAGTCTGAG ATCTGATTAC CTTAGGAAAC

TTATTCTAAG CCACGTGGAC CCAG-CTGAG GTCTGATTAC CTTAGGAAAG

TCTGTCTAAA CCACATGGGC TTGGTTTAAG AACTGATTAC CTTTGGAATT

TCATTCTCAA CCACATGCAC TCTGTTTGAG GTGTGATTAC CTTTGGAATT

GATGATAAAG AAGCAAAATT ATTTGCAAGA CTGATTTAAA TTATCTATAG

GATGATAAAG AAGCAAAATT ATTTGCAAGA CTGATTTAAA TTATCTATAG

GATGAGAAAG AAGCAAACTT GTCTGCAAGA CTGATTTAAA TTATCTATAG

GATGATAAAG AAGCAAACTT GTCTGCAAGA CTGATTTAAA TTATCTATAG

GATGATAAAG AAGCAAAATT ATCTTCAAGA CTGATTTAAA TTATCTATAG

GATGATAAAG AAGCAAAATT ACCTGCAAGA CTGATT-AAA TTATCTATAG

-ATGATAAAG ATGCAAAGTT GTCTACATGA CTGATTTGAA TTAGCTATAG

-ATGATAAAG ATTCGAAATT ATCTACATGA CTGATTTAAA TTAGCTATAG

GGTGATAAAG AAGCAAAATT ATCTGCAAGA CTGATTTAAA TTATCTATAG

GATGATAAAG AAGCAAACCT CTCTGCAAGA CTGATTTAAA TTATCTATAG

GATGATAAAG AAGCAAACCT ATCTGAAAGA CTGATTTAAA TTATCTATAG

GATGCTACAG AAGCAAACCT ATCTGCAAGA CTGATTTAAA TTATCTATAG

GATGATAAAG AAGCAAAACT CTTTGCAAGA CTGATTTAAT TTATCTATAG

GAGGATAGAG AAGCAAAGCT ATCTGCAAGC CTGATTTAAA TTATCTATAG

GATGAGAAAA GACGAAAACT ACCTGCTAGG CTGATTTAAA TTATCTATAG

GATGACAAAG GGCAAAAACG ATCTGC--GT CTGATTTAAA TTATCTATAG

TAGGATTCAG TGATGAATCT ATCTGCAGGG CTGATTTAAA TTATCTGTGG

TGGGCTACAG GAGTAAACCT ATCCGCATGG TGGATTTAAA TCCTCTATGG

ATGAGCTAAA TCTGTCCTAT AGATCAATTA AATGAATCAC TATTTTAATA

ATGAGCTAAA TCTGTCCTAT AGATCAATTA AATGAATCAC TATTTTAATA

ATGAGCTAAA TCTGTCCTAT AGATCAATTA AATGAATCAC TATTTTAATA

ATGAGCTAAA TCTGTCCTAT AGATCAATTA AATGAATCAC TATTTTAATA

ATGAGCTAAA TCTGTCCTAT AGATCAATTA AATGAATCAC TATTTTAATA

GTGAGCTAAA TCAGTCCTAT AGATCAATTA AAAGAATCAC TATTTTAATA

ATGAGCTAAA TCTGTCCTAT AGATCAATTA AAAGAATCAC TATTTTAATA

ATGAGCTAAA TCTGTCCTAT AGATCAATTA AAAGAATCGC TATTTCAATA

ATGAGCTAAA TCTGTCCTAT AGATCAATTA AAAGAATCAC TATTTTAATA

ATGAGCTAAA TTTGTCCTAT AGATCAATTA GAAGAATCAC TATTTTAATA

GTGAGCTAAA TCTGTCCTGT AGATCAATTA AAGGAATCAC TATTTTAATA

ATGAGCTAAA TCTGTCCTAT AGATCAATTA AAAGAATCAC TATTTTAATA

ATGAGCTAAA TCTGTCCTAT AGATCAATTA TAAGAATCAC TATTTTAATA

ATGAGCTAAA TCTGTCTTAT AGATCAATTA AAAGAATCAC TATTTTAATA

ATGAGCTAAA TCCGTCCTTC AGGTCAATTA GAAGAATCAC TATTTTAATA

ATGAGCTAAA TCTGTCCTAC AGGTCAATTA AAAGAATCAC TATTTTAATA

ATGAGTTAAA TCAATCCTAC AGATTAACAA AATGCATCAC TATTTTAATA

ATGATTTTAA TCATCCCTAT GAACGGATTA AAAAAATCAT TATTTTAAAC

TATCACATTC TTAATTACAC TGAATTAAAT ATAATTACAT ATTCACTGCA

TATCACATTC TTAATTACAC TGAATTAAAT ATAATTACAT ATTCACTGCA

TATCACATTC TTAATTACAC TGAATTAAAT ATAATTGCAT ATTCACTGCA

TATCACATTC TTAATCACAC TGAATTAAAT ATAATTGCAT ATTCACTGCA

TATCACATCC TTAATTACAC TGAATTAAAT ATAATTGCAT ATTCACTGCA

TATCACATCC TTAATTACAC TGAATTAAAT ATAATTGCAT ATTCACTGCA

TATCACATCC TTAATTACAC TGAATTAAAT GTAATTGCAT GCTCTCTTCA

TATCACATCC TTAATTACAC TGAATTAAAT GTAATTGCAT ACTTGCTTCA

TATCACATCC TTAATTACAC TGAATTAAAT GTAATTGCAT ATTCACTGCA

TATCACATAC TTAATTATAC TGAATTAAAT GTAATTGCAT ATTCACTGCA

TATCACATCC TTAATTATAC TGAATTAAAT GCAATTGCAT ATTCACTGCA

TATCACATCC TTAATTATAC TGAATTAAAT GTAATTGCAT ATTCACTGCA

TATCACAATC TTAATTACAC TGAATTAAAT ATAATTGCAT ATTCCCTGCA

TATCACATCC TTAATTACAC TGAATTAAAT GTAATTGCAT ATTCACAGCA

TACCATATCC TTAATTACAC TGAATTAAAC GTAATTGCAT ATTCACAGCA

TATCACATCC TAAGTTATAC CGAATTAAAT GTAATTGCAT ATTCACAGCA

TGCTGGTTCC TTAATTATGC TTAATTAAGT ACAATTACAC ACTCATGGAA

TATCAGCACT GCAATTATAG TTAATTAAAT GAACTTGCAT ATTCTCAGCA

TACATCATAA TGTTAATTAG GGGAAACATT TTGAATCAAA TAGACTTATT

TACATCATAA TGTTAATTAG GGGAAACATT TTGAATCAAA TAGACTTATT

TACATCATAA TGTTAATTAG GGGAAACATT TTGAATCAAA TAGACTTATT

TACATCATAA TGTTAATTAG GGGAAACATT TTGAATCAAA TAGACTTATT

TACATCATAA TGTTAACTAG GGGAAACATT TTGAATCAAA TAGACTTATT

TACATCATAA TGTTAATTAG GGGAAACATT TTGAATCAAA TAGACTTATT

CACATCACAG TGTTAATTGA GGGAAACATT TTGAGTCAAA CCGATTTATT

TACATCACAG TGTTAATTGA GGGAAACATT TTGAGTCAAA CCGACTTATT

TACATCATAA TGTTAACTAA GGGAAACATT TTGAATCAAA TAGACTTATT

TGCATCATAA TGTTAATTAG GGGAAACATT TTGAATCAAA TAGACTTATT

TGCATCATAA TGTTAATTAG GGAAAACAGT TTGAATCAAA TAGACTTATT

TGAATCATAA TGTTAATTAA GGGAAACATT TTGAATCAAA TAGACTTATT

TACATCATAA TGCTAATTAG GGGAAACATT CTGAATCAGA TA--------

TGCATCATGA TGTTAATTAA GGGAAACATT TTGAATTAAA TAGACTTATT

TGCATCGTAA TGTTAATTAG GGGAAACATT CTGAATCAAA TAGACTTATT

TGCATCGTGA TGTTAATTAG GGGAAACATT CCGAATAAAA TAGACTTATC

TGCATCATTA TGTGGATTAG GGAAAACATT TTGAATCTAA TAGACTTATT

TTTGTCATTA TGTTAATTAG GGGAAACATT TAGAATCAAA TAGACCTGTC

CATGGGTCAA TTCAGCGAAG TCCTCTTAAC ATCTATATCC ACCTTCATTA

CATGGGTCAA TTCAGCGAAG TCCTCTTAAC ATCTATATCC ACCTTCATTA

CATGGGTCAA TTCAGCGAAG TCCTCTTAAC ATCTATATCC ACCTTCGTGA

CATGGGTCAA TTCAGCGAAG TCCTCTTAAC ATCTATATCC ACCTTCGTGA

CGCAGGTCAA TTCAACAAAG TCCTCTTAAC GTCTATATCC ACCTTCATTA

CATGGGCCAA TTCAACCAAG TCCTCTTAAC ATCTATATCT ACTTTCATTA

CATGGGCCGG CTCAACCATG TACTCTTGAC GTCTATCTCT ACCTTCATCA

CATGGGCCGG CTTAACCAAG TGCTCCTGAC ATCCATCTCC ACCTTCATCA

CATGGGCCAG TTCAACCAAG TCCTCTTAAC ATCCATCTCC ACCTTCATTA

CACGGGCCAG TTCAACCAAG TCCTCTTAAC ATCTATATCC ACCTTTATCA

CATGGGCCAG TTCAACCAAG TCCTCTTAAC GTCTATATCC ACCTTCATCA

CATGGGCCAG TTCAACCGAG TCCTCCTGAC ATCTGTGTCG ACGTTCATTG

---------- ---------- ---------- ---------- ----------

TATGGGCCAG TTCAACCAAG TCCTTCTAAC ATCTATATCC ACCTTCATTA

CACGGGCCAG TTCAACCAGG TCCTCTTAAC ATCCATATCC ACCTTCATTA

CGCGGGCCAC TTCAGTCAAG TCCTCTTAAC TTCCATATCC ACCTTCATTA

CACAGGACAA TTCAAGCAAG TCCTTTTAAC ATCGATATCC ACCTTTATCA

CATGGGGAAG TTCAGTCATG TCCTCCTGAC GTCGATATCC ACCTTCATCA

AAGGAGACCT CACCATAGCT AATCTTGGGA CATCAGAGGG TCGCTTCATG

AAGGAGACCT CACCATAGCT AATCTTGGGA CATCAGAGGG TCGCTTCATG

AAGGAGACCT CACCATCGCT AATCTTGGGA CATCAGAGGG TCGCTTCATG

AAGGAGACCT CACCATCGCT AATCTTGGGA CATCAGAGGG TCGCTTCATG

AAGGAGACCT CACCATTGCT AATCTTGGGA CATCAGAGGG CCGCTTCATC

AAGGCGACCT CACCATTGCT AATCTTGGGA CATCAGAGGG TCGCTTCATG

AAGGTGACCT CACCATTGCT AATCTAGGGA CATCAGAAGG TCGCTTCATG

AAGGTGACCT CACCATTGCT AATCTAGGGA CGTCAGAAGG TCGCTTCATG

AAGGCGACCT CACCATTGCT AACCTCGGGA CCTCAGAGGG TCGTTTCATG

AAGGAGATCT CACCATCGCT AATCTCGGGA CATCAGAAGG TCGATTCATG

AAGGAGACCT CACCATTGCT AATCTTGGGA CGTCCGAGGG TCGCTTCATG

CAGGCGACCT CACCATCGCC AACCTCGGGA CGTCGGAGGG CCGCTTCATG

---------- ---------- ---------- ---------- ----------

AAGGTGACCT CACCATTGCT AATCTTGGGA CATCAGAGGG CCGTTTCATG

AAGGAAACCT CACGATTGCT AACCTTGGGA CCTCAGAGGG TCGTTTCATG

AGGGAGACCT CACGATTGCC AATCTCGGAA CGTCCGAGGG TCGCTTCATG

AAGGAAACCT AACTATTGCA AACCTTGGAA CCTCAGATGG CCGATTTATG

AAGGGAACCT CACCGTGGCC AATCTTGGCA CCGCCGAGGG GCGATTCATG

CAGGTACATT TATAATTTCT GTCACGTGAT TGGAAAATAA AATGAAACAA

CAGGTACATT CATAATTTCT GTCACATGAT TGGAAAATAA AATGAAACAA

CAGGTACATT CATAATTTCT GTCACATGAT TGGAAAATAA AATGAAACAA

CAGGTACATT CATAATTTCT GTCACATGAT TGGAAAATAA AATGAAACAA

CAGGTACGTT CATAATTTCT GTCACATGAT TGGAAAATAA AATGAAACAA

CAGGTACATT CACAATTTCT ATCACGTGAT TGGAAAATAA AATGAAACAA

CAGGTACATT CATAACTTTT ATCACATGAT TGGAAAGTAA AATGAAACAA

CAGGTACATT CATAACTTTT ATCACATGAT TGGAAAGTAA AAAGGAACAA

CAGGTGCATT CATAAATTCT GTCACATGTT TGGAAAGTAA AATGAAACAA

CAGGTACATT CATGATTTAA ATCACATGAT TGGAAAATAA AATGAAACAA

CAGGTACATT CATGATTTCT ATCACATGAT TGG--AATAA AATGAAACAA

CAGGTACATT CATGATTTCT ATCACATGAT TGGAAAATAA AATGAAACAA

---------- ---------- ---------- ---------- ----------

CAGGTACATT CATAATTTCT ATCACATGAT TGGAAAATAA AATGAAACAA

CAGGTACATT TATAATTTCT ATCACATGAT TGGAAAATAA AATGAAACAA

CAGGTACATT CATAATTTCT ATCACATGAT TGGAAAATAA AATGAAACAA

CAGGTACATT CACAATTTCC ACTACACAAA TGGAAAATAA CATGAAACAA

CAGGTACATT CACCATTTCG GCCCCATGAT AGAAAAATAA AATAAAACAA

AGCACTTCTA GGCAATTTTT TTTATTTCAA GGGAATCTTC ATGAGAAGAA

AGCACTTCTA GACAATTTTT TTTATTTCAA GGGAATCTCC ATGAGAAGAA

AGCACTTCTG GGCAATTTTT TTTATTTCAA GGGAATCTCC ATGAGAAGAA

AGCACTTCTG GGCAATTTTT TTTATTTCAA GGGAATCTCC ATGAGAAGAA

GGCAGTTCCA GACAATTTTT TCTATTTCAA GGGAATCTCC ATGAGAAGAC

AGCACTTCCA GGCGATGTTT CCTATTTCAG GGGAATCTCC ATGAGAAGAA

AGCACTTCCA GGCAATGTTT CCTATTTCAG GGGAATGTCC ATGAGAAGAG

AGCACTTCCA GGCAATGTTT CCTGTTTCAG GGGAATGTCC ATGAGAAGAG

AGTATTTCCA GGCCATGTTT CCTATCTCAA GGGAATCTCC GTGAGAAGAA

AGCACTTCCA GGCGATGTTT CCTATTTCAG GAGAATCTCC ATGAGAAGAA

AGCACTTCCA GGCGATGTTT CCTATTTCAA GGGAATCTCC ATGAGAAGAA

AGCACTTCCA GGCGATGTTT CCTATTTCAA GGGAATCTCC ATGAGAAGAA

---------- ---------- ---------- ---------- ----------

AGCACTTCCA GGCTATGTTT CCTATTACAA GGGAATCTCC ATGAGAAGAA

AGCACTTCCA GGCGATGTTT CCTATTTCAA GGGAATCTCC ATGAGACGAG

GGCACTTCCA GGCGCTGTTT CCTCTTTCAA GGGAACCTCC AGGAGAAGAA

AGCATTTCCA GGCAACATTT CCTATTTCAG GGGAATCTCT CTGAACAGAA

AGCCCTTCCA GCCAGTGTTT CCTACCCTAG GGGAATCTCC AGAAACAGAA

ACATTCACAT CATTGAGAAA TATTGGCAGG AGTTAGGAAG GGAAAGACAG

ACATTCACAT CACTGAGAAA TATTGGCAGG AGTTAGGAAG GGAAAGACAG

ACATTCACAT CATTGAGAAA TATTGGCAGG AGTTAGGAAG GGAAAGACAG

ACATTCACAT CATTGAGAAA TATTGGCAGG AGTTAGGAAG GGA-------

ACAATCACAT CATTGAGAAA TATTGGCAGG AGTTAGGAAG GGAAAGACAG

ACATTCACAT CATTGAGAAA TATTGGCAGG AGTTAGGAAG GGACAGACAG

GCATTCACAT CCCTGAGAGA CACTGGCAGG AATCAGAAAG GGAAAGGCAG

ACATTCACAT CCGTGAGAGA CACTGGCAGG AATCAGAAAG GGAAAGGCAG

ACATTCACAT CATTGAGAAA TATTGGCAGC AGTCAGGAAG GGAAAGACAG

ACATTCACAT CATTGAGAAA TATTGGCAGG CGTTAGGAAG GGAAAGACAG

ACATTCACAT CATTGAGAAG TATTGGCAGG AGTTAGGAAA GGAAAGACAG

ACATTCACAT CACTGAGAAA TGTTGGCAGC AGTTAGGAAG GGAAAAACAG

---------- ---------- ---------- ---------- ---AAGACAG

ACATTCACAT CATTGAGAAA TATTGGCAAG GGTTAGGAAG GGAAAGACAG

TCATTCACAT CGTTGAGAAG TACTGGCAGG AGTTAGGAAG GGGAAGACAG

ACGTTCCCAT CGTTGAGAAA TATGGGCAGG CGTCAGGAAG GGCAAGACAG

ATATTCACAT CATTGAGTGA AACTGGCTGG AATTAGGAAG GGAAAGACAG

ACGTTCACAG CCTTGAGAGC CGCCGGCTGA ACTTAGGGAA GGAAAGGCAG

CTGCCCTACA CAAAAGGGAA CAGAAAGATG ATTTTACTTC CTTCCTACCC

CTGCCCTACA CAAAAGGGAA CAGAAAAATG ATTTTACTTC CTTCCTACCC

CTGCCCTGCA CAAAAAGGAA CAGAAAAATG ATTTTACTTC CTTCCTACCC

---------- ---------- ---------- ---------- ----------

CTGCCTTACA CAAAAAGTAA CAGAAAAATG ATTTTACTTC CTTCCTACCC

CTGCCATACA TGAAAAGGAA CAGAAAAATG ATTTTACTTC CTTCCTATCC

CTGCCACACA TGAGAA-ACA CGGAGAAATG ATTTTACTTC CTTCCTACCC

CTGCTACACA TGAGAA-ACA CAGAGAAATG ATTTTACTTC CTTCCTACCC

CTGCCACACA TGAAAAGGAA CAGCAAAATG ATTTTATTTC CTTCCTACCC

CTGCCATACA TGAAAAGGAA CAGAAGAATG ATTTTACTTC CTTCCTACCC

CTGCCATACA TGAAAAGGAA CAGAAAAATG GTTTTACTTC CTTCCTACCC

CTGCCATACA TGAAAAGGAA AAGAAAAATG ATTTTACTTC CTTCCTAGCC

CTGCCCTGCA CGAAAAAGAA CAGAAAAATG ATTTTACTTC CTTCCTACCC

CTGCCATACA TGAAAAGGAA CAGAAAAATG ACTTTACTTC CTTCCTCCAC

CTGCCATACA TGAAAAGGAA CAGAAAAATG ATTTTACTTC CTTCCTACTC

CTGCCGT--- -GAAAAGGAA CAGAAAAATG ATTTTATTTC CTTCCTACAC

CTGCCATGCA TGAAAAGGAA TAGAAAAATT GTTTTACTTC CTTCCTAGGC

CTGCCAGGCC TGCAGAGGAA CACAAACACC ATTGGGTTTC CTTCCTGAGC

TGAAGCCTAA TGGCAAAGTT GCTTTATTTT TCTCCCAAGA GTTTGCAGCT

TGAAGCCTAA TGGCAAAGTT GCTTTATTTT TCTCCCAAGA GTTTGCAGCT

TGAAGCCTAA TGGCAAAGTT GCTTTATTTT TCTCCCAAGA GTTTGCAGCT

---------- ---------- ---------- ---------- ----------

TGAAGCCTAA TGGCAAAGTT GCTTTATTTT TCTCCCAAGA GTTTGCAGCT

TGCAGCCTAA CAGCAAAGTT GCTTTATTTT TCTCCCAAGA GTTTGCAGCT

TCAAGCCTAA TGGCCAAGTC GCTTAATTTT TATCCCAAGA GTTTGCAGCT

ACAAGCCTAA TGGCAAAGTT GCTTAATTTT TACCCCAAGA GTTTGCAGCT

TGAAGCCCAA TGGCAAGGTT GCTGTA-TTT TCTCCCAAGA GTTTGCAGCT

TGAAGCCTAA AGGCAAAGTT GCTTTATGTT TCTCCCAAGA GTTTGCAGCT

TGAAGCCTAA AGTCAAAGTT GCTTTATGTT TCTCTCAAGA GTTTGCAGCT

TGAAGCCTAA AGGCAAAGTT GCTTTATGCT TCTCCCAAGA GTTTGCAGCT

CGAAGCCTAA AAGCAAAGT- GCTTTTTTTT TCTCCCAAGA CTTTGCAGCT

TGAAGCCTAA AGGCAAAGTT GCTTTATTTT TTCCCCAAGA GTTTGCAGCT

TGAGGCCTAA AGCCAGAGTT GCTTTGTTTT TCTCCCAAGA GTTTGCAGCT

TGAGGCCTAA ATGCAG--TT ACTTTTTTTT TCTCCCAAGA GTTTGCAGCT

TAAAGTCTGA TGGCAACGTT GATTTTTTTT CCCCCAGAAA GTTTGCAGCT

TGAAGCTTAA TGGCAGAGTT GTTTATTTTT TCTCTCCAGC ATTTGCAGCT

GTTTCTGTCT TTCCTATTGT TTCAGCTCAG CTGTTGGGTG CAAAGTGAAT

GTTTCTGTCT TTCCTATTGT TTCAGCTCAG CTGTTGGGTG CAAAGTGAAT

GTTTCTGTCT TTCCTATTGT TTCAGCTCAG CTGTTGGGTG CAAAGTGAAT

---------- ---------- ---------- ---------- ----------

GTTTCTGTCT TTCCTATTGT TTCAGCTCAG CTGTTGGGTG CAAAGTGAAT

GTTTCTGCCT TTCCTATTGT TTCAGCTCAG CTGTTGGGTG CAAAGTGAAT

GTTTCTGCCC TTCCTATTGT CTCAACTCAG CTGTTGGGTG CAAAGTGAAT

GTTTCTGCCC TTCCTATTGT GTCAGCTCAG CTGTTGGGTG CAAAGTGAAT

GTTTCTGCCC TTCCTATTGT TTCAGCGCAG CTGTTGGGTG CAAAGTGAAT

GTTTCTGCCT TTCCTATTGT TTCAGCTCAG CTGTTGGGTG CAAAGTGAAT

GTTTCTGCCT TTCCTATTGT TTCAGCTCAG CTGTCGGGTG TAAAGTGAAT

GTTTCTGCCT TTCCTATTGT TTCAGCTCAG CTGTCGGGTG CAAAGTGAAT

GTTTCTGCCT TTCCTATTGT CTCAGCTCAG CTGTCGGGTG CAAAGTGAAT

GTTTCTGCCT CTCCTATTGT TTCAGCTCAG CTGTTGGGTG CACAGTGAAT

GTTTCTGCCT CTCCTATTGT TTCAGCTCAG CTGTTGGGCA CAAAATGGAT

GTCTCTGCCT TTCCTGTTGT TTGGGCTCAG CTGTTGGCCG CAAAATGAAT

GTTTCTGCCC TTCCTATTGT TTCAGTTCAG CTGTTGGGTG CAAAGTGAAT

GTTTCTGCCT CTCCTATTGT TTTGGTTCAG CTGTCGGGTA GACAATGAAT

CAGGTATTTC ACAGTGGTCA TTTGATTAGA AAGAATTATA TTGTTTAGCT

CAGGTATTTC ACAGTGGTCA TTTGATTAGA AAGAATTATA TTGTTTAGCT

CAGGTATTTC ACAGTGGTTA TTTGATTAGA AAGAATTATA TTGTTTAGCT

---------- ---------- ---------- ---------- ----------

CAGGTATTTC ACAGTGGTTA TTTGATTAGA AAGAATTCTG TTGTTTGGCT

CAGGTATTTC ACAGTGGTTA TTTGATTAGA AAGAATTATA TTGTTTAGCT

CAGGTATTTC ACAGTGGTTA TTTGATTAGA AAGAATTATA TTGTTTAGCT

CAGGTATTTC ACAGTGGTTA TTTGATTAGA AAGAATTATA TTGTTTAGCT

CAGGTATTTC ACAGTGGTTA TTTGATTAGA AAGAATTATA TTGTTTAGCT

CAGGTATTTC ACAGTGGTTA TTTGATTAGC AAGACTTATA TTGTTTAGCT

CAGGTATTTC ACAGTGGTTA TTTGGTTAGA AAGAATTATA TTGTTTAGCT

CAGGTATTTC ACAGTGGTTA TTTGATTAGA AAGAATTATA TTGTTTAGCT

CAGGTATTTC ACAGCGGTTA TTTGATTAGG AGGAATTATA TTGTTTAGCT

CAGGTATTTC ACAGTGGTTA TTTGATTAGA AAGAATTGTA TTGTTTAGCT

CAGGTATTTC ACAGTGGTTA TTTGATTAGG AAGAATTATA TTGTTTAGCT

CGGTTATTTC G--GGGGTTA TTTGATTAGG GAGAATTATA TTGTTTACCT

CAGGTATTTC ACAGTGGTTA TTTGATTAGA AAGAATTATA TTGTTTAACT

CAGGTATTTC ACAGTCATTA TTTGCTTAGA AAGAAAGATA TTGTTGCACT

AACATAGTGG GTGACTGGGT TTTAACCAAG TTATTTTCTG TTAGGTGTGA

AACATAGTGG GTGACTGGGT TTTAACCAAG TTATTTTCTG TTAGGTGTGA

AACATAGTGG GTGACTGGGT TTGAACCAAG TTATTTTCTG TTAGGTGTGA

----TAGTGG GTGACTGGGT TTGAACCAAG TTATTTTCTG TTAGGTGTGA

AATATAGTGA GTGACTGGGT TTTAACCAAG TTATTTTCTG TTAGGTCTAA

AACATAGTGG GTGACTGGGT TTTAAGCAAG TCATTTTCTG TTAGGTGTGA

AACACAGTGG GTAACTGGGC TTTAGTAAAA CCATGTTCTG CTAGGTGTGA

AACACAGTGG GTAACTGGGC TTTGGGAAAA TCATTTTCTG CTAGGTGTGA

AACATAGTGG GTGACTAGGT TTTAACCAAA TCCTTTCCTG TTAGGTGTGA

AACATACTGG GTGACTGGGT TTTAGCCAAA TCATTTTCTG TTAGGTGTGA

AACATGCAGG GTGACTGGAT TTTAACCAAA TCATTTTCTG TTAGGTGAAA

AACATG--GG GTGACTGGGT TTTAACCAAA TCAGTTTCTG TTGGGTGTGA

AACATACTGG GTGACTGGGT TTTAACCACA TCATTTTCTG TTAGGTGTGA

AACATACTGG GTAACTGGGT TTTAATCAAA TAATTTTCTG TTAGGTGTGA

AACATACTGG GTTAGTGGGC TTTAACTAAA TCATTTTCTG CTAGGTGTGA

AACA------ ---------- ---------- ---------- ----------

AATATACTGG GTGATGGTTC ATTAATTAAG TCATTTTTGT TTAAGCATAA

TGTATACTAA CTGCCTGGAC ATTAACCAGA TCATTTTGTA TTAAGTGTGA

CACAGTTATT AAGACTTAGC TTCAGCTGAG TCCATAAATC CCTCTAGGTT

CACAGTTATT AAGACTTAGC TTCAGCTGAG TCCATAAATC CCTCTAGGTT

CACGGTTATT AAGACTTAGC TTCAGCTGAG TCCATAAATC CCTCTAGGTT

CACGGTTATT AAGACTTAGC TTCAGCTGAG TCCATAAATC CCTCTAGGTT

CA--GTTATT AAGACTGAGC TTCAGCTGAG TCCATAAATC CCTCTAGGTT

CACAATTATT AAGACTTAGC TTCAGCTGAG TCCATAAATC CCTCTCGCTT

CACAGTTACC AAGCCTTGGC --GAGCTGAC TGTGTAAACC CCCCTCTGCC

CACAGTTGCC AAGCCTTGGC TTCAGCTGAG TCTACAAACC CCCCTCTGTC

CACAG----- -GGCCTTAGA --CAGCTGAG TCCATAAATC CCTTTCAGTT

CACAATTATT AAGCCTCAGC TTCAGCTGAG TCCATAAATC CCTTTTGGTT

CACAGTTATA AAGACTTAGC TTCAGCTGAG TCCATAAATC CCTTTCGGTT

CACAATTATT AAGACTTAGC TT-------- ---------- ------GGTT

CACTATTATT AAGATTTAGC TTCAGCTGAG TCCATAAATC CCTTTCGGTT

TACAATTTTT AAGACTTAAC TTCAGCTGAG TCTATAAATC CCTTTTGGTT

CACAGTTATG AAGACTGAGC TTCAGCTGAG TCCATAAATC CCTTTTGGTT

---------- ---------- ---------- ---------- ----------

CAGAATTAGT AAGACCAAGT TTCAGCTGAG AATATAAATC CCCTTTGCTT

CACAATTATT GAGACCCAGT TCCAGCTGAG TCTATAAATC CCTCTCAGTT

CATAGTTGTT AGGAAGATTT CATGCTGCAC CAGACTGTGA CAGTTACATT

CATAGTTGTT AGGAAGATTT CATGCTGCAC CAAACTGTGA CAGTTACATT

CATAGTTGTT AGGAAGATTT CATGCTGCAC TAGACCGTGA CAGTTACCTT

CATAGTTGTT AGGAAGATTT CATGCTGCAC TAGACCGTGA CAGTTACCTT

CATAGTTGTT AGGAAGATTT CATGCTGCAC TAGACCGTGA CAGTTACATT

CATAGCTGTT AGGAAGATTT CATGCTGCAC TAGACCGTGA CATTTACATT

CATAGCTGTT AGGAAGATTT CATGCCATGC TAGACCGTGA CATTGGCATT

CATAGCTGTT AGGAAGATTT CATGCCATAC TAGACCGTGA CATTGGCATT

CATAGATGTT AGGAAGATTT CATGCTGCAC TAGACCGTGA CATTTACATT

CATAGTTGTT AGGAAGATTT CATGCTGCAC TAGAGCATGA CATTTACATT

CATAGTTGTT AGGACGATTT CATGCTGCAC TAGACCGTGA CATTTACATT

CATAGTTCTT AGAAAGATTT CATGCTGCAC TAGAGCGTGA CATTCACATT

CATAGTTGTT AGGAAGATTT CATGCTGCAC TAGACCGTGA CATTTACATT

CATAGTTGTT AGGAAGATTT CATGCCTCAG TAAACCGTGA CATTTACATT

CATAGTTGTT AGGAAGATTT CATGCTGCAC TGGACCGTGA CATTTACATT

---------- ---------- ---------- ---------- ----------

CATAGTTGTT AGTGAGATTT CATGCTCTGC TAGACCATGA CGTTTACATT

CATAGTCATT AGGAAGATTT CACGCTCTGC TAGACTGTGA CGCTGACATT

CTTCCTGCAA AACATAAGAT CTTATGTGCT TAAATCTCTC CTACTGGATT

CTTCCTGCAA AACATAAGAT CTTATGTGCT TAAATCTCTC CTACTGGATT

CTTCCTGCAA GAAATAAGAT CTTAAGTGCT TCAATCTCTC CTACTGGATT

CTTCCTGCAA GAAATAAGAT CTTAAGTGCT TCAATCTCTC CTACTGGATT

CTTACTGCAA AAAATAAGAT CTTATCTGCC TAAATCTCTC CTACTGGATT

CTTACTGCAA AAAATAAGAT CTTATCTGCT TGAATCTCTC CTACTGGATT

CTTACTGCAA AAAATAAGAT TTT--CTGCC TCAATCTCTT CTGTTAGGAT

CTTACTGAAA AAAAAAAGAT TTT-TCTGCT TCAATCTGTC CTGCTGGTCT

CTTACTGCAA AAAGTAAGAT CTTATCTGCT TAAATCTCTC CTACTGGATT

CTTACTGCAA AGAATAAGAT CTTATCTGCT TAAATCTCTC CTACTGAATT

CTTACTGCAA AAAATAAGAT CTTATCTGCT TAAACCTCTC CTACTGGATT

CTTACTGCAA AAAATAAGAT CTTATCTGCT TAAATATCTC CTACTGGATT

CTTACTGAAA AAAAAAAGAT CTTATCTGCG TAAATCTCTC CTACTGAATT

CTTACTGCAA AAAATAAAAT CTTATCTGCT TAAATCTCTC CTACTGGATT

CTCGCTGCAA AAAATAAAAT CTTATCTGCT TAAATCTCTC CTACTGGATT

---------- ---------- ---------- ---------- ----------

CTTGCTGAAA AAAAAAAGAT CTAATCTGCT TAAATCTCTC CTACTGGATT

CTTACTGGCA AAAATAAAAT CTGATCCACT TAAATCTATC CCCCCGGATT

TCTACATAAT AATAAACAGA TTTTTCTAGT AGTTCTCATT TGAGGTAATT

TCTACATAAT AATAAACAGA TTTTTCTAGT AGTTCTCATT TGAGGTAATT

TCTACAGAAT AATAAACAGA TTTTTCTAGT AGTTCTCATT TGAGGTAATT

TCTACAGAAT AATAAACAGA TTTTTCTAGT AGTTCTCATT TGAGGTAATT

TCCACATAAT AATAAACAGA TTTTTCTAGC AGTTCTCATT TGAGGTAATT

TCTACATAAT AATAAACAGA TTTTTCTAGC AGTTCTCATT TGAGGTCATT

----TGCCCT AGTAAACAGA CCCCTCTAGC AGTTTTCATT TGAGGTAATT

GCCATATCAT AATAAACAGA TCCCCCTAGC AGTTCGCATT TGAGGTAGTT

TCTACATAAT AATAAACAGA TTTTTCTAGC AGTTCTCATT TGAGGTAATT

TCTACATAAT AATAAACAGA TTTTTCTAGC AATTCTCATT GGAGGTAATT

TCTGCAT--- ---------- ---------- --TTCTCATT T-AAGTGATG

TCTACATAAT AATAAACATA TTTCCCTAGC AGTTCTCATT TGAGGTAGTT

TCTACATAAT AATAAACAGA TTTCCCTAGC GGTTCTCATT TGAGGTAGTT

TCTACATAAT AATAAACAGA CTTTCCTAGC AGTTCTCTTT TGAGGTAATT

TCTACATCAT AATAAACAGA TTTTTTTAGC AGTTCTCATT TGAGGTAATT

---------- ---------- ---------- ---------- ----------

TCTACAAAAT AATAAATGGA TTTCTCTAGC AGTTTTCACT TGAGGTAATC

TCTACATAAT AATGAAAGGA TTCCTCCGGC GGTTTGCCTG TGAGGTAGCC

TTCAGGAAAG GCACAACAGT GTAATGGATT TTATGGGTAA AATGGCTATC

TTCAGGTAAG GCACAACAGT GTAATGGATT TTATGGGTAA AATGGCTATC

TTCAGGAAAG GCACAACTGT GTAATGGATT TTATGGGTAA AATGGCTATC

TTCAGGAAAG GCGCAACTGC GTAATGGATT TTATGGGTAA AATGGCTATC

TTCAGGAAAG GC-CAACAGT GTAATGGATT TTATGGGTAA AATGGCTATC

TTCATGAAAG GCACCACTGT GTAATGGATT TTATGGGTAA AATGGCTATC

TTCATGAAAG GCACAACTGT GTAGTGGA-T TTGTTTGTCA AACGGCGATT

TTCATGAAAG GCACAACTGT GTAATGGATT TTATGGGTAA AAAGGCGATT

TTCATGAAAG GCAC-ACTGT GTAATGGA-T TTATGGGTAA AATGGCTATC

TTCATGAAAG GCACAACTGT ATAATGGATT TCATGGGTAA AGTAGCTAT-

TTTGTGAAGG GCACAGGTGT GT----GATT TTATGGGTAA A---------

TTCATGAAAG ACACAACTGT ATAATGTATT TTATGGGTAA ATTAGCCATC

TTCATGAATG GCACAACTGT GTAATGGATT TTATGGGTAA AATAGCTATC

TTCATGAAAG GCACAACTGT GTAATGGATT TTATGGGTAA AATTGCTATC

TTCATGAAAG GCACAACTGT GTAATGGATT TTATGGGTAA AATGGCTATC

---------- ---------- ---------- ---------- ----------

TTTGTGGGGA AAAAAACTGT GTAATGGATT TTATGGATAA AATAGCAATC

CTCATGAAAG GCCCAACTGT GTAATGGATT TTTGGGGGGA AATAGCTATC

ATAAAAGCTT TAAGCTAAAA CTTTAAGGGG TGATAACGAG GGTACATTAA

ATAAAAGCTT TAAGCTAAAA CTTTAAGGGG TGATAACAAG GGTACGTTAA

ATAAAAGCTT TAAGCTAAAA CTTTAAGGGA TGATAACAAG GGTAAGTTAA

ATAAAAGCTT TAAGCTAAAA CTTTAAGGGA TGATAACAAG GGTAAGTTAA

ATAAAAACTT TAAGCTAAAA CTTTAAGGGG TGATAACAAG GGTGAGTTAA

ATAAAAGCAT TAAGCTAAGA CTTTAAGGGG TGGTAACAAG GGTAAGTTAA

AGAAAAGCAT TAGGCTGAGA CTTTAAGGGG TGGCAACAAG GGTGCGTTAA

ATAAAAGCAT TAAGCTGAGA CTTTAAGGGG TGGCAACAAG GGTGAGTTAA

ATAAAAGCAT TAAGCTAAGA CTTTAAGGGG TGGTAACAAG GGTAAGTTAA

ATAAAGGCAT TAGGCTAAGA CTTTAAGGGG TGACAACAAG GG-----TAT

---------- ---GCCAAGA CTTTAGGGAA TGGTAGCAAG GGTAAGTTAA

ATAAAAGCAT TAAGCTAAGA CTTTAAGGGG TGGTAACAAA GGTAAGTTAA

ATAAAAGCAT TAAGCTAAGA CTTTAAGGGG TGGTAACAAG GGTAAGTAAA

ATAAAAGCAT TAAGCTAAGA CTTTAAGGAG TGGTAACAAG GGTAAGTTCA

ATAAAAGCAT TAAGCTAAGA CCGTTGGGGG TGGTAACAAG GGTAAGTAAA

---------- ---------- ---------- ---------- ----------

ACTAAAGTTT TACTCTAAGA CCTTAAGGGG TGGTAACAAG TGTAAGTTAA

ACTCGAGCAT TAACCTGAGA CTCTTAGGGG TGGAAACAAG TGTAAGTTAA

GACTTTGGTT TGTTATTTAC TTAGGAAGGA TGGAAATGTT TGCAAGTTTG

GACTTTGGTT TGTTATTTAC TTAGGAAGGA TGGAAATGTT TGCAAGTTTG

GACTTTGGTT TGTTATTTAC TTAGGAAGGA TGGAAATGTT TGCAAGTCTG

GACTCTGGTT TGTTATTTAC TTAGGAAGGA TGGAAATGTT TGCAAGTCTG

AACTTTGGTT TGTTATTTAC TTAGGAAGGA TGGAAATATT TGCAAATCTG

GACTTTGGTT TGTTATTTAC TTAGGAAGGC TGGGAATGTT TGCAAATTTA

GGCATGGGTT TGTTACTTGC TTAGGGAGGG TGGGAATGTG TGCGAACCTG

GACATCGGTT TGTTATTTGC TTAGGAAGGA TGGGAATGTT TGCAAATTTG

GACTTTGGTT TGTTATTGAC TTAGGAAGGA TGGGAATGTT TGCAACTTTG

GACATTGGTT TGTTATTTAC TTGGGAGGGA TGGTAACATT TGCAGATTTG

GACTTTGGTT TGTTCTTTAC TTGGGATGGA TGGGAATGTT TCCAAATTTG

GACTTTGGTT TGTTATTTAC TTGGGAGGGA TAGGAATCTT TGTAAATTTG

GACATGGGTT TGTTATTTAC TTAGGCAGGC TGGGAATGTT TACAAATCAG

GATTTTGGTT TGTTATTTTC TTGGGAGGGA TGGGAGTGTT TGCAAATTTG

GACTTTGGTT TGTAATTTAC TCAGCAGGGA AGGGAATGTT TGCAAATTTG

---------- ---------- ---------- ---------- ----------

GCCTTTAGTT TGTTATTTAC TTGGAAGGGC TGGGAATGTT TGCAAATTTG

AACTTTTGTT TGTTGTTTAC TCGTGCGAGA CGGGAATGTT TGCAAATTTC

AATTTGGCTA AAAGCAAAAG AGCATTTCCT TTCCAATTGT CACAAAGCTG

AATTTGGCTA AAAGCAAAAG AGCATTTCCT TTCCAATTGT CACAAAGCTG

AATTTGGCTA AAAGCAAAAG AGCATTTCCT TTCCAATTGT CACAAAGCTG

AATTTGGCTA AAAGCAAAAG AGCATTTCCT TTCCAATTGT CACAAAGCTG

AATTTAGCTA AAAGCAAAAG AGCATTTCCT TTCCAATTGT CACAAAGCTG

AATTTTACTA AAAGTAAAAG AGCATTTCCT TTCCAATTGT CACAAAGCTG

AGTTCTTCTG GAAGCAAAAG AGCATTTCCT TTCCAATTGT CACACAGCTC

AGTTCTCCTG AAAGCAAAAG AGCATTTCCT CTCCAATTGT CACACGGCTC

CATTTGACTA AAAGCAAAAG AGTATTTCCT TTCCAATTGT CACAAGGCTG

AATTTTGCTA AAAGCAAAAG AGTATTTCCT TTCCAATTGT CACAAAGCTG

AATTTTGCTA AAAGCAAAAG AGCATTTCCT TTCCAATTGT GACAAAGCTG

AATTTTGCTA AAAGCAAAAC AGCATTTCCT TTCCAATTGT CACAAAGCTG

AATTTTGTTA AAAACAAAAG AGCATTTCCT TTCCAGTTGT CACAGAGGTG

AATTTTGCTA ACAGCAAAAG AGCATTTCCT TTCCAATTGT CATGAAGCTG

ACTCTTGCTA AAAGCAAAAG AGCATTTCCT TTCCAATTGT CCAAAAGTTG

---------- ---------- ---------- ---------- ----------

AATTTTGTTA AGAGTAAAAG AGCATTTCCT TTCAGATTGT CGCAAGGCTA

ATTTGTGTTA AAAGCGAATG AGCACTTCCT TTCAGATTGT CACCCAGCTT

CTGAGCTTAT TTTTAACTTG GAAATGTGTA TGTGGCTGGG ACTTTGGATT

CTGAGCTTAT TTTTAACTTG GAAATGTGTA TGTGGCTGGG ACTTTGGATT

CTGAGCTTAT TTTTAACTTG GAAATGTGTA TGTGGCTGGG ACTTTGGATT

CTGAGCTTAT TTTTAACTTG GAAATGTGTA TGTGGCTGGG ACTTTGGATT

CTGAGCTTAT TTTTAACTTG GAAATGTGTA TGTGGCTGGG ACTTTGGATT

CTGAGCTTAT TTTTAACTTG GAAATGTGTA TGTGGCTGGG ACTTTGGATT

CCCAGATTAT TTTTAACTTG GAAACGTGTA TGTGGCTGGG ACTTTGGATT

CCTGGCTTAT TTTTAACTTG GAAATGTGTA TGTGGCTGGG ACTTTGGATT

CTGGGCTTAT TTTTAACTTG CAAATGTGTC TGTGGCTGGG ACTTTGGGTT

CTGAGCTTAT TTTTAACATG GAAATTTGTT TGTGGCTGGG ACTTTGGATT

CTGAGCTTAT TTTTAACTTG GAAATTTGTA TGTGGCTGGG ACTTCGGATT

CTGAGCTTAT TTTTAACTTG GAAATTTGTA TGTGGCTGGG ACTTTGGATT

CTGAGCTTAT TTTTAACTTG GAAA----TA TGTGGCTGGG ACTTTGGATT

CTGAGCTTAT TTTTAACTTG GAAATGTGTC TGTGGTTGGG ACTTTGGATT

CTGAGCTTAT TTTTAACTTG GAAATTTGTA TGTGGCTGGG ACTTTGGATT

---------- ---------- ---------- ---------- ----------

TTCAGCTTAT TTTTGACTTG ATAATAtgTG TGTGGCTGGG ATTTTGGATT

CTGACCTTAT TTTTAACTTA GAAATTGATG TGTGGCTGGG ACTTCGGATT

TCGGAGGAAT AATAAATTTG ATTTAAAGAA AACTAGAGTT CTCCTTGGAA

TCGGAGGAAT AATAAATTTG ATTTAAAGAA AACTAGAGTT CTCCTTGGAA

TCGGAGGAAT AATAAATTTG ATTTAAAGAA AACTAGAGTT CTCCTTGGAA

TCGGAGGAAT AATAAATTTG ATTTAAAGAA AACTAGAGTT CTCCTTGGAA

TCGGAGGAAT AATAAATTCG ATTTAAAGAA AACTAGAGTT CTCCTTGGAA

CCGGAGGAAT AATAAATTTG ACTTAAAGAA AACCAGAGTT CTCCTTGGAA

CAAGAAGAAT AATAAATTTG ATTTAAGGAA AACCAAAGTT CTGCTTGGCA

CAGGAAGAAT AATAAATTTG ATTTAAGGAA AACCAAAGTT CTGCTTGGCA

CAGGAAGAAT AATAAATTTG ATTTAAAGAA AACCAAAGTC CTCCTTGGAA

CAAGAGGAAT AATAAATTTG ATTTGAAGAA AACCAGAGTT CTCCTTGGCA

CAGGAGGAAT AATAAATTTG ATTTAAAGAA AACCAAAGTT TTCCTTGGAA

CAGGAGGAAT AATAAATTTG ATTTAAAGAA AACCAGAATT CTGCTTGGAA

CCGGAGGAAT AATAAATTTG ATCTAAAGAA AACCAAGGTT CTCCTTGGAA

CAGGAGGAAT AATAAATTTG ATTTAAAGAA AACTCGAGTT CTCGTTGGAA

CAGGAGGAAT AATAAATTTG ATTTAAAGAA AACCAGAGTT CTCATTGGAA

---------- ---------- ---------- ---------- ----------

AAGAAAAAAT AATAAATTTG ATTTTAGAAA AACCAAAGTT CTCATTGGAA

CCAAAAGAAT AACAAGTTTG AGCTGAGGAA AACCAAGGTT CTCGTCGGCA

ATGAGAGCTG CACCTTTACG GTCCTATGGC TGGTGGCACT TTACTTACTT

ATGAGAGCTG CACCTTTACG GTCCTATGGC TGGTGGCACT TTACTTACTT

ATGAGAGCTG CACCTTTATG GCCCTATGGC TGGTGGCACT TTACTTACTT

ATGAGAGCTG CACCTTTATG GCCCTATGGC TGGTGGCACT TTACTTACTT

ATGAGAGCTG CACCTTTACG GTCCTATGTC TGGTGGCACT TTACTTACTT

ATGAGAGCTG TACCTTTATG GTCCTAAGGC CGGTGGTACT TTACTTACGT

ACGAGAGCTG TACCTTTATG GTCCTCATGC CGGAGGCACC TTACTCACTT

ACGAGAGCTG TACCTTTACG GCCCTCAGGC TGGAGGCACC TTACTCACTC

ATGAAAGCTG TGCCTTTATG GCCCCAAGGC TGGTGGCACT TTACTCACTT

ATGAGAGCTG CACCTTTATG GTCCAAAAAC TGGTGGCACT TTACTTACAT

ATGAGAGCTG CACCTTTATG GTCCCAAGAA TGGTGGCACC TTGCTCACTT

ATGAGAGCTG CACCTTTACG GTCCTAAGAC TGGTGGCACT TTACTTACTT

ATGAGAGCTG CACGTTTATG GCCCTCAGAC TGGCAGTACT TTACTTACTT

ATGAAAGTTG CACCTTTATG GTCCTAAGGC TGGTGGCACT TTACTTACCT

ATGATAGCTG CACCTTTATG GTCCTAAGGC TGGAGGCACT TTGGTTACTT

---------- ---------- ---------- ---------- ----------

ATGAGAGCTG CATATTTATG GTCCCAAGGC TGGTGGCACA TTACTTACCT

ACCAGAGCTG CACTCTTATG GTCCCATAGC TGGAGGAACT TTACTCacct

TAACTGGAAA TTACCTAAAC AGTGGGAATT CTAGACACAT TTCAATTGGT

TAACTGGAAA TTACCTAAAC AGTGGGAATT CTAGACACAT TTCAATTGGT

TAACTGGAAA TTACCTAAAC AGTGGGAATT CTAGACACAT TTCAATTGGT

TAACTGGAAA TTACCTAAAC AGTGGGAATT CTAGACACAT TTCAATTGGT

TAACCGGAAA TTACCTAAAC AGTGGGAATT CTAGACACAT TTCAATTGGT

TAATTGGAAA GTACCTAAAC AGTGGGAATT CGAGACACAT TTCAATTGGT

TGACTGGAAA ATACCTCAAC AGCGGCAATT CTAGACACAT TTCAATCGGA

TTACTGGGAA ATACCTCAAC AGTGGCAATT CTAGACACAT TTCAATTGGA

TAACTGGAAA ATACCTGGAC AGTGGAAATT CCAGACACAT TTCAATTGGT

TAACTGGAAA GCACCTAAAC AGTGGAAATT CAAGACACAT TTCAATTGGT

TAACTGGAAA ATACCTCAAC AGTGGGAATT CTAGACACAT TTCAATGGGT

TAACTGGAAA GTACCTAAAC AGTGGGAATT CTAGACACAT TTCAATTGGT

TAACTGGAAA ATACCTCAAC AGTGGAAATT CTAGACACAT TTCAATTGGT

TAACTGGAAA GTACCTAAAC AGTGGCAATT CTAGACATAT TTCAATTGGT

TAACTGGAAA GTACCTAAAC AGTGGGAACT CTAGACACAT TTCAATTGGT

---------- ---------- ---------- ---------- ----------

TAACTGGGAA ATATCTAAAC AGTGGAAATT CTAGATGTAT TTCAATTGGT

taactgggaa gtacctgaac tgtggaagtT CCAGACACAT TTCCATCGGC

GGAAAAACAT GTACTTTAAA AAGGTGTTGT AAATTTATTT TTTGTCATCT

GGAAAAACAT GTACTTTAAA AAGGTGTTGT AAATTTATTT TTTGTCATCT

GGAAAAACGT GTACTTTAAA AAGGTGTTGT AAATTTATTT TTTGTCATCT

GGAAAAACAT GTACTTTAAA AAGGTGTTGT AAATTTATTT TTTGTCATCT

GGAAAAACAT GTACTTTAAA AAGGTGTTGT AAATTTATTT TTTGTCATCT

GGAAAAACAT GTACTTTAAA AAGGTATTGT TCACTTATTT TTTGTCAGCT

GGGAAAACAT GTACTTTAAA AAGGTATTGT CGCT------ -----CAGCT

GGGAAAACAT GTACTTTAAA AAGGTATTGT AAAT------ -----CAGCT

GGAAAAACAT GTACTTTAAA AAGGTTTAAT AAATTTATTT ATTGTCAGCT

GGAAAAACAT GTACTTTAAA AAGGTGTTGT A-GTTTATCT TTTGTCAGCT

GGAAAAACAT GTACTTTAAA AAGGTGTTGT ATATTTATTT TTTGTCAGCT

GGAAAAACAT GTTCTTTAAA AAGGTGGTGT AAATTTATTT TTTGTCAGCT

GGAAAGACGT GTACCTTAAA AAGGT----- --------CT TTCATCAGCT

GGAAAAACAT GTACTTTAAA AAGGTATTGT AAATTTATTT TCTGTTAGCT

GGAAAAACGT GTACTTTAAA AAGGTGCTGT ACATGTATTT TCTGTCAGCT

---------- ---------- ---------- ---------- -----CAGCT

GGAAAATCGT GTATTTTAAA AAGGTGCTGT AAATATATTT CTTATCAGTT

GGAAAAACAT GCACCTTGAG GAGGTGCTGT GCTTCAGTCT TTAGTCACTT

TCAGTTACCG TGAAGATCCC ATTGTCTATG AAATTCATCC AACCAAATCT

TCAGTTACCG GGAAGATCCC ATTGTCTATG AAATTCATCC AACCAAATCT

TCAGTTACCG GGAAGACCCC ATTGTCTATG AAATTCATCC AACCAAATCT

TCAGTTACCG GGAAGACCCC ATTGTCTATG AAATTCATCC AACCAAATCT

TCAGTTACCG GGAAGACCCT ATTGTCTATG AAATTTATCC AACCAAATCT

TCAGTTACCA GGAAGATCCC ATCGTCTATG AAATTCATCC AATGAAATCT

TCAGTTACCG GGAAGACCCG GTTGTCTCTG AAATCCACCC GACCAAATCT

TCAGTTACCG GGAAGACCCC GTTGTCTATG AAATCCACCC GACCAAATCT

TCAGTTACCG GGAAGACCCC ATTGTGGATG AATTTTATCC AACCAAATCT

TCATTTACCG GGAAGACCCC ATTGTCTATG AAATACATCC CACCAAATCT

TCAGTTACCA GGAAGACCCC ATTGTCTATG CAATTCATCC AACGAAATCT

TCAATTACCG GGAAGACCCC ATTGTCGATG AAATTCACCC AACCAAATCT

TCAGTTACCG GGATGACCCC ATTGTCTATG AAATTCACCC AACCAAATCT

TCAGTTACCG AGAAGACCCC ATTGTCTATG AAATTCATCC AACCAAATCT

TCAGTTACCA GGAAGACCCT ACTGTCTATG AAATCCATCC AAACAAATCT

TCAGTTACCA GGAAAATCCC CTTGTCGTTG AAATCCATCC AACCAAATCT

TTACATACAA AGAGGACCCC GTTGTTCATG AAATCCATCC AACCAAATCT

TCATCTACAA GGAGGACCCC GTCATCCATG AGATTCACCC AACCAAATCA

TTTATTAGGT AAGTAGAAGT TTTCTATTTT GCTTTGCCAG TGGTGGGAGC

TTTATTAGGT AAGTAGAAGT TTTCTATTTT GCTTTGCCAG TGGTGGGAGC

TTTATTAGGT AAGTAGAAAT TTTCTATTTT TCTTTGCCAG TGGTGGGAGC

TTTATTAGGT AAGTAGCAAT TTTCTATTTT GCTTTGCCAG TGGTGGGAGC

TTTGTTAGGT AAGTAGAAAT TTTCTATTTC GATTTGCCAG TGGTGGGAGC

TTTGTTAGGT AAGTATGATT TTTCTGTTTT GTTTTGCCAG TGGTGGGAGC

TTTATCAGGT AAGTGAAAGT TTTCCGTTTT ATTTGACCAG TGGTGGAAGC

TTTATTAGGT AAGTAAAAGT TTTCTATTTT GTTTTACCAG TGGTGGAAGC

TTTATTAGGT AAGTAGAAAT TTTTTTTTTT TTTTTGCCAG TGGTGGAAGC

TTTATTAGGT AAGTAAAATT TTTCTATTTT ATTTTGCCAG TGGTGGGAGT

TTTATTAGGT AAGTATAATT TTTCTGTTTT ATTTTGCCAG TGGTGGGAGC

TTTATTAGGT AAGTAAAATT TTTCTGTTTT ATTTTGCTAG TGGTGGGAGC

TTTATCAGGT AAGTAAAACT TTTCTATTTT CCCTTACCAG TGGTGGGAGC

TTTCTCAGGT AAGTAAAATT TTTCAATTTT GTTTTACCAG CGGTGGCAGC

TTTATTAGGT ATGTAAAATT TTTCTATTTT ATTTTGCCAG TGGTGGAAGC

TTTGTCAGGT AAGTGAAATT TTCCTGTTTT ATTTTGCCAG CGGTGGAAGC

TTTCTTAGGT AAGTAAAACT TTTCTATTTT ATTTTATCAG TGGTGGAAGC

TTCCTTAGGT AAGGAGAATG TTTCCATTTT GTTTTGCCAG TGGCGGAAGC

ACAATAACAG GTGTTGGGAA AAACCTGAAT TCGATAGATT GAATTACAGC

ACAATAACAG GTGTTGGGAA AAACCTGAAT TCGATAGATT GAATTACAGC

ACAATAACAG GTGTTGGGAA AAACCTGCAT TCGATAGATT GAATTACAGC

ACAATAACAG GTGTTGGGAA AAACCTGCAT TCGATAGATT GAATTACAGC

ACAATAACAG GTATTGGGAA AAACCTGAAT TCGATAGATT GAATTACAGC

ACAATAACAG GTGTTGGAAA AAACCTGAAT TCGATAGATT GAATTACAGC

ACAATAACGG GGATTGGAAA GAACCTGAAT TCGGTAGATT GAATTATAGC

ACAATAACGG GTATTGGGAA GACCCTGAAC TCGGTAGATT GAATTATAGC

ACCATAACAG GTGTTGGAAA AAACCTGGAT TCGACAGATT GAATTAC-GC

ACAATAACAG GTGTTGGAAA AAACCTGAAT TCAATAGATT GAATTACAGC

ACAATAACAG CTGTTGGAAA AAACCTGAAT TCGATAGATT GAATTACAGC

ACAATAACAG GTGTTGGAAA AAACCTGAAT TCAAGAGATT GAATGACAGC

ACAATAACAG GTGTTGGAAA AAATCTGGAT TCGATAGATT AAATTACAGC

ACAATAACAG GTGTTGGGAA AAACCTGAAT TCGACCGATT GAATTACAGC

ACAATAACAG GTATTGGAAA AAACCTGAAT TCGATAGATT GAATTACAGC

ACAATAACAG TTGTTGGAAA AAATCTGAAT TCGATAGATT GAATTATGGT

ACAATAACAG GTTTTGGGAA AAACCTAAAT TCTCTTAATT GAATTACAGC

ACAATCACCG GATTTGGGAA AAACCTCAAC GGGGCAGGTT GAATTATGGG

ACTTCCTTTT AGATTTCCAT CCAATATTTC TTGAAGACTA AAGCCACAAT

ACTTCCTTTT AGATTTCCAT CCAATATTTC TTGAAGACTA AAGCCACAAT

ACTTCCTTTT AGATTCCCAT CCAATATTTC TTGAAGACTA AAGCCACAAT

ACTTCCTTTT AGATTCCCAT CCAATATTTC TTGAAGACTA AAGCCACAAT

ACTTCCTTTT AGATTCCCAT CCAATATTTC TTGAAGTCTA AAACCACAAT

ACTTTCTTTC AGAGTCCCAT CCAATATGTC TTGAAGACTA AAGCCACAGT

CCTTTCTTTG AAATTCCCAT CCAATATTTC TTAAGGACAA AAGCCACTCT

ACTTTCTTTG AAATTCCCAT CCAATATTTC ACAAGGACTA AAGCCACTCT

ATTTTCTTTT GGATTCCCAT CCAATGTGTC TTGAAAACCA AAGCCACAGT

ACTTCCTTTT AGATT-CCAT CCAATATTTC TTGAAGGCTA AGGCCGCAGT

ACTTTCTTTT AGATTTCCAT CCAATATTTC TTGAAGACTA AAGCCACAGT

ACTTTCTTTT AGATTCCCAT CCAATATTTC TTGAAGAGTA AGGC--CAGT

ACTCCCTTTT GCTTTCCCAT CCAACACTTC TCAAAGACTA AGGCCATAGT

CCTTTCTTTC AGATTCCCAT TCAATACATC TCCAAGACCC AAGCCATTGT

ACTTTCTCTT AGATTCTCAT CCAATATTTC TTGAAGACTA AAGCCACAGT

ACGCCCTCTT AGGTTTCCAT CTACTACTTC TTGATGGCTA AAGCCACGGT

ATTTCTTCTT AGATTTCTAT CCCAACATTT TTTAGAACTA AAGCCATCAT

ATTTCCCTTT TGGCTCCAAA CCAATACTTC TGAAAAACAA AAACCATCAA

GTTCTTTAAT TACAAAAACC AATTTGGGGC TAAAATGTCA AGCCAAATCA

GTTCTTTAAT TACAAAAACC AATTTGGGGC TAAAATGTCA ACCCAAATCA

GTTCTTTAAT TACAAAAACC AATTTGGGGC TAAAATGTCA AGCCAAATCA

GTTCTTTAAT TACAAAAACC AATTTGGGGC TAAAATGTCA AGCCAAATCA

GTTCTTTAAT TACAAAAGCC AATTTGGGGC TAAAATGTCA AGCTAAATCA

GTTCTTTAAT TACGAAAACC AATTTGGGGC TAAAATGTCA AGCCAAATCA

GGTCCTTAAT TACAGAAAGC AATTTGGGAC TAAAATGTCA AGCCAAATCA

GGTCCTTAAT TACAGAAAGC AATTTGGGAC TAAAATGTCA AGCCGAATCA

GTTCTTTAAT TACAAAAACC AATTTGGGGC TAAAATGTCA AGCCAAGTCC

GTGCTTTAAT TACAAAAACC AATTTGGGGC TAAAATGTCA AGCCAAATCA

GTTCTTTAAT TACAAAAACC AATTTGGGGC TAGAATGTCA AGCCAAATCA

GTTCTTTAAT TACAAAAAAC AATTTGGGGC TGAAATGTCA AGCC-AATCA

GTTCTTTAAT TACAAAAAAC AATTTGGGGC TAAAATGTCA AGCCAGATCA

GTTCTTTAAT TACAAAAACC AATTTTGCTC TAAAATGTCA AGCCATATCA

GTTCTTTAAT TACAAAAACC AATTTTGTGC TAAAATGTCA AGCCAAATCA

GTTCTTCAAT TACAAAAACC AATTTCGTGC TAAAATGTCA AGCCAAATCA

ATTCTTCAAT TACGGAGGCC AATTTTGTGC TAAAATGTCA AGCCAAATTA

TTTCTTCAGG GACTAAAGCC CCTCTTGGAC CCAAATGTTC TGTCAAATCC

CCAAATAATA AGGTCATTTC AAATAAGATT TTTCTTGGCC TTCTCAATCA

TCAAATAATA AGTTCATTTC AAATAAGATT TTTCTTGGCC TTCTCA-TCA

CCAAATAATA AGGTCATTTC AAATAAGATT TTTCTTGGCC TTCTCAATCA

CCAAATAATA AGGTCATTTC AAATAAGATT TTTCTTGGCC TTCTCAATCA

CCAAATAATA AGGTCATCTC AAATAAGATT TTTCTTGGCC TTCTCAATCA

CCAAACAATA AGGTCATCTC AAATGAGATT TTTCTTGGCC TTCTCAATCA

CCAAACACTG AGGTCATCTC AAATGAGATT CCTCTTGACC CTCTCAGTCA

CCAAACACTA AGGTCATCTC AAACGAGATT CCTCTTGACC CTCTCAGTCA

CCAAACAGTA AGGTCATTGC AAATGAGATT TTTCTTGACC TTCTCAATCA

CCAAACAATA AGGTCATCTC AAATAAGATT TTTCTTGACC TTCTCAATCA

CCAAACAATA AGGTCATCTC CAATGAGATT TTTCTTGACC TTCTCAATCA

CCAAACACTA AGGTCACCTC AAATGAGATT TTTCTTGACC TGCTCAGTCA

CCAAACAATA AGGTCATCTC AAATGAGATT TTTCTTGACC TTCTCAATCA

CCAAACAGTG AGGTCATCTC AAATGAGATT TCTCTTGACC TTCTCAATCG

CCAAACAATA AGGGCATCCC AAATGAGATT TTTCTTGACC TTCTCAATCA

CCAAACAATA AGGGCATCGC CAATGAGGTT TTTCTTGACC T-CTCAGTCA

CAAAACAATA AGGTCATCTC AAATGAGATT TCTCTTGACC TTCTCAATCA

CATAACATTA GGGTCACTTC AAATAAGATT TTTCTTGACC TCTTTAGTCA

GCCACATTGC ATCACCTCTA GAGTATTATT AGATTTTTAT CATTTCCTGA

GCCACATTGC ATCACCTCTA GAGTATTATT AGATTTTTAT CATTTCCTGA

GCCACATTGC ATCACCTCTA AAGTATTATT AGATTTTTAT CATTTCCTGA

GCCACATTGC ATCACCTCTA AAGTATTATT AGATTTTTAT CATTTCCTGA

GCTACATTGC ATCACCTCTA AAGTATTATT AGATTTTTAT CATTTCCTGA

GCCACATTGC ATCACCTCTA AAGTATTATT AGATTTTTAT CATTTCCAGA

TCCACATTGC ATCACCTCTA AAGTGTTATT AGATTCTTAC CATTTTCTGA

TCCACATTGC ATCACCTCTA AAGTGTTATT AGATTCTTAT CATTTTCCAA

GCCACATTGC ATCACCTCTA AGGTATTATT GGATTTTTAT CATTTCCTGA

GCCACATTGC ATCACCTCTA AAGTATTATT AGATTTTTAT AATTTCCTGA

GCCACATTGC ATCACCTCTA ATGTATTATT AGATTTTTAT CATTTCCTGA

GCCACATTGC ATCACCTCTA AAGTGTTATT AGATTTTTAT CATTTCTTGA

GCCACATTGC ATCACCTC-G AAGTGTTATT AGATTCTTAT CATTTCCTGA

GCCACATTGC ATCACCTCTA AAGTATTATT AGATTTTTAT CATTTCCTGA

GCCATATTGC ATCACCTCTA AAGTCTTATT AGATTTTTAT CATTTCCTGA

GCCACATTGC ATCATCTCTA TAGTCTTATT AGATTGTTAT CATTTCCTGA

GTCACATTGC ATCACCTCTG AAGTATTATT AGATTTTTAT CCTCTCCTGA

TTCCCATTAT GCCCCCTCTA AAGCCTGATT AGATTGTCAT CATTCTCAGA

GAGTTGTCCT TTTTATGTAT ATCCTGAGTC TTGTCAAAGA AAAGGAAATC

GAGTTGTCCT TTTTATGTAT ATCCTGAGTC TTGTCAAAGA AAAGGAAATC

GAATTGTCCT TTTTATGTAT ATCCTGAGTC TTGTCAAAGA AAAGGAAATC

GAATTGTCCT TTTTATGTAT ATCCTGAGTC TTGTCAAAGA AAAGGAAATC

GAGTTGTCCT TTTTATGTAT ATCCTGAGTC TTGCCAAAGA AAAGGAAATC

GAGTTGTGCT TTTTATGTAT ATCCTGAGTC TTGTCCAAGA AAAGGAAATC

GAG-TGTGCT TTTTATGTAT ATCCTGAGGC TTGTCAAAGA AAAGGAAATC

GAG-TGTGCT TTTTATGTAT ATCCTGAGGC TTGTCAAAGA AAAGGAAATC

GAGTTGTGCT TTTTATGTAT ATCCTGAGTT TTGTCAAAGA AAAGGAAATC

GAGTTGTGCT TTTTCTGTAT ATCCTGAGT- TTGTCAAAGA AAAGGAAATC

GAGTTGTGCT TTTTATGTAT ATCCTGAGTC TTGTCAAAGA AAAGGAAATC

GAGTTGTGCT TTTTATGTAT ATCCTGAGTC TTGTCAAAGA AAAGGAAATC

GAGTTGTGCT TTTTATGTAT ATCCTGAGTT TTGTCAAAGA AAAGGAAATC

GAGTTGTGCT TTTTATGTAC ATCCTGAGTC TTGTCAAAGA AAAGGAAATC

GAGTTGTGCT TTTTATGTAT ATCCTGAGTC TGGTCAAAGC AAAGGAAATC

GAG------- TGTTAGGTAT ATTCACAGTC TTGTCAAAAA AAGGGACATC

GAATTGAGCA TTTTATGTAT TTCCTAAGTC TTATATAAGA GAAAGAAATA

GGGCACTATA TCCTACAGAC ATTTAAAGTC TTACCAGAAA ACAGAATTTT

TAAGGTTTTC CTTGGGGGAA AAATGCATTA ACCTTATTAG TCTAAGTACA

TAAGGTTTTC CTTGGGGGAA AAATGCATTA ACCTTATTAG TCTAAGTACA

TAAGGTTTTC CTTGGGGGAA AAACGCATTA ACCTTATTAG TCTAAGTACA

TAAGGTTTTC CTTGGGGGAA AAACGCATTA ACCTTATTAG TCTAAGCACA

TAAGGTTTTC CTTGGGGAAA AAACGTATTA ACCTTATTAG TCTAAGTACA

TAAGGTTTTC CTTGGGGGAA AAATGCATTA ACCTTATGAG TCTAAGTACA

TAAGGTTTTC CTTGGGGGAG AAACACATTG ACTTTATT-- ------TACA

TAAGGTTTTC CTTGGGGGAG AAACACATTG ACTTTATTAG TC-AAGTACA

TAAGGTTTTC CTTGGGGGAA AAACACATTA ACATTATTAG TCTAAGTACA

TAAGGTTTTC CTTGGAGGGA AAATGCATTA ACCTTATTCG TCTAAGCACA

TAA-GTTTTC CTTGGGGGAA AAATGCATTA ACCTCATTAG TCTAAGCACA

TAAGGTTTTC CTTGGAAGAA AACCGCATTA ACCTTATTAG TCTAAGCACA

TAAGGTTTTC CTTGGGGGAA AAACTCATTA ACCTTATTAG TTGAAGCACA

AATGGTTTTC CTTAGGGGAA AAACTCATTA AACTTATCAG TGTAGTCACA

AAAGGTTTTC CTTGGGGGGA AAACTCATTA ACCTCATTAG TCCAAGCACA

AAAGGTTTTC CTTGGGGGGG GAACTCACTT ACTATA-TAG TCCAAGCACA

AAAGGTTGTA CTTTGGAAAA TCACTCATTG TACTTATTGA TCTAATCACT

TATTAaattc ctatggggaa aaattcaTGA ACTTTATTGG TCCAAGTGCC

ATCAAGACAG GCATGTCAAC ATCGCTCTAA TTCAGAGATA ATCTGTTGTA

ATCAAGACAG GCATGTCAAC ATCGCTCTAA TTCAGAGATA ATCTGTTGTA

ATCAAGACAG GCATGTCAGC ATCGCTCTAA TTCAGAGATA ATCTGCTGTA

ATCAAGACAG GCATGTCAGC ATCGCTCTAA TTCAGAGATA ATCTGCTGTA

ATCAAGACAG GCATGTCAGC ATCGCTCTAA TTCAGAGATA ATCTGCTGTA

ATCAGGACAG GCGTGTCAGC ATCGCTCTAA TGCAGAGATC CTCTGCTGCA

ATCAAGACAG GCGTGCCAAC ATCGCTCGAG TTCAGAGATC ATCTGCTGCA

ATCAAGACAG GCATGTCAGC ATCGCTCAAA TTCAGAGATC ATCTGCTGCA

ATCAAGACAG GCATGTCAGC ATCGGTCCAA CTCAGAGATC ATCTGCTGCA

ATCAAGACAG GCATGTCAAC ATCGCTCTAA TTCAGAGATA ATCTGCTGTA

ATCAAGACAG GCATGTCAAC ATCGCTCTAA TTCAGAGATA ATCTGTTGTA

ATCCAGACAG GCATGTCAAC ACCGCTCTAA TTCAGAGATA ATCTGCTGTA

ATCAAGACAG GCATGTCAAC ATCGCTCTAA TTCAGAGATA ATCTGCTGTA

ATCAAGGCAG GCCTGTCAAC ATCGCTCTAA TTCAGAGATA ATGTGCTGTA

ATCAAAAC-- ---------- ---------- ---------- ----------

ATCCAGACAG GCATGTCAAC AGCGCTCTAA TTCAGAGCTA ATCTGCTGTA

ATCAATATAG GCATGTAAAC AGCGCTCTAA TTCAGAGATA ATATGTTGTT

TGTGAAACAG GCATGTCAAA GTCGTTCTAA TGCAGAAATA ATCTGTTGTA

CCACTCCTTC CCTGCAACAG CTGAATCTGC AACTCCCCCT GAAAACCAAA

CCACTCCTTC CCTGCAACAG CTGAATCTGC AACTCCCCCT GAAAACCAAA

CCACTCCTTC CCTGCAACAG CTGAATCTGC AACTCCCCCT GAAAACCAAA

CCACTCCTTC CCTGCAACAG CTGAATCTGC AACTCCCTCT GAAAACCAAA

CCACTCCTTC CCTGCAACAG CTGAATCTGC AACTCCCCCT GAAAACCAAA

CCACTCCTTC ACTGCAACAG CTGAATCTGC AACTCCCCCT CAAAACCAAA

CCACTCCTTC CCTGCAACAG CTGGACCTGC AACTCCCCCT GAAGACCAAA

CTACTCCTTC ACTGAAACAG CTGGGCCTGC AACTCCCCCT GAAGACCAAA

CCACTCCTTC ACTGCAACAG CTGAATCTGC ACCTCCCCCT GAAAACCAAA

CAACTCCTTC AATAGAACAG CTGAACCTAC AACTCCCCCT GAAAACCAAA

CGACTCCTTC ACTGCAACAG CTGAATCTGC AACTCCCTCT GAAAACCAAA

CAACTCCTTC ACTCCAACAG CTGAATCTGC AGCTCCCCCT GACAACCAAG

CCACTCCTTC ATTGCAACAG CTGAATCTGC AGCTCCCCCT GACAACCAAG

CAACTCCTTC GCTGCAACAG CTGAATCTGC AACTTCCCCT GAAAACCAGA

---------- ---------- ---------- ---------- ----------

CGACTCCTTC ACTGCAACAG CTGGATCTGC AACTCCCCCT GAAAGCCACG

CAACTCCTTC CTTACAACAG CTAAATCTGC AACCCCCCTT GGAAACAAAA

CGACTCCTTC ACTGCAGCAA CTAAATCTGC CACCCCCCTT CAAAACGAAA

GCCTTTTTCA TGTTAGATGG GATCCTTTCC AAATACTTTG ATCTCATTTA

GCCTTTTTCA TGTTAGATGG GATCCTTTCC AAATACTTTG ATCTCATTTA

GCCTTTTTCA TGTTAGATGG GATCCTTTCC AAATACTTTG ATCTCATTTA

GCCTTTTTCA TGTTAGATGG GATCCTTTCC AAATACTTTG ATCTCATTTA

GCCTTTTTCA TGTTAGATGG GATCCTTTCC AAATACTTTG ATCTCATTTA

GCCTTTTTCA TGTTAGACGG GGTTCTTTCC AAATACTTTG ATCTCATTTA

GCCTTCTTCC TGCTGGACGG GATCCTTTCC AAACACTTTG ATCTCACTTA

GCCTTCTTCC TGTTAGACGG GATTCTTTCC AAACACTTTG ATCTCACTTA

GCCTTTTTCA TGTTGGATGG GATCCTTTCC AAACACTTTG ATCTCACTTA

GCCTTTTTCA TGTTAGATGG GATCCATTCC AAATACTTTG ATCTCATTTA

GCCTTTTTCA TGTTAGATGG GATCCATTCC AAATACTTTG ATCTCATTTA

GCCTTTTTCA TGTTAGACGG GATCCATTCC AAATACTTTG ATCTCATTTA

GCCTTTTTCA TGTTAGATGG GATCCATTCC AAATACTTTG ATCTCATTTA

GCCTTTTTCG TGTTAGATGG GATCTATTCC AATTACTTTG ATCTCATTTA

---------- ---------- ---------- ---------- ----------

GCCTTCTTCA TGCTAGATGG GATCCATTCC AGGGACTTTG ATCTCATTTA

GCTCTTTTCA TATTTGATGG TATCCATTCC ACTTACTTTG ATCTCATTTA

GCATTTTTTG TATTTGATGG GGTCCAGTCT GCTAACTTTG ACCTCATTTA

TGTACATAAT CCTGTGTTTA AGCCTTTTGA AAAGCCAGTG ATGATCTCAA

TGTACATAAT CCTGTGTTTA AGCCTTTTGA AAAGCCAGTG ATGATCTCAA

TGTACATAAT CCTGTGTTTA AGCCTTTTGA AAAGCCAGTG ATGATCTCAA

TGTACATAAT CCTGTGTTTA AGCCTTTTGA AAAGCCAGTA ATGATCTCAA

TGTACATAAT CCTGTGTTTA AGCCTTTTGA AAAGCCAGTG ATGATCTCAA

TGTACATAAT CCTGTGTTTA AGCCTTTTGA AAAGCCAGTG ATGATCTCAA

TGTACATGAT CCTATGTTTA AGCCTTTTGA AAAGCCAGTA ATGATCTCCA

TGTGCATAAT CCTGTGTTTG AGCCTTTTGA AAAGCCAGTA ATGATCTCAA

TGTACATAAT CCTGTGTTTA AGCCTTTTGA AAAGCCAGTG ATGATCTCAA

TGTACATAAT CCTGTGTTTA AGCCTTTTGA AAAGCCAGTG ATGATCTCAG

TGTACATAAT CCTGTGTTTA AGCCTTTTGA AAAGCCAGTG ATGATCTCAA

TGTACACGAT CCTGTGTTTA AGCTTTTTGA AAAGCCAGTG ATGATCTCAA

TGTACATAAT CCCGTGTTTA AGCCTTTTGA AAAGCCAGTG AAGATCTCAA

TGTACATAAT CCTGTGTTTA AGCCTTTTGA AAAGCCAGTG ATGATCTCAA

---------- ---------- ---------- ---------- ----------

TGTACCTAAT CCTGTGTTTA AGCTTTTTGA AAAGCCAGTG ATGATCTCAA

TGTCCATAAT CCTGTATTTA AGCCTTTTGA AAAGCCAGTG ATGATCTCAA

TGTGCACAAT CCAGTGTTTA AGCTGTTTGA AAAGCCAGTG ATGATCTCAA

TGGGCAATGA AAATGTACTG GAAATTAAGG TAAGAAATGC TTAACACCAG

TGGGCAATGA AAATGTACTG GAAATTAAGG TAAGAAATGC TTAACACCAG

TGGGCAATGA AAATGTACTG GAAATTAAGG TAAGAAATGC TTAACACCAG

TGGGCAATGA AAATGTACTG GAAATTAAGG TAAGAAATGC TTAACACCAG

TAGGCAATGA AAATGTACTG GAAATTAAGG TAAGAAATGC TTAACACTAG

TGGGCAATGA AAATATCCTG GAAATTAAGG TAAGAAAGGC TGAACATCAG

TGGGCAATGA GAATGTAGTG GAAATTAAGG TAAGAGTTGC TTAACATCAG

TAGGCAATGA AAATGTAGTG GAAATTAAGG TAAGAGTTGC TTAACATCAG

TGGGCAATGA AAATGTACTG GAAATTAAGG TAAGAAATGT TTAATATCAG

TAGGCAATGA AAATGTACTG GAAATTAAGG TAAGAAATGC TTAACATTAG

TAGGCAATGA AAATGTACTG GAAATTAAGG TAAGAAATGC TTAACATCAG

TAGGCAATGA AAATGTCCTG GAAATTAAGG TAAGAAATGC TTAACATCAG

TAGGCAATGA AAATGTACTG GAAATTAAGG TAAGTAATGC TTAACATTAG

TGGGCCGAGA AAATGTGCTG GAAATTAAGG TAAGAAATAC TTAACATCAA

---------- ---------- ---------- ---------- ----------

TGGGCAATGA GAATGTACTG GAAATTAAGG TAAGAAATGT TTACCATCAG

TGGGCAATGA AAATGTACTG GAAATTAAGG TAAGAAATGC TTAACATCAG

TGGGCAACAA ACATGTACTG GAAATTAAGG TAAGAAAAGC TCAACTTTGG

CAAATATATA AACTCCAAAT CTTTTTGGCA TAAAACTAAA GTGTTTTTAC

CAAATATATA AACTCCAAAT CTTTTTGGCA TAAAACTAAA GTGTTTTTAC

CAAATATATA AGCTCCAAAT CTTTTTGGCA TAAAACTAAA GTGTTTTTAC

CAAATATATA AGCTCCAAAT CTTTTTGGCA TAAAACTAAA GTGTTTTTAC

CAAATATCTA AGCTCCAAAT CTTTTTGGCA TAAAACTAAG GTGTTTTTAC

CAAATTTCTG AGCTCCAAAT CTTTTTGGCA TGAGACTAAA GTGTTTTTAC

CAAATATCAA AGCTC-AAAT CTTTTTGGCA GAAGACTAAA GCATTTCTAC

CAAATATCAA AGCTC-AAAT CTCTTTGGCA GAA-ACTAAA GCATTTTTAC

CAG-----TA AACTCGAAAT CTTTTTGGCA TAAGACTAAA GTGTTTTTAC

CTAATATCTA AGTTCCAAAT C-TTTTGACA TAGGACTAAA GTGTTTTCAC

CAAGTATCTA AGCTCCAAAT CTTTTTGGCA GAAGACTAAA GTGTTTTTAC

CAAATATCTA AGCTCCAAAT CTTTTGGGTA TAAGACTAAA GTGTTTTTAC

CAAATATATA AGCTCCAAAT CTTTGGGGCA TTAGACTAAA GTGTTTTTAC

CAAATATCTA AGCTCTAAAT CTTTTTGGCA TAAGACTAAA ATGTTTTTAC

---------- ---------- ---------- ---------- ----------

CCAATATCTA AGCTCCAAAT CTTTTTGGCA TAAGACTAAC ATATTTGAAT

CAAATATCTA AGCTCCAAAT CTTTTTGGCA TAACATTAAG GTGTTTTTGC

CAAGTATCTC AACTCCAAAT CTTTCTGGCA TTACACTAAA GTGTCTTTGC

AATAATTAAG CAAGCCCAGC ATAAAATTGA ATGGAAGCCC GCTTGCTGAC

AATAATTAAG CAAGCCCAGC ATGAAATTGA ATGGAAGCCC GCTTGCTGAC

GATAATTAAG CAAGCCCAGC ATGAAATTGA ATGGAAGCCC GCTTGCTGAC

GATAATTAAG CAAGCCCAGC ATGAAATTGA ATGGAAGCCC GCTTGCTGAC

AATAATTAAG CAAGCCCAGC ATGAAATTGA ATGGAAGCCC GCTTGATGAC

AATAATTAAG CAAACCCAGC ATGAAATTAA ATGGAAGCCT GCTTTCTGAC

AATAATTAAG TAAACCCAGC ATGAAATTAA ATGGAAGCCT GCTATCTGAC

AATAATTAAG TAAACCCAGC ATGAAATTAA ATGGAAGCCT GCTATCTGAC

AATAATTAAG CAAGCCCAGC ATGAAATTAA ATGGAAGCTT GCTTTCTGAC

AATAATTAAG CAAGCCCAGC ATGAAATTAA ATGGAAGCCT GCTTTCTGAC

AATAATTAAG CAAGCCCAGC ATGAAATTAA ATGGAAGCCT GCTTTCTGAC

AATAATTAAG CAAGCCCAGC GTGAAATGAA ATGGAAGCCT GCTTGCTGTC

AATAATTAAG CAAGCCCAGC ATGAAATTAA ATGGAAGCTT GCTTTCTGGC

AATAATTAAG CAAGCCTGGC ATGAAATTAA ATGGAAGCCT GTATTCTGAC

---------- ---------- ---------- ---------- ----------

GATAATTAAG CAAACCCAGC ATGAAATTAA ATGGAAGCCT GCTTTCTGAC

AATAATTAAA CAAGTGCAGC ATGAAATTAA ATGGTAGCCT GCTTTATGCT

AATAATTAAA CAAGCCCAGC ATGAAATGGA AGGGCAACCT GCTTTATAAT

TTTTATTTCA TTTTAGTGGA AGCAAGCAAT TTCTTCAACC GTCCTTGGAA

TTTTATTTCA TTTTAGTGGA AGCAAGCAAT TTCTTCAACC GTCCTTGGAA

TTTTATTTCA TTTTAGTGGA AGCAAGCAAT TTCTTCAACC GTCCTTGGAA

TTTTGTTTCA TTTTAGTGGA AGCAAGCAAT TTCTTCAACC GTCCTTGGAA

TTTTATTTCA TTTTAGTGGA AGCAAGCAAT TTCTTCAACC GTTCTTGGAA

TTTTAT--CA TTTTAGTGGA AGCAAGCAGT TTCTTCAACC GTCCTTGGAA

TTCTATTT-A TTCTAGTGGA AGCAAGCAGT CTCTTCAACT GTCCTTGGAA

TTCTATAT-A TTCCAGTGGA AGCAAGCAGT CTCTTCAACT GTTCTTGGAA

TTCTATTT-A CTTTAGTGGA AGCAAGCAGT TTCTTCAACT GTCCTTGGAA

TTTTATTTCA TTGTAGTGGA AGCAAGCAAT TTCTTCAACT GTTCTTGGAA

TTCTATTTCA TTTTAGTGGA AGCAAGCAGT TTCTTCAACC GTCCTTGGAA

TTTTCTTTCA TTTTAGTGGA AGCAAGCAGT TTCTTCAACT ATCCTTGGAA

TTTTCTGTTG TGCTAGTGGA AGCAAGCAGT TTCTTCAACT ATTCTTGGAA

TTTTATTTTG TTTTAGTGGA AGCAAGCAGT TTCTTCATAT ATCCTTGGAA

---------- ---------- ---------- ---------- ----------

TTTTCATTCA TTTCAGTGGA AGCAAGCAGT TTCTTCAACT GTCCTTGGAA

ATTTTTCTCA TTGTAGTGGA AGCAAGCAAT TTCTTCAACT GTCCTTGGAA

CATTCTTGTC TTGTAGTGGC AGCAGGCAGT CTCCTCGACC ATCCTTGGAA

AAGTAATAGT TCAACCAGAT CAGAATTTCA CAGGATTGAT TGCTGGTGTT

AAGTAATAGT TCAACCAGAT CAGAATTTCA CAGGATTGAT TGCTGGTGTT

AAGTAATAGT TCAACCAGAT CAGAATTTCA CAGGATTGAT TGCTGGTGTT

AAGTAATAGT TCAACCAGAT CAGAATTTCA CCGGATTGAT TGCTGGTGTT

AAGTAATAGT TCAACCAGAT CAGAATTTCA CAGGATTGGT TGCTGGTGTT

AAGTAATAGT TCAACCAGAT CAGAATTTCA CAGGGTTGAT TGTTGGTGTT

AAGTGATCGT TCAACCGGAT CAGAATTTTG CAGGATTGAT CATTGGTGCG

AAGTGATCGT TCAACCGGAT CAGAATTTTG CAGGATTGAT CATTGGTGCG

AAGTAATAGT TCAACCAGAT CAGAATTTTA TGGGATTGAT TGTTGGTGGT

AAGTGATAGT TCAACCAGAT CAGAATTTCA CAGGATTGAT TGTTGGTGTT

AAGTAATAGT TCAACCAGAT CAGAATTTCA CAGGATTGAT TGCTGGTGTT

AAGTAATAGT TCAACCAGAT CAGAATTTCA CAGGATTGAT TGTTGGTGTT

AAGTTGTAGT TCAACCAGAT CAGAATCTCA CGGGAATGAT TGTTGGTATT

AAGTAATAGT TCAATCAGAT CAGAGTTTCA CAGGAGTGAT TGTTGGTGTT

---------- ---------- ---------- ---------- ----------

AAGTAATAGT TCAGCCGGAT CAGAATTTCA CAGGATTGAT TGTTGGTGTT

AAGTGATAGT ACAACCAGAC CAGAATTTCA CAGGACTCAT TGTTGGAGTC

AGGTGATTGT TCTCCCTGAC CAGAATTTCA CAGGACTAAT CGCCGGAGTG

GTCTCAATAT CTTTAACAAG CTCTTTCTTT CTCTCTGTTT TAAGATCTGG

GTCTCAATAT CTTTAACAAG CTCTTTCTTT CTCTCTGTTT TAAGATCTGG

GTCTCAATAT CTTTAACAAG CTCTTTCTTT CTCTCTGTTT TAAGATCTGG

GTCTCAATAT CTTTAACAAG CTCTTTCTTT CTCTCTGTTT TAAGATCTGG

GTCTCAATCT CTTTAACAAG CTCTTTCTTT CTCTCTGTTT TAAGATCTGG

GTCTCAATAT CTTTAACAAG CTCCCTCTTT CTCTCTCTTT TAAGATCCAG

GTCTCAATAT CTT-AACAAG CTCTCTCTTT CTCTCTGTTT TAAGATCTGG

GTCTCAATAT CTTTAACAAG CTCTCTCTTT CTCTCTGTTT TAAGATCTGG

GTCTCCATAT CTTTAATAAG CTCTTTCTTT CTCTCTGTTT TAAGATCTGG

GTCTCAATAT CTTTAACGAG GTCTTTCTCT CTCT----TT TAAGATCTGG

ATCTCAATAT CTTTAACGAG CTCTTTCTCT CTCTCTGTTT TAAGATCTGG

ATCTCAATAT CTTTAACGAG CTCTCTCTCT CTCTCTGTTT TAAGATCTGG

GTTTTAATAT CTTTAACAAG CTCTCTCTCT CTCT--GTTT TAAGATCTGG

GTTGCAATAT CTTTAACAAG TGCCTTCTTT CTCTCTGTTT TAAGATCTGG

---------- ---------- ---------- ---------- ----------

GTCTCAATAT CCTTAACAAG TTCTTTCTTT CTCTCTGTTT GGAGATCTGG

GTCTTGATAT CTTTAACAAA CTTTTTCTTT CTGTTTGTTT TAAGATCTGG

GCCTCCATCT CCTTAACCAG CTGCTTCTTT CTCTCTGGTT TAAGATCTGG

GCAGTGAATT AGTTCGCTAC GATGCAAGAG TACACACTCC TCATTTGGAT

GCAGTGAATT AGTTCGCTAC GATGCAAGAG TACACACTCC TCATTTGGAT

GCAGTGAATT AGTTCGCTAT GATGCAAGAG TACACACTCC TCATTTGGAT

GCAGTGAATT AGTTCGCTAT GATGCAAGAG TACACACTCC TCATTTGGAT

GCAGTGAATT AGTTCGCTAT GATGCAAGAG TACACACTCC TCATTTGGAT

GCAGTGAATT AGTTCGCTAT GATGCAAGAG TACACACTCC TCATCTGGAT

GCAGTGAATT AGTTCGCTAT GACGCAAGAG TACACACTCC TCATTTGGAT

GCAGTGAATT AGTTCGCTAT GACGCAAGAG TACACACTCC TCATTTGGAT

GCAGTGAATT AGTTCGCTAT GATGCAAGAG TTCACACTCC TCATTTGGAT

GCAGTGAATT AGTTCGCTAT GATGCAAGAG TACACACTCC ACATTTGGAT

GCAGTGAATT AGTTCGCTAT GATGCAAGAG TACACACTCC TCATTTGGAT

GCAGTGAATT AGTTCGCTAT GATGCACGAA TACACACTCC TCACTTGGAT

GCAGTGAATT AGTTCGCTAT GATGCAAGAG TACACACTCC TCATTTGGAT

GCAGTGAATT AGTTCGTTAT GATGCAAGAG TACACACTCC TCATTTGGAT

---------- ---------- ---------- ---------- ----------

GCAGTGAATT AGTTTGCTAT GACGCAAGAG TCCACACTCC TCACTTGGAT

GCAGTGATTT AGTTCGCTAT GATGCAAGAG TACACACTCC TCACTTGGAT

GCAGTGAATT AGTTCGCTAT GACCCAAGAG TTCACACGCC TCACCTGGAC

AGGCTTGTAA GTGCCCGAAG TGTAAGCCCA ACTACAGAAA TGGTTTCAAA

AGGCTCGTAA GTGCCCGAAG TGTAAGCCCA ACTACAGAAA TGGTTTCAAA

AGGCTTGTAA GTGCCCGAAG TGTAAGCCCA ACAACAGAAA TGGTTTCAAA

AGGCTTGTAA GTGCCCGAAG TGTAAGCCCA ACAACAGAAA TGGTTTCAAA

AGGCTTGTAA GTGCCCGAAG TGTAAGCCCA ACTACAGAAA TGGTTTCAAA

AGGCTTGTAA GTGCCCGAAG TGTAAGCCCA ACTACAGAAA TGGTGTCAAA

AGGCTTGTAA GTGCCCGAAG TGTAAGCCCA ACTACAGAGA TGGTCTCAAA

AGGCTTGTAA GTGCCCGAAG TGTAAGTCCA ACTACAGAGA TGGTTTCAAA

AGGCTTGTAA GTGCCCGAAG TGTGAGCCCA ACTACAGAAA TGGTTTCAAA

AGGCTTGTCA GTGCCCGAAG TGTAAGCCCA ACTACAGAAA TGGTTTCCAA

AGGCTTGTAA GTGCCCGAAG TGTAAGCCCA ACTACAGAAA TGGTTTCAAA

AGGCTCGTAA GTGCCCGAAG TATAAGCCCA ACCACAGAAA TGGTCTCAAA

AGGCTTGTAA GTGCCCGAAG TGTAAGCCCA ACTACAGAAA TGGTTTCAAA

AGGCTTGTAA GTGCCCGAAG TGTAAGCCCA ACTACAGAAA TGGTTTCAAA

---------- ---------- ---------- ---------- ----------

CGGCTTGTAA GTGCCCGAAG TGTAAGCCCA ACTACGGAAA TGGTTTCAAA

AGGCTTGTAA GCGCCCGAAG TGTAAGCCCT ACCACGGAAA TGGTTTCCAA

CGCCTTGTAA GCGCCAGGAG TATCAGCCCC ACGGCGGAAA TGGTTTCCAA

TGAATCTGTA GACTACCGAG CTACTTTTCC AGAAGGTATA TTCAGTTTAT

TGAATCTGTA GACTACCGAG CTACTTTTCC AGAAGGTATA TTCAGTTTAT

TGAATCTGTA GACTACCGAG CTACTTTTCC AGAAGGTATA TTCAGTTTCT

TGAATCTGTA GACTACCGAG CTACTTTTCC AGAAGGTATA TTCAGTTTCT

TGAATCTGTA GACTACCGAG CTACTTTTCC AGAAGGTATA TTCAGTTTAT

TGAATCTGTA GACTACCGAG CTACTTTTCC AGAAGGTAAA ---AGT----

TGAGTCTGTA GACTACAGAG CTACTTTTCC AGAAGGTATA TTCAGTTTAC

TGAGTCTGTA GACTACAGAG CTACTTTTCC AGAAGGTATA TTCAGTTTAC

TGAATCTGTA GACTACAGAG CTACTTTTCC AGAAGGTATA TTCAGTTTAT

TGAATCTGTA GACTACCGAG CTACTTTTCC AGAAGGTATA TTCAGTTTAT

TGAATCTGTA GACTACCGAG CTACTTTTCC AGAAGGTATA TTCAGTTTAT

TGAATCTGTA GACTACCGCG CTACTTTTCC AGAAGGTATA TTCAGTTTTT

TGAATCTGTA GACTACAGAG CTACTTTTCC AGAAGGTATA TTTAGTTTAT

TGAATCTGTA GACTACCGAG CTACTTTTCC AGAAGGTATA TTCAGTTTAT

---------- ---------- ---------- ---------- ----------

TGAGTCCGTG GACTACCGGG CCACATTTCC AGAAGGTATG TTCCGTTTGT

TGAATCTGTA GACTACAGAG CAACATTTCC AGAAGGTATA TTCAGTTTAT

TGAATCTGTG GACTACCGAG CGACGTTCCC AGAAGGTATA TTCAGTTCCC

TGTTCTGAGA AATACCTATA CATATACCTC AGTGTTTCAT GTTGTTTTAT

TGTTCTGAGA AATACCTGTA CATATACCTC AGTGTTTGAT TCTGTTTTAT

TGTTCTGAGA AGTACCTATA CATACACCTC AGTGTTTCAC GTTGTTTTAT

TGTTCTGAGA AATACCTATA CATACACCTC AGTGTTTCAC GTTGTTTTAT

TGTTCTGAGA AATACCTATA CATATACCTC AGTGTTTCTT GTTGTTTTAT

------GATA AATACCTATA TGTATACCTC AGTGTTTCAT GATGTTTTAT

TATCCTGAGA AATATCTATA CATAGACCTC TGTGTTTCAC ATTGTTTTAG

TATCCTGAGA AATATCTATA CATAGACCTC TGTGTTTCAC ATTGTTTTAC

TGTTCTGAGA AATATATGTA CACCTACCTC AGTGTTTCAC ATTGTTATAT

TTTTCTGAAA AATACCTATA CATACACCTC AGTGTTTCAC ATTGTTTTAT

CATTCTAAGA AATACCTATA CATATACCTC AGTGTTTCAC ATTGTTTTAT

TGTTCTGAGA AATACCTGTG CATATACCTC AGTGTTTCAC ATTGTTTTAT

TGTTCTGAGA AAAACCTGTA CATATACCTC AGAG------ ----------

TGTTCTGAGA AATACCTATA CATATACCTC AGTGTTTCAC ATTGTTTCAT

---------- ---------- ---------- ---------- ----------

TGTTGTGAGA AACACCAGTG CAGCTCCCTC AGTGTTTCTC ATTGTTTCAC

TGTTCTGAGA AATACCTATA CATGTACTTC AATGCTTCAT ATTGTTTAAC

CGGTCTGAGA CCGGCCTGTT CACGCCGGCT AGTGTTTTGC ATTGTTTAGT

TTAAATCTCC TGGTAAAAGC AAAAGGTCTG TATTGTATTA TCTTTAGAAG

TTAA-TCTCC TGGTAAAAGC AAAAGGTCTG TATTGTATTA TCTTTAGAAG

TTAAATCTCC TGGTAAAAGC AAAATGTCTG TATTGTATTA TCTTTAGAAG

TTAAATCTCC TGGTAAAAGC AAAATGTCTG TATTGTATTA TCTTTAGAAG

TTAAATCTCC TGGTAAAAGC AAAATGTCTG TATTGTATTA TCTTTAAGAG

TTAAATCTCC TGGTAAACAC AAAATGTCTG TATTGTATTA TCTTTAGAAG

TTAAATGTCT TGCACAAAGT AAAATGTCTG TATTGTATTA TCTTTAGAAG

TTAAATGTCT TACACAAAGC AAAATGTCTG TATTGTATTA TCTTTAGAAG

TTACATCTCC TGGTGAAAGC AAAGTGTCTG TGTTGTATTA TCTTTAGAAG

TTAAATCTCC TGGTAAAGGC AGAATGTCTG TATTGTATTA TCTTTAGAAG

TTGAATCTCC TGGTAAAAGC AAAATGTCTG TATTGTATTA TCTTTAGAAG

CTAAATCTCC TGGTAAACGC AAAATGCCCG TATCATATTA TCTTTAGAAG

---------- ---------- ----CATCTG TATTGTATTA TCTTTAGAAG

TTAAATTTCC TGGTAAAAGC AAAATGTCTG TACTGTATTA TCTTTAGAAG

---------- ---------- ---------- ---------- ----------

TCAAGTCTCC TGGTCAAAGC AAAATGCCTG TATTGTATTA TCTCTAGAAG

TGAAATATGT TGGCAAAAGT ACAGTTTCTA CTTTTTATTA CCTTTAGAAG

TTGAATCTGT TGGCAAATAC CAAATTTCTG AATTGTATTA TCTTTAGAAG

TTACAGGATG TGAGTCAAGT ACAAGCATTT CCTTGGTTGA ATATTTACCA

TTACAGGATG TGAGTCAAGT ACAAGCATTT CCTTGGTTGA ATATTTACCA

TTACAGGATG TGAGTCAAGT ACAAGCATTT CCTTGGTTGA ATATTTACCA

TTACAGGATG TGAGTCAAGT ACAAGCATTT CCTTGGTTGA ATATTTACCA

TTACAGGATG TGAGTCAAGT ACAAGCATTT CCTTGGTTGA ATATTTACCA

TTACAGGACA TGAGTCAAGT TCAAGCATTT CCCTGGTTGA ATATTTACCA

TTACAGGACA TGAGTCAAGT GCAAGCACTT CCCTGTTGAA ATATCTACCA

TTACAGGACA TGAGTCAAGT ACAAGCACTT CCCTGTTGAA ATATCTACCA

TTACAGGACG TGAGTCAAGT ACAAGCATTT CCCTGGTTGA ATATTTACCA

TTACAGGACG TGAGTCAAGT ACAAGCATTT TCCTGGTTGA ATATTTACCA

TTCCAGGACG TGAGTCAAGT ACAAGCACTT CCCTGGTTGA ATATTTACCA

TTACAGGACG TGAGTCAAAT ACAAGCATTT CCCTGGTTGA ATATTTACCA

TTACAGGACA TGAGTCAAGT GCAAGCATTT TCCTGGTTGA ATATTTACCA

TTACAGGACA TGAGTCAAGT TCGAGTATTT CCCTGGTTGA ATATTTACCA

---------- ---------- ---------- ---------- ----------

TTACAGGACA TGAGTCAAGT ACAAGCACCT CCCTGGTTGA ATATTTACCC

TTTCAGGATA TGAGTCAACT GCAAGCGTTT TCCTGGTTGA ATATTTACCA

GTACAGGATG TGAGTCAACT CTAAGCATTT TTCTAGTTGA ATATTTACCA

TTGGACAAAT AAAATGAGTC ATCTTACAGA TCAGTTTCCT AATTCATCTC

TTGGACAAAT AAAATGAGTC ATCTTACAGA TCAGTTTCCT AATTCATCTC

TTGGACAAAT AAAATGAGTC ATCTTACAGA TCAGTTTCCT AACTCATCTC

TTGGACAAAT AAAATGAGTC ATCTTACAGA TCAGTTTCCT AATTCATCTC

TTGGACAAAT AAAATGAGTC ATTTTACAGA TCAGTTTCCT AACTCATCTC

TTGGACAAAT AAAATGAGTC ATCTTGCAGA CCAGTTTCCT AACTCATCTC

TTGGATAAAT AAAGTGAGTC ATCTTGCAGA CCAGTTTCCC AACTCCTCTC

TTGGATAAAT AAAGTGAGTC ATCTTGCAGA CCAGTTTCCC AACTCCTCTC

TTGGACAAAT AAAGTGAGTC ATCTTGCAGA CCAGTTTCCT AACTCATCTC

CTGGACAAAT AAAATGAGTC ATCTTGCAGA CCAGTTTCCT AATGCATCTC

TTGGACAAAT AAAATGAGTC ATCTTACAGA CCAGTTTCCT AATTCATCTC

CTGGACAAAC AAAATGAGTC ATCTTGCAGA CCAGTTTGCT AACTCATCCC

TTGAACAAAT AAAATGAGTC ATCTTACAGA CCAGTTTCCT AACTCATCTC

TTGGACAAAT AAAATGAGTC ATCTTGCAGA CCAATTTCCT AACTCATCTC

---------- ---------- ---------- ---------- ----------

TTGGACAAAT AAAATGAGTC ATCTTGCAGA CCAGTTTCCT AACTCATCTC

CTGGACAAAT ATAGTGAATC ATCTTGCAGA TCAGTTTCCA AATTCTTCCC

TAAGGCAAAT ATAGTGAGTC ACCTTCCAGA TCAGTTTCCC AATTCCTCTC

AGAACGGTTC ATGCCGACAA GTGCAGTATC CTCTAGTGGG GACTCTGATA

AGAACGGTTC ATGCCGACAA GTGCAGTATC CTCTAGTGGG GACTCTGATA

AGAACGGTTC ATGCCGACAA GTGCAGTATC CTCTAGTGGG GACTCTGATA

AGAACGGTTC ATGCCGACAA GTGCAGTATC CTCTAGTGGG GACTCTGATA

AGAATGGATC ATGCCGACAA GTGCAGTATC CTCTAGTGGG GACTCTGATA

AGAATGGATC ATGCAGACAA GTGCAGTATC CTCTAGCGGG GACTCTGATA

AGAATGGAGC CTGCAGACAA GTGCAGTATC CTCTAGTGGA GACTCTGATA

AGAATGGAGC ATGCAGACAA GTGCAATACC CTCTAGTGGA GACTCTGATA

AGAATGGATC ATGCAGACAA GTGCAGTATC CTCTAGTGGA GACTCTGATA

AGAATGGGTC ATGCAGACAA GTGCAGTATC CTCTAGTGGG GACTCTGATA

AGAATGGATC ATGCAGACAA GTACAATATC CTCTAGTGGG GACTCTGATA

AGAATGGATC CTGCAGACAA GTGCAGTGCC CTCTGGTGGA GACTCTGACA

AGAACGGATC TTGCAGACAA GTTCAGTATC CTCTAGTGGG GATTCTGATA

AGAATGGATC ATGCAGGCAA GTGCAGTATC CACTAGTGGG GACTCTGATA

---------- ---------- ---------- ---------- ----------

AGAACGGATC ATGCCGACAA GTGCAGTACC CCCTAGTGGG GACTCTGACA

AGAATGGATC CTGCAGACAA GCTCATTATC CTCTAGTGGA GACTCAGATC

AGAACGGATC ATGCAGACAA GCCCATTATC CCCTAGTGGG GACTCCAACC

TATCCAGTCC ATTACTGCAA AATACTGTCC ACATTGACCT CAGTGCTCTA

TATCCAGTCC ATTACTGCAA AATACTGTCC ACATTGACCT CAGTGCTCTA

TATCCAGTCC ATTACTGCAA AACACTGTCC ACATTGACCT CAGCGCTCTA

TATCCAGTCC ATTACTGCAA AACACTGTCC ACATTGACCT CAGCGCTCTA

TATCCAGTCC ATTACTGCAA AATACTGTCC ACATTGACCT CAGTGCTCTA

TATCCAGTCC ATTACTGCAA AATACTGTCC ACATTGACCT CAGTGCTCTA

TATCCAGCCC ATTACTACAA AACACTGTTC ACATTGACCT CAGCGCTCTA

TATCCAGCCC ATTACTACAA AATACTGTTC ACATTGACCT CAGTGCTCTA

TATCCAGTCC ATTACTGCAA AATACTGTCC ACATTGACCT CAGTGCTTTA

TATCCAGTCC ATTACTGCAA AATACCGTCC ACATCGACCT CAGTGCTCTA

TATCCAGTCC ATTATTGCAA AATACTGTCC ACATTGACCT CAGTGCTCTA

TATCCAGTCC GTTGCTGCAA AATACCGTTC GCATTGACCT CAGTGCACTG

TATCCAGTCC ATTACTGCAA AATAGTGTCC ACATTGACCT CAGTGCTCTA

TATCCAGTCC ATTACTGCAA AATGTTGTCC ACATTGATCT CAGTGCTCTA

---------- ---------- ---------- ---------- ----------

TCTCCAGTCC ATTACTGCAG AACACTGTCC ATATTGACCT CAGCGCTCTA

TCTCCAGTCC GCTGCTTCAG AATCACGTCC ACATTGATAT CAGCGCTTTA

CATCTAGCCC ACTTCTACAG AGCAACGTTC ACATTGACAT CAGCGCCCTG

AATCCAGAGC TGGTCCAGGC AGTGCAGCAT GTAGTGATTG GAGCCTGATT

AATCCAGAGC TGGTCCAGGC AGTGCAGCAT GTAGTGATTG GAGCCTGATT

AATCCAGAGC TGGTCCAGGC AGTGCAGCAT GTAGTGATTG GAGCCTGATT

AATCCAGAGC TGGTCCAGGC AGTGCAGCAT GTAGTGATTG GAGCCTGATT

AATCCAGAGC TGGTCCAGGC AGTGCAGCAT GTAGTGATTG GAGCCTGATT

AATCCAGAGC TAGTCCAGGC AGTCCAGCAC GTCGTGATTG GAGCCTGATT

AATCCAGAGC TGGTCCAAGC GGTGCAGCAC GTAGTGATTG GAGCCTGATT

AATCCAGAGC TGGTCCAAGC AGTTCAGCAC GTAGTGATTG GAGCCTGATT

AATCCAGAGC TGGTCCAAGC AGTCCAGCAC GTAGTGATTG GAGCCTGATT

AATCCAGAGC TGGTCCAGGC AGTTCAGCAC GTAGTAATTG GAGTCTGATT

AATCCAGAGC TGGTGCAGGC AGTCCAGCAT GTAGTGATTG GAGCCTGATT

AATCCGGAGC TGGTTCAGGC GGTCCAGCAC GTGGTGATTG GCGCCTGATT

AATCCAGAGC TGGTCCAGGC AGTGCAGCAC GTAGTAATTG GAGCTTGATT

AATCCAGAGC TGGTCCAAGA AGTCCAACAC GTAGTGATTG GAGCCTGATT

---------- ---------- ---------- ---------- ----------

AACCCTGAGT TGGTCCAAGC CGTCCAGCAC GTCGTGATCG GAGCCTGATT

AATCCTGAGC TGGTCCAAGC AGTTCAGCAT GTGGTAATTG GAGCCTGATT

AACCCAGATC TAGTCCAAGC CGTTCAGCAC GTGGTGATCG GAGCCTGATG

GTGCATTTCA ATGAAGTCAT AGGAAGAGGT AAGTATTTCC ACTATGTTAG

GTGCATTTCA ATGAAGTCAT AGGAAGAGGT AAGTATTTCC ACTATGTTAG

GTGCATTTCA ATGAAGTCAT AGGAAGAGGT AAGTATTTCC ACTATGTTAG

GTGCATTTCA ATGAAGTCAT AGGAAGAGGT AAGTATTTCC ACTATGTTAG

GTGCATTTCA ATGAAGTCAT AGGAAGAGGT AAGTATTTCC ACTATGATAG

GTGCATTTCA ATGAAGTCAT AGGAAGAGGT AAGTATTTCC ACTATGTTAG

GTGCATTTCA ATGAAGTCAT AGGAAGAGGT AAGTGTTTCC ACTGTGTTAG

GTGCATTTCA ATGAAGTCAT AGGAAGAGGT AAGTATTTCC A-TGTGTTAG

GTGCATTTCA GTGAAGTCAT AGGAAGAGGT AAGTATTTCC ACTATGTTAG

GTACATTTCA ATGAAGTCAT AGGAAGAGGT AAGTATTTCC ACTATGTTAG

GTGCATTTCA ATGAAGTCAT AGGAAGAGGT AAGTATTTCC ACTATGTTAG

GTGCATTTCA ATGAGGTCAT AGGAAGAGGT CAGTGTTTCC ACTATGTTAG

GTGCATTTCA ATGAAGTCAT AGGAAGAGGT AAGTATTTCC ACTATGTTAG

GTGCATTTCA ATGAAGTCAT AGGAAGAGGT AAGTATTTCC ACTATGTGGA

---------- ---------- ---------- ---------- ----------

GTGCATTTCA ATGAAGTCAT AGGAAGAGGT AAGTCTTTCC ACTGTATTAG

GTACATTTCA ATGAAATCAT AGGAAGAGGC AAGTATTCCA ACTATAATAC

GTGCATTTCA CTGAAATCAT AGGAAGAGGC AAGTGTTTTC CGTGTGTTAA

ACAATGGTGA AAGCAACTGA CAGAGCAGTG ATAACAACAT ATATCATCTT

ACAATGGTGA AAGCAACTGA CAGAGCAGTG ATAACAACAT ATATCATCTT

ACAATGGTGA AAGCAACTGA CAGAGCAGTG ATAACAACAT ATATCATCTT

ACAATGGTGA AAGCAACTGA CAGAGCAGTG ATAACAACAT ATATCATCTT

ACAATGGTGA AAGCAACTGA CAGAGCAATG ATAACGACAT ATATCATCTT

ACAATGGTGA AAGCAACTGA CAGAGCACTG ATAACCACAT ATGTCATCTT

ACAATGCTGA GGGCCCCTGA CAGAGC-CTG ATAATGACAT ATATCATCTT

ACAATGCTGA GGGCTCCTGA CAGAGC-CTG ATAA---CAT ATATCATCTT

ACAATGGTGA AAGCTACTGA CAGAGCACTG ATAACAACAT AGATCATCTT

ACAATGGTGA AAGCAACTGA CAGAGCAGTG ATAACAATAT ACATCATCTT

ACAATGGTGA ATGCAACTGA CAGAGCAGTG ATAACAACAT ATATCATCTT

ACAATGGTGA AAGCAACCGA CAGGACGGTG ATAACAACAT ATATCATCTT

ACAATGGGGA AAGTAACTGA CAGAACAGTG ATAACAACAC ATATCATCTT

ACAATGGTAA AAGCCACTGA CGAAGCATTG ATAACAACAT ATATCATCTT

---------- ---------- ---------- ---------- ----------

ACAATGGCCA AAGCCACTGA CCGCACAGTG ATAATAACAT ACATCATCTT

ATAATAATAA AAGCAAAGGA CAAAAAAGAA ATGAGAACAT ACATCATTTT

AGAACAACTG AAACCACTGA CAGGATAATG ATTAAAACAT AGATCATTTT

TCTTTATACA AAGATAAATG AATATTTGTA ATAAGCTAGA GGGCATTTTG

TCTTTATACA AAGATAAATG AATATTTGTA ATAAGCTAGA GGGCATTTTG

TCTTTATACA AAGATAAATG AATATTTGTA ATAAGCTAGA GGGCATTTTG

TCTTTATACA AAGATAAATG AATATTTGTA ATAAGCTAG- ----------

TCTTTATACA AAGATAAGTG AATGTTTGTA ATAAGCCAGA GGGCATTTTG

TCTGTATACA AAGAGGAATG AGTATTTGTA ATAAGCTAGA GGCCATTTTG

TCTTCACACA AA---GAATG ACTATTTGCA ATAAGCCAGA GGGCATTTTG

TCTTCACACA AA---AAATG ACTATTTGCA AGAAGCTAGA GGGCATTTTG

TATTTGTACA AAGAGGAATG AGTATTGGTA GTAAGCTAGA GGGCATTTTG

TCTTTATACA AAGAGGAATG AATATTTGTA TTAAGCCAGA GGACATTTTG

TCTTTATACA AAGAGG-ATG AATATTTGTA ATAAGCCAGA GGACATTTTG

TCTTTATACG GAGAGGAATG AATATTTGTA --AAGCCAGA GGACATTTTG

TCTTTCCACA AAGAGGGATG AATATTTGTA GCAAACCAGA GGACATTTTG

TCTTTACACA AAGAAGAGTG AATATTTGTA ATAAGCCAGA GGGCATTTTG

---------- ---------- ---------- ---------- ----------

GCTTTACACG GGGAGAAGGG GGTACTTGAA ATAAACCCTA GGCCATTTCG

CCTTTACACA AAGGGAAGCA GGTATTTGTA ATAAGGCAGA GGGCATTTTG

TCTTGACCCT CAGTGGAATG AATATCTACA CTCAGCCAGA GGTCATTTTG

GTTGTGTATA TCATGGGACT TTGTTGGACA ATGATGGCAA GAAAATTCAC

GTTGTGTATA TCATGGGACT TTGTTGGACA ATGATGGCAA GAAAATTCAC

GTTGTGTATA TCATGGGACT TTGTTGGACA ATGATGGCAA GAAAATTCAC

---------- ---------- ---------- ---------- ----------

GTTGTGTGTA TCATGGGACT TTGCTGGACA ATGATGGCAA GAAAATTCAC

GTTGTGTGTA TCACGGGACT TTGTTGGACA ATGATGGCAA GAAAATTCAC

GCTGTGTCTA TCATGGGACT TTGTTGGACA GTGACGGAAA GAAAATTCAC

GCTGTGTCTA TCATGGGACT TTGCTGGACA ATGACGGAAA GAAAATTCAC

GTTGTGTATA TCATGGGACT TTGTTGGACA ATGATGGCAA GAAAATTCAC

GGTGTGTATA TCATGGAACT TTGTTGGACA ACGATGATAA GAAAATTCAC

GGTGTGTATA CCATGGGACT TTGTTGGACA ATGACGACAA AAAAATTCAC

GTTGTGTGTA TCATGGGACT TTGTTGGATA ATGATGACAA GAAAATTCAC

GGTGTGTGTA TCATGGAACC TTGCTGGACA ATGATGACAA GAAAATCCAT

GGTGTGTATA TCATGGGACT TTGTTGGACA GTGACGACAA GAAAATTCAC

---------- ---------- ---------- ---------- ----------

GGTGTGTGTA TCATGGGACT CTGTTGGACA ATGATGACAG GAAAATCCAC

GGTGTGTATA TCATGGGACT TTATTGGATA ATGATGACAA GAAGATTCAT

GGTGTGTGTA TCACGGAACT CTGTTGGATA ATGATGACAA AAAAATCCAC

TGTGCTGTGA AATCCTTGAA CAGTAAGTGG CTAATATCAT TTAACTGTTT

TGTGCTGTGA AATCCTTGAA CAGTAAGTGG CTAATATCAT GTAACTGTTT

TGTGCTGTGA AATCCTTGAA CAGTAAGTGG CTAATATCAT TTAACTGTTT

---------- ---------- ---------- ---------- ----------

TGTGCTGTGA AATCCTTGAA CAGTAAGTGG CTAATATCAT TTAACTGTTT

TGTGCTGTGA AATCCTTGAA TAGTAAGTTA ATAATATCAC TCAACTGTTT

TGTGCTGTGA AATCCTTGAA CAGTAAGTTG CTAGTACCAT -----TGTTA

TGTGCTGTGA AATCCTTAAA TAGTAAGTTG CTACTACCA- -----CGTTC

TGTGCTGTGA AATCTTTGAA TAGTAAGTTA CTAATATTAC TTAACTGTTG

TGTGCTGTGA AATCCTTGAA TAGTAAGTTG TTAATATCAT TTAAATGTTT

TGTGCTGTGA AATCCCTGAA TAGTAAGTGA TTAATGTCAC TTAACTGTTT

TGTGCTGTGA AATCCTTGAA TAGTAAGTCA C---TATCAT TTAACTGTTT

TGTGCTGTGA AGTCCTTGAA TAGTAAGTTT CAAATATCGT TTAACTGTTT

TGTGCTGTGA AATCTTTGAA TAGTAAGTTA CTAATATCAT TTCACTGTTT

---------- ---------- ---------- ---------- ----------

TGTGCTGTGA AGTCCTTGAA TCGTAAGTTG CTAATATCGT TGAACTGTTT

TGTGCTGTGA AATCCTTGAA TAGTAAGTTA ATAATATAAT TTGACAGTCT

TGTGCTGTGA AGTCCTTGAA TAGTGAGTTA CTAATTGAAT TTGACTGTTT

GGTTTATAAT ATTTGTTTTC AAGTGACATT TCTGGAAACT ATTGGAAGCA

GGTTTATAAT ATTTGTTTTC AAGTGACATT TCTGGAAACT ATTGGAAGCA

GGCTTATAAT ATTTGTTTTC AAGTGACATT TATGGAAACT ATTGGAAGCA

---------- ---------- ---------- ---------- ----------

GGCTTATAAT ATTTGTTTTC AAGTGACAGT TAAGGAAACT ATTGGAAGCA

GGCTT-TAAT ATTTGTTTTC AAGTGACATT TATGGAAACT ATTGGAAGCA

GACTTATAAT ATTTCTTTTC AGATGACACT TATGGAGACT ATTGGAAGC-

AACTTATAAT ATTTGTTTTC AAATGACATT TATGGAAACT ATTGAAAGC-

GTCTTATAAT AATTGTTTTC AAGTGACATC TATGGAAAGT ATTGGAAGCA

GGCTTATAAT ATTTGTTTTC AAGTGACATT TATGGAAACT ATTGGAAGCA

GGCTTATAAT ACTTGTTTTC AAGTGACATT TGTGGAAACT ATTGGAAACA

GGCTTATAAT ATTTGTTATC AAGTGACATT TATGGAAAGT ATTGGAAGCA

GGCTTGTAGT ATTTGTTTTA AAGTGACATT TATGGAAACT ATTGGAAGCA

GGCTTATAAT ATTTGTTTTC AAATGACATT TATGGAAACT ATTGGAAGCA

---------- ---------- ---------- ---------- ----------

GACTTATAAT ATTTGCTTTC AAATGACATT TCTGGAAACT ATTGGAAGCA

AGCAGATGAT GTTTGTTTTC AAATGACATT TTTGGAAACT ATTAGAAACA

GGCTCCTAAC ATTTGTTTTC AAGGGAAATT TTTGGAAACT ATTAGAGGCA

TGTTGGCACC TACAGTAGAA TGACTCATTT ACCCAGAGGC ATTACTATAA

TGTTGGCACC TACAGTAGAA TGACTCATTT ACCCAGAGGC ATTACTAAAA

TGTTGGCACA TACAGTAGAA TGACTCATTT ACCCGGAGGC ATTACTATAA

---------- ---------- ---------- ---------- ----------

TGTTGGCGCA TACAGTAGAA TGACTCATTT ACCCAGAGGC ATTACTATAA

TGTTGGCACC TACATTAGAA TGATTCATTT ACCCAGAGGC ATTACTATAA

-ATTGGCACA TACCATAGAA TGGCTCGTTT GCCCAGAGAC ACGACTATAA

-GTTGGCACA TACCATAGAA TGACTCATTT GCCCAGAGAC ACTACTAC-A

TGTTGGCACA TTGAGTAGAA TGACTCATTT ACCCAGAGAC ATGGCAATAA

TTTTGGCGCA TACAGTAGAA TGACTCATGT ACCCAGAGGC ATTACTATAA

TGTTGGCACA TATAGTAGAA TGACTCATTT ACCCAAAGGA ATTACTATAA

TGTTGGCACA TACAATAGAA TGACTCATTT ACCCAGAGGC ATTACTATAA

TGTTGGCACA TACAGTCAAA TGACTCATTT ACCCAGAGGC ATTACTATAA

TGTTGGCACA TGCAGTAGTG TGACTCATTT ACCCGAAGGC ATTACTATAA

---------- ---------- ---------- ---------- ----------

TGTTGGCACG TGCAGTCAAA TGACTCATTT GCCCAGAGGC ATTACTATAT

TGTTGGCACA TAGAGTAAGA TGACTCATTC ATCCTCAGGC ATTACTGTAA

CGTTGGCACC CACAGCAGTG TGACTCATTT AACCTTGGGC ATGACTGTGA

TTTTTTTTAA CCACAACTTC CATTAAAAAG AAAAAATGAA ATAAGACAAA

TTTTTTTTAA CCACAACTTC CATTAAAAAG AAAAAATGAA ATAAGACAAA

TTTTTTTTAA CCACAACTTC CATTAAAAAG AAAAAATGAA ATAAGACAAA

---------- ---------- ---------- ---------- ----------

TTATTTTTAA CTACAACTTC CATTAAAAAG AAAAAATGAA ATAAGACAAA

TTTTTTTTAA CCACAACTTC CATTAAAAAT AAAAAAAGAA ACAAGACAAA

TTTTTCTGAA CCAGCACTTT CATTTTAGAG GAAGGATGGA ATAATACAAG

CTCTTTTTAA CCAGCAC-TC CATTTTAGAA AAACGATGAA GTAATACGAG

TTTTTTTTGA CCACAACTCC CATTAAAAAG AAAAA-TGTA ATAAGACAAA

TTTTCTTTAA CCACAACTTC CATCAAAAAG AAAAAATGAA ATAAGTCAAA

TTTTTTTTTA CCACAACTTT CATTAAAAAG AAAAAATGAA ATAAGACAAA

TTTTTTTTCA CCACAACTTC CATTAAAAAG AAAAAATGAA ATAAGACAAA

TTTTTTTCAA CCACAACTTC CATTAAAAAG AAAAAGTGAA ATAAGATTAA

TGTTTTTTAA CCACAATTCC CATTAGAAAG AAAAGGTGAA ATAAGACAAA

---------- ---------- ---------- ---------- ----------

TTTTTTTTAA CCACAACTCC CATTAAATAG AAAAAATGAA ATGAGACAAA

TTTTTAAAAA ATACAACTCC CATTAAAAAA GTGGAAAGAA ATAAGATAAA

CTTTCCTTAA TCACAACTCC CCTTACCAAG AGAAAATGGA CTGGGAGGAG

CAGGAGAATT GCTTTTCCTC TCTACTGCAG AATCCAACTG TAAAAGATCT

CAGGAGAATT GCTTTTCCTC TCTACTGCAG AATCCAACTG TAAAAGATCT

CAGGAGAATT GCTTTTCCTC TCTATTGCAG AATCCAACTG TAAAAGATCT

--------TT GCTTTTCCTC TCTATTGCAG AATCCAACTG TAAAAGATCT

CAGGAGAATT GCTTTTCCTT TCTATTGCAG AATCCAACTG TAAAAGATCT

CAGGAGAATT GCTTTTCCTC TCTATTGCAG AACCCAACTG TAAAAGATCT

CAGAGGAGTT CCTTTTCCTC TCTATTGCAG AACCCAACTG TGAAAGATCT

CAGGAGAGTT GCTTTTCCCC TCTATTGCAG AATCCAACTG TGAAAGATCT

CAGGAGAATT GCTTTTCCTC TCTATTTCAG AACCCGACTG TAAAAGATCT

CAAGAGAATT GGGTTTCTTC TCTATTACAG AACCCAACTG TAAAAGATCT

CAGGAGAATT GCTTTTTTTC TCTATTGCAG AACCCAACTG TAAAAGATCT

CAGGAGAATT GCGTTTCTTC TCTATTGCAG AACCCAACTG TAAAAGATCT

CAGGAGAGTT GCTTTTCTGC TCTATTGCAG AGTCCAACTG TAAAAGATCT

CAGGAGAATT GTTTTTCCTC TCTGCTGTAG AACCCAACTG TAAAAGATCT

---------- ---------- ---------- ---------- ----------

CAGGAGAACT GGTTGCCCCC CCTCTTGCAG AGCCCCACTG TGAAAGATCT

GAAGGGAATT ATTTATCCTT CCTATTTTAG AATCCAACAG TGAAGGACCT

CAGGAGAATT GCTTTTGCTC TCCGTTTCAG AATCCCACCG TGAAAGACCT

TATTGGCTTT GGTCTTCAAG TAGCCAAAGG CATGAAATAT CTTGCAAGCA

TATTGGCTTT GGTCTTCAAG TAGCCAAAGG CATGAAATAT CTTGCAAGCA

TATTGGCTTT GGTCTTCAAG TAGCCAAAGG CATGAAATAT CTTGCAAGCA

TATTGGCTTT GGTCTTCAAG TAGCCAAAGG CATGAAATAT CTTGCAAGCA

TATTGGCTTT GGTCTTCAAG TAGCCAAAGG CATGAAATAT CTTGCAAGCA

TATTGGCTTT GGTCTTCAAG TAGCCAAAGG CATGAAATAT CTTGCAAGCA

TATAGGATTC GGTCTTCAAG TAGCCAAGGG CATGAAATAT CTTGCCAGCA

TATAGGATTT GGCCTTCAAG TAGCCAAAGG CATGAAATAT CTTGCCAGCA

TATTGGCTTT GGTCTTCAAG TAGCAAAAGG CATGAAATAC CTTGCAAGCA

TATTGGCTTT GGTCTTCAAG TAGCCAAAGG CATGGAATAT CTTGCAAGCA

TATTGGCTTT GGTCTTCAAG TAGCCAAAGG CATGAAATAT CTTGCAAGCA

TATTGGCTTT GGCCTTCAAG TAGCCAAAGG CATGAAATAT CTTGCAAGCA

CATTGGCTTT GGTCTTCAAG TAGCCAAAGG CATGAAATAT CTTGCAAGCA

TATTGGCTTT GGTCTGCAAG TAGCCAAAGG CATGAAATAT CTTGCAAGCA

---------- ---------- ---------- ---------- ----------

CATTGGCTTT GGTCTTCAAG TCGCCAAGGG CATGAAATAT CTTGCCAGCA

CATTGGTTTT GGCCTTCAAG TAGCAAAAGG CATGAAATAC CTTGCAAGCA

CATCGGATTT GGCCTCCAAG TGGCCAAAGG TATGAAATAC CTTGCCAGCA

AAAAGTTTGT CCACAGAGAC TTGGCTGCAA GAAACTGTAT GTAAGTATCT

AAAAGTTTGT CCACAGAGAC TTGGCTGCAA GAAACTGTAT GTAAGTATCT

AAAAGTTTGT CCACAGAGAC TTGGCTGCAA GAAACTGTAT GTAAGTATCC

AAAAGTTTGT CCACAGAGAC TTGGCTGCAA GAAACTGTAT GTAAGTATCT

AAAAGTTTGT CCACAGAGAC TTGGCTGCAA GAAACTGTAT GTAAGTATCT

AAAAGTTTGT GCACAGAGAC TTGGCTGCAA GAAACTGCAT GTAAGTATCT

AAAAGTTTGT CCACAGAGAC TTAGCTGCAA GAAACTGCAT GTAAGTATCT

AAAAGTTTGT CCACAGAGAC TTAGCTGCAA GAAACTGCAT GTAAGTATCT

AAAAGTTTGT CCACAGAGAC TTGGCTGCAA GAAACTGTAT GTAAGTAGCT

AAAAGTTTGT CCACAGAGAC TTGGCTGCAA GAAACTGTAT GTAAGTATCT

AAAAGTTTGT CCACAGAGAC TTGGCTGCAA GAAACTGTAT GTAAGTATCT

AAAAGTTTGT CCACAGAGAC TTGGCCGCAA GAAACTGTAT GTAAGTATCT

AAAAGTTTGT CCACAGAGAC TTGGCTGCAA GAAACTGTAT GTAAGTATCT

AAAAGTTTGT CCACAGAGAC TTGGCTGCAA GAAACTGTAT GTAAGTATCT

---------- ---------- ---------- ---------- ----------

AAAAGTTTGT CCACAGAGAC CTGGCCGCCA GGAACTGTAT GTAAGTATCT

AAAAGTTCGT ACACAGAGAC TTGGCTGCAA GAAATTGTAT GTAAGTATCT

AAAAGTTTGT CCACCGAGAC TTGGCTGCCC GAAATTGTAT GTAAGTATCC

TAATGTTGTG GTTTCACACT GCATTAGTGT TGGGGAGGGA GAATTTGATC

TAATGTTGTG GTTTCACGCT GCATTAGTGT TGGGGAGGGA GAATTTGATC

TAATGTTGTG GTTTCACACT GCATTAGTGT TGGGGAGGGA GAACTTGATC

TAATGTTGTG GTTTCACACT GCATTAGTGT TGGGGAGGGA GAACTTGATC

TAATGTTGTG GTTTCACACT GCATTAGTGT TGGGGAGGCA GAATTTGATC

TAACGTTGTG GTTTCACACT GCATTAGTGT TTGGGAGGGA GAATTTGATC

TAGTGTTGTG GTTTCACACC GAATTAGTGA TTGGGAGAGA GAATTTGATC

TAGTGTTGTG GTTTCACACC GAATTAGTGA TTGGGAGAGA GAATTTGATC

TAGTGTTGTG GTTTCATGCC ACATTAGTGT TTGCTAGGGA GAATTTGATC

TAGTGTTGTG GTTTCACACT GCATTAGTGT TTGGAAGGGA CAATTCGATC

TAGTGTTGTG GTTTCACACT GCATTAGTGT TAGGGAGGGA GAATTTGATC

TAGTATTGTG GTCTCACGCT GCATTAGCAT TAGG-AGGTA GAATTTGA--

TAATGTTGTG GTTTCACACT GCATTAGAGT TAGGGAGGGA GAATTTGATC

TAATGTCGTG GTTTCACACT GCATTAGTGT TTGGGAGGGA GAATTTGATC

---------- ---------- ---------- ---------- ----------

TAGTGTGGTG GTTTCACACT GCATTAGTGT TTGGGAGGGA AAATTTGATC

TAATGTTGTG GTTTTATGCT ACATTAGCTT ACAGGAGGGA GAACTTGATC

TAATTTTGAG ATGTTATGCT AGATTCCTTT ACAGTAGGGA GAATATACTT

TTCAGCATTT GACAGTGAAA AGGATTCCCA AGAGTGGATT TGAAGCCAGC

TTCAGCATTT GACAGTGAAA AGGATTCCCA AGAGTGGATT TGAAGCCAGC

TTCAACATTT GACAGTGAAA AGGATTCCCA AGAGTGGATT TGAAGCCAGC

TTCAACATTT GACAGTGAAA AGGA------ ---------- ----------

TTCAGCATTT GACAGTGAAA AGGATTCCCA AGAGTGGATT TGAAGCCAGC

TTCAGCATTA TACAGTGAAA AGGATTCCCA AGAGTGGATT TGAAATCAGC

TCCAGCATTT GACAGTGAAA AAG------- --AGCAGCTT TGAAAGCAGC

TCCAGCATTT GACAGTGAAA GGG--TCCCA ACAGCAGCTC TGAAACCAGC

TTCAGCATTT TACAGTGAAA AGGATTCCCA AGAGTGGACT TGAAGTCAGC

TGCAGCATTT TACAGTGAAA CGGATTCCCA AGAGTGGATT TGAAGTCAGC

TTCAGCATTT TACAGTGAAA AGGATTCCTA AGACTGGATT TGAAGTCAGC

TTCAGCCTGT TACAATGAAA AGGATTCCCA AGGGTGGATC TGAAGTCAGC

TGCAACGTTT TACAATGAAA AGGATTCTCA AGAAAGGATT TGAAGTCAGC

TTCAGCATTT TATAGTGAAA AAGATTCCCA AGAGTGGATT TGAAGTCAGC

---------- ---------- ---------- ---------- ----------

TTCCACATTT TACGGTGAAA AGAATTCCCA AGAGTGGATT GGAAGTCAGC

TTCGGCATTC TACACTATAA AAGATGCCCA AGAGCTGATT TGGATTTGGA

TCCACCCTTC TTCATTGTAA AGGATTCCGG AGAATGAATT GGGGTTTAGG

CTGCAGAAGC CCTACCTGGT ATTTGGCAAT GAAATACAAA GACTTTATTT

CTGCAGAAGC CCTACCTGGT ATTTGGCAAT GAAATACAAA GACTTTATTT

CTGCAGAAGC CCTACCTGGT ATTTGGGAAT GAAATACAAA GACTTTATTT

---------- ---------- ---------- ---------- ---TTTATTT

CTGCAGAAGC CCTACCTGGT ATTTGGGAAT GAAATACAAA GACTTTATTT

CTGCAGAAGC CATACCTGGT TTTGGGGAAT GAAATACAAA GACTTTATTT

CTGCAGAAGC GCTACCTGGT CTTCGGGAAT GGAATACAAA GACTTTATTT

CTGCAGAAGC GCTACCTGGT CTTTGGGAAT GAAATACAAA GACTTTATTT

CTGCAGAAGC CCTACCTGGT ATTTGGGAAT GAAATACAAA GACTTTATTT

CTGCAGAAAC CCTACCTGGT ATTTGAGAAT GAAATACAAA GA-TTTATTT

CTGCAGAAGC CCTACCTGGT ATTTGGGAAT GAAATACAAA GACTTTATTT

CTGCAGAAGC CCTACCTGGT GTTTGGGAAT GAAATACAAA AG-C---TTT

CTGCAAAAGC CCTACCTGGT ATTTGGGAAT GAAATACAAA GACTTTATTT

CTGCTTAAGC CCTACCTGGT ATTTGGGAAT GAAATACAAA GACTTTATTT

---------- ---------- ---------- ---------- ----------

CTGCCGAAGC TCCACCCAGT GTCTGGGAAT GAAATACAAA GATGTTATTT

CTGGCTCAGT CCTACCTGGT ATTTAGGAAT GAAATACAAA GACTTTATTT

GTAGTTGAGC CCCGACTGGC AGTCAGAGAT GGGATATAGA GACCATATTC

GTGTCTGGCT GCCTGGCTTA CAACAGTCAA CAGTTTGTAA TTGAGTTCAT

GTGTCTGGCT GCCTGGCTTA CAACAGTCAA CAGTTTGTAA TTGAGTTCAT

GTGTCTGGCT GCCTGGCTTA CAACAGTCAA CAGTTTGTAA TTGAGTTCAT

GTGTCTGGCT GCCTGGCTTA CAACAATCAA CAGTTTGTAA TTGAGTTCAT

GTGTCTGGCT GCCTGGCTCA CAACAGTCAA CAGTTTGCAA TTGAGTTCAT

GTGTCTGGCT GCCTGGCTCA CAACAGTCAA CAGTTTGTAA TTGATTTAGT

GTGTCTGGCT GCCCGGCTCA CAACAGTCAA CAGTTTATAA TTGAATCCCG

GTGTCTGGCT GCCCGGCTCA CAACAGTCAA CAGTTTATAA TTGAGTCGCG

GTGTCTGGCT GCCTGGCTCA CAACAGTCAA CAGTTAGTAA TTGATTCAGT

GTGTCTGGCT GCCTGGCTTA CAACAGTCAA CAGTTGGTAA TTGATTCAGT

GTGTCTGGCT GCCTGGCTTT CAACAGTCAA CAGTTTGTAA TGGATTCAGT

GTACCTGGCT GTCTGGCTTG CAGCAGTC-- CAGTCTGTTA TTGATGCAGC

GTGTCTAGCT GCTGGGCTTA CAACAGTCAA CAGTTTGTAA TTGATTCAGT

GTGTCTGGCT GCCTGGTTTA CAACAGTCAA CAGTTTGTAA TTGATTCAGT

---------- ---------- ---------- ---------- ----------

GTGTCTGGCT GCCTGACTTA CAACAGTCAA CAGTTTGTCA TTGATTCAGT

GTGTCTGGCT GCCTGGCTTA CAACAGTCAA CAGTTAGTGA TTGATTCAAT

CTGCCTCAGT ACCAGGTCTG CTGAGGACAA CAGCTTCCGA TGGACTCAGG

GTTTTCCCTT GCATAGACTG ATTCATGACT TATGGTAATA ATTTTTGTCC

GTTTTCCCTT GCATAGACTG ATTCATGACT TATGGTAATA ATTTTTGTCC

GTTTTCCCTT GCATAGACTG ATTCATGACT TATGGTAATA ATTTTTGTCC

GTTTTCCCTT GCATAGACTG ATTCATGACT TATGGTAATA ATTTTTGTCC

GCTTTCCCTT GCATAGAGTG ATTCCTTACT TGTGGTAATA ATTTTTGTCC

GCTTT-CCTT GCATAGACTG ATTCATGACT TATGGTAATA ATTTTTGTCC

GCTTTCCCTT GCAGAGACTG ATTCATGGCT CGCAGTAATT -TTTTTCTC-

GCTTTCCCTT GCAGAGACTG ATCCATGGCT CGTGGTAATA ATTTTTCTC-

GCTTTCCCCT GCGCAGCCTG ATTCATGACT TATGGTAATT -TTTTTGTCC

GCTTTCCCTT CCATAGACTG ATTCATGACT TATGGTAATA ATTTTTGTTT

GCTTTCC-TC ACATAGACTG ATTCATGACT TATGGTAATA ATTTTTGTCC

CCTGG---TT GCGTAGACTC GTTCGTGACT CATGGCAATC ATTTTTGTCC

GCTTTCTCTT GCATAGACTG ATTCATGACT TATGG-AATA ATTTTTGTCT

GCTTTTCCTT GCATAGACTG ATTCATGACT TATGGTAATA ATTTTTGTCA

---------- ---------- ---------- ---------- ----------

ACTTTCCTTT GCGTGGGCTG ATTCATGACT TATGGTAATA CATTTTGTCC

AGTTTCCCTT TTATTGACTG ATTCATGACG TATGGTAATA ATTTTTGTCA

ACTGGCCCCT TTCTAGACTG ATTTAGGACT GGTGGTAATC AATTTTGTCA

TTTCTGTAGG CTGGATGAAA AATTCACAGT CAAGGTTGCT GATTTTGGTC

TTTCTGTAGG CTGGATGAAA AATTCACAGT CAAGGTTGCT GATTTTGGTC

TTTCTGTAGG CTGGATGAAA AATTCACAGT CAAGGTTGCT GATTTTGGTC

TTTCTGTAGG CTGGATGAAA AATTCACAGT CAAGGTTGCT GATTTTGGTC

TTTCAATAGG CTGGATGAAA AATTCACTGT CAAGGTTGCT GATTTTGGCC

TTTCTGTAGG CTGGATGAGA ATTTCACTGT CAAAGTTGCT GACTTTGGCC

---CTGTAGG TTGGATGAAA AATTCACTGT CAAGGTTGCT GATTTCGGTC

---CTGTAGG TTGGATGAAA AATTCACTGT CAAGGTTGCT GATTTCGGTC

ATTCTGTAGG TTGGATGAAA AATTCACTGT CAAAGTTGCT GATTTTGGTC

TTTCTGTAGG CTGGATGAAA AATTCACCGT CAAGGTTGCT GATTTTGGTC

TTTCTATAGG CTGGATGAAA AATTCACTGT CAAGGTTGCT GATTTTGGTC

TTTCTGTAGG CTGGATGAAA ACTTCACTGT CAAGGTTGCT GATTTCGGTC

TTTCTGTGTC TGCGACTGAG GGTAGACTGT TAAGGTTGCT GATTTTGGTC

TTTTTATAGG CTGGATGAAA AATTCACTGT TAAGGTGGCT GACTTTGGTC

---------- ---------- ---------- ---------- ----------

TTCCTGTAGG CTGGATGGAA AATTCACCGT CAAAGTTGCC GATTTTGGTC

TTTCTGTAGG CTGGATGAAA AATTTACTGT CAAGGTTGCT GATTTTGGGC

TTTCGATAGG CTGGATGAAA AATTCACTGT CAAGGTGGCT GATTTTGGCC

TTGCCAGAGA CATGTATGAT AAAGAATACT ATAGTGTACA CAACAAAACA

TTGCCAGAGA CATGTATGAT AAAGAATACT ATAGTGTACA CAACAAAACA

TTGCCAGAGA CATGTATGAT AAAGAATACT ATAGTGTGCA CAACAAAACA

TTGCCAGAGA CATGTATGAT AAAGAATACT ATAGTGTGCA CAACAAAACA

TTGCCAGAGA TATGTATGAT AAAGAATACT ATAGTGTACA CAACAAAACA

TTGCCAGAGA CATGTATGAT AAAGAATACT ACAGTGTACA TAACAAGACA

TTGCCAGAGA CATGTACGAC AAAGAGTATT ATAGCGTCCA CAACAAAACG

TTGCCAGAGA CATGTACGAT AAAGAGTACT ATAGTGTCCA CAACAAGACG

TTGCCCGAGA TATGTATGAT AAAGAATACT ATAGTGTACA CAACAAAACA

TTGCCAGAGA CGTGTATGAT AAGGAATACT ATAGCGTACA CAACAAAACA

TTGCCAGAGA CATGTATGAT AAAGAATACT ACAGCGTACA CAACAAAACA

TTGCCAGAGA CGTGTATGAT AAAGAATACT ACAGTGTCCA CAACAAAACA

TTGCCAGAGA TATATATGAT AAGGAATACT ATAGTGTCCA CAACAAAACA

TTGCCAGAGA TATGTATGAT AAAGAGTACT ATAGTGTGCA TAATAAAACA

---------- ---------- ---------- ---------- ----------

TTGCCCGGGA TATGTATGAT AAGGAATACT ACAGTGTGCA CAACAAAACC

TTGCCAGAGA TATGTATGAT AAAGAATATT ATAGTGTACA CAATAAAACA

TGGCCCGAGA TATGTACGAT AAAGAATATT ACAGCGTGCA CAATAAAACG

GGTGCAAGCT GCCAGTGAAG TGGATGGCTT TGGAAAGTCT GCAAACTCAA

GGTGCAAGCT GCCAGTGAAG TGGATGGCTT TGGAAAGTCT GCAAACTCAA

GGTGCAAGCT GCCAGTGAAG TGGATGGCTT TGGAAAGTCT GCAAACTCAA

GGTGCAAGCT GCCAGTGAAG TGGATGGCTT TGGAAAGTCT GCAAACTCAA

GGTGCAAGCT GCCGGTGAAG TGGATGGCTC TGGAAAGTCT ACAAACTCAA

GGTGCAAGCT GCCCGTGAAG TGGATGGCTC TGGAAAGTCT GCAGACGCAG

GGTGCAAACT ACCGGTGAAG TGGATGGCTT TAGAGAGTCT GCAGACGCAA

GGTGCAAGCT ACCAGTAAAG TGGATGGCTT TAGAGAGTCT GCAAACGCAG

GGTGCAAACT GCCAGTGAAG TGGATGGCTT TAGAAAGTCT GCAAACTCAG

GGTGCAAACT GCCAGTGAAA TGGATGGCTT TAGAAAGCCT GCAGACTCAG

GGCGCAAACT ACCAGTGAAG TGGATGGCTT TAGAAAGTCT GCAAACTCAG

GGCGCAAGCT GCCTGTGAAG TGGATGGCTT TGGAAAGTCT GCAGACTCAG

GGAGCAAACT GCCAGTGAAG TGGATGGCTT TAGAAAGTCT ACAAACTCAG

GGTGCAAACT GCCAGTGAAG TGGATGGCTT TGGAAAGTCT GCAAACTCAG

---------- ---------- ---------- ---------- ----------

GGGGCAAGCT GCCAGTGAAG TGGATGGCTC TGGAGAGCCT GCAGACGCAG

GGAGCAAACT GCCAGTGAAA TGGATGGCTT TAGAAAGTCT ACAAACTCAG

GGAGCAAGCT GCCAGTGAAA TGGATGGCTC TCGAAAGTCT GCAGACACAG

AAGTTTACCA CCAAGTCAGA TGTGGTAATG TATTGTTTTA CTAGTGGTCC

AAGTTTACCA CCAAGTCAGA TGTGGTAATG TATTGTTTTA CTAGTGGTCC

AAGTTTACCA CCAAGTCAGA TGTGGTAATG TATTGTTTTA CTAGTGGTCC

AAGTTTACCA CCAAGTCAGA TGTGGTAATG TATTGTTTTA CTAGTGGTCC

AAGTTTACCA CCAAGTCAGA CGTGGTAATG TATTGTTTTA CTAGTGGTCC

AAGTTCACCA CCAAGTCAGA CGTGGTAACG TGTTGTTTT- CTAGTGGTCC

AAGTTCACCA CCAAGTCAGA CGTGGTAATG TATTGTTTTA TTAGTGGTCC

AAGTTCACCA CCAAGTCAGA TGTGGTAATG TATTGTTTTA TTAGTGGTCC

AAGTTTACAA CCAAATCTGA TGTGGTAATG TATCATTTGA CTAGTGGTCC

AAATTTACCA CCAAGTCAGA TGTGGTAATG TATTGTTTGA CCAGTGGTCC

AAGTTTACCA CCAAGTCAGA TGTGGTAACG TATTGTTTGA CTAGTGGTCC

AAGTTCACCA CCAAGTCAGA TGTGGTAACG CATTGTTTGA CTAGTGGTCC

AAGTTCACCA CCAAGTCAGA TGTGGTAATG TACTGTTGTT CTAGTGGTCC

AAGTTTACCA CCAAGTCAGA TGTGGTAATG TATTGTTTCC CTAGTGGTCC

---------- ---------- ---------- --TTGTTTTA CTAGTGGTCC

AAGTTCACCA CCAAGTCAGA CGTGGTAAAG TGCTGTCCGA CTAGTGGTCC

AAGTTTACTA CAAAGTCAGA TGTGGTAATG TATTGTTTTT TCAGTGGTCG

AAGTTCACCT CCAAGTCAGA TGTGGTAATC AATTGTTTCA TTAGTGGTCC

TTTGGCGTGC TCCTCTGGGA GCTGATGACA AGAGGAGCCC CACCTTATCC

TTTGGCGTGC TCCTCTGGGA GCTGATGACA AGAGGAGCCC CACCTTATCC

TTTGGTGTGC TCCTCTGGGA GCTCATGACA AGAGGAGCCC CACCCTATCC

TTTGGTGTGC TCCTCTGGGA GCTCATGACA AGAGGAGCCC CACCCTATCC

TTTGGCGTAC TCCTCTGGGA GCTGATGACA AGAGGAGCCC CACCTTATCC

TTTGGTGTGC TCCTCTGGGA GCTGATGACG AGAGGAGCCC CACCTTACCC

TTCGGTGTGC TTCTCTGGGA GCTCATGACG AGAGGAGCCC CTCCTTATCC

TTTGGTGTGC TCCTCTGGGA GCTCATGACG AGAGGAGCCC CTCCTTATCC

TTCGGCGTGC TCCTGTGGGA GCTGATGACA AGAGGAGCCC CACCTTACCC

TTTGGCGTGC TTCTCTGGGA ACTGATGACA AGAGGAGCAC CACCTTATCC

TTTGGCGTGC TCCTCTGGGA ACTGATGACA AGAGGAGCAC CACCTTATCC

TTTGGCGTGC TCCTCTGGGA ACTGATGACA AGAGGTGCCC CGCCTTATCC

TTTGGTGTGC TCCTCTGGGA GCTGATGACA AGAGGGGCAC CGCCTTATCC

TTTGGTGTGC TGCTCTGGGA ACTGATGACA AGAGGCGCAC CACCTTATCC

TTTGGTGTGC TTCTCTGGGA ACTGATGACA AGGGGAGCTC CACCTTATCC

TTTGGTGTGC TCCTCTGGGA ACTGATGACA AGAGGAGCGC CCCCGTATCC

TTTGGAGTGC TTCTCTGGGA ATTAATGACC AGAGGAGCAC CTCCTTATCC

TTCGGCGTCC TTCTCTGGGA GCTGATGACA CGAGGCGCTC CCCCGTATCC

TGACGTAAAC ACCTTTGATA TAACTGTTTA CTTGTTGCAA GGGAGAAGAC

TGATGTGAAC ACCTTTGATA TAACTGTTTA CTTGTTGCAA GGGAGAAGGC

TGACGTGAAC ACCTTTGATA TAACTGTTTA CTTGTTGCAA GGGAGAAGGC

TGACGTGAAC ACCTTTGATA TAACTGTTTA CTTGTTGCAA GGGAGAAGGC

TGATGTGAAC ACTTTCGATA TAACTGTTTA CCTGTTGCAA GGGAGAAGGC

TGACGTCAAC ACCTTCGATA TAACTGTTTA CTTGTTACAA GGCAGAAGGC

TGACGTGAAC ACATTTGATA TCACTATATA CCTGTTGCAA GGCAGAAGAC

CGACGTGAAC ACATTTGATA TCACTATCTA CCTGTTGCAA GGCAGAAGAC

TGATGTGAAC ACCTTTGACA TAACCGTGTA CCTGCTGCAA GGCAGGAGAC

TGACGTCAAC ACCTTTGACA TAACAGTTTA CTTGTTGCAA GGAAGAAGGC

TGACGTCAAC ACCTTTGATA TAACAGTTTA CTTGTTGCAA GGCAGAAGGC

TGATGTCAAC ACCTTTGATA TAACAGTTTA CTTGTTGCAA GGCAGAAGGC

AGACGTCAAT ACATTTGACA TCACAATCTA CTTGTTGCAA GGCAGGAGGC

TGATGTGAAC ACATTTGACA TCACAGTTTA CTTATTGCAA GGCAGAAGGC

TGATGTAAAC ACCTTTGATA TAACAGTTTA CTTATTGCAA GGAAGGAGGC

CGACGTAAAC ACCTTCGATA TAACGGTTTA CTTATTGCAA GGAAGACGGC

TGATGTGAAT ACCTTTGATA TAACAGTTTA CTTATTGCAA GGTCGAAGGC

CGACGTGAAC ACTTTCGATA TAACGGTCTA CCTGCTGCAA GGAAGGAGGC

TCCTACAACC CGAATACTGC CCAGACCCCT TGTAAGCAGA TATGAAGTAA

TCCTACAACC CGAATACTGC CCAGACCCCT TGTAAGCAGA TACGAAGTAA

TCCTACAACC TGAATACTGC CCAGACCCCT TGTAAGCAGA TATGAAGTAA

TCCTACAACC CGAATACTGC CCAGACCCCT TGTAAGCAGA TATGAAGTAA

TCCTGCAACC CGAATACTGC CCAGACCCCT TGTAAGCAGA TATGAAGTCA

TCTTACAACC CGAATACTGC CCAGACCCCT TGTAAGCAGG TATGAAGTGA

TCTTGCAACC AGAGTACTGT CCAGACGCCT TGTAAGCAGG TATGAAGTGA

TCTTGCAACC AGAATACTGT CCAGACGCCT TGTAAGCAGG TACGAAGTGA

TCCTGCAACC CGAATACTGC CCAGATGCCT TGTAAGCAGG TACGAAGTGA

TCCTACAACC TGAATACTGC CCAGATCCCT TGTAAGCAGG TATGAAGTGA

TCCTGCAACC CGAATACTGC CCAGATCCCT TGTAAGTAGA TACGAAGTGA

TCTTGCAACC CGAGTACTGC CCAGATCACC TGTAAGCAGG TATGAAGTGA

TCTTACAACC TGAATACTGC CCAGATCCCT TGTAAGTAGG TATGAAGTGA

TCTTACAACC TGAATACTGC CCAGATCGCT TGTAAGCAGG TATGAAGTGA

TTTTACAACC TGAATACTGC CCCGATCCCT TGTAAGCAGG TATGAAGTGA

TCTTACAACC TGAATACTGC CCAGATCCCT TGTAAGCAGG TATGAAGTGA

TCTTGCAGCC TGAATACTGC CCAGATCCAC TGTAAGCAGG TATGAAGTCA

TGCTACAGCC AGAATACTGT CCGGACCCAC TGTGAGCAGA TTTGAAGTCA

TGCTAAAATG CTGGCACCCT AAAGCCGAAA TGCGCCCATC CTTTTCTGAA

TGCTAAAATG CTGGCACCCT AAAGCCGAAA TGCGCCCATC CTTTTCTGAA

TGCTAAAATG CTGGCACCCT AAAGCAGAAA TGCGCCCATC CTTTTCTGAA

TGCTAAAATG CTGGCACCCT AAAGCAGAAA TGCGCCCATC CTTTTCTGAA

TGCTAAAATG CTGGCACCCT AAAGCTGAAA TGCGCCCATC CTTTTCCGAA

TGCTAAAATG CTGGCACCCT AAAGCCGAAA TGCGCCCATC CTTTTCTGAA

TGCTAAAATG CTGGCACCCC AAAGCAGAAA TGCGCCCATC GTTTTCTGAA

TGCTAAAATG CTGGCACCCC AAAGCGGAAA TGCGCCCGTC CTTTTCCGAA

TGCTAAAGTG CTGGCACCCG AAAGCCGAAA TGCGCCCGTC CTTCTCAGAA

TGCTGAAATG CTGGCACCCT AAAGCTGAAC TGCGCCCATC CTTTTCTGAA

TGCTAAAATG CTGGCACCCT AGAGCTGAAC TGCGCCCATC TTTTTCTGAA

TGCTAAAATG CTGGCACCCG AAAGCTGAGC AGCGCCCTTC TTTTGCTGAG

TGTTAAAATG CTGGCACCCT AAAGCTGAAC TGCGCCCATC CTTCTCTGAA

TGCTAAAATG CTGGCACCCT AAAGCCGAAA TGCGCCCATC CTTTTCTGAA

TGCTAAAATG CTGGCATCCT AAAGCTGAAA TGCGTCCATC CTTTTCTGAA

TGCTTAAATG CTGGCACCCC AAGGCCGAAA TGCGCCCGTC CTTTACTGAG

TGCTAAAGTG CTGGCACCCC AAAGCTGAAA TGCGTCCCTC CTTCTCTGAA

TGCTAAAATG CTGGCATCCC AACGCTGAAA TGCGCCCGTC CTTCTCGGAA

CTGGTGTCCC GGATATCATC TTCTCTACTT TCATTGGGGA GCACTATGTC

CTGGTGTCCC GGATATCATC TTCTCTACTT TCATTGGGGA GCACTATGTC

CTGGTGTCCC GGATATCATC TTCTCTACTT TCATTGGGGA GCACTACGTC

CTGGTGTCCC GGATATCATC TTCTCTACTT TCATTGGGGA GCACTACGTC

CTCGTGTCCC GGATATCATC TTCTCTACTT TCATCGGGGA GCACTATGTC

CTGGTCTCTA GGATATCATC TTCTCTACTT TCATCGGAGA ACACTACGTC

CTGGTCTCCA GGATATCATC TTCTCCACTT TCATTGGCGA GCACTATGTC

CTGGTCTCCA GGATATCATC TTCTCCACGT TCATTGGGGA ACACTACGTC

CTGGTCTCCA GGATATCATC TTCTCTACTT TCATTGGGGA GCACTATGTC

CTGGTCTCCA GGATATCATA TTCTCTACTT TCATTGGGGA GCACTATGTC

CTGGTCTCCA GGATATCATA TTCTCTACTT TCATTGGGGA GCACTATGTC

CTGGTCTCCA GGATATCATA TTCTCTGCTT TCATCGGGGA GCATTATGTC

CTGGTCTCCA GGATATCATA TTCTCCACTT TCATCGGGGA ACATTATGTC

CTCGTCTCCA GGATATCATA TTCTCTACTT TCATCGGGGA ACACTACGTC

CTGGTGTCGC GGATATCATA TTCTCTACTT TCATCGGGGA GCACTATGTC

CTGGTCTCCC GGATCTCATA TTCTCCACAT TCATCGGGGA GCACTATGTC

TTGGTGTCAA AGATATCATA TTCTCTACAT TCATTGGAGA GCATTATGTT

CTGGTTTCAA AGATAGCATC TTCTCCACTT TCATCGGCGA GCATTACGTC

CATGTGAACG CTACTTATGT GAACGTAAAA TGTGTCGCTC CGTATCCTTC

CATGTGAACG CTACTTATGT GAACGTAAAA TGTGTCGCTC CATATCCTTC

CATGTAAACG CTACTTATGT GAACGTAAAA TGTGTCGCTC CATATCCTTC

CATGTAAACG CTACTTATGT GAACGTAAAA TGTGTCGCTC CATATCCTTC

CATGTGAACG CTACTTATGT GAACGTAAAA TGTGTCGCTC CATATCCTTC

CATGTGAATG CTACTTATGT GAATGTGAAA TGTGTTGCTC CATATCCCTC

CATGTGAACG CTACTTATGT GAATGTAAAA TGTGTTGCTC CATATCCTTC

CACGTGAACG CTACTTATGT GAATGTAAAA TGTGTTGCTC CATATCCTTC

CACGTGAATG CGACTTACGT GAACGTGAAA TGTGTGGCTC CCTATCCTTC

CATGTGAATG CTACTTACGT GAACGTCAAA TGTGTCGCTC CATATCCTTC

CATGTGAACG CCACTTATGT GAATGTCAAA TGTGTTGCTC CATACCCTTC

CACGTGAATG CCACCTATGT GAACGTCAAA TGTGTCGCTC CATACCCTTG

CATGTGAACA CTACTTATGT GAATGTCAAG TGTGTCGCTC CCTATCCTTC

CACGTGAATG CTACTTATGT GAATGTAAAA TGCATCGCTC CGTATCCTTC

CACGTGAACA CTACTTACGT GAATGTGAAA TGTGTCGCTC CATATCCTTC

CACGTGAACG CTACATACGT GAATGTCAAG TGTGTCGCTC CATACCCTTC

CACGTGAATG CAACCTACGT CAATGTGAAG TGTGTCGCTC CCTACCCTTC

CACGTGAACG CCACCTACGT CAACGTCAAG TGCGTGGCGC CCTACCCATC

TCTGTTGTCA GAAGATAATT CTGTGGAATT TTGTGCTTGC TACTGTATAG

TCTGTTGTCA GAAGATAATT CTGTGGAATT TTGTGCTTAC TACTGTATAG

TCTGTTGTCA GAAGATAATT CTGTGGAATT TTGTGCTTAC TACTGTATAG

TCTGTTGTCA GAAGATAATT CTGTGGAATT TTGTGCTTAC TACTGTATAG

TCTGTTGTCA CAAGATAACT CTGTGGAATT CCGTGCTTAC TACTGTCTAG

TCTGCTGTCA CAAGATAATT GGGTGGAATT TTGTGCTTAC TACTGTAGAG

TCTGTTGCCA CAAGACAACT CTGTGGAATT TTGTGCTTAC TACTGTATAG

TCTGTTGCCA CAAGACAACT CTGTGGAATT TTGTGCTTAC TACTGTATAG

TCTGCTGTCA CAGGATAATC CTGTGGAATT TTGTGCTTAC TACTGTATAG

TCTGTTGTCA CAAGATAATT CTGTGGGATT TTGTGCTTAC TACTGTATAA

TCTCTTGTCA CAAGATAATT CTGTGGGATT TTGTGCTTAC TACTGTATAG

TCTGTTGTCA CAAGATCATT CCGTGAGAGT TTGTGCTTAC TACTGTATAG

TCTTTTGTCC CAAGGTAATT CTGTGATATT TTGTGCTTAC TACTGTATAG

TCTCTTGTCA CAAGATGATT CTGTGGAATT TTGTGCTTAC TACTGTAGAA

TCTCTTGTCG CAAGATAGTT CTGTGGAATT TTGTGCTTAC TACTGTATAG

GCTCTTGTCA CACGACAC-- ---------- ---------- ----------

CCTTTTGTCA CAAGACAATT CTGTGGAATC TTGTGCCTAC TACTGTATAG

CCTCCTGTCC CAGGACGCTT CCTGGGGATT CTGTGCTGAC TACTGTAAAG

TGCATGTGGT GTAGGTTACT CTAACTGGTT TTGTACGTAA ACATTTAAAG

TGCATGTGGT GTAGGTTACT CTAACTGGTT TTGTATGTAA ACATTTAAAG

TGCATGTGGT GTAGGTTACT CTAACTGGTT TTGTATGTAA ACATTTAAAG

TGCATGTGGT ATAGGTTACT CTAACTGTTT TTGTATGTAA ACATTTAAAG

TGCATGTGGC GTAGGCTACT CTAACTGGTT TTGTATGTAA ACATTTAGAG

TGCATGTGGT GTAGCTTACT CTAACTGGTT TTGTATGTAA ACATTCAAAG

TGCATGTGGC TTAGGTTGCT GTAGCTGGTT TTGTGTGTAA ACATTGAAAG

TGCATGTGGC TTAGGTTACC CTAGCTGGTT TTGTGTGTAA ACATTGAAAG

TGCATGTGGC ATAGGTTACC CTAACTGGTT TTGTATGTAA ACATTGAAAG

TGCATGTGGC ACAGGATACT CAAACTGGTT TTGTATGTAA ACATTTAAAG

TGCATGTGGC ACAGGATACT ----CTGTTT TTGTATGTAA ACACTTAAAG

TGCACGGGGC ACAGAATACT CTAAGTGA-T TTGTATATGA ACatttaaag

TGCATGAGGC ACAGGATAAT GTAACTGGTT TTGTATGT-A ACatttaaag

TGCACGTGGC TTAGGATACT CGGACCGGTT TTGTATTTCA ACATTTAAAG

TGCATGTGGC ATGGGATACT CTACCTGGTT TTGTATGTAA ACatttaaag

---------- ---------- ---------- ---------- ----------

TGCATGTGGG ATAGGATATT ATAACTGGTT TTATATGTAA ACACTTAAAG

TGCATGTGGT GTAGGATATT ATCACAGGTT TTGTGTGTCG ACGCTTAAAG

TGttatattt ttataaaaat gtttattttt aatGATATGA GAAATTGTTA

TGttatattt ttataaaaat gtttattttt aatGATATGA GAAATTGTTA

TGttatattt ttataaaaat gtttattttt aatGACATGA GAAATTGTTA

TGttatattt ttataaaaat gtttattttt aatGACATGA GAAATTGTTA

TGttatattt ttataaaaat gtttattttt aatGATATGA GAAATTGTTA

TGttatattt ttataaaaat gtttattttt aatgatataa gaaattGTTA

TATTATATTT TTATAGAAAT GTTTATTTTT AATGATGTAT GAAATTGTTA

TATTATATTT TTATAGAAAT GTTTATTTTT AATGATGTAA GAAATCCTTA

tattatattt ttataaaaat gtttattttt aatgatataa gaaattGCTA

TATTATATTT TTATAAAAAG GTTTATTTTT AATGATGTGA GAAATTGTTA

tattatattt ttataaaaat gtttattttt aatGATATAA GAAATTGTTA

tattatattt ttataaaaat gtttattttt aatGATACAA GATATTGTTA

ttttatattt ttataaaaat gtttattttt aatGATATGA AAAATTGTTA

TGttatattt ttataaaaat gtttattttt aatGATAGAA GCAATTGTTA

tattatattt ttataaaaat gtttattttt aatGATATGA ACAATTGTTA

---------- ---------- ---------- ---------- ----------

tattatattt ttataaaaat gtttattttt aatGACATAC ACCATTGTTA

tattatattt ttataaaaat gtttattttt aatGACGTGT ATAATTGTTG

GGCCACAAAA CACTGCACTG TGAACATTTT AAAAAGGTAT TCAGACTGGG

GGCCACAAAA CACTGCACTG TGAACATTTT AAAAAGGTTT TCAGACTGGG

GGCCACAAAA CACTGCACTG TGAATATTTG AAAAAGGTAT TCAGACTGGG

GGCCACAAAA CACTGCACTG TGAATATTTG AAAAAGGTAT TCAGACTGGG

GGCCACAAAA CACTGCACTG TGAACATTTT AAAAAGGTAT TCAGACTGGG

GGCTACAAAA TACTGCACTG TGAACATTTC AAAAAGGTAT TCAGACGAAG

GGCCGCAGAA T--------- ---------- AGGGAGGTAT TGAGACTGGG

GGCCGCAGAA TCCTGCACTG TGAGCATTTC AAAGAGGTAT TGAGACCCAG

GGCCACAAAA TACTGCACTG TGAACATTTC AAGAAGGTAT TCAGACTCAG

GGCCACAAAA TACTGCACTG TGAACATTTC AAAAAGGTAT TCGAACTTGG

GGCCACGAAA TACTGCACTG TGAACATTTC AACAAGGTAT TCAGACTTGG

GGCCATAAAA TACTGCACTG TGAACATTTC AAAAAGGTAT TCAGACTTGG

GGCCACAAAA CACTGCACTG TGAACATTTC AAAAAGGAAT TCAGGCTTGG

GGCCACAAAA TACTGCACTG TGAACATTTC AAAAAGATAT TGTGACTTGG

GGCCACAAAA TACAGCACTG TGAACATTTC AAAAAGGTAT TCAGAGTTGG

---------- ---------- ---------- ---------- ----------

GGCCATGAAA AACTACACTG TGAACATTTC AAAAAGGTAT TCAGACTTGG

GGCCACAAAA TACTGCACTG TGAACATTTC CAAAGGTTCT TCGGACTCGG

ATTTGTAAAT TGCGATAAGG TTGCCAATAC ACCCCACCCT CATTACATCA

ATTTGTAAAT TGCGATAAGG TTGCCAATAC ACCCCACCCT CATTACATCA

ATTTGTAAAT TGTGATAAGG TTGCCAATAC ACCCCACCCT CATTACATCA

ATTTGTAAAT TGTGATAAGG TTGCCAATAC ACCCCACCCT CATTACATCA

ATTTGTAAAT TGTGATAAGG TTGCCAATAC ACCCCACCCT CATTACATCA

ATTTGTAAAT TGTGATAAGG TTGCCAGTAC ACCCCACCCT CATTACATCA

TTCTGTAAGT TATGATAAGG TTGCCAGTAC ACCCCACCCT CATTACGTCA

TTCTGTAAGT TATGATAAGG TTGCCAGTGC ACTCCACCCT CATTACGTCA

ATTTGTAAAT AGTGATAAGG TTGCCAGTAC ACCCCACCCT TATTACATCA

ATTTGTGAAT AGTGATAAGG CTGCCAGAAC TCCCCACCCT CATTACATCA

ATTTGTAAAT AGTGATAAGG TTGCCAGTAC ACCCCACCCT CATTACATCA

ATTTGTAAAT AGTGATAAGG TTGTCAGTAC ACCACACCCT CATTACATCA

ATTTATAAAT AGTGATAAGG TAGCCCGTAT ACCCCACCCT -ATTACATCA

ATTTGTAAAT AGTGATAAGG TTGCCAGTTC ACCCCACCCT CATTACATCA

ATTTGTACAT TGTGATAAGG TTGCCAGTAC ACTCCACCCT CATTACACCA

---------- ---------- ---------- ---------- ----------

ATTTGTAAAT AGTGATAAGG TTGCCAGTAC ACCCCACCCT CATGACATCA

ATTTGTTGAT CGCAATAAAG CGGCCAATAC CCTCCCCCTC CACTGGTTGC

TCAGGACTTG AAGCCAAGGG TTAACCCAGC AAGCTATGGG AGTAAGTGAT

TCAGGACTTG AAGCCAAGGG TTAACCCAGC AAGCTATGGG AGTAAGTGAT

CCAGGACTTG AAGCCAAGGG TTAACCCAAC AAGCTATGGG AGTAAGTGAT

CCAGGACTTG AAGCCAAGGG TTAACCCAAC AAGCTATGGG AGTAAGTGAT

CCAGGATTTG AAGCCAAGGG TTAACCCAGC AAGCTATGGG AGTAAGTGAT

CCAGGACCTG AAGCCAAGGG TTAACACAGC AAGC-AAGGG AGTAAGTGAT

CCGGGACTTG GAGCCAAGGG TTAATAGAGC AAGCTATGAG AGGAAGTCAT

CCAGGACTTG AAGCCAAGGG TTAAGAGGGC AAGCTATAAG AGTAAGTCAT

CCAGGACTTG AAGCCAAGGG TTAACATAGC AAGCTATGGA AGTAAGTGAC

CCAGGACTTG AAGCCAAGGG TTAACACAGC AAGCTATGGG AGTAAGTGAT

CCAGGACTTG AAGCCAAGGG TTAACACAGC AAGCTATGAG AGTAAGTGGT

-CAGGACTCG AAGCCAGGGG TTAACACA-C AAGCTATG-- --TGAGTGAA

CCAGGACTTA AAGCCAAGGG TTAACATAGC AAGCTATGGG AGTAAGTGAT

CCATGACTTG AAGCCAGAGG TTAATGCAGC AAGCTATGGG AATAAGTGAT

CCATGACTTG AAGCCCAGGG TTAACACAGC AAGCTATGGG AGTCAGTGAT

---------- ---------- ---------- ------TGGG AGTAAGTGAT

CCTTCACTTG AGGCCAAGGG TTAGCATAGC AAGCCATAAG AGTAAGTGAG

CCCTGGCTTG AACCCAAGGA TTAACTCCAC GAGCAATAAG AGTATGTGAT

TCTTCTAAGA ATTAGATACT TGTCATGCCT ATACCTGCAG CTGAACTGAA

TCTTCTAAGA ATTAGATACT TGTCATGCCT ATACCTGCAG CTGAACTGAA

TCTTCTAAGA ATTAGATACT TATCATGCCT ATACCTGCAG CTGAACTGAA

TCTTCTAAGA ATTAGATACT TATCATGCCT ATACCTGCAG CTGAACTGAA

TATTCTAAGA ATTAGATACT TGTCATGCCT ATACCTGCAG CTGAACTGAA

TCTTCTAGGA ATTAGATACT TGTCATGCCT ATAGCTGCAG TTGAGCTGAA

TCTCCTA--- ---AGATACT TGTCATGCCT TTACCTGCAC TTGAGCTGAA

TTTCCTAAGA ATTAGATATT TGTCATGCCT ATACCTGCAG TTGAGCTGAA

TCTTCCG--A ACTAGATGTT TGTCATGCCT ATACTTGCAG TTGAGCTGAA

TCTTCTAAGA ATTAGATACT TGTCATGCCT ATACCTGCAG TTGAGCTGAA

TCTTCTAAGA ATTAGATACT TGTCATGCCT ATACCTGCAG TTGAGCTGAA

GCCTCTACGA ATTAGATACT TGTCAAGCCC ATTCCTGCAA TTGAGCTGAA

TCTTCTAAGA ATTAGATACT TGTCATGCCT ATACCTGCAG TTGAACTGAA

TCTGCTAAGA ATTAGATACT TGTCATGCCT ATACCTGCAA TTGAGCTGAA

TCTTCTAAGA ATTAGCTACT TGTCATGCCT ATACCTGCAG TTGAGCTGAA

ACTTCTAAGA ATTAGCTACT TGTCGTGCCT GGACCTGCAG TGGAGCTGAA

TATTGTAAGA ATTTGATACT TTCCATGCCT ATGCCTGCAG ACGATCTGAA

TCTTGCCAGA ATTAAATGCT TGTCTTGTCT ATACCTGCAA ATGAGCTGAA

TGGTACTTCG TATGTTAATA GTTGTTCTGA TAAATCATAA GTGATCAACA

TGGTACTTTG TATGTTAATA GTTGTTCTGA TAAATCATAA GTGATCAACA

TGGTACTTTG TATGTTAATA GTTGTTCTGA TAAATCATAA GTGATCAACA

TGGTACTTTG TATGTTAATA GTTGTTCTGA TAAATCATAA GTGATCAACA

TGGTACTTTG TATGTTAATA GTTGTTCTGA TAAATCATAA GTGATCAACA

TGGTACTTTG TATGTTAATA CTTGTTCTGA GAAATCATAA GTCATTAACA

TGGTACTCCG TGTGTTAATA ATT---CTGG TAAATCATAA ATGACTAACA

TGGTACTCCA TGTGTTAATA ATT---CTGA TGAATCATAA GTGACTAACA

TGGTACTTTG TATGTTAATA GTTGTCCTGA TAAATCTTAA GTAATTAACA

TGGTACTTTG TATGTTAATA GTTGTTCGGA TAAATCATAA GTGATTATCA

TGGTACTTTG TATGTTAATA GTTGTTTTGA TAAATCATAA GTAATTATCA

TGGTACTTTG TATGTTAATA GTTGTTCTGA TCAATCAAAA ATAAATGTCA

TGGTACTTTG TATGTTAATA GTTGTTCTTA TCAATCATAA TTGTT---TA

TGGTACTTTG TATATTAATA GTTGTTCTGA TAAATCATAA GTGGTTAACA

TGGTACTTTG TATGTTAATA GTTGTTCTGA TAAATCATAA GTGATTAACA

TGGTACTTTG TATGTGAATA GTTGTTCTTA TAAACCATAA GTGATTAACA

TGGTACTTTG TATGTTTATA GTTGTTCTGA TAAATCATAA GAAATTAACA

TGGTACTCTG AATGTTTAGA TTTGGTTGGA TAAATCATAA GTGGTGAATA

TCTTGTATAC TGATAGTGGT TATTGCCTAT CTCAATTCCT TATCAAAAAC

TCTTGTATAC TGATAGTGGT TATTGCCTAT CTCAATTCCT TATCAAAAAC

TCTTGTATAC TGATAGTGGT TATTGCCTAT CTCAATTCCT TATCAAAAAC

TCTTGTATAC TGATAGTGGT TATTGCCTAT CTCAATTCCT TATCAAAAAC

TCTTGTATAC TGATAGTGGT TATTGCCTAT CTCAATTCCT TATCAAAAAC

TCTTGTGTAC AGAGAGTGAT TATTGCTCAT TTCAATTC-- TATCAAAAAT

TCTTGTATAC TGTGAGTGAC TGTAGCTTAT CTTAATTCCC TATCACAAAC

TCTTGTATAC TGTGAGTGGC TGTAGCTTAT CTTAATTCCC TATCACAAAC

TCTTGTATAC TGAGAGTTGT TATTGCTTGT CTCAATTCCC TATCACACAT

TCTTGTATAC TGAAAGTGGT TATTGTTTAT CTCAATTCCC TATCAAAAAC

TCTTGT-TCC TGAGAGTGGT TATTGCTTAT CTCGAGTCCC TATCAAAAAG

TCCTGTGTGC TGAGAGTGGT TATTGCTTAT TTCAATTCCT TATCAAAAAC

TCTTGTATCT TGAAAGTGGT TATTGCTTAT TTCAATTGCC ---CAAAAAC

TCTTGCATAC TGAAAGCAGT TATTGTTtat ctcagttccc TGTCAAAAAC

TCTTGTATAC TAAGAGCAGT TGTCGTTTAT CTCAATACCC TATCAAAAAC

TCTTGTAGCC TGAGAAC-GT TGTTGGTTAC CTCAAGCCGC TATCAAAAAC

TCCTATGTCT TCAGAGTGGT CCTTGCTTAT TTTAAATACT TTTTTGAAAG

TCATTCTTAC TGACACTGTT TATTGTGTAT CTTAAATGCC TATCAGAACC

TTCAGTCATA AACCAATGAA ATATTTCAGT TGGTGAATTG AAGGTAATAA

TTCAGTCATA AACCAATGAA ATATTTCAGT TGGTGAATTG AAGATAATAA

TTCAGTCATA AACCAATGAA ATATTTCAGT TGGTGAATTG AAGGTAATAA

TTCAGTCATA AACCAATGAA ATATTTCAGT TGGTGAATTG AAGGTAATAA

TTCAGTCATA AACCTATGAA ATATTTCAGT TGGTGAATTG AAGGTAATAA

TTCAGTCATC AACTGGTGAA ATATTTCAGT CGGTGAATTG AAGGTAATAA

TTCAGTCAT- AGCCAGTGAA ATATTTCAGT TGGTGAATTG AAGGTAATAA

TTCAGTCAT- AGCCAGTGAA ATATTTCAGC TGGTGAATTG AAGGTAATAA

GTCAGTCATA AACTAATGAA ATATTTCAGT TGGTGAATGG AAGGTAATAA

TTCAGTCATA AACCAGTGAA ATATTTCAGT TGGTGAATTG AAGGTAATAA

TTCGGTCATA AACCAGTGAA ATATTTCAGT TGGCGAAGTG AAGGTAATAA

CTCAGTCATA AACCAGTGAA ATATTTCAGT TGGTGAGCTG AAGGTAATAA

TTCAGTCATA AACCAGTGAA ATATTTCAGT TGGTGAATTG AAGGTAATAA

TTTAGTCATA AACCAGTGAG ATCTTTCAAT TGGTAAATTG ATGGCAATAA

TTCAGTCATA AACCAGTGAA ACGTTTCACT TGGTGAATTG AAGGTCATAA

TTCTGTCATA ATCCAGTGAA ATATTTCAGT TGGTGAATTG AAGGTAATAA

TTCAGTCATA AACCAGGGAA ATATTTCAGT TGGAGAATTG ACAGTAATAA

TTCAGTCATA AACCTGTGAA ATATTTCAGT TGGAGAATTG AGGGTAATAA

AATGGATGCT TGCCCAAACT GTCCTTTGTT GTAAATTATT CCAGAGAACT

AATGGATGCT TGCCCAAACT GTCCTTTGTT GTAAATTATT CCAGAGAACT

AATGGATGGT TGCCCAAACT GTCCTTTGCT GTAAATTATT CCAGAGATCT

AATGGATGGT TGCCCAAACT GTCCTTTGCT GTAAATTATT CCAGAGATCT

AACAGATGCT TGCCCAAACT GTCCTTTGCT GTAAATTATT CCAGAGATCT

AACAGAGGCT TGCCCAAACT GTCCTTTGTT GTAAATTATA CCAGAGGTCT

AACAGAGGC- TGCCCAAACT GTCCTTTGTT GTAAATCACG GCAAAGGTCT

AACAGAGGC- TGCCCAAACT GTCCTTTGTT GTAAATCACA GCAAAGGTCT

AATAGAGCCT TGCCCAAACT GTCCTTTGCA GTAAATTACA CCAGAGGTCT

AACAGAGGCT TGCCCAAACT GTCCTTTGTT GTAAATTACA CCAGAGGTCT

AACAGAGGCT CGTCCAAACT GTCCTTTGTA GTAAATTACA CCCGAGGCCT

AACAGAGCCT TACCCAAACT GTCCTTTGTT GTAAATTACA CCAGAGATCT

AACAGAGACT TGCTCAAACT GTCTTTTGTT GTAAATTATA CTAGAGGTCC

AACAGAGGCT TGCCCAAACT GTCCTTTGTT GTAAATTACA CCAGAGGTCT

AACAGAGGCT TGCCCATACT GTCTTTTGTT GTAAATTACA CCAGAGGTCT

AACAGAGGCT TGCCCAAACT GTCCTTTGTT GTAAATTATA CCAGAGGTCT

AACAGAGACT TGCCCCAACT GTCCTTTGTT GTACATTATA CCAGAGGTCT

AATAGAGGCC CGCACAAACT GTCCTTTGTT GTAAATTACA GCAGAGGTCC

AGATTACGTC AGCCAAAGAT ATTGATCACA ATGCTGAGAG GTTTATAACT

AGATTACGTC AGCCAAAGAT ATTGATCACA ATGCTGAGAG GTTTATAACT

AGATTACGTC AGCCAAAGAT ATTGATCACA ATGCTGAGAG GTTTATAACT

AGATTACGTC AGCCAAAGAT ATTGATCACA ATGCTGAGAG GTTTATAACT

AGATTACGTC AGCCAAAGAT ATTGATCACA ATGCTGAGAG GTTTATAACT

AGATTACGTC AGCCAAAGAT ATTGATCACA ACGCTGAGAG GTTTATAACT

AGATTACATC AGCCAAAGGT ATTGATCACA ACGCTGAGAG GTTTATAACT

AGATTACATC AGCCAGAGGT ATTGATCACA ATGCTGAGAG GTTTATAACT

CGATTACATC AGCCAAACGT ATTGATCACA ATGCTGAGAG GTTTATAACT

AGATTACGTC AGCCAAAGGT ATTGATCACA ATGCTGAGAG GTTTATAACT

AGATTACGTC AGCCTAAGGT ATTGATCACG ACGCTGAGAG GTTTATAACT

AGATTACGTC AGCCAAAGGA ATTGATCACA ATGCTGAGAG GTTTATAACT

AGATTACGTC AGCG-AAGGT ATTGATCACA ATGCTGAGAG GTTTATAACT

AGATTACGTC AGCCAAAGGT ATTGATCACA ACGCTGAGAG GTTTATAACT

AGATTACGTC AGCCAAAGGT ATTGATCCCA ACGTTGAGAG TTTTATAACT

AGATTACGTC AGCCAAAGGT ATTGATCTCG ACGCTGAGAG GTTTATAACT

AGATTACGTC AGCCAAAGAT ATTGATCACA TTGCTGAGAG GTTTATAACT

AGATTACGTC AGCCAAAGGT ATTGATCAAG TCCCTGAGAG GTTTATAACA

ACTATTAACT TACCATGTGA CCCCAGGGCA GAAATCTAGG AAAATTGCAA

ACTATTGACT TACCATGTGA CCCCAGGGCA GAAATCTAGG AAAATTGCAA

ACTATTGACT TACTATGTGA CCCCGGGGCA GAAATCTAGG AAAATTGCAA

ACTATTGACT TACTATGTGA CCCCGGGGCA GAAATCTAGG AAAATTGCAA

GCTATTGACT TACCATGTGA CCCTGGGGCA GAAATCTAGG AAAATTGCAA

ACTATTGACT TACCATGTGA CCCTGGAGCA GAAATCTAGG AAAATTGCAA

ACTATTGACT TACCATGTGA CCCTGGAACA GAAATCTAGG AAAATTGCAA

GCTATTGACT TGCCATGTGA CCCTGGAGCA GAAATCTAGG AAAACTGCAA

ACTCTTGACT TACTGTGTGA -CCTGGAGCA GAAATCCAAG AAAATCACAA

ACTATTGACT TACCATGTGA CCCTGGAACA GAAATCCAGG AAAATTGCAA

ACTATTGACT TACCGTGCGA CCCGGGAGCA GAAATCTAGG AAAATTGCAA

ACTATTGACT TACCATATGA CCCTGGAGAA GAAACCGAGG AAAATTGCAA

GCTATTGACT TACCATGTGA CCCCGGAGCA GAAATCCAGG AAAATTGCAA

ACTATTGACT TACCATGTGA CCCTGGAGCA GAACTCTAGG AAAACTGCAA

ACTATTGACT TACCATGTGA CCCTGGAGCA GAAATCTAGG AAAATTGCAA

ACTATTGACT TACCATGTGA CCCTGGAGCA GAAATCCAGG AAAATTGCAG

ACTATTGACT TGTCATCTGA CCTGGGAGCA GAAACCAGGG AAAATTGCAA

GCTATTGACT TGCCATGTGC CCCAGGAGCA GAGAACCGGG AAAATTGCAG

AAGCATATCT ACATTCTTTT CACCGCATGA GCCCAGGCTG AAACATCCAC

AAGCATATCT ACATTCTTTT CACCGCATGA GCCCAGGCTA AAACATCCAC

AAGCATATCT ACATTCTTTT CACCACATGA GTCCAGGCTG AAACATCCAC

AAGCATATCT ACATTCTTTT CACCACATGA GTCCAGGCTG AAACATCCAC

AAGCATATCT ACATTC-TTT CAGCACATGA GTCCAGGCTG AAACATCCAC

AAGCATATCT ACGCTCTTTT CACCACATGA GTCCAGGCTG AAACATCCAC

AAGCATACCT ACATTCTTCG TGCCACA-GA GTCCAGGCTG ACACATCCAG

AAGCATACCT ACATTCTTCG TACCACA-GA GTCCAGGCTA AAACATCCAG

GAGTATCTCT T--------- ---CACA-TG ATCCAGGCGG AAACATCCAC

AAGCATATCT ACGTTCTTTT CACCACATGA GTCCAGGCTG AAACATCCAC

AAGCATATCT CCATTCTTTT CACCACATGA GTCCAGGCTA AAACATCCAC

AAGCATATCT ACATTCTTTT CACCACATGA GTCCAGGCTG AAACATCCAC

AAGCATATCT ACATTCTTCT CACCACACGA GTCCAGGCTG AAAGAGCTAC

AAGCATATCT ACATTCTTTG TACCACATGA ATCCAGGCTG AAACATTGAC

AAGCATATCT ACATTCTTTT TACCACATGA GTCCAGGCTG AAACATCCAC

AAGCAGATCT A--------- ---------- ---------- ----------

AAGCATATCT ACATTCTTTA CACTACAAGA GTCCAGGCTC AGGCATCTAC

AAGCATATCT ACTTTGTCTC CGTCCCAGGA GCCCAAGCCC AGACAGCTAC

TAATCCTTAG ACCTCTTGGC TGGAACGACA AAAATGCATA AAATCTTACC

TAATCCTTAG ACCTCTTGGC TGGAACGACA AAAATGCATA AAATCTTACC

TAATCCTTAG ACCTCTTGGC TGGAACGACA AAAATGCATA AAATCTTACC

TAATCCTTAG ACCTCTTGGC TGGAACGACA AAAATGCATA AAATCTTACC

TAATCCTTAG ACCTCTTGGC TGGAACGACA AAAATGCATA AAATCTTACC

TAATCCTTAG ACCTCTCGGC TGGAACGACA AAAATGCATA AAATCTTACC

TAATCCTTAG ACCACTTGGC TGGAACTATA AAAATGCATA AAATCTTACC

TAATCCTGAG ACCACTTGGC TGGAACTACA AAAATGCATA AAATCTTACC

TAATCCTTAG ACCTCTGGAG TGGAACGACA AAAATGCGTA AAATCTTCCC

TAATCCTTAG ACCTCTGGAC TGGAACGACA AAAATGCATA AAATCTTACC

TAATCCTTAG ACCTCTGGGT TGGAACGACA AAAATGCATA AAATCTTACC

TAATTCTTAG ACCTCTGGGC TGGAACGACA AAAATGCATA AAATCTTACC

TAATCCTTAG ACCTCTGGGT TGGAACGACA AAAATGCATA AAATCTTACC

TAATTCTCAG ACCTCTCAGC TGGAAAGACA AAAATGCACA AAATCTAACC

TAATCCTCAG ACCTCTCAGC TGGAATGACA AAAATGCATA AAATCTTACC

---------- ---------- ---------- ---------- ----------

TAATCCCTAG ACCTCTCAGC TGGAACGACA AAAATGCATA AAATCTTAAT

TAATCCCTAG ACCTCTCTCC TGGAACGACA AAAATGCATC AAATCTTCCT

AGGCCCTTTA AAAATCTTTG GCATCCAAAC ATGTCAGAAC TGTTTTTCGC

AGGCCCTTTA AAAATCTTTG GCATCCAAAC ATGTCAGAAC TGTTTTTCGC

AGGCCCTTTA AAAATCTTTG GCATCCAAAC ATGTCAGAAC TGTTTTTCGC

AGGCCCTTTA AAAATCTTTG GCATCCAAAC ATGTCAGAAC TGTTTTTCGC

AGGCCCTTTA AAAATCCTTG GCATCCAAAA ATGTCAGAAC TGTTTTTTGC

AGGCCCTTTA AAAATCTTTG GCATCCAAAC ATGTCAGAAC TGTTTTTCAC

AGGCCCTCAA AAAATTTTTG GCATCCAAAC ATGTCAGAAT TGTTTTT---

AGGCCCTCTA AAAATTTTTG GCATCCAAAT ATGTCAGAAC TGTTTTT---

AGGCCCTTTA AAAATCTTTG GCATCCAAAC ATGTCAGAAC AGTTTTTCAC

AGGCCCTTTA AAAATCTTTG GCATCCAAAC ATGTCAGAAC TGTTTTTCAC

AGGCCCTTTA AAAATCTTTG GCATCCAAAC ATGTCAGAAC TGTTTTTCAC

AGGCCCTTTA AAAATCTTTG GCATCCCAAC ATGTCAGAAC TCTTTTTCAC

AGGCCCTTTA AAAATCTTTG GCATCCAAAC ATGTCAGAAC TGTTTTTCAC

AGGCCCTTTA AAAATCTTTG GCATTCAAAC ATGTCAGAAC TGTTTTCCAC

AGGCCCTTTA GAAATCTTTG GCATCCAAAC ATGTCAGAAC TGTTTTTCAC

---------- ---------- ---------- ---------- ----------

CAACTTTTTA AAAATCTGTG GCATCCAAAC ATGTTGAAAC TGTTTCTCAA

TGGCTCTTTA AAAATCCGTG GCATCCAAAC ATGTCCAAAC CATTGCTCAC

TGTTTTTGAA AACAAATTGA ATTGAAAAAT AATAATGATA ACATAATGAT

TGTTTTTTAA AACAAATTGA ATTGAAAAAT AATAATGATA ACATAATGAT

TGTTTTTGAA AACAAATTTA A-TGAAAAAT AGTAATGACA ACATAATGAT

TGTTTTTGAA AACAAATTTA A-TGAAAAAT AGTAATGACA ACATAATGAT

TGTTTTTGAA AACAAATTTA ATTGAAAAAT AATAATGATA ACATAATGAT

TGTTTTCGAA AATGAATTTA ACCGAAAAAT CATAATGATG ACATAATGAT

--TTCTT-AA GATGAACTTA AT--AAAAAT AACAGTAATA AGATAATGAT

--GCCTT-AA GATGAACTTA AT--AAAAAT AACAATAATA ACGTAATGAT

TGTTTTTGAA AATGAACtta attaaaaaa- -----taata ttATAATGAT

TGTTTTTGCA AATGAACTTA ATTGAAAAAC AATAATGATA ATATAATGAT

TGTTTTTGAA AATGAACTTA ATTGAAAACT AATAATGATG ATCTAATGAT

TGTTTTTGAA AATGAACTTA ATTGAAAAAT AATCATGATG ATATAATGAC

TGTTTTTGAA AGTGAGTTTA ATTGACaaat aaaaataata atataatGAT

TGTTTTTGAA AATTAGCTTG ATTGGGTGat aataataata atataataaC

GATTTT---- AATAAACTTA ATT------- ---GATAATA ATATGATGAT

--TTTTTCAA AATAAACTTA ATtggatgat gacgatgatg atatgatgat

TGTTTTCAAA TATTACTTTA AATGTATAAT CATTATTATT ACACCATGAG

TGtgtcTGAG GGTAAACCCA AATGGGttat tattattata atataatGAT

CTAATAGCTA ATGCTCTCAG TCTTGCTTAT TGAACTGCTA TTGTTTTAGT

CTAATAGCTA ATGCTCTCAG TCTTGCTTAT TGAACTGCTA TTGGTTTAGT

CTAATAGCAA ATGCTCTCAG TCTTGCTTAA TGAACTGCTA TTGGTTTAGT

CTAATAGCAA ATGCTCTCAG TCTTGCTTAA TGAACTGCTA TTGGTTTAGT

CTAATAGCTA ATGCTCTCAG TCTTGCTTAA TGAACTGCTA TTGGTTTAGT

CTCATAGCTA ATGCTCCCAG TCTTGCTTAA TGAAATGCTG TTGGTTTAGT

CTCATAGCTA ATGCTCTCAG TCTCGCTTAA TGAAATGCTG TTAGTTTAGT

CCCATAGCTA ATGCTCTCAG TCTCACTTAA TGAGATGCTG TTAGTTTAGT

CTCATAGCTA ATGCTCTGAG TCTTGCTTAA TGAAATGCTG TTAGTTTAGT

CTCATAGCTA ATGCTCTCAG TCTTGCTTAA TGAAATGCTG TTAGTTTAGT

CTCATAGCTA ATGCTCTCAG TCTTGCTTAA TGAAATGCTG CTTGTTTAGT

CTCATAGCTA ATGCTCTCAG TCTTGCTTAA TGAAATGCTG TTAGTTTAGT

CTCATAGCTA ATGCTCTCAG TCTTGCTTAA TGAAATGGTG TTAGTTTAGT

CTCATAGCTA ATGCTCTCTG TCCTGCTTAA TGAAATGCTG TTAGTTTAAT

CTCATAGCTA ATGCCCTCAG TCTGACTTAA TGCAATGCTG TTAGTTTAGT

CTCATAGCTA ATGCGCTCCA TCTTACTTAA TGCATTGCTG TTAGTTTAGT

CTCATAGCTA ATGCTTTAAT ACTGGACTAA TGAAATGCTG CTGGTTTAGT

CTCATAGCTA ATGCTCCCAT CCTTGCTTAA TGAAATGCTG TTCGTTTAGT

AAACGAATGC TTAAACTGCA GTGTTTTTCC AACATAATAA ACTGCCCTTT

AAACGAATGC TTAAACTGCA GTGTTTTTCC AACATAATAA ACTGCCCTTT

AAACGAATGC TTACAGTACA GTGTTTTTCC AACATAATAA ACTGCTCTTT

AAACGAATGC TTACAGTACA GTGTTTTTCC AACATAATAA ACTGCTCTTT

AAACGAATGC TTAAAGTGCA GTGTTTTTCC AACATAATAA ACTGCTCTTT

AAACGAATCC TCATAGTGCA GTGTTTTTCC AACATAAAAA ATTGCTCTTT

AAATGAATGC TTAAAATGCT GTGTTTTTCC AGCACAATTC ACTGCTCTTT

AAATGAATGC TTAAAATGCC GTGTTTTTCC AGTTCAATTC ATTGCTCTTT

AAACGAAAAC TTAAAATGCA GTGTTTTTCC AGCATTATAA ATTGTTCTTT

AAACGGATGC TTATAATGCA GTATTTTTCC AACATAATAA ATTACTCTTT

AAACGAATGC TTAAAATGCA GTGTTTTTCC AACATAATAA ATTACTCTTT

AAACGAACAC TTAAAATGCA GTGTTTTTCC AACATAATAA ATTGCTCTTT

AAATGAATGC TTAAAAT--- ---------- ---------- ----------

AAACGAATAT TTATACTGCA GTGTTTTTCC AACATAATAA ATTGTTCTTT

AAATGAATGT TTAAAATGCA GTGTTTTTCC AACATAATCC ATTGCCCTTT

AAATGAGTGT TTCCAATGCA GTGTTTTTCC AACAGCTTAT ATCGCTCTTT

AAACTAATAT TTTAAATGCA GTGTTTTTCC AACATTATGA ATTACCCTTT

AAACTAACGT TGAAAATGCA ATGTTTTTCC AACATAATTA ATGACTCTTT

GTTCAAATCA GAAACTGATT TCACTCGATT TTCCCAAATC GGCCCTGCCA

GTTCAAATCA GAAACTGATT TCACTCGATT TTCCCAAATC GGCCCTGCCA

GTTCAAATCA GAAACTGATT TCAGTCGATT TTCCCAAATC GGCCCTGCCA

GTTCAAATCA GAAACTGATT TCAGTCAATT TTCCCAAATC GGCCCTGCCA

GTTCAAATCA GAAACTGATT TCACTCGATT TTCCCAAATC GGCCCTGCCA

GTTCAAATCA GAAACTGAAT TCCCTTTATT TTCCAAAATC GGCCCTGCCA

GTTTAAATCA AA-----ACT TCATTTTATT TTCCACAAGC AGCCCTGCCA

GTTTAAATCA AA-----ACT TCATTTTATT TTCCAAAAGC AACCCTGCCA

GTTCAAATCA GAAACTGAAT TCACTTTATT TTCCAAAATC GGCCCTGCCA

GTTCAAGTCA GAAACTGAAT TCACTTTATT TTCCAAAATC GGCTCTGCCA

GTTTAAATCA GAAACTGAAT TCATTTTATT TTCCAAAATC GGCCCTGCCA

GTTCAAATCA GAAACTGAAT TCACTTTATT TTCCAAAATC GGCCCTGCCA

---------- ---------- ---------- ---------- ----------

GTCCAATTCA GAAACTGAAT TCACTTTATT TTCCAAATTC AGCCCTGCCA

GTTCAATGCA GAAACTGAAT TCACTTTATT TTCCAAAATC AGCCCTGCCA

GTTCAATTCT GAAACTGGGT TCACTTTATT TTCCAAAATC AGCCCTGCCA

GTTCAGTCCA GCTGCTGAAT TCATTTTATT TTCCAAAATC TACTGTGCCA

GTTCAGTTCA GCAACTGGAT TTGTTTTATT TCCCCAAATC TGTTCTGCCA

GGTTTCCTTG ATTTAATTGT ATATTTTTTG TCTGCTGACA ATGGAAAGCA

GGTTTCCTTG ATTTAATTGT ATATTTTTTG TCTGCTGACA ATGGAAAGCA

GGTTTCCTTG ATTTAATTGT ATATTTTTTG TCTGCTGACA ATGGAAAGCA

GGTTTCCTTG ATTTAATTGT ATATTTTTTG TCTGCTGACA ATGGAAAGCA

GGTTTCCTTG ATTTAATTGT ATATTTTTTC TCTGTTGACA ATGGAAAGCA

GGTTTCCTTG ATTTAATTGT ATCTTTTTTG TCTGCTGACA ATGGAAAGCA

GGTTTCCTTG ATTTAATTGC ATCTTTTTTG TCTGATGACA ATGGAGAACA

GGTTTCCTTG ATTTAATTGT ATCTTTTTTG TCTGATGACA ATGGAGAACA

GGTTTCCTTG ATGTAATTGT ATCTTTTTTG TCTGCTGACA ATGGAAAGCA

GGTTTCCTTG ATTTAATTGT ATCTTTTTTG TCTGCTGACA ATGGAAAGCA

GGTTTCCTTG ATTTAATTGT ATCTTTTTCG TCTGCTGACA ATGGAAAGCA

GATTTCCTTG ATTTAATTGT ATCTCTTTTG TCTGCTGACA ATGGAAAGCA

---------- ---------- ---------- ---------- ----------

GGTTTCCTTC ATTTAATTGT ATCTTTTTTG TCTGCTGACA ATGGAAAGCA

GGTTTCCTTG ATTTAATTGT ATCTTTTTTG TCTGCTGACA ATGGAAAGCA

GGTTTCCTTG ATTTAATTGT ATCTCTTTTG TCTGCTGACA ATGGAAAGGA

GGCTTCCTTG ATTTAATTGT GTCTTTTTTA TCTGCTGACA ATGGAAAGCT

GGTTTCCTTG ATTTGATTGT GTCTTTATTA TCTGCTGACA ATGGAGAGTT

GAAGAAAAAG GAAATGAAAT ATTACCAAGA AAAACAGGCA TTCTCACACT

GAAGAAAAAG GAAATGAAAT ATTACCAAGA AAAACAGGCA TTCTCACACT

GAAGAAAAAG GAAATGAAAT ACTACCAAGA AAAACAGGCA TTCTCACACT

GAAGAAAAAG GAAATGAAAT ACTACCAAGA AAAACAGGCA TTCTCACACT

GAAGAAAAAG GAAATGAAAT ATTACCAAGA AAAACAGGCA TTCTCACACT

GAAGAAAAAG GAAATGAAAT ATTACCAAGA AAAACAGGCA TTTTCACACT

GAAGAAAAAG GAAATGAAGT ATTACCAAAC ACAACAGTCA TTCTCCCACT

GAAGAAAAAG GAAATGAAAT ATTACCAAAA ACAACAAGCA TTCTCACACT

GAAGAAAAAG GAAATGAAAT ATTACCAAGA AAAACAGGCA TTCTCACACT

GAAGAAAAAG GAAATGAAAT ATGACCAAGA AAAACAGGCA TTCTCACACT

GAAGAAAAAG GAAATGAAAT ATTACCAAGA AAAACAGGCA TTCTCACACT

GAAGAAAAAG GAAATGAAAT ATGACCAAGA AAAACAGGCA TTCTCACACT

---------- ---------- ---------- ---------- ----------

GAAGAAAAAG GAAATGAAAT ATTACCAAGA AAAACAGACA TTCTCACACT

GAAGAAAAAG GAAATGAATT ATTACCAAGA AAAACAGGCA TTCTCACACT

GAAGAAAAAG GAAATGAAAT ATTACCAAGA AAAACAGATA CGCTCCCACT

GAAGAAAAAG GAAACGAAAT ATTACCAAGA ACAACAGGCA TTCTCACACT

TTGAAAAAAG GAAATGAAAT ATTAGTGGGG AAAATAGCCA TTCTAGAACT

GTGTGATCAA TCTACATGGC CCAAAATGGA AAGAAATTAA CTAGTTCATA

GTGTGATCAA TCTACATGGC CCAAAATGGA AAGAAATTAA CTAGTTCATA

GTATGATCAA TCTACATGGC CCAAAATGGA AAGAAATTAA CTAGTTCATA

GTATGATCAA TCTACATGGC CCAAAATGGA AAGAAATTAA CTAGTTCATA

ATATGATCAA TCTACATGGC CCAAAATGGA AAGAAATTAA CTAGTTCATA

GTATGATCAA TCTACATAAC CCAAAATGGA AAGAAATTAA CTAGTTCATA

ATAGGATCAA TCCGTATGGC CCAAAGTGGA AAGAAATTAA CCAGTTCATA

ATAGGATCAA TCCGTACGGC CCAAAGTGGA AAGAAATTAA CCGGTTCATA

GTATGATCAA TCTGCATGGC CCCATATGGA AAGAAATTAA CTAGTTCATA

ATATGATCAA TCTACACAGC CCAAAATGGA AAGAAATTAA CCAGTTCACA

GTATGATCAA TCTACATGCC CCAAAATGGA AAGAAATTAA CTAGTTCATA

GTATGATCAA TCTACATGGC CCAAAATGGG AAGAAATTAA CTAGTTCATA

---------- ---------- ---------- ---------- ----------

ATGTGATCAC TCTACATGGC CCAAAATGGA ATGAAGTTAA CTAGTTCATA

GTATGATCAA TCTACACAGC CCAAAATGGA AAGAAATTAA CTAGTTCATA

CTATGACCAA TCTACATGGC CCCAAATGGA AAGAAATTCA CTAGTTCATA

GTAAGATAAA GCTACTTGTC TTAAAATGGA AGGAGATCAA CCACTGAAGA

GTATGAATAA TCTACAGGTC TTGAAGTTTA TAAACACCAA GTCTCTCACA

AGGGTATTAA TTAGCATTAC TTTAGAATAA TGAAGCCTGT TCCATCCATT

AGGGTATTAA TTAGCATTAC TTTAGAATAA TGAAGCCTGT TCCATCCATT

AGGGTATTAA TTAGTGTTAC TTTAGAATAA TGAAGCCTGT TCCATCCATT

AGGGTATTAA TTAGTGTTAC TTTAGAATAA TGAAGCCTGT TGCATCCATT

AGGGTATTAA TCAGTGTTAC TTTAGAATAA TGAAGCCTGT TCTATCCATT

AGGGTATTAA TTAGTGTTAC TTTCGCATAA TGAAGACTAT TGCATCCATT

-GGGTATCAA TTAGTGTTCC TTTAGAATAA TGAAGCCTGC TCCATCCATT

AGGGTATCAA TTAGTGTTAC TTTAGAATAA TGAAGCCTGC TCCATCCATT

AGGGTATTAA TTAGTGCTAC TTTAGAATAA TGAAGCCTGT TCTATCCATT

AGGGTATTAA TTAGTGTTAC TTTAGAATAA TGAAGCCTGT TCAATCCATT

AGGGTATCC- CTGGGGCTGC TGCAGAAGCA CGACGCCTGT TCTACCCATT

AGGATATTAA TTAGTGTTAC TTTAGAATAA TGAAGCCTGT TCTATCCATT

------TTAA TTAGTGTTAC TTTAGAATAA TGAAGTCTGC TCCATCCATT

AGGGTATTAA TTAGTGCTAC TTTAGAATAA TGAAGCCTGT TCCATCCATT

AGGGTATTAA TTAGTGTTAC TTTAGAATAA TGAAGCCTGT TCCATCCATT

AGGGTACTTT GTCCTCTTAC TTTAGAATAG TGAAGCCTGC TCCATCCATT

TGGGCCCTGA TTGTTGTTTC TTCAGAATAG TGAAACCTGT TCTGTCCATT

AAGGTATCAA CTGATGTTTC TTTACCAGAA TGAAGCCCGG CCCAGCCGCT

AACAACATTT GATCTCCCCA TAGGTGGTTA AAAATTAATT ACTCTTCCAA

AACAACATTT GATCTCCCCA TAGGTGGTTA AAAATTAATT ACTCTTCCAA

AACAACATTT GATCTCCCCA TAGGTGGTTA AAAATTAATT ACTCTTCCAA

AACAACATTT GATCTCCCCA TAGGTGGTTA AAAATTAATT ACTCTTCCAA

AACAACATTT GATCTCCCTA TAGGTGGTTA AAAATTAATT ACTCTTCCAA

AACAACATTT GATCTCCCCA TAGATGGTTA AAAATTAATT ACTCTTCCAA

AACAACATTT GCTCTCCCCG TGGACGGTTA AAAATTAATT ACTCTTCCAA

AACAACATTT GCTCTCCCTG TGGATGGTTA AAAATTAATT ACTCTTCCAA

AACAACATTT GATCTCCCCA TGGACGGTTA AAAATTAATT ACTCTTCTAA

AACAACATTT GATCTCCCCA TGGACGGTTA AAAATTAATT ACTCTTCCAA

AACAACACTC CATCCCCCCA AGGATGGTTA AAAATTAATT ACTCTCCCAA

AACAACATTT GATCTCCCCA TGGATGGTTA AAAATTAATT GCTCTTCCAA

AACAACATTT GATCTCCTCA TGGATGGTTA AAAATTAATT ACTCCTCCAA

AACAACACTT GATCTCCCCA TGGATGGTTA AAAATTAATT ACTCTTCCAA

AACAACGTTT GATCTCCCCA TGGATGGTTA AAAATTAATT GCTCTTCCAA

AACGACATTC GATCTGCCTG TGGATGGTTA AAAATTAATT ACTCTTCCAA

ACGAACATTT GATCTCCCCA CGGACAGTTA AAAATTAATT ACTCTCCCAA

ATCAGTTTTC GGTGCCCATG TGACTGGTTA ATAATTATTT ACTCTTCCAA

CATATTGATT ACATGACTAT AAATATAAAA ACAGAACTAC AGAGTAACTA

CATATTGATT ACATGACTAT AAATATAAAA ACAGAACTAC AGAGTAACTA

CATATTGATT ACATGACTAT AAATATAAAA ACAGAACTAC AGAGCAACTA

CATATTGATT ACATGACTAT AAATATAAAA ACAGAACTAC AGAGCAACTA

CATATTGATT ACATGACTAT AAATACAAAA ACAGAACTAC AGAGCAACTA

CATATTGATT GCATGACTAT AAATATAAAA ACAGAACTAC AGAGCAACTA

CATATTGATT ACACGACTAT AAATATAAAA AC-------- --AGCAACTA

CATATTGATT ACACGACTAT AAATATACAA AC-------- --AGCAACTA

CATATTGATT ACATGACTAT AAATATAAAA ACAGGACTAC AGAGCAACTA

CATATTGATT ACATGACTAT AAATATAAAA ACAGAACTAC AGAGCAACTA

CATATTGATT ACAGGACTAT AAATATAAAA ACAGAACTCG GGAGCAACTA

CATATTGATT ACCTGGCTAT AAATATCAAA ACAGAACTAC AGAGCAACTA

CATATTGATT ACATGACTAT AAATATAAAA ACAGAACTAC AGAGCAACTA

CATATTGATT ACATGACTAT AAATATAAAA ACAGAACTAC AGAGCAACTA

CATATTGATT ACATGACTAT AAATATAAAA ACAAGGCTAC AGCGCAACTA

CATATTGATT ACATGACTAT AAATATAAAA ACAGGACTAC TGCGCAACTA

CATATTGATT ACATGACTAT AAAAGCAAAA ACAGAAGTCC AGAGCCATGA

CACGTTGACT GCATCCCTCT AAATATAAAA GCAGAAGTCC GGAACATGTG

CAGGGACCTT TGTTTAAATA GCATGTTTGC TCTTTAAAGG AGGTAATTCT

CAGGGACCTT TGTTTAAATA GCATGTTTGC TCTTTAAAGG AGGTAATTCT

CAGGGACCTT TGTTTAAATA GCATGTTTGC TCTTTAAAGG AGGTAATTCT

CAGGGACCTT TGTTTAAATA GCATGTTTGC TCTTTAAAGG AGGTAATTCT

-AGGGACTTT TGTTTAAATA GCATGTTTGC TCTTTAAAGG AGGTAATTTT

CAGGGACCTT TGTTTAAACA GCATGTTTGC TCTTTAAAGG AGGTAATTCT

CAGGGACCTT TGTTTAAACA GCTTGTTTGC TCTTTAAAGG GGAGAATTCT

TGGGGACCTT TGTTTAAACA GCTTGTTTGC TCTTTAAAGG GGAGAATTCT

CAGGGACCTT TGTTTAAATA GCTTGTTTGC TCTTTAAAGG AGGTAATTCT

GAGGGACCTT TGTTTAAATA GCATGTTTGC TCTTTAAAGG AGAGAATTCT

CCGGGACCTT TGTTTAAACA GCATGTTTGC TCCTTAAAAG AGGGAATTCT

CAGGGACCTT TGTTTAAACT GCATGTTTGC TCTTTAAAGG AGGGAATTCT

CAGGGACCTT TGTTTAAACA GCCTGTTTGC ACTTTCAAGG AGGTAATTCT

CAGGGACCTT TGTTTAAACA GCCCGTTTGC TCTTTAAAGG AGGTAATTCT

CAGGGACCTT TGTTTAAATA GCCTGTTTGC TCTTTCCAGG AGGTAATTCT

CGGGGACCTT TGTTTAAAGA GCGTGTTTGC TCTTCAGAGG AGGTAATGCT

TGGTGACCTT TGTTTAGAGA GCGTGTTTGC TTTCCAAAGC AAGTAATCAT

TGATGACCTT TGTTGGAAGA GCACGTTTGC TTTCCAGAGC AGGAATGTCC

TTTTCTATTG CCTTTATTTG AAACTATAAT TAAATAATGT CAAACTTGCA

TTTTCTATTG CCTTTATTTG AAACTATAAT TAAATAATGT CAAACTTGCA

TTTTCTATTG CCTTTATTTG AAACTATAAT TAAATAATGT CAAACTTGCA

TTTTCTATTG CCTTTATTTG AAACTATAAT TAAATAATGT CAAACTTGCA

TTTTCTATTT CCTGTATTTG AAACTATAAT TAATTAATGT CAAACTTGCA

TTTTCTATTG CCTTTATTTG AAACTATAAT TAAATAATGT TGAACTTGCA

TTTTCTATTG CCTTTGTTTG AAAC--TGAT TAAA-AATGT CAAACTTGCA

TTTTCTATTG CCTTTATTTG AAAC--TGAT TAAA-AATGT CAAACTTGCA

TTTTCTATTG CCTTTATTTG AAACCATAAT TAAGTAATGT CAAACTTGCA

TTTCCTGTTG TCCTTGTTTG AAACTGTAAT TAAGCAATGT CAAACTTGCA

TTTTCTGTTG CCTTTTTTTG AAGCTGTAAT TAAATAATGT CAAACTTGCA

TTTTCTGTTG CTTTTCTTT- GAACTATAAT TAAATAATGT CAGACTTGCA

TTTTCTGTTG CCTTTATTTG AAACCA-AAT TAGGTAATGT CAAACTTGCA

GTGTCTTTTG CCTTTGTTTG AAACTATAAT TAAATAATGC CAAACTTGCA

TTTTCTGTTG CCTTTATTTG AAACTGTAAT TAAATACTGT CAAGCTTGCA

TTTTCTGTTG CCTGTATTTG GAACCGCAAT TAAACAATGT CAAACTTGCA

TTTCCTGTTG CCAGTACTTG AAACTGTAAT TGAATAATGG TAAACTTGCA

TTTTCCTTCA CTTTTATGTG AGGCTGTAAT TGAATAATAC TATATTTACA

ACTATAACAA AATAGTATCT GCTCTTTGAA TATGATACAG GCCAAATTCT

ACTATAACAA AATAGTATCT GCTCTTTGAA TATGATACAG GCCCAATTCT

ACTATAACAA AATAGTATCC GCTCTTTGAA TATAATACAG GCCAAATTCT

ACTATAACAA AATAGTATCC GCTCTTTGAA TATAATACAG GCCAAATTCT

ACTATAAAAA AATAGTATCT GCTCTTTGAA TATAATACAG GCCAAATTCT

GCTATAACAA AACAGTATGT GCTGTTTGAA TATAATACAG GCCAAATCCT

GCTCTAACAA AATAATATCT GCTGTTTGAA TATAATACAG GCCAAATTGT

GCTCTAACAA AATAATATCT GCTGTTTGAA TATAGTACAG GCCAGATTGT

ACTATAACAA AATAGTATCT GCTGTTTGCA TATGATTCAG GCCAAATTCT

ACTGTAAGAA AATAATATCT GCTCTTTGAA TATAATACAG GCCAAGTTCT

AACATAACAA AATAGTATCT GCTGTTTGAA TATAATACAG GCCAAATTCT

ACTATAACAA AATAGTATCT GCTTTTTGCA TGTAATACAG GCCAAATTCT

AATATAACAA AATAGTATAT GCTGTCTGAA TATAATACAG GCCAAATTCT

AATATAACAA AATAGTATCA GCTGTTTGAA TATAATACAA GTCAAATTCC

ACTATAACAA AATAATATCT GCTGTTTGAA TATAATACAG GCCAAATTCT

ACTCTAACAA AATTGTATCT GTTGTTTGAA TAGAACAGGG GCCAAATTCT

GCTTTAACAA TGTGCAATCT GCTATTTGAA TGCAGTTCAA GTCAAATTCT

GCTAAAATAA CTTTCTGTCT GCTGTTTCAG TCTCCCACAG ACCATGTCCT

TAAACTTTAA TTAGCCCAAG CTTTCTTTTA GTTACCTCTG CCTTAAAGTA

TAAACTTTCA TTAGCCCAAG CTTTCTTTTA GTTACCTCTG CCTTAAAGTA

TAAACTTTAA TTAGCCCAAG CTTTCTTTTA GTTACCTCTG CCTTAAAGTA

TAAACTTTAA TTAGCCCAAG CTTTCTTTTA GTTACCTCTG CCTTAAAGTA

TAAACTTTAA TTAGGCCAAG CTTTCTTTTA GTTACCTCTG CCTTAAAATA

TAAACTTGAA TTAGGCCAAG CTTTCTTTTA GTTACCTCTG CCTTGAAATA

TAAACCTTAA TTAGGCTAAG CTTTATGTTA ATTACCTCTG CCTTAAAATA

TAAACCTTAA TTAGGCTAAG CTTTATGTTA ATTACCTTTG CCTTAAACTA

TAAACTTTAA TTAGGCCAAG CTTTCTTTTA ATTACCTCTG CCTTAAAATA

TAAACTTTAA TTAGGCCAAG CTTTCTTTTA ATTACCTCTG CCTTAAAATA

TAAACTTTAA TTAAGTCAAG CTTTCTTTTA ATTACCTCTG CCTTAAAATA

TAAACTTTAA TTAGGCCAAG CTTTCTTTTA ATTACCTCTT ACATAAAATA

TAAACTTAAA TTAAGCCAAG CTTTCTTTTA ATTACCTCTG CCTTAAAATA

TAAACTTTAA TTAAGCCAAG CTTTCTTTTA ATTACCTCTG CCTTACATTA

TAAACTTTAA TTAGGCCAAG CTT-CTTTTA ATTACCTCTT CCTTAAAATA

TAAACTTTAA TTAGGCCAAG CTTCCGTTTA ATTACCTCCG CCTTCAAATA

TATGCTTTAA TCAGGCCAAA CTTTATTTTA ATTACCTTTT CCCCCAAATA

CAGATTTTAA CTGTGAAAAC CTCTCTCTTA ATTATCTCGG CCACAAACTA

TGTAAGAATT TTCCCATTGC CTTTCTCACC AATACATGCA TAGCTGAACT

TGTAAGAATT TTCCCATTGC CTTTCTCACC AATACATGCA TAGCTAAACT

TGTAAAAATT TTCCCATTGC CTTTCTCACC AATACATGCA TAGCTAAACT

TGTAAAAATT TTCCCATTGC CTTTCTCACC AATACATGCA TAGCTAAACT

TGTAAGAATT GTCCCATTGC CTTTCTCACC AATACATGCA TAGCTAAACT

TGTAAGAATT TTCCCATTGT TTTTCTCACC AATACATAGA TAGCTAAACT

TGTAAGAGTT TTCC------ ------TACC AATACATGGA CAGCTAAACT

TGTAAGAGTT GTCC------ ------TACC AGTACATGGA T-GCTAAAAT

TGTAAGAATT TCCCCATTGC TTTTCTCACC AATACATGGA TAGCTAAACT

TGTAAGAATT TTCACATTGC TTTTCTCACC AATGCATGGA TAGCTAAACT

TGTAAGAATT TTCACATTGC TTTTCTCACC AATACACGGA TAGCTAAACT

CGTAAGAATT TTCACATCGC TTTTCTCACC AATGCATGGA TAGCTAAACT

TGTAAGAATT TTTACATTGC TTTTCTCACC AATGCATGAA TAGCTAAACT

TGTAAGAATT TTCATATCAT TTTTATCATC AATACATGGA TAGCTAAACT

TGTAAGAATT TTCACATTGC TTTTCTTCCC AATACATGGA TAGCTAAACT

TGTAAGGATT TTCACAGCGC GTTTCTCACC AATGCATGGA CAGCTAAACT

TGTAAAAGTT TTTACATCAG TTTTCTCACC AATAATTGGA CAACTAAACT

TGTAAGAACT TGTCCATAAA TTCACCCATC AGTACGTGGA CTATTCAGTC

AATAAAATGC TTAAATAGAT ACAAATCAAA CTTGAAAGGT TTAAACTGTT

AATAAAATGC TTAAATAGAT ACAAATCAAA CTTGAAAGGT TTAAACTGTT

AATAAAATGC TTAAATAGAT ACAAATCAAA CGTGAAAGGT TTAAACTGTT

AATAAAATGC TTAAATAGAT ACAAATCAAA CGTGAAAGGT TTAAACTGTT

AATAAAATGC TTAAATAGAT ACAAATCAAA CTCGAAAGGT TTAAACTGTT

AATAAAATGT TTAAATAGAT ACAAATCAAA CTTGAAAGGT TTAAACTGTT

AATAAAATGT TTAAATAGAT GCAAATTAAA CTTGAAAAGT TTAAACTGTT

AATAAAATGT TTAAATAGAT GCAAATTAAA CTTGAAAAGT TTAAACTGTT

AATCAAATGT TTAAATAGAA ACAAATCAAA CTTGAAGGGT TTAAACTGTT

AATAAAATGT TTAAATAGAT ACAAATCAAA CTTGAAAAGT TTAAACTGTT

AATA------ -------GAT ACAAATCAAA CTTGAAAGGT TTAAACTGTT

AATAAAATGT TTAAATAGAT ACAAATCAAA CTTGAAAGGT TTAAACTGTT

AATAAAAATT TTAAATAGAT ACAAATCAAA CTTGAAAGGT TTAAACTGTT

AATAAAATGT TTAAATAGAA ACAAATCAGA CTTGAAAGGT TTAAACTCTT

AATAAAATGT TTAAATAGTT ACAAATCAAA CTTGAAAGGC TTAAACTGTT

AATAAAACGT TTAAGTAGAT ACAAATCAAA CTTGAAAGGT TTAAACTGTT

AATAAAATGC TTAAATGGAT ACAAATCAAT CCTGAAGGGT TTAAACAGCT

AATATCATGT TTAAATAGAC CCAAATCCAA CTTGAAACAC TTAGAGGATT

GTTTTCATCC TGTAAGTCAG CTGAGAAAAA GCATACAGAC CGTAGTTCTA

GTTTTCATCC TGTAAGTCAG CTGAGAAAAA GCATACAGAC CGTAGTTCTA

GTTTTCATCC TGTAAGTCAG CTGAGAAAAA GTATACAGAC CGTAGTTCTA

GTTTTCATCC TGTAAGTCAG CTGAGAAAAA GTATACAGAC CGTAGTTCTA

GTGTTCATCC TGTAAGTCAG CTGAGAAAAA GCATACAGAC CGTAGTTCTA

GTTTTCATCC TGTAAGTCAG CTGAGAAAAA GCATACAGAC TGTAGTTCTA

GTTTTCATCC TGTAAGTCAG CTGAGAAAAA GCATACAGAC TGTGGTTCTA

GTTTTCATCC TGTAAGTCAG CTAAGAAATA GCATACAGAC TGTGGTTCTA

GTTTTCATCC TGTAATTCAG CTGAGAAAAA GCATGCAGAC TGTAGTTCTA

GTTTTCATCC TGTAAGTCAG CTGAGAAAAA GCATACAGAT TGTAGTTCTA

GTTTTCATCC TGTAAGTCAG CTGAGAAAAA GCATACAGAC TGCAGTTCTA

GTTTTCATCC TGTAAGTCAG CTGAGAAAAA GCATGCATAC TGTAGTTCTA

GTTTTCATCC TGTAAGTCAG CTGAGAAAAA GCATACAGAC TGTTGTTCTA

GTTTTCATCC TGTGAGTCAG ATGAGAAAAA GCATACAGTC TGTAGTTCTA

GTTTTCATCC TGTAAGTCAG CTGAGAAAAA GCATACAGTG TGTAGTCCTG

GTTTTCATCC TGTAAGTCAG CTGAGAAAAA GCATACAGTC TGTAGTTCT-

GTTTTCATCT TGTAAGACAG CTGAGAGTAC ACATACAAAC TACAGTCCTG

GttcccATTC TGCAAATCAG CCAAGGTAAA GAATGCAAAC AGCAGCCCTT

AATTATGTCC ATAGAGATTT CAAATCCAGT GAATTAACTC TTTTTTTTCC

AATTATGTCC ATAGAGATTT CAAATCCAGT GAATTAGCTC TTTTTTTTCC

AATTATGTCC ATAGAGATTT CAAATCCAGT GAATTAACTC TTTTTTTTCC

AATTATGTCC ATAGAGATTT CAAATCCAGT GAATTAACTC TTTTTTTTCC

AATTATGTCC ATAGAGATTT CACATCCAGT GAATTAACTC TTTTTTTTCC

AATTATGTCC GTAGAGATTT CAAATCCAGT GAATTAACTC TTTTTT-CCC

AATTATGTCC ACAGAGATTT CAAATCCAGC GAATTAACTC TTTCCTTGTC

AATTATGTCC ACAGAGATTT CAAATCCATC GAATTAACTC TTTCTTTGTC

AATTATGTCC ATAGAGATTT CAAATCCAGT GAATTAACTC TTTTTTTTCC

AATTATGTCC ATAGAGATTT CAAATCCAGT AAATTAACTC TTTTTTTCTC

AATTATGTCC ATAGAGATTT CAAATCCAGT GAATTAACTC TTTTTTTTTC

AATTATGTCC AGAGAGATTT CAAATTCAGT GAATTAACTC TTTTTTTT-C

AATTATGTCC ATAGAGATTT CAAATCTAGT GAATTAACTC TCTTTTTTCC

AATTATGTCT GTAGAGATTT CAAATGCAGT GACTTAACTC TTTTTTTCCC

AATTATGTCC ATAGAGATTT CAAGTCCAGT AAATTAACTC TTTTTCTTCC

AATTATGTCC ACAGAGATTT CAAATCTAGT GAATTAGCTC TTTTTTTTCC

AGTGATGTCC AGAGAGATTT CAAATCCAGT GAATTAGCTC TCTTTCCCCC

AATTATGTAT GTGGAGATTT CTAATCCATT GAATGAGCTC TTTTTTTCCC

CATTTGAGCT CATAAATGTT AACCTCAGAA GTGCCTCAGA TTCTTCCATT

CATTTGAGCT CATAAATGTT AACCTCAGAA GTGCCTCAAA TTCTTCCATT

CATTTGAGCT CATAAATGTT AACCTCAGAA GTGCCTCAAA TTCTTCCATT

CATTTGAGCT CATAAATGTT AACCTCAGAA GTGCCTCAAA TTCTTCCATT

CATTTGAGCT CATAAATGTT AACCTCAGAA GTGCCTCAAA TTCTTCCACT

CATTTGGGCT CATAAATGTT AACCTCAGAA GTGCCTCAAA TTCTTCTATT

CATTTGAGCT CATAAATGTT AACTTCAGAA GTGCTTTAGA TTCTTCCACT

CATTTGAGCT CATAAATGTT AACTTCAGAA GTGCTTCAGA TTCTTCCATT

CATTTGAGCT CATAAATGTT AACCTCAGAA GTGCCTCAAA TTCTTTCATT

TACTTGAGCT CATAAATGTT AACCTTAGAA GTGTCTCAAA TTCTTTCATT

TATTTGAGCT CGTAAATGTT AACCTCAGAA GTGCCTCAGA TTCTTCCATT

TATTTGAGCT CATAAATGTT AACCTCAGAA GTGCCTCAAA TTCTTCCATT

TATTTGAGCT CATAAATGTT AACCTCAGAA GTGCCTCAAA TTCTTTCATT

CATTTGAGCT CATAAATGTT AACCTCAGAA GTGCCTCAAA TTCTTCCATT

CATTCAAGCT CATAAATGTT AACCTCAGAA GTGCCTCAAA TTCTTCCATT

CTTTTGAGCT CATAAATGTT AACCTCAGAA GTGCCTCAAA TTCTTCCATT

CATTTGAGCT CATAAATGTT AACCTCATAA GTGCATCAAA TTCTTCCACT

TTTTCGAGTG TATAAATGGT AACCTCTTAA GTGTATCACA TTCCTCTATT

GAAATAAACT TGAACTGACA TAGCCAGATG ATGTAAAATT GAAACTGAAA

GAAATAAACT TGAACTGACA TAGCCAGATG ATGTAAAATT GAAACTGAAA

GAAATAAACT TGAACTGACA TAGCCAGATG ATGTAAAATT GAAACTGAAA

GAAATAAACT TGAACTGACA TAGCCAGATG ATGTAAAATT GAAACTGAAA

GAAATAAACT TGAACTGACA TAGCCAGATG ATGTAAAATA GAAACTGAAA

GAAATAAACT TGAACTGACA TAGCCAGATG ATGTAAAATT GAAACTGAAA

GAAATAAACG TGAACTGACA TAGCCAGATG ATGTAAAACT GGAACTGAAA

GAAATAAACT TGAACTGACA TAGCCAGATG ATGTAAAATT GGAACTGAAA

GAAATAAACT TGAACTGACA TAACCAGATG ATGTAAAATT GAAACTGAAA

GAAATAAAGT TGAACTGACA TAGCCAGATG ATGTAAAATT GAAACTGAAA

GAAATAAACT TGAACTGACA TAGCCAGATG ATGTAAAATT GAAACTGAAA

GAAATAAACT TGAACTGACA TAGCCAGATG ATGTAAAATT GAAACTGAAA

GAAATAAACT TGAACTGACA TAGCCAGATG ATGTAAAATT GAAACTGAAA

GAAATAAACT TGAACTGACA TAGCCAGATG ATGTAAAATT GAAACTGAAA

GAAATAAACT TGAACTGACA TAGCCAGATG ATGTAAAATT GAAACTGAAA

GAAATAAACT TGAACTGACA TAGCCAGATG ATGTAAAATT GAAACTGAAA

GAAATAAACC TGAACTGACA TAACCAGATG ATGTAAAATT GAAACTGAAA

AAAACAAACC TGTCATGTTA GAGGCAGATG ATGCAGAACC GAAACTGGAG

TTCTATCCTT GGCCCTCCCA TGTTCTTCCC CATAGGTATT AGAGGACTCA

TTCTATCCTT GGCCCTCCCA TGTTCTTCCC CATAGGTATT AGAGGACTCA

TTCTATCCTT GGCCCTCCCA TGTTCTTCCC CATAGGTATT AGAGGACTCA

TTCTATCCTT GGCCCTCCCA TGTTCTTCCC CATAGGTATT AGAGGACTCA

TTCTATCCTT GGCCCTCCCA TGTTCTTCCC CATAGGTATT AGAGGAATCA

TTCTATCCTT GGCCCTCCCA TGTTCTTCCC CGTAGGTATT AGAGGAATCC

TTCTATCCAC AGCCCTCCCA CATTCTTCCT CATAGGTATT AGAGGACTCA

CTCTATCCAT GGCCCTCCCA TGTTCTTCCT CATAGGTATT GGAGGACTCA

TTCTATCCTT GGCCCTCCCA TGTTCTTCCC CATAGGTATT AAAGGAATTG

TTCTATCCTT GGCCCTCCCA TGTTCTTCCC CCTAGGTATT AGAGGAATCA

TTCTATCCTT GGCCCTCCCA TGTTCTTCCC CATAGGTATT AGAAGAATCG

TTCTATCCTT GGCCCTCCCA TGTTTCTCCC CATAGGTATT AGAGGAATCA

TTCTATCCTT GGCCCTCCCA CGTTCTTACC CATAGGTATT AGAGGAATCA

TTCTATCCTT GGCCCTCCCA TGTACTTTCC CATAGGTATT AGAGGAATCA

TTCTATCCTT GGCCCTCCCA TGTTCTTCCC CATAGGTATT AGAGGAATCA

TCCTATCCTT GGCCCTCCCA TGCTCGTCCC CATAGGTATT AGAGGAATCA

TTCTATCCTT AGCCCTCCCA TCTTCTTTCC CATAGGTACT GGCAGAGCTG

TCCTGCTTCC AGTCCCCACA CATTTTGCCC AAAAGATATT TGAAGTGAAC

CAGGAGAGTT AGGTTTTCAC TCTTATTTGG GCAAAACAGG GCATATAAAT

TAGGAGAGTT AGGTTTTCAC TCTTATTTGG GCAAAACAGG GCATATAAAT

TAGAAGAGTT AGATTTTCAT TCTTATTTGG GTAAAAGAGG GCATATAAAT

TAGAAGAGTT AGATTTTCAT TCTTATTTGG GTAAAAGAGG GCATATAAAT

TAGGAGAGTT AGATTTTCAT TCTTATTTGG GCAAAACAGG GCATATAAAT

TAGGAGAGTT AGGTTTTCAT TCTTATTTGG GCAAAAGAGG GCATATAAAT

TAGGAAAGCC AGGTTTTCAT TCTTACATAG GCAAAGCGGG GCATATAAAT

TAGGAAAGCT AGGTTTTCAT TCTTATTTAG GCAAAGTGGG GCATATAAAT

TAGGAGAGTT AGGTTTTCAT TCTTATTTGG GCAAAACAGG GCATATAAAT

TAGGAAAGTT AAGTTTTCAT TGTTATTTGG GCAAAACAGG GCATATAAAT

TAGGAGAGTT AGGTTTTCAT TGTTATTTGG GCAAAGCAGG GCATATAAAT

TAGTCAAGTT AGGTTTTTAT TGTTATTTGG GCAAAACAGG GCATATAAAT

TAGGAGAGTT AGGTTTTTAT TGTTATTTGG GCAAAATA-G GCATATAAAT

TAGGAAAGTT AAGTTTTCGT TCTTATTTGG GCAAAACAGG GCATATAAAT

--GGAGAGTT AGGTTTTCAT TCATATTTGG GCAAAACAGG GCATATAAAT

CGGGAGAGTT GGGCTTTAGT TCGTATTTGG GCAAAACAGG GCATATAAGT

TAGAAGAGAT AGATTTTCAT ACTTATTTGG GTAGAATAGG GCATATAAAT

GGAAAGAGCC ATGTTTTCAC TCTTGTTCAG GGCATCGAAG ACCGATAAAC

TCTTGCTGCC AGGAGAAAGC AATTACCAAA AGATATTTAG AATGACTTAG

TCTTGCTGCC AGGAGAAAGC AATTACCAAA AGATATTTAG AATGACTTAG

TCTTGCTGCC AAGAAAAATC AGTTACCAAA AGATATTTAG AATGACTTAG

TCTTGCTGCC AAGAAAAATC AATTACCAAA AGATATTTAG AATGACTTAG

TCTTGCTGCC AGGAGAAAGC AATTACCAAA AGATATTTAG AATGACTTAG

TCTTGCTGCC AGGAGAAAGC AATTATCAGA AGATATTTAG AATGACTTCG

TCTTGCTGCC AGGAAAAAGC AATTAGCAAA GGATATTTAG AATGACTTGG

TCTTGCTGCC AGGAAAAAGC AATTAGCAAA GGATGTTTAG AATGACTTGG

TCTTGCTGCC AGGAGAAAGC AATTACCAAA AGATATTTAG AATGACTTAG

TCTTACTGCC AGAAGAAAGC AATTACCAAA ACATATTTAG AATGACTTAG

TCTGACTGCC AGGAGAAAGC AATTACCAAA AGATATTTAG AATGACTTAG

TCTTACTGCC AGGAGAAAGC AATTACCAAA AGATATTTAG AATGACTGGG

TCTTACTGCC AGGAGAAAGC AATTACCA-A AAATATCTAG AATGACTTGG

TCTTGCTGCC AGGAGAAAGC AATTACCAAA AGATATTTAG AATGACTTAA

TCTTGCTGCC AGGAGAAAGC AATTACCAAA AGATATTCAG AATGACTTAG

TCTCTCTGCC AGGAGAAAGC AATTACCAAA AGGTATTTAG AATGACTTAG

TCTTGCTGCC AGGAGAAAGC AATTACCAAA ACACATTCAG AATAGCTTGA

TCTTGCTGCC AGGAGAGAGC AAGTTTCAGA AATTTCTCCA AGTATTTTGT

CTGATAGAAA GGAATGTCTT TATTGTTGCC AAATGCTGAA TGACAGAGCT

CTGATAGAAA GGAACGTCTT TATTGTTGCC AAATGCTGAA TGACAGAGCT

CTGATAGAAA GGAATGTCTT TATTGTTGCC AAATGCTGAA TGACAGAGCT

CTGATAGAAA GGAATGTCTT TATTGTTGCC AAATGCTGAA TGGCAGAGCT

CTGATAGAAA GGAATGTCTT TATTGTTACC AAATGCTGAA TGACAGAGCT

CTTATAGAAA GAAATGTCTT TATTGTTGCC AGATGCTGAA TGACAGAGCT

CTTATAGAAA GGAATGTCTT TATTGTTGCC AGCTGCCTCA TGACAGAACT

CTTATAGAAA TGGATGTCTT TATTGTTGCC AGCTGCTTCA TGACAGAACT

CTTATAGAAA GGAATGTCTT TATTGTTGCC AAGTGCTGAA TGACAGA-TT

ATTATAGAAA GGAATATCTT TATTGTTGCC AAATGCTGAA TGATAGA--T

TTTATAGGAA GGAATGTCTT TATTGTTGCC AAATGCTGAA TGACAGGGCT

CTTATAGAAA GGAATGGCTT TATTGTTGCC AAATGCTGAA TG-CAGAGCT

CTTATAGAAA GGAATATCTT TATTGTTGCC AAATGCTGGA TGACAGAGAT

CTTATAGAAA GGAATATCTT TATGGTTGCC AAATGATAAA TGACAGAACT

CTTATAGAAA GAGCTGCCTT TATTGTTGCC AAGTGTTGAA TGACAGAGCT

CTTATAGAAA GGAATGCCTT TATTGCTGCC AAGGGTTGAA TGACAGAGCT

CTTTCATAAA GTAATGCTTT GATTGCTGCC AAGTGCTGGG TGATAGAGTT

TCTAATTCAA GGAATGAGTC TGTTGTTGCC AAGGTTTGAA TGACACGACT

TTAATTCTAA CTGCTTAATT AGAAATAATC TCAACTAGAA TCAAAATGAA

TTAATTCTAA CTGCTTAATT AGAAATAATC TCAACTAGAA TCAAAATGAA

TTAATTCTAA CTGCTTAATT AGAAATAATG TCAACTAGAA TCAAAATGAA

TTAATTCTAA CTGCTTAATT AGAAATAATG TCAACTAGAA TCAAAATGAA

TTAATTCTAA CCACTTAATT AGAAATAATA TCAACCAGAA TCAAAATGAA

TTAATTCTAA CAGCTTAATT AGAAATGGTG TCAACCAGAA TCAAAATGAA

TAAGTTCTAA CAGCTTAATT AGAAATGATG TCAGCCAGAA CCAAAGTGAA

TAAGTTCTAA CAGCTTAATT AGAAATGATG TCAGCCAGAA CCAAAGTGAG

TTAATTTTAA CAGCTTAATT AGAAATGATG TCAACCAGAA TCAAAGTGAA

TTAATTCTAA CAGCCTAATT AGAAATGATG TGAACCAGAA TCAGAATAAA

TTAATTCTAA CAGCCTAATT AGAGATGATG TCAACCAGAA TCAAAGTGAA

TTAATTCTAA CAGCCTAATT AGAAATGATG TCAACCAGAA TCAAAGTGAA

TTAATTCTAA CAGCCTAATT AGGAATGATG TCAACCAGAA TCAAAGTGAA

TTAATTCTAA CAGCCTATTT AGAGATGATG TCAACCAGAA TCAAAGTGAA

TTAATTCTAA CGGCCTAATT AGAAATGATG TCAACCAGAA TCAGAGTGAA

TTAATCCTAA CAGGCTAATT AGAAAGGGTG TCAACCAGAA TCAAAGTGAA

TTATCTAAAA AAAGCCAATT AGAGGTGACA TCAGCTGTGA TCAAAGTGAA

TTATCTCTAG AAGGCTAATT CGGAATGATG TGAGACGGAA TCAAAGTGAA

CCAGAACAAG ACATTGGTTA CAGAACATCA GCAATGTGTG GTAGTCTTTC

CCAGAACAAG ACATTGGTTA CAGAACATCA GCAATGTGTG GTAGTCTTTC

CCAGAACAAG ACATTGGTTA CAGAACATCA GCAATGTGTG GTAGTCTTTC

CCAGAACAAG ACATTGGTTA CAGAACATCA GCAATGTGTG GTAGTCTTTC

CCAGAACAAG ACATTGGTTA CAGAACATCA GCAATGTGTG GTAGTCTTTC

CCATAACAAG ACATTGGTTA CAGGACATCA AGAATGTGCG AGCGTCTCTC

CCAGAACAAG ACATTGGTTA CAGAACATCC ACAGTGTGTG GTTGTCTTCG

CCAGAACAAG ACATAAGTTA CAGAACATCC ACAGTGTGTG GGTGTCTTCG

CCAGAACAAG ACATTGGTTA CAGAATATCA ACAGTATGTG GTGGTCTTTC

CCAG-ACAAG ACATTGGTTG CAGAACATCA ACAATGTGTG ATAGTCTTTC

CCAGAACAAG ACATTGGTTA CAGAACATTA ACAATGCGTG GTAGTCTTTC

CCAGAACAAG ACATTGGTTA CAGAACATCA GCAATGTGTG GTAG-CTTTC

CCAGAACAAG AAATTGGTTC CAGAATATCA ACAATGTGTG GTAGTCTTTC

CAAGAACAAG ATATTGGTTA CAGAACATCA ACAACGTATG GTAGTCTTTC

CCAGAACAAG ATATTGGTTA CAGGACATCA ATAATGCGTG GGAGTCTTTC

CCAGAACAAG ACATTGGTTA CATAACATCT ACAATGTGTA GGAGTCTTTC

CCAGAGTGAA TAATTGTTTG CAGAACATCA GTAATTTGTG GCAGTCTTTC

CCAGGACATA ACATTGGCTG CAGCGCTTCA ATAACTTGTG GCAGACTTTA

ACCTCCTTTA TCAACATTTG GTACTAATAA CCTAATCATG GAAAATTACA

ACCTCCTTTA TCAACATTTG GTACTAATAA CCTAATCATG GAAAATTACA

ACTTCCTTTA TCAACATTTG GTACTAATAA CCTAATCATG GAAAATTACA

ACTTCCTTTA TCAACA---- ---------- ---------- ----------

ACTTCCTTTA TCAACATTTG GTACTAAAAA CCTAATCATG GAAAATTACA

GC-TCCTTCA CCAACATTTG ATACTAATAA CCTAATCATG GAAAATTACA

GCTTCCTTTA ATAGCA---- ---------- ---------- ----------

GCTTCCTTTA AGAGCATTTT GTACTAATAA CCCAATCATG GAAAATGACA

ACTTCCTTTA CTAACATTTC ATATTAATAA CCTAATCATG GAAAATTACA

ACTTCCTTTA CTAGCACTTG GTACTAATAA CCTAATTGTG GAAAATTACA

ACTTCCTTTA CTAGCATTTG GTACTAAGAA CCTAATAGTG GAAAATTACA

ACTTCCCTTC CCAGCATTTG GTACTAATAA CCTAATTGTG GAAAATTACA

ACTTCACATA CTAGCA---- ---------- ---------- ---------A

ACTTCCTTTA CTAACATTTG GTAGTAATAA CCTAATCATG GAAAATTACA

ACTTCCTTTC CTAACATTTG GTAGTAATAA TCTAATCGTG GAAAATTGCA

ACTTCCTGTG CTAACATTTG GTAGTAATAA CCTAATCGTG GAAAAAGACA

ATTTTCCTTT CCAACATTTT ATACTAATAA TGCACCTTTG GAAAATGACA

ACCTTCATTA CTGACATTTT ATATTTATGA TGTAAACTTG GAAAATTGCA

ACTTCCTGAG CTAGGTATAT CAGAGGTGGC CACAAAACTC TTTATGATGT

ACTTCCTGAG CTAGGTATAT CCGAGGTGGC CACAAAACTC TTTATGATGT

ACTTCCTGAG CTAGGTGTAT CAGAGGTGGC CACAAAACTC TTTATGATGT

---------- ---------- ---------- ---------- ----------

ACTTCCTGAG CTAGGTATAT CAGAGGTGGC CACAAAACTC TTTATGATGT

ACTTCCTGAG CTAGGTCTAT T-GTAGTAGC CACAAAAATC TTTATGATGT

---------- ---------- ---------- ---------- ----------

GTTTCCTGAG CCAC-TCGAC CAGAGGTAGC CACATAACTC TTTATGATGT

ACTTCCTGAG CTAAGTCTTT TGAAGATGGC CACAAAACTC TTTATGATGT

ACTTCCTGAG TTGGGTCTAT CAGAGGTGGC CACAAAACTC TTTATGATGT

ACTTCCTGAG CTGCGTCTAT CAGAGGTGGC CGCAAAACTC TTTATGATGT

ACTCTCTGAG CTGGGTCTAT CAGAGATGAC CACAAAACTC TTTATGATGT

ACATCCTGAG ATAGGTCTGT CAAAAATGAC CACAAAACTC --TATGATGT

ACTTCCTGAG CAGGGTCTAT CAGAGGTGGC CACAAAACTC TTTATGATGT

ACTTCCTGAG CTGGGTCTAT CAGAAGTGAC CACAAAACTC TTTATGATGT

GCTTCCTGAG CTAGGTCTAT CAAAAGTGGC CACAAAAATC TTTATGATGT

ACTTCCTGAG CTGGGTTGAG TAGAAGTGGC CACAACATAC TTTATGATGT

ACTTCCTGAG TTGACTATAT TGGACGCGGC CACAGGACAC TTTTTGAGAT

GGCCAAAAAT TCCTCCTACC ACCACAGCCT TGAAAATAGG TCAAAGACAC

GGCCAAAAAT TCCTCCTACC ACCACAGCCT TGAAAATAGG TCAAAGACAC

GGCCAAAAAT TCCTCCTACC ACCACAGCCT TGAAAATAGG TCAAAGACAC

---------- ---------- ---------- ---------- ----------

GGCCAAAAAT TCCTCCTACC ACCACAGTCT TGAAAGTAGG TCAAAGACAC

GGCCAAAAAT TCCTCCTGCT ACCACAGCCT TGAAAATAGG TCAAAG----

---------- ---------- ---------- ---------- ----------

GGCCAAAAAT TCCTCCTGCC ACCACAGCCT AGAAAATGGG TCAAAGACAT

AGCTAAAAAT CCCTCCTGCC ACCACAGCTC TGAAAATGGG TCAAAGACAC

GGCCAAAAAT TCTTCCTTCC ACCACAGCCT TGAAAATAGG TCAAAGACAC

GGCCAAAAAT TCCTCCTGCC ACCACAGCCT TAAAAATAGG TCAAAGATAC

GGCCAAAAAT TCCTCCTGCC ACCACAGCCT TAAAAATAGG TCAAAGACAC

GACCAAAAAT TCCTTCTGTC ACCACAGCCT TGAAAATAGG TCAAAGACAT

GGCCAAAAAT TCCTCCTGCC ACCACAGCCT TGAAAATAGG TCAAAGATGC

AGCCAAAAAT TCCTCCTACC ACCACAGCCT TAAAAATAGG TCAAAGACAC

GGCCAAAAAG TCCTCCTACC ACCACAGCCT TAAAAATAGG TCAAAGACGC

AGCCAAAAAT TCCTCCCACC ACCACAGCTC CCAAAATAGG TCAAAGACAT

GTGTAAAAAT TCCTCTGGCT ACCACAGCCC TGAAAATAGG TCACTGAGAT

CTTCTGAACT CTCAAAACAA GAACTTACTT TGGGCTTCAC AATCTCCTTC

CTTCTGAACT CTCAAAACAA GAACTTACTT TGGGCTTCAC AATCTCCTTC

CTTCTGAACT CTCAAAACAA GAACATACTT TGAGCTTCAC AATCTCCTTC

---------- ---------A GAACATACTT TGAGCTTCAC AATCTCCTTC

CTTCTGAACT GTCAAAACAA AAACCTACGT TGGGCTTCAC AATCTCCTTC

--TCTGAACT CTGAAAGCCA GAACCTACTT TGGGTTTTGC AATCTCCTTG

---------- ---------A GAACCTACTT TGGGCTTAGC AATCTCCTTG

CTTCTGAACT CTCCAAGCGA GAACCTACTT TGGGCTTAGC AATCTCCTTG

CTTCTGAACT CTCAAAGCAA GAACCTACCT TGGGTTTCGC AATCTCCTTG

CTTCTGAACT CTCAA---AA GAACTTACTT TGGGTTTTGC AATCTCCTTG

CTTCAGAACT CTTAA-GCAA GAACCTACTT TGGGTTTCGC GATCTCCTTG

CTTGTGAGCT CTCAGAGCAA GAACCTACTT TGGGTTTCGC GATCTCCTTG

CTTCAGAACT CTCAAAGCAA GAATCTACCT TAGGTTTTGC AATCTCCTTG

ATTCTGAACT CTCAAAGCAA AAACCTACTT TGGGTTTCGC AATCTCCTTG

GTTCTGAACT CTCAAAGCA- ---------- ---------- ----------

ATACTGAGCT CAAAAAGC-A GAACGTACTT TGGGCTTCGC AAGCTCCTTG

CTTTTGAATT ATCAAAACAA GAACTTACTT TAGGCTTGGA AATTTCCTTG

TATCTGAGTT ACCAAAACAA GGACCTACCT TGGGTTTAGC CAGCTCTTTA

ATCCTTTTGA TTTCCTCCTC AGACATGACA TTATAATACC TGATGATGTC

ATCCTTTTGA TTTCCTCCTC AGACATGACA TTATAATACC TGATGATGTC

ATCCTTTTGA TTTCCTCCTC AGACATGACA TTATAACACC TGATGATGTC

ATCCTTTTGA TTTCCTCCTC AGACATGACA TTATAACACC TGATGATGTC

ATCCTCTTGA TTTCCTCCTC AGACATGACA TCATAATACC TGATGATGTC

ATCCTCTCGA TTTCCTCGTC TGACATGACA TCGTAGTACC TGACGATGTC

ATCCTCTCGA TCTCTTCGTC CGACATCACA TCATAGTACC TGACGATGTC

ATCCTCTCGA TTTCTTCGTC GGACATCACA TCATAGTACC TGACGATGTC

ATCCTCTCGA TCTCCTCATC AGACATGACG TCGTAGTACC TGACGATGTC

ATCTTCCTGA TTTCCTTGTC AGACATGACA TCGTAGAACC TGACAATGTC

ATCCTCTCGA TTTCCTCATC AGACATGACA TCATAATACC TGACGATGTC

ATCTTCTCGA TTTCCTCATC AGACATGACG TCGTAGTACC TGACGATGTC

ATCCTCTCGA TTTCCTCATC AGACATGACA TCGTAGTACC TGACGATGTC

ATCCTCTCGA TTTCCTCGTC AGACATGATG TCATAGTACC GCACGATATC

---------- ---------- ---------- ---------- ----------

ATCCTCTCGA TTTCCTCATC AGACATGACA TCGTAGTACC TGACGATGTC

ATCTTCTCAA TCTCCTCATC TGATAAGACA TCGTAGTATC TCACGATGTC

ATCTTCTCAA TCTCTTCATC GGACAGGACG TCGTAATAGC GGACGATATC

CCACTCATCA TCCTCTTCCA AGTGAGTGAT GAGAAGCTGT GGAGCCCTCT

CCACTCATCA TCCTCTTCCA AGTGAGTGAT GAGAAGCTGT GGAGCCCTCT

CCACTCATCC TCCTCTTCCA AGTGGGTGAT GAGTAGCTGT GGAGCCCCCT

CCACTCATCC TCCTCTTCCA AGTGGGTGAT GAGTAG-TGT GGAGCCCTCT

CCACTCATCC TTCTCTTCCA AGTGGGTGAT GAGTAGCTGT GGACTTCTCT

CCACTCGTCC TCCTCTTTGA AGGGGGCGAT GAGCAGCTGT GGAGCCCTGT

CCACTCGTCT TCCTCTTTGA AGGGGGCAAT GAGGAGCTGT GGCACTCTGT

CCACTCGTCT TCCTCTTTGA AGGGGGCGAT GAGGAGCTGT GGCACTCTGT

CCACTCGTCC TCCTCCTTGA AGGGCGCGAT GAGCAGCTGC GGCGCCCCAT

CCATTCATCC TCCTCTTTGA AGGGGGCGAT GACCAGCTGG GGCATCCTGT

CCACTCATCC TCCTCTTTGA AGGGGGCGAT GAGCAGCTGT GGTGTCCTGT

CCACTCGTCC TCCTCTTTGA AGGGGGCAAT GAGCAGCTGT GGCGTCCTGT

CCACTCATCC TCCTCTTTGA AGGGGGCGAT GAGCAGCTGT GGCGCCCCGT

CCACTCATCT TCCTCTTTAA AGGGGGCGAT GAGCAGCTGG GGAGTCCTGT

---------- ---------- ---------- ---------- ----------

CCACTCGTCC TCCTCTTTGA AAGGGGCGAT GAGCAGCTGT GGGGTCCTGT

CCACTCGTCT TCCTCCTTAA AGGGTGCAAT CAGCAGCTGG GGTGTCTTAT

CCACTCATCC TCTTCCTTAA AGGGGGCGAT GAGCAGCTGG GGGGTTCTGT

TGCCATGGTA GTACCTACAG AAAAGCCTCT GCTGCTTCTG TGGTGCCTGG

TGCCATGGTA GTACCTACAG AAAAGCCTCT GCTGCTTCTG TGGTGCCTGG

TGCCATGGTA GTACCTACAG AAAAGCCTCT GCTGCTTCTG TCGTGCCTGG

TGCCATGGTA GTACCTACAG AAAAGCCTCT GCTGCTTCTG TCGTGCCTGG

TGCCAAAGCA GTACCTACAG AAAAGCCTCT GCTGTTTCTG TGGTGCCTGG

TGCCATGGTG GTACCTACAG AAGAGCCTCT TTTGCCTCCG GGGTGTCTGG

TTCCATGGTG GTATCTACAG AAAAGCCTCT TCTGCCTCCG GGGTGTCTGG

TTCCATGATG GTACCTACAG AAAAGCTTCT TCTGCCTCCG GGGTGTCTGC

TGCCATCGTG GTACCTGCAG AAAAGCCTCT TCTGTCTCCG GGGGGTCTGG

TGCCATAATG TAACCTACAG AAAAGCCTCT TCTGCCTCTG TGGTATCTGG

TGCCATGGTG GTACCTACAG AAAAGCCTCT TCTGCCTCCG GGGAGTCTAG

TGCCATGGTG GTACCTACAG AAAAGCCTCT TCTGCCTCCG GGGCGTCTGG

GGCCATGGTG GTACCTACAG AAAAGCCTCT TCTGCCTCCG GGGCGTCTGG

TGCCATGGTG ATATCTACAG AAAAGCCTCT TCTGCCTCCG GGGTGTCTGG

---------- ---------- ---------- ---------- ----------

GGCCATGATG GTATCTACAG AAAAGCCTCT TCTGCCTCCG GGGCGTCTGG

TGCTATCGTG GTATCTACAG AAAAGTCTCT TCCTTCTCTG AGGTGTCTGT

TGCCATCATG ATATCTGCAG AAAAGTCTCT TCTGCCTCCG GGGTGTCTGA

GGAGCACAGC AACATCTTTC TGGTAGGGAC ATTTTATTTT TGGTAAAGCC

GGAGCACAGC AACATCTTTC TGGTAGGGAC ATTTTATTTT TGGTAAAGCC

GGAGCACAGC AACATCTTTC TCGCAGGGAC ATTTTATTTT TGGTAAAGCC

GGAGCACAGC AACATCTTTC TCGCAGGGAC ATTTTATTTT TGGTAAAGCC

GGAGCACAGA AACATCTTTC TGGCAGGGAC ATTTTATTTT TGGTAAAGCC

GGAGCACAAC CACATCTTTC TGGCAGGGAC ATTTTATTTT TGGTAAAGCC

GGAGCACAGC ACAATCTTTC TGGCAGGAAC attttatttt TGGTAAAGCC

GGAGCACAGC ACAGTATTTC TGGCAGGAAC ATTTTATTTT TGGTAAAGCC

GGAACACAGT AGTGTCTTTC TGGCAGGGAC aatttttttt ttGTAAAGCC

GAAGCACAGC AACATCTTTC TGGCAGGGAC GTTTTATTTT -GGTAAAGCC

GGAGCACAGC AATATCTTTC CGGCAGGGAC ATTTTATTTT TGGTAAAGCC

GGAGCACAGC AACATCTTAC TGGCAGGGAC ATTTTATTTT TGGTAAAGCC

GGAGCAGAAT AACATCTTTT TGGCAGGGAC ATTTTATTTT TGGTAAAGCC

GAAGCAGAGC AACATCTTTT TGGCAGGGAC ATTTTATTTT TGGTAAAGCC

---------- ----TCTTTT TTGCAAGGAC ATTTTATTTT TGGTAAAACC

GAATCACAGT GACATCTTTT TAGCGAGGAC ATTTTATTTT TGGTAAAGCC

GGAGCACAGA AACATCTTTT GTATAGAAAT ATTTATTTTC CGGAAATTCC

GGTAGACACA CACATCTTTT TTGTAGGACC ATTTAATTTT TGTAAAACCC

ACATAGATAG AAATGCCATA AAAACAAACA TGTAAACAAG GTTCAGAACT

ACATAGATAG AAATGCCATA AAAACAAACA TGTAAACAAG GTTCAGAACT

ACATAGATAG AAATGCCATA AAAACAAACA TGTAAACAAG GTTCAGAACT

ACATAGATAG AAATGCCATA AAAACAAACA TGTAAACAAG GTTCAGAACT

ACATAGATAG AAATGCCATA AAAACAAACA TGTAAACAAG GTTCAGAACT

ACATTGACAG AAATGCCATA AAAACAAACA TGTAAACAAG GTTCAGAACT

ACACAAATAG AAATGTCATA AAAACAAACA TGTAAACA-- GTTCAGAACT

ACACAAATAG AAATGTGATA AAAACACACA TGTAAACA-- GCTCAGAACT

ACACTGATAG AAATGCCATA ---ACAAACA TGTAAACAAG GCTCAGAACT

ACATTAATAG AAATTCCATA AAAACAAGCA TGTAAACAAG GATCAGAACT

ACATTGATAG AAATGCCATA AAAACAAACA TGTAAACAAG GTTCAGAACT

ACATTAATAG AAATGCCATA AAAACAAACA TGTAAACAAG ATTCAGAACT

ACATTGACAG AAATGTCATA TAAACAAACA TGTAAACAAA TTTCAGAACT

ACACTCATAG AAATACCATA AAAACAATCA TGTAAACAAG GTTCAGAACT

ACATCGATGG AAATGTCATG AAAATAAATC TGTAAACAAG GTTCA-----

ATACTGATGA AAATGTCATA AAAACAAACA TGTAAACAAG ATCCAGCACT

GCAGCCATAG GAACACAATA AAATTAAATG AACACATGAT GTTTAAAACT

ACATCCATAG AAATGCAATA AAATTAAACA TACAAATAAC CTTCAAAATT

TTGGTTCACT GAAACATCTC ACACCTAAAA CACCTGAGGT ACAAGGCACT

TTGGTTCACT GAAACATCTC ACACCTAAAA CACCTGAGGT ACAGGGCACT

TTGGTTCATT GAAACATCTC ACACCTAAAA CACCTGAGGT ACAAGGCACT

TTGGTTCATT GAAACATCTC ACACCTAAAA CACCTGAGGT ACAAGGCACT

TTGGTTCATT GAAACACCTC ACACCTAAAA CACCTGAGGT ACAAGGCACT

TTGGTTTACT GAAACATCTC ACACCTAAAA CATCTGAGGT ACAAGGCACT

TTGGTTCACT GAAACATCGT CCACCTGAAA TATCTGAGGT ACAAGGCACT

TTGGTTCACT GAGACATCTT CTACCTGAAA TATCTGAGGT ACAAGGCACT

TTGGTTCACT GAAACATCTT ACACCTAAAA CATCTGAGGT ACAAGGCACT

TTGGTTCACT GAAACATCTC ACACCTAAAG CATCTGAGGT ACAAAGCACT

TTGGTTCACT GAAACACCTC ACACCTAAAA CATCTGAGGT ACAAGGCACT

TTGGTTCACT GAAACATCTC ACACCTAAAA CATCTGAGGT ACAAAGCACT

TTGGTTCACT GAAATGTCTC ACACCAAAAA CATCTGAGGT ACAAGGCACT

TTGGTTCACT GAGACATCCC ACACTTAAAA CATCTGAGGT ACCAGGCAAT

--GATTCACT AAAATATTTC ---------- ---------- ACAAGGCACT

TTGGTTCACT GAAATATCTC ACACCTAAAA CATCTGAGGT ACAAGGCACT

TTGGTTTGAG GAAATATCAA ATATCCAAAA CATCTGGGAA ATACATCACT

TTGGTTTGAT GAAATATTTA ACATCCATAG CATCTGAGGT ACAAGGCACT

CAGTCAACTT CTGTTGATCC ACAAGGTCTC AAGAACTCCT GTCCTCGTTC

CAGTCAACTT CTGTTGATCC ACAAGGTCTC AAGAACTCCT GTCCTCGTTC

CAGTCAACTT CTGTTGATCC ACAAGGTCTC AAGAACTCCT GTCCTCGTTC

CAGTCAACTT CTGTTGATCC ACAAGGTCTC AAGAACTCCT GTCCTCGTTC

CAGTCAACTT CTGTTGATCC ACAAGGCCTC AAGAACTCCT GTCCTCGTTC

CAGTCAACTT CTGTTGATCC ACAAGGCCTC AAGAACTCTT GTCCTCGTTC

CAATCGACTT CCGTTGTCCC ACAAGGCCTC AAGAACTCCT GTCCTCGCTC

CAATCAACTT CCGTTGTTCC ACAAGGTCTT AAGAACTCCT GTCCTCGCTC

CAGTCAACTT CTGTTGATCC ACAAGGTCTC AGGAACTCCT GTCCTCGTTC

CAGTCAACTT CTGTCGATCC ACACGGCCTC AAGAATTCCT GTCCTCGTTC

CAGTCAACTT CTGTTGATCC ACATGGCCTC AAGAATTCCT GTCCTCGTTC

CAGTCGACTT CTGTTGATCC ACACGGCCTC AAGAACTCCT GGCCTCGTTC

CAGTCAACTT CTGTTGATCC ACATGGCCTC AAGAACTCCT GTCCTCGCTC

CAGTCAACTT CTGTGGATCC ACAAGGCCGC AAGAATTCCT GTCCTCGTTC

CAGTCAACTT CTGTTGATCC A----GTCTT AAGAACTCTT GTCCTCACTC

CAGTCGACCT CTGTTGATCC ACACGGCCTC AAGAACTCCT GTCCTCGCTC

CAGTCGACTT CTGTTGATCC ACATCGTCTC AAGAACTCCT GCCCCCGCTC

CAATCAACTT CTGTGGATCC GCAAGGCCGC AAGAATTCCT GTCCTCGTTC

ATGGAACCAC TTATTGGAGA CTGTGAAAAG ACACTCACCC CACTTGCAGC

ATGGAACCAC TTATTGGAGA CTGTGAAAAG ACACTCACCC CACTTGCAGC

ATGGAACCAC TTATTGGAGA CTGTGAAAAG ACACTCACCC CACTTGCAGC

ATGGAACCAC TTATTGGAGA CTGTGAAAAG ACACTCACCC CACTTGCAGC

ATGGAACCAC TTATTGGAGA CTGTGAACAG ACACTCACCC CACTTGCAGC

ATGGAACCAC TTATTGGAGA CTGTGAACAG ATACTTACCC CACTTGCAGC

ATGGAACCAC TTATTGGAGA CTGTGAACAG ACACTCACCC CACTTGCAGC

ATGGAACCAC TTGTTGGAGA CTGTGAACAG ACACTCACCC CACTTGCAGC

ATGAAACCAC TTGTTGGAAA CTGTGAACAG A-ACTCACCC CACTTGCAAC

ATGGAACCAC TTATTGGAGA CTGTGAACAA ACACTCACCC CACTTGCAGC

ATGGAACCAC TTATTGGAGA CTGTGAACAG ACACTTACCC CACTTGCAGC

ATGGAACCAC TTATTGGAGA CTGTGAACAA ACACTCACCC CACTTGCAGC

ATGGAACCAT TTATTGGAGA CTGTGAACAG ACACTCACCC CACTTGCAGC

ATGGAACCAT TTATTGGAGA CTGTGAACAG ACACTCACCC CACTTGCAGC

ACAGAACCAC CTATTGGAAA CTGTGAACAG GCACTCACCT CACTCGTAGC

GTGGAACCAC TTATTGGAAA CTGCAAACAG ACACTCACCC CACTTGCAGC

ATGGAACCAT TTATTGGAAA CTGTGAATAA ACACTCACCC CATTTACTGC

ATGGAACCAT TTATTTGAAA CTGTGAACGG ACACTTACCC CACTTGCACC

CCACAAGCAC AGGGCAGGCA GCATGTCTTG TTCGGTAGTC ACCTTCCCCG

CCACAAGCAC AGGGCAGGCA GCATGTCTTG TTCGGTAGTC ACCTTCCCCG

CCACAAGCAC AGGGCAGGCA GCGTGTCTTG TTCGGTAGTC ACCTTCCCCG

CCACAAGCAC AGGGCAGGCA GCGTGTCTTG TTCGGTAGTC ACCTTCCCCG

CCACAAGCAC AGGGCAGGCA GCATGTCTTG TTCGGTAGTC ACCTTCCCCA

CCACAAGCAC AGGGCAGGCA GCATGTCTTG TTCGGTAGTC ACCTTCTCCA

CCACAAGCAC AGGGCAGGCC GCGTGCCTCG TTCGATAATC GCCTTCCCCG

CCACAAGCAC AGGGCAGGCT GCATGTCTCG TCCGATAATC ACCTTCCCCA

CCACAAGCAC AGGGCAGGCG GCGTGTCTTG TTCGGTAGTC ACCTTCCCCA

CCACAAGCAC AGGGCAGGCA GCATGTCTTG TTCGATAGTC ACCTTCCCCA

CCACAAGCAC AGGACAGGCA GCGTGTCTTG TACGGTAGTC ACCTTCCCCA

CCACAAGTAC GGGGCAGGCA GCGTGTCTCG TTCGGTAGTC ACCTTCCCCA

CCACAAGCAC GGGGCAGGCC GCATGCCTCG TCCGATAGTC CCCTTCCCCA

CCACAAGCAC GGGGCAGGCA GCATGTCTTG TCCGGTAGTC GCCTTCCCCA

CCACAAGCAC AAGGCAAGCA GCATGTCTCA TCAGATGGCT GCGTTTCTCA

CCACGAGCAC GGGGCAGGCA GCGTGTCTCG TCCGGTAGTC GCCTTCGCCA

CAACAAGCAC TGGACAGGCT GCATGTCTTG TCCGGTAGTC ACACTCCCCA

CTACTAGTAC CGGACAAGCC GCATGTCGTG TCCTGTAATC TCCCTCTCCG

CTCCGCAAGA GGTTGTACCA GAACACAGCT GTACCCTGGA AAGTGGCACC

CTCCGCAAGA GGTTGTACCA GAACACAGCT GTACCCTGGA AAGTGGCACC

CTCCGCAAGA GGTTGTACCA GAACACAGCT GTACCCTGGA AAGTGGCACC

CTCCGCAAGA GGTTGTACCA GAATACAGCT GTACCCTGGA AAGTGGCACC

CTCCGCAAGA GGTTGTACCA GAATACAGCT GTACCCTAGA AAGTGGCACC

CTCCGCAAGA GGTTGTACCA GAATACAGCT GTACCCTGGA AAGTGGCACC

CTCCGAAGAA GGTTATACCA AAATACAGCC GTGCCCTGGA AAGTGGCGCC

CTGCGAAGAA GGTTGTACCA GAATACAGCT GTGCCCTCGA AAGTGGCACC

CTCCGCAGAA GGTTGTACCA GAATACAGCT GTGCCCTCGA AAGTGGCACC

CTCCGTAGGA GGTTGTACCA GAATACAGCT GTGCCCTGGA AAGTGGCACC

CTCCGCAGAA GGTTGTACCA GAATACAGCT GTACCCTGGA AAGTGGCACC

CTCCTGAGGA GATTGTACCA GAATACTGCT GTGCCCTGGA AAGTGGCACC

CTCCGCAGGA GGTTGTACCA GAACACAGCG GTGCCCTGGA GAGTGGCACC

CTCCGCAGGA GGTTGTACCA GAACACGGCT GTGCCCTGGA AAGTGGCACC

CTCCACAGG- --TTATACCA GAACAAGCCT GTGCCCTAGA AG--------

CTCCGCAGGA GATTGTACCA GAACACAGCT GTGCCCTGTG CGGTGGCACC

CTGCGGAAGA GATTGTACCA GAATACTGAT GTGCCCTGAA GAGTGGCTCC

CTTCTGAACA GATTATACCA AAACACCGCT GTCCCCTGAA AGGTGGCACC

ACCAGCTTCT ACATCACTCA TCTGGAAATA TAACCTACTT TAGGTTTTGC

ACCAGCTTCT ACATCACTCA TCTGGAAATA CAACCTACTT TAGGTTTTGC

ACCAGCTTCT ACATCACTCA TCTGGAAATA TAACCTACTT TAGGTTTTGC

ACCAGCTTCT ACATCACTCA TCTGGAAATA TAACCTACTT TAGGTTTTGC

ACCAGCTTCT ACATCACTCA TCTGGAAATA TAACCTACTT TAGGTTTTGC

ACCAGCTTCT ACATCACTCA TCTGGAAACA TAACCTACTT TGGGTTTTGC

ACCAGCTTCA ACATCACTCA TCTGGGAACA TAACCTACTT TGGGCTTAGC

ACCAGCTTCG ACATCGCTCA TCTGGGAACA TAACCTACTT TGGGCTTAGC

ACCAGCTTCT ACATCACTCA TCTGGGAATA CAACCTACCT TGGGTTTCGC

ACCAGCTTCT ACATCACTCA TCTGGGAATA CAACCTACTT TGGGTTTCGC

ACCAGCTTCT ACATCACTCA TCTGAGAATA TAACCTACTT TGGGTTTCGC

CCCAGCTTCT ACATCACTCA TCTGGGAATA CAACCTACTT TGGGTTTCGC

CCCTGCTTCT ACATCACTCA TCTGGGAATG CAATCTACCT TAGGTTTTGC

ACCAGCTTCT ACATCACTCA TCTGGGAATA TAACCTACTT TGGGTTTCGC

---------- ---------- ---------- ---------- ----------

ACCAGCTTCC ACATCGCTCA TCTGGGAAGA TAACGTACTT TGGGCTTCGC

ACCAGCTTCA ACATCACTCA TCTAGGAAAA TAACTTACTT TAGGCTTGGA

ACCGGCTTCG ACGTCACTCA TCTACAAAGA CATCCTACCT TGGGTTTAGC

GATCTCCTTG ATCCTCTCGA TTTCCTCATC AGACATGACA TCGTAGTACC

GATCTCCTTG ATCCTCTCGA TTTCCTCATC AGACATGACA TCGTAGTACC

GATCTCCTTG ATCCTCTCGA TTTCCTCATC AGACATGACA TCGTAGTACC

GATCTCCTTG ATCCTCTCGA TTTCCTCATC AGACATGACA TCGTAGTACC

GATCTCCTTG ATCCTCTCGA TTTCCTCATC AGACATGACA TCATAGTACC

AATCTCCTTG ATCCTCTCGA TTTCCTCGTC TGACATGACA TCGTAGTACC

AATCTCCTTG ATCCTCTCGA TCTCTTCGTC CGACATCACA TCATAGTACC

AATCTCCTTG ATCCTCTCGA TTTCTTCGTC GGACATCACA TCATAGTACC

AATCTCCTTG ATCCTCTCGA TCTCCTCATC AGACATGACG TCGTAGTACC

AATCTCCTTG ATCCTCTCGA TTTCCTCGTC AGACATGACG TCGTAGTACC

GATCTCCTTG ATCCTCTCGA TTTCCTCATC AGACATGACA TCATAATACC

GATCTCCTTG ATCTTCTCGA TTTCCTCATC AGACATGACG TCGTAGTACC

AATCTCCTTG ATCCTCTCGA TTTCCTCATC AGACATGACA TCGTAGTACC

AATCTCCTTG ATCCTCTCGA TTTCCTCGTC AGACATGATG TCATAGTACC

---------- ---------- ---------- ---------- ----------

AAGCTCCTTG ATCCTCTCGA TTTCCTCATC AGACATGACA TCGTAGTACC

AATTTCCTTG ATCTTCTCAA TCTCCTCATC TGATAAGACA TCGTAGTATC

CAGCTCTTTA ATCTTCTCAA TCTCTTCATC GGACAGGACG TCGTAATAGC

TGACGATGTG CGGGCTGTCC CACTCGTCCT CCTCTTTGAA GGGGGCAATG

TGACGATGTG CGGGCTGTCC CACTCGTCCT CCTCTTTGAA GGGGGCAATG

TGACGATGTG TGGGCTGTCC CACTCATCCT CCTCTTTGAA GGGGGCGATG

TGACGATGTG TGGGCTGTCC CACTCATCCT CCTCTTTGAA GGGGGCGATG

TCACGATGTG CGGGCTGTCC CACTCGTCCT CCTCTTTGAA GGGGGCGATG

TGACGATGTG TGGGCTGTCC CACTCGTCCT CCTCTTTGAA GGGGGCGATG

TGACGATGTG TGGGCTGTCC CACTCGTCTT CCTCTTTGAA GGGGGCAATG

TGACGATGTG TGGGCTGTCC CACTCGTCTT CCTCTTTGAA GGGGGCGATG

TGACGATGTG CGGGCTGTCC CACTCGTCCT CCTCCTTGAA GGGCGCGATG

TGACGATGTG CGGGCTGTCC CACTCATCCT CCTCTTTGAA GGGGGCGATG

TGACGATATG CGGGCTGTCC CACTCATCCT CCTCTTTGAA GGGGGCGATG

TGACGATGTG CGGACTGTCC CACTCGTCCT CCTCTTTGAA GGGGGCAATG

TGACGATGTG CGGGCTGTCC CACTCATCCT CCTCTTTGAA GGGGGCGATG

GCACGATGTG TGGGCTATCC CACTCATCTT CCTCTTTAAA GGGGGCGATG

---------- ---------- ---------- ---------- ----------

TGACGATGTG TGGGCTGTCC CACTCGTCCT CCTCTTTGAA AGGGGCGATG

TCACGATGTG TGGGCTGTCC CACTCGTCTT CCTCCTTAAA GGGTGCAATC

GGACGATATG AGGGCTATCC CACTCATCCT CTTCCTTAAA GGGGGCGATG

AGCAGCTGTG GTTGCCATGG TGGTACCTAC AGAAAAGCCT CTTCTGTCTA

AGCAGCTGTG GTTGCCATGG TGGTACCTAC AGAAAAGCCT CTTCTGTCTA

AGCAGCTGTG GTTGCCATGG TGGTACCTAC AGAAAAGCCT CTTCTGTCTC

AGCAGCTGTG GTTGCCATGG TGGTACCTAC AGAAAAGCCT CTTCTGTCTC

AGCAGCTGTG ATTGCCATGG TGGTACCTAC AGAAAAGCCT CTTCTGCCTC

AGCAGCTGTG GTTGCCATGG TGGTACCTAC AGAAGAGCCT CTTTTGCCTC

AGGAGCTGTG GTTTCCATGG TGGTATCTAC AGAAAAGCCT CTTCTGCCTC

AGGAGCTGTG GTTTCCATGA TGGTACCTAC AGAAAAGCTT CTTCTGCCTC

AGCAGCTGCG GTTGCCATCG TGGTACCTGC AGAAAAGCCT CTTCTGTCTC

AGCAGCTGCG GTTGCCATGG TGATACCTAC AGAAGAGCCT CTTCTGCCTT

AGCAGCTGTG GTTGCCATGG TGGTACCTAC AGAAAAGCCT CTTCTGCCTC

AGCAGCTGTG GTTGCCATGG TGGTACCTAC AGAAAAGCCT CTTCTGCCTC

AGCAGCTGTG GTGGCCATGG TGGTACCTAC AGAAAAGCCT CTTCTGCCTC

AGCAGCTGGG GTTGCCATGG TGATATCTAC AGAAAAGCCT CTTCTGCCTC

---------- ---------- ---------- ---------- ----------

AGCAGCTGTG GTGGCCATGA TGGTATCTAC AGAAAAGCCT CTTCTGCCTC

AGCAGCTGGG GTTGCTATCG TGGTATCTAC AGAAAAGTCT CTTCCTTCTC

AGCAGCTGGG GTTGCCATCA TGATATCTGC AGAAAAGTCT CTTCTGCCTC

CGGGGTGTCT GCTCTCTTCC TCCAATAACT GCTCAAAGTA CCGCAGATTC

CGGGGTGTCT GCTCTCTTCC TCCAATAACT GCTCAAAGTA CCGCAGATTC

CGGGGTGTCT GCTCTCTTCC TCCAATAACT GCTCAAAGTA CCGCAGATTC

CGGGGTGTCT GCTTTCTTCC TCCAATAACT GCTCAAAGTA CCGCAGATTC

CGGGGTGTCT GCTTTCTTCC TCCAATAACT GCTCAAAGTA CCGCAGATTC

CGGGGTGTCT GCTTTCTTCC TCCAACAAGT GCTCAAAGTA CCGCAGATTT

CGGGGTGTCT GCTTTCTTCC TCTAACAGCC GCTCAAAGTA CCGCAGGTTC

CGGGGTGTCT GCTTTCTTCC TCTAACAACC GTTCAAAGTA CCGCAGATTC

CGGGGGGTCT GCTTTGTTCC TCCAACAACC GCTCAAAGTA GCGCAGATTC

CGGGGGGTCT GCTTTCTTCT TCCAACAACC GTTCAAAGTA GTGCAGATTC

CGGGGAGTCT ACTTTCTTCC TCCAGCAACC GTTCAAAGTA CCGCAGATTC

CGGGGCGTCT GCTTTCTTCC TCCAACAACC GTTCAAAGTA CCGCAGATTC

CGGGGCGTCT GCTCTCTTCC TCCAACAGTC GTTCGAAGTA CCGCAGATTG

CGGGGTGTCT GCTTTCTTCC TCTAACAACC GTTCAAAGTA CCGCAGATTC

---------- ---------- ---------- ---------- ----------

CGGGGCGTCT GCTTTCTTCT TCCAACAAGT GTTCAAAGTA CCGCAGGTTC

TGAGGTGTCT GCTTTCTTCC TCTATTAGCT TCTCAAAGTA CCGCAGATTC

CGGGGTGTCT GCTTTCTTCT TCGAGCAATT TCTCAAAATA TCGTAAGTTT

CCTCCAGCTC GTTCGTGGCT TGGGTCTAGA AAATGCAAGG AAAAGGAAAA

CCTCCAGCTC GTTCATGGCT TGGGTCTAGA AAAAGCAAGG AAAAGGAAAA

CCTCCAGCTC GTTCATGGCT TGGGTCTAGA AAAAGCAAGG AAAAGGAAAA

CCTCCAGCTC GTTCATGGCT TGGGTCTAGA AAAAGCAAGG AAAAGGAAAA

CCTCCAGCTC GTTCATGGCT TGGGTCTAGA AAAAGCAAGG AAAAAGAAAA

CCTCCAGCTC GTTCATGGCT CGGATCTAGA AAAAGCAAAG AAAAGGAAAA

CCTCCGGCTC GTTCATGGCT CGGGTCTAGA AAAAGGGAAA GA--------

CCTCCAGCTC GTTCGTGGCT TGGGTCTGGG AAAGGTGAAA GGAA---CAA

CCTCCAGCTC GCTCATGGCT TGGATCTAGA AAAAGCAAAG AAAAGGAAAA

CCTCCAGCTC GTTCATGGCT CGGGTCT-AC AATAGGAAAG AAAAGGAAAA

CCTCCAGCTC GTTCATGGCT TGGGTCTAGA AAGAGCAGAG AAAAGGGAAA

CCTCCAGCTC GTTCATGGCT TGGGTCTAGA AAAAGCACAG AAAAAGAAAA

CCTCCAGCTC GTTCGTGGCT CGGCTCTAGA AAAGGCAAAG GAAAGGAAGA

CCTCCAGCCC GTTCATGGCT TGGGTCTACA AAAAGCAAAG AAAAGGAAAA

---------- ---------- ---------- ---------- ----------

CCTCCAGCCC GTTCGTGGCT CGGGTCTGGA ACAAGCAGAG GGGCAGAAAA

CCCCCGGCTC TTTCATGATT GGGGTCTAGA AAAAGGAGAG AAGAGGACAA

CCTCCAGCTC GTTCGTGACT GGAGTCTAGA CAACAAAGAG GAAAAGAAAA

GGAAACAGAG AAATTGTGAG CTCCAGGGCA CGGTGCAGAT CACCCAACTG

GGAAACAGAG AAATTGTGAG CTCCAGGGCA CGGTGCAGAT CACCCAACTG

GGAAACAGAG AAATTGTGAG CTCCAGGGCA CGGTGCAGAT CACCCAACTG

GGAAACAGAG AAATTGTGAG CTCCAGGGCA CGGTGCAGAT CACCCAACTG

GGAAACAGAG AAATTGTGAG TTCCAGGGCA CGATGCAGGT CACCCAACTG

GGAAACAAGG AAATTGTGAG CTCCAGGGCA CGGTGCAGAT CACCCAACTG

--AAACACGG AGACTGTGAG TTCCACCGCA CGGTGCAGGT CACCCAGCTG

GGAAACACAG AAACTGTGAG TTCCACAGCA CGGTGCAGGT CACCCAGTTG

GGAAACAGAG GAATTGTGAG CTCCAGGGCA CGATGCAGGT CACCCAACTG

GGAAACAGAG AAATTGTGAG CTCCACGGCA CGGTGCAGGT CACCCAACTG

GGAAACAAAG ACATTGTAAG CTCCAGGGCA CGGTGCAGGT CACCCAACTG

GGAAACAGAG AAATTGTGAG CTCCAGGGCA CGGTGCAGGT CGCCCAGCTG

GGAAACAGAG AAATT----- ---------- ---------- ----------

GGAAACAGAG AAATTGTGAG CTCCAGGGCC CGGTGCATGT CTCCCAGCTG

---------- -----GTGAA GTCCACAATC CTGTGCAGGT -GCCCAAGCA

GGAAACAGAG ACCTTGTGAG CTCCAGGGCC CGGGGCAGGT CGCCCAGCTG

GGAAACAGAG AAATCGTGAA TTCTAGAGCC CTGTGAATGT CTCCAAGCTG

GGAAACAGGT AAGTCGTGAG CTCCACCGCT CTGTGTAGAT CTCCGAGCTG

GAAGACAGCA TAGCTGAGGT AGTCCAGCAC CTGTGACTTG TCCTCCCCGG

GAAGACAGCA TAGCTGAGGT AGTCCAGCAC CTGGGACTTG TCCTCCCCGG

AAAGACAGCA TAGCTGAGGT AGTCCAGCAC CTGGGACTTG TCCTCCCCAG

AAAGACAGCA TAGCTGAGGT AGTCCAGCAC CTGGGACTTG TCCTCCCCAG

GAAGACAGCA TAGCTGAGGT AGTCCAGCAC CTGGGACTTG TCCTCCCCAG

GAAGACAGCA TAGCTGAGGT AGTCCAGCAC CTGGGACTTG TCCTCCCCAG

GAAGACGGCA TAGCTCAGGT AGTCCAGCAC CAGGGATTTG TCCTCCCCAG

GAAGACAGCA TAGCTCAGGT AGTCCAGCAC CAGGGACTTG TCCTCCCCAG

GAAGACGGCA TAGCTGAGGT AGTCCAGCAC CTGAGATTTG TCCTCGCCAG

GAAGACAGCG TAGCTCAGAT AGTCCAGCAC CTGGGCCTTG TCCTCCCCAG

GAAGACAGCA TAGCTCAGGT AGTCTAGCAC CTGGGCCTTG TCCTCTCCAG

GAAGACAGCA TAGCTCAGGT AGTCCAGCAC CTGGGCCTTG TCCTCCCCAG

---------- ---------- ---------- ---------- ----------

GAAGACAGCA TAGCTGAGGT AGTCAAGCAC CTGGGCCTTG TCCTCCCCTG

GAAGCTGCCA TTACTGAGAT AATCCAGGAC TTAGGCCTTA TGCTCTCTTG

GAAGACGGCG TAGCTGAGGT AGTCCAGCAC CTGGGCCTTG TCCTCCTCCG

AAAGACAGCA TAGCTGAGGT AATCCAAGAT TTCGACTTTA TCCTCACCAG

AAAAACGGCA TAGCTGAGGT AATCCAAGAC CTCCACCTTG TCCTCCCCCT

CATCAAGCTG CTTTAGCACC TGCTCCATCC ACAACACCGT ATGATAATAG

CATCAAGCTG CTTTAGCACC TGCTCCATCC ACAACACCGT ATGATAATAG

CATCAAGCTG CTTTAGCACC TGCTCCATCC ACAACACCGT ATGATAATAA

CATCAAGCTG CTTTAGCACC TGCTCCATCC ACAACACCGT ATGATAATAG

CATCAAGCTG CTTTAGCACC TGCTCCATCC ACATCACCGT ATGATAATAG

CATCAAGCTG CTTGAGCACC TGCTCCATCC ATAGCACCGT GTGATAATAG

CATCCAGCTG CTTCAGCACC TGCTCCATCC ACAACACGGT GTGGTAATAG

CATCGAGCTG CTTCAGTACC TGCTCCATCC ACAGCACAGT ATGGTAATAG

CATCGAGCTG CTTCAGCACC TGCTCCATCC ACAGCACCGT GTGATAGTAG

CATCGAGCTG CTTTAGCACC TGTTCCATCC ACAACACCGT GTGGTAATAG

CATCGAGCTG CTTCAGTACC TGCTCCATCC ACAAGACTGT GTGGTAATAG

CATCAAGCTG CTTCAGCACC TGCTCCATCC ACAGCACCGT GTGGTAATAG

---------- ---------- ---------- ---------- ----------

CATCAAGCTG CTTCAGCACT TGCTCCATCC ACAAGACTGT ATGATAATAG

CATTCAGCTT CTTCACTGCC TATTATATCC AAAGCATTGA GTGATAAAAG

CGTCGAGCTG CTTCAGCACC TGCTCCATCC ACAGCACAGT GTGGTAGTAG

CATCGTGTTG CTTCAGTGCC TGCTGCATCC ACAGGACTGT GTGGTAATAG

GGTCGTGCTG CTTCAGGGCC TGCTGCATCC AAAGCACCGT GTGATAGTAA

TCCCCTTCAT TGTAGGCAAC TTTGAGAAAT GAAAAATTTT TATTTTTTAC

TCCCCTTCAT TGTAGGCAAC TTTGATAAAT GAAAAATTTT TATTTTTTAC

TCCCCTTCAT TGTAGGCAAC TTTGAGAAAT GAAAAATTTT TATTTTTTAC

TCCCCTTCAT TGTAGGCAAC TTTGAGAAAT GAAAAATTTT TATTTTTTAC

TCCCCTTCAT TATAGGCAAC TTTGAGAAAT GAAAAATTTT TATTTTTTAC

TCCCCTTCGT TGTAGGCAAC TTTGAGAAAT GAAAAATGTT TATTTTTTAC

TCTCCTTCGT TGTAAGCAAT TTTGAGaaat aaaaaatatt ttttttttaC

TCTCCTTCAT TGTAAGCAAT TTTGAGAAAT AAAAAATCTT TATTTTTTAC

TCCCCTTCAT TGTAGGCAAC TCTGAGAAAT GAAAAATCTT TATTTTTTAC

TCGCCTTCGT TGTAGGC--- ---------- ---------- ----------

TCACCTTCAT TATAGGCAAC TTTGAGAAAT GAAAAATCTT TATTTTTTAC

TCCCCCTCAT TGTAAGCAAC TTTGAGGAA- -AAAAATCTT TATTTTTTAC

---------- -------AAC TTTGAGAA-- -AGAAGTCTT TATTTTTTAC

TCTCCTTCAT TGTAGGCAAC TTTGAGAAAT GAAAAATCTT TATTTTTTTA

TCCTTGATAT GGTGGGCAAC TTTGAGAAAT GAAAAATCTT TATTTTTTAT

TCCCCCTCAT TGTGGAC--- ---------- ---------- ----------

TCCCCATTAT TGTAGGCAAC TTTGAAAATT GAGATATCTT TATTTTTTAC

TCCCCGTCAT TGTAGGCGTC TCTGAGATTT GAAAGGTCTT TATTTTTTAC

AAAAATTTTC ATAAATGCAT TTTATTTAAT ATGCATGCTA CTACAAAACT

AAAAATTTTC ATAAATGCAT TTTATTTAAT ATGCATGCTA TTACAAAACT

AAAAATTTTC ATAAATGCAT TTTATTTAAT ATACATGCTA TTACAAAACT

AAAAATTTTC ATAAATGCAT TTTATTTAAT ATACATGCTA TTACAAAACT

AAAAATTTTC ACAAATGCAT TTTGTTTGAT ATGCATGCTA TTACAAAACT

AAAAATTTTC ATAAATGCAT TTTACTCGAT ATGCATGCTA CTACAAAACT

AAAAGTTTCC GTCAGTGCAT TTTGTTTGCC GACCATGCTG TTACAAAAAT

AAAAGTTTCT GTAAGTGCAT TTTGTTTGCT GTTCATGCTG TTACAAAAAT

AAAAGTTTTC ATAAATGCAT TCTGTTTGAT ATGCATGCTA TTACAAAACT

---------- ---------- ---------- ---------- ----------

AAAAGTTTTC ATAAATGCAT TTTATTTGAT AGGCATGCTA TTACAAAACT

AAAAGTTTTC ATAAATGCAT TTTATTTGAT ATGCATGCTA TTACAAAACT

AAAAGTTTTC ATAAATGCAT TTTATTTAAT ATGCATGCTA CCataaaatt

CAAAATTTTC ATAAATGCAT TATATTTGAT ATGCATGCTA TTACAAAACT

AAAAGTTTTC ATAAATGCAT TTTGTTTGAT ATGCATGCTA TTACAAAACT

---------- ---------- ---------- ---------- ----------

AAAAGTTTTC ATAAATGCAT TTTATTCAAT ATACATGCTA CTACAAAACC

Aaaaattttc ataaatgctt ttttaatttt atacatgcta ttccaaAACT

GAAAATGTAT TACAGTTAAG ATACATGCAT ATTTGTAATA TTTTAATCCC

GAAAATGTAT TACAGTTAAG ATACATGCAT ATTTGTAATA TTTTAATCCC

GAAAATGTAT TACAGTTAAG GTACATGTAT ATTTGTAATA TTTTAATCCC

GAAAATGTAT TACAGTTAAG GTACATGTAT ATTTGTAATA TTTTAATCCC

GAAAATATAT TACAGTTAAG ATACATGCAT ATTTGTAATA TTTTAATCCC

GAAAATATAT TACAGTTAAG ATACATGCAT ATTTGTAATA TTTTAATCCC

GAAAATATAT TACAGTTAAG ATACATGCAT ATTTGTAATA TTTTAATCCA

GAAAATATAT TACAGTTAAG ATACATGCAT ATTTGTAATA TTTTAATTCC

GAAAATATAT TACAGTTA-- ---TATGCat atttttaata ttttaCTCCC

---------- ---------- ---------- ---------- ----------

AAAAATATAT TACAGTTAAG ATACATGCGT ATTTGTAATA TTTTAATCTC

-AAAATATAT TATAGTTAAG ATACATGCAT ATTTGTAATA TTTTAATCTC

aaaaatatat taCA-TGAAG ATACATGCAT ATTTGTAATC TTTTAATATC

GAAAATATAT TACCGTTAGG ATACATGCAT ATTTGTAATA TTTTAATCCC

TAAAATATAT TACAGTTAAG ACAAATGCAT ATTTGTAATA TTTTAATCCC

---------- ---------- ---------- ---------- ----------

AAAAATGTAT TACAATTAAG ACACGTACAT ATTTGTAATA TTTTAATCCC

GAAAAAGTAT TACAATTAAG ATATACAAAT ATTTGTCATA TTTTAATCTG

CTCGATTTTT AAAAATTGAA AGTGGAATAA GCAATTCTTC AAAAAATTAC

CTCAATTTTT AAAAATTGAA AGTGGAATTA GCAATTATTC AAAAAATTAC

CTCAATTTTT AAAAATTGAA AGTAGAATAA GAAATTCTTC AAAAAATTAC

CTCAATTTTT AAAAATTGAA AGTAGAATAA GCAATTCTTC AAAAAA----

CTCAATTTTT AAAAATTGAA AGTAGAATAA GCAATTCTTC AAAAAATTAC

CCCAATTTTT AAAAATTGAA AGTAGAATAA GCAATTCTTC AAAAAATTAC

TTCA-TTTTC AAAAGTTGAA AGTAGAGTAA GCAATTCTTC GAAAAATTAC

TTGA-TTTTC AAAAATTGAA AGTAGGATAA GCAATTCTTC AAAAAATTAC

CTCAATTTTC AAAAATTGAA AG---AATAA GCAGTTCTTC AAAAAATTAC

---------- ---------- ---------- ---------- ------TTAC

CTCAGTTTTT AGAAATTGAA AGTAGGATAA GCAATTCTTC AAAAAAGTAC

----ATTTTT AAAAATTGAA AGTAGCATAA GCAAGTCTTC AAAAAATTAC

----ATTTTA AAAAATTGAA ------ATAA GCAATTCTTC AAAAAA----

CTCATTTTAA AAAAATGTAA AGTAGAATAA GCAACTCTTC AAAAAATTAC

CTCAATTTTT AAAAATTGAA AGTAGAATAA GCAATTCTTC AAAAAA----

---------- ---------- ---------- ---------- ------TTAC

CTCACTTTTA AAAAAGTAAA ATTAGAATAA GCAAAACTTA CGAAAATTAC

TGCACTTTTT GGAAGTTGAA AATAGAATAA GCAATACTTA GGAAGATTAC

CTGGAAGTTC CCCTCTGGAA ATTGTGCCTG GGTCCAGCCT GTATGTGTCC

CTGGAAGTTC CCCTCTGGAA ATTGTGCCTG GGTCCAGCCT GTATGTGTCC

CTGGAAGTTC TCCTCTGGAA ATTGTGTCTG GGTCCAGCCT GTATGTGTCC

---------- ---------- ---------- ---------- ----------

CTGGAAGTTC TCCTCTGGAA ATTGTGTCTG GGTCCAGCCT ATATGTGTCC

CTGGAAGCTC CCCTCTGGAA ATTGTGTCTG GGTCCAGCCT GTATGTGTCC

CTGGAAGCTC CCCTCTAGAA ATCATGTCCG GATCCAGTTT GTAGGTGTCC

CTGGAAGTTC CCCTCTGGAA ATCGTGTCCG GATCCAGTTT GTACGTGTCC

CTGGAAGCTC CCCTCTGGAA ATCGTGGCTG GGTCCAGCTT GTAGGTGTCC

CTGGAAGTTC CCCTTTGGAA ATTGTATCTG GGTCCAGTTT GTATGTATCC

CTGGAAGTTG CCCTTTGGAA ATTGTGTCTG GATCCAGCTT GTATGTGTCC

CTGGAAGTTC CCCTTTGGAA ATCGTGTCTG GGTCCAGCTT GTATGTGTCC

---------- ---------- ---------- ---------- ----------

CTGGAAGCTC CCCTTTGGAA ATCGTGTCTG TGTCCAGCCG GTACGTGTCC

---------- ---------- ---------- ---------- ----------

CAGGAAGTGC CCCTTTGGAA ATGGTGTCCG GATCCAGTTT GTACGTGTCC

CTGGAAGATC CCCCTTGGAA ATAGTGTCAG GATCCAGTTT GTATGTGTCC

CTGGAAGGTC CCCTTTAGCA ATGGTGTCCG GGTCCAGTTT GTAAGTGTCT

TGAAGTCTCA TCAGGGCTTT GGCAGCTCCT ATCTCGTCCT CATCAGTGGG

TGAAGTCTCA TCAGGGCTTT GGCAGCTCCT ATCTCGTCCT CATCAGTGGG

TGAAGTCTCA TCAGGGCTTT GGCAGCTCCT ATCTCATCCT CGTCAGTGGG

---------- ---------- ---------- ---------- --TCATCGGG

TGAAGTCTCA TCAGGGCTTT GGCAGCTCCT ATCTCGTCCT CATCAGTGGG

TGAAGTCTCA TCAGGGCTTT GGCAGCCCCC ATCTCGTCCT CATCAGTAGG

TGAAGTCGCA TCAGGGCTCT GGCAGCTCCA GACTCGTCCT CGTCAGTGGG

TGAAGTCTCA TCAGGGCTCT GGCAGCTCCA GACTCGTCCT CATCAGTGGG

TGAAGTCTCA TCAGGGCCTT GGCGGCTCCA GCCTCATCCT CATCAGTGGG

TGAAGTCTCA TCAAGGCCTT GGCGGCTCCT GTTTCATCTT CATCGGTGGG

TGAAGTCTCA TCAGGGCCTT GGCGGCTCCC ATTTCGTCCT CGTCCGTGGG

TGAAGCCTCA TCAGGGCCTT GGCAGCTCCT GTTTCGTCCT CGTCAGTGGG

---------- ---------- ---------- ---------- ----------

TGAAGCCTCA TCAGGGCCTT GGCAGCTCCT GTTTCATCCT CGTCAGTGGG

---------- ---------- ---------- ---------- ----------

TGAAGCCTCA TGAGAGCCTT GGCAGCTCCC GTTTCATCCT CGTCTGTGGG

TGAAGGCGCA TTAATGCTAT TGCTGCTCCT GTTTCATCCT CTTCTGTGGG

TGCAGACGCA TTAAGGCCTT GGCTGCTCCG GTCTCATCCT CCTCCGTGGG

GAAGAACTGC CGCTGCACAG AGAGGTTGGC GATAAAACCT TGCCAGTCTG

GAAGAACTGC CGCTGCACAG AGAGGTTGGC GATAAAACCT TGCCAGTCTG

GAAGAACTGC CGCTGCACAG AGAGGTTGGC GATAAAACCT TGCCAGTCTG

GGAGA-CTGC CG-TGCCC-G AGAGGT-GGC GATAAA-CCT TGCCAGTCTG

GAAGAACTGC CGCTGCACAG AGAGGTTGGC GATAAAACCT TGCCAGTCTG

AAAGAACTGC CGCTGCACTG AGAGGTTGGC GATGAAACCT TGCCAGTCCG

GAAGAACTGC CGCTGAACTG AGAGGTTAGC CACAAAACCT TGCCAGTCTG

GAAGAATTGC CGCTGAACTG AGAGGTTAGC GACAAAACCT TGCCAGTCTG

AAAGAAGTGC CGCTGCATTG AGAGGTTGGC AATGAAACCT TGCCAGTCTG

GAAGAACTGT CGCTGCACTG ACAGGTTTGC AATAAAATCT TGCCAGTCCG

GAAGAACTGC CGCTGCACAG AGAGGTTGGC AATAAAACCT TGCCAGTCTG

GAAGAACTGT CGCTGTACAG AGAGGTTGGC AATAAAACCT TGCCAGTCTG

---------- ---------- ---------- ---------- -GCCAGTCTG

GAAGAATTGC CGCTGCACTG TGAGGTTGGC AAGAAAGCCT TGCCAGTCTG

---------- ---------- ---------- ---------- ----------

GAAGAACTGC CGCTGCACGG AGAGGTTGCC AATAAAGCCT TGCCAGTCTG

GAATAGCTGG CGCTGCATGG TGAAATTGGC AATAAAATCT TGCCAATCTG

GAAGAACTGT CGCTGCACTG TGAGGTTGGC GATGAAACCT GGCCAATCCG

TGTTTAGCCG CTTCACCAGT TTGTAGGCAT TCACAGGGTG AGCCAGGTAG

TGTTTAGCCG CTTCACCAGT TTGTAGGCAT TCACAGGGTG AGCCAGGTAG

TGTTTAGCCG CTTCACCAGT TTGTAGGCAT TCACAGGGTG GGCCAGGTAG

TGTTTAGCCG CTTCACCAGT TTGTAGGCAT TCACAGGGTG GGCCAGGTAG

TGTTTAGCCG CTTCACCAGT TTGTAGGCAT TCACAGGGTG GGCCAGGTAG

TATTGAGCCG CTTCACCAGT TTGTAGGCAT TCACAGGGTG AGCCAGGTAG

TGTTCAATCG CTTCACCAGC TTATAGGCGT TCACAGGATG GGCCAGGTAG

TGTTCAACCG CTTCACCAGC TTGTAGGCAT TCACAGGATG AGCCAGGTAG

AGTTCAGTCG CTTAACCAGT TTGTAGGCGT TCACAGGATG GGCAAGGTAG

TGTTTAGCCG CTTCACCAGT TTATAGGCAT TCACAGGGTG GGCCAGGTAG

TGTTGAGCCG CTTCACAAGT TTATAGGCAT TCACGGGGTG AGCCAGGTAA

TGTTCAGCCG CTTCACCAGT TTATAGGCAT TCACAGGGTG GGCCAGGTAG

TGTTCAGCCG CTTCACCAGT TTGTAGGCAT TCACGGGGTG GGCGAGGTAG

TGTTCAGCCG CTTCACCAGT TTGTAGGCAT TCACAGGGTG GGCCAGATAG

---------- ---------- ---------- ---------- ----------

TGTTCAGCCG CTTCACCAGT TTGTAGGCGT TCACCGGGTG TGCCAGGTAG

TATTTAGACG CTTCACCAGC TTGTAGGCAT TTACTGGATG AGACAAGTAG

TGTTTAGTCG CTTCACCAGC TTGTAGGCAT TCACCGGGTG TGCCAGGTAG

CCCTCAGCAT CAGCAGCTGA CTTGCTAGTC AAGGCTTCCA TTTTGTTGGC

CCCTCAGCAT CAGCAGCTGA CTTGCTAGTC AAGGCTTCCA TTTTGTTGGC

CCCTCAGCGT CAGCAGCTGA CTTGCTAGTC AAGGCTTCCA TTTTGTTGGC

CCCTCAGCGT CAGCAGCTGA CTTGCTAGTC AAGGCTTCCA TTTTGTTGGC

CCCTCAGCAT CAGCAGCTGA CTTGCTAGTC AAGGCTTCCA TTTTGTTGGC

CCCTCAGGGT CAGCAGCCGA CTTGCTGGTC AGGGCGTCCA TCTTGTTGGC

CCCTCGGGGT CAGCAGCTGA CTTGCTGGTC AGGGCCTCCA TCTTACTGGC

CCCTCGGGGT CGGCAGCTGA TCTGCTGGTC AGGGCTTCCA TCTTGCTGGC

CCCTCGGGGT CAGCAGCTGA CTTGCTGGTA TAGGCTTCCA TCTTGCTTGC

CCCTCAGGGT CAGCAGCCGA CTTGCTGGTC AGGGCTTCCA TTTTGTCAGC

CCTTCAGGGT CAGCAGCTGA CTTGCTGGTC AAGGCTTCCA TCTTGTTGGC

CCCTCGGGGT CAGCAGCCGA CTTGCTGGTC AGGGCTTCCA TTTTGTTGGC

CCCTCAGGGT CAGCTGCTGA CTTGCTGGTC AGGGCCTCCA TTTTGTTGGC

CCCTCAGGGT CAGCAGCTGA CTTGCTGGTG AGAGATTCCA ATTTGTTGGC

---------- ---------- ---------- ---------- ----------

CCCTCAGGGT CAGCCGTCGA CTTGCTGGTC AGCGCTTCCA TTTTGGTGGC

CCTTCAGGAT CAGAAGCTGA TTTGCTTGTC AGAATATCCA TTTTTTCAGC

CCCTCAGGAT CTGACGTGGA CTTGCTTGTC AGCAAGTCCA TTTTTTCAGC

CCAGCTACCT CTTAATCTTG GAAAGCTTGG CTTCCTCCAC AAGGATGTAC

CCAGCTACCT CTTAATCTTG GAAAGCTTGG CTTCCTCCAC AAGGATGTAC

CCAGCTACCT CTTAATCTTG GAAAGCTTGG CTTCCTCCAC AAGGATGTAC

CCAGCTACCT CTTAATCTTG GAAAGCTTGG CTTCCTCCAC AAGGATGTAC

CCAGCTACCT CTTAATCTTG GAAAGCTTGG CTTCCTCTAC AAGGATGTAC

CCAGCTACCT CTTAATCTTG GACAGCTTGG CTTCCTCCAC AAGGATGTAC

CCAGCTACCT CTTAATCTTG GAGAGCTTGG CTTCCTCTGC CAGGATGTAC

CCAGCTACCT CTTAATCTTG GCGAGCTTGG CTTCCTCCAC AAGGATGTAC

CCATCTACCT CTTGATCTTA GAGAGTTTGG CCTCCTCCGC AAGGATGTAC

CCAGCTACCT CTTAATCTTG GAGAGCTTGG CTTCCTCCAC CAGGATGTAC

CCAGCTACCT CTTAATCTTA GAGAGTTTGG CTTCCTCCAC AAGGATGTAC

CCAGCTACCT CTTAATCTTG GAGAGCTTGG CTTCCTCCTC AAGAATGTAC

CCAACTACCT CTTAATCTTG GAGAGTTTAG CTTCCTCCAC CAGGATGTAC

CCAACTACCT CTTGATCTTG GAAAGCTTGG CTTCTTCCAC AAGGATGTAC

---------- ---------- ---------- ---------- ----------

CCAACTACCT CTTAATCTGG GCGAGCTTGG CCTCCTCTGC TAGGATGTAC

CCAGCTACCT TTTAATCTTG AAGAGTCTGG CTTCTTCTGC TCTTATATAT

CCAGCTACCT TTTAATTTTC GAGAGCTTGG CCTCTTCCAC CTGGATGTAT

TCTTTCAGAG ACTGCACCAG CTCTTTCTCT GCATAAATCA GGTCAGTCAT

TCTTTCAGAG ACTGCACCAG CTCTTTCTCT GCATAAATCA GGTCAGTCAT

TCCTTCAGAG ACTGCACCAG CTCTTTTTCT GCATAAATCA GGTCAGTCAT

TCCTTCAGAG ACTGCACCAG CTCTTTTTCT GCATAAATCA GGTCAGTCAT

TCCTTCAGAG ATTGCACCAG CTCTTTCTCT GCATAAATCA GGTCAGTCAT

TCTTTCAGAG ACTGCACCAG ATCCTTCTCT GCATAAATCA GGTCAGTCAT

TCCTTCAGAG ACTGGACGAG GTCCTTCTCT GCATAAATCA GGTCGGTCAT

TCCTTCAGAG ACTGTACCAG GTCCTTCTCT GCGTAAATCA GATCGGTCAT

TCCTTCAGTG CGTGCACCAG GTCTTTCTCA GCATAGATCA GGTCGGTCAT

TCCTTCAGGG ACTGCACCAG GTCCTTCTCT GCATAAATCA GGTCTGTCAT

CCCTTCAGAG ACTGCACCAG GTCCTTCTCT GCATAAATTA GGTCAGTCAT

TCCTTCAGCG ACTGCACCAG GTCCTTCTCT GCATAAATCA GGTCCGTCAT

TCCTTCAGAG ACTGTACCAG GTCCTGCTCC GCATAAATCA GGTCTGTCAT

TCCTTCAGAG ACTGTACCAG GTCTTTCTCT GCATAAATCA GGTCTGTCAT

---------- ---------- ---------- ---------- ----------

TCCTTCAGGG ACTTCACCAG ATCCTTCTCT GCATAGATCA GGTCCGTCAT

TCTTTCAGAG ATTGAACCAG GTCTTTCTCT GCATAAATCA AGTCTGTCAT

TCCTTCAAAG ACTGCACCAG GTCTTTCTCT GCATAAATCA GGTCTGTCAT

GTGCCCTGAT TCATTCTTTT TTTCCTTAAG TCTGTTTCAT GCAAAAATAT

GTGCCCTGAT TCATTCTTTT TTTCCTTAAG TCTGTTTCAT GCAAAAATAT

GTGCCCTGAT TCATTCTTTT TTTCCTTAAG TCTGTTTCAT GCAAAAATAT

GTGCCCTGAT TCATTCTTTT TTTCCTTAAG TCTGTTTCAT GCAAAAATAT

GTGCCCTGAT TCAT--TTTT TTTCCTTAAG TCTGTTTCAT GCAAAAATAT

GTGGCCTGAT TCATTCTTTT TTTCCTTAAG TCTGTTTCAT GCAAAAATAT

GTGCCCTAAT TCATTCTTTG T--CCTTAAG TCTATTTCAT GCAAAAAAGA

GTGCCCTAGT TCATTCTTTG T--CCTTAAG TCTATTTCAT GCAAAAAAAA

GTGCCCTGAT TCATTCTTTT TTTCCTCAAG ACTGTTTCAT GCAAAAACAC

GTGTCCTG-- ---------- ---------- ---------- ----------

GTGCCCTGAT TCATTCTTTT TTTCCTTAAA TCTGTTTCAT GCAAAAATAT

GTGCCCTGAT TCATTCTTTT TTTCCTTAAG TCTGTTTCAT GCAAAAATAT

GTGCCCTGAT TCAT--TTTT TTCCCTTAAG TCTGTTTCAT GCAGAAATAT

GTGTCCTAAT TCATTCTTTC GTTCCTTAAA TCTGTTTCAT GCAAAAATAT

--------AT TCATTCTTTT TTTCCTCAGG TCTGCTTCAT GCAAAAATAT

GTGCCCTG-- ---------- ---------- ---------- ----------

CTGCCCTGCT TCATCATCTT TTTCCTTAGG TCCTGTTCAT TCAAAAATTT

TTGCCCTGAT TAATTCCATG CTTCCTTAGG TCTCTTTCAT GCAAAAATAT

TTTCCCATTC TTCACATTCa ttttaaagta ttttatattt taaataaCTA

TTTCCCATTC TTCACATTCa ttttaaagta ttttatattt taaataaCTA

TTTCCCATTC TTTACATTCa ttttaaagta ttttatattt taaataaCTA

TTTCCCATTC TTTACATTCa ttttaaagta ttttatattt taaataaCTA

TTTCCCATTC CTTACATTCA TTTTAACCTA TTTCATATTT TAAGTAACTA

TTTCCCATTC TTTACATTCA TTTTATAGCA GTTTATATTT TAAATAACTA

TTTCCCATTC TTTACATTCG TTTTCTAGTA TTTTGTATTT TAAATA---G

TTTCCCATTC TTTACATTCA TTTTCTAGTA TTTTATACCT TAAATAATTG

TTTCCCATTC TTTACATTCA TTTT------ ---------- ---ATA---G

---------- ---------- ---------- ---------- ----------

TTTTCCATTG TTTACATTCA TTTCATAGTA TTTTGTATTC TAAATAACTA

TTTTCCATTC TTTACATTCA TTTCATAGTA TTTTGTATTC TAAATAACTA

TTCCCCATTC TTTACATTCA TTTCACAGTA TTTTG----- -AAATAACTA

TTTCTCATTA TTTACATTCG TTTTATAGTA TTTTATGTTC TAAATAACTA

TTTCCTGTTC TTTACATTCA TTTTATAGTA TTTTATATTC TAAATAACTA

---------- ---------- ---------- ---------- ----------

TTCTCCTTTC TTTACATTCA TCTTAAAGGG TTTTATATTC AATACAAATA

TTCTACTTGT ATTACATGAA TATGTTTGTA CATACATATA TATATATATT

TATTGCATTT GTGCTTCATA CATTGGAAAA TAAAGGATAT TTCATTTTCT

TATCGCATTT GTGCTTCATA CATTGGAAAA TAAAGGATAT TTCATTTTCT

TATCACATTT GTGCTTCATA CATTGGAAAA TAAAGGATAT TTCATTTTCT

TATCACATTT GTGCTTCATA CATTGGAAAA TAAAGGATAT TTCATTTTCT

TATCACATTT GTGCTTCATA CATGGGAAAA TAAAGGATAC TTCATTTTCT

TATCACATTT GTGCTTTATA CATCGGAAAA TAAAGGCTAA TTCATTTCCT

TATCAGACTT GTGCTCAATA CAAGGAAAAA TAGAGAA--A TCCACTTA--

CATCAGACCT GTGCTTAATA CATGGAAAAA TAAAGAACAA TTCATTTA--

TATCACATTT GTGCTTTATG CATCGGAAAA TAAAA----A TTCATTTTCT

---------- ---------- ---------- ---------- ----------

TATCAC-TTT GTGCTTTATA CATTGGAAAA TAAAGAATAA TTCATTTTCC

TATCACATTT GTGCTTTATA CATTGGAAAA TAAAGAATAA TTCATTTTCT

TATCACATTG GTGTTTCATA TATTTGAAAA TAAAGAATAA TTAATTTTCT

TATCACATTT GTGCTTTATA CATTGGAAAA TAAAGAATGG TTCATTTTCT

TATCACATTT GTGCTTTATA TGCTGAAAAA TAAAGAATAA TTAGTTTTCT

---------- ---------- ---------- ---------- ----------

GATCAAAATT GTGATTTACA CACAGGAAAA TAAAGGTTAA GTTATTTTCT

TAGCAAATTT GTGCTTTACA CATGGAAAAA TAAAGGTCTC ATAATTTCCT

TTACTGCAGA AAAAAAACAT TACCTGATTG GAAAAATTCA ATGGAAAGTT

TTACTGCAGA AAAAAAACAT TACCTGATTG GAAAAATTCA ATGGAAAGTT

TTACTGCAGA AAAAAAACAT TACCTGATTG TAAAAATTCA ATGGAAAGTT

TTACTGCAGA AAAAAAACAT TACCTGATTG TAAAAATTCA ATGGAAAGTT

TTACTGCAGA AAAAAAACAT TACCTGATTG TAAAAATTCA ATGGAAAGTT

TTGCTGCAGA AAAAAAACAT TGTTTG---- -AAAAATCCG ATGGAAAGTT

TTATGGCTGA AAAAAAACGT TACCTGACTG TCAAACTCCA GTAGAGA---

TTATGGCAGA GAAAAAACAT TACCTGACTA TAAAACTCCA GTAGAGAAT-

TTACCATGGA AAAAAAATAT TACCTGATTA TAAAAATCTA ATGCACAGTT

---------- ---------- ---------- ---------- ----------

TTACTGCAGA AAAGAAACAT TACCTGACTA TAAAAATTGA ATGGGAAGTT

TTACTGCAGA AAAAAAGCAT TACCTGACTA TAAAAATAGA ATGGAAAGTT

TTAATTGGAA AAAATAATAT AACCTCACTA TGCAAATAGA ATGGAAATTT

TTACTGCAGA AATAAAGTAT TACCTGATTG TAAAAATCAA ATGGAAAGTT

TTACTGCAAA AAAAAAACAT TATCTGATTG TAAAAATCGA ATGCAAAGTT

---------- ---------- ---------- ---------- ----------

TCATAGCAGA AAAAAAACAT TACCCAGTTG TAAAAATTGA ATGAAAAAAT

TGATTCCAGA AGAAAAATGT TAACCCAATG AAGATATTCA GTGAGATGTT

CTATTTTTTC TCCTTGTAGA ATAAAGTTTC TCATTTCTTA ATTAAGTGGC

CTATTTTTTC TCCTTGTAGA ATAAAGTTTC TCATTTCTTA ATTAAGTGGC

CTATTTTTTC TCTTTGTAGA ATAAAGTTTC TCATTTCTTA ATTAAGTGGC

CTATTTTTTC TCCTTGTAGA ATAAAGTTTC TCATTTCTTA ATTAAGTGGC

CTATTTTAT- TCCTTGTAGA ATAAAGCTTC TCATTTATTA ATTAAGTGAC

CTATTTTTGC TCCTTGCAG- --AAAGTTTC TCATTTATTA ATTAAGTGGC

----TCTCCC CC-------G ATAAAACTT- TCTCTTGT-- ACTAGGTAGT

----TTTTTC CC-------G ATAAAGTTTC TTTCTTGT-- AGTGGGTAGT

CTACTTTTTC TCCTTGTAGA A-AAAGTTTC TCCTTAATTA ATTACGTGGA

---------- ---------- ---------- ---------- ----------

CTATTTTTCC TCCATCCAGA ATGAAGTTTC TCCTTTATTA ATTCAGCAGA

CTAATTTTTC TCC-----TA ATAAAGTTAC GCTTTTATTC ATTCAG----

CTA-TTTTTC TCCTTGAAAA ATAAAATTTT TCCTTTATTA ATT-AGTGGC

GTATTTTTTC TTCTTGCAGA ATAAAGTTTC TCCTTTATTA ATAAAGTGGA

TTATTTTTTC TCCTTGCAGA ATAATATTTT TCCTTTATTA TTTAAGTGAA

---------- ---------A ACCATATTTT TCCTTTATTA TTTAGGTGGA

TTATTTTTTT TTTTTGCAGG ATAAAGATTG TCTTTCATTA ATTAGGTAGT

TTATTTTCTC TTTTTCCAGa gtaatgTTTG TTCTGATTTA ATCTGCTGAA

ATTAAACAGA GAAGACATTT TAAGTATTGT TTATTCAGAA ACGCCTTTAA

ATTAAACAGA GAAGACATTT TAAGTATTGT TTATTCAGAA ACGCCTTTAA

ATTAAACAGA GAAGACATTT TAAGTATTGC TTATTCAGAA ACGCCTTTAA

ATTAAACAGA GAAGACATTT TAAGTATTGC TTATTCAGAA ACGCCTTTAA

ATTAAACAGA GAAGACATTT TAAGTATTGT TTATTCAGAA ATGCCTTTAA

ATTAAACAGA GAAGACATTT TAAGTATTGT TCATTCAGAA ATGCCTTTAA

GTTCTACAGA GGAG--CTTT CAAGTATTGC CTCACCAGAA ATGCATTTCG

GCTCTGCAGA GGA---CTTT CAATTATTGT CTTGCCAGAA ATGTATTTCT

GTT------- ---GACCTTT TAAATACCGT TTATTCAGAA ATGCCTTTAC

---------- ---------- ---------- ---------- ----------

GTTAAACAGA GGAGACATTT TTTTAATTGT TTTTTCAGAA CTGCCTTTAG

--TAAACAGA GGAGACATTT TTTAGATTGT TTATTCAGAA ATGCCTTTAA

ATTAAACAGA GAAGACATTT TTAGTATCAT T--------- ----CTTTA-

ATTAAACAAA GGAAACATTT TTAGTATTGT TTATTCAGAA ATGCCTTTAA

GGTAAACAGA GGAGACATTT TTAGTATTAT TTATTCAGAA ATGTCTTTCA

GCTACACAGA GGAGTCACAT TTAG-ATTAT TTATTCAGAA ATGTC--TCA

ATTAGTCGAA CGGTACATTT TGAGTATTTC TCATCCATAA ATGTAATTTG

ATTGAGTAAA GGGTACATTC GTGGTATTCC TTAATCAAAA ATGACTTTAA

TCAGTGTGTA TAGAACTAGC TCATTTCTTA ACTGTCAAAT TTAGAAGTGC

TCAGTGTGTA TAGAACTAGC TCATTTCTTA ACTGTCAAAT TTAGAAGTGC

TCAGTGTGTA TAGAACTAGC TCATTTCTTA AATGTCAAAT TTAGAAGTGC

TCAGTGTGTA TAGAACTAGC TCATTTCTTA AATGTCAAAT TTAGAAGTGC

TCAGTGTGCA TAGAACTAAC TCATTTATTA AATGTCAAAT TTATAAGTGC

-CAGTGGGCA TAGAACTAGC TCATTTATTA AATGTCAAAT TTaaaaaTGT

TTAATGCACA CAGCgctggC TCATTTAGTC AACGTCAAGT TAGTAGGTGC

TTAACACAAA CAgggctggC TCATTTAGTC AATGTCAGAT TAATAGGTGC

TTAATGTGTA TAGAACTAGC TTATTA---C AATGTCAAAT CTGTAAGTGT

---------- ---------- ---------- ---------- ----------

TTAATATGCA AAGACT-AGC ACATATACTC AAAGTCAGAT TTATAAGTGT

TTAAGGTGCT TAGAATGAGC TCCTTTACG- AAAGTCAAAT TTGTAAGTGT

TTAATGTTCA TAGAACTGGC TCATTTACTA AAAATGCAAT TTGTAAGTAC

TTAATGTGCA TATAACTAGT TGATTTGCTA AATGTCAAAT TTGTAAATTT

TTAATGTGCA TAAAACTAAC TCAGCTACTA AACGTCAAAT CCATAAGTGA

TTAATGTGCC TGTAACTCAT ---------- ---------- ---TAAGTGC

CCAGTCTGCA TACAGCTAGT TCATTCATTC TGTATTAAAC TTGCAAAAGC

CCAGTCGGTA TATAAGCAGT TCATTACTTC AATATTAAAC CTGTAAATGC

AACAGTGGGT CTTCAGAGAG ACATGATTCT ATACACCTAG CTTTAAGATT

AACAGTGGGT CTTCAGAGAG ACATGATTCT ATACACCCAG CTTTAAGATT

AACAGTGGGT CTTCAGAGAG ACATGATTCT GTACACCTAG CTTTAAGATT

AACAGTGGGT CTTCAGAGA- -CATGATTCT GTACACCTAG CTTTAAGATT

AACAGTGGGT CTTCAGAGAG ACATGATTCT GTACACCTAG CTTTAACATT

AGTAGTGGAT ATTTAGAGAG ACATGATTTT GTACACCTAG CTTTAAGATT

AACATTGAGT TTTCAGAGAG ATGTGACTTC CTACACCTTG GTTTCAGATC

AACAGTGAGT CTTCAGAAAG ACGTGGCTTC CTACAGCTTG CTTTAAGATC

AACAGTGGGT CTTTAGAGAG ACATAATTCT GTTTGCCTAG TTTTAGGATT

---------- ---------- ---------- ---------- ----------

AATAGAAGGT CTGCAGAAAG ACATGATTGT GTACACATGG TTTTAAGATT

AATAGTGGGT CTGCAAAGTG ACATGATTCT GTACACCTAG TTTCAAGATT

AATGATGGAT CCGAAGAAAG ACATGATTTT GTATACCTGT TTTTAAGAGT

AACAGTGGGT CTTCAGAAAG ACATGATTCT GTACACCTAG TTTTAAAATA

ACCAGTGGAT CTTCAGAAAG ACATGATTCT GTACACCTAA TTTTAAGATT

AACAGTGGGT CTTCAGAGAG ACATGATTCT GTATTCCT-- ------GACT

ATCAATGGAA TTTGAGAGAA ACAAGATTTT AAACACCAAA TTTAAAGACT

ATCAATGGAA TTTCAGAGAG ACGTGATTCA ATACATCCAG TCTTAAGATG

TAATTGTGAT TTTACATCAG TTACTGAATG AGGTAGTCCA CATTGCCTTT

TAATTGTGAT TTTACATCAG TTACTGAATG AGGTAGTCCA CATTGCCTTT

TAATTGTGAT TTTACATCAG TTATTGAATG AGGTAGTCCG CACTGCCTTT

TAATTGTGAT TTTACATCAG TTATTGAATG AGGTAGTCCG CACTGCCTTT

TAATTGTGAT TTTACGTCAG TTATTGAATG AGGTAGTCCA CATTGCCTTT

TCATTGTTAT TTTACGTAAG TTATTGAATG AGGTAGTCCA CATTGCCTTT

TAACTGTGAC TGTACATC-G TTATTTAATG AGGTAGTCCA CGTTGCCTTC

TAACTGTGAT GTTACATCAG TTATTTAATG AGGTAGTCCA CATTGCCTTC

TAACTGTGAT TTTACATCAG TGACTGAATG AGGTAGTCCA CATTGCCTTT

---------- ---------- ---------- ---------- ----------

TAACTGTGAT TCT--GACAG TTATTGAATG AGGTAGTCCA CATTGCCTTT

TCATTGTGAT TTTACAGCAG TTACTGAATG AGGTAGTCCA CTCTGCCTTT

TCATTGCGAC TTGACATCAG TTATTGCATG AGGTAGTCCA TATTGTCTTT

CAATTGTGAT TTTATATCAG TTATTGAATG AGGTAGTCCA CAATGCCTTT

TAACTGTGAT TTTACATCAG TTATTGAATG AGGTAGTCCA CATTGCCTTT

TAACTGAGAT T------CAG TTATTGAATG AGGTAGTCCA CATTGCCTTT

TACATGTGAT TTTTGGTATG TTATTAAATG AGGTAGACCA AATTGCCTAT

TGCTAGTAAT TTGACATAAA TTATTGAATG AGGTAGACCA CATTTTTTTT

GCTGCATCTG GTTACTGATT CACAAATAAT TAAGTAGAAT ATATCACTTA

GCTGCATCTG GTTACTGATT CACAAATAAT TAAGTAGAAT ATATCACTTA

ACTGCATCTG GTTACTATTT CACAAATAAT TAAGTACAAT ATGTCACTTA

ACTGCATCTG GTTACTATTT CACAAATAAT TAAGTACAAT ATATCACTTA

GCTGCATCTG GTTACTATTT CATAAATAAT TAAGTACAAT ATATCAGTTA

GCTGTATCTG GTTACTATTC CACAAATAAT TAAGTACAGT ATATCAGTTA

ACTGTACCTG GTTATTAGTC CACAAATAAT TAAGTACAAT ATATCGGTTA

ACTGTACCTG GTTATTACTC CACAAATAAT TAAGTACAAT ATATCAGTTA

GCTGTATCTG GTTTCTATTC CACAAATAAT TAAGTACAAT ATATCAGTTA

---------- ---------- ---------- ---------- ----------

GCTGTATCTG GTTACTATTC CACAAATAAT TAAGTACAAT ATATCAGTTA

GCTGTGTCTG GTTACTAGTC CACAAATAAT TAAGTACAAT GTATCAGTTA

GCTGTATCT- -TTACTATTC CACAAATAAT TAGGTACAAT ATATCAGTTA

GCTGTGTCTG GTTACTATTC CACAAATAAT TAACTACAAT ATATCAGTTA

CCTGTATCT- ------ATTC CACAAATAAT TAAGTACAAT ATATCAGTTA

GCTGTATCTG ATTTCTATTC CACAAATAAT TAAGTACAAT ATATCAGTTA

GTTTCATCTT GTGACTAGCT TGCAAATAAT TAGGTAGAAT ATTTCAATTA

CCTTTGTCTC GTTACtattt tataaataat taaataCAGC ATATCAATTA

TGTTTTTCCT TTATGTTTAA TAGTTCTGGA ACATTGACAA TATATTACTT

TGTTTTTCCT TTATGTTTAA TAGTTCTGGA ACATTGACAA TATATTACTT

TGTTTTTCCT TTATGTTTAA TAGTTCTGGA ACATTGAGAA TATATTACTT

TGTTTTTCCT TTATGTTTAA TAGTTCCGGA ACATTGAGAA TATATTACTT

TGTTTTTCCT TTATGTTTAA TAGTTCTGGA ACATTGAGAA TCTATTACTT

TATTTTTCCT TTATGTTTAA TAGTTCTGGA ACATTGAGAA TATATTACTT

TTTTTTTTCT TTATGTTTAA TAATTCTGGA ACATCGCGAA TATATTACTT

TGTTTTTTCT TTATGTTTAA TAATTCTGGA ACATTGCGAA TATATTACTT

TGTTTTTGCT TTATGTTTAA TAGTTCCGGA ACAT--AGAA TATATTACTT

---------- ---------- ---------- ---------- ----------

TATTTTTCCT TTATGTTTAA TAGTTCTGGA ACATTGAGAA TATATTACTT

TGGTTTTCCT TCATGTTTAA TAGTGCTGGA ACATTGAGAA TATATTTCTT

T--TTTTTCT TTATGTTTAA TAGTTCTGGA ACATTGAGAA TATATTACTT

TGTTTTTCCT TTATGTTTAA TAGTTGTGGA ACATTGAGAA TATATTACTT

TATTTTTCCT TTATGTTTAA TAATTCTGGA ACATTGAGAA TATATCACTT

TGTTTTTCCT TCATGTTTAA TAATTCTGGA ACATTGAGAA TATATTACTT

TATTTTCCCT CCATGTTTAA TATTTCTCGA ACATTGAGAA TATACTAATT

TGTTTTTCTT TTATGTTTAA TCATCTTGGA ACATGAAGAA TATATTACTT

TTAGTGGTAC ACAGTGAAAA GTCTTGATTT TTACATTGCC ATTTGTGATA

TTAGTGGTAC ACAGTGAAAA GTCTTGATTT TTACATTGCC ATTTGTGATA

TTAGTGGTAC ACAGTGAAAA GTCTTGATTT TTACATTGCC ATTTGTGATA

TTAGTGGTAC ACAGTGAAAA GTCTTGATTT TTACATTGCC ATTTGTGATA

TTAGTGGTAC ACAGTGAAAA GTCTTGATTT TTACATTGCC ATTTTTGATA

TTAGTGATAC ACAGTGAAAA ATCTGGATTT TTACATTGCC ATTTGTGATA

T-AGTGGTAC ACAGTGAAAG GTCTTGATTT GTACATTACC GTTTGCAATA

TTAGTGGTAC ACAGTGAAAG GTCTTGATTT GTACATTACC GTTTGCGATA

TTAGTGGTAC ACAGTGAAAA GTCTTGATTT ACACATTGCC ATTTGTGATA

---------- ---------- ---------- ---------- ----------

TTAGTGGTAC ACAGTGAAAA GTCTTGATTT TTACATTGCC ATTTGTGATA

TTAGCGGTAC ACAGGGAAAA GTCTTGAGTT TTACATTGCC ATTTGCAATA

TTAGTGGTAC ACAGTGAAAA GTCTTGATTT TTACATTGCC ATTTGCAATA

TTAGTGGTAC ACGGTGAAAA GTCTTGATTT TTACATTGCC ATTTGTGATA

TTAGTGATAC ACAGTGAAAA GTCTTGATTT TTACATTGCC ATTTATAATA

TTAGTGATAC ACAGTGAAAA ATCTTGATTT TTACATTGCC ATTTGTGATA

TTAGTGATAT ACCATGATAA GTCTTAATAT TTACATTGTC ATATGCCATA

TTTATGGAGC ACTGTGTGAA ATCTTGTTTT TTACATTATT GTTTGCCATA

TTTTATGATG ATAGAGAAAA TATTGTTAAA GACCTCTTGG GACAAAAGGC

TTTTATGATG ATAGAGAAAA TATTGTTAAA GACCTCTTGG GACAAAAGGC

TTTTATGATG ATAGAGAAAA TATTGTTAAA GACCTCTTGG GACAAAAGGC

TTTTATGATG ATAGAGAAAA TATTGTTAAA GACCTCTTGG GACAAAAGGC

TTTTATGATG ATAGAGAAAA TATTGTTAAA GACCTCTTGG GACAAAAGGC

TTTTATGATG ATAGAGAAAA TATTGTTAAA GACATCTTGG ATCAAAAGGG

TTTTATGATG GTAGAGAAAA TATTGTTAGA GACTTCTCAG GTCAAAAGGC

TTTTATGATG GTAGAGAAAA TATTGTTAGA GACTTCTTGG GTCAAAAGGC

TTTTATGATG ATAGAGAAAA TATTGTTAAA GATCTCTTGG AACAAGAGGC

---------- ---------- ---------- ---------- ----------

TTTTATGATG ATAGAGAAAA TATTGTTAAA GACCTCTTGG GACAAAAGGC

TTTTATGATG ATAGACAAAA TATTGTTAAA GACCTCTTGG GACAAAAGGT

TTTTATGATG ATAGAGAAAA TATTGTTAAA GACCTCTCGG GACAAAAGGC

TTTTATGATG ATAGAGAAAA TATTGTTAAA GACCTCGTGG AACAAAAGGC

TTTTATGATG ATAGAGAAAA TATTGTTAAA GACCTCTTGG GACAAAAGGC

TTTTATGATA ATAGAGAAAA TATTGTTAAA GACCTCTTGG GACAAAAGGC

TTGTATGATG ATAGAGAAAA TATTGTTAAA GATTTCTTGG AACAAAAGGC

TTTTATGATG ACAGAGAAAA TATTGTTAAA GACTTCTTGG GACAAAAAGC

TCATTTCAGC TGTGTCATTT GACTCATCTT TCAAGATAAC TATAATATTG

TCATTTCAGC TGTGTCATTT GACTCATCTT TCAAGATAAC TATAATATTG

TCATTTCAGA TGTGTCATTT GACTCATCTT TCAAGATAAC TATAATATTG

TCATTTCAGA TGTGTCATTT GACTCATCTT TCAAGATAAC TATAATATTG

TCATTTCACC TGTGTCATTT GACTCATCTT TCAAGATAAC TATAATATTG

TCATTTCAGT TGTGTCATTT GACTCATCTT TCAAGATAAC TATAATATTG

TCATCTGAGC TGTGGCATCT GACTCCTCTC TCAAGATAAT TAGAATATTG

TCATTTGAGC TGTGGCATCT GATTCCTCTC TCACGATAAC TATAATATTG

TCATTTCAGC TGTGTCATTT --CGTGTCTT TCAAGATAAC TATAATATTG

---------- ---------- ---------- ---------- ----------

TCATTTCGGC TGCATCATCT GATTCATCTT TCAAGATAAC TATAATATTG

TCATTTCAGC TGCGTCATCT GACTCCTCTT TCAGGATAAC TATGA-ATTG

T--TTTCAGC TGCGCCATCT GACTCATCTT TCAGGATAAC TATAATATTG

TCATTTCGAC TGTGTCATCT GACTCATCTT TCAAGATAAC TATAATATTG

TCATTTCAGC TGCATCATCT GAGTCATCTT TCAAGATAAC TA--ATATAA

TCATTTCAGC TGCATCATCT GATTCATTTT TCAAAATAAC TATAATATTG

TCATTTCAAA TGAATCATCT GACTGCTCTT TCAAGCTTAC TAGGATATTG

TCATTTCAAT TTCATCCTCT GTCTCTGCTT TCAAGATAAC TTTCATATTG

CTAGACAGTT CATTCACTTT CCTTGAACTT CACATGGAGA TTGAGTAAAG

CTACACAGTT CATTCACTTT CCTTGAACTT CACATGGAGA TTGAGTAAAG

CTACACAGTC CATTCACTTT CCTTGAACTT CACATGGAGA TTGAGTAAAG

CTACACAGTC CATTCACTTT CCTTGAACTT CACATGGAGA TTGAGTAAAG

CTACACAGTC CATTCATTTT CCTTGAACGT CACATGGAGA TTGAGTAAAG

CTATACAATC CATTGACTTC CCTTGAACTT CACATGGAGA TTGAGTAAAG

CTGCAAACTT TGCTCACC-T CCTTGAGCTT CACATGGAGA TTGAGTACAG

CTGCAAAGTT CATCCCCCCT CCTGGAGCTT CACACGGAGA CTAAGTAAAG

CTACACAGTC CATTCACTTT CCTTGAACTT CACACGGAGA TTGAGTAAAG

---------- ---------- ---------- ---------- ----------

CTACACAGTC CATTCACTTT CCTTGAACTT CACATGGAGA TTGAGTAAAG

CTACACGGTC CATTCACTTC CCTTGAACTT CACATGGAGG TTGAGTAAAG

CTACACAGTC CATTCACTTT CCTTGAATTT CATATGGAGA TTGAGTAAAG

CTATATAGTC CATTCACTTT TCTTGAACTT CACATGGAGA TTGAGTAAAG

CTACCGAGTC CATTCACTTT CCTTGAACTT CATACGAAGA CTGAATAAAG

CTACCCAGTC CGTTCACTTT CCTTGAACTT CATATGGAGC CTGAGTAAAG

CTACACCGTC CAGTCGCTGC CCTCAGACTT CACATTGAGG TTGAATAAAG

CTACAGAGTC CAATCACTTG TCTTGAACTT CACATGGAGG TTGAATAAAG

CTCTTCTATT TGTTTTTTGA AGTACTCTCT CAGCTCAGGT CTCTTAGCTT

CTCTTCTATC TGTTTTTTGA AGTACTCTCT CAGCTCAGGT CTCTTAGCTT

CTCTTCTATT TGTTTTTTGA AGTACTCTCT CAGCTCAGGT CTCTTAGCTT

CTCTTCTATT TGTTTTTTGA AGTACTCTCT CAGCTCAGGT CTCTTAGCTT

CTCTTCTATT TGTTTTTTGA AGTACTCTCT CAGCTCAGGT CTTTTAGCCT

CTCTTCTATT TGTTTTTTGA AGTACTCTCT CAGCTCAGGT CTCTTAGCCT

CTCTTCTATT TGCTTTTTGA AGTACTCTCT CAGCTCGGGC CTCTTAGCCT

CTCTTCTATT TGCTTTTTGA AGTACTCTCT CAGCTCCGGT CTCTTAGCCT

CTCTTCTATT TGCTTTTTGA AGTACTCCCT CAGCTCGGGT CTCTTAGCCT

---------- ---------- ---------- ---------- ----------

CTCTTCTATT TGTTTTTTGA AGTACTCTCT CAGCTCAGGT CTCTTAGCCT

CTCTTCTATT TGTTTTTTGA AGTACTCTCT CAGTTCAGGT CTCTTAGCCT

CTCTTCTATT TGTCTTTTGA AGTATTCTCT CAGCTCAGGT CTCTTAGCTT

CTCTTCTATT TGTTTTTTGA AGTACTCTCT CAACTCGGGT CTCTTCGCCT

GTCTTCTATT TGTGTTCTGA AGTACTCTCT CAGCTGGGGT CTCTTAGCCT

CTCTTCTATT TGCGTTCTGA AGTACTCTCT CAGTTGGGGT CTCTTAGCCT

CTCTTCTATT TGTTTTTTGA AGTAATCTCT CAGTTCTGGT CTCTTAGCCT

CTCTTCTATT TGTTTTTTGA AGTAATCTCT CAGTTCTGGT CTCTTAGCCT

TTAGTGTTGG TGTCAGCAAG CCATTTTGAA CTGAGAACAT GTCAGAATGG

TTAGTGTTGG TGTCAACAAG CCATTTTGAA CTGAGAACAT GTCAGAATGG

TTAGTGTTGG TGTCAGCAAG CCATTTTGAA CTGAGAACAT GTCAGAATGG

TTAGTGTTGG TGTCAGCAAG CCATTTTGAA CTGAGAACAT GTCAGAATGG

TTAGTGTTGG TGTCAGCAAG CCATTTTGAA CAGAGAACAT GTCAGAATGG

TTAGTGTTGG TGTCAGCAAG CCATTTTGAA CTGAGAACAT GTCAGAATGG

TTAGTGTTGG TGTCAGCAAG CCATTTTGAA CTGAGAACAT GTCACAATGG

TTAGTGTTGG TGTCAGCAGA CCATTTTGAA CAGAGAACAT GTCACAATGG

TTAGTGTTGG TGTCAGCAGG CCATTTTGAA CTGAGAACAT GTCAGAATGG

---------- ---------- ---------- ---------- ----------

TTAGTGTTGG TGTCAGCAAG CCATTCTGAA CTGAGAACAT GTCAGAGTGG

TTAGTGTCGG TGTCAGCAAG CCGTTTTGAA CTGAGAACAT GTCAGAATGG

TTAATGTTGG TGTCAGCAAG CCATTTTGAA CTGAGAACAT GTCACGGTGG

TTAGTGTTGG TGTCAGCAAG CCATTTTGAA CTGAGAACAT ATCAGAGTGG

TTAACGTTGG TGTCAGCAAG CCATTTTGAA CTGAGAACAT GTCAGTGTGG

TTAGTGTTGG TGTCAACAAG CCGTTTTGAA CTGAGAACAT GTCAGAATGG

TTAGTGTTGG TGTCAGCAAG CCATTTTGTA CTGAGAACAT GTCAGAGTGT

TTAATGTTGG TGTCAGCAAG CCGTTTTGCA CTGAAAACAT GTCAGAGTGG

ATGTGAATGG CTTTAACCTA AAAACCAAAT GCAGAAAACA TCTTTAACAG

ATGTGAATGG CTTTAACCTA AAAACCAAAT GCAGAATACA TCTTTAACAG

ATGTGAATGG CTTTAACCTA AAAACCAAAT GCAGAATACA TCTTTAACAG

ATGTGAATGG CTTTAACCTA AAAACCAAAT GCAGAATACA TTTTTAACAG

ATGTGAATGG CTTTAACCTA AAAACCAAAT GCAAAATAGC TCTTTAACAA

ATGTGAATGG CTTTAACCTA AAAACCAAAT GTAGAATACA TCTTTAACAG

ATGTAAATGG CTTTAACCTA AAAACCAAAC GCAGAATATA TCTTTAGTAG

ATGTAAATGG CTTTAACCTA AAAACCAAAG GCAGAACATA TCTTTAGTGG

ATGTGAATGG CTTTAACCTA AAAACCAAAT GCAGAACACA GCTTTAATAA

---------- ---------- ---------- ---------- ----------

ATGTGAATGG CTTTAACCTA AAAACCAAAT GCAGAATATG TCTTTATCAG

ATGTGAATGG CTTTAACCTA AAAACCAAAT GCAAAATACG TCTTTATCAG

ATGTGAATGG CTTTAACCTA AAAACCAAAT GAAGAATATA TCTTTATCAG

ATGTAAATGG CTTTAACCTA AAAACCAAAT ACAGAATACA TCTTTAACAG

ATGTGAATGG CTTTAACCTA AAAACCAAAT GCAGAATACA TCTTTAACAG

ATGTGAATGG CTTTAACCTA AAAACCAAAT GCAGAATACA TCTTTAACAG

ATGTAAATAG CTTTAACCTG AAAACAAGAC ACAAAACATA GCTTTAATTA

ATGTAAATAG CTTTAACCTA AAAAACAAAA GCAAAATCTA TTTTATACAG

GAATGAGCCC AAGCAGTCAG GCTCCTTCCG CTACTTGCAG GGCATGCTAG

GAATGAGCCC AAGCAGTCAG GCTCCTTCCG CTACTTGCAG GGCATGCTAG

GAATGAGCCC AAGCAGTC-- ---------- ---------- ----------

GAATGAGCCC AAGCAGTCAG GCTCCTTCCG CTACTTGCAG GGCATGCTAG

GCATGAGCCC AAGCAGTCAG GCTCCTTCCG CTACTTGCAG GGCATGCTGG

GAATGAGCCC AAGCAGTCAG GCTCCTTCCG CTACTTGCAG GGCATGCTAG

GAGT------ ---------- ---------- ---------- ----------

GAGTGAGCCG AAACAGTCAG GATCCTTCCG CTACCTGCAG GGCATGCTAG

GAAT------ ---------- ---------- ---------- ----------

----GAGCCC AAGCAGTCAG GCTCCTTCCG CTATTTGCAG GGCATGCTGG

GAATGAGCCC AAGCAGTCAG GCTCCTTCCG CTACTTGCAG GGCATGCTAG

GAATGAGCCC AAGCAGTCAG GCTCCTTCCG CTACTTGCAG GGCATGCTGG

GCATGAGCCC AAGCAGTCAG GCTCCTTCCG CTACTTGCAG GGCATGCTGG

GAATGAGCCC AAGCAGTCAG GCTCCTTCCG CTACCTGCAG GGCATGCTAG

GATT------ ---------- ---------- ---------- ----------

GAACGAACCC AAACAGTCCG GCTCCTTCCG ATACCTGCAG GGCATGCTAG

GAACGAGCCC AAGCAGTCTG GCTCCTTCAG ATACCTACAG GGCATGTTAG

GAATGCGCCC AAACAGTGTG GCTCCTTCAG ATATCTGCAG GGCAAGATGG

AGGCCGGCGA GGGCGGTAAG ATGCGGGACT ACGACGAGGT GATCGCCTTC

AGGCCGGCGA GGGCGGTAAG ATGCGGGACT ACGACGAGGT GATCGCCTTC

---------- ---------- ATGCGGGACT ACGACGAGGC GATCGCCTTC

AGGCCGGCGA GGGTGGTAAG ATGCGGGACT ACGACGAGGC GATCGCCTTC

AGGCCGGCGA GAGCGGTAAG ATGCGCGACT ACGACGAGGC GACCGCCTTC

AGGCCGGTGA GGGCGGTAAG ATGCGGGACT ACGACGAGGT GATCGCCTTC

---------- ---------- ATGCGGGACT ACGACGAGGT GACCGCCTTC

AGGCTGGCGA GGGCGGTAAG ATGCGGGACT ACGACGAGGT GACCGCCTTC

---------- ---------- ATGCGGGACT ACGACGAGGT GACCGCGTTT

AGGCGGGCGA GGGCGGTAGG ATGCAGGACT ACGACGAAGT GACCGCCTTC

AGGCCGGGGA GGGTGGTAAG ATGCGGGACT ACGACGAGGT GACCACCTTC

AGGCCAGCGA GGGCGGTAAG ATGCGGGACT ACGACGAGGT GACCGCCTTC

AGGCCGGCGA GGGGGGTAAG ATGCGGGACT ACGACGAGGT GACCGCCTTC

AGGCTGGGGA AGGCGGTAAG ATGCGTGACT ACGACGAGGC GATCGCCTTC

---------- ---------- ---------- ---------- ----------

AGGCTGGCCA GGGTGGTAAG ATGCGTGACT ACGACGAGGT GACCTCCTTC

AAGCTGGAGA GAATGGTAAG ATGCGGGACT ACGACGAGGT GACAGCCTTC

AGGCCGGGAG AGACCGCTCC ATGAGGGACT ACGACGAGGC GACGGCCTTC

CTGGGCGAGT GGGGGCCCTT CCAGCGCCTC ATCTTCTTCC TGCTCAGCGC

CTGGGCGAGT GGGGGCCCTT CCAGCGCCTC ATCTTCTTCC TGCTCAGCGC

CTGGGCGAGT GGGGACCCTT CCAGCGGCTC ATCTTCTTCC TGCTCAGCGC

CTGGGCGAGT GGGGACCCTT CCAGCGGCTC ATCTTCTTCC TGCTCAGCGC

CTGGGGGAGT GGGGGCCCTT CCAGCGCCTC ATCTTCTTCC TGCTCAGCGC

CTTGGCGAGT GGGGGCCCTT CCAGCGCCTC ATCTTCTTCC TGCTCAGCGC

CTGGGCGAGT GGGGGCCCTT CCAGCGCCTC ATTTTCTTCC TGCTCAGCGC

CTAGGCGAGT GGGGGCCCTT CCAGCGCCTC ATCTTCTTCC TGCTCAGCGC

CTGGGCGAGT GGGGGCCCTT CCAGCGCCTC ATCTTCTTCC TGCTCAGCGC

CTAGGCGAGT GGGGGCCCTT TCAGCGCCTC ATCTTCTTCC TGCTCAGCGC

CTGGGCGAGT GGGGGCCCTT CCAGCGCCTC ATCTTCTTCC TGCTCAGCGC

CTGGGCGAGT GGGGGCCGTT CCAGCGCCTC ATCTTCTTCT TGCTCAGCGC

CTGGGCCAGT GGGGGCCCTT CCAGCGCCTC ATCTTCTTCC TGCTCAGCGC

CTGGGCGAGT GGGGGCCCTT CCAGCGCCTC ATCTTCTTCC TGCTCAGCGC

---------- ---------- ---------- ---------- ----------

CTGGGCGAGT GGGGGCCCTT CCAGCGCCTC GTCTTCTTCC TGCTCAGCGC

CTGGGCGAGT GGGGACCCTT CCAGCGCCTC ATCTTCTTCC TGCTCAGCTC

CTGGGCGAGT GGGGCCCCTT CCAGCGGCTG GTCTTCTTCC TGCTGAGCGC

CAGCATCATC CCCAATGGCT TCAATGGTAT GTCAGTCGTG TTCCTGGCGG

CAGCATCATC CCCAATGGCT TCAATGGTAT GTCAGTCGTG TTCCTGGCGG

CAGCATCATC CCCAATGGCT TCAATGGTAT GTCAGTCGTG TTCCTGGCGG

CAGCATCATC CCCAATGGCT TCAATGGTAT GTCAGTCGTG TTCCTGGCGG

CAGCATCATC CCCAACGGCT TCAATGGTAT GTCAGTCGTG TTCCTGGCGG

CAGCATCATC CCCAACGGCT TCAATGGTAT GTCAGTCGTG TTCCTAGCTG

CAGCATCATC CCCAATGGCT TCAATGGTAT GTCTATCGTG TTCCTGGCGG

CAGCATCATC CCCAATGGCT TCAATGGTAT GTCCATCGTG TTCCTGGCGG

CAGCATCATC CCAAACGGCT TCAATGGGAT GTCAGTGGTG TTCCTGGCGG

CAGCATCATC CCCAACGGCT TCAATGGCCT GTCGTCCGTG TTCTTTACGG

CAGCATCATC CCCAACGGCT TCAATGGAAT GTCAGCCGTG TTCCTGGCCG

CAGCATCATC CCCAACGGCT TCAATGGTAT GTCAGCCGTG TTCCTGACGG

CAGCATCATC CCCAACGGCT TCAACGGGAT GTCAGTCGTG TTCCTGACGG

CAGCATCATC CCCAATGGCT TCAACGGTAT GTCAGCCGTG TTCCTGGCGG

---------- ---------- ---------- ---------- ----------

CAGCATCGTC CCCAACGGGT TCAACGGCCT GTCAGCCGTG TTCCTGACGG

CAGCATCATC CCCAACGGTT TCAATGGCAT ATCCGTAGTG TTCCTGGCGG

CAGCATCATC CCCAACGGCT TCAACGGCAT GTCGGCCGTC TTCCTGGCGG

GGACCCCGGA GCACCGCTGT CGAGTGCCTG GAGCAGGAGA GCTGCCTGGA

GGACCCCGGA GCACCGCTGT CGAGTGCCTG GAGCAGGAGA GCTGCCTGGA

GGACCCCAGA GCACCGCTGC CGGGTGCCTG GAGCAGGAGA GCTGCCTGGA

GGACCCCAGA GCACCGCTGC CGGGTGCCTG GAGCAGGAGA GCTGCCTGGA

GGACCCCAGA GCACCACTGC CGGGTGCCTG GAGCAGGAGA GCTGCCTGGA

GGACCCCGGA GCACCACTGC CGGGTGCCTG GAGCAGGAGA ACTGCCTAGA

GGACCCCGGA GCACCGTTGC CTTGTGCCTG GAGCAAGAGA ACTGCCTGGA

GGACCCCGGA GCACCGTTGC CTTGTGCCTG GAGCAGGAGA GCTGCCTGGA

GGACCCCCGA GCACCGCTGC CGGGTGCCTG GAGCAGGAGA GCTGCTTGGA

CCACCCCGGA ACACCACTGC CGGGTGCCTG GAGCAGGAGG GCTGCCTGGA

CCACCCCCGA GCACCGCTGC CGGGTGCCTG GAGCAGGAGA GCTGCCTGGA

GGACCCCGGA GCACCGCTGC CGGGTGCCTG GAGCAGGAGA GCTGCCTGGA

GGACCCCGGA GCACCGCTGC CGAGTGCCTG GAGCAGGAGG GCTGCCTGGA

GGACCCCCGA GCACCGCTGC CGGGTGCCTG GAGCAGGAGA GCTGCCTGGA

---------- ---------- ---------- ---------- ----------

CGACACCGCA GCACCGGTGC CGGGTGCCTG GAGCAGGAGA GCTGCCTGGA

GGATCCCCGA ACACCACTGC CGGGTGCCTG GAGCTGGAGC CCTGTCTGGA

GGACCCCCGA GCACCGGTGC TCGGTGCCTG GAGCAGGAGA AGTGTCTGGA

TGGCTGGGAG TTCAGCCAGG ACGTCTACTT ACCTGCTCAA AAGAATGGAG

TGGCTGGGAG TTCAGCCAGG ACGTCTACTT ACCTGCTCAA AAGAATGGAG

TGGCTGGGAA TTCAGCCAAG ACGTCTACTT ACCTGCTCAA AAGAATGGAG

TGGCTGGGAG TTCAGCCAAG ACGTCTACTT ACCTGCTCAA AAGAATGGAG

TGGCTGGGAG TTCAGCCAGG ACGTCTACTT ACCTGCTCAA AAGAATGGAG

TGGCTGGGAG TTCAGCCAGG ACGTCTATTT ACCTGTTCAA AAGAATGGAG

TGGCTGGGAG TACAACAAGG ACGTCTTCTT ACCTGTTCAA AAGAATGCAG

TGGCTGGGAG TACGACAAGG ACGTCTTCTT ACCTGTTCAA AAGAATGCAG

TGGCTGGGAG TACAGCCAGG ACGTGTACTT ACCTGTTCAA AAGAATGGAG

TGGCTGGGAG TTCAGCCAGG ACATCTAC-- ---------- ----------

TGGCTGGGAG TTCAGCCAGG ACGTCTACTT ACCTGTTCAA ATGAATGGAG

TGGCTGGGAG TTCAGCCAGG ACGTCTACTT ACCTGTTCAA AAGAATGGAG

TGGCTGGGAG TACAGCCAGG ACGTCTTCTT ACCTGTTCAA AAGAATGGAG

CGGCTGGGAG TTCAGCCAGG ACGTTTACTT ACCTGTTCAA AAGAATGGAG

---------- ---------- --------TT ACCTGTTCAA AAGAATAGAG

CGGCTGGGAG TTCAGCCAGG ACGTCTAC-- ---------- ----------

CGGCTGGGAA TACAGCCAGG ACACATACTT ACCTGTTCAA AGGAATGAAG

CGGCTGGGAC TACGGCCGCG ACGTCTACTT ACCTGTTCAA AAGAATGAAG

TCCACTTTCT TTTCCTAACC TCACCATATC TTCCAAAATG GCTTTCTTCA

TCCACTTTCT TTTCCTAACC TCACCATATC TTCCAAAATG GCTTTCTTCA

TCCACTTTCT TTTCCTAACC TCACCATATC TTCCAAAATG GCTTTCTTCA

TCCACTTTCT TTTCCTAACC TCACCATATC TTCCAAAATG GCTTTCTTCA

TCCACTTTCT TTTCCTAACC TCACCATATC TTCCAAAATG GCTTTCTTCA

TCCACTTTCT TTTCCTAACC TCACCATATC TTCCAATATG GCTTTCTTCA

TCCACTTTCT TTCCCCAACA TCACCATGTC ATCCAGAATG GCTTTCTTCA

TCCGCTTTCT TTCCCCAGCA TCACCATGTC ATCCAGAATG GCTTTCTTCA

TCCACTTTCT TTTCCTAACC TCACCAAATC TTCCAAAATG GCTTTCTTCA

---------- ---------- ---------- ---------- ----------

TCCGCTCTCT TTTCCTAACC TCACCATATC TTCCAAAATG GCTTTTTTCA

TCCGCTTTCT TTTCCTAACC TCATCATATC TTCCAAAATG GCTTTCTTCA

TCCACCTTCT TTTCCTAACC TCACCAAATC ATCCAAGATG TCCTTCTTCA

TCCACTTTCT TTTCCTAACC TCACCAGATC TTCCAAAATG GCTTTCTTCA

TCCACCTGCC TTTCCTAGCC TCACCAAATC GTCCAAAATG GCTTTCTTCA

---------- ---------- ---------- ---------- ----------

TCCACTTTCT TTTCCTAGTC TTACCATATC ATCCATTATA GCTTGCTTCA

TCCACTTTCT TTTCCTAACC TCACCAAGTC ATCCATTATA GCTTGCTTCA

GATCCTGAAT CAAACCATAT AAATTACAAA CCATGGCTGT ACAGACTGGC

GATCCTGAAT CAAACCATAT AAATTACAAA CCATGGCTGT ACAGACTGGC

GATCCTGAAT CAAACCATAT AAATTACAAA CCATGGCTGT ACAGACTGGC

GATCCTGAAT CAAACCATAT AAATTACAAA CCATGGCTGT ACAGACTGGC

GATCCTGAAT CAAACCATAT AAATTACAAA CTATGGCTAT ACAGACTGGC

GATCCTGAAT TGAACCACAT AAATTACAAA CCATGGCTGT GCAAACGGGC

ATTCCTGAAT TGAACCATGT AAACACTAAA CCATGGGCAT GCAGACTGGC

ATTCCTGAAT TGAACCATGT AAACAATAAA CCATGGGCAT GCAGACTGGC

GTTCCTGAAT TGAACCACAG AAATAAGCAA CCATGGGCAT GCAGACGGGC

---------- ---------- ---------A CCATGGCTGT GCAGACTGGC

GCTCCTGAAT TGAACCATAT AAATTACAAA CCATGGCTGT GCAGACTGGC

GCTCCTGAAT TGAACCATAG AAATTACAAA CCATGGCTGT ACAGACTGGC

GCTCCTGAAG TGAATCACAG AAATTACAAA CCATGGGCAT GCAGACAGGC

GCTCCTGAAT GGAACCATAT ATATTACAAA CCATGGCTGT GCAGACTGGC

CCTCCTGAAA TGAACCATGT CAATTACAA- ---------- ----------

---------- ---------- ---------A CCATGGCCGT GCAGACTGGC

GCTCCTAAAA TGAACCATAT CAGTTATAAA CAATGGCTGT GCAGACGGGC

GTTCCTAAAT TGAACCACAC AAATTACAAA CCATGGGCAT CCAGACGGGC

TTCAGCTTCC TGCAGATTTT CTCCATCAGC TGGGAGATGT TCACTGTGTT

TTCAGCTTCC TGCAGATTTT CTCCATCAGC TGGGAGATGT TCACTGTGTT

TTCAGCTTCC TGCAGATTTT CTCCATCAGC TGGGAGATGT TCACTGTGTT

TTCAGCTTCC TGCAGATTTT CTCCATCAGC TGGGAGATGT TCACTGTGTT

TTCAGCTTCC TGCAGATTTT CTCCATCAGC TGGGAGATGT TCACTGTGCT

TTCAGCTTCC TGCAGATTTT CTCCATCAAC TGGGAGATGT TCACCGCGCT

TTCAGCTTCC TGCAGCTCTT CTCTGTGAAC TTCGAGATGT TTACAGTGCT

TTCAGCTTCC TGCAGGTCTT CTCTGTGAAC TTCGAGATGT TTACAGTGCT

TTCAGCTTTG TGCAGGTCTT CTCAAAGAAC TTCGAGATGT TTGCCGTGCT

TTCAGCTTTC TGCAGGTTTT TTCCATCAAC TGGGAGATGT TCACTGTGTT

TTCAGCTTCC TGCAGATTTT CTCCATCAAC TGGGAGATGT TTACTGTGTT

TTCAGCTTCC TGCAGATTTT CTCCATCAAC TGGGAGATGT TCACTGTGTT

TTCAGCTTCC TGCAGGTCTT CTCGAAGAAC TTTGAGATGT TTTCCGTGCT

TTCAGCTTCC TGCAGATTTT CTCCATCAAC TGGGAGATGT TCACTATATT

---------- ---------- ---------- ---------- ----------

TTCAGCTTCC TGCAAATTTT CTCCGTCAAC TGGGAGATGT TCACTGTGCT

TTCAGTTTCC TACAGATCTT CTCCATCAAC TGGGAGATGT TCACTGTGCT

TTCAGCTTCG TGCAGGTTTT CTCGACGAGC TATGAGATGT TCTCGGTCCT

ATTTGTCATC GTGGGCATGG GCCAGATCTC CAACTATGTG GTAGCCTTCA

ATTTGTCATC GTGGGCATGG GCCAGATCTC CAACTATGTG GTAGCCTTCA

ATTTCTCATC GTGGGCATGG GCCAGATCTC CAACTATGTG GTAGCCTTCA

ATTTCTCATC GTGGGCATGG GCCAGATCTC CAACTATGTG GTAGCCTTCA

ATTTGTCATT GTGGGCATGG GCCAGATCTC CAATTATGTG GTGGCTTTCA

ATTTGTCATC GTGGGCGTGG GCCAGATCTC CAACTACGTG GTAGCCTTTA

TTTTGTCCTT GTTGGCATGG GTCAGATCTC CAACTACGTG GCAGCATTTG

TTTTGTCCTT GTTGGCATGG GTCAGATCTC CAACTACGTG GCAGCATTTG

GTTTGTCCTC GTGGGCATGG GCCAGATCTC CAACTACGTG GCAGCGTTTG

ATTTGTCATC GTCGGCATGG GCCAGATCTC CAACTACGTG GTGGCCTTCA

ATTTGTCATC GTTGGCATGG GCCAGATCTC CAACTATGTG GTGGCCTTCA

ATTTGTCATC GTCGGCATGG GCCAGATCTC CAACTATGTG GTGGCCTTCA

GTTTTTCCTT GTAGGCATGG GCCAGATCTC CAACTATGTG GCAGCATTTG

GTTCGTCATC GTGGGCATGG GCCAGATCTC CAACTACGTG GTGGCCTTCA

---------- ---------- ---------- ---------- ----------

GTTTGTCATC GTGGGCATGG GCCAGATCTC CAACTACGTG GTGGCCTTCA

GTTTGTCATC GTGGGCATGG GCCAGATCTC CAACTATGTA GTGGCCTTCA

TTTTCTCATC GTGGGCATGG GCCAGATCTC CAATTACGTG GCGGCTTTTG

TACTAGGTAA CCTTATTTGT GCAGAGATCT GCATATGTTC CTTCAATTCC

TACTAGGTAA CCTTATTTGT GCAGAGATCT GCATATGTTC CTTCAATTCC

TACTAGGTAA CCTTACTTGT GCAGAGATCT GCATATGTTC CTTCAATTCC

TACTAGGTAA CCTTACTTGT GCAGAGATCT GCATATGTTC CTTCAATTCC

TACTAGGTAA CCTTACTTGT GCAGAGATCT GCATATGTTC CTTCAATTCC

TACTAGGTAA CCTTACTTGT GCAGAGCTCT GCATATGTCC CTCCAATTCC

TCCTGGGTA- ---------- ---------- ---------- ----------

TCCTGGGTAA CCTTTTTCAT GCAGAGTTCC TGATAGGTCC CTTCGATTCC

TCCTGGGTAA CCTTATTTAT GCAGAGCTGT GTATAGGTCC CTTCAATCCC

TACTAGGTA- ---------- ---------- ---------- ----------

TTCTAGGTAA CCTTATTCGC ACAGAGCTCT GTGTATGAGC CTTCAATTCC

TACTCGGTAA CCTTATTGGC GCAGAGCTCC ACGTACGTCC CTTCAATGCC

TCCTGGGTA- ---------- ---------- ---------- ----------

TACTAGGTAA CCTTATTTGT GCAGAGCTCT GCATATGTTC CCTCAAATCC

---------A CCTTATTTGT ACAGAGCTCT TCATATGTCC CTTCAAATCC

TACTAGGTAA CCTTATTTGC ACAGAGCTCT TCATACGTCC CTTCAAATCC

TACTAGGTAA CCTTGTCTTG ACAGAGCTCT GCATACGTCC CATCAAATCC

TCCTCGGTAA CCTTATCTTC ACAGAGTTCT GCATATGCTC CTTCAAGGCC

TCTCTTCTGG GCCCAATAAT GATATAAAAA GCAATATTAA AGTGAGTTAA

TCTCTTCTGG GCCCAATAAT GATATAAAAA GCAATATTAA AGTGAGTTAA

TCTCTTCTGG GCCCAATAAT GATATAAAAA GCAATATTAA AGTGAGATAA

TCTCTTCTGG GCCCA---AT ACTATAAAAA --ATTAATAA AGCAAATTAA

TCTCTTCTGG GCCCAATAAT GATATAAAAA GCAACATTAA AATGAGTTAA

TCTCTTCTGG GCCCAGTAAT AATATAAAAA GCAATATTGA AGTGAGTTAA

---------- ---------- -----AAAAA GCAATGTCGA AGAGAGCAGA

TTTCTTCTGA GCCCA----- -----AAAAG GCAATGTTGA AGAGAGCAGA

CCTCTTCTGG GCCCAATGGT GATAGAAACA GCAATATTAA AGTGGATTAC

---------- -----GTAAT GATGCAAAAA GCAATATTAA AGTGAGTTAA

TCTCTTCTGG GCCCAGTAAT GATGTAAAAA GCAATATTGA AGTGAGCTAA

TCTCTTCTGG GCCCAGTAAT GATGTAAAAA GCAATATTAA AGTGAGTTAA

---------- -----GTAAT AATGTAAAGA GCAATGTTAA AGTGAGTGAA

TCTCTTCTGA GCCCAATAAT AATGTAAAGA TCAATATGAA AGTGAGCTAA

TTTCTTCTGG GCCCA----- ---------- ---------- ----------

TTTCTTCTGG GCCCAGTAAT AATGTGAAAC GCAATGATGA AGTGAGTGAA

TCTCTTCTTC GCCCAATAAT ATTGTAAAGA GCAAAAATAA AGTGAGTTAC

TCTCTTCTTT GCCCAATGAA ACTGTAAAGA GCAACGATGA AGTGAGTTAA

TATGCTAATA CTCCTCCCTT GTTTTGAACA GGAACAGAAA TTCTTGGCAA

TATGCTAATA CTCCTCCCTT GTTTTGAACA GGAACAGAAA TTCTTGGCAA

TATGCTAATA CTCCTCCCTT GTTTTGAACA GGAACAGAAA TTCTTGGCAA

ACTGCTAACT CGCCTCCCTT GTTTTGAACA GGAACAGAAA TTCTTGGCAA

AATGCTAATA CTCCTCCCTT GTTTTGAACA GGAACAGAAA TTCTTGGCAA

AATGCTAATA CTCCTCCCTT GTTTTGAACA GGAACAGAAA TTCTTGGCAA

AATGCTAATA CTGCCCCATC GTCTTGAACA GGGACTGAAA TCCTGAGCAA

AATGCTAATA CTGCC-CATT GTTTTGAACA GGAACTGAAA TCCTGAGCAA

AATGCTAATA CTCCTCCCTT GTTTTGAACA GGAACAGAAA TTCTTGGCAA

AATGCTAATA CTCCTCCCTT GTTTTGAACA GGAACAGAAA TTCTTGGCAA

AATGCTAATA CTCCTCCCTT GTTTTGAACA GGAACTGAAA TTCTGGGCAA

AATCCTAATA CTCCTCCCTT GTTTTGAACA GGAACAGAAA TCCTTGGCAA

AATGCTAACA CGCCTCCCTT GTTTTGAACA GGAACAGAAA TTCTTGGCAA

GATGCTAATA CTCCTCCCTT GTTTTGAACA GGAACAGAAA TTCTTGGCAA

---------- ---------- ---------- ---------- ----------

AGTGCTAATA CTCCTCCCTT GTTTTGAACA GGAACAGAAA TTCTTGGCAA

AATGCTAATA CTCCTCCCTT GTTTTGAACA GGAACAGAGA TACTTGGCAA

AATGCTAATA CTCCTCCCTT GTTTTAAACA GGAACAGAAA TTCTTGGCAA

GTCAGTTCGT ATTATATTCT CTACATTAGG AGTGTGCACA TTTTTTGCAG

GTCAGTTCGT ATTATATTCT CTACATTAGG AGTGTGCACA TTTTTTGCAG

GTCAGTTCGT ATTATATTCT CTACATTAGG AGTATGCACA TTTTTTGCAG

GTCAGTTCGT ATAATATTCT CTACGTTAGG AGTATGCATA TTTTATGCAT

GTCAGTTCGT ATTATATTCT CTACGTTAGG AGTATGCACC TTTTTTGCAG

GTCAGTTCGT ATTATATTCT CTACGTTAGG AGTGTGCACA TTTTTTGCAG

GTCGGTTCGC ATCCTCTTCT CCACATTAGG AGTCTGTACA TTTTTTGCCA

GTCGGTTCGC ATCATCTTCT CCACATTAGG AGTCTGTACA TTTTTTGCAA

GTCAGTTCGT ATTCTATTCT CTACGTTAGG AGTGTGCACA TTTTTTGCAG

GTCAGTTCGT ATTATATTCT CTACGTTAGG AGTGTGCACA TTTTTTGCAA

GTCAGTTCGT ATTATATTCT CTACATTAGG AGTGTGCACA TTTTTTGCAG

GTCAGTGCGT ATTATATTCT CTACGTTAGG AGTGTGCATA TTTTTTGCAG

GTCAGTTCGT ATTATATTCT CCACGTTAGG AGTGTGCACA TTTTTTGCAG

GTCAGTTCGT ATTATATTCT CCACGTTAGG CGTGTGCACA TTTTTTGCAG

---------- ---------- ---------- ---------- ----------

GTCAGTTCGT ATTATATTCT CCACGTTAGG AGTGTGCACA TTCTTTGCAG

ATCAGTTCGT ATTATATTCT CTACATTAGG AGTTTGCATA TTTTTTGCAA

ATCAGTTCGC ATTATATTCG CTACGTTAGG AATTTGCATA TTTTTTGCAA

TTGGCTATAT GCTGCTGCCA CTGTTTGCTT ACTTCATCAG AGACTGGCGG

TTGGCTATAT GCTGCTGCCA CTGTTTGCTT ACTTCATCAG AGACTGGCGG

TTGGCTATAT GCTGCTGCCA CTGTTTGCTT ACTTCATCAG AGACTGGCGG

TTGGCTACAT GGTGCTCCCA CTGTTTGCTT ACTTCATCCG AGACTGGCGA

TTGGCTATAT GCTGCTGCCA CTGTTTGCTT ACCTCATCAG AGACTGGCGG

TCGGCTACAT GCTGCTGCCA CTGTTTGCTT ACTTCATCAG AGACTGGCGG

TCGGCTACAT GGTGCTGCCG CTGTTTGCAT ACTTCATCCG AGACTGGAGG

TCGGCTACAT GGTCCTGCCA CTGTTTGCAT ACTTCATCAG AGACTGGAGG

TGGGCTACAT GCTGCTGCCA CTGTTTGCTT ACTTCATCAG AGATTGGCGG

TTGGCTACAT GCTGCTGCCA CTGTTTGCTT ACTTCATCAG AGACTGGCGG

TTGGCTACAT GCTGCTGCCA CTGTTTGCTT ACTTCATCAG AGACTGGCGG

TTGGCTACAT GCTGCTGCCA CTGTTTGCTT ACTTCATCAG AGACTGGCGG

TTGGCTACAT GCTGCTGCCC CTGTTTGCTT ACTTCATCAG AGACTGGCGG

TTGGCTACAT GCTGCTGCCG CTGTTTGCTT ACTTCATCAG AGACTGGCGG

---------- ---------- ---------- ---------- ----------

TTGGCTACAT GCTGCTGCCA CTGTTTGCTT ACTTCATCCG AGACTGGCGG

TTGGCTACAT GTTGCTGCCA CTGTTTGCTT ACTTCATCAG AGACTGGCGG

TTGGCTACAT GTTGCTGCCC CTATTTGCTT ACTTCATCAG AGACTGGCGG

ATGCTGCTGC TGGCGCTGAC GGTGCCGGGA GTGCTGTGTG TCCCGCTGTG

ATGCTGCTGC TGGCGCTGAC GGTGCCGGGA GTGCTGTGTG TCCCGCTGTG

ATGCTGCTGC TGGCGCTGAC GGTGCCGGGA GTGCTGTGCG TCCCGCTGTG

ATACTGCTGG TGGCACTGAC GATGCCAGGG GTGCTGTGCG TGGCACTCTG

ATGCTGCTGC TGGCGCTGAC GGTGCCGGGG GTGCTGTGTG TCCCGCTGTG

ATGCTGCTGC TGGCGCTGAC GGTGCCGGGG GTGCTGTGCG TCCCGCTGTG

ATGCTGCTGC TGGCACTGAC ACTGCCTGGG CTGTTCTGTG TCCCCCTGTG

ATGCTGCTGC TGGCCCTGAC ACTGCCTGGG CTATTTTGTG TCCCCCTGTG

ATGCTGCTGC TGGCGCTGAC GGTGCCGGGG GTGCTGTGTG TCCCGCTGTG

ATGCTGCTGC TGGCGCTGAC GGTGCCGGGG GTGCTGTGTG TCCCGCTGTG

ATGCTGCTGC TGGCGCTGAC GCTGCCGGGG GTGCTGTGCA TCCCTCTGTG

AAGCTGCTTC TGGCGCTGAC GGTGCCCGGG GTGCTGTGCG TCCCGCTGTG

ATGCTGCTGC TGGCGCTGAC GGTGCCGGGG GTGCTGTGCG TCCCGCTGTG

ATGCTGCTGC TGGCGCTGAC GGTGCCCGGG GTGCTGTGCG TCCCGCTGTG

---------- ---------- ---------- ---------- ----------

ATGCTGCTGC TGGCGCTGAC GGTGCCGGGG GTGCTGTGCG TCCCGCTGTG

ATGTTGCTAC TGGCTCTGAC GGTGCCGGGG GTGCTGTGTG TCCCGCTGTG

ATGTTGCTAC TGGCTCTTAC AATACCGGGG GTGTTCTGCG TGCCGCTGTG

GTGGTGAGTG TGTCACCTTT AAGCTGTCCC CATGGACATA GATTTGCGCC

GTGGTGAGTG TGTCACCTTT AAGCTGTCCC CATGGACATA GATTTGCGCC

GTGGTGAGTG TGTCACCTTT AAGCTGTCCC CATGTACATA GATTTGTGCC

GTGGTGAGTG TG-------- ---------- ---------- ----------

GTGGTGAGTG TGTCACCTTT AAGCTGTCCC CATGGACATA GATTTGTGCT

GTGGTGAGTG TGTCACCTTT AAACTGTCCC CATGGACGTA GACTTGTGCC

GTGGTGAGTA CATCACCTTT AAGCTGTCCC CATGGACGTA GATTTGCGCC

GTGGTGAGTA CATCACCTTT AAGCTGTCCC CGTGGACGTA GATTTGTGCC

GTGGTGAGTG TGTCACCTTT AAGCTGTCCC CATGGACATA GACCTGTGCC

GTGGTGAGTG TG-------- ---------- ---------- ----------

GTGGTGAGTG TGTCACCTTT AGGCTGTTCC CATGGACATA GACTTGTGCC

GTGGTGAGTG TGTCACCTTC AGGCTGTCCC CATGGACATA GATTTGTGCC

GTGGTGAGTG TGTCACCTTT AAGCTGTCCC CGTGGACATA GATTTGTGCC

GTGGTGAGTG TGTCACCTTT AAGCTGTCCC CGTGGACATA GATTTGTGCC

---------- --TCACCTTT AAACTGTCTC CATGGACATA TACTTGTGCC

GTGGTGAGTG CG-------- ---------- ---------- ----------

GTGGTGAGTG AGTTACCTGT AAGCTGTCTC CATGCACGTA CGTCTGAGCT

GTGGTGAGTT TATTACCTGT AAGCTGTCCC CGTGGACGTA AATCTGAGCA

ACAGGTTGGC TCCGGATGTA GATGTTCTCA ATCTTCTCGG GTGCAACATA

ACAGGTTGGC TCCGGATGTA GATGTTCTCA ATCTTCTCGG GTGCAACATA

ACAGGTTGGC TCCGGATGTA GATGTTCTCA ATCTTCTCGG GTGCAACATA

---------- ---------- ---------- ---------- ----------

ACAGGTTGGC TCCGGACGTA GATGTTCTCA ATCTTCTCGG GTGCAACATA

ACAGGCTGGC TCCGGATGTA GATGTTCTCA ATCTTCTCAG GTGCAACATA

ACAGGCTCAC TTCGGATGTA GATGTTCTCA ATCTTCTCGG GCGCAACGTA

ACAGGCTCAC TCCGGATGTA GATGTTCTCG ATCTTCTCTG GCGCAACATA

ACAGGCTCAC TTCGGATGTA GATGTTCTCA ATCTTCTCGG GTGCAACATA

---------- ---------- ---------- ---------- ----------

ACGGGCTCAC TCCGTATGTA GATGTTTTCA ATCTTCTCGG GTGCAATATA

ACAGGCTCAC TCCGGATGTA GATGTTCTCA ATCTTCTCGG GCGCAACATA

ACAGGTTCAC AACGGATGTA GATATTCTCG ATCTTCTCAG GTGCCACATA

ACAGGCTCAC TCCGGATGTA GATGTTCTCA ATCTTTTCCG GTGCAACATA

ACAGGTTCAC TCCGGATGTA GATGTTCTCA ATCTTCTCGG GTGCGACATA

---------- ---------- ---------- ---------- ----------

ACGGGTTCAC TTCGAATGTA GATATTTTCA ATCTTTTCTG GAGCAATGTA

ACTGGTTCAC TTCGGATGTA GATATTCTCA ATTTTTTCTG GGGCAATATA

TTCTCCCTGA GCAAGTTTAA ATATATGCTT TTTCCGATCA ATAATTTTAA

TTCTCCCTGA GCAAGTTTAA ATATATGCTT TTTCCGATCA ATAATTTTAA

TTCTCCCTGA GCAAGTTTAA ATATGTGCTT CTTCCGATCA ATAACTTTAA

---------- ---------- ---------- ---------- ----------

TTCTCCCTGA GCAAGTTTAA ATATGTGCTT TTTCCGATCA ATAATTTTAA

TTCCCCCTGA GCAAGTTTAA ATATGTGCTT TTTCCGATCA ATAATTTTAA

TTCCCCCTGA GCAAGTTTAA ATATGTGCTT TTTCCGATCG ATGATTTTAA

TTCCCCCTGA GCAAGTTTAA ATATGTGCTT TTTCCGATCA ATGATTTTGA

TTCTCCCTGA GCAAGTTTAA ATATGTGCTT TTTCCGGTCA ATAATTTTAA

---------- ---------- ---------- ---------- ----------

TTCTCCCTGA GCGAGTTTAA ATATGTGCTT TTTCCGATCG ATAATTTTAA

CTCTCCCTGA GCCAGTTTAA ATATGTGCTT CTTCCGATCA ATAATTTTAA

CTCTCCCTGA GCGAGTTTAA ATATGTGCTT TTTCCGATCA ATAATTTTAA

TTCTCCCTGA GCAAGTTTAA ATATGTGCTT TTTCCGATCA ATAACTTTAA

TTCTCCCTGA GCAAGTTTAA ATATGTGCTT TTTCCGATCA ATAATTTTAA

---------- ---------- ---------- ---------- ----------

TTCTCCCTGA GCAAGTTTAA ATATGTGCTT TTTCCGATCA ATAATTTTAA

TTCCCCCTGG GCAAGTTTAA ATATGTGCTT TTTTCGATCA ATAATTTTAA

GAGTTCCTGC CTGTCATGCG GGACTACGAC GAGGTGACCG CCTTCCTGGG

GAGTTCCTGC CTGTCATGCG GGACTACGAC GAGGTGACCG CCTTCCTGGG

GAGTTCCTGC CTGTCATGCG GGACTACGAC GAAGTGACTG CCTTCCTGGG

---------- ----CATGCG GGACTACGAC GAGGTGACCG CCTTCCTGGG

GAGTTCCTGC CTGTCATGCG GGACTACGAC GAGGTGACCG CCTTCCTGGG

GAGTTCCTGC CTGTCATGCG GGACTACGAC GAGGTGACCG CCTTCCTTGG

GCGTTCCCTC CTGTCATGCG GGACTACGAC GAGGTGACCG CCTTCCTGGG

GTGTTCCCTC CTGTCATGCG GGACTACGAC GAGGTGACCG CCTTCCTAGG

GAGTTCCTTC CTGT-ATGCG GGACTACGAC GAGGTGACCG CGTTTCTGGG

---------- ----CATGCA GGACTACGAC GAAGTGACCG CCTTCCTAGG

GAGTTCCTGC CTGTCATGCG GGACTACGAC GAGGTGACCA CCTTCCTGGG

GAGTTCCCGC CTATCATGCG GGACTACGAC GAGGTGACCG CCTTCCTGGG

GAGTTCCTGC CTATCATGCG GGACTACGAC GAGGTGACCG CCTTCCTGGG

GTGTTCCTGC CTGTCATGCG TGACTACGAC GAGGCGATCG CCTTCCTGGG

GTGTTCCTGC CTGT------ ---------- ---------- ----------

---------- ----CATGCG TGACTACGAC GAGGTGACCT CCTTCCTGGG

GAGTTCCTGT CTGTCATGCG GGACTACGAC GAGGTGACAG CCTTCCTGGG

GAGTTCCAGT CTGTGATGAG GGACTACGAC GAGGCGACGG CCTTCCTGGG

CGAGTGGGGG CCCTTCCAGC GCCTCATCTT CTTCCTGCTC AGCGCCAGCA

CGAGTGGGGG CCCTTCCAGC GCCTCATCTT CTTCCTGCTC AGCGCCAGCA

CGAGTGGGGG CCTTTCCAGC GCCTCATCTT CTTCCTGCTC AGCGCCAGCA

CGAGTGGGGG CCTTTCCAGC GCCTCATCTT GTTCCTGCTC AGCGCCAGCA

CGAGTGGGGG CCCTTCCAGC GCCTCATCTT CTTCCTGCTC AGCGCCAGCA

CGAGTGGGGG CCCTTCCAAC GCCTCATCTT CTTCCTGCTC AGCGCCAGCA

CGAGTGGGGG CCCTTCCAGC GCCTCATTTT CTTCCTGCTC AGCGCCAGCA

CGAGTGGGGG CCCTTCCAGC GCCTCATCTT CTTCCTGCTC AGCGCCAGCA

CGAGTGGGGG CCCTTCCAGC GCCTCATCTT CTTCCTGCTC AGCGCCAGCA

CGAGTGGGGG CCCTTTCAGC GCCTCATCTT CTTCCTGCTC AGCGCCAGCA

CGAGTGGGGG CCCTTCCAGC GCCTCATCTT CTTCCTGCTC AGCGCCAGCA

CGAGTGGGGG CCGTTCCAGC GCCTCATCTT CTTCTTGCTC AGCGCCAGCA

CCAGTGGGGG CCCTTCCAGC GCCTCATCTT CTTCCTGCTC AGCGCCAGCA

CGAGTGGGGG CCCTTCCAGC GCCTCATCTT CTTCCTGCTC AGCGCCAGCA

---------- ---------- ---------- ---------- ----------

CGAGTGGGGG CCCTTCCAGC GCCTCGTCTT CTTCCTGCTC AGCGCCAGCA

CGAGTGGGGA CCCTTCCAGC GCCTCATCTT CTTCCTGCTC AGCTCCAGCA

CGAGTGGGGC CCCTTCCAGC GGCTGGTCTT CTTCCTGCTG AGCGCCAGCA

TCATCCCCAA TGGCTTCATG GCGCAACCAC ACTGTCCCAC TGCGGCTGCG

TCATCCCCAA TGGCTTCATG GCGCAACCAC ACTGTCCCGC TGCGGCTGCG

TCATCCCTAA TGGCTTCATG GCGCAACAAC AGTGTCCCAC TGCGGCTGCG

TCATCCCTAA TGGCTTCATG GCGCAACCAC AGTGTCCCAC TGCGGCTGCG

TCATCCCCAA TGGCTTCATG GCGCAACCAC AGTGTCCCGC TGCGGCTGCA

TCATCCCCAA CGGCTTCATG GCGCAACCAC AGTATCCCGG TGCGGCTGCA

TCATCCCCAA TGGCTTCATG GCGCAACCAC AGTATCCCGT TGGAGACGAA

TCATCCCCAA TGGCTTCATG GCGCAACCAC AGTATCCCGT TGGAGACGAA

TCATCCCAAA CGGCTTCATG GCGCAACCAC AGTGTCCCGC TGCGGCTTCA

TCATCCCCAA CGGCTTCATG GCGCAACCAC AGTGTCCCCA TGCGGCTGCA

TCATCCCCAA CGGCTTCATG GCGCAACCAC AGTATCCCGC TGCGGCTGCA

TCATCCCCAA CGGCTTCATG GCGCAACCAC AGTGTCCCGC TGCGGCTGCA

TCATCCCCAA CGGCTTCATG GCGCAACCAC AGTATCCCGC TGCAGGTGCG

TCATCCCCAA TGGCTTCATG GCGCAACCAC AGTATCCCGC TGCGGCTGCA

---------- ---------- ---------- ---------- ----------

TCGTCCCCAA CGGGTTCATG GCGCAACCAC AGTGTCCCGC TGCGGCTGCA

TCATCCCCAA CGGTTTCATG GCGTAACCAT AGTATCCCGC TGCAGCTGCA

TCATCCCCAA CGGCTTCATG GCGGAACCAC AGCATCCCGC TGGAggcgcg

GGACGGCCGC GAGGTGCCCC ACAGCTGCCG CCGCTACCGG CTCGCAACTT

GGACGGCCGC GAGGTGCCCC ACAGCTGCCG CCGCTACCGG CTCGCAACTT

GGACGGCCGC GAGGTGCCCC ACAGCTGCCG CCGCTACCGG CTCGCAACTT

GGACGGCCGC GAGGTGCCCC ACAGCTGCCG CCGCTACCGG CTCGCAACTT

GGACGGCCGC GAGGTGCCCC ACAGCTGCCG CCGCTACCGG CTCGCAACTT

GGACGGTCGC GAGTTGCTCC ATAGCTGCCG CCGCTACCGG CTCGCAACTT

GGACGGACGA CAGGTGCCTC AGAGCTGCCG CCGCTACCGA CTGGCAACTT

GGACGGACGA CAGGTGCCTC AGAAATGCCG CCGCTACCGA CTGGCAACTT

GGACGGCCGC GAGGTGCCTC ACAGCTGCCG GCGCTACCGG CTCGCAACTT

GGACGGCCGC GAGGTGCCTC AGAGCTGCCG CCGCTACCGA CTCGCAACTT

GGACGGCCGC GAGGTGCCGC ACAGCTGCCG GCGCTACCGG CTCGCAACTT

GAACGGTCAT GAGGTGCCTC ACAGCTGCAG GCGCTACCGG CTCGCAACTT

GGACGGCCGC GAGGTGCCCC ACAACTGCCG CCGCTATCAG CTCGCAACTT

GGACGGCCGC GAGGTGCCCC ACAGCTGCCG CCGCTACCGG CTCGCAACTT

---------- ---------- ---------- ---------- ----------

GGACGGCCGC GAGGTGCCGC ACAGCTGCCG CCGCTACAAG CTGGCAACTT

GGACGGCCGG GAAGTGCCCA GACAGTGCCA GCGCTACCGG CTCGCAACTT

gggcgcgggg cgggggccca gccgctgccg ccgcTACCGG CTGCCAACTT

CTCGGCGCTT GGGCTGGAGC CGGGGCGCGA CGTGGACCTG GGGCAGCTGG

CTCGGCGCTC GGGCTGGAGC CGGGGCGCGA CGTGGACCTG GGGCAGCTGG

CTCGGCGCTC GGGCTGGAGC CGGGGCGTGA CGTGGACCTG GGGCAACTGG

CTCGGCGCTC GGGCTGGAGC CGGGGCGCGA CGTGGACCTG GGGCAACTGG

CTCGGCGCTC GGGCTGGAGC CGGGGCGCGA CGTGGACCTG GGGCAGCTGG

CTCAGCACTC GGGCTGGAAC CGGGGCGTGA CGTGGACCTG GAGCAGCTGG

CTCTGCGCTG GGGCTGGAGC CGGGACGGGA CGTGGACCTG GAGCAGCTGG

CTCTGAGCTA GGGCTGGAGC CGGGGCGGGA CGTGGACCTG GAGCAGCTGG

CTCGGCGCTG GGGCTGGAGC CCGGGCGCGA CGTGGACCTG GAGCAGCTGG

CTCGGAGCTC GGGCTGGAGC CGGGGCGCGA TGTGGACCTA GAGCAGCTGG

CTCGGCGCTC GGGCTGGAGC CGGAGCGCGA TGTGGACCTG GAGCAGCTGG

CTCGGCGCTC GGGCTGGAGC CGGGGCGCGA TGTGGACCTG GAGCAATTGG

CTCGGCGCTC GGGCTGGAGC CGGGGCGCGA TGTGGACCTG GAGCGGCTGG

CTCGGCGCTC GGGTTGGAGC CCGGGCGCGA CGTGGATCTG GCGCAGCTGG

---------- ---------- ---------- ---------- ----------

CTCGGCGCTC GGGCTGCTGC CGGGGCGCGA CGTGAACCTG ACGGAGCTGG

CTCCGCCATG GGGCTAGTGC CCGGTAGGGA CGTGAACCTG AGTCAGGTGG

CTCCGCCCTG GGGCTGGCGC CCGGCACCGA CGTCGACCTG GCCCGCCTGG

AGCAGGAGAG CTGTCTGGAT GGCTGGGAGT TCAGTCAGGA CGTCTACCTG

AGCAGGAGAG CTGTCTGGAT GGCTGGGAGT TCAGTCAGGA CGTCTACCTG

AGCTGGAGAG CTGCCTGGAT GGCTGGGAGT TCAGCCGGGA CGTCTATCTG

AGCTGGAGAG CTGCCTGGAT GGCTGGGAGT TCAGCCGGGA CGTCTATCTG

AGCTGGAGAG CTGCCTGGAT GGCTGGGAGT TCAGCCAGGA CGTCTACCTG

AGCAGGAGAA CTGCCTAGAT GGCTGGGAGT TCAGCCAGGA CGTCTACCTG

AGCAAGAGAA CTGCCTGGAT GGCTGGGAGT ACAACAAGGA CGTCTTCCTG

AGCAGGAGAG CTGCCTGGAT GGCTGGGAGT ACGACAAGGA CGTCTTCCTG

AGCAGGAGAG CTGCTTGGAT GGCTGGGAGT ACAGCCAGGA CGTGTACCTG

AGCAGGAGGG CTGCCTGGAT GGCTGGGAGT TCAGCCAGGA CATCTACCTG

AGCAGGAGAG CTGCCTGGAT GGCTGGGAGT TCAGCCAGGA CGTCTACCAG

AGCAGGAGAG CTGCCTGGAT GGCTGGGAGT TCAGCCAGGA CGTCTACCTG

AGCAGGAGGG CTGCCTGGAT GGCTGGGAGT ACAGCCAGGA CGTCTTCCTG

AGCAGGAGAG CTGCCTGGAC GGCTGGGAGT TCAGCCAGGA CGTTTACCAG

---------- ---------- ---------- ---------- ----------

AGCAGGAGAG CTGCCTGGAC GGCTGGGAGT TCAGCCAGGA CGTCTACCTG

AGCTGGAGCC CTGTCTGGAC GGCTGGGAAT ACAGCCAGGA CACATACCTG

AGCAGGAGAA GTGTCTGGAC GGCTGGGACT ACGGCCGCGA CGTCTACCTG

TCCACCATTG TGACCGAGGT GGGACCGGCA GCCATTTTCC GATGTCTCCA

TCCACCATTG TGACTGAGGT GGGACCGGCA GCCATTTTCC GATGTCTCCA

TCCACCATTG TGACCGAGGT GGGACCGGCA GCCATTTTCC GATGTCTCCA

TCCACCATTG TGACCGAGGT GGGACCGGCA GCCATTTTCC GATGTCTCCA

TCCACCATCG TGACCGAGGT GGGACCGGCA GCCATTTCCC AATATCTCCA

TCCACCATCG TGACCGAGGT GGGACCGGCA GCCACTTCCC GATGTCTCCA

TCCACCATCG TGACAGAGGT AGGACCGGCA GCCATTTTCC AATGTCTCCC

TCCACCATCG TGACAGAGGT AGGACCGGCA GCCATTTCCC AATGTCTCCA

TCCACCATCG TGACCGAGGT GGGACCGGAA GCCACTTGCC GATGTCTCCA

TCCACCATCG TCACCGAGGT GGG------- ---------- ----------

TCCACCATCG TGACCGAGGT GGGACCGGCA GCCACTTGCC AATGTCCCCA

TCCACCATCG TGACCGAGGT GGGACCGGCA GCCACTTTCC AATGTCCCCA

TCCACCATCG TCACCGAGGT GGGACCGGCA GCCACTTGCC AATGTCTCCA

TCCACCATCG TGACCGAGGT GGGACCGGCA GCCATTTCCC AATGTCTCCA

---------- ---------- ---ACCGGCA GCCATTTTCC AATGTCTCCA

TCCACCATCG TCACCGAGGT GGGACCGGCA GCCATCGTCC AATGTCTCCA

TCCACCATCG TCACTGAGGT GGGACAGGTA ACCACTTCCC AATATCTCCA

TCCACCGTCG TCACCGAGGT GGGACCGGTA ACCACTTTCC GATGTCTCCT

GTGTGAAGCC AGCCATCGCT GTCCAGGGCC TCCTTCGTCC TGTCGCAGTG

GTGTGAAGCC AGCCATCGCT GTCCAGGGCC TCCTTCGTCC TGTCGCAGTG

GTGTGAAGCC AGCCATCGCT GTCCAGGGCC TCCTTCGTCC TGTCGCAGTG

GTGTGAAGCC AGCCATCGCT GTCCAGGGCC TCCTTCGTCC TGTCGCAGTG

GTGTGAAGCC AGCCATCACT GTCCAGGGCC TCCTTTGTCC TGTCGCAGTG

GTGTGAAGCC AGCCATCACT GTCCAAGGCC TCATTTGTCC TGTCACAGTG

GTGTGAAGCC AGCCGTCACT GTCCAGGGCC TCCTTTGTCC TGTCACAGTG

GTGTGAAGCC AGCCGTCGCT GTCCAGGGCC TCCTTTGTCC TGTCACAGTG

GTGTGAAGCC AGCCGTCACT ATCCAGGGCC TCCTTCGTCC TGTTGCAGTG

---------- ---------- ---------- ---------- ----TCAGTG

GTGTGAAGCC AGCCGTCGCT GTCCAGGGCT TCCTTTGTCC TGTCACAGTG

GTGTGAAGCC AGCCGTCACT GTCCAGAGCC TCCTTTGTCT TGTCGCAGTG

GTGTGAAGCC AGCCGTCGCT GTCTAAGGCC TCTTTTGTCC TCTCTCAGTG

GTGTGAAGCC AGCCATCACT GTCCAGGGCC TCCTTTGTCT TCTCGTAGTG

GTGTGAAGCC AGCCATCGCT ATCCAGGGCC TCTTTCGTCT TCTC------

GTGTGAAGCC AACCATCACG GTCCAGGGCC TCTTTTGTCT TCTCGCAGTG

GTATGAAGCC AGCCATCCTT GTCCAGTGCT TCATCAGTCT TCTCACAGTG

GTGTGAAGCC AGCCTTCCTG GTCCAGAGCC TCATTAGTCT TCTCGCAGTG

GAACCTGGTG TGTGAGGACG ACTGGAAGGC CCCACTCACA ATCTCCTTGT

GAACCTGGTG TGTGAGGACG ACTGGAAGGC CCCACTCACA ATCTCCTTGT

GAACCTGGTG TGTGAGGACG ACTGGAAGGC CCCACTCACG ATCTCCTTGT

GAACCTGGTG TGTGAGGACG ACTGGAAGGC CCCACTCACG ATCTCCTTGT

GAACCTGGTG TGTGAGGACG ACTGGAAGGC CCCACTCACC ATCTCCTTGT

GAACCTGGTA TGTGAGGACG ACTGGAAGGC CCCGCTCACG ATCTCCATGT

GAACCTGGTG TGTAACGATG CCTGGAAAGC CCCACTCACC ACCTCCATGT

GGACCTGGTG TGTAAGGATG ACTGGAAAGC CCCACTCACC ACCTCCTTCT

GAACCTGGTG TGCGAGGATG ACTGGAAGAC TCCGCTCACC ACCTCCCTGT

GAACCTGGTG TGTGAGGACG ACTGGAAGGC CCCGCTCACA ATCTCCTTGT

GAACCTGGTG TGTGAGGACG ACTGGAAGGC CCCACTCACG GTCTCCTTGT

GAATCTGGTG TGTGAGGAAG ACTGGAAGAC GCCCCTCACC TCCTCCCTGT

GAACCTGGTA TGCGATGACG ACTGGAAGGC CCCGCTCACC ATCTCCTTAT

GAACCTGGTG TGCGACGATG ACTGGAAGAC GCCCCTCACC ACCTCCCTGT

---------- ---------- ---------- ---------- ----------

GAGCCTGGTG TGCGAGCACG ACTGGAAGAC GCCCCTCACC ACCTCCCTGT

GGACCTGGTC TGTGAGGATG ACTGGAAAGC TCCACTAACT ACCTCCTTAT

GAACCTGGTG TGTGATGATG ACTGGAAAGC TCCACTAACT ACCTCCTTAT

TCTTCGTGGG TGTGCTGTTG GGCTCCTTCA TTTCAGGGCA GCTGTCAGAC

TCTTCGTGGG TGTGCTGTTG GGCTCCTTCA TTTCAGGGCA GCTGTCAGAC

TCTTCGTGGG TGTGCTGTTG GGCTCCTTTG TTTCAGGGCA GCTGTCAGAC

TCTTCGTGGG TGTGCTGTTG GGCTCCTTCG TTTCAGGGCA GCTATCAGAC

TCTTCGTGGG TGTGCTGTTG GGCTCCTTCA TTTCGGGGCA GCTGTCAGAC

TTTTCGTGGG CGTGCTATTG GGCTCCTTCA TTTCAGGGCA GCTCTCAGAC

TTTACGTGGG TGTGCTCTTA GGCTCCTTCA TTTCGGGACA GCTCTCAGAC

TTTACGTGGG TGTGCTCTTA GGCTCCTTCA TTTCGGGACA GCTCTCAGAC

TCTTCGTGGG CGTGCTTATC GGCTCCTTCG TGTCCGGCCA GCTGTCCGAC

TTTTCGTGGG TGTGCTGATG GGCTCCTTCA TTTCGGGGCA GCTCTCAGAC

TTTTCGTGGG TGTGCTGGTG AGCTCCTTCA TTTCGGGGCA GCTCTCAGAC

TCTTTGTAGG CGTTCTCCTT GGTTCCTTTG TGTCCGGGCA GCTATCAGAC

TTTTCGGGGG CGTGCTCGTG GGCTCCTTCA TCTCAGGGCA GCTCTCAGAC

TCTTCGTAGG CGTGCTCCTT GGCTCCTTCG TGTCCGGGCA GCTCTCAGAC

---------- ---------- ---------- ---------- ----------

TTTTCCTGGG CGTGCTCTTC GGCTCCTTCG TGTCCGGGCA GCTGTCAGAC

TTTTTGTGGG TGTGCTTATC GGATCCTTTA TTTCAGGACA GCTCTCAGAC

TCTTTGTGGG TGTGCTTATC GGATCCTTTG TTTCGGGACA GCTCTCAGAC

AGGTAAGGAT CTTTCAAGTA GCCTTTGAAC ACATTTGGTC CTCTCACACA

AGGTAAGGAT CTTTCAAATA GCCTTTGAAC ACATTTGGTC CTCTCACACA

AGGTAAGGAT CTTTCAAGTA GCCTTTGAAC ACATTTGGTC CTCTCACACA

AGGTAAGGAT CTTTCAAGTA GCCTTTGAAC ACATTTGGTC CTCTCACACA

AGGTAAGGAT CTTTCAAGTA GCCTTTGAAC ACGTTTGGTC CTCTCACACA

AGGTAAGGAT CTTTCAAGTA GCCCTTGAAC ACATTTGGTC CTCTCACACA

AGGTAAGGGT CTTTTAAGTA GCCTTTGAAC ACATTTGGTC CTTTCACACA

AGGTAAGGGT CTTTTAAGTA GCCTTTGAAC ACATTTGGTC CTTTCACACA

AGGTAGGGAT CTTTCAAGTA GCCTTTGAAT ACATTTGGTC CTCTCACACA

AGGTAAGG-- ---------- ---------- ---------- ----------

AGGTAAGGAT CTTTCAAGTA ACCTTTGAAC ACATTTGGTC CTCTCACACA

AGGTAAGGAT CTTTCAAGTA GCCTTTGAAC ACATTTGGTC CTCTCACGCA

AGGTAAGGAT CTTTCAAGTA GCCCTTGAAT ACATTTGGTC CTCTCACACA

AGGTAAGGAT CTTTCAAGTA ACCTTTGAAC ACATTTGGTC CTCTCACACA

--------AT CTTTCAAGTA ACCTTTAAAC ACATTTGGTC CCCTCACACA

AGGTAAGGAT CTTTCAAGTA ACCTTTAAAC ACATTTGGTC CTCTCACGCA

AAGTAAAGGT CTTTGAGATA ACCTTTGAAA ACATTTGGGC CTTTCACACA

AGGTAAAGAT CTTTCAAATA ACCCTTGAAA ACATTTGGTC CCTTGACGCA

TATCTATGGC AGGTTTGGCC GGAAGAATGT GCTGTTCGTG ACCATGGGCA

TATCTATGGC AGGTTTGGCC GGAAGAATGT GCTGTTCGTG ACCATGGGCA

TATCTATGGC AGGTTTGGCC GGAAGAATGT GCTGTTCATG ACCATGGGCA

TATCTATGGC AGGTTTGGCC GGAAGAATGT GCTGTTCGTG ACCATGGGCA

TATCTGTGGC AGGTTTGGCC GGAAGAATGT GCTGTTTGTG ACCATGGGCA

TATCTGTGGC AGGTTCGGTC GGAAGAAAGT GCTGTTTGTA ACCATGGCCA

GATCTGTGGC AGGTTTGGTC GCAAGAATGT GCTGTTTTTG ACCATGGGCA

TATCTGTGGC AGGTTTGGTC GCAAGAATGT GCTGTTTTTG ACCATGGGCA

TATCTGCGGC AGGTTTGGTC GGAAGAACGT GCTCTTTGTC ACCATGGGCA

---------- AGGTTTGGTC GGAAGAACGT GCTGTTTGTG ACGATGGGCA

TATCTGTAGC AGGTTTGGCC GGAAGAATGT GCTCTTTGTG ACCATGGGCA

TATCTGCAGC AGGTTTGGTC GGAAGAACGT GCTGTTTGTG ACCATGGGCA

TATCTGTAGC AGGTTCGGCC GGAAGAATGT GCTGTTCGTG ACCATGGGCA

TATCTGCAGC AGGTTTGGCA GGAAGCGCAT CCTCTTTGCA ACCATGGCTG

TATCTGTGGC ---------- ---------- ---------- ----------

TATCTGTGGC AGATTTGGAC GGAAGAATGT GCTGTTAGTG ACCATGGGCA

GATCTGGATC AGGTTTGGAC GGAAGAATGT ACTATTTGTG ACAATGGGGA

TATCTGCAGG AGGTTTGGCC GGAAAAATGT CCTGTTTGTG ACCATGGGCA

TGCAGACAGG CTTCAGCTTC CTGCAGATCT TCTCGAAGAA TTTTGAGATG

TGCAGACAGG CTTCAGCTTC CTGCAGATCT TCTCGAAGAA TTTTGAGATG

TGCAGACAGG CTTCAGCTTC CTGCAGATCT TCTCAAAAAA CTTTGAGATG

TGCAGACAGG CTTCAGCTTC CTGCAGATCT TCTCAAAAAA CTTTGAGATG

TGCAGACAGG CTTCAGCTTC CTGCAGCTCT TCTCGAAGAA CTTTGAGATG

TGCAGACAGG CTTCAGCTTC CTGCAGGTCT TCTCGAAGAA CTTCGAGATG

TGCAGACTGG CTTCAGCTTC CTGCAGCTCT TCTCTGTGAA CTTCGAGATG

TGCAGACTGG CTTCAGCTTC CTGCAGGTCT TCTCTGTGAA CTTCGAGATG

TGCAGACGGG CTTCAGCTTT GTGCAGGTCT TCTCAAAGAA CTTCGAGATG

TGCAGACAGG CTTCAGCTTC CTGCAGATCT TCTCGAAGAA CTTTGAGATG

TGCAGACGGG CTTCAGCTTC CTGCAGATTT TCTCAAAGAA CTTTGAGATG

TGCAGACGGG CTTCAGCTTC CTGCAGGTCT TCTCCAAGAA CTTCGAGATG

TGCAGACAGG CTTCAGCTTC CTGCAGGTCT TCTCGAAGAA CTTTGAGATG

TGCAGACTGG CTTCAGCTTC CTGCAGATTT TCTCCATCAA CTGGGAGATG

---------- ---------- ---------- ---------- ----------

TACAGACGGG CTTCAGCTTC CTGCAGGTCT TCTCCACCAA CTTTGAGATG

TGCAGACAGG ATTTAGTTTT CTACAAATCT TCTCAACAAG CTTTGAGATG

TCCAGACGGG CTTCAGCTTC GTGCAGGTTT TCTCGACGAG CTATGAGATG

TTTGTCGTGC TGTTTGTCCT TGTAGGCATG GGCCAGATCT CCAACTATGT

TTTGTCGTGC TGTTTGTCCT TGTAGGCATG GGCCAGATCT CCAACTATGT

TTTGTCGTGC TGTTTGTCCT TGTAGGCATG GGCCAGATCT CCAACTATGT

TTTGTCATGC TGTTTGTCCT TGTAGGCATG GGCCAGATCT CCAACTATGT

TTTGTCGTGC TGTTTGTCTT TGTAGGCATG GGCCAGATCT CCAACTACGT

TTTATCGTGC TGTTTACCTT TGTAGGCATG GGCCAGATTT CCAACTATGT

TTTACAGTGC TTTTTGTCCT TGTTGGCATG GGTCAGATCT CCAACTACGT

TTTACAGTGC TTTTTGTCCT TGTTGGCATG GGTCAGATCT CCAACTACGT

TTTGCCGTGC TGTTTGTCCT CGTGGGCATG GGCCAGATCT CCAACTACGT

TTTACCGTGC TGTTTTTCCT TGTAGGCATG GGCCAGATCT CCAACTATGT

TTTACTGTGC TTTTTGTCCT TGTGGGCATG GGCCAGATCT CCAACTATGT

TTCACCGTGC TGTTTATCCT TGTAGGCATG GGCCAGATCT CCAACTATGT

TTTTCCGTGC TGTTTTTCCT TGTAGGCATG GGCCAGATCT CCAACTATGT

TTCACTATAT TGTTCGTCAT CGTGGGCATG GGCCAGATCT CCAACTACGT

---------- ---------- ---------- ---------- ----------

TATGCTGTGC TGTACTGCGA CGTGGGCATG GGCCAGATCT CCAACTACGT

TTCTCAGTGC TATTTGTCCT CGTGGGCATG GGACAGATCT CTAACTATGT

TTCTCGGTCC TTTTTCTCAT CGTGGGCATG GGCCAGATCT CCAATTACGT

GGCAGCATTT GTCCTGGGTA TGACTCCCTT GTTTTGAACA GGGACAGAAA

GGCAGCATTT GTCCTGGGTA TGGCTCCCTT GTTTTGAACA GGGACAGAAA

GGCAGCATTT GTCCTGGGTA TGGCTCCCTT GTTTTGAACA GGGACAGAAA

GGCAGCATTT GTCCTGGGTA TGGCTCCCTT GTTTTGAACA GGAACAGAAA

GGCAGCATTT GTCCTGGGTA TGGCTCCCTT ATTTTGAACA GGGACAGAAA

GGCAGCATTT GTCCTGGGTA TGGCTCCCTT GTTCTGAACA GGAACAGAAA

GGCAGCATTT GTCCTGGGTA TGGCTTCCTT GTTTTGAACA GGAACAGAAA

GGCAGCATTT GTCCTGGGTA TGGCTTCCTT GTTTTGAACA GGAACAGAAA

GGCAGCGTTT GTCCTGGGTA TGGCTCCCCT GTGTTGAACA GGAACCGAAA

GGCAGCATTT GTCCTGGGTA TGGCTCCCTT GTTTTGAACA GGAACAGAAA

GGCAGCATTT GTCCTGGGTA TGGCTTCCTT GTTTCAAACA GGAACAGAAA

GGCAGCATTT GTCTTGGGTA CGGCTCCCTT GTTTTGAACA GGAACAGAAA

GGCAGCATTT GTCCTGGGTA TGGCTCCCTT GTTTTGAACA GGAACAGAAA

GGTGGCCTTC ATACTAGGTA GGACTCCCTT GTTTTGAACA GGAACAGAAA

---------- ---------- ---------- ---------- ----------

GGCAGCGTTT GTCCTGGGTA TGGCTCTCTT GTCTTGAACA GGAACAGAAA

GGCTGCTTTT GTTCTTGGTA TGACTTTTTT TTTTTAAACA GGAACAGAAA

GGCGGCTTTT GTCCTCGGTA TGACTCCCTT GTTTTGAACA GGAACAGAAA

TTCTTGGCAA GTCAGTTCGT ATAATATTCT CTACGTTAGG AGTGTGCATA

TTCTTGGCAA GTCAGTTCGT ATAATATTCT CTACGTTAGG AGTGTGCATA

TTCTTGGCAA GTCAGTTCGT ATAATATTCT CTACGTTAGG AGTATGCATA

TTCTTGGCAA GTCAGTTCGT ATAATATTCT CTACGTTAGG AGTATGCATA

TTCTTGGCAA GTCAGTTCGT ATAATATTCT CTACGTTAGG AGTGTGCATA

TTCTTGGCAA GTCAATTCGT ATTATATTCT CCACGTTAGG AGTGTGCATA

TTCTTTCCAA GTCAATTCGA ATTATATTCG CCACCTTAGG AGTTTGCATA

TTCTTTCCAA GTCAATTCGA ATTATATTCG CCACCTTAGG AGTTTGCATA

TTCTCAGCAA GTCGATCCGG ATTGTGTTCT CCACACTGGG CGTGTGCATA

TTCTTGGCAA GTCAGTTCGT ATTATATTCT CTACGTTAGG CGTGTGCATA

TTCTTGGCAA ATCGGTTCGT ATTATATTCT CTACGTTAGG AGTGTGCATA

TCCTTGGCAA GTCAGTGCGT ATTATATTCT CTACGTTAGG AGTGTGCATA

TTCTTGGCAA GTCAGTTCGT ATTATATTCT CCACGTTAGG AGTGTGCACA

TTCTTGGCAA GTCAGTTCGT ATTATATTCT CCACGTTAGG CGTGTGCACA

---------- ---------- ---------- ---------- ----------

TTCTTGGCAA GTCAGCCCGC ATTATATTCT CCACGTTAGG AGTGTGCATA

TTCTGGGCAA ATCAATTAGA ATAATATTCT CCACATTGGG AGTTTGCATA

TTCTGGGCAA AGAGGTTCGC ATCATATACT CCACAATAGG TGTGTGCATA

TTTTATGCAT TTGGCTACAT GGTGCTGCCA CTGTTTGCTT ACTTCATCCG

TTTTATGCAT TTGGCTACAT GGTGCTGCCA CTGTTTGCTT ACTTCATCCG

TTTTATGCAT TTGGCTACAT GGTGCTGCCA CTGTTTGCTT ACTTCATCCG

TTTTATGCAT TTGGCTACAT GGTGCTCCCA CTGTTTGCTT ACTTCATCCG

TTTTATGCAT TTGGCTACAT GGTGCTGCCA CTGTTTGCTT ACTTCATCCG

TTTTATGCCT TCGGCTACAT GGTGCTGCCA CTGTTTGCTT ACTTCATCAG

TTTTATGCAT TTGGCTTCAT GGTGCTGCCT CTGTTTGCAT ACTTCATCAG

TTTTATGCGT TTGGCTTCAT GGTGCTGCCA CTGTTTGCAT ACTTCATCAG

TTCTACGCCG TGGGCTACAT GGTGCTGCCG CTGTTCGCCT ACTTCATCAG

TTTTATGCGT TTGGCTACAT GCTGCTGCCA CTGTTTGCTT ACTTCATCAG

TTTTATGCAT TTGGCTACAT GATGCTGCCA CTATTTGCCT ACTTCATCAG

TTTTATGCAG TTGGCTACAT GCTGCTGCCA CTGTTTGCTT ACTTCATCAG

TTTTTTGCAG TTGGCTACAT GCTGCTGCCC CTGTTTGCTT ACTTCATCAG

TTTTTTGCAG TTGGCTACAT GCTGCTGCCG CTGTTTGCTT ACTTCATCAG

---------- ---------- ---------- ---------- ----------

TTTTATTCCA TTGGCTACAT GCTGCTGCCA CTGTTTGCCT ACTTCATCCG

TTTTATGCAA TTGGCTACAT GCTCCTGCCT CTGTTTGCTT ATTTCATTCG

TTTTATGCCG TGGGCTACAT GTTGCTGCCC CTATTCGCTT ACTTCATCAG

AGACTGGCGG ATGCTGCTGG TGGCTTACCT CTCCCTCTCC TTTGCAGGCC

AGACTGGCGG ATGCTGCTGG TGGCTTACCT CTCCCTCTCC TTTGCAGGCC

AGACTGGCGA ATACTGCTGG TGGCTTACCT CTCCCTCTCC TTTGCAGGCC

AGACTGGCGA ATACTGCTGG TGGCTTACCT CTCCCTCTCC TTTGCAGGCC

AGACTGGCGG ATGCTGCTGC TGGCTTACCT CTCCCTCTCC TTTGCAGGCC

AGACTGGCGG ATGCTGCTGC TGGCTTACCT CTCCCTCTCC TTTGCAGGCC

AGACTGGAGG ATGCTGCTGC TTGCTCACCT CTCCTTCTCC TTTGCTGGTC

AGACTGGAGG ATGCTGCTGC TGGCTCACCT CTCCTTCTCC TTTGCAGGTC

AGACTGGCGG ATGCTGCTGC TGGCTTACCT CGCCCTCTCC TTTGCAGGTC

AGACTGGCGG ATGCTGCTGC TGGC------ ---------- ----------

AGACTGGCGG ATGCTGCTGC TGGCTTACCT CTCCCTCTCC TTTGCAGGTC

AGACTGGCGG AAACTGCTTC TGGCTTACCT CTCCCTCTCC TTTGTAGGTC

AGACTGGCGG ATGCTGCTGC TGGCTTACCT CGCCTTCGCC TTTGCTGGTC

AGACTGGCGG ATGCTGCTGC TGGCTTACCT CTCCCTCCCC TTTGCTGGTC

---------- ---------- ----TTACCT CTCCCTCTCC TTTGTAGGTC

AGACTGGCGG ATGCTGCTGC TGGC------ ---------- ----------

AGAATGGAGG ATGTTATTAC TGGCTTACCT CTCCCTCTCC TTTGGAGGCA

ACACTGGCGG ATGTTACTAC TGGCTTACCT CTCCCTCTCC TTTGGAAGCA

CAGTAGTTCA GTTCCTCAAC ATCAACGAGC TTGATATGAT TGCAGGGAAG

CAGTAGTTCA GTTCCTCAAC ATCAACGAGC TTGATATGAT TGCAGGGAAG

CAATAGTTCA GTTCCTCAAC ATCAACGAGC TTGATATGAT TGCAGGGAAG

CAATAGTTCA GTTCCTCAAC ATCAACGAGC TTGATATGAT TGCAGGGAAG

CAATAGTTCA GTTCCTCAAC ATCAACGAGC TTGATATGAT TGCAGGGAAG

CAGTAGTTCA GTTCTTCGAC ATCAACAAGC TTGATGTGAT TGCAAGGCAG

CAGTAGTTGA GTTCCTCTGC GTCGACCAGC TTGATGTGGT TGCAGGGCAG

CAGTAGTTGA GTTCCTCTGC ATCGACCAGC TTGATGTGGT TGCAGGGCAG

CAGTAGTTCA GTTCCTCAAC GTCGACAAGT TTGATGTGGT TGCAGGGCAG

---------- ---------- ---------- ---------- ----------

CAATAGTTCA GTTCCTCAAC ATCGATGAGC TTGATATGAT TGCAGGGCAG

CAATAGTTCA GTTCCTCAAC ATCGATGAGC TTGATGTGAT TGCAGGGCAA

CAATAGTTCA GTTCCTCCAC ATCAATCAGC TTGATATGAT TGCAGGGCAG

CAATAGTTCA GTTCCTCGAC ATCGATGAGC TTGATATGAT TGCAGGGCAG

CAATAGTTCA GCTCCTCAAC ATCCATGAGC TTGATATGAT TGCAAGGCAG

---------- ---------- ---------- ---------- ----------

AAATAGTTCA GTTCTTCCAC ATCTATCAGT TTGATGTAAT TGCAGGGTAA

AAATAGTTCA GTTCTTCCAC ATCTGTCAAC TTGATGAAAT TACAGGGCAA

TGGCGCCCCT ACGTGCCCTT CCTACCTGAG GTCCAGTCGC CAGGAGTGGT

TGGCGCCCCT ACGTGCCCTT CCTACCTGAG GTCCAGTCGC CAGGCGTGGT

CGGTGCCCCT ACGTGCCCTT CCTACCTGAG GTCCAGTCGC CAGGTGTGGT

CGGTGCCCCT ACGTGCCCTT CCTACCTGAG GTCCAGTCTC CAGGTGTGGT

TGGCGCCCCT ACGTGCCCTT CCTACCTGAG GTCCAGTCCC CAGGCGTGGT

TGGTGCCCCT ACATGCCCTT CCCACCTGAG GTCCAGTCTC CTGGCGTGGT

AGGGGCTCCC ACGTGCCCTT CCTACCTGAT GTCCAGTCCC CTGGCGTTGT

AGGTGCCCCT ACATGCCCTT CCTACCTGAT GTCCAGTCCC CTGGCGTTGT

AGGCGCCCCT ACGTGCCCT- ---------- ---------- ----------

---------- ---------- ---------- ---------- ----------

AGGTGCCCCT ACATGCCCTT CCTACCTGAG GTCCAATCCC CAGGTGTGGT

AGGCGCGCCT ACGTGCCCTT CCTACCTGAC GTCCAGTCCC CAGGTGTGGT

GGGAGCCCCA ACATGCCCTT CCTACCTGAG GTCCAGTCCC CGGGTGTGGT

AGGAGCCCCT ACATGCCCTT CCTACCTGAA GTCCAGTCCC CAGGTGTGGT

AGGTGCGCCG ACATGCCCTG CCTACCTGAG GTCCAGTCTC CGGGCGTGGT

---------- ---------- ---------- ---------- ----------

TGGTGCACCC ACATGACCTT CTTACCTGAG GTCCAGTCCC CTGGAGTTGT

AGGAGCCCCA ACGTGACCTT CTTACCTGAA GTCCAGTCAC CTGGAGTAGT

GAAGGTACAT CCAGCTGTGC ACTCAGTTTG GCCATAACCT TCATAAACCT

GAAGGTACAT CCAGCTGTGC ACTCAGTTTG GCCATAACCT TCATAAACCT

GAAGGTACAT CCAGCTGTGC ACTCAGTTTG GCCATAACCT TCATAAACCT

GAAGGTACAT CCAGCTGTGC ACTCAGTTTG GCCATAACCT TCATAAACCT

GAAGGTACAT CCAGCTGTGC ACTCAGTTTG GCCATAACCT TCATAAACCT

GAAGGTACAT CCAGCTGTGC ACTCAGTTTG GCCATAACCT TCATAAACCT

GAAGGTACAC CCAGCTGTGC ATTCAGTTTG CCCATAACCT TCATAGACCT

GAAGGTACAC CCAGCTGTGC ATTCAGTTTG CCCATAACCT TCATAGACCT

---------- ---------- ---------- ---------- ----------

---------- ---------- ---------- ---------- ----------

GAAGGTACAT CCAGCGGTGC ACTCAGTTTG GCCATAACCT TCATAAACCT

AAAGGTACAT CCAGCTGTGC ACTCGGTTTG GCCGTAACCT TCATAAACCT

AAAGGTACAT CCAGCTGTGC ACTCTGTTTG GCCATAACCT TCATAAACCT

GAAGGTGCAT CCAGCTGTGC ACTCAGTTTG GCCGTAACCT TCATAAACCT

GAAGGTACAT CCAGCTGTGC ACTCAGTTTG GCCGTAACCT TCATAAACCT

---------- ---------- ---------- ---------- ----------

GAAGGTACAC CCAGCTGTAC ACTCAGTTTG GCCATAACCT TCATATACCT

AAAAGTGCAT CCAGCAGTAC ACTCAGTTTG GCCATAACCT TCATAAACCT

TCAAACAAAA TACCTGGCAC CCTAGAGCTG CCCGGAGAAA TCCCAGAACT

TCAAACAAAA TACCTGGCAC CCTAGAGCTG CCCGGAGAAA TCCCAGAACT

TCAAACAAAA TACCTGGCAC CCTAGAGCTG CCCGGAGAAA TCCCAGAACT

TCAAACAAAA TACCTGGCAC CCTAGAGCTG CCCGGAGAAA TCCCAGAACT

TCAAGCAAAA TACCTGGCAC CCTAGAGCTG CCCGGAGAAA TCCCAGAACT

TCAAACAAAA TACCTGGCAC CCTAGAGCTG CCCGGAGGAA TCCTAGGACT

TCAAGCGAAA TACCTGGCAC CCCAGAGCTG CTCGTAGGAA TCCCAGGACT

TCAAGCAAAA TACCTGGCAC CCCAGAGCTG CTCGTAGGAA TCCCAGAACC

---------- TACCTGGCAG CCCAGAGCGG CCCGGAGGAA TCCCAGGACT

---------- ---------- ---------- ---------- ----------

TCAAACAAAA TACCTGGCAT CCTAGAGCAG CCCGGAGGAA TCCCAGGACT

TCCAGCAAAA TACCTGGCAG CCCAGAGCTG CACGGAGGAA TCCCAGGACG

TCAAGCAAAA TACCTGGCAT CCCAGAGCTG CTCGGAGGAA TCCCAGGACT

TCAAGCAAAA TACCTGGCAC CCCAGAGCTG CCCGGAGGAA TCCCAGGACT

TCAAGCAAAA TACCTGGCAC CCCAGAGCTG CCCGGAGGAA TCCCAGGACT

---------- TACCTGGCAC CCCAGAGCTG CTCTGAGGAA TCCCAAGACT

GCAAGAGAAA TACCTGACAC CCCAGGGCTG CACGAAGAAA ACCCAAAACT

GAAAACAATA TACTTGGCAC CCCAAAGCTG CCCGAAGGAA ACCGAGGACA

GTTGGTGATG CTGGGGCTGC TCCAGTAACA ATCATCCGCA CACACCCACC

GTTGGTGATG CTGGGGCTGC TCCAGTAACA ATCATCCGCA CACACCCACC

GTTGGTGATG CGGGGGCTGC TCCAGTAACG ATCATCCGCA CACACCCACC

GTTGGTGATG CGGGGGCTGC TCCAGTAACG ATCATCCGCA CACACCCACC

GTTGGTGATG CTGGGGCTGC TCCAGTAACA ATCATCCGCA CGCACCCACC

GCTGGTGATG CAGGGGCTGC TCCAGTAACA ATCATCCGCA CACATCCACC

GTTGGTGATG CGGGGGCTGC TCCGGTAACA ATCATTCTCA CGTGCCCTCC

GTTGGTGACG CGGGGGCTGC TCCAGTGACA ATCATCCTCA CATGCCCACC

GTTGGCGACG CGGGGGCTGC TCCAGTAACA ATCATCCGCA CACACCCACC

---------- ---------- ---------- ---------- ----------

GTCGGTGATG CAGGGGCTGC TCCGGTGACA ATCATCCTCA CGCATCCTCC

GCCGGCGATG CGGGGGCTGC TCCAGTCACT ATCATGCGTA CACACCCGCC

GCTGGAGAGG CGGGGGCTGC TCCAGTAACA ATCATCCTTA CGCTTCCCCC

GTTGGTGATG CGGGGGCTGC TCCAGTAACA ATCATCCGTA CGCACCCACC

GCTGGCGATG CAGGCGCTGC TCCAGTGACA ATCATCCGCA CGCGCCCGCC

GCTGGTGATG CGGGCGCCGC TCCAGTGACA ATCATTCGCA CGCACCCACC

GTGGGAGATG CAGGAGCTGC TCCAGTGACA ATCATTCTAA CACACCCACC

GTAGGAGATG CGGGAGCCGC ACCGGTGACA ATCATCCTTA CACAGCCCCC

AAGACTGGCC TGAATTACTT AGGACAATTT CTTCCAATTT CCATTTTAAT

AAGACTGGCC TGAATTACTT AGGACAATTT CTTCCAATTT CCATTTTAAT

AAGACTGGCC TGAATTACTT AGGACAATTT CTTCCAATTT CCATTTTAAT

AAGACTGGCC TGAATTACTT AGGACAATTT CTTCCAATTT CTATTTTAAT

AAGACTGGCC TGAATTACCT AGGACAATTT ATTCCAATTT CCATTATAAT

AAGACTGGCC TGAATTACTT AGAACAATTT CTCCCCACTT CCATTTTAAT

AAGACTGGCC TGAATTACTT AAGACAATTT TCTCCAGCTT CCATTTTAAT

AAGACTGGCC TGAATTACTT AAGACAATCT TCTCCAGCTT CCATTTTAAT

AAGGCTGGCC TGAATTACTT AGGACAATTT CTTCCAACTT CCATTTTAAC

---------- ---------- ---------- ---------- ----------

AAGACTGGCC TGAATTACTT AGGACAATTT CTTCCCACTT CCATTGTAAT

AAGACTGGCC TGAATTACTT AGGACAATTC CTTCCCACTT CCACCTTAAT

AAGACTGGCC TGAATTACTT AGGACAATTT CTTCCCACTT CCAGCTTAAT

AAGGCTGGCC TGAGTTACTT AGGATAATTT CTACCAACTT TCATTTTCAT

AAGGCTGGCC TGAATTACTT AGGACAATTT ATTCTAACTT TTATTTTAAT

AAGACTGGCC TGAATTACTT AGGACAATTC CTTCTAACTT CTATTTTAAT

AAGACTAGCC TGGCTGAGTT ATAAAAATTA CCTTGAATTT CTATGCTAAT

GAGGCTTGCC TGAGCTGAGT AGACTGATTT TTTCCTATTT CTATTTTGAT

GGCATTGCCT TTTTTCATGT ATAAATCAAT ATTACCTGAA TCTTATTAAA

GGCATTGCCT TTTTTCATGT ATAAATCAAT ATTACCTGAA TCTTATTAAA

GGCATTGCCT TTTTTCATGT ATAAATCAAT ATTACCTGAA TCTTATTAAA

GGCATTGCCT TTTTTCACGT ATAAATCAAT ATTACCTGAA TCTTATTAAA

GGCATTGCCT TTTTTCATGT ATAAATCAAT ATTACCTGAA TCTTATTAAA

GGCATGGCCT TTTTTCATTT GTAAATCAAT ATTACCTGAA TCTTATTAAA

GGCATCAGCT TTTTCTGTGT AGGAATCAAT ATTACCTGAA TCTTATTAAA

GGCATTGACT TTTTCTATGT AGAAATCAAT ATTACCTGAA TCTTATTAAA

ACTATTGTCT TATTTTGTAA ACAAATCAAT ACTACCTGGA TCTTTTTAAA

---------- ---------- ---------- ---------- ----------

GGCACAGCCT TTTCTCATTT TTAAATCAAT ATTACCTGAA TCTTATTAAA

GGCCTAGCCT TTTTTCATTT ATAAATCAAT ATTACCTGAA TCTTATTAAA

GGCATAATCA TTTTTCATGT AGAAATCAAT ATTACCTGAA TCTTATTAAA

GGTATTGCCT TTTTTCACTT CTAAATCAAT ATTACCTGAA TTTTGTTAAA

GGCATTGTCT TTCTTCATTT CTAAATCAAT GTTACCTGAA TTTTATTAAA

GGCTTTTCCT TTTCTCATGT CTAAATCAGT ATTACCTGAA TCTTATTGAA

GGCTTTGCTT TTTATAGTTT TCAGATCAAT ATTACCTGAA TCTTATTAAA

GACATTGCCT TTTCTTGATT CCTAATCAAT ATTACCTGAA TCTTATTAAA

GAAGAGTTCA TCCCAGATAC TATCATTCCT GATGATTCCA CTCCGGACCT

GAAGAGTTCA TCCCAGATAC TATCATTCCT GATGATTCCA CTCCGGACCT

GAAGAGTTCA TCCCAGATAC TATCATTCCT GATGATTCCA CTCCGCACCT

GAAGAGTTCA TCCCAGATAC TATCATTCCT GATGATTCCA CTCCGCACCT

GAAGAGTTCA TCCCAGATAC TATCATTCCT GATGATTCTA CTCCGGACCT

GAAGAGTTCA TCCCAGATAC TATCATTCCT GATGATTCCA CTCCGGATCT

GAAGAGTTCA TCCCAGATAC TATTGTTTCT GATGATTCCG CTCCGGACCT

GAAGAGTTCA TCCCAGATAC TATTGTTTCT GATGATTCCG CTCCGGACCT

GAAGAGCTCA TCCCAGATAC TGTCATTCCT GACGATCCCA CTCCGGACCT

---------- ---------- ---------- ---------- ----------

GAAGAGTTCA TCCCAGATAC TATCATTCCT GATGATTCCA CTCCGGACCT

GAAGAGTTCG TCCCAGATGC TATCATTCCT GATGATTCCA CTCCGGACCT

GAAGAGTTCA TCCCAGATAC TGTTGTTCCT GATGATTCCA CAGCGAATTT

GAAGAGTTCA TCCCAGATAC TATCATTTCT GATGATTCCA CTCCGAACCT

GAAGAGTTCA TCCCAGATAC TATCATTTCT GATGATTCCA CTCCGGACCT

GAAGAGTTCA TCCCAGATAC TATCATTTCT GATGATTCCG CTGCGGACCT

AAAAAGCTCA TCCCATATAC TATCATTTCT AATGATTCCA CTTCGAACTT

GAAAAGTTCA TCCCATATAC TGTCATTTCT AATGATTCCA CTCCGAACCT

CGGCTTGCTT ACGCTTTGCT GCAAACTCCA GGAGCCAGCG CTTTAATGGT

CGGCTTGCTT ACGCTTTGCT GCAAACTCCA GGAGCCAGCG CTTTAATGGT

CGGCCTGCTT ACGCTTTGCT GCAAACTCCA GGAGCCAGCG CTTTAATGGT

CGGCCTGCTT ACGCTTTGCT GCAAACTCCA GGAGCCAGCG CTTTAATGGT

CGGCTTGTTT ACGCTTTGCT GCAAACTCCA GGAGCCAGCG CTTTAATGGT

CAGCTTGCTT TCGCTTTGCT GCAAATTCCA GGAGCCAGCG CTTTAACGGT

CAGCCTGCTT GCGCTTTGCT GCAAACTCCA GGAGCCAGCG CTTCAGTGAG

CTGCCTGCTT GCGCTTTGCT GCAAACTCCA GGAGCCAGCG CTTTAGTGAG

CAGCCTGCTT GCGCTTGGCT GCAAACTCCA GGAGCCAGCG CTTTATCGGG

---------- ---------- ---------- ---------- ----------

CAGCCTGCTT ACGTTTTGCT GCAAACTCCA GGAGCCAGCG CTTTAGTCGT

CAGCCTGTTT ACGCTTCGCC GCAAACTCCA GGAGCCAACG CTTTAGTGGT

CAGCCTGTTT ACGCTTCGCA GCAAACTCCA GAAGCCAGCG CTTTAATGGT

CGGCCTGTTT ACGCTTTGCT GCAAACTCCA GGAGCCAGCG CTTTAATGGT

CGGCCTGCTT ACGCTTTGCC GCAAACTCCA GGAGCCAGCG CTTCAGTGGT

CGGCCTGTTT ACGCTTCGCC ACAAACTCCA GGACCCAGCG CTTTAGTGGC

CAGCCTTCTT ACGCTTTGCT GCAAATTCCA GGAGCCACCG TTTCAATGGA

CGGCCTTTTT ACGTTTTGCT GCAAATTCTA GAAGCCAGCG TTTTAGAGGC

GTGTTTGCCT GGCTGAAGAT CTGAGTTACC TTGTCGTACA TCCGGTTCAG

GTGTTTGCCT GGCTGAAGAT CTGAGTTACC TTGTCGTACA TCCGGTTCAG

GTGTTTGCCT GGCTGAAGAT CTGAGTCACC TTGTCGTACA TCCGGTTCAG

GTGTTTGCCT GGCTGAAGAT CTGAGTCACC TTGTCATACA TCCGGTTCAG

GTGTTTGCCT GGCTGAAGAT CTGAGTCACC TTGTCGTACA TCCGGTTTAG

GTGTTTGCCT GGCTGAAGAT CTGAGTCACC TTGTCGTACA TCCGGTTCAG

GTGTCTGCCT GGTGGAAGAT CTGAGCCACC TTGTCATACA TCCGATTCAG

GTGTCTGCCT GGTGGAAGAT CTGAGCCACC TTGTCATACA TCCGATTCAG

CCGTCCGCCT GGCTGAAGAT CTGAGTCACC TTGTCGTACA TCCGGTTCAG

---------- ---------- ---------- ---------- ----------

GTGTCTGCCT GGCTGAAGAT CTGAGTTACC TTGTCATACA TCCGGTTCAG

GTGTCTGCCT GGCTGAAGAT CTGAGTCACC TTGTCATACA TCCGGTTCAG

GTGTCTGCCT GACTGAAGAT CTGAGTCACC TTGTCATACA TCCGGTTCAG

GTGTCTGCCT GGCTGAAGAT CTGAGTCACC TTGTCATACA TCCGGTTCAG

GTATTTGCCT GGCTGAAGAT CTGAGTCACC TTGTCATACA TCCGGTTCAG

GTGTTTGCCT GGCTGAAGAT CTGAGTCACC TTGTCATACA TCCGGTTCAG

GTATTTGCTT GGCTGAAGAT CTGAGTCACC TTGTCATACA TTCTGTTCAG

GTATCAGCCT GGCTGAAGAT CTGAGTCACC TTATCATACA TTCGGTTCAA

CAGTCGTGGG ACCACAGGGA AGATGGTGGG GCATAGAGCC TTCATGTCAT

CAGTCGTGGG ACCACAGGGA AGATGGTGGG GCATAGAGCC TTCATGTCAT

CAGTCGTGGG ACCACAGGGA AGATGGTGGG GCATAGAGCC TTCATGTCAT

CAGTCGTGGG ACCACAGGGA AGATGGTGGG GCATAGAGCC TTCATGTCAT

CAGTCGTGGT ACCACAGGGA AGATGGTGGG GCATAGAGCC TTCATGTCAT

CAGTCGTGGG ACCACAGGGA AGATGGTGGG GTGTAGAGCC TGCATGTCAT

CAGCCGTGGG ACCACAGGGA AGATGGTGGG GCGGAGAGCT TTCATGTCGT

CAGCCGTGGG ACCACAGGGA AGATGGTAGG GCGGAGAGCC TTCATGTCAT

CAGTCGTGGC ACTACTGGGA AGATGGTGGG GCAGAGAGCC TTCATGTCAT

---------- ---------- ---------- ---------- ----------

CAATCGAGGG ACCACAGGGA AGATAGTGGG GCACAGAGCC TTCATGTCAT

CAGTCGTGGG ACCACAGGGA AGATGGTGGG GCATAGAGCT TTCATGTCAT

CAGGCGTGGG ACCACAGGGA AGATAGTGGG GCACAGAGCC TTCATGTCAT

CAGTCGTGGG ACCACAGGAA AGATGGTGGG GCACAGAGCT TTCATGTCAT

CAGTCGTGGG ACCACAGGGA AAATGGTGGG GCACAGAGCC TTCATGTCAT

CAGCCGTGGG ACCACTGGGA AGATGGTGGG GCACAGAGCC TTCATGTCGT

CAGTCTGGGG ACCACTGGGA AGATAGTGGG ATGTAGAGCT TTCATGTCAT

CAGCCTTGGG ACAACTGGAA AAATGGTGGG ACGCAGGGCT TTCATATCAT

CTGAGAGAAG GCGGATATCT CCCTGGAAGA AGCCAACACG CCCTCCGTGG

CTGAGAGAAG GCGGATGTCT CCCTGGAAGA AGCCAACACG CCCTCCGTGG

CTGAGAGGAG GCGGATGTCT CCCTGGAAGA AGCCAACGCG CCCTCCATGG

CTGAGAGGAG GCGGATGTCT CCCTGGAAGA AGCCAACGCG CCCTCCGTGG

CTGAGAGGAG GCGGATGTCT CCCTGGAAGA AGCCAACACG CCCTCCATGG

CTGAGAGGAG GCGGATGTCC CCTTGGAAGA AGCCAACACG TCCTCCGTGG

CTGAGAGGAG GCGGATGTCT CCCTGGAAGA AGCCCACACG CCCTCCATGG

CTGAGAGGAG GCGGATGTCT CCCTGGAAGA AGCCCACGCG CCCTCCGTGG

CCGAGAGGAG GCGGATATCT CCCTGGAAGA AGCCGACACG CCCTCCATGG

---------- ---------- ---------- ---------- ----------

CTGAGAGGAG ACGGATGTCT CCCTGGAAGA AGCCGACACG TCCCCCATGG

CTGAGAGGAG ACGGATGTCT CCCTGGAAGA AGCCAACACG CCCTCCATGG

CTGAGAGGAG ACGGATATCT CCCTGGAAGA AGCCAACGCG TCCTCCATGG

CTGAGAGGAG GCGGATGTCT CCCTGGAAGA AGCCGACGCG CCCTCCGTGG

CAGAGAGGAG GCGGATATCT CCCTGGAAGA AGCCGACGCG TCCTCCATGG

CAGACAGGAG GCGGATGTCT CCCTGGAAGA AGCCAACGCG CCCTCCGTGG

CTGACAAAAG GCGAATGTCT CCTTGGAAGA ATCCAACTCT TCCCCCATGG

CAGATAAAAG ACGAATATCT CCTTGAAAGA ATCCAACTCG TCCCCCATGG

CAATAGACGA CAGACTAGAA GGGTTATGGG TTCTAGGCAC AGAGTGCCTC

CAATAGACGA CAGACTGGAA GGGTTATGGG TTCTAGGCAC AGAGTGCCTC

CAATAGACGA CAGACTGGAA GGGTCATGGG TTCTAGGCAC AGAGTGCCTC

CAATAGACGA CAGACTGGAA GGGTCATGGG TTCTAGGCAC AGAGTGCCTC

CAATAGACAA CAGACTGGAA GGGTTATGGG TTCTAGGCAC AGACTGCCTC

CAGTAGACGA CAGACTGGAA GGGTTATGGG TTCTAGGCAC AGATTGCCTC

CAGTAGACAA CAGACTGG-G TAAGTGTAGG TTCTGGGCAC AGAGGGCCTC

CAGTAGACAA CAGACTGG-G TAAGTGTAGG TTCTGGGCAC GGAGGGCCTC

CAGTAGACAA CAGACTAGAA GGGTTACGGG TTCTAGGCAC AGACTGCCTC

---------- ---------- ---------- ---------- ----------

CAGTAGACAA CAGACTGGAA GGGTCCTGGG TTCTGGGCAC AGATTGCCTC

CAGTAGACAA CAGACTGGAA GGGTTCTGGG TTCTGGACAC AGATTGCCTC

CAGTAGACGA CAGACTGGAG GGGTTCTGGG TTCAGGGCAC AGACCGCCTC

CAGTAGACAA CAGACTGGAA GGGTTATGGG TTCTGGGCAC AGGTTGCCTC

CAGTAAACAA CAGACTGGGA GGGTTATGGG TTCTGGGCAC AGGCTGCCTC

CAGTACACAA CAGACT-GG- ---------- ---------- ----------

CAGTACACTA CTGACTGAAA GGGTTATTCG TTCTTGTCAC AGAAGACTTC

CAATAGACCA CGGACTGAAA TGGTTAGATT TTCTTGCAAT GGAAGGCTTC

CCTGATGCCA GGGCCTTACC TGCACCATTC GCTCAAACAT GTGTGCTAAA

CCTGATGCCA GGGCCTTACC TGCACCATTC GCTCAAACAT GTGTGCTAAA

CCTGATGCCA GGGCCTTACC TGCACCATTC GCTCAAACAT GTGTGCTAAA

CCTGATGCCA GGGCCTTACC TGCACCATTC GCTCAAACAT GTGTGCTAAA

CCTGATGCCA GGGCCTTACC TGCACCATTC GCTCAAACAT GTGTGCTAAA

CCTGATGCCA GGGCCTTACC TGCACCATTC GCTCAAACAT GTGTGCTAAA

CCTGATGCCA GGGCCTTACC TGCACCATTC GCTCAAACAT GTGTGCTAAA

CCTGATGCCG GGGCCTTACC TGCACCATGC GCTCAAACAT GTGTGCTAAA

CCTGATGCCA GGGCCTTACC TGCACCATTC GCTCAAACAT GTGTGCTAAA

---------- ---------- ---------- ---------- ----------

CCTGGAGCC- GGGCCTTACC TGCACCATTC GCTCAAACAT GTGTGCTAAA

CCTGAAACCA GGGCCTTACC TGCACCATTC GCTCAAACAT GTGTGCTAAA

CCTGAAACCA GGGCCTTACC TGCACCATTC GGTCAAACAT GTGTGCTAAA

CCTGATGCCA GGGCCTTACC TGCACCATTC GCTCAAACAT GTGTGCTAAA

CCTGATGCCA GGGCCTTACC TGTACCATTC GCTCAAACAT GTGTGCTAAA

---------- ---------- ---------- ---------- ----------

TCTGATGCTT GGGCCTTACC TGTACCATTC TCTCAAACAT GTGTGCTAAA

ACTGACACTA GGGCCTTACC TGTACCATTC TCTCAAACAT GTGTGCTAAA

GGCAAATAGG AAATGTGCAC ATCCGCACAA GTGGGAGCCC ACTGACTCTG

GGCAAATAGG AAATGTGCAC ATCCGCACAA GTGGGAGCCC ACTGACTCTG

GGCAAATAGG AAATGTGCAC ATCCGCACAA GTGGGAGCCC ACTGACTCTG

GGCAAATAGG AAATGTGCAC ATCCGCACAA GTGGGAGCCC ACTGACTCTG

GGCAAATAGG AAATGTGCAC ATCCGAACAA GTGGGAGCCC ACTGACTCTG

GGCAAATAGG AAATGTGCAC ATCCGCACAA GTGGGAGCCC ACTGACTCTG

GGCAAATAGG AAAAGTGTAC ATCCGCACAA GTGGGGGCCC ACTGACTCTG

GGCAAGTAGG AAAAGTGGAC GTCCGCACAG GTCGGGGCCC ACTGACTCTG

GGCAAATAGG AAAAGTGCAC ATCCGCACAA GTGGGAGCCC ACTGACTCTG

---------- ---------- ---------- ---------- ----------

GGCAAATAGG AAATGTGCAC ATCCGCACAA GTGGGAGCCC ACTGACTCTG

GGCAAATAGG AAATGTGTAC ATCCGCACAA GTGGGAGCCC ACTGACTCTG

GGCAAATAGG AAATGTGCAC ATCCGCACAC GTGGGAGCCC ACTGGCTCTG

GGCAAATAGG AAATGTGCAC ATCCGCACAA GTGGGAGCCC ACTGACTCTG

GGCAAATAGG AAATGTGCAC ATCCGTACAA GTGGGAGCCC ACTGACTCTG

---------- ---------- ---------- ---------- ----------

GGCAAATAGG AAATGTGTAC ATCCTCACAA GTGGGTGACC ACTGACTCTG

GGCAAATAGG AAATGTGTAC ATCCTCACAA GTGGGAGACC ACTGACTCTG

TAAGAAAAAA GAAGGAGCCC AGGCAAGGCC CGGTCAGTCA GTCTTACCTG

TAAGAAAAAA GAAGGAGCCC AGGCAAGGCC CGGTCAGTCA GTCTTACCTG

TAAGAAAAAA GAAGGAGCCC AGGCAAGGCC CCGTCAGTCA GTCTTACCTG

TAAGAAAAAA GAAGGAGCCC AGGCAAGGCC CCGTCAGTCA GTCTTACCTG

TAAGAAAAAA GAAGGAGCCC AGGCAAGGCC CGGTCAGTCA GTCTTACCTG

TAAGAAAACA GAAGGAGCCC AGGCAAGGCC CGGTCAGTCA GTCTTACCTG

CAAGAGAACA CAAGGAGCCC AGGCAGGTCC CAGTCAGCCA GGCTTACCTG

CAAGAGAACA CAGGGAGCCC AGGCGAGTCC CCGTCAGCCA GGCTTACCTG

TAAGAAAACA CAGGGAGCCC AGGCAAGTCC CAGTCAGCCA GGCTTACCTG

---------- ---------- ---------- ---------- ----------

TAAGAAAACA GAAGGAGCCC AGGCAAGGCC CGGTCAGTCA GTCTTACCTG

CAAGAAAAGA GAAGGAGCCC AGGCAAGGCC CGGTCAGTCA GTCTTACCTG

CAAGAAAACA CAAGGAGCCC GGGCAAGGCC C-GTCAGTCA GTCTTACCTG

CAAGAAAACA AAAGGAGCCC AGGCAAGGCC CGGTCAGTCA GTCTTACCTG

CAAGAAAAGA GAAGGAGCCC AGGCAAGGCC CGGTCAGTCA GTCTTACCTG

---------- ---------- ---------- ---------- ----------

TAAGAAAACA AAAGGAGCCC GAACCAGGCC CAGTCAGTCA GTCTTACCTG

GAAAAAAACA CAAGGAGCCC ACGCAAGGCC CGGTCAGTTA GTCTTACCTG

GATTACTCTC TCAAACATGT GAGCCAGAGG CAGGAAGGAG ATGAGCACAT

GATTACTCTC TCAAACATGT GAGCCAGAGG CAGGAAGGAG ATGAGCACAT

GATTACTCTC TCAAACATGT GAGCCAGAGG CAGGAAGGAG ATGAGCACAT

GATTACTCTC TCAAACATGT GAGCCAGAGG CAGGAAGGAG ATGAGCACAT

GATTACTCTC TCAAACATGT GAGCCAGAGG CAGGAAGGAG ATGAGCACAT

GATTACTCTC TCAAACATGT GAGCCAGAGG CAGGAAGGAG ATGAGCACAT

GATCACTCTC TCAAACATGT GAGCCAGAGG CAGGAAGGAG ATGAGCACAT

GATCACTCTC TCAAACATGT GAGCCAGAGG CAGGAAGGAG ATGAGCACAT

GATCACTCTC TCAAACATGT GAGCCAGAGG CAGGAAGGAG ATGAGCACAT

---------- ---------- ---------- ---------- ----------

GATTACTCTC TCAAACATGT GAGCCAGAGG CAGGAAGGAG ATGAGCACAT

GATCACTCTC TCAAACATGT GAGCCAGAGG CAGGAAGGAG ACGAGCACAT

GATCACTCTC TCAAACATGT GAGCCAGAGG CAGGAAGGAG ATGAGCACAT

GATTACTCTC TCAAACATGT GAGCCAGAGG CAGGAAGGAG ATGAGCACAT

GATTACTCTC TCAAACATGT GAGCCAAAGG CAGGAAGGAG ATGAGCACAT

---------- ---------- ---------- ---------- ----------

TATAACTCTT TCAAACATGT GAGCCAGAGG CAGGAAGGAG ATGAGCACAT

TATAACTCTT TCAAACATGT GAGCCAGAGG CAGGAAGGAG ATGAGCACAT

CGTCCTGTCT CGGAAAGATC ACTTTCTGCA GGCGACGGGC ATGGGAGACA

CGTCCTGTCT CGGAAAGATC ACTTTCTGCA GGCGACGGGC ATGGGAGACA

CGTCCTGTCT CGGAAAGATC ACTTTCTGCA GGCGACGGGC ATGGGAGACA

CGTCCTGTCT CGGAAAGATC ACTTTCTGCA GGCGACGGGC ATGGGAGACA

CGTCCTGTCT CGGAAAGATC ACTTTCTGCA GGTGACGGGC ATGGGAGACA

CGTCCTGTCT CGGAAAGATC ACTTTCTGCA GGTGACGGGC ATGGGAGACA

CGTCCTGTCT CGGAAAGATC ACTTTCTGCA GGTGACGGGC ATGGGAGACA

CGTCCTGTCT CGGAAAGATC ACTTTCTGCA GGTGACGGGC ATGGGAGACA

CGTCCTGTCT CGGAAAGATC ACTTTCTGCA GGTGACGGGC ATGGGAGACA

---------- ---------- ---------- ---------- ----------

CGTCCTGTCT CGGAAAGATC ACTTTCTGCA GGTGACGGGC ATGGGAGACA

CGTCCTGTCT CGGAAAGATC ACTTTCTGCA GGTGATGGGC ATGGGAGACA

CGTCCTGTCT CGGAAAGATC ACTTTCTGCA GGTGACGGGC ATGGGAGACA

CGTCCTGTCT CGGAAAGATC ACTTTCTGCA GGTGACGGGC ATGGGAGACA

CGTCCTGTCT CGGAAAGATC ACTTTCTGCA GGTGACGAGC ATGGGGGATA

---------- ---------- ---------- ---------- ----------

CGTCCTGTCT CGGAAAGATC ACTTTCTGCA GGTGAAGGGC ATGGGAGACA

CGTCCTGTCT CGGAAAGATC ACTTTCTGCA GGTGAAGGGC ATGGGAGACA

GAAAGGAAAG AAACACTGAC AGGAAGACGA CAAGAGAATC ATAAAACAAT

GAAAGGAAAG AAACACTGAC AGGAAGACGA CAAGAGAATC ATAAAACAAT

GAAAGGAAAG AAACACTGAC AGGAAGACGA CAAGAGAATC ATAAAACAAT

GAAAGGAAAG AAACACTGAC AGGAAGACGA CAAGAGAATC ATAAAACAAT

GAAAGGAAAG AAACACTGAC AGAAAGACAA CAAGAGAATC ATAAAACAAT

GAAAGGAAAG AAACACTGAC AGGAAGACGA CAAGAAAGTC ATAAAACAAT

GAAAGGAAAG AAACACTGAC AGGAAGACAA CGAGAGAGTC ATAAAACAAT

GAAAGGAAAG AAACACTGAC AGGAAGACAA CGAGAGAGTC ATCAAACAAT

GAAAGGAAAG AAACACTGAC AGGAAGACAA CGAGAGAGTC ATAAAACAAT

---------- ---------- ---------- ---------- ----------

GAAAGGAAAG AAACACTGAC AGGAAGACGA CAAGAGAGTC ATAAAACAAT

GAAAGGCAAG AAACACTGAC AGGAAGACAA CAAGAGAGTC ATAAAACAAT

GAAAGGAAAG AAACACTGAC AGGAAGACAA CAAGAGAGTC ATAAAACAAT

GAAAGGAAAG AAACACTGAC AGGAAGACGA CAAGAGAGTC ATAAAACAAT

GAAAGGAAAG AAACACTGAC AGGAAGACGA CAAGAGAGTC ATAAAACAAT

---------- ---------- ---------- ---------- ----------

GAAAGGAAAG AAACACTGAC AGGAAGACAA CAAGAAAGTC ATAAAACAAT

GAAAGGAAAG AAACACTGAC AGGAAGACAA CAAGAAAGTC ATAAAACAAT

AAAGAGAAAA TGGTCCTGGA GGGTAAACAG GAAGAGCAGC ATAGAAGCGG

AAAGAGAAAA TGGTCCTGGA GGGCAAACAG GAAGAGCAGC ATAGAAGCGG

AAAGAGAAAA TGGTCCTGGA GGGCAAACAG GAAGAGCAGC ATAGAAGCGG

AAAGAGAAAA TGGTCCTGGA GGGCAAACAG GAAGAGCAGC ATAGAAGCGG

AAAGAGAAAA TGGTCCTGGA GGGCAAACAG GAAGAGCACC ATAGAAGCGG

AAAGAGAAAA TGGTCCTGGA GGGCAAACAG GAagagCAGC ATAGAAGCGG

AAAGAGAAAA TGGTCCTGGG GGG--GAGGG GTGGGCCAGC ATAGAAGCGA

AAAGAGAAAA ATGGCCTGGg ga----aggg ggagggCAGC ATGGAAGCCA

AAAGAGAAAA CGGTCCTGAA GGGCAGATGG GAAGAG---- ----------

---------- ---------- ---------- ---------- ----------

ACAGAGAAAA TGGTCCTGGA GGGCAAATAG GAAGAGCAGC GTAGAGGCGG

AAAGAGAAAA TGGTCCTGGA GGGCAAACag gaagagCAGC ATAGAAGCGG

AAAGAGAAAA TGGTCCTAGA GGGCAAATAG GAagagCAGC ATAGAAGCGG

AAAGAGAAAA TGGTCCTGga gagcagacag gaagagCAGC GCAGAAGCGG

AAAGAGAAAA TGATCTTGGA GGGCAAACAG GAAGAGCGGC AGTGAA----

---------- ---------- ---------- ------CAGC CGTGAAGCGA

AAAGAGAAAA TGGTACCAGA GAGCAAACAG TAAAaagagg acagaagagg

AAAAGGAAAA TGGTACTAGA GAGTATACAG TAAAGGCAGC ATAGAAGCGA

AGTGTTAGGT CGCAGCGTCA TGGGTCTAGG TTATCTGCAA AGCAAAGAGC

AGTGTTAGGT CGCAGCGTCA TGGGTCTAGG TTATCTGCAA AGCAAAGAGC

AGTATTAGGT CGCAGCGTCA TGGGTCTAGG TTATCTGCAA AGCAAAGAGC

AGTATTAGGT CGCAGCGTCA TGGGTCTAGG TTATCTGCAA AGCAAAGAGC

AGTGTTAGGT CGCAGCGCCA TGGGTCTAGG TTATCTGCAA AGCAAAGAGC

AGTGTTAGGT CGCAGCGCCA TGGGTCTAGG TTATCTGCAA AGCAAAGAGC

AGTGTTAGGT CGCAGCGCCA TGGGTGTGGG TTATCTGCAA AGCAAAGAGG

AGTGTTAGGT CGCAGCGCCA TGGGTGTAGG TTATCTGCAA AGCAAAGAGC

---------- ---------- -------AGG TTATCTGCAA AGCAAAGAGC

---------- ---------- ---------- ---------- ----------

AGTGTTAGGG CGCA--GCCA TAGGTCTAGG TTATCTGCAA AGCAAAAAGC

AGTGTTAGGT CACAGCGCCA TGGGTCTAGG TTATCTGCAA AGCAAAGAGC

AGTGTTAGGT CGCAGCGCCA TGGGTCTAGG TTATCTGCAA AGCAAAGAGC

AGTGTTAGGT CGCAGCGCCA TGGGTCTAGG TTATCTGCAA AGCAAAGAGC

AGTGTTAGGT CGCAGCGCCA TGCGTCTAGG TTATCTGCAA AGCAAAGAGC

AGTATTAGGT CGCAGCGCCG TGGGTCTAGG TTATCTGCAA AGCAAAGCGC

agtgaaagat caaagagaca tagagaaagg agaaatagaa agaaaatgga

AGCGTTAGGT CACGACATCA TGAATCTAAG TTACTGGGAT AGTAAAGAGC

CAAATGAAGC ACACAGAAAC CAGCAGAAAG TTATGTCCTC AAGAGACACC

CAAATGAAGC ACACAGAAAC CAGCAGAAAG TTATGTCCTC AAGAGACACC

CAAATGAAGC ACACAGAAAC CAGCAGAAAG TTATGTCCTC AAGAGACACC

CAAATGAAGC ACACAGAAAC CAGCAGAAAG TTATGTCCTC AAGAGACACC

CAAATGAAGC ACACAGAAAC CAGCAGAAAG TTATGTCCTC AAGAGACAAC

CAAATGAAGC ACACATAAAC CAGCAGAAAG CTATGTCCTC AAGAGACACC

CACATGTAGC ACACATAAAC CAGCAGAGGG TTGTGTCCTT GAGAAAGGTC

CACAGGAAGC ACATTTAAAC CAGCAGAAAG TCACGTCCTG AAGAAAGGCC

CAAATGAAGC ACACATAAAC CAGCAGAGAG GTAACTCCCC TGGAGACACC

---------- ---------- ---------- ---------- ----------

CCAGTGAAGC ACACATAAAC CAGCAGA--- ---------- ----------

CAAATGAAGC ACACATAAAC CAGCAGAAAG TTATGTCCTC AAGAGACACC

CAAATGAAGC ACATAGAAAC CAGCAGAAAG TTATGTCCTC AAGAGACACC

CAAATGAAGC ACACATAAAC CAGCGGAAGG TTATGTCCTC AAGAGACACC

CAAATGAAGC ACACATAAAC CAGCGGAAGA TTATGTCCTC AAGAGACACT

CAAAGGAAGC AAACATAACC CAGCGGAAGA TTCTGTCCAC ----------

tggataaaga gttagaaaag cagaaaaAAA TTATGTCCTG GATGGGCGCC

AAAATGGAGC ACATATAAAA CAGCGGAAAG TTTTGTCCTG GATAGACATC

TGCAACATAA TGCTACTTTA TTCACCTCTG TCACTTTCAG AAAGCCTGAG

TGCAACATAA TTCTACTTTA TTCACCTCTG TCACTTTCAG AAAGCCTGAG

TGCAACATAA TTCTACTTTA TTCACCTCTG TCACTTTCAG AAAGCCTGAG

TGCAACATAA TTCTACTTTA TTCACCTCTG TCACTTTCAG AAAGCCTGAG

TGCAACACAA TTCTACTTTA TTCACCTCTG TCACTTTCAG AAAGCCTGAG

TGCATTATAA GTCTACTTTA TTCACCTCTG TCACTTTCAG AAAGCCTGAG

CCCA-TAGGA GTTTGCTTTA TTCACCTCTG TGACTTTCAG AAAGCCCGAG

TGTG-TAGAA GTTGGCTTTA TTCACCTCTG TCACTTTCAG AAAGCCCGAG

TGCAATGTCA GTTGACTTTA TTCACCTCTG TCACTTTCAG AAAGCCAGAG

---------- ---------- ---------- ---------- ----------

---------- ---------- ---------- ---------- ----------

TGCATTATGA GTCTACTTTA TTCACCTCTG TCACTTTCAG AAAGCCTGAG

TGCATTATGA GTCTATTCTA TTCACCTCTG TCACTTTTAG AAAGCCTGAG

TGCGTTAAAA GTCTACTATA TTCACCTCTG TCACTTTCAG AAAGCCTGAG

TGCATTATCA GTGTAATATA TTCACCTCTG TCACTTTCAG AAAGCCTGAG

---------- ------CATA TTCACCTCGG TCACTTTCAG AAAGCCTGAG

CACATTATGA TACTGCCATA TTCACCTCTG TGACTTTCAG AAAGCCTGAA

TGCTTTATGA AACTGCTGTA TTCACCTCTG TCACTTTCAA AAAGCCTGAA

AAATCAGCCA CCACGTTCCC ATGGGTGAGC ATCGCACCTT TTGGGTTCCC

AAATCAGCCA CCACGTTCCC ATGGGTGAGC ATCGCACCTT TTGGGTTCCC

AAATCAGCCA CCACGTTCCC ATGGGTGAGC ATCGCACCTT TTGGGTTCCC

AAATCAGCCA CCACGTTCCC ATGGGTGAGC ATCGCACCTT TTGGGTTCCC

AAATCGGCCA CCACGTTCCC ATGGGTGAGC ATCGCACCTT TTGGGTTCCC

AAATCAGCCA CCACGTTCCC ATGGGTGAGC ATCGCACCTT TTGGGTTCCC

AAATCGGCCA CCACGTTCCC GTGGGTGAGC ATCGCACCTT TGGGGTTCCC

AAATCGGCCA CCACGTTCCC GTGGGTGAGC ATTGCACCTT TGGGGTTCCC

AAATCAGCCA CCACGTTCCC ATGGGTGAGC ATCGCACCTT TTGGGTTCCC

---------- ---------- ---------- ---------- ----------

---------- ---------- ---------- ---------- ----------

AAATCAGCCA CCACGTTCCC ATGCGTGAGC ATCGCACCTT TTGGGTCCCC

AAATCAGCCA CCACGTTCCC ATGGGTGAGC ATCGCACCTT TTGGGTTCCC

AAATCAGCCA CCACGTTCCC ATGGGTGAGC ATCGCCCCTT TTGGGTTACC

AAATCAGCCA CCACGTTTCC ATGGGTGAGC ATCGCACCTT TTGGGTTCCC

AAATCAGCCA CCACGTTTCC ATGCGTGAGC ATCGCGCCTT TCGGGTTCCC

AAATCAGCAA CCACATTCCC ATGAGTGAGC ATCGCACCTT TCGGGTTCCC

AAATCAGCAA CGACATTTCC ATGGGTTAGC ATCGCACCTT TGGGGTTTCC

TATAAAAAGA AAGTAACCTT CAGAGGTGGA GGGCATGGGT GACACCTGGA

TATAAAAAGA AAGTAACCTT CAGAGGTGGA GGGCATGGGT GACACCTGGA

TATAAAAAGA AAGTAACCTT CAGAGGTGGA GGGCATGGGT GACACCTGGA

TATAAAAAGA AAGTAACCTT CAGAGGTGGA GGGCATGGGT GACACCTGGA

TATAAAAAGA AAGTAACCTT CAGAAGTGGA GGGCATAGGT GACACCTGGA

TGTAAAAAGA AAGTAACCTT CAGAGGCAGA GGGCATGGGT GACACCTGGA

TGGAAACAGA AGATAACCTT CAGAGGTGGA GGGCATGGGC GACACCTGCA

TGAAAACAGA AGATAACCTT CGGAGGTGGA AGGCATGGGT GACACCTGTA

TGGGGGAAAA A-GTAACCTT CAGAGGCGGA GGGCATGGGA GACACCTGGA

---------- ----AACCTT CAGAGGTGGA GGGCATGGGC GACACCTGAA

---------- ----AACCTT CAGAAGTGGA GGGCATAGGT GACACCTGGA

TGAAAAGAGA AAGTAACCTT CAGAGGTGGA GGGCATGGGC GACACCTGGA

TGAAAAGAGA AAGAAACCTT CAGAAGAGGA AGGCATAGGC GACACCTGGA

TGAAAAGAGA CAGTAACCTT CAGAGGTGGA GGGCATCGGC GACACCTGGA

TGAAAAGAGA AAGT------ ---------- ---------- ----------

TG--AAGAGA GAGTAACCTT CAGAAGTGGA AGGCATGGGC GACACCTGTA

TGAGAAGGAA AAATAACCAT CAGAGCTGGA GGGCAATGGC GACACCTGGA

TGGAAAGAGA AAATAATCTC CAGAGGAGCC GGGCTCTGGT GGCAAGGGGA

AGTGTACAGA TCACTGGTGC TGTCTCCTGC ATGTAGCCTG GAACTGTGTA

AGTGTACAGA TCACTGGTGC TGTCTCCTGC ATGTAGCCTG GAACTGTGTA

AGTGTACAGA TCACTGGTGC TGTCTCCTGC ATGTAGCCCG GAGCTGTGTA

AGTGTACAGA TCACTGGTGC TGTCTCCTGC ATGTAGCCCG GAGCTGTGTA

AGTGTACAGA TCACTGGTGC TGTCTCCTGC ATGTAGCCCG GAGTTGTATA

AGCGTACAGG TCACTGGTGC TATCTCCTGC AGATAGCCCT GAGCTGTGTA

AGTGTACAGA TCAGTGGTGC TGTCTCCTGC AGGTATCCCT GAGCTGTGTA

GGTATACAGA TCAGTGGTGC TATCTCCTGC AGGTAGCCCT GAGTGGTGTA

AGCGTACAGG TCACTGGTGC TGTCTCCTGA ATGTAGCCTT GGGCAGTGTA

AGTGTACAGG TCGCTGGTGC TGTCTCCTGC ATGTAGCTGC GAACTGTGTA

AGCGTAGAGG TCACTGGTGC TGTCTCCTGC ATGTAGCCGT GAGCTGTGTA

AATGTACAGG TCGCTGGTGC TATCTCCTGC ATGTAGCCCT GAGCTGTGTA

AGTGTACAGG TCACTGGTAC TGTCTCCTGC AGGTAGCCCT GAGCCGTGTA

AGTATACAGC TCACTGGTGC TGTCTCCTGC AGGTATCCCT GAGCTGTGTA

---------- ---------- ---------- ---------- ----------

AGTGTACAGG TCACTGGTGC TGTCTCCTCG AAGCAGACGT GAGCTGTGTA

AGGGTACATG TCATTGGTGC TATCTCCTGA GGATAAACCT GAGCTGTGTA

AGGGGAGAGC TCGCTGTTGC TGTCTCCTGG GCATAGTTGT GAGCTGTGTA

GCTGCTGTGG TCATCAGGCA GAGTGGAGCT GCTGAGTCCA TCAGAGAAGG

GCTGCTGTGG TCATCAGGCA GAGTGGAGCT GCTGAGTCCA TCAGAGAAGG

GCTGCTGTGG TCATCAGGCA GAGTGGAGCT GCTGAGTCCA TCAGAGAAGG

GCTGCTGTGG TCATCAGGCA GAGTGGAGCT GCTGAGTCCA TCAGAGAAGG

GCTGCTGTGG TCATCAGGCA GAGTGGAGCT GCTGAGTCCA TCAGAGAAGG

GCTGCTGTGG TCATCAGGCA GGGTGGAGCT GCTGAGGCCA TCAGAGAAGG

ACTGCTGTGG TCATCAGGCA GAGTAGAGCT GCTCAGTCCG TCAGATAAGG

ACTGCTGTGG TCATCAGGTA GGGTAGAGCT GCTGAGTCCA TCAGAGAAAG

GCCACTGTGG TCATCAGGCA GGGTGGAGCT GCTGAGTCCA TCAGAGAAGG

GTTGCTGTGG TCATCAGGCA GGGTGGAGCT ACTGAGTCCA TCAGAGAAGG

GCTGCTGTGG TCATCGGGCA GGGTGGAGGT GCTGAGTCCA TCAGAGAAGG

GCTGCTGTGG TCGTCAGGCA GGGTGGAGCT GCTGAGGCCA TCGGAGAAGG

GCTGCTGTGA TCGTCAGGCA GGGTGGAGCT GCTGAGCCCA TCAGACAAGG

GCTGCTGTGG TCATCAGGCA GGGTGGAACT GCTGAGTCCA TCAGAGAAGG

---------- ---------- ---------- ---------- ----------

GCCGCTGTGG TCATCGGGCA GAGTGGAGCT GCTGAGCCCG TCGGAGAAGG

TCCACTGTGG TCATCAGGCA GAGTTGAACT GCTAAGCCCA TCTGATTGGG

ACCGTTGTGG TCATCTGGTA GTGTGGAGCT GTTTAATGCT TCTGACTTGG

TATCACCTTC CTCTTGGCCT TGCTCTTAGC ATCTCGGCTG GACTTCGACT

TATCACCTTC CTCTTGGCCT TGCTCTTAGC ATCTCGGCTG GACTTCGACT

TATCACCTTC CTCTTGGCCT TGCTCTTAGC ATCTCGGCTG GACTTCGACT

TATCACCTTC CTCTTGGCCT TGCTCTTAGC ATCTCGGCTG GACTTCGACT

TATCACCTTC CTCTTGACCT TGCTCTT--- ATCTCGGCTG GACTTCGACT

TATCACCTTC CTTTTGGCCT TGCTCTTAGC ATCTCGGCTG GACTTGGACT

TGTCACCTTC CTCTTGGTTT TGCTCTTAGT GTCACGGCTG GACTTGGACT

TGTCACCTTC CTCTTGGTTT TGCTCTTAGT GTCTCGGCTG GACTTGGACT

TGTCACCTTC CTCTTGGCCT TGCCCTTAGC ATCTCGGGTG GACTTGGACT

TATCACCTTC TTCTTGGCCT TGCTCCTAGC GTCTCGGCTG GACTTGGACT

TGTCACCTTC CTCTTGGCCT TGCTCTTAGC ATCTCGGCTG GACTTGGACT

TATCACCTTC CTCTTGGCCT TGCTCTTAGT GTCTCGGCTG GACTTGGACT

CATC------ ---------- ---------- ---------- ----------

TATCACCTTC CTCTTGGTCT TGCTCTTAGC ATCTCGGCTG GACTTGGATT

---------- ---------- ---------- ---------- ----------

TATCACCTTC CTCTTGGCCT TGCTCTTGGT CTCTCGGCTA GACTTGGACT

TGTCACCTTT CTCTTGCTCT TGCTTCTAGA ATCTTTGGAT GACTTGGACT

CCTCACCTTT CTCTTATTCT TGTTTCTGGA GTCTCTTGAG GTCTTGGTCT

TTCTTTCTAT ACCTTTTCTC TGGTTCTTGG TGAGAGGTGG AAGCATCCGG

TTCTTTCTAT ACCTTTTCTC TGGTTCTTGG TGAGAGGTGG AAGCATCCTG

TTCTTTCTAT ACCTTTTCTC TGGTTCTTGG TGAGAGGTGG AAGCATCCGG

TTCTTTCTAT ACCTTTTCTC TGGTTCTTGG TGAGAGGTGG AAGCATCCTG

TTCTTTCTAT ACCTTTTCTC TGGTTCTTGT TGAGAGGTGG AAGCATCCGG

TTCTCTCTGT ACCTTTTCTC TGGTTCTTGG TGAGAGGTGG GAGCATCCGG

TTCTCT---- ---------- -----CTTGG TGAGGGGTGG CAGCATCCGG

TTCTCTCTCT ACCTTTCCTC TGGTTCCTGG TGAGGGGTGG CAGCATCCGG

TTCTCTCTGT ACCTTTCCTC TGGTTCCTGG TGAGGGGCGG GAGCATCCGG

TTCTCTCTGT ACCTTTCCTC TGGCTCTTGG TGAGGGGTGG GAGCATCCGG

TTCTCTCTAT ACCTTTTCTC TGGTTCTTGG TGAGGGGTGG GAGCATCCGG

TTCTCTCTAT ACCTTTCCTC TGGTTTTTGG TGAGGGGTGG GAGCATCCGA

---------- ---------- ---------- ---------- ----------

TCCTCTCTAT ACCTTTCCTC TGGTTCTTGG TGAGGGGTGG AAGCATCCGG

---------- ---------- ---------- ---------- ----------

TTCTTTCTAT ACCTTTCCTC TGGTTCTTGG TCAGCGGGGG GAGCATGCGG

TTCTCTCTAT ACCTTTCCTC TGGTTCTTGG TGAGAGGCGG GAGCATTCGG

TTCTCTCTAC ACCTTTTCTC TGGCTCTTGG ACAAAGGCGG AAGCATCCGA

TACACTCGCA CAGCTGAGCT GCCCTTGTTC CTGCTCTGGT CTTTCACCTC

TACACTCGCA CAGCTGAGCT GCCCTTGTTC CTGCTCTGGT CTTTCACCTC

TACACTCGCA CAGCTGAGCT GCCCTTGTTC CTGCTCTGGT CCTTCACCTC

TACACTCGCA CAGCTGAGCT GCCCTTGTTC CTGCTCTGGT CCTTCACCTC

TACACCCGCA CAGCTGAACT ACCCTTGTTC CTGCTCTGGT CCTTCACCTC

TACACCCGCA CGGCAGAGCT GCCCTTGTTC CTGCTCTGGT CCTTCACCTC

TACACGCGTA CAGCAGAGCT GCCCTTGTTC CTGCTCTGGT CCTTCACTTC

TACACCCGCA CAGCAGAGCT GCCCTTGTTC CTACTCTGAT CCTTCACTTC

TACACCCGCA CGGCCGAGCT GCCCTTGTTC CTGCTCTGGT CCTTCACCTC

TACACCCGCA CAGCTGAACT GCCCTTGTTC CTGCTCTGGT CCTTCACCTC

TACACCCGCA CAGCTGAGCT GCCTTTGTTC CTGCTCTGGT CCTTCACTTC

TACACCCGCA CAGCTGAGCT GCCTTTGTTT CTGCTTTGGT CCTTAACTTC

---------- ---------- ---------- ---------- ----------

TACACCCGCA CAGCTGAACT GCCCTTGTTC CTGCTCTGGT CCTTCACCTC

---------- ---------- ---------- ---------- ----------

TACACCCGCA CAGCTGAGCT GCCCTTGTTC CTGCTCTGGT CCTTCACCTC

TATACTCTCA CAGCTGAGCT TCCCTTGTTC CTGCTTTGGT CCTTCACTTC

TAGACGCGGA CGGCAGTGGA GCCTTTGTTC CTGCTCTGGT CCTTCACCTC

CTCGATATCT GGCAGGGAGT TCATGGCACA GCGAAAGTTG GCCTTCCACG

CTCGATATCT GGCAGGGAGT TCATGGCACA GCGAAAGTTG GCCTTCCACG

CTCAATATCT GGCAGGGAGT TCATGGCACA GCGAAAGTTG GCCTTCCACG

CTCAATATCT GGCAGGGAGT TCATGGCACA G--------- ---NTCCACG

CTCAATATCT GGCAGGGAGT TCATGGCACA GCGAAAGTTG GCCTTCCATG

CTCAATATCT GGCAGGGAGT TCATGGCACA GCGAAAGTTG GCCTTCCATG

CTCGATGTCT GGTAGGGAGT TCATGGCACA CCGGAAGTTT GCCTTCCAAG

CTCGATGTCT GGCAGGGAGT TCATGGCACA ACGGAAGTTT GCCTTCCATG

CTCGATGTCT GGCAGGGAGT TCATGGCACA GCGGAAGTTG GCCTTCCACG

CTCAATATCT GGCAGCGAGT TCATGGCGCA GCGAAAGTTG GCCTTCCAGG

CTCAATATCT GGCAGGGAGT TCATGGCACA GCGAAAGTTG GCCTTCCATG

CTCGATGTCC GGCAAGGAGT TCATGGCACA GCGGAAGTTG GCCTTCCATG

---------- ---------- ---------- ---------- ----------

CTCAATGTCT GGCAGGGAGT TCATGGCGCA GCGAAAATTG GCCTTCCACG

---------- ---------- ---------- ---------- ----------

CTCGATGTCC GGCAGCGAGT TCATGGCGCA GCGGAAGTTG GCTTTCCACG

CTCTATGTCA GGGAGGGAAT TCATGGCACA GCGGAAATTG GCCTTCCATG

CTCGATGTCG GGCAGGGAGT TCATGGCGCA GCGGAAGTTG GCCTTCCAGG

TCTTGGGATC TGGCTCCTTT TCCCCTGCTT TGTATCGGCC TACACTCACT

TCTTGGGATC TGGCTCCTTT TCCCCTGCTT TGTATCGGCC TACACTCACT

TCTTGGGATC TGGTTCCTTT TCCCCTGCTT TGTATCGGCC TACACTCACT

TCTTGGGATC TGGTTCCTTT TCCCCTGCTT TGTATCGGCC TACACTCACT

TTTTGGGGTC TGGCTCCTTT TCCCCTGCTT TGTATCGGCC TACACTCACT

TCTTGGGATC TGGCTCCTTT TCCCCTGCTT TGTATCGGCC TACACTCACT

TCTTGGGATC TGGCTCTTTT TCCCCTGCTT TGTATCGGCC TACACTCACT

TCTTGGGATC TGGCTCTTTT TCTCCTGCTT TGTATCGGCC TACACTCACT

TCTTGGGATC TGGCTCCTTT TCCCCAGCTT TGTATCGGCC TACACTCACT

TCTTGGGATC CGGCTCCTTT TCCCCTGCTT TGTATCGGCC TACACTCACT

TCTTAGGATC TGGCTCCTTT TCCCCGGCTT TGTATCGACC TACACTCACT

TCTTGGGATC TGGCTCCTTT TCCCCTGCTT TGTATCGGCC TACACTCACT

---------- ---------- ---------- ---------- ----------

TCTTGGGATC CGGCTCCTTT TCTCCTGCTT TGTATCGACC TACACTCACT

---------- ---------- ---------- ---------- ----------

TCTTGGGGTC TGTCTCCTTT TCCCCTGCTT TGTATCGGCC TGCACTCACT

TCTTAGGGTC TGGTTCCCTC TCCCCAACTT TGTAGCGACC TACTCTCACC

TCTTGGGGTC TGGCTCCTTT TCTCCGACCT TGTATCTCCC TGCATTCACC

TTATTAATCC AGATGAGCCC CGGGATTTGG TTGGAATTAA TCTGCATCTC

TTATTAATCC AGATGAGCCC CGGGATTTGG TTGGAATTAA TCTGCATCTC

TTATTAATCC AGATGAGCCC CGGGATTTGG TTGGAATTAA TCTGCATCTC

TTATTAATCC AGATGAGCCC CGGGATTTGG TTGGAATTAA TCTGCATCTC

TTATTAATCC AGATGAGCCC CGGGATTTGG TTGGAATTAA TCTGCATCTC

TTATTAATCC AGGTCAGCCC TGGGATTTGG TTGGAATTAA TCTGCATCTC

TTATTGATCC AGCTCAGCCC TGGAATTTGG TTGGAATTAA TCTGCATCTC

TTATTGATCC AGATCAGCCC TGGGATTTGG TTGGAATTAA TCTGCATCTC

TTATTAATCC AGATCAGCCC GGGGATCTGG TTGGAATTAA TCTGCATCTC

TTATTAATCC AGATCAGCCC TGGGATTTGG TTGGAATTAA TCTGCATCTC

TTATTAATCC AGATCAGCCC TGGGATTTGG TTGGAATTAA TCTGCATCTC

TTATTAATCC AGATCAGCCC TGGGATCTGG TTGGAATTAA TCTGCATCTC

---------- ---------- ---------- ---------- ----------

TTATTAATCC AGATCAGCCC CGGAATTTGG TTGGAATTAA TCTGCATCTC

---------- ---------- ---------- ---------- ----------

TTATTAATCC AGGTCAGCCC TGGGATTTGG TTGGAATTAA TCTGCATCTC

TTGTTAATCC AGGTCAGGCC TGGGATTTGG TTGGAATTAA TCTGCATCTC

TTATTGATCC ACGTAAGGCC TGGGATCTGA TTGGAATTGA TCTGCAACTC

TAGCCAGGGT CTCATGCGCA TCCGAGTGAT GGGCATGTTG GCTGTAAAGA

TAGCCAGGGT CTCATGCGCA TCCGAGTGAT GGGCATGTTG GCTGTAAAGA

TAGCCAGGGT CTCATGCGCA TCCGAGTGAT GGGCATGTTG GCTGTAAAGA

TAGCCAGGGT CTCATGCGCA TCCGAGTGAT GGGCATGTTG GCTGTAAAGA

TAGCCAGGGT CTCATGCGCA TCCGAGTGAT GGGCATGTTG GCTGTAAAGA

TAGCCAGGGT CTCATGCGCA TCCGAGTGAT GGGCATGTTG GCTGTGAAGA

TAGCCAGGGT CTCATTCGCA TCCGAGTGAT AGGCATGGTG GCTGGAGGGA

TAGCCAGGGT CTCATCCGCA TTCGAGTGAT TGGCATGGTG GCTGGAGGGA

TAGCCAGGGT CTCATGCGCA TCCGAGTGAT GGGCATGTTG CCTGTGAAGA

TAGCCAAGGT CTCATGCGCA TCCGAGTGAT GGGCATGTTG GCTGTGAAAA

TAGCCAGGGT CTCATGCGCA TCCGAGTGAT GGGCATGTTG GCTGTGAAGA

TAGCCAGGGT CTCATGCGCA TGCGAGTGAT GGGCATCTTG GCTGTGAAGA

---------- ---------- ---------- ---------- ----------

TAGCCAGGGT CTCATGCGCA TCCGAGTGAT GGGCATGTTG GCTGTAAAGA

---------- ---------- ---------- ---------- ----------

TAGCCAGGGT CTCATGCGCA TCCGAGTGAT GGGCATGTTG GCTGCAAAGA

TAGCCAGGGT CTCATGCGCA TTCTGGCAAT GGGCATGTTG CCTGTGAAGC

CAGCCAGGGT CTCATGCGCA TTCGTGTTGT CGGCATGTTG CCTGGGAAAT

GAACACAGAA GTACAGGGCG GCTGCTCCGC CCCACCTGCT TACCTGTCGT

GAACACAGAA GTACAGGGCG GCTGCTCCGC CCCACCTGCT TACCTGTCGT

GAAGACAGAA GTCCAGTAAG GGTGCTCCGC CCCACCTGCT TACCTGTTGT

GAAGACAGAA GTACAGGGCG GCTGCTCCGC CCCACCTGCT TACCTGTTGT

GAAGACAGAA GTACAGGGCG GCTGCTCCGC CCCACCTGCT TACCTGTTGT

GAAGACAGAA GTACAGGGCG GCTGCTCCTC CCCACCTGCT TACCTGTTGT

GAAGACAGAA GTACAAGGCG GCTGCGC-TC CCCACCGGCT TACCTGTTGT

AAAGACAGAA GTACAAGGCA GCTGTGC-TC CCCACCGGCT TACCTGTTGT

GAAGACAGAA GTACAGGGCG Gctgctcctc cccacc---- ----------

GAAGACAGAA GTACAGGGCG GCTGCCCCTC CCCACC---- ----------

GAAAACAGAA GTACAGGGCG GCTGCTCCTc cccaccTGCT CACCTGTTGT

GAAGACAGAA GTACAGGGCG GCAGCTCTTC CCCACCTGCT TACCTGTCGT

---------- ---------- ---------- ------AGCT TACCTGTGGT

GAAGACAGAA GTACAGGGCG GCTGCTCCTC CCCACCTGCT TACCTGTCGT

---------- ---------- ---------- ------TGCT TACCTGTTGT

GAAGATGAAA GTACAGGGCG GCTGCCTCTC CCCACC---- ----------

CAAGAAAAAA GTACAGAGcc tctttccctc ctctccTGGT TACCTGTGGT

GAGATTGGAA ATGTGTTCCG CCTAACCTTC CCCACTTGGT TACCTGTAGT

GCCGCTTGTG AAACACACAA TGGAGAGGTC ATCAGGCTCA CCTCCACGGC

GCCGCTTGTG AAACACACAA TGGAGAGGTC ATCAGGCTCA CCTCCACGGC

GCCGCTTGTG AAACACACAA TGGAGAGGTC ATCAGGCTCA CCTCCACGGC

GCCGCTTGTG AAACACACAA TGGAGAGGTC ATCAGGCTCA CCTCCACGGC

GCCGCTTGTG AAACACACAA TGGAGAGGTC ATCAGGCTCA CCTCCACAGC

GCCGCTTGTG AAACACACAA TGGAGAGGTC ATCAGGCTCA CCTCCATAGC

ACCGCTCGTG AAACACACAA TGGAGAGGTC ATCGGGCTCA CCTCTATAGC

ACCACTTGTG AAACACACGA TGGAGAGGTC ATCAGGCTCA CCTCTATAGC

---------- ---------- ---------- -------TCA CCTCCACAGC

---------- ---------- ---------- -------TCA CCTCCACATC

GCCGCTTGTA AAACACACAA TGGAGAGGTC GCTGGGCTCA CCTCCACAGC

GCCGCTTGTG AAACACACAA TGGAGAGGTC GCTGGGCTCA CCTCCACAAC

GCCGCTGGTG AAACACACAA TGGAGAGGTC GCAGGGC--- ----------

GCCACTTGTG AAACACACAA TTGAGAGGTC ATCTGGCTCA CCTCCACGGC

ACCGCTTGTG AAACATACAA TCGAGAGGTC ATCAGGCTCA CCTCCACAGC

---------- ---------- ---------- ---------- ----------

ACCACTAGTG AAACACACGA TTGAAAGGTC TTCTGGTTCA CCTCAACTTC

GCCACTGGTG AAACACACAA TTGACAGGTC TTCCGGCTCA CCTCGACTTC

CTGCATGGAC TTAATGACCA CCCCGCACTT CTGCCCTCTC TCTTTCAGTC

CTGCATGGAC TTAATGACCA CCCCACACTT CTGCCCTCTC TCTTTCAGTC

CTGCATGGAC TTAATGACCA CCCCGCACTT CTGCCCTCTC TCTTTCAGTC

CTGCATGGAC TTAATGACCA CCCCGCACTT CTGCCCTCTC TCTTTCAGTC

CTGCATGGAC TTAATGACCA CCCCGCACTT CTGCCCTCTC TCTTTCAGTC

CTGCATGGAC TTGACGACCA CCCCACACTT CTGCCCTCTT TCTTTCAGTC

CTGCATGGAC TTGATGTCCA CCCCGCACTT CTTTCCCCTC TCTCTCAGTC

CTGCATGGAC TTGATGTCCA CCCCGCACTT CTTTCCTCTC TCTCTCAGTC

CCGCATGGAC TTGAGGAGCA CCCCACACTT CTTCCCCCGC TCTCTCAGTC

CTGCATGGAC TTAATGACCA CCCCGCACTC TTGCCCTCTG TCTTTCAGTC

TTGCATGGAC TTAATGATCA CCCCGCACTC CTGCCCTCTT TCTTTCAGTC

CTGCATGGAC TTGATGACCA CCCCGCACTC CTGCCCTCTG TCTTTCAGTC

---------- ---------- ---------- ---------- ----TCAGTC

CTGCATGGAC TTAACGACCA CCCCGCACTC CTGCCCTTTC TCTTTCAGTC

CTGCATGGAC TTAATGACCA CCCCGCACTC CTGCCCTCTC TCTTTCAGTC

---------- ---------- ---------- ---------- ----------

TTTCATGGAC TGAATCTCCA CGCCACATTT CTTCCCTCTT TCTTTCAATC

CTGCATAGAC TGGATGAGCA CGCCACATTT TTTCCCTCGC TCCTTCAATT

CATGAGGATG ATCAGCTTGA GGCCTGGAGT CTCCTTCCTC TCCACATGCT

CATGAGGATG ATCAGCTTGA GGCCTGGAGT CTCCTTCCTC TCCACATGCT

CATGAGGATA ATCAGCTTGA GGCCTGGAGT CTCCTTCCTC TCCACATGCT

CATGAGGATA ATCAGCTTGA GGCCTGGAGT CTCCTTCCTC TCCACATGCT

CATGAGGATG ATCAGCTTGA GGCCTGGAGT CTCCTTCCTC TCCACATGCT

CATGAGGATA ATTAGCTTGA GGCCTGGAGT TTCCTTCCTC TCCACATGCT

CATGAGGATG ACCAGCTTGA GGCCCGGAGT CTCCTTCCTC TCCACGTGTT

CATGAGGATG ACCAGCTTGA GGCCCGGAGT CTCCTTCCTC TCCACGTGTT

CATGAGGATG ATCAGCTTGA GCCCTGGAGT CTCCTTCTTC TCCACGTGCT

CATGAGGATG ATCAGCTTGA GCCCTGGCGT CTCCTTCCTC TCCACGTGCT

CATGATGATG ATGAGCTTGA GCCCCGGAGT CTCCTTCCTC TCCACATGTT

CATGAGGATG ATAAGCTTGA GCCCTGGAGT CTCCTTCCTC TCCACGTGCT

CATGAGGATG ATGAGCTTGA GCCA-GGAGT CTCCTTCCTC TCCACATGCT

CATGAGGATG ATCAGCTTGA GCCCTGGAGT CTCGTTCTTC CGCACATGTT

CATGATGATG ATCAGCTTGA GCCCTGGAGT CTCCTTCTTC TCCACATGCT

---------- ---------- ---------- ---------- ----------

GATGAGAATA ATGAATTTCA GGCCAGGAAT TTCCTTTCTC TCCACATGTT

GATGAGGATA ATGAATTTCA GGCCCGGAGT GTCCTTTCTT TCCACGTGTT

CTAGCAGAAT TACCTGTATT GATGATGTAG CGGATAGCCC CAGGGCCCAG

CTAGCAGAAT TACCTGTATT GATGATGTAG CGGATAGCCC CAGGGCCCAG

CTAGCAGAAT TACCTGTATT GATGATGTAG CGGATAGCTC CAGGGCCCAG

CTAGCAGAAT TACCTGTATT GATGATGTAG CGGATAGCTC CAGGGCCCAG

GTAGCAGAAT TACCTGTATT GATGATGTAG CAGATAGCCC CAGGGCCCAG

CCAGCAGAAT TACCTGTATT GATGATATAG CGGATAGCCC CAGGGCCCAG

CTAGCAGGAT CACCTGTATT GATGATGTAG CGGATAGACC CTGGGCCCAG

CCAGCAGAAT CACCAGTATT GATGATGTAG CTGATAGACC CTGGGCCCAG

CCAGCAGTAT CACCTGTATT GATGATGTAG CGGATGGCCC CAGGGCCCAG

CCAGGAGAAT CACCTGTGTT GATGATGTAG CGGACTGCCC CAGGGCCCAG

CTAGCAGAAT CACCTGTGCT GATGATGTAG CGGATAGCAC CAGGGCCCAG

CCAGCAGAAT CACCTGTGTT GATGATGTAG CGTATAGCCC CGGGGCCCAG

CCAGCAGAGT CACCTGTATT GATGATGTAG CGGATGGCCC CAGGGCCCAG

CTAGCAGAAT CACCTGTGTT GATGATGTAG CGGATGGCCC CCGGGCCCAG

CTAGCAGAAC TACCTGTGTT GATGATGTAG TGGATGGCCC CGGGGCCCAG

---------- ---------- ---------- ---------- ----------

CCAGGAGAAT TACCTGTGTT GATAATGTAG CGAATGGCAC CGGGGCCTAA

CTAGGAGAAT TACCTGTGTT AATAATGTAG CGAATGGCTC CAGGCCCCAG

GGTGTCATAG GGACCACCAC CATGGAATAT GTGTAGCAGG CCAGCTCCAC

GGTGTCATAG GGACCACCAC CATGGAATAT GTGTAGCAGG CCAGCTCCGC

GGTGTCATAG GGACCACCAC CATGGAATAT GTGTAGCAGG CCAGCTCCGC

GGTGTCATAG GGACCACCAC CATGGAATAT GTGTAGCAGG CCAGCTCCAC

GGTGTCATAG GGACCACCAC CATGGAATAT GTGTAGCAGG CCAGCTCCGC

GGTGTCATAG GGACCACCAC CATGGAATAC GTGTAACAGG CCAGCTCTGC

TGTGTCATAG GTACCACCAC CATGGAATAT GTGTAGCAGG CCAGCTCAGC

GGTGTCGTAG GTACCACCAC CATGGAATAC GTGTAGCAGG CCAGCTCGGC

GGTGTCATAG GGACCACCAC CATAGAATAC GTGTAGCAGG CCAGCTCCGC

GGTGTCATAG GGACCACCAC CATAGAGTAT GTGTAACAGG CAAGCTCTGC

GGTGTCATAG GGACCACCAC CATAGAATAC GTGTAGCAGG CAAGCTCTGC

CGTGTCATAG GGACCACCAC CATGGAGTAG GTGTAGCAGG CAAGCTCAGC

GGTGTCATAG GGACCACCAC CATGGAATAT GTGTAGCAGG CCAGCTCCGA

GGTGTCGTAG GGACGACCAC CATGGAGTAC GTGTAGCAGG CCAGCTCTGC

GGTGTCATAG GGACCACCAC CATGGAATAT GTGTAGCAGG CTAGCTCTGC

---------- ---------- ---------- ---------- ----------

GGTGTCATAG GAACCACCAC CATGGAGTAG GTGTAGCAGG CCAGCTCCGA

TGTGTCGTAG GCACCACCAC CATGGAGTAG GTATAACAAG CCAACTCAGA

AATGATCCAC TGTGGAGATG CTTTACAATT GTGCTGGAGA AGTCCGGACC

AATGATCCAC TGTGGAGATG CTTTACAATT GTGCTGGAGA AGTCCGGACC

AATGATCCAC TATGGAGATG CTTTACAATT GTGCTGGAGA AGTCCAGACC

AATGATCCAC TATGGAGATG CTTTACAATT ATGCTGGAGA AGTCCAGACC

AATGATCCAC TGTGGAGATG CTTTACAATT GTGCTGGAGA AGTCCGGACC

AATGATCCAC TATGGAGATG CTTTACAGTT GTGCTGGAGC AGTCCGGACC

AATGATCCAC TGCAGAAGCA CTTTACAGTC ATGCTGGAGA AGTCCTGACC

AATGATCCAC TACAGAAACA CTTTACAGTC GTGCTGGAGA AGCCCCGACC

AATGATCCAC TGTGGAGATG CTTTACAATT GTGCCGCAGC AGTCCAGACC

GATGATCCAC TGTGGAGATG GTTTACAATT GTGCTGGAGA AGTCCAGACC

AATGATCCAC TGTGGAGACG GTTTACAATT GTGTTGGAGA AGTCCAGACC

AATGATCCAC TGTGGAGATG CTTTGCAATT GTGCTGGAGA AGTCCGGACC

GATGATCCAC TGTGGAGATG CTTTACAATT GTGCTGGAGA AGTCCAGACC

GATGATCCAC TGTGGAGATG ATTTACAGTT GTGCTGGAGA AGTCCAGACC

GATGATCCAC TGCAAAGATG CTTTACAATT GTGCTGGAGA AGTCCGGACC

---------- ---------- ---------- ---------- ----------

GATGATCCAC TGGTAAGATG GTTTACAGTC ATGTTGGAGG AGTCCTGATC

AATGATCCAC TGGGAAGATG GTTTACAGCC ATGGTGGAGC AGTCCTGACC

CCAGAAATTC AGCCCTGTCG GCCACCTCTC ACCTCCTGGT AGGACAGCCA

CCAGAAATTC AGCCCTGTCG GCCACCTCTC ACCTCCTGGT AGGACAACCA

CCAGAAATTC AGCCCGGTCG GCCACCTCTC ACCTCCTGGT AGGACAGC--

CCAGAAATTC AGCCCGGTCG GCCACCTCTC ACCTCCTGGT AGGACAGCCA

CCAGAAATTC AGCCCTGTCG GCCACCTCTC ACCTCCTGGT AGGACAGCCA

CCAGAAATTC AGCCCTGTCG GCCACCTCTC ACTTCCTGGT AGGACAGCCA

CCAGGAATTC AGCCCTTTTG GCCACCTCTC ACCTCTTGAT AGGACAGCCA

CCAGAAATTC AGCCCTTTTG GCCACCTCTC ACCTCTTGGT AGGACAGCCA

CCAGAAACTC AGCTCTGTCC ACCACCTCTC ACCTCCTGGT AGGACAGCCA

CTAGAAATTC AGCCCTGTCA GCCACCTCTC ACCTCCTGGT AGGACAGCCA

CTAGAAATTC AGCCCTGTCG GCCACCTCTC ACCTCCTGAT AGGACAGCCA

CTAGAAATTC AGCCCTGTCG GCCACCTCTC ACCTCCTGGT AGGACAGCCA

CCAGAAATTC AGCCCTGTCA GCCACCTCTC ACCTCCTGGT AGGACAGCCA

CCAGAAACTC AGCCCTGTCC GCCACCTCTC ACCTCCTGGT AGGACAGCCA

CTAGGAATTC AGCCCTGTCA GCCACCTCTC ACCTCCTGGT AGGACAGCCA

---------- ---------- ---------- ---------- ----------

CCAGAAACTC TGCTCTGTCT GCCACCTCTT ACCTCTCTGT AGGACAACCA

CCAAGAATTC TGCCCTTTTA GCCACCTCTT ACCTCTTTGT AGGATAACCA

CTGGTAAGGC TGCTTAGGCT TCCTGAAACC AAGACAGGGC CCATTCCCTG

CTGGTAAGGC TGCTTAGGCT TCCTGAAACC AAGACAGGGC CCATTCCCTG

---------- ---------- ---------- ---------- ----------

CTGGTAAGGC TGGTTGGGCT TCCTGAAACC AAGACAGGGC CCATTCCCTG

CTGGTAAGGC TGCTTGGGCT TCCTGAAACC AAGACAGGGC CCATTCCCTG

CTGGTAAGGC TGCTTGGGCT TCCTAAAGCC AAGACAGGGT CCATTCCCTG

CTGGTAAGGT TGCTCTGGCT TCCGGAAGCC AAGACAGGGT CCATTCCCTG

CTGGTAAGGT TGCTCTGGCT TCCGGAAGCC AAGACAGGGT CCATTCCCTG

CTGGTATGGC TGCTTGGGCT TTCTGAAGCC AAGACAGGGC CCGTTCCCTG

CTGATAAGGC TGCTTGGGAT TCCTGAAGCC AAGACAGGGT CCATTCCCTG

CTGATAAGGT TGTTTGGGCT TCCTGAAGCC AAGACAGGGT CCATTCCCTG

CTGATAAGGC TGCTTGGGCT TCCTGAAGCC AAGGCAGGGT CCATTCCCTG

CTGGTAAGGC TGCTTGGGCT TCCTGAAGCC GAGACATGGG CCATTTCCTG

CTGATAAGGC TGCTTGGGCT TCCTGGAGCC AAGGCAAGGC CCATTCCCTG

CTGGTAAGGC TGCTTGGGCT TCCTGGAGCC AAGGCAGGGC CCATTCCCTG

---------- ---------- ---------- ---------- ----------

CTGATATGGC TGCTTGGGCT TCCTGGATCC AAGGCAGGGA CCATCTCCTA

CTGATAAGGC TGCTTGGGTC TTCTGGATCC TAGACAAGGG CCATTCTCTG

TAGAGATCAC CTGAGATGCT AAGCCCACGG CGGAACACCT GGTACATGGT

TAGAGATCAC CTGAGATGCT AAGCCCACGG CGGAACACCT GGTACATGGT

------TCAC CTGAGATGCT AAGCCCACGG CGGAACACCT GGTACATGGT

CAGAGATCAC CTGAGATGCT AAGCCCACGG CGGAACACCT GGTACATGGT

CAGAAATTAC CTGAGATGCT AAGCCCACGG CGGAACACGT GGTACATGGT

CAGAGATCAC CTGAGATGCT AAGCCCACGG CGGAACACCT GGTACATGGT

TGGAAATTAC CTGAGATGCT AAGCCCACGT CGGAACACCT GGTACATCGT

CAGAGATTAC CTGAGATGCT AAGCCCACGG CGGAACACCT GGTACATGGT

CAGAGATCAC GTGAGATGCT GAGCCCTCGG CGGAACACGT GGTACATGGT

CAGAGATTAC CTGAGATCCT AAGCCCACGG TGGAACACCT CGTACATGGT

CAGAGATCAC CTGAGATGCT AAGCCCACGG CGGAACACCT GGTACATGGT

C--AGATCAC CTGAGATGCT AAGCCCACGA CGGAACACCT GGTACATAGT

CAGAGATCAC CTGAGATGCG AAGCCCACGA CAGAACACCT GGTACATGGT

TAGAGATCAC CTGAGATGCT AAGCCCACGG CGGAACACCT GGTACATGGT

CAGAGATCAC CTGAGATTCT AAGCCCACGG TGGAACACCT GGTACATGGT

---------- ---------- ---------- ---------- ----------

AAAAAGTCAC CAGAGATGCT CAGCCCCCTC TGGAAGACTT CATACATGGT

CAGAAATCAC CAGAAATGTT CAATCCCCGT CTGAAAACTT CGTACATGGT

CCGGGCATCA TCATAGTAGT GGGTAAGTAG CTCACCTCTA CTTCTTCTGA

CCGGGCGTCA TCATAGTAGT GGGTAAGTAG CTTACCTCTA CTTCTTCTGA

CCGGGCATCA TCATAGTAGT GGGTAAGTAG CTCACCTCTA CTTCTTCTGA

CCGGGCATCA TCATAGTAGT GGGTAAGTAG CTCACCTCTA CTTCTTCTGA

CCGGGCATCG TCGTAGTAGT GGGTAAGTAG CTTACCTCTA CTTCTTCTGA

CCGAGCATCA TCATAGTAGT GGGTAAGCAG CTCACCTCTA CTTCTTCCGA

CCGGGCATCG TCATAGTAGT GGGTAAGCAA CTCACCTCTA CTTCTTCCGA

CCGGGCATCG TCATAGTAAT GGGTAAGCAA CTCACCTCCA CTTCTTCCGA

CCGGGCGTCC TCGTAGTAGT GGGTGAGCAG ---------- ----------

CCTGGCATCG TCATAGTAGT GGGTGAACAG CTCACCTCCA CCTCTTCTGA

TCTGGCATCA TCATAGTAGT GGGTGAGCAA CTCACCTCCA CTTCCTCTGA

CCTGGCGTCA TCGTAGTAGT GGGTGAGCAG CTCACCTCCA CTTCTTCTGA

TCGGGCGTCG TCATAGTAGT GGGTCAGAAG CTCACCTCCA CTTCCTCTGA

CTGGGCATCT TCATAGTAGT GGGTAAGCAG CTCACCTCCA CTTCCTCCGA

TCGGGCGTCA TCATAGTAGT GGGTGAGGAG CTCACCTCCA CTTCCTCCGA

---------- ---------- ---------- ---------- ----------

CCGAGCATCA TCGTAGTAAT GGTTCAGCAG CTTACCTCTG CTTCTTCAGA

GCGAGCATCA TCATAGAAGT GAGTGAGCAG CTTACCTCCA CCTCTTTAGA

CTGCATCAGG AGGTTGCATG GCGGCTGCAA GGCCTTTGGC CGGTGAGTGA

CTGCATCAGG AGGTTGCATG GCGGCTGCAA GGCCTTTGGC CGGTGAGTGA

CTGCATCAGG AGGTTGCATG GTGGCTGCAA GGCCTTTGGC CGGTGAGTGA

CTGCATCAGG AGGTTGCATG GTGGCTGCAA GGCCTTTGGC CGGTGAGTGA

CTGCATCAGG AGGTTGCATG GTGGCTGCAA AGCCTTTGGC CGGTGAGTGA

CTGCATCAGG AGGTTGCAGG GAGGCTGCAA GGCCTTTGGC CGGTGAGTGA

CTGCATCAGG AGGTTGCATG GTGGTTGCAA GGCCTTTGGC CGGTGAGTGA

CTGCTTCAGG AGGTTGCATG GTGGTTGCAA GGCCTTTGGC CGGTGAGTGA

---------- ---------- ---------- ---------- ----------

CTGCATCAGG AGATTGCATG GTGGCTTTAA GGCCTTCGGT CGGTGAGTGA

CTGCATCAGG AGGTTGCATG GTGGTTGTAA GGCCTTTGGC CGGTGAGTGA

CTGCATCAGG AGGTTGCACG GTGGTTGCAA CGCCTTTGGC CGGTGAGTGA

CTGCATCAGG AGGTCACATG GCGGCTGCAA GGCCTTGGGC CGGTGAGTGA

CTGCCTCAGG AGGTCACACG GTGGTTGTAA GGCCTTTGGC CGATGAGTGA

CTGCCTCAGG AGGTCACATG GTGGTTGCAA GGCCTTTGGC CGATGAGTGA

---------- ---------- ---------- ---------- ----------

CTGCATCAGG AGGTCACATG GTGGCTTAAC AGCTTTTGGC CGATTAGCAA

CTGCATCAGG AGGTCACAGG GTGGCTTCAG GGCTTTGGGC CGGCTAGCGA

ACCAGTAGGC AAGGATGGCA GCCAGGGCAC CCATACTCAC GAGGGTGGTG

ACCAGTAGGC AAGGATGGCA GCCAGGGCAC CCATACTCAC GAGGGTGGTG

ACCAGTAGGC GAGGATGGCG GCCAGGGCAC CCATACTCAC GAGGGTGGTG

ACCAGTAGGC GAGGATGGCG GCCAGGGCAC CCATACTCAC GAGGGTGGTG

ACCAGTAAGC GAGGATGGCG GCCAGGGCAC CCATACTCAC AAGGGTGGTG

ACCAGTAGGC GAGGATGGCG GCCAGTGCAC CCATACTCAC GAGGGTGGTA

GCCAGTATGC AAGGATGGCA GCCAGTGCAC CCATACTCAC AAGGGTGGTG

GCCAGTAGGC AAGGACGGCA GCCAGTGCAC CCACACTCAC GAGGGTGGTA

---------- ---------- ---------- ---------- ----------

ACCAGTAGGC GAGGATGGCG GCTAGTGCAC CCATACTCAC GAGGGTGGTG

ACCAGTAGGC GAGGATGGCA GCCAGCGCAC CCACACTCAC GAGGGTAGTG

ACCAGTAGGC GAGGATGGCA GCCAACGCAC CCATACTCAC GAGGGTGGTG

ACCAGTAGGC GAGGAGGGCT GCCAGTGCGC CCATACTCAC GAGGGTGGTG

ACCAGTAGGC AAGGATGGCG GCCAGGGCAC CCATACTCAC GAGGGTGGTG

ACCAGTAGGC GAGGATGGCA GCCAGGGCAC CCATACTCAC GAGGGTGGTG

---------- ---------- ---------- ---------- ----------

ACCAATAGGC CAGCAGAGCA GCAAAGGCCC CGATACCCAC TAATGTGGTG

ACCAGTAGGC CAGGAGAGCA GCGAAGGCCC CGATGCCCAC CAGCGTGGTG

GCCGAGAGGC TGCGGAAAAA CTGTCCCAAG TCACCTAGCT CAGGCAGTCG

GCCGAGAGGC TGCGGAAAAA CTGTCCCAAG TCACCTAGCT CAGGCAGTCG

GCCGAGAGGC TGCGGAAAAA CTGGCCTAAG TCGCCTAGCT CAGGCAGTCG

GCCGAGAGGC TGCGGAAAAA CTGGCCTAAG TCGCCTAGCT CAGGCAGTCG

GCCGAGAGGC TGCGGAAAAA CTGGCCCAAG TCGCCTAGCT CAGGCAGCCG

GCTGAGAGGC TGCGGAAAAA CTGGCCCAAG TCACCCAGCT CGGGCAGCCG

GCTGAGAGGC TGCGGAAAAA CTGGCCCAAG TCCGATAGCT CGGGCAGCCG

GCTGAGAGGC TGCGGAAAAA CTGGCCCAAG TCCGATAGCT CGGGCAGCCG

---------- ---------- ---------- ---------- ----------

GCTGAGAGGC TGCGGAAAAA CTGGCCCAAG TCAGCCAGCT CGGGCAGCCG

GCTGAGAGGC TGCGGAAAAA TTGGCCCAAG TCACCCAGCT CGGGCAGCCG

GCTGAAAGAC TGCGGAAAAA CTGACCCAAG TCACCCAGCT CGGGCAGCCG

GCTGAGAAAC TGCGGAAAAA CTGGCCCAGG TCAGCCAGCT CGGGCAGCCG

GCTGAGAGGC TGCGGAAAAA CTGGCCCAAG TCACCCAGCT CGGGCAACCG

GCTGAGAGGC TGCAGAAAAA CTGGCCCAAG TCACCCAGCT CAGGCAGCTG

---------- ---------- ---------- ---------- ----------

GCCGGCAAGT TACGCAAAAA CTGGCTGAAA TCATCCAACT CTGGCAGTCG

GCCGGCAGGC TGCGGAAGAA CTGGCTGAAG TCATCGAACT CGGGCAGCCG

CAGTATCCTC AGGATCTCCT GTGTCTGCAT CTTCTCCAGA AGAATATTTT

CAGTATCCTC AGGATCTCCT GTGTCTGCAT CTTCTCCAGA AGAATATTTT

CAGTATCCTC AGGATCTCCT GTGTCTGCAT CTTCTCCAGA AGaatatttt

CAGTATCCTC AGGATCTCCT GTGTCTGCAT CTTCTCCAGA AGaatatttt

CAGTATCCTC AGGATCTCCT GTGTCTGCAT CTTCTCCAGA AGAACATTTT

CAGTGTCCTC AGGATCTCCT GGGTCTGCAT CTTCTCCAGA AGaatatttt

CAGGATCCTC AGGATCTCCT GGGTCTGCAT CTTCTCCAGA ACCATGTCCT

CAGGATCCTC AGGATCTCCT GGGTCTGCAT CTTCTCCAGA ACCATGCTTT

---------- ---------- ---------- ---------- -Gaatatttt

CAGCATCCTC AGGATCTCCT GAGTCTGCAT CTTCTCCAGA A---------

CAGCAACCTC AAGATCTCCT GTGTCTGCAT TTTCTCCAGA AGaatatttt

CAGCATCCGC AGGATCTCCT GTGTCTGCAT CTTCTCCAGA AGAATATTTT

CAGTATCTTT AGGATCTCCT GTGTCTGCAT CTTCTCCAGA A---------

CAACATCCTC AGGATCTCCT GTGTCTGCAT CTTCTCCAGC AGAATATTTT

CAGCATCCTG AGGATCTCCT GTGTCTGCAT CTTCTCCAGA AGaatatttt

---------- ---------- ---------- ---------- -GACTATTTT

CAGACTCCTC AGGATCTCCT GAGCTTGCAT CTTCTCCAGG AGAATATCtt

CAGGCTCCGC AAGATCTCCT GCGCTTGCAT CTTCTCCAGG AAAATGTCAT

AATTTATGAG TTTTGATAGC tttatttttt aagtatttat atatttataa

AATTTATGAG TTTTGATAGC tttattttta aaatatttat atatttataa

aatttatgag ttttaatagc tttatttttt aaatatttat atatttataa

aatttatgag ttttaatagc tttattttaa aaatatttat atatttataa

AATTTATGAG TTTTTATAGC tttattttta aaatatttat atatttataa

aatttatgag ttttaatagc tttatttttt taatatttat atatttataa

AACTTATGAG TTTTAATGGt tttatttttt taatatttat atatttataa

AACTTATGAA TTTTAATGGt ttta--tttt taatatttat atatttataa

aatttatgaa ttttaatggc tttatttttt taatatttat gtatttataa

---------- ---------- ---------- ---------- ----------

aatttatgag ttttaatagc tttattttaa aaatatttat atatttataa

AATTTATGAG TTTTAATTGC Cttattttaa aaatatttat atatttataa

---------- ---------- ---------- ---------- ----------

AATTTATAAG CTTTAATGCC tttattttaa aaatatttat atatttataa

aatttatgaa ttttaatagc tttatttttt taatatttat atatttataa

AATTTATGCA TT----TAGt tttatttttt taatatttat atatttataa

aatttataag tttcaatagt tatatatttt aaatatttat atatttataa

TATTTATGAG TTTCAAAAGt tatatatttt aaatatttat atatttataa

CTCATCATAA AAAGACTTAC CTTCTCCCTG TCTTTGGCCT TGCTTTCCCC

CTCATCATAA AAAGACTTAC CTTCTCCCTG TCTTTGGCCT TGCTTTCCCC

CTCATCATAA AAAGACTTAC CTTCTCCCTG TCTTTGGCCT GGCTTTCCCC

CTCATCATAA AAAGA----- ---------- ---------- ----------

ctcataataa aaAGACTTAC CTTCTCCCTG TCTTTGGCCT TGCTTTCCCC

tGCATCATAA AAAAACTTAC CTTCTCCCTG TCTTGGGCCG TGCCTTCCCC

ctctt---aa aaataCTCAC CTTCTACCGG TCTTTGGCCT GGCCTTTCCC

ttcat---aa aaATACTCAC CTTCGCCCCA TCTTTGGCCT TGCTTTCCCC

ttCATCATAA AAAGA----- ---------- ---------- ----------

---------- ---------- ---------- ---------- ----------

--cataataa aaaGACTTAC CTTCGCCCTG TCTTTGGCCT TGCTTTCCCC

CTCATCATAA AAAGACTTAC CTTCTCCCTG TCTTTGGCCT TGCTTTTCCC

---------- -----CTCAC CTTCTCCCTG TCTTGGGCCT TGCCTTTCCC

CTCATCATAA AAAAACTTAC CTTCTCCCAG TCTTTGGCCT TGCTTTCCCC

CTCATCATAA AAAGACTTAC CTTCTCCCTG TCTTTGGCCT TCCTTTTCCC

CTCACCCTGA AAAGACTTAC CGTCTCCCGG TCTTGGGCCT TCCTTTCCCC

tgtattataa aaGCTCTTAC CTTCTTCCAG TTTTTGGCCT TGACTTTCCC

GTGACCTTGG AATGActtac cttctccccG TTTTGGGCCT TGACTTTCCT

TTTGAAGTTC GTGGTCTTTC TAGTTTCATC AAAGACTGAC GCAGACTCTC

TTTGAAGTTC GTGGTCTTTC TAGTTTCATC AAAGACTGAC GCAGACTCTC

TTTGAAGTTC GTGGTCTTTC TAGTTTCATC AAAGACTGAC GCAGACTCTC

---------- ---------- ---------- ---------- ----------

TTTGAAGTTC GTGGTCTTTC TAGTTTCATC AAAGACTGAC GGAGACTCTC

TTAGAAGTCC GTGGCCTCTC TAATTTCATC AGAGACTGAC GTAGACTCTC

TTGGAGGTCC GTGGCCTTTC TAACTTCATC AGAGACTGAC GCAAACTCTC

TTGGAGGTCC GTGGCCTTTC TAACTTCATC AGAGACTGAC GCAGACTCTC

---------- ---------- ---------- ---------- ----------

---------- ---------- ---------- ---------- ----------

TTAGAAGTCC GTGGTCTCTC TAATTTCATC AAGGACTGCC GGAGACTCTC

TTTGAGGTCC GTGGCCTCTC CAATTTCATC AAGGACTGCC GCAGGCTCTC

TTTGAAGTTC GTGGCCGTTC TAATTTCATC AAAGACTGTC GCAGACTCTC

TTTGAAGTCC GTGGCCTTTC TAATTTCATC AAGGACTGCC GTAAGCTCTC

TTTGAAGTCC GTGGCCTTTC TAATTTCATC AAGGACTGCC GCAGGCTTTC

TTTGAAGTCC GTGGCCTTTC TAACTTCATC AGGGACTGCC GCAGACTCTC

TTTGAAGTGC GTGGCCTCTC TAATTTCATC AAAGATTGTC TGAGGCTTTC

TTCGAAGTCC TTGGCCTCTC CAGTTTCATC AGAGACTGCC TCAGACTTTC

CTCAGTATAG GCAAGATACA CGTGAGAAAG GTCCACCTCA AAGGGCTAAT

CTCAGTATAG GCAAGATACA CGTGAGAAAG GTCCACCTCA AAGGGCTAAT

CTCAGTATAG GCAAGATACA CATGAGAAAG GTCCACTTCA AAGGGC---T

---------- ---------- ---------- ---------- ----------

CTCAGTATAG GCGAGATACA CGTGAGAAAG GTCTACTTCA AAGGGCTAAT

CTCAGTATAG GCAAGATACA CGTGGGAAAG GTCCACTTCA AAGGGCTAAG

CTCTGTATAG GCGAGGTACA CGTGGGAAAG GTCTACTTCA AATGGC---T

CTCTGTATAG GCGAGGTACA CGTGGGAAAG GTCGACTTCA AATGGC---T

---------- ---------- ---------- ---------- ----------

---------- ---------- ---------- ---------- ----------

CTCAGTGTAG GCAAGATACA CGTGGGAAAG GTCCACTTCA AAGGGCTAAT

CTCAGTATAG GCAAGATACA CGTGGGAAAG GTCCACTTCA AAGGGCTAAA

TTCAGTATAG GCGAGATACA CATGAGAAAG GTCCACTTCA AAGGGCTAAG

CTCGGTATAA GCAAGATACA CATGGGAAAG GTCTACTTCA AAGGGCTAAT

CTCTGTGTAG GCGAGATACA CATGAGAAAG GTCCACTTCA AAGGGCTAAT

CTCTGTGTAG GCGAGATACA CATGGGAAAG GTCCACCTCG AAGGGCTACG

CTCAGTATAA GCTAAATACA CATGGGAAAG GTCTACTTCA AAAGGCTAAT

TTCTGTGTAG GCCAAGTACA CATGAGAAAG GTCCACTTCA AAGGGCTAAA

AAAAGAAACT TACATCTTTC TCCTTTTTAT CAGGTACTGG GGTTTGCTTC

AAAAGAAACT TACATCTTTC TCCTTTTTAT CAGGTACTGG GGTTTGCTTC

AAAAGAAACT TACATCTTTC TCCTTTTTAT CAGGTACTGG GGTTTGCTTC

---------- ---------- ---------- ---------- ----------

AAAAGAAACT TACATCTTTT TCCTTTTTAT CAGGTACTGG GGTTTGCTTC

AAAAGAAACT TACATCTTTC TCCTTTTTAT CAGGTACTGG GGTTTGCTTC

ACAAGAAACT TACGTCTCTC TCCTTCTTGT CAGGGACTGG AGTTTGTTTC

ACAAGAAACT TACGTCTCTC TCCTTCTTGT CAGGGACTGG AGTTTGTTTC

--------CT TACATCTTTC TCCTTTTTAT CAGGTACTGG AGTTTGCTTC

---------- ---------- ---------- ---------- ----------

AAAGGGAACT CACATCTTTC TCTTTTTTAT CAGGAGCTGG AGTCTGCTTC

AAAGGAGACT TACATCTTTC TCTTTTTTAT CAGGAACTGG AGTCTGCTTC

AAAGGAAACT TACATCTTTC TCTTTTTTAT CAGAAACTGG AGTCTGTTTC

AAAAGAAACT TACATCTTTT TCCTTTTTAT CAGGTACTGG AGTTTGCTTC

AAAAGAAACT TACATCTTTC TCCTTTTTAT CAGGTACTGG GGTCTGCTTC

AAAAGAAACT CACATCTTTC TCCTTTTTGT CAGGTACAGG GGTCTGTTTC

AAAAGAAACT TACATCTTTT TCCTTTTTAT CAGGTACTGG AGTCTGCTTT

AAAATAAACT TACATCCTTC TCCTTTTTGT CAGGTATTGG AGTCTGCTTT

CTCATGTTAT TTCCTGTATA AGCAACACAT TTCTCAAAAA ATACTCACTA

CTCATGTTAT TTCCTGTATA AGCAACACAT TTCTCAAAAA ATACTCACTA

CTCATGTTAT TTCCTGTATA GGCAACACAT TTCTCAAAAA ATACTCACTA

---------- ---------- ---------- ---------- ----------

CTCATGTTAT TTCCCGTGTA GGCAACACAT TTCTCAAAAA ATACTCACTA

CTCATGTTAT TTCCTGTGTA TGCAACACAT TTCTCAAAAA ATACTCACTA

CTCATGTTAT TTCCTGTGTA GGCCACACAT TTCT--GAAA ATACTCACCA

CTCATGTTAT TTCCTGTGTA GGCCACACAT TTCTTAAAAA ATACTCACTA

CTCATGTTAT TCCCTGTGTA GGCAACACAT TTCTCAAAAA ACACTCACCA

---------- ---------- ---------- ---------- ----------

CTCATGTTAT TTCCTGTGTA AGCAACACAT TTCTCAAAAA ATACTCACTA

CTCATGTTAT TTCCTGTGTA GGCAACACAT TTCTCAAAAA ATACTCACTA

CGCATATTAT TTCCTGTATA GGCAACACAT TTCTAAAAAA ATACTCACTA

CTCATGTTAT TTCCTGTGTA GGCAACACAT TTCTCAAAAA ATACTCACTA

CTCATGTTAT TTCCTGTGTA GGCAACACAT TTCTCAAAAA ATACTCACTA

CTCATGTTAT TTCCTGTATA AGCAACACAT TTCTCAAAAG ATACTCACTA

CTCATATTAT TTCCTGTGTA GGCAACACAT TTCTTGAAAA ATACTTAACA

CTCATGTTAT TTCCCGTGTA TGCAACGCAT TTCTAAATGG CTGCTTACCA

GCTGCCATTC TCCTATGTCT TCAACATAGT TTTCATCATT TCCTTTTAAA

GCTGCCATTC TCCTATGTCT TCAACATAGT TTTCATCATT TCCTTTTAAA

GCTGCCATTC TCCTATGTCT TCAACATAGT TTTCATCATT TCCTTTTAAA

---------- ---------- ---------- ---------- ----------

GTTGCCATTC TCCTATGTCT TCAACGTAGT TTTCATCATT TCCTTTTAAA

GCTGCCATTC TCCTATGTCT TCAACATAGT TTTCATCATT TCCTTTCAAA

GCTGCCATTC TCCTATGTCT TCAACGTAGT TTTCATCATT TCCTTCAAAA

GCTGCCATTC TCCTATGTCT TCAACGTAGT TTTCATCATT TCCTTCAGAA

ACTGCCATTC TCCAATGTCT TCAACATAAT TTTCATCATT TCCTTTAAAA

---------- ---------- ---------- ---------- ----------

GCTGCCATTC TCCTATGTCT TCAACATAGT TTTCATCATT TCCTTT--AA

GCTGCCATTC TCCTATGTCT TCAACATAGT TTTCATCATT TCCTTTTAAA

GCTGCCATTC TCCTATGTCT TCAACATAGT TTCCATCATT TCCTTTTAAA

ACTGCCATTC TCCTATGTCT TCAACATAGT TTTCATCATT TCCTTTTAAA

GCTGCCATTC TCCTATGTCT TCAACATAGT TTTCATCATT TCCTTTTAAA

GCTGCCATTC TCCTATGTCT TCAACATAAT TTTCATCATT TCCTTTTAAA

GCCATCAAAA TACTCTATTT TCAACATTTT TTTCATAATT TGAATTTTTA

ACTGCCATTC TCCGATGTCT TCAACATAAT TTTCATCATT TCCTGGGGAG

ATACCTGATA ATCCCGAGGT ATAAAGTTAT CAATAATAAG CATCTGAAGT

ATACCTGATA ATCCCGAGGT ATAAAGTTAT CAATAATAAG CATCTGAAGT

ATACCTGATA ATCCCGAGGT ATAAAATTAT CAATAATAAG CATCTGAAGT

---------- ---------- ---------- ---------- ----------

ATACCTGATA ATCCCGAGGT ATAAAGTTAT CAATAATAAG CATCTGAAGT

ATACCTGATA ATCCTGAGGT ATAAAGTTAT CAATAATAAG CATCTGAAGT

ACACCTGGTA GTCCTGAGGT ATGAAGTTAT CAATGATGAG CATCTGAAGT

ACACCTGGTA ATCCTGAGGT ATGAAGTTAT CAATGATGAG CATCTGAAGA

A--------- ---------- ---------- ---------- ----------

---------- ---------- ---------- ---------- ----------

ATACCTGATA ATCTTGAGGT ATAAAGTTAT CAATGATAAG CATCTGAAGT

ATACCTGATA ATCCTGAGGT ATAAAGTTAT CGATAATGAG CATCTGAAGT

ATACCTGATA ATCCTGGGGT ATAAAGTTAT CAATAATGAG CATCTGAAGT

ATACCTGATA ATCCTGAGGT ATAAAGTTAT CAATAATAAG CATCTGAAGT

ATACCTGATA ATCCTGAGGT ATAAAGTTAT CAATAATAAG CATCTGAAGT

ATACCTGGTA ATCCTGAGGT ATAAAGTTAT CGATGATAAG CATCTGAAGT

ATACCTGATA ATCCTGAGGA ATAAAGTTGT CAATAATAAG CATCTGAAGG

GTACCTGGTA GTCGTGAGGA ATGAAGTTGT CTATGATAAG CATTTGGAGT

CGAAGCTCCC GGCTAAGTTG CCGAATGTTC TCCAGTAGGC CTTCAATTTC

CGAAGCTCCC GGCTAAGTTG CCGAATGTTC TCCAGTAGGC CTTCAATTTC

CGAAGCTCCC GGCTAAGTTG CCGAATATTC TCCAGTAGGC CTTCAATTTC

---------- ---------- ---------- ---------- ----------

CGAAGCTCCC GGCTAAGTTG CCGAATATTC TCCAGTAGGC CCTCAATTTC

CGAAGCTCCC GGCTAAGCTG CCGAATGTTC TCCAGAAGGC CTTCAATTTC

CGGAGCTCAC GGCTGAGCTG CCGAATGTTC TCCAGGAGGC CTTCAATTTC

CGGAGCTCCC GGCTGAGCTG CCGGATGTTC TCCAGGAGGC CTTCGATCTC

---------- ---------- ---------- ---------- ----------

---------- ---------- ---------- ---------- ----------

CGGAGCTCCC GGCTAAGTTG TCGAATATTC TCCAGTAGGC CTTCAATTTC

CGAAGCTCCC TGCTAAGTTG TCGAATGTTC TCCAGCAGGC CTTCAATTTC

CGAAGTTCCC GACTAAGTTG TCGGATATTT TCCAATAGTC CTTCAATTTC

CGAAGCTCCC GGCTAAGTTG TCGAATGTTC TCCAGTAGGC CTTCAATTTC

CGAAGCTCCC GGCTAAGTTG TCGAATGTTC TCCAGTAGGC CTTCAATTTC

CGAAGCTCCC GACTAAGCTG TCGGATGTTC TCCAGTAAAC CTTCTATTTC

CGAAGTTCTC TGCTGAGCTG TCTAATGTTT TCCAGAAGGC CTTCAATTTC

CTCAGTTCCC TGCTGAGCTG TCGGATGTTC TCCAGGAGGC CTTCAATTTC

CCTCTGATGT TCTTGTTGGA GATCAGCCAT CTAACCAACC TCTGACTTTG

CCTCTGATGT TCTTGTTGGA GATCAGCCAT CTAACCAACC TCTGACTTTG

CCTCTGATGT TCCTGTTGAA GATCAGCCAT CTAACCAACC TCTGATTTTG

---------- ---------- ---------- ---------- ----------

CCTCTGATGT TCTTGTTGGA GATCAGCCAT CTAACCAACC TCCGACTTTG

CCTCTGATGT TCCTGTTGAA GATCAGCCAT CTAACCAACC TCTGACTTTG

CCTCTGATGC TCTTGCTGGA GATCAGCCAT CTAACCAACC TCTGACTTTG

CCTCTGATGC TCCTGCTGGA GATCAGCCAT CTAATCAACC TCTGACTTTG

---------- ---------- ---------- ---ACTGACC TCCGACTTTG

---------- ---------- ---------- ---------- ----------

CCTCTGATGC TCCTGTTGGA GATCAGCCAT CTAACCAACC TCTGATTTTG

CCGCTGGTGT TCCTGTTGGA GATCAGCCAT CTAACCAACC TCTGACTTTG

TCTCTGATGT TCTTGTTGAA GATCAGCCAT CTA------- ----------

TCGCTGATGT TCTTGCTGGA GATCGGCCAT CTAACCAACC TCTGACTTTG

TCTCTGATGT TCTTGTTGGA GATCGGCCAT CTAACCAACC TCTGACTTTG

CCTCTGATGT TCTTGCTGGA GATCCGCCAT CTAACCAACC TCAGATTTTG

CCTCTGATGT TCTTGTTGAA GATCTGCCAT CTGACTAACC TCTGACTTTG

TCGCTGATGT TCTTGCTGGA GATCTGCCAT CTGACTGACC TCTGACTTTG

CAGCCATCAG CATAGTCCAA ACTTTCTTTA ACTTCTTGGT CTTTCCCTGT

CAGCCATCAG CATAGTCCAA ACTTTCTTTA ACTTCTTGGT CTTTCCCTGT

CAGCCATCAG CATAGTCCAA ACTTTCTTTA ACTTCTTGGT CTTTCCCTGT

---------- ---------- ---------- ---------- ----------

CAGCCATCAG CATAGTCCAC ACTTTCTTTA ATTTCTTGGT CTTCCCCTGT

CAGCCATCAG CATTGTCCAA ACTTTCTTTA ACTTCTTAGT CTTTCCCTGT

CAGCCATCAG CATGGTCCAG ACTTTCTTTA ATTTCTTGGT CTTTCCTTGT

CAGCCATCAG CATGGTCCAG ACTTTCTTTA ATTTCTTGGT CTTTCCTTGT

CAGCCATCAG CATTGTCCAC ACTTTCTTTA ATTTCTTGGT CTTTCCCTGT

---------- ---------- ---------- ---------- ----------

CAGCCATTAG CATAGTCCAA ACTTTCTTTA ATTTCTTGGT TTTTCCCTGT

CAGCCATAAG CATAGTCCAA ACTTTCTTTA ATTTCTTGGT CTTTCCCTGT

---------- ---------- ---------- ---------- ----------

CAGCCATCAG CATAGTCCAA ACTTTCTTCA ATTTCTTCGT CTTTCCCTGA

CAGCCATCAA CATAGTCCAA ACTTTCTTTA ATTTCTTCGT CTTTCCCTGA

CGGCCATCAG CATAGTCCAG ACTTTCTTTA ACTTCTTGGT CTTTCCCTGA

CAGCCATTAG CATGGTCCAA ACTTTCTTCA ACTTCTTGGT CTTTCCCTGA

CAGCCATTAG CATCGTCTTT ATTTTCTTTA ATTTCTTGGT CTTGCCCTGA

GCTTCCTCTT GCAAACTGGT ATATTTTTCT TCAATATCCA AGCGTTCTTG

GCTTCCTCTT GCAAACTGGT ATATTTTTCT TCAATATCCA AGCGTTCTTG

GCTTCCTCTT GCAAGCTGGT ATATTTTTCT TCAATATCCA AGCGTTCTTG

---------- ---------- ---------- ---------- ----------

GCTTCCTCTT GCAAACTGGT ATATTTTTCT TCAATATCCA AACGTTCTTG

GCTTCCTCTT GCAAACTGGT ATATTTTTCT TCAATATCTA AGCGTTCTTG

GCCTCCTCTT GCAGACTGGT ATATTTTTCC TCGATGTCCA AGCGTTCTTG

GCCTCCTCTT GCAGACTGGT ATATTTTTCC TCGATGTCCA AGCGTTCTTG

GCTTCTTCTT GCAAACTGGT ATATTTTTCT TCAATATCCA AGCGTTCTTG

---------- ---------- ---------- ---------- ----------

GCTTCCTCTT GCAAGCTGGT ATATTTTTCT TCAATATCCA AGCGTTCTTG

GCTTCTTCTT GCAAACTGGT ATATTTTTCT TCAATATCCA AGCGTTCTTG

---------- ----ACTTGT TTGTTTTATT ATATTATTTA AGGCTTTTGG

GCCTCCTCTT GTAAACTGGT ATATTTTTCT TCAATATCCA AGCGTTCTTG

GCTTCCTCTT GTAAATTGGA AAACTTTTCT TCATTAAATC ATTATTCTAT

GCTTCCTCTT GCAAACGGGC ACGCTTTTCT TCACTAAATC ATCATTTTAT

GCTTCTTCCT GTAAACTAGT ATATTTCTCT TCAATATCCA GGCGTTCTTG

GCTTCCTCCT GCAGACTGGT GTATTTCTCT TCGATATCCA AACGTTCTTG

CTATGACAAA AATACCTCTT TTTCCTCAAG TTCTCTGCGA AGTTGCTCTG

CTATGACAAA AATACCTCTT TTTCCTCAAG TTCTCTGCGA AGTTGCTCTG

CTATGACAAA AATACCTCTT TTTCCTCAAG TTCTCTGCGA AGTTGCTCTG

---------- ---------- ---------- ---------- ----------

CTATGAAAAA AATACCTCTT TTTCCTCAAG TTCTCTGCGA AGTTGCTCTG

CTATGATAAA AATACCTCTT TTTCTTCAAG TTCTCTGCGA AGTTGCTCTG

CTATGATAGA AACACCTCTT TTTCCTCTAG TTCTTTCCGA AGTTGCTCAG

CTGTGATAAA AACACCTCCT TTTCCTCCAG CTCTTTCCGA AGCTGCTCAG

CTATGGCAAA AATACCTCTT TCTCCTCGAG TTCTTTGCGA AGTTGCTCTG

---------- ---------- ---------- ---------- ----------

CTGTGACAAA AATACCTCTT TTTCCTCAAG TTCTTTGCGA AGTTGCTCTG

CTATGACAAA AATACCTCTT TTTCCTCAAG TTCTCTGCGA AGTTGCTCGG

TGATGA-GCA GGTACCTCTT TTTCTTCAAG TTCTCTGCGA AGCTGCTCTG

CTATGACAAA AATACCTCTT TTTCCTCAAG TTCTCTGCGA AGCTGTTCGG

AAGTGATACA AATACCTCTT TTTCCTCAAG TTCTCTGCGA AGCTGCTCTG

TGATGGTACA AATACCTCTT TTTCCTCAAG CTCTCTGCGG AGCTGTTCTG

CTAAGGCAAA AATACCTCTT TTTCCTCAAG CTCTCTTCGA AGTTGTTCTG

CTGTGACAAA AATACCTCTT TTTCCTCCAG CTCTCTGCGA AGTTGCTCTG

CTCTTTTCCT CCTTTCTTCC AGTTCCATGT TAGATTCTTC AAGAAGTTTC

CTCTTTTCCT CCTTTCTTCC AGTTCCATGT TAGATTCTTC AAGAAGTTTC

CTCTTTTCCT CCTTTCTTCC AGTTCCATGT TAGATTCTTC AAGAAGTTTC

---------- ---------- ---------- ---------- ----------

CTCTTTTCCT CCTTTCTTCC AGTTCCATGT TAGATTCTTC AAGAAGTTTC

CTCTTTTCCT TCTTTCTTCC AGTTCCATAT TAGATTCTTC AAGAAGTTTC

CTCTTTTCCT CCGCTCCTCC AGCTCCATAT TGGACTCCTC GAGAAGCTTC

CTCGTCTCCT CCTCTCCTCC AGCTCCATGT TGGACTCCTC CAGAAGCTTC

CTCTTTTCCT CCTTTCTTCC AGCTCCATAT TAGATTCTTC AAGAAGTTTT

---------- ---------- ---------- ---------- ----------

CTCTTTTCCT CCGTTCTTCC AGTTCCATAT TAGATTCTTC AAGAAGTTTC

CTCTCTTCCT CCTTTCTTCC AGTTCCATAT TAGATTCTTC AAGCAGTTTC

CTCTTTTCCT CCTTTCTTCC AGTTCCATAT TGGATTCTTC AAGAAGTTTT

CTCTTTTCCT CCTTTCTTCC AGTTCCATAT TAGATTCTTC AAGAAGTTTC

CTCTTTTCCG CCTTTCTTCC AATTCCATAT TAGATTCTTC AAGAAGTTTC

CTCTTTTCCG CCTTTCTTCT AGCTCCATGT TAGATTCTTC AAGAAGTTTC

CTCTTTTCCT TCTTTCTTCT AGTTCCATGT TAGATTCCTC TAGAAGTTTC

CTCTTTTCCG CCTTTCCTCC AGCTCCATGT TGGATTCTTC CAGAAGCTTC

TCTTGTTCCT CAGCTTTGGC CAACAAGTCA ACCCCACCAA CAATTACCTT

TCTTGTTCCT CAGCTTTGGC CAACAAGTCA ACCCCACCAA CAATTACCTT

TCTTGTTCCT CAGCTTTGGC CAACAAATCA ACCCCACCAA CAATTACCTT

---------- ---------- ---------- ---------- ----------

TCTTGTTCCT CAGCTTTGGC CAACAAATCA ACCCCACCAA CAATTACCTT

TCTTGTTCCT CTGCTTTGGC CAACAAATCA ACACCACCAA CAATTACCTT

TCTTGCTCCT CAGCTTTGGC CAACAAGTCC ACACCACCGA CAATCACCTT

TCTTGCTCCT CAGCCTTGGC CAGCAGGTCC ACGCCGCCGA CAATGACCTT

TCTTGTTCTT CAGCTTTGGC CAACAAATCA ACACCACCAA CAATGACCTT

---------- ---------- ---------- ---------- ----------

TCTTGTTCCT CAGCTTTTGC CAACAAATCA ACACCACCAA CAATTACCTT

TCTTGTTCCT CAGCTTTCGC CAACAAATCA ACACCCCCAA CAATCACCTT

TCTTGTTCCT CGGCTTTTGC TAATAAATCT ACACCACCAA CAATCACCTT

TCTTGTTCCT CAGCTTTTGC TAACAAATCA ACACCACCAA CAATGACCTT

TCTTGTTCCT CAGCTTTTGC CAACAAATCA ACACCACCAA CAATGACCTT

TCTTGTTCCT CGGCTTTCGC CAGCAAATCA ACACCACCAA CAATCACCTT

TCTTGCTCCT CAGCTTTAGC CAACAAGTCA ACACCACCAA CAATTACCTT

TCTTGCTCCT CAGCCTTGGC CAACAAATCC ACACCACCAA CAATCACCTT

CTTTTCCAGG GCAGATAATT TTTCCAGCAA AGACTGATGC TCTTGTCTGT

CTTTTCCAGG GCAGATAATT TTTCCAGCAA AGACTGATGC TCTTGTCTGT

CTTTTCCAGG GCAGATAATT TTTCCAGCAA AGACTGATGC TCTTGTCTGT

---------- ---------- ---------- ---------- ----------

CTTTTCCAGG GCAGACAATT TTTCCAGCAA AGACTGATGT TCTTGTCTAT

CTTCTCCAGC GCAGATAATT TTTCTAGCAA AGACTGATGC TCTTGTCTGT

CTTCTCCAGC GCAGACAGTT TTTCCAGCAA AGACTGATGC TCTTGTCTGT

CTTTTCCAAA GCAGACAGCT TTTCCAGCAA AGACTGATGC TCTTGTCTAT

CTTCTCCAGT GCAGATAATT TTTCCAGCAA AGACTGATGC TCTTGTCTGT

---------- ---------- ---------- ---------- ----------

CTTCTCCAGG GCAGATAATT TCTCTAGCAA AGACTGATGT TCTTGTCTGT

CTTCTCCAGT GCAGATAATT TCTCTAACAA AGACTGATGC TCCTGTCTGT

CTTCTCCAAT GCAGATAACT TCTCTAGCAA AGTCTGATGC TCTTGTCTGT

CTTTTCAAGA GCAGACAACT TTTCCAACAA AGACTGATGC TCTTGTCTGT

CTTCTCCAGT GCAGATAACT TTTCCAGCAA AGACTGATGC TCTTGTCTGT

CTTCTCCAGT GCAGATAGC- TTTCCAGCAA AGACTGATGC TCTTGCCTGC

CTTTTCCAGT GCAGATAGTT TTTCCAAAAG TGACTGATGC TCTTGTCTAT

CTTCTCCAGG GCAGACAATT TTTCCAGCAA TGACTGATGT TCCTGTCTGT

TACTCACTGG GCTTTAAGAA GATCTTTTTC CCGTTTCTCT AATTCAGCTC

TACTCACTGG GCTTTAAGAA GATCTTTTTC CCGTTTCTCT AATTCAGCTC

TACTCACTGG GCTTTAAGAA GATCTTTTTC TCGTTTCTCT AATTCAGCTC

-ACTCACTGG GCTTTAAGAA GATCTTTTTC TCGTTTCTCT AATTCAGCTC

TACTCACTGG GCTTTAAGAA GATCTTTTTC CCGTTTCTCT AATTCAGCTC

TACTCACTGG GCTTTAAGTA GATCTTTTTC CCGTTTCTCT AGTTCAGCTC

-ACTCACTGG GCTTTCAGAA GGTCCTTCTC CCGTCGCTCC AGCTCAGCTC

-ACTCACTGG GCTTTCAGAA GGTCCTTCTC CCGTCTCTCT AGTTCAGCTC

---------- ---------- ---------- ---------- ----------

---------- ---------- ---------- ---------- ----------

TACTCACTGG GCCTTAAGAA GATCTTTCTC CCGTTTCTCT AATTCAGCTC

TACTCACTGG GCCTTAAGAA GATCCTTTTC CCGTTTCTCT AATTCAGCTC

TACTCACTGG GCCTTAAGAA GATCTTTTTC CCGTTTCTCT AATTCAGCTC

TACTCACTGA GCCTTAAGAA GATCTTTTTC CCGTTTCTCT AATTCAGCTC

TACTCACTGA GCCTTAAGAA GATCTTTTTC TCGTTTCTCT AACTCAGCTC

TACTCACTGC GCCTTAAGAA GATCCTTTTC TCGTTTCTCC AGCTCCGCTC

TACTCACTGG GCTTTAAGAA GGTCCTTCTC CCTTTTTTCC AGTTCAGCTC

TACTCACTGC GCTTTAAGCA GATCTTTCTC CCGTCTCTCC AGCTCAGCTC

TAGCCTTGTT TCTTTCTTCT TCTTCCATGT CGAGCTTTGT TTCAAGTGCT

TAGCCTTGTT TCTTTCTTCT TCTTCCATGT CGAGCTTTGT TTCAAGTGCT

TAGCCTTGTT TCTTTCTTCT TCTTCCATGT CAAGCTTTGT TTCAAGTGCT

TAGCCTTGTT TCTTTCTTCT TCTTCCATGT CGAGCTTTGT TTCAAGTGCT

TAGCCTTGTT TCTTTCTTCT TCTTCCATGT CAAGCTTTGT TTCAAGTGCT

TAGCCTTGTT TCTTTCTTCT TCTTCCATGT CAAGCTTTGT TTCAAGTGCT

TAGCCTTGTT TCTTTCTTCT TCCTCCATGT CGAGTTTGGT TTCCAGTGCT

TAGCCTTGTT TCTTTCTTCT TCTTCCATGT CAAGTTTGGT TTCCAGTGCT

---------- ---------- ---------- ---------- ----------

---------- ---------- ---------- ---------- ----------

TAGCCTTGTT TCTTTCTTCT TCTTCCATAT CAAGCTTTGT TTCAAGTGCT

TAGCCTTGTT TCTTTCTTCT TCTTCCATAT CGAGCTTTGT TTCAAGTGCT

TAGCCTTATT CCTTTCTTCT TCTTCCATGT CAAGCTTTGT TTCA------

TAGCCTTGTT TCTTTCTTCT TCTTCCATGT CAAGCTTTGT TTCAAGTGCT

TAGCTTTGTT TCTTTCTTCT TCCTCCATGT CAAGCTTTGT TTCAAGTGCT

TAGCCTTGTT CCGTTCTTCT TCCTCCATGT CGAGCTTTGT TTCAAGTGCT

TGGCTTTATT TCTTTCTTCT TCTTCCATAT CAAGTTTGGT TTCAAGTGCT

TAGCTTTGTT TCTTTCTTCT TCTTCCATGT CGAGTTTGGT CTCAAGGGCC

TTTCTCTCCT CATCAATTTT TGCTTGCATT TCAATCATCT TGTCTGGGGA

TTTCTCTCCT CATCAATTTT TGCTTGCATT TCAATCATCT TGTCTGGGGA

TTTCTCTCCT CATCAATTTT TGCTTGCATT TCAATCATCT TGTCTGGGGA

TTTCTCTCCT CATCAATTTT TGCTTGCATT TCAATCATCT TGTCTGGGGA

TTTCTCTCCT CATCAATTTT TGCTTGCATT TCAATCATCT TATCTGGGGA

TTTCTCTCTT CATCAATTTT TGCTTGCATT TCAACCATTT TATCTGGAGA

TTTCTCTCCT CATCAATTTT CGCTTGCATT TCCATCATCT TATCTGGGGA

TTTCTCTCCT CGTCAATTTT CGCTTGCATT TCCACCATCT TATCTGGGGA

---------- ---------- ---------- ---------- ----------

---------- ---------- ---------- ---------- ----------

TTTCTCTCCT CATCAATTTT TGCTTGCATT TCAACCATCT TATCTGGGGA

TTTCTCTCCT CATCAATTTT TGCTTGCATT TCAACCATCT TATCAGGGGA

---------- ---------- ---------- ---------- ----------

TTCCTCTCCT CATCAATTTT TGCTTGCATT TCAACCATCT TATCTGGGGA

TTCCTCTCCT CATCAATTTT TGCTTGCATT TCAACCATCT TATCTGGAGA

TTCCTCTCCT CTTCAATTTT TGCTTGCATT TCAACCATCT TATCTGGAGA

TTCCTTTCCT CATCAATTTT TGCTTGCATT TCAACCATCT TATCTGGAGA

TTCCTCTCCT CGTCGATCTT AGCTTGCATC TCCACCATCT TGTCTGGGGA

GACTTTCTTT TTTCCTATTT GAATCATTTA AGCTTTAAAC GTGATGAAAC

GACTTTCTTT TTTCCTATTT GAATCATTTA AGCTTTAAAC GTGATGAAAC

TACTTTCTTT TTTCCTATTT GAATCATTTA AGCTTTAAAC GTTATGAAAC

TACTTTCTTT TTTCCTATTT GAATCATTTA AGCTTTAAAC GTTATGAAAC

GACTTTCTTT TTACCTATTT GAATCATTTA AGCTTTAAAC ----------

AACTTTCTTT TTACCTATTT GAGTCATTTA ---------- GTTATGTGAC

AACTTTCTTT TTACCTATTT GAGTCATGTA AACTTTAAAA GTTCTGAAAC

AACTTTCTTT TTACCTATTT GAGTCATGTA ACCTC----G GTTCTGAAAC

---------- ---------- ---------- ---------- ----------

---------- ---------- ---------- ---------- ----------

CACTTTCTTT TTACCTATAT GAGTTATTTA AGGTTTAAAC GTTATGAAA-

AACTTTCTTT TTACCTATTT GAGTCATTTA AGCATTAAAC GTTATGAAAC

---------- ---------- ---------- AGCTTGAAAC GTTAAGAAAC

AACTTTCTTT TTACCTAATT GAGTTGTTTA AGCCTCAAAC GTAATGAAAC

AACTTTCTTT TTACCTATTT GAGTTATTTA AGCTT----- ---ATAAAAC

AACCTTCTTT TTACCTATGT AAGTTATTTA AGCCTTAAAC GTTATTAAAC

AACCTTCTTT TTACCTATGT GAGTTATTTA AACTTGAAGT GTTATGAAAA

AACCTTCTTT TTACCTATTT GAGTTATTTA AGCTCTAAAG ATTATGAAAC

TGAAGACAAT TCATTTCTGG TCATACAGTT TCGAAACATA TTCCTTCACA

TGAAGACAAT TCATTTCTGG TCATACAGTT TCCAAACACA TTCCTTCACA

TGAAGACAAT TAATTTCTGG TCATACAGTT TCGAAACATA TTCCTTCACA

TGAAGACAAT TCATTTCTGG TCATACAGTT TCGAAACATA TTCCTTCACA

---------- ---------- ---------- ---------- ----------

TGAAAAC--- --ATTTCTAG TAATATAATT TCGAAACATA TTCCTTCACA

TGATGACAAT TCATTTGTGG TTATGCAAGT TCGAAATGTG TTCCTTCA--

TGATGACAAT TCATTTGTGG TTATGCAAGT TCGAATTGTG TTCCTTC---

---------- ---------- ---------- ---------- ----------

---------- ---------- ---------- ---------- ----------

TGAATAT-AT TCATTTCTGG TAATACAACT TCGAAACATT TTTCTGTACA

TGAAGATAAT TCATTTCTGG TAATACAATT TCGAAGCATT TTTCCTTACA

AGAAGATGAT TTACTTTTTA TAACACAA-- -CGAAACATT TTCCTTCACA

TAAAGATAAT TCATTTCTGG TAATACTATT TCGAAACAGT TTCCTTCACA

TGAAGA---- TAATTTCTGG TAATGCAATT TCGAAACATT TTCCTTCACA

TGAAAAAAAT TCACTTCTGG TACTGCAATC TCGAAAAATT TTCCTTC---

AGAAGAAAAT CCAATCTTAG TTACACAATT TTGAAAGATT TTCCTTCGTA

TGAAGAAAAG TTTCTTCTTA GAACGTAAGT TCGAAACATG TCCCTCTAAA

AATGAAATAA AAGAGTAAAT AAAAGCCTGA TCCAATTCAC TTTGGTGTGG

AATGAAATAA AAGAGTAAAT AAAAGCCTGA TCCAATTCAC TTTGGTGTGG

AATGAAACAA AAGAGTAAAT AAAAGCCTGA TCCAATTCAC TTTGGTGTGG

AATGAAACAA AAGAGTAAAT AAAAGCCTGA TCCAATTCAC TTTGGTGTGG

---------- ---------- ---------- ---------- ----------

AATTAAATAA AAGAGTAAAT AAAAGCCTGA TTCAATTCAC TTTGGTGTGG

-------CCA AAGGGGAGCT GAGAGCGTGA TCCAGC---G GCTGGTGTGG

-------CCA ACGAGGAGCT CAGAGCGGGA CCCAG----G GCTGGGGTGG

---------- ---------- ---------- ---------- ----------

---------- ---------- ---------- ---------- ----------

AGTAAAA-AT AAGAGTAAAT AAGAGCCTGA TCCAATTCAC TTTGGTGTGG

AATAAAATAA AAGAGTAAAT AAAAGCCTGA TCCAATTCAC TTTGGTGTGG

AATAAAATAA AAGAGTAAAT CAAAGCTTGA TCCAATTCAC TCTGGTGTGG

AATAAAATAA AAGATGAAAT AAAAGCCTGA TCCAATTCAC TCTGGTGTGG

CATAAAATAA AAGATTAAAT AAAAGCCTGA TCCAATTCAC TCTGGTGTGG

---------- ---------- ---------- ---------- ----------

TATTACATAT AAAAGCAGAC TAAAATTTGA ATTAATGAAA TTTGGGTTAG

AAAAAAATGA TAAATGAAAT GACAGTTTGA TTCAACCCCT TTTGGTGTGG

TTGATTCATT GCATTCAAAG TCAGGATATA CTTACCTGGT AAAAAAACTA

TTGATTCATT GCATTCAAAG TCAGGATATA CTTACCTGGT AAAAAAACTA

TTGATTAATT GCATTCAAAG TCAGGATATA CTTACCTGGC aaaaaaagta

TTGATTAATT GCATTCAAAG TCAGGATATA CTTACCTGGC aaaaaaagta

---------- ---------- ---------- ---------- ----------

TTGATTCATT GCATTCAAAG TCAGGATATA CTTACCTTGC -aaaaaatta

TTGATTCACT GCACTC---- -CCCGAGATA CTTACCTTGt aaaaa--tta

TTGATTCACT GCACTC---G GCCCGAGATA CTTACCTTGt aaaaa--tta

---------- ---------- -------ATA CTTACCTTGt aaaaaaagta

---------- ---------- ---------- ---------- ----------

TTGATTCATT GCATTCAAAG TCAGGATATA CTTACCTTGt aaaaaaacta

TTGATTCATT GCATTCAAAG TCAGGATATA CTTACCTTGt aaaaa---ta

TTGATTCATT GCATTCAAAG TCAGGATATA CTTACCttat aaaatatttt

TTGATTCATT GCATTCGAAG TCAGGATATA CTTACCTTGT AAGaaaaaat

TTGATTCATT GCATTCGAAG TCAGGATATA CTTACCTTGT AAAAAAATTA

---------- ---------- -------ACA CTTACCTTGt taaaaaaaaa

TTGATATAAA GAATATAATG ATAGAATATA CTTACCTTGT AAATAAATTA

TTGATTCATT GCCATCGCAA ACAGGATATA CTTGCTTTAC GAATAGATTA

Ctaaatttta aaaataatgt atttaaaaAA ATAATTGTAC CATGTGGAGT

Ctaaatttta aaaataatgt atttaaaaAA ATAATTGTAC CATGTGGAGT

ttaaatttta aaaataatgt atttaaaaAA ATAATTGTAC CATGTGGAGT

ttaaatttta aaaataatgt atttaaaaAA ATAATTGTAC CATGTGGAGT

---------- ---------- ---------- ---------- ----------

ttaaatttta aaaataatgt atttaaaaa- --aattataC CACGTGGAGT

ttaaaataaa aaaatCAAGG ACTTAAAAGG GTAACTGCAC CAGATGAAGT

ttaaaataaa aaa--CAAGT ACTTAAAAGA GTGAGCGCAC CAGGTGAAGT

ttaaatttta aaaataatGT ATTCGAAAAA ATAATTGTAC CACGTGGAGT

---------- ---------- ---------- ---------- ----------

ttaaatttta aaaatAATGT AGTTAAAAAA ATAATTGTAC CATGTGGAGT

ttaaatttta aaaat----- -GTTAAGAAA ATAATTGTAC CACGTGGAGT

ttaaa--taa aaaatAATGA AGTT---AAA ATAACTGAAC CACGTGGAGT

ttaaataaaa aaaaaaatat aGTTAAAAAA ATAATTGTAC CATGTGGAGT

TTACATTTTA AAAGTAATGT AGTTAAAAAA ATAATTGTAC CATGTGGAGT

ttaaatttta AAAATAATGT AGTTAAAAAA ATAATTGTAC CGAGTGGAGT

TTCAATTTTA AAAAAAAAGA ACTTGAAACA GTAATTATAC CATGTGGAGT

TTCAAAATGA AAAATATTGA TGGAGAAAAG AAAATTGTAC GATATGGAGT

GCAAATCAAT CACACCAACC TGCTTGATCT TGCAATAAGA AGTAAGCAAA

GCAAATCAAT CACACCAACC TGCTTGATCT TGCAATAAGA AGTAAGCAAA

GCAAATCAAT CACACCAACC TGCTTGATCT TGCAATAAGA AGTAAGCAAA

GCAAATCAAT CACACCAACC TGCTTGATCT TGCAATAAGA AGTAAGCAAA

---------- ---------- ---------- ---------- ----------

GCAAATCAAT CACACCAACC TGCTTGATCT TGCAATAAGA AGTAAGCAAA

GCCAGCCAAT CACACCAACC TGCTTGGTCT TGCAA--AGA AGTAAGCGAG

GCCAGCCAAT CACACCAACC TGCTTGGTCT TGCAA--AGC AGTAAGCGAG

GCAAATCAAT CACACCAACC TGCTTGATCT TGCAATAAGA AGTAAGCAAA

---------- ---------- ---------- ---------- ----------

GCAAATCAAT CACACCAACC TGCTTGATCT TTCAGTAAG- AGTAAGCAAA

GCAAATCAAT CACACCAACC TGCTTGATCT TTCAATAAGA AGTAAGCAAA

GCAAATCAAT CACACCAACC TGCTTGATCT TTCAATAAGA AGTAAGCAAA

GCAAATCAAT CACACCAACC TGCTTGATCT TGCAATAAGA AGTAAGCAAA

GCAAATCAAT CACACCAACC TGCTTGATCT TGCAATAAGA AGTAAGCAAA

GCGAATCAAT CACACCAACC TGCTTGATCT TGCAATAAGA AGTAAGCAAA

GCAAATCAAT CACACCAACC TGCTTGATCT TGCAATAAGC AGTAAGTAAA

GCAAATCAAT CACACCAACC TGTTTGATCT TGCAATAAGA AGTAAGCAAA

TCGCAGACAG GAAGCATACG CACAGAGATG AGGAGGAAAA AAGAAACATT

TCGCAGACAG GAAGCATACG CACAGAGATG AGGAGGAAAA AAGAAACATT

TCGCAGACAG GAAGCATATG CACAGAGATG AGGAGGAAAA AAGAAACATT

TCGCAGACAG GAAGCATATG CACAGAGATG AGGAGGAAAA AAGAAACATT

---------- ---------- ---------- ---------- ----------

TCGTAGACAG GAAGCATATG CACAGAGATG AGGAGGAAAA AAGAAACATT

TCGCGGACAG GAAGCATGTG CACAGAGATA AGGAGGGAAG AAGAAACATT

CCGCAGACAG GAAGCGTGTG CACAGAGATG GGGAGGGAAG AAGAAACATT

TCGCAGACAG GAAGCGAACG CACAGAGATG AGGAGGAAAA AAGAAACATT

---------- ---------- ---------- ---------- ----------

TCGTAGACAG GAAGCATATG CACAGAGATG AAGAGGAAAA AAGAAACATT

TCGTAGACAG GAAGCATATG CACAGAGATG AGGAAGAAAA AAGAAGCATT

TCGTAGACAG GAAGCATATG CACAGAGATG AGGAGGAGAA AA-AAGCATT

TCGTAGACAG GAAGCATGAG CACAGAGATG AGGAGGGAAA AAGAAACATT

TCGTAGACAG GAAGCATGTG CACAGAGGTG AAGAGGAAAA AAAAAACATT

TCGCAGACAG GAAGCATGTG CACAGAGATG AGGAGGAAAA AAAAAACATT

TCGTAGACAG GAAGCACGTG CATAGAGATG AGGAGGAAAA AAGAAACATT

TCGTACACAG GAGGCATATG CACAGACATG AGGGGGAAAA AAGAAACATT

ATTTCTCTTG TGAAACAAAG AAAATATATA ATGCATTCAT TTAAGTGTAG

ATTTCTCTTG TGAAACAAAG AAAATATATA ATGCATTCAT TTAAGTGTAG

ATTTCTCTTG TAAAACAAAG AAAATACATA ATGCATTCAT TTAAGTGTAG

ATTTCTCTTG TAAAACAAAG AAAATACATA ATGCATTCAT TTAAGTGTAG

---------- ---------- ---------- ---------- ----------

ATTTCTTTTG TAAAACAAAG AAAATGCATA ATGCATTTAT TTAAGTACAG

ATTTCCTT-G TAAAACAGAG AAAATGTGTA ACGCATTCAT TTGAGCACAG

ATTTCCTTTG TAAAACAGAG AAAACGTGTA ACGCACTCAT TCGAGCACAG

ATTTTCTTTA TAAAACAAAG AAAATGTATA AT-------- ------ATAG

---------- ---------- ---------- ---------- ----------

ATTTCCTTTG TAAAACAAAG AAAATGTATA ATGCATTTAT TTAAGTATAG

ATTTCTTATG TAAAACAAAG AAAATGTATA TTGCATTTAT TTAAGTATAG

ATTTCTTTTG TAAAACAA-- AAAATGTATA AAGCATTCAT TTCAGTATAG

ATTTCTTTTG CAAAACAAAG AAAAAGTACA ATGCATTTAT TTAAGTATAG

ATTTCTTTTG TAAAACAAAG AAAATGTATA GTGCATTCAT TTAAGTACAG

ATTTCTTTCA TAAAACAAAG ACAAAGCACA ATGCATTCAT TT-AGTATAG

ATTTCTTTTA TAAAACAATA AAATTGTAAA AGGCATTAAT CTAACTTTAG

ATTTCTTTTG TAAAACAAAG AAAATGTGAA AAACATTCAT CTATCCACAA

AAATAAATCA AACCGTTATC AAAACTGAAA TAAAATGATA ACCAACAAAC

AAATAAATCA AACCGTTATC AAAACTGAAA TAAAATGATA ACCAACAAAC

AAGTAAATCA AATCGTTATC AAAACTGAAA TAAAATGATA ACCAACAAAC

AAGTAAATCA AATCGTTATC AAAACTGAAA TAAAATGATA ACCAACACAC

---------- ---------- ---------- TAAAATGATA ACCAACAAAT

AAATAAATCA AATCATTATC AAAACTAAAA TAAAATGATA ACCAACAAAC

ACATGAATCA AA---TAACC AAAACTGAAA TAAAATGATG ACCAGCAAAC

ACATGAATCA AA---TAATC AAAACTGAAA TAAAATGATG ACCAGCAAAC

AAATAAATCA CA---TTATC AAAACTGAAA TAAAATGATA ACCAAGATGC

---------- ---------- ---------- ---------- ----------

AAATAAATCA AATCATTATC AAAACTGAAA TAAAGTGATA ACCAACAAAC

AAAT-----A AATCGTTATC AAAACTGAAA CAAAGTGATA ACC---AAAC

GAAT------ ------CATC GAAATTGAAA TAAAGTGATA ACCAA-AACA

AAATAAACCA AATCATCATT GAAGCTGAAA TAAAGTGATA ACCAATGAAC

AAATAAATCA AACCACTATC AAAACTGAAA TAAAGTGACA ACCAACAAAC

AAACAAATCA AATCATTATC AAAACTGAAA TGAAGTGACA ACCAATAAAC

CAACAAATCA AACAATTATT AAACCAAAAG GAGAGCTATA CCCAACAAAC

AAGTAAACCA ATCAAACCAT GACACTGAAA TAACAAAATG ACCAACAAAT

TTACATTCCT TCTATTGCTT CAAGCTTTAT GTTTTTCTTT ATTTTTTAAT

TTACATTCCT TCTATTGCTT CAAGCTTTAT GTTTTTCTTT ATTTTTTAAT

TTACATTCCT TCTATTGCTT CAAGCTTTAC ATTTTTCTTT ATTTTTTAAT

TTACATT-CT TCTATTGCTT CAAGCTTTAC GTTTTTCTTT ATTTTTTAAT

TTACATTCCT TCTATTGCTT CAAGCTTCAC GTT----TTT ATTTT-----

TTACATTCAG TCTATTGCTT CAAACTTTTC ATTTTTCTTT ATTTTTTAAT

TGACATTC-A CCTAA-GCCG CAGGCCTCGC ACATCTCTTT ACTT------

TCCCATTCCG CCTAT-GCCT CGGGCTTCAC ACATCGCTTT ATCT------

TTACATTCAA TCTATC---- ---------- ---------- ----------

---------- ---------- ---------- ---------- ----------

TTGCATTCAA TTTATTGCTT CAAACTTTAC ATTTTTCTTT ATTTTTCAAG

TTGCATTCAA TCTATTGCTT CAAACTTTAC ----TTATTT ATTTTTCAAT

CTGCATTCAA TCTATTGCTT CAAATTTTAC TTCTTTCTTT ATTTTTCAAT

TTACATTCAG TCTATTGCTT CAAACTTTAC ATTTTTCTTT ATTTTTCAAT

TTACATTCAA TCTACTGCTT CAAACTTTAC ATTTTTTTTA ATTTTTCAAT

TTACATTCCA TCTACTGTTC CAAACTTCA- -TTTTTCTTA ATTTTTCTAT

TTATATTCAA CATATTACTT CAGAATTCAC ATTTCTCTAT ATTTTTTCAT

TTACAGTCAG AAGATTGCCT AAAGAGTCCC AATTTTTACT ATTTTCTAAC

CTTAAGCTTA TACAGCTTAC TAGCTCTCTT TACATTATCT CAATTGCAAA

CTTAAGCTTA TACAGCTTAC TAGCTCTCTT TACATTATCT CAATTGCAAA

CTTAAGCTTA TACAGCTTAC TAGCTCTCTT TACATTATCT CAATTGCAAA

CTTAAGCTTA TACAGCTTAC TAGCTCTCTT TACATTATCT CAATTGCAAA

-TTAAGCTTA TACAGCTTAC TAGCTCTCTT TACATTATCT CAATTGCAAA

CTTAAGCTTA TACAGCTTAC TAGGTCTCTT TACATTATCT CAATTGCAAA

--------CA CA--GCTTAC TAGCTCTCTT CACATTATCT CAGCGGCAAA

--------CA CACAGCTTAC TGGCTCTCTT -ACATTATCT CAGTGGCAAA

---------- ---------- ---------- ---------- ----------

---------- ---------- ---------- ---------- ----------

CTTAAGCTTA TACAGCTTAC TAGCTCTCTT TACATTATCT CAATGGCAAA

CTTAAGCTTG TACAGCTTAC TAGCTCTCTT TACATTATCT CGATTGCAGA

CTTAAGTTT- TACAGCTCAC TTGCTCTCTT TTTATTATCT CAATTGCAAA

CTTAAGCTTA TACAGCTTAC TAGCTCTCTT TACATTATCT CAATTGCAAA

CTTAAGCTTA TACAGCTTAC TAGCTCTCTT TACATTATCT CAATTGCAAA

CTTAAACT-A TCCAGCTTAC TAGCTCTCTT TACATTATCT CAATTGCAAA

CTAGAGCTTA TACAGCTTAC TAGTTCTCTT TACATTATCT CATTTGCAAA

CTCAAGCTCA CGCAGCTTAC TAGCTCTCTT TACATTATCT CAAATGCAAA

GCATGAATCT CTCTTCCATT CTCAAAATAA GCTAAGCATG TTTAACATGC

GCATGAATCT CTCTTCCATT CTCAAAATAA GCTAAGCATG TTTAACATGC

GCATGAATCT CTCTTCCATT CTCAAAATAA GCTAAGCATG TTTAACATGC

GCATGAATCT CTCTTCCATT CTCAAAATAA GCTAAGCATG TTTAACATGC

GCATGAATCT CTCTTCCATT CTCAAAATAA GCTAAGCATG TTTAACATGC

GCATGAATCT CTCTTCCATT CTCAAAACAA GCTAAGCATG TTTAACATGC

GCATGATTCT CTCTTTCACT CTCAAAATAG GCTCGGCATG TTTA-CATGC

GCATGAATCT CTCTCCCACT CTCAAAATAG GCTCGGCATG TTTA-CATGC

---------- ---------- ---------- ---------- ----------

---------- ---------- ---------- ---------- ----------

GCATGAACCT CTCTTCCATT CTCAAAATAA GCTAAGCACA TTTAACATGC

GCATGAATCT CTCTTCCATT CTCAAAATAA GCTTAGCATG TTTAACATGC

GCATGAATCT CTCTTCCATT CTCCAAAAAA ACTTAGCATG TTTAACATGC

GCATGACTCT CTCTTCCATT CTCAAAATAA GCTTAGCATG TTTAACATGC

GCATGAATCT CTCTTCCATT CTCAAAATAA GCTTAGCATG TCTAACATGC

GCATGAATCG CTCTTCCACT CTCAAGATCA GCTTAGCACG TTCAACATGC

GCATGAATTT ATAGACAATT CTCAAATTAA GCTTAGCATA TTTATTATGC

GCATGAATTT GTCTTCCATT CTCAAAATAA GCTTAGCATG TTTAACATGC

TTTGTTCTAC TGAGGAATCC AACATGTATT TTTAGGTGAC AGGGAGTGGT

TTTGTTCTAC TGAGGAATCC AACATGTATT TTTAGGTGAC AGGGAGTGGT

TTTGTTCTAC TGAGGAATCC AACATGTATT TTTAGGTGAC AGGGAGTGGT

TTTGTTCTAC TGAGGAAACC AACATGTATT TTTAGGTGAC AGGGAGTGGT

TTTGTTCTAC TGAGGAATCC AACATGTATT TGTAGGTGAC AGGGAGCGGT

TTTGTCCAAC TGAGAAGTCC AACATGTATT TTCAAGTGAC AGGAAACGGT

TT-GTTCTAC TG-GGAGCCC AACATGGACC ----AGTGAC AGGGAGTGGG

TT-GTTCTAC AG-GGAGCCC AACATGGACC ----AGTGAC AGGGAGTGGG

---------- ---------- ---------- ---------- ----------

---------- ---------- ---------- ---------- ----------

TTTGTTCTAC TGAGAAGTCC AACACGTATT TTTAAGTGAC GGGGAGCAGT

TCTGTTCTCC TGAGAAGTCC AACACGTATT TGTAAGTGAC AGGGAGCGGT

TTTGTTCTGC TGAGAAGTTA AACAAGTATT TTTAAGTGAG AGGGAGCAGT

TTTGGTCTAC TGAGAAATCC AATGTGTATT TTCAAGTGAC AGGGAACAGT

TTTGGTCTAC TGAGAAGTCC AACATGTACT CTTAAGTGAC AGGGAGCGGT

TT-GGTCTAC TGAGAAGTCC AACATGGATT GGTAAGTGAC AGGGAGCGGG

TATAGCTTAC TAAAATACTC AGTTTTTGTT TTTAAGTGAC AGGGAGCAGT

TTTGGCCTCT TCCAAAATCC AAAATATCTT TTCAAATGAC AGGGAGCAGT

GCAGCATTGC TCAAATGAAG CCCATGGGTT ATGGAAAGCC AAAGCTCTGT

GCAGCATTGC TCAAATGAAG CCCATGGGTT ATGGAAAGCC AAAGCTCTGT

GCAGCATTGC TCAAATGAAG CCCATGGGTT ATGGAAAGCC AAAGCTCTGT

GCAGCATTGC TCAAATGAAG CCCATGGGTT ATGGAAAGCC AAAGCTCTGT

GCAGCATTGC TCAAATGAAG CCCATGTGTT ATGGAAAGCC AAAGCTCTGT

ACAGCATATC TCAAATGAAG CCCATGGGTT ATGGAAAGCC AAAGCTCTGT

GCAGCATCTC CCAAATGAAG CCCATGAGTG ATGGAAAGCC AGAGCTCTGT

GCAGCATCTC CCAAATGAAG CCCATGAGTG ATGGAAAGCC AGAGCTCTGT

---------- ---------- ---------- ---------- ----------

---------- ---------- ---------- ---------- ----------

GCAGCATTTC TCAAATGAAG CCCATGGGTT ATGGAAAGCC AAAGCTCTGT

GCAGCATTTC TCAAATGAAG CCCATGGGTT ATGGAAAGCC AAAGCTCTGT

GCAGCAATTC TCAAATGAAG CCCATGGATT ATGGAAAGCC AAAGCTCTGT

GCAGCATTTC TCAAATGAAG CTCATGGGTT ATGGAAAGCC AAAGCTCTGT

GCAGCATTTC TCAAATGAAA CCCACGGGTT ATGGAAAGCC AGAGCTCTGT

GCAGCATTTC TCAGATGAAG CCCATGGGTT ATGGAAAGCC AAAGCTCTGT

GTAGCATTTA TAAAATGAAG CACAAAAGTT ATGGTAAGCC AAAGCTTTGT

GCAGCATTTA CAAAATGAGG TGTACAGGTT ATGGAAAGCC AGAGCTTTGT

TAGAGAATGT AAGAGCTACC ACTCACTAGG CAAGAACTTA TCCAGAGGTT

TAGAGAATGT AAGAGCTACC ACTCACTAGG CAAGAACTTA TCCAGAGGTT

TAGAGAATGT AAGAGCTACC ACTCACTAGG CAAGAACTTA TCCAGAGGTT

TAGAGAATGT AAGAGCTACC ACTCACTAGG CAAGAACTTA TCCAGAGGTT

TAGAGAACGT AAGAGCTACC ACTCACTAGG CAAGAACTTA TCCAGAGGTT

TAGAGAATTT AAGAGCTACC ACTCACTAGG CAAGAACTTA TCCAGAGGTT

TAGAGAAGGT GAGGGCCGCC ACTCACGAGG CAAGAACTCA TCCAGGGGCT

TAGAGAAGGT GAGGGCCGCC ACTCACGAGG CAAGAACTCA TCCAGGGGCT

---------- ---------- ---------- ---------- ----------

---------- ---------- ---------- ---------- ----------

GAGAGAATTT AAGAGCTACC ACTCACTAGG CAAGGACTTA TCCAGAGGTT

TAGAGGATTT AAGAGCTGCC ACTCACTAGG CAAGAACTTA TCCAGAGGTT

TAGAGAATTT AAGAGCTACC ACTCACTAGG CAAGAACTTA TCCAGAGGTT

TAGAGAATTT AAGAGCTACC ACTCACTAGG CAAGAACTTA TCCAGAGGTT

TAGAGAACTG AAGAGCTGCC ACTCACTAGG CAAGAACTTA TCCAGAGGTT

GAGAGAATGG ACGAGTTGCC ACTCACGAGG CAAGAACTTA TCCAGAGGTT

TAGAGGATTT AAGAGCTCCC ACTCACTAGT CAAGTACTTA TCCAGAGGTT

TAGAGGATTT AAGTGCTCCC ACTCACTAGT CAAGGAATTA TCCAGAGGTT

TCTCTATGAC AGAACATGTG GAGTCTGAAC TACTGCTGCT GCCTGGAAAA

TCTCTATGAC AGAACATGTG GAGTCTGAAC TACTGCTGCT GCCTGGAAAA

TCTCTATGAC AGAACATGTG GAGTCTGAAC TACTGCTGCT GCCTGGAAAA

TCTCTATGAC AGAACATGTG GAGTCTGAAC TACTGCTGCT GCCTGGAAAA

TCTCTATGAC AGAACATGTG GAGTCTGAAC TACTGCTGCT GCCTGGAAAA

TCTCTATGAC AGAACATGTG GAGTCTGAAC TACTGCTGCT GCCTGGAAGA

TCTCTATGAC AGAACATGTG GAGTCTGAGC TACTGCTGCT GCCTGGAAGA

TCTCTATGAC AGAACATGTG GAGTCTGAGC TACTGCTGCT GCCTGGAAGA

---------- ---------- ---------- ---------- ----------

---------- ---------- ---------- ---------- ----------

TCTCTATGAC AGAACATGTG GAGTCTGAAC TACTGCTGCT GCCTGGAAGA

TCTCTATGAC AGAACATGTG GAGTCTGAGC TACTGCTGCT GCCTGGAAGA

TCTCTATGAC AGAACATGTN NNNNNN---- ---------- ----------

TCTCTATGAC AGAACATGTG GAGTCTGAAC TACTGCTGCT GCCTGGAAAA

TCTCTATCAC AGAACATGTG GAGTCTGAAC TACTGCTGCT GCCTGGAAGA

TCTCTATCAC AGAGCATGTG GAGTCTGCAC TACTGCTGCT GCCTGGAAGA

TCTCTATGAC AGAGCATGTA GAATATGAAC TACTGCTGCT GCCTGGAAAA

TCTCTATGAC AGAACATGTG GAGTTTGAAC TACTGCTGCT GCCTGGAAAA

CAAAGAAATG AGCCCAACAG GAACCAAAGA AAGAGAAACA AGAAATGAAC

CAAAGAAATG AGCCCAACGG GAACCAAAGA AAGAGAAACA AGAAATGAAC

CAAAGAAATG AGCCCAACGG GAACCAAAGA AAGAGAAACA AGAAATGAAC

CAAAGAAATG AGCCCAACGG GAACCAAAGA AAGAGAAACA AGAAATGAAC

CAAAGAAATG AGCCCAACGG GAACCAAAGA AAGAGGAACA AGAAATGAAC

CAAAGAAATG AGCCCAACGG GAACCAAAGA AAGAGAAACA AGAAATGAAC

CAAAGAAACG AGCCCA-CGA CAACCAGAGA GAG-GGAACA AGATGTGAAC

CAAAGAAACG AGCCCA-CGG CAACCAGAGA GA--GGAACA AGAT-TGAAC

---------- ---------- ---------- ---------- ----------

---------- ---------- ---------- ---------- ----------

CAAAGAAATG AGCCCAACGG GAACCAAAGA AAGAGAAACA AGTAATGAAC

CAAAGACATG AGCCCAACGG GAACCACAGA AAAAGAAACA AGGAA-GGAG

---------- ---------- ---------- ---------- ------NNAC

CAAAGAAATG AGCCCAACGG GAACCAAATA AAGAGAAACA AGAAATGATC

CAAAGAAATG AGCCCAACCG GAGCCAAAGA AAGAGGAACA AGAAATGAAC

CAAAGAAATG AGCCCAACTG GAGCCAAAGG AAGAGGAGCG AGAAATGACC

CAAAGAAATG AGCCCAAGGG AAACCAAAGA AAGAAAAATA AGAAATGAAA

CAAAGAGATG CTCCCAACGG AAACCAGAGA AAGAGAAACA AGGATTGAAC

ACAGAGAAAA ATTCAGTTCT TACCCCTTCT TTTTTTCCTT TTCTCTCCAT

ACAGAGAAAA ATTCAGTTCT TACCCCTTCT TTTTTTCCTT TTCTCTCCAT

ACAAAGAAAA ATTCAGTTCT TACCCcttct ttttttcctt ttctctccat

ACAGAGAAAA ATTCAGTTCT TACCCcttct ttttttcctt ttctctccat

ACAGAGAAAA ATTCAGTTCT TACCCTTTC- TTTTTTCCTT TTCTCTCCAT

ACAGAG-AAA ATTCAGTTCT TACCTCTTCT TTTTTTCCTT TTCTCTCCAT

ACAGAGAAAA CCTGAGTTCT TACCCCTTCT CTTTTTCCTT TTTTCTCCAT

ACAGAGAAAA CCTGAGTTCT TACCCCTTCT CTTTTTCTTT TTTTCTCCAT

---------- --------CT TACCCCTTCT TTTTTTCCTT TTCTCTCCAT

---------- ---------- ---------- ---------- ----------

ACAGAGGAAA ATTCAGTTCT TACCCCTTCT TTTTTTCCTT TTCTCTCCAT

ACAGA--AAA ATTCAGTTCT TACCCCTCCT TTTTTTCCTT TTCTCTCCAT

ACAGAGAAAA ATTCAGTCCT TACCCCGtct ttttttcctt ttctctccat

ACAGAGAAAA ATTCAGTTCT TACCCCTTCT TTTTTTCCTT TTTTCTCCAT

ATGAAGAAAA ATTCAGTTCT TACCCcttct ctttttcctc ttctctccgt

ACGGGGGAAA G-TCACCTCT TAccccttct ttttttcctc ttctctccat

ACAGAGAAAA ATTCAGTTCT TACCCTTTTT CTTTTTCCTT TTTTCTCCAT

ATGCAGAAAA ATTCATTCTT TACCCCTTCG TTTCTTCCGC TTTTCTCCAT

CTTCTCCAAC CTCACCCTCA TCATCATCTT CCTCTGACCC ACTGATATCA

CTTCTCCAAC CTCACCCTCA TCATCATCTT CCTCTGACCC ACTGATATCA

cttctccaac ctcaccttca tcatcatctt cctctGACCC ACTGATATCA

cttctccaac ctcaccttca tcatcatctt cctctGACCC ACTGATATCA

CTTCTCCAAC CTCACCCTCA TCATCATCTT CCTCTGATCC ACTTATATCA

CTTCTCCAAT CTCACCATCA TCATCATCTT CCTCTGACCC ACTGATATCA

CTTCTCCAAG TTCACCCTCG TTGTCATCCT CCTCTGACCC ACTGATGTCA

CTTCTCCAAG TTCACCCTC- --GTCATCCT CCTCTGACCC ACTGATGTCA

CTTCTCCAAC CTCACCCTCA TCATCCTCCT CCCCTGAACC ACTGATATCA

---------- ---------- ---------- ---------- ----------

CTTCTCCAAC CTCACCCTCA TCATCCTCTT CCTCCGACCC ACTGATATCA

CTTCTCCAAC TTCACCCTCA TCATCATCCT CCTCTGACCC ACTGATATCA

cttctccaac ctcaccttct tcatcatctt cctctGAACC ACTGATATCA

CTTCTCCAAT CTCACCCTCA TCGTCTTCCT CCTCTGATCC ACTGATATCG

cttctccaac ttcatcctca tcatcttctt cctctGACTC ACTGATATCA

cctctccgac ttcaccctca tcctcctcct cctcTGACCC ACTGATATCA

GTTCTCCAGC CTCAGCCTCA TCTTGATCAT CTTCTGATCC ACTGACATCA

CTTCCCCAAC TTCCCCCTCG TCATCTTCCT CATCTGATCC ACTGATATCG

GAGCCTGATA TTTCTTCTCC TGGACAGAAA TGTATAAATA CCACCTAGTG

GAGCCTGATA TTTCTTCTCC TGGACAGAAA TGTATAAATA CCACCTAGTG

GAGCCTGATA TTTCTTCTCC TGGACAGAAA TGTATAAATA CCACCTAGTG

GAGCCTGATA TTTCTTCTCC TGGACAGAAA TGTATAAATA CCACCTAGTG

GAGCCTGATA TTTCTTCTCC TGGACAGAAA TGTATAAATA CCACCTAGTG

GAGCCTGATA TTTCTTCCCC TGAATAGAAA TGTATAAATA CCACCTAGTG

GAGCCTGACA CTTCTTCCCC TGGACAGAAA TGTATAAACG CCACCTAGTG

GAGCCTGACA CTTCTTCCCC TGGAGAGAAA TGTATAAATG CCACCTAGTG

GAGCCCGATA TTTCTTCCCC TGGATAGAGA TGTATAAATA CCACCTAGTG

---------- ---------- ---------- ---------- ----------

GAACCTGATA TTTCTTCCCC TGAATAGAAA TGTATAAATA CCACCTAGTG

GAGCCTGATA TTTCTTCCCC TGAATAGAAA TGTATAAATA CCACCTAGTG

GAGCCTGATA TTTCTTCCCC TGAGCAGGAA TGTATAAATA CCACCTAGTG

GAGCCTGATA TTTCTTCTCC TGGATAGAAA TGTATAAATA CCACCTAGTG

GATCCAGATA TTTCTTCCCC TGGACAGAAA TGTATAAATA CCACCTAGTG

GATCCTGATA TTTCTTCCCC TGGATAGAAA TGTTGACATA CCACCTAGTG

GAGCCTGAAA TCTCTTCCCC TGGATAGAAA TGTATAAATA CCACCTAGTG

GAGCCTGAGA TTTCTTCACC TAGAGAGGAA TGTTTAAGTA CCATCTAGTG

GTCACATTTA AAACACCAAA TTTCTTGAGG AAAGCTACCT TCTTCAAGCT

GTCACATTTA AAACACCAAA TTTCTTGAGG AAAGCTACCT TCTTCAAGCT

GTCACATTTA AAATACCAAA TTTCTTGAGA AAAGCTACCT TCTTCAAGTT

GTCACATTTA AAATACCAAA TTTCTTGAGA AAAGTTACCT TCTTCAAGTT

GTCATATTTA AAACACCAAA TTTCTTGAGA AAAGTTACCT TCTTCAAGCT

GTCACATATG AAACACCAAA CTTCTTGAGA AAAGTTACCT TCTTCGAGCT

ACCAT-TCAC AGATGGCGAC TTC-TTGTGA GAAGTTACCT TCCTCAAGCT

GCCAT-TTAC AGGTGGCCAG TTCCCTGTGA GAAGTTACCT TCCTCAAGCT

GTCAGATTTG AAACAACAAT TTTTTTGAGA AAAGTTACCT TCTTCAAGCT

---------- ---------- ---------- ---------- ----------

GCCATATTTA AAATACCAAA TTTCCTGAGA AAAGTTACCT TCTTCAAGCT

GTCACATTTA AAACACCAAA TTTCTTGA-A AAAGTTACCT TCTTCAAGCT

GTCACATTTA AAATACCAAA TTTTTTAAGA TTAGTTACCT TCCTCAAGTT

GTCACATTAA AAACACCAAA TTTCTTGAGA AAAGTTACCT TCTTCAAGCT

GTCACATTTA AAACACCAAA TTTCTTGATA AAAGTTACCT TCTTCAAGCT

GCCACATTTA AAACATCTAA TCTC------ -AAGTTACCT TCTTCAAGTT

GTCAAATTTA AAACCTTGAA CTTCATAAGA AACGTTACCT TCTTCAAGCT

GTCAGACTAA AAACTCAAAA TTTCTTGTGG AAAATTACCT TCTTCAAGCT

TCTTTTTCAG TTCTTCTATT TCTTTCTGGA ACTGACGCAG CAAAGCATCC

TCTTTTTCAG TTCTTCTATT TCTTTCTGGA ACTGACGCAG CAAAGCATCC

TCTTTTTCAG TTCTTCTATT TCTTTCTGGA ACTGACGCAG CAAAGCATCC

TCTTTTTCAG TTCTTCTATT TCTTTCTGGA ACTGACGCAG CAAAGCATCC

TCTTTTTCAG TTCTTCTATT TCTTTCTGGA ACTGACGCAG CAAAGCATCC

TTTTTTTCAG TTCTTCTATT TCTTTCTGAA ACTGACGAAG CAGAGCATCC

TTTTCTTCAG CTCTTCTATT TCTTTCTGAA ACTGGCGAAG CAGAGCGTCC

TTTTCTTCAG CTCTTCGATT TCTTTCTGAA ACTGGCGGAG CAGAGCATCC

TTTTCTTCAA TTCTTCTATT TCTTTCTGAA ACTGACGGAG CAAAGCATCC

---------- ---------- ---------- ---------- ----------

TCTTTTTCAG TTCTTCTATT TCTTTCTGAA ACTGGCGAAG CAAAGCATCT

TTTTTTTCAA TTCTTCTATT TCTTTCTGAA ATTGACGAAG CAAAGCATCT

TTTTTTTCAG TTCTTCTATT TCTTTCTGAA ATTGACGAAG CAAAGCATCC

TTTTTTTCAG TTCTTCTATT TCTTTCTGAA ACTGACGAAG TAAAGCATCC

TTTTTTTAAG TTCTTCTATT TCTTTCTGAA ACTGACGAAG CAAAGCATCC

TTTTTTTCAG TTCTTCTATT TCTTTCTGAA ATTGACGAAG TAAAGCATCC

TTTTTTTGAG TTCTTCTATT TCTTTCTGAA ACTGCCGAAG CAAAGCATCC

TTTTTTTGAG TTCTTCTATT TCTTTCTGAA ACTGGCGAAG CAAAGCGTCC

TTTGGATCTT CATTAATTCT AGCTTTATTT TTAATATTCT TAGCACGATT

TTTGGATCTT CATTAATTCT AGCTTTATTT TTAATATTCT TAGCACGATT

TTTGGATCTT CATTAATTCT AGCTTTATTT TTAATATTCT TAGCACGATT

TTTGGATCTT CATTAATTCT AGCTTTATTT TTAATATTCT TAGCACGATT

TTTGGATCTT CATTAATTCT AGCTTTATTT TTAATATTCT TAGCACGATT

TTTGGATCTT CATTAATTCT AGCTTTATTT TTAATATTCT TAGCACGGTT

TTTGGGTCTT CGTTAATTCT AGCTTTGTTT TTAATGTTCT TGGCGCGGTT

TTTGGGTCCT CATTGATTCT CGCTTTGTTT TTAATGTTCT TGGCGCGGTT

TTTGGATCTT CATTAATTCT AGCTTTATTT TTAATATTCT TAGCACGGTT

---------- ---------- ---------- ---------- ----------

TTTGGATCTT CATTAATTCT AGCTTTATTT TTAATATTCT TAGCACGGTT

TTTGGATCTT CATTAATTCT AGCTTTATTT TTAATATTCT TAGCACGGTT

TTTGGATCTT CATTAATTCT AGCTTTATTT TTAATATTCT TAGCACGGTT

TTTGGATCTT CATTAATTCT AGCTTTATTT TTAATATTTT TAGCACGGTT

TTTGGATCTT CATTAATTCT AGCTTTATTT TTAATATTTT TAGCACGGTT

TTTGGATCTT CATTAATTCT AGCTTTATTT TTGATATTTT TAGCACGGTT

TTTGGATCTT CATTAATTCT AGCTTTATTC TTAATATTTT TAGCACGGTT

TTTGGATCCT CATTAATTCT AGCCTTGTTC TTGATATTTT TAGCACGGTT

GGCATACCGT AATGTACTGA TAGTTTCATC ATAATTGTAA TCTGCTGGCC

GGCATACCGT AATGTACTGA TAGTTTCATC ATAATTGTAA TCTGCTGGCC

GGCATACCGT AATGTACTGA TCGTTTCATC ATAATTGTAA TCTGCTGGCC

GGCATACCGT AATGTACTGA TCGTTTCATC ATAATTGTAA TCTGCTGGCC

GGCATACCGT AATGTACTGA TAGTTTCATC ATAATTGTAA TCTGCTGGCC

AGCATACCGT AATGTACTGA TAGTTTCATC ATAATTGTAA TCTGCTGGCC

GGCATACCGC AAGGTACTGA TGGTTTCATC ATAATTATAA TCTGCTGGCC

GGCATACCGC AAGGTGCTGA TAGTTTCATC ATAATTATAA TCTGCTGGCC

GGCATACCGT AATGTACTAA TAGTTTCATC ATAATTATAA TCTGCTGGCC

---------- ---------- ---------- ---------- ----------

AGCATACCGC AATGTACTGA TAGTTTCATC ATAATTGTAA TCTGCTGGCC

AGCATACCGT AATGTACTGA TAGTTTCATC ATAATTGTAA TCTGCTGGCC

GGCGTACCGT AACGTACTGA TAGTTTCGTC GTAATTGTAG TCGGCTGGCC

GGCATACCGT AATGTACTGA TAGTTTCATC ATAATTGTAA TCTGCTGGCC

GGCATAGCGT AAAGTACTGA TAGTTTCATC ATAATTGTAA TCTGCTGGCC

GGCATACCGT AACGTACTGA TAGTTTCATC ATAATTGTAA TCTGCTGGCC

AGCATATCGC AATGTACTGA TAGTCTCATC ATAATTGTAG TCTGCAGGCC

AGCGTACCGT AATGTACTGA TGGTCTCGTC GTAATTGTAG TCTGCTGGAC

CAATATTTGC ACACTATTCT TACCATCATG GTTTTTGAAT TTCCTCCTAA

CAATATTTGC ACACTATTCT TACCATCATG GTTTTTGAAT TTCCTCCTAA

CAATATTTGC ACACTATTCT TACCATCATG GTTTTTGAAT TTCCTCCTAA

CAATATTTGC ACACTATTCT TACCATCATG GTTTTTGAAT TTCCTCCTAA

CAATATTTGC ACACTGTTCT TACCATCATA GTTTTTGAAT TTCCTCCTAA

CAATATTTGC ACACTGTTCT TACCATCATG GTTTTTGAAT TTCCTCCTAA

CAATATTTGC ACACTGTTCT TACCATCATG GTTTTTGAGT TCCCTCCTAA

CAATATTTGC ACACTGTTCT TACCATCATG GTTTTTGAGT TCCCTCCTAA

CAATATTTGC ACACTGTCCT TACCATCATG GTTTTTGAAT TTCCTCCTAA

---------- ---------- ---------- ---------- ----------

CAATATTTGC ACACTGTTCT TACCATCATG GTTTTTGAAT TTCCTCCTAA

CAATATTTGC ACACTGTTCT TACCATCATG GTTTTTGAAT TTCCTCCTAA

CAATATTTGC ACACTATTCT TACCATCATG GTTTTTGAAT TTCCTCCTAA

CAATATTTGC ACACTGTTCT TACCATCATG GTTTTTGAAT TTCCTCCTAA

CAATATTTGC ACACTGTTCT TACCATCATG GTTTTTGAAT TTCCTCCTAA

CAATATTTGC ACACTATTCT TACCATCATG GTTTTTGAGT TTCCTCCTAA

CAATATTTGC ACACTGTTCT TACCATCATG GTTTTTGAGT TTCCTCCTAA

CGATATTTGC ACACTGTACT TACCATCATG GTTTTTGAGT TGCCTCCTAG

GGAATCCTGA AGAAGACGAG TCAGTTTAGA GTTACGATAA GGCACATGAG

GGAATCCTGA AGAAGACGAG TCAGTTTAGA GTTACGATAA GGCACATGAG

GGAATCCTGA AGAAGACGAG TCAATTTAGA GTTACGATAA GGCACATGAG

GGAATCCTGA AGAAGACGAG TCAATTTAGA GTTACGATAA GGCACATGAG

GGAATCCTGA AGAAGACGAG TCAATTTCGA GTTACGATAA GGCACATGAG

AGAATCCTGA AGAAGACGTG TCAATTTAGA GTTACGATAA GGCACATGAG

GGAATCCTGC AGGAGACGTG TCAGTTTAGA GTTCCGATAC GGCACATGAG

GGAGTCCTGC AGGAGACGGG TCAGTTTGGA GTTCCGATAC GGCACGTGAG

GGAATCCTGA AGAAGTCGAG TCAATTTAGA GTTACGGTAA GGCACATGTG

---------- ---------- ---------- ---------- ----------

GGAATCCTGA AGAAGACGAG TCAGTTTTGA GTTGCGATAT GGAACATGAG

GGAGTCCTGA AGAAGACGAG TCAATTTAGA GTTACGATAA GGCACATGAG

GGAATCCTGA AGAAGACGGG TCAATTTAGA GTTACGATAA GGCACATGAG

GGAATCCTGA AGAAGACGAG TCAATTTAGA GTTACGATAA GGCACATGAG

GGAATCCTGA AGAAGACGAG TCAACTTAGA GTTACGATAA GGCACATGAG

AGAGTCTTGA AGGAGGCGAG TCAACTTAGA GTTACGATAC GGCACGTGGG

GGAGTCCTGA AGAAGGCGAG TCAATTTTGA ATTTCGATAT GGTACATGAG

GGAATCCTGA AGAAGACGGG TCAATTTTGA ATTACGATAG GGCACGTGGG

TGCTTTTTCC ATCAACCAAG GCAGAAATTA CATTACCAAG GGTGGAAAGT

TGCTTTTTCC ATCAACCAAG GCAGAAATTA CATTACCAAG GGTGGAAAGT

TGCTTTTTCC ATCAACCAAG GCAGAAATTA CATTACCAAG GGTGGAAAGT

TGCTTTTTCC ATCAACCAAG GCAGAAATTA CATTACCAAG GGTGGAAAGT

TGCTTTTTCC ATCAACCAAG GCAGAAATTA CATTACCAAG GGTGGAAAAT

TGCTTTTTCC ATCAACCAAG GCAGAAATTA CATTACCAAG GGTGGAAAGT

TGCTTTTTCC ATCCACCAAG GCAGAAATGA CATTGCCCAA GGTAGACAAT

TGCTTTTTCC ATCCACCAAG GCAGAAATAA CGTTGCCCAG GGTAGACAAT

TACTTTTCCC ATCAACCAAA GCAGAAATTA CATTGCCAAG CGTAGAAAGA

---------- ---------- ---------- ---------- ----------

TGCTTTTTCC GTCAACCAAG GCAGAAATTA CATTACCAAG GGTGGAAAGC

TGCTTTTTCC ATCAACCAAG GCAGAAATCA CATTGCCAAG GGTGGAAAGC

TGCTTTTTCC ATCAACCAAG GCAGAAATTA CATTACCAAG GGTAGAAAGT

TGCTTTTTCC ATCAACCAAG GCAGAAATTA CATTACCAAG GGTGGAAAGT

TGCTTTTTCC ATCAACCAAG GCAGAAATTA CATTACCAAG GGTGGAAAGT

TGCTCTTTCC ATCGACTAGC GCAGAGATGA CGTTCCCGAG GGTGGACAGG

TGCTTTTCCC ATCAACCAAG GCAGAAATTA CATTACCCAG AGTGGAAAGT

TACTTTTCCC ATCCACCAAA GCGGAGATTA CATTGCCAAG GGTGGAAAGG

GAAAGATTGA TTTTTGTAGC TTCCTTTAGG CGCTGTCCAG TAGCTCCAGT

GAAAGATTGA TTTTTGTAGC TTCCTTTAGG CGCTGTCCAG TAGCTCCAGT

GAAAGATTGA TTTTTGTAGC TTCCTTTAGG CGCTGTCCAG TAGCTCCAGT

GAAAGATTGA TTTTTGTAGC TTCCTTTAGG CGCTGTCCAG TAGCTCCAGT

GAAAGATTGA TTTTTGTAGC TTCCTTTAGG CGCTGTCCAG TAG-------

GAAAGATTGA TTTTTGTAGC TTCCTTTAGG CGTTGTCCAG TAGCTCCAGT

GAAAGGTTGA TTTTGGTAGC TTCTTTGAGG CGCTGCCCTG TGGCTCCAGT

GAAAGGTTGA TTTTGGTAGC TTCCTTGAGG CGCTGTCCTG TGGCTCCAGT

GAAAGATTAA TTTTTGTAGC TTCCTTTAAG CGCTGTCCAG TAGCTCCAGT

---------- ---------- ---------- ---------- ----------

GAAAGATTGA TTTTTGTGGC TTCTTTTAGG CGCTGTCCAG TTGCTCCAGT

GAAAGGTTGA TTTTTGTAGC TTCCTTCAGG CGCTGTCCAG TAGCTCCAGT

GAAAGATTGA TTTTTGTAGC TTCCTTTAGG CGCTGTCCAG TAGCTCCAGT

GAAAGATTGA TTTTTGTAGC TTCCTTTAGG CGCTGTCCAG TAGCTCCAGT

GAAAGATTGA TTTTTGTAGC TTCCTTTAGG CGTTGCCCAG TAGCTCCAGT

GAAAGATTGA TTTTTGTGGC TTCCTTTAGT CGCTGCCCAG TAGCTCCAGT

GAAAGGTTAA TTTTCGTTGC TTCTTTCAGT CGTTGTCCAG TAGCTCCAGT

GAAAGGTTAA TTTTGGTTGC TTCCTTTAGC CGCTGCCCTG TAGCTCCAGT

TTTTGCCTGT CTTTCTGAAC CCTACTATCC TGACATGCAT GTTACCATCA

TTTTGCCTGT CTTTCTGAAC CCTACTATCC TGACATGCAT GTTACCATCA

TTTTGCCTGC CTTTCTGAAC CCTACTATCC TGACATGCAT GTTACCATCA

TTTTGCCTGC CTTTCTGAAC CCT------- ---------- ----------

---------- ---------- ---ACTATCC TGACATGCAT GTTACCGTCA

TTTTGCTTGT CTTTCTGAAC CCTACTATCC TGACATGCAT GTTCCCATCA

TTTTGCTTGT CTTTCTGACC CCTACTATCC TGACGTGCAT GTTTCCATCC

Ttttgcttgt ctttctgacc cctACTATCC TGACGTGCAT GTTCCCGTCC

TTTTGCTTGT CTTTCTGAAC CCT------- ---------- ----------

---------- ---------- ---------- ---------- ----------

TTTTGCTTGT CTTTCTGAAC CCTACTATTC TGACATGCAT ATTACCATCA

TTTTGCTTGT CTTTCTGAAC CCTACTATCC GGACATGCAT GTTACCATCA

TTTTGCTTGT CTTTCTGAAC CCTACTATCC TGACATGCAT GTTACCGTCA

TTTTGCTTGT CTTTCTGAAC CCTACTATCC TGACATGCAT GTTACCGTCC

TTTTCCTTGC CTTTCTGAAC CCTACTATCC TGACATGCAT GTTACCATCA

TTTTGCTTGT CTTTCTGAAC CCTACTATCC TGACATGCAT ATTACCATCA

TTTTGCCTGT CTTTCTGAAC CCTGCAATTT AAATATGCAT TTTATCATTA

CTTCGCCTGC CTTTCTGATC CCTACTATTC GAACGTGCAT ATTGCCATCC

ATGCCTTTTT CACTGCATTC TAGTTCGTTC ATATTAGTTG CACCAACAGA

ATGCCTTTTT CACTGCATTC TAGTTCGTTC ATATTAGTTG CACCAACAGA

ATGCCTTTTT CACTGCATTC TAGTTCATTC ATATTAGTTG CACCAACAGA

---------- ---------- ---------- ---------- ----------

ATGCCTTTTT CACTGCATTC TAGTTCGTTC ATATTAGTTG CACCAACAGA

ACACCCTTCT CACTGCATTC TAGTTCATTC ATATTAGTTG CACCAACAGA

ACGCCTTTCT CGCTGCACTC GAGCTCGTTC ATATTGGTCG CACCAACGGA

ACGCCTTTCT CACTGCACTC TAGCTCGTTC ATATTAGTCG CACCGACAGA

---------- ---------- ---------- ---------- ----------

---------- ---------- ---------- ---------- ----------

ACGCCTTTTT CACTGCATTC TAGTTCATTC ATATTAGTTG CACCAACAGA

ACACCCTTTT CACTGCACTC TAGTTCGTTC ATATTAGTTG CGCCAACAGA

ACACCTTTCT CACTGCATTC TAGTTCGTTC ATATTAGTTG CACCAACAGA

ACACCTTTCT CACTGCATTC TAGTTCATTC ATATTAGTTG CACCAACAGA

ACGCCTTTTT CACTGCATTC TAGTTCATTC ATATTAGTTG CACCAACAGA

ACGCCCTTTT CACTGCACTC TAGCTCATTC ATATTAGTTG CACCAACAGA

AGGCATTCGT TACTAAATTA TAATTTATTC ATGTTAGTTC AATCAACGGT

ACTCCTTTCT CGCTGCATTC AAGTTCATTC ATATTGGTGG CGCCGACGGA

ACCTTTAATA AAACTTACGA TTTTTGTGGC CTAGCGTCAT AATTCTATCC

ACCTTTAATA AAACTTACGA TTTTTGTGGC CTAGCGTCAT AATTCTATCC

ACCTTTAATA AAACTTACGA TTTTTGTGGC CTAGTGTCAT AATTCTATCC

---------- --ACTTACGA TTTTTGTGGC CTAGTGTCAT AATTCTATCC

ACCTTTAATA AAACTTACGA TTTTTATGGC CTAGTGTCAT AATTCTATCC

ACCTTTAATA AAACTTACGG TTTTTGTGGC CTAGTGTCAT AATTCTATCC

ACCTTTAACA AAACTTACGG TTTTTGTGGC CCAGAGTCAT GATTCTATCC

ACCTTTAACA AAACTCACGG TTTTTGTGGC CTAGAGTCAT GATTCTATCC

---------- --ACTTACGA TTTTTGTGGC CTAGAGTCAT AATCCTATCC

---------- ---------- ---------- ---------- ----------

ACCTTGAAGG AAACTTACGA TTTTTGTGGC CTAGTGTCAT AATTCTATCC

ACCTTGAATA AAACTTACGA TTTTTGTGGC CTAGTGTCAT AATTCTATCC

ACCTTCAATA AAACTTACGA TTTTTGTGGC CTAGTGTCAT AATTCTATCC

ACCTTTAATA AAACTTACGA TTTTTGTGCC CTAGTGTCAT AATTCTATCC

ACCTTTAACA AAACTTACGA TTTTTGTGGC CTAGTGTCAT AATTCTATCC

ACCTTAAATA AAACTTACGA TTCTTGTGGC CTAATGTCAT AATCCTGTCC

TTTTATGCTG AAACTTACGA TTTTTGTGGC CTAATGTCAT AATTCTATCC

GCCTGTAACG TAACTTACGA TTTTTGTGGC CCAACGTCAT AATTCTATCC

ATATCATCAG CATTATTTAC CACATAAGCT GATAAATCTT TGATATAAAC

ATATCATCAG CATTATTTAC CACATAAGCT GATAAATCTT TGATATAAAC

ATATCATCAG CATTATTTAC CACATAAGCT GATAAATCTT TGATATAAAC

ATATCATCAG CATTATTTAC CACATAAGCT GATAAATCTT TGATATAAAC

ATATCATCAG CATTATTTAC CACATAAGCT GATAAATCTT TGATATAAAC

ATATCATCAG CATTATTTAC CACATAAGCT GATAAATCTT TGATATAAAC

ATGTCGTCAG CATTGTTGAC TACATATGCT GATAAATCTT TGATATATAC

ATGTCGTCAG CGTTGTTGAC TACATATGCT GATAAGTCTT TGATATATAC

ATATCATCAG CATTGTTTAC AACATAAGCT GATAAATCTT TGATATAAAC

---------- ---------- ---------- ---------- ----------

ATATCATCAG CATTATTTAC CACGTAAGCC GATAAATCTT TGATATAAAC

ATATCATCGG CATTATTTAC CACGTAAGCT GATAAATCTT TGATATAAAC

ATATCATCAG CATTATTTAC CACGTAAGCT GATAAATCTT TGATATAAAC

ATATCATCAG CATTATTTAC TACATAAGCT GATAAATCTT TGATATAAAC

ATATCATCAG CATTATTTAC CACGTAAGCT GATAAATCTT TGATATAAAC

ATATCATCGG CATTATTTAC CACGTAAGCT GACAAATCTT TAATATAAAC

ATATCATCTG CATTATTTAC CACATAAGCT GACAGATCTT TGATGTAAAC

ATATCATCTG CATTGTTCAC AACATAAGCT GACAGATCTT TGATGTAAAC

TCCCACATCA GGTCTTTCTT TAACCTATTA CCTCTAACCT TTGTGTCTGA

TCCCACATCA GGTCTTTCTT TAACCTATTA CCTCTAACCT TTGTGTCTGA

TCCCACATCA GGTCTTTCTT TAACCTATTA CCTCTAACCT TTGTGTCTGA

TCCCACATCA GGTCTTTCTT TAACCTATTA CCTCTAACCT TTGTGTCTGA

TCCCACATCA GGTCTTTCTT TAACCTATTA CCTCTAACCT TTGTGTCTGA

TCCCACATCA GGTCTTTCTT TAACCTATTA CCTCTAACCT TTGTGTCTGA

TCCCACGTCA GGCCTTTCTT TAACCTAATA CCTCCAACCT CTGTGTCTGA

TCCCACATCA GGCCTTTCTT TAACCTATTA CCTCTAACCT CTGGGTCTGA

TCCCACATCA GGTCTTTCTT TAACCTGTTA CCTCTAACCT CTGTGTCTGA

---------- ---------- ---------- ---------- ----------

TCCCACATCA GGTCTTTCTT TAACCTATTA CCTCTAACCT CTGTGTCTGA

TCCCACATCA GGTCTTTCTT TAACCTGTTA CCTCTAACCT TTGTGTCTGA

TCCTACATCA GGTCTTTCTT TAACCTATTA CCTCTAATCT CTGTGTCTGA

TCCCACATCA GGTCTTTCTT TAACCTATTA CCTCTAACCT CTGTGTCTGA

TCCCACATCA GGTCTTTCTT TAACCTATTA CCTCTAACCT CTGTGTCTGG

TCCCACATCA GGTCTTTCTT TAACCTATTA CCTCTAACCT CTGTGTTTGA

TCCTACATCA GGTCTTTCTT TAACCTATCA CCTCTAATCT TTGTGTCTGA

TCCCACATCA GGTCTTTCTT TAACCTATCA CCTCTAATCT CTGGGTCTGG

TCCTTGCCCA AAAGGTCACG AACTTCTTCA TTATATATTT CCAAATAAGA

TCCTTGCCCA AAAGGTCACG AACTTCTTCA TTATATATTT CCAAATAAGA

TCCTTGCCCA AAAGGTCACG AACTTCTTCA TTATATATTT CCAAATAAGA

TCCTTGCCCA AAAGGTCACG AACTTCTTCA TTATATATTT CCAAATAAGA

TCCTTTCCCA AAAGGTCACG AACTTCTTCA TTATATATTT CCAAATAAGA

TCCTTGCCCA AAAGGTCACG AACTTCTTCA TTATATATTT CCAAATAAGA

TCTTTACCCA GAAGGTCTCG GACTTCTTCA TTATATATCT CTAAGTATGA

TCTTTACCCA AAAGGTCTCG AACTTCTTCA TTATATATCT CCAAGTATGA

TCCTTGCCCA AAAGGTCACG AACTTCTTCA TTATATATTT CCAAATAAGA

---------- ---------- ---------- ---------- ----------

TCCTTGCCCA AAAGGTCACG GACTTCCTCA TTATATATTT CCAAATATGA

TCCTTGCCCA AAAGGTCACG AACTTCTTCA TTATATATTT CCAAATAGGA

TCCTTGCCCA AAAGATCACG AACTTCTTCA TTATATATTT CCAAATAAGA

TCCTTGCCCA AAAGGTCACG AACTTCTTCA TTATATATTT CCAAATAAGA

TCCTTGCCCA AAAGGTCACG AACCTCTTCA TTATATATTT CCAAATAAGA

TCCTTGCCCA AAAGGTCACG AACTTCTTCA TTATATATTT CCAAATAAGA

TCCTTTCCTA GAAGGTCACG CACTTCTTCA TTATAAATTT CCAAATAAGA

TCTTTTCCTA GCAGGTCACG CACTTCTTCG TTATAAATTT CCAAATAGGA

CACTCGAACC AAAAATCTAT AAACATTTTA AAATTTACCT TGTATCACCC

CACTCGAACC AAAAATCTAT AAACATTTTA AAATTTACCT TGTATCACCC

CACTCGAACC AAAAATCTAT AAACATTTTA AAATTTACCT TGTATCACCC

CACTCGAACC AAAAATCTAT AAACATTTTA AAATTTACCT TGTATCACCC

CACTCGAACC AAAAATCTAT AAACATTTTA AAATTTACCT TGTATCACCC

CACACGAACC AAAAATCTAT AAATATTTTA AAATTTACCT TGTGTCACCC

CACTCGAACC AGAAACCT-- --------TA AGGT--ACCT TGTGTCGCCC

CACTCGAACC AAAAACCT-- --------TA AGGT--ACCT TGTGTCGCCC

AACTCGAACC AAAAACCTAT AAACATTTTA AAATTTACCT TGTGTCACCC

---------- ---------- ---------- ---------- ----------

CACTCGAACC AAAAATCTAT AAACATT-TA AAATTTACCT TGTATCACCC

CACTCGAACC AAAAATCTAG AAACATTTTA AAATTTACCT TGTGTCGCCC

CACTCGAACC AAAAATCTAC aaatatttta aaa------- ----------

TACTCGAACC AAAAATCTAT AAACATTTTA AAATTTACCT TGTATCACCC

CACTCGAACC AAAAATCTGT AAATATTTTA AAAtttaCCT TGTATCACCC

TACTCGAACC AGAAATCtat aaatatttta aaaTTTACCT TGTATCGCCT

TACTCGAACC AAAAACCTGT GAATGTTTTA AaaTTTACCT TGTATCCCCC

TACTCGCACC AGAAATCTGT AAACATTTTG AAATTTACCT TGTGTCCCCC

TCCGCTTTTG CAATATGACC AAATATGTGA GCAAATGAAT TGGGAATTAT

TCCGCTTTTG CAATATGACC AAATATGTGA GCAAATGAAT TGGGAATTAT

TCTGCTTTTG CAATATGACC AAATATGTGA GCAAATGAAT TGGGAATTAT

TCTGCTTTTG CAATATGACC AAATATGTGA GCAAATGAAT TGGGAATTAT

TCTGCTTTTG CAATATGACC AAATATGTGA GCAAATGAAT TGGGAATTAT

TCTGCTTTTG CAATATGACC AAATATGTGA GCAAATGAAT TGGGAATTAT

TCTGCTTTTG CGATATGACC AAATATGTGA GCAAATGAGT TGGGGATGAC

TCTGCTTTTG CGATGTGACC AAATATGTGA GCAAATGAGT TGGGGATGAC

TCTGCCTTTG CAATATGACC AAATATATGA GCAAATGAAT TGGGAATGAT

---------- ---------- ---------- ---------- ----------

TCTGCTTTAG CAATGTGACC AAATATGTGA GCAAATGAAT TTGGAATAAT

TCTGCTTTTG CAATATGACC AAATATGTGA GCAAATGAAT TTGGAATGAT

---------- ---------- ---------- ---------- ----------

TCTGCTTTTG CAATATGACC AAATATGTGA GCAAATGAAT TTGGAATGAT

TCTGCCTTTG CAATATGACC AAATATGTGA GCAAATGAAT TTGGAATGAT

TCTGCTTTTG CAATATGACC AAATATGTGA GCGAATGAGT TTGGAATGAT

TCTGCTTTTG CAATATGACC AAATATATGA GCAAATGAAT TTGGGATAAT

TCTGCTTTTG CAATATGCCC AAAAATATGG GCAAATGAAT TTGGGATGAT

TCCTCTAAGT TCAGGAATAG CTCGAACACC TTCCATGGTA AAAGTTTTGC

TCCTCTAAGT TCAGGAATAG CTCGAACACC TTCCATGGTA AAAGTTTTGC

TCCTCTAAGT TCAGGAACAG CTCGAACACC TTCCATGGTA AAAGTTTTGC

TCCTCTAAGT TCAGGAACAG CTCGAACACC TTCCATGGTA AAAGTTTTGC

TCCTCTAAGT TCAGGAACAG CTCGAACACC TTCCATGGTA AAAGTTTTGC

TCCTCTAAGT TCAGGAACAG CTCGAACACC TTCCATGGTA AAAGTTTTGC

CCCTCTGAGC CCCGGCACTG CTCGAACACC TTCCATGGTG AAAGTCTTGC

CCCTCTGAGC CCCGGTACTG CTCGAACGCC TTCCATGGTG AAAGTCTTGC

TCCTCTAAGT TCAGGAACAG CTCGAACACC TTCCATGGTG AAAGTTTTGC

---------- ---------- ---------- ---------- ----------

TCCTCTAAGT TCAGGAACTG CTCGAACACC TTCCATGGTA AAAGTTTTGC

TCCTCTAAGT TCAGGAACAG CTCGAACACC TTCCATGGTA AACGTTTTGC

---------- ---------- ---------- ---------- ----------

TCCTCTAAGT TCAGGAACAG CTCGAACACC TTCCATGGTA AAAGTTTTGC

TCCTCTAAGT TCGGGAACAG CACGAACACC TTCCATGGTA AAAGTTTTTC

TCCTCTGAGT TCAGGAACAG CTCGAACACC TTCCATGGTG AAGGTTTTGC

TCCTCTAAGT TCAGGTACAG CTCGTACACC TTCCATGGTA AATGTTTTGC

TCCTCTAAGT TCAGGCACAG CTCGCACACC TTCCATTGTA AAGGTTTTGC

CTGTTCCGGT TTGTCCATAT GCAAAAATAG TCCCTGAAAT GAAGAACTTA

CTGTTCCGGT TTGTCCATAT GCAAAAATAG TCCCTGAAAT GAAGAACTTA

CTGTTCCAGT TTGTCCATAT GCAAAAATAG TCCCTGAAAT GAAGAACTTA

CTGTTCCAGT TTGTCCATAT GCAAAAATAG TCCCTGAAAT GAAGAACTTA

CTGTTCCAGT TTGTCCATAT GCAAAAATAG TCCCTGAAAT GACAAACTTA

CTGTTCCAGT TTGTCCATAT GCAAAAATAG TTCCTAAAAG GAAGAACTTA

CTGTCCCAGT CTGTCCATAT GCAAAAATGG TCCCTGAGAT GTAAGACTTA

CTGTCCCAGT CTGTCCATAT GCAAAGATGG TCCCTGAAAT GCAGAACTCA

CTGTTCCAGT TTGACCATAT GCAAAAATAG TCCCTGAAAT GAAGG-----

---------- ---------- ---------- ---------- ----------

CTGTTCCAGT TTGTCCATAT GCAAAAATAG TCCCTGAAAT TAAGAACTCA

CTGTTCCAGT TTGTCCATAT GCAAAAATAG TCCCTGAAAT TAAGAACTCA

---------- ---------- ---------- ---------- -----ACTCA

CTGTTCCAGT TTGTCCATAA GCAAAAATAG TCCCTGAAAT GAAGAACTTA

CTGTTCCAGT TTGTCCATAT GCAAAAATAG TTCCTGAAAT AAAGAACTTA

CTGTTCCCGT TTGTCCATAT GCAAAAATCG TTCCTGAAAT GAAGAAGCTA

CAGTTCCAGT CTGTCCATAT GCAAATATAG TACCTGAAAT AGAAAATTTA

CTGTTCCAGT CTGTCCATAT GCAAAAATAG TTCCTAAAAA AAAAAATTTA

CCATTGTAGC CTTCAAGTAC AGAATCAATA ATAGGTCTTG CAGTTAAGTT

CCATTGTAGC CTTCAAGTAC AGAATCAATA ATAGGTCTTG CAGTTAAGTT

CCATTGTAGC CTTCAAGTAC AGAATCAATA ATAGGTCTTG CAGTTAAGTT

CCATTGTAGC CTTCAAGTAC AGAATCAATA ATAGGTCTTG CAGTTAAGTT

CCATTGTAGC CTTCAAGTAC AGAATCAATA ATAGGTCTTG CAGTTAAGTT

CCATTGTAGC CTTCAAGAAC AGAATCAATA ATAGGTCTTG CAGTTAAGTT

CCATTGTAGC CTTCCAGGAC AGAATCAATA ATTGGTCTTG CAGTCAAGTT

CCATTGTAGC CTTCCAGAAC AGAATCAATA ATTGGTCTTG CAGTTAAGTT

---------- ---------- ---------- ---------- ----------

---------- ---------- ---------- ---------- ----------

CCATTGTAGC CTTCAAGAAC AGAATCAATA ATAGGTCTTG CAGTTAAGTT

CCATTGTAGC CTTCAAGCAC AGAATCAATA ATAGGTCTTG CCGTTAAGTT

CCATTGTAGC CTTCAAGAAC AGAATCAATA ATGGGTCTTG CAGTTAAGTT

CCATTGTAAC CTTCAAGAAC AGAATCAATA ATAGGCCTTG CAGTTAAGTT

CCATTGTAGC CTTCAAGAAC TGAATCAATA ATAGGCCTTG CGGTTAAGTT

TCACTA---- ---------- ---------- ---------- ----------

CCATTGTAGC CTTCTAGCAC AGAATCAATA ATGGGTCTTG CTGTTAGGTT

CCATTATAGC CTTCCAGAAC AGAGTCAATA ATAGGCCTTG CAGTCAAGTT

ATAAACATCA AGTTGTTTAC TCTCTGGTCC AAAAACAGTA TCAAAAGTAA

ATAAACATCA AGTTGTTTAC TCTCT-GTCC AAAAACAGTA TCAAAAGTAA

ATAAACATCA AGTTGTTTAC TCTCTGGTCC AAAAACAGTA TCAAAAGTAA

ATAAACATCA AGTTGTTTAC TCTCTGGTCC AAAAACAGTA TCAAAAGTAA

ATAAACATCA AGTTGTTTAC TCTCTGGTCC AAAAACGGTA TCAAAAGTAA

ATAAACATCA AGTTGTTTAC TCTCTGGTCC AAAAACAGTA TCAAAAGTAA

ATATACATCA AGTTGTTTAC TCTCTGGTCC AAAAACAGTA TCGAAAGTAA

ATACACATCA AGTTGTTTAC TTTCTGGTCC AAAAACAGTA TCGAAAGTAA

---------- ---------- ---------- ---------- ----------

---------- ---------- ---------- ---------- ----------

ATAAACATCA AGTTGTTTAC TCTCTGGTCC AAAAACAGTA TCAAAAGTAA

ATACACATCA AGCTGTTTAC TCTCTGGTCC AAAAACAGTA TCAAAAGTAA

ATAAACATCA AGTTGTTTAC TCTCCGGTCC AAAAACAGTA TCAAAAGTAA

ATAGACATCA AGTTGTTTAC TCTCTGGTCC AAAAACAGTA TCAAAAGTAA

ATAAACATCA AGTTGTTTAC TCTCTGGTCC AAAAACCGTA TCAAAAGTAA

---------- ---------- ------ggcc aacaagcaca ggaaaagtca

GTAAACATCA AGTTGTTTAC TATCTGGTCC AAAAACTGTG TCAAATGTAA

ATAGACATCG AGCTGTTTAC TCTCTGGGCC AAAAACAGTG TCAAACGTAA

ATGTCTTTGG AGGTTCATTG GAAGAATCAG TCTTATGTAC AGTGATAGTT

-TGTCTTTGG AGGTTCATTG GAAGAATCAG TCTTATGTAC AGTGATAGTT

ATGTCTTCGG AGGTTCATTG GAAGAATCAG TCTTATGTAC AGTGATAGTT

ATGTCTTCGG AGGTTCATTG GAAGAATCAG TCTTATGTAC AGTGATAGTT

ATGTCTTCGG AGGTTCATTG GAAGAGTCAG TTTTATGTAC AGTGATAGTT

ATGTCTTTGG AGGTTCATTG GAAGAATCAG TCTTGTGTAC AGTGATAGTT

ATGTCTTCGG AGGTTCGTTG GAAGAGTCGG TCTTATGGAC GGTGATAGTC

ATGTCTTTGG AGGTTCGTTG GAGGAATCGG TCTTATGGAC GGTGATAGTT

---------- ---------- ---------- ---------- ----------

---------- ---------- ---------- ---------- ----------

ATGTCTTTGG AGGTTCATTG GAAGAATCAG TCTTATGTAC AGTAATAGTT

ATGTCTTTGG AGGTTCATTG GAAGAATCAG TCTTATGCAC AGTGATAGTT

ATGTCTTTGG AGGTTCATTG GAAGAGTCAG TCTTATGAAC AGTGATAGTT

ATGTCTTCGG AGGTTCATTG GAAGAATCAG TCTTATGTAC AGTGATAGTT

ATGTCTTTGG AGGTTCATTG AAAGAATCAG TCTTATGTAC AGTGATAGTT

atgtcatcag ----tcattg aggagat--- ---------- ----------

ATGTCTTTGG AGGTTCGTTG GAAGAATCTG TTTTGTGCAC AGTAATAGTT

ATGTCTTTGG AGGTTCATTG GCAGAATCTG TTTTATTCAC TGTAATTGTT

CCCCTCATCT CATCCACACT GACAGCCTGT TTGTAGCACA TTGATTTCTC

CCCCTCATCT CATCCACACT GCCTGCATGT TTGTAGCACA TTGATTTCTC

CCCCTCATCT CATCCACACT GACAGCCTGT TTGTAGCACA TTGATTTCTC

CCCCTCATCT CATCCACACT GACAGCCTGT TTGTAGCACA TTGATTTCTC

CCCCTCATCT CATCCACACT GACAGCTTGT TTATAGCACA TTGATTTCTC

CCCCTCATCT CATCCACACT GACAGCCTGT TTGTAGCACA TTGATTTCTC

CCCCGCATTT CATCCACGCT GACGGCCTGT CTGTAGCACA TTGATTTCTC

CCCCTCATTT CATCCACGCT GACGGCCTGT CTATAGCACA TTGACTTCTC

---------- ---------- ---------- ---------- ----------

---------- ---------- ---------- ---------- ----------

CCCCTCATCT CATCGACACT GACAGCCTGT TTATAGCACA TTGATTTCTC

CCCCTCATCT CATCCACACT GACAGCCTGT TTGTAGCACA TTGATTTCTC

CCCCTCATTT CATCCACACT GACAGCCTGT CTGTAGCACA TTGATTTCTC

CCCCTCATCT CATCCACACT GACGGCCTGT TTGTAGCACA TGGATTTCTC

CCCCTCATCT CATCCACACT GACAGCCTGC TTATAGCACA TTGATTTCTC

---------- ---------- ---------- ---------- ----------

CCTCTCATCT CATCCACATT AACAGACAAT TTGTAGCACA TTGCTTTTTC

CCCCTCATCT CATCCACGCT AACGGAGAGT TTGTAACACA TAGCCTTCTC

TCTCTCATTG AGGGGCCGGC ACCTAACAAC AACCTTCACA TTATCGCAGC

TCTCTCATTG AGGGGCCGGC ACCGAACAAC AACCTTCACA TTATCGCAGC

TCTCTCATTG AGGGGCCGGC ATCTAACAAC AACCTTCACA TTATCGCAGC

TCTCTCATTG AGGGGCCGGC ACCTAACAAC AACCTTCACA TTATCGCAGC

TCTCTCATTG AGGGGCCGGC ACCTAACAAC AACCTTCACA TTATCGCAGC

TCTCTCATTG AGGGGCCGGC ACCTAACAAC GACCTTCACA TTATCACAGC

TCTCTCGTTG AGAGGTCGGC ACCTGACAAC TACCTTCACG TTGTCGCAGC

TCTCTCATTG AGAGGCCGGC ACCTAACCAC CACCTTCACA TTATCGCAGC

---------- ---------- ---------- ---------- ----------

---------- ---------- ---------- ---------- ----------

TCTCTCGTTG AGGGGCCGGC ACCTAACAAC GACCTTCACA TTATCACAGC

CCTCTCATTG AGGGGCCGAC ACCTAACCAC CACCTTCACG TTGTCACAGC

TCTCTCATTG AGGGGCCGGC ATCTAACAAC CACCTTCACA TTATCACAGC

TCTCTCATTG AGGGGCCGGC ACCTAACAAC GACCTTCACA TTATCGCAAC

TCTCTCATTG AGGGGCCGGC ACCTAACAAC AACCTTCACG TTGTCGCAGC

---------- ---------- ---------- ---------- ----------

TCTCTCGTTG AAGGGTCGAC ATCGAACAAC AACTTTGACA TTGTCGCAAC

CCGCTCATTA AACGGACGGC ATCGAACCAC AACTTTCACG TTGTCACAGC

TTTCTGGCTT CTCTGATTTA TTGATCTGTT GACAAAAATA GGGCTTTGGC

TTTCTGGCTT CTCTGATTTA TTGATCTGTT GATAAAAATA GGGCTTTGGC

TTTCTGGCTT CTCTGATTTA TTGATCTGTT GACAAAAATA GGGCTTTGGC

TTTCTGGCTT CTCTGATTTA TTGATCTGTT GACAAAAATA GGGCTTTGGC

TTTCTGGCTT CTCTGATTTA TTGATCTGTT GACAAAAATA GGGCTTTGGC

TTTCTGGCTT CTCTGATTTA TTGATCTGGT GACAAAAATA GGGCTTTTGC

TTTCTGGCTT CTCTGATTTA TTGATCTGGT GGCAAAAAGA AGGCTTTGGC

TTTCCGGCTT CTCCGACTTA TTGATCTGGT GACAAAAGTC AGGCTTTGGC

---------- ---------- ---------- --------TA GGGCTTTGGC

---------- ---------- ---------- ---------- ----------

TCTCAGGCTT CTCAGATTTA TTGATCTAGT GACAAAAACA GGGCTTTGGC

TCTCCGGCTT CTCTGATTTA TTGATCTGGT GACCAAAATA GGGCTTTGGC

TTTCTGGCTT CTCTGATTTA TTGATCTGGT GAT--AGATA GGACTTTGGC

TTTCCGGCTT CTCTGATTTG TTGATCTGGC GATAGGAATA GGGCTTTGGC

TTTCCGGTTT CTCTGACTTG TTGATCTGGT GACAAAAACA GGGCTTTGGC

---------- ---------- ---------- --------TA GGGCTTTGGC

TTTCAGGCTT CTCTGATTTG TTGATCTAAA CACAAAAATA GGAATTTATC

TTTCAGGTTT TTCTGGTTTT TCGATTTGTT GAGAAAAATA AGAATTTGGC

CTCTTTATTT TCTTTTTACA GTTTATTCCA CAAGTAAAAG ACTTCACAAA

CTCTTTATTT TCTTTTTACA GTTTATTCCA CAAGTAAAAG ACTTCACAAA

CTCTTTATTT TCTTTTTACA GTTTATTCCA CAAGTAAAAG ACTTCATAAA

CTCTTTATTT TCTTTTTACA GTTTATTCCA CAAGTAAAAG ACTTCATAAA

CTCTTTATTT CCTTTTTACA GGTAATGCCA C------AAG ACTTTATAAA

CTCTTTATTT TCATTTTACA GTTAATTCCA CATGTAAAAG ACTTCATAAA

CTCTTTATTT TTTTTTTACA GTCAATTCCA CGTGTAAAAG ACCTCATAAA

CTCtttattt tttttttaCA GTCAATTCCA CGTGTAAAAG ACGTCATAAA

CTCTTTATTT TCTTTTTACA GTTAATTCCA CATGTAAAAG ACTTCATAAA

---------- ---------- ---------- ---------- ----------

CTCTTTATTT TCTTTTTACA GTTAATTCCA CGCGTAAAAG ACTTCTTAAA

CTCTTTATTT TCTTTTTACA GTTAATTCCA CGCATAAAAG ACTTCATAAA

CTCTTTATTT TTTTTTAACA GGTAATTCCA CGCGTAAAAG ACTTCATAAA

CTTTTTATTT TCTTTTTACA GTTAATTCCA CGTGTAAAAG ACTTCATAAA

CTCTTTATTT TCTTTTTACA ATTAATTCCA CGCATAAAAG ACTTCATAAA

CACTTTATTT TCTTTTTACA GTTAATTCCA CATTTAAAAG ACTTCATTAA

TTTTTTATTT TCTCTTTACA GGTAATAGTA CCTGTGATGA TATTTGCTGT

TTCTTTATTT TTCTTTTACA GATAATACCG TCAGTCAAGA CACTTGCAGT

GCACTATGGC TCTATTCCCC GAATGAGCTG CTGAGGATGC TGGCTATTCT

GCACTATGGC TCTATTCCCC GAATGAGCTG CTGAGGATGC TGGCTATTCT

GCACTATGGC TCTATTCCCC GAATGAGCTG CTAAGGATGC TGGCTATTCT

GCACTATGGC TCTATTCCCC GAATGAGCTG CTAAGGATGC TGGCTATTCT

GCACTATGGC TCTATTCCCC GAATGAGCTG CTGAGGATGC TGGCTATTCT

GCACTATGGC TCTATTCCCC AAATGAGCTG CTGAGGATGC TGGCTATTCT

GCGT-GTGGC TCTGTACCCC GAATGAGCTG CTGAGGATGT TGGCTAATCT

GCGTTGTGGC TCTGTACCCC AAATGAGCTG CTGAGGATGT AGGCTATTCT

GCACTATGGC TCTATAGCCC CAGTGAGCTG CTGAGGATGC TGGCTATTCT

---------- ---------- ---------- ---------- ----------

GCACTATGGC TCTATAGCCC GAATGAGCTG CTGAGGATGC TGGCTATTTT

GCACTATGGC TCTATACCTC GAATGAGTTG CTGAGGATGC TGGCTATTTT

GCACTATGGC TGTATACCCC AAATGAGCTG TTGAGGATGC TGGCTA-TTT

GCACTATGGT TCTATACCCC GAATGAGCTG CTGAGGATGC TGGCTATTCT

GCGCTGTGGC TCTATGCCCT GAATGATCTG CTGAAGATGC TGGCTATTCT

GCACGATGGC TCTAGACCCC AGATGAGCTG CTGAAGATGC TGGCTATTCT

GTATCATCAC TGTACTCTCC TACAGAGCTG CTGAGAATGC AGGTCATCTT

GCGTTGTGGC TATGTTCTCC TACCGAGATT GTGAGGATAA GACTTATACT

GGACAGACTG CACTGGGACC TCTATATTGG GACGCCGCTG GACTTCTTGA

GGACAGACTG CACTGGGACC TCTATATTGG GACGCCGCTG GACTTCTTGA

GGACAGACTG CACTGGGACC TCTATATTGG GACGCCGCTG GACTTCTTGA

GGACAGACTG CACTGGGACC TCTATATTGG GACGCCGCTG GACTTCTTGA

GGACACATTG CACTGGGACC TCTATATCGG GACGCCGCTG GACTTCTTGA

GGACAAACTG CACTGGGACC TCTATATTGG GACGCCGCTG GATTTCTTGA

GGACAGACTT CATTAGAACC TCCACGTGGG AACGTCGCTG GATTTCTTGA

GGACAGACTC CATTAGGACC TCCACGCGGG AATGTCGCTG GGTTTCTTGA

GGACAGACTG CACTGGGACC TGTATCTCGC CACGCCACTG GATTTCTTGA

---------- ---------- ---------- ---------- ----------

GGACAAACTG CACTGGGACC TCTATATTGG GACGCCGCTG GACTTCTTGA

GGACAAACTG CACTGGGACC TCTATATTGG GACGCCGCTG GACTTCTTGA

GGACAAACTG CACTGGGACC TCTGAACTGG GACGCCGATG GAATTCTTGG

GGACGTACTG CAGTGGGACC TCTATATTGG GACGCCGCTG GACTTCTTGA

GGACAGACTG CACTGAGACC TCTATATCGG GATACCGCTG GACTTCTTGA

GGACAGAGTG CACCGAGACC TCTGTGTCGG GATGCCGCTG GACTGCAGGA

AGGCAATTTG CCCTAAGATC CTTTCACAGC CACAATGAGG GACTTCTTGT

AGATAAACTG GACTGGGACC TTTACACAGC AACACCGCTT GATTTCTTGA

CTATTGAGTA GGAGCAGCTG TTTACAACAT GGGATGGCAA AGCACAGGCG

CTATTGAGTA GGAGCAGCTG TTTACAACAT GGGATGGCAA AGCACAGGCG

CTATTGAGTA GGAGCAGCTG TTTACAACAT AGGACGGCAA AGCACAGGCG

CTATTGAGTA GGAGCAGCTG TTTACAACAT AGGACGGCAA AGCACAGGCG

CTGTTGAGTA GGAGCAGCTG TTTACAACAT AGGACGGCAA AGCACAGGCG

TTATTGAGTA GGAACAGCTG TTCACAACAT AGGACGGCAA AGCACAGGCG

TTATTGAGCA T---CAGCTG TTTACAACAC AGAACGGCAA GGCACAGGCG

TTATTGAGCA T---CAGCTG TTTACAACAC AGAACGGCAA GGCACAGGCG

CTCTTAAGCA CAAGCAGCTG TTCACA-CAC TGGACGGCCA AGCACAGGCG

---------- ---------- ---------- ---------- ----------

CCATTAAGTA CGAGCAGCTG TTCACAACAT AGGACGGCCA AGCACAGGTG

CCATTAAGTC CGAGCAGCTG CTCACAACAT AGGACGGCCA AGCACAGGCG

TCATTGAGTA TGAGCAGCTG TTCAC--CAC AGGACGG-CG GGCACAGGCG

CTATTGAGTA CGAGCAGCTG TTCACAACAT AGGACAGCAA AGCCCAGGCG

CTATTGAGTA CGAGCAGCTG TTCATCACAT AGGATGGCAA AGCACAGGCG

CTAGTGAGGA TGAGCAGCTG TTCACGACAT AGGACGGCAA AGCACGGGCG

TTGTTGAATA TAAGCAGCTA TTTACCAAGT AAGACTACAA AGCACAGATG

ACACTAAGCC TGAGCAGCCT ATGACCAAGT AAAACGAAGA AACAGAGGTA

TGCACACGCC CTTGCACAGA GTGAAAAGGT ATCTTAAATC CAACACAGTT

TGCACACGCC CTTGCACAGA GTGAAAAGGT ATCTTAAATC CAACACAGTT

TGCACACGCC CTTGCACAGA GTGAAAAGGT ATCTTAAATC CAACACAGTT

TGCACACGCC CTTGCACAGA GTGAAAAGGT ATCTTAAATC CAACACAGTT

TGCACACGTC CTTGCACAGA GTAAAAAGGT ATCTTAAATC CAACACAGTT

TGCACACGTC CTTGCACAGA GTGAAAATGT ATCTTAAATC CAACACAGTT

TGCACACGTC CTTGCACAGA GTGGAAAGTT ATCTTATACC CCACACCCTT

TGCACACGTC CTTGCACAGA GTGGAAAGTT ATCTTATACC CCACGCCATT

TGCACAGGTC CTCGCACAGA GTGAAAA--T GTGTTCGAGT CCA-------

---------- ---------- ---------- ---------- ----------

TGCACATGGC CTTGCACAGA GTGAAAATGT ATCGCAAATC CAACACAGTT

TGCACACTTC CTTGCACACA GTGAAAATGT ATCTTAAATT CAACACAGTT

AGCA------ ----CAC--- ---CAAATAG ATCTGCAACC CGGCACAGTC

TGCACACGTC TTTGCACAGA GTGAAAATGT ATCTCAAATC CAACACCGTT

TGCACACGTC CTTGCACAGA GTGAAAATGT ATCTCAAATC CAACACAGTT

TGCGCAAGTC CCCACACAGA ATGAAAATGT ATCACCAAGC CAACACAGTT

CACACAAATG TATGCACACA GTGAAAATAG ACCTAATATT CAAAATAATT

GACACCCCAC CCCACCCACA GTGAAAATGT ATTCACTATT CAAAATAATC

CCACCAGACT CCCACTTCTA AATTAACTTT CAAAGATTTT GACGGCACTT

CCACCAGACT CCCACTTCTA AATTAACTTT CAAAGATTTT GACGGCACTT

CCACCAGACT CCCACTTCTA AATTAACTTT CAAAGATTTT GACGGCACTT

CCACCAGACT CCCACTTCTA AATTAACTTT CAAAGATTTT GACGGCACTT

CCACCAGACT CCCACTTTTA AATTAACTTT CAAAGATTTT GACGGCACTT

CCACCAGACT CTCACTTCCA -ATTAATGTT CAAAGATTTT GATGGCACTT

CCACCAAATT CTCACGTCTA AGTTCACTTG CAAAGAATTT AACGGCACTT

CCACCAGACT CTCACGTCTA AGATCACTTG CAAAGAATTT AACGGCACTT

ACACCAAACT CCCGCTTCAA -GTTAACTTT CAAAGATTTT GACGGCACTC

---------- ---------- ---------- ---------- ----------

CCACCAGACT CCCACTTCTA AATTAACTTT TAAAGATTTT GACGGCACTT

TCACCAGACT CCCACTTCTA AATTAACTTT CAAAGATTTT GGCGGCACTT

CCGCCAGACA CCCATTCCCA AAACAACTTT CAGAGGTTTT GACGGCACTT

CCACCAGACT CCCACTTTCA AATTAACTTT CAAAGATTTT GACGGCACTT

CCACCAGACT CCCACTTCTA AATGAACTTT CAAAGGTTTT GACGGCACTT

CCACCAGACT CCCACTTGGA AATTAACTTT CAAAGGTTTC GACGGCACTG

CAACCATAAT CTCACtttta aattaacttt taaatatttt GACGGCACTT

CCACCAGACT CCCATTCTTA GACTAACTTT CAAAGATCTT TACAGCACTC

TATGGTGAGT TTCTCTTTTA AATACACACT GCAAAAATAT TTAACTTTCC

TATGGTGAGT TTCTCTTTTA AATACACACT GCAAAAATAT TTAACTTTCC

TATGGTGAGT TTCTCTTTTA AATACACACT GCAAAAATAT TTAACTTTCC

TATGGTGAGT TTCTCTTTTA AATACACACT GCAAAAATAT TTAACTTTCC

TATGGTGAGT TTCTCGTTTA AATACACACT GCAAAAATAT TTAACTTTCC

TATGGTGAGT TTCTCTTTTA AATACACACT GCAAAAATAT TTAACTTTCC

TATGGTCAGT TTCTCTCTTA GATACACACT GCAAAAATAT TTAACTTTCC

TATGGTCTGT TTCTCTCTTA GATACACACT GCAAAAATAT TTAACTTTCC

TATGGTGAAT TTCTCTTTTG AATACACACT GCAAAAATAT TTCACTTTGC

---------- ---------- ---------- ---------- ----------

TGTGGTGAGT TTTTCTTTTA AATACACACT GCAAAAATAT TTAGCTTTCC

TATGGTGAGT TTCTC--TTA AATACACACT GCAAAAATAT TTGACTTTCC

TATGGTGAAT CTCTCTTTTA AATACACGCT GCAAAAATAT TTAACTTTCC

TTTGGTGAGT TTCTCTTTTA AATACACACT GCAAAAATAT TTAACTTTCC

TATGGTGAGT TTCTCTTTTA AATATGCACT GCAAAAATAT TTCACTTTCC

TCCGGTGAGT TTCTCTTTTA AATACACACT GCAAAAATAG TTCACTTTCT

AATGATCAGT TTCTCTTTAA AATACACACT GCAAAAATAT TTACTTTTCC

TACAGTCAGT CTCTATTTTA AATGCACACT GCAAAAATAT TTAACTTTCC

ATTCTATGAA TACTGTACAT AAATATCTGT CTGCTTGCTA CACTTTACAC

ATTCTATGAA TACTGTACAT AAATATCTGT CTGCTTGCTA CACTTTACAC

ATTCTATGAA TACTGTACAT AAATATCTGT CTGCTTGCTA CACTTTACAC

ATTCTATGAA TACTGTACAT AAATATCTGT CTGCTTGCTA CACTTTACAC

ATTCTATGAA -----TACAT AAATATCTGT CTGCTTGCTA CACTTTACAC

ATTCTATGAA TACTGTACAG AAATATCTGT CTGCTTGCTA CACTTTACAC

ATTCTATGAA TATCGTATAT AAATATCTGT CTGCTTGCTA TACTTTACAC

ATTCTATGAA TATTGTATAT AAATATCTGT CTGCTTACTA CACTTTACAC

ATTCTGTGAC TATGGTACAT AAATAGCTAT CTGCTTGCT- CACTTTACAC

---------- ---------- ---------- ---------- ----------

ATTCTATGAA TACTGTACAT AAAAAGCTGT CTGCGTGCTA CACTTTACAC

ATTCTATGAA TACTGTACAT AAAAAGCTGT CTGCTTGCTG CACTTTACAC

ATTCTATGAA TACTGTACAT AAAAAGCTGT CATCTCGCTA CACTTTACAC

ATTCTATGAA TACTGTACAT AAAAAGCAGT CTGCTTGCTA CACTTTACAC

ATTCTATGAA TACTGTACAT AAAAAGCTAT GTGCTTACTA CACTTTACAC

AGTC--TGAC TGCTGTACAT AAAAAGCTCT CTGCTTGCAA CACTTTACAC

ATTCTAGTAA TACTGTAAAT AAAAAGCTAT CAGATTGCTA TAATTTACGC

ACTCTATTAA TACTGTACAT AAAAAGCCAT CTGTTTGCTA CATTTcacac

ACATTCACTT AGCTGTCTTT ATTCACCTAC ACACAATCCT TATTGAAGCT

ACATTCACTT AGCTGTCTTT ATTCACCTAC ACACAATCCT TATTGAAGCT

ACATTCACTT AGCTGTCTTT ATTCACCTAC GCACAATCCT TATTGAGGCT

ACATTCACTT AGCTGTCTTT ATTCACCTAC ACACAATCCT TATTGAGGCT

ACATTCACTT AGCTGTCTTT ATTCACCTAC ACACAATCCT TGTTGAGGCT

ACATTCACTT AGCTGTCTTT ATTCACCTAC ACACAATCCT CATTGAAGCT

ACATTCACTC AGCTGTCTTT ATTCACCTAC ACACAATCCT TATTGAGGCG

ACATTCACTT AGCTGTCTTT ATTCACCTAC ACACAATCCT TACTGAGGCG

ACATTCACTT AGCTGTCTCG ATTCATCTAC ACACAATCCT TATGGACGCT

---------- ---------- ---------- ---------- ----------

ACATTCACTT AGCTGTCTTT ATTCACCTAC ACACAATCCT TATTGAAGCT

ACATTCACTG AGCTGTCTTT ATTCACCTAC ACACAATCCT TATTGAGGCT

ACATTCACTG AGCTGTCTTT ATTCACCTAC ACACAATCCT TATTGAGGCT

ACATTCACTT GGCTGTCTTT ATCCATCTAC ACACAATCCT TATTGAAGCT

ACATTCACTT AGCTGTCTTT ATTTACCCAC ACACAATCCT TTTGGAGGCT

ACATTCACTT AGCTGTCTTT ATTCACCCAC ACACAACCCT TTTTGAGGCT

ACATTCTCTT AGCTGTCTTT ACTCACCCAT ATGTAACCTT TATTCAGGCT

acacacacac aACTGCCTTT GCTCACCCAC ACTTGACCCT TATTCCAGCT

TTAGACCATA TTGATCTGGC ACTTAAAAAA TTAAGGATAT GATTTGTTAG

TTAGACCATA TTGATCTGGC ACTTAAAAAA TTAAGGATAT GATTTGTTAG

TTAGACCATA TTGATCTGGC ACTTAAAAAA TTAAGGATAT GATTTGTTAG

TTAGACCATA TTGATCTGGC ACTTAAAAAA TTAAGGATAT GATTTGTTAG

TTAGACCATA TTGATCTGGC ACTTAAAAAA TTAAGGATAT GATTTGTAGT

TTAGACCATA TTGATCCGGC ACTTAAAAAA TTAAGGATAT GATTTGTTAG

TTAGACCGCA CTGATCTGGC ACTTAAAAAA TTCAGGATGT GCTT-GTTAG

TTAGACCGCA CTGATCTGGC ACTTAAAAAA TTCAGGATGT GCTT-GTTAG

TTAGACCATA TTGATCCGGC ACTTAAAAAA GTAGGGATAT GATTTATTAG

---------- ---------- ---------- ---------- ----------

TTAGACCATA TTGATCTGGC ACTTAAAAAA TTAAGGATAT GATTTGTTAG

TTAGACCATA TTGATCTGGC ACTTAAAAAA TTAAGGATAT GATTTGTTAG

TTAGACCATA TTGATCTGGC ACTTAAAAAA TTCAGGATAT GAT-TGTTAG

TTAGACCATA TTGATCTGGC ACTTGAAAAA TTAAGGATAT GATTTGTTAG

TTAGACCATA CTGATCTGGC ACTTAAAAAA TTAAGGATAT GATTTGTTAG

TTAGACCATA TCGATCTG-- ---------- -------TAT GATTTGTTAG

TTAGACCATA TTGATCTGGC ACTTAAAATA TTAGGGAAAT GATTTGTTAG

TTAGACCATT TTGATCTGGC ACTTACAAAA CCAAGGAGGT TATTTGTTAG

TAAGGCAAAA TTATGCAATG TGGAAATTCC CTGATGGAAA TTTAAATTGT

TAAGGCAAAA TTATGCAATG TGGAAATTCC CTGATGGAAA TTTGAATTGT

TAAGGC-AAA TTATGCAATG TGGAAATTCC CTGATGGAAA TTCAAATTGT

TAAGGC-AAA TTATGCAATG TGGAAATTCC CTGATGGAAA TTCAAATTGT

AAAGGC-AAA TTATGCAATG TGGAAATTCC CTGATGGAAA TTTAAATTGT

TAAGGCAAAA TTATGCAATA TGGAAATTCC CTGATGGAAA TTTAAATTGT

TAAGGCAAAA TTATGCAATG AGGAAATTCC CTGATGGAAA TCGAAACCGT

TAAGGCAAAA TTATGCAATG AGGAAATTCC CTGATGAAAA TCGAAACCGT

TAAGGCAAAA TTATGCAATG TGGAAATTCT CTGATGGAAA GTTAAGTTGT

---------- ---------- ---------- ---------- ----------

TAAGGCAAAA TTATGCAATG TGGAAATTCC CTGATGGAAA TTGAAATTGT

TAAGGCAAAA TTATGCAATG TGGAAATTCC CTGATGGAAA TTCAAATTGT

TAAGGCAAAA TTATGCAATG GGGCGATTCC CTGATGGAAA TGGAAATTGT

TAAGGCAAAA ATATGCAATG TGGAAATTCC CTGATGGAAG TTTAAATTGT

TAAGGCAAAA TTATGCAATG TGGAAATTCC CTGATGGAAA TTTAAATTGT

TAAGGCAAAA TTATGCAATG TGGCGATTTC CCGAAGGAGG CTGGCATCGT

TAAGGCAAAA TTATGCAACA TGAAAACTCC CTGATATAAA TCTGAACTGT

TAAGGCAAAA TTATGCAATA TGAAAATTCC CTGATGTGAA TGAAAATTGC

TTGCACTTTT GATCATTGAT ATAGGAAAAG TATGGCGTTT TCTGTTCTAA

TTGCACTTTT GGTCATTGAA ATAGGAAAAG TATGGGGTTT TCTGTTCTAA

TTGCACTGCT GGTCATTGAT ATATGAAAAG TATGGGGTTT TCTGTTCTAA

TTGCGCTTCT GGTCATTGAT ATATGAAAAG TATGGGGTTT TCTGTTCTAA

TTGCACTTTG GGTCATTGGT ATATGAAACG TATGGGGTTT TCTGTTCTAA

TTGCACTTTT GGTCATTGAT ATATGAAAAG TATGGGGTTT TCTGTTCTAA

TTGCACTTTT GGTCGTTGAT ATGTGAAAAC TAGAGGGGCC TCTGTCCTAA

TTGCACTTTT GGTTGTTGAT ATGTGAAAAC TAGAGGGGCC TCGGTCCTAA

GTGCACTTTT GGTCATTGAC ATAGGAAACG TATGGGGTTT TCTGTTCTAA

---------- ---------- ---------- ---------- ----------

TTGCATTTTT GGTCATTTAT CTATGAAAGG TAGGGGGTTT TGTGTTCTAA

TTGCACTTTT GGTCATTGAT ATATGAAAAG TATGGGGTTT TCTGTTCTAA

TTGCACTTTT GGTCATGGAT GTATGAAAAG TATGGGGGGT CCTGTTCTAA

TTGCACTTTT GGTCATTGAT ATATGAAAAG TATGGGGTTT TCTGTTCTAA

TTGCACTTTT GGTCAGTGAT ATATGAAAAG TATGGGGTTT TCTGTTCTGA

TTGCACTTTT GGTCACGGAC ATATGAAAAG GACGGGGTTT TCTGTTCTAA

TTGTACTTTG GGGTGATGAT ATGTTTAAAG TAAAGAGTTT GCTGATCTAA

TTACACTTTT GGTCCGTAAC ACATTCGAAG TATAAAATTC ACTGTTCTAA

CATTAAAAAC CCTCAGAGGA ATCCTTCCCT CCACGTCGCA TCCCTGACCA

CATTAAAAAC CCTCAGAAGA ATCCTTCCCT CCACCTCGCA TCCCTGACCA

CATTAAAAAC CCTCAGAGGA ATCCTTCCCT CCACGTCGCA TCCCTGACCA

CATTAAAAAC CCTCAGAGGA ATCCTTCCCT CCACGTCGCA TCCCTGACCA

CATTAAAAAC CCTCAGAGGA ATCCTTCCCT CCACGTCGCA TCCCTGACCA

CATTAAAAAC CCTCAGAGGA ATCCTTCCCG CCACGTTGCA TCCCTGACCA

CATTAAAAAA CCTCAGAGGA ATCCTTCCCT CCACGTCGCG TCCCTGACCA

CATTAAAAAC CCTCAGAGGA ATCCTTCCCT CCACGTCGCG TCCCTGACCA

CATTAAAAAT CCTCAGAGGA ATCCTTCCCT CCACGTCGCG TCCCTGACCA

---------- ---------- ---------- ---------- ----------

CATTAAAAAC CCTCAGAGGA ATCCTTCCCT CCACGTCGCG TCCCTGACCA

CATTAAAAAC CCTCAGAGGA ATCCTTCCCT CCACGTCGCG TCCCTGACCA

CATTAAAAAC CCTCAGAGGA AGCCTTCCCT TCACGTCGCG TCCATGAACA

CATTAAAAAC CCTCAGAGGA ATCCTTCCCT CCACGTCGCG TCCCTGACCA

CATTAAAAAC CCTCAGAGGA ATCCTTCCCG CCACGTCGCG TCCCTGACCA

CATTAAAAAC CCTCAGAGGA ATCCTTCCCG CCACGTCGTG TCCCTGACCA

TTTTAAAATC CCTCAGATGA ATCGTTCCTG CCCCGCCGGA TTCTTGTTCA

CTTTAAAAAC CCTCAGATGA ATCCTTCCCG CCACGTTGCG TTCTTGACCA

GGCAGCTGCA GCACTGTATG GCGGGCCACC AGCTGCTGCA GTTCAAGGGC

GGCAGCTGCA GCACTGTATG GGGGGCCCAC CACTGCTTCA GTTCAAGGGC

GGCAGCTGCA GCACTGTATG GCGGGCCACC AGCTGCTGCA GTTCAAGGGC

GGCAGCTGCA GCACTGTATG GCGGGCCACC AGCTGCTGCA GTTCAAGGGC

GGCAGCTGCA GCACTGTATG GCGGGCCACC AGCTGCTGCA GTTCAAGGGC

GACAGTTGCA GCACTGTATG GCAGGCCACC AGCTGCTGCA GTTCAAGGGC

GGCAGCTACA GCACTGGATG GCAGGCCACC AGCTGCTGCA GTTCAAGGGT

GGCAGCTGCA GCACTGGATG GCAGGCCACC AGCTGCTGCA GTTCAAGGGC

GGCAGCTGCA GCACTGTATG GCCGGCCACC AGCTGCTGCA GTTCAAGGGC

---------- ---------- ---------- ---------- ----------

GGCAGCTGCA GCACTGTATG GCGGGCCACC AGCTGCTGCA GTTCAAGGGC

GGCAGCTGCA GCACTGTATG GCGGGCCACC AACTGCTGCA GTTCAAGGGC

GGCACTTGCA GCACTGGCTG GCGGGCCACC GAGTGCTGCA ATTCAAGGGT

GGCAGCTGCA GCACTGTATG GCGGGCCACC AGCTGCTGCA GTTCAAGGGC

GGCAGCTGCA GCACTGTATG GCGGGCCACC AGCTGCTGCA GTTCAAGGGC

GGCAGCTGCA GCACTGGATG GCGGGCCACC AGCTGCTGCA GTTCAAGGGC

GGCAGTTGCA GCACTGTATG GCTTGCCACC AGCTACTGTA CTTTAAGGGC

GGCAGCTGCA GCACTGTATG GCATGCCACC AGCTATTGCA GTTCAAGGGC

TCCACACTGG CCTTGGTCAT CATCACCTTA GAGCTGGAGA GGTAGAAACA

TCCACACTGG CCTTGGTCAT CATCCCCTT- ---------- ----------

TCCACACTGG CCTTGGTCAT CATCACCTTA GAGCTGGAGA GGTAGAAACA

TCCACACTGG CCTTGGTCAT CATCACCTTA GAGCTGGAGA GGTAGAAACA

TCCACACTGG CCTTGGTCAT CATCACCTTA GAGTTGGAGA GGTAGAAACA

TCCACACTGG CCTTTGTCAT CATCACCTTA GAGTTGGAGA GGTAGAAACA

TCCACATTTG CTTTGGTCAT CATCACCTTA GAGCTGGAGA GGTAGAAACA

TCCACATTTG CTTTAGTCAT CATCACCTTA GAGTTGGAGA GGTAGAAACA

TCCACACTGG CCTTGGTCAT CATCACCTTA GAGTTGGAGA GGTAGAAACA

---------- ---------- ---------- ---------- ----------

TCCACACTGG CCTTGGTCAT CATCACCTTA GAGCTGGAGA GGTAGAAACA

TCCACACTGG CCTTGGTCAT CATCACCTTA GAGTTGGAGA GGTAGAAACA

TCCACAC--- ---------- ---------- ---------- ----------

TCCACACTGG CCTTGGTCAT CATCACCTTA GAGCTGGAGA GGTAGAAACA

TCCACACTGG CCTTGGTCAT CATCACCTTA GAGTTGGAGA GGTAGAAACA

TCCACACTGG CCTTGGTCAT CATCACCTTA GAGTTGGATA GGTAGAAACA

TCCACGCTGG ACCTTGTGAT CATCCCCTTA GCGCTGGAAA GGTAGAAACA

TCCACGCTGG CTTTGGTGAT CATCACCTTA GAGCTGGAGA GGTAGAAACA

CAGGTAGACA TCAACTTTGC CCCAGACCAG CCCAAAAAAG ACAAAAAACT

---------- ---------- ---------- CCCAAAAAAG ACAAAAAACT

CAGGTAGACA TCAACTTTGC CCCAGACCAG CCCAAAAAAG ACAAAAAACT

CAGGTAGACA TCAACTTTGC CCCAGACCAG CCCAAAAAAG ACAAAAAACT

CAGGTAGACA TCAGCTTTGC CCCAAACCAG CCCAAAAAAG ACAAGAAACT

CAGGTAGACA TCAACTTTGC CCCAGACCAG CCCAAAAAAG ACAAAAAACT

CAGGTAGACA TCAACTT--- ---------- CCCAAAAATG ACAAAAAACT

CAGGTAGACA TCAGCTC--- ---------- CCCAAAAATG ACAAAAAACT

CAGGTAGACA TCAACTTCGC CCCAGACCAG CCCAAAAAAG ACAAAAAACT

---------- ---------- ---------- ---------- ----------

CAGGTAGACA TCAACTTTGC TCCAGACCAG CCCAAAAAAG ACAAAAAACT

CAGGTAGACA TCAACTTTGC CCCAGACCAA CCCAAAAAAG ACAAAAAACT

---------- ---------- ---------- ---------- ----------

CAGGTAGACA TCAACTTTGC CCCAGACCAG CCCAAAAAAG ACAAAAAACT

CAGGTAGACA TCAACTTTGC CCCAGACCAG CCCAAAAAAG ACAGAAAACT

CAGGCAGACA TCAACTTTGC CCCAGACCAG CCAAAAAAAG ACAGAAAACT

TAAGTAGGAA TCAAAATCAC CCCAGACCAG CCCAAAAAAG ACAAAAACTA

CAGGTAGGGA TCAGATTCGC CCCAGACCCG CCCAAACCAA ACACGAAACT

CAAAATAAAT GCAAAATTTA ATGAAAAATA AGTACAAAGT GAATGGAAAA

CAAAATAAAT GCAAAATTTA ATGAAAAATA AGTACAAAGT GAATGGAAAA

CAAAATAAAT GCAAAATTTA ATGAAAAATG AGTACAAAGT GAATGGAAAA

CAAAATAAAT GCAAAATTTA ATGAAAAATG AGTACAAAGT GAATGGAAAA

CAAAATAAAT GCAAAATTTA ATGAAAAATG AGTACAAAGT GAATGGAAAA

CAAAATAAAT GCAAAATTTA ATGAAAAATG AGTACAAAGT GAATGGAAAG

CAAAATAAAT GCAAAATTTA ATGACAAATG AGTACAAAGT GGATGGAAAG

CAAAATAAAT GCAAAATTTA ATGACAAATG AGTACAAAGT GGATGGAAAA

CAAAAGAAAT GCAAAATTTA ATGAAAAAGG AGTACAAAGT GAATGGAAAA

---------- ---------- ---------- ---------- ----------

CAAAATAAAT GCAAAATTTA ATGAAAAAAG AGTACAAAGT GAATGGAAAG

CAAAATAAAT GCAAAATTTA ATGAAAAACA AGTACAAAGT GAGTGGAAAG

---------- ---------- ---------- ---------- ----------

CAAaataaat gcaaaattta atgataaatg agtacaaagt gaatggaaag

CAAaataaat gcaaaattta atgaaaaatg agtacaaagt gaatggaaag

CAAAATAAAT GCAAAATTTA ATGAAAAAAG ACTACAAAGT AAATGGAAAG

AAAAAAAAAA GTGAAATTTA ATAGGGAATG AATACAAGGT AAACAGAAAG

CAAAATAAAT GTGAAATTTA ATAAGAAATG AGTACAAAGT GAATGGGAAA

GAACAAAAAC AGTTGAAGCA ATTTAGAAAA GCATGTTATA ACAGACTAGC

GAACAAAAAC AGTTGAAGCA ATTTAGAAAA GCATGTTATA ACAGACTAGC

GAACAAAAAC AGTTGAAGCA ATTTAGAAAA GCATGTTATA ACAGACTAGC

GAACAAAAAC AGTTGAAGCA ATTTAGAAAA GCATGTTATA ACAGACTAGC

GAACAAAAAC AGTTGAAGCA ATTTAGAAAA GCAGGTTATA ACAGTCTAGC

GAACAAAAAC AGTTGAAGCA GTTTAGAAAA GCATGT--TA ACAGACTAGC

GAACAGAAAC AGTTGGAGTG GTTTAGACAT GCATGGGATA ACAGACAAGC

GAACAGAAAC AGTTGGAGTG GTTTAGAAAT GCATGGTATA ACAGAGAAGC

GAACAAAACC -GTTGCAGCA GTTCAGAAAA GC----TATG ACAGACAAGC

---------- ---------- ---------- ---------- ----------

GAACAAAAAC AGTTGAAGCA GTTTAGAAAA GCTTGTTATA ACAGACGAGC

GAACAAAAAC AGTTAAAGCA GTTTAGAAAA GCATATTATA ACAGACTAGC

---------- ---------- ---------- ---------- ----------

gaacaaaaac agttgaaGCA GTTTAGAAAA GCATGTTATA ACAGACTAGC

gaacaaaaac aattgaaGCA GTTTAGAAAA GCAAGTTATA ACAGACTAGC

GAACAAAAAC AGTTGAAGCT GTTTAGAAAA GCACATTATA ACAGACCAGC

ATACAAAAAC AGTTGAAGCA GTTTGGAAAA GCATGTTATT ACAAATCTGA

GAACAAAAAC GGTTGAAGCG GATTGGAGAA GTATCTTATT ACAAATCAGC

AAGCCACTGG TCTTGTTCAT GTTCCAATTC CATCCATAAA GAAATGCCAA

AAGCCACTGG TCTTGTTCAT GTTCCAATTC CATCCATAAA GAAATGCCAA

AAGCCACTGG TCTTGTTCAT GTTCCAATTC CATCCACAAA GAAATGCCAA

AAGCCACTGG TCTTGTTCAT GTTCCAATTC CATCCACAAA GAAATGCCAA

AAGCCACTGG TCTCGTTCAT GTTCCACTTC CATCCATAAA GAAATGCCAA

AAGCCACTGG TCTTGTTCAT GTCCCACTTT CATCCATAAA GAAATTCCAA

AAGCTGGTGG TCTCGTTCGG GGTCCACTTC TGTCCATAAA GAAGTCCCAA

AAGCTGGTGG TCTCAT-CAG GGTCCCCTTC TGTCCATAAA GAAGTCCCAG

AAGCCACTGG GCTTGTCCGG GTGCCCCTGT CATCCGTAAA GAAACTGCAA

---------- ---------- ---------- ---------- ----------

AAGCCACTGG TCTTGTTCAT GATCCACTTT CATCCGTAAA GAAATTCCAA

AAGCCACTGG TCTTATTCAT GTTCCACTTT CATCAGTAAA GAAATTGCAA

---------- ---------- ---------- ---------- ----------

AAGCCACTGG TCTTATGCAT GTTGTACTTT CATCCATAAA GAAATTCCGA

GAGCCACTAG TCTTGTTCAT GGTCCACTTT CATCGGTAAA GAAATTCCAA

AAGCCAC--- ---------- ---------- ------CAAA GAAATTCCAA

AAGTCACTGA CCCTGTTTAT GTATCACTTT TGCCAGTAAA GAAATTCCAA

AAGTCACTGA TCCTATTTAT GTTACAATCG TAATAGTAAA GACATGAAAA

ACACTGTCAT TACAGGAAGT TTATTACCCA CCTCTCCCTG TATTTATACA

ACACTGTCAT TACAGGAAGT TTATTACCCA CCTCTCCCTG TATTTATACA

ACACTGTCAT TACAGGAAGT TTATTACCCA CCTCTCCCTG TATTTATACA

ACACTGTCAT TACAGGAAGT TTATTACCCA CCTCTCCCTG TATTTATACA

ACACTGTCAT TACAGGAAGT TTATTACCCA CCTCTCCCTG TGTTTATACA

ACACTGTCAT TATAGGAAGT TTATTACCCA CCTCTCCCTA TATTTATACA

ACACTGGCAG AATAGGAACT TTATTCCCCA CCTCTCCCTG TGTCTCAATG

ACACTGGCAG AATAGGCACT TTATTCCCCG CCTCTCCCTA TAGTTCTATG

ACGCCGCCGT CACAGCAAGT TTATTGCCCA CCTCTCCCTA TAGTTATACA

---------- ---------- ---------- ---------- ----------

ACACTGTCAT TACAGGATGT TTATTGCCCA CCTCTCCCTA AATTTATACA

ACACTGTCAT GACAGGATGT TTATTACCCA CCTCTCCCTA TATTTATACA

---------- ---------- ---------- ---------- ----------

ACACTGTCAT TACAGGAAGT TTATTACCCA CCTCTCCCTA TATTTATACA

ACACTGTCAT TACAGGAAGT TTATTGCCCA CCTCTCCCTG TATTCATACA

ACACGGTCAT ----GGACGT TTATTCCTCG CTCT--CCTC TACTTACACA

GCACTGTCAT AATATGAAGT TTATTACCCA TGTCTCTGTG TATTAATACA

TCTCTGTCAT AACAGGTAGT TTATTACCTA TCTCTCTCTG CGCTTATACA

TAAGAAAGGT TTAGCTGATT TTTAGTTTTT AAAGGGAAGT GTTACTTATG

TAAGAAAGGT TTAGCTGATT TTTAGTTTTT AAAGGGAAGT GTTACTTATG

TAAGAAAGGT TTAGCGGATT TTTAGTTTTT AAAGGAAAGT GTTACTTATG

TAAGAAAGGT TTAGCGGATT TTTAGTTTTT AAAGGAAAGT GTTACTTATG

TAAAAAAGGT TTCGCGGATT TTTAGTTTTT AAAGGGAAGT GTTACTTATG

TAAGAAAGGT TTAGAGAATT TTTAGTTTTT AAAGGGAAGT GTTACTTATT

TAAGAA-GGT TTAGAGA-TC TTTAGTTTAC AAAGGGAAGT GTTACTTATG

TAAGAA-GGT TTAGAGA-TT TTTAGTTTTA AAAGGGAAGT GTTACTTATG

CGAGAAAGGT TTAGAGAA-- ---------- ---------- ----------

---------- ---------- ---------- ---------- ----------

TAAGAAAGGT TTAGAGAACT TTTAGTTTTT AAAGGGAAGG GTTACTTATG

TAAGAAAGGC TTAGAGAATT TTTAGTTCTT AAAGGGAAGT GTTACTTATG

---------- ---------- ---------- ---------- ----------

TAAGGAAGGT TTGGAGAAAT TTTAGTTTTT AAAGGGAAGT GTTACTTATG

TAAGAAAGGT TTAGAGAATT TTTAGTTTTT AAAGGGAAGT GTTACTTATG

TGAGAACCGT TTAGAGAGTT TTTAGTTTGT GAAGGAAGGT GTTACGTAGG

GAGGAAAAGC TTAGAGAATT TTTAGTTTTT AATGGGAAGT GTTACTGGTA

TACGGAACGT TTTTAGAAAA CTTAGTTTTT AAAGGGGATT GTTATTTATA

AAAATAATGG GAGAGGATAT TATTTGTCTT GACTGGTGCC AAAATAAATA

AAAATAATGG GAGAGGATAT TATTTGTCTT GACTGGTGCC AAAATAAATA

AAAATAATGG GAGAGGATAT TATTTGTCTT GATTGGTGCC AAAATAAATA

AAAATAATGG GAGAGGATAT TATTTGTCTT GATTGGTGCC AAAATAAATA

CAAATAATGG GAGAGGATAT TATTTGTCTT GAGTGGTGCC AAAATAAATA

AAAATAATGG GAGAGGATAT TATTTGTCTT TATTGGTGCC AAAATAAAAA

AAAATAACAG GAGAGGGTAT TATTTGTCTT TAGTGGTGCC AAAATGAAAA

AAAATAATGG GAGCGGATAT TATTTGTCTT TATTGGTGCC AAAATAAAAA

---------- ---------- ---------- ---------- ----------

---------- ---------- ---------- ---------- ----------

AAAATAATGG GAGAGGATAT TATCTGTCTT TATTTGTGCC AAAATAAAAA

AAAATAATGG GAGAGGATAT TATTCGTCTT TACTCGTGCC AAAATAAAAA

---------- ---------- ---------- ---------- ----------

AAAATAATGG GAGAGGATAT TCTCTGTCTT TATTCGTGCC aaaataaaaa

AAAATAATGG GAGAGGGTAT TATTTTTCTT TATTCATGCC AAAATAAAAA

AAAATAACGG TAGAGGGAAC AATTCATCTT TATTCATGCC AAAATAAAAA

AAAATAATGG GAAAGGATGT TACTTGGCTT TATTTGCATC AAGATACAAA

AAAATAATAG GAGGGGGTGT TATTCGACTT TATTTGCGCC AAGATGAAAA

TTTAGAGTTT TAAAGTTATT AAAATTAAGG CACAAACAGC TCCGTCAGGG

TTTAGAGTTT TAAAGTTATT AAAATTAAGG CACAAACAGC TCCGTCAGGG

TTTAGAGTTT TAAAGTTATT AAAATTAAGG CACAAACAGC TCCATCAGGG

TTTAGAGTTT TAAAGTTATT AAAATTAAAG CACAAACAGC TCCATCAGGG

TTTAGAGTTT TAAAGTTACT AAAATTAAGG CACAAACTGC TCCGTCAGGG

TTTAGAGTTT TTAAGTTATT AAAATTAGGG CACAAACTGC TCCGTCAAGG

TTTAGAGTTG AAAAGTTATT AAAATTAAGG CACAAGCTGC TCCGTCAAGG

TTTAGAGTTG AAAAGTTATT GAAATTAAGG CACAAACTGC TCCATCAAGG

---------- ---------- ---------- ---------- ----------

---------- ---------- ---------- ---------- ----------

TTTAGATTCT TAAAATTATT GAAATTAAGG CACAAACTGC TCCGTCAAGG

TTTAGAGTTT TAAAATTATT GAAATGAAGG CACAAACTGC TCCATCAAGG

---------- ---------- ---------- ---------- ----------

tttaaagttt taaaattatt gaaattaaGG CACAAACTGC TCCATCAAGG

TTTGGGGTTT TAAAATTATT GAAATTAAGG CACAGACTGC TCCGTCAAGG

TTTAGGGTTT TAAAATTATT GAAATGAAGG CCCAGACTGC TCCATCATGG

TTTAGACTTT TAGAATTAGG AAAGTTAAGG CACAAACTGC TCCATCAAGA

TTTAGACCCT TTTAATTACT GGAATTAAGA CCCAAACTGC TCAATCAATT

AATAGTGACA CTGTAAATGG AAGACCAGCC ACTGGCTCTG AAAAAAATAA

AATAGTGACA CTGTAAATGG AAGACCAGCC ACTGGCTCTG AAAAAAATAA

AATAGTGACA CTGTAAATGG AAGACCAGCC ACTGGCTCTG AAAAAAATAA

AATAGTGACA CTGTAAATGG AAGACCAGCC ACTGGCTCTG AAAAAAATAA

AATAGTGACA CTGTAAATGG AAGACCAGCC ACTGGCTCTG AAAAAAATAA

AATAGTGACA CTGTAAATGG AAGACCAGCC ACTGGCTCTG AAAAAAATAA

AATAGTGACA CTGTAAATGG AAGACCAGCC ACCGACTCTG GAAAAAATAA

AATAGTGACA CTGTAAATGG AAGACCAGCC ACCGGCTCTG GAAAAAATAA

---------- ---------- ---------- ---------- ----------

---------- ---------- ---------- ---------- ----------

AATAGTGACA CTGTAAATGG AAGACCAGCC ACTGGCTCTG AAAAAAATAA

AATAGTGACA CTGTAAATGG AAGACCAGCC ACTGGCTCTG AAAAAAATAA

---------- ---------- ---------- ---------- ----------

AATAGTGACA CTGTAAATGG AAGACCAGCC ACTGGCTCTG AAAAAAATAA

GATAGTGACA CTGTAAATGG AAGACCAGCC ACTGGCTCTG AAAAAAATAA

AATAGTGACA CTGTAAATGG AAGACCAGCC ACTGGCTCTG CAAAAAATAA

GATGGTGACA CTATAAATGG AAGACCAAGC ACTGGTTCTG AAAAAAATAA

GATGGTCACA CTATAAATGG AAGACCACCC ACTGGTTCTG AAAAAAATAA

ACCTCGTCAG TTTTGGTTCC AGCATTCTTT TACGTGTTTG CATAGAGAGG

ACCTCGTCAG TTTTGGTTCC AGCATTCTTT TACGTGTTTG CATAGAGAGG

ACCTCGTCAG TTTTGGTTCC AGCATTCTTT TACGTGTTTG CATAGAGAGG

ACCTCGTCAG TTTTGGTTCC AGCATTCTTT TACGTGTTTG CATAGAGAGG

ACCTTGTCAG TTTTGGTTCC AGCATTCTTT TACGTGTTTG CATAGAGAGG

ACCTTGTCAG TTTTGGTTCC AGCATTCTTT TACGTGTTTG CATAGAGAGG

ACCTTGTCAG TTTTGATTCC AGCATTCTTT GACGCATTTG CATAGAGAAG

ACCTTGTCAG TTTTGGTTCC AGCAGTCTTT GACGCATTTG CATAGAGAAG

---------- ---------- ---------- ---------- ----------

---------- ---------- ---------- ---------- ----------

ACTTTGTCAG TTTTGGTTCC AGCATTCTTT GATGTGTTTG CATAGAGAGG

ACCTCGTCAG TTTTGGTTCC AGCATTCTTT GACGTGTTTG CATAGAGAGG

---------- ---------- ---------- ---------- ----------

ACCTCGTCAG TTTTGGTTCC AGCATTCTTT TACATGATTG CATAGAGAGG

ACCTCGTCAG TTTTGGTTCC ATCATTCTTT TACATGTTTG CATAGAGAGG

ACCTCGTCAG TTT-GGCTCC AGCTTTCTTT TCCGTGTTTG CATAGAGATG

ACCTTGTCAG TTTCAGTTCC AACATTTTTT TTTGTCTTTG CTTGGGGGTG

ACCTCGTCAG TGCTGATCCC AACATGGTTT TGAGTCTCTG CATAGAGAAT

GGAAGGCTGT AAACACTGGA GTCTGTACCG GCTCCCAGTC CGTCCTCACA

GGAAGGCTGT AAACACTGGA GTCTGTACCG GCTCCCAGTC CGTCCTCACA

GGAAGGCTGT AAACACTGGA GTCTGTACCA GCTCCCAGTC CGTCCTCACA

GGAAGGCTGT AAACACTGGA GTCTGTACCA GCTCCCAGTC CGTCCTCACA

GGAAGGCTGT AAACACTGGA GTCTGTACCA GCTCCCAGTC CGTCCTCACA

GGAAAGCTGT AAACACTGGA GTTGGTACCG GCTCCCAGTC CGTCCTCACA

GGAAGGCTGT AAACACTGGA GTCTGTACGG GCTCCCAGTC CATCCTCACA

GAAAAGCTGT AAACACTGGA GTCTGTACCG GCTCCCAGTC CGTCCTCACA

--AAGGCTGT GAACCCTGGA GGCTGCACCG GCTCCCAGTC CGTCCTCGCA

---------- ---------- ---------- ---------- ----------

GGAAGGCTGT AAACACTGGA GTCTGTACCG GCTCCCAGTC CGTCCTCACA

GGAAGGCTGT AAACACTGGA GTCTGTACCG GCTCCCAGTC GGTCCTCACA

---------- ---------- ---------- ---------- ----------

GGAAGTCTGT AAACACCGGA GTCTGTACCG GCTCCCAGTC CGTCCGCACA

GGAAGGCTGT AAACACTGGA GTCTGTACCG GCTCCCAGTC CGTCCTCACA

G-AAGGCTGT AAACACTGGA GTCTGTACCA GCTCCCAGTC CGTCCTCACA

GGAAGGCTGT AAACACTGGA GTCTGTACTG GCTCTCAGTT TGTCCTCACA

AATAGGGTGT AAACACTGGA GCTGGTACTG GCTGTCTCTT TGCACTCACA

CCATGTTATG GGAGAAGGGT CACAAGGTGT AACTCAAGCA ACTTAACACA

CCATGTTATG GGAGAAGGGT CACAAGGTGT AACTCAAGCA ACTTAACACA

CCATGTTATG GGAGAAGGGT CACAAGGTGT AACTCAAGCA ACTTAACACA

CCATGTTATG GGAGAAGGGT CACAAGGTGT AACTCAAGCA ACTTAACACA

CCATGTTATG GGAGAAGGGT CACAAGGTGT AACTCAAGCA ACTTAACACA

CCATGTTATG GGAGAAGGGT CACAAGGTGT AACTCAAGCA ACTTAACACA

CCATGTTATG GGAGAAGGGT CACAAGGTGT AACTCAAGCA GTTTAACCCA

CCATGTTATG GGAGAAGGGT CACAAGGTGT AACTCAAGCG GTTTAACACA

CCAAGTTATG GGAGAAGGGC CACATGGCGT AACTCAAGCA AATTAACGCC

---------- ---------- ---------- ---------- ----------

CCATGTTATG GGAGAAGGGT CACAAGGTGT AACTCAAGGA ACTTAACACA

CCATGTTATG GGAGAAGGGT CACAAGGTGT AACTCAAGCA ACTTAACACA

---------- ---------- ---------- ---------- ----------

CCATGTTATG GGAGAAGGGT CACAAGCTGT AACTCAAGCA ATTTAACACA

CCATGTTATG GGTGAAGGGT CACAAGGTGT AACTCAAGCA ACTTAACACA

CCATGTTATG GGTGAAGGGT CACAAGGTGT GATTCACGCA ACTTAACACA

CCATGTTATG GGAGAAGGAT CACAAGGTGT AACTCAAGCA ACTTAACACA

CAATGCTATG ACAGAAGGAT CACAAGTTGG AACTCAAGCA ACTTAACACA

TGAAAGTCTA AAGTCCACTC GACATGTGCG TGCGTTAACT GAAAAATAAA

TGAAAGTCTA AAGTCCACTC GACATGTGCG TGCGTTAACT GAAAAATAAA

TGAAAGTCTA AAGTCCACTC GACATGTGCG TGCGTTAACT GAAAAATAAA

TGAAAGTCTA AAGTCCACTC GACATGTGCG TGCATTAACT GAAAAATAAA

TGAAAGTCTA AAATCCACTC GACATGTGCG TGCGTTAACT GAAAAATAaa

TGAAAGTCTA AAGTCCACTC GACATGTGCG TGCGTTAACT GAAAAATAAA

TGAAAGCCTA AAGTCCGCTC GACATGTGCG TGCTTTAACT GAAAAATAaa

TGAAAGCCTA AAGTCCGCTC GACATGTGCG TGCTTTAACT GAAAAATAAA

TGACAGTCTA AAGTCCGCTC GGCACGTGCG TGCGTTAACT GGAAAATAAA

---------- ---------- ---------- ---------- ----------

TGAAAGTCTA AAGTCCGCTC ----TGTGCG TGCGTTAACT GAAAAATAAA

TGAAAGTCTA AAGTCCGCTC GACATGTGCG TGCGTTAACT GAAAAATAAA

---------- ---------- ---------- ---------- ----------

TGAAAGTCTA AAGTCCGCTC GACATGTGTG TGCGTTAACT GAAAAATAAA

TGAAAGTCTA AAGTCCACTC GACCTGTGCG TGCGTTAACT GAAAAATAAA

TGAAAGGCTA AAGTCCACTC GACATGTGGG TGCGTTAACT GAAAAATAAA

TGAAAGTCTA AAGTCCATTC GACATGTGCG TGCTTTAACT GAAAAATAAA

TGACAGTAAA AAGTCCATTC GACATGTGCG TGCTTTAACT Gaaaaataaa

AATAAAAAAA AACAGACACA TTAAAAGATA TGCAGACCAA ATCAAACAGA

AATAAAAAAA AACAGACACA TTAAAAGATA TGCAGACCAA ATCAAACAGA

AATAAAAAAA AATAGACACA TTAAAAGATA TGCAGACCAA ATCAAACAGA

AATAAAAAAA AATAGACACA TTAAAAGATA TGCAGACCAA ATCAAACAGA

aataaaaaaa aacagacaca ttaaaagata cgcagaccaa atcaaacaga

AATAAAAAAA AACAGACACA TTAAAAGATA CGCAGACCAA ATCAAACATA

aataaaaaaa a-caGACACA TTAAAAGACA CGCAGACCAA ATCGAACAGA

AACAAAAAAA A-CAGACACA TTAAAAGATA CGCAGACCAA ATCAAACAGA

AATAAAAAAA AACAGACACA TTAAAAGACA AGCAGACCAA ACCAAACG--

---------- ---------- ---------- ---------- ----------

AATAAAAAAA AACAGACACA TTAAAAGGTA CGCAGACCAA ATCAAACAGA

AATAAAAAAA AACAGACACA TTAAAAGATA CGCAGACCAA ATCAAACAGA

---------- ---------- ---------- ---------- ----------

AATAAAAAGA AACAGACACA TTAAAAGATA CACAGACCAA ATCAAACAGA

AATAAAAAAA AACAGGCACA TTAAAAGATA CGCAGACCAA ATCAAACAGA

AACaaaaaaa acCAGACATG TTAAAAGATA CGCAGACCAA ATCAAACAGA

AATAAAAAAA GACACACACA TTAAAAGATA TGCAGACCAA ATCAAAGAGA

agcaaaaaaa aacaaaCCCA TTAAAAGATA CACACACAGA ATCCAACAGA

AGCAAAAGAC AGCAAGATGG ACACCGTGAA AGGGCAGTCA GTTTGTGACG

AGCAAAAGAC AGCAAGATGG ACACCGTGAA AGGGCAGTCA GTTTGTGACG

AGCAAAAGAC AGCAAGATGG ACACTGCGAA AGGGCAGTCA GTTTGTGACG

AGCAAAAGAC AGCAAGATGG ACACTGCGAA AGGGCAGTCA GTTTGTGACG

agcaaaagac agcaaGATAG ACACTGAGAA AGGGCAATCA GTTTGTGATC

AGCAAAAGAC AGCAAGATGG ACACCAGGAA AGGGCAGTCA GTTTGTGTCG

AGCAAAAGAC AGCAC--TGG GCACTGGGAA AGAGCAGTCA GTTTGTGACG

AGCAAAAGAC AGCAC--TGG GCACTGGGAA AGAGCAGTCA GTTTGTGACG

AGCAGGAGGC AGCGAGACGG ACACGGGGAG AGGGCAGTCA GTTTGTGAGG

---------- ---------- ---------- ---------- ----------

AACAAAAGA- --CAAGTTGG ACGCTGGGAA AGGGCAGTCA GTTTGTGACG

AGCAAAAGAC AGCAAGATGA ACACTGGGAA AGGGGATTCG GTTTGTCACA

---------- ---------- ---------- ---------- ----------

AGCAAAAGAC AGCAAAATGG ACACTGGGAA AGGGCAGTCA GTTTGTCACG

AGCAAAAGAC AGCAAGGTGG ACACTGGGAA AGGGCAGTCA GTTTGTGACG

AGCAAA---C AGCAAGGCGG GCACGGGGTA GGGGCAGTCA GTTTGTGACC

AGCAAAATAT GACGAGACAG ACGGTGGGAA AGAGCAGTCA GTTTGTGACG

AACAAAATGC GGCAAGGCGG ACAGTGGGAA AGGAGAGTCA GTTTTTGATG

AGCAAAGCAG AGAATTCACC TCAGTTTGTG GTGTCAAGAT CTGATTGGAA

AGCAAAGCAG AGAATTCACC TCAGTTTGTG GTGTCAAGAT CTGATTGGAA

AGCAAAGCAG AGAATTCACT TCCATTTGTG GCGTCAAGAT CTGATTAGAA

AGCAAAGCAG AGAATTCACT TCCGTTTGTG GCGTCAAGAT CTGATTGGAA

AGCAAAGCAG AGAATTCACT TCAGTTTGTG GCATCAAGAT CTGACTGGAA

AGCAAAGCAG AGAATTCACT TCGGTTTGTG GCATCAAGAT CTGATTGGAA

AGCAAAGCAG GGAACTCGCT TCAGTTTGTG GCGTCAAAAT CTGATTGGAA

AGCAAAGCAG GGAACTCGCT TCAGTTTGTG GCGTCAAAAT CTGATTGGAA

AGCAAAGCAC AGAGTTCGCA GCAGTGTGTG GCGTCAAGAT CTGACTGGAA

---------- ---------- ---------- ---------- ----------

AGCAAAGCAG AGAATTCGCT TCAGTTTGTG GCATCAAGAT CTGATTGGAA

AGCAAAGCAG AGAATTCGCT TCAGTTTGTG GCGTCAAGAT CTGATTGGAA

---------- ---------- ---------- ---------- ----------

AGCAAAGCAG GGAATTCACT TCAGTTTGTG GCGTCAAGAT CTGGTTGGAA

GGCAAACCAG AGACCTCGCT TCAGTTTGTG GCGTCAAGAT CTGGTTGGAA

AGCAAACCAG AGGACTCGCT GCAGTGTGTG GCGTCAAGAT CTGGTTGGAA

ACCAAATCAG AGAAATAGCT TCCATTTGTA ACATCATGAT CTGGTTGGAA

ACCGAATAAG AGAATCTGCT TCTGTTTGGG ACATCATGAT CTGGTTGGAG

GTAATTTTGC AAAAATGGAT TCAAATTTAC CTACTTCTTC TTGTCCTTGT

GTAATTTTGC AAAAATGGAT TCAAATTTAC CTACTTCTTC TTGTCCTTGT

GTAATTTTGC AAAAATGGAT TCAAATTTAC CTACTTCTTC TTGTCCTTGT

GTAATTTTGC AAAAATGGAT TCAAATTTAC CTACTTCTTC TTGTCCTTGT

GTAATTTTGC AAAAATGGAT TAAAATTTAC CTACTTCTTC TTGTCCTTGT

GTAATTTTGC AAAAATGGAT TAAAATTTAC CTACTTCTTC TTGTCCTTGT

GTAATTTCGC AAAAATTGAT TAAAATTTAC TTACTTCTTC TTGTCCTTGT

GTAATTTCGC AAAAATTGAT TAAAATTTAC TTACTTCTTC TTGTCCTTGT

GTAATGTTGC AAAAGTGGAT TAAAATGTAC CTACTTCTTC TTGTCCTTGT

---------- ---------- ---------- ---------- ----------

GTAATTTTGC AAAAATGGAT TAAAATTTAC CTACTTCTTC TTGTCCTTGT

GTAATTTTGC AAAAATGGAT TAAAATTTAC CTACTTCTTC TTGTCCTTGT

---------- ---------- ---------- ---------- ----------

GTAATTTTGC AAAAACGGAT TAAAATTTAC CTACTTCTTC TTGTCCTTGT

GTAATTTTGC AAAAACAGAT TAAAATTTAC CTACTTCTTC TTGTCTTTGT

GTAATTTTGC AAAAATGGAT TAACATTTAC CTACTTCTTC TTGTCTTTGT

ATAGTTTTGT AAAAACGGAT TAAGCGTTAC TTACTTTTTC TTGTCCTTGT

ATAGTTTTGC AAGAAGGGAT TATTAATTAC TTACTTTTTC TTGTCTTTGT

CCTTCCTCAG GGGCTGCTGC GAGGTGGCGT GCAAGGCCTG CGACTGCAGG

CCTTCCTCAG GGGCTGCTGC GAGGTGGCGT GCAAGGCCTG CGACTGCAGG

CCTTCCTCAG GGGCTGCTGC GAGGTGGCGT GCAAGGCCTG CGACTGCAGG

CCTTCCTCAG GGGCTGCTGC GAGGTGGCGT GCAAGGCCTG CGACTGCAGG

CCTTCCTCAG GGGCTGCTGC GAGGTGGCGT GCAAGGCCTG CGACTGCAGG

CCTTCCTCAG GGGCTGCTGT GAGGTGGCGT GCAAGGCCTG TGACTGCAGG

CTTTCCTCAG AGGCTGCTGT GAAGTGGCAT GTAAGGCCTG TGACTGCAGG

CTTTCCTCAG AGGCTGCTGT GAAGTGGCAT GCAAGGCCTG TGACTGCAGG

CCTTGCGCAG CGGCTGCTGC GAGGTGGCCT GCAGGGCCTG CGACTGAAGG

---------- ---------- ---------- ---------- ----------

CCTTCCTCAG AGGCTGCTGG GAGGTGGCAT GCAAAGCCTG GGACTGCAGG

CCTTCCTCAG AGGCTGCTGC AAGGTGGCGT GCAAGGCCTG AGACTGCAGG

---------- ---------- ---------- ---------- ----------

CTTTCCTCAG GGGCTGCTGC GAGGTGGCTT GCAAGGCCTG CGACTGCAGG

CCTTCCTCAG AGGCTGCTGT GAGGTGGCAT GAAAGGCCTG CGACTGCAGA

CTTTTCGCAG AGGCTGCTGC GAGGTGGCGT GGAAGGCCTG CGACTGCAGG

CTCTTCTCAA TGGTTGCTGT GAGGTGGCCT GTAAGGCTTG GACCTGCATG

CCTTTTTCAG GGGCTGCTGG GAGGTGGCTT GCAGGGACTG AGATTGCAGG

GCCTCCACCG CAGCCTTCCG GCGATTGAAG GCGTTGGTCT CCTCCTCCAG

GCCTCCACCG CAGCCTTCCG GCGATTGAAG GCGTTGGTCT CCTCCTCCAG

GCCTCCACCG CAGCCTTCCG GCGATTGAAG GCGTTGGTCT CCTCCTCCAG

GCCTCCACCG CAGCCTTCCG GCGATTGAAG GCGTTGGTCT CCTCCTCCAG

GCCTCCACCG CGGCCTTCCG GCGGTTGAAG GCATTGGTCT CCTCCTCCAG

GCCTCCACCG CAGCCTTCCT GCGGTTGAAG GCGTTGGTCT CCTCCTCCAG

GCCTCCATGG CTGCCTTCCG GCAGTTGAAG GCGTTGGTCT CCTCTTCCAG

GCCTCCATGG CCGCCTTCCG GCAGTTGAAG GCGTTGGTCT CCTCTTCCAG

GCCTCCA--- ---CCTTCCT GCGGCTGAAG GCGCGGGTCT TATGCAGCAG

---------- ---------- ---------- ---------- ----------

GCCTCCACAG CGGCCTTCCT GCGGTTGAAG GCGTTCGTCT CCTCCTCCAG

GCCTCCACCG CGGCCTTCCT GCGGTTGAAG GCGTTGGTCT CCTCCTCCAG

---------- ---------- ---------- ---------- ----------

GCCTCCACTG CGGCCTTCCT GCGGTTGAAG GCGTTGGTCT CCTCTTCCAG

GCCTCCACAG CTGCCTTTCT GCGATTGAAG GCATTGGTCT CCTCCTCCAG

GCCTCCACGG CTGCTTCTCT GCGGGTAAAG GCGCTGGTCT CCTCCTCCAG

GCCTCAACAG CTGCCTTCCT CCGATTGAAG GCAATTTTCT CCTCCTCAAG

GTTTCCACGG CCACTTTCCG CCGGTTGAAA GCGTTCAtct cctcctccag

TTCCCGGCGC TTTTCCTCCA CCTTGCGCTT CTCCTCCTGG TGGACCCGCT

TTCCCGGCGC TTTTCCTCCA CCTTGCGCTT CTCCTCCTGG TGGACCCGCT

TTCCCGGCGT TTTTCCTCCA CCTTGCGCTT CTCCTCCTGG TGGACCCGCT

TTCCCGGCGT TTTTCCTCCA CCTTGCGCTT CTCCTCCTGG TGGACCCGCT

CTCGCGGCGC TTTTCCTCCA CCTTGCGCTT CTCCTCCTGG TGGACCCGCT

TTCCCGGCGC TTCTCCTCCA CCTTGCGCTT CTCCTCCTGG TGGACCCGCT

CTCCCTGCGC TTCTCCTCTA CCTTGCGCTT CTCCTCCTGG TGGATCCGCT

CTCCCTGCGC TTCTCCTCTA CCTTGCGCTT CTCCTCCTGG TGGATTCGCT

CTCCCGGCGC TTCTCCTCCA CCTTGCGCTT CTCCTCC--- ----------

---------- ---------- ---------- ---------- ----------

CTCCCGCCGC TTCTCCTCCA CCTTGCGCTT CTCCTCCTGG TGGATCCGCT

CTCCCGGCGC TTCTCCTCCA CCTTCCGCTT TTCCTCCTGG TGGACCCGCT

---------- ---------- ---------- ---------- ----------

TTCCCGCCGC TTCTCCTCCA CTTTGCGCTT CTCCTCCTGA TGGACCCGCT

CTCCCGCCGC TTCTCCTCCA CCTTGCGCTT CTCCTCC-GG TGGACCCGCT

CTCCTGCCGC TTCTCCTCCA CCCTGCGCTT CTCCTCCGGG TGCACCCGCT

TTGCCGTCGT TTTTCTTCCA CCTTCCGCTT TTCTTCCTGG TGAGTCCTCT

ctcccttctc ttctcctcca ccttcctctt ctcctcctGG TGTATCCTCT

TCAGGTGCTC AAACTTCTCA TGGAGCTGGC ACATACCTCC CTTTCCTTCT

TCAGGTGCTC AAACTTCTCA TGGAGCTGGC ACATACCTCC CTTTCCTTCT

TCAGGTGCTC AAACTTCTCA TGGAGCTGGC ACATACCTCC CTTTCCTTCT

TCAGGTGCTC AAACTTCTCA TGGAGCTGGC ACATACCTCC CTTTCCTTCT

TCAGGTGCTC AAACTTCTCA TGAAGCTGGC ACATACCTCC CTTTCCTTCT

TCAGGTGCTC GAACTTCTCA TGGAGCTGGC ACACACCTCC CTTTCCTTCT

TCAGGTGCTC AAACTTCTCA TGGAGCTG-C ACATACCTCC CGTTCCTTCT

TCAGGTGCTC AAACTTCTCA TGGAGCTG-C ACACACCTCC CGTTCCTTCT

---------- ---------- ---------- ---------- ----------

---------- ---------- ---------- ---------- ----------

TAAGGTGCTC AAACTTCTCA TGGAGCTGGC ACACACCTCC CTCTCTTTCT

TGAGGTGCTC AAACTTCTCG TGGAGCTGGC ACCCACCTCC CTTTCCTTCT

---------- ---------- ---------- ---------- ----------

TGAGGTGCTC AAACTTCTCA TGGAGCTGGC ACACACCTCC CTTTCCTTCT

TCAGGTGCTC AAACTTCTCA TGAAGCTGGC ACACACCTCC CTTTCCTTCT

T--------- ---------- ---------- ---------- ----------

TTAAGAGCTC AAATTTCTCA TGAAGCTAGC ACTTACCTCC TTCTCTTTCT

TTAGGTGCTC AAACTTCTCG TGGAGCTGGC ACTCACCTCC CTCTCCTTCT

CCTTCAGCTC CAGCTCTGTC TCCTTCACTT TGTTGACAAA CATCTGCCTC

CCTTCAGCTC CAGCTCTGTC TCCTTCACTT TGTTGACAAA CATCTGCCTC

CCTTCAGCTC CAGCTCTGTC TCCTTCACTT TGTTGACAAA CATCTGCCTC

CCTTCAGCTC CAGCTCTGTC TCCTTCACTT TGTTGACAAA CATCTGCCTC

CCTTCAGCTC CAGCTCTGTC TCCTTCACTT TGTTGACAAA CATCTGCCTC

CCTTCAATTC CAGCTCTGTC TCCTTCACTT TGTTGACGAA CATCTGCCTC

CCTTCAATTC AAGCTCTGTC TCCTTCACTT TGTTGACAAA CATCTGCCTC

CCTTCAGCTC AAGCTCTGTC TCCTTGACTT TGTTGACAAA CATCTGTCGC

---------- ---------- ---------- ---------- ----------

---------- ---------- ---------- ---------- ----------

CCTTCAGCTC CAGTTCTGTC TCCTTCACTT TGTTGACAAA CATCTGCCTC

CCTTCAGCTC CAGTTCTGTC TCCTTCACTT TGTTGACAAA CATCTGCCTC

---------- ---------- ---------- ---------- ----------

CTTTCAACTC CAGCTCTGTC TCCTTCACTT TGTTAACAAA CATCTGCCTC

CCTTCAACTC CAGCTCCGTC TCCTTCACTT TGTTGACGAA CATCTGCCTC

---------- ---------- ---------- ---------- ----------

CTTTCAGTTC CAACTCTGTC TCCTTCACCT TGTTCACAAA CATCTGTCTC

CCTTCAGCTC CGACTCAGTC TCTTTCACTT TGTTGACAAA CATTTGCCTC

ATCTCTTCCT CCTTCCTCTG CAGCTCACTT AGGAACTCCT TCCTCTTGGC

ATCTCTTCCT CCTTCCTCTG CAGCTCACTT AGGAACTCCT TCCTCTTGGC

ATCTCTTCCT CCTTCCTCTG CAGCTCACTC AGGAATTCCT TCCTCTTGGC

ATCTCTTCCT CCTTCCTCTG CAGCTCACTC AGGAATTCCT TCCTCTTGGC

ATCTCTTCCT CCTTCCTCTG CAGCTCACTC AGGAACTCCT TCCTCTTGGC

ATCTCTTCCT CCTTCCTCTG CAGCTCACTC AGAAACTCCT TCCTCTTGGC

ATCTCTTCCT CCTTCCTCTG TAGCTCGCTC AGGAACTCCT TCCTCTTTGC

ATCTCTTCCT CCTTCCTCTG CAGCTCGCTT AGGAACTCCT TCCTCTTTGC

---------- ---------- ---------- ---------- ----------

---------- ---------- ---------- ---------- ----------

ATCTCCTCCT CCTTCCTCTG CAGCTCACTC AGGAACTCCT TGCGCTTGGC

ATCTCTTCCT CCTTCCTCTG CAGCTCACTC AGGAACTCCT TCCTCTTGGC

---------- ---------- ---------- ---------- ----------

ATCTCTTCCT CCTTCCTCTG CAGCTCACTC AGGAACTCCT TCCTCTTGGC

ATCTCTTCCT CCTTCCTCTG CAGCTCACTC AAGAACTCCT TCCTCTTGGC

---------- ---------- ---------- ---------- ----------

ATCTCTTCTT CCTTCTTCTG CAGCTCACCT AGGAACTCCT TTCTCCTGGT

ATTTCCTCCT CCTTCCGCTG CAGCTCCCCG AGAAACTCTT TCCTCTTCGT

CTCGTATGTC TCTTGTAGGC TGGTCACCTG AAGGGCTGGC TGTCACCATC

CTCGTATGTC TCTTGTAGGC TGGTCACCTG AAGGGCTGGC TGTCACCATC

CTCATATGTC TCTTGTAGGC TGGTCACCTG AAGGGCTGGC TGTCACCATC

CTCATATGTC TCTTGTAGGC TGGTCACCTG AAGGGCTGGC TGTCACCATC

CTCATATGTC TCTTGTAGGC TGGTCACCTG AAGGGCTGAC TGTCACCATC

TTCATATGTC TCTTGTAGAC TGGTCACCTG AAGGGCTGAC TGTCACCATC

CTCGTACGTC TCTTGGAGGC TGCTCACCTG AAGGGCTGGC TGTCACCATC

CTCATATGTC TCTTGGAGGC TGCAGACCTG AAGGGCTGGC TGTCACCGTC

---------- ---------- ---------- ---------- ----------

---------- ---------- ---------- ---------- ----------

CTCGTAGGTC TCCTGCAGGC TGGTCACCTG AAGGGCTGGC TGTCACCGTC

CTCGTAGGTC TCCTGTAGGC TGGTCACCTG AAGGGCTGGC TGTCCCCGTC

---------- ---------- ---------- ---------- ----------

CTCGTATGTC TCCTGTAGGC TGGTCACCTG AAGGGCTGGC TGTCACCGTC

CTCATATGTC TCCTGTAGGC TGGTCACCTG AAGGGCTGGC TGTCACCATC

---------- ---------- ---------- ---------- ----------

TTCATAGGTC TCCTGGAGGC TAGTCACCTA ACAGGCTTGC TCTCTGCAGC

CTCATATGTC TCTTGGAGGC TGGTCACCTG AACGGCTGGT TGTCCGGGTC

GCTGTCCTGA AAGCCCATCT CCTCCAACTT GCAGCGCCGG TAGAGCTCGT

GCTGTCCTGA AAGCCCATCT CCTCCAACTT GCAGCGCCGG TAGAGCTCGT

GCTGTCCTGG AAGCCCATCT CCTCCAACTT GCAGCGCCGG TAGAGCTCGT

ACTGTCCTGG AAGCCCATCT CCTCCAACTT GCAGCGCCGG TAGAGCTCGT

GCTGTCCTGA AAGCCCATCT CCTCCAACTT GCAGCGCCGG TACAGCTCAT

GCTGTCCTGG AAGCCCATCT CCTCCAACTT GCAGCGCCGG TAGAGCTCAT

ACTGTCCTGG AAGCCCATCT CCTCCAACTT GCAGCGCCGG TAGAGCTCGT

ACTGTCCTGG AAGCCCATCT CCTCCAACTT GCAGCGGCGG TAGAGCTCGT

---------- ---------- ---------- ---------- ----------

---------- ---------- ---------- ---------- ----------

GCTGTCCTGG AAGCCCATCT GCTCCAGCTT GCAGCGCCGG TAGAGCTCGT

GCTGTCCTGG AAGCCCATCT CCTCCAACTT GCAGCGCCTG TAGAGCTCAT

---------- ---------- ---------- ---------- ----------

GCTGTCCTGG AAGCCCATCT CCTCCAACTT GCAGCGCCGA TAGAGCTCGT

GCTGTCTTGG AAGCCCATCT CTTCCAATTT GCAGCGTCGG TAGAGCTCAT

---------- ---------- ---------- ---------- ----------

TGTGTCCTGG AAGCCCATCT CTTCAAGCTT ATGGCGCCGG TACAATTCAT

GGTGTCCTTG AAGCCCATCT CCTCCAGCTT GCAACGCCGG TAGAGCTCGT

AGTGCCGGCT GTGGGTCTGC TCGCGGAGGT CTTCCATGTT CACCCGGATC

AGTGCCGGCT GTGGGTCTGC TCGCGGAGGT CTTCCATGTT CACCCGGATC

AGTGCCGGCT GTGGGTCTGC TCGCGGAGGT CTTCCATGTT CACCCGGATC

AGTGCCGGCT GTGGGTCTGC TCGCGGAGGT CTTCCATGTT CACCCGGATC

AGTGCCGGCT GTGGGTCTGC TCACGGAGGT CCTCCATGTT CACCCGGATC

AGTGCCGACT GTGGGTCTGC TCCCGGAGGT CCTCCATGTT CACCCGGATC

AGTGCCGACT GTGGGTCTGC TCACGCAGGT CCTCCATGTT CACCCGGATC

AGTGCCGGCT GTGGGTCTGC TCGCGCAGGT CCTCCATGTT CACCCGGATC

---------- ---------- ---------- ---------- ----------

---------- ---------- ---------- ---------- ----------

AGTGCCGACT GTGGGTCTGC TCTCGGAGGT CCTCCATGTT CACCCGGATC

AGTGCCGACT GTGCGTCTGC TCGCGGAGGT CCTCCATGTT CACGCGGATC

---------- ---------- ---------- ---------- ----------

AGTGCCGGCT GTGGGTCTGC TCGCGGAGGT CCTCCATGTT CACCCTGATC

AGTGCCGACT GTGGGTCTGC TCACGGAGGT CTTCCATGTT CACCCGAATC

---------- ---------- ---------- ---------- ----------

AGTGTTTGCT GTGTGTCTGT TCTCGGAGAT CCTCCATGTT CACACGGATC

AGTGGCGGGT GTGGGTCTGT TCACGCAGAT CTTCCATGTT CACCCGGATC

AACATCTCCC GCAGCTTCAC GAAGTCGCAG TGATTCTCAT TCTCCACTGA

AACATCTCCC GCAGCTTCAC GAAGTCGCAG TGATTCTCAT TCTCCACTGA

AACATTTCCC GCAGCTTCAC GAAGTCGCAG TGATTCTCAT TCTCCACTGA

AACATTTCCC GCAGCTTCAC GAAGTCGCAG TGATTCTCAT TCTCCACTGA

AACATCTCCC GCAGCTTCAC GAAGTCGCAG TGATTCTCAT TCTCCACTGA

AACATCTCCC GGAGCTTCAC AAAGTCGCAG TGATTCTCAT TCTCCACTGA

AACATCTCCC GCAACTTCAC GAAGTCACAG TGGTTCTCGT TCTCCACTAA

AGCATCTCCC GCAGCTTCAC GAAGTCACAG TGATTCTCAT TCTCCACTAA

---------- ---------- ---------- ---------- ----------

---------- ---------- ---------- ---------- ----------

AGCATCTCCC GCAGCTTCAC AAAGTCGCAG TGATTCTCGT TCTCCACTGA

AGCATCTCCC GGAGCTTCAC GAAATCGCAG TGATTCTCAT TCTCCACTGA

---------- ---------- ---------- ---------- ----------

AGCATCTCCC GGAGCTTCAC GAAGTCACAG TGGTTCTCGT TCTCCACTGA

AGCATCTCCC GAAGCTTCAC AAAGTCACAG TGATTCTCGT TCTCCACTGA

---------- ---------- ---------- ---------- ----------

AGCATCTCCC GGAGTTTCAC AAAGTCACAG TGACTCTCAT TCTCCACTGA

AGCATCTCCC GCAGCTTCAC AAAGTCACAG TGACTCTCGT TCTCCACTGA

CTCACCCTGC ACCACTCCCC AGGGGTACTG CCGTGCTCGG ACCAGCTTGT

CTCACCCTGC ACCACTCCCC AGGGGTACTG CCGTGCTCGA ACCAGCTTGT

CTCACCCTGC ACCACTCCCC AGGGGTACTG CCGTGCTCGG ACCAGCTTGT

CTCACCCTGC ACCACTCCCC AGGGGTACTG CCGTGCTCGG ACCAGCTTGT

CTCACCCTGC ACCACTCCCC AAGGGTACTG CCGTGCTCGG ACCAGTTTGT

CTCACCCTGC ACCACTCCCC AAGGGTACTG CCGTGCTCGG ACCAGCTTGT

CTCACCCTGC ACCACGCCCC AGGGGTACTG TCGTGCTCGA ACCAGCTTGT

CTCACCCTGC ACCACACCCC AAGGGTACTG TCGTGCTCGA ACCAGCTTAT

---------- ---------- ---------- ---------- ----------

---------- ---------- ---------- ---------- ----------

CTTACCCTGC ACCACTCCCC AAGGGTACTG CCGTGCTCGG ACCAGCTTGT

CTCACCCTGC ACCACTCCCC ACGGGTACTG CCGCGCTCGC ACCAGCTTGT

---------- ---------- ---------- ---------- ----------

CTCACCCTGC ACCACTCCCC AAGGGTACTG CCGTGCTCGG ACCAGCTTGT

CTCACCCTGG ACTACTCCCC AAGGGTACTG CCGTGCTCGG ACCAGCTTGT

---------- ---------- ---------- ---------- ----------

CTCACCTTGC ACAACACCCC AGGGGTACTG CCGAGCTCGA ACCAGTTTGT

CTCACCTTGT ACGACCCCCC ACGGGTACTG TCGAGCCCGC ACCAGTTTGT

TCCCCACCTT CACCTCCTCG GTGCTGCCCA CCACGGCAAA GGGCAGATGT

TCCCCACCTT CACCTCCTCG GTGCTGCCCA CCACGGCAAA GGGCAGATGT

TCCCCACCTT CACCTCCTCG GTGCTGCCCA CTACGGCGAA GGGCAGATGT

TCCCCACCTT CACCTCCTCG GTGCTGCCCA CTACGGCGAA GGGCAGATGT

TCCCCACCTT CACCTCCTCG GTGCTGCCCA CCACGGCAAA GGGCAGGTGT

TTCCCACCTT CACCTCCTCG GTGCTGCCCA CCACAGCAAA GGGCAGGTGT

TCCCCACCTT CACCTCCTCT GTGCTGCCCA CCACGGCAAA AGGCAAGTGT

TCCCCACCTT CACCTCCTCT GTGCTGCCCA CCACGGCAAA AGGCAGGTGT

---------- ---------- ---------- ---------- ----------

---------- ---------- ---------- ---------- ----------

TCCCCACCTT CACCTCCTCA GTGCTGCCCA CCACAGCGAA GGGCAAGTGT

TCCCCACCTT CACCTCCTCG GTGCTGCCCA CCACGGCGAA GGGCAGGTGT

---------- ---------- ---------- ---------- ----------

TCCCCACCTT CACCTCCTCG GTGCTGCCAA CCACCGCAAA AGGCAGGTGT

TCCCCACCTT CACCTCCTCG GTGCTGCCCA CTACAGCAAA GGGCAGGTGT

---------- ---------- ---------- ---------- ----------

TCCCAACTTT TACTTCATCA GTACTGCCAA CCACAGCAAA GGGAAGATGG

TCCCGACTTT TACCTCTTCT GTGCTGCCGA CCACGGCGAA AGGTAGATGA

GCCTGGAACT CACATTCATG ACTGCGTTAA TCTCTGCAAC AGCCTCATCA

GCCTGGAACT CACATTCATG ACTGCGTTAA TCTCTGCAAC AGCCTCGTCA

GCCTGGAACT CACATTCATG ACTGCGTTAA TCTCTGCAAC AGCCTCGTCA

GCCTGGAACT CACATTCATG ACTGCGTTAA TCTCTGCAAC AGCCTCGTCA

GCCTGGAACT CACATTCATG ACTGCGTTAA TCTCTGCAAC AGCCTCGTCA

GCCTGGAACT CACGTTCATA ACTGCATTAA TCTCTGCAAC TGCCTCATCA

GCCTGAAACT CACATTCATG ACCGCATTAA TCTCGGCGAC AGCCTCATCG

GCCTGAAACT CACGTTCATG ACTGCATTAA TCTCGGCGAC AGCCTCGTCA

---------- ---------- ---------- ---------- ----------

---------- ---------- ---------- ---------- ----------

GCCTAGAACT CACATTCATG ACTGCGTTAA TCTCAGCAAC AGCCTCATCA

GCCTGGAACT CACATTCATG ACTGCGTTAA TCTCTGCAAC AGCCTCGTCA

--------CT CACATTCATG ACTGCATTGA TCTCTGCCAC AGCCTCGTCA

GCCTGGAACT CACATTCATG ACTGCGTTAA TCTCTGCAAC AGCCTCGTCA

GCCTGGAACT CACATTCATG ACTGCGTTTA TCTCTGCAAC AGCCTCATCA

---------- ---------- ---------- ---------- ----------

GCCTAAGACT TACATTCATT ACAGCATTGA TCTCTGCCAC AGCTTCATCA

GCCTAGAACT CACATTCATG ACGGCATTGA TCTCTGCTAC CGTCTCATCG

TCCGTGGGGA ACTGGTAGAT CTGGACCCCA TTGCTGACCA ACTCGCCCAT

TCCGTGGGAA ACTGGTAGAT CTGGACCCCG TTGCTGACCA ACTCGCCCAT

TCCGTGGGAA ACTGGTAGAT CTGGACCCCG TTGCTGACCA GCTCGCCCAT

TCCGTGGGAA ACTGGTAGAT CTGGACCCCG TTGCTGACCA GCTCGCCCAT

TCCGTGGGAA ACTGGTAGAT CTGGACCCCG TTGCTGACCA GCTCGCCCAT

TCTGTGGGAA ACTGGTAGAT CTGGACCCCG TTGCTGACCA GCTCACCCAT

TCGGTGGGAA ACTGGTAGAT CTGGACTCCA TTGCTGACCA GCTCACCCAT

TCGGTGGGAA ACTGGTAGAT CTGGACTCCG TTGCTGACCA GCTCACCCAT

---------- ---------- ---------- ---------- ----------

---------- ---------- ---------- ---------- ----------

TCAGTGGGAA ACTGGTAGAT CTGAACCCCA TTGCTGACCA GCTCGCCCAT

TCAGTGGGGA ACTGGTAGAT CTGGACCCCA TTGCTGACCA GCTCGCCCAT

TCGGTGGGGA ACTGGTAGAT CTGGACCCCG TTGCTAACCA GCTCGCCCAT

TCAGTGGGGA ATTGGTAGAT CTGGACCCCA TTGCTGACCA GCTCGCCCAT

TCAGTGGGAA ACTGGTAGAT CTGGACCCCA TTGCTGACCA GCTCGCCCAT

---------- -----TAGAT CTGGACCGCG TTGCAGACGA GCTCGCCCAT

TCTGTAGGGA ACTGATAGAT CTGGACTCCA TTTTGATCCA GTTCACTCAT

TCCGTGGGGA ACTGGTAGAT CTGGACACCG TTGCTGACCA GCTCACCCAT

GATCTTGATC TTGAACTTGT GGAGCTCGCT CTTGGAGATG GTGTCAGCCT

GATCTTGATC TTGAACTTGT GGAGCTCGCT CTTGGAGATG GTGTCAGCCT

GATCTTGATC TTGAACTTGT GGAGCTCGCT CTTGGAGATG GTGTCAGCCT

GATCTTGATC TTGAACTTGT GGAGCTCGCT CTTGGAGATG GTATCAGCCT

GATCTTGATC TTGAACTTGT GGAGTTCACT CTTGGAGATG GTGTCAGCCT

GATCTTGATC TTGAACTTGT GGAGCTCGCT CTTGGAGATG GTGTCGGCCT

GATCTTGATC TTGAACTTGT GGAGCTCGCT CTTGGAGATG GTGTCAGCCT

GATCTTGATT TTGAACTTGT GGAGCTCGCT CTTGGAGATG GTGTCAGCCT

---------- ---------- ---------- ---------- ----------

---------- ---------- ---------- ---------- ----------

GATCTTGATC TTGAACTTGT GGAGCTCACT CTTGGAGATG GTGTCAGCCT

GATCTTGATC TTGAACTTGT GGAGCTCACT CTTGGAGATG GTGTCAGCCT

GATCTTGATC TTGAACTTGT GGAGCTCACT CTTGGAGATG GTGTCGGCCT

GATCTTGATC TTGAACTTGT GGAGCTCGCT CTTGGAGATG GTATCAGCCT

GATCTTGATC TTGAACTTGT GGAGCTCACT CTTGGAGATG GTGTCAGCCT

GATCTCGATC TAGAACTTGT GAAGCTCGCT CTAGGAGATG GTGTCCGCCT

GATCTTGCTT TTGAACTTAT GCAGTTCACT CTTGGAGATG GTGTCAGCTT

GATCTTGATC TTGAACTTGT GCAACTCGCT CTTGGAAATG GTGTCGGCCT

TGGCGATGAT GGGAATAATG TTCACCTGAC CTTGCTGTCT AGTTTCTTCA

TGGCGATGAT GGGAATAATG TTCACCTGAC CTTGCTGTCT AGTTTCTTCA

TGGCGATGAT GGGAATAATG TTCACCTGAC CTTGCTATCT AGTTTCTTCA

TGGCGATGAT GGGAATAATG TTCACCTGAC CTTGCTATCT AGTTTCTTCA

TGGCGATGAT GGGAATAATG TTCACCTGAC CTTGCTGTCT AGTTTCTTCA

TGGCAATGAT GGGAATAATG TTCACCTGAC CTTGCTGTCG AGTTTCTTCA

TGGCAATGAT GGGGATTATG TTCACCTGAC CTTGCTATCT AGTTTCTTCA

TGGCAATGAT GGGGATTATA TTCACCTGAC CTTGCTATCT AGTTTCTTCA

---------- ---------- ---------- ---------- ----------

---------- ---------- ---------- ---------- ----------

TGGCAATGAT GGGAATAATG TTCACCTGGC CTTGCTGTCT AGTTTCTTCA

TGGCAATGAT GGGAATAATG TTCACCTGAC CTTGCTGTCC AGTTTCTTCA

TGGCAATGAT GGGGATGATG TTCACCTGAC CTTGCTGTCA AGCTTCTTCA

TGGCGATGAT GGGAATAATG TTCACCTGAC CTTGCTGTCC AGTTTCTTCA

TGGCGATGAT GGGGATAATG TTCACCTGAC CTTGCTGTCC AGTTTCTTCA

TGGCAATGAT GGGGATGATG TTCACCTGAC CTTGCTGTCC AGCTTCTTCA

TGGCAATGAT AGGGATAATG TTCACCTAAC CTTGCTATCC AGCTTCTTCA

TGGCGATTAT GGGGATGATG TTCACCTGAC CTTACTATCC AGTTTTTTCA

TGGTCACTAG ATCTAGAGAC TTCAGGGAGT GCCCTGTGGG CGTGATGAAG

TGGTCACTAG ATCTAGAGAC TTCAGGGAGT GCCCTGTGGG CGTGATGAAG

TGGTCACCAG ATCCAGGGAC TTCAGGGAGT GCCCCGTGGG CGTGATGAAG

TGGTCACCAG ATCCAGGGAC TTCAGGGAGT GCCCCGTGGG CGTGATGAAG

TGGTCACCAG ATCCAGGGAC TTCAGGGAGT GCCCCGTGGG CGTGATAAAG

TAGTCACCAG ATCAAGGGAC TTCAGGGAGT GCCCTGTGGG TGTGATGAAG

TGGTCACCAG ATCCAGGGAC TTCAGGGAGT GCCCGGTGGG TGTGATAAAG

TGGTCACCAG ATCCAGGGAC TTCAGGGAGT GCCCGGTGGG CGTGATGAAG

---------- ---------- ---------- ---------- ----------

---------- ---------- ---------- ---------- ----------

TGGTCACCAG ATCCAGGGAC TTGAGGGAGT GCCCTGTGGG TGTGATAAAG

TGGTCACCAG GTCCAGGGAC TTGAGGGAGT GCCCGGTGGG CGTGATGAAG

TGGTGACCAG ATCCAGGGAC TTGAGGGAGT GGCCGGTGGG GGTGATGAAG

TAGTCACCAG GTCCAGGGAC TTGAGGGAGT GCCCCGTGGG TGTGATGAAG

TGGTCACCAG GTCCAGGGAC TTGAGAGAAT GCCCCGTGGG CGTGATGAAG

TGGTCACCAG GTCCAGGGAC TTGAGGGAGT GGCCCGTGGG GGTGATGAAG

TGGTCACCAG GTCCAGCGAC TTGAGGGAAT GTCCAGTGGG TGTGATGAAA

TGGTCACCAA GTCCAGGGAC TTGAGTGAAT GTCCGGTGGG GGTGATGAAG

TAGAGGCAAA CGTGGATCCT TGTGTCATGG TAGTCGAAGA GCGAGCGGCG

TAGAGGCAAA CGTGGATCCT TGTGTCATGG TAGTCGAAGA GCGAGCGGCG

TAGAGGCAAA CGTGGATCCT TGTGTCATGG TAGTCGAAGA GCGAGCGGCG

TAGAGGCAAA CGTGGATCCT TGTGTCATGG TAGTCGAAGA GCGAGCGGCG

TAGAGGCAAA CGTGGATCCT CGTGTCATGG TAGTCGAAAA GCGAGCGGCG

TAGAGGCAAA CATGGATCCT AGTGTCATGG TAGTCGAAGA GCGAGCGGCG

TAGAGGCAAA CGTGGATCCT CGTGTCATGG TAGTCAAAGA GGGAACGGCG

TAGAGGCAAA CATGGATCCT CGTGTCGTGA TAGTCAAAGA GGGAACGGCG

---------- ---------- ---------- ---------- ----------

---------- ---------- ---------- ---------- ----------

TAGAGGCAGA CGTGGATCCT TGTGTCATGG TAGTCAAAGA GTGAGCGGCG

TAGAGGCACA CGTGGACCCT GGTGTCATGG TAGTCGAAGA GCGAGCGGCG

TAGAGGCACA CGTGGATCCT CGTGTCGTGG TAGTCGAAGA GTGAGCGGCG

TAGAGGCAAA CGTGGATCCT CGTGTCATGG TAGTCAAAGA GCGAGCGGCG

TAGAGGCAAA CGTGGATCCT CGTGTCATGA TAATCAAAGA GGGAACGGCG

TAGAGGCAGA CGTGGATCCT GGTGTCATGG TAGTCGAAGA GGGAGCGCCG

TAGAGACAGA CATGGATCCG CGTGTCATGA TAGTCAAATA GAGAGCGCTG

TAAAGGCAGA CGTGGATCCT GGTGTCGTGG TAGTTGAACA GGGCCCGCCG

GATCTTCAGC TCCTCCTGCA GATAATTTTC AAACTGCGCA TCGATGTAGT

GATCTTCAGC TCCTCCTGCA GATAATTTTC AAACTGCGCA TCGATGTAGT

GATCTTCAGC TCCTCCTGCA GATAATTTTC AAACTGCGCA TCGATGTAGT

GATCTTCAGC TCCTCCTGCA GATAATTTTC AAACTGCGCA TCGATGTAGT

GATCTTCAGC TCCTCCTGGA GATAGTTTTC AAACTGCGCA TCGATGTAGT

GATCTTCAGC TCCTCCTGGA GATAGTTTTC AAACTGCGCA TCGATGTAGT

AATCTTCAAC TCCTCCTGTA GATAGTTTTC AAACTGCGTG TCGATGTAAT

GATCTTCAAC TCCTCCTGCA GATAGTTTTC AAACTGCGCG TCGATGTAGT

---------- ---------- ---------- ---------- ----------

---------- ---------- ---------- ---------- ----------

GATCTTCAGC TCCTCCTGTA GGTAGTTTTC AAACTGCGCA TCGATGTAGT

AATCTTCAGC TCTTCCTGCA GATAGCTTTC AAACTGCGCA TCAATGTAGT

GATCTTCAGC TCCTCCTGCA GGTAGTTTTC AAACTGCGCA TCAATGTAGT

GATCTTCAGC TCCTCCTGTA GATAGTTTTC AAACTGCGCG TCGATGTAGT

GATCTTCAGC TCCTCTTGCA GATAGTTTTC AAACTGTGCG TCGATGTAAT

GATCTTCAGC TCCTCTTGCA GATAGTTTTC AAACTGCGCA TCAATGTAGT

AATCTTCAGC TCTTCCTGAA GGTAGTTTTC AAACTGCGTA TCAATGTAGT

GATCTTCAGT TCTTCTTGCA AGTAGTTTTC AAACTGCGTA TCAATGTAAT

CAACTATGGG CCTGTAACTA CAGACAGCTC CATCACCCGC CTCTTGCCTC

CAACTATGGG CCTGTAACTA CAGACAGCTC CATCACCCGC CTCTTGCCTC

CAACTATGGG CCTGTAACTA CAGACAGCTC CATCACCCGC CTCTTGCCTC

CAACTATGGG CCTGTAACTA CAGACAGCTC CATCACCCGC CTCTTGCCTC

CAACAATAGG CCTGTAACTA CAGACAGCTC CATCACCCGC CTCTTGCCTC

CAACTATGGG CCTGTAACTA CAGACAGCTC CATCACCCGC CTCTTGCCTC

CAACTATGGG CCTGTAACTA CAGACAGCTT TATCACCCGC CTCTTGCCTG

CAACTATGGG CCTGTAACTA CAGACAGCTT TATCACCCGC CTCTTGCCTG

---------- ---------- ---------- ---------- ----------

---------- ---------- ---------- ---------- ----------

CGACGATAGG CCTGTAACTA GAGACAGCTC CATCAcCCGC CTCTTGCCTC

CGACTATGGG CCTGTAACTA CAGACAGCTC CGTCACCCGC CTCTTGCCTC

CGACGATGGG CCTGTAACTA CAGACAACTC CGTCACCCGT CTCTTGCCTC

CAACGATGGG CCTGTAACTA CAGACGGCTC CATCACCCGC CTCTTGCCTG

CAACAATGGG CCTGTAACTA CAGACAGATC CATTACCTGC CTCTTGCCTC

CGACGATGGG CCTGTAACTA CAGAAAGATC CATCACCCGC CTCTTGCCTC

CAACTATAGG CCTGTAACTA CAAACAGCTC CGTCACACAC CACTTGCCTC

CAACTATTGG CCTGTAACTA CAAACAGATC CATTACCCCC CACTTGCCTC

TCATCCTTAT TGATCTGATC CCCAAAGCCC ACGGCATCCA CAATGGTCAG

TCATCCTTAT TGATCTGATC CCCAAAGCCC ACGGCATCCA CAATGGTCAG

TCATCCTTAT TGATCTGATC CCCAAAGCCC ACGGCATCCA CAATGGTCAG

TCATCCTTAT TGATCTGATC CCCAAAGCCC ACGGCATCCA CAATGGTCAG

TCATCCTTAT TGATCTGATC CCCGAAGCCC ACGGCGTCCA CAATGGTCAG

TCATCTTTGT TGATCTGATC CCCGAAGCCC ACAGCATCCA CAATGGTCAG

TCATCCTTAT TGATCTGATC CCCAAAGCCC ACAGCATCCA CGATGGTCAG

TCATCCTTAT TGATCTGATC GCCAAAGCCC ACAGCATCCA CGATGGTCAG

---------- ---------- ---------- ---------- ----------

---------- ---------- ---------- ---------- ----------

TCATCCTTAT TGATCTGATC CCCGAAGCCC ACAGCGTCCA CAATGGTCAG

TCATCCTTAT TGATCTGGTC CCCGAAGCCC ACAGCGTCCA CGATGGTCAG

TCATCCTTGT TGATCTGATC CCCGAAGCCC ACGGCATCCA CGATGGTCAG

TCATCCTTGT TGATCTGGTC CCCGAAGCCC ACGGCGTCCA CGATGGTCAG

TCATCCTTAT TGATTTGATC TCCGAAGCCC ACAGCGTCCA CAATGGTCAG

TCGTCCTTAT TGATCTGATC CCCGAAGCCC ACAGCGTCCA CGATGGTCAG

TCATCCTTAT TGATCTGGTC TCCAAAACCG ACTGCATCCA CAATGGTCAA

TCGTCCTTGT TGATCTGGTC TCCGAAGCCA ACGGCATCCA CGATGGTCAG

CTTGAGCTGC ACGTTGCTCT CCTGGAGGTC ATAGGTCTGG GGCCGCAGGC

CTTGAGCTGC ACGTTGCTCT CCTGGAGGTC GTAGGTCTGG GGCCGCAGGT

CTTGAGCTGC ACGTTGCTCT CCTGGAGGTC GTAGGTCTGG GGCCGCAGGC

CTTGAGCTGC ACGTTGCTCT CCTGGAGGTC GTAGGTCTGG GGCCGCAGGC

CTTGAGCTGC ACATTGCTCT CCTGGAGGTC ATAGGTCTGG GGCCGCAGGC

CTTGAGGTGC ACGTTGCTCT CTTGGAGGTC GTAGGTCTGG GGCCGCAGGC

TTTGAGATGA ACGTTACTCT CTTGGAGGTC ATAGGTCTGA GGCCGCAGGC

CTTGAGATGA ACGTTACTCT CTTGGAGGTC ATAGGTCTGA GGCCGCAGGC

---------- ---------- ---------- ---------- ----------

---------- ---------- ---------- ---------- ----------

CTTGAGGTGC ACATTGCTCT CTTGGAGGTC ATAGGTCTGG GGCCGTAGGC

CTTGAGCTGC ACGTTGCTCT CTTGGAGGTC ATAGGTCTGG GGCCGCAGGC

CTTGAGGTGC ACGTTGCTCT CCTGGAGGTC GTAGGTCTGG GGCCGCAGAC

CTTCAGCTGC ACGTTGCTCT CTTGCAGGGT GTAGGTCTGG GGCCGCAGGC

CTTGAGCTGC ACGTTGCTCT CCTGGAGGTC ATAGGTCTGG GGCCGCAGGC

CTTGAGCTGC ACGTTGCTCT CCTGGAGGTT GTATGTCTGG GGCCGCAGGC

TTTAAGGTGC ACATTGCTTT CCTGGAGATC ATAAGTGCGT GGCCGGAGGC

CTTGAGCTGC ACGCTGCTTT CCTGCAGGTC GTAGGTGCGC GGCTGCAGCC

GCACGCATGC CTCATGGTGA CTGGCTTCCT CAGTCTCGAA GGTCGTGTTG

GTACGCATGC CTCATGGTGA CTGGCTTCCT CAGTCTCGAA GGTCGTGTTG

GCACGCATGC CTCATGGTGA CTGGCTTCCT CAGTCTCGAA GGTCGTGTTG

ACACGCATGC CTCATGGTGA CTGGCTTCCT CAGTCTCGAA GGTCGTGTTG

GCACACACGC CTCATGGTGA CTGGCTTCCT CAGTCTCGAA GGTTGTGTTG

GCACACATGC CTCGTGGTGA CTGGCTTCCT CAGTCTCGAA GGTCGTGTTG

GTACACACTC TTCATGGTGA CTGGCTTCTT CAGTCTCAAA GGTCGTGTTG

GCACACACTC TTCATGGTGA CTGGCTTCTT CAGTCTCAAA GGTCGTGTTG

---------- ---------- ---------- ---------- ----------

---------- ---------- ---------- ---------- ----------

GCACACACTC CTCATGATGA CTGGCTTCTT CTGTCTCAAA GGTTGTGTTG

GCACACACTC CTCGTGGTGA CTGGCTTCCT CGGTCTCGAA GGTCGTGTTG

GCACGCACTC CTCGTGGTGA CTGGCCTCCT CGGTCTCGAA GGTGGTGTTG

GCACGCACTC CTCATGGTGG CTGGCTTCCT CAGTCTCGAA GGTCGTGTTA

GCACGCACTC CTCATGGTGA CTGGCTTCCT CGGTCTCAAA GGTTGTGTTG

GCACACACTC CTCATGGTGG CTGGCCTCCT CCGTCTCGAA TGTGGTGTTG

GTACACAGGT CTCATAGTGT CCAGACTCTT CTGTCTCAAA TATAGTGTTG

GGACGCAGTT CTCATAGTGG CTGGCCTCCT CCGTCTCAAA CGTAGTGTTG

AAGAGTGTGT TCATCAGTGT GGATTTGCCA ATGCCGGTCT CCCCTCACTC

AAGAGTGTGT TCATCAGTGT GGATTTGCCA ATGCCGGTCT CCCCTCACTC

AAGAGCGTGT TCATCAGTGT AGATTTGCCA ATGCCAGTCT CCCCTCACTC

AAGAGCGTGT TCATCAGTGT GGATTTGCCA ATGCCAGTCT CCCCTCACTC

AAGAGCGTGT TCATCAGTGT GGATTTGCCA ATGCCGGTCT CCCCTCACTC

AAGAGTGTGT TCATCAATGT GGATTTGCCA ATGCCAGTCT CCCCTCACGC

AAGAGCGTGT TCATTAGTGT GGACTTGCCA ATCCCAGTTT CCCCT-ACTT

AAGAGTGTGT TCATTAGCGT GGACTTGCCA ATCCCAGTTT CGCCT-ACTT

---------- ---------- ---------- ---------- ----------

---------- ---------- ---------- ---------- ----------

AAGAGTGTGT TCATCAGTGT GGACTTGCCA ATGCCAGTCT CCCCTCACAT

AAGAGTGTGT TCATCAGTGT GGACTTGCCA ATGCCAGTCT CCCCTCACTC

AAGAGCGTGT TCATCAGGGT GGACTTGCCG ATGCC----- -----CACTC

AAGAGCGTGT TCATCAGTGT GGACTTGCCA ATGCCAGTCT CCCCTCACTC

AAGAGCGTGT TCATCAGCGT GGATTTGCCA ATGCCAGTCT CCCCTCACTC

AAGAGCGTGT TCATCAGCGT GGACTTGCCA ATGCCAGTCT CCCCTCACTC

AAGAGTGTGT TCATCAGTGT AGACTTTCCA ATGCCTGTTT CCCCTCATTT

AACAGCGTGT TCATCAGGGT GGACTTCCCA ATGCCCGTCT CCCCTCACTT

ACCCACACAG AGGATGTTGA AGCTGAAGCC CTGAGTGACC GACTTGCTGA

ACCCACACAG AGGATGTTGA AGCTGAAGCC CTGAGTGACC GACTTGCTGA

ACCCACACAG AGGATGTTGA AGCTGAAGCC CTGAGTGACC GACTTGCTGA

ACCCACACAG AGGATGTTGA AGCTGAAGCC CTGAGTGACC GACTTGCTGA

ACCCACACAG AGTATGTTGA AGCTGAAGCC CTGAGTGACC GACTTGCTGA

ACCCACACAG AGGATGTTGA AGCTGAAGCC CTGAGTGACC GACTTGCTGA

ACCCACACAG AGAATGTTGA AGCTGAAGCC CTGAGTGACT GACTTGCTGA

ACCCACACAG AGTATGTTGA AGCTGAAGCC CTGAGTGACT GACTTGCTGA

---------- ---------- ---------- ---------- ----------

---------- ---------- ---------- ---------- ----------

ACCCACACAG AGGATGTTAA AGCTGAAGCC CTGTGTGACT GACTTGCTGA

ACCCACACAA AGGATATTGA AGCTGAAGCC CTGAGTGACC GACTTGCTGA

ACCCACACAG AGGATGTTGA AGCTGAAGCC CTGAGTGACC GCCTTGCTGA

ACCCACACAG AGGATGTTGA AGCTGAAGCC CTGAGTGACC GACTTGCTGA

ACCCACACAG AGGATGTTGA AGCTGAAGCC CTGAGTGACC GACTTGCTGA

ACCCACACAG AGGATGTTGA AGCTGAAGCC CTGAGTGACC GACTTGCTGA

ACCCACACAA AGGATGTGAA AGCTGAAGCC CTGAGATACG GACTTACTGA

ACCGACACAG AGGATGTTGA AGCTGAAGCC CTGGTTGACC GATTTGCTGA

CCAGCTGGTC GGGGAGGCTG TCGAAACCCA CATGGCCGCC CAGGGAGAGG

CCAGCTGGTC GGGGAGGCTG TCGAAACCCA CATGGCCGCC CAGGGAGAGG

CCAGCTGGTC GGGGAGGCTG TCAAAACCCA CATGGCCGCC CAGGGAGAGG

CCAGCTGGTC GGGGAGGCTG TCAAAACCCA CATGGCCGCC CAGGGAGAGG

CCAGCTGGTC AGGGAGGCTG TCAAAGCCCA CATGGCCGCC CAGGGAGAGG

CCAGCTGGTC GGGGAGGCTG TCAAAGCCCA CATGGCCGCC CAGGGAGAGG

CCAGCTGGTC AGGGAGGCTG TCGAAGCCCA CATGGCCACC CAGGGACAGG

CCAGCTGGTC AGGGAGGCTG TCAAACCCAA CATGGCCGCC CAGGGACAGG

---------- ---------- ---------- ---------- ----------

---------- ---------- ---------- ---------- ----------

CAAGCTGATC CGGGAGGCTG TCGAAGCCCA CATGACCACC AAGGGAGAGG

CCAGCTGGTC AGGGAGGCTG TCAAAGCCCA CGTGGCCGCC CAGGGAGAGG

CCAGCTGGTC GGGGAGGCTG TCGAAGCCCA CATGGCCGCC CAGGGAGAGG

CCAGCTGGTC CGGGAGGCTG TCGAAGCCCA CGTGGCCCCC CATGGAGAGG

CCAGCTGGTC GGGAAGGCTG TCGAAGCCCA CATGGCCCCC GAGGGAGAGG

CCAGCTGGTC GGGGAGGCTG TCGAAGCCCA CATGGCCCCC CAGGGAGAGA

CCAGCTGGTC TGGGAGGCTG TCAAAACCCA CATGGCCTCC CAGATTGAGG

CCAGCTGGTC CGGGAGGCTG TCAAAGCCAA CGTGGCCTCC GAGCGAAAGA

CTCCGTGGCT CTGCATTCTG GTGCTGGGCT TCCTGCAGGA GCACGGCGGG

CTCCGTGGCT CTGCATTCTG GTGCTGGGCT TCCTGCAGGA GCACG-----

CTCCGTGGCT CTGCATTCTG GTGCTGGGCT TCCTGCAGGA GCACGGCGGG

CTCCGTGGCT CTGCATTCTG GTGCTGGGCT TCCTGCAGGA GCACGGCGGG

CTCCGTGCCT CTGCATTCTG GTGCTGGGCT TCCTGCAGGA GCGCGGCGGG

CTCCGTGGCT CTGCACTCTG GTGCTGGGCT TCCTGCAAGA GCGCGGCGGC

CTCCGAGGCT CTGCGTTCTG GTGCTGGGCT TCCTGCGGGA GCACGGCGGG

CTCCGAGGCT CTGCGTTCTG GTGTTGGGCT TCCTGCGGGA GCACGGCGGG

---------- ---------- ---------- ---------- ----------

---------- ---------- GTGCTGGGTT TCCTGCAGGA GAACGGCGGG

TTCCGTGGCT CTGCATTCTG GTGCTGGGCT TCCTGCTGGA GCACGGCGGC

CTCCGTGGCT CTGCATTCTG GTGCTGGGCT TCCTGCAGGA GCACGGCGGA

GGCCGCGGCT CCGCGTTCTG ---------- ---------- ----------

TTCCGCGGCT CTGCATTCTG GTGCTGGGCT TCCTGCAGGA GCGCGGCGGC

CTCCGCGGCT CTGCATTCTG GTGCTGGGGT TCCTGCAGGA GCGCGGCGGG

TTTCGCAGTT CTGCATTCTG ---------- ---------- ----------

ATGCGCATCT CTTCTTTCTG GTGCTGGGCT TCCTGCAGGA GCGTGGGGGC

CTCCGCTTCT GTTCGCTCTG GCTGAGGGGT CCACGGAGGT CCAGAGGGGG

AAGGTGCGCA ACTCCGAGCT GCTGAGCCGC TTCAACGAga cccgcggggc

---------- ---------- ---------- ---------- ----------

AAGGTGCGCA ACTCCGAGCT GCTGAGCCGC TTCAACGAga cccgcggggc

AAGGTGCGCA ACTCTGAGCT GTTGAGCCGC TTCAACGAga cccgcggggc

AAGGTGCGCA ACTCGGAGCT GCTGAGTCGC TTCAAGGAga cccgcggggc

AAGGTGCCCA ACGCGGAACT GCTGAGTCGC TTCAAGGAGA CCCGCGCGGC

CAGGTGCGCA ACTCCGAGCT GCTGAGCCGC TTCAAGGAGA CCCACGGGGC

CAGGTGCGCA ACTCCGAGCT GCTGAGCCGC TTCAAGGAGA CCCACGGGGC

---------- ---------- ---------- ---------- ----------

AAGGTGCGCA ACTCTGAGCT GCTGAGCCGC TTCAAGGAGA CCCACGCGGC

CAGGTGCGCA GCTGCGACCT GCTGAGCCGC TTCAAGGAga cccgcgcggc

AAGGTGCGCA GCTCGGAGCT GGTGAGCCGC TTCAAGGAGA CCCGCGCGGC

---------- ---------- ---------- ---------- ----------

CAGGTGCGCA ACTCGGAGCT GCTGAGCCGC TTCAAGGAGA CCCGCAGGGC

AAGGTGCGTA ACTCCGAGCT GCTGAGTCGC TTCAAGGAGA CCCGCTCGGC

---------- ---------- ---------- -----CGAga cccgcgcggc

AAGGTACGCA ATTCGGAGCT CCTGAGCCGC TTCAAGGAGA cccgggcggc

AGGCCGAGGA GCTCGAagag gccgaggggc ccacggaagg tctgtgaggc

cgcgccgccc gcAGGGACCG GTGAAGGACG CTCCCCGCAC CTGAGGCGCC

---------- ---------- GTGAAGGACG CTCCCCGCAC CTGAGGCGCC

cgcgccgccc gcAGGGACCG GTGAAGGACG CTCCCCGCAC CTGAGGCGCC

cgcgccgccc gcAGGGACCG GTGAAGGACG CTCCCCGCAC CTGAGGCGCC

cgcgccgccc gcAGGGACCG GTAAAGGACG CTCCCCGCAC CTGCGGCGTC

CGAGCCGCCC GCAGGGACCG GTGAAGGACG CTCTCCGCAC CTGAGGCGCC

CGCGCGGCCC GTAGGGACCG GTGAAGGACG CTCCCCGCAT CTCAGGCGCC

CGCGCGGCCC GTAGGGACCG GTGAAGGACG TTCCCCGCAT CTCAGGCGCC

---------- ---------- --------CG CTCCCCGCAC CTGCGACGCC

CGCGCAGCCC GCAGGGACCG GTGAAGGACG CTCCCCTCAC CTGAGGCGCC

cgcgccgccc gcAGGGACCG GTGAAGGACG CTCCCCGCAC CTGAGGCGCC

CGCGCAGCCC GCAGGGACCG GTGAAGGA-- CTCCCCGCAT CTGAGACGCC

---------- ---------- --------CA CTCCCCGTAT TTGAGGCGCC

CGCGCCGCCC GCAGGGACCG GTGAAGGACG ATCCCCGCAC CTGAGGCGCC

CGCGCCGACC GCAGGGACCG GTGAAGGACG CTCCCCGCAC CTGAGGCGCC

cgcgcggccc gcAGGGACCG GTGAAGGACG CTCCCCGCAC CTGAGGCGCC

cgggccgccc gcAGGGACTG GTGAAAGACG GTCCCCTCAC CTCCGGCGCC

cgagcagccc acggaagtcg gccgaggaCG CTCCCCGGGC TTGAGGCGGC

TGTCGCGCGC CGGCCCGCGT CTGCTGAGCC CTGATGGCGG CCAAGCGCGA

TGTCGCGCGC CGGCCCGCGT CTGCTGAGCC CTGATGGCGG CCAAGCGCGA

TGTCGCGCGC CGGCCCGCGT CTGCTGAGCC CCGATGGCGG CCAAGCGCGA

TGTCGCGCGC CGGCCCGCGT CTGCTGAGCC CCGATGGCGG CCAAGCGCGA

TGTCGCGCGC CGGCCCGCGT CTGCTGAGCC CCGATGGCGG CCAAGCGCGA

TGTCGCGTGC TGGCCCGCGT TTGCTCAGCC CGGATGGCGG CCAAGCGCGA

TGTCGCGCGC CGGCCCGCGC CTGCTAAGTC CGGATGGCTG CCAAGCGCGA

TGTCCCGCGC CGGTCCGCGC CTGCTGAGTC CGGATGGCTG CCAAGCGCGA

TGTCGCGCGC CGGCCCCCGC CTGCTGAGCC CCGATGGCGG CCAAGCGCGA

TGTCGCGCGC CGGTCCGCGC CTGCTGAGCC CCGATGGCAG CCAAACGCGA

TGCCGCGCGC AGGCCCGCGC CTGCTAAGCC CGGATGGCGG CCAAGCGCGA

TGTCGCGCGC CGGCCCGCGC CTGCTGAGCC CCGATGGCGG CCAAGTGCGA

CGTCGCGCGC CGGCCCGCGG CTGCTGAGTC CCGATGGCGA CCAAGCGCGA

TATCGCGCGC CGGGCCGCGC CTGCTGAGCC CCGATGGCGG CCAAGCGCGA

TGTCGCGCGC AGGCCCGCGT CTCCTGAGCC CCGATGGCAG CCAAGCGCGA

TGTCACGGCC AAACGCGCGG CTGCTGAGCC CCGATGGCAG CCAAGCGCGA

TGTCCCGGGC CGGCCCGCGC CTGCTGAGCC CCGATGGCAA CCAAACGCGA

CCCTGCGGGC AGCTCCGCGC CTGCTGAGCC CCGATGGCGG CCAAGCGCGA

CTTCATGTCT GGCTTCACGG CCCTGCATTG GGCCGCCAAG AGCGGCGACG

CTTCATGTCT GGCTTCACGG CCCTGCATTG GGCCGCCAAG AGCGGCGACG

CTTCATGTCT GGCTTCACCG CCCTGCATTG GGCCGCCAAG AGCGGCAACG

CTTCATGTCT GGCTTCACGG CCCTGCATTG GGCCGCCAAG AGCGGCGACG

CTTCATGTCT GGCTTCACCG CCCTGCATTG GGCCGCCAAG AGCGGCGACG

TTTCATCTCT GGCTTCACGG CTCTACATTG GGCCGCCAAG AGCGGCGACG

CTTCATGTCG GGCTTCACCG CCCTACACTG GGCAGCCAAG AGCGGCGACG

CTTCATGTCC GGCTTCACCG CGCTACACTG GGCGGCCAAG AACGGCGACG

CTTCATGTCC GGCTTCACGG CCCTGCACTG GGCCGCCAAG AGCGGCGACg

CTTCATGTCT GGTTTCACAG CCCTGCACTG GGCCGCTAAG AGCGGCGACG

CTTCATGTCG GGCTTCACGG CCCTGCACTG GGCCGCCAAG AGCGGCGACG

CTTCATGTCT GGTTTCACGG CCCTGCATTG GGCCGCCAAG AGCGGCGACG

CTTCATCTCC GGCTTCACCG CCCTGCACTG GGCCGCCAAG AGCGGCGACg

CTTCATGTCG GGCTTCACGG CCCTGCATTG GGCCGCCAAG AGTGGCGACG

CTTCATATCG GGCTTCACTG CCCTGCACTG GGCCGCCAAG AGCGGCGACG

CTTCATCTCG GGCTTCACTG CCCTGCACTG GGCCGCCAAG AGCGGCGACg

CTTCATGTCG GGATTCACGG CCCTGCACTG GGCCGCCAAG AGCGGTGACG

CTTCACATCG GGCTTCACCG CCCTGCACTG GGCCGCCAAG AGCGGCGATG

TCGACGTGAA CGCACGCTCC CACGGCGGCT ACACGCCGCT GCACCTGGCG

TCGACGTGAA CGCACGCTCC CACGGCGGCT ACACGCCGCT GCACCTGGCG

TCGACGTGAA CGCGCGCTCC CACGGCGGTT ACACGCCGCT GCACCTGGCG

TCGACGTGAA CGCGCGCTCC CACGGCGGTT ACACGCCGCT GCACCTGGCG

TCGACGTGAA CGCGCGCTCC CACGGCGGCT ACACGCCTCT GCACCTGGCG

TCGATGTAAA TGCGCGCTCG CACGGCGGCT ACACACCCCT GCACCTGGCG

TGGACGTGAA CGCGCGCTCG CACGGTGGCT ACACGCCGCT GCACCTGGCG

TGGACGTGAA CGCGCGCTCT CACGGTGGCT ACACGCCGCT GCACCTGGCG

TCGACGTGAA CGCGCGCTCG CACGGCGGCT ACACGCCGCT GCACCTTGC-

TCGACGTCAA CGCGCGCTCG CACGGCGGCT ATACGCCGCT GCATTTGGCG

TGGACGTGAA CGCGCGCTCG CACGGCGGCT ACACGCCGCT GCACCTGGCG

TCGACGTGAA CGCGCGCTCA CATGGCGGCT ATACGCCCCT GCACCTGGCG

TGGACGTGAA CGCGCGCTCC CACGGCGGCT ACACGCCCCT GCACCTGGCG

TCGACGTGAA TGCGCGCTCG CACGGCGGGT ACACTCCGCT GCACCTGGCG

TGGACGTGAA CGCACGTTCG CATGGCGGCT ACACTCCACT GCATCTGGCG

TCGACGTGAA TGCGCGCTCC CACGGCGGCT ACACGCCGCT CCACCTGGCG

TCGACGTGAA CGCCCGTTCC CACGGAGGTT ACACTCCCTT GCACCTGGCG

TCGACGTCAA CGCCCGCTCC CACGGGGGCT ACACCCCGCT GCACCTGGCG

TACAGGTGGC CGCCACCATC CTCAGTTCCA CCACCAGTGC ATTTCTGGGC

TACAGGTGGC CGCCACCATC CTCAGTTCCA CCACCAGTGC ATTTCTGGGC

TGCAGGTGGC CGCCACCATC CTCAGTTCCA CCACCAGTGC ATTTTTGGGC

TGCAGGTGGC CGCCACCATC CTCAGTTCCA CCACCAGTGC ATTTTTGGGC

TGCAGGTGGC CGCCACCATC CTCAGTTCCA CCACCAGTGC ATTTCTAGGC

TGCAGGTTGC TGCCACCATC CTCGGTTCCA CCACCAGTGC GTTTCTGGGT

TGCAGGTGGC CGCCACCATC CTCGGCTCCA CCACTAGCGC GTTTCTGGGC

TGCAGGTAGC CGCCACCATC CTCAGCTCCA CCACTAGTGC GTTTCTGGGC

---------- ---------- ---------- ---------- ----------

TGCAGGTGGC GGCCACCATC CTCAGTTCCA CCACCAGCGC GTTTCTGGGC

TGCAGGTGGC CGCCACCATC CTCAGTTCCA CCACCAGCGC GTTCCTGGGC

TGCAGGTGGC CGCCACCATC CTCAGTTCCA CCACCAGCGC GTTCCTGGGT

TCCAGGTGGC CGCCACCATC CTCAGTTCCA CCACCAGCGC GTTCCTGGGC

TGCAGGTGGC TGCCACCATC CTCGGTTCCA CCACCAGCGC GTTTCTGGGC

TGCAGGTGGC CGCCACCATC CTCAGCTCCA CTACCAGCAC GTTTCTGGGC

TGCAAGTGGC CGCCACCATC CTCAGCAGCA CCACCAGCGC GTTTCTGGGC

TGCAGGTGGC CGCCTCCATT CTTAGCTCCA CTACTAGCGC CTTCCTGGGT

TCCAGGTGGC CGCCTCCATC CTTGGCTCCA CCACCAGCGC CTTCCTGGGT

GTCCTGGCTG ACGACAAATA CTTTGGAATT CTATTTAAAA ACACAGATAA

GTCCTGGCTG ACGACAAATA CTTTGGAATT CTATTTAAAA ACACAGATAA

GTCCTGGCCG ACGACAAATA CTTTGGAATT CTATTTAAAA ACACAGATAA

GTCCTGGCCG ACGACAAATA CTTTGGAATT CTATTTAAAA ACACAGATAA

GTCCTGGCCG ACGACAAATA TTTTGGAATT CTGTTTAAAA ACACAGATAA

GTCCTGGCGG ACGACAAATA TTTTGGAATT CTATTTAAAA ATGCAGATAA

GTCCTGGCCG ATGACAAATA TTTTGGAAAT CTGTCTTTAA ATATGGATAA

GTCCTGGCCG ATGACAAATA TTTTGGAAAT TTATCTTTAA ACATGGATAA

---------- ---------- ---------- ---------- ----------

GTCCTGGCTG ATGACAAATA TTTTGGAATT CTATTAAAAA ACACAG----

GTCCTGGCCG ACGACGAGTA TTTTGGAATT CTATTAAAAA CCACAG----

GTTCTGGCCG ACGACAAATA TTTTGGAATT CTATTTAAAA AGGCAG----

GTCCTCACCG AGGAC---TA TTTTGGAATT CTCTTTAAAT ACACAGATGA

GTCCTGGCAG ACGACAAATA TTTTGGAATT ATATTTAAAA ACACAGATAA

GTCTTGGCTG ACGACAAGTA TTTTGGAATT ATATTTAAAA ACACAGATAA

GTCCTGGCCG ATGACAAGTA TTTTTGAATT CTATTTTAAA ACACAGATC-

GTGCTGGCTG ATGACTAGCA TTTTAAACAC AGTTTTAAGG GCACAGACAA

GTCCTGGCCG ACGACTAACA TTTCAGACAG TGGTGGAAAA ACACAGACAG

CGATTGTGAC CATTTGAATG TAACTACAGT TGTACTGTGA ATGCTAAATA

CGATTGTGAC CATTTGAATG TAACTACAGT TGTACTGTGA ATGCTAAATA

CGATTGTGAC CATTTGAATG TAACTACAGT TGTACTGTGA ATGCTAAATA

CGATTGTGAC CATTTGAATG TAACTACAGT TGTACTGTGA ATGTTAAATA

TAATTGTGAC CATTTGAATG TAACTACAGT TGTACTGTGA ATGCTAAATA

TCATTGTGAC CATTTGAATG TAATTACAGT TGTACTGTGA ATGCTAAATA

CTATTGTGAG CCTATGAATG TAACTATAAT TATACTGTGA ATGTCAAATA

CTATTGTGAG CCTATGAATG TAACTACAAT TATACTGTGA ATGTCGAATA

---------- ---------- ---------- ---------- ----------

--AAAGTGAC CATTTGAATG TAATTACAGT TATACTGTGA ATGCTAAATA

--AATGTGAT CATTTGAATG TAATTCCAGT TGTACTGTGA ATGCTAACTA

--AATATAAC CATTTGAATG TA----CAGG TGTACTGTGA ATGCTAGATA

GCATTATGAC CATTTGAATG TAATTCCA-T TATACTGTGA ATGCGAAATA

CCATTGTGGC CATTTGAATG AAAGTACATT TGTACTGTGA ATGCTAAATA

CCATTGTGAC CATTTGAATG TAATTACAGC TGTACTGTGA ATGCTAAGTA

--------AC CGCTTGAATG TAATTCCGGT CGTCCTGTGA GCGCCAAATG

CCATTGTGAC CATTTGAATG TAATTACAGT TGTACTGTGA ATGTGAAATG

CCATTGTGAC CATTTGAATG TAATGCCAGT TCTACTGTGA ATGCTAAATG

TTTTTGAAAT TGTCTGGAAT GTTTTGATTG AGCAATGAAA TAAAGTCCTC

TTTTTGAAAT TGTCTGGAAT GTTTTGATTG AGCAATGAAA TAAAGTCCTC

TTTTTGAAAT TGTCTGGAAT GTTTTGATTG AGCAATGAAA TAAAGTCCTC

TTTTTGAAAT TGTCTGGAAT GTTTTGATTG AGCAATGAAA TAAAGTCCTC

TTTTTGAAAT TGTTTGGAAT GTTTTGACTG AGCAATGAAA TAAAGTCCTC

TTTTTGAAAT TGTCTGGAAT GTTTTGATTA AGCAATGAAA TAAAGTCCTC

TTTTTGAAAT CGTCTGAAAT G-----ATTA AACAATG--- -AAAGTTCCT

TTTTTGAAAT CGTCTGGAAC A-----AGTA AGCAATGAAA TAAAGTTTCT

---TTGAATT TATCTGGAAT GTTTTGATTA AGCAATGAAA TAAA---TCC

TTTCTGAAAT TGTCTGGAAT GTTTTGATTA ACCAATGAAA TAAATTTTCC

TT------AT TGCCTGGAAT GTTTTGATTA AGCACTAAAA TAAAGTCTCC

TTTCTGAAAT TGTCTGGAAT GTTTTGATTA AGCAATGAAA TAAAGTCTCC

CTTCTGA-AA TGTCTGGAAT GTTTTGAATA AGCAAGGAAA TAAAGCCTCC

TTTTTGGATT TATCTGAAAT GTTTTGAGTA AGCCCTGAAA TAAAGTTCGC

TTTTTGGAAT TGTCTGGAAT GCCTTGATTA AGCATTGAAA TAAAGTCTCC

TTTTTGAAAT TGTCTGGGAT ACTCTGATTA AGTGTTGGAA TAAAGTCCTC

TCTTTGAAAT TGTTTGGAAT GTTTTGATTC TGGAGTAAAT TAAAATACTT

ATTTTGAAAT TGTGTGGAAT GTTTTGGTTC AGAAATGAAT TAAAATACTT

ATTTTTTCTC TCTGCATAAA TTATTTAAAA TGCAAATTAA ATCTGCCTCT

ATTTTTTCTC TCTGCATAAA TTATTTAAAA TGCAAATTAA ATCTGCCTC-

ATTTTTTCTC TCTGCATAAA TTATTTAAAA TGCAAATTAA ATCTGCCTCT

ATTTTTTCTC TCTGCATAAA TTATTTAAAA TGCAAATTAA ATCTGCCTCT

ATTTTTTCTC TCTGCATAAA TTATTTAAAA TGCAAATTAA ATCTGCCTCT

----TTCCTC TTTGCATAAA TTATTTAAAA TGCAAATTAA ATCTGCCTCT

TTTTTTTCTC TCTGCATAAA TTATTTAAAA TGCAAATTAA ATCTGCCTCT

GTTTTTTCGT CCTGCATAAA TTATTTAAAA TGCAAATTAA ATCTGCCTCT

ATTTTT-CTC TCTGCAGAAA TTATTTAAAA TGCAAATGGA ATCTGCCTCT

ATTTTTCCTC GCTGCATAAA TTATTTAAAA TGCACATCAA ATCTGCCTCT

ATTTTTCTTC GCTGCATAAA TTATTTAAAA TGCAAATTAA A-CTGCCTCT

ATTTTTCCTC GCTGCATAAA TTATTTTAAA TGCAAATTAA ATCTGCCTCT

ATTTTTCTT- ---------- ---------- ---------- ----------

ATTTTTCCTC CCTGCATAAG TTATTTGAAA TGCAAATTAA ATCTGCCTCT

ATTTTTCCTC CCTGCATAAA TTATTTAAAA TGCAAATTAA TTCTGCCTCT

ATTTTTCCAT CCTGCATAAA TTATTTAAAA TGCAAATCAA TTCTACGGCT

CTTTTTCCTA CATGTAGAAA CTATTGAAAA TGCCCATTTA AAATGCTTCA

CTTTTCCCTA CTTGGACAAA GTAATTTGAA TGCAAATGAA ATCTGCCTCA

GCTGAAAAAT TTTCTAATTT ACAGCCTGAA GGTAAAAAGT ACACTGATTA

GCCGAAAAAT TTTCTAATTT ACAGCCTGAA GGTAAAAAGT ACACTGATTA

GCAGAAAAAT TTTCCAATTT ACAGCCTGAA GGTAAAAAGT ACACTGATTA

GCAGAAAAAT TTTCCAATTT ACAGCCTGAA GGTAAAAAGT ACACTGATTA

GCAGAAAAAT TTTCTAATTT ACTGCCTGAA GGTAAAAAGC ACATTGATTA

GCAGAAAAAT TTTCCAATTT ACAGTCTGAA GGTAAAAAGT ACACTAATTA

GCA------- TTTCCAATTT ACAGTATTAA AGTAAAAAGA ACACCAATTA

GCAGAAAAAA TTTCCAATTT ACAGTATTAA AGTAAAAAGT ACACCAATTA

GCAGGAAAAG ---------- ---------- ---------- ----------

GT-GAAAAAT TTTCCAATTT ACAGTCAGAA GGTAAAAAGT ACACTAATTA

GCAGAAAAAT TTTCCAATTG ACAGTCTGAA GGTAAAAAGT ACTCTAATTA

GCAGAAAAAT TTTTCAATTT GCTGTCTGAA GGTAAAAAGT ACACTAATTA

---------- ---------- ---GTCTGAA GGTGAACCGC ACATGAATCC

GCAGAAAAAT TTTCCAATTT ACAATCTGAA GGTAAAAAGT ACACCAATTA

GCAGAAAAAT TTTCCAATTT ACTGTCTGAA GGTAAAAAGT ACACTAATTA

GCGGGAAAAT TTTCCAATTG ACAGTGTGAA GGTAAAAAGT ACACTAATTA

CAAGAAAAGT TTTCCAATTT ACAGTTTAAA CGCAAAAAGT ACACAAATTA

GAAGAGAAAT CATCCAATTT ACTGTTTGAA TGTGCAAAGC GCATAAATTA

AATCCATGTT AGGAGGAGCA GCGGGGGCGG TAAAATTTAT AGACATGTGA

AATCCATGTT AGGAGGAGCA GCGGGGGCGG TAAAATTTAT AGACATGTGA

AATCCACATT AGGAGGAGCA GCGGGGGCGG TAAAATTTAT AGACATATGA

AATCCACATT AGGAGGAGCA GCGGGGGCG- ---------- ----------

AATCCATATT AGGAGGAGCA GCGGGGGCGG TAAAATTTAC AGACATGTGA

AATCCATATT AGGAGGAGCA GCGGGGGCGG TAAAATTGAC AGACATGTGA

AATTCTTAGG AGGAGGAGAA GAGGGGGCGG TAAAATTGAC AGATAGGTGA

AATTCTTAGG AGGAGGTGGA GCGGGGGCGG TAAAATTGAC AGACAGGTGA

---------- ---------- ---------- ---------- ----------

AATCCATATT AGGAGGAACC GCGGGGGCGG TAAAATTTAC AGACATGTGA

AATCCATATT AGGAGGAGCA GCGGGGGCGG TAAAATTTAC AGACATGTGA

CATCCATATT AGGAGCAGCA GCGGGAGCGG TAACATTTAC AGACATGTGA

AATCCTCAGC AAG-GGAGCC ACGGG-GCGG TCAAATTCAC GGCCATGAGA

AATCCATGTT GGGAGGAGCA GCGGGGGCGG TAAAGTTTAC AGACATGTGA

AATCCATATT AGGAGGAGAA GCGGGGGCGG TAAAATTTAC AGACATGTGA

CATCCATATT AGGGGGAGCT gcgcgggcgA CAACGGTGAC AGACATGCGG

AGCCCCAATT ATGAGAAAAG GTGGGGGTGG TGAAATTCAG AGCTATGTGG

AACCCATGTT AAgaggagag gtgggagggG TGAAGTTCAC AGCCATTTTA

ATACTTCTCT TGTTGGAGGC CACTAAAGGT TGAAGGTCAC ATCAATCTGA

ATACTTCTCT TGTTGGAGGC CACTAAAGGT TGAAGGTCAC ATCAATCTGA

ATACTTCTCT TGTTGGAGGC CACTAAAGGT TGAAGGTCAC ATCAATCTCA

---------- ---------- ---------- ---------- ----------

ACACTTCTCT TGTTGGAGGC CACTAAAGGT TGAAGGTCAC ATCAATCTCA

A---TCTTCT TGTTGGCGGC CACTAGAGGC TGAAGGTTAT GTCAATCTCA

ATGCTCCTCT CGCTTGAGGC CACCAGGGGC TGAAGGTCAC ATCCATCTCA

ATGCTCCTCT CGCTGGAGGT CACTAGGGGC TGAAGGTCAC ATCAATCTCA

---------- ---------- ---------- ---------- ----------

ATATTCCTCT TGTGGGAGGC CACCAGAGGC TCAAGGTCAC ATCAATCTCA

AGACTTCTCT TGTGCAAGGC CACTAGAGGC CGGAGGTCAC ATCAACCTCA

ATACTTCTCT TGTCAGAAGC CACTAGAGGC TGAAGGTCAC ATCAACCTCC

ATGCTTCTCT TGTTGGAGGC CACGAAAGGT TGAAGGTCAC ATCCATCTCG

ATACTTCTCT CGTTAGAGGC CACCAGGGGC TGAAGGTCAC ATCAAGCTCA

ATACTTTGTT TCTTGGAAGC AACCAGAGGC TGAAGGTCAC ATCAATCTCA

ATGTTTCGCT TGTTGGAGGC CACTAGAGGC TGCAGGTGAC ATCCATCTCG

ATATTTCTCT TGTTGGAGGC AACTAGAGGC TGAAGGTCAC ATCTATTTCA

ACACTCCTCT TGTTCGCAGC AATCAGAGGC CAAAGGTCAC ATCGATTTCA

ATCCATTTGT GTTTCTTTCC AAATTCAAAC GTGAAGAGCC GAACAGTGTT

ATCCATTTGT GTTTCTTTCC AAATTCAAAC GTGAAGAGCC GAACAGTGTT

ATCCATTTGT GTTTCTTTCC AAATTCAAAC GTGAAGAGCC GAACAGTGTT

---------- ---------- ---------- GTGAAGAGCC GAACAGTATT

ATCCACTTGT GTTTCTTTCC AAGTTCAAAC GTGAAGAGCC GAATAGTGTT

ATCCATTTAT GTTTCTTTTC AAATTCAAAC GTGAAGAGCC GAATAGTATT

ATCCACCTGT GTTTCCTC-- ---------- CTGAAGAGCC GGACGGTATT

ATCCATCTGT GTTTCTTC-- ---------- CTGAAGAGCC GGACGGTATT

---------- ---------- ---------- GTGAAGAGTC GAACTGTGTT

ATCCATTTGT ATTTCTTTCT AAATTCAAAC GTGAAGAGCC GAACAGTGTT

AACCATTTGT ATTTCTTTTT CAATTCAAAC GTGAAGAGTC GAACAGTGTT

ACCCACGTGT ACTTCTTTCT AAATTCAAAC GTGAATAGCC GAATGGTGTT

AGCCATTTGT GCTTCTTCTG AAATTCAAGC ---------- ----------

ATCCATTTGT GTTTCTTTCT AAATTCAAAC GTGAAGAGCC GAACAGTGTT

ATCCACTTGT GTTTCTTTCT AAATTCAAAC GTGAGGAGCC GAATAGTGTT

ATCCATTGGC GCTTCTTTCT AAATTCAAAC GTGAAGAGCC GAACAGTGTT

ATCCACTTGT GATTTTTTCT CAATTCAAAC GTGAAGAGAC GGACGGTGTT

ACCCACCTTC TCTTTTTTCT AAGTTTCAGG GTAAAGAGCC GAACTGTATT

GTAGAGGTGA CTTCTATTGG ATTTAGGAAT CCCTTCCTTG GTAGCATATG

GTAGAGGTGA CTTCTATTGG ATTTAGGAAT CCCTTCCTTG GTAGCATATG

GTAGAGGTGA CTTCTATTGG ATTTAGGAAT CCCTTCCTTG GTAGCGTATG

GTAGAGGTGA CTTCTATTGG ATTTAGGAAT CCCTTCCTTG GTAGCGTATG

GTAGAGGTGA CTTCTATTGG ATTTAGGAAT CCCTTCCTTC GTAGCATATG

GTAGAGGTGA CTTCTGTTGG ATTTGGGAAT CCCTTCCTTA GTAGCATATT

GTAGAGGTGA CTTCTGCTGG GTTTAGGAAC CCCCTCTTTG GTAGCATACG

GTAGAGGTGA CTTCTGCTGG GTTTGGGAAC CCCCTCTTTG GTAGCATACG

GTAGAGGTGA TTTCTGTTGG CTTTAGGAAT CCCCTCCTTG GTAGCGTAAG

GTAGAGGTGG CTTCTGTTGG ATTTAGGGGT CCCCTCCTTG GTAGCATATG

GTAAAGAGGG CTTCTGTTGG ATTTAGGGAT CCCCTCCTTG GTAGCACAGG

GGAGACGTGA CTTCTGTTGG ACTTAGGGAT CCCCTCCTTG GTAGCGTATG

---------- ---------- ---------- -----CTTTG GTTGCGTATG

GTAGAGGTGA CTTCTGTTGG ATTTAGGGAT TCCCTCCTTG GTAGCATATG

GTAGAGGTGA TGTCTGTCGG ATTTAGGGAT CCCCTCCTTG GTAGCATATG

GTAGAGGTGA CTTCTGGTGG ATTTAGGGAT CCCCTCCTTG GTAGCATATG

GTAGAGGTGA CTTCTGTCAG ACTTAGGGAT TCCTTCTTTG GTGGCATATG

GTAGAAGTGA CTCCTATTGG ACTTTGGGAT GCCTTCTTTG GAAGCATACA

TCTTATAGAG CTTCTTCATG TAGTGCAAAG CTCTGGAGTC TGGCTGCAGC

TCTTATAGAG CTTCTTCATG TAGTGCAAAG CTCTGGAGTC TGGCTGCAGC

TCTTATAGAG ATTCTTCATG TAGTGCAAAG CTCTGGAGTC TGGTTGCAGC

TCTTATAGAG ATTCTTCATG TAGTGCAAAG CTCTGGAGTC TGGTTGCAGC

TGTTATAGAG CTTCTTCATG TAGTGCAAAG CTCTGGAGTC TGGCTGCAGC

CCTTATAGAG CTTCTTCATG TATTGCAAGG CTCTGGAGTC TGGCTGTAGT

TCTTATAGAG CTTTTTCATG TAGTAGAGAG CTCTGGAGTC AGGCTGCAGC

TCTTATAGAG CTTTTTCATG TAGTAGAGTG CTCTGGAGTC AGGCTGCAGC

CTTTATAAAG CTTCTTCATG TACTGCAACG CTCTCGAGTC TGGCTGCAGC

CTTTATAGAG CCTCTTCATG TAGCTCAAAG CTCTGTCATC TGGCTGAAGC

CCTTATAGAG CCTCTTCATG TAGCGCAAAG CTCTA----- ----------

CCTTATAGAG CCTCTTCATG TAGCGCAAAT CTCTGGAGTC TGGCTGCAGC

CCTTATAGAG CCTCTTCATG TAGCGCAAAG CCCGGGAATC AGGCTGCAGC

CCTTATAGAG CCTCTTCATG TAGCGTAAAG CCCTGGTGTC TGGCTGCAGC

CCTTATAGAG CCTCTTCATG TAGCGCAAAG CTCTGGAGTC TGGCTGCAGC

CCTTATAGAG CCTCTTCATG TAGCGCAGAG CTCTCGAGTC TGGCTGCAGC

CTTTATAAAG CCTCTTCATG TAACGCAGGG ATCTTGAGTC AGGTTGTAGC

TCCGGTAGAG TCTCTTCATG AAGCCCAGGG CCGCGGAATC AGGCTGCAGT

CTTCAGGCCT CAAGTATGCC TAGCTGTTAT CAGCTCTATA GCATGGGCCG

CTTCAGGCCT CAAGTATGCC TAGCTGTTAT CAGCTCTATA GCATGGGCCG

CTTCAGGCCT CAAGTATGCC TAGCTGTTAT CAGCTGTATA GCATGGGCCG

CTTCAGGCCT CAAGTATGCC TAGCTGTTAT CAGCTGTATA GCATGGGCCG

CTTCAGGCCT CAAGTATGCC TAGCTGTTAT CAGCTCTATA GCATGGGTCG

CTTCAGGCCT CAAAGATGCC TAGCTGTTAT CAGCTCTATA ACATGGGCCG

TTTCAGACCT CAAGGATGCC TAGCTGTTAT CAGCTCTACA TCATGGGCCG

TTTCAGACCT CAAGCATGCC TAGCTGTTAT CAGCTCTACA TCATGGGCCG

CTTCAGACCT CAAGGACGCC TAGCTGTTAT CAGCTCTATG CCATGGGCCG

CTTCAGACCT TAAGGATGCT TAGCTGTTAT CA-------- --ATGGGCCG

--TCA-ACCT CAAGGACGCC TAGCTGTTGT CAGCTGTGCA ACATGGGCCG

CTTCAGACCT CAAGGATGCC TAGCTGTTAT CA-------- ----------

CTTCAGATCT AAAGAAGGCC TACCTGTTAT CAGGTGTATA ACATGGGCCG

CTTCAGCCCT CAAGGATGCC CAGCTGTAAT CAGTTCTGTA ACATGGGTCG

CTTTAGGCCT CAAGGATGCC TAGGTGTTAT CAACTCTATA ACATGGGCCG

CTTTAGACCT CAAGGGTGCC TAGCTGTTAT CAGCTCTATA GC--------

CTTTGGATGA TAAGGGTGCC CAGCTGTTGT CAGCTCTAGA ATATGGGCCG

GATTGGACCC CAAACGTGCC CAGCTGGGGT CAGCACTGCA GTATGGGTCG

CGAGTTTGGG AATCTGACGC GGATGCGGCA TGTGATCAGC TACAGCTTGT

CGAGTTTGGG AATCTGACGC GGATGCGGCA TGTGATCAGC TACAGCTTGT

CGAGTTTGGG AATCTGACGC GGATTCGGCA TGTGATCACC TACAGCTTGT

CGAGTTTGGG AATCTGACGC GGATTCGGCA TGTGATCACC TACAGCTTGT

CGAATTTGGG AATCTGACGC GGATGCGGCA TGTGATCTCC TACAGCTTGT

CGAGTTTGGG AACCTGACGC GGATGCGGCA TGTGATCACC TACAGCTTGT

CGAGTTTGGG AACCTGACGC GGATACGGCA TGTGATCTCC TACAGCTTGT

CGAGTTTGGG AACCTGGCGC GGATACGGCA CGTGATCTCC TACAGCTTGT

CGAGTTTGGG AACCTGACGC GGGTGCGGCA TGTGATCAGC TACAGCTTGT

CCAGTTTGGG CATCTGACAC GGGTGCGGCA TGTGATCACC TACAGCTTGT

CGAGTTTGGG AACCTGACCC GGATGCGGCA TGTGATCACC TACAGTTTGT

---------- ---------- ---------- ---------- ----------

CGAGTTCGGG CACCTGGCGC GGATGCGACA TGTGATCTCC TACAGCCTGT

CGAATTTGGG AACCTGACGC GGATGCGGCA TGTGATCACC TACAGCTTGT

CGAGTTTGGG AACCTGGCGC GGGTGCGGCA CGTGATCACC TACAGCTTGT

---------- ---------- ---------- ---------- ----------

CGAGTTTGGG AACCTAATCC GGATAAGGCA TATCATTTCC TACAGCCTGT

TGAGTTCGGA AACCTAATAA AAGTGAGGCA TATCATTTCC TATAGCCTGT

CACCGTTCGA GCAGCGCGCC TATCCGCACG TCTTCACTAA AGGAATCCCC

CACCTTTCGA GCAGCGCGCC TATCCGCACG TCTTCACTAA AGGAATCCCC

CACCTTTCGA GCAGCGCGCC TTGCCGCATG TTTTCAGTAA AGGAGTCCCC

CACCTTTCGA GCAGCGCGCC TTGCCGCACG TTTTCAGTAA AGGAGTCCCC

CACCTTTCGA GCAGCGCGCC TTCCCGCACG TCTTCTCTAA AGGAGTCCCC

CACCCTTCGA GCAGCGCGCT TTCCCGCACT ACTTCACTAA GGGAATCCCC

CGCCCTTTGA GCAGCGCGCC TTCCCGCACT ATTTCAGCAA AGGCATCCCC

CGCCCTTTGA GCAGCGCGCC TTCCCAAGCT ATTTCAGCAA AGGCATCCCC

CGCCCTTCGA GCAGCGCGCC TTCCCGCACT TCTTCAGCAA GGGCATCCCC

CGCCCTTCGA GCAGCGCGCC TTCCCGCACT ACTTCAGCAA GGGCATCCCC

CGCCCTTCGA GCAGCGCGCC TTCCCGCACT ACTTCAGCAA AGGCATCCCC

---------- ---------- ---------- ---------- ----------

CTCCCTTCGA ACAGCGCGCC TTCCCGCACT ACTTCAGCAA GGGCATCCCC

CACCCTTTGA GCAGCGCGCC TTTCCGCACT ACTTCAGCAA GGGTATCCCG

CGCCCTTCGA ACAGCGCGCT TTCCCTCACT ACTTCACCAA GGGCATCCCC

---------- ---------- ---------- ---------- ----------

CCCCGTTCGA GCAGAGGGCC TTCCCCAACT ACTTCTCAAA GGGCATCCCC

CGCCATTTGA GCAGAGGGCA TTCCCCAACT TCTTCATCAA AGGCATTCCG

AATGTTCTGC GCCGGAGTTC GAGAGATCCA AGAGGAAGAA TCCAGCTGCC

AACGTTCTGC GCCGGAGTTC GAGAGATCCA AGAGGAAGAA TCCAGCTGCC

AACATGCTGC GCCGGAGGTC GAGAAATCCA AGAGGAAGAA TCCAGCTGCC

AACATGCTGC GCCGGAGGTC GAGAAATCCA AGAAGAAGAA TCCAGCTGCC

AACGTGGTGC GCCGGAGTTT GAGCGATCCA AGAGGAAGAA TCCAGCTGAC

AACATGATTC GCCG------ ---------- ---------- ----------

AACGTGCTGC GCCGGAGTTT GCGCAGTCCA AACGGAAGAA TCCAGCTAAG

AACGTGCTGC GCCGGAGTTT GAGCAGTCGA AAAGGAAGAA TCCAGCCATG

AACCTGCTGC GCCGGAGTTT GAGAAATCCA AGAGGAAGAA TCCCGCCGCC

AACGTTCTGC GCCGGAGTTT GAGAAATCGA AGAGGAAGAA TCCAGCTGCC

AACGTGCTGC GCCGGAATTT GAGAAATCCA AGAGGAAGAA TCCAGCTGCC

---------- ---------- ---------- ---------- ----------

AACGTGCTGC GCCGGAATTT GAGAAATCCA AGAGGAAGAA TCCAGCCGAC

AACGTGCTGC GCCGGAGTTT GAGAAATCCA AGAGGAAGAA TCCAGCTGCC

AACGCGCTGC GCCGGAGTTT GAAAAATCCA AGAGGAAAAA TCCAGCCGAC

---------- ---------- ---------- ---------- ----------

AACATGATCC GCCGGAGTTT GAGAATTCAA AGAAGAAGAA GCCAGATGCC

AATGTCTTAC GGCGGAATTT GAAAAGTCCA AGAGGAAGAA CCCCGCTGAC

TATGAAAATG ACAAATGATT TCTGAAGACA TTATTGGATA TGGGTGATCT

TATGAAAATG ACAAATGATT TCTGAAGACA TTATTGGATA TGGGTGATCT

TATGAAAATG ACAAATGATT TCTGAAGACA TTATTGGATA TGGGTGATCT

TATGAAAATG ACAAATGATT TCTGAAGACA TTATTGGATA TGGGTGATCT

TATGAAAATG ACAAATGATT TCTGAAGACA TTATTGGATA TGGGTGATCT

---------- --------TT TCTGAAGACC TCATTAGATA TTAGTGATCT

TACGAGAATG ACAAGTGACT TCTGAAGATA TT---AGACA TGAACCATCT

TATGAAAATG ACAAGTAGCT CCTGAAGATA TT---AGAGA TTAACCATCT

TACGAACATG ACAAGTGATT TCTGAAGACA TTGTTAGATA TGAGTGATCT

TATGAAAATG ACAGATAATT TCTAAAGACA TTAGGAGATA TTAGTGATTT

TATGAAAATG ACAAATGA-- --------CA TTATGAGATA TTAGTGATCT

---------- --------TT TCTGAAGACA TTATTAGATA TTAGTGATCT

TATGAAAATG ACTCATGA-- ---------- ---------- ----------

TATGAAAATG ACAAATGATT TCTAAAGACA TTATTAGATA TTAATGATCT

TATGAAAATG ACAAGTGATT CCTGAAGACA TTATTAGATA TTAGTGATCT

---------- --------TT TCTGAAGA-- ---TTAAATA TTAATGATCT

TTTGAGGAAG ACAAGTAATT TTTAAAAAAA TTATGTGACA TTAGCAACTT

TATGAAAACG ACAAGTGATT TTTAAAGTAA TTACTGGGTA TTAGTGACCC

CACTGAGCTA TTTTGGAAAG AATGACCCCA TTTTGGAGAG GGGTTTCCCT

CACTGAGCTA TTTTGGAAAG AATGACCCCA TTTTGGAGAG GGGTTTCCCT

CACTGAGCTA TTTTGGAAAG AATGACCCCA TTTTGGAGAG GGGTTTCCCT

CACTGAGCTA TTTTGGAAAG AATGACCCCA TTTTGGAGAG GGGTTTCCCT

CACTGAGCTA TTTTGGAAAG AATGACCCCA TTTTGGAGAG GGGTTTCCCT

CACTGAGCTA TTTTGGAAAG AATGACCCCA TTTTGGAGAG GGGTTTCCCT

CACTGAGCTA TTTTGAAAAG AATGACCCCA TTTTGGAGAG GGGTTTCCCT

CACTGAGCTA TTTTGGAAAG AATGACCCCA TTTTGGAGAG GGGTTTCCCT

CATTGAGCTA TTTTGGAAAG AATGACCCCG TTTTGGAGAG GGGTCTCCCT

CACTGAGCTA TTTTGGAAAG AATGACCCCA TTTTGGAGAG CGGTTTCCCT

CACTGAGCTA TTTTGGAAAG AATGACCCCA TTTTGGAGAG GGGTTTCCCT

CACTGAGCTA TTTTAGAAAG AATGACCCCC TTTTGGAGAG GGGTTTCCCT

---------- --------AG AATGACACCG TTTTGGAGAG GGGTTTCCCT

CACTGAGCTA TTTTGGAAAG AATGACCCCA TTTTGGAGAG AGGTCTCCCT

CA-----CTA TTTTAGAACG AATGACCCCA TTTTGGAGAG GAGTGTCCCT

CATCAGGCTA TTTTGGACAG AATGACCCCG TTTTGGAGAG GAGTGTCCCT

CAGTGAATTA TTTTGAAAAG GATGACTCCC TTCTGGAGAG GTGTCTCCCT

CAGGGAACTA TTTTGGGAAG GATGACCCCG TTTTGGCGAG GGGTCTCCCT

CAGGCCTATT GGAGCCTCCT GCCGGGATGA TTCTGAGTGT ATCACAAGGC

CAGGCCTATT GGAGCCTCCT GCCGGGATGA TTCTGAGTGT ATCACAAGGC

CAGGCCCATT GGAGCCTCCT GCCGGGATGA TTCTGAGTGT ATCACAAGGC

CAGGCCCATT GGAGCCTCCT GCCGGGATGA TTCTGAGTGT ATCACAAGGC

CAGGCCCATT GGAGCCTCCT GCCGGGATGA TTCTGAGTGT ATCACAAGGC

CAGGCCCATT GGTGCCTCCT GTCGGGACAA TTCTGAGTGT ATCACAAGGC

CAGACCCATT GGTGCCTCAT GTCGGGATGA TTCGGAGTGT ATCACAAGAC

CAGGCCCATT GGTGCCTCGT GCCGGGATGA CTCTGAGTGT ATCACAAGAC

CAGGCCCATT GGAGCCTCGT GTCGGGACAA TGCTGAGTGT GTCACAAGGC

CAGGCCCATT GGAGCCTCCT GTCGGGATGA TTCTGAATGT ATCACAAGGC

AAGGCCCATT GGATCCTCCT GCCGGGACGA TTCTGAGTGT ATCACGAGGC

CAGGCCCATT GGAGCCTCTT GCCGGGACGA TTCTGAGTGT ATCACCAGGC

CAGGCCCATT GGAGCTTCCT GTCGGGACGA TTCTGAGTGC ATCACGAAAC

CAGGCGTATT GGAGCTTCCT GCATGGACAA TTCTGAATGT ACCACCAGGC

TAGGCCTATT GGAGCCTCCT GTCGGGATGA ATCTGAATGT ATCACAAGGC

TAGGCCTATT GGAGCCTCCT GCCGAGATGA TTCTGAATGT ACCACGAGAT

TAGGCCCATA GGGGCATCCT GTCGTGATGA CTCTGAGTGC ATCACAAGAC

CCGGCCCATT GGGGCCTCCT GCCGAGACAA CTCGGAATGC ATCACGCTGC

TATGCAGGTA CAATGCTCCG TTCTATGGAA TATTGATTAA CTGCATTTTG

TATGCAGGTA CAATGCTCCG TTCTATGGAA TATTGATTAA CTGCATTTTG

TATGCAGGTA CAATGCTCCG TTCTATGGAA TATTGATTAG CTGCATTTTG

TATGCAGGTA CAATGCTCCG TTCTATGGAA TATTGATTAG CTGCATTTTG

TATGCAGGTA CAATGCTCCG TTCTATGGAA TATTGATTAA CTGCATTTTG

TCTGCAGGTA CAATGCTCCG TTCTATGGAA TATTGACTAA CTGCATGTTC

TATGCAAGTA CAGTGCTCCG TTCTATGGAA TATTG-TTAA CTGCATTTTG

TATGCAGGTA CAGTGATCTG TTCTGGGGAA TATTG-TTAA CGACATTTTG

TATGCAGGTA CAATGCTCCG TTCTATGGAA TGTTG----A CTATGTTCTG

TATGCAGGTA CAATGCTCCG TTCTATGGAA TATTGATTAA CTGCA-TTTG

TGTGCAGGTA CAATGCTCCG TTCTATGGAA TATTGATTAA CTGCATTTTG

TATGCAGGTA CAATGCTCCG TTCTATGGAA TATTGAGTAA CTGCACTTTG

TATGCAGGTA CAATGCTCCG TTCTATGGAA TATTGACTAA C--CATTCTA

TATGCAGGTA TGATGCTCCA TTCTATGCAG TACCAATTAA CTGCATTTTG

TATGCAGGTA CAATGCTCCG TTCTATGGAA TATTGATTAA CTGCATTTTG

TGTGCAGGTG ---------- ---------- ---------- ----------

TATGCAGGTA CAATGCTCCG TTCTATGGAA TATTGATTAG CTGTATTCTT

TCTGCAGGTA CAATACTCCG TTCTATGGAA TATTGATTAC CTGCACTTGA

GCTGTTTTTA ATATTTAAAG GCAGATGTAC GCTTAATTAA ATGTCTCAAA

GCTGTTTTTA ATATTTAAAG GCAGATGTAT GCTTAATTAA ATGTCTCAAA

GCTGTTTTTA ATATTTAAAG GCAGATGTAT GCTTAATTAA ATGTCTCAAA

GCTGTTTTTA ATATTTAAAG GCAGATGTAT GCTTAATTAA ATGTCTCAAA

GCTGTTTTTA ATATTTAAAG GCAGATGTAT GCTTAATTAA ATGTCTCAAA

GTTGTTTTTA ATATTTAAAG ACAGATATAT GCTTAATTAA ATGTCTCACA

GTTGTTTTTA ATATTTAAAG ACAGATACTT GCTTAATTAA ATGTCTCAAA

GTT-TTTTTA ATATTTAAAG ACGGATACTT GCTTAATTAA ATGTCTCAAA

GCTGTTTTTA ATATTTAAAG ACAGAT---- GCTTAATTAA ATGTCTCA--

GCTGTTTTAA ATGTTTGAAG ACAGATATAC GCTTAATTAA ATGTCTCAAA

GCTGTTTTTA ATATTTAAAG ACAGATATGT GCTTAATTAA ATGTCTCAA-

GCTGTTTTTA AAATTTAAAG ACAGATGTAT GCTTAATTAA ATGTCTTAAA

GCTGTTTTTA ACATTTAAAG ACAGATGTAT GCCTAATTAA ATATCTCAAA

GCTGTTTGTA ATATTTAAAG ACAGATGTAT GCTTAATTAA ATGTTTCCAA

GCTGTTTTTA ATATTTAAAG ACAGATGTGT GCTTAATTAA ATGTCTCCAG

---------- ---------- ---------- ---------- ----------

ACTATATTTA ATATTTAAAG GTAGATTCAT ACCTATTTAA ACTTTAGAAA

ACTGTATTTA ATATTTAAAG ACAGATATAG GCTTGATTAA ATTTCCAAAA

TTGAAAGTCT GGATAAAGAG TACAGTGTTC AGTAACTTTA ATACAGAAAA

TTGAAAGTCT GGATAAAGAG TACAGTGTTC AGTAACTTTA ATACAGAAAA

TTGAAAGTTT GGATAAAGAG CACAGTGTTC ATTAACTTTA ATACAGAAAA

TTGAAAGTTT GGATAAAGAG CACAGTGTTC ATTAACTTTA ATACAGAAAA

TTGAAAGTCT GGATAAAGAG CACAGTGTTC ATTAACTTTA ATACAGAAAA

TTGAAAGTCT GGATAAAGAG CACAGTGTTT ATAAACTTTA ATACAGAAAA

TCTAAAGTCT GGA---AGAG CACAGTGCAT ATAAACGTTA -TACAGAAAA

TCTAAAGTCT GGA---AGAG CACAGTGCAT GCAAACGTAG -TACAGAAAA

TCGAAAGT-T GGA-----AG CACAGTGTAC ACAGACTTTA ATACAGAAAA

CTGAAAGCCT GGATAGACAG CACAGTGTTT ATAAACTTTA ATACAGAAAA

---AAAGTCT GGATAAAGAG CACAGTGTTT ATAAACTTTA ATACAGAAAA

TTGAAAGTCT GTATAAAGAG CACAGTGTTT ATGAAGTTTA ATACAGAAAA

C--AAAGTTT GGATAAAGAG CACAGGGGTT ATAAATTTTC ATACAGAAAA

TTTAAAGTCT GGATAAAGAG CACAGTGTTT ATAAACTTTA ATACAGAAAA

TTGAAAGTCT GGATAAAGAG CACAGTGTTT ATAAACTTTA ATACAGAAAA

---------- ---TAAAGAG CACAGTGTTT ATCAACTTTA ATACAGAAAA

TTAAGAGTCT GtaTAAACAG CATAGCATTT ATAAAGTTTA ATACAGAAAA

TTAAAAATTT GGATAAACAA TATGGTGTTT ATAAAGTTTA ATACAGAAAA

TCTATTTGAT ATTTATTAAA CTTAGTTTCT CCAACTATGG TTTGGCATTG

TCTATTTGAT ATTTATTAAA CTTAGTTTCT CCAACTATGG TTTGGCATTG

TCTATTTGAT ATTTATTAAA CTTAGTTTCT CCAGCTATGG TTTGGCATTG

TCTATTTGAT ATTTATTAAA CTTAGTTTCT CCAGCTATGG TTTGGCATTG

TCTATTTGAT ATTTATTAAA CTTAGTTTCT CCAGCTATGG TTTGGCATTG

TCTATTTGAT ATTTATTAAA CTTAGTTTCT CCAGCTATGG TTTGGCATTA

GCTATCTGAT ATTTATTAAA CTTAGTTTCT CCAGCTATGG TTTGGCATTA

GCTAGCTGAT ATTTATTAAA CTTAGTTTCT CCAGCTATGG TTTGGCATTA

TCTATTTGAT ATTTATTAAA CTTAGTTTCT CCAGCTATGG TTTGGCATTA

TCTATTTGAT ATTTATTAAA CTTAGTTTCT CCAGCTATGG TTTGGCATTA

TCTATTTGAT ATTTATTAAA CTTAGTTTCT CCAGCTATGG TTTGGCATTA

TCTATTTGAT ATTTATTAAA CTTAGTTTCT CCAGCTATGG TTTGGCATTA

TCTATTTGAT ATTTATTAAA CTTAGTTCTT CCAGCTATGG TTTGGCATTA

TCTATTTGAT ATTTATTAAA CTTAGTTTCT CCAGCTATGG TTTGGCATTA

TCTATTTGAT ATTTATTAAA CTTAGTTTCT CCAGCTATGG TTTGGCATTA

TCTATTTGAT ATTTATTAAA CTTAGTTTCT CCAGCTATGG TTTGGCATTA

TCTATTTGAT ATTTATTAAA CTTAGTTTTT CCAGTTATGG TTTTGCATTA

TCTATTTGAT ATTTATTAAA CTTAGTTTCT CCAGTTAAGG TTTTGCATTA

TACAAAGCAT TTAATGACAC AGTAGTTAAA AAGATCTTTA CAAATTGAAA

TACAAAGCAT TTAATGACAC AGTAGTTAAA AAGATCTTTA CAAATTGAAA

TACAAAGCAT TTAATGACAC AGTAGTTAAA AAGATCTTTA CAAATTGAAA

TACAAAGCAT TTAATGACAC AGTAGTTAAA AAGATCTTTA CAAATTGAAA

TACAAAGCAT TTAATGACAC AATAGTTAAA AAGATCTTTA CAAATTGAAA

TACAAAGCAT TTAATGACAC AGTAGTTAAA AAGATCTTTA CAAATTGAAA

TACAAAGCAT ATAATGACAC AGTAGTTAAA AAGAGTTTTA CAAATTGAAA

TACAAAGCAT GTAATGACAC AGTAGTTAAA AAGAGTTTTA CAAATTGAAA

TACAAAGCAT TTAATGACAC AGTAGTTAAA AAGATCTTTA CAAATTGAAA

TACAAAGCAT TTAATGACAC AGTAGTTAAA AAGATCTTTA CAAATTGAAA

TACAAAGCAT TTAATGACAC AGTAGTTAAA AAGATCTTTA CAAATTGAAA

TACAAAGCAT TTAATGACAC AGTAGTTAAA AAGATCTTTA CAAATTGAAA

TACAAAGCAT TTAATGACAC AGTAGTTAAA AAGATCTTTA CAAATTGAAA

TACAAAGCAT TTAATGACAC AGTAGTTAAA AAGATCTTTA CAAATTGAAA

TACAAAGCAT TTAATGACAC AGTAGTTAAA AAGATCTTTA CAAATTGAAA

TACAAAGCAT TTAATGACAC AGTAGTTAAA AAGATCTTTA CAAATTGAAA

TACAAAGCAT TTAATGACAC AGTAGTTAAA AAGATCTTTA CAAATTGAAA

TACAAAGCAT TTAATGACAC AGTAGTTAAA AAGATCTTTA CAAATTGAAA

TGTGATACCC TTGTCTCCAA ATATTTTAAT TGCCATAAAT TTGTTTAAAA

TGTGATACCC TTGTCTCCAA ATATTTTAAT TACCATAAAT TTGTTTAAAA

TGTGATACCC TTGTCTCCAA ATATTTTAAT TGCCATAAAT TTGTTTAAAA

TGTGATACCC TTGTCTCCAA ATATTTTAAT TGCCATAAAT TTGTTTAAAA

TGTGATACCC TTGTCTCCAA ATATTTTAAT TGCCATAAAT TTGTTTAAAA

TGTGATACCC TTGTCTCCAA ATATTTTAAT TGCCATAAAT TTGTTTAAAA

TGTGATACCC TTGTCTCCAA ATATTTTAAT TGCCATAAAT TTGTTTAAAA

TGTGATACCC TTGTCTCCAA ATATTTTAAT TGCCATAAAT TTGTTTAAAA

TGTGATACCC TTGTCTCCAA ATATTTTAAT TGCCATAAAT TTGTTTAAAA

TGTGATACCC TTGTCTCCAA ATATTTTAAT TGCCATAAAT TTGTTTAAAA

TGTGATACCC TTGTCTCCAA ATATTTTAAT TGCCATAAAT TTGTTTAAAA

TGTGATACCC TTGTCTCCAA ATATTTTAAT TGCCATAAAT TTGTTTAAAA

TGTGATACCC TTGTCTCCAA ATATTTTAAT TGCCATAAAT TTGTTTAAAA

TGTGATACCC TTGTCTCCAA ATATTTTAAT TGCCATAAAT TTGTTTAAAA

TGTGATACCC TTGTCTCCAA GTATTTTAAT TGCCATAAAT TTGTTTAAAA

TGTGATACCC TTGTCTCCAA ATATTTTAAT TGCCATAAAT TTGTTTAAAA

TGTGATACCC TTTTCTCCAA ATATTTTAAT CGCCATAAAT TTGTTTAAAA

TGTGATACCC TTTTCTCCAA ATATTTTAAT TGCCATAAAT TTGTTTAAAA

ATACACATTA AACGCATGTT TGACACTCTA AACTTTCTAT ACTCTCAATA

ATACACATTA AACGCATGTT TGACACTCTA AACTTTCTAT ACTCTCAATA

ATACACATTA AACGCATGTT TGACACTCTA AACTTTCTAT ACTTTCAATA

ATACACATTA AACGCATGTT TGACACTCTA AACTTTCTAT ACTCTCAATA

ATACACATTA AACGCATGTT TGACA--CTA AACTTTCTAT ACTCTCAATA

ATACACATTA AACGCATGTT TGACACTCTA AACTTTCTAT AATTTCAATA

ATACACATTA AACGCATGTT TGACACTCTA AACTCTCTAT AATCTCGATA

ATACACATTA AACGCATGTT TGACACTCTA AACTTTCTAT AATCTCAATA

ATACACATTA AACGCATGTT TGACACTCTA AACTTTCTAT AATCTCAATA

ATACACATTA AACGCATGTT TGACACTCTA AACTTTCTAT AATCTCAATA

ATACACATTA AACGCATGTT TGACACTCTA AACTTTCTAT AATCTCAATA

ATACACATTA AACGCATGTT TGACACTCTA AACTTTCTAT AATCTCAATA

ATACACATTA AACGCATGTT TGACACTCTA AACTTTCTAT AGTCTCAATA

ATACACATTA AACGCATGTT TGACACTCTA AACTTTCTAT AATCTCAATA

ATACACATTA AACGCATGTT TGACACTCTA AACTTTCTAT AATCTCAATA

ATACACATTA AACGCATGTT TGACACTCTA AACTTTCTAT AATCTCAATA

ATACACATTA AATGCATGTT TGACACTCTA AACTTTCTAT AATCTCAATA

ATACACATTA AACGCAGGTT TGACACTCTA AACTTTCTAT AATCTCAATA

CCACAAAATA CATATACTAT ACAGATGTGG AAAAATCTAG TTAGTATATA

CCACAAAATA CATATACTAT ACAGATGTGG AAAAATCTAG TTAGTATATA

CCACAAAATA CATATACTAT ACAGATGTGG AAAAATCTAG TTAGTATATA

CCACAAAATA CATATACTAT ACAGATGTGG AAAAATCTAG TTAGTATATA

CCACAAAATA CATATACTAT ACAGATGTGG AAAAATCTAG TTAGTATATA

CCACAAAATA CATATACTAT ACAGATGTGG AAAAATCTAG TTAGTATATA

CCACAAAATA CATATACTAT ACAGATGTGG AAAA-TCTAG TTAGTATGTA

CCACAAAATA CATATACTAT ACAGATGTGG AAAA-TCTAG TTAGTATATA

CCACAAAATA CATATACTAT ACAGATGTGG AAAAATCTAG TTAGTATATA

CCACAAAATA CATATACTAT ACAGATGTGG AAAAATCTAG TTAGTATATA

CCACAAAATA CATATACTAT ACAGATGTGG AAAAATCTAG TTAGTATATA

CCACAAAATA CATATACTAT ACAGATGTGG AAAAATCTAG TTAGTATATA

CCACAAAATA CATATACTAT ACAGATGTGG AAAAATCTAG TTAGTATATA

CCACAAAATA CATATATTAT ACAGATGTGG AAAAATCTAG TTAGTATATA

CCACAAAATA CATATACTAT ACAGATGTGG AAAAATCTAG TTAGTATATA

CCACAAAATA CATATACTAT ACAGATGTGG AAAAATCTAG TTAGTATATA

CCACAAAATA CATATACTAT ACAGATGTGG AAAAATCTAG TTAGTATATA

CCACAAAATA CATATGCTAT ACAGATGTGG AAAAATCTAG TTAGTATATA

ATACTTTGCT CACCATTAAC AGCTTTATCG TGTGAAATGC ACATACTGAC

ATACTTTGCT CACCATTAAC AGCTTTATCG TGTGAAATGC ACATACTGAC

ATACTTTGCT CACCATTAAC AGCTTTATCA TGTGAAATGC ACATACTGAC

ATACTTTGCT CACCATTAAC AGCTTTATCA TGTGAAATGC ACATACTGAC

ATACTTTGCT CACCATTAAC AGCTTTATCA TGTGAAATGC ACATACTGAC

ATACTTTGCT CACCATTAAC AGCTTTATTA TGTGAAATGC ACATACTGAC

ATACTTTGCT CACCATTAAC AGCTCG-TTA TGTGAA-TGC ACATACTGAC

ATACTTTGCT CACCATTAAC AGCTTA-TTA TGTGAAATGC ACATACTGAC

ATACTTTGCT CACCATTAAC AGCTTTCTTA TGTGAAATGC ACATACTGAC

ATACTTTGCT CACCATTAAC AGCTTTATTA CGTGAAATGC ACATACTGAC

ATACTTTGCT CACCATTAAC AGCTTTATTA TGTGAAATGC ACAT----AC

ATACTTTGCT CACCATTAAC AGCTTTATTA TGTGAAATGC ACATACTGAC

ATATTTTGCT TACCATTAAC AGCTTTATTA TGTGAAATGC ACATACTGAC

ATACTTTGCT CACCATTAAC AGCTTTATTG TGTGAAATGC ACATACTGAC

ATACTTTGCT CACCATTAAC AGCTTTATAA TGTGAAATGC ACACACTGAC

ATACTTTGCT CACCGTTAAC AGCTTTATAA TGTGAAATGC ACATACTGAC

ATACTTTGCT CACCATTAAC AGCTTTGTTA CGTGAAATGC ACATATTGAC

ATACTTTGCT CACCATTAAC AGCTTTATTA CGTGAAATGC ACATACTGAC

TTAGAAATCC TAGCTTTTGA GCCCCAAAGT ACCTCTGAAA TTGTAATGTA

TTAGAAATCC TAGCTTTTGA GCCCCAAAGT ACCTCTGAAA TTGTAATGTA

TTAGAAATCC TAGCTTTTGA GCCCCAAAGT ACCTCTGAAA TTTTAATGTA

TTAGAAATCC TAGCTTTTGA GCCCCAAAGT ACCTCTGAAA TTTTAATGTA

TTAGAAATCC TAGCTTTTGA GCCCCAAAGT ACCTTTGAAA TTTTAATGTA

TTAGAAATCC TAGCTTTTGA GCCCCAAAGT ACCTCTGAAA TTTTAATGTA

TTAGAAACCC TAGCTTTTGA GCCCCAAAGT ACCTCTGAAA TTT-AACATA

TTAGAAATCC TAGCTCTTGA GCCCCAAAGT ACCTCTGAAA TTT-AACGTA

TTAGAAATCC TAGCTTTTGA ACCCCAAAGT ACCTCTGAAA TTTTAATGTA

TTAGAAATCC TAGCTTTTGA GCCCCAAAGT ACCTCTGAAA TTTTAATGTA

TTAGAAATCC TAGCTTTTGA GCCCCAAAGT ACCTCTGAAA TTTTAATGTA

TTAGAAATCC TAGTTTTTGA GCCCCAAAGT ACCTCTGAAA TTTTAACGTA

TTAGAAATCC TAGCTTCTGA GCCCCAAAGT ACCTCTGAAA TTTTAATGTA

TTAGAAATCC TAGCTTCTGA GCCCCAAAGT ACCTCTGAAA TTTTAATGCA

TTAGAAATTC TAGCTTTTGA GCCCCAAAGT ACCTCTGAAA TTTTAATGTA

TTAGAAATTC TAGCTTTTGA GCCACAAAGT ACCTCTGAAA TCGTAATGTA

TTTAAAATCC TAACTTCTGA GCCCCAAACT ACCCTTGAAA TTTAAGTGTA

TTAGAAATCC TAACTTTTGA GCCCCAAAGT ACCCTTTAAA TTTAAATGTA

TTGCAACAAA TAAAATCACA GTATATAATT GAAATTCTGG TTGAAAATGT

TTGCAACAAA TAAAATCACA GTATATAATT GAAATTCTGG TTGAAAATGT

TTGCAACAAA TAAAATCACA GTATGTAATT GAAATTCTGG TTGAAAATGT

TTGCAACAAA TAAAATCACA GTATGTAATT GAAATTCTGG TTGAAAATGT

TTGCAACAAA TAAAATCACA GTATATAATT GAAATTTTGG TTGAAAATGT

TTGCAACAAA TAAAATCACA GTATATAATT GAAATTTTGG TTGAAAATGT

TTGCAACAAA TAAAATCACA GTATACAATT GAAATTTTGG TTCAAAGTGT

TTGCAACAAA TAAAATCACA GTATATAATT GAAATTTTGG TTGAAAGTGT

TTGCAACAAA TAAAAACACA GTATATAATT GAAATTTTGG TTGAAAATGT

TTGCAACAAA TAAAATCACA GTATATAATT GAAATTTTGG TTGAAAATGT

TTGCAACAAA TAAAATCACA GTATATAATT GAAATTTTGG TTGAAAATGT

TTGCAACAAA TAAAATCACA GTATATAATT GAAATTTTGG TTGAAAATGT

TTGCAACAAA TAAAATCACA GTATATAATT GAAATTTTGG TTAAAAATGT

TTGCAACAAA TAAAATCACA GTATACAATT GAAATTTTGG TTGAAAATGT

TTGCAACAAA TAAAATCACA GTATATAATT GAAATTTTGG TTGAAAATGT

TTGCAACAAA TAAAATCACA GTATATAATT GAAGTTTTGG TTGAAAACGT

TTGCAACAAA TAAAATCACA GTATATAGTT TAAGTTTTGG TTGAAAATAC

TTGCAACAAA TAAAATCACA GtatataatT AAAGTTTTGG TTGAAAATAT

CAGATGCCTA AATATTTTGG ATAAAAGAAA TGTAAAACAA AGATTTGGTT

CAGATGCCTA AATATTTTGG ATAAAAGAAA TGTAAAACAA AGATTTGGTT

CAGATGCCTA AATATTTTGG ATAAAAGAAA CGTAAAACAA AGATTTGGTT

CAGATGCCTA AATATTTTGG ATAAAAGAAA CGTAAAACAA AGATTTGGTT

CAGATGCCTA AATATTTTGG ATAAAAAAAA TGTAAAACAA AGATCTGGAT

CAGATGCCTA AATATTTTGG GTAAAAGAAA TGTAAAACAA AGATTTAAAT

CAGATGCCTA GATATTTTGG ATAAAAGAAA CATAAGACAA CGCT-TGGAT

CAGATGCCTA GATATTTTGG ATAAAAGAAA CCTAAGACAA AGAT-TGGAT

CAGATACCTA GGTATTTGAG ATAAAAGAAA TGTAGAACAA ACATCTGGGT

CAGATGCCTA AATATTTTGG ATAAAAGAAA TGTAAAACAA AGATTTGGAT

CAGATGCCTA AATATTTTGG ATAAAAGAAA TGTAAAACAA AGATTTGGAT

CAGATGCCTA AATATTTTGG ATAAAAGAAA TGTAAAACAA AGATGTGGAT

CAGATGCCTG AATATTTTGG ATGAAAGAAA TGTAAAACAA AGATTTGAAT

CAGATACCTA AATATTTTCG ATAAAAGAAA TGTAAAACAA AGATTTGGAC

CAGATGCCTA AATACTTTAG ATAGAAGAAA TGTAAAACAA AGATCTGGAC

AAGATGCCTA AATATTTTGG ATAAAAGAAA TGTAAAACAA AGATGTGGAT

CAGATGCCTA AATAATTTGG GTAAAACAAA TGTAAAACAG GGACTTGAGT

CAGACGCCTA AATATTCTGG AAAAAACAAA CGCAAAACAA GGGCTTGAGT

CATGTACAAA CAAAGGCATC CTATCAGTCA CTAACAGTAC CTTTCTGCAA

CATGTACAAA CAAAGGCATC CTATCAGTCA CTAACAGTAC CTTTCTGCAA

CATGTACAAA CAAAGGCATC CTATCAGTCA CTAACAGTAC CTTTCTGCAA

CATGTACAAA CAAAGGCATC CTATCAGTCA CTAACAGTAC CTTTCTGCAA

CATGTACAAA CAAAGGCATC CTATCAGTCA CTAACAGTAC CTTTCTGCAA

CATGTACAAA CAAAGGCATC CTATTAGTCA CTAACAGTAC CTTTCTGCAG

CATGTACAAG TAAAGGCCTC TC--CTACCA GCAACAGAGC CTTTCAGAAG

CATGTACAAG TAAAGGCCTC TCTCCCATCA GCAACAGCGC CCTTCAGGGG

CATGTACAAA CAAAGGCGTC CTCCCAGTCA CTAACAGTAC CTTTCCGCTG

CATGTATAAA CAAAGGCATC CTATCAGACA CTAACAGTAC CTTTCTGCAG

CATGTACAAA CAAAGGCATC CTATCAGACA GTAACAGTAC CTTTCTGCAA

CATGTACAAA CAAAGGCATC CAATCGGACA CTAACAGTAC CTCTCTGCAG

CATGTACAAA CAAAGGCATC CTATCAG--- ---------- ----------

CATGTACAAA CAAAGGCATC CTATCAGTCG CTAACAGTAC CTTTCTGCAG

CATGTACAAG CAAAGGCATC CTATCAGTCA CCAACAGTAC CTTTCTGCAG

CATGTACAAA TAAAGGCATC CTATCAGGTG CTAACAGTAC CTTTCAGAAG

CATGTAGAAA TAAAGACATC CTGTCAGTCA CTAACAGTAC TTTTCTTTAG

CATGTACAAA TAAAGACATC CCATCAATCA TTAACAGTAC CTTTCAGTAT

AATCAACAGG CATTTATTAT CTCCCCTCCA AAACCCTCCC CAAATATCAA

AATCAACAGG CATTTATTAT CTCCCCTCCA AAACCCTCCC CAAATATCAA

AATCAACAGG CATTTATTAC CTCCCCTCCA AAACCCTCCC CAAATATCAA

AATCAACAGG CATTTATTAC CTCCCCTCCA AAACCCTCCC CAAATATCAA

AATCAACAGG CATTTATTAC CACCCCTCCA AAACCCTCCC AAAATATCAA

AATCAATAGG CATTTATTAC CTCCCCTCCA AAGCCCTCCC CAAATATCAA

AGTCACCAGG CCTTTGTTAC TTCCCCTCCA AAACCCTCCC CAAATATCAA

AGTCATCAGG CATTTGTTAC TTCCC-TCCA AAACCCTCCC CAAATATCAA

AGTCAACAGG CACTTATCAT CTCCC-CTTA AAACCCTCCC CAAATATCAA

AATCAACAGG CTTTTATTAC CTCCCCTCCA AAACCCTCCC CAAATATCAA

AATCAACAGG CTTTTATTAC TTCCCCTCCA AAACCCTCCC CAAATATCAA

AATCAACAGG CTTTTGTTAC CTCCCCTCCA AAACCCTCCC CAAATAGCAA

---------- ---------- ---------- ---------- ----------

AATCAACAGG CTTTTATTAC CTCCCCTTCA AAACCCTCCC CAAATATCAA

AATTAACAGG CGTGTATTAC CTCCTCTCCA AAACCCTCCC CAAATATCAA

AATTAACAGG CATTTATTAC CTCCCCTCCA AAACCCTCCC CAAATATCAA

GATCAACGGG CAGTTCTTGC TTCCCTTCGA CAAATCCCCc aaaacaCTAA

GATCAACAAG GGTTTAATCC CTTCCCCCAG AGACCCCCCC CAAAAACCAA

ACAATTATAT ACCAAATAAT TTCAAATAAA AGGATTTAAT AAATATATAA

ACAATTATTT ACCAAATAAT TTCAAATAAA AGGATTTAAT AAATATATAA

ACAATTATTT ACCAAATAAT TTCAAATAAA AGGATTTAAT AAATATATAA

ACAATTATTT ACCAAATAAT TTCAAATAAA AGGATTTAAT AAATATATAA

ACAATTATTT ACCAAATAAT TTCAAATAAA AGGATTTAAT AAATATATAA

ACCATTACTT ACCAAATAAT TTCAAATAAA AGGATTTAAT AAATATATAA

ACCATTATTT ACCAAATAAT TTCAAATAAA AAGATCTAAT AAATATATAC

ACCATTATTT ACCAAATAAT TTCAAATAAA AAGATCTAAT AAATATATAA

ACCGTTATTT ACCAAATAAT TTCAAATAAA AGGATTTAAT AAATATATAA

ACCATTATTC ACCaaataat taaaaaaaaa aggatttaat aaatatataa

ACTGTTATTC ACCAAATAAT TTCAAATAAA AGGA--TAAT AAATATATAA

ACTGTTATTC ACCaaataat ttcaaataaa aggatttaat aaatatataa

---------- ---------- ---------- ---------- ----------

ACCATTAATC ACCAAATAAT TTCAAATAAA AGGACTTAAT AAATATATAA

ACCATTATTT ACCAAATAAT TTCAAATAAA AGGACTTAAT AAATATATAA

ACCATTATTC ACCaaataat ttcaaataaa aggatttaat aaatatataa

ACCCTTATTT ACCAAATAAT TTCAAATAAA AGGATTTAAT AAATATATAA

ACCCTTCTTC ACCAAATAAT TTCAAATAAA AGGATTTAAT AAATATATAA

GCATTTCATC CTCCATATAC AAAATTTCTT AAAACCATTC AAAACGGAGG

GCATTTCATC CTCCATATAC AAAATTTCTT AAAACCATTC AAAATGGAGG

GCATTTCATC CTCCATATAC AAAATTTCTT AAAACCATTC AAAACGGAGG

GCATTTCATC CTCCATATAC AAAGTTTCTT AAAACCATTC AAAACGGAGG

GCATTTCATC CTCCATATAT AAAATTTCTT AAAACCATTC AAAACGGAGG

GTATTTTATC CTCCATATAC AAAATTTCCT AAAACCATTC AAAACGGAGG

GCATTTCATC CTCCATATAT AAAATTTCTC AGAGTCATTC AAAACGGAGG

GCATTTCATC CTCCATATAT AAAATTTCTC AGAATCATTC AAAACGGAGG

GCATTTCATC CTCCATATAC AAAGTTTCTT AAAACCACTC AAAACGGAGG

gtattttGTC CTCCATATAC AAAATTTCTT AAAACCATTC AAAACGGAGG

GCATTTCATC CTCCATATAC AAAATTTCTT AAAACCA-TC AAAACGGAGG

GCATTTCATC CTCCATATAC AAAATTTCTT AAAACCATTC AAAACGGAGG

---------- ---------- ---------- ---------- ----------

GCATTTCACC CTCCATATAC AAAATTTCTT AAAATCTTTC AAAACGGAGG

GTATTTCATC CTCCATGTAC AAAATTTCTC AAAATCATTC AAAACGGAGA

GCATTTCATC CTCCATATAC AAAACTTCTT AAAACCGTTC AAAACGGAGG

AGCACTCATC CTCCATATAC AAAACCGTTT AAAATCATTC AAAAGGGAGA

GCACTTCATC CTCCATATAC AAAATTTCTT AAAACCATTC AATAGGGAGA

CTAGATAGAA ATTTTCATAA CTGTGCTTAC CAATACTGTT GAATGCTCAA

CTAGATAGAA ATTTTCATAA CTGTGCTTAC CAATACTGTT GAATGCTCAA

CTAGATAGAA ATTTTCATAA CTGTGCTTAC CAACACTGTT GAATGCTCAA

CTAGATAGAA ATTTTCATAA CTGTGCTTAC CAACACTGTT GAATGCTCAA

TTAGATAGAA ATTTTCATAA CTGTGCTTAC CAATACTGTT GAATGCTCAA

CTAGATAGAA ATTTTCATAA CTGTGCTTAC CAATACTGTT GAATGCAAAA

CTAGGTAGAA ATTTCCCTAA CTGTGCTTAC CAATACTGTC AAATGCTCAA

TTAGGTAGAA ATTTCCCTAA CTGTGCTTAC CAATACTGTT GAATGCTCAA

CTAGACAGAA ATTTTCATAA CTGTGCTTAC CAATACTGTT GAATGCTCAA

CTGGATAGAA ATTTTCATAA CTGTGCTTAC CAATACTGTT GAATGCTCAA

CTAGATAGAA ATTTCCATAA CCACGCTAAC CAATACTGTT GAACGCTCAA

CTAGATAGAA ATTTTCATGA CCGTGCTTAC CAATACTGTT GAATACTCAA

---------- ---------- ---------- ---------- ----------

CTAGATAGAA ATTTTTGTAA CTGTGCTTAC CAATACTGAT GAATGCTCAA

CTAGATAGAA ATTTTCCTAA CTGTGCTTAC CAATACTGTT GAATGCTCAA

CTAGATAGAA ATTTTCGTAA CCACGGTTCC CAAAACTGTT GAATGTGCAA

CCAGATACAA ATTATAGGAG CTGTGCTTGC AAATACTGAT GAATGCTCAA

CCAGATACAA ATTTGAGTAA CTGTGCTTGC AAATTCTGTG GAACTCTTGA

ATGATTTGCC AGGTGTGTGC AAAGTTGTAA AATGGGGACA CTTCTGGAAC

ATGATTTGCC AGGTGTGTGC AAAGTTGTAA AATGGGGACA CTTCTGGAAC

ATGATTTGCC AGGTGTGTGC AAAGTTGTAA AATGGGGACA CTTCTGGAAC

ATGATTTGCC AGGTGTGTGC AAAGTTGTAA AATGGGGACA CTTCTGGAAC

ATGATTTGCC AGGTGTGTGC AAAGTTGTAA AATGGGGACA CTTCTGGAAC

ATGATCTGCC AGGTGTGTGC AAAGTTGTAA AATGGGAACA CTTCTTAAAC

GTGATGTGCC AGGTGTGAGC AATG------ ----GGGACA CCTCTGAATC

GTGATGTGCC AGGTGTGAGC AAAG------ ----GGGACA CCTCTGGATC

GTGATTTGCC AGGTGCGTGC AAAGTTGTAA AATGGGGACA CTTCTTAAAC

ATGATCTGCC AGGTGTGTGC AAAGTTGTAA AATGGGGACA CTTCTTGAAC

ATGATTTGCC AGGTGTGTGC AAAGTTATAA AATGGGGACA CTTCTTGAAC

ATGATTTGCC AGGTGTGTGC GAAGTTGTAA AATGGGGACA CTTCTTGAAC

---------- ---------- ---------- ---------- ----------

ATGATTTGCT AAGTGTGTGC AAAGTTATAA GATGGGGATA CTTCTTGAAC

ATGATTTGCC AGGTGTGTGC AAAGTTGTAA GATGGGGACA CTTCTTGAAC

ATGATTATCC AGGTGTGTGC AACATTGTCT GATGGGGACA CTTCTTGTAC

CTTATTTGCT AGGGGTATGC AAAGTCACAA AACAGGGACA CTTTGAAAGT

ATGATTTGCC AGGGGTGTGC AAAATCACGA AACAAGGGCG CTTCCTGGAA

ACCAACAAAC TCAACAATAT GGATGATATG GCATTATTTG AAAGGCAGCA

ACCAACAAAC TCAACAATAT GGATGATATG GCATTATTTG AAAGGCAGCA

AGCAACAAAC TCAACAATAT GGAAAATATG GCATTATTTG AAAGGCAGCA

AGCAACAAAC TCAACAATAT GGAAAATATG GCATTATTTG AAAGGCAGCA

AGCAACAAAC TCAACAATAT GGATAATATG GCATTATTTG AAAGGCAGCA

AGCAACAAAC TCAACAGTAT GGATAACATG GCGTTATTTG AAAGGCAGCA

AGCGACAAGC TCAACCATAC GGACG-CGTG GCACTGTTTG AAAGGCAGCA

AGCAACAAGC TCAACTATAT GGACA-CATG GCACTGTGTG AAAGGCAGCA

AGCAACAAAC TCAACAGTAT AGGTAACACG GCACTATTTG AAAGGCAGTA

AGCAACAAAC TCAACAGTAT GGATAATATG GCATTATCTG AAAGCCAGCA

AGCAACAAAC TCAACAGTAT GGATAACATG GCATAATATG AAAGGCAGCA

AGCAACAAAC TCAACAGTAT GGATAATATG GCATTACTTG AAAGGCAGCG

---------- ---------- ---------- ---------- ----------

AACAACAAAC TCGACAGCAT GGATAATATG GCATTATTTG AAAGGCAGCA

AGCAACGAAC TCAACAGTAT GGATAATATG GCATTCTTTG AAAGGCAGCA

AGCAACAAAT GCAACGCTAT GGATCATACG GCATTATTTG AAAGGCAGTG

ATCACCAGGT GGAATAATAT GGGCCCTATG GCATAACTTG AAAGGCAGCA

GGTACCCAGT TCAATAGGAT GGATAGTATG CCATCATCTG AAAGATGACA

TTTCCATTTC AGAGAGCAGG TAGAAAATTC CAATGGTTAA AAGCTGAAAA

TTTCCATTTC AGAGAGCAGG TAGAAAATTC CAATGGTTAA AAGCTGAAAA

TTTCCATTTC AGAGACCAGG TAGAAAATTC CAATGGTTAA AAGCTGAAAA

TTTCCATTTC AGAGACCAGG TAGAAAATTC CAATGGTTAA AAGCTGAAAA

TTTCCATTTC AAAGAGCAGG TAGAGAATGC TAATGGTTAA AAGCTGAAAA

TTTCCATTTC AGAGAGCAGG TAGAAAATTC CAATGGTTAA AAGCTGAAAA

CTTCCACTTC AGAGAACAGG CAGAAAACCC CAGTGGCTAA AAGCTGAGAA

CTTCCACTTC AGAGAACAGG CAGAAAACCC CAGTGGCTAA AAGCTGAGAA

TTTTCATCTC AGAG------ CAGAAAACCC CAATGATTAA AAGCTGAAAA

TTTCCATTTC AGACAGCAGG TAGAAAATTC CAGTGGTAAA AAGCTGTAAA

TTTCCATTTC AGAGAGCAGG TAGAAAGTTC CAGTGGTTAA AAGCTGAAAA

TTTCCATTTC AGAGACCAGG TAGAAAATTC CAATGGTTAA AAGCTGAAAA

---------- ---------- ---------- ---------- ----------

TTTATATTTC AGAGAGCAGG TAGAAAATTC CAATGGTTAA AAGCTGAAAA

TTTCCATTTC AGAAAGCAGG TAAGAAATTC CAATGGTTAA AAGCTGAAAA

TTTCCGTTTC CGAGAGTGGG TAGAAAATTC CAATGGTTAA AAGTAGAAAA

TTTCACTTTC TGATAACAGG TAGAGAATTT CCAAGGTGAA TAAATAGAAT

TTTCCGTCCC AGGAATCAGA TCGAGACTCG GAAGGGTGAA AAGCTTTAAA

GAAGAATCTT TCCCACCCAA GAGCCCTAAC AGAAGGGCTC AAATACATTT

GAAGAATCTT TCCCACCCAA GAGCCCTAAC AGAAGGGCTC AAATACATTT

GAAAAATCTT TCCCACCCAA GAGCCCTAAC AGAAGGGCTC AAATACATTT

GAAAAATCTT TCCCACCCAA GAGCCCTAAC AGAAGGGCTC AAATACATTT

GAAAAATCTT TCCCACCCAA GAGCCCTAAT AGAAGTGCTC AAATACGTTT

GAAAAATCCT TCCCACCCAA GAGCCCTGAC AGAAGGACTC AAATATATTT

GAAAAATCTT TCCCACCCAA GAGCCCTAAC AGAAGGGCTC AAATAGATTT

GAAAAATCTT TCCCACCCAA GAGCCCTAAC AGAAGGGCTC AAATATATTT

GAAA------ ---------- ---------- ---------- ----------

GAAAAATCCT TCCCACCCAT GAGCCCTAAC GGAAGGGCTC AAATATATTT

GAAAAATCCT ACCCACCCAA GAGCCCTAAC AGAAGGGCTC AAATATATTT

GAAAAATCCT TCCCACCCAA GAGTCCTGAC AGAAGGGCTC AAATATATTT

----AATTCT TCCCACCCAA GAGCCCTAAC AGAAGGGCTC AAATATATTT

GCAAACTCCT TCCCACCCGA GAGCCCCAAC AGAGGGGCTC AAATATATTT

GAAACATCCT TTCCACCCAA GAGCCCTAAC AGAAGGACTC AAATATATTT

GAAA------ ---------- ---------- ---------- ----------

TAAAGATCCT CTCCACCCCA AAGCCCTGAA TAAAGGGCAC AAATACATTT

TGGAAATTCT TGTGACCAAA AAGCACTAAA TTAAGTATTT TAATATATTT

TAAATACTAC AAATAAGCTT TCAATCTAAA GGAATACACA TTGCACCCTT

TAAATACTAC AAATAAGCTT TCAATCTAAA GGAATACACA TTGCACCCTT

TAAATACTAC AAATAAGCTT TCAATCTAAA GGAATACACA TTGCACCCTT

TAAATACTAC AAATAAGCTT TCAATCTAAA GGAATACACA TTGCACCCTT

TAAATACTAC AAATAAGCTT TCAATCTAAA GGAATACACA TTGCACCCTC

TAAATACTAC AAATAAGCTT TCACTCTAAG GGAATACACA TTGCACCCTT

CAAATACTAC AAATAAGCTT TCAATCTAAA GGAATACACA TTGCATCCTT

CAAATACTAC AAATAAGCTT TCAATCTAAA GGAATACACA TTGCATCCTT

---------- ---------- ---------- ---------- ----------

TAAATACTAC AAATAAGCTT TCAATCTAAA GGAATACACA TTGCACCCTT

TAAATACTAC AAATAAGCTT TCAATCTAAA GGAATACACA TTGCACCCTT

TAAATACTAC AAATAAGCTT TCGATCTAAA GGAATACACA TTGCACCCTT

TAAATTCTAC AAATAAGCTT TCAATCTAAA GGAATACACA TTGCACCCTT

TAAATACTAC AAATAAGATT TCAATCTAAA GGAATACACA TTGCACCCTT

TAAATACTAC AAATAAGCTT TCAATCTAAA GGAATACACA TTACACCCTT

---------- ---------- ---------- ---------- ----------

TAGATACTGC AAATAAACTC TTAATCTAAA GGAATACACA TTGCACTCTT

TGGATATGAC AAGCAAGTCC CCAGTTCAAT GGAATACACA ATGCACTGTT

GAATAATATT CAAGGTGTCA ACAACTTAGA TCAATTTAAT AAATAATGTG

GAATAATATT CAAGGTGTCA ACAACTTAGA TCAATTTAAT AAATAATGTG

GAATAATATT CAAGGTGTCA ACAACTTAGA TCAATTTAAT AAATAATGTG

GAATAATATT CAAGGTGTCA ACAACTTAGA TCAATTTAAT AAATAATGTG

GAATAATATT CAAGGTGTCA ACAACTTAGA TCAATTTAAT AAATAATGTG

GAATAATGTT CAAGGTGTCA ACAACTTAGA TCAATTTAAT AAATAATGTG

GAATAATATT CAAGGTGCCA AGAACTTAGA TCAATTTAAT AAATAATGTG

GAATAATATT CAAGGTGCCA AGAACTTAGA TCAATTTAAT AAATAATGTG

---------- ---------- ---------- ---------- ----------

GAATAATATT CAAGGTGTCA ACAACTTAGA TC-----AAT AAATAATGTG

GAATAATATT CAAGGTGTCA ACAACTTAGA TCAATTTAAT AAATAATGTG

GAATAATATT CAAGGTGTCA ACAACTTAGA TCAATTTAAT AAATAATGTG

GAATAATATT CAAGGTGTCA ACAACTTAGA TCAATTTAAT AAATAATGTG

GAATAATATT CAAGGTGTCA ACAGCTTAGA TCAATGTAAT AAATAATGTG

GAATAATATT CAAGGTGTCA ACAACTTAGA TCAATTTAAT AAATAATGTG

---------- ---------- ---------- ---------- ----------

ATATACTATG CAAAATGTCA CTGACTTAGA TCAATTTAAT AAATAATGTG

AAAAAAAATG TGTAGTCCCA GCGACTGAGA TCTGCTGAAT AGTCAACATA

GAAATAATTT TAAGATCAAG TGCATACCCT TACACCTAGA AGAGTGCAAC

GAAATAATTT TAAGATCAAG TGCATACCCT TACACCTAGA AGAGTGCAAC

GAAATAATTT TAAGATCAAG TGCATACCCT TACACCTAGA AGAGTGCAAC

GAAATAATTT TAAGATCAAG TGCATACCCT TACACCTAGA AGAGTGCAAC

GAAATAATTT TAAGATCAAG TGCATACCCT TACACCTAGA AGAGTGCAAA

GAAATAATTT TAAGATCAAG TGCATACCCT CACACCTAGA AGAGTGCAAA

GAAATAATTT TAAGATCAAG TGCATACCCT CATACCTAGA --AGTGTAAA

GAAATAATTT TAAGATCAAG TGCATACCCT CATACCTAGA AGAGTGTAAA

---------- ---------- ---------- ---------- ----------

GAAATAATTT TAAAATCAAG TGCATACCCT CACACTTAGA AGAGTGCAAA

GAAATAATTT TAAAATCAAG CGCATACCCT CACACCTAGA AGAGCGCAAA

GAAATAATTT TAAAATCAAG TGCGTACCCT CACACCTGGA AGAGTGCAAA

GAAATAATTT TAAAATCAAG TGCATA-CCT CACACCTAAA AGAGTGCAAA

GAAATAATTT TAAAATCAAG TGCATACCCT CACACCTAGA AGAGTACAAA

GAAATAATTT TAAAATCAAG TGCATACCCT CACATCTAGA AGAGTACAAA

----TAATTT TAAAATCAAG TGCATACCCT CACACCTAGA AGAGTACAGA

GAAATAATTC TAAAGTCAAA CGCTCACTAT CACACCAAGA AGAGTGCAAA

AAAGTAACTC TATGCTTAAA TGTGTTCTAT CACAACAAAA AGAGTGAAAA

GTTTCTGTCA TTAAATACAA AATTAGCTGT CTGATCTGTC TGATTAAACT

GTTTCTGTCA TTAAATACAA AATTAGCTGT CTGATCTGTC TGATTAAACT

GTTTCTGTCA TTAAATACAA AATGAGCTGT CTGA--TGTC TGATTAAACT

GTTTCTGTCA TTAAATACAA AATGAGCTGT CTGATCTGTC TGATTAAACT

GTTTCTGTCA TTAAATACAA AATGAGCTGT CTTATCTGGC TGATTAAATT

GTTTCAGTCG TTAAATACAA AATGAACTGT CTGACCACGC TTATTAAACG

GTTTCAGCTG TTAAATACAA A-TGAGCTCT CTAATCTAGC TGATTAAACT

GTTTCAGCTG TTAAATACAA A-TGAGCTTT CTAATCTAGC TGATTAAACT

---------- ---------- ---------- ---------- ----------

GTCATTGTCA TTAAATACAA AATGAGCTGT CTGATCTAGC TGATTAGACT

GTTTCTGTCA TTAAATACAA AATGAGCTGT CTGATTTAGC TGATTAAATT

GTGTCTGCCA CTGAACACAA AATGAGCTGT CTGATCTAGC TGATTAAACT

GTTTCTGTCA TTAAATACAA AATGAGCTGA CTGATCTAGC TGATTAAACT

GTTTCTGTCT TTAAATACAA AACGAGCTGT CTGATCTAGC TGATTAAACT

GTTTCTGTCA TTAAATACAA AATGAGTTGT CTGATCTAGC TGATTAAACT

GTGGCTGTCA TTAAATACAA AATGAGCTTT CTGATCTAGC TGATTACACT

CTTGCAATCA GCCAATATAA AATTAGCTGT CTGAGCCAGC TGGTCTGATT

CTTTGGGCCA GTAAACCCCA AACCAACTGC TTGAACTAGC TGATTACACT

AATGAAAGAA AACAGAGTAA GTTTCTATAA TTAACCATAT ACTAAGTTTT

AATGAAAGAA AACAGAGTAA GTTTCTATAA TTAACCATAT ACTAAGTTTT

AATGAAAGAA AACAGAGTAA GTTTCTATAA TTAACCATAT ATTAAGTTTT

AATGAAAGAA AACAGAGTAA GTTTCTATAA TTAACCATAT ATTAAGTTTT

AATGAAAGAA AACAGAGTAA ATTTCTATAA TTAACCACAT GTTAAGTTTT

AATGAAAGAA AACAGAGTAA GTTTCTATAA TTAACTATAT ATTAAGTTTT

AATGAAAGAA AACTGAGTA- ATTTCTATCC CTAACTACAC AGTAGGTTTG

AATGAAAGAA AACTGAGCA- ATTTCTATTA CTAACTATAG AGTAAGTTTG

---------- ---------- ---------- ---------- ----------

AATGAAAGAA AACAGAACAA ATTTTTATAA TTAACTATA- -TTAAGTTTT

AATAAAAGAA AACAGAGTGA ATTTCTATAA TTAACTATAT ATGAAGTTTT

AATGAAAGAA GACAGAGTAA ATTTCTATAA TTAACTACGT ATTAAGTTTT

AATGAAACAA AACAGAATAA ATTTCTAGAA TTAACTATAT ATTGAGTTTT

AATGAAAGAT AACAGAGTAA ATTTCTATAA TTAACTACAT ATTAAGTTTG

AATGAAACAA AACAGAGTAA ATTTCTATAA TTAACTGTAT ATTAAGTTGT

AATGAAAGAA AAGAGAGTAA ATTTCTATTA TTAACTATAT AGTAAGTTTT

AATTAAAGAA GACTGCGTGA ATTTCTGAGA TGATCTATAT ATTCAGCTTT

AAGAAAAAAA AATGGATTAA ATCTTTACAA AGACCTAGGT ACTAGATTCT

TAGCCACTTA AATGCTTAGA AAAGAGGACA CCTAGGGAGG GAAGGAAGGA

TAGCCACTTA AATGCTTAGA AAAGAGGACA CCTAGGGAGG GAAGGAAGGA

TAGCCACTTA AATGGTTAGA AAAGAGGACA CCTAGGGAGG GAAGGAAGGA

TAGCCACTTA AATGGTTAGA AAAGAGGACA CCTAGGGAGG GAAGGAAGGA

TAGCCACTTA AATGGTTAGA AAAGAGGACA CCTAGGGAGG AATGGAAGGA

TAGCCACTTA AATGGTTAGA AAAGAGGACA CTTAGGGAGG AATGGAA--A

TAGCCACTTC AATGGCTAAA ACAAAGGGTG CCTAGGGGGG AGTGAGAAGA

TAGCCACTTA AATGGCTAAA AAAGAGGGCA CCTAGGGAGG GATGGGAGGA

---------- ---------- ---------- ---------- -----AAAGA

TAGCTACTTA AGTATTTAGA GAAGAAGACA CCTAAGGAGG AATGGGAGGG

TAGCCACTTA AATGGTTCGA GAAGAAGACA CCTAAGGAGG AATGGGAGGA

TAGCCACTTA AATGGTTAGA GAAGAAGTTA CCTAAGGAGG AATGGGAGGA

TAGCCACTTA AAAGATTAGA GAAGAAGACA CCTAAGAAGG AATAG-----

TAGCCACTTC AATGGTTAGA AAAGAAGACA CCTAGAGAGG AATGGGAGGA

TAGCTACTTA AATGATTAGA AAAGAAGACA CCTAGGGAGG AATGG-----

TAGCCTCTTA AATGGTTAGC AAAGAAGACA ACTAGGGAGG AACGGGAGGA

TTGTCATTTA AAAGTTCAAA AAACAAGAAA TTTAAGGTGG AATGCGAGGA

TTGCCATTTA AGCAGTTTGA AAAGGAGAGA ATCAGAGACG AATGAGAGGA

AGCAGCATTA CCCAACTGGT TGACTAAGTC ATACCAAAGC AATAGCTTTT

AGCAGCATTA CCCAGCTGGT TGACTAAGTC ATACCAAAGC AATAGCTTTT

AGCAGCATTA CCCAATTGGT TGACTAAGTC ATACCAAAGC AATAGCTTTT

AGCAGCATTA CCCAATTGGT TGACTAAGTC ATACCAAAGC AATAGCTTTT

AGCAGCATTA CCCAATTGGT CGACTAAGTC ATACCAAAGC AATAGCTTTT

AGCAGCATCA CCCAATTGGT TGACTAAGTC ATACTAAAGC ATTTGCTTTT

AGCAGCATTA CCCAATTGAT TCACTAAGTC ATATCAAAGT ACTAGCTTTT

AGCAGCATTA CCCAATTGAT TCACTAAATC ATACCAAAGT ACTAGCTTTT

AGCAACATTA CCCAACTGGT TGACTACGTC ATACCAAAGC ATTGGTTTTT

AGCAGCATTA CCCAATAGGT TGACTAAGTC ATACCAAAGC ATTAGCTTTT

AGCAGCATTA CCCAA----T CGACTAAGTC ATACCAAAGC ATTAACTTTT

AGCAGCATTA CCCAATAGGT TGACTAAGTC ATACCAAAGC ATTAGCTTTT

---------- ---------- ---------- ---------- ----------

AGCAGCATTA CCCAACAAGT TGACTAAGTC ATATCAAAGT ATTAGCTTTT

---------- ---------- -GACTAAGTC ATCCCAAAGT GTTAGCTTTT

AGCACTATCA CCCAATGTGT TGACTAAGTC ATTCCCAAGT GTTAGCTTTT

AGCAGCTTTA CTCACCAGAT TGAATAAGTC ATACTAAAGG GTTACCATTT

GGTGGAATTC TCCTGCCGCT TGAAGAAATC GCACCATGGA AATAGGATTT

CTGAAATAAA GAAAGATTTA AATGAAAGAA TTGAACATTA TTATTGTAGA

CTGAAATAAA GAAAGATTTA AACGAAAGAA TTAAACATTA TTATTGTAGA

CTGAAATAAA GAAAGATTTA AATGAAAGAA TTTAACATTA TTATTGTAGA

CTGAAATAAA GAAAGATTTA AATGAAAGAA TTTAACATTA TTATTGTAGA

CTGAAATAAA GAAAGATTTA AATGAAAGAA TTTAACATTA TTGTTGAAGA

CTGAAATAAA GGAAGATTTA AAGGAAAGAA TTTAACATTA TTACTGTAGA

CTGAAGTAAA GGAAGATTTA AATCAAAGAA TTTGAAATTA TTATTGTAGA

CTGAAGTAAA AGAAGATTTA AATCAAAGGA TTTGAAATTA TTATTGTAGA

TTGAAATAAG GGAAAATTTG AAGGAAAGAA TTTAACATTA CTAC---AGA

CTGAAATAAA GGACAACTGA AAGGAAAGAA TTTAACATTA TTACTACAGA

CTGAAATAAA CGAAGATTAA A---AAAGAA TTTAACATTA CTACTGCCAA

CTGAAATAAA GGAAGATTTA A---AAAGGA TTTAACAT-- -CGCTGCCGA

---------- ---------- ---------- ---------- ----------

CTGAATTAAA GGAAGATTTA AAGGAAAAAA TTTAACGTGA TTACTGGAGA

CTGAAATAAA GGAAGATTTA AAGAAAAGAA TTTAACATGA TTACTGCAGA

CTGAAATAAA AGAAGACAGA AAGAAAAAAA C----CATGA TTGCTGCCCA

ATAAAATAAA GAAACATTTA AAGGAAGAAA TTTTACCTAA CTACTGCACA

CCACGACAGA GAGACGCTCC CAGGAAGGAA TTTTACTAAA GTACTGCAGA

AGACCAAATC TGGTCCTAGT GTAAAACTTC ATTTGTAAAC AATCCATAGT

AGACCAAACC TGGTCCTAGT GTAAAACTTC ATTTGTAAAC AATCCATAGT

AGACCAAATC TGGTCCTAGT GTAAAACTTC AATTGTAAAC AATCCATAGT

AGACCAAATC TGGTCCTAGT GTAAAACTTC AATTGTAAAC AATCCATAGT

AGACCAAATC TGGTCCTAGT GTAAAACTTC ATTTGTAAAC AATCCATAGT

AGACCAAATC TGGTCCTAGT GTAAAACTTT ATCTGTAAAC AATCCACAGT

GGACCAAATC TGGTCCCAGC ATAAAACTTT ATCTGTAAAC AATCCATAGT

GGACCAAATC TGGTCCCAGC ATAAAACTTT ATCTGTAAAC AATCCATAGT

AGACCAAATC TGCTTCTAGT GTAAAACTTC ATCTGTAAAC AATCTGTAGT

AGACCAAATC TGCTCCTAGT GTAAAACTTC ATCTGTAAAC AATCCATAGT

AGACCAAATC TGGTCCTAGT GTAAAACTTC ATCTGTAAAC AATCCATAGT

AGACCAAATC TGGTCCTAGT GTAAAACTTC ATCTGTAAAC AGTCCATAGT

---------- ---------- ---------- ---------- ----------

AGACCAAACC TGGTCCTAGT GAAAAATGTC ATCTGTAAAC AATCCACAGT

AGACCAAACG TGCTCCTAGT GTAAAGCTTC TTCTGTAAAC AGTCCACAGT

AGACCAAAGC TGTTCCTCGT GTAAAACTTC ACCTGTAAAC AATC--CTGT

AAACCAGACC TGGTCCTCCT GTGATGCTTC GTCTGTAAAC AGTCCATAGT

AGACCGAACC TGGTCCTATT GTAAAACTTC ATCTGTAAAC AGTTCATAAT

CTTCATTTAA AGGTAAGATT AAGGCACACC AAACATGATA GGGGCATGAA

CTTCATTTAA AGGTAAGATT AAGGCACACC AAACATGATA GGGGCATGAA

CTTCATTTAA AGGTAAGATT AAGGCACACC AAACATGATA GGGGCATGAA

CTTCATTTAA AGGTAAGATT AAGGCACACC AAACATGATA GGGGCATGAA

CTTCATTTAA AGGTAAGATT AAGGCACACC AAACATGATA GGGGCATGAA

CTTCATTTAA AGGTAAGATT AAGGCACACC ATATATGATA GGGGCATTAA

TATCATTTAA AGTTAAGATT AAGGCACACC AGACATGACG GGGTCACTAA

TATCATTTAA AGTTAAGATT AAGGCACACC AGACATGACG GGGTCACTAA

TTTCATTTAA AGGTAAGATT AAGGCACACT AGAGAAGATA TGGGCACTAA

CTTCATTTAA AGGTAAGATT AAGGCACACC AGACATGATA GGGGCAC-TA

TTTCATTCAA AGGTAAGATT AAGGCACACC AGACATGATA GGGGCAC-AT

CTTCATTTAA GGGTAAGATT AAGGCACACC AGACATGATA GGGGCACTAA

---------- ---------- ---------- ---------- ----------

CTTCATCTAA AGGTAAGATT AAGGCACACC AGACATGATA GGGGCACTAA

CTTCATCTAA AGGTAAGATT AAGGCACACC AGACATGATA GTGGCACTAA

CTTCATCTAA AGGTAGGATT AAGGCACACC AGACATGATA GGGGCACTGA

CTTCATCTAA ATGTAAGATT AAGGCACACC AGACATGATA GGGGCACTAA

CTTCATCTAA GTGTAAGGAT AAGGCACACC AGACATGATA GGGGCACTAA

TTAGGTACTT TTCTAGTGTG ATTTTCTGAT TCCATCAGAA ACAATCATTG

TTAGGTACTT TTCTAGTGTG ATTTTCTGAT TCCATCAGAA ACAATCATTG

TTAGGTACTT TTCTAGTGTG ATTTTCTGAT TCCATCAGAA ACAATCATTG

TTAGGTACTT TTCTAGTGTG ATTTTCTGAT TCCATCAGAA ACAATCATTG

TTAGGTACTT TTCTAGTGTG ATTTTCTGAT TCCATCAGAA ACAATCATTG

TTAGGTACTT TCCTAGTGTG ATTTTCTGAT CCCATCAGAA ACAATCATTG

TTACATATTT TTCTAGTAT- -------AAT CCTGTCAAAA GTGATCGTTG

TTTGGTACTT TCCTAGTAT- -------GAT CCTGTCAAAA GCAATCATTG

TTAGGTACAT TTCTAGTGTG AATTTCTGAT CCCATCAGAA ACAATCATTG

TTAGGTACTT TTCTAGTGCG -TTTTCTGAT CCCATCAGAA ACAATCATTG

-CAGGTACTT TCCTAGTGTG ATCTTCTGAT ATCATCAGAA ACAATCATTG

TTAGGTACTT TTCTAGTGTG ATTTTCTGAT CCCATCAGAA AGAATCACTG

TTAAATGCTT TCGGTATCTG ATTTTCTGAT CCCATCAGAA ACAATTATTG

TTAGGTACTT TTCTAGTGTG ATATTCTGCT CCCATCAGAA ACAATCATTC

TTAGATACTT TCCTAGTGTG ATTTTCTGCT CCCATCAGAA ACAATCATTC

GTAGAGGCTT TCCTAGTGTG --TTCCTGAT CTCATCAAGA GCTCCAAACC

TAAGATACTT TTCTAAAGTG ATATTCTGCT CCCAACAGAA AGAATCATTA

TTAGATACTT CTCTAAAGTG ATATTCTACC CTCGTTGGAC ACACTCATTA

AAGAGGGCCT TCATAATGCA AAAGGGTAAT TCATCATCTC TACATCAGGC

AAGAGGGCCT TCATAATACA AAAGGGTAAT TCATCATCTC TACATCAGGC

AAGAGGGCCT TCATAATACA AAAGCGTAAT TCATCATCTC TACATCAGGC

AAGAGGGCCT TCATAATACA AAAGCGTAAT TCATCATCTC TACATCAGGC

AAGAGGGCCT TCATAATACA AAAGGGTAAT TCACCATCTC TACATCAGGC

ATAAGGGTCT TCATAATACA AAGGGATAAT TCATCATCTC TACATCAGGC

ATGAGGATCT TTGGAA---- ---------T TCATTATCTC CACATTAAAC

ATGAGGATCT TTGGAA---- ---------T TAATTTTCTC CACATTAGAC

ATGAGGGCCT TCATAACACA AAGGGGCAAT TCATCATCTC CACATTAGGT

ATGAAGGCTT TCAAAATACA AAGGTGTAAT TCATCATCTC TATATCAGGC

GTGAGGGCCT TCATAGTACA AAAGGGTCA- -CTTCATCTC TACCTCAGGC

ATGAAGGCCT TCATAATACA GAGGGGTAAT TCATCATCTC TACATCAGGC

ATGAGGGCCT TCATTATACG AAG--TTGAT TAATTATCT- ---ACCAGGC

ATGGAGGCCT TCATAATACA AAGGGGTAAT TCATCATCTC TTAATCAGGC

CTGGGGGCTC TCATAATACA AAGGGGTAAT TCACCATCTC TACATCAGGC

TCAGGGACAC TCTTAA---- ---------- ---------- ----------

CTGGGGGCCT TCATAATACA ATGGGGTAAT TCTATGTCTC CAGATCAGGT

ACGAGGGCCT TCATAATACA ATGGGGTAAT TCTACATCTC TACAGTACAT

ATTAGAACTG TAAGCTGTAT AGCAAAAGCA CACACCGAAG CTCCAGCCTA

ATTAGAACTG TAAGCTGTAT AGCAAAAGCA CACACCGAAG CTCCAGCCTA

ATTAGAACTG TAAGTTGTAT AGCAAAAGCA CACACCAAAG CTCCAGCCTG

ATTAGAACTG TAAGTTGTAT AGCAAAAGCA CACACCAAAG CTCCAGCCTG

ATTAGAACTG TAAGTTGTAT AGCAAAAGCA CACACCAAAG CTCCAGCCTA

ATTAGAACTG TAAGTTGTAT ACCAAAAGCA CACACCAAAG CTCCAGCCTA

ACTGGGAC-- ---------- ---------- ---------- ----ACCTCC

ATTGGGAC-- ---------- ---------- ---------- ----ACCTCC

ATTAGAACTG TAAGCTGTAT AGCAAAGCCA CACACCAAAG CTCCAGCCCA

ATTAGAACCC TAAGTTGTAT AGCAAAAGCA CACACCAAAG CTCCAGGCCA

ATTACAACCG TAAGTTGTAG AGCAAAAGCA CACAGCAAAG CTCCAGCCCA

ATTAGAACTC TAAGTTATAA AGCAAAAGCA CACACCAAAG CTCCAGCCCG

ATTAAAACTG TAAATTGTAC AGCAAAAGCA CACACCAAAG CTCTAGCCCA

ATTAGAACTA TAAATTGTAT AGCAAAAGCA CACACCAAAG TTCTAGCCCG

ATCAGAACTG TAAGCTGTAG AGCAAAAGCA CACACAAAAG CTCCAGCCTA

---------- ---------- ---------- ---------- ----------

TTTACAACTT TAAGTTGTAT GGTAAAATGA CACACCCAAA CTCCAACCCA

TTCACAACTT CAAACTGTAT AGTGAAATTC TGCATTCAAA CTCCAGCCCT

TTTCTGTATC AGTCATTGCA AACAAAAAAT AACATATGTT TTAATAAAAT

TTTCTGTATC AGTCACTGCA AACAAAAAAT AACATATGTT TTAATAAAAT

TTTCTGTATC AGTCACTGCA AACAAAAAAT AACATATGTT TTAATAAAAT

TTTCTGTATC AGTCACTGCA AACAAAAAAT AACATATGTT TCAATAAAAT

TTTCTGTATC AGTCACTGCA AATAAAAAAG AACATATGTT TTAATAAAAT

TTTGTACATC AGTCACTGTA AACAaaaaac aaaaCATGTT TTAATAAAAT

TTTCAGCTCC AGTTACTGCA AACAAAAAGG AATAGAAG-T TTAATGAGCT

TTTCAGCTCC AGTTCCTGAA AACAAAAAAG AATATAAGTT TTAACAAACT

TCTCCACATC AGTCGCTGCA AACAAAAAAG AATATACGTT TTAACAAAAT

TT-TTACATC AGTCACTGCA AGTACAACCA AACATATGTT -TAATAAAAT

TTCCTGCATT TGTCACTGTA AACA----AG AACACATGTT CTAATAAAAT

TTCCTACATC CGTCACTGCA AACA----AG AACACATGTT TTAATAAAAT

TTCCTACA-- --ACACTGCA AACA----AG AACATA-GTT TTAAT-AAAT

GTCCTACATC AGTCACTGCA AACAAAAAAG AACACATGAT TTAATAAAAT

TTCCTACATC AGTCACTGCA AACAAAAAAG AACATATCTT TTAATGAAAT

-----ACGTC AGCCACTGCA AATAGAAGCG --------CT TTAATGAAAT

TTCCCACAGC AGTCATTGCA AAAAACAAAG AGGATAAGTT TCAACAAAAC

ATCCCACAGT GAACATTGCA AACAAAAAAG AACACATGCT TTAACAAATA

CCGTAACGTT CAGGATTGTC CTTTTATGAA ATCTAGAAAG TTTGTCCTCC

CCGTAACGTT CAGGATTGTC CTTTTATGAA ATCTAGAAAG TTTGTCCTCC

CCGTAACGTT CAGGATTGTC CTTTTATGAA ATCTAGAAAG TTTGCCCTCC

CCGTAACGTT CAGGATTGTC CTTTTATGAA ATCTAGAAAG TTTGCCCTCC

CCATAAAGTT CAGGATTGTC C-TTTATGAA ATCTAGAAAG TTTGCCCACC

CCACAAAGTT CAAGATTGTC CTTTTATGAA ATCTACAAAG TTTGCCTGCC

C---ATAGTT TAAGATTGTC CTTT-A-GGA GTCCGCA--- -----ATCTG

C---ATAGTT TAAGATTGTC CTTT-ATGAA GTCCGCAAAT CT---ATCTG

CCATACAGTT CAAGATTGTC CTTTTATGAA ATCTGGAAAG TT-GCCTTTT

CCATAAGGTT CAAGATTGTC CTTTTATGAA ATCTAGAAAG TTTGCCTGCC

CCATGAAGTT CAAGATTGTC CATCTACAAA ATCTAGAAAG TTTGCCTGCC

CCATAAAGTT CAAGATTGTC CTTTTATGAT ATCTAGAAAG TCTGCCTGCC

CCATAAAGTT CAAGATTGTC CTTATATGAA ATCTAGAAAG TTTGCCTGCC

TGAGTAAGTC CAAGATTGTC CTTTTATGAA ATCTAGAAAG TTTGCTTGCC

CC-TAAAGTT CAAGATTGTC CCTTTATGAA ATCTAGAAAG CTTGCCTGCC

CCATAAAGTT CAAGATTGTC CCTTTATGAA A----GAAAG TCGGCCTGCC

TAAAAAGGTT CAAGATTGTC CTTTTATAAA ATCTAGAATG TTTACCTGAT

CAGAAAAGTA CAAGATTGTC CTTTTATGAA ATCTTGAAAG TTTACCTGAT

CTCTTTTGTA ATGCATTAAA TGCAAGCTAT AGTCACTGTG TGATTGCTGT

CTCTTTTGTA ATGCATTAAA TGCAAGCTAT AGTCACCGTG TGATTGCTGT

ATCTTTTGTA ATGCATTAAA TGCAAATTAT AGTCACCGTG TGATTGCTGT

CTCTTTTGTA ATGCATTAAA TGCAAGTTAT AGTCACCGTG TGATTGCTGT

CTCTTTTGTA ATGCATTAAA TGCAAGTTAT AGTCACCTTG TGATTGCTGT

CTCTTTTGTA ATGCATTAAA TGCAAGGAAT AGTCACCTTG TGATTGCTGT

C-TTTCTGTA ATGCATTAGA TGCAA--TGT AGTCACCTTG TGAGTGCTA-

CTTTTTTGTA ATGCATTAGA TGCAA--TGT AGTCACCTTG TGATTGCTA-

CTCTTCTGTA ATGCATTAAA TGCAAATCAT AGTCACCTTG TGATTGCTAT

CTCTTTTGTA ATGCATTAAA TGCAAGTTAC AGTCACCTTG TGATTGCTAT

CTCTTCTGTA ATGCATTAAA TGCAAGATAT AGTCACCTTG TGATTGCTAT

CTCTTTTGTA ATGCATTAAA TGCAAGTTAT AGTCACCTTG TGATTGTTAT

CTCATAAGTA ATGCATTAAA TGCAAGTTAT AGTCACCTTG TGATTGCTAT

CTCATCTGTA ATGCATTAAA TCCAAGTTAA AGTCACCTTA TGAGTGCTAT

CTCTTCTGTA ATGCATTCAA CGCAGGTTAC AGTCACCTTG TGATTGCTTT

CTCTTCTTTA ATGCATTAGA TGCACGTTAT AGTCACCTTG TGAAGGCTGT

CTCTTTTATA ACTCATTAAA TGTAAGTAAC TGTCACCGCG AGACTGCTAT

CTCCCTTGTA ACGCATTAAA TGCAAGTTAC TGTCATTCTG TGATTGTGAT

AGTGTTATCA CAGTTTTTGA AATAATGCAT ACCAAATCAG ACACATAGAA

AGTGTTATCA CAGTTTTTGA AATAATGCAT ACCAAATCAG ACACATAGAA

AGTGTTATCA CAGTTTTTGA AATAATGCAT ACCAAATCAG ACACATAGAA

AGTGTTATCA CAGTTTTTGA AATAATGCAT ACCAAATCAG ACACATAGAA

AGTGTTATCA CAGTTTTTGA AATAATGCAT ACCAAATCAG ACACATAGAA

AGTGTTATCA CAGTTTATGA AATAATGCAT ACCAAATCAG ACACATAGAA

-GTGTTATCA CAGTTTC-GA AATAATGCAT ACCAAACCAG ACCCATAGAA

-GTGTTATCA CAGTTTT-GA AATAATGCAT ACTAAACCAG ACCCACAGAA

AGTGTTATCA CAGTTTCTGA AATAATGCAC ACCAAATCAG ACACATACAA

AGTGTTATCA CAGTTCTTGA AATAATGCAT ACCAAATCAG AAACATAGAC

AGTGTTATCA CAGTTCTTGA AATAATGCAT ACCAAATCAG ACACATAGAA

AGTGTTATCA CAGTTCTTGA AATAATGCAT ACCAAATCAG ACACATAGAA

AGTGTTATCA CAGTTCTTGA AATAATGCAT ACCAAATTAG ACACATAGAA

AGTGTTAGTT CAGTTTGTGA AAAAATGTAC ACCAGATCAG ATACATAGAA

AGTGTTATCA CAGTTCCTGA AATAACGCAA ACCAAATGAG ACACATAGAG

AGTGTTATCA -AGTTTTTGA AATAATGCAT ACCAAATTGA ACTCATAGGG

AGTTATTTCA TGATTCTTGA AATAACGCAT CTTGAATCGA ACATGTAGAA

AGTGTTATCC TGGGTTTTGA TTTACCCCAA GGTAAATACA GCTAATAGTT

TGCCATTTTC ATATTTAAGT GATTTTTCAT GTAGAATATC CTTAATACTA

TGCCATTTTC ATATTTAAGT GATTTTTCAT GTAGAATATC CTTAATACTA

TGCCATTTTC ATATTTAAGT GATTTTTCAT GTAGAATATC CTTAATACTA

TGCCATTTTC ATATTTAAGT GATTTTTCAT GTAGAATATC CTTAATACTA

TGTCATTCTC ATATTTAAGT GATTTTTCAT GTAGAATATC CTTAATACTA

TGCCATCTTC ATATATAAGT GATTTTTCAT ATAGAATATC CTTAATACCA

TGCCATCTTC ATGCTTAAAT GACTTTTCAC ATAGAGCATC CTCAATATCA

TGCCGTCTTC ATGTTTAAAT GACTTTTCAC ATAGAGCATC CTTAATACCA

TGACATCTTC ATATTTAAGT GATTTTTCAT GTAGAATATC CTTAATATTA

TGCCATCTTC ATATCTAAGT GATTTTCCAT GTAGAATATC CTTAATACTA

TGCCATCTTC ATATTTAAAT GATTTTTCAT GTAGAGTATC CTTAATACTA

TGCTATCTTC CTATT----T GATTTTTCAT ACAGAATATC CTTAATACTA

TGCCATCTTC ATTTTTAAGT GATATTTCAT GTAGAATATC TTTAATACTA

TGCCATCTTC ATATTTAAGT GATTTTTCAT GTAGAATATC CTTAACCCTA

TGCCATCTTC ATATGTAAGT GATTTTTCAT GTAGACTACC TT-AATACTA

TGCCATCTCC ATACTTGAGT GATTTTTC-- --AGATTATC TTTAATACTA

TGCTATATTA ATATTTAAAT TTCTTTTCAC CTAGAATCTC CTTAACATTA

CTCCATTTCC ATATTTAAAT GATTTTCCAC ACAGAATATC CTAAATATTT

ACTGTAAATT CTGGAGTTAA GATTTCAAAT AAGGTCAGGT CAGGTGTAGA

ACTGTAAATT CTGGAGTTAA GATTTCAAAT AAGGTCAGGT CAGGTGTAGA

ACTGTAAATT CTGGAGTTAA GATTTCAAAT AAGATCAGGT TAGGTGTAGA

ACTGTAAATT CTGGAGTTAA GATTTCAAAT AAGATCAGGT TAGGTGTAGA

ACTGTAAATT CGAGAGTTAT CATTTCAATT A---TCAGGT TAGGTATAGA

----TAAAGT CTGTAGTTAA GATTTCAAAT AACATCAGGT TAGGTATAGA

AGCATAAACC CTGTAGT-AA GACTTAAAAG AACACAGGTG TAGCTGTAGA

AGCATAAATC CTGTAGT-AA GACTTGAAAG AACATAGGTG TAGCTGTAGA

AGCATCAATT CTGTAGTTAA GATCTGAAAC AGTATCAGGT TAGGTACAGA

AGTATAAATT CTGTACTTAA GATCTGAAGT AATATCAGGT TAGGTGTAGA

AGCATAAATT CTGTAGTTAA GATTTAAAAT AATATCAGGT TAGGTGTAGA

AGTATAATTT CTGTAGTTAA GATTTGAAAT ATTATCAGGT TAGGTGTTGA

AATAAAAATT CTGTAGTTAA GATTTGAAAT AACATCAGGT TAGGTGTAGA

AGTAGAAATT CTGAAGTTAA GATCTGAAAT AGTATCAGGT TAGGTGTATA

AATATGAATT CTGTAATTAA GATTTGAAAT AATATCAGGT TAGGTGTGGA

AATAAAAATT CTGTAGTTGA GATTTGAAAT AATATCGGGT CAGGTGTGGA

AGTACAAATT CTGTAGTTGT CATTTAAGAT AATAGGAGGC CAAACATGTT

AGTACAAATT CTGTAGTTGT CATTGAAGAT AATAGCAGAT TAGGCATGGA

AAGAATATGT CCTTATTTTA GAGAACCAAT TCTTACTCTT AGAACAGCTA

AAGAATATGT CCTTATTTTA GAGAACCAAT TCTTACTCTT AGAACAGCTA

AAGAATATGT CCTTATTTTA GAGAACCAAT TCTTACTCTT AGAACAGCTA

AAGAATATGT CCTTATTTTA GAGAACCAAT TCTTACTCTT AGAACAGCTA

AAGAATATGT CCTTATTTTA GAGAACCAAT TCTTACTCTT AGAACAGCTA

AAG---ATGT CCTTATTTTA GGGAACCAAT TCTTACTCTT AGAAGAGCTA

AAGAACAAGT CC-TGTCTTG GAAAGCCAAC TCTTA-TCTT AGAAGA----

GAGAACAAGT CC-TGTCTTG GAAAGCCAAC TCTTA-TCTT AGAAGA----

AAGAACAGGT TCATATTTTA GAGAACCAAC TCTTACTCTT AGAAGAGCTA

AAGAACATGT CCATATTCTA GAAAACCAAC TCTTACTCTT AGAAGAGTTA

AAGAACATGT CCATATTTTA GAGAACCCAC TCTTACTCTT AGAAGAGCTA

AAGTACATGT CCATATTTTA GAGAACCAAC TCTTACTCTT AGAAGAGCTA

AAGAATATGT CCATATTTTA GAGAACCAAC TCTTACTCTT AGAAGAGCTA

AAGAACATGT TCATATTTTA GAGAACCAAC TGTTACTTTT ACAAGAGCTA

AAGAATATGT CCATATTTAA GAGAACCAAC TCTTACTCTT TTAAGAGCTT

GCGGATCTGT CTATATCTAA GGGAACCAAG GCTTACTGTT TGAAGAGCTA

AAATACATGT CTCTACATTT GAGAATATAA TGATCCTTTT ATAAGCTCCA

AAGTACATGT CCCTATATTT TAGAATCACC CCTTATTCTT TTACAAGCTC

CCACAAAAGA TATTAGGTAA TAAAGCTCCC AAGGTATATA AATAAAAATG

CCACAAAAGA TATTAGGTAA TAAAGCTCCC AAGGTATATA AATAAAAATG

CCACAAAAGA TATTAGGTAA TAAAGCTCCC GAAGTATATA AATAAAAATG

CCACAAAAGA TATTAGGTAA TAAAGCTCCC GAAGTATATA AATAAAAATG

CCACAAAAGA CATTAGGTAA TAAAGCTCCC AAAGTATATA AATAAAAATG

CCATAAGACA TATTAGGTAA CAAACCTCCC AAAGTATATA AATAAAAATG

---------- CATTAAGTAA CACAGCTCCC AAAATATATA AATAAAA-TG

---------- CATTAAGTAA CACAGCTCCC AAAATATATA AATAAAA-TG

CCACAAAAGA TATTAGGTAA CAAACCTCCC AAAGTATATA AATAAAAATG

CCACAAAAGA CATTAGGTAA CAAAGCTCCC AAAGTATATA AATAAA---G

CCACAAAAGA CATTAGGTAA CAAAGCTCCC CAAGTATATA AATAAAAATG

CCACAAAAGA CATTAGGTAA CAAAGCTCCC GAAATATATA AATAAAAATG

CCACAAAAGA CATAAGGTAA CAAAGCTCCC AAAGTATATA AATAAAAACG

TCACAAAAGA CATTAAGTAG CAAAGCTCAC AAGGTATATA AATAAAAACA

CCACAAAACA TATTAGGTAA CAAAGCTCGC AAAGCATATA AATAAAAATG

CCACAAAAGA TATTAAGTAA CAAAGCTCGC AAAACATATA AATAAAAATG

CCACTGGAGA CATTAGAGAA CAAAAAGCTT AAAGTATATA AATAAAAGTG

CTACACAAGA CATTAAGTAA CAAAGCTCAC AAAGTATATA AATAAAAATG

TCTACATTAA ATTTATGCTA AATATTTAAA TTTATAGGCA GAAATAACTT

TCTACATTAA ATTTATGCTA AATATTTAAA TTTATAGGCA GAAATAACTT

TCTACATTAA ATTTATGCTA AATATTTAAA TTTATAGGCA GAAATAACTT

TCTACATTAA ATTTATGCTA AATATTTAAA TTTATAGGCA GAAATAACTT

TCTACATTAA ATTTATGCTA AATATTTAAA TTCATAGGCA GAAATAACTT

TCTACATTAA ATTTATGCTA AATATTTAAA TTTACAGACA GAAACAACTT

CTTACATTAA ATTTATGCTA AATATTTAAA TTTATAGGCA GAAATA-CTT

CCTACATTAA GTTTATGCTA AATATTTAAA TTTATAGGCA GAAATA-CTT

CCTACGTTAA ATTTATGCTA AATATT---- ---------- ----------

TCTACATTAC ATTTACATAA AATATTTAAA TTTATAGGCA GAAATAACTT

CCTACATTAA ATTTACGCTA AATTTTTAAA TTTATAGGCA GAAACTACTT

CCTAC----- -TTTATGCTA AATACTTAAA TTTATAGGCA GAAATAACTT

TCTACATTAA ACTTA-GCTA AATATTTAAA TTTATAGGCA GAAAAAACTT

CCTACATCAA AGTTATGCTA AATATTTAAG CTTATTGGTA GAAATAACTT

CCTACATTAC ATGTATGCTA AATGTTTAAA TTTATAGGTA GAAATAACTT

TCTACATTAA ATTTATGCTA AATAGTTTAA TTTGTAGGTA GAAATAACT-

TCTGCATGAA ATTTCTGCTA AGCATTTAAA TTTATAGGTA GAAATAATTT

TCTACATTGA ATTTCTGGTA AGTACTTAAA TTTATAGGAA GCAATAACTT

TAAACTAGCC CCAATCTGGG AACttaaaaa ttaaaataaa taatCAATTC

TAAACTAGCC CCAATCTGGG AACttaaaaa ttaaaataaa taatCAATTC

TAAACTAGCC CCAATCTGGG AACttaaaaa ttaaaataaa taatCAATTC

TAAACTAGCC CCAATCTGGG AACttaaaaa ttaaaataaa taatCAATTC

TAAACTAGCC CCAGTCTGGG AACttaaaaa ttaaaataaa taatCAATTC

TAAACTAGCC CCAATCTGGG AACTT-AAAA TTAAAATAAA TAGTCAACTG

TAAACAAGCT CCAATCTGGG AACTTGAAAA TGAAAATAAA TAGTTACTTG

TAAACAAGCT CCAATCTGGG AACTTGAAAA TGAAAATAAA TAGTTACTTG

---------- ---------- ---------- ---------- ----------

TAAACTAGCC CCAATCTGGG AACttaaaaa ttaaaataaa taGTCAACAA

TAAGCTAGCC CCAATCTGGG AACttaaaaa ttaaaataaa taGTCAATTG

TAAACTAGCC CCAATCTGGG AGCTT-AAAA TTAAAATAAA TAGTCAGTTG

TAAACTATTC CCAAACTGGG AACtt-aaaa ttaaaataaa ttatCA----

TAAACTAGTC CCAATCTGAG AACttaaaaa ttaaaataaa taGTAGATTG

TAAAGTAGCC CCAACCTGGG AACTAAAAAA CTAAAATAAA TAGTCAATTG

------AGCC CCAATCTGGG AACttt---- -ttaaataaa taatCAATTG

TAAAATAGTC CCAAACTGGA TAGttaaaaa ttaaaataaa tattaaattG

TAAAATAGCC CCAGACCGGG CAGTTTAAAA TTAAAATAAA TAGTAAACTG

ATTAATGAAT TGCAACTGGA ATTTCAATCT TATCGAAACC ATCTTATCAA

ATTAATGAAT TGCAACTGGA ATTTCAATCT TATCGAAACC ATCTTATCAA

ATTAATGAAT TGCAACTGGA ATTTCAATCT TATCGAAACC ATCTTATCAA

ATTAATTAAT TGCAACTGGA ATTTCAATCT TATCGAAACC ATCTTATCAA

ATTAATGAAT TGCAACTGGA ATTTCAATCT T---GAAACC ATCTTTTCAA

ATTAATGAAT TGCAACTGGA A-TTCAATCT TATTGAAACC ATCTTATCAA

ATTAATGAAT TGCAACTGGA ATTCCAATT- ----AAAACC ATCTTG-TGA

ATTAATGAAT TGCAACTGGA ATTCCAATT- ----AAAACC ATCTTGCTGA

---------- ---------- ---------- ---------- ----------

ATTAATGAAC TGCAACTGAA ATTTCAATCT TATTGAAACC AAC-TATCAA

ATCAATAAAT TGCAATTGGA ATTTCAATCT TATTAAAACC ATCTTATCAA

ATTAATGAAT TGCAACTGGA ATTTCAA--T TATTGAAACC ATCTTATCAA

ATTAATGAAT TACAACTGGA ATTTCAC--T CATTGAAACC ATCTTATTGA

AGTAATGAAT TGCAACTGGA ATTTCAATCT TATTGAAACC ATCTTATCAA

ATTAATGAAT TACAACTGGA ATTTCAATCT TATTGAAACC ATCTTATCAA

ATTAATGAAT TGCAACTGGA ATTTCAATCT TATTGAAACT ATCTCATCAA

ATTAATGCAT AACAACTAGA ATTCCAATCT TATTTAAAAC ATCTTATTAA

ATTAATGTAT TACAACTGGA ATCCCAATCC CAATGAGAAC ACTTTATTAA

TGTGCTGCAG ATTTAAAAGC ATTTAAATAA TCTTTCACAT TGGAAATATT

TGTGCTGCAG ATTTAAAAGC ATTTAAATAA TCTTTCACAT TGGAAATATT

TATGCTGCAG AATTAAAAGC ATTTAAATAA CTGTTCACAT TGGAAATATT

TATGCTGCAG AATTAAAAGC ATTTAAATAA CTGTTCACAT TGGAAATATT

TGTGCTGCAG ATTTAAAAGC ATTTAAATAA TTTTTCACAT TGGAAATATT

TGTGCTGCAG ATTTAAAAGC TTTTAAATAA TTTTTCACAT TGGGAATATT

TGTACTGCAT GTTTAGAA-C ATTTAAATA- CTAGTCACAT TAGAAATATT

TGTACTGAAG GTTTAGAAGC ATTTAAATA- CTTGTCACAT TGGGACTCTT

---------- ---------- ---------- ---------- ----------

CATGCTGCAG ATTTAAAAGT ATTTAAATAA -TTTTCTTAT TGGGAATATT

TGTGCTGCAG ATTTAAAAAC ATTTAAATAA TTTTTCATAT TGGGAATATT

TGTGCTGCAG ATTTAAAAGC ATTTAAATAA TTTTTCATAT TGGGAATATT

TGTGCTGCAT ATTTTAAAAC ATTTAAATAA TTCTTCAGAT TGGGAATATT

TGTGTTGTAG atttaaaaat atttaaataa tttttCATAT TGGGAACACT

TGTGCTGCAG ATTTAAAAGT ATTTAAATAA TTTTTCATAC TCAGAACATT

TGTGCTACAG GCTTGAAAGC ATTTAAATCA TTTTTCATGT TGGGAGCATT

TATGCTGCAG ATGTAAAAAT ACTTAAATAA TTTTCCAAAT AGGGAATACC

TATGCTGTAG atttaaaaat atttaaataa cttttaaaat GGGGAATACT

AAAGGGCCAA CTGACATGAT CGCTTTGGAT TATCAACAAC AACACATGAA

AAAGGGCCAA CTGACATGAT CGCTTTGGAT TATCAACAAC AACACATGAA

AAAGGGCCAA CTGACATGAT GGCTTTGGGT TATCAACAAC AACACATGAA

AAAGGGCCAA CTGACATGAT GGCTTTGGGT TATCAACAAC AACACATGAA

AAAGGGCCAA CTGACATGAT GGCTTTGGAT TATAAACAAC AACACATGAA

AAAGGGCCAA TCGACATGAC GGTGTTGGGT TATAA--AAC AACACATGAA

GAAGGGTCAA TTGATTAGAT GGCTTTGGGC TAT-AACAAC AACA--TGAA

CAAGGGTCAA TTGATATGAT AGCTTTGGGT TAT-AACAAC AACA--TAAA

---------- ---------- ---------- ---------- ----------

GAAGG-TCAA ATGA---CAT GACTTTGGGT TATTAAAAAA AATGCAGGAA

AAAGGCTCAA CTGACATCAT GGCTTTGGGT CATAAACAAG AACACATGAA

GAAGA-TCAA CTGACACGAT GGCTTTGGGG TATACACAAT AAAACATGAA

GAAGAGTCAA CTGACATGAT GGCTTTGGGT TTTTAAAAAC AATGCATGAA

GAAGGATCAA CTGACATGAT GGTTTTGGGC TATAAACATC AACACATGAA

AAAGGATCAA CTGACATGAT AGCTTGGGGT TATAAACAAC ACCACATGAA

GAAGGATCCA CTGACATGAT AGCTTGGGGT TATTAAAAAC AGCGCACGGA

TTAGAGTCCA TTAACATGAT GGCTTCAGGT Taaaaaaaac aATACATGAG

GAAGAATCCA TTAACATGAT GGCTTTGGGC gaaaaaaaac aacaaacgaa

CCAACGAGGA GAAAACATTC CTCATTCTTA GTGTCGACTA GGTGGTGTTA

CCAACGAGGA GAAAACATTC CTCATTCTTA GTGTCGACTA GGTGGTGTTA

CCAACGAGGA GAAAACATTC CTCATTCTTA GTGTCGACTA GGTGGCGTTA

CCAACGAGGA GAAAACATTC CTCATTCTTA GTGTCGACTA GGTGGTGTTA

CCAACGAGGA GAAAACATTC CTCATTCTTA GTGTCGACTA GGTGGTGTTA

CCAAGGAGGA GAAAACATTC CTTATTCTTA GTGTCGACTA GGTGGTGGTA

CC--AGAGGA GAGAACAGTC CTCATTCTTA GTGTCGACTA GGT-GTGTCA

CCAAAGAGGA GAAAACATTC CTCATTCTTA GTGTCGACTA GGTGGTGTTA

---------- ---------- ---------- ---------- ----------

CCAGGGAAGA GAAAACATTC CTCATTCTTA GTGTCAACTA GGTGGTGTTA

CCAGGGAGGA GAAAACATTC CTCATTCTTA GTGTCGACTA GGTGGTGTTA

CCAGGGAGGA GAAAACATTC CTCGTTCTTA GTGTCGACTA GGTGGTGTTA

TTAGGGAGGA GAAAACATTC CTCATTCTTA GTGTTGACTA GGTGGTGTTA

CCAGGGAGGA AAAAACATTC CTTATTCTTA GTGTCGACTA GGTGGTGTTA

CCAGGGAGGA GAAAACATTC CTCATTCTTA GTGTCGACTA GGTGGTGTTA

CGGGGAGGGA GGAAACATTC CTCAT-CTTA GTGTCGTCTA GCTGGTGGCA

ACAGTGAGGC AGAAGCATTC CTTGCTCTTA GTGTTGACTA AGCAGTGTTA

acaGAGATGA GAAAAGATTC CTTGCTCTTA GTGTCAACTA AGCAATGTTA

TGGCAGTTTT CCTTCGTGAT GTGACACAGT GTTGTGGAGA AAATCAGAGG

TGGCAGTTTT CCTTCGTGAT GTGACACAGT GTTGTGGAGA AAATCAGAGG

TGGCAGTTTT CCTTCGTGAT GTGACACAGT GTTGTGGAGA AAATCAGAGG

TGGCAGTTTT CCTTCGTGAT GTGACACAGT GTTGTGGAGA AAATCAGAGG

TGGCAGTTTT CCTTTGTGAT GTGACACAGT GTTGTGGAGA AAATCAGAGG

TGGCAGTTTT CCTTTGTGAT GTGACACAGT GTTGTGGAGA AAATCAGAGG

TGGCAGTTTT CCTTTGTGAT GGGACACAGT GTTGTGGAAA AAATCAGAGG

TGGCAGTTTT CCTTTGTGAT GTGACACAGT GTTGTGGAAA AAATCAGAGG

---------- ---------- ---------- GTTGTGG-AG AAATCAGAGG

TGGCAGTTTT CCTTCGTGAC ATGACACAGT GTTGTGGAGA AAATCAGAGG

TGGCAGTTTT CCTTCGTGAT GTGACACAGT GTTGTGGAGA AAATCAGAGG

TGGCAGTTTT CCTTCGCGAT GTAACACAGT GTTGTGGAGA AAATCAGAGG

TGGCAGTTTT CCTTCGTGAT GTGACACAGT GTTGTGGAGA AAATCAGAGG

TGGCAGTTTT CCTTCGTGAT GTGACACAGT GTTGTGGAGA AAATCAGAGG

TGGCAGTTTT CCTTCGTGAT GTGACACAGT GTTGTGGAGA AAATCAGAGG

TGGCAGTTTT CCTTCGTGAG GTGACACAGT GTTGTGGAGA AAATCAGAGG

TGGCAGTCTT CCTTATTGAT ATGACACAGT GTTGTGGAGA AAATCAGAGG

TGGCAGTCTT CCTTATTGAC ATGACACAGT GTTGTGGAGA AAATCAGAGG

CAACGAGAAT GTGTTCAGTT CAAGATATCA ACTTGGCATC CTGGCGAAGC

CAACGAGAAT GTGTTCAGTT CAAGATATCA ACTTGGCATC CTGGCGAAGC

CAACGAGAAT GTGTTCAGTT CAAGATACCA ACTTGGCATC CTGGCGAAGC

CAACGAGAAT GTGTTCAGTT CAAGATACCA ACTTGGCATC CTGGCGAAGC

CAACGAGAAT GTGTTCAGTT CAAGATATCA ACTTGGCATC CTGGCGAAGC

CAACGAGAAT GTGTTCAGTT CAAGATATCA ACTTGGCATC CTGGCGAAGC

CAACGAGAGT ATGTTCAGTT CAAGATATCA GCTTGGCATC CTGCCGAAGC

CAACGAGAGT ATGTTCAGTT CAAGATATCA GCTTGGCATC CTGCCGAAGC

CAACAAGAAT ACGTTCAGTT CAAGACTTGA ACTTGGTATC CTGGCGAAGC

CAATGAGATT GTGTTCAGTT CAGGATATCA ACTTGGCATC CTGGCGAAGC

CAACGAGAAT GTGTTCAGTT CAAGATATCA ACTTGGCATC CTGGCGAAGC

CAACGAGAAT GTGTTCAGTT CACGATATCA ACTTGGCATC CTGGCGAAGC

CAACGAGAAT GTGTTCAGTT CAAGATATCA ATTTGGCATC CTGGCGAAGC

CAACGAGAAT ATGTTCAGTT CAAGATATCA ATTTGGCGTC CTGGCGAAGC

CAACGAGAAT GTGTTCAGTT CAAGATATTA ACTTGGCATC CTGGCGAAGC

CAACGAGAAT GTGTTCTGTT CAAGAGATCA ACTTGGCATC CTGGCGAAGC

CAACGAGAAT ATGTTCAGTT CAAGATATTA ACTTGGCATC CAGACGAAGC

CAGCGAGAAT GTGTTCAGTT CAAGGTATCG ACTTGGCATC CTGACGCAAC

CAGTGCAGTC CCTGCCGGGT ATAACGAACT AGATCTGTCA TGATGCTTGC

CAGTGCAGTC CCTGCCGGGT ATAACGAACT AGATCTGTCA TGATGCTTGC

CAGTGCAGTC CCTGCCGGGT ATAACGAACT AGATCTGTCA TGATGCTTGC

CAGTGCAGTC CCTGCCGGGT ATAACGAACT AGATCTGTCA TGATGCTTGC

CAGTGCAGTC CCTGCCGGGT GTAACGAACT AGATCTGTCA TGATGCTTGC

CAGTGCAGTC CCTGCCGGGT ATAACGAACT AGATCTGTCA TGATGCTTGC

CAATGCAGTC CCTGCCGGGT GTAACGAGCC AGATCTGTCA TGATGCTTGC

CAATGCAGTC CCTGCCGGGT GTAACGAGCC AGATCTGTCA TGATGCTTGC

CAGTGCAGTC CCTGCCGGGT ATAACGAACC AGATCTGTCA TGATGCTTGC

CAGTGCAGTC CCTGCCGGGT GTAACGAACT AGGTCTGTCA TGATGCTTGC

CAGTGCAGTC CCTGCCGGGT GTATCGAACT AGGTCTGTCA TGATGCTCGC

CAGTGCAGTC CCTGCCGGGT GTAACGAACT AGGTCTGTCA TGATGCTTGC

CAGTGTAGTC CCTGCCGGGT ATAGCGAATT AGGTCTGTCA TGATGCTTGC

CAGTGTAATC CCTGCCGGGT GTAACGGACT AGGTCTGTCA TGATGCTTGC

CAGTGCAGTC CCTGCCGGGT GTAACGAACT AGGTCTGTCA TGATGCTTGC

CAGTGCAGTC CCTGCCGGGT GTAACGAACT AGGTCTGTCA TGATGCTTGC

CAGTGAAGTC CCTGCCGGGT GTAACGAACT AGGTCTGTCA TGATGCTTGA

CAGTGGAGTC CCTGCCGGGT GTAACGAACT AGGTCTGTCA TGATGCTTGA

ATTAAAGATG AGAGGGCCCA TTACTTTATC CAGTTCAGCA AAGAATTCTA

ATTAAAGATG AGAGGGCCCA TTACTTTATC CAGTTCAGCA AAGAATTCTA

ATTAAAGATG AGAGGGCCCA TTACTTTATC CAGTTCAGCA AAGAATTCTA

ATTAAAGATG AGAGGGCCCA TTACTTTATC CAGTTCAGCA AAGAATTCTA

ATTAAAGATG AGAGGACCCA TTACTTTATC CAGTTCAGCA AAGAATTCTG

ATTAAAGATG AGAGGACCCA TTACTTTATC CAGTTCAGCA AA--------

ATTAAAGATG AGAGGGCCCA TCACTTTGTC CAGTTCAGCA AAGAATTCTA

ATTAAAGATG AGAGGGCCCA TCACTTTGTC CAGTTCAGCA AAGAATTCTA

ATTAAAGATG AGCGGGCCCA TTACTTTATC CAGTTCAGCA AAGAATTCTA

ATTAAAGATG AGAGGGCCCA TTACTTTATC CAGTTCAGCA AAGAATTCTA

ATTAAAGATG AGAGGGCCCA TCACTTTATC CAGTTCAGCA AAGAATTCTA

GTTAAAGATG AGAGGGCCCA TTACTTTATC CAGTTCAGCA AAGAATTCTA

ATTAAAGATG AGAGGGCCCA TTACTTTATC CAGTTCAGCA AAGAATTCTA

ATTAAAGATG AGAGGTCCCA TTACTTTATC CAGTTCAGCA AAGAATTCTA

ATTAAAGATG AGAGGGCCCA TTACTTTATC CAGTTCAGCA AAGAATTCTA

ATTAAAGATG AGAGGGCCCA TTACTTTATC CAGTTCAGCA AAGAATTCTA

ATTAAAGATG AGAGGGCCCA TAACTTTATC CAGTTCAGCA AAGAACTCTG

ATTAAAGATG AGCGGGCCCA TAACTTTATC CAGTTCAGCA AAGAATTCTA

GAAAACCAAA AACATTCATT AAATTACCTT TTTGCTCTTT GGAAAGCTGT

GAAAACCAAA AACATTCATT AAATTACCTT TTTGCTCTTT GGAAAGCTGT

GAAAACCAAA AATATTCATT AAATTACCTT TTTGCTCTTT GGAAAGCTGT

GAAAACCAAA AATATTCATT AAATTACCTT TTTGCTCTTT GGAAAGCTGT

GAAAACCAAA AATATTCATT AAATTACCTT TTTGCTCTTT GGAAAGTTGT

---------- ---------- ---------- ---------- ----------

GAAAATTAAA AACATTCATT AAGTAACCTT TTTGTTCTTT GGAAAGCTGT

GAAAATTAAA AACATTCATT AAGTAACCTT TTTGTTCTTT GGAAAGCTGT

GAAA--CAAA GATATTTATT AAATTACCTT TTTGCTCTTT GGAAAGCTGT

GAAAACCAAA AACACTCATT AAATTACCTT TTTGCTCTTT GGAAAGCTGT

GAAAATCAAA AACAGTCATT AAATTACCTT TCTGCTCTTT GGAAAGCTGT

GAAAACCAAA AACACTCATT AAATTACCTT TTTGCTCTTT GGAAAGCTGT

GAAAATCAAA AAAACTCATT AAATCACCTT TTTGCTCTTT GGAAAGCTGT

GAAAATAAAA AAAATTCATT AAATTACCTT TTTGTTCTTT GGAAAGCTGT

GAAAACCAAA AACATTCATT AAATTACCTT TTTGCTCTTT GGAAAGCTGT

GAAAACCAAA AACATTCATT AAATTACCTT TTTGCTCTTT GGAAAGCTGT

GAAGAGCAAA AACATTAATT AAATTACCTT TTTGCTCCTT CAAAAGCTGT

GGATGACAAG AAAACTGATT AAATTACCTT TTTGCTCTTT GGAAAGCTGT

TCAGCTTGGT CCCAAATTTC GGTGGCATAG AGGAAGTTGG ATGTGACCTG

TCAGCTTGGT CCCAAATTTC GGTGGCATAG AGGAAGTTGG ATGTGACCTG

TCAGCTTGGT CCCAAATTTC GGTGGCATAG AGGAAGTTGG ACGTGACCTG

TCAGCTTGGT CCCAAATTTC AGTGGCATAG AGGAAGTTGG ACGTGACCTG

TCAGCTTGGT CCCAAATTTC GGTGGCATAG AGGAAGTTGG ATGTGACCTG

---------- ---------- ---------- ---------- ----------

TCAGCTTGGT CCCAAATTTC TGTGGCATAG AGGAAGTTAG ATGTAACCTG

TCAGCTTGGT CCCAAATTTC TGTGGCATAG AGGAAGTTAG ATGTAACCTG

TCAGCTTGGT CCCAGATTTC GGTGGCATAG AGGAAGTTGG ----------

TCAGCTTGGT CCCAAATTTC AGTGGCATAG AGGAAGTTGG ATGTAACCTG

TCAGCTTGGT CCCAAATTTC CGTGGCATAG AGAAAGTTAG ATGTAACTTG

TCAGCTTGGT CCCAAATTTC TGTGGCATAG AGGAAGTTGG ATGTGACCTG

TCAGCTTGGT CCCAAATTTC AATGGCATAG AGGAAGTTGG ATGTAACCTG

TCTGCTTGGT CCCAAATTTC AGTGGCATAG AGGAAGTTGG ATGTAACCTG

TCCGCTTGGT CCCAAATTTC AGTGGCATAG AGGAAGTTGG ATGTAACCTG

TCAGCTTGGT CCCAAATTTC AGTGGCATAG AGGAAGTTAG ATGTAACCTG

TCAGCTTGGT CCCAGATTTC AGTGGCATAA AGGAAGTTGG ATGTGACCTG

TCGGCTTGGT CCCAAATTTC AGTCGCGTAG AGGAAGTTGG AGGTGACCTG

AACATAGCTG GCTGCCATCT GGTGGATCTT CTGTGGAATG GTCACTGAAG

AACATAGCTG GCTGCCATCT GGTGGATCTT CTGTGGAATG GTCACTGAAG

AACATAGCTG GCTGCCATCT GGTGGATCTT CTGTGGAATG GTCACTGAAG

AACATAGCTG GCTGCCATCT GGTGGATCTT CTGTGGAATG GTCACTGAAG

AACATAGCTG GCTGCCATCT GGTGGATCTT CTGTGGAATG GTCACTGAAG

---------- ---------- ---------- ---------- ----------

GACATAGCTT GCTGCCATCT GGTGGATTTT CTGTGGAATG GTCACCGAAG

GACATAGCTT GCTGCCATCT GGTGGATTTT CTGTGGAATG GTCACTGAAG

---------- -------TCT GGTGGATCTT CTGTGGGATG GTAATGGATG

AACATAGCTG GCTGCCATGT GGTGAATCCT CTGTGGAATG GTCACTGAAG

AACATAGCTG GCTGCCATCT GGTGGATCCT CTGTGGAATG GTCACTGAAG

GACATAGCTG GCTGCCATGT GGTGGATCCT GTGTGGAATG GTCACTGAAG

AACATAGCTG GCTGCCATAT GGTGGATCCT CTGTGGAATG GTCACCGAAG

AACATAGCTG GCTGCCATCT GGTGGATCTT CTGTGGAATG GTCACTGAAG

AACATAGCTG GCTGCCATCT GGTGGATCCT CTGTGGGATG GTCACTGAAG

AACATAGCTG GCTGCCATCT GGTGGATCTT CTGTGGAATG GTCACGGAAG

AACATAGTTG GCTGCCATCT GGTGGATCTT CTGTGGAATA GTTACTGAAG

GACGTAGCTG GCCGCCATCT GGTGGATCCT CTGCGGAATT GTCACCGAAG

AACCACTTGC AGAAGCACTA CTGGCCCCAG ATGAATAATT TCCTGAATTG

AACCACTTGC AGAAGCACTA CTGGCCCCAG ATGAATAATT TCCTGAATTG

AGCCACTTGC AGAAGCACTA CTGGCCCCAG ATGAATAATT TCCTGAATTA

AGCCACTTGC AGAAGCACTA CTGGCCCCAG ATGAATAATT TCCTGAATTG

AACCACTTGC AGAAGCACTA CTGGCCCCAG ATGAGTAATT TCCTGAATTG

---------- ---------- ---------- ---------- ----------

AACCACTTGC TGAAGCACTA CTTCCCCCAG AAGAGTAACT TCCAGAATTG

AACCACTTGC TGAAGCACTA CTTCCCCCAG AAGAGTAACT TCCAGAATTG

AGGTGGTTGC TGAAGCACTA CTGGCCCCGG AGAATTCTTT TTTTGAATTG

AACCACTTGC TGAAGCACTA CTGGCTCCAG AAGAATAATT TCCTGAATTG

AACCACTTGC TGAAGCACTA CTGGCCCCTG AAGAATAATT TCCTGAATTG

AACCACTTGC TGAAGCACTA CTGGCCCCAG AAGAGTAACT TCCTGAATTG

AACCACTTGC TGAAGCACTA CTGGCTCCAG AAGAATAATT TCCTGAATTA

AACCACTTGC TGAAGCACTA CTGGCACCAG AAGAATAATT TCCTGAATTG

AGCCACTAGC TGAAGCACTA CTGGCTCCAG AAGAGTAATT TCCTGAATTG

ATCCACTTGC TGAAGCACCG CTGGCTCCAG AAGAGTAGTT TCCGGAATTG

AACTGCTCCC TGAAGTGCTG CTTGCCCCTG AAGTATAATT TCCTGAATTG

AACCGCTCCC CGAAGGGTTG CTGGCCCCTG AGGAGTAGTT TCCTGAATTG

CCTGGTGACA GCTTTGGAGA AACAGGGGAA GGCATCCCCA CAGCTTTGCT

CCTGGTGACA GCTTTGGAGA AACAGGGGAA GGCATCCCCA CAGCTTTGCT

CCTGGTGACA GCTTTGGAGA AACAGGGGAA GGCATCCCCA CAGCTTTGCT

CCTGGTGACA GCTTTGGAGA AACAGGGGAA GGCATCCCCA CAGCTTTGCT

CCTGGTGACA GCTTTGGAGA AACAGGGGAA GGCATCCCCA CAGCTTTGCT

---------- ---------- ---------- ---------- ----------

CCTGGTGACA GCTTTGGAGA AACAGGGGAG GGCATCCCAA CAGCTTTGCT

CCTGGTGACA GTTTTGGAGA AACAGGGGAG GGCATCCCAA CAGCTTTGCT

GCTGGTGACG TCTTTGGAGA AATTGGGGAA GGCATGCCAA CAGTTTTGCT

CCTGGTGACA GCTTTGGAGA AACAGGGGAA GGCATCCCAA CAGCTTTGCT

CCTGGCGACA ACTTTGGAGA AACAGGGGAA GGCATCCCAA CGGCTTTGCT

CCTGGAGACA GCTTTGGAGA AACAGGGGAA GGCATCCCAA CAGCTTTGCT

CCTGGTGACA GCTTTGGAGA AACAGGGGAT GGCATCCCAA CAGCTTTGCT

CCTGGCGATA GCTTTGGAGA AACAGGGGAA GGCATCCCAA CAGCTTTGCT

CCTGGTGACA GTTTTGGAGA AACAGGGGAA GGCATCCCAA CAGCTTTGCT

CCTGGTGACA GTTTTGGTGA CACAGGGGAA GGCATCCCAG CGGCTTTGCT

CCAGGAGAGA GCTTTGGAGA AACAGGGGAA GGCATCCCAA CAGCTTTGCT

CCCGGAGAAA GCTTGGGGGA GACAGGCGAG GGCATCCCGG CGGCTTTGCT

TAAAAAATTT ACCTACCTTC CCAAGCCAGG CGATGGTGCT TGAGAATTAT

TAAAAAATTT ACCTACCTTC CCAAGCCAGG CGATGGTGCT TGAGAATTAT

taaaaaattt aCCTACCTTC CCAAGCCAGG CGATGGTGCT TGAGAATTAT

taaaaaattt aCCTACCTTC CCAAGCCAGG TGATGGTGCT TGAGAATTAT

TAAAAAATTC ACCTACCTTC CCAAACCAGG CGATGGTGCT TGAGAATTAT

---------- ---------- ---------- ---------- ----------

AAAAAAATTT ACCTACCTCC CCAAGCCAGG TGAAGGTGCT TGAGAATTAC

GAAAAAACTT ACCTACCTCC CCAAGCCGGG TGAAGGTGCT TGAGAATTAC

taaaaaattt aCCTACCTTC CCAAGCCAGG GGATGGTGCT TGAGAATTAT

GAAAAAATTT ACCTACCTTC CCAAGCCTGG TGATGGTGCT TGAGAGTTAT

aaaaaaattt aCTTACGTTC CCAAGCCAGG TGATGGTGCT TGAGAATTAT

GAAAAAATTG ACCTACCTTC CCAAGCCAGG TGATGGTGCT TGAGAATTAT

---------- ---------- ---------- ---------- ----------

GAAAAAATTT ACCTACCTTC CCAAGCCAGG TGATGGAGCC TGAGAATTAT

GAAAAAGTTT ACCTACCTTC CCAAGCCAGG TGATGGAGCT TGAGAATTAT

GAATAAAGTT ACTTGCCTTC CCAAGCCAGG TGACGGAGCT TGAGAATTAT

GGAAAATTAT ACCTACCTTC CCATGCCAGG TGATGGAGCC TGAGAATTAT

AGAAAAATGG ATTTACCTTC CTATGCCAGG TGAGGGAGCT TGCGAATTAT

TATAAGAATT CTGTGGAAAA AGTAAATTAC CTTCAGGTGC TCTGTCAGTG

TATAAGAATT CTGTGGAAAA AGTAAATTAC CTTCAGGTGC TCTGTCAGTG

TATAAGAATT CTGTGGAAAA AATAAATTAC CTTCAGGTGC TCTGTCAGTG

TATAAGAATT CTGTGGAAAA AATAAATTAC CTTCAGGTGC TCTGTCAGTG

TGTAAGAATT CTGTGGAAAA AGTAAATTAC CTTCAGGTGC TCTGTCAGTG

---------- ---------- ---------- ---------- ----------

TGTAAGAATT CTGTGGGAAA AATACATTAC CTTCAGGTGC TCAGTCAGCG

TGTAAGAATT CTGTGGGAAA AA-AAATGAC CTTCAGGTGC TCAGTTAGCG

TATAAGAATT CTGTGGAAAA ATAAAATTAC CTTGAGGTGC TCTGTCAGTG

TGTAAGAATT CTGAGGAGAA AAGGAATTAC CTTCAGATGC TCTGTCAGTG

TGTAAGAATT CTGTGGa-aa aataaaTTAC CTTCAGGTGC TCTGTCAGTG

TATAAGAATT CTGTGGAGAA AATAAATTAC CTTCAGGTGC TCCGTCAGTG

---------- ---------- ------TTAC CTTCAGGTGC TCTGTCAGTG

TATAAGAATT CTGTGAAAAA AATAAATTAC CTTCAGGTGT TCTGTCAGTG

TGTAAGAATT CTGTGAAAAA AAAAAATTAC CTTCAGATGC TCCGTCAGTG

TGTAAGAATT CTGTGAAGAA AATAAATTAC CTTCAGATGC TCTGTCAGGG

TATAAGAATT CTAGGGAAAA AACAAATTAC CTTTAAATGT TCAGTTAATG

TATAGGAGTT CTGCGGAGGA AAAACATTAC CTTTAGATGT TCCGTCAGTG

TCTTTGAGTA CTTCAGAGCA TTTTCCTTCT TCAGTTTGAA CAGCCTCAGG

TCTTTGAGTA CTTCAGAGCA TTTTCCTTCT TCAGTTTGAA CAGCCTCAGG

TCTTTGAGTA CTTCAAAGCA TTTTCTTTCT TCAGTTTGAA AAGCCTCAGG

TCTTTGAGTA CTTCAAAGCA TTTTCTTTCT TCAGTTTGAA AAGCCTCAGG

TCTTTGAGTA CTTCAGAGCA TTTTCCTTCT TCAGTTTGAA TAGCCTCAGG

---------- ---------- ---------- ---------- ----------

TCTTGGAGTA CTTCAGAGCG TTCTCCTTCT TCAGTTTGAA CAGCCTCAGG

TCTTGGAGTA CTTGAGAGCG TTCTCCTTCT TCAGTTTGAA CAGCCTCAGG

TCTTTGAGTA CTTCAGAGCA TTCTCCTTCT TCAGCTTGAA TAGCCTCAGG

TCTTTGAATA CTTCAGAGCA GTTTCTTTCT TCAGTTTGAA GAGCCTCAGG

TCTTTGAGTA CTTCAGAGCA TTCTCCTTCT TCAGTTTGAA TAGCCTCAGA

TCTTGGAGTA CTTCAGAGCA TTTTCCTTCT TCAGTTTGAA TAGCCTCAGG

TCTTGGAGTA CCTCAAAGCA TTTTCCTTCT TCAGTTTGAA TAGCCTCAGG

TCTTGGAGTA CTTCAGAGCA TTCTCCTTCT TCAGTTTGAA TAACCTCAGG

TCTTTGAGTA CTTCAGAGCA TTTTCCTTCT TCAGTTTGAA TAGCCTCAGG

TCTTGGAGTA CTTGAGAGCA TTCTCCTTCT TCAGTTTGAA TAGC------

TTTTTGAGTA TTTCAAAGCA TTTTCTTTCT TCAGCTTAAA TAACCTCAGG

TTTTTGAGTA TTTCAACGCA CTCTCTTTCT TCAGCTTGAA CAACCTCAGG

TACAGCAAAG ACTCGCATCG CAGGCTAAAA CTCACCAAAG TACTGTGAGT

TACAGCAAAG ACTCGCATCG CAGGCTAAAA CTCACCAAAG TACTGTGAGT

TACAGCAAAG ACTCGCATCG CAGGCTAAAA CTCACCAAAG TACTGTGAGT

TACAGCAAAG ACTCGCATCG CAGGCTAAAA CTCACCAAAG TACTGTGAGT

TACAGCAAAG ACTCGCATCG CAGGCTAAAA CTCACCAAAG CACTGTGAGT

---------- ---------- ---------- ---------- ----------

TACAACAATG ACTGGCATCT CAGGCTAACA CTTACCAAAG TACTGTGAGT

TACAATAATG ACTGGCATCT CAGGCTAACA CTTACCAAAG TACTGTGAGT

TACAGCAAAG ACTGGCATCG CAGGCTGACA CTCACCAGAG CACCGTGAGT

TACAGCAAAG ACTGGCATCG CAGGCTAACA CTTACCAAAG CACTGTAAGT

TACAGCAAAG ACTGGCATCG CAGGCTAACA CTTACCAAAG TACTGTGAGC

TACAGCAAAG ACTCGCACCG TAGGCTAACA CTTACCAAAG TACTGTGAGT

TATAGCAAAG ACTGACACCG TAGGCTGACA CTTACCAAAG TACTGTAAGT

TAAAGCAAAG ACTGGCATCG TAGGCTAATG CTTACCAAAG TACTGTGAGT

TACAGCAAAG ACTGGCATCG TAGGCTAACA CTTACCAAAG TATTGTGAGT

---------- ---------- ---------- ---------- ----------

TACAATAAGG ACTGGCATCG AAGGCTAATA CACACCAAAG AACTGCCAGC

TACAACAGGG ACTGGCATCG CAGACTGATA CACACCATAG CACGGCTAGT

CGTTTATCTG CAGCTGTAGC ATCTGGTGCC AAGTAATTCT TTAGCTTCAT

CGTTTATCTG CAGCTGTAGC ATCTGGTGCC AAGTAATTCT TTAGCTTCAT

CGTTTATCTG CAGCTGTAGC ATCTGGTGCC AAGTAATTCT TTAGCTTCAT

CGTTTATCTG CAGCTGTAGC ATCTGGTGCC AAGTAATTCT TTAGCTTCAT

CGTTTATCTG CAGCTGTAGC ATCTGGTGCC AAGTAGTTCT TTAGCTTCAT

---------- ---------- ---------- ---------- ----------

CTCTTGTCTG CAGCTGTAGC ATCTGGCGCC AAATAATTCT TTAGTTTCAT

CTCTTGTCTG CAGCTGTAGC ATCTGGTGCC AAATAATTCT TTAGTTTCAT

CTCTTATCTG CAGCTGTGGC GTCTGGTGCT AAGTAACTCT TCAGCTTCAT

CTTTTATCTG CAGCTGTAGC ATCTGGTGCC AAGTAATTCT TTAGCTTCAT

CTTTTATCTG CAGCTGTAGC ATCAGGTGCC AAGTAATTCT TTAGCTTCAT

CTTTTATCTG CTGCTGTAGC ATCTGGTGCT AAGTAATTCT TTAGCTTCAT

CTTTTATCTG CAGCGGTAGC GTCTGGTGCC AAGTAACTCT TTAGTTTCAT

CTTTTATCTG CAACTGTAGC ATCTGGAGCC AAGTAATTCT TTAGCTTCAT

CTTTTATCTG CAGCTGTAGC ATCTGGCGCC AAGTAATTCT TTAACTTCAT

---------- ---------- ---------- ---------- ----------

CTTTTATCTG CAGATGTAGC ATCTGGTGCC AGGTAATTCT TCAGCTTCAT

CTTTTATCTG CAGCTGTGGC ATCTGGTGCC AAGTAATTCT TCAGCTTCAT

AGTGTATCTG TGAGTTACCA CTTACTTGAT GAGATCCACC GTCTCTGAAT

AGTGTATCTG TGAGTTACCA CTTACTTGAT GAGATCCACC GTCTCTGAAT

AGTGTATCTG TGAGTTACCA CTTACTTGAT GAGATCCACT GTCTCTGAAT

AGTGTATCTG TGAGTTACCA CTTACTTGAT GAGATCCACT GTCTCTGAAT

AGTGTATCTG TGAGTTACCA CTTACTTGAT GAGATCCACC GTCTCTGAAT

---------- ---------- ---------- ---------- ----------

AGTGTATCTA TGAGTTGACA CTTACTTAAT AAGCTCCACT GTGTCTGAGT

AGTGTACCTA TGAGTTGACA CTTACTTAAT GAGCTCCACC GTGTCTGAGT

AGTGTACCTG TGAGTC--CA CTTACTTGAT GAGCTCCACT GTCTCTGAAT

AGTGTATCTG TGAGATAACA CTTACTTGAT GAGCTCCACC GTTTCTGAAT

AGTGTATCTG TGAGTTAGCA CTTACTTGAT GAGCTCCACT GTCTCTGAAT

AGTGTATCTG TGAGTTAACA CTTACTTGAT GAGATCCACC GTCTCTGAAT

AGTGTATCTG TGAGTTAATA CTTACTTGAT GAGATCTGCT GTCTCTGAAT

AGTATATCTG TGAGTTAACA CTTACTTGAT AAGTTCCACC GTCTCTGAAT

AGTGTATCTG TGAGTTAACA CTTACTTGAT GAGATCCACC GTCTCTGAAT

---------- ---------- ---------- ---------- ----------

AGTATATCTA TAACATTACA CTTACTTAAT TAACTCCACA GTTTCTGAAT

AGTGTATCTG CAAGTAGATA CTTACTTAAT TAGTTCCACA GTTTCTGAAT

ACATAGGGAA TGGGGATTTG GATTCCTGAG CATTCTTCTC TAATGCATTC

ACATAGGGAA TGGGGATTTG GATTCCTGAG CATTCTTCTC TAATGCATTC

ACATAGGGAA TGGGGATTTG GATTCCTGAG CATTTTTCTC TAATGCATTC

ACATAGGGAA TGGGGATTTG GATTCCTGAG CATTTT---- ----------

ACATAGGGAA TGGGGATTTG GATTCCTGAG CATTTTTCTC TAATGCATTC

---------- ---------- ---------- ---------- ----------

ACATAGGGAA TGGAGACTTG GATTCCTGAG CGTTCTTCTC TAATGCATTC

ACATAGGGAA TGGGGACTTG GATTCCTGAG CGTTCTTCTC TAATGCATTC

ACATGGGGAA TGGGGATTTA GATTCCTGAG CATTTTTCTC CAAAGCGTTC

ACATAAGGAA TGGGGATTTG GATTCCTGAG CATTTTTCTC TAATGCATTC

ACATAGGGAA TGGGGATTTG GATTCCTGAG CATTTTTCTC TAATGCATTC

ACATAAGAAA TGGGGATTTG GATTCCTGAG CATTTTTCTC TAATGCATTC

ACATAGGGAA TGGGGACTTG GATTCCTGAG CATTTTTCTC TAACGCATTC

ACATAGGGAA TGGGGACTTG GATTCCTGAG CATTTTTCTC CAAGGCATTC

ACATAAGGAA TGGGGACTTG GATTCCTGAG CATTTTTCTC TAATGCATTC

---------- ---------- ---------- ---------- ----------

ACATAAGGAA TGGAGATTTG GATTCCTGAG CATTTTTCTC CAAAGCATTT

ACATAGGGAA CGGGGATTTG GATTCCTGAG CGTTTTTCTC CAATGCATTC

CCACATTCAA TGAAAGATAC CACAGCATCA AGATAGTATA CAGCTTTCTC

CCACATTCAA TGAAAGATAC CACAGCATCA AGATAGTATA CAGCTTTCTC

CCACATTCAA TGAAAGATAC CACAGCATCA AGATAGTATA CAGCTTTCTC

---------- ---------- ---------- ---------- ----------

CCACATTCAA TGAAAGATAC CACAGCATCA AGATAGTATA CAGCTTTCTC

---------- ---------- ---------- ---------- ----------

CCACATTCAA TGAAAGACAC TACGGCATCA AGGTAGTACA CAGCTTTCTC

CCACACTCAA TGAAAGACAC TACAGCATCA AGGTAGTACA CGGCTTTCTC

CCACATTCAA TGAAAGATAC CACAGCATCA AGATAGTACA CAGCTTTCTC

CCACATTCAA TGAAAGATAC CACAGCATCA AGATAGTATA CAGCTTTCTC

CCACATTCAA TGAAAGATAC CACAGCATCA AGATAGTATA CAGCTTTCTC

CCACATTCAA TGAAAGACAC CACGGCATCA AGATAGTACA CAGCCTTCTC

CCACATTCTA TGAAAGATAC CACAGCATCA AGATAGTACA CAGCTTTCTC

CCACATTCAA TGAAAGATAC CACAGCATCA AGATAGTATA CTGCTTTCTC

CCACATTCAA TGAAAGATAC CACAGCATCA AGATAGTACA CTGCTTTCTC

---------- ---------- ---------- ---------- ----------

CCACATTCAA TGAAAGATAC CACAGCATCA AGATAATAGA CAGCTTTCTC

CCACATTCAA TGAAAGACAC AACAGCATCA AGGTAGAAGA CCGCTTTCTC

AAACCTATCA GACTAAGGAG AATGCAAGTA ATGATCTACA TACCAATGCA

AAACCTATCA GACTAAGGAG AATGCAAGTA ATGATCTACA TACCAATGCA

AAACCTATCA GACTAAGGAG AATGCAAGTA ATGATTTACA TACCAATGCA

---------- ---------- ---------- ---------- ----------

AAACCTATCA GACTAAGGAG AATACAAGTA ATGATTTACA TACCAATGCA

---------- ---------- ---------- ---------- ----------

AAACCTATCA GACTAAAAAG GAAAACAGCA CGGATTTACA TACCAAAGCA

AAACCTGTCA GACT--AAAG GAAAACAGTG CTGATTTACA TACCAAAGCA

AAACCTATCA GACTAAGAAG AATAAAAATA GTGATTTACA TACCAATGCA

AAACCTATCA GACTAAGGAG AATAAAGGTA ATGatttaCA TACCAATGCA

AAACCTATCA GACTAAGGAG AATAAAGGTA ATGGTTTACA TACCAATGCA

AAACCTGTCA GACTAAGGAG AAAAAAGGGA ATGATTTACA TACCAAAGCA

AAACCTGTCA GACTAA--AG AATTAAGGTG ATGGTTTACG TACCAATGCA

AAACCTATCA GACTAAGGAG AATAAAAGTA GTGATTTACA TACCAATGCA

AAACCTATCA GACTAAGGAG AATAAAGGTA GTGATTTACA TACCAATGCA

---------- ---------- ---------- ---------- ----------

AAATCTATCA GACTAAGCAG TCAAGAGAAA ATGGCCAACA TACCAATGCA

AAACCTATCG GACTGAAAAG AAACAAAGGA AAGATTTACA TACCAACGCA

TCTGCATTGT GCTTTAGCTT TTTTGCTTCT TGTAAATAAT GGTCTGCTGA

TCTGCATTGT GCTTTAGCTT TTTTGCTTCT TGTAAATAAT GGTCTGCTGA

TCTGCATTGT GCTTTAGCTT TTTTGCTTCT TGTAAATAAT GGTCTGCTGA

---------- ---------- ---------- ---------- ----------

TCTGCATTGT GCTTTAGCTT TTTTGCTTCT TGTAGATAAT GGTCTGCTGA

---------- ---------- ---------- ---------- ----------

TCTGCATTGT GTTTGAGTTT TTTTGCTTCT TGTAAATAAT GGTCTGCTGA

TCTGCATTGT GTTTGAGTTT TTTTGCTTCT TGTAAATAAT GGTCTGCTGA

TCTGCATTGT GCTTTAACTT TTTTGCTTCT TGTAAATAAT GGTCTGCGGA

TCTGCATTGT GCTTTAGCTT TTTTGCTTCT TGTAAATAAT GGTCTGCTGA

TCTGCATTGT GCTTTAGCTT TTTTGCTTCT TGTAAATAAT GGTCTGCTGA

TCTGCATTGT GCTTTAGCTT TTTTGCTTCT TGTAAATAAT GGTCTGCTGA

TCGGCATTGT GCTTTAGCTT TTTTGCTTCT TGCAAATAAT GATCTGCTGA

TCTGCATTAT GCTTTAGCTT TTTTGCTTCT TGTAAATAAT GGTCTGCTGA

TCTGCATTGT GCTTTAGCTT TTTTGCTTCC TGTAAATAAT GGTCTGCTGA

---------- ---------- ---------- ---------- ----------

TCCGCATTGT GCTTTAGCTT TTTAGCTTCT TGTAAATAAT GGTCAGCTGA

TCTGCATTGT GCTTTAGTTT TTTTGCTTCT TGTAAGTAAT GATCTGCTGA

ATAATTTCTG CAAGACAAAT TCTCACCTGT CATCAAAGAC AAGCTTTGTT

ATAATTTCTG CAAGATAAAT TCTCACCTGT CATCAAAGAC AAGCTTTGTT

ATAATTTCTG CAAGGTAAAT TCTCACCTGT CATCAAAGAC AAGCTTTGTT

---------- ---------- -CTCACCTGT CATCAAAGAC AAGCTTTGTT

ATAATTTCTG CAAGGTAAAT TCTTACCTGT CATCAAAGAC AAGCTTTGTT

---------- ---------- ---------- ---------- ----------

ATAATTTCTG Caaaataaat tCTCACCTGT CATCAAAGGC GAGCTTCGTT

ATAATTTCTG CAAAACAATT TCTTACCTGT CATCAAAGGC AAGCTTTGTT

GTAACTTCTG CAATGGAAAT TCTCACCTGT CATCAAAGGC GAGC------

ATAATTTCTG CAATGTAAAT TCTTACCTGT CATCAAAGGC AAGCTTCGTT

ATAATTTCTG CAATGTAAAT TCTCACCTGT CATCAAAGGC AAGCTTTGTT

ATAATTTCTG CAATGTAAAT TCTCACCTGT CGTCAAAGAC AAGCTTTGTT

ATAATTTCTG CAATGTAAAT TCTCACCTGT CATCAAAGGC AAGCTTTGTT

ATAATTTCTG CAATGTAAAT TCTTACCTGT CGTCAAATGC AAGCTTTGTT

ATAATTCCTG CAATGGAAAC TCTCACCTGT CGTCAAAGGC AAGCTTTGTT

---------- ---------- ---------- ---------- ----------

GTAAATCCTG CAACAAAAAT TTTTACCTGT CATCAAAAGC AAGCTTTGCT

ATAGCTCCTG CAATAGAAAG TCTCACCTGT CATCAAAAGC AAGCTTTGCT

CTCCGAGGCT TAGAAGAATC AAGAGTTGGT GCAGATGGAG GACAGTTAGA

CTCCGAGGCT TAGAAGAATC AAGAGTTGGT GCAGATGGAG GACAGTTAGA

CTCCGAGGCT TAGAAGAATC AAGAGTTGGT GCAGATGGAG GACAATTAGA

CTCCGAGGCT TAGAAGAATC AAGAGTTGGT GCAGATGGAG GACAATTAGA

CTCCGAGGCT TAGAAGAATC AAGAGTTGGT GCAGATGGAG GACAGTGAGA

---------- ---------- ---------- ---------- ----------

CTTCGAGGCT TCGAAGAATC AGGAGTTGGT GTAGACGGAG GACAATTAGA

CTCCGAGGCT TCGAAGACTC AGAAGTTGGT GTAGACGGAG GACAATTAGA

---------- ---------- ---------- ---------- ----------

CTCCGAGGCT TAGAAGCATC AGGAGTTGGT GCAGATGGAG GACAGTTAGA

CTCCGAGGCT TAGAAGCATC AGGAGTTGGT GTAGATGAAG GACAGTTAGA

CTCCGAGGCT TAGAAGCATC AGGAGTTGGT GTAGATGGAG GACAGTTAGA

CTCCGAGGTT TAGAAGCTTC AGAAGTTGGT GTAGATGAAG GACAGTTTGA

CTCCGAGACT TAGACGCATC AGGAGTTGGT GTAGATGGAG GACAGTTAGA

CTCCGAGGCT TAGAAGCATC AGGAGTTGGT GTAGATGGAG GGCAGTTAGA

---------- ---------- ---------- ---------- ----------

CTTCGTGTTT TGGAGCCATC AGGAATTTGT GTAGCTGGAG GATAGTTCGA

CTTCGGGACT TGGAACTGTC AGGAGTTGTT GTAGATGGAG GACAATTTGA

GGAGCTACTT GGAGCCTTTT CCTTCCTTGG AGCTACTGGA AGTCTTCCCT

GGAGCTACTT GGAGCCTTTT CCTTCCTTGG AGCTACTGGA AGTCTTCCCT

GGAGCTACTT GGAGCCTTTT CCTTCCTTGG AACTACTGGA AGTCTTCCCT

GGAGCTACTT GGAGCCTTTT CCTTCCTTGG AACTACTGGA AGTCTTCCCT

GGAGCTACTT GGAGCCTTTT CCTTCCTTGG AGTTACTAGA AGTCTTCCCT

---------- ---------- ---------- ---------- ----------

GGAGCTATTT GGAGCCTTCT CCTTCTTTGG AGCTGCTGGA AACCTTCCCT

GGAGCTATTT GGAGCCTTCT CCTTCCTTGG AGCTGCTGGG ACCCTTGCCT

---------- ---------- ---TCCTTGG AGCTACTTGA GGTCTTCCCT

AGAACTATTT GGAGCTTTTT CCTTCCTTAG AGCTACTGGA AGTCTTCCCT

GGAGCTATTT GGAGCCTTTT CCTTCCTTGG AGCTACTGGA GGTCTTCCCC

GGAACTATTT GAAGCCTTTT CCTTCCTTAG AGCTACTGGA AGTCTTCCCT

GGAGCTATTT GAAGTCTTTT CCTTCCTTCG AGCTACCGGA CGTCTTCCCT

GGAGCTATTT GGAGCCTTTT CCTTCCTTGG AGTTACTGGA AGTCTTCCCT

GGAGCTATTT GGAGCCTTAT CCTTCCTTGG AGCTACTGGA AGTCTTCCCT

---------- ---------- ---TCCTTGG AGCTACTGGA GGTCTTCCCG

AGAACTACTT GGAGCCTTTT CCTTCCTTGG TACCACTAAA AACTTTCCCT

AGAGTTGTTT GGATTTTTTT CCTTCCTTGG AACTGGTAGA AACCTTTCCT

TCGGTCTTCT TCTGCTTTGA TGTGGAGGAA CTGTTTTTGC TCGTCTCCTT

TCGGTCTTCT TCTGCTTTGA TGTGGAGGAA CTGTTTTTGC TCGTCTCCTT

TCGGTCTTCT TCTGCTTGGA TGTAGAGGAA CTGTTTCTGC TAGTCTCCT-

TCGGTCTTCT TCTGCTTGGA TGTAGAGGAA CTGTTTCTGC TAGTCTCCT-

TCGGTCTTCT TCTGCTTGGA TGTGGAAGAA CTGTTTTTGC TTGTCTCCTT

---------- ---------- ---------- ---------- ----------

TCAGTCTTCT TCTGTTTTGC TGTGGAGGAA GTGCTCTTGC TGTTCTCTTT

TCAGTCTTCT TCTGCTTCGC TGTGGAGGAG GTGCTCTTGC TGTTCTCTTT

TCAGTCTTTT TCAGCTTTGA TGTGGAGGAA CTGCTTTTGC TGTTCTCCTT

TCAGTCCTCT TCTGCTTTGA TGCGGAGGAA CTGTTTTTGC TATTTTCCTT

TCAGTCTTCT TCTGCTTGGA TGTGGAGGAG CTGTTCCTGC TGTTCTCTTT

TCAGTTTTCT TCTGCTTTGA CGTGGAGGAA CTGTTTTTGC TGTTCTCCTT

TCAGTCTTCT TCTGCTTTGA TGCAGAGGAA CTGCTTTTGC TATTCTCCTT

TCAGTCTTCT TCTGCTTTGA TGTAGAGGAA CTGTTTTTGC TATTCTCCTT

TCAGTCTTCT TCTGCTTTGA TGTGGAGGAA CTATTTTTGC TATTCTCCTT

TCAGTCTTCT TCTGCTTGGA TGTGGAGGAG CCACTTTTGC TGTTCTCCTT

TCTGTCTTTT TCTGTTTGGA AGTAGAAGAA CTATTTTTAC TACTCTCCTT

TCTGTCTTTT TTTGCTTTGA AGAGGAAGAA CTGTTTTTAC TGCTCTCCTT

GTTGCTGTTA CTGCTTGACT TTAAGGAAGA AGACTGACTA ATAGTCCTCT

GGTGCTGTTA CTGCTTGACT TTAAGGAAGA AGACTGACTA ATAGTCCTCT

--TGCTGTTA CTGCTTGACT TTAAGGAAGA AGACTGACTA ATAGTCCTCT

--TGCTGTTA CTGCTTGACT TTAAGGAAGA AGACTGACTA ATAGTCCTCT

GTTGCTGTTA CTGCTTGACT TTAAGGAAGA AGACTGACTA ATAGTCCTCT

---------- ---------- ---------- ---------- ----------

GTTGCTGGTA CTGCTGGACT TTAAGGATGA TGACTGACTG ACAGTCCTCT

GCTGCTGGTC CCACTGGACT TTAAGGACGA CGACTGACTG ACAGTCCTCT

ATTGCTGTTA CTGCTTGACT TCAAGGAAGA TGACTGACTA ATGGTCCTCT

GTTGCTGTTA TTACTTGACT TTAAGGAAGA AGACTGACTA ACAGTCCTCT

GTTGCTGTTA GCACCGGACT TTAATGATGA TGACTGACTA ATAGTCCTCT

GTTGCTGTTA TTACTTGACT TTAAGGAAGA AGACTGACTA ATAGTCCTCT

GTTGCTGTTA TTACTTGACT TCAAAGAAGA AGACTGACTA ATAGTCCTCT

GTTGCTGTTA CTGCTTGACT TTAAGGAAGA AGACTGACTA ATAGTCCGCT

GTTGTTATTA TTGCTTGACT TTAGGGAGGA AGACTGACTA ATAGTCCTCT

GTTGCTATTA CTGCTAGACT TTAGGGAGGA AGACTGACTA ATAGTCCTCT

GTTGCTGCTA CTGGTAGATT TTAAAGACGA CGACTGGCTG ATTGTTCTCT

GATACTGTTG CTGCTAGATT TTAAGGAAGA CGATTGGCTG ACTGTCCTCT

TCCGAGAGCC ATGCTCTGTT TTTGGATCTT TTGAAGGAAC AGGCCCAGCG

TCCGAGAGCC ATGCTCTGTT TTTGGATCTT TTGAAGGAAC AGGCCCAGCG

TCCGAGAGCC ATGCTCTGTT TTTGGATCTT TTGAAGGAAC AGGCCCAGCA

TCCGAGAGCC ATGCTCTGTT TTTGGATCTT TTGAAGGAAC AGGCCCAGCA

TCCTAGAGCC ATGCTCTGTT TTTGGATCTT TTGAAGGAAC AGGCCCAGCG

---------- ---------- ---------- ---------- ----------

TCCGAGAGCC ATGCTCTGTT TTTGAATCTT TTGAAGGAAC AGGCCCAGCA

TCCGAGAACC ATGCTCTGTT TTTGAATCTT TTGAAAGAAT GGGCCCAGCA

TTCGAGAACT ATGCTCTGAT TTTGGATCTT TTGAAGGAAC AGGCCCAGCA

TCCGAGAGCC ATGCTCTGTT TTTGGATCTT TTGAAGGAAC AGGCCCAGCA

TCCGAGAGCC ATGCTCTGGT TTGGGGTCTT TTGAAGGAAC GGGCCCAGCA

TCCGAGAGCC ATGCTCTGTT TTTTGATCTT TTGAAGGAAC AGGCCCAGCA

TCCGAGAGCT ATGCTCAGTT TTTGGATCTT TTGAAGGAAC AGGAGCAGCA

TCCTAGAGCC ATGTTCTACT TTTGGATCTT TTGAAGGAAC AGGCCCAGCA

TCCGAGAGCT ATGCTCTGTT TTTGGATCTT TTGAAGGAAC AGGCGCAGCA

TCCGAGAGCC ATGCTCTGTT TTTGGATCTT TTGAAGGAAC AGGCCCGGCA

TCCTGGATCC ATGCTCAGTT TTTGAGTCTT TTATGGGAAT AGGTCCAGCA

TCCGGGACCC GTGCTCTGGT TTTGGATCTT TTGGGGGAAT AGGTGCAGCT

GGAGAAGGCA ACAAATCCTT TTCTTTTGCA GCACTCTGCT TAGATGACCT

GGAGAAGGCA ACAAATCCTT TTCTTTTGCA GCACTCTGCT TAGATGACCT

GGAGAAGGCA ACAAATCCTT TTCTTTTGCA GCACTCTGCT TAGATGACCT

GGAGAAGGCA ACAAATCCTT TTCTTTTGCA GCACTCTGCT TAGATGACCT

GGAGAAGGCA ACAAATCCTT TTCTTTTGCA GCACTCTGCT TAGATGACCT

---------- ---------- ---------- ---------- ----------

GGTGAAGGTA GCAAGTCCTT TTCTTTTGTA CTACTCTGCT TCGATGACCT

GGTGAAGGTA GCAAGTCCTT TTCTTTTGTA CTACTCTGCT TAGATGACCT

GGAGAAGGCA ACAAATCTTT TTCTTTTGCA GTACTCTGCT TAGATGACCT

GGAGAAGGCA ATAAATCTTT TTCTTTAGAA GCACTCTGTT TAGATGACCT

GGAGACGGCA ACAAATCCTT TTCTTTTGCA GCACTTTGCT TAGATGACCT

GGAGAAGGCA ACAAATCCTT TTCTTTTGCT GCACTCTGCT TAGACGACCT

GGAGAAGGCA ACAAATCCTT TTCTTTTGCA ACACTCTGCT TCGATGACCT

GGAGAAGGCA ACAAATCCTT TTCTTTTGCA GCACTTTGCT TAGAGGACCT

GGAGAAGGCA ACAAATCCTT TTCTTTTGCA GTGCTCTGCT TAGATGACCT

GGAGAAGGCA ACAAATCCTT TTCTTTTGCA GCACTCTGTT TGGATGACCT

GGAGAAGGTA ATGGATCCTT CTCTTTCACA GTACTCTGTT TGGATGACCT

GGGGAAGGCA AAAGATCCTT CTCTTTTATA GCACTTGGTT TGGATGACCT

GCCAAACCAA ACGAATGCCT TCATTTTCAC AAACCTTATG CTTCCTCTTG

GCCAAACCAA ACAAATGCCT TCATTTTCAC AAACCTTATG CTTCCTCTTG

GCCAAACCAA ACGAATGCCC TCATTTTCAC AAACCTTATG CTTCCTCTTG

GCCAAACCAA ACGAATGCCC TCATTTTCAC AAACCTTATG CTTCCTCTTG

GCCAAATAAA ACAAATGCCC TCATTTTCAC ACACCTTATG CTTTCTCTTG

---------- ---------- ---------- ---------- ----------

GTCAaaaaaa aaataTGCTC TCATTTTTAC AGACCTTGTG TTTCCTCTTG

GCCAAGAAAA AAAAATGCTC TTATTTTTAC AGACCTTGTG TTTCCTCTTG

GCCAAACCAA AAAA-TGTTC TTATTTTTAC ATACCTTATG TTTCCTCTTG

GCCAAACCAA ACAAATGCCC TCATTTTTAC AAACCTTATG TTTCCTCTTG

GCCAAACCAA ACAAATGCTC TCACTTTTAC AAACCTTATG TTTCCTCTTG

GCCAAACCAA ACAAATGCCC TCATTTTTAC AAACCTTATG TTTCCTCTTG

GTCAAACCCC AAAAAAGCCT TTATTT---- ---------- ----------

GCCAAATCAA ACAAATGCCC TCATTTTTAC AAACCTTATG TTTCCTCTTG

GCCAAATCAA ACAAATGCCC TCATTTTTAC AAACCTTATG TTTCCTCTTG

GTCAAACCAA ACACAT-CTC TTA-TT---- ---------- ----------

GCCAAATCAA ATAAATGCAA TTAGTTTTAC AAACCTTATG CTTCCTCTTT

GCCAAATCAA ATAAAGGTAC TGAGTTAGGC AAACCTTATG TTTCCTCTTG

CCTTTGTTGG AAACTTTTTC TGAGGCTTGT TTCTGAGCCT CTCTCGTGTG

CCTTTGTTGG AAACTTTTTC TGAGGCTTGT TTCTGAGCCT CTCTCGTGTG

CCTTTGTTGG AAACTTTTTC TGAGGCTTGT TTCTGAGCCT CTCTCGTGTG

CCTTTGTTGG AAACTTTTTC TGAGGCTTGT TTCTGAGCCT CTCTCGTGTG

CCTTTGTTGG AAACTTTTTC TGAGGCTTGT TTCTGAGCCT CTCTCGTGTG

---------- ---------- ---------- ---------- ----------

CCTTTGTTGG AAGCCTTTTC TGAGGCTTGC TTCTGCACCT CTCTTGAGTG

CCTTTGTTGG AAGCCTTTTC TGAGGCTTGC TTCTGCACCT CTCTCGAGTG

CCTTTGTTGG ACACTTTCTC TGAGGCTTGT TTCTGGGCCT CTCTTGTGTG

CCTTTGTTGG AAACTTTCTC TGAGGCTTGT TTCTGAGCCT CTCTTGTGTG

CCTTTGTTGG AAACTTTTTC TGAGGCTTGT TTCTGAGCCT CTCTTGTGTG

CCTTTGTTGG AAACTTTTTC TGAGGCTTGT TTCTGAGCCT CTCTTGTGTG

---------- ---------- ---------- ---------- ----------

CCTTTGTTGG AAACTTTTTC TGAGGCTTGT TTCTGAGCCT CTCTCGTGTG

CCTTTGTTGG AAACCTTTTC TGAGGCTTGT TTCTGAGCCT CTCTCGTGTG

---------- ---------- ---------- ---------- ----------

CCCTTGGTGG AACCTTTATC TGAGACTTGT TTCTGAGTTT CTCTTGTGTG

CCCTTGTTAG AGACTTTGTC TGAGGCTTGT TTCTGTGGGT CTCTCGGGTG

CTTTTCTGGC ACATTTTTCT TTTCCCCCTT GGGCGGCTCT GTTTCTTTGT

CTTTTCTGGC ACATTTTTCT TTTCCCCCTT GGGCGGCTCT GTTTCTTTGT

CTTTTCTGGC ACATTTTTCT TTTCCCCCTT GGGCGGCTCT GTTTCTTTGT

CTTTTCTGGC ACATTTTTCT TTTCCCCCTT GGGCGGCTCT GTTTCTTTGT

CTTTTCTGGC ACATTTTTCT TTTCCCCCTT GGGTGGCTCT GTTTCTTTGT

---------- ---------- ---------- ---------- ----------

TTTTTCTGGA ACATTTTTCT TTTCCCCCTT GGGTGGCTCT GTTTCTTTGT

TTTCTCTGGA ACATTTTTCT TTTCCCCCTT GGGTGGCTCT GTTTCTTTGT

CTTTTCTGGC ACATTTTTCT TTTCCCCCTT AGGTGGCTCT GTCTCTTTGT

CTTTTCTGGC ACATTTTTCT TTTCCCCCTT GGGTGGCTCT GTTTCTTTGT

CTTTTCTGGC ACATTTTTCT TTTCCCCCTT GGGTGGCTCT GTTTCTTTGT

CTTTTCTGGC ACATTTTTCT TTTCCCCCTT GGGTGGCTCT GTTTCTTTGT

---------- ---------- ---------- ---------- ----------

TTTTTCTGGA ATATTTTTCT TTTCTCCCTT GGGTGGTTCT GTTTCTTTGT

CTTTTCTGGC ACATTTTTCT TTTCCCCCTT GGGCGGCTCT GTTTCTTTGT

---------- ---------- ---------- ---------- ----------

CTTCTCAGGC ACATTTTTCT TTTCTATCTT GGGTGGCTCC ATTTCTTTGT

CTTTTCTGGC ACAGTTTTCT TTTCCACCTT AGGTGGCTCA GTTTCTTTGT

AAGGCTTTCC TGGTATTCTA GTCAAAAGAT TCAGGTCAAT CTTCACAATA

AAGGCTTTCC TGGTATTCTA GTCAAAAGAT TCAGGTCAAT CTTCACAATA

AAGGCTTTCC TGGTATTCTA GTCAAAAGAT TCAGGTCAAT CTTCACAATA

AAGGCTTTCC TGGTATTCTA GTCAAAAGAT TCAGGTCAAT CTTCACAATA

AAGGCTTTCC TGGTATTCTA GTCAAAAGAT TCAGGTCAAT CTTCACAATA

---------- ---------- ---------- ---------- ----------

AAGGCTTCCC GGGTATTCTA GTCAAAAGAT TCAGGTCTAT CTTCACAATG

AAGGCTTCCC GGGTATTCGA GTCAGAAGAT TGAGGTCAAT CTTCACAATG

AAGGCTTTCC TGGTATTCTA GTCAAAAGAT TCAGGTCAAT CTTCACAATA

AAGGCTTTCC TGGTATTCTA GTCAAGAGAT TCAGGTCAAT CTTCACAATA

AAGGCTTTCC TGGTATTCTG GTCAAAAGAT TCAGGTCAAT CTTCACAATA

AAGGCTTTCC TGGTATTCTA GTCAAGAGAT TCAGGTCAAT CTTCACAATA

---------- ---------- ---------- ---------- ----------

AAGGCTTTCC TGGTATTCTA GTTAAGAGAT TCAGGTCAAT CTTCACAATA

AAGGCTTTCC TGGTATTCTA GTCAAGAGAT TCAGGTCAAT CTTCACAATA

---------- ---------- ---------- ---------- ----------

AAGGCTTCCC TGGTATTCTT GACAAGAGAC TCAGGTCAAT CTTCACAATA

AAGGCTTCCC TGGTATTCTG GACAAGAGAT TCAGGTCAAT CTTCACAATG

AGTGGGTACC TGTCATCAGG CTCACTGAGG GGTGAAAGAA GTTCCTTCTC

AGTGGGTACC TGTCATCAGG CTCACTGAGG GGTGAAAGAA GTTCCTTCTC

AGTGGATACC TGTCATCAGG CTCACTGAGG GGTGAAAGAA GTTCCTTCTC

AGTGGATACC TGTCATCAGG CTCACTGAGG GGTGAAAGAA GTTCCTTCTC

AGTGGATACC TGTCATCAGG CTCACTGAGG GGTGAAAGAA GTTCCTTCTC

---------- ---------- ---------- ---------- ----------

AGTGGGTACC TGTCATCAGG GTCACTGAGT GGGGAAAGGA GCTCCTTCTC

AGTGGATACC TGTCATCAGG CTCACTGAGT GGGGAAAGGA GCTCCTTCTC

AGTGGATACC TGTCATCAGG CTCACTAAGG GGTGAAAGAA GTTCCTTCTC

AGTGGATACC TGTCATCAGG CTCACTGAGG GGTGAAAGAA GTTCCTTCTC

AGTGGATACC TGTCATCAGG CTCACTGAGG GGTGAAAGAA GTTCCTTCTC

AGTGGATACC TGTCATCAGG CTCACTAAGG GGTGAAAGAA GTTCCTTCTC

---------- ---------- ---------- ---------- ----------

AGTGGATACC TGTCATCAGG CTCACTGAGG GGTGAAAGAA GTTCCTTCTC

AGCGGATACC TGTCATCAGG CTCACTGAGG GGTGAAAGAA GTTCCTTCTC

---------- ---------- ---------- ---------- ----------

AGTGGATACC TGTCATCAGG ATCACTAAGT GGAGAAAGGA GTTCCTTCTC

AGAGGATACC TGTCGTCAGG CTCACTGAGT GGTGAAAGGA GTTCCTTCTC

TTCCATAGGA GAGAACATTC GTTGCCGAAA AAAGCTATCT TCTTCCTCCA

TTCCATAGGA GAGAACATTC GTTGCCGAAA AAAGCTATCT TCTTCCTCCA

TTCCATAGGA GAGAACATTC GTTGCCGAAA AAAGCTATCT TCTTCCTCCA

TTCCATAGGA GAGAACATTC GTTGCCGAAA AAAGCTATCT TCTTCCTCCA

TTCCATAGGA GAGAACATTC GTTGCCGAAA AAAGCTATCT TCTTCCTCCA

---------- ---------- ---------- ---------- ----------

TTCCATAGGA GAGAACATCC GCTGCCGGAA AAAGCTATCG TCTTCCTCCG

TTCCATAGGA GAGAACATTC GTTGCCGAAA AAAGCTATCT TCTTCCTCCA

TTCCATAGGA GAGAACATTC GCTGCCGAAA AAAGCTATCT TCTTCCTCCA

TTCCATAGGA GAGAACATTC GTTGCCGAAA AAAGCTATCT TCTTCCTCCA

TTCCATAGGA GAGAACATTC GTTGCCGAAA AAAGCTATCT TCTTCCTCCA

TTCCATAGGA GAGAACATAC GTTGCCGAAA AAAGCTATCT TCTTCCTCCA

---------- ---------- ---------- ---------- ----------

TTCCATAGGA GAGAACATTC GTTGCCGAAA AAAGCTGTCT TCTTCCTCCA

TTCCATAGGA GAGAACATTC GTTGCCGGAA AAAGCTGTCT TCTTCCTCTA

---------- ---------- ---------- ---------- ----------

TTCCATAGGA GAGAACATTC TTTGCCGAAA AAAGCTATCT TCCTCCTCTA

TTCCATAGGA GAGAACATTC TCTGCCGAAA AAAGCTGTCT TCCTCCTCCA

CTGAGGAGGG TTTAACAGGA GTCCTATTGC TCTCGGGGTA CTTAGGAGTT

CTGAGGAGGG TTTAACAGGA GTCCTATTGC TCTCGGGGTA CTTAGGAGTT

CTGAGGAGGG CTTAACAGGA GTCCTATTGC TCTCGGGGTA CTTAGGAGTT

CTGAGGAGGG CTTAACAGGA GTCCTATTGC TCTCGGGGTA CTTAGGAGTT

TTGAGGAGGG TTTAACAGGA GTCCTATTAC TCTCGGGGTA CTTAGGAGTT

---------- ---------- ---------- ---------- ----------

CCGAGGAGGG TTTGACAGGA GTCCTATTGC TCTCAGGGTA CTTAGGAGTC

CTGAGGAGGG TTTGACAGGA GTCCTGTTGC TTTCAGGGTA CTTAGGAGTC

CTGAGGAGGG TTTAACAGGA GTCCTATTGC TGTCAGGGTA CTTAGGAGTT

CTGAGGAGGG TTTAACGGGA GTCCTATTAC TCTCAGGGTA CTTAGGAGTT

CTGAGGAGGG TTTCACGGGA GTCCTGTTAC TCTCAGGGTA CTTAGGAGTT

CTGAGGAGGG CTTAACGGGA GTCCTATTAC TCTCAGGGTA CTTAGGAGTT

---------- ---------- ---------- ---------- ----------

CTGAGGAGGG TTTAACAGGA GTCCTATTGC TCTCAGGGTA CTTAGGTGTT

CTGAGGAAGG CTTAACAGGA GTCCTACTGC TCTCAGGATA CTTAGGAGTT

---------- ---------- ---------- ---------- ----------

CTGAGGAGGG TTTAACAGGA GTTCTGTTGC TCTCAGGATA TTTAGGAGTT

TGGAGGAGGG TTTAACAGGA GTCCTATTGC TCTCGGGATA TTTAGGAGTT

TGTGAGGAAG GAGGAAGGCT CTCACTTTCA TCTGAATCTG AGGATGAGGT

TGTGAGGAAG GAGGAAGGCT CTCACTTTCA TCTGAATCTG AGGATGAGGT

TGTGAGGAAG GAGGAAGGCT CTCGCTTTCA TCTGAATCTG AGGATGAGGT

TGTGAGGAAG GAGGAAGGCT CTCGCTTTCA TCTGAATCTG AGGATGAGGT

TGTGAGGAAG GAGGAAGGCT CTCACTTTCA TCTGAATCTG AGGATGAGGT

---------- ---------- ---------- ---------- ----------

TGGGAGGAGG GAGGGAGGCT CTCCCTCTCA TCTGAATCTG AGGAGGAGCT

TGTGAGGAGG GAGGCAGGCT CTCGCTGCCG TCCGAGTCTG AGGACGAGGT

TGAGAGGAAG GAGGAAGGCT CTCACTTTCA TCTGAATCTG AGGATGAGGT

TGTGAGGAAG GAGGAAGGCT CTCGCTTTCA TCCGAATCTG AGGATGAGGT

TGTGAGGAAG GAGGAAGGCT CTCGCTTTCA TCCGAATCTG AGGATGAGGT

TGTGAGGAAG GAGGAAGGCT CTCGCTTTCA TCCGAATCTG AGGATGAGGT

---------- ---------- ---------- ---------- ----------

TGTGAGGAAG GAGGAAGGCT CTCACTTTCA TCTGAATCTG AGGATGAGGT

TGTGAGGAGG GAGGAAGGCT CTCACTTTCA TCCGAATCTG AGGATGAGGT

---------- ---------- ---------- ---------- ----------

TGTGAAGAAG GAAAAAGGCC CTCACTTTCA TCAGAATCAG AGGATGAAGT

TGTGATGAAG GCGGGAGGCT CTCGCTTTCA TCAGAATCAG AGGATGAGGT

ATCTGTTTCT ATGATTTCCC TTGATTTCTG GGAAGATTTA CTTGTTGACT

ATCTGTTTCT ATGATTTCCC TTGATTTCTG GGAAGATTTA CTTGTTGACT

ATCTGTTTCT ATGATTTCCC TTGATTTCTG GGAAGATTTA CTTGTTGATT

ATCTGTTTCT ATGATTTCCC TTGATTTCTG GGAAGATTTA CTTGTTGATT

ATCTGTTTCT ATGATTTCCC TTGATTTCTG GGAAGATTTA CTTGTCGACT

---------- ---------- ---------- ---------- ----------

ATCTGTTTCT ATGATTTCCC GAGATTTCTG GGAGGATTTA CTTGCTGACT

GTCAGTCTCT ATGATCTCCC GAGACTTCTG GGACGGTTTA CTGGCTGACT

ATCTGTTTCT ATGATTTCCC TTGATTTCTG GGAAGACTTA CTCGTTGACT

ATCTGTTTCT ATAATTTCCC TTGATTTCTG GGAAGATTTA CTTGTTGACT

ATCTGTTTCT ATGATTTCCC TTGATTTCTG GGAAGATTTA CTCGTTGACT

ATCTGTTTCT ATGATTTCCC TTGATTTCTG GGAAGATTTA CTCGTTGACT

---------- ---------- ---------- ---------- ----------

ATCTGTTTCT ATGATTTCCC TTGATTTCTG GGAAGATTTG CTCGTTGACT

ATCTGTTTCA ATGATTTCCC TTGATTTCTG ACAAGATTTA CTCGTTGACT

---------- ---------- TCGATTTCTG GGAAGATTTG CTTGTTGACT

ATCTGTTTCT ATGATTTCCC TAGATTTTTG GGAAGATTTA CTTGTTGACT

ATCTGTTTCT ATGATTTCCC GAGATTTCTG GGAAGATTTG CTTGTTGACT

TATATTTCTT TTTCTCTGCT GTAGGTCGAG GGGAAGACTT AGACTCCTTC

TATATTTCTT TTTCTCTGCT GTAGGTCAAG GGAAAGACTT ACACTCCCTT

TATATTTCTT TTTCTCTGCT GTAGGTCGAG GGGAAGACTT AGACTCCTTC

TATATTTCTT TTTCTCTGCT GTAGGTCGAG GGGAAGACTT AGACTCCTTC

TATATTTCTT TTTCTCTGCT GTAGGTCGAG GGGAAGACTT AGACTCCTTC

---------- ---------- ---------- ---------- ----------

TGTATTTCTT TTTCTCTGCT GTAGGTCGAG GGGAAGATTT CGACTCCTTT

TGTACTTCTT CTTCTCTGCC GTAGGTCGAG GAGAAGATTT TGATTCCTTT

TGTATTTCTT TTTCTCTGCT GGAGGCCGAG GGGAAGATTT AGACTCCTTC

TGTATTTCTT TTTCTCTGCT GAAGGTCGAG GGGAAGATTT GGACTCCTTT

TGTATTTCTT CTTCTCTGCA GATGGTCGAG GGGAAGATTT GGACTCCTTT

TGTATTTCTT TTTCTCTGCT GAAGGTCGAG GGGAAGATTT GGACTCTTTT

---------- ---------- ---------- ---------- ----------

TGTATTTCTT TTTCTCTGCT GTAGGTCGAG GGGAAGATTT GGACTCCTTC

TGTATTTCTT TTTCTCTGCT GTAGGTCGAG GGGAAGATTT GGACTCCTTC

TGTATTTCTT CTTCTCTGCT GTCGGTCGAG GGGAAGATTT GGACTC-TTC

TATACTTCTT TTTTTCTGTT GTTGATCTTG GGGAAGATTT GGGTTCCTTT

TGTATTTCTT TTTCTCCGTC GTGGGCCTAG GGGATGACTT GGGTTCCTTC

TTTATATTGG GTTTCCTTGA GCCTTTGGTG GCTGCTTTGT GTCTGCTGGA

CTTTTATTGG GTTTCCTTGA CCCCTTGGTG GCTGCCTTGT GTCTGCTGGA

TTTATATTGG GTTTCCTTGA GCCTTTGGTG GCTGCTTTGT GTCTGCTGGA

TTTATATTGG GTTTCCTTGA GCCTTTGGTG GCTGCTTTGT GTCTGCTGGA

TTTATATTGG GTTTCCTTGA GCCTTTGGTG GCTGCTTTGT GTCTGCTGGA

---------- ---------- ---------- ---------- ----------

TTGATGTTAG GTTTCCTTGA GCCCTTGGTG GCTGCCTTAT GCCTGCTGGA

TTGATGTTGG GTTTCCTCGA GCCCTTGGTG GCTGCTTTGT GCCTGCTGGA

TTTATATTGG GTTTCCTTGA GCCTTTGGTG GCTGCTTTGT GTCTGCTGGA

TTTATATTGG GTTTTCTTGA GCCTTTGGTG GCCGCTTTGT GTCTGCTGGA

TTGATATTGG GTTTCCTTGA GCCTTTGGTG GCTGCTTTGT GTCTGCTGGA

TTTATGTTGG GTTTCCTTGA GCCTTTGGTG GCTGCTTTGT GTCTGCTGGA

---------- ---------- ---------- ---------- ----------

TTTATATTGG GTTTCCTTGA GCCTTTGGTG GCTGCTTTGT GTCTGCTGGA

TTTATATTGG GTTTCCTAGA GCCTTTGGTG GCTGCCTTGT GTCTGCTGGA

TTTATGTTGG GCTTCCTCGA GCCTTTGGTG GCTGCCTTGT GTCTGCTGGA

TTTATATTGG GTTTTCTTGA GCCTTTTGTG GCTGCCTTGT GCCTATTGGA

TTTATGTTGG GCTTCCTGGA GCCTTTTGTG GCTGCCTTGT GCCTGTTTGA

GGGCATGCTG CTAGCCAAGT CGTTTCACTT TCTATCTTCA GGCCTCCACG

GGGCATGCTG CTAGCCAAGT CGTTTCACTT TCTTTCTTCA GGCCCCCCAA

GGGCATGCTG CTAGCCAAGT CGTTTCACTT TCTATCTTCA GGCCTCCACG

GGGCATGCTG CTAGCCAAGT CGTTTCACTT TCTATCTTCA GGCCTCCACG

GGGCATGCTG CTAGCCAAGT CGTTTCACTT TCTATCTTCA GGCCTCCACG

---------- ---------- ---------- ---------- ----------

GGGCATGCTG GTAGCCATGT CGTCTCACTT TCTATCTTCA GGCCTCCTCG

GGGCATGCTG GCAGCCATGT CGTCTCACTT TCTATCTTCA GGCCTCCACG

GGGCATGCTG GCAGCCATGT CGTCTCACTT TCTATCTTCA GGCCTCCACG

GGGCATGCTG GTAGCCATGT CGTTTCACTC TCTATCTTCA GGCCTCCACG

AGGCATGCTG GTAGCCATGT CGTTTCGCTT TCTATCTTCA GGCCTCCCCG

AGGCATGCTG GTAGCCATGT CGTTTCACTT TCTATCTTCA AGCCTCCGCG

---------- ---------- ---------- ---------- ----------

GGGCATGTTG GTAGCCATGT CGTTTCACTT TCTATCTTCA GGCCTCCACG

GGGCATGCTG GTGGCCATGT CGTTTCACTT TCTATCTTCA GGCCTCCACG

GCGCATACTG GTGGCCATGT CGTTTCACTT TCGATCTTCA GGCCTCCTCG

GGGCATACTT GTTGTCATGT CGTTTCACTT TCTATTTTTA GGCCACCTCG

GGGCATGCTT GTGGCCATAT CGTTTCACTT TCTATTTTTA GTCCTCCCCT

AGGCTCTTCA GCAGCTGCCT TCTCAGCCTT TTTGGGTTGT TTTTTGCCTA

AGGCTCTTTA ACAACTGCCT TCTCCCCCTT TTTGGGTTTG TTTTTGCCCT

AGGCTCTTCA GCAGCTGTCT TCTCAGCCTT TTTGGGTTGT TTTTTGCCTA

AGGCTCTTCA GCAGCTGTCT TCTCAGCCTT TTTGGGTTGT TTTTTGCCTA

AGGCTCTTCA GCAGCTGCCT TCTCAGCCTT TTTGGGTTGT TTTTTGCCTA

---------- ---------- ---------- ---------- ----------

GGGCTCTTCA GCAGCTGACT TCTCAGTTTT TTTGGGTTGT TTTTTGCCAA

AGGCTCTTCA GCAGCTGACT TCTCAGGTTT TTTGGGTTGT TTTTTGCCGA

GGGTTCTTCA ACAGCTGCCT TCTCGGCCTT TTTGGGTTGT TTTTTGCCTA

GGGCTCTTCA GCAGCTGCCT TCTCAGTCTT TTTGGGTTGT TTTTTCCCTA

GGGCTCTTCA GTGGCTGCCT TCTCAGCCTT TTTGGGTTGT TTTTTGCCTA

GGGCTCTTCA GCGGCTGCCT TCTCAGCCTT TTTGGGTTGT TTTTTGCCTA

---------- ---------- ---------- ---------- ----------

AGGCTCTTCA GCAACTGCTT TCTCAGCTTT TTTGGGTTGT TTTTTGCCTA

GGGCTCTTCG GCAGCTGCTT TCTCAGCCTT TTTGGGTTGT TTTTTGCCTA

GGGCTCTTCT GCAGCTGCCT TCTCCGCCTT TTTGAGTTGT TTTTTGCCTA

AGGCTCCTCT ATACCTGCCT TCTCCGTTTT TTTGGGTTGT TTTTTTCCTA

AGGTTCCTCA ACAGCTGCTT TCTCTGCCTT TTTGGGCTGC TTCTTCCCCA

CAGTTCTTCT CTGTGTTGTG CTGTCACTCT GTGCAGGAGA TTTCTGCCTC

TAATTTTTTC C--------- ---------- ---------- ----------

CAGTTCTTCT CTGTGTTGTG CTGTCACTCT GTGCAGGAGA TTTCTGCCTC

CAGTTCTTCT CTGTGTTGTG CTGTCACTCT GTGCAGGAGA TTTCTGCCTC

CAGTTCTTCT CTGTGTTGTG CTGTCACTCT GAGCAGGAGA TTTCTGTCTC

---------- ---------- ---------- ---------- ----------

CAGTTCTCCT CTGTGTGGTA CTGTCACTCT GTGCAGGAGA CTTCTGCCTC

CAGTTCTCCT CTGAGTGGTA CTGTCGCTCT GTGCAGGAGA CTTCTGCCTC

CAGTTCTCCT CTGTGTTGTG CTGTCACTCT GTGCAGGAGA CTTCTGCCTT

CAGTTCTCCT CTGTGCTGTG CTGTCACTCT GGGCAGGAGA CTTTTGCCTC

CAGTTCTCCT CTGTGTTGTG CTGTCACTCT GTGCAGGGGA CTTCTGCCTC

CAGTTCTCCT CTGTGTTGTG CTGTCACTCT GTGCAGGAGA CTTCTGCCTC

---------- ---------- ---------- ---------- ----------

CAGTTCTCCT CTGTGTTGCA CTATCACTCT GTGCAGGAGA CTTCTGCCTC

CAGTTCTCCT CTGTGTTGTA CTGTCACTCT GTGCAGGAGA CTTCTGTCTC

CAGTTCGCCT CTGGGTTGTG CTGTCACTCT GGGCAGGAGA CTTCTGTCTC

CAGTTCTTCT CTGTGTAGTG TTATCACTCT GTGCTGGGGA CTTTTGTCTC

CAGTTCTTCT TTGGGTCGTG TTATCACTCT GGGCGGGAGA CTTTTGTCTG

CCACGCCCAC TTTCTGATCC CTTTTGGTTT TGGAGTCTCG TCCCGGAGTA

---------- ---------- ---------- ---------- ----------

CCACGCCCAC TTTCTGATCC CTTTTGGTTT TGGAGTCTCG TCCAGGAGTA

CCACGCCCAC TTTCTGATCC CTTTTGGTTT TGGAGTCTCG TCCAGGAGTA

CCACGCCCAC TTTCTGATCC CTTTTGGTTT TGGAGTCTCG TCCAGGAGTA

---------- ---------- ---------- ---------- ----------

CCACGGCCAC TTTCTGATCC TTTTTGGTTT TAGAGTCTCG TCCCGGAGTG

CCACGGCCAC TTTCTGATCC CTTTTGGTTT TAGAGTCTCG TCCTGGGGTG

CCGCGCCCAC TTTCTGATCC CTTTTGGTTT TAGAGTCTCG CCCAGGAGTA

CCACGCCCAC TTTCTGATCC CTTTTGGTTT TGGAGTCTCG TCCAGGAGTA

CCGCGCCCAC TTTCTGATCC CTTTTGGTTT TGGAGTCTCG TCCAGGAGTA

CCACGCCCAC TTTCTGATCC CTTTTGGTTT TGGAATCTCG TCCAGGAGTA

---------- ---------- ---------- ---------- ----------

CCTCGCCCAC TTTCTGATCC CTTTTGGTTT TGGAGTCTCG TCCAGGAGTA

CCTCGTCCAC CTTCTGATCC CTTTTGGGTT TGGAGTCTCG CCCAGGAGTA

CCTCGTCCAC TTTCTGATCC CTTTTGGTTT TGGAGTCTCG TCCTGGAGTG

CCACGACCAC TCTCTGATCC TTTTTGGTTT TGGAATCTCG TCCAGGAGTA

CCGCGACCAC CCTCGGATCC TTTTTGGTTT TGGAATCTCG GCCGGGAGTG

GAACTCGTTT CTTTAGGTCC ACTTGTATCA GTGTAGCTAT TCCCAGTGCC

---------- ---------- ---------- ---------- ----------

GAACTCGTTT CTTTAGGTCC ACTTGTATCA GTATAGCTAT TCCCAGTGCC

GAACTCGTTT CTTTAGGTCC ACTTGTATCA GTGTAGCTAT TCCCAGTGCC

GAACTCGTTT CTTTGGGTCC AGTCGTATCA GTGTAGCTAT TCCCAGTGCC

---------- ---------- ---------- ---------- ----------

GAGCTCGCTT CTTTGGCCCC ACCTGGATCT GTGTAGTTAC TGGCAGTGCC

GAGCTCGTTT CTTTTGTCCC CCCTGGATCT GTGTAGTTAC TGGCAGTGCC

GAACTTGTTT CTTTAGGTGC ACTCGTCTCC GTGTAGCTAT TCCCAGTGCT

GAACTTGTTT CTTTAGGACC ACCTGGATCT GTGTAGCTAT TCCCTGTGCC

GAACTTGTTT CTTTAGGTCC GCCTGGATCA GTATAGCTAT TCCCAGTGCC

GAACTAGTTT CTTTAGCTCC ACTTGGATCA GTGTAGCTAT TCCCAGTGCC

---------- ---------- ---------- ---------- ----------

GAACTTGTTT CTTTAGGTCC ACCTGGATCA GTGTAGCTAT TCCCCGTGCC

GAACTCGTTT CTTTAGGTCC ACCTGGATCA GTGTAGCTAT TCCCAGTGCC

GAACTCGTTT CTTTCGGTCC CCCTGTGTCA GGGTAGCTGT TCCCAGCACT

GAATTGGACT CCTTAGGTCC ATTTTGCTCA ATGTAGCCAC TTCCTGGTCC

GAGTTGGAAT CCTTAGGCCC TGTCTGATCG ACGTAGCCAC TCCCAGGGCA

CTGCTCTCGG CCTTCCTTTT TGTAGCCTTG AGATGATGGG ATGTTACTGT

---------- ---------- ---------- ---------- ----------

CTGCTCTCGG CCTTCCTTTT TGTAGCCTTG AGATGATGGG ATGTTACTGT

CTGCTCTCGG CCTTCCTTTT TGTAGCCTTG AGATGATGGG ATGTTACTGT

CTGCTCTCTG CCTTCCTTTT TGTAACCTTG AGATGATGGG ATATTACTGT

---------- ---------- ---------- ---------- ----------

CTGCTCTCGG CCTTCTTTCT TGTAGGCTTG AGACGATGGG ATGTTGCTAT

CTGCTCCCGG CCTTCTTTCT TGTAGGCTTG AGAAGATGGG ATGTTGCTGT

CTGTTCTCGG CCCTCCTTTT TGTAGCCTTG AGATGATGGG ATGTTACTAT

CTGTTCTCGG CCTTCCTTTT TATAGCCTTG AGATGATGGG ATATTACTGT

CTGTTCTCGG CCTTCCTTTT TATAGCCTTG AGATGACGGG ATGTTACTGT

CTGCTCTCGG CCTTCCTTTT TATAGCCTTG AGATGATGGG ATGTTACTGT

---------- ---------- ---------- ---------- ----------

CTGCTCTCGG CCTTCCTTTT TATATCCTTG AGATGATGGG ATGTTACTGT

CTGTTCTCGG CCTTCCTTTT TATAGCCTTG AGATGATGGG ATGTTACTGT

CTGTTCCCGG CCTTCCTTTT TATAGCCTTG AGATGACGGG ATGTTGCTAT

TTGCTCCCGA CCTTCCTTTT TATAACCTTG AGAAGATGGG ATATTGCTAT

TTGCTCCCGA CCCTCCTTCT TATAACCTTG TGAGGAGGGG ATGTTGCTGT

CCACTGAAGA GGCGGGTGAC ACTTTATGTG GGTTCACTTT ATTCAGCCAA

---------- ---------- ---------- ---------- ----------

CCACTGAAGA AGCGGGTGAC ACTTTATGTG GGTTCACTTT ATTCAGCCAA

CCACTGAAGA AGCGGGTGAC ACTTTATGTG GGTTCACTTT ATTCAGCCAA

CCACTGAAGA AGCGGGTGAC ACTTTATGTG GGTTCACCTT ATTCAACCAA

---------- ---------- ---------- ---------- ----------

CTACAGAGGA AGCTGGGGAC ACTTTATGTG GGTTCACTTT GTTCAACCAA

CTACAGAGGA AGCTGGGGAT ACTTTATGTG GGTTCACTTT ATTCAACCAA

CCACAGAAGA AGCTGGAGAC ACCTTATGCG GGTTCACTTT ATTCAACCAA

CCACAGAAGA AGCTGGTGAT ACTTTATGTG GATTTACTTT ATTCAGCCAG

CCACAGAAGA AGCTGGTGAC ACTTTATGTG GGTTCACTTT ATTTAACCAA

CCACAGAAGA AGCTGGTGAC ACTTTATGTG GGTTCACTTT ATTCAACCAA

---------- ---------- ---------- ---------- ----------

CCACGGAAGA AGCTGGTGAT ACTTTATGTG GGTTCACTTT ATTCAACCAA

CCACAGATGA AGCTGGTGAT ACTTTGTGTG GGTTCACTTT ATTCAACCAA

CCACAGAAGA CGCTGGAGAC ACTTTGTGGG GGTTCACTTT ATTCAACCAG

CTACAGAAGA AGCTGGTGAC ACTTTATGTG GATTCACTTT ATTCAGCCAA

CCACAGAGGA AGCAGGTGAC ACTTTGTGTG GATTCACCTT ATTCAACCAA

TTATCAAGTT GCCATTTGTT TGTTGGCGGG GGTTCAGGCT GAAAACACCT

---------- ---------- ---------- ---------- ----ACACCT

TTATCAAGTT GCCATTTGTT TGTTGGCGGG GGTTCAGGCT GAAAACACCT

TTATCAAGTT GCCATTTGTT TGTTGGCGGG GGTTCAGGCT GAAAACACCT

TTATCAAGTT GCCATTTGTT TGTTGGCGGG GGTTCAGGCT GAAAACACCT

---------- ---------- ---------- ---------- ----------

TTATCAAGTT GCCACTTGTT GGTTGGTGGT GGTTCAGGCT GAAAACACCT

TTATCAAGTT GCCACTTGTT TGTTGGTGGT GGTTCAGGCT GAAAACACCT

TTATCTAGTT GCCATTTGTT TGTTGGTGGT GGTTCAGGCT GAAAACACCT

TTATCAAGTT GCCATTTATT TGTGGGAGGT GGTTCAGGCT GAAAACACCT

TTATCAAGTT GCCATTTGTT TGTTGGTGGT GGTTCAGGCT GAAAACACCT

TTATCAAGTT GCCATTTGTT TGTAGGTGGT GGTTCGGGCT GAAAACACCT

---------- ---------- ---------- ---------- ----------

TTATCAAGTT GCCATTTGTT TGTTGGTGGA GGTTCAGGCT GAAAATACCT

TTATCCAGTT GCCATTTGTT TGTTGGTGGT GGTTCAGGCT GAAAACACCT

TTATCAAGCT GCCATTTGTT TGTTGGTGGT GGTTCAGGCT GAAAACACCT

TTGTCAAGTT GCCATTTATT TGTTGGTGGT GGTTCAGGCT AAAATTACCT

TTGTCCAGTT GCCATTTGTT GGTTGGGGGT GGCTCGGGCT GAAAGTACCT

CGGGAGATGC ACTCTGGGAT GGCTCATTTG CCTCACTGTC ACTGGAACTA

CGGGAGATGC ACTCTGGGAT GGCTCATTTG CCTCACTGTC ACTGGAACTA

CAGGAGATGC ACTCTGGGAT GGCTCATTTG CCTCACTGTC ACTGGAACTA

CAGGAGATGC ACTCTGGGAT GGCTCATTTG CCTCACTGTC ACTGGAACTA

CAGGAGATGC ACTCTGGGAT GGCTCATTTG CCTCACTGTC ACTGGAACTA

---------- ---------- ---------- ---------- ----------

CAGGGGATGC GCTCTGGGAT GGCTCATTTG CCTCACTGTC ACTGGAGCTG

CAGGAGATGC GCTCTGCGAC GGCTCATTAG CCTCACTGTC ACTGGAGCTG

CAGGAGATGC ACTCTGGGAT GGCTCATTTG CCTCACTATC ACTGGAACTA

CAGGAGATGC ACTCTGGGAT GGCTCATTTG CCTCACTGTC ACTGGAACTA

CAGGAGATGC ACTCTGGGAT GGTTCATTTG CCTCACTGTC ACTGGAGCTA

CAGGAGATGC ACTCTGGGAT GGCTCGTTTG CCTCACTGTC ACTGGAACTA

---------- ---------- ---------- ---------- ----------

CAGGAGATGC ACTCTGGGAT GGCTCATTTG CCTCACTGTC ACTGGAACTA

CAGGAGATGC ACTCTGGGAT GGCTCATTTG CCTCACTGTC ACTGGAACTA

CAGGAGATGC ACTCTGGGAT GGCTCATTGG CCTCACTGTC ACTGGAACTA

CAGGAGATGC ACTCTGTGAT GGTTCATTGG CCTCACTGTC ACTGGAACTA

CGGGAGACGC ACTCTGGGAT GGCTCGTTAG CCTCGCTGTC ACTGGAACTA

CTTTCACTCT CTGAGTCAGA TCCAGAGCTG CTTTCAGATC CACTGTGGCT

CTTTCACTCT CTGAGTCAGA TCCAGAGCTG CTTTCAGATC CACTGTGGCT

CTTTCACTCT CTGAGTCAGA TCCAGAGCTG CTTTCAGATC CACTGTGGCT

CTTTCACTCT CTGAGTCAGA TCCAGAGCTG CTTTCAGATC CACTGTGGCT

CTTTCACTCT CTGAGTCAGA TCCAGAGCTG CTTTCAGATC CACTGTGGCT

---------- ---------- ---------- ---------- ----------

CTTTCACTCT CGGAGTCGGA CCCAGAGCTG CTTTCAGACC CACTGTGGCT

CTCTCACTCT CCGAGTCAGA CCCAGAGCTG CTCTCAGATC CACTGTGGCT

CTTTCACTCT CGGAGTCAGA TCCAGAGCTG CTTTCAGACC CACTGTGGCT

CTTTCACTCT CTGAGTCAGA TCCAGAGCTG CTTTCAGATC CACTGTGGCT

CTTTCACTCT CTGAGTCAGA TCCAGAGCTG CTTTCAGATC CACTGTGGCT

CTTTCACTCT CTGAGTCAGA TCCAGAGCTG CTTTCAGACC CACTGTGGCT

---------- ---------- ---------- ---------- ----------

CTTTCACTCT CTGAGTCAGA TCCAGAGCTG CTTTCAGATC CACTATGGCT

CTTTCACTCT CGGAGTCAGA TCCAGAGCTG CTTTCAGATC CACTGTGGCT

CTTTCGCTCT CTGAGTCAGA TCCAGAGCTG CTTTCAGATC CACTATGGCT

CTTTCACTCT CCGAGTCAGA TCCAGAACTG CTTTCAGATC CACTATGGCT

CTCTCGCTTT CCGAGTCAGA TCCGGAACTG CTTTCGGATC CACTATGGCT

ACTAGAATCA TCCCTGGAGT TATCTGCTCC TTCACTATTA TGGTGTGAAG

ACTAGAATCA TCCCTGGAGT TATCTGCTCC TTCACTATTA TGGTGTGAAG

ACTAGAATCA TCCCTGGAGT TATCTGCTCC TTCACTATTA TGATGTGAAG

ACTAGAATCA TCCCTGGAGT TATCTGCTCC TTCACTATTA TGATGTGAAG

ACTAGAATCA TCCCTGGAGT TATCTGCTCC TTCACTATTA TGGTGTGAAG

---------- ---------- ---------- ---------- ----------

GCTTGAGTCA TCCCTGGAGT TATCTGCTCC CTCACTATTA TGGTGTGAAG

GCTCGAGTCA TCCCTGGAGT TGTCTGCTCC CTCACTGTTA TGGTGTGAAG

GCTAGAATCA TCTCTGGAGT TATCTGCCCC TTCACTATTA TGATGTGAAG

GCTAGAATCA TCCCTGGAGT TATCTGCCCC TTCACTATTA TGGTGTGAAG

GCTAGAATCA TCCCTGGAGT TATCAGCTCC TTCACTATTA TGGTGTGAAG

GCTGGAGTCA TCCCGTGAGT TTTCTGCCCC TTCACTGTTG TGGTGTGAAG

---------- ---------- ---------- ---------- ----------

GCTAGAATCA TCTCTAGAGT TATCTGCTCC TTCACTATTA TGGTGTGAAG

GCTAGAATCA TCCCTGGAGT TATCTGCTCC TTCACTATTA TGGTGTGAAG

GCTAGAATCA TCTCTGGAGT TATCTGCTCC TTCACTGTTA TGGTGTGAAG

ACTTGAGTCA TCCCTGGAAT TATCCGCTCC TTCGCTATTG TGGTGTGAAG

GCTCGAGTCA TCCCTGGAAT TATCTGCTCC TTCACTATTA TGGTGTGACG

GTTCAGAGTT ACTAAAAGAG ATGAAAAACA TCCAAATCGA ATGCCCACCT

GTTCAGAGTT ACTAAAAGAG ATGAAAAACA TCCAAATCGA ATGCCCACCT

GTTCAGAATT ACTAAAAGAG ATGAAAAACA TCCAAATCGA ATGCCCACCT

GTTCAGAATT ACTAAAAGAG ATGAAAAACA TCCAAATCGA ATGCCCACCT

GTTCAGAGTT ACTAAAAGAG ATGAAAAACA TCCAAATCTA ATGCTCACCT

---------- ---------- ---------- ---------- ----------

GCTCAGAGTT ACTGAAGGAG ATAAGCCCCA CTCAAATCTA ATCCCCACCT

GTTCAGAGTT ACTGAAGGAG ATAAACCCCA CGCAAATCTA ATCCCCACCT

GTTCAGAGTT ACTGAAAAAG AT--AAAACA TTCAAATCTA ATGTCTACCT

GTTCAGAGTT ACTAAAAGAA ATAATAAGCA TTCAAAGTTA ATGCCTACCT

GTTCAGAGTT ACTAAAAGAC ATAAAAAACA TCCAAATCTA ATGCATACCT

GTTCAGAGTT ACTAAAAGAG ATAAAAAAGA TCCAATTCTA ATGCCTACCT

---------- ---------- ---------- ---------- AAGCTTACCT

GTTCAGAATT ACTAAAAAAG ATAAAAAACA TCTAAATCTA ATGCCTACCT

GTTCAGAGTT ACTAAAAAAG AGAAAAGACA TCCAGATT-- AAGCCTACCT

GCTCAGAATT ACTGAAAAAG AGAAAAAGTA CCCAAATTAA AAGCCTACCT

GTTCAGAATT ACTAAAAGAA AGGAAATATG ACTGCTTTTA ATGCCTACCT

GTTCAGAATT ACTGAAAAAA AAAAAACACA CCCAAATTGG ATGCCTACCT

TCCTGGTGTA CTCCTCGGCA TTGTCTTATC ACAATCCTAA AATTAAAACA

TCCTGGTGTA CTCCTCGGCA TTGTCTTATC ACAATCCTAA AATTAAAACA

TCCTGGTGTA CTCCTCGGCA TTGTCTTATC ACAATCCTAA AATTAAAACA

TCCTGGTGTA CTCCTCGGCA TTGTCTTATC ACAATCCTAA AATTAAAACA

TCCAGGTGTG CTCCTTGGCA TTGTCTTATC ACAATCCTAA AATTAAAACA

---------- ---------- ---------- ---------- ----------

TCCTGGTGTA CTCCTTGGCA TTGTCTTATC ACAATCCTAA AAATGAAACA

TCCTGGTGTA CTCCTTGGCA TTGTCTTATC ACAATCCTAA AAATAAA---

TCCTGGTGTA CTTCTTGGCA TTGTTTTATC ACAATCCTAA AATAAAAACA

TCCTGGTGTA CTCCTTGGCA TTGTCTTATC ACAATCCTAA AATTAAAACA

TCCTGGTGTA CTCCTTGGCA TTGTCTTATC ACAATCCTAA AAATAAAACA

TCCTGGTGTA CTCCTTGGCA TTGTCTTATC ACAATCCTAA AATTAAAACA

CCCTGGTGTA CTCCTTGGCA TTGTCTTATC ACAATCCTAA AATGGAAACA

TCCTGGTGTA CTCCTTGGCA TTGTCTTATC ACAATCCTAA AATTAAAACA

TCCTGGTGTA CTCCGTGGCA TCGTCTTATC ACAATCCTAA AATTAAAACA

TCCTGGTGTG CTCCTTGGCA TGGTCTTATC ACAATCCTGA AATTAAAACA

TCCTGGTGTA CTCCTTGGCA CAGTCTTATC ACAATCCTAT AATTAAAACC

TCCAGGGGTA CTCCTTGGCA CTGTTTTATC ACAATCCTAC AATTAAAACA

CACCTGTTCC CCATCACTGT CTTCACTGCT GCTTAGTTTT AAGTCATCTT

CACCTGTTCC CCATCACTGT CTTCACTGCT GCTTAGTTTT AAGTCATCTT

CACCTGTTCC CCATCACTGT CTTCACTGCT GCTTAGTTTT AAGTCATCTT

CACCTGTTCC CCATCACTGT CTTCACTGCT GCTTAGTTTT AAGTCATCTT

TACCTGTTCC CCATCACTGT CTTCACTGCT GCTTAGTTTT AAGTCATCTT

---------- ---------- ---------- ---------- ----------

GACCTGTTCC CCATCACTGT CTTCACTGCT GCTTAGTTTT AAGTCATCTT

---CTGTTCC CCATCACTGT CTTCACTGCT GCTTAGTTTT AAGTCATCTT

TACCTGTTCC CCATCACTGT CTTCACTGCT GCTGAGTTTT AAGTCATCTT

CACCTGTTCT CCATCACTGT CTTCACTGCT GCTTAGCTTT AAGTCATCTT

CACCTGCTCC CCATCGCTGT CTTCACTGCT GCTTAGTTTT AAGTCATCTT

CACCTGTTCC CCATCACTGT CTTCACTGCT GCTTAGCTTT AAGTCATCTT

CACCTGTTCC CCATCACTGT CTTCACTGCT GCTTATTTTT AAGTCATCTT

CACCTGTTCC CCATCACTGT CTTCACTGCT GCTTAGTTTT AAGTCATCTT

TACCTGTTCC CCATCACTGT CTTCACTGCT GCTTAGTTTT AAGTCATCTT

CACCTGTTCC CCATCACTGT CTTCACTGCT GCTTAGTTTT AAGTCATCTT

TACCTGCTCT CCATCACTAT CTTCACTGCT GCTAAGTTTT AAATCATCCT

TACCTGTTCC CCATCACTGT CTTCACTGCT GCTGAGTTTT AAGTCATCTT

TTAACATACT AGAGGGAAAA ACGTTATGGT ATGCCAGAAC TAAATTCATT

TTAACATACT AGAGGGAAAA ACGTTATGGT ATGCCAGAAC TAAATTCATT

TTAACATACT AGAGGGAAAA GCGTTATGGT ATGCCAGGAC TAAATTCATT

TTAACATACT AGAGGGAAAA GCGTTATGGT ATGCCAGGAC TAAATTCATT

TTAACATACT A-AGAGAAAA GCGTTATGGT ATGCCAAAAC T----TTATT

---------- ---------- ---------- ---------- ----------

TTAACATACT AGAGGAAAAA GCATTACA-- --------GC TCGGTCCATT

TTAACATACT AGAGGGAAAA ACATTACACC AGGCCAGAAC TAAATCCATT

TTAACATACT AGAGGGAAAA GCGTTATGGC ATGCCAGAAT TAAAT-----

TTAACATACT AGAAGGAAAA AC-TTATGGC ATAGCAGAAT TAGATTTATT

TTAACATACT AGAGGGAAAA GCGTTATGGC ACACCACAAT TAAATTTATT

TTAACATACT GGAGGGAAAA GCGTTATGGC ATACCAGAAT TAAATTTATT

TTAACATACT AGAGGGAAAA GCGTTATGGC ATACCAGAAC TAAATTAAAC

TTAACATACT AGAGGGAAAA GCGTTATGGC ATACCAGAAT TAAATTTATT

TTAACATACT AGAGTGAAAA GAGTTATGGC AAACCAGAAT TAATT-----

TTAACATACT AGAGGGAAAA GTACTATGGC ATACCAGAAC TAAATTTATT

TTAACATACT AAAGGGAAAA ATATCATAGT ATATAAGAAT TAACTTAATT

TTAACATACT AGAGCAAGAG ACATAATAGT GAGCCAGaat ttaatTAATT

AAGCAATAAA CTTACGATTT AGACTGGTGC CCATTTGAAG TTTAAAGAAA

AAGCAATAAA CTTACGATTT AGACTGGTGC CCATTTGAAG TTTAAAGAAA

AAGCAATAAA CTTACGATTT AGACTGGTGC CCATTTGAAG TTTAAAGAAA

AAGCAATAAA CTTACGATTT AGACTGGTGC CCATTTGAAG TTTAAAGAAA

AAGCAATAAA CTTACGATTT AGACTGGTGC CCATTTGAAG TTTAAAGAAA

---------- ---------- ---------- ---------- ----------

AAACAATAAA CTTACGATTT TGACTGGTGT CCATTTGATG TTTAAATAAA

AAGCAATAAA CTTACGATTT TGACTGGTGT CCATTTGATG TTTAAAGAAA

---------- ---------- ---------- ---------- ---AAAAAAA

AAGCAATAAA CTTACGATTT AGACTGGTGT GCATTTGAAG TTTAAAGAAA

AAGCAATAAA CTTACGATTT AGACTGGTGT CCATTTGAAG TTTAAAGTAA

AAGCAATAAA CTTACGATTT AGACTGATGT CCATTTGAAG TTTAAAGAAA

AAA-AATAAA CTTACGATTT AGACTGGTGC CCATTTGAAG TTTAAAGTAA

TAAAAATATA CTTACGATTT AGACTGGTGT CCATTTGAAG TTTATAGAAA

---------- ---------- ---------- ---------- ----------

AAGAAATACA CTTACGATTT AGACTGGTGT CCATTTGAAG TCTAGAGAAA

AAGTAATTAA CTTACGATTT GGACTGATGC CCATTTGAAG TTTATAGAAA

ACTTGATTCA CTTACGATTT GGCCTGGTGA CCATTTGATG TTTCTAGAAA

TACTCACTTT GTTCTCCAGT GCCAAAATTG GACTGAGACT CCTAAGAAAA

TACTCACTTT GTTCTCCAGT GCCAAAATTG GACTGAGACT CCTAAGAAAA

TACTCACTTT GTTCTCCAGG GCCAAAATTG GACTGAGATT CCTAAG-AAA

TACTCACTTT GTTCTCCAGG GCCAAAATTG GACTGAGATT CCTAAG-AAA

TACTCACTTT GTTCTCCAGG GCCAAAATTG GACTGAGAAT CCTAAGAAAA

---------- ---------- ---------- ---------- ----------

TACTTACTTT GTTCTCCAGA GCCAAAGTTA GACTGAGACT CCTAGGAAAA

TACTTACTTT GTTCTCCAGG GCCAAAGTTA GATTGCGACT CCTAAGAGAA

TACTCACTTT GTTCTCCAGG ACCAAAGTTG GACTGAGGCT CCTAAGAAAA

TACTCACTTT GTTCTCCAGG GCCAAAACTG GACTGAGACT CCTGAGAAAA

TACTCACTTT GTTCTCCAGG GCCAAAACTG GACTGAGATT CCTAAGAAAA

CACTCACTTT GTTCTCCAGG ACCAAAGTTG GATTGAGATT CCTAAGACAA

TACTTACTTT GTTCTCCAGG GCCGAAATTG GACTGCGTTT CCTAAGAAAA

TACTCACTTT GCTCTCCAGG GCCAAAACTG GACTGAGACT CCTAAGAAAA

---------- ---------- ---------- ---------- ----------

TACTCACTTT GCTCTCCAGG GCCAAAATTA GATTGAGACT CCTAAGGAAA

TACTTACTTT GTTCTCCAGG GCCAAAATTT GATTGAGATT CCTAAAAAGG

TACTCACTTT GTTCTCCAGT ACCAAAATTG GACTGAGACT CCTGCAAATA

AGCTAACGAG AACGGATCTG ACAGAGCAGG ATTACCCACT CGGGAGCCGA

AGCTAATGAG AACGGATCTG ACAGAGCAGG ATTACCCACT CGGGAGCCGA

AGCTAACGAG AACGGATCTG ACAGAGCAGG ATTACCCACT CGGGAGCCGA

AGCTAACGAG AACGGATCTG ACAGAGCAGG ATTACCCACT CGGGAGCCGA

AGCTAACAAG AACGGATCTG ACAGAGCAGG ATTACCCACT CGGGAGCCGA

-GCTAACGAG AACGGATCTG ACAGAGCAGG ATTACCCACT CGGGAGCCGA

AGCTAACAGG GAAGGATCTG ACAGCACAGG ATTACCCACT CAGGAGCAGG

AGCTAACAAG GAAGGATCTG ACAGCACAGG ATTACCCACT CAGGAGCAGA

A--------- ---------- ---------- ---------- ----------

AGCTAACGAG AACGGATCTG ACAGAGCAGG ATTACCCACT CGGGAGCCGA

AGCTAACGAG AACGGATCTG ACAGAGGAGG ATTACCCACT CGGGAGCCGA

AGCTAAAGAG AACAGATCTG ACAGAGTAGG ATTACCCATT CGGGGGCCCA

A--------- ---------- ---------- ---------- ----------

AGCTAACGAG AGCGGATCTG ACAGAGCAGG ATTACCCTCG CGGGAGCTGA

-GCTAACGAG AACAGATCTG ACGGAGTTGG ATTACCCACT CAGGAGCTGA

A--------- ---------- ---------- ---------- ----------

GGCTAACGAG AACGGATCTG ACAGAGGAGG ATTACCCACT CGGGAGCTGA

GGCTAACGAG AACGGATCTG ACAGAGGAGG ATTACCCGCC CGGAAACCAA

GTGATTGTGT TTACTCCCAA ACCTGCCCTA TTTAGTGGTC TGTGTGCATC

GTGATTGTGT TTACTCCCAA ACCTGCCCTA TTTAGTGGTC TGTGTGCATC

GTGATTGTGT TTACTCCCAA ACCTGCCCTA TTTAGTGGTC TGTGTGCATC

GTGATTGTGT TTACTCCCAA ACCTGCCCTA TTTAGTGGTC TGTGTGCATC

GTGACTGTGT TTACTCCCAA ACCTGCCCTA TTTAGTGGTC TGTGTGCATC

GTGATTGTGT TTACTCCCAA ACCTGCCCTA TTTAGTGGTC TGTGTGCATC

GTGACTGT-- TTACTCCCAA ACCTGCCCTA TTTAGTGGTC TGTGTTCATC

GTGACTGTGT TTACTCCCAA ACCTGCCCTA TTTAGTGGTC TGTGTTCATC

---------- ---------- ---------- ---------- ----------

CTGATTGTGT TTACTCCCAA ACCTGCCCTA TTTAGTGGTC TGTGTGCATC

GTGATTGTGT TTACTCCCAA ACCTGCCCTA TTTAGTGGTC TGTGTGCATC

GTGATTGTGT TTACTCCCAA ACCTGCCCTA TTTAGTGGTC TGTGTGCATC

---------- ---------- ---------- ---------- ----------

GTGATTGTGT TTACTCCCAA ACCTGCCCTA TTTAGTGGTC GGCGTGCATC

GTGATTGTGT TTACTCCCAA ACCTGCCCTA TTTAGTGGTC TGTGTGCATC

---------- ---------- ---------- ---------- ----------

GTGATTGTGT TTACTCCCAA ACCTGCCCTA TTTAGTGGGC TGTGTGCATC

GTGATTGTAT TTACTCCCAA ACCGGCCCTA TTTAGTGGGC GGTGGGCATC

ATTAATCTTC TTCCACAATC CATTCCCCAT CTGCTCCCAC TTTTTAATGA

ATTAATCTTC TTCCACAATC CGTTCCCCAT CTGCTCCCAC TTTTTAATGA

ATTAATCTTC TTCCACAATC CGTTCCCCAT CTGCTCCCAC TTTTTAATGA

ATTAATCTTC TTCCACAATC CGTTCCCCAT CTGCTCCCAC TTTTTAATGA

ATTAATCTTC TTCCACAATC CGTTCCCCAT CTGCTCCCAC TTTTTAATGA

ATTAATCTTC TTCCACAATC CGTTCCCCAT CTGCTCCCAC TTTTTAATGA

ATTAATCTTC CTCCACAATC CGTTCCCCAT CTGCTCCCAC TTTTTAATGA

ATTAATCTTC CTCCACAATC CGGTCCCCAT CTGCTCCCAC TTTTTAATGA

---------- ---------- ---------- ---------- ----------

ATTAATCTTC TTCCACAATC CGTTCCCCAT CTGCTCCCAC TTTTTAATGA

ATTAATCTTC TTCCACAATC CGTTCCCCAT CTGCTCCCAC TTTTTAATGA

ATTAATCTTC TTCCACAATC CGTTCCCCAT CTGTTCCTAC TTTTTAATGA

---------- ---------- ---------- ---------- ----------

ATTAATCTTC TTCCACAATC CGTTCCCCAT CTGCTCCCAC TTTTTAATGA

ATTAATCTTC TTCCACAATC CGTTCCCCAT CTGCTCCCAC TTTTTAATGA

---------- ---------- ---------- ---------- ----------

ATTAATCTTC TTCTACAATC CGTTCCCCAT CTGCTCCTAC TTTTTAATGA

ATTAATCTTC TTCTGCGATC CGCTCCCCAT CTGCTCCCAC TTTTTAATGA

ACTGCCACTT GTCGCACTCA ATAAAATCAG CACTTTGCGA GCTGCCAGCT

ACTGCCACTT GTCGCACTCA ATAAAATCAG CACTTTGCGA GCTGCCAGCT

ACTGCCACTT GTCGCACTCA ATAAAATCAG CACTTTGAGA GCTGCCAGCT

ACTGCCACTT GTCGCACTCA ATAAAATCAG CACTTTGCGA GCTGCCAGCT

ACTGCCACTT GTCGCACTCA ATAAAATCAG CACTTTGCGA GCTGCCAGCT

ACTGCCACTT GTCGCACTCA ATAAAATCAG CACTTTGCGA GCTGCCAGCT

ACTGCCACTT GTCGCACTCA ATAAAATCAG TACTTTGCGA GCTACCAGCT

ACTGCCACTT GTCGCACTCA ATAAAATCAG TACTTTGCGA GCTACCTGCT

---------- ---------- ---------- ---------- ----------

ACTGCCACTT GTCGCACTCA ATAAAATCAG CCCTTTGCAA GCTGCCAGCT

ACTGCCACTT GTCGCACTCA ATAAAATCAG CACTTTGCGA GCTCCCAGCT

ACTGCCACTT GTCGCACTCA ATAAAATCAG CACTTTGCAA GCTGCCAGCT

---------- ---------- ---------- ---------- ----------

ACTGCCACTT GTCGCACTCA ATAAAATCAG CACTTGGCGA GCTGCCAGCT

ACTGCCACTT GTCGTACTCA ATAAAATCAG CACTTTGCGA GCTGCCAGCT

---------- ---------- ---------- ---------- ----------

ACTGCCACTT GTCGCACTCA ATAAAATCAG CACTTTGCGA GCTGGCAGCT

ACTGCCACTT GTCGCTCTCA ATAAAATCAC CACTTTGTGA ACATCCTGCT

CTGGGGATTC ATCTGATACT AGGCTATTAC CTTCTGAGCC AGCTGTCACT

CTGGGGATTC ATCTGATACT AGGCTATTAC CTTCTGAGCC AGCTGTCACT

CTGGGGATTC ATCTGATACC AGGTGATTAC CTCCTGAGCC AGCTGTCACT

CTGGGGATTC ATCTGATACC AGGTGATTAC CTCCTGAGCC AGCTGTCACT

CTGGGGATTC ATCTGATACC AGTCTATTAC CTCCTGAGCT GGCTGTCACT

CCGGGGATTC ATCCGATACC AGCCTATTAC CTCCTGAGCC AGCTGTCACT

CAGGGGACAT ATCCCACCCC GGGCTATTAC TTCCTGAGCC AGCTGTCACT

AAGGGGGCAT ATCCCACCCC GGGCTATTAC TTCCTG---- ----------

---------- ---------- ---------- ---------- ----------

CTAGGGATTC ATCCGATACC AGGCTATTAC CTCCTGAGCC AGCTGTCACT

CTGGGGATTC GCCCGATACC AGGCTATTAC CTCCTGAGCC AGCTGTCACT

CTGGGAATTC ATCCGATACC AGGCTATTAT GTCCTGAGCC AGCTGTCACT

---------- ---------- ---------- ---------- ----------

CTGGGGATTC ATCCGCTACC AGGCTATTAC CTCCTGAGCC AGCTGTCACT

CTGGGGATTC ATCTGATACC AGGCTATTAC CTCCTGAGCC AACTGCCACC

---------- ---------- ---------- ---------- ----------

CTGGGGATTC ATCCGATACC AGGCTATTAC CTCCTGAGTC AGCTGTCACT

TGGGAGCATC AGCCGAGACC AGGCTCTCCC TCTCTTAGTC AACTGTCACT

TAGGCACTTA CAGGCAGAAC TGTGGAAAAA CCAAACCACT GTTATTACAC

TAGGCACTTA CAGTCAGAAC CGTGGAAAAA CCAAACCACT GTTATTATAC

TAGGCACTTA CAGGCAGAAC CGTGGGAAAA CCAAACCACT GTTATTACAC

TAGGCACTTA CAGACAGAAC CGTGGAAAAA CCAAACCACT GTTATTACAC

TAGGCACTTA CAGGCAGAAC CGTGGAAAAA CCAAACCACT GTTATTATAC

TAGGCACTTA CAGGCAGAAC CGCGGAAAAA CCAAACCACT GTTATTACAC

TAGGCACTTA TGGGCAGAAC CGCGGAAAAA CCAAACCACT GTTATTACAC

---------- ---------- ---------- ---------- ----------

---------- ---------- ---------- ---------- ----------

TAGGCACTTA CAGGCAGAAC TGTGGAAAAA CCAAACCACT GTTATTACAC

TAGGCACTTA CAGGCAGAAC CGTGGAAAAA CCAAACCACT GTTATTACAC

TAGGCACTTA CAGGCAGAAC CGCAGAAAAA CCAAACTACT GTTATTACAC

---------- ---------- ---------- ---------- ----------

TGGGCACTTA CAGGCAGAAC CGCGGAAAAA CCAAACCACT GTTATTACAC

TAGGCACTCA CAGGCAGAAC CGTGGAAAAA CCAAACCACT GTTATTACAT

---------- ---------- ---------- ---------- ----------

TAGGCACTTA CAAGCAGAAC CGCGGAAAAA CCAAACCACT GTTATTACAC

TAGGCACTTA CGGGCAGAAC CGCGGAAAAA CCAAACCACT GTTATTACAC

TTAATAACCA GTTTCCACAT CATCAACTCA TTGGAGTCTG ACAGGCGCTG

TTAATAACCA GTTTCCACAT CATCAACTCA TTGGAGTCTG ACAGGAGCTG

TTAATAACCA GTTTCCACAT CATCAACTCA TTGGAGTCTG ACAGGCGCTG

TTAATAACCA GTTTCCACAT CATCAACTCA TTGGAGTCTG ACAGGCGCTG

TTAATAACCA GTTTCCACAT CATCAACTCA TTGGAGTCTG ACAGGCGCTG

TTAATAACCA GTTTCCACAT CATCAACTCA TTGGAGTCTG ACAGGCACTG

TTAATAACCA GTTTCTACCT CATCAACTCA TTGGCGTCTG ACAGGCGCTG

---------- ---------- ---------- ---------- ----------

---------- ---------- ---------- ---------- ----------

TTAATAACTA GTTTCCACAT CATCAACTCA TTGGAGTCTG AGAGGCGCTG

TTAATAACCA GTTTCCACAT CATCAACTCA TTGGAGTCTG ACAGGCGCTG

TTAATAACCA GTTTCCACAT CATCAATTCA TTGGAGTCTG ACAGGCGTTG

---------- ---------- ---------- ---------- ----------

TTAATAACCA GTTTCCACCT CATCAATTCA TTGGCGTCTG ACAGGCACTG

TTAATAGCCA GTTTCCACGT CATCAACTCA TTGGAGTCTG ACAGGCGCTG

---------- ---------- ---------- ---------- ----------

TTAATAACCA GTTTCCACAT CATCAACTCA TTGGAGGCTG ACAGGAGCTG

TTAATAACCA GTTTCCACAT CATCAACTCA TCGGCGTTTG ACAGGGGCTG

TTAGGGTGTC AGCTCCCTCA GCCTAACAAG TCACAGGAAT CAATGCAATC

TTAGGGTGTC AGCTCCCTCG GCCTAACAAG TCACAGGAAT CAATGCAATC

TTAGGATGTC AGCTCCCTCG GCCTAACAAG TCACAGGAAT CAATGCAATC

TTAGGATGTC AGCTCCCTCG GCCTAACAAG TCACAGGAAT CAATGCAATC

TTAGGGTGTC AGCTCCCTCG GCCTAACAAG TCACAGGAAT CAATGCAATC

TTAGGGTGTC AGCTCCCTCG GCCTAACAAG TCACAGGAAT CAATGCAATC

TTAGGGTGCC AGCGCCTTTG GCCTAACAAG TCACAGGAAT CAATGCAATC

---------- ---------- ---------- ---CAGGAAT CAATGCAATC

---------- ---------- ---------- ---CAGGAAT CAATGCAATC

TTAGGGTGTC AGCTCCCTCA GCCT--CAAG TCACAGGAAT CAATGCAATC

TTAGGGTGTC AGCTTCCTTG GCCTAACAAG TCATAGGAAT CAATGCAATC

TTAGGGTGTC AGCTCCCTTG GCCTAACAAG ---CGGGAAT CAATGCAATC

---------- ---------- ---------- ---------- ----------

TTAGGGTATC AGCTCCCGCG GCCCCACGAG TCACAGGAAT CAATGCAATC

TCAGGGCATC AGCTCCCTCG GCCTAACAAG TCACTGGAAT CAATGCAATC

---------- ---------- ---------- ---------- ----------

TTAGAGTGTC AGCTCGCCTG GTCTAACAAG TCATAGGAAT CAATGCAATC

TTATGGCGTC AACTCGCACG GCCTGACAAG TTGCAAGAAT CACTGCAATC

CCCTAAAAGC TCATAACTTG GAGGTAATTA TAAGAAGCAT CCCCAGCAAT

CCCTAAAAGC TCATAACTTG GAGGTAATTA TAAGAAGCAT CCCCAGTAAT

CCCTAAAAGC TCATAACTTG GAGGTAATTA TAAGAAGCAT CCTCAACAAT

CCCTAAAAGC TCATAACTTG GAGGTAATTA TAAGAAGCAT CCTCAACAAT

CCCTAAAAGC TCATAACTTG GAGGTAATTA TAAGAAGCAT CCCCAGCAAT

CCCTAAAAGC TCATAACTCG GAGGTAATTA TAAGAAGCAT CCCCAGCAAT

CCCTAAAAGC TCATAACTCG GAGGTAATTA TAAGAAGCAT CCCCAGCAAT

CCCTAAAAGC TCATAACTCG GAGGTAATTA TAAGAAGCAT CCCCAGCAAT

CTCTAAAAGC TCATAACTCG GAGGTAATTA TAAGAAGCAT CCCCAGCAAT

CCCTAAAAGC TCATAACTCG GAGGTAATTA TAAGAAGCAT CCCCAGCAAT

CCCTAAAAGC TCATAACTCG GAGGTAATTA TAAGAAGCAT CCCCAGCAAT

CCCTAAAAGC TCATAACTCG GAGGTAATTA TAAGAAGCAT CCCCAGCAAT

---------- ---------- ---------- ---------- ----------

CCCTAAAAGC TCATAACTTG GAGGTAATTA TAAGAAGCAT CCCCAGCAAT

TCCTAAAAAC TCATAACTCG GAGGTAATTA TAAGAAGCAT CCCCAGCAAT

---------- ---------- ---------- ---------- ----------

CCCTAAAAGC TCATAACTCG GAGGTAATTA TAAGAAGCAT CCCCAGCAAT

TCCTAAAAGT TCAGAACTCG AAGGGAAACC TAAGTGGAGT CCCAGGGAGC

GTGGTGATTT TACAATGTAA ACAAAACCTC TCTCCATTCA TAGATGCCCA

GTGGTGATTT TACAATGTAA ACAAAACCTC TCTCCATTCA TAGATGCCCA

GTGGTGATTT TACAATGTAA ACAAAACCTC TCTCCATTCA TAGATGCCCA

GTGGTGATTT TACAATGTAA ACAAAACCTC TCTCCATTCA TAGATGCCCA

GTGGTGATTT TACAATGTAA ACAAAACCTC TCTCCATACA TAGATGCCCA

GTGGTGATTT TACAATGTAA ACAAAACCTC TCTCCATTCA GAGATGCCCA

GTGGTGATTT TACAATGTAA ACAAAACCTT GCTCCATTCG TAGATGCCCA

GTGGTGATTT TACAATGTAA ACAAAACCTC GCTCCATTCA TAGATGCCCA

GTGG-GATTT TACA-TGTAA A--------- ---------- ----------

GTGGTGATTT TACAATGTAA ACAAAACCTC TCTCCATTCA TAGATGCCCA

GTGGTGATTT TACAATGTAA ACAAAACCTC TCTCCATTCA TAGATGCCCA

GTGGTGATTT TACAATGTAA ACAAAACCTC TCTCCATTCA TAGATGCCCA

---------- ---------- ---------- ---------- ----------

GTGGTGATTT TACAATGTAA ACAAAACCTC TCTCCATTCA TAGACGCCCA

GTGGTGATTT TACAATGTAA ATAAAACCTC TCTCCATTCA TAGATGCCCA

---------- ---------- ---------- ---------- ----------

GTGGTGATTT TACAATGTAA ACAAAACCTC TCTCCATTCA TAGATGCCCA

GTGGTGACCG TACAATGGAA ACTAAGCCTG TTTCCTTTCA CAGACCCTCA

TTCAGCCATA TTTGTTTGTT GCTATCACAC AGATGCAGAG TATTACATTG

TTCAGCCATA TTTGTTTGTT GCTATCACAC AGATGCAGAG TATTACATTG

TTCAGCCATA TTTGTTTGTT GCTATCACAC AAATGCAGAG TATTACATTG

TTCAGCCATA TTTGTTTGTT GCTATCACAC AAATGCAGAG TATTACATTG

TTCAGCCATA TTTGTTTGTT GCTATCACAC AAATGCAGAG TATTACATTG

TTCAGCCATA TTTGTTTGCT GCTATCACAC AAATGCAGAG TATTACATTG

TTCAGCCATA TTTGTTTGTT GCTATCACAC GAATGCAGAG TATTACATAG

TTCAGCCATA TTTGTTTGTT GCTATCACAC GAATGCAGAG TATTACATAG

---------- ---------- ---------- ---------- ----------

TTCATCCATA TTTGTTTGTT GCTATCACAC AGATGCAGAG TATTACATTG

TTCAGCCATA TTTGTTTGTT GCTATCACAC AAATGCAGAG TATTACATTG

TTCAGCCATA TTTGTTTGTT GCTATCACAC AAATGCAGAG TATGACATCG

---------- ---------- ---------- ---------- ----------

TTCAGCCATA TTTGTTTGTT GCTATCACAC AAATGCAGAG TTTTACATTG

TTCAGCCATA TTTGTTTGTT GCTATCACAC AAATGCAGAG CATTACATTG

---------- ---------- ---------- ---------- ----------

TTCAGCCATA TTTGTTTGTT GCTATCACAC AAATGCAGAG TATTACATTG

GCCGGTCACA CTTGTTTATT GGACTCTccc aagcacttag tactgtgctg

TAGGGAATAA TTGAATCAAT ACAGTAGAAT CTCAGCGCCT AACCAACAGT

TAGGGAATAA TTGAATCAAT ACAGTAGAAT CTCAGCGCCT AACCAACAGT

TAGGGAATAA TTGAATCAAT ACAGTAGAAT CTCAGTGCCT AACCAACAGT

TAGGGAATAA TTGAATCAAT ACAGTAGAAT CTCAGTGCCT AACCAACAGT

TAGGGAATAA TTGAATCAAT ACAGTAGAAT CTCAGCGCCT AACCAACAGT

TAGGGAATAA TTAAATCAAT ACAGTAGAAT CTCAGCGCCT AACCAACAGT

CAGGGAATAA TTGAATCAAT ATAAGAGAAT CTCAGCGCTT AACCAACAGT

CAGGGAATAA TTGAATCAAT ATAAGAGAAT CTCAGCGCTT AACCAACAGT

---------- ---------- -----AGAAT CTCAGCGCCT AACCAACAGT

TAGGGAATAA TTGAATCAAT ATAGTAGAAT CTCAGCGCCT AACCAACAGT

TAGGGAATAA TTGAATCAAT ACAGTGGAAT CTCAGCGCCT AACCAACAGT

TAGGGAATAA TTGAATCAAT ATAGTGGAAT CTCAGCGCCT AACCAACAGT

---------- ---------- ---------- ---------- ----------

TAGGGGATAA TTGAATCAAT ACAGCGGAAT CTCAGCGCTT AACCAACAGT

TAAGGAATAA TTGAATCAAT GCAGTGGAAT CTCAGCGCCT AACCAACAGT

---------- ---------- -----GGAGT CTCTGCACCT AACCAATAGT

TAGGGAATAA TTGAATCAAT ATAGTGGAAT CTAGGCGCCT AACCAACAGT

tacacagtaa gtgaataaat acaatAGAGT CATGGCGCCC AAGCCATAAA

ACACACAACT AAATAAAAAT CCATTTTCCA AGGAAAATTC TATTCTTATC

ACACACAACT AAATAAAAAT CCATTTTCCA AGGAAAATTC TATTCTTATC

ACACACAACT AAATAAAAAT CCATTTTCCA AGGAAAATTC TATTCTTATC

ACACACAACT AAATAAAAAT CCATTTTCCA AGGAAAATTC TATTCTTATC

ACACACAACT AAATAAAAAT CCATTTTCCA AGGAAAATTC TATTCTTATC

ACACACAACT AAATAAAAAT CCATTTTCCA AGGAAAATTC TATTCTTATC

ACACACAACT AAATAAAAAT CCATTTTCCA AGGAAAATTC TATTCTTATC

ACACACAACT AAATAAAAAT CCATTTTCCA AGGAAAATTC TATTCTTATC

ACACACAACT AAATAAAAAT CCATTTTCCA AGGAAAATTC TATTCTTATC

ACACACAACT AAATAAAAAT CCATTTTCCA AGGAAAATTC TATTCTTATC

ACACACAACT AAATAAAAAT CCATTTTCCA AGGAAAATTC TATTCTTATC

ACACACAACT AAATAAAAAT CCATTTTCCA AGGAAAATTC TATTCTTATC

---------- ---------- ---------- ---------- ----------

ACACACAACT AAATAAAAAT CCATTTTCCA AGGAAAATTC TATTCTTATC

ACACACAACT AAATAAAAAT CCATTTTCCA AGGAAAATTC TATTCTTATC

ACACACAACG AAATACAAAT CCATTTTCCA AGGAAAATTC TATTCTTATC

ACACACAACT AAATAAAAAT CCATTTTCCA AGGAAAATTC TATTCTTATC

ATCCACAGTT AAACAGAAAT CCATTTCCCG AGGAAAAGGG GCTTGCTATC

AGAGTTTTGC AAGAGGGTTG CATTAATTGA AGCTTTGATG TTTGTAGGAG

AGAGTTTTGC AAGAGGGTTG CATTAATTGA AGCTTTGACG TTTGTAGGAG

AGAGTTTTGC AAGAGGGTTG CATTAATTGA AGCTTTGATG TTTGTAGGAG

AGAGTTTTGC AAGAGGGTTG CATTAATTGA AGCTTTGATG TTTGTAGGAG

AGAGTTTTGC AAGAGGGTTG CATTAATTGA AGCTTTGATG TTTGTAGGAG

AGAGTTTTGC AAGAGGGTTG CATTAATTGA AGCTTTGATG TTTGTAGGAG

AGAGTTTTGC AAGAGGGTTG CGTTAATTGA AGCTTTGATG TT-GTGGGAA

AGAGTTTTGC AAGAGGGTTG CGTTAATTGA AGCTTTGATG TT-GTGGGAA

AGAGTTCTGC AAGAGGGCTG CGTTAATTGA AGCTTTGATG TTTGTAGGAG

AGAGTTTTGC AAGAGGGTTG CATTAATTGA AGCTTTGATG TTTGTAGGAG

AGAGTTTTGC AAGAGGGTTG CATTAATTGA AGCTTTGATG TTTGTAGGAG

AGAGTTTTGC AAGAGGGTTG CATTAATTGA AGCTTTGATG TTTGTAGGAG

---------- ---------- ---------- ---------- ----------

AGAGTTTTGC AAGAGGGTTG CATTAATTGA AGCTTTGATG TTTGTGGGAG

AGAGTTTTGC AAGAGGGTTG CATTAATTGA AGCTTTGATG TTTGTAGGAG

AGAGTTTTGC AAGAGGGTTG CATTAATTGA AGCTTTGATG TTTGC---GG

AGAGTTCTGC AAGAGGGTTG CATTAATTGA GGCTTTGATG TTTCTTGGAG

AGGCCGCTGA AAGAGGCCTG CATTAATTGG AGTGTTGATG GACCCTGGAG

GAGGCCTCTA ATCATCCTCC AGCAAATCAA GCATCCCCTT CTTGGGATGT

GAGGCCTCTA ATCATCCTCC AGCAAATCAA GCATCCCCTT CTTGGGATGT

GAGGCCTCTA ATCATCCTCC AGCAAATCAA GCATCCCCTT CTTGGGATGT

GAGGCCTCTA ATCATCCTCC AGCAAATCAA GCATCCCCTT CTTGGGATGT

GAGGCCTCTA ATCATCCTCC AGCAAATCAA GCATCCCCTT CTTGGGATGT

GAAGCCTCTA ATCATCCTCC AGCAAATCAA GCATCCCCTT CTTGGGATGT

GAGGCCTCTA ATCATCCTCC AGCAAACCAA GCATCCC-TT CTTGGGATGT

GAGGCCTCTA ATCATCCTCC AGCAAACCAA GCATCCCCTT CTTGGGATGT

GAGGCCTCTA ATGATCCTCC AGCAAATCAA GCATCCCCTT CTTGGGATGT

GAGGCCTCTA ATCATCCTCC AGCAAATCAA GCATCCCCTT CTTGGGATGT

GAGGCCTCTA ATCATCCTCC AGCAAATCAA GCATCCCCTT CTTGGGATGT

GAAGCCTCTA ATCATCCTCC AGCAAATCAA GCATCCCCTT CTTGGGATGT

---------- ----TCCCCC AGCAAATCAA GCATCCCCTT CTTGGGATGT

GAGACCTCTA ATCATCCTAC AGCAAATCAA GCATCCCCTT CTTGGGATGT

GAGGCCTCTA ATCATCCTCC AGCAAATCAA GCATCCCTTT CTTGGGATGT

GAGGCTTGTA ATCA------ ---------- ---------- ----------

GAGGCCTCTA ATCATCCTCC AGCAAATCAA GCATCCCCTT CTTGGGATGT

GAAGGCCTCC ACCACCCTGC AGCCCACCCG ACCTCCCCTC CTTGGGACGT

TTTCAATCTG TTTTATGTTG ATTTTAATTA AAGAATTCGA AACTGAACAA

TTTCAATCTG TTTTATGTTG ATTTTAATTA AAGAATTCGA AACTGAACAA

TTTCAATCTG TTTTATGTTG ATTTTAATTA AAGAATTCGA AACTGAACAA

TTTCAATCTG TTTTATGTTG ATTTTAATTA AAGAATTCGA AACTGAACAA

TTTCAATCTG TTTTATGTTG ATTTTAATTA AAGAATTCGA AACTGAACAA

TTTCAATCTG TTTTATGTTG ATTTTAATTA AAGAATCCGA AACTGAACAA

TTTCAATCTG TTTTATGTTG ATTTTAATTA AAGAATTCGA AACTGAACAA

TTTCAATCTG TTTTATGTTG ATTTTAATTA AAGAATTCGA AACCGAACAA

TTTCAATCTG TTTTATGTTG ATTTTAATTA AAGAATCCGA AACTGAACAA

TTTCAATCTG TTTTATGTTG ATTTTAATTA AAGAATTCGA AACTGAACAA

TTTCAATCTG TTTTATGTTG ATTTTAATTA AAGAATCCGA AACTGAACAA

TTTCAATCTG TTTTATGTTG ATTTTAATTA AAGAATCAGA AACTGAACAA

TTTCAATCTG TTTTATGTTG ATTTTAATTA AAGAATCCGA AACTGAACAA

TTTCAATCTG TTTTATGTTG ATTTTAATTA AAGAATCCGA AACTGAACAA

TTTCAATCTG TTTTATGTTG ATTTTAATTA AAGAATCCGA AACTGAACAA

---------- ---------- ---------- ---------- ----------

TTTCAATCTG TTTTATGTTG ATTTTAATTA AAGAATCTGA AACTGAACAA

TTCCAATCTG CTCTGTGTTG ATTATAATTA ACGGATCCGA AACTGAACAA

ACAGTTCCTA GCAGGTGGCC ATACAAGTGT GAAATAAGCA AACTGTTTTA

ACAGTTCCTA GCAGGTGGCC ATACAAGTGT GAAATAAGCA AACTGTTTTA

ACAGTTCCTA GCAGGTGGCC ATACAAGTGT GAAATAAGCA AACTGTTTTA

ACAGTTCCTA GCAGGTGGCC ATACAAGTGT GAAATAAGCA AACTGTTTTA

ACAGTTCCTA GCAGGTGGCC ATACAAGTGT GAAATAAGCA AACTGTTTTA

ACAGTTCCTA GCAGGTGGCC ATACAAGTGT GAAATAAGCA AACTGTTTTA

ACAGTTCCTA GCAGGTGGCC ATACAAGTGT GAAATAAGCA AACTGCTTTA

ACAGTTCCTA GCAGGTGGCC ACACAAGTGT GAAATAAGCA AACTGTTTTA

ACAGTTCCTA GCAGGTGGCC ATACAAGTGT GAAATAAGCA AACTGTTTTA

ACAGTTCCTA GCAGGTGGCC ATACAAGTGT GAAATAAGCA AACTGTTTTA

ACAGTTCCTA GCAGGTGGCC ATACAAGTGT GAAATAAGCA AACTGTTTTA

ACAGTTCCTA GCAGGTGGCC ATACAAGTGT GAAATAAGCA AACTGTTTTA

ACAGTTCCTA GCAGGTGGCC ACACAAGTGT GAAATAAGCA AACTGTTTTA

ACAGTTCCTA GCAGGTGGCC ATACAAGTGT GAAATAAGCA AACTGTTTTA

ACAGTTCCTA GCAGGTGGCC ATACAAGTGT GAAATAAGCA AACTGTTTTA

---------- ---------- ---------- ---------- ----------

ACAGTTCCTA GCAGGTGGCC ATACAAGTGT GAAATAAGCA AACTGTTTTA

ACAGCCCCTA GCAGGTGGCC ACACGAGTGT GAAATAAGCA AACTGTTTTA

TCTGCCACAA ACTCTGCTAG TAAATTTCTT CAAATTAATC CCGCTAAGCA

TCTGCCACAA ACTCTGCTAG TAAATTTCTT CAAATTAATC CCGCTAAGCA

TCTGCCACAA ACTCTGCTAG TAAATTTCTT CAAATTAATC CCGCTAAGCA

TCTGCCACAA ACTCTGCTAG TAAATTTCTT CAAATTAATC CCGCTAAGCA

TCTGCCACAA ACTCTGCTAG TAAATTTCTT CAAATTAATC CCGCTAAGCA

TCTGCCACAA ACTCTGCTAG TAAATTTCTT CAAATTAATC CCGCTAAGCA

TCTGCCACAA ACTCTGCTAG TAAATTTCTT CAAATTAATC CCGCTAAGCA

TCTGCCACAA ACTCTGCTAG TAAATTTCTT CAAATTAATC CCGCTAAGCA

TCTGCCACAA ACTCTGCTAG TAAATTTCTT CAAATTAATC CCGCTAAGCA

TCTGCCACAA ACTCCACTAG TAAATTTCTT CAAATTAATC CAGTTAAGCA

TCTGCCACAA ACTCTGCTAG TAAATTTCTT CAAATTAATC CCGCTAAGCA

TCTGCCACAA ACTCTGCTAG TAAATTTCTT CAAATTAATC CCGCTAGGCA

TCTGCCACAA ACTCCGCTAG TAAATTTCTT CAAATTAATC CCACGCAGCA

TCTGCCACAA ACTCTGCTAG TAAATTTCTT CAAATTAATC CCGCCAAGCA

TCTGCCACAA ACTCTGCTAG TAAATTTCTT CAAATTAATC CCGCAAAGCA

---------- ---------- ---------- ---------- ----------

TCTGCCACAA ACTCTGCTAG TAAATTTCTT CAAATTAATC CCCCTAAGGA

TCTGTCGCAA ATTCCTCGAG GAGATTTCTT CAGATGAATC CGCCCAAGCT

ATCCTAGCCT CCAAATATTT GCTTTCTGTA TACGGGCTTC TGCAAACACA

ATCCTAGCCT CCAAATATTT GCTTTCTGTA TACGGGCTTC TGCAAACACA

ATCCTAGCCT CCAAATATTT ACTTTCTGTA TACGGGCTTC TACAAACACA

ATCCTAGCCT CCAAATATTT ACTTT----- ---------- ----------

ATCCTAGCCT CCAAATATTT GCTTTCTGTG TACCAGCTTC TGCAAACACA

ATCCCAGCCT CCAAATATTT GCTTTCTGTG TACAGGCTTC TGCAAACACA

ATCCGGGCCT CCAAATATTT GCTTTCTGTA CACAGGCTTC TGCAAACACA

ATCCCAGCCT CCAAATATTT GCTTTCTGTA CACAGGCTTC TGCAAACACA

ATCCCAGCCT CCAAATATTT GCTTT----- ---------- --CAAACACC

ATCCCAGCCT CCAAATATTT GCTTTCTGTA TACAGGCTTG TGCAAACACA

ATCCCAACCT CCAAATATTT GCTTTCTGTA TACAGGCTTC TGCAAACACA

ATCCCAGCCT CCAAATATTT GCTTT----- ---------- ----------

ATCCCGACCT CCAAATATTT GCTCTCGGGG TGCGGGCTTC TGCAAACACA

ATCCCAACCT CCAAATATTT GCCATCTGTA TACAGGCTTC GGCAAACACA

ATCCCAGCCT CCAAATATTT GGTTTCTGTA TACAGGCTTC TGCAAACACA

---------- ---------- -----CTGTA CACAGGCTTC TGCAAACACA

ATTTCAATTT CCTATTGTTT GCCTTCTGTA TACAGGCTTC TGCAAACACA

CATCGGCCCC CCAAACATTT GTCTTCTGTC AAAAGACCCT AACAAACAGC

ATCATCTCCT CAGTTATTAA TGGGCCCGCT CGCTGCCTCT TCATTAGTGC

ATCATCTCCT CAGTTATTAA TGGGCCCGTT CGCTGCCTCT TCATTAGTGC

ATCATCTCCT CAGTTATTAA TGGGCCCGCT CGCTGCCTCT TCATTAGTGC

---------- ---------- ---------- ---------- ----------

ATCATCTCCT CAGTTATTAA TGGGCCCGCT CGCTGCCTCT TCATTAGTGC

ATCATCTCCT CAGTTATTAA TGGGCCCGCT CGCTGCCTCT TCATTAGTGC

ATCACCTCCT CAGTTATTAA TGGGCTTGCT CGCTGCCTCT TCATTAGTGC

ATCACCTCCT CAGTTATTAA TGGGCTCGCT CGCTGCCTCT TCATTAGTGC

ATCATCTCAT CAGTA--TAA TGAGCT-GCT CGCTGCCTCT TCATTAGTGC

ATCATCTCCT CCGTTATTAA TGGGCCTGCT CACTGCCTCT TCATTAGTGC

ATCATCTCCT CAGTTATTAA TGGGCCCGCT CGCTGCCTCT TCATTAGTGC

---------- ---------- ---------- ---------- ----------

ATCATCTCCT CCGTTATTAA TGGGCCCGCG CACTGCCTCT TCATTAGTGC

ATCATCTCCT CCGTTATTAA TGGGCCCACT CGCTGCCTCT TCATTAGTGC

ATCATCTCCT CAGTTATTAA TGGGCCCGCT CGCTGCCTCT TCATTAGTGC

ATCATCTCTT CAGTTATTAA TGGGCCCGCT CGCTGCCTCT TCATTAGTGC

ATCATCTCCT CAGTTATTAA TGGGCCCACT CGCTGCCTCT TCATTAGCAC

ATCTCTTCCA CGGTCGCTAA TGGGCCCTCA CACAGGCTCT TTATTAACGC

TCAAATATCC ACAGAAAACG TTTACTTACC ATGGGCTCCT GCTCGCACCG

TCAAATATCA CCAGAAAACG TTTACTTACC ATGGGCTCCT GCTCGCACCG

TCAAATATCA ACAGAAAACG TTTACTTACC ATGGGCTCCT GCTCGTACCG

---------- ---------- ---------- ---------- ----------

TCAAATATCA ACAGAAAACG TTTACTTACC ATGGGCTCCT GCTCGCACCG

TCAAATATCA ACAGAAAACG TTTACTTACC ATGGGCTCCT GCTCGCACCG

TCAAATATCA ACAGAAAACG TTTACTTACC ATGGGTTCCT GCTCTCGCCG

TCAAATATCA ACAGAAAACG TTTACTTACC ATGGGTTCCT GCTCGCGCCG

TCAAATATCA ACAGAAAACG TTTACTTACC ACGAGCTCCT GCTTGCGCCG

TCAAATATCA ACAGAAAACG TTTACTTACC ATGGGCTCCT GCTCGCGCCG

TCAAATATCA ACAGAAAACG TTTACTTACC ATGGGCTCCT GCTCGCGCCG

---------- ---------- ---------- ---------- ----------

TCAAATACCA ACAGAAACCG TTTACTTACC ACGGGTTCCT GCTCGCGCCG

TCAAATATCA ACAGAAAACG TTTACTTACC ATGGGCTCCT GCTCTCGCCG

TCAAATATCA ACAGAAAACA TTTACTTACC ATAGGCTCCT GCTCGCACCG

TCAAATATCA ACAGAAAACA TTTACTTACC ATGGGCTCCT GCTCACACCG

TCAAATATCA ACAGAAAACG TTTACTTACC ACAGGCTCCT GCTCACACCG

TCCAAGATCA GCAGAGGACG CTTGCTCTAT GCCGTTGTCT GCGCAAGTCG

AGCAAATTTG GACAGGGCTA GTCAAACAGG GAGCTTGTTG TCGGGCTAAT

AGCAAATTTG GACAGGGCTA GTCAAACAGG GAGCTTGTTG TTAGGCTAAT

AGCAAATTTG GACAGGGCTA GTCAAACAGG GAGCTTGTTG TCGGGCTAAT

---------- ---------- ---------- ---------- ----------

AGCAAATTTG GACAGGGCCA GTCAAACAGG GAGCTTGTTG TCAGGCTGAT

AGCAAATTTG GACAGGGCTA GTCAAACAGG GAGCTTGTTG TTGGGCTAAT

AGCAAATTTG GACAGGGCTA GTCAAACAGG GAGCTTGTTG CTGGGCTAAT

AGCAAATTTG GACAGGGCTA GTCAAACAGG GAGCTTGTTG TTGGGCTAAT

AGCAAATTTG GACAGAGCTA GGCAAACAGA GAGCTTGTTG TTGAGCTAAT

AGCAAATTTG GACAGGGCTA GTCAAATAGG GAGCTTGTTG TTGGGCTAAT

AGCAAATTTG GACAGGGCTA GTCAAACAGG GAGCTTGTTG TTGGGCTAAT

---------- ---------- ---------- ---------- ----------

AGCAAATTTG GACAGGGCTA GTCAAACAGG GAGCTCGCGG CT-GGCTAAT

AGCAAATTTG GACAGGGCTA GTCAAACAGG GAGCTTGTTG TCGGGCTAAT

AGCAAATTTG GACAGGGCTA GTCAAACAGG CAGCTTGTTG TTAGGCTAAT

AGCAAATTTG GACAGGGCTA GTCAAACAGG GAGCTTGTTG TTGGGCTAAT

AACAAATTTG GACAGGGCTA GTCAAACATG GAGCTTGTTG CTGAGCTAAT

AGCAAATTTG GACAGTGCCA GTCAAACATG GGGTTCGGGG CTGGGCTAAT

GACAGAGCCA GTGTAAAGTT TAGCGACTCA CATGAACAAT TCCAGGCAGG

GACAGAGCCA GTGTAAAGTT TAGCGACTCA CATGAACAAT TCCAGGCAGG

GACAGAGCCA GTGTAAAGTT TAGCGACTTA CATGAACAAT TCCAGGCAGG

---------- ---------- ----GACTTA CATGAACAAT TCCAGGCAGG

GACAGAGCCA GTGTAAAGTT TAGCGACTCA CATGAACAAT TCCAGGGAGG

GACAGAGCCA GTGTAAAGTT TagcGACTCA CATGAACTAT CCCAGGCAGG

GACAGAGCCA GTGTAAAGTT TAGCGACTCA CATGAACTAT TCCAGGCAGG

GACAGAGCCA GTGTAAAGTT TAGCGACTCA CATGAACTAT TCCAGGCAGG

GACAGAGCCA GTGTAAAGTT TAGC------ -ATGAACAAT TGCATGTAGA

GACAGAGCCA GTGTAAAGTT TAGCGACTCA CATGAACAAT CCCAGGCAGG

GACAGAGCCA GTGTAAAGTT TAGCGACTCA CATGAACAAT CCCAGGCAGG

---------- ---------- ---------- ---------- ----------

GACAGAGCCA GTGTAAAGTT TAGCGACTCA CATGAACAAT CCCAGGCAGG

GACAGAGCCA GTGTAAAGTT TAGCGACTCA CATGAACAAT CCCAGGCAGG

GACAGAGCCA GTGTAAAGTT TAGCGACTCA CATGAACAAT CCCAGGCAGG

GACAGAACCA GTGTAAAGTT TAGC------ ---------- ----------

GACAGAGCCA ATGTAAAGTT TAGAGACTCA CATGAACAAT CCCAGGTAGG

TGCCCAGCCA GAGTAAAGTT TGGAGACTTA CGTGAACGAT CCCGGGCAGG

GCTGCCACAT TGCCAATCTG TTTCATGGCT GGCAGGAAGC CACCAACACC

GCTGCCACAT TGCCAATCTG TTTCATGGCT GGCAGGAAGC CACCAACACC

GCTGCCACGT TGCCAATCTG TTTCATGGCT GGCAAGAAGC CACCAACACC

GCTGCCACGT TGCCAATCTG TTTCATGGCT GGCAAGAAGC CACCAACACC

GCTGCCACAT TGCCAATCTG TTTCATGGCT GGCAGGAAGC CACCAACACC

GCTGCCACGT TGCCAATCTG TTTCATGGCT GGCAAGAAGC CACCAACACC

GCTGCCACAT TGCCAATCTG CTTCATGGCT GGCAGGAAGC CACCAACACC

GCTGCCACAT TGCCAATCTG CTTCATGGCT GGCAGAAAGC CACCAACACC

GTTTCTACAT TGTCAATCTG TTTCATGGAT GGCACTAACT CACCAACAC-

GCGGCCACAT TGCCAATTTG TTTCATGGCT GGCAGGAAGC CACCAACACC

GCTGCCACGT TGCCAATTTG TTTCATGGCT GGTAGGAAGC CACCAACACC

---------- ---------- ---------- ---------- ----------

GCTGCCACGT TGCCGATCTG CTTCATAGCA GGCAGGAAGC CTCCAACCCC

GCGGCCACAT TGCCAATCTG TTTCATGGCT GGCAGGAAGC CACCAACACC

GCTGCCACAT TGCCAATCTG TTTCATGGCT GGCAGGAAGC CACCAACACC

---------- ---------- ---------- ---------- ----------

GCTGCCACAT TGCCAATCTG TTTCATTGCT GGCAGGAAGC CACCGACACC

GCGGCCACGT TGCCGATCTG CTTCATGGCC GGCAGGAAGC CACCAGCACC

TGAAGTACTT ACCACCACCT CGACAGGCAT TCCTTAATTC CTCAAACATC

TGAAGTACTT ACCACCACCT CGACAGGCAT TCCTTAATTC CTCAAACATC

TGAAGTACTT ACCACCACCT CGACAGGCAT TCCTTAATTC CTCAAACATC

TGAAGTACTT ACCACCACCT CGACAGGCAT TCCTTAATTC CTCAAACATC

TGAAATACTT ACCACCACCT CGACAGGCAT TCCTTAATTC CTCAAACATC

TGAAGTACTT ACCACCACCA CGACAGGCAT TCCTTAATTC CTCAAACATC

TGAAGTACTT ACCACCACCT CGACAGGCAT TCCGTAACTC CTCAAACATG

TGGGGTACTT ACCACCACCT CGACAGGCGT TCCTTAATTC CTCAAACATT

---------- ---------- ---------- ---------- ----------

TGAATTACGT ACCACCACCT CGACAGGCAT TCCTTAATTC TTCAAACATT

TGAAGCACTT ACCACCACCT CGACAGGCAT TTCTTAATTC CTCAAACATT

---------- ---------- ---------- ---------- ----------

TGAAATACAC ACCACCACCT CGGCAGGCAT TCCGCAATTC TTCGAACATT

TGAAGTACTT ACCACCACCT CGACAGGCGT TCCTTAACTC CTCAAACATT

TGGAGTACTC ACCACCACCT CGGCAGGCAT TCCGCAACTC CTCAAACATT

---------- ---------- ---------- ---------- ----------

TGAAGTACTT ACCCCCACCT CGGCAGGCAT TTCTTAACTC CTCAAACATT

TGAATCACTC ACCACCACCT CGGCAGGCAT TTCTTAACTC CTCAAACATG

AATTTCTCCA GAGCATCATT CACATAGAAA ACACCTTCAA CCTAATAGAA

AATTTCTCCA GAGCATCATT CACATAGAAA ACACCTTCAA CCTAATAGAA

AATTTCTCCA GAGCATCATT CACGTAGAAA ACGCCCTCAA CCTGATAGAA

AATTTCTCCA GAGCATCATT CACGTAGAAA ACGCCCTCAA CCTGATAGAA

AGTTTCTCCA GAGCATCATT CACATAGAAA ACGCCTTCAA CCTGATAGAA

AATTTCTCCA GAGCATCATT CACATAGAAG ACTCCTTCAA CCTGATAGAA

AGCTTTTCCA GAGCGTCATT CACATAAAAC ACCCCTTCAA CCTGATAGAA

AGTTTTTCCA GAGCATCATT CACATAAAAC ACTCCTTCAA CCTGTTAGAA

---------- ---------- ---------- ---------- ----------

AATTTTTCCA GAGAATCATT CACATAGAAA ACTCCTTCAA CCTGATAGAA

AATTTTTCCA GAGCATCATT CACATAGAAA ACGCCTTCCA CCTGATAGAA

---------- ---------- ---------- ---------- ----------

AATTTTTCCA GAGAATCGTT CACATAGAAA ACGCCTTCAA CCTG------

AACTTTTCCA GAGCATCATT CACATAGAAA ACTCCTTCAA CCTGATAGAA

AATTTTTCCA AGGCATCATT CACATAGAAA ACTCCTTCCA CCTGATAGAA

---------- ---------- ---------- ---------- ----ATAGAA

AATTTCTCAA GTGGATCATT TACATAGAAC ACTCCTTCCA CCTGATGAGG

AGCTTCTCTA GAGGATCGTT CACATAGAAT ACTCCTTCCA CCTGGTAGAA

AACATTTTAA TTAAACACAG AAGATAGTAA CAGTGAATAT AAAACATGAA

AACATTTTAA TTAAACACAG AAGATAGTAA CAGTGAATAT AAAACATGAA

AACATTTTAA TTAAACACAG AAGACAGTAA CAGTGAATAT AAAACATGAA

AACATTTTAA TTAAACACAG AAGACAGTAA CAGTGAATAT AAAACATGAA

AACATTTTAA GTAAACACAG AAGACAGTAA CAGTGAATAT AAAATGTTAA

CACATTTTAA TCAAACACAG GGGACAGTAA CAGCAAATAT AAAACATGAC

AACATTTTAA TTAAGCACAG GACACAGTAA TAGTAAACAT AAAACACAAA

AACATTTTAA TTAAGCACAG GACA--GTAA TAGTAAACAT AAAACATAAA

---------- ---------- ---------- ---------- ----------

AACATTTTAA TTAAATACAG GAGACAGTAG CAGTAAATAT AAAACATAAA

AACAGTTTAA TTAAATACAA GGGGCAGTAG CTGTAAATAT AAAACATAAA

---------- ---------- ---------- ---------- ----------

---------- ---------- ---------- ---------- ----------

AACATTTTAA TTAAATAAAG GAAATAGTAA CAGTAAATAT AAAACAGAAA

AACATTTTTA TTAAATAAAG GGCATAGTAA CAGTAAATAC AAAACATAAA

AACATTTTAA TTAAATAAAG GGCACAGTGG CAGTAAATAT AAAACACAAA

AGAATTTTAA TTAAATATGG GAGAATAAAA TATGAAGTAC AAAATACAAA

AATATTTTAA TTAAAGGGCG GGCACAGTAA TGGCGGGTAC AAAACACAAA

AACCCTTACC ATTGGCCAGT GGGAGGACTC CAAAGTGAAG AATGGACGAT

AACCCTTACC ATTGGCCAGT GGGAGGACTC CAAAGTGAAG AATGGAGGAT

ATCCCTTACC ATTGGCCAGT GGGAGGACTC CAAAGTGAAG AATAGAGGAC

ATCCCTTACC ATTGGCCAGT GGGAGCACTC CAAAGTGAAG AATAGAGGAC

ATCCCTTACC ATTTGCCAGT GGGAGGACTC CAAAGTGAAG AATAGAGGAC

ATCCCTTACC ATTGGCCAGT GGGAGGACTC CGAAGTGAAG AATGGAGGAC

GTCCCTTACT GTTGGCCAGT GGGAGGACTC CGAAGTGAAG AATGGAAGAC

GTCCCTTACT GTTGGCCAGT GGGAGGACGC CGAAGTGAAG AATGGAAGAC

---------- ---------- ---------- ---------- ----------

ATCCCTTACC ATTGGCTAGT GGGAGGACTC CAAAGTGGAG AATGGAGGAC

ATCCCTTACC GTTGGCCAGT GGGAGGATTC CAAAGTGGAG AATGGAGGAC

---------- ---------- ---------- ---------- ----------

---------- ---------- ---------- ---------- ----------

ATCCCTTACC ATTGGCCAGT GGGAGGACGC CAAAGTGAAG AATGGTGGAC

ATCCCTTACC GTTGACCAGT GGAAGCACTC CAAAGTGAAG AATTGAAGAC

AACCCTCACC ATTGGCCAGC GGCAGCACTC CGAAGTGAAG GATGGAAGAC

ATCCCTTACC ATTTGCCAAT GGGATGACTC CAAAGTGAAG GATAGAGGAG

GTCCCTTACC GTTGGCCAGC GGGATGAGCC CGAAATGCAG GACGGAGGAC

AGAATATTTT CAAACCTCAA GACCTAAAAT AAACTTACCT GTTCAGAGAC

AGAATATTTT CGAACCTCAA GACCTAAAAT AAACTTACCT GTTCAGAGAC

AGAATATTTT CAAACCTCAA GACCTAAAAT AAACTTACCT GTTCAGAGAC

AGAATATTTT CAAACCTCAA GACCTAAAAT AAACTTACCT GTTCAGAGAC

AGAATATTTT CAAACCTCAA GACCTAAAAT AAActtacct gttcagAGAC

AGAATGTTTT CAAACCTCAA GACCTAAAAT AAACTTACCT GTTCAGAGAC

AGGATATTCT CAAATCTCAC AACCTAGAAT GCACTTACCT GTTCAAAGAT

AGGATATTCT CAAATCTCAC AACCTGGAAT GCACTTACCT GTTCAAAGAT

---------- ---------- ---------- ---------- ----------

AGAATGTTCT CAAACCTCAA GACCTAAAAT GAACTTACCT GTTCAGAGAT

AGAATATTCT CAAACCTCAA GACCTAAAAT GAACTTACCT GTTCAGAGAC

---------- ---------- ---------- ---------- ----------

---------- ---------- ---------- ---CTTACCT GTTCAGAGAC

AGAATATTCT CAAATCTCAA CACCTAAAAT GAACTTACCT GTTCAGAGAC

AGAATATTCT CAAACCTCAA GACCTAAGAT GAGCTTACCT GTTCAGAGAT

AGGATGTTCT CAAACCTCAG GACCTAAGAT CAACGTACCT GTTCAGCGAT

AGAATATTCT CAAATCTCAG GACCTGAAAC AAACTTACCT GTTCAGAGAT

AGAATATTCT CAAATCTCAG CATCTGAAAC AAACTTACCT GTTCAGAGAT

AAGGAGCAGA CCAGTCTTTG TCCCAAAATA ACCAGGCCAA CACTGGTACT

AAGGAGCAGA CCAGTCTTTG TCCCAAAATA ACCAGGCCAA CACTGGTACT

AAGGAGCAGA CCAGTCTTTG TCCCAAAATA ACCAGGCCAA CACTGGTACT

AAGGAGCAGA CCAGTCTTTG TCCCAAAATA ACCAGGCCAA CACTGGTACT

AAGGAGCACA CCAGTCTTTG TCCCAAAATA ACCAGGCCTA CACTCGTACT

AAGGAGCAGA TCAGTCTTTG TCCCAAAATA GCCAGGCCAA CACTGGTACT

AAGGAGCAGA TCAGTTTTTG TCCCAAAATA GCCAGGCCAA CACTGGTACT

AAGGAGCAGA TCAGTTTTTG TCCCAAAATA GCCAGGCCAA CACTGGTACT

---------- ---------- ---------- ---------- ----------

AAAGAGCAGA TCAGTCTTTG TCCCAAAATA GCCAGGGCAA CATTGGTACT

AACGAGCAGA CCAGTCTTTG TCCCAAGATA ACCAGGCCAA CACTAGTACT

---------- ---------- ---------- ---------- ----------

AAGGAGCAGA TCAGTTTTTG TCCCAAGATG ACGAGGCCCA CACTGGTGCT

AAGGAGCAGA TCAGTCTTTG TCCCAGTATA ACCAGACCAA CACTGGTACT

AAGGAGCAGA CCAGTCTTTG TCCCAAAATC ACCAGGCCGA CACTGGTACT

AAGGAGCAGA CCAGCCTTTG TCCCAGGATA ACCAGGCCGA CGCTGGTACT

AGGGAGCAGA TCAACCTTTG TCCAAAAATA GCCAGGTCTA CACTGGTACT

AAGGAGCAGA TCAGCTTCTG TCCTAAAATA ACCAGATCAA CGCTGGTACT

AGCAACCTCT CACCTCTTCG GTGGAGAGAG TGACATTGAA AACCCCAGCT

AGCAACCTCT CACCTCTTCG GTGGAGAGAG TGACATTGAA AGCCCCAGCT

AGCAACCTCT CACCTCTTTG GTGGAGAGAG TGACATTGAA AGCCCCAGCT

AGCAACCTCT CACCTCTTTG GTGGAGAGAG TGACATTGAA AGCCCCAGCT

AGCAACCTCT CACCTCTTTG GTGGAGAGAG TGACATTGAA AGCCCCAGCT

AGCAACCTCT CACCTCTCTG GTGGAGAGCA TGACGTCGAA AGCCCCAGCA

AGCAACCT-- ---------- ---------- ---------- ----------

AGCAACCTCT TACCTCCCTA GTGGACAGCG TGGTACCAAG AGCCCCAGAT

---------- ---------- ---------- ---------- ----------

AGCAACCTCT CACCTCTTTT GTGGTGAGAG TGAAGCTGAA AGCCCCAGCT

AGCAACCTCT CACCTCTTTC ATGGAGAGAG TGACGTTGAA GGCCCCAGCT

---------- ---------- ---------- ---------- ----------

GGCAACCTCT CACCTCTTCC GTGGACAGGG TG-------- ----------

AGCAACCTCT CACCTCTTTT GTGGAGAGCG TGACATTGAA AGCCCCGGCT

AGCAACCTCT CACCTCCTTT GTGGAGAGTG TGACATTGAA AGCCCCAGCT

AGCAACCTCT CACCTCCTTG GTGGAGAGCG TGACATTGAA AGCCCCAGCC

TGCCATCTCT CACCTCTTTT GTGGTCAGAG TGATGTCGAA GGCCCCAGTG

GGCTACCTCT CACCTCTTTC GTCGTCAAGG TGATGTTGAA GACCCCTGCT

GTGAAATGAG CAAAGGACGC AGATTTAAAG AAATACTCGG CGATTCCAAT

GTGAAATGAG CAAAGGACGC AGATTTAAAG AAATACTCGG CGATTCCAAT

GTGAAATAAG CAAAGGACGC AGATTTAAAG AAATACTCGG CGATTCCAAT

GTGAAATAAG CAAAGGACGC AGATTTAAAG AAATACTCGG CGATTCCAAT

GTGAAATAAG CAAATGATGC AGATTTAAAG AAATACTCGG CGATTCCTAT

ATGAAATAAG CAAAGGAAGC CGACTTAAAG AAATACTCGG AGATTCCAAT

---------- ---------- ---------- --GTACTCAG AGACGCCAAT

ACATAGTGAG CAAATGACGC AGATTTAAAG AAGTACTCAG AGATGCCAAT

---------- ---------- ---------- ---------- ----------

GTAAAATAAG CATAGGATGC AGATTTAAAG AAATACTCGG AGATTCCAAT

ATAAAGTAAG CAAAGGATGC AGATTTAAAG AAAAACTCGG AGATTCCAAC

---------- ---------- ---------- ---------- ----------

---------- ---------- ---------- ---------- ----------

GTGAAATAAG CAAGGGATGC AGATCGAAAG AAATATTCAG AGATTCCGAT

GTAAAGTGAG CAAAGGATGC AGATTTAAAG AAATAGTCGG AGATCCCAAC

ATGAAGTGGG CGAAGGATGC GGATTTAAAA AAATACTCGG AGATGCCCAG

GTGAAATATG CGAATGATGC AGACTTAAAG AAATACTCTG AGATTCCAAG

GTGAAATAGG CAAATGATGC AGACCTAAAG AAATATTCTG AGATTCCGAT

GTAGAGCATA AGTTACCTTC AAGTTCAGGT CAAGGTAGTT CTCAGTAATT

GTAGAGCATA AGTTACCTTC AAGTTCAGGT CAAGGTAGTT CTCAGTAATT

GTAGAGCATA AGTTACCTTC AAGTTCAGGT CAAGGTAGTT CTCAGTAATT

GTAGAGCATA AGTTACCTTC AAGTTCAGGT CAAGGTAGTT CTCAGTAATT

GTAGAGCATA AGTTACCTTC AAGTTCAGGT CAAGGTAGTT CTCAGTAATT

GTAGAGCATA AGTTACCTTC AAGTTCAGGT CAAGGTAGTT TTCAGTAATT

GTAGAGCATA AGTTACCTTC AAATGAAAGT CAAGGTGGTT CTCGGTGACT

GTAGAGCATA AGTTACCTTC AAATTAAAGT CAAGGTGGTT CTCGGTGATT

---------a aGTTACCTTA AAATTCACGT CAAGGGAGTT CTCAGTAATT

GTAGAGCATA AATTACCTTC AAATTGAGGT CAAAGTAATT CTCAGTAATT

ATAGAGCATT AGTTACCTTC AAATTCAGCT CGATATAATT CTCAGTAATT

---------- ---------- ---------- ---------- ----------

---------A AGTTACCTTC AAATTCAGAT CCATGTAATT CTCAGCAACT

GTAGAGCATA AGTTACCTTC AAATTCAGGT CAATGGAGTT CTCATGAACA

GTAGAGCATA AGTTACCTTC AAATTCAGGT CCATGTAATT CTCAGTAATT

GTAGAGCATC AATTACCTTC AGATTCAGGT CCATGTAGTT CTCAGTAATC

GTAAAGCATA AATTACCTTC AATTGCAGGT CAATGGATGA CTGGGTGATT

GTACACCATA AATTACCTTC AAATCCATGT CGATGGCTGA CTCTTGAATT

TCTGGAGAAC TGATTAGGGA GTAATCCAGC AGAGTGTAGT TGTCAATCTT

TCTGGAGAAC TGATTAGGGA GTAATCCAGC AGAGTGTAGT TGTCAATCTT

TCTGGAGAAC TGATTAGGGA GTAATCCAAC AGAGTGTAGT TGTCAATCTT

TCTGGAGAAC TGATTAGGGA GTAATCCAAC AGAGTGTAGT TGTCAATCTT

TCTGGAGAAC TGATTAGGGA GTAATCCAGC AGAGTATAGT TGTCAATCCT

TCTGGAGAAT CGATTAGGGA ATAATCCAGC ACAGTATAGT TGTCGATCTT

TCCGGAGGAC TGATCAGAGA ACAATCCAGC ACAGTGTAGT TGTCAATCTT

TCTGGAGGAC TGATCAGGGA ACAATCCAGC ACTGTGTAGT TGTCAATCTT

TCTGGAGAAT CGATTATGGA ATAATCCAGA AGAGTGTAGT TGTCAATCTT

TCTGGGGAAC TGATTAGGGA ATAATCCAGC AGAGTATAGT TGTCAATCTT

TCCGGAGAAC TGATGAGGGA ATAATCCAGC AGAGTATAGT TGTCAATCTT

---------- ---------- ---------- ---------- ----------

TCTGGAAAAC TGATTAGGGA ATAATCCAGG ATAGTATAGT TGTCGATCTT

GCTGGAGAAT CGATTAGGGA AAAATCCAGC AGAGTATAGT TGTCAATCTT

TCTGGAGAAT TGATCAGGGA ATAATCCAGC AGAGTATGGT TGTTGATCTT

TCTGGAGCAT CGAGTAGGGA ATAATCCAGC AGAGTGTGGT TGTTGATCCT

TCTGGAGGCT CCAAAAGGGA ATAATCTAGC ACAACATCCT CACCAAACTT

TCCGGGGAGT TGATGAGAGA ATAATCTAAC AGAGTATTTT CATCAATCCT

GGTTAAAACT AAACTCACCA TTTCATTTAA GTTCTTTAAA ATGGGTTTCT

GGTTAAAACT AAACTCACCA TTTCATTTAA GTTCTTTAAA ATGGGTTTCT

GGTTAAAACT AAACTCACCA TTTCATTTAA GTTCTTTAAA ATGGGTTTCT

GGTTAAAACT AAACTCACCA TTTCATTTAA GTTCTTTAAA ATGGGTTTCT

GGTTAAAACT AAACTCACCA TTTCATTTAG GTTCTTTAAA ATGGGTTTCT

GGTTAAAACT AAACTTACCA TTTCATTTAA GTTCTTTAAA ATGGGTTTTT

GGTTAAAACT AAACTCACCA TTTCATTTAA ATTCTTCAGA ATGGGTTTCT

GGTTAAAACT AAACTCACCA TTTCATTTAA ATTCTTTAGA ATGGGTTTCT

GGTTAGGACT ACACTTACCA TTTCATTTAA GTTCTTTAAA ATGGGTTTCT

TCTTAAAACT AAACTTACCA TTTCATTTAA TTTCTTTAAA ATGGGTTTCT

GGTTAAAACt aaaCTTACCA TTTCATTTAA GTTCTTTAAA ATGGGTTTTT

---------- ---------- ---------- ---------- ----------

GGTTAACACT AAACTTACCA TTTCATTTAA ATTCTTTAAA ATGGGTTTCT

GGTTAAAACT AAGCTTACCA TTTCATTTAA ATGCCTTAAA ATGGGTTTCT

GGTTAGAACT AAACTTACCA TTTCATTTAA GTTCTTTAAA ATGGGTTTCT

GGTTAGAACT AAA------- ---------- ---------- ----------

GGTTACAACT AAACTTACCC TTTTATTCAA AGTCTTCAGA ATAGGTTTCT

GGTTAGGACT GCACTTACTT TTTCATTCAA GTTCTTCAGG ATGGGTTTCT

CCATGGGCTC AGCAAAGGAG TTATAACCTG AGTTCTCCGG AAAATGAGAC

CCATGGGCTC AGCAAAGGAG TTATAACCTG AGTTCTCCGG AAAATGAGAC

CCATGGGCTC AGCAAAGGAG TTATAACCTG AGTTCTCCGG AAAATGAGAC

CCATGGGCTC AGCAAAGGAG TTATAACCTG AGTTCTCCGG AAAATGAGAC

CCATGGGTTC AGCAAATGAG TTATAACCTG AGTTCTCCGG AAAATGAAAC

CCATGGGGTC AGCAAAGGAG TTATAACCTG AGCTCTCCAG AAAATGAGAC

CCATGGGCTC AGCAAAGGAG TTATAACCTG AGGCCTCCAG AAAATGAGAC

CCATGGGCTC AGCAAAGGAG TTATAACCTG AGACTTCCGA AAAATGAGAC

CCATGGGCTC AGCAAAGGAG TTATAACCTG AACTGTCCAA AAAATGAGAC

CCATGGGCTC AGCGAAGGAG TTATAACCTG AGCTCTCCAG AAAACGAGAC

CCATGGGCTC AGCAAAGGAG TTGTAACCTG AGTTCTCCAG AAAATGACAC

---------- ---------- ---------- ---------- ----------

CCATGGGTTC AGCGAAGGAG TTATAACCTG AGCTCTCCGG AGAATGTGAC

CCATGGCCCC AGCAAAGGAG TTATAACCTG AGATCTCCGG AAAAGGAGAC

CCATGGGCTC AGCAAAGGGG TTATAACCTG AGTTCTCCAG AGAACGAGAC

---------- ---------- ---------- ---------- ----------

CCATTGGATG TGCAAAGGAG TTATAACCTG AGTTCTCCTG AAAATGATAT

CCATTGGCTC AGAGAAGGAG TTATAACCTG AGTTCTCCTG AAAACGCAAT

GTGGGCATGG CTCAGTTGGG CGTAGCAATC TTGGAGCTTC AGGGTAGGAT

GTGGGCATGG CTCAGTTGGG CGTAGCAATC TTGGAGCTTC AGGGTAGGAT

ATGGGCATGG CTCACTTGGG CGTAGCAATC TTGGAGCTTC AGTGTAGGAT

ATGGGCATGG CTCACTTGGG CGTAGCAATC TTGGAGCTTC AGTGTAGGAT

GTGGGCGTGG CTCACTTGGG CATAGCAATC TTGGAGCTTC AGGATAGGAT

ATGGGCGTGG CTCACTTGGG CGTAGCAGTC TTGGAGCCTC AGGATTGGAT

TCGGGCGTGG CTCACTTGTA CATGGCAGTC TTGCAGTTCC AGAGCTGGGT

TCGGGCATGG CTCACTTGAA CATGGCAGTC TTGGAGTTCC AGAGCTGGGT

GTGGGCATGG CTCACTTGGG CATAGCAGTC TTCAAGCTTC AGGAGAGGCT

GTCAGCATGG CTCACTTGAG CATAGCAGTC TTGGAGCTTC AGGATCGGGT

GTGGGCATGG CTCACTTGGG CATAACAGTC TTGGAGCTTC AGGATTGGGT

---------- ---------- ---------- ---------- ----------

GTGGGCGTGG CTCACTTGGG CGTGGCAGTC CTGGAGTTTC AGGATTGGGT

GTGGGCATGG CTCACTTGAG CGTAGCAGTC TTGGAGCTTC AGGGTTGGTT

ATGGGCGTGG CTCACTTGAG CATGGCAGTC TTGGAGCTTC AGGGTTGGGT

---------- ---------- ---------- ---------- ----------

GTGGGCATGA CCAACTTGAA CATGGCAATC TACTAAATGC ATGCTTGGGT

GTGGGCATGA CTGATGTGAA CGTAACAGTC TTCGAGCTTC ATGGTCGGGT

GACCAAAGTC ATTTCGGGTT ATACTCACAA AAGTGGAGAC TCGAACCCCC

GACCAAAGTC ATTTCGGGTT ATACTCACAA AAGTGGAGAC TCGAACCCCC

GACCAAAGTC ATTTCGGGTT ATACTCACAA AAGTGGAGAC TCGAACTCCC

GACCAAAGTC ATTTCGGGTT ATACTCACAA AAGTGGAGAC TCGAACTCCC

GACCAAAGTC GTTTCGGGTT ATACTCACAA AATTGGAGAC TTGAATTCCC

GCCCAGAGTC ATTTCGGGTC ATACTCACAA AAGTGGAGAC TTGATTTCCC

GACCAAAGTC ATTCCGGGTG ATACTCACAA AAGTGGAGCC TTGACGTCCC

AACCAAAGTC ATTACGGGTA ATACTCACAA AAGTGGAGCC TTGACGCTCC

GACCAAAGTC ATTTCGG--- ---------- ---------- ----------

GACCAAAGTC ATTTCGGGAC ATACTCACAA AAGTGGAGAC TTGACTTCCC

GACCAAAGTC ATTTTGGGAG ATACTCACAA GAGTGGAGAT TTGACATCCC

---------- ---------- ---------- ---------- ----------

GGCCAAAGTC ATTCCGGGTC ACACTTACAA GAGTGGAGAC TTGAATTCCC

TACCGAAGTT ATTTCGGGAC ATACTCACAA AAGTGGAGAG CTGACCGCCC

GACCAAAGTC ATTTCGGGAC ATACTTACAA AAGTGGAGAC TTGACTTCCC

---------- ---------- -TACTCACAA GAGCGGAGAC TTGACGTCCC

GGCCTGTATC ACTCCGGGAC ATACTTACAA AAGTGGAGAC ATTATTTCCC

GGCCCATGGC GCTCCGGGAC ATACTTACAA AAGTGGGGAC TTTATTTCCC

AGTCTGTGCT GATGTTGGCA GTGCCATGGT TGGTTAGGAG GTATTTGGAA

AGTCTGTGCT GATGTTGGCA GTGCCATGGT TGGTTAGGAG GTATTTGGAA

AGTCTGTGCT GATGTTGGCA GTGCCGTGGT TGGTTAGGAG GTATTTGGAA

AGTCTGTGCT GATGTTGGCA GTGCCGTGGT TGGTTAGGAG GTATTTGGAA

AGTCTGTGCT GATGTTGGCA GTGCCATGGT TGGTTAGGAG GTATTTGGAA

AGTCTGTGCT GATATTGGCA GAGCCGTGGT TGGTCAGGAC GTATTTGGAA

AGTTGGTGCT GATGTTGGCA GTGCCGTGAT TGCTCAGGAG GTGTTTGGGA

AGTTGGTGCT GATGTTGGCA GTGCCGTGGT TGCTCAGGAG GTATTTGGGA

---------- ---------- ---------- -GGTCAGGAA GTATTTGGAA

AGTCTGTGCT GATGTTGGTA GTTCCATGGT TGGTCAGGAA GTATTTGGAA

AGTTGGTACT GATGTTGGCT GTGCCATGGT TGGTCAGGAA GTATTTGGAA

---------- ---------- ---------- ---------- ----------

AGTCGGTGCT GATGTTGGCG GTGCCGTGGT TGGTCAGGAC GTGTTTGGAA

AGTCGGTGCT GATGTTGGCA GTGCCGTGGT TGGTCAGGAA GAATTTGGAA

AGTGTGTGCT GATGTTGGCT GTGCCGTGAT TGGTGAGGAA GTATTTGGAA

AGTGGGTGCT GATGTTGGCC ATGCCATGAT TGGTGAGGAA GTGTTTGGAA

ATCCCATGCT GATGTTTGTT GAACCATGAT TGGTTAAGAA ATATTTGGAA

AGTCTGTGCT GATGTTTGCA GAACCGTGAT TGGTTAAGAA GTATTTGGAA

ATGAAAAGGC ACTGATTTTT ATACTGTAAA ACCAGAAACa aaatgctctt

ATGAAAAGGC ACTGATTTTT ATACTGTAAA ACCAGAAACa aaatgctctt

ATGAAAAGGC CCTGATTTTT ATACTGTAAA ACCAGAAACa aaatgctctt

ATGAAAAGGC CCTGATTTTT ATACTGTAAA ACCAGAAACa aaatgctctt

ATGAAAAGGC ATTGATTTTT ATACTGTAAA ACCAGAAACa aagtgctctt

ATGAAAAGGC ATTGATTTTG ATGCTGTAAA ACAAGAAACA AAATGCC---

ATGAAAAGGC ATTGATTTTT ATGCTGTAAA ACAAGAAACA gcaTGTGCCA

ATGAGAAGGC ATTGATTTTT ATGCTGTAAA ACAAGAGGCA ACATGCTCCA

ATGAAAAGGC ATTGATTTTT ATGCTGTAAA ACAAGAAAG- ----------

ATGAAAAGGC ATTGATTTTT ATGCTATAAA ACAAGAAACA AAATGCTCTT

ATGAAAAGAC ATTGATTTTT ATGCTGTAAA A-AACAAACA AAATACTCTT

---------- ---------- ---------- ---------- ----------

ATGAAAAGGC ATTGATCGAT ATGCTGTGAA ACAAGAAACA AATTGCTCTT

ATGAAAAGGC ATTGATTTTT ATGCTGTAAA ACAAGAAACA AAATGAACTT

ATGAAAAGGC CTTGATTTTT ATGCTGTAAA ACAAGAAACA AAATGCTCTC

ATGAAAAAGC CTTGATTTTT ATGCTGCAAA ACAAGAAACA AAATGCTCTC

ATGAGAACAT GTTGATTTTT ATGCTATAAA AAAAGAAACA GAATGATCTT

ATGAAAAGGC ACTGAATCGA ATGCTATAAA ACGGAAAACA TAATACCCTG

ctttacttaC TTTGAAAAAT TGTAGTTTAC ATAATCAACT TTTAGAAATT

ctttacttaC TTTGAAAAAT TGTAGTTTAC ATAATCAACT TTTAGAAATT

ctttacttaC TTTGAAAAAT TGTAGTTTAC GTAATCAACC TTTAGAAATT

ctttacttaC TTTGAAAAAT TGTAGTTTAC GTAATCAACC TTTAGAAATT

ctttacttaC TTTGAAAAAT TATAGTTCAC ATAATCAACC TTTAGAAATT

--TTACTTAC TTTGAAAAGT TGTAGTTCAC ATAATCAACC TTTAGGAAGT

CT---CTTAC TTTGAGAAGT TGTACTTTAC ATAGTCGATC TTCAGAAACT

CT---CTTAC TTTGAGAAGT TGTACTTTAC ATAGTCAATC TTCAGAAATT

---------- ---------- ---------- ---------- ----------

CTTTACTTAC TTTGTAAAAT TGTAGTTCAC ATAATCGATT TTCACAAATT

CT--ACTTAC TTTGAAAAAT TATAGTTCAC ATAATCAACC TTCAGAAATT

---------- ---------- ---------- ---------- ----------

CTCAACTTAC TTTGAAAAGT TGTAGTCCAC ATAATCAACC TTCAGAAATT

CTTTACTTAC TTTGAAAAAT TGTAGTCCAC ATAATCCACC TTCAGAAATT

TTTTACTTAC GTTGAAAAAT TGTAGCCCAC ATAATCAACC TTCAGAAATT

TCTCACTTAC TTTGAAAAGT TGTAGTTCAC ATAATCCAGC TGCAGAAACT

TTTCACTTAC TTTGAAAAAT TATAGTCAAC ATAGTCAACC TTTAAAAAGT

CTTCACTCAC TTTGAAAAAT TGTAGTCGAT GTATTCAACT TTCAGAAAAT

CAAGAGACTC AGAACCGCTT AAATCTGGGA CTCACCATAG TCAAGTGCCC

CAAGAGACTC AGAACCACTT AAATCTGGGA CTCACCATAG TCAAGTGCCC

CAAGAGACTC AGAACCGCTT AAATTTGGGA CTCACCATAG TCAAGTGCCC

CAAGAGACTC AGAACCCCTT AAATCTGGGA CTCACCATAG TCAAGTGCCC

CAAGAGACTC AGAACCGCTT AAATCTGGGA CTCACCATAG TCAAGTGCCC

CAAGAGACTC GGAACCATTT ATATCTGGGA CTCACCATAG TCCAGTGCGC

TAAGAGACTC AGAACCGTTC AAATCTGGGA CTCACCATAG TCGAGTGCAC

TAAGAGACTC AGAACCGTTC AAATCTGGGA CTCACCATAG TCTAGTGCAC

---------- ---------- ---------- ---------- ----------

CAAGAGACTC AGAACCTTTT AAATCTGGGA CTCACCATAG TCCAGGGCCC

CTAGAGATTC AGAACCTTTT AGGTCTGGGA CTCACCATAG TCCAGAGCCC

---------- ---------- ---------- ---------- ----------

CCAGGGTTTC CGAGCCTTTT AAGTCCGGGA CTCACCATAG TCCAGAGCCC

CAAGAGACTC AGAACCATTT AAATCTGGCA CTCACCATAG TCCAGCAACC

CAAGAGATTC AGAACCTTTT AAATCTGGGA ---------- ----------

CAAGAGACTC AGAACCGTTT AAACTGGGGA CTCACCATAG TCCAGTGCCC

CAAGAGATTC AGAGCCTTTG AAATCCGGAA CTTACCATAG TCCAGGCCCT

CGAGGGTTTC AGAGCCTTTA AAATCTGGTA CTTACCATAG TCAAGGGCCT

TCTGAGTAAT CCTTGCCTTG ATTCCAGGGT CTTTGAAGCT ATGTGTTCTC

TCTGAGTAAT CCTTGCCTTG ATTCCAGGGT CTTTGAAGCT ATGTGTTCTC

TCTGAGTAAT CCTTGCCTTG ATTCCAGGGT CTTTGAAGCT ATGTGTTCTC

TCTGAGTAAT CCTTGCCTTG ATTCCAGGGT CTTTGAAGCT ATGTGTTCTC

TCTGAGTAAT CCTTGCCTTG ATTCCAGGGT CTTTGAAGCT ATGTGTTCTC

GCTGAGTAGC TCTCACCTTG ATTCCAGGGT CTTTGAAGCC ATGTGTTCTC

GCTGGGTGAT CCTCGCCTTG ATTCCAGGGT CTTCGAAGCA GAGTGTTCTC

GCTGGGTGAT CCTCGCCGTG ATTCCAGGGT CTTTGAAGCA GAGTGTTCTC

---------- ---------- ---------- ---------- ----------

GCTGAGTAGC CCTCGCCTTG ATTCCAGGGT CTTTGAAGTT ATGTGTTCTC

GCTGAGTAAC CCTGGCCTTG ATTCCAGGGT CTTTGAAACT ATGTGTTCCC

---------- ---------- ---------- ---------- ----------

GCTGTGTAAT CCTGGCCTTG ATTCCAGGGT ---------- ----------

TCTGAGTAGC CCTCGCCTTG ATTCCTGGGT CTTTGAAGCC TTGTGTTCTT

---------- ---------- ---------- CTTTGAAGCC GAGCGTTCTC

GCTGGGTGAT CCTTGCCTTG ATTCCAGGGT ctgggaagcc ctgtgttctc

TCTGAGTAAT CCTCACTTTG ATTCCAGGGT CTTTGAAGCC GGGTGTTGAG

TCTGAGTGAT CCTCACTTTG ATCCCTGGCT CTTTGAAGCC ATATTCTTTC

CACTTGCGCC TTAAACTCAG CTGCTAAAAA GCCAAGATAA TTAAGTCTGA

CACTTGTGCC TTAAACTCAG CTGCTGAAAA GCCAAGATAA TTAAGTCTGA

CACTTGCGCC TTAAACTCAG CTGCTGAAAA GTCAAGATAA TTAAGTCTGA

CACTTGCGCC TTAAACTCAG CTGCTGAAAA GCCAAGATAA TTAAGTCTGA

CACTTGCGCC TTAAACTCAG CTGCTGAAAA GCCAAGATAA TTAAGTCTGA

CACTTGCG-C TTCAACTCAG CTGCCGAAAA GCCAAGATAA TTAAGTCTGA

TACGTGCACC TTGAACGCAG AAGCCGAAAC ATCAAGATAA TTAAGTCTGA

TGCGTGCTCC TTAAGCGCAG AAGCCGAAAC ATCAAGATAA TTAAGTCTGA

---------- ---------- ---------- ---------- ----------

CACTGGAGCC TTGAACTCAT CTGCTGCAAA GAT--GATAA TTAAGTCTGA

CACTTGAGCC TTGAACCCAG CTGCTGAAAA GACAAGATAA TTAAGTCTGA

---------- ---------- ---------- ---------- ----------

---------- ---------- ---------- ---------- ----------

CACTTGTGCC TTGAACTCAG CTGCTGCAAA GCCACGGTAA TTAAGTCTGA

CACTTGTGCC TTGAACTCAG TTGCTGCAAA GCCAGGGTAA TTACTTCTGA

cacctgtgcc ttgaactcag ctgctgtaaa gccaagagaa tgacatctga

CACTTGTGCC TGAAACTCAA CTGTAATCGA GCCAAGATAA TTAGGTCTGA

CACTTGCGTC TTAAACTCAA GTGTAAAAAA GCCAAGGTAA TTAGGCCTGA

CTCATCTAGT TTTTTTAATG CCTCTTTCAA AATGTGATAA TAATTGCTTC

CTCATCTAGT TTTTTTAATG CCTCTTTCAA AATGTGATAA TAATTGCTTC

CTCATCTAGT TTTTTTAATG CCTCTTTCAA AATGTGATAA TAATTGCTTC

CTCATCTAGT TTTTTTAATG CCTCTTTCAA AATGTGATAA TAATTGCTTC

CTCATCTAGT TTTTTTAATG CCTCTTTCAA AATGTGATAA TAATTGCTTC

ATCATCTAGT TTTTTTAATG CCTCTTTCAA AAGGTGATAG CAATTGCTTC

GTCATCTAGC TTTTTTGATG ACTCTTTCAA AATGTGATAA TAATTGCCTC

GTCATCTAGC TTTTTTGATG ACTCTTTCAA AATGTGATAA TAATTGCCTC

---------- ---------- ---------- ---------- ----------

ATCATCTAGT CTTTTTAAAG CCTCTTTCAA AATGTGACAA TAATTGCTTC

ATCATCTACT TTTTTTAATG CCTCTTTCAA AATGTGATAA TAATTGCTAC

---------- ---------- ---------- ---------- ----------

---------- ---------- ---------- ---------- ----------

ATCATCTAGT TTTTTTAAAG CCTCTTTCAA AATGTGATAA TAAGTGCTTC

ATCATCTAGT TTTTTTAATG CCTCTTTCAA AATGTGATAA CAATTGCTTC

atcatcttgt tctcttAATG CCTCTTTCAA AACATGATGC CAGTTGCTTC

ATCATCTTGT TTTTTTTAAG CCTCTTTCAA AATACAATAA TAATTACTTC

ATAATTTTGT TTTTTTCAAG TCTCTTTCAA AATGTAATAG TAATTGCTTA

ACTACAATTA CCCAATGGCA TGAATGAGTC TATGCTTATC TGTCTTTTTG

ACTACAATTA ---------- --------TC TATGCTTATC TGTCTTTTTG

ACTACAATTA CCCAATGGCA TGAATGAATC TGTGCTTATC CATCTTTTTG

ACTACAATTA CCCAATGGCA TGAATGAATC TGTGCTTATC CATCTTTTTG

ACTACAATTA CCCAATGGCA TGAATGAATC TATGCTTATC CATCTTTTTG

ACTTCAATTA CCCAATGGCT TGAATGAATC TATCCTTATC CGTCTTTTTG

ACTACAATTA CCCAATGGCA TGAATGAATC TATGCTCATC CGTCTTTTTG

ACTACAATTA CTCAATGGCA TGAATGAATC TATGCTCATC CATCTTTTTG

---------- ---------- ---------- ---------- ----------

ACTTCAGTTA CCCAACAACA TGAATGAA-- ---------- ----------

ACTTCAATTG CCCAATAGCA TGAATGAATC TATCCTTATC CATCTTTTTG

---------- ---------- ---------- ---------- ----------

---------- ---------- --------TC TATCCTTATC CGTCTTTTTG

ACCACAGTTA CCCAATGGCA TGAATGAATC TGTCCTTATC CATCTTTTTG

ACCACAATTA CCCAATGGCA TGAATGAATC TATCCTTATC CGTCTTTTTG

ACCACAATTA CCCAATGGCA TGAATGAATC CGTCCTTATC CGTCTTTTTG

ATTGCAATTA AGCAATGGCA TGAATGAATC TTTCCTTATC CATTTCTTTG

AATGCCATTA CCCAATAGCT TGGTTGAATC TGTCCATCTC CACCATTTTC

GCAGTTCTAA TACCCGATTT ACAATTACAT TGAATACAAG GATCCCCTCA

GCAGTTCTAA TACCCGATTT ACAATTACAT TGAATACAAG GATCCCCTCA

GCAGTTCTGA TACCCGATTT ACAATTACAT TGAATACAAG GATGCCCTCA

GCAGTTCTGA TACCCGATTT ACAATTACAT TGAATACAAG GATGCCCTCA

GCAGTTCTGA TACCCGATTT GCAATTACAT TGAAAACAAG GATGCCCTCA

GCAGTTCTGA TACCCGATTT GCAATTACAT TGAAAACAAG GATGCCCTTA

GCAGTTCTGA TACCCGATTT GCAATTACAT TGAAAACAAG GATGCCCTCA

GCAGTTCTGA TACCCGATTT GCAATTACAT TGAAAACAAG GATGCCCTCA

---------- ---------- ---------- ---------- ----------

---------- ---------- ---------- ---------- ----------

GCAGTTCTGA TACCCGGTTT GCAATCACAT TGAAAACAAG GATGCCCTCA

---------- ---------- ---------- ---------- ----------

GCAGTTCCGA TGCCCGGTTT GCAATTACAT TGAAAACAAG GATGCCCTCA

GCAGTTCTGA TACCCGGTTT GCAATTACGT TGAAAAGAAG GATGCCCTCA

GCAGTTCTGA TACCCGGTTT GCAATTACAT TGAAAACAAG GATGCCCTCA

GCAGTTCTGA TACCCGGTTT GCAATTACGT TGAAAACAAG GATGTCCTCA

GCAGTTCTGA CATCCAGTTT GCAGTAACAT TGAAAGCAAG GATGCCCTCA

GCAGCCCTGA TACCCGTTTT TCAATAACAC TGAAAAGAAA GATGCCCTCA

CTGGAGATGA AGAGACCTTG GCTTCATATG GGATTGTTTC TGGGGACTTG

CTGGAGATGA AGAGACCTTG GCTTCATATG GGATTGTTTC TGGGGACTTG

CTGGAGATGA AGAGACCTTG GCTTCCTATG GGATTGTTTC TGGGGACTTG

CTGGAGATGA AGAGACCTTG GCTTCCTATG GGATTGTTTC TGGGGACTTG

CTGGAGATGA AGAGACCTTG GCTTCATATG GGATTGTTTC TGGGGACTTG

CTGGAGATGA AGAGACCTTG GCTTCATATG GGATTGTTTC TGGGGACTTG

CTGGAGATGA AGAGACCTTG GCTTCATATG GGATTGTTTC TGGGGACTTG

CTGGAGATGA AGAGACCTTG GCTTCATATG GGATTGTTTC TGGGGACTTG

---------- ---------- ---------- ---------- ----------

---------- ---------- ---------- ---------- ----------

CTGGAGATGA AGAGACCTTG GCTTCATATG GGATTGTTTC TGGGGACTTG

---------- ---------- ---------- ---------- ----------

CTGGAGATGA AGAGACCCTG GCTTCATATG GGATTGTTTC TGGGGACTTG

CTGGCGATGA AGAGACTTTG GCTTCATATG GGATTGTTTC TGGGGACTTG

CTGAAGATGA AGAGACCTTG GCTTCATATG GGATTGTTTC TGGGGACTTG

CTGGAGATGA AGAGACCTTG GCTTCCTTTG GGATTGTTTC TGGGGACTTG

CTGAAGATGA AAAGACCTTG GCGGAATATG GGATTGTTTG TGGGGACTTG

CTGAAGATAA CGAGACCTTG GCTTCATACG GAATTGTCTG CGGGGACCTG

ATATGTTTGA TTCTTCAAGA TGACAATATA CCTTCATCAG ATTCAGAGCA

ATATGTTTGA TTCTTCAAGA TGACAATATA CCTTCATCAG ATTCAGAGCA

ATATGTTTGA TTCTTCAAGA TGACAATTTA CCTTCATCAG ATTCAGAGCA

ATATGTTTGA TTCTTCAAGA TGACAATTTA CCTTCATCAG ATTCAGAGCA

ATATGTTTGG TTCTTGAAGA TGCCAATTTA CCTTCATCAG ATTCAGAGCA

ATATGTTTGA TTCTTGAAGA TGCCAATTTA CCTTCATCAG ATTCAGAACA

ATATGTTTGG TTCTTGAAGA TGAAAACTTA CCTTCATCAG ATTCAGAGCA

ATATGTTTGG TTCTTGAAGA TGACAACTTA CCTTCATCAG ATACAGAGCA

---------- ---------- ---------- ---------- ----------

---------- ---------- ---------- ---------- ----------

ATATGTTTGA TTCTTGAAGA TGCCAACTTA CCTTCATCAG ATTCAGAGCA

---------- ---------- ---------- ---------- ----------

ATATGTTTGA TTCTTGAAGA TGCCAATTTA CCTTCATCAG ATTCAGAGCA

ATATGTTTGA TTCTTGAAGA TGCCAACTTA CCTTCATCAG ATTCAGAGCA

ATATGTTTGA TTCTTGAAGA TGCCAACTTA CCTTCATCAG ATTCAGAGCA

CTATGTTTGG TTCTTGAAGA TGCCAACTTG CCTTCATCAG ATTCAGAGCA

ATATGTTTAG TTCTTGAAGA GGCCAACTTG CCTTCATTAG ATTTAGCACA

ATATGTGTAG TCCTCGAAGA TGCCAGCCTC CCTTCACCAG ATTCAGCTCT

TTCTTCACTC CAGAATAATG AGCAACCCTC TTTGGCCACC AGAGCACTGA

TTCTTCACTC CAGAATAATG AGCAACCCTC TTTGGCCACC AGAGCACTGA

TTCTTCACTC CAGAATAATG ACCAACCCTC TTTGGCCACC AGAGCACTGA

TTCTTCACTC CAGAATAATG ACCAACCCTC TTTGGCCACC AGAGCACTGA

TTCTTCACTC CAGAATAATG ACCAACCCTC TTTGGCCACT AGAGCACTGA

TTCCTCACTC CAGAATAATG ACCAACCCTC TTTGGCCGCC AGAGCACTGA

TTCCTCTCTC CAGAATAATG ACCAACCTCC TTTGGCTGCC ACAGTGCTGA

TTCCTCACTC CAGGATAATG ACCAACCTTC TTTGGCTGCC ACAGTACTGA

---------- ---------- ---------- ---------- ----------

---------- ---------- ---------- ---------- --AGCACTGA

TTCCTCACTC CAGAATAATG ACCCACCCTC TTTGGCCGCC AGAGCCCTGA

---------- ---------- ---------- ---------- ----------

TTCCTCACTC CAGAATAATG ACCAACCCTC TCTGGCCGCC AGAGCACTGA

TCCCTCACTT CAGAGTAGTG ACCCATCTTC TTTGGCTGCC AGAGCACTAA

TTCCTCACTC CAGAATAACG ACCAACCCTC TTTGGCCGCC AG--------

TTCCTCACTC CAGAATAACG ACCAAGCCCC TTTGGCCGCC AGAGCCCTGA

TTCCTCATTC GCGAGTAATG ATCAGCCCTC TTTGGCCACC AGAGCTTTAA

TTCCGGACTT CAGGATAGCG AGTCTCCCTC CTTGACCGCC AGAGCCCTGA

ACCTACCAGA TGTATTTGGG TTGGTCGTCC TCCCATTGGA ACTGAAACTA

ACCTACCAGA TGTATTTGGG TTGGTCGTCC TCCCATTGGA ACTGAAACTA

GCCTACCAGA TGTATTTGGG TTGGTCGTCC TCCCCTTGGA ACTGAAACTA

GCCTACCAGA TGTATTTGGG TTGGTCGTCC TCCCGTTGGA ACTGAAACTA

ACCTACCAGA TGTATTTGGT TTGGTCGTCC TCCCATTGGA ACTGAAACTA

ACTTGCCAGA CGCATTTGGG CTGGTTGTCC TTCCATTGGA GTTAAAACTA

ACCTCCCAGA TGTGTTTGGG CTGGTTGTCC TTCCACTGGA GCTGAAACTA

ACCTTCCAGA TGTGTTTGGG CTGGTTGTCC TTCCACTGGA GCTGAAACTA

---------- ---------- ---------- ---------- ----------

ACCTACCAGA TGTATTCGGG TTGGTAGTCC TCCCGTTGGA GCTGAAACTG

ACCTGCCAGA CGTATTTGGG TTGGTGGTCC TCCCATTGGA GCTGAAGCTG

---------- ---------- ---------- ---------- ----------

GCCTGCCGGA TGTATTTGGG CTGCTGGTCC TTCCACTGGA GCTGAAACTA

ACTTACCAGA TGTATTTGGG TTAGTAGTGC TCCCATTGGA GCTGAAACTG

---------- ---------- ---------- ---------- ----------

ACCTCCCAGA TGCGTTTGGG CTGCTAGTCC TCCCACTGGA GCTGAAGCTG

ACCTGCCTGA TGTGTTTGGT TTAGTCGTCC TTCCATTGGA ATTGAAACTC

ACCTCCCTGA TGTTTTTGGC TTGGTGGTAC TCCCTCTGGA ACTGAAACTC

CGGATCTTCC GACTTCTGGA TGTTCGTTCC GTCTTGTCTT TGTCTGCGGT

CGGATCTTCC GACTTCTGGA TGTTCGTTCC GTCTTGTCTT TGTCTGCGGT

CGGATCTTCC GACTTCTGGA TGTTCGTTCC GTCTTGTCTT TGTCTGCAGT

CGGATCTTCC GACTTCTGGA TGTTCGTTCC GTCTTGTCTT TGTCTGCAGT

CGGATCTTCC GACTCTTGGA TGTTCGTTCC ATCCTGTCTT TGTCTGCAGT

CGGATCTTCC GACTTTTGGA TGTTCGTTCT GTCCTGTCTT TGTCTGCGGT

CGCATCTTCC GACTTCTGGA TGTTCATTCC GTCCTGGCTC TGTCTGCAGT

CGCATCTTCC GACTTTTGGA TGTTCATTCT GTCCTGGCCC TGTCTGCAGT

---------- ---------- ---------- ---------- ----------

CGGATCTTCC GACTTTTGGA CGTTCGTTCT GTCCTGTCTC TGTCTGCAGT

CGGATCTTCC GACTTTTGGA TGTTCGTTCT GTTCTCTCTT TGTCTGCGGT

---------- ---------- ---------- ---------- ----------

CGGATATTTC GACTTCTGGA TGTTCGCTCG GTTCTGTCCT TGTCTGCAGT

CGGATCTTTC GACTTTTGGA TATTCGTTCC GTCCTCTCTT TGTCTGCAGT

---------- ---------- ---------- ---------- ----------

CGCATCTTCC GACTTTTGGA TGTCCGCTCT GTCCTGTCCC TGTCGGCCGT

CGGATTTTCC GGCTCTTAGA TGTCCGTTCA GTCCTGGCTC TGTCTGCTGT

CGGATTTTCC GCCTGCTGGA CGTCCGTTCT GTCCTTTCTC TGTCCGCTGT

TTGTCGTGAC CTCTTCAAAT GACCCACTCC TGTGGAGGTT TTTATATCTG

TTGTCGTGAC CTCTTCAAAT GACCCACTCC TGTGGAGGTT TTTATATCTG

TTGTCGTGAC CTCTTCAAAT GACCCGCTTC TGTGGAGGTT TTTATATCTG

TTGTCGTGAC CTCTTCAAAT GACCCACTTT TGTGGAGGTT TTTGTATCTG

TTGTCGTGAC CTCTTCAAAT GACCCACTCC TGTGGAGGTT TTTATATCTT

TTGTCGTGAC CTCTTCCAAT GACCCACTCC TGTGGAGGTG TTTGTATCTG

CTGCCATGAC CTCCTCAAAT GACCCACTGC TGTGGAGGTG CTTGTATCTG

CTGTCATGAC CTCCTCAAAT GACCCACTGC TGTGGAGGTG TTTGTATCTG

---------- ---------- ---------- ---------- ----------

TTGTCGTGAC CTCTTCAAAT GATCAACTTC TGTGGAGGTG TTTGTATCTG

TTGTCGTGAC CTCCTCAAAT GACCAGCTGC TCTGGAGGTG TTTATATCTG

---------- ---------- ---------- ---------- ----------

TTGCCACGAT CTCTTCCAAC GACCAGCTCC TTTGGAGGTG CTTGTATCTG

TTGTCGTGAT CTCTTCAAAT GACCAACTCC TGTGGAGGTG TTTGTATCTG

---------- ---------- ---------- ---------- ----------

GTGTCGTGAC CTCTTCCAAT GACCAGCTCC TGTGGAGGTA TTTGCTTCTG

TTGTCGTGAC CTTTTCAAAT GACCAGCTCC TGTGGAGGTT TATGTACTTA

CTGCCGAGAC CTCTTCCAAT GACCAGCTCT TGTGGAGGTT TATGTATCTT

CGTGATTTTC GAGGTGAACT CACATGTAAG CCTTGTAGTT GGATCCAATT

CGTGATTTTC GAGGTGAACT CACATGTAAG CCTTGTAGTT GGATCCAATT

CGTGATTTTC GAGGTGAACT CACATGTAAG CCTTGTAGTT GGATCCAATT

CGTGATTTTC GAGGTGAACT CACATGTAAG CCTTGTAGTT GGATCCAATT

CGTGATTTTC GAGGTGAACT CACATGTACG CCTTGTAGTT GGATCCAATT

CGGGATTTTC GAGGTGA--- ---------- ---------- ----------

CGGGATTTTC GAGGTGAACT TACATGTATG CCTTGTAGTT GGATCCGATC

CGGGATTTTC GAGGTGAACT TACATGTATG CCTTGTAGTT GGATCCAATC

---------- -------ACT CACATGTAGG CCTTGTAGTT GGCCCCGATT

CGGGATTTTC GAGGTGAACT CACATGTAGG CCTTGTAGTT GGACCCGATC

AGGGATTTTC GAGGTGAACT CACATGTAAG CCTTGTAGTT GGATCCGATT

---------- ---------- ---------- ---------- ----------

CGGGATTTCC GAGGTGAACT CACATGTAGG CTTTGTAGTT GGATCCGATT

CGTGATTTTC GAGGTGAACT CACATGTAAG CTTTGTAGTT GGATCCGATT

---------- ---------- ---------- ---------- ----------

CGGGATTTTC CAGGTAAACT CACATGTAAG CCTTGTAGTT GCAGCCGATT

AGAGATTTTC GAGGTGAACT CACATGTAGG CCTTGTAATT GTTGCCAATT

CGGGACTTTA GAGGTGAACT CACATGTAGG CCTTGTAGTT ACTGCCAATT

TTCTGGATGC GGATGTCGTA CTTGGAGTCG ATGAAGGCCT CGGTGGTGGC

TTCTGGACGC GGATGTCGTA CTTGGAGTCG ATGAAGGCCT CGGTGGTGGC

TTCTGGACGC GGATGTCGTA CTTGGAGTCG ATGAAGGCCT CGGTGGTGGC

TTCTGGACGC GGATGTCGTA CTTGGAGTCG ATGAAGGCCT CGGTGGTGGC

TTCTGGACAC GGATGTCATA CTTGGAGTCG ATGAAGGCCT CGGTGGTGGC

---------- ---------- ---------- ---------- ----------

TTCTGTATGC GGATGTCATA CTTGGAGTCA ATAAAGGCCT CCGTGGTGGC

TTCTGTATGC GGATGTCATA CTTGGAGTCA ATAAAGGCCT CCGTGGTGGC

TTCTGGATGC GGATGTCGTA CTTGGAGTCG ATGAAGGCCT CGGTGGTGGC

TTCTGGATGC GGATGTCATA CTTGGAGTCG ATGAAGGCCT CGGTGGTGGC

TTCTGGATGC GGATGTCGTA CTTGGAGTCA ATGAAGGCCT CAGTGGTAGC

---------- ---------- ---------- ---------- ----------

TTCTGGATGC GGATGTCATA CTTGGAGTCA ATGAAGGCTT CGGTGGTGGC

TTCTGAATGC GGATATCGTA CTTGGAGTCG ATGAAGGCCT CGGTGGTGGC

---------- ---------- ---------- ---------- ----------

TTCTGGATGC GGACGTCGTA CTTGGAGTCA ATGAAGGCCT CAGTGGTGGC

TTCTGGATCC GAATGTCGTA CTTGGAATCG ATGAAGCTTT CTGTGGTGGC

TTCTGGATCC GAATGTCATA TTTGGAGTCA ACAAAGGTCT CTGTGGTGGC

GTAGGTTTTG GCCATGGCGA CCACGCTGGT GATGTCCTGG AAGTCAAGCT

GTAGGTTTTG GCCATGGCGA CCACGCTGGT GATGTCCTGG AAGTCAAGCT

GTAGGTTTTG GCCATGGCGA CCACGCTGGT GATGTCCTGG AAGTCAAGCT

GTAGGTTTTG GCCATGGCGA CCACGCTGGT GATGTCCTGG AAGTCAAGCT

ATAGGTTTTG GCCATGGCGA CCACACTGGT GATGTCCTGG AAGTCAAGCT

---------- ---------- ---------- ---------- ----------

ATAGGTTTTG GCCATGGCCA CCACGCTGGT GATGTCCTGG TAGTCGTGCT

ATAGGTTTTG GCCATGGCCA CCACGCTGGT GATGTCCTGG TAGTCGTGCT

GTAGGTCTTG GCCATGGCCA CCACGCTCGT GATGTCCTGG TAGTCGTGCT

ATAGGTTTTG GCCATAGCCA CCATGCTGGT GACGTCCTGG TAGTCGTGCT

ATAGGTTTTG GCCATAGCCA CCATGCTGGT GATGTCCTGG TAGTCATGCT

---------- ---------- ---------- ---------- ----------

GTAGGTCTTG GCCATGGCCA CGATGCTGGT GATGTCCAGA TAGTCGTGCT

GTAGGTTTTG GCCATGGCGA CCACGCTGGT GATGTCCTGA TAGTCATGCT

---------- ---------- ---------- ---------- ----------

GTAGGTTTGG GCCATGGCGA CCACGCTGGT GATGTCCTGG TAGTCATGCT

GTAAGTCTTG GCCATAGCCA CAATGCTGGT GATGTCCTGG TAATCGTACT

ATAAGTCTTG GCCATGGCCA CCACGCTGGC GATGTCCTGG AAGTCATACT

GGTTTTCCAC TTTGATCTTC ATACCTTTCC CATTCCAGCG TGGGCATGTC

GGTTTTCCAC TTTGATCTTC ATACCTTTCC CATTCCAGCG TGGGCATGTC

GGTTTTCCAC TTTGATCTTC ATACCTTTCC CATTCCAGCG TGGGCATGTC

GGTTTTCCAC TTTGATCT-- ---------- ---------- ----------

GGTTTTCCAC TTTGATCTTC ATACCTTTCC CATTCCAGCG TGGGCATGTC

---------- ---------- ---------- ---------- ----------

GGTTTTCTAC TTTGATCT-- ---------- ---------- ----------

GGTTTTCTAC TTTGATCTTC CTACCTTTCC CATGCCAGCA TGGGCATGTC

GGTTTTCCAC TTTGATCTTC TTACCTTTCC CATCCCGGCG TGGGCGTGTC

GGTTTTCTAC TTTGATCTTC ATACCTTTCC CATTCCGGCG TGGGCATGTC

GGTTTTCTAC TTTGATCTTC ATACCTTTCC CATTCCGGCA TGGGCGTGTC

---------- ---------- ---------- ---------- ----------

GGTTTTCCAC CTTGATCTTC TTACCTTTCC CATGCCGGCA TGGGCATGAC

GGTTTTCCAC TTTGATCTTC CTACCTTTCC CATTCCAGCG TGGGCATGCC

---------- --------TC TTACCTTTCC CATTCCAGCG TGGGCATGTC

GGTTTTCCAC CTTGATCT-- ---------- ---------- ----------

GGTTTTCCAC TTTCACCTTC TTACCTTCCC CATCCCAGCA TGAGCATGTC

GGTTTTCCAC TTTTACCTCC TTACCTTTCC CATTCCAGCG TGAGCATGTC

CCAGCTTGAC TACCACCGGG AACACCAGAG GACAAAAAAC TTGCCGGCTA

CCAGCTTGAC TACCACCGGG AACACCAGAG GACAAAAAAC TTGCCGGCTA

CCAGCTTGAC CACCACCGGG AACACCAGAG GACAAAAAAC TTGCCGGCTA

---------- ---------- --CACCAGAG AACAAAAAAC TTGCTGGCTA

CCAGCTTGAC CACCACCGGG AACACCAGAG GACAAAAAAC TTGTCGGCTA

---------- ---------- --CACCGGAG GACAAAAAAC TTGTGGGCTA

---------- ---------- ---------- ---------- ----------

CCAGCTTGAT CACCACGGGG AACACCAGAG GACAGAAAAC TTGTTGGCTA

CCAGCTTGAC CACCACGGGG AACACCGGAG GACAAAAAAC TTGTCGGCTA

CCAGCTTAAC CACCACGGGG AACACTGAAG GACAAAAAAC TTGTCAGCTA

CCAGCTTGAC CACCACAGGG AACACCAGAG GACAAAAAAC TTGTCGGCTA

---------- ---------- ---------- ---------- ----------

CCAGCTTCAC CACCACGGGG AA-------- ---------- ----------

CCAGCTTGAC CACCACGGGG AACACTGGAG GACAAAAAAC TTGTCGGCTA

CCAACTTGAC CACCACAGGG AACACCAGAG GACAAAAAAC TTGTGGGCTA

---------- ---------- ---------- ---------- ----------

CCATCTTAAC TACCACTGGA AACACGGCAA GAAAAAAAAA TTGTTGGCTA

CTAGTTTGAC CACCACAGGG AACACCAAAG GAAAAAAAAC TCATTGGCTA

TTTTCTCTTC CCTTCCCAAC AATAGCTTTA TTTAGTTTTC CATTATCTTA

TTTTCTCTTC CCTTCCCAAC AATAGCTTTA TTTAGTTTTC CATTCTCTTA

TTTTCTCTTC CTTTCCCAAC AATAGCTTTA TTTAGTTTTC CATTCTCTTA

TTTTCTCTTC CCTTCCCAAC AATAGCTTTA TTTAGTTTTC CATTCTCTTA

TTTTCTCTTC CTTTCCCAAC AATAGCTTTA TTTAGTTTTC CTTTCTCTTA

TTTTCTCTTC CCTTCCCAAC AATAGCTTTA TTTAGTTTTC CTTTCTCTTA

---------- ---------- ---------- ---------- ----------

TTTTCTCTTC CCTTTGCAAC AATAGCTTTA TTTAGTTTTC CTTTTTCTTA

TTTTCTCTTC CTTCCCCAAC AATAGCTTTA TTTAGCATTC CTTTCTCTTA

TTTTCTCTTC CCTTCCCAAC AATAGCTTTA TTTAGTTTTC CTTTTTCTTA

TTTTCTCTTC TCTTCCCAAC AATAGCTTTA TTTAGTCTTC CTTTCTCTTA

---------- ---------- ---------- ---------- ----------

---------- ---------- ---------- -------TTC CCTTCTTGTA

TTTTCTCTTC CCTTCCCAAC AATAGCTTTA TTTAGTTTTC CTTTCTCTTA

TTTTCCCTTC CCTTCTCTAC AATAGCTTTA TTTAGTTTTC CTTTCCCTTA

---------- ---------- ---------- -------TTC CTTTCTCTTA

TTTTCTTTTC CCTTCCCAAC AATGGTTTTA TATAGTTTTC CTTTTTCTCA

TTTTCTCTTT CCTTCCTAAC AATAGTTTTA TTTAGTTTTT TCTTTCCTTG

CATTTCCTGT TTCCCTGAGA AACGGAATAG TCAAAAATAA ACTGATAGGA

CATTTCCTGT TTCCCTGAGA AACGGAATAG TCAAAAATAA ACTGATAGGA

CATTTCCTGT TTCCCTGAGA AACGGAATAG TCAAAAATAA ACTGATAGGA

CATTTCCTGT TTCCCTGAGA AACGGAATAG TCAAAAATAA ACTGATAGGA

CATTTCCTGT TTCCCTGAGA AACGGAATAG TAAAAAATAA ACTGATAGGA

CATTTCCTGT TCCCCTGAGA AACGGAATGG TCAAAAATAA ACTGATAGGA

---------- ---------- ---------- ---------- ----------

CATTTCCTGT TTCCCTGAGA AGTGCAATAG TCAGAAATGA ACCCACAGGC

CATTTCCTGT TTCCCTGAGA AACGGAATAG TCAAAAATAA ACTGAGAGGA

CATTTCCTGT TTCCCTGAGA AACGGAACAG TCAAAAATAA ACCGATAGGA

CATTTCCTGT TTCCCTGAGA AACGGAATTG TCAAAAATAA ACCGATAGGA

---------- ---------- ---------- ---------- ----------

CATTTCCTGT TTCCCTGAGA AACGGAACAG TGGGAAATAA ACCCCTCGGA

TATTTCCTGT TTCCCTGAGA AACGGAATAG TCAAAAATAA ACCGATAGGA

CATTTCCTGT TTCCCTGAGA AACGGAATAG TCAAAAATAA ACCGATAGGA

CAGTTCCTGT TTCCCTGAGA AACGGAATAG TCAAAAATAA ACTGATAGTA

CATTTCCTGT TTCCTTGAGA AACGGAATTG TCAGAAATAA ACAGATTGGA

CATTTTCTCT TTCTCAGAAA AATGGAATTG TCAAAAATAA ACCCATTGGA

TGCAGGAAAA AGCAATTTCC TCCCAGCACG AGAGCTTCCA AAAAGGCAGT

TGCAGGAAAA AGCAATTTCC TCCCAGCACG AGAGCTTCCA AAAAGGCAGT

TGCAGGAAAA AGCAATTTCC TCCCAGCACA AGAGCTTCCA AAAAGGCAGT

TGCAGGAAAA AGCAATTTCC TCCCAGCACA AGAGCTTCCA AAAAGGCAGT

TGAAGGAAAA AGCAATTTCC TCCCAGCACA AGAGCTTCCA AAAAGGCAGT

TGCAGGAAAA AGCAATTTCC TCTCAGCACA AGAGCTTCCT AAAAGGCCGT

---AGGACAA AGCCATTTCC TCTCAGCTCC AAAGCAT-CA AAGAGGCATG

TGCAGGACAA AGCCATTTCC TCTTAGCTCC AAAGCAT-CA AAGAGGCATG

CGCAGGAAAA AGCAATTTCC TCCCAGCCCA CGCGCTTCCA AAAAGCCCTT

TGCAGGAAAA AGCAATTTCC TCTCAGCACA AGAGCTTCCA AAAAGGCAGT

TGCAGGAAAA AGAAATTTCC TTTCAGTGCA AGAGCTTCCA AAAAAGCAGT

---------- ---------- ---------- ---------- ----------

TGC------- ---------- ---------- ---------- ----------

TGCAGGAAAA AGCAATTTCC TCTCAGCACA AGAGCTTCCA AAAAGGCAGT

TGCAGGAAAA GGCAATTTCC TTTTAGCATG AGAGCTTCCA AAAAGGTGGT

TGC------- ---------- ---------- ---------- ----------

TACGAAAAAA AATAATTGCT TGTTAACAAA GAAAATTTTA AACAATCAGT

GACAGGAAGT GGCGGTTTCT TGCCAATTCA TGGGCTGCCA AACAATCAGG

GGTTTAAAAA AGCTTCTAGA TGCTTTTAAA TTAGGCAAAT GAGGCTGGGC

GGTTTAAAAA AGCTTCCAGA TGCTTTTAAA TTAGGCAAAT GAGGCTGGGC

GGTTTAAAAA AGCTTCTAGA TGCTTTTAAA TTAGGCAAAT GAGGCTGGGC

GGTTTAAAAA AGCTTCTAGA TGCTTTTAAA TTAGGCAAAT GAGGCTGGGT

GGTTTAAAAA AGCTTCTAGA TGCTTTTAAA TTAGGCAAAT GAGGCTGGGC

GGTTTAAAAA AGCTTCTGGA TGCTTTTAAA TTAGGCAAAT GAGGCTGGGC

GCTTTAAAAA AGCTTCTAGA TGCTTTTAAA TCAGGCAAAT GAGGCTGGGT

GCTTTAAAAA AGCTTCTAGA TGCTTTTAAA TCAGGCAAAT GAGGCTGGGT

GGTTTAAAAA AGCCCCTAGA TGCTTTTCAA TTAGGCAAAT GAGGCTGGGC

GGTTTAAAAA AGCTTCTAGA TGCTTTTGAA TTAGGCAAAT GAGGCGGGAT

GATTTAAAAA AGCTTCTTGA TGCTTTTAAA TTAGGCAAAT GAGGCTGGGC

---------- ---------- ---------- ---------- ----------

-----GAGAA AGCTTCTAGA GGCTTTTAAC TTAGGCAAAG GAG-------

GGTTTAGAAA AGCTTCTAGA TGCTTTTAAA TTAGGCAAAT GAGGCTGGGC

GGTTTAAAAA AGCTTCTAGA TGCTTTTAAA TTAGGCAAAT GAGGCTGGGC

---------- ---------- ---------- ---------- ---GCTGGGT

GGTTTAAAAA GGAATTTAAA TGGTATCAAA TTAGGCAAAT GAGGCTGGGC

GGTTTAAAGA AGAGTCCGAA TGCTATTCAA TTAAGCAAAT AAGGTTGGGC

ATCCGAGCTG GCCACAGCAT TTGAACATTT GGTATTTACT TCTCAGTACT

ATCCGAGCTG GCCACAGCAT TTGAACATTT GGTATTTACT TCTCAG----

ATCCGAGCTG GCCGCAGCAC TTGAACATTT GGTATTTACT TCTCAGTACT

ATCCGAGCTG GCCCCAGCAC TTGAACATTT GGTATTTACT TCTCAGTACT

ATCCGAGCTG GATACAGCAT TTGAACATTT GGTATTTACT TCTCAGTATT

ATCCGAGCTG GCCATGGCAT TTGAACATTT GGTATTTATT TCTCAG----

ATCCAAGCTG CCCACAGCAT TTGAACATTT GGTGTTTATT TCTCAGTTAC

ATCCAAGCTG CCTGCAGCAC TTGAACATTT GATGTTTATT TCTCAG--AC

ACCCGAGCTG GCCGCAGCAT TTGAACATTT GGTGTTTATT TCCCAG----

ATCCGAGCTG GCCGCAGCAT TTGAACATTT GGTATTTATT T-TCAGAATT

ATCCGAGCTG GCCGCAGCAT TTGAACATTT GGTATTTATT TCTCAGCATT

---------- ---------- ---------- ---------- ----------

---------- ---------- ---------- ---------- ------TATT

ATCCGAGCTG GCCGCAGCAT TTGAACATTT GGTATTTATT TCTCAGCATT

ACCCGAGCTG GCGGCAGGAT TTGAACATTT GGTCCTTCTT TCTTAGCATT

GTCCGAGCTG G--------- -TGAACACT- GGTATTTACT TCTCAG----

GTTACAGATG GCCACAGCAT TTGAACATTT GGTATTTGTT TTTCATCATT

GTCGCAACTG GGCACAGCAT TTGAACATTT GGTGTGCATT TTCCTAGATT

CTTCAGTGGC CCGAACCTGT AACTTGATCT TGATTGTTCT GGATGTCCTG

---------- ---------- ---------- ---------- ----------

CTTCAGTGGC CCGAACCTGT AACTTGATCT TGATTGTTCT GGATGTCCTG

CTTCAGTGGC CCGAACCTGT AACTTGATCT TGATTGTTCT GGATGTCCTG

TTTCAGTGGC CCGAACCTGT AACTTGATCC TGATTGTTTT GGATGTCCTG

---------- ---------- ---------- ---------- ----------

CCTCAGTGGC CCGAACGTGT AATTTGATCT TGATTGTTCT GGATGTCCTA

CCTCAGTGGC CCGAACGTGT AATTTGATTT TGATTGTTCT GGATGTCCTA

---------- ---------- ---------- ---------- ----------

CTTCAGTGGC CAGAACGTGT AACTTGATCT TGATTGTTCT GGATGTCCTG

CTTCAGTGGC CCGAACATGT AACTTGATCC TTATTGTTCT GGATGTCCTG

---------- ---------- ---------- ---------- ----------

CTTTAGTGGC CCGGACCTGT AACTTGATTC TGATTGTTCT GGATGTTCTG

CTTCAGTGGC CCGAACCTGT AACTTGATCC TGATTGTTCT GGATGTCCTT

CTTCAGTGGC TCGAACCTGT AACTTGATCC TGATTGTTCT GGATGTCCTG

---------- ---------- ---------- ---------- ----------

CTTCAGTGGC ACGGACCTGT AATTTAATTC TGATTGTTCT GGATGTGCTG

CCTCAGTGGC TCGAACCTGT AACTTGATCC TGATTGTCCT GGATGTACTG

AAACCTTTGG GACATAAGAA GATAATTGAA AATGAGCTGG AAGGCTTTGG

---------- ---------- ---------- ---------- ----------

AAACCTTTGG GACATAAGAA GATAATTGAA AATGAGCTGG AAGGCTTTGG

AAACCTTTGG GACATAAGAA GATAATTGAA AATGAGCTGG AAGGCTTTGG

AAACCCTTGG GACATAAGAA GATAATTGAA AATGAACTGG AAGGCTTTGG

---------- ---------- ---------- ---------- ----------

AAACCCTTGG GGCACAAGAA GATAATTGAA AATGAGTTGG AAGGCTTTGG

AAACCCTTGG GACATAAGAA GATAATTGAA AATGAGTTGG AAGGCTTTGG

---------- ---------- ---------- ---------- ----------

AAACCCTTGG GACATAAGAA GATAATTGAA AATGAGCTGG AAGGCTTTGG

AAACCCTTGG GACATAAAAA GATAATTGAA AATGAGCTGG AAGGCTTTGG

---------- ---------- ---------- ---------- ----------

AAACCCTTGG GACATAAGAA GATAATTGAA AATGAGCTGG AAGGCTTTGG

AAACCCTTGG GGCATAAGAA GATAATTGAA AATGAGCTGG AGGGTTTTGG

AAACCCTTGG GACATAAGAA GATAATTGAA AACGAGCTGG AAGGCTTTGG

---------- ---------- ---------- ---------- ----------

AAGCCGCTGG GACACAAGAA GATCATCGAG AATGAGCTCG AGGGCTTTGG

AAGCCCCTCG GACACAAGAA AATCATTGAG AATGAACTGG AAGGCTTTGG

CATTCGCTTG AACAGCAAAC CCCCCAACAT TGGCTTTAAG AAGAAGGACA

---------- ---------- ---------- ---------- ----------

CATTCGCTTG AACAGCAAAC CCCCCAACAT TGGCTTTAAG AAGAAAGACA

CATTCGCTTG AACAGCAAAC CCCCCAACAT TGGCTTTAAG AAGAAAGACA

CATTCGCTTG AACAGCAAAC CCCCCAACAT TGGCTTTAAG AAGAAGGACA

---------- ---------- ---------- ---------- ----------

GATTCGTTTG AACAGCAAAC CCCCCAACAT TGGCTTTAAG AAGAAAGACA

GATTCGCTTG AACAGCAAAC CCCCCAACAT TGGCTTTAAG AAGAAAGACA

---------- ---------- ---------- ---------- ----------

CATTCGCTTG AACAGCAAAC CCCCCAACAT TGGCTTTAAG AAGAAGGATA

CATTCGTTTG AACAGCAAAC CCCCCAACAT TGGCTTTAAG AAGAAGGATA

---------- ---------- ---------- ---------- ----------

TATTCGCTTA AACAGCAAAC CCCCTAACAT TGGCTTTAAG AAAAAGGATA

CATTCGCTTG AACAGCAAGC CTCCCAACAT CGGCTTTAAG AAGAAGGATA

TATTCGCTTG AACAGCAAAG CCCCCAACAT TGGCTTTAAG AAGAAGGACA

---------- ---------- ---------- ---------- ----------

GATCCGCTTA AACAGCAAGC CCCCCAACAT AGGCTTTAAG AAGAAGGACA

AATCCGTTTG AACAGCAAAC CTCCCAACAT TGGCTTTAAG AAGAAGGACA

AGGGAGGCAT TAATCTCACA GCCACTGTAA GTTGTGACAA CACAGGATGA

---------- ---------- ---------- --TGTGACAA CACAGGATGA

AGGGAGGCAT TAATCTCACA GCCACTGTAA GTTGTGACAA CACAAGATGA

AGGGAGGCAT TAATCTCACA GCCACTGTAA GTTGTGACAA CACAAGATGA

AGGGAGGCAT TAATCTCACA GCTACTGTAA GTTGTGACAA CACAGGATGA

---------- ---------- ---------- --TGTGACAT CACAGGGTGA

AGGGAGGCAT TAATCTCACA GCCACTGTAA GTAGTGACAT CACAGGGTGA

AGGGAGGCAT TAACCTCACA GCCactgtaa gtAGTGACAT CACAGGGTGA

---------- ---------- ---------- --GGTGACAT CACAGGGTGA

AAGGAGGCAT TAATCTCACA GCCACTGTAA GTTGTGACAT CATGGGATGA

AAGGAGGCAT TAATCTCACG GCCACTGTAA GTTGTGACAT CACAGGATGA

---------- ---------- ---------- ---------- ----------

AAGGAGGCAT TAATCTCACA GCCACTGTAA GT-------- ----------

AAGGAGGCAT TAATCTCACA GCCACTGTAA GTTGTGATCT CATAGGATGA

AGGGAGGCAT TAATCTCACA GCCACCGTAA GTTGTGACAT CACAGGATGA

---------- ---------- ---------- ---------- ----------

AAGGAGGCAT CAACCTCACG GCCACGGTGA GTTGTGACAT CATTGAATGA

AAGGAGGCAT CAATCTCACA GCTACTGTAA GGTGGGACAT GCCAGAATCA

CTCAGGCAGG GGGTGGCCGG ACAGGGAAGC CCTGGTTAAA ACCTCAGTAA

CTCAGGCAGG GGGTGGCCAG ACAGGGAAGC CCTGGTTAAA ACCTCAGTAA

CTCAGGCAGG GGGTGGCTGG ACAGGGAAGC CCTGGTTAAA ACCTCAGTAA

CTCAGGCAGG GGGTGGCTGG ACAGGGAAGC CCTGGTTAAA ACCTCAGTAA

CTCAGGCAGG GGGTGGCTGG ACAGGGAAAC CCTGGTTAAA ACCTCAGTAA

CTCAGGCAGG AGGTGGCAGG ACAGGGAAGC CCTGGTTAAA ACCTCAGTAA

CTCATGCTGG AGATGGCGGG ACAGGGAAGC CCTGGTTAAA ACCTCAGTAA

CTCAGGCTGG AGATGGTAGG ACAGGGAAGC CCTGGTTAAA ACCTCAGTAA

CTCAGGCAGG AGGTGGCGGG ACAGGGAAGC CCTGGTTAAA ACCTCAGTAA

CTCAGGCAGG AGGTGGCAGG ACAGGGAAGC CCTGGTT-AA ACCTCAGTAA

CTCAGGCAGG AGGTGGTAGG ACAGGGAAGC CCTGGTTAAA ACCTCAGTAA

---------- ---------- ---------- ---------- ----------

---------- ---------- ---------- ---------- ----------

CTCAGGTAGG AGGGGGCAGA ACAGGGAAAC CCTGGTTAAA ACCTCAGTAA

CTCAGGCAGG AGGTGGCAGG ATGGGGAAGC CCTGGTTAAA ACCTCAGTAA

---------- ---------- ---------- ------TAAA -CCTCAGTCA

CTCTGGCAGG AGACAGCAGG ACTTGGAAGA CATGATTATG ACCTCAGTAA

ATGAGGCCGG AGGTGGCTGT TGAGGGAAGC CCCGACTTGG ATCTCTGTAA

ATAGGGACAG GGCGGTAACA TTAGTAGACA GGGCTGGGCC ACAGAATCGG

ATAGGGACAG GGCGGTAACA TTAGTAGACA GGGCTGGGCC ACAGAATCGG

ATAGGGACAG GGCGGTAACA TTAGTAGACA GGGCTGGGCC ACAGAATTGG

ATAGGGACAG GGCGGTAACA TTAGTAGACA GGGCTGGGCC ACAGAATTGG

ATAGGGATAG GGCGGTAACA TTAGTAGACA GGGCTGGGCC ACAGAATTGG

ATAGGGACAG GGCGGTAACA TCAGCAGACA GGGCTGGGCC ACAGCCTTGG

ATAGGGACAG GGCGGTAACG TTAGTAGACA GGGATGGGCT ACAGAATCGG

ATAAGGATGG GGCGGTAACA TTAGTAGACA GGGATGGGCC ATAGAATTGG

ATAGGGACAG GGCGGTAACA TTAGTAGACA AGGCTGGGCC ACAGA-TTGG

ATAGGAACAG GGCGGTAACA TTAGTAGACA GGGTTGGGCC ACAGAATTGG

ATAGGGACAG GGTGGTAACA TTAGTAGACA GGGCTGGGTC ACAGAATCGG

---------- ---------- ---------- ---------- ----------

---------- ---------- ---------- ---------- ----------

ATAAGGAGAG GGCAGTAACA TTAGTAGACA GGGTTGGGCC ACAGAATTGG

ATAGGGACAG GGTGGTAACA TTAGTAGACA GGGTTGGGTC ACAGAATTGG

ATAAGGGCAG GGCAGTAACA TTAGTAGACA GGGCTGGACC ACAGCATTGG

ATAGAGATGG AGCAATCCCT TTATAAAAAG GGGAAGGGCT GGTGGGATGG

ATAAGGATGA AGCAGTGACA TTATTCTAAG GGGCAGCCCA GTACGCTGGA

TGGTTTAGGA CAAATAGACT TTTTATTTTG GAAATTTTCC CAAGTGTCTC

TGGTTTAGGA CAAATAGACT TTTTATTTTG GAAATTTTCC CAAGTGTCTC

TGGTTTAGGA CAAATAGACT TTTTATTTTG GAAATTTTCC CAAGTGTCTC

TGGTTTAGGA CAAATAGACT TTTTATTTTG GAAATTTTCC CAAGTGTCTC

TGGTTTAGGA CAAATAGACT TTTTATTTTG GAAATTTTCC CAAGTGTCTC

TGGCTTAGGT CAAATAATCT TTTTATTTTG GAAATTTTCC CAAGTGTCTC

TGGTTTGAGA AAAATAAGCT TTTTATTTTG GAAATTTTCC CAAGTGTCTC

TGGTTTGAGA CAAATAAACT TTTTATTTTG GAAATTTTCC CAAGTGTCTC

TGGTTTAGGT CAAATAGACT TTTTATTTTG GAAATTTTCC CAAGTGTCTC

TGGTTTGGGT CAAATAGACT TTTTATTTTG GAAATTTTCC CAAGTGTCTC

TGGTTTGGGT CAAATAGAC- TTTTATTTTG GAAATTTTCC CAAGTGTCTC

---------- ---------- ---------- ---------- ----------

---------- ---------- ---------- ---------- ----------

TGGTTTGGGT CAAATGGACT TTTTATTTTG GAAATTTTCC CAAGTGTCTC

TGGTTTGGGT CAAGTACACT TTTTATTTTG GAAATTTTCC CAAGTGTTTC

CGGTTTGGGT CAAATCGACT TTTTATTTTG GAAATTTTCC CACATGTCTC

TGGTGTGGGA GAAATGTACT CTTCATTTTG GAAATCTTCC CGAGTCTCTC

TTGTTCGGGC CACATGGATC CTCTCTTTTT GAACTTCTCT TCAGTgtctg

ACTGGAACAA GTGGAGAGCC CCTCTTGAAG GAAGTGATTC AGAAACCATG

ACTGGAACAA GTGGAGAGCC CCTCTTGAAG GAAGTGATTC AGAAACCATG

ACTGGAACAA GTGGAGAGCC CCTCTTGAAG GAAGTGATTC AGAAACCATG

ACTGGAACAA GTGGAGAGCC CCTCTTGAAG GAAGTGATTC AGAAACCATG

ACTGGAACAA GTGGAGAGCC CCTCTTGAAG GAAGTGATTC AGAAACCACG

ACTGGAATAA GCAGAGAGCC CCTCTTGGAG GAAGTGATTC AGAAACCACG

ACTGGAACGA GCGCAGGTCC CCTCTTGAAG GAAATGATTC AGAAACCATG

ACTGGGACAA GCTCAGAGTC CCTCTTGAAG GAAATGATTC AGAAACCACG

ACTGGAACAA GTGGAGAGCC CCTCTCGGAG GAAGTGATTC AGAAACCGCC

ACTGGAACAA GTGGAGAGCC TCTCTTGGAG GAAGTGATTC AGAAACCACG

ACTGGAACAA GTGGAGAGCC CCTCTTGGAG GAAGTGATTC AGAAACCGCG

---------- ---------- ---------- ---------- ----------

---GGAACAA GTGGAGAGCC CCTCTTAGAG GAAGTGATTC AGAAACCGCG

ATTGGAACAA GTGGAGAGCC CCTTTTGGAG GAAGTGATTC AGAAACCACG

ACTGAAACAA GTGGGGAGCC CCTCTTGGAG GAAGTGATTC AGAAACCACG

ACTGGAACAA GTGGA----- ------GGAG GAAGTGAT-- ----------

ATTGGAACAA GTGGAGAACT CCTGGCGGAG GAAATGATTC AGAAGCCACT

tctGGAACAA GTGGACAGAG CCTCACGGAG GAAGTGATTC AGAGGCTCTG

ATGGCCTGTG ACTCACAGCA CATCAATGGG GGAATTGTCT GTAGAAGCCG

ATGGCCTGTG ACTCACAGCA CATCAATGGG GGAATTGTCT GTAGAAGCCG

ATGGCCTGTG ACTCACAGCA CATCAATGGG GGAATTGTCT GTGGAAGCTG

ATGGCCTGTG ACTCACAGCA CATCAATGGG GGAATTGTCT GTGGAAGCTG

ATGGCTTGTG ACTCATAGCA CATCAATGGG GGAATTGTCT GTGGAAGCGG

ATGGCCTGTG ACTCACAGCA CATCAATGGG GGAATTGTCT GTGGAGGCTG

CTGGTCTGTA ATTCACAACA CATCAATGGG GGAATTATGT GTAG-GGCTG

GTGGCCTGTA ATTCATAGTA CATCAATAGG GGAATTATGT GTAG-GGCTG

ATGGCCTGTG ACTCACAGCA CATCAAT-GG GGAATTGTGT GTGGAGGCTG

ATGGCCTGTG ACTCACTACA CATCAATGGA GGAATTGTCT GTGGAGGCTG

ATGGCCTGTG ACTCACAGCA CATCAATGGG GGAATTGTCT GTGGAGGCTG

---------- ---------- ---------- ---------- ----------

ATGGCCTGTG ACTCACAGCA CATCAATGGG GGAATTGTCT GTGGGGGC-G

ATGGCCTGTG ACTCACAGCA CATCAATGGG GGAATTGTCT GTGGAGGCTG

ATGGCCTGTG ACTCACAGCA CATCAATGGG GGAATTGTCT GTGGAGGCTG

---------- ---------- ---------- ---------- ----------

ATGGCCTGTG ACTCACAAGA CTTCAATGGG GGAATTGTCT GGGGAGGCTG

AAGGCCCGTG ACTCATAAGA CATCAATGGG AGAATTGTCT GGAGAGGCTG

AGAATTCCAA AGCTCTtttc tttcctTTGT GCAACAGCAG GGCTGCCCAG

AGAATTCCAA AGCTCTtttc tttcctTTGT GCAACAGCAG GGCTGCCCAG

AGAATTCCAA AGCTCTtttc tttcctTTGT GCAACAGCAG GGCTGCCCAG

AGAATTCCAA AGCTCTtttc tttcctTTGT GCAACAGCAG GGCTGCCCAG

AGAATACCAA AGCTCTtttc tttcctTTGT GCAACAGCAG GGCTGCCCAA

AGAATACCAG AGCTCTtttc ttgcctTTGT GCAACACCAA GACTGTCCAG

AGAATAGCAA AGCTCAtttc tttcctTAGA GAAATTCCAT GGCAGCCCAG

AGAACACCAA AGCTCATTTC TTTCCTTAAA TCAATTCCAA GGCAGCCCAG

AGAATACCAA AGCGCTTTTC TTTCCTTTGT GCAACACCA- GGCTGCCCAG

AGAATACCAA AGCTcttttc tttcctTTGC GCAACACCAG AGCTGCCCAG

AGAATACCAA AGCtcttttc tttcctCTGC GCAACACCAG GGCTGCCCAG

---------- ---------- ---------- ---------- ----------

AGGAGACCAA AGCN------ ---------- ---------- ----------

AGAATACCAA AGCTCTtttc tttcctTTGA GCAACACCAG GGCTGCCCAG

AGAATACCAA AGCTCttttc tttcctTTGC GCAACAACAG GGCTGCCCAG

---------- ---------- ------TTGT GCAACAGCAG GGCCTCCCCG

AGAATACCAA AGCTCCTTTC TTTGCTTTGT GCAACAACCC AGCTACTCAA

AGAGTACCAA GAGTCTTTTA TTTTCTTTGC ACAACACTTC TGCTGCTCAG

AAACATGTGG TTTTCATCAT GTAGCTATTT CAGATCCAAT CACGTCAAGC

AAACATGTGG TTTTCATCAT GTAGCTATTT CAGATCCAAT CACGTCAAGC

AAACATGTGG TTTTCATCAT GTAGCTATTT CAGATCCAAT CACGTCAAGC

AAACATGTGG TTTTCATCAT GTAGCTATTT CAGATCCAAT CACGTCAAGC

AAACATGTGG TTCTCATCAT GTAGCTATTT CAGATCCAAT CACGTCAAGC

-AGCATGTGT TTCTCATTA- ---------- ---------- ----------

AAGCACGTGC TTCTCATTAT GTAGCTGTTC CAGATCCCCT CACATCAAGC

---CACATGC TTCTCATTAT GTAGCTGTTC CAGATCCGTT CACATCAAGC

AGACATGTGT TTCTCATCAT GTAGCTATTT CAGATCCAGT CACGTCAAGC

AAACATGTGT TTCTCATCAT GTAGCTATTT CAGGCCCAAT CATGTCAAGC

AAACATGTGT TTCTCATCAT GTAGCTATTT CAGACCCAAT CACGTCAAGC

---------- ---------- ---------- ---------- ----------

---------- ---------- ---------- ---------- ----------

AAACATGTGT TTCTCATCAT GTAGCTATTT CAGATCCAAT CACATCAAGT

AAACATGTGT TTCTCATCAT GTAGTTATTT CAGATCCAAT CACGTCAAGC

AGACGTGTGT TTTTCTTCC- ---------- ---------- ----------

AAACATGTGT TTCTCATCAT GTACCTCTTC CTGGTCCAAT CAAGTCTTGT

AAAAATGTGT TTCCCATTAC ATAGCCACCC CTGTTCCAAG CAAATCACAT

CCAGACAGAG ATTTAAGGCG ACTGAGCCGA CAATCTGTTT CTCATTAGCT

CCAGACAGAG ATTTAAGGCG ACTGAGCCGA GAATCTGTTT CTCATTAGCT

CCAGACAAAG ATTTGAGGCG ACTGAGCCGA GAATCTGTTT CTCATTAGCT

CCAGACAAAG ATTTGAGGCG ACTGAGCCGA GAATCTGTTT CTCATTAGCT

CCAGACAAAG ATTTGAGGCG ACTGAGCCGA GAATCTGTTT CTCATTAGCT

---------- ---------- ---------- ---------- ----------

TCAGACAGAG ATCTGAGGCA ACTGAGTCGA GAGCCTGTTT CTCATTAGCT

TCAGCCGGAG ATCTGAGGAG ACTGAGCCGG GAGCCTGTTT TTCATTAGCT

CCAGACAGAG ATCCGAGGCA ACA------- ---------- ----------

CCAGACAAAG ATCTAAGGCG ACTGAGCCGA GAATCTGTTC CTCATTAGCT

CCAGACAGAG ATCTAAGACG ACTGAGCCGA AAATCTGTTT CTCATTAGCT

---------- ---------- ---------- ---------- ----------

---------- ---------- ---GAGCCGA GAATCTGTTT CTCATTAGCT

CCAGACAGAG ATGTGAGGTG ACTGAGCCGA GAATCTGTTT CTCATTAGCT

CCAGACAGAG ATCTGAGGCA ACTGAGCCGC AAATCTGTTT CTCGTTAGCT

---------- ---------- ---------- ---------- ----------

CCAGACGGAG ATCTGAAGGG ACTGAGCCAG GAATCTGTTT TCCATTAGCT

CCAAGCAGAG ACTTGGAAGA GTTAAGCCAA GAATCTGTTT TTCATTAGCT

GTACTTTTGC TGTGAAATGA AGTGCTCTTT GAACCTGTGG GAGCAGGAAA

GTACTTTTGC TGTGAAATGA AGTGCTCTTT GAACCTGTGG GAGCAGGAAA

GTACTTTTGC TGTGAAATGA AGTGCTCTTT GAACCTGTGG GAGCAGGAAA

GTACTTTTGC TGTGAAATGA AGTGCTCTTT GAACCTGTGA GAGCAGGAAA

GTACTTTTGC TGTGAAATGA AGTGCTCTTT GAACCTGTGG AAGCAGGAAA

---------- ---------- ---------- ---------- ----------

GTACTTTTCC TGTACAATGA AGTGCTCTTT GAACCTGTAG GAGCAAGAAA

GTACTTTTCC TGTACAATGA AGTGCTCTTT GAACCTGTAG GAACAAGAAA

---------- ---------- ---------- ---------- ----------

GTACTTTTGC TGTGAAATGA AGTGCTCTTT GAACCTGGGG GAGCAGGAAA

GTACTTTTGC TGTGAAATGA AGTGCTCTTT GAACCTGTGG GAGCAGGAAA

---------- ---------- ---------- ---------- ----------

GTACTTTTGC TGTGAGATGA AGTGTTCTTT GAACCTGCAG GAGCAAAAAA

GTACTTTTGC TGTGAAATGA AGTGCTCTTT GAACCGGTGG GAGCAGGAAA

GTACTTTTGC TGTGAAATGA ACTGCTCTTT GAACCTGTGG GAGCAGAAAA

---------- ---------- ---------- ---------- ----------

GTACTTTTCC CGGAGACTGA ATCGTTCTTT GAACCTGTAG AAGCAGGAAA

GTACTTTTGC TGGAAACCGA ATTGCTCTTT GAACCTACGG GAGCAGGAAG

CCCATTTACA CCCTGAGAAG ACAGGCAGCC ATATCACACA ATTATGATGA

CCCATTTACA CCCTGAGAAG ACAGGCAGCC ATATCACACA ATTATGATGA

GCCATTTACA CCCTGAGAAG ACAGGCAAAC ATATCACACA ATTATGATGA

GCCATTTACA CCCTGAGAAG ACAGGCAAAC ATATCACACA ATTATGATGA

GCCATTTACA CCCTGAGAAG ACAGGCAGCC ATATCACACA ATTGTGATGA

---------- ---------- -----CAGCC ATGTTACACA ATGATGATGA

GTCATTGACA CTCAGAGAAG ACAGGCAGCC ATTTTACACA ATTATGATGA

GTCATTTACA CTCTGAGAAG ACCGGCAGCC ATATTACACA ATTATGATGA

---------- ---------- -----TAGCC GTATTACACA GCTTTGATGA

GCCATTTACA CCCCGAGAGG ACAGGTAGCC ATATTACACA ATTATGATGA

GCCATTTACA CCCCGAGGAG ACAGGCAGCC ATATTACACA ATTATGATGA

---------- ---------- -----CAGCC ATATTACACA ATTATGATGA

GCCATTTACA CCCCGAGAAG ACAGGCAGCC ATATTACACA ATTATGATGA

GCCATTTACA CCCCGAGAGG ACAGGCAGCC ATATTACACA ATTACGATGA

GCCATTTACA CCCCGAGAGG ACAGGCAGCC ATATTATACA ATCGTGATGA

---------- ---------- -----CAGCC ATATTATACA ATTATAATGA

CCCATTAAAA CCCTGAGAGG ACAGGCATTC GTGTTGTACA ATAATGATGA

CCCATTAAAA CCCTGACAGG ACAGGCGGTT ATATTGCACA ATAACGATGA

TGCATAGATA TTCTTGGGCC TAAAATGCTG CTCAAGTCCT GAGGGGAACA

TGCATAGATA TTCTTGGGCC TAAAATGCTG CTCAAGTCCT GAGGGGAACA

CGCATAGACC TTCTTGGGCC TAAAATGCTG CTCAAGTCCC AAGGGGAACA

CGCATAGACC TTCTTGGGCC TAAAATGCTG CTCAAGTCCC AAGGGGAACA

CGCATAGACC TTCTTGGGC- -AAAATGCTG CTCAAGTCCC GAGGGAAACA

CGCACAGACC TTCTTGGGCC TGAAATGCTT CCCAAGTCCC GAGGGCAATA

CGCACGGGCC TTCTTGGGCC TAAAATGCTT CCCGAGTCCC GAGGGGAGTC

CGCACGGGCC TTCTTGGGCC TAAAATGCTT CCCGAGTCCC GAGGGGAGTC

CGCACAGACC TTCTAGGGCC TCAAATGCTT CCCCAGTCCC GAGGGGAGTA

CGCACAGACC TTCTTGGGCC TAAAATGCTT CCCAAGTCCC GAGGGGAACA

CGCACGGACC TTCTTGGGCC TAAAAGACTT CCCAAGTCCC GAGGGGAATA

CGCACAGACC TTCTTGAGCC TAAAATGCTT CACAAGTCCC GAGGGGAATA

CGCATGGACC TTCTTGCGCC TGAAATGCTT CCCTAGTCCC GAGGGGGGTA

CGCACAGACC TTCTTGGGCC TAAAATGCTT CCCAAGTCCC GAGGGGAGCA

CGCACAGACC TTCTTGGGCC TAAAATGCTT CCCAAGTCCT GAGAGGAACT

CGCACGGGCC TTCTTAGGCC TAAAATGCTT CCCCAGTCCC GAGGGGAACT

TGTAAAGTCC TTCTTGGGAC TAAACTGCGT CCTAAGCCCC ATGGGAAAAC

CGCAAAAAAC CTCTCGGATC TAAAATGCTG CCCTCCACCC TAGGGGAAAA

GCATGAATAC CTCATTGTAC CCATGGAGGA GGAACTAACA AAGACGTCTT

GCATGAATAC CTCATTGTAC CCATGGAGGA GGAACTAACA AAGACGTCTT

GCATGAATAC CTCATTGTAC CCATGGAGGA GAAACTAACA AAGATGTCTT

GCATGAATAC CTCATTGTAC CCATGGAGGA GGAACTAACA AAGATGTCTT

GCATGAATAC CTCATTGTAC CCATGGAGGA GGAACTAACA AAGACGTCTT

GCATGAATAC CTCATTGTAC CCATGGAGGA GGAACAAACA AAGACTTCTT

GCATGAATAC CTCATTGTGC CCACAGAGGA GGAAGTAACA AAGACTTCTT

GCATGAATAC CTCATTGTGC CCACAGAGGA GGAAGTAACA AAGACTTCTT

GCATGAATAC TTCATTGTGC CCACAGAGGA GGCCCTAACA AAGACTTCTT

GCATGAATAC CTCATTGTAC CCATGGAGGA GGGACTAACA AAGACTTCTT

GCATGAATAC CTCATTGTAC CCATGGAGGA GGAACTAACA AAGACTTCTT

GCATGAATAC CTCATTGTAC CCAGGGAGGA GGAACTAACA AAGGCTTCTT

GCATGAATAC TGCATTGTGC CCAAGGAGGA GGAACTAACA AAGACTTCTT

GCATGAATAC CTCATTGTAC CCATGGAGGA GGAACTAACA AAGACTTCTT

GCATGAATAC CTCATTGTAC CCATGGAGGA GAAACTAACA AAGACTTCTT

CCATGAATAC CTCATTGTAC CCATAGAGAA GGACCAAACA AAGACTTCTT

CCATGAATAC CTCATTGTGT CCACAGAGGA GGGAATCACA AAGAGTTCTT

AGATGAATAC CTTATTGTAC CCACAGAGGA TGAAAGAACA AAGAACTCTT

ATCTTCCAGC TTCCTATTCC AGGATCAGAA TTATCTCTGA CATTCTGAAA

ATCTTCCAGC TTCCTATTCC AGGATCAGAA TTATCTCTGA CATTCTGAAA

ATCTTCCAGC TTCCTATTCC AGGATCAGAA TTATCTCTGA CATTCTGAAA

ATCTTCCAGC TTCCTATTCC AGGATCAGAA TTATCTCTGA CATTCTGAAA

ATCTTCCAGC TTCCTATTCC AGGATCAGAA TTATCTCTGA CATTCTGAAA

ATCTTCCAAC TTCCTATGCC AGGGTCAAAA TTATCTTTGA CATTCTGAAA

ATCTTGCAGC TCGCCATGCC AGGACCAGAA TTATCTTTGA CATTCTGAAA

ATCTTGCAGC TCACCATGCC AGGACCAGAA TTATCTTTGA CATTCTGAAA

ATCTTCCTGT TGCCTATGGC AGGAGCAGTC TTATCTCTCA CATCCTGAGA

ATCTTCCAGC TTCCTATGCC AGGATCAGAA TTATCTCTGA CATTCTGAAA

ATCTTCCAGA TTCTTATGCC AGGATCAGAA TTATCTCTGA CATTCTGAAA

ATCTTCCAGC TTCCAATGCC AGGATCAGAA TTATCTCTGA CATTCTGAAA

CTCATCCAGC TTCCTATGCC AAGGTCAGAA TTATCTCTGA CATTCTGAAA

ATCTTCCAAC TTCCTATGCC AGGATTAGAA TTATCTCTGG CATTCTGAAA

ATCTTCCAGC TTTCTATGCC AGGATCAGAA TTATCCATGA CATTCTGAAA

ATCTTCCAGC TTCCTGGGCC AGGATCAGAA TTATCTCTGA CATTCTCAAA

AACTGCCAGA TATCTAGGGC AATACCAGGA CTTTCATGGA CACTCTGAAA

AACTTCTAGC TTGCAGTGCT GATAACAGCA TTTTTGCAGA TTCTCAGAAA

ATGCAAAGCA TTCCAGCCAT TTTAATTACT GGGCATTGCA TAAGCCAGCA

ATGCAAAGCA TTCCAGCCAT TTTAATTGCT GGGCGTTGCA TAAGCCAGCA

ATGCAAAGCA TTCCAGCCAT TTTAATTACT GGGCGTTGCA TAAGCCAGCA

ATGCAAAGCA TTCCAGCCAT TTTAATTACT GGGCGTTGCA TAAGCCAGCA

ATGCAAAGCA TTCCAGCCAT TTTAATTACT GGGCATTGCA TAAGCCAGCA

ATGCAAAGCA TTCCAGCCAT TTTAATTACT GGGCGTTGTA TAAGCCAGCA

ATTCTAAGTA TTCCAGCCAT TTTAATTACT GGGAGTTGCA TAAGCCAGAA

ATGCAAAGTA TTCCAGCGAT TTTAATTACT GGGAGTTGCA TAAGCCAGAA

ATGCGAGGCA TTCCAGCCAT TTTAATTACT GGGCGTTGCA TAAGCCAGCG

ATGCAAAGCA TTCCAGCCAT TTTAATTACT GGGTGTTGCA TAAGCCAGCA

ATGCAAAGCA TTCCAGCCAT TTTAATTACT GGGCGTTGCA TAAGCCAGCG

ATGCAAAGCA TTCCAGCCAT TTTAATTACT AGGCGTTGCA TAAGCCAGCA

ATGTGAAGCA TTCCAGTCAT TTTAATTACT GGGCGTTGCA TAAGCCAGCA

ATGCGAAGCA TTCCAGCCAT TTTAATTACT GAGCGTTGCA TAAGCCAGCG

ATGCAAAGCA TTCCAGCCAT TTTAATTACC GGGCGTTGCA TAAGCCAGCA

ATGCAAGGCA TTCCAGCCAT TTTAATTACT G---GTTGCA TAAGCCAGTA

ATGCAAAGCA TTCCAGTCAT TTTAATTACT GGGCGGTGCG CACACCAGAA

ATGTAAAGCA TTCAAGCCAT TTTAATTACC AGCCACTGCA TAAGCCAGAA

ACCAGTTCAT ACAAAGGAGC AATAGACAAA AAATGATTTT TCAACATGCT

ACCAGTTCAT ACAAAGGAGC AATAGACAAA AAATGATTTT TCAACATGCT

ACCAGTTCAT ACAAAGGAGC AACAGACAAA AAATGATTTT TCAACATGCT

ACCAGTTCAT ACAAAGGAGC AACAGACAAA AAATGATTTT TCAACATGCT

ACTAGTTCGT ACAAAGGAGC AACAGACAAA AAATGATTTT TCAACATGCT

ACCAGTTCAT ACAAAGGAAC AACAGACAAA AAATGATTTT TCAACATGCt

ACCAGTTTAT TCAAAGGAAC AAAAGACAAA AAAAATATTT TCACCATGCt

ACCAGTTTAT TCAAAGGAAC AGGAGATAAA AAAAATGTCT TCACCATGCT

ACCGGTTCAT ACAAAAGAAC AACAGACAAA AAATGATTTT TCAACATGCT

ACCAGTTCAT ACAAAGGAAC AACAGACAAA AAATGATTTT TCAACATGCT

ACCAGTTCAT ACAAAGGAAC AACAGACAAA AAATGATTTT TCAACATGCT

ACCAGATCAT ACAAAGGAAC AACAGAC-AA AAATTATTTT TCAACATGCC

ACCGGTTCAT ACAAAAGAAT GACAGGCAAA AAGATATTTT TCAGTATGCT

AGCAGTTCAT ACAAAGGAAC AACAGACAAA AAATGATTTT TCAACATGCT

ACCAGTTCAT ACAAAGGAAC AACAGACAAA AAATGATTTT TCAACATGCT

ACCAGTTCAT ACAAAGGAAC AACAGACCCA AAATGATTTT TCAACATGCC

ACTGGTTCAT ACAAAGAAAC AACAGACGAA AAATGATTTT CAAGCATACT

ACCAGCTCGT ACAAAGGGAC AACAGAGAGA AAATTATTTT TCAAAATCCC

TTCTCTAAAT ACTTGAGGCA AAAGCAGTTC CAGCCACCTT CAAGTGTTTT

TTCTCTAAAT ACTTGAGGCA AAAGCAGTTC CAGCCACCTT CAAGTGTTTT

TTCTCTAAAT ACTTGAGGCA AAAGCAGTTC CAGCTACCTT CAAGTGTTTT

TTCTCTAAAT ACTTGAGGCA AAAGCAGTTC CAGCTACCTT CAAGTGTTTT

TTCTCTAAAT ACTTGAGGCA AAAGTAATTC CAGCTACCTT CAAGTGTTTT

ctctGTAAAT ACTTGAGGCA AAAGCAGTTC CAGCTACCTT CAAGTGGTTT

ttttGTAAAT ACTTGAGGTA GAAGCAGTTC CAGGCACCTT CAAGCCGTTT

TTTTGTAAAT ACTTGAAGCA GAAGCAGTTC CCCGCACCTT CAAGCAGTTT

TTTTCTAAAT ACTTGAGGCC AAAGCAGTCC CAGCTAGCTT GGAGCGGTTT

TTCTCTAAAT ACTTGAGGCA GAGGCAGTTC CAGCTACGAT CAAGTGGTTT

TTTTCTAAAT ACTTGAGGCA GAGGCAGTTC TAGCTACCTT CAAGCAGTTT

TTCTCTAAAT ACTTGCGGCA GAAGCAGTTC CAGCTACCTT CAAGCAGTTC

TTCTTTGAAT TCTTGAGACA GAGGCAGTTC CAGCCACTTT CAAGCGGTTT

TTCTTGAAAT ACTTGAGGCG AAAGCAGTTC CGGCTACCTT GGAGCAGTTT

TTCTCTAAAT ACTTGAGGCA GAAGCAGTTC CAGCTACCTT GAAGCAGTTT

TCCTCTGAAT ACTTGAGGCC CGAGTAGTTC C--------- ----CAGCAT

GTCTCCTGAT ACTTGTGTTA GAAGCAGTTG AAGCCACCTA AAAGCAGTTG

CCCTCCACAT ACCTTGGCTG CAAGCGGTTC CAACAGCCTC AGAGCAGATT

GCCCGCGGAG GGCCGATGAG GTAATGCGGC TCTGCCATTG GTCGAGGGGG

GcccgcGGAG GGCCGATGAG GTAATGCGGC TCTGCCATTG GTCGAGGGGG

GcccgcGGAG GGCCGATGAG GTAATGGGGC TCTGCCATTG GTCGAGGGGG

GcccgcGGAG GGCCGATGAG GTAATGCGGC TCTGCCATTG GTCGAGGGGG

GcccgcGGAG GGCCGATGAG GTAATGCGGC TCTGCCATTG GTCGAGGGGG

GCCCGCGGAG GGCCGATGAG GTAATGCGGC TCTGCGATTG GTCGAGGGGG

GcccgcGGAG GGCCGATGAG GTAATGCGGC GCTGCCATTG GTCGAGGGGG

GcccgcGGAG GGCCGATGAG GTAATGCGGC GCTGCCATTG GTCGAGGGGG

GcccgcGGAG GGCCGATGAG GTAATGCGGC TCTGCCATTG GTCGAGGGGG

GcccgcGGAG GGCCGATGAG GTAATGCGGC TCTGCTATTG GTCGAGTCGG

GcccgcGGAG GGCCGATGAG GTAATGCGGC TCTGCCATTG GTCCAGGGGG

GCCCGCGGAG GCCCGATGAG GTAATGCGGC CCCGCGATTG GTCGAGG-GG

G--------- ---------- ---------- ---------- ----------

GcccgcGGAG GGCCGATGAG GTAATGCGGC TCTGCCATTG GTCGAGGGGG

GcccgcGGAG GGCCGATGAG GTAATGCGGC TCTACCATTG GTC-------

GcccgcGGAG GGCCGATGAG GTAATGCGGG TCTGCCATTG GTCGGCGGGG

GCCCGCGGGG GGGCGATGAG GTAATGCGGC TCTCCGATTG GTCGTggggg

ACCTGCGTGG GGCGGGGGCG GGGGGATGGA TGTGCACCGG GGCGGGAGGG

CGGGCCCCAT GACCCCTTGG CTCGGGCTCA TCGTGCTCCT GGGCAGCTGG

CGGGCCCCAT GACTCCTTGG CTCGGGCTCA TCGTGCTCCT GGGCAGCTGG

CGGGCCCCAT GACTCCTTGG CTCGGGCTCA TCGTGCTCCT GGGCAGCTGG

CGGGCCCCAT GACTCCTTGG CTCGGGCTCA TCGTGCTCCT GGGCAGCTGG

CGGGCCCCAT GACTCCCTGG CTCGGGCTCG TCGTCCTCCT GGGCAGCTGG

CGGGTCCCAT GACTCCCTGG CTCGGGCTGG TCGTGCTCCT GGGCATCTGG

CGGACCCCAT GACTCCCTGG CTTGGGCTTG TCCTGCTCCT GAGCTGCTGG

CGGACCCCAT GACTCCCTGG CTTGGGCTTG TCGTGCTCCT GAGCTGCTGG

CGG-CCCCAT GACTCTGTGG CTCGGCCTCG TGGTGCTCCT GGGCAGCTGG

CGGGCCCCAT GACTCCCTGG CTCGGGCTCG TCGTGCTCCT GGGCAGCTGG

CGGGCCCCAT GACTCCCTGG CTCGGGCTAG TGGTGCTTCT GGGCAGCTGG

CGGGCCCCAT GACTCCGTGG CTCGGGCTCG TCGTGCTCCT GGGCAGCTGG

---------- ---------- ---------- ---------- ----------

CGGGCCGCAT GACGCCCTGG CTCGGGCTCG TCGTGCTCCT GGGCAGCTGG

---------- ---------- ---------- ---------- ----------

CGGGCCCCAT GACTCCCTGG CTGGGACTCG TAGTGCTCCT GGGCAGCTGG

cggccctcAT GAGCCCGTGG CTCAGTCTCG TGGTGCTTCT CAGCAGCTGG

TGGCCCCCAT GGCCCTCTGG CTCAGCCTCG TCATGCTCCT GGGCAGCTGG

AGCCTGGGGG ACTGGGGCGC CGAGGCGTGC ACATGCTTTT GCTCCTCAGG

AGCCTGGGGG ACTGGGGCGC CGAGGCGTGC ACATGCTTTT GCTCCTCAGG

AGCCTGGGGG ACTGGGGCGC CGAGGCGTGC ACATGCTTTT GGTCCTCAGG

AGCCTGGGGG ACTGGGGCGC CGAGGCGTGC ACATGC---- ----------

AGCCTGGGGG ACTGGGGCGC CGAGGCGTGC ACATGCTTTT GCTCCTCAGG

AGCCTGGGGG ACTGGGGCGC TGAGGCGTGC ACATGC---- ----------

AGCCTTGGGC ACTGGGGAAC GGAAGCGTGC ACATGCTTCT GCTCCTCAGA

AGCCTTGGGC ACTGGGGCGC GGAAGCGTGC ACATGCTTTG GCTCCTCAGA

GGACTGGGGG ACTGGGGCGC CGAGGCGTGC ACATGCTTCT GTTCCTCAGG

AGCCTGGGGG ACTGGGGCGC TGAGGCGTGC ACATGCTTGT GCTCCTCAGG

AGCCTGGGGC ACTGGGGCGC CGAGGCGTGC ACATGCTCCT --TCCTCAGA

AGCTTGGGGG ACTGGGGTGC CGAGGCGTGC ACGTGCTTTT GCTTCTCAGA

---------- ---------- ---------- ------TTTT GCCTCTCAGA

AGCCTGGGGC ACTGGGGCGC CGAGGCGTGC ACATGCTTTT GCTCCTCAGA

---------- ---------- ---------- ------TTTT GTTCCTCAGA

AGCCTGGGCG ACTGGGGCGC CGAGGCGTGC ACATGCTTTT GCTCTTCAGA

AGCCTGAGGG AGCTGGGGGT AGACGCGTGC ACTTGTTTTT GCTGATTAGA

ACCCTCGACG ACTCGGGGGC GGAGGCCTGC ACTTGCATTT CCCACCTAGA

TATTACCTGT ACAACCTGAT GTTCCAGACG TGGAAGAGGT ATTTTTCTGG

TATTACCTGT ATAACCTGAT GTTCCAGACC TGGAAGAGGT ATTTTTCTGG

TATTACCTGT ATAACCTGAT GTTCCAGACC TGGAAGAGGT ATTTTTCTGG

---------- ---------- ---------- -------GGT ATTTTTCTGG

TATTACCTGT ACAACCTGAT GTTCCAGACC TGGAAGAGGT ATTTTTCTGG

---------- ---------- ---------- -------AGC ATTTTTCTGG

TACTACTTGT ACAACCTGAT TTTCCAGAAC TGGAAGTGGT ATTTTTCTGG

TACTACTTGT ACAACCTGAT TTTCCAGAAC TGGAAGAGGT ATTTTTCTGG

TACTACTTGT ACAACCTGAT GTTCCAGAAC TGGAAGATGG ATTTT-CTGG

TATTACTTGT ACAGCCTGAT GTTCCAGACC TGGAAGAGGT ATTTTTCTGG

TATTACTTGT ACAACCTGAT GTTCCAGACT TGGAGGAGGT ATTTTTCTGG

TATTACTTGT ACAATCTGAT GTTCCAGACC TGGAAGAGGT ATTTTTCTGG

TATTACTTGT ACAACCTGAT GTTCCAGACT TGGAAGCCGT GCTTTTCTGG

TACTACTTGT ACAACCTGAT GTTCCAGACC TGGAAGAGGT ATTTTTCTGG

TACTACTTGT ACAGTTTGAT GTTCCAGACC TGGAAGAGGT ATTTTTCTGG

TATTACCTGT ACAACCTGAT GTTCCAGACC TGGCAGA--- ----------

TATTTCTTGT ACAACCTGAT GTTCCAAACC TGGCAGAGGT GTTTTTCTGG

TATTTCTTGT ACAACCTGAT GTTCCAGTCC TGGAAGAAGT ATTTTTATGC

GCACTGCTTC AAAGGGGCTG TGGTTGAAGA CCATGCTGCT ATTCTGGTGG

GCACTGCTTC AAAGGGGCTG TGGTTGAAGA CCATGCTGCT ATTCTGGTGG

GCACTGCTTC AAAGAGGCTG TGGTTGAAGA CCATGCTGCT ATTCTGGTGG

GCACTGCTTC AAAGAGGCTG TGGTTGAAGA CCATGCTGCT ATTCTGGTGG

GCACTGCTTC AAAGGGGCTG TGGTTGAAGA CCATGCTGCT ATTCTGGTGG

GCACTGCTTC AAAGGGGCTG TAGCTGAAGA CCACACTGCT ATTCTGGTGG

GCATTGCTTC AAAGGGGCCA AGGTTGAAGA CCAGGCTGCT ATTCTGGTGG

GCACTGCTTC AAAGGGGCTG TGGTTGAAGA CCGGGCTGCT ATTCTGGTGG

GCACTGCTTC AAAGGGGCTG TGGTTAAGGA CCAGGCTGCT TTTCTGGTGG

GCATTGCTTC AAAGGGGCCA TAGTTTAAGA CCAGGCTGCT ATTCTGGTAG

GCACTGCTTC AAAGGGTCCA TGGTTGAAGA CCAGGCTGCT ATTCTGGTGG

GCACTGCTTC AAAGGGGCCG TGGTTGAAGA CCAGGCTGCT ATTCTGGTGG

GCGCCGCTTC AAAGGGGCTC TGGTTGAAGA CCAGGCCGCT ATTCTGGCTG

GCACTGCTTC AAAGGGGCTG GGGTTGAAGA CCAGGCTGCT ATTCTGGTGG

GCACTGCTTC AAAGGGGCTG TGATTGAAGA CCAGGCTGCT ATTCTGGTGG

---------- ---------- ---------- ---------- ----------

GCAGGGCTTC AAAGAGACTG TGGTTTAAAA ATATGCGGCT ATTTCAGTGA

GCACCACTTC AAAGAGGCTG TGGTTTCGAA ATATGCTGCT ATTTTGGTGA

CCACTTGTGT GGGATAAAGT TTCCCATGGG CCCCCGTGGA GGGCTTTTAA

CCACTTGTGT GGGATAAAGT TTCCCATGGG CCCCCGTGGA GGGCTTTTAA

CCACTTGTGT GGGATAAAGT TTCCCATGGG CCCCTGTGGA GGGCTTTTAA

CCACTTGTGT GGGATAAAGT TTCCCATGGG CCCCTGTGGA GGGCTTTTAA

CCACTTGTGT GGGATAAAGT TTCCCATGGG CTCCTGTGGA GGGCTTCTAA

CCACTTGTGC GGGATAAAGT TTCCCATGGG CCCCTGGGGA GGGCTTTCAA

CCACTTGTGT GGGATAAAGT TTCCCATGGG GCCATGGGAA GGGCT---AA

CCACTTGTGT GGGATAAAGT TTCCCATGGG CCCATGGGAA GGGTT---AA

CCACTTCTGT GGGATGAAGT TTCCCAGGGC CCCCCGGGAG GGCTTTTTAA

CCTCTTGTGT GGGATAAAGT TTCCCATGGG CCCCTGGGGA GGGCTTTTAA

CCTCTTGTGT GGGATAAAAT TTCCCATGGG CCCCTGGGGA GGGCTTTTAA

CTTCTTGTGT GGGATAAAGT TTCCCATGGG CCCCTGGGGA GGGCTTTTAA

CCTC-TGTGT GCT-CCGAGT TTCCCATGGG CCCCTGGGGA -GGCT-----

CTGCTTGTGT GGGGTAAAGT TTCCCATCGG CCCCTGGGGA GGGCTTTTAA

CCACTTGTGT GGGATAAAAT TTCCCATGGG CCCCTGGGGA GGGCTTTTAA

---------- ---------- ---------- ---------- -----CTTAA

CCACTTGTGT GGGATAAAGT TTCTCATGGG AGCCCAGGGA GGATTTTTAA

CCACTTGTGT GGGATAAAAT TTCCCATCGG CCTCTGGGGA GGGCATTTAA

TAGCTTTCCC TGAGATGGAA ACTTGGTTGA AATGATTTGC AGCTTTCCAG

TAGCTTTCCC TGAGATGAAA ACTTGGTTGA AATGATTTGC AGCTTTCCAG

TAGCTTTCCC TGAGATGGAA ACTTGGTTGA AATGATTTGC AGCTTTCCAG

TAGCTTTCCC TGAGATGGAA ACTTGGTTGA AATGATTTGC AGCTTTCCAG

TAGCTTTCCC TGAGATGGAA ACTTGGTTGA AATGATTTGC AGCTTTCCAG

TAGCTTTCCC TGAGATGGAA ACTTGGTTGA AATGATTTGC AGCTTTCCAG

TGGCATCCCC TGAGATGGAA --TTGGCTGA AATGATTTGC AGCTTTCCAG

TGGCCTTCCC TGAGATGGAA --TTGGCTGA AATGATTTGC AGCTTTCTAG

TAGCTTTCCC TGAGATGGAA ACTTGGTTGA AATGATTTGC AGCTTTCCAG

TAGCTTTCCC TGAGATGGAA ACTTGGTTGA ATTGATTTGC AGCCCTCCAG

TAGCTTTCTC TGAGATGGAA ACTTGGTTGA AATGATTTGC AGCTCTCCAG

TAGCTTTCCC TGAGATGGAA ACTTGGTTGA AATGATTTGC AGCTCTCCAG

---------- ---------- ---------- ---------- ----------

TAGCTTTCCC TGAGATGGAA ACTTGGTTGA AATGATTTGC AGCTCTCCAG

TAGCTTTCCC TGAGATGGAA ACTTGTCTGA AATGATTTGC AGCTCTCCAG

TGGCTTCCCC TGCCATGGAA ACTTGGCTGG GGTGATTAGC AGTGTTCCAG

TAGCTTTTGT GGAGATGGAA ACTTGGCTCA AATGACTTGC ATCTCTCCAG

TAGCTTTAAT GTCGATGGAA ACTTGGTTGA AAGGACTTGA CTCCCTCCAG

ATTTTCTGAG TGTGTGTCTT TGGGTTTATT TTTAGCCACA GGATTCTCTT

ATTTTCTGAG TGTGTGTCTT TGGGTTTATT TTTAGCCACA GGATTCTCTT

ATTTTCTGAG TGTGTGTCTT TGGGTTTATT TTTAGCCACA GGGTTCTCTT

ATTTTCTGAG TGTGTGTCTT TGGGTTTATT TTTAGCCACA GGGTTCTCTT

ATTTTCTGAG TGTATGTCTT TGGGTTTATT TTTAGCCACA GGGTTCTCTT

ATTTTTTGAG TGTGTGTATT TGGATTTATT TTTAGCCACA GGGTTCTCTT

ATTTTCTAAG CGTGTGTGAT TGGGTTTATT TTTAGCCGCA GGATTCTCTT

ATTTTCTAAG CGTGTGTGAT CGGGTTTATT TTTAGCCACA GGATTCTCTT

ATTTTTtgtg tgtgtgttTT GGGTTTTATT TTTAGCCGCA GGGTTCTCTT

ATTTTTtgtg tgtgtgtATT CGGGTTTATT TTTAGCTGCA GGGTTTTCTT

ATTTTTTGTG TGTGTGTATT TGGGTTTATT TTTAGCCACA GGGTTCCCGT

ATTTTTTGTG TGTGTGTATT TGGGTTTATT TTTAGCCGCA AGGTTCTCTT

---------- ---------- ---------- ---------- ----------

ATTTTCTGAG TGTATGTATT GGGGTTTATT TTTAGCCGCA GGGTTTCTTT

ATTTTC-GTG TGTGTGTGTT TGGGTTTATT TTTAGCCACA GGGTTCTCTT

ATTTCCtgtg tgtgtgtATT GGGGTTTATT TTTAGCCTTG AGGTTCTCTT

ATTTTTCTTT TTTGAATGTT TGGATTTATT TTTAGCCTCT TGGTTCTCTT

ATTTTTTGTG TGTGTGTGTT TAGATTTATT TTTAGCCTCA GGGTTCTCTT

GCCAAAAAAA CCTGCTTAAA TAAAACCAAG ATTAATATTT TTACTTAACG

GCCAAAAAAA CCTGCTTAAA TAAAACCAAG ATTAATATTT TTACTTAACG

GCCAAAAAAA CCTGCTTAAA TAAAACCAAC ATTAATATTT TTACTTAACG

GCCAAAAAAA CCTGCTTAAA TAAAACCAAC ATTAATATTT TTACTTAACG

GCCAAAAAAA CCTGCTTAAA TAAAACCAAG ATTAATATTT TTACTTAACG

GCC-AAAAAA CCTGCTTAAA TAAAACCAAG ATTAATATTT TTACTTAACG

GCCAAAAAAA CCTGCTTAAA TAAAACCAAG ATTAATATTT TTACTTAACG

GCCAAAAAAA CCTGCTTAAA TAAAACCAAG ATTAATATTT TTACTTAACG

GCC-AAAAGA CCTGCTTAAA TAAA-CCAAG ATTAATATTT TT--------

GCCAAAAAAA CCTGCTTAAA TAAAACCAAG ATTAATATTT TTACTTAAAG

GCCAAAAAAA CCTGCTTAAA TAAAACCAAG ATTAATATTT TTACTTAACG

GCCAAAAAAA CCTGCTTAAA TAAAACCAAG ATTAATATTT TTACTTAGCG

---------- ---------- ---------- ---------- ----------

GCCAAAAAAA CCTGCTTAAA TAAAACCAAG ATTAATATTT TTACTTAACG

GCCAAAAA-C TCTGCTTAAA TAAAACCAAG ATTAATATTT TTACTTAACG

GCCAAAAAGC TCTGCTTAAC TCAAACCAAG ATGAATATTT GTA-TTAACA

ATCAAAAAAA TCTGCTTAAA TAAAACCAAG ATTAATATTT TTACTTAATG

AGCTGAAGAA TCCACTGAAA TAAAACAAAG ATTAATATTT TTACTTAACG

GAAAGAGTTA GTCAGGAAGG CTTTGAACTG GGACTCCTTA TCTTATGGGG

GAAAGAGTTA GTCAGGAAGG CTTTGAACTG GGACTCCTTA TCTTATGGGG

GAAAGAGTTA GTCAGGAAGG CTTTGAACTG GGACTCCTTA TCTTATGGGG

G-AAGAGTTA GTCAGGAAGG CTTTGAACTG GGACTCCTTA TCTTATGGGG

GAAAGAGTTA GTCAGGAAGG CTTTGAACTG GGACTCCTTA TCTTATGGGG

GAAAGAGTTA GTCAGGAAGG CTTTGAACTG GGACTCCTTA TCTTATTGGG

GAAAGAGTTA GTCAGGGAGG CTTTGAACTG GGCCTGCTTA TCTTACGGGG

GAAAGAGTTA GTCAGGAAAG CTTTGAACTG GGCCTGCTTA TCTTACGGGG

---------- ---------- ---------- ---------- ----------

GAAAGAGTTA GTCAGGAAGG CTTTGAACTG GGACTCCCTA TCTTATTGGG

GAAAGAGTTA GTCAGGAAGG CTTTGAACTG GGACTCCCTA TCTTATTGGG

GAAAGAGTTA GTCAGGAGGG CTTTGAACTG GGGCTCCCTA TCTTATTGGG

---------- ---------- ---------- ---------- ----------

GAAAGAGTTA GTCAGGGAAG CTTTGAACTG GGACTCCTTA TCTTATGGGG

GAAAGAGTTA GTCAGGGAAG CTTTGAACTA GGACTCTTTA TCTTATTGGG

GAAAGAGTTC ACCAGGGAGA CTTTGAACTG CGACTCCGTA TCTCACGGGG

GAAAGAGTTA GTCAGCGAGG CTTTGAACTC CAAGCTTATA TTTTATACTA

GAAAGAGTTA GTCAGTGAAG CTTTGAACTG TGACTCCTTA TCTTCTGCAG

AGTGCATGTT AACGGCGTTG ACATCAACGA AAAGGGCCTT TGAAACGGAC

AGTGCATGTT AACGGCTTTG ACATCAACAA AAAGGGCCTT TGAAACGGAC

AGTGCATGTT AACGGCTTTG ACATCAACAA AAAGGGCCTT TGAAACGGAC

GGTGCATGTT AACGGCTTTG ACATCAACAA AA--GGCCTT ------GAAC

AGTGCATATT AATGGCTTTG ACATCAACAA AAAGGGCCTT TGAAATGGAC

AATGCATTTT AACGGCTTTG ACATCAACAA AAAGGGCCTT TGAAACTGAC

AGTGCATTTT AAATGCTTCG ACATCAACCG AAAGCGCCTT TGAAACTGAC

AGTGCATTTT AAATGCTTCA ACATCAACAA AAAGGGCCTT TGAAACTGAC

---------- ---------- ---------- ---------- ----------

AGTGCATCTG AACAGCTTTG ACATCAACAA AAAGGGCCTT TGAAATAGAC

AGTGCATCTC CAT-GCTTTG ACAGCAACAA AAAAGGCCTT TGAAACTGAC

GGTACATCTT AACTGCTTTG ACTTCAACAA AAAGGGCCTT TGAAACTAAC

---------- ---------- ---------- ---------- ----------

AGTGCATTTG AACTGCTCTG ACATCAACAG AAGGGGCCTT TGAAATTGAC

AGTACATTTT AACTGCTTGT ACAACAACAA AAAGAGTCTT TGAAAATGAC

AACGCATTGC AACTGCTTGG ACATCCACCA AAAGGGTCTT TGAAATTGAC

AGTGTATATT AAATGCTTTG AAATCAATAA AAAGGATCTG AGAAACTGAC

AATGGATTTT AAAAGCTTTG AGATCAACAA AAAGGGTCTT TGAATCTGAC

AAGAGAGAAG GGCCCTGCAG TGATCCGGGC CAAGGTGGTG GGGAAGAAGC

GAGAGAGAAG GGCCCTGCAG TGATCCGGGC CAAGGTGGTG GGGAAGAAGC

AAGAGAGAAG GGCCCTGCAG TGATCCGGGC CAAGGTGGTG GGGAAGAAGC

GACAGAGAAG G--CCTGCAG TGATCCGGGC CAAGGTGGTG GGGAAGAAGC

AAGAGAGAAG GGCCCTGCAG TGATCCGGGC CAAGGTGGTG GGGAAGAAGT

AAGAGAGAAG GGTCCTGCAG TGATCCGGGC CAAGGTGGTG GGGAAGAAGC

AAGGGAGAAG AGCCCTACAG TGATCCGGGC CAAAGTGGTG GGAAAGAAGC

AAGGGAGAAG AGCCCTACAG TGATCCGGGC CAAAGTGGTG GGAAAGAAGC

---------- ------GCAG TGATCCGGGC CAAGGTGGTG GGGAAGAAGC

-AGAGAGAAG TGCCCTCCAG TGATCCGAGC CAAGGTGGTA GGGAAGAAAC

-AAGGAGAAG TGCCCTGCAG TGATCCGGGC CAAGGTGGTA GGGAAGAAGC

AAGAGAGAAG TGCCCTGCAG TGATCCGGGC CAGGGTGGTG GGAAAGAAGC

---------- ------CCAG TGATCCGGGC CAAGGTGGTG GGGAAGAAGC

AGGAGAGAAC GGCCCTGCAG TGATCCGGGC CAAGGTGGTG GGGAAGAAGC

AGGAGAGAGG G-CCCTACAG TGATCCGGGC CAAGGTAGTG GGGAAGAAGC

TGGGGAGAGG GACCCTGCAG TGATCCGGGC CAAGGTGGTG GGGAAGAAGC

AAGAGCAAAG AATCTTCCAG TCATCCGTGC CAAGGTGGTG GGAAAGAAGC

AAGAGAGAAG AGCCCTACAG TGATACGTGC CAAGGTGGTG GGGAAAAAGC

TGGTAAAGGA GGGGCCCTTC GGCACGCTGG TCTACACCAT CAAGCAGATG

TGGTAAAGGA GGGGCCCTTC GGCACGCTGG TCTACACCAT CAAGCAGATG

TGGTAAAGGA GGGGCCCTTC GGCACGCTGG TCTACACCAT CAAGCAGATG

TGGTAAAGGA GGGGCCCTTC GGCACGCTGG TCTACACCAT CAAGCAGATG

TGGTAAAGGA GGGGCCTTTT GGCACACTGG TCTACACCAT CAAGCAGATG

TGGTGAAGGA GGGGCCCTTT GGCACACTGG TCTACACCAT CAAGCAGATG

TGGTGAAGGA AGGGCCCTTT GGCACTCTGG TCTACACTAT TAAGCAAATG

TGGTGAAGGA GGGGCCCTTT GGCACTCTGG TCTACACTAT TAAGCAGATG

TGGTGAAGGA AGGGCCCTTC GGCACGATGG TCTACACCGT CAAGCAGATG

TGCTGAAGGA GGGGCCCTTT GGCACGATGG TCTACACCAT CAAGCAGATG

TGGTGAAGGA GGGGCCCTTT GGCACGCTGG TCTATACCAT CAAGCAGATG

TGGTGAAGGA GGGGCCGTTT GGTACGCTGG TCTACACCAT TAAGCAGATG

TGGTGAAGGA GGGACCCTTC GGCACGATGG TCTACACCAT CAAACAGATG

TGGTGAAGGA GGGGCCCTTT GGCACACTGG TCTACACCAT CAAGCAGATG

TGGTGAAGGA TGGGCCCTTT GGCACGCTGG TCTACACCGT CAAGCAGATG

TGGTGAAGGA AGGGCCCTTT GGCACGCTGG TCTACACCAT CAAGCAGATG

TGGTGAAGGA GGGACCCTTT GGGACACTGA TTTACACCAT CAAGCAGATG

TTGTGAAGGA GGGGCCTTTT GGAACCCTGA GATACACCAT CAAGCAAATG

AAGGTAGGAA CCTGTTGCTT TGGATTCAAT TTTGCAAACA TCCACAGCCC

AAGGTAGGAA CCTGTTGCTT TGGATTCAAT TTTGCAAACA TCCACAGCCC

AAGGTAGGAA CCTGTTGCTT TGGATTCAAT TTTGCAAACA TCCACAGCCC

AAGGTAGGAA CCTGTTGCTT TGGATTCAAT TTTGCAAACA TCCACAGCCC

AAGGTAGGAA CCTGTTGCTT TGGATTCAAT TTTGCAAACA CCCACAGCCC

AAGGTGAGAA CCTGTTGCTT TGGATTCAAT GTTGGAAACA TCCACAGCCC

AAGGTGAGGA CCTGTTGCTC TGGCTTCGAT TCTGGAAACA CTCCCAGGCT

AAGGTGAGAA CCTGTTGCTC TGGCTTCAAT TCTGGAAACA GTCCCAGGCT

AAGGTAAGAA CCTGTTGCTT TGGATTCGAT TTTGGAAACA TCCACAGCCC

AAGGTGAGAA CCTGTTGCTT TGGATTCAAT TTTGGAAACG TCCACAGCCC

AAGGTAAGAA CCTGTTGCTT TGGATTAAAC TTTGGAAATA TCCACAGCCC

AAGGTGAGAA CCTGTCACTT TGGATTCAGT TTTGGAAACA TCCACGGCCC

AAGGTGAG-- ---------- ---------- ---------- ----------

AAGGTGAGAA TCTGTTGCTT TGGATTCAAT TTTGGAAACA TCCACAGCCC

AAGGTGAG-- ---------- ---------- ---------- ----------

AAGGTGGGAA CCTGTTGCTT TGGATTCGAT TTTGGAAACA TCCACAGCCC

AAGGTAAGAA GCTGCTCCTT TGAGTTCATT TCTGGAAATA CCCATTGACC

AAGGTGAGAA CCTGCTGCTT TGGAAAGAAT TTTTGAAGAG CCAATTGACC

TGGTTATGTA ATGTCCAGAG TCAGCCCTGG CCAGTTGCCC TTGGCTTGCC

TGGTTATGTA ATGTCCAGAG TCAGCCCTGG CCAGTTGCCC TTGGCTTGCC

TGGTTATGTA ATGTCCAGAG TCAGCCCTGG CCAGTTGCCC TTGGCTTGCC

TGGTTATGTA ATGTCCAGAG TCAGCCCTGG CCAGTTGCCC TTGGCTTGCC

TGGTTATGTA ATGTCCAGAG TCAGCCCTGG CCAGTTGCCC TTGGCTTGCC

TGGTTATGTA ATGTCCAGAG TCAGCCCTGG CCAGCTGCCC TTGGCTTGTT

TGGTTATGTA ATGTTCAGAG TCAGCCCTGG CCAGTTGCCC TTGGCTTGCC

TGGTTATGTA ATGTTCAGAG TCAGCCCTGG CCAATTGCCC TTGGCTTGCC

TGGTTATGTA ATGTCCACAG TCAGCCCTGG CCAGTTGCCC TTGGCT----

TGGTTATGTA ATGTCCAGAG TCAGGCCTGG ATGGCTGCCC TTGGCTTGCC

TGGTTATGTA ATGTCCAGAG TCAGCCCTGG CTAGGTGCCC TTGGCTTGCC

TGGTTATGTA ATATCCAGAG TCAGCGCTGG CCTGTTGCCC TTGGCTTGCC

---------- ---------- ---------- ---------- ------TGCC

TGCTTATGTA ATGTCCAGAG TCGGCCCTGG CCAGTTGCCC TTGGCTTGCC

---------- ---------- ---------- ---------- ------TGCC

TGGTTATGTA ATGTCCAGAG TCAGCCCTGG CCAGCTGCCC TTGGCTCACC

TTGGTATGTA ATATTAAAAA TCCATCCTGG CTTGCTTCCC TTGGCCTCCC

TTGTTATGTA ATGTTAAAAG TCAGCCCTGG ATTGTTTTCC TTGGCTTTCC

CACAGATGTA CCGAGGCTTC ACCAAGATGC CCCATGTGCA GTACATCCAT

CACAGATGTA CCGAGGCTTC ACCAAGATGC CCCATGTGCA GTACATCCAC

CACAGATGTA CCGAGGCTTC ACCAAGATGC CCCACGTGCA GTACATCCAC

CACAGATGTA CCGAGGCTTC ACCAAGATGC CCCACGTGCA GTACATCCAC

CACAGATGTA CCGAGGCTTC ACCAAGATGC CCCATGTGCA GTACATCCAC

GACAGATGTA CCGAGGCTTC ACCAAGATGC CCCATGTGCA GTACATCCAC

CACAGATGTA CCGAGGATTC AGTAAGATGC CCCATGTGCA GTACATTCAC

CACAGATGTA CCGAGGCTTC AGTAAGATGC CCCACGTGCA GTACATTCAC

---------- ---------- ---------- ---------- ----------

CACAGATGTA CCGAGGATTC ACCAAGATGC CCCATGTGCA GTACATCCAC

CACAGATGTA CCGAGGCTTC ACCAAGATGC CCCACGTGCA GTACATCCAC

CACAGATGTA CCGAGGCTTC ACCAAGATGC CCCACGTGCA GTACATCCAC

CGCAGATGTA CCGAGGCTTC ACCAAGATGC CCCACGTGCA GTACATCCAC

CACAGATGTA CCGCGGCTTC ACCAAGATGC CCCATGTGCA GTACATCCAC

CACAGATGTA CCGAGGCTTC AGCAAGATGC CCCATGTACA ATACATCTAC

CACAGATGTA CCGAGGCTTC ACCAAGATGC CCCACGTGCA GTACATCTAC

CACAGATGTA CAGGGGCTTT AGCAAGATGC CCCATGTGCA GTATATCCAC

CACAGATGTA CCGAGGCTTC AGCAAGATGC CTCACGTGCA GTACATCTAC

ACGGAAGCTT CCGAGAGTCT CTGTGGCCTT AAGCTGGAGG TCAACAAGTA

ACGGAAGCTT CCGAGAGTCT CTGTGGCCTT AAGCTGGAGG TCAACAAGTA

ACGGAAGCTT CCGAGAGTCT CTGTGGCCTT AAGCTGGAGG TCAACAAGTA

ACGGAAGCTT CCGAGAGTCT CTGTGGCCTT AAGCTGGAGG TCAACAAGTA

ACAGAGGCTT CTGAGAGTCT CTGTGGCCTT AAGCTGGAGG TCAACAAGTA

ACAGAAGCTT CCGAGAGTCT CTGTGGCCTT AAGCTGGAGG TCAACAAGTA

ACAGAAGCCT CTGAAAGTCT CTGTGGCCTT AAGCTAGAAG TCAACAAATA

ACGGAAGCCT CTGAAAGTCT TTGTGGCCTC AAGCTAGAAG TCAACAAATA

---------- ---------- ---------- ---------- ----------

ACAGAAGCTT CTGAAAGTCT CTGTGGCCTT AAGCTTGAGG TCAACAAGTA

ACAGAAGCTT CTGAAAGTCT CTGTGGCCTT AAGCTAGAGG TCAACAAGTA

ACGGAAGCTT CCGAAAGTCT CTGTGGCGTT AAGCTGGAGG TCAACAAGTA

ACGGAAGCTT CCGAGAGCCT CTGTGGCCTG AAGCTGGAGG TGAACAAGTA

ACAGAAGCTT CTGAAAGTCT CTGTGGCCTT AAGCTGGAGG TCAACAAGTA

ACGGAAGCTT CCGAAAGTCT CTGTGGCCTT AAGCTGGAGG TCAACAAGTA

ACGGAAGCCT CTGAGAGTCT GTGCGGCCTG AAGCTGGAAG TGAACAAGTA

ACAGAAGCTT CCGAGAGCCT TTGTGGGCTG AAGTTGGAAG TCAACAAGTA

ACGGAAGCTT CTGAAAGTCT TTGTGGGCTG AAGCTGGAGG TGAACAAGTA

CCAGTACCTG CTGACAGGTA AAGGTCGCGT CTATGATGGC AAGATGTACA

CCAGTACCTG CTGACAGGTA AAGGTCGCGT CTATGATGGC AAGATGTACA

CCAGTACCTG CTGACAGGTA AAGGTCGCGT CTACGATGGC AAGATGTACA

CCAGTACCTG CTGACAGGTA AAGGTCGCGT CTACGATGGC AAGATGTACA

CCAGTACCTG CTGACAGGTA AAGGTCGTGT CTATGATGGC AAGATGTACA

CCAGTACCTA CTGACAGGTA AAGGCCGCGT CTATGATGGA AAGATGTACA

CCAGTACCTG CTGACAGGTG AAGGGCGCGT GTATGAAGGC AAGATGTACA

CCAGTACCTG CTGACAGGTA AAGGGCGCGT GTATGAAGGC AAGATGTACA

---------- ---------- ---------- ---------- ----------

CCAGTACCTG CTGACAGGTA AAGGCCGAGT CTATGATGGC AAGATGTACA

CCAGTACCTG CTGACAGGTA AAGGCCGCGT CTATGATGGT AAGATGTACA

CCAGTACCTG CTGACAGGTA AAGGCCGTAT CTACGATGGT AAGGTGTACA

CCAGTACCTG CTCACAGGTA AAGGTCGTGT CTATGATGGG AAGATGTACA

CCAGTACCTA CTGACAGGTA AAGGCCGTGT CTACGATGGC AAGGTATACA

CCAGTACCTG CTGACAGGTA AAGGTCGTGT CTATGATGGC AAGGTATATA

CCAGTACCTG CTGACAGGTA A--------- ---------- ----------

CCAGTATCTG CTGACAGGTA AAGGTCGGGT ATATGATGGC AAGATGTACA

CCAGTACCTG CTGACAGGTA AAGGCCGTGT TTATGACGGG AAGGTGTACA

CGGGGCTGTG CAACTTCGTG GAGAGGTGGG ACCAGCTCAC CCTCTCCCAG

CAGGGCTGTG CAACTTCGTG GAGAGGTGGG ACCAGCTCAC CCTCTCCCAG

CAGGGCTGTG CAACTTTGTG GAGAGGTGGG ACCAGCTCAC CCTCTCCCAG

CAGGGCTGTG CAACTTTGTG GAGAGGTGGG ACCAGCTCAC CCTCTCCCAG

CAGGACTGTG CAACTTTGTG GAGAGGTGGG ACCAGCTCAC CCTCTCCCAG

CAGGACTGTG CAACTTTGTG GAGCGGTGGG ACCAGCTCAC CCTCTCCCAG

CAGGGCTGTG CAACTTTGTG GAGAGGTGGG ACCACCTCAC ACTGTCCCAG

CAGGACTGTG CAACTTTGTG GAGAGGTGGG ACCACCTCAC ACTGTCCCAG

---------- ---------- ---------- ---------- ----------

CAGGACTGTG TAACTTTGTA GAGAGGTGGG ACCAGCTCAC CCTCTCCCAG

CAGGACTATG CAACTTTGTG GAGAGGTGGG ACCAGCTCAC CCTCTCCCAG

CAGGACTCTG CAACTTCGTG GAGCGGTGGG ACCAGCTCAC CATCTCACAG

CGGGCCTGTG TAACTTCGTC GAGAGATGGG ACCAGCTCAC CCTCTCGCAG

CCGGGCTGTG CAACTTTGTG GAGAGATGGG ACCAGCTCAC GCTCTCCCAG

CAGGGCTGTG CAACTTTGTG GAGAGATGGG ACCAGCTCAC CCTCTCCCAG

---------- ---------- ---------- ---------- ----------

CAGGCTTGTG TAACTTTGTG GAGCGGTGGG ACCGGCTCAC CCTCTCCCAG

CTGGCCTGTG TAACTTCGTT GAGCGATGGG ACCAGCTGAC CCTCTCCCAG

CGCAAGGGGC TGAACTATCG GTATCACCTG GGTTGTAACT GCAAGGTAAG

CGCAAGGGGC TGAACTATCG GTACCACCTG GGTTGTAACT GCAAGGTAAG

CGCAAGGGGC TGAACTATCG GTATCACCTG GGTTGTAACT GCAAGGTAAG

CGCAAGGGGC TGAACTATCG GTATCACCTG GGTTGTAACT GCAAGGTAAG

CGCAAGGGGC TGAACTATCG GTACCATCTG GGTTGTAACT GCAAGGTAAG

CGCAAGGGCC TGAACTATCG GTATCACCTG GGCTGTAACT GCAAGGTAAG

CGCAAGGGCC TCAATTACCG CTACCACCTG GGTTGCAATT GCAAGGTGAG

CGCAAGGGCC TCAATTACCG CTACCACCTG GGTTGCAATT GCAAGGTGAG

---------- ---------- ---------- ---------- ----------

CGCAAGGGGC TGAACTATCG ATATCACCTG GGCTGTAACT GCAAGGTAAG

CGCAAGGGGC TCAACTACAG GTATCACCTG GGCTGTAACT GCAAGGTAAG

CGCAAGGGGC TGAACTATCG GTATCACCTG GGTTGTAATT GCAAGGTAAG

CGCAAGGGGC TAAACTACCG CTACCACCTG GGCTGTAACT GCAAGGTGAG

CGCAAGGGGC TGAACTATCG TTATCACCTG GGTTGCAACT GCAAGGTAAG

CGCAAGGGGC TGAATTATCG GTATCATCTG GGCTGTAACT GCAAGGTGAG

---------- ---------- ---------- ---------- ----------

CGCAAGGGTC TAAATTATCG GTACCACTTG GGCTGCAACT GCAAGGTAAG

CGCAAAGGCC TGAATTATCG TTACCATCTG GGCTGCAACT GCAAGGTAAG

ATTGCAGATC AAGTCCTGCT ACTACCTGCC TTGCTTTGTG ACTTCCAAGA

ATTGCAGATC AAGTCCTGCT ACTACCTGCC TTGCTTTGTG ACTTCCAAGA

ATTGCAGATC AAGTCCTGCT ACTACCTGCC TTGCTTTGTT ACCTCCAAGA

ATTGCAGATC AAGTCCTGCT ACTACCTGCC TTGCTTTGTT ACTTCCAAGA

ATTGCAGATC AAGTCCTGCT ACTACCTGCC TTGCTTTGTG ACTTCCAAGA

ATTGCAGATC AAATCCTGCT ACTACCTGCC TTGCTTTGTG ACCTCCAAGA

ATTACAGATC AAGTCCTGCT ACTACTTGCC TTGCTTTGTG ACCTCCAAGA

ATTACAGATC AAGTCCTGCT ACTACTTGCC TTGTTTTGTG ACCTCCAAGA

ATTGCAGATC AAGTCCTGCT ACTACCTGCC CTGCTTCGTG ACCTCCAAGA

ACTGCAGATC AAATCCTGCT ACTACCTGCC TTGCTTTGTA ACCTCCAAGA

ATTGCAGATC AAATCCTGCT ACTACCTGCC TTGCTTTGTG ACCTCCAAGA

ATTGCAGATC AAGTCCTGCT ACTACCTGCC TTGCTTTGTG AGCTCCAAGA

GTTGCAGATC AAGTCCTGCT ---------- ---------- ----------

ATTACAGATC AAGTCCTGCT ACTATCTGCC TTGCTTTGTG ACCTCCAAGA

ATTACAGATC AAGTCCTGCT ACTACCTGCC TTGCTTTGTG TCCGCCAAGA

---------- ---------- ---------- ---------- ----------

ATTACAGATT AAGTCATGTT ATTACCTGCC TTGCTTTGTG ACTTCCAAGA

ACGGCAGATC AAATCCTGCT ACTACCTGCC TTGTTTCGTG ACCTCCAAGA

ACGAGTGTCT CTGGACCGAC ATGCTCTCCA ATTTCGGTTA CCCTGGCTAC

ACGAGTGTCT CTGGACCGAC ATGCTCTCCA ATTTCGGTTA CCCTGGCTAC

ACGAGTGTCT CTGGACCGAC ATGCTCTCCA ATTTCGGCTA CCCTGGCTAC

ACGAGTGTCT CTGGACCGAC ATGCTCTCCA ATTTCGGCTA CCCTGGCTAC

ACGAGTGTCT CTGGACCGAC ATGCTGTCCA ATTTCGGCTA CCCTGGCTAC

ACGAGTGTCT CTGGACCGAC ATGCTGTCCA ACTTCGGCTA CCCCGGCTAT

ATGAATGTCT CTGGACCGAC ATGCTCTCCA ATTTCGGGTA CCCTGGCTAT

ATGAGTGTCT CTGGACCGAC ATGCTCTCCA ATTTTGGGTA CCCTGGCTAT

ATGAGTGCCT TTGGACCGAC ATGCTCTCCA ACTTCGGCTA CCCCGGCTAC

ACGAGTGTCT CTGGACCGAC ATGCTCTCCA ATTTCGGCTA CCCTGGCTAC

ACGAGTGCCT CTGGACCGAC ATGCTCTCCA ATTTTGGCTA CCCTGGCTAC

ATGAGTGTCT CTGGACCGAC ATGCTCTCCA ATTTCGGCTA CCCCGGCTAC

---------- ---------- ---------- ---------- ----------

ATGAGTGTCT TTGGACCGAC ATGCTTTCCA ACTTCGGATA CCCTGGCTAC

ACGAGTGTCT CTGGACCGAC ATGCTATCCA GCTTCGGCCA CTCTGGCTAC

---------- ---------- ---------- ---------- ----------

ATGAGTGTCT GTGGACGGAC ATGCTCTCCA ACTTTGGCTA CCCTGGGTAC

ACGAGTGCCT CTGGACGGAC ATGCTCTCCA ACTTCGGCTA TCCCGGATAC

CAGTCCAAAC ACTACGCCTG CATAGAAATA TAATCTCTAT TTTTTTAGGA

CAGTCCAAAC ACTACGCCTG CATAGAAATA TAATCTCTAT TTTTTTAGGA

CAGTCCAAAC ACTATGCCTG CATAGAAATA TAATCTCTAT TTTTTTAGGA

CAGTCCAAAC ACTATGCCTG CATAGAAATA TAATCTCTAT TTTTTTAGGA

CAGTCCAAAC ATTATGCCTG CATAGAAATA TAATCTCTAT TTTTTTAGGA

CAGTCCAAAC ACTACGCCTG CATAGAAATA TAATCTCTAT TTTTTTAGGA

CAGTCCAAAC ACTACGCCTG CATAGAA-TA TAATCTATAT TTTTTTAGGA

CAGTCCAAAC ACTACGCCTG CATAGAA-TA TAATCTATAT TTTTTTAGGA

CAGTCCAAAC ACTATGCCTG CAT----ATA TAATCTCTAT TTTTTTAGGA

CAGTCCAAAC ACTACGCTTG CATAGAAATA TAATCTATAT TTTTTTAGGA

CAGTCCAAAC ATTATGCCTG CATAGAAATA TAATC--TAT TTTTTTAGGA

CAGTCCAAAC ACTACGCATG CATAGAAACG TAATCTATAT TTTTTTAGGA

---------- ---------- ---AGAACTA TAATCTCTA- TTTTTTAGGA

CAGTCCAAAC ACTATGCCTG CATAGAAATA TAATCTATAT TTTTTTAGGA

CAGTCCAAAC ATTATGCCTG CATAGAAATA TAATCTATAT TTTTTTAGGA

---------- ---------- ---------- ---------- ----------

CAGTCGAAGC ATTATGCCTG CATCGAAATA TAATCTATAT TTTTTTAGGA

CAGTCCAAGC ACTATGCTTG CATAGAAATA TAATCTATAT TTTTTTAGGA

AAACAAAAAT GAAAAACTAC TCCATTTGAG GATTGTAATA AGACGAGGTC

GAACAAAAAT GAAAAACTAC TCCATTTGAG GATTGTAATA AGACGAGGTC

AAACAAAAAT GAAAAACTAC TCCAGTTGGG GATTGTAATA AGACGAGGTC

AAACAAAAAT GAAAAACTAC TCCAGTTGGG GATTATAATA AGACGAGGTC

AAACCGAAAT TAAAAACTAT TCCATTTGGG CATTGTAATA AGACGAGGTC

AAACAAAAAC GAAAAACTAC CCCCCTTGGG CATTGTAGTA AGACGAGATC

AAACAGAAAT GAAAAACTAC CCCATTGGGG CATT-TAATA AGACGAGTTC

AAACAGAAAT GAAAAATTAT CCCATTGGGG CATT-TAA-A AGGCGGGTTC

AAACAGAAAT GAAAAACTAT CCCATCCTGG CATTGTCATA AGAC------

AAACAAAAAT GAAAAACTAC CCGATTAGGG CATTGTAATA AGACGAGGTC

AAACAAAAAT GAAAAACTAT CCCATTTGGG CATTGTAACA AGACGAGGTT

AAACAAAAAT ACACAACTGT CCCATTTGGG CATTGTCCTA AGACGAGGTC

CAACAAAAAT GAAAAACCAC CCCACTTAGG CATTGTCATA AGACGAGGTC

AAACAAAAAT GAAAAACTGC CCCATTCGGG CGCTGTAATA AGACGAGGTC

AAACAAAAAT GAGAAACTAC CCCATTTGGG CATTGTAATA AGACGAGGTC

---------- ---------- ---------- ---------- ----------

AAACAAAAAT TAAAAACTAT CCCAGTTGGG CATTGTAATA ATGCAGAGCT

AAACAAAAAT TAAAAACTAT CCCAGTTGGG CATTGTAATA AGAGGAGGCC

AGGAGGCAAT TTGCCTGCCT TGAAAAGCTC AGGTGAAAGA CTGAGACACT

AGGAGGCAAT TTGCCTGCCT TGAAAAGCTC AGGTGAAAGA CTGAGACACT

AGGAGGCAAT TTGCCTGCCT TGAAAAGCTC AGGTGAAAGA CTGAGACACT

AGGAGGCAAT TTGCCTGCCT TGAAAAGCTC AGGTGAAAGA CTGAGACACT

AGGAGGCAAT CTGCCTGCCT TGAAAACCTC AGGTGAAAGA CTGAGACACC

AGGAGGTAAT TGGCCTGCCT TGAAAGGCTC AGGTGAAAGA CTGAGACACT

AGCAGGCAAC CTGCCTGTTT -GAAAAACTC AGACGAAGGG CTGAGACACC

AGCAGGCAAC CTGCCCGTCT -GAAAAACTC A----AAGGG CTGAGACACT

AGGAGGGGAC ---------- --AAAGGCTC GGGAGCCAGA CAGAGACGCT

AGGAGGCAAT G----TGCCT TAAAAAGCTC AGGTGAGAGA CTAAGACACT

AGGAGGCAAT TTGCCTGCCT TGAAAAGCTC AGGTGAAAGA CTGAGACACT

CGGAGGCAAT GTGCCTGCCT TGAGAAGCTC AGGTGAAAGA TTGAGACACT

AGAAGGCAAG TTGCTTGCCA TG---AGCTC AGGCGAGAGA C-GAGACACC

AGGAGGCAAT TTGCCTGCCT TGGAATGCTC AGGTGAAAAA CTGAGACACT

AGGAGGCAAT TTGCCTGCCT TGAAAAGCTC AGGTGAAAGA CTGAGACACT

---------- ---------- ---------- ---------- ----------

TGGAGGAAAT GTACCTGCAT CTAGCAGACC AGGTGAAAGG CTGAGACACA

GGGGGGCAAT TATCCTGCCT TGAAGAGCCC AGGTGAAGCT CTGAGAcaca

ATCCACAGAT ATAGCCAAGT AGATTTGGGT AGAGGATACT ATTTCCAAAT

ATCCACAGAT ATAGCCAAGT AGATTTGGGT AGAGGATACT ATTTCCAAAT

ACCCACAGAT ATAGCCAAGT AGATTTGGGT AGAGGATACT ATTTCCAAAT

ATCCACAGAT ATAGCCAAGT AGATTTGGGT AGAGGATACT ATTTCCAAAT

ATCCACAGAT ATAGCCAAGT AGACTTGGGT AGAGGATATT ATTTCCAAAT

ATCCACAGAT ATAGCCAAGT AGATTTGGGT AGAGAATACT ATTTCCAAGT

ATCCACAGAT ATAGTCAAGT AGATTTGGGT AGAGAATACT ATTTCCAAGT

ATCCACAGAT ATAGTCAAGT AGATTTGGAT AGAGAATACT ATTTCCAAGT

ATTCACAGTT AGAGCCCCAG AGGTGTGGGT TGAGAATACT ATTTCCAAGT

ATCCACAGAT AGAGACAAGT AGATTTGGGT AGAGGATATT ATTTCCAACT

ATCCACAGAT ATAGCTAAGT AGATTTGGGT AGAGAGTACT ATTTCCAAGT

ATCCACAGGT ATAGCCAAGT AGACTTGGAC AGAGAATACT ATTTCCAAGT

ATCCACCGAT ATAGCCAAGT AGATTTGGGT AGAGAATACT ATTTCCAAGT

ATCTGCAAAT ATAGCCAAGT AGATTTGAGT AGAAAATACT ATTTCCACGT

ATCCACAGAT ATAGCCAAGT AGATTTGGAT AGAGAATACT ATCTCCAAGT

---------- ---------- ---------- ---------- ----------

ATTCTCAGCT AGAGTCAATT AGAGTTGCTT AGAGGATACC ATTGCCACTT

GTCTACAGAT CTAGTTAAGT AGAGCTGGGG AAAGGGTTTT GTTGCCAACT

AGTGTTTAGC TCACCTAGGG GATATGTTTG TATACACATT TGCATACACA

AGTGTTTAGC TCACCTAGGG GATATGTTTG TATACACATT TGCATACACA

AGTGTTTAGC TCACCTAGGG GATATGTTTG TATACACATT TGCATACACA

AGTGTTTAGC TCACCTAGGG GATATGTTTG TATACACATT TGCATACACA

AATGTTTAGC TCACCTAGGG AATATGTTTG TATACACATT TCCATACACG

AGCATTTAGC TCACCTAGGG GATGTGTTTG TACACACATC TGCATACACA

AGTGTTTAGC TCATCTAGGG GATGTGTTTA TATATACATT TGCATACACA

AGTGTTTAGC TCATCTAGGG GATGTGTTTA TATATACATT TGCATACACG

AGTGCTTAGC TCACCTAGGG GATGTGTTTG TACACGCCTT GGCATACACA

AGATTTTAGT TCACCTAGGG GGTGTGTTTG CATACACATT TACATACACA

AGGGTTTAGC TCACCTAGGG GATGTGTTTG TATACACATT TGCATACACA

AGCATTTAGC TCACCGAGGG GGTGTGTTTG CATACACATT TGCATACACA

AGCCGTTAGC TCACCTAGGA GATGTGTCTT CATACACATT TGCATATACA

AGCGTTTAGC TCACCTAGGG GATGTGTTTG AATACATATT TGCATATACA

AATTTTTAGC TCACCTAGGG GATTTGTTTG TATACACATT TGCATACATA

---------- ---------- ---------- ---------- ----------

AGACTTCAGC TCAAAGGGGG GGCGTATTTA GGTGCACATA TACCTACACA

AGTATTTAGC TCAACTAGAG AATGTGCTCA TATACACACA TTCATACAGA

TGGGGACATA AGCTAATTTT TTACAGGACA CAGAATTGCT GTTAAAAATC

TGGGGACATA AGCTAATTTT TTACAGGACA CAGAATTGCT GTTAAAAATC

TGGGGACATA AGCTAATTTT TTACAGGACA CAGAATTGCT GTTAAAAATC

TGGGGACATA AGCTAATTTT TTACAGGACA CAGAATTGCT GTTAAAAATC

TGGGGACATA AGCTAATTTT TTACAGGACA CAGAATTGCT GTTACAAATC

CGGGGACATA AGCTAATTTT TTATAGGACA CAGAATTGCT GTTAAAAATC

GGGGATTATG AGCTAATTTT TTATAGGACA CAGAATTGCT GTTAAAAATC

GGGGATTATG AGCTAATTTT TTATAGGACA CAGAATTGCT GTTAAAAATC

TGGGAC-ATA AGCTAATTTT TTATAGGACA CAGAATTGCT GTTAAAAATC

TGGGGACATA AGCTAATTTT TTACAGGACA CAGAATTGCT GTTAAAAATC

CGGGGACATA AGCTAATTTT TTACAGGACA CAGAATTGCT GTTAAAAATC

CAAGGACATA AGCTGATTTT TTACAGGACA CAGAATTGCT GTTAAAAAGC

CGGGGACATA AGCTAATTTT TTACAGGACA CAGAATTGCT GTTAAACATT

CGGGGACATA TGCTAATTTT TTACAGGACA CAGAATTGCT GTTAAAAATC

TGGGGACATG AACTAATTTC TTACAGGACA CAGAATTGCT ATTAAACTCC

---------- ---------- ---------- ---------- ----------

CAGAGGTATA AGCTAAGATT TTACAGGACA CAGAATTGCT GTTGCAATCC

CAGGGATATA AGCTAATTTT TTACAGGACC CACAGTTGTT GATAAAAATC

TCTTTTTTTG AAGAAAATCA TGACATTCCA AGTTTGTTGT ttttttgaaa

TCTTTTTTTG AAGAAAATCA TGACATTCCA AGTTTGTTGT ttttttgaaa

TCTTTTTTTG AAGAAAATCA TGACATTCCA AGTTTGTTGT tttttttaaa

TCTTTTTTTG AAGAAAATCA TGACATTCCA AGTTTGTTGT ttttt--aaa

TCTC---TTG AAAAAAATCA TGACATTCCA AGTTTGTTGT TGTTT-----

TCTTTTTTTG AAGAAAATAA TGACATTCCA AGTTTGTTGT TTTTTTGAAA

TCTTTTTTTG AGGGAAATTG TGACATTCCA AGTTTGTTGT TTTTT-TAAA

TTTTTTTTTG AGGGAAATTG TGACATTCCA AGTTTGTTGT TTTTT--AAA

TCTCTTTTTG AAGAAAATCA TGACATTCCA AGTTTATTGT TCTTT-GAAA

TCTTTTTTTG AAGAAAATCA TGACATTCCA AGTTTGTTGT TTTTTTGAAA

TCTTTTTTTG AAGAAAATCA TGACATTCCA AGTTTGTTGT TTTTTTGAAA

TCTTTTTTTG AAGAAAATCA AGACATTCCA AGTTTGTCGT TTTTTTGAAA

TCTTTTTTGG AAGAAAATCA TGACATTCCA AGTTTGTTGT TTTTTTGGAC

TCTTTTTTTG AAGAAAATCA TGACATTCCA AGTTTGTTGT TTTTATCAAA

TCTTTTTTTG AAGAGAATCA TGACATTCCA AGTTTGTTGT ttttttaaaa

---------- ---------- ---------- ---------- ----------

TCTTTTTTTA AAGAAAACCC TGACATTCCA AGTTTGTTGT tttttttaaa

TTTTGTTTTA AAGGAAACCC TGACATTCCA AGTTGTTGGC tttttttaaa

taaaactata atataaattc tctaaataaa attattttaa GTTTTAGTGT

taaaactata atataaattc tctaaataaa attattttaa GTTTTAGTGT

taaaactata atataaattc tctaaataaa attattttaa GTTTTAGTGT

taaaactata atataaattc tctaaataaa attattttaa GTTTTAGTGT

------GGTA ATATAAGTTC TCTGAATAAA ATTATTTTAA GTTTTGGTGT

TAAAACTATG ATTTAAATTC TCTAAATGAA ATGATTTTAA GTTTTAGTGT

TAAAAGTATA GTATAAATTT TCTAAATAAG ATTATTTTAA GTTTTACTGT

TAAAAGTACA GTATAAATTT TCTAAATAAG ATTATTTTAA GTTTTAGTGT

TAAAACTATA ATATAAATTC TCGAAATAAG ATCATTTT-- ----TAGTGT

TAAAACTATA ATATAAATTC TCTAAATAAA ATTATTTTAA GTTTTAGTGT

TAAAACTATA ATATAAGTTA TCTAAATAAA AGTATTTTAA GTTTAAGTGT

TAAAACTATA ATATAAATTC TCGaaataaa attattttat aatttaGTGC

CGAGACTATA ATATAAATAC CCTCAATAAA ATTCTTTTCG GTGTTAGTGT

TAAAACTATA ATATAAATTC CCtaaataaa attattttaa GTTTTAGTGT

taaaactata atataaattC TCTAAATAAA ATTATTCTAA GTTTTAGTGT

---------- ---------- ---------- ---------- ----------

gaaaactata atataaattc tctaaataaa attattttaa gttttaatGT

gaaaactata atataaattc tctaaataaa attattttaC GTTTTAATGT

CAAGAGGGCG GTTTGAGCTT TCTATAAGCT TTGTTTATTT CACCCGTTCA

CAAGAGGGCG GTTTGAGCTT TCTATAAGCT TTGTTTATTT CACCCGTTCA

CAAGAGGGCG GTTTGAGCTT TCTATAAGCT TT-TTTATTT CACCCGTTCA

CAAGAGGGCG GTTTGAGCTT TCTATAAGCT TT-TTTATTT CACCCGTTCA

CAAGAGGGCG GTTTGAGCTT TCTATAAGCT TTGTTTATTT CACCTGTTCA

TAAGAGGGCG GTTTGAGCTT TCTATAAGCT TTGTTTATTT CACCTGTTCA

CAAGAGAGCC GTTTGCGCTT TCAATAAGCT TTGTTTCTTT CTCCTGTTCA

CAAGAGAGCG GTTTGAGCTT TCAATAAGCT TTGTTTATTT CACCTGTTCA

TGGGAGGGCG GTTTGAGCTT TCTATAAGCT TTGTTTCTTT CACCTGTTCA

TGAGAGGGCG GTTTGAGCTT TCTATAAGCT TTGTTTATTT CACCTGTTCA

TGAGAGGGCG GTTTGAGCTT TCTATAAGCT TTGTTTATTT CACCTGTTCA

TGAGAGGGCG GTTTGAGCTT TCTATAAGCT TTGTTTATTT CACCTGTTCA

TGAGAGGGCG GTTTGAGCTT TCTATAAGCT TTGTTTATCT CACCTGTTCA

CAAGAGGGCG GTTTGAGCTT TCTATAAGCT TTGTTTATTT CACCTGTTCA

CGAGAGGGTG GTTTGAGCTT TCTATAAGCT TTGTTTATTT CACCTGTTCA

---------- ---------- ---------- ---------- ----------

TAAAAGGGTG AACTGAGCTT GCTATAAGCT TTGTTGATTT TATCTGTTCA

TAAGAGGGCA GCTTAAGCTT TTCGTGAGCT TTGTGTATTT TACCTGTTCA

CTTACTGTAT AATTTAAAAT CATTTATGTA GCTGAGACAC TTCTGTATTT

CTTACTGTAT AATTTAAAGT CATTTATGTA GCTGAGACAC TTCTGTATTT

CTTACTGTAT AATTTAAAGT CATTTATGTA GCTGAGACAC TTCTGTATTT

CTTACTGTAT AATTTAAAGT CATTTATGTA GCTGAGACAC TTCTGTATTT

CTTACTGTAT AATTTAAAGT CATTTATGTA GCTGAGACAC TTCTGTATTT

CTTACTGTAT AATTTAAAGT CATTTATGTA GCTGAGACAC TTCCATATTT

CTTACTGTAT AATTTAAAGT CATTTATGTA GCTGAGACAC TTTGTTATTT

CTTACTGTAT AATTTAAAAT CATTTATGTA GCTGAGACAC TTTGATACTT

CTTACTGTAT AATTTAAAGT CATTTATGTA GCTGAGACAC TTCTGTATTT

CTTACTGTAT AATTTAAAGT CATTTATGTA GCTGAGACAC TTCCGTATTT

CTTACTGTAT AATTTAAAAT CATTTATGTA GCTGAGACAC TTCCGTATTT

CTTACTGTAT AATTTAAAGT CATTTATGTA GCTGAGACAC TTCTGTATTT

CTTCCTGTAT AATTAAAAGT CATTTATGTA GCTGAGGCAC TTCCGTATTT

CTTACTGTAT AATTTAAAAT CATTTATGTA GCCGAGACAC TTCAGTATTT

CTTACTGTAT AATTTAAAAT CATTTATGTA GCTGAGACAC TTCAGTATTT

---------- ---------- ---------- ---------- ----------

CTTAATGTAT AATTCAAGGT CATTTATAGA GCTGAAATAT CTCAGTACTT

CTTACTGTAT ATTTTATGGT CTTTTAATTA GCTGAGACAC TTTGGAATTT

CAATCTATTT TTTTTTGCTA AATCTTGTGT CATGTGTAGG CTGTAATATG

CAATCTATTT TTTTTTGCTA AATCTTGTGT CATGTGTAGG CTGTAATATG

CAATCTATTT TTTTTTGCTA AATCTTGTGT CATGTGTAGG CTGTAATATG

CAATCTATTT TTTTTTGCTA AATCTTGTGT CATGTGTAGG CTGTAATATG

CAATCTATTT TTTTTTGCTA AAACTTGTGT CATGTGTAGG CTGTAATATG

CAATTTATTT TTTTTTGCTA AATCTTGTGT CATGTGTAGG CTGTAATATG

CAATCTATTT TTTTTTGCTA AATCATGTGC CATGTGTAGG CTGT--CGTG

CAATTTATTT TTTTTTGCTA AATCATGTGC CATGTGTAGG CTGT--TGTG

CAATCTATTT TTTTTTGCTA AATCTTGTGT CATGTGTAGG CTGTGATATG

CATTCTTTTT TTTTTTGCTA AATCTTATGT CATGTGTAGG CTGTAATATG

CAATCTATTT TTTTTTGCTA AATCTTGTGT CATGTGTAGG CTTTAATATG

CAATCTATTT TTTTTTGCTA AATCTTATGT CATGTGTAGG CTGTCATATG

CAATCTATTT TCTTTTGCTA AATCTTGTGT CATGCGTAGG CTGTTATATG

CAATCTATTT TTTTTTGCTA AATCTTTGGT CATATGTAGG CTGTAATATG

CAATCTATTT TTTTTTGCTA AATCTTGTGT CATGTGTAGG CTGTAATATG

---------- ---------- ---------- ---------- ----------

CAAATTATTT TTTTTTGCTA AGTTCTGTGT TGTATGTAGG TTGTAATATC

GAATTAATCT TTTTTGGCTA GCTCATATGT CCTAAGTAGG CTGTAATATT

TGTACATTGT GTTTAAGATG AAACCCACAT GCCGCCATTT TCCATCAAAG

TGTACATTGT GTTTAAGATG AAACCCACAT GCCGCCATTT TCCATCAAAG

TATACATTGT GTTTAAGATG AAACCCACAT GCCGCCATTT TCCATCAAAG

TGTACATTGT GTTTAAGATG AAACCCACAT GCCGCCATTT TCCATCAAAG

TGTACATTGT GTTTAAGATG AAACCCACAT GCCACCATTT TCCATCAAAC

TATACATTGT GTTTAAGAAA AAACCCACAT GCCACCATTT TCCATCAAAG

TGTACACTGT GTCTAAGAA- AACCCCACAT GCCATTTTTT TCCTTCAAAA

TGTACACTGT GTCTaagaag aaaCCCACAT GCCATTTTTT TTCTTCAAAA

TGTCCATTGT GTGTAAGA-- ---------- ---------- ----------

TGTACACTGT GTTTAAGACA AAACCCACAT GCCACCATTT TTCATCAAAG

TGTACATTGT GTTTAAGATG AAACCCACAT GCCGCCATTT TCCATCAAAG

TGTACATCGT GTTTAAGATG AAACCCACAT GCCGCCATTT TCCATCAAAG

TGTACATTGT GCTTAAGACG AAACCC-CAT GCCACCATGT TCCCTCAAAG

TGTACATTGT GTTTAAGATT AAATCCACAT GCCGCCATTT TCCATCAAAG

TATACATTGT GTTTAAAATT AAACCCACAT GCCGCCATTT TCCATCAAAG

---------- ---------- ---------- ---------- ----------

TGCACAATTC TTCAAAGCTA GACTCCCCAC TCCCCCACAT GCCACCAAAA

TCCGCACCTG GTCGAAAAAA AACTCCACAC GCTATCGCTT TCCTCCAAAG

GAATGAGCCA GTCTGCTGTC CTGAAAACCA ACAAGAGAGA ACCCTGTCTA

GAATGAGCCA GTCTGCTGTC CTGAAAACCA ACCAGAGAGA ACCCTGTCTA

GAATGAGCCA GTCTGCTGTC CTGAAAACCA ACCAGAGAGA ACCCTGTCTA

GAATGAGCCA GTCTGCTGTC CTGAAAACCA ACCAGAGAGA ACCCTGTCTA

GAATGAGCCA GTCTGCTGTC CTGAAAACCA ACTAGAGAAA ACCCTGTCTA

GAAAGAGCCA GTCTGCTGTC CTGAAAACCA ACCAGAGAAA ACCCTGTCTA

GAATGAACAA GTCAGCTGTG CTGAACTCCA GCCAGAGAAA AACCCATCTA

GAATGAGCAA GCCAGCTGTG CTGAACACCA GCCAGAGAAA ACCCCCTCTA

-----AGCCA GTCTGCTGCC CTGCAAACCA ACCAGAGAAA GCCCTGTCTG

GAATGAGCCG GTCTGCTGTC CTGAAAATCA ACTAGAGAAA ACCCTGTCTG

GAATGAGCCA GTCTGCTGTC CTGAAAACCA ACCAGAGAAA ACCCTATCTG

GAATGAGCCA GTCTGCTGTC CTGAAAACCA ACCAGAGAAA ACCCTGTCTA

GAATGAGCCA GTCTGCTGCC CTGAAAACCA ACCAGAGGAA ACCCTGTCTG

GAAAGAGCCA GTCTGCTGCC CTGAAAACCA ACCAGAGAAA ACCCTGTCTA

GAACGAGCCA GTCTGCTGTC CTGAAAACCA ACCAGAGAAA ATCCTGTCTG

------GACT GCCTGCTGTC CTAGATACCA TCCAGAGAGA ACCC-GCCTG

CTTTGAGCCA ATCTGCTGTC CTCAAAACCA CCAGGAGAAA ACCTTGTCTG

GGATGAGCCA GTGGACCGTC CTGAAAACCA CCCAGAGAAA ACCTTGTCTG

GAAGGAATGT ATTTGTTGCT AAATTTCGTA GCACTGTTTA CAGTTTCCTC

GAAGGAATGT ATTTGTTGCT AAATTTCGTA GCACTGTTTA CAGTTTCCTC

GAAGGAATGT ATTTGTTGCT AAATTTTGTA GCACTGTTTA CAGTTTCCTC

GAAGGAATGT ATTTGTTGCT AAATTTTGTA GCACTGTTTA CAGTTTCCTC

GAAGGAATGT ATTTGTTGCT AAATTTTGTA GCACTGTTTA CAGTTTCCTC

GAAGGAATGT ATTTGTTGCT AAATTTTGTA GCACTGTTTA CAGTTTCCTC

GAAGGAATGT ATTTGTTGCT AAATTTTGTA GCACTGTTTA CAGTTTCCTC

GAAGGAATGT ATTTGTTGCT AAATTTTGTA GCACTGTTTA CAGTTTCCTC

GAAGGAATGT ATTTGTTGCT AAATTTTGTA GCACTGTTTA CAGTTTCCTC

GAAGGAATGT ATTTGTTGCT AAATTTTGTA GCACTGTTTA CAGTTTCCTC

GAAGGAATGT ATTTGTTGCT AAATTTTGTA GCACTGTTTA CAGTTTCCTC

GAAGGAATGT ATTTGTTGCT AAATTTTGTA GCACTGTTTA CAGTTTCCTC

GAAGGAATGT ATTTGTTGCT AAATTTTGTA GCACTGTTTA CAGTTTCCTC

GAAGGAATGT ATTTGTTGCT AAATTTTGTA GCACTGTTTA CAGTTTCCTC

GAAGGAATGT ATTTGTTGCG AAATTTTGTA GCACTGTTTA CAGTTTCCTC

GAAGGAATGT ATTTGTTGCG AAATTTTGTA GCTCTGTTTA CAGTTTCCTC

GAAGGAATGT ATTTGTCGCT AAATTTTGTA GCACTGTTTA CAGTTTCCTC

GAAGGAATGT ATTTGTTGCT AAATTTTGTA GCACTGTTTA CAGTTTCCTC

CATGTTATTT ATGAATTTTA TATTCCGTGA ATGTATATTG TCTTGTAATG

CATGTTATTT ATGAATTTTA TATTCCGTGA ATGTATATTG TCTTGTAATG

CATGTTATTT ATGAATTTTA TATTCCGTGA ATGTATATTG TCTTGTAATG

CATGTTATTT ATGAATTTTA TATTCCGTGA ATGTATATTG TCTTGTAATG

CATGTTATTT ATGAATTTTA TATTCCGTAA ATGTATATTG TCTTGTAATA

CATGTTATTT ATGAATTTTA TATTCCGTGA ATGTATATTG TCTTGTAATG

CATGTTATTT ATGAATTTTA TATTCCGTGA ATGTATATTG TCTTGTAATG

CATGTTATTT ATGAATTTTA TATTCCGTGA ATGTATATTG TCTTGTAATG

CATGTTATTT ATGAATTTTA TATTCCGTGA ATGTATATTG TCTTATAATG

CATGTTATTT ATGAATTTTA TATTCCGAGA ATGTATATTG TCTTGTAATG

CATGTTATTT ATGAATTTTA TATTCCGTGA ATGTATATTG TC-TGTAATG

CATGTTATTT ATGAATTTTA TATTCCGTGA ATGTATATTG TCTTGTAATG

CATGTTATTT ATGAATTTTA TATTCCGTGA ATGTATATTG TCTTGTAATG

CATGTTATTT ATGAATTTTA TATTCCGTGA ATGTGTATTG TCTTGTAATG

CATGTTATTT ATGAATTTTA TATTCCGTGA ATGTATATTG TCTTATAATG

CATGTTATTT ATGAATTTTA TATTCCGTGA ATGTATATTG TCTTGTAATG

CATGTTATTT ATGAATTTTA TATTCCGTGA ATGTATATTG TCTTGTAATG

CATGTTATTT ATGAATTTTA TATTCAGTGA ATGTATATTG TCTTGTAATG

TTGCATAATG TTCACTTTTT ATAGTGTGTC CTTTATTCTA AACAGTAAAG

TTGCATAATG TTCACTTTTT ATAGTGTGTC CTTTATTCTA AACAGTAAAG

TTGCATAATG TTCACTTTTT ATAGTGTGTC CTTTATTCTA AACAGTAAAG

TTGCATAATG TTCACTTTTT ATAGTGTGTC CTTTATTCTA AACAGTAAAG

TTGCATAATG TTCACTTTTT ATAGTGTGTC CTTTATTCTA AACAGTAAAG

TTGCATAATG TTCACTTTTT ATAGTGTGTC CTTTATTCTA AACAGTAAAG

TTGCATAATG TTCACTTTTT ATAGTGTGTC CTTTATTCTA AACAGTAAAG

TTGCATAGTG TTCACTTTTT ATAGC-TGTC CTTTATTCTA AACAGTAAAG

TTGCATAATG TTCACTTTTT ATAGTGTGTC CTTTATTCTA AACAGTAAAT

TTGCATAATG TTCACTTTTT ATAGTGTGTC CTTTATTCTA AACAGTAAAG

TTGCATAATG TTCACTTTTT ATAGTGTGTC CTTTATTCTA AACAGTAAAA

TTGCATAATG TTCACTTTTT ATAGTGTGTC CTTTATGCTA AACAGTAAAG

TTGCATAATG TTCACTTTTT ATAGTGTGTC CTTTATTCTA AACAGTAAAG

TTGCATAATG TTCACTTTTT ATAGTGTGTC CTTTATTCTA AACAGTAAAG

TTGCATAATG TTCACTTTTT ATA--GTGTC CTTTATTCTA AACAGTAAAG

TTGCATAATG TACACTTTTT ATAGTGTGTC CTTTATTCTA AACAGTAAAG

TGGCATAATG TTCACTTTTT ATAGTGTGTC CTTTATTCTA AACAGTAAAG

TTGCATAATG TTCACTTTTT ATAGGGTGTC CTTTATTCTA AACAGTAAAA

TGGTTTATTT CTATCACGCA CTCACCATTG GCTTATGGTT GGGGAAAAAT

TGGTTTATTT CTATCACGCA CTCACCATTG GCTTATGGTT GGGGAAAAAT

TGGTTTATTT CTATCACGCA CTCACCATTG GCTTATGGTT GGGGAAAAAT

TGGTTTATTT CTATCACGCA CTCACCATTG GCTTATGGTT GGGGAAAAAT

TGGTTTATTT CTATCACGCA CTCACCATTG GCTTATGGTT GGGGAAAAAT

TGGTTTATTT CTATCACGTA CTCACCATTG GCTTATGGTT GGGAAAAAAT

TGGTTTATTT CTATCACGCA CTCACCATTG GCTTGTGGTT GGGGAAAAAT

TGGTTTATTT CTATCACGCA CTCACCATTG GCTTGTGGTT GGGGAAAAAT

TGGTTTATTT CTATCACGCA CTCACCATTG GCTTGTGGTT GGCAAAAAAC

TGGTTTATTT CTATCACGTA CTCACCATTG GCTTGTGATT GGGGAAAAAT

TGGTTTATTT CTATCACGCA CTCACCATTG GCTTATGGTT GGGGAAGAAT

TGGTTTATTT CTATCACGCA CTCACCATTG GCTTATGGTT GGGGAAAAAC

TGATTTATTT CTATCACGCA CTCACCATGG GCTTGTGGTT GGGGAAGAAG

TGGTTTATTT CTATCACGCA CTCACCATTG GCTTATGGTT GGGGAAAAAT

TGGTTTATTT CTATCACGCA CTTACCATTG GCTTATGGTT GGGGAAAAAT

TGGTTTATTT CTATCACGTA CTCACCATTG GCTTGTGATT GGGGAAGAAC

TGGTTTATCT CTATCACGTA CTCACCATTT GCTTGTGGTT GGGGAAAAAT

TGGTTTATTT CTATCACGTA CTCACCATCT GTTTATGGTT GGGGAAAAAC

GTTTGCTCCA CAAGCGGGAA CTTCTCAGGA CCCAGGGAAT GGAAGATCTT

GTTTGCTCCA CAAGCGGGAA CTTCTCAGGA CCCAGGGAAT GGAAGATCTT

GTTTGCTCCA CAAGCGGGAA CTTCTCAGGA CCCAGGGAAT GGAAGATCTT

GTTTGCTCCA CAAGCGGGAA CTTCTCAGGA CCCAGGGAAT GGAAGATCTT

GTTTGCTCCA CAAGTGGGAA CTTCTCAGGA CCCAGGGAAT GGAAGATCTT

GTTTGCTCCA CAAGCGGGAA CTTCTCAGGA CCCAGGGAAT GGAAGATCTT

GTTTGCTCCA CTAGTGGAAA CTTCTCAGGA CCCAAGGAAT GGAAGATCTT

GTTTGCTCCA CTAGTGGAAA TTTCTCAGGA CCCAAGGAAT GGAAGATCTT

GTTTGCTCCA CCAGCGGGAA CTTCTCAGGG CCCAAGGAGT GAAAGATCTT

GTTTGCTCCA CAAGCGGGAA CTTCTCAGGA CCCAGGGAAT GGAAGATCTT

GTTTGCTCCA CAAGTGGGAA CTTCTCAGGA CCCAGGGAAT GGAAGATCTT

GTTTGCTCCA CAAGCGGGAA CTTCTCAGGA CCCAGGGAAT GTAAGATCTT

GTCTGTTCCA CCAGCGGAAA CTTCTCAGGA CCCAGGGAGT GGAAGATCTT

GTTTGCTCCA CAAGCGGGAA CTTCTCAGGA CCCAGGGAAT GAAAGATCTT

GTCTGCTCCA CAAGCGGGAA CTTCTCAGGA CCCAGGAAAT GGAAGATCTT

GTTTGCTCCA CCAGCGGGAA CTTCTCGGGC CCCAGGGCAT GGAAGATCTT

GTTTGTTCTA CAAGTGGAAA CTTCTCTGGT CCCAATGAAT TGAAGATCTT

GTCTGCTCCA CAAGAGGAAA CTTCTCTGGA CCCAGCGAAT TGAAGATCTT

AATGAGCTGA GAGAACTAGG ACACCTACCA CCCAGGGCTT GCTGCAGAAG

AATGAGCTGA GAGAACTAGG ACACCTACCA CCCAGGGCTT GCTGCAGAAG

AATGAGTTGA GAGAACTAGG ACACCTACCA CCCAGGGCTT GCTGCAGAAG

AATGAGTTGA GAGAACTAGG A--------- ---------- ----------

AATGAGTTGA GAGAACTAGG ACACCTACCA CCCAGGGCTT GCTGCAGAAG

AATGAGTTGA GAGAACTAGA ACACTTACCA CCCAGGGCTT GCTGCAGAAG

AATGAGCTGA GAGAACTGGG ATACCTACCA CCCAAGGCTT GCTGCAGAAG

AATGAGCTGA GAAAACTGGG AAACCTACCA CCCAAGGCTT GCTGCAGAAG

AATGAGCTGA GAGAACTAGG ACACCTACCA CCCAGGGCTT GCTGCAGAAG

AATGAGCTGA GAGAACTAGG ACACCTACCA CCCAGGGCTT GCTGCAGAAA

GATGAGTTGA GAGAACTTGA ACACCTACCA CCCAGGGCTT GCTGCAGAAG

AATGAGCTGA GAGAACTAGG ACACTTACCA CCCAGGGCTT GCTGCAGAAG

GATGAGCTGC GAGAACTAGG ACACTTACCA CCCAGGGCTT GCTGCAGAAG

AATGAGCTGC GAGAACTAGG ACACCTACCA CCCAGGGCTT GCTGCAGAAG

AATAAGCTGA GAGAACTAGG ACACCTACCA CCCAGGGCTT GCTGCAGAAG

GATGAGCTGT GAGAACTAGG ACACCTACCA CCCAGGGCTT GCTGCAGAAG

AATTAACTGG GAGAACTGGA ACACTTACCA CCCAGGGCTT GCTGCAGAAG

AATGAGTTGG GAGAACTGAA ATACCTACCA CCCAGGGTTT GCTGCAGAAG

TTGTAGACGG AGTAGAGAGA GTTGACAGCA GGCAGCCCTC CATACTGCAG

TTGTAGACGG AGTAGAGAGA GTTGACAGCA GGCAGCCCTC CATACTGCAG

TTGTAGACAG AGTAGAGAGA GTTGACAGCA GGCAGCCCTC CATACTGCAG

---------- ---------- ---------- ---------- ----------

TTGTAGACAG AGTAGAGAGA GTTGACAGCA GGCAGTCCTC CATACTGCAG

TTGTAGACGG AGTAGAGAGA GTTGACGGCG GGGAGCCCTC CGTACTGCAG

TTGTATACGG AGTAGAGAGA GTTGACAGCA GGAAGCCCTC CATACTGGAG

TTGTATACGG AGTAGAGAGA GTTGACAGCA GGAAGCCCTC CATACTGGAG

TTGTAGACAG AGTAGAGGGA GTTGACGGCG GGCAGCCCTC CGTACTGCAG

TTGTAGACAG AGTAGAGGGA GTTGACGGCA GGCAGCCCTC CATACTGGAG

TTGTAGACAG AGTAGAGGGA GTTGACGGCA GGCAACCCTC CATACTGAAG

TTATAGACGG AGTAGAGGGA GTTGACGGCG GGCAGCCCTC CGTACTGGAG

TTGTAGACAG AGTAGAGGGA GTTGACGGCA GGCAGCCCCC CGTACTGCAG

TTGTAGACGG AGTAGAGGGA GTTGACGGCA GGCAGCCCTC CGTACTGGAG

TTGTAGACGG AGTAGAGGGA GTTGACAGCG GGCAGCCCTC CATACTGGAG

TTGTAGACGG AGTAGAGGGA GTTGACAGCA GGCAGCCCTC CATACTGGAG

TTGTAAATGG AATACAAGGA GTTCACACTA GGGAGTCCGC CATATTGGAG

TTGTAGATGG AGTACAGAGA GTTCACACTG GGCAGGCCGC CATACTGGAG

GCCGATGACC AGGCTGCGGT AGTCTTCCCC CAGGGCCATG CTGTAGGCAT

GCCGATGACC AGGCTGCGGT AGTCCTCCCC CAGGGCCATG CTGTAGGCAT

GCCGATGACC AGGCTGCGGT AATCTTCCCC CAGGGCCATG CTGTAGGCGT

---------- ---------- ---------- ---------- ----------

GCCGATGACC AAGCTGCGGT AGTCTTCCCC CAGGGCCATG CTGTAGGCGT

GCCGATGACC AGGCTGCGGT AGTCCTCGCC CAGGGCCATG CTGTAGGCAT

GCCGATGACC AGGCTTCGGT AGTCCTCAGC CAGGGCCATG CTATAGGCAT

GCCGATGACC AGGCTCCGGT AGTCCTCAGC CAGGGCCATG CTGTAGGCAT

GCCGATGACC AGGCTGCGGT AGTCTTCGCC CAGGGCCATG CTGTAGGCGT

GCCGATGACC AGGCTGCGGT AGTCTTCGCC CAGGGCCATG CTGTAGGCGT

GCCAATGACC AGGCTTCGGT AGTCTTCACC CAGAGCCATG CTGTAGGCAT

GCCAATGACC AGGCTGCGGT AATCTTCTCC CAGGGCCATG CTGTAGGCAT

GCCGATGAGC AGGCTGCGAT AGTCCTCCCC CAGTGCCATG CTGTAGGCGT

GCCGATGACC AGGCTGCGAT GGTCTTCGCC CAGGGCCATG CTATGGGCGT

GCCGATGACC AGGCTGCGGT AATCTTCGCC CAGGGCCATG CTGTAGGCGT

GCCGATGACC AGGCTGCGGT AGTCTTCGCC CAGGGCCATG CTGTAGGCGT

ACCAATGACC AGGCTTCGGT AATCCTCTGC CAGGGTCATG CTGTAAGCAT

GCCGATGATC AGGCTGCGGA AGTCTTCCCC AAGAGTCATG CTGTAGGCAT

GCTGGCGGAC CAGGATGAAG TCTGGCTTGA AGGATCTGCT GAGAAAAAGC

GCTGGCGGAC CAGGATGAAG TCTGGCTTGA AGGATCTGCT GAGAGAAAGC

GCTGGCGGAC CAGGATGAAG TCTGGCTTGA AGGATCTGCT GAGAGAAAGC

---------- ---------- ---------- ---------- ----------

GCTGGCGGAC CAAGATGAAG TCTGGCTTGA AGGATCTGCT GAGAA-AAGC

GCTGGCGGAC CAGGATGAAG TCTGGTTTGA AGGATCTGCT GAGAGAAGGC

GCTGTCTAAC CAGAATGAAG TCAGGTTTGA AGGACCTGCC GAGAGAAGGC

GCTGTCGGAC CAGGATGAAG TCAGGTTTGA AGGACCTGCC AAGAGAAGGC

GCTGGCGGAC CAGGATGAAG TCTGGTTTGA AGGATCTGCC GAGAGAAGGC

GCTGGCGGAC AAGGATGAAG TCCGGCTTGA AGGATCTGCC AAGAGAAAGT

GCTGACGGAC TAGGATGAAG TCTGGCTTGA AGGATCTGCA AAGAGAAGGC

GCTGACGGAC CAGGATGAAG TCCGGCTTGA AGGATCTGCC GAGAGAAGGC

GCTGGCGGAC CAGGATGAAG TCCGGCTTGA AGGATCTGCC AAGAGA----

GCTGGCGCAC CAGGACGAAG TCTGGCTTGA AGGACCTACC AAGAGAAGGC

GCTGGCGCAC CAGGATGAAG TCTGGCTTGA AGGACCTGCC AAGAGAAGGC

GCTGGCGCAC CAGGATGAAA TCGGGCTTGA ATGACCTGCC AAGAGA----

GCTGCCGAAC CAAGACAAAG TCAGGTTTGA ATGATCTGAG AAGAGAAGGC

GTTGTCTGAC CAGGACAAAG TCGGGCTTGA AGGATCTGAA ATGAGAAGGC

TGGGGCCAGA GGTGTGACTC TTACCATTGG AGAAGTGTTA ATTCTCTCAA

TGGGGCCAGA GGTGGGACTC TTACCATTGG AGAAGTGTTA ATTCTCTCAA

TGGGGCCAGA GGTGTGACTC TTACCATTGG AGAAGTGTTA ATTCCCCCAA

---------- ---------- ---------- ---------- ----------

TGGGGCCAGA GGTGTGACTC TTACCATTGG AGAAGTGTTA ATTCCCTCAA

AGGGGCCAGC AGCTTGACTT TCGCCACTGG AGAAGTGTTA ATTCCCTCAA

AGGGGCCCAA GCCTTCGCTC TGACCACTGG AAAAGTGTTA ATTCCTTTAA

AGGGGCCCAA GACTTCACTC TGACC-CTGG AGAAGTGTTA ATTCCTTCAG

AGGGGCCAAA GGCATGACTT TTACCACCGG AGAAGTGTTA ATTCCCTCAA

GGGGGCCAAA GGCTTCACTT TTATCACTGG AGAAGCTTTA ATTCCCTCAA

AGGCGCCAAA GGCTTGACTT TTACCATCAG AGAAGTATTG TTTCCCTCAA

AGGGGCCAAA GGCTTGACTT TTACCATCAC AGAAGTATTA ATGCCCTCCC

---------- ---------- ---------- ---------- ----------

AGGGGCCAAA AGCTTGACTT TCACCATCTG AGAAGTGTCG ATTCCCCCAA

AGGGGCTGAA GGCTTGATTT TCACCATCTG AGAAGTACTA ATTCCCTCAA

---------- ---------- ---------- ---------- ----------

AGGGGCCAAA AGTTTGACTT CTACCATCTG AGATGTGTTA CGTCCTCCAG

AGAGGCCAAA GGCCTGACTT TTACCACCTG AGAAGTGTTA ATTCCCTCAA

ATATGCAAGG CCTCCTGCCA GCTGCTGCTC GGGGAAGGGG AACATTATTC

ATATGCAAGG CCTCCTGCCA GCTGCTGCTC GGGGAAGGGG AACATTATTC

ATATGCTAGG CCTCCTGCCA GCTGCTGCTT GGGGAAGGGG AACATTATTC

--------GG CCTCCTGCCA GCTGCTGCTT GGGGAAGGGG AACATTATTC

ATATGCAAGG CCTCCTGCCA GCTGCTGCTT GGGGAAGGAG AACATTATTC

ATATGCAAGG CCTCCTGCCA GCTGCTGCTC GGGGAAGGGG AACATTATTC

CTATGGAGGG CCTCCTGCCA ACCACTGCTT AGGGAAGGGG -ACATTATTC

CTATGTAGGG CCTCCTGCCA CCCACTGCTT GGGGAAGGGG -ACATTATTC

CTATGCAAGG CCTCCTGCCA GCTGCCGCTC GGGGAAGGGG TGCATTATTC

-TATGCAAGG CCTCCTGCCA GCTGCCTCTG CGGGAAGGGG AACATTATTC

ATATGCAAGG CCTCCTGCCA GCTGCTGCTC AGGGAAGGGG AACATTATTC

ATAGGCAAGG CCACCTGCCA GCTGCCGCTG CGGGAAGGGG GACATTATTC

--------GG CCTCGTGCCA GCCGCGGC-- GGGGA--GGG AACATCATTC

ATATGCAAGG CCTCCTGCCA GAGGCCGCTG GGGGAAGGGG AACATTATTC

ATATGCAAGG CCTCCTGCCA GCTGACTCTC GGGAAAGGGG AACATTATTC

--------GG CCTCCTGCCC GCTGCCCCTC GGGAA-GGGA AACATTATTC

CTCTGCCAGG CCGCCCGCCA GCTGCTGCAA AGCGAAGGGG GACATTATTC

ATATCCACGG CTGCCTGCCA GCTGCTGCGA GGCGAAGAGG GACATTATTC

ATCGGAGAGA TAAGGCAGCT GATGAGAGAT GGGCCTCAGA GCAGCTGTTC

ATCGGAGAGA TAAGGCAGCT GATGAGAGAT GGGCCTCAGA GCAGCTGTTC

ATCCGAGAGA TAAGGCAGCC GATGAGAGAT GGGCCTCAGA GCAGCTGTTC

ATCCGAGAGA TAAGGCAGCC GATGAGAGAT GGGCCTCAGA GCAGCTGTTC

ATTGGAGAGA TAAGGCAGCT GATGAGAGAT GGGCCTCAGA GCAGCTGTTC

ATCGGGGAGA TAAGGCAGCT GATGAGAGAT GGACCTCCAG GCAGCTGTTC

ATCCAGGAGA TAAGGCAGCT GATGAGAGGT GGGCCTCTGA GCAGCTGTTC

ATCCAGGAGA TAAGGCAGCT GATGAGAGGT GGGCCTCCGA GCAGCTGTTC

ATCGGGGAGA TAAGGCAGCT GATGAGAGAT GGGCC-CGGA GCAGCTGTTC

ATCAGGGAGA TAAGGCAGCT GATGAGAGAC GGGCCTCCAA GCAGCTGTTC

ATCAGGGAGA TAAGGCAGCT GATGAGAGAC GGACCTCAGA GCAGCTGTTC

ATCAGGGAGA TAAGGCAGCT GATGAGAGAT GGGCCTCAGA GCAGCTGTTC

A-CAGGGAGA TAAGGC-GCC GATGAGAGAT GGGC-TCCGA GCAGCTGCTC

ATCCGGGAGA TAAGGCAGCT GATGAGAGAC GGGCCTCTGA GCAGCTGTTC

ATTGGGGAGA TAAGGCAGCC GATGAGAGAT GGGCCTCAGA GCAGCTGTTC

AC-GGGGAGA TAAGGCAGCT GATGAGAGAC GG-CGTCCGC ACAGCTGTTC

ATCTGGGAGA TAAGGCAGCC GATGAGAGAA GCCCCTCTAA GCAGCTGTTC

ATGTGGGAGA TAAGCCGGCC GACGAGAGAG GCGCCTCGAA GCAGCTGTTG

CTCAGCGGCT GGAACGGATG CTAGATGCAA AGCAAAAGAT GCGACAGGCC

CTCAGCGGCT GGAACGGATG CTAGATGCAA AGCAAAAGAT GCGACAGGCC

CTCAGCGGCT GGAACGGATG CTAGATGCAA AGCAAAAGAT GCGACAGGCC

CTCAGCGGCT GGAACGGATG CTAGATGCAA AGCAAAAGAT GCGACAGGCC

CCCAGCGGCT GGAACGGATG CTAGATGCAA AGCAAAAGAT GCGACAAGCC

CTCAGCGGCT GGAACGGATG ---------- ---------- ----------

CTCGGTGGCT GGAACGGATG CCAGATATGA AACAGAAGAT GCGCCACGCC

CT---TGGCT GGAACAGATG CTAGATACGA AGCAGAAGAT GTGCCAAGCC

CTCAGCGGCT GGAGCGGATG CTAGATGCAA AGCAAAAGAT GCGACAGGCC

CTCAG-GGTT GGAGCGGATG CTAGATGCAA AGCGAAATAT GCGACGGGCC

CGC---GGCT GGAACGGATG CTAGATGCAA AGCAAAAGAT GCGGCAGGCC

CTCAGCAGCT GGAACGGATG CTAGATGTAA AGCAAAAGAT GCGACAGGCC

CT-------- ---------- ATAGATGCAA AACAGAAGAT GCGACACGCC

CTCAGCCGCA GGAACGGATG CTAGATGCAA AGCAAAAGAT GCGCTGGGCC

CA-------- --ACCGGATG TTAGATGCAA AGCGAAAGAT TCGCCAAGCC

CACAGCAGCT GGAACGGATG ---------- ---------- ----------

GGCAGCAGCT GGAAAAGATG TCAGCGGCCA AGCATAAGCT GCAGCTGGCC

AACAGCAGCT GGAATGGATG CTAGCGGCAA AACAGAGGAT TCACCGGGCA

TGGAAGTCCT GGTTGATCTA CGTGGTTGTT CGTAGGACCA AACTTCAGAT

TGGAAGTCCT GGTTGATCTA CGTGGTTGTT CGTAGGACCA AACTTCAGAT

TGGAAGTCCT GGTTGATCTA CGTGGTTGTT CGTAGGACCA AACTTCAGAT

TGGAAGTCCT GGTTGATCTA CGTGGTTGTT CGTAGGACCA AACTTCAGAT

TGGAAGTCTT GGTTGATCTA CGTGGTTTTT CGTAGGACCA AACTTCAGAT

---------- ---------- ---------- ---------- ----------

TGGAAGTCCT GGTTAATATA CATGGTTGCT CGTAGGACCA AATTTCGCAT

TGGAAGTCCT GGTTGATATA CATGGTTTCT CGTAGGACCA AACTTCACAT

TGGAAGTCCT GGTTGATCTA TGTGATTTTT CGGCAAACCA AACTTGAGAT

TGGAAGTCCT GGTTGGTCTA CGTGGTTTTC CGTAGGACCA AACTTGAGAT

TGGAAGTCCT GGCTGATCTA CGTGGTTTTT CGTAGAACCA AACTTCATAT

TGGAAGTCCT GGTTGATCTA TGTGGTTTTT CGAAGAACCA AACTTCAGAT

TGGAAATCCT GGTTGATCTA CGTGGTTTTT CGTAGAACCA AACATGAGAT

TGGAAGTCCT GGTTGATCTA CGTGGTTGTT GGCAGGACCA AACTTCAGAT

TGGAAGTCCT GGTTGATCTA CGTGGTGGTT CGTAGGACCA AACTTCAGAT

---------- ---------- ---------- ---------- ----------

TGGAAACACT GGCTCATCTA CGTGGACGTT CGTCGGACCA AACGGGGGAT

TGGCAGCACT GGCTGATCTA TGTGGAAGTT CGTAGGACCA AAGCTCAGAT

GCGCTCATGG ACCATCGGCC TTGTAAAGAA TGTGAGGTGC AATGGGCAGC

GCGCTCATGG ACCATCGGCC TTGTAAAGAA TGTGAGGTGC AATGGGCAGC

GCGCTCATGG ACCGTCGGCC TTGTAAAGAA TGTGAGGTGC AATGGGCAGC

GCGCTCATGG ACCGTCGGCC TTGTAAAGAA TGTGAGGTGC AATGGGCAGC

GCGCTCATAG ACCATCGGCC TTGTAAAGAA TGTGAGGTGC AGTGGGCAGG

--GCTCATGG ACCATCAGCC TTGAAAAGAA TGTGAGGTGC AGTGGGCAAC

GAGCTCCTGG ACCATCAGCC TTGCAAAGAA TGTGCCTTTC AGCGGGCAGC

GAGCTCCTGG ACCATCGGCC TTGCAAAGAA TGTGTCTTCC AGCGGGCAGC

GCGCTCCTGG ACCGTCGGCC TTGCAAAGAA TGTGAGGTGC AGTGGGCAGC

GCGC--ATGG ACCATTGGCC TGGAAAAGAA TGTGAGGTGC AATGGGCAGC

GCGCTCATGG ACCATCGGCC GTGAAAAGAA TGTGAGGTGC CATGGGCAGC

GCGCTCATGG ACCGTCGGCC TTGAAAAGAA TGTGAGGTGT AATGGGCAGC

GCGCTCATGG ACGGCTGACT TTGCAAAGAA TGTGAGGTGC AACGGGCATC

GCGCTCATGG ACCATCGGCC TTGAAAAGAA TGTGAGGTGC AACGGGCAGC

GCGCTCATGG ACCATCGGCC TTGAAAAGAA TGTGAGGTGC AATGGGCAGC

---------- ---------- ---------- ---------- ----------

GCGCTCATGG ATGAAATGCC TTGCCAGTAA TGTGAGGTGC ACCCAGCAGC

GCGCTAATGG ACCAAGGGCC TCAGAGAGAA CTTATGTCAT AAATGGCATT

CTTCCAGGTA CCCAAGAAGG GAGGCTTCAT CCTTTCAGGA TGTCTCAGGC

CTTCCAGGTA CCCAAGAAGG GAGGCTTCAT CCTTTCAGGA TGTCTCAGGC

CTTCCAGGGA CCCAAGAAGG GAGGCTTCAT CCTTTCAGGA TGTCTCAGGC

CTTCCAGGGA CCCAAGAAGG GAGGCTTCAT CCTTTCAGGA TGTCTCAGGC

CTTCCAGGGA CCCAAGAAGG GAGGCTTCAT CCTTTCAGGA TGTCTCAGGC

CTTCCAGAGA CCCAAGAAGG GAGGCCTCAT CCTTTCAGGA TGTCTCAGGC

CTTCCAGCGA CCCGAGAAGG GAG-CCTCAT CCTTTCAGGA CGTCTC-AAC

CTTCCAGCGA CCCGAGAAGG GAG-CCTCAT CCTTTCAGGA CGTCTC-GAC

CATCCAGAGA CCCAGAAGGG AGG-CCTCAT CCTTTCAGGA TGTCTCAGGC

CTTCCAGGGA CCCAAGAAGG GAGGCCTCAT CCTTTCAGGA TGTCCCTGGC

CTTCCAGGGA CCCAAGAAGG GAGGCCTCAT CCTTTCAGGA TGTCTCAGGC

CTTCCAGGGG CCCCCGAAGG GAGGCCTCAT CCTTTCAGGA GGTTTCAGGC

CTTCCAGGGA CCCGAGAAGG GAGGCCTCAT CCTTTCAGGA TGTCTCAGGC

CTTCCAGGGA CCCAAGAAAG GAGGCCCCAT CCTTTCAGGA TGTCTCGGGC

CTTCCAGGGA CCCAAGAAGG GAGGCCTCAT CCTTTCAGGA TGTCTCAGGC

---------- ---------- ---------- ---------- ----------

CTTCCAAGGA ACCAAGAGAA AACTCATCAG CTTTTCAAGA CATCTCCTTT

CTTCCAAAGA ACTAAGAAAA TACGCATAAT ATTTCTGAGA TACCTGGCTT

TTCAGATAAA ACCCTTCCTG TTCCAGCTCA TTAGGTGTTT TATCTTTCTC

TTCAGATAAA ACCCTTCCTG TTCCAGCTCA TTATGTGTTT TATCTTTCTC

TTCAGATAAA ACCCTTCCTG TTCCAGCTCA TTATGTGTTT TATCTTTCTC

TTCAGATAAA ACCCTTCCTG TTCCAGCTCA TTATGTGTTT TATCTTTCTC

TTCAGATAAA ACCCTTCCTG TTCCAGCTCA TTATGTGTTT TATCTTTTGC

TTCAGATAAA ACCCTTCCTG TTTCAGCTCA TTATGTGTTT TATCTTTTGC

TACAGATAAA ATCCTTCCTG TCCCAGCTCA TTCTGCATTT TATCTTTCAT

TACAGATAAA ATCCTTCCTG TCCCAGCTCA TTCTGCATTT TATCTTTCGT

TCCAGATAAA ACCCTTCCTG TTGCGGCTCG CCATGCGCTT TATCTCTCGC

TTCAGATAAA ACCCTTCCTG TTTCAGTTCA TTATGTGTTT TATCTTTTGC

TTCAGATAAA ACCCTTCCTG TTCCAGCTCA TTATGTGTTT TATCTTTTGC

TTCGGATAAA ACCCTTCCTG TTCTAGTTCA TTATGTGTTT TATCTTTTGC

TTCAGATAAA ACCCTTCCTG TTCCCGCTC- ---------- ----------

TTCAGATAAA ACTCTTCCTG TTTCAGCTCA TTATGTGTCT TATCTTTTGC

TTCAGATAAA ACCCTTCCTG TCCCAGCTCA TTATGTGTTT CATCTTTAGC

---------- ---------- ---------A TTATGTGCTT TATCTTCAGC

TGCAGATAAA ACTTTTCCTG TTCTAACTTC CAACGTGTTT TATCTCAGAC

TTACAATAAA ATCCTTGCTG TTCTAACTTG CCGTGCGATT GATCGCTTGC

TCCCAGGGTC TAATAAACAA CGTCCATCCA GTTTTTAAAA GACACTCTGG

TCCCAGGGTC TAATAAACAA TGTCCATCCA GTTTTTAAAA GACACTCTGG

TCCCAGGGTC TAATAAACAA TGTCCATCCA GTTTTTAAAA GACACTCTGG

TCCCAGGGTC TAATAAACAA TGTCCATCCA GTTTTTAAAA GACACTCTGG

TCCCAGGGTC TAATAAACAA TGTCCATCCA GTTTTTAAAA GACACTCTGG

TCCCAGGGTT TAATAAACAA TGTCCATCCA GTTTTTCAAA GACACTCTGG

TCCCAGCGCC TAATAAACAA TGTCCATCCA GTTTTTAAAA GGTGCTCTGG

TCCCAGTACC TAATAAACAA TGTCCATCCA GTTTTTAAAA GACGCTCTGG

TCCCAGGGTC TAATAAACAA TGTCCAGCCC GCTTTTAAA- GCCACTCCGG

TCCCAGGGTC TAATAAACAA TGTCCATCCA GCTTTTAAAA GACACTCTGG

TCCCAGGGTC TAATAAACAA TGTCCATCCA GTTTTTAAAA GACACTCTGG

TCCCAGGGTC TAATAAACAA TGTCCATCCG GTTTTTAAAA GACACTCTGG

---------- ---------- ---------- ---------- ----------

TCCCAGGGTC TAATAAACAA TGTCCATCCA GTTTTTAAAA GAAACTCTGG

TCCCAGGGTC TAATAAACAA TGTCCATCCA GTTTTTAAAA GAAACTCTGG

TCCCCGGGCC TAATAAACAA TGTCCATCCC GTTTTTAAAA GAAACTCTGG

TTCCAGGGTC TAGTCATGAA TGCCCATGGG GGTCTCCAGA GATTCTTTGG

TTTCAGGGTC TAATTAACAA GGTCCGACAG GTCCCCGAAA GAATCTCTGG

CCTGAATTTC CAAACACTGT TGGTTTAGCA TTTCTCAAAT GTATCCCATT

CCTGAATTTC CAAACACTGT TGGTTTAGCA TTTCTCAAAT GTATCCCATT

CCTGAATTTC CAAACACTGT TGGTTTAGTA TTTCTCAAAT GTATCCCATT

CCTGAATTTC CAAACACTGT TGGTTTAGTA TTTCTCAAAT GTATCCCATT

CCTGAATTTC CAAACACTGC TGGTTTAGCA TTTCTCAAAT GTATCCCATT

CCTGAATTTC CAAACACTGT TGGTTTAGCA TTTCCCAAAT GTATCCCATT

CCTGAATCTC CAGA------ ---------- -----CAAAT GGATCCCACA

CCTGAATCTC CAACAC-TGT TGATTTAGCA TTTATCAAAT GAATCCCACG

CTGGAATGTC CAAACCCTGT TGGTTTAGTA TTTCTCAAAT ATATCCCATT

CCTGAATTTC CAAACACTGT TGGTTTAGCA TTTCTCAAAT GTACCCCATT

CCTGAATTTC CAAACACTGT TGGTTTAGCA TTTCTCAAAT GTATCCCATT

CCTGACCTTC CGAAGACTGT TGGTTTAGCA TTTCTCAAAC GTATCCTGTT

---------- ---------- ---------- ---------- ----------

CCTGAATTTC CAAACACTGT TGGTTTAGCA TTTCTTAAAT GTATCCCATT

CCTGAATTTC CAAACACTGT TGGTTTAGCA TTTCTCAAAT GTATCCCATT

CCTGAATTTC CAAACACTGT TGGTTTAGCA TTTCTCAAAT GTACCCCATT

CTCTTCTTGC CAGACTCTAT TTGTTTAACA TCTTTCAAAT GTATTCCATT

CCAGAATTTC TAAATAACAT TTATGAAACA CATCTCGAAT GGATCCCATT

AACACTATGA ACCTGTCAGC CTTACCTCAC CACTTTGGTC CCATTTCTCA

AACACTATGA ACCTGTCAGC CTCACCTCAC CACTTTGGTC CCATTTCTCA

AACACTATGA ACCTGTCAGC CTTACCTCAC CACTTTGGCC CCATTTCTCA

AACACTATGA ACCTGTCAGC CTTACCTCAC CACTTTGGCC CCATTTCTCA

AACACTATGA ACCTGTCAGC CTTACCTCAC CACTTTGGTC CCATTTCTCA

AACACCAGGA ACCC-TCAGC CTTACCTCAC AACCTTGGTC CCGTTCCTCA

AACCCTGGGT ACCTGTCGGC CTTACCGCAC AATTTTGGTG CCATTTCTCA

AATCCTGGGT ACCTGTCAGC CTTACCGCAC AATTTTGGTG CCATTTCTCA

AACACCCTGA ACCTGTCAGC CTTACCTCAC AACCTTGGTC CCGTTTCTGA

AACACTAGGA ACCGGTCAGC CTTACCTCAC AACCTTGGTT CCATTTCTCA

AACACTATGA ACCTGTCAGC CTTACCTCAC AACCTTGGTC CCATTTCTCA

AACACTAGGA ACCTGTTAGC CTTACCTCAC AACCTTGGTC CCATTTCTCA

---------- ---------- CTTACCTTAC CACCTTGGTC CCA-TTCTCA

AACATTATGA ATTTGTCAGC CTTACCTCAC AACCTTGGTT CCATTTCTCA

AACATGATGA ACCTGTCAGT CTTACCTCAC AACCTTGGTT CCATTTCTCA

AACATGATGA ACCTGTCAGC ---------- ---------- ----------

AGCATTATGG ACCTGTCAGC CTCACCTCAC AACTTTTGTC CCATTCCTCA

TGCATTATGA ATCTGTCAGC CTTACTTCAC AACTTTGGTC CCGTTCCTGA

CGACCTGCAT GTCCACCATG CAGCCCCCGG TCACATAGGC AGCTAGGTTC

CGACCTGCAT GTCTACCATG CAGCCCCCGG TCACATAGGC AGCTAGGTTC

CGACCTGCAT GTCCACCATG CAGCCCCCGG TAACATAGGC TGCTAGGTTC

CGACCTGCAT GTCCACCATG CAGCCCCCGG TAACATAGGC TGCTAGGTTC

CAACCTGCAT GTCCACCATG CAGCCCCCGG TCACATAGGC TGCTAGGTTC

CGACCTGCAT GTCCACCATA CAGCCCCCGG TCACATAGGC GGCAAGGTTC

CAACTTGCAT GTCCACCATG CAGCCTCCGG TGACATAAGC AGCCAGGTTC

CAACTTGCAT GTCCACCATG CAGCCTCCGG TAACATAAGC AGCCAGGTTC

TGACCTGCAT GTCCACCACA CAGCCCCCGG TCACATAGGC AGCCAGGTTC

TGACTTGCAT GTCCACCATA CAGCCCCCAG TAACATAGGC GGCTAGATTC

TGACTTGCAT GTCCACCATA CAGCCCCCAG TAACATAGGC AGCTAGGTTC

CGACCTGCAT GTCCACCATG CAGCCCCCAG TAACATAGGC GGCGAGGTTC

CGACTTGCAT GTCCACCATA CAGCCCCCCG TGACATAAGC GGCCAGATTC

TGGCTTGCAT GTCCACCATA CAGCCTCCAG TAACATAGGC AGCTAGGTTC

TGACTTGTAT GTCCACCATA CAGCCCCCAG TAACATAGGC AGCTAGGTTT

---------- ---------- ---------- ---------- ----------

TGACCTGCAT GTCCACCATA CAGCCCCCGG TGACATAGGA GGCCAGGTTC

TGACCTGCAC ATCCACCATA CAGCCCCCCG TGACATAGGC CGCCAGGTTC

AACTCTGAGA ATTCAGCCTG AAGAATAAAG CAAATTCAGT GCCCCCAGAG

AACTCTGAGA ATTCAGCCTG AAGAATAAAG CAAATTCAGT GCCCCCAGAG

AACTCTGAGA ATTCAGCCTG AAGAAGAAAG CAAATTCAGT GCCCCCAGAG

AACTCTGAGA ATTCAGCCTG AAGAAGAAAG CAAATTCAGT GCCCCCAGAG

AACTCTGAGA ATTCAGCCTG AAGGAGAAAG CAAATTCAGT GCCCCCAGAG

AATTCTGAGA ATTCAGCCTG CAGGTAA--- -CAA------ ----------

AGCTCAGAGA ACTCAGCCTG GAGAAGAAAG CAAATGCAGT GTCCTCAGAG

AGCTCAGAGA ACTCAGCCTG CAGAAGAAAG CAAATGCAGT GTCCTCAGAG

AACTCGGAGA ATTCAGCCTG --GGAGAAAG CAAA------ ----------

AACTCTGAGA ATTCGGCCTA GAAGAGAAAG CAAATTCAGT GCCCTCAGAG

AACTCTGAGA ATTCAGCCTG AAGGAGAAAG CAAATTCAGT GCCCTCAGAG

AACTCTGAGA ATTCAGCCTG AAGGAGAGAG CAAA------ ----------

AACTCAGAGA ATTCTGCCTG AAGGAGAAAG CAAATTCAGT GCCCTCAGAG

AACTCTGAGA ATTCAGCCTG CAGGAGAAAG CAAATTCAGT GCCCACAAAG

AACTCTGAGA ATTCAGCCTG AAGGAGAAAG CAAATTCAGT GCCCTCAGAG

---------- ---------- ---------- ---------- ----------

AGCTCTGAGA ATTCAGCCTA GAGAAAAAAC AAAACCCAGT GCCCTCAGAG

AACTCTGAAA ATTCAGCCTG GAAGGAAAAA aaaaTTCAGT GCCCTCAGAG

TGAGCTGGAT GCTGAAACTG TGAAGAGCAT TCTGGCTGAA TACAAGATTC

TGAGCTGGAT GCTGAGACTG TGAAGAGCAT TCTGGCTGAA TACAAGATTC

TGAGCTAGAT GCTGAAACTG TGAAGAGCAT TCTGGCCGAA TACAAGATTC

TGAGCTAGAT GCTGAAACTG TGAAGAGCAT TCTGGCCGAA TACAAGATTC

TGAGCTGGAT GCTGAAACTG TGAAAAGCAT TCTGGCCGAA TACAAGATTC

---------- ---------- ---------- ---------- ----------

TGAACTAGAT GCTGAAACGG TGAAGAGCAT TCTGGCTGAA TATAAAATTC

TGAACTGGAT GCTGAAACAG TGAAGAGCAT TCTGGCTGAA TATAAGATTC

---------- ---------- ---------- ---------- ----------

TGAGCTGGAT GCTGAAACTG TGAAGAGCAT TCTGGCTGAG TACAAAATCC

TGAGCTGGAT GCTGAGACTG TGAAGAGCAT TCTGGCTGAA TACAAAATTC

---------- ---------- ---------- ---------- ----------

TGAGCTGGAT GCTGAAACTG TGAAGAGCAT TCTGGCTGAA TACAAAATTC

TGAGCTGGAT GCTGACACTG TGAAGAGCAT CTTGGCGGAA TACAAAATTC

TGAGCTGGAT GCTGAAACTG TGAAGAGCAT TCTGGCTGAA TACAAAATTC

---------- ---------- ---------- ---------- ----------

TGAGCTCGAT GCTGACACCG TGAAGAGTAT CCTGGCAGAG TACAAAATCC

TGAACTGGAT GCAGAGACAG TGAAGAGCAT TTTAGCAGAA TATAAAATCC

ATAATGCCGA TGTGACTCTA CGTAGTGATG CTACAGCTGA TGACCTCATT

ATAATGCCGA TGTGACTCTA CGTAGTGATG CTACAGCTGA TGACCTCATT

ATAATGCTGA TGTGACTCTA CGTAGTGATG CTACAGCTGA TGACCTCATT

ATAATGCTGA TGTGACTCTA CGTAGTGATG CTACAGCTGA TGACCTCATT

ATAATGCTGA TGTGACTCTA CGTAGTGATG CTACAGCTGA TGACCTCATT

---------- ---------- ---------- ---------- ----------

ATAATGCTGA TGTAACTTTG CGTAGTGATG CCACAGCTGA TGACCTCATC

ATAATGCTGA TGTGACTTTG CGTAGTGATG CCACAGCTGA CGACCTCATT

---------- ---------- ---------- ---------- ----------

ATAATGCTGA TGTGACTCTG CGTAGTGACG CCACAGCAGA TGACCTCATT

ACAATGCTGA TGTGACGCTG CGTAGTGATG CCACAGCAGA CGACCTCATC

---------- ---------- ---------- ---------- ----------

ATAATGCTGA TGTGACTCTG CGTAGCGATG CCACAGCAGA TGACCTCATT

ATAATGCTGA TGTGACTCTG CGTAGTGATG CCACAGCTGA TGACCTCATT

ATAATGCCGA TGTGACTCTG CGAAGCGATG CCACAGCTGA TGACCTCATT

---------- ---------- ---------- ---------- ----------

ACAACGCTGA TGTGACCCTG CGCAGCGACG CCACGGCCGA CGACCTCATT

ACAATGCTGA TGTGACCCTA CGCAGCGATG CCACTGCCGA TGACCTCATT

GATGTGGTGG AAGGAAACAG GTACTGAGAA AGGAGATATT TCCTTCCCCT

GATGTGGTGG AAGGAAACAG GTACTGAGAA AGGAGATATT TCCTTCCCCT

GATGTGGTGG AAGGAAACAG GTACTGAGAA AGGAGATACT TCCTTCCCCT

GATGTGGTGG AAGGAAACAG GTACTGAGAA AGGAGATACT TCCTTCCCCT

GATGTGGTGG AAGGAAACAG GTACTGAGAA AGGAGATATT TCCTTCCCCT

---------- ---------- -------GAA AGGAGATACT TCCTTCCCCC

GACGTGGTTG AAGGAAACAG GTACTTAGAG AGGAGATATT TCCTCTCCTT

GACGTGGTCG AAGGGAACAG GTACTCAGAG AGGAGATATT TCCTTTCCTT

---------- ---------- ---------- ---------- ----------

GATGTGGTGG AAGGAAACAG GTACTGAGAA AGGAGATATT TCCTTCCCCT

GATGTGGTGG AAGGAAACAG GTACTGAGAA AGGAGATACT TCCTTCCCCT

---------- ---------- -------GAA AGGAGATATT TCCTTCCCCT

GATGTAGTGG AAGGAAACAG GTACTGAGAA AGGAGATATT TCCTTCCCTT

GATGTGGTGG AAGGAAACAG GTACCCAGAA AGGAGATATT TCCTCTCCTT

GATGTGGTGG AAGGAAATAG GTACTGAGAA AGGAGGTATT TCCTTCCCCT

---------- ---------- -------GGG AGGAGACAAC CCCTTTCTCA

GATGTGGTGG AAGGCAACAG GTGCTGAGAA AGGAGACATT TCTTTTCCCT

GATGTGGTGG AAGGCAACAG GTACCGAGAA CTGAGACACT ACATGGCCCA

GGCTGACACA CCTGATGCAG GAAGCAGGAT GGCTTAGCAC TTCTCCTGTG

GGCTGACACA CCTGATGCAG GAAGCAGGAT GGCTTAGCAC TTCTCCTGTG

GGCTGACACA CCTGATGCAG GAAGCAGGAT GGCTTAGCAC TTCTCATGTG

GGCTGACACA CCTGATGCAG GAAGCAGGAT GGCTTAGCAC TTCTCATGTG

GGCTGACACA CCTGATGCAG GAAGCAGGAT GGCTTAGCAC TTCTCATGTG

TGCAGACATA CCTGATGCAG GAAGCAGGAT GGCTTAGCAC TTCTCCTGTG

GGCTCACACA CCTGCCACAG GAAGCAGGAT GGCTTAGCGC GTCCCAAGCT

GGCTCACACA CCTGCCACAG GAAGCAGGAT GGCTTAGCAC GTCCCAAGCT

---------- ---------- ---------- ---------- ----------

G-CTGACACA CCTGATGCAG GAAGCAGGAT GGCTTAGCAC TTCCCATGTC

GGCTGACACA CCTGATGCAG GAAACAGGAT GGCTTAGCAC TTCCCATGTT

GGCTGACACA CCTGATGCAG GAAGCAGGAT GGCTTAGCAC TTGCCATGTT

GGCTCACACA CTTGGTGCAG GAAGCAGGAT GGCTTAGCGC ATCCCAGGAT

GGCTGGCACA CCTGATGCAG GAAGCAGGGT GGCTTAGCAC TTGCCATGTT

GGCTGACACA CCTGAAGCAG GAAGCAGGAT GGCTTAGCAC TTCCCATGTT

GGTTGACACA CCTGTGGCTG GAAGCAGG-T GGCTTAGCAA TCCCCGTGTC

AATAGAGCTG CCTGTATCAG GAAGGAAAAT GCCCTACCAA TTTCCGTGTT

ATTGGACACA TCTGAAACTG GAATTAAAAT TCCCCAGCAC TTTCCATGCT

AGTTTCTGAA GCTACCGAAC AGCCTAAGGC CCCATCTGTT TTCAATCTTG

AGTTTCTGAA GCTACTGAAC AGCCTAAGGC CCCATCTGTT TTCAATCTTG

AGTTTCTGGA GCTACTGAAC AGCCTAAGGC CCCATCTGTT TTCAATCTTG

AGTTTCTGGA GCTACTGAAC AGCCTAAGGC CCCATCTGTT TTCAATCTTG

AGTTTCTGGA GCTACTGAAC AGCCTAAGG- CCCATCTGTT TTCAATCTTG

AGTTTCTGGA GCTACTGAAG CGCCTAAGGC CCCATCTGTT TTCAATCTTG

AGTTTTTGGA GCTACTGAAT CGCCTAAGGC CCCATCTGTT TTCAATCTCG

AGTTTTTGGA GCTACTGAAT AGCCTAAGGC CCCATCTGTT TTCAATCTTG

---------- ---------- ---------- ---------- ----------

AGTTTCTGGA GCTACTGAAC AGCCTAAGGC CCCATCTGTT TTCAATCTTG

CATTTCTGGA GCTACTGAAC AGCCTGAGGC CCCATCTGTT TTCAATCTTG

AGTTCCTGGA GCTACTGAAC AGCCGAAGAC CCCATCTGTT TTCAATCTTG

AGTTCCCGGC GCTACTGAAC AGCCTCGGGC TCCATCTGTT TCTCATCTTG

GGTTTCTGGA GCTACTGAAC AGCCTCAGGC CACATCTGTT TTCAATCTTG

CATTTCTGGA GCTACCGAAC AGCCCAAGGC CCCATCTGTT TTCAATCTCG

TGTCTCTGGA GCTGCGGTGG AG-------- CCCATCTGTT TCCAATCTTG

AGTTTTCTGA GCTACTGAAT GGCTTCCATC ACCATCTGCT TTCAATCTTT

AGTTTCCTGA GCTACTGAAT ATCTTAAACA CCCATCTGTT TTCAATCTTG

AAGGGCCTCA TGTTGGCGCC TATTTAAAAT AATGCACTTT TTAAAAGCAG

AAGGGCCTCA TGTTGGCGCC TATTTAAAAT AATGCACTTT TTAAAAGCAG

AAGGGCCTCA TGTTGGCGCC TATTTAAAAT AATGCACTTT TTAAAAGCAG

AAGGGCCTCA TGTTGGCGCC TATTTAAAAT AATGCACTTT TTAAAAGCAG

AAGGGCCTCA TGTTGGCGCC TATTTAAAAT AATGCACTTT TTTAAAGCAG

AAGGGCCTCA TGTTGGCGCC TATTTAAAAT AATGCACTTT CTAAAAGCAG

AAGGGCCTCG TGCTCGTGCC TGTTTAAAAT AACGCACTTT CTAAAAGTAG

AAGGGCCTCA TGCTGGCGCC TATTTAAAAT AACGCACTTT CTAAAAGTAG

---------- ---------- ---------- ---------- ----------

AAGGGCCTCG TGTTGGCGCC TATTTAAAAT AATGCACTTT CTAAAAGCAG

AAGGGCCTCG TGTTGGCGCC TATTTAAAAT AATGCACTTT CTAAAAGCAG

AAGCGCCTCA TGCTGGCGCC TATTTAAAAT AATGCACTTT CTAAAAGCAG

AAGGGCCTCG TGTTGGTGCC TATTTAAAAT AATGCACTTT CTAAGAg---

AAGGGCCTCG TGTTGATGCC TATTTAAAAT AATGCACTTT CTAAAAGCAG

AAGGGCCTCG TGTTGGCGCC TATTTAAAAT AATGCACTTT CTAAAAGTGG

AGGGGCCTCG AGGCGGTGCC TGTTTAAAAC GACGCACTTT GAAAACGTGA

AAGGGCCTCA TGTTAAGGTT TGTTGCAAAT AATGCTCTTT TTAAAAATAG

AAGTGCCTCA AGTTAAGTCC CATTTAAAAT AAGGCATttt ttttttATGG

CTTATGTGTT TATTTTGGAG CAAGCTTTGG AGCAGACACA GCTAATTTCT

CTTATGTGTT TATTTTGGAG CAAGCTTTGG AGCAGACACA GCTAATTTCT

CTTATGTGTT TATTTTGGAG CAAGCTTTGG AGCAGACACA GCTAATTTCT

CTTATGTGTT TATTTTGGAG CAAGCTTTGG AGCAGACACA GCTAATTTCT

CTTATGTGTT TATTTTGGAG CAAGCTCTGG AGCAGACACA GCTAATTTCT

CTTATGTGTT TACTTTGGAG CAAGCTTTGG GACAGACATA GCTAATTTCT

CTTATGTGTT TATTTTGGAG CATGCATTGG GACAGACATA GCTAATTTCT

CTTATGTGTT TATTTTGGAG CAGGCATTGG GACAGACATA GCTAATT--T

---------- ---------- ---------- ---------- ----------

CTTATGTGTT TACTTTGGAG CTGGTTTCGG GACAGACACA GCTAATTTCT

CTTATGTGTT TATTTTGGAG CAGGCTTTGG GACAGACATA GCTAATTTCT

CTTACGTGTT TGTTTTGGAG CAGGCTTTGG GACAGAC--- ---AATTGCT

---------- ---------- ---------- ---------- ----------

CTTATGTGTT TACTTTGGAG CAGGCTTTGG GACACACATA GCTAATTTCT

CTTATATGTT TATTTTGGAG CAGGCTTTGG GACAGACATA GCTAATTTTT

CTGCTCTGTT GACTTTGGAG CCGGCTTTGG GACAGACGCT GCTCA-GTCT

TAGATGTGTT TTCCTTGGAA CTGGTCTTGA AGCGGTCATA GCTAATTTCT

TCTGTGTGTT TATCTTGGAA CAGAACTTGG GACAGACAGA GCTAATTTCT

CTTTCCCCAT AGGCTTTTGC TCCAAGCATT TGTCCTGTTG ATATGTCAGC

CTTTCCCCAT AGGCTTTTGC TGCAAGCATT TGTCCTGTTG ATATGTCAGC

CTTTCCCCAT AGGCTTTTGC TCCAAGCATT TGTCCTGTTG ATATGTCGGC

CTTTCCCCAT AGGCTTTTGC TCCAAGCATT TGTCCTGTTG ATATGTCGGC

CTTTCCCCAT AGGCTTTTGC TCCAAGCATT TGTCCTGTTT ATATGTCAGC

CTTTCCCCAT AGGCTCTCAC TCCAAGCATT TGTCCTGTTT ATGTGTCATC

CTTTCCC--T AGGCTTTTGC TCCGAGCATT TGTCCTGTTT ATGTGTCAGT

CTTTCCC--T AGGCTTTTGC TTCAAGCATT TGTCCTGTTT ATGTGTCGGC

---------- ---------- ---------- -GTCCTGTTT ATGCGCCCGC

CTTTCCCCAC AGGCTTTTGC TCCAAGCATT TGTCCTGTTT ATGTGCCGGC

CTTTCCCCAT AGGTTTCTGC TCCAAGCCTT TGTCCTGTTT ATGTGCGGGC

CTTTCCCTGT AGGTTTCTGC TCCAAGCATT TGTCCTGTTT CTGTGTCGGC

---------- ---------- ---------- -GTTCTGTTT ATGTGTCGGC

CTTTCCCCGT AGGCTTCTGC TCCAAGCATT TGTCCTGTCA TTGTGTTGGC

CTTTCCCCAC AGGGTTTTGC TCCAAGCCTG TGTCCTGTTT ATGTGTCAGC

CTTTCCCCAC AGGGTTTTGC TGCAGGTGTT TGTCCTGTGG ATGTGTCCGC

CCTTCTCCAG TGGCTTTTGC TCCATTAATT TGTTCTGTTT ATGTGTCAGT

GTTCCCCCTT GGGCTTTCAC TCTTTGGATT TGTTCTATTT ATGGGTCAGC

TCACTCCCCT CCTAGGAGAC TCTGCTTAGA GTGGGCTGGG AGATTCCTTA

TCACTCCCCT CCTAGGAGAC TCTGCTTAGA GTGGGCTGGG AGATTCCTTA

CCACTCCCCT CCTAGGAGAC TCTGCTTAGA GTGGGCTAGG AGACTCCTTA

CCACTCCCCT CCTAGGAGAC TCTGCTTAGA GTGGGCTAGG AGACTCCTTA

CCACTCCCTT CCTAGGAGAC TAT--TTCAA GTGGGCTGGG AGATTCCTTA

CCACTCCACT CCTAGGAGAC TCTGCTTAGG GTGGGCTGGG AGATTCCTTA

CCACTCCCTT CCTGGGAGAC -ATGCTCAGG GTGGGCTGGG AGATTCCTTA

CCACTCCCTT CCTAGGAGAC -ATGCACAGG GTGGGCTGGG AGATTCCTTA

CCTCTCGCCT TCTCTGAGAC TCTGCCCAGA GTGGGCTGGG AGACTCCTTA

CAACTCCCCA CCTAGGAGAT TCTGGTCAGG GTGGGCTGGG AGATTCCTTA

CTACTTCCCT CCTGGGAGAT TCTGGTCAGG GTGGGGTGGG AGATTCCTTA

CCACTCCCCT CCTAGGAGAT TCTGGTCAGG GTGGGCTGGG AGATTCCTTA

CCCCCTCTGT CCTGGGGGCT TCTGCTCAGG GTGGGCT-GG AGATTCCTGA

CCACTGTCCT CCTGGGAGAC TCTGGCCAGG GTGGGTTGGG AGATTCCTTA

CCACTCCCCT TCTAAGAGAC TCTGGTCATG GTGGGCTGGG AGAGTCCTTA

CCCCTCCTCT CCTCGGAGGC TCTGGTCCCG GCGGGCTGGG AGATTCCTTA

CCCCACCCCT CCTAAGAGAC TCCAGGCAGG GTGGGTTGAA CCATACCCAA

TCACTCCCCT TCTTGGAGAC CCGAATCAGG GTGGATTCGA CCGTTTCTTA

GACAATCTAA TAGATTAGTT TCCAGTTTAG CATAACAGCC AGACAGATTT

GACAATCTAA TAGATTAGTT TCCAGTTGAG CATAACAGCC AGACAGATTT

GACAATCTAA TAGATTAGTT TCCAGTTTAG CATAACAGCC AGACAGATTT

GACAATCTAA TAGATTAGTT TCCAGTTTAG CATAACAGCC AGACAGATTT

GACAATCTAA TAGATTAGTT TCCAGTTTAG CATAACAGCC AGACAGATTT

GACAATCTAA TAGATTAGTT TCCAGTTTAG CATAACAGCC AGACAGATTT

GACAGTATAA TAGATTCATT TCCAGTTTAG CATAACAGCC AGACACAGTT

GACAGTATAA TAGATTCATT TCCAGTTTAG CATAACAGCC AGACACGGTT

GACAATCTAA TAGATTAGTT TCTAGTTTAG CATAACAGCC AGACAGAGCT

GACAATCTAA TAGATTAGTT TCCAGTTTAG CATAACAGCT AGACAGATTT

GACAATCTAA TAGATTAGTT TCCAGTTTAG CATAACAGCT AGACAGATTT

GACAATCTAA TAGATTAGTT TCCAGTTTAG CATAACAGCC AGACAGATTT

GACCATCGAA TAGATGAGTT TCCAGTTTAG CATAACAGCC AGACAGACTT

GAAAATCTAA TAGATCAGTT TCCAGTTTAG CATAACAGCC AGACAGATTT

AACAATCTAA TAGATTAGTT TCCAGTTTAG CATAACAGCC AGACAGATTT

GACAATCTAA TAGATCAGTT TCCCGTTTAG CATAACAGCC AGACAGATTT

GACAATCTCA TAGATTAGTT TCACGTTTAG CATAGCAGCC AGACAGATTT

GACAATCCAA TAGATTAATT TCAAGTTTAG CATAACAGCC AGACAGATTT

TTGCAGTGAA GTCATCCAAG TCAATTAAAA GGCATTTTTC CCTGCTGTGA

TTGCAGTGAA GTCATCCAAG TGAATTAAAA GGCATTTTTC CCTGCTGTGA

TTGCGGTGAA GTCATCCAAG TCAATTAAAA GGCATTTTTC CCTGCTGTGA

TTGCGGTGAA GTCATCCAAG TCAATTAAAA GGCATTTTTC CCTGCTGTGA

TTGCAGTGAA GTCATCCAAG TCAATTAAAA GGCATTTTTC CCTGCTGTGA

TTGCGGTGAA GTCATCCAAG TCAATTAAAA GGCATTTTTC CCTGCTGGGA

CTGCCGTGAA GTCATCCAAG TCAATTAAAT GGTGTTTTTC CCTGCCGGGC

CTGCCGTGAA GTCATCCAAG TCAATTAAAT GGTGTTTTTC CCTGCTGGGC

CTGTGGTGAA GTCATCCGAG TCAATTACCA GGCGCTTTTC CCCGCTGCGA

CTGCAGTGAA GTCATCCAAG TCAATTAAAA GGCATTTTTC CCTGCTGTGA

TTGTGGTGAA GTCATCCCAG TCAATTAAAA GGCATTTTTC CCTGCTGCGA

TTGCGGTGAA GTCATCCAAG TCAATTAAAA AGCATTTTTC CCTGCTGTGA

TTGTGGTGAA GTCATCCAAG TCAATTAAAA GACATTTTTC CCTGCTGGGA

TTGTGGTGAA GTCATACAAT TCAATTAAAA GGCATTTCTG CCTGCTGTGA

TTGTGGTGAA GTCATCCAAT TCAATTAAAA GGCATTTTTC CCTGCTGTGA

TTGCCGTGAA GTCATCCAAT TCAATTAAAA GGCGgccccc cccGCTGAGC

GTGTAGTGAA GTCATCCAAT GGCCTCCAAG ACTGCATTTC CCTGCTGTGA

TTGCGGTGAA GTCATCTAAT TCAATTAAAA GGCATTTTTC CCTGCTGTGA

GTTCAGAAGT CCCTTTGGTT CATTCCAGTT TTCTGGATGT TTCAATAGGT

GTTCAGAAGT CCCTTTGGTT CATTCCAGTT TTCTGGATGT TTCAATAGGT

GTTCAGAAGT CTCTTTGGTT CATTCCAGTT TTCTGGATGT TTCAATAGGT

GTTCAGAAGT CTCTTTGGTT CATTCCAGTT TTCTGGATGT TTCAATAGGT

GTTCAGAAGT CCCTTTGGTT CATTCCAGTT TTCTGGATGC TTTAATAGGT

GTTCAGAAGT CCCTTTGGTT CGTTCCAGTT TTCTGAATGT TTCAATAAGT

ATTTAGAAGT CCCTTTGGTG CCTTCTGGTT TTCTGGCTGT TTCAATAGGT

ATTTAGACGT CCTTTTGGTT CCTTCTGGTT TTCTGGCTGT TTCAATAGGT

GTTCAGAAGT CCCTCCGGTT CCTTCCAGCT TTGGGGATGT TTCAACAGGT

GTTCAGAAGT CCCTTTGGTT TGTTCCAGCT TTCTGGATGT TTCAATAGGT

GTTCAGAAGT CCCTTTGGTT TGTTCCAGCT TTCTGAATGT TTCAATGGGC

ATTCAGAAGT CCCTTTGGTT TGTTCCAGCT TTCTGGATGT TTCAATAGGT

GTTCCAAAGT CCCTCCGGTT GGTTCCAGCT TTCTGGATGT TTCCCTCGGC

GTTCAGCAGT CCCTTTGGTT TGTTCCAGCT TTCTGGATGT TTCAATAGGC

GTTCAGAAGT CCCTTTGGCT CCTTCCAGCT TTCTTGATGT TTCAATAGGT

GTTCAGAAGG CTCTTTGACT CGTGCCAGC- ---------T TTCAATAGGT

GTTCTGAACC TCTTCTGGTT CGCTCCAACT TTCTGGCTGT TTCCAGAGGT

GTTCAGAAGC TCCTCAGGGG TGTTCCGACT TTCTGGATGT TTCCATGGGT

TCCACCCTAG GTTAATTAAT AACCCTTGAT TCCCACTGAA ATGGCTGGTT

TCCACCCTAG GTTAATTAAT AACCCTTGAT TCCCACTGAA ATGGCTGGCT

TCCACCCTAG GTTAATTAAT AACCCTTGAT TCCCACTGAA ATGGCTGGTT

TCCACCCTAG GTTAATTAAT AACCCTTGAT TCCCACTGAA ATGGCTGGTT

TCCACCATAG GTTAATTAAT AACCCTTGAT TCCCACTGAA ATGGCTGGTT

TCCACCCTGG GTTAATTAAT AACCCTGGAT TCCCACTGAA ATGGCTGGTT

TCCACCCTGA ATTAATTAAT AACCCCGAGG TCCCGCTAGA ATGGCTGGTT

TCCGCCCTGA ATTAATTAAT AACCCTGGGA TCCCACTAGA ATTGCTGCTT

TCCACCCCGG -TTAATGAAT AACCCTGGAA TCCCACTGAA ATGGCTGGTT

TCCACCCTGG GTTAATTAAT AACCCTTGAT TCCTACTGAA ATGGCTGTTT

TCCACCCTGG GTTAATTAAT AACCCTTGAT TCCT------ ---GCTGGTT

TCCACCCCGG GTTAATTAAT AACCCTTGAT TCTTACTGAA ATGGCTGGTT

TCCACCCTGG GTTAATTCCT AACTCTGGAT TCCTCCTGAA AGGGCTGGTT

TCCACCCTGG GTTAATTAAT AACTCTTGAC TCCTACTGAA AGGGCTGGTT

TCCACCCTGG CTTAATTAAT AACCCTTGAT TCCTACTGAA ATGGTTGGTT

TCCGCCCTGG GTTAATTAAT AACCCTTGAT TCCAACGGAA AGG-------

TCCAGCCTGG GCTAATTAAT AACCCTTCAT TTTCACTCCA ATGGTTAGTT

TCCGGGCTGG GTTAATTAAT AACCCTTAAT TCCTACTCAA ATGTTTAGTT

CTTAGATTTG AAAAGAAAAA GGCCTCCATG GAGACCTGCA CGCTTTTAAA

CTTAGATTTG AAAAGAAAAA GGCCTCCATG GAGACCTGCA CGCTTTTAAA

CTTAGATTTG AAAAGAAAAA GGCCTCCATG GAGACCTGCA CGCTTT----

CTTAGATTTG AAAAGAAAAA GGCCTCCATG GAGACCTGCA CGCTTTTAAA

CTTAGATTTG AAGAGAAAAA GGCCTCCATG GAGACCTGCA CGCTTTTAAA

CTTAGATTTG AAAAGCAAAA GGCCTCCATG GAGACCTGCA CGCTTTTAAA

CTTAAATTTG AAGAGAAAAG GGCCTCCATG GAGACCCGCA CACTTTTAAA

CTTAAATTTG AAGAGAAAAG GGCCTCCAAG GAGATCTGCA CACTTTTAAA

CTTCGATTCG AAAAGAAACA GGCCTCCATG GAGACCTGCA CGCTTTTCAA

CTTAAATTTG AAAAGAAAAA AACCTCCATG GAGACCTGCA CGCTTTTAAA

CTTAGATTTG AAAAGAAAAA GGCCTCCATG GAGACCTGCA CGCTTTTAAA

CTTAGATTTG AAAAGAAAAA GGCTTCCATG GAGACCTGCA CGCTTTTAAA

CTTAGATTGG AAGAGAAAAA GGCCTCCATG GAGACCTGCA CGCTTTTAAA

CTTAGATTTG AAAAGAAAAA GGCCTCCATG GAGACCTGCA GGCTTTTAAA

CTTAGATTTG AAAAGAAAAA TGCCTCCATG GAGACCTGCA CGCTTTAAAA

---------- ---------- ---------- ---------- ----------

CTTTGATTTA CAAAGAAAAA AGCTTCGATA GAGACATGCA GGGCTTTAAA

TTTAGATCCA AAATGAAAAA TGCAACCATG GAGACCTGGA TGTCTTTTCA

ATAATACAAA ACAGAACATC TGAGGTCAGG GTAAGCCAAG CCAAACAAAT

ATAATACAAA ACAGAACGTC TGAGGTCAGG GTAAGCCAAG CCAAACAAAT

-TAATACAAA ACAGAACGTC TGAGGTCAGG GTAAGCCAAG CCAAACAAAT

ATAATACAAA ACAGAACGTC TGAGGTCAGG GTAAGCCAAG CCAAACAAAT

ATAATACAAA ACAGAATGTC TGAGGTCAGG GTAAGCCAAG CCAAACAAAT

ATAATACAAA ACAGAACGTC TGAGGTCAGG GTAAGCCAAG CCAAACAAAT

ATAACACAAA ACAGAACATC TGAGGTCAGG GTTAGCCAAG CCAAACAAAT

ATAACACAAA ACAGAACATC TGAGGTCACG GTAATCCAAG CCAAACAAAT

ATAATACAAA ACAGAATGTC CAAGGTCAGG GGAAGCCAAG CCAAACAAAT

ATAATACAAA ACAGAACGTC TGAGGTCAGG GTAAGCCAAG CCAAACAAAT

ATAATACAAA ACAGAATGTC TGAGGTCAGG GTAAGCCAAG CCAAACAAAT

ATAATACAAA ACAGAACGTC TGAGGTCAGG GGAAGCCAAG CCAAACAAAT

ATAATACAAA ACAGAATGTC TGAGGTCAGG GCAAGCCAAG CCAAACAAAT

ATAATACAAA ACAGAATGTC TGAGGTCAGA GTAAGCCAAG CCAAACAAAT

ATAATACAAA ACAGAACGTC TGAGGTCAGG GTAAGCGAAG CCAAACAAAT

---------- ---------- ---------- ---------- ----------

GCAACACAAA ATGAGCCCTC TTGGGTCAGA GGAAGCCAAG GCAAACAAAT

ACAATACAAA ACAGGCCCCT TAAGTCAAAG GGAAGCCAAG CCAAACAAAT

AAATCTTGCA GGATTGTGTT AACAAAACAA GGCTTAGTGG ATCAGAGCAG

AAATCTTGCA GGATTGTGTT AACAAAACAA GGCTTAGTGG ATCAGAGCAG

AAATCTTGCA GGATTGTGTT AACAAAACAA GGCTTAGTGG ATCAGAGCAG

AAATCTTGCA GGATTGTGTT AACAAAACAA GGCTTAGTGG ATCAGAGCAG

AAATCTTGCA GGATTGTGTT AACAAAACAA GGCTTAGTGG ATCAGAGCAG

AAATCTTGCA GGATTGTGTT AACAAAACAG GGCTTAGTGG ATCAGAGCAG

AAATCTTGGA GGGTTGTATT AACAAAACAA GGCTTACTGG ATCAGTGCAG

AAATTTTGCA GGATTGTATT AACAAAACAA GGCTTACTGG ATCAGTGCAG

CAATCTTGCA GGATCGTGTT AACAAAACAA GACTTAGCGG ATCAGAGCAG

AAATCTTGCA GGATTGTGTT AACAAAACAA GGCTTAGTGG ATCAGAGCAG

AAATCTTGCA GGATTGTGTT AACAAAACAA GGCTTAGTGG ATCAGAGCAG

AAATCTTGCA GGATTGTGTT AACAAAACAA GGCTTAGTGG ATCAGAGCAG

GAATCTTGCA GGCCTGTGTT AACAAAACAA GACCGGGAGG ATCAGAGTAG

AAATCTTGCA GGATTGTGTT AACAAAACAA GGCTTAGCGG ATCAGAGCAG

AAATCTTGTA GGACTGTGTT AACAAAGCAA GGCTTAGTGG ATCAGAGCAG

---------- ---------- ---------- ---------- ----------

AAATCTTGCA TGATTGTGTT AACCAAATAG TGCTGTGCGG ATCAGTACAA

ATTTCTTGGG TTGTTACGTT AACAAAATAG AACTCCGTGG ATCAGAACAG

TCCTGCAGAG AACACCAAGC TCTGGTGAAT TTCAAACACA AGGAGGTCAC

TCCTGCAGAG AACACCAAGC TCTGGTGAAT TTCAAACACA AGGAGGTCAC

TCCTGCAGAG AACACCAAGC TCTGGTGAAT TTCAAACACA AGGAGGTCAC

TCCTGCAGAG AACACCAAGC TCTGGTGAAT TTCAAACACA AGGAGGTCAC

TCCTCCAGAG AACACCAAGC TCTGGTGAAT TTCAAACACG AGGAGGTCAC

CCCTGCTGAG AACGCCAAGC TCTGGTGAAT TTCAAACACG AGGAGGTCAC

CTCTGTAGAG AACGCCAAGT TCTGGTGAGT TTCAAACACA GGGAGGTCAC

CTCTGTAGAG AATGTCAAGC TCTGGTGAGT TTCAAACCCA GGGAGGTCAT

CCCTGCCGAG AACACCAAAC TCCAGTGAAT TCCAAACACA GGCCAGTCAC

CCCTGCAGAG AATACCAAGC TCTGGTGAAT TTCAAACACA AGGAGGTCAC

CCCTGCAGAG AACGCCAAGC TCTGGTGAAT TTCAAACACA AGAAGGTCAT

CCCTGCAGAG AACGCCAAGC TCTGGTGAAT GTCAAACACA AGGAGGTCAC

CCCCGCCGAA AACGCCAGG- ---------- ---------- ----------

CCCTGCAGAG AACGCCAAGC TCTGGTGAAT TTCAAACACA AGGAGGTCAC

CTCTGCAGAG ACCGCCAAGC TCTGGTGACT TTCAAACACA AGGAGGTCAC

---------- ---------- ---------- ---------- --------AC

CCCTGCACAG AATGCCAAGC TCTGGCTAAT GTCAAATGCT AGGAGGTCAC

CCCTCTGCAG AATGCCAAGC TCTGGATAAC ATCAGATACT CGGAGATCAC

AGAGGGGTGA CTTGTGCCTG TGGAGAAAAG TGCAATTATC TGGTTAATTT

AGAGGGGTAA CCTGTGCCTG TGGAGAAAAG TGCAATTATC TGGTTAATTT

AGAGGGGTGA CTTGTGCCTG TGGAGAAAAG TGCAATTATC TGGTTAATTT

AGAGGGGTGA CTTGTGCCTG TGGAGAAAAG TGCAATTATC TGGTTAATTT

AGAGGGGTGA CTTGTGCCTG TGGAGAAAAG TGCAATTATC TGGTTAATTT

AGA-GGGTGA CTTGTGCCTG TGGAGAAGAG TGCAATTATC TGGTTAATTT

CGAGGAGTGA CTTGTGTCTG TGGAAAAGTG TACAATTATC TGACTAATCT

AGAGGAGTGA CTTGTGTCTG TGGAAAAGTG CACAATTATC TGGCTAATCT

AGAG-CGTGA CTTGTGCCTG TGGAGAAGAG TGCAATTATC TGGTTAATTT

AGAGGGGTGA CTTGTACCTG TGGAGAAAGG TGCAATTATC TGGTTAATTT

TGAGGGGTGA CTTGTACCTA TGGAGAAAAG TGCAATTATC TGGTTAATTT

AGAGGGGTGA CTTGTACCTG TGGAGAAAAG TGCAATTAGC TGGTTAATTT

---------- ---------- ---------- ---------- ----------

AGTGGAGTGA CTTGTGCCTG GGGAGAAAAG TGCAATTTTC TAGTTAATTT

AGAGGGGTGA GTTGTGCCTG TGGAGAAAAG TGCAATTATC TGGTTAAGTT

CGAGGGGTGA TGTGTGCCTG TGGAGAAAAG GGCAATGATC TGGGTAAATT

AGGGGGGGGG CATGGGCTTG TGGAGGAGAG AGCAATCCTT TAGCAAATCC

AGAGGAGTGG CTCGAAGAAG GGGAGAAGAG TGCGATTATC TGGTTAATTT

GGCCAGCTCA GCTAAACAGT GGCTTCCACC TACCTGCTCC ACTCGGATCT

GGCCAGCTCA GCTAAACAGT GGCTTCCACC TACCTGCTCC ACTCGGATCT

GGCCAGCTCA GCTAAACAGT GGCTTCCACC TACCTGCTCC ACTCGGATCT

GGCCAGCTCA GCTAAACAGT GGCTT----- ---------- ----------

GGCCAGCTCA GCTAAACAGT GGCTTCCACC TACCTGCTCT ACTCGGATCT

GGCCAGCTCA GCTAAACAGT GGCTTCCACC TACCTGCTCC ACTCGAATCT

GGACAGTTTA GCTAAACAGT GGCATCCACG TACCTGTTCC ACCCGGATCT

GGCCAGCTTA GCTAAACAGT GGCTTCCACG TACCTGTTCC ACTCGGATCT

GGCCAGCTCA GCTAAACAGT -----CCACC TACCTGTTCC ACTCGGATCT

GGCCAGCTCA GCTAAACAGT GGCTTCAACC TACCTGTTCC ACTCGGATCT

GGCCAGCTCA GCTAAACAGT GGCTTCAACC TACCTGCTCC ACTCGGATCT

GGCCAGCTCA GCTAAACAGT GGCTTCAACC TACCTGCTCC ACTCGGATCT

---------- ---------- ---------- ---------- ----------

GTCCAGGTCA GCTAAACAGT GGTTTCCACC TACCTGTTCC ACTCGGATCT

GGCTAGTGCA GATAAATGGT GGCTTCCACC TACCTGTTCC ACTCGAATCT

GGATGGCTCG GCCAAACAGT GGCCTCCACC TACCTGTTCC ACTCGGATCT

AGCTGGTTCA GTTCAACAGA GGCTTTTACT AACCTGTTCC ACTCGAATCT

GGCCGGTTCA GCTAAAGAGC AGCTTTCACT GACCTGTTCC ACTCGGATCT

CAATCTCTCC ATTCACCTTC TTCCCATGGA AATACTTCGA CCTGTAAAGG

CAATCTCTCC ATTCACCTTC TTCCCATGGA AATACTTTGA CCTGTAAAGG

CAATCTCTCC ATTCACCTTC TTCCCATGGA AATACTTTGA CCTGTAAAGG

---------- ---------- ---------- ---------- ----------

CAATCTCTCC ATTCACCTTC TTCCCATGGA AATACTTGGA CCTGTAAAGG

CGATCTCCCC GTTCACCTTC TTTCCCTGGA AATACTTTGA CCTGTAAAGG

CAATGTCGCC ATTCACTTTC TTCCCATGAA AATATTTCGA CCTG-AAAGG

CAATGTCGCC ATTCACTTTC TTCCCATGAA AATACTTTGA CCTG-AAAGG

CAATCTCTCC ATTCAGCTTC TTGCCCTGGA AATACTTAGA CCTGCAAAGG

CAATCTCTCC ATTCACCTTC TTTCCATGGA AATACTTGGC CCTGTGAAGG

CAATCTCTCC ATTCACCTTC TTTCCATGAA AATACTTGGT CCTGTGAAAG

CAATCTCTCC ATTCACCTTC TTTCCATGGA AATACTTGGC CCTGTGAAGT

---------- ---------- ---------- ---------- ----------

CAATGTCTCC ATTAACCTTC TTGCCATGGA AGTACTTGGT CCTGTGAAGG

CAATCTCTCC ATTCACTTTC TTTCCATGGA AATATTTGGC CCTGTGAAGT

CAACCTCTCC ATTCACCTTC TTCCCATGAA AATACTTGGC CCTGTGAAGG

CAATCTCTCC GTTCACCTTC TTTCCATAGA AATATTTGGC CCTTTGAGAA

CCAGCTCTCC ATTCGTCCTC TTTGCGTGGA AATACTTGGC CCTGTGAGAG

GGAAGAACAT CTGAGGCCCC AGGAAGAAGA ATATTATAAA ACCTTACCAG

GGAAGAACAT CTGA------ -------AGA ATATTATAAA ACCTTACCAG

GGAAGAACAT CTGAGGCCCC AGGAAGAAGA ATATTATAAA ACCTTACCAG

------ACAT CTGAGGCCCC AGGAAGAAGA ATATTATAAA AC--------

AGAAGAACAT CTGAGGCCCT AGGAAGAAGA ATATCATAAA ACCTTACCAG

AGAGGA---- ---------- ---------- ---------- --CTTACCAG

AGAAGAACAT TGAAGGCAGC AGGAAGGAGA ATATTGCAAA CTCTTACCAG

AGAAGAACAT TAAAGGCAGC AGGAAGGAGA ATATTACAAG CTCTTACCAG

CGAAGAACAT CAGAGGCACC AGGAAGGGGG ATATTAGAAA CCCTTACCAG

AGAAGAACAT CAGAGGCACC AGGAAGGAGA ATATTATAAA ACCTTACCAG

AGAAGAACAT CAGAGGCACC AGGAAGGAGA ATATTATAAA ACCTTACCAA

AGAAGAACAT CAGAGGCACC AGGAAGGAGA ATATTATAAA ACCTTACCAG

---------- ---------- ---------- ---------- ----------

AAAAGAACAT CAGAGGCACC AGGAAGGAGA ATATTATAAA ACCTTACCAG

AGAAGAACAT CAGAGATACC AGGAAGGAGA ATATTATAAA ACCTTACCAG

AGAAAGACAT CAGA------ ------GAGA ATCTTACACA GCCTTACCAG

AAAAGAATAT TATAGAAGGT CGGAAGGTGA GAATTAGCCC ATCTTACCAG

AAGAGGACAT CAGAGAGATC ATCATAGAGG GATTTGTAAA ACCTTACCAG

TCTGTATGGG CATCATCGAT CACCAACAGG ATCCTGGGTC TTTGAAGCCT

TCTGTATGGG CATCATCGAT CACCAACAGG ATCCTGGGTC TTTGAAGCCT

TCTGTATGGG CATCATCGAT CACCAACAGG ATCCTGGGTC TTTGAAGCCT

---------- ---------- ---------- ---------- ----------

TCTGTATGGG CATCATCAAT TACCAACAGG ATCCTGGGTC TTTGAAGCCT

TCTGTGTGTG CGTCATCAAT CACCAACAGG ATCCTGGGTT TTTGAAGTCT

TCTGTGTGGG CATCATCAAT GACCAACAAG ATCCTGGGTC TTTGAAGCCT

TCTGTATGGG CGTCATCGAT AACCAACAAG ATCCTGGGTC TCTGAAGCCT

TCTGTGTGGG TGTCGTCAAT CACCAGCAAG ATCCTGGGTC TTTGAAGTCT

TCTGTGTGGG CATCGTCGAT CACCAGCAGG ATCCTGGGCC TCTGAAGCCT

TCTGTGTGGG CATCATCGAT CACCAACAAG ATCCTGGGCC TTTGAAGCTT

TCTGTGTGGG CATCATCGAT CACCAACAGG ATCCTGGGCT TTTGAAGCCT

---------- ---------- ---------- ---------- ----------

TCTGTATGGG CATCGTCGAT CACCAGCAGG ATCCTGGGCC TTTGAAACTT

TCCGTATGGG CATCATCAAT CACCAGTAGG ATCCTAGGCC TTTGAAGCCT

TCGGTGTGGG CATCATCAAT CACCAGGAG- ---------- ----------

TCTGTATGCG CATCATCCAC CACCAACAGG ATCTTGGGTT TCTGGAGCCT

TCTGTGTGGG CATCATCCAC TACCAACAGG ATCTTAGGTT TCTGAGGCTT

GAGGGGCCTG CTTCATGGCA CTGGAGAGGG AGCTAAAAAG GCTGGATCCT

GAGGGGCCTG CTTCATGGCA CTGGAGAGGG AGCTAAAAAG GCTGGATCCT

GAGGGGCCTG CTTCATGGCA CTGGAGAGGG AGCTAAAAAG GCTGGATCCT

---------- ---------- ---------- ---------- ----------

GAGGGGCCTG CTTCATGGCA CTGGAGAGGG AGCTAAAAAT GCTGGATCCT

GTGGGGTCTG CTTCATGGCA CTGGAGAGGG AGCTAAACAG GCTGGATCCC

GTGGGGTCTG TTTCATGGCA CTGGAGAAGG AGCTGAACAG GCTGGATCCT

GTGGGGTCTG TTTCATGGCA CCGGAGAAGG AGCTGAACAG GCTGGATCCT

GAGGGGTCTG CTTCACAGCA CTGGAGAAGG AGCTAAAAAG GCTGGATCCT

GTGAGGTCTG CTTCATGGCA CTGGAGAAGG AGCTAAAAAG GCTGGATCCT

GTGAGGTCTG CTTCATGGCA CTGGAGAAGG AGCTAAAGAG ACTGGATCCT

GCGAGGTCTG CTTCATGGCA CTGGAGAAGG AGCTAAAAAG GCTGGATCCT

---------- ---------- ---------- ---------- ----------

GTGAGGTTGG CTTCATGGCA CTGGAGAAGG AGCTAAAAAG GCTGGATCCT

GTGAGGTCGG CTTCATAGCA CTGGAGAAGG AGCTAAAAAG GCTGGATCCT

---------- ---------- ---------- ---------- ----------

GTGTGGTCTG CTTCATGGCA CTAGAGAGGG AACTGAAGAG GCTGGAGCCA

GTGCGGTCTG TTTCATGGCG CTCGAGAATG AGCTGAAGAG ACTGGGCCCC

GGAGAGGAGA AGGAGGCAGC CAGGGGCTGG GGCCTCCTCT CCATGGCGGG

GGAGAGGAGA AGGAGGCAGC CAGGGGCTGG GGCCTCCTCT CCATGGCCGG

GGAGAGGAGA AGGAGGCAGC CAGGGGCTGG GGCCTCCTCT CCATGGCCGG

---------- ---------- ---------- ---------- ----------

GGAGAGGAGA AGGAGGCAAC CAGGGGCTGG GGCCTCCTCT CCATGGCCGG

GGAGAGGAGA AGGAGGCGGC CAGGGGCTGC GGCCTCCTCT CCATGGCCGG

GGAGAAGAGA AGGAGGCAGC CAGGGGCTGG GGCCTTCTCT CTGTGGCTGG

GGAGATGAGA AGGAGGCAGC CAGGGGCTGG GGCCTCCTCT CTGTGGCCGG

GGTGAGGAGA AGGAGGCTGC CAGGGGCTGG GGCCTCCTCT CCATGGCTGG

GGAGAGGAGA AGGAGGCTGC CAGGGGCTGA GGCCTCCTCT CCATGGCGGG

GGAGAGGAGA AGGAGGCTGC CAGGGGCTGG GGCCTCCTTT CCATGGCTGG

GGAGAGGAGA AGGAGGCTGC CAGGGGCTGG GGCCTCCGCT CCATGGCCGG

---------- ---------- ---------- ---------- ----------

GGAGAAGAGA AGGAGGCTGC CAGGGGCTGG GGCCTCCTCT CCATGGCTGG

GGGGAAGAGA AAGAGGCGGC TAGGGGCTGG GCTCTTCTCT CCATGGCCGG

---------- ---------- ---------- ---------- ----------

GAGGAGGAGG AGGAGGCAGA CAAAGGCTGT GGCCTTCTCT CCATCGCTGG

GAGGAGGATG AAGACGGTGT GGTAGGCTGA AGCCTCCTCT CAGTTGCCGG

GGAAGCAGGT GAGCTGGTGG AGCTATCTGG GCGTTGCAGG TCCGTCATAT

GGAAGCAGGT GAGCTGGTGG AGCTATCTGG GCGTTGCAGG TCCGTCATAT

GGAAGCAGGT GAGCTGGTGG AGCTATCTGG GCGCTGCAGG TCCGTCATAT

---------- ---------- ---------- ---------- ----------

GGAAGCAGGT GAGCTGGACG AGCTATCTGG GCGTTGCAGG TCCGTCATGT

GGAGGCAGGG GAGCTGGTGG AGCTGTCTGG GCGCTGCAAG TCCGGCATGT

GGATGCTGGG GAGCTACTGG AGCTCTCTGG GCGCTGCAGG TCCGGCATGT

GGATGCTGGA GAGCTACTGG AGCTCTCTGG GCGCTGCAGG TCCGGCATGT

GGAGGCCGGG GAGCTGGTGG AGCTATCTGG GCGTTGAAGG TCCGCCATGT

GGATGCAGGT GAGCTGGTGG AGCTGTCTGG GCGCTGCAGG TCCGTCATGT

GGAGGCAGGG GAGCTGGTGG AGCTGTCTGG GCGCTGCAGG TCTGTCATGT

GGAAGCAGGG GAGCTGGTGG AGCTATCTGG GCGCTGCAGG TCCGTCATGT

---------- ---------- ---------- ---------- ----------

GGAGGCGGGG GAGCTGGTGG AGCTTTCTGG GCGCTGCAGG TCTGTCATGT

GGAGGCAGGG GAGCTGGTGG AGCTATCTGG GCGCTGCAGG TCTGTCATGT

---------- ---------- ---------- ---------- ----------

GGAAACGGGG GAGCTGGTGC AACTGCTGGG ACGTTGCAGG TCCATCATGT

GGAAACCGGA GAGCTGGTGG AGCTGTCGGG ACGTTGCAGG TCCATCATGT

AGCCATTAGG CAGGTTGGCC ATGAAGCTGC TGTCAGAGAG ACGTCGCCGG

AGCCATTAGG CAGGTTGGCC ACGAAGCTGC TGTCAGAGAG ACGTCGCCGG

AGCCATTAGG CAGGTTGGCC ACGAAGCTGC TGTCAGAGAG ACGTCGCCGG

---------- ---------- ---------- ---------- ----------

AGCCATTAGG CAGGTTGGCC ACGAAGCTGC TGTCAGAGAG ACGTCGACGG

AGCCATTGGG CAGGTTGGTC ACGAAGCTGC TGTCGGAGAG ACGCCGCCGG

AGCCATTGGG CAGGTTGGCC ACAAAGCTGC TGTCTGACAG CCGCCTCCGG

AGCCATTGGG CAGGTTGGCC ACAAAGCTGC TGTCAGACAG CCGCCTCCGG

AGCCATTGGG CAGGTTGGCC ACAAAGCTGC TGTCTGAGAG ACGCCGCCGG

AGCCGTTAGG CAGGTTGGCC ACAAAGCTGC TGTCAGAGAG GCGCCGCCGG

AGCCATTAGG CAGGTTGGCC ACAAAACTGC TGTCAGAGAG GCGCCGCCGG

AGCCATTAGG CAGGTTGGCC ACGAAGCTGC TGTCAGAGAG CCGCCGCCGG

---------- ---------- ---------- ---------- ----------

AGCCATTAGG CAGGTTGGCC ACGAAGCTGC TGTCGGAGAG GCGCCGCCGG

AGCCATTAGG CAGGTTGGCC ACGAAGCTGC TGTCAGACAG GCGCCGCCGG

---------- ---------- ---------- ---------- ----------

AGCCGTTGGG CAAGTTGGCC ACGAAGCTGC TGTCAGAGAG GCGGCGCCGG

AGCCATTGGG CAAGTTGGCC ACGAAGCTGC TGTCCGACAG ACGTCGTCTG

AGGAAATTCA TGGCTGTTAG CTGCTGCACA GGTTCTGGAA CCTCTGGCGG

AGGAAATTCA TGGCTGTTAG CTGCTGCACA GGTTCTGGAA CCTCTGGCGG

AGGAAATTCA TGGCTGGCAG CTGCTGCACA GGTTCTGGAA CCTCTGGCAG

---------- ---------- ---------- ---------- ----------

AGGAAATTCA TGGCTGTTAG CTGCTACACA GGTTCTGGAA CCTCTGGTGG

AGGAAATTCA TGGCTG---- ---------- ---------- ----------

AGGAAGTTCA TGGCTGGAAG CTGCTCCACA GGTTCTGGAA CCTCTGGCAG

AGGAAGTTCA TGGCTG--AG TTGCTTCACA GGTTCTGGAA CCTCTGGCAG

AGGAAGTTCA TGGCTGGCAG CTGATGCGCA GGTTCTGGGA CCTATGGCAG

AGGAAATTCA TGGCTGTCAG CTGCTTCACA GGGTCTGGAA CCTCTGGCAG

AGGAAATTCA TGGCTATCAG CTACTACACA GGTTCTGGAA CCTCTGGCAG

AGGAAATTCA TGGCTGTCAG CTGCTGCACA GGTTCTGGAA CCTCTGGCAG

---------- ------TCAG CTGCTGCGTA GTTTCTGGAA CCTCTGGCAA

AGGAAATTCA TGGCTGTCAG GTGCAGCGCA GGTTCTGGAA CCTCTGGCAG

AGGAAATTCA TGGCTGGCAG CTGCTGCGCC GGTTCTGGGG CTCCTGGCAG

---------- ---------- ---------- ---------- ----------

AGGAAGTTCA TGGCTGCCAG CTTTTGCAGA GGTTCTGGCA CCGCTGGTGT

AGGAAATTCA TGATGGTCAG TTGTTGCAGA GGTTCTGGAA TTGTTGGCAA

TCTCAGATTG AGCAGAAAAA GGATAGAGTT TATATCCCCT GTATCTATGT

TCTCAGATTG AGCAGAAAAA GGATAGAGTT TATATCCCCT GTATCTATGT

TCTCAGATTG AGGAGAAAAA GGATAGAGTT TATATCCCCT GTATCTATGT

---------- ---------- ---TAGAGTT TATATCCCCT GTATCTATGT

TCTCAGATTG AGAAGAAAAA GGATAGAGTT TATATCCCCT GTATCTATGT

---------- ---------- ---------- ---------- ----------

TCTCGAGTTG AACAGAGGGA GGATAGAGTT TATATTCCCT GTATCTATGT

TCTCGAATTG AGCAGAGGGA GGATAGAGTT TATATTCCCT GTATCTATGT

TGTCGGATTG AGCAGAAGGA GGA------- ---------- ----------

TCGCGGCTGG AGCAGAGGGA GGATAGAGTC TATATCCCCT GTATCTATGT

TCTCGGATTG AGCAGAGGGA AGATAGAGTT TATATCCCTT GTATCTATGT

TCTCGGATGG AGCAGAGGGA GGA------- ---------- ----------

TCTCGAATTG AGCAGAAGGA GGA------- ---------- ----------

TCTCGGATTG AGCAGAGTGA GGATAGAGTT TATATCCCCT GTATCTATGT

TCTCTGCTTG AGCAGAGGGA GGATAGAGTT TATATCCCCT GTATCTATGT

---------- ---------- ---------- ---------- ----------

GCTCGGGCTG ACCAGAGAGA GGACAGAGTT TACATCCCTT GTATCTACGT

TATCGGGTAG AACAGAAGGA AGACAGAGTT TACATCCCTT GCATTTATGT

GTTAAATAAG ATTGACCAAA TCTCCATTGA GGAATTGGAT ATCATCTATA

GTTAAATAAG ATTGACCAAA TCTCCATTGA GGAATTGGAT ATCATCTATA

GTTAAATAAG ATTGACCAAA TCTCCATTGA GGAATTGGAT ATCATCTATA

GTTAAATAAG ATTGACCAAA TCTCCATTGA GGAATTGGAT ATCATCTATA

GTTAAATAAG ATTGACCAAA TCTCCATTGA GGAATTGGAT ATCATCTATA

---------- ---------- ---------- ---------- ----------

ATTAAATAAG ATTGATCAGA TCTCCATTGA GGAGTTGGAT ATTATATATA

ATTAAATAAG ATTGATCAGA TCTCCATTGA GGAGTTGGAT ATTATCTATA

---------- ---------- ---------- ---------- ----------

GTTAAATAAG ATTGATCAGA TTTCCATTGA GGAGTTGGAT ATTATCTATA

GTTGAATAAG ATTGATCAGA TCTCCATTGA GGAATTGGAT ATCATCTATA

---------- ---------- ---------- ---------- ----------

---------- ---------- ---------- ---------- ----------

GTTAAATAAG ATTGACCAGA TCTCCATTGA GGAATTGGAT ATCATCTATA

GTTAAATAAG ATTGATCAGA TCTCCATTGA GGAATTGGAT ATCATCTATA

---------- ---------- ---------- ---------- ----------

GCTGAACAAG ATCGACCAGA TCTCCATCGA GGAGCTGGAC ATCATCTATA

GCTGAATAAG ATTGATCAGA TCTCCATCGA GGAGCTCGAC ATTATCTACA

AGGTGCCTCA CTGTGTACCC ATCTCTGCCC ATCACCGCTG GAATTTTGAT

AGGTGCCTCA CTGTGTACCC ATCTCTGCCC ATCACCGCTG GAATTTTGAT

AGGTGCCTCA CTGTGTACCC ATCTCTGCCC ATCACCGCTG GAATTTTGAT

AGGTGCCTCA CTGTGTACCC ATCTCTGCCC ATCACCGCTG GAATTTTGAT

AGGTGCCTCA CTGTGTACCC ATCTCTGCCC ATCACCGTTG GAATTTTGAT

---------- ---------- ---------- ---------- ----------

AGGTGCCTCA CTGTGTACCC ATCTCTGCTC ATCACCGCTG GAATTTTGAT

AGGTGCCTCA CTGTGTACCC ATCTCTGCTC ATCACCGCTG GAATTTTGAT

---------- ---------- ---------- ---------- ----------

AGGTGCCACA CTGTGTACCC ATCTCTGCCC ATCACCGCTG GAATTTTGAT

AGGTGCCTCA CTGTGTACCC ATTTCTGCCC ATCACCGCTG GAATTTTGAT

---------- ---------- ---------- ---------- ----------

---------- ---------- ---------- ---------- ----------

AGGTACCTCA CTGCGTACCC ATCTCTGCCC ACCACCGTTG GAATTTTGAT

AGGTACCTCA CTGTGTACCC ATCTCTGCCC ACCACCGCTG GAATTTTGAT

---------- ---------- ---------- ---------- ----------

AGGTGCCGCA CTGTGTGCCC ATCTCTGCTC ACCACCGCTG GAATTTTGAT

AGGTGCCTCA TTGCGTGCCG ATCTCCGCCC ATCACCGTTG GAACTTTGAT

GACCTATTGG AAAAGATCTG GGACTATCTG AAACTAGTGA GAATGTAAGT

GACCTATTGG AAAAGATCTG GGACTATCTG AAACTAGTGA GAATGTAAGT

GACCTATTGG AAAAGATCTG GGACTATCTG AAACTAGTGA GAATGTAAGT

GACCTATTGG AAAAGATCTG GGACTATCTG AAACTAGTGA GAATGTAAGT

GACCTATTGG AAAAGATCTG GGACTATCTG AAACTAGTGA GAATGTAAGT

---------- ---------- ---------- ---------- ----------

GACCTTTTGG AGAAGATCTG GGACTATCTG AAACTAGTGA GAATGTGAGT

GACCTTTTGG AGAAAATCTG GGACTATCTG AAACTAGTGA GAATGTGAGT

---------- ---------- ---------- ---------- ----------

GACCTGTTGG AAAAGATCTG GGACTATCTG AAACTAGTGA GGATGTAAGT

GACCTATTGG AAAAGATCTG GGACTATCTG AAACTAGTGA GGATGTAAGT

---------- ---------- ---------- ---------- ----------

---------- ---------- ---------- ---------- ----------

GACCTGTTGG AAAAGATCTG GGACTATCTG AAACTAGTAA GGATGTAAGT

GACCTGTTGG AAAAGATCTG GGACTATCTG AAACTAGTGA GGATGTAAGT

---------- ---------- ---------- ---------- ----------

GACCTGCTCG AGAGAATCTG GGACTACCTG CGCCTAGTGA GGATGTAAGT

GACCTGCTGG AGAGGATTTG GGATTACTTG AGACTAGTGC GAATGTAAGT

CTTTCTAGTT ACACCAAACC CAAAGGCCAG TTACCAGATT ACACATCCCC

CTTTCTAGTT ACACCAAACC CAAAGGCCAG TTACCAGATT ACACATCCCC

CTTTCTAGTT ACACCAAACC CAAAGGCCAA TTACCAGATT ACACATCCCC

CTTTCTAGTT ACACCAAACC CAAAGGCCAA TTACCAGATT ACACATCCCC

CTTTCTAGTT ACACCAAACC CAAAGGCCAG TTACCAGATT ACACATCCCC

---------- ---------- ---------- ---------- ----------

CTTTCTAGTT ACACTAAACC CAAAGGCCAG TTACCAGATT ACACGTCTCC

CTTTCTAGTT ACACCAAACC GAAAGGCCAG TTACCAGATT ACACATCTCC

---------- ---------- ---------- ---------- ----------

CTTTCTAGTT ACACCAAACC CAAGGGCCAG TTGCCCGATT ACACGTCTCC

CTTTCTAGTT ACACCAAACC CAAAGGCCAG TTGCCTGATT ATACATCTCC

---------- ---------- ---------- ---------- ----------

--TTCTAGTT ATACAAAACC CAAAGGGCAG TTGCCAGATT ACACATCTCC

CTTTCTAGTT ACACCAAGCC CAAAGGCCAG TTACCAGACT ACACATCCCC

CTTTCTAGTT ACACAAAACC CAAAGGCCAG TTACCAGACT ACACATCCCC

---------- ---------- ---------- ---------- ----------

CTTCCTAGCT ACACCAAGCC CAAGGGGCAG CTGCCAGACT ACACCTCCCC

CTTTACAGCT ACACCAAACC CAAAGGCCAG TTACCAGACT ATACATCCCC

AGTGGTGCTT CCTTACTCCA GGACCACAGT GGAGGATTTC TGCATGAAGA

AGTGGTGCTT CCTTACTCCA GGACCACAGT GGAGGATTTC TGCATGAAGA

AGTGGTACTT CCTTACTCCA GGACCACAGT GGAGGATTTC TGCATGAAGA

AGTGGTACTT CCTTACTCCA GGACCACAGT GGAGGATTTC TGCATGAAGA

AGTGGTGCTT CCTTACTCCA GGACCACAGT GGAGGATTTC TGCATGAAGA

---------- ---------- ---------- ---------- ----------

AGTAGTCCTG CCTTATTCTA GGACCACGGT GGAAGATTTC TGCATGAAGA

AGTAGTGCTG CCTTATTCTA GGACCACTGT GGAAGATTTC TGCATGAAGA

---------- ---------- ---------- ---------- ----------

AGTGGTGCTG CCTTACTCCA GGACCACGGT GGAGGATTTC TGCATGAAGA

AGTGGTGCTG CCTTATTCCA GGACTACAGT GGAGGATTTC TGCATGAAGA

---------- ---------- ---------- ---------- ----------

AGTGGTGCTG CCTTATTCCA GGACCACAGT AGAGGATTTC TGCATGAAGA

AGTGGTGCTG CCTGACTCCA GGACCACAGT GGAGGATTTC TGCATGAAGA

GGTTGTGCTG CCTGACTCCA GGACCACGGT GGAGGATTTC TGCATGAAGA

---------- ---------- ---------- ---------- ----------

AGTGGTGCTG CCTGACTCCC GGACCACAGT GGAGGATTTC TGTATGAAGA

AGTGGTGCTG CCCGACTCCC GGACTACAGT AGAGGATTTT TGCATGAAAA

TTCACAAAAA TCTTATCAAA GAATTTAAAT AGTAAGTGGA GGCAGAAGAT

TTCACAAAAA TCTTATCAAA GAATTTAAAT AGTAAGTGGA GGCAGAAGAT

TTCACAAAAA TCTTATCAAA GAATTTAAAT AGTAAGTGGA GGCAGAAGAC

TTCACAAAAA TCTTATCAAA GAATTTAAAT AGTAAGTGGA GGCAGAAGAC

TTCACAAAAA TCTTATCAAA GAATTTAAAT AGTAAGTGGT GGCAGAAGAT

---------- ---------- ---------- ---------- ----------

TTCACAAAAA TCTGATCAAA GAATTTAAAT AGTAAGTGGA GGCAGAAGGT

TTCACAAAAA TCTCATCAAA GAATTTAAAT AGTAAGTGGA GGCAGAAGAT

---------- ---------- ---------- ---------- ----------

TTCACAAAAA TCTTATCAAA GAATTTAAAT AGTAAGTGGT GTCAGAAGAT

TTCATAAAAA TCTTATCAAA GAATTTAAAT AGTAAGTGGT GGCAGAAGAT

---------- ---------- ---------- ------TGGT GGCAGAAGAT

TTCATAAAAA TCTTATTAAA GAATTCAAAT AGTAAGTGGT GTCAGAAGAT

TTCACAAAAA TCTTATCAAA GAATTTAAAT AGTAAGTGGT GGCAGAAGAT

TTCACAAAAA TCTTATCAAA GAATTTAAAT AGTAAGTGGT GGCAGAAGAT

---------- ---------- ---------- ------TGGC AGCAGAAGAT

TCCATAAAAA CCTCATCAAA GAATTCAAGT AGTGAGTGGT GGCAGAAGGC

TTCACAAGAA TCTCATCAAA GAATTCAAGT AGTAAGTGGT GGCAGAGGAT

GTTTCAGCAT CGAGAAAACC GCCTGGCAGA GAGAATGGTA AGAGGTGGCA

GTTTCAGCAT CGAGAAAACC ACCTGGCAGA GAGAATGGTA AGAGGTGGCA

GTTTCAGCAT CAAGAAAACC GCCTGGCAGA GAGAATGGTA AGAGGTGGCA

GTTTCAGCAT CAAGAAAACC GCCTGGCAGA GAGAATGGTA AGAGGTGGCA

GGTACAGCAT CGAGAAAACC GCTTGGCAGA GAGAATGGTA AGAGGTGGCA

---------- ---------- ---------- ---------- ----------

GTCTCAGCAT CGAGAAATCC GCTTGGCTGA GAGAATGGTG AGAGGTTGCA

GTCTCAGCAT CGAGAAAACT GCTTGGCAGA GAGAATGGTG TGAGGTGGCG

---------- ---------- ---------- ---------- -GAGGTGACA

GTCTCAGCAT CGAGAACACC GCTTGGCAGA GAGGATGGTG AGAGGCGGCT

GTTTCAGCAT CGAGAAAACC GCTTGGCAGA GAGAATGGTA AGAGGTGGCA

GTTTCAGCAT CGAGAAAACC GCTTGGCAGA GAGAATGGTA AGAGGTGGCA

GTTTCAGCAT CAAGAAAACC GTTTGGCAGA GAGAATGGTA A---------

GTTTCAACGT CGAGAGAACC GCTTGGCAGA GCGAATGGTG AGACGTGGCA

GTCTCAGCAT CGAGAAAACC GCTTGGCAGA GAGAGTGGTA AGAGGTGGCA

GCATCAGCAC CGAGAGAGCC GCTTGGTAGA GAGAGTGGTA AGAGGTGGCG

GTATCGGCAG CGAGAAGTGC GATTGTCTGA GAGGATGGTA ACACGTGGCA

GCGTCAGCAG CAAGAAAACC ATTTGGCAGA GAGAATGGTA ACATGTAGCT

GCCAGGGAGA GCCAGCACAA CAGGCAGCTG CTGCGGTGAC CAGGTCCTGT

GCCAGGGAGA GCCAGCACAA CAGGCAGCTG CTGCGGTGAC CAGGTCCTGT

GCCAGGGAGA GCCAGCACAA CAGGCAGCTG CTGCGGTGAC CAGGTCCTGT

GCCAGGGAGA GCCAGCACAA CAGGCAGCTG CTGCGGTGAC CAGGTCCTGT

GCCAAGGAGA GCCAGCACAA CAGGCAGCTG CTGCGGTGA- ----------

---------- ---------- ---------- ---------- ----------

GCCAGGGAAA GACAGCACAA CAGGCAGCTG CTGTGGTGAC CAGGTCATGC

GCCAGGGAGA GACAGCACAA CAGGCAGCTG CTGTGGTGAC CAAGTCATGC

GCCAGGGAGA GGCGGCACCA CAGGCAGCTG CTGCGGTGA- ----------

GCCAGGGAGA GCCGGCACAA GAGGCGGCTG CTGCGGTGAC CAGGTCCTGC

GCCAGGGAGA GCCAGCACAA GAGGCGGCTG CTGCGGTGAC CAGGTCCTGC

GCCAGGGAGA ACCAGCACAA GAGGCAGCTG CTGCGGTGAC CAGGTCCTAC

---------- ---------- ---------- ---------- ----------

GCCAGGGAGA GCCGGCACGC CAGGCAGCTG CTGCGGTGAC CAGGTCCTGC

GCCAGGGAGA GCCAGCACAA AAGGCAGCTG CTACGGTGAC CAGGTCCTGC

GCCAGGGAGA GCCAGCACAA CAGGCGCTTG CTGCGGTGAC TAGCTCCTGC

GAGAGAGAAG AGTGGCATCG CCGGGAGCTC CTGCGGTAGC CAGGTCCTGG

GAGAAGGAGA AACAGCATAA TAGGCAACTG TTAAGGTAAC TAGGTCTTGG

CGGCGGCAAG CGAGCAGCCT GCGCAGGTGG CTGGAGCTGA ACTTTTTTAT

CGGCGGCAAG CGAGCAGCCT GCGCAGGTGG CTGGAGCTGA ACTTTTTTAT

CAGCGGCAAG CGAGCAGCCT GCGCAGGTGG CTGGAGCTGA GCTTTTTTAT

CAGCGGCAAG CGAGCAGCCT GCGCAGGTGG CTGGAGCTGA GCTTTTTTAT

---------- ---------- ---------- ---------- --TTTTTTAT

---------- ---------- ---------- ---------- ----------

CAGCGACAAG CCAACAGCCT GCGTAGGTGG CTGGAGCTAA GCTGTTTAAT

CAGCAACAAG CCAACAGCCT GCGTAGGTGG CTGGAGCTAA GCTTTTTAAT

---------- ---------- ---------- ---------- ----------

CAGCGGCAAG CACACAGCCT GAGGCGGTGG CTAGAGCTGA GCTTT---AT

CAGCGGCAAG CAAGAAGCCT GCGCAGGTGG CTGGAGCTGA GCTTTTTTAT

CAGCGGCAAG CGAGCAGCCT GCGCCGGTGG CTGGAGCTGA GCTTTTTTAT

---------- ---------- ---------- ---------- ----------

GAGCGGCAAG CCAGCAGCCT GCGCAGGTGG CTGGCGCTGA GCTTTTTTAT

CAGCGGCAAG CGAGCAGCCT GCGCAGGTGG TTGGAGCTGA GCTTTTTTAT

CAGCGGCAAG CCAGCATCCT GCGTAGGTGG CTGGAGCTGA GCTTTTTTAT

CAGAGACAGG CGGACAGCCT GAGGAGGTGG TTGGCGCTGA GCTTTTTTAT

AAAAGGCAGG CAAATAGCTT GCGCAGATGG CTGGAACTGA GCTCTTTTAT

TTCAAAATGT TGCAATTAAA TGAATTACTG TTCAGAAGTC TCCCACTTTC

TTCAAAATGT TGCAATTAAA TGAATTACTG TTCAGAAGTC TCCCACTTTC

TTCAAAATGT TGCCATTAAA TGAATTACTG TTCAGAAGTC TCCCACTTCC

TTCAAAATGT TGCCATTAAA TGAATTACTG TTCAGAAGTC TCCCACTTCC

TTCAAAATGT TGCAATTAAA TGAATTACTG TTCAGAAGTC TCCCACTTTC

---------- ---------- ---------- ---------- ----------

CTCAAAATGT TGCAATTAAA TGAATTACTG TTCAGAAGTC TCCTATTTTC

TTCAAAATGT TGCAATGAAA TGAATTACTG TTCAGAAGTC TCCTACTTTC

---------- ---------- ---------- ---------- ----------

TTCAAAATGT TGCAATTAAA TGAATCACTG TTCAGAAGTC TCCCACTTTC

TTCAAAATGT TGCAATTAAA TGAATTACTG TTCAGAAGTC TCCTACTTTC

TTCAAAATGT TGCAATTAAA TGAATTACTG TTCAGAAGTC TCCCACTTTC

---------- ---------- ---------- ---------- ----------

TTCAAAATGT TGCAATTAAA TGAATTACTG TTCAGAAGTC TCCCACTTTC

TTCAAATTGT AGTAATTAAA TGAATTACTG TCCAGAAGTC TCCCACTTTC

TTCAAAATGT TGCAATTAAA TGAATTACTG TTCAGAAGTC TCCCGCTTTC

TGGAAAATGT TGCAATTAAA TGAGTTCCCG TCCCCACGTC TTGGGCTGAC

TTCAAAAGTT TGCAATTAAA TGAGTAACCG TTCATAAGTC TTACACTTTC

ATACAAAATA CTGTGCTAGA TACAGGAGCG GGAGGGCGCC GAAGGGCAGG

ATACAAAATA CTGTGCTAGA TACAGGAGCG GGAGGGCGCC GAAGGGCAGG

ATACAAAATA CTGTGCTAGA TACAGGAGCG GGAGGGCGCC GAAGGGCAGG

ATACAAAATA CTGTGCTAGA TACAGGAGCG GGAGGGCGCC GAAGGGCAGG

ATACAAAATA CTGTGCTAGA TACAGGAGCG GGAGGGCGCC GAAAGGCAGG

---------- ---------- ---------- ---------- ----------

ATACAAAATA CTGTATTAGA TACAGGAGGG GGAGGGCACC GAAGGGCAGG

ATACAAAATA CGGTACTAGA TACAGGAGGG GGAGCGCACG GAAGGGCAGG

---------- ---------- ---------- ---------- ----------

ATACAAAATA CTGTGCTAGA TACAGGAGGG GGAGGGCGCC GAGGGGCAGG

ATACAAAATA CTGTGCTAGA TACAGGAGCG GGAGGGCACC GAAGGGCAGG

ATACAAAATA CTGTGCTAGA TACAGGAGTG GGAGGGCACC GAAGGGCAGG

---------- ---------- -----GAGCG GGAGGGCGCC CAGGGGCAGG

GTACAAAATA CTGTGCTAGA TACAGGAGCG GGAGGGCGCC GAAGGGCAGG

ATACAAAATA CTGTGGTAGA TACAGGAGCG GTAGGGCTCC AAAGGGTAGG

ATACAAAATA CTGTGCTAGA TACAGGAGCG GGAGGGCGCC GAAGGGCAGG

ATACAAAATA CTGGGCTAGA CACAGGAGCG GGAGGCGACG AGAGGAAAAG

ATACAAAATA CTGTGCTAGA TACAGCAGGG GAAATGCACC GAAAGGCAGA

AGGTAGTAGA GGACGGTCAA GGGCCAGGAG CGCAGTTTCA GAGCGGGTCT

AGGTAGTAGA GGACAGTCAA GGGCCAGGAG CGCAGTTTCA GAGCGGGTCT

AGGTAGTAGA GGACGGTCAA GGGCCAGGAG CGCAGTTTCA GGGCGGGTCT

AGGTAGTAGA GGACGGTCAA GGGCCAGGAG CGCAGTTTCA GGGCGGGTCT

AGGTAGTAGA GGACAGTCAA GGGCCAGGAG CGCAGTTTCA GAGCAGGTCT

---------- ---------- ---------- ---------- ----------

AGGTAGTAGA GGATGGTCAG GGGCCAGGAG CGCAGTTTCA GAGCAGGTCT

AGGTAGTGGA GGATGGTCAG CGGCCAGGAG CGCAGTTTCA GAGCTGGTCT

---------- ---------- ---------- ---------- ----------

AGATAGTAGA GGACCGTCAG GGGCCAGGAG CGCAGTTTCA GGGCAGGTTT

AGGTAGTAGA GGACCGTCAG GGGCCAGGAG CGCAGTTTCA GGGCAGGTCT

AGGTAGTAGA GAACCGTCAG GGGCCAGGAG CGCAGTTTCA GGGCAGGTCT

AGGTAGTAGA GCACGGTCAG GGGCCAGGAG CGCAGTTTCA GGGCAGGTCT

AGGTAGTAGA GGACCGTCAA GGGCCAGGAG CGCAGTTTCA GGGTGGGTCT

AGGTAGTAGA GGACGGTGAG GGGCCAGGAG CGCAGTTTCA GAGCGGGTCT

AGGTAGTAGA GGACGGTGAG GGGCCAGGAG CGCAGTTTCA GGGCGGGTCT

AGGAAGTCGT CATTGTGCTC TGACCCTCGG CGCTGCTTCT GGGAGCCTCT

AGGTAATAGA GAATTGTCAG AGGCCAGGAG CGGAGTTTCA GGGCAGGCCT

GGACATGCAG CTCAGCTGCC CCAGCCTCCG GCAACCTGCT ACCTCCCAGT

GGACATGCAG CTCAGCTGCC CCAGCCTCCG GCAACCTGCT ACCTCCCAGT

GGACATGCAG CTCAGCTGCC CCAGCCTCCG GCAACCTGCT ACCTCCCAGT

GGACATGCAG CTCAGCTGCC CCAGCCTCCG GCAACCTG-- ----------

GGACATGCAG CTCAGCTGCC CCAGCCTCCT GCAACCTGCT ACCTCCCAGT

---------- ---------- ---------- ---------- ----------

GGACATGCAG CTCAGCTGGC CTAGCCTCCG ACAACCTGCT ACCTCCCAGT

GGACATGCAG CTCAGCTGGC CTAGCCTCCG ACAACCTGCT ACCTCCCAGT

---------- ---------- ---------- --------CT ACCTCCCAGT

GGACATGCAG CTCAGCTGTC CCAGCCTCCG GCAACCTGCT ACCTCCCAGT

GGACATACAG CTCAACTGTC CCAGCCTCCG GCAACCTGCT ACCTCCCAGT

GGACATGCAG CTCAGCTGTC CCAGCCTCCG GCAACCTGCT ACCTCCCAGT

GGACATGCAG CTCAACTGTC CCAGCCTCCG GCAACCTGCT ACCTCCCAGT

GGACATGCAG CTCAACTGTC CCAGCCTCCG GCAACCTGCT ACCTCCCAGT

GGACATACAG CTCAACTGCC CCAGCCTCCG GCAACCTG-- ----------

GGACATACAG CTCAGCTGTC CCAGCCTCCG GCA-----CT ACCTCCCATT

GAGGAGGCAG CTCAGCCGCC CCTGACGCCG TCCTCCATCT ACCTCCCATT

AGACATACAG CTTAGCTGGC CCAACCTCCG GCAACCTGCT ACCTCCCATT

GACCAGCAAG TTTGGGTGGA ATCTCCAATC CCAGCTTCTC CAGCTCTCGC

GACCAGCAAG TTTGGGTGGA ATCTCCAATC CCAGCTTCTC CAGCTCTCGC

GACCAGCAAG TTTGGGTGGA ATCTCCAATC CCAGCTTCTC CAGCTCTCGC

---------- ---------- ---------- ---------- ----------

GACCAGCGAG TTTGGGTGGA ATCTCCAATC CCAGCTTCTC CAGCTCTCGC

---------- ---------- ---------- ---------- ----------

GACTAGCAAG CTTGGGCGGG ATCTCCAACC CCAGCTTCTC GAGCTGCCGC

GACTAGCAAG TTTGGGCGGG ATCTCCAAGC CCAGCTTCTC AAGCTTCCGT

GACCAGCAAG TTTGGGTGGA ATCTCCAGTC CCAGCCTCTC CAGCTCTCGC

GACCAGCAAG CTTGGGCGGG ACGTCCAAGC CCAGCTTCTC CAGCTCTCGC

GACCAGCAAG TTTGGGTGGA ATCTCCAACC CCAGCTTCTC CAGCTCTCGC

GACCAGCGAG TTTGGGTGGA ATCTCCAATC CCAGCTTCTC CAGCTCTCGC

GGCCAGCGAT CCTGGGTGGA ATCTCCACTC CCATCTTCTC CAGCTCTTGC

GACCAGCAAG TTTGGGTGGA ATCTCCAATC CCAGCTTCTC CAGCTCTTGC

---------- ---------- ---------- ---------- ----------

CACCAGCAAG TCTGAGTGGA ATTTCCAGTC CCAGCTTCTC CAGCTCCCGC

CTCCCGCAAG CCTGGGTGGC ACCTCCAGTC CCAGCCTCTC CAGCTCTCGA

CGCCTGCAAT TTGGGGTGGT ATTTCAATTC CCAGTTTCTC CAACTGATGA

TCCCTGTACT TCTCATACTG CCGGTACCCT GCATACTTTT TCCCTTAGTG

TCCCTGTATT TCTCATACTG CCGGTACCCT GCATACTTTT TCCCTTAGTG

TCCCTGTACT TCTCATACTG CCGGTACCCT GCATACTTTT TCCCTTAGTG

---------- ---------- ---------- -----CTTTT TCCCTTAGTG

TCCCTGTACT TCTCATACTG CCGGTACCCT GCATACTTTT TCCCTTAGTG

---------- ---------- ---------- ---------- ----------

TCCCTGTACT TCTCGTACTG CCGGTACCCA GCATACTTTA TTCCTTAGTG

TCCCTGTACT TCTCATACTG CCGGTACCCG GCATACTTTA TTCCTCAGTG

TCCCTGTACT TCTCATACTG CCGGTACCCT GCATACTTTC TTCCTCAGCG

TCCCGGTACT TCTCGTACTG CCGGTACCCT GCATACTTTT TTTCTTAGTG

TCCCTGTACT TCTCATACTG CCGGTAACCT GCATATTTTT TTTCTTAGCG

TCCCTGTACT TCTCATACTG CCGATAACCT GCATA----- ----------

TCCCTGTACT TCTCATACTG CCGGTAGCCT GCATACTTTT TTTCTTAGTG

TCCCTGTACT TCTCATACTG CCGGTACCCT GCATACTTTT TTTCTTAGTG

---------- ---------- ---------- -----CTTTT TTCCTTAGCG

TCCCTGTACT TCACATACTG CCGGTACCCT GCATA----- ----------

TCTCTGTACT TCTCGTACTG GCGGTACCCT ACATACTCTC TCTCCTAGTG

TCTCGGTATT TCACATACTG ACGGTATCCT GCATAAACCT TCTCTTAGTG

CTCTGGTCTG GGGTCTCTCT GTGAAACACA ATCCTCAGAA AGTGGGTAAA

CTTTGGTCTG GGGTCTCTCT GTGAAACACA ATCCTCAGAA AGTGGGTAAA

CTCTGGTCTG GGGTCTGTCT GTGAAACACA ATCCTCAGAA AGTGGGTAAA

CTCTGGTCTG GGGTCTGTCT GTGAAACACA ATCCTCAGAA AGTGGGTAAA

CTCTGGTCTG GGGTCTCTCT GTGAAACACA ATCCTCAGAA AGTGGGTAAA

---------- ---------- ---------- ---------- ----------

CTTTGGTCTG GGGTCTCTCT GTGAAACATA ATCCTCAGAA AGTGGGTAAA

CTTTGGTCTG GGGTCTGTCT GTGAAACATA ATCCTCAGAA AGTGGGTAAA

CTCTGGTCTG GGGTCTCTCT GTGAAGCACA ACCCTCAGAA AGTGGGTAAA

CTCTGGTGTG GGGTCTGTCT GTGAAACACA ATCCTCAAAA AGTGGGTAAA

CTTTGGTTTG GGGTCTGTCT GTGAAACATA ATCCTCAAAA AGTGGGTAAA

---------- ---------- ---------- ---------- ----------

CTCTGGTCTG GGGTCTCTCT GTGAAACATA ATCCTCAAAA AGTGGGTAAA

CTCTGGTCTG GGGTCTCTCT GTGAAGCACA ATCCTCAGAA AGTGGGTAAA

CTCTGGTCTG GGGTCTGTCT GTGAAACACA ATCCTCAGAA AGTGGGTAAA

---------- ---------- ---------- ---------- ----------

CGCTGGTCTG GGGTCTGTCT GTGAAGCACA ACCCTCAGAA GGTGGGGAAA

CTCTGGTATG GGGTCTGTCT GTGAAGCATA ATCCCCAGAA AGTAGGCAAA

GACCATACGT TGGAGGATGA GGATGTCATT CAAATTGTGA AGAAGTGAAG

GACCATACGT TGGAGGACGA GGATGTCATT CAAATTGTGA AGAAGTGAAG

GACCATACGT TGGAGGACGA GGATGTCATT CAAATTGTGA AGAAGTGAAG

GACCATACGT TGGAGGACGA GGATGTCATT CAAATTGTGA AGAAGTGAA-

GACCATACGT TGGAGGATGA GGATGTCATT CAAATTGTGA AGAAGTGAAG

---------- ---------- ---------- ---------- ---------G

GACCATACGT TGGAAGATGA AGATGTCATT CAGATCGTGA AGAAGTGAAG

GACCACACAT TGGAAGATGA AGACGTCATT CAGATTGTGA AGAAGTGAAG

GACCACACGC TGGAGGATGA GGACGTCATT CAGATTGTGA AGAAGTGAA-

GACCATACGT TGGAGGACGA GGATGTCATT CAGATTGTGA AGAAGTGATG

GACCATACGT TGGAGGACGA GGATGTCATT CAGATTGTGA AGAAGTGAAG

---------- ---------- ---------- ---------- ----------

GACCATACGT TGGAGGATGA GGATGTCATT CAGATTGTGA AGAAGTGAAG

GACCATACAT TGGAGGACGA GGATGTCATT CAGATTGTGA AGAAGTGAAG

GATCATACAT TGGAAGACGA GGATGTCATT CAGATTGTGA AGAAGTGAAG

---------- ---------- ---------- ---------- ----------

GACCACACAT TGGAAGACGA GGACGTCATT CAGATCGTCA AGAAGTGAAG

GATCACACAA TGGAGGACGA AGATGTCATT CAGATTGTGA AGAAATAAAG

AGAAGGAGCT CACCTTTTGG GTCGAGGAAG GCCCATGGGG GTAAGATTAG

AGAAGGAGCT CACCTTTTGG GTCGAGGAAG GCCCATGGGG GTAAGATTAG

AGAAGGAGCT CACCTTTTGG GTCGAGGAAG GCCCATGGGG GTAAGATTAG

---------- ---------- ---------- ---------- ----------

AGAAGGAGCT CACCTTTTGG GTCGAGGAAG GCCCATGGGG GTAAGATTAG

AGAAGCAGCT CACCTTTTGG GTCGAGGAAG GCCCATGGGA GTAAGATTAG

AAAAA---CT CACCTTTTGG GCCGAGGCAG ACCCATTGGG GTAAGGTTGG

AGAAG---CT CACCTTTTGG GCCGAGGCAG GCCCATTGGG GTAAGGTTGG

---------- ---------- ---------- ---------- ----------

AGAAGGCGCT CACCTTTTGG GTCGAGGAAG GCCCATGGGC GTGAGGTTAG

AGAAGGAGCT CACCTTTTGG GTCGAGGAAG GCCCATGGGG GTAAGGTTAG

--AAAGAGCT CACCTTTTGG GTCGAGGAAG GCCCATGGGG GTAAGATTAG

AGAAGGGGCT CACCTTTTAG GTCGTGGAAG GCCCATCGGG GTAAGGTTAG

AGAGGGAGCT CACCTTTTGG GTCGAGGAAG GCCCATGGGG GTAAGATTAG

AGAAGGAGCT CACCTTTTGG GTCGAGGGAG GCCCATGGGG GTAAGGTTAG

---------- ---------- ---------- ---------- ----------

AGAAAGAGCT TACCTTTTAG GTCGAGGCAG TCCCATAGGG GTAAGGTTGG

AGAAGGAGCT CACCTTTTGG GTCGAGGCAG CCCCATGGGG GTGAGGTTCG

GATCTGGCCT AGGAATGGTT TTCCGGATGC GAATCTGTGA GACTTTCTTT

GATCTGGCCT AGGAATGGTT TTCCGGATGC GAATCTGTGA GACTTTCTTT

GATCTGGTCT AGGAATGGTT TTCCGGATGC GAATCTGTGA GACTTTCTTT

---------- ---------- ---------- ---------- ----------

GATCTGGCCT AGGAATAGTT TTCCGGATGC GAATCTGTGA GACTTTCTTT

GATCTGGCCT AGGAATGGTT TTCCGGATGC GAATCTGCGA GACTTTCTTT

GATCTGGTTT AGGAATGGTT TTCCGGATGC GGATCTGTGA GACTTTCTTT

GATCTGGTTT AGGAATGGTT TTCCGGATGC GGATCTGTGA GACTTTCTTT

---------- ---------- ---------- ---------- ----------

GGTCTGGCTT AGGAATGGTT TTCCGGATGC GAATCTGTGA GACTTTCTTC

GGTCTGGCTT AGGAATGGTT TTCCGGATAC GAATCTGTGA GACTTTCTTT

GGTCTGGCTT AGGAATGGTT TTCCGGATGC GAATCTGTGA GACTTTCTTT

GGTCTGGCTT AGGGATGGTT TTCCGGATTC GAATCTGTGA CACTTTCTTT

GGTCAGGCTT AGGAATGGTT TTCCGGATGC GTATCTGTGA GACTTTCTTT

GGTCTGGCTT AGGAATAGTT TTCCGGATGC GAATCTGTGA GACTTTCTTT

---------- ---------- ---------- ---------- ----------

GATCTGGCCT AGGAATAGTT TTCCGGATTC GAATCTGTGA GACTTTTTTT

GGTCTGGCTT AGGAACGGTC TTCCGGATCC GGATTTGAGA GACCTTCTTG

GGCCTCTTCT CTGGCTCAGC CTATTTTTAA GCACGAACCT GGAACTCAGA

GGCCTCTTCT CTGGCTCAGC CTATTTTTAA GCAGGAACCT GGAACTCAGA

GGCCTCTTCT CTGGCTCAGC CTATTTTTAA GCAGGAACCT GGAACTCAGA

---------- ---------- ---------- ---------- ----------

GGCCTCTTCT CTGGCTCAGC CTATTTTTAA GCAGGAACTT GGAACTCAGA

GGCCTCTTCT CTGGCTCAGC CTATTTTTAG GCAGGAACTT GGAAGTCAGA

GGTCTCTTCT CTGGCTCGGC CTGTTTTTAA ACAGGAACCT GGAGCTCCGA

GGTCTCTTCT CTGGCTCAGC CTGTTTTTAA ACAGGAACTG CGAGCTCAGA

---------- ---------- ---------- -CAGGGACTT GGAGCTCGGA

GGCCTCTTCT CCAGCTCAGC CTATTTTTAA GCGGGAACCG GGAACTCTGA

GGCCTCTTCT CTGGCTCAGC CTATTTTTAA GCAGGAACCT GGAACTCGGA

GGCCTCTTCT CTGGCTCAGC CTATTTTTAA GCAGGAACCT GGAATTCAGA

GGCCTCTTCT CTGGCTCTGC CTATTGCAAG GCAGGAACCT GGAACTCAGA

GGCCTCTTCT CTGGCTCAGG CTATTTATAA GCAGGAACCT GGAACTCAGA

GGCCTCTTCT CTGGCTCAGC CTATTTTTAA GCAGGAACCT GGAACTCAGA

---------- ---------- ---------- -CAGAGACCT GGAACGCAGA

GGCCGCTTCT CTGGTTCAGT CTATTTTTAT GCAGGAATCT GGTAGTCAGA

GGCTTCTTCT TGGGCTCCAT CTATTTTCAA GGAGGAACCT GGAAGTTGGA

GGCTGAAAGC AGGAGCTTGC TGACAGCTGA TTCTGCTACC AAGCAAGACT

GGCTGAAAGC AGGAGCTTGC TGACAGCTGA TTCTGCTACC AAGCAAGACT

GGCTGAAAGC AGGAGCTTGC TGACAGCTGA TTCTGCTACC AAGCAAGACT

---------- ---------- ---------- ---------- ----------

GGCGGAAAGC AGGAGCTTGC TGACAGCTGA TTCGGCTACC AAGCAAGACT

GGCTGAAAGC AGGAGCTTGC TCACAGCTGA TTCTGTTACC AAGCAAGACC

GGCCGAGAGC AGGAGCGTGC TGACAGAGGG TTCTGTTACC AAGCAAGACT

GGCAGAGAGC AGCAGTGTGC TGACAGCGGC TTCTGTTACC AAGCAAGACT

GGCTGAAAGC AGGAGTTGGC TGACAGCTGG CTCCGTGACC AAGCAAGACT

GGCTGAGAAC AGGAGCTTGC TGACGGCTGA GTCTGTTACC AGGTGAGACT

GGCTGAAAGC AGGAGCTTGC TGACAGCTGA ATCTGCTACC AAGCAAGACT

GGCTGACAGC AGGAGCTTGC TGACAGCTGA GTCTGCTACC AAGCAAGACT

GGTTGAAAGC AGGAGTTTGC TGACAGTCGA GTCTGTTACC AAGCAAGACT

GGCTGAAAGG AGGAGCTTGC TGACAGCTGA TTCTGTTACC AAACAGGACT

GGCTGAAAGC AGGAGCTTGC TGACAGCTGT TTCTGTTACC AAGCAAGACT

GGCTGAAAGC AGTAGGTTGC TGACAGCTGT CTCTGTTACC ACGCAAGGCT

GGCTGAAAGT CGGAGCACAC TGACCGCTGG TTCAAAGGCT AAGCAGGACT

GGCTGAAAGT CTGTGTGTTA TTAAAGTTGG TTCTGGAGCT AAACACGACT

TTGGTACCAA GGCAGGGAAT GGAGGCTCGT TGCCGCTTGC AGTGAGGGGA

TTGGTACCAA GGCAGGGAAT GGAGGCTCGT TGCCGCTTGC AGTGAGGGGA

TTGGTACCAA GGCAGGGAAT GGAGGCTCGT TGCCGCTTGC AGTGAGTGGA

---------- ---------- --------GT TGCCGCTTGC AGTGAGTGGA

TTGGTACCAA GGCAGGGAAT GGAGGCTCAT TGCCGCTTGC TGTGAGTGGA

TTGGTACCAA GGCAGAGAAT GTAGGTTCTT TGCTGCTTGC TGGGAGTAGA

TTGGCACCAA GGCAGGAAAC ACAGGCTCCC TGCTGCTTGC TATGAGGAGA

TTGGCACCAA GGCAGGAAAC ACAGGCTCCC TGCTGCTTGC TAGGGGTGGA

TTGGTACCAA GGCAGCAAAC GAAGGCTCGC TGCTCCTTGC TGCGAGTGGA

TTGGTACCAA GGCAGGGAAT GGAGGCTCAC TGCCGCTTGC TGTGAGAGGG

TTGGTACCAA GGCAGGGAAT GGAGGCTCGC TGCCGCTTGC TGTGAGCGGA

TTGGTACCAA GGCAGGAAAT GGAGGTTCGC TGCCGCTTGC TGTGAGTGGA

TTGGTACCAA GGCAGGAAAT GGAGACTCGC TGCTCCTCGC TGTGAGTGGA

TTGGTACCAA GGCAGAGAGG GCAGGCTCGC TGCCGCGTGC TGTGATTGGA

TTGGTACCAA GGTAGGGAAT GGAGGCTCgc tgcCACTTGC TGTGACTGGA

TTGGTACCAA GGCAGAGAAG GAAGGCTCGC TGCCACTTGC TGTAATTAGA

TTGGTGCCAA AGCAGAGCAA GGAGGTCCGC GGCCACTTGC TGTGTTTGGA

TTGGTAGCAG AGCAGAGAAT GGAGGTTCGC TGCCACTCGC TGCGATTGGA

ATGAAGAGAA GAGATTTCGA AGGCAGAAGG ACCATGCTGG CTCCAAATGG

ATGAAGAGAA GAGATTTCGA AGGCAGAAGG ACCATGCTGG CTCCAAATGG

ATGAAGAGAA GAGATTTCGA AGGCAGAAGG CCCATGCTGG CTCCAAATGG

ATGAAGAGAA GAGATTTCGA AGGCAGAAGG CCCATGCTGG CTCCAAATGG

ATGAAGAGAA GAGATTTCGA AGGCAGAAGG CCCATGGTGG CTCCAAATGG

ATGAAGAGCA GAGATTTCGA AGGCAGAAGG CCCATGCTGA CTCCAAATGG

ATGAAGAGCT GGCAATTTGA AGG-AGAGGG TCCGTGCTGG CTCCAAATGG

ATGAAGAGCT GGCAATTTGA AGG-AGAGGG CCCGTGCTGG CTCCAAAGGG

CTGAAAAGCA GAGATTTCAA AGGCAGAAGG TCCATGCTGG CTCCAAATGG

TGGAAGGGCA GAGATCTCCA AGGCGGAAGG CCCATGCTGG CTCCAGATGG

ATGAAGAGTA GAGATTTCGA AGGCAGAAGG CCCATGCTGG CTCCAAATGG

ATGAAGAGCA GAGATTTCGA AGGCAGAAGG TCCATGCTGG CTCCAAATGG

ATGGAGAGAA GAGATTTTGA AGGAAGAAGG TCCATGCTGG CTCCAAATGG

ATGAAGAGCA GTGACTTCGA AGGCAGAGGG CCCATGCTGG CTCCAAATGG

ATGAAGAGCA GAAATTTCGA GGGTAGAAGG ACCATGCTGG CTCCAAATGG

ATGAAGAGCA GAGATTTCCA GGGCAGAAGG GCCATGCTGG CTCCAAATGG

ATGGAGAGTA GTGATTTCGA AGGCAGAAGG ACCATGCTGG CTCCATATGG

ATGGAGAGTA GTGATTTTGA AGGTAGGAGG ACCATGCTGG CTCCATATGG

ATAGGACTCT GCCCACCGAG TAGGGCAGAG AACAGAAGAC CTTGTCTGCT

ATAGGACTCT GCCCACCGAG TAGGGCAGAG AACAGAAGAC CTTGTCTGCT

ATAGGACTCT GCCCACCGAG TAGGGCAGAG AACAGAAGAC CTTGTCCGCT

ATAGGACTCT GCCCACCGAG TAGGGCAGAG AACAGAAGAC CTTGTCCGCT

ACAGCACTCT GCCCACCGAG TAGGGAAGAG AACAGAAGAC CTTGTCTGCT

ACAGCACTCT GCCCACTGAG TAGGGCAGAG AACAGAAGAC CTTGTCTGCT

ACAACACTCG GCCCACCGAG TAGGGCAGAG AAGAGAAGAC CCTATCGGCT

ACAACACCCG GCCCACCGAG TAGGGCAGAG AACAGAAGAC CTTATCTGCT

ACAGCACTCT GCCCACCGAG TAGGGCAGAG AACAAAAGAT CTTGTCTGCT

ACAGCACCCG GCCCACCGAG TAGGGCAGGG AGCAGAAGAC CTTGTCAGCT

ATAGCACTCG GCCCACCGAG TAGGGAAGAG AACAGAAGAC CTTGTCTGCT

AGAGCACTCG GCCCACCGAG TAGGGCAGAG AACAGAAGAC CTTGTCAGCT

ACAGCACTCG GCCCACGGAG TAGGGCAGAG AGCAGAAGAT CTTGTCGGCG

ACAGCACTCT GCCCACAGAG TAGGGCAGAG AACAGAAGAC CTTGTCTGCT

ACAGCACCCT ACCCACTGAG TAGGGCAGAG AACAGAAGAC TTTGTCTGCT

ACAGCACCCT GCCCACTGAG TAGGGCAGGG AACAGAAGAC TTTGTCTGCT

ACAGTACCCT GCCCACTGAG TAGGGCAGAG AAGGGAAGAG GCTGTTGGAC

AGAGGACCCT TCCCAGGGAG TAGGGCAGTG AAGAGAAGAG ATCATCTGAT

ATGGAGGCTG GAGACCGTAA CCCTTTCAGG CCCTGTGTAA ACTGGGTCAT

ATGGAGGCTG GAGACCGTAA CCCTTTCAGG CCCTGTGTAA ACTGGGTCAT

ATGGAGGCTG GAGACCGTAA CCCTTTCAAG CCCTGTGTAA ACTGGGTCAT

ATGGAGGCTG GAGACCGTAA CCCTTTCAAG CCTTGTGTAA ACTGGGTCAT

ATGGAAGATG GAGAGCGCAA CCCTTTCAAA CCCTGTGTAA ACTGGGTCAT

ATGGAGGCTG GAGACCTCAA CCCTTTCAGG CCCTGTGTAA ACTGGGTCAT

ATAGAGGCTG GAGACCTTAA CCCTTTTAGG CCCTGTGTAA ACTGGGTCAA

ATAGAGGCTG GAGACCTTAA CCCTTTTAGG CCCTGTGTAA ACTGGGTCAG

ATGGAGGCTG GAGACCTCAA CCCCTTTAAG CCCTGTGTAA ACTGGGTCAG

ATGGAGGCGG GAGACCGCAG CCCTTTGAGG CCCTGTGTTA ACTGGGTCAT

ATGGAGGCTG GAGATCTTAA CCCTTTTAAG CCCTGTGTAA ACTGGGTCAT

ATGGAGGCTG GAGACCTTAA CCCTTTCAAG CCCTGTGTAA ACTGGGTCAT

ATGGAGGCAG GGGACCTTAA GCCTTTCAAG CCCTGTGTAA ACTGGGTCAT

ATGGATGCTG GAGACCTTAA CCCTTTCAAG CCCTGGGTAA ACTGCGTCAT

ATGGATGCTG GAGGCTTTAA TCCTTTCAAG CCCTGTGTAA ACTGTGTCAT

ATGGATGCAG GAGACCTTAA CCCCTTCAAG CCCTGTGTAA ACTGTGTCAT

AGAGATGCTG GAAGCCTTAA CCCTTTGAAG CCCTGTGTAA ACTGAGTCAT

ATGGAAGTAG GAGATCTTAA CCCCTTTAAG CCCTGTGAAA ACTGTGTCAA

GAAGAGCCTC TGCATGGTAG GCATGTGGCT GGAGAAGCTC CGTTTTTTTG

GAAGAGCCTC TGCATGGTAG GCATGTGGCT GGAGAAGCTC CGTTTTTTTG

GAAGAGCCTC TGCATGGTAG GCATGTGGCT GGAGAAGCTC CGTTTTTTTG

GAAGAGCCTC TGCATGGTAG GCATGTGGCT GGAGAAGCTC CGTTTTTTTG

GAAGAGCCTC TGCAGAGTAG GCATGTGGCT GGAGAAGTTC CGTTTTTTTG

GAAGAGCCTC TGCATGGAAG GCATGTGACT GGAGAAGCTC CGTTTTTTTG

GAGGAGCCTC TGCATGGTAG GCATATGACT GGAGAAGTTC CGTCTTTTTG

GAAGAGCCTC TGCATGATAG GCATATTACT GGAGAAGTTC CGTCTTTTTG

GAAAAGCCTC TGCACAGTAG GCATGTGGCT GGAGAAGCTC CGTTTTTTTG

GAAGAGCCTC TGCATGGCAG GCGTGTGGCT GACGCAGCTC CGTTTCTTCG

GAAGAACCTC TGCATGGTAG GCATGTGACT GGAGAAGTTC CGTTTTTTTG

GAAAAGCCTC TGCATGGTAG GCATGTGACT GGAGAAGCTC CGTTTTTTTG

GAAGAGCCTC TGCACGGTAG GCATGTGGTT GGAGAAGCTC CGTTTCTTTG

GAAGAGCCTC TGTATGGTGG GCATGTGACT GGAGAAACTC CGTTTTTTTG

GAAGAGCCTC TGTATGGTAG GCATGTGGTT AGAGAAGCTC CATTTTTTTG

GAAGAACCTT TGTATGGTAG GCATGTGGCT GGAGAAGTTC CGTTTTTTTG

GAAGAGCCTC TGGATGGTAG GCAAATGACT GGATAAGCTC CTTCTTCTCG

GAAGAGCCTC TGGATGGAAG GGGTGTGACT GGAGAAGTTC CTTCTTCTTG

TCCAGATTCG GTAACATCCT GGGGAACAAA GTGCTAGAAG TGTGTGAAGG

TCCAGATTCG GTAACATCCT GGGGAACAAA GTGCTAGAAG TGTGTGAAGG

TCCAGATTCG GTAACATCCT GGGGAACAAA GTGCTAGAAG TGTGTGAAGG

TCCAGATTCG GTAACATCCT GGGGAACAAA GTGCTAGAAG TGTGTGAAGG

TCCAGATTCG GTAACATCCC GGGGAACAAA GTGCTAAAAG TGTGTGAAGG

TCCAGATTCG GTAACATCCT GGGGAACAAA GTGCTAGAAG CGTGTGAAGG

TCCAGATTCG ATAACATCCG GGTGAACAAA GTGCTAGAAG TGTGTGAAGG

TCCAGATTCG GTAACATCCG GGTGAACAAA GTGCTAGAAG TGTGTGAAGG

TCCAGATTCG ATAACATCCT GGTGAACAAA GTGCGAGAAG CGTGTGAAGG

TCCAGATCCG GTAGCATCCC GGGGAGCAGA GTGCTAGGAG CGTGTGGAGC

TCCAGATTCG GTAACATCCA GGAGAACAAA GTGCTAGAAG TGTGTGAAGG

TCCAGATTCG GTAACATCCA GGGGAACAAA GTGCTAGCAG TGTGTGAAGG

TCCAGATTCG GTAGCATCCA GGGGAACAAA GTGCTAGCAG TGTATGAAGG

TCCAGATTCG GTAACACCCG GGGGAACAAA GTGCGAGAAG CGTGTGAAGG

TCCAGATCCG GTAACATCCA GGGGAACAAA GTGCTAGCAG CGTTTGAAGG

TCCAGATCCG GTAACATCCA GGGGAACAAA GTGCTAACAG TGTATGAAGG

TCCAGATCCT ATAACATCCA GGGGAAGAAA GTGCTAAAAC TGTATGAAGG

TCCAGATCCT GTAACATCCA GGGGAGGAAA GTGCTAAAAA GGTATGAAGG

CCAAGGACAG AGCAGTCTGC TCTGGTCTGG AGCTTGATTT CACATGAAAG

CCAAGGACAG AGCAGTCTGC TCTGGTCTGG AGCTTGATTT CACATGAAAG

CCAAGGACAG AGCAGTCTGC TCTGGTCTGG AGCTTGATTT TACATGAAAG

CCAAGGACAG AGCAGTCTGC TCTGGTCTGG AGCTTGATTT TACATGAAAG

CCAAGGACAG AGCAGTTGGC TCTGGTCTGG AGCTTGATTT CACATGAAAA

CCAAGGACAG AGCAGTCTGC TCTGGTCTGG AGCCTGATTT CATGTGAAAG

CCAAGGCCAG AGCAGTCTGC TCTGGTCTGG ATGCTGACTT CACATGAAAG

CCAAGGCCAG AGCGGTCTGC CCTGGTCTGG ACACTGATTT CACATGAAAG

CCAAGGACAG AGCAGTCTGC TCTGGGCTGG AGCCCGACTT CACAGGGAAG

CCGAGGACGG AACAGCCCCC TCTGGTCTGG GGCCCAGCTT CACACGGAAG

CCAAGGACAG AGCAGCCTGC TCTGGTCTGG ATCCCGATTT CATGTGAAAA

CCAAGGACAG AGCGGCCTGC TCTGGTCTGG AGCCCGATTT CACATGAAAG

CCCAGGATGG AGCAGCCTGC TCTGGTCTG- ---------- ----------

CCAAGGACAG AGCGACCTGC TCTAGTCTGG AGCCTGATTT CACCTGAAAT

CCGAGGACAG AGAAACCTGC TCTGGTCTGG AGCCTGAGTT CATGTGAAAG

CCAAAGACTG AGAAACCTGC TCTGGTCTGG AACCTGAGTT CACATGGAAG

CCAAGGACAG AGCAACCAGC TCTGACCTTG AGTCCAGGTT TATGTGAAAG

CCAAGCACAG AGCAAGTTGC TCTGTTTGTG ATTCCAGTTT TATATGAAAG

GATACTGGGA AGATTTCAGA ACCAAACAGC AGGGACGGCA TCTTGTTGCT

GATACTGGGA AGATTTCAGA ACCAAACAGC AGGGACGGCA TCTTGTTGCT

GATACTGGGA AGATTTCAGA ACCAAACAGC AGGGACGGCA TCTTGTTGCT

GATACTGGGA AGATTTCAGA ACCAAACAGC AGGGACGGCA TCTTGTTGCT

GATACTGGGA AGATTTCAGA ACCAAACAGC AGTGACGGCA TC---TTGCT

GATACTGGCA AGATTTCAGA ACCAAAGAGC AGCGATGGCA TCTTGTTGCT

GAAAACGGGG CGTTTTCGGT ACCAAGCAGC AGTGACGGCA TCTTGTTGCT

GAAAATGGGA CGTTTTCCGT ACCAAACAGC AGTGATGGCA TCTTGTTGCT

GACACTGGGA AGATTCTGGA ACCAAACAGC AGTGATGGCA TCCTGTTGCT

GATACCGGGA AGGTTTCGGA ACCGAGCAGC AGCGATGGCA TCTTGTCACT

GATACCGGGA AAATTTCAGA ACCAAACAGC AGTGACGGCA CCTTGTTGCT

GATACCGGGA AGATTTCGGA ACCGAACAGC AGGGACGGCA TCTTGTTGCT

---------- ---------- ---------- ---------- ----------

GACACTGGGA AGATTTCAGA ACCAAACAGT AGTGATGGCA TCTTGTTGCT

GATACCGGAA AGATTTCCGT ACCAAACAGC AGAGATGGCA TCTTGTTGCT

TATATCGGAA GGATTTCGGC ACCACCCAAC AGGGACGGCA TCTTGTTGCT

GATATTGGGA AGATTTGGGC ATTGAACTGC AATGATGGCA TTTTGCTAAT

GAAATTGGGA AGATCGGGGT ATCGAAAAGC AACGAGGGTG TTTTGTTATT

CAAATCTATG AAGGTCATCT TGATGGATTC GAGAGAATAA AGTGCCATGG

CAAATCTATG AAGGTCATCT TGATGGATTC GAGAGAATAA AGTGCCATGG

CAAATCTATG AAGGTCATCT TGATGGATTC GAGAGAATAA AGTGCCACAG

CAAATCTATG AAGGTCATCT TGATGGATTC GAGAGAATAA AGTGCCACGG

CAAATCTATG AAGCTCATCT TGATGGATTC GAGAGAATAA AGTGCCATGG

CAAATCTATG AAGCTCATTT TGATGGCTTC AAGAGAATAA AATGTCAACG

CAAATCTATG AAGCTTATTT TAACAGCTTC AAGAGAATAA AGTGCCAAGG

CAAATCTATG AAGCTCATTT TAACAGCTTC AAGAGAATAA AGTGCCAAGG

CAGGTCGATG AAGCTCATCT TGATGCCTTC AAGAGAATAA AGTGCCAGGG

CACATCTATG AAGCTCATTT TGACAGTTTC GAGCGAATAA AGCATCAGGG

CAAATCTATG AAGCTCATTT TGATGGCTTC AAGCGAATAA AGTGTCAAGG

CAAATCTATG AAGCTCATTT TGATGGCTTC CAGCGGGTAA AGTGTCAAGG

---------- ---------T TGATGGTTTC GAGCGAATAA AGAGTCCAGG

CAAATCTATG AAGTTCATTT TGATGGGCTC AAGAGAATAA AGTGCCGAGG

CAGATCTATG AAGCTCATTT TGATGGCTTC AAGAGGATAA AGTGCCACGG

CAGATCTATG AAGCTCA-TT TGATGGCTTC AAGAGAGTAA AGTGCCATAG

CAAGTCTAAG AAGCCTATTC TGAGGGCTTC AAGGGGATAA AGTGCCAAAG

CAAGTCTAAG AAGCGTATTT GGATGGCTTC GGTGGGATAA AGTGCCAGAG

GCTTACTGGA AAATCCGTCC AGGAGTCTTT TATATCTGAG GCGCTTGTAG

GCTTACTGGA AAATCCATCC AGGAGTCTTT TATATCTGAG GCGCTTGTAG

GCTTACTGGA AAATCCATCC AGGAGCCTTT TATATCTGAG GCGCTTGTAG

GCTTACTGGA AAATCCATCC AGGAGCCTTT TATATCTGAG GCGCTTGTAG

GCTTACTGGA AAATCCATCC AGGAGCCTTT TGTATCTGAG GCGCTTAAAG

GCTTACTGGA AAATCCATCC AGGAACCGTT TATATCTGAG GCGCTTGTAG

GCCTACTGGA AAATCCATCT AGGAGCTTTT TGTATCGGAG GCGCTTGTAG

GCCTACTGGA AAATCCATCT AGGAGCTTTT TATATCTGAG GCGCTTGTAG

GCTTACTGGA AAACCCATCG AGGAGCCGTT TATATCTGAG CCGCTTGAAG

GTTTACTGGA GAAGCCGTCC AGGAGCCTCC GGTATCGGAG CTGCTTGTAG

GCTTACTGGA AAATCCATCC AGAAGTCTTT TGTATCTGTG CTGCTTATAG

GTTTACTAGA AAATCCATCC AGGAGCCTTT TATATCTGAG CCGCTTGTAG

GCTTACTGGA AAACCCGTCC AGGAGCCTGG TGTATCTGAA TCGCTTGCAG

GCTTACTGGA AAATCCATCC AAGAACCTTC TGTATCTGAA CCTTTTGTAG

GCTTATTGGA AAATCCATCC AGAAATCTTT TATATCTGAG CCGCTTGTAG

GCTTACTG-- ---------- ---------- ---------- ----------

TCTGACTGGG AAATTCATCC TGGAGCTTTT TAAACCTGAG CCGTTTGTAG

GCTGAAAGGA AAATACATCC AGAAGATTTT TAAATCTGAG CTGGTTGTAT

CTTTTAGCCA TTGGAAGAAG TTCAGAGGAA CAGTTTGGAG GCAAGGATGG

CTTTTAGCCA TTGGAAGAAG TTCAGAGGAA CAGTTTGGAG GCAAGGATGG

CTTTTAGCCA TTGGAAGAAG TTCAGAGGAA CAGTTTGGAG GCAAGGATGG

CTTTTAGCCA TTGGAAGAAG TTCAGAGGAA CAGTTTGGAG GCAAGGATGG

CTTTTAGCCA TTGGAAGAAG TTCAGAGGAA CAGTTTGGAG GCAAGGATGG

GTTTTAGCCA CTGGAAGAAG TTCAGAGGAA CAGTCTAGAG GCAAGGATGG

TTTTTAGCCA GTGGAACAAG GGAAGAGGAA CAGTTTGGAG GCCAGGACAG

TTTTTAGCCA ATGGAACAAG GGAAGAGGAA CAGTCTGGAG GCAAGGACAG

CTTTTAGCCG CTGGAACAAG TTCAGAGGAA GAGTTTGGAG GCAAGGACAG

CTTTTAGCCA CGGGGAGAAG GTCCGAGGAG CAGTCTGGAG GCGAGGATAG

CTTTTAGTCA CTGGAAGAAG TTCAGAGGAA CAGTTTGGAG GCAAGAATGG

CTTTTAGCCA CTGGAAGAAG TTCAGAGGAA CAGTTTGGAG GCAAGGATGG

CTTTTGGCCA CGGGAAGGAG TTCTGAGGAA CAGTTTGGAG GCGAGGATGG

CTTTTAGCCA CAGGAAGCAG TTCAGAGGAA CAGTTTACAG GCAAGGATAG

CTTTTAGCCA CTGGAAGGAG TTCAGAGGAA CAGTTTACAT GCGAGGATGG

---------- ---------- ---------- ---------- ----------

GATTCAGCCA CGGGAAGAAG TTCAGTAGAG CAGCTTCCTG GCCAGAACAG

CGTTCGGCCA CTGGAAGAGC TCTGGAGGAA CAGTTTACTG GCAAGGACGG

ACAGCTTGTT TAGTAAGGCT GATTCTTTGG TAGTCTTCTG AACGTGTACA

ACAGCTTGTT TAGTAAGGCT GATTCTTTGG TAGTCTTCTG AACGTGTACA

ACAGCTTGTT TAGTAAGGCT GATTCTTTGG TAGTCTTCTG AATGTGTAAA

ACAGCTTGTT TAGTAAGGCT GATTCTTTGG TAGTCTTCTG AATGTGTAAA

ACAGTTTGTC TAGTAAGGCT GATTCTTTGG TAGGCTTCTG AACATGTAAA

ACAGCTTGTT CAATAAGGCT GCTTCTTTGG TAGGCTTCTG AATGTCTAAA

ACAGCTTGTC TAGTAAGGCT GATTCTTTGG TAGGCTTCTC AAGGTTTAAA

ACAGCTTGCT TAGTAAGGCT GATTCTTTGG TAGGCTTCTG AAGGTTTAAA

ACAGCTTGTT TAGTAAGGCT GATTCTTTGG TAGGCTTCTG AGTATCTGAA

ACAGCTTGCT GAGTAAGGCC AATTCTTTGG TAGGCATCTG AACATCTAAA

ACAGCTTGTT GAGTAAGGCT GATTCTTTGG TAGGCTTCTG AATGTCTAAC

ATAGCGTGGT GAGTAAGGCC GATTCTTTGG TAGGCGTCTG AATGTCTAAA

ACAGCTTTCG GAGTAAGGCT GAGTCTTTGG TAGGCTT--- ----------

ACAGCTTGTT TAGTAAGGCT AATTCTTTGG TAGGCTTCTG AATGTCTAAA

ACAGCTTGGT TAGTAACGCT GATTCTTTGG TAGGCTTCTG AATGTCTGAA

---------- ---------- ---------- --------TG AATGTCTGTA

ACAATTTGTT TAGCAAAGCT GAGTCTTTGG TAGATTTCTG AATTTCTGAA

ACAACTTCTT CAGCAGGGCC GGCTCTTTGG TAGAGTCCtg cttttctaaa

GTAATTTCTT CAGTGTTGGT GATTCTTCTT TAGGAGTTGC TTCTGTTAAT

GTAATTTCTT CAGTGTTGGT GATTCTTCTT TAGGAGTTGC TTCTGTTAAT

GTAATTTCTT CAGTTTTGGT GATTCTTCTT TAGGAGTTGC TTCTGTTAAT

GTAATTTCTT CAGTTTTGGT GATTCTTCTT TAGGAGTTGC TTCTGTTAAT

GTAATTTCTT CAG------- ----CTTCTT TAGGAGTTGC TTCTGTTAAT

GTTATTTCTT CAGTGTTGAT GATTCCTCGT TAGAAGTTGC TTCTGTTAAT

GTAATTTCTT CAGGGCTGGT GATTCTTCTT TAAGAGTTGC TTTTGTTAAT

GTAATTTCTT CAGTGCTGGT GATTCTTGTT TAAGAGTTGC TTTT------

GTGACTTCTT CAGTGTTGCT GACTCTTCCT TAGGAGTTGC TTCTGTTAAT

GTGATTTCTT TAGTGTTGGT GATTTTTCTT TTGGATTTGC TTCTGTTAAT

GTGTTTTCTT CAGTGTTGGT GATTCTTCTT TAGAAGTTGC TTCTGTTATT

GTGATTCCTT CAGTGTTGGT GATTCTTCTT TAGGAGTTGC TTCTGTTAAT

---------- ---------- ---------- ---------- ----------

GTGATTACTT CAGTGTTGAT GATTCTTCTT TAGAAGTTGC TTCTCTTAAT

GTCATTTCTT CAGTGTTGGT GATTCTTCTT TAGAAGTTGC TTCTGTTAAT

GTGATACCTT CAG------- ---------- ---------- ----------

GGGCTTTCTT CAGTGTTGGT GATTCTTCTT TTGAAGGCGC TTTTGTCAAT

atgatTCATT CGGACTTGCT GTTTCTTCTT CGGAAGGTGC TTCTGTTAAC

ACAGGGTCCA CAAAGGTTAA AGGTTCTGGG GTAACTAATG TGCTGGTTTC

ACAGGGTCCA CAAAGGTTAA AGGTTCTGGG GTAACTAATG TGCTGGTTTC

ACAGGGTCCA CAAAGATTAA AGGTTCTGGG GTAACTAATG TGCTGGTTTC

ACAGGGTCCA CAAAGGTTAA AGGTTCTGGG GTAACTAATG TGCTGGTTTC

CCAAGGTCCA CAAAGGTTAA TGGTTCTAGG GTAACTAACG TGCTGGTTTC

CCAGGGTCCA CGAAGGTTAA AGGCTCTAGG GTAACTAATC TGTTGGTTTC

TCAGGGTCCA CAAAGGTTAC AGGCTCTGT- GTACCTAAAA CTCTGGTTTC

-----GTCCA CAAAGGTTAC AGGTTCTGTG GTAATGAAAG CTATGTTTTC

CCAGGGTCCA TAAAATTTAA AGGCTTTAGG GAAATTAACG TGCTGGTTTC

CCAGGGTCCA CAATGTTTAA AGGCTCTAGA GAAACTAAGG TGCTGGACTC

CCAGGGTCCA CAAAGGTTAA AGGCTCTAGG GAAACTAATG TGCTGGTTTC

CCAGGGTCCA CAAAGTTTAA AGGCTCTAGG GAAACTAATG TGCTGGTTTC

---------- ---------- ---------- ---------- ----------

CCAGGGTCCA CAAGGATTAA AGGTTCTAGG GAAACTAATG TGCTCTTTTC

CCAGAGTCCA CAAAGGTTAA AGGCTCTAGG GAAACTAACG TGCTGGTTTC

---------- ---------- ---------- ---------- ----------

TCAGGCTCCA CAGAGGTTAA AGGCTCTAGG GAAACTAGGG TGCTGGTTTC

CCAGCTTCTG CAAAGGTTAA AGGCTCTGGG GAAACTAAGG TGCTGGTTTC

TGAAACCTCA TCACTGGTCA TGATTTTGTC TTTTACTCAC ATTGCCATTT

TGAAACCTCA TCACTGGTCA TGATTTTGTC TTTTACTCAC ATTGCCATTT

TGAAACCTCA TCACTGTTCA TGATTTTGTC TTTTACTCAC ATTGCCATTT

TGAAACCTCA TCACTGTTCA TGATTTTGTC TTTTACTCAC ATTGCCGTTT

TGAAACCTGA TCACTGGTCA CGATTTTGTC TatcgatcaC ATTGCCATTT

TGAAACCTCA CCACTGGTCA TGATTTTGTT TTTTACTCAC ATTGCCATTC

CCAAAGCTCA CTACTGGTCA TGCTCCTGTC TCTGACTCAC AGTGCCAC-C

CCAAAACTCA TTGCTGGTCA TGCTCCTATC TTTGTCTCAC AGTGCCAC-C

TGAAACCTCA CCACTGGCCA TGGTTCTGTC TTTTACTCAC ATTGCCGTTC

TGAAACCTCA GCACTGGTCA TGATTTCGTC TCTGACTCAC ACGGCCATTC

TGAAACCTCA CCACTGGTCA TGATTTTGTC TTTTACTCAC ATTGCCATTC

TGAAACCTCA CTACTGGTCA TGATTTTGTC TTTTACTCAC ATTGCCATTC

---------- ---------- ---------- ---------- ----------

TGAAACCTCA CCACTGGTCA AAATTTTGTC TTTTACTCAC ATTGCCATTC

TAAAACTTCA CCACTGGTCA TGATTTTGTC TTTTACTCAA GTTGCCATGC

---------- ---------- ---------- ---------- ----------

TGAACCCTCA CCACTGGTCA TGATTTTGTC TTTTACTCAC TTTGTCATTC

TGAAACCTCA CCAGTGGCCA TGCTTTTGTC TTCTACTCAC TTTGTCACTC

TTATCCGTCT TAGTCCCATT GACCTTCATA CCTTGTACTT CGGCGGATTT

TTATCCGTCT TAGTCCCATT GACCTTCATA CCTT-TACTT CGGTGGATTT

TTATCCGTCT TAGTCCCATT GACCTTCATA CCTTGTACTT CAGTGGATTT

TTATCTGTCT TAGTCCCATT GACCTTCATA CCTTGTACTT CAGTGGATTT

TTATCCATCT TAGTCCCACT GGTCTTCATA CCTTGTACTT CAGTAGATTT

TTATCCATCT TAGTGCCACT GACCTTCATA CCTTGTACTT CAATGGATTT

CAGTCCCC-- TAGTTACTCT GGCTTTCATA CCTCGTCCTT CAGTGGCTTC

CCGTCCCCCT TGGTTCCTCT GGCTTTCATA CCTTGTCCTT CTGTGAGTTC

CTGTCCGTCT CAGTCCCATT GACCTTCATA CCTTGTACTG CAGGGGGTTC

TCGTCCACCT TAGTTCCATG GACCTTCATA ACTTGGACCT CAGTAGATGT

TAATCCGTCT TAGTCCCATT GACCTTCATA CCTTGTACTT CAGTAGATTT

TTATCCGTCT TAGTCCCATT GACCTTCATA CCTTGTACTT CAGTAGATTT

---------- ---------- ---------- ---------- ----------

TTATCCGTCT TAGTCCCATT GACCTTCATA CCTTGTACTT CTGGGGATTT

TTATCCATCT TAGCCTCATT GACCTTCATA CCTTGTATTT CAATGGATTT

---------- ---------- ---------- ---------- ----------

CTAATGGTCT CAGTCCCATT GACCTTCATA CCTTGTTCTT CAGTAGGTTT

TTAATCGTCC TAGTCCCATT GACCTTGGTA CCTTGTACTT CAATAGGTTT

TAGCAGAGTT TCAGCTGTAA TCTGTACATT TCCTTTCACA CACTTAGCAT

TAG------- ---------- ---------- ----TTCACA CACTTAGCAT

TAGCAGAGTT TCAGCTGTAA TCTGTACATT TCCTTTCACA CACTTAGCAT

TAGCAGAGTT TCAGCTGTAA TCTGTACATT TCCTTTCACA CACTTAGCAT

TAGCAGAGTT TCAGCTGTAA TCTGTACATT TCCTTACACA CACTTAGCAT

TGGCAGAGTT TTGGTTATAA TTTGTACATT TCCT------ ----------

TGGCAGAGTT CCAGCTGTAT TGTGTACATT TCCTTTCACA CGCGTGGCAT

TGGCAGAGCT GCAGTTGTAT TGTGTACATT TCCTTTCACA CACGTGGCAT

TACCACGCTT TCATTTATAA TCTGCACACT TTGCTCCACA CACTGGGCAT

CAGAAGAGTT TCAGCTGTGA TTTGTACATT TCCTTTCACA CACTTAGCAT

TAGCAGAGTT CCAGTTGTAA GTTGTACATT TTCTTTCACA CACTTAGCAT

TAGGAGAGTT TCAGTTGTAA TTTGTACATT TCCT------ ----------

---------- ---------- ---------- ----TTCACG CAATTAGCAT

TAGCAGAGTT TCAGTTGTAA TCTGTACATT CCCTTTCACA CACTTAGCAT

TAGTAGAGTT TCAGTTGTAA TCTGTACATT TCCTTTCACA CACTTAGCAT

---------- ---------- ---------- ----TTCACA CACTTAGCAT

TGGCAGAGTT TCTGTTGTAA CCTGTACGTT TCCTCTCACA CGCTTGGCAT

TAATGGAGTT TCTGTTGTAA CCTGCTCGCT TCCTTCCACA CACTTCGCAT

ATGCAATTTT AATCAACCAA AAGAAAATCC CAAATGTACA AGTGAAAAAA

ATGCAATTTT AATC------ ---------- ---------- ----------

ATGCAATTTT AATCAACCAA AAGAAAATCC CAAATGTACA AGTGAAAAAA

ATGCAATTTT AATCAACCAA AAGAAAATCC CAAATGTACA AGTGAAAAAA

ATGCAATTTT AATCAACCAA AAGAAAATCC CAAATGTACA AGTGAAAAAA

---------- ---------- ---------- ---------- ----------

ATGCAACTTT AATAAACCAA GAAAAAGTCC CAAATATACA AGTGAAAAAA

ATGTAACTTT AATAAACCAA GAAAAAGTCC CAAATATTCA AGTGAAAAAA

ATGCAACAG- AATCAACCAA AAAAAAGTCC CAAATGTACA AGAAAAAAAA

ATGCAACTTT AATCAACCAA GAAAAATCCC CAACTGTACA AGTGAAAAAA

ATGCAACTTT AATCAACCAA GAAAAAGTCC CAAATGTACA AGTGAAAAAA

---------- ---------- ---------- ---------- ----------

ATGCAACTTT AATCAACCAA GAAAAAGTCC CAAATGTACA AGTGAAAAAA

ATGCAACTTT AATCAACCAA AAAAAAGTCC CAAATGTACA AGTGAAAAAA

ATGCAACTTT AATCAACCAA AAAAGAGTCC CAAATGTACA AGTGAAAAGA

ATGCAACTTT AATCAACCAA AAAAAAGTCC CAAATGTACA AGTGAAAAAA

ATGCAACTTT AATCAAACAA GGGAAAAACC CAAATGTACA TGAATGAAAG

ATGCAACTTT AATTAACAAA AAGAAAGTCC CACATGTACA ACAAAAAAAA

ATCCAAACTG TTGACACAGG CCTAACTAAT GCTACTTTAT GTACAGCTGA

---------- ---------- ---------- --------AT GTACAGCTGA

ATCCAAACTG TTGACACAGG CCTAACTAAT GCTACTTTAT GTACAGCTGA

ATCCAAACTG TTGACACAGG CCTAACTAAT GCTACTTTAT GTACAGCTGA

ATCCAAACTG TTGACACAGG CTTAACTAAT GCTACTTTAT GTACAGCTGA

---------- ---------- ---------- ---------- ----------

ATCCAAACAG TTGACACAGG CCTAACTGAT GTTACTTTAT GTACAGCTGA

ATCCAAACAG TTGACACAAG CCTAACTGAT GTTACTTTAT GTACAGCTGA

ATCCAAACTG TCGACACAGG ---------- ---------- ----------

ATCCAAAATG TTGACACAGG CTTACCTAAC GCTACTTTAT GTACAGCTGA

A----AAATG TTGACACAGG CTTAACTAGT GCTACTTTAT GTACAGCTGA

---------- ---------- ---------- ---------- ----------

ATCCAAACTC TTGACACAGA CTTAACTATT GCTACTTTAT GTACAGCTGA

ATCCAAACTG TTGACACAGG CTTAATTAAT GCTACTTTAT GTACAGCTGA

ATCCAAACTG TTGACACAGG CTTAACTAAT GCTACTTTAT GTACAGCTGA

ATCCAACTT- -TGACACAGG CTTAACTAGT GCTACTTTAT GTACAGCTGA

TTCCCGAGTG CAGACACAAC CTTAACGGAG GCTACTTTTT GTACAGCTGA

ATCCAAACTG TAGGCACATC TTTAACTATT GCTACTTTAT GTACAGCTGA

GTTCAAAAAA GCTATCCTAT ATGTATATAC AAAAGTTTAT ACACAGGTCT

GTTCAAAAAA GCTATCCTAT ATGTATATAC AAAAGTTTAT ACACAGGTCT

GTTCAAAAAA GCTATCCTAT ATGTATATAC AAAAGTTTAT ACACAGGTCT

GTTCAAAAAA GCTATCCTAT ATGTATATAC AAAAGTTTAT ACACAGGTCT

GTTCAAAAAA G-----CTAT ATGTATATAC AAAAGTTTAT ACACAGGTCT

---------- ---------- ---------- ---------- ----------

GTTCAAAAAA GCTATCCTAT TTGTATGTAC AAAAGTTTAT ACACAGTTCT

GTTCAAAAAA GCTACCCTAT TTGTATGTAC AAAAGTTTAT ACACAGGTCT

---------- ---------- ---------- ---------- ----------

TTTCAAAAAA GCTATCCTAT ATGTATGTAC AAAAGTTTAT ACACAGGTCT

GTTCAAAAAA GCTATCCTAT ATGTATATAC AAAAGTTTAT ACACAAGTCT

---------- ---------- ---------- ---------- ----------

GTTCAAAAAA GCTATCCTAT ATGTATATAC AAAAGTTTAT ACACAGGTCT

GTTCAAAAAA GCTATCCTAT ATGTATATAC AAAAGTTTAT ACACAGGTCT

GTTCAAAAA- GCTATCCTAT ATGTATATAC AAAAGTTTAT ACACAGGTCT

GTTCAAAAAA GCTATCCTAT ATGTATATAC AAAAGTTTAT ACACAAGTCT

GTTCAAAAAA GCCATCCTGT CGGTATGTAC AAAAGTGTAT ACACAAGTCT

GTTCAAAAAA GCTATTCTAT ATTTATATAC AAAAATTTAT ACACAAATCT

GTACATAAGG GTCTATACAT TTATTTCCTC AGAACCCTTA GGTGCCACCT

GTACATAAGG GTCTATACAT TTATTTCCTC AGAACCCTTA GGTGCCACCT

GTACATAAGG GTCTATACAT TTATTTCCTC AGAACCCTTA GGTGCCACCT

GTACATAAGG GTCTATACAT TTATTTCCTC AGAACCCTTA GGTGCCACCT

GTACATAAGG GTCTATACAT TTATTTCCTC AGAACCCTTA GGTGCCACCT

---------- ---------- ---------- ---------- ----------

GTACATAAGG GTCTATACAT TTATTTTCTC AGAACCCTTA GGTGTCACCT

GTACATAAGG GTCTATACAT TTATTTTTTC AGAACCCTTA GGTGTCACCT

---------- ---------- ---------- ---------- ----------

GTACATAAGG GTCTATACAT TTATTTCCTC AGAACCCTTA GGTGCCACCT

GTACAT-AGG GTCTATACAT TTATTTCCTC AGAACCCTTA GGTGCCACCT

---------- ---------- ---------- ---------- ----------

GTACATAAGG GTCTATACAT TTATTTCCTC AGAACCCTTA GGTGCCACCT

GTACATAAGG GTCTATACAT TTATTTCCTC AGAACCCTTA GGTGCCACCT

GTACATAAGG GTCTATACAT TTAATTCCTC AGAACCCTTA GGTGCCACCT

GTACATAAGG GTCTATACAT TTATTTCCTT AGAACCCTTA GGTGCCACCC

GTACATGAGG GTCTATACAT TTATTTCTGC AGAACCCTTA GGTGCCACCT

GTACACCAGG TTCTATACAC TTATTTCTGC AGAACCTTTA GCTGCTACCT

CTTGAGGACA CCAACACTTC ATTCACATAT CTTACAAAAG AAAGACATTG

CTTGAGGACA CCAACACTTC ATTCACATAT CTTACAAAAG AAAGACATTG

CTTGAGGACA CCAACACTTC ATTCACATAT CTTACAAAAG AAAGACATTG

CTTGAGGACA CCAACACTTC ATTCACATAT CTTACAAAAG AAAGACATTG

CTTGAGGACA CCAACACTTC ATTCACATAT CTTACAAAAG AAAGACATTG

---------- ---------- ---------- ---------- ----------

CTAGAAGACA CCAACACTTC ATTCACATAT TTTATAAAAG AAAGACACTG

CTAGAAGACA CCAACACTTC ATTCACATAT TTTATAAAAG AAAGACACTG

---------- ---------- ---------- ---------- ----------

CTTGAAGACA CCAACACTTC ATTCACATAT CTTAC---AA AAAGACACTG

TTTGAAGACA CCAACACTTC ATTCACATAT CTTAC--AAA AAAGACATTG

---------- ---------- ---------- ---------- ----------

TTTGAAGACA CCAACACTTC ATTCACATAT TTTACAAAAA AAAAACATTG

CTTGAAGACA CCGACACTTC ATTCACATAT CTTACAAAAG AAAGACGTTG

CT--AAGACA CCAACACTTC ATTCACATAT CTTACAAAAG AAAGACATTG

TCTGAGGGCA CCAACACTTC ATTCACATAT CTTACAAAAG AAAGACATCG

CTCAAGGTCA CCTGCACTTC ATTCACATAT CTTACAAAAC AAAAACAAAG

TTTCAAGACA TCAGCAATTC ATTCACATAT CTTACAAAAA AAAAAGATTG

ACATCACTGT CGGTATTCCA ATTCATCTAC ACTGATAACT TTGGCGGGCA

ACATCACTGT CGGTATTCCA ATTCATCTAC ACTGATAACT TTGGCGGGCA

ACATCACTGT CGGTATTCCA ATTCATCTAC ACTGATAACT TTGGCAGGCA

ACATCACTGT CGGTATTCCA ATTCATCTAC ACTGATAACT TTGGCAGGCA

ACATCACTGT CGGTATTCCA ATTCATCTAC ACTGATAACT TTGGTGGGCA

---------- ---------- ---------- ---------- ----------

ACATCACTGT CTATATTCCA GTTCATCTAC ACTGATGACT TTGGTGGGCA

ACATCACTGT CTATATTCCA GTTCATCTAC ACTGATGACT TTGGTGGGCA

---------- ---------- ---------- ---------- ----------

ACATCACTGT CGGTACTCCA GTTCATCTAC ACTGATGACT TTGGTGGGCA

ACATCATTGA CGGTACTCCA ATTCATCTAC ACTGATGACT TTGGCAGGCA

---------- ---------- ---------- ---------- ----------

ACATCACTGT CGGTATTCCA ATTCATCTAC ACTGATGACT TTGGCGGGCA

ACATCACTGT CGGTATTCCA GTTCATCTAC ACTGATGACT TTGGCGGGCA

ACATCACTGT CGGTATTCCA ACTCATCTAC GCTGATGACT TTGGCGGGCA

ACATCACGGT CGGTACTCCA GCTCGTCTAC ACTGATGACT TTGGCAGGCA

ACATCACTGC CTATACTCCA GCTCATCCAC GCTGATGACT TTGGAGGGCA

AGCTCACTGA CGATATTCCA GTTCATCTAC GCTGATAACT TTGGAGGGCA

TGGAGGGCAG GGGTTGCTGT AGCACATCTG AGCCAAACCA TTTGGCAAGG

TGGAGGGCAG GGGTTGCTGT AGCACATCTG AGCCAAACCA TTTGGCAAGG

TCGAAGGCAG GGGTTGCTGT AGCACATCTG AGCCAAACCA TTTGGCAAGG

TCGAAGGCAG GGGTTGCTGT AGCACATCTG AGCCAAACCA TTTGGCAAGG

TGGAGGGCAG GGGTTGCTGT AGCACATCTG AGCCAAACCA TTTGGCGAGT

---------- ---------- ---------- ---------- ----------

TGGAGGGCAG AGGCTGCTGT AGCACATCTG AGCCAAACCA CTTGGCAAGA

TGGAGGGCAG AGGCTGCTGT AGCACATCTG AGCCAAACCA TTTGGCAAGG

---------- ---------- ---------- ---------- ----------

TGGAGGGCAG GGGCTGCTGT AGCACATCTG AGCCAAACCA TTTGGCGAGG

TGGAGGGCAG GGGTTGCTGT AGCACATCTG AGCCAAACCA CTTAGCGAGG

---------- ---------- ---------- ---------- ----------

TGGAGGGCAG AGGCTGCTGT AACACATCCG AGCCAAACCA TTTGGCGAGG

TAGAGGGCAG GGGCTGCTGC AGCACGTCTG AACCAAACCA CTTGGCGAGG

TAGAGGGGAG GGGCTGCTGC AGCACATCTG AGCCAAACCA TTTGGCAAGG

TAGAAGGCAA GGG-TGCTGC AGCACATCTG AGCTAAAC-- ----------

TCGAGGGCAG GGGCTGCTGC AGCACGTCTG AGCCGAACCA CTTGGCCAAG

TAGAAGGGAG AGGTTGTTGC AAGACATCCG AGCCAAACCA TTTTGCAAGG

CCCACAGGGG AGCTGCTCCT CTGGCTGGGG CGATGCTCCA GCTGGGAGTG

CCCACAGGGG AGCTGCTCCT CTGGCTGGGG CGATGCTCCA GCTGGGAGTG

CCCACAGGGG AGCTGCTCCT CTGGCTGGGG CGATGCTCCA GCTGGGAGTG

CCCACAGGGG AGCTGCTCCT CTGGCTGGGG CGATGCTCCA GCTGGGAGTG

CCCACGGGGG AGCTGCTCCT CTGGCTGGGG CGATGCTCCA GCTGGGAGTG

---------- ---------- ---------- ---------- ----------

CCCACGGGGG AGCTGCTCCT TTGGCTGGTG CGATGCTCCA GCTGGGAGTG

CCCACAGGGG AGCTGCTCCT TTGGCTGGTA CGATGCTCCA GCTGGGAGTG

---------- ---------- ---------- ---------- ----------

CCCACAGGGG AGCTGCTCCT CTGGCTGGGG CGGTGCTCGA GCTGGGAGTG

CCTACTGGGG AGCTGCTCCT CTGGCTGGGG CGATGTTCCA GCTGGGAGTG

---------- ---------- ---------- ---------- ----------

CCCACTGGGG AGCTGCTCCT CTGGCTGGGG CGGTGCTCCA GCTGGGAGTG

CCCACAGGGG AGCTGCTCCT CTGGCTGGTG CGGTGCTCCA GCTGAGAGTG

CCCACCGGGG AGGTGCTCCT CTGGTTGGAG CGGTGCTCCA GCTGGGAGTG

---------- ---------- ---------- ---------- ----------

CCCACAGGGG AGGTGCTCCT CTGGCTGGGG CGGTGCTCCA GCTGTGGGGG

CCAACAGGAG AACCACTTCG CTGGCTGGAG CGATGGTCGA GCTGCGAGTG

CATGTGGGGC AGGCCTGACC GGCTGGGCAC GTTCTGAGGG GTCTGAACGC

CATGTGGGGC AGGCCTGACC GGCTGGGCAC GTTCTGAGGG GTCTGAACGC

CATGTGGGGC AGGCCCGACC GGCTGGGCAT GTTCTGAGGA GTCTGAACGC

CATGTGGGGC AGGCCCGACC GGCTGGGCAT GTTCTGAGGA GTCTGAACGC

CATGTGGGGC AGGCCTGACC GGCTGGGCAC ATTCTGAGGG GTTTGAACAC

---------- ---------- ---------- ---------- ----------

CATATGGGGC AGGCCTGACC GGCTGGGTAC ATTCGGAGGA GTCTGAACGT

CATGTGGGGC ATGCCCGACC GGCTGGGTAC ATTCTGAGGA GTCTGAACGC

---------- ---------- ---------- ---------- ----------

CACATGCGGC AGGCCTGACC GGCTGGGCAC ATTCTGAGGA GTCTGAACGC

CACGTGGGGC AGGCCTGACC TGGTGGGCAC ATTCTGAGGG GTCTGAACGC

---------- ---------- ---------- ---------- ----------

CATGTGGGGC AGGCCTGACC GACTGGGCAC ATTCTGAGGG GTCTGAATGC

CATGTGGGGC AGGCCTGACC GGCTGGGCAC ATTCTGGGGG GTCTGAACGC

CACGTGGGGC AGGCCTGACC GGCTGGGCAC ATTTTGGGGA GTCGGAATGC

---------- ---------- ---------- ---------- ----------

AATGTGGGGC AGGCCCGGCC GGCCCGACAG GTTCTGGGGG GTCTGGACAC

TACATGAGGC AGTCCAGAAC GACCAGGCGT GTTCTGGGAG GTTTGTCCAC

TGACAGCTGC TGCATGGGAA CCAGAGCCTG GAGGATGCAG AACTGTGGAC

TGACAGCTGC TGCATGGGAA CCAGAGCCTG GAGGATGCAG AACTGTGGAC

TGACAGCTGC TGCCTGGGAA CCAGAGCCTG GAGGATGCAG AACTGTGGAC

TGACAGCTGC TGCCTGGGAA CCAGAGCCTG GAGGATGCAG AACTGTGGAC

TGACAGCTGC TGCCTGGGAA CCAGAGCCTG GAGGATGCAA AACTGTGGAC

---------- ---------- ---------- ---------- ----------

TGATAGCTGC TGCCTGGGAA CTAGAGCCTG GAGGATGCAG AACTATGGAC

TGATAGCTGC TGCCTGGGAA CTAGAGCCTG GAGGATGCAG AACTATGGAC

---------- ---------- ---------- ---------- --------AC

TGACAGCAGC TGCCTGGGAA CCAGAGCCTG GAGGATGAAG AACTGTGGAC

TGACAGCTGC TGCCGGGGAA CCAGAGCCTG GAGGATGAAG AACTATGGAC

---------- ---------- ---------- ---------- ----------

TGACAGCTGC TGCCTGGGAA CCAGAGCCTG GAGGATGAAG AACTGTGGAC

CAACGGCTGC TGCCTGGGAA CCAGAACCTG GAGGATGCAG AACTTTGGGC

TGACAGCTGC TGCCTGGGAA CCGGAGCCTG GAGGATGCAG AACTGCGGGC

---------- ---------- ---------- ---------- --------GC

CAATGGCTGT TCCCTGTGAG CCAGAGCCCG GGGGATGGAG GACTGTGGAC

CACCAGTCAT TCCCTGGGAG CTGGAGCTTG GAGAATGTAA GACTGTTACC

CTACCTGAGC GCTGTAGCTG CTGTTGCACC ATTGCCAGAT GCAGAGGTGT

CTACCTGAGC GCTGTAGCTG CTGTTGCACC ATTGCCAGAT GCAGAGGTGT

CTACCTGAGC GCTGTAGCTG CTGTTGCACC ATTGCCAGAT GCAGGGGTGT

CTACCTGAGC GCTGTAGCTG CTGTTGCACC ATTGCCAGAT GCAGAGGTGT

CTACCTGAGC GCTGTAGCTG CTGTTGCATC ATTGCCAGAT GCAGAGGTGT

---------- ---------- ---------- ---------- ----------

CTACCTGAGC GCTGTAGCTG CTGCTGCATC ACTGCCAAAT GCAGAGGGGT

CTACCTGAGC GCTGTAGCTG CTGCTGCATC ACCGCCAAAT GCAGAGGGGT

CTACCTGAGC GCTGTAGCTG CTGCTGCATC ATTGCCAGAT GCAAAGGTGT

CTACCTGAGC GCTGTAGCTG CTGCTGCATC ATTGCCAGAT GCAGAGGTGT

CTACCTGAGC GCTGTAGTTG CTGCTGCATC ATTGCCAGAT GCAGAGGTGT

---------- ---------- ---------- ---------- ----------

TTACCTGAGC GCTGTAGCTG CTGTTGCATC ACTGCCAGAT GCAAAGGTGT

CTACCTGAGC GCTGTAGCTG CTGTTGCATC ATTGCCAGAT GCAGTGGTGT

CTACCTGAGC GCTGTAACTG CTGCTGCATC ATTGCCAGAT GCAAAGGTGT

TCACCTGAGC GCTGTAACTG CTGCTGCATC ATTGCCAGAT GCAGAGGTGT

CTACCTGACC GCTGCAGCTG CTGCTGCATC ACCGCGAGCT GCAGGGGGGT

TTACCTGACC GCTGGAGTTG TTGTTGCATC ATTGCCAGCT GCAGGGGTGT

TCCAGGACGA GGGTTTAAGA GAGGGTGACT AGCAGCAGGT AAAGGGTAAA

TCCAGGACGA GGGTTTAAGA GAGGGTGACT AGCAGCAGGT AAAGGGTAAA

TCCAGGACGA GGGTTTAAGA GAGGGTGACT AGCAGCAGGT AAAGGGTAAA

TCCAGGACGA GGGTTTAAGA GAGGGTGACT AGCAGCAGGT AAAGGGTAAA

TCCAGGACGA GGGTTTAAGA GAGGGTGACT AGCAGCAGGT AAAGGGTAAA

---------- ---------- ---------- ---------- ----------

CCCAGGACGA GGGTTTAGGA GAGGGTGACT AGCAGCAGAC AAAGGGTACA

CCCAGGACGA GGGTTTAGGA GAGGGTGACT AGCAGCAGAC AAAGGGTACA

CCCAGGACGA GGGTTTAGGA GAGGGTGACT AGCAGCAGGC AGAGGGTAAA

CCCAGGACGA GGGTTTAGGA GAGGGTGACT AGTGGCAGGT AGAGGGTAAA

CCCAGGGCGA GGGTTTAGGA GAGGGTGACT AGCCGTAGGT AAAGGGTAAA

---------- ---------- ---------- ---------- ----------

CCCAGGACGA GGGTTTAGGA GAGGGTGGCT AGGGGCAGGT ATAGGGTAAA

CCCTGGACGA GGGTTTAGGA GAGGGTGACT AGTGGTGGGT AAAGGGTAAA

CCCAGGACGA GGGTTTAGGA GAGGGTGACT AACAGCGGGT AAAGGGTAAA

CCCGGGACGA GGGTTTAGTA GAGGGTGACT AGCAGCGGGT AAAGGGTAAA

CCCCGAGCGA GGGGTCAAGA GAGGGTGACT GGCTGCAGGG AGGGGGTAAA

ACCAGAGCGT GGGTTCAAGA GGGGGTGGCT AGCAGTGGGT AATGGGTAAA

AGGGCTGACC CAGGATGGGG CCAGATATTC CCTGTAAATG ACTCAAGTCC

AGGGCTGACC CAGGATGGGG CCAGATATTC CCTGTAAATG ACTCAAGTCC

AGGGCTGACC CAGGATAGGG CCAGATATTC CCTGTAAATG AGTCAAGTCC

AGGGCTGACC CAGGATAGGG CCAGATATTC CCTGTAAATG AGTCAAGTCC

AGGGCTGACC CAGGATAGGG CCAGATATTC CCTGTAAATG AGTCAAGTCC

---------- ---------- ---------- ---------- ----------

AGGGTTGACC TAGGAGTGGG CCAGATAGTC CCTGTAAAGG AGCTATGTCT

AGGGTTGACC CAGGAGTGGG CCAGATAGCC CCTGTAAAGG AGCCATGTCT

AGGGCTGACC CAGAATTGGG CCAGATAATC CCTGTAAATG AGTCAGGTCC

AGGGTTGACT CAGGATTGGG CCAGACAGTC CCTGTAAATG GGTCAGGTCC

AGGGTTGACT CAGGATTGGG CCAGATATTC CCTGTAAAGG AGTCAGGTCG

---------- ---------- ---------- ---------- ----------

ATGGTTGACC CAGAATTGGG CCAGATAGTC CTTGTAAATG AGTCAGATCC

AGGGCTGACC CAGGATTGGA CCAGATATTC CCTGTAAATG AGTCAGGTCC

AAGGCTGACC CAGGATAGGG GCAGATATTC CTTGTAAATG AGTCAGGTCC

AAGGCTGACC CAGGATTGGG CCAGATATAC CCTGTAAATG CGTCAGGTCC

AGGGTTGCCC CAGGAGAGGG CCAGAGATTC CCTGCAAATG AGACAAGTCC

ATGGTTGCCC CAGAATGGGA CCAGATATTC CTTGCAAGTG AGACAAGTCC

ATCCCAGGAG GAAGCACACC TCTATCCAAG ATCCCAGATG CCACAGAACC

ATCCCAGGAG GAAGCACACC TCTATCCAAG ATCCCAGATG CCACAGAACC

ATCCCAGGAG GAAGCACACC TCTATCCAAG ATCCCAGATG CCACAGAACC

ATCCCAGGAG GAAGCACACC T--------- ---------- ----------

ATCCCAGGAG GAAGCACGCC TCTATCCAAG ATCCCAGATG CCACAGAACC

---------- ---------- -CTATCCAAG ATCCCAGAAG CCACAGAGCC

ATCCCAGGAG GAAGCACACC TCTATCCAAG ACCTCATAAG CCATAGGACC

ATCCCAGGAG GAAGCACACC TCTATCCAAG ATCTCATAAG CCATAGGATC

ATCCCAGGAG GAAGCACACC TCTATCCAAG ATCCCAGAAG CCAGAGAACC

ATCCCAGGGG GAAGCACACC TCTATCCAGG CTCCCAGGAG CCACAGGACC

ATTCCAGGAG GAAGCACACC TCTATCCAAG ATCCCAGAAG CCACAGAACC

---------- ---------- -CTAGCCAAG ATCCCAGAAG CCACAGAACT

ATCCCAGGAG GAAGCACACC TCTATCCAAG ATGCCAGAAG CTAAAGAACC

ATCCCTGGAG GAAGCACACC TCTATCCAAA ATCCCAGAAG CCACAGAGCC

ATCCCAGGAG GAAGCACACC TCTATCCAAG ATGCCAGAAG CCACAGAGCC

ATCCCTGGTG GAAGCATACC TCTATCCAAG ATCACAGACG CCATAGCACC

ATCCCAGGAG GAAGCATACC TTTCTCAAAG ACTCCTGAAG CCACAGCATC

ACCCCAGGAG GAATCATGCC TCTGTCAAAG TCTCCAGAAG CCACCGGTTC

TCCTGCTGTG GAGTCCACTA GCCCACATCG CCCAGGCTCA CTCCCATGAT

TCCTGCTGTG GAGTCCACTA GCCCACATCG CCCAGGCTCA CTCCCATGAT

TCCTGCTGTG GAGTCCACTA ACCCACATTG CCCAGGCTCA CTCCCAGGAT

---------- ---------- ---------- ---------- ----------

TCCTGCTGTG GAGTCCACTA GCCCATATCG CCCAGGCTCA CTCCCATGAT

TCCTGCTGTG GAGTCCACTA GCCCACACTG CCCAGGCTCA CCCCCATGAT

TCTTG---TA GGGTCCACTA GTCCACACCA CCCAGTCTTG CCCCCATAAT

TCTTG---TA GGATCCACTA GTCCGCACCA CCCATTCTTG CCCCGACAAT

TCCTGCTGTG GGGTCCACTA GCCCACACCT CCCAGGCGCA CCCCCACGAT

TTCTGTTGAG GGGTCCACCA GCCCACACTG CCCAGGCTCA CCCCCATGGT

TCCTGCTGTG GAGTCCACCA GCCCACACCG CCCAGGCTCA CCCCCATGAT

GCCTCCTGTG GAGTCCACCA GCCCACACCG CCCAGGCTCA CCCCCATGAT

TCGTGCTGTG GAGTCCACCA GCCCACACCG CCTAGGCTCA GCCCCATGGT

TCCTGATGTG CAGTCCACCA GCCCACACCG CCCAGGCACA CCCCCATCAT

TCCTGCTGTG GAGTCCACCA GCCCACACCA CCCAGGCTCA CCCCCATCAT

TCCTGCTGTA GAGTCCACCA TCCCACACCT CCCAGGCTCA CCCCCATCAT

TCTGGCTGGG GAGCCTGCCA GTCCACAACA TCTTGGTTTC CCCCTAACAT

TCTGGCTGTG GATTCTGCCA GCTTAGGACA CCCAGCTTTG TCCCCGTGGT

CCAAGGTTTT CTTCACAACT CTCCACATGA GTCCTCTGCA GCTCCAAGGG

CCAACGTTTT CTTCACAACT CTCCACATGA GTCCTCTGCA GCTCCAAGGG

CCAAGGTTTT CTTCACAACT CTCCACACGA GTCCTCTGCA GCTCCAAGGG

---------- ---------- ---------- ---------- ----------

CCAACGTTTT CTTCACAACT CTCCGCATGA ATCCTCTGCA GCTCCAAGGG

TCAAGGTTTT CTTCACAACA TTTTGCATGA GTCCTCTGCA GCTCCAAGGG

CCACGGTTTT CTTCACAACC TTCTGCATGA GTCCTCTGCA AGCCCTGGGG

CCAAGGTTTT CTTCTCAACC TTCTGCATGA GTCCTCTGCA ACCCCAGGGG

CCAGGCTTTT CTTCACAGCG TTCCGCGTGA GTCCTCTGCA GCTCCAAGGG

CCGAGGTCTT CTTCACAACT GTCCGCAGGA GTCCTCTGCA GCTCCGAGGG

CCAAGGTTTT CTTCACAACT TTCCGCACGA GTCCTCTGCA GCTCCAAGGG

CCGAGTTTTT CTTCGCAACT TTCCGCATGA GTCCTCTGCA GCTCCAAGGG

CCAAGGTTTT CTTCGCAACT TTCCGAACGG GTCCTCTGCA GCTCCAAGGG

CCAAGGTTTT CTTCACAACT TTCCACATGA GTCCTCTGTA GCTCCAAGGG

CCAAGGTTTT CTTCACAACT TTCCACATGA GTCCTCTGCT GCTCCAAGGG

CCAAGGTTTT CTTCAC---T TTTCACACGA GTCCTCTGCT GTTCCAGAGG

CCAGGGTTTT CTGGACAATT TTCCACATGA GTTCTCTGGG GCTCTACGGG

CCAGAGTTTT TTGAGCATTT TTCACCATGA GTCTCGGGCT GCTTTGCTGG

CAATGCCCTA AGAGGCTCCA GGTTTTACAT CTGGAATCAC ACTTGGCTCA

CAATGCCCTA AGAGGCTCCA GGTTTTACAT CTGGAATCAC ACTTGGCTCA

CAATGCCCTA AGAGGCTCCA GGTTTTACAT TTGGAATCAC ACTTGGCTCA

---------- ---------- ---------- ---------- ----------

CAATGCGCTA AGACGCTCCA GGTTTTACAT CTGGAATCAC ACTTGGCTCA

CAATGCCCTA AGAGGCTCCA GGTTTTACAT CCGGAATCAC ACTTGGCTCA

TGATGCCCTG AGAGGCTCCA GGCTTTAGAT CTGGAGTCAT ACTCGGCTCC

TGAGGCCCTG AGAGGCTCCA GGCTTTACAT CTGCAATCTT ACTTGGCTCC

CAATGCCCTA AGGGGCTCCA GGTTTCCTAT CTGGAATCAC ACTCGGCTCA

CACAGCCCTA AGAGGCTCCG GGTTTTACAT CTGGAGTTGC ACTCGGCTCG

CACTGCCCTA AGAGGCTCCA GGTTTTACAT CTGGAATCAC ACTTGGCTCA

CACTGCCCTA AGAGGCTCCA GGTTTTACAT CTGGAATCAC ACTTGGCACA

CACCGCCCTA AGAGGCTGCA GGTTTTACAT CTGGAATCAC ACTTGGCTCA

CAATGTCCTA AGAGACTCCG GGTTTTACAT CTGGTATCAC ACTTGGTTCA

CAGTGTCCTA AGAGGCTCCG GGATTTACAT CTGGAATCCC ACTTGGCTCA

TAATGTCCTA AGAGGCTCTG GGTTTTACAT GTGGAATCCC ATTTGGCTCA

CAAAGTCCGG AGAGGCTTCA GGTTTTGCAT CTAGAACAAT ACTTGGCTCA

CAATTTACTG AGGGGCTTCA GGTTTTGCAT CTGGATTAAC ACTTGGCTCA

GGGTCAGCAT GAAGCTCTCT GGAGACACTT ACTGGGAGTT CAGAGTATAA

GGGTCAGCAT GAAGCTCTCT GGAGACACTT ACTGGGAGTT CAGAGTATAA

GGATCAGCAT GAAGCTCTCT GGAGACACTT ACTGGGAGTT CAGAGTATAA

---------- ---------- ---------- ---------- ----------

GGGTCAGCAT GAAGCTCTTT GGAGACGCTT ACTGGGAGTT CAGAGTATAA

GAATCAGCAT GAAGCTCTTT GGAGACACTT ACTGGGAGTT CAGAGTATAA

TGCTCAGCAT GAAGCTCTTT GGAGACACTT ACTGAGAGTC CAGAGTACAA

TGCTCAGCAT GAAGCTCTTT GGAGACACTT ACTGAGAGTT CCGAGTACAA

GAGTCAGCAT GAGGCTCTTT GGAGACACTT ACTGGGAGTT CAGAGTATAA

GGGTCAGCAT GAAGCTCTTT GGAGGCACTC CCTGGGAGTT CAGAGTATAA

GGGTCAGCAT GAAGCTCTTT GGAGACACTC ACAGGGAGTT CAGAGTATAA

GGGTCAGCAT GAAGCTCTTT GGAGACACTT ACTGGGAGTT CAGAGTATAA

TGGTCAGCAT GAAGCTCTTT GGAGACACTT ACTGGGAGGT CAGCGTATAA

GAGTCAGCAT GAAGCTCTTT GGAGACACTT ACTGGGAGTT CAGAGAATAA

GAGTCAGCAT GAAGCTCTTT GGAGACACTC ACTGGGAGTT CAGAGTATAC

GAATCAGCAT GAAGCTCTTT GGAGACACTT ACTGGGAGTT CAGAGTCTAA

AACTCGGCAG AAAGCTCCGG GGAGACGCTC ACTGGAAGTT CCGGGTACAA

CGCTCAGCAG GCAGCTCAGG GGAGATGCTG ACAGGAAGTT CATTATACAA

TTCCTGTACC ACGGCAGACA TGGAGGAATC AAGTGGAACT GGCTGAGTCT

TTCCTGTACC ACGGCAGACA TGGAGGAATC AAGTGGAACT GGCTGAGTCT

TTCCTGTACC ACGGCAGACA TGGAGGAATC AAGTGGAACT GGCTGAGTCT

---------- ---------- ---------- ---------- ----------

TTCCTGTACC ACGGCAGACA TGGAGGAATC AAGTGGAACT GGCTGAGTCT

TTCCTGTACC ACGGCAGACA TGGAGGAATC AAGTGGAACT GGCTGAGTCT

CTCCTGTACC ACAGCAGACA TGGAGGAACC AAGTGGAACT GGCTGAGTCT

TTCCTGTACC ACAGCAGACA TGGAGGAATC AAGTGGAACT GGCTGGGTCT

TTCCTGTACC ACGGCAGACA TGGAGGAATC AAGCGGAACT GGCTGAGTCT

TTCCTGCACC TCAGCAGACA TGGAGGAATC AAGTGGAACG GGCTGGGTCT

TTCCTGTACC ACGGCAGACA TGGAGGAATC AAGTGGAACT GGCTGAGTCT

TTCCTGTACC ACGGCAGACA TGGAGGAATC AAGTGGAACT GGCTGAGTCT

TTCCTGTACC ACGGCAGACA TGGAGGAATC AAGTGGAACT GGCTGAGTCT

TTCCTGTACC ACGGCAGACA TGGAGGAATC AAGAGGAGCT GGCTGAGTCT

TTCCTCTACC ACTGCAGACA TGGAGGAATC AAGTGGAACT GGCTGAGTCT

TTCCTGTACC ACAGCAGACA TGGAGGAATC AAGTGGAACT GGCTGAATCT

CTCCTGGGCC CCTGCAGACA TGGGGGAATC AAGAGGGACT GGCTGCGTCT

TTCTTGTGCC ACGCCAGACA TGGAGGACTC AAGTGGGGCT GGCTGGGTCC

CTACTCCAGA TGACAGCATT AGGACAGATG CAGATTATGT TCACCCTGAC

CTACTCCAGA TGACAGCATT AGGACAGATG CAGATTATGT TCACCCTGAC

CTACTCCAGA TGACAGCATT AGGGCAGATG CAGATTATGT TCACCCTGAC

---------- ---------- ---------- ---------- ----------

CCACTCCCGA TGACAGCATT AGGGCAGATG CAGATTATGT TCACCCTGAC

CTATTCCGGA TGACAGCATT AGGGCAGATG TAGATTTTGT TCACCCTGAC

CTACTCCAGA TGACAGCATT AGGGCAGATG CAGATTATGT TTACCCTGAC

CTACTCCAGA TGACAGCATT AGGGCAGATG GAGATTATGT TTACCCTGAC

CTATTCCGGA TGACAGCATT GGGGCAGATG CAGATTATGT TCACCCTGAC

CCACGCCGGA TGACAGCATG GGGGCAGGTG CAGA-TATGT TCACCCTGAC

CTACTCCGGA TGACAGCATT AGGGCAGATG GAAGATATGT TCACCCTGAC

CTACTCCGGA TGACAGCATT AGGGCAGACT GTAGATATGT TCACCCTGAC

CCACTCCAGA TGACAGCATT GGGGCAGATG AAGAGTATGT TCACCCTAAC

CTACTCCCGA TGACAGCATT AGGGCAGACG TAGATTATGC TCACCCTGAC

CTACTCCGGA TGACAGCATT AGGGCAGACG TAGATTATGT TCACCCTGAC

CTACTCCGGA TGACAGCATT GGGGCAGATG CAGATTATGT TCAGCCTGAC

CTATGCCAGA TGACAGCATG AGGGCTGATG AGGACAGTAC TCACCCTAGC

CGGCTCCGGA TGTCAGCATT AGAGCAGATG GTGACCACAC TCACCCTGAA

ACAAATCACA GGAACTCACC AGTTTGGAGC AAACTTGGAA GATGCTGTGG

ACAAATCACA GGAACTCACC AGTTTGGAGC AAACTTGGAA GATGCTGTGG

ACAAATCACA GGAACTCACC AGTTTGGAGC AAACTTGGAA GATGCTGTGG

---------- ---------- ---------- ---------- ----------

ACAAATCAAA GGAACTCACC AGCTTGGAGC AAATTCGGAA GATGCTGTGG

ACAAATCAAA GG-------- ---------- ---------- ----------

ACAAATCAAA GGAACTCACC AGCTTGGAGC AAACCTGGAA GATGCTGTGG

ACAAATCAAA GGAACTCACC AGCTTGGAGC AAACTTGGAA GATGCTGTGG

ACAAATCAAA GGAACTCACC AGCTTGCAGC AGGTTTGGGA GATGCTGTGG

ACAAATCAAA GGAACTCACC AGCTTGGAGC AAACTTGGAA GATGCTGTGG

ACAAATCAAA GGAACTCACC AGCCTGGAGC AAACTTGGAA GATGCTGTGG

ACAAATCAAA GG-------- ---------- ---------- ----------

ACAAATCAAA GGAACTCACC AGCTTGGAGC AAATTTGTAA GATGCTGTGG

ACAAATCAAA GGAACTCACC AGCTTGGAGC AAACTTGGAA GATGCTGTGG

ACAAATCAAA GGAACTCACC AGCTTGGAGC AAACTTGGAA GATGCTGTGG

ACAAATCAAA GGAACTTACC AGCTTGGAGC AAACTTGGAA GATGCTGT--

ATAGACCAAA AAAACTCACC GGCTTGAAGC AGCGTTGGGA GATGCTGAGG

ACAGAGCAAA AGTACTTACC AGCCTGCAGT AAAGATGGAA GATGTTGTGG

ATGTACTCCC TGGGCCAGCA TCCTCTGTAC CAACCCTGGG TGAAGCTGGT

ATGTACTCCC TGGGCCAGCA TCCTCTGTAC CAACCCTGGG TGAAGCTGGT

ATGTACTCCC TGGGCCAGCA TCCTCTGTAC CAACCCTGGG TGAAGCTGGT

---------- ---------- ---------- ---------- ----------

ATGTACTCCC TGTGCCAGCA TTCTCTGTAC CAATCCTGGG TGAAGCTGGT

---------- ---------- ---------- ---------- ----------

ATGCACCCCT TGGGCTATCA ACCTCTGGAC TAATCCTGGG TGAAGCTGAT

ATGTACCCCT TGGGCTATCA ACCTTTGGAC TAATCCTGGG TGAAGCTGGT

ATGGACTCCT TGGGCCAGCA TCCTCTGAAC CAGCCCCGGG TGAAGCTGGT

ATGTACTCCC TGGGCCAGCA TCCTTTGGAC CAACCCTGGG TGAAGCTGGT

ATGTACTCCC TGGGCCAGCA TCCTCTGGAC CAACCCTGGG TGAAGCTGGT

---------- ---------- ---------- ---------- ----------

ATGTACTCCC TGGGCCAGCA TCCTTTGGAC CAATCCTGGG TGGAGCTGGT

ATGTACTCCC TGGGCCAGCA TCCTTTGGAC CAAGCCTGGG TGAAGCTGAT

GTGTACTCCC TGGGCAAGCA TCCTTTGGAC CAGCCCTGGG TGAAGCTGGT

---------- TGAATATGCA TCCTTTGGAC CAGGCCTGGG TGAAGCTGGT

ATGGACCCCC TGGGCCAGCA TCCTTTGGAC CAGTCCCGGG TGAAACTGAT

ATGCACTCCC TGTGCCAACA TTCTTTGGAC CAGCCCTGGG TGCAATTGGT

GAGCAGGCCT AACCATAGGG ACATGGGGGA CAAGGGGAAC TTGGTGGACA

GAGCAGGCCT AACCATAGGG ACATGGGGGA CAAGGGGAAC TTGGTGGACA

GAGCAGGCCT AACCATAGGG ACATGGGGGA CAAGGGGAAC TTGGTGGACA

---------- ---------- ---------- ---------- ----------

GAGCAGGCCG AACCATAGGG ACGTGGGGGA CAAGGGGAAC TTGGTGGACA

---------- ---------- ---------- ---------- ----------

GTGCAGGTCG AACAATAGGA ACATGAGGGA CGAGGGGAAC TTGGTGGGTA

GTGCAGGTCG AACAATAGGA ACATGAGGGA CAAGGGGAAC TTGGTGGGTA

GAGCAGGCCG GACAATAGGG ACGTGGGGAA CGAGGGGAAC TTGGTGGACG

GAGCGGGCCG AACGATGGGG ACGTGGGGGA CCAGGGGGAC TTGGTGGACA

GAGTAGGCCG AACGATTGGG ACATGGGGGA CAAGGGGAAC TTGGTGGACA

---------- ---------- ---------- ---------- ----------

GAGCAGGCCG AACAATTGGA ACATGGGGGA CAAGGGGAAC TTGATGGACA

GCGCAGGCCG AACGATTGGT ACATGGGGGA CAAGGGGAAC TTGGTGGACA

GAGCAGGCCG AACGATCGGT ACATGGGGGA CAAGGGGAAC TTGGTGGACA

GAGCAGGTCG AACGATTGGT ACATGGGGGA CCAGGGGGAC TTGGTGGACA

GAGCAGGCCG AACAATTGGA ACATGGGAGA CAAGCGGGAC CTGGTGGACA

GGGAAGGGCG AACGACTGGA ACGTGGGACA CAAGCGGCAC CTGGTGAACC

GGGCGGAGAA AAGGTGTTGT AGGAACTGGG GATGCCAAGG TGGGTGTTTT

GGGCGGAGAA AAGGTGTTGT AGGAACTGGG GATGCCAAGG TGGGTGTTTT

GGGCGGAGAA AAGGTGTTGT AGGAACTGGG GATGCCAAGG TGGGTGTTTT

---------- ---------- ---------- ---------- ----------

GGGCGGAGAA AGGGTGTTGC AGGAACTGGG GATGCCAAGG TGGGTGTTTT

---------- ---------- ---------- ---------- ----------

GGGCGGAGAA AAGGTGTTCC TGGGACTGGG GATGCCAAGG TAGGTGTTTT

GGGCGGAGAA AAGGTGTTCC TGGGACTGGG GATGCCAAGG TAGGTGTTTT

GGGCGGAGGA AAGGTGTTCC TGGAACCGGG GACGCCAAGG TGGGCGTTTT

GGGCGGAGAA AAGGTGTTCC TGGAACGGGG GATGCCAGGG TGGGTGTCTT

GGTCGGAGAA AAGGTGTTCC TGGAACTGGG GATGCCAAGG TGGGGGTTTT

---------- ---------- ---------- ---------- ----------

GGGCGGAGAA AAGGTGTTCC TGGAACTGGT GATGCCAAAG TAGGAGTTTT

GGGCGGAGAA AAGGTGTTCC TGGAACTGGG GAAGCCAAGG TGGGGGTTTT

GGGCGGAGAA AAGGTGCTCC TGGAACTGGG GAAGCCAAGG TGGGTGTTTT

GGGCGGAGAA AAGGTGCTCC TGGAACTGGG GAGGCCAAGG TGGGTGTTTT

GGACGTAGGA AAGGTGCCCC TGGCACTGGG GAGGCCAGGG TGGGAGTCTT

GGCCGGAGAA AAGGAGTCCC TGGTATTGGG GATGCCATCG TAGGAGTCTT

TCTCCCAGTT GCTTTAGGTC GATAATCTTG TTCTTTGGTG TAACGGTTGG

TCTCCCAGTT GCTTTAGGTC GATAATCTTG TTCTTTGGTG TAACGGTTGG

TCTCCCAGTT GCTTTAGGTC GATAATCTTG TTCTTTGGTG TAACGGCTGG

---------- ---------- ---------- ---------- ----------

TCTCCCAGCT GCTTTAGGTC GATAATCTTG TTCTTTGGTG TAACGGTTGG

---------- ---------- ---------- ---------- ----------

CCTCCCAGCT GCTTTAGGGC GATAATCTTG TTCTTTGGTA TAACGGTTGG

CCTTCCTGCT GTTTTAGGTC GATAATCTTG TTCTTTGGTA TAACGGCTGG

CCTCCCAGTT GCTTTAGGGC GGTAATCTTG TTCTTTGGTG TAACTGGTGG

CCTCCCCGTT GCTTTAGGTC GATAATCTTG TTCTTTGGTG TAACGGTTGG

TCTCCCAGTT GCTTTAGGTC GATAATCTTG TTCTTTGGTG TAACGGTTGG

---------- ---------- ---------- ---------- ----------

TCTCCCAGTT GGTTTAGGTC GGTAATCTTG TTCTTTGGTG TAGCGGTTGT

TCTCCCAGTT GTTTTAGGTC GATAATCTTG TTCTTTGGTG TAACGGTTGG

TCTCCCAGTT CCCTTAGGTC GATAATCTTG TTCTTTGGTG TAACGGTTGG

CCTCCCGGCT GCTTTAGGTC GATAATCTTG TTCTTTGGTG TAACGGTTGG

TCTGCCTGCT GCCTTAGGCC GATAGTCCTG TTCTTTGGTG TAGCGGCTGG

CCGACCAGTT GATTTTGGCC TGTAGTCTTG CTCTTTGGTG TAACGGTTGG

CCTGGGACAG TGGGGTGGAA CACGAAGACC TCTGTAATGC TGACAGTTTG

TCTGGGACAG TGGGGTGGAA CACGAAGACC TCTGTAATGC TGACAGTTTG

TCTGGGACAG TGGGGTGGAA CACGAAGACC TCTGTAACGC TGACAGTTTT

---------- ---------- ---------- ---------- ----------

TCTGGGACAG TGGGGTGGAA CACGAAGACC TCTGTAACGC TGATAGTTTC

---------- ---------- ---------- ---------- ----------

TCTGGGACAG TGGGGTGGAG CATGAAGACC GCTGTAATGT TGAAAGTTTA

TCTGGGAGAG TGGGGTGGAA CATGAAGACC GCTGTAATGT TGAAAGCTTA

TCTGGGACAG TGGTGTGGAA CACGAAGACC GCTGTAACGC TGACAGTTTG

TCTGGGACAG TGGGGTGGAA CAGGAAAACC GCTGCAGGGT TGACAATTTG

TTTGAGACAG TGGGGTAGAA CACGAAGACC GCTGCAACGT TGAAAGTTTG

---------- ---------- ---------- ---------- ----------

TCTGGGACAG TGGAGTGGAA CATGAAG--- GTCGCAATGT TGAAAGTTTG

TCTGGGATAG TGGGATGGAA CATGAAGACC GCTGCAACGT TGACAGTTTG

TTTGAGACAG TGGGGTAGAA CACGAAGACC GCTGCAACGT TGACAGCTTG

TTTGGGACAG AGGGGTAGAG CAGGAAGACC GCTGCAACGT TGACATCTTG

CCTGGGACAG GGGGGTGGAA GATGCAGATC GCTGCAGCGT GGAGAGTTTC

TTTGAGACAG TGGGGTGGAA CATGCCGAGC GCTGAAGTGC TGACAGTTTG

GAATTTGTAG TGGGAGAAGA GTCTCGATCG GCACTGGGTA CAGAGCTTGA

GAATTTGTAG TGGGAGAAGA GTCTCGATCG GCACTGGGTA CAGAGCTTGA

GGATTTGTAG TGGGAGAAGA GTCTCGATCG GCACTGGGTA CAGAGCTGGA

---------- ---------- ---------- ---------- ----------

GGATTTGTAG TGGGAGAAGA GCCTCGATCA GCACTGGGTA GAGAGCTTGA

---------- ---------- ---------- ---------- ----------

GGATTTGCCG TAGGAGAAGA GTCTTGATCA GAATTGGATA TGGAATTTGA

GGATTTGTCG T---AGAAGA GTCTTGATCA GTATTGGGTA TGGGATTTGA

GGATTTGTAG TGGGCGAGGA GTCCCGATCA GCACTGGGTA GAGAGTTCGA

GGATTTGTGG TGGGAGAAGA GTCCCGATCA GCAGTGGGCA CAGAGCTTGA

GGATTTGTAG TAGGAGAAGA GTCACGATCA GCGCTGGGCA CAGAGTTTGA

---------- ---------- ---------- ---------- ----------

GGATTTGTAG TAGGAGAAGA GTCTCGATCA GCACTGGACA GAGAGTTTGA

GGATTTGTAG TGGGAGAAGA GTCTCTATCA GCATTGGGCA CAGAGTTTGA

GAGTTTGTAG TGGGAGAAGA GTCTCGATCA GCAGTGGGCA CAGAGGTTGA

GGATTTGTCG TGGGAGAAGA GTCTCGATCA GCACTGGACA TGGGGTTTGA

GGGTTCATGG TAGGGGAAGT GTCTCGGTCA GAATTCGCCA TGGAACTCAA

GTACTTAGGG GGGGGATAGC ATCTTGATCC GAGTTCACTA CGGAACTCGA

CAGGAGGTTT TCTGTGACAA GATGAGAACA ACAAAGGTCT ACAAACTCGT

CAGGAGGTTT TCTGTGACAA GATGAGAACA ACAAAGGTCT ACAAACTCGT

CAGGAGGTTC TCTGTGACAA GATGAGAACA ACAAAGGTCT ACAAACTCGT

---------- -------CAA GATGAGAACA ACAAAGGTCT ACAAACTCGT

CAGGAGGTTC TCTATGA--- ---------- ---------- ----------

---------- -------CAA AATGAGAACA ACTAAGGTCT ACAAACTCGT

CAGCAGGTTT TCTGAAACAA AATGAGAACC ACAAAGGTCT ACAAACTTGT

CAGCAGGTTC TCTGTAACAA AATGAGAACC ACAAAGGTCT ACAAACTTGT

CAGGAGGTTT TCTGTAA--- ---------- ---------- ----------

CAGGAGGTTC TCTGTAACAA GATGAGAACA ACAAAGGTCT ACAAACTCGT

CAGCAGATTC TCTGTAACAA GATGAGAACA ACAAAAGTCT ACAAACTTGT

---------- -------CAA GATGAGAACA ACAAAAGTCT ACAAACTGGT

CAGGAGATTC TCTGTGACAA AATGAGAACA ACAAAGGTCT ACAAACTTGT

CAGGATGTTC TCTGTAACAA GATGAGAACA ACAAAGGTCT ACAAACTTGT

CAGGCGGTTC TCTGTATCAA GATGAGAACA ACGAAAGTCT ACAAACTCGT

CAGCAGGTTG TCTGTAA--- ---------- ---------- ----------

CAGAAGGTTC TCTGTAACAA GATGAGGACC AATAAGGTCT ACAAGCTCGT

AAGGAGGTGA TCTGTAACAA GATGAGAACC ACGAAGGTCT ACAAACTCGT

CATCCACAAG AAGGGCTTTG GGGGCAGTGG TCAGTATAGT ACCTTCACTG

CATCCACAAG AAGGGCTTTG GGGGCAGTGG TCAGTATAGT ACCTTCACTG

CATCCACAAG AAGGGCTTTG GGGGCAGTGG TCAGTATAGT ACCTTCACTG

CATCCACAAG AAGGGCTTTG GGGGCAGTGG TCAGTAT--- ----------

---------- ---------- ---------- -------AGT ACCTTCACTA

CATCCACAAG AAGGGTTTTG GGGGCAGTGG TCAGTAT--- ----------

CATCCACAAG AAGGGCTTTG GGGGCAGTGG TCAGTATAGT ACCTTCGCTG

CATCCACAAG AAGGGCTTTG GGGGCAGTGG TCAGTATAGT ACCTTCGCTG

---------- ---------- ---------- -------GGT ACCTTCACTG

CATCCACAAG AAGGGCTTCG GGGGCAGTGG TCAGTATAGT ACCTTCACTG

CATCCACAAG AAGGGCTTTG GGGGCAGTGG TCAGTATAGT ACCTTCACTA

CATCCACAAG AAGGGCTTTG GGGGCAGTGG TCAGTAT--- ----------

CATCCACAAG AAGGGCTTCG GGGGCAGTGG TCAGTATAGT ACCTTCATTG

CATCCACAAG AAGGGCTTTG GGGGCAGTGG TCAGTATAGT ACCTTCACTG

CATCCACAAG AAGGGCTTTG GGGGCAGTGG TCAGTATAGT ACCTTCACTG

---------- ---------- ---------- -------AGT ACCTTCACTG

CATACACAAG AAGGGATTTG GGGGCAGCGG TGAGCATAGT ACCTTCATTT

TATACACAAG AAGGGCTTTG GGGGTAGTGG TCAGTATTGT ACCTTCATTT

GCCTTCTGAG TATCCTCTTT ACTGTCACCA GCTGCTTTTC CAGTTGTCTT

GCCTTCTGAG TATCCTCTTT ACTGTCACCA GCTGCTTTTC CAGTTGTCTT

GCCTTCTGAG TATCCTCTTT ACTGTCACCA GCTGCTTTTC CAGTTGTCTT

---------- ---------- ---------- ---------- ---TTGTCTT

GACTTCTGAG TATCCTCTTT ACTGTCACCA GCTGCTTTTC CAG-------

---------- ---------- ---------- ---------- ---TTATCTT

GCCTTCTGAG CATCCTCTTT ACCATCACCA ACCACTGTTC CGGTTGTCTT

GTCTTCTGAG TATCCTCTTT ACCATCACCA ACCACCATTC CAGTTGTCTT

GCCTTCTGCG CATCCTCTTT ACTCTCACTC GCGGCTTTCC CGGTTGTCTT

GGCTTCTGGG TGTCCTCTTT ACCGTCACCA GCTGCTTTTC CAGTTGTCTT

GCCTTCTGAG TGTCTTCTTT ACTGTCACCA GCTGCTTTTC CAGTTGTCTT

---------- ---------- ---------- ---------- ---TT-TCTT

GTCTTCTGTG TATCTTCTTT GCAATCGCCA GCTGTTTTTC CAGTTGCCTT

GCCTTCTGAG TGTCCTCTTT GCTGTCACCA GTCGCTTTTC CAGCTGTCTT

GCCTTGTGAG TGTCCTCTTT ACTATCACTG GCTGCTTTTC CAGTTTTCTT

GCCTCCTGAG TGCCCTCTTT ATGGTCACCA GCGGCTTTCC CAGTTACCTT

GCCCTCTGGC CTTCTTCTTT CCCATCACTG GCTGGTTTTC CAGTTGTCTT

GTCCTTGAAT TTTCATCTTT CCCATCACCT GTTGGTTTTC CAGTTGTCTT

TCAGATGATG AGCTAGTTGT GAACCCCAAA GTGTTCCCTC ACATCAAGCT

TCAGATGATG AGCTAGTTGT GAACCCCAAA GTGTTCCCTC ACATCAAGCT

TCAGATGATG AGCTAGTTGT GAACCCCAAA GTGTTTCCTC ACATCAAGCT

TCAGATGATG AGCTAGTTGT GAACCCCAAA GTGTTTCCTC ACATCAAGCT

---------- ---------- ---------- ---------- ----------

TCAGATGATG AGCTAGTTGT GAACCCAAAA GTGTTTCCTC ACATCAAGCT

TCAGATGATG AGCTGGTCGT GAACCCTAAA GTCTTTCCTC ACATCAAACT

TCAGATGATG AGCTAGTCGT GAACCCTAAA GTATTTCCTC ACATCAAACT

TCAGACGATG AGCTGGTTGT GAACCCCAAA GTGTTTCCTC ACATCAAGCT

TCAGACGATG AGCTAGTTGT GAACCCCAAA GTATTTCCAC ACATCAAGCT

TCAGATGATG AGCTAGTCAT GAACCCTAAA GTGTTTCCTC ACATCAAGCT

GCAGATGATG AGCTGGTAAT GAACCCCAAA GTCTTTCCTC ACATCAAGCT

TCAGATGATG AGTTGGTTAT GAACCCCAAA GTGTTTCCTC ACATCAAGCT

TCAGATGATG AGTTAGTCGT GAACCCCAGA GTGTTTCCTC ATATCAAGCT

TCAGACGATG AGCTGGTTGT GAACCCCAAA GTGTTTCCCC ACATTAAGCT

TTAGATGATG AGCTGGTTGT AAACCCCAAA GTGTTTCCTC ACATCAAGCT

GCAGATGATG AGCTCATGGT GAACCCCAAA GTGTTCCCCC AAATTAAGCT

TCAGATGATG AGCTGATGGT GAACCCCAAA GTCTTTCCAC AGATTAAACT

TGGAGACATT GTAGAGATTG CACACCCCAA CGATGAATAC AGGTGATCCT

TGGAGACATT GTAGAGATTG CACACCCCAA CGATGAATAC AGGTGATCCT

TGGAGACATT GTAGAGATTG CACACCCCAA CGATGAATAC AGGTGATCCT

TGGAGACATT GTAGAGATTG CACACCCCAA CGATGAATAC AGGTGA----

---------- ---------- ---------- ---------- ------TCCT

TGGAGACATT GTAGAGATTG CTCACCCCAA CGATGAATTC AGGTGA----

TGGAGACATT GTGGAGATCG CTCACCCCAA TGATGAATAC AGGTGATCCT

TGGAGACATT GTGGAGATTG CTCACCCCAA TGATGAGTAC AGGTGATCCT

TGGAGACTTA TTGGAGATTG CTCACCCCAA TGACGAATAC AGGTGATCCT

TGGAGACATC GTGGAGATTG CCCACCCCAA CGATGAGTAC AGGTGATCCT

TGGAGACATT GTGGAGATTG CTCACCCCAA TGATGAATAC AGGTAATCCT

TGGAGACATT GTGGAGATTG CTCACCCCAA CGATGAGTAC AGGTTA----

TGGGGACATC GTGGAGATTG CTCACCCCAA CGATGAATAC AGGTGATCCT

TGGAGATATT GTAGAGATTG CTCACCCCAA CGATGAATAC AGGTGATCCT

CGGAGACATT GTGGAGATTG CTCACCCCAA CGATGAATAC AGGTGATCCT

GGGAGACATC GTGGAGATTG CTCACCCCAA CGATGAGTAC AGGTGATCCT

GGGGGACATT GTGGAGATCG CACACCCCAA TGACGAGTAC AGGTAATCTT

GGGAGACATT GTGGAAATTG CACACCCTAA TGATGAGTAC AGGTGATCCT

CCTTGCTTTT CTCTTTGCTC TCGTACATCT TACGAATCAC TGAGGTAGGG

CCTTGCTTTT CTCTTTGCTC TCGTACATCT TACGAATCAC TGAGGTAGGG

CCTTGCTTTT CTCTTTGCTC TCGTACATCT TACGAATCAC TGAGGTAGGG

---------- ---------- ---------- ---------- ----------

CCTTGCTCTT CTCTTTGCTC TCGTACATCT TACGAATCAC TGAGGTAGGG

---------- ---------- ---------- ---------- ----------

CCTTGCTTTT CTCTCTGCTT TCATACATCT TGCGGATCAC TGAGGTAGGG

CCTTGGTTTT CTCTCTGCTT TCATACATCT TCCGGATCAC TGAGGTAGGG

CCTTGGTTTT CTCTTTGCTC TCGTACATCT TACGGATCAC TGAGGTAGGG

CCTTGCTTTT CTCTTTGCTC TCATACATCT TACGAATCAC TGAGGTAGGA

CCTTGCTTTT TTCTTTGCTC TCATACATCT TACGAATCAC TGAGGTAGGG

---------- ---------- ---------- ---------- ----------

CTTTGCTTTT CTCTTTGCTC TCATACATCT TACGAATCAC TGAGGTAGGG

CCTTGCTTTT CTCTTTGCTC TCGTACATCT TACGAATCAC TGAGGTAGGG

CCTTGCTTTT CTCTTTGCTC TCGTACATCT TACGAATCAC TGAGGTAGGG

CCTTGCTTTT CTCTTTGCTC TCGAACATCT TTCGAATCAC CGAGGTAGGG

CTTTGTTCTT CTCTTTGCTT TCATACATCC TGCGGATCAC TGAGGTAGGA

CTTTACTTTT CTCTTTGCTT TCATACATCT TTCGTATCAC TGAGGTAGGG

GTAAAGGAAG GAGAAAGCTG CAGAGAAAAA CATACTGACC ATGCTTGTGA

GTAAAGGAAG GAGAAAGCTG CAGAGAAAAA CATACTGACC ATGCTTGTGA

GTAAAGGAAG GAGAAAGCTG CAGAGAAAAA CATACTGACC ATGCTTGTGA

---------- ---------- ---------- ---------- ----------

GTAAAGGAAG GAGAAAGCTG CAGAGAAAAA CATACTGACC ATGCTTGTGA

---------- ---------- ---------- ---------- ----------

GTGAAGGAAG GAGAAAGCTG CAAAGAAGAG AGTACTGACC ATGCTCGTGA

GTGAAGGAAG GAGAAAGCTG CAGAGAA--G AGTACTGACC ATGCTTGTGA

GTGAAGGAAG GAGAGAGCTG CAGAGAGAAA CATACTGACC ATGCTTGTGA

GTGAAGGAAG GAGAAAGCTG TAGAGGAAAA CATACTGACC ATGCTTGTGA

GTGAAGGAAG GAGAAAGCTG CAGAGAAAAA CATACTGACC ATGCTTGTGA

---------- ---------- ---------- ---------- ----------

GTGAAGGAAG GAGAAAGCTG CAGGGAAATA CATACTGACC ATGCTTGTAA

GTGAAGGAAG GAGAAAGCTG CAGAGAAAAA CGTACTGACC ATGCTTGTGA

GTGAAGGAAG GAGAAAGCTG CAGAGAAAAA CATACTGACC ATGCTTGTGA

GTGAAGGAGG GAGAAAGCTG CAGAG-AAAA CATACTGACC ATGCTTGTGA

GTGAAGGAAG GAGATAGCTA CAAACAAAAG CATACTCACC ATGCTGGTGA

GTAAAGGAAG GAGACAGCTA CAAGCAAGAA AATACTAACC ATGCTAGTGA

TGGAGGCAGC AGGGGCAGGG GAAGAGGAAT TCCCTCGATG CACGGGTGCT

TGGAGGCAGC AGGGGCAGGG GAAGAGGAAT TCCCTCGATG CACGGGTGCT

TGGAGGCAGC AGGGGCAGGG GAAGAGGAAT TCCCTCGATG CACTGGTGCT

---------- ---------- ---------- ---------- ----------

TGGAGGCAGC AGGGGCGGGG GAAGAGGAAT TCCCTCGATG CACTGGTGCT

---------- ---------- ---------- ---------- ----------

TGGAGGCCGC GGGAGCTGGG GAAGAGGAAT TTCCTCGGTG CATGGGTGCT

TGGAGGCAGC AGGAGCTGGG GAAGAGGAAT TTCCACCATG CATGGGTGCT

TGGAGGCAGA AGGCGCGGGG GAAGAGGAAT TCCCTCGGTG CACGGGTGCT

TGGAGGCGGC TGGGGCAGGG GAAGAGGAAT TCCCTCGGTG CACGGGTGCT

TGGAGGCAGC AGGGGCAGGG GAAGAGGAAT TCCCTCGGTG CACGGGTGCT

---------- ---------- ---------- ---------- ----------

TGGAGGCGGC AGGGGCAGGG GAAGAAGAAC TCCCTCGGTG CACAGGTGCT

TGGAGGCAGC AGGGGCTGGA GAAGAGGCAT TCCCTCGGTG CACAGGTGCT

TGGAGGCAGA GGGGGCAGGG GAAGAGGAAT TCCCTTGATG TACGGGTGCT

CGGAGGCCGA GGGAGCAGGG GAAGAGGAAT TCCCTTGATG CATGTTTGCT

CAGCAGCAGT GGGAGCAGGA GATGCTGAAT TCCCACGATG CACAGGGGCT

TGGATGCACT GGGAGCAGGA GAAGCTGAAT TTCCTCGATG TCCAGGTGCA

GGTGACTTGG TCACTCGCTG TTGCCTGTTT TCAGAGCACA GGCTGGTGAA

GGTGACTTGG TCACTCGCTG TTGCCTGTTT TCAGAGCACA GGCTGGTGAA

GGTGACTTGG TCACTCGCTG TTGCCTGTTT TCAGAGCACA GGCTGGTGAG

---------- ---------- --------TT TCAGAGCACA GGCTGGTGAG

GGTGACTTGG TCACTCGCTG TTGCCTGTTT TCAGAGCACA GGCTGGTGAG

---------- ---------- --------TT TCAGGGCACA AGCTGGTGAG

GGTGACTTTG TCATTCGCTG TTGCCTGTTT TCAGGGCACA GGCTGGTGAG

GGTGACTTTG ACATTCGCTG TTGCCTGTTT TCAGGGCACA GGCTGGTGAG

GGTGACTTGG TCACTCGCTG CTGCCTGT-- ---------- ----------

GGTGACTTGG TCACTCGCTG TTGCCTGTTT TTAGGGCACA GGCTGGTGAG

GGTGACTTGG TCACTCGCTG TTGCCTGT-T TCAGGGCACA GGCTGGTGAG

---------- ---------- --------TT TTAGGGCACA GGCTGGTGAG

GGTGACTTGG TCACTCGCTG TTGCCTGT-- ---------- ----------

GGCGATTTGG TCACCCGCTG CTGCCTGTTT TCAGGGCACA GGCTGGTGAA

GGCGACTTGG TCACTCGCTG CTGCCTGTTC TCAGGGCACA GGCTGGTGAG

GGCGACTTGG TCACTCGCTG TTGCCTGT-- ---------- ----------

GGCGACTTGG CCACACGCTG CTGCCTGTTT TCAGGGCCCA GGCAGGTGAG

GGAGATTTAA TCACTCTCTG TTGTCTATTT TCAGGGCACA GGCTGGTGAA

CTGTGGGTTA AGAATGAGAA GGTCATGTGT GGCTACATCA GTGAAGATAC

CTGTGGGTTA AGAATGAGAA GGTCATGTGT GGTTACATCA GTGAAGATAC

CTGTGGGTTA AGAATGAGAA GGTCATGTGT GGTTACATCA GTGAAGATAC

CTGTGGGTTA AGAATGAGAA GGTCATGTGT GGTTACATCA GTGAAGATAC

CTGTGGGTCA AGAATGAAAA GGTCATGTGT GGTTACATCA GTGAAGATAC

CTATGGGTCA AGAATGAAAA AGTCATGTGT GGTTATATTA GCGAAGATAC

CTGTGGGTCA AGAATGAGAA GGTCATGTGT GGTTACATTA GTGAAGAGAC

CTGTGGGTCA AGAATGAGAA GGTCATGTGT GGTTACATTA GTGAAGAGAC

---------- ---------- ---------- ---------- ----------

CTGTGGGTCA AGAATGAGAA GGTCATGTGT GGTTATATCA GCGAAGATAC

CTGTGGGTCA AGAATGAGAA AGTCATGTGT GGTTATATCA GTGAAGATAC

CTGTGGGTCA AGAATGAGAA GGTTATGTGT GGTTATATCA GTGAAGATAC

---------- ---------- ---------- ---------- ----------

CTATGGGTCA AGAATGAGAA GGTCATGTGT GGTTATATCA GTGAAGATAC

CTGTGGGTCA AGAATGAGAA GGTCATGTGT GGTTATATCA GTGAAGATAC

---------- ---------- ---------- ---------- ----------

CTGTGGGTGA AGAGTGAAAA AGTCATGTGT GGGTACATCA GTGAAGACAC

TTTGGGGTTA AAAGTGAAAA GGTCATGTGT GGGTACATCA GTGAGGACAC

CAGGGTAAGA TTTTTTCAGG TGGTGTTTCG TTCTACGTCG GCTATGGTTT

CAGGGTAAGA TTTTTTCAGG TGGTGTTTCG TTCTACGTCG GCTATGGTTT

CAGGGTAAGA TTTTTTCAGG TGGTGTTTCG TTCTACGTCG GCTATGGTTT

CAGGGTAAGA TTTTTTCAGG TGGTGTTTCG TTCTACGTCG GCTATGGTTT

CAGGGTAAGA TTTTTTCAGG TGGTGTTTCG TTCTACATCA GCTATGGTTT

CAGGGTAAGA TTTTTTCAGG TGGTGTTCCG TTCTACGTCG GCTATGGTTT

CAGGGTAAGA TTTTTACAGG TGGTGTTCCG TTCTACGTCG GCTATGGTTT

CAGGGTAAGA TTTTTACAGG TGGTGTTCCG TTCTACGTCG GCTATGGTTT

---------- TCTTTCTAGG TGGTGTTCCG TTCTACGTCA GCTATGGTTT

CAGGGTGAGG TTTTTGCAGG TGGTGTTCCG TTCTACGTCG GCTATGGTTT

CAGGGTGAGA TTTTTTCAGG TGGTGTTCCG TTCTACGTCG GCTATGGTTT

CAGGGTAAGA TTTTTTCAGG TGGTGTTCCG TTCTACGTCG GCTATGGTTT

---------- TTTTTTCAGG TGGTGTTTCG CTCTACGTCA GCTATGGTTT

CAGGGTAAGA TTTTTTCAGG TGGTATTCCG TTCTACGTCG GCTATGGTTT

AAGGGTAAGC TTTTTTCAGG TGGTGTTTCG TTCTACATCG GCTATGGTTT

---------- ---------- ---------- ---------- ----------

ACGGGTAAGA TTTTTTCAGG TGGTGTTCCG CTCGACTTCA GCGATGGTTT

AAGGGTAAGA TTTTTTAAGG TGGTGTTCCG CTCAACTTCA GCTATGGTCT

ACATATTTAT TCAGATGAGC TGTGAAATGT GGGATTTTGA TATTTATGGT

ACATATTTAT TCAGATGAGC TGTGAAATGT GGGATTTTGA TATTTATGGT

ACATATTTAT TCAGATGAGC TGTGAAATGT GGGATTTTGA TATTTATGGT

ACATATTTAT TCAGATGAGC TGTGAAATGT GGGATTTTGA TATTTATGGT

ACATATTTAT TCAGATGAGC TGTGAAATGT GGGATTTTGA TATTTATGGT

ACATATTTAT TCAGATGAGC TGTGAAATGT GGGATTTTGA TATTTATGGT

ACATATTTAT TCAGATGAGC TGTGAAATGT GGGATTTTGA TATTTATGGT

ACATATTTAT TCAGATGAGC TGTGAAATGT GGGATTTTGA TATTTATGGT

ACATATTTAT TCAGATGAGC TGTGAAATGT GGGATTTTGA TATTTATGGT

ACATATTTAT TCAGATGAGC TGTGAAATGT GGGATTTTGA TATTTATGGT

ACATATTTAT TCAGATGAGC TGTGAAATGT GGGATTTCGA TATTTATGGT

ACATATTTAT TCAGATGAGC TGTGAAATGT GGGATTTTGA TATTTATGGT

ACATATTTAT TCAGATGAGC TGTGAAATGT GGGACTTCGA TATTTATGGT

ACATATTTAT TCAGATGAGC TGTGAAATGT GGGATTTTGA TATTTATGGT

ACATATTTAT TCAGATGAGC TGTGAAATGT GGGATTTTGA TATTTATGGT

---------- ---------- ---------- ---------- ----------

ATATATTTAT TCAGATGAGC TGTGAAATGT GGGATTTCGA TATTTATGGT

ATATATTTAT CCAGATGAGC TGTGAAATGT GGGATTTTGA TATTTATGGT

ATTTCTTTTC AGGGGATTTG TATTTTGAGA AAGCTGTGAA TGGTTTCCTT

ATTTCTTTTC AGGGGATTTG TATTTTGAGA AAGCTGTGAA TGGTTTCCTT

ATTTCTTTTC AGGGGATTTG TATTTTGAGA AAGCTGTGAA TGGTTTCCTT

ATTTCTTTTC AGGGGATTTG TATTTTGAGA AAGCTGTGAA TGGTTTCCTT

ATTTTTTTTT AGGGGATTTG TATTTTGAGA AAGCTGTGAA TGGTTTCCTT

ATTTCTTTTC AGGGGATCTG TATTTCGAGA AAGCTGTGAA TGGTTTCCTT

ACTTCTTTTC AGGGGATCTG TACTTTGAGA AAGCTGTGAA TGGTTTCCTT

ACTTCCTTTC AGGGGATCTG TACTTTGAGA AAGCTGTGAA CGGTTTCCTC

ATTTATTTTC CGTGGAGGGG TATTTTGAGA AAGCTGTGAA TGGTTTCCTT

ATTTCTTTTC AGGGGATCTG TATTTTGAGA AAGCTGTGAA TGGTTTCCTT

ATTGCTTTTC AGGGGATCTA TATTTTGAGA AAGCTGTGAA TGGTTTCCTT

ATTTCTTTTC AGGAGATCTA TATTTTGAGA AAGCTGTGAA TGGTTTCCTT

ATTTCTTTTC AGGGGATTTG TATTTTGAGA AAGCTGTGAA TGGTTTCCTT

ATTTCTTTTC AGGGGATCTG TATTTTGAGA AAGCTGTGAA TGGTTTCCTT

ATTTCTTTAC AGGAGATTTG TATTTTGAGA AAGCTGTGAA TGGTTTCCTT

---------- ---------- ---------- ---------- ----------

ATTCCTTTTC AGGAGATTTG TATTTTGAAA AAGCTGTGAA TGGTTTCCTT

ATTTCTTTGC AGGAGACCTG TATTTTGAGA AAGCTGTGAA TGGTTTTCTT

GCTGATCTAT TTACCAAGTG GAAGGTAAGG AGAAGAACTG TAGTCATGAA

GCTGATCTAT TTACCAAGTG GAAGGTA--- ---------- ----------

GCTGATCTAT TTACCAAGTG GAAGGTA--- ---------- ----------

GCTGATCTAT TTACCAAGTG GAAGGTAAGG AGAAGAACTG TAGTCATGAG

GCTGATCTAT TTACCAAGTG GAAGGTAAGG AGAAGAACTG TAGTCATGAA

GCTGATCTAT TTACCAAATG GAAGGTAAGG AAAAGAATTG TAGTCATGAA

GCCGACCTGT TTACTAAGTG GAAGGTAAGG AGAAGAACTG TAGCCATGAA

GCCGACCTGT TTACTAAATG GAAGGTAAGG AGAAGAACTG TAGTCATGAA

GCTGATCTGT TTACCAAGTG GAAGGGAAGG AGAAGAATTG TAGTCATGAA

GCTGATCTCT TTACCAAGTG GAAGGTAAGG AGAAGAACTG CAGTCATGAA

GCTGATCTCT TTACCAAGTG GAAGGTAAGG AGAAGAACTG TAGTCATGAA

GCTGATCTCT TTACCAAGTG GAAGGTAAGG AGAAGAACTG TAGTCATGAA

GCTGACCTTT TTACCAAGTG GAAGGTAAGG AGAAGAACTG TAGTCATGAA

GCTGATCTCT TTACCAAGTG GAAGGTAAGG AGAAGAACTG TAGTCATGAA

GCTGATCTCT TTATCAAGTG GAAGGTAAGG ACAAGAACTG TAGTCATGAA

---------- ---------- -------AGG AGAAGAACTG TAGCCATGAA

GCTGACCTCT TTACCAAGTG GAAGGTAAGG AAAAGAACTG TAGCCACGAA

GCTGATCTCT TTACTAAGTG GAAGGTAAGG AAAAGAATTG TAGTCACGAA

GTGACAGTGG TCCTGTTTTC TAGAACTTTC TATGATGCAA AATCTGTTGG

---------- ---------- ---------- ---------- ----------

---------- ---------- ---------- ---------- ----------

GTGACAGTGG TCCTGTTTTC TAGAACTTTC TATGATGCAA AATCTGTTGG

GTGACAGTGG TCCTGTTTTC TAGAACTTTC TATGATGCAA AATCTGTTGG

GTAACAGTGG TCCTATTTTC CAGAACTTTC TATGATGCAA AATCTATTGG

GTGACTGTGG TTCTGTTTTC TAGAACTTTT TATGATGCAA AATCTATTGG

GTGACTGTGG TCCTGTTTTC CAGAACTTTC TATGATGCAA AATCTATTGG

GTGACGGTGG TCCTGTTTTC TAGAACTTTC TATGATGCAA AATCTACTGG

GTGACAGTGG TCCTGTTTTC TAGGACTTTT TATGATGCGA AATCTCTTGG

GTGACAGTGG TCCTATTTTC TAGAACTTTT TATGATGCAA AATCTCTTGG

GTGACAGTGG TCCTATTTTC TAGAACTTTC TATGATGCAA AATCTGTTG-

GTGACAGTGG TCCTGTTTTC TAGAACTTTC TATGATGCAA AATCTCTTG-

GTGACAGTGG TCCTGTTTTC TAGAACTTTC TATGATGCAA AATCTATTGG

GTGACAGTGG TACTATTTTC TAGAACATTC TATGATGCAA AATCTATTGG

GTGACAGTGG TCCTGTTTTC TAGAACTTTC TATGATGCGA AATCTATTGG

GTGACCGTGG TCCTGTTTTC CAGGACATTC TATGATGCCA AGTCCATTGG

GTCACAGTGG TGCTATTCTC TAGGACCTTC TATGATGCAA AATCAATAGG

TGAGTTATTT TTTCTCTATT TCAGATGAAT TTCCTGAAAT AAACCGAGCC

---------- ---------- ---------- ---------- ----------

---------- ---------- ---------- ---------- ----------

TGAGTTCTTT TTTCTCTATT TCAGATGAAT TTCCTGAGAT AAACCGAGCC

TGAGTTCTTT TTTCTCTATT TCAGATGAAT TTCCTGAAAT AAACCGAGCC

TGAGTTCTTT TTTCTCTATT TCAGATGAAT TTCCTGAAAT AAACCGAGCC

TGAGTTTTTT TTTCTTTATT TCAGATGAAT TTCCTGAAGT AAACCGAGCT

TGAGTTTTTT TTTCTTTATT TCAGATGAAT TTCCTGAAAT AAACCGAGCT

TGAGTCCTTT TTTGTCTATT TCAGATGAAT TTCCTGAAGT AAACCGAGCC

TGAGTTCTTT TTTCTCTATT TCAGATGAAT TTCCTGAAAT AAACCGAGCC

TGAGTTCTTT TTTCTTT--T TCAGATGAAT TTCCTGAAAT AAACCGAGCC

---GTTCTTT TTTCTCTATT TCAGATGAAT TTCCTGAAGT AAACCGACCC

---GTTC--- TTTATCTATT TCAGATGAAT TTCCTGAAAT AAACCGAGCC

TGAGTTCTTT TATCTCTATT CCAGATGAAT TTCCTGAAAT AAACCGAACC

TGAGTTCTTT TTTCTCTGTT TTAGATGAGT TTCCCGAAGT AAGCCGAGCC

TGAGTTCTTT TTTCTTTATT TCAGATGAGT TTCCTGAAGT AAACCGAGCC

TGAGTCCTCT TCTTTCCTCT TTAGATGAGT TTCCCGAGAC ACACCGAGCC

TGAGGCTTAT TTTCTATATT TCAGATGAGT TTCCAGAAAT ACATCGAGCT

TCAATTCGAC AGGATCACAA GGGGAGATTC TATGAAGACT TTTACAAGTA

---------- ---------- ---------- ---------- ----------

---------- ---------- ---------- ---------- ----------

TCAATTCGAC AGGATCATAA GGGGAGATTC TATGAAGACT TTTACAAGTA

TCAATTCGAC AGGATCACAA AGGGAGATTC TATGAAGACT TTTACAAGTG

TCAATTCGAC AAGATCACAA GGGACGGTTC TATGAAGACT TTTACAAGTA

TCAATTCAAC AGGATCATAA AGGGAGATTC TACGAGGACT TTTACAAGTA

TCAATTCAAG AGGATCACAA AGGGAGATTC TATGAGGACT TTTACAAGTA

TCAATTAGAC AGGACCACAA GGGGCGATTC TATGAAGACT TTTACAAGTA

TCAATTAGGC AGGATCACAA GGGGCGATTC TATGAAGACT TTTACAAGTA

TCAATTCGAC AGGATCACAA GGGACGGTAC TATGAAGACT TTTATAAGTA

TCAATTCGAC AGGATCACAA GGGACGATTC TATGAAGACT TTTATAAGTA

TCAATTCGAC AGGATCACAA GGGTCGATTC TATGAAGACT TTTATAAGTA

TCAATTCGAC AGGATCACAA GGGTCGATTC TATGAAGACT TCTACAAGTA

TCAATTCGAC AGGATCACAA AGGACGATTC TATGAAGACT TTTACAAGTA

TCAATTAGAC AGGATCACAA GGGGCGATTC TATGAAGATT TTTACAAGTA

TCAATTCGCC AGGATCATGA AGGCCGATTC TATGAAGACT TTTACAAGTA

TCAATTCGAC AGGATCATGA AGGCAGATTC TATGAAGACT TTTACAAGTA

TGTTTTTTCA GAGTGGTGGT GCAGAATGAG AGAAGAGAAG AATGGACTTC

-----TTTCA GAGTGGTGGT GCAGAATGAG AGAAGAGAAG AATGGACTTC

---------- ---------- ---------- ---------- ----------

TGTTTTTTCA GAGTGGTGGT GCAGAATGAG AGAAGAGAAG AATGGACTTC

TGTTTTTTCA GAGTAGTGGT ACAGAATGAG AGAAGAGAAG AATGGACTTC

TGTTTTTTTA GAGTGGTGGT GCAGAATGAG AGAAGAGAAG AATGGACTTC

TGTTTTTCCA GAGTGGTGGT GCAGAACGAG AGAAGGGAAG AGTGGACGTC

TGTTCTTTCA GAGTGGTGGT GCAGAATGAG AGAAGGGAAG AGTGGACTTC

TGTTATTTCA GAGTAGTGGT GCAGAATGAG AGAAGAGAAG AGTGGACCTC

TGTTTTTTCA GGGTGGTGGT GCAGAACGAG AGGAGGGAAG AGTGGACCTC

TGTTTTTTCA GAGTGGTGGT GCAGAATGAG AGGAGAGAAG AGTGGACGTC

TGTTTTTTCA GAGTGGTGGT ACAGAATGAG AGGAGAGAAG AGTGGACTTC

TGTTTTTTCA GAGTGGTGGT GCAGAACGAG AGGAGAGAAG AGTGGACGTC

TGTTTTTTCA GAGTGGTGGT GCAAAATGAG AGAAGAGAAG AGTGGACTTC

TGTTTTTTCA GAGTGGTGGT GCAGAATGAG AGAAGAGAAG AATGGACTTC

CGCTT-TTTA GAGTGGTGGT GCAGAATGAG AGACGGGAAG AGTGG-----

GGTTTTTTCA GAGTAGTGGT TCAGAACGAG AGACGAGAAG AGTGGACGTC

TGGATTTTCA GGGTGGTGGT TCAGAATGAG AGGCGAGAAG AGTGGACGTC

ACTTCTCGTA ACCATTAAAA AACTCTTCAT CCAGTATCCA GTGTTGGTGC

ACTTCTCGTA ACCATTAAAA AACTCTTCAT CCAGTATCCA GTGTTGGTGC

---------- ---------- ---------- ---------- ----------

ACTTCTCGTG ACCATTAAAA AACTCTTCAT CCAGTATCCA GTGTTGGTGC

ACTTCTTGTG ACCATTAAAA AACTCTTCAT CCAGTATCCA GTGTTGGTGC

GCTTCTTGTG ACCATTAAAA AACTCTTCAT CCAGTATCCA GTGTTGGTGC

ACTCCTTGTG ACCATTAAGA AACTCTTCAT CCAGTATCCA GTGTTGGTTC

ACTCCTCGTG ACCATTAAAA AACTCTTCAT CCAGTATCCA GTGTTGGTGC

CCTCCTTGTG ACCATTAAAA AGCTCTTCAT CCAGTATCCA GTGTTGGTGC

ACTCCTCGTG ACCATCAAAA AGCTCTTCAT CCAGTACCCA GTGCTGGTGC

ACTCCTTGTG ACCATTAAAA AACTCTTCAT CCAGTATCCA GTGTTGGTAC

ACTCCTTGTG ACCATTAAAA AACTCTTCAT CCAGTATCCA GTGTTGGTGC

ACTGCTTGTG ACCATTAAAA AACTCTTCAT CCAGTATCCA GTGTTGGTGC

ACTCCTTGTG ACCATTAAAA AACTCTTCAT CCAGTATCCA GTGTTGGTGC

ACTTCTTGTG ACCATTAAAA AACTCTTCAT CCAGTATCCA GTGTTGGTGC

---------- ---------- ---------- ---------- ----------

GCTCTTAGTG ACCATTAAGA AGCTCTTTGT CCAGTACCCA GTGCTGGTGC

ACTGCTCGTG ACTATTAAAA AACTCTTCAT CCAGTACCCG GTGCTGGTAC

GACTGGAACA GGCAGGTACC TTTTGCTTCA GAGGGCTTTC CTCAAGGAGA

GACTGGAACA GGCAGGTACC TTTTGCTTCA GAGGGCTTTC CTCAAGGAGA

---------- ---------- ---------- ---------- ----------

GACTGGAACA GGCAGGTACC TTTTGCTTCA GAGGGCTTTC CTCAAGGAGA

GGCTGGAACA GGCAGGTACC TTTTGCTTCA GAGGGCTTTC CTCAAGGAGA

GCCTGGAGCA GGCAGGTACC TTTTGTTTCA GAGGGCTTTC CTCATGGAGA

GACTGGAACA GGCAGGTACC TTTTGCTTCA GGGGGCTTTC CTCAAGGAGA

GACTGGAACA GGCAGGTACC TTTGGCTTCA GGGGGCTTTC CTCAAGGAGA

GACTGGAACA GGCAGGTACC TTTTGTTTCA GAGGGCTTTC CTCAAGGAGA

GACTGGAACA GGCAGGTACC TTCTGCTACA GAGGGCTTTC CCCAGGGAGA

GACTGGAACA GGCAGGTACC ATTTGCTTCA GAGGGCTTTC CTCAAGGCGA

GGCTGGAACA GGCAGGTACC TTTTGCTTCA GAGGGCTTTC CTCAAGGAGA

GACTGGAACA GGCAGGTACC ---------- ---------- ----------

GACTGGAACA GGCAGGTACC TTTTGCTTCA GAGGGCTTTC CTCAAGGAGA

GACTGGAACA GGCAGGTACC TTTTGCTGCA GAGGACTTTC CTCAAGGAGA

---------- ---------- ---------- ---GGCTTTC CTCAAGGAGA

GGCTGGACCG GGCAGGTACC TTTGTGTGCA GAGGGCTTTC CTCAGGGTGA

GATTGGAACA GGCAGGTACT TCATGCTCCA GAGGGCTTTC CTCGAGGTGA

TAATTCTACC TCAGCACAAG GAAACTACCT GGAGGCCATC AATCTGTCAT

TAATTCTACC TCAGCACAAG GAAACTACCT GGAGGCCATC AATCTGTCAT

---------- ---------- ---------- ---------- ----------

CAATTCTACC TCAGCACAAG GAAACTACCT GGAGGCCATC AATCTGTCAT

CAATTCTACC TCAGCACAAG GAAACTACCT GGAGGCCATC AATCTGTCAT

TAATTCTACC TCAGCACAAG GAAACTACCT AGAGGCCATC AATCTGTCAT

CAATTCTACC TCAGCACAAG GAAACTACCT AGAGGCCATC AACTTGTCAT

CAATTCTACC TCAGCACAAG GAAACTACCT AGAGGCCATC AACTTGTCAT

CAATTCTACC TCAGCCCAAG GAAACTACCT AGAGGCCATC AACCTGTCAT

CAATGCTACC TCAGCACAAG GAAACTACCT GGAGGCCATC AACCTGTCCT

CAATTCTACC TCAGCACAAG GAAACTACCT AGAGGCCATC AATTTGTCAT

CAATTCTACC TCAGCACAAG GAAACTACCT AGAGGCCATC AATCTGTCAT

---------- ---------- ---------- ---------- ----------

CAATTCTACC TCAGCACAAG GGAACTACCT AGAGGCCATC AATCTGTCAT

CAATTCTACC TCAGCACAAG GAAACTACCT AGAGGCCATC AATCTGTCAT

CAATTCTACC TCAGCACAAG GAAACTACCT AGAGGCCATC AATCTATCAT

CAATTCTACC TCGGCCCAAG GGAACTACTT GGAGGCCATC AACCTGTCAT

CAATTCTACC TCAGCTCAAG GGAACTACTT AGAGGCCATA AATCTGTCGT

TCAATGGTGA GTACCTGTTT CTGAATCCAT CTCTGGTTCT GTCCACCTGT

TCAATGGTGA GTACCTGTTT CTGAATCCAT CTCTGGTTCT GTCCACCTGT

---------- --ACCTGTTT CTGAATCCAT CTCTGGTTCT GTCCACCTGT

TCAATGGTGA GT-------- ---------- ---------- ----------

TCAATGGTGA GTACCTGTTT CTGAATCCAT CTCTGGTTCT GTCCACCTGT

TCAATGGTGA GT-------- ---------- ---------- ----------

TCAATGGTGA GTACCTGTTT CTGAGCCCAT CTCTGGTTCT GTCCACTTGA

TCAATGGTGA GTACCTGTTT CTGAGCCCAT CTCTGGTTCT GTCCACTTGT

TCAATGGTGA GTACCTGTTT CTGAATCCAT CTCTGGTTCT GTCCACCTGT

TCAACGGTGA GTACCTGTTT CTGAATCCAT CTCTGGTTCT GTCCACCTGC

TCAATGGTGA GTACCTGTTT CTGAATCCAT CTCTGGTTCT GTCCACTTGT

TCAATGGTGA GT-------- ---------- ---------- ----------

---------- --ACCTATTT CTGAACCCAT CTCGGGTTCT GTCCATCTGT

TCAATGGTGA GTACCTGTTT CTGAATCCAT CTCTGGTTCT GTCCACCTGT

TCAATGGTGA GTACCTGTTT CTGAATCCAT CTCTGGTTCT GTCCACCTGT

TCAATGGTGA GTACCTGTTT CTGTATCCAT CTCTGGTTCT GTCCACTTGT

TCAATGGTGA GTACCTGTTC CTAAAGCCAT CTCTGCTTTT GTCCATTTGG

TCAATGGTGA GTACCTGTTC TTAAAGCCAT CTCTGTTTTT GTCAATCTGG

GGTTTGCCAA AACCAGGCTG GTAGAAGTTT GCTGCCTGTA CAGCAAGGTC

GGTTTGCCAA AACCAGGCTG GTAGAAGTTT GCTGCCTGTA CAGCAAGGTC

GGTTTGCCAA AACCAGGCTG GTAGAAGTTT GCTGCCTGTA CAGCAAGGTC

---------- ---------- ---------- ---------- ----------

GGTTTGCCAA AACCAGGTTG ATAGAAGTTT GCTGCCTGTA CAGCCAGATC

---------- ---------- ---------- ---------- ----------

GGTTTGCTAA AACCAGGCTG GTAGAAGGGT GTGGTCTGTA CTGCAAGGTC

GGTTTGCTAA AACCAGGCTG GTAGAAGGGT GCTGTCTGGA CTGCAAGGTC

GGCTTGCCAA GACCAGGCTG GTAGAAGCTT GCAGCCTGCA CTGCAAGGTC

GGTTTGCCAA AACCAGGCTG GTAGAAGTTT GCTGCCTGCA GCGCGAGGTC

GGCTTGCCAA AACCAGGTTG GTAAAAGTTT GCTGCCTGCA TTGCAAGGTC

---------- ---------- ---------- ---------- ----------

GGTTTGCCAA AACCAGGCTG ATAGAAATTT GCTGCTGGCA CTGTAAGGTC

GGTTTGCCAA AACCAGGCTG GTAGAAGTTT GCTGCCTGCA TTGCAAGGTC

GGTTTGCCAA AACCAGGCTG GTAGAAGTTT GCTGCCTGCA TTGCAAGGTC

GGTTTGCCAA AACCAGGCTG GTAGAAGTTT GCTGTCTGCA TTGCAAGGTC

GGTTTGCCGA AGCCAGGTTG GTAAAAGCTT GCTGCCTGCA TGGCTACCTC

GATTTGCCAA AGCCAGGCTG GTAGAAATTA GTCGTCTGCA TTGCTAGTTC

ATGTGGCAAG GCCAGCCCTT CTAAAGCTGC CTGTTGCAAC TCCAGCTGGC

ATGTGGCAAG GCCAGCCCTT CTAAAGCTGC CTGTTGCAAC TCCAGCTGGC

ATGTGGCAAG GCCAGCCCTT CTAAAGCTGC CTGTTGCAAC TCCAGCTGGC

---------- ---------- ---------- ---------- ----------

ATGTGGCAAG GCCAACCCCT CTAAAGCTGC CTGTTGTATC TCCAGCTGGC

---------- ---------- ---------- ---------- ----------

ATGTGGCAGG GTCAGCCCTT CTAAAGCAGC TTGCTGCAGT TCTAGCTGGC

ATGTGGTAGG GCCAGCCCCT CCAAAGCAGC TTGCTGCAAT TCTAGCTGGC

GTGAGGCAGG GCCAGCCCCT CCAGGGCAGA CTGCTGCAAC TCCAGCTGGC

ATGTGGCACG GCCAGCCCCT CCAAAGCTGC CTGTTGCAAT TCCAGCTGGC

GTGTGGCAGG GCCAGCCCCT CCAAAGCTGC CTGTTGCAAT TCTAGCTGGC

---------- ---------- ---------- ---------- ----------

ATGTGGTAAG GCCAGTCCCT CCAAAGCTGC CTGCTGCAGT TCCAGCTGGC

ATGAGGCAAG GCCAACCCCT CCAAAGCGGC CTGTTGCAAC TCCAGCTGGC

ATGTGGCAAA GCCAGCCCCT CCAAAGTGGC CTGTTGCAAC TCCAGCTGGC

ATGTGGCAAG GCCAGCCCCT CCAGAGCGGC CTGTTGCAAC TCCAACTGGC

ATGAGGCAGG TTGAGTCCCT CCAAGGCAGG GTGCTGCAGC TCCAGCTGGC

ATGAGGCAAC GCGAGCCCTT CTAGTGCAGC CTGCTGCAAC TCTAACTGAC

TCATCTGCTG AATTTCAGTG TTTGATAAGC ACTACATCAA CCGCAACTTT

TCATCTGCTG AATTTCAGTG TTTGATAAGC ACTACATCAA CCGCAACTTT

TCATCTGCTG AATTTCAGTG TTTGATAAGC ATTACATCAA CCGCAACTTT

---------- --TTTCAGTG TTTGATAAGC ATTACATCAA CCGCAACTTT

TCATCTGCTG AATTTCAGTG TTTGATAAGC ACTACATCAA CCGCAATTTT

---------- --TTTCAGTG TTTGACAAGC ACTACATCAA CCGCAACTTT

TCATCTGATC AATTTTAGTG TTTGACAAGC ACTACATCAA CCGAAACTTT

TCATCTGATC AATTTTAGTG TTTGACAAGC ACTACATCAA CCGAAACTTT

TCATCTGCGG AC-------- ---------- ---------- ----------

TCATCTGCTG GATTTCAGTG TTTGACAAGC ACTACATCAA CCGCAACTTT

TCATCTGCTG AATTTGAGTG TTTGACAAGC ACTACATCAA CCGCAACTTT

---------- --GTTCAGTG TTTGACAAGC ACTACATCAA CCGCAACTTT

TCATCTGCTA AATTTCAGTG TTTGACAAGC ACTACATCAA CCGCAACTTT

TCATCTGCTA AATTTCAGTG TTCGACAAGC ACTATATCAA CCGCAACTTT

TCATCTGCTG AATTTCAGTG TTTGACAAGC ATTACATCAA CCGAAACTTT

TCATCTGATA AATTTTAGTG TTTGACAAGC ATTACATCAA CCGCAACTTT

TCATCTGCTC AATTTCAGTG TTTGACAAGC ACTATATCAA CCGTAACTTT

TCATCTGCTG AATTTTAGTG TTTGACAAGC ATTACATCAA CCGGAACTTT

GACCGAACTG GGCAGATGTC AGTGGTGATC ACGCCCGGGG TGGGTGTCTT

GACCGAACTG GACAGATGTC AGTGGTGATC ACGCCCGGGG TGGGTGTCTT

GACCGAACGG GGCAGATGTC AGTGGTGATC ACGCCCGGGG TGGGTGTCTT

GACCGAACGG GTCAGATGTC AGTGGTGATC ACGCCCGGGG TGGGTGTCTT

GACCGAACGG GGCAGATGTC AGTGGTAATC ACGCCTGGGG TGGGTGTCTT

GACCGAACTG GACAGATGTC TGTGGTGATC ACGCCTGGGG TGGGTGTCTT

GACCGAACTG GGCAGATGTC CGTGGTGATC ACGCCGGGGG TGGGTGTCTT

GACCGAACTG GGCAGATGTC TGTGGTGATC ACGCCGGGGG TGGGTGTCTT

---------- ---------- ---------- ---------- ----------

GACCGAACCG GGCAGATGTC TGTGGTGATC ACACCCGGGG TGGGCGTGTT

GACCGAACTG GGCAGATGTC TGTGGTGATC ACGCCTGGGG TGGGTGTTTT

GACCGAACTG GGCAGATGTC CGTGGTGATC ACACCTGGGG TGGGTGTGTT

GACCGGACGG GCCAGATGTC TGTGGTGATC ACCCCTGGGG TGGGTGTGTT

GACCGGACCG GACAGATGTC TGTGGTGATC ACGCCCGGAG TGGGTGTGTT

GACCGGACGG GGCAGATGTC TGTGGTGATC ACACCTGGGG TGGGTGTGTT

GACCGGACGG GGCAGATGTC TGTGGTGATT ACACCCGGAG TGGGTGTGTT

GACCGGACGG GGCAGATGTC GGTGGTCATC ACCCCCGGGG TCGGCGTGTT

GACCGGACTG GACAGATGTC TGTTGTTATC ACGCCCGGGG TAGGAGTGTT

TGAAGTGGAC CGCCTACTCA TGATCCTGAC CAAGCAGCGG ATGATAGATA

TGAAGTGGAC CGCCTACTCA TGATCCTGAC CAAGCAGCGG ATGATAGATA

TGAGGTGGAC CGCCTACTCA TGATCTTGAC CAAGCAGCGG ATGATAGATA

TGAGGTGGAC CGCCTACTCA TGATCTTGAC CAAGCAGCGG ATGATAGATA

TGAAGTGGAC CGCCTACTCA TGATCTTGAC CAAGCAGCGG ATGATAGATA

TGAAGTGGAC CGCCTACTCA TGATCCTGAC CAAGCAGAGG ATGATAGATA

TGAAGTGGAC CGCCTCCTCA TGATCTTGAC CAAGCAGCGG ATGATCGATA

TGAAGTGGAC CGCCTACTCA TGATCTTGAC CAAGCAGCGG ATGATTGATA

---------- ---------- ---------- --AACAGCGG ATGATAGATA

CGAGGTGGAC CGGCTGCTCA TGATCCTGAC CAAGCAGCGG ATGATAGATA

TGAAGTGGAC CGTCTACTTA TGATCTTGAC CAAGCAGCGG ATGATAGATA

CGAAGTAGAC CGTCTCCTCA TGATCTTGAC CAAGCAGCGG ATGATAGATA

TGAAGTGGAC CGCCTGCTCA TGATCCTGAC CAAGCAGCGG ATGATAGACA

TGAAGTGGAC CGATTACTCA TGATCTTGAC CAAGCAGCGG ATGATAGATA

TGAAGTGGAC CGCTTACTCA TGATCTTGAC CAAGCAGCGG ATGATAGATA

TGAAGTAGAT CGCTTACTCA TGATCTTGAC CAAGCAGCGG ATGATAGATA

TGAGGTCGAC CGGCTGCTCA TGATCCTGAC CAAGCAGCGG ATGATCGACA

TGAAGTTGAC CGGTTACTCA TGATTCTGAC CAAACAGCGC ATGATAGACA

ATGGTAATTT TTAGGAATTG GTGTGGATTT GGTGTGCATG GGAGAGCAAC

ATGGTAATTT TTAGGAATTG GTGTGGACTT GGTGTGCATG GGAGAGCAAC

ATGGTAATTT TTAGGAATTG GTGTGGACTT GGTGTGTATG GGAGAGCAAC

ATGGTAATTT TTAGGAATTG GTGTGGACTT GGTGTGTATG GGAGAGCAAC

ATGGTAATTT TTAGGAATTG GCGTCGACTT GGTGTGCATG GGAGAGCAAC

ATGGTACTTT TTAGGAATTG GTGTGGACTT GGTATGCATG GGAGAGCAAC

ATGGTAATTT T-AGGAATTG GTGTGGACTT AGTGTGCATG GGAGAGCAGC

ATGGTAATTT T-AGGAATTG GTGTGGACTT AGTGTGCATG GGAGAGCAGC

ACGGTAATTT TTAGGAATTG GCGTGGACTT GGTGTGCATG GGAGAGCAAC

ACGGTAATCC TTAGGAATCG GTGTGGACCT GGTGTGCATG GGGGAGCAGC

ATGGTAATTT TTAGGAATTG GTGTGGACTT GGTGTGCATG GGGGAGCAAC

ACGGTAATTT TTAGGAATTG GTGTCGACTT GGTGTGCATG GGGGAGCAGC

ATGGTAA--- ---------- ---------- ---------- ----------

ATGGTAATTT CTAGGAATTG GAGTAGACTT GGTGTGCATG GGGGAGCAAC

ATGGTAATTT T--GGAATTG GTGTGGACTT GGTGTGCATG GGGGAGCAAC

ACGGTAATTT TTAGGAATTG GTGTGGACTT AGTATGTATG GGAGAGCAAC

ACGGTAACTC TTAGGGATCG GCGTGGACTT GGTGTGCATG GGGGAGCAGC

ACGGTAACCT TCAGGAATTG GTGTGGATTT GGTGTGCATG GGGGAGCAAC

CGATTCTCGT CTGGGCGATG ACTATAATAT CCCTCACTGG ATAAACCACA

CGATTCTCGT CTGGGCGATG ACTATAATAT CCCTCACTGG ATAAACCACA

CGATTCTCGT CTGGGCGATG ACTATAATAT CCCTCACTGG ATAAACCACA

CGATTCTCGT CTGGGCGATG ACTATAATAT CCCTCACTGG ATAAACCACA

CGATTCTCGT CTGGGCGATG ACTATAATAT CCCTCACTGG ATAAACCACA

CGATTCTCGT CTGGGTGATG ACTATAATAT TCCTCACTGG ATAAACCACA

CGATTCTCGG CTGGGCGATG ACTATAATAT TCCTCACTGG ATAAACCACA

CGATTCTCGA CTGGGTGATG ATTATAATAT TCCTCACTGG ATAAACCATA

CGATTCTCGT CTGGGCGATG ACTATAATAT TCCTCACTGG ATAAACCACA

CGACTCCCGT CTGGGGGATG ACTACAACAT CCCTCACTGG ATAAACCACA

CGATTCTCGT CTGGGCGATG ACTATAATAT TCCTCACTGG ATAAACCACA

CGATTCTTGT CTGGGTGACG ACTATAATAT TCCTCACTGG ATAAACCACA

---------- ---------- ---------- ---------- ----------

CGACTCTCGT CCAGGTGATG ACTACAATAT TCCTCACTGG ATAAACCACA

CGATTCTCGT CTGGGTGATG ACTATAATAT TCCTCACTGG ATAAACCACA

CGACTCTCGT CTGGGTGATG ACTATAATAT TCCTCACTGG ATAAACCACA

CGATTCCCGC CTCGGGGACG ACTACAACAT CCCTCACTGG ATAAACCACA

CGATTCCCGC TTAGGAGATG ACTATAATAT TCCTCACTGG ATAAATCATA

GGTTTTTTCA GCCCGCCTCT GAGAAAGCAA AAAATGGCCG TGATACATGT

GGTTTTTTCA GCCCGCCTCT GAGAAAGCAA AAAATGGCCG TGATACGTGT

GGTTTTTTCA GCCTGCCTCT GAGAAAGCAA AAAATGGCCG TGATACATGT

GGTTTTTTCA GCCTGCCTCT GAGAAAGCAA AAAATGGCCG TGATACATGT

GGTTTTTTCA GCCTGCCTCT GAGAGAGCGA AAAATGGCCG TGATACATGT

GGTTTTTACA GCCTGCCTCT GAGAAAGCAA AAAATGGCCG TGATACATGT

GGTCTTTTCA GTCTGCCACT GAGAAAACCA AAAATGGCCG TGATACATGT

GGTTTTTTCA GTCTGCCTCT GAGAAAACCA AAAATGGTCG TGATACATGT

GGT------- ---------- ---------- ---------- ----------

GGTCTTTACA GCCCACCTGT GAGAAAGCAA AAAATGGCCG TGATACATGT

GGTTTTTACA GCCTGCCTCT GAGAAAGCAA AAAATGGCCG TGATACATGT

GGTTTTTACA GCCTGCCTCT GAGAAAGCAA AAAATGGCCG TGATACGTGT

---TTTTACA GCCTGCCTCT GAGAAAGCAA AAAATGGCCG GGATACATGT

GGTTTTTACA GCCTGCCTCT GAGAAAGCAA AAAATGGCCG TGATACATGT

GGTTTTTACA GCCTACCTCT GAGAAAACAA AAAATGGCCG TGATACGTGT

GGTTTTTACA GTGTGCTTCT GAGAAAGCAA AGAATGGCCG TGATACTTGT

GGTTTTTACA GCCTGCAACC GAAAAGGCAA AAAACGGCCG GGATACCTGT

GGTTTTTTCA GCCAGCTGCA GAGAAAGCAA AAAACGGCCG GGATACCTGT

GAGTATTTTG AGAAAGCTCA GCCTGACCTG GGCTGTGATG GGGCTCATGG

GAGTATTTTG AGAAAGCTCA GCCTGACCTG GGCCGTGATG GGGCTCATGG

GAGTATTTTG AAAGAGCTCA GCCTGACCTG GGCCGTGATG GGGCTCATGG

GAGTATTTT- ---------- ---------- ---------- ----------

AAGTATTTTG AAAGAGCTCA GCCTGACCTG GGCCGTGATG GGGCTCATGG

GAGTACTTT- ---------- ---------- ---------- ----------

GAGTATTTTG AAAGAGCTCA GC-TGACCTG GGCTGTGACA GGACTCATGG

GAGTATTTTG AAAGAGCTCA GCCTGACCTG GGCTGAGACA GGACTCATGG

---------G GGAAAGCACA GGCTGACCTG GGCCGAGACC GGGCTCGTGG

GAGTATTTTG AAAAA-CTCA GCCAGACCTG GGCCGTGACT GGGCTCATGG

GAGTATTTTG AAAAAGTTCA GACAGACCTG GGCCGTGACC GGGCTCATGG

GAGTATTTT- ---------- ---------- ---------- ----------

GAGTATTTTG AAAAAGTTCA GCCAGACCTG GGCTGTAACA GGGCTCATGG

GAGTATTTTG AAAGAGTTTG GCCTGACCTG GGCTGCAACA GGGCTCATAG

GAGTATTTTG AAAGAGCTCA GCCTGACCTG GGCTGTGACA GGACTCACGG

GAGTATTTTG AAGGAGCATG GTCTGACCTG GACTGTGATG GGGCTCATGG

AAGTATTCTC AAAGACCTCA ATCTGACCTG GGCAGCAACA GGACTCATCG

AAGTATTCTG AAAGAGTTTA GCCTAACCTG TGCAGCAACA GGGCTCATTG

GTTTGCGCAT GCCTTGGAAT GGATCTCCGA GTAGCTGCTG TGGTCCTGGT

GTTTGCGCAT GCCTTGGAAT GGATCTCCGA GTAGCTGCTG TGGTCCTGGT

GTTTGCGCAT GCCTTGGAAT GGATCTCCGA GTAGCTGCTG TGGTCCTGGT

---------- ---------- ---------- ---------- ----------

GTTTGCGCAT GCCTTGGAAT GGATCTCCGA GTAGCTGCTG TGGTCCTGTT

---------- ---------- ---------- ---------- ----------

GCTTGCGCAT GCCTTGGAAT GGATCTCCAA GTAGTTGCTG GGGTCCTGAT

GCTTGCGCAT GCCTTGGAAT GGATCTCCAA GTAGTTGCTG GGGTCCTGAA

GCTTGCGTGT GCCTTGGAAT GGGTCTCCAA GTAGCGGCTG TGGTCCCGGC

GCTTGCGCAT GCCTTGGAAT GGATCTCCGA GAAGCTGCTG TGGTCCTGAC

GCTTGCGCAT GCCTTGGAAT GGATCTCCAA GTAGCTGCTG TGGTCCTGGT

---------- ---------- ---------- ---------- ----------

ACTTGCGCAT GCCTTGAAAT GGATCTCCAA GTAGTTGCTG TGGTCCTGCT

GTTTGCGCAT GCCTTGGAAT GGATCTCCGA GCAGCTGCTG TGGCCCTGGG

GCTTGCGCGT GCCTTGGAAC GGGTCTCCGA GCAGCTGCTG TGGCCCTGCT

GCTTGCGCAT GCCTTGGAAC GGGTCCCCAA GAAGCTGCGG TGGTCCTGGT

ATTTGCGCAT TCCATGAAAT GGATCTCCAA GCAACTGCTG AGAACTGGGT

GTTTCCGCAA TCCTTGAAAT GGATCCCCAA GCAACTGTTG AGAGCTTGGT

GTGAAACCTA TATTATTTGT GTATTTCAGC TCTCGGGAGT CCAAAAGAAT

GTGAAACCTa tattatttgt gtatttcagc tctcGGGAGT CCAAAAGAAT

GTGAAACCTA TGTTATTTGT GTATTTCAGC TCTCGGGAGT CCAAAAGAAT

---------A TGTTATTTGT GTATTTCAGC TCTCGGGAGT CCAAAAGAAT

GTGAAACCTA TATTATTTGT GTATTTCAGC TCTCGGAAGT CCAAAAGAAT

---------- --TTATTTGT GTATTTCAGC TCTCGGGTCT CCAAAAGAAT

GGGAAACCTG TATTATTTGT GTTTTGCAGC TCTTGGGGCT CCAAAGGAAT

GGGAAACCTG TATTATTTGT GTTTTGCAGC TCTCGGGACT CCAAAGGAAT

GTGAAAGCTA TGTTCCCTGT GTGTTTCAGC TCTCGGAACT CCAAAAGAAT

GTGAAACCTA TATTTTTTAT GTGTTTCAGC TCTCGGGACC CCCAAAGAAT

GTGAAACCTA TGTGATTTAT GCATTTCAGC TCTCGGGACT CCAAAAGAAT

---------G TA---TTTAT GTATTTCAGC TCTTGGGACG CCGAAAGAAT

GTGAAACCTG CA---TTTGT GTTTTTCAGC TCTCGGAACT CCGAAAGAGT

GTGAAACCTG TATTACTTGT GTGTTTCAGC CCTCGGGACT CCCAAAGATT

GTAAAACCTG CATTACTTGT GTGTTTCAGC TCTGGGGACT CCCAAAGAAG

GGGAAACCTG CATTATT--- GTATTTCAGC TCTGGGGACT CCCAAAGAGG

CCAAAACCTT CCTTATTTGT GCATTGCAGC CATCGGGACC CCAAAGGAAT

CCAAAGCCTA TATTTCTCAT GTGTTTCAGC TCTTGGGACT CCAAAGGAAT

CTGAGAACGC CCTTCCCATC CAAGTAGATT ATGACGCCTA TGACGCTCAA

CTGAGAACGC CCTTCCCATC CAAGTAGATT ATGACGCCTA TGACGCTCAA

CTGAGAACGC CCTTCCCATC CAAGTAGATT ATGATGCCTA TGACGCTCAA

CTGAGAACGC CCTTCCCATC CAAGTAGATT ATGATGCCTA TGACGCTCAA

CTGAGAACGC CCTTCCCATC CAAGTAGATT ATGACGCCTA TGACGCTCAA

CTGAGAACGC CCTTCCCATC CAAGTAGATT ATGATGCCTA TGATGCTCAA

CTGAAAACAC CCTTCCCATC CAAGTAGATT ATGATGCCTA CGATGCCCAA

CTGAAAACAC CCTTCCCATC CAAGTAGATT ATGATGCCTA CGATGCTCAA

CTGAGAACGC CCTTCCCATC CAAGTGGATT ACGATGCCTA CGATGCGCAA

CTGAGAATGC CCTTCCCATC CAGGTGGATT ACGATGCTTA CGATGCACAA

CTGAGAACGC CCTTCCCATC CAAGTAGATT ATGATGCCTA TGATGCCCAA

CTGAGAACGC CCTTCCCATC CAAGTAGATT ATGATGCCTA CGACGCTCAA

CTGAGAATGC CCTTCCCATC CAAGTGGACT ACGATGCCTA TGATGCACAA

CTGAGAATGC CCTTCCCATC CAAGTAGATT ATGATGCCTA CGACGCTCAA

CTGAGAACAC CCTTCCCATC CAAGTAGATT ATGATGCCTA CGATGCTCAA

CTGAGAACGC GCTTCCCATC CAAGTAGATT ATGATGCCTA CGACGCCCGA

CAGAGAATGC CCTGCCCATC CAGGTGGACT ATGATGCATA CGATGCCCAA

CTGAAAATGC TCTTCCCATC CAAGTGGATT ATGACGGCTA CGATGCCCAA

GTGTTCAGGC TGCCCGGCCC ATCCCGGGCC CAGTGCCTCA CCACCTGCAG

GTGTTCAGGC TGCCCGGCCC ATCCCGGGCC CAGTGCCTCG CCACCTGCAG

GTGTTCAGGC TGCCCGGCCC ATCCCGGGCC CAGTGCCTCG CCACCTGCAG

GTGTTCAGGC TGCCCGGCCC ATCCCGGGCC CAGTGCCTCG CCACCTGCAG

GTGTTCAGGC TGCCCGGCCC ATCCCGGGCT CAGTGCCTCG CCACCTGCAG

GTGTTCAGGC TGCCCGGCCC ATCCCGGGCT CAGCGCCTCG CCACCTGCAG

GTATTCCGGC TGCCTGGTCC ATCCCGGGCC CAGCGACTCG CCACCTGCAG

GTGTTCAGGC TGCCTGGCCC ATCCCGGGCT CAGCGCCTCG CCACCTGCAG

GTGTTCAGGC TGCCTGGCCC GTCCCGGGCT CAGCGCCTCG CCACCTGCAG

GTCTTCAGGC TCCCTGGCCC CTCCCGGGCT CAGCGCCTCA GCACCTGCAG

GTGTTCAGGC TGCCAGGCCC ATCCCGGGCT CAGCGCCTCG CCACCTGCAG

GTGTTCAGGC TGCCAGGCCC ATCCCGGGCT CAGCGCCTCG CCACCTGCAG

GTGTTCAGGC TGCCTGGTCC ATCTCGGGCT CAGCGCCTCG CCACCTGCAG

GTGTTCAGGC TTCCAGGCCC ATCCCGAGCT CAGCGCCTTG CCACCTGCAG

GTGTTTAGGC TGCCAGGCCC ATCCCGAGCA CAGCGCCTCG CCACCTGCAG

GTGTTCAGGC TGCCAGGCCC AGCCCGGGCA CAGCGCCTCG CCACTTGCAG

GTGTTCAGGC TGCCTGGCCC GGCCCGTGCC CAGCGCCTCA CCACCTTCAG

GTGTTCAGAC TGCCTGGCCC ATCACGAGCC CAGCGCCTCG TCACCTTCAG

GTTCGAAAGA GTGCCAGCTC CTGTGATGTT TCATCCAGCC CTTCCCTACC

GTTCGAAAGA GTGCCAGCTC CTGTGATGTT TCATCCAGCC CTTCCCTACC

GTTCGAAAGA GTGCCAGCTC CTGTGATGTT TCATCCAGCC CTTCCCTACC

GTT------- ---------- ---------- ---------- ----------

GTTCGAAAGA GTGCCAGCTC CTGTGATGTT TCATCCAGCC CTTCCCTACC

GTTCGAAAGA GCGCCAGCTC CTGCGATGTC TCATCCAGCC CTTCCCTGCC

GTCCGCAAAA GTGCCAGCTC CTGTGACGTC TCCTCCAGCC CTTCCTTGCC

GTTCGCAAAA GTGCAAGCTC CTGTGATGTC TCATCCAGCC CTTCCCTGCC

GTTGGAAAGA GTGCCAGCTC CTGTGACGTC TCATGCAGCC CTTCCCTGCC

GTTCGGAAGA GCGCCAGCTC CTGCGACGTT TCATCCAGCC CTTCGCTGCC

GTTCGAAAGA GTGCCAGCTC CTGTGATGTC TCATCCAGCC CTTCCCTGCC

GTTCGAAAGA GTGCCAGCTC CTGTGACGTC TCATCCAGCC CTTCCATGCC

GTTCGAAAGA GCAGCAGCTT TTGTGACGTC TCCTCCAGCC CTTCCCTGCC

GTTCGAAAGA GCGCCAGCTC CTGCGATGTC TCATCCAGCC CTTCGCTGCC

GTTCGAAAGA GCGCCAGCTC CTGTGATGTC TCCTCCAGCC CTTCCCTGCC

GTTCGAAAGA GTGGCAGCTT CTGCGATGTC TCCTCCAGCC CCTCCATGCT

GTCCGGAAGA GCTCCAGCAG CTTCGACGTG TCATCCAGCC CCTCGGTGCA

GTCCGGAAGA GCTCCAGCTC CTACGACGTC TCGTCCAGCC CTTCGCTGCA

AAGCCGGAGG AAGTGAGGAG CCAGGCTTCT GACGACAGCT CCCTAGGCAA

AAGCCGGAGG AAGTGAGGAG CCAGGCTTCT GACGACAGCT CCCTAGGCAA

AAGCCGGAGG AAGTGAGGAG CCAGGCTTCT GATGACAGCT CCCTGGGCAA

---------- ---------- ---------- ---------- ----------

AAGCCGGAGG AAGTGAGGAG CCAGGCTTCT GACGACAGCT CCTTGGGCAA

CAGCCGGAGG AAGTGAGAAG CCAGGCTTCT GATGACAGCT CCCTGGGCAA

AAGCCGGAGG AAGTGAGGAG CCAGGCTTCA GACGACAGCT CCCTGGGCAA

AAGCCGGAGG AAGTGAGGAG CCAGGCTTCT GATGACAGCT CCCTGGGCAA

TAGCCGGAGG AAGTGAGGAG CCAGGCTTCC GACGACAGCT CCCTGGGCAA

CGGCCGGAGG AGGTGAGGAG CCAGGCGTCT GACGACAGCT CGCTGGGCAG

CAGCCGGAAG AAGTGAGGAG CCAGGCTTCT GACGACAGCT CCCTGGGCAG

CAACCGGAGG AAGTGAGGAG CCAGGCTTCT GATGACAGCT CCCTGGGCAG

CAGCCGGAGG AAGTGAGGAG CCAGGCTTCT GACGACAGTT CCCTGGGCAG

AAACCGGAGG AAGTGAGGAG CCAGGCTTCT GATGACAGCT CCCTGGGCAG

AAGCCGGAGG AAGTGAGGAG CCAGGCTTCT GATGATAGCT CCCTGGGCAG

GAGCCGGAGG AAGTGCGCAG CCAGACCTCT GACGACAGCT CTCTGGGCAG

GAGCCGGAGG AGGGGAGGAG CCAGGCCTCA GACGACAGCT CCCTGGGCAA

GAGCCGGAGG AGGTGAGGAG CCAGGCCTCC GACGACAGCT CCTTGGGAAA

GAGTGCCAAC ATCCTGATGA TCCCACACCC CCACCTGCAC CAGTATGAAG

GAGTGCCAAC ATCCTGATGA TCCCACACCC CCACCTGCAC CAGTATGAAG

GAGCGCCAAC ATCCTGATGA TCCCACACCC CCACCTGCAC CAGTATGAAG

---------- ---------- ---------- ---------- ----------

GAGTGCCAAC ATCCTGATGA TCCCACACCC CCACCTGCAC CAGTACGAAG

GAGTACCAAC ATCCTGATGA TCCCACACCC CCACTTGCAC CAGTATGAAG

GAGCACCAAC ATCCTGATGA TCCCTAACCC CCATCTGCAC CAGTATGAAG

GAGCACCAAC ATCCTGATGA TCCCTAACCC CCACCTGCAC CAGTATGAAG

GAGTGCCAAC ATCCTGATGA TCCCACACCC CCACCTGCAC CAGTATGAAG

GAGCGCCAAC ATCCTGGTGA TCCCTCACCC CCACCTGCAC CAGTACGAAG

GAGTGCCAAT ATCCTGATGA TCCCACACCC CCACCTGCAC CAGTATGAAG

GAGTGCCAAC ATCCTGATGA TCCCACACCC CCACCTGCAC CAGTATGAAG

GAGCGCCAAC ATCCTGATGA TCCCACACCC GCACCTGCAC CAGTATGAAG

GAGCGCCAAC ATCCTGATGA TCCCACACCC CCACCTGCAC CAGTATGAAG

GAGTGCCAAC ATCCTGATGA TCCCACACCC CCACCTGCAC CCGTATGAAG

GAGCGCCAAT ATCCTGATGA TCCCACACTC CCATCTGCAC CAGTATGAAG

GGGCTCCCAC ATCCTGATGA TCCCCCACTC CCACCTGCAC CAGTATGACG

GAGCTCCAAC ATTCTGATGA TTCCCCACTC TCACCTCCAC CAGTACGAAG

TCAGCAGCTC CTTGGGATAC ACCAGCACTC GAGGTAAGCA GATGTCCTGG

TCAGCAGCTC CTTGGGATAC ACCAGCACTC GAGGTAAGCA GATGTCCTGG

TCAGCAGCTC CTTGGGATAC ACCAGCACTC GAGGTAAGCA GATGTCCTGG

---------- ---------- ---------- --------CA GATGTCCTGG

TCAGCAGCTC CTTGGGCTAC ACCAGCACTC GAGGTCAGCA GATATCCTGG

TCAGCAGCTC CCTGGGCTAC ACCAGCACTC GAGGTCAGCA GATGTCCTGG

TCAGCAGCTC ACTGGGCTAC ACCAGCACCC GAGGTC--CA GATGTCCTGG

TCAGCAGCTC ACTGGGCTAC ACCAGCACCC GAGGTCAGCA GATGTCCTGG

TCAGCAGCTC CTTGGGCTAC ACCAGCACTC GAGGTCAGCA GACGCCCTGG

TCAGCAGCTC CCTGGGCTAC ACCAGCACTC GAGGTCAGCA GACGTCCTGG

TCAGCAGTTC CTTGGGCTAT ACCAGCACTC GAGGTCAGCA GATGTCCTGG

TCAGCAGCTC CTTGGGCTAC ACCAGCACTC GAGGTCAGCA GACATCCTGG

TCAGCAGCTC CTTGGGTTAC ACCAGCACTC GAGGTCAGCA GACGTCCTGG

TTAGCAGCAG CTTGGGCTAT ACCAGCACTC GAGGTCAGCA GATGTCCTCG

TCAGCAGCTC CTTAGGCTAC ACCAGCACTC GAGGTCAGCA GATGTCCTGG

TCAGCAGTTC TTTGGGCTAC ACCAGCACTC GAGGTCAGCA GATGTCCTGG

TCAGCAGCTC TCTCGGCTAC CCCAGCACCC GAGGTCAGCA GATGTCCTGG

TCAGCAGCTC CTTGGGCTAC ACCAGCACCC GAGGTCAGCA GATGTCCTGG

AGAACATGAT GGAGCCACCA CAGCGAGACT CCAGTGCACC AGGGAGGTTT

AGAACATGAT GGAGCCACCG CAGCGAGACT CCAGTGCACC AGGGAGGTTT

AGAACATGAT GGAGCCACCA CAGCGAGACT CCAGTGCACC AGGGAGGTTC

AGAACATGAT GGAGCCACCA CAGCGAGACT CCAGTGCACC AGGGAGGTTC

AGAACATGAT GGAGCCACCA CAGCGAGACT CCAGTGCACC AGGGAGGTTT

AGAACATGAT GGAGCCACCA CAGAGAGATT CCAGCGCACC AGGGAGGTTC

AGAACATGAT AGAACCACCA CAGCGGGACT CTAGTGCACC TGGAAGGTTC

AGAACATGAT AGAACCCCCA CAGAGGGACT CCAGTGCACC AGGAAGGTTC

AGAACATGAT GGAGCCGCCG CAGCGAGACT CCAGCGCACC AGGGAGGTTC

AGAACATGAT GGAGCCACCA CAGCGGGATT CCAGCGCACC GGGCAGGTTC

AGAACATGAT GGAGCCACCA CAGCGAGACT CTAGCGCACC AGGGAGGTTC

AGAACATGAT GGAGCCATCG CAGCGAGACT CCAGCGCGCC GGGGAGGTTC

AGAACATGGT GGAGCCACCA CAGCGAGACT CCAGCGCGCC AGGGAGGTTC

AGAACATGAT GGAGCCACCA CAGCGAGATT CCAGTGCACC AGGGAGGTTC

AGAACATGAT GGAGACTTCA CAGCGAGACT CCAGCGCTCC AGGGCGGTTC

AGAACCTGAT GGAGGCCTCG CAGCGAGATT CCAGTGCTCC GGGCCGGTTC

AGAACCTGAT GGAGTCCCAG CAGCGAGACT CAAGTGCGCC TGGGAGGTTC

AGAACATGAT GGAGACGCAA CAGCGGGACT CCAGTGCACC AGGGAGGTTC

CACGTTGGCA GTGCAGAATC CATGCTGCAT GTTCGACCTG GTGGATACAC

CACGTTGGCA GTGCAGAATC CATGCTGCAT GTTCGACCTG GTGGATACAC

CACGTTGGCA GTGCAGAATC CATGCTGCAT GTTCGACCTG GTGGATACAC

CACGTTGGCA GTGCAGAATC CATGCTGCAT GTTCGACCTG GTGGATACAC

CACGTTGGCA GCGCAGAATC CATGCTGCAT GTACGACCTG GTGGATACAC

CATGTGGGCA GCGCAGAATC CATGCTCCAT GTCCGGCCTG GTGGATACAC

CACGTGGGCA GTGCAGAGTC CATGCTACAT GTTCGACCTG GAGGCTACAC

CATGTGGGTA GTGCAGAGTC CATGCTCCAT GTCCGACCTG GAGGATACAC

CACGTAGGCA GTGCAGAGTC CATGCTACAC GTTCGACCTG GCGGATACAC

CACGTGGGCA GCGCAGAGTC CATGCTGCAT GTCCGCCCCG GGGGGTACAC

CATGTAGGCA GTGCGGAATC CATGCTGCAT GTTCGACCCG GGGGATACAC

CACGTGGGCA GCGCAGAGTC CATGCTGCAT GTTCGACCTG GGGGATACAC

CACGTAGGCA GCGCGGAATC CATGCTGCAC GTTCGACCTG GGGGATACAC

CATGTAGGCA GCGCTGAATC CATGCTACAT GTGCGGCCTG GTGGCTACAC

CATGTAGGCA GTGCGGAGTC CATGCTACAC GTTCGGCAGG GTGGATATAC

CATGTAGGTA GTGCGGAGTC CATGCTGCAC GTCCGGCCTG GTGGATACAC

CACGTGGGCA GTGCCGAGTC CATGCTGCAC GTGCGTCCTG GCGGCTACAC

CACGTGGGCA GTGCAGAATC CATGCTGCAT GTCCGTCCTG GCGGATTCAC

GCCCCAGAGA GCACTGATTA ACCCCTTCGC TCCCTCATGC CCATGAAGCT

GCCCCAGAGA GCACTGATTA ACCCCTTCGC TCCCTCATGC CCATGAAGCT

GCCCCAGAGA GCACTGATTA ACCCCTTCGC TCCCTCATGC CCATGAAGCT

GCCCCAGAGA GCACTGATTA ACCCCTTCGC TCCCTCATGC CCATGAAGCT

GCCTCAGAGA GCACTGATTA ACCCCTTCGC CCCCTCATGC CCATGAAGCT

CCCCCAGCGA GCGCTGATTA ACCCCTTTGC CCCCTCATGC CCATGAAGCT

CCCTCAGAGA GCACTGATTA ACCCCTTCGC CCCCTCATGC CCATGAAGCT

GCCTCAGAGA GCGCTGATTA ACCCCTTCGC CCCCTCATGC CCATGAAGCT

GCCGCAGCGA GCGCTGATTA ACCCCTTCGC CCCCTCATGC CCATGAAGCT

GCCCCAGCGG GCACTGATTA ACCCCTTTGC GCCCTCATGC CCATGAAGCT

GCCCCAGCGG GCGCTCATTA ACCCCTTTGC CCCCTCATGC CCATGAAGCT

ACCCCAGCGG GCGCTCATCA ACCCCTTTGC CCCCTCATGC CCATGAAGCT

GCCGCAGCGG GCGCTGATTA ACCCTTTTGC CCCCTCATGC CGATGAAGCT

ACCCCAACGA GCACTGATTA ACCCCTTTGC CCCCTCATGC CCATGAAGCT

ACCCCAGCGA GCACTGATTA ACCCCTTCGC CCCCTCATGC CCATGAAGCT

ACCCCAGCGG GCGCTGATTA ACCCCTTCGC CCCCTCATGC CGATGAAACT

GCCCCAGCGA GCGCTGATCA ACCCCTTTGC TCCGTCATGC CCATGAAGCT

CCCTCAGCGG GCTCTGATCA ATCCCTTTGC GCCGTCATGC CAATGAAGCT

TACGTCCAAC AGAAGGCGCT GGATGCACAC TTTTCCTGTG GGTAAGCAGT

TACATCCAAC AGAAGGCGCT GGATGCACAC TTTTCCTGTG GGTAAGCAGT

TACGTCCAAC AGAAGGCGCT GGATGCACAC TTTTCCTGTG GGTAAGCAGT

TACGTCCAAC AGAAGGCGCT GGATGCACAC TTTTCCTGTG GGTAAGCAGT

CACGTCCAAC AGAAGGCGGT GGATGCACAC ATTTCCTGTG GGTAAGCAGT

CACATCCAAC AGAAGGCGCT GGATGCACAC TTTTCCTGTG GGTAAGTAGT

CACCTCCAAC AGAAGGCGCT GGATGCATAC TTTTCCTGTA GGTAAGCATT

CACCTCCAAC AGAAGGCGCT GGATGCACAC TTTTCCTGTA GGTAAGCAGT

GACGTCCAAC AGAAGGCGCT GGATGCACAC CTTTCCTGTG GGTGAGCAGT

CACGTCCAAC AGGAGGCGCT GGATGCACAC TTTCCCTGTG GGTAAGCAGT

CACATCCAAC AGAAGACGCT GGATGCACAC TTTTCCTGTG ggtaagCAGT

CACATCCAAC AGAAGACGCT GGATGCACAC TTTTCCTGTG GGTAAGCAGT

CACGTCCAAC CGAAGACGCT GGATGCACAC GTTTCCTGTG GGTAAGCAGT

CACTTCCAAC AGAAGACGCT GGATGCACAC TTTTCCTGTG GGTAAGCAGT

GACTTCCAAC AGAAGGCGCT GGATGCATAC CTTCCCTGTG GGTAAGCAGT

GACTTCCAAC AGAAGGCGCT GGATGCACAC CTTCCCTGTG GGTAAGCAGC

GACCTCCAAC CGGAGGAGGT GGATGCACAC CTTCCCCGTG GGTAAGCAGT

TACTTCTAAC AGAAGACGCT GGATGCACAC CTTCCCTGTT GGTGAGTATT

GGTTCTTTAT CTGGGTAGGG CCATCCGGAG AAGCCATCCA GATCCACCAC

GGTTCTTTAT CTGGGTAGGA CCATCCGGAG AAGCCATCCA GATCCACCAC

GGTTCTTTAT CTGGGTAGGA CCATCCGGAG AAGCCATCCA GATCCACCAC

GGTTCTTTAT CTGGGTAGGA CCATCCGGAG AAGCCATCCA GATCCACCAC

GGTTCTTTAT CTGGGTAGGA CCATCCGGAG AAGCCATCCA GATCCACCAC

AGTTCTTTGT CTGGGTAGGA CCATCCGGAG AGGCCATCCA GATCCATCAC

GGTTCTTTGT CTGGGCAGGA CCATCTGGAG AGGCCATCCA GATCCATCAT

GGTTCTTTGT CTGCCTAGGC CCATCTGGAG AGGCCATCCA GATCCATCAT

GGTTGTCTGT CTGGGTAGGA CCATCCGGGG AGGCCATCCA GATCCATCAC

GGGTCTCTGT CTGGATAGGA CCGTCGGGAG AGGCCATTCA GATCCATCAC

GGATCTTTGT CTGGGTAGGA CCATCCGGAG AGGCCATCCA GATCCATCAC

GGATCTTTAT CTGGGTAGGA CCGTCCGGAG AGGCCATCCA GATCCATCAC

GGCTTTTTGT CTGTGTAGGA CCATCGGGAG AGGCCATCCA GATCCACCAC

GGCTCTTTGT CTGAGTAGGA CCTTCCGGAG AAGCCATCCA GATCCATCAC

GAATCTTTGT CTGGGTAGGA CCATCCGGAG AGGCCATCCA GATCCATCAT

GGATCTTTGT CTGGATAGGA CCCTCCGGAG AGGCCATCCA GATCCATCAC

GGCTGCTTGC CTGGACAGGT CATCTGGGAG AAGCCATCCA GATCCATCAC

GGATGCCTGT CTGCGTAGGT CCATCCGGAG AAGCCATTCA GATTCATCAC

CAGACCCGAC AGAATATGGC GGAGCTACAA GGCAGCGGGC AGAGGGACAC

CAGACCCGAC AGAATATGGC GGAGCTACAA GGCAGCGGGC AGAGGGACAC

CAGACCCGAC AGAATATGGC GGAGCTGCAA GGCAGTGGGC AGAGGGACAT

CAGACCCGAC AGAATATGGC GGAGCTGCAA GGCAGTGGGC AGAGGGACAT

CAGACCCGAC AGAATATGGC CGAGCTGCAG GGCAGCGGGC AGAGGGACAC

CAGACCCGAC AGAATATGGC GGAGCTGCAG GGCAGCGGGC AGAGGGACAC

CAAACCCGGC AAAACATGGC AGAGCTGCAG GGCAGCAGGC AGCGGGACAC

CAGACCCGGC AAAACATGGC AGAGCTGCAG GGCAGCAGGC AGAGGGACAC

CAGACCCGGC AGAATATGGC GGAGCTGCAG GACAGCGGGC AGAGGGACAC

CAGACCCGAC AGAATATGGC AGAGCTGCAG GGCAGCGGGC AGAGGGACAT

CAGACCCGAC AGAATATGGC GGAGCTACAG GGCAGCGGGC AGAGGGACAC

CAGACCCGAC AGAATATGGC GGAGCTGCAG GGCAGCGGGC AGAGGGACAC

CAGACCCGGC AGAATATGGC AGAGCTGCAG GGCAGCGGGC AGCGGGACAT

CAGACCCGAC AGAACATGGT GGAGCTGCAG GGCAGCAGGC AGAGGGACAC

CAGACCCGAC AGAACATGGC GGAGCTGCAG GGCAGCGGGC AGAGGGACAC

CAGACCCGGC AGAACATGGT GGAGCTGCAG GGCAGCGGGC AGAGAGACAC

CAGACTCGCC AGACATGGGT GGAGCTCCAG GGCAGGGGGC AGCGGGACAC

CAGACTCGCC AGAACATGGC AGAACTTCAA GGCAGTGGGC AGAGGGACAC

TCCTCTGCAG AGCTGCTGGA GTTAGCATAT CATGAAGCTG CTGGAAGGTG

TCCTCTGCAG AGCTGCTGGA GTTAGCATAT CATGAAGCTG CTGGAAGGTG

TCCTCTGCAG AGCTGCTGGA GTTAGCATAT CATGAAGCTG CTGGAAGGTG

TCCTCTGCAG AGCTGCTGGA GTTAGCATAT CATGAAGCTG CTGGAAGGTG

TCCTCTGCAG AGCTGCTGGA GTTAGCATAT CACGAAGCTG CTGGAAGGTG

TCCTCTGCAG AGCTGCTGGA GTTAGCATAT CATGAAGCTG CTGGAAGGTG

TCCTCTGCAG AACTGTTGGA GCTGGCATAT CATGAAGCCG CAGGAAGGTG

TCCTCTGCAG AGCTGTTGGA GTTGGCATAC CATGAAGCTG CCGGGAGGTG

TCCTCGGCAG AGCTGCTGGA GTTAGCGTAT CACGAAGCTG CTGGAAGGTG

TCCTCCGCGG AGCTGCTGGA GCTGGCATAC CACGAGGCCG CCGGCAGGTG

TCCTCTGCAG AGCTGCTGGA GTTAGCATAT CAGGAAGCTG CTGGAAGGTG

TCCTCTGCAG AGCTGCTGGA GTTAGCATAT CATGAAGCTG CTGGAAGGTG

TCCTCTGCAG AGCTGCTGGA GTTAGCGTAT CATGAAGCTG CTGGAAGGTG

TCCTCTGCAG AGCTGCTGGA GTTAGCCTAC CATGAAGCTG CTGGAAGGTG

TCCTCTGCCG AGCTGCTGGA GTTAGCGTAT CATGAAGCTG CTGGAAGGTG

TCCTCTGCAG AGCTGCTGGA GCTAGCATAT CACGAAGCTG CCGGAAGGTG

TCATCTGCAG AGCTGCTGGA GCTGGCATAC CACGAAGCGG CAGGAAGGTG

TCTTCTGCGG AGCTGCTGGA GTTAGCGTAT AATGAAGCGG CTGGCAGGTG

AATTTACCAA TTGGTGATGG TATTCTTGGG CGAAGGTAGT CAGCTGAGGC

AATTTACCAA TTGGTGATGG TATTCTTGGG CGAAGGTAGT CAGCTGAGGC

AATTTACCAA TTGGTGATGG TATTCTTGGG CGAAGGTAGT CAGCTGAGGC

A--------- ---------- ---------- ---------- ----------

AACTTACCAA TTGGTGATGG TATTCTTGGG CGAAGGTAGT CTGCTGAGGC

A--------- ---------- ---------- ---------- ----------

AAGTTACCAA TGGGTGATGG TATCCTTGGA TGAAGGTAGT CTGCTGAGGC

AAGTTACCTA TTGGTGATGG TATCCTTGGA TGAAGGTAGT CTGCTGAGGC

AACTCACCAA TCGGTGACGG TATTCTTGGG CGAAGGTAGT CTGCTGAGGC

AATTTACCAA TTGGTGATGG TATTCTGGGA CGAAGATAGT CTGCTGAGGC

AATTTACCAA CTGGTGATGG TATTCTAGGA CGAAGGTAGT CTGCTGAAGC

AATTTACCAA TTGGTGATGG TATTCGAGGA CGAAGGTAGT CTGCTGAGGC

AATTTACCAA TAGGTGATGG TATTCTAGGA CGCAGGTAAT CTGCCGAGGC

AATTTACCAA CTGGTGATGG TATTCTGGGG CGAAGGTAGT CTGCTGAAGC

AATTTACCAA CTGGTGAAGA TATTCTAGGG CGAAGGTAGT CTGCCGAGGC

AGCTTACCAA CTGGTGAAGA TATTCTAGGG CGAAGGTAGT CTGCCGAGGC

AACTTACCAA TGGGTGAGGG TATCCTAGGG CGCAAGTAGT CTGCAGAGGC

AGCTTACCAA GTGGTGAAGG AATTCGGGGT CGAAGATAGT CTGCTGAAGA

TGCTCGAGTT TGAAACACCT GTGACAAGGG AGGAGAGGGT GCTCTTTGGC

TGCTCGAGTT TGAAACACCT GTGACAAGGG AGGAGAGGGT GCTCTTTGGC

TGCTCGAGTT TGAAACACCT GTGACAAGGG AGGAGAGGGT GCTCTTTGGC

---------- ---------- ---------- ---------- ----------

TGCTCGAGTT TGAAACACCT GTGACAAGGG AGGAGAGGGT GCTCTTTGGC

---------- ---------- ---------- ---------- ----------

GGCTTGAGTT CGAAACACCT GTGACATTGG AGGAGAGGGT GCTCTTTGGC

TGCTTGAGTT CGAAATACCT GTGACATTGG AGGAGAGGGC GCTCTTTGGC

TGCTCGAGTT TGAAACACCT GTGACAAGGG GGGAGACGGT GCCCTCTGGC

TGCTCTCGTC TGAAACACCT GTGACAATGG AGGAGAGGGT GCTCTCTGGC

TGCTCGTGTT TGAAACACCT GTGACAAGGG AGGAGAGGGT GCTCTTTGGC

TGCTCGTGTC TGAAACACCT GTGACAGGGG AGGAGAGGGT GCCCTTTGGC

TGCTCGTGTC TGAAACACCT GTGACAAGGG AGGAGAAGGT GCTCTTTGGC

TGCTCGTGTC TGAAACACCT GTGACAAGGG AGGAGAAGGT GCTCTTTGGC

TGCTCGCGAT TGAAACACCT GTGATAAGGG AGGAGTGGGT GCTCTTTGGC

TGCTCGAGTT TGAAATGCCT GTGACAAGGG AGGAGAGGGT GCCCTTTGGC

TGCTCGAGTT GGAAACACCT GTGAAATGGG AGGAGAGGGG GCTCTCTGGC

AGCACGAGTT GGAAACACCT GTGAGATGGG GGGAGAGGAT GCTCTTTGGC

CCAGTAAAGA TGTTGTAGGC TCCAAGCTCC CCATAAGGCC ACTCAGAAGA

CCAGTAAAGA TGTTGTAGGC TCCAAGCTCC CCATAAGGCC ACTCAGAAGA

CCAGTAAAGA TGTTGTAGGC TCCAAGCTCC CCATAAGGCC ACTCAGAAGA

---------- ---------- ---------- ---------- ----------

CCAGTAAAGA TGTTGTAGGC TCCAAGCTCC CCATAAGGCC ACTCAGAAGA

---------- ---------- ---------- ---------- ----------

TCAATAAAGA TGCAGTAGGC TCCAAGCTCC CCATAAGGCC ACTGAGAAGA

TCAATAAAGA TGCAGTAGCC TCCAAGCTCC CCATAAGGCC ACTGAGAAGA

CCAGGATCGA TGTCGTAGGC TCCAAGCTTC CCATGAGGCC ACTCAGAAGA

CCAGTAATGA TGTCGTAGGC TCCAGGCTCC CCATAAGGCC ACTCAGGAGA

CCAGTAAAGG TGTTGTAGGT TCCAAACTCC CCATAAGGCC ACTCAGAAGA

CAAGCAAAGA TGTTGTGGGC TCCAAGCTCC CCATAAGGCC ACTCAGAAGA

CCAGTAAAGA TGTGGTATGC TCCAAGCTCC CCATAAGGCC ACTCAGAAGA

CCAGTAAAGG TGCTGTAGGC TCCAGGCTCC CCATAAGGCC GCTCAGAAGA

CTAGTAAAGA TGTTGTAGGC TCCAAGCTCC CCATAAGGCC ACTCAGAAGA

CCAGTAAAGA TGCTGTAGGC TCCAAGCTCC CCATCAGGCC ACTCAGAAGA

CCAGCAAGGA TGCTGTTGGT TCCAGACTCC CCATAAGGCC ACTCAGGAGA

CTAGTAAATT TGCAGTAGGT TCCAAACTCC CCATTAGGCC ACTAAGAAGA

TTGGAAGAAG CAGGTCTCTG AACTGGTTGA CCCAGAAGTT CCTAAAGGCA

TTGGAAGAAG CAGGTCTCTG AACTGGTTGA CCCAGAAGTT CCTAAAGGCA

TTGGAAGAAG CAGGTCTCTG AACTGGTTGA CCCAGAAGTT CCTAAAGGCA

---------- ---------- ---------- ---------- ----------

TTGGAAGAAG CAGGTCTCTG AACTGGTTGA CCCAGAAGTT CCTAAAGGCA

---------- ---------- ---------- ---------- ----------

TTGGAAGAAG CAGGTCTCTG AACCGGTTGA CCCAGAAGTT CCTATAGGCA

TTGGAAGAAG CGGGTCTCTG AACCGGTTGA CCCAGAAGTT CCTATAGGCA

TTGGAAGACG CAGGTCTCTG AACGGGCTGA CCCAGGAGTT CCTAGAGGCA

TTGGAAGAAG CAGGTCTCTG AACAGGTGGG CCCAGAAGTT CCTAAAGGCA

TTGGAAGAAG CAGGTCTCTG AACTGGTGGA CCCAGAAGTT CCTAAGGGCA

TTGGAAGAAG CAGGTCTCTG AACTGGTGGC CCCAGTAATT CCTACAGGCA

TTGGAAGACG CAGGTCTCGG AACTGGTGGA CCCAGAAGTT CCTAAA-GCA

TTGGAAGAAG CAGGTCTCTG GACTGGTGGA CCCAGAAGTT CCTAAAGGCA

TTGGAAGACG CAGGTCTCTG AACTGGTGGA CCCAGAAGTT CCTAAAGGCA

TTAGAAGAAG CCGGTCTCTG AACTGGTGGA CCCAGAAGTT CCTGAAAGCA

TGAGAAGAAG CAGGTCTTGT AACTGGTGGA CCCAGAAGCT CCTGGAGGGG

TTAGATGAAG CAGGTCTGGT GACTGGTGGA CCTAGAAGTT CCTAAGGAGA

GAAAAGTCTG TTCTCTGCAG GCACAGCAAT TCCCGCCAGC CTGGTGACGG

GAAAAGTCTG TTCTCTGCAG GCACAGCAAT TCCCGCCAGC CTGGTGACGG

GAAAAGTCTG TTCTCTGCAG GCACAGCAAT TCCCGCCAGC CTGGTGACGG

------TCTG TTCTCTGCAG GCACAGCAAT TCCCGCCAGC CTGGTGACGG

GAAAAGTCTG TTCTCTGCAG GCACAGCAAT TCCTGCCAGC CTGGTGACGG

------TCTG TTCTCTGCAG GCACAGCACT TCCCACCAGC CTGGTGACGG

GAAATATTTG TTCTCTGCAG GCACAGCACT TCCCGACAGC CTGGTGACAG

GAAATGTTTG TTCTCTGCAG GCACAGCACT TCCCGCCAGC CTGGTGACAG

GAGAAGTCTG TTCTCTGCAG GCACAGCACA TCCCGCCAGC CTGGCGACGG

GAAAAGTCTG TTCTCTGCAG GCACAGCACT TCCCGCCAGC CCGGCGACGG

GAAAAGTCTG TTCTCTGCAG GCACAGCACT TCCCGCCAGC CTGGTGACAG

GAAAAGTCTG TTCTCTGCAG GCACAGCACC TCCCGCCAGC CTGGTGACGA

GAAAAATCTG TTCTCTGCAG GCACAGCACT TCCCGCCAGC CTGGTGACGG

GAAAAGTCTA TTCTCTACAG GCACAGCACG TCCCGCCAGC CAGGTGACCG

GAAAAGTCTA TTCTCTGCAG GCACAGCTCT TCCCGCCAGC CTGGTGACAG

GAAAAGTCTG TTCTTTGCAG GCACAGCACT TCCCGCCAGT CTGGTGACAA

AAGGAGTTTG CTCTCTGCAG GCATCTCCCT TCCCGCCAGC CTGGGGACAG

AGATAGTACT TTCTCTGCAG GCACATCGCT TCTCGGCAGC CTGGCGACAG

CATGTCCTTC TTGAACTTCA GTGGAACAGA GGAGCTTTCT GTCGGCCTTA

CATGTCCTTC TTGAACTTCA GTGGAACAGA GGAGCTTTCT GTCGGCCTTA

CATGTCCTTC TTGAACTTCA GTGGAACAGA AGAACTTTCT GTCGGTCTTA

CATGTCCTTC TTGAACTTCA GTGGAACAGA AGAACTTTCT GTCGGTCTTA

CATGCCTTTC TTGAACTTCA GTGGAACAGA AGAGCTTTCT GTCAGCCTTA

CATGTCCTTC TTGAACTTCA GTGGAACAGA AGAGCTTTCT GTCAGCCTTA

CATGTC---C TTGAACTTCA GTGGAACGGA AGAGCTTTCT GTCAGCCTTA

CATGTC---C TTGAACTTCA GTGGAACGGA AGAGCTTTCT GTCAGCCTTA

CCTGTCTTTC CTGAGCTTCA GCGGAACAGA -GAGCTT-CT GCCAGCCTTA

CACGTCCTTC TTGAGCTTCG CTGGGACGGA GGAGCTCTCG GTTAGCCTCC

CATGTCCTTC TTGAGCTTCA GTGGAACAGA AGAGCTTTCT GTCAGCCTTA

CATGTCCTTC TTGAACTTCA GCGGAACTGA AGAACTTTCT GTCAACCTTA

CATGTCCTTC TTGAACTTCA GTGGAACGGA AGAGCTCTCT GTCGGCCTTA

CATGTCCTTC TTGAACTTCA ATGGAACAGA AGAGCTTTCT GTCAGCCTTA

CATGTCCTTC TTGAACTTCA GTGGAACAGA AGAGCTTTCT GTCAGCCTTA

CATGTCCTTC TTGAACTTCA GTGGAACAGA AGCGCTCTCT GTCAGCCTTA

CGCATCCTTC CTGAATTTCA GTGGATCTAA CGAGTTTTCT GCCCGCCTCA

CTTGTCCTTC CTGAGCTTCA GTGGAACAGA AGAATTTTCT GTCAGCCTTA

GCAACAGTGG TGCAGGTAAT CTATTTTGAG ATCATTTCAG CCACATAATG

GCAACAGTGG TGCAGGTAAT CTATTTTGAG ATCATTTCAG CCACATAATG

GCAACAGTGG TGCAGGTAAT CTATTTTGGG ATCATTTCAG CCACATAATG

GCAACAGTGG TGCAGGTAAT CTATTTTGGG ATCATTTCAG CCACATAATG

GCAACAGTGG TGCAGGTAAT CTATTTTGGG ATCATTTCAG CCACATAATG

GCAACAGTGG TGCAGGTAAT CTATTTTGAG ATCATTTCAG CCACGTAATG

GCAACAGCGG CACAGGTAAT CTATTTTGGG ATCTTTCCAG CCACGTAATG

GCAACAGCAG TACAGGTAAT CTATTTTGGG ATCTTTTCAG CCACATAATG

GCA-CAGCGG CACAGGTAA- ---------- ---------- -----TAATG

GTGGCAGTGG TGCAGGTAAT CTATTTTGGG ATCATTTCAG CCACATAATG

GCAACAGTGG TGCAGGTAAT CTATTTTGGG ATCATTTCAG CCACATAATG

GCAACAGTGG TTCAGGTAAT CTATTTTGGG ATCATTTCAG CCACATAATG

GCAACAATGG TGCAGGTAAT CTATTTCGGG AGCGTTTCAG CGACATAATG

GCAACAGTGG TGCAGGTAAT CTATTTTGGG ATCATTTCAG CCACGTAATG

GCAACAGTGG TGCAGGTAAT CTATTTTAGG ATCATTTCAG GCACATAATG

GCAACAGCTG TGCAGGTAAT GTATTTGGGG ATTGTTTCTA TACTATAATG

GCAATAGTGC GACAGGTAAT CTATCTTTAG ACTCTCTGAG ACACATCATT

GCAACCACAG TGCAGGTAAT CTCTCTTTGG GCTGCCTCAG CTATATAGTC

CATGGAATTA AAAATGGGCT GAAGAGAAGT ACCTGCAGGA TGTTCTTAGG

CATGGAATTA AAAATGGGCT GAAGAGAGGT ACCTGCAGGA TGTTCTTAGG

CATGGAATTA AAAATGGGCT GAAGAGAAGT ACCTGCAGGA TGTTCTTAGG

CATGGAATTA AAAATGGGCT GAAGAGAAGT ACCTGCAGGA TGTTCTTAGG

CATGGAATTA TAAATGGGCT GAAGAGAAGT ACCTGCAGAA TGTTCTTAGG

CATGGAATTA TAAATGGGCT G--------- ---------- ----------

CATAGGATTA TAAATGGGCC GAAGAAACAT ACCTGTAGGA TGTTTTTAGG

CATGGGATTA TAAATGGGCT GAAGAAACAC ACCTGTAGGA TGTTTTTAGA

CATGGAATTA TAAATGGGCG A--------- ---------- ----------

CATGGAATTA TAAATGGGCT GAAGATAAGT ACCTGCAAGA TGTTCTTAGG

CATGGAATTA TAAATGGCCT GAAGAGAAGT ACCTGCAGGA TGTTCTTAGG

CATGGCATTA TAAATGGGCT GAAGAGAAGT ACCTGCAGGA TGTTCTTAGG

CATGGAATTA TAAATGGACT GAAGAAAAGT ACCTGCAAGA TGTTCTTAGG

CATGGAATTA TAAATGGGCT GAAGAGAAGT ACCTGCAGGA TGTTCTTCGG

CATGGAATTA TAAATGGGCT GAAGAGAAGT ACCTGGAGGA TGTTCTTAGG

CATGGGATTA TAAATGGGCT G--------- ---------- ----------

AATGGTGCTC TAAGTGGGCC GAAGAGAAGT ACCTGTAAGA TGTTCTTAGG

CAGGGAACTA TAAAATGGGC GAAAAAAAAT ACCTGCAAGA TGTTCTTTGG

GACAGGCTGG CCTGGAATCT CAGCAGGGGA CATCAAATGG CTTTCAAGGT

GACAGGCTGG CCTGGAATCT CAGCAGGGGA CATCAAATGG CTTTCAAGGT

GACAGGCTGG CCTGGAATCT CAGCAGGGGA CATCAAATGG CTTTCAAGGT

GACAGGCTGG CCTGGAATCT CAGCAGGGGA CATCAAATGG CTTTCAAGGT

GACAGGCTGG CCTGGAATCT CAGCAGGGGA CATCAAATGG CTTTCAAGGT

---------- ---------- ---------- ---------- ----------

GACAGGCTGG CCTGGAATCT CAGCAGGAGC CAACAAGTGA CTTTCAAGAT

AACAGGCTGG CCTGGAATCT CAGCAGGGGC CAATAAGTGA CTTTCAACGT

---------- ---------- ---------- ---------- ----------

GACAGGCTGG CCTGGAATCT CAGCAGGGGA CATCAAATGG CTTTCAAGAG

GACAGGCTGG CCTGGAATCT CAGCAGGGGA CATCAAATGG CTTTCAAGGT

GACAGGCCGG CCCGGACTCT CGGCAGCGGA CAGCAAATGG CTTTCAAGGT

GACAGGCTGG CCTGGAATCT CAGCAGGGGA CATCAAGTGG CTTTCAAGGT

AACAGGCTGG CCTGGGATCT CAGCAGGGGA CATCAAATGG CTTTCAAGGT

GACAGGCTGG CCTGGTATCT CCGCAGGGGA CATCAAATGG CTTTCAAGGT

---------- ---------- ---------- ---------- ----------

GAGAGGTTGC CCGGGAATCT CAGCATGGGA CATCAAATGG CTTTCAAGGT

AAGAGGCTGA CTTGGGATCT CTGGATGGGA CATCAAATGA CTTTCGAGAT

TTCGCTAAAA TCAAAAGAAA ACTGGTTTTA ACTTTAACTC ACTTTCTTTC

TTCGCTAAAA TCAAAAAAAA ACTGGTTTTA ACTTTAACTC ACTTTCTTTC

TTCGCTAAAA TCAAAAGAAA ACTGGTTTTA ACTTTAACTC ACTTTCTTTC

TTCGCTAAAA TCAAAAGAAA ACTGGTTTTA ACTTTAACTC ACTTTCTTTC

TTCGCTAAAA TCAAAGGAAA ACTGGTTTTA ACTTTAACTC ACTTTCTTTC

---------- ---------- ---------- ---------- ----TCTTTC

TTCGCtaaaa aaaaaataaa aCGGTTTTCA GTTTTAACAC AATTTCTTTC

TTCGCTaaaa aaaaaagaaa aaaGTTTTCA ACTTCAACAC ACTTTCTTTC

---------- ---------- ---------- ---------- ----TCTCTC

TTCGCTAAAA TCAAAGGAAA ACTGGTTTTA ACCTTCACAT ACTTTCTTTC

TTCGCTAAAA TCAAAGGAAA ACTGGTTTTA ACCTTTACAC ACTTTCTTTC

TTCGCTAAAA GCAGAGGAAA ACTGGTTTTA ACCTTTACAC ACTTTTTTTC

TTCGCTAAAA TCAAAGAAAA ACTGATTTTA ACCTTAAC-C ACTTTCTTCC

TTCGCTAAAA TCAAAGGAAA ACTGGTTTTA ACTTTTACAT ACTTTCTTTT

TTCGCTAAAA TCAAAGGAAA ACTGGTTTTA ACCTTTACAC ACTTTCTTTC

---------- ---------- ---------- ---------- ----------

TTCGCTAAAA TCAGAGGAAA ATGTCATTTA ACTTTCACAT CATTTCTTTC

TTCGCTATGA TCAAAGAAAA ACTTAGGTTA ACCTCCTCCC CCTCTCTTTT

CATAGGTCCT AGGACCCAGA ATAAGGATTC TCTAGAGGAC AGTGTTTCTA

CATAGGTCCT AGGACCCAGA ATAAGGATTC TCTAGAGGAC AGTGTTTCTA

CATAGGTCCT AGGACCCAGA ATAAGGATTC TCTAGAGGAC AGTGTTTCTA

CATAGGTCCT AGGACCCAGA ATAAGGATTC TCTAGAGGAC AGTGTTTCTA

AATAGGTCCT AGGACCCAGA ATAAGGATTC TCTAGAGGAC AGTGTTTCTA

CATAGGTCCT AGGACCCAGA ATAAGGATTC TCTAGAGGAC AATGTTTCTA

TCTAGGTCCT AGGAACCAGA ATAAGGACTC TCTAGAGGAT GGTGTTTCTA

TCTAGGTCCT AGGACCCAGA ACAAGGACTC CCTAGAGGAC AGTGTTTCTA

CACAGCTCCT AGGACCCAGA ATAAGGATTC GTTAGAGGAC AATGTTTCTA

CATAGATCCT CGGACGCAGA ATAAGGATTC TCCAGAGGAC AGTGTGTCTA

CATAGGTCCT AGAACTCAGA ATAAGGATTC TCTAGAGGAC AGTGTTTCTA

CATAGGTCCT AGGACCCAGA ATAAGGATTC TCTAGAGGAC AATGTTTCTA

CATAGGTCCT AGGACCCAAA ATAAGGATTC GCTAGAAGAC AGTGTTTCTA

CCTAGGTCCT AGGACTCAGA ATAAGGATTC TCTGGAGGAC AATGTTTCTA

CATAGGTCCT AGGACCCAGA ATAAGGATTC TCTAGAGGAC AGTGTCTCTA

---------- ---------- ---------- ---------- ----------

TGCAGGACCC AGGAGCCAGA ACAAGGATTC TCTAGAAGAC AGTGTGTCCG

CTTAGGTCCT AGGACCCAGA ACAAGGACTC TGTAGAAGAC AATGTATCGG

CCGACCCAAG TAAGAGGGGT AACTTTCCCA GACTCCCCCC TTTTGAATCT

CCGACCCAAG TAAGAGGGGT AACTTTCCCA GACTCCCCCC TTTTGAATCT

CCGACCCAAG TAAGAGGGGT AACTTTCCCA GACTCCCACC TTTTGGATCT

CCGACCCAAG TAAGAGGGGT AACTTTCCCA GACTCCCACC TTTTGGATCT

CCGACCCAAG TAAGAGGGGT AACTTTCCCA GACTCCCCCC TTTTGGATCT

CCGAACCAAG TAAGAGGAGT AACATTCCCA GGCTCCT-CT TTTTAGATCT

CCGACCCAAG TAAGAGGGGT AGCTGTCC-- GACTCCTTCC TTCTGAAGCT

CCGACCCAAG TAAGAAGGGT AACTGTCCCA GACTCCTTCC T---GAATCT

CCGACCCAAG TAAGAGGGGT AACTTTCCCA GACTCCTCCC TTTGGGATCT

CCGACCCAAG TAAGAGGGGT AACTTTCCCA GATTCCTCCC TGATGGATCT

CCGACCCAAG TAAGAGGGGT AACTT-CCCA GACTCCTCCC TTTTGGATCT

CCGACCCAAG TAAGTGGGG- ---------- --CTCCTCCC TTTTGGATCT

CCGACCCAAG TAAGAGGGGC AACTGCCCCA GACGTGTTCC TTTTGGATCT

CCGACCCAAG TAAGAGGGGT AACTTTCTCA GATTCCCCCC TTTTGGACCT

CCGACCCAAG TAAGAGGGGT AACTTTCCCA GACTTCTCCC TTTTGGATCT

---------- ---------T AACTTCCCCC AACTCCCCCC TTTTAGATTG

CCGATCCTAG TAAGAGGGAG AGCTGTCTGG CGCTTTTCCC TTTCGGATCT

CCGATCCAAG TAAGAGGGAT GACTTTACAG GATGCCCCTC TTTTGGATCT

GTTACTTTTG CAGTGCTTTC TGCAGCTCAC AGTCAGCAGA AATGAAAAAT

GTTACTTTTG CAGTGCTTTC TGCAGCTCAC AATCAGCAGA AATGAAAAAT

GTTACTTTTG CAGTGCTTTC TGCAGCTCAC AATCAGCAGA AATGAAAAGT

GTTACTTTTG CAGTGCTTTC TGCAGCTCAC AATCAGCAGA AATGAAAAGT

GTTACTTTTG CGGTGCTTTC TGCAGCTCAC GATCAGCAGA AATGAAAAGT

GTTACTTTGG TGATTCTTTC TGCAGCTCAC AATCAGCAGA AATGAAACAT

ATT------G TGATTGTCTC TGCA-CTCAT GATCAGCAGA AATGAAAAAT

ATTAC--TTG TGATTCTTTC TGCAGCTCAT GATCAGCAGA AATGAAAAAT

GTTACTATTG CCATTCTTTC TGC------- ---------- ----------

GTGACTTTT- -------TTC TGCAGCTCAC GATCAGCAGA AATGAAAAGT

ACGACTTTTG CAATTCTTTC TGCAGCTTAA GATCAGCAGA AATGAAAAGT

GTGACTTTTG CAATTCTTTC TGCAGCTTAC GATCAGCAGA AATGAAAAGT

GTGACTTTTG CAATTCTTTC TGCAGCTTAC AATCAGCAGA GATGAGACGT

GCTACTTTTG CAATTCTTTC TGCAGCTTAC GATCAGCAGA AATTGAAAGT

GTTACTTTTG CAGTTCTTTC TGCAGCTTAA GGGCAGCAGA AATTAAACGT

ATTCCTTTTA CAATTCTTTT TGC------- ---------- ----------

GTCGCGTGGC CGCCTCCGTC TGCAGCTTGT AACCGACAGT GATTCTCAGA

GTCCCTTTTA TAACTCTCTG TGCAGCTTAC AATCAACAGC AATTAAAAAT

ATCAGCTGTG AATCCATTGC ATGATGGGAT TGGATAAGCA TGTGCACTGC

ATCAGCTGTG AATCCATTGC ATGATGGGAT TGGATAAGCA TGTGCACTGC

ATCAGCTGTG AATCCATTGC ATGATGGGAC TGGATAAGCA TGTGCACTGC

ATCAGCTGTG AATCCATTGC ATGATGGGAC TGGATAAGCA TGTGCACTGC

ATCAGCTGTG AATCCATTGC ATGATGGGAT TGGATAAGCA TGTGCACTGC

ACCAGCTGTG AATCCATTGC ATGATGGGAT TGGATAAGCA TGTGCACTGC

AGCGGCTGTG AA-CCATTGC ATGATGGGAT TGGATAAGCA TGTTT--TGC

AGCGGCTGTG AATCCATTGC ATGATGGGAT TGGATAAGCA TGTGT--TGC

---------- ---------- ---------- ---------- ----------

GTCAGCTGCG ACTCCATTGC ATGGTGGGAT CGGATAAGCA C------TGC

ATCAGCTGTG AATCCATTGC ATGATGGGAT TGGATAAGCA CGTGCACTGC

ATCAGCTGTG AATCTATTGC ATGATGGGAT TGAATAAGCA CGCGCACTGC

ATCAGCTGTG GATCCATTGC ATGATGGGAT TGGATGAGCA CGTGCACTGC

ATCAGCTGCG CATCCATTGC ATGATGGGAT TGGATAAGCA TGTGCACTAT

ATCAGCTGTG AATCCATTGC ATGATGGGAT TGGATAAGCA TGTGCACTGC

---------- ---------- ---------- ---------- ----------

ATTAGCCAAG CGGCCACCGC ATGATGGGCC TCAAGACGCA TGTGCATTGC

ATTAGTTGTG AATCCATTGC ATGATGGGAT TGGATAAGCA TGTGCACTGC

ATTGTCTAAC AAACTCTGTT TCTCTCTCTA GGCAGTTCTG ACACTGTCTG

ATTGTCTAAC AAACTCTGTT TCTCTCTCTA GGCAGTTCTG ACACTGTCTG

ATTGTCTAAC AAACTCTGTT TCTCTCTCTA GGCAGTTCTG ACACTGTCTG

ATTGTCTAAC AAACTCTGTT TCTCTCTCTA GGCAGTTCTG ACACTGTCTG

ATTGTCTAAC AAACTCTGtt tctctctctA GGCAGTTCTG ACACTGTCTG

ATTGTCTAAC AAATTCTGTT TCTTTCTCTA GGCAGTTCTG ACACTGTCTG

GTTATCTAAC AAACTCTGTT TCTCTCTCTA GGCAGTTCTG ACACTGTCTG

GTTATCTAAC AAActctgtt tctctctctA GGCAGTTCTG ACACTGTCTG

---------- ---------- ---------- ---------- ----------

GTTGTCTAAC AGCCTCTGTT tctctctctA GGCAGTTCTG ACGCTGTCTG

ATTGTCTAAC AAACTCTGTT TCTCTCTCTA GGCAGTTCTG ACACTGTCTG

ATTGTCTAAC AAA--CTGTT TCTTTCTCTA GGCAGTTCTG ACACTGTCTG

ATTGTCTAAC AGACtctgtt tctctttttA GGCAGTTCTG ACACTGTCTG

ATTGTCTAAC AAACTCTATT TCTCTCTCTA GGCAGTTCTG ACACTGTCTG

ATTGTCTAAC AAACTCTGTT TctctttctA GGCAGTTCTG ACACTGTCTG

---------- ---------- ---------- ---------- ----------

CCTGGTTAAC AGActctgtc tctctctccC GGCAGTTCTG ACGCTGTCTG

ATTATTTAAC AAACTCTGTT TTTCTCTCCA GGCAGTTCTG ACACTGTCTG

CTCCCCCTGT AGTGCCAGGC TTCTGTTGCA CAGTTGGAGT GGACTGGAAG

CTCCCCCTGT AGTGCCAGGC TTCTGTTGCA CAGTTGGAGT GGACTGGAAG

CTCCCCCTGT AGTGCCAGGC TTCTGTTGCA CAGTTGGAGT GGACTGGAAG

CTCCCCCTGT AGTGCCAGGC TTCTGTTGCA CAGTTGGAGT GGACTGGAAG

CTCCCCCTGT AGTGCCAGGC TTCTGTTGCA CAGTTGGAGT GGACTGGAAG

CTCCCCCTGT AGTGCCAGGC TTCTGTTGCA CAGTTGGAGT GGACTGGAAG

CTCCCCCTGT AGTGCCAGGC TTCTGTTGCA CAGTTGGAGT GGATTGGAAG

CTCCCCCTGT AGTGCCAGGC TTCTGTTGCA CAGTTGGAGT GGATTGGAAG

---------- ---------- ---------- ---------- ----------

CTCCCCCTGT AGTGCCAGGC TTCTGTTGCA CAGTTGGTGT GGACTGGAAG

CTCCCCCTGT AGTGCCAGGC TTCTGTTGCA CAGTTGGAGT GGACTGGAAG

CTCCCCCTGT AGTGCCAGGC TTCTGTTGCA CAGTGGGAGT GGACTGGAAG

CTCCCCCTGT AGTGCCAGGC TTCTGTTGCA CAGTTGGAGT GGACTGGAAG

CTCCCCCTGT AGTGCCAGGC TTCTGTTGCA CAGTTGGAGT GGACTGGAAG

CTCCCCCTGT AGTGCCAGGC TTCTGTTGCA CAGTTGGAGT GGACTGGAAA

---------- ---------- ---------- ---------- ----------

CTCCCCCTGT AGTGCCGGGC TTCTGTTGCA CAGTGGGAGT GGACTGGAAG

CTCCCCCTGT AGTGCCAGGC TTCTGTTGCA CAGTTGGAGT GGACTGGAAG

TCTCTCACTA CTCCGGCGTG CCTCCCCCTT ACCACCGACT ACTTCCCTGA

TCTCTCACTA CTCCGGCGTG CCTCCCCCTT ACCACCGACT ACTTCCCTGA

TCTCTCACTA CTCCGGCGTG CCTCCCCCTC ACCACCGACT ACTTCCCTGA

TCTCTCACTA CTCCGGCGTG CCTCCCCCTC ACCACCGACT ACTTCCCTGA

TCTCTCACTA CTCCGGCGTG CCTCCCCCTC ACCACCGACT ACTTCCCTGA

TCTCTCACTA CTCCGGCGTG CCTCCCCCTC ACCACCGACT ACTTCCCTGA

TCTCTTACTA CTCCTGCATG CCTTCCCCTC ACCACTGACT ACTTCCCGGA

TCTCTTACTA CTCCTGCATG CCTTCCCCTC ACCACTGACT ACTTCCCGGA

---------- ---------- --TCCCCCTC ACCACCGACT ACTTCCCTGA

TCACTCACGA CTCCCGCGTG CCTCCCCCTC ACCACGGACT ACTTCCCCGA

TCTCTCACTA CTCCGGCGTG CCTCCCCCTC ACCACCGACT ACTTCCCCGA

TCTCTCACTA CTCCGGCGTG CCTCCCGCTC ACCACCGACT ACTTCCCCGA

TCTCTCACTA CTCCGGCTTG CCTCCCCCTC ACCACCGACT ACTTCCCTGA

TCTCTCACTA CTCCGGCGTG CCTCCCCCTT ACCACCGACT ACTTCCCTGA

TCTCTCACTA CTCCGGCATG CCTGCCCCTC ACCACCGACT ACTTCCCCGA

---------- ---------- ---------- ---------- ----------

TCTCTCACGA CTCCGGCGTG CCTGCCCCTC ACCACCGACT ACTTCCCAGA

TCTCTCACTA CCCCGGCCTG CCTCCCCCTC ACTACGGACT ACTTCCCGGA

CCGCCAGGGC CTGCAGAATG ACTACACAGA GGGCTGTTAT GATCTCCTTC

CCGCCAGGGC CTGCAGAATG ACTACACAGA GGGCTGTTAT GATCTCCTTC

CCGCCAGGGC CTGCAGAATG ACTACACAGA GGGCTGTTAT GATCTCCTTC

CCGCCAGGGC CTGCAGAATG ACTACACAGA GGGCTGTTAT GATCTCCTTC

CCGCCAGGGC CTGCAGAATG ACTACACAGA GGGCTGTTAT GATCTCCTCC

CCGCCAGGGC CTCCAGAACG ACTACACAGA GGGCTGTTAT GATCTCCTCC

CCGTCAGGGC CTGCAGAATG ACTACACAGA GGGCTGCTAT GATCTCCTCC

CCGTCAGGGC CTGCAGAATG ACTACACAGA GGGCTGCTAT GATCTCCTCC

CCGCCAGGCC CTGCAGAACG ACTACACCGA GGGCTGCTAC GACCTGCTCC

CCGCCAGGGC CTACAGAATG ACTACACGGA GGGCTGCTAC GATCTCCTCC

CCGCCATCGC CTGCAGAACG ACTACACAGA GGGCTGTTAC GATCTCCTTC

CCGCCAGGGC TTGCAGAACG ACTATACAGA GGGCTGTTAT GACCTCCTCC

CCGCCAGGGC CTACAGAACG ACTACACAGA GGGCTGTTAT GATCTTCTCC

CCGCCAGGGC CTGCAGAATG ACTACACTGA AGGCTGTTAT GATCTCCTCC

CCGCCAGAGC CTGCAGAACG ACTACACGGA AGGCTGCTAC GATCTCCTCC

---------- ---------- ---------- ---------- ----------

CCGCCAGGGC CTTCAGAACG ACTACACCGA GGGCTGTTAC GACCTCCTCC

TCGCCAGGGC CTGCAAAATG ATTACACCGA AGGCTGTTAC GACCTGCTCC

CAGAAGCAGA CATCGACAGG TTTTAGGAGG GACGAAGATG GTGTGCAGAT

CAGAAGCAGA CATCGACAGG TTTTAGGAGG GACGAAGATG GTGTGCAGAT

CAGAAGCAGA CATCGACAGG TTCTAGGAGG GACGAAGACG GTGTGCAGAT

CAGAAGCAGA CATCGACAGG TTCTAGGAGG GACGAAGACG GTGTGCAGAT

CAGAAGCAGA CATCGACAGG TTTTAGGAGG GATGAAGAGG GTGTGCAGAT

CGGAAGCAGA TATGGACAGG TTTTAGGAGG GACGAGGAGG GTGTACAGAT

CAGAAGCAGA CATGGACAGG TTCCAGGAGG GACGAGGAGG GCGTTCAAAT

CAGAAGCAGA CATGGACAGG TTCCAGGAGG GATGAGGAGG GTGTTCAAAT

CGGAGGCGGA CATCGACAGG TTCCAGGAGG GACGAGGAGG GCGTGCAGAT

CGGAGGCTGA CATCGACAGG TTTTAGGAGG GATGAGGAGG GCGTGCAGAT

CAGAAGCAGA TATTGACAGG TTCTAGGAGG GATGAGGAGG GTGTGCAGAT

CGGAAGCGGA CATGGACAGG TTCTAGGAGA GACGAGGAGG GTGTGCAGAT

CGGAAGCAGA CATGGACAGG TTTTAGGAGG GATGAGGAGG GTGTACAGAT

CAGAAACGGA CACAGACAGG TTTTAGGAGG GACGAGGAAG GTGTGCAGAT

CAGAAACAGA CATTGACAGG TTTTAGGCGG GATGAGGAGG GCGTGCAGAT

---------- ---------- -TTTAGGAGG GATGAGGAGG GTGTGCAGAT

CGGAGGCCGA CATGGACAGG TTTTAGGAGG GATGAAGAGG GCATGCAGAT

CGGAGGCGGA TATTGACAGG TTTTAGGAGA GATGAAGAGG GTGTGCAGAT

GACAGCCCAG CAGGTATTTG AAGAGTTTAT TTGCCAACGT CTCATGCAGG

GACAGCCCAG CAGGTGTTTG AAGAGTTTAT TTGCCAACGT CTCATGCAGG

GACAGCCCAG CAGGTGTTTG AAGAGTTTAT TTGCCAGCGT CTCATGCAGG

GACAGCCCAG CAGGTGTTTG AAGAGTTTAT TTGCCAGCGT CTCATGCAGG

GACAGCCCAG CAGGTGTTTG AAGAGTTTAT TTGCCAACGT CTCATGCAGG

GACGGCCCAG CAGGTGTTTG AAGAATTCAT TTGCCAGCGT CTCATGCAGG

GACAGCCCAG CAAGTGTTTG AAGAGTTCAT CTGCCAGCGT CTCATGCAGG

GACAGCCCAG CAAGTGTTTG AAGAGTTCAT CTGCCAGCGT CTCATGCAGG

GACGGCCCAG CAGGTGTTTG AAGAGTTCAT ATGTCAGCGC CTCATGCAGG

GACGGCCCAG CAGGTATTTG AAGAGTTCAT CTGCCAGCGG CTCATGCAGG

GACGGCCCAG CAGGTGTTTG AAGAGTTCAT CTGCCAGCGT CTCATGCAGG

GACGGCTCAG CAGGTGTTTG AAGAGTTCAT TTGCCAGCGT CTCATGCAGG

GACAGCCCAG CAAGTGTTTG AGGAGTTCAT CTGCCAGCGC CTCATGCAGG

GACAGCTCAG CAGGTGTTTG AGGAGTTTAT TTGCCAGCGT CTCATGCAGG

GACAGCCCAG CAGGTGTTTG AAGAGTTCAT TTGCCAGCGG CTCATGCAGG

GACAGCCCAG CAGGTGTTTG AAGAGTTCAT TTGCCAGCGG CTCATGCAGG

GACTGCCCAG CAGGTGTTCG AGGAGTTTAT TTGCCAACGT CTCATGCAGG

GACAGCTCAG CAAGTGTTTG AAGAATTTAT TTGCCAGCGT CTTATGCAGG

GCTACCAAAT CATAGTGCAG CCCAAGACAC AGAAACCTAG GCCTTGTGTC

GCTACCAAAT CATAGTGCAG CCCAAGACAC AGAAACCTAG GCCTTGTGTC

GCTACCAAAT CATAGTGCAG CCCAAGGCAC AAAAACCTAG GCCTTGTGTC

GCTACCAAAT CATAGTGCAG CCCAAGGCAC AAAAACCTAG GCCTTGTGTC

GCTACCAAAT CATAGTGCAG CCCAAGGCAC AGAAACCTAG GGCTTGTGTC

GGTATCAAAT CATAGTGCAG CCCAAGGCAC AGAAACCTAG GTCTTGTTTC

GTTACCAAAT CATAGTACAG CCAAAGACCC AGAAGCCCAG GCCTTGTGTC

GTTACCAAAT CATAGTACAG CCAAAGACCC AGAAACCTAG GCCTTGTGTC

GCTACCAGAT CATAGTGCAG CCCAAGGCCC AGAAGCC--- ----------

GTTACCAGAT CATCGTTCAG CCCCGCGCAC AGAAGCCTAG GCCTTGTGTC

GCTACCAAAT CATAGTGCAG CCCAAGGCAC AAAAATCTAG GCCTTGTGTC

GCTATCAAAT CATAGTGCAG CCCAAGGCCC AGAAAGCTAG GCCTTGTGTC

GCTACCAGAT CATAGTGCAG CCCAAAGCCC CCAAACCCAG GTCTCGTGTC

GCTACCAGAT CATAGTGCAG CCCAGGGCGC CGAGACCTAG GCCTTGTGTC

GCTACCAAAT CATAGTGCAG CCCAAGGCAC AGAAATCTAG GCCTTGTGTC

GCTACCAAAT CATAGTGCAG CCCAAGGCCC AGAAATC--- ----------

GCTACCAGAT CATCGTACAG CCAAAGCCCC AGAAACCTAG GCCTCGTGTC

GCTACCAGAT TATAGTTCAG CCTAAACCAC AGAAACCCAG GGCTTGTGTC

CCGAAACCGC CCTGAGGAGG AGGACCAGTA TTGGCTGAGT ATGGGCAGAA

CCGAAACCGC CCCGAGGAGG AGGACCAGTA TTGGCTGAGT ATGGGCAGAA

CCGAAACCGC CCCGAGGAGG AGGACCAGTA TTGGCTGAGT ATGGGCAGAA

CCGAAACCGC CCCGAGGAGG AGGACCAGTA TTGGCTGAGT ATGGGCAGAA

CCGAAACCGC CCTGAGGAGG AGGACCAGTA TTGGCTGAGT ATGGGCAGAA

CCGAAACCGC CCTGAGGAAG AGGACCAGTA TTGGCTGAGT ATGGGCAGAA

CCGAAACCGC CCTGAAGAGG AGGGCCAGTA TTGGCTGAGT ATGGGCAGAA

CCGAAATCGC CCTGAGGAGG AAGGCCAGTA TTGGTTAAGT ATGGGCAGAA

---------- ---------- ---------- ---------- ----------

CCGAAACCGC CCGGAAGAGG AAGACCAGTA CTGGCTGAGC ATGGGCAGAA

CCGAAACCGC CCTGAGGAGG AGGACCAATA CTGGCTGAGT ATGGGCAGAA

CCGAAACCGC CCTGAGGAAG AGGACCAGTA CTGGTTGAGT ATGGGCAGAA

CCGTAACCGC CCCGAGGAAG AAGACCAGTA TTGGCTGAGT ATGGGCAGAA

CCGAAACCGC CCTGAAGAGG AAGATCAGTA CTGGCTGAGT ATGGGCAGAA

CCGAAACCGC CCCGAGGAGG AGGACCAGTA CTGGCTGAGT ATGGGCAGAA

---------- ---------- ---------- ---------- ----------

CCGAAACCGT CCTGAGGAGG AAGACCAGTA TTGGCTGAGT ATGGGTCGCA

TCGTAACCGC CCCGAGGAGG AAGACCAATA CTGGCTGAGC ATGGGTCGTA

CGTTCCACAA AGTGACGCTG AAGGATAAGA TGATCACAGT GACGCGATAC

CGTTCCACAA AGTGACGCTG AAGGATAAGA TGATCACAGT GACGCGATAC

CGTTCCACAA AGTGACGCTG AAGGATAAGA TGATCACAGT GACGCGATAC

CGTTCCACAA AGTGACGCTG AAGGATAAGA TGATCACAGT GACACGATAC

CGTTCCACAA AGTGACACTG AAGGATAAGA TGATTACAGT GACACGATAC

CTTTCCACAA AGTGACACTG AAGGATAAGA TGATCACAGT GACACGGTAC

CCTTCCATAA GGTGACACTT AAGGACAAGA TGATCACAGT GACTCGTTAC

CTTTCCATAA GGTGACACTC AAGGACAAGA TGATCACAGT AACTCGTTAC

---------- ---------- ---------- ---------- ----------

CCTTCCACAA AGTCACACTG AAAGACAAGA TGATCACAGT GACCCGCTAC

CGTTCCACAA AGTGACGCTG AAAGATAAGA TGATCACAGT GACACGTTAC

CTTTCCACAA AGTGACACTG AAGGATAAGA TGATCACAGT GACACGGTAC

CTTTCCACAA GGTGACACTG AAGGATAAGA TGATCACAGT CACACGCTAT

CTTTCCACAA AGTGACGCTG AAGGATAAGA TCATCACAGT GACGCGATAC

CTTTCCACAA AGTGACCCTG AAGGATAAGA TCATCACAGT GACACGATAC

---------- ---------- ---------- ---------- ----------

CTTTCCACAA AGTGACGTTG AAGGACAAAA TCATCACAGT CACCCGCTAC

CTTTTCACAA AGTAACGCTG AAGGATAAAA TCATCACCGT CACCCGGTAC

CTTCCCAAGT GAGTTTATTT TGCAGGTATC CTTATGAATC TGCCCAGATC

CTTCCCAAGT GAGTTTATTT TGCAGGTATC CTTATGAATC TGCCCAGATC

CTTCCCAAGT GAGTTTATTT TGCAGGTATC CTTATGAATC TGCCCAGATC

CTTCCCAAGT GAGTTTATTT TGCAGGTATC CTTATGAATC TGCCCAGATC

CTTCCCAAGT GAGTTTATTT TCCAGGTATC CTTATGAATC TGCCCAGATC

CTTCCCAAGT GAGTTTA--- --CAGGTACC CTTATGAATC TGCCCAGATC

CTTCCCAAGT GAGTTTGTTT TTCAGGTACC CTTATGAATC TGCCCAGATC

CTTCCCAAGT GAGTTTGTTT TTCAGGTACC CTTATGAATC TGCCCAGATC

---------- ----TCCTTT TCCAGGTACC CTTATGAATC CGCCCAGATC

CTTCCCAAGT GAGT---TTT TTCAGGTACC CGTACGAGTC CGCCCAGATC

CTTCCCAAGT GAGTTTGTTT TTCAGGTACC CTTATGAATC TGCCCAGATC

CTTCCCAAGT GAGTTTTTTC CCCAGGTACC CTTATGAGTC TGCCCAGATC

CTTCCCAAGT GAGTTTATTT TTCAGGTACC CTTATGAATC TGCCCAGATC

CTCCCCAAGT GAGTTTATTT TTCAGGTATC CTTATGAATC TGCTCAGATC

CTTCCCAAGT GAGTTTGTTT TTCAGGTACC CTTATGAATC TGCCCAGATC

---------- ---------- ---------- ---------- ----------

CTGCCCAAGT GAGTCTTTGT TGCAGGTATC CCTACGAGTC TGCCCAGATC

CTACCCAAGT GAGTTTTATT TTCAGGTACC CCTATGAATC TGCCCAGATC

CACTACACCT ACAGCCTCTG TCCTTCCCAC TCAGACTCAG AGTTCGTCTC

CACTACACCT ACAGCCTCTG TCCTTCCCAC TCAGACTCAG AGTTCGTCTC

CACTATACCT ACAGCCTCTG TCCTTCCCAC TCAGACTCAG AGTTCGTCTC

CACTATACCT ACAGCCTCTG TCCTTCCCAC TCAGACTCAG AGTTCGTCTC

CACTACACCT ACAGCCTCTG TCCTTCACAC TCAGACTCAG AGTTCGTCTC

CACTACACCT ATAGCCTCTG CCCTTCCCAC TCAGACTCAG AGTTTGTATC

CATTACACCT ACAGCCTCTG CCCTTCCCAC TCTGACTCAG AGTTTGTCTC

CATTACACCT ACAGCCTCTG CCCTTCCCAC TCTGACTCGG AGTTTGTCTC

CACTACGCCT ACAGCCTCTG CCCTTCCCAT TCGGACTCGG AGTTCGTCCC

CACTACACCT ACAGCCTCTG CCCCTCCCAC TCCGATTCAG AGTTCGTCTC

CACTACACCT ACAGTCTCTG CCCTTCCCAC TCAGACTCGG AGTTCGTCTC

CACTACACCT ACAGCCTCTG CCCTTCCCAC TCAGACTCGG AGTTTGTCTC

CACTACACCT ACAGCCTCTG CCCCTCCCAC TCTGACTCAG AGTTTGTCTC

CACTATACCT ATAGCCTCTG CCCTTCCCAC TCAGACTCAG AATTCGTCTC

CACTACACAT ATAGCCTCTG CCCCTCCCAC TCAGACTCAG AGTTCGTCTC

---------- ---------- ---------- ---------- ----------

AACTATACCT ACAGCCTGTG CCCCTCGCAC TCCGACTCAG AGTTTGTGTC

AACTACACCT ACAGCTTGTG TCCCTCCCAT TCAGACTCCG AATTTGTCTC

CTGCTGGGTG GAATTCTCCC ACGAACGGCT GGAGGAGTAC AAGTGGAATT

CTGCTGGGTA GAATTCTCCC ACGAACGGCT GGAGGAGTAC AAGTGGAATT

CTGCTGGGTG GAATTCTCCC ATGAACGGCT GGAGGAGTAC AAGTGGAATT

CTGCTGGGTG GAATTCTCCC ATGAACGGCT GGAGGAGTAC AAGTGGAATT

CTGCTGGGTG GAATTCTCCC ATGAACGGCT GGAGGAGTAC AAGTGGAATT

CTGTTGGGTG GAATTCTCCC -CGAACGGCT GGAGGAGTAC AAATGGAATT

CTGTTGGGTG GAATTCTGCC ATGAACGGCT AGAGGAATAC AAGTGGAATT

CTGTTGGGTG GATTTCTGCC ACGAACGGCT CGAGGAATAC AAGTGGAATT

CTGCTGGGTG GAGTTCTCCC ACGAGCGGCT GGAGGAGTAC AAGTGGAATT

CTGTTGGGTG GAATTCTCGC ATGAGCGACT GGAGGAGTAC AAGTGGAATT

TTGTTGGGTG GAATTCTCCC ATGAGCGGCT GGAGGAGTAC AAGTGGAATT

CTGTTGGGTG GAATTCTCCC ACGAGCGGCT GGAGGAGTAC AAGTGGAATT

TTGCTGGGTG GAATTCTCTC ACGAACGGCT GGAGGAGTAC AAGTGGAATT

TTGCTGGGTG GAATTCTCCC ATGAACGGCT GGAGGAGTAC AAGTGGAATT

CTGTTGGGTA GAATTCTCCC ACGAACGGCT GGAGGAGTAC AAGTGGAATT

---------- ---------- ---------- ---------- ----------

CTGCTGGGTG GAGTTCTCCC ACGAACGCCT GGAAGAATAC AAGTGGAATT

TTGCTGGGTG GAGTTCTCCC ATGAACGACT GGAGGAGTAT AAGTGGAATT

ACTTAGATCA GTATATCTGT TCTGCCGGCT CTGAAGACTT CAGGTCAGAG

ACTTAGATCA GTATATCTGT TCTGCCGGCT CTGAAGACTT CAGGTCAGAG

ACTTAGATCA GTATATCTGT TCTGCCGGCT CTGAAGATTT CAGGTCAGAG

ACTTAGATCA GTATATCTGT TCTGCCGGCT CTGAAGATTT CAGGTCAGAG

ACTTAGATCA GTATATCTGT TCTGCAGGCT CTGAAGACTT CAGGTCAGAG

ACTTAGATCA GTATATCTGT TCTGCAGGCT CCGAGGACTT CAGGTCAGAG

ACTTAGATCA GTACATTTGT TCTGCCGGCT CCGAAGATTT CAGGTTAGAG

ACTTAGATCA GTATATTTGT TCTGCTGGCT CTGAAGACTT CAGGTGAGAC

ACTTGGACCA GTACATCTGC TCTGCCGGCT CCGAGGACTT CAGGTCAGAG

ACTTAGATCA GTATATCTGC TCTGCCGGCT CTGAGGACTT CAGGTCAGAG

ACTTAGATCA GTATATCTGT TCTGCTGGCT CTGAGGACTT CAGGTCAGAG

ACTTAGATCA GTATATCTGT TCCACCGGCT CTGAGGATTT CAGGTCAGAG

ACTTAGATCA GTATATTTGC TCTGCTGGCT CCGAGGACTT CAGGTCAGAG

ACCTCGATCA GTATATTTGC TCCGCTGGCT CTGAGGACTT CAGGTCAGAG

ACTTGGATCA GTATATCTGT TCCGCTGGCT CCGAGGACTT TAGGTCAGAG

---------- ---------- ---------- ---------- ----------

ACCTGGATCA GTACATCTGC TCGGCGGGCT CCGAGGACTT caggtcagag

ATTTAGATCA ATATATCTGC TCTGCTGGCT CCGAGGACTT CAGGTCAGAT

AAGCTTAATT GAGTCCCTGA AGTTCTGGAG GACCCGCTTC CTGCTGCTGC

AAGCTTAATT GAGTCCCTGA AGTTCTGGAG GACCCGCTTC CTGCTGCTGC

AAGCTTAATT GAGTCTCTGA AGTTCTGGAG GACCCGCTTC CTGCTGCTGC

AAGCTTAATT GAGTCTCTGA AGTTCTGGAG GACCCGCTTC CTGCTGCTGC

AAGCTTAATT GAGTCCCTAA AGTTCTGGAG GACCCGATTC CTGCTGCTGC

AAGCTTAATT GAGTCCCTGA AGTTCTGGAG GACCCGCTTT CTGCTGCTGC

AAGCTTAATC GAGTCTCTGA AGTTCTGGAG AACCCGATTT CTACTACTGC

AAGCTTAATC GAGTCTCTGA AGTTCTGGAG AACCCGATTT TTACTACTGC

AAGCTTAATT GAGTCTCTGA AGTTCTGGAG AACCCGCTTC CTGCTGCTGC

AAGCTTAATC GAGTCCCTGA AGTTCTGGAG GACACGCTTC CTGCTGCTGC

GAGCTTAATT GAGTCTCTGA AGTTCTGGAG GACCCGCTTC CTGCTGCTGC

AAGCTTAATT GAGTCTCTGA AGTTCTGGAG GACCCGATTC CTGCTGCTGC

A--------- ---------- ---------- ---------- ----------

AAGCTTAATT GAGTCCCTGA AGTTCTGGAG GACCCGCTTC CTGCTGCTGC

AAGCTTAATT GAGTCGCTGA AGTTCTGGAG GACCCGCTTC CTGCTGCTGC

---------- ---------- ---------- ---------- ----------

gAGTTTAATT GAGTCGCTGA AATTCTGGAG AACGCGCTTC CTGCTGCTCC

AAGCTTAATT GAGTCTCTGA AGTTCTGGAG GACCCGCTTC CTGCTGCTTC

CAGCCTGTGT CACCGCCACC AAGCGCATCA CGGAGGGGGA GGCCCACTGC

CAGCCTGTGT CACCGCCACC AAGCGCATCA CGGAGGGGGA GGCCCACTGC

CAGCCTGTGT CACCGCCACC AAGCGCATCA CAGAGGGGGA GGCCCACTGT

CAGCCTGTGT CACCGCCACC AAGCGCATCA CAGAGGGGGA GGCCCACTGC

CAGCCTGTGT CACCGCCACC AAGCGCATCA CGGAGGGAGA GGCCCACTGC

CAGCCTGTGT CACTGCCACC AAGCGCATCA CAGAAGGGGA GGTACACTGC

CAGCCTGTGT CACTGCCACC AAGCGCATCA CAGAAGGGGA AGTGCACTGT

CAGCCTGTGT TACTGCCACC AAGCGCATCA CAGAAGGGGA GGTGCACTGT

CAGCCTGCGT CACTGCCACC AAGCGTATCA CAGAGGGCGA GGCCCACTGC

CGGCCTGCGT CACTGCCACC AAGCGCATCA CGGAGGGAGA GGCGCACTGC

CAGCCTGTGT CACTGCCACT AAGCGCATCA CAGAAGGGGA GGCCCACTGC

CAGCCTGTGT CACTGCCACC AAGCGCATCA CAGAGGGGGA GGCCCACTGC

---------- ---------- ---------- ---------- ----------

CAGCCTGTGT CACCGCCACC AAGCGCATCA CAGAGGGGGA AGCCCACTGC

CAGCCTGTGT CACTGCCACC AAGCGCATCA CAGAGGGGGA AGCCCACTGC

---------- ---------- ---------- ---------- ----------

CAGCATGCGT CACTGCTACC AAACGCATCA CAGAAGGGGA GGCGCACTGT

CTGCCTGCAT CACCGCCACC AAGCGCATCA CTGAGGGCGA GTCCCACTGT

GACATCTATG GGGAGACGAG GACGAGTGGC AACTCCTGGA TGGTTTTGTC

GACATCTATG GGGAGACGAG GACGAGTGGC AACTCCTGGA TGGTTTTGTC

GACATCTATG GGGAGACGAG GACGAATGGC AACTCCTGGA TGGCTTTATC

GACATCTATG GGGAGACGAG GACGAATGGC AACTCCTGGA TGGCTTTATC

GACATCTATG GGGAGACGAG GATGAGTGGC AACTCCTGGA TGGCTTCATC

GACATCTATG GGGAGATGAG GACGAGTGGC AACTTCTGGA TGGTTTTATC

GACATCTACG GGGAGATGAA GATGAGTGGC AGCTTCTAGA TGGCTTTATT

GACATCTATG GGGAGATGAA GATGAGTGGC AGCTTCTAGA TGGCTTTATT

GACATCTATG GGGAGACGAG GACGAGTGGC AGCTTCTGGA CGGCTTTATC

GACATCTACG GGGAGACGAG GATGAGTGGC AGCTCCTGGA CGGCTTCATC

GACATCTATG GGGAGACGAG GAGGAATGGC AGCTCTTGGA TGGTTTTATC

GACATCTACG GGGAGATGAG GAGGAGTGGC AGCTCCTGGA TGGCTTTATC

---------- ---------- ---------- ---------- ----------

GACATCTACG GGGAGATGAG GAGGAGTGGC AGCTCCTAGA CGGTTTCATC

GACATCTATG GGGAGATGAG GAAGAGTGGC AGCTCCTCGA CGGCTTTATC

---------- ---------- ---------- ---------- ----------

GACATCTACG GGGAGATGAG GAAGAGTGGC AGCTCTTGGA TGGCTTCATC

GACATCTACG GGGAGATGAG GAAGAGTGGC AGCTCTTAGA TGGCTTTATC

CGCTTTGTGG AGGGCTTGAA TCGCATTCGC AGGCGGCATC GCTCGGATCG

CGCTTTGTGG AGGGCTTGAA TCGCATTCGC AGGCGGCATC GCTCGGATCG

CGCTTTGTGG AGGGCTTGAA TCGCATTCGC AGGCGGCATC GCTCGGATCG

CGCTTTGTGG AGGGCTTGAA TCGCATTCGC AGGCGGCATC GCTCGGATCG

CGCTTTGTGG AGGGCTTGAA CCGCATTCGC AGGCGGCATC GCTCAGATCG

CGCTTTGTGG AAGGCTTGAA TCGGATCCGC AGGCGGCATC GGTCAGATCG

CGCTTTGTAG AGGGCTTGAA CCGCATCCGC AGGCGCCACC GGTCAGACCG

CGCTTTGTAG AGGGCTTAAA TCGCATCCGC AGGCGCCACC GCTCAGACCG

CGCTTTGTGG AGGGCTTGAA CCGGATCCGC AGGCGGCACC GCTCAGACCG

CGCTTCGTGG AGGGCCTGAA CCGCATCCGC CGGCGGCACC GCTCGGACCG

CGCTTCGTGG AGGGTTTGAA CCGGATCCGC AGGCGACATC GCTCAGATCG

CGCTTTGTGG AGGGCTTGAA CCGGATCCGC AGGCGCCATC GCTCCGATCG

---------- ---------- ---------- ---------- ----------

CGCTTTGTGG AGGGTTTGAA CCGGATCCGC AGGCGGCATC GCTCAGATCG

CGCTTTGTGG AGGGCCTAAA CCGGATCCGC AGGCGGCACC GCTCTGATCG

---------- ---------- ---------- ---------- ----------

CGCTTCATGG AAGGCTTGAA TCGCATCCGG CGGCGCCACC GGTCAGACCG

CGTTTCATGG AAGGTTTGAA CCGGATTCGA AGGCGTCATC GATCAGATCG

CATGATGCGG GTAAGGCCCC CAGTGGGGAA GAAGGGAACC TCAGCTCTCT

CATGATGCGG GTAAGGCCCC CAGTGGGGAA GAAGGGAACC TCAGCTCTCT

CATGATGCGG GTAAGGCCCC CAGTGGGGAA GAAGGGAACC TCGGCTCTCT

CATGATGCGG GTAAGGCCCC CAGTGGGGAA GAAGGGAACC TCGGCTCTCT

CATGATGCGG GTAAGGCCCC CAGTGGGGAA GAAGGGAACC TCAGCTCTCT

CATGATGCGG GTGAGGCCTC CGGTTGGGAA GAAGGGAACC TCAGCTCTGT

CATGATTCGG GTAAGGCCCC CAGTCGGGAA GAAAGGAACC TCAGCTCTGT

CATGATTCGG GTAAGGCCTC CAGTCGGCAA GAAAGGAACC TCAGCTCTGT

CATGATGCGG GTGAGGCCCC CTGTCGGGAA GAAGGGAGCC TCAGCTCTGT

CATGATGCGG GTGAGGCCCC CGGTTGGGAA GAAGGGAACC TCAGCTCTGT

CATGATGCGG GTAGGGCCCC CAGTTGGGAA GAAGGGAACC TCAGCTCTGT

CATGATGCGG GTGAGGCCCC CGGTTGGGAA GAAGGGAACC TCAGCTCTGT

---------- ------CCTC CTGTTGGGAA GAAGGGGACC TCAGCGCTGT

CATGATACGG GTGAGGCCCC CGGTTGGAAA GAAGGGGACA TCCGCTCTGT

CATGATGCGG GTGAGGCCCC CAGTTGGGAA GAAGGGAACC TCAGCTCTGT

---------- ------CCCC CGGTTGGGAA GAAGGGAACG TCCGCTCTGT

CATGATACGG GTGAGTCCAC CGGTTGGAAA GAAGGGGACG TCTGTCCTCT

CATGATTCGG GTGAGGCCAC CAGTCGGGAA GAAGGGAACC TCAGCGCTCT

CTGCCCTGTT GGAGATGGAG GCCAGTCAGA AGTAAGTGAG AGCAGCAGCG

CTGCCCTGTT GGAGATGGAG GCCAGTCAGA AGTAAGTGAG AGCGGCAGCG

CTGCCCTGTT GGAGATGGAG GCCAGTCAGA AGTAAGTGAG AGCAGCAGCG

CTGCCCTGTT GGAGATGGAG GCCAGTCAGA AGTAAGTGAG AGCAGCAGCG

CTGCCCTGTT GGAGATGGAG GCCAGTCAGA AGTAAGTGAG AGCAGCAGCG

CTGCCCTGCT GGAGATGGAG GCCACTCAGA AGTAAGTGAG AGCAGCAGTG

CTGCCCTGCT GGAGATGGAG GCTAGTCAGA AGTAAGTGAG AACAGCAGTG

CTGCCCTGCT GGAGATGGAA GCTAGTCAGA AGTAAGTGAG AACAGCAGTG

CCGCCCTGCT GGAGATGGAG GCCAGCCAGA AGTAAGTGAG AGCGGCAGCG

CTGCCCTGCT GGAGATGGAG GCCAGTCAGA AGTAAGTGAG AGTGGCGGTG

CCGCCCTGTT GGAGATGGAG GCCAGTCAGA AGTAAGTGAG AGCAGCAGCG

CTGCCCTGTT GGAGATGGAG GCCAGTCAGA AGTAAGTGAG AGCAGCAGCG

CTGCCCTGTT GGAGATGGAG GCCAGTCAGA AGTAAGTGAG AGCAGTAGCG

CCGCCTTGCT GGAAATGGAG GCCAGTCAGA AGTAAGTGAA AGCGGCAGTG

CTGCCTTGTT GGAGATGGAG GCCAGTCAGA AGTAAGTGAG AGCAGCAGTG

CTGCTCTGTT GGAGATGGAG GCCAGTCAGA AGTAAGTGAG AGCAACAGTG

CGGCTTTGCT GGAAATGGAA GCCAGTCAGA AGTGAGTGAG AATAGTGGTG

CTGCCTTGCT GGAAATGGAA GCCAGTCAGA AGTGAGTGAG AGTAGCAGTA

TTGCCATGAC TCCCACCTAC ATGGACAGCC CACGAAAGGT AAATCCTCCT

TTGCCATGAC TCCCACCTAC ATGGACAGCC CACGAAAGGT AAATCCTCCT

TCGCCATGAC TCCCACCTAC ATGGACAGCC CACGAAAGGT AAATCCTCCT

TCGCCATGAC TCCCACCTAC ATGGACAGCC CACGAAAGGT AAATCCTCCT

TTGCCATGAC TCCCACCTAC ATGGACAGCC CACGAAAGGT AAATCCTCCT

TTGCCATGAC TCCCACCCAT GTGGACAGCC CACGGAAGGT AAATCCTCCT

TTGCCATGAC TCCCACCTAT GTGGACAGCC CACGCAAGGT AAGTCCTCCT

TTGCCATGAC TCCCACCTAT GTGGACAGCC CACGCAAGGT AAATCCTCCT

TTGCCATGAC TCCCACCTAC GTGGACAGCC CACGAAAGGT AAATCCTCCT

TAGCCATGAC TCCCACCTAT GTGGACAGCC CGCGAAAGGT AAATGCCGCT

TTGCCATGAC TCCCACCTAC GTGGACAGCC CACGAAAGGT AAATCCTCCT

TTGCCATGAC TCCCACCTAC GTGGACAGCC CACGAAAGGT AAATCCTCCT

TTGCTATGAC TCCCACCTAC GTGGATAGCC CACGAAAGGT AAA-------

TTGCCATGAC TCCCACCTAC GTGGACAGCC CACGAAAGGT AAATCCTCCT

TTGCCATGAC ACCCACATAC GTGGACAGCC CACGAAAGGT AAGTCCTCCT

TTGCCATGAC ACTCACCTAC GT-GACAGCC CACGAAAGGT AAGTGCTCCT

TGGCCCTCAC ACCGACTTAC CTGGACAGCC CCCGAAAGGT GAGTCCTCCA

TTTCTATCAC ACCCACTTAC CTGGACAGTC CTCGCAAGGT AAATCCTCTG

GTGACTTCAG GACGGGGCCT TCTTTATGGA GTTTGTCCGC AGCCCACGCA

CTGACTTCAG GACGGGGCCT TCTTTATGGA GTTTGTCCGC AGCCCACGCA

CTGACTTCAG GACGGGGCCT TCTTTATGGA GTTTGTCCGC AGCCCACGCA

CTGACTTCAG GACGGGGCCT TCTTTATGGA GTTTGTCCGC AGCCCACGCA

CTGACTTCAG GACGGGGCCT TCTTTATGGA GTTTGTCCGC AGCCCACGCA

CTGACTTCAG GACAGGGCCT TCTTTATGGA GTTTGTCCGC AGCCCACGCA

CTGACTTCAG GACGGGGCCT TCTTTATGGA GTTTGTCCGC AGCCCACGCA

CTGACTTCAG GACGGGGCCT TCTTTATGGA GTTTGTCCGC AGCCCACGCA

CTGACTTCAG GACGGGGCCT TCTTTATGGA GTTTGTCCGC AGCCCACGCA

CTGACCTCAG GACGGGGCCT TCTTTATGGA GTTTGTCCGC AGCCCGCGCA

CTGACTTCAG GACGGGGCCT TCTTTATGGA GTTTGTCCGC AGCCCTCGCA

CTGACTTCAG GATGGGGCCT TCTTTATGGA GTTTGTCCGC AGCCCGCGCA

---------- ---------- TCTTTATGGA GTTTGTCCGC AGCCCTCGAA

CTGACTTCAG GACGGGGCCT TCTTTATGGA GTTTGTCCGC AGCCCACGCA

CTGACTTCAG GATGGGGCCT TCTTTATGGA GTTTGTCCGC AGCCCACGCA

CTGACTTCAG GATGGGGCCT TCTTTACGGA GTTTGTCCGC AGCCCACGCA

CCGCCTCCAG GAGGGGGCCT TCTTTACGGA GTCTGTCCGC AGCCCACGTA

ATGCCTTCAG GATGGGGCCT TCTTTATGGA GTTTGTTCGC AGCCCACGTG

CAGCATCGTC CGCCTTCTAC CCTCAGGTTA GTGTCAGATA CTTACGCTGA

CAGCATCGTC CGCCTTCTAC CCTCAGGTTA GTGTCAGATA CTTACGCTGA

CAGCATCGTC CGCCTTCTAC CCTCAGGTTA GTGCCAGATA CTTACGCTGA

CAGCATCGTC CGCCTTCTAC CCTCAGGTTA GTGCCAGATA CTTACGCTGA

CAGCATCGTC CACCTTCTAC CCTCAGGTTA GTGCCAGATA CTTACGCTGA

CAGCATCGTC CACCTTCTAC CCTCAGGTTA GT-------- ----------

CAGCATCATC TGCCTTCTAC CCTCAGGTTA GTACCGAATA CTTACACTGA

CAGCATCATC TGCCTTCTAC CCTCAGGTTA GTACTGAATA CTTACACTGA

CGGCATCGTC CGCCTTCTAC CCTCAGGTCA GTGCCAAATA CTTACGCTGA

CAGCATCATC CGCCTTCTAC CCGCAGGTCA GTGCCAAATA CTTACACTGA

CAGCGTCATC CGCCTTCTAC CCACAGGTCA GTGCCAAATA CTTACGCTGA

CAGCATCGTC CGCCTCCTAC TCTCAGGTCA GTGCCAAATA CTTACGCTGA

CAGCATCGTC TACCTTCCAC TCACAGGTCA GTACCAAATA CTCACGCTGA

CAGCATCGTC TGCCTTCTAC CCACAGGTTA GTGCCAAATA CTTACGCTGA

CAGCATCGTC TGCCTTCTAC CCACAGGTTA GTGCCATATA CTTACATTGG

CAGTATCGGC CGCCTTCTAC CTGCAGGTTA GTGC-GCACA CTCACACTGG

CAGCGCCATC TGCCTTCTAC CCCCAGGTCA GTGTGTACTA CTTACATTGA

CCGCCTCTTC CACCTTCTAC CCACAGGTCA GTGCCATGTA CTTACATTGA

CTTTGGGCTG AGAAGGCAAA GTCCCACTTG CCTTCATTGT GCTCACTAGC

CTTTGGGCTG AGAAGGCAAA GTCCCACTTG CCTTCATTGT GCTCACTAGC

CTTTGGGCTG AGAAGGCAAA GTCCCACTTG CCTTCATGGT GCTCACTAGC

CTTTGGGCTG AGAAGGCAAA GTCCCACTTG CCTTCATTGT GCTCACTAGC

CTTTGGGCTG AGAAGGCAAA GTCCCACTTG CCTTCATTGT GCTCACTAGC

---------- ---------- ---------- ---------- ----------

CTTTGGGCTG GGTAGGCAAA GTCCCACTTG CCTTCATGGT GTTCACTAGT

CTTTGGGCTG GGTAGGCAAA GTTCCACTTG CCTTCATGGT GTTCACTAGC

CTTTGGGCTG AGAAGGCAGA GTCCCGCTCG CCTTCATGGT GCTCACTAGC

CTTTAGGCTG AGAAGGCAAA GTCCCACTTG CCTTCATGGT GCTCACTAGC

CTTTGGGCTG AGAAGGCAAA GTTCCACTTG CCTTCATGGT GCTCACTAGT

CTTTGGGCTG AGAAGGCAAA GTCCCACTTG CCTTCATGGT GCTCACTAAC

TTTTGGGCTG AGAAGGCAAA GTCCCACTTG CCTTCATGGT GCTCACAAGT

CTTTGGGCTG TGAAGGCAAA GTCCCACTTG CCTTCATGGT GCTCACCAGC

CTTTGGGCTG TGAAGGCAAA GTCCCACTTG CCTTCATGGT GCTCACTAGT

CTTTGGGCTG CGAGGGCAGA GTCCCGCTTG CTTTCATGCT GCTCACTAGC

CTTTGGGCTG GGATGGCAGA GTGCCACTGG CCTTCATGGT GCTCACGAGC

CTTTGGGCTG TGAAGGTAAA GTCCCACTTG CCTTCATGCT GCTCACTAGC

TTGTTGAACG CAGTCATGTC TCCGTCTTTC TTCAGTTGTC GATTGTTGGT

TTGTTGAACG CAGTCATGTC TCCGTCTTTC TTCAGTTGTC GATTGTTGGT

TTGTTGAACG CAGTCATGTC TCCGTCTTTC TTGAGTTGTC GATTGTTGGT

TTGTTGAACG CAGTCATGTC TCCGTCTTTC TTGAGTTGTC GATTGTTGGT

TTGTTGAATG CAGTCATGTC TCCATCTTTC TTGAGTTGTC GATTGTTGGT

---------- ---------- ---------- ---------- ----------

TTGTTGAATG CAGTCATATC TCCATCTTTC TTGAGCTGCC GATTGCTAGT

TTGTTGAATG CAGTCATATC TCCATCTTTC TTGAGCTGTC TATTGCTACT

TTGTTGAAAG CAGTCATATC GCCGTCTTTC TTGAGTGGCC GACTGCTGGT

TTGTTGAACG CAGTCATGTC TCCGTCTTTT TTGAGCTGTC GATTGCTGGC

TTGTTGAATG CAGTCATATC TCCATCTTTC TTAAGCTGTC GATTGCTGGT

TTGTTGAATG CAGTCATATC TCCATCTTTT TTGAGCTGTC GATTGCTGGT

TTGTTGAAAG CAGTCATATC TCCATCTTTC TTGAGCTGCC GATTGCTGGT

TTGTTGAAAG CAGTCATATC TCCATCCTTT TTGAGCTGCC GATTGCTGGC

TTGTTGAACG CAGTCATATC TCCATCCTTT TTGAGCTGCC GACTGCTTGT

TTGTTGAATG CAGTCATATC GCCGTCCTTT CTGAGCTGCC GGTTGCTTGT

TTGTTAAAGG CAGACATGTC ACCGTCTTTT GTGAGCGGCC GGGAGCTGGC

TTGTTAAATG CAGACATATC ACCATCTTTT TTGAGTTGTC TAGAGCTAGC

TACGGCACTC AAGGTCTCTT CTAGGTGTTC TGCCATGAAG GGAGTTGAAT

TACAGCACTC AAGGTCTCTT CTAGGTGTTC TGCCATGAAG GGAGTTGAAT

TACAGCACTC AAGGTCTCTT CTAGGTGTTC TGCCATGAAG GGAGTTGAAT

TACAGCACTC AAGGTCTCTT CTAGGTGTTC TGCCATGAAG GGAGTTGAAT

TACAGCATTC AAGGTCTCTT CTAGGTGTTC TGCCATGAAG GGAGTTGAAT

---------- ---------- ---------- ---------- ----------

CACAGCACTC AGGGTCTCCT CTAAATGTTC TGCCATGAAG GGAGTTGAAT

CGCAGCACTC AGGGTCTCCT CTAAATGTTC TGCCATGAAG GGAGTTGAAT

TCCGGCACTC AGGGGCTCCT CTAAGTGCTC TGCCATGAAG GGAGTCGAGT

CACAGTACTC AGGGTCTCCT CTAAATGTTC TGCCATAAAG GGGGTCGAAT

TACAGCACTC AAGGTCTCCT CTAAGTGTTC TGCCATGAAG GGAGTCGAAT

TACAGCACTC AGGGTCTCCT CTAAGTGTTC TGCCATGAAG GGAGTTGAAT

TACAGCACTC AGAGTCTCCT CTAACTGTTC TGCCATGAAG GGAGTTGAAT

TACAGCACTC AGGGTCTCCT CTAAGTGTTC TGCCATGAAG GGAGTTGAAT

TACAGCATTT AGAGTCTCCT CTAAATGTTC TGCCATGAAG GGAGTTGAAT

TACAGCACTC AGGGTCTCCT CTAAATGTTC TGCCATGAAA GGAGTTGAAT

CACGGCACTC AGGGCCTCCT CTGCACGCTC AGCCAGGAAG GGTGGGCCAC

CACTGCAGTT AGCGCCTCCT CCAAATGCTC TGCCATGAAA GGTGTAGAGA

TCTTCACTTG CTGGTCAACC TTCAAGCCCT TCAGACCTGC TTCTACCTCC

TCTTCACTTG CTGGTCAACC TTCAAGCCCT TCAGACCTGC TTCTACCTCC

TCTTCACTTG CTGGTCCACC TTCAAGCCCT TCAGACCTGC TTCTACCTCC

TCTTCACTTG CTGGTCCACC TTCAAGCCCT TCAGACCTGC TTCTACCTCC

TCTTCACTTG CTGGTCCACC TTCAAGCCCT TCAGCCCTGC TTCTACCTCT

---------- ---------- ---------- ---------- ----------

TCTTTATTTG CTGGTCCACC TTCAAGCCCT TCAGCCCGGC TTCTACCTCT

TCTTCATTTG CTGGTCCACT TTCAAGCCCT TCAGCCCTGC TTCTACCTCT

TCTTCACTTG CTGGTCCACC TTCAGGCCCG TGAGCCCTGC TTCCACCTCT

TCTTCACTTG CTGGTCAACC TTCAAGCCCT TGAGCCCTGC TTCCACCTCC

TCTTCACTTG CTGGTCCACC TTCAAGCCCT TGAGCCCTGC TTCTACCTCT

TTTTCACTTG CTGGTCCATC TTCAAGCCCT TGAGCCCTGC TTCTACCTCT

TCTTCATTTG TTGATCCACT TTCAAGCCCT TGAGCCCAGC TTCTACCTCT

TCTTCACTTG CTGGTCCACC TTCAAGCCCT TAAGCCCTGC TTCGACTTCT

TCTTCACTTG CTGGTCCACC TTCAAGCCCT TGAGCCCTGC TTCTACCTCT

TCTTCATGTG CTGGTCCACC TTCAAGCCCT TGAGCCCTGC TTCTACCTCT

TCTTCCCTTG CTGGTCTACT CTCAGGCCCT TGAGCCCAGC TTCCACCTCT

TTTTCCCCTG CTGATCCACT TTCAGGCCTT TCAGCCCAGC TTCTACTTCT

TCCACTGAAA GCACAACCCC TGAATGTGCT GAGAAGTGAG AATGGGCATA

TCCACTGAAA GCACAACCCC TGAATGTGCT GAGAAGTGAG AATGGGCGTA

TCCACGGAAA GCACAACCCC TGAATGTGCT GAGAAGTGAG AATGGGCATA

TCCACGGAAA GCACAACCCC TGAATGTGCT GAGAAGTGAG AATGGGCATA

TCCACTGAAA GCACGACCCC TGAATGTGCT AAGAAGTGAG AATGGGCATA

---------- ---------- ---------- ---------- --TGGGGGTA

TCCACTGAAA GTACAACCCC TGAATGTGCT GAGAAGTGAG AATAGGGGTC

TCCACTGAAA GTACAACCCC TGAATGTGCT GAGAAGTGAG AATAGGGGTC

TCCACGGAAA GCACGACCCC CGAATGTGCT GGAAAATGAG AATGGGGATA

TCCACGGAAA GCACAACCCC TGAATGTGCT GAAAGGTGAG AATGGGGGTG

TCCACTGACA GCACAACTCC TGAATGTGCT GAAAGATGAG AATAGGGGTA

TCCACTGAAA GCACAACCCC TGAATGTGCT GAAAGGTGAG AATGGGGGTA

TCCACCGAAA GTACAACTCC TGAGTGTGCT GAAAGGTAAG AATGGGGATA

TCCACAGAAA GCACAACTCC TGAATGTGCT GAAAAGTGAA AATGGGGGTA

TCCACTGAAA GCACGACCCC TGAATGCGCT GAAAAGTGAA AATGGGGGTA

TCCACTGAGA GCACAACCCC TGAATGTGCT GAAAAGTGAA AATGGGGCTA

TCCACCGAAA GCACAACCCC TGTATGCGCT AAAGAGGGAG AATGGGCATC

TCCACTGACA GCACAACTCC GGAATGCGCT GCTGATTAAC AATGGGGATA

TGCACAGGCC AATCCATGGA CAGAGGCAAC AGCCAGACCT TTGGGAACTC

TGCACAGGCC AATCCATGGA CAGAGGCAAC AGCCAGACCT TTGGGAACTC

AGCACAGGCC AATCCGTGGA CAGAGGCAAC AGCCAGACCT TTGGGAACTC

AGCACAGGCC AATCCGTGGA CAGAGGCAAC AGCCAGACCT TTGGGAACTC

AGCACAGGCC AATCTGTGGA CAGAGGCAAC AGCCAGACCT TTGGGAACTC

AGCACAGGCC AATCCGTGGA CAGAGGCAAC AGTCAGGCCT TTGGGAACTC

AGCACGTGCC AGTCCGTGGA TAGAGGCAAT AACCAGACCT TTGGGAACTC

AGCACAGGCC AGCCCATGGA TAGAGGCAAC AACCAGACCT TTGGGAACTC

ACCATGGGCC AGTCTGTGGA CAGAGGCAAC AGCCAGACCC TGGGGAACGC

AGCACAGGCC AGTCCGTGGA CAGGGGCAAC AGCCAGACCT TCGGGAACTT

AGCACAGGCC AGTCTGTGGA CAGAGGCAAC AGCCAGGCCT TTGGGAACTC

AGCGCAGGTC AATCCGTGGA CAGAGGCAAC AGCCAGACCT TTGGGAACTC

AGCACAGGCC AGTCCATGGA CAGAGTCAAC AGCCAGACCG TTGGGAACAC

AGCACTGGCC AATCCATGGA CAGAGGCAAC AGCCAGACCT TTGGGAACTC

AGCACAGGCC AATCCATGGA CAGAGGCAAC AGCCAGACCT TTGGGAACTC

AGCACAGGCC AGTCCGTGGA CAGAGGCACC AGCCAGACCT TTGGGAACTC

AGCGTGGGCC AGCCCATGGA CAGAGGCAGC ACGCAAGCCT TTGGGAATGC

AATGCAGGCC AGTCAACTGA TAGAAGCAGC ACCCAGCCCT TTGGGAGCTC

CCAGAACATA GGAGAACAGG GTCCTAGAAA ATGCTGGAAG AGCAGCTCAT

CCAGAACATA GGAGAACAGG GTCCTAGAAA ATGCTGGAAG AGCAGCTCAT

CCAGAACATA GGAGAACAGG GTCCTAGAAA ATGCTGGAAG AGCAGCTCAT

CCAGAACATA GGAGAACAGG GTCCTAGAAA ATGCTGGAAG AGCAGCTCAT

CCAGAACATA GGAGAACAGG GTCCTAGAAA ATGCTGGAAG AGCAGCTCAT

CCAGAACATA GGAGAACAGG GTCCTAGAAA ATGCTGGAAG AGCAGTTCAT

CCAGAACATA G---AACAGG CTCCTAGAAA ATGCTGGAAG AGCAGCTCAT

CCAGAACATA G---AACAGG CTCCTAGAAA ATGCTGGAAG AGCAGCTCAT

ACAGAATGTG GGGGAACAGG GTCCTAGAAA ATGCTGGAAG AGCAGCTCAT

GCAGAGCTTG GGGGAACAGG GTCCTAGAAA ATGCTGGAAG AGCAGCTCAT

CCAGAACTCA GGGGAACAGA GTCCTAGAAA ATGCTGGAAG AGCAACTCAT

TCAGAACTCA GGGGAACAGG GTCCCAGAAA ATGCTGGAAG AGCAGCTCAT

ACAGAGCTCA GGGGAACAGG GTCCTAGAAA ATGCTGGAAG AGCAGCTCAT

CCAGAATGCT GGGGAACAGG GTCCTAGAAA ATGCTAGAAG AGCAGCTGAT

CCAGAATGCA GGGGAACAGG GTCCTAGAAA ATGCTAGAAG AGCAACTCAT

CCAGAACGCA GGGGAACAGG CCTTTAGAAA ATGCTGGAAG AACAACTCAT

CCAGGCTGCC GGAGAGCAGG GTCTTAGAAA ATGCTGGAGG AGCAGCTCAT

CCAGAATTTA GGGGAACAGG GTCTTAGAAA ATGTTGGAGG AGCAGCTGAT

CACACATGCA TCTGGCGAAG CCTGGCGGAC CTTCATCTAC GGCTTCTATT

CACACATGCA TCTGGCGAAG CCTGGCGGAC CTTCATCTAC GGCTTCTATT

CACACACGCA TCTGGCGAAG CCTGGCGGAC CTTCATCTAC GGCTTCTATT

CACACACGCA TCTGGCGAAG CCTGGCGGAC CTTCATCTAC GGCTTCTATT

CACACACGCA TCTGGCGAAG CCTGGCGGAC CTTCATCTAC GGCTTCTATT

CATGCATGCA TCTGGGGAAG CCTGGCGGAC CTTCATCTAT GGCTTCTATT

AGCGCATGCA TCTGGAGAAG CATGGCGAAC CTTCGTCTAC GGCTTCTATT

AACGCATGCA TCTGGTGAAG CATGGCGGAC CTTCATCTAT GGCTTCTATT

CACACACGCA TCTGGGGAAG CCTGGCGGAC CTTCATCTAC GGCTTCTACT

TACACACGCC TCTGGTGAAG CCTGGCGGAC CTTCATCTAC GGCTTCTATT

CACACACGCA TCTGGTGAAG TCTGGCGGAC CTTCATCTAT GGCTTCTATT

CACGCACGCA TCTGGGGAAG TCTGGCGGAC CTTCATCTAC GGCTTCTATT

CACGCACGCG TCTGGTGAAG TCTGGCGGAC CTTTATCTAT GGCTTCTATT

CACGCACGCA TCTGGTGAAG TCTTGCGGAC CTTCATCTAC GGCTTCTACT

CACACATGCC TCCGGTGAAG TTTTGCGGAC CTTCATCTAC GGTTTCTATT

CATACATGCA TCTGGCGAAG CTTTGCGGAC CTTCATCTAT GGCTTCTATT

TACCCACGCC TCGGGGGAGG CCCTGCGCAC CTTCATCTAC GGCTTCTACT

CACACATGCC TCTGGGGAAG CCCTGCGAAC CTTCATTTAT GGATTCTATT

TCTACAAGAT AGTCACTTAC AGCTTTCTTT AAGTTTTTCT TTATTTGCAG

TCTACAAGAT AGTCACTTAC AGCTTTCTTT AAGTTTTTCT TTATTTGCAG

TCTACAAGAT AGTCACTTAC AGCTTTCTTT AAGTTTTTCT TTATTTGCAG

TCTACAAGAT AGTCACTTAC AGCTTTCTTT AAGTTTTTCT TTATTTGCAG

TCTACAAGAT AGTCACTTAC AGCTTTCTTT AAGTTTTTCT TTATTTGCAG

TCTACAAGAT CGT------- ---------- ---------- ----------

TCTACAAGAT AGTTACTTAC AGCTTTCTTT AAGTTTTTCT TTATTTGCAG

TCTACAAGAT AGTTACTTAC AGCTCTCCTT AAGTTTTTCT TTATTTGCAG

TCTACAAGAT CGTCACTTAC AGCTCTCCTT AAGTTTTTCT TTATTTGCAG

TCTACAAGAT CGTCACTTAC AGCTCTCTTT AAGCTTTTCT TTATTTGCAG

TCTACAAGAT AGTCACTTAC AGCTTTCCTT AAGCTTTTCT TTATTTGCAG

TCTACAAGAT AGTCACTTAC AGCTCTCTTT AAGTTTTTCT TTATTTGCAG

TCTACAAGAT AGTAACTTAC AGCTCTCTTT AAGTTTTTCT TTATTTGCAG

TCTACAAGAT AGTCACTTAC AACTTTCTTT AAGTTTTTCT TTATTTGCAG

TCTACAAGAT AGTCACTTAC AGCTTTCTTT AAGTTTTTCT TTATTTGCAG

TCTACAAGAT AGCTACTTAC AGCTTTCTTT AAGCTTTTCT TTATTTGCAG

TCTATAAGAT CGTTACTCAC AGCTTTCTTT CAGCTTTTCC TTGTTGGCAG

TCTACAAGAT AGTCACTTAC AGCTCTCTTT AAGCTTTTCC TTGTTGGCGG

AAAGGCTGGA AAGAAGAGGT TTCAAATCCA CTTTGGCTTT CTGTAGCATT

AAAGGCTGGA AAGAAGAGGT TTCAAATCCA CTTTGGCTTT CTGTAGCATT

AAAGGCTAGA AAGAAGAGGT TTCAAATCCA CTTTGGCTTT CTGTAGCATT

AAAGGCTAGA AAGAAGAGGT TTCAAATCCA CTTTGGCTTT CTGTAGCATT

AAAGGCTGGA AAGAAGCGGT TTCAAATCCA CTTTGGCTTT CTGTAGCATT

---------- ---------- ---------- ---------- ----------

AAAGGCTAGA AAGAAGGGGT TTTAAATCCA CTTTGGCTTT CTGTAGCATT

AAAGGCTAGA AAGAAGAGGT TTTAAATCTA CTTTGGCTTT CTGTAGCATT

AAAGGCTGGA AAGAAGAGGT TTTAAATCCA CTTTGGCTTT CTGTAGCATT

AAAGGCTGGA AAGAAGAGGC TTTAAATCCA CTTTGGCTTT CTGTAGCATT

AAAGACTGGA AAGAAGAGGT TTTAAATCCA CTTTGGCCTT CTGCAGCATT

AAAGGCTGGA AAGAAGAGGT TTTAAATCCA CTTTGGCTTT CTGTAGCATT

AAAGGCTGGA AAGAAGAGGT TTTAAATCCA CTTTGGCTTT CTGTAGCATT

AAAGGCTGGA GAGAAGAGGT TTTAAGTCCA CTTTGGCTTT CTGTAGCATT

AAAGGCTGGA AAGAAGAGGT TTTAAGTCCA CTTTGGCTTT CTGTAGCATT

AAAGGCTGGA GAGAAGAGGT TTTAAGTCCA CTTTGGCTTT CTGTAGCATT

AAAGGTTGGA GAGGAGGGGC TTTAGGTCCA CTTTGGCCTT CTGAAGCATT

AGAGACTTGA GAGGAGAGGT TTTAAGTCTA CTTTTGCTTT CTGTAGCATT

TCTAAAATAT CCACTTTATT TTCAGCATGG TCTTCCAATG GTATAGGAGC

TCTAAAATAT CCACTTTATT TTCAGCATGG TCTTCCAATG GTATAGGAGC

TCTAAAATAT CCACTTTATT TTCAGTATGG TCTTCCAATG GTATAGGAGC

TCTAAAATAT CCACTTTATT TTCAGTATGG TCTTCCAATG GTATAGGAGC

TCTAAAATAT CCACTTTATT TTCAGTATGG TCTTCCAATG GTATAGGAGC

---------- ---------- ---------- ---------- ----------

TCTAAAATGT CCACTTTATT TTCAGCATGG TCTTCTGATG GTATAGGAGC

TCTAAAATGT CCACTTTATT TTCAGCATGG TCTTCCGATG GTATAGGAGC

TCTAAAATGT CTACTTTATT TTCAGCATGG TCTTCCAATG GTATAGGAGC

TCTAGAATAT CCACTTTAGT TTCAGCATGG TCTTCTAATG GTATAGGAGC

TCTAAAATAT CCACTTTATT TTCAGTGTGG TCTTCCAGTG GGATAGGAGC

TCTAAAATAT CCACTTTATT TTCAGCATGG TCTTCCAATG GTATAGGAGC

TCTAAAATAT CTACTTTATT TTCAGCATGA TCTTCCAATT GTATAGGAGC

TCTAAAATAT CCACTTTATT TTCAGCATGG TCTTCCAATG GTATAGGAGC

TCTAAAATAT CCACCTTATT TTCAGCATTA TCTTCCAATG GTATAGGAGC

TCTAAAATAT CCACTTTATT TTCAGCATGA TCTTCTGATG GTATAGGAGC

TCTAGAATGT CCACCTTGTT TTCTGCATGG TCTTCTAATG GGATAGGAGC

TCTAGAATAT CCACTTTGTT TTCAGAGTGA TCTTCCAATG GTATAGGAGC

AAAGTAATTC CCCGAGTTCT GTCCAGGAGA GAGGATGGCT TGCTCCAGAC

AAAGTAATTC CCCGAGTTCT GTCCAGGAGA GAGGATGGCT TGCTCCAGAC

AAAGTAATTC CCCGAGTTCT GTCCAGGAGA GAGGATGGCT TGCTCCAGAC

AAAGTAATTC CCCGAGTTCT GTCCAGGAGA GAGGATGGCT TGCTCCAGAC

AAAGTAATTC CCCGAGTTCT GTCCAGGAGA GATGATGGCT TGCTCCAGAC

---------- ---------- ---------- ---------- ----------

AAAATAATTC CCCGAGTTCT GTCCAGGAGA GAGGACGGCT TGCTCCAAAC

AAAATAATTC CCCGAGTTCT GTCCAGGAGA GAGGACGGCT TGCTCCAAAC

AAAATAATTC CCCGAGTTCT GTCCAGGAGA GAGGATGGCT TGCTCCAGAC

AAAATAATTC CCCGAGTTCT GTCCAGGAGA GAGTATGGCT TGTTCCAGAC

AAAATAATTC CCCGAGTTCT GTCCAGGAGA GAGGATTGCT TGTTCCAGAC

AAAATAATTC CCCGAATTCT GCCCAGGAGA GAGGATGGCT TGCTCCAGAC

AAAATAATTC CCCGAATTCT GTCCAGGAGA GAGTATGGCT TGCTCTAGAC

AAAATAATTT CCTGAGTTCT GTCCAGGAGA GAGGATGGCT TGCTCTAGAC

AAAGTAATTC CCCGAGTTCT GTCCAGGAGA GAGGATGGCT TGCTCCAGAC

AAAGTAATTC CCCGAGTTCT GTGCAGGAGA GAGGATGACC TGCTCCAGAC

AAAGTAATTT CCTGAGTTCT GGCCTGGGGA TAGAATTGCT TGTTCCAAAC

AAAGTAGTTT CCCGAGTTCT GGCCTGGGGA GAGAATAGCT TGCTCCAGAC

CTGATATTGC AAATGGCACA CAGCAGGAGT GGACGACTTC GCCAGCTTCC

CTGATATTGC AAATGGCACA CAGCAGGAGT GGACGACTTC GCCAGCTTCC

CTGATATTGC AAATGGCACA CAGCAGGAGT GGACGACTTC GCCAGCTTCC

CTGATATTGC AAATGGCACA CAGCAGGAGT GGACGACTTC GCCAGCTTCC

CTATTATTGC AAATGGCACA CAGCAGGAGT GGATGACTTC GCCAGCTTCC

---------- ---TGGCACA CAGCAGGGGG AGACGACTTC GCCAGCTTCC

CTGATTTTGT AAATGGCACA CAGCAGGAGT GGATGACTTT GCCAGCTTCC

CTGATTTTGT AAATGGTACA CAGCAGGAGC GGATGACTTT GCCAGCTTCC

CTGATATTGC AAATGGCACA CGGCGGGGGT GGACGACTTT GCCAGCTTCC

CTAACATGGC AAGTGGCACA CGACGGCAGT GGACGACTTC GCCAGCTTCC

CTAATATTAC AAGTGGCACA CGACGGCGGG GGAGGACTTC GCCAGCTTCC

CTAATATTTC AAGTGGCACA CCGCAGCGGT GGGCGACTTC GCCAGCTTCC

CT---ATCAC AAGTGGCACA CGACAGCGGT GGACGACTTC GCCAGCTTCC

CTGATATTGC AAATGGCACA CGACAGCGGT GGACGACTTC ACCAGCTTCC

CTGGCATTGC AAATGGCACA CGACAGCAGT AGACGACTTC ACCAGCTTCC

CTAGTGT--- -GATGGCACA CGACAAGCAA GGATGACTTC GCCAGCTTCC

CTGGAAGAAC AAATGGCACT CGGCGGCCAT GGAGGACTTC ACGGCCTTCC

CTGAAATAGC AAATGGCACA CAGCAGCAGT GGATGATTTT ACCACCTTCC

AGCGCAAGTG GTTTGAGGTG GCCTTTGTGG CAGAAGAGCT TGCACTCTGA

AGCGCAAGTG GTTTGAGGTG GCCTTTGTGG CAGAAGAGCT TGCACTCTGA

AGCGCAAGTG GTTTGAGGTG GCCTTTGTGG CAGAAGAGCT TGCACTCTGA

AGCGCAAGTG GTTTGAGGTG GCCTTTGTGG CAGAAGAGCT TGCACTCTGA

AGCGCAAGTG GTTTGAGGTG GCCTTTGTGG CAGAAGAGCT TGCACTCTGA

AGCGTAAGTG GTTTGAGGTG GCCTTTGTGG CAGAAGAACT TGCACTCCGA

AACGCAAGTG GTTTGAGGTG GCCTTTGTGG CAGAAGAGCT TGCACTCCGA

AACGCAAGTG GTTTGAGGTG GCCTTTGTGG CAGAAGAGCT TGCACTCTGA

AGCGCAAGTG GTTTGAGGTG GCCTTCGTGG CCGAAGAGCT TGCACTCCGA

AGCGCAAGTG GTTCGAGGTG GCCTTCGTGG CAGAGGAGCT TGCACTCGGA

AGCGCAAGTG GTTTGAGGTG GCCTTTGTGG CCGAAGAGCT TGCACTCCGA

AGCGCAAGTG GTTCGAGGTG GCCTTTGTGG CTGAAGAGCT TGCACTCTGA

AGCGCAAGTG GTTTGAGGTG GCCTTTGTGG CCGAAGAGCT TGCACTCGGA

AGCGAAAGTG GTTTGAGGTA GCCTTTGTGG CCGAAGAGCT TTCACTCTGA

AGCGGAAGTG GTTCGAGGTG GCCTTTGTGG CAGAAGAGCG TGCACTCGGA

AGCGGAAGTG GTTCGAGGTA GCCTTTGTGG CAGAAGAGCG TGCACTCTGA

AGCGGAAGTG GTTCGAGGTG GCCTTCGTGG CCGAGGAGCT TGCACTCGGA

AGAGGAAGTG GTTTGAGGTA GCGTTTGTAG CAGAAGAGCT TGCACTCTGA

GATTCCTGCC TTTCTCCTGC CCTGGCTGCC TAGCCGGCCA GCCTCCTATG

GATTCCTGCC TTTCTCCTGC CCTGGCTGCC TAGCCGGCCA GCCTCCTATG

GATTCCTGCC TTTCTCCTGC CCTGGCTACC CAGCCGGCCA GCCTCCTATG

GATTCCTGCC TTTCTCCTGC CCTGGCTACC CAGCCGGCCA GCCTCCTATG

GATTCCTGCC TTTCTCCTGC CCTGGCTGCC CAGCCGGCCA GCCTCTTATG

GATTCCTGCC TTCCTCCTGC CCTGGCTGCC GAGCCGGCCA GCCTCATATG

GATTCCTGCC TTCCTCCTGC CCTGGCTACC TAGCCGGCCA GCCTCTTACG

GATTCCTGCC TTCCTCCTGC CCTGGCTACC TAGCCGGCCA GCCTCTTATG

GATCCCCGCC TTCCTCCTGC CCTGGCTGCC CAGCCGGCCA GCCTCCTACG

GATCCCCGCC TTCCTGCTGC CCTGGCTGCC CAGCCGGCCA GCCTCCTACG

GATCCCTGCT TTCCTCTTGC CCTGGCTGCC CAGCCGACCA GCCTCCTATG

GATTCCCGCC TTCCTCCTGC CCTGGTTGCC CAGCCGGCCA GCCTCCTATG

GATTCCGGCT TTCCTGCTGC CCTGGCTGCC CAGCCGACCT GCCTCCTATG

GATCCCCGCC TTCCTCCTGC CCTGGCTGCC CAGCCGGCCA GCCTCCTATG

GATCCCTGCC TTCCTCCTGC CCTGGCTGCC CAGTCGACCA GCCTCCTACG

GATTCCCGCC TTCCTCTTGC CCTGGCTGCC CAGCCGGCCG GCCTCCTACG

GATCCCGGCC TTCCTGCTGC CCTGGCTGCC CAGCCGCCCC GCGTCCTACG

AATCCCAGGA TTCCTCCTGC CTTGGCTGCC CAGCCGCCCA GCCTCTTACG

CAAGTAGGCA CAGCTCCTTT AGCCGAAGTT TTGGAGGACG GAGCCAGGCG

CAAGTAGGCA CAGCTCCTTT AGCCGAAGTT TTGGAGGACG GAGCCAGGCG

CAAGTAGGCA CAGCTCCTTT AGCCGAAGTT TTGGAGGACG GAGCCAGGCA

CAAGTAGGCA CAGCTCCTTT AGCCGAAGTT TTGGAGGACG GAGCCAGGCA

CAAGTAGGCA CAGCTCCTTT AGCCGAAGTT TTGGAGGACG GAGCCAGGCA

CAAGCAGGCA CAGCTCCTTC AGCCGCAGTT TTGGAGGACG GAGCCAGGCA

CAAGCAGGCA CAGCTCCTTT AGCCGAAGTT TTGGAGGACG GAGCCAGGCA

CAAGCAGACA CAGCTCCTTT AGCCGAAGTT TTGGAGGACG GAGCCAGGCA

CAAGCAGGCA CAGCTCCTTT AGCCGCAGCT TTGGAGGACG GAGCCAGGCA

CCAGCAGGCA CAGCTCCTTT AGCCGCAGCT TTGGGGGCCG CAGCCAGGCC

CCAGCAGGCA CAGCTCCTTC AGCCGCAGCT TTGGAGGGCG GAGCCAGGCA

CAAGCAGGCA CAGCTCCTTC AGCCGCAGCT TTGGAGGACG GAGCCAGGCA

CAAGCAGGCA CAGCTCCTTC AGCCGCAGCT TCGGCGGCCG GAGCCAGGCT

CAAGCAGGCA CAGCTCCTTT AGCCGCAGTT TTGGAGGACG GAGCCAGGCA

CAAGCAGGCA CAGCTCCTTC AGCCGCAGCT TTGGAGGACG GAGCCAGGCA

CAAGCAGGCA CAGCTCCTTC AGCCGCAGCT TCGGGGGACG GAGCCAGGCA

CCAGCCGCCA CAGCTCCTTC AGCCGCAGCT TCGGCGGGAG GAGCCAGGCG

CAAGTAGGCA CAGCTCCTTC AGTCGCAGTT TTGGAGGACG GAGCCAAGCA

GCAGCACTTT TAGGTAATTT TACCTGCAAG TCTCTCTAGC TCTTCATGTG

GCAGCACTTT TAGGTAATTT TACCTGCAAG TCTCTCTAGC TCTTCATGTG

GCAGCACTTT TAGGTAATTT TACCTGCAAG TCTCTCTAGC TCTTCATGTG

GCAGCACTTT TAGGTAATTT TACCTGCAAG TCTCTCTAGC TCTTCATGTG

GCTGCACTTT TAGGTAATTT TACCTGCAAG TCTCTCTAGC TCTTCGTGTG

GCCGCGCTCT TAGGTG---- ---------- ---------- ----------

GCTGCACTGT TAGGTAATCT TACCTGCCAG TCTCTCAAGT TCTTCATGGG

GCTGCGCTGT TAGGTAATCT TACCTGCCAG TCTCTCAAGT TCTTCATGGG

GCTGCGCTTC TAGGTAATGT TACCTGCAAG TCTCTCCAGC TCTTCATGTG

GCCGCGCTCT TGGGTAACTG TACCTGCAAG TCTCTCCAGT TCTTCATGAG

GCTGCACTTT TAGGTAATTT TACCTGCTAG TCTCTCCAGT TCTTCATGTG

GCTGCCCTTT TAGGTAATT- TACCTGCAAG TCTCTCCAGT TCTTCATGTG

GCTGCGCTCT TAGGTA---- ---------- ---------- ----------

GCCGCACTTT TAGGTAATTT TACCTGCAAG TCTCTCCAGT TCTTCATGTG

GCCGCACTTT TAGGTAATGT TACCTGCAAG CCTCTCCAGT TCTTCATGTG

GCCGCACTTT TAGGTAGTTT TACCTGCAAG TCTCTCCAGT TCTTCATGTG

GCCGCACTCC TAGGTAATGT TACCTGCAAG TCTCTCCAAT TCCTCATGAG

GCTGCACTAT TAGGTAACTT TACCTGCTAG TCTTTCTAAT TCCTCATGCG

GTGTTGACCC AAGACTGCTG GATCGGCTTC CTGATCTGCT CGGGTTAGAG

GTGTTGACCC AAGACTGCTG GATCGGCTTC CTGATCTGCT CGGGTTAGAG

GTGTTGACCC AAGACTGCTG GATCGGCTTC CTGATCTGCT CGGGTTAGAG

GTGTTGACCC AAGACTGCTG GATCGGCTTC CTGATCTGCT CGGGTTAGAG

GTGTTGACCC AAGACTGCTG GATCGGCTTC CTGATCTGCT CGGGTTAGAG

---------- ---------- ---------- ---------- ----------

GTGTGGATCC GAGGCTGCTG GATCTGCTCC CTGACCGGCT TGGGTTAGAG

GTGTGGATCC AAGGCTGCTG GATCTGCTCC CTGACCGGCT TGGGTTAGAG

GCGTCGACCC AAGGCTGCTG GATCGGCTCC CTGACTGGCT CGGGTTGGAG

GTGTTGACCC AAGGCTGCTG GATCGGCTTC CTGATCGGCT CGGGTTAGAG

GTGTTGACCC AAGACTGCTG GATCGGCTTC CTGATCGGCT CGGATTAGAG

GTGTTGACCC AAGACTGCTG GATCGGCTTC CTGATCGGCT CGGGTTAGAG

---------- ---------- --TCGGCTTC CTGATCGACT TGGGTTAGAG

GCGTTGACCC AAGACTGCTG GACCGGCTTC CTGATCGGCT TGGGTTAGAG

GTGTTGACCC AAGACTGCTG GACCGGCTTC CTGATCGGCT CGGGTTAGAG

GTGTTGACCC AAGACTGCTG GACCGGCTTC CTGATCGGCT GGGGTTAGAA

GTGTTGACCC AAGGCTGCTG GATCGACTTC CTGATCGACT AGGATTGGAA

GTGTTGATCC TAAACTACTG GACCGGCTTC CGGATCGACT AGGATTGGAG

AACCACCTAC TGAACCGACT GGCAGAGACT GACCCTTCTC CCAAAACATC

AACCACCTAC TGAACCGACT GGCAGAGACT GACCCTTCTC CCAAAACATC

AACCACCTAC TGAACCGACT GGCAGAGACT GACCCTTCTC CCAAAACATC

AACCACCTAC TGAACCGACT GGCAGAGACT GACCCTTCTC CCAAAACATC

AACCACCTAC TGAACCGACT GGCAGAGACT GACCCTTCTC CCAAAACATC

---------- ---------- ---------- ---------- ----------

AACCACCTAC TGAAGCGGCT GGCAGAGACT GATCCTTCTC CCAGAACATC

AACCACCTAC TGAAGCGGCT GGCAGAGACC GATCCTTCCC CCAGAACATC

AACCACCTAC TGAACCGACT GGCAGAGACG GAGCCTTCTC CCAAGACGTC

AACCACCTAC TGAACCGACT GGCAGAGACT GAACCTTCTC CCAAAACATC

AACCATCTAC TGAACCGACT TGCAGAGACT GATCCTTCTC CCAAAACATC

AACCACCTAC TGAACCGACT GGCAGAGACG GACCCTTCTC CCAAAACATC

AACCACCTAC TGAACCGACT GGCAGAGACA GACCCTTCTC CCAAAACGTC

AACCACCTAC TAAACCGACT GGCAGAGACT GACCCTTCTC CCAAAACATC

AACCACCTAC TGAACCGACT GGCAGAGACT GATCCTTCCC CCAAAACATC

AACCACCTAC TAAAGCGGCT GGCCGAGACG GACCCTTCCC CTAGAACATC

AACCACCTAC TAAATCTGCT GGCAGAGACG GAACCTTCTC CGAGAACATC

AACCACCTAC TGAACCTGCT GGCAGAGACT GAACCTTCTC CCAGAACGTC

TTCTATCATC TGAAAGCTGC CACTGTCCCA GAGCAGAGGA CTGTGACCCT

TTCTATCATC TGAAAGCTGC CACTGTCCCA GAGCAGAGGA CTGTGACCCT

TTCTATCATC TGAAAGCTGC CACTGTCCCA GAGCAGAGGA CTGTGACCCT

TTCTATCATC TGAA------ ---------- ---------- ----------

TTCTATCATC TGAAAGCTGC CACTGTCCCA GAGCAGAGGA CTGTGACCCT

---------- ----AGCTGC CACTGTCCCA GAGCAGAGGA CTGTGACCCT

TTCTATCATC TGATAGCTGC CACTGTCCCA GAGCAGAGGA CTGTGACCCT

TTCTATCATC TGATAGCTGC TACTGTCCCA GAGCAGAGGA CTGTGACCCT

TTCTATCATC TGAAAGCCGC CACGGTCCCA GAGCAGAGGA CCGTCACCCT

TTCTATCATC TAAAAGCTGC CACTGTCCCG GAGCAGAGGA CCGTGACCCT

TTCTATCATC TAAAAGCTGC CACTGTCCCA GAGCAGAGGA CTGTGACCCT

TTCTATCATC TAAAAGCGGC TACAGTCCCG GAACAGAGGA CTGTGACCCT

TTCGATCATC TAAAAGCTGC CACCGTCCCT GAGCAAAGGA CAGTCACCCT

TTCTATCATC TAAAAGCTGC CACTGTCCCA GAGCAGAGGA CTGTGACCCT

TTCTATTATC TAAAAGCTGC CACTGTCCCA GAGCAGAGGA CGGTGACCCT

TTCTATCATC TGCAAGCTGC CACTGTCCCA GAGCAGAGGA CCGTGACCCT

TTCTATCATC TAAAAGCAGC CACTGTGCCC GAGCAGAGAA CGGTGACCCT

TTCAATCATC TAAAAGCTGC CACAGTACCA GAGCAGCGGA CAGTGACCCT

GGATGTTGAC GTGAACAACC GCACAGACCG GCTGGAGTGG TGCAGCTGTT

GGATGTTGAC GTGAACAACC GCACAGACCG GCTGGAGTGG TGCAGCTGTT

GGATGTTGAT GTGAACAACC GCACAGACCG GCTGGAGTGG TGCAGCTGTT

---------- ---------- ---------- ---------- ----------

GGATGTTGAT GTGAACAACC GCACAGACCG GCTGGAGTGG TGCAGCTGTT

GGATGTTGAT GTGAACAACC GTACAGACCG GCTGGAGTGG TGCAGCTGTT

GGATGTCGAT GTGAACAACC GAACGGACCG GCTGGAGTGG TGCAGCTGTT

GGATGTTGAT GTGAACAACC GAACGGACCG GCTGGAATGG TGCAGCTGTT

GGACGTCGAC GTGAACAACC GCACGGACCG GCTGGAGTGG TGCAGCTGCT

GGACGTGGAC GTGAACAACC GCACGGACCG GCTGGAGTGG TGCAGCTGTT

GGATGTGGAC GTGAACAACC GCACAGACCG GCTGGAGTGG TGCAGCTGTT

GGACGTGGAC GTGAACAACC GCACAGACCG GCTGGAGTGG TGCAGCTGTT

GGATGTCGAT GTGAACAACC GCACAGACAG GCTGGAATGG TGTAGCTGCT

GGATGTCGAT GTGAACAACC GCACAGACCG GCTGGAGTGG TGCAGCTGTT

GGACGTCGAT GTGAACAACC GCACAGACCG GCTCGAGTGG TGCAGCTGCT

AGATGTTGAT GTGAACAATC GCACAGATCG GCTGGAGTGG TGCAGCTGCT

GGACGTGGAC GTGAACAACC GCACCGACCG GCTGGAGTGG TGCAGCTGCT

GGATGTCGAT GTGAACAACC GCACAGACCG GCTGGAGTGG TGTAGTTGTT

ATTACCATGG CAACTTTTCT CTGAATGCAG CCTTTGAGAT CAAGCTGCAC

ATTACCATGG CAACTTTTCT CTGAACGCAG CCTTTGAGAT CAAGCTGCAC

ATTACCATGG CAACTTTTCT CTGAACGCAG CCTTTGAGAT CAAGCTGCAC

---------- ---------- ---------- ---------- ----------

ATTACCATGG CAACTTTTCT CTGAACGCAG CCTTTGAGAT CAAGCTGCAC

ATTACCATGG CAACTTCTCC CTGAATGCAG CCTTCGAGAT CAAGCTGCAC

ACTACCACGG AAACTTCTCT CTCAACGCAG CCTTTGAAAT CAAGCTACAC

ACTACCATGG AAACTTCTCT CTCAATGCCG CCTTTGAGAT CAAGCTACAC

ACTACCATGG CAACTTCTCC CTGAACGCAG CCTTCGAGAT CAAGCTGCAC

ACTACCACGG CAACTTCTCT CTGAACGCGG CCTTCGAGAT CAAGCTGCAC

ATTATCATGG CAATTTCTCC CTGAATGCAG CTTTTGAGAT CAAGCTCCAC

ATTACCATGG CAACTTCTCC TTGAATGCAG CCTTCGAGAT CAAGCTCCAC

ATTACCATGG AAACTTCTCC CTGAACGCAG CCTTTGAGAT CAAGCTCCAC

ATTACCATGG CAACTTTTCC CTGAACGCAG CTTTCGAGAT CAAGCTACAC

ATTACCATGG CAACTTCTCC CTGAATGCGG CTTTCGAGAT CAAGCTACAC

ATTACCACGG CAACTTTTCC TTGAATGCAG CTTTTGAGAT CAAGCTCCAC

ACTACCACGG GAACTTCTCC CTCAACGCCG CCTTCGAGAT CAAGCTGCAC

ATTATCACGG CAACTTCTCC CTGAATGCTG CCTTTGAGAT CAAACTTCAC

TGGATGGCGG TGACCGCAGC AGTACTCTTC GAGATGGTGA GAGGTCCAAG

TGGATGGCGG TGACCGCAGC AGTACTCTTC GAGATGGTGA GAGGTCCAAG

TGGATGGCGG TGACCGCAGC AGTACTCTTC GAGATGGTGA GAGGTCCAAG

---------- ---------- ---------- ---------- ----------

TGGATGGCAG TGACCGCAGC AGTACTCTTC GAGATGGTGA GAGGTCCAAG

TGGATGGCAG TGACCGCTGC GGTACTCTTT GAGATGGTGA GAGGTCCAAG

TGGATGGCAG TGACTGCCAC AGTGCTGTTT GAGATGGT-- -AGGTCCAAG

TGGATGGCAG TGACTGCCAC AGTGCTGTTT GAGATGGTGA GAGGTCCAAG

TGGATGGCAG TGACTGCGGC AGTGCTCTTC GAGATGGTGA GAGGTCCAGG

TGGATGGCTG TGACCGCCGC CGTGCTCTTT GAGATGGT-- GAGGTTCAAG

TGGATGGCTG TGACTGCTGC GGTCCTCTTC GAGATGGTAA GAGGTCCAGG

TGGATGGCGG TGACTGCTGC GGTCCTCTTC GAGATGGTGA GAGGTCCAAG

TGGATGGCGG TGACCGCCGC AGTCCTCTTT GAGATGGTGA GAGGTCCAAG

TGGATGGCAG TGACAGCGGC AGTGCTCTTC GAGATGGTGA GAGGTCCAAG

TGGATGGCAG TGACCTCTGC AGTGCTTTTT GAGATGGTGA GAGGTCCAAG

TGGATGGCGG TGACAGCTGC CGTGCTCTTT GAGATGGTGA GAGGTCCAAG

TGGATGGCCG TGACGGCCGC CGTGCTCTCC GAGATGGTGA GAGGTTCAGG

TGGATGGCGG TGACTGCTGC TGTACTCTTT GAGATGGTGA GAGGTTCAAG

GTTGGCATCG GAAAGCCACC TCCTGTGGCT TCTTGTTAGT CCCAGTTTTG

GTTGGCATCG GAAAGCCACC TCCTGTGGCT TCTTGTTAGT CCCAGTTTTG

GTTGGCATCG GAAAGCCACC TCCTGTGGCT TTTTGTTGGT CCCAGTTTTG

---------- ---------- ---------- ---------- ----------

GTTGGCATCG GAAAGCCACC TCCTGTGGTT TTTTGTTGGT CCCGGTTTTG

GTTGGCATCG GAAAGCTACC TCCTGTGGCT TTTTGTTGGT CCCGGTTTTA

GTTGGCATCG AAAAGCTACA TCCTGCGGCT TCTTGTTAGT CCCGGTTTTG

GTTGGCATCG AAAAGCTACC TCCTGTGGCT TCTTGCTGGT CCCAGTTTTG

GCTGGCACCG GAAGGCCACC TCCTGTGGCT TTTTGTTGGT CCCGGTGTTG

GTTGGCACCG CAAGGCCACC TCCTGTGGCT TTTTGTTGGT GCCGGTTTTG

GTTGGCATCG CAAAGCCACT TCCTGCGGCT TTTTATTGGT CCCTGTTCTG

GTTGGCATCG AAAAGCCACC TCTTGTGGCT TTTTATTGGT CCCGGTTTTG

GCTGGCATCG CAAGGCCACC TCTTGTGGCT TTTTGCTAGT CCCGGTGTTG

GCTGGCATCG AAAGGCCACC TCCTGTGGCT TCCTGCTGGT CCCCGTTTTG

GTTGGCATCG AAAAGCTACC TCCTGTGGTT TTCTGCTGGT CCCAGTTTTG

GTTGGCATCG AAAAGCCACC TCCTGTGGTT TTCTGCTGGT CCCGGTCTTG

GTTGGCACCG GAAGGCCACG TCCTGTGGCT TTTTGTTAGT GCCAGTTCTG

GTTGGCATCG GAAAGCCACC TCCTGCGGCT TCTTATTGGT TCCAGTTTTG

GAGGGGCCTT TTGCACTGCC CAGTTACCTG TATGGCGACC CCCTTCGTGC

GAGGGGCCTT TTGCACTGCC CAGTTACCTG TATGGCGACC CCCTTCGTGC

GAGGGGCCTT TTGCACTGCC CAGTTACCTG TATGGCGACC CCCTTCGTGC

---------- ---------- ---------- ---------- ----------

GAAGGGCCTT TTGCACTGCC CAGTTACCTG TATGGTGACC CACTTCGTGC

GAGGGGCCTT TCGCACTGCC CAGCTACCTG TATGGAGACC CCCTACGAGC

GAGGGTCCTT TTGCACTGCC CAGTTACCTG TACGGTGACC CCTTGAGGGC

GAGGGTCCTT TTGCTCTGCC CAGTTACCTG TATGGTGATC CCCTGAGGGC

GAGGGTCCTT TTGCGCTGCC CAGTTACCTG TACGGTGACC CGCTGCGAGC

GAGGGGCCCT TTGCTCTGCC CAGTTACCTG TACGGCGACC CGCTCCGGGC

GAGGGTCCCT TTGCGCTGCC CAGTTACCTG TATGGCGATC CACTTCGAGC

GAAGGTCCCT TTGCTCTGCC CAGTTACCTG TATGGCGATC CGCTCCGAGC

GAGGGCCCCT TTGCGCTGCC CAGTTACCTG TACGGCGACC CGCTGCGAGC

GAAGGTCCTT TTGCACTGCC CAGTTACCTT TATGGCGATC CCCTTCGTGC

GAGGGACCCT TTGCACTGCC CAGTTACCTG TATGGTGATC CCCTTCGTGC

GAAGGTCCCT TCGCACTGCC CAGCTACCTG TACGGCGACC CCCTTCGGGC

GAAGGCCCGT TTGCCCTGCC CAGCTACCTG TATGGAGACC CGCTGCGAGC

GAAGGCCCAT TTGCACTACC CAGTTACCTC TATGGCGACC CACTTCGAGC

CCAGCTCTTC ATCCCACTCA ACATCAGCTG CTTGCTCAAG GAGGGCAGCG

CCAGCTCTTC ATCCCACTCA ACATCAGCTG CTTGCTCAAG GAGGGCAGCG

CCAGCTCTTC ATCCCACTCA ACATCAGCTG CTTGCTCAAG GAGGGCAGCG

---------- ---------- ---------- ---------- ----------

CCAGCTCTTC ATCCCACTCA ACATCAGCTG CTTGCTAAAG GAGGGCAGCG

TCAGCTCTTC ATCCCGCTCA ACGTCAGCTG CTTGCTCAAG GAGGGCAGTG

CCAGCTCTTC ATCCCGCTCA ACCTCGGCTG CTTGCTCAAG GAGGGCAGCG

CCAGCTCTTC ATCCCGCTCA ACCTCAGCTG CTTGCTCAAG GAGGGCAGCG

CCAGCTCTTC ATCCCGCTCA ACATCAGCTG TCTGCTCAAG GAGGGCAGCG

CCAGCTGTTC ATCCCACTCA ACATCAGCTG CCTGCTCAAG GAGGGCAGCG

CCAGCTCTTC ATCCCACTCA ACACCAGCTG CCTGCTCAAG GAGGGCAGTG

CCAGCTCTTC ATCCCACTCA ACGTCAGCTG TCTGCTCAAG GAGGGCAGTG

CCAGCTCTTC ATCCCGCTCA ACATCAGCTG CCTGCTCAAG GAAGGCAGCG

CCAGCTCTTC ATCCCACTCA ACATCAGCTG CCTGCTCAAG GAGGGCAGTG

CCAGCTCTTC ATCCCACTCA ACATCAGCTG TCTGCTCAAG GAGGGCAGTG

CCAGCTCTTC ATCCCACTCA GCGTCAGCTG CCTGCTCAAG GAGGGCAGTG

CCAGCTGTTT ATCCCACTGA ACATCAGCTG CCTGCTGAAG GAGGGCAGCG

CCAGTTGTTC ATTCCGCTCA ACATCAGCTG CTTACTGAAG GAAGGCAGTG

AGCACCTGTT TGATAGTAAG ATTTTCCTTT CAGGCTTTGA ACCCGAAACG

AGCACCTGTT TGATAGTAAG A--------- ---------- ----------

AGCACCTGTT TGATAGTAAG ATTTTCCTTT CAGGCTTTGA ACCGGAAACG

---------- ---------- -TTTTCCTTT CAGGCTTTGA ACCGGAAACG

AGCACCTGTT TGATAGTAAG ATTTTTTTTT CAGGCTTTGA ACCAGAAACA

AGCACCTGTT CGATAGTAAG ATTTTCCTTT TAGGCTTTGA ACCAGAAACA

AGCACCTGTT CGATAGTAAG ATATTCCTTT CAGGCTTTGA ACCAGAGACA

AGCACCTGTT CGATAGTAAG ATATTCCTTT CAGGCTTTGA ACCAGAGACA

AGCACCTGTT TGACAGTAAG A--------- ---------- ----------

AGCACCTGTT TGACGGTAAG ATTTTCCTTG CAGGCTTTGA ACCAGAAACA

AGCACCTGTT TGATAGTAAG ATTCTCCTTT CAGGCTTCGA GCCAGAAACA

AGCACCTGTT TGACAGTAAG A---TCCTTT CAGGCTTTGA ACCGGAGACA

AGCACCTGTT TGACAGTAAG ATTTCCCTTT CAGGTTTTGA GCCAGAAACA

AGCACCTGTT TGACAGTAAG ATTTTCCTTT CAGGCTTTGA ACCCGAAACA

AGCACCTGTT TGATAGTAAG ATCTTTCTTT CAGGCTTTGA ACCAGAGACA

AGCACCTGTT TGATAGTAAG AGCTTTCTTT CAGGCTTTGA ACCAGAGACA

AGCACCTGTT TGACAGTAAG ATTTCTCCCC TAGGCTTTGA ACCAGAGACT

AACACCTCTT CGATAGTAAG ATTTTTATTT CAGGTTTTGA ACCCGAAACT

TACTGGGATC GAATGCACCT CTTCCAGGAA GCCATTGCAC ACAGGTTTGT

---------- ---------- ---------- ---------- ----------

TACTGGGATC GAATGCACCT CTTCCAGGAA GCCATTGCAC ACAGGTTTGT

TACTGGGATC GAATGCACCT CTTCCAGGAA GCCATTGCAC ACAGGTTTGT

TACTGGGATC GAATGCATCT CTTTCAGGAA GCCATTGCAC ACAGGTTTGT

TACTGGGATC GAATGCACCT TTTCCAGGAA GCCATTGCAC ACAGGTTTGT

TATTGGGATC GAATGCACCT TTTCCAGGAA GCCATTGCAC ACAGGTTTGT

TATTGGGATC GAATGCATCT TTTCCAGGAA GCCATTGCAC ACAGGTTTGT

---------- ---------- ---------- ---------- ----------

TACTGGGACC GAATGCACCT TTTCCAGGAA GCCATCGCGC ACAGGTTTGT

TACTGGGATC GAATGCACCT TTTCCAGGAA GCCATTGCAC ACAGGTTTGT

TACTGGGATC GAATGCACCT TTTCCAGGAA GCCATCGCAC ACAGGTTTGT

TACTGGGATC GAATGCACCT TTTCCAGGAA GCCATAGCAC ACAGGTTTGT

TACTGGGATC GAATGCACCT TTTCCAGGAG GCCATTGCAC ACAGGTGTGT

TACTGGGATC GAATGCACCT TTTCCAGGAA GCCATTGCAC ACAGGTTTGT

TACTGGGATC GGATGCATCT TTTCCAGGAA GCCATTGCAC ACAGGTTTGT

TACTGGGATC GGATGCACCT CTTCCAAGAA GCTGTGGCTC ACAGGTATGT

TACTGGGATC GAATGCATCT CTTTCAGGAG GCCATTGTTC ACAGGTATGT

CTAGGTTTGG GTTTGTACAA GATAAATATT CTGCCTCTGC TTTTAACTTC

CTAGGTTTGG GTTTGTACAA GATAAATATT CTGCCTCTGC TTTTAACTTC

CTAGGTTTGG GTTTGTACAA GATAAATATT CTGCCTCTGC TTTTAACTTC

---------- ---------- ---------- ---------- ----------

CTAGGTTTGG ATTTGTACAA GATAAATATT CTGCTTCTGC CTTTAACTTC

TTAGGTTTGG GTTTGTACAG GATAAATACT CTGCCTCTGC CTTTAACTTT

CTAGGTTTGG ATTTGTGCAA GATAAATATT CTGCCTCCGC GTTTAACTTC

CTAGGTTTGG ATTTGTGCAA GATAAATATT CTGTCTCCGC TTTTAACTTC

TCAGGTTTGG CTTCGTACAA GATAAATACT CTGCCTCCGC CTTTAACTTC

TTAGGTTTGG GTTTGTTCAA GATAAGTATT CTGCGTCCGC TTTCAACTTC

CTAGGTTTGG ATTTGTGCAA GATAAATATT CCGCCTCTGC TTTTAACTTC

CTAGGTTTGG GTTTGTACAA GATAAATATT CTGCGTCTGC TTTTAACTTT

---------- ----GTGCAT GACAAATATT CTGCCTCTGC TTTTAACTTT

CTAGGTTTGG GTTTGTACAA GATAAATATT CTGCCTCTGC TTTTAACTTC

CTAGGTTTGG GTTTGTACAA GATAAATATT CTGCCTCTGC TTTTAACTTC

---------- ---------- ---------- ---------- ----------

CCAGATTCGG ATTTGTACAA GACAAATATT CTGCCTCCGC CTTCAACTTC

TCAGGTTTGG ATTCGTGCAG GATAAATACT CTGCTTCTGC TTTCAACTTT

CCTGCTGAGA ACAAGCCTCA GTATATCCAC GTTACAGGTC AGGAACAGTG

CCTGCTGAGA ACAAGCCTCA GTATATCCAC GTTACAGGTC AGGAACAGTG

CCTGCTGAGA ACAAGCCTCA GTACATCCAC GTTACAGGTC AGGAACAGTG

---------- ---------- ---------- ---------- ----------

CCTGCTGAGA ACAAGCCTCA GTACATCCAC GTTACAGGTC AGGAACAGTG

CCTGCTGAGA ACAAGCCTCA GTACATCCAT GTTACAGGTC AGGAACGGTA

CCTGCTGAGA ATAAACCACA GTATATCCAC GTCACAGGTC AGGAACAGTG

CCTGCTGAGA ATAAGCCACA GTATATCCAT GTCACAGGTC AGGAACGGTG

CCAGCT---- ---------- ---------- ---------- ----------

CCAGCCGAGA ACAAGCCCCA GTACATCCAT GTCACAGGTC AGGGACAGTG

CCTGCTGAGA ACAAGCCTCA GTATATCCAT GTTACAGGTC AGGAACAGTG

CCAGCTGAGA ACAAGCCTCA GTACATCCAC GTTACAGGTC AGGAACAGTG

CCTGCCGAGA ACAAGCCTCA GTACATTCAC GTTACAGGTC AGGAACAGTC

CCTGCTGAGA ACAAGCCTCA GTACATCCAT GTTACAGGTC AGGGACAGTG

CCTGCTGAGA ACAAGCCTCA GTACATCCAC GTTACAGGTC AGGAACAGTG

---------- ---------- ---------- ---------T AGGAACAGTG

CCTTCTGAGA ACAAGCCCCA GTACATCCAC GTCACAGGTC AGGGACTGTC

CCATCAGAGA ATAAACCACA GTATATCCAT GTTACAGGTC AGGGACCGTC

TTTCTGCAGC TGCCCTACTC CAAGCGCAAG TTCTCAGGGC AGCAGCGGCG

TTTCTGCAGC TGCCCTACTC CAAGCGCAAG TTCTCAAGGC AGCAGCGGCG

TTTCTGCAGC TGCCCTATTC CAAGCGCAAG TTCTCAGGGC AGCAGCGGCG

---------- ---------- ---------- ---------- ----------

TTTCTGCAGC TGCCCTACTC CAAGCGCAAG TTCTCGGGGC AGCAGCGGCG

TTTCTGCAGC TGCCGTACTC CAAGCGCAAG TTCTCAGGGC AGCAGCGGCG

TTTCTTCAGC TGCCGTACTC CAAACGCAAG TTTTCTGGGC AGCAGCGGCG

TTCCTTCAGC TGCCGTACTC CAAACGCAAG TTTTCAGGGC AGCAGCGGCG

---------- ---------- ---------- ---------- ----------

TTCCTGCAGC TGCCCTACTC CAAGCGCAAG TTCTCAGGGC AGCCGCGGCG

TTCCTGCAGC TGCCTTACTC CAGGCGCAAG TTCTCCGGGC AGCAGCGGCG

TTCCTGCAGC TGCCCTACTC CAAGCGCAAG TTCTCCGGGC AGCAGCGGCG

TTCCTGCAAC TGCCCTACTC CAAGCGCAAG TTCGCGGGCC AGCCGCGGCG

TTTCTGCAGC TGCCCTACTC CAAGCGCAAG TTCTCGGGAC AACAGCGGCG

TTTCTGCAGC TGCCCTACTC CAAGCGGAAG TTCTCAGGGC AGCAGCGTCG

TTTCTGCAGC TGCCCTACTC GAAGCGGAAG TTCTCAGGAC AGCAGCGGCG

TTCCTGCAGC TGCCCTACTC CAAGCGCAAG TTCTCGGGCC AGCAGCGGCG

TTCCTGCAAC TACCCTACTC CAAGAGGAAG TTCTCTGGAC AGCAACGGCG

GCGGCGGAAC TCCACCAGCT CCACCAACCA GAACATGTTC TGCGAGGAGC

GCGGCGGAAC TCCACCAGCT CCACCAACCA GAACATGTTC TGCGAGGAGC

GCGGCGGAAC TCCACCAGCT CCACCAACCA GAACATGTTC TGCGAGGAGC

---------- ---------- ---------- ---------- ----------

GCGGCGGAAC TCCACCAGCT CCACCAACCA GAACATGTTC TGCGAGGAGC

GCGGCGGAAC TCCACCAGCT CCACCAACCA GAACATGTTC TGCGAGGAGC

GCGACGGAAC TCTACCAGCT CCACCAACCA AAACATGTTC TGTGAGGAGC

GCGACGGAAC TCTACCAGCT CCACCAACCA AAACATGTTC TGTGAGGAGC

---------- ---------- ---------- ---------- ----------

GCGCCGCAAC TCCACCAGCT CCGCCAGCCA GACCCTGTTC TGCGAGGAGC

GCGGCGGAAC TCCACCAGTT CCACCAACCA GAACTTGTTC TGTGAGGAGC

ACGGCGGAAC TCCACCAGCT CCACCAACCA GAACATGTTC TGCGAGGAGC

GCGACGGAAT TCCACCAGCT CCACCAGCCA GAACATGTTC TGCGAGGAGC

GCGGCGGAAC TCCACCAGTT CCACCAACCA GAACATGTTC TGCGAGGAGC

GCGGCGGAAC TCCACCAGCT CTACCGGCCA GAACATGTTC TGCGAGGAGC

GAGGCGGAAC TCCACCAGCT CCACCAGCCA GAACATGTTC TGCGAGGAGC

CAGGCGCAAC TCGACCAGCT CCACCGGCCA GAACATGTTC TGCGAGGAGC

CCGACGGAAT TCCACCAGCT CCACCAACCA GAACATGTTC TGTGAGGAGC

GGGTCGGCTA CAACTGGGCC TACAACACCA TGCTCACCAA AACATGGCGC

GGGTCGGCTA CAACTGGGCC TACAACACCA TGCTCACCAA AACATGGCGC

GGGTCGGCTA CAACTGGGCC TACAACACCA TGCTAACCAA GACATGGCGC

---------- ---------- ---------- ---------- ----------

GGGTCGGCTA CAACTGGGCC TACAACACCA TGCTCACCAA GACATGGCGC

GGGTTGGCTA CAACTGGGCC TACAACACGA TGCTGACCAA GACATGGCGC

GGGTTGGCTA CAACTGGGCC TATAACACCA TGCTGACCAA GACATGGCGC

GGGTTGGCTA CAACTGGGCC TACAACACCA TGCTGACCAA GACATGGCGC

---------- ---------- ---------- ---------- ----------

GCGTGGGCTA CAACTGGGCC TACAACACCA TGCTCACCAA GACCTGGCGC

GTGTTGGCTA CAACTGGGCC TACAACACCA TGCTGACCAA GACATGGCGT

GGGTCGGCTA TAACTGGGCC TACAACACCA TGCTGACCAA GACGTGGCGC

GGGTGGGCTA CAACTGGGCC TACAACACCA TGCTGACCAA GACGTGGCGC

GGGTTGGTTA CAACTGGGCC TACAACACCA TGCTGACCAA GACGTGGCGC

GGGTTGGCTA CAACTGGGCC TATAACACCA TGCTGACCAA GACATGGCGC

GGGTGGGCTA CAACTGGGCC TACAACACCA TGCTGACCAA GACCTGGCGC

GCGTGGGCTA CAACTGGGCC TACAACACCA TGCTGACCAA GACGTGGCGC

GGGTGGGTTA CAACTGGGCC TACAACACTA TGCTGACTAA AACCTGGCGC

TCCAGCGCCA CAGGGGATGA AAAGTTTGCT GATCGGCTGC TGAAGGACTT

TCCAGCGCCA CAGGGGATGA AAAGTTTGCT GATCGGCTGC TGAAGGACTT

TCCAGTGCCA CAGGGGATGA AAAGTTTGCT GATCGGCTGC TGAAGGACTT

---------- ---------- ---------- ---------- ----------

TCCAGTGCCA CAGGGGATGA AAAATTTGCT GATCGGCTGC TGAAGGACTT

TCCAGTGCCA CGGGGGATGA GAAATTTGCC GACCGGCTGC TGAAGGACTT

TCTAGTGCCA CGGGGGATGA GAAGTTTGCC GATCGGCTGC TGAAGGACTT

TCTAGTGCCA CAGGGGACGA GAAGTTTGCT GATCGGCTGC TGAAGGACTT

---------- ---------- ---------- ---------- ----------

TCCAGCGCCA CCGGCGACGA GAGGTTCGCC GACCGGCTGC TGAAGGACTT

TCTAGTGCCA CGGGGGATGA GAAGTTTGCT GATCGGCTGC TCAGGGATTT

TCCAGCGCCA CAGGGGACGA GAAGTTTGCT GATCGGCTGC TGAAGGACTT

TCCAGCGCCA CGGGGGACGA GAAGTTTGCC GACCGGCTGC TGAAGGACTT

TCCAGCGCCA CGGGAGACGA GAAGTTTGCT GACCGGCTGC TGAAGGACTT

TCCAGTGCCA CAGGGGACGA GAAGTTTGCC GACCGGTTGC TGAAGGACTT

TCCAGTGCCA CCGGGGACGA GAAGTTTGCC GACCGGCTGC TGAAGGACTT

TCCAGCGCCA CCGGGGACGA GAAGTTTGCC GACCGGCTGC TCAAGGACTT

TCCAGCGCCA CGGGCGACGA GAAGTTTGCC GATAGGCTGC TGAAGGACTT

CACGGACTTC TGCATCAACC GTGACAACCG GCTGGTGTGC TGCTACACTC

CACGGACTTC TGCATCAACC GTGACAACCG GCTGGTGTGC TGCTACACTC

CACAGACTTC TGCATCAACC GTGACAACCG TCTGGTGTGC TGCTACACTC

---------- ---------- ---------- ------GTGC TGCTACACTC

CACAGACTTC TGCATCAACC GTGACAACCG GCTGGTGTGC TGCTACACTC

CACAGACTTC TGCATTAACC GTGACAACCG GCTGGT---- ----------

CACGGATTTC TGTATCAACC GTGACAACCG CCTGGTGTGT TGTAACACTC

CACGGATTTC TGCATCAACC GTGACAACCG ACTGGTGTGT TGTTACACTC

---------- ---------- ---------- ---------- ----------

CACAGACTTC TGTGGCAACC GCGACAGCCG GCTGGTATGC TGCTACACTC

CACGGACTTC TGCATCAACC GCGACAACCG GCTGGTGTGC TGCTACACTC

CACGGATTTC TGTATCAACC GCGACAACCG ACTCGTGTGC TGCTACACTC

CACGGACTTT TGCGTCAATC GTGACAACCG CCTGGT---- ----------

CACAGACTTC TGCATCAACC GAGACAACCG GCTGGTGTGC CGCTACACTC

CACGGACTTC TGTGTCAACC AAGACAACCG GCTGGTGTGC TGCTACACTC

CACCGACTTC TGTGTCAACC GAGACAACCG GCTGGT---- ----------

CACCGACTTC TGCGTCAACC GCGACAACCG CCTCGTGTGT TGGTTCACTC

TACGGAGTTC TGTGTTAACC GAGACAACAG GCTGGTGTGC GGGTTCACTC

ACCGAAGCCA AGCATGGCAC CTTATCAAGG TTAAAGAACT CATTAAAGTC

ACCGAAGCCA AGCATGGCAC CTTATCAAGG TTAAAGAACT CATTAAAGTC

ACCGAAGCCA AGCATGGCAC CTTATCAAGG TTAAAGAACT CATTAAAGTC

ACCGAAGCCA AGCATGGCAC CTTATCAAGG TTA------- ----------

ACCGAAGCCA AGCCTGGCAC CTTATCAAGG TTAAAGAACT CGTTAAAGTC

---------- ---------- ---------- ---------- ----------

ACCGAAGCCA AGCATGGCAC CTTATCAAGG TTAAAGAATT CATTAAAGTC

ACCGAAGCCA AGCATGGCAC CTTATCAAGG TTAAAGAATT CATTAAAGTC

---------- ---------- ---------- ---------- ----------

ACCGAAGCCA AGCATGGCAC CTTATCAAGG TTAAAGAATT CATTAAAGTC

ACCGAAGCCA AGCCTGGCAC TTTATCAAGG TTAAAGAACT CATTAAAGTC

ACCGAAGCCA AGCACGGCAC CTTATCAAGG TTAAAGAACT CATTAAAGTC

---------- ---------- ---------- ---------- ----------

ACCGAAGCCA AGCATGGCAC CTTTTCAAGG TTAAAGAACT CATTAAAGTC

ACCGAAGCCA AGCACGGCAC CTTATCAAGG TTAAAGAACT CATTAAAGTC

---------- ---------- ---------- ---------- ----------

ACCGAGGCCA GGCCTGGCAC CTTATCAAGG TTAAAGAACT CATTAAAATC

ACCGAAGCCA AACCAGGGAC TTTGTCGAGG TTAAAGAACT CATTGAAATC

AAAGTCTCCT GGGGACTGCT CAGGCAAGAC AGCATCCCTT GGCACTTCCT

AAAGTCTCCT GGGGACTGCT CAGGCAAGAC GGCATCCCTT GGCACTTCCT

AAATTCTCCT GGGGACTGCT CAGGCAAGAT GGCATCCCTT GGCACTTCCT

---------- ---------- ---------- ---------- ----------

AAATTCTCCT GGGGACTGCT CAGGCAAGAC AGCATCCCTT GGCACTTCTT

---------- ---------- ---------- ---------- ----------

AAATTCTCCT GGGGACTGCT CAGGTAGGAT GACATCCCTT GGAACTTCCT

AAATTCTCCT GGGGACTGCT CAGGTAGGAT GACATCCCTT GGAACTTCCT

---------- ---------- ---------- ---------- ----------

AAATTCTCCC GGGGACTGCT CGGGCAAGAC GGCCTCCCTT GGCACTTCCT

AAATTCTCCT GGGGACTGCT CAGGCAAGAC GGCGTCCCTT GGCACTTCCT

AAATTCTCCT GGGGATTGCT CAGGCAAGAC GGCGTCCCTC GGCACTTCCT

---------- ---------- ---------- ---------- ----------

AAATTCTCCT GGGGACTGCT CGGGCAAGAC GGCATCCCTT GGCACTTCCT

AAATTCTCCT GGGGACTGCT CAGGCAAGAC GGCGTCCCTT GGCACCTCCT

---------- ---------- ---------- ---------- ----------

AAATTCTCCT GGCGACTGTT CAGGCAGCAC TGCTTCCCTG GGCACTTCCT

GAATTCTCCA GGTGCTTGTT CTGGCAGCAC TGCTTCTCTG GGCACCTCCT

GATCAGCCGC AGGCTCCTGT GCAAGGATGA CCTCCACTTC ATCCTCTTCG

GATCAGCCGC AGGCTCCTGT GCAAGGATGA CCTCCACTTC ATCCTCTTCG

GATCAGCTGC AGGCTCCTGT GCAAGGATGA CCTCCACTTC ATCTTCTTCG

---------- ---------- ---------- ---------- ----------

GATCAGCCGC AGGCTCCTGT GCAAGGATGA CCTCTACCTC ATCCTCTTCG

---------- ---------- ---------- ---------- ----------

GATCAGCAGA GGGCTCCTGG GCCAGAATGA CTTCCACCTC GTCCTCTTCA

GATCAGCAGA GGGCTCCTGG GCCAGAATGA CTTCCACCTC GTCCTCTTCA

---------- ---------- ---------- ---------- ----------

GATCAGCAGC AGGCTCCTGT TCGAGGATGA CCTCCACCTC ATCCTCTTCA

GATCAGCAGC AGGCTCCTGT GCGAGGATGA CCTCCACCTC ATCCTCTTCG

GATCAGCAGC AGGCTCGTGT GCGAGGATGA CCTCCACCTC GTGCTCTTCG

---------- ---------- ---------- ---------- ----------

GATCAGCAGC AGGCTCCTGT GCCAGGATGA CCTCCACCTC ATCCTCTTCG

GATCAGCAGC AGGCTCCTGT GCAAGAATGA CCTCCACCTC ATCCTCTTCG

---------- ---------- ---------- ---------- ----------

GATCTGCAGC GGGCTCCTGG GCAAGAATGA CCTCCACTTC ATCCTCTTCT

GATCGGCTGC TGTCTCCTGA GCAAGAATGA CCTCCACTTC ATCCTCTTCT

GCCACTCCTC CATTGCACTC TACTATACCT GAAAGCAAGG AAGGAGTCGG

GCCACTCCTC CATTGCACTC TACTATACCT GAAAGCAAGG AAGGAGTCGG

GCCACTCCTC CATTGCACTC TACTATACCT GAAAGCAAGG AAGGAGTCGG

---------- ---------- ---------- --------GG AAGGAGTCGG

GCCACTCCTC CATTGCACTC TATTATACCT GAAAGCAAGG AA-------G

---------- ---------- ---------- --------GG AAGGAGTCAG

GCCACCCCTC CATTGCACTC CACTATGCCT GAAAACAAGG AATGAGTTGG

GCCACCCCTC CATTGCACTC CACTATGCCT GAAAACAAGG AATGAGTTGG

---------- ---------- ---------- ---------- ----------

GCCACTCCTC CATTGCACTC GACTATGCCT GAAAGCAA-- ----------

GCCACTCCTC CATTGCACTC GACTATGCCT GAAAGCAAAG AA--AGCCTG

GCCACTCCTC CATTGCACTC GACTATGCCT GAAAGCAAGG AAGGAGTCAG

---------- ---------- ---------- ---------- ----------

GCCACTCCTC CATTGCACTC GATTATGCCT GAAAGCAAGG AAGGAATCCA

GCCACTCCTC CATTGCACTC GACTATGCCT GAAAGCAAGA AAGCAGTCAG

---------- ---------- ---------- --------GG AAG-------

GCAACGCCTC CATTGTATTC TACTATTCCT TAAGGCAAGG AAGAAGCCAG

GCCACTCCAC CATTGCATTC TACTATTCCT TAGGATGAGG AAGGAGCCAG

AGAGGAGGGG TTTTTTGGGG TTTTGCAGGT GACAGAGCTG AATGAACCTC

AGAGGAGGGG TTTTTTGGGG TTTTGCAGGT GACAGAGCTG AATGAACCTC

AGAGGAAGGG TTTCTTGGGG TTTTGCAGGT GACAGAGCTG AATGAACCTC

AGAGGAAGGG TTTCTTGGGG TTTTGCAGGT GACAGAGCTG AATGAACCTC

AGAGGAGGGG TTTTTTGGGG TTTTGCAGGT GACAGAGCTG AATGAACCTC

AGAAGAAGAG TTTTTT-GGG TTTTGCAGGT GACAGAGTTG AATGAACCTC

ACAGGAAGAG TTTCTAGATG TTTTGCAGGT GACAGAGCTG AATGAACCTC

ACAAGAAGAG TTTCT-GATG TCTTCCAGGT GACAGAGCTG AATGAACCAC

---------- ------AATG TTTTGCAGGT GACCGAGCTG AATGAACCCC

---------- ---------- ---------- ---------- ----------

AGAGGAAGGG TTTTTGGGGA TTTTGCAGGT GACAGAGCTC AATGAACCTC

AGGGGAAGGG TTTTTTGGGG TTTTGCAGGT GACAGAGCTC AATGAACCTC

---------- ---------- ---------- ---------- ----------

AGAGGAAGGG TTTTTTGGGG TTTTGCAGGT GACGGAGCTC AATGAACCTC

AGAGGAAGGG TGTTTTGGGG TTTTGCAGGT GACAGAGCTC AATGAACCTC

----GAAGGG TTTTTTGGGG TTTTGCAGGT GACAGAGCTC AACGAACCTC

AGAAGAAGGA CTTCTTGGGT CTTTGCAGGT GACTGAGCTC AATGAGCCAC

AGAGGAAGAA TTTCTGTGGG TTTTACAGGT GACCGAACTG AATGAGCCCC

TCTCCAATGA AGATCGAAAT CTCCTCTCTG TGGCCTACAA GAATGTGGTT

TCTCCAATGA AGATCGAAAT CTCCTCTCTG TGGCCTACAA GAATGTGGTT

TCTCCAATGA AGATCGAAAT CTCCTCTCTG TGGCCTACAA GAATGTGGTT

TCTCCAATGA AGATCGAAAT CTCCTCTCTG TGGCCTACAA GAATGTGGTT

TCTCCAATGA AGATCGAAAT CTCCTCTCTG TGGCTTACAA GAATGTAGTT

TCTCCAATGA AGATCGAAAT CTCCTCTCTG TGGCCTACAA GAATGTAGTT

TATCTAATGA AGATAGAAAT CTCCTCTCTG TGGCCTACAA GAATGTAGTT

TATCTAACGA AGATCGAAAT CTCCTCTCTG TGGCCTACAA GAATGTAGTT

TCTCCAATGA AGATCGAAAC CTCCTGTCCG TGGCCTACAA GAACGTGGTT

---------- ---------- ---------- ---------- ----------

TTTCCAATGA AGATCGAAAC CTCCTCTCTG TGGCCTACAA GAATGTGGTT

TCTCCAATGA AGATCGAAAC CTCCTCTCCG TGGCTTACAA GAATGTGGTT

---------- ---------- ---------- ---------- ----------

TCTCCAATGA AGATCGCAAT CTTCTCTCTG TGGCCTACAA GAATGTGGTT

TTTCCAATGA AGATAGAAAT CTCCTCTCTG TGGCCTACAA GAATGTGGTT

TCTCCAACGA AGATCGAAAT CTGCTCTCCG TGGCCTACAA GAATGTAGTT

TGTCCAACGA GGACCGCAAT CTCCTGTCTG TCGCCTACAA GAACGTGGTC

TCTCAAATGA AGATCGAAAC CTGCTCTCTG TTGCTTACAA GAATGTGGTT

GGTGCCAGGC GATCTTCCTG GAGGGTCATT AGCAGCATTG AGCAGAAAAC

GGTGCCAGGC GATCTTCCTG GAGGGTCATT AGCAGCATTG AGCAGAAAAC

GGTGCCAGGC GATCTTCCTG GAGGGTCATT AGCAGCATTG AGCAGAAAAC

GGTGCCAGGC GATCTTCCTG GAGGGTCATT AGCAGCATTG AGCAGAAAAC

GGTGCCAGGC GATCTTCCTG GAGGGTCATT AGCAGCATTG AGCAGAAAAC

GGTGCCAGGC GATCTTCCTG GAGGGTCATT AGCAGCATTG AGCAGAAAAC

GGTGCCAGGC GATCTTCTTG GAGGGTTATT AGTAGCATTG AGCAGAAAAC

GGTGCCAGGC GATCTTCTTG GAGGGTTATT AGTAGCATTG AGCAGAAAAC

GGTGCCAGGC GATCCTCTTG GCGGGTCATC AGCAGCATTG AGCAGAAAAC

---------- ---------- ---------- ---------- ----------

GGTGCCAGGC GATCTTCCTG GAGGGTCATC AGCAGCATTG AGCAGAAAAC

GGTGCCCGGC GATCTTCCTG GAGGGTCATC AGCAGCATTG AGCAGAAAAC

---------- ---------- ---------- ---------- ----------

GGTGCCAGGC GATCTTCCTG GAGGGTGATT AGCAGCATTG AACAGAAAAC

GGTGCCAGGC GATCTTCCTG GAGGGTTATC AGCAGCATTG AGCAGAAGAC

GGTGCCCGGC GGTCTTCTTG GAGGGTCATC AGCAGCATTG AGCAAAAAAC

GGGGCTAGGC GGTCCTCCTG GCGAGTCATC AGCAGCATCG AGCAGAAGAC

GGTGCTAGGC GATCTTCTTG GAGAGTCATC AGTAGTATTG AGCAGAAAAC

CATGGCTGAT GGAAACGAAA AGAAATTGGA GAAAGTTAAA GCTTACCGGG

CATGGCTGAT GGAAACGAAA AGAAATTGGA GAAAGTTAAA GCTTACCGGG

CATGGCTGAT GGAAACGAAA AGAAATTGGA GAAAGTGAAA GCTTACCGGG

CATGGCTGAT GGAAACGAAA AGAAATTGGA GAAAGTGAAA GCTTACCGGG

CATGGCTGAT GGAAACGAAA AGAAATTGGA GAAAGTT-AA GCTTACCGGG

CATGGCTGAT GGAAATGAAA AGAAATTGGA GAAAGTTAAA GCTTACCGGG

CATGGCAGAT GGGAATGAGA AGAAGCTGGA GAAAGTCAAA GCCTATCGGG

CATGGCAGAT GGGAATGAGA AGAAGTTGGA GAAAGTTAAA GCCTACCGGG

CATGGCGGAT GGAAATGAAA AGAAGCTGGA GAAAGTGAAA GCCTACCGGG

---------- ---------- ---------- ---------- ----------

CATGGCTGAT GGAAACGAAA AGAAATTGGA GAAAGTTAAA GCTTACCGGG

CATGGCTGAT GGGAATGAAA AGAAATTGGA GAAGGTTAAA GCTTACCGGG

---------- ---------- ---------- ---------- ----------

CATGGCTGAT GGAAATGAAA AGAAATTGGA GAAGGTTAAA GCTTACCGGG

AATGGCTGAT GGAAATGAAA AGAAATTGGA GAAGGTTAAA GCTTACCGGG

AATGGCTGAT GGAAATGAGA AGAAGTTGGA GAAGGTAAAG GCTTACCGGG

CATGGCGGAC GGGAATGAGA AGAAGCTGGA GAAGGTGAAG GCCTACCGGG

CATGGCTGAT GGCAATGAGA AGAAGCTGGA AAAGGTCAAG GCCTATAGGG

AGAAGATTGA GAAGGAGCTG GAGACAGTTT GCAATGATGT CCTGTCTCTG

AGAAGATTGA GAAGGAGCTG GAGACAGTTT GCAATGATGT CCTGTCTCTG

AGAAGATTGA GAAGGAGCTG GAGACAGTTT GCAATGATGT CCTGTCTCTG

AGAAGATTGA GAAGGAGCTG GAGACAGTTT GCAATGATGT CCTGTCTCTG

AGAAGATTGA GAAGGAGCTG GAGACATTTT GCAATGATGT TCTGTCTCTG

AGAAGATTGA GAAGGAGCTG GAGACAGTTT GCAATGATGT CCTGGCTCTG

AGAAGATTGA GAAGGAGCTG GAGACAGTTT GCAATGATGT CTTGGCTCTG

AGAAGATTGA AAAGGAGCTG GAGACAGTTT GCAATGATGT CTTGGCTCTG

AGAAGATCGA GAAGGAGCTG GAGACCGTCT GCAACGATGT CCTGGCTCTG

---------- ---------- ---------- ---------- ----------

AGAAGATTGA GAAGGAGCTG GAAACAGTGT GCAATGATGT CCTGGCTCTG

AGAAGATTGA GAAGGAGCTG GAAACAGTGT GCAATGATGT GCTGGCTCTG

---------- ---------- ---------- ---------- ----------

AGAAGATTGA GAAGGAGTTG GAGACAGTTT GCAATGATGT CCTGGCTCTG

AGAAGATTGA GAAAGAGCTG GAGACAGTTT GCAATGATGT CCTGGCTCTG

AGAAGATTGA GAAAGAGCTG GAGACCGTTT GCAATGATGT CCTGGCTCTG

AGAAGATCGA GAAGGAGCTG GAGACCGTCT GCAACGACGT GCTGGCGCTG

AGAAGATAGA AAAGGAGCTG GAGACAGTCT GCAACGACGT CCTGGCTCTG

CTTGACAAGT TCCTGATCAA GAACTGCAAT GATTTCCAGT ATGAGAGCAA

CTTGACAAGT TCCTGATCAA GAACTGCAAT GATTTCCAGT ATGAGAGCAA

CTTGACAAGT TCCTGATCAA GAACTGCAAT GATTTCCAGT ATGAGAGCAA

CTTGACAAGT TCCTGATCAA GAACTGCAAT GATTTCCAGT ATGAGAGCAA

CTTGACAAGT TCCTGATCAA GAACTGCAAT GATTTCCAGT ATGAGAGCAA

CTTGACAAGT TCCTCATCAA GAACTGCAAT GATTTCCAGT ACGAGAGCAA

CTCGACAAGT TCCTTATCAA GAACTGCAAT GATTTTCAGT ACGAGAGCAA

CTTGACAAGT TCCTTATCAA GAACTGCAAT GATTTTCAGT ATGAGAGCAA

CTCGACAAGT TCCTCATCAA GAACTGCAAT GATTTCCAGT ATGAGAGCAA

---------- ---------- ---------- ---------- ----------

CTTGACAAGT TCCTCATCAA GAACTGCAAT GATTTCCAGT ATGAGAGCAA

CTTGACAAGT TCCTCATCAA GAACTGCAAT GATTTCCAGT ATGAGAGCAA

---------- ---------- ---------- ---------- ----------

CTTGACAAGT TCCTCATCAA GAACTGCAAT GATTTCCAAT ATGAGAGCAA

CTTGACAAGT TCCTCATCAA GAACTGCAAT GATTTCCAGT ACGAGAGCAA

CTCGACAAAT TCCTCATCAA GAACTGCAAT GATTTCCAGT ATGAGAGCAA

CTCGACAAGT TCCTCATTAA GAACTGCAAC GACTTCCAGT ACGAGAGCAA

CTGGATAAAT TCCTAATCAA GAATTGCAAT GACTTCCAGT ATGAGAGCAA

GGTGTTTTAC CTGAAAATGA AGGGTGATTA CTACCGCTAC TTAGCAGAGG

GGTGTTTTAC CTGAAAATGA AAGGTGATTA CTACCGCTAC TTAGCAGAGG

GGTGTTTTAC CTGAAAATGA AAGGTGATTA CTACCGCTAC TTAGCAGAGG

GGTGTTTTAC CTGAAAATGA AAGGTGATTA CTACCGCTAC TTAGCAGAGG

GGTGTTTTAC CTGAAAATGA AAGGTGATTA CTACCGCTAC TTAGCAGAGG

GGTATTTTAC CTGAAAATGA AAGGTGATTA CTATCGCTAC TTGGCAGAGG

GGTGTTCTAC CTGAAAATGA AGGGCGATTA CTACCGCTAC CTGGCAGAGG

GGTGTTCTAC CTGAAAATGA AGGGCGATTA CTACCGCTAC CTGGCAGAGG

GGTGTTTTAC CTGAAAATGA AGGGCGATTA CTACCGCTAC CTGGCCGAGG

---------- ---------- ---------- ---------- ----------

GGTATTTTAT CTGAAAATGA AAGGCGATTA CTACCGCTAC TTGGCAGAGG

GGTATTTTAC CTGAAAATGA AGGGTGATTA CTACCGCTAC TTGGCGGAGG

---------- ---------- ---------- ---------- ----------

GGTGTTTTAC CTGAAAATGA AAGGCGATTA CTACCGCTAC TTGGCGGAGG

GGTGTTTTAC CTGAAAATGA AAGGTGATTA CTACCGCTAC TTGGCAGAGG

GGTGTTTTAC CTGAAGATGA AAGGCGATTA CTACCGCTAC TTGGCAGAGG

AGTGTTCTAC CTGAAGATGA AGGGCGACTA CTACCGCTAC CTGGCCGAGG

GGTCTTCTAC CTGAAAATGA AAGGCGATTA CTACCGTTAC CTGGCAGAAG

TCGCTTCTGG GGAGAAGAAA AACAGTGTGG TCGAAGCTTC TGAAGCTGCC

TCGCTTCTGG GGAGAAGAAA AACAGTGTGG TCGAAGCTTC TGAAGCTGCC

TCGCTTCTGG GGAGAAGAAA AACAGTGTGG TCGAAGCTTC TGAAGCTGCC

TCGCTTCTGG GGAGAAGAAA AACAGTGTGG TCGAAGCTTC TGAAGCTGCC

TCGCTTCTGG GGAGAAGAAA AACAGTGTGG TCGAAGCTTC TGAAGCTGCC

TAGCTTCTGG GGAGAAGAAA AACAGTGTGG TGGAAGCTTC TGAAGCTGCC

TGGCTTCTGG GGAGAAGAAA AACAGTGTGG TTGAAGCTTC TGAGGCAGCG

TGGCTTCTGG GGAGAAGAAA AACAGTGTGG TTGAAGCTTC TGAGGCCGCG

TGGCTTCCGG GGAGAAGAAG AACAGCGTGG TCGAAGCATC TGAGGCCGCC

---------- ---------- ---------- ---------- ----------

TAGCTTCCGG GGAGAAGAAA AACAGTGTGG TCGAAGCTTC TGAGGCTGCC

TAGCTTCCGG GGAGAAGAAA AACAGTGTGG TCGAAGCTTC TGAGGCGGCC

---------- ---------- ---------- ---------- ----------

TAGCTTCTGG GGAGAAGAAA AACAGTGTGG TTGAAGCTTC TGAGGCCGCT

TAGCTTCTGG GGAGAAGAAA AACAGTGTGG TCGAAGCTTC TGAGGCTGCC

TAGCTTCGGG GGAGAAGAAA AACAATGTGG TTGAAGCTTC TGAGGCTGCC

TGGCCGCCGG CGAGAAGAAG AGCAGCGTGG TGGAGTCCTC CGAGGCCGCC

TTGCTGCAGG TGAGAAGAAG AACAGTGTCG TGGAAGCTTC CGAGGCCGCC

TACAAGGAAG CCTTTGAAAT CAGCAAAGAG CAGATGCAAC CCACGCATCC

TACAAGGAAG CCTTTGAAAT CAGCAAAGAG CAGATGCAAC CCACGCATCC

TACAAGGAAG CCTTTGAAAT CAGCAAAGAG CAGATGCAGC CCACGCACCC

TACAAGGAAG CCTTTGAAAT CAGCAAAGAG CAGATGCAGC CCACGCACCC

TACAAGGAAG CCTTTGAAAT CAGCAAAGAG CAGATGCAGC CCACGCACCC

TACAAGGAAG CCTTTGAAAT CAGCAAAGAG CAAATGCAGC CCACACATCC

TATAAGGAAG CCTTCGAAAT CAGCAAAGAG CACATGCAGC CAACACACCC

TATAAGGAAG CCTTCGAAAT CAGCAAAGAG CACATGCAGC CAACACACCC

TACAAGGAAG CCTTTGAGAT CAGCAAAGAG CACATGCAGC CGACGCACCC

---------- ---------- ---------- ---------- ----------

TACAAAGAAG CCTTTGAAAT CAGCAAAGAG CACATGCAGC CAACGCACCC

TATAAGGAAG CCTTTGAAAT CAGCAAGGAG CACATGCAGC CGACGCACCC

---------- ---------- ---------- ---------- ----------

TACAAGGAAG CCTTTGAAAT CAGCAAAGAG CATATGCAGC CAACACATCC

TATAAGGAAG CTTTTGAAAT CAGCAAAGAG CACATGCAAC CGACGCATCC

TATAAAGAAG CCTTTGAAAT CAGCAAAGAG CACATGCAGC CTACACATCC

TACAAGGAGG CCTTCGAGAT CAGCAAGGAG CACATGCAGC CCACCCACCC

TACAAAGAGG CCTTTGAAAT CAGCAAAGAG CACATGCAGC CGACTCACCC

CATCCGGCTG GGCCTGGCCC TCAACTTCTC CGTGTTCTAC TATGAGATCC

CATCCGGCTG GGGCTGGCCC TCAACTTCTC CGTGTTCTAC TATGAGATCC

CATCCGGCTG GGCCTGGCCC TCAACTTCTC CGTGTTCTAC TATGAGATCC

CATCCGGCTG GGCCTGGCCC TCAACTTCTC CGTGTTCTAC TATGAGATCC

TATTCGGCTG GGCCTGGCCC TCAACTTCTC TGTGTTCTAC TATGAGATCC

CATCCGGCTG GGCCTGGCCC TCAACTTCTC TGTGTTCTAC TATGAGATCC

CATCCGGCTT GGCCTGGCCC TCAATTTTTC TGTGTTCTAC TATGAGATCC

CATCCGGCTT GGCCTGGCCC TCAATTTTTC TGTGTTCTAC TATGAGATCC

CATCCGGCTG GGCCTGGCCC TCAACTTCTC GGTGTTCTAC TACGAGATCC

---------- ---------- ---------- ---------- ----------

CATCCGGCTG GGTCTGGCCC TCAATTTCTC CGTGTTCTAC TATGAGATCC

CATCCGCCTG GGCCTGGCCC TCAACTTCTC TGTGTTCTAC TACGAGATCC

---------- ---------- ---------- ---------- ----------

CATTCGGCTG GGTCTGGCCC TCAACTTCTC TGTGTTCTAC TATGAGATCC

TATCCGGCTG GGCCTGGCCC TCAACTTCTC CGTGTTTTAC TACGAGATCC

CATCCGGCTG GGCCTGGCCC TCAACTTCTC CGTGTTCTAC TATGAGATCC

CATCCGGCTG GGGCTGGCGC TCAACTTCTC CGTCTTCTAC TACGAGATCC

AATTAGGCTG GGCTTGGCGC TCAATTTCTC CGTGTTCTAC TACGAAATCC

AGAATGCACC TGAGCAAGCC TGCCTCTTAG CCAAACAAGC CTTCGATGAT

AGAATGCACC TGAGCAAGCC TGCCTCTTAG CCAAACAAGC CTTCGATGAT

AGAATGCACC CGAGCAAGCC TGCCTCTTAG CCAAACAAGC CTTCGATGAT

AGAATGCACC CGAGCAAGCC TGCCTCTTAG CCAAACAAGC CTTCGATGAT

AGAATGCACC TGAGCAAGCC TGCCTCTTAG CCAAACAAGC CTTCGATGAT

AGAATGCACC TGAGCAAGCC TGCCTCTTAG CCAAACAAGC CTTCGATGAT

AGAATGCACC AGAGCAGGCC TGCCTCTTAG CCAAACAAGC CTTCGATGAT

AGAATGCACC AGAGCAAGCC TGCCTCTTAG CCAAACAAGC CTTCGATGAT

AGAATGCTCC GGAGCAGGCC TGCCTCTTAG CCAAACAAGC CTTTGATGAC

---------- ---------- ---------- ---------- ----------

AGAATGCACC CGAGCAGGCC TGCCTCTTAG CCAAACAAGC CTTCGATGAT

AGAATGCACC CGAGCAGGCC TGCCTCTTAG CCAAACAAGC CTTTGATGAT

---------- ---------- ---------- ---------- ----------

AGAATGCACC TGAGCAGGCC TGCCTCTTAG CCAAACAAGC CTTTGATGAT

AGAATGCGCC TGAACAGGCG TGCCTCCTAG CCAAACAAGC CTTTGATGAT

AGAATGCGCC TGAGCAAGCT TGCCTCCTAG CCAAACAAGC CTTTGATGAT

AGAACGCTCC CGAGCAGGCC TGCCTCCTGG CCAAGCAAGC CTTCGACGAC

AGAATGCCCC CGAGCAGGCC TGCCTTTTAG CCAAACAAGC CTTCGATGAC

GCCATAGCTG AGCTGGACAC ACTAAACGAG GATTCCTATA AGGACTCCAC

GCCATAGCTG AGCTGGACAC ACTAAACGAG GATTCCTATA AGGACTCCAC

GCCATAGCTG AGCTGGACAC ACTAAACGAG GATTCCTATA AGGACTCCAC

GCCATAGCTG AGCTGGACAC ACTAAACGAG GATTCCTATA AGGACTCCAC

GCCATAGCTG AGCTGGACAC ACTAAATGAG GATTCCTATA AGGACTCCAC

GCGATAGCTG AGCTGGACAC ACTAAACGAG GATTCCTATA AGGACTCCAC

GCTATAGCTG AGCTGGACAC ATTAAACGAG GATTCCTATA AGGACTCCAC

GCTATAGCTG AGCTGGACAC ATTAAACGAG GATTCCTATA AGGACTCCAC

GCCATAGCTG AGCTGGACAC GCTAAACGAG GATTCCTATA AGGACTCCAC

---------- ---------- ---------- ---------- ----------

GCCATAGCTG AGCTGGACAC ACTAAACGAA GATTCCTATA AGGACTCCAC

GCCATAGCTG AGCTGGACAC TCTAAACGAG GATTCCTATA AGGACTCCAC

---------- ---------- ---------- ---------- ----------

GCCATAGCTG AGCTGGACAC ACTAAATGAG GATTCCTATA AGGACTCCAC

GCCATAGCAG AGCTGGACAC ACTAAACGAG GATTCCTATA AGGACTCCAC

GCCATAGCAG AGCTGGACAC ACTAAACGAG GATTCCTATA AGGACTCCAC

GCCATCGCCG AGCTGGACAC GCTGAACGAG GATTCCTATA AAGACTCCAC

GCCATAGCAG AGCTGGACAC ACTAAATGAG GATTCCTATA AGGACTCCAC

GCTGATCATG CAGTTGCTGC GAGACAACCT CACCCTCTGG ACGAGCGACC

GCTGATCATG CAGTTGCTGC GAGACAACCT CACCCTCTGG ACGAGCGACC

GCTCATCATG CAGTTGCTGC GAGACAACCT CACCCTCTGG ACGAGCGACC

GCTCATCATG CAGTTGCTGC GAGACAACCT CACCCTCTGG ACGAGCGACC

GCTCATCATG CAGTTGCTGC GAGACAACCT CACCCTCTGG ACGAGCGACC

ACTCATCATG CAATTGCTGC GAGACAACCT CACCCTCTGG ACAAGCGACC

TCTCATCATG CAGTTGCTGC GAGACAACCT CACCCTCTGG ACGAGCGACC

TCTCATCATG CAGTTGCTGC GAGACAACCT CACCCTCTGG ACGAGCGACC

GCTCATCATG CAGTTGCTGC GAGACAACCT CACCCTCTGG ACGAGCGACC

---------- ---------- ---------- ---------- ----------

GCTCATCATG CAGTTGCTGC GAGACAACCT CACCCTCTGG ACGAGCGACC

ACTCATCATG CAGTTGCTGC GAGACAACCT CACCCTCTGG ACAAGCGACC

---------- ---------- ---------- ---------- ----------

ACTCATCATG CAGTTGCTGC GAGACAACCT CACCCTCTGG ACGAGCGACC

ACTCATCATG CAGTTGCTGC GAGACAATCT CACCCTCTGG ACAAGCGACC

GCTCATCATG CAGTTGCTGC GAGACAACCT CACTCTCTGG ACCAGTGACC

TCTGATCATG CAGCTGCTGC GAGACAACCT CACTCTCTGG ACGAGCGACC

TCTTATCATG CAGTTACTGC GAGACAACCT CACTCTCTGG ACAAGCGACC

AGCAGGATGA AGAAGCAGGA GAAGGCAACT GAATCATCAC CGATTCTTCC

AGCAGGATGA AGAAGCAGGA GAAGGCAACT GAATCATCAC CGATCCTTCC

AGCAGGATGA AGAAGCAGGA GAAGGCAACT GAATCATCAC CGATTCTTCC

AGCAGGATGA AGAAGCAGGA GAAGGCAACT GAATCATCAC CGATTCTTCC

AGCAGGATGA AGAAGCAGGA GAAGGCAACT GAATCATCAC TGATTCTTCC

AGCAGGATGA AGAAGCAGGA GAGGGCAACT GAATCATCAC CAATTCTTCC

AGCAGGATGA AGAAGCCGGA GAAGGCAACT GAATTATCAC TGATTCTTCC

AGCAGGATGA AGAAGCAGGA GAAGGCAACT GAATTATCAC TGATTCTTCC

AGCAGGATGA AGACGCCGGA GAAGGCAACT GAATCATCAC CGATTCTTCC

---------- ---------- ---------- ---------- ----------

AGCAGGATGA AGAAGCAGGA GAAGGCAACT GAATCATCAC TGATTCTTCC

AGCAGGATGA AGAAGCAGGA GAAGGCAACT GAATCATCAC CGATTCTTCC

---------- ---------- ---------- ---------- ----------

AGCAGGATGA AGAAGCAGGA GAAGGCAACT GAATCATCAC CGATTTTTCC

AGCAGGATGA AGAAGCAGGA GAAGGCAACT GAATCATCAC TGATTCTTCC

AGCAGGATGA AGAAGCGGGA GATGGCAATT GAATCATCAC CAACTTTTCC

AACAGGACGA GGAAGCAGGA GAAGGCAACA GAGTCATCAT CGACACTCCC

AGCAGGATGA GGAAGCAGGA GAGGGCAACT GAATCATCAC CAATACTTCC

TTGCCACAAT CACTAAATAT CTAGTGCTAA ACCTATCTGT ATTGGCAGCA

TTGCCACAAT CACTAAATAT CTAGTGCTAA ACCTATCTGT ATTGACAGCA

TTGCCACAAT CACTAAATAT CTAGTGCTAA ACCTATCTGT ATTGGCAGCA

TTGCCACAAT CACTAAATAT CTAGTGCTAA ACCTATCTGT ATTGGCAGCA

TTGCCACAAT CACTAAATAT CTAGTGCTAA ACCTATCTGT ATTGGCAGCA

TTGCCACAAT CACTAAATAT CTAGTGCTAA ACCTATCTGT ATTGGCAGCA

TTGCCACAAT CACTA--TAT CTAGTGCTAA ACCTATCTGT ATTGGCAGCA

TTGCCACAAT CACTA--TAT CTAGTGCTAA ACCTATCTGT ATTGGCAGCA

TTGCCACAAT CACTAAGTAT CTAGTGCTAA ACCTATGTGT ATTGGCAGCA

---------- ---------- ---------- ---------- ----------

TTGCCACAAT CACTAAATAT CTAGTGCTAA ACCTATCTGT ATTGGCAGCA

TTGCCACAAT CACTAAATAT CTAGTGCTAA ACCTATCTGT ATTGGCAGCA

---------- ---------- ---------- ---------- ----------

TTGCCACAAT CACTAAATAT CTAGTGCTAA ACCTATCTGT ATCGGCAGCA

TTGCCACAAT CACTAAATAT CTAGTGCTAA ACCTATCTGT ATTGGCAGCA

TTGTCACAGT CACTAAATAT CTCGTGCTAA ACCCATCTGT ATCAGCGGCA

CCGCCACGGT CCCTTGATAT CTAGTGCTAA ACATATCTGT ATTGTGCGCA

TTGCCACGAT CACTAAATAT CTAGTGCTAA ACATGTCTGT ATTGTCAGCA

CAGCTACTCA GATCTGCACT CCGGAAGCAG TTTCAGATAA ATCATGGGCA

CAGCTACTCA GATCTGCACT CCGGAAGCAG TTTCAGATAA ATCATGGGCA

CAGCTACTCA GATCTGCACT CCGGAAGCAG TTTCAGATGA ATCATGGGCA

CAGCTACTCA GATCTGCACT CCGGAAGCAG TTTCAGATAA ATCATGGGCA

CAGCTACTCA GATCTGCACC CTGGAAGCAG TTTCAGATAA ATCATGGGCA

CAGCTACTCA GATCTGCTCT CCGAAAGCAG TTTCAGATAA ATCTTGGGCA

CAGCTATTCA GATCTGCCCT CCGGAAGCAG TTTCAGATAA ATCATGGGCA

CAGCTATTCA GATCTGCCCT CCGGAAGCAG TTTCAGATAA ATCATGGGCA

CAGCTACTCC GGGCTGCTCT CCGGAAGCAG CTGCAGCTAA ATCATGGGCA

---------- ---------- ---------- ---------- ----------

CAGCTACTCA GAGCTGCTCT CCGGAAGCAG TTTCAGATAA ATCATGGGCA

CAGCTACTCA GATCTGCTCT CCGGAAGCAG TTTCAGATAA ATCATGGGCA

---------- ---------- ---------- ---------- ----------

CAGCTACTCA GATCTGCTTT CCGGAAGCAG TTTCAGATAA ATCATGGGCA

CAGCTATTCA GACCTGCTCT CCGGGAGTAG TTTCAGATAA GTCAT-GGCA

CAGCTACTCA GATCTACTCT CCGGGAGCAG GTTCAGACAA CTCACAAGCA

CAGCGACTCC AAGCTGCCCT CCGGAAGCGG CCTCAGACAC CCCATGGGCA

CAGCTCTTCA GATTTGCTCT CCGGAAGCAA TTTCAGATAT GTCAAGGGCA

TTGCTGGACT GATGGCTTTT GAATTGTGTG GAGAAGTGTG GAGGCATTTT

TTGCTGGACT GATGGCTTTT GAATTGTGTG GAGAAGTGTG GAGGCATTTT

TTGCTGGACT GATGGCTTTT GAATTGTGTG GAGAAGTGTG GAGGCATTTT

TTGCTGGACT GACGGCTTTT GAATTGTGTG GAGAAGTGTG GAGGCATTTT

TTGCTGGACT GATGGCTTTT GAATTATGTA GAGCAGTGTA GAGGCATTTT

TTGCTGGATT CATGGCTTTT GAATTGTGCA GAGAAGTG-- GAGGCATTTT

TTGCTGGACT GATGGCTTTT GAATTGTGCA GAGAAGTGTG GAGGCATTCT

TTGCTGGACT GATGGCTTTT GAATTGTGCA GAGAAGTGTG AAGGCATTCT

TTGCTGGACT GATGGCTTGT GAGTTGTGCA GAGAAGTGAG GGGGCATTTG

---------- ---------- ---------- ---------- ----------

TTGCTGGATT GATGGCTTTT GAATTGTGCA GACAAGTGCG GAGGCATTTT

TTGCTGGGTT GATGGCATTT GAATTGTGCA GACAAGTGCG GAGGCATT-T

---------- ---------- ---------- ---------- ----------

TTGCTGGATC GATGGCTTTT GAATTGTGCT GACAAGTGTG GAGGCATTTT

TTGCTGGATT GATGGCTTTT GGATTGTGCA GACAAGTGCA GAGGTATTTT

TTGCTGGATT GATGGTTTTT GGGGTGTGCA GACAAGCTCA -AGGCATTTT

TTGCTGGATG GATGGCTTCA GAATTGTGCA GGCAGGAGAG GAGGTGCCTT

TTGCTGGATC GATGGCCACT GAATTGTTCA GAAGGGTACG GAGGCGTTTT

GATATTTAAA ACAAGCTGAT AGTGTTTCGT TAAGCAGTAC ATCTTGTGCA

GATATTTAAA ACAAGCTGAT AGTGTTTTGT TAAGCAGTAC ATCTTGTGCA

GATATTTAAA ACAAGCTGAT AGTGTTTCGT TAAGCAGTAC ATCTTGTGCA

GATATTTAAA ACAAGCTGAT AGTGTTTCGT TAAGCAGTAC ATCTTGTGCA

GATATTTAAA ACAAGCTGAT AGTGTTTCTT TAAGCAGTAC ATCTTGTGCA

GATATTTAAA ACAAGCTGAT AGTGTTTTGT TAAGCAGTAC ATCTTGTGCA

GCTATTTAAA ACAAGCTGAT AGTGTGTTGT TAAGCAGTAC ATCTCGTGCA

GCTATTTAAA ACAAGCTGAT AGTGTTTTGT TAAGCAGTAC ATCTTGTGCA

GATATTTAAA ACAAGCTGAT AGTGTTTTGT TAAGCAGTAC ATCTTGTGCA

---------- ---------- ---------- ---------- ----------

GATATTTAAA ACAAGCTGAT AGTGTTTTGT TAAGCAGTAC ATCTTGTGCA

GATATTTAAA ACAAGCTGAT AGTGTTTTGT TAAGCAGTAC ATCTTGTGCA

---------- -CAAGCTGAT AGTGTTTTGT TAAGCTGTAC ATCTTGTGCA

GATATTTAAA ACAAGCTCAT AGTGTTTTGT TAAACAGTAC ATCTTGTGCA

GATATTTAAA ACAAGCTGGT AGTGTTTTGT TAAGCAGTAC ATCTTGTGCA

GATATTTCAA ACAAGCTGAT AGTGTTTTGT TAAGCAG--- ---TTGTGCA

GCAATTTCTG ATGAGCTAAT AGTGTTTTGT TGAGCAGTCC GTCATCTGCA

GATATTTAAT ACGAGCTGAT AGTGTTTTGT TAAGCAGTAC ATCTTGTGCA

TGCAAAATGA ATTTCTTCAA TTAATGGAAA ACTGTTAAGG GAAGCTGATA

TGCAAAATGA ATTTCTTCAA TTAATGGAAA ACTGTTAAGG GAAGCTGATA

TGCAAAATGA ATTTCTTCAC CTAATGGAAA ACTGTTAAGG GAAGCTGATA

TGCAAAATGA ATTTCTTCAC CTAATGGAAA ACTGTTAAGG GAAGCTGATA

TGCAAAATGA ATTTCTTCAA CTAATGGAAA ACTGTTAAGG GAAGCTGATA

TGCAAAACGA ATTTCTTCAG CTAATGGAAA ACTGTTAAGG GAAGCTGATA

TGCAAAATGA ATTCTTTCAG CTAATGGAAA ---------- ----CTGACA

TGCAAAATGA ATTCCTTCAG CTAATGGAAA ---------- ----CTGACA

TGCAAAATGA ATTTCCTCAG CTAATGGGAA ACTGTTAAGG GAAGCTGATA

---------- ---------- ---------- ---------- ----------

TGCAAAATGA ATTTCTTCAG CTAATGGAAA ACTGTTAAGG GAAGCTGATA

TGCAAAATGA ATTTCTTCAG CTAATGGAAA ACTGTTAAGG GAAGCTGATA

TGCAAAATGA ATTT-TTCAG CTAATGGAAT ACTGTTAAGG GAAGCTGATA

TGCAAAATGA ATTTCTTCAG CTAATGGAAA ACTGTTAAGG GAAGCTGATA

TGCAAAATGA ACTTCTTCAG CTAATGAAAA ACTGTTAAGG GAAGCTGATA

TGCAAAATGA TCTTCTTCAG CTAATGGAAA AC--TTAAGG GAAGCCAATA

TGCGAAACGT GTTTCTTCCG CGAACGGGAA ACAGGGACAG GAAGCTGACT

TGCAAAATAA ATTTCTTTAG CTGTTGGGAA ACAGTGAGTG GAAGCTGTTG

CAGAGACAAC TTGCTCCTTT CCATCAGCTT ATAATAAACT GTTTAAGTGA

CAGAGACAAC TTGCTCCTTT CCATCAGCTT ATAATAAACT GTTTAAGTGA

CAGAGACAAC TTGCTCCTTT CCATCAGCTT ATAATAAACT GTTTAAGTGA

CAGAGACAAC TTGCTCCTTT CCATCAGCTT ATAATAAACT GTTTAAGTGA

CAGAGACAGC TTGCTCCTTT CCATCAGCTT ATAATAAACT GTTTAAGTGA

CAGAGACAAC TTGCTCCTTT CCATCAGCTT ATAATAAACT G-TTAAGTGA

CA-CGACAAC TTGTTCCTTC CCATCAGCTT AT---AAACT GTTTCTGTGA

CAGCGACAAC TCATTCCTTC CCATCAGCTT AT---AAACT GTTTCTGTGA

CAGAGACAAC TTGCTCCTTC CCGTCAGCTT ATGATAAACT GTTTAAGTGA

---------- ---------- ---------- ---------- ----------

AAAAGACCAC TTGCTCCTTT CCATCAACTT ATAAGAAACT GTTTAAGTGA

CAAAGACCAC TTGCTCCTTT CCATCAGCTT GTAATAAACT G-TTAAGTGA

AAAAGAC--C TTGCTCCTTT TCATCAGCTT ATAATAAACT GTCTAAGTGA

CAAAGTCAAC TTGCTCCTTT CCATCAGCTT ATAATAAACT GTTTAAGTGA

CAGAGACAGC TCGCTCCTTC CCATCAGCTT ACAATAAACT GTTTAAGTGA

CGAAGACAAC TTGCTCCTTC CCATCAGCTT ATAATAAACT GTTTAAGTGA

CCAAGATGCC CCGATCCTCG CCATCAGCTT ACAATGAACT GTCTAAGTGA

CTGACACAGT TTGCCCCTCT GCACCAGCTT ACTATAAACT GTTTAAGTGA

GGTTTCAGTA GCTCCTTGTT TTGCCTCTTT AAATTATGTG CACAAACCTT

GGTTTCAGTA GCTCCTTGTT TTGCCTCTTT AAATTATGTG CACAAACCTT

GGTTTCAGTA GCGCCTTGTT TTGCCTCTTT AAATTATGTG CACAAACCTT

GGTTTCAGTA GCGCCTTGTT TTGCCTCTTT AAATTATGTG CACAAACCTT

GGTTTCAGTA GCTCCTTGTT TTGCCTCTTT AAATTATGTG CACAAACCTT

GGTTTCAGTA GCGCCTTGTT TTGCCTCTTT AAATTATGTG CACAGACCTT

GCTTTCAGCG GC-CCCTGCT GTGCCTCTTT AAATTATGTG CGCACACCTT

GCTTTCAGCA GC-CCTTGCT GTGCCTCTTT AAATTATGTG CACACACCTT

GGTTTCAGTA GCGCCTTGTT TTGCCTCTTT AAATTATGTG CACAAACCTT

---------- ---------- ---------- ---------- ----------

GGTTTCAGTA GCGCCTTGTT TTGCCTCTTT AAATTATGTG CACAAACCTT

GGTTTCAGTA GCGCCTTGTT TTGCCTCTTT AAATTATGTG CACAAACCTT

GGTTTCAGTA GCGCCTTGTT TTGCCTATTT AAATTATGTG CACAAACCTT

GGTTTCAGTA GCGCCTTGTT TTGCCTCTTT AAATTATGTA CACAAACCTT

CGTTTCGGTA GCGCCTTGTT TTGCCTCTTT AAATTATGTG CGCAAACCTT

AGTTTCAGTA GAGCCTCGTT TTGCCTCTTT AAATTATGTG CACAAACCTT

GGTTTCAGTA ATGCCTTGTT TTGCCTCTTT AAATTATGTG CACAAACCTT

GGTTTCAGTA GCGCCCTGTT CTGCCTCTTT AAATTATGTG CACAAACCTT

CTTTTAATGC AATGCATCTA AAGTTTTGAT ACTTGTAACT TTTTTTTTGG

CTTTTAATGC AATGCATCTA AAGTTTTGAT ACTTGTAACT TTTTTTTTGG

CTTTTAATGC AATGCATCTA AAGTTTTGAT ACTTGTAACT TTTTTTTTGG

CTTTTAATGC AATGCATCTA AAGTTTTGAT ACTTGTAACT TTTTTTTTGG

CTTTTAATGC AATGCATCTA AAGTTTTGAT ACTTGTAACT TTTTTTTTGG

CTTTTAATGC AATGCATCTA AAGTTTTGAT ACTTGTAACT TTTTTTTTGG

CTTTTAATGC AATGCATCAA GGTTTTTGAT ATGTGTAAC- TTTTTTTTGG

CCTTTAATGC AATGCATCAA AGTTTTTGAT ATGTGTAAC- TTTTTTTTGA

CTTTTAATGC AATGCATCTA AGTTTT-GAT ACGTGTAACT CTTTTTTTGG

---------- ---------- ---------- ---------- ----------

CTTTTAATGC AATGCATCTA AAGTTTTGAT ACCTGTAACT TTTTTTTTGG

CTTTTAGTGC AATGCATCTA AAGTTTTGAT ACCTGTAACT TTTTTTTTGG

CTTTTA-TGC AATGCATCTA AAGTTTTGAT ACTTGTAAC- ----TTTTGG

CTTTTAATGC AATGCATCTA AAGTTTTGAT ACTTGTAACT TTTTTTTTGG

CTTTTAATGC AATGCATCTA AAGTTTTGAT ACTTGTAACT TTTTTTTTGG

TTT---AAGC AATGCATCTG AAGTTTTGAT ATTTGTAACT TTTTTTTTGG

GTTTTAATGC AATGCCTCTA AAGTTTGGAT ACTTGTAACT TTGTTTTTGG

CTTTTAATGC AATGCACCTA AAGTTTTGAT ACTTGTAACT TTTTGTTGGG

TTGCAATTGT TTAAGAATCA TGGATTTATT TTTTGTAACT CTTTGGCTAT

TTGCAATTGT TTAAGAATCA TGGATTTATT TTTTGTAACT CTTTGGCTAT

TTGCGATTGT TTAAGAATCA TGGATTTATT TTTTGTAACT CTTTGGCTAT

TTGCGATTGT TTAAGAATCA TGGATTTATT TTTTGTAACT CTTTGGCTAT

TTGCAATTGT TTAAGAATCA TGGATTTATT TTTTGTAACT CTTTGGCTAT

TTGCGATTGT TTAAGAATCA TGGATTTATT TTTTGTAACT CTTTGGCTAT

TTGTGATT-- --AAGAATCA TGGATTTATT TTTTGTAACT CTTTGGCTAT

TTGTGATT-- --AAGAATCA TGGATTTATT TTTTGTAACT CTTTGGCTAT

TTGCGATTGT TTAAGAATCA TGGATTTATT TTTTGTAACT CTTTGGCTAT

---------- ---------- ---------- ---------- ----------

TTGCGATTGT TTAAGAATCA TGGATTTATT TTTTGTAACT CTTTGGCTAT

TTGCGATTGT TTAAGAATCA TGGATTTATT TTTTGTAACT C--TGGCTAT

TTGCAATTGT TTAAGAATCA TGGATTTATT TTTTGTAACT CCTCAGCTAT

TTGCGATTGT TTAAGAATCA TGGATTTATT TTTTGTAACT CTTTGGCTAT

TTGTGATTGT TTAAGAATCA TGGATTTATT TTTTGTAA-- CTTTGGCTAT

TTGTGATGGT TTAAAAATCA TGGATTTATT TTTTGTAACT CTTTGGCTAT

TTGCGATTGT TCAAGAATCA TGGATttaTT TTTGGTAACT CTTTGGCCGT

TTGCAGTTGT TTAAGAATCA TGGATTTATT TTTTGTAACT CTTTGGCTGT

TGTCCTTGTG TATCCTGACA GCGCCATGTG TCAGCCCATA TCAAGATGGG

TGTCCTTGTG TATCCTGACA GCGCCATGTG TCAGCCCATA TCAAGATGGG

TGTCCTTGTG TATCCTGACA GCGCCATGTG TCAGCCCATA TCAAGATGGG

TGTCCTTGTG TATCCTGACA GCGCCATGTG TCAGCCCATA TCAAGATGGG

TGTCCTTGTG TATCCTGACA GCGCCATGTG TCAGCCCATA TCAAGATGGG

TGTCCTTGTG TAT-CTGACA GCACCATGTG TCAGCCCATA TCAAGATGGG

TGTTCTTGTG TACCCTGACA GCATCATGTG TCAACCTGTA TCATGATGGG

TGTTCTTGTG TACCCTGACA GCATCATGTG TCAACCTGTA TTATGATGGG

TGTCCTTGTG TATTCTGACC GCGCCATGTG TCAGCCCATA TCAAGATGGG

---------- ---------- ---------- ---------- ----------

TGTCCTTGTG TATCCTGACA GCGCCATGTG TCAGCCCACA TCAAGATGGG

TGTCCTTGTG TATCCTGACA GCGCCATGTG TCAGCCCACA TCAAGATGGG

TGTCCTTGTG TATCCTGACC AGGCCATGTG TCAGCCCATA TCAAGATGGG

TGTCCTTGTG TATCCTGACA GCACCATGTG TCCACCCATA TCAATATGGG

TGTCATTGTG TATCCTGACA GCGCCATGTG TCAGCCCATA TCAAGATGGG

TGTCCTTGTG TATCCTGATA GCGCCATGTG TTAACCCATA TCAAGATGGG

TGTCCTTGTG TATTCTGACA GCGCCATGTG TCAGCCCATA TCAAGGTGGA

TGTCTCTGTG TATTCTGACA GCACCATGTG TCAGCCCATA TCAAGATGGA

TGATTATGAA ATGCCAGACT TCTAAAATAA ATGTTTTGGA ATTCAATGGG

TGATTATGAA ATGCCAGACT TCTAAAATAA ATGTTTTGGA ATTCAATGGG

TGATTATGAA ATGCCAGACT TCTAAAATAA ATGTTTTGGA ATTCAATGGG

TGATTATGAA ATGCCAGACT TCTAAAATAA ATGTTTTGGA ATTCAGTGGG

TGATTATGAA ATGCCAGACT TCTAAAATAA ATGTTTTG-- ----------

TGATTATGAA ATGCCAGACT TCTAAAATAA ATGTTTTGGA ATTCAATGGG

TGGTTATGAA ATGCCAGATT GCTAAAATAA ATGTTTTG-G ACTTAAAGAG

TGGTTATGAA ATGCCAGACT GCTAAAATAA ATGTTTTG-G ACTTAAAGAG

TGATTATGAA ATGCCAGACT TCTAAAATAA ATGTTTGG-A ATCCAATGGG

---------- ---------- ---------- ---------- ----------

TGATTATGAA ATGCCAGACT TCTAAATTAA ATGTTTTGGA ATTCAGTGGG

TGATTATGAA ATGCCAGACT TCTAAATTAA ATGTTTTGGA ATTCAATGGG

TGATTATGAA ATGCCAGACT TCTAAATTAA ATGTTTTGGA TTTCAATAGG

TGATTATGAA ATGCCAGACT TCTAAATTAA ATGTTTTGGA ATTCAGTGGG

TGATTATGAA ATGCCAGACT TCTAAATTAA ATGTTTG-GA ATTCAGTGGG

TGATTATGAA ATGCCAGACT TCTAAATTCA ATGTTTTGGG AATCCATGGG

TGATTGTGAA ACATCAGACT TCTAAAATAA GTGTTTTGGA ATTCAGTGGA

TTATAATGAA ATGCCAGACT TCTAAATTAA ATGTTTTGGA ATTAAATGGA

TAAATAAATG CTGCTTTGGG GATATTATCT CTGTTTGGTC TTTTCTGACC

TAAATAAATG CTGCTTTGGG GATATCATCT CTGTTTGGTC TTTTCTGACC

TAAATAAATG CTGCTTTGGG GATATTATCT CTGTATGGTC TTTTCTGACC

TAAATAAATG CTGCTTTGGG GATATGATCT CTGTATGCTC TTTT------

---------- ---------- ---------- ---------- ----CTGACC

TAAATAAATG CTGCTTTGGG GATATTATTT CTGTATGGTC TTTT------

TAAATAAATG CTGCTTTGGG GATATTATCT -TGCCTGGCC TTTCCTGACC

TAAATAAATG CTGCTTTAGG GATATTATCT -TGCCTGGCC TTTTCTGACC

TAAATAAATG CTGCTTTGGG GATATTGTCC ATGTATGGTC TTTTCTGACC

---------- ---------- ---------- ---------- ----CTGACC

TAAATAAATG CTGCTTTGGG GATATTCTGT CTGTGTGGTT TTTTCTTACC

TAAATAAATG CTGCTTTGGG GATATTTTCT CTGTACAGTC TTTTCTGACC

TAAATAAATG CTGCTTTGGG GATATTCTTT CTGTATGGTC TTTTCTGACC

TAAATAAATG CTGCGTAGGG GATATTATCT ATGTATGGTC TTTTCTGACC

TAAATAAATG CTGCTTTGGG GATATTATCT CTGTGTGGTC TTTTCTGACC

TAAATAAATG CTGCTTTGGG GATAATATCT CTGTGTGGTC TTTT------

TAAATAAATG CTGCTTTGGG GATATTATCG CTGCATAGTC TTTTCTGACC

TAAATAAATG CCGCTTTGGG GATATTAACT CTGCCTTGTC TTTTCTAACC

TTCCTTCACA GAGGCTGTCC GTCGCCTTGT TCTTTTTCTC CCTTTGTGAT

TTCCTTCACA GAGGCTGTCC GTCGCCTTGT TCTTTTTCTC CCTTTGTGAT

TTCCTTCACA GAGGCTGTCC GTCGCCTTGT TCTTTTTCTC CCTTTGTGAT

---------- ---------- ---------- ---------- ----------

TTCCTTCACA GAGGCTGTCC GTCGCCTTGT TCTTTTTCTC CCTTTGTGAT

---------- ---------- ---------- ---------- ----------

TTCCCTCACA GAGGCAGTCC GTCGCCTTGT TCGTTTTCTC CCTTTGTGAT

TTCCTTCACA GAGGCAGTCC GTCGCCTTGT TCTTTTTCTG CCTTTGTGAT

TTCCTTCACC GAGGCCGTCC TCCGTCTCGT CCTTTTTCTC CCCTTGTGAT

TTCCTTCACA GAGGCTGTTC GTCGCCGCGT TCTTTTTCTC CCCTTATGGT

TTCCTTCACG GAGGCCGTCC TTCGCCTTGT TCTCTTTCTC CCCTTGTGAT

TTCCTTCACA GAGGCTGTCC GTCGCCTTGT TCTTTTTCTC CCTTTGTGAT

TTCTTTCACA GATGCTGTCC GTCGCCTCGT TCTTTTTCTC CCCTTGTGAT

TTCCTTCATA GAGGCTGTCC GTCGCCTTGT TCGTTTTCTC CCTTTGTGAT

TTCCTTCACA GAGGCTGTCC GTCGCCTTGT TCTTTTTCTC CCTTTGTGAT

---------- ---------- ---------- ---------- ----------

TTCCTTCACA GACATGGCAC GTCGCCGCGT TCTTTTTCTC CCTTTGTGAT

TTCCTTCACG GATGCTGTGC GCCGTCTTGT TCGTTTTCGT CCTTTATGAT

CTTCTTCTAG TATCTTATCA TCAAAGCCAG TCAGTTCGAT GGTTTCAGAC

CTTCTTCTAG TATCTTATCA TCAAAGCCAG TCAGTTCGAT GGTTTCAGAC

CTTCTTCTAG TATCTTATCA TCGAAGCCAG TCAGTTCGAT GGTTTCAGAC

---------- ---------- ---------- ---------- ----------

CTTCTTCTAG TATCTTATCA TCAAAGCCAG TCAGTTCGAT GGTTTCAGAC

---------- ---------- ---------- ---------- ----------

CCTCCTCTAG AATCTTATCA TCAAAGCCAG TCAGCTCTAT GGTTTCAGAC

CCTCCTCTAG AATCTTATCA TCAAAGCCAG TCAGCTCTAT GGTTTCAGAC

CTTCTTCTAG TATCTTATCA TCAAAGCCAG TAAGTTCAAT GGTTTCAGAC

CTTCTTCCAG TATCTTATCA TCAAAGCCAG TCAGCTCAAT GGTTTCGGAC

CTTCTTCCAG TATCTTATCA TCAAAGCCAG TCAGCTCAAT GGTTTCAGAT

CTTCTTCCAG TATCTTATCA TCAAAGCCAG TCAGCTCAAT GGTTTCAGAC

CTTCTTCCAG TATCTTATCA TCGAAACCAG TCAGTTCAAT GGTTTCAGAC

CTTCTTCTAA TATCTTATCA TCAAAGCCAG TCAGCTCAAT GGTTTCTGAC

CTTCTTCTAG TATCTTATCA TCAAAGCCAG TAAGCTCAAT GGTTTCAGAC

---------- ---------- ---------- ---------- ----------

CTTCTTCCAG AATTTTGTCA TCAAAGCCAG TGAGCTCAAT GGTTTCAGAC

CTTCTTCTAG TATTTTATCA TCAAATCCTG TAAGCTCAAT AGTTTCAGAC

TGACTTGTGG GTCCAGCAGA GAACCACTCT GGTTCTTCTT CTGTGTAAGA

TGACTTGTGG GTCCAGCAGA GAACCACTCT GGTTCTTCTT CTGTGTAAGA

TGACTTGTGG GTCCAGCAGA GAACCACTCT GGTTCTTCTT CTGTATAAGA

---------- ---------- ---------- ---------- ----------

TGACTTGTGG GTCCAGCTGA GAACCACTCT GGTTCTTCTT CTGTGTAAGA

---------- ---------- ---------- ---------- ----------

TGACTGGTGG GTCCAGCTGA AAACCATTCT GGTTCTTCCT CAGTGTATGA

TGACTGGTGG GTCCAGCTGA AAACCATTCT GGTTCTTCCT CAGTGTATGA

TGACTCGTGG GTCCAGCTGA GAACCACTCT GGTTCTTCTT CCGTGTAAGA

TGACTCGTGG GTCCAGCTGA GAACCATTCT GGTTCTTCTT CTGTGTAGGA

TGACTTGTGG GTCCAGCTGA GAACCATTCT GGTTCTTCTT CTGTGTAGGA

TGACTTGTGG GTCCGGCTGA GAACCATTCG GGTTCTTCTT CTGTGTAGGA

TGACTTGTGG GTCCAGCTGA GAACCATTCT GGTTCTTCCT CTGTGTAGGA

TGACTTGTGG GTCCAGCTGA GAACCATTCT GGTTCTTCTT CTGTGTAGGA

TGACTTGTAG GTCCAGCTGA GAACCATTCT GGTTCTTCTT CCGTGTAAGA

---------- ---------- ---------- ---------- ----------

TGACTTGTGG GTCCAGCTGA AAACCACTCT GGCTCTTCTT CTGTATAGGA

TGACTTGTCG GTCCAGCAGA GAACCATTCA GGTTCCTCTT CAGTATAGGA

ATCATTTCTT CTACGCTCAC CAAAGACACG CTTACTATCT CCAAACTCTC

ATCATTTCTT CTACGCTCAC CAAAGACACG CTTACTATCT CCAAACTCTC

ATCATTTCTT CTACGCTCGC CAAAGACACG CTTACTATCT CCAAACTCTC

---------- ---------- ---------- ---------- ----------

ATCATTTCTT CTGCGTTCAC CAAAGACACG CTTACTATCC CCAAACTCTC

---------- ---------- ---------- ---------- ----------

ATCGTTTCTT CTACGCTCAC CAAAGACACG TTTACTATCT CCAAACTCCC

ATCGTTTCTT CTACGCTCAC CAAAGACACG TTTACTATCT CCAAACTCCC

ATCATTCCTT CTACGCTCGC CAAAGACACG CTTACTATCC CCAAACTCCC

ATCGTTTCTT CTACGCTCAC CAAAGACACG CTTACTATCC ACAAACTCCC

ATCATTTCTT CTTCGCTCAC CAAAGACACG CTTACTATCC CCAAACTCCC

ATCATTTCTT CTACGCTCAC CAAAGACACG CTTACTATCG CCAAACTCCC

ATCATTTCTT CGACGTTCAC CAAAGACACG CTTACTATCC CCAAACTCCC

ATCATTTCTT CTACGTTCAC CAAAGACACG CTTACTATCC CCAAACTCCC

ATCGTTTCTT CTACGCTCGC CAAAGACTCG CTTACTATCC CCAAACTCTC

---------- ---------- ---------- ---------- ----------

ATCATTTCTT CTGCGCTCTC CAAATATACG TTTGCTGTCA CCAAACTCTC

ATCATTTCTT CTACGTTCTC CAAAGACACG CTTACTTTCC CCAAACTCTC

TCTGGAAAAG aagacaaaCC CTGAAACGCT TGTCCTTGAA GTCCCTCTCT

TCTGGAAAAG aagacaaaCC CTGAAACGCT TGTCCTTGAA GTCCCTCTCT

TCTGGAAGAG GAGACAAACC CTGAAACGCT TGTCCTTGAA GTCCCTCTCT

---------- ---------- ---------- ---------- ----------

TCTGGAAAAG AAGACAAACC CTGAAACGCT TGTCCTTGAA GTCCCTCTCT

---------- ---------- ---------- ---------- ----------

TCTGAAAAAG AGGACAAACC CTGAAACGCT TGTCCTTGTA GTCCCTCTCC

TCTGAAAAAG AGGACAAACC CTGAAACGTT TGTCCTTGTA GTCCCTCTCT

TCTGGAAAAG AAGACAAACC CGGAAGCGCT TGTCCTTGTG GTCCCTCTCG

TCTGGAAAAG GAGACAAACC CTGAAGCGCT TGTCCTTGTA GTCCCTCTCG

TCTGGAAAAG AAGACAAACC CTGAATCGCT TGTCCTTATA GTCTCTCTCT

TCTGGAAAA- AA-ACAAACC CTGAAACGCT TGTCCTTGTA GTCCCTCTCT

TCTGGAAAAG AAGACAAACC CGAAACCGCT TGTCCTTGTA GTCTCTCTCT

TCTGGAAAAG AAGACAAACC CTGAAACGCT TGTCCTTGTA GTCCCTCTCG

TCTGGAAAAG AAGACAAACC CTAAAACGCT TGTCCTTGTA GTCCCTCTCA

---------- ---------- ---------- ---------- ----------

TCTAAAAAAG AAAACACACC CGGAAACGCT TGTCCTTATA ATCTCTCTCC

TCTGGAAAAG AGAACTAACC CTAAAACGTT TGTCCTTGTA GTCCCTCTCG

CGGTCTCTGT CTCTTCCTTA TCGCTACGGT GATCCTTCTC AGAGATTATC

CGGTCTCTGT CTCTTCCTTA TCGCTACGGT GATCCTTCTC AGAGATTATC

CGGTCTCTGT CTCTTCCTTA TCGCTACGGT GATCCTTCTC AGAGATTATC

---------- ---------- ---------- ---------- ----------

CGGTCTCTGT CTCTTCCTTA TCGCTACGGT GATCCTTCTC AGAGATTATC

---------- ---------- ---------- ---------- ----------

CTGTCTCGGT CTCTTCCTTA TCGCTACGGT GATCCTTCTC AGAAATTATT

CTGTCTCGGT CTCTTCCTTA TCACTGCGGT GATCCTTTTC AGAAATTATC

CGGTCTCTGT CTCTTCCTTC TCGCTGCGGT GGTCCTTCTC AGAGACTATC

CGGTCTCTCT CTCTTCCTTC TCGCCACGGT GATCCTTCTC AGAGATGATC

CGGTCTCTGT CTCTTCCTTA TCACTACGGT GATCCTTCTC AGAGATTATC

CGGTCTCTGT CTCTTCCTTA TCGTTACGGT GATCCTTTTC AGAGATTATC

CGATCTCTGT CTCTTCCTTA TCACTACGGT GATCCTTCTC AGAGATAATC

CGGTCTCTGT CTCTTCCTTA TCACTACGGT GATCCTTCTC AGAGATTATT

CGGTCTCTGT CTCTTCCTTA TCACTACGGT GATCCTTCTC AGAGATTATT

---------- ---------- ---------- ---------- ----------

CGATCTCTGT CTCTTCTTTG TCCCCACGGT GGTCTTTGTA AGAGATGATC

CGATCTCTGT CTCTTCCTTT TCTCCACGGT GATCTTTATC AGATATTATT

CTCCCACTGC CAATCCTACG TCCACCAAGC AGACGAAGCC CATCACTATC

CTCCCACTGC CAATCCTACG TCCACCAAGC AGACGAAGCC CATCACTATC

CTCCCACTGC CAATCCTACG TCCACCAAGC AGACGAAGCC CATCATTATC

---------- ---------- ---------- ---------- ----------

CTCCCACTGC CAATCCTACG TCCACCAAGC AGACGAAGCC CATCACTGTC

---------- ---------- ---------- ---------- ----------

CTTCCACTGC CAATTCTTCG TCCACCAAGC AGGCGAAGCC CATCACTGTC

CTTCCACTGC CAATTCTTCG TCCACCAAGC AGGCGAAGTC CATCACTGTC

CTTCCACTGC CGATGCGACG CCCGCCAAGC AGGCGAAGTC CATCGCTGTC

CTCCCGCTGC CGATCCTGCG TCCGCCAAGC AGGCGGAGCC CGTCGCCGTC

CTCCCACTGC CAATCCTTCG TCCGCCAAGC AGGCGAAGCC CATCATTTTC

CTCCCACTGC CAATCCTTCG TCCCCCAAGA AGGCGGAGCC CATCACTGTC

CTTCCGCTCC CAATCCTCCT TCCACCAAGC AGGCGAAGTC CATCACTATC

CTCCCACTGC CAATCCTGCG TCCGCCAAGC AGACGAAGTC CATCATTATC

CTCCCACTGC CAATCCTGCG TCCACCAAGC AGGCGAAGCC CATCACTGTC

---------- ---------- ---------- ---------- ----------

CTCCCACTGC CGATCCTACG ACCTCCAATC ATTCGAACAC CATCATTGTC

CTTCCACTGC CAATTCTACG CCCTCCAATA ATTCGCACAC CATCATTTTC

TTTCTCTAAT GGACTTCCTG AGCGCCGGGA GCTAACAGCG GCTGTCACGT

TTTCTCTAAT GGACTTCCTG AGCGCCGGGA GCTAACAGCA GCTGTCACGT

TTTCTCTAAT GGACTTCCTG AGCGCCGGGA GCTAACAGCA GCTGTCACGT

---------- ---------- ---------- ---------- ----------

TTTTTCTAAT GGACTTCCTG AGCGCCGAGA ACTAACAGCA GCTGTCACGT

---------- ---------- ---------- ---------- ----------

TTTCTCCAAT GGACTGCCAG AGCGCCGGGA GCTAACAGCA GCTGTTACAT

TTTCTCCAAT GGACTGCCAG ATCGTCGGGA GCTAACAGCA GCTGTCACAT

TTTCTCTAAG GGACTGCCTG AGCGCCGGGA GCTAACGGCA GCTGTTACGT

TTTCTCCAGC GGGCTTCCTG AGCGCCGGGA GCTGACGGCC GCCGTCACGT

TTTTTCCAAC GGGCTTCCTG AGTGCCGGGA ACTAACAGCA GCCGTCACGT

TTTCTCCAAC GGGCTTCCTG AGCGCCGGGA GCTAACAGCA GCCGTCACGT

TTTTTCCAGA GGGCTTCCTG AGCGCCGGGA GCTAACAGCT GCTGTCACGT

TTTCTCCAAT GGGCTTCCTG AGCGTCGGGA GCTAACAGCA GCCGTCACAT

TTTCTCCAAT GGGCTTCCTG AGTGCCGGGA GCTAACGGCA GCTGTCACGT

---------- ---------- ---------- ---------- ----------

CTTTTCCAAT GGGCTCCCTG ATCTGCGGGA GCTGACAGAG GTTGTGACAT

TTTTTCCAAA GGACTCCCTG ATCGTCGTGA GCTAACGGTT GTTGTTACAT

GGCAGCCCCC TCCAAAGCTC CGTCTCTGAG GGCTGAGAAC AACATCTAAG

GGCAGCCCCC TCCAAAGCTC CGTCTCTGAG GGCTGAGAAC AACATCTAAG

GGCAGCCCCC TCCAAAGCTC CGTCTCTGAG GGCTGAGAAC AACATCTAAG

---------- ---------- ---------- ---------- ----------

GGCAGCCCCC TCCAAAGCTC CGCCTCTGAG GGCTGAGAAC AACATCTAAG

---------- ---------- ---------- ---------- ----------

GGCAGCCACC GCCAAAACTT CGTCTCTGTG GGCTGAGAAC AACATCTAAG

GGCAGCCACC TCCAAAGCTT CGTCTCTGTG GGCTGAGAAC AACATCTAAG

GGCAGCCGCC GCCAAAGCTG CGTCTCTGTG GGCTGAGAAC AACATCTAAG

GGCAGCCCCC TCCAAAGCTG CGTCTCTGTG GGCTGAGAAC AACATCTAAG

GGCAGCCCCC TCCAAAGCTT CGTCTTTGTG GGCTGAGAAC AACATCTAAG

GGCAGCCCCC TCCAAAGCTT CGTCTCTGTG GGCTGAGAAC AACATCTAAG

GGCAGCCCCC ACCAAAGCTT CGTCTCTGTG GGCTGAGAAC AACATCTAAG

GGCAGCCCCC CCCAAAGCTT CGTCTCTGTG GGCTGAGAAC AACATCTAAG

GGCAGCCTCC TCCAAAGCTT CGTCTCTGTG GACTGAGAAC AACATCTAAG

---------- ---------- ---------- ---------- ----------

GACAGCCCCC TCCAAAACTG CGCCTCTGGG GACTTAGAAC AACGTCTAAA

GACAACCTCC CCCAAAACTG CGCCTCTGGG GGCTTAAGAC TACATCCAAA

TCATCTTCTT TCACACGCTC TCGTGGATCT GGAAGGACTC ACCTACTATC

TCATCTTCTT TCACACCCTC TCGTGGATCT GGAAGGACTC ACCTACTATC

TCATCTTCTT TCACACGCTC TCGTGGATCT GGAAGGACTC ACCTACTATC

---------- ---------- ---------- ---------- ----------

TCATCTTCTT TTACACGCTC TCGTGGATCT AGAATGACTC ACCTACTATC

---------- ---------- ---------- ---------- ----------

TCATCTTCTT TCACTCGCTC TCGTGGATCT GAAAAGACTT ACCTGCTATT

TCATCTTCTT TCACTCGCTC TCGTGGATCT GAAAAGACTT ACCTGCTATT

TCGTCTTCTT TCACGCGCTC CCGTGGGTCT GGAAATA-TC ACCTACTATC

TCATCTTCCT TCACACGCTC TCGTGGATCT GCAAAGACTT ACCTACTATC

TCATCTTCTT TCACGCGCTC TCGTGGATCT GGAAAAACTC ACCTGCTATT

TCATCTTCTT TTACGCGCTC TCGTGGATCT GCAAAGACTC ACCTACTATC

TCATCTTCTT TCACACGTTC CCGTGGATCT GGAAAGACTC ACCTACTATC

TCATCTTCTT TCACGCGCTC TCGTGGATCT GGAAAGACTC ACCTACTATC

TCGTCTTCTT TCACACGCTC TCGTGGATCT GTAAAGACTC ACCTACTATC

---------- ---------- ---------- ---------- ----------

TCATCTTCTT TAACCCGCTC TCTTGGATCT GTAAAAACTT ACCTGCTGTC

TCATCATCTT TTACTCGCTC TCTGGGATCT GCAGAAGCTC ACCCACTAGG

CTGCGCACCA GGGAAGGCCG GTCTGTATCC AACTCTTTCT TCAGACTTTC

CTGCGCACCA GGGAAGGCCG GTCTGTATCC AACTCTTTCT TCAGACTTTC

CTGCGCACCA GGGAAGGCCG GTCTGTATCC AACTCTTTCT TCAGACTTTC

---------- ---------- ---------- ---------- ----------

CTGCGCACCA GGGAAGGCCG GTCTGTATCC AACTCTTTCT TCAGACTTTC

---------- ---------- ---------- ---------- ----------

CTGCGCACCA GAGAAGGCCG ATCTGATTCC GGCTCTTTCT TCAGACTTTC

CTGCGCACCA GAGAAGGTCG ATCGGATTCT GACTCTTTCT TCAGACTTTC

CTGCGCACCA GGGAAGGTCG GTCTGTTTCC AACTCTTTCT TCAGACTTTC

CTGCGCACCA GGGAAGGCCG GTCCGTGTCC AGCTCTTTCT TCAGGCTTTC

CTGCGCACAA GAGAAGGCCG CTCTGCATCC AACTCTTTCT TCAGACTTTC

CTGCGCACCA GGGAAGGCCG GTCTGTATCC AACTCTTTCT TCACACTTTC

TTGCGCACCA GGGAAGGCCG GTCTGTATCC AAATCTTTCT TCAGACTTTC

CTGCGCACCA GGGAAGGCCG GTCTATATCC ACCTCTTTCT TTAGACTTTC

CTACGTGCCA GAGAAGGCCG GTCTGTATCG AGCTCTTTCT TCAGACTTTC

---------- ---------- ---------- ---------- ----------

CTTCGCATCA GAGGAGGTCG CTCCAAGTCC AGCTCTTTCT TGAAGCTTTC

CGACGCATAA GGGGAGTCCG GTCTGAATCC AAATCTTTTT TGAAGCTTTC

CACTGGTGAG CTCCGCCCTG AAGCTGGGTA GAGAGAGGCA TGCCACTTCT

CACTGGTGAG CTCCGCCCTG AAGCTGGGTA GAGAGAGGCA TGCCACTTCT

CACTGGCGAG CTTCGCCCTG AAGCTGGGTA GAGAGAGGCA TGCCACTTCT

---------- ---------- ---------- ---------- ----------

CACTGGTGAG CTCCGCCCTG AAGCCGGGTA GAGAGAGGCA TGCCACTTCT

---------- ---------- ---------- ---------- ----------

CACTGGTGAG CTGCGCCCTG ACGCTGGGTA AAGAGAGGCA TGCCACTTCT

CACTGGTGAG CTCCGCCCCG AGGCTGGGTA GAGAGAGGCA TGCCACTTCT

CACCGGTGAG CTTCGCCCTG AAGCTGGGTA GAGAGAAGCG TGCCACTTCT

CACCGGTGAG CTCCGCCCTG AAGCTGGGTA GAGGGAGGCG TGCCACTTCT

CACTGGCGAG TTCCGCCCTG AAGCTGGGTA GAGAGAGGCA TGCCACTTTT

CACTGGTGAG CTCCGTCCTG AAGCTGGGTA AAGAGAGGCA TGCCACTTCT

CACTGGTGAA CTCCGCCCTG AAGCTGGATA GAGAGAGGCA TGCCACTTCT

CACTGGTGAG CTCCGTTCTG AAGCTGGGTA GAGAGAGGCA TGCCACTTCT

CACTGGCGAG CTCCGCCCTG AAGCTGGGTA GAGAGAGGCA TGCCACTTCT

---------- ---------- ---------- ---------- ----------

TACTGGAGAA CTTCTCCCTG AGCTTGGGTA AAGAGATGCA TGCCACTTCT

CACTGGAGAA CTTCGCCCCG AACTTGGGTA GAGAGAGGCA TGCCATTTCT

CAGGGTCCCA GACACCATCA CTACAGGTCA AAAAACATTC GCAATGCAGC

CAGGGTCCCA GACACCATCA CTACAGGTCA AAAAACATTC GCAATGCAGC

CAGGGTCCCA GACACCATCA CTACAGGTCA GAAAACATTC GCAATGCAGC

---------- ---------- ---------- -----CATTC GCAATGCAGC

CAGGGTCCCA GACACCATCA CTACAGGTCA AAAAG-ATCC AAAATGCTGC

---------- ---------- ---------- -----CATCC GCAATGCGGC

CAGGGTCCCA GACACCATCA CTACAGGTTA GAGGACATTC GCAATGCGGC

CAGGGTCCCA GACACCATCA CTACAGGTTA GAGGACATTC GCAATGCGGC

CAGGGTCCCA GACACCATCG CTGCAGGTCA AGAGGCATCC GCAATGCGGC

CAGGGTCCCA GACACCATCA CTGCAAGTCA AAGGGCATCC GCAATGCAGC

CAGGGTCCCA GACACCGTCA CTACAAATCA AAAGGCATCC GCAATGCCGC

CGGGGTCCCA GACGCCATCA CTATAAGTCA AAGGGCATCC GCAATGCGGC

CAGGGTCCCA GACACCATCA CTACAGGTCA AAGGGCATCC GCAATGCGGC

CAGGGTCCCA GACGCCATCA CTATGTGTCA AAAGGCATCC GCAATGCGGC

CAGGGTCCCA GACGCCATCG CTGCATGTCA AAAGGCATCC GCAATGCCGC

---------- ---------- ---------- -----CATCC GCAATGCCGC

CAGGATCCCA GACGCCATCG CTACAGATCA AGAGAAATCA AGAATGCCGC

CAGGGTCCCA GACACCATCA CTAAAAGTAA AAAAACATCA GGAACCCCGC

CGATATCTCC ATCATCGTTA TCTACTTCGT GGTAGTGATG GCCGTCGGAC

CGATATCTCC ATCATCGTTA TCTACTTCGT GGTAGTGATG GCCGTCGGAC

TGACATCTCT GTCATCGTTA TCTACTTCGT GGTGGTGATG GCCGTCGGAC

TGACATCTCT GTCATCGTTA TCTACTTCCT GGTGGTGATG GCCGTCGGAC

TGACATCTCC GTCATTGTCA TCTATTTTCT GGTGGTGATG GCTGTTGGGC

CGATATCTCC GTGATTGTCA TCTACTTCGT GGTGGTGATG GCCGTCGGGC

TGACATATCA GTCATCGTCA TCTACTTCGT GGTGGTGATG GCTGTCGGGC

TGACATCTCA GTCATCGTCA TCTACTTCGT GGTGGTGATG GCTGTCGGGC

CGACATCTCC GTCATCGTCA TCTACTTCTT GGTGGTGATG GCCGTCGGGC

TGACATCTCT GTCATCGTCA TCTACTTCGT GGTGGTGATG GCTGTCGGGC

CGACATCTCT GTCATCGTCA TCTACTTTGT GGTGGTGATG GCTGTGGGGC

CGACATCTCT GTCATCGTTA TCTACTTCGT GGTGGTGATG GCTGTCGGGC

CGACATCTCC GTCATCGTCA TCTACTTCGT GGTGGTCATG GCTGTGGGAC

CGACATCTCC GTCATCGTTG TCTACTTCGT GGTGGTGATG GCTGTCGGGC

CGACATCTCG GTCATCGTCA TCTACTTCTT AGTGGTGATG GCCGTCGGGC

CGACATCTCG GTCATCGTCG TTTACTTCGT GGTGGTGATG GCTGTCGGGC

AGACATCTTT GTAATTGTCA TCCACTTCTT GGTGGTCTTG GCTGTAGGAA

CAACATTGCC GTCATTGTCA TCTACTTCTT TGTCATCATG GCCACGGGGC

TGTGGGTATG ATACCTGTCA TATTTTTCAG AAAGGCATGA AGGCCTCTGT

TGTGGGTATG ATACCTGTCA TATTTTTCAG AAAGGCACGA AGGCCTCTGT

TGTGGGTATG ATACCTGTCA TATTTTTCAG AAAGGCACGA AGGCCTCTGT

TGTGGGTATG ---------- ---------- ---------- ----------

TGTGGGTAAG ATACCTGTCA TATTTTTCAG AAAGGCATGA AGGCCTCTGT

TGTGGGTATG ---------- ---------- ---------- ----------

TGTGGGTATG ATACCTGTCA TATTTTTCAG AGAGGCATGA TGGCCTCTGT

TGTGGGTATG ATACCTGTCA TATTTTTCAG AGAGGCATGA TGGCCTCTGC

TGTGGGTATG ---------- ---------- ---------- ----------

TGTGGGTAGG ATACCTGTCA TATTTCTCAG AGAGGCACGA AGGCCTCTGT

TGTGGGTATG ATACCTGTCA TATTTTTCAG AGAGGCATGA TGGTCTTTGT

TGTGGGTATG --ACCTGTCG TATTTTTCAG AGAGGCATGA AGGCCTTTGT

TGTGGGTAAG ---------- ---------- ---------- ----------

TGTGGGTATG ATACCTGTCA TATTTTTCAG AGAGGCATGA TGGCCTTTGT

TCTGGGTATG ATACCTGTCG TATTTTTCAG AGAGGCATGA AGGCCTCTGT

TCTGGGTATG ---------- ---------- ---------- ----------

TATAGGTATG ATACCTGTCA TATTTCTCAG AAAGGCACGA AGGCCTCTGT

TCTGGGTACG ATACCTGTCA TATTTTTCAG AAAGACATGG AGGTCTCTGC

TTGGAATGGG GGAGTTCTTT TATATCCAAG AGTTCTTCCT AAAAAGAGAA

TTGGAATGGG GGAGTTCTTT TATATCCAAG AGTTCTTCCT AAAAAGAGAA

TTGGAATGGG GGAGTTCTTT TATATCCAAG AGTTCTTCCT AAAAAGAGAA

---------- ---------- ---------- ---------- ----------

TTGGAATGGG GGAGTTCTTT TATATCCAAG AGTTCTTCCT AAAAAGAGAA

---------- ---------- ---------- ---------- ----------

TTGGAGTATG GGCGTTCTTT TATATCCAAG AGCTCCTCCT AAAAAGAGAA

TTGGAGTATG GACGTTCTTT TATATCCAAG AGCTCCTCCT AAAAAGAAAA

---------- ---------- ---------- ---------- ----------

TTGGAATGAG GGCGTTCTTT AATATCCAAG AGCTCCTCCT AGAAAGAGAA

TTGGAATGAG GGCGTTCTTT TATATCCAAG AGCTCCTCCT aaaaaaagaa

TTGGAATGAG GGCGTTCTTT TATATCCAAA AGCTCCTCCT AGAAAGAGAA

---------- ---------- ---------- ---------- ----------

TTGGAATGGG GGCGTTCTTT TATATCCAAG AGTTCCTCCT AAAAAGAGAA

TTCGAATGGG GATGTTCTTT TATATCCAAG AGCTCCTCCT GAAAAGAGAA

---------- ---------- ---------- ---------- ----------

TTAGAGTGAG GACGTTCTTT AATGTCCAAA AGTTCCTCCT GAAAAAAGGA

TTAGAGTGGG GGCGTTCTTT AATATCCAAG AGTTCTTCCT AGAAAACAAA

AGGCTATGTT TTCCACCAAT CGTGGGACTG TTGGAGGCTT CTTCCTGGCA

AGGCTATGTT TTCCACCAAT CGTGGAACTG TTGGAGGCTT CTTCCTGGCA

AGGCTATGTT TTCCACCAAT CGTGGAACTG TTGGAGGCTT CTTCCTGGCA

AGGCTATGTT TTCCACCAAT CGTGGAACTG TTGGAGGCTT CTTCCTGGCA

AGGCAATGCT GAGGACCAAC CGAGGCACTA TAGGAGGCTT CTTCCTCGCT

AGGCTATGTT TTCTACTAAT CGTGGCACGG TTGGCGGCTT TTTCCTGGCA

AGGCCATGTT TTCCACTAAT CGTGGGACTG TTGGAGGCTT CTTCCTGGCA

AGGCCATGTT TTCCACTAAT CGTGGTACCG TTGGAGGCTT CTTCCTGGCA

AGGCAACACT GAAGACAAAC CGCAGCACTG TAAGAGAATT CTTCTTGGCT

AGGCCATGTT TTCCACTAAC CGTGGGACCG TTGGAGGCTT CTTCCTGGCA

AGGCTATGTT TTCCACTAAT CGTGGGACTG TTGGAGGCTT CTTCTTGGCA

AGGCTATGTT TTCCACTAAT CGTGGGACCG TTGGAGGATT CTTCCTGGCA

AGGCTATGTT TTCCACCAAC CGTGGGACTG TTGGAGGTTT CTTCCTGGCA

AGGCTATGTT TTCCACTAAT CGTGGGACAG TTGGAGGCTT CTTCCTGGCA

AGGCTATGTT TTCCACTAAT CGTGGGACTG TTGGAGGCTT CTTCCTGGCA

AGGCTATGTT TTCCACGAAT CGTGGGACCG TTGGAGGCTT CTTTCTGGCA

AGGCCTTGCT GAAAACAAGT CGAGGAACAG TGGGAGGCTT TTTCCTGGCT

AGGCCATGCT GTCGACCAAT CGGGGAACAG TTGGAGGCTT CTTCCTGGCT

GGCCGAAGTA TGGTGTGGTG GCCGGTAAGT GGCAAACCTT ACTTTTGTAT

GGCCGAAGTA TGGTGTGGTG GCCGGTAAGT GGCAAACCCT ACTTTTGTAT

GGCCGAAGTA TGGTGTGGTG GCCGGTAAGT GGCAAACCCT ACTTTTGTAT

GGCCGAAGTA TGGTGTGGTG GCCGGTAAGT GGCAAACCCT ACTTTTGTAT

GGTCGTGATG TGGCCTGGTG GCCGGTAAGT AACAAAGCGT ACTTTTGTAT

GGCCGAAGTA TGGTGTGGTG GCCGGTAAGT ---------- ----------

GGCCGAAGTA TGGTGTGGTG GCCGGTAAGT AACAAAACTT ACTTTCGTAT

GGCCGAAGTA TGGTGTGGTG GCCGGTAAGT AACAAAACTT ACTTTCGTAT

GGTCGAGACG TGGCCTGGTG GCCGGTGAGT AACAACGCCT ACTTTCGTGT

GGTCGAAGTA TGGTGTGGTG GCCGGTAAGT AACAAAGCCT ACTTTTGTAT

GGACGGAGTA TGGTGTGGTG GCCGGTAAGT AACAAAACTT ACTTTTGTAT

GGACGAAGTA TGGTGTGGTG GCCGGTAAGT AACAAAGCCT ACTTTTGTAT

GGCCGAAGTA TGGTGTGGTG GCCGGTAAGT TACAACGCCT ACTTTTGTAT

GGCCGAAGTA TGGTGTGGTG GCCGGTAAGT AACAAATCCT ACTTTTGTAT

GGTCGAAGCA TGGTGTGGTG GCCGGTAAGT ATCAAAGCCT ACTTTTGTAT

GGCCGGAGTA TGGTGTGGTG GCCGGTAAGC ---------- ----------

GGCCAGGACA TGGTGTGGTG GCCATTAAGT AGCAAAGCCT ACTTTTGTGT

GGCAGGAGTA TGGTGTGGTG GCCGGTGAGT AATTAAACCT ACTTTCGTGT

AGCGATGGGG GCATTTGGGC TTCTTCAGGT CAAGGAAAGC ATCTCCACTT

AGCGATGGGG GCATTTGGGC TTCTTCAGGT CAAGGAAAGC ATCTCCACTT

AGCGATGGGG GCATTTGGGC TTCTTCAGGT CAAGGAAAGC ATCTCCATTT

AGCGATGGGG GCATTTGGGC TTCTTCAGGT CAAGGAAAGT ATCTCCATTT

AGCGATGGGG GCATTTGGGC TTCTTCAGGT CAAGGAAAGC ATCTCCATTT

---------- ---------- ---------- ---------- ----------

AGCGGTGGGG GGATTTGGGC TTCTTCAGGT CAAGGAAAGC ATCTCCATTT

AGCGATGGGG GGATTTGGGT TTCTTCAGCT CAAGGAAAGC ATCTCCATTT

AACGATGCGG TGATTTGGGC TTCTTCAGGT CAACGAAAGC ATCTCCATTT

AGCGATGGGG GGATCTGGGC TTCTTCAGGT CGAGAAAAGC ATCTCCATTT

AGCGATGGGG GGATTTGGGC TTCTTCAGGT CAAGGAAAGC ATCTCCGTTT

AGCGATGGGG GGATTTCGGC TTCTTCAAGT CAAGGAAAGC ATCTCCATTT

AGCGATGGGG GGATTTGGGC TTCTTCAGGT CAAGGCAAGC ATCTCCATTT

AGCGATGGGG GGATTTGGGC TTCTTCAGGT CAAGGAAGGC ATCTCCATTT

AGCGATGGGG GGATTTGGGC TTCTTCGGGT CAAGGAAAGC ATCTCCATTT

---------- ---------- ---------- ---------- ----------

AACGATGGGG GGACTTGGGC TTCTTCAGGT CCAGGAAGGC TTCGCCATTT

AACGATGGGG GGATTTGGTC CTCTTCAGGT CAAGGAAGGC ATCACCATTT

TCTGTTTCAC CCATACTTCT CCTATCCATG GCTCCTTGGT CTACAATGCT

TCTGTTTCAC CCATACTTCT CCTATCCATG GCTCCTTGGT CTACAATGCT

TCTGTTTCAC CCACACTTCT CCTATCCATG GCTCCTTGGT CTACAATGCT

TCTGTTTCAC CCACACTTCT CCTATCCATG GCTCCTTGGT CTACAATGCT

TCTGTTTCAC CCACACTTCT CCTATCCATG GCTCCTTGGT CTACAATGCT

---------- ---------- ---------- ---------- ----------

TCTGTTTCAT CCACACTTCT CTTCTCCATG GCTTCTTGGT GTACAATTTT

TCTGTTTCAG CCACACTT-- -TTCTCCATG GCTTCTTGGT GTACAATTTT

TCTGTTTCCC CCACACTTCT CCTATCCATG GCTCCCTGGT CTAGAATTCT

TCTGTTTCAC CCACACTTCT ---ATCCATG GCTCCTTGGT TGACAGTTTT

TCTGTTTCAC CAACACTTCT CCTATCCATG GTTCCTTGGT CTACAATTCT

TCTGTTTCGC CCACACTTCT CCTATCCATG GCTCCTTGGT CTACAATTCT

TCCGTTTCAC CCAAACTTTT CCTATCCATG GCTTCTTGGC TAACAATTCT

TCTGTTTCAC CCACACTTCT CCTATCCATG GCTCCTAGGT CTACAATACT

TCTGTTTCAC CCACACTTCT CCTATCCATG GCTCCTCGGT CTACAATTCT

---------- ---------- ---------- ---------- ----------

TCCGTGTCTC CCACCCCTCT TCTGTCCATG GTTGTTTGGT CCTTAACTCT

TCTGTTTCTC CCCCACCTTT CCTCTCCATG GCCATTTGAT TTTAAACCCT

CTGCACCTGG AACCACCTAC CCACAGCCCA ACAGCCATCA CCACCAGAAA

CTGCACCTGG AACCACCTAC CCACAGCCCA ACAGCCATCA CCACCAGAAA

CTGCACCTGG AACCACCTAC CCACAGCCCA ACAGCCATCA CCACCAGAAA

CTGCACCTGG AACCACCTAC CCACAGCCCA ACAGCCATCA CCACCAGAAA

CTGCACCTGG AACCACTTAC CCACAGCCCA ACAGCCATCA CCACCAGAAA

---------- --CCACATAC CCACAGCCCG ACAGCCATTA CTGCCACAAA

TTGCACCTGG AACCACATAC CCACACTCCA ACAGCCATCA CCACCACAAA

TTGCACCTGG AACCACCTAC CCACACTCCA ACAGCCATGA CCACCACAAA

TCGCACCTGA AACCACGTAC CCACATCCCA ACAGTCATCA CCACTATGAA

TTGCACCTGT AACTACATAC CCACAGTCCA ACGGCCATCA CCAACACGAA

TTGCACCTGG GACTACGTAC CCACAGCCCA ACAGCCATCA CCACCACGAA

TTGCACCTGG AACTACATAC CCACAGCCCG ACAGCCATCA CCACCACGAA

TTGCACCTGG AA-CACTTAC CCACAGTCCC ACAGCCATGA CCACCACGAA

TTGCACCTGG AACCACATAC CCACAGCCCG ACAGCCATCA CCACCACGAA

TTGCACCTGG AA--ACATAC CCAGAGCCCG ACGGCCATCA CCACTAAGAA

---------- --CCACATAC CCAGAGCCCG ACAGCCATCA CCACCACGAA

ATGCACCTGA AACCACATAC CTATATTCCT ACAGCCAAGA CCACCAAGAA

ACCCACCTGG AATCCCGTAC CCAGAGCCCC GTGGCCATGA TGACAAAGAA

ATAGATGACA ATGACTGAGA TGTCAGCAGC ATTCTATCCT TTGATCACAG

ATAGATGACA ATGACTGAGA TGTCAGCAGC ATTCTATCCT TTGATCACAG

ATAGATGACA ATGACTGAGA TGTCAGCAGC ATTCTATCCT TTGATCACAG

ATAGATGACA ATGACTGAGA CGTCAGCAGC ATTCTATCCT TTGATCACAG

ATAGATGACA ATGACGGAGA TGTCAGCAGC ATTCTATCCT TTGATCACAG

ATAGATGACA ATCACAGAGA TGTCTGCAGG GTTCTACCCT TTGATAACCG

GTAGATGACA ATGACAGAGA TATCAGCAGG ATTCTATCCT TTAATAACAG

ATAGATGACG ATGACAGAGA TGTCAGCAGC ATTCTACCCT TTAATAACAG

GTAGACGACA AGGACAGAGA TGTCAGCAGC ATT------- ----------

ATAGATGACA ATGACGGAGA TGTCAGCAGC GTTCTATCCT TTGATAACAG

ATAGATGACA ATGACAGAGA TGTCAGCAGC ATTCTATCCT TTGATCACAG

ATAGACAACA ATGACGGAGA TGTCAGCAGC GTT------- ----------

GTAGATGACG ATGACGGAGA TGTCGGCCGC ATTCTACCCT TTGATCACAG

GTAGACAACG ATGACGGAGA TGTCGGCCGC ATTCTAGCCT TTGATAACAG

GTAGATGACG ATGACCGAGA TGTCGGCGGC ATTCTAGCCT TTGATAACAG

GTAAACGACG ATGACCGAGA TGTCGGCGGC ATT------- ----------

GTGGATGACA ATTACAAAGA TGTCTGCGGC ATTTTAGCCT TTGATAACTG

GTAGATGACA ATGACGGCAA TGTTGGCGGG GTTCTAGCCT TTGATAACAG

CAATTGGTCT CAGTTTAATG GCTTTCTTGC TGATTCCAGC ATCATGGCAG

CAATTGGTCT TAGTTTAATG GCTTTCTTGC TGATTCCAGC ATCATGGCAG

CAATTGGTCT CAGTTTAATG GCTTTCTTGC TGATTCCAGC ATCATGGCAG

CAATTGGTCT CAGTTTAATG GCTTTCTTGC TGATTCCAGC ATCATGGCAG

CAATTGGTCT CAGTTTAATG GCTTTCTTGC TGATTCCAGC ATCATGGCAG

CGACTGGTCT CAGTTTAATG GCTTTCTTGC TGATCCCAGC GTCATGGCAG

CAATTGGTCT CAGTTTAATG GCCTTCTTGC TGATCCCAGC ATCATGGCAA

CAATTGGTCT CAGTTTAATG GCCTTCTTGC TGATCCCAGC ATCATGGCAG

---------- ---------- ---------- ---------- ----------

CAATTGGCCT CAGTTTAATG GCCTTCTTGC TGATTCCGGC ATCATGGCAG

CAATCGGTCT CAGTTTAATG GCCTTCTTGC TGATTCCAGC ATCATGGCAG

---------- ---------- ---------- ---------- ----------

CGATCGGTCT GAGTTTAATG GCCTTCTTGC TGATGCCGGC ATCATGGCAG

CAATCGGTCG CAGTTTAATG GCCTTCTTGC TGATTCCAGC GTCATGGCAG

CAATTGGCCT CAGTTTGATG GTCTTCTTAC TGATTCCAGC ATCATGGCAG

---------- ---------- ---------- ---------- ----------

CAATTGGTCT GAGTTTGATG GCCTTCTTGC TGATTCCAGC ATCATGGCAA

CGATAGGTCT CAGTTTAATG GCTTTCTTAC TAATGCCGGC ATCGTGACAG

GTATTTACCA CATCTGTCAC ATTCTTATAG GACTCAGGAG CCTGCAGGAT

GTATTTACCA CATCTGTCAC ATTCTTATAG GACTCAGGAG CCTGCAGGAT

GTATTCACCA CATCTGTCAC ATTCTTATAG GACTCAGGAG CCTGCAGGAT

GTATTCACCA CATCTGTCAC ATTCTTATAG GACTCAGGAG CCTGCAAGAT

GTATTCACCA CATCTGTCAC ATTCTTATAG GACTCAGGAG CCTGCAGGAT

GTGTTCACCA CATCTGTCAC GTTCTTATAG GACTCAGGAG CCTGCAGGAT

GTGTTCACGA CGTCTGTCAC ATTCTTATAT GATTCTGGAG CCTACAGGAT

GTGTTCACGA CGTCTGTCAC ATTCTTATAG GACTCTGGTG CCTACAGAAC

---------- ---------- ---------- ---------- --------AT

GTGTTCACCA CATCCGTCAC GTTCTTATAG GACTCAGGGG CCTGTAGGAT

GTGTTCACCA CATCCGTCAC ATTCTTATAG GACTCAGGAG CCTACAGGGC

---------- ---------- ---------- ---------- ----------

GTGTTCACCA CGTCGGTCAC ATTCTTATAG GACTCTGGAG CCTGCGGG--

GTGTTGACCA CGTCGGTCAC GTTCTTGTAG GATTCAGGAG CCTGCAGGAT

GTGTTCACCA CATCTGTTAC ATTCTTATAG GATTCAGGAG CCTACAAGAT

---------- ---------- ---------- ---------- ----------

GTATTCACCA CATCTGTCAC GTTTTTATAG GATTCTGGAG CCTAAAAGAT

GTATTCACCA CATCTGTGAC GTTTTTATAG GATTCCGGAG CCTGCAAGTC

TTCACTTACC TCTTCCATAA CCAGTTTGGG TGAGGCAACA CGGATCGCAA

TTCACTTACC TCTTCCATAA CCAGTTTGGG TGAGGCAACA CGGATCGCAA

TTCACTTACC TCTTCCATAA CCAGTTTGGG TGAGGCAACA CGGATTGCAA

TTCACTTACC TCTTCCATCA CCAGTTTGGG TGAGGCAACA CGGATTGCAA

TTCACTTACC TCTTCCATAA CCAGTTTGGG TGAAGCAACA CGGATTGCAA

TTCACTTACT TCTTCCATAA CCAGTTTGGG TGAGGCAACA CGGATTGAGA

GCCACTTACC TCTTCCATAA CCAGCTTGGG GGACGCAACC CGGATGGCGA

GCCACTCACC TCTTCCATAA CCAGCTTGGG GGAAGCAACC CGGATTGCAA

TCCACTTACC TCTTCCATAA CCAGTTTGGG CGAGGCAACA CGGATTGCGA

TTCACTTACC TCTTCCATTA CCAGCTTGGG TGAGGCGACA CGGATTGCAA

TTCACTTACC TCTTCCATAA CCAGTTTGGG TGAGGCAACT CGGATTGCGA

---------- ---------- ---------- ---------- ----------

---------- ---------- ---------- ---------- ----------

TTCTCTTACC TCTTCCATAA CCAGTTTGGG TGAGGCAACA CGGATTGCAA

TTCACTTACC TCTTCCATTA CTAGTTTGGG TGAGGCCACA CGGATTGCAA

---------- ---------- ---------- ---------- ----------

CTTACTTACC TCCTCCATCA CCAATTTAGG GGAAGCAACC CTGATGGCGA

TCCACTAACC TCTTCCATCA CCAGTTTGGG TGAAGCAACG CGGATCGCGA

TTCCCATATC TGCCAATTTG TCTAAGACAT CCTGGAAATC TAAATTACGT

TTCCCATATC TGCCAATTTG TCTAAGACAT CCTGGAAATC TAAATTACGT

TTCCCATATC TGCCAATTTG TCTAAAACAT CCTGGAAATC TAAATTACGT

TTCCCATATC TGCCAATTTG TCTAAAACAT CCTGGAAATC TAAATTACGT

TTCCCATATC TGCCAATTTG TCTAAGACAT CCTGGAAATC TAAATTACGT

TTCCCATATC TGCCAATTTG TCTAAGACAT CCTGGAAATC TAAATTACGT

TTCCCATGTC TGCCAGCTTG TCTAAGACAT CTTGGAAATC TAAATTACGA

TTCCCATGTC TGCCAGTTTG TCTAAGACAT CTTGGAAATC TAAGTTACGA

TCCCCATATC TGCCAGTTTG TCTAAGACAT CCTGGAAATC TAGATTACGC

TTCCCATGTC TGCCAATTTG TCGAGGACAT CCTGGAAATC TAAATTACGT

TTCCCATATC TGCCAGTTTG TCCAAGACAT CCTGGAAATC TAAATTACGA

---------- ---------- ---------- ---------- ----------

---------- ---------- ---------- ---------- ----------

TCCCCATATC TGCCAATTTG TCCAAGACAT CCTGGAAATC TAAATTACGT

TTCCCATATC TGCCAATTTA TCCAAGACAT CCTGGAAGTC TAAATTACGC

---------- ---------- ---------- ---------- ----------

TGCCCATGTC AGCCAATTTA TCTAGAACAT CCTGGAAGTC TAAGTTACGT

TTCCCATGTC TGCCAGTTTA TCTAAAACGT CCTGGAAGTC CAAATTTCGT

CGAGATTTTG CTCGGGACAA TGCACGGCCC TAGATCCTTA CCGCTCCATG

CGAGATTTTG CTCGGGACAA TGCACGGCCC TAGATCCTTA CCGCTCCATG

CGAGATTTTG CTCGGGACAA TGCACGGCCC TAGATCCTTA CCGCTCCATG

CGAGATTTTG CTCGGGACAA TGCACGGCCC TAGATCCTTA CCGCTCCATG

CGAGATTTTG CTCGGGACAA TGCACGGCCC TAGATCCTTA CCGCTCCATG

CGAGATTTTG CCCGGGACAA TGCACGGCCC TAGATCCTTA CCGCTCCATG

CGTGATTTTG CTCTGGACAA AGCACGGCCC TAGATCCTTA CCGCTCCATG

CGTGATTTTG CTCTGGACAA AGCACGGCCC TAGATTCTTA CCGCTCCATG

CGAGATTTTG CTCGGGACAA TGCACGGCCC TAGA------ ----------

CTTGACTTTG CTCGGGACAG TGCACGGCCC TAGGTCCTCA CCGCTCCATG

CGAGATTTTG CTCGGGACAA TGCACGGCCC TAGATCCTTA CCGCTCCATG

---------- ---------- ---------- ---------- ----------

---------- ---------- ---------- ----TCCTTA CCGCTCCGTG

CGTGATTTAG CTCGGGACAA TGCACGGCCC TACATCCTTA CCGCTCCATG

CGAGATTTCG CTCGAGACAA TGCACGGCCC TAGATCCTTA CCGCTCCGTG

---------- ---------- ---------- ---------- ----------

CGAGATTTTG CTCGGGACAA TGCACGACCC TGGATACTTA CTGCTCCATG

CGAGATTTTG CTCGGGACAG TGCACGGCCC TGGGTACTTA CAGCTCCGTG

ACAGGTTGTT CCAAAGGTCT CAGTCATGCC CTGTTCAGTG CCAGTAAGAA

ACAGGTTGTT CCAAAGGTCT CAGTCATGCC CTGTTCAGTG CCAGTAAGAA

ACAGGTTGTT CCAAAGGTCT CAGTCATGCC CTGTTCAGTG CCAGTAAGAA

ACAGGTTGTT CCAAAGGTCT CAGTCATGCC CTGTTCAGTG CCAGTAAGAA

ACAGGTTGTT CCAAAGGTCT CAGTCATGCC CTGTTCAGTG CCAGTAAGAA

ACAGGTTGTT CCAAAGGTCT CAGTCATGCC CTGCTCAGTG CCTGTAAGAA

ACAGGTTGTT CCAAAGGTCT CAGTCATGCC TTGTTCAGTG CCAGTCAGAA

ACAGGTTGTT CCAAAGGTCT CAGTCATGCC TTGTTCAGTG CCAGTCAGAA

---------- ---------- ---------- ---------- ---------A

ACAAGTTGTT CCAAAGGTTT CAGTCATGCC CTGCTCAGTA CCAGTAAGAA

GCAAGTGGTC CCAAAGGTCT CCGTCATGCC CTGCTCGGTG CCCGTAAGAA

---------- ---------- ---------- ---------- ----------

GCAGGTTGTT CCAAAGGTCT CAGTCATGCC CTGTTCGGTG CCAGTCAGGA

ACATGTTGTT CCAAAGGTTT CTGTCATGCC CTGTTCCGTG CCAGTAAGAA

ACAGGTGGTT CCAAAGGTTT CAGTCATGCC CTGTTCAGTG CCAGTAAGTA

---------- ---------- ---------- ---------- ----------

GCAGGTTGTT CCAAAGGTCT CAGTCATGCC CTGCTCAGTC CCAGTAAGGA

GCAGGTTGTT CCGAAGGTCT CGGTCATGCC CTGCTCGGTG CCGGTGAGTA

CATAACTACA GGTTCCCATG GTGCCACCAA TGAGCACTGG CTGTCCAGTG

CGTAACTACA GGTTCCCATG GTGCCACCAA TGAGCACTGG CTGTCCAGTG

CATAACTACA GGTTCCCATG GTGCCACCAA TGAGCACTGG TTGTCCAGTG

CATAACTACA GGTTCCCATG GTGCCACCAA TGAGCACTGG TTGTCCAGTG

CATAACTACA GGTTCCCATG GTGCCACCAA TGAGTACTGG TTGTCCAGTG

CGTAACTACA GGTTCCCATG GTGCCACCAA TGAGTACTGG TTGTCCAGTG

CATAACTACA GGTCCCCATG GTGCCACCGA TAAGCACTGG TTGTCCAGTG

CGTAACTACA GGTCCCCATG GTGCCACCAA TAAGCACTGG TTGTCCTGTG

CGTAACTGCA GGTTCCCATG GTGCCACCAA TGAGCACTGG TTGTCCAGTG

CATAGCTACA GGTTCCCATG GTGCCACCAA TGAGCACTGG TTGTCCGGTA

CGTAACTGCA GGTGCCCATG GTGCCACCAA TGAGCACCGG CTGTCCAGTG

---------- ---------- ---------- ---------- ----------

CATAACTACA GGTCCCCATG GTGCCACCAA TGAGCACTGG CTGTCCCGTG

CGTAACTACA GGTTCCCATG GTGCCACCAA TGAGTACAGG CTGTCCCGTG

CATAACTGCA GGTTCCCATG GTGCCACCGA TGAGCACCGG CTGTCCAGTG

---------- ---------- ---------- ---------- ----------

CATAGCTACA GGTTCCCATG GTTCCCCCAA TGAGGACTGG CTGTCCAGTG

CATAGCTGCA GGTCCCCATG GTCCCACCGA TCAGCACGGG CTGTCCGGTC

AGCTGAGGAT GCACAACTTG GTAATCAACA GCAATGAGGG GATGGTGAGG

AGCTGAGGAT GCACAACTTG GTAATCAACA GCAATGAGGG GATGGTGAGG

AGCTGAGGAT GCACAACTTG GTAATCAACA GCTATGAGGG GATGGTGAGG

AGCTGAGGAT GCACAACTTG GTAATCAACA GCTATGAGGG GATGGTGAGG

AGCTGAGGAT GCACAACTTG GTAATCAACA GCAATGAGTG GATGGTGAGG

AGCTGAGGAC GTACAACTTG GTAATCTACA GCAATGAGGG GATGGTGAGG

AGCTGAGG-- GTACAACCTG GTAATCAACA GCAATGAGGG GATGGTGAGG

AGCTGAGG-- GTACAACTTG ATAATCCACA GCAATGAGGG GGTGGTGAGG

AGCTGAGAAT GCACAACCTG GTAATCAACC GCGATGAGGG GATGGTGAGG

AGCTGAGGAA GCACAACTTG GTAATCAACC GCAATGAGGG GATGGTGAGG

AGCTGAAGGC ACACAACTTG GTAATCCACA GCAATGAGGG GATGGTGAGG

---------- ---------- ---------- ---------- ----------

AGCTGAGGAA GCACA----- ---------- ---------- ----------

AGCTGAGGAT GCACAACCTG GTAATCAACA GCAATGAGGG GGTGATGAGG

AGCTGAGGAT GCACAACCTG GTAATCCACG GCAATGAGGG GGTGGTGAGG

---------- ---------- ---------- ---------- ----------

AGCTGAGGAT GTAAAACCTG GTAATCCACA GCAATGAGGG GATGGTGTGG

AGCTAAGGAT ACAAAACCTG ATAATCAACA GCAATGAGGG GATGGTGAGG

AGGGAAAGCG CGGGTGGATC CCTTCCTGTG TACTAACAGT GTCCGTTCCT

AGGGAAAGCG CGGGTGGATC CCTTCCTGTG TACTAACAGT GTCCGTTCCT

AGGGAAAGCG CGGGTGGATC CCTTCCTGTG TACTAACAGT GTCCGCTCCT

AGGGAAGGCG CGGGTGGATC CCTTCCTGTG TACTAACAGT GTCCGCTCCT

AGGGAACGCG CGGGTGGATC CCTTCCTGTG TACTAACAGT GTCCGTTCCT

AGGGAAGGCG CGGGTGGACC CCTTCCGGTG CACTAACAGC GTCCGCTCCT

AGGGAAAGCT CGGGTGGACC CTTTCCTGTG CACCAACAGC GTCCGCTCCT

CGGGAAAGCA CGGGTGGATC CCTTCCTGTG CACCAGCAGC GTCCGTTCCT

AGGGAAAGCC CGGGTGGATC CCTTCCTGTG CACCAGCAGC GTCCGCTCCT

AGGGAAGGCT CGGGTGGACC CCTTCCTGTG TACTAACAGA GTCCGCTCCT

AGGGAAAGCC CGAGTGGATC CCTTCCTGTG TACCAACAGT GTCCGTTCCT

---------- ---------- ---------- ---------- ----------

---------- ---------- ---------- ---------- ----------

AGGGAATGCA CGGGTGGACC CCTTCCTGTG CACCAGCAGC GTCCGTTCCT

CGGGAAAGCC CGGGTGGATC CCTTCCTGTG CACCAACAGC GTCCGCTCCT

---------- ---------- ---------- ---------- ----------

GGGGAAGGCC CGGGTGGAGC CCTTCCTGTG CACCAACAAT GTCCGCTCTT

AGGGAAAGCC CTGGTGGACC CTTTCCTGTG TACCAGAAGA GTTCGCTCTT

TTCCGTCCAC CACATGCTGC TCCACTTTGG CAATGTTGTG AGAAACATCA

TTCCGTCCAC CACATGCTGC TCCACTTTGG CAATGTTGTG AGAAACATCA

TCCCGTCCAC TACATGCTGC TCCACTTTGG CAATGTTGTG AGAAACATCA

TCCCGTCCAC TACATGCTGC TCCACTTTGG CAATGTTGTG AGAAACATCA

TTCCGTCCAC CACATGCTGC TCCACTTTGG CAATGTTGTG AGAAACATCA

TCCCGTCCAC CACATGCTGC TCCACTTTGG CAATGTTGTG AGAAACATCA

TTCCGTCTAC CACGTGCTGC TCCACTTTGG CGATGTTGTG AGAAACATCA

TCCCATCCAC CACGTGCTGC TCCACTTTGG CGATATTGTG CGACACATCA

TCCCGTCCAC CACGTGCTGC TCGACCTTGG CGATGTTGTG AGAGACATCA

TCCCGTCCAC CACATGCTGT TCTACTTTGG CAATATTGTG AGAAACATCA

TTCCATCCAC CACGTGCTGT TCTACTTTGG CAATATTGTG AGAAACATCA

---------- ---------- ---------- ---------- ----------

---------- ---------- ---------- ---------- ----------

TTCCATCCAC CACATGCTGC TCTACTTTGG CAATATTGTG AGAAACGTCG

TTCCGTCCAC AACGTGCTGC TCTATCTTGG CAATATTGTG AGAAACATCA

---------- ---------- ---------- ---------- ----------

TCCCATCCAC CACATGCTGT TCCACCTTGG CGATGTTGTG GGAGACGTCA

TTCCATCCAC CACATGCTGT TCTACCTTGG CAATATTGTG AGATACATCA

TAGATCACAT GTAGGTCCAA GTCATCAGGG GTTGTGTTGA AGACCTTGGC

TAGATCACAT GTAGGTCCAA GTCATCAGGG GTTGTGTTGA AGACCTTGGC

TAGATCACGT GCAGGTCCAA GTCATCAGGG GTTGTGTTGA AGACCTTGGC

TAGATCACGT GCAGGTCCAA GTCATCAGGG GTTGTGTTGA AGACCTTGGC

TAGATCACAT GGAGGTCCAA GTCATCAGGG GTTGTGTTGA AGACCTTGGC

TAGATCACGT GCAGGTCCAA GTCATCAGGG GTTGTGTTGA AGACCTTGGC

TAAATCACAT GCAGGTCCAG GTCGTCAGGG GTTGTGTTGA AGACTTTGGC

TAGATCACAT GCAGGTCCAG GTCATCAGGG GTTGTGTTGA AGACTTTGGC

TAGATCACGT GCAGGTCCAA GTCATCAGGG GTTGTGTTGA AGACCTTGGC

TAGATCACAT GCAGGTCCAA GTCATCAGGG GTTGTGTTGA AGACCTTGGC

TAGATCACAT GTAGATCCAA GTCATCAGGG GTTGTGTTGA AGACCTTGGC

---------- ---------- ---------- ---------- ----------

---------- ---------- ---------- ---------- ----------

TAGATCACGT GTAGATCCAA GTCATCAGGG GTTGTGTTGA AGACTTTGGC

TAGATCACAT GTAGGTCCAA GTCATCAGGG GTTGTGTTGA AGACCTTGGC

---------- ---------- ---------- ---------- ----------

TAGATCACGT GGAGGTCCAG GTCATCAGGA GTTGTGTTGA AAACCTTGGC

TAAATCACAT GTAGGTCCAG GTCATCAGGG GTTGTGTTGA AGACTTTGGC

GAAAGCCTAT TCCCTTCAGA TAGTCTTGAC CCTCGAAGCG ATTCGAGCAC

AAAAGCCTAT TCCCTTCAGA TAGTCTTGAC CCTCGAAGCG ATTCGAGCAC

AAAAGCCTAT TCCCTTCAGG TAGTCCTGAC CCTCGAAGCG ATCCGAGCAC

AAAAGCCTAT TCCCTTCAGG TAGTCCTGAC CCTCGAAGCG ATCCGAGCAC

AAAAGCCTAT TCCCTTTAGG TAGTCTTGAC CCTCGAAGCG ATTCGAGCAC

AAAAGCCTAT TCCCTTCAGG TAGTCCTGAC CCTCGAAGCG ATTCGTGCAC

AAAAGCCTAT TCCCTTTAGA TAGTCTTGTC CCTCGATGCA ATCCGAGCAC

AAAAGCCTAT TCCCTTTAGA TAGTCTTGTC CCTCGATGCA ATCCGAGCAC

AAAAGCCTAT TCCCTTCAGG TAGTCCTGAC CTTCGAAGCG ATTCGTGCAC

AAAAGCCTAT TCCCTTCAGG TAGTCCTGAC CCTCGAAGCA ATTCGAGCAC

AAAAGCCTAT CCCCTTCAGG TAGTCCTGAC CCTCGAAGCA ATCCGAGCAC

---------- ---------- ---------- ---------- ----------

---------- ---------- ---------- ---------- ----------

AAAAGCCTAT TCCCTTCAGG TAGTCCTGAC CCTCGAAGCG ATTCGGGCAC

AAAGGCCTAT TCCCTTCAGG TAGTCCTGAC CCTCGAAGCG ATTCGAGCAC

---------- ---------- ---------- ---------- ----------

AAAAGCCTAT CCCCTTTAGG TAATCCTGCC CCTCGAGCTG ATCCTCGCGC

AAAGGCCTAT TCCCTTCAGG TAGTCCTGTC CCTCGAGGAG ATTCGGGCGC

AAGCCAACTG CCGATCATTG ACTATAATCT TGTCTCTCTT CATGGCCTTC

AAGCCAACTG CCGATCATTG ACTATAATCT TGTCTCTCTT CATGGCCTTC

AAGCCAACTG CCGATCATTG ACTATAATCT TGTCTCTCTT CATGGCCTTC

AAGCCAACTG CCGATCATTG ACTATAATCT TGTCTCTCTT CATGGCCTTC

AGGCCAACTG CCGATCATTG ACTATAATCT TGTCTCTCTT CATGGCCTTC

AAGCCAACTG CCGGTCGTTG ACTATAATCT TGTCTCTCTT CATGGCCTTT

ACGCCAGCTG CCGGTCATTG ACTATAATCT TGTCTCTCTT CATGGCTTTC

AAGCCAACTG CCGGTCATTG ACTATAATCT TGTCTCTCTT CATGGCCTTT

AAGCCAGCTG ACGGTCATTT ACGATGATCT TGTCTCTCTT CATGGCTTTT

AAGCCAACTG ACGGTCATTG ACTATAATCT TGTCTCTCTT CATGGCTTTT

AAGCCAACTG CCGGTCGTTG ACTATAATCT TGTCTCTTTT CATGGCTTTT

---------- ---------- ---------- ---------- ----------

---------- ---------- ---------- ---------- ----------

AAGCCAGCTG GCGGTCATTG ACTATGATTT TGTCTCTCTT CATGGCTTTT

ATGCCAGCTG ACGGTCATTG ACTATAATCT TGTCTCTCTT CATGGCTTTT

---------- ---------- ---------- ---------- ----------

ATGCTAACTG ACGGTCATTG ACAATAATCT TGTCTCTCTT CATAGCTTTT

AGGCCAGCTG GCGGTCATTC ACTATGATCT TGTCCCGTTT CATAGCCTTT

TCCATAGCTA CCAGCGCATC TGTACCTGTG GCTACTTGGT GGCCCAAGCC

TCCATAGCTA CCAGCGCATC TGTACCTGTG GCTACTTGGT GGCCCAAGCC

TCCATAGCTA CCAGTGCATC TGTACCTGTG GCTACTTGGT GGCCCAAGCC

TCCATAGCTA CCAGTGCATC TGTACCTGTG GCTACTTGGT GGCCCAAGCC

TCCATAGCTA CCAGAGCATC TGTACCTGTG GCTACTTGGT GGCCCAGGCC

TCCATAGCTA CCAGCGCATC TGTACCTGTG GCTACTTGGT GGCCCAAGCC

TCCATAGCTA CCAGTGCGTC TGTACCTGTA GCTACTTGAT GGCCCAAGCC

TCCATAGCTA CCAGTGCATC TG--CCTGTA GCTACTTGGT GGCCCAAGCC

TCCATAGCCA CCAGTGCATC TA-------- ---------- ----------

TCCATAGCTA CAAGTGCATC TGTACCTGTG GCAACTTGGT GGCCCAAGCC

TCCATAGCTA CCAGTGCATC TGTACCTGTG GCTACTTGGT GGCCCAAGCC

---------- ---------- ---------- ---------- ----------

---------- ---------- --TACCTGTG GCGACCTGGT GGCCCAAGCC

TCCATAGCTA CCAGTGCATC TGTACCTGTG GCTACTTGGT GGCCCAAGCC

TCCATGGCTA CCAGTGCATC TGTACCTGTG GCTACTTGGT GGCCCAAGCC

---------- ---------- ---------- ---------- ----------

TCCATCGCTA CCAGTGCATC TGTACCTGTA GCTACTTGAT GGCCCAGGCC

TCCATAGCCA CCAGGGCATC TGTACCTGTA GCGACCTGAT GGCCCAGACC

TCTGCTTCCA CTGTGGATCA TCACACACAC CTGTCCCTTA TGGTCGATGC

TCTGCTTCCA CTGTGGATCA TCACACACAC CTGTCCCTTA TGGTCGATGC

TCTGCTTCCA CTGTGGATCA TCACACACAC CTGTCCCTTA TGGTCGATGC

TCTGCTTCCA CTGTGGATCA TCACACACAC CTGTCCCTTA TGGTCGATGC

TCTGCTTCCA CTGTGGATCA TCACACACAC CTGTCCCTTA CGGTCGATGC

TCTGCTTCCA CTGTGGATCA TCACACACAC CTGTCCCTTA TGGTCGATGC

TCTGCTCCCG CTGTGGATCA TCACGCACAC CTGTCCCTTA TGGTCGATGC

TCTGCTTCCA CTGTGGATCA TCACACACAC CTGTCCCTTA TGGTCGATGC

---------- ---------- ---------- ---------- ----------

TCTGCTTCCA CTGTGGATCA TCACACACAC CTGTCCCTTA TGGTCAATGC

TCTGCTTCCA CTGTGGATCA TCACACACAC TTGTCCCTTA TGGTCGATGC

---------- ---------- ---------- ---------- ----------

CCGGCTTCCG CTGTGGATCA TCACACACAC CTGCCCCTTG TGGTCAATGC

TCTGCTGCCA CTGTGGATCA TCACACACAC CTGTCCCTTG TGGTCGATGC

TCTGCTTCCG CTGTGGATCA TCACACACAC CTGTCCCTTG TGGTCGATGC

---------- ---------- ---------- ---------- ----------

TCTGCTGCCA CTGTGGATCA TCACACACAC CTGTCCCTTG TGGTCAATGC

TCTGCTGCCG CTGTGGATCA TCACGCACAC TTGCCCTTTG TGGTCGATGC

CCATTTTTTT AGCAGCATAC TCATTGAAAA TCTCATCCAC AACCTGGATT

CCATTTTTTT AGCAGCATAC TCATTGAAAA TCTCATCCAC AACCTGGATT

CCATTTTTTT AGCAGCGTAC TCATTGAAAA TCTCATCCAC AACCTGGATT

CCATTTTTTT AGCAGCGTAC TCATTGAAAA TCTCATCCAC AACCTGGATT

CCATTTTCTT AGCAGCGTAC TCATTGAAAA TCTCATCCAC AACCTGGATT

CCATTTTCTT AGCAGCATAC TCATTGAAAA TCTCATCCAC AACCTGGATT

CCATCTTCTT GGCAGCATAC TCGTTGAAAA TCTCATCTAC AACCTGGATC

CCATCTTCTT GGCGGCATAC TCATTGAAAA TCTCATCTAC AACCTGGATT

---------- ---------- ---------- ---------- ----------

CCATTTTCTT AGCAGCATAC TCGTTGAAAA TCTCATCCAC AACCTGGATT

CCATTTTCTT AGCAGCATAC TCATTGAAAA TCTCATCCAC AACCTGGATT

---------- ---------- ---------- ---------- ----------

CCATCTTCTT GGCGGCGTAC TCATTGAAGA TTTCATCCAC AACCTGGATT

CCATTTTCTT AGCAGCATAC TCATTGAAAA TCTCATCCAC AACCTGGATT

CCATTTTCTT AGCAGCATAC TCGTTGAAAA TCTCATCCAC AACCTGGATT

---------- ---------- ---------- ---------- ----------

CCATCTTCTT GGCAGCGTAC TCATTATAAA TATCATCCAC AACTTGGATT

CCATTTTTTT GGCGGCATAC TCGTTAAAAA TCTCATCCAC CACCTGGATT

TCTGCATAAT GGTTGCCTGC TCCCAGGGTC CCCAACTGTT ACCTGAGGAA

TCTGCATAAT GGTTGCCTGC TCCCAGGGTC CCCAACTGTT ACCTGAGGAA

TCTGCATAAT GGTTGCCTGC TCCCAGGGTC CCCAACTGTT ACCTGAGGAA

TCTGCATAAT GGTTGCCTGC TCCCAGGGTC CCCAACTGTT ACCTGAGGAA

TCTGCATAAT GGTTGCCTGC TCCCAGGGTC CCCAACT--- ----------

TCTGCATAGT GATTGCCTGC TCCCAGCGTC CCCAACT-TT ACCTGAGGAA

TCTGCATAAT GGTTGCCTGC TCCCAGGGTC CCCAACTCTT ACCTGAGGAA

TCTGCATAAT GGTTGCCTGC TCCCAGGGTC CCCAACTGTT ACCTGAGGAA

---------- ---------- ---------- ---------- -------GGA

TCTGCATAGT GGTTGCCTGC TCCCAGAGTC CCCAACTGTT ACCTGGGGAA

TCTGCATAGT GGTTGCCTGC TCCCAGGGTT CCCAACT-TT ACCTGAGGAA

---------- ---------- ---------- ---------- ----------

TCTGCATAGT GGTTGCCTGC GCCCAGGGTC CCCAACTGTT ACCTGAGGCA

TCTGCGTAAT GGTTGCCTGC TCCTAAGGTC CCCAGCTGTT ACCTGAGGAA

TCTGCATAAT GGTTGCCAGC TCCCAGGGTC CCCAACTCTT ACCTGAGGGA

---------- ---------- ---------- ---------- ----------

TCAGCATAAT GATTTCCTGC TCCCAGGGTC CCAAGCTTTT ACCTGAGGAA

TCGGCATAGT GGTTTCCCGC TCCCAGGGTC CCTAGCTGTC ACCTGGGGCA

GGCCTCTTTT CTTCGCCCTT GCAGAAACTT TATTGGGGTC AGCCTGCAGC

GGCCTCTTTT CTTAGCCCTT GCAGAAACCT TATTGGGGTC AGCTTGCAGC

GGCCTCTTTT CTTAGCCCTT GCAGAAACCT TATTGGGGTC AGCTTGCAGC

GGCCTCTTTT CTTAGCCCTT GCAGAAACCT TATTGGGGTC AGCTTGCAGC

---------- ---------- ---------- ---------- ----------

GGCCTCTTTT CTTAGCCCTT GCAGAGACCT TGGTGGGGTC AGCTTGCAGC

GGCCCCTTTT CTTTGCCCTG GGTGAGACCT TATTGGGGTC GGCTTGGAGC

GGCCCCTTTT CTTTGCCCTG GGTGAGACCT TATTGGGGTC GGCTTGCAGC

GAATCTATTT TTTGACACTC AGAGATACAA TATTTGAGTT ATCTTGAAGA

GGCCTCTTTT TTTAGCCCTG GCTGAGACTT TATTGGGATC AGCTTGCAGC

GGCCTCTTTT CTTAGCCCTT GCAGAGACTT TATTGGGGTC GGCCTGCAAC

---------- ---------- ---------- ---------- ----------

GGCCTCTTTT CTTGGCCCTG GCAGACACCT TGTTGGGGTC GGCCTGCAGC

GGCCTCTTTT TTTGGCCCTT GCAGAAACTT TATTGGGGTC AGCTTGCAGC

GGCCCCTTTT TTTGGCCCTT GCAGAAACCT TGTTGGGGTC AGCCTGCAGC

---------- ---------- ---------- ---------- ----------

GGCCTCTCTT TTTAGCCCTG GCTGAGACCT TATTGGGGTC AGCTTGCAGC

GGCCTCTCTT CTTAGCCCGT GCGGACACCT TGTTGGGGTC AGCTTGCAGC

ATCCTTCCGT ACTCCTCGCA GTGCTCCTTG TCTTCAGCCC AGGCATACCC

ATCCTTCCGT ACTCCTCGCA GTGCTCCTTG TCTTCAGCCC AGGCATACCC

ATCCTTCCAT ACTCCTCGCA GTGCTCCTTG TCTTCAGCCC AGGCATACCC

ATCCTTCCAT ACTCCTCGCA GTGCTCCTTG TCTTCAGCCC AGGCATACCC

---------- ---------- ---------- ---------- ----------

ATCCTTCCGT ACTCCTCACA GTGCTCCTTG TCTTCAGCCC AGGCATAGCC

ATCCTTCCAT ACTCCTCACA GTGCTCCTTG TCCTCAGCCC AGGCATAGCC

ATCCTTCCAT ACTCCTCACA GTGCTCCTTG TCTTCAGCCC AGGCATAGCC

ATCCTTCTAC ATTCTTCATC GTCTTCTCTG CACTCAGCCT AGGCGTAACC

ATCCTTCCAT ACTCCTCACA GTGCTCCTTG TCCTCTGCCC AGGCATAGCC

ATCCTGCCGT ACTCCTCACA GTGCTCCTTG TCCTCAGCCC AGGCATAGCC

---------- ---------- ---------- ---------- ----------

ATCCTCCCGT ACTCCTCACA GTGCTCCTTG TCCTCCGCCC AGGCGTAGCC

ATCCTTCCAT ACTCCTCACA GTGCTCCTTG TCTTCAGCCC AGGCATACCC

ATCCTTCCAT ACTCCTCACA GTGCTCCTTG TCTTCGGCCC AGGCATAGCC

---------- ---------- ---------- ---------- ----------

ATACGCCCAT ATTCCTCACA ATGCTCCTTA TCCTCAGCCC AGGCATACCC

ATCCGTCCGT ACTCCTCACA GTGCTCCTTG TCCTCGGCCC AGGCATAGCC

TTCTCTTAAG GACCAGTCCA CCCCCATCTC CAAGGCCTCC TCCAAGTCTC

TTCTCTTAAG GACCAGTCCA CCCCCATCTC CAAGGCCTCC TCCAAGTCTC

TTCTCTTAAG GACCAGTCCA CCCCCATCTC CAAGGCCTCC TCCAAGTCTC

TTCTCTTAAG GACCAGTCCA CCCCCATCTC CAAGGCCTCC TCCAAGTCTC

---------- ---------- ---------- ---------- ----------

TTCTCTCAGG GACCAGTCCA CACCCATCTC CAAGGCCTCC TCCAAGTCTC

TTCTCTCAGG GACCAGTCCA CACCCATCTC CAATGCCTCC TCCAAGTCTC

TTCCCTCAGG GACCAGTCCA CCCCCATCTC CAATGCCTCC TCCAAGTCTC

TTCTCTTTGG GGCCAGTCGA TACCCATCTC CAGGTTATCC TTTAAGTCTT

TTCTCTCAGG GACCAGTCCA CACCCATCTC CAAGGCCTCC TCCAAGTCTC

TTCCCTCAGG GACCAATCCA CACCCATCTC TAAGGCCTCC TCCAAGTCTC

---------- ---------- ---------- ---------- ----------

TTCCCGCAGG GACCAGTCCA CGCCCATCTC CAAGGCCTCC TCCAGGTCTC

TTCTCTCAAG GACCAGTCCA CACCCATCTC CAAGGCCTCC TCCAAGTCTC

TTCTCTCAAG GACCAGTCCA CACCCATCTC CAAGGCCTCC TCCAAGTCTC

---------- ---------- ---------- ---------- ----------

TTCTCGTAGA GACCAGTCCA CCCCCATCTC CAGAGCCTCC TCCAAGTCCC

CTCTCGTAAG GACCAGTCCA CACCCATCTC CAGGGCCTCT TCTAGGTCCC

TGCTTACTTG GCATTCATTG GGATGACACC TTTTGACCCC ACCCCAACAG

TGCTTACTTG GCATTCATTG GGATGACACC TTTTGACCCC ACCCCAACAG

TGCTTACTTG GCATTCATTG GGATGACACC TTTTGATCCC ACCCCAACAG

TGCTTACTTG GCATTCATTG GGATGACACC TTTTGATCCC ACCCCAACAG

--CTTACTTG GCATTCATTG GGATGACACC TTTTGATCCT ACCCCAACAG

TGCTTACTTG GCATTCATTG GGATGACGCC TTTTGATCCC ACCCCAACAG

TGCTTACTTG GCATTCATTG GAATGACACC TTTCGATCCC ACCCCGACAG

TGCTTACTTG GCATTCATTG GAATGACACC TTTTGATCCC ACCCCAACAG

CG-----TCG GTGTTCATTG TGTTGACACC TCTTTATTTC ACCTCAACAA

TGCTTACTTG GCATTCATTG GGATGACACC TTTTGACCCC ACTCCCACAG

TGCTTACTTG GCACTCATGG GGATGACACC TTTTGATCCC ACTCCAACAG

---------- ---------- ---------- ---------- ----------

TGCTTACTTG GCATTCATCG GGATGACGCC TTTGGAGCCG ACACCGACAG

TGCTTACTTG GCATTCATTG GGATGACACC TTTTGATCCT ACTCCAACAG

TGCTTACTTG GCATTCATTG GGATGACACC TTTTGATCCC ACCCCAACAG

---------- ---------- ---------- ---------- ----------

TGCTCACTTG GCATTCATTG GGATAACGCC TTTTGATCCC ACCCCAACAG

TGCTTACTTG GCATTCATGG GGATCACCCC CTTAGATCCC ACCCCGACGG

GAATGTGGTC AAACATAGCT TGGGCAAGTT GCTCCTTCAC AGGCTGGACA

GAATGTGGTC AAACATAGCT TGGGCAAGTT GCTCCTTCAC AGGCTGGACA

GAATGTGGTC AAACATAGCT TGGGCAAGTT GCTCCTTCAC AGGCTGGACA

GAATGTGGTC AAACATAGCT TGGGCAAGTT GCTCCTTCAC AGGCTGGACA

GAATGTGGTC AAACATAGCT TGGGCAAGTT GCTCCTTCAC AGGCTGGACA

GAATGTGGTC AAACATAGCT TGGGCAAGTT GCTCCTTCAC AGGCTGGACA

GGATGTGGTC AAACATAGCT TGGGCAAGTT GTTCCTTCAC AGGCTGTACA

GGATGTGGTC AAACATAGCT TGGGCAAGTT GTTCCTTCAC AGGCTGTACA

GTATATGGTA AAACATAGCT TGGGTAAATT GATCCTTAGC AAGATTGACA

GAATGTGGTC AAACATAGCT TGGGCAAGTT GCTCTTTCAC AGGCTGAACA

GAATATGGTC AAACATAGCT TGGGCAAGCT GCTCCTTCAC AGGCTGGACA

---------- ---------- ---------- ---------- ----------

GGATGTGGTC AAACATGGCC TGGGCCAGCT GCTCCTTGAC GGGCTGCACG

GAATGTGGTC AAACATAGCT TGGGCAAGCT GCTCCTTCAC AGGCTGGACA

GAATGTGGTC AAACATAGCT TGGGCAAGTT GCTCCTTCAC AGGCTGAACA

---------- ---------- ---------- ---------- ----------

GAATGTGGTC AAACATGGCC TGGGCTAGTT GTTCCTTCAC AGGCTGGACA

GGATGTGGTC AAACATGGCC TGGGCGAGCT GCTCCTTCAC CGGCTGGACG

TCACTTTCAT CTAAATTGGT TCTTAGCAAG CGGACACCAC AGTTGATGTC

TCACTTTCAT CTAAATTGGT TCTTAGCAAG CGGACACCAC AGTTGATGTC

TCACTTTCAT CTAAATTGGT TCTTAGCAAG CGGACACCAC AGTTGATGTC

TCACTTTCAT CTAAATTGGT TCTTAGCAAG CGGACACCAC AGTTGATGTC

TCACTTTCGT CTAAATTGGT TCTGAGCAAG CGGACACCAC AGTTGATGTC

TCACTTTCAT CTAAATTGGT TCTTAGCAAG CGGACACCAC AGTTGATATC

TCGCTCTCAT CTAAATTGGT TCTTAGCAAG CGGACACCAC AGTTAATATC

TCGCTCTCAT CTAAATTGGT TCTTAGCAAG CGGACACCAC AGTTAATATC

TCATTTTCAT CTAAATTGGT TCCTAGCAAG AATACACTAT TGTTGATGTC

TCACTTTCAT CTAAATTGGT TCTCAGCAAG CGGACACCAC AGTTAATGTC

TCGCTTTCAT CTAAATTGGT TCTTAGCAAG CGGACACCAC AGTTGATGTC

---------- ---------- ---------- ---------- ----------

TCGCTCTCGT CCAGGTTGGT TCTCAGCAGG CGCACCCCGC AGTTGATATC

TCACTTTCAT CTAAATTGGT TCTTAGCAAG CGGACACCAC AGTTTATGTC

TCACTTTCAT CTAAATTGGT TCTCAGCAAA CGGACACCAC AGTTGATATC

---------- ---------- ---------- ---------- ----------

TCACTTTCAT CAAGGTTGGT TCTCAGCAAT CGGACACCAC AGTTGATATC

TCACTTTCAT CCAAGTTGGT TCTCAGCAAG CGGACGCCAC AGTTGATATC

AAACCCGACA CCACCTCTTA CCTGGGGATA CTACTGCTTC AGGGTCATTC

AAACCCGACA CCACCTCTTA CCTGGGGATA CTACTGCTTC AGGGTCATTC

AAACCCGACA CCACCTCTTA CCTGGGGATA CCACTGCTTC AGAGTCATTC

AAACCCGACA CCACCTCTTA CCTGGGGATA CCACTGCTTC AGAGTCATTC

AAACCCGACA CCACCTCTTA CCTGGGGATA CCACTGCTTC AGGGTCATTC

AAATCCGACA CCACCTCTTA CCTGGGGATA CCACTGCTTC AGGGTCATTC

AAATCCGACA CCACCTCTTA CCTGGGGATA CAACTGCCTC AGGGTCATTC

AAATCCGACA CCACCTCTTA CCTGGGGATA CAACGGCCTC AGGGTCATTC

AAA-ACAATA CTACCT---- -----GGATA CTACTGCTTA AAGGTTATTA

AAACCCAACA CCACCTCTTA CCTGGGGATA CCACTGCTTC AGGGTCGTTC

AAATCCGACA CCACCTCTTA CCTGGGGATA CCACTGCTTC GGGGTCATTC

---------- ---------- ---------- ---------- ----------

GAAGCCGACA CCACCTCTCA CCTGGAGACA CCACTGCTTC GGGGTCGCTC

AAATCCGACA CCCCCTCTTA CCTGGGGATA CCACTGCTTC AGGGTCACTC

AAATCCGACG CCACCTCTTA CCTGGTGAAA CCACTGCGTC AGGGTCATTC

---------- ---------- ---------- ---------- ----------

AAATCCGACA CCACCTCTTA CCTGGTGACA CCACAGCTTC TGGGTCACTC

AAATCCGACG CCACCTCTTA CCTGGGGACA CCACCGCTTC TGGGTCACTC

ATATCAAAGG CTGCCATGTT CCCAATAGCA AACCCATATC CTGAATGGAC

ATATCAAAGG CTGCCATGTT CCCAATAGCA AACCCATATC CTGAATGGAC

ATATCAAAGG CTGCCATGTT CCCAATAGCA AATCCATATC CTGAATGGAC

ATATCAAAGG CTGCCATGTT CCCAATAGCA AATCCATATC CTGAATGGAC

ATATCAAAGG CTGCCATATT TCCAATGGCA AACCCATATC CTGAATGGAC

ATATCAAAGG CTGCCATATT CCCAATGGCA AACCCATAGC CTGAATGGAC

ATATCAAAGG CAGCCATGTT CCCTATGGCA AACCCGTAGC CTGAGTGGAC

ATATCAAAGG CAGCCATGTT CCCTATGGCA AACCCATAGC CTGAATGGAC

ATTTCAAATG GCACACTATT CCTTAAAGCA AATCCATTCC CTGAATTGAA

ATATCAAAGG CTGCCATATT TCCAATAGCA AACCCATAAC CTGAATGGAC

ATATCGAAGG CTGCCATATT CCCAATAGCA AACCCATAAC CTGAATGGAC

---------- ---------- ---------- ---------- ----------

ATGTCAAAGG CCGCCATGTT GCCAATAGCG AACCCGTACC CTGAATGGAC

ATATCAAAAG CTGCCATATT CCCAATAGCA AACCCATAAC CGGAATGGAC

ATGTCAAAGG CTGCCATATT CCCAATAGCA AACCCATAGC CTGAGTGGAC

---------- ---------- ---------- ---------- ----------

ATATCAAAAG CAGCCATGTT CCCAATTGCG AATCCATAAC CAGAATGGAC

ATGTCAAAGG CGGCCATATT CCCAATGGCA AATCCATAAC CAGAGTGGAC

ATCAGGAAGC CCAATAGATC GCTGAAAAAA CCTGTAACCT GAGGCATACA

ATCAGGAAGC CCAATAGATC GCTGAAAAAA CCTGTAACCT GAGGCATACA

ATCAGGAAGC CCAATAGATC GCTGAAAGAA CCTGTAACCC GAGGCATACA

ATCAGGAAGC CCAATAGATC GCTGAAAGAA CCTGTAACCC GAGGCATACA

ATCAGGAAGC CCAATAGATC GCTGAAACAA CCTGTAACCC GAGGCATACA

ATCAGGAAGC CCAATTGATC GCTGTAAGAA CCTATACCCC GAGGCATAGA

ATCAGGAAGC CCAATAGACC GCTGGAAGAA CCTGTAACCA GATGCATACA

ATCAGGAAGC CCGATAGACC GCTGCAAGAA CCTGTAACCA GACGCATACA

ATCAGGAAGC CAAGTGGATC AATGAACAAA CCTGTACCCC GAGGCGTACA

ATCAGGAAGA CCGATGGATC GCTGAAAGAA CCTGTAACCC GAGGCGTACA

ATCAGGAAGC CCAATAGATC TCTGAAAGAA CCTGTAGCCC GAGGCATACA

---------- ---------- ---------A CTTGTAACCC GAGGCATACA

ATCTGGAAGC CCGATAGATC TCTGAAAGAA CCTGTAGCCC GAGGCGTAGA

ATCAGGAAGT CCAATAGATC GCTGAAAGAA CCTGTAGCCG GAGGCATACA

GTCTGGAAGA CCAATGGAAC GCTGAAAGAA CCTGTAGCCT GAGGCATACA

---------- ---------- ---------- ---------- ----------

ATCAGGAAGC CCAATAGATC TCTGAAAGAA CTTGTAGCTT GAAGGATTCA

GTCGGGAAGC CCAATAGATC GCTGAGGGAA CTGGTAGCCC GAGGCGAACA

GGGACTCACA GATGAGCTTC ATGTGGTTGT TCAAAATGTG AAAGATCACT

GGGACTCACA GATGAGCTTC ATGTGGTTGT TCAAAATGTG AAAGATCACT

GGGACTCACA GATGAGCTTC ATGTGGTTGT TCAAAATGTG AAAGATCACT

GGGACTCACA GATGAGCTTC ATGTGGTTGT TCAAAATGTG AAAGATCACT

GGGACTCACA GATGAGCTTC ATGTGGTTGT TCAAAATGTG ACAGATCACT

GGGCCTCACA AATAAGCTTC ATGTGGTTGT TCAAAATGGG AAAGATCACT

GGGATTCACA GAGGAGCTTC ATGTGGTTGT TCAAAATGTG TAAGATCACT

GGGACTCACA GAGGAGCTTC ATGTGGTTGT ACAAAATGCG TAAGATCACT

GGGACTCACA GATGAGCTTC ATGTGGTTGT TCATAATGCG ACAGATCGCT

GGGACTCACA GATGAGCTTC ATGTGGTTAT TCAAAATGGG AAAGGTCACT

GGGACTCACA GATGAGCTTC ATGTGGTTAT TTAAAATGGG AAAGATCGCT

GGGACTCACA GATGAGCTTC ATGTGGTTAT TCAAAATGGG AAAGATCACT

GGGCCTCGCA GAGCAGCTTC ATGTGGCTGT TGAAGGTGTG CCAGGTCGCT

GGGCCTCGCA GATGAGCTTC ATGTGGTTAT TCAAAATGTG ACAGGTCACT

GGGACTCGCA GATGAGCTTC ATGTGGTTAT TCAAAATGGG AAAGATCGCT

---------- ---------- ---------- ---------- ----------

ATGATTCATA AATCAGCTTC ATATGGCTAT TCAAAATGAG AAAGGTCACC

GCGACTCGTA GAGCACCTTC ATGTGATTGT TGAAGGTGGG CCAGGTCGCT

TGCTGTCCGC AATAATATGG CCTCGAAAAC TTGAAATATT AACATCGTCT

TGCTGTCCGC AATAATATGG CCTCGAAAAC TTGAAATATT AACATCGTCT

TGCTGTCCGC AATAATATGG CCTCAAAAAC TTGAAATATT AACACCGTCT

TGCTGTCCGC AATAATATGG ACTCAAAAAC TTGAAATATT AACACCGTCT

TGCTGTCCGC AACAATATGG CCTCGAAAAC TTGAAATATT AATATCATCT

TGCTGTCCTC AACAATATGG CTTCGAAAAC TTGAAATATT AATAGCATCT

GGCTGTCCTC AGTAATATGG CCTCAAAAGC TTGAAATATT AACAGCACCT

GGCTGTCCTC AGTAATATGG CCTCGAAAGC TTGAAATATT AACAGCATCT

GGCTGTCCTC AGCAGGATGG CTTCCAAAAC TTGAAATA-- ----------

GGCTGTCCGC AATAATATGG CCTCGAAAAC TTGAAATACT AAAAGCATCT

CGCAGTCCGC AACAGTATGG CCTCAAAAAC TTGAAATATT AACAGCATCT

TGCGGTCCGT AATAATATGG CCTCAAAAAC TTGAAATATT AATAGCATCT

GGCCGTCCGC AGCAGGATGG CCTCCAGCGC CTGGAAGACC AGCAGCATCT

AGCTGTTCGC AATAAGATGG CCTCCAATGC TTGAAATGTT GACAGCATCT

TGCCGTCCGC AACAATATGG CCTCGAAAGC TTGAAATATT AATAGTATCT

---------- ---------- ---------- ---------- ----------

TGCTGTTCGA AGTAATATGG CTTCTAAAGC TTTAAATATT AATAGCCGCT

GGCCGTGCGC AGCAGGATGG CTTCCAGGGC CTGCAGGATC AGCATCACCT

GCAAAGATCA GGTCCATAGC TCTTCGGTTC TTCCAAATTT GTAGATCAGA

GCAAAGATCA GGTCCATAGC TCTTCGGTTC TTCCAAATTT GTAGATCAGA

GCAAAGATCA GGTCCATAGC TCTTCAGTTC TTCCAAATTT GTAGATCAGA

ACAAAGATCA GGTCCATAGC TCTTCAGTTC TTCCAAATTT GTAGATCAGA

GCAAAGATCA GGTCCATAAC TCTTCGGTTC TTCCAAATTT GTAGATCAGA

GCAAAGATCA GGTCCATAGC TCTTCAGTTC TTCCAAATTT ATAGATCAGA

GCAAAGGTCA GGCCCAGAGC TCTTCAGTTC TTCCGAATTT GTAGATCAGA

GCAAAGGTCA GGCCCAGAGC TCTTCAGTTC TTCCAAATTT GTAGATCAGA

-------TCA GGTCCATAGC TCTTCAGTTC TTCCAAACTT GTAGATCAGA

GCAAAGATCA GGTCCACAGC TCTTCAGTTC TTCCAAATTT GTAGATCAGA

GCAA---TCA GGTCCAAAGC TCTTCAGTTC TTCCAAATTT GTAGATCAGA

GCAAAGATCA GGTCCATAGT TCTTCAGTTC TTCCAAATTT GTAGATCAGA

GCAAAGCTCA GCTCCACAGC TCCTCCGTCC TTCCGAATTT GTAGATCAGA

GTGAAGATCA GGTCCACAGC TCCTCAGTTC TTCCAAATTT GTAGATCAGA

GCAAAGATCA GGTCCATAGC TCCTCAGTTC TTCCAAACTT GTAGATCAGA

---------- ---------- ---------- ---------- ----------

GAAAAAATTA TCCCCATAAT TCTTCTGTTC GTCCAAATTT GTAGATGAGG

GCAAAAATCC CGCTCATAAT TCTTCTAGTC TTCCAAACTT GTAGATTAGA

GTGCTCTTAC CTAAAAAATA TAAGTGCATT TTGAAAAAAC ACAGCTAGTC

GTGCTCTTAC CTAAAAAATA TAAGTGCATT TTGAAAAAAC ACAGCTAGTC

GTGCTCTTAC CTAAAAAATA TAAGCGCATT TTGAAAAAAC ACAGCTAGAC

GTGCTCTTAC CTAAAAAATA TAAGTGCATT TTGAAAAAAC ACAGCTAGAC

GTGCTCTTAC CTAAAAAATA TAAGTGCATT TTGAAAAAAC ACAGCTAGTC

GTGCTCTTAC CTAAAAAATA TAAGTGCATT TTGAAAAAAC ACAGCTAGTC

GTGCTCTTAC CTAAAAAATA TAAGTGCATT TTGAAAGAAC ACAGCTAGTC

GTGCTCTTAC CTAAAAAATA TAAGAGCATT TTGAAAGAAC ACAGCTAGTC

GTGCTCTTAC CTAAAAAATA TGAGTGCATT TTGAAAAAAC ACAGCTAGTC

GTGCTCTTAC CTAAAAAATA TAAGTGCATT TTGAAAAAAC ACAGCTAGTC

GTGCTCTTAC CTAAAAAATA TAAGTGCATT TTGAAAAAAC ACAGCTAATC

GTGCTCTTAC CTAAAAAATA TAAGTGCATT TTGAAAAAAC ACAGCTAGTC

GTGCTCTCAC CTAAAAAAAA TCAGCGCATT TTGAAAGAAC ACAGCTAGTC

GTGCTCTTAC CTAAAAAATA TAAGAGCATT TTGAAAAAAC ACAGCTAGTC

GTGCTCTTAC CTAAAAAATA TAAGTGCATT TTGAAAAAAC ACAGCTAGTC

-----CTTAC CTAAAAAATA TCAGTGCATT ---------- ----------

GTGCTCTTAC CTAAAAAATA TAAGTGCATT CTGAAAAAAT ACAGCTAGTC

GTACTCTTAC CTAAAAAATA TCAGCGCATT TTGGAAAAAC ACAGCCAGGC

CCGGATAAAC ATCAGTATCT ACAAGAATAC TTACTTTGGG TAACATATGC

CCGGATAAAC ATCAGTATCT ACAAGAATAC TTACTTTGGG TAACATATGC

CCGGATAAAC ATCAGTATCT ACAAGAATAC TTACTTTGGG TAACATATGC

CCGGATAAAC ATCAGTATCT ACAAGAATAC TTACTTTGGG TAACATATGC

CCGGATAAAC ATCAGTATCT ACAAGAATAC TTACTTTGAG TAACATATGC

CTGGATAAAC ATCAGTATCT GCAAGAATAC TTACTTTGGG TAACATATGC

CAGGGTAAAC ATCAATATCT GCAAGAGCAC TTACTTTGAG TAACATATGC

CAGGGTAAAC ATCAATATCT GCAagaACAC TTACTTTGAG TAACATATGC

CTGGATAAAC ATCAGTATCT GCAAGAACAC TTACTCTGGG TAACGTATGC

CTGGATAAAC ATCAGTATCT GCAATAATAC TTACTCTGGG TAACATAGGC

CTGGATAAAC ATCAGTATCT GCAAGAATAC TTACTTTGGG TAACATATGC

CTGGATAAAC ATCAGTATCT GCA--AATAC TTACTTTGGG TAACATATGC

CTGGATAAAC ATCGGTATCT GCAAGAATAC TTACTTTGGG TAACATATGC

CTGGATACAC ATCAATATCT GCAAGAACAC TTACTTTGGG TAACATAGGC

CTGGATACAC ATCCGTATCT GCAAGAATAC TTACTTTGGG TAACATATGC

---------- ---------- ------ATAC TTACTTTGGG TAACATACGC

CTGGATAAAC ATTAGTATCT GCACCAATAC TTACTTTGAG TAACATAGGC

CTGGATAGAC GCTTATATCT GCAAGAACAC CTACTTTGGG TTACATAGGC

ACCAAAAAGA ATCCACATGG AAGCAATAAG TGACCCAAAC ATCAACATGA

ACCAAAAAGA ATCCACATGG AAGCAATAAG TGACCCAAAC ATCAACATGA

ACCAAAAAGA ATCCACATGG AAGCAATGAG TGACCCAAAC ATCAACATGA

ACCAAAAAGA ATCCACATGG AAGCAATAAG TGACCCAAAC ATCAACATGA

ACCAAAAAGA ATCCACATGG AAGCAATAAG TGACCCAAAC ATCAACATGA

ACCAAAAAGA ATCCACATGG AAGCAATAAG TGACCCAAAC ATCAACATGA

ACCAAAAAGG ATCCACATGG AGGCGATAAG CGATCCGAAC ATCAGCATGA

ACCAAAAAGG ATCCACATGG AGGCGATGAG TGACCCGAAC ATCAGCATGA

ACCAAAAAGA ATCCACATGG AAGCAATGAG TGACCCAAAC ATCAGCATGA

ACCAAAGAGA ATCCACATAG AAGCAATCAG TGACCCAAAC ATCAACATGA

ACCAAAGAGA ATCCACATGG AAGCAATAAG TGATCCAAAC ATCAACATGA

ACCAAAAAGA ATCCACATGG AAGCAATAAG TGACCCAAAC ATCAACATGA

ACCAAAGAGA ATCCACATGG AGGCAATTAG TGACCCGAAC ATCAACATGA

ACCAAAAAGA ATCCACATGG AAGCAATAAG TGACCCAAAC ATCAACATGA

ACCAAAAAGG ATCCACATGG AAGCAATAAG TGACCCAAAC ATCAACATGA

ACCAAAAAGA ATCCACATGG AAGCAATAAG TGACCCAAAC ATCAACATGA

ACCAAAAAGA ATCCACATGG AAGCGATAAG CGACCCAAAC ATCAACATGA

GCCAAAAAGG ATCCACATTG AAGCGATGAG GGAACCAAAC ATCAGCATGA

AACCAATGAA AAGCCAAACT CGAGCACCTC CTTACTTCAC TTTCAGGTCG

AACCAATGAA AAGCCAAACT CGAGCACCTC CTTACTTCAC TTTCAGGTCG

AACCAATGAA AAGCCAAACT CGAGCACCTC CATACTTCAC TTTCAGGTCG

AACCAATGAA AAGCCAAACT CGAGCACCTC CATACTTCAC TTTCAGGTCG

AACCAATGAA AAGCCAAACT CGAGCACCTC CATACTTCAC TTTCAGGTCG

AACCAATAAA AAGCCAAACT CGAGCACCTC CATACTTCAC TTTCAGGTCG

AACCAATGAA GAGCCAGACC CGAGCTCCTC CCTACTTCAC TCTCGGGTCG

ACCCAATGAA GAGCCAGACC CGAGCACCTC CCTACTTCAC TCTCGGGCCG

AACCAATGAA GAGCCAAATC CGAGCACCTC CATACTTCAC TTTCGGGCCG

AACCAATGAA GAGCCAAACT CGAGCACCTA CGTACTTCAC TTTCAGGGCG

AACCAATGAA GAGCCAAACT CGAGCACCTT CGTACTTCAC TTTCAGGTCG

AACCAATGAA GAGCCAAACC CGAGCACCTT CATACCTCAC TTTCAGGTCG

AACCGATGAA GAGCCACACT CGAGCACCTC C---CCTCGC TTTCAGGCCG

AACCAATGAA AAGCCAAACT CGAGCACCTC CATACCTCAC TTTCTGGTCG

AACCAATGAA AAGCCAAACT CGAGCACCTC CAGACCTCAC TTTCAGGTCG

AACCAATGAA AAGCCAAACT CGAGCGCCT- ---------- ----------

AACCAATGAA GAGCCAAACT CGAGCACCTT CTTACTTCAT GTTCAGTTCT

AACCAATGAA GAGCCAGACA CGGGCCCCTC CTCACCTCCC TCTCCGGCCT

TTTCTCTCCA CTTAGGAGCT GGAAAATTTC GACACACTCA ACAGAAATCT

TTTCTCTCCA CTTAGGAGCT GGAAAATTTC GACACACTCA ACAGAAATCT

TTTCTCTCCA CTTAGGAGCT GGAAAATTTC TGCACACTCA ACAGAAATCT

TTTCTCTCCA CTTAGGAGCT GGAAAATTTC TGCACACTCA ACAGAAATCT

TTTGTCTCCA CTCAGGAGCT GGAAAATTTC GGCACACTCA ACAGAAATCT

CTTCTCTCCA CTCAGAAGCT GGAAAATTTC AGCACACTCA ACAGAAATCT

CTTCTCTCCA CTCAGGAGCT GGAAAATTTC TGCACACTCC ATGGAAATCT

CTTCTCCCCA CTCAGGAGCT GGAAAATCTC TGCACATTCC ATGGAAATCT

CTTCTCCCCA CTCAGGAGCT GGAAGACCTC GGCGCACTCG ACGGAGATCT

CTTCTCCCCA CTCAGGAGCT GGAAAATCTC GGCACACTCC ACAGAAATCT

CTTCTCCCCA TTCAGGAGTT GGAAGATCTC TGCACACTCA ACAGAAATCT

CTTCTCCCCA CTCAGGAGCT GGAAAATCTC TGCACACTCC ACAGAAATCT

CTTCTCCCCA CTCAGGAGCT GGAAGATCTC GGTGCACTCC ACGGAGATCT

CTTCTCTGCA CTCAGGAACT GGAAAATTTC TGCACACTCC ACAGAAATCT

CTTCTCGCCA CTCAGGAGCT GGAAAATTTC TGCACACTCA ACGGAAATCT

---------- ---------- ---------- ---------- ----------

TTTCTCTCCA CTTAGAAGCT GGAAAATTTC TGAACACTCA ACAGAAATCT

CTTCTCTCCA CCGAACAGCT GGAAGATCTC CGCGCACTCC ACCGAGATCT

TTATATACCC TTCCACAACA TCATACAAGG CAAACATCCT GTGTCTGCTT

TTATATACCC TTCCACGACA TCATACAAGG CAAACATCCT GTGTCTGCTT

TTATATACCC TTCCACAACA TCATACAAGG CAAACATCCT GTGTCTGCTT

TTATATACCC TTCCACAACA TCATACAAGG CAAACATCCT GTGTCTGCTT

TTATGTAACC TTCCACAACA TCATATAAGG CAAACATCCT GTGTCTGCTT

TTATATAACC TTCCACAACA TCATACAAGG CAAGCATCCT GTTTCTGCCT

TGATGTAGCC CTCGACAACA TCGCACAAGG CAAACATCCT GTGTCTGCCT

TGATGTAGCC CTCGACAACA TCACACAAGG CAAACATCCT GTGTCTGCCT

TTATGTACCC TTCCACCACA TCATACAAGG CAAACATCCT GTGTCTGCCT

TGATGTACCC CTCCACAACA TCGTACAAGG CAAACATCCT GTGTCTGCCT

TTATGTACCC TTCCACAACA TCATATAAGG CAAACATCCT GTGTCTGCTT

TGATGTACCC TTCCACGACA TCATACAAGG CAAACATCCT GTGTCTGCCT

TGATGTAGCC CTCCACAATG TCGTACAAGG CAAACACCCT GTGTCTGCTT

TTATATATCC TTCCACAACA TCATACAAGG CAAACATCCT GTGTCTGCCT

TTATGTATCC TTCCACGACA TCATACAAGG CAAACATCCT GTGTCTGCCT

---------- ---------- ---------- ---------- ----------

TTATATAACC TTCCACGACA TCATATAAGG TAAACATCCT GTATCTGCAT

TGACGTACTC TTCCACGACA TCACACGAGG TAAACATCCC GTATCCGCAC

TGATGGAGAT CTGTAATAAA AGAAGGTGGC AACCACCTGA ATTTCTATTG

TGATGGAGAT CTGTAATAAA AGAAGGTGGC AACCACCTGA ATTTCTATTG

TGATGGAAAT CTGTAATAAG AGAAGGTGGC AACCACCTGA ATTTCTGTTG

TGATGGAAAT CTGTAATAAG AGAAGGTGGC AACCACCTGA ATTTCTGTTG

TGATGGAAAT CTGTAATAAG AGAAGGTGGC AACCACCTGA ATTTCTATTG

TGATGGAAAT CTGTAATAAG AGAAGGTGGC AACCACCAGA ATTTCTCTTG

TGATGGAAAT CTGTAATAAG AGAAGGTGGC AACCACCTGA ATTTCTCTTG

TGATGGAAAT CTGTAATAAG AGAAGGTGGC AACCACCTGA ATTTCTCTTG

TGATGGAAAT CTGTAACAAG AGAAGGTGGC AACCACCTGA ATTTCTTTTG

TGATGGAAAT CTGTAATAAG AGAAGGTGGC AACCACCTGA ATTTCTCTTG

TGATGGAAAT CTGTAATAAG AGAAGGTGGC AACCACCAGA ATTTCTCTTG

TGATGGAAAT CTGTAATAAG AGAAGGTGGC AACCACCTGA ATTTCTCTTG

TGATGGAAAT CTGTAATAAG AGAAGGTGGC AACCACCAGA ATTTCTCTTG

TGATGGAAAT CTGTAATAAG AGAAGGTGGC AACCACCTGA ATTTCTCTTG

TGATGGAAAT CTGTAATAAG AGAAGGTGGC AACCACCTGA ATTTCTCTTG

---------- ---------- ---------- ---------- ----------

TGATGGAGAT CTGTAATAAA AGAAGGTGGC AACCACCTGA ATTTCTCTTG

TGATGGAGAT CTGTAATAAA AGGAGATGGC AGCCGCCTGA ATTCCTCTTG

GTCCATGATA GTGGCCCTGA TCATCGCAAA CATTTTCTCT TTAGGGTAAA

GTCCATGATA GTGGCCCTGA TCATCGCAAA CATTTTCTCT TTAGGGTAAA

GTCCATGATA GTGGCCCTGA TCATCGCAAA CATTTTCTCT TTAGGGTAAA

GTCCATGATA GTGGCCCTGA TCATCGCAAA CATTTTCTTT TTAGGGTAAA

GTCCATGATA GTGGCCCTGA TCATCGCAAA CATTTTCTCT TTAGGGTAAA

GTCCATGATA GTGGCCCTGA TCATCGCAAA CATTTTCTCT TTAGGGTAAA

GTCCATGACA GTGGCCCTGA CCATCGCAAA CACTTCCTCT TTAGGGTAAA

GTCCATGACA GTGGCCCTGA CCATCGCAAA CATTTCCTCT TTAGGGTAAA

GTCCATGATA GTGGCCCTGA TCATCGCAAA CATTTTCTCT TTAGGGTAAA

GTCCATGATA GTGGCCCTGA TCATCGCAAA CATTTTCTCT TTAGGGTAAA

GTCCATGATA GTGGCCCAGA TCATCGCAAA CATTTTCTCT TTAGGGTAAA

GTCCATGATA GTGGCCCTGA TCATCGCAAA CATTTTCTCT TTAGGGTAAA

GTCCATGATA GTGGCCCTGA TCACCGCAAA CATTTTCTCT TTAGGGTAAA

GTCCATGATA GTGGCCCTGA TCATCGCAAA CATTTTCTCT TTAGGGTAAA

GTCCATGATA GTGGCCCTGA TCATCGCAAA CATTTTCTCT TTAGGGTAAA

---------- ---------- ---------- ---------- ----------

GTTCATGATA GTGGCCCTGA TCATCGCAAA CACTTTCTCT TTAGGGTAAG

GTGCACGACA GTGGCCCCGA TCACCGCAAA CATTTCCTCT TTAGGGTGAG

TATGAATTTC TATGGGTTTT AATTACTAGC ATTTAGTGAA AAATACAAAG

TATGAATTTC TATGGGTTTT AATTACTAGC ATTTAGTGAA AAATACAAAG

TATGAATTTC TATGGGTTTT AATTACTAGC ATTTAGTGAA AAATACAAAG

TATGAATTTC TATGGGTTTT AATTACTAGC ATTTAGTGAA AAATACAAAG

TATGAATTTC TATGGGTTTT AATTACTAGC ATTTAGTAAA AAATACAAAG

TATG------ TATGGGTTTT AATTACTAGC ATTTAGTGAA AAATACAAAC

TATGAACTTA TATGGGGTTT GGTTATTAGA ATTTAGTGAA AAATACAAAG

TATGAACTTA TGTGGGGTTT GATTTTTAGA ATTTAGTAAA AAATACAAAG

TATGAATTTA TATGGGTTTT AGTT-CTAGC ATTTAGTGAA AAATAAAAAG

TATGAATTTT TATGGGTTTT AGTTACTAGC ATTTAGTGAA AAATACAAAG

TATGAATTTC TGTGGGTTTT AGTTACTAGC ATTTAGTGAA AAATACAAAG

TATGAATTTC TATGGGTTTT AATTACTAGC ATTTAGTGAA AAATACAAAG

TATACGTTTT TATGGGtatt a-taattaGC ATTTAGTGAA AAATACAA--

TATGAATTTC TATGGGTTTT AGTTATTAGC ATTTAGTAAA AAATACAAAG

TATGAATTTC TATGGGTTTT AGTTGTTAGC ATTTAGTGAA AAATACAAAG

---------- ---------- ---------- ---------- ----------

CATGCATTTC TATGGGTTTT GATAACTAGA TATTAGTGAA AAATACAAAG

CATGAATTTC TGTCGGTTGT GATGACTAGA TGTTAGAGAA AAATAAAAAG

CTAATAGATA AAAGTTTGAT GCTGTTCTTa ttgtatgttt ttcttgaata

CTAATAGATA AAAGTTTGAT GCTGTTCTTa ttgtatgttt ttcttgaata

CTAATAGATA AAAGTTTGAT GCTGTTCTTa ttgtatgttt ttcttgaata

CTAATAGATA AAAGTTTGAT GCTGTTCTTa ttgtatgttt ttcttgaata

CTAATAGATA AAAGTTTGAT GCTGTTCTTa ttgtatgttt ttcttgaata

CTAATAGATA AAAGTTTGAT GCTGTTCTTa ttgtatgttt ttcttgaata

CTAATAAATA AAAGTTTGAT GCTGTTCTTa ttgtatgttt ttcttgacta

CTAATAGATA AAAGTTTGAT GCTGTTCTTa ttgtatgttt ttcttgaata

CTAATAGATA AAAGTTTGAT GCTGTTCTTa ttgtatgttt ttcttgaata

CTAATAGATA AAAGTTTGAT GCTGTTCTTa ttgtatgttt ttcttgaata

CTAATAGATA AAAGTTTGAT GCTGTTCTTa ttgtatgttt ttcttgaata

CTAATAGATA AAAGTTTGAT GCTGTTCTTa ttgtatgttt ttcttgaata

--AATAGATA AAAGTTTGAT GCTGTTCTTa ttgtatgttt ttcttgaata

CTAATAAATA AAAGTTTGAT GCTGTTCTTa ttgtatgttt ttcttgaata

CTAATAGATA AAAGTTTGAT GCTGTTCTTa ttgtatgttt ttcttgaata

---------- ---------- ---------- ---------- ----------

CTTATAGATA AAAGTTTGAT GCTGTTCTTa ttgtatgttt ttcttgaata

CTCATAGATA AAAGTTTGAT GCTGTTCTTa ttgtatgttt ttcttgaata

ggtattgaga aatggagccc ttaccagccc aattgtatgt ttttcttgaa

ggtattgaga aatggagccc ttaccagccc aattgtatgt ttttcttgaa

ggtattgaga aatggagccc ttaccagccc aattgtatgt ttttcttgaa

ggtattgaga aatggagccc ttaccagccc aattgtatgt ttttcttgaa

ggtattgaga aatggagccc ttaccagccc aattgtatgt ttttcttgaa

ggtattgaga aatggagccc ttaccagccc aattgtatgt ttttcttgaa

ggtattgaga aatggagccc ttaccagccc aattgtatgt ttttcttgaa

ggtattgaga aatggagccc ttaccagccc aattgtatgt ttttcttgaa

ggtattgaga aatggagccc ttaccagccc aattgtatgt ttttcttgaa

ggtattgaga aatggagccc ttaccagccc aattgtatgt ttttcttgaa

ggtattgaga aatggagccc ttaccagccc aattgtatgt ttttcttgaa

ggtattgaga aatggagccc ttaccagccc aattgtatgt ttttcttgaa

ggtattgaga aatggagccc ttaccagccc aattgtatgt ttttcttgaa

ggtattgaga aatggagccc ttaccagccc aattgtatgt ttttcttgaa

ggtattgaga aatggagccc ttaccagccc aattgtatgt ttttcttgaa

---------- ---------- ---------- ---------- ----------

ggtattgaga aatggagccc ttaccagccc aattgtatgt ttttcttgaa

ggtattgaga aatggagccc ttaccagccc aattgtatgt ttttcttgaa

taggtattga taaatggaag cgcttaccag cccaGCTTTG CCAGCCCTAA

taggtattga taaatggaag cgcttaccag cccaGCTTTG CCAGCCCTAA

taggtattga taaatggaag cgcttaccag cccaGCTTTG CCAGCCCTAA

taggtattga taaatggaag cgcttaccag cccaGCTTTG CCAGCCCTAA

taggtattga taaatggaag cgcttaccag cccaGCTTTG CCAGCCCTAA

taggtattga taaatggaag cgcttaccag cccaGCTTTG CCAGCCCTAA

taggtattga taaatggaag cgcttaccag cccaGCTTTG CCAGCCCTAA

taggtattga taaatggaag cgcttaccag cccaGCTTTG CCAGCCCTAA

taggtattga taaatggaag cgcttaccag cccaGCTTTG CCAGCCCTAA

taggtattga taaatggaag cgcttaccag cccaGCTTTG CCAGCCCTAA

taggtattga taaatggaag cgcttaccag cccaGCTTTG CCAGCCCTAA

taggtattga taaatggaag cgcttaccag cccaGCTTTG CCAGCCCTAA

taggtattga taaatggaag cgcttaccag cccaGCTTTG CCAGCCCTAA

taggtattga taaatggaag cgcttaccag cccaGCTTTG CCAGCCCTAA

taggtattga taaatggaag cgcttaccag cccaGCTTTG CCAGCCCTAA

---------- ---------- ---------- ---------- ----------

taggtattga taaatggaag cgcttaccag cccaGCTTTG CCAGCCCTAA

taggtattga taaatggaag cgcttaccag cccaGCTTTG CCAGCCCTAA

TAAGAAGCAT GCTAAAGCCA CAGCAGCTAC TGTGGTTCTT CAAGCAATGG

TAAGAAGCAT GCTAAAGCCA CAGCAGCTAC TGTGGTTCTT CAAGCAATGG

TAAGAAGCAT GCTAAAGCCA CAGCAGCTAC TGTGGTTCTT CAAGCAATGG

TAAGAAGCAT GCTAAAGCCA CAGCAGCTAC TGTGGTTCTT CAAGCAATGG

TAAGAAGCAT GCTAAAGCCA CAGCAGCTAC TGTGGTTCTT CAAGCAATGG

TAAGAAGCAT GCTAAAGCCA CAGCAGCTAC TGTGGTTCTT CAAGCAATGG

TAAGAAGCAT GCTAAAGCCA CAGCAGCTAC TGTGGTTCTT CAAGCAATGG

TAAGAAGCAT GCTAAAGCCA CAGCAGCAAC TGTGGTTCTT CAAGCAATGG

TAAGAAGCAT GCTAAAGCCA CAGCTGCTAC TGTGGTTCTT CAAGCAATGG

TAAGAAGCAT GCTAAAGCCA CAGCAGCTAC TGTGGTTCTT CAAGCAATGG

TAAGAAGCAT GCTAAAGCCA CAGCAGCTAC TGTGGTTCTT CAAGCAATGG

TAAGAAGCAT GCTAAAGCCA CAGCAGCAAC TGTGGTTCTT CAAGCAATGG

TAAGAAGCAT GCTAAAGCCA CAGCAGCTAC TGTGGTTCTT CAAGCAATGG

TAAGAAGCAT GCTAAAGCCA CAGCAGCTAC TGTGGTTCTT CAAGCAATGG

TAAGAAGCAT GCTAAAGCCA CAGCAGCTAC TGTGGTTCTT CAAGCGATGG

---------- ---------- ---------- ---------- ----------

TAAGAAGCAT GCTAAAGCCA CAGCAGCTAC TGTGGTTCTT CAAGCAATGG

TAAGAAGCAT GCTAAAGCCA CAGCAGCTAC TGTGGTTCTT CAAGCAATGG

GCCTTGTACC AAAGGACCTC ATGGCTAATG CCACTTGCTT CAGGAGTGCC

GCCTTGTACC AAAGGACCTC ATGGCTAATG CCACTTGCTT CAGGAGTGCC

GCCTTGTACC AAAGGACCTC ATGGCTAATG CCACTTGCTT CAGGAGTGCC

GCCTTGTACC AAAGGACCTC ATGGCTAATG CCACTTGCTT CAGGAGTGCC

GCCTTGTACC AAAGGACCTC ATGGCTAATG CCACTTGCTT CAGGAGTGCC

GCCTTGTACC AAAGGACCTC ATGGCTAATG CCACTTGCTT CAGGAGTGCC

GCCTTGTACC AAAGGACCTC ATGGCTAATG CCACTTGCTT CAGGAGTGCC

GCCTTGTACC AAAGGACCTC ATGGCTAATG CCACTTGCTT CAGGAGTGCC

GCCTTGTACC AAAGGACCTC ATGGCTAATG CCACTTGCTT CAGGAGTGCC

GCCTTGTACC AAAGGACCTC ATGGCTAATG CCACTTGCTT CAGGAGTGCC

GCCTTGTACC AAAGGACCTC ATGGCTAATG CCACTTGCTT CAGGAGTGCC

GCCTTGTACC AAAGGACCTC ATGGCTAATG CCACTTGCTT CAGGAGTGCC

GCCTTGTACC AAAGGACCTC ATGGCTAATG CCACTTGCTT CAGGAGTGCC

GCCTTGTACC AAAGGACCTC ATGGCTAATG CCACTTGCTT CAGGAGTGCC

GCCTTGTACC AAAGGACCTC ATGGCTAATG CCACTTGCTT CAGGAGTGCC

---------- ---------- ---------- ---------- ----------

GCCTTGTACC AAAGGACCTC ATGGCTAATG CCACTTGCTT CAGGAGTGCC

GCCTTGTACC AAAGGACCTC ATGGCTAATG CCACTTGCTT CAGGAGTGCC

TCACGTAGAT AGATTGAGGT TTTATAATAA TCATTTCAGA ATTTTACTCT

TCACGTAGAT AGATTGAGGT TTTATAATAA TCATTTCAGA ATTTTACTCT

TCACGTAGAT AGATTGAGGT TTTATAATAA TCATTTCAGA ATTTTACTCT

TCACGTAGAT AGATTGAGGT TTTATAATAA TCATTTCAGA ATTTTACTCT

TCACGTAGAT AGATTGAGGT TTTATAATAA TCATTTCAGA ATTTTACTCT

TCACGTAGAT AGATTGAGGT TTTATAATAA TCATTTCAGA ATTTTACTCT

TCACGTAGAT AGATTGAGGT TTTATATTAA TCATTTCAGA ATTTTACTCT

TCACGTAGAT AGATTGAGGT TTTATATTAA TCA-TTCAGA ATTTTACTCT

TCACGTAGAT AGATTGAGGT TTTATATTAA TCATTTCAGA ATTTTACTCT

TCACGTAGAT AGATTGAGGT TTTATATTAA TCATTTCAGA ATTTTACTCT

TCACGTAGAT AGATTGAGGT TTTATATTAA TCATTTCAGA ATTTTACTCT

TCACGTAGAT AGATTGAGGT TTTATAATAA TCATTTCAGA ATTTTACTCT

TCACGTAGAT AGATTGAGGT TTTATATTAA TCATTTCAGA ATTTTACTCT

TCACGTAGAT AGATTGAGGT TTTATATTAA TCATTTCAGA ATTTTACTCT

TCACGTAGAT AGATTGAGGT TTTATAGTAA TCATTTCAGA ATTTTACTCT

---------- ---------- ---------- ---------- ----------

TCACGTAGAT AGATTGAGGT TTTATATTAA TCATTTCAGA ATTTTACTCT

TCACGTAGAT AGATTGAGGT TTTATATTAA TCATTTCAGA ATTTTACTCT

GCATCACAAT GTATTTCCTC TTTAATGTTG TAAATATTTG GCAATTTAGA

GCATCACAAT GTATTTCCTC TTTAATGTTG TAAATATTTG GCAATTTAGA

GCATCACAAT GTATTTCCTC TTTAATGTTG TAAATATTTG GCAATTTAGA

GCATCACAAT GTATTTCCTC TTTAATGTTG TAAATATTTG GCAATTTAGA

GCATCACAAT GTATTTCCTC TTTAATGTTG TAAATATTTG GCAATTTAGA

GCATCACAAT GTATTTCCTC TTTAATGTTG TAAATATTTG GCAATTTAGA

GCATCACAAT GTATTTCCTC CTTAATGTTG TAAATATTTC ACAATTTAGA

GCATCACAAT GTATTTCCTC CTTAATGTTG TAAATATTTC ACAATTTAGA

GCATCACAAT GTATTTCCTC TTTAATGTTG TAAATATTTG GCAATTTAGA

GCATCAAAAT GTATTTCCTC TTTAATGTTG TAAATATTTG GCAATTTAGA

GCATCAAAAT GTATTTCCTC TTTAATGTTG TAAATATTTG GCAATTTAGA

GCATCAAAAT GTATTTCCTC TTTAATGTTG TAAATATTTG GCAATTTAGA

GCATCAAAAT GTATTTCCTC TTTAATGTTG TAAATATTTG GCAATTTAGA

GCATCAAAAT GTATTTCCTC TTTAATGTTG TAAATATTTG GCAATTTAGA

GCATCAAAAT GTATCTCCTC CTTAATGTTG TAAATATTTG GCAATTTAGA

---------- ---------- ---------- ---------- ----------

GCATCAAAAT GTATTTCTTC TTTAATGTTG TAAATATTTG GCAACTTAGA

GCGTCCCAAA ATAGTTCCTC TCGACTGTTG TAAATATTTG GCAACTTAGA

CATTGTGTAA AAAGCAATCT GTAAAAACAT CTCCAGGCTT TGATTTTGTA

CATTGTGTAA AAAGCAATCT GTAAAAACAT CTCCAGGCTT TGATTTTGTA

CATTGTGTAA AAAGCAATCT GTAAAAACAT CTCCAGGCTT TGATTTTGTA

CATTGTGTAA AAAGCAATCT GTAAAAACAT CTCCAGGCTT TGATTTTGTA

CATTGTGTAA AAAGCAATCT GTAAAAACAT CTCCAGGCTT TGATTTTGTA

CATTGTGTAA AAAGCAATCT GTACAAACAT CTCCAGGCTT TGATTTTGTA

CATTGTGTAA AAAGCAATCT GTACAAACAT CTCCAGGCTT T-GTTTTGTA

CATTGTGTAA AAAGCAATCT GTACAAACAT CTCCAGGCTT TCGTTTTGTA

CATTGTGTAA AAAGCAATCT GTACAAACAT CTCCAGGCTT TGATTTTGTA

CATTGTGTAA AAAGCAATCT GTACAAACAT CTCCAGGCTT TGATTTTGTA

CATTGTGTAA AAAGCAATCT GTACAAACAT CTCCAGGCTT TGATTTTGTA

CATTGTGTAA AAAGCAATCT GTATAAACAT CTCCAGGCTT TGATTTTGTA

CATTGTGTAA AAAGCAATCT GTACAAACAT CTCCAGGCTT TGATTTTGTA

CATTGTGTAA AAAGCAAGCT GTACAAACAT CTCCAGGCTT TGATTTTGTA

CACTGTGTAA AAAGCAATCT GTACAAACAT CTCCAGGCTT TGATTTTGTA

---------- ---------- ---------- ---------- ----------

CATTGTGTAA AAAGCAATCT GTACAAACAT CTCCAAGTTT TGGTTTTATA

CATCGTGTAA AAAGCAACCT GTACAAACAT CTCCCGGCTT TGGTTTTGTA

CCATGGAAAT TGTATTTAAC CATACAGGGT TTTGGTATGT TTATATTGTT

CCATGGAAAT TGTATTTAAC CATACAGGGT TTTGGTATGT TTATATTGTT

CCATGGAAAT TGTATTTAAC CATACAGGGT TTTGGTATGT TTATATTGTT

CCATGGAAAT TGTATTTAAC CATACAGGGT TTTGGTATGT TTATATTGTT

CCATGGAAAT TGTATTTAAC CATACAGGGT TTTGGTATGT TTATATTGTT

CCATGGAAAT TGTATTTAAC CATACAGGGT TTTGGTATGT TTATATTGTT

CCATGGAAAT TGTATTTAAC CACACAGGGT TTTGGTATGT TTATATTGTT

CCATGGAAAT TGTATTTAAC CATACAGGGT TTTGGTATGT TTATATTGTT

CCATGGAAAT TGTATTTAAC CATACAGGGT TTTGGTATGT TTATATTGTT

CCATGGAAAT TGTATTTAAC CATACAGGGT TTTGGTATGT TTATATTGTT

CCATGGAAAT TGTATTTAAC CATACAGGGT TTTGGTATGT TTATATTGTT

CCATGGAAAT TGTATTTAAC CATACAGGGT TTTGGTATGT TTATATTGTT

CCATGGAAAT TGTATTTAAC CATACAGGGT TTTGGTATGT TTATATTGTT

CCATGGAAAT TGTATTTAAC CATACAGGGT TTTGGTATGT TTATATTGTT

CCATGGAAAT TGTATTTAAC CATACAGGGT TTTGGTATGT TTATATTGTT

---------- ---------- ---------- ---------- ----------

CCATGAAAAT TGTATTTAAC CATACAGGGT TTTGGTATGT TTATATTGTT

CCATGGAAAA TGTATTTAAC CATACAGGGT TTTGGTATGT TTATATCGTT

TACCTTAGTG ATGTATTTGT TTAAGTGGCT AACATCCAAA CGACTGTTTG

TACCTTAGTG ATGTATTTGT TTAAGTGGCT AACATCCAAA CGACTGTTTG

TACCTTAGTG ATGTATTTGT TTAAGTGGCT AACATCCAAA CGATTGTTTG

TACCTTAGTG ATGTATTTGT TTAAGTGGCT AACATCCAAA CGATTGTTTG

TACCTTAGTG ATGTATCTGT TTAAGTGGCT AACATCCAAA CGATTGTTTG

TACCTTAGTG ATGTATTTGT TTAAGTGGCT AACATCCAAA CGATTGTTTG

TACCTTAGTG ATGTATTTGT TTAAGTGGCT AACATCAAAA CAATTGTTTG

TACCTTAGTG ATGTATTTGT TTAAGTGGCT AACATCCAAA CAATTGTTTG

TACCTTAGTG ATGTATTTGT TTAAGTGGCT AACATCCAAA CAGTTGTTTG

TACCTTAGTG ATGTATTTGT TTAAGTGGCT AACATCCAAA CGATTGTTCG

TACCTTAGTG ATGTATTTGT TTAAGTGGCT AACATCCAAA CGATTGTTTG

TACCTTAGTG ATGTATTTGT TTAAGTGGCT AACATCCAAA CGATTGTTTG

TACCTTAGTG ATGTATTTGT TTAAGTGGCT AACATCCAAA CAATTGTTTG

TACCTTAGTG ATGTATTTGT TTAAGTGGCT AACATCCAAA CGATTGTTTG

TACCTTAGTG ATGTATTTGT TTAAGTGGCT AACATCCAAA CGATTGTTTG

---------- ---------- ---------- ---------- ----------

TACCTTAGTG ATGTATTTGT TTAAGTGGCT AACATCGAAA CAATTGTTTG

TACCTTAGTG ACGTATCTGT TTAAGTGGCT AACGTCGAAA CGACCGTTCG

AAGGCATCAG TAATCTTCAG TGTGGAATGT TAAATAACGC TTTTATACTG

AAGGCATCAG TAATCTTCAG TGTGGAATGT TAAATAACGC TTTTATACTG

AAGGCATCAG TAATCTTCAG TGTGGAATGT TAAATAACGC TTTTATACTG

AAGGCATCAG TAATCTTCAG TGTGGAATGT TAAATAACGC TTTTATACTG

AAGGCATCAG TAATCTTCAG TGTGGAATGT TAAATAACGC TTTTATACTG

AAGGCATCAG TAATCTTCAG TGTGGAATGT TGAATAACGC TTTTATACTG

AGGGCATCAG TAATATTCAG TGTGGAATGT TAAATAACGC TTTTATACTG

AGGGCATCAG TAATATTCAG TGTGGAATGT TAAATAACGC TTTTATACTG

AAGGCATCAG TAATCTTCAG TGTGGAATGT TAAATAACGC TTTTATACTG

AAGGCATCCG TAATCTTCAG TGTGGAATGT TAAATAACGC TTTTATACTG

AAGGCATCCG TAATCTTCAG TGTGGAATGT TAAATAACGC TTTTATACTG

AAGGCATCCG TAATCTTCAG TGTGGAATGT TAAATAACGC TTTTATACTG

AAGGCATCCG TAATCTTCAG TGTGGAATGT TAAATAACGC TTTTATACTG

AAGGCATCAG TAATCTTCAG TGTGGAATGT TAAATAACGC TTTTATACTG

AAGGCATCAG TAATCTTCAG TGTGGAATGT GAAATAATGC TTTTATATTG

---------- ---------- ---------- ---------- ----------

AAGGCATCAG TAATCCTAAG TGTGGAATGT TAAATAACGC TTTTATACTG

AAGGCATCAG TAATCTTCGG TGTGGAATGT TAAATCCCGC TTTTATACTG

TATTTTGTAC TATGATGTAA CTCCCTCCTT ATGGCTAGGC TACTGTAACA

TATTTTGTAC TATGATGTAA CTCCCTCCTT ATGGCTAGGC TACTGTAACA

TATTTTGTAC TATGATGTAA CTCCCTCCTT ATGGCTAGGC TACTGTAACA

TATTTTGTAC TATGATGTAA CTCCCTCCTT ATGGCTAGGC TACTGTAACA

TATTTTGTAC TATGATGTAA CTCCCTCCTT ATGGCTAGGC TGCTGTAACA

TATTTTGTAC TATGATGTAA CTTCCTCCTT ATGGCTAGGC TGCTGTAACA

TATTTTGTAC TATGATGTAA CTTCCTCCAT ACGGCTAGGC TGCTGTAACA

TATTTTGTAC TATGATGTAA CTTCCTCCTT ACGGCTAGGC TGCTGTAACA

TATTTTGTAC TATGATGTAA CTCCCTCC-T ATGGCTAGGC TGCTGTAACA

TATTTTGTAC TATGATGTAA CTCCCTCCTT ATGGCTCGGC TGCTGTAACA

TATTTTGTAC TATGATGTAA CCTCCTCCTT ATGGCTAGGC TGCTGTAACA

TATTTTGTAC TATGATGTAA CTCCCTCCTT ATGGCTAGGC TGCTGTAACA

TATTTTGTAC TATGATGTAA CTCCCTCCTC -TGGCTAGGC TGCTGTAACA

TATTTTGTAC TATGATGTAA CTCCCTCCTT ATGGCTAGGC TGCTGTAACA

TCTTTTGTAC TATGCTGTAA CTCCCTCCCT GTGGCTAGGC TGCTGTAACA

---------- ---------- ---------- ---------- ----------

TATTTTGTAC TATGATGTAA TTCTTCCCTT ATGGCTAGGC TGCTGTAACA

TATTTTGTAC TACGACGTGA CTCCCCCCTC GCGGCCAGGC CGCTCTAACG

CTTGCACAGA TAATGGGCCA GTGTTAGTCC TGAAGCCACT TGGGTCATAA

CTTGCACAGA TAATGGGCCA GTGTTAGTCC TGAAGCCACT TGGGCCATAA

CTTGCACAGA TACTGGGCCA GTGTTAGTCC TGAAGCCACT TGGGTCATAA

CTTGCACAGA TACTGGGCCA GTGTTAGTCC TGAAGCCACT TGGGTCATAA

CTTGCACAGA TATTGGGCCA GTGTTAGTCC TGAAGCCACT TGGGTCATAA

CTTGCACAGA TACTGGGCCA GTGTTAGTCC TGAAGCCACT TGGGTCA---

CTTGCACAGA TACTGGGCCA GTGTTAGTCC TGAAGCCACT TGGGTCATAA

CTTGCACAGA TACTGGGCCA GTGTTAGTCC TGAAGCCACT TGGGTCATAA

CTTGCACAGA TACTGGGCCA GTGTTAGTCC TGAAGCCACT TGGGTCATAA

CTTGCACAGA TACTGGGCCA GTGTTAGTCC TGGAGCCACT TGGGCCATGA

CTTGCACAGA TACTGGGCCA GTGTTAGTCC TGAAGCCACT TGGGCCATGA

CTTGCACAGA TACTGGGCCA GTGTTAGGCC TGAAGCCACT TGGGCCATGA

CTTGCACAGA TACTGGGCCA GTGTTAGTCC TGAAGCCACT TGGGCCATGA

CTTGCACAGA TACTGGGCCA GTGTTAGTCC TGAAGCCATT TGGGCCATAA

CTTGCACAGA CTCTGGGCCA GTGTTAGTCC TGAAGCCACT TGGGCCATAA

---------- ---------- ---------- ---------- ----------

CTTGCACAGA TATTGGGCCA GTGTTAGTCC TAAAGCCACT GATGCCATAA

CTCGCACGGA GGCCGGGCCG GCGTCGGTCC CGGAGCCACC AACGCCGGGA

GAAGGGAGTA AAAATGAAGT CTGACTAGAA TTATTGCAGA GGCCAGTACA

GAAGGGAGTA AAAATGAAGT CTGCCTAGAA TTATTGCAGA GGCCAGTACA

GAAGGGAGTA AAAATGAAGT CTGACTAGAA TTATTGCAGA GGCCAGTACA

GAAGGGAGTA AAAATGAAGT CTGACTAGAA TTATTGCAGA GGCCAGTACA

GAAGGGAGTC AAAATGAAGT CTGACTAGAA TTATTGCAGA GGCCAGTACA

-AAGGGAGTC -AAATGAAGT CTGACTAGAA TTACTGCAGA AGCCAGTACA

GAAAGAAGTC AAAAGGAAGT CCAACTAGAA TTACTTCAGA GGCCAGTACA

GAAGGAAGTC AAAAGGAAGT CCA------- --ACTTCAGA GGCCAGTACA

GAGTGGA--- ----GGAAGT CTGACTAGAA T-TCTGCAGA GGCCAGTATG

GAAGGGAGTC AAAATGAAGT CTGACTAGAA TTACTGCAGA GGCCAGTACA

GAAGGGAGTC AAAATGAAGT CTGATTAGAA TTACTGCAGA GGCCAGTACA

GAAGGGAGTC AGAATGAAGT CCGACTAGAA TT-CTGCAGA GGCCAGTACA

GAAGGGAGTT AAAATGAAGT CCTATTAGAA TT-CTGCAGA GGCCAGTACA

GAAGGGAGTC AAAATGAAGT CTGACTAGAA TTACTGCAGA GGCCAGTACA

GAAGGGAGTC AAAATGAAGT CTGAGTAGGA TTTCTGCAGA GGCCAGTCCA

---------- ---------- ---------- ---------- ----------

GAAGGAAGTC CAAATGAAGA CCCACTAGAA TTAATGCAGA CGCCAGAAAA

GGAGGAGATC CGAAGGAAGA TCGACTAGAA TCCCCGCGGA AGCCAGCCGA

TAGTATGGCA TTGAGTTGTG ATATAGTTTT ACTTTGATGT GCATTTTGAA

TAGTATGGCA TTGAGTTGTG ACATAGTTTT ACTTTGATGT GCATTTTGAA

TAGTATGGCA TTGAGTTGTG ATATAGTTTT ACTTTGATGT GCATTTTGAA

TAGTATGGCA TTGAGTTGTG ATATAGTTTT ACTTTGATGT GCATTTTGAA

TAGTATGGCA TTGAGTTGTG ATATAGTTTT ACTTTGATGT GCATTTTGAA

TAGTATGGCA TTGAGTTGTG ATATAGTTTT ACTTTGATGT GCATTTTGAA

TAGTATGGC- TTGAGTTGTG ATATAGTTTT ACTTTGATGT GCATTTTGAA

TAGTATGGC- TTCAGTTGTG ATATAGTTTT ACTTTGATGT GCATTTTGAA

TAGTATGGCA TTGAGTTGTG ATATAGTTTT ACTTTGATGT GCATTTTGAA

TAGTATGGCA TTGAGTTGTG ATATAGTTTT ACTTTGATGT GCATTTTGAA

TAGTATGGCA TTGAGTTGTG ATATAGTTTT ACTTTGATGT GCATTTTGAA

TAGTATGGCA TTGAGTTGTG ATATAGTTTT ACTTTGATGT GCATTTTGAA

TAGTATGGCA TTGAGTTGTG ATATAGTTTT ACTTTGATGT GCATTTTGAA

TAGTATGGCA TTGAGTTGTG ATATAGTTTT ACTTTGATGT GCATTTCGAA

TAGTATGGCA TTGAGTTGTG ACATAGTTTT ACTTTGATGT GCATTCTGAA

---------- ---------- ---------- ---------- ----------

TGGAATGGCA TTGAGTTGTG ATGTAGTTTT ACTTTAATGT GCATTTTGAA

TAGTATGGCG TTGAGTTGTG ATGTAGTTTT ACTTTCGTGT GCATTTTGAA

TTTCAGCTAC ACCTAGATAG ACGTAAAATA ATTAAAATGC TGTAACCAAC

TTTCAGCTAC ACCTAGATAG ACGTAAAATA ATTAAAATGC TGTAACCAAC

TTTCAGCTAC ACCTAGATAG ACGTAAAATA ATTAAAATGC TGTAACCAAC

TTTCAGCTAC ACCTAGATAG ACGTAAAATA ATTAAAATGC TGTAACCAAC

TTTCAGCTAC ACCTAGATAG ACGTAAAATA ATTAAAATGC TGTAACCAAC

TTTCAGCTAC ACCTAGATAG ACGTAAAATA ATTAAAATGC TGTAACCAAC

TTTCAGCTAC ACCTAGATAG ACGTAA-ATA ATAAAAATGC TGTAACCAAC

TTTCAGCTAC ACCTAGATAG ACGTA----A ATAAAAATGC TGTAACCGAC

TTTCAGCTAC ACCTAGATAG ATGTAAAATA ATTACAATGC TGTAACCAAC

TTTCAGCTAC ATCTAGATAG ACGTAAAATA ATTAAAATGC TGTAACCAAC

TTTCAGCTAC ACCTAGATAG ACGTTAAATA ATTAAAATGC TGTAACCAAC

TTTCAGCTAC ACCTAGATAG ACGTAAAATA ATTAAAACGC TGTAACCAAC

TTTCAGCTAC ATCTAGATAG ACGTAAAATA ATTAAAATGC TGTAACCAAC

TTTCAGCTAC ACCTAGATAG ACTTAAAATA ATTAAAATGC TGTAACCAAC

TTTCAGCTAC ACCTAGGTAG ATTAAAAATA ATTAAAATGC TGTAACCAAC

---------- ---------- ---------- ---------- ----------

TTTCAGCTAC AATTAGATAG ACTCAAGATA ATGCAAATGC TGTAACCAAC

TTGCAGCTAC AACTAGATAG ACTCGAAATA CTTTAAACGC TGTAACCGAC

TTTAATAAAA TTGGCAACCA GCCACTATTT TGTTGACTAT GAGAAAAAGT

TTTAATAAAA TTGGCAACCA GCCACTATTT TGTTGACTAT GAGAAAAAGT

TTTAATAAAA TTGGCAACCA GCCACTATTT TGTTGACTAT GAGAAAAAGT

TTTAATAAAA TTGGCAACCA GCCACTATTT TGTTGACTAT GAGAAAAAGT

TTTAATAAAA TTGGCAACCA GCCACTATTT TGTTGACTCT GAGAAAAAGT

TTTAATAAAA TTGGCAACCA GCCACTATTT TGTTGACTAT GAG--AAAAT

TTTAATAAAA TTGGCAACCA GCCGCTATTT TGTTGAC--T GAGAATAAGT

TTTAATAAAA TTGGCACCCA GCCGCTATTT TGTTGAC--T GAGG--AAGT

TTTAATAAAA TTGGCAACCA GCCACTATTT TGTTGACTAT GAGA--AAGT

TTTAATAAAA TCGGCACCCA GCCACTATTT TGTTGACTAT GAGAATAAGT

TTTAATAAAA TCGGCACCCA GCCACTATTT TGTTGACTAT GAGAATAAGT

TTTAATAAAA TCGGCACCCG GCCACTATTT TGTTGACTAT GAGAATAAGT

TTTAATAAAA TTGGCACCCA GCCACTATTT TGTTGACTAT GAGAATAAGT

TTTAATAAAA TCGGCACCCA GCCACTATTT TGTTGACTAT GAGAATAAGT

TTTAATAAAA TCGGCACCCA GCCACTATTT TGTTGACTAT GAGAATAAGT

---------- ---------- ---------- ---------- ----------

TTTAATAAAA TCAGCACCAG GCCACTATTT TGTTGACTGC AAAAATAAGT

Ctaaataaaa tCAGCACCAG GCCACTATTC TGTCGACTAT GCGGATAAGT

TTATGTTTTA GGGTCTATAG AATATTTCAT GTGTATTACA GTGGTATTCA

TTATGTTTTA GGGTCCATAG AATATTTCAT GTGTATTACA GTGGTATTCA

TTATGTTTTA GGGTCCATAG AATATTTCAT GTGTATTACA GTGGTATTCA

TTATGTTTTA GGGTCCATAG AATATTTCAT GTGTATTACA GTGGTATTCA

TTATGTTTTA GGGTCCATAG AATATTTCAT GTGTATTACG GTGGTATTCA

TTATGTTTTA GGGTCTATAG AATATTTCAT GTGTATTACA GTGGTATTCA

TTATGTTTTA GGGTCTATAG AATA-TTCAT GTGTATTACA GTGGTATTCA

TTATGTTTTA GGGTCTATAG AATA-TTCAT GTGTATTACA GTGGTATTCA

TTATGTTTTA GGGTCTATAG AGTATTTCAT GTGTATTACA GTGGTATTCA

TTATGTTTTA GGGTCTATAG AATATTTCAT GTGTATTACA GTGGTATTCA

TTATGTTTTA GGGTCTATAG AATATTTCAT GTGTATTACA GTGGTATTCA

TTATGTTTTA GGGTCTATAG ACTATTTCAT GTGTATTACA GTGGTATTCA

TTCTGTTTTA GGGTCTATAG AATATTTCAT GTGTATTACA GTGGTATTCA

TCATGTTTTA GGGTCTATAG AATATTTCAT GTGTATTACA GTGGTATTCA

TCATGTTTTA GGGTCTCTAG ACTATTTCAT GTGTATTACA GTGGTATTCA

---------- ---------- ---------- ---------- ----------

TCATGTTTTA GGGTCTATAG AATATTTCAT GTGCATTATA GTGGTACTCA

TCGTGTTTTA GGGTCTATAG AATATTTCAT GTGCATTATA GTGGTACTCA

TATGCTGTCT CTAAACTTTA TTTTCAAAAG CTTAAGGCCC AAATACAAAC

TATGCTGTCT CTAAACTTTA TTTTCAAAAG CTTAAGGCCC AAATACAAAC

TATGCTGTCT CTAAACTTTA TTTTCAAAAG CTTAAGGCCC AAATACAAAC

TATGCTGTCT CTAAACTTTA TTTTCAAAAG CTTAAGGCCC AAATACAAAC

TATGCTGTCT CTAAACTTTA TTTTCAAAAG CTTAAGGCCC AAATACAAAC

TATGCTGTCT CTAAGCTTTA TTTTCAAAAG TTTAAGGCCC AAATACAAAC

TATGCTGTCT CTAAACTTTA TTTTCAAAAG CTTAAGGCCC AAATACAAAC

TATGCTGTCT CTAAACTTTA TTTTCAAAAG CTTAAGGCCC AAATACAAAC

TATGCTGTCT CTAAACTTTA TTTTCAAAAG CTTAAGGCCC AAATACAAAC

TATGCTGTCT CTAAACTTTA TTTTCAAAAG CTTAAGGCCC AAATATAAAC

TATGCTGTCT CTAAACTTTA TTTTCAAAAG CTTAAGGCCC AAATATAAAC

TATGCTGTCT C--AACTTTA TTTTCAAAAG CTTAAGGCCC AAATATAAAC

TATGCTGTCT CTAAACTTTA TTTTCAAAAG CTTAAGGCCC AAATATAAAC

TATGCTGTCT CTAAACTTTA TTTTCAAAAG CTTAAGGCCC AAATACAAAC

TATGCTGTCT CTAAACTTTA TTTTCAAAAG CTTAAGGCCC AAATACAAAC

---------- ---------- ---------- ---------- ----------

TATGCTGTCT TTAAACTTTA TTTTAAAATG CTTAAGGCCC AAATACAAAC

TATGCTGTCT TTCAACTTTG TTTTAAAAAG CTTAAGGCCC AAATACAGAC

TTCTGGAATA AACGTGGTGT TTTATTTTCT GAACTTCAAG AATCTTTCAG

TTCTGGAATA AACGTGGTGT TTTATTTTCT GAACTTCAAG AATCTTTCAG

TTCTGGAATA AACGTGGTGT TTTATTTTCT GAACTTCAAG AATCTTTCAG

TTCTGGAATA AACGTGGTGT TTTATTTTCT GAACTTCAAG AATCTTTCAG

TTCTGGAATA AATGTGGTGT TTTATTTTCT GAACTTCAGG AACCTTTCAG

TTCTGGAATA AATGCGTTGT TTTATTTTCT GAACTTCAAA AATCTTTCAG

TTCTGGAATA AATGTGGTGT CTTATTCTCT GAAAGCCAGA GATCTTTCAG

TTCTGGAATA AATGTTGTGT CTTATTCTCT GAAAGCCAGG GATCTTTCAG

TTCTGGAACA AACGTGGTGG TTTATTTTCT GAAGGCCAAA GATCTTTCAG

TTCTGGAATA AATGTGGTGT TTTATTTTCT GAAAGCCAAA TATCTTTCAG

TTCTGGAATA AATGTGGTGT TTTATTTTCT GAGAGCCAAA GATCTTTCAG

TTCTGGAATA AATGTTGTGT TTTATTTTCT GAAAGCCAAA GATCTTTCAG

TTCTGGAATA AATGTGGTGT TTTATTTTCT GATAGCCAAA GATCTTTCAG

TTCTGGAATA AATGTGGTGT TTTATTTTCT GAAAGCCAAA GATCTTTCAT

TTCTGGAATA AATGTGGTGT TTTATTTTCT GAAAGCCACT CATCTTTCAG

---------- ---------- ---------- ---------- ----------

TTCTGGAATA AATGTGGTGT TTTATTCTTT CAATGTTGAG AATCTTTCAA

TTCTGGAATA AGCGTGGTGT TTTATTGCGT GAATAAAGGA GATGTTTCAG

GATCTCCAAT TATAAATGTA GTCTCTCAGC ACCATTAAAT ACAGCAGTTG

GATCTCCAAT TATAAATGTA GTCTCTCAGC ACCATTAAAT ACAGCAGTTG

GATCTCCAAT TATAAATGTA GTCTCTCAGC ACCATTAAAT ACAGCAGTTG

GATCTCCAAT TATAAATGTA GTCTCTCAGC ACCATTAAAT ACAGCAGTTG

GATCTCCAGT TATAAATGTA GTCTCTCATC ACCATTAAAT ACAGCAGTTG

GATGTCCAAT TATAAACGTA GTCTCTCATT TCAATTAAAT ACAGCAGTTG

GATCTCCAAT TTACAATGTA GTCACTCATT TCCATTGAAT ACGGCAGTTG

GATCTCCAAC TTAAAATGTA GTCACTCATT TCCATTGAAA ACTGCAGTTG

GATCTCCAAT TATAAACGTA GTCACTCATT TCCATTAAAT ACAGTAGTTG

GATCTCCAAT TATAAATGTA GTCACTCATT TCCATTAAAT ACAGCAGTTG

GATCTCCAAT TATAAACGTA GTCATTCATT TCCATTAAAT ACAGCAGTTG

GATCTCCAAT TATAAATGTA GCCACTCATT TCCATTAAAT ACAACAGTTG

GACCTCCAAT TATAGATGTA GTCATTCATT TCCATTGAAC ACAGCAGTTG

GATCTCCAAT TATAAATGTA GTCACTCATT TCCATTAAAT ACAGCAGTTG

GATCTCCAAT TATAAGTGTA GTCACTCATT TCGATTAAAT ACAGCAGTTG

---------- ---------- ---------- ---------- ----------

GACCTCCAAT TACAAATGTA ATTATCCATT TCCATTAAAT ACAGATGTTG

GCTCTCCAGC TGTAGTTGTA ATTGTCCACT TGGATTAAAT ACGTTGGTCG

GCTCGTGTGG ATACAGTACT GCAGAGAAAG ATCCACTCTG TGAAGATTTG

GCTCGTGTGG ATACAGTACT GCAGAGAAAG ATCCACTCTG TGAAGATTTG

GCTCGTGTGG ATATAGTACT GCAGAGAAAG ACCCACTCTG TGAAGATTTG

GCTCGTGTGG ATATAGTACT GCAGAGAAAG ACCCACTCTG TGAAGATTTG

GTTCATGTGG ATATAGTAGT GCAGAGAAAG ACCCGCTCTG TGAAGATTTG

GTTCATGTGG ATACAGTCCG ACACAGAAAG CCCCACTCTG TGAAGATCTC

GCTCATGTGC ATACAATCCC GCAGAGAATG AGCCTCTCTG TGAAGATCGG

GCTCGTGTGC ATACAGTCCT GCAGAGAAGG ACCCTCTCTG TGAAGATCGG

GCTCATGTGG ATAGAGTCCT GCCGAGAAGG ACCCGCTTTG TGAAGACTGG

GCTCATGTGT ATACAGTCCT GCAGAAAACG ACCCATTCTG TGAAGACTGG

GCTCATGTGT ATATAGTCCA GCAGAAAATG ACCCATTCTG TGAAGATCTG

GCTCATGGGT ATACAGCCCT GCAGAAAATG ACCCACTCTG TGAAGATTTG

GCTCATGTGT ATACAGTCCT GCAGTAAATG ACCCACTCTG TGCAGATCTG

GCTCATGAGT ATACAATCCT GAAGAAAATG ATCCACTCTG CGAAGATCTG

GCTCATGTGT ATACAGTCCT GCTGAAAAAG ACCCAGCCTG TGAAGATTTG

---------- ---------- ---------- ---------- ----------

GTTCATGAGT ATATAATCTT GCCGAAAAAG ATCCTTTTTG TGCAGATTTG

GTTCATGAGA ATACAGTCCG GCAGAGAAAG AGCCCTTCTG TGCAGACTTG

AGCAGCATGG TCAGTGAATG CAGAGAATGA GGCATGATAG GACCTGTAAT

AGCAGCATGG TCAGTGAATG CAGAGAATGA GGCATGATAG GACCTGTAAT

AGCAGCATGG TCAGTGAATG CAGAGAATGA GGCATGATAG GACCTGTAAT

AGCAGCATGG TCAGTGAATG CAGAGAATGA GGCATGATAG GACCTGTAAT

AGCAGCATGG TCAGTGAGTG CAGGCAATGA GGCATGATAG GACCTGTAAT

AGTAGCATGG TTACAGAATG TAGAGAATGG GGCATGACAG GACCTGTGAT

AGTAGCATGG CCACAGAATG CAAAGCGTGA GGCATGACAG GACCTGTGAT

AGTAGCATGC TCATAGAATG CAATGCATGA GGCATGACAG GCCCTGTGAT

AGCAGCATGG TGACGGAATG CACACAATGA GGCAGGATAG GACCTGTAAT

AGTAGCATCG TCACAGAGTG TAGAGAATGA GGCATAATGG GGCCTGTAAT

AGTAGCATGG TTACTGAATG TAGAGAATGA GGCATGATAG GACCCGTAAT

AGTAGCGTGG TCATAGAATA TAGACAATGA GGCATGATAG GTCCTGTAAT

AGAAGCATCA TCACAGAATG GAGACAGTGA GGCAGGACAG GACCGGTGAT

AGTAGTGTTG TCACAGAATG TAGAGAATGA GGCATGACAG GCCCTGTAAT

AGTAGCATGG TCACAGAATG GAGAGAATGT GGCATGACAG GACCTGTAAT

---------- ---------- ---------- ---------- ----------

AGCAGCATAG TCAATGAGTG CAGAGAATGA GGCATGACAG GGCCTGTAAT

AGTAGCATAG TCAGTGAATG CAGAGAGTGA GGCATGATGG GGCCTGTAAT

CTCCAAACTA AATTGGTCTC TGTATCCAGA AAGAGCTTGT GTCTTCACAT

CTCCAAACTA AATTGGTCTC TGTATCCAGA AAGAGCTTGT GTCTTCACAT

CTCCAAACTA AACTGGTCTC TGTATCCAGA AAGAGCTTGT GTCTTCACAT

CTCCAAACTA AACTGGTCTC TGTATCCAGA AAGAGCTTGT GTCTTCACAT

CTCCAAACTA AATTGGTCTC TGTATCCAGA AAGAGCTTGT GTCTTCACAT

TTCCAAACTA AACTGAGCTT TATATCCAGA AAGAGCTTGT GTCTTCACAT

CTCCAAACTA AACTTATCTC TGTATCCAAA AAGAGCTTGT GTCTTTACAT

ATCCAAACTA AACTTATCTC TGTATCCAGA AAGAGCTTGT GTCTTTACAT

CTCCAAACTA AACTGATCTT TGTATCCAGA AGGAGCTTGG CTCTTCACAT

CTCCAAGCTA AACTGATTTC TGTATCCAGA AAGAGCTTGT GTCTTCACAT

CTCCAAACTA AATTGATCTT TGTATCCAGA AACAGCTTGT GTCTTCACAT

CTCCAAACTA AATTGATCTT TGTATTCAGA AAGACCTTGT GCCTTCACAT

CTCCAAACTA AACTGATCTT TATAACCAGC GTGGGCATGT GCCTTCACAT

CTCCAAACTA AATTGATCCT TGTATCCAGA ATGAACTTGT GTCTTCACAT

CTCCAAACTA AATTGATCTT TGTATCCAGA AAGAGCTTGC GTCTTCACAT

---------- ---------- ---------- ---------- ----------

CTCCAAACTA AACTGATCTT TATAACCAGA ATAAACTGGT GTCTTCACAT

CTCCAAACTA AATTGATCTT TAAAACCAGC ATCTACTTGT CTTTTCACAT

TCACACTCCG TGCCTTTTAT TACCTTAAGC ATTTGCATTG TGGCACCTCG

TTACACTCCG TGCCTTTTAT TACCTTAAGC ATTTGCATTG TGGCACCTCG

TTACACTTCG TGCCTTTTAT TACCTTAAGT ATTTGCATTG TGGCACCTCG

TTACACTTCG TGCCTTTTAT TACCTTAAGT ATTTGCATTG TGGCACCTCG

TTACACTTCG TGCCTTTTAT TACCTTAAGC ATTTGCATTG TGGCACCTCG

TTACACTTCG TGCCTTTTGT TACCTTAAGC ATTTGCATTG TGGCACCTCG

TAATGCTTCG TGCCTACACT TACCTTAAGC ATTTGCATTG AGGCACCTCG

TACTGCTCCG TGCCTACACT TACCTTAAGC ATTTGCATTG AGGCACCTCG

TCACACTTCG TGCCTTAAAT TACCTTAAGC ATTTGCATCG TGGCACCTCG

TCACACTTCG AGCCTTTTAT TACCTTAAGC ATTTGCATTG TGGCACCTCG

TTACACTTCT TGCCTTTCAT TACCTTTAGC ATTTGCATTG TGGCACCTCG

TTATACTTCG TGCCTTTTAT TACCTTAAGC ATTTGCATTG TGGCTCCTCG

TCACACTTCG TGCCT---GT TACCTTTAGC ATTTGCATTG TGGCACCTCG

TTATACTTCG TGCCTTTTAT TACCTTAAGC ATTTGCATTG TGGCACCTCG

TGACACTTCG TGCCTTTTAT TACCTTAAGC ATTTGCATTG TGGCACCTCT

---------- ---------- ---------- ---------- ----------

TCACCGTTCG TGCCTTTTTT TACCTTGAGC ATTTGCATGG TGGCACCCCG

TTATAGTTCG AGCCTTCTTT CACCTTGAGC ATCTGCATCG TGGCTCCTCG

GAAAGCAACA GGGGACAAGA GGGTTGGAGG AAGTCCTGCC TGTGGACCTG

GAAAGCAACA GGGGACAAGA GGGTTGGAGG AAGTCCTGCC TGTGGACCTG

GAAAGCAACA GGGGACAAGA GGGTTGGAGG AAGTCCTGCC TGTGGACCTG

GAAAGCAACA GGGGACAAGA GGGTTGGAGG AAGTCCTGCC TGTGGACCTG

GAAAGCAACA GGGGATAAGA GGGTTGGAGG AAGGCCTGCC TGTGGACCTG

GAAAGCCACA GGGGCTAAGA TGGTTGGTGG AAGTCCTGCC TGTGGACCTG

AAAGGCAACT GGGGATAAGA GAGTGGGTGG GAGACCTGCC TGTGCGCCTG

GAAGGCTATT GGGGATAAGA GAGTGGGTGG GAGACCTGCC TGTGCACCTG

GAAAGCTATA GGGGACAGCA GGGTTGGTGG AAGTCCTGCC TGCGGGCCTG

GAAAGCTACT GGGGATAAGA GGGTTGGTGG AAGTCCTGCC TGTGGACCTG

GAAAGCTACA GGTGATAAGA GGGTTGGTGG AAGTCCTGCC TGCGGACCTG

GAAAGCTACT GGGGATAAGA GGGTTGGTGG AAGTCCTGCC TGTGGACCTG

AAAGGCTACA GGGGATAAGA GGGTTGGTGG CAGTCCTGCC TGAGGACCAG

AAAAGCTATA GGGGATAACA ATGTTGGTGG AAGTCCTGCC TGTGGACCAG

GAAAGCTACA GGGGATAACA GGGTGGGTGG AAGTCCTGCT TGTGGACCTG

---------- ---------- ---------- ---------- ----------

GAATGCTGCT GGAGACAATA GTGTTGGGGG GAGGCCTGCC TGAGGCCCCG

GAAAGCAGTA GGGGACAGCA GGGTTGGTGG GAGTCCTGCC TGAGGACCCG

AGGTAGCAAC TAAACTCTTA GAGTTAATCA AAAAATTGAG CAATGTAAAG

AGGTAGCAAC TAAACTCTTA GAGTTAATCA AAAAATTGAG CAATGTAAAG

AGGTAGCAAC TAAACTCTTA CAGTTAATCA AAAAATTGAG CAATGTAAAG

AGGTAGCAAC TAAACTCTTA CAGTTAATCA AAAAATTGAG CAATGTAAAG

AGGTAGCAAC TAAACTCTTA GAGTTGATCA AAAAATTGAG CAATGTAAAG

AGGTAGCAAC TAAACTCTTA CAATTCATCA AAAAATTGAG CAACGTGAAG

AGGTAGCAAC TAAACTCTTG CAATTGATCA AAAAGTTGAG CAGTTTGAAG

AGGTAGCAAC TAAACTCTTG CAGTTGATCA AAAAATTGAG CAGTTTGAAG

AGGTTGCAAC TAAACTCTTG CAGTTGATCA AAAAATTGAG TAACGTAAAA

AGGTAGCAAT TAAACTCTTA CAGTTGATCA AAAAATTTAG CAGTGTAAAG

AACTAGCAAT TAAACTCTTA GAGTTGATCA AGAAATTCAG CAATGTAAGG

AAGTAGCAAT TAAACTCTTA CAGTTGATCA AAAAATTGAG CAATGTAAAG

AGCTAGCAAC TAAACTCTTA CAGTTGATCA AAAAATTCAG CAACATCAAA

AGGTAGCAAC TAAACTTTTA CAGTTGATCA AGAAATTGAG CAAGGTAAAG

AGGTGGCAAC TAAACTTTTA CAGTTGATCA AAAAATTGAG CAACGTAAAG

---------- ---------- ---------- ---------- ----------

ACGTAGCAAC TACACTCTTG CAATTTATCA GGAAGTTCAG CAGGGTAAAG

CAGAGGCTAC TAGGCTCTTA CAGTTTATTA AGAAGTTCAA CAACCGGAAA

GTGTTGATTC CTTTTACCAA CACAACAGAT TCAGGTCTGT GATCCATTTG

GTGTTGATTC CTTTTACCAA CACAACAGAT TCAGGTCTGT GATCCATTTG

GTGTTGATTC CTTTTACCAA CACAACAGAC TCAGGTCTGT GATCCATCTG

GTGTTGATTC CTTTTACCAA CACAACAGAC TCAGGTCTGT GATCCATCTG

GTGTTGATTC CTTTCATGAA CACAACAGAC TCAGGTCTGT GATCCATTTG

GTGTTGATTC CTTTCACCAA CACAACAGAC TCAGGTCTAT GATCCATTTG

GTATTGAGTC CTTTCACCAG TACGACAGAC TCTGGTCGGT GATCCATCTG

GTGTTGAGTC CTTTCACCAG TACGACAGAC TCAGGTCTGT GGTCCATTTG

GTGTTGATTC CTTTCACCAA CACAACAGAC TCAGGCCTGT GGTCCATTTG

GTATTCATTC CCTTCACCAA CACAACAGAC TCTGGTCTGT GATCCATTTG

GTATTCATTC CCTTCACCAA CACAACAGAC TCAGGTCTGT GATCCATTTG

GTGTTCGTTC CCTTCACCAG CACAACAGAC TCAGGTCTGT GATCCATTTG

GTGTTCATTC CCTTCACCAA CACGACAGAC TCAGGTCGAT GATCCATTTG

GTGTTGATTC CTTTCACCAA TACAACTGAC TCAGGTCTGT GATCCATTTG

GTGTTGATTC CTTTCACCAA TACAACAGAC TCAGGCCTGT GATCCATTTG

---------- ---------- ---------- ---------- ----------

GTGTTGATTC CTTTCACCAG CACAACAGAC TCGGGCTTGT GATCCAGCTG

GTGTTCATTC CTTTCACCAG GACGACAGAC TCGGGTTTGT GATCCATCTG

TACTTCATGT TTCTCTTTTC TGGCTTTTTA ATTTTATCTT GCACACCCAT

TACTTCATGT TTCTCTTTTC TGGCTTTTTA ATTTTATCTT GCACACCCAT

TACTTCATGT TTCTCTTTTC TGGCTTTTTA ATTTTATCTT GCACACCCAT

TACTTCATGT TTCTCTTTTC TGGCTTTTTA ATTTTATCTT GCACACCCAT

TACTTCATGT TTCTCTTTTC TGGCTTTTTA ATTTCATCTT GCACTCCCAT

TACTTCATGT TTCTCTTTTC TGGTTTTTTA ATTTTATCTT GCACACCCAT

TACTTCATGT TTTTCTTTTC TGGCTTCTTA ATTTGATCCT GCACACCGAT

TACTTCATGT TTTTCTTTTC TGGCTTCTTA ATTTGATCTT GCACGCCTAT

TACTTCATGC TTCTCTTTTC TGGCTTTTTA ATTTTATCTT GCACACCCAT

TACTTCATGT ATCTCTTTTC TGGTTTTTTA ATTTTATCTT GGACACCCAT

CACTTCGTGT TTCTCTTTTC TGGTTTTTTA ATTTTATCTT GCACACCCAT

TACTTCATGT TTCTCTTTTC TGGTTTTTTA ATTTTATCTT GCACACCCAT

CACTTCATGT TTTTCTTTTC TGGCTTTTTA ATTTTATCTT GCACACCCAT

TACTTCATGT TTCTCTTTTC TGGTTTTTTA ATTTTATCTT GCACACCCAT

TACTTCGTGT TTCTCTTTTC AGGTTTTTTA ATTTTATCTT GCACACCCAT

---------- ---------- ---------- ---------- ----------

CACTTCCTTT TTCTCTTTTC TGGTTTTCTT ATCTTATCCT GTACACCCAT

CACCTCGTGT TTCTCTCTTC CGGTTTCTTA ATTTTATCTT TTACTCCGAT

CTCTTCCAGC CAGGAAAAAC TTTCCTCTTC ATCCTCATCA CTTTTTACCT

CTCTTCCAGC CAGGAAAAAC TTTCCTCTTC ATCCTCATCA CTTTTTACCT

CTCTTCCAGC CAGGAAAAAC TTTCCTCTTC GTCCTCATCA CTTTTTACCT

CTCTTCCAGC CAGGAAAAAC TTTCCTCTTC GTCCTCATCA CTTTTTACCT

CTCTTCCAGC CAGGAAAAAC TTTCCTCTTC GTCCTCATCA CT-TTTACCT

CTCTTCCAGC CAGGAAAAAC TTTCCTCTTC ATCTTCATCA CTTTTTACCT

CTCTTCCAGC CAAGACAAAC TTTCCTCTTC ATCTTCGTCA CTTATTACCT

CTCTTCCAGC CAAGAAAAAC TCTCCTCTTC ATCTTCGTCA CTTATTACCT

TTCTTCCAGC CAGGAAAAAC TTTCCTCTTC ATCCTCATCA CTGTTTACCT

CTCTTCCAGC CAGGAAAAAC TTTCTTCTTC ATCCTCATCA CTTTTTACCT

CTCTTCCAGC CAGGAAAAGC TCTCATCTTC ATCCTCATCA CTTTTTACCT

CTCTTCTAGC CAGGAAAAAC TTTCTTCTTC ATCCTCATCA CTTTTTACCT

CTCTTCCAGC CAGGAAAAAC TTTCATCTTC ATCCTCATCA CT---TACCT

CTCTTCCAGC CAGGAAAAAC TTTCTTCTTC ATCTTCATCA CTTGTTACCT

CTCTTCCAGC CAGGAAAAAC TTTCCTCTTC ATCCTCATCA CTTTTTACCT

---------- ---------- ---------- ---------- ----------

CTCTTCCAAC CAGGAAAAAC TTTCCTCTTC ATCCTCATCA CTATTTACCT

CTCTTCGAGC CAAGAATAAC TTTCCTCTTC ATCTTCATCA CTTTTTACCT

TCATTTCTCA TAGCTTCTCT TAAACCTCGA GTTGTTGGAG ATATGAGAGC

TCATTTCTCA TAGCTTCTCT TAAACCTCGA GTTGTTGGAG ATATGAGAGC

TCATTTCTCA TAGCTTCTCT TAAACCTCGA GTTGTAGGAG ATATGAGAGC

TCATTTCTCA TAGCTTCTCT TAAACCTCGA GTTGTAGGAG ATATGAGAGC

TCATTTCTCA TAGCTTCTCT TAAACCTCGA GTTGTAGGAG ATACAAGAGC

TCATTTTTCA TAGCTTCTCT TAAACCTCGA GTTGTAGGAG ATATGAGAGC

TCACTTTTCA TAGCTTCTCT TAAGCCTCTA GTTGTAGGGG TGATGAGCGC

TCGTTTCTCA TAGCTTCTCT TAAGCCTCTT GTCGTAGGGG ATATGAGCGC

TCATTTTTCA TGGCTTCTCT CAGACCTCTA GTTGTAGGAG AAATAAGAGC

TCGTTTTTCA TAGCTTCTCT TATACCTCTA GTTGTAGGAG ATATGAGAGC

TCATTTTTCA TAGCTTCTCT TATGCCTCTA GTTGTAGGAG AGATGAGAGC

TCATTTTTCA TAGCTTCTCT TATACCTCTA GTTGTAGGAG ATATGAGAGC

TCATTTTTCA TCGCTTCTCT GATTCCTCTG GTGGTAGGAG AGATGAGAGC

TCATTTTTCA TAGCTTCTCT TAAACCTCTA GTTGTGGGAG ATAGGATAGC

TCATTTTTCA TAGCTTCCCT TAAACCTCTA GTTGTGGGAG ATATGACAGC

---------- ---------- ---------- ---------- ----------

TCATTTTTCA TAGCTTCTCT TAAGCCTCTG GTAGTGGGTG ACAAGACAGC

TCGTTCTTCA TAGCTTCTCT TAAACCTCTG GTAGTGGGGG ACATGACAGC

TGTGATTAAG TCACTTCCAG CTAATCCTGC TGCTCGGAAC AGGACAGTAA

AGTGATTAAG TCACTTCCAG CTAATCCTGC TGCTCGGAAC AGGACAGTAA

TGTGATTAAG TCACTTCCAG CTAATCCTGC TGCTCGGAAG AGGACAGTAA

TGTGATTAAG TCACTTCCAG CTAATCCTGC TGCTCGGAAG AGGACAGTAA

TGTGATTAAG TCATTTCCAG CTAATCCTGC TGCTCGGAAC AGGACAGTAA

TGTGATCACA TCACTTCCTG CTAATCCTGC TGCTCGGAAT AGGATAGTAA

TGTGATCACA CTGCTTCCTG CTAATCCTGC TGCCCGGAAC AGGACCGTAA

TGTGATCACA CTGCTTCCGG CCAGTCCTGC TGCCCGGAAC AGGACGGTGA

CGTGACTGCA TCACTTCCTG CTAACCCTGC TGCTCGAAAC AGGACCGTGA

CGTGATTACA TCATCTCCTG CTAACCCTGC TGCACGGAAC AGGATAGTAA

TGTGATTACA TCATTTCCTG CTAATCCTGC TGCTCGGAAC AAGATCGTAA

TGTGATTACA TCATTTCCTC CTAATCCTGC TGCACGGAAC AGGACAGTAA

AGTGAGGACG TCACTGCCGG CCAGTCCTGC TGCACGGAAC AGGATCGTAA

TGTGATTACA TCATTTCCTG CTAATCCTGC TGCACGGAAG AGGACAGTAA

TGTGATGACA TCACTTCCTG CAAATCCTGC TGCCCGGAAA AGGACAGTGA

---------- ---------- ---------- ---------- ----------

TGTAATTACA TCACTTCCTG CTAGCCCTGC TGCACGAAAC AGCACAGTGA

TGTGATTACA TCACATCCTC CTAATCCAGC AGCACGGAAC AGCACGGTAA

ACTGATAGGT ACAAACGTAG AAATAGGGGC AAAGTTTTGT CTTCAGCAAA

ACTGATAGGT ACAAACGTAG AAATAGGGGC AAAGTTTTGT CTTCAGCAAA

ACTGATAGGT ACAAACGTAG AAATAGGGGC AAAGTTTTGT CTTCAGCAAA

ACTGATAGGT ACAAACGTAG AAATAGGGGC AAAGTTTTGT CTTCAGCAAA

ACTGATAGGT ACAAACGTAG AAATAGGGGC AAAGTTTTGT CTTCAGAAGA

ACTGATAGGT ACAAACGTAG AAATAGGGGC AAAGTTTTGT CTTCAGCAGA

ACTGATAGGA ACAAACGTAG AAATAGGGGC AAAGCTTCGT CTTCAGCAGG

ACTGATAGGA GCAGACATAG AAATAGGGAC AAAGCTTTGT CTTCAGCAGG

ACTGGTAAGT GCAAACGTAG AAATAGGGGC AGAGCTTGGT CTTCAGTAGG

ACTGATAGGT ACAAACGTAG AAATAAGGGC AAAGTTTTGT CTTCAGCAGG

ACTGATAGGT ACAAACGTAG AAATATGGGC AAAGCTTTGT CTTCAGTAGA

ACTGATAGGT ACAAATGTAG AAATAGGGGC AAAGTTTTGT CTTCAGCAGA

ACTGATACGT GCAAACGTAG AAATAGGGGC AGAGTTTGGT CTTCACCAGA

ACTGATAGGT ACAAACGTAG AAATAGGGGC AAAGTTTTGT CTTCAGTAGA

ACTGGTAAGT ACAAACGTAG AAATAGGGGC AAAGCTTTGT TTTCAGCAGA

---------- ---------- ---------- ---------- ----------

ATTGGTAGGT ACAAACATAG AAATAGGGAC AAAGTTTTGT CTTCAGTAGA

ATTGATAGGT GCACACATAA AAATAGGGGC AAAGCTTTGT CTTCAGCAGG

TTATATAGAG AAGTAAAGCT CACAGACCTA GTCAACAATC ACCATTTTGG

TTATATAGAG AAGTAAAGCT CACAGACCTA GTCAACAATC ACCATTTTGG

TTATATAGAG AAGTAAAGCT CACGGACCTA GTCAACAATC ACCATTTTGG

TTATATAGAG AAGTAAAGCT CACGGACCTA ---------- ----------

TTATATAGAG AAGTAAAGCT CACAGACCTA GTCAACAATC ACCATTTTGG

TTATAGAGAG AAGTAAAGCT CACAGACCTG GTCAACAATC AGCACTTCGG

TTATATAAAG ACGTAAAACT CACAGACCTA GTCGATAATT ACCACTTCGG

TTATACAGAG ATGTGAAACT CACAGACCTA GTCGATAATT ACCACTTCGG

CTGTACAGAG AAGTAAAGCT CACAGACCTA GTCAACAACC ACCATTTCGG

TTATATAGAG AAGTAAAGCT CACAGACCTA GTCAACAACC ACCATTTCGG

TTATAGAGAG AAGTGAAGCT CACAGACCTA GTCAACAATC ACCATTTCGG

TTATATAGAG AAGTGAAGCT CACAGACCTA GTCAACAATC ACCATTTTGG

TTATAAAGAG AGGTAAAGCT CACAGACCTA GTCAACAACC ACCATTTCGG

TTATATAGAG AAGTAAAACT CACAGACCTA GTCAACGACC ACCATTTTGG

TTATACAGAG AAGTAAAGCT CACAGACCTA GCCAACAATC ACCACTTCGG

---------- ---------- ---------- --------TC ACCATTTCGG

TTATACAAAG AAGTGAAGCT GATGGACCTA GTCAACAATC ACCATTTTGG

TTATAGAGAG AAGTGAAACT GATGGACCTA GTGAGCGATC ACCAGTTCGG

AAGCCCCTGT TTGCCAGGCG TGAAAAAATA CCTCACTGTC TTACCAGTCA

AAGCCCCTGT TTGCCAGGCG TGAAAAAATA CCTCACTGTC TTACCAGTCA

AAGCCCCTGT TTGCCAGGTG TGAAAAAATA CCTCACTGTC TTACCAGTCA

---------- ---------- ---------- ---------C TTACCAGTCA

AAGCCCCTGT TTGCCAGGCG TGAAAAAATA CCTCACTGTC TTACCAGTCA

AAGCCCCTGT CTGCCAGGAG TGAAAAAATA CCTCACTGTC TTACCAGTCA

AAGCCCATGT CTTCCCGGAG TGAAAAAATA CCTCACTGTC TTACCAGTCA

AAGCCCCTGT CTTCCAGGAG TGAAAAAATA CCTCACTGTC TTACCAGTCG

AAGCCCCTGT CTGCCAGGAG TGAAAAAATA CCTCACTGTC TTACCAGTCA

AAGCCCCTGT CTGCCAGGAG TGAAAAAATA TCTCTCGGTC TTACCAGTCA

GAGCCCCTGT CTGCCAGGAG TGAAAAAATA CCTCACTGTC TTACCAGTCA

AAGCCCCTGT CCGCCAGGAG TGAAAAAATA CCTCACCGTC TTACCAGTCA

GAGCCCCTGC CTGCCAGGAG TGAAGAAATA CCTCACAGTC TTACCAGTCA

AAGCCCCTGT CTGCCAGGAG TGAAAAAACA CCTCACTGTC TTACCAGTCA

AAGCCCCTGT CTGCCAGGAG TGAAAAAATA CCTCACCGTC TTACCAGTCG

AAGCCCCTGT CTGCCCGGAG TGAAAAA-TA CCTCACAGT- ----------

AAGTCCTTGT CTGCCAGGAG TGAAAAAATA CCTCACTGTT TTACCATTCA

AAGCCCGTGT CTGCCGGGAG TGAAGAAGTA CCTCACAGTC TCACCATTCG

CTCATTAAAA CATGCTGCAG GGTTGCATCA TTTGACCAAG GACTTGTCTT

CTCATTAAAA CATGCTGCAG GGTTGCATCA TTTGACCAAG GACTTGTCTT

CTCATTAAAA CATGCTGCAG GGTTGCATCA TTTGACCAAG GACTTGTCTT

CTCATTAAAA CATGCTGCAG GGTTGCATCA TTTGAACAAG GACTTGTCTT

CTCATCAAAA CATGCTGCAG GGTTGCATCA TTTGACCAAG GACTTGTCTT

CTCATTAAAA TTTGTTGTAG GGTTTCATCA TTTGACCATG GGCTTGTCTT

CTCATTAAAG CATGCTGCAG GCTTTCATCC GTTGACCATG GACTTGTCTT

CTCATTAAAG CATGCTGCAG GGTTTCATCA GCTGACCACG GACTTGTCTT

CTCATTAAAA TGTGTTGTAG GGTTTCATCA TTTGACCAAG GACTTGTCTT

CTCATTAACA CATGCTGCAG GGTTTCATCA TTTGACCACG GGGTTGTCTT

CTCATTAAAA TGTGTTGCAA GGTTTCATCA TTTGACCAAG GGCTTATCTT

CTCATTAAAA CGTGTTGCAG GGTTTCATCG TTTGACCAAG GGCTTGCCTT

CTCATTAAAA TATGCTGCAG CTCTTCGTCA GTGGCCCAGG GGCTGTTCTT

CTCATTAAAA TATGTTGTAG GGTTTCATCA TTTGACCAAG GGCTTGTCTT

CTCATTAAAA CGTGTTGTAG GGTTTCATCA TTTGACCATG GGCTGGTCTT

---------- ---------- ---------- ---------- ----------

CTCATTAAAA TTTGTTGTAG TTCCTCATCA TTTGACCAAG GACTTGTTTT

CTCATCAGCA CTTGCTGCAG CGTCTCATCC TGGGACCAAG GACTGTTCTT

TCCAGCCATT TTTCTATCAG CTCCAATACG AGGGAACAGT GGTAGCCAGC

TCCAGCCATT TTTCTATCAG CTCCAATACG AGGGAACAGT GGTAGCCAGC

TCCAGCCATT TTTCTATCAG CTCCAATACG AGGGAACAGT GGTAGCCAGC

TCCAGCCATT TTTCTATCAG CTCCAATACG AGGGAACAGT GGTAGCCAGC

TCCAGTCATT TTTCTATCAG CTCCAATACG AGGGAACAGT GGTAGCCAGC

TCCAGTCATT TTCCTATCAG CTCCAATACG AGGGAACAGT GGTAGCCAGC

TGCAGCCATT TTTCTATCAG CTCCAATTCG AGGGAAGAGT GGCAGCCAGC

TGCAGCCATT TTCCTATCAG CTCCAATTCT AGGGAAGAGT GGCAGCCAGC

TCCAGCCATT TTCCTATCAG CTCCAATGCG AGGAAACAGT GGTAGCCAGC

TCCAGCCATT TTTCTGTCAG CTCCGATACG AGGGAACAAT GGCAGCCAGC

TCCAGCCATT TTTCTATCAG CTCCAATGCG AGGGAACAGT GGTAGCCAGC

TCCAGCCATT TTTCTATCAG CTCCAATCCG AGGGAACATT GACAGCCAGC

GCCGGCCATC TTCCTCTCGG CACCAATCCG TGGGAAGAGC GGGAGCCAGC

TCCAGTCATT TTTCTATCAG CTCCAATACG AGGGAACAGA GGTAGCCAGC

TCCAGCCATT CTTCTATCCG CTCCTATACG AGGGAATAGT GGCAGCCAGC

---------- ---------- ---------- ---------- ----------

TCCAGCCATC TTTCTGTCAG CTCCAATGCG TGGGAAAAGC TGTAACCAGA

CCCGACCATT TTCCTATCGG CCCCAATCCG TGGAAACAAA TGCATCCAGA

AGGGTGGAGC CAATAGATAA GGCTCTGCTG GAAGGTACAA CGGAGCTCAG

AGGGTGGAGC CAATAGATAA GGCTCTGCTG GAAGGTACAA CGGAGCTCAG

AGGGTGGAGC CAATAGATAA GGCTCTGCTG GAAGGCACAA CGGAGCTCAG

AGGGTGGAGC CAATAGATAA GGCTCTGCTG GAAGGCACAA CGGAGCTCAG

AGGGTGGAGC CAATAGACAA GGCTCTGCTG GAAGGTACAA CGGAGCTCAG

AGGGTGGAGC CAATAAATAA GGCTCTGTTG GAAGGCACAC CGGAGTTCAG

AGGGTGAAGC CAGTAGACCA GGGTCTGCTG GAAGGCACAA CGTAGCACAG

AGGGTGAAGC CAGTAGACCA GAGCCTGCTG GAAGGCGCAG CGCAGCGCAG

AGGGTGGAGC CAATAGATAA GGCTCTGTTG GAAGGCACAG CGGAGCTCAG

AGGGTGGAGC CAATAGATGA GGCTCCGCTG GAAAGCACAG CGGAGCTCAG

AGGGTGCAAC CAATAGATTA GGCTCTGCTG AAAGGCACAA CGGAGCTGAG

AGGGTGGAGC CAATAGATAA GGCTCTGTTG GAAGGCACAA CGAAGCTCAG

CGGGTGCAGC CAGTACACGA GGCTCTGCTG GAAGGCGCAG CGCAGCTCTG

AGGATGGAGC CAATAGATAA GACTCTGCTG GAAGGCACAA CGGAGCTCAG

AGGGTGAAGC CAATAGAAGA GGCTCTGCTG GAAGGCACAG CGGAGCTCGG

---------- ---------- ---------- ---------- ----------

GGGATGAAGC CAATAAATAA GGTTCTGCTG GAACGCACAA CGAAGTTCAG

CGGGTGGATC CAATACACGA GGCTCTGCTG GAAGGCACAG CGAAGCTCGG

AGGAGAGTTT GGGATCCTAA AATTACCTGT ATACTTTTAG GCAAAGTAAC

TGGAGAGTTT GGGATCCTAA AATTACCTGT ATACTTTTAG GCAAAGTAAC

TGGAGAGTTT GGGATCCT-A AATTACCTGT ATACTTTTAG GCAAAGTAAC

TGGAGAGTTT GGGATCCT-A AATTACCTGT ATACTTTTAG GCAAAGTAAC

TGGAGAGTTT GGGATCCTAA AATTACCTGT ATACTTTTAG GCAATGTAAC

TGGAGAGTTT GGGATCCTAA AATTACCTGT ATACTTTGAG GCAAAGTAAC

TGGACAGTTT GGGATCCTAA ATTTACCGGT ATACTCTGTG GTAATGTAAC

TGGACAGTTT GGGATCCTAA ATTTACCTGT ATACTCTGGG GTAAAGTCAC

TGGATAGCTT GGGATCCTGA AATTACCTGT ATACTCTGAG GCAAAGTAAC

TGGAGAGTTT GGGATCCTAA AATTACCTGT ATACTTTGAG GCAAAGTAAC

TGGAGAGTCT GGGATCCTAA AATTACCTGT ATACTTTGAG GCAAAGTAAC

TGCAGAGTTT GGGGTCCTAA AATTACCTGT ATACTTTGAG GCAGAGTAAC

TGCACAGCTT GGGGTCCTGC AATTACCTGT ATACTCAGGG GTAAACTCAC

TAGACAATTT AGGATCCTAA AATTACCTGT ATTCTTTGAG GCAAAGTAAC

TGGAGAGCCT GGTATCCTGA AATTACCTGA ATATGTTCAG GTAAGGTGAC

---------- ---------- --TTACCTGT ATCCTCTGAG GTAAGGTAAC

CAGAGAGTCG TGGCTCCTAA AAGTACCTGT ATATTCTCAG GCAGATCAAC

TGGAGAGTTT GGGGTCCTAG AAATACCTGT ATGCTATGAG GCAAACTAAT

TTCTGTTGCC CTACAATGCT GGACAAGACC TTGAGCTTCT TCCTGTGCTT

TTCTGTTGCC CTACAATGCT GGACAAGACC TTGAGCTTCT TCCTGTGCTT

TTCTGTTGCC CTACAATGCT GGACAAGACC TTGAGCTTCT TCCTGTGCTT

TTCTGTTGCC CTACAATGCT GGACAAGACC TTGAGCTTCT TCCTGTGCTT

TTCTATTGCC CTACAATGCT GGACAAGACC TTGAGCCTCT TCCTGTGCTT

TTCAGTTGCC CTACAATGTT GGATGAGACC TTGGGCCTCT TCCTGTGCTT

TTCTGTTGCC CTACAATGCT GGACAAGTCC TTGGGCTTCT TCCTGTGCTT

TTCTGTTGCC CTACAATGCT GGACAAGTCC TTGGGCTTCT TCCTGTGCTT

TTCTGTTGCC CTACAATGCT GGACAAGACC CTGGGCTTCT TCCTGTGCTT

TTCTGTTGCC CTGCAATGCT GGATGATACC TTGAGCCTCT TCCTGGGCTT

TTCTGTTGCC CTACAATGCT GGATGACACC TTGGGCCTCT TCTTGTGCTT

TTCTGTTGAC CTACAATGCT GGACGACACC TTGAGCCTCT TCCTGGGCTT

TTCTGTGGCC CGGCAGTGCT GGACAAGTCC CTGGGCCTCC TCCTGGGCTT

TTCCGTTGCC CTACAGTGCT GGACAAGACC TTGAGCCTCT TCCTGTGCTT

TTCTGTCCCC CTACAGTGCT GGACAAGACC TTGAGCCTCT TCATGTGCTT

TTCTGTTGCC CGGCAGTGCT GTACGAGACC TTGAGCCTCT TCGTGTGCTT

TTCTGTTGCT CTGCAGTGTT GGACAAAGCC TTGTGCTTCT TCTTGAGCCT

TGCTGTTGCT CTACAGTGCT GGATAAATCC TCTAGCTTCT TCTTGTGCTT

TCAAATGATC TGCCCAGGTA AAGGGTTGAG AAGAGGTGAA AAGGAGTCGC

TCAAATGATC TGCCCAGGTA AAGGGTTGAG AAGAGGTGAA AAGGAGTCGC

TCAAATGATC TGCCCAGGTA AAGGGTTGAG AAGAGGTGAA AAGGAGTCGG

TCAAATGATC TGCCCAGGTA AAGGGTTGAG AAGAGGTGAA AAGGAGTCGG

TCAAATGATC TGCCCAGGTA AAGGGTTCAG AAGAGGTGAA AAGGAGTCGG

TCAAATGCTC TGCCCAGGTA AAGGGTTGAG AAGAGGTGAA AAGGAGCCGA

TCAAATGTTC TGCCCAGGAA AAGGGTTGAG AAGAGGTGAA AAGGAGGCGG

TCAGATGATC TGCCCAGGAA AAGGGTTGAG AAGAGGTGAA AAGGAGTCGG

TCAAATGATC CGCCCACGCA AAGGGTTGAG AAGAGGTGAA GAGGAGTCGA

TCAAATGATC TGCCCAGGTA AAGGGTTGAG AAGAGGTGAA AAGGAGTCGT

TCAAGTGATC TGCCCAGGTA AAGGGTTGAG AAGAGGTGAA AAGGAGTCGA

TCAAATGATC TGCCCAGGTA AAGGGTTGAG AAGAGGTGAA AAGGAGTCGA

TCAAATGCTC TGCCCAGGTG AAGGGGTGAG AAGAGGTGAA AAGGAGTCGG

TCAAATGATC TGCCCAGGTA AAGGGTTGAG AAGAGGTAAA AAGGAGTCGA

TCAGATGATC TGCCCAGGCA AAAGGCTGAG AAGAGGTGAC AAGGAGTCGA

TCAAATGATC TGCCCAGGCA AAGGGTTGAG AGGAGGTGAA AAGAAGTCTA

TCAAATGATC TGCCCAGGTA AAAGATTGGG AAGAGGTAAA AAGGAGGCGC

TCAAATGATC TGCCCAGGTG AAAGGTTGGG AAGAGGTGAA AAGGAGGCGA

GTTTTAATAC TCCAGTCCAC AGGTAACTCT TTCAGGAAAC TTCTCTTCCC

GTTTTAATAC TCCAGTCCAC AGGTAACTCT TTCAGGAAAC TTCTCTTCCC

GTTTTAATAC TCCAGTCCAC AGGTAACTCT TTCAGGGAAC TTCTCTTCCC

GTTTTAATAC TCCAGTCCAC AGGTAACTCT TTCAGGGAAC TTCTCTTCCC

GTTTTAATAC TCCAGTCCAC AGGTAACTCT TTCAGGAACC TTCTCTTCCC

GTTTTAATAC TCCAGTCCAC AGGTAACTCT TTCAGGCAAT GTCTCTTCCC

GTTTTAATAC TCCAGTCCAC AGGTAACTCT TTCATGAGAT GCCTCCTCCC

GTTTTAATAC TCCAGTCCAC AGGTAACTCT TTCATGAGAT GATTCCTCCC

gttttaatac tccagtccac aggtaactcT TTCAGGACTC ATCTCATCCC

GTTTTAATGC TCCAGTCCAC AGGTAACTCT TTCAGGAACC TTCTCTTCCC

GTTTTAATAC TCCAGTCCAC AGGTAACTCT TTCAGGAAAC TTCTCCTCCC

GTTTTAATAC TCCAGTCTAC AGGTAACTCT TTCAGGAAAC TTCTCTTCCC

GTTTTAATAC TCCAGTCTGC CGGTAACTC- ---------- ----------

GTTTTAATAC TCCAGTCCAC AGGTAACTCT TTCAGGCAAC TTCTCGTCCC

GTTTTAATAC TCCAGTCCAC AGGTAACTCT TTCAGGAAAC TTCTCTTCCC

GTTTTAATAC TCCAGTCCAC GGGTAATTT- ---------- ----------

GTTTTAATAC TCCAATCCAC AGGAAACTCT TTCGGAAAAC TCTTCTTTCC

GTTTTAATAC TCCAGTCTAC CGGAAGCTCT TTCTGAAAGC TCTTCTTCCC

ATAGCAAATC ATTTTCTTGA TTAGAATCTA AAAACTGAGG TTAAATATTT

ATAGCAAATC ATTTTCTTGA TTAGAATCTA AAAACTGAGG TTAAATATTT

ATAGCAAATC ATTTTCTTGA TTAGAATCTA AAAACTGAGG TTAAAAATTT

ATAGCAAATC ATTTTCTTGA TTAGAATCTA AAAACTGAGG TTAAAAATTT

GTAGCACATC ATTTTCTTGA TTAGACTCTA AAGGCTGAGG TTAAAAATTT

ATAGCAAATC ATTTTCTTGA TGAGAATCTA AAAACTGAGG TTAAAAAAtt

ACAGCAAGTT ATTTTCTCCA TTAGAATCTA GAAGCTGAGG TTAAAAAATT

ACAGCAAGTT ATTTTCTTCA TTAGAATCTA GAAGCTGAGG TTAAGAAATT

ACTGCAAATC AT-------- ---------- ---------- --------TT

AGAGCAAATC ATTTTCTTGA TTAGAATCTA AAAACTGAGG GAAAAAAATT

ACAGCGAATC ATTTTCTTGA TTAGAATCTA CAAACTGAGG GGAAAACGTT

AAAGCAAGTC ATTCTCTTGA TTGGAATCTA AAAACTGTGG AAAAAAAATT

---------- ---------- ---------- ---------- ----------

ACAACAAATC ATTTTCTTGA TTAGAATCTA CAAGCTGAGG TTAAAAAAtt

ATAGCAAATC ATTTTCTTGA TTAGAATCCA AAACCTGAGG TTAAAAAATT

---------- ---------- ---------- ---------- --------T-

AGGTCACATT ATTTTCTTGA TTAAAATCTA AAAACTGAGA TGAAAGAATT

AGGCAAAATC ATTTTCTTGA TTAGAATCTG AATTCTAAGG AAAAAAAATT

TAAAATTGAA CAACTTGCTT TTTTCTTCCT TGATTTTTCT GAACAGCTTC

TAAAATTGAA CAACTTGCTT TTTTCTTCCT TGATTTTTCT GAACAGCTTC

TAAAATTGAA CAACTTGCTT TTTTCTTCCT TGATGTTTCT GAACAACTTC

TAAAATTGAA CAACTTGCTT TTTTCTTCCT TGATGTTTCT GAACAGCTTC

TAAAACTGAA CAACTTGCTT TTTTCTTCCT TGATTTTTCT GAACAGCTTC

taaaattgaa caacttgctt ttttcttcct tgatttttct gaacaatttc

TAAAATTGAA CAACTTGCTT TTTTCTTTCT TGACTTTTCT GAACAACTTC

TAAAATTGAA CGACTTGCTT TTTTCTTTCT TGATTTTTCT GAACAACTTC

TAAAATTGAA CAACTTGCTT TTTTCTTCCT TGATTTTTCT GAACAACTTC

TAAAACTGAA CAACTTGCTT CTTTCTTCCT TGATTTTTCT GAACAACTTC

TAAAATTGAA CAACTTGCTT TTTTCTTCCT GGATTTTTCT GAACAACTTC

TAAAATTGAA CAACTTGCTT TTTTCTTCCT TGATTTTTCT GAACAACTTC

---------- ---------- ---------- ---------- ----------

taaaattgaa caacttgttt ttttcttcct tgatttttct gaataacctc

TAAAATTGAA CAACCTGTTT TTTTCTTCCT TCATTTTTTT GAACAACTTC

CAAAACTGAA CAACTTGTTT TTTTCTTTCT TGATTTTTTT GAACAACTTC

CAAAACTGGA TAACTTGTTT TTTTCTTGCT TGACTTTTCT GAACAATTTC

TACAACTGAA TAACTTGTCT TTTTCTTGCT TGATTGCTCT GAACAACTTC

CATGGAAACT TTTGCCTCAT ACAGTGGAAT GGGTTCATCC AACTGAGGTC

CATGGAAACT TTTGCCTCAT ACAGTGGAAT GGGTTCATCC AACTGAGGTC

CATGGAAACT TTTGCCTCAT ACAGTGGAAT GGGTTCATCC AACTGAGGTC

CATGGAAACT TTTGCCTCAT ACAGTGGAAT GGGTTCATCC AACTGAGGTC

CATGGAAACT TTTGCCTCAT ACAGTGGAAT GGGTTCATCC AACTGAGGTC

catggaaact tttgcctcgt acagtggaat ggcttcatcc atatgaggtc

CATGGAAACT TTGGCCTCAT ACAGTGGAAT GGGTTCATCA AGCAGAGGTC

CATGGAAACC TTTGCCTCAT ACAGTGGGAT GGGCTCATCG AGCAGAGGCC

CATGGAAACT TTGGCTTCAT AGAGTGGGAT GGGTTCTTCC AACTGTGGTC

CATGGTAACT TTTGCCTCGT ATAGTGGAAT GGGTTCATCC AACTGAGGTC

CATGGAAACT TTCGCTTCAT ACAATGGAAT GGGTTCATCC AACTGAGGCC

CATAGAAACT TTTGCTTCAT ATAGTGGAAT GGGTTCATCC AACTGAGGCC

---------- ---------- ---------- ---------- ----------

catggaaact ttggcctcat atagtggaat aggttcatcc agctgaggtc

CATGGAAACT TTGGCCTCAT ATAGTGGAAT GGGTTCATCT AGCTGAGGTC

CATGGAAACT TTGGCTTCAT ACAGTGGAAT GGGTTCATCC AACTGAGGTC

CATGGAAACT TTGGCATCAT ATGGTGGCAC AGGTTCATCC AAAAGAGGTC

CATGGTCCCT TTGGCTTCAT ACAGAGGAAC AGGTTCATCC AACATAGGCC

TGAGAAGGAA TGAAATTAGA AATGTACCTG AAAACACCAG TTGATAACTT

TGAGAAGGAA TGAAATTAGA AATGTACCTG AAAACACCAG TTGATAACTT

TGAGAAGGAA TGAAATTAGA AATGTACCTG AAAACACCAG TTGATAACTT

TGAGAAGGAA TGAAATTAGA AATGTACCTG AAAACACCAG TTGATAACTT

TGAGAAAGAA TGAAATTAGA AATGTACCTG AAAACACCAG TTGATAACTT

taaggaagaa ggaaaTTAGA AATGTACCTA AAAACACCAT TTGATAACTT

TGAGGAGGAA AATGATTAGA CATGTACCTG AACACACCAG CTGATAGCTT

TGAGGAGGGA AATGATTCGA GATGTACCTG AAAACACCAG CTGATAACTT

TGAAGAAGGA AATGAGTAGA AATGT----- ---------- ----------

TGAGGAAGAA TGAAATTAGA AATGTACCTA AAAACACCAG TTGATAACTT

TGAGGAAGAA TGGTATTAGA AAAGTACCTA AAAACTCCAG TTGATAGCTT

TGAGGAAGAA TGAAATTA-- -----ACCTA AAAACACCAG TCGATAACTT

---------- ---------- ---------- ---------- ----------

tgaggaagaa AGAAATTAAA AATGTACCTA AAAACACCAG TTGATAACTT

TAAGGAAGAA TGAAATTAGA AATGTACCTA AAAACACCCG TTGATAGCTT

TATGGAAGAA TTAAATTAGA AATGTAACTG AAAACACCAT TTGATAGCTT

TAAAGAAAAA TGAAATTAGA AGTGAACCTG AAAATACCAG TTGATAGCTT

TGAGGAAAAA AGAAAAAAGA AATGAACCTG AAGATGCCAT TTGATAAGTT

CTCCATCACA TCCTTTAAGA TACGTGCTGG AAGGATTGTT AAGGTAGGAT

CTCCATCACA TCCTTTAAGA TACGTGCTGG AAGGATTGTT AAGGTAGGAT

CTCCATCACA TCCTTTAAGA TACGTGCTGG AAGGATTGTT AAGGTAGGAT

CTCCATCACA TCCTTTAAGA TACGTGCTGG AAGGATTGTT AAGGTAGGAT

CTCCATCACA TCCTTTAAGA TACGTGCTGG CAGGATTGTT AAGGTAGGAT

CTCCATCACA TCTTTTAAGA TACGTGCTGG AAGGATTGTT AAGGTAGGAT

CTCCATCACG TCTTTTAAGA TGCGTAATGG AAGAACTGTT AAGGTAGGAT

CTCCATCACA TCTTTTAAGA TACGTAATGG AAGGATTGTT AAGGTAGGAT

---------- ---------- ---------- ---------- -----AGGAT

CTCCATCACA TCTTTTAAGA TACGTGCTGG AAAGACTGTT AAGGTAGGAT

CTCCATCACA TCTTTTAGGA TACGTGCTGG AAAGATTGTT AAGGTAGGAT

CTCCATCACA TCTTTTAAGA TACGTGCTGA AAGGATTGTT AAGGTAGGAT

---------- ---------- ---------- ---------- -----AGGAT

CTCCATCACA TCCTTTAAGA TACGTTCTGG AAGGATAGTT AAGGAAGGAT

CTCCATCACA TCTCTTAAGA TACGTGCTGG AAGGATTGTT AAGGTAGGAT

CTCCATCACA TCTTTTAAGA TACACG---- ---------- -----AAGAC

CTCCATTACA TCTTTTAAGA TGCGTCTTGA AAGGTCTGTT AAGTTAGGAT

TTCCATTACA TCTTTTAAGA TACGTTCTGA GAGTACGGTT AAGGTAGGAT

ATAAAGATAT TTTCCCTGTT GTACATTTGA CAAATTCCAA ACTTCATCAT

ATAAAGATAT TTTCCCTGTT GTACATTTGA CAAATTCCAA ACTTCATCAT

ATAAAGATAT TTTCCCTGTT GTACATTTGA CAAATTCCAA ACTTCATCAT

ATAAAGATAT TTTCCCTGTT GTACATTTGA CAAATTCCAA ACTTCATCAT

ATAAAGATAT TTTCCCTGTT GTACATTTGA CAAATTCCAA ACTTCATCAT

ATAAAGATAT TTTCCCTGTT GTACATTTGA CAAATTCCAA ACTTCATCAT

ATAAAGATAT TTTCCCTGTT GTGCATTTGA CAAATTCCAA ACTTCATCAT

ATAAAGATAT TTTCCCTGTT GTGCATTTGA CAAATTCCAA ACTTCATCAT

ATAAAGATAT TTTCCCTGTT GAACGTTGGA CAAATTCCAG ACTTCATCGT

ATAAAGATAC TTTCCCTGTT GTACATTTGA CAGATTCCAA ACTTCATCAT

ATAAAGATAT TTTCCCTGTT GCACATTTGA CAAATTCCAA ACTTCGTCAT

ATAAAGATAT TTTCCTTGCT GTACATTTGA CAAATTCCAA ACTTCATCGT

ATAAAGGTAT TTCCCCTGTT GTGCATTTGA CAAATTCCAC ACCTCCTCGT

ATAAAGATAT TTCCCCTGTT GTACATTTGA CAAATTCCAA ACTTCATCAT

ATAAAGGTAT TTTCCCTGTT TTACATTTGA CAGATTCCAA ACTTCATCAT

ACAGAGGTAG GGTCCCTGTT GTGCATTTGG AAGATTCCAC ACTTCTTCGT

ATAAAGATAC TTTCCTTGTT GCACATTCGA CAAATTCCAC ACTTCATCAT

ATAAAGATAC TTGCCCTGCT GCACATTTGA CAAGTTCCAC ACTTCATCAT

TCAGGAAAGC TATTCTGATA GTACCTGAAG GTTTTCTTCT GTTGTTACCC

TCAGGAAAGC TATTCTGATA GTACCTGAAG GTTTTCTTCT GTTGTTACCC

TCAGGAAAGC TATTCTGATA ATACCTGAAG GTTTTCTTCT GTTGTTACCC

TCAGGAAAGC TATTCTGATA ATACCTGAAG GTTTTCTTCT GTTGTTACCC

TCAGGAAAGC TATTCTGATA ATACCTGAAG ATTTTCTTCT GTTGTTATCC

TAAGGAAAGC CATTCTGACA GTACCTGAAG GTTTTCTTCT GTTGTTACCC

TAAGGAAAGC CATTCTGATA ATACCTGAAG GTTTTCTTCT GTCGTTATCC

TAAGGAAAGC CATTCTGATA ATACCTGAAG GTTTTCTTCT GTTGTTACCC

TAAGGAAAGC CATTCTGATA ATACCTGAAG GTTTTCTTCT GTTGTTACCC

TAAGGAAAGC CATTCTGATA ATACCTGCAG GTTTTCTTCT GTTGTTACCC

TAAGGAAAGC CATTCTGATA ATACCTGCAG GTTTTCTTCT GTTGTTACCC

TCAGGAAAGC CATTCTGATA ATACCTGCAG TTTTTCTTCT GTTGTTACCC

TAAGGAAAGC CA-------- ---------- ---------- ----------

TAAGGAAAGA CATTCTGACA ATACCTGAAG GTTTTCTTCT GTTGTTACCC

TAAGGAAAGA CA--AAGATA ATACCTGAAG GTTTTCTTCT CTTGTTACCC

TAAGGAAAGA CATTCTGACT GTACCTGAAG GCTTTCTTCT CTTGTCACCC

TAAGGAAAGA CATTGTGATA ATACCTGAAG ATTTTCTTCT GTGGTGACCC

TGAGGAAAGA GATAGTGATA TTACCTGAAG GTTTTTTTCT GAGGTGATCC

AGTGGCCTCC AACACCAAGA AGTGTGATGA TATCATGTTT ATGTGGTAGT

AGTGGCCTCC AACACCAAGA AGTGTGATGA TATCATGTTT ATGTGGTAGT

AGTGGCCTCC AACACCAAGA AGTGTGATGA TATCATGTTT ATGTGGTAGT

AGTGGCCTCC AACACCAAGA AGTGTGATGA TATCATGTTT ATGTGGTAGT

AGTGGCCTCC AACACCAAGA AGTGTGATGA TATCATGTTT ATGTGGCAGT

AATGGCCTCC AACACCAAGA AGTGTGATAA CATCATGTTT ATGTGGTAGT

AATGGCCTCC AACACTGAGA AGTGTGATGA TGTCATGCTT ATGTGGTAG-

AATGGCCTCC GACACCAAGA AGTGTGATGA TGTCATGCTT ATGTGGCAG-

AATGGCCTCC AACACCAAGA AGTGTGATGA TATCATGTTT ATGTGGTAG-

AGTGGCCTCC AACACCAAGA AGGGTGATGA TATCATGTTT ATGTGGTAGT

AATGGCCTCC AACACCAAGA AGTGTGATGA TATCATGTTT ATGAGGTAGT

AATGGCCTCC GACACCAAGC AATGTGATGA TATCATGTTT ATGTGGTAGT

---------- ---------- ---------- ---------- ----------

AATGACCTCC AACACCAAGA AGTGTGATGA TATCATGCTT ATGAGGCAGC

AGTGGCCTCC AACACCTAGA AGTGTGACGA TGTCATGCTT ATGTG---GT

AGTGGCCTCC GACAGCCAGA AGTGCGATGA TATCATGCTT ATGCGGCAGT

AGTGACCCCC AACACCAAGA AGTGTGATGA TATCATGCTT GTGTGGTAGT

AGTGCCCACC GACACTCAAA AGCAGGATGA TATCATGCTT GTGTGGAAGT

AGTTGTAGTT TTACAGCTGG TTCATCATCT TTACTATATA ATCTCACTTA

AGTTGTAGTT TTACAGCTGG TTCATCATCT TTACTATATA ATCTCACTTA

AGTTGTAGTT TTACAGCTGG TTCATCATCT TTACTATACA ATCTCACTTA

AGTTGTAGTT TTACAGCTGG TTCATCATCT TTACTATACA ATCTCACTTA

AGTTGGAATT TTACATCTGG TTCATCATCT TTACTATATA ATCTCACTTA

AATTGTAATT TTGCAGCTGG TTCATCATCT TTACTATACA ATCTCACTTA

--TTGTTGTT TTACAGCTGG TTCATCATCT TTACTATATA ATCTCACTTa

--ATGTAACT TTACAGCTGG TTCATCATCT TTACTATATA ATCTCACTTA

--CTGTAACT TCATGGATGG CTCATCATCT TTATTATACA ATCTCACTTA

AGTTGTAATT TTTCAGCTGG TTCGTCATCT TTACTATATA ACCTCACTTA

AGGTGTAATT TTACAGCTGG TTCATCATCT TTACTATATA ATCTCACTTA

AGTTGTAATT TTACATCTGG TTCATCACCT TTACTATATA ATCTCACTTA

---------- ---------- ---------- ---------- ---------A

AGTTGTAATT TTATAGCTGG TTCATCATCT TTACTATATA ATCTCACTTA

AGTTGTAATT TGACAGTGGG TTCATCAACT TTACTATATA ATCTCACTTA

AGTTGCGGTT TTAAAGCAGG CTCATCATCT TTACTGTACA ATCTCACTT-

AGTTGTAATT TTAAAGCAGG CTCATCATCT TTACTATATA ATCTCACTTA

AGTTGTAACT TTGAAGGTGA CTCATCTTCT TTACTATACA GTCTCACTTA

CCTCCAGCAT CTAGGACAAT ATCTACTCCC AGGCCACCTG TTTCTTCCAA

CCTCCAGCAT CTAGGACAAT ATCTACTCCC AGGCCACCTG TTTCTTCCAA

CCTCCAGCAT CTAGGACAAT ATCTACTCCC AGGCCACCTG TTTCTTCCAA

CCTCCAGCAT CTAGGACAAT ATCTACTCCC AGGCCACCTG TTTCTTCCAA

CCTCCAGCAT CTAGGACAAT ATCTACTCCC AGGCCACCTG TTTCTTCCAA

CCTCCAGTAT CTAGGACAAT ATCTACTCCT AGGCCACCTG TTTCTTCCAA

cctcctgcat ctATGACAAT ATCTACTCCC AGGCCACCTG TTTCTTCCAA

CCTCCTGCAT CTATGACAAT ATCTACTCCC AGGCCACCTG TTTCCTCCAA

CCTCCAGCAT CTAGGACAAT GTCGACTCCC AGGCCACCCG TTTCTTCCAA

CCTCCAGCAT CTAGGACAAT GTCCACTCCG AGGCCACCTG TTTCTTCTAA

CCTCCAGCAT CTAGGACAAT ATCTACTCCC AGGCCACCTG TTTCCTCTAA

CCTCCGGCAT CCAGGACAAT ATCTACTCCC AGGCCACCTG TTTCTTCTAA

CCTCCAGCAT CTAGGACGAT ATCTACCCCC AAGCCACCTG TTTCTTCTAA

CCTCCAGCAT CTAGGACAAT ATCTACTCCC AGGCCACCTG TTTCTTCTAA

CCTCCAGCAT CTAGGACAAT ATCTACTCCC AGGCCACCTG TTTCTTCCAA

-CTCCAGCAT CTAGGACAGT ATCAACTCCT AGGCCACCTG TCTCTTCCAA

CCTCCAGCAT CTAGGATAAT ATCTACTCCC ATGCCACCTG TTTCTTCCAA

CCGCCAGCAT CTAGAACGAT ATCGACTCCC AGGCCGCCCG TCTCTTCCAA

ACAGCTTTCA GCAACATGAA CTTTCCCATT AGATACATCA ATCACTCGGG

ACAGCTTTCA GCAACATGAA CTTTCCCATT AGATACATCA ATCACTCGGG

ACAGCTTTCA GCAACATGAA CTTTCCCATT AGATACATCA ATCACTCGGG

ACAGCTTTCA GCAACATGAA CTTTCCCATT AGATACATCA ATCACTCGGG

ACAGCTTTCA GCAACAAGAA CTTTCCCATT AGATACATCA ATCACTCGGG

ACAGCTTTCA GCAACATGGA CTTTACCATT AGATACATCA ATCACTCGGG

GCACCTTTCA GCAACATGGA CTTTCCCACT AGACACATCA ATCACTCGGG

ACAGCTTTCA GCAACATGGA CTTTCCCATT AGACACATCA ATCACACGGG

ACAGCTTTCA GCAACATGGA CTTTCCCATT AGATACATCA ATTACTCGGG

ACAGCTTTCA GCCACATGGG CTTTCCCGTT AGAAACATCG ATCACTCGAG

ACAGCTTTCA GCAACATGGG CTTTCCCACT AGAAACGTCG ATGACTCGGG

ACAGCTTTCA GCAACATGGG CTTTCCCATT AGATAAATCG ATCACTCGGG

ACAGCTTTCA GCAATATGGG CTTTCCCGTT AGAAATATCC ACCACGCGGG

ACAGCTTTCG GCAACATGAA TCTTTCCATT AGATACATCA ATCACACGGG

ACAGCTTTCA GCAACATGGA CTTTCCCATT AGATACATCA ATCACTCGGG

ACAGCTTTCA GCCACGTGGA CTTTCCCAGT AGAGATGTCG ATCTCTCGGG

ACAGCTTTCA GCAATGTCAA CTTTTCCATT AGATACATCT ATCACTCGAA

ACAGCTTTCA GCAACGGCAA CCTTCCCGTT AGACACATCT ATCACTCGAG

CTGTAAAGGA CAGGAAATCA GGCAATTACC CACCTATGGG AGGTCTGAAT

CTGTAAAGGA CAGGAAATCA GGCAATTACC CACCTATGGG AGGTCTGAAT

CTGTAAAGGA CAGGAAATCA GGCAATTACC CACCTATGGG AGGTCTGAAT

CTGTAAAGGA CAGGAAATCA GGCAATTACC CACCTATGGG AGGTCTGAAT

CTGTAAAGGA CAGGAAATCA GGCAATTACC CACCTATGGG AGGTCTGAAT

CTGTAAA-GA CAAGAAATCA GGCAATTACC TACCTATAGA AGGCCTAAGT

CTGTAAAGGA CAGGAAATGA AGCAATTACG CACCTAGAGA AGGCCTAAGC

CTGTAAAGGA CAGGAAATGA AGCAATTACT CACCTATAGA AGGCCTAAGC

CTGTGAAGGA CAGGAGAT-A GGAAATTACC CACCTACAGG AGGCCTAAGT

CTGCAAAGGA CAGCAAGTCA GGCGATTACT CACCTATAGG AGGCCTAAAT

CTGTAAAGGA CAGGAAGTCA GGCAATTACT CACCTATAGA AGGCCTAAGT

CTGCAAAGGA CAGGAAGTCA GGCAATTACC CACCTATAGA AGGCCTAAGT

CTGTAAAGGA CAGAAAGTCA -GTGATT--- ---------- ----------

CTGTAAAGGA CAGGAAATTA GGCAATTACT CACCTATAGC AGGCCTAAAT

CTGTGAAGGA CAGGAAACCA GGTTATTACC CACCTATAGT AGGCCTAAGG

CTATA----- ---------- ---------- ---CTATAGT AGGCCTACCT

CTATAAAGGA CAAAGAATTA GGTAATTACT TACCTATTGC AGGCCTAAGT

CTGGAAAGGA TGGAAGATTG GGCGGTTACT TACCAACAGT TGGCCTCAGC

CTTTCAAGGC ACTGCTTATC TTCAAGGCTG CATGCTGTTG AAATCACTTT

CTTTCAAGGC ACTGCTTATC TTCAAGGCTG CATGCTGTTG AAATCACTTT

CTTTCAAGGC ACTGCTTATC TTCAAGGCTG CATGCTGTTG AAATCACTTT

CTTTCAAGGC ACTGCTTATC TTCAAGGCTG CATGCTGTTG AAATCACTTT

CTTTCAAGGC ACTGCTTATC TTCAAGGCTG CATGCTGTTG AAATCACTTT

CTTTCAAGGA ACTGCTTGTC TTCAAGGCTG CATGCTGTTG AAATCACCTT

CTTTCAAGAT GTTGCTTGTC CTCCAGGCTG TGTGCTGTTG AGATCACCTT

CTTTCAAGAT GTTGCTTGTC CTCCAGGCTG TGTGCTGTTG AGATAACCTT

CTTTCGAGGC ACTGCTTGTC TTCCAGGCTG CATGCTGTTG AAATCACTTT

CTTTCAAGAA ACTGCTTGTC TTCAAGGCTG CAGGCTGTTG AAATCACTTT

CTTTCAAGGC ATTGCTTGTC TTCAAGGCTG CCTGCTGTTG AAATCACTTT

CTTTCCAGGT ACTGCTTGTC TTCAAGGCTG CACGCTGTCG CAATCACTTT

---------- ---------- ---------- ---------- ----------

CTTTCAAGGT GCTGCTTGTC TTCAAGGCTG CACGCTGTTG AAATCACTTT

CTTTCAAGGC ACTGCTTGTC TTCCAGGCTG CACGCTGTGG AAATCACCTT

CTTGCAAGGA ACTGCTTGTC TTCCAGGCTG CATGCTGTTG AAATCACTGG

CTTTCAAGAT ACTGTTTATC TTCCAGACTG TAGGCTGTTG AAATAACTTT

TTTTCAAGGT ACTCTTTGTC TTCTGGACTG TATGCTGTAG AAATAACTTT

GGCTCCTCTA TGATGTGCTA ACTGAATAGC TATTGTACCA AATGCCTGCA

GGCTCCTCTA TGATGTGCTA ACTGAATAGC TATTGTACCA AATGCCTGCA

GGCTCCTCTA TGATGTGCTA ACTGAATAGC TATTGTACCA AATGCCTGCA

GGCTCCTCTA TGATGTGCTA ACTGAATAGC TATTGTACCA AATGCCTGCA

GGCTCCTCTA TGATGTGCTA ACTGAATAGC TATGGTACCA AATGCCTGCA

GGCTCCCCTA TGGTGTGCTA ACTGAATAGC TATTGTACCA AATGCCTGCA

GGCTCCTCTG TGGTGTGCTA ACTGAATGGC TATTGTGCCA AATGCCTGGA

GGCTCCTCTG TGGTGGGCTA ACTGAATGGC TATTGTGCCA AATGCCTGGA

GGCTCCTCTA TGGTGTGCTA ACTGAATAGC TATTGTGCCA AACGCCTGCA

GGCTCCTCTG TGGTGTGCTA ACTGAATGGC TATTGTACCA AGTGCCTGTA

GGCTCCTCTA TGGTGTGCTA ACTGAATAGC TATCATACCA AGTGCCTGCA

GGCTCCTCTA TGGTGTGCTA ACTGAATAGC TATCGTACCA AGTGCCTGCA

---------- ---------- ---------- ---------- ----------

GGCTCCTCTA TGGTGTGCTA ACTGAATGGC TATTGTACCA AAGGCCTGCA

GGCTCCTCTG TGGTGTGCTA ACTGAATAGC TATTGTTCCA AATGCCTGCA

GGCTCCTCTG TGGTGGGCTA ACTGAATAGC TATCATCTCC AAAGCTTGCA

GGCCCCTCTA TGGAGTGCCA ACTGAATAGC TATTGTACCA AATGCCTGTA

AGCTCCTCTA TGGTGTGCTA ACTGAATAGC TATTATACCA AGCGACTGTA

CCTACACTTG CTCCATCCAT TATCAGCACT GATTTTCCAG GAGAGAGATG

CCTACACTTG CTCCATCCAT TATCAGCACT GATTTTCCAG GAGAGAGATG

CCTACACTTG CTCCATCCAT TATCAGCACT GATTTTCCAG GAGAGACATG

CCTACACTTG CTCCATCCAT TATCAGCACT GATTTTCCAG GAGAGACATG

CCTACACTTG CTCCATCCAT TATCAGCACT GATTTCCCAG CAGAGAGATG

CTTACACTCG CTCCATCCAT GATCAGCACT GACTTTCCTG GAGAGAGCTG

CTCACACTTG CCCCATCCAT GATCAGCACG GACTTCCCGG GAGAGAGCTG

CTCACACTTG CCCCGTCCAT GATCAGCACA GACTTTCCAG GAGAGAGTTG

CCTACACTTG CTCCATCCAT GATCAGCACT GATTTTCCTG GAGAGAGATG

CCTACACTTG CTCCGTCCAT TATCAGCACG GATTTTCCAG GCGAGAGATG

CCTACACTGG CTCCATCCAT TATCAGCACT GATTTTCCAG GAGAGAGATG

CCTACACTTG CTCCATCCAT TATCAGCACT GATTTTCCTG GAGAGAGCTG

---------- ---------- ---------- ---------- ----------

CCTACACTTG CTCCATCCAT TATCAGCACT GATTTTCCAG GAGAGAGATG

CCCACACTTG CTCCGTCCAT TATCAGCACC GACTTTCCTG GCGAGAGATG

TCTCCACTTG TTCCATCCAT GATCAGCACC GAGTTCCCAG GAACAAGATG

CCCACACTTG CTCCGTCCAT TATAAGCACG GATTTTCCAG GACAGAGATG

CTTACACTGG CTCCGTCCAT TATCAGCACT GATTTTCCAG GAGAAATATG

AGAAAGATAA TGCAGAGCTG TATAGGCACG CACTCCATCC CGAATGCTTC

AGAAAGATAA TGCAGAGCTG TATAGGCACG CACTCCATCC CGAATGCTTC

AGAAAGATAA TGCAGAGCTG TATAGGCGCG CACTCCATCG CGAATGCTTC

AGAAAGATAA TGCAGAGCTG TATAGGCGCG CACTCCATCC CGAATGCTTC

AGAAAGATAA TGCAGAGCTG TATAGGCACG CACTCCATCC CGAATGGTTC

AGAAAGGTAA TGCAGTGCTG TGTAGGCTCG AACTCCATCC CGAATGGTTC

AGAAAGGTAA TAGAGGGCTG TGTAGGCTCG GACTCCATCC CGGATGACTC

AGAAAGATAA TACAGAGCTG TGCAGGCTCG GACTCCGTCC CGGATGACGC

AGAAAGATAA TGCAGAGCGG TGTAGGCTCG CACTCCGTCT CGGATGGTTC

AGAAAGATAA TGCAGGGCTG TATAGACTCG TAATCCATCC CGGATGGTTC

AGAAAGATAA TGCAGAGCTG TATAGGCTCG TACTCCATCC CGAATGGTTC

AGAAAGATAA TGCAGAGCTG TGTAGGCTCG TACTCCATCC CGAATGGTTC

---------- ---------- ---------- ---------- ----------

AGAAAGATAA TGCAGAGCTG TATAGGCTCG TACTCCATCC CGAATGGTTC

AGAAAGGTAA TGCAGAGCAG TATAGGCCCG CATTCCATCC CGAATGGTTC

AGAAAGGGAA TGGAGAGCTG TGTAGGCTCG TAGTCCATCC CGAATGGTTC

GGAAAGAGAA TGAAAAGCTG TATAGGAACG CAGCCCATCG CGGATGACTC

AGAAAGATAA TGCAGAGCTG TATAGGCACG AACTCCATCA CGGATCGTTC

CTGCTGCTTC CGTCCATGTG ACCTTTTCTG GTTTATGAAC TAAGTAATGC

CTGCTGCTTC CGTCCATGTG ACCTTTTCTG GTTTATGAAC TAAGTAATGC

CTGCTGCTTC CGTCCATGTG ACCTTTTCTG GTTTATGAAC TAAGTAATGC

CTGCTGCTTC CGTCCATGTG ACCTTTTCTG GTTTATGAAC T---------

CTGCTGCTTC TGTCCATGTA ACCTTTTCTG GTTTATGAAC TAAGTAATGC

CAGCTGCTTC TGTCCAAGTG ACCTTTTCTG GTTTATGAAC TAAGTAATGC

CAGCGGCTTC CGTCCATGGC ACCTTTTCTG GCTTATGAAC TAGGTAATGC

CAGCAGCTTC CGTCCATGAC ACCTTTTCTG GCTTATGAAC TAGGTAATGC

CAGCTGCTTC TGTCCACGTG ACCTTTTCCG GCTTATGAAC T---------

CAGCAGCTTC TGTCCACGTG ACTTTTTCTG GTTTGTGAAC TAAGTAATGC

CAGCAGCTTC TGTCCATGTA ACTTTTTCTG GTTTGTGAAC TAAGTAATGC

CAGCAGCTTC TGTCCATGTG ACTTTTTCTG GCTTGTGAAC TAAGTAATGC

---------- ---------- ---------- ---------- ----------

CAGCTGCTTC TGTCCATGTA ACCTTTTCTG GTTTGTGAAC TAAACAGTGC

CAGCCGCTTC TGTCCATGTA ACCTTTTCTG GTTTATGAAC T---------

CAGCCGCTTC TGCCCACGTA ACCTTTTCTG GTTTATGAAC TAAGTAATGC

CTGCTGCGTC GGCCCACGTG ACCTTTTCTG GTTTATGAAC TAAGTAATGC

CCGCTGCTTC GACCCAAGTG ACTTTTTCTG GCTTATGAAC TAAGTAATGC

TCATGTACTC TAACAACTTC ACAAAGCAGG GTCTTCAGAG TCCAGGGGCA

TCATGTACTC TAACAACTTC ACAAAGCAGG GTCTTCAGAG TCCAGGGGCA

TCATGTACTC TAACAACTTC ACAAAGCAGG GTCTTCAGAG TCCAGGGGCA

---------- ---------- ---------- ---------- ----------

TCATGTACTC TAACAACTTC ACAAAACAGG GTCTTCAGAA TCCAGAGGCA

TCATGTACTC TAACAACTTC ACAAAGCAGA GTCTTCAGAG TCCAATGGCA

TCCTGTACTC GGATAACTTC GCAGAGCGGG GTCTTCCGAA TCCAAAGGCA

TCATGTACTC GAATAACCTC ACAGAGCGGG GTCTTCGGAA TCCAAAGGCA

---------- ---------- ---------- ---------- ----------

TCATGGACTC GAACAGCCTC ACAAAGCAGG GTCTTCAGAA TCCAGGGGCA

TCGTGCACTC TAACAACTTC ACAAAGCAGG GTCTTCAGAA TCCAGGGGCA

TCATGTACTC TAACAACTTC ACAAAGCAGG GTCTTCAGAA TCCAGGGGTA

---------- ---------- ---------- ---------- ----------

TCATGTACCC TAAGCACTTC ACAGAGCAGG GTCTTCGGAA TCCAGGGGTA

---------- ---------- ---------- ---------- ----------

TCGTGGACTC TATCGGCACC ATGAAGCTGA GTCTTCAGAT CCCAGGGGTA

TCATGTATTC TAACAATTTC ACAGAGCAGA TTCTTCAGAA TCCAGTGGTA

TCGTGAACTC TGATCACTTC ACAAAGCAGA TTCTTCAGAA TCCATCGGTA

AAATAACTTA CCAACTACTT CATCATCTGG TTGAAAGAAT GATACCTTGC

AAATAACTTA CCAACTACTT CATCATCTGG TTGAAAGAAT GATACCTTGC

AAATAACTTA CCGACTACTT CATCATCTGG TTGAAAGAAT GATACCTTGC

----AACTTA CCGACTACTT CATCATCTGG TTGAAAGAAT GATACCTTGC

AAATAACTTA CCGACTACTT CATCATCTGG TTGAAAGAAT GATACCTTGC

AAATAACTTA CCGACTACTT CATCATCTGG TTGAAAGAAT GATACCTTGC

AAATAACTTA CCGACGACTT CATCATCTGG CTGAAAGAAC GATACCTTCT

GAATCACTTA CCGACCACTT CGTCGTCTGG CTGAAAGAAC GTTACCTTCC

----AACTTA CCAACTACTT CATCATCTGG CTGAAAGAAT GACACCTTTT

GAATAACTTA CCCACTACTT CATCATCTGG TTGAAAGAAT GATACCTTGC

GAATAACTTA CCGACGACTT CATCATCTGG TTGAAAGAAT GATACCTTGC

AAATAACTTA CCGACTACTT CATCATCTGG TTGAAAGAAT GATACCTTGC

---------- ---------- ---------- ---------- ----------

AAATAACTTA CCAACTACTT CATCATCTGG TTGAAAGAAC GATACCTTGC

---------- ---------- ---------- ---------- ----------

CAATAACTTA CCTACTACTT CATCATCTGG TTGAAAGAAT GAGACTTTGC

AAATAACTTA CCTACTACTT CATCATCTGG ATGAAAGAAT GATACTTTGC

AAATAACTTA CCAACCACTT CATCATTTGG TTGAAAGAAG GATACTTTAT

TTCCAACTAA AGAGACTTAC CATCTAATAC AATTCCAGCA ATTTCTCTCC

TTCCAACTAA AGAGACTTAC CATCTAATAC AATTCCAGCA ATTTCTCTCC

TTCCAACTAA AGAGACTTAC CATCTAATAC AATTCCAGCA ATTTCTCTCC

TTCCAACTGA AGAGACTTAC CATCTAATAC AATTCCAGCA ATTTCTCTCC

TTCCAACTAA AGAGACTTAC CATCTAATAC AATTCCAGCA ATTTCTCTCC

TTCCAACTAG AGAGACTTAC CTTCTAACAC AATTCCAGCA ATTTCTCTCC

TGCCAACTGA AGAG-----C CATCTAATAC AATCCCAGCG ACTTCTCTCC

TGCCAACTAA AGAGACTTAC CCTCTAATAC AATTCCAGAA ACTTCTCTTC

TCCCAACTAT AAAG-----C CATCTAACAC AACTCCAGCA ATTTCTCTGC

TTCCAACTAA AATGACTTAC CATCTAACAC AATTCCAGCA ACTTCTCTCC

TTCCAACTAA AGAGACTTAC CATCTAACAC AATTCCAGCA ATTTCTCTCC

TTCCAACTAA AGAAACTTAC CATCTAACAC AATTCCAGCA ATTTCTCTCC

---------- ---------- ---------- ---------- ----------

TTCCAACTAA AGAAACTCAC CATCTAACAC AATTCCAGCA ACCTCTCTTC

---------- ---------- ---------- ---------- ----------

GTCCAACTAA AAAAACTTAC CATCTAACAC AGTTCCAGCA ACTTCTCTCC

TTCCAACTGA AAAGACTTAC CCTCTAGTAC AACTCCAGCA ATTTCTCTCC

TTCCAACTGC AAAGACTTAC CTTCCAAAAC TATTCCTGCA ATTTCTCTCC

CAACAGGAAA TAAATCCTTT TTCTTTGTAT TTATCTGGCT CAGAGACAAG

CAACAGGAAA TAAATCCTTT TTCTTTGTAT TTATCTGACT CAGAGACAAG

CAACAGGAAA TAAATCCTTT TTCTTTGTAT TTATCTGGCT GAGAGACAAG

CAACAGGAAA TAAATCCTTT TTCTTTGTAT TTATCTGGCT GAGGGACAAG

CAACAGGAAA GAAATCCTTT TTCTTTGTAT TTATCTGGCT CAGAGGCAAG

CAACAGGAAA GAATTCCTTC TTCTTCGTAT TTATCTGGCT CAGAGACAAG

CAACAGGGAA GAATTCCTTT TCCTTTGTGT TTATGTGGCT CAGGGACAGG

CAACAGGGAA GAAATCCTTT TCCTTTGTGT TTATGTGGCT CAGGGACAAG

CAACAGGAAA GAACTCCTTT TCCTTCGTAT TTATCTGGCT TAGAGACAAG

CAACAGGAAA GAATTCCTTT TCCTTTGTAT TTATCTGGCT CAGAGACAAG

CAACAGGAAA GAAATCTTTC TCCTTTGTAT TTATCTGGCT TAGAGACAAG

CAACAGGAAA GAAATCCTTC TCCTTTGTAT TTATCTGGCT CAGAGACAGG

---------- ---------- ---------- ---------- ----------

CAACAGGAAA GAAATCCTTC TCCTTTGTAT TTATCTGGCT CAGAGACAAG

---------- ---------- ---------- ---------- ----------

CAACAGGAAT AAAATCTTTT TCCTTTGTGT TTATCTGGCT CAGAGACAAG

CAACAGGAAA AAATTCCTTC TCCTTTGTAT TTATCCAGCT TAGAGACAAG

CCACAGGGAA AAAATCCTCC TCCTCTGCAT TTATCCAGCT TAGAGACAGG

CTTTAACTTG AAGTTTCACA AAGTTATCCT TACCTTTCTT GAAATACAAA

CTTTAACTTG AAGTTTCACA AAGTTATCCT TACCTTTCTT GAAATACAAA

CTTTAACTTG AAGTTTTACA AAGTTATCCT TACCCTTCTT GAAATACAAA

CTTTAACTTG AAGTTTTACA AAGTTATCCT TACCCTTCTT GAAATACAAA

CTTTAACTTG AAGTTTCACA AAGTTGTCCT TACCCTTCTT GAAATACAAA

CTTTAACTTG AAGTTTCACG AAATTATCCT TACCCTTCTT GAAATACAAA

CTTTAACTTG CACTCTCACA AAGTTATCCT TACCTTTCTT GAAATACAAA

CTTTAACTTG AACTCTCACA AAGTTATCCT TACCTTTCTT GAAATACAAA

CTTTTACTTG AAGTTTCACA TAGTTATC-- ---------- ----------

CTTTAACTTG AAGTTTCACA TAGTTATCCT TACCCTTCTT GGAACACAAA

CTTTAACTTG AAGTCTCACA TAGTTATCCT TACCCTTCTT GAAATACAAA

CTTTAACTTG AAGTTTCACA TAGTTATCCT TACCCTTCTT GAAATACAAA

---------- ---------- --------CT TACCCTTCTT GAAATACAAA

CTTTAACTTG AAGTTTCACA AAGTTATCCT TACCCTTCTT GAAATACAAA

---------- ---------- ---------- ---------- ----------

TTTTAATTTG AAGTTTCACA AAGTTATCCT AACCCTTCTG AAAATATAAA

CTTTTACTTG AAGCTTCACA TAGTTATCCT CACCCTTCTT GAAATACAAA

CTTTAACTTG AAGTGTCACA TAATTGTCCT CACCCGTCTT GATACACAAA

TGTTATTTCT TCATCTGTGG AACTCTGTGG AAATATAAGC CTTTCATAGT

TGTTATTTCT TCATCTGTGG AACTCTGTGG AAATATAAGC CTTTCATAGT

TGTTATTTCT TCATCTGTGG AACTCTGTGG AAATATAAGC CTTTCATAGT

TGTTATTTCT TCATCTGTGG AACTCTGTGG AAATATAAGC CTTTCATAGT

TGTTATTTCT TCATCTGTGG AACTCTGTGG AAATATAAGC CTTTCATAGT

TGTTATTTCT TCATTTGTGG AACTTTGTGA AAATATAAGC CTTTCATAGT

TGTTACTTCT TCATTTGTAG AACTCTGTGA AAATACAAGC CTTTCATAGT

TGTTACTTCT TCATTTGTAG AACTCTGTGA AAATACAAGC CTTTCATAGT

---------- ---------- ---------- ---------- ----------

TGTTATTTCT TCATTTGTAG AACTTTGTGA AAATACAAGC CTTTCATAGT

TGTTATTTCT TCATTTGTAG AACTCTTTGA AAATATAAGC CTTTCATAGT

TGTTATTTCT TTATTTGTAG AACTCTGTGA AAATATAAGC CTTTCATAGT

TGTGATTTCT TCATTTGCGG AACTCTGTGA AAATACAATC CTTTCATTGT

TGTTATCTCT TCATTTGTGG AAGTCTGTGA AAATATAAGC CTTTCATAGT

---------- ---------- ---------- ---------- ----------

TGTCATTTCC TCATTTGTAG AATTCTGTGA AAATATAAGC CTTTCATAGT

TGAAATTTCT TCTCCTGGTG AACTCTGTGA AAATATAAGC CTTTCATAGT

TTTCACTTCA TCTTCTGAAG AAGCTCGTGG ACATATAAGC CTTTCATAGT

CACCtaaaaT GGTACCGTCA CAATTATTCT GAGTAAGAGA GGAAATGAAC

CACCtaaaaT GGTACCGTCA CAATTATTCT GAGTAAGAGA GGAAATGAAC

CACCTAAAAT GGTACCTTCA CAATTATTCT GAGTAAGAGA GGAAATGAAC

CACCTAAAAT GGTACCTTCA CAATTATTCT GAGTAAGAGA GGAAATGAAC

GACCTAAAAT GGTACCTTCA GAATTATTCT GAGTAAGAGA GGAGATGAAC

CACCTAAAAT GGTACCTTCC CCATTATTGT G----ATAGA GGACATGAAC

TACCTATACC GGTACTGCCA CAATTATTCT GAGTAAGAGA GGAACTGAGC

TACCTATACT GGTACCGCCA CAATTATTCT GAGTAAGAGA GGAAATGAAC

---------T GATACCTCCA CAATTATTCT GAGTAGGAGA GGAAATGAAC

CACCTAAAAT GGTACCTTCA CAATTATTCT GAGTAAGAGA GGAAATGAAC

CACCTAAAAT GGCACCTTCA CAATTATTCT GAGTAGGAGA GGAAATGAAC

CACCTAAAAT GGTACCTTCA CAATTATTCT AAGTAAGAGA GGAAATGAAC

CACCTAAAAC AGTACCTTCA CAATTATTCT GAGCGAGGGA GGAAATGAAC

TGCCTACAAT GGTACCTTCA CAATTATTCT GAGTAAGAGA GGCAATGAAC

---------- ---------- ---------- ---------- ----------

GACCTAAACT GGTACCTTCA AGATCATTTT GGGTAAGAGA GGAAATGAAC

GACCTGAAAT AGTACCTTCA AAATTATCCT GGTTAGGAGA GGAAATTCAC

GACCTGAAAT AGTACTTCCA TAATTATTCC GAACGAGTGG GGAAGCGAGC

AAATTTTTAA TGTTGTGAAA TAGGGCATAA AATGAGTGGT AGAGTTCTGT

AAATTTTTAA TGTTGTGAAA TAGGGCATAA AATGAGTGGT AGAGTTCTGT

AAATTTTTAA TGTTGTGAAA TAGGGCATAA AATGAGTGGT AGAGTTCTGT

AAATTTTTAA TGTTGTGAAA TAGGGCATAA AATGAGTGGT AGAGTTCTGT

AAATTTTTAA TGTTGTGAAA TAGGGCATAA AATAG--GGT AGAGTTCTGT

AAAGTTTTAA TGTTGTGAAA TAGGGCATAA AAGGAGTGGT AGAGTTCCCT

ACATTTCTAA TGTTGTGAAA TAGGGCATGT AATGAGTGG- GGAGTTCCCT

ACATTT-TAA TGTTGTGAAA TAGGGCATAA AATGAGTAGC GGAGTTCCCT

ACATTTTTAA TGTTGTGAAA TAGGGCATAA AATGAGTGCT AGAGTTCTGG

ACATTTTTGA TGTTGTGAAA TAGGGCATAA AATGAGTGGT AGAGTTCCCT

AAATTTTTGA TGTTGTGAAA TAGGGCATAA AATGAGTAGT AGAGTTCCCT

AAATTTTTGA TGTTGTGAAA TAGGGCATAA AATGA---AT AGAGTTCCGT

ACATTTTTGA TGTTGTGAAA TAGGGCATAA AATGAGTGGT AGAGTTCCCT

AAATTTTTAG TGTTGTGAAA TAGGGCATAA AATGAGTGGT AGAGTTTCAT

---------- ---------- ---------- ---------- ----------

ACATTTTTAA TGTTGTGAAA TAGGGGATAA AATGAGTGGT AGAGTTCCGT

AAATATTCAG AATtatgtgt caggccctat actaagtgct ggagtgcaaa

AAATCTTGAA TGTAATGCAA TGGGTCCTAA AACTAGGAGT AGTCTTCCGT

GAAATTAAAA TATTTCTTTT TGGCTTTTAG TTCCCAAGAA CATACTCACA

GAAATTAAAA TATTTCTTTT TGGCTTTTAG TTCCCAAGAA CATACTCACA

GAAATTAAAA TATTTCTTTT CGGCTTTTAG TTCCCAAGAA CATACTCACA

GAAATTAAAA TATTTCTTTT CGGCTTTTAG TTCCCAAGAA CATACTCACA

GAAAGTAAAA TGTTTCTTTT TGGCTTTTAG TTCCCAAGAA CATACTCACA

GAAATTAAAA TATTTCTTTT TGGCTTTTAG TTCCAAAGAA CATCCTTGCA

GAAATTAAAA TACTTCTTTT TGGTTTTTAG TTCCCAAGAA CATACTCACG

GAAATAAAAA TACTTCTTTT TGTTTTTTAG TTCCCAAGAA CATACTCACG

GAAGTTAAAA TCGTTCTCT- --GGTTTCAG TTCCCAAGAG CATACTCTCA

GAAACTAAAA TATCTCTTTT TGGTTTTCAG TTCCCAAGAA TATACTCACC

GAAATTAAAA TATTTCTTTT TGGCTTTTAG TTCCCAAGAA CATACTCACA

GAAATTACAG TATTTCTTTT TGGGTTTTAG TTCCCAAGAA CATACTCACA

GAAA------ ---------- ---------- ---------- ----------

GAAATTAAAC TCTTTCTTTT TGGTTTTTAG TTCCCAAGAA CATACTTACA

----TTAAAA TATTTTTTCT TGGTTTTTAG TTCCCAAGAA CATCCTCGCA

GAAAttaaaa cattttatta ggggctTTAG TTACCCAATC CATACACACA

gtaaCAAAAA TTTCTCTTTT TTATTTTTAG TTCCCAAAAA CATACTTACA

GCGAGAAAAT TCTTTCTTTT TCATTTCTAG TTCCCAAACA CATACTTTCT

GCGATTGATC CAGCCATTGC TAATCTAAAA CCTTCTTTGA AGCAGAAAGA

GCGATTGATC CAGCCATTGC TAATCTAAAA CCTTCTTTGA AGCAGAAAGA

GCGATTGATC CAGCCATTGC TAATCTAAAA CCTTCTTTGA AGCAGAAAGA

GCGATTGATC CAGCCATTGC TAATCTAAAA CCTTCTTTGA AGCAGAAAGA

GCGATCGATC CAGGCATTGC TAATCTAAAA CCTTCTTTGA AGCAGAAAGA

GCGATTGATC CAGCTGTTGC TAATCTAAAA CCTTCTTCGA AGCAGAAAGA

GCGGTTGACC CAGCCGTTGC TAACCTAAAT CCCTCTCTGA ---AGAAAGA

GCAGTGGATC CGGCTGTTGC TAATCTAAAT CCTTCTCTGA ---AGAAAGA

GCGATCGACC CAGCCGTCGT CGACCTCAGA CCCTCTCTGG AGCAGGAAGA

GCAATTGACC CATCAGTTGC TAATCTAAAA CCTTCTGTGA AGCAGAAAGA

GCGATCGATC CAGCCATTGT TAATCTAAAA CCTTCTTTGA AGCAGAAAGA

GCAATTGATC CAGCTATTGC TAATCTGAAG CCTTCTCTGA AGCAGAAAGA

---------- ---------- ---------- ---------- ----------

GCAATTGATC CAGCTGTTGC TAATCTAAAA CCTTCTTTGA AGCAGAGAGA

GCAATTGATC CAGCTGTTGC TAATCTAGAA CCTTCTTTGA AGAAGAAAAA

TCAATTG--- ---------- ---------- ---------- ----------

GCGGTTGATC CAATGGTCTC TAATCTAAAC CCTTCTTTGA AGCAGAAAGA

GCTATCGATC CAATGGTTTC CCTGCTAAAT CCTACTTGGA AGCAGAAAGA

TGGTGAATAT GGTAATTTTT ATGGCTTATA CCTTTGTTTT CAGTGTGGAT

TGGTGAATAT GGTAATTTTT ATGGCTTATA CCTTTGTTTT CAGTGTGGAT

TGGTGAATAT GGTAATTTTT ATGGCTTGTA CCTTTGTTTT CAGTGTGGAT

TGGTGAATAT GGTAATTTTT ATGGCTTGTA CCTTTGTTTT CAGTGTGGAT

TGGTGAATAT GGTAATTTTT ATGGCTTATA CCTTTGTTCT CAGTGTGGAT

CACTGAATAT GGTAATTTTT ATGGTTTGTA CCTTTGTTTT CAGTGTGGAT

CGATGAATAC GGTAATTTCG ACGGCTTGTA CCTTTGTTTT CAGAGTGGAC

TGATGAACAC GGTAATTTCT ATGGCTTGTA CCTTTGTTTT CAGTGTGGAC

TGGTGCGCAC GGTAATGTCC ATGCCTTGTA CCTTTGTTTT CAATGTGGAC

TGGCGACTAT GGTAATGTTT ATGGCTTGTA CCTTTGTTTT CAGTGTGGAC

TGGTGAATAT GGTAATTTTT ATGGCTTGTA CCTTGGTTTT CAGCGTGGAT

TGATGAATAT GGTAATTTTT -TGGCTTGTA CCTTTGTTTG CAGGGTGGAT

---------- ---------- -------CAA CCTTTGTTTT CAGCGTGGAC

TGGTGAACAT GGTAAGTTTT ATGGCTTTTA CCTTTGTTTT CAGTGTAGAT

TGGGGAATAT GGTAATTTTT ATGGCTTGTA CCTTTGTTTT CAGAGTGGAT

---------- ---------- ---------- ---------- ----------

TGGTGACTCT GGTAATATTT ATGGCTTATA CCTTGGTTCC CAGGGTGGAC

CGGTGACTAC GGTAATTTCC GTTTCTTGCA CCTTGGATCT CAGGGTGGAC

AATAAAATAT TGATGGTAGA GATCCTATCT TCCTTTATCC CTGAGCTAAA

AATAAAATAT TGATGGTAGA GATCCTATCT TCCTTTATCC CTGAGCTAAA

AATAAAATAT TGATGGTAGA GATCCTATCG TCCTTTATCC CTGAGCTAAA

AATAAAATAT TGATGGTAGA GATCCTATCT TCCTTTATCC CTGAGCT-AA

AACAAAATAT TGACGGTAGA GATCCTATCT TCCTTTATCC CTGAGCTAAA

AACAAAATAT TGACAGTAGA AATCCTATCC TCCTTTATGC CTGAGCTAAA

AACAAAATGT TGATAGTAGA AATCTTATCT TCCTTTATGC CTGAGCTGAA

AATAAAATGT TGATGGTAGA AATCTTATCC TCCTTTATAC CTGAGCTAAA

AACAAAATAT TGATGGTAGA AATCCTATCT TCCTTTATGC CTGAGCTAAA

AACAGGATAC TGATGGTGGA GATCCTATCT TCCTTGATGC CTGAGCTAAA

AACAAAATAT TGATGGTAGA AATCCTGTCT TCCTTTATGC CTGAGCTAAA

AACAAAATAT TGATGGTAGA AATCCGATCT TCCTTTATGC CTGAGCCAAA

AGCAACATAT TGACGGTAGA GATCCTGTCC TCCTTGATGC CCGAGCTGAA

AACAAAAGAT TGACGGTAGA AATCCTATCT TCCTTTATGC CTGAGCTAAA

AACAAGAGAC TGACGGTAGA AATCCTGTCT TCCTTTACGC CTGAGCTGAA

---------- ---------- ---------- ---------- ----------

AACAAAAGAT TGATAGTAGA AATTCTATCT TCCTTCAAGC CAGTACTAAA

AGCAGGAGGT CGATGGTGGA GATTCTGTCC TCTCTCAAGC CGGTGCTGAA

AATGCAAGGA ATAAATTCTG AAAGTAAAAG TTTCAAAGCA TTAAATAGCA

AATGCAAGGA ATAAATTCTG AAAGTAAAAG TTTCAAAGCA TTAAATAGCA

AATGCAAGGA ATAAATTCTG AAAGTAAAAG TTTCAAAGCA TTAAATAGCA

AATGC-AGGA ATAAATTCTG AAAGT-AAAG TTTCAAAGCA TTAAA-----

AATGCAAGGA ATAAATTCTG AAAGTAAAAG TTTCAAAGCA TTAAATAGCA

GATGCAAGGA ATAAATTCTG AAAGTAAAAG TTTTAAAGCA TTAAATAGCA

AATGCAGGGG ATGAACTCTG AAAATAAATG TTGCAAAGAA TTAAATAGCA

GATGCAGGGG ATAAATTCTG AAAATGAATG TTGCAAAGAA TTAAACAGCA

AATGCAAGGA ATAAACTCTG AAAATAAAAG GTTCAAAGCA TTAAATAGCA

AATGCAAGGA ATAAATTCTG AAAGTAAAAA GTT-----CG TTAGATAGCA

GATGCAAGGA ATAAATTCTG AAAGTAAAAG GTTCACAGCA TTAAATAGCA

AATGCAAGGA ATAAATTCTG AAAGTAAAAG TTTCAAAGCA TTAAATAGCA

GATGCAGGGG ATGAACTCTG ATGGCAAAAG GTC------- -----TAGCA

AATGCAAGGA ATAAATTCTG AAAGGAAAAG TTTCAAAGTA TTAAATAGCA

GATGCAAGGT ATGAACTCTG AAAGCCAAAG TTTCAGAATA TTAGA-----

---------- ---------- ---------- ---------- -----CAGCA

AATGACAGGG AGGAATTCTG AAAACAAAAT TTTCAAAGAA TTCAATAGCA

AATGGCCGGG ATGAAGTCTG CGGACGGAAG TTTCGCAAGG CTGAGTAGGA

AGGTAAAAGT AACAGAACCA TGGCTCAGTT TCCAACACCT TTTGGTGGTA

AGGTAAAAGT AACAGAACCA TGGCTCAGTT TCCAACACCT TTTGGTGGTA

AGGTAAAAGT AACAGAACCA TGGCTCAGTT TCCAACACCT TTTGGTGGTA

---------- ---------- ---------- ---------- ----------

AGGTAAAAGT AACAGAACCA TGGCTCAGTT TCCAACACCT TTTGGTGGTA

AGGTAAAAGT AACAGAACCA TGGCTCAGTT TCCAACACCT TTTGGTGGTA

AGGTAAAGGT AACAGAACCA TGGCTCAGTT TCCGACACCT TTTGGTGGTA

AGGTGAACGT AATAGAACCA TGGCTCAGTT TCCCACACCT TTCGGTGGTA

AGGTAAAAGC AACGGAACCA TGGCTCAGTT TCCAACACCT TTTGGTGGTA

AGGTGAAAGT AACAGAACCA TGGCTCAGTT TCCCACACCC TTTGGTGGTA

AGGTAAAAGT GACAGAACCA TGGCTCAGTT TCCAACACCT TTTGGAGGTA

AGGTAAAAGT AACAGAACCA TGGCTCAGTT TCCAACACCT TTTGGTGGTA

AGGTAAAAGT GACAGAACCA TGGCTCAGTT CCCAGCACCT TTTGGAGGTA

AGGTGAAAGT AACAGAACCA TGGCTCAGTT TCCAACACCT TTTGGTGGTA

---------- ---------- ---------- ---------- ----------

AGGTAAAAGT AACAGAACCA TGGCTCAATT TCCAACACCT TTTGTGGGTA

AGCTAAAAGT GATAGAACCA TGGCTCAGTT TCCAACATCT TTTGGTGGTA

AGCCAAAAGT GACGGAACCA TGGCTCAGTT TCCGGCTCCT TTCGGGGGTA

AGTTTAGGCA GCCTGGATAT CTGGGCCATA ACTGTAGAGG AAAGAGCGAA

AGTTTAGGCA GCCTGGATAT CTGGGCCATA ACTGTAGAGG AAAGAGCGAA

AGTTTAGGCA GCCTGGATAT CTGGGCCATA ACTGTAGAGG AAAGAGCGAA

----TAGGCA GCCTGGATAT CTGGGCCATA ACTGTAGAGG AAAGAGCGAA

AGTTTAGGCA GCCTGGATGT CTGGGCCATA ACTGTAGAGG AAAGAGCGAA

AGTTTAGGCA GCCTGGATAT CTGGGCCATA ACTGTAGAGG AAAGAGCGAA

AGTTCAGGGA GCCTGGACAT CTGGGCCATA ACCGTGGAGG AAAGAGCCAA

AGTTCAGGTA GCCTGGATGT CTGGGCCATA ACTGTGGAGG AAAGGGCCAA

AGTT------ ---------- ---GGCCATC ACTGTGGAGG AAAGGGCGAA

AGTTTAGGCA GCCTGGATAT TTGGGCCATA ACTGTAGAGG AAAGAGCAAA

AGTTTAGGCA GCCTGGATAT CTGGGCCATA ACTGTAGAAG AAAGAGCAAA

AGTTTAGGCA GCCTGGATAT CTGGGCCATA ACTGTAGAGG AAAGAGCGAA

AGATTAGGCA GCCTGGACAT CTGGGCCATC ACCGTAGAGG AGAGAGCGAA

AGTTTAGGCA GCCTGGATAT CTGGGCCATA ACTGTGGAGG AAAGAGCAAA

---------- ---------- ---------- ---------- ----------

AGGTTAGGTA GCCTGGACAT CTGGCCCATA ACTGTAGAAG AAAGAGCAAA

AGTGCAGGCA GCCTGGATAT TTGGGCCATC ACTGTAGAAG AACGAGCAAA

AGTTTAGGCA CCCTGGATAT CTGGGCCATA ACCGTGGAGG AAAGAGCAAA

GCATGATCAG CAGTTCCATA GTTTAAAGCC AATATCTGGA TTCATTACTG

GCATGATCAG CAGTTCCATA GTTTAAAGCC AATATCTGGA TTCATTACTG

GCATGATCAG CAGTTCCATA GTTTAAAGCC AATATCTGGA TTCATTACTG

GCATGATCAG CAGTTCCATA GTTTAAAGCC AATATCTGGA TTCATTACTG

GCATGATCAG CAGTTCCATA GTTTAAAGCC AATATCTGGA TTTATTACTG

GCATGATCAG CAGTTCCATA GTTTAAAGCC AATATCTGGA TTTATTACTG

GCATGACCAG CAGTTCCAGA GTTTGAAGCC GATATCTGGA TTCATCACTG

GCATGACCAG CAGTTCCTTA GCCTGAAGCC GATAGCGGGA TTTATTACTG

GCATGACCAG CAGTTCCATA GCCTAAAGCC AAGATCGGGA TTCATTACTG

GCATGATCAA CAGTTCCATA GTTTAAAGCC AATATCTGGA TTTATTACTG

GCATGATCAA CAGTTCCATA GTTTAAAGCC AATATCTGGA TTTATTACTG

GCATGATCAA CAGTTCCATA GTTTAAAGCC AATATCTGGA TTTATTACTG

GCACGATCAG CAGTTCCACA GTTTAAAGCC AATATCTGGA TTTATTACTG

GCATGATCAG CAGTTCCACG GTTTAAAGCC AATATCTGGA TTTATTACTG

---------- ---------- ---------- ---------- ----------

GCATGATCAA CAATTCCACA GTTTAAAGCC AATATCTGGA TTTATTACTG

GCATGATCAG CAGTTCCATA GTCTCAAGCC AACATCGGGG TTCATTACTG

GCACGATCAG CAGTTTCACA GTCTCAAACC CACATCTGGA TTTATCACCG

GTAATTTTTA CAGGTGATCA AGCTAGAAAC TTTTTTTTTC AATCTGGGTT

GTAATTTTTA CAGGTGATCA AGCTAGAAAC TTTTTTTTTC AATCTGGGTT

GTAATTTTTA CAGGTGATCA AGCTAGAAAC TTTTTTTTTC AATCTGGGTT

GTAATTTTTA CAGGTGATCA AGCTAGAAAC TTTTTTTTTC AATCTGGGTT

GTAATTTTTA CAGGTGATCA AGCTAGAAAC TTTTTTTTTC AATCTGGGTT

GTAA------ ---------- ---------- ---------- ----------

GTAATTTTTA CAGGTGATCA AGCAAGGAAC TTCTTTTTCC AATCTGGGTT

GTAATTTTTA CAGGTGATCA AGCGAGGAAC TTTTTTTTCC AATCTGGGTT

GTAATTATTA CAGGTGATCA AGCAAGAAAC TTTTTTTTTC AATCTGGGTT

GTAATTTTTA CAGGTGATCA AGCTAGAAAC TTTTTTTTTC AATCTGGGTT

GTAATTTTTA CAGGTGATCA AGCTAGAAAC TTTTTTTTTC AATCTGGGTT

GTAATTTTTG CAGGTGATCA AGCTAGAAAC TTTTTTTTTC AATCTGGGTT

GTAATATTTA CAGGTGATCA AGCTAGAAAC TTTTTTTTTC AATCTGGGTT

GTAATTTTTA CAGGTGATCA AGCTAGAAAC TTTTTTTTTC AGTCTGGGTT

---------- ---------- ---------- ---------- ----------

GTAATTTTTG CAGGTGATCA AGCTAGAAAC TTTTTTTTTC AATCTGGGTT

GTAGTTTTTG CAGGTGATCA AGCCAGAAAC TTTTTTTTCC AATCTGGGTT

GTAATTTTTA CAGGTGATCA AGCTAGGAAC TTTTTTTTTC AATCTGGGTT

ACCTCAACCT GTTTTAGCAC AGATATGGTA AGTTGGAATT TTTGCAGGGC

ACCTCAACCT GTTTTAGCAC AGATATGGTA AGTTGGAATT TTTGCAGGGC

ACCTCAACCT GTTTTAGCAC AGATATGGTA AGTTGGAATT TTTGCAGGGC

ACCTCAACCT GTTTTAGCAC AGATATGGTA AGTTGGAATT TTTGCAGGGC

ACCTCAACCT GTTTTAGCAC AGATATGGTA AGTTGGAATT TTTGCAGGGC

---------- ---------- ---------- ---------- -TTGCAGGGC

ACCTCAGCCC GTCTTAGCAC AAATATGGTA AGTTGGGATT TTTGCAGGGC

ACCTCAGCCT GTCTTAGCAC AAATATGGTA AGTTGGAATT TTTGCAGGGC

ACCTCAACCT GTTTTAGCAC AGATATGGTA AGTTGGAATT TTTGCAGGGC

ACCTCAGCCT GTTTTAGCAC AGATATGGTA AGTTGGAGCT TCTGCAGGGC

ACCTCAACCT GTTTTAGCAC AGATATGGTA AGTTGGAACT TTTGCAGGGC

ACCTCAACCT GTTTTAGCAC AGATATGGTA AGTTGTAACT TTTGCAGGGC

ACCTCAGCCT GTTTTAGCAC AGATATGGTA AGTTGGAACT TTTGCAGGGC

ACCTCAACCT GTTTTAGCAC AGATATGGTA AGTTGGAATT TTTGCAGGGC

---------- ---------- ---------- ---------- ----------

ACCGCAACCT GTTTTAGCAC AGATATGGTA AGTTGGAATT TTTGCAGGGC

ACCTCAGCCT GTTTTAGCAC AGATATGGTA AGCTGAAAAT GTTGCAGGGC

ACCTCAGCCC GTTTTAGCAC AGATATGGTA AGTTGAAAGT TTTTCAGGGC

ACTAGCTGAC ATGAATAATG ATGGAAGAAT GGATCAAGTG GAGTTTTCCA

ACTAGCTGAC ATGAATAATG ATGGAAGAAT GGATCAAGTG GAGTTTTCCA

ACTAGCTGAC ATGAATAATG ATGGAAGAAT GGATCAAGTG GAGTTTTCCA

ACTAGCTGAC ATGAATAATG ATGGAAGAAT GGATCAAGTG GAGTTTTCCA

ACTAGCTGAC ATGAATAACG ATGGAAGAAT GGATCAAGTG GAGTTTTCCA

ACTAGCTGAC ATGAATAATG ATGGAAGAAT GGATCAAGTG GAGTTTTCCA

GCTGGCTGAC ATGAATAAGG ACGGAAGGAT GGATCAGGTG GAGTTTTCCA

GCTAGCGGAC ATGAATAACG ATGGAAGGAT GGATCAAGTG GAATTTTCCA

ACTAGCTGAC ATGAATAACG ATGGAAGGAT GGATCAAGTG GAGTTTTCCA

GCTCGCTGAT ATGAATAATG ATGGAAGGAT GGATCAAGTG GAGTTTTCCA

GCTAGCTGAC ATGAACAATG ATGGACGAAT GGATCAAGTG GAGTTTTCCA

ACTGGCTGAT ATGAATAATG ATGGAAGAAT GGATCAAGTG GAGTTTTCCA

ACTGGCCGAC ATGAATAACG ACGGCAGGAT GGATCAGGTG GAGTTCTCCA

ACTAGCTGAC ATGAATAATG ATGGAAGAAT GGATCAAATG GAGTTTTCCA

---------- ---------- ---------- ---------- ----------

GCTAGCTGAC ATGAATAATG ATGGAAGAAT GGATCAAGTG GAGTTTTCCA

ATTAGCTGAC ATGAACAATG ATGGAAGAAT GGATCAGATA GAATTTTCCA

CCTGGCTGAC ATGAACAATG ATGGTAGAAT GGATCAGATG GAGTTTTCCA

TAGCTATGAA ACTTATCAAA CTGAAGCTAC AAGGATATCA GCTACCCTCT

TAGCTATGAA ACTTATCAAA CTGAAGCTAC AAGGATATCA GCTACCCTCT

TAGCTATGAA ACTTATCAAA CTGAAGCTAC AAGGATATCA ACTACCCTCT

TAGCTATGAA ACTTATCAAA CTGAAGCTAC AAGGATATCA ACTACCCTCT

TAGCTATGAA ACTTATCAAA CTCAAGCTAC AAGGATATCA GCTACCCTCT

TAGCTATGAA ACTTATCAAA CTGAAGCTGC AAGGATATCA GCTCCCCTCC

TAGCCATGAA GCTCATCAAA CTGAAGCTGC AAGGATATCA GCTCCCCCCC

TAGCCATGAA GCTTATCAAA CTGAAGCTAC AAGGATATCA GCTCCCCTCC

TAGCTATGAA ACTTATCAAA CTGAAGCTGC AAGGCTATCA GCTCCCCTCT

TTGCTATGAA ACTCATCAAG CTGAAGCTCC AAGGATATCA GCTCCCCTCT

TAGCTATGAA ACTCATCAAA CTCAAGCTGC AAGGATATCA ACTTCCTTCT

TAGCTATGAA ACTTATCAAA CTGAAGCTAC AAGGCTATCA ACTCCCCTCT

TCGCCATGAA GCTCATCAAG CTGAAGCTGC AGGGCTACCA GCTGCCCGCC

TTGCTATGAA ACTTATTAAA TTGAAGCTGC AAGGATATCA GCTCCCCTCC

---------- ---------- ---------- ---------- ----------

TCGCCATGAA ACTTATCAAA TTGAAGCTAC AAGGCTATCA GCTCCCCTCT

TAGCAATGAA ACTTATCAAA CTGAAGCTCC AAGGTTATCA GTTACCTGCT

TTGCGATGAA ACTGATCAAA CTGAAGCTCC AAGGCTATCA GCTTCCCTCT

GCACTTCCCC CTGTCATGAA ACAGCAACCA GTTGCTATTT CTAGCGCACC

GCACTTCCCC CTGTCATGAA ACAGCAACCA GTTGCTATTT CTAGCGCACC

GCACTTCCCC CTGTCATGAA ACAGCAACCA ---GCTATTT CTAGCGCACC

GCACTTCCCC CTGTCATGAA ACAGCAACCA ---GCTATTT CTAGCGCACC

ACACTTCCCC CTGTCATGAA ACAGCAACCA GTTGCTATTT CTAGTGCACC

ACACTTCCGC CTGTCATGAA ACAGCAACCA GTTGCTATTT CCAGTGCACC

GCACTTCCCC CTGTCATGAA GCAGCAGCCA GCGGCCATCT CTAGTGCACC

ACACTTCCCC CTGTCATGAA ACAGCAACCA GTGGCTATTT CCAGTGCACC

GCACTTCCCC CTGTCATGAA ACAGCAGCCA GTTGCGATTT CTAGTGCACC

GCACTTCCCC CTGTCATGAA ACAGCAACCA GTTGCTATTT CTAGTGCACC

GCACTTCCCC CCGTCATGAA ACAGCAACCA GTTGCTATTT CTAGCGCACC

GCGCTCCCCC CTGTCATGAA ACAGCAACCA GTTGCTATTT CTAGTGCACC

ACGCTGCCCC CCGCCATGAA GCAGCAGCCC GTCGCTCTTC CCACCGCGCC

GCTCTTCCCC CTGTCATGAA ACAGCAGCCA GTTGCTATTT CTAGTGCCCC

---------- ---------- ---------- ---------- ----------

GCACTTCCCC CTGTCATGAA ACAGCAGCCA GTTGCTATTT CTACTGCACC

GCACTTCCTC CTGTCATGAA ACAGCAACCT GTTGCTATTT CTGGTGCACC

GCACTTCCTC CCATCATGAA ACAGCAACCC ATTGCTATTT CCGGTGCACC

AGCATTTGGT AAGACAGCTG TTGCTCCAGT GCCAATGGGA TCCATTCCAG

AGCATTTGGT AAGACAGCTG TTGCTCCAGT GCCAATGGGA TCCATTCCAG

AGCATTTGGT AAGACAGCTG TTGCTCCAGT GCCAATGGGA TCCATTCCAG

AGCATTTGGT AAGACAGCTG TTGCTCCAGT GCCAATGGGA TCCATTCCAG

AGCATTTGGT AAGACAGCTG TTGCTCCAGT GCCAATGGGA TCCATTCCAG

AGCATTTGGT AAGACAGCTG TTGCTCCAGT GCCAATGGGA TCCATTCCAG

AGCGTTTGGT AAGACAGCTG TTGCTCCCGT GCCAATGGGC TCCATCCCAG

AGCATTTGGT AAGACAGCTG TTGCTCCTGT GCCAATGGGC TCCATTCCAG

AGCATTTGGT AAG------- ---------- ---------- ----------

AGCCTTTGGT GAGACCGCTG TTGCTCCTGT ACCAATGGGC TCCATTCCCG

AGCCTTTGGT AAGACAGCTG TTGCTCCAGT GCCAATGGGA TCCATTCCAG

AGCGTTTGGT AAGACAGCTG TTGCTCCAGT GCCAATGGGA TCCATTCCAG

CACGTTCGGT AAGACAGCCG TAGCTCCCGT GCCAATGAGC TCCATCCCGG

AGCGTTTGGT AAGACAGCTG TTGCTTCTGT GCCAATGGGA TCCATTCCAG

---------- ---------- ---------- ---------- ----------

AGCATTTGGT AAGACAGCAG TTGCTCCAGT GCCAATGGGG TCCATTCCCG

CACATTTGGT AAGACAGCTG TTGCTCCAGT CCCAATGGCA TCAATTCCAG

AGCATTTGGT AAGACAGCTG TTGCCCCAGT GCCAATGCCA TCCATTCCAG

TTGTTGGAAT GTCTCCAACC CTAGTATCTT CTGTCAGCAG CTGTGCCCCC

TTGTTGGAAT GTCTCCAACC CTAGTATCTT CTGTCAGCAG CTGTGCCCCC

TTGTTGGAAT GTCTCCACCC CTAGTATCTT CTGTCAGCAG CTGTGCCCCC

TTGTTGGAAT GTCTCCACCC CTAGTATCTT CTGTCAGCAG CTGTGCCCCC

TTGTCGGAAT GTCTCCACCC CTAGTATCTT CTGTCAGCAG CTGTGCCCCC

TTGTTGGAAT GTCTCCACCC CTAGTATCTT CTGTCAGCAG CAGTGCCTCC

TTGTTGGAAT GTCTCCGCCC TTAGTATCTT CTGTAAGCAG CAGTGCCTCC

TTGTTGGAAT GTCTCCACCC TTAGTATCTT CTGTCAGCAG CAGTGCCTCC

---------- ---------- ---------- ---------- ----------

TTGTTGGGAT GTCTCCCCCA CTAGTGTCAT CTGTCAGCAG GGGTGCCCCC

TTGTCGGAAT GTCTCCACCT CTAGTATCTT CTGTCAGCAG CAGTGCCTCC

TTGTCGGAAT GTCTCCACCC CTAGTATCTT CTGTCAGCAG CAGTGCCTCC

TCGTAGGAAT GTCCCCACCC CTCGTCTCCT CCGTCAGCAG CAGTGCCTCC

TTGTTGGAAT GTCTCCACCC CTAGTATCTT CAGTCGGCAG CAGTGCCTCC

---------- ---------- ---------- ---------- ----------

TGGTCGGGAT GTCTCCACCC CTCGTTTCTT CTGTCAGCAG CAGTGGCTCC

TAGTAGGAAT GTCCCCACCT CTCGTATCTT CTGTCAGCAG CAGTTCCTCC

TAGTCGGAAT GTCCCCGCCT CTAGTATCTT CCGTCAGCAG CAGCGCCTTC

CCTGGCTAAC GGGGCTCCCC CTGTTATACA ACCTCTGCCT GCATTTGCTC

CCTGACTAAC GGGGCTCCCC CTGTTATACA ACCTCTGCCT GCATTTGCTC

CCTGGCTAAC GGGGCTCCCC CTGTTATACA ACCTCTGCCT GCATTTGCTC

CTTGGCTAAC GGGGCTCCCC CTGTTATACA ACCTCTGCCT GCATTTGCTC

GCTGGCTAAC GGGGCTCCCC CTGTTATACA ACCTCTGCCT GCATTTGCTC

CCTGGCTAAC GGGGCTCCCC CTGTCATACA ACCTCTGCCT GCATTTGCTC

CCTGGCTAAC GGGGCCCCTC CTGTCATACA GCCTCTGCCT GCATTTGCTC

CCTGGCTAAC GGGGCTCCTC CCGTCATACA GCCTCTGCCT GCGTTTGCGC

---------- ---------- ---------- ---------- ----------

CCTGGCCAAT GGGGCGCCCC CGGTCATACA GCCTCTGCCT GCATTTGCTC

CCTGGCTAAC GGGGCTCCCC CTGTTATACA ACCTCTGCCT GCATTTGCTC

CCTGGCTAAC GGGGCCCCCC CTGTTATACA ACCTCTGCCT GCATTTGCTC

CTTGGCCAAC GGGGCGCCCC CTGTGATCCA GCCCCTCACG GCCTTCGCTC

CATGGCTAAC GGAGCCCCCC CAGTCATACA ACCTCTGCCT GCATTTGCAC

---------- ---------- ---------- ---------- ----------

CCTGGCGAAT GGGGCTCCCC CTGTCATACA ACCTCTGCCT GCATTTGCTC

CCTGGCTAAT GGCACTCCTT CTGTCATACA ACCTCTGCCT GCTTTTGCTC

CCTGGCTAAC GGCACGTCTT CGGTCATACA GCCTCTGCCT GCTTTTGCTC

ATCCTGGTAT GTGCAGAGTG GGCTGTTCCT CAGTCATCAA GACTGAAATA

ATCCTGGTAT GTGCAGAATG GGCTGTTCCT CAGTCATCAA GACTGAAATA

ATCCTGGTAT GTGCAGAATG GGCTGTTCCT CAGTCATCAA GACTGAAATA

ATCCTGGTAT GTGCAGAATG GGCTGTTCCT CAGTCATCAA GACTGAAATA

ATCCTGGTAT GTGCAGAATG GGCTGTTCCT CAGTCATCAA GACTTAAATA

ATCCCGGTAT GTGCAGAATG GGCTGTTCCT CAGTCATCGA GACTGAAATA

ATCCTGGTAC GTGCAGAATG GGCTGTGCCT CAGTCGTCAA GACTGAAATA

ATCCTGGTAC GTGCAGAATG GGCTGTGCCT CAGTCATCAA GGCTGAAATA

---------- ---------- ---------- ---------- ----------

ATCCTGGTAT GTGCTGAGTG GGCTGTTCCT CAGTCATCCA GGCTGAAATA

ATCCTGGTAC GTGCAGAATG GGCTGTCCCT CAGTCATCCA GACTTAAATA

ATCCTGGTAT GTGCAGAATG GGCCGTTCCT CAGTCATCCA GACTGAAATA

ACCCCGGTAC GTGCAGAATG GGCCGTCCCG CAGTCATCCA GGCTCAAGTA

ATCCTGGTAT GTGCAGAATG GGCTGTTCCT CAGTCATCAA GACTGAAATA

---------- ---------- ---------- ---------- ----------

ATCCTGGTAT GTGCAGAATG GGCTGTTCCC CAGTCATCGA GACTGAAATA

ATCCTGGTAT GCGCAGAATG GGCTGTCCCT CAGTCATCAA GACTGAAATA

ATCCCGGTAC GCGCGGAATG GGCTGTCCCT CAGTCATCTC GGTTGAAGTA

CAGGCAATTA TTCAATAGTC ATGACAAAAC TATGAGTGGA CACTTAACAG

CAGGCAATTA TTCAATAGTC ATGACAAAAC TATGAGTGGA CACTTAACAG

CAGGCAATTA TTCAATAGTC ATGACAAAAC TATGAGTGGA CACTTAACAG

CAGGCAATTA TTCAATAGTC ATGACAAAAC TATGAGTGGA CACTTAACAG

TAGGCAGTTA TTCAATAGTC ATGACAAAAC TATGAGTGGA CACTTAACAG

CAGACAATTA TTCAATAGTC ATGACAAAAC TATGAGTGGA CACTTAACAG

CAGGCAGTTA TTCAACAGTC ACGACAAGAC CATGAGTGGA CACTTAACAG

CAGGCAGTTA TTCAACAGCC ACGACAAAAC TATGAGTGGA CACTTAACAG

---------- ---------- ---------- ---------- ----------

CAGACAGCTC TTCAATAGTC ATGACAAAAC CATGAGTGGA CACCTAACAG

CAGACAGTTA TTCAACAGTC ATGACAAAAC TATGAGTGGA CACTTAACAG

CAGACAGTTG TTCAATAGTC ATGACAAAAC TATGAGTGGA CACTTAACAG

CCGGCAGTTG TTCAACAGCC ACGACAAGAC CATGAGCGGA CACCTGACAG

CAGGCAATTA TTCAATAGTC ATGACAAAAC TATGAGTGGA CACTTAACAG

---------- ---------- ---------- ---------- ----------

CAGACAGTTA TTCAATAGTC ATGACAAAAC TATGAGTGGA CATCTCACAG

TAGGCAACTG TTCAATAGCC ACGACAAAAC AATGAGTGGA CATTTAACAG

TAGGCAGCTG TTCAATAGCC ATGACAAAAC GATGAGCGGC CACCTCACAG

GTAAGTTAGG TCCCCAAGCA AGAACTATTC TTATGCAGTC AAGTTTACCA

GTAAGTTAGG TCCCCAAGCA AGAACTATTC TTATGCAATC AAGTTTACCA

GTAAGTTAGG TCCCCAAGCA AGAACTATTC TTATGCAATC AAGTTTACCA

GTAAGTTAGG TCCCCAAGCA AGAACTATTC TTATGCAATC AAGTTTACCA

GTAAGTTAGG TCCCCAAGCA AGAACTATTC TTATGCAATC AAGTTTACCA

GTAAGTTAGG TCCCCAAGCA AGAACTATTC TTATGCAATC AAGTTTACCA

GTGAGGTAGG TCCCCAGGCA AGAACTATTC TCATGCAATC AAGTTTACCC

GTAAGGTAGG TCCCCAGGCA AGAACTATTC TCATGCAATC AAGTTTACCC

----GTTAGG TCCACAAGCA AGAACTATTC TCATGCAATC AAGTTTACCA

GTAAATTAGG TCCCCAAGCA AGAACTATTC TTATGCAATC AAGTTTACCA

GTAAGTTAGG TCCACAAGCA AGGACTATTC TTATGCAATC AAGTTTGCCA

GTAAGTTAGG TCCCCAAGCA AGAACTATTC TTATGCAATC AAGTTTACCG

GTA-GTTAGG CCCCCAAGCA AGAACTATCC TTATGCAATC AAGTCTACCG

GTAAGTTAGG TCCCCAAGCA AGAACAATTC TTATGCAATC AAGTTTACCA

---------- ---------- ---------- ---------- ----------

GTAAATGAGG TCCCCAAGCA AGAACTATTC TTATGCAATC AAGTTTACCA

GTAATTTAGG TCCTCAAGCA AGAACTATTC TTATGCAATC AAGTTTACCA

GTAAGTCAGG TCCCCAAGCA AGAACTATCC TTATGCAATC AAGTTTACCC

CAGGCTCAGC TGGCTTCAAT ATGGTAAGGT TTTGTTGAAT AGGAATCTTT

CAGGCTCAGC TGGCTTCAAT ATGGTAAGGT TTTGTTGAAT AGGAATCTTT

CAGGCTCAGC TGGCTTCAAT ATGGTAAGGT TTTGTTGAAT AGGAATCTTT

CAGGCTCAGC TGGCTTCAAT ATGGTAAGGT TTTGTTGAAT AGGAATCTTT

CAGGCTCAGC TGGCTTCAAT ATGGTAAGGT CTTGTTGAAT AGGAATCTTT

CAGGCTCAGC TGGCTTCAAT ATGGTAAGGT TTTGTTGAAT AGGAATCTTT

CAGGCTCAGC TGGCTTCAAT CTGGTAAGGT TCTGTTGATT AGGAATCTTT

CAGGCTCAGC TGGCTTCAAT ATGGTAAGGT TCTGTTGATT AGGAATCTTT

CAGGCTCAGT TGGCTTCAAT CTGGTGAGGT TCTGTTGACT AGGAATCTTT

CAGGCTCAGC TGGCTTCAAT ATGGTAAGGT TCTGTTGAAT AGGAATCTTT

CAGGCTCAGC TGGCTTCAAT ATGGTAAGGT TTTATTGAAT AGGAATCTTT

CAGGCTCAGC TGGCTTCAAT ATGGTAAGGT TTTGTTGAAT AGGAATCTTT

CAGGCTCAGC TGGCTTCAAT ATGGTTAGGT TTCCCTGCCC AGGAACCTCT

CAGGCTCAAC TGGCTTCAAT ATGGTAAGGT TTTGTTCCAT AGGAATCTTT

---------- ---------- ---------- ---------- ----------

CAGGCTCAGC TGGCTTCAAT ATGGTAAGGT TTTGTTGAAT AGGAATCTTT

CAAACTCAGC TGGCTACAAT ATGGTAAGAT TTGGCTCCAT AGGAATCTTT

CAGGCTCAGC TGGCTACGAT ATGGTAAAGT TTTATTAAAT AGGAATCTTT

CTGACATTGA TCAAGATGGA AAACTTACAG CAGAGGAATT TATCCTGGCA

CTGACATTGA TCAAGATGGA AAACTTACAG CAGAAGAATT TATCCTGGCA

CTGACATTGA TCAAGATGGA AAACTTACAG CAGAAGAATT TATCCTGGCA

CTGACATTGA TCAAGATGGA AAACTTACAG CAGAAGAATT TATCCTGGCA

CTGACATTGA TCAAGATGGA AAACTTACAG CAGAAGAATT TATCCTGGCT

CTGACATTGA TCAAGATGGA AAACTCACAG CAGAAGAATT TATCCTGGCT

CCGACATTGA TCAAGATGGA AAGCTCACCG CAGAAGAATT CATCCTAGCG

CTGACATTGA TCAAGATGGA AAACTCACTG CAGAAGAATT TATCCTAGCT

CTGACATTGA TCAAGATGGA AAACTCACAG CAGAAGAATT TATCCTGGCT

CTGACATTGA TCAAGATGGA AAACTCACAG CAGAAGAATT TATCCTGGCT

CTGACATTGA TCAAGATGGA AAACTCACAG CAGAAGAATT TATCTTGGCT

CTGACATTGA TCAAGATGGA AAACTTACAG CAGAAGAATT TATCCTGGCT

CCGACATCGA CCAGGACGGG AAGCTCACGG CCGAAGAGTT CATCCTGGCC

CTGACATTGA TCAAGACGGA AAACTCACAG CAGAAGAATT TATCCTGGCT

---------- ---------- ---------- ---------- ----------

CCGACATTGA CCAAGACGGA AAGCTCACAG CAGAAGAATT TATCCTGGCT

CAGATATTGA TCAAGATGGG AAACTAACAG CAGAAGAATT TATTCTGGCA

CGGACATCGA TCAAGATGGA AAACTCACAG CAGAAGAATT TATCCTGGCT

ATGCACCTCA TTGATGTAGC TATGTCTGGC CAACCACTGC CACCTGTCCT

ATGCACCTCA TTGATGTAGC TATGTCTGGC CAACCACTGC CACCTGTCCT

ATGCACCTCA TTGATGTAGC TATGTCTGGC CAACCACTGC CACCTGTCCT

ATGCACCTCA TTGATGTAGC TATGTCTGGC CAACCACTGC CACCTGTCCT

ATGCACCTCA TTGATGTAGC TATGTCTGGC CAACCACTGC CACCCGTCCT

ATGCACCTAA TCGATGTGGC TATGTCTGGC CAACCACTGC CACCTGTCCT

ATGCACCTGA TTGATGTTGC TATGTCTGGT CAGCCACTGC CGCCTGTCCT

ATGCACCTAA TTGATGTTGC CATGTCTGGT CAGCCACTGC CGCCCGTCCT

ATGCACCTAA TAGATGTAGC TATGTCTGGC CAACCGCTGC CACCTGTCCT

ATGCACCTCA TTGATGTAGC TATGTCTGGT CAACCACTGC CACCGGTCCT

ATGCACCTAA TTGATGTAGC TATGTCTGGC CAACCACTGC CACCTGTCCT

ATGCACCTAA TTGATGTAGC TATGTCTGGC CAACCACTGC CACCTGTCCT

ATGCACCTCA TCGACGTGGC CATGTCGGGC CAGCCGCTGC CCCCTGCCCT

ATGCACTTAA TTGATGTAGC TATGTCTGGC CAACCACTGC CACCTGTCCT

---------- ---------- ---------- ---------- ----------

ATGCACCTAA TTGATGTGGC TATGTCTGGC CAGCCCCTGC CACCGGCTCT

ATGCACTTAA TTGATGTGGC TATGTCTGGC CAGCCCCTAC CACCAGCACT

ATGCACTTAA TCGACGTGGC CATGTCCGGC CAACCCCTGC CTCCAGTATT

GCCTCCAGAA TACATTCCAC CTTCTTTTAG GTAAGGACAT CTGTAGATCA

GCCTCCAGAA TATATTCCAC CTTCTTTTAG GTAAGGACAT CTGTAGATCA

GCCTCCAGAA TACATTCCAC CTTCCTTTAG GTAAGGACAT CTGTAGATCA

GCCTCCAGAA TACATTCCAC CTTCCTTTAG GTAAGGACAT CTGTAGATCA

GCCTCCAGAA TACATTCCAC CTTCCTTTAG GTAAGGACAT CCGTAGATCA

TCCTCCTGAA TACATTCCAC CTTCCTTTCG GTAAAGACAT CTGCAGATCA

GCCTCCAGAA TACATCCCTC CTTCCTTTAG GTAAGTGCAT CTGCAGACCA

GCCTCCAGAA TACATCCCTC CTTCCTTCAG GTAAGTACGT CTGTGGATCA

GCCTCCGGAA TACATTCCAC CTTCCTTTAG GTAAGGACAT CTGTAGACCA

GCCCCCAGAA TACATTCCAC CTTCCTTTAG GTAAGGATAT CGGTGGATCA

GCCTCCAGAG TACATTCCAC CTTCCTTTAG GTAAGGACAG CAGTAGATCA

GCCTCCAGAA TACATTCCAC CTTCCTTTAG GTAAGGACAT CAGTAGATCA

GCCCCCAGAA TACATCCCGC CTTCCTTCCG GTAAGG---T CCGTGGACCA

GCCTCCAGAA TATATTCCAC CTTCATTTAG GTAAGGAGAT CTGTAGATCA

---------- ---------- ---------- ---------- ----------

GCCTCCAGAG TATATCCCAC CTTCCTTTAG GTAAGAACAT CTGTCGATCA

GCCTCCAGAA TATATTCCTC CTTCATTTAG GTAAGAACAT CTGTAGACCA

GCCTCCAGAG TATATTCCAC CTTCATTTAG GTAAGTGGAT CTGTGGATCA

GAGGCTACCA GAGGAACCAG TTTTAGAAGA TGAACAACAA CAATTAGAAA

GAGGCTACCA GAGGAACCAG TTTTAGAAGA TGAACAACAA CAATTAGAAA

GAGGCTACCA GAGGAACCAG TTTTAGAAGA TGAACAACAA CAATTAGAAA

GAGGCTACCA GAGGAACCAG TTTTAGAAGA TGAACAACAA CAATTAGAAA

GAGGCTACCA GAGGAACCAG TTTTAGAAGA TGAACAGCAA CAATTAGAAA

GAGGCTACCA GAGGAACCAG TTTTGGAAGA TGAACAGCAA CAATTAGAAA

GCGGCTGCCC GAGGAGCCAT CGTCAGAGGA TGAGCAGCAG G---TGGAAA

GAGGCTGCCT GAGGAGCCGT CGTCAGAGGA TGAGCAGCAG C---CAGAGA

GAGGTTACCA GAGGAACCAG TGTTAGAAGA CGAACAGCAA CAATTGGAAA

GAGGTTGCCA GAGGAACCAG TTTTGGAAGA TGAACAGCAA CAACTAGAAA

GAGGTTACCA GAGGAACCAG TTTTAGAAGA TGAACAACAA CAACTAGAAA

GAGGTTACCA GAGGAACCAG CCTTAGAAGA CGAACAACAA CAAATAGAAA

GCGGCTGCCG GAGGAGCCTG CACTGGAGGA TGAACAGCAG CAGCTGGAAA

AAGGTTACCA GAAGAACCAG TTTTAGAAGA TGAACAGCAA CAGCTAGAGA

---------- ---------- ---------- ---------- ----------

GAGGTTACCA GAGGAACCAG TTTTAGAAGA CGAACAGCAG CAACTGGAAA

AAGGTTACCA GAGGAACCAG TGTTAGAAGA GGAGCAGCAA CAGCTGGAAA

GAGGTTACCA GAGGAACCAG CATTAGAAGA AGAGCAGCAG CAGCTGGAAA

AGAAATTACC TGGTAAGAGG AATCTCAGCA ACAGCCCACC CGGAGAGAGC

AGAAATTACC TGGTAAGAGG AATCTCAGCA ACAGCCCACC CGGAGAGAGC

AGAAATTACC TGGTAAGAGG AATCTCAGCA ACAGCCCACC CGGAGAGAGC

AGAAATTACC TGGTAAGAGG AATCTCAGCA A--------- ----------

AGAAATTACC TGGTAAGAGG AATCTCAGCA ACAGCCCACC CCGAGAGAGC

AGAAATTACC TGGTAAGAGG AATCTCAGCA GCAGCCCATC CCGAGAGAGC

AGAAGCTGCC TGGTGAGAGG AATCTCAGCA GCAGCCCACC CCGAGAGAGC

AGAAACTGCC TGGTGAGAGG AATCTCAGCA ACAGCCCACC CAGAGAGAGC

AGAAATTACC TGGTAAGAGG AGTCTCAGCA GCAGCGCACC CCGAGAGAGC

AGAAACTACC TGGTAAGAGG AGTCCCAGCA A--------- ----------

AGAAATTACC TGGTAAGAGG AAGCCCAGCA ACAGCCCACC CAGAGAGAGC

AAAAATTGCC TGGTAAGAGG AGTCTCAGCA ACAGCCCACC CTGAGAGAGC

AGAAACTGCC GGGTAAGAGG AGTCCCAGCA GCAGCCCACC CGGAGAGAGC

AGAAATTACC TGGTAAGAAG AGTCCCAGCA ACAGCCCACC CCGAGAGGGC

---------- ---------- ---------- -CAGCCCACC CCGAGAGAGC

AGAAGCTACC TGGTAACAGG AATCTCAGCA ACAGCCCACC CCGAGAGAGC

AGAAGTTACC TGGTGAGAGG AATCTCAGCA ACAGCTCACT CAGAAAGGGC

AGAAATTGCC AGGTGAGAGG AGTCTCAGCA GAAGCACACC CCGAAAGGGC

TGCCCTGCTG TTCGTGTCCA GTGTCTGCAT CGGCCTGGCC CTCACACTGT

TGCCCTGCTG TTCGTGTCCA GTGTCTGCAT CGGCCTGGCC CTCACACTGT

TGCCCTGCTG TTCGTGTCCA GTGTCGGCAT CGGCCTGGCC CTCACATTGT

---------- ---------- ---------- ---------- ----------

TGCCCTGCTG TTTGTGTCCA GCGTCTGCAT CGGCCTGGCC CTCACATTGT

TGCCCTGCTG TTCGTGTCCA GTGTTTGCAT TGGCTTGGCC CTCACACTGT

TGCTCTGCTG TTCGTGTCTA GTGTCTGCAT CGGCCTGTTC CTCACACTGT

CGCTCTGCTG TTCATGTCCA GTGTCTGCAT CGGCCTGCTC CTCACACTGT

CGCCCTGCTG TTCGTGTCCA GCGTGTGCGT CGGCCTGGTG CTCACCCTGT

---------- ---------- ---------- ---------- ----------

AGCCCTGCTC TTCGTGTCCA GTGTCTGCAT CGGTCTGGCG CTCACGTTGT

TGCCCTGCTG TTCGTGTCCA GTGTCTGCAT CGGTCTGGCA CTCACGTTGT

GGCCCTGCTG TTCGTGTCCA GCGTCTGCGT GGGCCTGGCG CTCACGCTGT

CGCCCTGCTG TTCGTGTCCA GCGTCTGCCT CGGCCTGGTG CTCACCCTGG

CGCCCTGCTG TTCATGTCCA GTGTCTGCAT CGGCCTGGCG CTCACGATGT

CGCCCTGCTG TTTGTGTCCA GTGTCTGCAT TGGCCTGGTC CTCACCTTGT

TGCTCTGCTG TTTGTGTCCA GTGTTTGTAT TGGCCTAATC GTCACCCTGT

GGCCCTGCTG TTCGTGTCCA GCGTCTGCCT CGGGTTGGTC GTCACGCTGG

GCGCCCTGGT CATCAGAGAG TCCTGTGCCA AGGACAATAG GAGATTCACT

GCGCCCTGGT CATCAGAGAG TCCTGTGCCA AGGACAATAG GAGATTCACT

GTGCCCTGGT CATCAGAGAG TCCTGTGCCA AGGACAATAG GAGATTCACT

---------- ---------- ---------- -----AATAG GAGATTCACT

GTGCCTTGGT TATCAGAGAG TCCTGCACCA AGGATAATAG GAGATTCACT

GTGCCTTGGT CATCAAAGTG TCCTGCACCA AAGACAATAG GAGATTCACT

GTGCCCTGGT CATCAGAGTG TCCTGCACCA AGGACAATAG GAGATTCACT

GTGCCCTGGT CATCAGGGTG TCCTGTACGA AGGACAATAG GAGACTCGCT

GCGCCCTGGT CATCCAGCTG TCCTGCGCCA AGGACAACAG GAGATTCACT

---------- ---------- ---------- -----AACAG GGGATTCACT

GTGCCTTGGT CATCAGAGTG TCCTGCGTCA AGGACAATAG GAGATTCACT

GCGCCTTGGT TGTCAGAGGG TCCTGCACCA AAGACAACAG GAGATTCACT

GCGCCTTGGT CATGCGTGTG TCCTGCGCCC GGGACACCAG GCGATTCCCT

GTGCCCTGGT CGTCAGAGTG TCCTGCACCA AGGACAATAG GAGATTCACT

GTGCCTTGGT CATCAGAGTG TCCTGCACCA AGGAC----- ----------

GCGCCCTGGT CGTCAAAGTG TCCTGCACCA AGGACAATAG GAGATTCACT

TTGCTGTGGT GATCAGAGTA TCATGCACCA AAGACAATAG GAGATTCTCT

GCGCGGTGGT GGCCCGAGGG TCGTGCCCCA AAGACAACAG GAGATTCACT

TGTTACACTT AAAAGAGCCT TAGAAGCAAA AGAACTAGCT CGGCAGCACC

TGTTACACTT AAAAGAGCCT TAGAAGCAAA AGAACTAGCT CGGCAGCAGC

TGTTACACTT AAAAGAGCCT TAGAAGCAAA AGAACTAGCT CGGCAGCAGC

TGTTACACTT AAAAGAGCCT TAGAAGCAAA AGAACTAGCT CGGCAGCAGC

TGTTACACTT AAAAGAGCCT TAGAAGCAAA AGAAATAGCT CGACAGCAGC

TCTTACACTT AAAAGAGCCT TAGAAGCAAA AGAACTAGCT CGGCAACAGC

TCTTACCCTC AAAAGAGCCT TGGAAGCAAA GGAACTGGCC CGGCAGCAGC

TCTTACCCTC AAAAGAGCCT TGGAAGCAAA GGAGCTGGCC CGGCAGCAGC

TCTTACACTC AAAAGAGCCC TAGAAGCGAA AGAACTAGCT CGGCAACAGC

TCTCACACTC AAAAGAGCCT TAGAAGCGAA AGAACTGGCT CGGCAGCAGC

GCTTACACTC AAAAGAGCGT TAGAAGCAAA AGAACTAGCT CGGCAGCAGC

TCTTACACTC AAAAGAGCCT TAGAAGCAAA AGAACTAGCT CGGCAGCAGC

CCTCACACTC AAAAGAGCAC TAGAGGCCAA GGAGATCCTC CGGCAGCAGC

TCTTACACTC AAAAGAGCCT TAGAAGCAAA AGAACTAGCT CGGCAACAGC

---------- ---------- ---------- ---------- ----------

TCTTACGGTC AAAAGAGCCC TAGAAGCAAA AGAACTGGCT CGGCAGCAGC

TCTTACACTT AAAAGAGCCT TAGAAGCAAA AGAACTTGCT CGTCAGCAAC

TCTCACCCTG AAGAGAGCTT TAGAAGCAAA AGAAGTAGCC CGTCAGCAGC

TACGAGACCA ACTGGATGAA GTGGAGAAAG AAACTAGATC AAAACTACAG

TACGAGACCA ACTGGATGAA GTGGAGAAAG AAACTAGATC AAAACTACAG

TACGAGACCA ACTGGATGAA GTGGAGAAAG AAACTAGATC AAAACTACAG

TACGAGACCA ACTGGATGAA GTGGAGAAAG AAACTAGATC AAAACTACAG

TACGAGACCA ACTGGATGAA GTGGAGAAAG AAACTAGATC AAAACTACAG

TACGAGACCA GCTGGATGAA GTGGAAAAAG AAACAAGATC AAAGCTACAG

TTCGAGAGCA GCTGGACGAG GTGGAGAAAG AGACCAGGTC AAAGCTGCAG

TCCGGGAGCA GCTGGACGAG GTGGAGAGAG AGACCAGGTC AAAGCTGCAG

TTCGAGACCA ACTAGATGAA GTGGAAAAAG AAACGAGATC AAAACTGCAG

TACGAGACCA ACTGGATGAA GTGGAGAAAG AAACTAGGTC AAAATTACAG

TACGCGATCA ACTGGATGAA GTTGAGAAAG AAACCAGATC AAAACTACAG

TACGAGACCA ATTAGATGAA GTTGAGAAAG AAACTAGATC AAAACTACAG

TGCGAGACCA GCTGGACGAC GTGGAGAAGG AGACGAGATC CAAACTCCAG

TACGAGACCA ACTGGATGAA GTTGAGAAAG AAACTAGATC AAAACTACAG

---------- ---------- ---------- ---------- ----------

TCCGGGACCA GCTGGATGAG GTTGAGAGAG AAACTAGATC AAAGCTGCAG

TGAGAGACCA GCTGGATGAG GTTGAGAAGG AAACTAGATC AAAGCTACAG

TTCGAGACCA GCTGGATGAA GTTGAGAAAG AAACCAGATC CAAGTTGCAG

GAGATTGATA TTTTCAATAA TCAGCTGAAG GTAAGAGCTC GCAGAAAGGA

GAGATTGATA TTTTCAATAA TCAGCTGAAG GTAAGAGCTC GCAGAAAGGA

GAGATTGATA TTTTCAATAA TCAGCTGAAG GTAAGAGCTC GCAGAAAGGA

GAGATTGATA TTTTCAATAA TCAGCTGAAG GTAAGAGCTC GCAGAAAGGA

GAGATTGATA TTTTCAATAA TCAGCTGAAG GTAAGAGCTC GCAGAAAGGA

GAGATTGATA TTTTCAATAA TCAGCTAAAG GTGAGAGCTT GCAGAAAGGA

GAGATTGATG TTTTCAACAA CCAGCTGAAG GTGAGAGCTG GCAGAAAGGA

GAGATTGATG TTTTCAACAA CCAGCTGAAG GTGAGAGCTG GCGGAAAGGA

GAGATTGATA TTTTCAATAA TCAGCTGAAG GTAAGAACTG GCCGAGAGGA

GAGATTGATA TTTTCAACAA TCAGCTGAAG GTACGAGTTG GCAGAAAGGA

GAGATTGATA TTTTCAATAA TCAGCTGAAG GTAAGAGCTG GCAGAAAGGA

GAGATTGATA TTTTCAATAA TCAACTGAAG GTAAGAGCTG GCAGAGAGGA

GAGATCGATA TTTTCAACAA CCAGCTGAAG GTACGAGCTG GCGGAAAGGA

GAGATTGATA TTTTCAATAA TCAGCTGAAG GTAAGAGCTG GCGGAGAGGA

---------- ---------- ---------- ----GAGCTA GCAGAAAGGA

GAGATTGATA TTTTCAATAA TCAGCTGAAG GTAAGAGCTT GCAGAGAGGA

GAGATTGATA TTTTCAATAA TCAGCTAAAG GTAAGAGATG GCCGAAAGGA

GAGATTGATA TTTTCAACAA TCAGCTGAAG GTAAGACCTG GCCGAGAGGC

TTGAGCGCAG GGAGCAAATC ATTCAGGAAA TATGGATGAA CAGTGGTTTG

TTGAGCGCAG GGAGCAAATC ATTCAGGAAA TATGGATGAA CAGTGGTTTG

TTGAACGCAG GGAGCAAATC ATTCAGGAAA TATGGATGAA CAGTGGTTTG

TTGAACGCAG GGAGCAAATC ATTCAGGAAA TATGGATGAA CAGTGGTTTG

TTGAGCGCAG GGAGCAAATA ATCCAGGAAA TATGGATGAA CAGTGGTTTA

TTGAGCGCAG GGAACAAATC ATTCAGGAAA TATGGATGAA CAGTGGTTTG

TTGAGCGCAG GGAGCAAATC ATTCAAGAAA TATGGATGAA CAGTGGCCTA

TTGAACGCAG GGAGCAAGTC ATTCAAGAGA TATGGATGAA CAGTGGCCTG

TCGAGCGCAG GGAACAGATC ATCCAGGAGA TATGGATGAA CAGCGGTCTG

TTGAGCGCAG GGAGCAGATC ATCCAGGAAA TATGGATGAA CAGTGGCTTG

TTGAGCGCAG GGAGCAAATC ATCCAGGAAA TATGGATGAA CAGTGGTTTG

TTGAGCGCAG GGAGCAAATC ATCCAGGAAA TATGGATGAA CAGTGGTTTG

TTGAGCGGCG GGAACAAATC ATCCAGGAAA TCTGGCTGAA CAGCGGCTTG

TCGAGCGCAG GGAGCAGATC ATTCAGGAAA TATGGATGAA CAGTGGCTTG

TTGAGCGCAG AGAGCAAATC ATTCAGGAAA TATGGATGAA CAGTGGTTTG

TCGAGCGCAG AGAGCAAATC ATTCAGGAAA TATGGCTGAA CAGTGGCTTG

TAGAGCGTAG AGATCAGATC ATTCAGGAAA TTTGGATGAA CAGTGGTTTG

TGGAGTGGCG GGACCAGATC ATCCAGGAGA TATGGCTGAG CAGTGGCTTG

GATTATAGGA ACTAAGAGAA ATACACAATA AGCAACAACT CCAGAAGCAA

GATTATAGGA ACTAAGAGAA ATACACAATA AGCAACAACT CCAGAAGCAA

GATTATAGGA ACTAAGAGAA ATACATAATA AGCAGCAACT CCAGAAGCAA

GATTATAGGA ACTAAGAGAA ATACATAATA AGCAGCAACT CCAGAAGCAA

GATTATAGGA ACTAAGAGAA ATACATAGTA AGCAGCAACT CCAGAAGCAA

GATAATAGGA ACTAAGAGAA ATACATAATA AGCAGCAACT CCAGAAGCAA

GATTCTAGGA ACTGAGAGAG ATACACAGTA AACAGCAGCT CCAGAAGCAG

GAGTCTAGGA ACTGAGAGAG ATACATAGCA AACAGCAACT CCAGAAGCAG

GATTACAGGA ACTAAGAGAA ATGCA----- ---------- ----------

GATTATAGGA ACTTAGAGAA ATACATAATA AGCAGCAGCT CCAGAAACAG

GATTATAGGA ACTAAGAGAA ATACATAGTA AGCAACAACT CCAGAAACAA

GATTATAGGA ACTAAGAGAA ATACATAATA AGCAGCAACT CCAGAAACAA

GA-------- ---------- ---------- ---------- ----------

GATTGTAGGA ACTAAGAGAA ATACACAATA AGCAGCAGCT CCAGAAACAA

GA-------- ---------- ---------- ---------- ----------

GATTGTAGGA ACTCAGAGAA ATACATAATA AGCAACAGCT CCAGAAACAA

GATTTTAGGA ACTAAGAGAA ATACATAACA AGCAACAGCT TCAaaaacaa

GATTGTAGGA ACTAAGAGAA ATCCATAGCA AGCAACAACT CCAGAAACAG

AAGTCCATGG AGGCTGAACG ACTGAAACAG AAAGAACAAG AACGAAAGAT

AAGTCCATGG AGGCTGAACG ACTGAAACAG AAAGAACAAG AACGAAAGAT

AAGTCGATGG AGGCTGAACG ACTGAAACAG AAAGAACAAG AGCGAAAGAT

AAGTCGATGG AGGCTGAACG ACTGAAACAG AAAGAACAAG AGCGAAAGAT

AAGTCCCTGG AGGCGGAACG ACTGAAACAG AAAGAACAAG AGAGAAAGAT

AAGTCCATAG AGGCTGAACG ACTGAAACAG AAAGAACAAG AGCGAAAGAT

AGGTCCATCG AGGCGGAGAG GCTAAAGCAG AAAGAGCAGG AGAGGAAGAG

AGGTCCCTGG AGGCAGCGCG ACTGAAGCAG AAAGAGCAGG AGAGGAAGAG

---------- ---------- ---------- ---------- ----------

AAGTCCATAG AGGCTGAACG ACTGAAACAG AAGGAACAAG AACGAAAGAT

AAGTCCATAG AGGCCGAACG TCTGAAACAG AAAGAACAAG AGCGAAAAAC

AAATCCATAG AAGCTGAACG ACTAAAACAG AAAGAGCAAG AGCGAAAGAT

---------- ---------- ---------- ---------- ----------

AAGTCTATAG AGGCTGAACG ACTGAAACAG AAAGAACAAG AGAGAAAGAT

---------- ---------- ---------- ---------- ----------

AAATCCATAG AGGCCGAACG CCTGAAACAG AAAGAACAAG AGCGAAAGAT

aagaatttag aagcggaaag gttgaaacag aaagaaaaag agaggaaaac

AAGAACTTAG AAGCTGAAAG ATTGAAACAG AAAGAGCAAG AGAAAAAGAC

CATAGAATTA GAAAAACAAA AAGAAGAAGC CCAAAGGTGA GTGTAGATAA

CATAGAATTA GAAAAACAAA AAGAAGAAGC CCAAAGGTGA GTGTAGATAA

CATAGAATTA GAAAAACAAA AAGAAGAAGC CCAAAGGTGA G-GTAGATAA

CATAGAATTA GAAAAACAAA AAGAAGAAGC CCAAAGGTGA GTGTAGATAA

CGTAGAATTA GAAAAACAAA AAGAAGAAGC CCAAAGGTGA GTGTGGATAA

CATAGAATTA GAAAAGCAAA AAGAAGAAGC CCAAAGGTGA GTGTAGATAA

CCTGGAGTTG GAGAAGCAAA AGGAAGAAGG TCAGAGGTGA G-GTAGACAA

CCTGGAGTTA GAGAAGCAAA AGGAAGACGC TCAGAGGTGA G-GTAGACAA

---------- ---------- ---------- ---------- ----------

CCTGGAATTA GAAAAACAAA AAGAAGAAGC CCAAAGGTGA GTGTAGATAA

CATAGAATTA GAAAAACAAA AAGAAGAAGC CCAAAGGTGA GTGTAGATAA

CATAGAATTA GAAAAACAAA AAGAAGAAGC CCAAAGGTGA GTGTAGATAA

---------- ---------- ---------- ---------- ----------

CATAGAATTA GAAAAACAAA AAGAGGAAGC CCAAAGGTGA GTGTAGATAA

---------- ---------- ---------- ---------- ----------

CCTAGAATTA GAAAAACAAA AAGAGGAAGC CCAAAGGTGA GTGTAGAAAG

agtagaacta gaaaagcaaa aagaggaaag ccaaaggtaa gTGTCGACTC

AGTAGAATTA GAGAAGCAGA AAGAAGACGT CCAAAGGTAA GTGTAGATTT

TGGAATTATA CAAGGAAATT GGGACACTTG GTGTGCTCAT TTGCATGCTA

TGGAATTATA CAAGGAAATT GGGACACTTG GTGTGCTCAT TTGCATGCTA

TGGAATTATA CAAGGAAATT GGGACACTTG GTGTGCTCAT TTGCATGTTA

TGGAATTATA CAAGGAAATT GGGACACTTG GTGTGCTCAT TTGCATGTTA

TGGAATTATA CAAGGAAATT GGGACACTTG GTGTGCTCAT TTGCATGCTA

TGGAATTATA CAAGGAAATT GGGACACTTG GCGTGCTCAT TTGCATGCTA

TGGAAGTAGA CAAGAAAATT GGGACACTCG GTTAGTTCAT TTGCATGCCA

TGGAAGTAGA CAGGAAAATT GGGACACTTG GTTAGTTCAT TTGCATGCCA

---------- ---------- ---------- ---------- ----------

TGGAATTACA CAAGGAT-TT GGGCCAATTG ATATGCCTGT TTGCATGCCA

TGGAATTATA CAGGGAAATT AGGACACTTG GTCTGCTCAT TTGCATGCCA

TGGAATTATA CAAGGAAATT GGGACACTTG GTAGGCTCAT TTGCATGCCA

---------- ---------- ---------- ---------- ----------

TGGAATTAGA CAAGGCAGTT GGAACACTTG GTATGCTCAT TTGCATGCCA

---------- ---------- ---------- ---------- ----------

TGGAATTATA CAATGAAATT GGGACACTTG GTATGCTCGT TTGCATGTCA

TGGAATTCTA CAAGGAAACA AGAATGCTTG GTTTGCTCCT TTGCATGCTG

GGGAATCATA CAAGGAAATT GGGGCGGTCG GGGTGCTCAT TTGCATCCCA

ATGGCCAGTT CACAGCCTTT TACTAAGCAG ACGAGCTCAG GACAAGCAGT

ATGGCCAGTT CACAGCCTTT TACTAAGCAG ACGAGCTCAG GACAAGCAGT

ATGGCCAGTT CACAGCCTTT TACTAAGCAG ACGAGCTCAG GACAAGCAGT

ATGGCCAGTT CACAGCCTTT TACTAAGCAG ACGAGCTCAG GACAAGCAGT

ATGGCCAGTT CACAGCCTTT TAGTAAGCAG ACGAGCTCAG GACAAGCAGT

ACGACCAGTT CACAGCCTTT TAACAAGCAG ACGCGCACAG GACAGGCAGT

ATGACCAGTT CACAGCCTCC TAATAAGCAG ACGAGTTCAG GACAAGCAAT

ATGACCAGTT CACAGCCTCC TAATAAGCAG ACGAGTTCAG GACAAGCAAT

---------- ---------- ---------- ---------- ----------

ATGACCAGTT CACAGCCTTT TAATAAACAG ACGAGCTCAG GACCAGCAGT

ATGACCAGTT CACAGCCTTT TCATAAGCAG ACGAGCTCAG GACAAGCAGT

ATGACCAGTT CACAGCTTTT TAATAAGCAG ACGAGCTCAG GATAAGCAGT

---------- ---------- -------CAG ACGAGCTCAG GACAAGCCGT

ATGACCAGTT CACAGCCTTC TAATAAGCAG ACGCGCTCAG GACAAGCAGT

---------- ---------- ---------- ---------- ----------

ATGACCAGTT CACAGCCTTT TAATAGGCAG ACGAGCTCAG GACAAACAGT

AGAACCAGTT CACAGCTTTT TAATAAGCAG ACGTTCCCAG GACAAGCAGT

ATAACCAGTT CACAGCTTTT TAATAAGCAG GCGAGCGCAA GAGAAACCGT

GGCTGGAGCA TGTGCAGCAG GAGGACGATG TCATTAAACA TTATTTTGCC

GGCTGGAGCA TGTGCAGCAG GAGGACGATG TCATTAAACA TTATTTTGCC

GGCTGGAGCA TGTGCAGCAG GAGGACGATG TCATTAAACA TTATTTTGCC

GGCTGGAGCA TGTGCAGCAG GAGGACGATG TCATTAAACA TTATTTTGCC

GGCTGGAGCA TGTGCAGCAG GAGGATGATG TCATTAAACA TTATTTTGCC

GGCTGGAGCA CGTGCAGCAG GATGAGGATT TCATTAAATA TTATTTTGCC

GGCAGGAGCA TGTGCAGCAG GAG---GATT TCATTAAACA -TCTTTTGCC

GGCTGGAGCA TGTGCAGCAG GAG---GATT TCATTAAACA -TCTTTTGCC

---------- ---------- ---------- ---------- ----------

GGCTGGAGCG CGTGCATCAG GAGGATGATC TCATTAAACA TTGTTTTGCC

GGATTGAGCA TGTGCAGCAG GACGATGAT- TCATTAAACA TT--TTTGCC

GGCCTGAGCA TGTGCCACAG GACGAGGATT TCATTAAATA TTATTTTGCC

GGCTGGAGCG CGTGCAGCGG GACGAGGATT GCATTAACCC TT-CTTTGCC

GGCTTGAGCA TGTGCAGCAG GAAGATGACT TCATTAAACA TTATTTTGCC

---------- ---------- ---------- ---------- ----------

GGCTGGAGCA TGTGCAGCAG GAGGATGATT TCATTAAACA TTATTTTGCC

GGCTTGACAG AGTTCAACAA GATGAAGATT TTGTGAAACA TTATATTGCC

GGCCGGACCG GGCGCAGCAG GACGAAGAGT CTGTGAAACC TTATTTTGCC

TTGCTTTCAA ACCTTTGCTT TCCTGGTGGA GGTTAAAGGG GAATGGGTAA

TTGCTTTCAA ACCTTTGCTT TCCTGGTGGA GGTTAAAGGG GAATGGGTAA

TTGCTTTCAA ACCTTTGCTT TCCTGGTGGA GGTTAAAGGG GAATGGGTAA

TTGCTTTCAA ACCTTTGCTT TCCTGGTGGA GGTTAAAGGG GAATGGGTAA

TTGCTTTCAA ACCTTTGCTT TCCTGGTGGA GGTTAAAGGG GAATGGGTAA

TTGCTTTCAA ACCTTTGCTT TCCTGGTGGA GGTTAAAGGG GAATGGGTAA

TTGCTTTTAA ACCTTTGCTT TCCTGGTGGA GGTTAAAGGG GAATGGGTAA

TTGCTTTTAA ACCTTTGCTT TCCTGGTGGA GGTTAAAGGG GAATGGGTAA

---------- ---------- ---------- ---------- ----------

TTGCTTTCAA ACCTGTGCTT TCCTGGTGGA GGTTAAAGGG GAATGGGTGA

TTGCTTTCAA ACCTTTGCTT TCCTGGTGGA GGTTAAAGGG GAATGGGTAA

TTGCTTTCAA ACCTTTGCTT TCCTGGTGGA GGTTAAAGGG GAATGGGTAA

TTGCTTTCGA ACCTTTGCTT TCCCGGTGGA GGTTAAAGGG GAATGGGTAA

TTGCTTTCAA ACCTTTGCTT TCCTGGTGGA GGTTAAAGGG GAATGGGTAA

---------- ---------- ---------- ---------- ----------

TTGCTTTCAA ACCTTTGCTT TCCTGGTGGA GGTTAAAGGG GAATGGGTAA

TTACTTTCAA ATCCTTGCTT TCCTTGTGGA GGTTAAAGGG GAATGGGTAA

TTACTTTCAA ATCTTTGCTT TCCTGGTGGA GGTAAAAGGG GTCTGGGTAA

GTGAGGAAGT CTGCATCTTA TTCAATGTTT TTCACTTGAA TTTGCAGCAT

GTGAGGAAGT CTGCATCTTA TTCAATGTTT TTCACTTGAA TTTGCAGCAT

GTGAGGAAGT CTGCATCTTA TTCAATGTTT TTCACTTGAA TCTGCAGCAT

GTGAGGAAGT CTGCATCTTA TTCAATGTTT TTCACTTGAA TCTGCAGCAT

GTGAGGAAGT CTGCATCTGA TTCAATGTTT TTCACTTGAA TTTGCAGCAT

GT-AGGAAGT CTGCATCTTA TTAAATGTTT TTCACTTTAA TTTGCAGCAT

GTGAGGAAGT CCACCTCCCA CTCTGTGTCT TTCACTTTAA -TTACAGCAT

GTGAGGAAGT CCGCCTCCCA CTCCGTGTCT TTCACTTTAA -TCACAGCAT

---------- ---------- ---------- ---------- -TTTCAGCGT

GTGAGGAAGT CTGTATCTTA TTAAATGTTT TTGACTTTAA TTTCCAGCAT

GT-AGGAAGT CTGCATCTTA TTAAATGTTT TTCACTTTAA TTTTCAGCAT

GTGAGGAAGT CTGCATCTTT TTAAATGTTT TTCACTTTGA TTTTCAGCAT

GTG------- ---------- ---------- ---------- ----------

GTGAGGAAGT CTGCATCTTA TTAAATGTTT TTCACTTTAG TTTTCAGCAT

---------- ---------- ---------- ---------- -TTTCAGCGT

GTGAGGAAGT CTGCATCTTC TTCAGTGTTT TTCGCTTTAA T---------

GTGTGGAAGT CTGTATTTTC TTATATGTAT TTCATTTTAA TCTTAAGCAT

GTCAGGAAGT CTACATCTTC TTAAATGTAT TTCATTTAAC TCTCCAGCAT

GGATTCTGGT TCTTCAGAGA GTCCTGCTAG TCTAAAGCGA GTAGCCTCTC

GGATTCTG-- ---------- ---------- ---------- ----------

GGAGTCTGGT TCTTCAGAGA GTCCTGCTAG TCTAAAGCGA GTAGCCTCTC

GGAGTCTGGT TCTTCAGAGA GTCCTGCTAG TCTAAAGCGA GTAGCCTCTC

GGAGTCTGGT TCCTCCGAGA GTCCTGCTAG TCTAAAGCGA GTAGCCTCTC

GGAGTCTGGT TCTTCAGAAA GTCCTGCTAG TCTAAAGAGA GTGGCTTCCC

CGATACTGGC CCTACGGAAG CTCCCTCTAG TCTAAAGAGA GTGGCTTCCC

CGATACTGGC CCTACTGAAA GTCCTGCTAG TCTAAAGAGA GTGGCTTCCC

TACTAGAGGT CCTTCAGATA GTCCCGCCAG CCGAAAAAGA G-GCCTTCCG

GGATTCTGGT CCCTCAGAAA GTCCTGCAAG TCTAAAGAGG GTAGCTTCCC

GGATTCTGGT TCTTCAGAAA GTCCCGCTAG TCTAAAGAGA GTAGCTTCCC

GGATTCTGGT TCTTCAGAAA GTCCGGCTAG TCTAAAGAGA GTAGCTTCCC

---------- ---------- ---------- ---------- ----------

GGATTCGGGT TCTTCAGAAA GTCCTGCAAG TCTAAAGAGA GTAGCTTCCC

GGAGCCCGGC TCTTCAGAAA GTCCTGGGAG TCTGAAGAGA GTAGCTTCCC

---------- ---------- ---------- ---------- ----------

GGATTCAGGT TCTTCAGAAA GTCCTGCTGT TCTAAAGAGA GTAGCATCAC

GGATTCAGGT TCTTCAGAAA GCCCCGCTAG TCTAAAGAGG GTGTCATCAC

CAGCAGCCAA GAACTTCAAA GCTGTCTGGG ATTTCTTAAC AATCAAGCTC

---------- -AACTTCAAA GCTGTCTGGG ATTTCTTAAC AATCAAGCTC

CAGCAGCCAA GAACTTCAAA GCTGTCTGGG ATTTCTTAAC AATCAAGCTC

CAGCAGCCAA GAACTTCAAA GCTGTCTGGG ATTTCTTAAC AATCAAGCTC

CAGCAGCCAA GAACTTCAAA GCTGTCTGGG ATTTCTTAAC AATCAAGCTC

CAGCAGCCAA GAACTTCAAA GCTGTCTGGG ATTTCTTAAC AATCAAGCTC

CAGCCGCCAA GAACTTCAAA GCTGTCTGGG ATGCCGTAAC AATCACGCTT

CGGCCGCCAA GAACTTCAAA GCTGTCTGGG AT--TGTAAC AATCAAGCTT

CGAAGGCCA- -AACCTCAAA GCTGTCTGGG ATTTCTTAAC AATCAAGCTC

CAGCAGCCAA GAACTTCAAA GTTGTCTGGG ATTTCTGAAC AATCAAGCTC

CAGCAGCCAA GAACTACAAA GCTGTCTGGG ATCTCTGAAC AATCAAGCTC

CAGCAGCCAA GAACTTCAAA GCTGTCTGGG ATTTCTGAAC AATCAAGCTC

---------- -AACCTCGAA GCGGTCTGGG ATCTCTGAAC AATCAAGCTC

CAGTAGCCAA GAACTTCAAA GCTGTCTGGG ATTTCTTAAC AATCAAGCTC

CAGCAGCCAA AAACTTCAAA GCTGTCTGGG ATTTCTTAAC AGTCAAGCTC

---------- ---------- ---------- ---------- ----------

CAGCAGCTAA AAGATTTATA CATGTATACC ATTTCTCAAA AGTTAAGCAC

CAGCTTCCAA AGATTACAGA GCCGTTAAGG ATTTCTGGAA ATTCCATTTC

TCTCCGTTAT TTCTCCCCCC AGAATTTATT GCCATGTACA CTTACGAGAG

TCTCCGTTAT TTCTCCCCCC AGAATTTATT GCCATGTACA CTTACGAGAG

TCTCCGTTAT TTCTCCCCCC AGAATTTATT GCCATGTACA CTTACGAGAG

TCTCCATTAT TTCTCCCCCC AGAATTTATT GCCATGTACA CTTACGAGAG

TCTCCGTTAT TTCTCCCCCC AGAATTTATT GCCATGTACA CTTACGAGAG

TCTCCGTTAT TTCTCCCCCC AGAATTTATT GCCATGTACA CTTACGAGAG

TCTCCGTTAT TTCT----CC AGAATTTGTT GCTATGTACA CTTACGAGAG

TCTCCGTTAT TTCT----CC AGAGTTTATT GCCATGTACA CATACGAGAG

TCTCCATTAT TTCTCCCCCC AGAGTTTATT GCCATGTACA CTTACGAGAG

TCTCCGTTAT TTCTCCCCCC AGAATTTATT GCCATGTACA CTTACGAGAG

TCTCCGTTAT TTCTCCCCCC AGAATTTATT GCCATGTACA CTTACGAGAG

TCTCCGTTAT TTCTCCCCCC AGAATTTATT GCCATGTACA CTTACGAGAG

TCTCCGTTAT CTCTCCCCCC AGAGTTTATT GCCATGTACA CTTACGAGAG

TCTCCGTTAT TTCTCCCCCC AGAATTTATT GCCATGTACA CTTACGAGAG

TCTCCGTTAT TTCTCCCCCC AGAATTTATT GCCATGTACA CTTACGAGAG

---------- ---------- ---------- ---------- ----------

GCGTCATCAT TTCTCCCCCC AGAATATATT GCCATGTACA CTTATGAGAG

TTCCCATCAT TTGCCCCTCC AGAATTTATC GCCATGTACA CCTACGAAAG

TTCTGAGCAA GGAGATTTAA CCTTTCAGCA AGGGGATGTG ATTTTGGTTA

TTCTGAGCAA GGAGATTTAA CCTTTCAGCA AGGGGATGTG ATTTTGGTTA

TTCTGAGCAA GGAGATTTAA CCTTTCAGCA AGGGGATGTG ATTTTGGTTA

TTCTGAGCAA GGAGATTTAA CCTTTCAGCA AGGGGATGTG ATTTTGGTTA

TTCTGAGCAA GGAGATTTAA CCTTTCAGCA AGGGGATGTG ATTTTGGTTA

TTCTGAGCAA GGAGATTTAA CCTTTCAGCA AGGGGATGTG ATTTTGGTTA

TTCTGAGCAT GGAGATTTAA CCTTTCAGCA AGGGGATGTG ATTGTGGTTA

TTCTGAGCAA GGAGATTTAA CCTTTCAGCA AGGGGATGTG ATTGTGGTTA

TTCTGAGCAA GGAGACTTAA CCTTTCAGCA AGGGGATGTG ATTTTGGTTA

TTCTGAGCAA GGCGATTTAA CCTTTCAGCA AGGGGATGTG ATTTTGGTTA

TTCTGAGCAA GGCGATTTAA CGTTTCAGCA AGGGGATGTG ATTTTGGTTA

TTCTGAGCAA GGCGATTTAA CCTTTCAGCA AGGGGATGTG ATTTTGGTTA

TTCTGAGCAA GGCGATTTAA CCTTTCAGCA AGGGGACGTG ATTCTGGTCA

TTCTGAGCAA GGAGATTTAA CCTTTCAGCA AGGGGAAGTG ATTTTGGTTA

TTCTGAGCAA GGAGATTTGA CCTTTCAGCA AGGGGATGTG CTTTTGGTTA

---------- ---------- ---------- ---------- ----------

TTCTGAACAA GGCGACTTAA CCTTTCAGCA AGGAGATGTG ATTTTGGTGA

CTCCGAACAG GGAGATTTAA CCTTTCAGCA AGGGGACGTG ATCTTGGTCA

CCAAGAAAGA TGGTGACTGG TGGACAGGAA CAGTGGGCGA CAAGGCCGGA

CCAAGAAAGA TGGTGACTGG TGGACAGGAA CAGTGGGCGA CAAGGCCGGA

CCAAGAAAGA TGGTGACTGG TGGACAGGAA CAGTGGGCGA CAAGTCCGGA

CCAAGAAAGA TGGTGACTGG TGGACAGGAA CAGTGGGCGA CAAGTCCGGA

CCAAGAAAGA TGGTGACTGG TGGACAGGAA CAGTGGGCGA CAAGTCCGGA

CCAAGAAAGA TGGTGACTGG TGGACAGGAA CAGTGGGCGA CAAGTCCGGA

CCAAGAAGGA TGGTGACTGG TGGACGGGCA CAGTGGGCGA GACGTCCGGA

CCAAGAAAGA TGGTGACTGG TGGACGGGAA CGGTGGGCGA CAAGTCCGGA

CCAAGAAAGA TGGTGACTGG TGGACGGGAA CAGTGGGCGA CAAGTCCGGA

CCAAGAAAGA TGGTGACTGG TGGACAGGAA CAGTGGGCGA CAAGTCCGGC

CCAAGAAAGA TGGTGACTGG TGGACAGGAG CAGTGGGCGA CAAGTCCGGA

CCAAGAAAGA TGGTGACTGG TGGACAGGAA CAGTGGGCGA CAAGTCCGGA

CCAAGAAGGA TGGTGACTGG TGGACAGGAG CGGTGGGCGC CAAGACCGGA

CCAAGAAAGA TGGTGACTGG TGGACAGGAA CAGTGGGCGA CAAATCCGGA

CCAAGAAAGA CGGTGACTGG TGGACAGGAA CAGTGGGCGA CAGATCCGGA

---------- ---------- ---------- ---------- ----------

CAAAGAAGGA TGGTGATTGG TGGACAGGCA CTGTGGCTGA CAAATCTGGA

CAAAGAAAGA TGGCGACTGG TGGACAGGAA CAGCGGGTGA CAAATCCGGA

GTCTTCCCTT CTAACTATGT GAGGCTTAAA GATTCAGAGG TAATGCAAAT

GTCTTCCCTT CTAACTATGT GAGGCTTAAA GATTCAGAGG TAATGCAAAT

GTCTTCCCTT CTAACTATGT GAGGCTTAAA GATTCAGAGG TAATGCAAAT

GTCTTCCCTT CTAACTATGT GAGGCTTAAA GATTCAGAGG TAATGCAAAT

GTCTTCCCTT CTAACTATGT GAGGCTTAAA GATTCAGAGG TAATGCAAAT

GTCTTCCCTT CTAACTATGT GAGGCTTAAA GATTCAGAGG TAATGCAAAT

GTCTTCCCTT CTAACTATGT GAGGCTTAAA GACTCAGAGG TAATGCCAAT

GTCTTCCCTT CTAACTATGT GAGGCTTAAA GATTCAGAGG TAATGCCAAT

GTCTTCCCTT CTAACTATGT GAGACTTAAA GATTCAGAGG TAA-------

GTCTTCCCTT CTAACTATGT GAGGCTTAAA GATTCGGAGG TAATGCAAAT

GTCTTCCCTT CTAACTATGT GAGGCTTAAA GATTCAGAGG TAATGCAAAT

GTCTTCCCAT CTAACTATGT GAGGCTTAAA GATTCAGAGG TAATGCAAAT

GTCTTCCCTT CTAACTATGT GAGGCTTAAG GACGCCGAGG TGATGCAAAT

GTCTTCCCTT CTAACTATGT GAGGCTTAAA GATTCAGAGG TAATGCAAAT

GTCTTCCCTT CTAACTATGT GAGGCTGAAA GACTCAGAGG TAATGCAAAT

---------- ---------- ---------- ---------- ----------

GTCTTTCCTT CTAACTACGT GAGGCTTAAA GACTCCGAGG TAATATAAAA

GTCTTCCCTT CTAACTACGT GCGGCTCAAG GATTCCGAGG TAATTCAAAT

GAGACCTTTT GCTCTGTTTT AGGGCTCTGG AACTGCTGGG AAAACAGGGA

GAGACCTTTT GCTCTGTTTT AGGGCTCTGG AACTGCTGGG AAAACAGGGA

GAGACCTTTT GCTCTGTTTT AGGGCTCTGG AACTGCTGGG AAAACAGGGA

GAGACCTTTT GCTCTGTTTT AGGGCTCTGG AACTGCTGGG AAAACAGGGA

GAGACCTTTT GCTCTGTTTT AGGGCTCTGG AACTGCTGGG AAAACAGGGA

GAGACCTTCT GCTCTGTTTT AGGGCTCTGG AACTGCTGGG AAAACAGGGA

GAGACCTTTT GCTCTGTTTT AGGGCTCTGG AACTGCGGGG AAAACAGGGA

GAGACCTTCT GCTCTGTTTT AGGGCTCTGG AACTGCTGGG AAAACAGGGA

---------- ---------- ---------- ---------- ----------

GAGACCTTCT GCTCTGTTTC AGGGCTCTGG AACTGCTGGG AAAACAGGGA

GAGACCTTTT GCTCTGTTTT AGGGCTCTGG AACTGCTGGG AAAACAGGGA

GAGACCTTTT GCTCTGTTTT AGGGCTCTGG AACTGCTGGG AAAACAGGGA

GCGACCTTCT GCTCTCTTTG AGGGTGCCGG GACCTCCGGG AAAGCCGGGA

GAGACCTTTT GCTCTGTTTT AGGGCTCTGG AACTGCTGGG AAAACAGGGA

AAGACCTTTT GCTCTGTTTT AGGGCTCTGG AACCGCTGGG AAAACAGGGA

---------- ---------- ---------- ---------- ----------

TTCATCTTTC CATCTGTTTT AGGTCCCTGG AGCTTCTGGG AAAGCAGGGA

GAAATCTTCT CCTCTGTCCC AGGTCCCCGG AACCGCTGGG AAAACAGGAA

GTTTAGGAAA AAAACCTGGT AAGTTACAAT TTGATCTTTT TTTCAGAAAT

GTTTAGGAAA AAAACCTGGT AAGTTACAAT TTGATCTTTT TTTCAGAAAT

GTTTAGGAAA AAAACCTGGT AAGTTACAAT TTGATCTTTT TTTCAGAAAT

GTTTAGGAAA AAAACCTGGT AAGTTACAAT TTGATCTTTT TTTCAGAAAT

GTTTAGGAAA AAAACCTGGT AAGTTACAAT TTGATCTTTT TTTCAGAAAT

GTTTAGGAAA AAAACCTGGT AAGTTACAAT TTGGTCTTTT TTTCAGAGAT

GTTTAGGAAA AAAACCTGGT AAGTCACAAT CTGGTCTTTT TTTCAGAAAT

GTTTAGGAAA AAAACCTGGT AAGTTGTAAT TTGATCCTTT TTTCAGAAAT

---------- ---------- ---------- ---------- ----------

GTTTAGGAAA AAAACCTGGT AAGTGACAAT TTGGTCTTTT TTCCAGAAAT

GTTTAGGAAA AAAACCTGGT AAGTTGCAAT TTGATCTTTT TTTCAGAAAT

GTTTAGGAAA AAAACCTGGT AAGTTACAAT TGGATCTTTT TTTCAGAAAT

GTTTGGGGAA GAAGCCGGGT AAGT------ ---------- ----------

GTTTAGGAAA AAAACCTGGT AAGTTACAAT TTGATCTTTT TTTCAGAAAT

GTTTAGGAAA GAAACCTGGT AAGTTACAAT TTGGTCTTTT TTTCAGAAAT

---------- ---------- ---------- ---------- ----------

GTTTAGGAAA GAAACCTGGT AAGTTGTAGT TTTATTTTAT TTTCAGAAAT

GTTTGGGAAA GAAACCAGGT GAGTTACAAT TTTATTTTTA TTCCAGAAAT

TGCCCAGGTT ATTGCCTCAT ACACCGCCAC CGGCCCCGAG CAGCTCACTC

TGCCCAGGTT ATTGCCTCAT ACACCGCCAC CGGCCCCGAG CAGCTCACTC

TGCCCAGGTT ATTGCCTCAT ACACCGCCAC CGGCCCCGAG CAGCTCACTC

TGCCCAGGTT ATTGCCTCAT ACACCGCCAC CGGCCCCGAG CAGCTCACTC

TGCCCAGGTT ATTGCCTCAT ACACCGCCAC CGGCCCCGAG CAGCTCACTC

TGCCCAGGTT ATTGCATCAT ACACTGCTAC TGGTCCTGAG CAGCTCACTC

CGCCCAGGTC ATTGCTTCCT ACACTGCTAC GGGTCCTGAA CAGCTCACCC

TGCCCAGGTT ATTGCTTCCT ACGCTGCTAC TGGTCCCGAA CAACTCACCC

---------- ---------- ---------- ---------- ----------

CGCGCAGGTC ATTGCCTCCT ACACAGCTAC GGGTCCTGAA CAGCTTACCC

TGCGCAGGTT ATCGCCTGCT ACACAGCTAC TGGTCCCGAG CAGCTCACCC

CGCCCAGGTT ATTGCTTCGT ACACCGCGAC GGGTCCCGAG CAGCTTACCC

---------- ---------- ---------- ---------- ----------

TGCCCAGGTT ATCGCCTCAT ACACCGCTAC CGGTCCTGAG CAGCTGACTC

TGCCCAGGTC ATTGCCTCGT ACACTGCTAC CGGCCCTGAG CAGCTCACCC

---------- ---------- ---------- ---------- ----------

TGCCCAGGTT ATTGCATCTT ATACTGCTAC TGGACCTGAA CAGCTTACCT

TGCCCAGGTT ATTGCCTCTT ACACAGCCAC CGGCCCCGAA CAACTCACCC

TCGCCCCTGG TCAGCTGATT TTGATCCGAA AAAAGAACCC AGGTGGATGG

TCGCCCCTGG TCAGCTGATT TTGATCCGAA AAAAGAACCC AGGTGGATGG

TCGCCCCTGG TCAGCTGATT TTGATCCGAA AAAAGAACCC AGGTGGATGG

TCGCCCCTGG TCAGCTGATT TTGATCCGAA AAAAGAACCC AGGTGGATGG

TTGCCCCTGG TCAGCTGATT TTGATCCGAA AAAAGAACCC AGGTGGATGG

TGGCCCCTGG TCAGCTGATT TTGATCCGAA AAAAGAACCC AGGCGGATGG

TGGCTCCTGG TCAGCTGATT TTGATCCGAA AAAAGAACCC CGGTGGATGG

TGGCTCCTGG GCAGCTGATT CTGATCCGGA AAAAGAACCC AGGTGGATGG

---------- ---------- ---------- ---------- ----------

TGGCCCCCGG TCAGCTGATC CTGATCCGGA AAAAGAACCC AGGTGGATGG

TGGCCCCTGG TCAGCTGATT TTGATCCGAA AAAAGAACCC AGGCGGTTGG

TGGCCCCTGG TCAGCTGATT TTGATCCGAA AAAAGAACCC AGGTGGATGG

---------- ---------- ---------- ---------- ----------

TGGCCCCTGG TCAGCTGATT TTGATCCGAA AAAAGAATCC AGGTGGATGG

TGGCCCCTGG TCAGCTGATT TTGATCCGAA AAAAGAATCC AGGAGGATGG

---------- ---------- ---------- ---------- ----------

TGGCACCTGG ACAACTCATT TTGATCCGGA AGAAGAATCC AGGAGGATGG

TGGCACCCGG TCAACTGATT CTGATCCGCA AGAAGAATCC CGGAGGATGG

TGGGAAGGAG AGCTGCAAGT CAGTAGGCAC GTGGGAAAAA GCGCCAGATA

TGGGAAGGAG AGCTGCAAGT CAGT------ ---------- ----------

TGGGAAGGAG AGCTGCAAGT CAGTAGGCAC GTGGGAAAAA GCGCCAGATA

TGGGAAGGAG AGCTGCAAGT CAGT------ ---------- ----------

TGGGAAGGAG AGCTGCAAGT CAGTAGGCCC GTGGGAAAAA GCGCCAAATA

TGGGAAGGAG AGCTGCAAGT TAGTAGGCAC GTGGGAAAAA ACGCCAGATA

TGGGAAGGAG AACTACAAGT CAGTAGGCTC GAGGGAAAAA GCGCCAGATA

TGGGAAGGAG AACTGCAAGT TAGTAGGCTC GAGGGAAAAA GCGCCAGATA

---------- ---------- ----AGGCAC GTGGAAAAAA GCGCCAGATA

TGGGAAGGAG AGCTACAGGT TAGTAGGCAC GTGGGAAAAA GCGCCAGATA

TGGGAAGGAG AGCTGCAAGT TAGTAGGCCC GTGGGAAGAA GCGCCAGATA

TGGGAAGGAG AGCTGCAAGT TAGTAGGCAC GTGGGAAAAA GCGCCAGATA

---------- ---------- ---------- ---------- ----------

TGGGAAGGAG AGCTGCAAGT TAGTAGGCAC GTGGAAAGAA GCGCCAGATA

TGGGAAGGAG AGCTGCAGGT TAGTAGGCAC GTGGGAAAAA GCGCCAGATA

---------- ---------- ---------- ---------- ----------

TGGGAAGGAG AACTTCAAGT TAGTAGGCTC GTGGGAAAAA GCGTCAGATA

TGGGAAGGAG AGCTTCAAGT TAGTAGGCAC GTGGGAAAAA ACGACAGATC

GGCTGGTTCC CAGCTAATTA TGTAAAGCTT CTAAGCCCTG GGACGAGCAA

---------- ---------- ---------- ---------- ----------

GGCTGGTTCC CAGCTAATTA TGTAAAACTT CTAAGCCCTG GGACGAGCAA

---------- ---------- ---------- ---------- ----------

GGCTGGTTCC CAGCTAATTA TGTAAAACTT CTAAGCCCTG GGACGAGCAA

GGCTGGTTCC CAGCTAATTA TGTAAAACTT CTGAGCCCTG GGACGAGCAA

GGGTGGTTCC CAGCAAATTA TGTCAAACTT CTAAGCCCTG GAACAAGCAA

GGGTGGTTTC CAGCAAATTA TGTCAAACTT CTAAGCCCCG GAACAAGCAA

GGCTGGTTCC CAGCTAATTA TGTAAAACTT CTAAGTCCTG GGACGAGCAA

GGCTGGTTTC CTGCTAATTA CGTCAAACTT TTAAGCCCTG GGACAAGCAA

GGCTGGTTCC CCGCGAATTA TGTAAAACTT TTGAGCCCTG GCACGAGCAA

GGCTGGTTTC CTGCTAATTA TGTAAAACTT TTAAGCCCTG GGACGAGCAA

---------- ---------- ---------- ---------- ----------

GGCTGGTTCC CAGCTAATTA TGTAAAACTT TTAAGCCCTG GGTCGAGCAA

GGCTGGTTCC CAGCTAATTA TGTCAAACTT TTAAGCCCTG GGACGAGCAA

---------- ---------- ---------- ---------- ----------

GGCTGGTTCC CAGCTAATTA TGTAAAACTT TTAAGTCCTG GCACCAGCAA

GGCTGGTTCC CGGCAAATTA CGTCAAACTC CTGAGCCCCG GCACCAGCAA

AATCACTCCA ACAGAGCCAC CTAAGCCTTC AACAAGGGCC AGATCATCAT

---------- ---------- ---------- ---------- ---------T

AATCACTCCA ACAGAGCCAC CTAAGCCTTC AACAAGGGCC AGATCATCAT

---------- ---------- ---------- ---------- ---------T

AATCACTCCA ACGGAGCCAC CTAAGCCTTC AACAAGGGCC AGATCATCAT

AATCACTCCA ACAGAGCCAC CCAAGCCTTC AACAAGGGCC AGATCATCAT

AATCACCCCA ACTGAGCTAC CCAAGCCTTC AGCAAAGGCC AGATCATCAT

AATCACCCCA ACTGAGCTAC CCAAGCCTTC AGCAAAGGCC AGATCATCAT

AATCACTCCA ACGGAGCCAC CCAAG----- ---------- ----------

AATCACTCCA ACAGACCCAC CGAAGCCTTC AGCAAGGGCC AGGTCATCAT

AATCACTCCA ACGGAGCCAC CTAAGCCTTC AGCAAGGGCC AGATCATCAT

AATCACTCCA ACCGAGCCCC CTAAGCCTTC AACAAGGGCC AGCTCATCAT

---------- ---------- -----CTTTC AGCAAGGGCG TGATTCTGGT

AATCACTCCA ACAGAGCCAC CTAAGCTTTC AGCAAGGGGA AGATTTTGGT

AATCACCCCA ACAGAGCCAC CGAAGCCTTC AGCAAGGGCC AGATCATCAT

---------- ---------- ---------- ---------- ----------

AGTCACTCCA ACGGAGCCAC CGAAGCATTC AACAAAGGCC AGATCATCAT

GATCACACCA ACCGAGCCCC CTAAGCGTTC GGCAAGGGCC AGATCATCAT

CAACAAGGAG GACCCTGACT GGTGGAAAGG AGAAGTCAAT GGACAAGTGG

TACCAAGAAA GATGGTGACT GGTGGACAGG AACAGTGGGC GACAAGGCCG

CAACAAGGAG GACCCCGACT GGTGGAAGGG GGAAGTCAAT GGACAAGTGG

TACCAAGAAA GATGGTGACT GGTGGACAGG AACAGTGGGC GACAAGTCCG

CAACAAGGAG GACCCCGACT GGTGGAAAGG AGAAGTCAAT GGACAAGTGG

CAACAAGGAG GACCCCGACT GGTGGAAAGG AGAAGTGAAT GGACAAGTGG

CAGCAAGGAG GACCCGGACT GGTGGAAAGG AGAAGTCAGT GGGCAGGTTG

CAACAAGGAG GACCCGGACT GGTGGAAAGG AGAAGTCAGT GGGCAAGTTG

---------- ---------- ---------- ---------- ----------

CAACAAGGAG GACCCCGACT GGTGGAAGGG TGAAGTCCAC GGGCAGGTGG

CAACAAAGAC GACCCGGACT GGTGGAAGGG CGAGGTCAAC GGGCAAGTGG

CAACAGGGAG GACCCTGATT GGTGGAAAGG AGAAGTCAGT GGCCAAGTGG

CACCAAGAAG GATGGTGACT GGTGGACAGG AGCGGTGGGC GCCAAGACCG

TACCAAGAAA GATGGTGACT GGTGGACAGG AACAGTGGGC GACAAATCCG

CAACAAGGAA GACCCCGACT GGTGGAAAGG AGAAGTCAGC GGACAAGTGG

---------- ---------- ---------- ---------- ----------

TAATAAGGAA GATCCTGATT GGTGGAAAGG AGAAGTCAAT GGACAAGTGG

CAACAAGGAA GACCCCGACT GGTGGAAAGG GGAGGCGAAC GGGCAAGTGG

GGCTCTTCCC ATCCAATTAT GTGAAGCTTT GTGTTTTCCA GGGTGTTCAG

GAGTCTTCCC TTCTAACTAT GTGAGGCTTT GTGTTTTCCA GGGTGTTCAG

GGCTCTTCCC ATCCAATTAT GTGAAGCTTT GTGTTTTCCA GGGTGTTCAG

GAGTCTTCCC TTCTAACTAT GTGAGGCTTT GTGTTTTCCA GGGTGTTCAG

GACTCTTCCC ATCCAATTAT GTGAAGCTTT GTGTTTTCCA GGGTGTTCAG

GGCTCTTCCC GTCCAACTAT GTGAAGCTTT GTGTTTTCCA GGGTGTTCAG

GGCTCTTCCC GTCCAATTAT GTGAAGCTTT GTGTCTTCCA GGGTGCTCAG

GGCTCTTCCC ATCCAATTAT GTAAAGCTTT GTGTCTTGCA GGGTGCTCAG

---------- ---------- --------TT GTGTCTTCCA GGGTGTTCAG

GGCTCTTCCC GTCCAACTAC GTGAAGTTTT GTGTTTTGCA GGGTGCTCAG

GGCTCTTCCC CTCCAACTAC GTGAAGCTTT GTGTTTTCCA GGGTGCTCAG

GGCTCTTCCC ATCCAATTAT GTGAAGCTTT GTGTTTTCCA GGGTGTTCAG

GAGTCTTCCC TTCTAACTAT GTGAGGCTCC GTGCGTTGCA AGGTGCTCCG

GAGTCTTCCC TTCTAACTAT GTGAGGCTTT GTGTTTTCCA GGGTGTTCCG

GGCTCTTCCC GTCCAATTAT GTGAAGCTTT GTGTTTTCCA GGGTGTTCAG

---------- ---------- --------TT ACTTTTTCCA GGGTGTTCAG

GGCTCTTCCC ATCCAATTAT GTGAAAATTT GTATTTTCCA GGGTGTGCAG

GCCTCTTCCC CTCCAACTAC GTGAAGCTTT GTGTCTTCCA GGGTGCGCGG

ACTTACATCT CTTGGATATG TTGACCCCAA CTGAAAGAAA GCGACAAGGA

ACTTACATCT CTTGGATATG TTGACCCCAA CTGAAAGAAA GCTACAAGGA

ACTTACATCT CTTGGATATG TTGACCCCAA CTGAAAGAAA GCGACAAGGA

ACTTACATCT CTTGGATATG TTGACCCCAA CTGAAAGAAA GCGACAAGGA

ACTTACATCT CTTGGATATG TTGACCCCAA CTGAAAGAAA ACGACAAGGG

ACTTGCATCT CTTGGATATG TTGACCCCAA CTGAGAGGAA GAGACAAGGG

ACTTACATCT CTTAGATATG TTGACCCCGA CTGAGAGGAA GAGGCAAGGC

ACCTGCATCT CTTAGATATG CTGACCCCGA CTGAGAGGAA GCGGCAAGGC

ACTTGCATCT CCTGGATATG TTGACCCCGA CGGAGAGAAA GCGACAAGGC

ACTTACACCT CTTGGATATG CTGACCCCAA CTGAAAGAAA GCGACAGGGG

ACTTACACCT CTTGGATATG TTGACCCCAA CGGAGAGAAA GCGACAAGGA

ACTTACATCT CTTGGATATG TTGACCCCAA CTGAAAGAAA GCGACAAGGG

ACCTCCACCT CCTGGACATG CTGACGCCCA CCGAGCGGAA GCGGCAGGGC

ACTTACATCT CTTGGATATG CTGACCCCAA CTGAAAGAAA GCGGCAAGGC

ACTTACATCT CTTGGATATG TTGACCCCAA CGGAGAGAAA ACGACAAGGG

ACCTACATCT CTTGGATATA TTGGCCCCCA CGGAAAGAAA GCGACAAGGA

ATTTACATCT TTTGGATATG TTGACACCAA CAGAAAGAAA ACGACAGGGA

ACTTGCATCT GCTGGACATG CTGACCCCTA CGGAAAGGAA GCGCCAGGGC

TACATCCACG AGCTCATTGT CACAGCTAAC TGCTTTCTGA ACTGCTTCGT

TACATCCACG ACCTCATTGT CACAGCTAAC TGCTTTCTGA ACTGCTTCGT

TACATCCACG AGCTCATTGT CACAGCTAAC TGCTTTCTGA ACTGCTTCGT

TACATCCACG AGCTCATTGT CACAGCTAAC TGCTTTCTGA ACTGCTTCGT

TACATCCACG AGCTCATTGT CACAACTAAC TGCTTTCTGA ACTGCTTCGT

TACATCCACG AACTCATTGT CACAGCTAAC TGCTTTCTCA ACTGCTTCGT

TACATCCATG AACTCATTGT CACAGCTAAC TGCTTTCTGA ACGGCTTCAT

TACATCCATG AACTCATTGT CACAGCTAAC TGCTTTCTGA ACGGCTTCGT

TACATCCATG AGCTCATTGT CACCGCTAAT TGCTTTCTGA ACTGCTCCGT

TACATCCACG AGCTCATCGT CACAGCTAAT TGCTTTCTGA ACTGCTTCGT

TACATCCATG AGCTCATTGT CACAGCTAAT TGCTTTCTGA ACTGCTTCGT

TACATCCACG AACTCATTGT CACAGCTAA- TACTCTCTGA ACTGCTTCGT

TACATCCACG AGCTCATCGT CACAGCTAAC TGCTTTCTGA ACCGCTTCGT

TACATCCACG AGCTCATCGT CACAGCTAAT TGCTTTCTGA ACCGCTTCGT

TACATCCACG AACTCCTTGT CACAGCTAA- --CTTTCTGA ACTGCTTCGT

TACATCCACG AACTCATCGT CACAGCTAAT TGCTTTCTGA ACTGCTTCGT

TATATCCATG AGCTACTTGT TACAGCTAAT TGCTTACTGA ATTGCTTCCT

TACATCCACG AGCTGATCCT GACATGGAAT TTCGCGGTGT CGTGCTTTGT

TTTCTGCATC TGTAGATTTT TCAAAAACCC CTGATGGAGT CTGAGCTGCT

TTTCTGCATC TGTAGATTTT TCAAAAACCC CTGATGGAGT CTGAGCTGCT

TTTCTGCATC TGTAGATCTT TCAAAAACCC CTGATGGAGT CTGAGCTGCT

TTTCTGCATC TGTAGATCTT TCAAAAACCC CTGATGGAGT CTGAGCTGCT

TTTCTGCATC TGTAGATCTT TCAAAAACCC CTGATGGAGT CTGAGCTGCT

TTTCTGCATC TGTAGATCTT TCAGAAACCC CTGATGGAGT CTGAGCTGCT

TTTCTACACC TGTAGATCTT TCAGAAACCC CTGACGGAGT CTGAGCTGCT

TTTCTACACC TGTAGATCTT TCAGAAACCC CTGACGGAGT CTGAGCTGCT

TTTCTGCATC TGTAGATCTT TCAGAAACTC CTGATGGAGT CTGAGCTGCT

TTTCTGCATC TGTAGATCTT TCAGAAACCC CTGATGGAGT CTGAGCTGCT

TTTCTGCACC TGTAGATCTT TCAGAAACCC CTGATGGAGT CTGAGCTGCT

TTTCTGCATC TGTAGATCTT TCAGAAACCC CTGATGGAGT CTGAGCTGCT

TTTCTGCACC TGTAGATCTT TCAGAAACCC CTGATGGATT CTGAGCTGCT

TTTCTGCATC TGTAGATCTT TCAGAAACCC CTGATGGAGT CTGAGCTGCT

TTTCTGCACC TGTAGATCTT TCAGAAACCC CTGATGGAAT CTGAGCTGCT

TTTCTGCGTC TGTAGATCTT TCAGAAACCC CTGATGGAAT CTGAGCTGCT

TTTCTGCATC TATAGATCTT TCAGAAACCG CTGATGGAGT CTGAGCTGCT

TTTCTGCCCC TGCAGGTCTT TCAGAAACCC CTAATGGAAT CCGAGCTGCT

GACAGAAAAA GAGGTTGCTA TGATTTTTGT GAACTGGAAG GAGCTGATTA

GACAGAAAAA GAGGTTGCTA TGATTTTTGT GAACTGGAAG GAGCTGATTA

GACAGAGAAA GAGGTTGCTA TGATTTTTGT GAACTGGAAG GAGCTGATTA

GACAGAGAAA GAGGTTGCTA TGATTTTTGT GAACTGGAAG GAGCTGATTA

GACAGAAAAA GAGGTTGCTA TGATTTTTGT GAACTGGAAG GAGCTGATTA

GACAGAGAAA GAGGTTGCTA TGATTTTTGT CAACTGGAAG GAGCTGATTA

GACAGAAAAA GAGGTTGCTA TGATTTTTGT TAACTGGAAG GAGCTGATTA

GACAGAAAAA GAGGTTGCTA TGATTTTTGT TAACTGGAAG GAGCTGATCA

GACAGAAAAA GAGGTTGCTA TGATTTTTGT TAACTGGAAG GAGCTGATTA

GACAGAAAAA GAGGGTGCTA TGATTTTTGT TAACTGGAAG GAGCTGATTA

GACAGAAAAA GAGGGTGCTA TGATTTTTGT TAACTGGAAG GAGCTGATTA

GACAGAAAAA GAGGGTGCTA TGATTTTTGT TAACTGGAAG GAGCTGACTA

GACAGAGAAA GAGGGTGCTA TGATTTTTGT TAACTGGAAG GAGCTGATTA

GACAGAAAAA GAGGTTGCTA TGATTTTTGT TAACTGGAAG GAGCTGATTA

GACAGAAAAA GAGGTTGCTA TGATTTTTGT TAACTGGAAG GAGCTGATTA

GACAGAAAAA GAGGTTGCTA TGATTTTTGT TAACTGGAAG GAGCTGATTA

GACAGAAAAA GAGGTGGCTA TGATTTTTGT TAACTGGAAG GAGCTGATAA

GACGGAAAAG GAGGTGGCCA TGATTTTTGT CAACTGGAAG GAGCTGATAA

TGTGTAATAT CAAACTACTA AAGTAAGCGT GTTTCTGCAG AGCGCTGAGA

TGTGTAATAT CAAACTACTG AAGTAAGCGT GTTTCTGCAG AGCGCTGAGA

TGTGTAATAT CAAACTACTG AAGTAAGCGT GTTTCTGCAG AGCGTTGAGA

TGTGTAATAT CAAACTACTG AAGTAAGCGT GTTTCTGCAG AGCGCTGAGA

TGTGTAATAT CAAACTACTG AAGTAAGCGT GTTTCTGCAG AGCGCTGAGA

TGTGTAATAT CAAACTGCTG AAGTAAGCGT GTTTCTGCAG AGCACTGCGA

TGTGTAATAT CAAACTGCTG AAGTAAGCGT GTTTCTGCAG GGCGCTGAGA

TGTGTAATAT CAAACTGCTG AAGTAAGCAT GTTTCTGCAG AGCGCTGAGA

TGTGTAATAT CAAACTACTG AAGTAAGCGT GTTTCTGCAG GGCGCTGCGG

TGTGTAATAT CAAACTACTG AAGTAAGCGC GTTTCTGCAG AGCGCTGAGG

TGTGTAATAT CAAACTATTG AAGTAAGCGT GTCCCCGCAG AGCGCTGAGG

TGTGTAATGT CAAACTGCTG AAGTAAGCCT GTTTCTGCAG AGCGCTGAGA

TGTGTAATAT CAAACTACTG AAGTAAGCGC GTTTCTGCAG GGCGCTGAGG

TGTGTAATAT CAAACTACTG AAGTAAGCGC GTTTCTGCAG AGCGCTGAGG

TGTGTAATAT CAAGCTACTG AAGTAAGCTT GTTTCTGCAG GGCACTGAGG

TGTGTAATAT CAAACTACTG AAGTAAGCTT TCCCCTGCAG AGCGCTGAGG

TGTGTAATAT CAAACTGCTG AAGTAAGCTT TCTGCTGCAG AGCCCTGAGA

TGTGTAACAT CAAACTACTG AAGTAAGCCC GTTCCTGCAG GGCCCTGAGG

GTCCGCAAGA AGATGTCCGG GGAGAAGATG CCTGTGAAGA TGATTGGGAC

GTCCGCAAGA AGATGTCCGG GGAGAAGATG CCTGTGAAGA TGATTGGGAC

GTCCGTAAGA AGATGTCCGG GGAGAAGATG CCCGTGAAGA TGATTGGGAC

GTCCGTAAGA AGATGTCCGG GGAGAAGATG CCCGTGAAGA TGATTGGGAC

GTCCGCAAGA AGATGTCCGG GGAGAAGATG CCCGTGAAGA TGATTGGGAC

GTGCGCAAGA AGATGTCGGG GGAGAAGATG CCAGTGAAGA TGATCGGGAC

GTCCGCAAGA AGATGTCCGG AGAGAAGATG CCGGTGAAGA TGATCGGGAC

GTCCGCAAGA AGATGTCTGG GGAGAAGATG CCGGTGAAGA TGATTGGGAC

GTCCGCAAGA AGATGTCTGG GGAGAAGATG CCGGTGAAGA TGATCGGGAC

GTCCGCAAGA AGATGTCTGG GGAGAAGATG CCCGTGAAGA TGATCGGGAC

GTCCGCAAGA AGATGTCGGG GGAGAAGATG CCCGTGAAGA TGATCGGGAC

GTTCGCAAGA AGATGTCTGG AGAGAAGATG CCTGTGAAGA TGATCGGGAC

GTCCGCAAGA AGATGTCGGG GGAGAAGATG CCCGTGAAGA TGATCGGGAC

GTCCGCAAGA AGATGTCCGG GGAGAAGATG CCCGTGAAGA TGATCGGGAC

GTCCGCAAGA AGATGTCTGG GGAGAAAATG CCGGTGAAGA TGATCGGGAC

GTTCGCAAGA AGATGTCTGG GGAGAAAATG CCCGTGAAGA TGATCGGGAC

GTTCGAAAGA AGATGTCTGG TGAGAGGATG CCCGTGAAGA TGATCGGGAC

GTGCGGAAGA AGATGTCGGG GGAGAGGATG CCCGTGAAAA TGATCGGGAC

ATCCTGAGCG CACAGCTGCC GCACATGCAG CCCTACATCC GCTTCTGCAG

ATCCTGAGCG CACAGCTGCC GCACATGCAG CCCTACATCC GCTTCTGCAG

ATCTTGAGCG CCCAGCTGCC GCACATGCAA CCCTACATCC GCTTCTGCAG

ATCTTGAGCG CCCAGCTGCC GCACATGCAA CCCTACATCC GCTTCTGCAG

ATCCTGAGCG CCCAGCTGCC GCACATGCAG CCCTACATCC GCTTCTGCAG

ATCCTGAGCG CTCAGCTGCC ACACATGCAG CCCTACATCC GCTTCTGCAG

ATCCTGAGCG CCCAGCTGCC CCACATGCAG CCTTACATCC GCTTCTGCAG

ATCCTGAGCG CCCAGCTGCC GCACATGCAG CCTTACATCC GCTTCTGCAG

ATCTTGAGCG CCCAGCTGCC GCATATGCAG CCGTACATTC GCTTCTGCAG

ATCCTGACGG CCCAGCTGCC ACACATGCAG CCCTACATCC GCTTCTGCAG

ATCCTGACGG CGCAGCTGCC GCACATGCAG CCTTACATCC GCTTCTGCAG

ATCCTGACGG CCCAGCTGCC ACATATGCAA CCCTACATCC GCTTCTGCAG

ATCCTCACGG CCCAGCTGCC GCACATGCAG CCCTACATCC GCTTCTGCAG

ATCCTGACGG CCCAGCTGCC CCACATGCAG CCCTACATTC GCTTCTGCAG

ATCCTGAGCG CCCAGCTGCC CCACATGCAG CCATACATCC GCTTTTGTAG

ATCCTGACCG CCCAGCTGCC CCACATGCAG CCATATATCC GCTTCTGCAG

ATCTTGACTG CCCAGTTGCC CCACATGCAG CCTTACATCC GGTTCTGTAG

ATCCTCACGG CCCAGCTGCC GCACATGCAG CCGTACATCC GCTTCTGCAG

CCGCCAGCTC AACGGGGCTG CCCTGATCCA GCAGAAGACG GATGAGGCCC

CCGCCAGCTC AACGGGGCTG CCCTGATCCA GCAGAAGACG GACGAGGCCC

CCGCCAGCTC AACGGGGCTG CCCTGATCCA GCAGAAGACA GACGAGGCCC

CCGCCAGCTC AACGGGGCTG CCCTGATCCA GCAGAAGACA GACGAGGCCC

CCGCCAGCTC AACGGGGCTG CCCTGATCCA GCAGAAGACA GACGAGGCCC

CTGCCAGCTC AACGGGGCTG CCCTGATCCA GCAGAAGACG GACGAGGCCC

CTGCCAGCTC AATGGGGCCG CCCTCATCCA GCAGAAGACA GATGAGGCTC

CTGCCAGCTC AATGGGGCTG CCCTCATCCA GCAGAAGACG GACGAGGCTC

CTGCCAGCTC AACGGAGCCG CCCTGATCCA GCAGAAGACG GACGAGGCTC

CTGCCAGCTC AACGGGGCCG CCTTGATCCA GCAGAAGACT GACGAGGCCC

CTGTCAGCTC AACGGGGCTG CCCTCATCCA GCAGAAGACC GACGAGGCCC

CTGCCAGCTG AACGGGGCAG CCCTGATCCA GCAGAAGACG GATGAAGCCC

CTGCCAGCTC AACGGGGCCG CCCTCATCCA GCAGAAGACT GACGAGGCCC

CTGCCAGCTC AACGGGGCGG CCCTGATCCA GCAAAAGACA GACGAGGCCC

CTGCCAGCTA AATGGGGCAG CCCTGATCCA GCAGAAGACG GACGAAGCTC

CTGTCAGCTC AACGGGGCAG CCCTGATCCA GCAGAAGACG GACGAGGCCC

CTGCCAGCTA AACGGGGCAG CCCTCATCCA GCAGAAGACG GATGAAGTCC

CTGCCAGCTC AACGGGGCCG CCCTCATCCA GCAGAAGACC GACGAAGTCC

CAGACTTCAA GGAGTTCGTC AAAGTAAGTT TCAGAGATTG GCAATGGATC

CAGACTTCAA GGAGTTTGTC AAAGTAAGTT TCAGAGATTG GCAATGGATC

CAGACTTCAA GGAGTTCGTC AAAGTAAGTT TCAGAGATTG GCAATGGATC

CAGACTTCAA GGAGTTCGTC AAAGTAAGTT TCAGAGATTG GCAATGGATC

CAGACTTCAA GGAGTTTGTC AAAGTAAGTT TCAGAGACTG GCAATGGACC

CCGACTTTAA GGAGTTTGTC AAAGTAAGTT TCAGAGATTG GCAATGGACC

CAGACTTCAA GGAGTTCGTC AAAGTAAGTT TCAGAGACTG GCAATGGACC

CAGACTTCAA GGAGTTCGTC AAAGTAA-TT TCAGAGACTG GCAATGGACC

CGGACTTCAA GGAGTTTGTC AAGGTAAGTT TCAGAGACTG GCAATGGAGC

CAGACTTCAA GGAGTTTGTC AAAGTGAGTT TCAGAGACTG GCCATGGACC

CGGACTTCAA GGAGTTTGTC AAAGTAAGTT TCAGAGGCTG GCAATGGACC

CGGACTTCAA GGAGTTTGTC AAAGTAAGTT TCAGAGATTG GCAATGGACC

CTGACTTCAA GGAGTTCGTC AAGGTACGTT GCAGAGACTC GCCATGGACC

TGGACTTCAA GGAGTTTGTC AAGGTAAGTT TCAGAGATTG GCAATGGACC

CAGACTTCAA GGACTTTGTC AAAGTAAGTT TCAGAGGTTG GCAATGGACC

CGGACTTCAA GGAATTTGTC AAAGTAAG-- ---------- ----------

CCGAGTTCAA GGATTTTGTC AAAGTAAGTT TCAGAGATTA GCTATGGATC

CCGAGTTCAA GGAGTTCGTC AAGGTAAGTT ACAGAGACTG GCCATGGACC

CTCGGTGTAA AGGGATGCCA CTCTCTAGTT TTATACTGAA GCCTATGCAA

CTCGGTGTAA AGGGATGCCA CTCTCTAGTT TTATACTGAA GCCTATGCAA

CTCGGTGTAA AGGGATGCCA CTCTCTAGTT TTATACTGAA GCCTATGCAA

CTCGGTGTAA AGGGATGCCA CTCTCTAGTT TTATACTGAA GCCTATGCAA

CTCGGTGTAA AGGGATGCCA CTCTCTAGTT TTATACTGAA GCCTATGCAA

TGCGCTGTAA AGGGATGCCA CTCTCTAGCT TTATTCTCAA GCCTATGCAA

CTCGGTGCAA AGGCATGCCA CTGTCCAGCT TCATACTGAA ACCTATGCAG

CTCGGTGCAA AGGAATGCCT CTGTCCAGCT TTATACTGAA GCCTATGCAG

CCAGATGTAA AGGGATGCCT CTTTCCAGTT TTATACTGAA GCCCATGCAG

CACGCTGTAA GGGAATGCCC CTGTCTAGTT TTATACTGAA GCCTATGCAA

CTCGATGTAA AGGGATGCCA CTCTCTAGTT TTATCCTGAA GCCTATGCAA

CTCGATGTAA AGGGATGCCT CTCTCTAGTT TTATACTGAA GCCTATGCAA

CCCGCTGCAA AGGCATGCCG CTCTCCAGCT TCATCCTGAA GCCCATGCAG

CTCGATGTAA AGGGATGCCA CTCTCTAGTT TTATACTGAA GCCGATGCAA

CTCGATGTAA AGGGATGCCG CTCTCTAGCT TTATACTGAA GCCTATGCAG

---------- ---------- ---------- ---------- ----------

CTCGATGTAA AGGGATGCCA CTCTCTAGTT TTATATTGAA GCCTATGCAA

CCCGCTGTAA AGGGATGCCC CTGTCCAGCT TCATCCTGAA GCCCATGCAG

CGGGTAACAA GATACCCACT GATCATTAAA AATGTAAGTA GATCCTGGAA

CGGGTAACAA GATACCCACT GATCATTAAA AATGTAAGTA GATCCTGGAA

CGGGTAACAA GATACCCACT GATCATTAAA AATGTAAGTA GATCCTGGAA

CGGGTAACAA GATACCCACT GATCATTAAA AATGTAAGTA GATCCTGGAA

CGGGTAACAA GATACCCACT GATCATTAAA AATGTAAGTA GATCCTGGAG

CGGGTAACAA GATACCCACT GATCATTAAA AATGTAAGTA GATCCTGGAA

CGTGTCACAA GATACCCACT GATCATTAAA AACGTAAGTA GATCCTGGAA

CGTGTCACAA GATACCCGCT GATCATTAAA AACGTAAGTA GATCCTGGAA

CGGGTGACAA GATACCCCCT GATCATTAAA AATGTAAGT- ----------

CGAGTAACGA GATACCCACT GATCATTAAA AATGTAAGTA GATCCTGGAA

CGCGTAACGA GATACCCACT GATCATTAAA AATGTAAGTA GATCCTGGAA

CGGGTAACAA GATACCCACT GATCATTAAA AATGTAAGTA GATCCTGGAA

CGCGTCACCA GATACCCCCT CATCatcaag aacgtgagtA GATTCTCGAG

CGGGTAACCA GATATCCACT GATCATTAAA AATGTAAGTA GATCCTGGAA

CGGGTGACAA GATACccact gatcatcaaa aacgtaagtA GATCCTGGAA

---------- ---------- ---------- ---------A GATCCTGGAA

CGGGTAACAA GATACCCACT AATTATTAAA AATGTAAGTA GATTTTAGAA

CGGGTGACCA GATACCCCCT CATCATTAAA AACGTAAGTA GATCCTGGAA

AACACCCCTG AAAACCACCC GGACCACAGC CACTTGAAGC ACGCCCTGGA

AACACCCCTG AAAACCACCC GGACCACAGC CACTTGAAGC ACGCCCTGGA

AACACCCCTG AAAACCACCC GGACCACAGC CACTTGAAGC ACGCTCTGGA

AACACCCCTG AAAACCACCC GGACCACAGC CACTTGAAGC ACGCTCTGGA

AACACCCCTG AAAACCACCC GGACCACAGC CACTTGAAGC ACGCCCTGGA

AACACTCCTG AAAACCACCC CGACCACAGC CACTTGAAGC ATGCCCTGGA

AACACTCCTG AAAACCACCC CGACCACAGC CACCTGAAGC ACGCCCTGGA

AACACTCCCG AGAACCATCC AGACCACAGC CACCTGAAGC ATGCCCTGGA

---------- ---------- ---------- ---------- ----------

AACACTCCTG AAAACCACCC AGACCACAGC CACCTGAAGC ACGCCCTGGA

AACACTCCTG AGAACCACCC CGACCACAGC CACCTGAAGC ACGCCCTGGA

AACACTCCTG AAAATCACCC CGACCATAGC CACCTGAAGC ACGCCCTGGA

AACACCCTGG AAAACCACCC TGACCACAGC CACCTGAAGC ACGCCCTGGA

AACACTCCTG AAAACCACCC TGACCACAGT CACCTAAAAC ATGCCTTGGA

AACACTCCTG AAAACCACCC TGACCACAGC CACTTGAAAC ACGCCCTGGA

AACACACCTG AAAACCACCC CGACCACAGC CACTTACAAC ACGCCCTGGA

AACACTCCTG AAAACCACCC TGATTACAGC CACTTGAAGC ATGCTCTCGA

AACACGCCCG AAAACCACCC CGACCACTGC CACCTGAAGC TGGCCCTGGA

GAAGGCGAAG AGCTCTGTTC CCAGGTGAAC GAAGGGGTGC GGGAGAAGGA

GAAGGCGAAG AGCTCTGTTC CCAGGTGAAC GAAGGGGTGC GGGAGAAGGA

GAAGGCGAGG AGCTCTGTTC CCAGGTGAAC GAAGGGGTGC GGGAGAAGGA

GAAGGCGAGG AGCTCTGTTC CCAGGTGAAC GAAGGGGTGC GGGAGAAGGA

GAAGGCGAAG AGCTCTGCTC CCAGGTGAAC GAAGGGGTGC GGGAGAAGGA

GAAGGCGAAG AGCTGTGTTC CCAGGTGAAC GAAGGTGTTC GTGAGAAGGA

GAAAGCGAAG AGCTGTGCTC CCAAGTGAAC GAGGGAGTTC GAGAGAAGGA

AAAGGCGAGG AGCTATGCTC CCAGGTGAAC GAGGGAGTTC GAGAGAAGGA

---------- ---------- ---------- ---------- ----------

GAAGGCGAGG AGCTGTGCTC CCAGGTGAAT GAAGGGGTGC GGGAGAAGGA

GAAGGCGAAG AGCTGTGTTC ACAGGTGAAC GAAGGGGTGC GCGAGAAGGA

GAAGGCGAAG AGCTGTGTTC CCAGGTGAAT GAAGGGGTGC GGGAGAAGGA

GAAGGCGAAG AGCTGTGTTC CCAGGTGAAC GAAGGAGTGC GGGAGAAGGA

GAAAGCGAAG AGTTGTGTTC TCAGGTGAAC GAAGGGGTAC GCGAGAAGGA

AAAGGCGAAG AGCTGTGTTC CCAGGTGAAT GAAGGAGTGC GCGAGAAGGA

GAAGGCGAAG AGCTGTGTTC CCAGGTGAAC GAAGGAGTGC GTGAGAAGGA

GAAGGCGAAG AATTGTGTTC CCAAGTCAAC GAAGGTGTGC GTGAGAAGGA

GAAGGCGAGG AGCTGTGCTC CCAAGTCAAC GAGGGCGTGC GGGAGAAGGA

GAACTCGACC GGCTGGAGTG GATCCAGGCC CACGTGCAGT GTGAAGGCCT

GAACTCGACC GGCTGGAGTG GATCCAGGCC CACGTGCAGT GTGAAGGCCT

GAACTCGACC GGCTGGAGTG GATCCAGGCC CATGTGCAGT GTGAAGGCCT

GAACTCGACC GGCTGGAGTG GATCCAGGCC CATGTGCAGT GTGAAGGCCT

GAACTCGACC GGCTGGAGTG GATCCAGGCC CATGTGCAGT GCGAAGGCCT

GAACTCGACC GGCTGGAGTG GATCCAGGCC CATGTGCAGT GCGAGGGCCT

GAACTCGACC GGCTGGAGTG GATCCAGGCG CATGTGCAGT GCGAAGGCCT

GAACTCGACC GGCTGGAGTG GATCCAAGCC CACGTGCAGT GTGAAGGCCT

---------- ---------- ---------- ---------- ----------

GAACTCGACC GACTGGAGTG GATCCAGGCC CACGTGCTGT GTGAAGGCTT

GAACTCGACC GCCTCGAGTG GATCCAGGCG CACGTGCAGT GCGAAGGCCT

GAACTCGACC GGCTGGAGTG GATCCAAGCC CACGTGCAGT GTGAAGGCCT

GAACTCGACC GGCT-GAGTG GATCCAAGCC CACGTGCAGT GTGAAGGCCT

AAACTCGACC GGCTGGAGTG GATCCAAGCT CACGTGCAGT GTGAAGGCTT

GAACTCGACC GGCTGGAGTG GATCCAAGCC CATGTGCAGT GTGAAGGCCT

GAACTCGACC GACTGGAGTG GATCCAAGCC CACGTGCAGT GCGAAGGCCT

GAATTCGACC GGTTGGAGTG GATCCAAGCC CATGTACAGT GTGAAGGCCT

GAACTCGACC GGCTGGAGTG GATCCAGGCC CACGTACAGT GTGAAGGCCT

GTCTGAGGTA TTGTTTCTTT CTCAGCAACT TGTGTTCAAT TCAGTGACCA

GTCTGAGGTA TTGTTTCTTT CTCAGCAACT TGTGTTCAAT TCAGTGACCA

GTCTGAGGTA TTGTTTCTTT CTCAGCAACT TGTGTTCAAT TCAGTGACCA

GTCTGAGGTA TTGTTTCTTT CTCAGCAACT TGTGTTCAAT TCAGTGACCA

GTCTGAGGTA TTGTTTCTTT CTCAGCAACT TGTGTTCAAT TCTGTGACCA

GTCTGAGGTA TTGTTTCTTT CTCAGCAACT TGTGTTCAAT TCAGTGACCA

TTCTGAGGTA TGGTTGTTTT CT-AGCAACT GGTGTTCAAT TCGGTGACCA

TTCTGAGGTA TGGTTGTTTT CT-AGCAACT GGTGTTCAAT TCAGTGACCA

---------- TGTTTTCTTT CTCAGCAACT TGTGTTCAAT TCAGTGACCA

GTCAGAGGTA TTGTTTCTTC CTCAGCAACT TGTGTTCAAT TCCGTGACCA

GTCCGAGGTA TTGTTTCTTT CTCAGCAACT TGTGTTCAAT TCAGTGACCA

GTCTGAGGTT TTGTTTCTTT CTCAGCAACT TGTGTTCAAT TCAGTGACCA

GTCGGAGGTG TTGTTTCTCT CTCAGCAACT TGTGTTCAAT TCAGTGACCA

GTCTGAGGTA TTGTTTCTTC CTCAGCAACT TGTGTTCAAT TCCGTGACCA

GTCTGAGGTA TTGTTTCTTC CTCAGCAACT TGTGTTCAAT TCAGTGACCA

GTCGGAGGTA TTGTTTCTTC CTCAGCAACT TGTGTTCAAT TCAGTGACCA

GTCTGAGGTG TTGTTTCTTC ATCAGCAACT TGTCTTCAAC TCAGTCACCA

CTCGGAGGTA CTGTTTCATT CTCAGCAACT GGTCTTCAAC TCGGTGACCA

ATTGCTTGGG GCCGCGCAAA TTTCTGCACA GTGGGAAGCT CTACAAGGCC

ATTGCTTGGG GCCACGCAAA TTTCTGCACA GTGGGAAGCT CTACAAGGCC

ACTGCTTGGG GCCGCGCAAA TTTCTGCACA GCGGGAAGCT CTACAAGGCC

ACTGCTTGGG GCCGCGCAAA TTTCTGCACA GTGGGAAGCT CTACAAGGCC

ATTGCTTGGG GCCACGCAAA TTTCTGCACA GTGGGAAGCT CTACAAGGCC

ATTGCCTGGG GCCAAGGAAG TTTCTGCACA GTGGGAAACT CTACAAGGCC

ACTGCTTGGG ACCACGCAAG TTTCTGCACA GCGGGAAGCT CTACAAGGCC

ACTGCTTGGG ACCACGCAAG TTTCTGCACA GCGGGAAGCT CTACAAGGCC

ACTGCTTGGG GCCACGCAAG TTTCTGCACA GCGGGAAACT CTACAAGGCC

ACTGCCTGGG GCCGCGCAAG TTCCTGCACA GTGGGAAGCT CTACAAGGCC

ATTGCTTGGG GCCACGCAAG TTTCTGCACA GTGGGAAACT CTACAAGGCC

ATTGCTTGGG GCCACGCAAG TTTCTGCACA GTGGGAAACT CTACAAGGCC

ACTGCTTGGG GCCGCGCAAG TTCCTGCACA GCGGCAAGCT CTACAAGGCC

ACTGCTTGGG GCCACGCAAG TTTCTGCACA GTGGGAAACT CTACAAAGCC

ATTGCTTGGG GCCACGCAAG TTTCTGCACA GTGGGAAACT CTACAAAGCC

ATTGCTTGGG GCCTCGCAAG TTTCTGCACA GTGGGAAACT CTACAAAGCC

ATTGCTTGGG ACCCCGGAAG TTTCTGCACA GTGGGAAATT GTACAAAGCC

ACTGCCTGGG GCCCCGCAAG TTCCTGCACA GTGGGAAGCT GTACAAGGCC

AAGAGCAACA AGGAGCTGTA TGGCTTCCTT TTCAACGACT TCCTCCTGCT

AAGAGCAACA AGGAGCTGTA TGGCTTCCTT TTCAACGACT TCCTCCTGCT

AAGAGCAACA AGGAACTGTA TGGTTTCCTT TTCAACGACT TCCTCCTGCT

AAGAGCAACA AGGAACTGTA TGGTTTCCTT TTCAACGACT TCCTCCTGCT

AAGAGCAACA AGGAGCTGTA CGGTTTCCTT TTCAACGACT TCCTCCTGCT

AAGAGCAACA AGGAGCTGTA CGGTTTCCTC TTCAACGACT TCCTCCTGCT

AAGAGCAACA AAGAGCTGTA TGGCTTCCTC TTCAATGACT TCCTCCTGCT

AAGAGCAACA AAGAACTGTA TGGCTTCCTC TTCAACGACT TCCTCCTGCT

AAGAGCAACA AGGAGCTCTA CGGTTTCCTG TTCAACGACT TCCTCCTGCT

AAGAGCAACA AGGAGCTGTA TGGCTTCCTC TTCAACGACT TCCTGCTGCT

AAGAGCAACA AGGAGCTGTA TGGCTTCCTC TTCAACGACT TCCTCCTGCT

AAGAGCAACA AGGAGCTGTA CGGCTTCCTC TTCAACGATT TCCTCCTGCT

AAGAGCAACA AGGAGCTGTA CGGCTTCCTC TTCAACGACT TCCTGCTGCT

AAGAGCAACA AGGAGCTGTA TGGCTTCCTC TTCAACGACT TCCTCCTGCT

AAGAGCAACA AGGAACTGTA TGGCTTCCTC TTCAACGACT TCCTCCTGCT

AAGAGCAACA AGGAGCTGTA CGGCTTCCTC TTCAACGACT TCCTCCTGCT

AAGAGCAACA AGGAACTGTA TGGCTTCCTT TTCAATGACT TTCTCCTTCT

AAGAGCAACA AGGAGCTGTA CGGCTTCCTC TTCAACGACT TCCTCCTGCT

GACTCAGATC ACGAAGCCTT TGGGGTCTTC TGGCACCGAC AAAGTCTTCA

GACTCAGATC ACGAAGCCCT TGGGGTCTTC TGGCACCGAC AAAGTCTTCA

GACTCAGATC ACAAAGCCCT TGGGATCTTC TGGCACCGAC AAAGTCTTCA

GACTCAGATC ACGAAGCCCT TGGGATCTTC TGGCACCGAC AAAGTCTTCA

GACTCAGATC ACCAAGCCCT TGGGGTCATC TGGCACCGAC AAAGTCTTCA

GACTCAAATC ACAAAGCCCT TGGGGTCTTC TGGCACCGAC AAAGTCTTCA

GACCCAAATC ACAAAGCCCT TAGGCTCTTC CAGCACTGAC AAAGTCTTCA

GACCCAAATC ACAAAGCCCT TAGGCTCTTC CGGCACCGAC AAAGTCTTCA

GACCCAGATC ACCAAGCCTT TGGGGTCTTC CGGCACCGAC AAAGTCTTCA

GACCCAAATC ATCAAGCCCC TGGGCTCCTC GGGCACCGAC AAAGTCTTCA

GACCCAAATC ATAAAGCCCT TGGGCTCTTC CGGCACCGAC AAAGTCTTCA

GACCCAAATC ATAAAGCCCC TGGGCTCTTC TGGCACTGAC AAAGTCTTCA

CACCCAGATC ATCAAGCCGC TGGGCTCCTC GGGCACCGAC AAGGTCTTCA

GACTCAGATC ATAAAGCCCT TGGGGTCTTC TGGCACCGAC AAAGTCTTCA

GACCCAAATC ATAAAGCCCT TGGGGTCTTC TGGCACCGAC AAGGTCTTCA

CACCCAAATC ATAAAGCCCC TCGGGTCTTC CGGCACCGAC AAGGTCTTCA

GACCCAGATA ATAAAGCCTT TGGGTTCTTC TGGGACTGAC AAGGTCTTCA

GACCCAGATC ATCAAGCCGC TCGGCTCCTC GGGCACCGAC AAAGTCTTCA

GCCCCAAATC AAACCTGCAG TATAAAATGT ATAAAACAGT CTTTCCAGCC

GCCCCAAATC AAACCTGCAG TATAAAATGT ATAAAACAGT CTTTCCAGCC

GCCCCAAATC AAACCTACAG TATAAAATGT ACAAAACTGT CTTTGCAGCC

GCCCCAAATC AAACCTGCAG TATAAAATGT ACAAAACTGT CTTTGCAGCC

GCCCCAAATC AAACCTGCAG TATAAAATGT ACAAAACAGT CTTTCCAGCC

GCCCCAAATC TAACCTGCAG TACAAAATGT ACAAAACAGT CTTTCCAGCC

GCCCCAAATC TAACCTGCAG TATAAAATGT ACAAAACGGT TTTTGCAGCC

GCCCCAAATC TAACCTTCAG TATAAAATGT ACAAAACGGT TTTTGCAGCC

GCCCCAAATC CAACCTGCAG TATAAAATGT ACAAAACCGT TTTTCCAGCC

GCCCCAAATC AAACCTGCAG TATAAAATGT ACAAAACAGT TTTTGCAGCC

GTCCCAAATC TAACCTGCAG TATAAAATGT ACAAAACAGT TTTTTCAGCC

GCCCCAAATC TAACCTGCAG TATAAAATGT ACAAAACAGT TTTTTCAGCC

GCCCCAAGTC CAACCTGCAG TACAAGATGT ACAAGACGGT TTTTCCAGCC

GCCCTAAATC TAACCTGCAG TATAAAATGT ACAAAACAGT TTTTTCAGCC

GTCCCAAATC TAACCTGCAG TATAAAATGT ACAAAACAGT ----------

GCCCCAAATC TAACCTGCAG TATAAAATGT ACAAAACAGT TTTTTCAGCC

GCCCCAAATC GAATCTGCAG TATAAAATGT ATAAGACGGT TTTTTCAGCC

GCCCCAAGTC GAACCTGCAG TACAAGATGT ACAAAACGGT TCTTCCAGCC

TATTTTCCTA AATGAGGTTC TAGTAAAATT ACCCACCGAC CCTTCTGGAG

TATTTTCCTA AATGAGGTTC TAGTAAAATT ACCCACCGAC CCTTCTGGAG

TATTTTCCTA AATGAGGTTC TAGTAAAATT ACCCACTGAC CCTTCTGGAG

TATTTTCCTA AATGAGGTTC TAGTAAAATT ACCCACCGAC CCTTCTGGAG

TATTTTTCTA AATGAGGTTC TAGTAAAATT ACCCACGGAT CCTTCTGGAG

TATTTTCCTA AACGAGGTTC TAGTAAAGTT ACCCACTGAC CCTTCTGGAG

CATTTTCTTA AATGAGGTTC TAGTAAAATT GCCCACGGAC CCTTCTGGAG

CATTTTCTTA AATGAGGTTC TAGTAAAATT GCCCACGGAC CCTTCTGGAG

TATTTTCCTA AACGAGGTTC TAGTGAAATT ACCCACGGAC CCTTCTGGAG

TATTTTCCTA AATGAGGTTC TAGTGAAGTT GCCCACTGAC CCTTCTGGGG

TATTTTCCTG AATGAGGTTC TAGTAAAATT ACCCACTGAC CCTTCTGGAG

TATTTTCCTC AATGAGGTCT TAGTAAAATT ACCCACTGAC CCCTCTGGAG

CATTTTCCTC AATGAGGTTC TGGTGAAGCT CCCCACCGAC CCCTCTGGTG

CATTTTCCTA AATGAGGTTC TCGTAAAATT ACCCACTGAC CCTTCTGGAG

---------- ---------- ---------- ---------- ----------

GATTTTCCTC AATGAGGTTC TAGTAAAATT GCCCACCGAC CCTTCTGGAG

CATTTTCCTC AATGAAGTCC TAGTAAAATT ACCCACTGAC CCTTCTGGAG

CATTTTCCTG AACGAGGTCC TGGTCAAGCT GCCGACCGAC CCCTCCGGAG

ACGAGCCCAT CTTCCACATC TCCCACATTG ACCGCGTCTA TACTCTCCGA

ACGAGCCCAT CTTCCACATC TCCCACATTG ACCGCGTCTA TACTCTCCGA

ACGAGCCCAT CTTCCACATC TCCCACATTG ACCGCGTCTA CACCCTCCGA

ACGAGCCCAT CTTCCACATC TCCCACATTG ACCGCGTCTA CACCCTCCGA

ATGAGCCCAT CTTCCACATT TCCCACATTG ACCGAGTCTA TACCCTCCGG

ATGAACCCAT CTTCCACATT TCCCACATTG ATCGAGTCTA CACTCTCCGA

ATGAACCTAT CTTTCACATT TCGCACATTG ACCGAGTCTA CACGCTCCGA

ATGAGCCTAT CTTCCACATT TCCCACATCG ACCGGGTCTA CACCCTCCGA

ATGAACCCAT CTTCCACATT TCCCACATCG ACCGGGTCTA CACCCTCCGA

ATGAGCCCAT CTTCCACATT TCCCACATTG ACCGAGTCTA CACCCTCCGA

ATGAGCCCAT CTTCCACATC TCCCACATTG ACCGAGTCTA CACCCTCCGA

ATGAACCCGT CTTCCACATT TCCCACATCG ACCGCGTCTA CACCCTCCGA

ACGAGCCCAT CTTCCACATC TCACACATCG ACCGGGTCTA CACCCTGCGG

ATGAACCCAT CTTCCACATT TCCCACATCG ACAGGGTCTA CACCCTCCGT

---------- ---------- ---------- ---------- ----------

ATGAGCCCAT CTTTCACATT TCCCACATCG ACCGAGTCTA CACCCTCCGG

ATGAGCCCAT CTTCCACATT TCCCACATCG ATAGAGTCTA CACACTCCGT

ACGAGCCCAT CTTCCACATC TCCCACATCG ACCGAGCTTC AGCTGCTCGA

GCAGAGTCCG TTCCCAAAGG GCAACAGGCA TTGGAAGGTT GATGGTGAAC

GCAGAGTCCG TTCCCAAAGG GCAACAGGCA TTGGAAGGTT GATGGTGAAC

GCAGAGTCCG TTCCCAAAGG GCAACAGGCA TTGGAAGGTT GATGGTGAAC

GCAGAGTCCG TTCCCAAAGG GCAACAGGCA TTGGAAGGTT GATGGTGAAC

GCAGAGTCCG TTCCCAGAGG GCAACAGGCA TTGGAAGGTT GATGGTGAAC

GCTGAGTGCG TTCCCAAAGG GCCACAGGCA TTGGAAGGTT GATGGTGAAC

GCAGAGTCCG TTCCCAGAGA GCGACCGGTA TTGGAAGATT GATGGTGAAC

GCAGAGTCCG TTCCCAGCGG GCGACCGGTA TTGGAAGGTT GATGGTGAAC

GCAGAGTGCG GTCCCAGAGG GCAACGGGCA TTGGAAGGCT GATGGTGAAC

GCAGAGTCCG CTCCCAAAGA GCCACAGGCA TCGGAAGGTT GATGGTGAAC

GCCGAGTCCG TTCCCAAAGG GCGACAGGTA TTGGAAGGTT GATGGTGAAC

GCCGAGTCCG TTCCCAAAGG GCAACAGGGA TTGGAAGGTT GATGGTGAAT

GCAGAGTTCG CTCCCAGAGG GCCACCGGCA TCGGGAGGCT GATGGTGAAC

GCAGAGTCCG TTCCCAGAGG GCAACAGGTA TCGGCAGGTT GATGGTGAAC

----AGTCCG TTCCCAAAGG GCAACAGGTA TTGGAAGGTT GATGGTGAAC

GCAGAGTTTG CCCCCAAAGG GCAACAGGTA TTGGGAGGTT GATGGTGAAC

GCAGAGTGCG CTCCCAAAGA GCAACAGGTA TCGGGAGGTT GATGGTGAAC

GATGAGTGCG CTCCCAGAGG GCCACAGGCA TTGGCAGGCT GATGGTGAAC

GTGGTTGAAG GCATCGAGTT GAAACCCTGT CGGTCACATG GTAAGAGGAA

GTGGTTGAAG GCATTGAGTT GAAACCCTGT CGGTCACATG GTAAGAGGAA

GTGGTTGAAG GCATTGAGTT GAAACCCTGT CGGTCACATG GTAAGAGGAA

GTGGTTGAAG GCATTGAGTT GAAACCCTGT CGGTCACATG GTAAGAGGAA

GTGGTCGAAG GCATTGAGCT GAAACCCTGT CGGTCACATG GTAAGAGGGA

GTGGTTGAAG GCATTGAGCT GAAGCCCTGC CGGTCACACG GTAAGAGGAA

GTAGTAGAAG GCATTGAGCT GAAGCCCTGT CGGTCACATG GTAAGAGGAA

GTGGTAGAAG GCATTGAGCT GAAGCCCTGT CGGTCACATG GTAAGAGGAA

GTGGTAGAGG GCATTGAGCT GAAGCCCTGC CGGTCACACG GTGAGAGGAA

GTGGTTGAAG GCATTGAGCT GAAACCCTGT CGCTCCCATG GTAAGAGGAA

GTCGTTGAGG GCATTGAGCT GAAACCCTGT CGATCACATG GTAAGAGGAA

GTTGTTGAAG GCATTGAGCT GAAACCCTGT CGGTCACACG GTAAGAGGGA

GTGGTGGAGG GGATCGAGCT GAAGCCGTGT CGCTCTCATG GTAAGAGGGA

GTCGTCGAAG GCATCGAGCT GAAGCCTTGT CGCTCACATG GTGAGAGGAA

ATTGTTGAAG GCATTGAGCT GAAGCCCTGC CGCTCACACG GTAAGAGGAA

GTGGTTGAAG GCATTGAGCT GAAGCGTGG- ---TCACGTG GTAAGAGGAA

ATTGTTGAAG GCATTGAATT AAAGCCCTGT TGGTCACATG GTAAGAGGTA

GTGGTGGAAG GAATTGAGTT AAAGCCCTGC CGCTCACACG GTAAGAGGAA

AGAGCAACCC GTACTGTGAG GTGACCATGG GTTCCCAGTG CCACATCACC

AGAGCAACCC GTACTGTGAG GTGACCATGG GTTCCCAGTG CCACATCACC

AGAGCAACCC CTACTGCGAG GTGACCATGG GTTCCCAGTG CCACATCACC

AGAGCAACCC CTACTGCGAG GTGACCATGG GTTCCCAGTG CCACATCACC

AGAGCAACCC ATACTGCGAG GTGACCATGG GTTCCCAATG CCACATCACT

AGAGCAACCC GTACTGTGAA GTGACCATGG GCTCCCAGTG CCACATCACC

AGAGCAACCC GTACTGTGAG GTGACCATGG GCTCTCAGTG CCACATCACC

AGAGCAACCC GTACTGTGAG GTGACCATGG GCTCTCAGTG CCACATCACC

AGAGCAACCC CTACTGCGAG GTGACCATGG GCTCCCAGTG CCACATCACC

AGAGTAACCC GTACTGTGAG GTGACCATGG GCTCCCAGTG TCACATCACC

AGAGTAACCC GTACTGTGAG GTGACCATGG GTTCCCAGTG TCACATCACC

AGAGTAACCC GTACTGCGAG GTGACCATGG GCTCCCAGTG TCACATCACC

AGAGCAACCC ATACTGCGAG GTGACCATGG GCTCACAATG CCACATCACT

AGAGCAACCC ATACTGTGAG GTGACCATGG GCTCCCAGTG CCACATCACC

AGAGCAACCC TTACTGCGAG GTGACCATGG GCTCCCAGTG TCACATCACC

AGAGTAACCC ATACTGCGAG GTGACCATGG GCTCCCAGTG CCACATCACC

AGAGTAACCC CTATTGTGAA GTGACAATGG GCTCCCAGTG TCATATTACC

AGAGCAACCC TTACTGCGAG GTGACCATGG GCTCCCAGTG CCACATCACC

AAGACGATCC AGGACACTCT GAACCCCAAG TGGAATTCCA ACTGCCAGTT

AAGACGATCC AGGACACTCT GAACCCCAAG TGGAATTCCA ACTGCCAGTT

AAGACGATCC AGGACACTCT GAACCCCAAG TGGAATTCCA ACTGCCAGTT

AAGACGATCC AGGACACTCT GAACCCCAAG TGGAATTCCA ACTGCCAGTT

AAGACAATCC AGGACACTCT AAACCCCAAG TGGAACTCCA ACTGCCAGTT

AAGACAATCC AGGACACGCT GAACCCCAAG TGGAATTCCA ACTGCCAGTT

AAGACAATCC AGGACACGCT GAACCCTAAG TGGAATTCTA ACTGCCAGTT

AAGACAATCC AGGACACGCT AAACCCCAAG TGGAATTCTA ACTGCCAGTT

AAGACGATCC AGGACACCCT GAACCCCAAG TGGAATTCCA ACTGCCAGTT

AAGACGATCC AGGACACTCT GAATCCCAAG TGGAATTCCA ACTGCCAGTT

AAGACGATCC AGGACACTCT GAATCCCAAG TGGAATTCCA ACTGCCAGTT

AAGACGATCC AGGACACTCT GAATCCCAAG TGGAATTCCA ACTGCCAGTT

AAGACCATCC AGGACACCTT GAACCCCAAG TGGAACTCCA ACTGCCAGTT

AAGACCATCC AGGACACGCT GAACCCCAAG TGGAACTCCA ACTGCCAGTT

AAGACCATCC AGGACACGCT CAACCCCAAG TGGAACTCCA ATTGCCAGTT

AAGACCATCC AGGACACGCT CAACCCCAAG TGGAATTCCA ACTGCCAGTT

AAGACGATAC AGGACACGCT GAATCCTAAG TGGAATTCCA ACTGCCAGTT

AAGACCATCC AGGACACGCT GAGCCCCAAG TGGAACTCCA ACTGCCAGTT

CTTCATCCGA GACCTGGAGC AGGAAGTCCT CTGCATCACT GTGTTCGAGA

CTTCATCCGA GACCTGGAGC AGGAAGTCCT CTGCATCACT GTGTTCGAGA

CTTCATCCGA GACCTGGAGC AGGAAGTCCT CTGCATCACT GTGTTCGAGA

CTTCATCCGA GACCTGGAGC AGGAAGTCCT CTGCATCACT GTGTTCGAGA

TTTCATCCGA GACCTGGAGC AGGAAGTCCT CTGCATCACT GTGTTTGAGA

CTTCATCAGA GACCTGGAGC AGGAGGTTCT TTGTGTCACT GTGTTTGAGA

CTTCATCAGA GACCTGGAAC AGGAGGTTCT CTGCATCACT GTGTTTGAGA

CTTCATCAGA GACCTGGAGC AGGAGGTTCT CTGCATCACA GTGTTTGAGA

CTTCATCAGA GACCTGGAGC AGGAAGTCCT GTGCATCACC GTGTTCGAGA

CTTCATCAGA GACCTGGAGC AGGAGGTGCT GTGCATCACT GTGTTTGAGA

CTTCATCAAA GACCTGGAGC AGGAGGTCCT GTGCATCACC GTGTTTGAGA

CTTCATCAAA GACCTGGAGC AGGAGGTCCT CTGCATCACG GTGTTTGAGA

CTTCATCAGA GACCTGGAGC AGGAAGTCCT GTGTATCACC GTATTTGAGA

CTTTATCAAG GACCTGGAGC AGGAAGTCCT CTGTATCACT GTGTTTGAGA

CTTCATCAGA GACCTGGAGC AGGAAGTCCT CTGCATCACA GTGTTTGAGA

CTTCATCCGA GACCTGGAGC AGGAAGTGCT CTGCGTCACG GTCTTTGAGA

CTTTATCAAG GACCTAGAGC AGGATGTTCT CTGCATAACG GTGTTTGAGA

CTTCATCAAA GACCTGGAGC AGGACGTGCT GTGCATCACC GTCTTCGAGC

GGGACCAGTT CTCACCAGAT GGTGAGTTTT CAGATTTTTT GGGTCGGACG

GGGACCAGTT CTCACCAGAT GGTGAGTTTT CAGATTTTTT GGGTCGGACG

GGGACCAGTT CTCACCAGAT GGTGAGTTTT CAGATTTTTT GGGTCGGACG

GGGACCAGTT CTCACCAGAT GGTGAGTTTT CAGATTTTTT GGGTCGGACG

GGGACCAGTT TTCACCAGAT GGTGAGTTTT CAGATTTTTT GGGCCGGACG

GAGACCAGTT CTCACCAGAC GGTGAGTTTT CAGATTTTTT GGGGCGGACG

GGGACCAATT CTCGCCAGAC GGTGAGTTTT CAGATTTTTT GGGTCGGACA

GGGACCAGTT CTCGCCTGAT GGTAAGTTTT CAGATTTTTT GGGTCGGACA

GGGACCAGTT CTCACCCGAT GGTGAGTTTT CAGATTTCTT GGGTCGAACA

GGGACCAGTT CTCGCCAGAT GGTGAGTTTT CAGATTTTTT GGGTCGGACG

GGGACCAGTT CTCTCCAGAC GGTGAGTTTG CAGATTTTTT GGGTCGGACA

GGGACCAGTT CTCTCCAGAT GGTGAGTTTT CAGATTTTTT AGGTCGGACG

GGGACCAGTT CTCTCCTGAT GGTGAGC--- ---------- ----------

GGGACCAGTT CTCCCCGGAT GGTGAGTTTT CAGATTTTTT GGGTCGGACA

GGGACCAGTT CTCCCCCGAT GGTGAGTTTT CAGATTTTTT GGGTCGGACA

GGGACCAGTT CTCCCCCGAC GGTGAGT--- ---------- ----------

GGGACCAGTT CTCCCCTGAC GGTGAGTTTC CAGATTTTTT GGGTCGGACA

GGGACCAGTT CTCCCCGGAC GGTGAGGTTT CAGACTTTCT GGGTCGGACG

GAGATCCGTG TGGCGGACAT CAAGAAAGAC CAGGGCTCCA AAGGTCCTTC

GAGATCCGCG TGGCGGACAT CAAGAAAGAC CAGGGCTCCA AAGGTCCTTC

GAGATCCGTG TGGCGGACAT CAAAAAGGAC CAGGGCTCCA AGGGTCCTTC

GAGATCCGTG TGGCGGACAT CAAAAAGGAC CAGGGCTCCA AGGGTCCTTC

GAGATCCGCG TGGCGGACAT CAAGAAAGAC CAGGGCTCCA AGGGTCCTTC

GAGATCCGTG TGGCCGACAT CAAGAAAGAC CAGGGCTCCA AGGGGCCTTC

GAGATCCGAG TGGCCGACAT CAAGAAAGAC CAGGGCTCCA AGGGGCCTTT

GAGATCCGAG TGGCCGACAT CAAGAAAGAC CAGGGCTCCA AGGGGCCTTC

GAGATCCGTG TGGCTGACAT CAAGAAAGAC CAGGGCTCCA AGGGGCCTTC

GAGATCCGCG TGGCCGACAT CAAGAAGGAC CAGGGCTCCA AGGGTCCTTC

GAGATCCGTG TGGCCGACAT CAAGAAGGAC CAGGGTTCCA AGGGTCCTTC

GAGATCCGCG TAGCAGACAT CAAGAAGGAC CAGGGTTCCA AGGGTCCTTC

---------- ---------- ---------- ---------- ----------

GAGATCCGCG TGGCTGACAT CAAGAAAGAT CAAGGTTCCA AAGGTCCTTC

GAGATCCGTG TGGCCGACAT CAAAAAAGAT CAGGGTTCCA AGGGCCCTTT

---------- ---------- ---------- ---------- ----------

GAAATTCGTG TGGCCGACAT CAAGAAAGAC CAGGGGTCCA AGGGGCCTTC

GAGATCCGTG TGGCCGACAT CAAGAAGGAC CAGGTCTCCA AGGGCCCTTC

AAGCAAAGTG TGTGTTGGAA GGACAACAAA GTTTACATTT CATACTTTTA

AAGCAAAGTG TGTGTTGGAA GGACAACAAA GTTTACATTT CATACTTTTA

AAGCAAAGTG TGTGTTGGAA GGACAACAAA GTTTACATTT CATACTTTTA

AAGCAAAGTG TGTGTTGGAA GGACAACAAA GTTTACATTT CATACTTTTA

AAGCAAAGTG TGTGTTGGAA GGACAACAAA GTTTACATTT CATACTTTAA

AAGCAAAGCG TGTGTTGGAA GGACAACAAA GTTTACATTT CATACTTTTA

GAGTGATGTG TACGGGGCAA GGACAAC--A GTTTACATTT CATACTTCCG

AAGTGACGTG TATGGGGCAA GGACAAC--A GTTTACATTT CATACTTCCA

AATCAAAGTG TGTGTTGGAA GGACAACAAA GTTTACATTT CATACTTTTA

AAGCAATGTA TGTGTCGGAA GGGCAACAAA GTTTACATTT CATACTTTTA

AAGCAATGTG TGTGTTAGAA GGGCAACAAA GTTTACATTT CATACTTTTA

AAGCAATGTG TGTGTTGGAA GGGCAACAAA GTTTACATTT CATACTTTAC

---------- ---------- ---------- ---------- ----------

AAGCAACGCT TATGTTGGAA GGACAACAAA GTTTACATTT CATACTTTTA

AAGCAGAGCC TATGTTGGAA GGACAACAAA GTTTACATTT CATACTTTTA

---------- ---------- ---------- ---------- ----------

AAGCAAATTG TATGTTGGAA GAACAACAAA GTTTACATTT CATACTTCGA

AAGCGGAACG TACGGTGGAG AGGAGCGCGA GGTTGCATTT CATGCTGTGA

AGAAACTTTA TTATTTATTT ATTGAAGATA GTGTAGAATT TTGTATCAAG

AGAAACTTTA TTATTTATTT ATTGAAGATA GTGTAGAATT TTGTATCAAG

AGAAACTTTA TTATTTATTT ATTGAAGATA GTGTAGAATT TTGTATCAAG

AGAAACTTTA TTATTTATTT ATTGAAGATA GTGTAGAATT TTGTATCAAG

AGAAACCTTA TTATTTATTT ATTGAAGATA GCGTAGAATT TTGTATCAAG

AGAAACTTTA TTATTTATTT ATTGAAGATA GTGTAGAATT TTATATCAAG

AGA--CTTCG -TATTTATTT ATGGAAAATA GTGTTGACTT TTGTATCAAG

AGAAGCTTAG -AGTTTATTT ATTGAAGATA GTGTTGACTT TTGTATCAAG

AGAAACTTTA TTATTTATTT ATTGAAAATA GTGTAGAATT TTGTATTGAG

AGATACTTTA TTATTTATTT ATTAAAGATA GTGTAGACTT TTGTATCAAG

AGAAACTTTA TTATTTATTT ATTGAAGATA GTGTAGACTT TTGTATCGAG

AAAAACTTTA TTATTTATTT ATTGAAGATA GTATAGAATT TTGTATCAAG

---------- --ATTTATTT ATGGGAAATA GCATAGACTT TTGTATCCAG

AGAAACTTTA TTATTTATTT ATTGAATATA GTGTAGAATT TTGTATCGAG

AGAAACTTTA TTATTTATTT ATTGACGATA GTGTAGAATT TTGTATCAGG

---------- ---------- ---------- ---------- ----------

AGAAAtttta ttatttattt attaaaGATA GTGTAAAATT TTGTATCAGA

AGGAACGTGA TGATGTATTT ATGGGAGATA GGGTAGCATG TGGTGTCAAA

AACAACAGAC ATAAGTATTT TTTGAAACTT AAGTTTATAT TTTTTTAAAT

AACAACAGAC ATAAGTATTT TTTGAAACTT AAGTTTATAT TTTTTTAAAT

AACAACAGAC ATAAGTATTT TTTGAAACTT AAGTTTATAT TTTTTTAAAT

AACAACAGAC ATAAGTATTT TTTGAAACTT AAGTTTATAT TGTTTTAAAT

AACAACAGAC ATACGTATTT TTTGAAACTT --GTTTATAT TTTT-----T

AATAACAGAC ATAAGTATTT TTGGAAACTT AAGTTTATAT TTTCTTAAAT

AACAACAGAC ACGCCTATTT TTTTAAACAC ACGTTTATGT TTTTTTAAAT

AACAACAGAC ACACCTATTT TTTGAAACAC ACATTCATGT TTTTTTAAAT

GACAACAGAC ATAAGTATTT TTTGAAACTT AAGTTTATAT TTTTTCAAAT

AACAACAGAC ATAAGTATTT TTTGAAACTT AAGTGTATAG TTTTTTAAGT

AACAACAGAC --AAGTATTT TTTAAAACTT ACGTTTATAT TTTGTGAGAT

AACAACAGAC ATCCGTATTT TTTGAAACTT ACGTTTATAT TTTTTTAAAT

AGCCCC---C ACATGTATTT TTCGGGACtt ttgtttttgt ttTTGTAA--

AACAACAGAC ATACGTATTT TTTGAAACTT AAGTTTATAT TTTTTTAAAT

AGCAACAGGC GTAAGTATTT TTTGAAATTT AAGTTTATAT TTTTTTAAAT

---------- ---------- ---------- ---------- ----------

AATAAGAGAC ATAAGTATTT TTTGAACCTT CAGTTTATAT TTTTTGGAAC

GGTAACAGGC GTAAGTATTT TTTGAACCTT AAGTTTCCGT CTTTTCGGAT

TAAATCTATA AATGCTGTTA AATGTTGCAT AAACTAACAC AGATTCTGTA

TAAATCTATA AATGCTGTTA AATGTTGCAT AAACTAACAC AGATTCTGTA

TAAATCTATA AATGCTGTTA AATGTTGCAT AAACTAACAC AGATTCTGTA

TAAATCTATA AATGCTGTTA AATGTCGCAT AAACTAACAC AGATTCTGTA

TAAATCTATA AATGCTGTTA AATGTTGCAT AAACTAACAC AGATTCTATA

TAACTCTATA AATGCTGTTA AATGTTGCAT AAACTAACAC AGATTCTGTA

GAAACCTATA AATGCTGTTC AATGTTGCAT AAACA---AC AGATTCTCTA

GATACCTATA AATGCTGTTC AATGTTGCAT AAACT---TA ACAGTCTCTA

TAAATCTATA AATGTTGTTA AATGTTGCAA ACACTAACAC AGATTCTGTA

GAAACCTATG AATGCTGTTA AGTGTTGCAT AAACTAATGC AGATTCTGTA

TAAATCTATA AATGCTGGTA AATGTTGCAT AAACTAACAC AGATTCTGTA

TAAATCTATA AAT-CTGTTA AATGTTGCAA AAACTAA--C AGATTCTGTA

---------- ---------- ---GTTGCAT AAACTAACCC AGACTTTGTA

TAAATCTATA AGTGCTGTTC AACGTTGCAT AAACTAATAC AGATTCTGTG

TAAATCTATA CATGGTGTTA AATGTTGCAG AAGCTAACAT GGATTCTGTA

---------- ---------- ---------- ---------- ----------

CAAATCTATA AATACTGTTA AATAATGCAC AAACTAATAA ATATACTGTA

TAAACCTACA GACGTTGTCA AATATCGCAT AAACTAACAC AGATACCGTA

GTCAAAATAT TTAACTTGTT ATTGTAAAAT GTGATTTGTC ATTGAGAGAT

GTCAAAATAT TTAACTTGTT ATTGTAAAAT GTGATTTGTC ATTGAGAGAT

GTCAAAATAT TTAACTTGTT ATTGTAAAAT GTGATTTGTC ATTGAGAGAT

GTCAAAATAT TTCACTTGTT ATTGTAAAAT GTGATTTGTC ATTGAGAGAT

GTCAAAATAT TTAACTTGTT ATTGTAAAAT GTGATTTGTC ATTGAGAGAT

GTGAAAATAT TTAACTTGTT ATTGTAAAAT GTGATTTGTC ATCGAGAGAT

GTCTGAATAC TTAACTTGTT ATTGTAAAAT GTGATTTCTC ATTGAGAGAT

GTCTGAATAT CTAACTTGTT ATTGTAAAAT GTGATTTCTC ATCGAGAGAT

GTCCAAATAT TTAACTTGTT ATTGTAAAAT GTGACTTGTC ATTGAGAGAT

GTTTAAATAA TTGACTTGTT ATTGTAAAAT GTGATTTGTC ATTGAGAGAT

GTC-AAATAT TTAACTTGTT ATTGTAAAAT GTGATTTGTC ATGGAGAGAT

GTCAAAATAT TTAACTTGTT ATTGTAAAAT GTGATTTGTC ATTGAGAGAT

GTCAAACTAT TTAACTTGCT ATTGTAAAGT GTGATTTGTC ATTGAGAGAT

GTCAAAATAT TTAACTTGTT ATTGTACAAT GTGACTTGTC ACTGAGAGAT

GTCAAAATAT TTAACTTGTT ACTGTAAAGC GTGATTTGTC ATTGAGAGAC

---------- ---------- ---------- ---------- ----------

GGCATCATAT TTTACTTGTT ACTTTCAAAT GTGAATTGTC CTGGAGAGAT

GGCATACTAT TTTACTTGTT ATTTTAAACT GTGAATCGTC CTTGAGAGAT

TATTTTTCTG TATGTATATG AGAGTATTTT AGGAATCTTA AGTGCATAAA

TATTTTTCTG TATGTATATG AGAGTATTTT AGGAATCTTA AGTGCATAAA

TATTTTTCTG TATGTATATG AGAGTATTTT AGGAATCTTA AGTGCATAAA

TATTTTTCTG TATGTATATG AGAGTATTTT AGGAATCTTA AGTGCATAAA

TATTTTTCTG TATGTATATG AGAGTATTTT AGGAATCTTA AGTGGATAAA

TATTTTTCTG TATGTATATG AGAGTATTTT AGGAATCTTA AGTGCATAAA

TATTTTTCTG TATGTATATG AGAGTATTTT AGGAATCTTA AGTGCATAA-

TATTTTTCTG TATGTATATG AGAGTATTTT AGGAATCTTA AGTGCATAA-

TATTTTTCTG TATGTATATG AGAGTATTTT AGGAATCTTA AGTGCATACT

TATTTTTCTG TATGTATATG AGAGTATTTT AGGAATCTTA AGTGCATAAA

TATTTTTCTG TATGTATATG AGAGTATTTT AGGAATCTTA AGTGCATAAA

TATTTTTCTG TATGTATATG AGAGTATTTT AGGTATCTTA AGTGCATAAA

TATTTTTCTG TATGTATATG AGAGTATTTT AGGAATC-TA AGTGCATAAA

TATTTTTCTG TAAGCATAGG AGAGTATTTT AGGAATATTA AGTGCATAAA

TATTTTTCTG TATGCAGACA GGAGTATTTT AGGAATCTTA AGTGCATAAA

---------- ---------- ---------- ---------- ----------

TATTTTTCTG TATGTATATC AAAATATTTT TAGGATCTTA AGTGCATTTA

TATTTTTCTG TATATATATG AGACTATTTT TAGGATTTTA AGTGCATAAA

ACTATAGAAA AAATACTTCT GTTGCCAAAT AATGGTACTG TTTAATTTTC

ACTATAGAAA AAATACTTCT GTTGCCAAAT AATGGTACTG TTTAATTTTC

ACTATAGAAA AAATACTTCT GTTGCCAAAT AATGGTACTG TTTAATTTTC

ACTATAGAAA AAATACTTCT GTTGCCAAAT AATGGTACTG TTTAATTTTC

ACTATAGAAA AAATACTTCT GTTGCCAAAT AATGGTACTG TTTAATTTTC

ACTATAGAAA AAATACTTCT GTTGCCAAAT AATGGTACTG TTTAATTTTC

ACTATAGAAG AAACCTATTT GTTGCCAAGT AACAGTAC-- ----------

ACTATAGAAG AAACCTGCCT GTTGCTAGAT AACAGTAC-- ----------

ACTATAGAAA AAATACTTCT GTTGCCAAAC GATGGAATGG TTCAGTTTTC

ACTATAGAAA AAGTACTTCT GTTGCCAAAT AACGGTATTG TTTAATTTCC

ACTATAGAAA AAATACTTCT GTTGCTGAAT AATGGTACTG TTTAATTTTC

ACTATAGAAA ATATACTTCT GTTGCCAAAT AATGGTACTG TTTAATTTTC

CTTATAGAAA AAATACTTCT GTTGCCAAAT AAAAGTACTG TTCAATTTTC

AGCATAGAGA AGATACTTCT GTTGCCAAAC AATGGTACTG TTTAATTTTC

ACTATAGAGA AAGTACTTCT GTTGCCAAAT AATGGTACTG TTTAATTCTC

---------- ---------- ---------- ---------- ----------

ACTGTAGGAC AAATAATGCT GTAAACAGCC AGAGGAGTCT TCCCTTTTTC

ACTTTAGCAA AAAGTTTTCT GTTGCCAAAT AATGGTATCG TATTGTTTCC

TGCCCAGCTT CTGAATCTTG GTCTTGACAG ACATGTCAAC ATATTTCTCG

TGCCCAGCTT CTGAATCTTG GTCTTGACAG ACATGTCAAC ATATTTCTCG

TGCCCAGCTT CTGAATCTTG GTCTTGACAG ACATGTCAAC ATACTTCTCT

TGCCCAGCTT CTGAATCTTG GTCCTGACAG ACATGTCAAC ATACTTCTCT

TGCTCAGCTT CTGAATCTTG GTCTTGGCAG ACATGTCCAC GTATTTCTCT

TGGTCAGCTT CTGAATCTTT GTTTTTGCAG ACATGTCCAC ATATTTCTCT

TGCTGAGCTT CTGAATCTTG CTCTTTGCAG ACATATCAAC GTATTTCTCT

TGCTGAGCTT CTGAATCTTG CTCTTTGCAG ACATATCAAC GTACTTTTCC

TGCTCAGCTT CTGAATCTTG GTTTTTGCAG ACATATCGAC GTACTTCTCT

TGCTCAGCTT CTGAATCTTG GTTTTTGCAG ACATATCAAC GTATTTCTCT

TGCTCAGCTT CTGAATCTTG GTCCTTGCAG ACATATCAGC ATATTTCTCT

TACTCAGCTT CTGAATCTTG GTTTTTGCAG ACATATCAAC ATATTTCTCT

TGCTCAGCTT CTGGATCTTG GTCCTGGCAG ACATGTCCAC GTACTTCTCT

TGCTCAGCTT CTGAATCTTG GTTTTTGCAG ACATATCAAC ATACTTCTCT

TCTTCAGCTT CTCAATCTTG GATTTTGCAG ACATATCAAC ATATCTCTCG

TGCTCAGCTT CTGAATCTTG GACTTGGCCG ACATATCCAC GTACTTCTCG

TGCTTAGCTT CTGGATCTTG GTTCTTGCAG ACATATCCAC ATATTTCTCT

TGCTCAGCTT CTGAATCTTG GTTCTTGCAG ACATATCGAC AAATTTCTCC

CCAATGCGCA CAATCATTCC ACCCAAGATT GACGGATCAG TCTACAAAAG

CCAATGCGCA CAATCATTCC ACCCAAGATT GACGGATCAG TCTACAAAAG

CCAATGCGCA CAATCATTCC ACCCATGATT GACGGATCAG TCTACAAAAG

CCAATGCGCA CAATCATTCC ACCCATGATT GACGGATCAG TCTACAAAAG

CCAATACGCA CAATCATTCC ACCCATGATT GATGGATCAG TCTACAAAAG

CCAATTCGAA CAATCATTCC ACCCATGATC GCTGGATCAG TCTAGGAAAG

CCAATTCGGA CAATCATCCC ACCCATGATT GATGGGTCAG TCTAGGAAAG

CCAATTCGGA CAATCATCCC GCCCATGATT GACGGGTCAG TCTAGGAAAG

CCAATGCGGA CAATCATCCC ACCCATGATC GACGGGTCCG TCTAGGG---

CCAATACGGA CAATCATTCC ACCCATGATC GACGGATCAA TCTAGGAAAG

CCAATACGGA CAATCATTCC CCCCATGATT GACGGATCAA CCTAGGAAAG

CCAATACGGA TAATCATTCC ACCCATGATT GATGGATCAG TCTAGGAAAG

CCGATGCGGA CGATCATCCC CCCCATGATG GACGGGTCGG TCTGGGGAAG

CCAATACGGA CAATCATCCC ACCCATGATT GACGGATCAG TCTAGGAAAG

CCAATACGGA CAATCATTCC GCCCATGATT GATGGATCCG TCTAGGAAAG

CCAATGCGGA CAATCATTCC ACCCATGATT GATGGGTCAG TCTAGGAAGT

CCTATACGAA CAATCATTCC TCCCATGATT GAAGGATCAG TCTGAAAAGA

CCAATGCGGA CAATCATCCC TCCCATGATT GACGGGTCCG TCTGAGAAGA

AAGAGGAAGC TCTTGAGGAC AGTTTTTAAT TCAGAGAGTG TGGCTTCTTC

AAAAGGAAGC TCTTGAGGAC AGTTTTTAAT TCAGAGAGTG TGGCTTCTTC

AAAAGGAAGC TCTTGAGGAC AGTTTTTAAT TCAGAGAGTG TGGCTTCTTC

AAAAGGAAGC TCTTGAGGAC AGTTTTTAAT TCAGAGAGTG TGGCTTCTTC

AAAAGGAAGC TCTTCAGGAC TGTTTTTAAT TCAGAGAGTG TGGCTTCTTC

AAAAGGAAGC TTTTCAGGAC TGTTTTTAAT TCAGTGAGAG TAGCTTCTTC

AAAAGGAAGC TATTCAGCAC TGTCTTTAAC TCAGAGAGAA CAGCTTCATC

AAAAGGAAGC TCTTCAGCAC CGTCTTTAAC TCAGAGAGAA CAGCGTCATC

---------- ---------- ---------- ---------- ----------

AAAAGGAAGC TCTTCAGGAC CGTTTTCAAT TCAGTCAGAG TGGCTTCATC

AAAAGGAAGC TCTTCAGGAC CGTTTTTAGT TCAGTAAGAG TGGCTTCATC

AAAAGGAAGC TCTGCAGCAC CGTTTTTAAT TCAGAAAGAG TGGCCTCATC

AACAGGAAGC TCTGCAGGAC TGTTTT---- ---------- ----------

AAAAAGAAAC TCTTCAGGAC TGTTTTTAAT TCAGAAAGAG TAGCTTCATC

AAAAGGAAGC TGTTCAGGAC GGTTTTTAAT TCAGAAAGAG AGGTGTTGTC

AAAAGGAAGC TGTTCAGGAC CGTTTTCAAT TCAGAGAGAG AGGTGTTGTC

GAAAGGAAGC TGTTCAGGAC TGCTTCTAAT TCTGAAAGAG TTGCGCTATC

AAAAGGAAGC TGTTGAGGAC AGTCTTGAGC TCTGTCAGCG TGGCACTGTC

TAAAGGTCAG CATTAAAGAC AGATATATTT ATTTGACAGC ACGTTTCACA

TAAAGGTCAG CATTAAAGAC AGATATATTT ATTTGACAGC ACGTTTCACA

TAAAGGTCAG CATTAAAGAC AGATATCTTT ATTTGACAGC ACGTTTCACA

TAAAGGTCAG CATTAAAGAC AGATATATTT ATTTGACAGC ACGTTTCACA

TAAAGGTCAG CATTAAAGAC AGATATATTT ATTTGACAGC ACATTTCACA

CAGAGGTCAG CATTAAAGAC AAGTATATTT ATTTGACAGC ACATTTCACA

TAAAGGTCAG CGTTAAAGAC A----TATTT ATTTGACAGC ACATTTCACA

TAGAGGTCAG CGTTAAAGAC A----TATTT ATTTGACAGC ACATCTCACA

-----GTCAG CATTAAATAC AGGTGTATTT ATTTGACAGC ACATTGCACA

TAAAGGTCAG TATTAAAGAC AGGTATATTT ATTTGACAGC AAATTTCACA

CAAAGGTCAA CACTAAAGAC AGGTATATTT ATTTGACAGC ACATTTCACA

TAAAGGTCAA TATTAAAGAC AGGTATATTT ATTTGACAGC ACATTTCACA

-------CAG TATTAAAGAC AGGTATATTT ATTTGACGGC ACATTTCACA

TAAAGGTCAA CATTAAAGAC TGGTATATTT ATTTGATAGC ACATTTCACA

TAAGGGTCAA CATTAAAGAC AAGTGTATTT ATTTGACAGC ACATTTCACA

TAAGG----- ---------- ---------- ---------- ----------

TAAAGCTCAA CATTAAAGAC AGGTATTTTT AATTGATGCC ACATTTCACA

TAGGGGTCAA CATCAAAGAC AGGTATATTT ATTCAATGCC ACATCGCACA

GGATATGTAC AGAATGTACC ACTGACTTTA ATACTTCTAT AAAGTTTAGT

GGATATGTAC AGAATGTACC ACTGACTTTA ATACTTCTAT AAAGTTTAGT

GGATATGTAC AGAATGTACC ACTGACTTTA ATACTTCTAT AAAGTTTAAT

GGATATGTAC AGAATGTACC ACTGACTTTA ATACTTCTAT AAAGTTTAAT

GGATATGTAC AGAATGTACC ATTGACTTTA ATACTTCTAT AAAGTTTAGT

GGATATGTAC ---ATATATC ACCGACTTTA ATACTTCTAT AAAGTATAGT

GGATATGTAC AGAATATACC AGCGACTTTA ATACTTCTAT AAAGTATAGT

AGATATGTAC AGAATATACC AGCAACTTTA ATACTTCTAT AAAGTATAGT

GGATATGTAC AGAATATACC ATTGACTTTA ATACTTCTAT AAAGTATAGT

GGATATGTTC AG--TATGCC ACTGACTTTA ATACTCCTAT AAAGTACAGT

GGATATGTAC AGAATATACC ACTGACTTTA ATACTTCTAT AAAGTATAGT

GGATATGTAC AGACTATACC ACTGACTTTA ATACTTCTAT AAAGTATAGT

GGATATGTAC AGAAGATACC ACTGACTTTC ATACTTCTAT AAAGGGTAGT

GAATATGTAC ATAATATAGC ACTGACTTTA ATACTTCTAT AAAGTGTAGT

GGATATGTAC AGAATATACC ACTGACTTTA ATACTTCTAT AAAGTATAGT

---------- ---------- ---------- ---------- ----------

GGATATGCAC AGAACAGACC ATTGCATTTA ATACTTTTGT AAAGTGTAGT

GGATATGTAC AGAATTTACC ACGGGCTATA ACACTTCGGA AAAATATAGT

TATAAATAGT ATGCCACAAG CAATAAAATT CTTACATATA AACAGCAATC

TATAAATAGT ATGCCACAAG CAATAAAATT CTTACATATA AACAGCAATC

TATAAATAGT ATGCCACAAG CAATAAAATT CTTACATATA AACAGCAATC

TATAAATAGT ATGCCACAAG CAATAAAAT- CTTACATATA AACAGCAATC

TATAAATAGT ATGCCACAAG CAATAAAATT CTTACATATA AACAGCAATC

TATAAATAGT ATGCCACAAG CAATAAAATT CTTACATATA AACAGCAATC

TATAAATAGT ATGCCACAAG CAATAAACCT CTTACATATA AACAGCAATC

TATAAATAGT ATGCCACAAG CGATAAACCT CTTACATATA AACAGCAATC

TATAAATAGT ATGCCACAAG CAATAAAATT CTTACATATA AACAGCAATC

TATAAATAGT GTGCCACAAG CAATAAAGTT CTTACCTATA AACAGCAATC

TATAAATAGT ATGCCACAAG CAATAAAATT CTTACATATA AACAGCAATC

TATAAATAGT ATGCCACAAG CAATAAAATT CTTACATATA AACAGCAATC

TATAAATAGG ATGCCACAAG CAATAAAATA CTTACATATA AACAGAAATC

TATAAATAGT ATGCCACAAG CAATAAAATT CTTACATATA AACACCAATC

TATAAATAGT ATGCCACAAG CAATAAAACT TTTACATATA AACAGCAAAC

---------- ---------- ---------- ---------- ----------

TATCCATAGT GTGCCACAAG CAATAACATC ATTACAAAGA AATAGCAACT

TATAAATAGT AGGCCACAAG CCACAAAACT GCTACAGATG AACAGCAATT

TAATATAGAA CACAGAGTTC ACAGAGATCC TTAGTGTCTA ACTTCTGCTC

TAATATAGAA CACAGAGTTC ACAGAGATCC TTAGTGTCTA ACTTCTGCTC

TAATATAGAA CACAGAGTTC ACAGAGAT-C TTAGTGTCTA ACTTCTGCTC

TAATATAGAA CACAGAGTTC ACAGAGAT-C TTAGTGTCTA ACTTCTGCTC

TACTATAGA- -ACAGAGTTC ACAGAGATCC TTAGTGTCTA ACTTCTGCTC

TACTATAGAA CAAAGAATTC ACAGAGATCC TTAGTGTCTA ATTTCTGTTC

TACTAGAGAA CAGAG-ACTC ACAAAGATTC TTAGTGTCTG TTTTCTGCTT

TACTAGAGAA CAGAG-ACTC ACAGAGATTC TTAGTGTCTG TTTTCTGCTT

TACTACAGAA CAAAGAATTC ACAGAGATCC TCAGTGTCTA ATTTCTGCTC

TACTATAGAA CAAAGAACTC ACAGAGATCC TTAGTGTCTA GTTTCTGCTC

TACTATAGAA CAAAGAATTC ACAGAGATCC TTAGTGTCTA ATTTCTGCTC

TACTATAGAA CAAAGAATTC ACAGAGATCC TCAGTGTCTA ATTTCTGCTC

TACTATAGAA CAAGTAATTC ACAGAGATTC TTAGTGTCTA ATTCCTGCTC

TACTGTAGAA CAAAGAATTC ACAGAGATCC TTAGTATCTA ATTTCTGCTC

TACTATAGAA CAAAGAATTC ACAGAGAGCC TTAGTGTCTA ATTTCTGCTC

---------- ---------- ---------- ---------- ----------

TACCACAAAA CAAAGAATTA GTAAAGTTTC TTGATTTTGT ACTTCATTTT

CACTACAGAA TGCGGAACTC GGAAACGCGT TTAGCGTTTC ATCTCGGTGC

TGCTTTAAAG AACTAGTAAA TATTTAATAA TACATAGATA ATGGCTAGTT

TGCTTTAAAG AACTAGTAAA TATTTAATAA TACACAGATA ATGGCTAGTT

TGCTTTAAAG AACTAGTAAA TATTTAATAA TACACAGATA ATGGCTAGTT

TGCTTTAAAG AACTAGTAAA TATTTAATAA TACACAGATA ATGGCTAGTT

TGCTTTAAAG AACTAGTAAA TATTTAATAA TACTCAGATA ATGGCAAGTT

TGCTCTAAAG AACTGCTAAA TATTTAATAA TACACAG--A CTGGCTACTT

TG----ACAG AGCTGGTA-- --------AA TACAC--ACA ATGTTGAGTT

TG----ACAG ACCTGGCA-- --------AA TACAC--ACG ATGTCGAGTT

TGCTTTAAAG AACTGGTAAA TATTTAATAA TACACAGATA ATGACTAGTT

TGCTTTAAAG AACTGGTCCA TATTTAATAA TATGTAGATA ATGGCTAGTT

TGCTTTAAAG AGCTGGTAAA TATTTAATAA CAC--AGATA ATGGCTAGTT

TGCTTTAAAG AACTGGTAAA TATTCAATAA TACACAGATA ACGGCTAGTT

TGCTTTAAAG AGCTGGTAGA TACTTAATAA TACACAGATA ATGGCTAGTT

TGCTTTAAAG AACTGGtaaa tatttaataa tatataGATA ATGGCTAACT

TGCTTTAAAG AACTGGTAAA GATTCAATAA TACACAGAAA GTGGCTAGTT

---------- ---------- ---------- ---------- ----------

TGTTTTAAAG AAATATTAGT TATTTACTAA CACATATACA CTGGTTAGCT

TGTTTCAAAG GACGGCTAGT TATTCGGCAA GACACGGATA GTGGTTAGCT

ATTTCAGCAT ACCTTTAATG AGCAAATACC TATACACTAG TCACATGGAT

ATTTCAGCAT ACCTTTAATG AGCAAATACC AATACACTAG TCACATGGAT

ATTTCAGCAT ACCTTTAATG AGCAAATACC TATACACTAG TCACATGGAT

ATTTCAGCAT ACCTTTAATG AGCAAATACC TATACACGAG TCACATGGAT

ATTTCAGCAT ACCTTTAATG AGCAAATACC TATGCACTAG TCACGTGGAT

ATTTCAGCAT ACCTTTAATG AGCAAATACT TATACACTAG TCACATGGAT

CTTTTAGCAA AC-TTTAATG AGTGGATACC TACACACTAC TCACACAGAT

CTTTTAGCAA AC-TTTAATG AGTGGATACC TACACACTAC TCACACAGAT

ATTTCAGCAT ACTTTTAATG AGCAAATACC TATACACTAA TCACACAGAT

ATTTCAGTGT ACCTTTAACG AGCAAATACC TATACACTAA TCACACAGAT

ATTTCAGCAT ACCTTTAATG AGCAAATACC TATACACTAA TCACATAGAT

ATTTCAGCAT ACCTTTAATA AGCAAATACC AATACACTAA TCACACAGAT

ATTGCAGCAG ACCTTTAATG AGCAAATACC TGTACACTAA TCACAGAGAT

ATCTCAGCAT ACCTTTAATG AGCAAATACC TATATACTAA TCACATAGAT

GTTTGAGTGT ACTTTTAATG AGCAAGGACC TATACACTAA TCACGTAGAT

---------- ---------- ---------- ---------- ----------

ACTTCAGAAT TCCTCTAGTA AGCATGTATC CAAACACTGA TCACACCGAT

TTGTCAGCAC ACCTTGATTG AGCATACGCC AAGATGCTAA GTACACAGAT

GTTAAAGCCA CAGTTTCAAC AAGTTAATTC ACATTTGAAG CAACTGCTTA

GTTAAAGCCA CAGTTTCAAC AAGTTAATTC ACATTTGAAG CAACTGCTTA

GTTAAAGCCA CAATTTCAAC AAGTTAATTC ACATTTGAAG CAACTGCTTA

GTTAAAGCCA CAATTTCAAC AAGTTAATTC ACATTTGAAG CAACTGCTTA

GTTACAGCCA CAGTTTCAAC -----AATTC ACATTTGAAG CAACTGCTTA

GTCAAAGCCA CAATTTCAAC AAGTTAATTC ACATTTGAAG CAACTGCTTA

GTTACAGCCA CAATCTCCAC AAATTAATTC ACATTTGAAG TAACTGCTTA

GTTACAGCCA CAATCTCAAC AAGTTAATTC ACATTTGAAG CAACTGCTTA

GTTAAAGCCA CAGTTTCAAC AAGTTAATTC ACATTTGAAG CAACTGCTTG

GTTAAAGCCA CAGTTTCAAC AAGTTAATTC ACATTTGAAG CAATTGCTTA

GTTAAAGCCA CAGTTTCAAC AAGTTAATTC ACATTTGAAG CAACTGCTTA

GTTAAAGCCA CAGTTTCAAC AAGTTAATTC ACATTTGAAG CAACTGCTTA

GGTAAAGCCA CGGGTTCAAC AAGTTAGTTC ACATTCGAAG CAACTGCT--

GTTAAAGCCA CAGTTTCAAC AAGTTAATTC ACATTTGAAG TAACTGCTTA

GTTAAAGCCA CAGTTTCAAC AAGTTAATTC ACATTTGAAG CAGATGCTCT

---------- ---------- ---------- ---------- ----------

GTTGGAGCCA CAATCTGAAC AAGTTAATTC ACATATGAAG CAACTGCTGA

GTTAACGACA CAGCTTGGAC AGACTTATCC ACATACGAAA CAGCCGCTTA

GTAATACTGC TGTTTACGCT ACTGCTAATG GATTCAAAAT ACTACATTCT

GTAATACTGC TGTTTACGCT ACTGCTAATG GATTCAAAAT ACCACATTCT

GTAATACTGC TGTTTACACT ACTGCTAATG GATTCAAAAT ACTACATTCT

GTAATACTGC TGTTTACACT ACTGCTAATG GATTCAAAAT ACTACATTCT

GTAATACTGC TTTTTACA-- ACTACTAATG GATTCAAAAT ACTACATTCT

GTAACACTGC TGTTTATACT ACTGCTAATG GATTCAAAAT ACTACATTCT

GTAATACTGC AGTTTCTACT ACTGCTAATG GATTCAAAAT ACTATGtttt

GTAATACTGC AGTTTCTACT ACTGTTAATG GATTCAAAAT ACTAtgttTT

GTAATACT-- -GTTTATACT ACTGCTAATG GATTCAAAAT ACTACATTCT

GTAATACTGC TGTTTATACT ACTGCTAATG GATTCAAAAT ACTACATTCT

GTAATACTGC TGTTTATACT ACTGCTAATG GATTCAAAAT ACTACATTGT

GTAATACTGC TGTTTATACC AATGCTAATG GATTCAAAAT ACTACATTCT

-TAATACTGT TGTTTATACT ACCGCTAATG GCTTCAAAAT ATAACATTCT

GTAATACTGC TCTTTATACT ACTGCTAACG GATTCAAAAT ACTACATTAG

GTAATACTGC TGTTTATACT ACTGCTAATG GCTTCAA--T ACCATGCTCT

---------- ---------- ---------- GATTCAAAAT ACTAAGTTCT

GTAATACTGC TGTTTGTACA AATACTAAAG GATTCAAAAT ACCCCATTTT

GTGATGCTGC TGTTTGTACG ACCGTTAATG GACCCGCAAA AGCCCATTCT

GTAAAAATTC TGTGTACAAT AAAATATACA CATAGTAACT CTGTACACAT

GTAAAAATTC TGTATACAAT AAAATATACA CATAGTAACT CTGTACACAT

GTAAAAATTC TGTGTACAAT AAAATATACA CATATTAACT CTGTACACAT

GTAAAAATTC TGTGTACAAT AAAATATACA CATATTAACT CTGTACACAT

GTAAAAATTC TGTATA-AAT AAAATATACA CATATTAACT CTGTACACAT

GTAAAAATTC TGTGTACAAT AAAATATACA CATATTAACT CTGTATACAC

gtAAAAATTC CATGTATAAT AAGATATACA CAAACTAGCT TTGTCCACAT

GTAAAAATTC CATGTATAAT AAGATATACA CAAATTAGCT TTCTCCACAT

GTAAAAATTC TGTATACAAT AAAATATACA CATATTAACT TTGTACATAT

CTATAAGTC- -ATGAACAAT AAGATATACA CATATTAACT CTGTACACAT

GTAAAAATTC TGTGAACAAT AAAATATACA CATATTAACT CTGTACACAT

GTAAAAATCC TGTGTACAAT AAAATATACA CATATTAACT TTGTACACAT

GT-------- -------AGG AAAATATACA CATATTAGCT CTGTACACAT

GTAAAAATTC TGTGTACGAT AAAATATACA CATATTAGCT TTGTACACAT

GTAGAAATCC TGTATATAAG AAAATATACA CATATTAGCT TTGTACACAT

GTAAAAATCC TGTGGACAAT AAAATGTGCA CTTATTAACT TTGTATACAT

GTATAAATTT TGCACACAAG AAAAAATATA CATATTAAAT TTGTACACAT

GTCTAAATTC CGTGCATAAG AAGATATATA CAGATTAAAC TTTTACACAT

CAAATAGTGG TGTTTTGTGA AGATATCAGT GCACTTATTA TTGCACACTG

CAAATAGTGG TGTTTTGTGA AGATATCAGT GCACTTATTA TTGCACACTG

CAAATAGTGG TGTTTTGTGA AGGTATCAGT GCACTTATTA TTGCACACTG

CAAATAGTGG TGTTTTGTGA AGATATCAGT GCACTTATTA TTGCACACTG

CAAATAGTGG TGTATTGTGA AGATATCAGT GCACTTATTA TTGCACACTG

CAAATAGTGG TATTTCATGA AGATATCAGT GCACTTATTA TTGCACACTG

CCTCTAGTGG TATTTTATAT AGATATCAGT GCACTTCTTA TTGCACACTG

CCTCCAGTGG TATTTTATAT AAATATCAGT GCTCTTCTTA TTGCACACTG

CAAATAGTGG TATATCATAA AGATATCAGT GCACTTATTA TTGCACGCTG

TAAATAGTGG TATTTCATGA CAACATCAGT GCACTTATTA TTGCACACTG

TAAATAGTGG TATTTCATGA CAACGTCAGT GCACTTATTA TTGCACGCTG

TAAATAGTGG TATTTCATGA CGACATCAGT GCACTTATTA TTGCACACTG

TCAATAGTGG TATC--GTGA AGATATCAGT GCACTAATTA TTGCACACTG

TAAATAGTGG TATTTTATGA AGATATCAGT GCTTGTATTA TTGCACACTG

TAAATAGTGG TATTTTATGA AGATATCAGT GCGTTCGTTA TTGCACACTG

TAAATAGTGG TATTTTTTGA AGGTA-CAGT GCGTTCGTTA TTGCACA---

TAAAGAGTGG TATTTCATGA AGATATCAGT GCATTTGGTA TTGCACACTG

TACATAGTGG TATTTGATGG ATATATCAGT GCATTTATTA TTGCACAGTG

AGCCTTTGAT ACAAGCAGAT TAAATGACAG GTCAATCTTT AATGAGCATT

AGCCTTTGAT ACAAGCAGAT TAAATGACAG GTCAATCTTT AATGAGCATT

AGCCTTTGAT ACAAGCAGAT TAAATGACAG GTCAATCTTT AACGAGCATT

AGCCTTTGAT ACAAGCAGAT TAAATGACAG GTCAATCTTT AACGAGCATT

AGCCTTTGAT ACAAGCAGAT TAAATGACAG GTCAATCTTT AATGAGCATT

AGCCTTTGAT ACAAGCAGAT TAAGTGATAG GTCACTCTTT AATGAGCATT

AGCCTTGGAT ACAAGCACAC TAAGTGAGAG GTCACTCTTC AATGGGCATT

AGCCTTTGAT ACAAGCACAC TAAGTGAGAG GTCACTCTTC AATGGGCATT

AGCCTTTGAT ACAAGCAGAT TAAATGACAG GTCACTCTTT AATGAGCATT

AGCCTTTGAT ACAAGCAGGT TCAATGACAG GTCACTCTTT AATGAGCATT

AGCCTTTGAT ACAAGCAGAT TAAATGACAG GTCACTCTTT AATGAGCATT

AGCCTTTGAT ACAAGCAGAT TAAATGACAG GTCACTCTTT AATGAGCATT

AGTCTTGGAG ACAAGCAGGT TCAATGACAG GTCACTCTTG AACGAGCATT

AGTCTTTGAT ACAAGCAGAT TAAATGACAG GTCACTCTTT AATGAGCATT

AGCCTTTGAT ACAAGCAGAT TAAATGACAG GTCACTCTTT AATGAGCATT

---------- ---------- ---------- ---------- ----------

AGGCTTTGAT GCAATCGGAT TAAATGAAAG GTCACTGCTT AGTTAGCATT

ACACTTCGAT AAAAGCGGCT TAAGAGACAG GACGTGGTTT AATTAACATT

AGCAAGAACA ACTACATGGT TCCTAAAATC CAAAATACTA AGACGGATGA

AGCAAGAACA ACTACATGGT TCCTAAAATC CAAAATACTA AGACGGATGA

AGCAAGAACA ACAACATGGT TCCTAAAATC CAAAATACTA AGACGGATGA

AGCAAGAACA ACAACATGGT TCCTAAAATC CAAAATACTA AGACGGATGA

AACAAG---- ACTAAATGGC TCCTAAAATC CAAAATACTA AGATGGATGA

AGCAAGAACA ACTAAATGGT TCCTAATATC CAAAATGCTA AGACAGATGA

AGCAAA---A ACTATATGAT TCCTAAAACC CAAAGTACTA AGACAGATGA

AGCAAG---A ACTATATGAT TCCTAAAACC CAAAGTACTA AGATGGGTGG

AGCAAGAACA ACTAAATGGC TCCTAAAATC CAAAATGCTG AGACAGATGA

AGCAAGAATA ACTAAATGGT TCCTAAGATT CAAAATACTA AGACAGATGG

AACAAGAA-- -CTAAATGGT TCCTAAAATT TGAAATACTA AGACAGATGA

AGCAAGAA-- --CAAATGGT TCCTAAAATT CAAAATACTA AGACAGATGG

AGCTAGAATA ACTCGATGGT TCCTAAAACT CAAAATCCTA AGACAGATGA

AGAAAGAACA ACTAAATGAT TCCTAAAATC CAAAATACTA AGACAGATGG

AGCAAGAAAG ACTAAATGGT TCCTAAAATT CGAAATAGTA AGACGGATGG

---------- ---------- ---------- ---------- ----------

TGTAAATACA ACTAAAAGAC TCCTGAAATC TAAAATGCTA AGACAGATGC

TTATAATGCC ACTATACTGT TCCTGTAGTT CCGAAGGCTC AGACAGATGA

AAACACATGT AAGATTGATA ATTGTCAGAT ACCTGTTACC CTGATGGTTG

AAACACATGT AAGATTGATA ATTGTCAGAT ACCTGTTACC CTGATGGTTG

AAACACATGT AAGATTGATA ATTGTCAGAT ACCTGTTACC CTGATGGTTG

AAACACATGT AAGATTGATA ATTGTCAGAT ACCTGTTACC CTGATGGTTG

AAACACATGT AAGATTGATA ACAGTCAGAT ACCTGTTACC CTGATGGTTG

AAACACATGT AAGACTGATA ATAATCAGAT ACCTGTTACC CTGATGGTTG

AAGCACATGG AAGGCGGATA AGAGTCAGGT ACCTGTTACC CTGTTGGTTG

AAGCACATGG AAGGCTGATG AGAGTCAGGT ACCTGTTACC CTGTTGGTTG

AAGCACATGT AAGATTGATA ATAGTCAGAT ACCTGTTACC CTGATGGTTG

AAGCACATAT AAGATTGAGA ATAATCCGAT ACCTGTTACC CTGATGGTTG

AAGCACAGAT AAGATTGAGA AGAATCCAAT ACCTGTTACC CTGATGGTTG

AAGCACATAG AAGACGGAGA ATAATCCAGT ACCTGTTACC CTGATGGTTG

AAGCACAAGT AAGACTGAGA ATAATCCAAT ACCTGTTACC CTGACGGTTG

AAGCACATAT AAGATTGATA ATAGTCAGAT ACCTGTTACC CTGATGGTTG

AAGCACAGAG AAGATTGATA ATAATCAGAT ACCTGTTACC CTGAAGGGTG

---------- ---------- ---------- ---------- ----------

GAGCACATAG AAGATTGATA TTAATCAGAT ACCTGTTACC CTGATGGTTG

GAGCACATAG AAGATTGGTA ATAATAAGGT ACCTGTTACC CTGATGGTTG

CTCCTGCTTT GAGAAAGGAA ACAGACATCA AAGGAAAGAG TTAGGAGGAC

CTCCTGCTTT GAGAAAGGAA ACAGACATCA AAGGAAAGAG TTAGGAGGAC

CTCCTGCTTT GAGAAAGGAA ACAGACATCA AAGGAAAGAG TTAGGAGGAT

CTCCTGCTTT GAGAAAGGAA ACAGACATCA AAGGAAAGAG TTAGGAGGAT

CTCCTGCTTT GAGAAAGGAA AAAGACATCA AAGGAAAGAG TTAGGAGGAC

CTCCTGCTTT GAGAAAGGAA ACAGACATTA AAGGAAAGAG TTAGGAGGAC

CTCCTGCTTT GAGAAAAGGA ACAGACATCA AAGTAAAGAG TT---AGGAC

CTCCTGCTTC GAGAAAAGGA ACAGACATCA AAGGAAAGAG TT---AGGAC

CTCCTGCTTC GAGAAAGGGA ACAGACATTA AAGGAAAGAG TTAGGAGGAT

CTCCTGCTTT GAGAAAAGAA ATAGACATCA AGGGAAAGAG TTAGGAGGAC

CTCCTGCTTT GAGAAAGGGA ACAGACATCA AAGGAAAGAG TTAGGAGAAC

CTCCTGCTTT GAGAAAGGGA ACAGACATCA AAGGAAAGAG TTAGGAGGAC

CTCCTGCTTT GAGAAAAGGA ACAGATGTTA AAGGCAAGAG TTAGGAGGAC

CGCCTGCTTT GAGAAAGGGA ACAGACATCA AAGGAAAGAG TTAGGAGCAC

CTCCTGCTTT GAGAAAGAGA ACAGACATCA AAGGAGAGAA TTAGGAGGAC

---------- ---------- ---------- ---------- ----------

CTCCTGCTTT AAGAAAGGAG ACAGACATCA AAGGAAAGAG TTAGGAGGAA

CTCCTGCTTG GAGAAAGGAG ACAGGCATCA AAGGACAGAG TTAGGAGGGA

AATCTTTTAC CTATATTATC TTTCCTTGTT GTTCCAGGGC TTTTGGGTGC

AATCTTTTAC CTATATTATC TTTCCTTGTT GTTCCAGGGC TTTTGGGTGC

AATCTTTTAC CTATGTTATC TTTCCGTGTT GTTCCAGGGC TTTTGGGTGC

AATCTTTTAC CTATGTTATC TTTCCGTGTT GTTCCAGGGC TTTTGGGTGC

AATCTTTTAC CTA---TATC TTTCCGTGTT GTTCCAGGGC TTTTGGGTGC

AATCTTTTAC CTATATTATC TTTCCGTGTT GTTCCAGGGC TTTTGGGTGC

AATTTTTTAC CTATATTATC TTTCCGTGCT GTTCCCGGGC TTTTGGGTGC

ATTTTTTTAC CTATATTATC TTTCCGTGCT GTTCCAGGGC TTTTGGGTGC

AATCTTTTAC CTATATTATC CTTCCGTGTT GTCCCAGGGC TTTTGGGTGC

AATCTT--AC CTAGATTATC TCTCCGGGTC GTTCCTGGGC TTTTGGGTGC

AATCTTTTAC CTATAGTATC TTTCCGTGTT GTTCCAGGGC TTTTGGGTGC

AATCTTTTAC CTATATTATC TTTCCGTGTT GTTCCAGGGC TTTTGGGTGC

AATTTTTTAC CTATATTATC TTTCCGCGTC ACTCCGGGGC TTTTGGGAGC

AATCTTTTAC CTATATTATC TTTCCGTGTC ATTCCAGGGC TTTTGGGTGC

AATCTTTTAC CTA---TATC TTTCCGTGTC ATTCCAGGGC TTTTGGGTGC

----TTTTAC CTATATTATC TTTCCGTGAC ATTCCAGGGC TTTTGGGTGC

AATCTTTTAC CTGAACTATC TTTTTTTATT GTTCCAGGGC TTTTTGGTGC

AACCCGTTAC CTAAATTCTC TTTCCTTGAA GTCCCAGGGC TTTTGGGGGC

TTTGAACAAG AAGGGAAAAA ATGTTACCTT CCATCTCTCT CCTAGCTACC

TTTGAACAAG AAGGGAAAAA ATGTTACCTT CCATCTCTCT CCTAGCTACC

TTTGAACAAG AAGGGAAAAA ATGTTACCTT CCATCTCTCT CCTAGCTACC

TTTGAACAAG AAGGGAAAAA AT-------- ---------- ----------

TTTGAACAGG AAGGGAAAAA ATGTTACCTT CCATCTCTCT CCTAGCTACC

-TTAAACAGG AAGGAAAAAA ATGTTACCTT CCATCTCTCT CCTAGCCACC

TTTGAACAGA AAAGAAAAAG ATGTTACCTT CCATCTCTCT CCTAGTAACC

TTTGAACAGA AAGGAAAAAG ATGTTACCTT CTATCTCTCT CCTAGCAACC

TTTGAACAGG AAGGGAAAAG ATGTTACCTT CCATCTCTCT CCTAGCCACC

TTTGAACAGG AAGGAAAAAA ATGTTACCTT CCATCTCTCG CCTAGCTACC

TTTGAACAAG AAGGAAAAAA ATGTTACCTT CCATCTCTCG CCTAGCTGCC

TTTGAACAGG AAGA-AAAAC ATGTTACCTT CCATCTCTCG CCTAGCTACC

TTTGCACAGG AAGGAAAGAG ATGTTACCTT CCATCTCTCG CCTAGCTACC

TTTGAACAGG AAGGAAAAAA ATGTTACCTT CCATCTCTCT CCTAGCTACC

TTTGAACAGG AAGGAAAAAA ATACTTTCCT CCATCTGTCT CCTAGCTACC

TTTGAACAGG AAGGAAAAAA --GTTACCTT CCATCTCTCT CCTAGCTACC

TTTTAACAGG AAGGAAAAGA ATGTTACCTG CTATATCTTT CTTAGATACC

TTTGCGCAGG AAAAACAAAG ATGTTACCTT CCATCTCTCT CCTAGCCGCC

CCAGGACTGG GAGGGGCTCC AATACCTTTT TAGAGAAAGA ACAGAAACTA

CCAGGACTGG GAGGGGCTCC AATACCTTTT TAGAGAAAGA ACAGAAACTA

CCAGGACTGG GAGGGGCTCC AATACCTTTT TAGAGAAAGA ACAGAAGCTA

---------- ---------- ---------- ---------- ----------

CCAGGACTGG GAGGGGCTCC AATACCTTTT CAGAGAAAGA ACAGAAGCTA

CCAGGACTGG GAGGGGCTCC TGTACCTTTT TAGAGAAAGA ACAAAAGCTG

CCGGGACTGG GAGGGGCTCC GACACCTTTT TAGAAAAAGA ACAAAAGCTA

CCGGGACTGG GAGGGGCTCC AACACCTTTT TAGAAAAAGA ACAAAAGCTA

CCAGGACTGG GAGGGGCTCC AATACCTTTT CAGAGAAAGA ACAGAAGCTA

CCCGGACTGG GAGGGGCTCC AACACCTTTT TAGAGAGAGA ACAGAAGCTG

CCAGGACTGG GAGGGGCTCC AACACCTTTT TAGAGAAAGA ACAGAAGCTG

CCAGGACTGG GAGGGGCTCC AATACCTTTT TAGAAAAAGA ACAGAAGCTG

CCGGGACTGG GAGGGGCTCC AACACCTTTT CAGAGAAAGA ACAAAAGCTG

CCAGGACTGG GAGGGGCTCC AACACCTTTT TAGAGAAAGA ACAGAAGCTG

CAAGGACTGG GAGGGGCCCC TACACCGTTT TAAAGAGAGA ACAGAAGCT-

CCAGGACTGG GGGGGGCTCC AGCACCTTTT TAAAGAAAGA ACAGAAGCTA

CCAGGGCTGG GAGGGCCTCC AAGACCTTTT TAGAGAAAGA ACAGAAGCTA

CCAGGGCTCG GTGGGGCTCC CAGACCTTTT TAGAAGAAGA ACAGAAGCCA

TATATTGCAG AACAAATGTA AATGGTTACC TCCAAATTCC TTTCTAGTGG

TATATTGCAG AACAAATGTA AATGGTTACC TCCAAATTCC TTTCTAGTGG

TATATTGCAG AACAAATGTA AATGGTTACC TCCAAATTCC TTTCTAGCGG

---------- AACAAATGTA AATGGTTACC TCCAAATTCC TTTCTAGCGG

TATATTGCAG AACAAATGTA AATGGTTACC TGCAAATTCT TTTCTAGCGG

TATATGGCAG AAAAAATGTA AGTGGTTACC TCCAAATTCT TTTCTAGCAG

TATATTGCAG AGTGAATGTA AATGGTTACC TCCAAATTCT TTTCTAGCAG

TATATTGCAG AGCAAATGTA AATGGTTACC TCCAAATTCT TTTCTAGCAG

TATATTGCAG AATAAATGTA AATGGTTACC TCCAAATTCT TTTCTAGCAG

TGTATTGCAG AACAAATATA AATGGTTACC TCCAAACTCT TTTCTAGCAG

TATATTGCAG AACAAATATA AATGGTTACC TCCGAATTCT TTTCTAGCAG

TATATTGCAG GACAAATATA AATGGTTACC TCCAAATTCT TTTCTAGCAG

TATATTGCAG AACAAAGAGA AACGGTTACC TCCAAATTCT TTTCTAGCAG

TATATTGCAG AACAAATGTA AATGGTTACC TCCAAATTCT TTTCTAGCAG

-----TGCAG AACAAATGTA AACAGTTACC TCCAAGTTCT TTTCTAGCAG

TGTATTGCAG AACAA-TGTA AACTATTACC TCCAAATTCT TTTCTAGCAG

TAAAGCACAG AACAAATATA AACTGTTACC TCCAAATTCC TTTCTAGAAG

GATATTGCAG aaaaaaagaA CACGTTTACC TCCAAATTCT TTTCTAGCGG

GTGCAGGACT CCTAGCCCCT TATAGTTCAT AAGAAAATAG GCAGCAGAAA

GTGCAGGACT CCTAGCCCCT TATAGTTCAT AAGAAAATAG GCAGCAGAAA

GTGCAGGACT CCTGGCCCCT TATAGTTCAT AAGAAAATAG GCAGTAGAAA

GTGCAGGACT CCTGGCCCCT TATAGTTCAT AAGAAAATAG GCAGTAGAAA

GTGCAGGACT TCTAGCCCCT TATAGTTCAT AAGAAAATAG GCAGCAGAAA

GTGCAGGACT CCTAGCCCCT TATAGTTCAT AAGAAAATAT GCAGCAGAAA

GTGCCGGACT CCTAGCCCCT TATAGTTCAT AAGAAATTAG GCAGCAGAAA

GCGCAGGACT CCTAGCCCCT TATAGTTCAT AAGAAATTAG GCAGCAGAAA

GTGCAGGACT CCTAGCCCCT TATAGTTCAT GAGAAA-TAT GCAGCAGAAA

GTGCAGGACT CCTAGCCCCT TATAGTTCAT AAGGGAATAT GCAGCAGAAA

GTGCAGGACT CCTAGCCCCT TATAGTTCAT AAGAAAATAT GCAGCAAGAA

GTGCAGGACT CCTAGCCCCT TATAGTTCAT AAGAAAATAT GCAGCAGGAA

GTGCAGGACT CCTAGCCCCT GAGTGTTCAT AAGGAAATGT GCGGCAGAAA

GTGCAGGACT CCTAGCCCCT TATAGTTCAT AAGAAAGTGT GCAGCAGAAA

GTGCAGGACT CCTAGCCCCT TATAGTTCAC AAGAAAACAC GCAGCAGAAA

GTGCAGGACT CCTAGCCCCT TATAGTTCAT AAGAAAATAC GCAGCAGAAA

GTGCAGGACT CCTATTCCCT TTAAATTCAT AAGAAAGTAC GCAACGCAAA

GGGCAGGACT CCTACCTCCT TATGATTCAT CAGAAAATAT GCAGCACAAA

GGAAATGACT CACCTGAAGG CGGAGGAGGT CTCTGTGGGG GAGCCGGGCG

GGAAATGACT CACCTGAAGG CGGAGGAGGT CTCTGTGGGG GAGCCGGGCG

GGAAATGACT CACCTGAAGG CGGAGGAGGT CTCTGTGGGG GAGCCGGGCG

GGAAATGACT CACCTGAAGG CGGAGGAGGT CTCTGTGGGG GAGCCGGGCG

GGAAATTACT CACCTGAAGG CGGAGGAGGT CTCTGTGGGG GAGCGGGGCG

GGAAATGACT CACCTGAAGG TGGAGGTGGC CTCTGTGGGG GAGCAGGACG

GGGAATGACT TACCTGAAGG TGGAGGTGGC CTCTGTGGTG GGGCAGGACG

GGGAATGACT TACCTGAAGG TGGAGGTGGT CTCTGTGGTG GGGCAGGACG

GGAAATGACT TACCTGAAGG CGGAGGTGGT CTCTGTGGGG GCGCTGG---

GGAAATGACT CACCTGAAGG CGGAGGTGGT CGTTGTGGAG GAGCTGGACG

GGAAAGGACT TACCTGAAGG TGGAGGTGGT CTCTGGGGGG GCGCAGGGCG

GGAAATGACT TACCTGAAGG TGGAGGTGGT CTTTGTGGGG GAGCTGGACG

GGAGATGACT TACCTGAAGG CGGAGGTGGT CTCTGCGGGG GAGCAGGCCG

GGAAATGACT TACCTGAAGG CGGAGGTGGT CTCTGGGGGG GAGCTGGACG

GGAAATGACT TACCTGACGG TGGAGGTGGT CTTTGTGGGG GAGCTGGACG

GAAAATGAC- ---------- ---------- ---------- ----------

GGAAATGACT TACCTGAAGG AGGGGGTGGT CTTTGTGGTG GAGCTGGACG

GGAAATGAGG TACCTGAAGG CGGAGGCGGC CTCTgcggcg gggccgggcg

TGTGGGAGGG GCGTAGGTGA CTGGCAGGGA CTAGTTCGGG GTGAAGAGCT

TGTGGGAGGG GCGTAGGTGA CTGGCAGGGA CTAGTTCGGG GTGAAGAGCT

TGTGGGAGGG GCGTAGGTGA CTGGCAGGGA CTAGTTCGGG GTGAAGAGCT

TGTGGGAGGG GCGTAGGTGA CTGGCAGGGA CTAGTTCGCG GTGAAGAGCT

TGTGGGAGGA GCGTAGGTGA CTGGCAGGGA CTAGTCCGGG GTGAAGAGCT

GGTGGGAGGA GCGTAGGTGA TTGGCAGGGA CTAGTCCGGG GTGAGGAGCC

TGCTGGAGGA GCGTGGGTGA CTGGCAGGGA CTGGTCCGGG GCGAAGAGCT

TGCCGGAGGA GCGTGGGGGA CTGGCAGGGA CTGGTCCGGG GTGAAGAACT

---------- ---------- ---------- ---------- ----------

TGCAGGAGGA GCGTAGGTGA CTGGCAGGGA CTGGTCCGGG GTGAAGAACC

TGCGGGGGGA GCGTCGGTGA CTGGCAGGGA CTGGTCCGGG GTGAAGAGCC

TGTGGGAGGA GCGTAGGTGA CTGGCAGGGA CTCGTACGGG GTGAAGAGCT

GGCTGGAGGA GCGTGGG-GA CTGGCAGGGG CTGGTCCGGG -TGAAGAGCT

TGCTGGAGGA GCGTAGGAGA CTGGCAGGGA CTGGTCCGGG GTGAAGAGCT

CGTAGGAGGA GCGTAGGTGA CTGGCAGGGA CTGGTCCGGG GTGAAGAGCT

---------- ---------- ---------- ---------- ----------

TGCAGGTGGG GCGTAGGGGA CTGGCAGGGA CTGGACCGAG GTGAACAGCT

ggcgggcggA GCGTAGGCGA CTGGCAGGGA CTGGAGCGAG GCGACGAGCT

GGGGGAAGTA CCAAGGCCGG AACTTGAAGA TGGCTGGAGA TGCTGAGGAA

GGGGGAAGTA CCAAGGCCGG AACTTGAAGA TGGCTGGAGA TGCTGAGGAA

GGGGGAAGTA CCAAGGCCGG AACTTGAAGA TGGCTGGAGA TGCTGAGGAA

GGGGGAAGTA CCAAGGCCGG AACTTGAAGA TGGCTGGAGA TGCTGAGGAA

GGGGGAAGTG CCAAGACCGG AACTTGAAGA TGGCTGGAGA TGCTGGGGAA

TGGGGAAGTG CCAAGGCCAG AACTTGATGA TGGCTGGAGA TGCTGAGGAA

TGGAGAGGTG CCGAGGCCAG AGCTGGAGGA TGGCTGGAGG TGCTGAGGAA

TGGAGAAGTG CCCAGGCCAG AACTTGAGGA CGGCTGCAGA TGCTGAGGAA

--GGGGAGTG CCGAGGCCAG AACTTGAGGA GGGCTGGAGA TGCTGAGGAA

GGGGGACGTG CCAAGGCCAG AACTGGAAGA CGGCTGGAGA TGCTGAGGAA

TGGGGAAGTG CCCAGGCCAG AACTTGAAGA TGGCTGGAGG TGCTGGGGAA

CGGGGAAGTG CCGAGGCCAG AACTTGAAGA TGGCTGGAGA TGCTGAGGAA

CGGCGAGGTA CCAAGGCCAG AGCTGGAGGA GGGCTGGAGA TGCTGAGGAA

TGGGGAAGTG CCAAGGCCAG AACTTGAAGA TGGCTGGAGA TGCTGGGGAA

TGGCGAAGCA CCCAGGCCAG AGCTTGAAGA CGGCTGGAGA TGCTGAGGAA

---------- ---------- ---------- ---------- ----------

CGGGGAAGTT CCAAGACCAC AGCTTGAAGT TGGCTGTAGA TGCTGAGGAA

CGGCGAGGTC CCGGGGCCAG AGCCGGGGGA CGGCTGCAGG TGCTGAGGTA

GAAGTTCCTC CACTTCAGCA CTATAGTCAT CAACATCACC TATTTTCTCT

GAAGTTCCTC CACTTCAGCA CTATAGTCAT CAACATCACC TATTTTCTCT

GAAGTTCCTC CACTTCGGCA CTATAGTCAT CAACATCACC TATTTTCTCT

GAAGTTCCTC CACTTCGGCA CTATAGTCAT CAACATCACC TATTTTCTCT

GAAGCTCCTC CACTTCAGCA CTGTAGTCAT CAACATCACC TATTTTCTCT

GAATTTCCTC CACTTCTGCA CTGTAGTCAT CAACATCACC TATTTTCTCT

GAAGCTCCTC CACCTCAGCA CTGTAGTCGT CGACATCACC TATTTTCTCT

GAAGCTCCTC TACTTCAGCG CTGTAGTCAT CGACGTCACC TATTTTCTCT

GCAGCTCCTC TACTTCTGCA CTGTAGTCAT CAACATCACC TATTTTCTCT

GGATCTCCTC TACCTCAGCA CTATAGTCAT CCACATCACC TATTTTCTCT

GAATTTCCTC CACTTCAGCA CTGTAGTCAT CGACATCACC TATTTTCTCT

GAATTTCCTC TACTTCAGCA TTATAGTCAT CAACGTCCCC TATTTTCTCT

GAATTTCCTC CACTTCGGCA CTATAGTCAT CGACGTCGCC T---------

GAATTTCCTC TACTTCAGCA CTATAGTCAT CGACATCACC TATTTTCTCT

GGATTTCCTC TACTTCTGCA CTGTAGTCAT CAACATCACC TATTTTCTCC

---------- ---------- ---------- ---------- -ATTTTCTCC

GAATTTCTTC TACTTCTGCA CTCTCATCAT CAACATCACC TATTTTCTCC

GACTCTCTTC AACTTCCACA TTGTCGTCAT CGATGTCTCC TATCTTCTCC

AAACTCATTT CTTCTTCCAA ATTTTTGATC CAGTCTGGAC TTTTTAAAGC

AAACTCATTT CTTCTTCCAA ATTTTTGATC CAGTCTGGAC TTTTTAAAGC

AAACTCATTT CTTCTTCCAA ATTTTTGATC CAGTCTGGAC TTTTTAAAGC

AAACTCATTT CTTCTTCCAA ATTTTTGATC CAGTCTGGAC TTTTTAAAGC

AAACTCATTT CTTCTTCCAA ATTTTTGATC CAGTCTGGAC TTTTTAAAGC

AAACTCATTT CTTCTTCCAA ATTTTTGATC CAGTCTGGAC TTTTTAAAGT

AAACTCATCT CTTCTTCCAA AGTTTTGATC CAGTCTGGAC TTTTTAAAGT

AAACTCATCT CTTCTTCCAA ATGTTTGATC CAGTCTGGAC TTTTTAAAGT

AGACTCATTT CTTCTTCCAA ATTTTTGATC CAGTCTGGAC TTTTTAAAGT

AAGCTCATTT CTTCTTCCAA AGTTTTGATC CAGTCTGGAC TTTTTAAAGT

AAACTCATTT CTTCCTCCAA ATTTTTGATC CAGTCTGGAC TTTTTAAAGT

AAACTCATTT CTTCTTCCAA AGTTTTGATC CAGTCTGGAC TTTTTAAAGT

---------- ---------- ---------- ---------- ----------

AAACTCATTT CTTCTTCCAA AGTTTTGATC CAGTCTGGAC TTTTTAAAGT

AAACTCATTT CTTCCTCCAA AGTTTTGATC CAGTCTGGGC TTCTTAAAGT

AAACTCATTT CTTCTTCCAA AGTCTTGATC CAGTCTGGAC TTTTTAAAGT

AAATTTATTT CTTTCTCCAA ATCAGCAATC CAGTTTGGGT TTTTTAAGGT

AGACTCATTT CTTCTTCCAG ATTGGCGATC CAGTCCGGGC TTTTTAAGGT

AATAGTTATA GTCCGATTCA ATAACTACCT TACCTCTTTA CCATTTAGGC

AATAGTTATA GTCCGATTCA ATAACTACCT TACCTCTTTA CCATTTAGGC

AATAGTTATA GTCCGATTCA ATAACTACCT TACCTCTTTA CCATTTAGGC

AATAGTTATA GTCCGATTCA ATAACTACCT TACCTCTTTA CCATTTAGGC

AATAGTTATA GTCCGATTCA ATAACTACCT TACCTCTTTA CCATTTAGGC

AATAGTTATA GTCCGATTCA ATAACTA--- ---------- ----------

AATTGTTATA GTCCGATTTA ATAACTACCT TACCTCTTTA CCATTTAGGC

AATTGTTATA GTCCGATTTA ATAGCTACCT TACCTCTTTC CCATTTAGGC

AATAGTTATA GTCCGATTCA ATAACTATTT TACCTCTTTC CCATTTAAGC

AATTGTTATT GTCCTACCCA GTAACTGCTT TACCTCTTTC CCATTCAGGT

AATAGTTATA GTCCGACCCA ATAACTACCT TACCTCTTTA CCATTTAGGT

GATAGTTATA GTCCGACCCA ATAACTAACT TACCTCTTTA CCATTTAGGC

---------- ---------- -------CCT TACCTGTGTT CCATTCAGGC

AATGGTAATA GCTCGACCCA ATAACTACAT TACCTCTTTA CCATTTAGGC

GATAGTTATA GTCCGACCCA GTAACTACAT TACCTCTTTA CCATTTAGGT

AATAGTTATA GTCCGACCCA GTAACTG--- ---------- ----------

AATACTTATG AGTCGATCTA AAAACTGCAT TACCTCTTTA CCATTTAGGT

AATATTTATA ACTCTGCCCA ACAGCTGCAT TACCTCAGTT CCGTGTAGAC

TCAGAACATT CAAGGCAGAG CTTCCCTCCA AAAATGTAAC CCACATTTTA

TCAGAACATT CAAGGCAGAG CTTCCCTCCA AAAATGTAAC CCACATTTTA

TCAGAACATT CAAGGCAGAG CTTCCCTCCA AAAATGTAAC CCACATTTTA

TCAGAACATT CAAGGCAGAG CTTCCCTCCA AAAATGTAAC CCACATTTTA

TCAGAACATT CAAGGCAGAG CTTCCTTCCA AAAATGTAAC CCACATTTTA

---------- ---------- ---------- ---------- ----------

TCAGAACATT CAAGGCAGAG CTCCCCTCCA AAAATGTAAC CCACATTTTA

TCAGAGCATT CAAGGCAGAG CTTCCCTCCA AAAATGTAAC CCACATTTTA

TCAGAGCACT CAAGGCAGAA CTTCCCTCCA GAAACGTAAC CCACATCTTA

TCAGAACATT CAAGGCAGAG CTTCCCTCTA AAAACGTAAC CCACATTTTA

TCAGAACACT CAAGGCAGAG CTTCCCTCCA AAAATGTGAC CCACATTTTA

TTAGAACACT CAAGGCAGAA CTTCCCTCCA AAAATGTAAC CCACATTTTA

TGAGAACACT CAAGGCAGAG CTGCCCTCCA AAAATGTAAC CCACATTTTA

ACAGAGCATT CAAGGCAGAG CTTCCTTCCA AAAATGTAAC CCACATTTTA

TCAGAACATT CAAGGCAGAG CTTCCCTCCA AAAATGTAAC CCACATTTTA

---------- ---------- ---------- ---------- ----------

TCAGAACTTT CAGGGCAGAG CTTCCTTCCA AAAATGTTAC CCACATCTGG

TCAGAACGCT CAAGGCCGAG CTTCCTTCCA GAAACGTTAC CCACATCTTG

TCTTCTACAA ATCTTTAAGA AACCTTATAA GTATAACTTC ACCAAAACTT

TCTTCTACAA ATCTTTAAGA AACCTTATAA GTATAACTTC ACCAAAACTT

TCTTCTACAA ATCTTTAAGA AACCTTATAA GTATAACTTC ACCAAAACTT

TCTTCTACAA ATCTTTAAGA AACCTTATAA GTATAACTTC ACCAAAACTT

TCTTCTACAA ATCTTTAAGA AACCTTATAA GTATAACTTC ACCAAAACTT

---------- ---------- ---------- ---------- ----------

TCTTCTACGA ATCTTTAAGA GACCTTATAA GTATAACTTC GCCGAAGTGT

TCTTCTACAA ATCTTTAAGA GACCTTATGA GTATAACTTC ACCAAAGTGT

TCTTCTACAA ATC-TGAAGA GACGTTATGA GTATAACTTC ACCAAAATTT

TCTTCCACAA ATCTTTAAGG AACCTTATGA GTATAACTTC ACCGAAATTT

TCTTCCACAA ATCTTCAAGA AACCTTATGA GGATAACTTC ACCAAAATTT

TCTTCTACAA ATCTTCAAGA AACCTTATGA GTATAACTTC ACCAAAATTT

TTTTCTACAA ATCTTCAAGA AACCTTATGA GTATGACTTC GCCATAATTT

TCTTCCACAA ATCTTTAAGG AACCTTATGA GTATAACTTC CCCAAAATTT

TCTTCTACAA ACCtttaaaa aACCTTATGA GTATAACTTC ACCGAAACTT

---------- ---------- -ACCTTATAA GTATAACTTC CCCAAAATTT

TCTTCTACAA ACCTTTGAGA AACCTTATGA GGATAACTTC ACCATGGTTT

TCTTCTACAA ACCTGCAAGA AACCTGATGA GTATGACTTC ACCATAGTTG

GCAAACTGCT GCAGAAGCTC ATCAATCAAG GCATCATCAA AAAAATTATT

GCAAACTGCT GCAGAAGCTC ATCAATCAAG GCATCATCAA AAAAATTATT

GCAAACTGCT GCAGAAGCTC ATCAATCAAG GCATCATCAA AAAAATTATT

GCAAACTGCT GCAGAAGCTC ATCAATCAAG GCATCATCAA AAAAATTATT

GCAAACTGCT GCAGAAGCTC ATCAATCAAG GCATCATCGA AAAAATTATT

---------- ---------- ---------- ---------- ----------

GCAAACTGCT GCAGAAGCTC ATCGATCAAA GCATCGTCAA AGAAAGTATT

GCAAACTGCC GCAGAAGCTC ATCAATCAAA GCATCATCAA AAAAAGTACT

GCAACTGGCC CTAG-GGTTC ATCAATCAAA GCATCATCAA AGAAATTATT

GTAAACTGCT GTAAAAGCTC ATCAATCAAA GCATCGTTGA AAAAATTATT

GTAAACTGCT GCAGAAGCTC ATCAATCAAA GCATCATCGA AAAAATTATT

GTAAACTGCT GCAGAAGCTC ATCGATCAAA GTATCATCAA AGAAATTATT

GTAAACTGCT GCAGAAGCTC ATCAATCAGA GCATCATCAA AAAAGTTACT

GCAAACTGCT GGAGAAGCTC ATCAATCAGA CCATCATCGA AAAAATTATT

GCAAACTGCT GGAGAAGCTC ATCAATCAAA GCATCATCAA AAAAATTATT

GCAAATTGCT GGAGAAGCTC ATCAATCAAA GCATCATCAA AAAAGTTATT

CCAAACTCCT GAAGAAGCTC ATTAATCAAG CTATCGTCGA AAAAATTATT

GCAAATTGCT GGAGAAGTTC ATCGATCAAG ATGTCATCAA AGAAATGATC

TTCTGGTAAA GAACTTTTGA TTGAGACCAA TACTGTACCA TCTGGTGGAC

TTCTGGTAAA GAACTTTTGA TTGAGACCAA TACTGTACCA TCTGGTGGAC

TTCTGGTAAA GAACTTTTGA TTGAGACCAA TACCGTACCA TCTGGTGGAC

TTCTGGTAAA GAACTTTTGA TTGAGACCAA TACCGTACCA TCTGGTGGAC

TTCTGGTAAA GAACTTTTGA TTGAGACCAA TACCGTACCA TCTGGTGGAC

---------- ---------- ---------- ---------- ----------

CTCTTGTGCA GAACTTTTGA TTGAGACCAG TACTGTGCCG TCCGGTGGGC

TTCTTGTGCA GAACTTTTGA TTGAGACCAG CACTGTGCCA TCTGGTGGGC

T--------- ---------- ---------- ---------- ----------

TTCTGGTAAA GAGCTTTTGA TTGAGACCAA CACCGTACCA TCTGGTGGAC

TTCTTGGACA GAACTTTTGA TTGAGACCAA TACTGTACCA TCTGGTGGGC

TTCTGGTACA GAACTTCTGA TTGAGACCAA CACCGTACCA TCTGGTGGAC

TTCTGGGGAG GAAGTTTTGA TTGAGACCAA CACGGTCCCG TCGGGTGGCC

TTCTGGTAAA GAACTTCTGA TTGAGACCAA TACTGTACCA TCTGGTGGGC

TTCTGGTAAA GAACTTTTGA TTGAGACCAG TACTGTGCCA TCTGGGGGAC

ATCTGACAGA GAACTTTTGA TTGAGACCAA TACTGTGCCA TCAGGTGGAC

TTCTGATGAT GACCTTTTGA TGGTGACCAA TACTGTGCCA TCTGTTGGTC

TTCCACAGAA GAACTTTTGA TAGAGACCAA CACCGTCCCA TCTGGGGGGC

CCTGAACTGC AATTACTTCT TTATAAATGT TTTGCCTCTC TTCAGCTTCA

CCTGAACTGC AATTACTTCT TTATAAATGT TTTGCCTCTC TTCAGCTTCA

CCTGAACTGC AATGACTTCT TTATAAATGT TTTGCCTCTC TTCAGCTTCA

CCTGAACTGC AATTACTTCT TTATAAATGT TTTGCCTCTC TTCAGCTTCA

CCTGAACTGC AATTACTTCT TTATAAATGT TTTGCCTCTC TTCAGCTTCA

---------- ---------- ---------- ---------- ----------

CCTGGACTGC AATAACTTCT TTATAAATTT TTTGTCTCTC TTCAGCTTCA

CCTGGACGGC AATAACTTCT TTATAAATTT TTTGTCTCTC TTCAGCTTCA

---------- ---------- ---------- ---------- ----------

CCTGAACTGC AATTACTTCT TTATAAATGT TTTGCCTCTC TTCAGCTTCA

CCTGAACTGC GATTACTTCT TTATAAATGT TTTGCCTCTC TTCAGCTTCA

CCTGAACTGC AATTACTTCT TTATAAATGT TTTGTCTCTC TTCAGCTTCA

CCTGCACGGC AATGACCTCC TTATAAATGT TCTGCCTCTC TTCCGCTTCA

CCTGTACTGC AATTACTTCT TTATAAATAT TTTGCCTTTC TTCAGCTTCA

CCTGAACTGC AATCACTTCC TTATAAATGT TTTGCCTTTC TTCAGCTTCA

CCTGCACCGC AATCACTTCT TTATAAATGT TTTGCCTTTC TTCAGCTTCA

CTTGAGTGGC AATGACTTCC TTATAAACAA TTTGTCGTGT TTTAGCTTCC

CCTGGAGAGC GATCACTTCT TTATAAACGT TCTGTCTCTC TTCAGCTTCC

ACTTCAAATA TATCTATATC AATCAGGGCA ACGACAGGCC TACCTGTGGT

ACTTCAAATA TATCTATATC AATCAGGGCA ACGACAGGCC TACCTGTGGT

ACTTCAAATA TATCTATATC AATCAGGGCA ACGACAGGCC TACCTATGGT

ACTTCAAATA TATCTATATC AATCAGGGCA ACGACAGGCC TACCTATGGT

ACTTCAAATA TATCTATATC AATCAGGGCA ACGACAGGCC TACCTATGGT

---------- ---------- ---------- ---------- ----------

ACTTCAAATA TATCTATATC GATCAGGGCG ACAACGGGCC TACCTGTGGT

ACTTCAAATA TATCTATGTC AATCAAGGCA ACGACGGGCC TACCTATGGT

---------- ---------- ---------- ---------- ----------

ACTTCAAATA TATCAATATC GATCAGGGCA ACAACAGGCC TACCTATGGT

ACTTCAAATA TATCTATATC AATCAGGGCA ACGACAGGCC TACCTATGGT

ACTTCAAATA TATCTATATC AATCAAGGCA ACGACAGGCC TACCTATGGT

ACTTCAAAGA TATCGATATC GATCAGGGCA ATGACAGGCC TACCTGTGGT

ACTTCAAATA TATCTATATC AATCATGGCA ATGACAGGCC TACCTATGAT

ACTTCAAATA TATCTATATC AATCAGGGCA ACGACAGGCC TACCTATGGT

ACTTCAAATA TATCTATATC AATCAGCGCA ATGACTGGCC TACCTGTGAT

ACTTCAAATA TATCTATATC GATCAGTGCA ACAACAGGTC TACCTGTGAT

ACTTCAAATA TATCTACATC CATCAGGGCC ACCACAGGCC TACCTGTGAT

CAGAAGTCTT CAGCTCAGCT CTTCCATAGT GCAGCAAAGT GCCTGGAGTC

CAGAAGTCTT CAGCTCAGCT CTTCCATAGT GCAGCAAAGT GCCTGGAGTC

CAGAAGTCTT CAGCTCAGCT CTTCCATAGT GCAGCAAAGT GCCTGGAGTC

CAGAAGTCTT CAGCTCAGCT CTTCCATAGT GCAGCAAAGT GCCTGGAGTC

CAGAGGTCTT CAGCTCAGCT CTTCCATAGT GCAACAAAGT GCCTGGAGTC

---------- ---------- ---------- ---------- ----------

CAGAAGTCTT CAGCTCGGCT CTTCCATAGT GCAGCAAGGT GCCAGGAGTC

CAGAAGTCTT CAGCTCGGCT CTTCCATAGT GCAGCAAGGT GCCAGGGGTC

---------- ---------- ---------- ---------- ----------

CAGAAGTCTT CAGCTCAGCC CTTCCGTAGT GCAGCAAAGT GCCAGGAGTC

CAGAAGTCTT CAGCTCAGCT CTTCCATAGT GCAGCAAAGT GCCAGGAGTC

CGGAGGTCTT CAGCTCCGCT CTGCCATAGT GCAGCAAAGT ACCCGGAGTC

CTGAAGTCTT CAGCTCTGCG CGCCCATAGT GCAGAAGCGT GCCAGGAGTC

CAGAAGTCTT CAGCTCGGCT CTTCCATAAT GGAGCAAAGT GCCAGGAGTC

CCGAAGTCTT CAGCTCAGCC CGTCCGTAGT GCAGCAGCGT GCCAGGAGTC

CAGACGTCTT TAGCTCGGCC CTTCCATAGT GCAGCAAAGT GCCAGGGGTC

CAGAAGTCTT CAGCTCAGCT CTTCCATAAT GCAGCAAAGT GCCAGGATTC

CCGAGATCTT CAACTCGGCT CTTCCGTAGT GCAACAGAGT GCCGGGCGTC

CACGTGTATT TGCTTTCATC TTGAAAACTA GCATTTAGAA GATCTAGATC

CACGTGTATT TGCTTTCATC TTGAAAACTA GCATTTAGAA GATCTAGATC

CACGTGTATT TGCTTTCATC TTGAAAACTA GCATTTAGAA GATCTAGATC

CACGTGTATT TGCTTTCATC TTGAAAACTA GCATTTAGAA GATCTAGATC

CACGTGTATT TGCTGTCATC TTGAAAACTA GCATTTAGAA GATCTAGATC

---------- ---------- ---------- ---------- ----------

CATGTATATT TACTTTCATC TTGGAAACTA GCATTCAGAA GATCTAAGTC

CATGTATATT TACTTTCATC TTGGAAACTA GCATTCAGGA GATCTAAATC

---------- ---------- ---------- ---------- ----------

CATGTGTATT TGCTTTCATC TTGAAAACTA GCATTTAGGA GATCTAAATC

CATGTGTATT TGCTTCCATC TTGAAAACTA GCATTTAGAA GATCTAAATC

CAGGTGTATT TGCTTCCATC TTGAAAACTA GCATTTAGAA GATCTAAATC

CACGTGTATT TGCTTCCATC CTGAAAGCTC GCATTGAGAA GATCTAAGTC

CAGGTGTATT TGCTTTCATC TTGAAAACTA GCATTTAGAA GATCTAAATC

CAGGTGTATT TGCTTTCATC CTGAAAACTC GCACTTAGAA GATCTAAATC

CAAGTGTATT TGCTTCCATC TTGAAAACTA GCATTCAGAA GGTCTAAGTC

CAAGTGTATT TGCTGTCATC TTGAAAGCTA GCATTCAGAA GATCTAAATC

CAAGTGTATT TACTTTCATC TTGAAAGCCG GCATTCAGAA GATCGAGGTC

TTCAGCTGCA AACCTGATCT ATCAAAAGGC CATTTCCTCC TTCTCCAAAG

TTCAGCTGCA AACCTGATCT ATCAAAAGGC CATTTCCTCC TTCTCCAAAG

TTCAGCTGCA AACCTGATCT ATCAAAAGGC CATTTCCTCC TTCTCCAAAG

TTCAGCTGCA AACCTGATCT ATCAAAAGGC CATTTTCTCC TTCTCCAAAG

TTCAGCTGCA AACCTGATCT ATCAAAAGGC CATTTCCTTC TTCTCCAAAG

---------- ---------- ---------- ---------- ----------

TTCAGCTGCA CACCTGATCT GTCAAAAGGC CACTTCCTCC TTCTCCAGAG

TTCAGCTGCA AACCTGATCT GTCAAAAGGC CACTTCCTCC TTCTCCAGAG

---------- AACCTGATCT ATCAAAAGGC CACTTCCTCC TTCTCCAGAG

TTCAGCTGCA -ACCTGATCT ATCAAAAGGC CACTTCCGTC TTCTCCAGAG

TTCAGCTGTA AACCTGATCT GTCAAAAGGC CACTTCCTTC TTCTCCAGAG

TTCAGCTGCA CACCTGATCT GTCAAAAGGC CATTTCCTTC TTCTCCAGAG

TTCAGCTGC- AACCTGATCT ATCAAAAGGC CATTTCCTCC TTCTCCAGAG

TTCAGCTGCA AACCTGATCT ATCAAAAGGC CATTTCCTTC TTCTCCAGAG

TTCAGCTGCA AACCTGATCT ATCAAAAGGC CACTTCCTTC TTCTCCAGAG

TTCAGCTGCA ---------- ---------- ---------- ----------

TTCAGCTGCA AACCTGATCT ATCAAAGGGC CATTTCCTTC TTCTCCAAAG

TTCGGCTGCA CACCTGATCT ATCAAATGGC CATTTCCTTC TCCTCCAGAG

GACACGGTCT GTCCAGCAGG GGTGCGGCAC TTTTCACTGG TGTCATAGTC

GACACGGTCT GTCCAGCAGG GGTGCGGCAC TTTTCACTGG TGTCATAGTC

GACACGGTCT GTCCAGCAGG GGTGCGGCAC TTTTCACTGG TGTCATAGTC

GACACGGTCT GTCCAGCAGG GGTGCGGCAC TTTTCACTGG TGTCATAGTC

GACACGGTCT GTCCAGCAGG GGTGCGGCAC TTTTCACTGG TGTCATAGTC

---------- ---------- ---------- ---------- ----------

GACTCGGTCT GTCCAGCAGG GGTGCGGCAC TTTTCACTGG TGTCATAGTC

GACACGGTCT GTCCAGCAGG GGTGCGACAC TTCTCACTGG TGTCATAGTC

GACACGATCT GTCCAGCAGG GGTACGGCAC TTCTCACTGG TATCATAGTC

GACACGGTCT GTCCAGCAGG GGTGCGGCAC TTTTCACTAG TGTCGTAGTC

GACACGGTCT GTCCAGCAGG GGTGCGGCAC TTTTCACTGG TGTCATAGTC

GACACGGTCT GTCCAGCAGG GGTGCGGCAC TTTTCACTCG TGTCATAGTC

GACACGGTCT GTCCAGCAGG GGTGCGGCAT TTCTCACTGG TATCATAGTC

GACACGATCT GTCCAGCAGG GGTGCGGCAC TTTTCACTGG TGTCATAATC

GACACGGTCC GTCCAGCTGG GGTGCGGCAC TTTTCACTGG TGTCATAGTC

---------- ---------- ---------- ---------- ----------

AATGCGATCT GTCCAGCTGG AGTGCGACAT TTCTCACTGG TATCATAGTC

GATGCGATCC GTCCAGCCGG GGTGCGGCAT TTTTCACTGG TATCGTAGTC

GTCAGAAAAC AAGTCATACT TATATGTCGG AGCAAAGGTT ACCTTTCCTT

GTCAGAAAAC AAGTCATACT TATATGTCGG AGCAAAGGTT ACCTTTCCTT

GTCAGAAAAC AAGTCATACT TATATGTCGG AGCAAAGGTT ACCTTTCCTT

GTCAGAAAAC AAGTCATACT TATATGTCGG AGCAAAGGTT ACCTTTCCTT

ATCAGAAAAC AAGTCATACT TATATGTTGG AGCAAAGGTC ACCTTTCCCT

---------- ---------- ---------- ---------- ----------

TTCGGAAAAC AAGTCATATT TATAGGTTGG AGCAAACGTT ACTTTTCCCT

TTCAGAAAAC AAGTCATATT TATAGGTTGG AGCAAACGTT ACTTTTCCCT

ATCAGAAAAC AAATCATATT TGTACGTCGG AGCAAAGGTA ACTTTTCCCT

ATCAGAAAAC AAGTCGTATT TATACGTTGG AGCAAAGGTC ACTTTTCCTT

GTCAGAAAAC AAGTCATATT TGTATGTCGG AGCAAAGGTC ACTTTTCCTT

TTCAGAAAAC AAATCATATT TATATGTCGG AGCAAAGGTT ACTTTTCCTT

GTCCGAAAAT AAATCATATT TGTATGTTGG AGCAAAGGTT ACTTTTCCCT

ATCAGAAAAC AAATCATATT TATATGTAGG AGCAAAAGTC ATTTTTCCTT

ATCAGAAAAC AAATCATACT TATATGTCGG AGCAAAAGTT ACTTTCCCTT

---------- ---------- ---------- ---------- ----------

ATCAGAAAAC AAATCATACT TGTAGGTAGG AGCAAAAGTT ATTTTTCCTT

ATCAGAAAAC AAGTCATATT TGTACGTAGG AGCGAAAGTT ACTTTTCCCT

CTAAAAATCC TCTAAAAACC TAAAATAAAC ATATACCTGT CCAGCATTTT

CTAAAAATCC TCTAAAAATC TAAAATAAAC ATATACCTGT CCAGCATTTT

CTAAAAATCC TCTAAAAATC TAAAATAAAC ATATACCTGT CCAGCATTTT

CTAAAAATCC TCTAAAAATC TAAAATAAAC ATATACCTGT CCAGCATTTT

CTAAAAATCC TCTAAAAATC TAAAATAGAC ATATACCTGT CCGGCATTTT

---------- ---------- ---------- ---TACCTGT CCAGCATTTT

CTAAAAATCC TCTAAAGATC TAAAATAAAC ATATACCTGT CCTGCATTTT

CTAAGAATCC TCTAAAGATC TAAAATAAAC ATATACCTGT CCTGCATTTT

CTAAAAATCC TCTGAAAATC TAAAATAAAC ACA------- ----------

CTAAAAATCC TCTAAAAATC TAAAATAAAC ATATACCTGT CCAGCATTTT

CTAAAAATCC TCTAAAAATC TAAAACAAAC ATATACCTGT CCAGCATTTT

CTAAAAATCC TCTAAAAATC TATAATAAAC ATATACCTGT CCAGCATTTT

CCAAGAATCC TCTAAAAATC TAAAACAAAC ACA------- ----------

CTAAAAATCC TCTAAAAATC TAAAATAAAC ATATACCTGT CCAGCATTTT

CTAAAAATCC TCTAAAAATC TAAAATAAAC ATATACCTGT CCAGCATTCT

--AAAAACCC TCGAAAGATC TAAAAGAAAC ATATACCTGT CCAGTATTTT

CTAAAAATCC TCTAAAGATC TATGGCAAAC ATATACCTGT CCAGCATTTT

CTAAGAATCC TCTAAAAATC TAAAGGAAAC AAATACCTGG CCAGCATTTT

TCTGATTGAT AAGTTGATCT CCTGCTATAA GAGAATCCCA ATTTTGCTGT

TCTGATTGAT AAGTTGATCT CCTGCTATAA GAGAATCCCA ATTTTGCTGT

TCTGATTGAT AAGTTGATCT CCTGCTATAA GAGAATCCCA ATTTTGCTGT

TCTGATTGAT AAGTTGATCT CCTGCTATAA GAGAATCCCA ATTTTGCTGT

TCTGATTGAT AAGTTGATCT CCTGCTATAA GAGAATCCCA GTTTTGCTGT

TCTGATTGAT AAGTTGATCT CCTGCTATAA GAGAATCCCA ATTTTGCTGT

TCTGATTGAT GAGTTGATCT CCAGCTATAA GAGAATCCCA ATTTTGCTGT

TCTGGTTGAT GAGCTGATCC CCAGCGATCA GAGAATCCCA GTTTTGTTGT

---------- ---------- ---------- ---------- ----------

TCTGATTGAT AAGCTGATCT CCTGCGATAA GAGAATCCCA GTTTTGCTGT

TTTGATTGAT AAGCTGATCT CCTGCTATAA GAGAATCCCA ATTTTGCTGT

TCTGGTTGAT AAGTTGATCT CCTGCTATAA GAGAATCCCA ATTTTGCTGT

---------- ---------- ---------- ---------- ----------

TCTGATTGAT AAGCTGATCT CCTGCTATAA GAGAATCCCA ATTTTGTTGT

TCTGATTGAT TAGCTGATCT CCTGCTATAA GAGAATCCCA ATTTTGCTGT

TCTGATTGAT TAGTTGATCT CCTGCTATAA GAGAATCCCA ATTTTGCTGT

TCTGATTGAT AAGCTGATCT CCTGCTATAA GTGAATCCCA GTTCTGGTGT

TCTGATTGAT AAGTTGGTCT CCCGCGATAA GGGAATCCCA GTTCTGCTGC

CTTATGAGCT CTTTAACTTC TTCGTTAGGG AGATCGATTC GATAGTTGAA

CTTATGAGCT CTTTAACTTC TTCGTTAGGG AGATCGATTC GATAGTTGAA

CTTATGAGCT CTTTAACTTC TTCATTAGGG AGATCAATTC GATAGTTGAA

CTTATGAGCT CTTTAACTTC TTCATTAGGG AGATCAATTC GATAGTTGAA

CTTATGAGCT CTTTAACTTC TTCGTTAGGC AGATCGATTC GATAGTTGAA

CTTATAAGCT CTTTAACTTC TTCATTAGGG AGATCGATTC GATAGTTGAA

CTTATGAGCT CTTTAACTTC TTCATTAGGA AGATCGATTC GATAGTTGAA

CTTATGAGCT CTTTAACCTC TTCATTCGGA AGGTCAATTC GATAGTTGAA

---------- ---------- ---------- ---------- ----------

CTGATAAGCT CTTTCACTTC CTCATTAGGG AGATCGATTC GGTAGTTGAA

CTTATGAGCT CTTTAACTTC TTCATTAGGG AGATCAATCC GATAGTTGAA

CTTATGAGCT CTTTAACTTC TTCATTAGGG AGATCAATTC GATAGTTGAA

---------- ---------- ---------- ---------- ----------

CTTATGAGCT CTTTAACTTC TTCATTAGGG AGATCGATTC GATAGTTGAA

CTTATAAGCT CTTTAACTTC TTCGTTAGGG AGATCGATTC GGTAGTTGAA

CTTATGAGCT CTTTGACTTC TTCATTAGGG AGATCAATTC GATAGTTGAA

TTTATTAATT CCCTAACTTC TTCATTTGGT AGATCAATAC GATAATTAAA

CGTATCAGCT CCTTCACTTC TTCGTTGGGG AGATCGATAC GGTAATTAAA

ATCACCACAC CAAAATACAT AGTCATGGGA AAATAGCATC CTTCCCTGAA

ATCACCACAC CAAAATACAT AGTCATGGGA AAACAGCATC CTTCCCTGAA

ATCACCACAC CAAAATACAT AGTCATGGGA AAACAGCATC CTTCCCTGAA

ATCACCACAC CAAAATACAT AGTCATGGGA AAACAGCATC CTTCCCTGAA

ATCACCACAC CAAAATACAT AGTCATGGGA AAACAGCATT CTTCCCTGAA

ATCGCCACAC CAAAATACAT AGTCATGGGA AAAAAGCATC CTTCCCTGAA

ATCACCACAC CAGAATACGT AATCATGGGA GAAGAGCATT CTTCCCTGGA

ATCACCACAC CAAAATACAT AGTCATGGGA GAAGAGCATT CGTCCCTGGA

---------- ---------- ---------- ---------- ----------

ATCGCCACAC CAAAACACAT AGTCATGGGA GAAGAGCAGC CTTCCCTGGA

ATCACCACAC CAAAATACGT AGTCATGGGA AAACAGCATC CTTCCCTGAA

ATCACCACAC CAAAATACAT AGTCATGGGA AAACAGCATC CTTCCCTGGA

---------- ---------- ---------- AAACAGCATC CTTCCCTGCA

ATCGCCACAC CAAAACACAT AATCATGAGA AAACAGCATC CTTCCCTGGA

ATCTCCACAC CAAAACACAT AATCGTGGGA AAACAGCATC CTTCCCTGGA

ATCGCCACAC CAAAATACAT AATCATGGGA AAACAGCAGC CTCCCCTAGA

ATCACCACAC CAAAATACAT AGTCATGTGA TAACAACATC CTCCCCTGGA

GTCACCGCAC CAAAACACGT AGTCGTGGCA GAAGAGCATC CGTCCCTGGA

AGCAAAGCTT ACCATAGGAA AACTCAATTT TCGTGCTATT TCTATAAAAT

AGCAAAGCTT ACCATAGGAA AACTCAATTT TCGTGCTATT TCTATAAAAT

AGCAAAGCTT ACCATAGGAA AACTCAATTT TCGTGCTATT TCTACAAAAT

AGCAAAGCTT ACCATAGGAA AACTCAATTT TCGTGCTATT TCTACAAAAT

AGCAAAGCTT ACCATAGGAA AACTCAACTT TCGTGCTATT TCTACAAAAT

AGCAAAGCTT ACCATAGGAA AACTCAATTT TCGTGCTATT TCTACAAAAT

AAC-AAGCTT ACCATGGGGA AACTCAATTT TCGTGCTATT TCTACGAAAT

AAC-AAGCTT ACCATGGGGA AACTCAGTTT TCGTGCTATT TCTACAAAAT

---------- ---------- ---------- ---------- ----------

AGAAGAGTTT ACCATTGGAA AACTCAACTT CCGCGCTATT TCTACAAAAT

GGCAAAGCTT ACCATTGGAA AACTCAACTT CCGTGCAATT TCTACAAAAT

AGCAAGACTT ACCATAGGGA AACTCAGTTT CCGTACTATT TCTACAAAGT

AGCAATGCTT ACCATGGGAA AACTCAGCTT GCGTGCTATT TCAACAAAGT

AGCAAAGCTT ACCATAGGAA AACTCAATTT GCGAGCTATT TCTACAAAAT

AGCAAAGCTT ACCATGGGAA AACTCAGTTT CCGCGCTATC TCTACGAAAT

AGCAAACCTT ACCATGGGAA AACTCAATTT TC-------- ----------

AAAAAAGCTT ACCATAGGAA AGCTCAATCT TCTGGCAATT TCTACAAAAT

ACCAAAGCTC ACCATGGGAA ATGTTAGTTT TCGAGCTATT TCCCCAAAGT

CTTCATTTCT TTCTTTGACT TGTGACTGCC CTGCAGCAAA GTGGCTACAG

CTTCATTTCT TTCTTTGACT TGTGACTGCC CTGCAGCAAA GTGGCTACAG

CTTCATTTCT TTCTTTGACT TGTGACTGCC CTGCAGCAAA GTGGCTACAG

CTTCATTTCT TTCTTTGACT TGTGACTGTC CTGCAGCAAA GTGGCTACAG

CTTCATTTCT TTCTTTGACT TGTGACTGCC CTGCAGCAAA GTGACTACAG

CTTCATTTCT TTCTTTGACT TGTGACTGCC CTGCGGCAAA GTGGCTACAG

CTTCATTTCT CTCTTTGACT TGGGACTGGC CTGCAGCAAA GTGACTGCAG

CTTCATTTCT CTCTTTGACT TGGGACTGCC CTGCAGCAAA GTGGCTGCAG

---------- ---------- ---------- ---------- ----------

CATCATTTCG TTCTTTGACT TGGGATTGTC CCGCAGCGAA GTGGCTGCAG

CTTCATTTCT TTCTTTGACT TGTGATTGCC CTGCAGCAAA GTGGCTACAG

CTTCATTTCT TTCTTTGACT TGTGATTGTC CCGCAGCAAA GTGGCTGCAG

CTTCATTTCT TTCTTTGACC TGTGACTGGC CCGCGGCAAA GTGGCTACAG

CTTCATTTCT TTCTTTAACT TGTGATTGCC CTGCAGCAAA GTGGCTACAG

CCTCATTTCT CTCTTTCACC TGTGACTGCC CCGCAGCGAA GTGGCTGCAC

---------- ---------- ---------- ---------- ----------

CTTCATTCCT TTCTTTGACT TGTGATTGGC CTGCAGCAAA GTGACTACAG

CTTCATTTCT CTCCTTGACC TGCGACTGCC CCGCGGCGAA GTGACTACAG

ACGAAGCAAA GGCTGGTTGT ATGGAAGAGC ATTCGGATTG CAACTGCTCC

ACGAAGCAAA GGCTGGTTGT ATGGAAGAGC ATTCGGATTG CAACTGCTCC

ACAAAGCAAA GGCTGGTTGT ATGGAAGAGC ATTCGGATTG CAACTGCTCC

ACAAAGCAAA GGCTGGTTGT ATGGAAGAGC ATTCGGATTG CAACTGCTCC

ACGAAGCAAA GGCTGGTTGT ATGGAACAGC ATTCGGATTG CAACTGCTCC

ACGAAGCAAA GGCTGGTTGT ATGGAACAGC ATTCGGATGG CAACTGCTCC

ACAAAGCAAA GGCTGGTGGT GTGGAAGAGC ATCCGGATTG CAACAGCGCC

ACGAAGCAAA GGCTGGTGGT GTGGAAGAGC ATCCGAATTG CAACGGCGCC

---------- ---------- ---------- ---------- ----------

ACAAAGCAGA GGCTTGTGGT GTGGAACAGC ATTCGTATTG CAACTGCTCC

ACGAAGCAGA GGCTTGTTGT ATGGAATAGC ATTCGGATTG CAACTGCTCC

ACGAAGCAAA GACTTGTCGT GTGGAATAAC ATTCGGATTG CGACCGCTCC

ACAAAGCACA GGCTCGTGGT ATGGAACAGC ATGCGGATTG CCACTGCTCC

ACGAAGCAAA GGCTTGTTGT GTGGAATAAC ATTCGAATTG CAACTGCTCC

ACGAAGCAGA GGCTGGTGGT GTGGAACAGC ATCCGGATGG CAACTGCCCC

---------- ---------- ---------- ---------- ----------

ACAAAGCAAA GGCTAGTGGT GTGGAATAAC ATCCGTATTG CAACTGCTCC

ACGAAACAAA GGCTGGTCGT ATGGAACAGC ATCCGTATGG CGACTGCCCC

CTTATTTCCA GTTGCACCTC CCATTCCAGT CTTCACAGTA TCAACTGCAA

CTTATTTCCA GTTGCACCTC CCATTCCAGT CTTCACAGTA TCAACTGCAA

CTTATTTCCA GTTGCACCTC CCATTCCAGT CTTCACAGTA TCAACTGCAA

CTTATTTCCA GTTGCACCTC CCATTCCAGT CTTCACAGTA TCAACTGCAA

CTTATTTCCA GTTGCACCTC CCATTCCAGT CTTCACAGTA TCAACTGCAA

CTTATTGCCA GTTGCACCTC CCATTCCAGT CTTCACTGTA TCAACTGCAA

CTTATTTCCA GTCGCGCCTC CCATCCCAGT TTTCACAGTA TCGACTGCAA

CTTGTTTCCA GTTGCACCTC CCATGCCAGT TTTCACAGTA TCAACTGCAA

---------- ---------- ---------- ---------- ----------

CTTATTTCCA GTTGCGCCTC CCATTCCAGT TTTCACAGTA TCAACTGCAA

CTTATTTCCA GTGGCACCTC CCATTCCAGT CTTCACAGTA TCAACTGCAA

CTTATTTCCA GTGGCACCTC CCATTCCGGT CTTCACAGTA TCAACTGCGA

CTTATTTCCA GTGGCGCCGC CCATCCCAGT CTTCACGGTA TCGACTGCAA

CTTATTTCCA GTTGCACCTC CCATTCCAGT CTTTACAGTA TCAACTGCAA

CTTATTCCCA GTTGCACCTC CCATCCCAGT CTTCACAGTA TCAACTGCAA

---------- ---------- ---------- ---------- ----------

TTTATTTCCA GTAGCACCTC CCATTCCAGT TTTAACGGTA TCAAATCCAA

TTTATTCCCT GTCGCGCCTC CCATGCCAGT TTTCACCGTA TCAACTGCAA

CATCCCTTAC TGACCTGATA AAAGGAGCAT GCTGTGGTCT GATAAAAACA

CATCCCTTAC TGACCTGATA AAAGGAGCAT GCTGTGGTCT GATAAAAACA

CATCCCTTAC TGACCTGATA AAAGGAGCAT GCTGTGGTCT GATAAAAACA

CATCCCTTAC TGACCTGATA AAAGGAGCAT GCTGTGGTCT GATAAAAACA

CATCCCTTAC TGACCTGATA AAAGGAGCAT GCTGTGGTCT GATAAAAACA

CATCCCTTAC TGACCTGATA AAAGGAGCAT GCTGTGGTCT GATAAAAACA

CATCCCTAAC TGACCTGATA AAGGGAGCAT GCTGCGGTCT GATAAAGACA

CATCCCTAAC TGACCTGATA AAAGGAGCGT GCTGTGGTCT GATAAAGACA

---------- ---------- ---------- ---------- ----------

CATCCCTTAC TCACCTGATG AAGGGAGCGT GCTGTGGTCT GATAAAAACA

CATCCCTTAC TGACCTGATG AAAGGAGCAT GCTGTGGTCT GATAAAAACA

CATCCCTTAC TGACCTGATG AAAGGAGCAT GCTGAGGTCT GATGAAAACA

CATCCCTTAC TGACCTGATG AACGGAGCAT GCTGGGGCCT GATAAAAACG

CATCCCTTAC TGACCTGATG AAGGGAGCAT GCTGTGGTCT GATAAAAACA

CATCCCTTAC TGACCTGATA AAAGGAGCAT GCTGTGGTCT GATAAAAACA

---------- ---------- ---------- ---------- ----------

CATCCCTTAC TGACCTGATG AAAGGAGCAT GTTGGGGTCT GATAAAAACG

CATCCCTTAC TGACCTGATA AAGGGGGCGT GCTGGGGGCG GATGAAGACG

AACAAACAGA CGCCCACCAA CTGTTCAGAA GCCAGCAGCA CATACTTGTT

AACAAACAGA CGCCCACCAA CTGTTCAGAA GCCAGCAGCA CATACTTGTT

AACAAACAGA CGCCCACCAA CTGTTCAGAA GCCAGCAGCA CATACTTGTT

AACAAACAGA CGCCCACCAA CTGTTCAGAA GCCAGCAGCA CATACTTGTT

AACAAACAGA CGCCCACCAA CTGCTCAGAA GCCAGCAGCA CATACTTGTT

AACAGACAGA CGCCCACCAA CTGTTCAGAA GCCAGCAGCA CATACTTATT

AACAGGCAGA CACCCACCAA CTGCTCCGAG GCCAGGAGAA CATACTTGTT

AACAGGCAGA CACCCACCAG CTGCTCCGAG GCCAGCAGGA CATACTTGTT

---------- ---------- ---------- ---------- ----------

AACAGACAGA CGCCCACCAA CTGCTCAGAG GCCAGCAGCA CATACTTGTT

AACAGGCAGA CGCCCACCAG CTGCTCAGAA GCCAGCAGCA CATACTTGTT

AACAGGCACA CGCCCACCAG CTGCTCAGAA GCCAGCAGCA CGTACTTGTT

AACAGGCAGA CGCCTACCAA CTGCTCGGAT GCCAGCAGCA CATACTTGTT

AAGAGACAGA CGCCCACCAA TTGCTCAGAA GCCAGCAGCA CATATTTGTT

AAGAGGCAGA CGCCCACCAG CTGCTCAGAG GCCAGCAGCA CATACTTGTT

---------- ---------- ---------- ---------- ----------

TAGAGACAGA TGCCTACCAA TTGCTCTGAA GCCAGCAGCA CATACTTTCT

AAGAGGCAGA CCCCCACCAG CTGCTCGGAC GCCAGCAGCA CGTACTTGTT

GTCTCTGGAG ATTGTCTTCT GAAGTTCTAC AGCCCAGAGC TTCTGATTTG

GTCTCTGGAG ATTGTCTTCT GAAGTTCTAC AGCCCAGAGC TTCTGATTTG

GTCTCTGGAG ATTGTCTTCT GAAGTTCTAC AGCCCAGAGC TTCTGATTTG

GTCTCTGGAG ATTGTCTTCT GAAGTTCTAC AGCCCAGAGC TTCTGATTTG

GTCTCTGGAG ATTGTCTTCT GAAGTTCTAC AGCCCAGAGC TTCTGATTTG

GTCTCTGGAG ATTGTCTTCT GAAGTTCCAC AGCCCAGAGC TTCTGATTTG

GTCTCTGGAG ATGGTCTTCT GCAGTTCCAC TGCCCACAGC TTCTGGTTTG

GTCCCTGGAG ATGGTCTTCT GCAGCTCCAC TGCCCACAGC TTCTGGTTGG

---------- ---------- ---------- ---------- ----------

GTCTCTGGAG ATGGTCTTCT GAAGTTCCGC AGCCCAGAGC TTCTGATTTG

GTCTCTGGAG ATGGTCTTCT GCAGTTCCAC AGCCCAGAGC TTCTGATTGG

GTCTCTGGAG ACGGTCTTCT GAAGTTCCAC AGCCCAGAGC TTCTGATTTG

GTCCCTGGAG ATGGTCTTCT GAAGTTCCAC AGCCCAGAGC TTCTGATTTG

GTCTCTGGAG ATAGTCTTTT GAAGTTCCAC AGCCCAGAGC TTCTGATTAG

GTCTCTGGAG ACTGTCTTCT GAAGTTCCAC GGCCCAGAGC TTCTGATTAG

---------- ---------- ---------- ---------- ----------

ATCTCTGGAG AGAATCTTCT GTAGTTCAAC AGCCCATAAT TTCTGATTAG

ATCTCTGGAG ATGGTCTTCT GCAGTTCGGC AGCCCAGAGC TTCTGGTTCG

TTGTGCTCTT ACCTCTCACA ATGTTTCCAG CATTCAATTC TACCATTTCT

TTGTGCTCTT ACCTCTCACA ATGTTTCCAG CATTCAATTC TACCATTTCT

TTGTGCTCTT ACCTTTCACA ATGTTTCCAG CATTCAATTC TACCATTTCT

TTGTGCTCTT ACCTTTCACA ATGTTTCCAG CATTCAATTC TACCATTTCT

TTGTGCTCTT ACCTTTCACA ATGTTTCCTG CATTCAGTTC TACCATTTCT

TTGTGCTCTT ACCTTTCACA ATGTTTCCAG CATTCAACTC TACCATTTCT

TTGTGCTCTC ACCTTTCACA ATGTTCCCAG CATTCAGCTC CACCATTTCT

TTGTGCTCTT ACCTTTCACA ATGTTCCCAG CATTCAGCTC CACCATTTCT

---------- ---------- ---------- ---------- ----------

TTGTGCTCTT ACCTTTCACA ATGTTTCCAG CATTCAGCTC TACCATTTCC

TTGTGCTCTT ACCTTTCACG ATGTTTCCAG CATTTAACTC AACCATTTCT

TTGTGCTCTT ACCTTTCACA ATGTTTCCAG CATTCAACTC TACCATTTCT

TGGTGCTCTT ACCTTTCACA ATGTTTCCAG CATTCAGTTC TACCATTTCT

TTGTACTCTT ACCTTTCACT ATGTTTCCAG CATTCAACTC TACCATTTCT

TTGTGCTCTT ACCTTTCACA ATGTTTCCAG CATTCAACTC CACCATTTCT

-------CTT ACCTTTCACA ATGTTTCCAG CATTCAACTC TACCATTTCT

TTGTACTTTT AAATCTCACA ATGTTTCCAG CAAGTAATTC TACCATTTCT

TCGTGCTCTT ACCTTTCACT ATATTTCCTG CATTTAATTC CACCATTTCT

TCAAAACCAA TTGCAAATAT ATCAGTTGGC TTACTTCTTT TATCTAAAAT

TCAAAACCAA TTGCAAATAT ATCAGTTGGC TTACTTCTTT TATCTAAAAT

TCAAAACCAA TTGCAAATAT ATCAGTTGGC TTACTTCTTT TATCTAAAAT

TCAAAACCAA TTGCAAATAT ATCAGTTGGC TTACTTCTTT TATCTAAAAT

TCAAAACCAA TTGCAAATAT ATCAGTTGGT TTACTTCTTT TATCTAAAAT

TCAAAACCAA TTGCAAATAT ATCAGTTGGC TTACTTCTTT TATCTATAAT

TCAAAGCCAA TTGCAAATAT ATCAGTTGGC TTACTTCTCT TATCTACAAT

TCAAAGCCAA TTGCAAATAT ATCAGTTGGC TTACTTCTCT TATCTACAAT

---------- ---------- ---------- ---------- ----------

TCAAAACCAA TTGCAAATAT ATCCATTGGT TTACTTCTTT TATCTAAAAT

TCAAAACCAA TTGCAAATAT ATCTGTTGGC TTACTTCTTT TATCTACAAT

TCAAAACCAA TTGCAAATAT ATCCGTTGGC TTACTTCTTT TATCTACAAT

TCAAAGCCAA TTGCAAATAT ATCCGTTGGC TTACTTCGTT TGTCTGCAAT

TCAAAACCAA TTGCAAATAT ATCGGTTGGC TTACTTCTTT TATCTACAAT

TCAAAACCAA TTGCAAATAT ATCAGTTGGC TTACTTCTTT TATCTATAAT

TCAAAACCAA TTGCAAATAT ATCGGTTGGC TTACTTCTTT TATCTGTAAT

TCAAAACCAA TTGCAAATAT GTCAGTAGGC TTATCTCTTT GATCTAAAAT

TCAAAGCCGA TTGCAAATAT GTCCGTAGGC TTGCTTCTCT GATCTATAAT

TAACATCCAA GAAAACCTTG AAACTCCTGG ATGCCAGCTA ACTTGGGTGC

TAACATCCAA GAAAACCTTG AAACTCCTGG ATGCCAGCTA ACTTGGGTGC

TAACATCCAA GAAAACCTTG AAACTCCTGG ATGCCAGCTA ACTTGGGTGC

TAACATCCAA GAAAACCTTG AAACTCCTGG ATGCCAGCTA ACTTGGGTGC

TAACATCCAA GAAAACCTTG AAACTCCTGG ATGCCAGCTA ACTTGGGTGC

TAACATCCAA TAAAACCTTG AAACTCCTGG ATGCCAGCTA ACTTGGGTGC

TAACATTCAA -AAAACCTTG AAACTCCTGG ATGCCAGCTA ACTTGGGTGC

TAACATCCAA -AAAACCTTG AAACTCCTGG ATGCCGGCTA ACTTGGGAGC

---------- ---------- ---------- ---------- ----------

TAACATCCAA GAAAACCTTG AAACTCCTGG ATGCCAGCTA ACTTGGGGGC

TAACATCCAA GAAGACCTTG AAATTCCTGG ATGCCAGCTA ACTTAGGTGC

TAACATCCAA AGAAACCTTG AAACTCCTGG ATGCCAGCTA ACTTGGGTGC

TAACATCCAA GAAAACCTTG AAATTCCTGG ATGCCAGCCA ACTTGGGTGC

TAACATTCAA GAAAACCTTG AAACTCTTGG ATGCCAGCTA ATTTGGGTGC

TAACACCCAA GAAAACCTTG GAATCCCTGG ATGCCAGCTA GCTTGGGCGC

TAACATCCAA GAA------- ---------- ---------- ----------

TAATATTTGA GAAAACCTTG AAGTTCTTGG ATACTAGTTA ACACTGGAGT

TAACATCGGA GAACACCTTG GAACTCCTGG ACCCCGGCCA GTTTGGGGGC

ATCAAGAAGC CAGTCAGTGA GTGTCTGATT CTTAAAAGCT ATGCTGCGAA

ATCAAGAAGC CAGTCAGTGA GTGTCTGATT CTTAAAAGCT ATGCTGCGAA

ATCAAGAAGC CAGTCAGTGA GTGTCTGATT TTTAAAAGCT ATGCTGCGAA

ATCAAGAAGC CAGTCAGTGA GTGTCTGATT TTTAAAAGCT ATGCTGCGAA

ATCAAGAAGC CAGTCAGTGA GTGTCTGATT CTTGAAAGCT ATGCTGCGAA

ATCAAGAAGC CAGTCAGTCA GCGTCTGATT CTTAAAAGCT ATACTGCGAA

ATCAAGAAGC CAGTCGGTGA GGGTCTGGTT CTTGAATGCT ATGCTGCGGA

GTCCAGAAGC CAGTCTGTGA GCGTCTGGTT CTTGAACGCT ATGCTGCGGA

---------- ---------- ---------- ---------- ----------

ATCAAGAAGC CAGTCCGTAA GGGTCTGATT CTTAAAAGCT ATGCTGCGAA

ATCAAGAAGC CAGTCGGTGA GTGTCTGATT CTTAAAAGCT ATACTGCGAA

ATCAAGAAGC CAGTCGGTGA GTGTCTGATT CTTAAAAGCT ATACTGCGAA

GTCCAGAAGC CAGTCAGTGA GCGTTTGGTT CTTAAAAGCT ATGCTGCGAA

ATCAAGCAGC CAGTCAGTGA GTGTCTGATT CTTAAAAGCT ATGCTGCGAA

ATCCAGCAGC CAGTCAGTGA GCGTCTGATT CTTGAAAGCT ATGCTGCGAA

---------- ---------- ---------- ---------- ----------

CTCTAGTAGC CAATCAGTAA GTGTCTGATT CTTAAAGGCA ATGCTTCGAA

GTCGAGCAGC CAGTCGGTGA GGGTCTGGTT CTTAAAAGCG ATGCTGCGGA

ATTGCTTCCC ACCATTCACA TTCCAGGTTC CGACACATAC TCGAATTTTC

ATTGCTTCCC ACCATTCACA TTCCAGGTTC CGACACATAC TCGAATTTTC

ACTGCTTCCC ACCATTCACA TTCCAGGTTC CGACACATAC TCGAATTTTC

ACTGCTTCCC ACCATTCACA TTCCAGGTTC CGACACATAC TCGAATTTTC

ATTGTTTCCC ACCATTCACG TTCCAGGTTC CAACACATAC TCGAATTTTC

ACTGCTTCCC ACCATTCACA TTCCAGGTTC CGACACATAC TCGAATTTTC

ACTGTTTCCC GCCGTTCACA TTCCAGGTTC CAACACACAC TCGGATCTTC

ACTGCTTCCC GCCGTTCACA TTCCAGGTGC CAACACACAC TCGGATCTTC

---------- ---------- ---------- ---------- ----------

ACTGCTTCCC GCCATTCACG TTCCATGTGC CCACACATAC TCGAATTTTC

ACTGCTTCCC ACCATTCACG TTCCAGGTTC CAACACATAC TCGAATTTTC

ACTGTTTCCC ACCGTTCACA TTCCAGGTTC CAACACATAC TCGGATTTTC

ACTGCTTCCC GCCATTCACG TTCCAGGTGC CAACACACAC ACGGATTTTC

ACTGCTTCCC TCCATTCACA TTCCAGGTTC CAACACATAC TCGAATTTTC

ACTGCTTCCC TCCGTTCACG TTCCAGGTCC CAACACACAC TCGGATTTTC

---------- ---------- ---------- ---------- ----------

ACTGTTTCCC ACCATTCACA TTCCAGGTTC CAATACACAC TCGAATTTTC

ACTGCTTGCC CCCGTTGACG TTCCACGTTC CGGCGCACAC TCGGATCTTC

TTAGGCTTTG AATATTTGTA GAAATTCTCA CACATGCTCT TTAGTACTTT

TTGGGCTTTG AATATTTGTA GAAATTCTCA CACATGCTCT TTAGTACTTT

TTGGGCTTTG AATATTTGTA GAAATTCTCA CACATGCTCT TTAGTACTTT

TTGGGCTTTG AATATTTGTA GAAATTCTCA CACATGCTCT TTAGTACTTT

TTCGGCTTTG AATATTTGTA GAAATTCTCA CACATGCTCT TTAGTACTTT

CTGGGCTTTG AATACTTGTA AAAATTCTCA CACATGCTCT TTAGTACTTT

TTGGGCTTTG AGTACTTGTA GAAGTTCTCA CACATGTTCT TTAAGACTTT

TTGGGCTTTG AGTATTTGTA GAAGTTCTCA CACATGTTCT TTAAGACTTT

---------- ---------- ---------- ---------- ----------

TTGGGCTTTG AATACTTGTA GAAATTCTCA CACATACTCT TTAGTACTTT

TTGGGCTTTG AATACTTGTA GAAATTCTCA CACATGCTCT TTAGTACTTT

TTGGGCTTTG AATACTTGTA GAAATTCTCA CACATGTTCT TTAGTACTTT

TTGGGCTTCG AATACTTGTA GAAATTCTCA CACATGCTCT TTAGGACTTT

TTGGGTTTTG AATACTTATA GAAATTCTCA CACATGCTCT TTAGTACTTT

TTGGGCTTTG AATACTTGTA GAAATTCTCA CACATGCTCT TTAGGACTTT

---------- ---------- ---------- ---------- ----------

TTGGGTTCTG TATATTTATA GAAGTTCTCA CACATACTCT TTAGTATCTT

CTAGGCTTGG AATACTTGTA GAAGTTCTCA CACATACTCT TCAGTACTTT

AGAAGATGCT AATCAAGAGA AGAATTTCTA TACTGTACCT GACTGTAATG

AGAAGATGCT AATCAAGAGA AGAATTTCTA TACTGTACCT GACTGTAATG

AGAAGATGCT AATCAAGACA AGAATTTCTA TACTGTACCT GACTGTAATG

AGAAGATGCT AATCAAGACA AGAATTTCTA TACTGTACCT GACTGTAATG

AGAAGATGCT AAACGAGACA AGAATTTCTA TACTGTACCT GACTGTAATG

AGAAGATGCT AATCAAAACA AGAATTTCTA TACTGTACCT GACTGTAATG

GGAAGATGCT AGACAAAACA AGAATTTCTA TACTGTACCT GACTGTAATG

GGAAGATGCT AATCAAAACA AGAATTTCTA TACTGTACCT GACTGTAATG

---------- ---------- ---ATTTCTA TACTGTACCT GACTGTAATG

AGAAGATGCT GGT-AAAACA AAAATTTCTA TACTGTACCT GACTGTAACG

GGAAGATGCT AATCAAAACA AAAATTTCTA TACTGTACCT GACTGTAATG

AGAAGAGGCT AAT-AAAACA AAAATTTCTA TACTGTACCT GACTGTAATG

AGACGACGCT AATCAAAACA AAAATTTCTA TACTGTACCT GACTGTAATG

AGAAGATGCT ATTCAAAACA AAAGTTTCTA TACTGTACCT GACTGCAATG

AGAAGATGCT GTTCAAAACA AAAATTTTGA TACTGTACCT GACTGTAACG

---------- ---------- ---ATTTCTA TACTGTACCT GACTGTAATG

TGGAGATGCT GTGTAAAACA AAAATTGCTG TACTATACCT GACTGCAATG

TGTGGATGCT GTTCAAAACA ACAGTTACTG TACTATACCT GACTGTAACG

TCTGCTCAGA AACTATGGAT GCAACAAGAA GAAAACAAAG AATCATTAGT

TCTGTTCAGA AACTATGGAT GCAACAAGAA GAAAACAAAG AATCATTAGT

TCTGTTCAGA AACTATGGAT GCATCAAGAA GAAAAAAAAG AATCATTAGT

TCTGTTCAGA AACTATGGAT GCATCAAGAA GAAAACAAAG AATCATTAGT

TCTGTTCAGA AACTATGGAT GCATCAAGAA GAAAACAAAG AATCATTAGT

TCTGTTCAGA AACTATGGAT GCATCAAGAA GAAAACAAAG AATCATTAGT

TCTGTTCAGA CACTATGGAT GCATCAAGAA GAAAACAAAG AATCATTAGT

TCTGTTCAGA AACTATGGAT GCATCAAGAA GAAAACAAAG AATCATTAGT

TCTGTTCAGA AACTATGGAT GCATCAAGAA GAAAACAAAG AGTCATTAGT

TCTGTTCAGA AACTACGGAT GCATCAAGAA GAAAACAAAG AGTCATTAGT

TCTGTTCAGA AACTATGGAT GCATCAAGAA GAAAACAAAG AATCATTAGT

TCTGTTCAGA AACTATCGAT GCATTAAGAA GAAAACAAAG AATCATTAGT

TCTGTTCAGA AACTAGGGAT GCAGCAAGAA GAAAACAAAG AATCATTAGT

TCTGTTCAGA AACTACGGAT GCATCAAGAA GAAAAAAAAG AGTCATTAGT

TCTGTTCAGA AACTATGGAT GCACCAAAAA GAAAACAAAG AGGCATTAGT

TCTGTTCAGA AACTATGGAT GCATCAAGAA GGAGAGAAAG ATTCATTAGT

TCTGCTCAGA AACTTTGAAT GCATCAAGAA AAAAACAAGA AGACATTAGT

TCTGCTCAGA AACTATGGGT GCATCAAGAA GAAAACAAGA AGAAATTAGT

AAAACATACC ACGCAAACTT CCAGTAGTTA AAAGTGCTCG AGCTTTGTCA

AAAACATACC ACGCAAACTT CCAGTAGTTA AAAGTGCTCG AGCTTTGTCA

AAAACATACC ACGCAAACTT CCAGTAGTTA AAAGTGCTCG AGCTTTGTCA

AAAACATACC ACGCAAACTT CCAGTAGTTA AAAGTGCTCG AGCTTTGTCA

AAAACATACC ACGCAAACTT CCAGTAGTTA AAAGTGCTCG AGCTTTGTCA

AAAACATACC ACGCAAACTT CCAGTAGTTA AAAGAGCTCG AGCTTTGTCA

AAAACATACC ACGCAAACTC CCAGTAGTTA GAAGTGCCCG GGCTTTGTCG

AAAACATACC ACGCAAACTT CCAGTAGTTA GAAGCGCTCG GGCTTTGTCA

AAAA------ ---------- ---------- ---------- ----------

AACACCTACC ACGCAAACTT CCAGTAGTTA AAAGGGCTCG AGCTTTGTCA

AAAACATACC ACGCAAACTT CCTGTAGTTA GAAGTGCTCG AGCTTTGTCA

AAAACATACC TCGCAAACTT CCAGTTGTTA AAAGTGCTCG AGCTTTGTCA

CAAACATACC ACGCAAACTT CCAGTAGTTA AAAGTGCTCT AGCTTTGTCA

AAAACATACC ACGCAAACTT CCAGTAGTTA AGAGTGCTCG AGCTTTGTCA

AAAACATACC TCGCAGACTT CCAGTAGTTA GGAGCGCTCG AGCTTTGTCA

AAAA------ ---------- ---------- ---------- ----------

AAAACATACC TCTTAAGCCT CCAGTAGTTA AGAGTGCTCG AGCTTTATCT

TAAACGTACC ACGGAGGCTT CCGGTGGTTA GGAGCGCTCG GGCTTTATCG

GCTAAATCAC TATTCAGAGT ATTTCCCAGA GCAAAACATC AATGGCCTCT

GCTAAATCAC TATTCAGAGT ATTTCCCAGA GCAAAACATC AATGGCCTCT

GCTAAATCAC TATTCAGAGT ATTTCCCAGA GCAAAACATC AATGGCCTCT

GCTAAATCAC TATTCAGAGT ATTTCCCAGA GCAAAACATC AATGGCCTCT

GCTAAATCAC TATTCAGAGT ATTTCCCAGA GCAAAACATC AATGGCCTCT

GCTAAGTCAC TATTTAGAGT ATTTCCCAGA GCAAAACATC AATTGCCTCT

GCTAAGTCGC TGTTCAGAGT GTTTCCCAGA GCAGAACATC AATGGCTTCT

GCTAAATCGC TGTTGAGAGT ATTTCCCAGA GCAGGACATC AATGGCTTCC

---------- ---------- ---------- ---------- ----------

GCTAAATCAC TATTTAGAGT ATTTCCCAGA GCAAAACATC AATTGCTTCT

GCTAAATCAC TATTTAGAGT ATTTCCCAGA GTAAAACATC TATGGCCTCT

GCTAAATCAC TATTTAGAGT ATTTCCCAGA GCAAAATGTC AATGGCCTCT

GCTAAGTCAC TATTTAGAGT ATTTCCCAGA GCAAAACATC AATGGCCTCT

GCTAAATCGC TATTTAGAGT ATTTCCAAGA GCAAAACATC AATGGCCTCT

GCTAGATCAC TGTTCAGAGT GTTTCCCAGA GCAAAATGTC AATGGCCTCT

---------- ---------- ---------- ---------- ----------

GCCAAGTCAC TATTTAAAGT ACTCCCCAAA GCAAAATATC AATGGCTTCT

GCCAGGTCGC TGTTGAGCGT ATTCCCCAGA GCAAAACGTC GATGGCCTCT

TGCTTGGAGC TGTCAAAGAA GTTATTCTGA ATTGTTCGGG TAACAGAGCG

TGCTTGGAGC TGTCAAAGAA GTTATTCTGA ATTGTTCGGG TAACAGAACG

TGCTTGGAGC TGTCAAAGAA GTTATTCTGA ATTGTCCGGG TAACAGACCG

TGCTTGGAGC TGTCAAAGAA GTTATTCTGA ATTGTCCGGG TGACAGACCG

TGCTTAGAGC TGTCAAAGAA GTTATTCTGA ATTGTTCTGG TAACAGAACG

TGCTTGGAGC TGTCAAAGAA GTTATTTTGA ATTGTTCTGG TTACAGAACG

TGCTTGGAGC TGTCGAAGAA GTTATTCTGA ATGGTTCTGG TGACAGATCG

TGCTTGGAGC TGTCGAAGAA GTTATTCTGG ATGGTTCTGG TGACAGATCG

---------- ---------- ---------- ---------- ----------

TGCTTGGAGC TGTCAAAGAA GTTATTCTGA ATTGTTCTAC TAACTGAACG

TGCTTGGAGC TGTCAAAGAA GTTATTCTGA ATCGTTCTAG TAACAGAACG

TGCTTGGAGC TGTCAAAGAA GTTATTTTGA ATCGTTCTAG TAACAGAACG

TGCTTGGAGC TGTCAAAGAA GTTATTCTGA ATTGTTCGAG TCACAGAACG

TGCTTGGAGC TGTCAAAGAA ATTATTCTGA ATTGTTCTAG TAACAGAACG

TGCTTGGAGC TGTCAAAGAA GTTGTTCTGA ATTGTTCTGG TAACAGAGCG

---------- ---------- ---------- ---------- ----------

TGCTTGGAGC TGTCAAAGAA GTTGTTCTGA ATTGTTCTGG TTACAGAACG

TGCTTGGAGC TGTCAAAGAA GTTATTCTGA ATCGTTCTGG TTACGGAGCG

AGCACCATCT TTTAACTTTC CAGCCTGAAA AGAACTCACC ATTATCAGGC

AGCACCATCT TTTAACTTTC CAGCCTGAAA AGAACTCACC ATTATCAGGC

AGCACCATCT TTTAACTTTC CAGCCTGAAA AGAACTCACC ATTATCAGGC

AGCACCATCT TTTAACTTTC CAGCCTGAAA AGAACTCACC ATTATCAGGC

AGCACCATCT TTTAACTTTC CAGCCTGAAA AGAACTCACC ATTATCAGGC

AGCACCATCT TTTAACTTTC CAGCCTGAAA AGAACTCACC ATTATCAGGC

CGCACCATCT TTTAACTTTC CAGCCTGAAA GAAACTCACC ATTATCAGGC

CGCACCATCT TTTAACTTTC CAGCCTGAAA GAGACTCACC ATTATCAGGC

---------- ---------- ---------- ---------- ----------

AGCACCATCT TTTAACTTTC CAGCCTGAAA AGGACTCACC ATTATCAGGC

AGCACCATCT TTTAACTTTC CAGCCTGAAA AGAACTCACC ATTATCAGGC

AGCGCCATCT TTTAACTTTC CAGCCTGAA- -GTACTCACC ATTATCAGGC

AGCACCATCT TTTAACTTTC CAGCCTGAAA AGGACTCACC ATTATCAGGC

AGCACCATCT TTTAACTTTC CAGCCTGAAA AGAACTCACC ATTAACAGGC

AGCTCCATCT TTTAACTTCC CACCCTGAAA AGAACTCACC ATT-TCAGGC

---------- ---------- -------AAA AGAACTCACC ATTATCAGGC

AGCACCATCT TTTAACTTTC CAGACTGAAA AGGACTCACC ATTGTCAGGC

AGCGCCATCC TTCAGCTTCC CAGCCTGAAA AAAACTCACC ATTATCAGGC

AATTAATCAA AAGGAACCCA GAGAAAGGAA AAAAAGAGCA GCAGATGTTA

AATTAATCAA AAGGAACCCA GAGAAAGGAA AAAAAGAGCA GCAGATGTTA

AATTAATCAA AAGGAACCCA GAGAAAGGAA AAAAAGAGCA GCAGATGTTA

AATTAATCAA AAGGAACCCA GAGAAAGGAA AAAAAGAGCA GCAGATGTTA

AA-TAAT--- AAGGAACCCA GAGAAAGGAA AAAAAGAGCA GCAGATGTTA

AATTAGTCAA AAGGAACCCA GAGAAAGGAA AAAAAGAGCA GCAGATGTTA

AATTAATCAA AAGGAGCCGA GAGAAAGGAA AAAA--AGCA GTGGATGTTA

AATTAATCAA AAGGAGCCGA GAGAAAGGAA AAAA--AGCA GCAGATGTTA

---------- ---------- ---------- ---------- ----------

AATTAATCAA AAGGAACCCA GAGAAAGGAA AAAAAGAGCA GCAGATGTTA

AATTAATCAA AAGGAACCCA GAGAAAGGAA AAAAAGAGCA GCAGATGTTA

AATTAATCAA AAGGAACCCA GAGAAAGGAG AAAAAGAACA GCAGATGTTA

AATTAACCAA AAGGAACCCA GAGAAAGGAA AAAAAGAGCA GCAGAAGTTA

AATTAATCAA AAGGAGCCCA GAGAAAGGGA AAAAAGAGCA GCAGATGTTA

AATTAATCAA AGGGAACCCA GAGAAAGGAA AAAAAAAGCA GCAGATGTTA

AATTAATCAA AAGGAACCCA GAGAAAGGAA AAAAAGAGCA GCAGATGTTA

AGTTAATCAA AAGGAACCCA GAGAAAGCAA AAAAAGAGCA GCAGAGATTA

AATTAATCGA AAGGAACGCG GAGAAAGCAA AAAGAGAGCA AGAGATGTTA

GATTTAGTTA ATTCAGGCaa tatctattat GTTAAAGATA CCTTCGCTTT

GATTTAGTTA ATTCAGGCaa tatctattat GTTAAAGATA CCTTCGCTTT

GATTTAGTTA AATCAGACAA TATCTGTTAT GTTAGAGATA CCTTCGCTTT

GATTTAGTTA ATTCAGACAA TATCTGTTAT GTTAGAGATA CCTTCGCTTT

GATTTAGTTA ATTCAGACAA TAT----TAT GTTAAAGATA CCTTCGCTTT

GATTTAGTTA AATCAGACAA TATCTATTAT GCT----ATA CCTTAGCCTT

GATTTAGCTA ATTCAGACAA TATCTATTAT GTTAGAGATA CCTTGGCCTT

GATTTAGCTA ATTCAGACAA TATCTATTAT GTT--AGATA CCTTGGCCTT

---------- ---------- ---------- ---------- ----------

GATTTAGGTA ATTCAGACAA TATCTAGTTT GTTAG---TA CCTTAGCCTT

GAGTTAGTTA ATTCAGGCAA TTTCTATTAT GTTAGAGATA CCTTAGCCTT

GAGTTAGTTA ATACAGACAA TATCTATCAG GTTAGAGATA CCTTAGCCTT

GATTTAGTTA ATCCAAACAA TATCTACT-T GTTAGAGATA CCTTTGCCTT

GATTTAGTTA GATCAGACAA TATCTATTAT GTTAAAGATA CCTTGGCCTT

GACTTAGT-A ATTCAGACAA TACCTAT-AC ATTACTGATA CCTTGGCCTT

GATTTAGTTC ATTCAGACAA TAGCTATTAC ATTAAAGATA CCTTGGCCTT

GAATTAGGAA GTCAAGATAA TATTTAAAAT GTTTTAGATA CCTTTGCTTT

GATTTGACCG GTTCAGAGAA CATTTATTAT GCCGAAGATA CCTTGGCCTT

CCCTTCAAGA GCTCCAGTTC CTGCATATAT CTTACTGATT GAATCACCAT

CCCTTCAAGA GCTCCAGTTC CTGCATATAT CTTACTGATT GAATCACCAT

CCCTTCAAGA GCTCCAGTTC CTGCATATAT CTTACTGATT GAATCACCAT

CCCTTCAAGA GCTCCAGTTC CTGCATATAT CTTACTGATT GAATCACCAT

CCCTTCAAGA GCTCCAGTTC CTGCATATAT CTTACTGATT GAATCACCAT

TCCTTCAAGA GCACCAGTCC CTGCATATAT CTTACTGATG GAATCACCAT

CCCTTCCAGG GCTCCGGTGC CTGCATATAT TTTACTGATT GAATCGCCAT

CCCTTCCAGG GCCCCGGTGC CTGCATATAT TTTACTGATT GAGTCGCCAT

---------- ---------- ---------- ---------- ----------

TCCTTCAAGG GCTCCAGTTC CTGCATAAAT CTTACTGATT GAATCACCAT

TCCTTCAAGA GCTCCAGTCC CTGCGTATAT CTTACTGATG GAATCACCAT

TCCTTCCAGA GCCCCAGTTC CGGCATATAT CTTACTGATT GAATCACCAT

TCCTTCGAGA GCGCCAGTTC CCGCATATAT CTTACTGATC GAATCACCAT

CCCTTCAAGA GCTCCGGTTC CTGCATAAAT CTTACTGATT GAATCGCCAT

CCCTTCAAGC GCTCCAGTTC CTGCGTATAT CTTACTGATG GAATCACCAT

CCCTTCAAGG GCTCCAGTCC CTGCATATAT CTTACTGATG GAGTCACCAT

GCCTTCCAAA GCTCCAGTGC CTGCATAAAT TTTGCTGATT GAGTCACCAT

GCCTTCAAGA GCTCCGGTTC CCGCGTAGAT TTTGCTGATG GAATCTCCAT

TCACGGACCA CATTGACCGA AAAACTTCTT GAAAGCGAGT CACCAACTGA

TCACGGACCA CATTGACCGA AAAACTTCTT GAAAGCGAGT CACCAACTGA

TCACGGACCA CATTGACCGA AAAACTTCTT GAAAACGAGT CACCAACTGA

TCACAGACCA CATTGACCGA AAAACTTCTT GAAAGCGAGT CACCAACTGA

TCACAGACCA CATTGACCGA AAAACTTCTT GAAAGCGAGT CACCAACTGA

TCACAGACCA CATTGACCGA AAAACTTCTT GAAAGCGAGT CACCAACTGA

TCACAGACCA CATGGACCGG AAAACTTCTT GAAAGCGAGT CACCAACTGA

TCACAGACCA CATGGACCGG AAAACTTCTT GGAAGCGGGT CACCAGCTGA

---------- ---------- ---------- ---------- ----------

TCACAGACCA CATTGATCGA AAAACTTCTT GAAAGCGAGT CACCAACTGA

TCACAGACCA CATTGACCGA AAAACTTCTT GAAAGCGAGT CACCAACTGA

TCACAGACCA CATTGATCGA AAAACTTCTT GAAAGCGAGT CACTAACTGA

TCACAGACCA CATGGACCGA AAAACTTCTT GAAAGCGAGT CACCAGCTGA

TTACAGACCA CATTGATCGA AAAACTTCTT GAAAGCGAGT CACCAACTGA

TCACAGACCA CATTGACCGG AAGACTTCTT GAAAGCGGGT CACCAACTGA

TCACAGACCA CATTGACCTG AAGACTTCTT GAAAGCGGGT CACCAACTGA

TCATAGACCA CATTGATCGG AAAACTTCTA GGAAACGAGT CATCAACTGA

TCATAGACCA CATCGACCGG AAAACCTCCT GGAAGCGAGT GATCAACTGC

GGCTTTTCAG CTAAACCAAG AGCTTCCAAC TGTTTAGCTA GCATCTTAAA

GGCTTTTCAG CTAAACCAAG AGCTTCCAAC TGTTTAGCTA GCATCTAAAA

GGCTTTTCAG CTAAACCAAG AGCTTCCAAC TGTTTAGCTA GCATCTAAAA

GGCTTTTCAG CTAAACCAAG AGCTTCCAAC TGTTTAGCTA GCATCTAAAA

GGCTTTTCAG CTAAACCAAG AGCTTCCAAC TGTTTTGCTA GCATCTAAAA

GGCTTTTCAG CTAAGCTGAG AGCTTCCAAC TGTTTAGTTA GCATCTGTAA

GGCTTTTCAG CCAAGCCCAG AGCTTCCAGC TGTTTAGCTA GCATCTGAAA

GGCTTCTCAG CCAAGCCCAG AGCTTCCAGC TGCTTAGCGA GCATCTGAAA

---------- ---------- ---------- ---------- ----------

GGCTTTTCAG CTAAACCAAG AGCTTCCAAC TGTTTAGTCA GCATCTAAAA

GGCTTTTCAG CCAAGCCGAG AGCTTCCAGC TGTTTAGCTA GCATCTAAAA

GGCTTTTCAG CCAAGCCAAG AGCTTCCAAC TGTTTAGCGA GCATCTAAAA

GGTTTCTCAG CTAAGCCCAG AGCTTCCAAC TGTCTAGCTA GCATCTGAAA

GGCTTTTCAG CCAACCCAAG GCCTTCCAAC TGTTTAGCTA GTATCTGGAA

GGCTTTTCAG CTAAGCCAAA AGCTTCCAAC TGCTTAGCTA GCATCTGAAA

GGCTTTTCAG CTAAGCCAAG CGCTTCTAAC TGTTTAGCTA GCATCTGAAA

GGCTTTTCAG CTAAGCCAAG GGCTTCTAAT TGTTTGGGTA GAACCTGAAA

GGCTTCTCAG CTAAACCCAG GGCCTCCAGT TGTTTGGCCA GCATCTGAAA

GTTTTACAGT TGCACATCAG GTTGGCCAAA CTGTAGTCAC AATCCCGGAT

GTTTTACAGT TGCACATCAG GTTGGCCAAA CTGTAGTCAC AATCCCGGAT

GTTTTACAGC TGCACATCAG GTTGGCCAAA CTGTAGTCAC AATCCCGGAT

GTTTTACAGC TGCACATCAG GTTGGCCAAA CTGTAGTCAC AATCCCGGAT

GTTTTACAGC TGCACATCAG GTTGGCCAAA CTGTAGTCAC AATCCCGGAT

GTTTTACAGC TGCACATCAG GTTGGCCAAA CTGTAGTCAC AGTCCCGGAT

GACTTACAGC TGCACATCAG GTTGGCCAAA CTGTAGTCAC AGTCCCGGAT

GACTTACAGC TGCATATCAA GTTAGCCAAA CTGTAGTCAC AGTCCCTGAT

GTTTTGCAGC TGCACATCAG GTTGGCCAAA CTGTAGTCAC AGTCCCGGAT

GTTTTACAGC TGCACATCAG GTTGGCCAAA CTGTAGTCAC AGTCGCGGAT

GTTTTGCAGC TGCACATCAA GTTGGCCAAA CTGTAGTCAC AGTCCTGGAC

GTTTTACAGC TGCACATTAG GTTGGCCAGA CTGTAATCAC AGTCCCAGAT

---------- ---------- ---------- ---------- ----------

GTTTTGCAGC TGCACATCAG GTTGGCCAAA CTGTAGTCAC AGTCCTGGAT

---------- ---------- ---------- ---------- ----------

GTTTTACAGC TGCACACCAG GTTGGCCAAG CTGTAGTCAC AGTCTTGGAT

GTTTTACAAG TGCACATCAG ACTGGCCAAA CTGTAATCAC AATCCCGTAT

GTCCGGCAGG AGCACAGCAG GTTGGCCAGG CCGTAGTCAC AGTCCCGGAC

GTCGAACAGC TGCAGTTCCG AATGGTGTTT TCCCTTGTGA AGATGAGCGT

GTCGAACAGC TGCAGTTCCG AATGGTGTTT TCCCTTGTGA AGATGAGCGT

GTCGAACAGC TGCAGTTCCG AATGGTGTTT TCCTTTGTGA AGATGAGCGT

GTCGAACAGC TGCAGTTCCG AATGGTGTTT TCCTTTGTGA AGATAAGCGT

GTCGAGCAGC TGCAGTTCCG AATGGTGTTT TCCTTTGTGA AGATGAGCGT

GTCGAGCAGC TGCAGTTCCG AATGGTGTTT TCCCTTGTGA AGATGAGCGT

GTCGGGCAGC TGCAGTTACG AATGGTGTTT CCCTTTGTGA AAATCAGTGT

GTCGGGCAGC TGCAGTTGCG AATGGTATTT CCTTTTGTGA AAATCAGTGT

GTCGAGCAGG TGCAGTTCCG AATGGCGTTT TCCCGGGCGA GAATCAGGGT

GTCGAGCAGC TGCACTTCCG AATGGTGTTT TCCTTGGTGA AAATGAGCGT

ATCGAGCAAC TGCAGTTCCG AATGATGTTT TCCTTTGTGA AAATGAGTGT

GTCGAGCAGC TGCAGTTCCG AATGGTGTGT TCCTTTGTGA AAATTAGTGT

---------- ---------- ---------- ---------- ----------

GTCGAGCAGC TGCAGTTCCG AATGGTGTTT TCCGTGGTGA AAATTAGGGT

---------- ---------- ---------- ---------- ----------

GTCGGGCAGC TACAGTTCCG AATGGTGTGT TCCTTTGTGA AAATCAGCGT

ATCGAGCAGC TGCAGTTTCG AATGGTGTTT TCCTTGGTAA AAATCAACGT

GGCGGGCAGC TGCAGTTGCG GACGGTGTCC TCCCTGGTGA AGATCAGCGT

GCTGTTCTGA AACATTCCTT TTGAAGCCAA GTATAAACAT TATCTAAATG

GCTGTTCTGA AACATTCCTT TTGAAGCCAA GTATAAACAT TATCTAAATG

GCTGTTCTGA AACATTCCTT TTGAAGCCAA GTATAAACAT TATCTAAATG

GCTGTTCTGA AACATTCCTT TTGAAGCCAA GTATAAACAT TATCTAAATG

GCTGTTCTGA AACATTCCTT TTGAAGCCAG GTATAAACGT TATCTTAACA

GCTGTTCCGA AGTATTCCTT TTGGAGCCAA GTATAAACAT TATCTAAATG

CCTG---CGA GACAGTCCTT TTAGAGCCAA GTATAACTAT GATCTAAACG

GTTG---TGA GACAGTCCTT CTGGAGCCAA CTCTAACCAT GCTCTAAACG

GCTGTTCT-- ---------- ---------- ---------- ----------

GCCGTTCCGA AACGTTCCTT TTGGAGCCAA GTATAAACAT TATCTAAATT

GCTGTTCTGA AACATTCCTT TTGGAGCCAA GTATAAACAT TATCTAAAAG

GCTGTTCTGA AACATTCCTT TTGGAGCCAA GTATAAACAT TATGTAAATG

--------GG AACATTCCTT TGGGGGCCAA GTGTAAACAT TATCTAAATG

ACCGTTCTGA AACATTCCTC TTAGAGCCAA GTATAAACAT TATCTAAATG

--------GA AACATTCCTT TCGGAGTCAA GTGTAAACAT TATCTAAATG

GCTGTTCAGA AACATTCCTT TTGGAGTCAA GCA-AAACAC TCTCTACATG

GTTGTTCTAA CACATTCCTT TTGGAGAAAG ATATAAACAT TATCTAAATG

GCCGTTTCGA AGCATGCTTT CTGGAGGGGC AGAGGAGCAC TATCTGACCG

TTGTGTAATC CTAGATTGAG AAGGAATCTG TGTGGAATTT GTTTTGAATC

TTGTGTAATC CTAGATTGAG AAGGAATCTG TGTGGAATTT GTTTTGAATC

TTGTGTAATC CTAGATTGAG AAGGAATCTG TGTGGAATTT GTTTTGAATC

TTGTGTAATC CTAGATTGAG AAGGAATCTG TGTGGAATTT GTTTTGAATC

TTATGTAATC CTAGATTGAG AAGGAATC-- TGTGGAATTT GTTTTGAATC

TTGTGTAATC CTAGATTGAG GAGGAATCTG TGTGGAATTT GTTTTGAATC

TTGTGTAACC CTAGATTGAG GAGGAATCTC TGTGGAATTT GTACTGAATC

TAGTGTAACC CTAGATTGAG AAGGAATCTC TGTGGAATCT GTGTTGAATC

---------- ---------- ---------- ---------- -------ATC

TTGTGTAATC CTAGATTGGG AAGGAATCTG TGTGGAATTT GTTTTGAATC

TTGTGTAATC CTAGATTGGG GAGGAATCTG TGTGGAACTT GTTTTGAATC

TTGTGTAATC CTAGATTGAG -AGGAATCTG TGTGGAATTT GTTTTGAATC

TTGTGTAATC CTAGATTGGG AAGGAATCTG TGTGGGATTT GTTTGGAATC

TTGTGTAATC CTAGATTGAG AAGGAATCTG TGTGGAATTT GTTTTGAATC

TTGTGTAATC CTAGATTGGA AAGGAATCTG TGCGGAATTT GTTTTGAATC

TTGGAAACTC CTGGATTGAG GAAGAATCAG TGTGGAATTT GTTTTGAA--

CTGCTTAATC CTAGATTTAG AAGGAATCTG TATGGAATTT GTTTTGAATC

GCGTGTAATC CTACGCCGGG GAGGAGGCTG TGCGGAATTT GTTTCGAATC

CAACACCTGC ACTATAGTGC TGTCATCACC CGCAATTAAA AAGGAGAGAG

CAACACCTGC ACTATAGTGC TGTCATCACC CGCCATTAAA AAGGAGAGAG

CAACACCTGC ACTATAGTGC TGTCATCACC CGCAATTAGA AAGGAGAGAG

CAACACCTGC ACTATAGTGC TGTCATCACC CGCAATTAGA AAGGAGAGAG

CAACACTTGC ACTATAGTGC TGTCATCACC CGCAATTAGA AAGGAGAGAG

CAGCACCTGC ACTATAGTGC TATCATCACC AGCAATTAAA AAGGAAAGGG

CAGCACTTGC CCTATAGTGT TGTCATCACC TGCAATGAGG AAGGAAAGGG

CAGGACTTGT CCAATGGTGT TGTCATCACC CGCAATTAGG AAGGAAAGGG

CAGCACCTGC GCTATAGTGC TGTCGTCACC AGCAATCAAG AAGGACAGAG

CAGCACTTGC GTGATCGTGC TGTCATCACC GGCAATCAGA AAGGAAAGGG

CAGCACTTGC ATGATCGTGC TGTCATCGCC GGCAATCAGA AAGGAAAGGG

CAGCACCTGC ACTATGGTGC TGTCATCACC GGCAATCAGA AAGGAGAGGG

CAGCACCTGC ACCACTGTGC TGGTGTCACC CGTGACAAGG AAGGAGAGCG

CAGCACTTGC ACGATGGTGC TGTCGTCACC TGCGATTAAA AAGGAAATGG

GAGCACTTGC ACGATGGTGC TGTCGTCACC GGCAATCAAG AAGGAAAGGG

---------- ---------- ---------- ---------- ----------

CAATACTCGT GCAATTGTAT TATCATCACC AGCAATTAAG AAGGAAAGTG

CAGCACCTGC GCGATGGTGT TGTCGTCCCC GGCGATGAGG AAGGAGAGGG

CAAACTGAAC GTAGGCCTGC CGAACGTCCA ACATGTTTAC AAGATGTGTG

CATACTCCCC CTCCGCCTGC CGAACGTCCA ACATGTTTAC AAGATGTGTG

CAAACTGAAT GTAGGCCTGC CGAACGTCCA ACGTGTTTAC AAGATGTGTA

CAAACTGAAT GTAGGCCTGC CGAACGTCCA ACATGTTTAC AAGATGTGTA

CAAACTGAAC ATAGGCCTGC CGGATGTCCA ACATGTTTAC AAGATGGGTA

CAAACTGAAT GTAGGCCAGC CGGACATCCA ACATGTTTAC AAGATGTGTA

CAAACTGGAT ATAGGCCAGC CGTACGTCCA ACATGTTTAC AAGCTGTGTG

CAAACTGGAT GTAGGCCAGC CGGACGTCCA ACATGTTTAC AAGCCGTGTG

CAAACTGGAT GTAGGCCAGC CGGACGTC-- ---------- ----------

CGAACTGAAT GTAGGCCAGC CGGACATCCA ACATGTTTAC AAGATGTGTA

CAAACTGAAT GTAGGCCAGC CGGACATCCA ACATGTTTAC AAGATGTGTA

CGAACTGGAT GTAGGCCAGC CGGACGTCCA ACATGTTTAC AAGATGTGTA

CGAACTGGAT ATAGGCCTGC CGGACGTC-- ---------- ----------

CGAACTGGAT GTAGGCCAGC CTGACGTCCA ACATGTTTAC AAGATGTGTA

CAAACCGGAT GTAGGCCAGC CGGACGTC-- ---------- ----------

---------- ---------- --------CA ACATGTTTAC AAGATGTGTA

CAAACTGGAT ATATGCCAAT CGAATATCCA ACATGTTTAC AAGATGTGTA

CGAACTGAAC GTACGCCATC CGGACGTCCC ACATGTTTGC CGGGTGGGTA

GCTATTAGCA ACATCTTACA AAGAAAGAGG ACCAAATTGC ATCAGTGAAA

GCTATTAGCA ACATCTTACA AAGAAAGAGG ACCAAATTGC ATCAGTGAAA

GCTATTAGCA ACATCCTACA AAGAAAGAGG ACCAAATTGC ATCAGTGAAA

GCTATTAGCA ACATCCTACA AAGAAAGAGG ACCAAATTGC ATCAGTGAAA

GCTATTAGCA ACATCTTACA AAGAAAGAGG ACCAAATCAC ATCAGTGAAA

GCTATTAGCA ACATCTTACA AAGAAAGAGA GCCAAATTGC ATCAGTGGAA

GCTATTAGCA ACATCTTATA AAGAAAGAGG GCCAAATTGC ATCAGTGAAA

GCTATTAGCA ACATCTTATA AAGAAAGAGG GCCAAATTGC ATCAGTGAAA

---------- ---------- ---------- ---------- -------AAA

GCTATTAGCA ACATCTTACA CAGAAAGAGG GCCAAATTGC ATCAGTGGAA

GCCATTAGCA ACATCTTACA AAGAAAGAGG GCCAAATTGC ATCAGTGAAA

GCTATTAGCA ACATCTTACT AAGAAAGAGG GCCAAATTGC CTCAGGGAAA

---------- ---------- ---------- ---------- ----------

GCTATTAGCA ACATCTTACA AAGAAAGAGG GCCAAATTGC ATCAGTGAAA

---------- ---------- ---------- ---------- ----------

GCTATTAGCG ACATCTTACA CACGAAGAGG GCCAAATTGC ATCAGTGAAA

GCTATTAGCA ACATTTCACA AAGAAAGAGC ACTAAATTGC ATCAGCAAAA

GCTATTAGCA ACGTTTTTCA aggAGAGGGC TCTCCGTTGC ATCAGCAAAA

TAACTTGTGA AAAGAATTGC TAAGTGACTG ATAAATAGGC TTAAGTTACT

TAACTTGTGA AAAGAATTGC TAAGTGACTG ATAAATAGGC TTAAGTTACT

TAACTTGTGA AAAGAATTGC TAAGTGACTG ATAAATAGGC TTAAGTTACT

TAACTTGTGA AAAGAATTGC TAAGTGACTG ATAAATAGGC TTAAGTTACT

TAACTTGTGA GAAGAATTGC TAAGTAACTG ATAAATAGGC TTAAGTTACT

TAACTTGTGA GAAGAATTGC TAAGTGACTG ATAAATAGGC TTAAGTTACT

TAGCTTGTGT AAAGAATCGC TGACTGACT- -TAAATAGGC TTAAGTAACT

TAGTTTGTGT AAAGAATTGC TGAGTGACT- -TAAATAGGC TTAAGTAACT

TAGCTTGTGA GAAGAATTGC TAAGTGACTG ATAAATAGGC TTAAGTTACT

TCACTTGTGA AAAGAATTGC TAAGTGACTG ATAAATAGGC TTAAGTTACT

TAACTTGTGA AAAGAATTGC TAAGTGGCTG GTAAATAGGC TTAAGTTACT

AAACTTGTGA AAAGAATTGC TAAGTGACTG ATAAATAGGC TTAAGTTACT

-----TGTGA AAAGAATCAC TAAGTGACTG ACAAATAGGC TTAAGTGACT

TAACTTGTGA AAAGAATTGC TAAGTGACTG ATAAATAGGC TTAAGTTACT

---------- ---------- ---------- ---------- ----------

TAACTTGTGA ACAGAATCGC TAAGCGACTG ATAAACAGGC TTCCGTTGCT

TAGCTTGTGA AAAGAATTGC TAAGTAACTG ATAAATAGGC TTAAGTTACT

TAAATTGCAC AAAAGATCGC TAAGAGATGG AGAAATAGAC TTTGGTCACT

TTGCATCATC TAAATTTCTA AATACCTGGA GTATATTTGT AAACATGGCA

TTGCATCATC TAAATTTCTA AATACCTGGA GTATATTTGT AAACATGGCA

TTGCATCATC TAAATTTCTA AATACCTGGA GTATATTTGT AAACATGGCA

TTGCATCATC TAAATTTCTA AATACCTGGA GTATATTTGT AAACATGGCA

TTGCATCATC TAAATTTCTA AATACCTGGA GTATATTTGT AAACATGGCA

TTGCATCATC TAAATTTCTA AATACCTGGA GTATATTTGT AAACATGGCA

CTGTATCGTC TAAATTTCTA AATACCTGGC ATAGATTTGT AAATGCGGCA

CTATATCATC TAAATTTCTA AATACCTGGC ATATATTTGT AAACACGGCA

TGCCATCATC TGAATTTCTA AATACCTGGA GTATATTTGT AAACATGGCA

TTGCATCATC TAAATTTCTA AATACCTGGA GTATATTTGT AAACATGGCA

CTGCATCATC TAAATTTCCA AATACCTGGA GTATATTTGT AAACATGGCA

TTGCATCATC TAAATTTCTA AATACCTGGA GTATATTTGT AAACATGGCA

TTGCCTCAGC TAAATTTCTA AATACCTGCA GTATATTTGT AAACATGGCA

TTGCATCATC TAAATTTCTA AATACCTGGA GTATATTTGT AAACATGGCA

---------- ---------- ---------- ---------- ----------

TTGCATCAGC TAAATTTCTA AATACCTGGA GTATATTTGT AAACATGGCA

TTGTATCATC TAAATTTCTA AATACCTGTA GTATATTTGT AAACATGGCA

TTATAGCATC TAAATTTCCA AAAACCTGGG AGAAATTTGT CAACAAGGCA

ATCTAACATG TTATTGAACA GCACATTCAT ATTCAAATTA GCATAAACGA

ATCTAACATG TTATTGAACA GCACATTCAT ATTCAAATTA GCATAAACGA

ATCTAACATG TTATTGAACA GCACATTCAT ATTCAAATTA GCATAAATGA

ATCTAACATG TTATTGAACA GCACATTCAT ATTCAAATTA GCATAAATGA

ATCTAACATG TTACTGAACA GCACATTCAT ATTCAAATGA GCATAAATGA

ATCTAACATG TTATTGAACA GCACATTCAT ATTCAAATTA GCATAAATGA

GTCTAATGTG TTATTGGGCA GC-AATTCGA GTTTAAATGA GCATGTGGGA

GTCTAACGTG TTATTGAACA GC-CATTCAT ATTTAAATGA GCACATGGGA

ATCTAACATG TTATTGAAGG GCGCAGTCCT ATTCAAATTA GCATACACGA

ATCTAACATG TTATTGAACA GCACATTCAT ATTCAAATGA GCATAAATGA

ATCTAACGTG TTATTGAACA GCACATTCGT ATTCAAATTA GCATAAATGA

ATCTAACATG TTATTGAACA GCACATTCAT ATTCAAATTA GCATAAATGA

ATCTAACAGG TTATTGAACG GCACATTCGT GTGCAAATTA GCATAAATGA

ATCTAACATG TTATTGAACA GCACATTCAT ATTCAAATTA GCATAAATGA

---------- ---------- ---------- ---------- ----------

ATCCAACGTG TTCCCGAACA GCACATTCAG ATTCAAATGA GCACAAACGA

ATCTAACATT TTATTGAATG GCACATTGAT ATTCAAATGA GCACAAATGA

CTCTAACATG CTAGATAGCA GCAGGTTGGC ATTCCAGGGA ACCTAAACGA

GGATTTAACA GCTGATTGCA CAGCGTGAAT GATAGTAACT AATTGTCAAG

GGATTTAACA GCTGATTGCA CAGTGTGAAT GATAGTAACT AATTGTCAAG

GGATTTAACA GCTGATTGCA CAGTGTGAAT GATAGTAACT AATTGTCAAG

GGATTTAACA GCTGATTGCA CAGTGTGAAT GATAGTAACT AATTGTCAAG

GGATTTAACA GCTGATTGCA CAGTGTGAAT GATAGTAACT AATTGTCAAG

GGATTTAACA GCTGATTGCA CAGGGTGAAT GATAGTAACT AATTGTCGAG

TGGTTTAACA GCTGATTGCA CAGCGTGAAT GACTGCAGCG AACTGTCAGG

CAGTTTAACA GTTGACTGCA CAGTGTGAA- ---TGCAGCA AACTGTCAGG

GGATTTAACA GCTGATTGCA CAGCGTGAAT GACAGCGACT AATTGTCAAG

GGATTTAACA GCTGACTGCA CAATGTGAAT GATAGTAACT AATTGTCAAG

GGATTTAACA GCTGATTGCA CAGCGTGAAT GATAGTAACT AATTGTCAAG

GGATTTAACA GCTGATTGCA CAGTGTGAAT GATAGTGACT AATTGTCAAG

GGATTTAACA GCTGATTGCC CAGCGTGAAT GATAGGAACT AATTGTCAGG

GGATTTAACA GCCGATTGCA CAGTGTGAAT GATAGTAACT AATTGTCAAG

---------- ---------- ---------- ---------- ----------

GGATTTAACA GCTGATTGCA CAGCGTGAAT GATAGTGACT AATTGTCAAG

GGATTTAACA GCTGATTGCA CAGTGTGAAT GATAGTGACT AATTGTCCAG

GGCTTGTAGA GCCCCTCGCT CACGGTGAAT GATGGTCCCt aatagtcggg

AAGAGAGCCA CACATAGATA CCAAAGAGTT AATTTGCGTT TTTTAAACCT

AAGAGAGCCA CACATAGATA CCAAAGAGTT AATTTGCGTT TTTTAAACCT

AAGAGAGCCA CACATAGATA CCAAAGAGTT AATTTGCGTT TTTTAAACCT

AAGAGAGCCA CACATAGATA CCAAAGAGTT AATTTGCGTT TTTTAAACCT

AAGAGAGTCA CACATAGATA CCAAAGAGTT AATTTGCGTT TTTTAAACCT

AAGAGAGCCA CACGTAGATA CCAAAGAGTT AATTTGCGTT TTTTAAACCT

GACGCAGCCA CACATAGATA CCGAAGAGTT AATTTGCGTT TTTTAAACCC

GACACAGCCA CACATAGATA CCGAAGAGTT AATTTGCGTT TTTTAAACCC

AAGACAGCCA CACATAGATA GCAAAGAGTT AATTTGCGTT TGTTAAGCCT

GAGAGAACCA CACAGAGATA CCAAAGAGTT AATTCTCGTT TTTTAAACCT

AAGAGAGCCA CACAGAGATA CCAAAGAGTT AATTTGCGTT TTTTAAACCT

AAGAGAGCCA CACAGAGATA CCAAAGAGTT AATTTGCGTT TTTTAAACCT

---------- ---------- ---------- ---------- ----------

AAGAGAGCCA AACATAGATA GCAAAGAGTT AATTTGTGTT TTTTCAGCCT

AAGACAGCCA AACATAGATA CCAAAGAGTT AATTTGCGTT TTCTAAACCT

AAGACAGCCA AACA-AGATA CCAAAGAGTT AATTTGCGTT TTTTAAACCT

AAAGAAGCCA TACATAGATA GCAAAGAGTT AATTTGCGTT TTGAAAACCT

AAGAGAACCA GACACAGAGC CCAAAGAGTT AATTTGCGTT TTTCCAACCT

TCTTCCCAAA GCCTGCTCTC ATTCCTGCAT AAAATCCCAT GTCCAGACAT

CCTTCCCAAA GCCTGCTCTC ATTCCTGCAT AAAATCCCAT GTCCAGACAT

CCTTCCCAAA GCCTGCTCTC ATTCCTGCAT AAAATCCCAT GTCCAGACAT

CCTTCCCAAA GCCTGCTCTC ATTCCTGCAT AAAATCCCAT GTCCAGACAT

CCTTCCCAAA GCCTGCTCTC ATTCCTGCAT AAAATCCCAT GTCCAGACAT

CCTTCCCAAT GCCTGCTCTC ATTCCTGCAT AAAATCCCAT GTCCAGACAT

CCTTTCCAAT GCCCGCTCTC ATTCCT--AT AAAATCTCAT GCCCAGACAT

CCTTTCCAAT GCCCGCTCTC ATTCCT--AT AAAATCTCAT GCCCAGACAT

CCTTCCCCAT GCCAGCTCTC ATTCCTGCAT AAAATCCCAC GTCCAGACAT

CCTTCCCAAT GCCCGCTCTC ATTCCTGCAT AAAATCCCAT GTCCAGACAT

CCTTCCCAAT GCCCGCTCTC ATTCCTGCAT CAAATCCCAT GTCCAGACAT

CCTTCCCAAT GCCCACTCTC ATTCCTGCAT AAAATCCCCT GTCCAGACAT

---------- ---------- ---------- ---------- ----------

CCTTCCCAAT GCCTGCTCTC ATTCCTGCAT AAAATCCCAG GTCCAGACAT

GCTTCCCAAT GCCTGCTCTC ATTCCTGCAT AAAACCCCAT GTCCAGACAT

CTTTCCCAAT GCCCCCTCTC ATTCCTGCAT AAAACCCCAT GTCCAGACAT

CCTTCCCAAT ATCCTCTCTC ATTCCTGCAT GAAATCCCAT GTCCAGACAT

CCTTCCCAAT ATCCTCTCTC ATTCCTGCAT AAAATCCCAT GTCCAGACAT

CAAATCAGTA AGCCCTGTTG CCTGTCAGAG AATACAATGT CAGGCCATTA

CAAATCAGTA AGCCCTGTTG CCTGTCAGAG AATACAATGT CAGGCCATTA

CAAATCAGTA AGCCCTGTTG CCTGTCAGAG AATACAATGT CAGACCATTA

CAAATCAGTA AGCCCTGTTG CCTGTCAGAG AATACAATGT CAGACCATTA

CAAATCAGTA AACCCTGTTG CCTGTCAGAG AATACAATGT CAGGCCATTA

CAAATCAGTA AGCCCTGTTG CCTGTCAGAG AATACAATGT CAGGCCATTA

CAAATCAGTA AGCCCTGTTG CCTGTCAGTG AATACAATGT CAGGCCATTA

CAAATCAGTA AGCCCTGTTG CCTGTCAGTG AATACAATGT CAGGCCATTA

CAAATCAGTA AGCCCTGTTG CCTGTCAGGG AATACAATGT CAGGCCATTA

CAAATCAGTA AGCCCTGTTG CCTGTCAGAG AATACAATGT CAGGCCATTA

CAAATCAGTA AGCCCTGTTG CCTGTCAGAG AATACAATGT CAGGCCATTA

CAAATCAGTA AGCCCTGTTG CCTGTCAGAG AATACAATGT CAGGCCATTA

---------- ---------- ---------- ---------- ----------

CAAATCAGTA AGCTCCGTTG CCTGTCAGAG AATACAATGT CAAGCCATTA

CAAATCAGTA AGCCCTGTTG CCTGTCAGAA AATACAATGT CAGCCCATTA

CAAATCAGTA AGCCCTGTTG CCTGTCAGGA AATACAATGT CAGGCCATTA

CAAATCAGTA AACCCTGTTG CCTGTCAGAG AATACAATGT CAGGCCATTA

CAAATCAGCA AACTCTGGAG CTTGTCAGAG AATACAATGT CAGACCATTA

GTATGTTAAT AAGTCCGGGG GCGACAGGTG GCCTACTAGA CAGCTCAATT

GTATGTTAAT AAGTCCGGGG GCGACAGGTG GCCTACTAGA CAGCTCAATT

GTATGTTAAT AAGTCCGGGG GCGACAGGTG GCCTACTAGA CAGCTCAATT

GTATGTTAAT AAGTCCGGGG GCGACAGGTG GCCTACTAGA CAGCTCAATT

GTATGTTAAT AAGTCCGGGG GCGACAGGTG GCCTACTAGA CAGCTCAATT

GTATGTTAAT AAGTCCGGGG GCGACAGGTG GCCTACTAGA CAGCTCAATT

GTATGTTAAT AAGTCCGGGG GCGACAGGTG GCCTACTAGA CAGCTCAATT

GTATGTTAAT AAGTCCGGGG GCGACAGGTG GCCTACTAGA CAGCTCAATT

GTATGGTAAT AAGTTCGGGG GCGACAGGTG GCCTACTAGA CGGCTCAATT

GTATGTTAAT AAGTCCGGGG GCGACAGGTG GCCTACTAGA CAGCTCAATT

GTATGTTAAT AAGTCCGGGG GCGACAGGTG GCCTCCTAGA CAGCTCAATT

GTATGTTAAT AAGTCCG-GG GCGACAGGTG GCCTACTAGA CAGCTCAATT

---------- ---------- ---------- ---------- ----------

GTATGTTAAT AAGTCCGGGG GCGACAGGTG GCCTACTAGA CAGCTCAATT

GTATGTTAAT AAGTCCGGGG GCGACAGGTG GCCTACTAGA CAGCTCAATT

GTATGTTAAT AAGTCCGGGG GCGACAGGTG GCCTTCTAGA CAGCTCAATT

GTATGTTAAT AAGTCCGGGG GTGACAGGTG GCCTACTAGA CAGCTCAATT

GTATGTTAAT GTGTCCAGGG GTGACAGGTG GTCTACTAGA CAGCTCAATT

AAGCTGCTCA TTAAACACAC GCTAACGAAC AGCGAGGCAG GGCCTGAACA

AAGCTGCTCA TTAAACACAC GCTAACGAAC AGCGAGGCAG GGCCTGAACA

AAGCTGCTCA TTAAACACAC GCTAACGAAC AGCGAGGCAG GGCCTGAACA

AAGCTGCTCA TTAAACACAC GCTAACGAAC AGCGAGGCAG GGCCTGAACA

AAGCTGCTCA TTAAACACAC GCTAACGAAC AGCGAGGCAG GGCCTGAACA

AAGCTGCTCA TTAAACACAG GCTAACGAAC AGCACGGCAG GGCCTGAACA

AAGCTGCTCA TTAAACACAC GCTAACGAAC AGCAAGGCAG GGCCTGAACA

AAGCTGCTCA TTAAACACAC GCTAACGAAC AGCAAGGCAG GGCCTGAACA

AAGCTGCTCA TTAACCACAG GCTAACGAAC AGCAAGGCAG GGCCTGAACA

AAGCTGCTCA TTAAACACAC GCTAACGAAC AGCCAGCCAG GGCCTGAGCA

AAGCTGCTCA TTAAACACAC GCTAACGAAC AGCCAGGCAG GGCCTGAACA

AAGCTGCTCA TTAAACACAC GCTAACGAAC AGCCAGGCAG GGCCTGAACA

---------- ---------- ---------- ---------- ----------

AAGCTGCTCA TTAAACACAC GCTAACGAAC AGCAAGGCAG GGCCTGAACC

AAGCTGCTCA TTAAACACAC GCTAACGAAC AGCAAGGCAG GGCCTGAACA

AAGCTGCTCA TTAAACACAC GCTAACGAAC AGCGAGGCAG GGCCTGAACA

AAGCTGCTCA TTAAACACAC GCTAACGAAC AGCAAGGCAG GGCCTGAACA

AAGCTGCTCA TTAAACACAC GCTAACGAAC AGCAAGGCAG GGCCTGAACA

CACCATTCTG TAATGACATG ATGTCAGAGT GAACAAATGA GGAAAAATAA

CACCATTCTG TAATGACATG ATGTCAGAGT GAACAAATGA GGAAAAATAA

CACCATTCTG TAATGACATG ATGTCAGAGT GAACAAATGA GGAAAAATAA

CACCATTCTG TAATGACATG ATGTCAGAGT GAACAAATGA GGAAAAATAA

CACCATTCTG TAATGACATG ATGTCAGAGT GAACAAATGA GGAAAAATAA

CACCATTCTG TAATGACATG ATGTCAGAGT GAACAAATGA GGAAAAATAA

CACCATTCTG TAATGACATG ATGTCAGAGA GAACAAATGA GAAAAAATAA

CACCATTCTG TAATGACATG ATGTCAGAAG GAACAAATGA GGAAAAATAA

CACCATTCTG TAATGACATG ATGTCAGAGT GAACAAACGA GGAAAAATAA

CACCATTCTG TAATGACATG ATGTCAGAGT GAACAAATGA GGAAAAATAA

CACCATTCTG TAATGACATG ATGTCAGAGT GAACAAATGA GGAAAAATAA

CACCATTCTG TAATGACATG ATGTCAGAGT GAACAAATGA GGAAAAATAA

---------- ------CATG ATGTCAGAGC GAACAAATGA GGAAAAATAA

CACCACTCTG TAATGACATG ATGTCAGAGA GGACAAATGA GGAAAAATAA

CACCATTCTG TAATGACATG ATGTCAGAGC GAACAAATGA GGAAAAATAA

CACCATTCTG TAATGACATG ATGTCAGAGC GGACAAATGA GGAAAAATAA

CACCATTCTG TAATGACATG ATGTCAGAGC GAACAAATGA GGAAAAATAA

CACCATTCTG TAATGACATG ATGTCAGAGC GAACAAATGG GGAAAAATAA

CAAAAACATT ACAGCAGGGA CAGACGCCAC TAATAAGGAC CCCTCGCACT

CAAAAACATT ACAGCAGGGA CAGACGCCAC TAATAAGGAC CCCTCGCACT

CAAAAACATT ACAGCAGGGA CAGACGCCAC TAATAAGGAC CCCTCGCACT

CAAAAACATT ACAGCAGGGA CAGACGCCAC TAATAAGGAC CCCTCGCACT

CAAAAACATT ACAGCAGGGA CAGACGCCAC TAATAAGGAC CCCTCGCACT

CAAAAACATT ACAGCAGGGA CAGACGCCAC TAATAAGGAC CCCTCGCACT

CAAAAACATT ACAGCCGGGA CAGACGCCAC TAACAAGGAC CCCTCGCACT

CAAAAACATT ACAGCCGGGA CAGACGCCAC TAACAAGAAC CCCTCGCACT

C-AAAACATT ACTGCAGGCA CAGACGCCAC TAATAGGGGA CTCTCGTACT

CAAAAACATT ACAGCAGGGA CAGACGCCAC TAATAAGGAC CCCTCACACT

CAAAAACATT ACAGCAGGGA CAGACGCCAC TAATAAGGAC CCCTCGCACT

CAAAAACATT ACAGCAGGGA CAGACGCCAC TAATAAGGAC CCCTCGCACT

CAAAAACATT ACAGCAGGGA CAGACGCCAC TAATAAGGAC CCCTCGCACT

CAAAAACATT ACAGCAGGGA CAGACGCCCC TAATAAGAAC CCCTCGCCCT

CAAAAACATT ACAGCAGGGA CAGACGCCCC TAATAAGGAC CCCTCGCGCC

CAAAAACATT ACAGCAGGGA CAGACGCCAC TAATAAGGGC CCCTCGCACT

CAAAAACATT ACAGCAGTGA CAGACGCCAC TAATAAGGAC CCCTTGCACT

CAAAAACATT ACAGCGATGG CAGACGCCAC TAATAAGGAC TCCTCACACT

ACATCAGCCC GCAAATCAAT GAGTCACCGG GATCAAAAAA CTCGACCCAA

ACATCAGCCC GCAAATCAAT GAGTCACTGG GATCAAAAAA CTCGACCCAA

ACATCAGCCC GCAAATCAAT GAGTCACCGG GATCAAAAAA CTCGACCCAA

ACATCAGCCC GCAAATCAAT GAGTCACCGC GATCAAAAAA CTCGACCCAA

ACAGCAGCCC GCAAATCAAT GAGTCACCGG GATCAAAAAA CTCGACCCAA

ACATCAGCCC GCAAATCAAT GAGTCACAGG GATCAAAAAA CTCGACCCAA

ACATCAGCCC CCAAATTAAT GAGTCACAGG GATCAAAAAA CTCGACCCAA

ACATCAGCCC CCAAATTAAT GAGTCACAGG GATCAAAAAA CTCGACCCAA

ACAGCGGCCG GTGAATCATT GAGTCGCGGG GATCAAAAAA CCCGACCCAA

ACATCAGCCC GCAAATCAAT GAG-CACAGG GATCAAAAAA CTCAACCCAA

ACATCAGCCC GCAAATCAAT GAGCCACAGG GATCAAAAAA CTCGACCCAA

ACATCAGCCC GCAAATCAAT GAGCCACAGG GATCAAAAAA CTCGACCCAA

ACATCAGC-- --AAATCAAT GAGCCACGGG GATCAAAAAA CTCGACCCAA

ACATGAGCCC CCAAATCAAT GAGCCACGGG GATCAAAAAA CTCGACCCAA

ACGTC-GCCC GCGAATCAAT GAGCCACAGG GATCAAAAAA CTCGACCCAA

ACATCAGCCC GCAAATCAAT GAGCCACAGG GATCAAAAAA CTCGACCCAA

ACATCAGCCC TCAAATCAAT GAGCAACAGG GATCAAAAAA CTCGACCCAA

ACATCAGCCC TTAAATCAAT GAGCCACCGG GATCAAGAAA CTCGACCCAA

CATTCTCAGG AGACTGAGAT TTTTTTGGTC ACTACTCTGG GTTTAAATGA

CATTCTCAGG AGACTGAGAT TTTTTTGGTC ACTACTCTGG GTTTAAATGA

CATTCTCAGG AGACTGAGAT TTTTTTGGTC ACTACTCTGG GTTTAAATGA

CATTCTCAGG AGACTGAGAT TTTTTTGGTC ACTACTCTGG GTTTAAATGA

CATTCTCAGG AGACTGAGAT TTTTTCGGTC ACTACtCTGG GTTTAAATGA

CATTCTCAGG AGACTGAGAT TTTTTTGGTC ACTACTTTGG GTTTGAATGA

CATTCTCAGG AGGCTGAGAT TTTTTTGGTC ACTATTT-GA ATTTGAATGG

CATTCTCAGG AGGCTGAGAT TTTTTTGGTC ACTATTTGGA ATTTGAATGA

CATTCTCAGG AGACTCCGAT TTTTTTGGTC ACTACTTTGG GTTTGAATGA

CATTCTCAGG AGACTGAGAT TTTTTTGGTC AGTACTTTGA GTTTGAATCA

CATTCTCAGG AGACTGAGAT TTTTTTGGTC AGTACTTTGA GTTTGAATGA

CATTCTCAGG AGACTGAGAT TTTTTTGGTC AGTACTTTGA GTTTGAATGA

CATTCTCAGG AGACTGAGAT TTTTGTGGTC AGTCCTTTGA GTTTGAATGG

CATTCTCAGG AGACTGAGAT TTTTTTGGTC ACGACTTGGG GTTTGAATGA

CATTCTCAGG AGGCTGAGAT TTTTTTGGTC ACTACTTTGG GGTTGAATGA

CATTCTCAGG AGACTGAGAT TTTTTTGGTC ACTACTTTGG GGCTGAATGA

CATTCTCAGG AGACTGAGAT TTTTTTGGGC TGTGCTTTGG GTTTGAATGA

TATCCTCGGG GGACTTAGAT TTTGTTGGTC AGTTTTATGG GTTGGAATGA

AAATCATTAT TTAATGGGAT CATTATTTAC ATTTGATTGT TTCTTAGGTG

AAATCATTAT TTAATGGGAT CATCATTTAC ATTTGATTGT TTCTTAGGTG

AAATCATTAT TTAATGGGAT CATCATTTAC ATTTGATTGT TTCTTAGGTG

AAATCATTAT TTAATGGGAT CATCATTTAC ATTTGATTGT TTCTTAGGTG

AAATCATAAT TTAATGGGAT CATCATTTAC ATTTGATTG- TTCTTAGGTG

AAATCATTAT TTAATGGGAT CATCATTTAC ATTTGATTGT TTCTTAGGCA

AAATCATTAT TTAATGAGAT CATCATTGAC ATTTGATTGC TTCTCAGGTG

AAATCATTAT TTAATGAGAT CATCATTGAC ATTTGATTGC TGCTCAGGTG

AAATCATTAT TTAATGGGCT CATCATTTGC GTTTGATTGT TGCTTAGGTG

AAATCATTTT TTAATGGGAT CATCATTTAC ATTTGATTGT TTCTTAGGTG

AAATCATTAT TTAATGCGAT CATCATTTAC ATTTGATTGT TTCTTAGGTG

AAATCATTAT TTAATGGGAT CGTCATTTAC ATTTGATTGT TTCTTAGGTG

AAATCATTAT TTAATGGGTT CATCATTTAC ATTTGATTG- -----AGGTG

CAATCATTAT TTAATGGGAT CATCATTTAC ATTTGATTGT TTCTTAGGTG

AAATCATTAT TTAATGGGAT CATCATTTCT ATTTGATTGT TTCTTAGGTG

AAATCATTAT TTAATGGGAT CATCATTTAC ATTTGATTGT GTCTTCGGTG

AAATCATTCT TTAATGGGAT CATCATTTAC ATTTGATTGT TTCTTAGGTG

AAATCATTAT TTAATGGGAG CGTCATTTAC ATTTGATTGT TCCTCAAGTG

AAGATTCTGC TTGTCATTAT TTTAAGAGAA GGGACTTCCA GCAGCTGCTA

AAGATTCTGC TTGTCATTAT TTTAAGAGAA GGGACTTCCA GCAGCTGCTA

AAGATTCTGC TTGTCATTAT TTTAAGAGAA GGGACTTCCA GCAGCTGCTA

AAGATTCTGC TTGTCATTAT TTTAAGAGAA GGGACTTCCA GCAGCTGCTA

AAGATTCTGC TTGTCATTAT TTTATGAGAA GGGACTTCCA GCAGCTGCTA

AAGATTCTGC TTGTTATTAT TTTAAGAGAA GGGACTTCCA GCAGCTGCTA

AGGGTCCTAC TTGTTATGGA TGCAAACCAA AGGACTTCCA GCGGCTGGTA

AGGCTCCTAC TTGTTAGGGA TGTAAGCCCC GGGACTTCCA GCTGCTGCTA

AAGGTTCTAC TTGTTACTAA -GAAAGAGAA GAGACTTCCG GCAGCTGCTA

AAGATTCTAC TTGTTATCAT TTGAAAAGAA GAGATTTCCA GAGGTTGCTA

AAGATTCTTC ---TTATTAT TTTAAAAGAA GGGATTTCCA GCAGTTGCTA

AAGATTCTAC ---TTATTAT TGTAAAAGAA GGGGTTTCCA GCGGTTGCTA

AAGAGTCTCC TGGCCGTCAT TGTTAAAGCA GGGATCCCCA GCAGTTGTTG

AAGATTCTAC TTCTTCTTAT TTTAAGAGAA GGGACTTCCA GCAGCTGCTA

GAAA--CTAC TTGTTATTAT TTTAAGAGAA GGAACTTCCA GCAGCTG-CA

GAGCTTCTAC TTGTCAGCAT TTTAAGAGAA GGGATTTCCA GCAGCTGTTA

GAGATTCTAC TTATCATTAT TTTAAGAGAA GGGACTACCA GCAGCTGCTA

AAGACTCCAC TTATTACAGC TTTAAGAGAA GGAGCTGGTG TCCGCCGAGA

GAGGTTTCTC TCATTTTCAA GTAAGGCGCT TCCTGAATAA TAAAATCAAA

GAGGTTTCTC TCATTTTCAA GTAAGGCGCT TCCTGAATAA TAAAATCAAA

GAGGTTTCTC TCATTTTCAA GTAAGGCGCT TCCTGAATAA TAAAATCAAA

GAGGTTTCTC TCATTTTCAA GTAAGGCGCT TCCTGAATAA TAAAATCAAA

GAGGTTTCT- TCATTTTCAA GTAAGGCACT TCCTGAATAA TAAAATCAAA

GAGGTTTCCC TCATTTTCAA GTAAGGGGCT TCCTGAATAA TGAAATCAAA

GAAGTTTCTC TCATTTTCTA GTAAGTAGCT TCCTG----- ---AACCAAA

GGCG-TTATC TCATTTCCTA AGAAGTAGCT TCCAG----- ---AAGCAAA

GAGGTGTGTC TTGGCTTCAA GTGAGAGTCT ACCTGAACAA CAAAATCAAA

GAGGTTTCTC TCATTTTCAA GTAAGGGGCT TCCTGAATAA TAAAATCAAA

GAGGTTGCTC TCATTTTCAA GTAAGGGGCT TCCTGAATAA TAAAATCAAA

GAGGTCTCTC TCACTTTCAA GGGATGGGCT CCCTGAATAA TAAAATCAAA

GAGGTTGCTC TTATTTGTAG GGAAGGGGTT TCTCaaataa taaaatttCA

GAGCTTTCTC TCATTTTCAA GTAAGGGGCT TCCTGAATAA TAAAATCAAA

GAGGCTTCTC TCATTCTCAA GTAAGGGGCT TCCTGAATAA TAAAATCAAA

GAGGTTTCTG CCATTTTCAT GTGAGGGGCT TCCTGAATAA TAAAATCCCA

AAGGCTTCTC CAATTTTCAA GTAAAGGAAT TTCTTCATCA CAAAATCAAA

AAGACACCTC TCCTTTTCAA GGCAGGGGCT TCCCGGTTAA TAAAATCAAA

CCTCAAACTA ATTGTAACAA GTGGTTGGTG AAACGCAGGC TTCCGAAGGC

CCTCtAACTA ATTGTAACAA GTGGTTGGTG AAACGCAGGC TTCCGAAGGC

CCTCAAACTA ATTGTAACAA GTGGTTGGCG AAACGCAGGC TTCCGAAGGC

CCTCAAACTA ATTGTAACAA GTGGTTGGCG AAACGCAGGC TTCCGAAGGC

CCTCTAACTA ATTGTAACAA GTGGTTGGTG AAACGCAGGC TTCCGAAGGT

CCTCAAACTA ATTGTAACAA GTGGCTGGTG AAACCCAGGC TTCCGAAGGC

CCTCAAACTA ATTGTAATAA GTGGTTGGTG AAACGCAGGC TTCCTCAGGC

CCTCAAACTA ATTGTAATAA GTGGTTGGTG AAACGCAGGC TTCCTCAGGC

CCTCAAACTA ATTGTAACAA GTGGTTGATG AAATGTAGGC TTCCTAAGGC

TCGCAAACTA ATCATAACAA GTGGTTGGTG AAACGCAGGC TCCCTAAGGC

TCTCAAACTA ATTGTAACAA GTGGT-GGTG AAAC-CAGGC TTCCTATGGC

TCTCAAACTA ATTGTAACAA GTGGTTGGAG AAACGCAGGC TTCCTAAGGC

TCTCAAACTA ATTGAAACAA GCGGTTGGTG AAAAACAGGC TTCCTAACCA

CCTCTAACTA ATTGTAATAA GGGGTTGGTG AAATGCAGCC TTCCTAAAAC

CCTCGAACTA ATTGTAAC-- TTGGCTGGTG AAACGCAGAC TTCCTAAGGC

CCTCAAACTA ATTGTAACAA GTGGTTGGTG AAAGACAGGC TTCCCTAGCT

TATAAAAATA AATGAAACAA ATGGATGACT AGACACAGTC TTCCTAAGTG

CCCCAAACTA ATTGTTACAA GTGGATCGCA AAACCCAGCC TCTCTCCGCT

TGCCCTTCCA GTGACCCATA CCCTGACCTG GACAGACCAC CTTCTGTTTT

TGCCCTTCCA GTGACCCATA CCCTGACCTG GACAGACCAC CTTCTGTTTT

TGCCCTTCCA GTGACCCATA CCCTGACCTG GACAGACCAC CTTCTGTTTT

TGCCCTTCCA GTGACCCATA CCCTGACCTG GACAGACCAC CTTCTGTTTT

TGCCCTTCCA GTGACCCATA CCCTGACCTG GACGGACCAC CTTCTGTTTT

TGCCCTCGCG ATGACCCATG CCCTGACCTG GACAGACCAC CTTCTGTTTT

CGCCCTTCCA CTGACCCATA CCCTGACCTG CCCAGACCAC CTTCTGTTTT

CGCCCTCCCA GTGACCCATA CCCTGACCTG CCCAGACCAC CTTCTGTTTT

TGCCCTCCCA GTGACCCATG CCCTGACCTG GACAGACCAC CTTCTGTTTT

TGCCCTTCCA ATGACCCATA CCCTGACCTG GACAGACCAC CTTCTTTTTT

TGCCCTTCCA ATGACCCATA CCCTGACCTG GACAGACCAC CTTCTGTTTT

TGCCCTTCCA ATGACCCATA CCCTGACCTG GACAGACCAC CTTCTGTTTT

TGCCCTTCCA ATGACCTCTA TCCTGACCTG CACAGACCAC CTTCTGTTTT

CGCCCTGCCG ACGACCCATT CCCTGACCTG GATAGACCAC CTTCTGTTGT

TGCCATCTCA GTGACCCACA CGCTGACCTG CACAGACCAC CTTCTGTTTT

TGACCTTTCA ATGACCCCTA CCCTGACCTG GACAGACCAC CTTCTGTTTT

TAGCCTTCTA AGGACATCTA ACCTGACCCA GAAAGATCAC CTGCTTTTTT

TGCCCTTCCA GTGACCTATG ACCTGCCCTG GACAGCTCCC CTTCTGTTTG

TGTTCTATGA ATCTTAAAGA GTTCTGAAGA GCGAAAGTTA TTGCCCTTCC

TGTTCTATGA ATCTTAAAGA GTTCTGAAGA GCGAAAGTTA TTGCCCTTCC

TGTTCTATGA ACCTTAAAGA GTTCTGAAGA GCGAAAGTTA TTGCCCTTCC

TGTTCTATGA ACCTTAAAGA GTTCTGAAGA GCGAAAGTTA TTGCCCTTCC

TGTTCTATGA ACCTTAAAGA GTTCTGAAGA G-GAAAGTTA TTGCCCTTCC

TGTTCTATGA ACCTTAAAGG GTTCTGAAGA GCAAAAGTTA TTGCCCTTCC

TGTTCAATAA ACCTTAAAGA GCTCCGAAGA GCGAAACTCA CCGCCCTCCC

TGTTCCATAA ACCTTAAAGA GCTCCGAAGA GCgagactga ccgcccTCCC

TGTTCCATGA A----AAGGA GCTCTGCAGA GCGAAAGTTC TTGCCCTTCC

TGTTCCATGA ACCTTAAAGA GCTCTGAAGA GCAGAAGTTA TTGCCCTTGC

TGTTCCATGA ACCTTAAAGA GCTCTGAAGA GCAAGAGTTA TTGCCCTTCC

TGTTCCATGA ACCTTAAAGA -CTCTGAAGA GCAAAAGTTA TTGCCCTTCC

TGTTCCATGA ACCTT----- -TTCTGAAGG GCAAAAGTTA TTGCCCTTGC

TGTTTTATGA ACCTTAAAGA GCCCTGAAGG GCAAAAGTTA TTGCCCTGCC

TGTTCCATGA ACCTTAAAGA GCTCTGAAGG GCAAAAGTTA TTGCCCTTCC

TGTTTCATGG ACCTTAAAGA GCTCTGAAGA GCAAAAGTTA TTGCCCTTCC

TGTTTCATTA ACCTTAAAGG GCTCTAAAGA GCAAAAGTTA TTGCCCCTCC

GTTTCCAGGA GCTTTGGAGG GAGCGGAAAA GCAAAAGTTA TTGCCCGCCC

AAATGCCAGT TTGTTTTTTG ATTTTCCACG GTCCAGCCCC GTATTTAACA

AAATGCCAGT TTGTTTTTTG ATTTTCCACG GTCCAGCCCC GTATTTAACA

AAATGCCAGT TTGTTTTTTG ATTTTCCACG GTCCAGCCCC ATATTTAGCA

AAATGCCAGT TTGTTTTTTG ATTTTCCACG GTCCAGCCCC GTATTTAGCA

AAATGCCAGT TTGTTTTTTG ATTTGCCACA GTCCAGCCCT GTATTTAGCA

AAATGCCAGT TTGTTTTTTG ATTTTCCACA GTCCAGCCCT GTAGTTAGCA

AAAGGCCAGT TTGTTTTTTG ATTTTCCACG GTCCGGCCCT GTGTTTAGCA

AAAGGCCAGT TTGTTTTTTG ATTTTCCACG GTCCAGCCCT GTGTTTAGCA

AAAGGCCAGT TTGTTTTTTG ATTTTCCACG GTCCAGCCCT GCGTTTAGCA

GAATGCCAGT TTGTTTTTTG ATTTTCCACG GTCCAGCCCT GTATTTAGCA

AAATGCCAGT TTGTTTTTTG ATTTTCCACG GTCCAGCCCT GTATTTAGCA

AAACGCCAGT TTGTTTTTTG ATTTGCCACG GTCCAGCCCT ATATTTAGCA

AAATGCCAGT TTGTTTTTTG ATTTTCCACG GTCCAGCCAC GTATTTAGCA

AAATGCCAGT TTGTTTTTTG ATTTTCCACA GTCCGGCCCT GTATTTAGCA

AAATGCCGGT TTGTTTTTTG ATTTTCCACG GTCCAGCCCT GCGTTTAGCA

AAATGCCAGT TTGTTTTTTG ATTTTCCACG GTCCAGCCCT GTATTTAGCA

AAATGCCAGT TTGTTTTTTG ATTTTCCACG GTCTGGCCCT GTATTTAGCA

AAATGCGAGT TTGTTTTTTG ATTCTCCACG GCCCGGCCCT GTATTTAGCA

CAGAAAGATG CAACACTGAT AATAACCCCA AATATACACA AAGACAAAGC

CAGAAAGATG CAACACTGGT AATAACCCCA AATATACACA AAGACAAAGC

CAGAAAGATG CAACACTGGT ACTAACCCCA AATATACACA AAGACAAAGC

CAGAAAGATG CAACACTGGT AATAACCCCA AATATACACA AAGACAAAGC

CGGGAAGACA CAACACTGGA AATAACCCCA AATATACACA AAGACAAAGC

CAGAAAGACA CGACACCGGT AATAACCCCA AATAGACACA AGGACAAAAC

CAGAAAGACA CAACACTTGT CGTCCTCTCC AATACACACA AGGACAATGC

CAGAAAGACA CAACACTTGT CATCCCCGCA AATACACACA AGGACAAAGC

CAGAAAGCCG CAAC-CCGGT AATAACCATA AATATGCACC CGCAGAAAGC

CAGAAAGCCA CGACACTGAT AATAACCCCC AACATACACG AGGACAAAGC

CAGAAAGACA CGACACCGAT AACAACCCCA AATATCCACC GGGACAATGC

CAGAAAGACA CGACACTAAT AACAACCCCA AATATACACA AGGACAAAGC

CAGAAAGACA CGACATGGAG AAGCATGCCC AGTGTCCCCA AGGACAAAGC

CAGAAAGACA CGACATCGAT AATAACCCCA AATAAACACC AGGACAAAGC

CAGAAAGACA CGACACTGGT AATAACCCCA AATATAAACA AGGGCAAAGC

CAGAAAGACA CGACACCAGT AATAACCCCA AATAGACACA AGGACAAAGC

CAGAAAGACA CGACACTGAT AATAACCCTA AATATACACA AGGACAAAGC

CAGAAAGACA TGACACCCAT AATCACCCTA AACACACAAG GCGACAAGGC

AGAGGGCATG CCTTTTTGTT TATGAGATGA TATACCAGAA AACAACACAT

AGAGGGCATG TCTTTTTGTT TATGAGATGA TGTACCAGAA ACCAACACAT

AGAGGGCATG TCTTTTTGTT TATGAGATGA TGTACCAGAA ACCAACACAT

AGAGGGCATG TCTTTTTGTT TATGAGATGA TGTACCAGAA ACCAACACAT

GGAGGGCATG CCATTTTGTT TATGAGATGA TGTACCAGAA ACCAGCACAT

AGAGGGCATA TCTTTTTGTT CATGAGAGGA TGTACCAGAA ACCAACACAT

AGGGGGCATG TCTCTTTGTT ------AAGA TGCACTGGAA AGCTA-GTAT

AGGGGGCATG TCTCTTTGTT T-----AAGA TGAACTGGAA AGCTA-GGGT

AGGG------ ---------- ---------- ---------- ----------

AGAAGG---- --CTTTTGTT TATGAGACG- --CGCCAGAG GCCAACACAT

AGAAGGCCTG GCCTTTTGTT TATGAGATGA TGTGCTAGAA -CCAGCACAT

AGAAGA-CAT GCCTTTTGTT TATGAGATGA TGTCCTAGAA ACCAACAGAT

AGAAGGCCTG GCCTTTTGTT TCCTGGCGGA AGGACTAGAA GCCA------

GGAGGGCATG CCTTCTTGTT TATGAGGTCA TTTACTAGAA AGGAACACAG

TGAGGGCATG TCTTTTTGTT TATGAGATGA TGTACTAGAA GTGAACGCAT

CGAGGGCTTG TCTATTTGTC CAAGA----- TGTGCAGGAA AAGCATCCAC

AAGGGGCACA TCTTTTTGTT CATGAGATGA TGTACTAGAA ACCAACACAT

AAAGGACTCG TCTTGTGTAT TATGAGACGA TGTACTAGAA ACTAACGCAT

CTGACCAAGA AGTCTCTGAA GTTGCAGCCT TTGTATTACA CTTTTGAGCT

CTGACCAAGA GGTCTCTGAA GTTGCAGCCT TTGTATTACA CTTTTGAGCT

CTGACCGAGA GGTCTCTGAA GTTGCAGCCT TTGTATTACA CTTTTGAGAT

CTGACCGAGA GGTCTCTGAA GTTGCAGCCT TTGTATTACA CTTTTGAGAT

CTGACCAAGA GGTCTCTGAA GTTGCAGCCT TTGTATTACA CTTTTGAAAT

CTGACC-AGC GGTCTCTGAA GTTGCAGCCT TTGTATTACA CTTTGGAAGT

CTGACCAAGA GGTCTCTGAG GCAGCAGCCT TTGTGTTATA CATTGGGAAT

CTGACCAAGA GGTCTCTGA- -----AGCC- ---------- ------AAAT

---------- ---------- ---------- ---------- ------GAAT

GTGACCAAGA GGTCTCTGAA GTTGTAGCCT TTGTATTACA CTTTTGAAAT

CCGACCAAGA GGTCTCTGAA GTTGTAGGCT TTGTATTACA CGTTTGAAAT

CCGATCAACA GA--TCTGAA GTCTCAGGCT TTGTATTACA CTTTTGAAAT

------AGGG GCTCTCTGAA GTTGCAGCCT TTGACCTACA CTTTGTAAAT

CTGACCAAGA GGTCTCTGAA GTTGCAGGCT TTGTATTACA CTTTTGAAAT

CTGACCAAAA GGTCTCTCAA GTTGCAGCCT TTGTATTACA CTTTTGAAAT

CTGACCAAGG GCTCTCTGAA GTTGCAGCCT TTGTATTACA CTTTTGAAAT

CTGACCCAGA AGCCTCTGAA GTTGCAGCCT TTGTATTACA CTTTTGAAAT

CTCACCAAGA AGTCTCTGAA GTTGCAGTCT TTGTATTACA CTTTTGAAAT

ATCTCCATTG TGATGCAAAT CTCGATTTTC AATCAAAATT GGCAGTTCTG

ATCTCCATTG TGATGCAAAT CTCGATTTTC AATCAAAATT GGCAGTTCTG

ATCTCCATTG TGATGCAAAT CTCGATTTTC AATCAAAATT GGCAGTTCTG

ATCTCCATTG TGATGCAAAT CTCGATTTTC AATCAAAATT GGCGGTTCTG

ATCTTCATTG TGATGCAAAT CTTGATTTTC AATCAAAATT GGCAGTTCTG

ATCTCTATTG TGATGCAAAT CCTGATTTTC AATCAAAATT GGCAGTTCTG

ATCTCCATCG TGGTGCAAGT CTTAAATTTC AATCAAAATG GTCA------

ATCTCCATTG TGATGCAAAT CTTAATTTTC AATCAAAATT GTCA------

ATCTCCATTG TGATGCAAAT CTCGATTTTC AATCAAAATT GGCAGTTCTG

ATCTCTATTG TGATGCAAAT CTTGATTTTC AATCAAAATT GGCAGTTCTG

ATCCCTATTG TGATGCAAAT CTTGATTTTC AATCAAAATT AGCAGTTCTG

ATCTCCATTG TGATGCAAAT C--GATTTTC AATCAAAATT GGCAGTTCTG

TTCCCTATTG TGATGCAAAT CTTGAGTTAC A--TCAAATT GGCAGTCTGA

ATCTCTATTG TGATGCAAAT CTTGATTTTC CATCAAAATT GGCAGTCCTG

ATCTCCATTG TGATGCAAAT CTTGATTTTC AATCAAAATT GGCAGTTCTG

GACTCCATTG TGATGCAAAT CTTGATTTTC CATCAACGTC GGCAGCTC-G

ATTTCTATTG TGATGCAAAT TCTGATTTCC AATCAAAATT GGCAATTCTG

ATCTCCATTG TGATGCAAAT GATGATTTTC ACTCAAAACT GGCGGCTCTG

AAGGAGCATT TCTCCTGCCA AAATACACAC ACAGCCTGGG ATTAAAAGGG

AAGGAGCATT TCTCCTGCCA AAATACACAC ACAGCCTGGG ATTAAAAGGG

AAGGAGCATT TCTCCTGCCA AAATACACAC ACAGCCTGGG ATTAAAAGGG

AAGGAGCATT TCTCCTGCCA AAATACACAC ACAGCCCGGG ATTAAAAGGG

AAGGAGCATT TCTCCTGCCA AAGTACACAC ACAGCCTGGG ATTAAAAGGG

AAGGAGCATT TCTCCTGCCA AAATACACAC ACAGCCTGGG ATTAAAAGGG

-----GTGTT TCTCCTGCCA AAATACACAC ACAGCCTGGG ATTAAAGGGG

-----GCGTT TCTCCTGCCA AAATACACAC ACAGCCCAGG ATTAAAGGCG

AAGGGGCATT TCTCCTGCCA AAATACACAC ACAGCCTGGG ATTAAAAGGG

AGGAAGCATT TCTCCTGCCA AAATACACAC A--GCCTGGG ATTAAAAGGG

AAGGAGCATT GCTCCTGCCA AAATACACAC ACAGCCTGGG ATTAAAAGGG

AGGGAGCATT TCTCCTGCCA AAATACACAC ACAGCCTGGG ATTAAAAGGG

AAGAAACATT TCTCCTGCCA AAATACACAC ACAGTCTGGG ATTAAAAGGC

AAGGAGCGTT TCTGCTGCCA AAATACACAC ACAGCCTGGG ATTAAAAGGG

AAGGAGCATT GCTCCTGCCA AAATACACAC ACAGCTTGGG ATTAAAAGGG

AAGGAGCCTT CCTCCTGCCA AAGTACACAC ACAGCCTGGG ATGAAAAGGG

AAGGAACATT TCTGCTGCCA AAATACACAC ACAGTCTGGG ATTAAAAGGG

AGGGGACAGT TCTCCTGCCA AAATACACAC ACAGTCTGGG ATTAAAAGGG

CTGAAACCTA CCAGCAAGTT CTTAACGCCC TCCCACAATC ATTAGACCAA

CTGAAACCTA CCAGCAAGTT CTTAACGCCC TCCCACAATC ATTAGACCAA

CTGAAACCTA CCAGCAAGTT CTTAACGCCC TCCCACAATC ATTAGACCAA

CTGAAACCTA CCAGCAAGTT CTTAACGCCC TCCCACAATC ATTAGACCAA

CTGAAACCTA CCAGCAAGTT CTTAACGCCC TCCCACAATC ATTAGACCAA

CTGAAACCTA CCAGCAAGTT CTTAACGCCC TCCCACAATC ATTAGACCAA

CTGAAACCTA CCAGCAAGTT CTTAGCGCCC TCCCACAATC ATTAGACCAA

CTGAAACCTA CCAGCAAGTT CTTAACGCCC TCCCACAATC ATTAGACCAA

CTGAAACCTA CCAGCAAGTT CTTACCGCCC TCCCACCATC ATTACACCAA

CTGAAACCTA CCAGCAAGTT CTTACCGCCC TCCCACAATC ATTAGACCAA

CTGAAACCTA CCAGCAAGTT CTTAACGCCC TCCCACAATC ATTAGACCAA

CTGAAACCTA CCAGCAAGTT CTTAACGCCC TCCCACAATC ATTAGACCAA

TTGAAACCTA CCAGCAAGTT CTTAACGCCC TCCCACAATC ATTAGACCAA

CTGAAACCTA CCAGCAAGTT CCGAACGCCC TCCCACAATC ATTAGACCAA

CTGGAACCTA CCAGCAAGTT CTTAACGCCC TCCCACAATC ATTAGACCAA

CTGGAACCTA CCAGCAAGTT CTTAACGCCC TCCCACAATC ATGAGACCAA

CTGAAACCTA CCACCAAGTT CTTAACGCCC TCCCACAATC ATTAGACTAA

CTGAAACCTA CCACCAAGTC CTTAACGCCC TCCCACAATC ATTAGACTAA

ATCCACCATC ATTCCAGATT CATTTTTCCC TTATTCATCT GTCCAACTAA

ATCCACCATC ATTCCAGATT CATTTTTCCC TTATTCATCT GTCCAACTAA

ATCCACCATC ATTCCAGATT CATTTTTCCC TTATTCATCT GTCCAACTAA

ATCCACCATC ATTCCAGATT CATTTTTCCC TTATTCATCT GTCCAACTAA

ATCCACCATC ATTCCAGATT CATTTTTCCC TTATTCATCT GTCCAACTAA

ATCCACCATC ATTCTAGATT CATTTTTCCC TTATTCATCT GTCCAACTAA

ATCCA-CATC ATTCTAGATT CATTTTTCCT TTATTCATCC GTCCAACTAA

ATCCA-CATC ATTCTAGATT CATTTTTCCT TTATTCATCC GTCCAACTAA

ATCCAGCGTC ATTCTAGATT CATTTTTCCC GTATTCATCT GTCCAACTAA

ATCCACCATC ATTCTAGATT CATTTTTCCC TTATTCATCT GTCCAACTAA

ATCCACCATC ATTCCAGATT CATTTTTCCC TTATTCATCT GTCCGACTAA

ATCCACCATC ATTCTAGATT CATTTTTCCC TTATTCAACT GTCCAACTAA

ACCCACCTTC ATTCTAGATT CATTTTCCCC TTATTCATCT GCCCAACTAA

ATCCACCATC ATTCCAGATT CATTTTTCCC TTATTCATCT GTCCAACTAA

ATCCACCATC ATTCCAGATT CATTTTTCCC TTATTCATCT GTTCAACTAA

ATCCGCCATC ATTCCAGATC CATTTTTCCC TTATTCATCT GTCCAACTAA

ATCCGCCATC ATTCCAGATT CATTTTTCCC TTATTCATCT GTCCAACTAA

ATCTGCCATC ATTCCAGATT CATTTTTCCC TTATTCATCT GTCCAACTAA

TTCCTGCCAC ATTCACCAGG CTTATATCTT AATCTTTTCT GTTACATAGA

TTCCTGCCAC ATTCACCAGG CTTATATCTT AATCTTTTCT GTTACATAGA

TTCCTGCCAC ATTCACCAGG CTTATATCTT AATCTTTTCT GTTACATAGA

TTCCTGCCAC ATTCACCAGG CTTATATCTT AATCTTTTCT GTTACATAGA

TTCCTGCCAC ATTCACCAGG CTTATATCTT AATCTTTTCT GTTACATAGA

TTCCTGCCGC ATTCACCAGG CTTATATCTT AATCTTTTCT GTTACATAGA

TTCCTCCCGC ATTCACCGGG CTTATATCTT AATCTTTTCT GTTACATAGA

TTCCCCCCGC ATTCACCAGG CTTATATCTT AATCTTTTCT GTTACATGGA

TTCCTGCCAC ATTCAGCGGG CTTATATCTT AATCTTTTCT GTTACATAGA

TTCCTGCCGC ATTCACCAGG CTTATATCTT AATCTTTTCT GTTACATAGA

TTCCTGCCGC ATTCACCAGG CTTATATCTT AATCTTTTCT GTTACATAGA

TTCCTGCCGC ATTCACCAGG CTTATATCTT AATCTTTTCT GTTACATAGA

TTCCTGCCGC ATTCACCGGG CTTATATCTT AATCTTTTCT GTTACATAGA

TTCCTGCCGC ATTCACCAGG CTCACATCTT AATCTTTTCT GTTACATAGA

TTCCTGCGGC ATTCACCAGG CTTACATCTT AATCTTTTCT GTTACATAGA

TTCCTGCCGC ATTCACCGGG CTTACATCTT AATCTTTTCT GTTACATAGA

TTCCTGTTGC ATTCAGCAGG CTTACATCTT AATCTTTTCT GTTACATAGA

TTCCTGCTGC ATTCAGTAGG CTTACATCTT AATCTTTTCT GTTACATAGA

GAATACATAA TGAAAAGCTG CATTTTCTGG GTACATGCAC ACATTTGGAG

GAATACATAA TGAAAAGCTG CATTTTCTGG GTACATGCAC ACATTTGGAG

GAATACATAA TGAAAAGCTG CATTTTCTGG GTACATGCAC ACATTTGGAG

GAATACATAA TGAAAAGCTG CATTTTCTGG GTACATGCAC ACATTTGGAG

GAATACATAA TGAAAAGCTG CATTTTCTGG GTACATGCAC ACATTTGGAG

GAATACATAA TGAAAAGCTG CATTTTCTGG GTACATGCAC ACATTGGGAG

GAATACGTAA TGAAAAGCTG CATTTTCTGG GTACATGCAC ACATTTGGAA

GAATACATAA TGAAAAGCTG CATTTTCTGG GTACATGCAC ACATTGGGAA

GAAGACACAA TGAAAAGCTG CATTTTCTGG GTACATGCAC ACATTGGGAG

GAATACATAA TGAAAAGCTG CATTTTCTGG GTACATGCAC ACATTTGGAG

GAATACATAA TGAAAAGCTG CATTTTCTGG GTACATGCAC ACATTTGGAG

GAATACATAA TGAAAAGCTG CATTTTCTGG GTACATGCAC ACATTTGGAG

GAATACATCA TGAAAAGCTG CATTTTCTGG GTACATGCAC ACATTTGGAG

GAATACATCA TGAAAAGCTG CATTTTCTGG GTACATGCAC ACATTTGGAG

GAATACATAA TG-AAAGCTG CGTTTTCTGG GTACATGCAC ACATTTGGAG

GAATACATCA TGAAAAGCTG CATTTTCTGG GTACATGCAC ACATTTGGAG

GAATACATAA TGAAAAGCTG CATTTTCTGG GTACATGCAC ACATTTGGAG

GAATACATAA TGAAAAGCTG CATTTTCTGG GTACATGCAC ACATTTGGAG

CGGATGAACC ATATGTGAAG CGACATACAA TGCTGAATGC CGGCTGAATG

CGGATGAACC ATATGTGAAG CGACATACAA TGCTGAATGC CGGCTGAATG

CGGATGAACC ATATGTGAAG CGACATACAA TGCTGAATGC CGGCTGAATG

CGGATGAACC ATATGTGAAG CGACATACAA TGCTGAATGC CGGCTGAATG

CGGATGAACC ATATGTGAAG CGACATACAA TGCTGAATGC CGGCTGAATG

CGGATGAACC ATATGTGAAG CGACATACAA TGCTGAATGC CGGCTGAATG

CGGATGAACC ATATGTGAAG CGACATACAA TGCCGAATGC CGGCTGAATG

CGGATGAACC ATACGTGAAG TGACATACAA TGCTGAATGC TGGCTGAATG

CGGGTGAACC ATATGTGAAG TGACATACAA TGCTGAATGC CCGCTGAATG

CGGATGAACC ATATGTGAAG TGACATACAA TGCTGAATGC AGGCTGAATG

CGGATGAACC ATATGTGAAG GGACATACAA TGCTGAATGC GGCCTGAATG

CGGATGAACC ATATGTGAAG TGACATACAA TGCTGAATGC GGGCTGAATG

TGGATGAACC ATATGTGAAA GGACATACAA TGCTGAAGGC GGGTTGAATA

CGGATGAACC ATATGTGAAG TGACATACAA TGCTGAATGC CGGCTGAATG

CGGATGAACC ATACGTGAAG TGACATACAA TGCCGAATGC TGCCTGAATG

CGGATGAACC ATATGTGAAA GGACATACAA TGCTGAATGC CGCCTGAATG

CGGATGAACC ATATGTGAAG TGACATACAA TGCTGAATGG TGGCTGAATG

CGGATGAACC ATATGTGAAG TGACATACAA TGCTGAATGG GGGCTGAATG

GCGTCCAGCG CTAAAAACAC CGCGAGCGCA GGAACAAAAA GGCCTCTGCT

GCGTCCAGCG CTAAAAACAC CGCGAGCGCA GGAACAAAAA GGCCTCTGCT

GCGTCCAGCG CTAAAAACAC CGCGAGCGCA GGAACAAAAA GGCCTCTGCT

GCGTCCAGCG CTAAAAACAC CGCGAGCGCA GGAACAAAAA GGCCTCTGCT

GCGTCCAGCG CTAAAAACAC CGCAAGCGCA GGAACAAAAA GGCCTCTGCT

GTGTCCAGCG CT-AAAACAC TGTGAGCGCA GGAACAAAAA GGCCTCTGCT

GTGTCCAGCA CTAAAAACAC TGCGAACCCA GGAACAAAAA GGCCTCTGCG

GTCTCCAGCA CTAAAAACAC GGCGAACCCA GGAACAAAAA GGCCTCCGCA

GCGGCCGGTG CTGCAAACAC TATGAGTGCA GAGACAAAAA GGCCTCTGCT

GTGTCCAGCG CTAAAAACAC TGCGAAGGCA GGAACAAAAA GGTCTCCACT

GTGTCCGGCG CTAAAAACAC TGCGGACGTC CGAACAAAAA GGCCTCTGCT

GTGTCCAGCG CTAAAAACTC TGCGAACGCA GGAACAAAAA GGCCTCTGCT

GTGTCCAGCG CTAAAAACAC AGCGAAGGCA GAAACAAAAA GGCCCCGGCT

GTGTCCAGCG CCAAAAACAC TGCGCAGGCA GGAACAAAAA GGCCTCTGCT

GGGTCCAATG CTGAAAACAC TGTGGACGCG GGAACAAAAA GGCCGCGGCT

GTGTCCAGCG CGAAAAACAC TGCGGACGCA GGAACAAAAA GGCCGCGACT

GTATCCAGTG CTAAAAACAC TGCAAACCCA GGAACAAAAA GGCCTCTGCT

GTGTCCAGTG CTAAAAACAC TGCAAACCCA GGAACAAAAA GGCCTCTGCT

GGAACACCAG GGGCTTCGCC GCGAAAACAT GGGTATAATT TCACTGTTTT

GGAACACCAG GGGCTTCGCC GCGAAAACAT GGGTATAATT TCACTGTTTT

GGAACACCAG GGGCTTCGCC GCGAAAACAT GGGTATAATT TCACTGTTTT

GGAACACCAG GGGCTTCGCC GCGAAAACAT GGGTATAATT TCACTGTTTT

GGAACACCAG GGGCTTCGCC GCAAAAACAT GGGTATAATT TCACTGTTTT

AGAACACCAG GGGATTCGCC GCGAAAACAT GGGTATAATT TCACTGTTTT

AGGACACCGG GGGATTCGCC GGGAAAACAT GGGTATAATT TCACTGTTTT

AGGACACCAG GGGATTCGCC GGGAAAACAT GGGTATAATT TCAGTGTTTT

CCAGCGCCAG GGGACTCGCT GCGAAAACAT GGGTATAATT TCACTGTTTT

AGAACACCAG GGGATTCGCC GCTAAAACAT GGGTATAATT TCACTGTTTT

AGAACACCAG GGGATTCGTC GCCGAAACAT GGGTATAATT TCACTGTTTT

GGAACACCAG GGGACTCGCC GCTAAAACAT GGGTATAATT TCACTGTTTT

CGAACACCAG GGGGCTCGCC GCTAAAACAT GGGTATAATT TTACTGTTTT

CAAACACCAG GGGACTGGCC ACTAAAACAT GGGTATAATT TCACTGTTTT

GGAACACCAG GGGACTGGCT GCTAAAACAT GGGTATAATT TCACTGTTTT

GGAACACCAG GGGACTGGCC GCTGAAACAT GGGTATAATT TCACTGTTTT

TGAACACCAG GGGATTCACC ACTAAAACAT GGGTATAATT TCACTGTTTT

TAAACACCAG GGGACTCGCC AGTAAAACAT GGGTATAATT TCACTGTTTT

CACTCACACA AGCTGTGGGA AAGGAGTCAC CATCTGATGT TTGATTGTGT

CACTCACACA AGCTGTGGGA AAGGAGTCAC CATCTGATGT TTGATTGTGT

CACTCACACA AGCTGTGGGA AAGGAGTCAC CATCTGATGT TTGATTGTGT

CACTCACACA AGCTGTGGGA AAGGAGTCAC CATCTGATGT TTGATTGTGT

CACTCACACA AGCTGTGGGA AAGGAGTCAC CATCTGATGT TTGATTGTGT

CACTCACACA AGCTGTGGGA AAGGAGTCAC CATCTGATGT CTGATTGTGT

CACTCACGCA AGCTGTGGGA AAGGAGTCAC CATCTGATGT TTGATTGTGT

CAATCACGCA AGCTGTGGGA AAGGAGTCAC CATCTGATGT TTGATTGTGT

CAGTCACACA AGCTGTGGGA AGGGAGTCAC CATCTGATGT TTGATTGTGG

CACTCACACA AGCTGTGGGA AAGGAGTCAC CATCTGATGT TTGATTGTGT

CACTCACACA AGCTGTGGGA AAGGAGTCAC CATCTGATGT TTGATTGTGT

CACTCACACA AGCTGTGGGA AAGGAGTCAC CATCTGATGT TTGATTGTGC

CACTCACACA AGCTGTGGGA AAAGAGTCAC CATCTGATGT TTGATTGTGT

CACTCACACA AGCTGTGGGA AAGGAGTCAC CATCTGATGT TTGATTGTGT

CACTCACACA AGCTGTGGGA AAGGAGTCAC CATCTGATGT TTGATTGTGT

CACTCACACA AGCTGTGGGA AAGGAGTCAC CATCTGATGT CTGATTGTGT

CACTCACACA AGCTGTGGGA AAGGAGTCAC CATCTGATGT TGGATTGTGA

CACTCACACA AGCTGTGGGA ATGGAGTCAC CATCTGATGT TTGATTGTGC

GGGATATGTT TTTGGCAGTG GCCTTGTATT CCTCATTGAC aaaaaaaagg

GGGATATGTT TTTGGCAGTG GCCTTGTATT CCTCATTGAC aaaaaaaaGG

GGGATATGTT TTTGGCAGTG GCCTTGTATT CCTCATTGAC aaaaaaagGG

GGGATATGTT TTTGGCAGTG GCCTTGTATT CCTCATTGAC aaaaaaagGG

GGGATATGTT TTTGGCAGTG GGCTTGTATT CCGCATTGAC aaaaaaagGG

GGGATATGTT TTTGGCAGTG GCCTTGTATT CTTCATTGAC CaaaaaaaGG

GGGGTATGTT TTCGGCAGTG GCCTTGTATT CTGCATCGAC aaaaaaaaAG

GGGATATGTT TTTGGCAGTG GCCCTGTATT CTGCATTGAC aaaaaaaaAG

GGGATATGTT TTTGGCAGTG GCCTTGTAGT CTGCATTCAT gaaaaaaagg

GGGATATGTT TTTGGCAGTG GCCTTGTATT CCGCATTGAC AAAAAAGAGA

GGGATATGTT TTTGGCAGTG GCCTTGTATT CCACATTgac aagaaaaagg

GGGATATGTT TTTGGCAGTG GCCTTGTATT CTGCATTGAC aaaaaaagGG

GGGATATGTT TTTGGCAGTG GCCTTGTATT CTGCACTGaa aaaaaaaaGG

GGGATATGTT TTTGGCAGTG ACCTTGTATT GCGCATTGAC aaaaaaaagg

GGGATATGTT TTTGGCAGTG GCCTTGTATT CTGCATTGAC AAAAAAAGGG

GGGATATGTT TTTGGCAGTG GTCTTGTATT CCGCATTGAC AAAAAAAGGA

GGGATATGTT TTTGGCAGTG GCCTTGTATT CTACATTGAA GGGGAGAGGA

GGGATATGTT TTTGGCAGCG GCCTTGTATC CTGCATTGAG GGGAAAAAGG

aaaataaaaa agaaTAGAGC TACATTTGCT GTTTTCAATT TGCAGTCTGG

AAAATAAAAA AGAATAGAGC TACATTTGCT GTTTTCAATT TGCAGTCTGG

AAAATAAAAA AGAATAGAGC TACATTTGCT GTTTTCAATT TGCAGTCTGG

AAAATAAAAA AGAATAGAGC TACATTTGCT GTTTTCAATT TGCAGTCTGG

AAAGTGAAAA AGAATAGAGC TACATTTGCT GTTTTCAATT TGCGGTCTGG

AAAAGGAAAA AGAATAGAGC TACATTTGCT ATTTTCAATT TGCTGTCTGG

GAAATAAAAA GGAATCCAGC TACATTTGCT GTTTTCAATT TGCTGCTTGG

GAAATAAAAA AGAATGCAGT TACATTTGCA GTTTTCAATT TGCTGCTTGG

aaaatgaaaa agaaCAGAGC TACAATCGCT GTTTTCAATG TGCTCTCTGG

AAAAAAAAAA AGAATAGAGC TACATTTGCT GTTTTTAATT TGCCGTCTGG

aagataaTCA AGAATAGAGC TACATTTGCT GTTTTTAATT TGTAGTCTGG

AAAATAAAAA AGAATAGAGC TACATTTGCT GTTTTTAATT TGCCGTCTGG

AAAAGGAGAA AGAATAGAGC TACATTTGCT GTTTTTAATT TGCCGTCTGG

aaaagaaaaA GGAATAGAGC TACATTTGCT GTTTAGAATT TGGCATCTGG

-AAAATAAAA AGAATAGAGC TACATTTGCT GTTTTGAA-T TGGCGTCTGT

AAAAAAAAAA AGAATAGGGC TACATTTGCT GTTTTGAATT TGGTGTCTGG

AAAATAAAAA AGAATAGAGC TACATTTGCT GTTTTGAATT TGGCATCTGG

AAAATAAAAA AGAATAGAGC TACATTTGCC GTTTTGAATT TGCCGTCTGT

GGACAGGCAC AGCTGATAAT GATGGAGCAA TATATCTACA ATAAATTAAG

GGACAGGCAC AGCTGATAAT GATGGAGCAA TATATCTACA ATAAATTAAG

GGACAGGCAC AGCTGATAAT GATGGAGATA TATATCTACA ATAAATTAAG

GGACAGGCAC AGCTGATAAC GATGGAGCCA TATATCTACA ATAAATTAAG

GGACAGGCAC AGCTGAAAAT GATGGAGCAA TATATCTACA ATAAATTAAG

GGACAGGCAC AGCTGATAAT GATGGAGCAA TATATCTACA ATAAATTAAG

GGACAGACAC AGCTGATAAT GGCGCAGCAA TAGACCTACA ATAAATTAAG

GGACAGACAC AGCTGATAAT GACCCCGCAA TAGACCTACA ATAAATTAAG

GCACAGGCAC AGCTGATAAT GATGGAGCAA TGTATCTCCA ATAAATTAAG

GGCCAGGCAC AGCTGATAAT GACGGAGCAA TATATCTACA ATAAATTAAG

GGACAGGCAC AGCTGATAAT GACGGAGCAA TATATCTACA ATAAATTAAG

GGACAGACAC AGCTGATAAT GACGGAGCAA TATATCGACA ATAAATTAAG

GGACCAGCCC AGCTGA---- ---------- ---------- ----ATTAGG

GGACGGGCAC AGCTGATAAT TATCGAGCAA TATATCTACA ATAAATTAAG

GGCCAGGCAC AGCTGATAAT TATAGAGCAA TATAGCTACA CTAAATTAAG

GG-------- ------TAAT TATAGAGCAA TATATCTACA CTAAATTAAG

GGGCGGGCAC AGCTGATAAT TATGGAGCAA TATATCTACA ATAAATTAAG

GGAAGGGCAC AGCTGATAAT TACGCGGCAA TATATCCGCA ATAAATTAAG

ACTGCTTTGC AATTAGTCCG ATGATCACAG CTGCGGAGAC AAATGGCATA

ACTGCTTTGC AATTAGTCCG ATGATCACAG CTGCGGAGAC AAATGGCATA

ACTGCTTTGC AATTAGTCCG ATGATCGCAG CTGTGGAGAC AAATGGCATA

ACTGCTTTGC AATTAGTCCG ATGATCACAG CTGTGGAGAC AAATGGCATA

ACTGCTTTGC AATTAGTCCG ATGATCACAG CTGCGGAGAC AAATGGCATA

ACTGCTTTGT AATTAGTCCG ATGATCACAG CTGTGGAGAC AAATGGCATA

GCTGCATTGC AATTAGCCCA ATGATCACAG CTGCC-CCAC AAATGGCATA

GCTGCATTGC AATTAGCCCA ATGATCACAG CTGCCGCCAT AAACGGCATA

GCTGCTTTGC AATTAGTCCG ATGATCACAG CTGCGGAGAC AAATGGCATA

ACTGCTTTGC AATTAGTCCG ATGATCACAG CTGTGGAGAC AAATGGCATA

ACTGCTTTGC AATTAGTCCA ATGATCACAG CTGTGGAGAC AAATGGCATA

ACTGCTTTGC AATTAGTCTG ATGATCACAG CTGTGGAGAC AAATGGCACA

GCGGCTTTTC CATTAGCACG ATGATCACAC TGGTGGAGAC AAATGGCATA

ACGGCTCTGC AATTAGTCCG ATGATCACAG CTGTGGAGAC AAATGGCATA

ACTGCTTTGC AATTAGTCCG ATGATCACAG CTGTAGAGAC AAATGGCCTA

ACTGCTTTGC AATTAGGCTG ATGATCGCAG CTGTGGAGAC AAATGGCAAA

GCTGCTTTGC AATTAGTTGG AGGAACGCAG CTGTGGAGAC AAATGACATA

GCCGCTTGGC AATTAGTTGG GCGCACGCAG CTGGGGCGAC AAACGGCATA

TGTGCTAATT AAATAATTTG TCACTTCACT GTTAAACAAA CAATTCAGCC

TGTGCTAATT AAATAATTTG TCACTTCACT GTTAAACAAA CAATTCAGCC

CGTGCTAATT AAATAATTTG TCACTTCACT GTTAAACAAA CAATTCAGAC

TGTGCTAATT AAATAATTTG TCACTTCACT GTTAAACAAA CAATTCAGCC

TGTCCTAATT AAATAATTTG TCACTTCACT GTTAAACAAA CAATTCGGCC

TGTGCTAATT AAATAATTTG TCACTTGACT GTTAAACAAA CAATTCAGCC

TGCACTAATT AAATAATTTG TCACTTGACT GTTAAACAAA CAACTCAGCC

TGCACTAATT AAATAATTTG TCACTTGACT GTTAAACAAA CAACTCAGCC

TGTGCTAATT AAATAATTTG TCGCTCGACT GTTAAACAAA CAATTCAGC-

TGTGCTAATT AAATAATTTG TCACTTGACT GTTAAACAAA CAATTCAGGC

TGTGTTAATT AAATAATTTG TCACTTGACT GTTAAACAAA CAATTCAGCC

TGTGCTAATT AAATAATTTG TCACTTGATT GTTAAACAAA CTATTCAACC

TGTGCTAATA AAATAATTTG TCACTTGACT GCTAAACAA- ---TTCGGGC

TGTGCTAATT AAATAATTTG TCACTTGACT GTTGAACAAA CAATTCAGCC

CATGTTAATT AAATAATTTG TCACTTGACT GTTAAACAAA CAATCCAGCC

TGCACTAATT AAATAATTTG TCACTTGACT GTTAAACAAA CAATTCAGCC

TGTGCTAATT AAATAATTTG TCACTTGACT ATTAAACAAA CAATTCAGCC

CGTGCTAATT AAATAATTTG TCACTTGACT GTTAAACAAA CAAGCCGGCC

TCTTTATATT ACAAACTGCC ATCTCCAGAA CAAACCAAGT AGCATCCATT

TCTTTATATT ACAAACTGCC ATCTCCAGAA CAAACCAAGT AGCATCCATT

TCTTTATATT ACAAACTGCC ATCTCCAGAA CAAACCAAGT AGCATCCATT

TCTTTATATT ACAAACTGCC ATCTCCAGAA CAAACCAAGT AGCATCCATT

TCTTTATATT ACAAACTGCC ATCTCCAGAA CAAACCAAGT AACATCCATT

TCTTTATATT ACAAACTGCC ATCTCCAGAA CAAACCAAGT AGCATCTATT

TCTTTACGTT ATACTCTGCC ATTTCCAGCA C-AACCGAGT A---TCCATT

TCTTTATATT ACACTCTGCC ATTTCCAGCA CAAACCGAGT AGCTTCCATT

-CTTTACATT GCAGCCTGCC ACTTGCAGAA GAAACCAAGG CACTTCCATT

TCTTTACATT ACAAATTGCC ATCTCCAGAG CAAACCAAGT AACATCCATT

TCTTTACATT ATAAACTGCC ATCTCCAGAA CGAACCAAGT AGCATCCATT

TCTTTACATT ACAAACTGCC ATCTCCAGAA CAAACCAAGT AGCATCCATT

TCTTTCCATT ACAAAGTGCC ATCTCTGGAA CAACCCAGGT AGCAGCCACT

TCTTTACATT ACAAACTGCC ATCTCCAGCG CGAACCAAGG AGCATCCATT

TCTTTACATT ACCAACTGCC ATCTCCAGAA CAAATCAAGG AGCATCCACT

TC--TACATT CCAAACTACC ATCTCCAGAG CCAGCCAAGG CTCATCCATT

TCTTTCCATG ACAAACTAGC ATCTCTCGAA CAAACTAAGT AGCATCTATT

TCTTTGCATC CCGGCCTAGC GTCTCCGGAA TAAGCAAAGG AGCATCTATT

CAGTTTCTCT AATCAGCTTC AAAAGAAGTC GATTTCTTCC TGCATTCAGA

CAGTTTCTCT AATCAGCTTC AAAAGAAGTC GATTTCTTCC TGCATTCAGA

CAGTTTCTCT ACTCAGCTTC AAAAGAAGTT GATTTCTTCC TGCATTCAGA

CAGTTTCTCT ACTCAGCTTC AAAAGAAGTT GATTTCTTCC TGCATTCAGA

CAGTTTCTCT AATCAGCTTC GAAAGAAGTC GATTTCTTTC TGCATTCAGA

CAGTTTCTCT AATCAGCTTC AAAAGAAGTC GATTTCTCCC TTCATTCAGA

CGGTTTCTCT AATCAGCTTG AAAGGGACTG TATTCTCCCA AGCATTCGGA

CAGTTTCTCT AATCGGCTTG AAAAGAACTG TATCTCTCCG GGCACTCAGA

CAGTTTCTCT AATCAGCTTC AAAAGAAGTC GCTCTCTTCC TGCATTGCTT

CAGTTTCTCT AATCAGCTTC AAAAGAAGTC GATTTCTTCC CACATTCAGA

CAGTTTCTCT AATCAGCTTC AAAAGAAGTT GATTTCTTCC TGCATTCAGC

CAGTTTCTCT AATCAGCTTC AAAAGAAGTC GATTTCTTCC TGCATTCAGA

CGCTTGCTCT GATCGGTGTC AGC-GAGGTC AGCGCCTTCC TGCCTTCAGG

CAGTTTCTCT AATCAGCTTC AAAAGAAGTC TGTTTCTTCC TGCATTCAGA

CAGTTTCTCT AATCAGCTTC AAAGAAAATC TGTTTCTCCC ATCGTTCAGA

CAGTTTCTCT AATCAGCTTC AAGGAAAGTC TGTTTCTCCC CGCATTCAGC

GGGTTTCTCT AATCAGTCTC AATAGAAATC TATTTCGTCT TGCATTCAGA

CAGTCTCTCT AATCAGTTTC AAAAGAAGTC TATTTCGTCT AGCATTCAGG

CCTCGTCTGC CCCTGAGTGG TGCAGATTAC ATGAGGTAAT ACGGCAGTTA

CCTCGTCTGC CCCTGAGTGG TGCAGATTAC ATGAGGTAAT ACGGCAGTTA

CCTCATCTGC CCCTGAGTGG TGCAGATTAC ATGAGGTAAT ACGGCAGTTA

CCTCATCTGC CCCTGAGTGG TGCAGATTAC ATGAGGTAAT ACGGCAGTTA

CCTCATCTGC CCCTGAATGG TGCAGATTAC ATGAGGTAAT ACGGCAGTTA

CCTCATCTGC CCTTGAATGG TGGAGCTTAC ACTAGGCAAC AT-GCAGTTC

TCTCCTCTGC CCGGGAAGGG TGGTGATGAC AAG--GGAAC TGGGCAGTTC

TCTCATCTGC CCCCAAATGG CAGCTATTAC AAG--GTAAC AGGGCATTTC

TCAGACCTGC CCGCTCACTG TGCAAGTTAC AGGAGGGGAT GGGGCAG---

CCTCAGCTGC CCCTGAATGG TGGAGATTAC AGGAGGTAAT ACCGGCGTTA

CCTCAGCTGC CCCTGAATGG TGGAGATTAC ATGAGGTAAT ACGGCAGTTA

CCTCAGCTGC TCCTGAATGG TGGAGATTAC ATGAGGTAAT AATACCGTTA

CCT------- ---------- ---------- ---------- ---------A

CCTCATCCGC CCCTGAATGG TGGCGATTCC AGGAGGTAGT ACAGCAGCTA

CCTCATCTGC CCCTGAGTGG CAGAGATTAC ATGAGGTAAC ACAGCAGTTA

CCTCCTCTGG CCCTGAATGG CGGAGATTGC ATGAGGTAAC ACACTAGTTA

CCTAATCTGA CCCTGAATGG TGGAGATTAC ATGAGGTAAT ACAGCAGTTA

CCTAATCTGA CCCTGAATGG TGGAGATTAC ATGAGGTAAT ACAGCAGTTA

TTTCTACCTG CTGCAGTTCA TCAGCTCCAT CTCCAGCTGA TAGCAATCTG

TTTCTACCTG CTGCAGTTCA TCAGCTCCAT CTCCAGCTGA TAGCAATCTG

TTTCTACCTG CTGCAGTTCA TCAGCTCCAT CTCCAGCTGA TAGCAATCTG

TTTCTACCTG CTGCAGTTCA TCAGCTCCAT CTCCAGCTGA TAGCAATCTG

TTTCTACCTG CTGCAGTTCA TCAGCTCCAT CTCCAGCTGA TAGCAATCTG

TTTCTACCTG CTACAG-CCA TCAGCTCCTT CTCCAGCTGA TAGCAATCTG

TTTCTACCTA TTGTAGCTCA CCAGCTCCAT CTCCAGCTAA TGGCAGCCTG

TTTCTACCTA TTGTAGCTCA TCAGCTCCAT CTCCAGCTGA TGGCAGCCTG

-------CTA CTG-----CC TCAGCTCCCT CC-------- ---------G

TTTCTACCTG CTGTAGTCCA TCAGCTCCAT CTCCAGCTGA GAGCAATCTG

TTTCTACCTG CTGTAGTTCA TCAGCTCCAT CTCCAGCTGA TAGCAATCTG

TTTCTACCTG CTGCAGTCCA TCAGCTCCAT CTCCAGCTGA TAGCAATCTG

TCTCTACCTG CC-CAGAGTG CCAGCTCCAC CTCCAGCTGA CAGTGAGCTG

TTTCTACTTA CTGTAGTTCA TCTGTGCCAT CTCCAGCTGA CAGAAGTCTG

TTTCTACCTG CTCTAGTTCA TCAGCTCCTT CTCCAACG-- ---AAATCTG

TTTCTACCTG CTGCAGTTCA TCAGCTCCTG CTCCAGCAGA TAGAAATCTG

TTTCTACCTG CTACAGTTCA TAAGCTCCAT CTCCAGCTGA CAGAAAGCGG

TTTCTACCTG CTGTAGTTCA TAAACTCCGT CTCCAGCTGA CAGAAATCTG

GATGTTGCCT ACACTTTGAG AAAGGTTTTT GAAGAAAGAT TAGCAGGATT

GATGTTGCCT ACACTTTGAG AAAGGTTTTT GAAGAAAGAT TAGCAGGATT

GATGTTGCCT ACACTTTGAG AAAGGTTTTT GAAGAAAGAT TAGCAGGATT

GATGTTGCCT ACACTTTGAG AAAGGTTTTT GAAGAAAGAT TAGCAGGATT

GATGTTGCCT ACACTTTGAG AAAGGTTTTT GAAGAAAGAT TAGCAGGATT

GATGTTGCCT ACACTTTGAG AAAGGTTTTT GAAGAAAGAT TAGCAGGATT

GATGCTGCCT ACAGTGGGAG AAAGTTTTTT GAAGAAAGAT TAGCAGGATT

GTTGCTGCCT ACAGTGGGAG AAAGTTTTTT GAAGAAAGAT TAGCAGGATT

GACGTTGCCT ACACTTTGAG GACGGTTTTT GAAGAAAGAT TAGCAGGATT

GATGTTGCCT ACACTTTGAG AAAGGTTTTT GAAGGCAGAT TAGCAGGATT

GATGTTGCCT ATACTTTGAG AAAGGTTTTT GAAGAAAGAT TAGCAGGATT

GATGTTGCCT ACACTTTGAG AAAGGTTTTT GAAGAAAGAT TAGCAGGATT

GATGTTGCC- GCACTTGGAG AAAGGTTTCT GAAGGGAGAT TAGCAGGATT

GATGTTGCCT ACACTTTGAG AAAAGTTTTT GAAGACAGAT TAGCAGGATT

GATGTTGCCT GCACTTTGAG AAAGGTTTTT GAAGAAAGAT TAGCAGGATT

GATGTTGCCT ACACTTTGAG AGAGGTTTTT GAAGACAGAT TAGCAGGATT

TATGTTGCCT GCACTTTGAG AAAGGTTTTT GAAGAAAGAT TAGCAGGATT

GATGTTGCCT ACACTTTGAG AAAGGTTTTT GAAGGAAGAT TAGCAGGATT

AGGAAAGGTT CACATATGTT CAGCCTCACT CTTGGTATTC AAGACATCAC

AGGAAAGGTT CACATATGTT CAGCCTCACT CTTGGTATTC AAGACATCAC

AGGAAAGGTT CACATATGTT CAGCTTCACT CTTGGTATTC AAGACATCAC

AGGAAAGGTT CACATATGTT CAGCTTCACT CTTGGTATTC AAGACATCAC

AGGAAAGGTT CACATATGTT CAGCCTCACT CTTGGTATTC AAGACGTCAC

AGGAAAGGTT CACATATGTT CAGCCTTTCT CTTGGTATTC AAGACATCAC

AAGAAAGGTT CACATATGTT CAGC------ ---------- ----------

AAGAAAGGTT CACATATGTT CAGCCTTACT CTTGGTGTTC AAGATATCAC

AGGAAGGGTT CACATATGTT CAGCCTCACT CTTGGTATTC AAGACATCAC

AGGAAAGGTT CACATATGTT CAGCCTCACT CTTGGTATTC AAGACATCAC

AGGAAAGGTT CACATATGTT CAGCCTCACT CTTGGTATTC AAGACATCAC

AGGAAAGGTT CACATATGTT CAGCCTCACT CTTGGTATTC AAGACATCAC

AGGAAAGGTT CACATATGTT CCGCCTCATT CTTGGTATTC AGGACGCCAC

AGGACAGGTT CACATATGTT CAGCCTCAAT CCTGTTATTC AGGCGGTCAC

AGGGAAGATT CACATATGTT CAGCCTCACT CTTGGTATTC AAGACATCAC

AGGAAAGGTT CGCATATGTT CAGCCTCACT CTTGGTATTC AAGACATCAC

AGGAAAGGTT CACATATGTT CAGTTTCACT ATTGGTATTC AAGACATCAC

AGGAAAGATT CACATATGTT CAGCTTTACT ATTGGTATTC AAGACATCAC

AACCCTGCAC CACCCAGGAA GCTAATTACT ATTTAGGTTG CCTGGTTCCC

AACCCTGCAC CACCCAGGAA GCTAATTACT ATTTAGGTTG CCTGGTTCCC

AACCCTGTAC CACCCAGGAA GCTAATTACT ATTTAGGTTG CCTGGTCCCC

AACCCTGTAC CACCCAGGAA GCTAATTACT ATTTAGGTTG CCTGGTTCCC

AACCCTGCAC CACCCAGGAA GCTAATTACT ATTTAGGTTG CCTGGTTCCC

AACCCTGTGC CACCCAGGAA GCTAATTATT ATCTGGGTTG CCTGGTTCCC

---CTCCAAC CACCCAGGAA GCTCATTACC ATGCAGGTTG CCCGCGTCCC

AGGCTCCAGC CACCCAGGAA GCTCATTACT GGGCAGGTTG CCCGCGTCCC

AACCCCGCGC CACCCAGGAA GCTAATTATC ATCTGGGTTG CTTGGTTCCC

AACCCTGCAC CACCCAGGAA GCCCATTATT GTCTAGGTTG CTTGGTTCCC

AACCCTGCAC CACCCAGGAA GCTAATTACT ATCTGGGTTG CCTGGTTCCC

AACCCTGCAC CACCCAGGAA GCTAATTACT ATCTAGGTTG CCTGGTTCCC

AGCCCCGCAC CACCCAGGA- GCTAATTACT AGCTGGGTGG CCTGGTTCCC

AACGCTGCAC CACCCAGGAA GCCCATTATC ATCTGGGTTG CCTGCTTCCC

AACCCTACAC CACCCAGGAA GCTAATTACT ATCTAGGTTG CCTGGTTCCC

AGCCCTGCAC CACCCAGGAA GCTAAATACT ATCTAGGTTG CCTGGCTCCC

AACTCTGCAC CACCCAGGAA GCTAATTACT GTTTGGGTTG CTGGCTTCCC

AACTCCACAC CACCCAGGAA GCTAATTACT GTCTGGGCTG GCGGCTCCCT

TATCCTTATA ATGAGTAAGA TATTTAGAAT CCCGAACTGT TTATGTGACA

TATCCTTATA ATGAGTAAGA TATTTAGAAT CCCGAACTGT TTATGTGACA

TATCCTTATA ATGAGTAAGA TATCTAGAGT CCCGAACTGT TTATGTGACA

TATCCTTATA ATGAGTAAGA TATTTAGAGT CCCGAACTGT TTATGTGACA

TATCCTTATA ATGAGTAAGA TATTTAGAGT CCCGGACTGT TTATGTGACA

TACCCTTATA GTAAGTAAGA TATTTAGAAT CCTGAACTGT TTATGTGACA

TCTCTGGATA ATGAGTAAGC TATTTAGAAT CTCGAACTGT TTATGTGACA

TCTCTGGATA ATGAGTAAGC TATTTAGAAT CTCGAACTGT TTATGTGACA

TATCCTTATA ATGAGTAAGA TATTTCGAGT CCCGAACTGT TTATGTGACA

TATCCTTTTA ATGAGTAAGA TATTTAAAGT CCCGAACTGT TGATGTGACA

TATCCTTATA ATGAGTAAGA TATTTAGAGT CC-GAACTGT TTATGTGACA

TATCCTTATA ATGAGTAAGA TATTTAGAGT CCTGCACTGT TTATGTGACA

TCTCCCTATA ATGAGTAAGA TATTTTGAAT CTCGAACTGT TTATGTGACA

CAGCCCTGTA ATGAGTAAGA TATTTAGAGT CGCGAACTGT TTATGTGACA

TATTTTTCTA ATGAGTAAGA TTTTTAAAGT CCTGAGCTGT TTATGTGACA

TATCCTTATA ATGAGTAAGA TATTTAAAGT CCCGAACTGT TTATGTGACA

TATCCCTCTA ATGAGTAAGA TTGTTAAAGT CCCGAACTAT TTATGTGACA

TCCCCCTGTA ATGAGTAAGA TATTTGAAGG CCCGAACTGT TTATGTGACA

TTTCAATAAG TAATGCAGAG ATTTGACAAC ATTTCCATAT TCCTCTTTTA

TTTCAATAAG TAATGCAGAG ATTTGACAAC ATTTCCATAT TCCTCTTTTA

TTTCAATAAG TAATGCAGAG ATTTGACAAC ATTTCCATAT TCCTCTTTTA

TTTCAATAAG TAATGCAGAG ATTTGACAAC ATTTCCATAT TCCTCTTTTA

TTTCAATAAG TAATGCAGAG ATTTGACAAC ATTTCCATAT TCCTCTTTTA

TTTCAATAAG TAATGCAGAG ATTTGACAAC ATTTCCATAT TCCTCTTTTA

TTTCAATAAG TAATGCAGAG ATTCAACAAC ATTTCCATAT TCCTCTTTTA

TTTCAATAAG TAATGCAGAG ATTCAACAAC ATTTCCATAT TCCTCTTTTA

TTTCAATAAG TAATGCTCAG ATTTGACAAC ATTTCCATAT TCCTCTTTTA

TTTCAATAAG TAATGCAGAG ATTCAACAAC ATTTCCATAT TCCTCTTTTA

TTTCAATAAG TAATGCAGAG ATTTGACAAC ATTTCCATAT TCCTCTTTTC

TTTCAATAAG TAATGCAGAG ATTTGACAAC ATTTCCATAT TCCTCTTTTA

TTTCAGTAAG TAATGCAGAG ATTTGACAGC ATTTCCATAT TCCTCTTTTA

TTTCAATAAG TTATGCAGAG ATTTGACAAC ATTTCCATAT TCCTCTTTTA

TTTCAATAAG TGATGCAGAG ATTTGACAAC ATTTCCATAT TCCTCTTTTA

TTTCAATAAG TAACGCAGA- -TTTCACAAC ATTTCCATAT TCCTCTTTTA

TTTCAATAAG CAATGCAGAA ATTGAACAAC ATTTCCATAT TCCTCTTTTA

TTTCAATAAA TAATGCAGAG ATTTGACAAC ATTTCCATAT TCCTCCTTTA

AAGTCTGATG CCTCTGCCTT AATCCAAGCT CCTTGGCATT TATTACTGTT

AAGTCTGATG CCTCTGCCTT AATCCAAGCT CCTTGGCATT TATTACTGTT

AAGTCTGATG CCTCTGCCTT AATCCAAGCT CCTTGGCATT TATTACTGTT

AAGTCTGATG CCTCTGCCTT AATCCAAGCT CCTTGGCATT TATTACTGTT

AAGTCTGATG CCTCTGCCTT AATCCAGGCT CCTTGGCATT TATTACTGTT

AAGTCTGATG CCTCTGCCTT AATCCAAGCT CCTTGGCATT TATTACTGTT

AAGCCCGAAG CCCCTGCCTT AATCCCAGCT CTTTGGCATT TATTACTGTT

AAGCCTGAAG CCCCTGCCTT AATCCAAGCT CTTTGGCATT TATTACTGTT

AAGTCTGATG CTCCTGCCTT AATCCAAGCT CCTTGGCATT TATTACTGTT

AAGTCTGGTG CCTCTGCCTT AATCCAAGCT CCTTGGCATT TATTACTGTT

AAGTCTGATG CCTCTGCCTT AATCCAAGCT CCTTGGCATT TATTACTGTT

AAGTCTGGTG CCTCTGCCTT AATCCAAGCT CCTTGGCATT TATTACTGTT

AAGTCTGATG CCTCTGCCTT AATCCAGGCT CCTTGGCATT TATTACTGTT

AAGTCTGATG CCTCTGCCTT AATCCAAGCT CCTTGGCATT TAGTGCTGTT

AAGTCTGATG TCTCTGCCTT AATCCGAGCT CATTGGCATT TATTACTGTT

AAGTCTGATG CCTCTGCCTT AATCCAAGCT CCTTGGCATT TATTACTGTT

ATGTCTGATG TCTCTGACTT AATCCCAGTT CCTTGGCATT TATTACTGTT

AAGTCTGAAG TCTCCGGCTT AATCCAAGTT CCTTGGCATT TATTACTGTT

TGTTACAATA ACAAGTAAGA TTATGAGAAT TCTAATGAAG GCTTCCAGTG

TGTTACACTA ACAAGTAAGA TTATGAGAAT TCTAATGAAG GCTTCCAGTG

TGTTACACTA ACAAGTAAGA TTATGAGAAT TCTAATGAAG GCTTCCAGTG

TGTTACACTA ACAAGTAAGA TTATGAGAAT TCTAATGAAG GCTTCCAGTG

TGTTACACTA ACAAGTAAGA TTATGAGAAT TCTAATGAAG GCTTCCAGTG

TGTTACACTA ACAAGTAAGA TTATGAGAAT TCTAATGAAG GCTTCCAGTG

TGTCACACTA ACAAGTAAGA TTACGGGAAT CCTAATGAAG GCTTCCAGCG

TGTTACACTA ACAAGTAAGA TTATGGGAAT CCTAATGAAG GCTTCCAGTG

TGTTACACTA ACAAGTAAGA TTATGAGAAT TCTAATGAAG GCTTCCAGTG

TGTTACACTA ACAAGTAAGA TTACGAGAAT TCTAATGAAG GCTTCCAGTG

TGTTACACTA ACAAGTAAGA TTATGAGAAT TCTAATGAAG GCTTCCCGTG

TGTTACACTA ACAAGTAAGA TTATGAGAAT TCTAATGAAG GCTTCCAGTG

TGTTACACTA ACAAGTAAGA TTATGAGAAT TCTAATGAAG GCTTCCAGTG

TGTTACACTA ACAAGTAAGA TTATGAGAAT TCTAATGAAG GCCTCCAGTG

TGTTACACTA ACAAGTAAGA TTATGAGAAT TCTAATGAAG GCTCCCCGTG

TGTTACACTA ACAAGTAAGA TTATGAGAAT TCTAATGAAG GCTTCCCGTG

TGTTACACTA ACAAGTAAGA TTATGAGAAT TCTAATGAAG GCTTCCAGTG

TGTTACACTA ACAAGTGAGA TTATGAGAAT TCTAATGAAG GCTTCCGGGG

AAAAACTCTG TGATTAAAAT GCCATAATAA CAGTTGCATG CATTTATGTG

AAAAACTCTG TGATTAAAAT GCCATAATAA CAGTTGCATG CATTTATGTG

AAAAACTCTG TGATTAAAAT GCCATAATAA CAGTTGCATG CATTTATGTG

AAAAACTCTG TGATTAAAAT GCCATAATAA CAGTTGCATG CATTTATGTG

AAAAACTCTG TGATTAAAAT GCCATAATAA CAGTTGCATG CATTTATGTG

AGAAACTCTG TGATTAAAAT GCCATAATAA CAGTTGCATG CATTTATGTG

AAAAAGTCCG TGATTAAAAT GCCATAATAA CAGTTGTGTG CATTTACGTG

AAAAAGTCCG TGATTAAAAT GCCATAATAA CAGTTGCGTG CATTTATGTG

AAAAACTCTG TGATTAAAAT GCCATAATAA CAGTTGCATG CATTTATGCG

AAAAACTCTG TGATTAAAAT GCCATAATAA CAGTTGCATG CATTTATGTG

AAAAACTCTG TGATTAAAAT GCCATAATAA CAGTTGCATG CATTTATGTG

AAAAACTCTG TGATTAAAAT GCCATAATAA CAGTTGCATG CATTTATGTG

AAAAACTCTG TGATTAAAAT GCCATAATAA CAGTTGCATG CATTTATGTG

GAAAACTCTG TGATTAAAAT GCCATAATAA CAGTTGCATG CATTTATGTG

AAAAAGTCTG TGATTAAAAT GCCATAATAA CAGTTGCATG CATTTATGTG

AAAAAGTCTG TGATTAAGAC GCCATAATAA CAGTTGCATG CATTTATGTG

AAAAACTCTG TGATTAAAAT GCCATAATAA CAGTTGTCTG CATTTATGTG

AAAAACTCTG TGATTAAAAT GCCATAATAA CAGTTGCATG CATTTATGTG

CCTGATTACC ATAATCTATT TAGCACAAGT AAACATACAA ACAGAAGGAG

CCTGATTATC ATAATCTATT TAGCACAAGT AAACATACAA ACAGAAGGAG

CCTAATTAGC ATAATCTATT TAGCACAAGT AAACATACAA ACAGAAGGAG

CCTAATTAGC ATAATCTATT TAGCACAAGT AAACATACAA ACAGAAGGAG

CCTGATTACC ATAATCTATT TAGCACAAGT AAACATACAA ACAGAAGGAG

CCTGATTACC ATAATCTATT TAGCACAAGT AAACATACAA ACAGAAGGAG

CCTGATTACC ATAATCTATT TAGCACAAGT AAACACACAA ACAGAAGGAG

CCTGATTACC ATAATCTATT TAGCACAAGT AAACACACAA ACAGAAGGAA

CCTGATTACC ATAATCTATT TAGCACAAGT AAACACACAA ACAGAAGGAG

CCTGATTACC ATAATCTATT TAGCACAAGT AAACACACAA ACAGAAGGAG

CCTGATTACC ATAATCTATT TAGCACGAGT AAACATACAA ACAGAAGGGG

CCTGATTACC ATAATCTATT TAGCACAAGT AAACATACAA ACAGAAGGAG

CCTGATTACC ATAATCTATT TAGCACAAGT AAACAGACAA ACAGAAGGAG

CCTGATTATC ATAATCTATT TAGCACAAGT AAACATACAA ACAGAAGGAG

CCTGATTATC ATAATCTATT TAGCACAAGT AAACACACAA ACAGAAGGAG

CCTGATTATC ATAATCTATT TAGCACAAGT AAACACACAA ACAGAAGGAG

CCTGATTATC ATAATCTATT TAGCACAAGT AAACATACAA ACAGAAGGAG

CCTGATTATC ATAATCTATT TAGCACAAGT AAACACACAA ACAGACGGAG

AAAAGCAAGC CCCCTGACAC TTCAGCTAAA GGAGAATTTG AAATGTGGAG

AAAAGCAAGC CCACTGACAC TTCAGCTAAA GGAGAATTTG AAATGTGGAG

AAAAGCAAGC CCACTGACAC TTCAGCTAAA GGAGAATTTG AAATGTGGAG

AAAAGCAAGC CCACTGACAC TTCAGCTAAA GGAGAATTTG AAATGTGGAG

AAAAGCAAGC CCACTGGCAC TCCAGCTAGA GGAGAATTTG AAATGTGGAG

AAAAGCAAGC TCACTGACAC TTCAGCTAAA GGAGAATTTG AAATGCGG-G

AA-----AGC CCACTGACAC TTCGGCT--G GGGGGGTGGG GGATGCGAGG

GA-----AGC CCACTGACAC TTCGGCTAAG GGGGAATTTT AAATGCGAGG

AGAAGCCAGC CCACTGACAC TTCAGCTGAA GGAGAATTCG CAATGCGAAG

AAAAGCAAGC CCACTGACTC TCCAGCTAAA GGAGAATTTG AAATGCGAGA

AAAAGCAAGC CCACTGACTC CTCAGCTAAA GGGGAATTTG AAATGCGAGG

AAAAGCAAGC CCACTGACAC TTCAGCTAAA GGAGAATTTG AAATGCGAGG

AAAAGCTAGT CCACTGACAC TTCAGCTAGA GGGGAATTTG AAATGCAAGT

AAAAGCAAGC CCACTGACAC TTGAGCTAAA GGAGAATTTG AAATGCGGGG

AAAAGCAAAC CCACTGATAC TTCAGCTAAA GGACAATCTG CAATGCGGGG

AAAAGTAAGC CCACTGACAC TTCAGCTAAA GGAGAATTTG AAATGCGGGG

AAAAGCAAGC ACACTCAAAC TCTAGCTAAA GGCGAATTTT AAATGTGGGG

AAAAGCAAGC CCATGGACAC TTGAGCTGAA GGAGAATGGA GAATGAGCGG

GAGACAATGT AAATATGTAA ATGTCTGTGT ATTCTTTTTA TCTCTCTCCT

GAGACAATGT AAATATGTAA ATGTCTGTGT ATTCTTTTTA TCTCTCTCCT

GAGACAATGT AAATATGTAA ATGTCTGTGT ATTCTTTTTA TCTCTCTCCT

GAGACAATGT AAATATGTAA ATGTCTGTGT ATTCTTTTTA TCTCTCTCCT

GAGACAATGT AAATATGTAA ATGTCTGTGT Attcttttta tctctctcct

GACACAATGT AAATATGTAA ATGTCTGTGT ATTCTTTTCA TCTCTCTCCT

GAGACAATGT AAATATGTAA ATGTCTGTGC ATTCTTTTCA TCTCTCTCCT

GAGACAATGT AAATATGTAA ATGTCTGTGT ATTCTTTTCA TCTCTCTCCT

GAGACAATGT AAATATGTAA ATGTCTGTGT ATTCTTTTCA TCTCTCTCCT

GAGACAATGT AAATATGTAA ATGTCTGTGC Attcttttca tctctctcct

GAGACAATGT AAATATGTAA ATGTCTGTGT ATTCTTTTCA TCTCTCTCCT

GAGACAATGT AAATATGTAA ATATCTGTGT ATTCTTTTCA TCTCTCTCCT

GAGACAATGT AAATATGTAA ATGTCTGTGT ATTCTTTTCA TCTCTCGCCT

GAGACAATGT AAATATGTAA ATGTCTGTGT Attcttttca tctctttcct

GAGACAATGT AAATATGTAA ATGTCTGTGT ATTCTTTTCA TCTCTTTCCT

GAGACAATGT AAATATGTAA ATGTCTGTGT ATTCTTTTCA TCTCTCTCCT

AAGACAATGT AAATATGTAA ATGTCTGTGT ATTCTTTTTA GCTTTTCCCT

GAGACAATGT AAATATGTAA ATGTCTGTGG ATTCTTTTTA TCTGGTTCTT

TTCTCATCCT TGCCTTTTTT TTTTTTCTGT TTGCTAATGA GAAGACTATG

TTCTCATCCT TGCCTTTTTT TTTTTTCTGT TTGCTAATGA GAAGACTATG

TTCTCATCCT TGCCTTTTTT TTTTTCCTGT TTGCTAATGA GAAGACTATG

TTCTCATCCT TGCCTTTTTT TTTTTCCTGT TTGCTAATGA GAAGACTATG

ttctcatcct tgcctttttt ttttttctgt ttgctAATGA GAAGACTATG

TTCTCATCCT TGCC---TTT TTTTTTCTGT TTGCTAATGA GAAGACTATG

TCCTCATCCC CGCTTTCTAT TCTTTTCTGT TTGCTAATGA GGAAAC-ATG

TTCTCAT-CC TGCTTTCTAT TCTTTTCTGT TTGCTAATGA GGAGAC-ATG

TTCTCATCCC TGCTTTTTTT TTTTTTCCAT TTGCTAATGA GAAGGCTATG

ttctcatcct tgcctgtttt ttttttctgt tTGCTAATGA GAAGACTATA

TTCTGCTCCT TGCCtttttt tttttTCTGT TTGCTAATGA GAAGACTATA

TTCTCATCCT GGCCTTTTTT TTTTTTCTGT TTGCTAATGA GAAGACTATA

TTCTCATCCT GGCCTTTTTT TTTTCTCTGT TTGCTAATGA GAAGACTATG

ttctcatcct tgcctttttt ttttttCTGT TTGCTAATGA GAAGCCTATG

TTCTCATCCT GGtttttttt ttttttCTGT TTGCTAATGA GAAGACTATA

TTCTCATCCT GGCTTTTTTT TTTTTTCTGT TTGCTAATGA GAAGACTATG

GTCTCCTCCT AGCCTTTTTT TCTCTTCTGT TTGTTAATGA GGCATCTATG

CTCTCATCCT CGCTGTTTTG TTTTTTCTGT TTGCTAATGA GACGTCCAGG

TCATCCCATG TGGTCTCTGC TTTACCGGCT GAGGTGCATT TGGGGCCATC

TCATCCCATG TGGTCTCTGC TTTACCGGCT GAGGTGCATT TGGGGCCATC

TCATCCCATG TGGTCTCTGC TTTACCGGCT GAGGTGCATT TGGGGCCATC

TCATCCCATG TGGTCTCTGC TTTACCGGCT GAGGTGCATT TGGGGCCATC

TCATCCCATG TGGTCTCCGC TTTACCGGCT GAGGTGCATT TGGGGCCATC

CCATCCCATG TGGTCCCTGC TCTACCGGCT GAGGTGCATT TGGGGCCATC

CCATCCCATG TGGTCCCTGC TCTATCGGCT GAGGTACATT TGGGGCCATC

CCATCCCATG TGGTCCCTGC TCCATCGGCT GAGGTACATT TGGGGCCATC

CCATCCCATG TGGTCCCTGC TCTACCGGCT GAGGTGCATT TGGGGCCATC

CCATCCCATG TGGTCCCTGC TCAACCGGCT GAGGTACATT TGGGGCCATC

CCATCATATG TGGTCCTCGC TCTATCGGCT GAGGTGCATT TGGGGCCATC

CCATCACATG TGGTCCCTGC TCTACCGGCT GAGGTGCATT TGGGGCCATC

CCATCACATG TGGTCCCCCT TCTACCGCCT GAGGTGCATT GGGGACCATC

CCATCACATG TGGTCCCCGC TCCACCGGCT GAGGTGCATT TGGGGCCATC

CCATCACATG TGGTCCCTGC TCCACCGGCG GAGGTGC--T TGGGGCCATC

CCATCACATG TGGTCCCCGC TTCTCCGGCG GAGGTGCATT TGGGGCCATC

CCCTCACATG TGGTACAGGC TCTACTGGCG AGGGTGCATT TGGGGCCATC

CTTCCCCGCA CGGGGACGGG CCCGCCGGCC GAGGTGCGCT CGGGACCATC

TGCTGGGCCA GCATCCTAAA CAATGCCACC CAGAAAGCGG GCAGAGAGTA

TGCTGGGCCA GCATCCTAAA CAATGCCACC CAGAAAGCGG GCAGAGAGTA

TGCTGGGCCA GCATCCTAAA CAATGCCACC CAGAAAGCGG GC--AGAGTA

TGCTGGGCCA GCATCCTAAA CAATGCCACC CAGAAAGCGG GC--AGAGTA

TGCTGGGCCA GCACCCTAAA CAATACCACC CAGAAAGCGG GCAGAGAGTA

TGCTGGGCCA GCATCCTAAA CAATGCCACC CAGAAAGCGG GCAGAGAGTA

TGCTGGGCCA GCATCCTACA CAATGCCACC AAGAAAGTGG GCAGAGTGTA

TGCTGGGCCA GCATCCTAAA CAATGCCACC CAGAAAGTGG GCAGAGTGTA

TGCTGGGCCA GCGTCCTAAA CAATGCCACC CAGAAAGCGG GCCGAGAGTA

TGCTGGGCCA GAATCCTAAA CAATGCCACC CAGGGAGCGG GCAGAGAGTA

TGCTGGCCCA GCCTCTTAAA CAATGCCACC CAGAAAGCGG GCAGAGAGTA

TGCTGGGCCA GTATCCTAAA CAATGCCACC CAGAAAGCGG GCAGAGAGTA

TGCTGGGCCA GCACCCTCAA CAATGCCACC CAGAGAGC-- ----------

TGCTGGTCCA GCATTGTAGA CAATGCCACC CAGAAAGCGA GCAGAGAGTA

TGCTAGGCCA GGTTCCCAAA CAATGCCACC CAGAAAGCGG GCAGAGAGTA

TGCTGGGCCA GCATCCCAGA CAATGCCACC CAGAAAGC-- ----------

TGCTGGGACA ATATCCTAAA CAAAGCCACC TAGAAAGCGG GCAGAGATTA

TGCTGGGCCG ACACCCCAAA CAATGCCACC CACCATGCGG GCAGAGATTA

TCATCTGTTT TATTTTTGTA AAAACAAAGT GCTAAATAAT ATTTATTACT

TCATCTGTTT TATTTTTGTA AAAACAAAGT GCTAAATAAT ATTTATTACT

TCATCTGTTT TATTTTTGTA AAAACAAAGT GCTAAATAAT ATTTATTACT

TCATCTGTTT TATTTTTGTA AAAACAAAGT GCTAAATAAT ATTTATTACT

TCATCTGTTT TATTTTTGTA AAAACAAAGT GCTAAATAAT ATTTATTACT

TCATGTGTTT TATTTTTGTA AAAACAAAGT GCTAAATAAT ATTTATTACT

TCACTTGTTT TATTTTTGTA AAAAAGAAGT GCTAAATAAT ATTTATTACT

TCACCTGTTT TATTTTTGTA AAAAAGAAGT GCTAAATAAT ATTTATTACT

TCATCTGTTT TATTTTTGTA AAAAAAAAGT GCTAAATAAT ATTTATTACT

TCATCTGTTT TATTTTTGTA AGATAAAAGT GCTAAATAAT ATTTATTACT

TCATCTGTTT TATTTTTGTA AGAAAAAAGT GCTAAATAAT ATTTATTACT

TCATCTGTTT TATTTTTGTA AGAAAAAAGT GCTAAATAAT ATTTATTACT

---------- ---------- ---------- ---------- ----------

TCATCTGTTT TATTTTTGTA AAAAAAAAGT GCTAAATAAT ATTTATTACT

TCATCTGTTT TATTTTTGTA GAAAAAAAGT GCTAAATAAT ATTTATTACT

---------- ---------- ---------- ---------- ----------

TCATCTGTTT TATTTTTGTA AAAAAAAAAT GCTAAATAAT ATTTATTACT

TCATCCTTTT TATTTTTGTA GAAAAAGAGT GCTAAATAAT ATTTATTACT

TGTTGGTTGC AAAAACGGAA TAAATGACTG AGTGTTGAGA TTTTAAATAA

TGTTGGTTGC AAAAACGAAA TAAATGACTG AGTGTTGAGA TTTTAAATAA

TGTTGGTTGC AAAAACGAAA TAAATGACTG AGTGTTGAGA TTTTAAATAA

TGTTGGTTGC AAAAACGAAA TAAATGACTG AGTGTTGAGA TTTTAAATAA

TGTTGGTTGC AAAAACGAAA TAAATGACTG AGTGTTGAGA TTTTAAATAA

CGTTGGTTGC AAAAACGAAA TAAATGATCG AGTGTTGAGA TTTTAAATAA

TGTTGGTTGC AAAAACGAAA CAAACGATGG AGTGTTGAGA TTTTAAATAA

TGTTGGTTGC AAAAACGAAA CAAATGATGG AGTGTTGAGA TTTTAAATAA

TGTTGGTTGC AAAAACGAAA TAAATGATGG AGTGTTGAGA TTTTAAATAA

TGTTGGTTGC AAAAATGAAA TAAATGACCG AGTGTTGAGA TTTTAAATAA

TGTTGGTTGC AAAAACGAAA TAAATGATCA AGTGTGGAGA TTTTAAATAA

TGTTGGTTGC -AAAACGAAA TAAATGATCG AGTGTTCAGA TTTTAAATAA

---------- ---------- ---------- ---------- ----------

TGTTGGTTGC AAAAACGAAA TAAATGATCG AGTGTTGGAA TTTTAAATAA

TGTTGGTTGC AAAAACGAAA TAAATGATCG AGTGTTGAGA TTTTAAATAA

---------- ---------- ---------- ---------- ----------

TGTTGGTTGC AAAAACAAAA TAAATGATCA ACTGTTGAAA TTTTAAATAA

TGCTGGTTGC AGAAACAAAA TAAATGACGG TCGATGGAGG TTTTAAATAA

AACAATTCTA ATATATTTCA ATATATTCAT TATCATGCAC TAAAAGAGGA

AA-------- ---------- ---TATTCAT TATCATGCAC TAAAAGAGGA

AACAATTCTA ATATATTTCA ATATATTCAT TATCATGCAC TAAAAGAGGA

AACAATTCTA ATATATTTCA ATATATTCAT TATCATGCAC TAAAAGAGGA

AACAATTCTA ATATATTTCA ATATATTCAT TATCATGCAC TAAAAGAGGA

AACAATTCTA ATATATTTTA ATGTATTCAT TATTATGCAC TAAAAGAGGA

AACAATTCTA ATATATTTCA ATATATTCAT TATTGTCTAT TAAAAAA-GA

AACAATTCTA ATATATTTCA ATATATTCAT TATTGTCCAT TAAAAAA-GA

CACAATTCTA ATATATCGCA ATATATTCAT TATCATGCAC CAAAAGAGGA

AACAATTCTA ATATATGTCA ATATATTCAT TACCGTGCCC TGAAAGCGGA

AACAATTTTA ATATATTTCA ATATATTCAT TATCATGCAC TAAAAGAGGA

AACAATTCTA ATATATTTCA ATATATTCAT TATCATGCAC TAAAAGAGGA

---------- ---------- ---------- ---------- ----------

AACCATTCTA ATATATTTTA ATATATTCAT TATCATGCAC CAAAAGAGGG

AACAATTCTA ATATATTTAA ATATATTCAT CATCACACAC TAAAAGAGGA

--CGAGTCTA ATCTATTTAG ACATATTCAT CATCAGGCGC TAATATAGGA

AATAATTCGA ATATGTTTTA ATATATTCAT CATAATGCAT TAAAAGAGTA

AAcatctcAA GAATATTTTA GAATATTCTT TATGACGCCT TAAGGGGGAA

GACAGCCTTG TTTGTAACAG CCCCGGGCAT AAGCAATCAT TGTGTAGCTT

GACAGCCTTG TTTGTAACAG CCCCGGGCAT AAGCAATCAT TGTGTAGCTT

GACAGCCTTG TTTGTAACGG CCCCGGGCAT AAGCAATCAT TGTGTAGCTT

GACAGCCTTG TTTGTAACGG CCCCGGGCAT AAGCAATCAT TGTGTAGCTT

GACAGCCTTG TTTGTAACAG CCCCGGGCAT AAGCAATCAT TGTGTAGCTT

GACAGCCTTG TTTGTAGCAG CCCCGGGCAG AAGCAGGCAT TGTGTAGCTT

GACAGCCTGG TTTGTCACAG CCCAGGGCAC AAGCAATCAT TGTGTTGCTT

GACAGCCTGG TTTGTCACAG CCCAGGGCAT AAGCAATCAT TGTGTGGCTT

GACAGCCTCG TTTGTAACAG CCCAGGGCAT AAGCCAGCAT TGTGTAGCTT

GACAGCCTTG TTTGTGACAG CCCCGGGCAT AAGCAATCAT TGTGTAGCTT

GACAGCCTCG TTTGTGACAG CCCCGGGCAT AAGCAATCAT TGTGTCGCTT

GACAGCCTTG TTTGTGACAG CCCCGGGCAT AAGCAATCAT TGTGTCGCTT

---------- ---------- ---------- ---------- ----------

GACAGCCTTG TTGGGAACAG CCCCGGGCTC CAGCCGTCAT TGTGCGGCTT

GACAGCCTCG TTTGTCACAG CCCCGGGCAT AAGCAATCAT TGTGTCGCTT

G--------- --TGTAGCCG CCCCGGGCGT AAGCCATCAT TGTGTCCCTT

AACAGTCCTG TCTGCAAGTG CCCTGGGTAG GAACAATCAT TGTGTCATTT

GACAGCCTTG TTGGCCGCTG CCCCGGGTAG AAGCTATCAC TGTGCAACTC

AGATATTCGC TGCAGATAAA GGCAACGCAA GTGCGAGCTC TCCCAAGGAC

AGATATTCGC TGCAGATAAA GGCAACGCAA GTGCGAGCTC TCCCAAGGAC

AGATATTCGC TGCAGATAAA GGCAACGCAA GTGCGGGCTC TCCCAAGGAC

AGATATTCGC TGCAGATAAA GGCAACGCAA GTGCGGGCTC TCCCAAGGAC

AGATATTCGC TGCAGATAAA GGCAGCGCAA GTGCGGGCTC TCCCAGGGAC

AGATATTCGC TGCAGATAGA GGCAGCGCAA GTGCAGGCTC TCCCAAGGAC

AGCTATTCGC TGCAGAT-AA CACAGCACAA GTGTGAACGC TCCCAAGGAC

AGCTATTCGC TGCAGAT-AA CGCAGCACAA GTGTGAGCGC TCCCAAGGAC

AGATATTCGC --CAGATCAA GGCATCGCAA GTGCAGGCTC TCCCAAGGAC

AGATATTCGC TGCAGATAAA GGCAGCACAA GTGCGAGCTC TCCCAAGGAC

AGCTATTCGC TGCAGATAAA AGCAGCACAA GTGCAAGCTC TCCCAAGGAC

AGATATTCGC TGCAGACAGA GGCAGCACAA GTGTAAGCTC TCCCAAGGAC

---------- ---------- ---------- ---------- ----------

AGATATGCGC TGCGGATAAA GGCGGCGCAA GTGCGAGCTC TCCCAAGGCC

AGATATTCGC TTCAGA-GAA AGCCGCGCAA GTGCAGGGTC TCCCAAGGAC

AGATATTCGC TTCAGACGGA GGCAGCGCAA GTGCGCGCTC TCCCAAGGAC

AGATATTCAT TTGAAATACA AACAGAACAA GTGCAAGCTC TCCCAAGGAC

AGCTATTCGT CAGAAATCGA GTCGGAGCAA GTGCTGGCTC TCCCAACGCC

CTGTGGCCAG CCGGATTCAC CGATTGGTTC CAGTTTATTA ATAGCCTATG

CTGTGGCCAG CCGGATTCAC CGATTGGTTC CAGTTTATTA ACAGCCTATG

CTGTGGCCAG CCGGATTCAC CGATTGGTTC CAGTTTATTA ACAGCCTATG

CTGTGGCCAG CCGGATTCAC CGATTGGTTC CAGTTTATTA ACAGCCTATG

CTGTGGCCAG CCGGATTCAC CGATTGGTTC CAGTTTATTA ACATCCTATG

CTGCGGCCAG CCGGATTCAC CGATTGGTTC CAGTTTATTA ACAGCCTATG

CTGCGTCCAG C---GGTCAC CAATTGGTTC CAGTTTATTA ACAGCCTATA

CTGCGTCCAG C---GGTCAC CAATTGGTTC CAGTTTATTA ACAGCCTATA

CTGCGGCCAG CCGGATTCAC CAATTGGTTC CGGTTTATTA ACAGCCTATG

CCGCGGCCAG CCGGATTCAC CGATTGGTTC CAGTTTATTA ACAGCCTATG

CTGCGGCCAG CCGGATTCAC CGATTGGTTC CAGTTTATTA ACAGCCTATG

CTGTGGCCGG TGGGATTCAC CAATTGGTTC CAGTGTATTA ACAGCCTATG

---------- ---------- ---------- ---------- ----------

CTGCGGCCAG CCGGGGCCGC CGATCGGTTC CGGCCTATTA ACAGCCTATG

CTGCGGCCAG CCGGATCCAC CAATTGGTTC CAGTTTATTA ACAGCTTATG

CTGCGGCCAG CCGGATTCAG CGATCGGTTC CGGTGTATTA ACAGCCTATG

CTGTGGCTGG ACCGATTCAT GGATTGGTTA CAGTTTATTA ACAGCCTATG

CCGCGGCCAG GCGGATTCAC GGATTGGTTC CGGCGTATTA AGGGCCTACG

AACTTCTCAT CTGCTCCCTA GGATGTCACC TGCAACAGTT TAGCAGCTCA

AACTTCTCAT CTGCTCCCTA GGATGTCACC TGCAACAGTT TAGCAGCTCA

AACTTCTCAT CTGCTCCCTA GGATGTCACC TGCAACAGTT TAGCAGCTCA

AACTTCTCAT CTGCTCCCTA GGATGTCACC TGCAACagtt tagcagctca

AACTTCTCAT CTGCTCCTTG GGATGTCACC TGCAACAGTT TAGCAGCTCA

AACTTCTCAT CTGCTCCTTA GGATGTCACC TGCAACagtt tagcagctca

AACTTCTCAT CTGATCCATA GGATGTCACC TGCAACGGTT TGGCAGCCCG

AACTTCTCAT CTGTTCCATA GGATGTCACC TGCAACGGTT TGGCAGCCCG

AACTTCTCAT CTGTTTCCTT GGATGTCACC TGCAGCagtt cagcagctca

AACTTCTCAT CGGCTCCCCA GGATGTCCCC TGCAACagtt tagcagctca

AACTTCTCAT CAGCTCCTCA GGACGTCACC TGCAACAGTA TAGCAGCTCA

AACTTCTCAT CGGCTCCGCA GGATGTCACC TGCAACagtt tagcagctca

---------- ---------- ---------- ---------- ----------

AACTTCTCAT CTGCTGCCTC CGACGTCCCC TGCAACagtt tagcagctca

AACTTCTCAT CTGCTCCTTA GGGTGTCACC TGCAACGGTT TAGCAGCCCA

AACTTCTCAT CTGCTCCCGA GGATGTCACC TGCAACagtt tagcagctca

AACTTCTCAT CTGCTCCTTA GGATGTCACC TGCAACAGTT GAGCAGCTCA

AACCTCTCAT CTGCTTGTTC GGGTGTCACC TGCGGCGGTT CACCAGCTCA

GCGCCCGGCC TTCTCCATTC TCTCCCCAAA CACATCGGCC TGTTTGTTCT

GCGCCCGGCC TTCTCCATTC TCTCCCCAAA CACATCGGCC TGTTTGTTCT

GCGCCCGGCC TCCTCCATTC TCTCCCCAAA CACATCGGCC TGTTTGTTCT

gcgcccggcc ttctccattc tcTCCCCAAA CACATCGGCC TGTTTGTTCT

GCGCCCGGCT TTCTCCATTC TCTCCCCAAA CACATCGGCC TGTTTGTTCT

gcgcccggcc ttctccattc tcTCCCCAAA CACATCGGCC TGTTTGTTCT

AAGCCCCGCC CTCTCCATTC TCTCCCCGAA CACATCAGCC CGTTTGTTCC

ATGCCCCGCC CTCTCCATTC TCTCTCTGAA CACATCAGCC CGTTTGTTCC

ggccccggcc ttctccattc tcTCCGCAAA CACATCGGCC TGTTTGTTCT

gagcccggcc ttctccattc tcTCCCCAAA CACATCAGCT TGTTTGTTCC

GCGCCCGGCC TTCCCCATTC TCTCCCCAAA CACATCGGCC TATTTGTTCT

gtgcccaggc ttctccattc tcTCCCCAAA CACATCGGCT TGTTTGTTCT

---------- ---------- ---------- ---------- ----------

gtgcccggcc gtctccattc tcTCCCCAAA CACATCGGCC CGTTTGTTCT

GTGCCAAGC- -TCCCGGTTC TCTCCCCAAA CACATCGACC CGTTTGTTCT

gtgcccggcc ttccccattc ttcCCCCAAA CACATCGACC TGTTTGTTCT

GTGCCCTGTC TTTTCCATTC TCTCCCCTAG TACATCGGCT TGTTTGTTCT

GCCCCCTGCC TTCTCCATTT TCTCCCCTAA CACATCAGAT TGTCGGTTCT

CTTTTTCTTC CTGTTGCATT CTGCCGGTTC AATGGGGAAG GAGTGAAACC

CTTTTTCTTC CTGTTGCATT CTGCCGGTTC AATGGGGAAG GAGTGAAACC

CTTTTTCTTC CTGTTGCATT CTGCCGGTTC AATGGGGAAG GAGTGAAACC

CTTTTTCTTC CTGTTGCATT CTGCCGGTTC AATGGGGAAG GAGTGAAACC

CTTTTTCTTC CTGTTGCATT CTGCCGGTTC AATGGGGAAG GAGTGAAACC

CTTTTTCTTC CTGTTGCATT CTGCCCGTTC AATGGGGAAA GAGTGAAACC

CTTGTTCTTC CTGTCGCATT CTGCCTGTTC AATGGGGAAA GAGTGGAACC

CTTGTTCTTC CTGTTGCATT CTGCCTGTTC AATGGGGAAA GAGTGGAACC

CTTTTTCTTC CTGTTGCATT CTGCCCGTTC AATGGGGAAA GAGTGGAACC

CTTTTTCTTC CTGTCGCATT CTGCCTGTTC AATGGGGAAA GAGTGAAACC

CTTTTTCTTC CTGTTGCATT CTGCACATTC AATGGGGAAA GAGTGAAACC

CTTTTTCTTC CTGTTGCATT CTGCCTGTTC AATGGGGAAA GAGTGAAACC

---------- ---------- ---------- ---------- ----------

CTTTTTCTTC CTGTCGCATT CTGCCCGCTC AATGGGGAAA GAGCGAAACC

CTTTTTCTTC CTGTCGTATT CTGCCCGTTC AATGGGGAAA GAGTGAAACC

TTCA-TCTTC CGGTCGCATT CTGCCCGTTC AATGGGGAAA GAGTGAAACC

CTTTTTCTTC CTGTCGCATT TTGCCCATTC AATAGAGAAA TAGTGAAACC

TTCTTTCTTC CTGCCGCATT CTGTCCATTC AATGGGGAAA GAGTGAAACC

GGATGGCTGG ACCCAGTGCG GGGAAATGAC TTCAAGCCAC CTTTGTTTCC

GGATGGCTGG ACCCAGTGCG GGGAAATGAC TTCAAGCCAC CTTTGTTCCC

GGATGGCTGG ACCCAGTGCG GGGAAATGAC TTCAAGCCAC CTTTGTTTCC

GGATGGCTGG ACCCAGTGCG GGGAAATGAC TTCAAGCCAC CTTTGTTTCC

GGATGGCTGG ACCCAGTGCG GGGAAATGAC TTCAAGCCAC CTTTGTTTCC

GGATGGCTGG ACCCAGTGCG GGGAAATGAC TTCAAGCCAC CTTTGTTTCC

GGATGGCTGG ACCCAGTGCA GGGAAGTGAC TTCA-ACCAC CTTTGTTTCC

GGATGGCTGG ACCCAGTGCA GGGAAGTGAC TTCA-GCCAC CTTTGTTTCT

GGATGGCTGG ACCCAGCACA GGGAAATGAC TTCAAGCCAC CTTTGTTCCC

GGATGGCTGG ACCCAGTGCG GGGAAATGAC TTCAAGCCAC CTTTGTTTCC

GGATGGCTGG ACCCAGTGCG GGGAAATGAC TTCAAGCCAC CTTTGTTTCC

GGATGGCTGG ACCCAGTGCG GGGAAATGAC TTCAAGCCAC CTTTGTTTTC

---------- ---------- ---------- ---------- ----------

GGATGGCTGG ACCCAGCGCG GGGAAGTGAC TTCAAGCCAC CTTTGTTTCC

GGATGGCTGG ACCCAGTGTG GGGAAATGAC TTCAAGCCAC CTTTGTTCCC

GGATGGCTGG ACCCAGTGCG GGGAAATGAC TTCAGGCCAC CTTTGTTCCC

GGATGGCTGG ATTCAGTACA GGGAAATGAC TTCAAGCTAC CTTTGTTTCC

GGATGGCGAG ATTCAGTGCG GGGAAATGAC TTCAAGCTAC CTTTGTCTTC

CTCATTCCTT TGAGCGCACT TGGCCGAAAG GGCTTGTTTG AACCATTCGG

CTCATTCCTT TGAGCGCAAT TGGCCGAAAG GGCTTGTTTG AACCATTCGG

CTCATTCCTT TGAGCGCACT TGGCCGAAAG GGCTTGTTTG AACCATTCGG

CTCATTCCTT TGAGCGCACT TGGCCGAAAG GGCTTGTTTG AACCATTCGG

CTCATTCCTT TGAGCGCACT TGGCCGAAAG GGCTTGTTTG AACCATTCGG

CTCATTCCTT TGAGCACACT TGGCCGAAAG GGCTTGTTTG AACCATTTGG

CTCATTCCTT TGAGCACACT TGGCCGAAAG GGCTTGTTTG AACCATTTGG

CTCATTCCTT TGAGCACACT TGGCCGAAAG GGCTTGTTTG AACCATTTGG

CTTATTCCTT TGAGCACACT TGGCCGAAAG GGCTTGTTTC AACCGTTTGG

CTCATTCCTT TGAGCACACT TGGCCGAAAG GGCTTGTTTG AACCATTTGG

CTCATTCCTT TGAGCACACT TGGCCGAAAG GGCTTGTTTG AACCATTTGG

CTCATTCCTT TCAGCACACT TGGCCGAAAG GGCTTGTTTG AACCATTTGG

---------- ---------- ---------- ---------- ----------

CTCATTCCTT TGAGCGCACT TGGCCGAAAG GGCTTGTTTG AAGCATTGGG

CTCATTCCTT TGAGCACACT TGGCCGAAAG GGCTTGTTTG AACCACTTGG

CTCATTCCTT TGAGCGCACT TGGCCGAAAG GGCTTATTTG AACCATTTGG

CTCATTTCTT TGAGCACACT TGGCTGAAAG GGCTTGTTGA AACCATTTGG

CTCGTTCCTT TGAGTACACT TGGCCGAAAG AGCCCATCTC AACGGTCGGG

AACATCCAGG CCAGAACCGC TCGCATCTTT TCAGGCTGCC TGAGACTGGG

AACATCCAGG CCAGAACCGC TCGCATCTTT TCAGGCTGCC TGAGACTGGG

AACATCCAGG CCAGAACCGC TCGCATCTTT TCAGGCTGCC TGAGACTGGG

AACATCCAGG CCAGAACCGC TCGCATCTTT TCAGGCTGCC TGAGACTGGG

AACATCCAGG CCAGAACTGC TCAAATCTTT TCAGGCTGCC TGAGACTGGG

AACATCCAGG CCAGAATCGC TCACATCTTT TCAGGCTGCC TGAGACTGGG

AACATCCAGG CCAGAACCGC TCACATCTTT TCAGGCTGCC TGAGCCTGAG

AACATCCAGG CCAGAATCGC TCGCCTCTTT TCAGGCTGCC TGAGACTGGG

GGCATCCAG- ---------- ---------- ---------- ----------

AACATCTCGG CCAGAATCGC TCACATCTTT TCAGGCTGCC TGAGACTGGG

AACATCTAGG CCAGAATCGC TCGCATCTTT TCAGGCTGCC TGAGACTAGA

AACATCTAGG CCAGAATCAC TCGCATCTTT TCAGGCTGCC TGAGACTGGG

---------- ---------- ---------- ---------- ----------

AGCATCCAGG CCAGGATCCC TCCCAGCTTT TCAGGCTGCC TGGGACTGGG

AACATCCAGG CCAGAATCCC TCGCAGCTTT TCAGGCTGCC TGAGACTGGG

AACATCCAGG CCAGAATCGC TCGCGGCTTT TCAGGCGGCC TGAGACGGAG

AACATCCAGG CCAGACTCAC TCACATCTTT TCAGGTTGAC TGAGACTGAG

AACAACCACG CCAGAATCGC TCCCATCTTT TCAGGCCTTC TGAGGCCGCA

TTAAACTGCC AAATTTTTGC AACAAAACTA ACCATGACAT TGGACATTGT

TTAAACTGCC AAATTTTTGC AACAAAACTA ACCATGACAT TGGACATTGT

TTAAACTGCC AAATTTTTGC AACAAAACTA ACCATGACAT TGGACATTGT

TTAAACTGCC AAATTTTTGC AACAAAACTA ACCATGACAT TGGACATTGT

TTAAACTGCC AAATTTTTGC AACAAAACTA ACCATGACAT TGGACATTGT

TTAAACTGCC AAATTTTTGC AACAAAACTA ACCATGACAT TGGACATTGT

TGAGGCTGCC AAATTTTTGC AACAAAACTA ACCATGACAT TGGACATTGT

TTAAGCTGCC AAATTTTTGC AACAAAACTA ACCATGACAT TGGACATTGT

---------- ---------- ---------- ---------- ----------

TGAAACTGCC AAATTTTTGC AACAAAACTA ACCATGACAT TGGACATTGT

TTAAACTGCC AAATTTTTGC AACAAAACTA ACCATGACAT TGGACATTGT

TTAAACTGCC AAATTTCTGC AACAAAACTA ACCATGACAT TGGACATTGT

---------- ---------- ---------- ---------- ----------

TTAAACTGCC AAATTTTCGC AACAACACTA ACCATGACAT TGGACATTGT

TTAAACTGCC AAATTTTTGC AACAAAACTA ACCATGACAT TGGACATTGT

GTAAACTGCC AATTTTTTGC AACAAAACTA ACCATGACAT TGGACATTGT

TTAAACTGCC AAATTTTTGC AACAAAACTA ACCATGACAT TGGACATTGT

TTACCCTGCC CCATTTTGGC AACCAAACGG GCCCTGACAA GGGGCATTGT

GTTATAAATT AGCCACAGCC TTCCAAGCAA ATTGTCTCTT TTTATTGTAT

GTTATAAATT AGCCACAGCC TTCCAAGCAA ATTGTCTCTT TTTATTGTAT

GTTATAAATT AGCCACAGCC TTCCAAGCAA ATTGTCTCTT TTTATTGTAT

GTTATAAATT AGCCACAGCC TTCCAAGCAA ATTGTCTCTT TTTATTGTAT

GTTATAAATT AGCCACAGCC TTCCAAGCAA ATTGTCTCTT TTTATTGTAT

GTTATAAATT AGCCACAGCC TTCCAAGCAA ATTGTCTCTT TTTATTGTAT

GTTATAAATT AGCCGCAGCC TTCCAAGCAA ATTGTATCTT TTCGTTGTCT

GTTATAAATT AGCCACAGCC TTCCAAGCAA ATTGTATCTT TTCGTTGTCT

---------- ---------- ---------- ---------- ----------

GTTATAAATT AGCCACAGCC TTCCAAGCAA ATTGTCTCTT TTCATTGTGT

GTTATAAATT AGCCACAGCC TTCCAAGCAA ATTGTCTCTT TTCATTGTAT

GTTATAAATT AGCCACAGCC TTCCAAGCAA ATTGTCTCTT TTCATTGTAT

---------- ---------- ---------- ---------- ----------

GTTATAAATT AGCCACAGCC TTCCAAGCAA ATTGTCTCTT TTTATTGTAT

GTTATAAATT AGCCACAGCC TTCCAAGCAA ATTGTCTCTT TTTATTGTAT

GTTATAAATT AGCCACAGCC TTCTAAGCAA ATTGTCTCTT TTTATTGTAT

GTTATAAATT AGCCACAGCC TTCCAAGCAA ATTGTCTCTT TTTATTGTAT

GTTAAAAATT AGCCACAGCC TTCCAAGCAA ATTGTCTCTT TTTATTGTAT

CAGGATTTTG AGTGGAAAAG TAGTTCCACT TGTCAATCAG CTACGTTGCA

CAGGATTTTG AGTGGAAAAG TAGTTCCACT TGTCAATCAG CTACGTTGCA

CAGGATTTTG AGTGGAAAAG TAGTCCCACT TGTCAATCAG CTACGTTGCA

CACGATTTTG AGTGGAAAAG TAGTCCCACT TGTCAATCAG CTACGTTGCA

CAGGATTTTG AGTGGAAAAG TAGTCCCACT TGTCAATCAG CTATGTTGCA

CAGGATTTTG AGTGGAAAAG TAGTCCCACT TGTCAATCAG CTAAGTTGCA

CAGGGTTTTC AGTGGAAAAG TAGTCCTGCT TGTCAATCAG CTAAGTTCCA

CAGGGTTTTC AGTGGAAAAG TGGCCCTGCT TGTCAATCAG CTAAGTCCCA

-TGGATTTTG AGTGGAAAAG TAGTTCCCCT TGTCAATCAG CTAAGTTGCC

CAGGATTTTG AGTGGAAAAG TAGTCCCACT TGTCAATCAG CTAAGTTGCC

CAGGATTTTG AGTGGAAAAA TAGTCCCACT TGTCAATCAG CTAAGTTGCA

CAGGATTTTG AGTGAAAAAG TAGTCCCGCT TGTCAATCAG CTAAGTTGCA

-CAGATTTTG AGTGAAAAAG TAGATCCACT TGTCAATCAG TTAAGTCGCA

CAGGATTTTG AGTGGAAAAG TAGTCCCACT TGTCAATCAG CTAAGTTGCA

CAGGATTTTG AGAGGAAAAG CAGTCTCACT TGTCAATCAG CTAAGTTGCA

C--------- ---------- ---------- ---------- ----------

CAGGGTTTGG AGGAGCCAAG TAGCTCCACT TGTCAATCAG CTAAGTTGCA

CCCGGTTCCG AGCGGAGGAG GAATCCTGCT TGTCGATCAG CTAATTTGCC

GGGCTAATAG ATTCTGGTGT TTAGGGAGTC AACACGGAAT TTAATTTAAC

GGGCTAATAG ATTCTGGTGT TTAGGGAGTC AACACGGAAT TTAATTTAAC

GGGCTAATAG ATTCTGGCGT TTAGGGAGTC AACACAGAAT TTAATTTAAC

GGGCTAATAG ATTCTGGCGT TTAGGGAGTC AACACAGAAT TTAATTTAAC

GGGCTAATAG ATTCTGGCAT TTAGGGAGTC AACACAGAAT TTAATTTAGC

GGGCTAATAG ATTCTGGCAT TTAGGGGGTC AACACAGAAT TTAATTTAAC

GGGCTAATAG ATTCCGGTGT TTGGGGGATC AACACAGAAT TTAATTTAAC

GGGCTAATAG ATTCCGGTGT TTGGGGAGTC AACACAGAAT TTAATTTAAC

AGGCTAATAG ATTCTGGGGT TTGGGAGGTC AACACAGAAT TTAATTTAAC

GGGCTAATAG ATTCTGGTGT TTAGGGGGTT GACACAGAAT TTAATTTAAC

GAGCTAATAG ATTCTGGTGT GTAGGGGGTC AACACGGAAT TTAATTTAAC

GGTCTAATAG ATTCTGGTGT TTGGGGGGTC AACACAGAAT TTA--TTAAC

GGGCTGATAG AGTCTGGTGT TTGGGGGGTC AACACAGGAT TTAATTTAAC

AGCCTAATAG ATTCTGGTGT TTAGGGGGTC AACACAGAAT TTAATTTAAC

GGGCTAATGG ATTCCGGTGT TTAGGGGGTC AACACAGAAT TTAATTTAAC

---------- ---------- ---------- ---------- ----------

GTGCTAATAG ATTCTGGTGT TTAGGGGGTC AACACAGAAT TTAATTTAAC

GGACTAATAG AATCTGGCAT TTGGGGGGTC GACACCGAAT TTAATTTAGC

ATCTCACAAT ACACCCACAT GCCCAGAAAA TGTGGACACT TGATAGGGTT

ATCTCACAAT ACACCCACAT GCCCAGAAAA TGTGGACACT TGATAGGGTT

ATCTCACAAT ACACCCACAT GCCCAGAAAA TGTGGACACT TGATAGGGTT

ATCTCACAAT ACACCCACAT GCCCAGAAAA TGTGGACACT TGATAGGGTT

ATCTCACAAT ACACCCACAT GCCCAGAAAA TGTGGACACT TGATAGGGTT

ATCTCACAAT ACACCCACAT GCCCAGAAAA TGTGGACACT TGATAGGGTT

ATCTCACAAT ACATCCACAT GCCCAGAAAA TGTGGACACT TCATAGGGCG

ATCTCACAAT ACATCCACAT GCCCAGAAAA TGTGGACACT TCACGGGGCG

ATCTCACAAT ACACCCACAT GCCCAGAAAA TGTGACCACT TGATAGGGTT

ATCTCACAAT ACGCCCACAT GCCCAGAAAA TGTGGACACT TGATGGGGTT

ATCTCACAAT ACGCCCACAT GCCCAGAAAA TGTGGACACT TGATGGGGTT

GTCTCACAAT ACGCCCACAT GCCCAGAAAA TGTGGACACT TGATGGGGTT

ACCTCACAAT ACGTCCACAT GCCCAGAAAA TGTGGACAAT TGATAGGGTT

ATCTTACAAT ACGCCCACAT GCCCAGAAAA TGTGGACACT TGATAGGGTT

ATCTTACAAT ACGCCCACAT GCCCAGAAAA TGTGGACACT TGATAGGGTT

---------- ---------- ---------- ---------- ----------

GACTTACAAT ACACCCACAT GCCCAGAAAA TGTGGACACT TGATAGGGTT

GACTTACAAT ACGCCCACAT GCCCAGAAAA TGTGGATCCT TGATAGAGTT

CACTGAGCCA AAGAAGCAAG ACCCAAACAG TGCATTAATG TATAGGGAAG

CACTGAGCCA AAGAAGCAAG ACCCAAACAG TGCATTAATG TATAGGGAAG

CACTGAGCCA AAGAAGCAAG ACCCAAACAG TGCATTAATG TATAGGGAAG

CACTGAGCCA AAGAAGCAAG ACCCAAACAG TGCATTAATG TATAGGGAAG

CACTGAGCCA AAGAAGCAAG ACCCAAACAG TGCATTAATG TATAGGGAAG

CACTGAGCCA AAGAAGTAAG ACCCAAACAG TGCATTAATG TATAGGGAAG

CAGTGAGGCA AAGAAGCAAG GCCCAAACGG GGCAGTAATG TATAGGAGAG

CAGTGAGGCA AAGAAGCAAG GCCCAAACGG TGCATTAATG TATAGGAGAG

CGCTGAGCCA AAAAAGCAAG ACGCAAACAG TGCATTAATG TATAGGGAAG

CACTGAGCCA AAGAAGCAAG ACCCAAACAG TGCATTAATG TATAGGGAAG

CACTGAGCCA AAGAAGCAAG ACCCAAACAG TGCATTAATG TATAGGGAAG

CACTGAGCCA AAGAAGCAAG ACCCAAACAG TGCATTAATG T-TTGGGAAG

CACTGAGCCA AAGAAGCAAG ACCCAAACAG TGCATTAATG TATAGGGAAG

CACTGAGCCA AAGAAGCAAG ACCCAAACAG TGCATTAATG TATAGGGAAG

CACTGCGCCA AAGAAGCAGG ACCCAAACAG TGCGTTAATG TATAGCAAAG

---------- ---------- ---------- ---------- ----------

CACTGAGCCA AAGAAGTAAG ACCAAAACAG TGCACTAATG TATAGGGAAG

CAGCAAGCCA AAGGAAGAAG AGGAAAACAG TGCATTAATG TATAGGAGAG

AATAGTTGCA ATCAGCCCCT TGTGAATTGG ACAGAGGGCT TGGAGATGAA

AATAGTTGCA ATCAGCCCCT TGTGAATTGG ACAGAGGGCT TGGAGATGAA

AATAGTTGCA ATCAGCCCCT TGTGAATCGG ACAGAGGGCT TGGAGATGAA

AATAGTTGCA ATCAGCCCCT TGTGAATTGG ACAGAGGGCT TGGAGATGAA

AATAGTTGCA ATCAACCCCT TGTGAATTGG ACAGAGGGCT TGGAGATGAA

AATAGTTGCA ATCAGCCCCT TGTGAATTGG ACAGAGGGCT TGGAGATGAA

AATAGTTGTA ATCAGCC-CT TGTGAATTGG ACAGAGGGCT TGGAGATGAA

AATAGTTGCA ATCAGCC-CT TGTGAATTGG ACAGAGGGCT TGGAGATGAA

AATGCTTGCA ATCAGCCACT CGTGAATCGG ACAGAGGGCT TGAAGACCAA

AATGGTTGCA ATCAGCCCCT TGTGAATTGG ACAGAGGGCT TGGAGATGAA

AATAGTTGCA ATCAGCCCCT TGTGAATTGG ACAGAGGGCC TGGAGATGAA

AATACTTGTA ATCAGCGCCC TGTGAATTGG ACAGAGGGCT TGGAGATGAA

AATAGCCCCA ATCAGCCCCT TGTGAATCGG ACAGAGGGCT TGGGGATGAA

AATAGTTGCA ATCAGCCCCT TGTGAATTGG ACAGAGGGCT TGGAGATGAA

AATGGCTGCA ATCAGCCCCT TGTGAATTGG ACAGAGGGCC TGGAGATGAA

---------- ---------- ---------- ---------- ----------

AATAGTTGCA ATCAGCCCCT TGTGAATTAG AAAGAGAACT TGGAGATGAA

AATGGTAGCA ATCAGGGCTC TGTGAATTGG ACAGAGAACT TGGAGATGAA

GCAATTATCA GCCATCGGGG ATTAATAGTA AAGTGTGAAT ACCAAGCTGT

GCAATTATCA GCCATCGGGG ATTAATAGTA AAGTGTGAAT ACCAAGCTGT

GCAATTATCA GCCATCGGGG ATTAATAGTA AAGTGTGAAT ACCAAGCTGT

GCAATTATCA GCCATCGGGG ATTAATAGTA AAGTGTGAAT ACCAAGCTGT

GCAATTATCA GCCATCGGGG ATTAATAGTA AAGTGTGAAT ACCAAGCTGT

GCAATTACCA GCCATCGGGG ATTAATAGTA AAGTGTGAAT ACCAAGCTGT

GCAATTACCA GCCATCGGGG ATTAATAGTG AAGTGTGAAT GCCAAGCTGT

GCAATTATCA GCCATCGGGG ATTAATAGTG AAGTGTGAAT ACCAAGCTGT

ACAATTATCA GCCAACGGGG ATTAATGGTA AAGTGTGAAT GCCAGGCTGT

GCAATTATGA GCCATCGGGG ATTAATAGTA AAGTGTGAAT ACCAAGCTGT

GCAATTATCA GCCATCGGGG ATTAATAGCA AAGTGTGAAT GCCAAGCTGT

GCAATTATCA GCCATCGGGG ATTAATAGTA AAGTGTGAAT ACCAAGCTGT

GCAATTATCA GGCATGGGGG ATTAATAGTA AACCGTGAAT ACCAAGCTGT

GCAATTATCA GCCATCGGGG ATTAATAGTA AAGTGTGAAT ACCAAGCTGT

GCAATTATCA GCCATCGGGG ATTAATAGTA AACGGTGAAT ACCAGGCTGT

---------- ---------- ---------- ---------- ----------

GCAATTATCA GCCATCGGGG ATTAATAGAA AAGTGTGAAT ACCAAGCTGT

GCAATTATCA GCCACTGGGG ATTAATAGTA AAGAGTGAAT ACCAAGCTGT

ATATGTTAGG GGCAGAATAT GTTGCCTATA GAAAACAACA AAGGACGTTT

ATATGTTAGG GGCAGAATAT GTTGCCTATA GAAAACAACA AAGGACGTTT

ATATGTTAGG GGCAGAATAT GTTGCCTATA GAAAACAACA AAGGACGTTT

ATATGTTAGG GGCAGAATAT GTTGCCTATA GAAAACAACA AAGGACGTTT

ATATGTTAGG GGCAGAATAT GTTGCCTATA GAAAACAACA AAGGACGTTT

ATATGTTAGG GGCAGAATAT GTTGCCTATA GAAAACAACA AAGGACGTTT

ATATGTTAGG GGCAGAATAT GTTGCCTATA GAAAACAACA AAGGACGTTT

ATATGTTAGG GGCAGAATAT GTTGCCTATA GAAAACAACA AAGGGCGTTT

ATATGTTAGG GGCAGAATAT GTTGCCTATA GAAAACAACA AAGGACGTGT

ATATGTTAGG GGCAGAATAT GTTGCCTATA GAAAACAACA AAGGACGTTC

ATATGTTAGG GGCGGAATAT GTTGCCTATA GAAAACAACA AAGGACGTTT

ATATGTTAGG GGCAGAATAT GTTCCCTGTA GAAAACAACA AAGGACGTTT

ATATGTTAGG GGCCGAATAT GTTGCCTATA GAAAACCACA AAGGACGCGT

ATATGTTAGG GGCAGAATAT GTTGCCTATA GAAAACAACA AAGGACATTT

ATATGTTAGG GGCAGAATAT GTTGCCTATA GAGAACAACA AAGGACGTTT

---------- ---------- ---------- ---------- ----------

ATATGTTCGG GACAGAATAT GTTGCCTATA GAAAACAACA AAGAGCGTTT

ATATGTTAGG GGCAGAATAT GTTGCCTATA GAAAACAACA AAGAGCGTGT

CTTACTATTA AGTGAGATTA AACGTAATGG CCTGATTTAA TCACAAAGGC

CTTACTATTA AGTGAGATTA AACGTAATGG CCTGATTTAA TCACAAAGGC

CTTACTATTA AGTGAGATTA AACGTAATGG CCTGATTTAA TCACAAAGGC

CTTACTATTA AGTGAGATTA AACGTAATGG CCTGATTTAA TCACAAAGGC

CTTACTATTA AGTGAGATTA AACGTAATGG CCTGATTTAA TCACAAAGGC

CTTACTATTA AGTGAGATTA AACGTAATGG CCTGATTTAA TCACAAAGGC

CTTACTATTA AGTGAGATTA AACGTAATGG CCTGATTTAA TCACAAAGAC

CTTACTATTA AGTGAGATTA AACGTAATGG CCTGATTTAA TCACAAAGAC

CTTACTATTA AGTGAGATTA AACGTAATGG CCTGATTTAA TCACAAAGGC

CTTACTATTA AGTGAGATTA AACGTAATGG CCTGATTTAA TCACAAAGGC

CTTACTATTA AGTGAGATTA ATCGTAATGG CCTGATTTAA TCACAAAGGC

CTTACTATTA AGTGAGATTA AACGTAATGG CCTGATTTAA TCACAAAGGC

CTTACTATTA AGTGAGATTA AACGTAATGG CCTGATTTAA TCACAAAGGC

CTTACTATTA AGTGAGATTA AACGTAATGG CCTGATTTAA TCACAAAGGC

CTTACTATTA AGTGAGATTA AACGTAATGG CCTGATTTAC TCACAAAGGC

---------- ---------- ---------- ---------- ----------

CTTACTATTA AGTGAGATTA AACGTAATGG CCTGATTTAA TCACAAAAGC

CTTACTATTA AGTGAGATTA AACGTAATAG CCTGATTTCA TCACAAAAGC

AGAATTAAAT TCAGATGGCA ACTTCAAAGT GGACACAGGA ACACAGGCCA

AGAATTAAAT TCAGATGGCA ACTTCAAAGT GGACACAGGA ACACAGGCCA

AGAATTAAAT TCAGATGGCA ACTTCAAAGT GGACACAGGA ACACAGGCCA

AGAATTAAAT TCAGATGGCA ACTTCAAAGT GGACACAGGA ACACAGGCCA

AGAATTAAAT TCAGATGGCA ACTTCAAAGT GGACACATGA ACACAGGCCA

AGAATTAAAT TCAGATGGCA ACTTCAAAGT GGACACATGA ACACAGGCCA

AGAATTAAAT TCAGATGGCG ACTTCAAAGT GGACACATGA GCACAGGCCA

AGAATTAAAT TCAGATGGCA ACTTCAAAGT GGACACATGA GCGCAAGCCA

AGAATTAAAT TCAGATGGCA ACTTCAAAGT GGACACATGA ACAGGGGCCA

AGAATTAAAT TCAGATGGCA ACTTCAAAGT GGACACAGGA ACACAGGCCA

AGAATTAAAT TCAGATGGCA ATTTCAAAGT GGACGCATGA ACACAGACCA

AGAATTAAAT TCAGATGGCA ACTTCAAAGT GGTCACATGA ACACAGGCCA

AGAATTAAAT TCAGATGGCA ACTTCAAAGT GGACACAGGA GCACCGGCCA

AGAATTAAAT TCAGATGGCA ACTTCAAAGT GGACACAGGA ACACAGGCCA

AGAATTAAAT TCAGATGGCA ACTTCAAAGT GGACACATGA ACACAGACCG

---------- ---------- ---------- ---------- ----------

AGAATTAAAT TCAGATGGCA ACTTCAAAGT GGCCACATGC ACACAGGCTA

AGAATTAAAT TCAGATGGCG ACTTCAAAGT GGACACAGGC GTACAGGCTA

CTGCTTACAT TTACATATAG CTGGCATAGC TTGCACAATA GGATAAAGTT

CTGCTTACAT TTACATATAG CTGGCATAGC TTGCACAATA GGATAAAGTT

CTGCTTACAT TTACATATAG CTGGCATAGC TTGCACAATA GGAT--AGTT

CTGCTTACAT TTACATATAG CTGGCATAGC TTGCACAATA GGAT--AGTT

CTGCTTACAT TTACATATAG CTGGCATAGC TTGCACAATA GGATAAAGTT

CTGCTTACAT TTACATATAG CTGGCATAGC TTGCACAATA GGATAAAGTT

CAGCTTGCAT TTACATATAG CTGGTATGGC TTGCACAATA GGGTAAAGTT

CTGCTTGCAT TTACATATAG CTGGCATAGC TTGCACAATA GGATAAAGTT

CTGCTTGCAT TTACATATAG CTGGCATAGC TTGCACAATA GGATAAAGTT

CTGCTTACAT TTACATATAG CTGGCATAGC TTGTACAATA GGATAAAGTT

CTGCTTACAT TTACATATAG CTGGCATAGC TTGCACAATA GGATAAAGTT

CTGCTTACAT TTACATATAG CTGGCATAGC TTGCACAATA GGATAAAGTT

CTGCCCGCAT TTACATATAG CTGGCATCGC TCGCACAATA GGCCAAACTT

GTGCTTACAT TTACATATAG CTGGCATAGC TTGCACAATA GGATAAAGTT

CTGCTTGCAT TTACATATAG CTGGCATAGC TTGCACAATA GGATAAAGTT

---------- ---------- ---------- ---------- ----------

CTGCATACAT TTACATATAG CTGGTCCAGC TTGCACAATG GGATAAAGTT

GTGCATAGAT TTACATATAG CTGGCATAGC TCGCTCAATG GGATAAAGTT

TCTTTGGCCA CTTAAACAAt tttctattct tcaccttttT TGCATTTAGT

TCTTTGGCCA CTTAAACAAT TTTCTATTCT CCACCttttT TGCATTTAGT

TCTTTGGCCA CTTAAACAAT TTTCTATTCT TCACcttttT TGCATTTAGT

TCTTTGGCCA CTTAAACAAT TTTCTATTCT TCACcttttT TGCATTTAGT

TCTTTGGCCA CTTAAACAAT TTTCTATTCT TCACCTTTTT TGCAT----T

TCTTTGGCCA CTTAAACAAT TTTCTATTCT TCACTTTTTT TGCATTTAGC

TCTTTGGCCA CTTAAACAAT TTTCTATTCT TCACTTTTCT TGCGTTTAGC

TCTTTGGCCA CTTAAACAAT TTTCTATTCT TCCCTTTTTT TGCATTCAGC

TCTTTGGCCA CTTAAACAAT TTTCTATTCT TCACTTTTTT TGCATTTAGT

TCTTTGGCCA CTTAAACAAT TTTCTATTCT TCACTTTTTT TGCATTTAGT

TCTTTGGCCA CTTAAACAAT TTTCTATTCT TCACTGTTTT TGCATTTAGT

TCTTTGGCCA CTTAAACAAT TTTCTATTCT TCACtttttT TGCATTTAGT

TCTTGGGCCA CTTAAACAAT TTTCTATTCT TCACTTTTTC TGCATTTTGT

TCTTTGGCCA CTTAAACAAT TTTCTTTTCT TCACTTTTTT TGCATTTAGT

TCTTTGGCCA CTTAAACAAT TTTCCATTCT GCACTTTTTT TGCATTTAGT

---------- ---------- ---------- ---------- ----------

TCTTTGGCCA CTTAAACAAT TTTCTATTAT TTATTTTTTT TGCATTGAGT

TCTTTGGCCA CTTAAACAAT TTTCCATTCT TTGCTTTTTT TGCGTGCGGT

TAGTAAGAAG GAGCTAAGTG TTATATTTAT TCATGGAGGC TATAACTGTT

TAGTAAGAAG GAGCTAAGTG TTATAGTTAT TCATGGAGGC TATAACTGTT

TAGTAAGAAG GAGCTAAGAG TTATATTTAT TCATGGAAGC TGTAATTTTT

TAGTAAGAAG GAGCTAAGAG TTATATTTAT TCATGGAAGC TGTAATTTTT

TAGTAAGAAA GAGCTAAGAG TTATATTTAT TCATAAAGGC TATAATTTTT

TAATAACAAG GAGCTAAGAG TTATATTTAT TCATGGAGGC TATAATTTTT

TGGTGAGAAG GAGCTGGGAG TTATTTTTAT TCACGGATGC TATAATTTTT

TGATGAGAAG GAGCCGAGAG TTATTTTTAT TCACGGATGC TATAATTTTT

TAATAAGAAG AGCCTAAGAG TTGTATGTAT TCATGG---- ----------

TAATAAGAAG GAGCTAAGAG TTCTATTTAT TTGTGGATGC TATAATTTTT

CAGCAAGAAG GAGCTAAGAG TTGGATTTAT TCATGGACGC TAGAATTTTT

TAATAAGAAG GACCTCGGAG TTATATTTAT TCATGGATAC TATAATTTTT

TCGTAAGGGG AAGCTAATCG CGATATGTCT CCGTGGGTGC TATCATTTTT

TAAGAAGATG GAGCTAAGAG TTACATTTAT TCATGGATGC TGCAATTTTT

TAATAAGAAG GAGCTAACAC TTATATTTAT TCGTGGGTGC TGTGATTTTT

---------- ---------- ---------- ---------- ----------

TACGAAGATG GAGCGAAGAG TTACATTGAT TCATAGAAGC TGTACTTTTT

TAATAAGGCG GAGCGGAGAG TTATGCTTAT TCATGGAAGC TGTACTTTTT

AATTCTTCCA AATAAAGCCA GGCCACCCGT TCAGGAATGT GGGTTAATTC

AATTCTTCCA AATAAAGCCA GGCCACCCGT TCAGGAATGT GGGTTAATTC

AATTCTTCCA AATAAAGCCA GGCCACCCCT TCAGGAATGT GGGTTAATTC

AATTCTTCCA AATAAAGCCA GGCCACCCGT TCAGGAATGT GGGTTAATTC

AATTCTTTCA AATAAAGCCA GGCCACCCGT TCAGGAATGT GGGTTAATTC

AATTCTTCCA AATAAAGCCA GGCCACCCGT TCAGGAATGT GGGTTAATTC

AATTCTTTCC AAGAAAGCCA GGCCACCCGT TCAGGAATGT GGGTTAATTC

AATTCTTTCA AAGAAAGCCA GGCCACCCGT TCAGGAATGT GGGTTAATTC

---------- ---AAAGCCA GGCCACCCGT TCAGGAATGT GGGTTAATTC

AATTCTTTCA AATAAAACCA G-CTACCGGT TCAGGAATGT GGGTTAATTC

GATTCTTTCG AATAAAGCCA GGCCACCCGT TCAGGAATGT GGGTTAATTC

AATTCTTTCA AATAAAGCCA GGCCACCCGT TCAGGAATGT GGGTTAATTC

AATTCTTGCA AAT------- ---------- ---------- ----------

AATTCTTTCA AATAAAGCCA GGCCACCCGT TCAGGAATGT GGGTTAATTC

AATTCTTTGA AATAAAGCCA GGCCACCCGT TCAGGAATGT GGGTTAATTC

---------- ---------- ---------- ---------- ----------

CATTCTTTCT CGTAAAGCAA AGCCACGCGC TCAGGAATGT GGGTTAAGGC

AATTCTTTCA AATACAGACC GGCCGCCAGT TAAGGAATGT GGGCTCATAC

GCCACCCTTT TTACCCCCAC AGCTCTGTTT TTGTGAGGTT GGCAGAAACT

GCCACCCTTT TTACCCCCAC AGCTCTGTTT TTGTGAGGTT GGCAGAAACT

GACACCCTTT TTACCCCCAC AGCTCTGTTT TTGTGAGGTT GGCAGAAACT

GACACCCTTT TTACCCCCAC AGCTCTGTTT TTGTGAGGTT GGCAGAAACT

GCCACCCTTT TTACCCCCAC AGCTCTGTTT TTGTGAGGTT GGCAGAAACT

GCCACCCTTT TTACCCCCAC AGCTCTGTTT TTGTGAGGTT GGCAGAAACT

GCCACCCTTT TTACCCCCAC AGCTCTGTTT TTGTGAGGTT GGCAGAAACT

GCCACCCTTT TTACCCCCAC AGCTCTGTTT TTGTGAGGTT GGCAGAAACT

GCCACCCTTT GTACCCCCAC AGCTCTGTTT CTGCGAGGCT GGCAGAAACT

GCCACCCTTT TTACCCCCAC AGCTCTGTTT TTGTGAGGTT GGCAGAAACT

GCCACCCTTT ATACCCCCAC AGCTCTGTTT TTGTGAGGTT GGCAGAAACT

GCCACCCTTT TTACCCCCAC AGCTCTGTTT TTGTGAGGTT GGCAGAAACT

---------- ---------- ---------- ---------- ----------

GCCACCCTTT TTACCCCCAC AGCTCTGTTT TTGTGAGGCT GGCAGAAACT

GCCACCCTTT TTACCCCCAC AGCTCTGTTT TTGTGAGGTT GGCAGAAACT

---------- ---------- ---------- ---------- ----------

GCCACCTTTA TTACCCCCAC AGCTCTGTTT TTGTGAGGTT GGCAGAAACT

GCCACCCTTT CTAACCCCCC GACTTGGTTT TGGGGAGGTT GGCAGAAACT

GACACGTTTT CTTTTGCTTA ATTAAGTTGT TCAGTAGAAG GCTGGTCAAA

GACACGTTTT CTTTTGCTTA ATTAAGTTGT TCAGTAGAAG GCTGGTCAAA

GACACGTTTT CTTTTGCTTA ATTAAGTTGT TCAGTAGAAG GCTGGTCAAA

GACACGTTTT CTTTTGCTTA ATTAAGTTGT TCAGTAGAAG GCTGGTCAAA

GACACGTTTT CTTTTGCTTA ATTAAGTTGT TCAGTAGAAG GCTGGTCAAA

GACACGTTTT CTTTTGCTTA ATTAAGTTGT TCAGTAGAAG GCTGGTCAAA

GACACGTTTT CTTTTGCTTA ATTAAGTTGT TCAGTAGAAG GCTGGTCAAA

GACACGTTTT CTTTTGCTTA ATTAAGTTGT TCAGTAGAAG GCTGGTCAAA

GACACGTTTT CTTTTGCTTA ATTAAGTTGT TCAGTAGAAG GCTGGTCAAA

GACACGTTTT CTTTTGCTTA ATTAAGTTGT TCAGTAGAAG GCTGGTCAAA

GACACGTTTT CTTTTGCTTA ATTAAGTTGT TCAGTAGAAG GCTGGTCAAA

GACACGTTTT CTTTTGCTTA ATTAAGTTGT TCAGTAGAAG GCTGGTCAAA

---------- ---------- ---------- ---------- ----------

GACACGTTTT CTTTTGCTTA ATTAAGTTGT TCAGTAGAAG GCTGGTCAAA

GACACGTTTT CTTTTGCTTA ATTAAGTTGT TCAGTAGAAG GCTGGACAAA

---------- ---------- ---------- ---------- ----------

GACACTTTTT CTTTTGCTTA ATTAAGTTGT TCAGAGAAAG GCTGGTCAAA

GACACTTTTT CTTTGGCTTA ATTAAGTCGT TCAGAAGGAG GCTGGTCAAA

CAGTTGCATC TACGTGGGGA GTCTGGAGAT GCACAGAAAG CCGTGGGCGT

CAGTTGCATC TACGTGGGGA GTCTGGAGAT GCACAGAAAG CCATGGGCGT

CAGTTGCATC TACGTGGGGA GTCTGGAGAT GCACAGAAAG CCGTGGGCGT

CAGTTGCATC TACGTGGGGA GTCTGGAGAT GCACAGAAAG CCGTGGGCGT

CAGTTGCATC TACGTGGGGA GTCTGGAGAT GCACAGAAAG CCGTGGGCGT

CAGTTGCATC TATGTGGGGA GTCTGGAGAT GCACAGAAAG CCGTGGGCGT

CAGTTGCATC TACGTGGGGA GTCTGGGGAT GCGCAGAAAG CCGTGGGCGT

CAGTTGCATC TACGTGGGGA GTCTGGGGAT GCGCAGAAAG CCGTGGGCGT

CAGTTGCATC GACGTGGGGA GTCTGCAGTT GCTCAGAAAG CCGTGGGCGT

CAGTTGCGTC TACGTGGGGA GTCCGAAGAT ACACAGAAAG CTGTGGGCGT

CAGTTGCATC TACGTGGGGA GTCTGCAGAT ACAGAGAAAG CCGTGGGCGT

CAGTTGCATC TACGTGGGGA GTCTGCAGAT ACACAGAAAG CCGTGGGCGT

---------- ---------- ---------- ---------- ----------

CAGTTGCGTC TACGTGGGGA GTCTGGAGAC GCACAGAAAG CCGTGGGCGT

CAGTTGCCAC TACGTGGGGA GTCTGGGGAG GCACAGAAAG CCGTGGGCGT

---------- ---------- ---------- ---------- ----------

CAGTTGCATC TACGTGGGGA GTCTTGAAAT GAACAGAAAG CAGTGGGCGT

CAGTTGCATC TACGTGGGGA GTCCCGAAAT GAACAGAAAG CAGAGGGCGT

TTCCAACATA AAAAGGGGGA TTCTGTTATT GTTTGGGTGT AGACGGGTCA

TTCCAACATA AAAAGGGGGA TTCTGTTATT GTTTGGGTGT AGACGGGTCA

TTCCAACATA AAAAGGGGGA TTCTGTTATT GTTTGGGTGT AGACGGGTCA

TTCCAACATA AAAAGGGGGA TTCTGTTATT GTTTGGGTGT AGACGGGTCA

TTCCAACATA AAAAGGGGGA TTCTGTTATT GTTTGGGTGT AGACGGGTCA

TTCCAACATA AAAAGGGGGA TTCTGTTATT GTTTGGGTGT AGACGGGTCA

TTCCAACATA AAAAGGGGGA TTCTGTTATT GTTTGGGTGT AGAC-GGTCA

TTCCAACATA AAAAGGGGGA TTCTGTTATT GTTTGGGTGT AGACGGGTCA

GCCCGACATA GAAAGGGGGA TTCTGTTATT GTTTGGGTGT AGACGGGTCA

CTCCAACATG AAAAGGGGGA TTCTGTTATT GTTTGGGTGT AGACGGGTCA

TTCCAACATA AAAAGGGAGA TTCTGTTATT GTTTGGGTGT AGACGGGTCA

TTCCAACATA AAAAGGGGGA TTCTGTTATT GTTTGGGTGT AGACGGGTCA

---------- ---------- ---------- ---------- ----------

TTCCAACATA AAAAGGGGGA TTCTGTTATT GTTTGGGTGT AGACGGGTCA

TTCCAACATA AAAAGGGGGA TTCTGTTATT GTTTGGGTGT AGACGGGTCA

---------- ---------- ---------- ---------- ----------

TGCCAACATA AAAAGGGGGA TTCCGTTATT GTTTGGGTGT AGACGGGTCA

TGCCAACATA AAAAGGGGGA TTCTGTTATT GTTACGGCGC GGATGGGTCA

TTTTTTCAAA GTTACTGCTG CCTGAGTGAC CACAACCCTG TGCGCAGCCC

TTTTTTCAAA GTTACTGCTG CCTGAGTGAC CACAACCCTG TGCGCAGCCC

TTTTTTCAAA GTTACTGCTG CCTGAGTGAC CACAACCCTG TGCGCAGCCC

TTTTTTCAAA GTTACTGCTG CCTGAGTGAC CACAACTCTG TGCGCAGCCC

TTTTTTCAAA GTTACTGCTG CCTGAGTGAC CACAACCCTG TGCGCAGCCC

TTTTTTCAAA GTTACTGCTG CCTGAGTGAC CACAACCCTG CACACAGCCC

TTTTTTCAAA GTTACTGCTG CCTGAGTGAC CACAACCTTG TGCAGAGCCC

TTTTTTCAAA GTTATTGCTG CCTGAGTGAC CACAACCTTG CGCAGAGCCC

TTTTTTC--A GTTACTGCTG CCTCGGTGAC CACAACCCGG CGCACAGCCC

TTTTTTCAAA GTTACTGCTG CCTGAGTGAC CACAGCCCTG CACACAGTCC

TTTTTTCAAA GTTACTGCTG CCTGAGTGAC CACAACCCAG CGCACAGCCC

TTTTTTCAAA GTTACTGCTG CCTGAGTGAC CACAACCCTG CACGCAGCCT

---------- ---------- ---------- ---------- ----------

TTTTTTCAAA GTTACTGCTG CCTGAGTGAC CACAGCCCTG CACGCAGCCC

TTTTTTCAAA GTTACTGCTG CCTGAGTAAT CACAACCCTG GGCACAGCCC

---------- ---------- ---------- ---------- ----------

TTTTTTCAAA GTTACTGCTG CCTGAGTGGC CACAACCCTG CAAGAAGGAG

CTTTTTCAAA GTTACTGCTG GCTGAGTGAC CCAAACCCTG CAAGCATCCG

TGAAAGGACA TTAAAGCTGC TTAAATACAA CTACCTTCGG TTTACTTAAA

TGAAAGGACA TTAAAGCTGC TTAAATACAA CTACCTTCGG TTTACTTAAA

TGAAAGGACA TTAAAGCTGC TTAAATACAA CTACCTTGGG TTTATTTAaa

TGAAAGGACA TTAAAGCTGC TTAAATACAA CTACCTTGGG TTTATTTAAA

TGAAAGGACA TTAAAGCTGC TTAAATACAA CTACCTTGGG TTTATTTAAA

TGAAAGGACA TTAAAGCCGC TTAAACACAA CTACCTTGGG TTTATTTAAA

TGAAAGGACG TTAAAGCTGC TTAAACACAA CCACCTTGGG TTtatttaag

TGAAAGGACA TTAAAGCTGC TTAAACACAA CCACCTTGGG CTTGTTTAAA

TGAAAGGACC TTAACGCCAC TTAAATGCAA CTACCTTGGG TTTACTTAAA

TGAAAAGACA TTAAAGCTGG TTCAATACGA CTACCTTGGG TTTATTTAAA

TGAAAGGAAA TTAAAGCTGC TTAAATACAA CTACCTTGGG TTTATTTAAA

TGAAAAGACA TTAAAGCTGC TTAAATACAA CCACCTTGGG TTTATTTAAA

---------- ---------- ---------- ---------- ----------

TGAAAGGACA TTAAAGCCGC TTAAATACAA CTACCTTGGG TTTATTCgaa

TGAAAGGACG TGAAAGCTGC TTAAGCACAA CCGCCTTGGG CTTCTTTAAA

---------- ---------- ---------- ---------- ----------

TGAAAGGAAG TTAAAGCTGC TTAAATACAA CTACCTTGGG TTTATTTAAA

TGAAAGACCA TTAAAGCGGC TTAAACTCGG CTACCTTGGA CTCATTTAAA

GGTCCTGCAG ACAGAGGGGA GGATTCACAA GGGCTGGCCA GGAGCCATCC

GGTCCTGCAG ACAGAGGGGA GGATTCACAA GGGCTGGCCA GGAGCCATCC

GGTCCTGCAG ACAGAGGGGA GGATTCACAA GGGCTGGCCA GGAGCCATCC

GGTCCCGCAG ACAGAGGGGA GGATTCACAA GGGCTGGCCA GGAGCCATCC

GGTCCTGCAG ACAGAGGGGA GGATTCACAA GGGCTGGCCA GGAGCCATCC

GGTCCTGGGG ACAGAGGGGA GGATTCACAA GGGCTGGCCG AGAGCCATCC

GGGTCTTGGG ACAGAGGGAA GGATTCACAA GAGCTGGCCA GGAGCCATGC

GGGCCTTGGG ACAGAGGGGA GGATTCACAA GAGCTGGCCA GAAGCCACGC

GGTCCTGGAG ACGGAGGGGA GGATTCAC-A GGGCTGGCCA GGAGCCATGC

GGTCCCGGAG ACAGAGGGGA GGACTTGCAA GGGCTGGCCG GGAGCCATCC

GGTCCTGGAG ACGGAGGG-A GGACTCACAA GGGCTGGCCA CGAGCCATCT

GGTCCTGGAG ACAGAGGGGA TGACTCACAA GGGCTGGCCA GGAGCCATCC

---------- ---------- ---------- ---------- ----------

GGCCCCGGAG ACAGAGGGAA GGATTCACAA GGGCTGGCCG CGAGCCATCC

GGTCCTGGAG ACAGAGGGGC AGATTCACAA GGGCTGGCCA GGAGCCATCC

---------- ---------- ---------- ---------- ----------

GGTCCTAAAG ACAGAGGAAA AGATTCACAA GGGCTGGCTG GGAGCCATCT

AGTCCTAAAG ACAGAGGGAA GGATGCACAA GGGCTGGCCT CGGGCCAGCT

CACTAGCCCT GGCACTCCAC ATGGAGCTCT GCGGCTCTTC AGGACAAAGA

CACTAGCCCT GGCACTCCAC ATGGAGCTCT GCGGCTCTTC AGGACAAAGA

CACTAGCCCT GGCACTCCAC ATGGAGCTCT GCGGCTCTTC AGGACAAAGA

CACTAGCCCT GGCACTCCAC ATGGAGCTCT GCGGCTCTTC AGGACAAAGA

CACTAGCCCT GGCACTCCAC ATGGAGCTCT GCGGCTCTTC AGGACAAAGA

CACTATCCCT GGCACTCCAC ACGGAGCTCT GCGGCTCTTC AGGACAAAGA

CACTATCCC- GGCACTCCAC ACGGAGCTGT GCGGCTCCTA GGGACAAAGA

CACTATCCC- GGCCCTCCAC ACGGAGCTGT GCGGCTCCTA GGGACAAAGA

CACTATCCCT GGCACTCTGT ATGGAACTCT GCGGCTGGTG CGGACAAAGA

CCCGATTCCT GGCACTCCAC ATGGAGCTCT GCG-CTCTGC AGGACAAAGA

TACAATCCCT GGCACTCCAC ATGGAGCTCT GCGGCTCTTC AGGACAAAGA

CACTATCCCT GGCACTCCAC ATGGAGCCCT GCAGCTCTAC AGGACAAAGA

---------- ---------- ---------- ---------- ----------

CGCGCTCCCT GGCACTCCAC CCGGAGCTTT GCGGCTCTCC GGGACAAAGG

CACTATCCCT GGTACTCCAC ACAGAGTGTT GTGGCTCTTC AGGACAAAGA

---------- ---------- ---------- ---------- ----------

TACTATTCCT GGCATTCCAC ATGGAGCTTT GCAGCTTGTC AGGACAAAGA

CTCTCGCCTC GGCCCTCCAC ATGGAGGTTT GAGACTCGGC GGGACAAAGA

GGAGTGTTTT TATCAGCCCA ACGTTCTGCT GACAAATGCA TAATAAACTG

GGAGTGTTTT TATCAGCCCA ACGTTCTGCT GACAAATGCA TAATAAACTG

GGAGTGTTTT TATCAGCCCA ACGTTCTGCT GACAAATGCA TAATAAACTG

GGAGTGTTTT TATCAGCCCA ACGTTCTGCT GACAAATGCA TAATAAACTG

GGAGTGTTTT TATCAGCCCA ACGTTCTGCT GACAAACGCA TAATAAACTG

GGAGTGTTTT TATCAGCCCA ACGTGCTGCT GACAAATGCA TAATAAACTG

GGGGTGTTTT CACCAGTCCA ACGTTCTGCT GACAAATGCA TAATAAACTG

GGGGTGTTTT CAGCAGCCCG ACGTTCTGCT GACAAATGCA TAATAAACTG

AGAGCGtttt CAGCAGCCCT GCGTTCTGCT GACAAACGCA TAATAAACTG

GGAGCGTTTT TATCCGCCCC GAGTTCTGCT GACAAACGCA TAATCAACTG

GGAGCGTTTT TATCAGCCCA ACGTTCTGCT GACAAATGCA TAATAAACTG

GGAGCGTTTT TATCAGCCCA ACGTTCTGCT GACAAATGCA TAATAAACTG

---------- ---------- ---------- ---------- ----------

TGCGCGTTTT CACCGGCCCG ACGTTCTGCT GACAAACGCA TAATCGAGTG

GAAGCGTTTT TACCAGCCCC GTGTTTTGCT GACAAATGCA TAATAAACTG

---------- ---------- ---------- ---------- ----------

GTACAGTTTT TATCAGCCCA ACATTCTGCT GACAAATGCA TAATAAACTG

GCGCAGTTTC GACGAGCCCC GCGTTCCGCT GACAGACGCA GAATCGACTG

ACAGAAAGCG ACCAAGCAGC ATTTCAAAAC CCCAGTTACG GGGAGGGGAG

ACAGAAAGCG ACCAAGCAGC ATTTCAAAAC CCCAGTTACG GGGAGGGGAG

ACAGAAAGCG ACCAAGCAGC ATTTCAAAAC CCCAGTTACG GGGAGGGGCG

ACAGAAAGCG ACCAAGCAGC ATTTCAAAAC CCCAGTTACG GGGAGGGGCG

ACAGAAAGCG ACCAAGCAGC ATTTCAAAAC CCCAGTTACG GGGAGGGGAG

ACAGAAAGCA ACCAAGCAGC ATTTCAAAAC CCCAGTTACG AGGAGGGGGG

ACAGA----- ---AAGCAGC ATTTCAAACC CCCAGTTACG GGGAGGGGGG

ACAGAAAGCA ACCAAGCAGC ATTTCAAAGC CCCAGTTACG GGGAGGGGGG

ACAGAAAGCC ACCAAGCCAC ATCTCCAAAC CCCCGTTCCA GGGAGGGGGG

ACAGAAAGCG ACGGAGCAGC GTTTCCAAAC CCCAGTTACG GGGAGGGGGG

ACAGAGAACC ACCAAGCAGC ATTTCAAAAC CCCAGTTACC GGGAGGGGGG

ACAGAAAGCG ACCAAGCAGC ATTTCAAAGC CCCAGTTATG GGGAGG-GGG

---------- ---------- ---------- ---------- ----------

ACAGAGCGCG GCCGCGCGGC ATTTCCAAAG CCTGGTTCCG GGGAGCAGGG

GCAGAAAGCG ACCAAGCAGC ATTTCAAAAC CCCGGTTACG GGGAGGGGGG

---------- ---------- ---------- ---------- ----------

ACAGAAAGAG ACCGAGCAGC ATTTCAAAAC CCCAGTTACT GGGAGGGGGG

ATGGAGAGGG CCCGAGCCGC CTTTCAAAAA GCCAGTGACA GGGAGGGGGG

ATGGTCTTTC TGCAGGTGTT CGGCCAGTGT CCCTGCATTT GCATGCATTT

ATGGTCTTTC TGCAGGTGTT CGGCCAGTGT CCCTGCATTT GCATGCATTT

ATGGTCTTTC TGCAGGTGTT CGGCCAGTGT CCCTGCATTT GCATGTATTT

ATGGTCTTTC TGCAGGTGTT CGGCCAGTGT CCCTGCATTT GCATGCATTT

ATGGTCTTTC TGCAGGTGTT CGGCCAGTGT CCCTGCATTT GCATGCATTT

ATGGTCTTTC TGCAGGTGTT CGGCCAGTGT CCCCACATTT GCATGCATTT

ATGGTCTTTC TGCAGGTGTC AGGCCAGTGT CCCCACATTT GCATGCATTT

ATGGTCTTTC TGCAGGTGTC AGGCCAGTGT CCCTGCATTT GCATGTATTT

ATGGTC-TTC TGCAGGTGTT GGGCCAGTGT CCCCACATTT GCATGCATTT

ATGGTCTTTC TGCAGGTGTT CGGCCAGTGT CCCCTCATTT GCATGCATTT

ATGGTCTTTC TGCAGGTGTT AGGCCAGTGT CCCTGCATTT GCATGCATTT

ACGGTCTTTC TGCAGGTGTT CGGCCAGTGT CCCTGCATTT GCATGCATTT

---------- ---------- ---------- ---------- ----------

ATGGTCTCCG TGCAGGTGTT GGGGCCGCGT CTCTGCATTT GCATACATTT

ATGGTCTTTC TGCAGGTGTT CGGCCAGTGT CCCTGCATTT GCATACATTT

---------- ---------- ---------- ---------- ----------

ATGGTCTTTC TGCAGGTGTT CGTCCAGTGT CCCTGAATTT GCATTTATTT

ATGGTCTTTC TGCAGGTGTT CGCCCACTGT CCCCGCATTT GCATGCATTT

GCATATACTG TCAAACAGTT GGCTGAAGAA AGTCTGAGGA GGTCTGGACA

GCATATACTG TCAAACAGTT GGCTGAAGAA AGTCTGAGGA GGTCTGGACA

GCATATACTG TCAAACAGTT GGCTGAAGAA AGTCTGAGGA GGTCTGGACA

GCATATACTG TCAAACAGTT GGCTGAAGAA AGTCTGAGGA GGTCTGGACA

GCATATACTG TCAAACAGTT GGCTGAAGAA AGTCTGAGGA GGTCTGGACA

GCATATACTG TCAAACAGTT GGCTGAAGAA AGTCTGAGGA GGTCTGGACA

GCATAGACTG TCAAGCAGTT GGCTGCGGAG AGTCTGAGGA GGTCTGGACA

GCATAGACTG TCAAACAGTT GGCTGCGGAG AGTCTGAGGA GGTCTGGACA

GCATAGACTG TCAAACAGTT GGCTGAAGAG AGTCTGAGGA GGCCTGGACA

GCATTGACTG TCAAACAGTT GGCTGAAGAA AGTCTGAGGA GGTCTGGACA

GCATAGACTG TCAAACAGTT GGCTGAAGAA AGTCTGAGGA GGTCTGGACA

GCATATACTG TCAAACAGTT GGCTGAAGAA AGTCTGAGGA GGCCTGGACA

---------- ---------- ---------- ---------- ----------

GCATAGACTG TCAAACAGTT GGCTGAAGGA AGTCTGAGGG GGTCTGGACA

GCATAGACTG TCAAACAGTT GGCGGAGGAG AGTCTGAGGA GGTCTGGACA

---------- ---------- ---------- ---------- ----------

GCATATACTG TCAAACAGTT GGCTGAAGAA ACTCTGAGAA GGTCTGGACA

GCATAGACTG TCAAACAGTT GGCTGAAGAA ACTCTGAGTA GCTCTGGACG

CAGCCCAAAC CTGTCAGGGC TTTCTCCTTG CCAGTCAAGG TTGGCTGTGG

CAGCCCAAAC CTGTCAGGGC TTTCTCCTTG CCAGTCAAGG TTGGCTGTGG

CAGCCCAAAC CTGTCAGGGC TTTCTCCTTG CCAGTCAAGG TTGGCTCTGG

CAGCCCAAAC CTGTCAGGGC TTTCTCCTTG CCAGTCAAGG TTGGCTCTGG

CAGCCCAAAC CTGTCAGGGC TTTCTCCTTG CCAGTCAAGG TTGGCTCTGG

CAGCCCAAAC CTGTCAGGGC TTTCTCCTTG CCAGTCAAGG TTGGCTTTGG

CAGCCCAAAC CTGTCAGGGC TTTCTCCTTG CCAGTCAAGG TTGGCTTTAG

CAGCCCAAAC CTGTCAGGCC TTTCTCCTTG CCAGTCAAGG TTGGCTTTCG

CAGCCCAAAC CTGTCAGGGC TTTCTCCTTG CCAGTCAAGG TTGGCTCCGG

CAGCCCAAAC CTGTCAGGGC TTTCTCCTTG CCAGTCAAGG TTGGCTTGGG

CAGCCCAAAC CTGTCAGGGC TTTCTCCTTG CCAGTCAAGG TTGGCCTGGG

CAGCCCAAAC CTGTCAGGGC TTTCTCCTTG CCAGTCAAGG TTGGCTTGGG

---------- ---------- ---------- ---------- ----------

CAGCCCAAAC CTGTCAGGGC TTTCTCCTTG CCAGTCAAGG TTGGCTTTGC

CGGCCCAAAC CTGTCAGGGC TTTCTCCTTG CCAGTCAAGG TTGGCTTTGG

---------- ---------- ---------- ---------- ----------

GAGCCCAAAC CTGTCAGGGC TTTCTCCTTG CCAGTCAAGG TTGGCTTTGG

CGGCCCAGAC CTGTCAGGGC TTTCTCCTTG CCGGTCAAGG TTGGCTCCGA

CTGCAATTAA GGCAGAAACG AATACTTCTG ACATTGCAAT ATACCAGCCA

CTGCAATTAA GGCAGAAACG AATACTTCTG ACATTGCAAT ATACCAGCCA

CTGCAATTAA GGCAGAAACG AATACTTCTG ACATTGCAAT ATACCAGCCA

CTGCAATTAA GGCAGAAACG AATACTTCTG ACATTGCAAT ATACCAGCCA

CTGCAATTAA GGCAGAAACG AATACTTCTG ACATTGCAAT ATACCAGCCA

CTGCAATTAA GGCAGAAACG AATCCTTCTG ACATTGCAAT ATACCAGCCA

CTGCAATTAA AACAGAAATG AATACTTCTG ACATTGCAAT AGACCAGCCA

CTGCAATTAA AACAGAAATG AATACTTCTG ACATTGCAAT AGACCAGCCA

CTGCAATTAA AGCAGAAGCG GAGCCTTCTG ACATCGCCAT AGCCTAGCCA

CTGCAATTAG AGCAGAAACG AATACTTCTG ACACTGCAAT ATACCAGCCA

CGGCAATTAA AGCGGAAACG CGTACTTCTG ACATTGCAAT ATACCCGCCA

CTGCAATTAA GGCAGAAACG AATACTTCTG ACATTGCAAT ATACCCACCG

---------- ---------- ---------- ---------- ----------

CCGCAATTAG AGCTGGAACG ACTACTTGTG ACATTGCAGC ATCCCGGCCA

CTGCAATTAA AGTTGAAACC AATACTTCTG ACATTGCAAT ATACCAGCCA

---------- ---------- ---------- ---------- ----------

CTGCAATTAA AATGGAAAGC AATACTTCTA ACATTGTACT GTAGAAGCCA

CCTCAATTAA AACGGAGAGG AAAACTTCTG ACGCCGCGGT CGGGAAGTCA

CAGCTGGGAG ATGTTGTTTT CAGATTCTTT AAGCGAGACC TAACAATTTA

CAGCTGGGAG ATGTTGTTTT CAGATTCTTT AAGCGAGACC TAACAATTTA

CAGCTGGGAG ATGTTGTTTT CAGATTCTTT AAGCGAGACC TAACAATTTA

CAGCTGGGAG ATGTTGTTTT CAGATTCTTT AAGCGAGACC TAACAATTTA

CAGCTGGGAG ATGTTGTTTT CAGATTCTTT AAGCGAGACC TAACAATTTA

CAGCTGGGAG ATGTTGTTCT CAGATTATTT AAGCGAGACC TAACAATTTA

CAGCTGGGAG GTGCTGTGTT TAGCAGCTTT AAGCAAGACC TAACAATTTG

CAGCTGGGAG GTGCTGTGTT TAGCAGCTTT AAGCAAGACC TAACAATTTG

CAGCTGGGAG ATGTTGTCTT CAGATGCCTT AAGCGAGACC CAACAATTTA

CAGCTGGGAG ATGTTGTTTC CAGATTCTTT AAGCGAGACC TAACAATTTA

CAGCTGGGAG ATGTTGTTTC --GATTCTTT AAGCGAGACC TAACAATTTA

CAGCTGGGGG ATGTTGTTTC CAGATTCTTT AAGCGAGACC TAACAATTTA

---------- ---------- ---------- ---------- ----------

CAGCTGGGAG ATGTTGTTTT CCAATTCCCT AAACGAGACC TAACAATTTA

CAGCTGGGAG ATGTGGTTTT CATCTTCTTT AAGCGAGACC TCACAATTTC

---------- ---------- ---------- ---------- ----------

CAGCTGGGAG TTGTTATTTT CATATTCTTT TACTGAGCCA TAACAATTTA

CAGCTGGGAG TTATTGTTCC CAGATTCCCT AACCGAGCCA TAACAATTTA

TTAAATACTT CATCTCTAAA CAGTGGTGGG CACTAGAGTT TCATGAGAGC

TTAAATACTT CATCTCTAAA CAGTGGTGGG CACTAGAGTT TCATGAGAGC

TTAAATACTT CATCTCTAAA CAGTGGTGGG CACTAGAGTT TCATGAGAGC

TTAAATACTT CATCTCTAAA CAGTGGTGGG CACTAGAGTT TCATGAGAGC

TTAAATACTT CATCTCTAAA CAGTGGTGGG CACTAGAGTT TCATGAGAGC

TTAAATACTT CATCTCTACA CAGTGGTAGG CACTAGAGTT TCATGAGAGT

TTAAATACTT CATCTCCAAA CAGCGGTGGG CACTACAGTT TCATGGGAGC

TTAAATACTT CATCTCCAAA CAGTGGTGGG TACTACAGTT TCATGAGAGC

TTAAATACTT CATCTCCAAA CAGCGGTGAG CACTAGAGTT TCATGAGAGC

TTAAATACTT CATCTCTCAA CAGTGGTGGG CACTAGAGTT TCATGAGCGG

TTAAATACTT CATCTCTAAA CAGTGGTGGG CACTAGAGTT TCACGAGAGC

TTAAATACTT CATCTCTAAA CAGTGGTGGG CACTAGAGTT TCATGAGAGC

---------- ---------- ---------- ---------- ----------

TTAAATACTT CATCTCTCAA CAGTGGTGGG CACTAGAGTT TCATGAGAGC

TTCAATACTT CATCTCTACA CAGTGCTGGG CACTAGAGTT TCATGAGGGC

---------- ---------- ---------- ---------- ----------

TTAAATACTT AATCTCTCAA CAGTGGTGGG CACTAGAGTT TCATGAGAAC

TTAAATGCTT CATCTTTAAA CAGGGGTGGG CACTACAGTT TCATGAGGCC

AAACAATAGA TTCTGTTTAT TCGCCAACAA TACAGACACT GTTTAGCTCC

AAACAATAGA TTCTGTTTAT TCGCCAACAA TACAGACACT GTTTAGCTCC

AAACAATAGA TTCTGTTTAT TCGCCAACAA TACAGACACT GTTTAGCTCC

AAACAATAGA TTCTGTTTAT TCGCCAACAA TACAGACACT GTTTAGCTCC

AAACAATAGA TTCTGTTTAT TCGCCAACAA TACAGACACT GTTTAGCTCC

GAACAATAGA TTCTGTTTAT TCGTCAACAA TACAGACACT GTTTAGCTCC

AGATAATAGA TTCTGTTTAT TTGCCAACAA TACCAACACT GTTCAGTCCC

AGATAATAGA TTCTGTTTAT TCGCCAACAA TACCGACACT GTTTCGCCCC

ACACAATAGC TTCTGTTGAT TCGCCAACAA TACAGACACT GTTCGGCTCC

AGACAATAGA TTCTGTTTAT TCGCCAACAA TACAGATACT GTTTAGCTCT

ACACAATAGA TGCTGTTTAT TCGCCAACAA TACAGACTCT GTTTAGCTCT

AGACAATAGA TTCTGTTTAT TCGCCCACAA TACAGACACT GTTTAGTTCT

---------- ---------- ---------- ---------- ----------

GGACAATAGA TTCTGTTTAT TCGCCGACAA TACAGACACT GTTTTGCTCG

GGACAATGGA TTCTGTTTAT TCGCCAACAA TACAGACACT GTTTCACTCT

---------- ---------- ---------- ---------- ----------

AGACAATAGA TTCTGTTTTT TCATCAACAA TAAAGACACT GTTTAGCTCG

GGACAATAGG TTCTGTTTAT TCGCCAACAA TAAAGAGATT GTTTGACTGG

GGACTGCAAG TGGCTGAGCC ATCTGGCTCA TATCTCCACA GTAAAACATT

GGACTGCAAG TGGCTGAGCC ATCTGGCTCA TATCTCCACA GTAAAACATT

GGACTGCAAG TGGCTGAGCC ATCTGGCTCA TATCTCCACA GTAAAACATT

GGACTGCAAG TGGCTGAGCC ATCTGGCTCA TATCTCCACA GTAAAACATT

GGACTGCAAG TGGCTAAGCC ATCTGGCTCA TATCTCCACA GTAAAACATT

GGACTGCAAG TGGCTGAGCC ATCTGGCTCA TATCTCCACA GTAAAACATT

GGACTGCAAG TGGCTGAGCC ATCTGGCTCA CATCTCCACA GTAAAACATT

GGACTGCAAG TGGCTGAGCC ATCTGGCTCC TATCTCCACA GTAAAACATT

GGACTGCAAG TGGCTGAGCC ATCTGGCTCG TATCTCCACA GTAAAACATT

GGACTGCAAG TGGCTGAGCC ATCTGGCTCA TATCTCCACA GTAAAACATT

GGACTGCAAG TGGCTGAGCC ATCTGGCTCA TATCTCCACA GTAAAACATT

GGACTGCAAG TGGCTGAGCC ATCTGGCTCA TATCTCCACA GTAAAACATT

---------- ---------- ---------- ---------- ----------

GGACTGCAAG TGGCTGAGCC ATCTGGCTCA TATCTCCACA GTAAAACATT

GGACTGCAAG TGGCTGAGCC ATCTGGCTCA TATCTCCACA GTAAAACATT

---------- ---------- ---------- ---------- ----------

GGACTGCAAG TGTCTGAGCC ATCTGGCCCA TATCTCCACA GTAAAACATT

AGGCTGCAAG TGACTGAGCC ATCTGGCCCA TATCACCACA GTAAAACTTT

GTCCGTACAC AAAAAATGAC TACATCCCCA GGACAAACTA GTGTGACCTC

GTCCGTACAC AAAAAATGAC TACATCCCCA GGACAAACTA GTGTGACCTC

GTCCGTACAC AAAAAATGAC TACATCCCCA GGACAAACTA GTGTGACCTC

GTCCGTACAC AAAAAATGAC TACATCCCCA GGACAAACTA GTGTGACCTC

GTCCGTACAC AAAAAATGAC TACATCCCCA GGACAAACTA GTGTGACCTC

GTCCGTACAC AAAAAATGAC TACATCCCCA GGACAAACTA GTGTGACCTC

GTCCGTACAC AAAAAATGAC TACATCCCCA GGACAAACTA GTGTGACCTC

GTCCGTACAC AAAAAATGAC TACATCCCCA GGACAAACTA GTGTGACCTC

GTCCGTACAC AAAAAATGAC GACATCCCCA GGACAAACTA GTGTGACCCC

GTCTGCACAC AAAAAATGAC TACATCCCCA GGACAAACTA GTGTGACCTC

GTCTGTACAC AAAAAATGAC TACATCCCCA GGACAAACTA GTGTGACCTC

GTCTGTACAC AAAAAATGAC TACATCCCCA GGACAAACTA GTGTGACCTC

---------- ---------- ---------- ---------- ----------

GTCCGTACAC AAAAAATGAC TACATCCCCA GGACAAACTA GTGTGACCTC

GTCCGTACAC AAAAAATGAC AACATCCCCA GGACAAACGA GTGTGACCTC

---------- ---------- ---------- ---------- ----------

GTCTGTACAC AAAAAATGAC TACATCCACA GGACAAACTA ATGTGACCTC

TTCGGTCTAC AAAAAATGAC TACATCCCCA GGACCGACTA ATGCGAGGCC

TAAATAAAGC CTTCCTCTGA AGCTATGATA TCACACTGCA CAGGGGACAC

TAAATAAAGC CTTCCTCTGA AGCTATGATA TCACACTGCA CAGGGGACAC

TAAATAAAGC CTTCCTCTGA AGCTATGATA TCACACTGCA CAGGGGACAC

TAAATAAAGC CTTCCTCTGA AGCTATGATA TCACACTGCA CAGGGGACAC

TAAATAAAGC CTTCCTCTGA AGCTATGATA TCACACTGCA CAGGGGACAC

TAAATAAAGC CTTCCTCTGA AGCTATGATA TCATACTGCA CAGGGGACAC

TAAATAAAGC CTTCCTCTGA AGCTATGATA TCACACTGCA CAGGGGACAC

TAAATAAAGC CTTCCTCTGA AGCTATGATA TCACACTGCA CAGGGGACAC

TACATAAAGC CTTCCTCTGA AGCTATGATA TCATACTGCC CAGGAGACAC

TAAATAAAGC CTTCCTCTGA AGCTATGATA TCACACAGCC CAGGGGACAC

TAAATAAAGC CTTCCTCTGA AGCTATGATA TCACACTGCA CAGGGGACAC

TAAATAAAGC CTTCCTCTGA AGCTATGATA TCACACTGCA CAGGGGACAC

---------- ---------- ---------- ---------- ----------

GAAATAAAGC CTTCCTCTGA AGCTATGATA TCACACTGCA CGGGGGACAT

TAAATAAAGC CTTCCTCTGA AGCTATGATA TCACACAACC CAGGGGACAC

---------- ---------- ---------- ---------- ----------

TACATAAAGC CTTCTTCTGA AGCTATGATA TCATACTGCC TAGGGGACAC

CAAATAAAGC CTCGCTCTGA CGCCGTGATA TCACACTGCA CCGGGGACTC

CAGCACAATT GAATCTGGTC ACCAAAAGCC AAGTGAGATA GGACTATGTA

CAGCACAATT GAATCTGGTC ACCAAAAGCC AAGTGAGATA GGACTATGTA

CAGCACAATT GAATCTGGTC ACCAAAAGCC AAGTGAGATA GGACTATGTA

CAGCACAATT GAATCTGGTC ACCAAAAGCC AAGTGAGATA GGACTATGTA

CAGCACAATT GAATCTGGTC ACCAAAAGCC AAGTGAGATA GGACTATGTA

CAGCACAATT GAATCTGGTC ACCAAAAGCC AAGTGAGATA GGACTATGTA

CGGCACAATT GAATCTGGTC ACCAAAAGCC AAGCGAAATA GGACTATGTA

CAGCACAATT GAATCTGGTC ACCAAAAGCC AAGTGAGATA GGACTATGTA

CGGCACAATT GAATCTGCTC ACCAAAAGCC AAGTGAGATA GGACTATGTA

CAGCACAATT GAATCTGGTC ACCAAAAGCC AAGTGAGATA GGACTATGTA

CAGCACAATT GAATCTGGTC ACCAAAAGCC AAGTGAGATA GCACTATGTA

CAGCACAATT GAATCTGGTC ACCAAAAGCC AAGTGAGATA GGACTATGTA

---------- ---------- ---------- ---------- ----------

CAGCACAATT GAATCTGGTC ACCGAAAGCC AAGGGAGAGA GCACTATGTA

CAGCGCAATT GAAGCTGGCC ACCAAAAGCC AAGTGAGATA GGACTGTGTA

---------- ---------- ---------- ---------- ----------

CAGCACAATT GAATCTGGTC ACCAAGAGAC AAGTGAGATA GGACTATTTA

CAATACAATT GAATCTGGTC ACCGAGAAAC AAGTGAGAAA AGACTATTTA

AATGGAATTT TATAGGAGAC TTTTGTCCTC CGTGGCAAAC CATCCAGATC

AATGGAATTT TATAGGAGAC TTTTGTCCTC CGTGGCAAAC CATCCAGATC

AATGGAATTT TATAGGAGAC TTTTGTCCTC CGGGGCAAAC CATCCAGATC

AATGGAATTT TATAGGAGAC TTTTGTCCTC CGGGGCAAAC CATCCAGATC

AATGGAATTT TATAGGAGAC TTTTGTCCTC CGTGGCAAAC CATCCAGATC

AATGGAATTT TATAGGAGAC TTTTGTCCTC CGTGGCAAAC CATCCAGATC

AATGGGATTT CATAGGAGAC TTTTGTCCTC CGTGGCGAAC CATCGAGATC

AATGGGATTT TATAGGAGAC TTTTGTCCTC TGT-GCAAAC CATCGAGATC

AATGGGATTT TATAGGAGAC TTTTGTCCTC CGTGGCAAAC CATCCAGATC

AATGGAATTT TATAGGAGTC TTTTGTCCTC CGTGGCAAAC CACCCAGATC

AATGGAATTT TATAGGAGAC TTTTGTCCTC CGTGGCAAAC CATCCAGATC

AATGGAATTT TATAGGAGAC TTTTGTCCTC CGTGGCAAAC CATCCAGATC

---------- ---------- ---------- ---------- ----------

AATCGAATTT TATAGGAGAC TTTTGTGCTC CATGGCAAAC CGTCCAGATC

AATCAGATTT TACAGGAGAC TTTTGTCCTC CGTGGCAAGC CATCCAGATC

---------- ---------- ---------- ---------- ----------

AACCCAATTT TATAGGAGAC TTTTGTCCTC TGTGGCAAAC CATCCAGATT

AAGGCCATTT TACAGGAGAC TTTTGTCCTC CGGGCCAAGC CATCCAGATC

CGTCCACAGC ACTGAAACAT CTTTTTCATT CACCCCTGGG ACAACAGTGT

CGTCCACAGC ACTGAAACAT CTTTTTCATT CACCCCTGGG ACAACAGTGT

CGTCCACAGC ACTGAAACAT CTTTTTCATT CACCCCTGGG ACAACAGTGT

CGTCCACAGC ACTGAAACAT CTTTTTCATT CACCCCTGGG ACAACAGTGT

CGTCCACAGC ACTGAAACAT CTTTTTCATT CACCCCTGGG ACAACAGTGC

CGTCCACAGC ACTGAAACAT CTTTTTCATT CACCCTTGGG ACAACAGTGC

CGTCCACAGC ACTGAAACAT CTTTTTCATT CACCCTGAGG ACAACAGTGC

CGTCCACAGC ACTGAAACAT CTTTTTCATT CACCCTGAGG ACAACAGTGT

CGTCCACAGC ACTGAACCAT CTTTTTCATT CACCCTGGGG ACAACAGTGT

CATCCACAGC ACTGAAACAT CTTTTTCATT CACCGCTGGG ACAACACTGC

CGTCCACAGC ACTGAAACAT CTTTCTCATT CACCCTTGGG ACAACAGTGA

CGTCCACAGC ACTGAAACAT CTTTTTCATT CACCCTTGGG ACAACAGTGC

---------- ---------- ---------- ---------- ----------

CGACCACAGC ACTGAAACAT CTTTTTCATT CACCCTCGGG AAAACAGTGC

CGACCACAGC ACTGAAACAT CTTTCTCATT CACCCTTGGG ACAACAGTGT

---------- ---------- ---------- ---------- ----------

CGACTCCAGC ACTGAAACAT CTTTTTTATT CACTCTTAAG GCAACAGTGC

CGACTACGGG CCTGAAACAT CTTTTGCATT CTCTCTTCAG GCCACAGTGC

GGTTCTCTTT CTCCTCCTGC CAGGGAGAAG GCCAAGTTTG CCAGCTTCTT

GGTTCTCTTT CTCCTCCTGC CAGGGAGAAG GCCAAGTTTG CCAGCTTCTT

GGTTCTCTTT CTCCTCCTGC CAGGGAGAAG GCCAAGTTTG CCAGCTTCTT

GGTTCTCTTT CTCCTCCTGC CAGGGAGAAG GCCAAGTTTG CCAGCTTCTT

GGTTCTCTTT CTCCTCCTGC CAGGGAGAAG GCCAAGTTTG CCAGCTTCTT

GGTTCTCTTT CTCCTCCTGC CAGGGGGAAG GCCAAGTTTG CCAGCTTCTT

GGTTCTCTTT CTCTACCTGC CAGGGAGAGA GCCAAGTTTG CCAGATTCTT

GCTTCTCTTT CTCTTCCTGC CAGGGAGAAG GCCAAGCTTG CCAGCTTCTT

GGCTCTCTTT CTCCTCCTGC CAGAGAGAAG GCCAAGGCTG CCAGCATCTT

GGTTCTCTTT TTCCTCCTGC CAGAGAGGAG GCCAAGTTTG CCAGCTTCTT

GGTTCTCTTT CTCCTCCTGC CAGGGAGCAG GCCACGTTTG CCAGCTTCTT

GGTTCTCTTT GTCCTCCTGC CAGGGAGAAG GCCAAGTCTG CCAGCTTCTT

---------- ---------- ---------- ---------- ----------

GGTTCTCTTT CTCCTCCAGC CAGAGAGAAT GCCACGGTTG CCAGCTTCTT

GGTTCTCTTT CTCCTCCTGC CAGGGAGAAG CCCGAGTTTG CCAGCTTCTT

---------- ---------- ---------- ---------- ----------

GGTTCTCTTT CTCTTCCTGC CAGTTAGTAG GCCAAGTCTG CCAGCTTCAC

CGTTCTCTTT CTCCTGCTGC CAGCGGGAGA GCCGGGCCTG CCAGGCTCTT

AAATAGCCAA ACAAGTCACT CTCAGCCTTG CGCCACCTGA ATGCCCGCTT

AAATAGCCAA ACAAGTCACT CTCAGCCTTG CGCCACCTGA ATGCCCGCTT

AAATAGCCAA ACAAGTCACT CTCAGCCTTG CGCCACCTGA ATGCCCGCTT

AAATAGCCAA ACAAGTCACT CTCAGCCTTG CGCCACCTGA ATGCCCGCTT

AAATAGCCAA ACAAGTCACT CTCAGCCTTG TGCCACCTGA ATGCCTGCTT

AAATAGCTGA GCAAGTCACT CGCAGCCTTG CGCCACCTGA ATGCCCGCTT

AGATAGCTGA ACAAGTCACC AGCAGCCTTG AGCCACCTGA ATGCCCACTT

AAATAGCTGA ACAAGTCACC AGCAGCCTTG TACCACCTGA ATGCTCGCTT

AAATAGGGGA ACAAGTCACT CGCAGCTTTG TACCACCTGA ATGCCCGCTT

AAATAGCCGA ACAAGTCACT CGCAGCATTG CGCCACCTGA ATGCCCGCTT

AAACAGCC-- -CAAGTCACC CGCAGCATTG CGCCACCTGA ATGCCCGCTT

AAATAGCCCA ACAAGTCACT TGCAGCATCG CGCCACCTGA ATACCCGCTT

---------- ---------- ---------- ---------- ----------

AAACAGCCGA ACAAGTGACT CACAGCATTG CACCACCTGA ATACCCGCTT

AAATAGCCCA ACAAGTCACT CACAGCACTG AGCCACCTGA ATGCCTGCTT

AAACAGCCCA ACAAGTCACT CACAGCATTG AGCCACCTGA ATGCCTGCTT

AAATAGCTGA ACGAGTCACT CACAGCACTG CGTTACCTGA ATGGCTGCTT

AAAGGGCAGA ACAAGCCCCT CTCAGGGCCG ACCCACCTGA TTGGCAGCTT

CACGAGGCAA ACAATTCTAG TGCTTTTTCT CCACCAAACC AACTTAAGCT

CACGAGGCAA ACAATTCTAG TGCTTTTTCT CCACCAAACC AACTTAAGCT

CACGAGGCAA ACAATTCTAG TGCTTTTTCT CCACCAAACC AACTTAAGCT

CACGAGGCAA ACAATTCTAG TGCTTTTTCT CCACCAAACC AACTTAAGCT

CACGAGGCAA ACAATTCTAG TGCTTTTTCT CTACCAAACC AACTTAAGCT

CACGAGGCAA ACAATTCTAG TGCCTTTTCT CCACCAAACC AACTTAAGCT

CATGAGGCAA ACAATTCCAG TGC-----CC TCCCCCAATC AACTTAAGCT

CATGAGGCAG GCAATTCCAG TGC-----CC TCCCCGAACC AACTTAAGCT

CACGAAGCCA ACCA------ ---------- ---------- ----------

CACTGGGCAA ACAATTCTAG TGCCTTTTTT CCACCAAACC AACTTAAGCT

CACTAGGCCA ACA-TTCTAG TGCCTTTT-G CCACCAAACC AACTTAAGCT

CACTAGACAA ACAATTCTAG TGCCCTTTCT CCACCAAACC GACTTAAGCT

---------- ----TTCTAG TGCCTTTT-- ---------- ----------

CCCAAGGCAA ACAATTCTAG TGCCTTTTCT CCACCAAACC AGCTTAAGCT

CACGAGGCGA ACAATTCTCC TGTCTTTTTT CCACCGAACC AACTTAAGCT

CATGCGGCAA ACAATTCTAG TGCCCTTTTT CCACCAAACC AACGTAAGCT

CATGAAGCAA ACAATTCTAG TGCTTCTTCT CCACCAAACC AACTTAAGCT

CGTGATGCAT GCACGATAAG GGTTCTTTTC CAACCGAGTC ATCTTAGGCT

GATTACTGCC ATCTCAAACA TCACTGGGCT CCAGATATAT CCATTTAATA

GATTACCGCC ATCTCAAACA TCACTGGGCT CCAGATATAT CCATTTAATA

GATTACCGCC ATCTCAAACA TCATTGGGCT CCAGATATAT CCATTTAATA

GATTACCGCC ATCTCAAACA TCATTGGGCT CCAGATATAT CCATTTAATA

GATTACCGCC ATCTCAAGCA TCACTGGGCT CCAGATATAT CCATTTAATA

GATTACCGCC ATCTCAAACA TCACTGGGCT CTAGATATAT CCATTTAATA

AACT------ -------AAC TCACTGAGTT CTGGGGATAT CCATTTAATA

GACTGCT--- -------ACC TCACTGAGTT CTGGGTATAT CCATTTAATA

---------- ---------- ---------- ---------- ----------

GATTACCGCC ATCTCAAACA TCACTGGGCT CTAGATATAT CCATTTAATA

GATTACCGCC ATCTCAAACA TCGCTGGGCT CTAGATATAT CCATTTAATA

GATTACAGCC ATCTCAAACA TCACTGGGCT CTAGATATAG CCATTTAATA

---------- ---------- ---------- ---------- ----------

GATTACCACC ACCTCCAGCA TCGCCGGGCC CTCGATATGG CCGTTTAATA

GATTACCACC ATCTCAAACA TCGGCTGGCT CTAGATATGG CCATTTAATA

GATTACCGCC ATCTCAAACA TCGCCCGGCT CTGGATATGG C-GTTTCGTA

GATTATCGTA ATCTAAAGCA TCACTTGCTT CTAGATATGT CCATTTAATA

GATCCTCACC CTCTCCAACG CCCCCGGCTT CCAGCTATCC CCATTCGACA

TCCAAGACAA GCAGCCATCT ATTCACTTTC AAAGTAGCTC TCCACGGCTT

TCCAAGACAA GCAGCCATCT ATTCACTTTC AAAGTAGCTC TCCACGGCTT

TCCAAGACAA GCAGCCATCT ATTCACTTTC AAAGTAGCTC TCCACGGCTT

TCCAAGACAA GCAGCCATCT ATTCACTTTC AAAGTAGCTC TCCACGGCTT

TCCAAGACAA GCAGCCATCT ATTCACTTTC AAAGTAGCTC TCCACGGCCT

TCCAGGACAA GCAGCCATCT ATTCACTTTC AAAGTAGCTC TCCACGGCTT

TCC-GGACAA GCAGCCATCT ATTCCCTTTC AAAGTAGCTC TCCCTGGCTT

TCC-AGACAA GCAGCCATCT ATTCACTTTC AAAGTAGCTC TCCATGGCTT

---------- ---------- ---------- ---------- ----------

TCCAGGACAA GCAGCCATCT ATTCACTTTC AAAGTAGCTC TCCACGGCTT

TCCAAGACAA GCAGCCATCT ATTCACTTTC AAAGTAGCTC TCCACGGCTT

TCCAAGACAA GCAGCCATCT ATTCACTTTC AGAGTAGCTC TCCCGGGCTT

---------- ---------- ---------- ---------- ----------

TCCAAGACAA GCAGCCATCT ATTCACTTTC AAAGTAGCTC TCCACGGCTT

TCCACGACAA GCAGCCATCT ATTCACTTTC AAAGTAGCTC TCCTCGGCTT

TCTAGGACAA GCAGCCATCT ATTCACTTTC AAAGCAGCTC TCCACGGCAT

TCCAATACAA GCAGCCATCT ATTCACTTTC AAAGTAGCTC TCCATGGTTT

TCCAACACGG AAGACCATCT ATTCGCTTCC TAAGGGGCAC TCCCAGACCT

TCCCCCATTC TGACACCTTT CAATGATTTG TTGACATCTG AAGAGTTATG

TCCCCCATTC TGACACCTTT CAATGATTTG TTGACATCTG AAGAGTTATG

TCCCCCATTC TGACACCTTT CAATGATTTG TTGACATCTG AAGAGTTATG

TCCCCCATTC TGACACCTTT CAATGATTTG TTGACATCTG AAGAGTTATG

TCCCCCATTC TGACACCTTT CAATGATTTG TTGACATCTG AAGAGTTATG

TCCCTCATTC TGACACCTTT CAATGATTTG TTGACATCTG AAGAGTTATG

TCTCTCGCTC TGACCTCTTT CAATGATTTG TTGACATCTG GAGAGTTATG

TCTCTCACTC TGACCTCTTT CAGTGATTTG TTGACATCTG GAGAGTTATG

---------- ---------- ---------- ---------- ----------

TCCCCCATTC TGACACCTTT CAATGATGTG TTGACATCTG AAGAGTTATG

TCCCTCATTC TGACACCTTT CAATGATTTG TTGACGTCTG AAGAGTTATA

TCCCTCATTC TGACACCTTT CAATGATGTG TTGACATCTG AAGAGTTATG

---------- ---------- ---------- ---------- ----------

TCCCTCATTC TGACACCTCT CAATGATTTG TTGACATCTG AAGAGTTACG

TCCCTCATTC TGACGCCACT CAGTGATTTG TTGACATCTG AAGAGTTACG

TCCCTCATTC TGACACCGCT CAATGATTTG TTGACATCTG AAGAGTTATA

TCCCTCCTTC TGACACCTTT CAATGACTTG TTGACATCTG AAGAGATATG

CCCCTCTTCC CGACGTTTCT TACCGACTGG CTGGCTGCTG TAAATTAGTG

GGCAATCTTA GAAGAGGCAG CTGGACTTTC CGCCCCAAAG AACAAGCACA

GGCAATCTTA GAAGAGGCAG CTGGACTTTC CGCCCCAAAG AACAAGCACA

GGCAATCTTA GAAGAGGCAG CTGGACTTTC CACCCCAAAG AACAAGCACA

GGCAATCTTA GAAGAGGCAG CTGGACTTTC CACCCCAAAG AACAAGCACA

GGCAATCTTA GAAGAGGCAG CTGGACTTCC CACCCCAAAG AACAAGTACA

GGCAATCTTA GAAGAGGCAG CTGGACTTTC CACCCTGAAG AACAAATACA

GGTGAGCTCA GAAGGGCCAG CTGGACTTTC TATCCTGGAG AAC----ACA

GGCGAGCTCA GAAGGGCCAG CTGGACTTTC TATCCCGGAG AAC----TCG

---------- ---------- ---------- ---------- ----------

GGCAATCTTA GAAGAGGCAG CTGGACTTTC TACCCCAAAG AACAAATACA

GGCAATCTCA GAAGAGGCAG CTGGACTTTC CACCCCAAAT A--------A

AGCAATCTTA GAAAAGGCAG CTGGACTTTC CACCCCAAAG AACACATACA

---------- ---------- ---------- ---------- -ACAAATGCA

TGTAATCTTC TAAGAGGCAG CTGGACTTCC CACCCCCAAG AACAAACACA

TGCCATCTTA CAAGAGGCAG CTGGACTTTC CGCCCCAGAG AATACAGACA

TGCAATCTTA CAAGGGGCAG CTGGACTTTG CACCCCAAAG AGCAAACACA

TGCAGTCTTA CAAGAGGCAG CTGGACTTTC TACCCCAAAG AACAAATACA

TGCAATTGTG GACAAAGCAC CTGGTCCTTC CACCCCAGTG GATCAGTACA

AAAAGGATTC CCATACAGCA TCTGAAATAA CTTTGCAAAT GGTAATTTTA

AAAAGGATTC CCATACAGCA TCTGAAATAA CTTTGCAAAT GGTAATTTTA

GAAAGGATTC CCATACAGCA TCTGAAATAA CTTTGCAAAT GGTAATTTTA

GAAAGGATTC CCATACAGCA TCTGAAATAA CTTTGCAAAT GGTAATTTTA

AAAAGGATTC CCATACAGCA TCTGAAATAA CTTTGCAAAT GGTAATTTTA

AAAAAGATTC CCATACAGCA TCTGAAATAA CTTTGCACAT GGTAATTTTA

GGAGAGATTC CCTTGCAGCA TCAGAAATAA CTTTGCAAAG GGTCATTTCA

GGAAGGATTC CCATGCAGCA TCAAAAATAA CTTTGCAAAG GGTCATTTTG

---------- ---------- ---------- ---------- ----------

GAAAGGATTC CCATACAGCA TCTGAAATAA CTTTGCAAAT GGTGATTTTA

GAAAGGATTC CCATACAGCA TCTGAAATAA CTTTGCAAAT GGTCA-----

GAAAGGATTC CCGTATAGCA TCTGAAATA- -TCTGCAAAT GGTAATTTTA

GAAAGGATTC CCATGCAGCA TCTGAAGTGA CTTTGCAAAT GG--------

AAAAGGATTC CCATACAGCA TCTGAAATAA CTTTGAAAAT GGTAATTTCA

AAAAGGATTC CCATACAGCG TCTGAAATAA CTCTGCAAAC GGTAGTTTTA

AAAGGGATTC CCATACAGCA TCTGAAATAA CTTTGCAAAT GGTAATTTGA

AAAAGGATTC CCATACAGCA TTTGAAATAA CTTTGCAAAT GATAATTTTA

AGGACCATTC CCGACAAGAG GGAAAAATGA GCCTACCAGC CGTAATTTTA

TCTCCGTCTG CACCTCATTA CTCTGTGGTA GCTTGTTTCG TGCAAACAAT

TCTCCGTCTG CACCTCATTA CTGTGTGGTA GCTTGTTTCG TGCAAACAAT

TCTCCGTCTG CACCTCATTA CTGTGTGGTA GCTTGTTTCG TGCAAACAAT

TCTCCGTCTG CACCTCATTA CTGTGTGGTA GCTTGTTTCG TGCAAACAAT

TCTCCGTCTG CACCTCATTA CTGTGTGGTA GCTTGTTTTG TGCAAACAAT

TCTCCGTCTG CACCTCATTA CTGTGTGGTA GCTTGTTTCG TACAAACAAT

CCTCTGGCTG CACCTCATTA CCGTGTGGTA GCTCCTTTTG TGCAAACAGC

CCTCTGGCTG CACCTCATTA CCGTGTGGTA GCTCCTTACG TGCAAACAAC

---------- ---------- ---------- ---------- ----------

TCTCCGTCTG CACCTCATTA CTGTGTGGTA GCTTGTTT-- ----------

-CTCTGTCTG CACCTCATTA CTGTGTGCTA GCTTGTTTTG TGCAAACAAT

TCTCCGTCTG CACCTCATTA CTGTGTGGTA GCTTGCTTCG TGCAAACAAC

---------- ---------- --------TA CCGCGTCTCC TGCAAACAAT

TCTCCGCCTG CACCTCATTA TTGTGTGGGA GCTCGTTTCG TGCAAACAAC

CCTCTGTCTG CAC-TCATTA CTGTCGGGGA GCTTGTTTCG TACAAACAAT

TCTCCGTCTG CACCTCATTA CTGTCTGGGA GCTTGCTCCG TACAAACAGT

TCTCCGTCTC TGCCTCATTA CTGTGCAGTA GCTTGTTTCA TGCAAACAAT

TCTCTGTCCG GGACTCATTA CCGTGGCGGG GCTCATTGCT CGCAAACAAT

TGCTGGTTTC TAGCATAACA AGGGTGGTGT CTGTTATTCC TCAGAGAATG

TGCTGGTTTC TAGCATAACA AGGGTGGTGT CTGTTATTCC TCAGAGAATG

TGCTGGTTTC TAGCATAACA AGGGTGGTGT CTGTTATTCC TTAGAGAATG

TGCTGGTTTC TAGCATAACA AGGGTGGTGT CTGTTATTCC TTAGAGAATG

TGCTGGTTTC TAGCATAACA AGGATGGTGT CTGTTATTCC TCAGAGAATG

TGCTGGTTTC TAGCATAACA AGGGTGGTGT CTGTTATTCC TCAGAGAATG

CGCTGGTTTC TAGCATAACA AGGGTGGTGT CTGTTATTCC TCGGAGAATG

CGCTGGTTTC TAGCATAACA AGGGCGGTGT CTGTTATTCC TCGGAGAATG

---------- ---------- ---------- ---------- ----------

---------C TAGCATAACA AGGGTGGTGT CTGTTACTCC TTAGAGAATG

TGCCGGTTTC TAGCATAACA AGGATGGAGT CTGTTATTCC TCTGAGAATG

CCCTGGTTTC TAGCATAACA AGGGTGGTGT CTGTTATTCC TCAGAGAATG

CCCCGGTTTC CAGCATAACA AGGGTGGTGT CTGTTATTCC TCAGAGAAC-

TGCTGGTTTC TAGCATAACA AGGGCGGTGT CTGCCGGTCC TCAGAGGCCA

TGCTGGTTTC TAGCATAACA AGGGTGGCGT CTGTTATTCC TCAGAGAATG

GGCTGGTTCC TAGCATAACA AGGGTGGCGT CTGTTATTCC TCGGAGAACG

TGCTGGTTTC CAGAATAACA AGAGTGGTGT CTGTTATTCC TTAGAGAATG

CGCCGGTTTT CAGGATAACA AGGGTGGTGT CTGTAATTCC TTAGAGAGCG

AGAAATCTCC AACCGGGAAA GAGGAGATTC TTGTTAAGGC CTGGAAATAG

AGAAATCTCC AACCGGGAAA GAGGAGATTC TTGTTAAGGC CTGGAAATAG

AGAAA-CTCC TACCGGGAAA GAGGAGATTC TTGTTAAGGC CTGGAAATAG

AGAAA-CTCC TACCGGGAAA GAGGAGATTC TTGTTAAGGC CTGGAAATAG

AGAACTCTCC AACCGGGAAA CAGGAGATTC TTGTTAAGCT CTGGAAATAG

AGAAATCTCC AACCGGGAAA GAGGAGATTC TTGTTAAGCC CTGGAAATAG

AGAAATCTCC GACCAGGGAA GGGGAGAGCC TTGTTAGGCG CTGGAAAGAG

AGAAATCTCC AACCTGGGAA GAGGAGATCC TTGTTAAGCG GTGGAGAGAG

---------- ---------- ---------- ---------- ----------

AGAAATCTCC AACCGGGAAA GAGGAGATTC TTGTTAAGCC CTGGAAATAG

AGAAATCTCC AACCGGGAAA GAGGAGATTC TTGTTAAGCC CTGGAAATAG

AGAAATCGCC AACCGGGAAA GAGAAAATTC TTGTTAAACC CTAGAAATAG

-----CCTCC AAGCGGGGAA GGGGAGACTC TTGTTAAACC CTGGAAAGCG

CGAAACCGCC AACCGGGAAA GAGGAGCTTC TAGTTAAGCC CTGGAAAGAG

AGAAACCTCC AGCAGGGAAA GAGGAGATTC TTGTTAAGCT CTGGAAATAG

AGAACGCGCC AACCGGGAAA GAGGAGATTC TCGTTAAGCC CTGGAAATAG

GGAAATCTCC AGCTGGGAAA GAGGGGATTC TTGTTAAGCA CTGGAAATAG

AGACGTCTCC AGCTGAGAAA GGTGGATTCC TTGTTAAGCG CTGAAAATAG

GCTGAAATGT AAATGTGTGT TCCCTTCCAC AACGTGCCTA ATTGCCCATA

GCTGAAATGT AAATGTGTGT TCCCTTCCAT AACATGCCTA ATTGCCCATA

GCTGAAATGT AAATGTGTGT TCTCTTCCAC AACGTGCCTA ATTGCCCATA

GCTGAAATGT AAATGTGTGT TCTCTTCCAC AACGTGCCTA ATTGCCCATA

GCTGAAATGT AAATGTGTGT TCCCTTCCAC AACGTGCCTA ATTGCCCATA

GCTGAAATGT AAATGTGTGT TCCCTTCTAC AACGTGCCTA ATTGCCCATA

GCTGGAATGC GCATGCGCGT CGCCTTCCAG TAC-TGCCTA ATTGCCCATA

ACTGAGATGC GCATGCGCGT CACCTTCCAG CAC-TGCCTA ATTGCCCATA

---------- ---------- ---------- ---------- ----------

GCTGAAATGT GAATCTGCGT CCCCTTCCA- CACGTGCCTA ATTGCCCATA

GCTGACATAT AAATGTGCG- CCCCTGCCA- CACGTGCCTA ATTGTCCATA

GCTGAAATGT AAATGTGTGT TCCCTTCCA- CACGTGCCTA ATTGCCCATA

GCTGGACTGT GAATGCGACT TGCAGCCCAC CACGTGCCTA ATTGCCCATA

GCTAGAATGG AAATGCGGGT TCCCTTCCAC AGCGCGCCTA ATTGCCCATA

GCCGAAACGT AACCGTGTGC TCCCTTCCAC GGCGTGCCTA ATTGCCCATA

GCCGAAATGG AAATGTGTGC TCCCTTCGAC AACGTGCCTA ATTGCCCATA

GCTGCAATGT AAATGTGTGC TCATTTTTAC ACCATTCCTA ATTGCCCATA

CCCGAAATGG AAATGTGTGT TCCTTTCTTC AACGTGCTTA ATTGCCCATA

AATGTCATTA AGTCAAGAAA CATTTTCCAC CAAATTAACT TTGACTCATG

AATGTCATTA AGTCAAGAAA CGTTTTCCAC CAAATTAACT TTGACTCATG

AATGTCATTA AGTTGAGAAA TGTTTTCCAC CAAATTAACT TTGACTCATG

AATGTCATTA AGTTGAGAAA TGTTTTCCAC CAAATTAACT TTGACTCATG

AATGTCATTA AGTTGAGAAA TGTTTTCCAC CAAATTAACT TTGACTCATG

AATGTCATTA AGTTGAGAAA TGTTTTCCAC CAAATTAACT TTGACTCATG

AATGTCATTA AGTTGAGAAA TGTTTTCCAC CAAATTAGCT TCAACTCATG

AATGTCATTA AGTTGAGAAA TGTTTTCCAC CAAATTAGCT CCAACTCATG

---------- ---------- ---------- ---------- ----------

AATGTCATTA AATTGAGAAA TGTTTTCCAC CAAATTAACT TTGACTCATG

AATGTCATTA GGTTGAGAAA TGTTTTCCAC CAAATTAACT TTGACTCATG

AATGTCATTA CGTTGAGAAA TGTTTTCCAC CAAATTAACT TTGACTCATG

AATGTCATTA AGTTGAGAAA TG-TTTCCAC CAAATTAACT TTCGCTCATG

AATGTCATTA AGTTGAGAAA TGTTTTCCAC CAAATTAACT TTGACTCATG

AATGTCATTA AGTTGAGAAA TGTTTTCCAC CAAATTAACT TTGACTCATG

AATGTCATTA AGTTGAGAAA TGTTTTCCAC CAAATTAACT TTGACTCATG

AATGTCATTA CATTGAGAAA TGTTTTCCAC CAAATTAACT TTGACTCATA

AATGTCATTA AGTCCAGAAA TGTTTTCCAC CTAATAAACT TGGACTCATG

ATGTCTAATT AGCAGGAGAA GCTGGAAGAT AACTTATCAG TCACTGTTTA

ATGTCTAATT AGCAGGAGAA GCTGGAAGAT AACTTATCAG TCACTGTTTA

ATGTCTAATT AGCAGGAGAA GCTGGAAGAT AACTTATCAG TCACTGTTTA

ATGTCTAATT AGCAGGAGAA GCTGGAAGAT AACTTATCAG TCACTGTTTA

ATGTCTAATT AGCAGGAGAC GCTGGAAGAT AACTTATCAG TCACTGTTTA

ATGTCTAATT AGCGGGAGAA GCTGGAAGAT AACTTATCAG TCACTGTTTA

ATGTCTAATT ATCTGGGGAA GCTGGAAGAT AACGTATCAG CCTGCGTTTG

ATGTCTAATT AGCTGGGGAA GCTGAAAGAT AACTTATCAG CCGGTGTTTG

---------- ---------- ---------- ---------- ----------

ATGTCTAATT AGCAGGAGAA GCTGGAAGAT AACTTATCAG TCACTGTTTA

TTGTCTAATT AGCAGCAGAA GCTGGAAGGT AATTTATCAG CCG-TGTTTA

ATGTCTAATT AGCAGGAGAA GCTGGAAGAT AACTTATCAG TCACTGTTTA

ATGTCTCATT AGCGGCGGCC GCTGGAAGAT AACTCACTAG TCACTGTTTA

ATGTCTAATT AGCGGGAAAA GCTGGAAGAT AACTTCTCAG TCACTGTTTA

ATGTCTAATT AGCGGGAGAA GCTGGAAGAT AACTTATCAG TCACTGTTTA

ATGTCTAATT AGTGGGAGAA GCTGGAAGAT AAGGTATCAG TCACTGTTTA

ATGTCTAATT AGCTGGAGAA ACTGAAAGAT AACTTATCAG TCACTGTTTA

ATGTCTAATT AGCCCGAGAA GCTGAAAGAT AACTTACCGG TCTCTGTTGA

AGAAAACATG TTTAACGGGT GACTTTTGGC TAGCAGAATG GCAGTTTGAC

AGAAAACATG TTTAACGGGT GACTTTTGGC TAGCAGAATG GCAGTTTGAC

AGAAAACATG TTTAACGGGT GACTTTTGGC TAGCAGAATG GCAGTTTGAC

AGAAAACATG TTTAACGGGT GACTTTTGGC TAGCAGAATG GCAGTTTGAC

AGAAAACATG TTTAACGGGT GACTTTTGGC TAGCAGAATG GCAGTTTGAC

AGAAAACATG TTTAACGGGT GACTTTTGGC TAGCAGAATG GCAGTTTGAC

AGAAAACATG TTTAACGGGT G--------- CAGCAGAGTG GCAGTTTGAC

AGAAAACATG TTTAACGGGT GACTTTGGGC CAGCAGAGTG GCAGTTTGAC

---------- ---------- ---------- ----AGAATG GCAGTTTGAC

AGAAAACATG TTTCACGGGT GACTTCTGGC TAGCAGAATG GCAGGTTGAC

AGGAAACATG TTTTGTGGGT GACTTTTGGC TAGCAGAATG GCAGTTTGAC

AGAAAACATG TTTAGCGGGT GACTTTTGGC TAGC------ ----------

GGAAAACATG GTTAGTGGCT GACTTTTGGC TAGC------ ----------

AGAAAACATG TTTAGCGGGT GACTTTTGGC TAGCAGAATG GCAGGTTGAC

GGAAAACATG TTTAACGGGT GACTTTTAGC TAGCAGAATG GCAGTTTGAC

AGAAAACATG GTTAGC-GGT GA-------- ---------- ----------

AGAAAACATG TTTAATGGGT GACTTTTTGC TTGCTGGATG GCAGTTTGAC

AGAAAACATG TTGAATGGGT GACTTTCAGC TCGCAGAGTG GCAGTTTGAC

ACTGCACTGT CACAGTTAAA ATGGGCCCTG CGCGTGACCC AGGCTTGGAC

ACTGCACTGT CACAGTTAAA ATGGGCCCTG CGCGTGACCC AGGCTTGGAC

ACTGCACTGT CACAGTTAAA ATGGGCCCTG CGCGTGACCC AGGCTTGGAC

ACTGCACTGT CACAGTTAAA ATGGGCCCTG CGCGTGACCC AGGCTTGGAC

ACTGCACTGT CACAGTTAAA ATGGGCCCCG CGCGTGACCC AGGCTTGGAC

ACTGCACTGT CACCGCTAAA ACGGGCCCTG CGCGTGACCC AGGCTTGGAC

ACCGCACTGT CACAGTTAAA ATGGGCCCGG CGCATGACCC GGGCTCGGAC

ACCGCACTGT CACAGTTAAA ATGGGCCTGG CGCGTGACCC GGGCTCGGAC

ACTGCACTGT CACAGTTAAA ACGGACCCTG CGCGTGACCC AGGCCTGGAC

ACTGCACTGT CACTGTGAAA ATGGGCCCTG CGCCTGACCC GGGCTTGGAC

ACTGCACTGT CACAGTTAAA ACGGGCCCTG CGCGTGACCC AGGCTTGGAC

---------- ---------- ---------- ---------- ----------

---------- ---------- ---------- ---------- ----------

ACTGCACTGT CACAGGGAAA ACGGGCCCTT CGCGTGACCC AGGCTTGGAC

ACTGTACTGT CACAGTTAAA ACGGGCCCTT CGCATGACCC AGGCCTGGAC

---------- ---------- ---------- ---------- ----------

ACTCTACTGT CACAATTAAG ACGGCTCCTC CGTGTGACCC AGGCCTGGAT

ACCGGACTGT CACCGTTAAA GCGGGTCCTC CGTGTGACCC AGGCCCGGCC

CGTGCCCAAT CACAGCTGAG GCTGCATCTG CCACGTTCAC GGACCCGCGC

CGTGCCCAAT CACAGCTGAG GCTGCATCTG CCACGTTCAC GGACCCGCGC

CGTGCCCAAT CACAGCTGAG GCTGCATCTG CCACGTTCAC GGACCCGCGC

CGTGCCCAAT CACAGCTGAG GCTGCATCTG CCACGTTCAC GGACCCGCGC

CGTGCCCAAT CACAGCTGAG GCTGCATCTG CCACGTTCAC GGACCCGCGC

CGTGCCCAAT CACAGCTGAG GCTGCATCTG CCACGTTCAT GGACCCGCGC

CGTGCCCAAT CACAGCTGAG CTTGCATCTG CCACGTTCAC GG--CCGCGC

CGTGCCCAAT CACAGCTGAG GTTGCATCTG CCATGTTCAC GG--CCGCGC

CCTGCCCAAT CACAGCTGAG CCAGCATCTG CCACGCTCGC AG--CCGCGC

CGTGCCCAGT CACAGCTGAG GCTGCATCTG TCACGTTCAC GGACCTGCGC

CGTGCCCAAT CACAGCTGAG GCTGCATCTG CCACGTTCAC GGACCCGCGC

---------- ---------- ---------- ---------- ----------

---------- ---------- ---------- ---------- ----------

CGCGCCCGAT CGCAGCTGCG GCCGCATCTG CTGCGTCCGC GGCCCGGCGC

CGTGCCCAAT CACAGCTGAG GCTGCATCTG CCAGGTTCAC GGACCCGCGC

---------- ---------- ---------- ---------- ----------

CGTACCCAAT CACAGCTGAA GCTGCATCTG CTGTGTTCAT GGGCCTGCAC

CGTACCCAAT CACAGCTGAA GGGGCATCTG CCGCGTTCCC GGGCCTGCGC

TCCATGCTGG GGGACCAGAT GCCTCTGTTT ATTCCAGGTT ACCTAACCAA

TCCATGCTGG GGGACCAGAT GCCTCTGTTT ATTCCAGGTT ACCTAACCAA

TCCATGCTGG GGGACCAGAT GCCTCTGTTT ATTCCAGGTT ACCTAACCAA

TCCATGCTGG GGGACCAGAT GCCTCTGTTT ATTCCAGGTT ACCTAACCAA

TCCATGCTGG GGGACCAGAT GCCTCTGTTT ATTCCAGGTT ACCTAACCAA

TCCATGCTGG GGGACCAGAT GCCTCTGTTT ATTCCAGGTT ACCTAACCAA

TGCATGCTGG GGGACCAGAT GCCTCTGTTG ATTCCAGGTT ACTTAACCAA

TCCATGCTGG GGGACCAGAT GCCTCTGTTG ATTCCAGGTT ACTTAACCAA

TCCATGCTGG GGGACCAGAT GCCTCTGTTT ATTCCAGGTT ACCTAACCAA

TCCATGCTGG GGGACCAGAT GCCTCTGTTT ATTCCAGGTT ACCTAACCAA

TCCATGCTGG GGGACCAGAT GCCTCTGTTT ATTCCAGGTT ATCTAACCAA

---------- ---------- ---------- -TTCCAGGTT ACCTAACCAA

---------- ---------- ---------- ---------- ----------

GCCATGCTGG GGAACCAGAT GCCTCTGTTT ATTTCAGGTT ACCTAACGAA

TCCATGCTGG GGGACCAGCT GCCTCTGTTT ATTCCAGGTT ACCTAACCAA

---------- ---------- ---------- ---------- ----------

TCCATGCTGT GCAGCCAGAT GCCTCTGTTT ATTCTAGGTT ACCTAACCAA

TCCATGCTGA GAAACCAGCT GCCTCTGTTT ATTCCAGGCT ACCTCAGCAA

ACTCCTGCAA AACCACACCA CCTATGCCTG TGATGGGGAC TATTTGAATC

ACTCCTGCAA AACCACACCA CCTATGCCTG TGATGGGGAC TATTTGAATC

ACTCCTGCAA AACCACACCA CCTATGCCTG TGATGGGGAC TATTTGAATC

ACTCCTGCAA AACCACACCA CCTATGCCTG TGATGGGGAC TATTTGAATC

ACTCCTGCAA AACCACACCG CCTATGCCTG TGATGGGGAC TATTTGAATC

ACTCCTGCAA AACCACACCG CCTATGCCTG TGACGGGGAC CATTTGAGCC

ACTCCTGCAA AACCACACCG CCTATGCCTG TGACGGGGAC TATTTGAATC

ACTCCTGCAA AACCACACTG CCTATGCCTG TGACGGGGAC TATCTGAATC

ACTCCTGCAG AACCACACGG CCTATGCATG TGACGGGGAC CACTTGAACC

ACTCCTGCAA AACCACACCG CCTATGCCTG TGACGGGGAC CATTTGAATC

ACTCCTGCAA AACCACACCG CCTATGCCTG TGACGGGGAC CATTTGAATC

ACTCCTGCAA AACCACACCG CCTATGCCTG TGATGGGGAC CATTTGAATC

---------- ---------- ---------- ---------- ----------

GCTCCTGCAG AACCACACCG CCCACGCCTG CGACGGGGAC CGTTTGCTTC

ACTCCTGCAA AACCACACCG CCTATGCCTG CGATGGGGAC CACTTGAATC

---------- ---------- ---------- ---------- ----------

ACTTCTGCAA AACCACACCG TGTACGCTTG TGACGGTAAC CGTTTGAGTC

GCTGCTGCAG AACCACACGG CCCTGGCGTG TGACGGGGAC CGCCTGAGCC

TACAGTGCCC TCGGCATTCT ACGATAAGTG TCCAATCGGC ATTTTATGGG

TACAGTGCCC TCGGCATTCT ACGATAAGTG TCCAATCGGC ATTTTATGGG

TACAGTGCCC TCGGCATTCT ACGATAAGTG TCCAATCGGC ATTTTATGGG

TACAGTGCCC TCGGCATTCT ACGATAAGTG TCCAATCGGC ATTTTATGGG

TACAGTGCCC TCGGCATTCT ACGATAAGTG TCCAATCAGC ATTTTATGGG

TCCAGTGCCC TCGGCATTCT ACGATAAGTG TTCAATCGGC ATTTTATGGG

TGCAGTGTCC TCGGCATTCA ACAATAAGTG TGCAGTCGGC ATTTTATGGG

TACAGTGCCC TCGGCATTCA ACAATAAGTG TCCAGTCGGC ATTTTATGGG

TGCAGTGCCC TCGGCATTCC ACCATCAGTG TCCAATCGGC GTTTTACGGG

TGCAGTGTCC TCGGCATTCC ACGATAAGTG TCCAGTCGGC ATTTTATGGG

TGCAGTGTCC TCGGCATTCC ACGATAAGTG TCCAATCGGC ATTTTATGGG

TACAGTGCCC TCGGCATTCT ACAATAAGTG TGCAATCGGC ATTTTACGGG

---------- ---------- ---------- ---------- ----------

TGCAGTGCCC TCGGCACTCC ACCATCAGCG TCCAGTCAGC CTTTTACGGG

TCCAGTGTCC ACGGCATTCT ACGATAAGTG TCCAGTCGGC ATTTTATGGG

---------- ---------- ---------- ---------- ----------

TTCAGTGCCC TCGACATTCG ACAATAAGTG TCCAGTCAGC CTTTTATGGT

TGCAGTGCCC ACGACACTCC TCCATCAGCG TCCAGGCGGC CTTCTACGGG

CAAGATTACC AAATGTGTAT ATGTATGTTT CTTTCATCTA ATTAGGTAAT

CAAGATTACC AAATGTGTAT ATGTATGTTT CTTTCATCTA ATTAGGTAAT

CAAGATTACC AAATGTGTAT ATGTATGTTT CTTTCATCTA ATTAGGTAAT

CAAGATTACC AAATGTGTAT ATGTATGTTT CTTTCATCTA ATTAGGTAAT

CAAGATTACC AAATGTGT-- ---------- ---------- ----------

CAAGATTACC AAATGTGTAT GTGTATGTTT CTTTCATCTA ATTAGGTAAT

CAAGATTTCC AAAGGTGTAT GTGTATGTTT CTCTCATCTA ATTAGGTAAT

CAAGATTACC AAATGTGTAT GTGTATGTTT CTCTCATCTA ATTAGGTAAT

CAAGATTTCC GAATGTGTAT GTGTATGTTT CTTTCGTCTA ATTAGGCAAT

CAAGATTACC AAAAGTGTAT GTGTATGTTT CTGTCATCTA ATTAGGTAAT

CAAGATTACC AAATGTGTAT GTCTATGTTT CTGTCATCTA ATTAGGTAAT

CAAGATTACC GAGCATGTAT GTGTATGTGT CTGTCATCTA ATTAGGTAAT

---------- ---------- ---------- ---------- ----------

CAAGACAACC ACACGTGTAT GTGTATGTTT CTTTCATCTA ATTAGGTAAT

CAAGATGATC AAATGTGTAT GTGTATGTTT CTTTCATCTA ATTAGGGAAT

---------- --------AT GTGTATGTTT CTTTCATCTA ATTAGGTAAT

CAAGATTACC AAAGGTGTGT GTATGTGTTC CTTTCATCTA ATTAGGTAAT

CCCGACTCCA AGAGGTGTAT GGCTAAGTTT CTTTCATCTA ATTAGGTAAT

CTGCCCAGGT GTTTAGGACC AAGGCCTCTT TTAATTAGAA AAGTCTAGGA

CTGCCCAGGT GTTTAGCACC AAGGCCTCTT TTAATTGGAA AAGTCAAGGA

CTGCCCAGGT GTTTAGCACC AAGGCCTCTT TTAATTGGAA AAGTCAAGGA

CTGCCCAGGT GTTTAGCACC AAGGCCTCTT TTAATTGGAA AAGTCAAGGA

---------- ---------- ---------- ---------- ----------

CTGCCCAGGT GTTTAGTACC GAGGCCTCTT TTAATTGGAA AAGTCAAGGA

CTGCCCAGGT GTTTAGCACC AAGCACTTGT TTAATTGGAA AAGTTAAGGA

CTGCCCAGGT GTTTAGCACC AAGCACTCGT TTAATTGGAA AAGTTAAGGA

CTGCCCAGGT GTTTAGCACC AAAGCCTCTT TTAATGGGAA CAGTCGAGAA

CTGCCCAGGT GTTTAGCACC AAGGCCTCTT TTAATTGGAA AAGTCAAGGA

CTGCCCAGGT GTTTAGCACC AAGTCCTCTT TTAATTGGAA AAGTCAAGGA

CTGCCCAGGT GTTTAGCACC CAGGCCTCTT TTAATTGGAA AAGTCAAGGA

---------- ---------- ---------T TTA---TGAA AAGTCAAGGA

CTGCCCAGGT GTTTAGCACC AAGGTTTCTT TTAATTGGAA AAGTCAAGGA

CTGCCCAGGT GTTTAGCACC AAGGCCTCTT TTAATTGGAA AAGTCAAGGA

CTGCCCAGGT GTTTAGCACC AAGGCCTCGT TTAATTGGAA AAGTCAAGGA

CTGCCCAGGT GTTTAACACC AAGGCATCTC TTAATTGGAA AAGTCAAGGA

CTGCCCAGGT GTTTAGCACC AAAGCACCCG GTAATTGGAA AAGTCAAGGA

CAGAAACTAA TTAGCAGCAT TATTCCAAAC CAGAAGTCTA ATGATATTAA

CAGAAACTAA TTAGCAGCAT TAATCCAAAC CAGAAGTCTA ATGATATTAA

CAGAAACTAA TTAGCAGCGT TAATCCAAAC CAGAAGTCTA ATGATATTAA

CAGAAACTAA TTAGCAGCGT TAATCCAAAC CAGAAGTCTA ATGATATTAA

---------- ---------- ---------- ---------- ----------

CAGAAACTAA TTAGCAGCCT TAATCCAAAC CAGAAGTCTA ATGATATTAA

CAGAAACTAA TTAGCAGCCA TAATCCAAAC CAGAAGCGTA ATGATATTAA

CAGAAACTAA TTAGCAGCCA TAATCCAAAC CAGAAGTCTA ATGATATTAA

CAGAAACTAA TTAGCAGCAT TAATCCAAAC CAGAAGTCTA ATGATATTAA

CAGAAACTAA TTAGCAGCAT TAAACCAAAC CAGAAGTCTA ATGACATTAA

CAGAAACTAA TTAGCAGCAT TAATCCAAAC CAGAAGTCTA ATGATATTAA

CAAAAACTAA TTAGTAGCAT TAATCCAAAC CAGAAGTCTA ATGATATTAA

CTGAAACTAA TTAGCAGCAT TAATCCTAAC CAGAAGTCTA ATGATA-TAA

CAGAAACTAA TTAGCAGCAT TAATCCAAAC CAGAAGTCTA ATGATATTAA

CAGAAACTAA TTAGCACCAT TAATCCAAAC CAGAAGTCTA ATGATAGAAA

CAGAAACTAA TTAGCAGCAT TAATCCAAAC CAGAAGTCTA ATGATATTAA

CAGAAACTAA TTAGTGGTAT TAACCCAAAA CAAAAGTTTA ATGATATTAA

TGGAAACTAA TTAGCAGTGG TAATTCAAAG CAGACATCTA ATGGTATTAA

CCACAAATCG CTATTGCAAT CAGGTCCCAC CTTGATAAAA AAACACACCC

CCACAAATCA CTATTGCAAT CAGGTCCCAC CTTGATAAAA AAACACACCC

CCACAAATCA CTATTGCAAT CAGGTCCCAC CTTGATAAAA AAACACGCCC

CCACAAATCA CTATTGCAAT CAGGTCCTAC CTTGATAAAA AAACACGCCC

---------- ---------- ---------- ---------- ----------

CCGCAAATCA CCATTACAAT CAGGTCCCAC CTTGATAAAA AGACACACCC

CCACAAGTCA CCATGGCTAT CAGGTCCCAC CTTGATACCA GGACACACCC

CCACAAGTCA CCATGGCTAT CAGGTCCCAC CTTGATACCA GGACACACCC

-CACAAGTCA CCATTGCAAT CAGGTCCCAC CTTGATAAAA AAACACACCC

CCACCAGTCA CCATTGCAAT TGGGTCCCAC CTTGACAAAA AAACACACCC

CCACAAATCA CCATTACAAT CAGGTCCCAC CTTGATAAAA AAacacacCC

CCGCCAATCA CCACTGCAAT CAGGTCCCAC CTTGATAAAA AAACACACCC

ACACAAGTCA CTACCGCAAT CAGGTCCCAC CTTGATGAAA AA--------

CCACAAATCA CCATTGCAAT CAGGTCCCAC CTTGATAAAA AAACACACCC

CCACAAATCA CCGTGGCATT CAGGTCCCAC CTTGACAAAA AAAAAA---C

CCACAAATCA CCATTGCAAT CAGGTCCCAC CTTGATTTAA AAAACCACCC

CCACATATTA CTATTATAAT CAAGATCCAC CTTGATTAAA AAATCCACCT

CCACAAATCA CCATTACAAT GGGGTCCCAC CTTGGCCAGA AAACACACCC

ATTTCATTTA GACTGTATAC TAAGAATAAT TGCCACCAAT ATTGATTGGC

ATTTCATTTA GACTGTATAC TAAGAATAAT TGCCACCAAT ATTGATTGGC

ATTTCATTTG GACTGTATAC TAAGAATAAT TGCCACCAAT ATTGATTGGC

ATTTCATTTG GACTGTATAC TAAGAATAAT TGCCACCAAT ATTGATTGGC

---------- ---------- ---------- ---------- ----------

ATTTCATTTG GACTGTGTAC TAAGAATAAT TGCCACCAAT ATTGATTGGC

ACTGCATTTG GGCCCTGTGC TAAGAATAAT TGTCACCAAT ATTGATTGGC

ACTGCATTTG GGCCCTGTGC TAAGAATAAT TGTCACCAAT ATTGATTGGC

ATTTCATTTG GACTGTGTAC TAAGAATAAT TGCCACCAAT ATTGATTGGC

ATTTCATTTG GACTGTGTAC TAAGAATAAT TGCCACCAAT ATTGATTGGC

ATTTCATTTG GACTGTGTAC TAAGAATAAT TGCCACCAAT ATTGATTGGT

ATTTCATTTG GACTGTGTAC TAAGAATAAT TGCCACCAAT ATTGATTGGC

---------- ---------- ---------- ---------- ----------

ATTTCATTTG GACTGTGTAC TAAGAATAAT TGCCACCAAT ATTGATTGGC

ATTTCATTTG GACTGTGTAC TAAGAATAAT TGCCACCAAT ATTGATTGGC

ATTTCACTTG GACTGTGTAC TAAGAATAAT TGCCACCAAT ATTGATTGGC

ATTTTCTTTG GGCTGTGTAC TAAGAATAAT TGCCACCAAT ATTGATTGGT

CCGTGCTTTG ACTTGGAAGC TAAGAATAAT TGCCATCGAT ACTGATTGGG

CTTTTGGGAT CCAGCTGAAT TTAAAAATTG CAGACTGTGA AATCCCAGCT

CCTTTGGGAT CCAGCTGAAT TTAAAAATTG CAGACTGTGA AATCCCAGCT

CCTTGGAGAC CCAGCTGAAT TTAAAAATTG CAGACTGTGA AATCCCAGCT

CCTTGGAGAC CCAGCTGAAT TTAAAAATTG CAGACTGTGA AATCCCAACT

---------- ---------- ---------- ---------- ----------

CCTTGGGGAC CTGGCTGAAT TTAAAAATTG CAAACTGTGA AATCCCAGCT

CCTTGGGGAC TCGGCTGAAT TTAAAAACTG CAGACTGTGA AATCCCAGCT

CCTTGGGGAC TCGGCTGAAT TTAAAAACTG CAGACTGTGA AATCCCAGCT

CCTTGGGGAC CCGGCTGAAG GT-AAATTTG CACACGGCGA AATCCCAGCT

CCTTGGGGAC CCAGCTGAAT TTAAAAATTG CAGACTGTGA AATCCCAGCT

CCTTGGGGAC CCAGCTGAAT TTAAAAACGG CAGGC--TGA AATCCCAGCT

CCTTGGGGAC ACGGCTGAAT TTAAAAATTG CAGGCTATGA AATCCCAGCT

---------- ---------- ---------- ---------- ----------

CCTTGGGGAG CCGGCTGAAT TTTAAAACTG CAGCCTGAGA AATCCCAGCT

CCTTGGGGAC ACGGCCGAAT GTAAAAACTG TGGTCTGTGA AATCCCAGCC

CCTTGGGGAC AGGGCTGAAT GTAAAAATTG CAGACTGTGA AATCCCAGCT

CCTTGGGGAC AGGGTTAAAT TTTAAAATTG CAGACTGTGA AATCCCAACT

CCAGCGGGAC GGGGTTGCAG TTAAAAATGA CACACTGCGA AATCCCGGCG

GTTTTCAAGC CTGTCCCTTT ACCCCTCTTC TAGAAGGTGC TGGACGAATG

GTTTTCAAGC CTGTCCCTTT ACCCCTCTTC TAGAAGGTGC TGGACGAATG

GTTTTCAAGC CTGTCCCTTT ACCCCTCTTC TAGAAGGTGC TGGACGAATG

GTTTTCAAGC CTGTCCCTTT ACCCCTCTTC TAGAAGGTGC TGGACGAATG

-TTTTCAAGT CTATCCCTTT ACCCCTCTTC TAGAAGGTGC TGGACGAATG

GTTTTCAAGC CTATCCCTTC ACTCCTCTTC TAGAAGGTGC TGGATGAATG

GTTTACAAGC CTATCCCTTT ACCCCTCTTC CAGAAGGTGC TGGACGAGTG

GTTTACAAGC CTAT-CTTTT ACCCCTCTTC CAGAAGGTGC TGGACGAGTG

G--------- ---------- ---------- ---------- ----------

GTTCTCAAGC CTGTCCCTTT AC-CCTCTTT TAGAAGGTGC TGGACGAGTG

GTTCTCAAGC CTATCCTTTC ACTCCTCTTT TAGAAAGTGC TGGACGAATG

G--------- ---------- ---------- ---------- ----------

---------- ---------- ---------- ---------- ----------

GTTTTCAAGT CTATCCCTTT ACCCCTCTTT TAGAAGGTGC TGGACGAGTG

CTTTTCACGT CTGTCCCTTT ACCCCTCTTT TAGAAGGTCC TGGATGAGTG

G-----ACGT CTGTCC-TTT ACCCCCCTTT TAGAAGGTGC TGGATGAGTG

GTTTTCAAGG CTATCCTTTT ACTTATTTTT TAGAAAGTTT TGGATGAGTG

GACGTCAGGG TTGCCCGCTC GCCCCTCTTT CAGAAAGTGT TGGACGAGTG

CCAGAACCAG CGGGCCTGCC ACCTCCTGGT CAATAGCCGT GTTTTTGGAC

CCAGAACCAG CGGGCCTGCC ACCTCCTGGT CAATAGCCGT GTTTTTGGAC

CCAGAACCAG CGGGCCTGCC ACCTCCTGGT CAATAGCCGT GTTTTTGGAC

CCAGAACCAG CGGGCCTGCC ACCTCCTGGT CAATAGCCGT GTTTTTGGAC

CCAGAACCAG CGGGCCTGCC ACCTCCTGGT CAATAGCCGT GTTTTTGGAC

CCAGAACCAG CGGGCCTGCC ACCTCCTGGT CAATAGCCGT GTATTTGGAC

CCAGAACCAG CGGGCCTGCC ACCTCCTGGT CAATAGCCGA GTCTTCGGAC

CCAGAACCAG CGGGCCTGCC ACCTCCTGGT CAATAGCCGA GTCTTCGGAC

---------- ---------- ---------- ---------- ----------

TCAGAACCAG CGGGCCTGCC ACCTCCTGGT CAATAGCCGT GTTTTTGGAC

CCAGAACCAG CGGGCCTGCC ACCTCCTGGT CAATAGCCGT GTTTTTGGAC

---------- ---------- ---------- ---------- ----------

---------- ---------- ---------- ---------- ----------

CCAGAACCAA CGGGCCTGCC ACTTCCTGGT CAATAGCCGT GTTTTTGGAC

CCAGAACCAG CGGGCCTGCC ACCTCCTGGT CAATAGCCGT GTTTTTGGAC

CCAGAACCAG CGGGCCTGCC ACCTCCTGGT CAATAGCCGT GTCTTTGGAC

CCAGAACCAG AGAGCCTGCC AACTTCTTGT CAATAGTCGT GTTTTTGGAC

CCAGGACCGG AGGGCCTGCC AGCTGCCCGT CAACAGCCGC CTCTTCGGAC

CTGACCTTTG TCCAGGAAGC AGTAAATACC TCCTGGTCTC CTTTAAATGC

CTGACCTTTG TCCAGGAAGC AGTAAATACC TCCTGGTCTC CTTTAAATGC

CTGACCTTTG TCCAGGAAGC AGTAAATACC TCCTGGTCTC CTTTAAATGC

CTGACCTTTG TCCAGGAAGC AGTAAATACC TCCTGGTCTC CTTTAAATGC

CTGACCTTTG TCCAGGAAGC AGTAAATACC TCCTGGTCTC CTTTAAATGC

CTGACCTTTG TCCAGGAAGC AGTAAATACC TCCTGGTCTC CTTTAAATGC

CTGACCTTTG TCCAGGAAGC AGTAAATACC TCCTGGTCTC CTTTAAATGC

CTGACCTTTG TCCAGGAAGC AGTAAATACC TCCTGGTCTC CTTTAAATGC

---------- ---------- ---------- ---------- ----------

CTGACCTTTG TCCAGGAAGC AGTAAATACC TCCTGGTCTC CTTTAAATGC

CTGACCTTTG TCCAGGAAGC AGTAAATACC TCCTGGTCTC CTTTAAATGC

---------- ---------- ---------- ---------- ----------

---------- ---------- ---------- ---------- ----------

CTGACCTTTG TCCTGGAAGC AGTAAATACC TCCTGGTCTC CTTTAAATGC

CTGACCTTTG CCCAGGAAGC AGTAAATACC TCCTGGTCTC CTTTAAGTGC

CTGACCTTTG TCCAGGAAGC AGTAAATACC TCCTGGTCTC CTTTAAATGC

CTGACCTTTG TCCAGGAACG ACTAAATATC TCCTGGTCTC CTTTAAATGC

CGGACCCTTG TCCAGGCGTG GCCAAGTCCC TCCTGGTGTC CTTCAAATGC

CAACCTAGTA AGTAAGGAAT GGTACCACCT CCCGAAAATG TCAGAATGAA

CAACCTAGTA AGTAAGGAAT GGTACCACCT CCCGAAAATG TCAGAATGAA

CAACCTAGTA AGTAAGGAAT GGTACCACCT CCTGAAAATG TCAGAATGAA

CAACCTAGTA AGTAAGGAAT GGTACCACCT CCTGAAAATG TCAGAATGAA

CAACCTAGTA AGTAA----- ---------- ---------- ----------

CAGCCTAGTA AGTAAGGAAT GGTGCCACCT CCTGAAAATG TCAGAATGAA

CAACCTAGTA AGTAA----- ---------- ---------- ----------

CAACCTAGTA AGTAAGGAAT GATTCCACCC CCTGAGAAGG TCAGAATGAA

---------- ---------- ---------- ---------- ----------

CAACCTAGTA AGTAAGGAAT GGTTCCACCT CCTGAAAATG TCAGAATGAA

CAGCCTAGTA AGTAAGGGCT GGTGCCACCT CCTAAAAACG TCAGAATGAA

---------- -----GGAAT GGTGCCTCCT CCTGAAAACG TCAGAATGAA

---------- -----GCAGT GGTGCCACCC CCTGAAAATC TCCACATGCA

CAACCTAGTA AGTAAGGAAT GGTGCCGCCT CCTGAAAATG TCAGGATGAA

CAACCTAGTA AGTAAGGAAT GGTGCCTCCT CCGGAAAACG TCAAAATGAA

CAACCTAGTA AGTAAGGCAT GGTGCCTCCT CCCCAAAATG TCAGAATGAA

AAACCTAGTA AGTAAGGAAT TCTTCCCAAG CCCCGGAACG TAAGAATGAA

AAGCCCGGTG AGTGGGGAGC AGTGCCAAAG CCCGAAAATG TCAGGATGAA

TTCTGTTAAT TTCAAGAACA TTCTACAGTG GGAGTCACCT GCTTTTGCCA

TTCTGTTAAT TTCAAGAACA TTCTACAGTG GGAGTCACCT GCTTTTGCCA

TTCTGTTAAT TTCAAGAACA TTCTACAGTG GGAGTCACCT GCTTTTGCCA

TTCTGTTAAT TTCAAGAACA TTCTACAGTG GGAGTCACCT GCTTTTGCCA

---------- ---------- ---------- ---------- ----------

TTCAGTTAAT TTCAAGAACA TTCTACAGTG GAAGTCACCT GCTTTTCCCA

---------- ---------- ---------- ---------- ----------

TTCAGTTAAT TTCAAGAACA TTCTACAGTG GGAGGTACCT GCTTTCCCCA

---------- ---------- ---------- ---------- ----------

TTCAGTTAAT TTCAAGAATA TTCTACGATG GGAGTCACCT GCTTTTCCCA

GTCAGTTAAT TTCAAGAACA TTCTCCAGTG GGAGTTGCCT GCTTTTCCCA

TTCAGTTAAT TTCCGGAATA TTCTACAATG GGAGTCGCCT GCTTCTCCCA

TTCACTTAAC TTCAAGAACA TTCTCCGGTG GAAGTCACCT GCTTTTCCCA

CTCAGTTAAT TTCAAGAACA TTCTACAGTG GGAGTCACCT GCTTTTCCCA

TTCAGTTAAT CTCAAGAACG TTCTTCAGTG GGAGCCTCCT GCCTTCCCCA

TTCTGTTAAC TTCAAGAACA TCCTACAGTG GGAGCCGCCA GCTTTTGCCA

TTCAGTTAAT TTGAAGAACA TTCTGCAGTG GGACCCACCT GCTTTCTATC

TTCGGTGAAT TTGAAGAACA TCTTAGAGTG GGAGTTGGCC CCTTTACCCT

AAGGGAACCT GACTTTCACA GCTCAGGATG AGCATTCAGA CTGGGTAAAC

AAGGGAACCT GACTTTCACA GCTCAGGATG AGCATTCAGA CTGGGTAAAC

AAGGGAACCT GACTTTCACA GCTCAGGATG AGCATTCAGA CTGGGTAAAC

AAGGGAACCT GACTTTCACA GCTCAGGATG AGCATTCAGA CTGGGTAAAC

---------- ---------- ---------- ---------- ----------

AAGGGAATCT GACTTTCACA GCTCAGGATG AGCATTCAGA CTGGGTAAAC

---------- ---------- ------GATG AACATTCGGA GTGGGTAAAC

AAACGAACCT GACTTTCACA GCTCAGGATG AACATTCGGA GTGGGTCAAT

---------- ---------- ------GAGG AGCACTCGGA CTGGGTGCAG

AGGGGAATCT GACGTTCACA GCTCAG---- ---------- ----------

AAGGGACTCT GACTTTCACA GCCCAGGATG AGCATTCTGA CTGGGTAAAC

AAGGGAATCT GACTTTCACA GCTCAGGATG AATATTCAGA CTGGGTAAAC

GAGGGAACCT CACTTTCACA GTTCAGGCTG AGCACTCAGA ATGGGTCACC

AAGAGAATCT GACTTACACA GCTCAGGACG AGCACTCAGA CTGGGTAAAC

AAGGGAATCT GACTTACACA GCTCAGGATG AGCATTCAGA CTGGGTAAAC

AAGGGAACCT GACTTACACA GCTCAGGATG AATATTCAGA CTGGATAAAC

AAGGAAATGT GACTTACAGA GCCCAGGATG AACAGTCAGA GTGGGTAAAC

ATGAAAATGT GACCTTCACA GCTCAAGGAG GCCATTCCGA CTGGGTGAAC

ATCACCTTCT GTCCTGTGGA TGACAGTAAG TTTTTATTCA AATCAAAATG

ATCACCTTCT GTCCTGTGGA TGACAGTAAG TTTTTATTCA AATCAAAATG

ATCACCTTCT GTCCTGTGGA TGACAGTAAG TTTTTATTCA AATCAAAATG

ATCACCTTCT GTCCTGTGGA TGACAGTAAG TTTTTATTCA AATCAAAATG

---------- ---------- ---------- TTTTTATTCA AATCAAAACG

ATCACCTTCT GTCCTGTGGA TGACAGTAAG TTTTTATTCA AATCAAAATG

GTCACCTTCT GTCCCGTGGA AGACAGTAAG TTTTTATTCA AATCAAAACA

GTCACCTTCT GCCCCGTGGA AGACAGTAAG TTTTTATTCA AATCCAAAGA

GTCGGCTTCT GCCCTGTGGA TGATAGTAAG TTTTTATTCA AATCAAAATG

---------- ---------- ---------- TTCTTATTCA AATCAAAATG

ATCACCTTTT GTCCTGTGGA TGACAGTAAG TTTTTATTCA AATCAAAATG

ATCACCTTCT GTCCTGTGGA TGACAGTAAG TTCTTATGCA AATCAAAATG

ACCGTCTTCT GTCCCGTGGA TGACAGTAAG TTCTTGTTCA AATCGAAGAG

ATCACCTTTT GTCCTGTGGA TGACAGTAAG TTTGTATTCA AATCAAAATG

ATTACCTTTT GTCCTGTGGA TGACAGTAAG TTTTTATTCA GATCAAAGTG

ATTACCTTTT GCCCTATGGA TGACAGTAAG ---------- ----------

CTCACCTTTT GTCCTTTGGA AGACAGTAAG TTCTTATTGA AGTCAAAATG

GTCACCTTCC ACCCGGTGGA GGACAGTAAG TTCTTGTTGA AGTCGAAATG

GCTGCAGACG TCCCTGGCAG CTTCCGGACC CTGGGTCACC ATGGCGGTCA

GCTGCAGACG TCCCTGGCAG CTTCCGGACC CTGGGTCACC ATGGCGGTCA

GCTGCAGACA TCCCTGGCAG CTTCCGGACC CTGGGTCACC ATGGCGGCCA

GCTGCAGACA TCCCTGGCAG CTTCCGGACC CTGGGTCACC ATGGCGGCCA

GCTGCAGACG TCCCTGGCAG CTTCCGGACC CTGGGTCACC AGGGCAGCCA

GCTGCAAACA TCTCTGGCAG CTTCGGGACC CTGAGTCACC ATGGCAGCCA

GCTGCACACA TCTCTGGCAG CTTCAGGGCC CTGGGTCACC ACAGCAGTCA

GCTGCACACA TCTCTGGCAG CTTCAGGACC CTGGGTCACC ATGGCAGTCA

GCCGCAGACG TCCCTGGCAG CTTCAGGACC CTGGGTCACC ATGGCGGCCA

GCTGCAGACG TCCCTGGCGG CCTCGGGACC CTGGGTCACC ATGGCAGCCA

ACTGCAGACA TCCCTGGCAG CCTCAGGGCC CTGGGTCACC ATGGCGGCCA

GCTGCAGACA TCCCTGGCGG CCTCAGGACC CTGGGTCACC ATGGCGGCCA

GCTAGAGACG TCCCTGGCGG CCTCGGCCCC CTGGGCCACC ATGGCGGTCA

ACTGCAGACG TCCCTGGCCG CTTCGGGACC CTGGGCCACC AGGGCGGACA

GCTGCAGACA TCCCTGGTAG CTTCGGCTCC CTGGGCCACC AGGGCCGCCA

---------- ---------- ---------- ---------- ----------

ACTATAGAAA TCCCTGGCAG CATCTGGACC CTGTGCCACC ATAGCTGACA

GCCGTAGACG TCGCGGGCGG CATCCGGCCC CTGGGCCACC ATCGCCGCCA

TCAGGCTCAG GCAGGCGCGA GCCAACCTGA TAAAATTTAT AAACTCTCAC

TCAGGCTCAG GCAGGCGCGA GCCAACCTGA TAAAATTTAT AAACTCTCAC

TCAGGCTCAG GCAGGCGCGA GCCAACCTGA TAAAATTTCT AAACTCTCAC

TCAGGCTCAG GCAGGCGCGA GCCAACCTGA TAAAATTTCT AAACTCTCAC

TCAGGCTCAG GCAGGCACGA GCCAACCTG- ---------- ----------

TCAGATTCAG GCAGGCTCGA GCCAACCTGA TACAATTTAT GAACTCTCAC

TCAGGTCCAG GCAGGCTCGA GCCATCCTGA AAAGATATCA AAGCTCTTGC

TCAGGTCCAG GCAGGCTCGA GCCATCCTGA AAAGATACCA GAACTCTTGC

TCATGTTGAG GCAGGCTCGA GCCATCCTG- ---------- ----------

TCAGGTTCAG GCAGGCTCGA GCCATCCTGA TAAAATTTAT AAACTGTCAC

ACAGGTTCAG GCAGGCTCGA GCCATCCTGA TAAAATTTAT AATCTCTCAC

TCAGGTTCAG GCAGGCTCGA GCCATCCTG- ---------- ----------

GCAGGTTGAG GCAGGCGCGA GCCATCCTG- ---------- ----------

TCAGGTTCAG GCAGGCTCGA GCTGTCCTGA TAAGATTCAT AAGCTTTCAC

TCAGGTTCAG GCAGGCTCGA GCCAACCTG- ---------- ----------

---------- ---------- ---------G TAAAATTTAT AAACTCTCAC

TGAGGTTCAA GCAGGTTTGA GTCATTCTAA TAAAATTAAT GATCTCACAC

TGAGGTTCAG GCAGGCCCGG GACATCCTGA TGAAATTCAT AATCTGGCAC

CAGAGACTAC TTATTGTCTA AAAGTATATG TCTTCTTTCC ATCACTTAAA

CAGAGACTAC TTATTGTCTA AAAGTATATG TCTTCTTTCC ATCACTTAAA

CAGAGACGAC TTATTGTTTA AAAGTATATG TCTTCTTTCC ATCACTTAAA

CAGAGACTAC TTATTGTTTA AAAGTATATG TCTTCTTTCC ATCACTTAAA

---------- ---------- ---------- ---------- ----------

CAGAGACTAC TTATTGTTTA AAAGTATATG TGTTCTTCCC ATCACTTAAA

CAGAGACTAC CTACTGTTTA GAAGTATACG TGTTCTCCCC ACCACTCAAG

CAGAGACTAC TTACTGTTTA GAAGTACATG TGTGCTTCCC ACCACTCAAG

---------- ---------- ---------- ---------- ----------

CAGAGATTAC TTATTGTTTA AAAGTATATG TGTTCTTTCC ATCAAGTAAA

CAGAGACCAC TTACTGTTTA AAAGTATATG TGTTCTTTCC ATCAAGTAAA

---------- ---------- ------TATG TGTTCTTTCC ATCAAGTAAA

---------- ---------- ------TTTG TTTATTGTCC ACCAAGTAAG

CAGAGACTAT GTATTGTTTA AAAGTATATG TGTTTTTTCC ATCACGTAAA

---------- ---------- ---------- ---------- ----------

CTGAAACTAC TTACTGTTTA CAAGTATATG TAGTCCCCCT TTGAGAGAAA

CAGAAACTAC TTATTGTTTA AAAGTACATA TATTCTCTCC ATCTTGGAAA

CGGAGACCAC TTACTGTTTG AAAGTATATG TGTTCTATCC ATCGTGCAAA

CCTTCTTCCA GTATAGATGA GGTATGTCTT ACATTTCTGG AGAGGACATG

CCTTCTTCCA ATATAGATGA GGTATGT--- ---------- ----------

CCTTCTTCCA ATATAGATGA GGTATGTCTT ACATTTCTGG AGAGGACATG

CCTTCTTCCA ATATAGATGA GGTATGTCTT ACATTTCTGG AGAGGACATG

---------- ---------- -------CTT ACATTTCTGG AGAGGACATG

CCTTCCTCCA ACATAGATAA GGTACGTCTT ACATTTCTGG AGAGGACATG

CCTCCCTGCA GTATCGACGA GGTACGTCTT ACATTTCTGG AGACGACATG

CCTCCCCGCA GTATTGATGA GGTATGTCTT ACATTTCTGG AGACGACATG

---------- ---------- -------CTT ACATTTCTGG AGAAGACATG

CCTCCTTCCT CTGTTGACGA GGTAGGT--- ---------- ----------

CCTCCATCCA CTATAGATGA GGTATGTCTT ACATTTCCGG AGAGGACATG

CCTCCTTCCA CTATAGATCA GGTATGTCTT ACATTTCCGG AGAGGACATG

CCTCCTTCCA CCGTAAATGA GGTATGCCTC ACATTTCTTG CGAGGCCATG

CCTCCTTCCA GTATAGATGA GGTATGTCTT ACATTTCAGG GGAGGACATG

---------- ---------- ---------- ---------- ----------

CCGCCGCCCT GTATCCAGGA GGTATGTCTT ACATTTCCGG AGAGGACATG

CCTCCTTCAA GTATGAGTGA GGTATGTCTT ACATTTCTGG CGAGGACAAG

CCCCCTTCAA TTATAGGGGA GGTAGGTCTT ACATTTCCGG GGAGGAGAAG

GGCCGCTTGT TGGCTGGCTT GGTGCCAGAG CTGTCTTTAC TGGTTTCTGA

---------- ---------- ---------- ---------- ---------A

GGCCGCTTGT TGGCCGGCTT GGTGCCAGAG CTGTCTTTAC TGGTTTCTGA

GGCCGCTTGT TGGCCGGCTT GGTGCCAGAG CTGTCTTTAC TGGTTTCTGA

GGCCGTTTGC TGGCCGGCTT GGTGCCAGAG CTGTCTTTAC TGGTTTCTGA

GGCCGCTTGC TGGCAGGCTT GGTGCCAGAG CTGTCTTTGC TGGTTTCTGA

GGCCGCTTGC TAGCTGGCTT GGTGCCAGAG CTGTCTTTAC TGGTTTCTGA

GGCCGCTTGC TGGCCGGCTT GGTGCCAGAG CTGTCTTTAC TAGTTTCTGT

GGCCGCTTGC TGGCCGGCTT GCTGCCAGAG CCGTCTTTAC TGGTTTCTAA

---------- ---------- ---------- ---------- ----------

GGCCGCTTGC TGGCTGGCTT GGCGCCAGAG CTGTCTTTAC TGGTTTCTGA

GGCCGCTTGC TGGCTGGCTT GGTGCCAGAG TTGTCTTTAC TGGTTTCTGA

GGCCGCTTGC TGGCCGGCCT GGTGCCAGAG CTGTCCTTGT TGGTTTCTG-

GGCCGCTTAC TGGCCGGCTT GGTGCCAGAA CTGTCTTTAC TGGTCTCTGA

---------- ---------- ---------- ---------- ----------

GGCCGTTTGC TGGCTGGCTT GGCGCCAGAG CTGTCTTTGC TGGTTTCTGA

GGACGCTTGC TGGCAGGTTT TGAAGTGGAG CTGTCTTTGC TGGATTCTGA

GGCCTCTTGC TCGCAGGCTT TGTTCCAGAG CCGTCTTTAC TTGACTCTGA

CTCACTTTGC TGATTTTAAT GGCACAAGGG GCCCCTGGGA AACCAGGCAG

CTCACTTTGC TGATTTTAAT GGCACAAGGG GCCCCTGGGA AACCAGGCAG

CTCACTTTGC TGATTTTAAT GGCACAAGGG GCCCCTGGAA AACCAGGCAG

CTCACTTTGC TGATTTTAAT GGCACAAGGG GCCCCTGGGA AACCAGGCAG

CTCACTTTGC TGATTTTAAT GGCACAAGGG GCCCCTGGGA AACCAGGCAG

CTCACTTTGC TGATTTTAAT GGCACAAGGG GCCCCTGGGA AACCAGGTAG

CTCACCTTGC TGATTTTAAT AGCACAAGGA GCCCCTGGAA AACCAGGCAG

CTCACCTTGC TGATTTTAAT AGCACAAGGA GCCCCTGGGA ACCCAGGCAG

CTCACTTTGC TGATTTTAAT GGCACAAGGG GCCCCTGGGA AACCAGGCAG

---------- ---------- ---------- ---------- ----------

CTCACTTTGC TGATTTTAAT GGCACAGGGG GCCCCTGGGA AACCAGGCAG

CTCACTTTGC TGATTTTAAT GGCACAGGGG GCCCCTGGGA AACCAGGCAG

-----TTTGC TGATTTTAAT GGTGCAGGGG GCCCCCGGGA AACCGGGCAG

CTCACTTTGC TGATTTTAAT GGCACAAGGC GCCCCTGGGA AGCCAGGCAG

---------- ---------- ---------- ---------- ----------

CTCACTTTGC TTATCTTAAT GGCACAAGGA GCCCCCGAGA AGCCGGGCAA

CTCACTTTAC TGATTTTAAT GGCACAAGGA GCCCCAGGAA AGCCAGGAAG

CTGACCTTGC TGATTTTTAT GGCACAGGGG GCTCCGGGGA AGCCTGGCAG

GCACGTCTTA AAGGCTGAGA TTTCGCTGAA GGGCCCCCGG CCACAGGCAT

GCACGTCTTA AAGGCTGAGA TTTCGCTGAA GGGCCTCCGG CCACAGGCAT

ACACGTCTTA AAGGCTGAGA TTTCGCTGAA GGGCCCCCGG CCACAGGCAT

ACACGTCTTA AAGGCTGAGA TTTCGCTGAA GGGCCCCCGG CCACAGGCAT

ACATGTCTTA AAGGCTGAGA TTTCACTGAA AGGCCCCCGG CCGCAGGCGT

ACACGTCTTA AATGCTGAGA TCTCACTGAA GGGCCCCCGG CCACAGGCGT

ACAAGTCTTA AAGGCTGAGA TCTCACTGAA GGGGCCCCGG CCACAAGCAT

ACAAGTCTTA AAGGCTGAGA TCTCACTGAA GGGTCCCCGG CCACAAGCAT

ACACGTCTTA AAGGCTGAGA TCTCGCTGAA CGGCCCCCGG CCACAGGCGT

---------- ---------- ---------- ---------- ----------

ACACGTCTTA AAGGCCGAGA TCTCGCTGAA GGGCCCTCGG CCACAGGCAT

ACACGTCTTA AAAGCTGAGA TCTCGCTGAA GGGCCCCCTG CCACAGGCAT

GCACGTCTTG AAGGCCGAGA TCTCGCTGAA CGGGCCCCGG CCACAGGCGT

ACACGTCTTA AAGGCGGAAA TCTCACTGAA GGGCCCCCGG CCACAGGCGT

---------- ---------- ---------- ---------- ----------

GCATGTCTTG AAGGCTGAGA TCTCGCTGAA GGGTCCCCGG CCGCAGGCAT

ACAAGTCTTA AAGGCAGAGA TCTCGCTGAA GGGCCCCCGG CCACAGGCAT

ACACGTCTTG AAAGCTGAGA TTTCACTGAA GGGGCCCCGC CCACAGGCAT

TGATTCCGGC AACACGAAAC TTATAGGCTG TGCCTGGCTG CAGCTCCTGC

TGATTCCTGG CACACGAAAC TTATAGGCTG TGCCTGGCTG CAGCTCCTGC

TGATTCCGGC AACACGAAAC TTATAGGCTG TGCCTGGCTG CAGCTCCTGC

TGATTCCGGC AACACGAAAC TTATAGGCTG TGCCTGGCTG CAGCTCCTGC

TGATTCCGGC AACACGAAAC TTATAGGCTG TGCCTGGCTG CAGCTCCTGC

TGATCCCGGC AACACGAAAT TTATAGGCGG TGCCTGGCTG CAGTTCCTGC

TGATTCCAGC AACTCGAAAT TTATAAGCTG TGCCTGGCTG CAGCTCCTGC

TGATTCCAGC AACTCGAAAT TTGTAAGCCG TGCCTGGCTG TAGCTCCTGC

TGATCCCAGC CACACGAAAC TTATAGGCTG TGCCCGGCTG CAGCTCCTGC

---------- ---------- ---------- ---------- ----------

TGATCCCGGC AACACGGAAC TTATAGGCTG TGCCCGGCTG CAGCTCCTGC

TGATCCCGGC AACACGAAAC TTATAGGCTG TGCCGGGCTG CAGTTCCTGC

TGATGCCCGC CACGCGAACC TTGTAGGCGG TGCCCGGCTG CAGCTCTTGC

TGATCCCAGC CACCCGGAAC TTATAGGCCG TGCCCGGCTG CAGCTCTTGC

---------- ---------- ---------- ---------- ----------

TGACCCCTGC TACACGGAAC TTATAGGCTG TGCCTGACTG CAGCTCCTGC

TGATGCCTGC GACACGGAAC TTGTAGGCTG TGCCCGGCTG GAGCTCTTGC

TGATCCCAGC CACACGGAAC TTGTACGCTG TTCCGGGCTG CAGTTCTTGC

TTCTTCAGCT GGTTATAGTC AGGTCATCTG GTGGCAGGAA ATAGTGTGTC

TTCTTCAGCT GGTTATAGTC ---TCATCTG GTGGCAGGAA ATAGTGTGTC

TTCTTCAGCT GGTTATAGTC AGGTCATCTG GTGGCAGGAA ATAGTGTGTC

TTCTTCAGCT GGTTATAGTC GGGTCATCTG GTGGCAGGAA ATAGTGTGTC

TTCTTCAGCT GGTTATAGTC AGGTCGTCTG GTGGCAGAAA ATAGTGTGTC

TTCTTCAGCT GGTTATAGTC AGGTCATCTG GTGGCAGGAA ATAGTGTGTC

TTCTTTAGCT GGTTATAGTC TGGTCATCTG GTGGCAGAAA ATAGTGTGTC

TTCTTTAGCT GGTTATAGTC TGGTCATCTG GTGGCAGAAA ATAGTGTGTC

TTCTTCAGTT GGTTATAGTC AGGTCATCTG GCGGCAGGAA ATAGTGTGTC

---------- ---------- ---------- ---------- ----------

TTCTTTAGCT GGTTGTAGTC GGGTCATCTG GTGGCAGGAA ATAGTGTGTC

TTCTTCAGCT GCGTATAGTC TGGTCATCTG GTGGCAGGAA ATAGTGTGTC

TTCTGCAGCT CGTGGTAGTC GGATCGTCCG GCGGCAGGAA GTAGTGGGTC

TTCTTCAGCT GGTTGTAGTC TGGTCGTCTG GCGGCAGGAA ATAGTGTGTC

---------- ---------- ---------- ---------- ----------

TTCTTCAGCT GGGTGTAGTC AGGTCATCAG GGGGCAGAAA ATAGTGTGTC

TTCTTTAGCT GGTTGTAGTC AGGTCGTCTG GGGGCAGAAA ATAGTGCATG

TTCTTTAGCT GGTTGTAGTC GGGTCGTCAG GTGGTAGGAA GTAGTGGGTC

ACCATTACAT TGGTGCCCTT AATGACTCCC ACATCAAACC ACTGGTTTTC

ACCATTACGT TGGTGCCCTT AATGACTCCC ACATCAAACC ACTGGTTTTC

ACCATTACAT TGGTGCCCTT AATGACCCCT ACATCAAACC ACTGGTTCTC

ACCATTACAT TGGTGCCCTT AATGACCCCG ACATCAAACC ACTGGTTCTC

ACCATTACAT TGGTGCCCTT AATGACCCCC ACATCAAACC ACTGGTTTTC

ACCATTACAT TGGTGCCCTT AATGACTCCC ACATCAAACC ATTGGTTCTC

ACCATTACAC TGGTACCTTT AATGACCCCC ACATCAAACC ACTGGTTCTC

ACCATTACAC TGGTACCCTT AATGACCCCC ACATCAAACC ACTGGTTCTC

ACCATTACAT TGGTGCCCTT GATGACCCCC ACGTCAAACC ACTGATTCTC

---------- ---------- ---------- ---------- ----------

ACCATTACGT TGGTACCCTT AATGACCCCC ACATCAAACC ACTGATTCTC

ACCATTACAT TAGTGCCCTT GATCACTCCC ACATCAAACC ACTGGTTCTC

ACCGTGACAT TGGTGCCTTT CACGACCCCC ACGTCGAACC A---------

ACCATCACGT TGGTGCCCTT GATGACTCCC ACGTCGAACC ACTGGTTCTC

---------- ---------- ---------- ---------- ----------

ACCATTACAT TGGTGCCCTT AATGACTCCC ACGTCAAACC ACTGGTTCTC

ACCATCATGT TGGTGCCTTT GATGACCCCA ACATCGAACC ACTGGTTTTC

ACCATCATAT TGGTGCCTTT GATGACGCCC ACGTCGAACC ACTGGTTCTC

CTTCTTCATG GGGGCTTTAC TCACCACACC CAGGGACTCG ATGCCATTGG

CTTCTTCATG GGGGCTTTAC TCACCACACC CAGGGACTCG ATGCCATTGG

CTTCTTCATG GGGGCTTTAC TCACCACACC CAGGGACTCG ATGCCATTGG

CTTCTTCATG GGGGCTTTAC TCACCACACC CAGGGACTCG ATGCCATTGG

CTTCTTCATG GGGGCTTTAC TCACCACACC CAGGGACTCG ATGCCATTGG

CTTCTTTACT GGTGCTTTAC TCACCACACC CAGGGACTCA ATGCCATTGG

CTTTTTTATA GGGGCTTTAC TTACCACACC CAGGGACTCA ATGCCATTTG

CTTTTTCACA GGGGCTTTAC TCACCACACC CAGGGACTCA ATGCCATTGG

CTTCTTCACG GGAGCTTTAC TCACCACACC GAGGGACTCG ATGCCATTGG

---------- ---------- ---------- ---------- ----------

CTTCTTCACG GGGGCTTTAC TCACCACGCC CAGAGACTCA ATGCCGTTGG

TTTCTTCACA GGGGCTTTAC TCACCACACC CAGGGACTCG ATGCCATTGG

---------- ---------- ---------- ---------- ----------

CTTCTTCACG GGGGCTTTAC TCACCACCCC GATAGACTCG ATGCCGTTGG

---------- ---------- ---------- ---------- ----------

CTTTTTCACA GGGGCTTTAC TCACCACCCC AATGGTTTCA ATGCCGTTGG

CTTCTTGGCA GTAGCTTTAC TCACCCCCAC TGGTGACTCG ATGCCATTAG

CTTCTTAACC GGAACTTTAC TTACCGCTGC TGTGGACTCA AGGCCGTTGG

CCACTTCGGT CAGGGTAGCT GCAGCCTGCA GCTTGGCTGG GCTGGCCACC

CCACTTCGGT CAGGGTAGCT GCAGCCTGCA GCTTGGCTGG GCTGGCCACC

CCACTTCAGT CAGGGTAGCT GCAGCCTGCA GCTTGGCTGG GCTGGCCACC

CCACTTCAGT CAGGGTAGCT GCAGCCTGCA GCTTGGCTGG GCTGGCCACC

CCACTTCAGT CAAGGTAGCT GCAGCCTGCA GCTTGGCTGG GCTGGCCACC

CCACTTCGGT TAGGGTTGCT GCAGCCTGCA GCTTCGCTGG GCTGGCTACC

CCACTTCAGT AAGGGTTGCT GCAGCCTGCA TCTTTGCTGG ACTGGCTACA

CCACTTCAGT AAGGGTAGCT GCAGCCTGCA TCTTTGCTGG ACTAGCTACA

CTACTTCAGT CAGAGTAGCT GCAGCCTGCA GCTTGGCTGG GCTGGCTACC

---------- ---------- ---------- ---------- ----------

CCACCTCGGT CAGGGTGGCT GCAGCCTGCA GCTTTGCGGG GCTTGCTACC

CCACCTCAGT CAGGGTAGCG GCGGCCTGCA GCTTTGCGGG ACTGGCTCCC

---------- ---------- ---------- ---------- ----------

CCACTTCCGT CAGGGTGGCC GCAGCCTGCA GCTTTGCCGG GCTGGCGACC

---------- ---------- ---------- ---------- ----------

CTACTTCGGT CAGGGTGGCT GCTGCCTGCA GCTTCGCCGG GCTGGCAACC

CTACCTCAGC CAGGGCAGCA GCTGCCTGGA GCTTCGCCGG ACTGGGCGCC

CCACCTCAGC TAGTGTGGCA GCAGCCTGCA GCTTGGCGGG ACTGGCCACC

ACCGGCTGGG GGGCCACAAA TGTGTTGGAT GGAGCCAGGC CTATCAGTCA

ACCGGCTGGG GGGCCACAAA TGTGTTGGAT GGAGCCAGGC CTA-------

ACCGGCTGGG GGGCCACAAA TGTGTTGGAT GGGGCCAGGC CTGTCAGTCA

ACCGGCTGGG GGGCCACAAA TGTGTTGGAT GGGGCCAGGC CTGTCAGTCA

ACCGGCTGGG GGGCCACAAA TGTGTTGGAT GGAGCCAGGC CTGTCAGTCA

ACTGGCTGGG GGGCCACAAA TGTGTTGGAT GGAGCCAGGC CTGTCAGTCA

ACAGGCTGAG GAGCCACAAA TGTGTTAGAT GGAGCCAAAC CTGTCGATAC

AC-GGCTGGG GAGCCACAAA TGTGTTAGAT GGAGCCAGGC CTGTCAGTCA

ACCGGCTGGG GGGCCACAAA CGTGTTGGAT GGGGCCAGGC CTGTCAGTAA

---------- ---------- ---------- ---------- ----------

ACGGGCTGGG GGGCCACAAA GGTGTTGGAG GGGGCCAGGC CTGTCACTCA

ACTGGCTGGG GGGCCACAAA CGTGTTGGAA GGAGCCAGGC CTGTCAGTCA

---------- ---------- ---------- ---------- ---TCAGTCT

ACTGGCTGGG GGGCCACAAA CGTGTTCGAC GGTGCCAAAC CTGTCAGTCA

---------- ---------- ---------- ---------- ----------

ACCGGCTGTG GGGCCACGAA TGTGTTAGAT GGGGCCAGGC CTGTCAGTCT

ACTGGCTGAG GAGCCACAAA TGTGTTGGAT GGTGCCAAAC CTGTCAGTTG

ACCGGCTGGG GAGCCACAAA CGTACTGGAC GGAGCTAGGC CTGTCAACCT

ATTTCCTCTT GTTTTGTCCC TCATTTAGCC TGGGCAGCTG CTTCGTCACT

---------- ---------- -------GCC TGGGCAGCTG CTTCGTCACT

ATTTCCTCTT GTTTTGTTCC TCATTTAGCC TGGGCGGCTG CTTCGTCACT

ATTTCCTCTT GTTTTGTTCC TCATTTAGCC TGGGCGGCTG CTTCGTCACT

ATTTCCTC-- -TTTTGTCCC TCATTTAGCC TGGGCGGCTG CTTCGTCACT

ATTTCTTCTT GTCTTGTCCC TTGTTTAGCC TGGGCAGCTG CTTCGTTACT

TCTGCCTCTC CTTTGGTTTT TTCTT--GCT TGAGCAGCTG CTTCGTCACT

GTTGCTTCTT GTTTTGTTTC TCATT--GCT TGAGCAGCTG CTTCGTCACT

ATTTCTTCTT GTCTTGTCCC TCATTCAGCC TGGGCAGCTG CTTCGTCACT

---------- ---------- ---------- ---------- ----------

AATTTGTCTT CTCTTGTCCT TCATTTAGCC TGGGCAGCCG CTTCGTCACC

GTTTTGTCTT CTCTTGTCCT TCATTTAGCC TGGGCAGCTG CTTCGTCACC

GTATTATCTT CTCTTGTCTT TCATTTA--- ---GTGGCAG CTTTATCACC

AATTTTTCTT GTTTTGTCCC TCATTTAGCC TGGGCGGCCG CCTCGTCACC

---------- ---------- ---------- ---------- ----------

AGTTTGTCTT GTCTTGTCCC TCATTGAGCC TGGGCAGCTG CATCGTCACT

GCTTTTTCTG TGCTCGTTTC GGACTTGGCC TGGGCTGCAG CCTCGTCACT

TTTCCATCTT GTCTTTTTCC CCAGCTGGCC TGGGGAGTGG CCCCGCCGCC

CCAGCTCCTC GGGGGTGAGC CCCGTTACCA TGAGGGTGGT GGTGCCAGCT

CCAGCTCCTC GGGGGTGAGC CCCGTTACCA TGAGGGTGGT GGTGCCAGCT

CCAGCTCCTC AGGGGTGAGC CCCGTTACCA TGAGGGTGGT GGTGCCAGCT

CCAGCTCCTC AGGGGTGAGC CCCGTTACCA TGAGGGTGGT GGTGCCGGCT

CCAGCTCTTC GGGGGTGAGC CCCGTTACCA TGAGGGTGGT GGTGCCGGCC

CCAGTTCCTC AGGGGTGAGC CCTGTCACCA TGAGGGTGGT GGTGCCTGCC

CCAGCTCCTC TGGAGTGAGC CCTGTTACCA TAAGGGTTGT GGTGCCCGCC

CCAGCTCCTC TGGAGTGAGC CCTGTTACCA TAAGGGTTGT GGTGCCCGCC

CCAGCTCCTC AGGGGTGAGC CCTGTTACCA TGAGGGTCGT GGTGCCCGCC

---------- ---------- ---------- ---------- ----------

CCAGCTCCTC AGGGGTGAGG CCCGTCACCA TGAGGGTGGT GGTGCCCGCC

CCAGCTCCTC AGGGGTGAGC CCTGTTACCA TGAGGGTGGT GGTGCCTGCC

CCAGCTCTTT AGGGATGAAC TTTGGTAGGA TGAGGGTA-- ----------

CCAGCTCCTC GGGGGTGAGC CCCGTTACCA TGAGGGTGGC GGTGCCTGCT

---------- ---------- ---------- ---------- ----------

CAAGCTCCTC AGGGGTGAGC CCCGTCACCA TCAGGGTGGT GGTGCCCGCC

CCAACTCCTC TGGGGTAAGG CCTGTAACCA TAAGGGTTGT GGTGCCAGCC

CCCGCTCCTC CACGGCCGGC TCCGGAACCC TGTGCTTGGG GCTGGCCGCC

TGGGCCTCGG CCATTAGCTC TTGGGGAAGT GATAACTGTC TCAGTCATTG

TGGGCCTCGG CCATTAGCTC TTGGGGAAGT GATAACTGTC TCAGTCATTG

TGGGCCTCGG CCATTAACTC TTGGGGAAGT GATAACTGTC TCAGTCATTG

TGGGCCTCGG CCATTAACTC TTGGGGAAGT GATAACTGTC TCAGTCATTG

TGGGCCTCAG CCATTAGCTC TTGGGGAAGT GATAACTGTC TCAGTCATTG

TGAGTCTCGG CCATCAGCTC TTGGGGAAGT GATAACTGTC TCAGTCACTG

TGGGCTTCAG CCATCAGCTC TTGGGGAAGT GATAACTGTC TCAGTCAATG

TGGGCTTCAG CCATCAGCTC TTGGGGAAGT GATAACTGTC TCAGTCAATG

TGGGCCTCGG CCATCAGCTC TTGGGGAAGT GACAACTGTC TCAGTCACTG

---------- ---------- ---------- ---------- ----------

TGGGCCTCAG CCATCAGCTC TTGAGGAAGG GACAACTGCC TCAGTCATTG

TGGGCCTCGG CCATCAGCTC TTGGGGAAGT GACAACTGTC TCAGTCACGG

---------- ---------- ---------- ---------- ----------

TGGGCCTCGG CCATCAGCTC CTGGGGCAGC GACAACTGTC TCAGTCACGG

---------- ---------- ---------- ---------- ----------

TGGGCCTCAG CCATCAGCTC TTGGGGCAGT GCCAACTGTC TCAGTCATTG

TGAGCCTCAG CCATCAGCTC TTGAGGCAGA GATAGTTGTC TCCGTCATCG

TGCGCCCCCG ACGTTGGCCG TCATGGTGGT GCCGGTGGTC ACAGTCATCG

ATGAGATCTT CTAGAAAGGG AAGTGACAGT GGTGGCCGTG TGAGTGGTGC

ATGAGATCTT CTAGAAAGGG AAGTGACAGT GGTGGCCGTG TGAGTGGTGC

ATGAAATCTT CTAGAAAGGG AAGTGACAGT GGTGGCCGTG TGAGTGGTGC

ATGAAATCTT CTAGAAAGGG AAGTGACAGT GGTGGCCGTG TGAGTGGTGC

ATGATATCTT CTAGAAAGGG AAGTGACAGT GGTAGCTGTG TGAGTGGTGC

ACGAGATCTT CTAGAAAGGG AAGTGACAGT GGTAGCCGTG TGTGTAGTGC

ATGAGATGTT CTAGAAAGGG AGGTGACAGT GGTGGCTGTG TGCGTTGTAC

ATGAGATGTT CTAGAAAGGG AGGTGACAGT GGTGGCTGTG TGCGTGGTAC

ATGAGATCTT CTAGAAAGGG AGGTGACAGT GGTGGCTGTG TGTGTGGTGC

---------- ---------- ---------- ---------- ----------

ATGAGATCTT CTAGAAAGGG AGGTGACTGT GGTGGCTGTG TGCGTGGTGC

ATGAGATCTT CTAGAAAGGG AGGTGACAGT AGTGGCCGTG TGCGTGGTGC

---------- ---------G AGGTGACAGT GGTGGCCGTG TGCGTGGTGC

ATGAGATCCT CTAGAAAGGG AGGTGACGGT GGTGGCTGTG TGCGTGGTGC

---------- ---------- ---------- ---------- ----------

ATGAGATCCT CTAGAAAGGG AGGTGACGGT GGTGGCCGTG TGCGTGGTGC

CTGAAATGTT CTACAAGGTG AGGTGGCAGT GGTGGCCGTA TGTGTGGTGC

TGGTGCCGGT CGAGCTAGTG AGGTGGCGGT GGTGGCGGTG TTGGTGGTGC

CCGTCTCGTG GGTCTCACAG GGGGGGTTGG AGCACACCCT CTGTGTTGGG

CCGTCTCGTG GGTCTCACAG GGGGGGTTGG AGCACACCCT CTGTGTTGGG

CCGTCTCGTG GGTCTCACAG GGGGGGTTGG AGCACACCCT CTGCGTTGGG

CCGTCTCGTG GGTCTCACAG GGGGGGTTGG AGCACACCCT CTGCGTTGGG

CTGTCTCGTG GGTCTCACAG GGGGGGTTGG AGCATACCCT CTGTGTTGGA

CCGTCTCATG GGTCTCACAG GGAGGATTGG AGCACACCCT CTGTGTTGGG

CTGTCTCGTG GGTCTCGCAA GGAGGATTGG AGCATACCCT CTGTGTTGAG

CTGTCTCGTG GGTCTCGCAA GGAGGGTTGG AGCATACCCT CTGTGTTGGG

CCGTCTCGTG GGTCTCACAC GGGGGATTGG AGCACACCCT TTGCGTTGGG

---------- ---------- ---------- ---------- ----------

CCGTCTCGTG CGTCTCACAA GGGGGGTTGG AGCAGACCCT CTGTGTCGGG

CGGTCTCGTG GGTCTCACAG GGAGGGTTGG AGCACACCCT CTGCGTTGGA

CCGTCTCGTG GGTCTCACAC GGCGGGTTGG AACAGACCCG CTGTGTGGGG

CCGTCTCGTG GGTCTCACAG GGGGGGTTGG AGCAGACCCG CTGGGTTGGG

---------- ---------- ---------- ---------- ----------

CCGTCTCATG GGTCTCACAA GGTGGGTTGG AGCAGACCCG CTGGGTGGGA

CAGTCTCGTG GGTCTCACAG GGTGGGTTGG AGCACACCTG CTGGATCGAG

CCGTCTCGTG GGTTTCACAG GGAGGATTGG AGCAGACCCG AGGGGTTGGG

AAAGGAGCCA GCAGTGTTGG TGGTGCCTGT CTCGTGGGTC TCACATGGTG

AAAGGAGCCA GCAGTGTTGG TGGTGCCAGT CTCGTGGGTC TCACAGGGTG

AAAGGAGCCA GCAGTGTTGG TGGTGCCTGT CTCGTGGGTC TCACATGGTG

AAAGGAGCCA GCAGTGTTGG TGGTGCCTGT CTCGTGGGTC TCACATGGTG

AAAGGAGCCA GCAGTGTTGG TAGTGCCTGT CTCATGGGTC TCACATGGTG

AAAGGAGCCA GCAGTGTGGG TGGTGCCTGT CTCGTGGGTC TCACATGGTG

AAAGAGGCCA GCAGTGTTGG TGGTACCCGT CTCATGGGTC TCACATGGTG

AAAGAGGCCA GCAGTGTTGG TGGTACCCGT CTCATGGGTC TCACATGGCG

AACGGAGCAA GCAGTATTGG TGGTGCCCGT CTCATGGGTC T---------

---------- ---------- ---------- ---------- ----------

AAAGGAGCCA GCAGTGTTGG TGGTGCCCGT CTCGTGGGTC TCGCAGGGTG

AAAGGAGTCA GCAGTGTTGG TGGTGCCTGT CTCGTGGGTC TCGCACGGCG

AAGGGGGCCA GCAGTGCTGG TGGTGCCCGT CTCGTGGGTC TCGCACGGTG

AAAGGCGCCA GCAGTGTGCG TGGTGCCCGT CTCGTGGGTC TCGCACGGGG

---------- ---------- ---------- ---------- ----------

AAAGGTGCCT GAAGTATTGG TAGTCCCTGT CTCGTGGGTC TCGCACGGCG

GTAGTGGCTG TGTGGGCCCG TAGAACCTGT GGCGGTGGTC TCACAGGATG

CACAACGCCT CTGGTGCTGG TTGAGCTAGT TTGGTGGGTT TGGCATGGGG

GGTTGGAGCA GACTGGCAGT CATGGTGGTG CTGGTCGCGC TGGTCTGGCG

GGTTGGAGCA CACTGGCAGT CATGGTGGTG CTGGTCGCGC TGGTCTGGCG

GGTTGGAGCA GACTGGCAGT CATGGTGGTG CTGGTCGTGC TGGTCTGGCG

GGTTGGAGCA GACTGGCAGT CATGGTGGTG CTGGTCGTGC TGGTCTGGCG

GGTTGGAACA GACTGGCAGT CATGGTGGTG CTGGTCGCGC TGGTCTGGCG

GGTTGGAGCA GACTGGCAGT CATGGTGGTG CTGGTTGCAC TGGTCTGGCG

GGTTGGAGCA GACTGGCAGT CATGGTGGTG CTGGTCATGT TGGTCTGCTG

GGTTGGAGCA GACTGGCAGT CATGGTGGTG GTGGTCATGT TGGTCTGCTG

---------- -ACTTGCAGT CATGGTGGTA CTGGTTGCAC TGGTCTGGCG

---------- ---------- ---------- ---------- ----------

GGTTGGAGCA GACTGGCAGT CATTGTGGTG CTGGTCACAC TGGTCTGGCG

GGTTGGAGCA GACTGGCAGT CATGGTGGTG CTGGTTGCAC TGGTCTGGCG

GGTTGGAGCA GATTGGCGGT GGTGGCCGTG TTGGTGGTGC CTGTCTAATG

GGTTGGAGCA GACCGGCAGT CATGGTGGTG CTGGTGGAGC TGGTCTGGCG

---------- ---------- ---------- ---------- ----------

GGTTGGAGCA GACCTGCAGT CATGGTGGTG CCAGTGGTGG TGGTCTGGTG

GACTGGGGCA GACTGGCCAT Ggtggccgtg ttggtggtac ccgtttcatg

GCTTGGAGGG GACTGGCCGT GGTGGCCGTG CTGGTGGTGC CCGTCTCGTG

GGTTTGGCAC TGGGACTGTG GCAGTGTTGG TGGTGCCCGT CTCGTGGGTC

GGTTTGGCAC TGGGGCTGTG GCAGTGTTGG TGGTGCCCGT CTCGTGGGTC

GGTTTGGCAC TGGGGCTGTG GCAGTGTTGG TGGTGCCCGT CTCATGGGTC

GGTTTGGCAC TGGGGCTGTG GCAGTGTTGG TGGTGCCCGT CTCATGGGTC

GGTTTGGCAC TGGGGCTGTG GCAGTGTTGG TGGTGCCCGT CTCGTGGGTC

GGTTTGGCAC TGGGGCTGTG GCAGTGTTGG TGGTGCCTGT CTCATGGGTC

GGTTTGGCAC GGAGGCTGTG GCAGTGCTGG TGGTACCTGT CTCATGAGTC

GGTTTGGCAC TGAGGCTGTG GCAGTGCTGG TGGTGCCTGT CTCATGAGTC

GGTTTGGCAT TGGGGCTGTG GCGGTGCTGG TGGTGCCGGT CTCGTGGGTC

---------- ---------- ---------- ---------- ----------

GGTTTGGCAC AAGGGCTGTG GCGGTGCTGG TGGTGCCCGT CTCGTGGGTC

GGTTTGGCAT GAGGCTTGAG GCGGTGTTGG TGGTGCCCGT CTCGTGGGTT

GGTCTCACAC GGAGGGTGTG GCAGTGCTGG TGGTGCCCGT CTCGTGGGTC

GGTCTGGCAC GGGGGCTgtg gccgtgctgg tggtgcccgt ctcgtgggtc

---------- ---------- ---------- ---------- ----------

TGTCTCGCAG GGGCGGCGTG GCGGTATTGG TAGTCCCTGT CTCGTGGGTC

ggtctcacat gggggatgtg gctgtgttgg tggtacctgt ctcatgggtc

GGTCTCGCAG GGTGGGTGTG GCCGTGTTGG TGGTGCCCGT CTCGTGCACC

TCGCAGGGTG GGTTTGAGCA GCCCaggtgg cggtggtggc ggtgttggtg

TCGCAGGGTG GG-TTGAGCA GCCC------ ------TGGT GGTGCTGGTC

TCGCAGGGTG GGTTTGAGCA GCCCaggtgg cggtggtggc ggtggtggtg

TCGCAGGGTG GGTTTGAGCA GCCCaggtgg cggtggtggc ggtgttggtg

TCGCAGGGCG GGTTTGAGCA GCCCaggtgg cggtggtggc ggtgttggtg

TCACAGGGTG GGTCTGAGCA GCTCAGGTGG CAGTGGTGGC AGTATTGGTG

TCGCAGGGAG GATTCGAGCA GCCCAGGTGG CTGTTGTGGC AGTGTTGGTG

TCACAGGGGG GGTTCGAGCA GCCCAGGTGG CTGTTGTGGC AGTGTTGGTA

TCACAGGGCG GGTTCGAGCA GCCCaggtgg cggtggtggc ggtgttggtg

---------- ---------- ---------- ---------- ----------

TCGCACGGCG GGTTGGAGCA GCTCACGTGG CGGTGGTGGC CGTGTGCGTG

TCACAGGGCG GGTTGGAGCA GCCCAGGTGG CAGTGGTGGC GGTATTGGTG

TCGCACGGTG GGTTGGAGCA GCCC------ ---------- ----------

tcgcagggcg ggttggagca gaccacatgg cggtggtggc cgtgtgcgtg

---------- ---------- ---------- ---------- ----------

TCGCACGGCG GGTTGGAGCA GACTCGGTGG CAGTGGTGGC CGTGTGCGTG

tcacagggag ggttggagca CACTCACTGA CCACAGTGGG AGTGTTGGTG

TCGCAGGGTG GGTTGGAGCA CACGCGCTGA CCACTGTGGG CGTGTTGGTG

gtgCCCGTCT CGTGGGTCTC GCAGGGCGGG TTCGAACAGA CCTCCAGTGG

GCGCTGGTCT GGCGGGTTTG GCACTGGGGC TTAGAGC--- -CTCCGGTGG

gtgCCCGTCT CATGGGTCTC GCAGGGTGGG TTTGAGCAGA CCTCCGGTGG

gtgCCCGTCT CATGGGTCTC GCAGGGTGGG TTTGAGCAGA CCTCCGGTGG

gtgCCCGTCT CATGGGTCTC GCAGGGTGGG TTCGAGCAGA CGTCCGGTGG

GTGCCCGTTT CATGGGTCTC GCAAGGAGGG TTCGAGCAGA CTTCTGGTGG

GTGCCTGTCT CATGGGTTTC ACAGGGGGGG TTTGAACAGA CCTCCGGTGG

GTGCCCGTCT CATGGGTCTC ACAGGGGGGG TTTGAACAGA CCTCCGGTGG

gtgCCAGTCT CGTGGGTCTC GCAGGGCGGG TTGGAGCAGA CCTCCGGTGG

---------- ---------- ---------- ---------- ----------

GTGCCCGTCT CATGGGTCTC GCACGGCGGG TTGGAGCAGA CCTCCGGTGG

GTGCCTGTCT CATGGGTCTC GCACGGTGGG TTGGAGCAGA CGTCCGGTGG

---------- ---------- ---------- ---------- -ATCTGCTGG

gtgcccgtct cgtgggtctc acaggggggg ttggagcaga cCTCCGGTGG

---------- ---------- ---------- ---------- ----------

GTGCCGGTCT CGTGGGTCTC ACAGGGCGGG TTGGAGCAGG CCTCCGGTGG

GTGCCTGTCT CATGGATCTC ACAGGGGGGG TTTGAACAGA CCTCTTGTTG

GTGCCCGTCT CATGGGTCTC ACATGGTGGA TTAGAGCAGA CCTCCGGTGG

TGGCCGTGTT GGTGGTGCCA GTCTCGTGGG TCTCACAGGG TGGGTTGGAG

TGGCCGTGTT GGTGGTGCCA GTCTCGTGGG TCTCACAGGG TGGGTTGGAG

TGGCTGTGTT GGTGGTGCCA GTCTCGTGGG TCTCACAGGG TGGGTTGGAG

TGGCTGTGTT GGTGGTGCCA GTCTCGTGGG TCTCACAGGG TGGGTTGGAG

TGGCCGTGTT GGTGGTGCCA GTCTCGTGGG TCTCACAGGG TGGGTTGGAG

TAGCTGTGTT GGTGGTGCCC GTCTCATGGG TCTCACATGG TGGGTTGGAG

TAGCTGTGTT GGTGGTGCCT GTTTCATGGG TTTCACAGGG TGGGTTGGAA

TAGCTGTGTT GGTGGTGCCT GTTTCATGGG TTTCACAGGG TGGGTTGGAA

TGGCTGTGTT GGTGGTGCCA GTCTCGTGAG TCTCACAGGG TGGGTTGGAG

---------- ---------- ---------- ---------- ----------

TGGCCGTGTT GGTGGTGCCC GTCTCGTGGG TCTCGCAGGG CGGGTTGGAG

TGGCCGTGTT GGTGGTGCCT GTCTCGTGGG TCTCACAAGG CGGGTTGGAG

GAGGT----- ----GTCCCC ATCTCCTGGG TCTCACCGGG CAGGCTAGAG

TGGCCGTATT GGTGGTGCCC GTCTCGTGAG TCTCACAGGG CGGGTTGGAG

---------- ---------- ---------- ---------- ----------

TGGCGGTATT GGTAGTCCCT GTCTCGTGGG TCTCGCACGG CGGGTTGGAG

TGGCTGTATT AGTAGTGCCC GTCTCATGGG TCTCGCAGGG GGGGTTGGAG

TGGCCGTATT GGTGGTGCCT GTCTCATGGG TCTCACAGGG CGGGTTGGAG

CACACCAAGG TGACAGTGCC GCTGTCAGCG TGGTCTGTGC GGCCGACACA

CACACCAAGG TGACAGTGCC GCTGTCAGCG TGGTCTGTGC GGCCGACACA

CACACCAAGG TGACAGTGCC GCTGTCAGCG TGGTCTGTGC GGCAGACACA

CACACCAAGG TGACAGTGCC GCTGTCAGCG TGGTCTGTGC GGCAGACACA

CACACCAAGG TCACGGTGCC GCTGTCAGCG TGGTCTGTGC AGCTGACACG

CATACCAGTG TCACAGTGCC GCTGTCAGTG TGGTCTGTGC AGCTGACACG

CACACCAGTG TCACAGTGCA GCTGTTAGTG TAGTCTGTGC AGCTGACACT

CACACCAGTG TCACAGTGCA GCTGTTAGTG TAGTCTGTGC AGCTGACACT

CACACTAGTG TCACAGTGCT GCCGTCAGCG TGGTCTGTGC AGCCGACACT

---------- ---------- ---------- ---------- ----------

CACACCAGCG TCACGGTGCC GCCGTCAGTG TGGTCTGCGC GGCCGAAACT

CAGACCAGCG TCACGGTGCC GCTGTCAGCG TGGTCTGTGC GGCCGACACG

CACATCAACG TCATCACGCC GCCGTGAGCG TGGTCTGCGC GGCCGACACG

CAGACCAGCG TCACTGTGCC GCGGTCAGCG TGGTCTGGGC GGCCGAGACG

---------- ---------- ---------- ---------- ----------

CAGACCTGCG GCACAGTGCG GCAGTCAGCG TAGTCTGAGC AGCGGAGACG

CACACTAGGG TCACAGTGCC GCTGTCAGAG TGGTCTGGGC AGCTGACACA

CACACCAGGG TCACGGTGCT GCCGTCAGGG TAGTTTGGGC AGCTGAGACC

GTGATGGCAG TGGGGTTGAT CACCTGGCTT GAGAGGGTGG CAATGGTGCC

GTGATGGCAG TGGGGTTGAT CACCTGGCTT GAGAGGGTGG CAATGGTGCC

GTGATGGCAG TGGGGTTGAT CACCTGGCTT GAGAGGGTGG CGATGGTGCC

GTGATGGCAG TGGGGTTGAT CACCTGGCTT GAGAGGGTGG CGATGGTGCC

GTGATGGCAG TGGGGTTGAT CACCTGGCTG GAGAGGGTGG CGATGGTGCC

GTGATAGCAG TGGGGTTGAT CACCTGGCTT GAGAGGGTGG CGATAGTACC

GTGATAGCAG TAGGGTTGAT CACCTGACTT GAGAGAGTAG CAATGGTGCC

GTGATAGCAG TAGGGTTGAT CACCTGGCTT GAGAGCGTAG CAATAGTGCC

GTGATGGCAG TGGGGTTGAT CACTTGGCTC GACAGGGTGG CAATGGTGCC

---------- ---------- ---------- ---------- ----------

GTGATGGCAG TGGGGTTGAT CACTTGGCTT GAGAGGGTGG CAATGGTACC

GTGATGGCAG TGGGGTTGAT CACCTGGCTC GAGAGGGTGG CAATGGTGCC

GTGATGGCCG TGGGGTTGAT CACCTGGCTT GAGAGAGTGG CAATGGTGCC

GTGATGGCTG TGGGGTTGAT CACTTGGCTG GAGAGGGTGG CGATGGTCCC

---------- ---------- ---------- ---------- ----------

GTGATGGCCG TGGGATTGAT CACCTGGCTT GAGAGGGTGG CGATGGTCCC

GTAATGGCTG TAGGATTGAT CACCTGGCTC GAAAGAGTGG CAATGGTCCC

GTGATGGCGG TCGGGTTGAT CACCTGACTT GAGAGGGTGG CAATGGTACC

CAAGGTGGTG ATGGGCGTGG CCAGGGAAGC ACTAGTGCTG TGGCCCCCCG

CAAGGTGGTG ATGGGCGTGG CCAGGGAAGC ACTAGTGCTG TGGCCCCCCG

CAAGGTGGTG ATGGGCGTGG CCAGGGAAGC ACTGGTGCTG TGGCCCCCTG

CAAGGTGGTG ATGGGCGTGG CCAGGGAAGC ACTGGTGCTG TGGCCCCCTG

CAAGGTGGTG ATGGGCGTGG CCAGGGACGC ACTGGTGCTG TGGCCCCCCG

CAAGGTGGTG ATGGGCGTGG CCAGGGAGGC ACTAGTGCTG TGGCCCCCGG

CAAAGTAGTG ATAGGTGTAG CCAGAGAAGC ACTGGTGCTA TGTGCCCCAG

CAAGGTAGTG ATAGGTGTAG CCAGGGAAGC ACTGGTGCTA TGTGCCCCAG

CAAAGTGGTG ATGGGCGTGG CCAGAGAGGC GCTGGTACTG TGGCCCCCAG

---------- ---------- ---------- ---------- ----------

CAAGGTGGTG ATGGGTGTGG CCAGGGAGGC GCTGGTACTG TGGCCCCCAG

TAAGGTGGTG ATGGGTGTGG CCAGGGAAGC ACTGGTACTG TGGCCCCCAG

CAGGGTGGTG ATGGGCGTGG CCAGGGAGGC ACTGGTGCTG TGGC------

CAGGGTGGTG ATAGGCGTGG CCAGGGAGGC GCTGGTGCTG TGCCCCCCTG

---------- ---------- ---------- ---------- ----------

CAAAGTGGTG ATAGGCGTGG CCAGTGAGGC GTTGGTGCTA TGACCGCCAG

CAGGGTGGTG ATAGGAGTGG CCAGAGAGGC ATTGGTGTTA TGCCCGCCTG

CAGGGTGGTG ATGGGAGTGG CCAGAGAGGC ATTGGTGTTA TGGCCGCCAG

CCCCGGCAAG GCTGGTGGAG ACGGTGCCTG TCACTGTGCC TAGGGTCGTG

CCCCGGCAAG GCTGGTGGAG ACGGTGCCTG TCACTGTGCC TAGGGTCGTG

CCCCGGCAAG GCTGGTGGAA ACCGTGCCTG TCACTGTGCC TAGGGTCGTG

CCCCGGCAAG GCTGGTGGAG ACCGTGCCTG TCACTGTGCC TAGGGTCGTG

CCCCAGCAAG GCTGGTGGAG ACTGTGCCTG TCACTGTGCC TAGGGTTGTG

CTCCAGCTAG GCTGGTGGAC ACTGTGCCTG TGACTGTGCC CAACGTCGTG

CTCCGGCAAG GCTGGTGGAG ACAGTGCCTG TCACTGTGCC TAGTGTTGTA

CTCCGGCCAG GCTGGTGGAG ACAGTGCCTG TCACTGTGCC TAGCGTTGTA

CTCCAGCGAG GCTGGTAGAG ACAGTGCCTG TCACCGTGCC TAAAGTTGTG

---------- ---------- ---------- ---------- ----------

CTCCGGCCAG GCTGGTGGAA ACGGTGCCTG TCACTGTACC GAGGGTTGTG

CTCCAGCAAG GCTGGTGGAA ACGGTACCTG TCACGGTGCC CAGGGTTGTG

---------- ---------- ---------- ---------- ---GGTCACT

CTCCAGCGAG GCTGGTAGAA ACAGTCCCGG TCACCGTGCC CAGGGTTGTG

---------- ---------- ---------- ---------- ----------

CACCAGCCAG GCTGGTAGAG ACAGTGCCTG TCACAGTGCC CAAGGTGGTG

CTCCAGCAAG GCTGGTAGAC ACTGTGCCTG TCACAGTGCC AAGAGTTGTA

CGCCAGCGAG GCTGGTGGAC ACGGTGCCAG TCACAGTGCC CAGGGTAGTT

ACACCTACCT GTGGTGCCTT CGGTGACGGG TGTGACCAGG CGAACACCCC

ACACCTACCT GTGGTGCCTT CGGTGACGGG TGTGACCAGG CGAACACCCC

ACACCTACCT GTGGTGCCTT CGGTGACGGG TGTGACTAGG CGAACACCCC

ACACCTACCT GTGGTGCCTT CGGTGACGGG TGTGACTAGG CGAACACCCC

ACCCCTACCT GTGGTGCCTT CGGTGACGGG GGTGACCAGG CGGACGCCCC

ACACCTACCT GTGGTGCCCT CAGTGACAGG GGTGACCAGG CGGACGCCCC

ACACCTACCT GTGGTGCCCT CGGTGACAGG GGTGACCAGG CGAACGCCGC

ACACCTACCT GTGGTACCCT CGGTGACAGG GGTGACCAGG CGAACGCCGC

ACACCTACCT GTGGTGCTCT CAGTGACGGG GGTAACCAGG CGAACACCCC

---------- ---------- ---------- ---------- ----------

ACACCTACCT GTGGTGCCCT CAGTGACAGG GGTGACCAGG CGGACACCCC

ACACCTACCT GTGGTGCCCT CAGTGACGGG GGTGACCAGG CGGACGCCCC

GTGCCCACCT GTAGTGCCCT CGGTGACGGG GGTCACCAGG CGGACACCCC

ACACCTACCT GTGGTGCCCT CGGTGACAGG AGTGACCAAG CGAACGCCAC

---------- ---------- ---------- ---------- ----------

ACGCCTACCT GTAGTGCCCT CGGTAACTGG AGTGACCAAG CGCATGCCAC

ACTCCTACCT GTGGTACCCT CTGTGACTGG GGTGACAAGC CGCACCCCAC

ACGCCTGGAG GTCGTGCACT TCGTGATCAG GGTCACCGGG CTGAGACACG

CCATGGGCAC AGTGCGGAGG AGCACCACCT GGACACCAGA TCACCTGGGC

CCATGGGCAC AGTGCGGAGG AGCACCACCT GGACACCAGA TCACCTGGGC

CCATGGGCAC AGTGCGGAGA AGCACCACCT GGACACCAGA TCACCTGCGC

CCATGGGCAC AGTGCGGAGA AGCACAACCT GGACACCAGA TCACCTGCGC

CCATGGGCAC AGTGCGGAGG AGCACCACCT GGACACCAAA TCACCTGGGC

CCATGGGCAC GGTGCGGAGG AGCACCACCT GGACACCAGA TCACCTGGGC

CCATGGGCAC AGTGCGGAGG AGCACCACCT GGACACCAGA TCACCTGGGC

CCATAGGCAC AGTACGGAGG AGCACCACCT GGACAGCAGA TCACCTGGGC

CCATGGGCAC GGTGCGGAGG AGCACCACCT GGATAGCAAA TCACCTGGGC

---------- ---------- ---------- ---------- ----------

CCATGGGCAC GGTGCGGAGG AGCACCACCT GGATGCCAGA TCACCTGAGC

CCATGGGCAC GGTGCGGAGG AGCACCACCT GGACAACAGA TCACCTGGGC

CCATGGGCAC TGTGCGCAGG AGCACCACCT GGACACCAGA TCACCTGGGC

CCATGGGCAC CGTGCGGAGG AGCACCACCT GGATGCCAGA TCACCTGGGC

---------- ---------- ---------- ---------- ----------

CCATGGGCAC CGTGCGAAGG AGCACCACCT GGACACCAGA TCACCTGGGC

CCATGGGAAC TGTTCGGAGG AGCACCACCT GCACACCAAA TTACCTGGGC

GGCTGGGAGC AGAGAGGAAG AGAGTCAACG GCACCCCAAG TCTCGTGAGC

CAGTGGCAAT TTTGGGGACA GAGTTCCTGA AGTCATCACC TTGGGGTGAT

CAGTGGCAAT TTTGGGGACA GAGTT----- ---------- ----------

CAGTGGCAAT TTTGGGGACA GAGTTCCTGA AGTCATCACC TTGGGGTGAT

CAGTGGCAAT TTTGGGGACA GAGTTCCTGA AGTCATCACC TTGGGGTGAT

CAGTGGCAAT TTTGGGGACA GAGTTCCTGA AGTCATAACC TTGGGGTGAT

CAGTAGCAAT TTTGGGGACA GAGTTCCTGA AGTCATAACC TTGGGGTGAT

CAGTAGCAAT CTTGGGGACA GTGTTCCTGA AGTCATCACC TTGGTGTGAT

CAGTAGCAAT CTTGGGGACA GTGTTCCTGA AGTCATCACT TTGGTGTAAT

CAGTGGCAAT CTTGGGGACA GAGTTCCTGA AGTCATAACC TTGGGGTGAT

---------- ---------- ---------- ---------- ----------

CAGTGGCGAT TTTGGGGACA GAGTTCCAGA AGTCATCACC TTGGGGTGAT

CAGTGGCAAT TTTGGGAACA GAGTTCCCGA AGTCATAACC TTGGGGTGAT

CGGTGGCAAT CTTGGGGACA GAGTGCCAGA AGTCATGACC TTGGGGTGAT

CAGTGGCAAT CTTGGGGACA GGGTTCCTGA GGTCATGACC TTGGGGTGAT

---------- ---------- ---------- ---------- ----------

CAGTGGCAAT CTTAGGGACA GAGTTCCTGA TGTCATAACT TTGGGGTGAT

CAGTAGCGAT TTTGGGGACA GTGTGCCAGA TGTCATGACC TTTGGGTGAT

AACTTGGGTT TTTGGGGAGT GTGCCGCTCA AGCAATTACC CTGGGGAAAT

GGGGGACTTG ATGCCAGGAC TGCTGGTCAC ACCTGCCTGG GTGATGATGG

---------- ---------- ---------- ----GCCTGG GTGATGATGG

GGGGGACTTG ATGCCAGGAC TGCTGGTCAC ACCTGCCTGG GTGATGATGG

GGGGGACTTG ATGCCAGGAC TGCTGGTCAC ACCTGCCTGG GTGATGATGG

GGGGGACTTG ATGCCAGGAC TGCTGGTCAC ACCTGCCTGG GTGATGATGG

GGGGGACTTG ATGCCAGGAC TGCTGGTCAC GCCTGCCTGG GTGATGATGG

GGGGGACTTA ATGCCAGGAC TGCTGGTAAC ACCTGCCTGG GTGATAATTG

TGGGGACTTA ATGCCAGGAC TGCTGGTAAC ACCTGCCTGG GTGATAATGG

GGGGGACTTG ATTCCAGGAC TGCTCGTCAC ACCTGCCTGG GTGATGATGG

---------- ---------- ---------- ---------- ----------

GGGGGACTTG ATGCCAGGAC TGCTGGTCAC ACCTGCCTGT GTGATGATGG

AGGGGACTTG ATGCCAGGAG TACTGGTCAC ACCTGCCTGC GTGATGATGG

TGGGGACTTG ATGCCGGGAG TGCTGGTCAC ACCTGCCTGC GTGATGATGG

GGGGGACTTG ATGCCGGGGC TGCTGGTCAC GCCTGCCTGC GTGATGATGG

---------- ---------- ---------- ---------- ----------

AGGGGATTTG ATGCCCGGAC TGCTGGTCAC ACCTGCCTGG GTAATGATGG

TGGGGATTTG ATGCCAGGGC TGCTGGTTAC ACCTGACTGG GTGATGATAG

GGGAGAGCCA ATCACACGAG GGATGGTCAA ATCTGCCTGC ATGGTGATGG

CCGACATGGG GATGGTTTTG ATGATGGTGG TCGTGCCGGG CTTGGTGGTA

CCGACATGGG GATGGTTTTG ATGATGGTGG TCGTGCCGGG CTTGGTGGTA

CCGACATGGG GATGGTTTTG ATGATGGTGG TCGTGCCGGG CTTGGTGGTA

CCGACATGGG GATGGTTTTG ATGATGGTGG TTGTGCCAGG CTTGGTGGTA

CCGACATGGG GATGGTTTTG ATGATGGTGG TCGTGCCAGG CTTGGTGGTA

CTGACATGGG TATGGTCTTG ATGATGGTGG TCGTGCCCGG CTTGGTGGTG

CTGACATGGG AATGGTCTTA ATGATGGTAG TTGTGCCGGG TTTGGTGGTG

CCGACATAGG AATGGTCTTA ATAATGGTAG TTGTGCCAGG TTTGGTGGTG

CTGACATGGG GATGGTCTTG ATGATGGTGG TCGTGCCGGG TTTGGTAGTA

---------- ---------- ---------- ---------- ----------

CTGACATGGG GATGGTCTTG ATGATGGTGG TCGTGCCAGG TTTGGTGGTG

CTGACATGGG GATGGTCTTG ATGATGGTGG TGGTGCCGGG CTTGGTGGTG

CGGACATGGG AATGGTCTTG ATGATGGTGG TCGTGCCAGG CTTGGTGGTG

CCGACATGGG GATGGTCTTG ATGATGGTGG TGGTGCCAGG CTTGGTGGTG

---------- ---------- ---------- ---------- ----------

CCGACATGGG GATGGTCTTG ATGATAGTGG TGGTGCCGGG CTTGGTGGTG

CCGACATGGG GATGGTTTTG ATGATGGTGG TAGTGCCCGG TTTGGTGGTG

CTGCCGTCAG GGTAGTTTGG GCGACCGTGA TGGCGGTCGG GTTGATCACA

CTGGGGGAGA CGCTGCTGAT GCCCAGGATG GTGGGCTTGG TCCCCGCCCC

CTGGGGGAGA CGCTGCTGAT GCCCAGGATG GTGGGCTTGG TCCCCGCCCC

CTGGGGGAGA CGCTACTGAT GCCCAGGATG GTGGGCTTGG TCCCCGCCCC

CTGGGGGAGA CGCTACTGAT GCCCAGGATG GTGGGCTTGG TCCCCGCCCC

CTGGGGGAGA CGCTGCTGAT GCCCAGGATG GTGGGCTTGG TCCCCGCCCC

CTGGGGGACA CGCTGCTGAT GCCCAGGATG GTGGGCTTTG CCCCTGCCCC

CTAGGAGAGA CACTACTGAT GCCCAGGATA GTGGGCTTGG TCCCTGCCCC

CTGGGAGAAA CACTACTGAT GCCCAGGATA GTGGGCTTGG TCCCTGCCCC

CTGGGGGAGA CGCTGCTGAT GCCCAGGATG GTGGGCTTGG TTCCCGCCCC

---------- ---------- ---------- ---------- ----------

CTTGGGGACA CGCTGCTGAT GCCCAGAATG GTGGGCTTTG TCCCAGCCCC

CTGGGGGACA CGCTGCTGAT GCCCAGGATG GTGGGCTTAG TCCCAGCCCC

CTGGGGGACA CACTGCTGAT GCCCCAGGAT GTAGGTTTAG TCCCGCCCCC

CTGGGGGAGA CGCTGCTGAT GCCCAGGATG GTGGGCTTGG TCCCCGCCCC

---------- ---------- ---------- ---------- ----------

CTGGGCGAGA CGCTGCTGAT ACCCAGGATG GTCGGCTTAC TGCCTGCCCC

TTGGGGGAGA CACTGCTGAT GCCCAAGATG GTGGGTTTGG TTCCAGTCCC

CTTGAGAGGG TGGCAATGGT ACCCAGGGTG GTGGGAGTGG CCAGAGAGGC

ACTGGCCTGC GTGGTAGTGA TGATGGTGGT GGGCTTGCCA GTCACCAGCT

ACTGGCCTGC GTGGTAGTGA TGATGGTGGT GGGCTTGCCA GTCACCAGCT

GCTGGCCTGC GTGGTAGTGA TGATGGTGGT GGGCTTGCCA GTCACCAGCT

ACTGGCCTGC GTGGTAGTGA TGATGGTGGT GGGCTTGCCA GTCACCAGCT

GCTGGCCTGT GTGGTAGTGA TGATGGTGGT GGGCTTGCCA GTCACCAACT

ACTGGCCTGT GTGGTGGTGA TGATGGTAGT AGGCTTGCCA GTCACCAGCT

ACTAGCCTGT GTGGTGGTGA TGATGGTTGT GGGCTTGCCA GTCACCAACT

ACTAGCCTGT GTGGTGGTAA TGATGGTTGT GGGCTTGCCA GTCACCAGCT

ACTGGCCTGC GTGGTAGTGA TGATGGTGGT AGGCTTGCCA GTCACCAGCT

---------- ---------- ---------- ---------- ----------

ACTGGCCTGC GTGGTGGTGA TGATGGTGGT GGGCTTGCCA GTCACTAGCT

GCTGGCCTGT GTGGTAGTGA TGATGGTGGT GGGCTTGCCA GTCACCAGTT

ACTGGCCTGT GTGGTAGTGA TGATGGTGGT GGGCTTGCCA GTCACTAGCT

GCCGGCCTGC GTGGTGGTGA TGATGGTGGT GGGCTTGCCG GTCACCAGCT

---------- ---------- ---------- ---------- ----------

ACTGGCCTGT GTGGTGGTGA TGATGGTGGT GGGCTTGCCG GTCACCAGCT

GCTGGCCTGG GTGGTGGTGA TGATAGTGGT AGGCTTGCCA GTTACCAGCT

ATTGGTCCGC GCGCCAGCGA GGCTGGTGGA CACGGTGCCA GTCACAGTGC

TCAGGATCTG GATGATCTGA GTCACAGGAC CCGTGGACGC CTGGCCTGTG

TCAGGATCTG GATGATCTGA GTCACAGGAC CCGTGGACGC CTGGCCTGTG

TCAGGATCTG GATGATCTGA GTCACAGGAC CCGTGGACGC CTGGCCTGTG

TCAGGATCTG GATGATCTGA GTCACAGGAC CCGTGGACGC CTGGCCTGTG

TCAGGATCTG GATGATCTGA GTCACAGGAC CTGTGGACGC CTGGCCTGTG

TCAGGATCTG GATGATCTGA GTCACAGGGC CTGTGGATGC CTGGCCAGTG

TCAGGATCTG GATGATCTGA GTCACAGGAC CTGTAGATGC TTGGCCTGTC

TCAGGATCTG GATGATCTGA GTCACAGGAC CTGTAGATGC TTGGCCTGTC

TCAGGATCTG GATGATCTGA GTCACGGGGC CCGTAGATGC CTGACCTGTA

---------- ---------- ---------- ---------- ----------

TCAGGATCTG GATGATCTGA GTCACCGGGC CCGTAGATGC CTGGCCTGTG

TCAGGATCTG GATGATCTGG GTCACCGGGC CTGTAGATGC CTGGCCTGTG

TCAGAATCTG AATGATCTGG GTCACTGGGC CTGTGGATGC CTGGCCCGTG

TGAGGATCTG GATGATCTGC GTCACGGGGC CCGTCGAGGC CTGGCCCGTG

---------- ---------- ---------- ---------- ----------

TCAGAATCTG GATGATCTGT GTCACTGGGC CAGTTGAGGC CTGGCCCGTT

TCAGAATCTG GATGATCTGA GTCACAGGGC CAGTAGAAGC CTGGCCTGTG

CCAGGGTCTG GATGATTTGA GTCACGGGGC CGGTCGAAGC CTGGCCCGTG

ACTGCTGAAG TCTGAACTGG TTTGGTCTGG ACCACCGACA TCACTTTGCC

ACTGCTGAAG TCTGAACTGG TTTGGTCTGG ACCACCGACA TCACTTTGCC

ACTGCTGAAG TCTGAACTGG TTTGGTCTGG ACCACCGACA TCACTTTGCC

ACTGCTGAAG TCTGAACTGG TTTGGTCTGG ACCACCGACA TCACTTTGCC

ACTGCTGAAG TCTGAACTGG TTTGGTCTGG ACCACTGACA TCACTTTGCC

ACTGCTGAAG TCTGAACTGG CTTGGTCTGG ACCACTGACA TCACTTTGCC

ACTGCTGAAG TCTGAACTGG TTTGGTCTGG ACCACTGACA TCACTTTTCC

ACTGCTGATG TCTGAACTGG TTTGGTCTGG ACCACCGACA TCACTTTTCC

ACCGCTGAAG TCTGAACTGG TTTGGTCTGG ACTACTGACA TCACTTTGCC

---------- ---------- ---------- ---------- ----------

ACCGCTGACG TTTGAACTGG TTTGGTCTGA ACCACTGACA TCACTTTGCC

ACTGCCGAAG TCTGAACTGG TTTGGTTTGC ACCACTGACA TCACTTTGCC

ACTGCGGATG TCTGAACAGG TTTGGTCTGG ACCACAGACA TCACCTTGCC

ACCGCTGAGG TCTGAACAGG CTTGGTCTGC ACCACTGACA TCACCTTGCC

---------- ---------- ---------- ---------- ----------

ACGGCGGATG TCTGCACGGG CTTGGTCTGC ACAACAGACA TGACTTTGCC

ACTGCTGAGG TCTGAACCGG TTTGGTCTGA ACAACTGACA TCACTTTGCC

ACTGCAGAGG TCTGGACCGG CTTGGTCTGA ACAACCGACA TCACTTTGCC

CAGATTGGAA ATCTAAAAAG AAGGGCTCAC CAGAGCACTG CCTCCTGGGA

CAGATTGGAA ATCTAAAAAG AAGGGCTCAC CAGAGCACTG CCTCCTGGGA

CAGATTGGAA ATCTAAAAAG AAGGGCTCAC CAGAGCGCTG CCTCCTGGGA

CAGATTGGAA ATCTAAAAAG AAGGGCTCAC TAGAGCGCTG CCTCCTGGGA

AAGATTGGAA ATCTAAAAAA AAAGGCTCAC CAGAGCGCTG CCTCCTGGGA

CAGATTAGAA ATCTGAAAAG AAAGGCTCAC CAAAGCACTG CCTCCTGGGA

AAGATTGGAA ATCTAAAAAG AAAGGCTCAC CAGAGCACTG CCTCCCGGGA

CAGATTGGAA ATCTAAAAAG AAAGGCTCAC CAGAGCACTG CCTCCTGGGA

CAGATTGGAA ATCTAGAAAG AAAGACTCAC CAGAGCACTG CCTCCTGGGA

---------- ---------- ---------- ---------- ----------

AAGGTTGGAA ATCTAAAAGG AAAGGCTCAC CAGAGCGCTG CCTCCTGGGA

CAGGTTGGAA ATCTAAAAGG AAAGGCTCAC CAGAGCACTG CCTCCTGGGA

GAGATTAGAA ATCTGCAAAG GAAGGCTCAC CAAAGCACTG CCTCCGGGGA

GAGGTTAGAG ATCTGCGGAG GGAGGcTCAC CAGGGCGCTG CCTCCCGGGA

---------- ---------- ---------- ---------- ----------

GAGGTTGGAG ATCTACACAG AAAGGCTCAC CAGAGCACTG CCTCCGGGAA

CAGATTAGAA ATCTGTAAAG AAATGCTTAC CAGTGCACTG CCCCCTGGGA

CAGATTGGAA ATCTGGAAAG AAAGGCTCAC CAGAGCACTG CCCCCTGGGA

CAGAGATGGG GCTCTTCACC AGGGTGATGG TCTTGGTGAC CCCGCCCACA

CAGAGATGGG GCTCTTCACC AGGGTGATGG TCTTGGTGAC CCCGCCCACA

CAGAGATGGG GCTCTTCACC AGGGTGATGG TCTTGGTGAC CCCGCCCACG

CAGAGATGGG GCTCTTCACC AGGGTGATGG TCTTGGTGAC CCCGCCCACG

CAGAGATGGG GCTCTTCACC AGAGTGATGG TCTTGGTGAC CCCACCCACG

CAGAGATGGG GCTTTTCACC AAGGTGATGG TCTTGGTGAC CCCGCCCACC

CAGAGATGGG GCTCTTCACT AGAGTGATGG TCTTGGTGAC TCCGCCTACC

CAGAGATGGG GCTCTTCACT AGGGTGATGG TCTTGGTGAC TCCACCTACC

CAGAGATGGG GCTCTTCACG AGGGTGATGG TCTTGGTGAC CCCACCCACC

---------- ---------- ---------- ---------- ----------

CAGAGATGGG GCTCTTCACC AGGGTGATGG TCTTGGTGAC TCCACCCACC

CGGAAATGGG GCTCTTCACC AGGGTGATGG TCTTGGTGAC CCCACCCACG

CAGAGATGGG ACTCTTCACC AGGGTGATGG TCTTGGTGAC TCCACCCACC

CCGAGATGGG GCTCTTCACC AGGGTGATGG TCTTGGTGAC ACCACCCACC

---------- ---------- ---------- ---------- ----------

CCGAGATGGG GCTCTTCACC AAAGTGATGG TCTTGGTCAC GCCACCCACC

CAGAGATGGG GCTCTTGACC AGAGTGATGG TTTTGGTGAC CCCACCGACC

CGGAGATGGG GCTCTTCACT AGCGTGATGG TTTTGGTGAC TCCGCCCACT

ACTGTGGTCA CCACCTGGGC TTGCTGGGCC ACTGTCACAG TGCCTGACTT

ACTGTGGTCA CCACCTGGGC TTGCTGGGCC ACTGTCACAG TGCCTGACTT

ACTGTGGTCA CCACCTGGGC TTGCTGGGCC ACCGTCACAG TGCCTGACTT

ACTGTGGTCA CCACCTGGGC TTGCTGGGCC ACTGTCACAG TGCCTGACTT

ACTGTGGTCA CCACCTGGGC TTGCTGGGCC ACTGTCACAG TGCCTGACTT

ACCGTGGTGA CCACCTGGGC TTGCTGGGCC ACTGTCACAG TCCCTGACTT

ACCGTGGTCA CCACCTGGGC TTGCTGGGCC ACTGTTACAG TCCCTGATTT

ACCGTGGTCA CCACCTGGGC TTGCTGGGCC ACTGTTACAG TTCCTGATTT

ACTGTGGTCA CCACCTGGGC TTGCTGGGCC ACTGTCACAG TCCCCGACTT

---------- ---------- ---------- ---------- ----------

ACCGTGGTCA CCACCTGGGC TTGCTGGGCC ACAGTCACAG TCCCTGATTT

ACTGTGGTCA CCACCTGGGC TTGCTGGGCC ACAGTCACAG TCCCGGACTT

ACTGTGGTCA CGACCTGGGC TTGCTGGGCT ACGGTTACGG TCCCGGACTT

ACGGTGGTCA CCACCTGGGC TTGCTGGGCG ACCGTCACCG TCCCCGACTT

---------- ---------- ---------- ---------- ----------

ACAGTGGTCA CCACCTGGGC TTGCTGTGCC ACGGTGACTG TCCCGGATTT

ACGGTGGTCA CCACCTGGGC TTGCTGGGCC ACAGTCACAG TGCCAGATTT

ACAGTGGTCA CCACCTGGGC TTGCTGGGCC ACTGTCACGG TCCCCGACTT

GTGCACTGTG ATGATAGGGC GGGTAGACGT GTTGGGTCCC CACCTGGGCG

GTGCACTGTG ATGATAGGGC GGGTAGACGT GTTGGGTCCC CACCTGGGCG

GTGCACTGTG ATGATAGGGC GGGTAGACGT GTTGGGTCCC CACCTGGGCA

GTGCACTGTG ATGATAGGGC GGGTAGACGT GTTGGGTCCC CACCTGGGCA

GTGCACCGTG ATGATAGGGC GGGTAGATGT GTTGGGTTCC CACCTGGGCA

GTGCACCGTG ATGATAGGGC GGGTAGACGT GTTGGGTTCC CACCTGGGCG

ATGTACTGTG ATGATAGGGC GGGTAGATGT GTTGGGTCCC CACTTGGGCA

GTGTACTGTG ATGATAGGGC GAGTAGATGT GTTGGGTCCC CACTTGGGCA

GTGCACCGTG ATGATAGGGC GGGTAGATGT GTTGGGTCCC CACCTGGGCA

---------- ---------- ---------- ---------- ----------

GTGGACTGTG ATGATGGGGC GAGTGGATGT GTTGGGTCCC CACCTGGGCG

GTGCACTGTG ATGATGGGGC GAGTGGATGT GTTGGGTCCC CACCTGGGCG

GTGCACCGTG ATGATAGGGC GCGTGGATGT GTTGGGTCCC CACCTGGGCG

GTGCACCGTG ATGATGGGGC GGGTGGACGT GTTGGGTGCC CACCTGGGCG

---------- ---------- ---------- ---------- ----------

GTGCACAGTG ATGATAGGTC GAGTGGACGT GTTGGGTCCC CACCTGGGCG

ATGGACGGTG ATGATGGGGC GGGTGGGAGT ATTAGGTGCC TACTTGGGCT

GTGCACGGTG ATGATGGGTC GCGTGGGCGT ATTAGGTGCC CACCTGAGCC

GCTGCAGTCT TCAGCATGCG AGTGGCAGGG TTGCTCACCT CTCACCATGA

GCTGCAGTCT TCAGCATGCG AGTGGCAGGG TTGCTCACCT CTCACCATGA

GCTGCAGTCT TCAGCATGCG AGTGGCAGGG TTGCTCACCT CTCACCATGA

GCTGCAGTCT TCAGCATGCG AGTGGCAGGG TTGCTCACCT CTCACCATGA

GCTGCTGTCT TCAGCATGCG AGTGGCAGGG TTGCTCACCT CACACCATGA

GCTGCAGTCT TCAGCATTCG GGTGGCTGGG TTGCTCACCT CTCACCATGA

GCTGCAGTCT TTAGCATTCG AGTAGCTGGG TTGCTCACCT CTCACCATAA

GCTGCAGTCT TTAGCATTCG AGTGGCTGGG TTGCTCACCT CTCACCATGA

GCTGCAGTCT TCAGCATGCG GGTGGCCGGG TTGCTCACCT CTCACCATGA

---------- ---------- ---------- ---------- ----------

GCTGCAGTCT TCAGCATTCG AGTGGCTGGG TTGCTCACCT CTCACCATGA

GCTGCAGTCT TTAGCATGCG AGTGGCTGGG TTGCTCACCT CTTACCATGA

GCGGCAGTCT TCAGCATGCG TGTGGCCGGG TTGCTCACCT CTCACCATGA

GCAGCGGTCT TGAGCATGCG TGTGGCCGGG TTGCTCACCT CTCACCATGA

---------- ---------- ---------- ---------- ----------

GCAGCAGTCT TCAGCATGCG GGTGGCAGGG TTGCTCACCT CTCACCATGA

GCTGCTGTCT TCAACATTCT GGTGGCTGGG TTGCTCACCT CTCACCATGA

GCTGCTGTCT TCAACATCCG GGTGGCTGGG TTGCTCACCT CTAACCATGA

CTGGCGAGGA GGCCACCTTC ACAGTGGCTG GGAGGGTGGT AGTGCCAGGT

CTGGCGAGGA GGCCACCTTC ACAGTGGCTG GGAGGGTGGT AGTGCCAGGT

CTGGCGAGGA GGCCACCTTC ACAGTGGCTG GGAGGGTGGT AGTGCCAGGT

CTGGCGAGGA GGCCACCTTC ACAGTGGCTG GGAGGGTGGT AGTGCCAGGT

CTGGCGAGGA GGCCACCTTC ACAGTGGCTG GGAGGGTGGT AGTGCCAGGT

CTGGTGAGGA GGCCACCTTC ACAGTGGCTG GGAGGGTAGT AGTGCCAGGT

CAGGTGAGGA GGCCACCTTC ACAGTGGCTG GAAGAGTGGT TGTACCAGGT

CAGGGGAGGA GGCCACCTTC ACAGTGGCTG GAAGAGTGGT CGTGCCAGGT

CTGGCGAGGA GGCCACCTTC ACAGTAGCCG GGAGGGTGGT AGTGCCGGGT

---------- ---------- ---------- ---------- ----------

CTGGTGAGGA GGCCACCTTT ACAGTAGCCG GGAGGGTCGT GGTGCCAGGT

CTGGTGAGGA GGCCACCTTC ACAGTGGCCG GGAGGGTGGT AGTGCCAGGT

CAGGTGAGGA GGCCACCTTC ACCGTGGCGG GGAGGGTAGT GGTGCCAGGC

CGGGAGAGGA GGCCACTTTC ACCGTGGCCG GGAGTGTGGT GGTGCCGGGG

---------- ---------- ---------- ---------- ----------

CGGGTGAGGA GGCCACCTTC ACCGTGGCTG GCAGGGTGGT GGTGCCGGGA

CTGGGGAGGA GGCCACCTTC ACTGTGGCTG GAAGTGTGGA GGTGCCAGGA

CGGGAGAGGA GGCCACCTTA ACGGTGGTTG GGAGCGTGGT GGTTCCAGGC

GTCACAGCCA TGGTCTTCAC GATGGTGGTA CCCGCTGGGA CACTCAGCAC

GTCACAGCCA TGGTCTTCAC GATGGTGGTA CCCGCTGGGA CACTCAGCAC

GTCACAGCCA TGGTCTTCAC GATGGTGGTG CCCGCTGGGA CACTCAGCAC

GTCACAGCCA TGGTCTTCAC GATGGTGGTG CCCGCTGGGA CACTCAGCAC

GTCACAGCCA TGGTCTTCAC GATGGTGGTG CCCGCGGGGA CACTCAGAAC

GTCACAGCCA CAGTCTTGAC AATGGTGGTA CCTGCTGGGA CACTCAGCAC

GTCACAGCCA CTGTCTTGAC AATGGTGGTG CCTGCTGGGA CACTTAGCAC

GTCACAGCCA CTGTCTTGAC GATGGTGGTC CCTGCTGGGA CACTCAGCAC

GTCACGGCCA CGGTTTTGAC AATGGTGGTG CCTGCTGGGA CACTCAGCAC

---------- ---------- ---------- ---------- ----------

GTCACGGCCA CGGTCTTGAC GATGGTGGTG CCCGCCGGGA CGCTCAGCAC

GTTACGGCCA CGGTTTTCAC AATGGTGGTG CCTGCTGGGA CACTCAGCAC

GTCACGGCTA CAGTCTTAAC GATCGTGGTG CCTGCCGGGA CGCTCAGCAC

GTCACTGCCA CGGTTTTGAC GATGGTGGTG CCCGCTGGCA CGCTCAGCAC

---------- ---------- ---------- ---------- ----------

GTCACAGCCA CAGTTTTCAC GATGGTAGTG CCGGCAGGCA CACTAAGCAC

GACACTGCTA CAGTTTTGAC AATGGTGGTC CCTGCAGGGA CACTCAGTAC

GACACTGCCA CAGTTTTGAC GATGGTGGTG CCTGCAGATA CACTCAACAC

CGTGGGTGCC GAGGAAGGGG GGATCTTCTG GGTGGCAGCG GCCGCAGCGG

CGTGGGTGCC GAGGAAGGGG GGATCTTCTG GGTGGCAGCG GCCGCAGCGG

CGTGGGTGCC GAGGAAGGGG GGATCTTCTG GGTGGCAGCA GCCGCAGCGG

AGTGGGTGCC GAGGAAGGGG GGATCTTCTG GGTGGCAGCA GCCGCAGCGG

TGTGGGTGCC GAGGAAGGGG GTATCTTCTG GGTGGCAGCA GCCGCAGCAG

TGTGGGCGCT GAGGAAGGCG GGATCTTCTG GGTGGCAGCA GCTGCAGCTG

TGTGGGTGCT GAGGAAGGAG GGATTTTCTG TGTGGCAGCA GCAGCAGCAG

CGTGGGTGCT GAGGATGGAG GGATTTTCTG TGTGGCAGCA GCAGCAGCAG

TGTGGGCGCC GAGGAAGGAG GGATCTTCTG GGTGGCAGCG GCAGCAGCTG

---------- ---------- ---------- ---------- ----------

AGTGGGTGCT GAGGAAGGAG GGATCTTCTG GGTGGCGGCA GCTGCAGCTG

TGTGGGCGCC GAGGAAGGAG GGATCTTCTG AGTGGCGGCT GCTGCAGCCG

TGTGGGAGCC GAGGACGGAG GGATCTTCTG GGTGGCCGCA GCAGCAGCCG

CGTGGGGGCC GAGGAAGGAG GGATCTTCTG TGTGGCAGCC GCGGCCGCCG

---------- ---------- ---------- ---------- ----------

GGTGGGTGCT GAGGAGGGCG GGATCTTCTG GGTGGCCGCT GCTGCGGCTG

TGTGGGAGCT GAGGAAGGAG GGATCTTCTG GGTGGCTGCA GCTGCTGCGG

AGTGGGGGCT GAGGAAGGAG GGATTTTTTG GGTGGCAGCT GCTGCTGCTG

CCAGTGCGGC CATCCCACTC ATCTGTGGGC TACTGCCAAT CACCTCTCAC

CCAGCGCGGC CATCCCACTC ATCTGTGGGC TACTGCCAAT CACCTCTCAC

CCAGTGCGGC CATCCCACTC ATCTGTGGGC TACTGCCAAT CACCTCTCAC

CCAGTGCGGC CATCCCACTC ATCTGTGGGC TACTGCCAAT CACCTCTCAC

CCAGTGCGGC CATCCCACTC ATCTGTGGGC TACTGCCAAT CACCTCTCAC

CCAGCGCAGC CATCCCACTC ATCTGCGGGC TGCTACCGAT CACCTCTCAC

CCAATGCAGC CATCCCACTC ATCTGTGGGT TGCTGCCAAT CACCTCTTAC

CCAATGCGGC CATCCCACTC ATCTGTGGGT TGCTGCCGAT CACCTCTCAC

CCAGCGCAGC CATCCCGCTC ATCTGGGGGT TACTGCCGAT GACCTCTCAC

---------- ---------- ---------- ---------- ----------

CCAGCGCAGC CATGCCACTC ATCTGTGGGC TGCTGCCAAT CACCTCTCAC

CCAACGCAGC CATGCCACTC ATCTGTGGGT TGCTGCCAAT CACCTCTCAC

CCAGGGCAGC CATCCCACTC ATCTGTGGGT TGCTGCCAAT CACCT-----

CCAGTGCAGC CATCCCACTC ATCTGCGGGC TGCTGCCGAT CACCTCTCAC

---------- ---------- ---------- ---------- ----------

CCAGCGCTGC CATTCCGCTC ATCTGTGGGT TGCTGCCAAT CACCTCTCAC

CCAGGGCTGC CATGCCACTC ATCTGAGGGT TGCTGCCAAT CACCTCTCAC

CCAGAGCTGC CATGCCACTC ATTTGAGGGC TGCTGCCAAT CACCTCTCAC

CGTTCCCTGG GCACTCTGTG TTGGCACAAC CATCCGCACT CCGGCGGGAA

CGTTCCCTGG GCACTCTGTG TTGGCACAAC CATCCGCACT CCGGCGGGAA

CGTCCCCTGG GCACTCTGTG TTGGCACAAC CATCCGCACT CCAGCGGGCA

CGTCCCCTGG GCACTCTGCG TTGGCACAAC CATCCGCACT CCAGCAGGCA

CGTCCCCTGG GCACTCTGTG TTGGCACAAC CATCCGTACT CCGGCAGGCA

CGTCCCCTGG GCACTCTGTG TGGGCACAAC CATTCTCACA CCAGCGGGCA

CGTGCCCTGG GCACTCTGAG TGGGTACAAC CATTCTCACA CTGGCAGGCA

CGTCCCCTGG GCACTCTGTG TGGGTACAAC CATTCGAACA CTGGCAGGCA

CGTCCCTTGG GCACTCTGCG TTGGCACAAC CATTCGCACG CCAGCGGGAA

---------- ---------- ---------- ---------- ----------

CGTCCCCTGG GCGCTCTGCG TTGGCACAAC CATTCGCACG CCTGCAGGAA

CGTGCCCTGG GCACTCTGAG TCGGCACGAC CATCCGCACG CCTGCAGGAA

---------- ---------- ---------- ---------- ----------

CGTGCCCTGG GCACTCTGGG TTGGCACAAC CATTCGCACC CCCGCAGGCA

---------- ---------- ---------- ---------- ----------

CGTGCCCTGG GCACTCTGTG TGGGCACGAC CATTCGCACC CCCGCAGGCA

TGTTCCCTGG GCACTTTGTG TCGGTACAAC CATTCGGACA CCGGCGGGCA

AGTTCCCTGG GCACTCTGCG TCGGCACAAC CATCCGTACG CCGGCGGGCA

GGGAGGTCAC GGTGACAGGG GCTTTCCCAG CCTGGCTGGC AGGTCGCATG

GGGAGGTCAC GGTGACAGGG GCTTTCCCAG CCTGGCTGGC AGGTCGCATG

GGGAGGTCAC GGTGACAGGG GCTTTCCCGG CCTGGCTGGT AGGTCGCATG

GGGAGGTCAC GGTGACAGGG GCTTTCCCGG CCTGGCTGGT AGGTCGCATG

GGGAGGTCAC GGTGACGGGG GCTTTCCCGG CCTGGCTGGC AGGTCGCATG

GGGAGGTCAC AGTGACAGGG GCTTTCCCGG CCTGGCTGGC AGGTCGCATG

GGGAAGTCAC AGTGACAGGA GCTTTTCCAG CCTGGCTTGC AGGTCTCATG

GGGAAGTCAC AGTGACAGGA GCTTTTCCAG CCTGGCTTGC AGGTCTCATG

GGGAGGTCAC AGTGACAGGG GCTTTCCCAG GTTGGCTGGC AGGTCGCATA

---------- ---------- ---------- ---------- ----------

GGGAGGTCAC GGTGACGGGG GCTTTCCCAG CCTGGCTGGC AGGTCGCATG

GGGAGGTCAT GGTGACGGGG GCTTTCCCAG CCTGGCTGGC AGGTCGCATT

---------- ---------- ---------- ---------- ----------

GGGAGGTCAC AGTGACGGGG GCTTTCCCAG CCTGGCTGGC GGGCCGCATA

---------- ---------- ---------- ---------- ----------

GGGAGGTCAC AGTGACAGGA GCTTTCCCAG CCTGACTGGC AGGGCGCATA

GCGAAGTCAC AGTAACTGGA GCTTTTCCAG GTTGGCTGGC TGGTCGAACA

GCGAGGTCAC AGTAACCGGG GCTTTGCCAG CCTGGCTAGC TGGCCTCACC

GTGACCAATG GAGTTCCTGT TGTAGCCTGA GGACCGGTCA CTTTGAGAAC

GTGACCAATG GAGTTCCTGT TGTAGCCTGA GGACCGGTCA CTTTGAGAAC

GTGACCAATG GAGTTCCTGT TGTAGCCTGA GGACCGGTCA CTTTGAGAAC

GTGACCAATG GAGTTCCTGT TGTAGCCTGA GGACCGGTCA CTTTGAGAAC

GTGACCAATG GTGTTCCTGT TGTAGCCTGA GGACCGGTCA CTTTGAGAAC

GTGACCAACG GGGTTCCTGT TGTAGCCTGA GGACCGGTTA CCTTGAGAAC

GTGACCAGTG GTGTTCCTGT TGTAGCTTGA GGACCAGTCA CTTTGAGAAC

GTAACCAGTG GTGTTCCTGT TGTAGCTTGA GGACCAGTCA CTTTGAGAAC

GTGACCAATG GGGTTCCTGT TGTAGCCTGA GGTCCAGTCA CTTTGAGAAC

---------- ---------- ---------- ---------- ----------

GTGACCAATG GGGTTCCTGT TGTAGCCTGA GGACCGGTGA CTTTGAGAAC

GTGACCAACG GGGTTCCTGT TGTAGCCTGA GGACCGGTAA CTTTGAGAAC

---------- ---------- ---------- ---------- ----------

GTGACCAGCG GGGTTCCTGT GGTAGCCTGA GGACCAGTCA CTTTGAGGAC

---------- ---------- ---------- ---------- ----------

GTGACCAGGG GGGTTCCTGT GGTAGCCTGA GGCCCGGTGA CTTTGAGCAC

GTGACTAGGG GAGTTCCTGC TGGAGCCTGG GGGCCAGTCA CTTTGAGAAC

GTCACCAGGG GGGCTCCTGT GGCCGCCTGG GGACCGGTCA CTTTGAGGAC

AGCAGGGACA CCTGAGGCAA GACCTGGATG GTGGTGGTGG TCGGGGGTGC

AGCAGGGACA CCTGAGGCAA GACCTGGATG GTGGTGGTGG TCGGGGGTGC

AGCAGGGACA CCTGAGGCAA GACCTGGATG GTGGTGGTGG TCGGGGGCGC

AGCAGGGACA CCTGAGGCAA GACCTGGATG GTGGTGGTGG TCGGGGGCGC

AGCAGGGACA CCTGAGGCAA GACCTGGATG GTGGTGGTGG TCGGGGGCGC

AGCAGGGACA CCTGAGGCAA GACCTGGATG GTGGTGGTGG TCGGGGGCGC

AGCAGGGACA CCTGAGGCAA GACCTGGATG GTGGTTGTGC TTGGGGGTGC

AGCAGGGACA CCTGAGGCAA GACCTGGATG GTGGTTGTGC TTGGGGGTGC

AGCAGGAACA CCTG-GGCAA GACCTGGATG GTGGTGGTGG TTGGGGGCGC

---------- ---------- ---------- ---------- ----------

AGCAGGGACA CCTGGGGCAA GACCTGGATG GTGGTGGTGG TCGGGGGCGC

AGCAGGGACA CCTGGGGCAA CACCTGGATG GTGGTGGTGG TCGGAGGCGC

---------- ---------- ---------- ---------- ----------

AGCAGGGACA CCTGAGGCAA GACCTGGATG GTGGTGGTGG TCGGGGGCGC

---------- ---------- ---------- ---------- ----------

AGCAGGGACA CCTGGGGCAA GACCTGGATC GTGGTGGTGG TCGGGGGTGC

AGCAGGGACA CCTGCGGCAA GACCTGGATG GTGGTGGTGG TTGGGGGGGT

AGCGGGAACA CCTGCGGCAA GACCTGGATg gtggtggcgg caggaggggt

GGGGGCAGCC TGGGGCAGGA GCGTGATGCC TACTTGGGTC AGCGGCTGCA

GGGGGCAGCC TGGGGCAGGA GCGTGATGCC TACTTGGGTC AGCGGCTGCA

GGGGGCAGCC TGGGGCAGGA GCGTGATGCC TACTTGGGTT AGCGGCTGCA

AGGGGCAGCC TGGGGCAGGA GCGTGATGCC TACTTGGGTC AGCGGCTGCA

GGGGGCAGCC TGGGGCAGGA GCGTGATGCC TACTTGGGTC AGTGGCTGCA

GGCGGCAGCC TGGGGCAGGA GCGTGATGCC TACTTGGGTT AGCGGCTGCA

AGCGGCAGCC TGGGGCACAA GTGTGATGCC TACTTGGGTC AGTGGTTGTA

AGTGGCAGCC TGGGGCACAA GTGTGATGCC TACTTGGGTC AGTGGCTGTA

AGCAGCAGCC TGGGGCAGGA GCGTGATGCC TACTTGGGTT AGCGGCTGCA

---------- ---------- ---------- ---------- ----------

GGTGGCAGCC TGGGGCAGGA GCGTGATGCC TACTTGGGTC AGCGGCTGCA

GGCAGCAGCC TGGGGCAGGA GCGTGATGCC TACTTGGGTC AGCGGCTGCA

---------- ---------- ---------- ---------- ----------

GGCGGCAGCC TGGGGCAGGA GCGTGATGCC TACTTGGGTC AGTGGCTGTA

---------- ---------- ---------- ---------- ----------

GGTGGCAGCT GGGGGCAGAA GTGTGATGCC CACCTGGGTC AGGGGCTGCA

TGCAGCTGCC TGTGGGAGCA GTGTGATGCC AACCTGGGCC AGTGGCTGCA

ggccgcggcC TGTGGGAGGA GCGTAATGCC CACCTGGGCA AGTGGCTGCA

CAGCAGGTGC GGCTGCTGCT GGGGCAGGGC TCTTGGGAGG GTTGGCAGGC

CAGCAGGTGC GGCTGCTGCT GGGGCAGGGC TCTTGGGAGG GTTGGCAGGC

CAGCAGGTGC GGCTGCTGCT GGGGCAGGGC TCTTGGGAGG GTTGGCAGGC

CAGCAGGTGC GGCTGCTGCT GGGGCAGGGC TCTTGGGAGG GTTGGCAGGC

CAGCGGGTGC GGCTGCTGCC GGGGCAGGGC TCTTGGGAGG GTTGGCAGGC

CAGCAGGTGC AGCTGCTGCT GGGGCAGGGC TCTTGGGAGG GTTGGCAGGC

CAGCAGGTGC AGCTGCTGCT GGCGCAGGGC TCTTGGGAGG GTTGGCAGGC

CAGCAGGTGC AGCTGCTGCT GGCGCAGGGC TCTTGGGAGG GTTGGCAGGC

CAGCAGGCGC AGCTGCTGCT GGGGCAGGGC TCTTGGGAGG GTTGGCGGGC

---------- ---------- ---------- ---------- ----------

CAGCAGGTGC GGCTGCTGCT GGGGCAGGGC TCTTGGGAGG GTTGGCAGGC

CAGCAGGTGC GGCTGCTGCC GGGGCAGGGC TCTTGGGAGG GTTGGTAGGC

---------- ---------- ---------- ---------- ----GCAGGC

CAGCAGGGGC CGCTGCCGCA GGGGCGGGGC TCTTGGGAGG GTTGGCAGGC

---------- ---------- ---------- ---------- ----------

CAGCCGGAGC CGCTGCTGCA GGTGCAGGAC TCTTGGGAGG ATTGGCAGGC

CAGCAGGGGC TGCTGCAGCT GGGGCAGGGC TCTTGGGGGG ATTGATAGGC

CGGCAGGGGC GGCAGCCGCC GGAGCGGGAC TCTTTGGGGG GTTGACTGGC

ACAGATGGGA CCGGATTGGG GGGAGGTGGC AGTAGCAGCC GTGGGAATGT

ACAGATGGGA CCGGATTGGG GGGAGGTGGC AGTAGCAGCC GTGGGAATGT

ACAGATGGGA CCGGATTGGG GGGAGGTGGC AGTAGCGGCC GTGGGAATGT

ACAGATGGGA CCGGATTGGG GGGAGGTGGC AGTAGCGGCC GTGGGAATGT

ACAGATGGGA CTGGATTGGG GGGAGGTGGC AGTAGCGGCC GTGGGAATGT

ACAGATGGGA CTGGATTGGG GGGAGGTGGC AGTAGCAGCC GTGGGAATGT

ACAGACGGGA CTGGATTGGG GGGAGGTAGC CGTAGCAGCT GTGGGAATGT

ACAGACGGGA CTGGATTGGG GGGAGGTAGC CGTAGCAGCT GTGGGAATGT

ACAGACGGGA CTGGATTGGG GGGAGGTGGC AGTAGCAGCC GTGGGAATGT

---------- ---------- ---------- ---------- ----------

ACAGACGGGA CTGGATTGGG GGGAGGTGGC AGTAGCAGCC GTGGGAATGT

ACAGATGGGA CTGGATTGGG GGGAGGTGGC AGTAGCAGCC GTGGGAATGT

ACAGAAGGAA CGAGGTTGGG GGCTGCTGTC CGGAGTAGTG GTTGGTAGGT

ACCGACGGGA CGGGGTTGGG GGGAGGCGGC AGTGGCTGCG GCGGGAATGT

---------- ---------- ---------- ---------- ----------

GCCGACGGGA CCGGGTTGGG AAGAGGTGAC AGTGGCAGCA CTGGGAATGT

ACAGATGGAA CAGGGTTGGG GTGAggtggc agtggcagcg gcggGAATAT

ACTGACGGGA CGGGGTTGGG GAGAGGCGGC AGCAGCAGCA GCGGGAATGT

CATATTTCTG GAGCTGGAGA AGGTAGCTGT CGGCTGTTGC CACTGCCCCC

CATATTTCTG GAGCTGGAGA AGGTAGCTGT CGGCTGTTGC CACTGCCCCC

CATATTTCTG GAGCTGGAGA AGGTAGCTGT CGGCTGTTGC CACTGCCCCC

CATATTTCTG GAGCTGGAGA AGGTAGCTGT CGGCTGTTGC CACTGCCCCC

CATATTTCTG GAGCTGGAGA AGGTAGCTGT CGGCTGTTGC CACTGCCCCC

CATATTTCTG GAGCTGCAGA AGGTAACTGT CGGCTGTCGC CACTGCCCCC

CATATTTCTG GAGTTGTAGA AGGTAACTGT CGGCTGTTGC CACTGCACCC

CATATTTCTG GAGTTGTAGA AGGTAACTGT CAGCTGTTGC CACTGCACCC

CATATTTCTG GAGCTGTAGA AGGTAACTGT CGGCTGTTGC CACTGCCCCC

---------- ---------- ---------- ---------- ----------

CATATTTCTG GAGCTGCAGA AGGTAACTGT CGGCTGTCGC CACTGCCCCC

CGTATTTCTG GAGCTGCAGA AGGTAACTGT CGGCAGTTGC CACTGCCCCC

CGTATTTCTG AAGCTGCAGA AGGTAACTGT CAGCGGTTGC CACCGCCCCC

CATACTTCTG GAGCTGCAGC AGGTAACTGT CGGCTGTGGC CACCGCCCCC

---------- ---------- ---------- ---------- ----------

CATACTTCTG GAGCTGCAGG AGGTAACTGT CGGCTGTGGC CACGGCCCCC

CATACTTTTG GAGCTGCAGG AGATAACTGT CAGCCGTTGG CACTGCCCCC

CGTACTTCTG GAGCTGGAGC AGGTAGCTGT CGGCTGTTGG CACTGCTCCC

CAGCTCACCT CCAGGGAGTT GGTGTTGGCG CGTACCAGTT GTACTCGGGC

CAGCTCACCT CCAGGGAGTT GGTGTTGGCG CGTACCAGTT GTACTCGGGC

CAGCTCACCT CCAGGGAGTT GGTGTTGGCG CGTACCAGCT GTACTCGGGC

CAGCTCACCT CCAGGGAGTT GGTGTTGGCG CGTACCAGCT GTACTCGGGC

CAGCTCACCT CCAGGGAGTT GGTGTTGGCG CGTACCAGTT GTACTCGGGC

CAGCTCACCT CCAGGGAGTT GGTGTTGGCT CGTACCAGCT GTACCCGGGC

CAGCTAACCT CCAGTGAGTT GGTGTTGGCT CGTACTAGTT GAACTCGGGC

CAGCTAACCT CCAGTGAGTT GGTGTTGGCT CGTACTAGTT GTACTCGGGC

CAGCTCACCT CCAGGGAGTT GGTGTTGGCT CGTACCAGTT GCACCCGGGC

---------- ---------- ---------- ---------- ----------

CAGCTCACCT CCAGGGAGTT GGTGTTGGCT CGCACCAGCT GTACCCGGGC

CAGCTCACCT CCAGGGAATT GGTGTTGGCT CGTACCAGTT GTACTCTGGC

CAGCTCACCT CTAACGAACT GGTGTTGGCT CGAACGAGCT GCACCCGGGC

CAGCTCACTT CCAGGGAGTT GGTGTTGGCG CGGACCAGCT GGACCCGGGA

---------- ---------- ---------- ---------- ----------

CAGCTGACCT CCAGGGAAGT GGTGTTGGCG CGCACCAGCT GCACCCGGGA

CAGCTCACCT CCAGGGAGTT GGTGTTGGCT CGGACCAGCT GCACCCGGGA

CAGCTCACCT CCAGCGAGTT GGTGTTGGCT CGCACTAGCT GCACCCGGGA

TGGGGGTGGT GGCTTTTCTG GCCTACCTGT CTCTAGGTAC CAGAGGTCCT

TGGGGGTGGT GGCTTTTCTG GCCTACCTGT CTCTAGGTAC CAGAGGTCCT

TGGGGGTGGT GGCTTTTCTG GCCTACCTGT CTCTAGGTAC CAGAGGTCCT

TGGGGGTGGT GGCTTTTCTG GCCTACCTGT CTCTAGGTAC CAGAGGTCCT

TGGGGGTGGT GGCTTTTCTG GCCTACCTGT CTCCAGGTAC CAGAGGTCCT

TGGGGGTGGT GGCTTTTCTG TCTTACCTGT CTCCAGGTAC CAGAGGTCCT

TGGGGGTGGT GGCTTTTCTG CCTTACCTGT CTCCAGATAC CACAGGTCCT

TGGGGGTGGT GGCTTTTCTG CCTTACCTGT CTCCAAATAC CACAAGTCCT

TAAAGGAGGT GGCTTTTCTG TCTTACCTGT CTCCAGGTAC CACAGGTCCT

---------- ---------- ---------- ---------- ----------

GGGGGGCGGT GGCTTTTCTG CCTTACCTGT TTCCAGGTAC CAGAGATCCT

CGGGGGCGGT GGCTTTTCTG CCTTACCTGT TTCCAGGTAC CAGAGGTCCT

TGGGGGTGGC GGTTTTTCTG ---------- ----AGGTAC CAGAGGTCCT

CGGGGCTGGT GGCTTTTCTG CCTCACCTGT TTCGAGGTAC CAGAGGTCCT

---------- ---------- ---------- ---------- ----------

GGGCGGTGGC GGCTTTTCTG CCTCACCTGT TTCCAAGTAC CAGAGATCCT

TGGGGCAGGA GGCTTTTCTG GCTCACCTGT CTCTAGGTAC CAAAGGTCCT

TGGGGCAGGC GGTTTTTCTG ACTCACCTGT CTCCAGGTAC CACAGGTCCT

TGCAGCAGAC CTGGTTGTTC CAGGCCTTGC GGTAGCCGTC ACGCCCACTC

TGCAGCAGAC CTGGTTGTTC CAGGCCTTGC GGTAGCCGTC ACGCCCACTC

TGCAGCAGAC CTGGTTGTTC CAGGCCTTGC GGTAGCCGTC ACGCCCACTC

TGCAGCAGAC CTGGTTGTTC CAGGCCTTGC GGTAGCCGTC ACGCCCACTC

TGCAGCAGAC CTGGTTGTTC CAGGCCTTGC GGTAGCCATC GCGCCCACTC

TGCAGCACAC CTGGTTGTTC CAGGCCTTGC GGTAGCCATC GCGCCCACTC

TGCAGCAGAC CTGATTGTTC CAGGCCTTGC GATAGCCATC ACGGCCACTC

TGCAGCAGAC CTGGTTGTTC CAGGCCTTGC GGTAGCCATC ACGGCCACTC

TACAGCATAC TTGGTTGTTC CAGGCCTTGC GGTAGCCATC GCGCCCACTC

---------- ---------- ---------- ---------- ----------

TGCAGCAGAC CTGGTTGTTC CAGGCCTTTC GGTAGCCGTC GCGCCCACTC

TACAGCACAC CTGGTTGTTC CAGGCCTTTC GGTAGCCATC ACGCCCACTC

TGCAACAGAC CTGGTTGTTC CAGGCTTTTC GATAGCCATC ACGCCCACTC

TGCAGCAGAC CTGGTTGTTC CAGGCTTTGC GGTAGCCGTC GCGGCCGCTC

---------- ---------- ---------- ---------- ----------

TGCAGCAAAC CTGGTTGTTC CAGGCTTTCC GGTAGCCGTC CCGCCCGCTC

TACAGCAGAC TTGGTTATTC CATGCTTTTC GGTAACCGTC TCGCCCACTC

TGCAGCAGAC CTGATTGTTC CACGCTTTGC GGTAGCCATC CCGCCCGCTC

CAAATGTACA GGCGGGTGTT GATGGCGACT GCGCAGTGGC CAGCCCGACA

CAAATGTACA GGCGGGTGTT GATGGCGACT GCGCAGTGGC CAGCCCGACA

CAAATGTACA AGCGAGTGTT GATGGCGACT GCGCAGTGGC CAGCCCGACA

CAAATGTACA AGCGAGTGTT GATGGCGACT GCGCAGTGGC CAGCCCGACA

CAAATGTACA GGCGGGTGTT GATGGCGACT GCGCAGTGGC CGGCCCGACA

CAAATGTAGA GACGGGTGTT GATGGCAACT GCACAGTGGC CAGCTCGACA

CAAATATACA GGCGAGTATT GATGGCAACA GCACAGTGGC CTGCTCGACA

CAAATATACA GACGAGTATT GATGGCAACA GCACAGTGGC CTGCTCGACA

CAAATGTACA AGCGGGTGTT GATGGCAACA GCACAGTGGC CGGCTCGATA

---------- ---------- ---------- ---------- ----------

CATATGTATA GGCGGGTATT GATGGCTACG GCACAGTGGC CAGCTCGGTA

CAAATGTATA GGCGGGTATT GATTGCTACA GCGCAGTGGC CGGCTCGGTA

CAAATGTACA AACGGGTATT GATCGCGACA GCACAGTGGC CAGCCCGG--

CAAATGTATA GGCGTGTGTT GATGGCCACG GCGCAGTGGC CGGCTCGACA

---------- ---------- ---------- ---------- ----------

CAGATGTACA GGCGGGTGTT GATGGCGACG GCACAGTGCC CAGCTCGACA

CAGATATAGA GTCGGGTGTT AATGGCCACT GCACAGTGGC CAGCTCGACA

CAGATGTACA GGCGGGTGTT GATGGCCACG GCGCAGTGCC CGGCCCTTCA

CTTACTTATT TCCGATGGTG GTTGCCGAGT GGAGACTGCG AGGAAGAGGC

CTTACTTATT TCCGATGGTG GTTGCCGAGT GGAGACTGCG AGGAAGAGGC

CTCACTTATT TCCGATGGTG GTTGCCGAGT GGAGACTGCG AGGAAGAGGC

CTCACTTATT TCCGATGGTG GTTGCCGAGT GGAGACTGCG AGGAAGAGGC

CTCACTTATT TCCAATGGTG GTTGCTGAGT GGAGACTGCG AGGAAGAGGC

CTCACTTGTT TCCAATGGTG GTTGCAGAGT GAAGACTACG AGGAAGAGGT

CTCACTTGTT TCCTATGGTG GTTGCAGAGT GGAGGCTGCG AGGAAGGGGT

CTCACTTGTT TCCTATGGTG GTTGCAGAGT GGAGGCTGCG AGGAAGGGGT

CTCACTTGTT TCCTATTGTA GTTGCCGAGT GGAGACTGCG AGGAAGAGGC

---------- ---------- ---------- ---------- ----------

CTCACTTGTT TCCTATGGTC GTGGCTGAGT GAAGACTTCG AGGAAGAGGT

CTCACTTGTT GCCGATGGTC GTGGCCGAGT GGAGGCTGCG AGGAAGAGGT

-----TTGTT TCCTATGGTG GTGGCTGAAT GGAGACTCCG AGGCAAAGGT

CTCACTTGTT TCCAATCGTC GTGGCCGAGT GGAGACTGCG AGGGAGAGGG

---------- ---------- ---------- ---------- ----------

CTCACTTATT TCCAATGGTC GTGGCTGAGT GAAGACTGCG AGGCAACGGT

CTCACTTGTT TCCTATGGTG GTGGCAGAAT GAAGACTTCG AGGGAGAGGA

CTCACTTGTT TCCTATGGTT GTAGCAGAGT GAAGGCTGCG GGGGAGAGGA

GCCACCCCGC TGAGACTGGG CTTATTCCAC GTCAGGGTGT CAGACAATAT

GCCACTCCGC TGAGACTGGG CTTATTCCAC GTCAGGGTGT CAGACAATAT

GCCACCCCGC TGAGACTGGG CTTATTCCAC GTCAGTGTGT CAGACAATAT

GCCACCCCGC TGAGACTGGG CTTATTCCAC GTCAGTGTGT CAGACAATAT

GCCACCCCGC TGAGACTGGG CTTATTCCAT GTCAGTGTAT CAGACGATAT

GCCACCCCGC TGAGACTGGG CTTGTTCCAC GTCAGAGTCT CAGACAATGT

GCCACCCCAC TAAGGCTGGG CTTATTCCAT GTCAGTGTCT CA--CAATGT

GCCACCCCAC TAAGGCTGGG CTTATTCCAT GTCAGTGTCT CA--CAATGT

GCCACCCCAC TGAGACTGGG CTTATTCCAT GTCAGGGTTT CG--CAATAT

---------- ---------- ---------- ---------- ----------

GCCACCCCAC TGAGACTGGG TTTATTCCAC GTCAGGGTCT CAGACAATAT

GCCACCCCGC TGAGACTGGG CTTATTCCAT GTCAGAGTCT CAGACAATAT

GCTAGCCCAC TGAGAGTGGG CTTATTCCAC GTCAAAGTCT C---CAATGT

GCTACCCCGC TGAGGCTGGG CTTGTTCCAC GTCAGAGTCT CAGGCGATAT

---------- ---------- ---------- ---------- ----------

GCCACGCCGC TGAGGCTGGG CTTGTTCCAT GTCAGTGTGT CACACAATAT

GCCACCCCAC TGAGAGTAGG TTTATTCCAG GTCAGGGTCT CACACAATAT

GCCACGCCAC TGAGACTGGG TTTATTCCAG GTGAGCGTTT CAAACAATGT

CTAGGGTCCA CAGGTCCCCC AGCCTGCAGC CACTCATCCC GCCGTAGATC

CTAGGGTCCA CAGGTCCCCC AGCCTGCAGC CACTCATCCC GCCGTAGATC

CTAGGGTCCA CAGGTCCCCC AGCCTGCAGC CACTCATCCC GCCGTAGATC

CTAGGGTCCA CAGGTCCCCC AGCCTGCAGC CACTCATCCC GCCGTAGATC

CTAGGGTCCA CAGGTCTCCC AGCCTGCAGC CACTCATCCC GCCGTAGATC

CAAGGGTCCA GAGGTCCCCC AGCCTGCAGC CACTCATCCC TCCGTAGATC

CCAGGGTCCA AAGGTCCCCT AGCCTGCAGC CACTCATCCC TCCATAGATC

CCAGGGTCCA AAGGTCCCCT AGCCTGCAGC CACTCATCCC TCCATAGATC

CCAGGGTCCA GAGGTCGCCC AGCCTGCAGC CACTCATCCC TCCATAAATC

---------- ---------- ---------- ---------- ----------

CCAGGGTCCA GAGGTCCCCC AGCCTGCAGC CACTCATCCC TCCGTAGATC

CCAGGGTCCA CAGGTCCCCG AGCCGGCAGC CACTCATCCC TCCATAGATG

CCAGGGTCCA GAGATCTCCT AGCCTGCAGC CACTCATCCC TCCATAGATC

CGAGGGTCCA GAGGTCCCCC AACCTGCAGC CACTCATCCC GCCGTAGATG

---------- ---------- ---------- ---------- ----------

CAAGGGTCCA GAGGTCCCCC AGCCTGCAGC CACTCATCCC TCCATAGATG

CTAGGGTCCA GAGGTCCCCA AGCCTGCAGC CACTCATTCC CCCATAGATA

CCAGCGTCCA GAGATCTCCC AACCTGCAGC CACTCATGCC TCCGTAGATG

ACCAGCTTGG ACTTCTTATT GTCTTTTTCG GTGTAGACCA CGGCAGTATG

ACCAGCTTGG ACTTCTTATT GTCTTTTTCG GTGTAGACCA CGGCAGTATG

ACCAGCTTGG ACTTCTTGTT GTCTTTCTCG GTGTAGACCA CGGCAGTATG

ACCAGCTTGG ACTTCTTGTT GTCTTTCTCG GTGTAGACCA CGGCAGTATG

ACCAGCTTGG ACTTCTTGTT GTCTTTTTCG GTGTAGACCA CGGCAGTATG

ACCAGCTTGG ATTTTTTGTT ATCTCTTTCA GTGTAGACCA CAGCAGTATG

ACCAGCTTGG ATTTCTTGTT ATCTTTTTCA GTGTAGACCA CAGCAGTATG

ACCAGCTTGG ATTTCTTGTT ATCTTTTTCA GTGTAGACCA CAGCAGTATG

ACCAGCTTGG ATTTCTTGTT ATCCCTTTCA GTGTAGACCA CAGCAGTATG

---------- ---------- ---------- ---------- ----------

ACCAGCTTGG ACTTCTTATT GTCTTTCTCA GTATAGACCA CAGCCGTATG

ACCAGCTTGG ACTTCTTGTT GTCTTTCTCC GTGTACACCA CGGCAGTGTG

ACCAGCTTGG ACTTCTTCCT GTCCTTTTCC GTGTAGACTA CTGCCGTATG

ACCAGCTTGG ATTTCTTGTT GTCTTTCTCA GTGTAGACCA CAGCCGTGTG

---------- ---------- ---------- ---------- ----------

ACCAGCTTGG ATTTCTTACT GTCTTTCTCA GTGTAGACCA CAGCAGTATG

ACCAACTTGG ATTTCTTGTT GTCTTTTTCT GTATAGACTA CAGCCGTGTG

ACCAACTTGG ATTTCTTATT GTCCTTCTCG GTGTAGACCA CTGCGGTGTG

TGACTCCCGG GGTGGTGGTA GGACCCCGTA AGTGATGGGA ATGTCCCAGG

TGACTCCCGG GGTGGTGGTA GGACCCCGTA AGTGATGGGA ATGTCCCAGG

TGACTCCCGG GGTGGTGGTA GGACCCCATA AGTGATGGGG ATATCCCAGG

TGACTCCCGG GGTGGTGGTA GGACCCCATA AGTGATGGGG ATATCCCAGG

TGACTCCCGA GGTGGTGGTA GGACCCCGTA AGTGATAGGG ATGTCCCAGG

TGACTCCCGG GGTGGAGGCA GGACCCCATA AGTGATGGGG ATGTCCCAGG

TGACTCCCGG GGTGGAGGAA GGACACCATA AGTGATGGGA ATGTCCCAAG

TGACTCCCGG GGTGGAGGCA GGACACCGTA AGTGATGGGG ATGTCCCAAG

CGACTCCCGG GGTGGGGGCA GCACCCCATA AGTGATGGGG ATGTCCCAGG

---------- ---------- ---------- ---------- ----------

TGACTCCCGG GGTGGAGGCA GGACGCCGTA AGTGATGGGG ATGTCCCAGG

CGACTCCCGG GGTGGGGGCA GGACGCCGTA GGTAATGGGA ATGTCCCAGG

CGACTCCCGG GGTGGGGGCA GGACCCCATA AGTGATGGGG ATGTCCCAGG

TGACTCCCGC GGAGGGGGCA GGACCCCGTA AGTGATGGGG ATGTCCCAGG

---------- ---------- ---------- ---------- ----------

GGACTCTCGT GGTGGGGGCA GAACCCCGTA AGTGATGGGG ATGTCCCAGG

GGACTCCCGG GGGGGCGGCA GGACCCCATA AGTGATGGGG ATGTCCCAGG

TGATTCCCGG GGCGGCGGGA GGACGCCATA AGTGATAGGG ATGTCCCAGG

CTACCACTCC AGAGCCTGGC CGTAATTCCA GGATATATAA GTCATTCAGG

CTACCACTCC GGAGCCTGGC CGTAATTCCA GGATATATAA GTCATTCAGG

CTACCACTCC GGAGCCTGGC CGTAATTCCA GGATATATAA GTCATTCAGG

CTACCACTCC GGAGCCTGGC CGTAATTCCA GGATATATAA GTCATTCAGG

CTACCACTCC GGAGCCTGGC CGTAATTCCA GGATGTATAA GTCATTCAGG

CTACCACTCC GGAGCCTGGC CGCAATTCCA GGATGTATAA GTCATTCAGG

CTACCACTCC AGAGCCTGGC CGTAGTTCGA GAATATATAA GTCATTCAGG

CTACCACTCC AGAGCCTGGC CGTAGTTCGA GAATATATAA GTCATTCAGG

CTACCACTCC AGAGCCTGGG CGTAATTCCA GGATATACAA GTCATTGAGG

---------- ---------- ---------- ---------- ----------

CTACGACTCC GGAGCCTGGC CGCAATTCCA GGATATATAA GTCATTCAGG

CTACCACTCC GGAGCCGGGC CGCAACTCCA GGATGTATAA GTCATTCAGG

CGACCACTCC TGAGCCCGGC CGCATTTCTA GGATGTACAA GTCATTCAGG

CTACCACCCC GGAGCCTGGT CGCAATTCCA GGATATATAA GTCATTCAGG

---------- ---------- ---------- ---------- ----------

CCACCACACC GGAGCCTGGC CGTAATTCCA GGATGTACAG GTCATTCAGG

CTACCACACC AGAGCCTGGT CGTAACTCCA GAATATATAG GTCATTCAGG

CGACTACTCC GGAGCCTGGG CGCAATTCCA GGATGTACAG GTCATTCAGG

TACCTTTGCC CACAAGGGAG AAGCTGTGCC CGAGTCGAGG ACACGGAGGG

TACCTTTGCC CACAAGGGAG AAGCTGTGCC CGAGTCGAGG ACACGGAGGG

TACCTTTGCC CACAAGGGAG AAGCTGTGCC CGAGCCGAGG ACATGGAGGG

TACCTTTGCC CACGAGGGAG AAGCTGTGCC CGAGCCGAGG ACATGGAGGG

TACCTTTGCC CACAAGGGAG AAGCTGTGCC CGAGCCGAGG ACATGGAGGG

TACCTTTGCC AACAAGGGAG AAGCTGTGCC CAAGCCGAGG ACATGGAGGG

TACCTTTGCC CACAAGGGAG AAACTGTGTC CAAGCCGAGG ACACGGAGGA

TACCTTTGCC CACAAGGGAG AAGCTATGTC CAAGCCGAGG ACATGGAGGA

TACCTTTGCC CACAAGGGAG AAGCTGTGGC CAAGTCGAGG ACACGGAGGG

---------- ---------- ---------- ---------- ----------

TACCTTTGCC CACAAGGGAG AAGCTGTGCC CAAGCCGAGG ACATGGAGGG

TACCTTTGCC CACAAGGGAG AAGCTGTGCC CGAGCCGAGG ACATGGAGGG

TACCTTTGTC CACCAGGGAG AAGCTGTGGC CCAGTCGAGG GCACGGAGGG

TACCTTTGCC CACGAGGGAG AAGCTATGCC CGAGCCGAGG ACACGGAGGG

---------- ---------- ---------- ---------- ----------

TACCTTTGCC CACAAGGGAG AAGCTGTGGC CAAGCCGAGG ACAGGGAGGG

TACCTTTGCC CACAAGAGAA AAGCTGTGCC CAAGTCGGGG GCATGGAGGC

TACCTTTGCC TACTAAGGAG AAGCTGTGAC CAAGCCTGGG GCAGGGAGGT

GGCCCGTTTT TGGGCGTCTT TGCTTTGAGT CTCTTCCACT CCCACCGGCT

GGCCCGTTTT TGGGCGTCTT TGCTTTGAGT CTCTTCCACT CCCACCGGCT

GGCCCGTTTT TGGGCGTCTT TGCTTTGAGT CTCTTCCACT CCCACCGGCT

GGCCCGTTTT TGGGCGTCTT TGCTTTGAGT CTCTTCCACT CCCACCGGCT

GGCCCATTTT TGGGTGTCTT TGCTTTGAGT CTCTTCCACT CCCACCGGCT

GGCCCATTTT TGGGTGTCTT TGCTTTGAGT CTCTTCCACT CCCACCGGCT

GGCCCGTTTT TGGGTGTCTT TGCCTTCAGT CTCTTCCATT CCCAGCGACT

GGCCCATTTT TGGGTGTCTT TGCCTTCAGT CTCTTCCATT CCCAACGACT

GGCCCATTTT TGGGCGTCTT TGCTTTGAGT CTCTTCCACT CCCATCGGCT

---------- ---------- ---------- ---------- ----------

GGCCCATTTT TGGGCGTCTT GGCTTTGAGT CTCTTCCATT CCCACCGGCT

GGCCCATTTT TGGGCGTCTT TGCTTTGAGT CTCTTCCACT CCCACCGGCT

GGCCCGGTTA GGGGCGTTTT TGCTTTGAGT TTCTTCCACT CCCAGCGGCT

GGCCCGTTCT TGGGCGTCTT TGCTTTGAGC CTCTTCCACT CCCAACGACT

---------- ---------- ---------- ---------- ----------

GGTCCATTTT TGGGGGTCTT TGCTTTGAGT CTCTTCCACT CCCAGCGACT

GGCCCATTTT TGGGGGCTTT TGCTTTGAGC CTTTTCCATT CCCACCTGCT

GGCCCATTTT TGGGTGTCCT GGCTTTGAGT CTCTTCCATT CCCACCGACT

CGCCTGCATA CCTGGAGTTC GTAGAGGTCA TTGCTGTATT TCCCATACTC

CGCCTGCATA CCTGGAGTTC ATAGAGGTCA TTGCTGTATT TCCCATACTC

CGCCTGCATA CCTGGAGCTC GTAGAGGTCA TTGCTGTATT TCCCATACTC

CGCCTGCATA CCTGGAGCTC GTAGAGGTCA TTGCTGTATT TTCCATACTC

CGCCTGCATA CCTGCAGCTC GTAGAGGTCA TTGCTGTATT TCCCATACTC

GGCCTGAATA CCTGGAGCTC ATAGAGGTCA TTGCTGTATT TCCCATATTC

TGCCTACATA CCTGGAGCTC ATAGAGGTCG TTGCTGTATT TTCCATACTC

TGCCTACATA CCTGGAGTTC ATAGAGGTCG TTGCTGTATT TTCCATACTC

TGCCTGCATA CCTGGAGCTC GTAGAGATCG TTGCTGTATT TCCCATATTC

---------- ---------- ---------- ---------- ----------

TGCCTGTATA CCTGGAGCTC ATAGAGGTCG TTGCTGTATT TCCCATACTC

TGCCTGCATA CCTGGAGCTC ATAGAGGTCA TTGCTGTATT TCCCATACTC

GGCCTGCA-- CCTGCAGCTC GTAGAGGTCG TTGCTGTACT TGCCGTACTC

CGCCTGTATA CCTGAAGCTC GTAGAGGTCG TTGCTGTATT TCCCATACTC

---------- ---------- ---------- ---------- ----------

GGCCTGCATA CCTGGAGCTC ATAGAGGTCG TTGCTGTATT TTCCATATTC

TGCCTGCATA CCTGCAACTC GTAGAGGTCG TTGCTGTACT TCCCATATTC

TGCCTGCATA CCTGCAGTTC ATACAGGTCG TTGCTGTATT TTCCATACTC

CACCATCCCA CCAAACACCA GGAGGCGAGT CCCGTCACAC ACGAAGCCAT

CACCATCCCA CCAAACACCA GGAGGCGAGT CCCGTCACAC ACGAAGCCAT

CACCATCCCG CCAAACACCA GGAGGCGAGT CCCGTCACAC ACGAAGCCAT

CACCATCCCG CCAAACACCA GGAGGCGAGT CCCGTCACAC ACGAAGCCAT

CACCATCCCG CCAAACACCA GGAGGCGAGT TCCATCACAA ACAAAGCCAT

CACCATCCCA CCAAACACCA GCAGGCGAGT CCCGTCACAC ACAAAGCCAT

TACCATTCCA CCAAACACCA GCAGGCGAGT ACCATCACAT ACAAAGCCAT

TACCATTCCA CCAAACACCA ATAGGCGAGT ACCATCACAC ACAAAGCCAT

CACCATCCCA CCAAACACCA GCAGGCGAGT ACCGTCACAC ACGAAGCCAT

---------- ---------- ---------- ---------- ----------

CACCATCCCA CCAAACACTA GCAGGCGGGT CCCGTCACAC ACGAAGCCAT

GACCATCCCA CCGAACACCA GCAGGCGGGT CCCATCGCAC ACAAAGCCAT

CACCATGCCG CCGAACACCA GCAGGCGACT CCCGTCGCAC ACGAAGCCAT

CACCATTCCG CCAAACACCA ACAGGCGAGT CCCATCGCAC ACGAAGCCGT

---------- ---------- ---------- ---------- ----------

CACCATTCCA CCAAACACCA GTAGGCGGGT CCCGTCACAC ACAAAGCCAT

CACCATCCCA CCAAACACCA GCAGTCGAGT CCCATCACAC ACAAAGCCAT

CACCATGCCG CCAAAAACGA GCAGGCGGGT TCCATCACAC ACAAAGCCAT

AGGCTGCACA CCCAGGGGGA ATGTCCCCCC TCACGGCTGG GATGAACCAC

AGGCTGCACA CCCAGGGGGA ATGTCCCCCC TCACGGCTGG GATGAACCAC

AGGCTGCACA CCCAGGGGGA ATGTCCCCCC TCACGGCTGG GATGAACCAC

AGGCTGCACA CCCAGGGGGA ATGTCCCCCC TCACGGCTGG GATGAACCAC

AGGCTGCACA CCCCGGAGGG ATGTCCCCCC TCACAGCTGG GATGAACCAC

AGGCTGCACA CCCAGGCGGG ATGTCCCCTC TCACAGCAGG AATGAACCAC

AGGCTGCACA CCCTGGAGGG ATATCCCCTC TCACAGCTGG GATGAACCAC

AGGCTGCACA CCCTGGAGGG ATATCCCCTC TCACAGCTGG GATGAACCAC

AGGCGGCACA TCCAGGGGGG ATGTCCCCTC TCACAGCCGG AATGAACCAC

---------- ---------- ---------- ---------- ----------

AGGCTGCACA CCCAGGGGGG ATGTCCCCTC TCACGGCTGG GATGAACCAC

AGGCTGCACA CCCAGGGGGG ATGTCCCCTC TCACGGCTGG GATGAACCAC

AAGCTGCGCA CCCCGGGGGG ACGTCCCCCC GCACGGTGGG GCTGAACCAC

AGGCTGCGCA GCCCGGGGGG ATGTCCCCTC GCACTGCCGG GATGAACCAC

---------- ---------- ---------- ---------- ----------

AGGCTGCACA CCCAGGGGGA ATGTCCCCCC TCACTGCAGG AATGAACCAC

AGGCAGCACA GCCTGGAGGG ATGTCCCCTC GAACAGCAGG GATAAACCAC

ACGCAGCACA GCCCGGTGGG ATGTCTCCTC GCACAGCAGG AATGAACCAC

TGGTTGGTTG CTGGAGCTGT CTACAGAAAA GGTGTATTCG GGAGTATCAA

TGGTTGGTTG CTGGAGCTGT CTACAGAAAA GGTGTATTCG GGAGTATCAA

TGGTTGGTTG CTG------- ---------- ---------- ----------

TGGTTGGTTG CTGGAGCTGT CTACAGAAAA GGTGTATTCG GGAGTATCAA

TGGTTGGTTG CTGGAGCTGT CTACAGAAAA GGTGTATTCG GGAGTATCAA

TGATTGGTTG CTG------- ---------- ---------- ----------

TGGTTGGTCG CT-GAGCTGT CTACAGAAAA GGTGTATTCG GGAGTATCAA

TGGTTGGTTG CT-GAGCTGT CTACAGAAAA GGTGTATTCG GGAGTATCAA

TGGTTAGTTG CTGGAGCTGT CTACAGAAAA GGTGTATTCG GGAGTATCAA

---------- ---------- ---------- ---------- ----------

TGGTTAGTTG CTGGAGCTGT CTACAGAAAA GATGTATTCG GGAGTATCAA

TGGTTGGTGG CTGGAGCTGT CTACAGAAAA GATGTATTCG GGAGTATCAA

TGGTTTGGTG ---GGGCTGT CTACAGAAAA GATGTATTCG GGAGTATCAA

TGGTTGGTTG CTGGAGCTGT CTACAGAAAA GATGTATTCG GGAGTATCAA

---------- ---------- ---------- ---------- ----------

TGGTTGGTAG CTAGAGCTGT CTACAGAGCA GGTGTATTCG GGAGTATCAA

TGGTTGGTTG CTGGAGCTGT CTACAGAAAA GATGTGTTCG GGAGTATCAA

TGGTTGGTGG CTGAAGTTCT CCAGGGCCAT GGTGTGTGCG GGAGTATAAA

GCAGAACTGG TGAGCTGTAT TTTTATAGTT GAAAGCCCCG GCTATTTGGG

GCAGAACTGG TGAGCTGTAT TTTTATAGTT GAAAGCCCCG GCTATTTGGG

---------- ---------- ---------- ---------- ----------

GCAGAACTGG TGAGCTGTAT TTTTATAGTT GAAAGCCCCG GCTATTTGGG

GCAGAACTGG TGAGCTGTAT TTTTATAGTT GAAAGCCCCG GCTAGTTGGG

---------- ---------- ---------- ---------- ----------

GCAGAACTGG TGAGCTGTAT TTTTATAGTT GAAAGTCCCG GCTTTTTGGA

GCAGAACTGG TGAGCTGTAT TTTTATAGTT GAAAGTCCCG GCTTTTTGGA

GCAGAACTGG TGAGCTGTAT TTTTATAGTT GAAAGCCCCG GCTATTTAGG

---------- ---------- ---------- ---------- ----------

GCAGAACTGG TGAGCTGTAT TTTTATAGTT GAAAGCCCCG GCTATTTGGG

GCAGAACTGG TGAGCTGTAT TTTTATAGTT GAAAGCCCCG GCTATTTGGG

GCAGAACTGC TGAGCTGTAT TTTTATAGTT GAAAGTCCCG GTGATTCAGG

GCAGAACTGG TGAGCTGTAT TTTTATAGTT GAAAGCCCCG GCTATTTGGG

---------- ---------- ---------- ---------- ----------

GCAGAGCTGA TGAGCTGTAC TTTTATAGTT GAAAGTCCCG GCAACTTGGG

GCAGAACTGG TGATCTGTGT TTTTATAGTT GAAAGCCCCG GCTATTTGGA

GCAGAACTGG TGTTCTCTAT TTTTGTAGTT GAAAACCCCG GCTATTTGGG

ACATAGTACT CGCGCGCGCC GCCGACGCCG GGACCCCGCA CGGCCGACGT

ACATAGTACT CGCGCGCGCC GCCGACGCCG GGACCCCGCA CGGCCGACGT

---------- ---------- ---------- ---------- ----------

ACATAGTACT CGCGCGCGCC GCCGACGCCG GGACCCCGCA CGGTCGACGT

ACATA-TACT cgcgcgcgcc gccgacgccg ggaccccgca cggccgacgt

---------- ---------- ---------- ---------- ----------

ACATAGTACT -GCGCGCGNN NNCNNTGCCG GNNCCCCNNN NNGCCGANGT

ACATCATACT CGCGCGCGCA ACCGATGCCG GGACCCCGCA CGGCAGACGT

ACATAATACT ---------- ---------- ---------- ----------

---------- ---------- ---------- ---------- ----------

ACATAGTGCT CGCGCGCGCC GCCGACGCCG AGACCCCGCA CGGCCGACGT

ACATAGTACT CGCGCGCGCC GCCGACGCCG GGACTCCGCA CGGTCGACGT

ACACAGTACA ---------- ---------- ---------- ----------

ACATGGTACT TGCGCGCGCC GCCGACGCCG AGACCCCGCA CGGCCGACGT

---------- ---------- ---------- ---------- ----------

ACATAGTGCT TGCGCGCGCC ACCGACGCCG GGACCCCGCA CGACCGACGT

GTATAATTCT CGCGCGCGCC GCCGACACTG GGAGCGCGCG CGGTCTCCGC

GTATAAGACT CGCGCGCGCC GCCGACACCT TGGGCAcgcg cgcccgacgt

CGCGCCCCGC CCTCCGAGGG GCAGGCACAC AAGGTGGAGA CAAAGACCAA

CGCGCCCCGC CCTCCGAGGG GCAGGCACAC AAGGTGGAGA CAAAGACCAA

---------- ---------- ---------- ---------- ----------

cgcgccccgc cctcTGAGGG GCAGGCACAC AAGGTGGAGA TAAAGACCAA

cgcgccccgc cctcCAAGGG GCAGGCACAC AAGGTGGAGA CAAAGACCAA

---------- ---------- ---------- ---------- ----------

CGCGCCCCGC CCTCCAAAGG GCAAGAACAC AATGTAGAGA CCACGACCAA

CGCGCCCCGC CCTCCAAGGG GCAAGAACAC AATGTAGAGA CCACGACCAA

---------- ----CAAGGG GCAGGCACAC ACGGTGGAGA CGAGGACCAG

---------- ---------- ---------- ---------- ----------

CGCGCCCCGC CCTCCAAGGG GCAGGAGCAC ACAGTGGAGA TGAAGACGAG

CGCGCCCCGC CCTCCAAGGG GCAGGTGCAC ACAGTGGCGA TGAAGACGAG

---------- ---------- ---------- ---------- ----------

CGCGCcccgc cctcCAAGGG GCAGGCGCAC ACGATGGACA CGGAGACCAA

---------- ---------- ---------- ---------- ----------

CGCGCCCCGC CCTCCCAGGG GAAAGCGCAC TTGGTGGAGA TGCAGACCAA

CACGCCACGC CCTCCAAGTG GCAGCTACAG ATGGTAGACA CGCATATGAG

ctcgccccac ccccCAAGGG GCAGCAGGAC ATGCTAGTGA TGCAGACCAG

GAGGACGGTT GCCAGTGAGA GGCGCGGACT CAGGACGAGG GGCAGGCACA

GAGGACAGTT GCCAGTGAGA GGCGCGGACT CAGGACGAGG GGCAGGCACA

---------- ---------- ---------- ---------- ----------

GAGGACGGTT GCCAGTGAGA AGAGCAGACT CACGATGAGG GGCAGGCACA

GAGGACGGTG GTCAGTGAGA GGAGCGGTCT CAGGACAAGG GGCAGGCACA

---------- ---------- ---------- ---------- ----------

GAGGATAGTG GTCAGAGTGA GAAGCAGGCT TATGACAAAG GGCAAGAACA

GAGGATAGTG GTCAGAGTGA GAAGCAGGCT TATGACAAGG GGCAAGAACA

GAGGACAGTG GCCAGCGTGA GGAGCAGGCT CATGACAAGG GGCAGGCACA

---------- ---------- ---------- ---------- ----------

GAGGATGGTA GCCAGTGTGA GGAGCAGGCT CATGACAAGG GGCAGGAGCA

GAGGATGGTG GCCAGTGTAA GGAGTAGGTT CCCGACAAGG GGCAGGTGCA

---------- ---------- ---------- ---------- ----------

GAGGATGGTG GCCAGCGTGA GGAGCAGGCT CATGACAAGG GGCAGGCGCA

---------- ---------- ---------- ---------- ----------

GAGGATGGTG GCCAGAGTGA GGAGCAGGCT GATGACCAGG GGAAAGCGCA

GACGATGGTG GTCAAAGTTA GGAGCAAGCT CATGACAAGT GGCAGCTACA

GAGGATGTTG GTCAAAGTGA CGAGTAGGTT CATGACAAGG GGCAGCAGGA

CAAGGTGGAG ACAAAGACCA AGAGGACGGT TGCCAGTGAG AGGCGCGGAC

CAAGGTGGAG ACAAAGACCA AGAGGACAGT TGCCAGTGAG AGGCGCGGAC

---------- ---------- ---------- ---------- ----------

CAAGGTGGAG ATAAAGACCA AGAGGACGGT TGCCAGTGAG AAGAGCAGAC

CAAGGTGGAG ACAAAGACCA AGAGGACGGT GGTCAGTGAG AGGAGCGGTC

---------- ---------- ---------- ---------- ----------

CAATGTAGAG ACCACGACCA AGAGGATAGT GGTCAGAGTG AGAAGCAGGC

CAATGTAGAG ACCACGACCA AGAGGATAGT GGTCAGAGTG AGAAGCAGGC

CACGGTGGAG ACGAGGACCA GGAGGACAGT GGCCAGCGTG AGGAGCAGGC

---------- ---------- ---------- ---------- ----------

CACAGTGGAG ATGAAGACGA GGAGGATGGT AGCCAGTGTG AGGAGCAGGC

CACAGTGGCG ATGAAGACGA GGAGGATGGT GGCCAGTGTA AGGAGTAGGT

---------- ---------- ---------- ---------- ----------

CACGATGGAC ACGGAGACCA AGAGGATGGT GGCCAGCGTG AGGAGCAGGC

---------- ---------- ---------- ---------- ----------

CTTGGTGGAG ATGCAGACCA AGAGGATGGT GGCCAGAGTG AGGAGCAGGC

GATGGTAGAC ACGCATATGA GGACGATGGT GGTCAAAGTT AGGAGCAAGC

CATGCTAGTG ATGCAGACCA GGAGGATGTT GGTCAAAGTG ACGAGTAGGT

TCAGGAAAAG GTGGAGACAA AGACCAAGAG GACGGTTGCC AGTGAGAGGC

TCAGGAAAAG GTGGAGACAA AGACCAAGAG GACAGTTGCC AGTGAGAGGC

---------- ---------- ---------- ---------- ----------

TCACGAAAAG GTGGAGATAA AGACCAAGAG GACGGTTGCC AGTGAGAAGA

TCAGGAAAAG GTGGAGACAA AGACCAAGAG GACGGTGGTC AGTGAGAGGA

---------- ---------- ---------- ---------- ----------

TTATGAAAAT GTAGAGACCA CGACCAAGAG GATAGTGGTC AGAGTGAGAA

TTATGAAAAT GTAGAGACCA CGACCAAGAG GATAGTGGTC AGAGTGAGAA

TCATGAAACG GTGGAGACGA GGACCAGGAG GACAGTGGCC AGCGTGAGGA

---------- ---------- ---------- ---------- ----------

TCATGAGACA GTGGAGATGA AGACGAGGAG GATGGTAGCC AGTGTGAGGA

TCCCGAAACA GTGGCGATGA AGACGAGGAG GATGGTGGCC AGTGTAAGGA

---------- ---------- ---------- ---------- ----------

TCATGAAACG ATGGACACGG AGACCAAGAG GATGGTGGCC AGCGTGAGGA

---------- ---------- ---------- ---------- ----------

TGATGAATTG GTGGAGATGC AGACCAAGAG GATGGTGGCC AGAGTGAGGA

TCATGAAATG GTAGACACGC ATATGAGGAC GATGGTGGTC AAAGTTAGGA

TCATGAAATG CTAGTGATGC AGACCAGGAG GATGTTGGTC AAAGTGACGA

GCGGACTCAG GAACTTTTGC TGTAGCATCT CCAGCTTGGA TATTCGGCTC

GCGGACTCAG GAACTTTTGC TGTAGCATCT CCAGCTTGGA TATTCGACTC

---------- ---------- ---------- ---------- ----------

GCAGACTCAC GAACTTTTGC TGTAGCATCT CCAGCTTGGA TATTCGGCTC

GCGGTCTCAG GAACTTTTGC GGTAGTGTCT CCAGCTTGGA TATTCGGCTC

---------- ---------- ---------- ---------- ----------

GCAGGCTTAT GAATCTTTGC TGTAGAGTTT CTAGCTTTGT TATTCTGTTC

GCAGGCTTAT GAATCTTTGC TGTAGAGTTT CTAGCTTGGT TATTCTGCTC

GCAGGCTCAT GAAGATTCGC TCCAGAGCTT CTAGTCTGGA GATTCGGCTC

---------- ---------- ---------- ---------- ----------

GCAGGCTCAT GAGCCGTTGT TGCAGAGTTT CAAGCTTAGC TATACGGTTC

GTAGGTTCCC GAATCGTTGC TGTAGCGTTT CGAGCTTGGA TATACGACTC

---------- ---------- ---------- ---------- ----------

GCAGGCTCAT GAACTTTTGC TGTAGTGTCT CCAGCTTGGC TATCTGGCTC

---------- ---------- ---------- ---------- ----------

GCAGGCTGAT GAACTTCTGC TGGAGGTTCT CCAGCTTAGT GACCCGGCTC

GCAAGCTCAT GAACCTTTGC TGTTGGGTCT CCAGCTTGGA AATTCGACTC

GTAGGTTCAT GAACTTTTGC TGCAGAGCCT CTAGTTTGGA AATGCGGGTC

TTGAAGGTCT CCGTGATCTC CTAGCTCCGG AGCACCTGCC CCAGGGCCAG

TTGAAGGTCT CCGTGATCTC CTAGCTCCGG AGCACCTGCC CCAGGGCCAG

---------- ---------- -TAGCTTCGG AGCACCTGCC CCAGGGCCAG

TTGAAGGTCT CCATGATCTC CTAGCTTCGG AGCACCTGCC CCAGGGCCAG

TCGAAGGTCT CCATGATCTC CTAGCTCCGG AGCACCTGCC CTAGGGCCAA

---------- ---------- -TAGCTCCGA AGCACCTGCC CCAGAGCCAG

TTGAAAGTCT CCATTACATC CTAGCTTCGG AGGGCCTGCT CCAGGGCCAA

TTGAAAATCT CCATTACATC CTAGCTCCGG AGGGCCTGCT CCAGGGCCAA

TTGAAAGTTT CCATGACTTC CTAGCTCCGG AGCGCCTGCC CCAGGGCCAA

---------- ---------- ---------- ---------- ----------

TTGAAAGTCT CCATGACCTC CTAGCTCCGG AGCACCTGCC CCAGGGCTAA

TTGAAAGTCT CCATGACCTC CTAGCTGCGG AGCACCTGCC CCAGGGCCAA

---------- ---------- -TAGCTCCTC AGCGCCTGGC CCAGGGCCAA

TTGAAAGTCT CCATGACCTC CTAGCTCCGG AGCACCTGGC CCACAGCCAA

---------- ---------- ---------- ---------- ----------

TGGACCGTCT CCATGACCTC CTAGCTCCGG AGCAGCTGGC CTAGGGCCAG

TGGAAGGTCC TCATCACCTC CTAGCCACCA AGAAACCTGC TCAAGGCCAG

TGAAAGGTTT CCATCACCTC CTAGCTCTTC AGCACCTTGC CCAATGCCAG

GTGGAACCCT GTGTCACAGC ACTGGTAGTA GTTACTGACA GCAGCGTTGA

GTGGAACCCT GTGTCACAGC ACTGGTAGTA GTTACTGACA GCAGCGTTGA

GTGGAACCCT GTGTCGCAGC ACTGGTAGTA GTTACTGACA GCGGCGTTGA

GTGGAACCCT GTGTCGCAGC ACTGGTAGTA GTTACTGACA GCGGCGTTGA

GTGGAACCCT GTGTCGCAGC ACTGGTAGTA GTTCCTGACA GCAGCATTGG

GTGGAACCCC ATGTCACAGC ACTGGTAGTA GTTGCTGACA GCGGCATTGA

GTGGAAACCT GTGTCACAGC ACTGGTAGTA GTTGCTGACA GCAGCATTGA

ATGGAAACCT GTGTCACAGC ACTGGTAGTA GTTGCTGATG GCGGCATTGA

GTGGAAGCCT GTGTCACAGC ACTGGTAGTA GTTGCTGACA GCAGCGTTGA

---------- ---------- ---------- ---------- ----------

GTGGAATCCT GTGTCGCAGC ACTGGTAGTA GTTGCTGACA GCAGCATTGA

GTGGAAGCCC GTGTCACAGC ACTGGTAGTA GTTGCTGACC GCAGCGTTGA

GTGGAAGCCC GTGTCGCAGC ACTGGTAGTA GTTGCTGACA GCAGCGTTGA

GTGGAAGCCG GTGTCACAGC ACTAGTAGTA GTTGCTGACG GCGGCGTTGA

---------- ---------- ---------- ---------- ----------

GTGGAACCCT GTGCCGCAGC ACTGGTAGTA GTTTCTAACG GCTGCGTTCA

GTGGAAACCC AGATCATTGC ACTAATAGTA GTTGCTGACG GCTGCGTTTG

GTGGAATCCC ACGTCACAGC ACTGATAGTA GTTGTTGACG GCGGCATTAA

CACTAGCCAG GCTAAGCAGG TACTCGTTGC GCGCCTTTGT GCACTTGAGT

CACTGGCCAG GCTAAGCAGG TACTCGTTGC GCGCCTTTGT GCACTTGAGT

CGCTGGCCAG GCTAAGCAGG TACTCATTGC GCGCCTTTGT GCACTTGAGT

CGCTGGCCAG GCTAAGCAGG TACTCGTTGC GCGCCTTTGT GCACTTGAGT

CGCTGGCCAG GCTCAGCAGG TACTCGTTGC GTGCCTTTGT GCACTTGAGT

CACTGGCCAG GCTGAGCAGG TACTCATTGC GTGCTTTTGT GCACTTGAGT

CACTGGCCAG GCTGAGCAGG TACTCATTTC GGGCCTTGGT GCATTTAAGT

CACTGGCCAG GCTGAGCAGG TACTCATTTC GGGCCTTGGT GCACTTAAGT

CGCTGGCCAG GCTCAGCAGG TATTCATTCC GTGCCTTTGT GCACTGGAGT

---------- ---------- ---------- ---------- ----------

CACTGGCCAG GCTGAGCAGG TACTCGTTGC GCGCCTTTGT GCACTTGAGT

CACTGGCCAG GCTGAGCAGG TACTCGTTGC GCGCTTTCGT GCACTTCAGT

CGCTCGCCAG GCTCAGCAGG TATTCGTTCC GCGCCTTCGT GCACTTGAGC

CGCTGGCCAG GCTGAGCAGG TACTCGTTCC GCGCCTTCGT GCACTTGAGT

---------- ---------- ---------- ---------- ----------

CACTGGCCAG GCTGAGCAGG TACTCGTTCC GAGCCTTTGT GCACTTGACC

CACTGGGCAG ACTGAGCAAG TACTCATTCC GGGCCTTGGT GCACTTCAGT

CGCTGGCCAA GCTGAGCAGG TACTCGTTCC GGGCCTTGGT GCACTTCAGC

TTGTGCTCCA TGAACTTGGC CTGCCGCTAG CCTGCCCGCT TCTCCTCCTG

TTGTGCTCCA TGAACTTGGC CTGCCGCTAG CCTGCCCGCT TCTCCTCCTG

TTGTGCTCCA TGAACTTGGC CTGCCGCTAG CCTGCCCGCT TCTCCTCCTG

TTGTGCTCCA TGAACTTGGC CTGCCGCTAG CCTGCCCGCT TCTCCTCCTG

TTGTGCTCCA TGAACTTGGC CTGCCGCTAG CCTGCCCGCT TCTCCTCCTG

TTGTGCTCCA AGAACTTGGC CTGACGCTGG CCAGCCCGCT TCTCCTCCTG

TTATGTTCCA AGAACTTGGC CTGACGCTGG CCTGACCGCT TCTCCTCTTG

TTATGCTCCA AGTACTTGGC CTGACGCTGG CCTGACCGCT TCTCCTCTTG

TTGTGCTCCA GGAACTTGGC GTGGCGCTGC CCTGCCCGCT TCTCCTCTTG

---------- ---------- ---------- ---------- ----------

TTGTGCTCCA AGAACTTGGC CTGACGCTGG CCCGCCCGTT TTTCCTCCTG

TTGTGCTCCA AGAACTTGGC CTGCCGCTGG CCTGTCCGCT TTTCTTCCTG

TTGTGTTCCA AGAACTTGGC CTGACGCTGG CCTGCCCGCT TCTCCTCCTG

TTGTGCTCCA GGAATTTGGC CTGGCGCTGg cccgcccgcT TCTCCTCCTG

---------- ---------- ---------- ---------- ----------

TTGTGCTCCA GGTACTTGGC CTGCCGCTGG CCTGCTCTCT TCTCCTCCTG

TTGTGCTCGA AGTACTTGAC CTGGCGCTGG CCGGCCCGCT TCTCTTCTTG

TTGTGCTCCA GGTATTTGGC CTGGCGCTGC CCACCTCTCT TCTCCTCCTG

CCGCTCGGCC TCCCGGAGCT TGGCCTCGGC ATTCACGCTC TCTCACCGTG

CCGCTCGGCC TCCCGGAGCT TGGCCTCGGC ATTCACGCTC TCTCACCGTG

CCGCTCAGCC TCCCGGAGCT TGGCCTCAGC ATTCACGCTC TCTCACCGTG

CCGCTCGGCC TCCCGGAGCT TGGCCTCAGC ATTCACGCTC TCTCACCGTG

CCGCTCGGCC TCCCGGAGCT TGACCTCAGC ATTCACACTC TCTCACCGTG

TCGCTCAGCT TCACGGAGCT TGTTCTCAGC ATTCATGCTC TCTCACCGTG

CCTCTCAGCT TCCCGAAGTT TAGTCTCAGC ATTCATGCTC TCTTACTGTG

CCGCTCCGCT TCCCGAAGTT TAGTCTCAGC ATTCATGCTC TCTTACTGTG

TCGCTCAGCT TCTCGGAGCT TGGCCTCGGC GTTCACGCTC TCTCACCGTG

---------- ---------- ---------- ---------- ----------

CCGTTGGGCT TCCGGGAGCT TGGCCTCAGC ATTCACGCTC TCTCACCGTG

CCGCTCGGCT TCCCGGAGCT TGGCCTCAGC ACTCATGCTC TCTCACCGTG

CCGTTCGGCT TCGCGGAGCT TGGCCTCAGC ATTCAGGCTC TCTCACCGTG

CCTCTCGGCT TCCCGGAGCT TGGTCTCGGC GTTCACGCTC TCTCACCGTG

---------- ---------- ---------- ---------- ----------

GCGCTCGGCT TCTCGGAGCT TGGTCTTGGC GTTCACGCTT TCTCACCGTG

CCTTTCAGCT TCTCTCAGCT TGCCCTCAGA GCTTACGCTC TCCCATTCCG

CCGTTCTGCC TCACGCAGCT TGTTCTCGGC ATTCACGCTC TCTGACCATG

GAGCTCTGAG ACCACCTCCA GGAGCTCATC CTGGAGGAGA AGCGCTCGGC

GAGCTCTGAG ACCACCTCCA GGAGCTCATC CTGGAGGAGA AGCGCTCGGC

GAGCTCTGAG ACCACCTCCA GGAGCTCGTC CTGGAGGAGA AGCGCTCGGC

GAGCTCTGAG ACCACCTCCA GGAGCTCGTC CTGGAGGAGA AGCGCTCGAC

TAGCTCTGAG ACCACCTCCA GGAGCTCGTC CTGGAGGAGA AACGCTCAGC

GAGCTCTGAG ACCACCTCCA TGAGCTCATC CTGGAGGAGA AGCGCTCGGC

GAGTTCTGAG ACCACCTCCA GGAGCTCATC CTGGAGGTAA AGCGTTCAGC

GAGTTCTGAG ACCACTTCCA GGAGCTCAGC CTGGAGGTAA AGCGTTCAGC

CAGCTCTGAC ACCACCTCCA GGAGCTCCTC CTAGAGGAGA AGCGCTCTGC

---------- ---------- ---------- ---------- ----------

AAGCTCCGAC ACCACCTCCA GCAGCTCGTC CTGGAGGAGA AGCGCTCGGC

AAGCTCTGAC ACCACCTCCA GGAGCTCCTC CTGGAGGAGA AGCGCTCGGC

CAGCTCCGAC ACCACCTCCA GGAGTTCCTC CTGGAGGAGA AGCGCTCAGC

GAGCTCCGAC ACCACCACCA GCAGTTCCTC CTGGAGGAGA AGCGTTCGGC

---------- ---------- ---------- ---------- ----------

CAGCTCCGAG ACCACCTCCA GGAGCTCCTC CTGGAGGAGA AGCGCTCAGC

GGGCTCAAAT ACCATCCTAG CTaccttggc atGAAGAGGA ACCTGTCGCT

GACCCCAGAC AGAACCACCC CGTGATCCCC CTGGAGGAGA AGCGTTCAGC

CAGCTTTTCC AGGCCCCGGG AGTATTCCAG CTCCACCTCC GCCGCATGAA

CAGCTTTTCC AGGCCCCGGG AGTATTCCAG CTCCACCTC- ----------

CAGCTTTTCC AGGCCCCGGG AGTACTCCAG CTCCACCTCC GCCGCATGAA

CAGCTTTTCC AGGCCCCGGG AGTACTCCAG CTCCACCTCC GCCGCATGAA

CAGCTTTTCC AGGCCCCGAG AGTACTCCAG CTCCACCTCC GCCGCATGAA

TAGCTTGTCC AGGCCACGGG AGTACTCCAG CTCCACCTCC GCCGCATGAA

CAACTTGTCC AGGCCCCGAG AATACTCCAG CTCCACCTCC GCCGCATGAA

CAACTTGTCC AGGCCCCGAG AATACTCCAG TTCTACCTCC GCCGCATGAA

CAACTTCTCC AGGCCCCGGG AGTACTCGAG CTCCACCTCC GACGCATGAA

---------- ---------- ---------- ---------- ----------

CAGCTTGTCC AAGCCCCGGG AGTACTCCAG CTCCACCTCC GCCGCATGAA

CAGCTTGTCC AGGCCACGGG AGTACTCCAG CTCCACATCC GCCGCATGAA

CAGGCGCTCC AGGCCCCGGG AGTACTCGAG CTCCACGTCC GCCGCATGAA

CAGCTTCTCC AGGCCCCGGG AGTACTCCTG CTCCACCTCC GCCGCATGAA

---------- ---------- ---------- ---------- ----------

CAGCTTGTCC AGGCCTCGCG AGTACTCGAG CTCCACCTCC TCCTCATGAA

GAGCTTCTCC AGTCCCTTGG AGTATTCCAG CTCCACCTCC GCCGCATGAA

CAGCCTTTCC AGTCCACGCG AGTACTCCAG CTCCACTTCC GACGCAGGAA

CTCTGCCAGC TCCTGCAGCA ACTCCCGCCG CAGCTCGCCC TGCAGCTCCA

---------- ---------- ---------- ---------- ----------

CTCTGCCAGT TCCTGCAGCA GCTCCCGCCG CAGCTCACCC TGCAGCTCCA

CTCTGCCAGT TCCTGCAGCA GCTCCCGCCG CAGCTCACCC TGCAGCTCCA

CTCTGCCAAC TCCTGCAGCA GCTCCCGACG CAGCTCGCCC TGCAGCTCCA

TTCAGCCAGC TCCTGCAGCA ACTCCCGCCG CAGCTCACCC TGTAGCTCTA

CTCAGCTAGC TCCAGCAGCA AATCCCGCCG CAGCTCTCCC TGAAGCTCCA

CTCAGCTAGC TCCTGCAGCA AATCCCGCCG AAGCTCTCCC TGAAGCTCCA

CTCGGCTAGC TCCTGCAGCA GCTCCCGCCG AAGCTCGCCC TGCAGCTCCA

---------- ---------- ---------- ---------- ----------

CTCGGCCAGC TCCTGCAGCA GCTCCCGCCG CAGCTCGCCC TGCAGCTCCA

CTCAGCCAGC TCCTGCAGCA GCTCCCGCCG CAGCTCACCC TGCAACTCCA

CTCGGCCAGC TCCTGCAGCA GTTCCCGCCG CAGCTCCCCC TGCACCTCCA

CTCGGCCAGC TCCTGCAGCA GCTCCCGCCG GATGTCGCCC TGCAGCTCCA

---------- ---------- ---------- ---------- ----------

CTCCGCCAGC TCCTGCACCA GCTCCCGTCG TAGGTCCCCC TGCAGCTCCA

CTCGCCCAGA TCCTGGAGCA GTTCTCGCCG AAGCTCCCCT TGACCATCCA

TTCGCTCAGA TCCTGCAGCA ATTCCCGGCG CAGTTCTCCC TGCACCTCCA

GGCAGCGCAG CTGCTCGCTC AGCTGCCAGC GCATCTCTGC CATTCCAGGA

---------- ---------- ---------- ---------C CATTCCAGGA

GGCAGCGCAG CTGCTCGCTC AGCTGCCAGC GCATCTCTG- ----------

GGCAGCGCAG CTGCTCGCTC AGCTGCCAGC GCATCTCTGT CATTCTAGGA

GGCAGCGTAG CTGTTCGCTC AGCTGCCAGC GCATCTCTGT AATTCTAGGA

GGCAGCGCAG CTGCTCACTC AGCTGCCAGC GCATCTCTG- ----------

GGCAATGAAG CTGCTCACTC AACTGCCCGC GCATCACTG- -----CAGGA

GGCAATGAAG CTGCTCACTC AACTGCCAGC GCATCTCTGC TATTT----A

GGCAGCGCAG CTGCTCGCCC AGCTGCCCGC GCATCTCTGC CATTC----A

---------- ---------- ---------- ---------- ----------

GGCAGCGCAG CTGCTCGCTC AGCTGCCAGC GCATCTCTGC CATTTTAGGA

GGCAGCGCAG TTGCTCGCTC AGCTGCCAGC GCATCTCTGC CATTCTAGGA

GGCAGCGCAG CTGCTCGCTC AGCTGGGAGC GCATCTCTGC CATTCTAGAA

GGCAGCGCAG CTGCTCGCTC AGCTGCCAGC GCATCTCTGC TGTTCTAGGA

---------- ---------- ---------- ---------T CCTTTCAGGA

GGCAGCGCAG CTGCTCACTC AGCTGCCAGC GGATCTCTGT CCCTTCAGGA

GGCATCGGAG CTGTTCTGCC AACTGACATC GGATCTCTGT CATTCTAGGA

GGCAGCGCAA TTGCTCTCCC AGCTGAGAAC GGATCTCTGA AATATCAGGA

CAGGTTTCAT TAATCTTGAC AAGCCCTCTA ACCCCTCTTC CCATGAGGTG

CAGGTTTCAT TAATCTTGAC AAGCCCTCTA ACCCCTCTTC CCATGAGGTG

---------- ---------- ---------- ---------- ----------

CAGGTTTCAT TAATCTTGAC AAGCCCTCTA ACCCCTCTTC CCATGAGGTG

CAGGTTTCAT TAATCTCGAC AAGCCCTCTA ACCCCTCTTC CCATGAGGTG

---------- ---------- ---------- ---------- ----------

CAGGTTTCAT TAATCTTGTC AAGCCCTATA ACCCCTCTTC CCATGAGGTG

CCGGTTTCAT TAATCTTGAC AAGCCCTCTA ACCCCTCTTC CCATGAGGTG

CAGGTTTCAT TAATCTTGAC AAGCCCTCCA ACCCCTCTTC CCATGAGGTG

---------- ---------- ---------- ---------- ----------

CAGGTTTTAT TAATCTTGAC AAGCCCTCCA ACCCCTCTTC CCATGAGGTG

CAGGTTTCAT TAATCTTGAC AAGCCCTCCA ACCCCTCTTC CCATGAGGTG

CAGGTTTCAT TAATCTCGAC AAGCCCTCCA ATCCCTCTTC CCATGAGGTG

CAGGTTTCAT TAACCTTGAC AAGCCATCCA ACCCCTCTTC CCATGAGGTG

CAGGTTTCAT TAACCTTGAC AAGCCTTCTA ACCCCTCTTC CCATGAGGTG

CAGGTTTCAT TAACCTGGAC AAGCCCTCCA ACCCGTCTTC ACATGAAGTA

CAGGTTTCAT TAACCTTGAC AAACCCTCCA ATCCCTCTTC CCATGAGGTA

CCGGCTTCAT TAACCTCGAC AAGCCCTCCA ACCCCTCTTC CCATGAAGTG

GTAGCCTGGA TTCGACGGAT ACTTCGGGTG GAGAAGACAG GGCACAGTGG

GTAGCCTGGA TTCGACGGAT ACTTCGGGTG GAGAAGACAG GGCACAGTGG

---------- ---------- ---------- ---------- ----------

GTAGCCTGGA TTCGACGGAT ACTTAGGGTG GAGAAGACAG GGCACAGTGG

GTAGCCTGGA TTCGACGGAT ACTTAGGGTG GAGAAGACAG GGCACAGTGG

---------- ---------- ---------- ---------- ----------

GCAGCCTGGA TCCATCGAAT ACTTCAGGTA GAGAAGACTG ACCACAGTGG

GTAGCCTGGA TCCGACGAAT ACTTCGGGTA GAGAAGACTG GCCACAGTGG

GTAGCCTGGA TCCGACGAAT CCTTCGGGTG GAGAAGACAG GACATAGCGG

---------- ---------- ---------- ---------- ----------

GTAGCCTGGA TCCGCCGAAT ACTTCGGGTG GAGAAGACGG GGCACAGTGG

GTAGCCTGGA TCCGACGGAT ACTTCGGGTG GAGAAGACAG GACACAGTGG

GTAGCCTGGA TTCGACGGAT ACTTCGGGTA GAAAAGACTG GACACAGTGG

GTAGCCTGGA TCCGACGAAT ACTTCGGGTT GAGAAGACAG GACACAGTGG

GTAGCCTGGA TCCGGCGAAT ACTCCGTGTT GAGAAGACAG GACACAGCGG

GTGGCTTGGA TCCGGCGGAT ACTTCGTGTA GAGAAAACAG GACACAGTGG

GTTGCATGGA TCCGGCGAAT ACTCCGGGTT GAGAAGACGG GCCACAGTGG

GTCGCTTGGA TCCGGCGAAT CCTGCGGGTG GAGAAGACAG GACACAGTGG

TACGCTGGAT CCCAAGGTGA CTGGTTGTTT ATCCACAGCT CGTTCATGGC

TACTCTGGAT CCCAAGGTGA CTGGTTGTTT ATCCACAGCT CGTTCATGGC

---------- ---------- ---------- -TCCACAGCT CGTTCATGGC

TACTCTGGAT CCCAAGGTGA CTGGTTGTTT ATCCACAGCT CGTTCATGGC

TACTCTGGAT CCCAAGGTGA CTGGTTGTTT ATCCACAGCT CGTCCATGGC

---------- ---------- ---------- -TCCACAGCT CATTCATGGC

CACACAGGAT TCCAAAGTGA CTGATTGTTT ATCCACAGTT CATTCATGGC

CACACTAGAT CCCAAAGTGA CTGGTTGTTT ATCCACAGTT CATTCATGGC

TACTCTGGAT CCCAAGGTGA CTGGCTGTTT A--------- ----------

---------- ---------- ---------- ---------- ----------

TACTCTGGAT CCCAAGGTGA CTGGTTGTTT ATCCACAGCT CGTTCATGGC

TACTCTAGAC CCTAAGGTGA CTGGCTGTTT ATCCACAGCT CGTTCATGGC

TACTCTGGAT CCCAAGGTGA CCGGCTGTTT A--------- ----------

TACCTTGGAT CCCAAGGTGA CAGGCTGTCT ATGCCCAGCT CGTCCATGGC

TACTCTGGAT CCCAAGGTGA CTGGCTGTCT G--------- ----------

GACACTGGAC CCCAAGGTGA CCGGCTGTCT GGCCATAGCT CGTCCATGGC

TACTTTGGAT CCGAAGGTGA CTGGCTGTTT ATCCACAGCT CATCCATGGC

AACTCTAGAT CCCAAAGTGA CGGGCTGCTT GTCCAGAGTT CATCCATGGC

CATGGTGTAG AAACACTCAC TGAAGATGGT TCGCTGCACC TTGACCGGGC

CATGGTGTAG AAACACTCAC TGAAGATGGT TCGCTGCACC TTGACCGGGC

CATGGTGTAG AAACACTCGC TGAAGATGGT CCGCTGCACC TTGACCGGAC

CATGGTGTAG AAACACTCGC TGAAGATGGT CCGCTGCACC TTGACCGGAC

CATAGTGTAG AAACACTCGC TGAAGATGGT CCGCTGCACC TTGACCGGGC

CATGGTGTAG AAGCACTCGC TGAAGATGGT CCGCTGCACC TTGACTGGGC

CATGGTGTAA AAGCACTCGC TGAAAATGGT CCGCTGCACC TTCACTGGAC

CATGGTGTAG AAGCACTCGC TGAAAATGGT CCGCTGCACC TTCACTGGCC

---------- ---------- ---------- ---------- ----------

---------- ---------- ---------- ---------- ----------

CATGGTGTAG AAACACTCGC TGAAGATGGT TCGCTGCACC TTGATGGGCT

CATAGTGTAG AAACATTCAC TAAAGATGGT TCGCTGCACC TTGACAGGGC

---------- ---------- ---------- ---------- ----------

CATGGTGTAG AAACACTCGC TGAAGATGGT CCGCTGCACC TTGACCGGGC

---------- ---------- ---------- ---------- ----------

CATGGTGTAA AAACACTCGC TGAAGATGGT GCGCTGCACC TTGACTGGGG

CATAGTGTAG AAGCACTCGC TGAAAATGGT TCGCTGAATT TTGATGGGGC

CATGACATAG AAGCACTCGC TGAAGATGGT ACGCTGGACT TTGACGGGCC

GGCCGTCCCG AGTCAGCACA AAGGCACACT TACCTGCCTC CCCTGCAGCC

GGCCGTCCCG AGTCAGCACA AAGGCACACT TACCTGCCTC CCCTGCAGCC

GGCCGTCCCG AGTCAGCACA AAGGCACACT TACCTGCCTC CCCTGCAGCC

GGCCGTCCCG AGTCAGCACA AAGGCACACT TACCTGCCTC CCCTGCAGCC

GGCCGTCGCG AGTCAGCACA AAGGCACACT TACCTGCCTC CCCTGTAGCC

GGCCATCCCG AGTTAGCACA AAGGCACACT TACCTGCCTC CCCTGAAGCC

GGCCGTCCTG GGTCAGCACA AAAGCACATT TACCTGCCTC CCCTGCAGCC

GGCCATCCCG AGTCAGCACA AAAGCACATT TACCTGCCTC CCCTGCAGCC

---------- ---------- ---------- ---------- ----------

---------- ---------- ---------- ---------- ----------

GGCCGTCCCG CGTCAGCACA AAGGCGCACT TACCTGCCTC CCCTGCAGCC

GGCCATCCCG CGTCAGCACA AAGGCACACT TACCTGCCTC CCCTGCAGCC

---------- ---------- ---------- ---------- ----------

GGCCGTCCCG CGTCAGCACG AAGGCGCACT TACCTGCCTG CCCTGCAGCC

---------- ---------- ---------- ---------- ----------

AGCCATCTCG AGTCAGCACG AAAGCACATT TACCTGCCTC CCCTGCAGCC

GGCCGTCCCG AGTCAGTACG AAGGCGCACT TACCTGCCTC CCCTGCAGCC

GGCCATCCCG AGTCAGCACA AAGGCACACT TACCTGCCGG CCTTGCAACC

ACACATACTT GAGGTCATCA TACACCCGCC CCTCGTTATT TCAAAAAAAG

ACACATACTT GAGGTCATCA TACACCCGCC CCTC------ ----------

ACACGTACTT GAGGTCATCA TACACCCGTC CCTCGTTATT TCAAAAAAAG

ACACGTACTT GAGGTCATCA TACACCCGTC CCTCGTTATT TCAAAAAAAG

ACACATACTT GAGGTCATCA TACACCTGCC CATCGTTATT TCAAAAAAAG

AGACATACTT GAGGTCATCA TACACCTGCC CGTCGTTATT TCAAAAAAAG

AGACATATTT GAGGTGATCA TATACCTGCC CATCGTTATT TCAAAAAAAG

AAACATATTT GAGGTGATCA TATACCTTCC CATCGTTATT TCAAAAAAAG

---------- ---------- ---------- ----GTTATT TCAAAAAAAG

---------- ---------- ---------- ---------- ----------

AGACGTACTT GAGATCATCA TACACCTGCC CGTCGTTATT TCAAAAAAAG

AGACATACTT GAGGTCGTCA TACACCCGCC CATCGTTACT TCAAAAAAAG

---------- ---------- ---------- ---------- ----------

AGACGTATTT GAGGTCATCA TACCTCTGTC CATCGTTATT TCAAAAAAAG

---------- ---------- ---------- ---------- ----------

ACACGTACTT GAGGTCGTCA TACACTTGCC CATCGTTATT TCAAAAAACG

AGACATACTT GAGGTCGTCG TACACTTGGC CCTCGTTATT TCAAAAAAAC

ACACATACTT GAGGTCATCA TACACCTGGC CATCATTATT TCCAAAAAAT

TCTTTAATTG TTCAAAATAG CACAAACGAC ATCGCACTAT GGTAATATTG

---------- ---------- ---------- ---------- ----------

TCTTTAATTG TTCAAAATAG CACAAACGAC ATCGCACTAT GGTAATATTG

TCTTTAATTG TTCAAAATAG CACAAACGAC ATCGCACTAT GGTAATATTG

TCTTTAATTG TTCAAAATAG CACAAACGAC ATCGCACTAT GGTAATATTG

TCTTTAATTG TTCAAAATAG CACAAACGAC ATCGCACTAT GGTAATATTG

TCTTTAATTG TTCAAAATAG CACAAACAAC ATCGCACTAT GGTAATATTG

TCTTTAATTG TTCAAAATAG CACAAACAAC ATCGCACTAT GGTAATATTG

TCTTTAATTG TTCAAAATAG CACAAACGAC ATCGCACTAT GGTAATATTG

---------- ---------- ---------- ---------- ----------

TCTTTAATTG TTCAAAATAG CACAAACGAC ATCGCACTAT GGTAATATTG

TCTTTAATTG TTCAAAATAG CACAAACGAC ATCGCACTAT GGTAATATTG

---------- ---------- ---------- ---------- ----------

TCTTTAATTG TTCAAAATAG CACAAACGAC ATCGCACTAT GGTAATATCG

---------- ---------- ---------- ---------- ----------

CCTTTAATTG TTCAAAATAG CACAAACGAC ATCGCACTAT GGTACTATGG

GTATTTATTG TTCAAAATAG CACAAACAAC ATAGCACTAG GGTAATATTG

GTCTTAATTG TTCAAAATAG CACAAACGAC ATAGCACTAT GGTAATATTG

AGTCCAGGGG TTACTCTACA ATAGTGATAG GGACTAAACG TGACATTTTC

---------- ---------- -------TAG GGACTAAACG TGACATTTTC

AGTCCAGGGG TTACTCTACA ATAGTGATAG GGACTAAACG TGACATTTTC

AGTCCAGGAG TTACTCTACA ATAGTGATAG GGACTAAACG TGACATTTTC

AGTCCAGGGG TTACTCTACA ATAGTGATAG GGACTAAACG TGACATTTTC

AGTCCAGG-G TTACTCTACA ATAGTGATAG GGACTAAACG TGACAGTTTC

AGTCCAAGGG TTACTCTACA ATAGTGATAT TGACTAAACA TGAC--TTTC

AGTCCAAGGG TTACTCTACA ATAGTGATAT TGACTAAACA TGAC--TTTC

AGTCCAAGGG TTACTCTACA ATAGTGATAG GGACTAAACG TGACGTCTTC

---------- ---------- ---------- ---------- ----------

AGTCCGGGGG TTACTCTACA ATAGTGATAG GGACTAAACG TGACATTTTC

AGTCC-GGGA TTACTCTACA ATAGTGATAG GGACTAAACG TGACATTTTC

---------- ---------- ---------- ---------- ----------

AGTCCAGGGG TTACTCTACA ATAGTGGTAG GGACTAAACG TGACATTTCC

---------- ---------- ---------- ---------- ----------

AGTCCGGGGC TTCCTCTACA ATAGTGGCAG GGACCAAGCG TGACATTTCC

AGTCCAGGGG GGGCTCTACA ATGGAAGTAG GAATGGAGTC TAAGAGACTA

AGTCCGGGGA TTACTCTACA ATGGAGATAA AGACTAAACG GTAAAGTTTC

CACAGCGAAA TATACTATAA TACAAAGTCA GAGGCTGGGC TGGCCAGGGT

CACAGCGAAA TATACTATAA TACAAAGTCA GAGGCTGGGC TGGCCAGGGT

CACAGCGAAA TATACTATAA TACAAAGTCA GAGGCTGGGC TGGCCAGAGT

CACAGCGAAA TATACTATAA TACAAAGTCA GAGGCTGGGC TGGCCAGAGT

CACAGCGAAA TATACTATAA TACAAAATCA GAGGGTGGGC TGGCTAGGGT

CACAGTGAAA TATACTATAA TACAAAATCA AAGGGTGGGC TGGCTAGGGT

CACAGAGA-- ---AATATAA TACAAAGTCA AAGGGTGGGC TAGCTAGGGT

CACAGA---- ---AATATAA TACAAAGTCA AAGGGTGGGC TGGCCAGGGT

CACACGAA-A TGTACTATAA TACAA----- ---------- ----------

---------- ---------- ---------- ---------- ----------

CACAGCGAAG TATACTATAG TACAAAGTCA AATGGGGGAC TGGCTAGGGT

CACAGCGAAA TATACTATAA TACAT----- ---------- ----------

---------- ---------- ---------- ---------- ----------

CACGGCGAAA TATACTATAA TACAAAGTCA AAGGGGGGGC TGGCTAGGGT

---------- ---------- -----AGTCG AAGGGGGAGC TGGCCAAGGT

CACAGAAAGA TGTACTATAA TACAA----- ---------- ----------

CACAGTGAAA TTTTCTATAA TACAAAGTCA AAGGGAGGGC TGGCCAGAGT

CACAGCGAAA TCCACTATAA TACAAAGTCA AAGGGAGGAC TGACCAGGCC

CGGGTAGGGC AGCAGTTTGT CTGGACCCCG AGAAACCCAA CTGGAATCCA

CGGGTAGGGC AGCAGTTTGT CTGGACCCCG AGAAACCCAA CTGGAATCCA

TGGGTAGGGC AGCAGTTTAT CTGGACCCCG AGAAACCCAG CTGGAATCCA

TGGGTAGGGC AGCAGTTTAT CTGGACCCCG AGAAACCCAG CTGGAATCCA

CGGGTAGGGC AGCAGTTTGT CTGGACCCCG AGACACCCAA CTGGAATCCA

AGGGTAGGGC AGCAGTTTAT CTGGCCCCCG AGAAACCCAA CTGGAATCCA

AGGGTAGGGT AGCAATTTGT CTGGCCCTCG GGAAACCCAA CTTGAATCCA

AGGGTAGGGT AGCAATTTGT CTGGCTCTCG GGAAACCCAA CTTGAATCCA

---------- ---------- ---------- ---------- ----------

---------- ---------- ---------- ---------- ----------

GGGGTAGGGC AGCAGTTTGT CTGGCCCCCG AGAAACCCAA CTGGAGTCCA

---------- ---------- ---------- ---------- ----------

---------- ---------- ---------- ---------- ----------

GGGGTATGGT AGGAGTTTGT CTGGCCCCCG AGAAACCCAG CTTGAATCCA

GGGGTAGGGC AGCAGTTTGT CTGGCCCCCG AGACACCCAG CTTGAATCCA

---------- ---------- ---------- ---------- ----------

GGGGTAGGAG AGCAGTTTGT CAGGGCCTCG GGCTGCCCAG CCTGTGGTGA

GGGATACTGG AGCAGCTTGT CAGAGCCCCG GGAGGCCCAG CTCGAGCCCA

GGGCCTCATC TGCTTCAAAG CCAAAGTCTT CCTCAACCTT AATCTGTCTT

GGGCCTCATC TGCTTCAAAG CCAAAGTCTT CCTCAACCTT AATCTGTCTT

GGGCCTCATC TGCTTCAAAG CCAAAGTCTT CCTCGACCTT AATCTGTCTT

GGGCCTCATC TGCTTCAAAG CCAAAGTCTT CCTCGACCTT AATCTGTCTT

GGGCCTCGTC TGCTTCAAAG CCAAAGTCTT CCTCGACCTT AATCTGTCTT

GAGCCTCATC TGCTTCAAAG GCAAAGTCTT CCTCAACCTT AATCTGTCTT

AGGCCTCATC TGCTTCAAAG CCAAAGTCTT CCTCGACCTT AATCTGTCTT

AGGCCTCATC TGCTTCAAAG CCAAAGTCTT CCTCAACCTT AATCTGTCTT

---------- ---------- ---------- ---------- ----------

---------- ---------- ---------- ---------- ----------

GGGCCTCATC TGCTTCGAAG CCAAAGTCTT CCTCGACCTT AATCTGTCTT

---------- ---------- ---------- ---------- ----------

---------- ---------- ---------- ---------- ----------

GAGCCTCGTC TGCTTCAAAG CCAAAGTCTT CCTCGACCTT AATCTGTCTT

GGGCCTCATC CGCTTCGAAG CCAAAGTCTT CCTCGACCTT GATCTGTCTT

---------- ---------- ---------- ---------- ----------

GGGCCTCATC TGGCTCGAAG CTGAAGTCAT CCTCAACTTT GATCTGTCCT

GCGTTTCTTC GGGTTCAAAG CCAAAATCGT CTTCGGTTTT GATCTGTCCC

TCTCTGGAGG CTCATTCTCC GCATTGCCTG GGGTGGGGGC ATCCGTGCCC

TCTCTGGAGG CTCATTCTCA GCATTGCCTG GGGTGGGGGC ATCCGTGCCC

TCTCTGGAGG CTCATTCTCA GCATTGCCTG GGGTGGGAGC ATCCGTGCCC

TCT------- ---------- ---------- ---------- ----------

TCTCTGGAGG CTCATTCTCA GCATTACCTG GGGTGGGAGC ATCTGTGCCC

TCTCTGGGGG TTCATTCTCG GCATTGCCTG GGGTGGGGGC ATCTGTGCCT

TCTCTGGAGG TTCATTCTCA ACATTGCCTG GGGTAGGGAC ATCAGTGCCT

TCTCTGGAGG TTCATTCTCA GCATTGCCTG GGGTAGGGAC ATCTGTGCCT

---------- ---------- ---------- ---------- ----------

---------- ---------- ---------- ---------- ----------

TCTCTGGGGG CTCATTCTCA GCATTGCCGG GAGTGGGAGC ATCTGTGCCC

---------- ---------- ---------- ---------- ----------

---------- ---------- ---------- ---------- ----------

TCTCTGGAGG CTCATTCTCA GCATTGCCTG GGGTGGGGGC ATCTGTGCCC

TCTCAGGAGG CTCGTTCTCA GCATTGCCTG GGGTGGGGGC ATCAGTGCCC

---------- ---------- ---------- ---------- ----------

TTTCAGGGGG CTCATTCTCT GCATTTCCCG GGGTGGGGGC ATCAGGGCCT

GCTCAGGCGG CTCATTCTCT GCGTTGCCTG GGGTTGGGGC GTCAGGACCG

TGGCTGCCCT CATCCTGGCT CACCTCCAGC ACAATGGTGA ACTGGCTGGC

TGGCTGCCCT CATCCTGGCT CACCTCCAGC ACAATGGTGA ACTGGCTGGC

TGGCTGCCCT CATCCTGGCT CACCTCTAGC ACAATGGTGA ACTGGCTGGC

---------- ---------- ---------- ---------- ----------

TGGCTGCCCT CATCCTGGCT CACCTCCAGC ACAATGGTGA ACTGGCTGGC

TGGCTGCCCT CATCCTGGCT CACCTCCAGC ATGATGGTGA ACTGGCTGGC

TGGCTGCCAT CATCCTGGCT CACCTCCAAC ACAATGGTGA ACTGGCTATC

TGGCTGCCAT CATCCTGGCT CACCTCCAAC ACAATGGTGA ACTGGCTATC

---------- ---------- ---------- ---------- ----------

---------- ---------- ---------- ---------- ----------

TGGCTGCCCT CGTCCTGGCT CACCTCCAGG ACTATGGTGA GCTGGCTGGC

---------- ---------- ---------- ---------- ----------

---------- ---------- ---------- ---------- ----------

TGGCTGCCCT CATCCTGGCT CACCTCCAGC ACAATGGTGA GCTGGCTGGT

TGGCTGCCCT CGTCCTGGCT CACCTCCAGC ACGATAGTGA ACTGGCCGGC

---------- ---------- ---------- ---------- ----------

TGGCTCCCCT CATCCTAGCT CACCTCCAGG ACAATAGTGA ACTGGCTCTT

TGACTGCTCT CATCCTGGCT CACCTCCAGG ACGATGGTGA ACTGACTTTT

CCGGTAGTCA TCCCCGTAGG AGTCCAGCAC TCTCATGAGG AACCTGATGT

CCGGTAGTCA TCCCCGTAGG AGTCCAGCAC TCTCATGAGG AACCTGATGT

CCGGTAGTCA TCCCCATAGG AGTCCAGCAC TCTCATGAGG AACCTGATGT

---------- ---------- ---------- ---------- ------ATGT

CCGGTAGTCA TCCCCGTAGG AGTCCAGCAC TCTCATGAGG AACCTGATGT

CCGGTAATCA TCCCCATAGG AGTCGAGCAC TCTCATGAGG AACCTAATGT

CCGGTAGTCA TCCCCGTATG AGTCCAGCAC TCGCATGAGG AATCTGATGT

TCGGTAGTCA TCCCCGTAGG AGTCCAGCAC CCTCATGAGG AACCTGATGT

---------- ---------- ---------- ---------- ----------

---------- ---------- ---------- ---------- ----------

ACGGTAGTCA TCGCCATAAG CATCCAGCAC TCTCATGAGG AACCTGATGT

---------- ---------- ---------- ---------- ----------

---------- ---------- ---------- ---------- ----------

TCGGTAGTCG TCCCCGTAAG AGTCCAGCAC TCTCATGAGG AACCTGATGT

CCGGTAGTCA TCCCCGTAGG AGTCCAGCAC TCGCATGAGG AACCTGATGT

---------- ---------- ---------- ---------- ----------

CCTGTAGTCA TCTCCATAGG AATCCAGGAC TCGCATGAGA AACCTGATGT

CCTGTAGTCG TCACCATAAG AGTCGAGGAC TCTCATGAGG AACCTGATGT

CTCTGGCAGA TGAGCTCTTA GCTGATCTCg aagaggcagc agaagaggag

CTCTGGCAGA TGAGCTCTTA GCTGATCTCg aagaggcagc agaagaggag

CTCTGGCAGA TGAGCTCTTA GCTGATCTCg aagaggcagc agaagaggag

CTCTGGCAGA CGAGCTCTTA GCTGATCTCg aagaggcagc agaagaggag

CTCTAGCAGA TGAGCTCTTA GCTGATCTCg aagaggcagc agaagaggag

CTCTAGCAGA TGAGCTCTTA GCTGACCTag aagaggcagc agaagaggaa

CGCTGGCAGA TGAGCTCTTG GCTGACCTTg aggaggcagc agaagaagaa

CTCTGGCTGA TGAGCTCTTG GCTGACCTCG AGGAGGCAGC AGAAGAAGAG

---------- ---------- ---------- ---------- ----------

---------- ---------- ---------- ---------- ----------

CTCTGGCAGA TGAGCTTCTG GCCGACCTTg aggaggcggc agaggaggaa

---------- ---------- ---------- ---------- ----------

---------- ---------- ---------- ---------- ----------

CTCTGGCAGA TGAGCTCTTG GCTGACCTCg aggaggcggc tgaagaggag

CTCTGGCAGA CGAGCTCTTG GCTGACCTCg aggaggcggc tgaagaggag

---------- ---------- ---------- ---------- ----------

CGCTTGCGGA TGAGCTCCTG GCTGACCTGG AGGAGGCAGC TGAGGAGGAA

CCCTGGCAGA TGAGCTCCTG GCAGACCTgg aggaggcggc ggaggaggag

gaaggaggaa gctatgggga ggaagaagag gagccagcga tcgaggatgt

gaaggaggaa gctatgggga ggaagaagag gagccagcga tcgaggatgt

gaaggaggaa gctatgggga ggaagaggag gagCCAGCCA TCGAGGATGT

gaaggaggaa gctatgggga ggaagaggag gagCCAGCCA TCGAGGATGT

gaaggaggaa gctatgggga ggaggaagag gagccagcga tcgaggatgt

gaaggaggaa gctatgggga ggaagaagag gaGCCAGCGA TTGAGGATGT

gaaggaggaa gctatggaga agaagaggag gaGCCAGCAA TTGAGGATGT

GAAGGAGGAA GCTATGGAGA AGAGGAGGAG GAGCCAGCAA TTGAGGATGT

---------- ---------- ---------- ---------- ----------

---------- ---------- ---------- ---------- ----------

gaaggaggaa gctatgggga ggaagaagag gagccagcaa tcgaggatgt

---------- ---------- ---------- ---------- ----------

---------- ---------- ---------- ---------- ----------

gaaggaggaa gctatgggga ggaagaagag gagccagcga tcgaggatgt

gaaggaggaa gctatgggga ggaagaagag gagCCAGCAA TTGAGGATGT

---------- ---------- ---------- ---------- ----------

GAGGGTGGGA GCTTTGGGGA GGAGGAAGAA GAACCTCCCA TTGAAGATGT

gagggaggca gctacgggga ggaagaagag gaACCGGCCA TCGAGGATGT

gcaggaggag ACACAGCTGG ATCTTTCCGG GGATTCAGTC AAGACCATCG

gcaggaggag ACACAGCTGG ATCTTTCCGG GGATTCAGTC AAGACCATCG

GCAGGAGGAG ACACAGCTGG ATCTTTCCGG GGATTCGGTC AAGACCATCG

GCAGGAGGAG ACACAGCTGG ATCTTTCCGG GGATTCGGTC AAGACCATCG

gcaggaggag ACACAGCTGG ATCTTTCCGG GGATTCGGTC AAGACCATCG

TCAGGAAGAG ACACAACTGG ATCTTTCTGG GGATTCAGTC AAGAGCATCG

ACAAGAGGAG ACACAACTGG ATCTTTCTGG AGATTCAGTC AAGAGCATTG

ACAGGAGGAG ACCCAGCTGG ATCTTTCTGG AGATTCAGTC AAGAGCATTG

---------- ---------- ---------- ---------- ----------

---------- ---------- ---------- ---------- ----------

gcaggaggag ACGCAGCTGG ATCTTTCTGG GGATTCAGTC AAGAGTATCG

---------- ---------- ---------- ---------- ----------

---------- ---------- ---------- ---------- ----------

gcaggaggaA ACACAGCTGG ATCTTTCTGG GGATTCGGTC AAGAGCATTG

GCAGGAGGAA ACACAGCTGG ACCTTTCTGG GGACTCAGTC AAGAGCATCG

---------- ---------- ---------- ---------- ----------

CCAGGAGGAG ATGCAGCTGG ACCTCTCCGG GGACTCGGTC AAGAGCATTG

GCAGGAGGAA ATGCAGCTGG ACCTGAGCGG GGACTCGGTC AAGAGCATCG

CCAAGCTATG GGATAGTAAG ATGGTAAGAG TTTGCTGAGA TTATGATGAA

CCAAGCTATG GGACAGTAAG ATGGTAAGAG TTTGCTGAGA TTATGATGAA

CCAAGCTATG GGACAGTAAG ATGGTAAGAG TTTGCTGAGA TTATGATGAA

CCAAGCTATG GGACAGTAAG ATGGTAAGAG TTTGCTGAGA TTATGATGAA

CCAAGCTATG GGACAGTAAG ATGGTAAGAG TTTGCTGAGA TCATGATGAA

CCAAGCTATG GGACAGCAAG ATGGTAAGAG TTTGCTGAGA TTATGATGAA

CCAAGCTATG GGATAGCAAG ATGGTGAGAG TTTGCTGAGA TCATGATGAA

CCAAGCTATG GGACAGCAAG ATGGTGAGAG TTTGCTGAGA TCATGATGAA

---------G GGACAGCAAG ATGGTGAGAG TTCGCGGAGA TCATGATGAA

---------- ---------- ---------- ---------- ----------

CCAAGCTGTG GGACAGCAAG ATGGTGAGAG TTTGCTGAGA TCATGATGAA

---------- ---------- ---------- ---------- ----------

---------- ---------- ---------- ---------- ----------

CCAAACTATG GGACAGCAAG ATGGTGAGAG TTTGCTGAAA TTATGATGAA

CCAAGCTATG GGACAGCAAG ATGGTGAGAG TTTGCTGAGA TCATGATGAA

---------- ---------- ---------- ---------- ----------

CCAAGTTGTG GGACAGCAAG ATGGTGAGAG TTTGCTGAGA TCATGGTGAA

CCAAGCTGTG GGACAGCAAG ATGGTGGGAG TTCGCGGAGA TCATGGTGAA

GATTGAGGAG TATATCAGCA AGCAAGCCAA AGCTTCAGAA GGTAGTGATG

GATTGAGGAG TATATCAGCA AGCAAGCCAA AGCTTCAGAA GGTAGTGATG

GATTGAGGAG TATATCAGCA AGCAAGCCAA AGCTTCAGAA GGTAGTGATG

GATTGAGGAG TATATCAGCA AGCAAGCCAA AGCTTCAGAA GGTAGTGATG

GATTGAGGAG TATATCAGCA AGCAAGCCAA AGCTTCCGAA GGTAGTGATG

GATTGAGGAG TATATCAGCA AGCAAGCCAA AGCTTCAGAA GGTAGTGATG

GATTGAGGAG TATATCAGCA AGCAAGCCAA AGCTTCAGAA GGTAGTGATG

GATTGAGGAG TATATCAGCA AGCAAGCCAA CGTGTCGGAA GGTAGTGATG

GATCGAGGAG TACATCAGCA AGCAGGCGAA GGCGGCGGAA GGTAGTGATG

---------- ---------- ---------- ---------- ----------

GATTGAGGAG TATATCAGCA AGCAGGCCAA GGCCTCGGAA GGTAGTGATG

---------- ---------- ---------- ---------- ----------

---------- ---------- ---------- ---------- ---AGTGATG

GATTGAGGAG TATATCAGCA AGCAAGCCAA AGCTTCAGAA GGTAGTGATG

GATCGAGGAG TATATAAGCA AGCAAGCCAA AGCTTCAGAA GGTAGTGATG

---------- ---------- ---------- ---------- ---AGTGATG

GATTGAAGAG TATGTCAGTA AACAGGCCAA GGCTTCTGAA GGTAGTGACG

GATCGAAGAG TACATCAGTA AGCAAGCCAA GGCTTCCGAA GGTAGTGCTG

GGACCAGTGG AGGCCGCGCC TGAATACCGC GTCATCGTGG ATGCCAACAA

GGACCAGTGG AGGCAGCACC TGAATACCGC GTCATCGTGG ATGCCAACAA

GGACCGGTGG AGGCAGCGCC TGAATACCGC GTCATCGTGG ATGCCAACAA

GGACCGGTGG AGGCAGCGCC TGAATACCGC GTCATCGTGG ATGCCAACAA

GGGCCAGTGG AGGCAGCCCC TGAATACCGC GTCATTGTGG ATGCCAACAA

GGGCCAGTGG AGGCAGCCCC TGAGTACCGG GTCATTGTGG ATGCCAACAA

GGACCGGTGG AGGCAGCCCC TGAGTACCGA GTCATTGTGG ATGCCAACAA

GGACCAGTGG AGGCAGCTCC TGAGTACCGA GTCATTGTGG ATGCCAACAA

GGACCGGTGG AGGCGGCCCC TGAGTACCGT GTCATCGTGG ACGCCAACAA

---------- ---------- ---------- ---------- ----------

GGACCTGTTG AGGCGGCCCC TGAATACCGA GTCATCGTGG ATGCTAACAA

---------- ---------- ---------- ---------- ----------

GGGCCGGTGG AGGCGGCCCC CGAGTACCGG GTCATCGTGG ACGCCAACAA

GGCCCGGTGG AGGCGGCCCC TGAGTACCGC GTCATCGTGG ATGCCAACAA

GGCCCAGTAG AGGCGGCCCC TGAATACCGG GTCATCGTGG ACGCCAACAA

GGCCCGGTAG AGGCGGCCCC TGAATACCGG GTCATCGTGG ATGCCAACAA

GGGCCAGTGG AGGCAGCCCC TGAGTATCGG GTCATCGTGG ATGCCAACAA

GGCCCTGTGG AAGCCGCCCC TGAATATCGA GTCATCGTGG ACGCCAACAA

CCTGACCGTG GAGATCGAAA ACGAGCTGAG TGAGTAGGTG TGGCCGGCTC

CCTGACCGTG GAGATCGAAA ATGAGCTGAG TGAGTAGGTG TGGCCGGCTC

CCTGACCGTG GAGATCGAAA ACGAGCTAAG TGAGTAGGTG TGGCCGGCTC

CCTGACCGTG GAGATCGAAA ACGAGCTGAG TGAGTAGGCG TGGCCGGCTC

CCTGACCGTG GAGATCGAAA ACGAGCTGAG TGAGTAGGTG TGGCCGGCTC

CCTGACTGTG GAGATCGAGA ATGAGCTGAG TGAGTAGGTG TGGCTGGCTC

CCTGACTGTG GAGATAGAAA ACGAGCTGAG TGAGTAGGTG TGGCTGGCTC

CCTGACTGTG GAGATAGAAA ATGAGCTGAG TGAGTAGGTG TGGCTGGCTC

CCTGACCGTG GAGATCGAGA ACGAGCTGAG TGAGT----- ----------

---------- ---------- ---------- ---------- ----------

CCTGACCGTG GAGATCGAGA ATGAGCTGAG TGAGTAGGGG TGGCTGGCTC

---------- ---------- ---------- ---------- ----------

CCTGACGGTG GAGATCGAGA ATGAGTTGAG TGAGTAGGGG TGGCCGGCTC

CCTGACCGTG GAGATTGAGA ACGAACTGAG TGAGTAGGGG TGGCCGGCTC

CCTGACCGT- GAGATCGAGA ATGAACTGAG TGAGTAGGAG TAGCTGGCTC

CCTGACCGTG GAGATCGAGA ACGAGCTTAG TGAGTAGGCG TGGCTGGCTC

CCTGACTGTG GAGATTGAga atgagctgag tgagtAGGGG TGGGGTGCTT

CCTGACCGTG GAGATTGAGA ATGAGCTGAG TGAGTAGGCG TGGCAGGCTC

GTCTACCTCA GTGCTGCCCC ACACCGGCTA CATCTACCAC AGTGACATCG

GTCTACCTCA GTGCTGCCCC ACACCGGCTA CATCTACCAC AGTGACATCG

GTCTACCTCA GTGCTGCCCC ACACCGGCTA CATCTACCAC AGTGACATTG

GTCTACCTCA GTGCTGCCCC ACACCGGCTA CATCTACCAC AGTGACATTG

ATCCACCTCA GTGCTGCCCC ATACCGGCTA CATCTACCAC AGCGACATCG

ATCTACCTCC GTGCTGCCCC ACACTGGTTA CATCTACCAC AGTGACATCG

TTCTACCTCA GTGCTGCCTC ACACTGGCTA CATCTACCAC AGTGACATTG

TTCTACCTCA GTGTTGCCTC ATACTGGTTA TATCTACCAC AGTGACATTG

---------- ---------- ---------- ---------- ----------

---------- ---------- ---------- ---------- ----------

GTCCACGTCT GTGCTGCCCC ACACTGGTTA CATCTACCAC AGTGACATTG

---------- ---------- ---------- ---------- ----------

GTCCACATCA GTGCTCCCAC ACACCGGCTA CATCTACCAC AGCGACATCG

GTCTACCTCG GTACTGCCGC ACACCGGCTA CGTCTACCAC AGCGACATCG

GTCAACCTCT GTGCTGCCCC ACACGAGCTA CATCTACCAC AGCGACATTG

GTCCACTTCG GTGCTGCCCC ACACGGGCTA CATCTACCAC AGTGACATCG

CCCCAGGTGG GCTACGACCC TCAAGGACGA GATTGAGCGC AATGACAAGT

CAGCACCTCG GTGCTGCCCC ACACGGGCTA CATCTATCAC AGTGACATCG

TGCAGTCCCT GCCACCGGTA GATCGAGGAG GACGCCTACC AGGAGGACCT

TGCAGTCCCT GCCACCGGTA GATCGAGGAG GACGCCTACC AGGAGGACCT

TGCAGTCCCT GCCCCCGGTA GATTGAGGAG GACGCCTACC AGGAGGACCT

TGCAGTCCCT GCCCCCGGT- ---------- ---------- ----------

TGCAGTCCCT GCCCCCGGTA GATCGAGGAG GACGCCTACC AGGAGGACCT

TGCAGTCGCT GCCCCCGGTA GATTGAGGAG GATGCCTACC AGGAGGACCT

TGCAGTCTCT GCCCCCAGTA GATTGAGGAG GATGCCTATC AAGAAGACCT

TGCAGTCTCT GCCCCCAGTA GATTGAGGAG GATGCCTATC AGGAAGACCT

---------- ---------- ---------- ---------- ----------

---------- ---------- ---------- ---------- ----------

TGCAGTCTCT GCCCCCGGTA GATCGAGGAA GACGCCTACC AAGAGGACCT

---------- ---------- ---------- ---------- ----------

TGCAGTCGCT GCCCCCGGT- ---------- ---------- ----------

TGCAGTCCCT GCCTCCGGTA GATCGAGGAG GACGCCTACC AGGAGGACCT

TGCAGTCCCT GCCCCCAGT- ---------- ---------- ----------

TGCAGTCCCT GCCCCCGGTA GATCGAGGAG GACGCCTACC AGGAGGACCT

GGCAGGAGCC GCCGCCCGTA GATTGAGGAG GACGCCTACC AGGAGGATTT

TCCAGTCTCT GCCCCCGGTA GATCGAGGAG GACGCCTACC AGGAGGACCT

GGGATTCAGC CTGGGCCACC TGGGCAAGTC GGGCAGTGGG CGTGTGCGGC

GGGATTCAGC CTGGGCCACC TGGGCAAGTC GGGCAGTGGG CGTGTGCGGC

GGGATTCAGC CTGGGCCACC TGGGCAAGTC GGGCAGTGGG CGCGTGCGGC

---------- ---------- ---------- ---------- ----------

GGGATTCAGC CTGGGCCACC TGGGCAAGTC GGGCAGTGGG CGCGTGCGGC

GGGCTTCAGT CTGGGCCACC TGGGCAAGTC AGGGAGTGGG CGTGTGCGGC

GGGCTTTAGC CTGGGCCACT TGGGCAAGTC AGGCAGTGGG CGTGTGCGGC

GGGCTTTAGC CTGGGCCACT TGGGCAAGTC AGGCAGTGGG CGTGTGCGGC

---------- ---------- ---------- ---------- ----------

---------- ---------- ---------- ---------- ----------

GGGCTTCAGC CTGGGCCATC TGGGCAAATC AGGCAGCGGG CGGGTACGGC

---------- ---------- ---------- ---------- ----------

---------- ---------- ---------- ---------- ----------

GGGCTTCAGC CTGGGCCACC TGGGCAAATC AGGCAGTGGG CGTGTCCGGC

---------- ---------- ---------- ---------- ----------

GGGCTTCAGC CTGGGGCACC TGGGCAAGTC GGGCAGCGGG CGAGTGCGGC

GGGCTTCAGC CTGGGCCACC TGGGCAAGTC GGGCAGCGGG CGCGTGCGGC

CGGCTTCAGC CTGGGCCACC TGGGCAAGTC GGGGAGCGGG CGGGTGCGGC

AGACACAGGT AAACGAGGCC ACCAAGGCCA GGATCTCCAA GACGCTGCAG

AGACGCAGGT AAACGAGGCC ACCAAGGCCA GGATCTCCAA GACGCTGCAG

AGACGCAGGT CAACGAGGCC ACCAAGGCGA GGATCTCCAA GACGCTGCAG

---------- ---------- ---------- ---------- ----------

AGACACAGGT CAACGAGGCC ACCAAGGCCA GGATCTCCAA GACGCTGCAG

AGACACAGGT GAACGAGGCC ACCAAGGCCA GGATCTCTAA GACATTGCAG

AGACACAGGT GAATGAGGCC ACCAAGGCTA GGATCTCCAA GACACTACAG

AGACCCAGGT GAATGAGGCC ACCAAGGCCA GGATCTCCAA GACACTGCAG

---------- ---------- ---------- ---------- ----------

---------- ---------- ---------- ---------- ----------

AGACACAGGT GAACGAGGCC ACCAAAGCCA GGATCTCTAA GACACTGCAG

---------- ---------- ---------- ---------- ----------

---------- ---------- ---------- ---------- ----------

AGACGCAAGT GAATGAGGCC ACCAAAGCCA GGATCTCCAA GACGCTACAG

---------- ---------- ---------- ---------- ----------

AGACGCAGGT GAACGAGGCC ACCAAAGCCA GGATCTCCAA GACGCTGCAG

AGACCCAAGT CAACGAAGCC ACCAAGGCCC GCATCTCCAA GACGCTGCAG

AGACACAGGT CAACGAGGCC ACTAAAGCCC GGATCTCCAA GACCTTACAG

GTAAGGGCCT GGAGATTGTG AACCCACAGG CGGCAGAGAA GAAGGTGGCT

GTAAGGGCCT GGAGATTGTG AACCCACAGG CGGCAGAGAA GAAGGTGGCT

GTAAGGGCCT GGAGATTGTG AACCCGCAGG CGGCAGAGAA GAAGGTGGCT

---------- ---------- ---------- ---------- ----------

GTAAGGGCCT GGAGATTGTG AACCCACAGG CCGCGGAGAA GAAGGTGGCT

GTTAGGGCTT GGAGATTGTG AACCCACAAG CAGCAGAGAA AAAGGTGGCT

GTAAGGGTCT GGAGATTGTG AACCCACAAG CAGCCGAGAA GAAGGTGGCA

GTAAGGGTCT AGAGATTGTG AACCCACAAG CAGCCGAGAA GAAGGTGGCA

---AGGGCCT GGAGATCGTG AACCCACAGG CAGCCGAGAA GAAGGTGGCG

---------- ---------- ---------- ---------- ----------

GTAAGGGCCT AGAGATTGTG AACCCACAAG CAGCTGAGAA GAAGGTGGCT

---------- ---------- ---------- ---------- ----------

---------- ---------- ---------- ---------- ----------

GTAAGGGCCT GGAGATCGTG AACCCACAAG CTGCCGAGAA GAAGGTGGCC

---AGGGCTT GGAGATTGTG AACCCACAGG CGGCCGAGAA GAAGGTGGCT

GTAAGGGCCT GGAGATCGTA AACCCGCAGG CGGCGGAGAA GAAGGTGGCC

GTAAGGGCTT GGAGATCGTG AACCCACAGG CGGCTGAGAA GAAGGTGGCT

GTGAGGGCTT GGAGATCGTG AATCCGCAGG CAGCGGAGAA GAAGGTAGCC

GAGGCCAACC AGAAGTATTT CTCCAGCATG GCTGAGTTCC TCAAGGTCAA

GAGGCCAACC AGAAGTATTT CTCCAGCATG GCTGAGTTCC TCAAGGTCAA

GAGGCCAACC AGAAGTATTT CTCTAGCATG GCTGAGTTCC TCAAGGTCAA

---------- ---------- ---------- ---------- ----------

GAGGCCAATC AGAAGTATTT CTCCAGCATG GCAGAGTTCC TCAAGGTCAA

GAGGCCAACC AGAAGTACTT CTCCAGCATG GCCGAGTTCC TCAAGGTCAA

GAGGCCAATC AGAAGTATTT CTCTAGCATG GCCGAGTTCC TCAAGGTCAA

GAGGCCAACC AGAAGTATTT CTCTAGCATG GCCGAGTTCC TCAAGGTCAA

GAGGCCAACC AGAAGTACTT CTCCAGCATG GCCGAGTTCC TCAAGGTCAA

---------- ---------- ---------- ---------- ----------

GAAGCCAACC AGAAGTATTT CTCCAGCATG GCCGAGTTCC TCAAGGTCAA

---------- ---------- ---------- ---------- ----------

---------- ---------- ---------- ---------- ----------

GAGGCCAACC AGAAGTACTT CTCCAGCATG GCCGAGTTCC TCAAGGTCAA

GAGGCCAACC AGAAGTACTT CTCCAGCATG GCCGAGTTCC TCAAGGTCAA

GAGGCTAACC AGAAGTACTT CTCCAGCATG GCCGAGTTCC TCAAGGTGAA

GAGGCCAACC AGAAGTACTT TTCCAGCATG GCCGAGTTCC TCAAGGTCAA

GAGGCCAATC AGAAATACTT CTCCAGCATG GCCGAGTTCC TTAAAGTCAA

GGGCGAGAAG AGTGGCCTTA TGTCCACCTG AGCAGCGTCC GTCTGAGAGT

GGGCGAGAAG AGTGGCCTTA TGTCTACCTG AGCAGCGTCA GTCTGAGAGT

GGGCGAGAAG AGTGGCCTCA TGTCCACCTG AGCAGCGTCC GTCTGAGAGT

---------- ---------- ---------- -GCAGCGTCC GTCTGAGAGT

GGGCGAGAAG AGTGGCCTCA TGTCTACCTG AGCAGCGTCC GTCTGAGAGT

GGGTGAGAAG AGTGGCATCA TGTCCACCTG AGCAGTGTCC GTCTGAGAGT

GGGTGAGAAA AGTGGCACCA TGTCTACTTG AGCAGCGTCC GTTTGAGAGT

GGGTGAGAAA AGTGGCACCA TGTCTACCTG AGCAGCGGCC ATTTGAGAGT

GGGCGACAAG AGCGGCATCA TGTCCACCTG AGCAGCGTCC GTCTGGGAGT

---------- ---------- ---------- ---------- ----------

GGGCGAGAAA AGCGGCATCA TGTCCACCTG AGCAGCGTCC GTCTGAGAGT

---------- ---------- ---------- ---------- ----------

---------- ---------- ---------- ---------- ----------

GGGCGAGAAG AGCGGCCTCA TGTCCACCTG AGCAGCGTCC GACTGAGAGT

GAGCGAGAAG AGTGGCATCA TGTCTA-TTG AGCAGCGTCC GTCTGAGAGT

GAGCGAGAAG AGCGGCATCA TGTCCACCTG A--------- ----------

GGGGGAGAAG AGTGGGGTGC TGTCCACGTG AGCAGTATCT GACTGGGAGC

GGGCGAGAAG AGCGGCATCA TGTCCACCTG AGCAGCGTCC GGCTGGGAGT

ATGAAGAGAG TGCGTCTGTA GGGCAGGAAG ATGGCGGACA AGCGCAAACT

ATGAAGAGAG TGCGTCTGTA GGGCAGGAAG ATGGCGGACA AGCGCAAACT

ATGAAGAGAG TGCGTTTGTA GGGCAGGAAG ATGGCGGACA AGCGCAAACT

ATGAAGAGAG TGCGTCTGTA GGGCAGGAAG ATGGCGGACA AGCGCAAACT

ATGAAGAGAG TGCGTCTGTA GGACAGGAAG ATGGCGGACA AGCGCAAACT

ATGAAGAGAG TGCGTCTGTA GGGCAGGAAG ATGGCGGATA AGCGCAAACT

ATGAAGAGAG TGCGTCTGTA GGGCAGGAAG ATGGCGGACA AGCGCAAACT

ATGAAGAGAG TGCGTCTGTA GGGCAGGAAG ATGGCGGACA AGCGCAAACT

ATGAAGAGAG TGCGTCTGTA GGGCCGGAAG ATGGCGGACA AGCGCAAACT

---------- ---------- ---------- ---------- ----------

ATGAAGAGAG TGCGTCTGTA GGGCAGGAAG ATGGCGGACA AGCGCAAACT

---------- ---------- ---------- ---------- ----------

---------- ---------- ---------- ---------- ----------

ATGAAGAGAG TGCGTCTGTA GGGCAGGAAG ATGGCGGACA AGCGCAAACT

ATGAAGAGAG TGCGTCTGTA GGGCAGGAAG ATGGCGGACA AGCACAAACT

---------- ---------- ---------- ---------- ----------

ATGAAGAGCA GATATCTCTA GGCCAGAAAG ATGGCGGATA AGCGAAAACT

ATGAAGACGG CTCCACTTTA GGGCAGAAAC ATGGCGGACA AGCGCAAACT

CCAAGGTTCC CAGGTGAGAT TGATCGCTGC CTCAAGAAGG TGTCCGAGGG

CCAAGGTTCC CAGGTGAGAT TGATCGCTGC CTCAAGAAGG TGTCCGAGGG

CCAAGGTTCC CAGGTGAGAT TGATCGCTGC CTCAAGAAGG TGTCCGAGGG

CCAAGGTTCC CAGGTGAGAT TGATCGCTGC CTCAAGAAGG TGTCCGAGGG

CCAAGGTTCC CAGGTGAGAT TGATCGCTGC CTCAAGAAGG TGTCCGAGGG

CCAAGGTTCC CAGGTGAGAT TGATCGCTGC CTCAAGAAGG TGTCTGAGGG

CCAAGGTTCC CAGGTGAGAT TGATCGCTGC CTCAAGAAGG TGTCCGAAGG

CCAAGGTTCC CAGGTGAGAT TGATCGCTGC CTCAAGAAGG TGTCCGAAGG

CCAAGGTTCC CAGGTGAGAT TGACCGCTGC CTCAAGAAGG TGTCCGAGGG

---------- ---------- ---------- ---------- ----------

CCAAGGTTCC CAGGTGAGAT TGATCGCTGC CTCAAGAAGG TGTCCGAGGG

---------- ---------- ---------- ---------- ----------

---------- ---------- ---------- ---------- ----------

CCAAGGTTCC CAGGTGAGAT TGATCGCTGC CTCAAGAAGG TGTCCGAGGG

CCAAGGTTCC CAGGTGAGAT TGATCGCTGC CTCAAGAAGG TGTCCGAGGG

---------- ---------- ---------- ---------- ----------

CCAAGGTCCC CAGGTGAGAT TGATCGCTGC CTCAAGAAGG TGTCCGAAGG

TCAAGGTTTT CAGGAGAGAT CGACCGCTGC CTCAAGAAAG TGTCCGAGGG

CGTGGAGCAG TTTGAAGATA TTTGGCAGAA GGTCAGACCT GGAGATCAGC

CGTGGAGCAG TTTGAAGATA TTTGGCAGAA GGTCAG---- ----------

CGTGGAGCAG TTTGAAGATA TTTGGCAGAA GGTCAGACCT GGAGATCAGC

CGTGGAGCAG TTTGAAGATA TTTGGCAGAA GGTCAGACCT GGAGATCAGC

CGTGGAGCAG TTTGAAGATA TTTGGCAGAA GGTCAGACCT GGAAATCAGC

TGTGGAGCAG TTTGAAGATA TTTGGCAGAA GGTTAGACCT GGAGATCAGC

TGTGGAGCAG TTTGAAGATA TTTGGCAAAA GGT-AGACCT AGAGACGATC

GGTGGAGCAG TTTGAAGATA TTTGGCAAAA GGT-AG---- ----------

CGTGGAGCAG TTTGAAGACA TCTGGCAGAA GGT------- ----------

---------- ---------- ---------- ---------- ----------

CGTGGAACAG TTTGAGGATA TTTGGCAGAA GGTCAGACCT GGAGATTAAG

---------- ---------- ---------- ---------- ----------

---------- ---------- ---------- ------ACCT GGAGATCAGC

TGTGGAGCAG TTTGAAGATA TCTGGCAGAA GGTCAGACCT GGAGATCAGC

CGTGGAGCAG TTTGAGGATA TTTGGCAGAA GGTCAGACCT GGAGAACAGC

---------- ---------- ---------- ---------- ----------

TGTTGAGCAG TTCGAGGACA TTTGGCAGAA GGTACGACAG GGAGATCAAT

CGTCGAGCAG TTTGAGGATA TCTGGCAGAA GGTGAAACCT GGCCAGGAGG

AGAAGGGGCA CAGCAAAAGC CTGGGTCCCC AGGAAGAAGA GTCAGCAGAT

---------- ---------- ---------- ---------- GTCAGCAGAT

AGAAGGGGCG CAGCAAAAGC CTGGGTCCCC AGGAAGAAGA GTCAGCAGAT

AGAAGGGGCG CAGCAAAAGC CTGGGTCCCC AGGAAGAAGA GTCAGCAGAT

AGAAGGGGTA CAGCAAAAGC CTGAGTCCCC AGGAAGAAGA GTCAGCAGAT

AGAAGGGGCA CAGCAAAAGC CTGGGTTCCC AGGAAGAAGA GTCAGCAGGT

AGAAGGGGTA CAGTAAAAGC CTGGGTTCCC AGAAAGTAGA ATCAGCAGAT

---------- ---------- ---------- ---------- ATCAGCAGAT

---------- ---------- ---------- ---------- ----------

---------- ---------- ---------- ---------- ----------

AGAAGAGGCA CAGCAAAAGC CTGGGTCCCC AGGAAAAAGA GTCAGCAGAT

---------- ---------- ---------- ---------- GTCAGCAGAT

AGAAGGGGTA CAGCAAAGGC CTGGGTGCCC AAGAAGAAGA GTCAGCAGAT

AGAAGGGGCA CAGCAAAAGA CTGGGTCCCC AGGAAGAGGA GTCAGCAGGT

AGAAGGGGCA CAGCAAAGGC CTGGGTCCCC AGGAAGAAGA GTCAGCAGAT

---------- ---------- ---------- ---------- ----------

TGTGGCGGGG AGTTAGAGAG CTGAGGGCCT TGGAGGAGGT GTCAGCAGGT

AATAGCGGGA CCGCAAACGT CTGGGTCCCC AGGAAGAAAA GTCAGCAGGT

CAAAGAGCAG AAGTTTGTAC ATTTCCTGGC CCAGGACAGT CTCCCAGCAC

CAAAGAGCAG AAGTTTGTAC ATTTCCTGGC CCAGGACAGT CTCCCAGCAC

CAAAGAGCAG AAGTTTGTAC ATTTCCTGGC CCAGGACAGT CTCCCAGCAC

CAAAGAGCAG AAGTTTGTAC ATTTCCTGGC CCAGGACAGT CTCCCAGCAC

CAAAGAGTAG GAGCTTGTAC ATTTCCTGGC CCACGAGAGT CTCCCAGCAC

CAAAAAGCAG AAGCTTGTAC ATCTCTTGGC CAAGCCGAGT CTCCCAGCAC

CAAAGAGCAC AAGTTTGTAC ATCTCCTGGC CCAGTCGAGT CTCCCAGCAC

CAAAGAGTAC AAGTTTGTAC ATCTCCTGGC CCAGCCGAGT CTCCCAGCAC

---------- ---------- ---------- ---------- ----------

---------- ---------- ---------- ---------- ----------

CAAAGAGCAG GAGTTTGTAC ATCTCCTGCC CCAGCCGGGT CTCCCAGCAC

CAAAAAGCAG GAGTTTGTAC ATCTCTTGCC CCAGCCGAGT CTCCCAGCAC

CAAAGATCAG AAGTTTGTAC ATCTCCTGTC CCATACGTGT CTCCCAGCAC

CAAACAGCAG GAGTTTGTAC ATCTCCTGCC CCAGCCGAGT CTCCCAGCAC

CAAAAAGCAT GAGTTTGTAC ATCTCCTGCT CTAGCCGGGT CTCTCAGCAC

---------- ---------- ---------- ---------- ----------

CAAACAGCAG CAGCTTGTAC ATCTCCTGCC CTAGCCGAGT CTCCCAACAC

CAAACAGCAG CAGTTTATAC ATCTCCTGGC CCAGCATGGT CTCCCAGCAC

TGCCCCCACA AGTGATCTGA TTCCAGAGAG AGAAGAGCAG GACCACCAGG

TGCCCCCACA AGTGATCTGA TTCCAGAGAG AGAAGAGCAG GACCACCAGG

TGCCCCCACA AGTGATCTGT TTCCAGAGAG AGAAGAGCAG GACCACCAGG

TGCCCCCACA AGTGATCTGT TTCCAGAGAG AGAAGAGCAG GACCACCAGG

TGCCCCCACA AGTGATCTGA TTCCAGAGAG AGAAGAGCAG GACCACCAGG

TGCCCCCACA GGTAATCTGA TTCCAGAGGG AGAAGAGCAG GACCACCAAG

T-CCTCCACA CGTGATCTGA TTCCACAGAG AGAACAGAAG AAACACCAGG

TGCCCCCACA CGTGATCTGA CTCCACAGAG AGACCAGAAG GAACACCAGC

---------- ---------- ---------- ---------- ----------

---------- ---------- ---------- ---------- ----------

TGCCCCCACA AGTGATCTGA TTCCAGAGGG AGAAGAGCAG GACTAGCAGG

TGCCCCCGCA AGTGATCTGC TTCCAGAGAG AGAAGATCAG GACCAGCAGG

TGCCCCCACA AGTGATCTTA TTCCAGAGGG AGATGATGAG GACCACCAGG

TGCCCCAGCA AGTGATCTTA CTCCAGAGGG AGAAGAGCAG AACCAGCAGG

TGCCCCCGCA AGTGATCTGA TACCAGAGAG AGATGAGCAG GACCAGGAGA

---------- ---------- ---------- ---------- ----------

TGCCCCCACA GGTGATCTGC TTCCAGAGGG ACCCGAGCAG GACCACGAGC

TGCCCTTACA GGTGATCTTG GCCCACAGGG TGAGGAGGAG GACCACCAGG

GAGGCGAGGC GAAGAAACAC GGTCCTACCT GAGCAGGATA AAAACGATCT

GAGGCGAGGC GAAGAAACAC GGTCCTACCT GAGCAGGATA AAAACGATCT

GAGGCCAGGC GAAGAAACAC GGTCCTACCT GAGCAGGATA AAAACGATCT

GAGGCCAGGC GAAGAAACAC GGTCCTACCT GAGCAGGATA AAAACGATCT

GAGGCCAGGC GAAGAAACAG GGTCCTACCT CAGCAAGATT AAAATGATCT

GAGGTGAGAC GGAGAAACAC AGTCCTACCT GAGCAAAATA AAAACGATCT

GAGGCCAGAC GCAAAAACAC AGTCCTACCT AAGCAGGATC AAGACGATCT

GAGGCCAGAC GCAAAAACAC AGTCCTACCT AAGCAGGATC AAGACGATCT

---------- ---------- ---------- ---------- ----------

---------- ---------- ---------- ---------- ----------

GATGCCAGGC GGAGAAACAC GGTCCTACCT GAGCAGGATA AAAGCAATCT

GAGGCCAGGC GGAGAAATAT AGTCCTACCT GAGCAGTATT AAAACGATCT

GAAGCCAGGC GGAGGAATAC AGTCCT---- ---------- ----------

GAGATGAGGC GTAGAAACAC GGTCCTACCT GAGCAGGACT AAAACTATCT

GAGAAAAGGC GGAGAAACAC AGTCCTACCT GAGCAGGATT AAAACGATCT

---------- ---------- ---------- ---------- ----------

GAGGCCAGCC GGAGAAACAC GGTCCTCCAC TAGAAGAAGG ATGAAGACCT

GAAGCGAGTC GGAGGAACAC GGTCCTACCT GAGCAGGATG AACAGGACCT

GGCGACTCCG AGTGTAGCCC TCGGGCAGCA CAAAATTGAC CCCAGCGATG

GGCGACTCCG AGTGTAGCCC TCGGGCAGCA CAAAATTGAC CCCAGCGATG

GGCGACTTCT AGTGTAGCCT TCGGGCAGCA CAAAGTTGAC CACAGCGATG

GGAGACTTCT AGTGTAGCCT TCGGGCAGCA CAAAGTTGAC CACAGCGATG

GGCGACTCCG GGTGTAGCCC TCGGGCAGCA CAAAGTTAAC CCCGGCGATG

GGCGGCTCCG AGTATAGCCC TCGGGGAGCA CAAAGTTGAC CACAGAGATG

GACGGCTGTG AGTGTAGCCC TCGGGCAGCA CAAAGTTGAA CACGGAAATG

GTCGGCTTTG AGTGTAGCCC TCGGGCAGCA CAAAGTTGAA CAGAGAAATG

---------- ---------- ---------- ---------- ----------

---------- ---------- ---------- ---------- ----------

GGTGGCTCCT GGTGTAGCCC TCGGGCAGCA AGAAGTTAAC CCCAGAGATG

GGCGGCTCCT GGTATAGCCC TCGGGCAGCA CAAAGTTGAC CCCGGCGATG

---------- ---------- ---------- ---------- ----------

GGCGGCTCCG AGTGTAGCCC TCGGGCAGCA GGAAGTTGAC CACGGAGATG

GGCGGCTTCG GGTGTAGCCC TCGGGCAGCA CGAAGTTGAA TACGGCAATG

---------- ---------- ---------- ---------- ----------

GGCGGCTCCG AGTGTAGCCC TCGGGCAGAA TCAGGTTAGC ACCGGACATG

GGCGGCTCAG GGTATACCCC TCAGGCAGGA TCAGGTTGAC CGCGGAGATG

AAGATGGACG GAAGGTAATT CACCCTCACC GCAGGACATA GGAACGGGCA

AAGATGGACG GAAGGTAATT CACCCTCCCC GCGGGACATA GGAA-GGGTA

AAGATGGACG GAAGGTAATT CACCCTCACC GCAGGACATA GGAACGGGCA

AAGATGGACG GAAGGTAATT CACC------ ---------- ----------

AAGATGGACG GAAGGTAATT CACCCTCACC GCAGCACATA GGAGCGGGTC

AAGATGGACG GAAGGTAATT CACCCTCACC TTAAAACATA GGAGCGGAAA

AAGATAGATG GAAGATAATT CACCCTCACC TCAATACGTA GGAGCGGAAA

AAGATGGATG GTAGATAATC CACCCTCACC TCAAAACGTA GGAGCGGAAA

---------- ---------- ---------- ---------- ----------

---------- ---------- ---------- ---------- ----------

AAGATGGATG GAAGGTAATC CACCCTCACC TGAGAACATA GGAGCGGGCA

AAAATGGATG GAAGGTAATC CACCCTCACC TGAGGACGTA GGAGCGGGCG

---------- ---------- ---------- ---------- ----------

AAGATGGACG GAAGGTAATC CACCCCCACC TCAGCACGTA GGAGCGGGTG

AAGATGGATG GAAGGTAATC CACC------ ---------- ----------

---------- ---------- ----CTCACC TCAGCACGTA GGAGCGCACG

AAGATGGACG GCAGATAGGC GACCCTCACC GAAGCACGTA GGAGCGGGCC

AAGATGGACG GGAGGAAGTC CACTcTCACC GCAGCACGTA GGAGCGGGCC

GGTGCGCTCT TGTAGATATA CTGCGCCAGC CACCACTGCA CCGTCATGTT

GGTGCGCTCT TGTAGATATA TTGCGCCAGC CACCACTGCA CCGTCATGTT

GGTGCGCTCT TGTAGATATA CTGCGCCAGC CACCACTGCA CCGTCATGTT

---------- ---------- ---------- ---------- ----------

GGTGCGCTCT TGTAGATGTA CTGCGCCAGC CACCACTGCA CCGTCATGTT

GGTGCGCTCT TGTAGATATA ATGTGCCAGC CACCATTGCA CCGTCATGTT

GGTGCACTCT TGTAGATGTA CTGTGCCAGC CACCACTGCA CGGTCATGTT

GGTGCGCTCT TGTAGATGTA CTGTGCCAGC CACCACTGCA CGGTCATGTT

---------- ---------- ---------- ---------- ----------

---------- ---------- ---------- ---------- ----------

GGTGCGCTCT TGTAGATATA CTGTGCCAGC CACCACTGCA CTGTCATGTT

GGCGCGCTCT TGTAGACGTA CTGCGCCAGC CACCACTGCA CCGTCATGTT

---------- ---------- ---------- ---------- ----------

GGCGCGCTCT TGTAGACGTA CTGGGCCAGC CACCACTGCA CCGTCATGTT

---------- ---------- ---------- ---------- ----------

GGTGCGCTCT TGTAGATATA CTGCGCCAGC CACCACTGCA CCGTCATGTT

GGTGCCGTCT TGTAGATGTA TTGGGCCAGC CACCACTGCA CCGTCATGTT

GGAGCGCTCT TGTAGATGTA CTGGGCCAGC CACCACTGCA CCGTCATGTT

CCAGTACCGC ATGCCATCGC GCACCCGCAC GCAGAAATCT GTGCTGTAGC

CCAGTACCGC ATGCCATCGC GCACCCGCAC GCAGAAATCT GTGCTGTAGC

CCAGTACCGC ATGCCATCGC GCACCCGCAC GCAGAAATCT GTGCCGTAGC

---------- ---------- ---------- ---------- ----------

CCAGTACCGC ATGCCCTCGC GCACCCGCGT GCAGAAATCC GTGCCATAGC

CCAGTACCGC ATGCCATCCC GCACCCGCAC GCAGAAGTCT GTGCCATAGC

CCAGTACCGC ATGCCATCCC GCACACGCAC GCAGAAGTCT GTGCCATAGC

CCAGTATCGC ATGCCATCCC GCACACGCAC GCAGAAGTCT GTGCCATAGC

---------- ---------- ---------- ---------- ----------

---------- ---------- ---------- ---------- ----------

CCAGTACCGC ATGCCATCCC GCACCCGCAC GCAGAAGTCA GTGCCATAGC

CCAGTACCGC ATGCCTTCTC GCACCCGCAC GCAGAAGTCG GTGCCGTAGC

---------- ---------- ---------- ---------- ----------

CCAGTAGCGC ATGCCATCCC GCACCCGCAC ACAGAAGTCC GTGCCGTAGC

---------- ---------- ---------- ---------- ----------

CCAGTACCGC ATGCCATCCC GCACGCGCAC GCAGAAGTCT GTGCCGTAGC

CCAGTACCGC ATCCCGTCTC GGACCCGGAC GCAGAAGTCG GTCCCGTGAC

CCAGTAGCGC ATCCCGTCCC GCACCCGGAC GCAGAAGTCG GTGCCGTGGC

AGTCGATGTT GCGGATGGTC TCATAGTCAT ACTCCACTGA CCTGCTGGGG

AGTCGATGTT GCGGATGGTC TCATAGTCAT ACTCCA---- ----------

AGTCGATGTT GCGGATGGTC TCATAGTCAT ACTCCACTGA CCTGCTGGGG

---------- ---------- ---------- ---------- ----------

AGTCGATGTT GCGGATGGTC TCATAGTCAT ACTCCACTGA CCTGCTGGGG

AGTCGATGTT GCGGATGGTC TCATAGTCGT ACTCCACTGA CCTGCTGGGG

AGTCGATGTT ACGGATGGCC TCATAGTCAT ACTCCACTGA CCTGCTAGGG

AGTCGATGTT ACGGATGGTC TCATAGTCAT ACTCCACTGA CCTGCTAGGG

---------- ---------- ---------- ------CTGA CCTGCTGGGG

---------- ---------- ---------- ---------- ----------

AGTCGATGTT GCGGATGGTC TCATAGTCGT ACTCCACTGA CCTGCTGGGG

AGTCGATGTT GCGAATGGTC TCATAGTCGT ACTCCACTGA CCTGCTGGGG

---------- ---------- ---------- ------CTGA CCTGCTGGGG

AGTCGATGTT GCGGATGGTC TCGTAGTCGT ACTCCACTGA CCTGCTGGGG

---------- ---------- ---------- ---------- ----------

AGTCGATGTT GCGGATGGTC TCATAGTCGT ACTCCACTGA CCTGCTGGGG

AGTCGATGTT GCGGATGGTC TCGTAGTCAT ACTTGGATGA CCTGTCGGGG

AGTTGATGTT CCGGATCGTC TCGTAATCGT ACTCCACTGA CCTGTCGGGG

GGTGGGCATT GGAGGGTGGG GCCGCCTCCG GCCCGGGCTT TGGCGGCCAC

---------- ---------- ---------- ---------- ----------

GGTGGGCATT GGAGGGTggg gccgcccccg gcccgggcTT TGGCGGCCAC

---------- ---------- ---------- ---------- ----------

GGTGGGCATT GGAGGGTGGG GCCACCCCCG GCCCGGGCTT TGGCGGCCAC

GGTGGGCATT GGAGGGTggg gccgcccccg gcccgggcTT TGGCGGCCAC

GGTGGGCATT GGAGGGTGGG GCCGCCACCG GCCCGGGCTT TGGCAGCCAC

GGTGGGCATT GGAGGGTggg gccgcccccg gcccgggcTT TGGCAGCCAC

GGTGGGCATT GGAGGGTggg gccgcccccg gcccgggcTT TGGCGGCCAC

---------- ---------- ---------- ---------- ----------

GGTGGGCATT GGAGGGTggg gccgcccccg gcccgggccT TGGCGGCCAC

GGTGGGCATT GGAGGGTggg gccgcccccg gcccgggcTT TGGCGGCCAC

GGTGGGCATT GGAGGGTggg gccgcccccg gcccgggcTT TGGCGGCCAC

GGTGGGCATT GGAGGGTggg gccgcccccg gcccgggcTT TGGCGGCCAC

---------- ---------- ---------- ---------- ----------

GGTGGGCATT GGAGGGTggg gccgcccccg gcccgggcTT TGGCGGCCAC

GCGGGGCAGG GCCGGGTGGG GCCGCCTCCC GCCCTCGACT TGGCCCCCAC

GGAGGACATT CGACGGTCGG GCCGCCCCCG GCCCTGGCCT TGGCCCCGAC

GGGGTAGGCC CCAAAGCCGG CGGCAATGCA GCCGCACTCG GCGGCAATCC

---------- ---------- ---------- ---------- ----------

GGGGTAGGAA CCGAAGCCGG CGGCAATGCA GCCGCACTCG GCGGCAATCC

---------- ---------- ---------- ---------- ----------

GGGGTAGGCC CCGAAGCCGG CGGCAATGCA GCCGCACTCG GCGGCAATCC

GGGGTAAGCC CCGAAGCCAG CGGCAATGCA GCCGCACTCA GCTGCAATCC

AGGGTAGGCC CCGAAGCCCG CGGCAATGCA ACCGCACTCG GCCGCAATCC

AGGGTAGGCC CCGAAGCCCG CGGCAATGCA ACCGCACTCG GCCGCAATCC

GGGGTAGGCC CCGAAGCCGG CGGCAATGCA GCCGCACTCG GCGGCGATCC

---------- ---------- ---------- ---------- ----------

AGGGTAGGCC CCGAAGCCGG CAGCAATGCA GCCGCACTCA GCGGCAATCC

CGGGTAGGCC CCGAAGCCGG CGGCAATGCA GCCGCACTCG GCCGCAATCC

GGGGTAGGCC CCGAAGCCCG CGGCAATGCA GCCGCACTCG GCGGCAATCC

GGGGTAGGCC CCGAAGCCGG CGGCAATGCA GCCGCACTCG GCGGCAATCC

---------- ---------- ---------- ---------- ----------

CGGGTAGGCC CCAAAGCCAG CGGCAATGCA GCCGCACTCG GCTGCAATCC

AGGGTAGGCC CCGAAGCCAG CTGCTATGCA GCCACACTCG GCTGCGATCC

GGGGTAGGCC CCGAAGCCGG CCGCGATGCA GCCGCACTCG GCCATGATCC

AGGCCACGTA GAAGCGCATG CGGAAGGCGA AGAAGACGGG GATCATGTAG

---------- ---------- ---------- ---------- ----------

CGTACACGTA GAAGCGCATG CGGAAGGCGA AGAAGACGGC CTTTATGTAG

---------- ---------- ---------- ---------- ----------

AGGCCACGTA GAAGCGCATG CGGAAGGCGA AGAAGACGGG GATCATGTAG

AGGCCACGTA GAAGCGCATG CGGAAGGCGA AGAAGACGGG GATCATGTAG

AGGCCACGTA GAAGCGCATG CGGAAGGCGA AGAAGACCGG GATCATGTAG

AGGCAACGTA GAAGCGCATG CGGAAGGCGA AGAAGACCGG GATCATGTAG

AGGCCACGTA GAAGCGCATG CGGAAGGCGA AGAAGACCGG GATCATGTAG

---------- ---------- ---------- ---------- ----------

AGGCCACGTA GAAGCGCATG CGGAAGGCGA AGAAGACGGG AATCATGTAG

AGGCCACGTA GAAGCGCATG CGGAAGGCGA AGAAGACGGG GATCATGTAG

AGGCCACGTA GAAGCGCATG CGGAAGGCGA AGAAGACGGG GATCATGTAG

AGGCCACGTA GAAGCGCATG CGGAAGGCGA AGAAGACGGG GACCATGTAG

---------- ---------- ---------- ---------- ----------

AGGCCACGTA GAAGCGCATG CGGAAGGCGA AGAAGACGGG GATCATGTAG

AGGCGACGTA GAAGCGCATC CGGAAGGCAA AGAAGATGGG CAGCATGTAG

AGGCCACGTA GAAGCGCATG CGGAAGGTGA AGAAGACGGG GGTCATGTAG

AAGAGGCGGG CGGGCAGCGG GCGGGCGTAG AAGGCGTCCT CGCGCACGGC

---------- ---------- ---------- ---------- ----------

AAGAGGCGGG CGGGCAGCGG GCGGGCGTAG AAGGCGTCCT CGCGCACGGC

---------- ---------- ---------- ---------- ----------

AACAGGCGGG CGGGCAGCGG GCGGGCGTAG AAGGCGTCCT CGCGCACGGC

AATAGGCGGG CAGGCAGCGG GCGGGCGTAG AAGGCGTCCT CACGCACGGC

AAGAGGCGGG TGGGCAGCGG GCGGGCGTAG AAGGCATCCT CACGCACAGC

AAGAGGCGGG TGGGCAGCGG GCGGGCGTAG AAGGCGTCCT CACGCACAGC

AAGAGGCGGG CGGGCAGCGG GCGGGCGTAG AAGGCGTCCT CGCGCACGGC

---------- ---------- ---------- ---------- ----------

AAGAGGCGGG CGGGCAGCGG GCGGGCGTAG AAGGCGTCCT CGCGCACGGC

AAGAGGCGGG CAGGCAGCGG GCGGGCGTAG AAGGCGTCCT CGCGCACGGC

AAGAGGCGGG CAGGCAGCGG GCGGGCGTAG AAGGCGTCCT CTCGCACGGC

AAGAGGCGCG CGGGCAGCGG GCGGGCGTAG AAGGCGTCCT CGCGCACGGC

---------- ---------- ---------- ---------- ----------

AAGAGGCGCG CGGGCAGCAG GCGGGCGTAG AAGGCGTCCT CGCTCCCGGC

AAGAGGCGGA AGGCCAGGCT GCGGCTGTAG AAGGCATCTT CCCGCACGGC

AAGAGCCGGA AGGCGAAGGA GCGGGCGTAG AAGGCCTCCT CCCGCACGTA

CTCCAGCGGC TGACCTTCAG CGTCAGCAGC AGCTGGACGG CATTGGTGAA

---------C TGACCTTCAG CGTCAGCAGC AGCTGGACGG CATTGGTGAA

CTCCAGCGGC TGACCTTCAG CGTCAGTAGC AGCTGGACGG CATTGGTGAA

---------- ---------- ---------- ---------- ----------

CTCCAGCGGc TGACCTTCAG CGTGAGCAGC AGCTGGACGG CATTGGTGAA

CTCCAGAGGC TGACCTTCAA TGTTAGCAGC AGCTGGACAG CATTGGTGAA

TTCCAGTGGC TCACCTTCAG TGTCAACAGC AGCTGGACAG CATTGGTGAA

TTCCAGTGG- ---------- ---------- ---------- ----------

---------- ---------- ---------- ---------- ----------

---------- ---------- ---------- ---------- ----------

CTCCAGCGGC TGACCTTGAG TGTGAGCAGC AGCTGAACCG CATTGGTGAA

CTCCAGCGGC TGACCTTCAG TGTCAGCAGC AGCTGGACCG CATTG-----

---------- ---------- ---------- ---------- ----------

CTCCAGCGGC TGACCTTCAG TGTCAGCAGC AGCTGGACGG CATTGGTGAA

---------C TGACCTTCAG TGTGAGCAGC AGCTGTACGG CATTGGTGAA

CTCCAGCGG- ---------- ---------- ---------- ----------

CTCCAGCGGC TCACCTTGAG CGTGAGCAGC AGCTGCACGG CATTGGTGAA

GTCCAGCGGC TGACCTTGAG CGTGAGCAGC AGCTGAATGG CATTGGTGAA

GGGCGTGGGA GTGGGCAGGC CCAGCGGAAG AACAGGAGAT AGGAGAAAGT

GGGCGTGGGA GTGGGCAGGC CCAGCGGAAG AACAGGAGAT AGGAGAAAGT

GGGCGTGGGA GTGGGCAGGC CCAGCGGAAG AACAGGAGAT AGGAGAAGGT

---------- ---------- ---------- ---------- ----------

GGGCGTGGGA GTAGGCAGGC CCAGCGGAAG AACAGGAGAT AGGAGAAAGT

GGGCGTGGGA GTGGGCAGGC CCAGCGGAAG AACAGGAGAT AGGAGAAGGT

GGGTGTGGGA GTGGGCAGGC CCAGCGGAAG AAGAGAAGAT AGGAGAAGGT

---------- ---------- ---------- ---------- ----------

---------- ---------- ---------- ---------- ----------

---------- ---------- ---------- ---------- ----------

GGGTGTGGGG GTGGGGAGGC CCAGCGGAAG AACAGCAGGT ACGAGAAGGT

---------- ---------- ---------- ---------- ----------

---------- ---------- ---------- ---------- ----------

GGGCGTCGGG GTGGGCAGGC CCAGCGGAAG AACAGAAGAT AGGAGAAGGT

CGGTGTGGGT GTGGGCAGGT CTAGCGGAAG AACAGTAGAT AGGAGAAGGT

---------- ---------- ---------- ---------- ----------

AGGCGTGGGG GCCGGGAGCC CAAACGGAAG AAGAGGAGGT AGAGGAAGGT

GGGCGTGGGG GCCGGCAGGC CAAACGAAAG AAGAGCAGAT AAGAGAACGT

CCAGGCCAGA GCCAGGGCGT GGCAGGAGCT TCACCAGGGC TGGGCCTGAA

CCAGGCCAGA GCCAGGGCGT GGCAGGAGCT TCACCAGGGC TGGGCCTGAA

CCAGGCCAGA GCCAGGGCGT GGCAGGAGCT TCACCACGGC TGGGCCTGAA

---------- ---------- ---------- ---------- ----------

CCAGGCCAGG GCCAGAGCGT GGCAGGAGCT TCACCAGGGC TGGGCCTGAA

CCAGGCTAGA GCCAGGGCGT GGCAGGAGCT TCACCAGGGC TGGGCCTGAA

CCAGGCAAGA GCCAGCGCGT GGCAGGAGCT TCACCAGGGC TGGGCCTGTA

---------- ---------- ---------- ---------- ----------

---------- ---------- ---------- ---------- ----------

---------- ---------- ---------- ---------- ----------

CCAGGCCAGG GCGAGGGCGT GGCAGGAGCT TCACCAGGGC TGGGCCTGGA

---------- ---------- ---------- TCACCAGGGC TGGGCCTGAA

---------- ---------- ---------- ---------- ----------

CCAGGCCAGG GCCAGGGCGT GGCAGGAGCT GCACCAGGGC TGTGCTTGGA

TCAGTCTAGT GCCAAGGCGT GGCAGGAGCT ---------- ----------

---------- -----GGCGT GGCAGGAGCT TCACCAGGGT TGGGCCTGAA

CCAGGCCAGT GCCAGGGCAT GGCAGGCCCT TTACCTGGGC CGGATCTCCA

CCAGCCCAAG GCCAGGAAGT GGCAGGACCT TCACCTGGGC TGGACCTTCA

TGAGGGCCCA GGTCCCGAGG ATGGTGACCA GAGAATGCAA AGTGTGGGGG

TGAGGGCCCA GGTCCCGAGG ATGGTGACCA GAGAATGCAA AGTGTGGGGG

TGAGGGCCCA GGTCCCAAGG ATGGTGATCA GAGAATGCAA AGTGTGGGGG

---------- ---------- ---------- ---------- ----------

TGAGGGCCCA GGTCCCGAGG ATGGTGATCA GAGAATGCAA AGTGTGGGGG

TGAGGGCCCA GGTCCCCAGG ATGGTGATCA GAGAATGCAA AGTGTGGGGG

TGAGGGCCCA GGTTCCCAAG ATGGTGATCA GAGAATGCAA ACTGTGGGGG

---------- ---------- ---------- ---------- ----------

---------- ---------- ---------- ---------- ----------

---------- ---------- ---------- ---------- ----------

TGAGGGCCCA GGTCCCAAGG ATGGTGATCA GAGAATGCAA AGTGTGGGGG

TGAGGGCCCA GGTCCCAAGG ATGGTGACCA GAGAATGCAA AGTGTGGGGG

---------- ---------- ---------- ---------- ----------

CCAGGGCCCA GGTCCCGAGG ATGGTCACCA GAGAATGCAA GGTGTGGGGG

---------- ---------- ---------- ---------- ----------

TAAGGGCCCA GGTCCCGAGG ATGGTGACCA AAGAATGCAA GCTGTGAGGT

TGATGATCCA GGTGCCAAGG ATGGTGACCA GCGAGTGGAG GATGTGGGCT

TGATGCCCCA CGTCCCCAGG ATGGTGACCA GGGAGTGCAG GACGTGAGGG

CCACAGGTGA ACAGGGTGAG CCCCAGGCCC ACAGCAGCGC CCCGCGTGGA

CCACAGGTGA ACAGGGTGAG CCCCAGGCCC ACAGCAGCGC CCCGCGTGGA

CCACAGGTGA ACAGGGTGAG CCCCAGGCCC ACAGTAGCGC CCCGCGTGGA

---------- ---------- ---------- ----TAGCGC CCCGCGTGGA

CCACAGGTGA ATAGGGTGTG CCCCAGGCCC ACAGCAGCGC CCCGCGTGGA

CCACAGGTGA ACAAGGTGAG CCCCAGGCCC ACAGTAGCGC CCCGCGTGGA

CCACAGGTGA ATAAGGTGAG CCCCAGGCCC ACAGCAGCGC CCCGCGTGGA

---------- ---------- ---------- ---------- ----------

---------- ---------- ---------- ---------- ------TGGA

---------- ---------- ---------- ---------- ----------

CCGCAGGTGA ACAAGGTGAG CCCCAGGCCC ACGGCAGCGC CCCGCGTGGA

CCACAGGTGA ACAAGGTGAG CCCCAGGCCC ACAGCAGCGC CCCACGTAGA

---------- ---------- ---------- ----CTGCGC CCCGCGTAGA

CCACAGGTGA ACAGGGTCAG CCCCAGGCCC ACCGCAGCGC CCCGCGTGGA

---------- ---------- ---------- ----CAGCGC CCCGCGTGGA

CCGCAGGTGA ACAAGGTGAG CCCCAGGCCC ACAG------ ----------

CTACAGGTGA GCAGCGTCAG GGCCAGCCCC ACCCCACAGC CCCGCGTGGA

CCGCAGGTCA GCAACGTCAA GCCCAGCCCT ACAGcagagc gctggaTGGA

CTGGCGTGTC CAGTCTAAAG ACTGGCCCCA CGCCGGCCGC CCTGCCCACG

CTGGCGTGTC CAGTCTAAAG ACTGGCCCCA CGCTGGCCGC CCTGCCCACG

CTGGCGTGTC CAGTCTAAAG ACTGGCCCCA CGCCGGCCGC CCTGCCCATG

CTGGCGTGTC CAGTCTAAAG ACTGGCCCCA CGCCGGCCGC CCTGCCCATG

CTGGCGTGTC CAGTCTAAAG ACTGGCCCCA CGCCGGCCGC CCTGCCCATG

CTGGCGTGTC CAGTCCAAAG ACTGGCCCCA CGCTGGCCGG CCTGCTCATG

CTGGCGTGTG CAGTCCAAAG ACTGGCCCCA TGCTGGCCGT CCTGCTCATG

---------- ---------- ---------- ---------- ----------

CTGGCGCGTG CAGTCCGAGG ACTGGCCGCA CGCCGGCCGG CCTGCCCACG

---------- ---------- ---------- ---------- ----------

TTGGCGTGTC CAGTCCAAAG ACTGGCCGCA TGCTGGCCGT CCTGCCCATG

CTGGCGTGTC CAGTCCAAAG ACTGGCCCCA CGCTGGCCGT CCAGCCCATG

CTGGCGTGTC CAGTCCCAAG ACTGGCCTCA TGCTGGCCGG CCCGCCCATG

CTGGCGAGTC CAGTCCAAAG ACTGGCCCCA CGCTGGCCGT CCAGCCCATG

CTGGCGTGTC CAGTCCAAAG ACTGGCCCCA TGCTGGCCGT CCCACCCACG

---------- ---------- ---------- ---------- ----------

CTGGCACCAG CCATCACCAG ATTGGCCCTA CGCTGGAGAC CCAAGGCATG

CTGGCGCGTC CCCTCCCCGG ACTGGCCCCA CGCCGGCCGT CCCTCCCACG

AGCTGCGCTA CAGTATCTAC AGAGACCTGT GGGAGCGAGG CTTCTTCCTC

AGCTGCGCTA CAGTATCTAC AGAGACCTGT GGGAGCGAGG CTTCTTCCTC

AGCTGCGCTA CAGCATTTAC AGAGACCTGT GGGAGAGAGG CTTCTTCCTC

AGCTGCGCTA CAGCATTTAC AGAGATCTGT GGGAGAGAGG GTTCTTCCTC

AGCTGCGCTA CAGCATCTAC AGAGACCTGT GGGAGCGGGG CTTCTTCCTC

AGCTGCGCTA CAGCGTCTAC AGAGACCTGT GGGAGCGGGG CTTCTTCCTC

AGCTTCGCTA CAGCATCTAC AGAGACCTGT GGGAGAGAGG CTTCTTCCTC

---------- ---------- ---------- ---------- ----------

AGCTGCGCTA CAGCATCTAC AGGGACCTGT GGGAGCGCGG CTTCTTCCTC

---------- ---------- ---------- ---------- ----------

AGCTTCGCTA TAGCATCTAC AGAGACCTGT GGGAACGAGG CTTCTTCCTC

AGCTACGCTA CAGCATCTAC AGAGACCTGT GGGAACGAGG CTTCTTCCTC

AGCTGCGCTA CAGCATCTAC AGAGACCTGT GGGAGCGAGG CTTCTTCCTC

AGCTGCGCTA CAGCATCTAC AGAGACCTGT GGGAGCGAGG CTTCTTCTTA

AGCTGCGCTA TAGCATTTAC AGAGACCTAT GGGAGCGAGG CTTCTTCCTC

---------- ---------- ---------- ---------- ----------

AGCTGCGGTA CCGAGTCTAC AAGGACCTGT GGGAGAGGGG TTACTTCCTC

AGCTCCGATA CCGCGTCTAC CGTGACCTGT GGCAGCGGGG CTACTTCCTC

AGTGCGGCTG GCAAGTTCGG AGGTGACTTC CTGGTCTATC CTGGTGAGGA

AGTGCGGCTG GCAAGTTCGG AGGTGACTTC CTGGTCTATC CTGGTGAGGA

AGTGCGGCTG GCAAGTTCGG AGGTGACTTC CTGGTCTATC CTGGTGAGGA

AGTGCGGCTG GCAAGTTCGG AGGTGACTTC CTGGTCTATC CTGGTGAGGA

AGCGCGGCTG GGAAGTTTGG AGGTGACTTC CTGGTCTATC CGGGTGAGGA

AGTGCCGCTG GCAAGTTCGG GGGGGACTTC TTGGTCTATC CTGGTGAGGA

AGCGCAGCTG GGAAGTTTGG TGGTGACTTC CTGGTCTATC CTGGTGAGGA

---------- ---------- ---------- ---------- ----------

AGCGCCGCGG GCAAGTTCGG CGGCGACTTC CTGGTCTACC CAGGTGAGGA

---------- ---------- ---------- ---------- ----------

AGTGCAGCTG GCAAGTTCGG GGGTGACTTC CTGGTCTATC CTGGTGAGGA

AGTGCTGCTG GCAAGTTCGG GGGCGACTTC CTGGTCTATC CCGGTGAGGA

AGTGCCGCGG GCAAGTTTGG GGGCGACTTC CTGGTCTATC CTGGTGAG--

AGTGCCGCTG GCAAGTTTGG GGGAGACTTC CTGGTCTATC CTGGTGAGGA

AGTGCTGCTG GCAAGTTCGG GGGCGACTTC CTGGTCTATC CTGGTGAGGA

---------- ---------- ---------- ---------- --------GA

ACCTCTGGGG GCAAGTTTGG GGGTGACTTT CTGGTCTATC CAGGTGAGGA

ACGGCCGGGG GCAAGTTCGG CGGGGACTTC CTGGTCTACC CCGGTGAGGA

CACCATCCCA CTCCAAGACC TGGTTGCTGC TGGGCGCCTT GGAACCAGCG

CACCATCCCA CTCCAAGACC TGGTTGCTGC TGGGCGCCTT GGAACCAGCG

CCCCATCCCA CTCCAGGACC TGGTTGCTGC TGGGCGCCTT GGAACCAGCG

CCCCATCCCA CTCCAGGACC TGGTTGCTGC TGGGCGCCTT GGAACCAGCG

CCCCATCCCA CTCCAGGACC TGGTTGCTGC TGGGCGCCTT GGAACCAGCG

CCCCATCCCA CTCCAGGACC TGGTCTCTGC TGGCCGCCTT GGGACCAGCG

CCCCATCCCA CTTCAGGACC TGGTCTCTGC AGGCCGCCTG GGAACCAGTG

---------- ---------- ---------- ---------- ----------

CCCCATCCCC CTGCAGGACC TGGTCTCTGC CGGCCGCCTG GGCACCAGCG

---------- ---------- ---------- ---------- ----------

CCCCATTCCA CTCCAGGACC TGGTTTCTGC TGGCCGCCTG GGGACCAGCG

CCCCATCCCA CTCCAGGACC TGGTTTCTGC TGGACGCCTC GGAACCAGTG

---------- ---------- ---------- ---------- ----------

CCCGATCCCC CTCCAGGACC TGGTTTCTGC TGGCCGCCTC GGAACCAGCG

CCCAATCCCA CTCCAGGACC TGGTTTCTGC TGGCCGCCTC GGAACCAGCG

CTCCATCCCA CTCCAGGACC TGGTCTCAGC CGGCCGCCTC GGAACCAGTG

CCCCCTCCCA CTGCCAGACC TTGTCTCTGC TGGCCGCCTG GGGACCAACG

GCCCCTTCCG CTCCGGGACC TGGTCTCCGC CGGACGATTG GGCACCAACG

TCAGAAAGAC CCTGCTCCTC TGTTCTCCGC AGCCTGATGG TAAGGTGGTC

TCAGAAAGAC CCTGCTCCTC TGTTCTCCGC AGCCTGATGG TAAGGTGGTC

TCAGAAAGAC CCTGCTCCTC TGTTCTCCGC AGCCTGATGG TAAGGTGGTC

TCAGAAAGAC CCTGCTCCTC TGTTCTCCGC AGCCTGATGG TAAGGTGGTC

TCAGAAAGAC CCTGCTCCTC TGCTCTCCGC AGCCTGATGG TAAGGTGGTC

TCAGAAAGAC CCTGCTCCTG TGTTCACCGC AGCCTGATGG TAAGGTGGTC

TCAGAAAGAC TCTGCTTCTC TGCTCCCCTC AGCCTGATGG GAAGGTGGTT

---------- ---------- ---------- ---------- ----------

TGAGGAAGAC CGTGCTGCTC TGCTCCCCAC AGCCCGGCGG CAAGGTGGTC

---------- ---------- ---------- ---------- ----------

TCAGAAAGAC GCTGCTGCTC TGTTCCCCTC AGCCTGATGG TAAAGTGGTC

TCAGGAAGAC GCTGCTCCTC TGTTCTCCGC AGCCTGATGG TAAGGTGGTC

---------- ---------- ---------- ---------- ----------

TCAGAAAGAC ACTGCTGCTC TGTTCCCCAC AGCCTGACGG TAAGGTGGTC

TCAGAAAGAC CCTGCTCCTT TGTTCACCGC AGCCTGATGG TAAGGTGGTG

TCAGAAAGAC CCTGCTGCTG TGTTCCCCGC AGCCGGACGG GAAGGTGGTG

TTCGGAAGAC TCTGCTTCTG TGCTCCCCTC TGCCCGACGG CTCCGTGGCC

TCCGCAAGAC GGTGCTCCTG TGCTCGGCCC GCCCCGACGG CTCCGTCTGC

TACACCTCCC TGCAATGGGC CAGCCTGCAG TGAACTCAGG CGAGTATGGG

TACACCTCCC TGCAATGGGC CAGCCTGCAG TGAACTCAGG CGAGTATGGG

TACACCTCCC TGCAATGGGC CAGCCTGCAG TGAACTCAGG CGAGTATGGG

TACACCTCCC TGCAATGGGC CAGCCTGCAG TGAACTCAGG CGAGTATGGG

TACACCTCCC TGCAGTGGGC CAGCCTGCAG TGAACTCAGG CGAGTATGGG

TACACCTCCC TGCAGTGGGC CAACCTGCAA TGAACTCAGG CGAATATGGG

TATACTTCCC TGCAGTGGGC CAGCCTGCAG TGAGC--AGG AGAGTATGGG

---------- ---------- ---------- ---------- ----------

TACACCTCCC TGCAGTGGGC CGGCCTGCAG TGAGCGCAGG CGAGTACGGG

---------- ---------- ---------- ---------- ----------

TACACCTCCC TGCAGTGGGC CAGCCTACAG TGAACTCAGG CGAGTACGGG

TACACCTCCC TTCAGTGGGC CAGCCTGCAG TGAACTCAGG AGAGTATGGG

---------- ---------- ---------- -------AGG CGAGTATGGG

TACACCTCCC TGCAGTGGGC CAGCCTGCAG TGAACTCAGG CGAGTATGGG

TACACCTCCC TGCAGTGGGC CAGCCTGCAG TGAACTCAGG CGAGTATGGT

TACACCTCCC TGCAGTGGGC CAGCCTGCAG TGAG------ ----------

TACACGTCTC TGCAGTGGGC TGGCCTGCCG TGACCAGAGG TGAGTACGGG

TACACCTCGC TGGAGTGGGC CGGGCTGGCC TGAGCCCAGG CGAGTACGGG

CTCCGGAACA AACGTGAGGT CTGGAGGGTC AAATTTACCC TGGCCAAGAT

CTCCGGAACA AACGTGAGGT CTGGAGGGTC AAATTTACCC TGGCCAAGAT

CTCCGGAACA AACGTGAGGT CTGGAGGGTC AAATTTACCC TGGCCAAGAT

CTCCGGAACA AACGTGAGGT CTGGAGGGTC AAATTTACCC TGGCCAAGAT

CTTCGGAACA AACGTGAGGT CTGGAGGGTC AAGTTTACCC TGGCCAAGAT

CTCCGGAACA AACGTGAGGT CTGGAGGGTC AAGTTTACCT TGGCCAAAAT

CTCCGGAACA AACGTGAGGT GTGGAGGGTC AAATTTACCC TGGCGAAGAT

---------- ---------- ---------- ---------- ----------

CTGCGGAACA AGCGCGAGGT GTGGAGGGTC AAGTTCACGC TGGCCAAGAT

---------- ---------- ---------- ---------- ----------

CTCCGGAACA AACGTGAGGT CTGGAGGGTC AAGTTTACCC TGGCCAAGAT

CTTCGGAACA AACGTGAGGT CTGGAGGGTC AAATTTACAC TGGCCAAGAT

CTCCGTAACA AGCGAGAGGT CTGGCGGGTC AAGTTTACTC TGGCCAAGAT

CTCCGGAACA AGCGTGAGGT CTGGAGGGTC AAGTTTACTC TGGCTAAGAT

CTCCGGAACA AACGTGAAGT CTGGAGGGTC AAGTTTACTC TGGCCAAGAT

---------- ---------- ---------- ---------- ----------

CTCCGCAACA AGCGCGAAGT GTGGCGAGTC AAGTTCACGC TGGCCAAGAT

CTCCGCAACA AGCGCGAGGT CTGGAGGGTC AAGTTCACCC TGGCCAAGAT

CCGCAAGGCC GCCCGGGAAC TGCTGACGCT TGATGAGAAG GACCCACGGC

CCGCAAGGCC GCTCGGGAAC TGCTGACGCT TGATGAGAAG GACCCACGGC

CCGCAAGGCC GCCCGGGAAC TGCTGACGCT TGATGAGAAG GACCCACGGC

CCGCAAGGCC GCCCGGGAAC TGCTGACGCT TGATGAGAAG GACCCACGGC

CCGCAAGGCC GCCCGAGAGC TGCTGACACT TGACGAGAAG GATCCACGGC

CCGCAAGGCC GCCCGGGAGC TGCTGACGCT GGACGAGAAG GACCCGCGGC

TCGTAAGGCT GCCCGGGAGC TGTTGACGCT GGACGAGAAG GATCCTCGGC

---------- ---------- ---------- ---------- ----------

CCGCAAGGCC GCCCGCGAGC TGCTGACGCT GGACGAGAAG GACCCGCGCC

---------- ---------- ---------- ---------- ----------

CCGCAAGGCC GCCCGGGAGC TGCTGACGCT GGACGAAAAG GACCCGCGGC

CCGCAAGGCT GCCCGGGAGC TGCTGACGCT GGATGAGAAA GACCCACGGC

CCGTAAGGCC GCCCGGGAGC TGCTGACGCT GGACGAGAAG GACCCGCGGC

CCGCAAGGCT GCCCGGGAGC TGCTGACGCT GGACGAGAAG GACCCACGGC

CCGCAAAGCT GCCCGGGAGT TGCTGACGCT GGACGAGAAG GACCCACGGC

---------- ---------- ---------- ---------- ----------

CCGCAAGGCG GCCCGGGAGC TGCTCACTCT CGATGAGAAG GACCCGCGGC

CCGCAAGGCG GCCCGGGAGC TGCTCACGCT GGACGAGAAG GACCAGAGGC

GTCTGTTCGA AGGTCTCTGG AAGGGGTACC TGAACAGCAG CTTATTGCCC

GTCTGTTCGA AGGTCTCTGG AAGGGGTACC TGAACAGCAG CTTATTGCCC

GTCTGTTCGA AGGTCTCTGG AAGGGGTACC TGAACAGCAG CTTATTGCCC

GTCTGTTCGA AGGT------ ---------- ---------- ----------

GTCTTTTCGA AGGTCTCTGG AAGGGGTACC GGAACAGCAG CTTATTGCCC

GTCTGTTTGA AGGTCTCTGG AAGGGGTACC TGAACAGCAG CTTATTGCCC

GTCTGTTTGA AGGTCTCTGG AAGGGATACC TGAAGAGCAG CTTATTGCCC

---------- ----CTCTGG AAGGGGTACC TGAAGAGCAG CTTATTGCCC

GGCTGTTCGA AGGT------ ---------- ---------- ----------

---------- ----CTCTGG AAAGGGTACC TGAACAGCAG CTTATTGCCC

GCCTGTTCGA AGGT------ ---------- ---------- ----------

GTCTGTTTGA AGGTCTCTGG AAGGGGTACC TGAACAGCAG TTTATTGCCC

GTCTCTTCGA AGGT------ ---------- ---------- ----------

GGCTGTTTGA AGGTCGCTGG AAGGGGTACC TGAACAGCAG CTTATTGCCC

GTCTGTTTGA AGGT------ ---------- ---------- ----------

---------- ----CTCTGG AAGGGGTACC TGAACAGCAG CTTATTGCCC

GTCTGTTCga gggtCTCTGA AATGGGTACC TGAATAGTAA CTTATTGCCC

GGCTGTTTGA AGGTCGCTGG AAGGGGTATC TGAAGAGCAG CTTGTTGCCC

CTGCTCCCCG AGCTCACCAG AATCACGCTG ATGGGGCTGG TGTTGTCCCG

CTGCTCCCCG AGCTCACCAG AATCACGCTG ATGGGGGTGG TGTTGTCCCG

CTGCTCCCCG AGCTCACCAG AATCACGCTG ATGGGGCTGG TGTTGTCCCG

---------- ---------- ---------- ---------- ----------

CTGCTCCCCG AGCTCACCAG AATCACGCTG ATGGGGCTGG TGTCGTCCCC

CTGCTCCCCG AGCTCACCAG AATCACGCTG ATGGGACTGG TGTTGTCCCC

CTGCTGCCCG AGCTCACCAG GATCACGCTG ATGGGGCTAG TGTTGTCCCC

CTGCTGCCCG AGCTCACCAG GATCACGCTG ATGGGGCTGG TATTGTCCCC

---------- ---------- ---------- ---------- ----------

CGGCTCCCCG AGCTCACAAG AATCACGCTG ATGGGGCTGG TGTTGTCCCC

---------- ---------- ---------- ---------- ----------

CTGCTCCCCG AGCTCACCAG AATCACGCTG ATGGGGCTGG TGTTGTCCCC

---------- ---------- ---------- ---------- ----------

CGGCTGCCCG AGCTCACCAG AATCACGCTG ATGGGGCTGG TGTTGTCCCC

---------- ---------- ---------- ---------- ----------

CTGCTCCCCG AGCTCACCAG AATCACGCTG ATGGGGCTCG TGTTGTCCCC

CTGCTCCCTG AACTGACCAG GATCACACTG ATGGGGCTGG TGTTCTCCCC

CTGCTCCCCG AGCTGACCAG GATGACGCTG ATGGGGCTGC TGTTGTCCCC

CATCGCCATG TCTCTGACCA AGACTGAGAG GACCATCATT GTGTCCATGT

CATCGCCATG TCTCTGACCA AGACTGAGGG GACCATCATT GTGTCCATGT

CATCGCCATG TCTCTGACCA AGACTGAGAG GACCCTCATT GTGTCCATGT

---CGCCATG TCTCTGACCA AGACTGATAG GACCCTCATT GTGTCCATGT

CATTGCTGTC TCCC------ --GCCGACAA GAGCAACGTC AAGGCCGCCT

CATCACCATG TCTCTGACCA AGACTGAGAG GGCCATCATT GTGTCCATGT

CATCACCATG TCTCTGATGA AGAATGAGAG AGCTATCATC ATGTCCATGT

CATCACCATG TCTCTGATGA AGAATGAGAG AGCTATCATC ATGTCCATGT

---CACCATG TCTCTGACCA AGAGCCAGAG GACCATCATC ATGTCCCTCT

CATCACCATG TCTCTGACCA GAGCTGAGAG GACCATCATC GTGTCCATGT

---CACCATG TCTCTGACCA AGGCCGAGAG GACCATCATC CTGTCCATGT

CATCACCATG TCTCTGACCA AGGCTGAGAG GACCATCATC ACATCCATGT

---CACCATG TCGCTGACCA AGGCCGAGAG GACCATCATC CTGTCCATGT

CATCGCCATG TCGCTGACCA AGGCCGAGAG GACCATCATC GTGTCCATGT

---CTCCATG TCTCTGACCA AGGTGGAGAG GACCATCATC ACGTCCATGT

CAT------- ---------- ---------- ---------- ----------

CATCGCCATG TCTCTGACCA AGACCGACAA GGCTATCATC CTCACCATCT

CATCACCATG TCTCTGACCA AGGCCGACAA GACCGCAGTC ATCTCCCTCT

GGGCCAAGAT CTCCACGCAG GCCGACACCA TCGGCACCGA GACTCTGGAG

GGGCCAAGAT CTCCACGCAG GCCGACACCA TCGGCACCGA GACTCTGGAG

GGGCCAAGAT CTCCACCCAG GCCGACACCA TTGGCACCGA GACTCTGGAG

GGGCCAAGAT CTCCACTCAG GCCGACACCA TCGGCACCGA GACTCTGGAG

GGGGTAAGGT CGGCAGCCAC GCTGGCGACT ATGGTGCCGA GGCCCTGGAG

GGGGCAAGAT CTCCACACAG GCCGACACCA TTGGCACTGA GACCCTGGAG

GGGACAAAAT GGCGCCTCAT GCAGAGCCCA TTGGCACTGA GACTCTGGAG

GGGAGAAGAT GGCTGCTCAG GCCGAGCCCA TTGGCACTGA GACTCTAGAG

GGGACAAGGT CTCCTCCCAG TCTGAGGACA TTGGCGCAGA GGCCCTGGAG

GGAGCAAGAT CTCCACACAG GCGGACCTCA TTGGCACCGA GACCCTGGAG

GGGGCAAGAT CTCCACCCAG GCGGATGCTA TTGGCACCGA GGCCCTGGAG

GGAACAAGAT CTCCACACAG GCGGACGCCA TCGGCACCGA GGCCCTGGAG

GGGGCAAGAT CTCCACACAG GCGGATGTCA TC-------- ----------

GGGGCAAGAT CGCCTCGCAG GCCGACGCCA TCGGCACCGA GGCCCTGGAG

GGGGCAAGGT CTCCACACAG GCTGACAGCA TCGGCACCGA GGCCCTGGAG

---------- ---------- ---------- ---------- ----------

GGAACAAGAT CGCCACTCAG GCTGACTCTG TTGGTACTGA GGCCTTGGAA

GGGGAAAGGC GGCCACCCAG GCTGACGCCA TCGGGGCCGA GTCTCTGGAG

AGGTGAGGGG TCCGCGCAGT TGCGCGCGCA CGGCTCCAAG GTGGTGGCCG

AGGTGAGGAC GCGACGCAGC TGCTGAGCCA CGGGCAGCGC ATGCTGGCGG

AGGTGAGGGG TCCGCGCAGC TGCAAGCGCA CGGCTCCAAG GTGGTGGCCG

AGGTGAG--- ---------- ---------- ---------- ----------

AGGTGAGGGC TCCGCGCAGG TCAGAGCGCA CGGCCAGAAG GTGGCGGACG

AGGTGAGGGG TCGGCGCAGC TGCGTGCGCA CGGCTCAAAG GTGGTGGCCG

AGGTAAGGGG TCTCAGCAGT TGCGTGCCCA CGGCTCCAAG ATCTTGGCCG

AGGTAAGGGG TCTCAGCAGT TGCGGGCCCA CGGCTTCAAG ATCATGACCG

AGGTAGGGGC TCCGCGCAGC TGCGCGCTCA CGGCGCCAAG GTGGCGGCCG

AGGTGAGGGC TCCGCGCAGC TGCGCGCGCA CGGCTCCAGG GTGGTGGCCG

AGGTCAGGGC TCGGCGCACC TGAGGGCGCA CGGCGCCAAG GTGGTGGCCG

AGGTGAGGGC TCCGCGCAGC TGCGCGCGCA TGGCTCCAAG GTGGTGGCCG

-------GGC TCGGCGCAGC TGCGCGCGCA CGGCTCCAAG GTGGTGGCCG

CGGTGAGGGC TCGGCGCAGC TGCGGGCGCA CGGCTCCAAG GTGGCGGCCG

AGGCGAGGGC TCGGCGCAGC TGCGCGCGCA CGGCTCCAAG GTGGTGGCCG

-------GGC TCAGCCCAGG TCAAGGCGCA CGGCAAAAAG GTGGCCGCCG

CGGTAAGGGC TCGGCTCAAA TCAAGGGTCA AGGCCAGAAA ATCGCCGACG

AGGTGAGGGC TCCGCTCAGA TCAAGGCTCA TGGCAAGAAG GTGGCCGACG

CCGTGGGCGA CGCCTGAGCG AGCTGCACGC CTACATCCTG CGCGTGGACC

CTGTGGGCGC GGCCTGGCGG ACCTGCACGC GCTCGTGCTG CGCGTGGACC

CCGTGGGCGA CGCCTGAGCG AGCTGCACGC CTACGTCCTG CGCGTGGACC

---------- ---------- ---------- ---------- ----------

CGCTGAGCCT CGCCTGAGCC ACCTGCACGC GCGCCAGCTG CGAGTGGACC

CCTTGGGCGA CGCCTGAGCG AGCTGCATGC CTACATCCTG CGGGTGGACC

CTGTGGGTGA TGCTTGAGCG AGCTACACGC CTACATCCTG CGTGTGGACC

CCGTAGGGGA TGCCTGAGCG AGCTGCATGC CTACATCCTG CGTGTGGATC

CGCTGGACAA AGCCTGAGCG AGCTGCACGC CTACGTGTTG CGTGTGGACC

CCGTGGGCGA CGCCTGAGCG AGCTGCACGC CTACGTGCTG CGCGTGGACC

-CGCTAACGA CGCCTCAGCG ACCTGCACGC CTACAAGCTG CGCGTGGACC

CCGTGGGCGA CGCCTGAGCG AGCTGCACGC CTACATCCTG CGCGTGGACC

CCGTGGGCGA CGCCTGAGCG AGCTGCACGC CTACGTCCTG CGCGTGGACC

CCGTGGGCGA GGCCTGAGCG AGCTGCACGC CTACGTGCTG CGCGTGGACC

CTGTGGGTGA CGCCTGAGCG AGCTGCACGC CTACATCCTG CGCGTGGACC

CACTGACCCA AGCCTGAGTG ACCTACACGC GCACAGGCTC CGAGTGGATC

CCGTGTCCTT GGCCTGAGCG ATCTGCACGC CCACAACCTG AAGGTGGATC

CTCTGTCCAC GGCCTGAGCG ACCTGCACGC CCACAAGCTG AGAGTGGACC

CGGTCAACTT CAAGGTGCAC GGCTCGGTGG TGGCCGCCGT GGGCGACGCG

CAGCCAACTT TCCGGT-CAC GGGCACATGC TGGCGGCTGT GGGCGCGGCG

CGGTCAACTT CAAGGTGCAC GGCTCGGTGG TGGCCGCCGT GGGCGACGCA

---------- ---------- ---------- ---------- ----------

CGGCCAGCTT CCAGGTGCAC GGCCAGGTGG CGGACGCGCT GAGCCTCGCG

CGGTCAACTT CAAggtgCAC GGCTCGGTGG TGGCCGCCTT GGGCGACGCT

CGGTCAACTT CAAGGTGCAC GGCTCGATCT TGGCCGCTGT GGGTGATGCG

CGGTCAACTT CAAGGTGCAC GGCTTGATCA TGACCGCCGT AGGGGATGCG

CGGTCAACTT CAAGGTGCAC GGCGCGGTGG CGGCCGCGCT GGACAAAGCG

CGGTCAACTT CAAGGTgCAC GGCTCGGTGG TGGCCGCCGT GGGCGACGCG

CCGTCAACTT CAAGGTGCAC GGCGCGGTGG TGGCCGCGCT -AACGACGCG

CGGTCAACTT CAAGGTGCAT GGCTCGGTGG TGGCCGCCGT GGGCGACGCG

CGGTCAACTT CAAGGTGCAC GGCTCGGTGG TGGCCGCCGT GGGCGACGCG

CCGTCAACTT CAAGGTgCAC GGCTCGGTGG CGGCCGCCGT GGGCGAGGCG

CGGTCAACTT CAAGGTgCAC GGTTCGGTGG TGGCCGCTGT GGGTGACGCG

CGGTCAACTT CCAGGTGCAC GGCAAGGTGG CGGATGCGCT GACCCTCGCC

CCGTCAACTT CAAGGTGCAC GGCAAATTGG CTGCCGCGCT GACTTTGGCA

CGGTCAACTT CAAAGTGCAC TGCCCGGCAC TGGCCTCAGT GCCCATGCCC

GTGAAGAGCA TCGACGACAT CTGAGCGAGC TGCACGCCTA CATCCTGCGC

GTGCGGCACA TGGACAACCT CTGGCGGACC TGCACGCGCT CGTGCTGCGC

GCGAAGAACA TCGACAACAT CTGAGCGAGC TGCACGCCTA CGTCCTGCGC

---------- ---------- ---------- ---------- ----------

GTGGAACGCC TGGACGACCT CTGAGCCACC TGCACGCGCG CCAGCTGCGA

GTCAAGAACA TCGACAACAT CTGAGCGAGC TGCATGCCTA CATCCTGCGG

GTTAAGAACA TCGACAACCT TTGAGCGAGC TACACGCCTA CATCCTGCGT

GTTAAGAGCA TCGACAACCT CTGAGCGAGC TGCATGCCTA CATCCTGCGT

GTCAAGAACA TCGACAACTT CTGAGCGAGC TGCACGCCTA CGTGTTGCGT

GTCAAGAGCA TTGACAACGT CTGAGCGAGC TGCACGCCTA CGTGCTGCGC

GTGCGCAGCC TCGACGACGT CTCAGCGACC TGCACGCCTA CAAGCTGCGC

GTCAAGAGCA TCGATAACAT CTGAGCGAGC TGCACGCCTA CATCCTGCGC

GTCAAGAGCA TCGACAACAT CTGAGCGAGC TGCACGCCTA CGTCCTGCGC

GCGGGCAGCG TGGACGACGT CTGAGCGAGC TGCACGCCTA CGTGCTGCGC

GTCAAGAGCA TCGATAATAT CTGAGCGAGC TGCACGCCTA CATCCTGCGC

GTCGGCCACA TGGACGACCT CTGAGTGACC TGCACGCCTA CAAGCTGAGG

GTCAGCCACC TGGATGACCT CTGAGCGATC TCCATGCCCA CAGACTCAAG

CTGACAGGTC CCGCTCTCTC CTTTCCCCAC CACAGACCTA CTTCTCCCAC

GTGGACCCGG TCAACCTGAG GAAGCTGCAT GTCCACGAGC TGTGGGTGGA

GTGGACCCAG CCAAC----- ---------- ---------- ----------

GTGGACCCGG TCAACCTGAG GCAACTGCAC GTCAACGAGC TGTGGGTGGA

---------- ---------- ---------- ---------- ----------

GTGGACCCGG CCAGCCTCAG CGACCTGCAC GCGCACAAGC TTCGGGTGGA

GTGGACCCGG TCAACCTGAG TGACCTGCAT GCGCACAAGC TGAGAGTGGA

GTGGACCCGG TCAACCTGAG CGACCTGCAT GCCCACAAAC TGCGTGTGGA

GTGGATCCGG TCAACCTGAG CGACCTGCAT GCCCACAAGC TGCGTGTGGA

GTGGACCCGG TCAACCTCAG CGACCTGCAC GCGCACAAGC TGCGGGTGGA

GTGGACCCGG TCAACCTGAG TGACCTGCAC GCTCACAAGC TGCGTGTGGA

GTGGACCCCG TCAACCTCAG CGACCTGCAC GCCTACAAGC TGCGCGTGGA

GTGGACCCGG TCAACCTGAG CGACCTGCAC GCCCACAAGC TGCGTGTGGA

GTGGACCCGG TCAACCTGAG CGAGCTGCAC GCCTACGTCC TGCGCGTGGA

GTGGACCCCG TCAACCTGAG CGACCTGCAC GCCCACAAGC TGCGGGTGGA

GTGGACCCGG TCAACCTCAG CGACCTGCAC GCTCACAAGC TGAGGGTGGA

GTGGACCCCG TCAACCTGAG TGACCTGCAC GCCTACAAGC TGAGGGTGGA

GTGGATCCCA CCAACCTGAG CGATCTCCAT GCCCACAGAC TCAAGGTGGA

TTCGACCTGA GCCACCTGAG CGACCTGCAC GCCCACAAGC TCAGAGTGGA

CCCAGGCAAC ATCAGGGAGA GTTCTAAAAC TCACAGCTAG CTCCAAAACA

---------- ---------- -TTCTAAAAC TCACAGCTAG CTCCAAAACA

CCCAGGCAAC ATCAGGGTGA GTTCTAAAAC TCACAGCTAG CTCCAAAACA

---------- ---------- -TTCTAAAAC TCACAGCTAG CTCCAAAACA

CCCGGTCAAC TTCAAGGTGA GTTCTAAAAC TCACAGCTAG CTCCAAAACA

CCCGGTCAAC TTCAAGGTGA GCTCTAAAAC TCACAGTTAG CTCCAAAACA

TCCTGTCAAC TTCAAGGTGT G-TCTAAAAC TCACAGCTAG GGCCAAA-CA

TCCCGTCAAC TTCAAGGTAT GCTCTAAAAC TCACAGCTAG GACCAAA-CA

CCCGGTGAAT TTCAAGGTGA GCTCTACGAC TCACAGCTAG CTCCAAAACA

CCCGGTCAAC TTCAAGGTGA GCTCTAAAAC TCACAGCTAG CTCCAAAACA

CCCCGTCAAC TTCAAGGTGA GCTCTAAAAC TCACAGCTAG CTCCAAAACA

TCCCGTCAAC TTCAAGGTGA GCTCTAAAAC TCACAGCTAG CTCCGAAACA

CCCGGTCAAC TTCAAGGTGA G--------- ---------- ----------

CCCGGTCAAC TTCAAGGTGA gCTCTAAAAC TCACGGCTAG CTCCAAAACA

CCCTGTCAAC TTCAAGGTGA GCTCTAAAAC TCACTGCTAG CTCCAGAACA

CCCTGTCAAC TTCAAGGTGA GCTCTG---- ---------- ----------

TCCCACCAAC TTCAAAGTTA GCTCTAAAAC TCACAGCTAT CTTCAAAACA

CCCGATCAAC TTCAAAGTGA GCTAGAAAAC CGGTGACGAT CATTGAGACA

ATAGAAATTT TAAACTACAA AAGATGAGTT GTATTCAGCA AATATAAAGG

ATAGAAATTT TAAACTACAA AAGATGAGTT GTATTCAGCA AATATAAAGG

ATAGAAATTT TAAACTACAA AAGATGAGTT GTATTCAGCA AATATAAAGG

ATAGAAATTT TAAACTACAA AAGATGAGTT GTATTCAGCA AATATAAAGG

ATAGAAATTT TAAACTACAA AAGATGAGTT ATATTCAGCA AATATAAAGG

ATAGAAATTT TAAACTACAA AAGATGAATT GTATTCAGCA AATATAAAGG

ATAG-AAATT TTAACTACAA AAGATGAGTT GTAGTCAGCA AATATAAAGG

ATAG-AAATT TTAACTACAA AAGATGAGTT GTA-TCAGCA AATATAAAGG

ACAGCAAGTT TAAACTACAA AAGATGAGCT GTATTCAGCA AATAGAAAGG

ATAGAAATGT TAAACTACAA AAGATTAGCT GTATTCAGCA AATAGAAAGG

ATAGAAATCG TAAACTACAA AAGATGAGTT GTATTCAGCA AATATAAAGG

ATAGAAATTT TAAACTACAA AAGATGAGTT GTATTCAGCA AATATAAAGG

---------- ---------- ---------- ---------- ----------

ATAGAAACTT TGAACTACAA AAGGTGAGTT GTATTCAGCA AATATGAAGG

ATATAAAGTT TAAACTACAA AAGGTGAGTT GTAGTCAACA AATATAAAGG

---------- ---------- ---------- ---------- ----------

ATAAAAATTT AAATCTACAA AAGATGAGTT GTATTCGGCA AATATAAATG

ATAAAAACCC CAAGCTAAGA AAGATGAGCT GTGCTCGGCA GAAAGGAACA

GTAACTCCTG TTGCAGTACC GAAGGAGTCA GAGTAGTTAC TTGTATTTCG

GTAACTCCTG TTGCAGTACC GAAGGAGTCA GAGTAGTTAC TTGTATTTCG

GTAACTCCTG TTGCAGTACC GAAGGAGTCA GAGTAGTTAC TTGTATTTCG

GTAACTCCTG TTGCAGTACC GAAGGAGTCA GAGTAGTTAC TTGTATTTCG

GTAACTCCTG TTGCAGTACC GAAGGAGTCA GAGTAGTTAC TTGTATTTCG

GTAACTCCTG CTGCAGTACT GAAGGAGTCA GAGTAGTTAC TTGTATTTCG

GTAACTTCTG CTGCAGTACT -GAGGAGTCA GAGTAGTTAC TTGTATTTTG

GTAACTCCTG TTGAAGTACT -GAGGAGTCA GAGTAGTTAC TTGTATTTTG

GTAGCTCCTG TGGCAGTACC CAAGGAGTCA TGGTAGTTAC TTGTATTTCG

GTAACCTCCG CTGCAGTACT AAAGGAG-CA GAGTAGTTAC TTGTATTTCG

GTAACTCCTG TTGCAGTACT GAAGGATTCA GAGTAGTTAC TTGTATTTCG

GTAACTCCTG TTGCAGTACT GAAGGAGTCA GAGTAGTTAC TTGTATTTCG

---ACTCCTG TTGCAGTACT GAAGGAGTCA GAGTAGTTAC TTGTATTTCG

GTGCCTCCTG CTGCAACACT GAACAAGTCA GAGTAGTTAC TTGTATTTCG

GTGACTCCTG CTGCAGTACC GAAGGACTCA GGT-AGTTAC TTGTATTTCG

---------- ---------- --AGGAGC-- -AGAAGTTAC TTGTGTCTCG

GTAACTCCTG TTGCTGTACT GAAGGAGTCA GAGTAGTTAC TTGTGTTTCG
[truncated: 6,662,449 more chars]
